# Evolutionary History of Mammalian Transposons Determined by Genome-Wide Defragmentation

**DOI:** 10.1371/journal.pcbi.0030137

**Published:** 2007-07-13

**Authors:** Joti Giordano, Yongchao Ge, Yevgeniy Gelfand, György Abrusán, Gary Benson, Peter E Warburton

**Affiliations:** 1 Department of Genetics and Genomic Sciences, Mount Sinai School of Medicine, New York, New York, United States of America; 2 Department of Neurology, Mount Sinai School of Medicine, New York, New York, United States of America; 3 Center for Translational Systems Biology, Mount Sinai School of Medicine, New York, New York, United States of America; 4 Laboratory for Biocomputing and Informatics, Boston University, Boston, Massachusetts, United States of America; 5 Departments of Computer Science and Biology, Boston University, Boston, Massachusetts, United States of America; Fred Hutchinson Cancer Research Center, United States of America

## Abstract

The constant bombardment of mammalian genomes by transposable elements (TEs) has resulted in TEs comprising at least 45% of the human genome. Because of their great age and abundance, TEs are important in comparative phylogenomics. However, estimates of TE age were previously based on divergence from derived consensus sequences or phylogenetic analysis, which can be unreliable, especially for older more diverged elements. Therefore, a novel genome-wide analysis of TE organization and fragmentation was performed to estimate TE age independently of sequence composition and divergence or the assumption of a constant molecular clock. Analysis of TEs in the human genome revealed ∼600,000 examples where TEs have transposed into and fragmented other TEs, covering >40% of all TEs or ∼542 Mbp of genomic sequence. The relative age of these TEs over evolutionary time is implicit in their organization, because newer TEs have necessarily transposed into older TEs that were already present. A matrix of the number of times that each TE has transposed into every other TE was constructed, and a novel objective function was developed that derived the chronological order and relative ages of human TEs spanning >100 million years. This method has been used to infer the relative ages across all four major TE classes, including the oldest, most diverged elements. Analysis of DNA transposons over the history of the human genome has revealed the early activity of some MER2 transposons, and the relatively recent activity of MER1 transposons during primate lineages. The TEs from six additional mammalian genomes were defragmented and analyzed. Pairwise comparison of the independent chronological orders of TEs in these mammalian genomes revealed species phylogeny, the fact that transposons shared between genomes are older than species-specific transposons, and a subset of TEs that were potentially active during periods of speciation.

## Author Summary

Transposable elements (TEs) are interspersed repetitive DNA families that are capable of copying themselves from place to place; they have literally infested our genome over evolutionary time, and now comprise as much as 45% of our total DNA. Because of their great age and abundance, TEs are important in evolutionary genomics. However, estimates of their age based on DNA sequence composition have been unreliable, especially for older more diverged elements. Therefore, a novel method to estimate the age of TEs was developed based on the fact that as TEs spread throughout the genome, they inserted into and fragmented older TEs that were already present. Therefore, the age of TEs can be revealed by how often they have been fragmented over evolutionary time. We performed a genome-wide defragmention of TEs, and developed a novel objective function to derive the chronological order of TEs spanning >100 million years. This method has been used to infer the relative ages of TEs from seven sequenced mammalian genomes across all four major TE classes, including the oldest, most diverged elements. This age estimate is independent of TE sequence composition or divergence and does not rely on the assumption of a constant molecular clock. This study provides a novel analysis of the evolutionary history of some of the most abundant and ancient repetitive DNA elements in mammalian genomes, which is important for understanding the dynamic forces that shape our genomes during evolution.

## Introduction

The most abundant type of DNA in the human genome consists of the four major classes of interspersed transposable elements (TEs), comprising ∼45% of our total DNA [1]. Short interspersed repeat elements (SINEs), long interspersed repeat elements (LINEs), and retrovirus-like long terminal repeat (LTR) retrotransposons propagate by reverse transcription of an RNA intermediate. DNA transposons move by a direct “cut and paste” mechanism [2]. TEs have been active in mammalian genomes for hundreds of millions of years, and have had a huge impact on our genomic structure [3,4]. Each TE has had a distinct period of transpositional activity in which it has spread through the genome, followed by inactivation and accumulation of mutations. Both SINE and LINE transpositions have been associated with insertional mutations causing human disease and pseudogene formation [1]. TEs may actively influence the expression of nearby genes, usually due to the regulatory promoter and terminator sequences found in LTRs [5].

TEs in the human and other genomes have been classified into a comprehensive database, called Repbase [6]. A program called Repeat Masker [7] was developed in order to identify all known repeat elements based on homology to the derived consensus sequences curated in Repbase. Repeat Masker has proven to be extremely valuable in gene identification and genome annotation, primarily by “masking” transposable elements in query sequences during homology searches so that the presence of a common transposon does not lead to many spurious, biologically uninteresting matches. Repeat Masker also provides a wealth of information regarding the classification, genome position, length, fragmentation, and divergence of each repeat element.

Each copy of a particular TE in a genome is derived from an active sequence that, once transposed, has accumulated mutations randomly and separately from other copies [3]. Consensus sequences of the original active copies, found in Repbase [8], have been derived from multiple sequence alignments of the present-day diverged copies. The age of these elements can be inferred from the average sequence divergence of the copies from the consensus sequence, and such classification has been applied to both Alu [9,10] and L1 [11] elements, permitting assignment of approximate ages [3]. However, these divergence-based classifications are limited by the assumption that the mutation rate, or molecular clock, has been constant both over time and between the different classes of transposable elements [12,13]. Substitution rates will depend on the original sequence of the element, especially the CpG frequency, because of its higher mutation rate. Substitution rates are known to change significantly during evolution and to differ between species, chromosomes of the same species, and even regions of the same chromosome [14–16]. Furthermore, the variance in percent divergence within a TE family will be dependent on both the length and age of the element. Hence, while estimates of the age of younger TE subfamilies have been presented [9–11], this has not been possible with older, more diverged elements.

Nevertheless, the apparent age of TEs is increasingly being used to obtain reference points in phylogenomic analysis [17]. Schueler et al. relied on the relative ages of LINE elements to date different parts of the human X chromosome centromeric alpha satellite arrays [18,19]. Specific insertions of MLT1A0 and L1MA9 elements were used as evidence for the sister–taxon relationship of primates and rodents [20,21]. Recently, evidence has been presented that some individual TEs have been exapted for use as conserved, functional, noncoding elements in mammalian genomes, which places these particular elements under selective pressure [22–25].

This study presents a novel genomic analysis of TE evolution and its impact on genomic organization, which will greatly facilitate the analysis of TEs for use in phylogenomics. A genome-wide defragmentation of TEs in the human and other mammalian genomes was performed, and the number of times that each TE has inserted into each other TE was compiled in a matrix. A novel computational method was developed that uses the age information implicit in the patterns of TE insertions to determine the relative chronological age of TEs in the human and other genomes spanning over 100 million years, independent of sequence divergence and the molecular clock. This method confirms the relative ages of TEs within classes, and was used to determine the relative ages of TEs between different classes and for older elements for which sequence divergence is particularly unreliable. This study also provides the methodological framework for the analysis of the patterns of interruptions of TEs by TEs on a genome-wide level, which represents a large, essentially untapped genomic dataset that is of fundamental importance regarding TE classification and organization. The data and analysis tools supplied here will provide a rich source of genomic information for data mining to further explore transposon biology and genome evolution.

## Results

### Transposon Defragmentation

**Figure 1:**
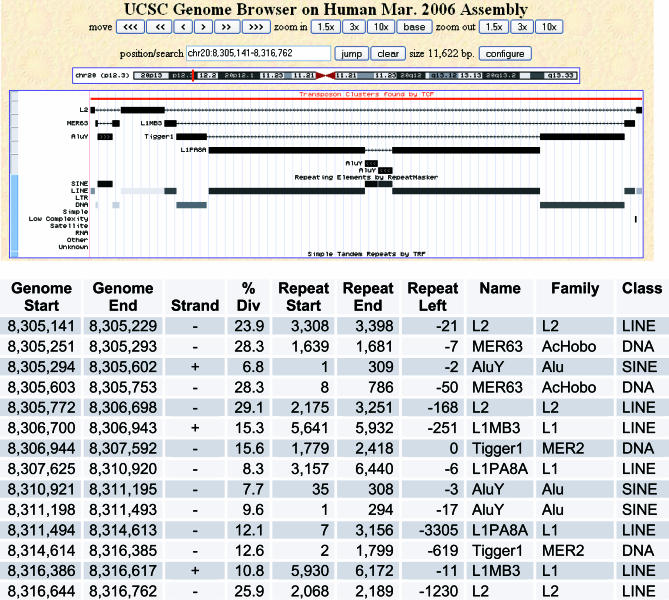
Defragmentation of an ∼11 kb TE Cluster by TCF A window from the UCSC genome browser showing an 11-kb transposon cluster from Chromosome 20p12.3. The uploaded custom track output provided by TCF and the output from the Repeat Masker track are shown. An L2 LINE (on the minus strand) has been interrupted by two TEs, a DNA transposon MER63, and a LINE L1MB3. The MER63 element has in turn been interrupted by an AluY element. The L1MB3 has been interrupted by a DNA transposon Tigger1 element, which has in turn been interrupted by a LINE L1PA8, which in turn has been interrupted by two Alu Y elements. This analysis reveals the evolutionary history of this region of the genome by defragmentation of TE clusters. The cluster table (bottom) shows Repeat Masker data for each TE fragment collected by TCF. Columns in the table are Genome Start, Genome End (starting and ending hg18 genomic coordinates for each TE fragment), Strand, % Divergence (of TE fragment from consensus), Repeat Start, Repeat End, Repeat Left (repeat indices relative to the derived consensus), and Name, Family, and Class (of each TE fragment).

The constant bombardment of the human genome by different TEs over millions of years has resulted in the high density of TEs in the human genome. During this time, many TEs have directly inserted into the sequence of other TEs that were already present, splitting the original TE into two noncontiguous TE fragments. We define the occurrence of TEs that interrupt other TEs as “transposon clusters.” Large transposon clusters can reveal the evolutionary history of regions of the human genome (Figure 1) resulting from the succession of transposition events over time. The relative age of the TEs in transposon clusters is implicit in their organization, where newer TEs have interrupted older TEs that were already present.

We have developed a software package called Transposon Cluster Finder (TCF; available at http://www.mssm.edu/labs/warbup01/paper/files.html) that identifies transposon clusters in the human genome by defragmentation of TEs and identification of TEs that have inserted into them. TCF starts with the collection of TE fragments provided by Repeat Masker [6,7]. Potential transposon clusters were initially identified by collecting sets of transposon fragments that (1) had the same name, (2) were on the same strand, and (3) were separated by ≤500 bp of nontransposon (not Repeat Masked) DNA sequence. Within these potential clusters, TE pairs were defragmented based on the difference in repeat indicies (ΔRI; see Materials and Methods). TCF provides a custom track to visualize all TE clusters in the human genome on the University of California Santa Cruz (UCSC) genome browser (http://genome.ucsc.edu/cgi-bin/hgGateway), as shown for the examples in Figures 1 and S1.

TCF identifies common family-specific variations in patterns of TE occurrence that do not represent independent transposition events [26]. TCF identified 3,101 examples of intact LTR transposons in which two LTRs with the same name precisely flank a full-length internal LTR element in the same orientation, and the second LTR was not counted as an independent transposition event (Dataset S1; example in Figure S1). TCF also identified 2,273 examples of L1 LINE elements that contain a 5′ inversion, proposed to be due to the twin priming mechanism [27], and were counted as a single interruption (Dataset S2; example in Figure S1). Both the intact LTR and the 5′ L1 inversions are detected regardless of whether they have undergone subsequent fragmentation. Human endogenous retroviruses (HERVs) such as HERV-H show recurrent patterns of specific deletions due to transposition complementation in *trans,* where transpositionally inactive elements can nevertheless be packaged together with active elements in viral particles and be propagated in the genome [28]. These elements were properly defragmented by TCF and counted as single interruptions (Figure S1).

The genome also contains many types of tandem repeats that have been generated from TE fragments that have spread by processes, including replication slippage or unequal crossing-over, that appear as spurious clusters or interruptions. Clusters that contained many interruptions of the same TE were screened for possible tandem repeats. A total of 40 clusters were found that contained tandemly repeated TE fragments, often amplified internal portions of a larger more complete TE (Dataset S3; example in Figure S1). In addition, several larger arrays of tandem repeats contained TE clusters that were duplicated in each repeat unit, or contained spurious clusters because of defragmentation of TEs in adjacent tandem repeats (Dataset S4; example in Figure S1). Spurious interruptions seen in tandem repeats were removed from the dataset by exclusion of the genomic regions. Finally, regions of segmental duplications in the human genome (hg18) were searched to identify clusters that had been duplicated one or more times, and only a single copy of each was included in the dataset (Dataset S5). The custom track provided by TCF (available for upload from the Datasets) shows all clusters, but indicates which were excluded from the final dataset as not representing independent transposition events.

Running TCF on hg18 after removing the interruptions described above yielded 307,412 clusters, which contain 636,125 interruptions and cover 542Mbp (or ∼19% of the genome and >40% of all transposon base pairs). The largest cluster observed covers 91 kb on chromosome Xq13.3 (Figure S1), found in a region of the X chromosome previously noted for a high density of LINEs [29]. A 21-kb cluster in chromosome band Xq11.22 contained 86 interruptions, the most observed in any single cluster (Figure S1). A Web-based Cluster Browser (http://sungene-bk.genetics.mssm.edu/cluster/index.html) is available that permits the user to query for any TEs interrupting any other TEs, with wild cards available, across the human genomes, and provides cluster tables (as in Figure S1) and links to custom tracks on the UCSC genome browser.

### Interruptional Matrix Analysis

TE dispersal in mammalian genomes can be characterized by a period of transpositional activity during which copies of the TE are spread throughout the genome, followed by gradual inactivation by loss of transpositional ability due to accumulated damage. The remnant copies of the TE remain behind and become further degraded over time by mutational events, including fragmentation by the insertion of newer TEs. The result, over eons, is that older TEs will be heavily interrupted by newer elements, but will not have inserted into newer elements. In contrast, newer elements, with a relatively recent period of activity, will have inserted into older elements that were present in the genome, but will not be interrupted by older elements. Elements of intermediate age will have both inserted into older elements and been themselves fragmented by newer elements.

The TCF analysis presented above provided an accurate count of the number of times every TE interrupts every other TE, and takes into account the most common family-specific variation that does not represent independent transposition events [26]. Therefore, computational methods were developed to take this unique dataset provided by TCF and determine the relative age of TEs in the genome based on interruptions of TEs into each other.

Many of the 908 types of TEs in the human genome are found in very few copies, and therefore interact with none or very few other TEs (interactions defined as either getting interrupted by or interrupting another TE; Table S1). Therefore, a method was developed to identify a subset of TEs that interacted with a certain percentage of other TEs, which was defined as percent connectedness (see Materials and Methods). For our initial analysis, the percent connectedness was set at 29% (each TE interacts with at least 29% of all other TEs). This provided a set of 360 TEs for further analysis, which nonetheless represented >95% of all TEs and >92% of all interruptions found in clusters by TCF.

**Figure 2:**
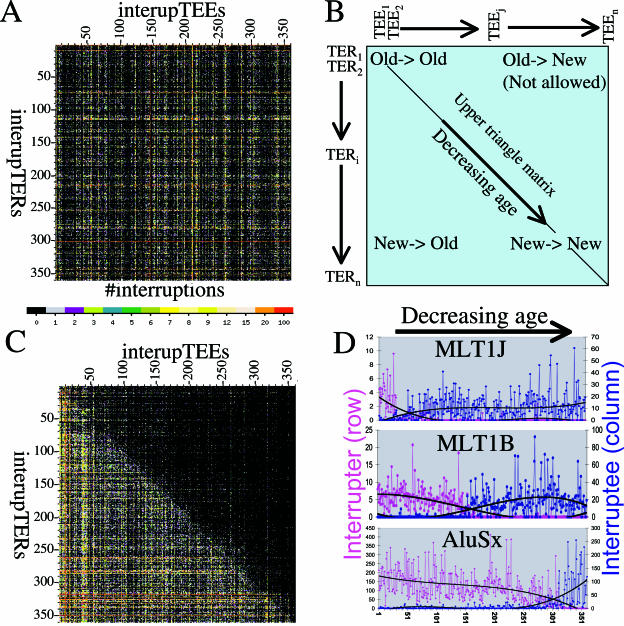
Interruption Matrix Analysis of the Chronological Order of TEs (A) A 360 × 360 adjacency matrix showing the number of times that each of the 360 human TEs interrupt each of the other 360 TEs, with values represented as a heat map as indicated. The TEs are shown in the same order on both the horizontal and vertical axes. This matrix shows a random TE order, and has an upper triangle matrix penalty score of ∼45,000. (B) Schematic of the matrix with TEs arranged in the correct chronological order from oldest to youngest (decreasing age) on both the horizontal axis (left to right) and the vertical axis (top to bottom). The corners of this matrix will contain points that represent old into old TEs (top left), new into old TEs (bottom left), and new into new TEs (bottom right). New into old TEs (top right) are not expected. This forms the basis for the objective function, which minimizes the upper triangle matrix by element repositioning (see text). (C) The 360 × 360 adjacency matrix after performing the repositioning algorithm. This represents one solution from one starting order, with a penalty of ∼7,800. There are 360! possible orders, which represents a state space that is far too large (∼10^500^ orders) to search completely. (D) Graphical illustration of the results for three TEs of different relative ages. For each TE, the pink graph shows the amount that the TE has interrupted the other elements (interrupTER row in the matrix), and the blue graph shows the amount that the TE has been interrupted by other TEs (interrupTEE column in matrix). The TEs are arranged along the horizontal axis in the final chronological order as determined by IMA. The MLT1J element (top) is relatively old (position 32), and interrupts only a few relatively old elements (pink), but is interrupted by many newer elements (blue). The MLT1B element (middle) is of intermediate age (position 154), and gets interrupted by newer elements (blue) and interrupts older elements (pink) in similar amounts. The AluSx (bottom) is relatively new (position 317), and interrupts many older elements (pink) but is only interrupted by a few newer elements (blue). The values in these graphs have been normalized as described in Materials and Methods. A polynomial trend line of power 3 (black curve) is fitted to each set of points.

The number of times that each of these 360 TEs interacts with every other TE was displayed as an *n* × *n* (360 × 360) adjacency matrix (Figure 2A). Each point in the matrix shows the number of times that the TE on the vertical axis (the interrupTER) has transposed into the TE on the horizontal axis (the interrupTEE) (Figure 2A). We realized that a hypothetical matrix where the TEs are arranged in the correct chronological order of decreasing in age on both the horizontal (left to right) and vertical (top to bottom) axes (Figure 2B) would have certain properties as follows. The top left corner of the matrix represents old TEs interrupting old TEs; the bottom left corner represents new TEs interrupting old TEs; and the bottom right corner represents new TEs interrupting new TEs. The top right corner represents old TEs interrupting new TEs, which should not be observed. Thus, in theory, the region of the matrix above the diagonal, the upper triangle submatrix, should be mainly populated by zeros (no interruptions). Nonzeros will, however, be found above the diagonal when pairs of TEs have both interrupted each other, which indicates that these TEs had overlapping periods of activity (were contemporaneous). Additional nonzeros above the diagonal might also represent defragmentation errors, cluster misidentification, or other mutational events that give the appearance of TE insertion. Notably, interruptions of the same type of TEs into themselves (which would be recorded directly on the matrix diagonal) are not scored due to the fact that they are difficult to confidently identify and do not affect the ordering analysis.

**Figure 3:**
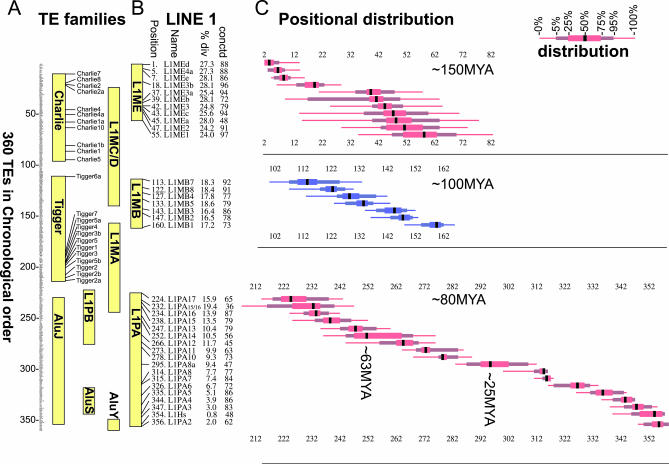
Chronological Ordering of Human TEs (A) Chronological order of 360 human TEs as derived by IMA. The individual names of the elements are not visible in Figure 3 (see Table S2 for full dataset). The range of positions of several TE families is shown (yellow bars) to illustrate the agreement with previous phylogenetic age analyses. The names and positions of the Charlie and Tigger families of DNA transposons are shown. (B) Position of individual elements from the L1 subfamilies L1ME, L1MB, and L1PA. Also shown is the median percent divergence from the Repeat Masker–derived consensus sequence (Table S2), and the percent connectedness of this TE in the matrix (see text and Table S2). In general, the percent divergence agrees well with the relative age of the element as determined by IMA. (C) The positional distribution for the TEs listed in (B) is displayed on the horizontal axis, relative to the overall chronological order of 360 elements on the vertical axis. (Thin line, range of the lowest 5% or highest 5% of positions calculated; thicker line, range of the next lowest 20% or next highest 20% of positions calculated; thickest line, range of middle 50% of positions calculated; black bar, median position). The chronological order in (A) was derived by ordering the medians of the positional distributions.

Therefore, we developed a computational method called interruptional matrix analysis (IMA) that performs systematic repositioning of all elements on the axes of the *n* × *n* matrix, and searches for an ordering that minimizes the summation of nonzero entries (hereafter called the penalty score) in the upper triangle matrix, selecting a new order when the penalty score decreases. Instead of direct summation of nonzero entries, the penalty score uses a continuous log function for values greater than 3 to prevent TEs with large numbers of interruptions from dominating the results (see Materials and Methods). Starting from a random order (with an initial penalty score of ∼45,000; e.g., Figure 2A), approximately seven rounds of repositioning each element were required to reach a minimum penalty score (of ∼7,800), where changing the position of any element either does not change or increases the penalty score (e.g., Figure 2C). Note that in the final ordering, the oldest, newest, and intermediate age elements follow the expected patterns of fragmentation and insertion described at the beginning of this section (Figure 3D).

The IMA algorithm is a version of hill climbing. A single run of IMA will find a penalty score that represents a local minima from that random starting order, but this is not guaranteed to be the ordering with the overall lowest possible minimum penalty score for the entire matrix. Furthermore, inevitable errors in the defragmentation data preclude using any single result of IMA as a final solution. Therefore, in order to optimize the objective function over the very large (360! or ≈10^500^) number of possible orders, we chose to estimate the correct ordering from many independent runs of the method. IMA was run 100,000 times, starting each time from a different randomized order of TEs, which resulted in a distribution of possible positions for each of the 360 TEs in chronological order (see below). A chronological order was obtained by ordering the TEs by their median positions, resolving ties using their mean positions (Figure 3 and Table S2).

A subset of this final matrix is shown for the L1PA family of primate specific LINEs (including L1Hs) in Table 1. The L1PA elements are shown in the final chronological order derived by IMA (Figure 3), with decreasing age running from top to bottom and left to right. The ordering of these elements is in remarkable agreement with published chronologies, in that numerical order (e.g., L1PA15, L1PA14, etc) reflects relative age (Figure 3B) [11,30]. The number of times each L1PA element has inserted into each other L1PA element is shown (Table 1). As expected, the older elements are heavily interrupted by younger elements, indicated by the relatively large positive values below the diagonal of the matrix. Conversely, the newer elements are not interrupted by the older elements, indicted by the abundance of zero values above the diagonal of the matrix. Several larger values appear above but near the diagonal, which represent bona fide interruptions of contemporary elements into each other (e.g., 12 interruptions of L1PA15 into L1PA16, and six interruptions of L1PA16 into L1PA15). A notable discrepancy is the placement of L1Hs, the newest and only remaining active L1 element in the human genome, slightly before the inactive L1PA2 in the chronological order (Figure 3).

**Table 1:**
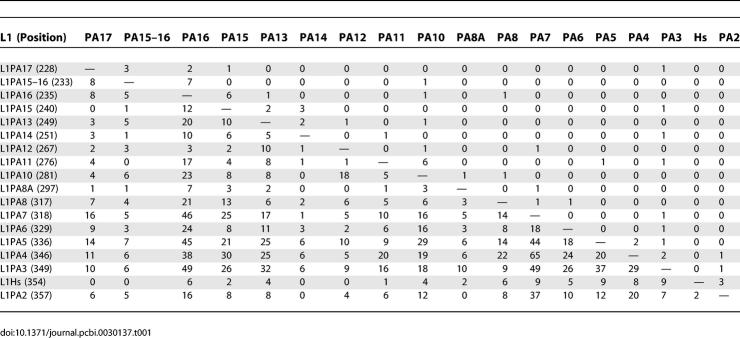
Interruptions of the LINE L1PA Family into Each Other

### Chronological Order of Human TEs

The chronological order derived from this IMA method agreed very well not only with the L1PA elements, but also with the other families of TEs for which limited phylogenetic analyses has been performed (Figure 3A). For example, the oldest TEs found by this method include LINE L3 and LINE L2, and the MIR elements that were dependent on them for transposition [26,31]. The different subfamilies of LINE1 are in remarkable agreement with published chronologies based on sequence divergence [11], including an overlap between the L1M (mammalian) and L1PA and L1PB (primate) elements [32]. The radiations of the Charlie and later Tigger families of DNA transposons are also observed (however, see below for further analysis) [3]. The relative age of the Alu element families is also consistent with published reports [9]. Note that the chronological order in general agrees with the median percent divergence. Thus, we conclude that our novel method of determining transposon chronology is accurate and robust, and can be used to infer the relative age of the human TEs both within and between different classes.

Running IMA from 100,000 random starting orders resulted in a distribution of possible positions for each of the 360 TEs in chronological order (Figure 3 and Table S2). The width of this distribution represents an estimate of the confidence of the position of each TE in the chronological order. Since TEs had a distinct period of activity and did not occur at a single point in time, these positional distributions may represent useful estimates of the relative timespan of transpositional activity of each individual TE. The positional distributions for the LINE 1 subfamilies L1ME, L1MB, and L1PA are shown in Figure 3C. The fact that these positional distributions overlap within each subfamily support the continuous evolution of these elements over time, suggested previously by derived phylogenetic trees [30,32]. We suggest that overlapping distributions represent TEs that were contemporaneous with each other in time (Figure 3C), even though there may not be any examples where they actually transposed into each other. The width and overlaps of the positional distributions of older elements may be somewhat extended, because TEs with a high percent divergence from the consensus may be more prone to Repeat Masker misidentifcation of specific elements within subfamiles.

### Analysis of DNA Transposons in the Human Genome

**Figure 4:**
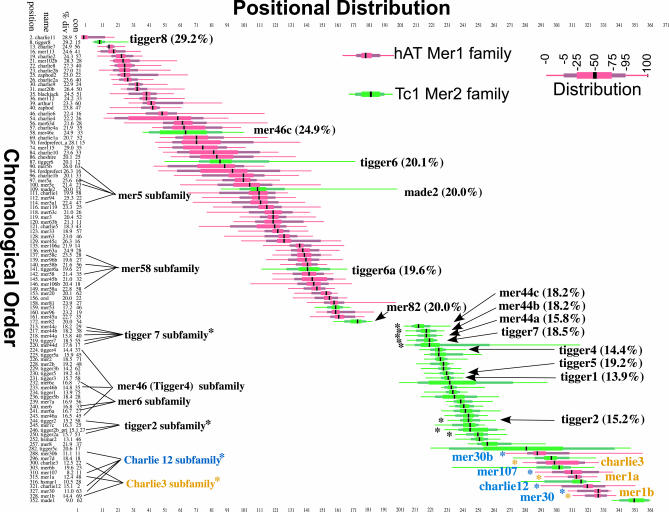
Analysis of DNA Transposon Activity in the Human Genome The chronological order for MER1 (red) and MER2 (green) families of DNA transposons, taken from the 405 elements used in this run of IMA, is shown on the left (vertical axis) from oldest (top) to youngest (bottom). The positional distribution is shown on the horizontal axis. The name of each element, its position in the chronological order (out of 405 elements), the median percent divergence (Table S2), and the percent connectedness (Table S2) are shown. The two major radiations of MER1 and MER2 DNA transposons can be seen. Subfamilies of DNA transposons containing autonomous parental elements and dependent nonautonomous elements are indicated. The following DNA transposons were not included in this figure because of either low connectedness and/or long positional distribution, for space and clarity (position name; % divergence; connectedness): (37 MER102a- 27.9; 5), (41 MER69b- 25.1; 20), (45 MER91- 23.9; 6), (59 MER91b- 25.8; 9), (72 MER97c- 23.6; 33), (85 MER117- 28.1; 15), (95. MER69a- 24.3; 10), (103 MER91a- 30.4; 12), (105, MER91c- 26.5; 9), (106 MER58d- 19.9; 10), (110 MER97a- 22.6; 7), (117 MER97b- 24.4; 5), (143 Tigger6b- 19.0; 5), (127 MER4–5 21.4; 15), (125 MER45-r 21.0; 13), and (210 pMER- 15.9; 0).

Examination of the results obtained above for the 360 human TEs (Figure 3) showed agreement with the two major radiations of Charlie and Tigger DNA transposons in the human genome [3]. However, we observed that the Tigger6a element was placed significantly earlier in the chronological order (position 111) than the eleven other Tigger elements, which clustered tightly together from positions 185 to 213 (Figure 3A), suggesting that Tigger6a was active at an earlier time than the other Tigger elements. We used TCF and IMA to further investigate the evolutionary history of DNA transposons in the human genome. The Charlie and Tigger DNA transposons belong to the hAT medium reiterated sequence 1 (MER1) and the Tc1-like MER2 families, respectively, which are distinguished by the structure of the target site duplication and the terminal inverted repeat [33]. IMA was run using an interruption matrix that included the 45 additional human DNA transposons from the MER1 and MER2 families, as classified by Repeat Masker, for a total of 405 TEs (Table S2). Figure 4 shows the chronological order and positional distribution of the MER1 (red) and MER2 (green) DNA transposons from this run of IMA, which again shows the two major radiations of MER1 followed by MER2. However, several additional MER2 elements were apparently active quite early, especially Tigger8 (position 8) and MER46c (position 58). Tigger6 (position 87) is also positioned early, suggesting that the Tigger6 subfamily (Tigger6 and Tigger6a) occurred earlier than most of the other Tigger elements. The majority of MER2 activity occurred following the MER1 activity, with the remaining Tigger elements active during this period. However, another period of MER1 activity apparently occurred following the MER2 activity. Importantly, the median percent divergence for these elements is consistent with the periods of activity found by IMA.

Many DNA transposons are found as internal deletion products of larger intact transposons. These nonautonomous elements retain intact terminal inverted repeat sequences but are dependent on transposases from autonomous “parental” transposons for their transposition [33]. The second period of activity of MER1 elements consist of two distinct subfamilies of Charlie elements and their nonautonomous deletion products, the Charlie12 element and deletion products MER30, MER30b, and MER107, and the Charlie3 and deletion products MER1a and MER1b. The nonautonomous members of transposon subfamilies would be expected to be “active” only when the parental autonomous transposon is active, and IMA has independently grouped these elements together in time with no a priori consideration of their sequence structure or subfamily classification. Additional subfamilies are also grouped together, including Tigger7 (position 219) and its nonautonomous elements MER44a, MER44b, MER44c, and MER44d (positions 218, 217, 213, and 220, respectively), and others (Figure 4). However, not every subfamily appeared to group correctly, such as MER46c (position 58), which did not group with Tigger4 (position 224) and its other nonautonomous family members MER46a and MER46b (positions 243 and 233, respectively). However, the relatively high median divergence (24.9%) of MER46c compared with the 14%–15% divergence of the other Tigger4 elements supports the finding of IMA, and suggests that MER46c may be derived from another Tigger element that was active earlier than Tigger4. These overall results are consistent with a recent analysis of DNA transposons in human and primate lineages [34].

### Analysis of Additional Mammalian Genomes

**Figure 5:**
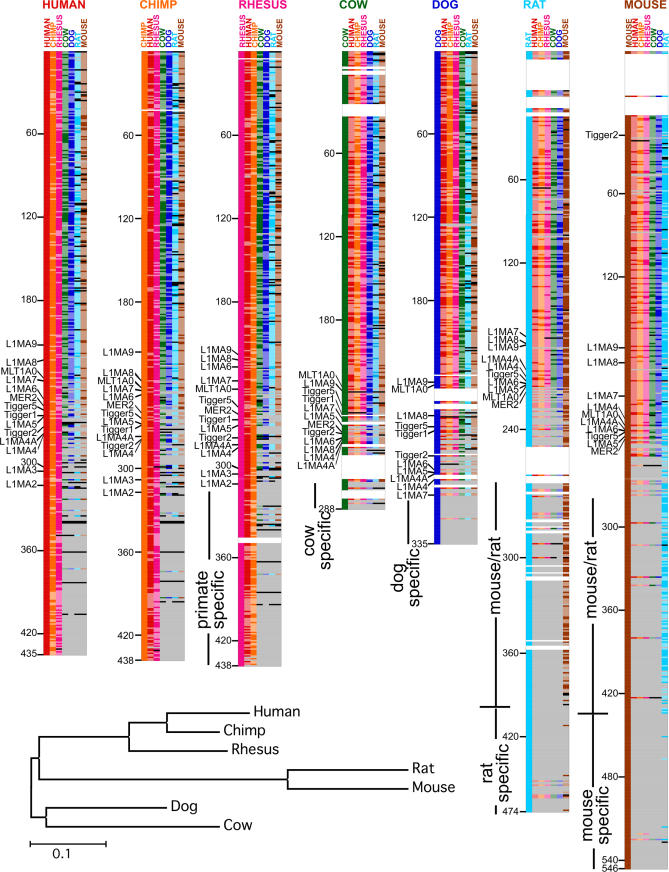
Comparison of Transposon History in Seven Mammalian Genomes A colorimetric pairwise comparison of the chronological order of the TEs from seven mammalian genomes. Each of the seven species is indicated by a different color. Each species is shown in turn as the reference genome (indicated on top), with the chronological order derived from IMA shown on the left. For each reference genome, the TEs from the other six genomes are shown aligned to the reference genome. TE names in reference genomes have been replaced in this figure by solid-color bars due to space considerations (see Figure S2 for TE names and full details). When a TE in the aligned genome matches the position in the chronological order of the reference genome (based on the criteria described in Materials and Methods), it is indicated by a solid-color bar corresponding to the species, and when it does not match the position (but is found in the reference genome), it is indicated by a lighter (stippled) color bar. A gray bar indicates that the TE was in the reference genome, but is not found at all in the aligned genome. A black bar indicates the TE was in both the reference and aligned genome, but was not ordered in the aligned genome (not in the set used for IMA analysis; see Materials and Methods). The total count of the matching (solid), not matching (stippled), not in genome (gray), and not in set (black) bars are shown in Table S4 for each reference genome. Subsequent to this pairwise comparison, the list of TEs from each genome were aligned to the human genome by insertion of spaces (white bars), which helps to maintain the position of TEs down the lists in order to allow comparison across the different species. After the point of divergence of the mouse and rat clade from the rest of the mammalian genomes, the rat genome is aligned to the mouse. Neighbor-joining tree of the seven mammals is shown at the bottom. Note that the tree is unrooted.

To provide further insight into transposon history across multiple mammalian species, TCF was run on six additional mammalian genomes for which Repeat Masker data were available (from the UCSC genome browser; Figure 5 and Table S3). Each species contained a distinct set of TEs, including elements that were either species-specific or shared between two or more genomes (see Materials and Methods). An independent chronological order for the set of TEs found in each genome was derived using IMA. These chronological orders from different species were compared in a pairwise manner to examine the extent of agreement and the regions of overlap and divergence (Figure 5 and Tables S4 and S5). The position of each element in the chronological order is represented by the positional distributions calculated by IMA (e.g., Figure 3), so that the order of elements between two species need not be exactly the same to represent significant agreement. Elements were considered to be in matching positions if the positional distributions were significantly overlapping (see Materials and Methods). These pairwise comparisons are shown in Figure 5, where each genome is represented by a different color. See Figure S2 to access the original Excel file with full details. Matching TEs are shown by a solid-color bar, nonmatching TEs are shown by a lighter stippled bar, and TEs not present are shown by a gray bar (see Figure 5 legend for details).

Older TEs (starting at the top of the chronological orders) are shared between the different species. The mouse and rat share fewer old TEs with the other species, consistent with a higher mutation rate in the rodent lineages, making the older TEs less recognizable by Repeat Masker [35,36]. The point of divergence of rat and mouse from the other species is visible as the position where the majority of TEs are no longer shared, after which rat and mouse share many additional TEs (Figure 5, blue and brown bars) and form a rodent clade. Most species-specific TEs in the cow, dog, rat, and mouse are found in the newer positions at the bottom of the chronological order. Human, chimp, and rhesus show the best agreement between the chronological orders (Figure 5 and Table S4), forming a primate clade with few species-specific elements. The cow and dog genomes also show a similar period of overlap with the primates and rodents, followed by a series of species-specific elements. These results further confirmed the ability of TCF and IMA to accurately age TEs across multiple mammalian species.

The pairwise comparison of the chronological orders of TEs provides a novel method for constructing a phylogenetic tree containing these seven mammalian species by computing a distance matrix based on the degree of matching between species (see Materials and Methods). The oldest elements (older than position 49 in the human) were not included in this analysis because many of them are no longer recognizable in the rodent species and thus would overestimate the distance between rodents and other species. A neighbor-joining tree constructed using this distance matrix was in good agreement with the current view of mammalian evolution [17,20,37].

TEs in those parts of the chronological orders where species diverge are informative and may be useful for phylogenomic analysis. For example, the L1MA family of elements is the youngest of the LINE1 elements shared by mammals, and are found near the points of divergence of the different species (Figure 5). MLT1A0 also appears in this region. Both L1MA9 and MLT1A0 have been observed in clade-specific insertions and used to support phylogenies (see Discussion) [20,21]. However, we suggest that many of the other TEs found in the region of divergence, such as MER2, and Tigger1, Tigger2, and Tigger5, will also prove useful for further phylogenomic studies. Furthermore, several TEs in the most recent region of the rat chronological order that do not match in the other genomes (Figure 5, light-colored bars) were the MIRb, L3, and MIR3 elements, which are among the oldest TEs in the mammalian genomes, suggesting that these may represent relatively new rat-specific elements that have been misidentified by Repeat Masker in the rat genome.

## Discussion

Although mammalian TEs represent almost half the DNA sequences in mammalian genomes, they are disproportionately understudied. We have described in this report a unique genome-wide evolutionary analysis of TEs that takes advantage of the completed human and other genome sequences and consider all TEs in the genomes on a comprehensive basis. A software package called TCF has been developed that performs a genome-wide defragmentation of all TEs in the human and other genomes. This defragmentation is based on a simple parsimonious tenet that fragments from the same TE in the same orientation, relatively close together, and with successive repeat indices are most likely from the same original transposon (Figures 1 and S1). Importantly, the defragmentation events that TCF identifies includes all the more sophisticated defragmentations performed by Repeat Masker itself, which assesses by homology to derived consensus sequences whether fragments initially identified as different elements could be from the same element which has been fragmented. However, TCF finds many additional defragmentation events. After attempting to consider other genomic parameters in the defragmentation, such as genomic distance between fragments and difference in percent divergence from the Repbase consensus sequence, extensive analysis of resulting clusters showed that simply using repeat indices provided the most reasonable TE defragmentation with recognizable insertions of TEs into other TEs. When more stringent conditions for defragmentation were used, many clusters contained TE fragments that appeared to originate from the same TE but were not defragmented. However, keeping the amount of distance of non–Repeat Masked sequences between fragments considered for possible defragmentation to ≤500 bp prevented very large inaccurately defragmented clusters from being identified.

TCF is completely dependent on Repeat Masker data, and obviously as Repeat Masker data improves, our defragmentation data will improve. One could in theory improve the data presented here by a manual evaluation of each defragmentation event, especially those found in the upper triangle matrix after IMA, which may not be consistent with the derived chronological order, and by rejection of those that do not appear accurate (Figure 2C). Such an analysis would be greatly facilitated by the Web-based Cluster Browser made available in this study that allows the user to perform specific queries of clusters where a particular TE interrupts another TE. The analysis presented here will also improve the Repeat Masker output by refining TE subfamily classifications, such as removing MER46c from the Tigger4 subfamily (Figure 4), removal of outliers such as the ancient insertion of MLT1F1 into L1MC3 (see Materials and Methods), and identification of TEs that show wide disagreement between species, such as the “MIR” and “L3” elements, which in the rat fall within the most recent elements (Figure 5).

Uniquely, TCF also records the number of times that each TE interrupts each other TE and provides these data in an adjacency matrix, or an interruptional matrix. TCF identified and excluded from this matrix certain types of transposon organization seen in the human genome that do not represent independent transposition events [26] (Datasets S1–S5). The relative age of TEs in individual transposon clusters is implicit in their organization (Figure 1). Nevertheless, TCF does not provide the means to arrange the TEs in the interruptional matrix in an overall chronological order. Therefore, we developed a computational method called IMA that approximates the ideal matrix of elements arranged in chronological order (Figure 2B) by searching for an order that minimizes an objective function (the penalty score) (Figure 2C). The robustness of the chronological order derived by this method was confirmed in several ways. (1) The position of different subfamilies of human LINEs, DNA transposons, and SINE elements (Figures 3A and 4) were consistent with approximate ages based on limited phylogenetic analysis [3,9–11]. (2) Analysis of human DNA transposons showed that the transpositional activity of nonautonomous elements coincided in the chronological order with the autonomous elements on which they depended for transposition (Figure 4) [33,34]. (3) Analysis of six additional mammalian genomes showed that clade- and species-specific TEs were found in the most recent positions of the chronological orders (Figure 5).

Because the rate of sequence divergence (the molecular clock) may not be constant over time or between lineages, the age estimates of TEs based on percent divergence may not be entirely reliable, especially for the older, more diverged elements. Our method to determine relative ages of TEs is not dependent on the percent divergence from derived consensus sequences or on an assumption of a constant molecular clock, and hence can be applied to all TEs in a given genome that have interacted with (inserted into or been interrupted by) enough TEs. Furthermore, this analysis is independent of the actual DNA sequence of the elements. Hence, the relative ages are determined across different classes and subfamilies of TEs. This method is as applicable to the older elements as it is to the younger elements. This to our knowledge is the first method to derive age and chronological information that does not rely on divergence of DNA sequence. Nevertheless, our relative age estimates are consistent for the most part with average percent divergence (Figures 3 and 4). One could specifically examine elements that show a disagreement between the derived chronological order and the percent divergence to find elements that may be undergoing positive or negative selection at the sequence level.

A total of seven mammalian genomes were analyzed using our method, and the chronological orders were aligned and compared, which showed older elements shared between species and newer elements, primarily species- or clade-specific. Phylogenetic trees derived from this type of TE data may be suitable to help resolve phylogenetic issues concerning the evolution of mammals [17,20,37] and other species with sufficient numbers of TEs. Analysis of elements found within regions of divergence of these chronological orders provided a set of TEs that may be phylogenomically informative, including MLT1A0 and L1MA9. Thomas et al. [20] observed three insertions of MLT1A0 elements that were shared between rodents and primates, but not between carnivores (dog) and artiodactyls (cow), and one MLT1A0 and two L1MA9 insertions that were shared between carnivores and artiodactyls, but not between rodents and primates. These clade-specific TE insertions were used as evidence for placing rodents and primates in one sister group and the carnivore and artiodactyl in another sister group, and supported the idea that these TEs were active around the time of divergence of these sister groups. The analysis presented in Figure 5 provides many additional TEs for use in intergenomic examination of TE insertion and phylogenetic relationships, such as several of the more recent Tigger elements (e.g., Tigger1, Tigger2, and Tigger5) as well as MER2 and MLT1A1.

Thus, we have performed the first genome-wide transposon defragmentation analysis of the human genome, and used the overall age information implicit in these fragmentation events to derive relative ages of TEs. This interruptional analysis of TEs represents an essentially untapped genomic dataset that represents as much as 45% of the genome. The rich and complex nature of the data presented in this report will provide a great potential for genomic data mining to further understand the evolutionary history and impact of TEs in mammalian genomes.

## Materials and Methods

### TCF analysis.

TCF scans Repeat Masker data collected from the UCSC genome browser, and only considers TEs, not low complexity, satellite, or simple repeats from the Repeat Masker input. TCF scans the Repeat Masker data and looks for transposon fragments that could be combined into a unit. To be considered for defragmentation, two fragments *X* and *Y* must be the same transposon (have the same TE name), on the same strand, and separated in the genome by no more than 500 bp of nonrepeat masked sequence. Note that additional TE fragments may lie between *X* and *Y*, but the lengths of those fragments (which would be masked by Repeat Masker) are not counted toward the 500 bp. TCF then checks any TE fragments found between fragments *X* and *Y*, and looks for transposon fragments that could be combined with them using the same criteria as for fragments *X* and *Y*. In this way, TCF collects a list of TE fragments that contain possible pairs for defragmentation and additional fragments between them. TCF closes this list when no more TE fragments on the list have possible pairs for defragmentation.

Once the list is closed, TCF determines which TE pairs to defragment into units based on the difference between the repeat indices (ΔRI). TCF joins TE fragments together from the whole list in order of increasing ΔRI (fragments with the most closely matching consecutive repeat indices get defragmented first). TE pairs that overlap (e.g., appear to have a duplication of a portion of the transposon) are allowed to be defragmented only when they overlapped by ≤50% of the size of the smaller of the two fragments, in which case the ΔRI is the amount of overlap. This overlap rule was important because many TE fragment pairs showed an overlap of one or very few base pairs, due to Repeat Masker often extending the homology match of both fragments to the consensus by several base pairs. Any fragment that does not pair up becomes its own unit. Additional TE fragments can be added onto defragmented pairs, but only on their free ends. Note that for any fragment order *X W Y T*, once *X* and *Y* are combined, *W* and *T* are not allowed to combine, because the two units *XY* and *WT* would imply that each fragmented the other (see Figure S1I).

Once all the units in a list are defragmented, TCF checks for units that fall between fragments in another unit (interruptions). Units that are interrupted by another unit are clusters, which always consist of two or more units and at least three fragments. Note that several different clusters can result from the initial list of fragments.

After the units are constructed, TCF examines them to detect L1 5′ inversions and intact LTRs. If a unit is an L1, TCF examines the next unit in the cluster. If it has the same name or is from the same L1 subfamily, is within 6 bp, is on the opposite strand, and has repeat indices within 25 bp, it will be tagged as a 5′ L1 inversion, not be considered a separate unit, and not counted as an interruption. If a unit is an LTR, TCF examines the next two units in the cluster. If the next unit is an LTR–internal sequence, and the next unit is an LTR with the same name as the first LTR unit, and all three are on the same strand, than it might constitute an intact LTR. The first LTR must have ≤10 bp missing from its end, and the last LTR must have ≤10 bp missing from its start. This is tagged an intact LTR, and the second LTR element is not counted as an interruption.

TCF writes the custom track file in the 12-column BED format used by the UCSC genome browser, which is stored as a compressed gzip file on our server (http://www.mssm.edu/labs/warbup01/paper/files.html) for downloading. TCF generates text files containing descriptions of all the clusters. TCF generates tab-delimited files to populate a MySQL database, which is used by Cluster Browser. Queries of TE interruptions run with Cluster Browser are processed by a Java servlet that accesses the MySQL database and returns the relevant clusters in an html format with links to the UCSC Genome Browser.

TCF produces an *n* × *n* interruption matrix, where *n* is the number of types of TEs under consideration. The cell for row *i* and column *j* stores the number of times that TE *i* was found to interrupt TE *j* (Figure 2). For each TE, the percent connectedness in the matrix is defined as the fraction of other TEs that have either interrupted or been interrupted by the TE. For TE *i*, it is the number of other TEs *j* (*j ≠ i*) such that cell (*i, j*) or cell (*j, i*) is nonzero, divided by *n* − 1. To determine the set of TEs with a minimum connectedness (e.g., 29% for the human TEs in Figure 3), the connectedness of each TE is initially calculated for the entire matrix (as shown in Table S1 for all human TEs). If any TEs had a connectedness less than the minimum cutoff, then the TE with the lowest connectedness is removed, and the connectedness of each remaining TE is recalculated. This process was iterated until every remaining TE was at or above the minimum cutoff (e.g., Table S2, 306 elements). TCF then generates an interruption matrix for those features, which is submitted to IMA as described below. When additional elements are added back to an existing set (e.g., the additional 45 DNA transposons; Figure 4) or the overlapping sets of elements between different species (Figure 5), the percent connectedness is recalculated for each element in the final set used, and a corresponding table is included in Tables S2 and S5.

### Interruption matrix analysis.

IMA seeks to determine a chronological ordering of the TEs that minimizes the interruption of newer TEs by older TEs (Figure 2B). It defines an ordering penalty score as the summation of nonzero entries in the upper triangle of the interruption matrix (Figure 2); i.e., in all cells (*i,j*) with *j ≥ i*. Before the summation, the nonzero values are transformed by a continuous function *τ*(*x*) = *x* for *x* ≤ 3 and *τ*(*x*) = 3 + log(*x +* 1) / 4 for x > 3. The median of nonzero entries is three in the upper triangle matrix, and the log part of function *τ*(*x*) moderates the effects of the large nonzero entries on the penalty score. This transformation results in a penalty score in a randomly ordered matrix of about 45,000, even though there are ∼650,000 interruptions.

IMA searches for an ordering of the TEs that minimizes the penalty score by repositioning TEs in the interruption matrix. IMA starts at the first TE (top of the matrix), and moves it to the position that results in the greatest decrease in the penalty score. A new interruption matrix is generated by moving the rows and columns of the matrix appropriately. Since in the adjusted matrix the first TE is now different, IMA checks the first TE again. When repositioning of the first TE no longer results in a decrease in the penalty score, IMA checks the second TE in the matrix, and when it can no longer be repositioned to decrease the penalty score, it checks the third TE, and so on until it reaches the last TE. This constitutes one round of processing. IMA then repeats the process from the first TE until it reaches a minimum penalty score, where repositioning of any element does not result in a decrease in the penalty score. Approximately seven to ten rounds of repositioning were required from each random starting order to reach the local minima from that random starting order. IMA iterates this procedure multiple times (100,000 times) and records the ordering of TEs after each local minimum is produced. For each TE, the distribution of its positions across all iterations is recorded and displayed as an interval (e.g., positional distribution in Figure 3), with the interval divided into lowest 5% and highest 5% of positions, next lowest 20% and next highest 20% of positions, the middle 50% of positions, and the median position.

### Graphic and outlier analysis.

The individual graphs showing the numbers of interrupTEEs and interupTERs for each TE (Figure 2D) were generated using Excel Visual Basic (Microsoft, http://www.microsoft.com), and have been normalized as follows. InterrupTER values (pink) are normalized for the target size of the fragmented TE (interruptions per Mbp of the fragmented TE). InterrupTEE values (pink) are normalized for the total number of each inserting element (interruptions per 10,000 elements of the inserting TE; a factor of 10,000 is used to put the numbers on an integral scale). Some additional TE clusters contained interruptions that did not represent independent transposition events. These were identified by analysis of the individual graphs showing the numbers of interrupTEEs and interupTERs for each TE (Figure 2D). For the graph of each TE, any element that was greater than three standard deviations from the mean of either the interrupTERs or interrupTEEs values was identified, and that pair of TEs was examined for unusual or spurious transposition events using Cluster Browser and by consulting Repbase. For example, the LTR MLT1F1 was seen to interrupt both LINE L1MC3 and L1MD3 114 and 93 times, respectively, which was much more frequently than it interrupted other elements, and indeed Repbase [6] described this as an ancient insertion that has subsequently been propagated by transposition of these LINEs. Several similar putative ancient insertions were identified in this manner, including LTR8 into MER4A1-int (107 times). Additional outliers identified were LTR37A into MER31-int (22 times), LTR49 into MER4A1-int (23 times), MER112 into L1ME3b (34 times), and MER77 into MER21c (75 times). These pairs of elements were removed from the adjacency matrix, and the chronological order was recalculated (the final order after removing these outliers is included in Figures 2 and 3). These outliers still appear in the custom tracks and in Cluster Browser queries so that they may be examined.

### Multiple species analysis.

For each additional genome—chimp (panTro2), rhesus (rheMac2), cow (bosTau2), dog (canFam2), rat (rn4), and mouse (mm8)—TE defragmentation was performed by TCF. The same conditions were used for excluding intact LTR elements and 5′ L1 inversions as for the human genome; these datasets are available by request. To determine the set of elements for consideration in this analysis, the percent connectedness was set at a value that accounted for approximately 95% or greater of the total TEs in the genome (29% for human, 30% for chimp and rhesus, and 10% for cow, dog, rat, and mouse). The overlap of these sets was further maximized by subsequently adding back to each set any elements that were present in two or more of the genomes analyzed (but not within the original percent connectedness threshold). After initially running 40,000 iterations of IMA on these sets of TEs, elements were excluded whose positions within the chronological order were not-well supported because they showed a very low connectedness and a very large positional distribution. IMA was rerun for 40,000 iterations, which generated a chronological order and positional distributions for this set of TEs for each of the genomes (Table S5).

The chronological orders from each genome were compared pairwise. Elements that were found in only one of the two genomes under comparison were identified, and all elements below it and up were shifted up in the chronological order (by subtracting 1 from the position of all TEs below it), which maintained the alignment of the remainder of the elements that were in common between the two species. Subsequently, the degree of agreement between the chronological orders of each genome in a pairwise manner was determined. A TE was considered to be matching in position in the two chronological orders if the mean of the positional distribution of the TE in both genomes fell between the central 90% of positions calculated for that TE in the other genome (yielding a solid-color bar in Figure 5).

Each genome, assigned a different color (Figure 5), was compared pairwise to each other genome, and a set of matrices where each genome in turn is the reference genome was produced. These matrices contained the set of TEs used in the IMA analysis for each reference genome (in rows), with the alignment of the TEs from each of the other genomes (the aligned genomes) in the columns. A value and color was assigned to each position to indicate whether the TE in the reference genome was: (1) gray, not present in the aligned genome (as included in the set of TEs used for IMA analysis; Table S4); (2) solid color, matching in the aligned genome; (3) lighter stippled color, not matching in the aligned genome; and (4) black, present in the aligned genome but not found in theset used for IMA analysis. An Excel Visual Basic macro was used to give each cell its color depending on its value. See Figure S2 for full details.

Figure 5 also included a secondary alignment of the matrices of reference and aligned genomes (all genomes were aligned to the human, and a portion of the rat genome was aligned to the mouse; see Figure 5). This used an Excel Visual Basic script to maintain the alignment of TEs that match between the two species by inserting additional spaces (white). This was especially important for comparisons between the human and rodent species, where many TEs found in the human genome were not in the rodent genomes. This served to keep the genomes aligned so that they could be more easily compared across Figure 5.

The phylogenetic tree (Figure 5, bottom) was constructed for the seven mammalian genomes by calculating pairwise distances with the following formula: mismatches / (matches + mismatches). The oldest elements were excluded above position 49 in the human order (Figure 5) because this is the position where the transposon alignments are continuous in all species examined. The resulting distance matrix was used to build a neighbor-joining phylogenetic tree, using the program T-Rex (http://www.labunix.uqam.ca/~makarenv/trex.html). Although this method is suitable to determine the correct topology of the tree, the branch lengths may not be accurate because of differences in transposon activity over time and in different species.

### Web site and URLs.

The Warburton lab Website (http://www.mssm.edu/labs/warbup01/paper/files.html) contains this manuscript, with its figures, tables, and supporting information. It also contains a link to automatically upload the TCF custom track onto the UCSC genome browser, and a link to the Cluster Browser. To manually upload the custom track, upload the following URL onto the UCSC genome browser (http://www.mssm.edu/labs/warbup01/tracks/tcftrack.gz). Cluster Browser is available at http://sungene-bk.genetics.mssm.edu/cluster/index.html.

## Supporting Information

Datasets S1–S5 provide lists of clusters with interruptions that were not considered independent transposition events, with links to the UCSC genome browser for each cluster. Each dataset contains a different category of transposon organization that was not considered an independent tranposition event. To view TCF custom tracks for each cluster, upload the custom track file using the link included.

Dataset S1 Intact LTR Transposons (3,101 Examples) (9.3 MB HTML)

Dataset S2 L1 5′ Inversions (2,273 Examples) (5.6 MB HTML)

Dataset S3 Tandem Repeats (40 Examples) (282 KB HTML)

Dataset S4 Large Tandem Arrays (360 KB HTML)

Dataset S5 Segmental Duplications (9.6 MB HTML)

Figure S1 Transposon Clusters in the Human Genome A selection of transposon clusters from the human genome identified by TCF, highlighting clusters that were excluded from the matrix analysis because they were not independent transpositional events. The cluster table below each browser window shows Repeat Masker data for each TE fragment collected by TCF. Columns in the table are Genome start, Genome end (starting and ending hg18 genomic coordinates for each TE fragment), Strand, % div (of TE fragment from consensus), Repeat start, Repeat end, Repeat left (repeat coordinates relative to the derived consensus), and Name, Family, and Class (of each TE fragment). Additional table columns from TCF output (not shown in Figure 1) are Size (genomic size of TE fragment), Space (genomic space between TE fragments), Div (difference in divergence between next fragment in unit), Rep (difference in repeat indices), and Unit (defragmented unit number).
(A) An intact THE1B LTR retrotransposon interrupting an L2 element, counted as a single transpositional event.
(B) An intact MSTA LTR retrotransposon, which has itself been interrupted by several Alu elements.
(C) A 5′ L1 inversion in an L1PA4 element, counted as a single transpositional event.
(D) A HERV-H element which shows a common pattern of deletions indicative of *trans* complementation (see text). Note that this HERV-H, flanked by LTR7 elements, is an intact LTR element.
(E) A HERV element showing an internal tandem amplification of 12- to ∼80-bp pieces, which are not counted as independent transpositional events. This is also an intact LTR element.
(F) A 22-kb LTR array cluster containing tandem ∼3 kb repeats from Chromosome 9 (Warburton, unpublished data), which is excluded from the matrix of interruptions.
(G) Largest transposon cluster.
(H) Transposon cluster with the most interruptions (86 interruptions).
(I) an example of a defragmentation of MLT2C1 that excluded the defragmentation of an L1MB8 (shaded portion; see Materials and Methods).
(858 KB PDF)

Figure S2 Comparison of Transposon History in Seven Mammalian Genomes The full dataset from Figure 5 (as an Excel file) showing all information, including names and chronological positions of TEs.
(526 KB XLS)

Table S1 Statistics of All 908 TEs in the Human Genome This table contains the data as processed by TCF for all human TEs after removal of excluded clusters and outliers. This Table is available as an Excel file for sorting purposes.
Data columns: (1) name of TE, (2) number of fragments, (3) number of units, (4) number that gets interrupted, (5) number of interrupts, (6) total interactions (number that gets interrupted plus number of interrupts), (7) ratio of number that gets interrupted to number of interrupts, (8) percent connectedness (this is the initial value for all TEs), (9) percent divergence distribution (0%–5%–25%–50%–75%–95%–100%) for all units in genome.
(148 KB XLS)

Table S2 Chronological Order for 360 and 405 Human TEs This table contains the data for the 360 human TEs that fell above the cutoff of ≥29% connectedness (Figure 3), and for the 405 TEs including all DNA transposons (Figure 4). The width of the positional distribution is also presented. Table S2 is available as an Excel file for sorting purposes.
Data columns: (1) position in chronological order, (2) name of TE, (3) number of fragments, (4) number of units, (5) number that gets interrupted, (6) number of interrupts, (7) total interactions (number that gets interrupted plus number of interrupts), (8) ratio of number that gets interrupted to number of interrupts, (9) percent connectedness (all TEs ≥29% connectedness; see Materials and Methods), (10) percent divergence (0%–5%–25%–50%–75%–95%–100%), (11) positional distribution 0%–5%–25%–50%–75%–95%–100%, (12) width of positional distribution, (13) sorted by TE family in columns (L3,L2,MIRS, Hs) L1ME, L1MB, L1PB, L1MC/D, L1PA, L1MA, AluJ, Charlie, Tigger, AluS, and AluY (as in Figure 3).
(304 KB XLS)

Table S3 TCF Results for Seven Mammalian Genomes (34 KB XLS)

Table S4 Comparisons of IMA Results for Seven Mammalian Genomes (36 KB XLS)

Table S5 TCF and IMA Results for Seven Mammalian Genomes Each table on a separate worksheet within an Excel file. Data columns are the same as Table S1 (statistics) and Table S2 (chronological order).
Human 435: Chronological Order of 435 Human TEs Analyzed by IMA; Chimp 929: Statistics of All 929 TEs in the Chimp Genome; Chimp 438: Chronological Order of 438 Chimp TEs Analyzed by IMA; Rhesus 907: Statistics of All 907 TEs in the Rhesus Genome; Rhesus 438: Chronological Order of 438 Rhesus TEs Analyzed by IMA; Cow 421: Statistics of All 421 TEs in the Cow Genome; Cow 288: Chronological Order of 288 Cow TEs Analyzed by IMA; Dog 502: Statistics of All 502 TEs in the Dog Genome; Dog 335: Chronological Order of 335 Dog TEs Analyzed by IMA; Rat 748: Statistics of All 748 TEs in the Rat Genome; Rat 474: Chronological Order of 474 Rat TEs Analyzed by IMA; Mouse 871: Statistics of All 871 TEs in the Mouse Genome; Mouse 546: Chronological Order of 546 Mouse TEs Analyzed by IMA.
(1.7 MB XLS)

## Abbreviations

HERV: human endogenous retrovirus
IMA: interruptional matrix analysis
LINE: long interspersed repeat element
LTR: long terminal repeat
MER: medium reiterated sequence
SINE: short interspersed repeat element
TCF: Transposon Cluster Finder
TE: tranposable element
