# Supplementary material for: Evolutionary History of Mammalian Transposons Determined by Genome-Wide Defragmentation
Source: PLoS Comput Biol. 2007 Jul 13;3(7):e137. doi: 10.1371/journal.pcbi.0030137 (PMC1914374; doi:10.1371/journal.pcbi.0030137)
Supplement: Supplementary file 1 [file pcbi.0030137.sg001.pdf]

Figure S1A: An Intact LTR

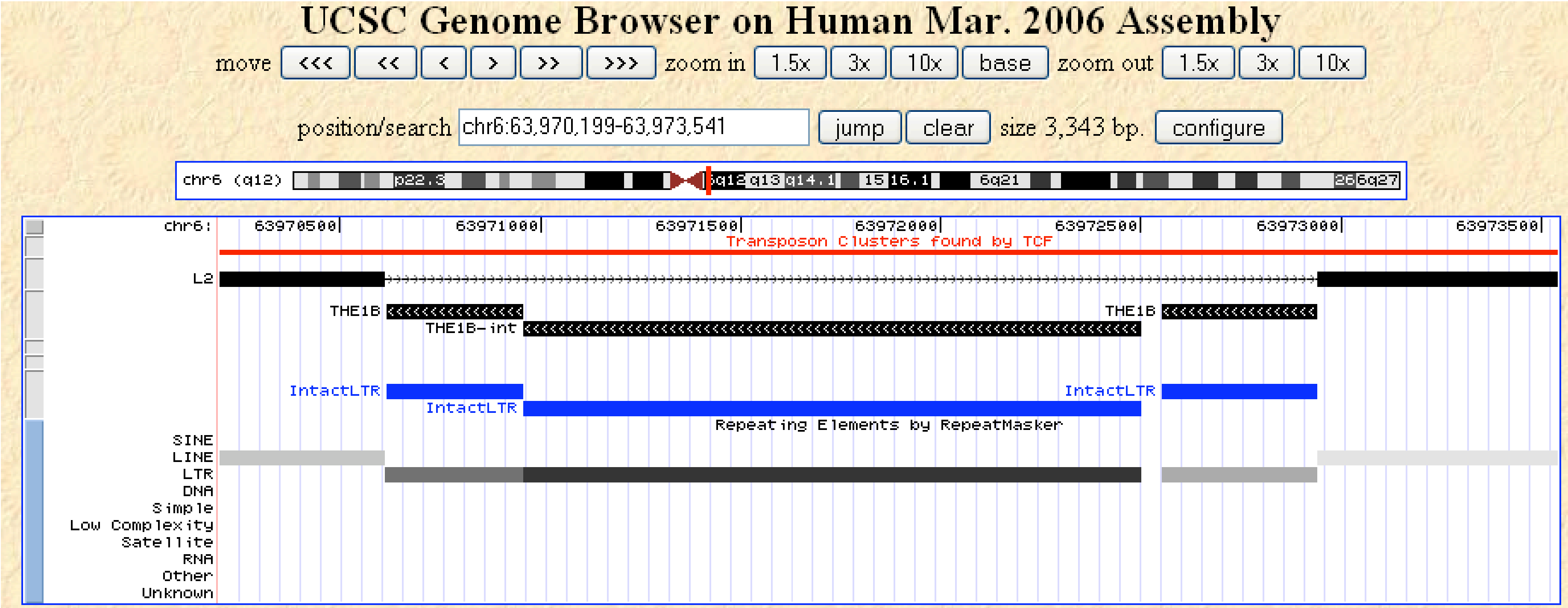

Genome Start	Genome End	Strand	%Div	Repeat Start	Repeat End	Repeat Left	Name	Family	Class	Size	Space	Div	Rep	Unit
63,970,199	63,970,613	+	31.0	2,115	2,542	-877	L2	L2	LINE	415	-	-	-	1
63,970,614	63,970,956	-	15.1	1	364	0	THE1B	MaLR	LTR	343	0	-	-	2
63,970,957	63,972,502	-	10.2	31	1,580	0	THE1B-int	MaLR	LTR	1,546	0	-	-	3
63,972,553	63,972,940	-	19.0	1	363	-1	THE1B	MaLR	LTR	388	50	-	-	4
63,972,941	63,973,541	+	25.4	2,538	3,329	-90	L2	L2	LINE	601	0	5.6	-4	1

Figure S1B: An Intact LTR with Interruptions

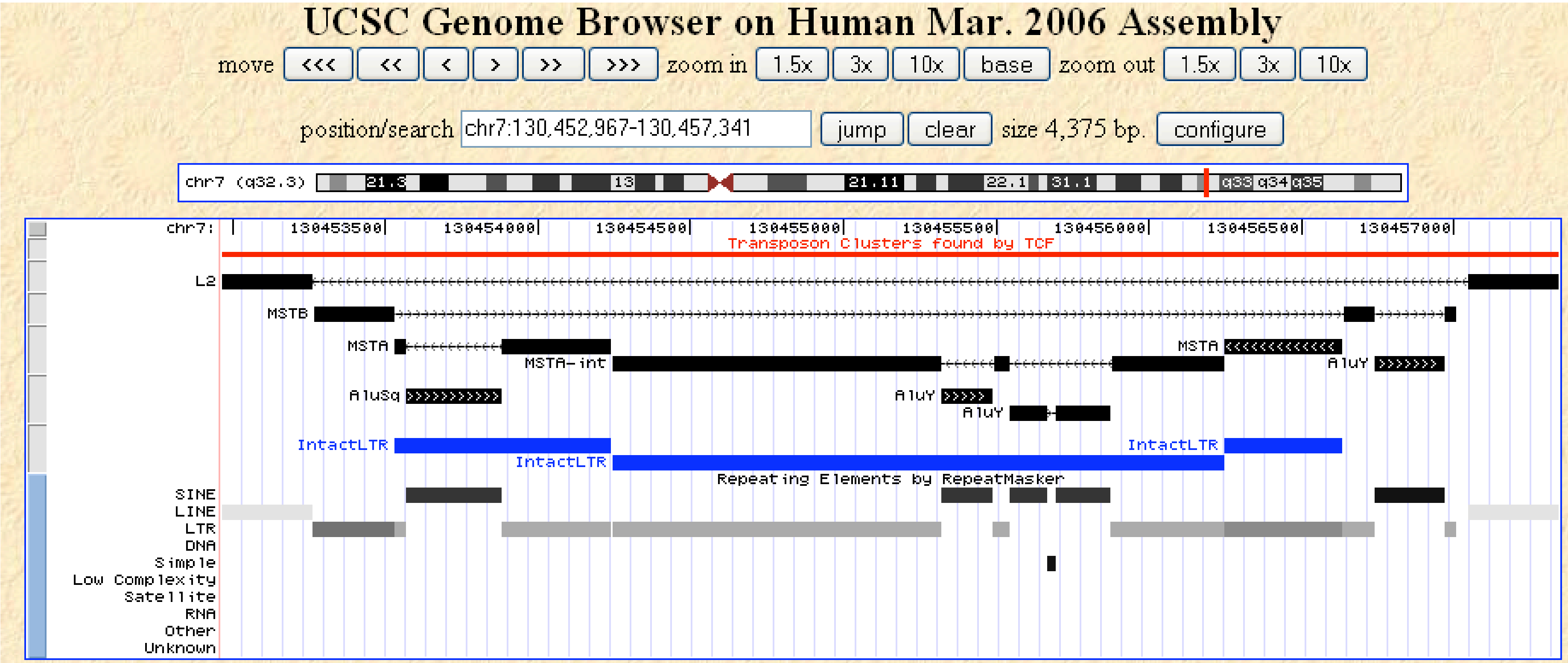

Genome Start	Genome End	Strand	%Div	Repeat Start	Repeat End	Repeat Left	Name	Family	Class	Size	Space	Div	Rep	Unit
130,452,967	130,453,264	-	36.0	2,475	2,786	-633	L2	L2	LINE	298	-	-	-	1
130,453,265	130,453,528	+	14.8	1	281	-145	MSTB	MaLR	LTR	264	0	-	-	2
130,453,529	130,453,570	-	14.3	402	428	0	MSTA	MaLR	LTR	42	0	-	-	3
130,453,571	130,453,882	+	9.9	1	313	0	AluSq	Alu	SINE	312	0	-	-	4
130,453,883	130,454,236	-	14.3	1	401	-27	MSTA	MaLR	LTR	354	0	0	0	3
130,454,243	130,455,319	-	12.8	426	1,573	-7	MSTA-int	MaLR	LTR	1,077	6	-	-	5
130,455,320	130,455,491	+	7.1	135	303	-8	AluY	Alu	SINE	172	0	-	-	6
130,455,492	130,455,542	-	12.8	372	425	-1,155	MSTA-int	MaLR	LTR	51	0	0	0	5
130,455,543	130,455,667	+	8.5	1	124	-187	AluY	Alu	SINE	125	0	-	-	7
130,455,697	130,455,876	+	8.5	125	308	-3	AluY	Alu	SINE	180	29	0	0	7
130,455,877	130,456,246	-	12.8	3	369	-1,211	MSTA-int	MaLR	LTR	370	0	0	2	5
130,456,249	130,456,635	-	14.4	1	428	0	MSTA	MaLR	LTR	387	2	-	-	8
130,456,636	130,456,737	+	16.3	277	386	-40	MSTB	MaLR	LTR	102	0	1.5	-4	2
130,456,738	130,456,967	+	7.0	83	311	0	AluY	Alu	SINE	230	0	-	-	9
130,456,968	130,457,006	+	16.3	387	414	-12	MSTB	MaLR	LTR	39	0	0	0	2

130,457,046	130,457,341	-	28.3	2,049	2,421	-998	L2	L2	LINE	296	39	7.7	53	1

Figure S1C: An L1 5’ Inversion

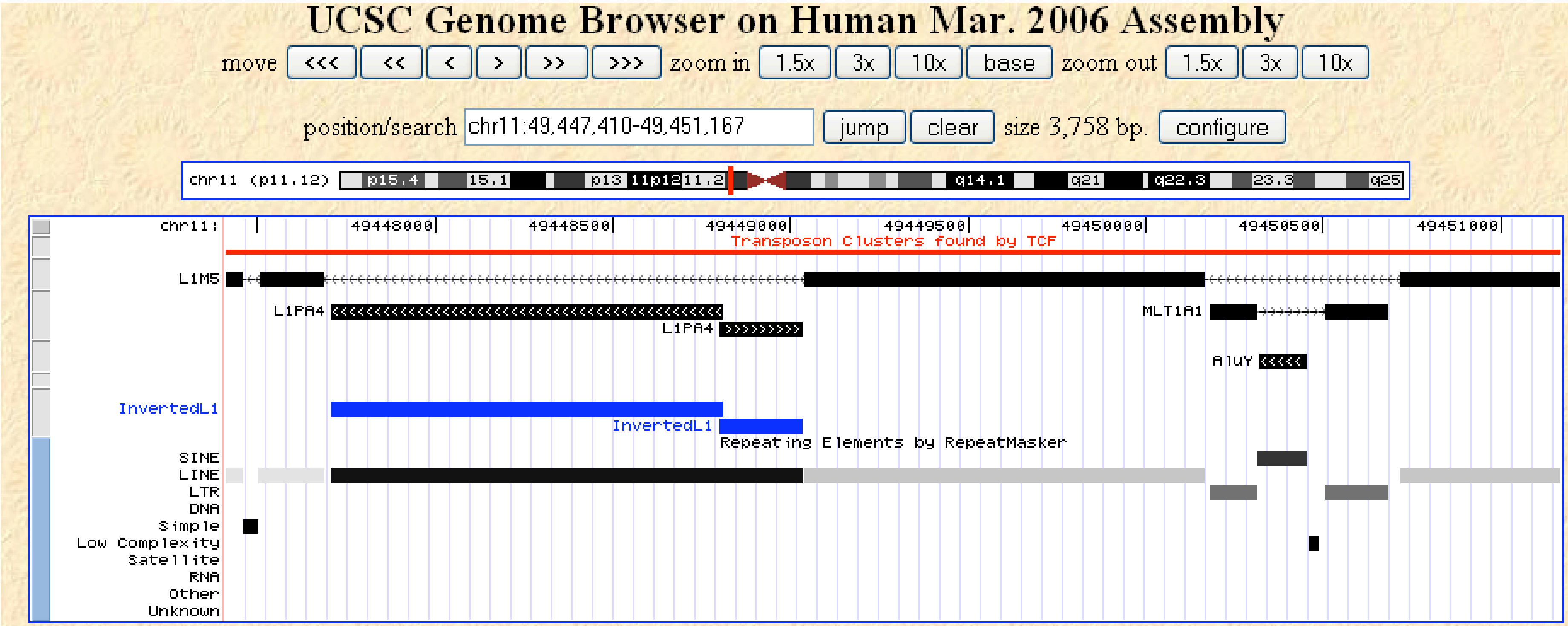

Genome Start	Genome End	Strand	%Div	Repeat Start	Repeat End	Repeat Left	Name	Family	Class	Size	Space	Div	Rep	Unit
49,447,410	49,447,459	-	31.4	5,795	5,844	-326	L1M5	L1	LINE	50	-	-	-	1
49,447,503	49,447,688	-	31.4	5,599	5,794	-376	L1M5	L1	LINE	186	43	0	0	1
49,447,705	49,448,810	-	5.0	5,051	6,155	0	L1PA4	L1	LINE	1,106	16	-	-	2
49,448,797	49,449,036	+	5.0	4,827	5,066	-1,080	L1PA4	L1	LINE	240	-14	-	-	3
49,449,038	49,450,166	-	30.5	4,374	5,552	-612	L1M5	L1	LINE	1,129	1	0.9	46	1
49,450,181	49,450,316	+	20.0	3	138	-227	MLT1A1	MaLR	LTR	136	14	-	-	4
49,450,317	49,450,455	-	8.6	171	309	-2	AluY	Alu	SINE	139	0	-	-	5
49,450,506	49,450,681	+	16.8	228	408	0	MLT1A1	MaLR	LTR	176	50	3.2	89	4
49,450,718	49,451,167	-	26.1	3,836	4,313	-1,833	L1M5	L1	LINE	450	36	4.4	60	1

Figure S1D: A Defragmented HERV

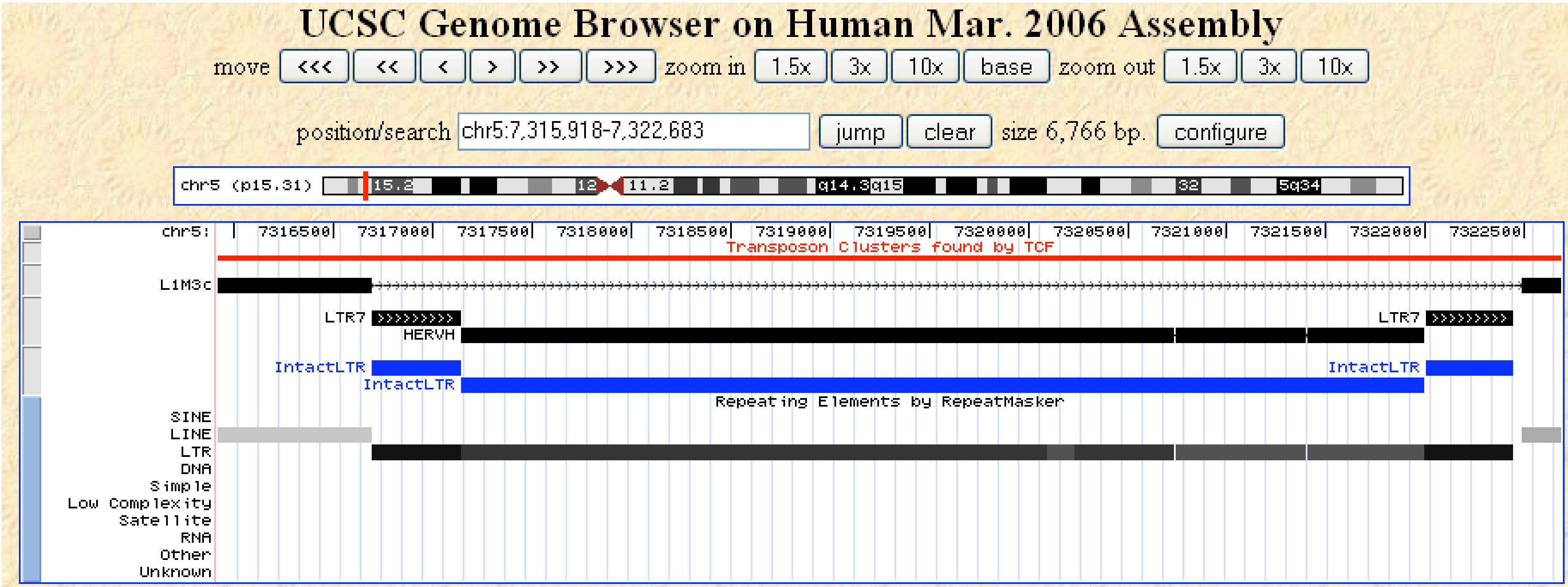

Genome Start	Genome End	Strand	%Div	Repeat Start	Repeat End	Repeat Left	Name	Family	Class	Size	Space	Div	Rep	Unit
7,315,918	7,316,692	+	25.6	2,315	3,119	-3,091	L1M3c	L1	LINE	775	-	-	-	1
7,316,693	7,317,141	+	6.9	1	448	-2	LTR7	ERV1	LTR	449	0	-	-	2
7,317,144	7,320,101	+	8.9	1	2,987	-4,726	HERVH	ERV1	LTR	2,958	2	-	-	3
7,320,096	7,320,242	+	12.9	3,140	3,290	-4,423	HERVH	ERV1	LTR	147	-6	4	152	3
7,320,233	7,320,736	+	11.4	3,492	3,993	-3,720	HERVH	ERV1	LTR	504	-10	1.5	201	3
7,320,739	7,321,401	+	14.8	4,488	5,149	-2,564	HERVH	ERV1	LTR	663	2	3.4	494	3
7,321,403	7,321,618	+	14.8	5,600	5,819	-1,894	HERVH	ERV1	LTR	216	1	0	450	3
7,321,619	7,321,996	+	8.3	7,324	7,713	0	HERVH	ERV1	LTR	378	0	6.5	1504	3
7,321,997	7,322,444	+	4.0	1	448	-2	LTR7	ERV1	LTR	448	0	-	-	4
7,322,488	7,322,683	+	28.0	3,129	3,332	-2,878	L1M3c	L1	LINE	196	43	2.4	9	1

Figure S1E: HERVH Fragments Tandemly Repeated

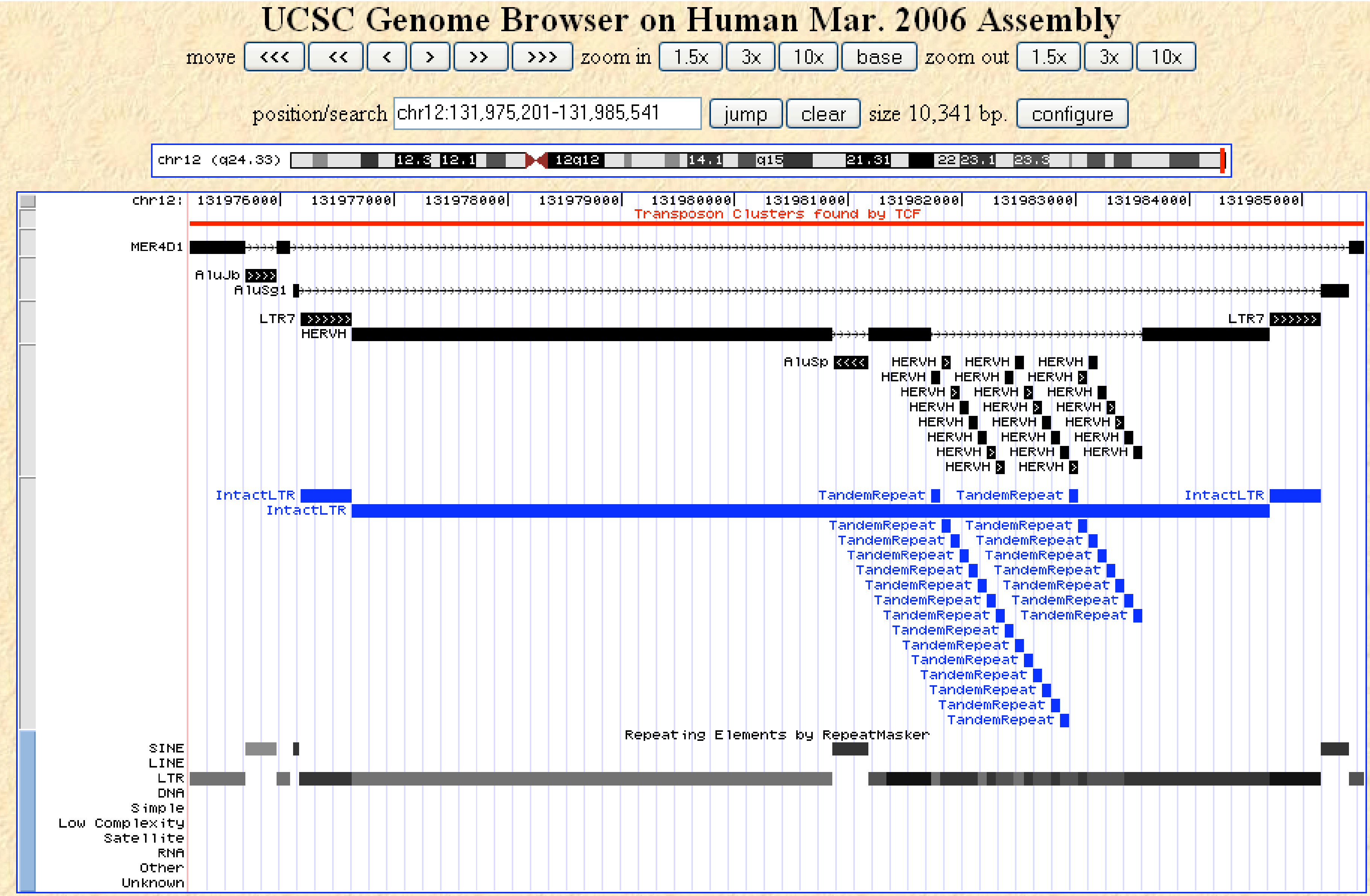

Genome Start	Genome End	Strand	%Div	Repeat Start	Repeat End	Repeat Left	Name	Family	Class	Size	Space	Div	Rep	Unit
131,975,201	131,975,682	+	12.7	1	533	-367	MER4D1	ERV1	LTR	482	-	-	-	1
131,975,683	131,975,967	+	15.8	1	306	-6	AluJb	Alu	SINE	285	0	-	-	2
131,975,968	131,976,083	+	12.7	534	659	-241	MER4D1	ERV1	LTR	116	0	0	0	1

131,976,107	131,976,169	+	9.5	1	63	-255	AluSg1	Alu	SINE	63	23	-	-	3
131,976,170	131,976,628	+	7.8	1	448	-2	LTR7	ERV1	LTR	459	0	-	-	4
131,976,631	131,980,865	+	18.3	1	4,017	-2,512	HERVH	ERV1	LTR	4,235	2	-	-	5
131,980,866	131,981,174	-	8.1	-2	311	2	AluSp	Alu	SINE	309	0	-	-	6
131,981,175	131,981,342	+	7.9	4,015	4,181	-2,372	HERVH	ERV1	LTR	168	0	10.4	-2	5
131,981,337	131,981,734	+	7.8	6,283	6,680	-1,033	HERVH	ERV1	LTR	398	-6	0.1	2101	5
131,981,735	131,981,812	+	19.2	6,600	6,678	-1,035	HERVH	ERV1	LTR	78	0	-	-	7
131,981,815	131,981,895	+	13.6	6,600	6,680	-1,033	HERVH	ERV1	LTR	81	2	-	-	8
131,981,896	131,981,976	+	13.6	6,600	6,680	-1,033	HERVH	ERV1	LTR	81	0	-	-	9
131,981,977	131,982,057	+	13.6	6,600	6,680	-1,033	HERVH	ERV1	LTR	81	0	-	-	10
131,982,058	131,982,138	+	13.6	6,600	6,680	-1,033	HERVH	ERV1	LTR	81	0	-	-	11
131,982,139	131,982,216	+	19.2	6,600	6,678	-1,035	HERVH	ERV1	LTR	78	0	-	-	12
131,982,219	131,982,299	+	13.6	6,600	6,680	-1,033	HERVH	ERV1	LTR	81	2	-	-	13
131,982,300	131,982,380	+	14.8	6,600	6,680	-1,033	HERVH	ERV1	LTR	81	0	-	-	14
131,982,381	131,982,461	+	16.1	6,600	6,680	-1,033	HERVH	ERV1	LTR	81	0	-	-	15
131,982,462	131,982,541	+	18.8	6,600	6,680	-1,033	HERVH	ERV1	LTR	80	0	-	-	16
131,982,542	131,982,622	+	13.6	6,600	6,680	-1,033	HERVH	ERV1	LTR	81	0	-	-	17
131,982,623	131,982,703	+	16.1	6,600	6,680	-1,033	HERVH	ERV1	LTR	81	0	-	-	18
131,982,704	131,982,784	+	16.1	6,600	6,680	-1,033	HERVH	ERV1	LTR	81	0	-	-	19
131,982,785	131,982,865	+	13.6	6,600	6,680	-1,033	HERVH	ERV1	LTR	81	0	-	-	20
131,982,866	131,982,945	+	17.5	6,600	6,680	-1,033	HERVH	ERV1	LTR	80	0	-	-	21
131,982,946	131,983,026	+	14.8	6,600	6,680	-1,033	HERVH	ERV1	LTR	81	0	-	-	22
131,983,027	131,983,107	+	12.3	6,600	6,680	-1,033	HERVH	ERV1	LTR	81	0	-	-	23
131,983,108	131,983,187	+	15.0	6,600	6,680	-1,033	HERVH	ERV1	LTR	80	0	-	-	24
131,983,188	131,983,268	+	14.8	6,600	6,680	-1,033	HERVH	ERV1	LTR	81	0	-	-	25
131,983,269	131,983,349	+	16.1	6,600	6,680	-1,033	HERVH	ERV1	LTR	81	0	-	-	26
131,983,350	131,983,430	+	14.8	6,600	6,680	-1,033	HERVH	ERV1	LTR	81	0	-	-	27
131,983,431	131,983,510	+	11.2	6,600	6,680	-1,033	HERVH	ERV1	LTR	80	0	-	-	28
131,983,511	131,983,590	+	12.5	6,600	6,680	-1,033	HERVH	ERV1	LTR	80	0	-	-	29
131,983,591	131,984,707	+	8.5	6,600	7,713	0	HERVH	ERV1	LTR	1,117	0	0.7	-80	5
131,984,708	131,985,158	+	6.1	1	448	-2	LTR7	ERV1	LTR	451	0	-	-	30
131,985,161	131,985,407	+	9.9	59	301	-8	AluSg1	Alu	SINE	247	2	0.4	-4	3
131,985,408	131,985,541	+	17.2	765	900	0	MER4D1	ERV1	LTR	134	0	4.5	105	1

Figure S1F: An LTR Array

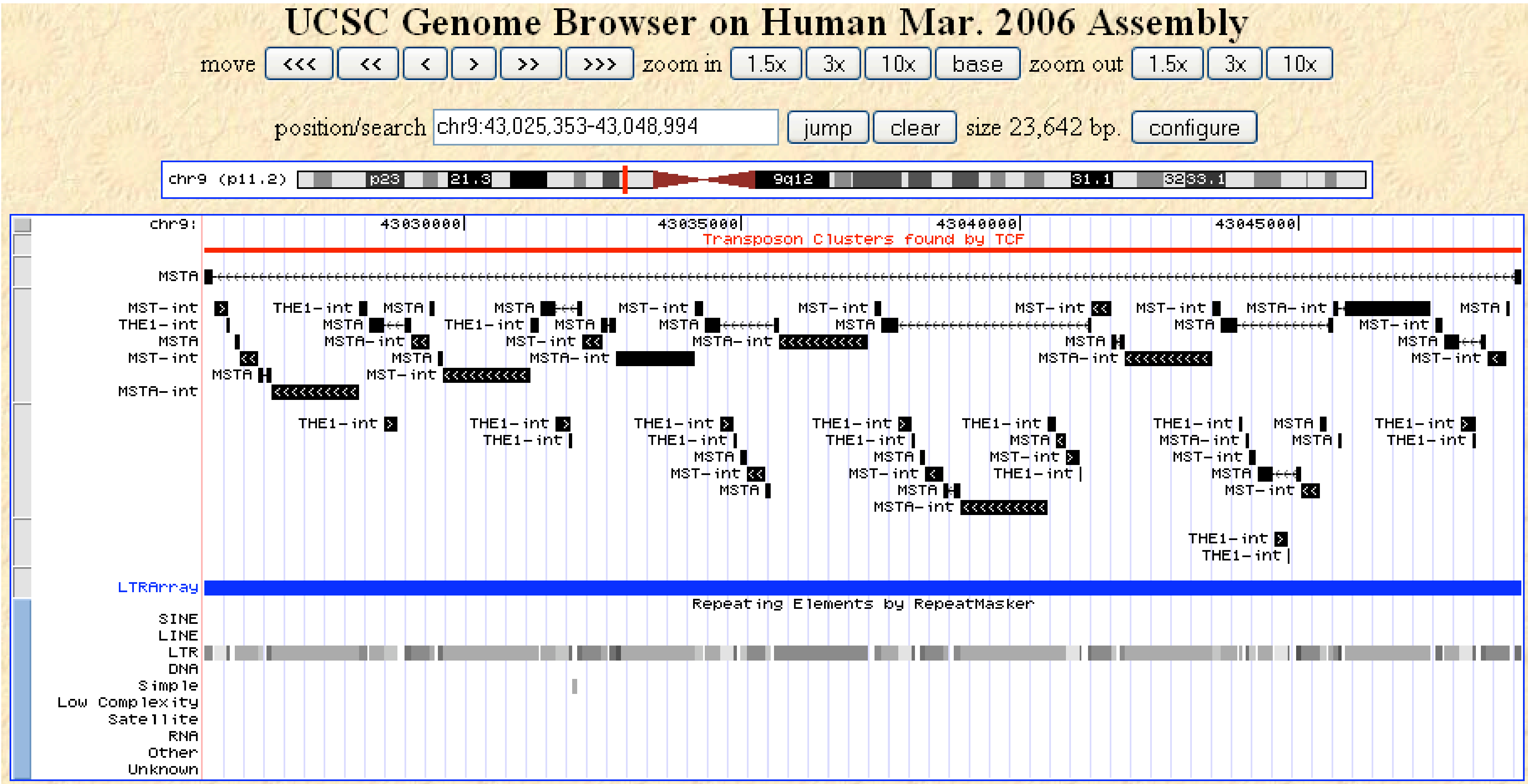

Genome Start	Genome End	Strand	%Div	Repeat Start	Repeat End	Repeat Left	Name	Family	Class	Size	Space	Div	Rep	Unit
43,025,353	43,025,496	-	25.2	128	274	-154	MSTA	MaLR	LTR	144	-	-	-	1
43,025,525	43,025,766	+	23.2	976	1,256	-395	MST-int	MaLR	LTR	242	28	-	-	2
43,025,755	43,025,810	-	19.6	911	967	-613	THE1-int	MaLR	LTR	56	-12	-	-	3
43,025,894	43,025,995	-	22.4	1	106	-322	MSTA	MaLR	LTR	102	83	-	-	4
43,025,996	43,026,315	-	24.8	1,317	1,650	-1	MST-int	MaLR	LTR	320	0	-	-	5
43,026,314	43,026,410	-	24.7	60	171	-257	MSTA	MaLR	LTR	97	-2	-	-	6
43,026,460	43,026,557	-	21.6	1	99	-329	MSTA	MaLR	LTR	98	49	3.1	-40	6
43,026,558	43,028,138	-	23.2	28	1,651	0	MSTA-int	MaLR	LTR	1,581	0	-	-	7
43,028,137	43,028,276	+	25.9	466	608	-972	THE1-int	MaLR	LTR	140	-2	-	-	8
43,028,313	43,028,578	-	20.8	76	368	-60	MSTA	MaLR	LTR	266	36	-	-	9

43,028,583	43,028,826	+	18.6	907	1,191	-389	THE1-int	MaLR	LTR	244	4	-	-	10
43,028,954	43,029,048	-	17.9	1	97	-331	MSTA	MaLR	LTR	95	127	2.9	-22	9
43,029,049	43,029,394	-	21.7	1,318	1,651	0	MSTA-int	MaLR	LTR	346	0	-	-	11
43,029,402	43,029,495	-	28.6	65	159	-269	MSTA	MaLR	LTR	94	7	-	-	12
43,029,546	43,029,640	-	21.1	1	97	-331	MSTA	MaLR	LTR	95	50	-	-	13
43,029,641	43,031,211	-	23.4	34	1,651	0	MST-int	MaLR	LTR	1,571	0	-	-	14
43,031,214	43,031,367	+	28.0	464	617	-963	THE1-int	MaLR	LTR	154	2	-	-	15
43,031,384	43,031,668	-	23.4	74	376	-52	MSTA	MaLR	LTR	285	16	-	-	16
43,031,668	43,031,911	+	18.4	904	1,191	-389	THE1-int	MaLR	LTR	244	-1	-	-	17
43,031,900	43,031,956	-	21.4	911	967	-613	THE1-int	MaLR	LTR	57	-12	-	-	18
43,032,053	43,032,147	-	17.9	1	97	-331	MSTA	MaLR	LTR	95	96	5.5	-24	16
43,032,148	43,032,484	-	20.5	1,317	1,651	0	MST-int	MaLR	LTR	337	0	-	-	19
43,032,483	43,032,583	-	19.8	60	171	-257	MSTA	MaLR	LTR	101	-2	-	-	20
43,032,633	43,032,730	-	20.6	1	99	-329	MSTA	MaLR	LTR	98	49	0.8	-40	20
43,032,731	43,032,846	-	15.7	1,535	1,651	0	MSTA-int	MaLR	LTR	116	0	-	-	21
43,032,843	43,034,160	-	25.1	32	1,389	-262	MSTA-int	MaLR	LTR	1,318	-4	9.4	145	21
43,034,164	43,034,304	+	24.1	508	670	-981	MST-int	MaLR	LTR	141	3	-	-	22
43,034,334	43,034,613	-	24.0	76	372	-56	MSTA	MaLR	LTR	280	29	-	-	23
43,034,618	43,034,858	+	19.5	907	1,191	-389	THE1-int	MaLR	LTR	241	4	-	-	24
43,034,847	43,034,900	-	18.9	914	967	-613	THE1-int	MaLR	LTR	54	-12	-	-	25
43,034,980	43,035,082	-	20.0	1	104	-324	MSTA	MaLR	LTR	103	79	-	-	26
43,035,083	43,035,422	-	23.1	1,317	1,651	0	MST-int	MaLR	LTR	340	0	-	-	27
43,035,421	43,035,520	-	28.0	60	171	-257	MSTA	MaLR	LTR	100	-2	-	-	28
43,035,570	43,035,666	-	23.7	1	99	-329	MSTA	MaLR	LTR	97	49	0.3	-24	23
43,035,667	43,037,257	-	22.0	28	1,651	0	MSTA-int	MaLR	LTR	1,591	0	-	-	29
43,037,383	43,037,495	+	22.7	506	661	-963	MST-int	MaLR	LTR	113	125	-	-	30
43,037,512	43,037,801	-	20.1	66	376	-52	MSTA	MaLR	LTR	290	16	-	-	31
43,037,806	43,038,045	+	24.2	907	1,187	-393	THE1-int	MaLR	LTR	240	4	-	-	32
43,038,038	43,038,093	-	21.4	911	967	-613	THE1-int	MaLR	LTR	56	-8	-	-	33
43,038,190	43,038,285	-	19.8	1	97	-331	MSTA	MaLR	LTR	96	96	-	-	34
43,038,288	43,038,626	-	22.7	1,318	1,649	-2	MST-int	MaLR	LTR	339	2	-	-	35
43,038,634	43,038,725	-	26.7	65	159	-269	MSTA	MaLR	LTR	92	7	-	-	36
43,038,816	43,038,921	-	19.6	1	105	-323	MSTA	MaLR	LTR	106	90	7.1	-41	36
43,038,922	43,040,498	-	24.3	27	1,651	0	MSTA-int	MaLR	LTR	1,577	0	-	-	37
43,040,496	43,040,631	+	28.9	466	605	-975	THE1-int	MaLR	LTR	136	-3	-	-	38
43,040,636	43,040,819	-	25.3	76	266	-162	MSTA	MaLR	LTR	184	4	-	-	39
43,040,825	43,041,064	+	24.5	976	1,256	-395	MST-int	MaLR	LTR	240	5	-	-	40
43,041,053	43,041,106	-	18.9	914	967	-613	THE1-int	MaLR	LTR	54	-12	-	-	41
43,041,226	43,041,288	-	19.1	1	65	-363	MSTA	MaLR	LTR	63	119	1	0	31
43,041,289	43,041,628	-	21.5	1,317	1,651	0	MST-int	MaLR	LTR	340	0	-	-	42
43,041,627	43,041,727	-	27.7	60	171	-257	MSTA	MaLR	LTR	101	-2	-	-	43

43,041,777	43,041,873	-	23.7	1	99	-329	MSTA	MaLR	LTR	97	49	4	-40	43
43,041,874	43,043,451	-	24.1	28	1,651	0	MSTA-int	MaLR	LTR	1,578	0	-	-	44
43,043,447	43,043,582	+	19.3	504	661	-990	MST-int	MaLR	LTR	136	-5	-	-	45
43,043,609	43,043,912	-	24.7	75	385	-43	MSTA	MaLR	LTR	304	26	-	-	46
43,043,917	43,043,986	+	30.0	907	977	-603	THE1-int	MaLR	LTR	70	4	-	-	47
43,044,035	43,044,100	-	19.7	36	102	-1549	MSTA-int	MaLR	LTR	66	48	-	-	48
43,044,107	43,044,244	+	21.2	507	670	-981	MST-int	MaLR	LTR	138	6	-	-	49
43,044,274	43,044,541	-	27.2	96	374	-54	MSTA	MaLR	LTR	268	29	-	-	50
43,044,567	43,044,801	+	20.0	907	1,191	-389	THE1-int	MaLR	LTR	235	25	-	-	51
43,044,790	43,044,845	-	19.6	911	967	-613	THE1-int	MaLR	LTR	56	-12	-	-	52
43,044,942	43,045,037	-	15.6	1	97	-331	MSTA	MaLR	LTR	96	96	11.6	-2	50
43,045,038	43,045,385	-	22.1	1,317	1,651	0	MST-int	MaLR	LTR	348	0	-	-	53
43,045,379	43,045,486	-	22.4	58	180	-248	MSTA	MaLR	LTR	108	-7	-	-	54
43,045,532	43,045,627	-	28.1	1	100	-328	MSTA	MaLR	LTR	96	45	3.4	-26	46
43,045,628	43,045,705	-	18.7	1,574	1,651	0	MSTA-int	MaLR	LTR	78	0	-	-	55
43,045,708	43,045,768	+	18.0	317	377	-51	MSTA	MaLR	LTR	61	2	-	-	56
43,045,841	43,047,367	-	22.9	15	1,583	-68	MSTA-int	MaLR	LTR	1,527	72	4.2	-10	55
43,047,471	43,047,583	+	19.1	506	661	-963	MST-int	MaLR	LTR	113	103	-	-	57
43,047,600	43,047,889	-	20.1	66	376	-52	MSTA	MaLR	LTR	290	16	-	-	58
43,047,894	43,048,172	+	24.9	907	1,218	-362	THE1-int	MaLR	LTR	279	4	-	-	59
43,048,126	43,048,181	-	21.4	911	967	-613	THE1-int	MaLR	LTR	56	-47	-	-	60
43,048,278	43,048,373	-	20.8	1	97	-331	MSTA	MaLR	LTR	96	96	0.7	-32	58
43,048,376	43,048,721	-	22.1	1,318	1,649	-2	MST-int	MaLR	LTR	346	2	-	-	61
43,048,726	43,048,793	-	20.6	95	166	-262	MSTA	MaLR	LTR	68	4	-	-	62
43,048,888	43,048,994	-	20.4	1	104	-324	MSTA	MaLR	LTR	107	94	4.8	23	1

Figure S1G: The Largest Cluster Found

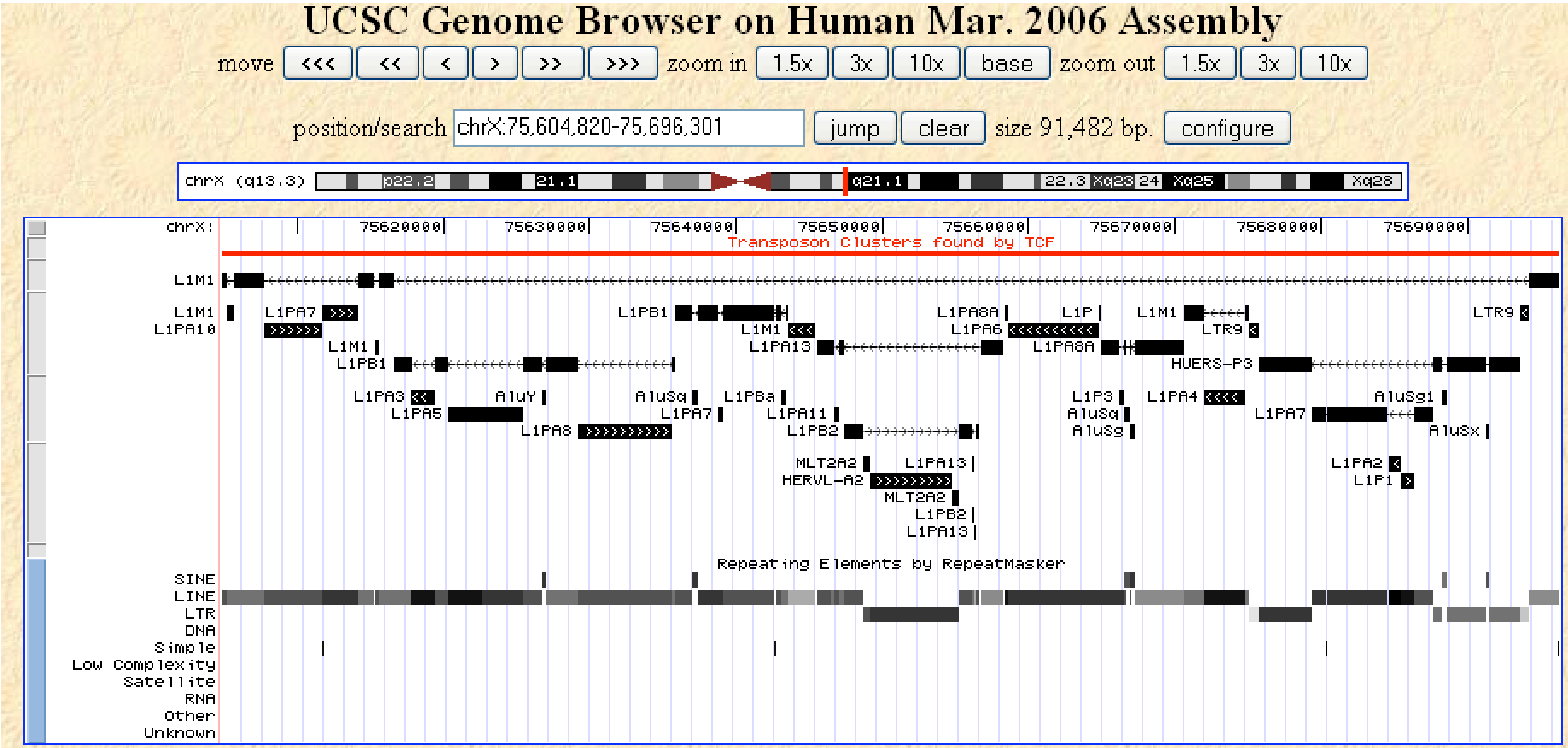

Genome Start	Genome End	Strand	%Div	Repeat Start	Repeat End	Repeat Left	Name	Family	Class	Size	Space	Div	Rep	Unit
75,604,820	75,605,206	-	14.2	6,804	7,200	-523	L1M1	L1	LINE	387	-	-	-	1
75,605,203	75,605,679	+	16.4	6,318	6,797	-768	L1M1	L1	LINE	477	-4	-	-	2
75,605,680	75,607,737	-	15.9	4,152	6,231	-1,334	L1M1	L1	LINE	2,058	0	1.7	572	1
75,607,738	75,611,668	+	13.6	4	3,989	-2,472	L1PA10	L1	LINE	3,931	0	-	-	3
75,611,710	75,614,179	+	10.6	3,990	6,468	-1	L1PA7	L1	LINE	2,470	41	-	-	4
75,614,178	75,615,261	-	16.9	3,061	4,170	-3,395	L1M1	L1	LINE	1,084	-2	1	-19	1
75,615,265	75,615,587	-	14.2	2,282	2,609	-3,537	L1M1	L1	LINE	323	3	-	-	5
75,615,585	75,616,566	-	17.8	2,088	3,064	-4,501	L1M1	L1	LINE	982	-3	0.9	-4	1
75,616,589	75,617,832	-	13.5	5,691	6,867	0	L1PB1	L1	LINE	1,244	22	-	-	6
75,617,828	75,619,354	-	3.2	4,615	6,141	-14	L1PA3	L1	LINE	1,527	-5	-	-	7
75,619,354	75,620,325	-	13.8	4,728	5,701	-1,161	L1PB1	L1	LINE	972	-1	0.3	-11	6

75,620,350	75,622,710	-	6.4	3,793	6,154	0	L1PA5	L1	LINE	2,361	24	-	-	8
75,622,702	75,625,504	-	9.7	1	2,803	-3,343	L1PA5	L1	LINE	2,803	-9	3.3	989	8
75,625,505	75,626,703	-	14.7	3,544	4,744	-2,118	L1PB1	L1	LINE	1,199	0	0.9	-17	6
75,626,704	75,627,006	-	7.7	1	300	-11	AluY	Alu	SINE	303	0	-	-	9
75,627,007	75,629,162	-	13.5	1,113	3,543	-3,319	L1PB1	L1	LINE	2,156	0	1.2	0	6
75,629,171	75,635,598	+	12.3	5	6,487	0	L1PA8	L1	LINE	6,428	8	-	-	10
75,635,597	75,635,870	-	16.4	863	1,125	-5,738	L1PB1	L1	LINE	274	-2	2.9	-13	6
75,635,868	75,637,032	-	14.5	5,708	6,866	-1	L1PB1	L1	LINE	1,165	-3	-	-	11
75,637,033	75,637,330	-	12.5	1	296	-17	AluSq	Alu	SINE	298	0	-	-	12
75,637,331	75,638,816	-	10.7	4,213	5,707	-1,155	L1PB1	L1	LINE	1,486	0	3.8	0	11
75,638,817	75,639,139	+	9.3	5,826	6,148	-6	L1PA7	L1	LINE	323	0	-	-	13
75,639,140	75,642,661	-	13.2	691	4,212	-2,650	L1PB1	L1	LINE	3,522	0	2.5	0	11
75,642,690	75,643,132	-	13.7	256	690	-6,172	L1PB1	L1	LINE	443	28	0.5	0	11
75,643,147	75,643,397	-	17.3	634	879	-5,984	L1PBa	L1	LINE	251	14	-	-	14
75,643,398	75,643,593	-	20.9	3	200	-6,663	L1PB1	L1	LINE	196	0	7.2	55	11
75,643,599	75,645,477	-	23.2	62	2,109	-5,456	L1M1	L1	LINE	1,879	5	-	-	15
75,645,489	75,646,462	-	15.5	2,586	3,576	-3,190	L1PA13	L1	LINE	974	11	-	-	16
75,646,439	75,646,751	-	18.6	1,963	2,283	-4,502	L1PA13	L1	LINE	313	-24	3.1	302	16
75,646,752	75,647,032	+	12.7	5,905	6,174	0	L1PA11	L1	LINE	281	0	-	-	17
75,647,033	75,647,396	-	18.6	1,600	1,962	-4,823	L1PA13	L1	LINE	364	0	0	0	16
75,647,394	75,648,671	+	16.1	3,678	4,966	-1,180	L1PB2	L1	LINE	1,278	-3	-	-	18
75,648,672	75,649,119	+	13.2	1	453	0	MLT2A2	ERVL	LTR	448	0	-	-	19
75,649,120	75,654,778	+	10.5	1	5,654	0	HERVL-A2	ERVL	LTR	5,659	0	-	-	20
75,654,779	75,655,229	+	9.8	1	453	0	MLT2A2	ERVL	LTR	451	0	-	-	21
75,655,231	75,656,152	+	16.0	4,963	5,881	-270	L1PB2	L1	LINE	922	1	0.1	-3	18
75,656,145	75,656,183	+	5.1	405	443	-6,342	L1PA13	L1	LINE	39	-8	-	-	22
75,656,180	75,656,273	+	17.4	5,804	5,895	-256	L1PB2	L1	LINE	94	-4	-	-	23
75,656,247	75,656,410	+	14.6	401	592	-6,193	L1PA13	L1	LINE	164	-27	-	-	24
75,656,414	75,656,683	+	12.6	5,876	6,151	0	L1PB2	L1	LINE	270	3	3.4	-5	18
75,656,701	75,658,282	-	22.9	13	1,587	-5,198	L1PA13	L1	LINE	1,582	17	4.3	12	16
75,658,342	75,658,637	-	6.4	5,387	5,683	-448	L1PA8A	L1	LINE	296	59	-	-	25
75,658,639	75,664,815	-	10.0	5	6,152	-2	L1PA6	L1	LINE	6,177	1	-	-	26
75,664,825	75,664,874	-	10.0	5,352	5,401	-771	L1P	L1	LINE	50	9	-	-	27
75,664,899	75,666,219	-	10.1	5,000	6,287	-29	L1PA8A	L1	LINE	1,321	24	-	-	28
75,666,215	75,666,537	+	10.2	3,809	4,131	-2,015	L1P3	L1	LINE	323	-5	-	-	29
75,666,529	75,666,559	-	11.0	3,269	3,293	-3,038	L1PA8A	L1	LINE	31	-9	0.9	1706	28
75,666,560	75,666,868	-	14.0	1	307	-6	AluSq	Alu	SINE	309	0	-	-	30
75,666,869	75,666,911	-	11.0	3,237	3,268	-3,063	L1PA8A	L1	LINE	43	0	0	0	28
75,666,912	75,667,205	-	11.6	1	292	-18	AluSg	Alu	SINE	294	0	-	-	31

75,667,206	75,670,671	-	16.3	3	3,236	-3,095	L1PA8A	L1	LINE	3,466	0	5.3	0	28
75,670,675	75,672,000	-	16.8	2,514	3,831	-3,734	L1M1	L1	LINE	1,326	3	-	-	32
75,671,997	75,674,873	-	5.6	3,279	6,155	0	L1PA4	L1	LINE	2,877	-4	-	-	33
75,674,857	75,675,106	-	15.6	2,086	2,346	-5,219	L1M1	L1	LINE	250	-17	1.2	167	32
75,675,107	75,675,741	-	28.6	1	612	0	LTR9	ERV1	LTR	635	0	-	-	34
75,675,742	75,678,290	-	11.4	6,366	8,919	0	HUERS-P3	ERV1	LTR	2,549	0	-	-	35
75,678,343	75,679,384	-	11.2	5,225	6,275	-2,644	HUERS-P3	ERV1	LTR	1,042	52	0.2	90	35
75,679,385	75,680,366	-	9.1	5,493	6,466	-3	L1PA7	L1	LINE	982	0	-	-	36
75,680,401	75,684,563	-	11.5	1,321	5,492	-969	L1PA7	L1	LINE	4,163	34	2.4	0	36
75,684,659	75,685,449	-	2.3	5,362	6,152	-3	L1PA2	L1	LINE	791	95	-	-	37
75,685,447	75,686,354	+	3.1	2,172	3,083	-3,063	L1P1	L1	LINE	908	-3	-	-	38
75,686,351	75,687,665	-	12.7	1	1,339	-5,122	L1PA7	L1	LINE	1,315	-4	1.2	-19	36
75,687,668	75,688,287	-	15.6	4,704	5,233	-3,686	HUERS-P3	ERV1	LTR	620	2	4.4	-9	35
75,688,288	75,688,593	-	14.2	1	282	-27	AluSg1	Alu	SINE	306	0	-	-	39
75,688,594	75,691,248	-	15.6	2,065	4,703	-4,216	HUERS-P3	ERV1	LTR	2,655	0	0	0	35
75,691,249	75,691,556	-	13.1	1	306	-6	AluSx	Alu	SINE	308	0	-	-	40
75,691,557	75,693,563	-	15.6	1	2,064	-6,855	HUERS-P3	ERV1	LTR	2,007	0	0	0	35
75,693,634	75,694,244	-	29.2	1	581	-31	LTR9	ERV1	LTR	611	70	-	-	41
75,694,246	75,696,196	-	25.6	110	2,088	-5,477	L1M1	L1	LINE	1,951	1	7.8	0	1
75,696,222	75,696,301	-	25.6	15	109	-7,456	L1M1	L1	LINE	80	25	0	0	1

Figure S1H: The Cluster with the Most Interruptions

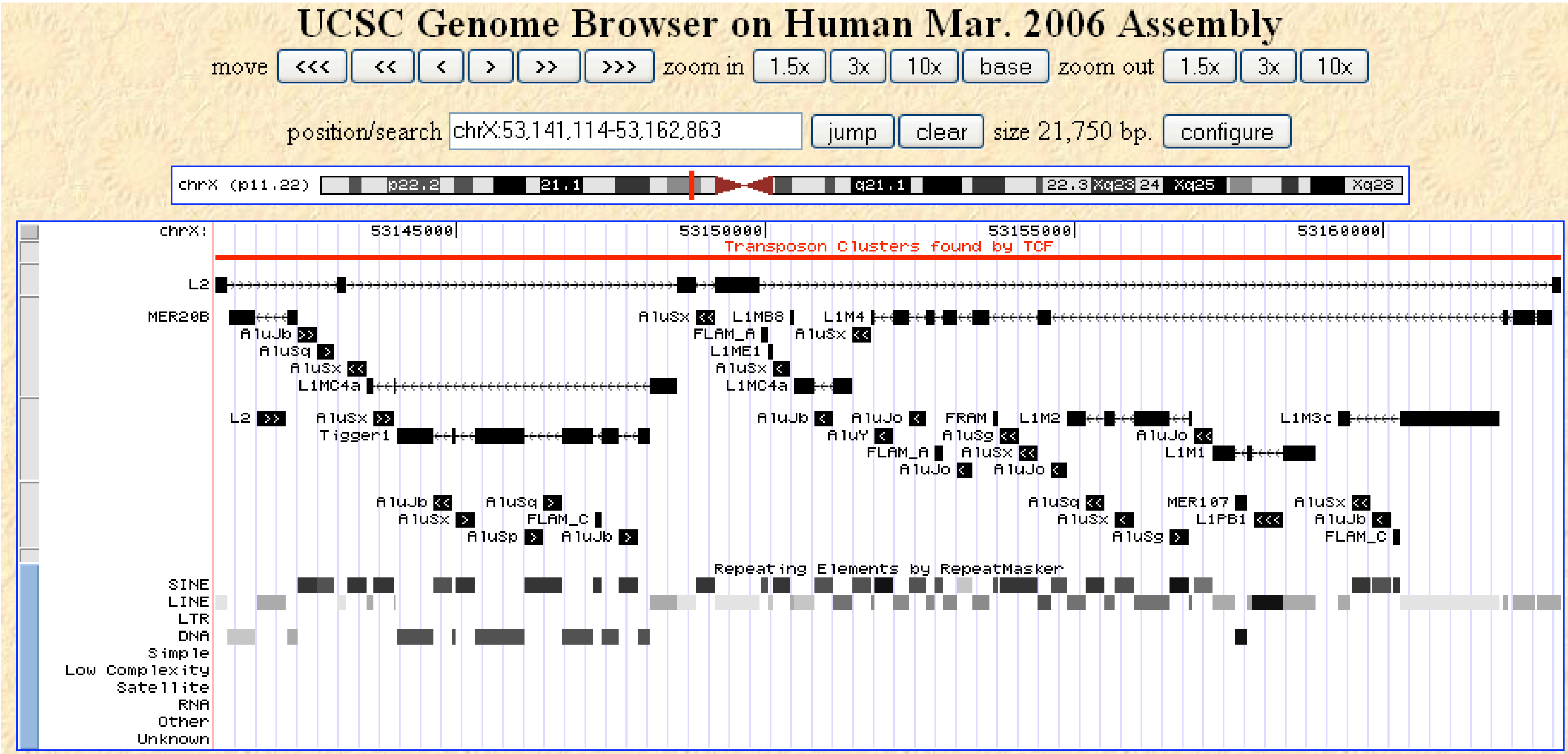

Genome Start	Genome End	Strand	%Div	Repeat Start	Repeat End	Repeat Left	Name	Family	Class	Size	Space	Div	Rep	Unit
53,141,114	53,141,313	+	24.8	218	455	-2,964	L2	L2	LINE	200	-	-	-	1
53,141,322	53,141,764	-	24.4	303	781	-2	MER20B	MER1_type	DNA	443	8	-	-	2
53,141,769	53,142,260	+	25.0	2,913	3,418	-1	L2	L2	LINE	492	4	-	-	3
53,142,268	53,142,447	-	27.0	51	238	-545	MER20B	MER1_type	DNA	180	7	2.6	64	2
53,142,451	53,142,751	+	12.0	1	303	-9	AluJb	Alu	SINE	301	3	-	-	4
53,142,754	53,143,034	+	7.6	1	298	-15	AluSq	Alu	SINE	281	2	-	-	5
53,143,086	53,143,227	+	25.2	451	613	-2,806	L2	L2	LINE	142	51	0.4	-4	1
53,143,247	53,143,559	-	10.6	1	310	-2	AluSx	Alu	SINE	313	19	-	-	6
53,143,566	53,143,675	-	20.0	7,712	7,808	0	L1MC4a	L1	LINE	110	6	-	-	7
53,143,676	53,143,985	+	10.0	1	310	-2	AluSx	Alu	SINE	310	0	-	-	8
53,143,986	53,144,039	-	20.0	7,662	7,711	-97	L1MC4a	L1	LINE	54	0	0	0	7
53,144,047	53,144,631	-	10.6	1,791	2,402	-16	Tigger1	MER2_type	DNA	585	7	-	-	9

53,144,632	53,144,931	-	16.1	1	301	-11	AluJb	Alu	SINE	300	0	-	-	10
53,144,932	53,144,995	-	10.6	1,786	1,790	-628	Tigger1	MER2_type	DNA	64	0	0	0	9
53,144,996	53,145,290	+	11.6	1	293	-19	AluSx	Alu	SINE	295	0	-	-	11
53,145,291	53,146,105	-	10.6	967	1,785	-633	Tigger1	MER2_type	DNA	815	0	0	0	9
53,146,106	53,146,417	+	9.9	1	313	0	AluSp	Alu	SINE	312	0	-	-	12
53,146,425	53,146,728	+	8.9	1	306	-7	AluSq	Alu	SINE	304	7	-	-	13
53,146,729	53,147,230	-	10.6	463	962	-1,456	Tigger1	MER2_type	DNA	502	0	0	4	9
53,147,231	53,147,363	+	12.8	1	133	0	FLAM_C	Alu	SINE	133	0	-	-	14
53,147,364	53,147,622	-	10.6	230	462	-1,956	Tigger1	MER2_type	DNA	259	0	0	0	9
53,147,623	53,147,924	+	11.0	1	299	-13	AluJb	Alu	SINE	302	0	-	-	15
53,147,925	53,148,143	-	10.6	2	229	-2,189	Tigger1	MER2_type	DNA	219	0	0	0	9
53,148,144	53,148,588	-	22.1	7,179	7,660	-148	L1MC4a	L1	LINE	445	0	2.1	1	7
53,148,589	53,148,879	+	26.0	625	905	-2,514	L2	L2	LINE	291	0	0.8	11	1
53,148,880	53,149,182	-	10.2	1	312	0	AluSx	Alu	SINE	303	0	-	-	16
53,149,183	53,149,899	+	26.0	906	1,802	-1,617	L2	L2	LINE	717	0	0	0	1
53,149,947	53,150,045	-	18.2	35	133	-9	FLAM_A	Alu	SINE	99	47	-	-	17
53,150,046	53,150,126	-	27.4	6,066	6,132	-34	L1ME1	L1	LINE	81	0	-	-	18
53,150,127	53,150,396	-	10.7	38	307	-5	AluSx	Alu	SINE	270	0	-	-	19
53,150,397	53,150,463	-	20.4	6,004	6,070	-108	L1MB8	L1	LINE	67	0	-	-	20
53,150,464	53,150,792	-	22.0	5,598	6,001	-1,994	L1MC4a	L1	LINE	329	0	-	-	21
53,150,793	53,151,090	-	15.1	1	299	-13	AluJb	Alu	SINE	298	0	-	-	22
53,151,091	53,151,397	-	14.3	5,282	5,597	-587	L1MC4a	L1	LINE	307	0	7.7	0	21
53,151,398	53,151,706	-	14.8	1	299	-13	AluSx	Alu	SINE	309	0	-	-	23
53,151,707	53,151,753	-	16.0	5,241	5,281	-865	L1M4	L1	LINE	47	0	-	-	24
53,151,754	53,152,063	-	2.9	1	310	-1	AluY	Alu	SINE	310	0	-	-	25
53,152,064	53,152,314	-	16.0	4,998	5,240	-906	L1M4	L1	LINE	251	0	0	0	24
53,152,315	53,152,603	-	15.6	1	295	-17	AluJo	Alu	SINE	289	0	-	-	26
53,152,604	53,152,744	-	16.0	4,856	4,997	-1,149	L1M4	L1	LINE	141	0	0	0	24
53,152,745	53,152,870	+	19.1	2	127	-6	FLAM_A	Alu	SINE	126	0	-	-	27
53,152,871	53,153,091	-	16.0	4,635	4,855	-1,291	L1M4	L1	LINE	221	0	0	0	24
53,153,092	53,153,348	-	18.0	2	302	0	AluJo	Alu	SINE	257	0	-	-	28
53,153,349	53,153,625	-	16.0	4,352	4,634	-1,512	L1M4	L1	LINE	277	0	0	0	24
53,153,671	53,153,759	-	19.1	69	157	-19	FRAM	Alu	SINE	89	45	-	-	29
53,153,793	53,154,095	-	8.3	1	302	-8	AluSg	Alu	SINE	303	33	-	-	30
53,154,102	53,154,409	-	12.7	1	307	-5	AluSx	Alu	SINE	308	6	-	-	31
53,154,410	53,154,614	-	16.5	4,169	4,371	-1,775	L1M4	L1	LINE	205	0	0.5	-20	24
53,154,615	53,154,884	-	17.1	25	293	-19	AluJo	Alu	SINE	270	0	-	-	32
53,154,885	53,155,176	-	12.6	4,421	4,708	-1,435	L1M2	L1	LINE	292	0	-	-	33
53,155,177	53,155,488	-	9.3	3	313	0	AluSq	Alu	SINE	312	0	-	-	34
53,155,489	53,155,649	-	12.4	4,268	4,420	-1,723	L1M2	L1	LINE	161	0	0.2	0	33
53,155,650	53,155,955	-	13.4	7	312	0	AluSx	Alu	SINE	306	0	-	-	35

53,155,956	53,156,549	-	12.6	3,630	4,267	-1,876	L1M2	L1	LINE	594	0	0.2	0	33
53,156,550	53,156,840	+	7.2	1	291	-19	AluSg	Alu	SINE	291	0	-	-	36
53,156,841	53,156,909	-	12.6	3,558	3,629	-2,514	L1M2	L1	LINE	69	0	0	0	33
53,156,919	53,157,228	-	16.6	1	304	-8	AluJo	Alu	SINE	310	9	-	-	37
53,157,232	53,157,581	-	23.6	2,987	3,354	-4,211	L1M1	L1	LINE	350	3	-	-	38
53,157,582	53,157,774	+	6.7	1	195	-2	MER107	MER1_type	DNA	193	0	-	-	39
53,157,775	53,157,883	-	23.6	2,902	2,986	-4,579	L1M1	L1	LINE	109	0	0	0	38
53,157,884	53,158,381	-	6.5	5,652	6,151	0	L1PB1	L1	LINE	498	0	-	-	40
53,158,382	53,158,904	-	23.6	2,366	2,901	-4,664	L1M1	L1	LINE	523	0	0	0	38
53,159,249	53,159,459	-	25.2	1,961	2,176	-4,861	L1M3c	L1	LINE	211	344	-	-	41
53,159,471	53,159,787	-	8.8	1	307	-5	AluSx	Alu	SINE	317	11	-	-	42
53,159,816	53,160,109	-	13.2	3	290	-22	AluJb	Alu	SINE	294	28	-	-	43
53,160,135	53,160,252	-	12.8	2	118	-25	FLAM_C	Alu	SINE	118	25	-	-	44
53,160,254	53,161,860	-	18.2	6	1,958	-5,072	L1M3c	L1	LINE	1,607	1	7	2	41
53,161,910	53,162,015	-	21.8	1,242	1,347	-5,557	L1M4	L1	LINE	106	49	5.3	2821	24
53,162,093	53,162,437	-	28.5	897	1,237	-5,667	L1M4	L1	LINE	345	77	6.7	4	24
53,162,470	53,162,714	-	27.6	16	598	-6,306	L1M4	L1	LINE	245	32	0.9	298	24
53,162,720	53,162,863	+	25.7	1,783	1,931	-1,488	L2	L2	LINE	144	5	0.3	-19	1

Figure S1I: Defragmentation of MLT2C1 Excluding Defragmentation of L1MB8

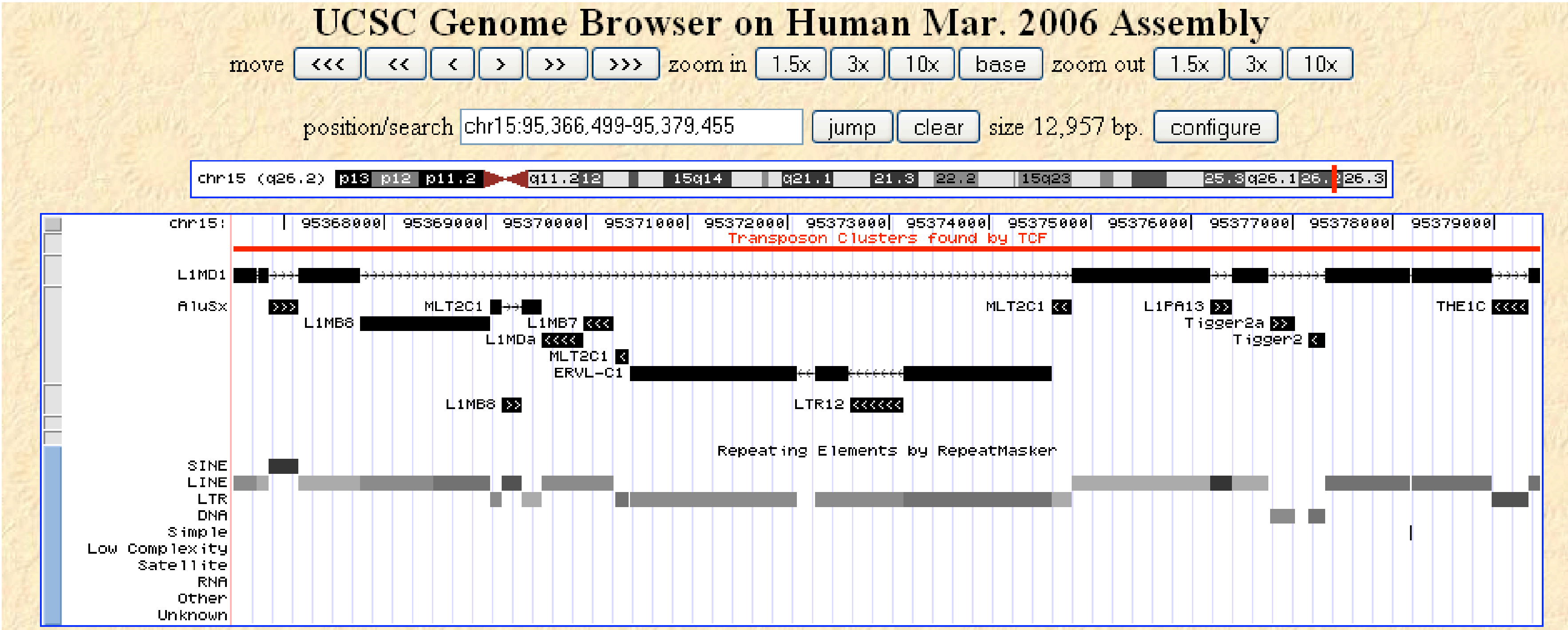

Genome Start	Genome End	Strand	%Div	Repeat Start	Repeat End	Repeat Left	Name	Family	Class	Size	Space	Div	Rep	Unit
95,366,499	95,366,736	+	27.4	125	362	-6,378	L1MD1	L1	LINE	239	-	-	-	1
95,366,738	95,366,847	+	23.0	934	1,055	-5,487	L1MD1	L1	LINE	110	1	4.4	571	1
95,366,848	95,367,147	+	8.9	1	292	-20	AluSx	Alu	SINE	300	0	-	-	2
95,367,148	95,367,749	+	23.0	1,056	1,656	-4,886	L1MD1	L1	LINE	602	0	0	0	1
95,367,756	95,368,476	+	22.8	4,023	4,767	-1,379	L1MB8	L1	LINE	721	6	-	-	3
95,368,474	95,369,036	+	19.6	5,259	5,821	-363	L1MB8	L1	LINE	563	-3	3.2	491	3
95,369,043	95,369,154	+	24.3	112	224	-173	MLT2C1	ERVL	LTR	112	6	-	-	4
95,369,160	95,369,350	+	18.0	5,989	6,177	-1	L1MB8	L1	LINE	191	5	-	-	5
95,369,351	95,369,550	+	23.6	213	397	0	MLT2C1	ERVL	LTR	200	0	0.7	-11	4
95,369,551	95,369,964	-	21.6	1,647	2,062	-4,571	L1MDa	L1	LINE	414	0	-	-	6
95,369,972	95,370,274	-	18.5	5,844	6,175	-9	L1MB7	L1	LINE	303	7	-	-	7
95,370,277	95,370,419	-	17.5	1	146	-273	MLT2C1	ERVL	LTR	143	2	-	-	8
95,370,429	95,372,083	-	21.7	4,002	5,704	-10	ERVL-C1	ERVL	LTR	1,655	9	-	-	9
95,372,272	95,372,604	-	21.0	3,559	3,909	-1,805	ERVL-C1	ERVL	LTR	333	188	0.7	92	9
95,372,605	95,373,138	-	13.3	1	600	-226	LTR12	ERV1	LTR	534	0	-	-	10

95,373,139	95,374,611	-	19.9	2,087	3,555	-2,159	ERVL-C1	ERVL	LTR	1,473	0	1.1	3	9
95,374,612	95,374,805	-	21.5	122	310	-87	MLT2C1	ERVL	LTR	194	0	-	-	11
95,374,806	95,376,189	+	18.9	3,208	4,477	-2,167	L1MD1	L1	LINE	1,384	0	4.1	1551	1
95,376,190	95,376,397	+	9.1	5,949	6,163	0	L1PA13	L1	LINE	208	0	-	-	12
95,376,398	95,376,767	+	19.1	4,478	4,871	-1,773	L1MD1	L1	LINE	370	0	0.2	0	1
95,376,772	95,377,026	+	15.7	1	259	-175	Tigger2a	MER2_type	DNA	255	4	-	-	13
95,377,158	95,377,325	-	16.7	2,535	2,708	0	Tigger2	MER2_type	DNA	168	131	-	-	14
95,377,326	95,378,160	+	20.5	4,940	5,795	-945	L1MD1	L1	LINE	835	0	1.4	68	1
95,378,190	95,378,969	+	18.5	5,796	6,618	-122	L1MD1	L1	LINE	780	29	2	0	1
95,378,970	95,379,332	-	10.1	1	375	0	THE1C	MaLR	LTR	363	0	-	-	15
95,379,333	95,379,455	+	18.5	6,619	6,737	-3	L1MD1	L1	LINE	123	0	0	0	1
