# Supplementary material for: Evolutionary History of Mammalian Transposons Determined by Genome-Wide Defragmentation
Source: PLoS Comput Biol. 2007 Jul 13;3(7):e137. doi: 10.1371/journal.pcbi.0030137 (PMC1914374; doi:10.1371/journal.pcbi.0030137)
Supplement: Supplementary file 2 [file pcbi.0030137.st003.pdf]

Supplementary Table S3: TCF results for seven mammalian genomes

	Human	Chimp	Rhesus	Cow	Dog	Rat	Mouse
Transposons (TEs)	908	929	907	421	502	748	871
TEs BP/Genome BP	46.71	39.50	42.61	41.93	34.39	36.09	37.34
Clusters	307,412	294,435	283,742	297,651	217,550	139,579	144,654
Cluster MBp	542	491	477	422	336	249	258
%Cluster BP/Genome BP	18.97	15.19	16.69	14.74	13.30	8.91	9.77
Interruptions	636,125	577,495	559,715	602,896	440,611	228,546	254,513
TEs Analyzed by IMA	435	438	438	288	335	474	546
%Units Analyzed by IMA	97.04	96.54	96.53	99.13	97.52	96.85	97.02
Interruptions Analyzed by IMA	601,694	546,073	529,172	600,177	437,061	225,762	251,332
