# Supplementary material for: Evolutionary History of Mammalian Transposons Determined by Genome-Wide Defragmentation
Source: PLoS Comput Biol. 2007 Jul 13;3(7):e137. doi: 10.1371/journal.pcbi.0030137 (PMC1914374; doi:10.1371/journal.pcbi.0030137)
Supplement: Supplementary file 3 [file pcbi.0030137.st004.pdf]

Table S4: Comparison of IMA results for seven mammalian genomes

Aligned Genomes	Human 435 TEs				Chimp 438 TEs				Rhesus 438 TEs				Cow 288 TEs			
	M	NM	NS	NG	M	NM	NS	NG	M	NM	NS	NG	M	NM	NS	NG
Human					352	83	0	3	323	110	0	5	100	168	2	18
Chimp	352	83	0	0					314	121	0	3	126	142	2	18
Rhesus	323	110	2	0	314	121	2	1					131	137	2	18
Cow	100	168	16	151	126	142	16	154	131	137	15	155				
Dog	146	123	9	157	171	98	9	160	162	107	8	161	159	108	1	20
Rat	46	186	36	167	63	169	36	170	55	176	36	171	57	170	28	33
Mouse	52	194	35	154	63	185	35	155	52	195	35	156	70	172	27	19

Reference Genomes

Aligned Genomes	Dog 335 TEs				Rat 474 TEs				Mouse 546 TEs			
	M	NM	NS	NG	M	NM	NS	NG	M	NM	NS	NG
Human	146	123	2	64	46	186	1	241	52	194	1	299
Chimp	171	98	2	64	63	169	1	241	63	185	1	297
Rhesus	162	107	2	64	55	176	2	241	52	195	2	297
Cow	159	108	5	63	57	170	6	241	70	172	5	299
Dog					70	157	2	245	68	174	2	302
Rat	70	157	29	79					252	120	11	163
Mouse	68	174	27	66	252	120	12	90				

M – Matching
NM – Not Matching
NS – Not in Set
NG – Not in Genome
