# Supplementary material for: Evolutionary History of Mammalian Transposons Determined by Genome-Wide Defragmentation
Source: PLoS Comput Biol. 2007 Jul 13;3(7):e137. doi: 10.1371/journal.pcbi.0030137 (PMC1914374; doi:10.1371/journal.pcbi.0030137)
Supplement: Supplementary file 4 [file pcbi.0030137.sd001.htm]

Clusters with Intact LTRs

**Supplementary Table S1a: Intact LTR Elements**  
  
To load the custom track into the UCSC Genome Browser, click here. The custom track must be loaded in order to view the clusters in Supplementary Table S1. It may take a minute to load, so please be patient.  
  
Jump to:  
chr1  
chr2  
chr3  
chr4  
chr5  
chr6  
chr7  
chr8  
chr9  
chr10  
chr11  
chr12  
chr13  
chr14  
chr15  
chr16  
chr17  
chr18  
chr19  
chr20  
chr21  
chr22  
chrX  
chrY  
  


|  |  |  |  |  |  |  |  |  |  |
| --- | --- | --- | --- | --- | --- | --- | --- | --- | --- |
| chr1:343159-345974 | | | 2812 bp | | |  |  |  |  |
| Genome Start | Genome End | Strand | Div | Repeat Start | Repeat End | Repeat Left | Name | Family | Class |
| 343159 | 343452 | - | 29.0 | 252 | 542 | -13 | MLT1H1 | MaLR | LTR |
| 343453 | 343812 | + | 15.0 | 3 | 364 | 0 | THE1B | MaLR | LTR |
| 343817 | 345376 | + | 30.2 | 4 | 1735 | 0 | MLT-int | MaLR | LTR |
| 345377 | 345734 | + | 18.6 | 1 | 364 | 0 | THE1B | MaLR | LTR |
| 345735 | 345974 | - | 29.0 | 3 | 251 | -304 | MLT1H1 | MaLR | LTR |

  

|  |  |  |  |  |  |  |  |  |  |
| --- | --- | --- | --- | --- | --- | --- | --- | --- | --- |
| chr1:623446-626243 | | | 2794 bp | | |  |  |  |  |
| Genome Start | Genome End | Strand | Div | Repeat Start | Repeat End | Repeat Left | Name | Family | Class |
| 623446 | 623684 | + | 29.8 | 2 | 249 | -306 | MLT1H1 | MaLR | LTR |
| 623685 | 624042 | - | 18.1 | 1 | 364 | 0 | THE1B | MaLR | LTR |
| 624043 | 625602 | - | 31.2 | 4 | 1735 | 0 | MLT-int | MaLR | LTR |
| 625607 | 625966 | - | 16.1 | 3 | 364 | 0 | THE1B | MaLR | LTR |
| 625967 | 626243 | + | 29.8 | 250 | 524 | -31 | MLT1H1 | MaLR | LTR |

  

|  |  |  |  |  |  |  |  |  |  |
| --- | --- | --- | --- | --- | --- | --- | --- | --- | --- |
| chr1:2860129-2865399 | | | 5101 bp | | |  |  |  |  |
| Genome Start | Genome End | Strand | Div | Repeat Start | Repeat End | Repeat Left | Name | Family | Class |
| 2860129 | 2860682 | + | 33.2 | 601 | 1178 | -5138 | L1MEd | L1 | LINE |
| 2860845 | 2861014 | + | 28.6 | 1335 | 1508 | -4641 | L1MEd | L1 | LINE |
| 2861015 | 2861388 | - | 12.1 | 3 | 375 | 0 | THE1C | MaLR | LTR |
| 2861391 | 2862958 | - | 30.0 | 2 | 1735 | 0 | MLT-int | MaLR | LTR |
| 2862961 | 2863335 | - | 10.4 | 1 | 375 | 0 | THE1C | MaLR | LTR |
| 2863336 | 2864196 | + | 28.6 | 1509 | 2387 | -3762 | L1MEd | L1 | LINE |
| 2864201 | 2864646 | + | 25.7 | 3804 | 4295 | -1834 | L1MEd | L1 | LINE |
| 2864647 | 2864934 | - | 14.2 | 1 | 289 | -23 | AluSx | Alu | SINE |
| 2864935 | 2865399 | + | 25.7 | 4296 | 4802 | -1327 | L1MEd | L1 | LINE |

  

|  |  |  |  |  |  |  |  |  |  |
| --- | --- | --- | --- | --- | --- | --- | --- | --- | --- |
| chr1:4860711-4863233 | | | 2521 bp | | |  |  |  |  |
| Genome Start | Genome End | Strand | Div | Repeat Start | Repeat End | Repeat Left | Name | Family | Class |
| 4860711 | 4860793 | + | 29.9 | 13 | 93 | -175 | MIRb | MIR | SINE |
| 4860794 | 4861159 | - | 14.7 | 1 | 364 | 0 | THE1B | MaLR | LTR |
| 4861160 | 4862729 | - | 30.1 | 2 | 1735 | 0 | MLT1A0-int | MaLR | LTR |
| 4862732 | 4863086 | - | 14.5 | 3 | 364 | 0 | THE1B | MaLR | LTR |
| 4863087 | 4863233 | + | 29.9 | 94 | 252 | -16 | MIRb | MIR | SINE |

  

|  |  |  |  |  |  |  |  |  |  |
| --- | --- | --- | --- | --- | --- | --- | --- | --- | --- |
| chr1:4877680-4880457 | | | 2776 bp | | |  |  |  |  |
| Genome Start | Genome End | Strand | Div | Repeat Start | Repeat End | Repeat Left | Name | Family | Class |
| 4877680 | 4877949 | + | 25.6 | 2 | 279 | -226 | MLT1D | MaLR | LTR |
| 4877950 | 4878314 | - | 13.4 | 1 | 360 | -4 | THE1B | MaLR | LTR |
| 4878315 | 4879867 | - | 31.1 | 2 | 1735 | 0 | MLT-int | MaLR | LTR |
| 4879870 | 4880235 | - | 14.4 | 1 | 364 | 0 | THE1B | MaLR | LTR |
| 4880236 | 4880457 | + | 25.6 | 280 | 502 | -3 | MLT1D | MaLR | LTR |

  

|  |  |  |  |  |  |  |  |  |  |
| --- | --- | --- | --- | --- | --- | --- | --- | --- | --- |
| chr1:5004106-5013313 | | | 9168 bp | | |  |  |  |  |
| Genome Start | Genome End | Strand | Div | Repeat Start | Repeat End | Repeat Left | Name | Family | Class |
| 5004106 | 5004715 | - | 27.6 | 1020 | 1683 | -3151 | L1MEe | L1 | LINE |
| 5004716 | 5005155 | + | 5.9 | 1 | 448 | -2 | LTR7 | ERV1 | LTR |
| 5005158 | 5008188 | + | 9.4 | 1 | 2995 | -4718 | HERVH | ERV1 | LTR |
| 5008168 | 5008314 | + | 13.6 | 3140 | 3290 | -4423 | HERVH | ERV1 | LTR |
| 5008304 | 5012569 | + | 7.6 | 3491 | 7713 | 0 | HERVH | ERV1 | LTR |
| 5012570 | 5013018 | + | 5.8 | 1 | 448 | -2 | LTR7 | ERV1 | LTR |
| 5013089 | 5013313 | - | 23.9 | 699 | 902 | -3952 | L1MEe | L1 | LINE |

  

|  |  |  |  |  |  |  |  |  |  |
| --- | --- | --- | --- | --- | --- | --- | --- | --- | --- |
| chr1:5419979-5422784 | | | 2804 bp | | |  |  |  |  |
| Genome Start | Genome End | Strand | Div | Repeat Start | Repeat End | Repeat Left | Name | Family | Class |
| 5419979 | 5420107 | + | 26.2 | 3 | 127 | -340 | MLT1C | MaLR | LTR |
| 5420108 | 5420461 | - | 9.8 | 1 | 364 | 0 | THE1B | MaLR | LTR |
| 5420462 | 5422107 | - | 30.6 | 2 | 1735 | 0 | MLT-int | MaLR | LTR |
| 5422110 | 5422465 | - | 11.3 | 1 | 364 | 0 | THE1B | MaLR | LTR |
| 5422466 | 5422784 | + | 26.2 | 128 | 464 | -3 | MLT1C | MaLR | LTR |

  

|  |  |  |  |  |  |  |  |  |  |
| --- | --- | --- | --- | --- | --- | --- | --- | --- | --- |
| chr1:5500802-5503977 | | | 3121 bp | | |  |  |  |  |
| Genome Start | Genome End | Strand | Div | Repeat Start | Repeat End | Repeat Left | Name | Family | Class |
| 5500802 | 5500886 | - | 21.2 | 305 | 390 | 0 | MLT1B | MaLR | LTR |
| 5500892 | 5501296 | + | 14.7 | 1 | 423 | -25 | MER83 | ERV1 | LTR |
| 5501345 | 5501379 | - | 18.1 | 268 | 302 | -88 | MLT1B | MaLR | LTR |
| 5501380 | 5501743 | - | 11.3 | 1 | 364 | 0 | THE1B | MaLR | LTR |
| 5501744 | 5503346 | - | 31.0 | 2 | 1735 | 0 | MLT-int | MaLR | LTR |
| 5503349 | 5503713 | - | 9.3 | 1 | 364 | 0 | THE1B | MaLR | LTR |
| 5503714 | 5503977 | - | 18.1 | 7 | 267 | -123 | MLT1B | MaLR | LTR |

  

|  |  |  |  |  |  |  |  |  |  |
| --- | --- | --- | --- | --- | --- | --- | --- | --- | --- |
| chr1:5510928-5518340 | | | 7219 bp | | |  |  |  |  |
| Genome Start | Genome End | Strand | Div | Repeat Start | Repeat End | Repeat Left | Name | Family | Class |
| 5510928 | 5511424 | - | 31.5 | 2465 | 3047 | -372 | L2 | L2 | LINE |
| 5511431 | 5511850 | + | 12.0 | 1 | 428 | 0 | MSTA | MaLR | LTR |
| 5511853 | 5513456 | + | 28.2 | 2 | 1735 | 0 | MLT-int | MaLR | LTR |
| 5513459 | 5513876 | + | 16.4 | 3 | 428 | 0 | MSTA | MaLR | LTR |
| 5513905 | 5514062 | - | 32.9 | 1 | 158 | -932 | MER97c | MER1\_type? | DNA |
| 5514083 | 5515435 | - | 28.0 | 878 | 2409 | -1010 | L2 | L2 | LINE |
| 5515438 | 5515932 | - | 29.4 | 81 | 594 | -1 | MLT1G1 | MaLR | LTR |
| 5515991 | 5516270 | - | 24.9 | 330 | 718 | -754 | MLT1H-int | MaLR | LTR |
| 5516271 | 5516844 | - | 10.1 | 1 | 576 | 0 | LTR24B | ERV1 | LTR |
| 5516845 | 5516990 | - | 27.1 | 167 | 329 | -1143 | MLT1H-int | MaLR | LTR |
| 5517002 | 5517089 | - | 22.7 | 1360 | 1462 | -1957 | L2 | L2 | LINE |
| 5517093 | 5517162 | - | 17.1 | 1 | 70 | -1402 | MLT1H-int | MaLR | LTR |
| 5517171 | 5517570 | - | 26.3 | 75 | 559 | -8 | MLT1F1 | MaLR | LTR |
| 5517625 | 5518340 | - | 27.1 | 19 | 882 | -2537 | L2 | L2 | LINE |

  

|  |  |  |  |  |  |  |  |  |  |
| --- | --- | --- | --- | --- | --- | --- | --- | --- | --- |
| chr1:11491079-11496567 | | | 5454 bp | | |  |  |  |  |
| Genome Start | Genome End | Strand | Div | Repeat Start | Repeat End | Repeat Left | Name | Family | Class |
| 11491079 | 11491287 | - | 22.7 | 3550 | 3774 | -2763 | L1MEc | L1 | LINE |
| 11491288 | 11491540 | - | 12.4 | 6047 | 6302 | 0 | L1MA1 | L1 | LINE |
| 11491541 | 11492472 | - | 22.7 | 2270 | 3231 | -2988 | L1MD | L1 | LINE |
| 11492473 | 11492768 | + | 9.6 | 11 | 307 | 0 | AluY | Alu | SINE |
| 11492769 | 11493334 | - | 15.3 | 2082 | 2689 | -4029 | L1MEc | L1 | LINE |
| 11493335 | 11493444 | + | 13.6 | 2 | 114 | -198 | AluJo | Alu | SINE |
| 11493445 | 11493803 | - | 9.6 | 1 | 355 | 0 | THE1A | MaLR | LTR |
| 11493804 | 11495395 | - | 8.4 | 1 | 1580 | 0 | THE1A-int | MaLR | LTR |
| 11495396 | 11495753 | - | 6.8 | 1 | 355 | 0 | THE1A | MaLR | LTR |
| 11495754 | 11495950 | + | 17.4 | 110 | 305 | -7 | AluJo | Alu | SINE |
| 11495953 | 11496332 | - | 19.9 | 1599 | 1999 | -4634 | L1MEc | L1 | LINE |
| 11496366 | 11496567 | - | 17.5 | 337 | 571 | -6147 | L1MEc | L1 | LINE |

  

|  |  |  |  |  |  |  |  |  |  |
| --- | --- | --- | --- | --- | --- | --- | --- | --- | --- |
| chr1:13759233-13771804 | | | 12315 bp | | |  |  |  |  |
| Genome Start | Genome End | Strand | Div | Repeat Start | Repeat End | Repeat Left | Name | Family | Class |
| 13759233 | 13759579 | + | 11.0 | 1 | 317 | -4832 | HERVIP10FH | ERV1 | LTR |
| 13759586 | 13759729 | + | 23.8 | 288 | 792 | -7420 | HERVIP10FH | ERV1 | LTR |
| 13759923 | 13760310 | + | 4.4 | 1 | 464 | 0 | LTR7B | ERV1 | LTR |
| 13760311 | 13763189 | + | 20.5 | 1 | 2830 | -3792 | HERVH | ERV1 | LTR |
| 13763190 | 13763994 | + | 14.7 | 3357 | 4094 | -3619 | HERVH | ERV1 | LTR |
| 13763984 | 13765623 | + | 9.5 | 4370 | 6016 | -1697 | HERVH | ERV1 | LTR |
| 13765617 | 13766189 | + | 12.4 | 7157 | 7713 | 0 | HERVH | ERV1 | LTR |
| 13766190 | 13766576 | + | 5.7 | 1 | 464 | 0 | LTR7B | ERV1 | LTR |
| 13766639 | 13767127 | + | 21.5 | 565 | 1010 | -4092 | HERVIP10FH | ERV1 | LTR |
| 13767142 | 13767603 | + | 7.7 | 736 | 1197 | -3905 | HERVIP10FH | ERV1 | LTR |
| 13767604 | 13767892 | + | 6.6 | 1 | 288 | -23 | AluY | Alu | SINE |
| 13767893 | 13771804 | + | 7.7 | 1198 | 5102 | 0 | HERVIP10FH | ERV1 | LTR |

  

|  |  |  |  |  |  |  |  |  |  |
| --- | --- | --- | --- | --- | --- | --- | --- | --- | --- |
| chr1:14044164-14053315 | | | 9101 bp | | |  |  |  |  |
| Genome Start | Genome End | Strand | Div | Repeat Start | Repeat End | Repeat Left | Name | Family | Class |
| 14044164 | 14044599 | - | 17.9 | 4689 | 5111 | -1426 | L1MDa | L1 | LINE |
| 14044600 | 14044894 | - | 9.6 | 1 | 292 | -18 | AluSg | Alu | SINE |
| 14044895 | 14046550 | - | 17.9 | 3011 | 4688 | -1849 | L1MDa | L1 | LINE |
| 14046551 | 14046927 | + | 14.4 | 1 | 381 | 0 | THE1D | MaLR | LTR |
| 14046930 | 14048463 | + | 30.4 | 2 | 1735 | 0 | MLT-int | MaLR | LTR |
| 14048464 | 14048518 | + | 10.9 | 1 | 55 | -373 | MSTA | MaLR | LTR |
| 14048519 | 14048874 | + | 13.6 | 1 | 364 | 0 | THE1B | MaLR | LTR |
| 14048875 | 14050157 | + | 10.8 | 1 | 1284 | -296 | THE1B-int | MaLR | LTR |
| 14050158 | 14050331 | - | 17.9 | 2 | 175 | -27 | MER6C | MER2\_type | DNA |
| 14050336 | 14050771 | + | 27.3 | 1980 | 2425 | -187 | Charlie7 | MER1\_type | DNA |
| 14050801 | 14051111 | + | 10.9 | 1269 | 1580 | 0 | THE1B-int | MaLR | LTR |
| 14051112 | 14051469 | + | 13.7 | 1 | 364 | 0 | THE1B | MaLR | LTR |
| 14051470 | 14051797 | + | 13.4 | 50 | 381 | 0 | THE1D | MaLR | LTR |
| 14051799 | 14051983 | - | 18.4 | 2824 | 3014 | -3523 | L1MDa | L1 | LINE |
| 14051999 | 14052504 | - | 18.2 | 2062 | 2588 | -4045 | L1MDa | L1 | LINE |
| 14052505 | 14053020 | - | 20.9 | 1 | 505 | 0 | MLT1D | MaLR | LTR |
| 14053021 | 14053315 | - | 18.2 | 1763 | 2061 | -4572 | L1MDa | L1 | LINE |

  

|  |  |  |  |  |  |  |  |  |  |
| --- | --- | --- | --- | --- | --- | --- | --- | --- | --- |
| chr1:14053512-14057897 | | | 4384 bp | | |  |  |  |  |
| Genome Start | Genome End | Strand | Div | Repeat Start | Repeat End | Repeat Left | Name | Family | Class |
| 14053512 | 14053789 | - | 8.8 | 5886 | 6158 | -3 | L1PA14 | L1 | LINE |
| 14053790 | 14054149 | + | 16.0 | 1 | 381 | 0 | THE1D | MaLR | LTR |
| 14054152 | 14055761 | + | 30.5 | 2 | 1735 | 0 | MLT1L-int | MaLR | LTR |
| 14055762 | 14056106 | + | 11.7 | 1 | 381 | 0 | THE1D | MaLR | LTR |
| 14056107 | 14057897 | - | 8.5 | 4106 | 5885 | -276 | L1PA14 | L1 | LINE |

  

|  |  |  |  |  |  |  |  |  |  |
| --- | --- | --- | --- | --- | --- | --- | --- | --- | --- |
| chr1:14314107-14316695 | | | 2587 bp | | |  |  |  |  |
| Genome Start | Genome End | Strand | Div | Repeat Start | Repeat End | Repeat Left | Name | Family | Class |
| 14314107 | 14314387 | - | 21.2 | 95 | 374 | 0 | MLT1A | MaLR | LTR |
| 14314388 | 14314718 | + | 13.4 | 1 | 364 | 0 | THE1B | MaLR | LTR |
| 14314721 | 14316290 | + | 31.1 | 2 | 1735 | 0 | MLT-int | MaLR | LTR |
| 14316291 | 14316626 | + | 15.1 | 1 | 364 | 0 | THE1B | MaLR | LTR |
| 14316627 | 14316695 | - | 21.2 | 25 | 94 | -280 | MLT1A | MaLR | LTR |

  

|  |  |  |  |  |  |  |  |  |  |
| --- | --- | --- | --- | --- | --- | --- | --- | --- | --- |
| chr1:16301101-16307949 | | | 6821 bp | | |  |  |  |  |
| Genome Start | Genome End | Strand | Div | Repeat Start | Repeat End | Repeat Left | Name | Family | Class |
| 16301101 | 16301213 | + | 12.7 | 5785 | 5898 | -280 | L1MB7 | L1 | LINE |
| 16301215 | 16301371 | - | 19.1 | 133 | 290 | -22 | AluJb | Alu | SINE |
| 16301392 | 16301702 | - | 13.8 | 1 | 308 | 0 | AluSx | Alu | SINE |
| 16301703 | 16301892 | + | 13.2 | 5908 | 6113 | -71 | L1MB7 | L1 | LINE |
| 16301896 | 16302410 | - | 9.4 | 1 | 511 | 0 | MER9 | ERVK | LTR |
| 16302411 | 16304600 | - | 6.2 | 3708 | 5916 | 0 | HERVK9 | ERVK | LTR |
| 16304599 | 16307381 | - | 6.2 | 1 | 2786 | -3130 | HERVK9 | ERVK | LTR |
| 16307382 | 16307894 | - | 9.2 | 1 | 511 | 0 | MER9 | ERVK | LTR |
| 16307901 | 16307949 | + | 10.2 | 6117 | 6165 | -19 | L1MB7 | L1 | LINE |

  

|  |  |  |  |  |  |  |  |  |  |
| --- | --- | --- | --- | --- | --- | --- | --- | --- | --- |
| chr1:17542500-17544974 | | | 2473 bp | | |  |  |  |  |
| Genome Start | Genome End | Strand | Div | Repeat Start | Repeat End | Repeat Left | Name | Family | Class |
| 17542500 | 17542627 | + | 36.2 | 1 | 141 | -127 | MIRb | MIR | SINE |
| 17542628 | 17542974 | + | 10.2 | 1 | 355 | 0 | THE1A | MaLR | LTR |
| 17542977 | 17544550 | + | 29.6 | 2 | 1735 | 0 | MLT-int | MaLR | LTR |
| 17544551 | 17544897 | + | 8.1 | 1 | 355 | 0 | THE1A | MaLR | LTR |
| 17544898 | 17544974 | + | 36.2 | 142 | 214 | -54 | MIRb | MIR | SINE |

  

|  |  |  |  |  |  |  |  |  |  |
| --- | --- | --- | --- | --- | --- | --- | --- | --- | --- |
| chr1:20190774-20206939 | | | 16023 bp | | |  |  |  |  |
| Genome Start | Genome End | Strand | Div | Repeat Start | Repeat End | Repeat Left | Name | Family | Class |
| 20190774 | 20192428 | - | 9.7 | 2638 | 4338 | -2123 | L1P3 | L1 | LINE |
| 20192464 | 20195007 | - | 13.5 | 74 | 2637 | -3824 | L1P3 | L1 | LINE |
| 20195009 | 20196490 | + | 9.0 | 1 | 1502 | -773 | L1PA6 | L1 | LINE |
| 20196491 | 20197003 | + | 10.8 | 1 | 511 | 0 | MER9 | ERVK | LTR |
| 20197004 | 20197594 | + | 6.8 | 1 | 592 | -5324 | HERVK9 | ERVK | LTR |
| 20197595 | 20201946 | + | 7.3 | 1268 | 5641 | -275 | HERVK9 | ERVK | LTR |
| 20202033 | 20202294 | + | 6.5 | 5635 | 5916 | 0 | HERVK9 | ERVK | LTR |
| 20202299 | 20202807 | + | 11.8 | 5 | 511 | 0 | MER9 | ERVK | LTR |
| 20202808 | 20206159 | + | 7.4 | 1498 | 4843 | -1303 | L1PA6 | L1 | LINE |
| 20206172 | 20206850 | + | 5.0 | 5476 | 6154 | 0 | L1PA6 | L1 | LINE |
| 20206856 | 20206939 | - | 10.7 | 6 | 89 | -6372 | L1P3 | L1 | LINE |

  

|  |  |  |  |  |  |  |  |  |  |
| --- | --- | --- | --- | --- | --- | --- | --- | --- | --- |
| chr1:20210587-20217104 | | | 6502 bp | | |  |  |  |  |
| Genome Start | Genome End | Strand | Div | Repeat Start | Repeat End | Repeat Left | Name | Family | Class |
| 20210587 | 20210784 | - | 15.9 | 6295 | 6492 | -1070 | L1M1 | L1 | LINE |
| 20210785 | 20211187 | - | 16.2 | 4 | 408 | 0 | MLT1A1 | MaLR | LTR |
| 20211192 | 20212934 | - | 24.7 | 1 | 1734 | -1 | MLT1A1-int | MaLR | LTR |
| 20212935 | 20213334 | - | 16.6 | 1 | 408 | 0 | MLT1A1 | MaLR | LTR |
| 20213335 | 20215410 | - | 15.9 | 4174 | 6294 | -1268 | L1M1 | L1 | LINE |
| 20215407 | 20215495 | - | 18.0 | 3844 | 3935 | -3627 | L1M1 | L1 | LINE |
| 20215500 | 20215554 | - | 9.1 | 2583 | 2637 | -71 | Tigger2 | MER2\_type | DNA |
| 20215557 | 20215759 | + | 13.9 | 2510 | 2704 | -4 | Tigger2 | MER2\_type | DNA |
| 20215770 | 20216252 | - | 24.1 | 2886 | 3388 | -4177 | L1M1 | L1 | LINE |
| 20216253 | 20216538 | - | 13.6 | 1 | 290 | -22 | AluSx | Alu | SINE |
| 20216539 | 20217104 | - | 24.1 | 2291 | 2885 | -4680 | L1M1 | L1 | LINE |

  

|  |  |  |  |  |  |  |  |  |  |
| --- | --- | --- | --- | --- | --- | --- | --- | --- | --- |
| chr1:25509086-25514001 | | | 4656 bp | | |  |  |  |  |
| Genome Start | Genome End | Strand | Div | Repeat Start | Repeat End | Repeat Left | Name | Family | Class |
| 25509086 | 25509172 | - | 24.1 | 6059 | 6152 | -18 | L1ME3A | L1 | LINE |
| 25509175 | 25509257 | - | 16.9 | 5922 | 6011 | -159 | L1ME3A | L1 | LINE |
| 25509262 | 25509620 | + | 22.0 | 5 | 394 | -2 | MSTD | MaLR | LTR |
| 25509625 | 25509767 | + | 21.0 | 2 | 144 | -1591 | MSTD-int | MaLR | LTR |
| 25509769 | 25510079 | + | 11.6 | 2 | 311 | -2 | AluSq | Alu | SINE |
| 25510080 | 25510348 | + | 21.0 | 140 | 395 | -1340 | MSTD-int | MaLR | LTR |
| 25510346 | 25510445 | + | 30.0 | 558 | 657 | -1078 | MSTD-int | MaLR | LTR |
| 25510451 | 25510631 | + | 8.3 | 130 | 310 | -1 | AluY | Alu | SINE |
| 25510632 | 25511542 | + | 23.6 | 652 | 1671 | -64 | MSTD-int | MaLR | LTR |
| 25511547 | 25511838 | + | 15.7 | 7 | 293 | -19 | AluJo | Alu | SINE |
| 25511845 | 25512027 | + | 15.3 | 1 | 176 | 0 | FRAM | Alu | SINE |
| 25512030 | 25512098 | + | 13.0 | 1655 | 1735 | 0 | MSTD-int | MaLR | LTR |
| 25512099 | 25512468 | + | 23.7 | 1 | 396 | 0 | MSTD | MaLR | LTR |
| 25512469 | 25512593 | - | 16.1 | 5797 | 5926 | -314 | L1ME3A | L1 | LINE |
| 25512651 | 25512785 | - | 25.8 | 415 | 551 | -71 | MER74B | ERVL | LTR |
| 25512951 | 25513001 | - | 19.6 | 3 | 53 | -569 | MER74B | ERVL | LTR |
| 25513015 | 25513566 | - | 21.4 | 5202 | 5803 | -367 | L1ME3A | L1 | LINE |
| 25513567 | 25513881 | - | 17.5 | 1 | 305 | -7 | AluSx | Alu | SINE |
| 25513882 | 25514001 | - | 15.9 | 5081 | 5201 | -945 | L1ME3A | L1 | LINE |

  

|  |  |  |  |  |  |  |  |  |  |
| --- | --- | --- | --- | --- | --- | --- | --- | --- | --- |
| chr1:30387682-30390846 | | | 3143 bp | | |  |  |  |  |
| Genome Start | Genome End | Strand | Div | Repeat Start | Repeat End | Repeat Left | Name | Family | Class |
| 30387682 | 30387845 | + | 17.9 | 1 | 151 | -239 | MLT1C | MaLR | LTR |
| 30387856 | 30388225 | - | 9.6 | 1 | 355 | 0 | THE1A | MaLR | LTR |
| 30388226 | 30389783 | - | 9.5 | 21 | 1580 | 0 | THE1A-int | MaLR | LTR |
| 30389789 | 30390142 | - | 12.2 | 1 | 355 | 0 | THE1A | MaLR | LTR |
| 30390150 | 30390551 | - | 14.3 | 1 | 426 | 0 | MSTB | MaLR | LTR |
| 30390552 | 30390846 | + | 21.4 | 157 | 467 | 0 | MLT1C | MaLR | LTR |

  

|  |  |  |  |  |  |  |  |  |  |
| --- | --- | --- | --- | --- | --- | --- | --- | --- | --- |
| chr1:30445876-30458031 | | | 12097 bp | | |  |  |  |  |
| Genome Start | Genome End | Strand | Div | Repeat Start | Repeat End | Repeat Left | Name | Family | Class |
| 30445876 | 30447045 | - | 24.6 | 3849 | 5063 | -1156 | L1MEc | L1 | LINE |
| 30447046 | 30447361 | - | 11.9 | 1 | 311 | -2 | AluSp | Alu | SINE |
| 30447362 | 30447501 | - | 24.6 | 3731 | 3848 | -2371 | L1MEc | L1 | LINE |
| 30447502 | 30447895 | - | 19.3 | 1 | 408 | 0 | MLT1A1 | MaLR | LTR |
| 30447896 | 30448052 | - | 24.6 | 3577 | 3730 | -2489 | L1MEc | L1 | LINE |
| 30448053 | 30448360 | - | 15.3 | 1 | 307 | -5 | AluJo | Alu | SINE |
| 30448361 | 30448953 | - | 24.6 | 2967 | 3576 | -2643 | L1MEc | L1 | LINE |
| 30448970 | 30449111 | - | 23.2 | 2645 | 2796 | -3423 | L1MEc | L1 | LINE |
| 30449112 | 30449499 | + | 5.2 | 1 | 464 | 0 | LTR7B | ERV1 | LTR |
| 30449500 | 30449955 | + | 18.6 | 1 | 459 | -7254 | HERVH | ERV1 | LTR |
| 30450014 | 30452069 | + | 13.1 | 791 | 2830 | -4883 | HERVH | ERV1 | LTR |
| 30452070 | 30453367 | + | 18.3 | 3357 | 4719 | -1935 | HERVH | ERV1 | LTR |
| 30453358 | 30453618 | + | 13.0 | 5756 | 6016 | -1697 | HERVH | ERV1 | LTR |
| 30453612 | 30454163 | + | 11.7 | 7157 | 7713 | 0 | HERVH | ERV1 | LTR |
| 30454164 | 30454547 | + | 6.0 | 1 | 464 | 0 | LTR7B | ERV1 | LTR |
| 30454548 | 30454569 | - | 23.0 | 2632 | 2649 | -3570 | L1MEc | L1 | LINE |
| 30454570 | 30455028 | + | 8.9 | 1 | 463 | -318 | MER4E1 | ERV1 | LTR |
| 30455029 | 30455323 | - | 9.6 | 1 | 293 | -16 | AluSc | Alu | SINE |
| 30455324 | 30455638 | + | 8.9 | 464 | 781 | 0 | MER4E1 | ERV1 | LTR |
| 30455639 | 30455894 | - | 23.0 | 2366 | 2631 | -3588 | L1MEc | L1 | LINE |
| 30455895 | 30456200 | - | 18.0 | 1 | 308 | -4 | AluJb | Alu | SINE |
| 30456201 | 30456916 | - | 19.1 | 1732 | 2459 | -3941 | L1MEc | L1 | LINE |
| 30456921 | 30457071 | + | 20.7 | 1391 | 1524 | -4978 | L1MEc | L1 | LINE |
| 30457072 | 30457583 | + | 7.4 | 1 | 453 | 0 | MLT2A2 | ERVL | LTR |
| 30457584 | 30457824 | + | 23.2 | 1415 | 1689 | -4837 | L1MEc | L1 | LINE |
| 30457823 | 30458031 | - | 22.4 | 12 | 219 | -6181 | L1MEc | L1 | LINE |

  

|  |  |  |  |  |  |  |  |  |  |
| --- | --- | --- | --- | --- | --- | --- | --- | --- | --- |
| chr1:32670305-32678514 | | | 8164 bp | | |  |  |  |  |
| Genome Start | Genome End | Strand | Div | Repeat Start | Repeat End | Repeat Left | Name | Family | Class |
| 32670305 | 32670502 | + | 16.7 | 1 | 199 | -321 | MLT2B1 | ERVL | LTR |
| 32670525 | 32670937 | + | 8.0 | 1 | 448 | -2 | LTR7 | ERV1 | LTR |
| 32670940 | 32673931 | + | 9.3 | 1 | 2987 | -4726 | HERVH | ERV1 | LTR |
| 32673926 | 32674073 | + | 12.8 | 3140 | 3290 | -4423 | HERVH | ERV1 | LTR |
| 32674064 | 32674566 | + | 10.6 | 3492 | 3993 | -3720 | HERVH | ERV1 | LTR |
| 32674569 | 32675230 | + | 13.5 | 4488 | 5149 | -2564 | HERVH | ERV1 | LTR |
| 32675232 | 32675450 | + | 10.2 | 5600 | 5819 | -1894 | HERVH | ERV1 | LTR |
| 32675449 | 32675825 | + | 9.9 | 7322 | 7713 | 0 | HERVH | ERV1 | LTR |
| 32675826 | 32676226 | + | 8.0 | 1 | 448 | -2 | LTR7 | ERV1 | LTR |
| 32676237 | 32676318 | - | 16.7 | 230 | 307 | -5 | AluJo | Alu | SINE |
| 32676319 | 32677034 | + | 10.7 | 1 | 900 | 0 | MER4B | ERV1 | LTR |
| 32677037 | 32677240 | - | 19.6 | 10 | 231 | -81 | AluJo | Alu | SINE |
| 32677266 | 32677289 | + | 22.1 | 223 | 246 | -274 | MLT2B1 | ERVL | LTR |
| 32677290 | 32677589 | - | 8.0 | 3 | 303 | -10 | AluSp | Alu | SINE |
| 32677590 | 32677746 | + | 22.1 | 247 | 430 | -90 | MLT2B1 | ERVL | LTR |
| 32677747 | 32678438 | - | 16.1 | 1 | 727 | 0 | LTR8A | ERV1 | LTR |
| 32678439 | 32678514 | + | 22.1 | 478 | 564 | -3 | MLT2B1 | ERVL | LTR |

  

|  |  |  |  |  |  |  |  |  |  |
| --- | --- | --- | --- | --- | --- | --- | --- | --- | --- |
| chr1:33563297-33566241 | | | 2886 bp | | |  |  |  |  |
| Genome Start | Genome End | Strand | Div | Repeat Start | Repeat End | Repeat Left | Name | Family | Class |
| 33563297 | 33563380 | - | 32.1 | 123 | 210 | -58 | MIRb | MIR | SINE |
| 33563405 | 33563752 | + | 30.4 | 33 | 406 | -136 | MLT1F | MaLR | LTR |
| 33563764 | 33563846 | + | 17.9 | 1228 | 1325 | -147 | MLT1H-int | MaLR | LTR |
| 33563848 | 33563971 | + | 28.2 | 416 | 542 | 0 | MLT1F | MaLR | LTR |
| 33563972 | 33564859 | + | 23.0 | 1 | 900 | -589 | MLT1F-int | MaLR | LTR |
| 33564860 | 33565155 | + | 11.8 | 1 | 301 | -11 | AluSx | Alu | SINE |
| 33565156 | 33565650 | + | 23.0 | 901 | 1479 | -10 | MLT1F-int | MaLR | LTR |
| 33565663 | 33566145 | + | 22.5 | 6 | 482 | -60 | MLT1F | MaLR | LTR |
| 33566157 | 33566241 | - | 18.1 | 8 | 98 | -170 | MIRb | MIR | SINE |

  

|  |  |  |  |  |  |  |  |  |  |
| --- | --- | --- | --- | --- | --- | --- | --- | --- | --- |
| chr1:33989426-33993303 | | | 3756 bp | | |  |  |  |  |
| Genome Start | Genome End | Strand | Div | Repeat Start | Repeat End | Repeat Left | Name | Family | Class |
| 33989426 | 33989826 | - | 30.6 | 1802 | 2208 | -1211 | L2 | L2 | LINE |
| 33989827 | 33990112 | - | 11.5 | 1 | 281 | -28 | AluSc | Alu | SINE |
| 33990113 | 33990124 | - | 30.6 | 1792 | 1801 | -1618 | L2 | L2 | LINE |
| 33990145 | 33990270 | - | 30.6 | 1675 | 1791 | -1628 | L2 | L2 | LINE |
| 33990294 | 33990373 | + | 24.9 | 10 | 93 | -167 | MER112 | MER1\_type | DNA |
| 33990374 | 33990776 | + | 15.2 | 3 | 426 | 0 | MSTB | MaLR | LTR |
| 33990779 | 33992319 | + | 26.9 | 2 | 1735 | 0 | MLT-int | MaLR | LTR |
| 33992320 | 33992748 | + | 17.4 | 1 | 424 | -2 | MSTB | MaLR | LTR |
| 33992749 | 33992875 | + | 24.9 | 94 | 229 | -32 | MER112 | MER1\_type | DNA |
| 33992953 | 33993303 | - | 32.7 | 1274 | 1656 | -1763 | L2 | L2 | LINE |

  

|  |  |  |  |  |  |  |  |  |  |
| --- | --- | --- | --- | --- | --- | --- | --- | --- | --- |
| chr1:34639856-34643304 | | | 3161 bp | | |  |  |  |  |
| Genome Start | Genome End | Strand | Div | Repeat Start | Repeat End | Repeat Left | Name | Family | Class |
| 34639856 | 34639970 | - | 26.0 | 481 | 585 | -6 | MLT1K | MaLR | LTR |
| 34639971 | 34640196 | - | 15.3 | 4 | 219 | 0 | MER20 | MER1\_type | DNA |
| 34640197 | 34640251 | - | 26.0 | 424 | 480 | -111 | MLT1K | MaLR | LTR |
| 34640527 | 34640888 | - | 16.9 | 1 | 396 | 0 | MSTD | MaLR | LTR |
| 34640889 | 34642744 | - | 23.2 | 2 | 1735 | 0 | MSTD-int | MaLR | LTR |
| 34642755 | 34643106 | - | 24.6 | 3 | 388 | -8 | MSTD | MaLR | LTR |
| 34643110 | 34643304 | - | 30.2 | 27 | 225 | -366 | MLT1K | MaLR | LTR |

  

|  |  |  |  |  |  |  |  |  |  |
| --- | --- | --- | --- | --- | --- | --- | --- | --- | --- |
| chr1:38624800-38630319 | | | 5425 bp | | |  |  |  |  |
| Genome Start | Genome End | Strand | Div | Repeat Start | Repeat End | Repeat Left | Name | Family | Class |
| 38624800 | 38625193 | + | 18.6 | 1896 | 2303 | -4097 | L1MEc | L1 | LINE |
| 38625194 | 38625562 | - | 10.9 | 1 | 364 | 0 | THE1B | MaLR | LTR |
| 38625563 | 38626695 | + | 23.8 | 2226 | 3344 | -2875 | L1MEc | L1 | LINE |
| 38626689 | 38627260 | + | 23.2 | 5382 | 5976 | -190 | L1ME1 | L1 | LINE |
| 38627264 | 38627653 | - | 17.7 | 1 | 426 | 0 | MSTB | MaLR | LTR |
| 38627654 | 38627708 | - | 14.6 | 1596 | 1651 | 0 | MSTB-int | MaLR | LTR |
| 38627711 | 38627863 | + | 20.9 | 5757 | 5920 | -244 | L1ME2 | L1 | LINE |
| 38627882 | 38628470 | - | 17.7 | 1003 | 1587 | -64 | MSTB-int | MaLR | LTR |
| 38628468 | 38629071 | - | 16.1 | 1 | 612 | -1039 | MSTB-int | MaLR | LTR |
| 38629072 | 38629432 | - | 15.8 | 1 | 426 | 0 | MSTB | MaLR | LTR |
| 38629434 | 38629582 | + | 22.5 | 5982 | 6275 | -59 | L1MA10 | L1 | LINE |
| 38629662 | 38629869 | + | 29.8 | 865 | 1090 | -5029 | L1MEd | L1 | LINE |
| 38629872 | 38630319 | + | 22.6 | 3452 | 3954 | -2265 | L1MEc | L1 | LINE |

  

|  |  |  |  |  |  |  |  |  |  |
| --- | --- | --- | --- | --- | --- | --- | --- | --- | --- |
| chr1:40578109-40582922 | | | 4485 bp | | |  |  |  |  |
| Genome Start | Genome End | Strand | Div | Repeat Start | Repeat End | Repeat Left | Name | Family | Class |
| 40578109 | 40578677 | + | 29.3 | 2122 | 2764 | -655 | L2 | L2 | LINE |
| 40578690 | 40578870 | - | 18.7 | 188 | 365 | -140 | MLT1D | MaLR | LTR |
| 40578871 | 40579166 | - | 9.8 | 1 | 299 | -14 | AluSq | Alu | SINE |
| 40579167 | 40579344 | - | 18.7 | 1 | 187 | -318 | MLT1D | MaLR | LTR |
| 40579352 | 40580151 | - | 24.4 | 621 | 1482 | -7 | MLT1D-int | MaLR | LTR |
| 40580152 | 40580314 | - | 17.4 | 131 | 296 | -16 | AluJo | Alu | SINE |
| 40580344 | 40580451 | - | 17.4 | 19 | 130 | -182 | AluJo | Alu | SINE |
| 40580452 | 40580743 | - | 24.4 | 305 | 620 | -869 | MLT1D-int | MaLR | LTR |
| 40580744 | 40581051 | - | 12.7 | 1 | 309 | -3 | AluSx | Alu | SINE |
| 40581052 | 40581440 | - | 21.1 | 24 | 429 | -942 | MLT1D-int | MaLR | LTR |
| 40581449 | 40581518 | - | 19.0 | 438 | 505 | 0 | MLT1D | MaLR | LTR |
| 40581519 | 40581826 | + | 9.1 | 1 | 308 | -1 | AluSc | Alu | SINE |
| 40581827 | 40582291 | - | 19.0 | 8 | 437 | -68 | MLT1D | MaLR | LTR |
| 40582296 | 40582513 | + | 32.9 | 2761 | 2984 | -435 | L2 | L2 | LINE |
| 40582783 | 40582922 | + | 29.5 | 3268 | 3419 | 0 | L2 | L2 | LINE |

  

|  |  |  |  |  |  |  |  |  |  |
| --- | --- | --- | --- | --- | --- | --- | --- | --- | --- |
| chr1:41130906-41133841 | | | 2930 bp | | |  |  |  |  |
| Genome Start | Genome End | Strand | Div | Repeat Start | Repeat End | Repeat Left | Name | Family | Class |
| 41130906 | 41131026 | - | 22.3 | 5237 | 5363 | -783 | L1M4 | L1 | LINE |
| 41131027 | 41131096 | - | 12.5 | 322 | 381 | 0 | THE1D | MaLR | LTR |
| 41131097 | 41131407 | + | 11.9 | 1 | 310 | 0 | AluSg | Alu | SINE |
| 41131408 | 41131750 | - | 12.5 | 1 | 321 | -60 | THE1D | MaLR | LTR |
| 41131755 | 41133338 | - | 12.9 | 3 | 1576 | -4 | THE1D-int | MaLR | LTR |
| 41133341 | 41133710 | - | 13.8 | 1 | 381 | 0 | THE1D | MaLR | LTR |
| 41133715 | 41133795 | - | 14.8 | 5153 | 5237 | -909 | L1MC | L1 | LINE |
| 41133792 | 41133841 | - | 22.0 | 4747 | 4796 | -1350 | L1M4 | L1 | LINE |

  

|  |  |  |  |  |  |  |  |  |  |
| --- | --- | --- | --- | --- | --- | --- | --- | --- | --- |
| chr1:42327373-42335686 | | | 8197 bp | | |  |  |  |  |
| Genome Start | Genome End | Strand | Div | Repeat Start | Repeat End | Repeat Left | Name | Family | Class |
| 42327373 | 42328097 | + | 29.6 | 778 | 1583 | -4758 | L1MEb | L1 | LINE |
| 42328098 | 42328640 | + | 25.3 | 5 | 567 | 0 | MLT1F1 | MaLR | LTR |
| 42328641 | 42328914 | + | 28.5 | 1475 | 1869 | -4472 | L1MEb | L1 | LINE |
| 42328934 | 42330322 | + | 26.6 | 1917 | 3770 | -2551 | L1MEb | L1 | LINE |
| 42330352 | 42330588 | + | 26.2 | 3771 | 4022 | -2299 | L1MEb | L1 | LINE |
| 42330596 | 42331018 | - | 12.1 | 1 | 425 | -7 | MSTB1 | MaLR | LTR |
| 42331019 | 42331974 | - | 18.1 | 682 | 1651 | 0 | MSTB1-int | MaLR | LTR |
| 42331983 | 42332370 | - | 15.6 | 41 | 426 | 0 | MSTB | MaLR | LTR |
| 42332410 | 42334128 | - | 3.6 | 4441 | 6155 | 0 | L1PA3 | L1 | LINE |
| 42334129 | 42334166 | - | 7.9 | 1 | 38 | -388 | MSTB | MaLR | LTR |
| 42334176 | 42334820 | - | 18.3 | 5 | 668 | -983 | MSTB1-int | MaLR | LTR |
| 42334825 | 42335261 | - | 15.0 | 1 | 432 | 0 | MSTB1 | MaLR | LTR |
| 42335264 | 42335686 | + | 26.3 | 4021 | 4456 | -1865 | L1MEb | L1 | LINE |

  

|  |  |  |  |  |  |  |  |  |  |
| --- | --- | --- | --- | --- | --- | --- | --- | --- | --- |
| chr1:42859001-42871877 | | | 12787 bp | | |  |  |  |  |
| Genome Start | Genome End | Strand | Div | Repeat Start | Repeat End | Repeat Left | Name | Family | Class |
| 42859001 | 42859247 | - | 21.2 | 279 | 524 | -339 | MER21B | ERV1 | LTR |
| 42859248 | 42859401 | - | 13.1 | 284 | 437 | 0 | LTR23 | ERV1 | LTR |
| 42859407 | 42859564 | - | 18.9 | 7568 | 7721 | -1 | L1MA4A | L1 | LINE |
| 42859565 | 42859740 | - | 15.6 | 134 | 312 | 0 | AluJo | Alu | SINE |
| 42859741 | 42859755 | - | 7.4 | 284 | 311 | 0 | AluY | Alu | SINE |
| 42859787 | 42860068 | - | 7.4 | 2 | 283 | -28 | AluY | Alu | SINE |
| 42860069 | 42860204 | - | 15.6 | 2 | 133 | -179 | AluJo | Alu | SINE |
| 42860205 | 42860795 | - | 18.9 | 7031 | 7567 | -155 | L1MA4A | L1 | LINE |
| 42860796 | 42861034 | - | 11.6 | 1 | 227 | -83 | AluSg | Alu | SINE |
| 42861035 | 42862736 | - | 15.4 | 5174 | 7030 | -692 | L1MA4A | L1 | LINE |
| 42862737 | 42863029 | - | 19.9 | 9 | 301 | -11 | AluJo | Alu | SINE |
| 42863030 | 42864827 | - | 14.1 | 3349 | 5173 | -2389 | L1MA4A | L1 | LINE |
| 42864828 | 42865183 | + | 8.5 | 1 | 355 | 0 | THE1A | MaLR | LTR |
| 42865184 | 42866744 | + | 9.6 | 1 | 1580 | 0 | THE1A-int | MaLR | LTR |
| 42866745 | 42867098 | + | 9.3 | 1 | 355 | 0 | THE1A | MaLR | LTR |
| 42867100 | 42867879 | - | 13.2 | 2547 | 3352 | -4213 | L1MA4A | L1 | LINE |
| 42867880 | 42868163 | - | 8.8 | 9 | 308 | -2 | AluSg | Alu | SINE |
| 42868164 | 42868227 | - | 13.2 | 2513 | 2546 | -5019 | L1MA4A | L1 | LINE |
| 42868228 | 42868532 | - | 12.5 | 1 | 305 | -7 | AluSx | Alu | SINE |
| 42868533 | 42868628 | - | 13.2 | 2430 | 2512 | -5053 | L1MA4A | L1 | LINE |
| 42868629 | 42868939 | - | 12.5 | 1 | 312 | 0 | AluSx | Alu | SINE |
| 42868940 | 42870579 | - | 13.2 | 754 | 2429 | -5136 | L1MA4A | L1 | LINE |
| 42870582 | 42870728 | + | 15.7 | 140 | 286 | -26 | AluSg/x | Alu | SINE |
| 42870740 | 42871040 | + | 8.6 | 1 | 305 | -8 | AluSp | Alu | SINE |
| 42871080 | 42871367 | - | 13.9 | 1 | 291 | -21 | AluSx | Alu | SINE |
| 42871369 | 42871596 | - | 15.1 | 1 | 216 | -221 | LTR23 | ERV1 | LTR |
| 42871597 | 42871877 | - | 20.9 | 1 | 282 | -581 | MER21B | ERV1 | LTR |

  

|  |  |  |  |  |  |  |  |  |  |
| --- | --- | --- | --- | --- | --- | --- | --- | --- | --- |
| chr1:43343546-43349877 | | | 6255 bp | | |  |  |  |  |
| Genome Start | Genome End | Strand | Div | Repeat Start | Repeat End | Repeat Left | Name | Family | Class |
| 43343546 | 43343966 | - | 14.8 | 5029 | 5456 | -728 | L1M4 | L1 | LINE |
| 43344042 | 43344210 | - | 18.3 | 135 | 305 | -7 | AluJb | Alu | SINE |
| 43344212 | 43344296 | - | 17.6 | 4957 | 5041 | -1105 | L1M4 | L1 | LINE |
| 43344297 | 43344602 | - | 13.8 | 77 | 428 | 0 | MSTA | MaLR | LTR |
| 43344603 | 43346335 | - | 8.4 | 1 | 1578 | 0 | LTR12C | ERV1 | LTR |
| 43346336 | 43346413 | - | 13.8 | 1 | 76 | -352 | MSTA | MaLR | LTR |
| 43346414 | 43347500 | - | 18.8 | 536 | 1651 | 0 | MSTA-int | MaLR | LTR |
| 43347501 | 43347862 | + | 14.3 | 1 | 364 | 0 | THE1B | MaLR | LTR |
| 43347863 | 43348397 | - | 18.8 | 3 | 535 | -1116 | MSTA-int | MaLR | LTR |
| 43348399 | 43348780 | - | 14.3 | 1 | 428 | 0 | MSTA | MaLR | LTR |
| 43348781 | 43348967 | - | 17.8 | 4773 | 4961 | -1185 | L1M4 | L1 | LINE |
| 43348968 | 43349268 | - | 8.0 | 1 | 301 | -10 | AluY | Alu | SINE |
| 43349269 | 43349877 | - | 17.8 | 4131 | 4772 | -1374 | L1M4 | L1 | LINE |

  

|  |  |  |  |  |  |  |  |  |  |
| --- | --- | --- | --- | --- | --- | --- | --- | --- | --- |
| chr1:45471990-45477779 | | | 5789 bp | | |  |  |  |  |
| Genome Start | Genome End | Strand | Div | Repeat Start | Repeat End | Repeat Left | Name | Family | Class |
| 45471990 | 45472031 | + | 21.1 | 4345 | 4380 | -1766 | L1ME2 | L1 | LINE |
| 45472032 | 45472327 | + | 12.5 | 1 | 296 | -16 | AluSx | Alu | SINE |
| 45472328 | 45472731 | + | 21.1 | 4381 | 4773 | -1373 | L1ME2 | L1 | LINE |
| 45472732 | 45473047 | + | 14.8 | 1 | 312 | 0 | AluJb | Alu | SINE |
| 45473048 | 45473144 | + | 21.1 | 4774 | 4871 | -1275 | L1ME2 | L1 | LINE |
| 45473145 | 45473555 | + | 16.3 | 1 | 426 | 0 | MSTB | MaLR | LTR |
| 45473557 | 45473745 | + | 27.6 | 1 | 252 | -1483 | MLT-int | MaLR | LTR |
| 45473746 | 45474047 | + | 9.7 | 1 | 300 | -10 | AluSg | Alu | SINE |
| 45474048 | 45475463 | + | 27.6 | 253 | 1735 | 0 | MLT-int | MaLR | LTR |
| 45475464 | 45475932 | + | 14.2 | 1 | 424 | -2 | MSTB | MaLR | LTR |
| 45475933 | 45476235 | + | 21.1 | 4872 | 5195 | -951 | L1ME2 | L1 | LINE |
| 45476236 | 45476542 | + | 18.0 | 1 | 306 | -6 | AluJo | Alu | SINE |
| 45476543 | 45476831 | + | 20.4 | 5196 | 5480 | -684 | L1ME2 | L1 | LINE |
| 45476832 | 45477122 | + | 8.6 | 1 | 296 | -17 | AluSp | Alu | SINE |
| 45477123 | 45477779 | + | 20.1 | 5481 | 6154 | -10 | L1ME2 | L1 | LINE |

  

|  |  |  |  |  |  |  |  |  |  |
| --- | --- | --- | --- | --- | --- | --- | --- | --- | --- |
| chr1:46622516-46631762 | | | 9201 bp | | |  |  |  |  |
| Genome Start | Genome End | Strand | Div | Repeat Start | Repeat End | Repeat Left | Name | Family | Class |
| 46622516 | 46622881 | - | 4.7 | 4735 | 5102 | 0 | HERVIP10FH | ERV1 | LTR |
| 46622893 | 46624039 | - | 8.2 | 1933 | 3088 | -2014 | HERVIP10FH | ERV1 | LTR |
| 46624041 | 46624363 | - | 4.6 | 1 | 324 | -456 | LTR17 | ERV1 | LTR |
| 46624368 | 46624663 | - | 7.1 | 6836 | 8621 | -5 | HERV17 | ERV1 | LTR |
| 46624678 | 46627415 | - | 4.5 | 3284 | 6017 | -2609 | HERV17 | ERV1 | LTR |
| 46627416 | 46627717 | - | 8.4 | 1 | 299 | -12 | AluY | Alu | SINE |
| 46627718 | 46629050 | - | 4.5 | 1953 | 3283 | -5343 | HERV17 | ERV1 | LTR |
| 46629039 | 46629080 | - | 14.3 | 2 | 43 | -8583 | HERV17 | ERV1 | LTR |
| 46629081 | 46629585 | - | 5.4 | 253 | 780 | 0 | LTR17 | ERV1 | LTR |
| 46629602 | 46630480 | - | 6.5 | 913 | 1810 | -3292 | HERVIP10FH | ERV1 | LTR |
| 46630481 | 46630772 | - | 5.8 | 1 | 291 | -20 | AluY | Alu | SINE |
| 46630773 | 46631047 | - | 6.5 | 624 | 912 | -4190 | HERVIP10FH | ERV1 | LTR |
| 46631060 | 46631762 | - | 21.0 | 1 | 649 | -4832 | HERVIP10FH | ERV1 | LTR |

  

|  |  |  |  |  |  |  |  |  |  |
| --- | --- | --- | --- | --- | --- | --- | --- | --- | --- |
| chr1:47839998-47850090 | | | 9555 bp | | |  |  |  |  |
| Genome Start | Genome End | Strand | Div | Repeat Start | Repeat End | Repeat Left | Name | Family | Class |
| 47839998 | 47840486 | + | 33.5 | 693 | 1221 | -4994 | L1MC4a | L1 | LINE |
| 47840488 | 47841469 | + | 11.3 | 1 | 983 | -5172 | L1P1 | L1 | LINE |
| 47841466 | 47843350 | + | 4.8 | 4305 | 6190 | 0 | L1PA5 | L1 | LINE |
| 47843352 | 47844277 | + | 29.2 | 1212 | 2107 | -4108 | L1MC4a | L1 | LINE |
| 47844314 | 47844673 | + | 29.2 | 2108 | 2492 | -3723 | L1MC4a | L1 | LINE |
| 47844676 | 47844917 | - | 22.1 | 326 | 565 | -2 | MLT1F1 | MaLR | LTR |
| 47844937 | 47845110 | - | 28.3 | 43 | 223 | -339 | MLT1F1 | MaLR | LTR |
| 47845121 | 47845299 | + | 31.6 | 2501 | 2679 | -3535 | L1MC4a | L1 | LINE |
| 47845460 | 47845562 | + | 14.6 | 6148 | 6250 | -50 | L1MA4 | L1 | LINE |
| 47845676 | 47846040 | - | 15.0 | 1 | 379 | -2 | THE1D | MaLR | LTR |
| 47846041 | 47847655 | - | 13.8 | 3 | 1580 | 0 | THE1D-int | MaLR | LTR |
| 47847658 | 47848024 | - | 13.8 | 1 | 381 | 0 | THE1D | MaLR | LTR |
| 47848048 | 47848174 | + | 28.0 | 2929 | 3060 | -3154 | L1MC4a | L1 | LINE |
| 47848350 | 47850090 | + | 29.6 | 4200 | 5427 | -787 | L1MC4a | L1 | LINE |

  

|  |  |  |  |  |  |  |  |  |  |
| --- | --- | --- | --- | --- | --- | --- | --- | --- | --- |
| chr1:48712172-48716393 | | | 4005 bp | | |  |  |  |  |
| Genome Start | Genome End | Strand | Div | Repeat Start | Repeat End | Repeat Left | Name | Family | Class |
| 48712172 | 48712508 | - | 30.2 | 5261 | 5631 | -609 | L1M5 | L1 | LINE |
| 48712613 | 48712711 | - | 21.9 | 206 | 302 | 0 | AluSg/x | Alu | SINE |
| 48712712 | 48713061 | + | 13.2 | 1 | 355 | 0 | THE1A | MaLR | LTR |
| 48713064 | 48714620 | + | 30.6 | 2 | 1735 | 0 | MLT-int | MaLR | LTR |
| 48714621 | 48714854 | + | 10.0 | 1 | 235 | -120 | THE1A | MaLR | LTR |
| 48714855 | 48715188 | + | 3.9 | 5821 | 6152 | -2 | L1PA7 | L1 | LINE |
| 48715189 | 48715348 | + | 10.0 | 236 | 355 | 0 | THE1A | MaLR | LTR |
| 48715349 | 48715558 | - | 15.7 | 1 | 210 | -102 | AluJb | Alu | SINE |
| 48715663 | 48715823 | + | 16.8 | 130 | 298 | -14 | AluJb | Alu | SINE |
| 48715831 | 48716393 | - | 28.9 | 4506 | 5085 | -1061 | L1M5 | L1 | LINE |

  

|  |  |  |  |  |  |  |  |  |  |
| --- | --- | --- | --- | --- | --- | --- | --- | --- | --- |
| chr1:50043241-50046501 | | | 3228 bp | | |  |  |  |  |
| Genome Start | Genome End | Strand | Div | Repeat Start | Repeat End | Repeat Left | Name | Family | Class |
| 50043241 | 50043869 | - | 25.4 | 1672 | 2334 | -1085 | L2 | L2 | LINE |
| 50043878 | 50044234 | + | 19.7 | 5 | 361 | -4 | MLT1A0 | MaLR | LTR |
| 50044243 | 50045965 | + | 21.2 | 5 | 1727 | -8 | MLT1A0-int | MaLR | LTR |
| 50045976 | 50046335 | + | 19.0 | 3 | 360 | -5 | MLT1A0 | MaLR | LTR |
| 50046343 | 50046501 | - | 28.9 | 1509 | 1670 | -1749 | L2 | L2 | LINE |

  

|  |  |  |  |  |  |  |  |  |  |
| --- | --- | --- | --- | --- | --- | --- | --- | --- | --- |
| chr1:50461745-50465833 | | | 3929 bp | | |  |  |  |  |
| Genome Start | Genome End | Strand | Div | Repeat Start | Repeat End | Repeat Left | Name | Family | Class |
| 50461745 | 50461942 | - | 21.3 | 3199 | 3419 | 0 | L2 | L2 | LINE |
| 50461948 | 50462290 | + | 16.4 | 3 | 388 | -40 | MSTA | MaLR | LTR |
| 50462291 | 50462593 | + | 11.9 | 1 | 303 | -9 | AluSx | Alu | SINE |
| 50462612 | 50462639 | - | 9.8 | 284 | 310 | 0 | AluSg | Alu | SINE |
| 50462674 | 50462956 | - | 9.8 | 1 | 283 | -27 | AluSg | Alu | SINE |
| 50462957 | 50462997 | + | 16.4 | 389 | 427 | -1 | MSTA | MaLR | LTR |
| 50463000 | 50464643 | + | 13.6 | 3 | 1567 | -13 | MSTA-int | MaLR | LTR |
| 50464650 | 50465030 | + | 13.9 | 2 | 428 | 0 | MSTA | MaLR | LTR |
| 50465040 | 50465267 | - | 24.9 | 2924 | 3192 | -227 | L2 | L2 | LINE |
| 50465354 | 50465756 | + | 29.6 | 74 | 521 | -2 | LTR33A | ERVL | LTR |
| 50465757 | 50465833 | - | 18.2 | 2833 | 2911 | -508 | L2 | L2 | LINE |

  

|  |  |  |  |  |  |  |  |  |  |
| --- | --- | --- | --- | --- | --- | --- | --- | --- | --- |
| chr1:50989240-51003032 | | | 13287 bp | | |  |  |  |  |
| Genome Start | Genome End | Strand | Div | Repeat Start | Repeat End | Repeat Left | Name | Family | Class |
| 50989240 | 50989284 | - | 4.5 | 3985 | 4028 | -2191 | L1MEc | L1 | LINE |
| 50989351 | 50989582 | + | 19.8 | 3888 | 4111 | -2058 | L1MD | L1 | LINE |
| 50989583 | 50989901 | - | 13.2 | 1 | 310 | 0 | AluSg | Alu | SINE |
| 50989931 | 50990224 | - | 24.9 | 3575 | 3876 | -2343 | L1MEc | L1 | LINE |
| 50990225 | 50990531 | - | 11.5 | 1 | 305 | -7 | AluSx | Alu | SINE |
| 50990532 | 50990934 | - | 24.9 | 3165 | 3574 | -2645 | L1MEc | L1 | LINE |
| 50990937 | 50991243 | - | 12.1 | 1 | 308 | -4 | AluSx | Alu | SINE |
| 50991272 | 50991757 | - | 24.6 | 5646 | 6161 | -5 | L1ME1 | L1 | LINE |
| 50991795 | 50992014 | - | 20.8 | 937 | 1154 | -5246 | L1MEc | L1 | LINE |
| 50992015 | 50992315 | - | 12.3 | 1 | 301 | -9 | AluSg | Alu | SINE |
| 50992316 | 50992746 | - | 19.9 | 476 | 936 | -5464 | L1MEc | L1 | LINE |
| 50992732 | 50992801 | - | 25.7 | 45 | 114 | -6286 | L1MEc | L1 | LINE |
| 50992812 | 50992937 | - | 27.8 | 3032 | 3161 | -3058 | L1MEc | L1 | LINE |
| 50992945 | 50993296 | - | 11.8 | 1 | 364 | 0 | THE1B | MaLR | LTR |
| 50993297 | 50994861 | - | 29.6 | 2 | 1735 | 0 | MLT-int | MaLR | LTR |
| 50994864 | 50995213 | - | 9.2 | 1 | 364 | 0 | THE1B | MaLR | LTR |
| 50995214 | 50996186 | - | 28.8 | 2112 | 3029 | -3190 | L1MEc | L1 | LINE |
| 50996188 | 50996477 | - | 16.9 | 1 | 292 | -20 | AluSx | Alu | SINE |
| 50996478 | 50996612 | - | 10.4 | 2 | 136 | -176 | AluSq/x | Alu | SINE |
| 50996618 | 50997089 | - | 23.6 | 1489 | 2114 | -4286 | L1MEc | L1 | LINE |
| 50997119 | 50997370 | - | 9.7 | 37 | 288 | -23 | AluY | Alu | SINE |
| 50997407 | 50998359 | - | 3.1 | 5202 | 6155 | 0 | L1PA3 | L1 | LINE |
| 50998356 | 51001208 | + | 3.1 | 2397 | 5250 | -896 | L1PA3 | L1 | LINE |
| 51001234 | 51001527 | + | 12.4 | 1 | 292 | -20 | AluSx | Alu | SINE |
| 51001564 | 51001943 | - | 27.4 | 1117 | 1495 | -4905 | L1MEc | L1 | LINE |
| 51001971 | 51002496 | + | 18.0 | 2046 | 2663 | -98 | Charlie1 | MER1\_type | DNA |
| 51002682 | 51003032 | - | 30.6 | 22 | 383 | -6017 | L1MEc | L1 | LINE |

  

|  |  |  |  |  |  |  |  |  |  |
| --- | --- | --- | --- | --- | --- | --- | --- | --- | --- |
| chr1:51169177-51173331 | | | 3986 bp | | |  |  |  |  |
| Genome Start | Genome End | Strand | Div | Repeat Start | Repeat End | Repeat Left | Name | Family | Class |
| 51169177 | 51169503 | - | 26.5 | 2628 | 2901 | -3924 | L1M4 | L1 | LINE |
| 51169609 | 51169849 | - | 10.1 | 119 | 355 | 0 | THE1A | MaLR | LTR |
| 51169850 | 51170146 | + | 10.4 | 5 | 294 | -17 | AluY | Alu | SINE |
| 51170189 | 51170263 | - | 10.1 | 50 | 118 | -237 | THE1A | MaLR | LTR |
| 51170278 | 51170405 | - | 8.7 | 1 | 128 | -227 | THE1A | MaLR | LTR |
| 51170406 | 51171958 | - | 31.1 | 2 | 1735 | 0 | MLT-int | MaLR | LTR |
| 51171961 | 51172313 | - | 10.1 | 1 | 355 | 0 | THE1A | MaLR | LTR |
| 51172316 | 51172520 | - | 24.4 | 2399 | 2632 | -4193 | L1M4 | L1 | LINE |
| 51172525 | 51172700 | - | 25.4 | 2145 | 2310 | -4515 | L1M4 | L1 | LINE |
| 51172701 | 51172982 | + | 15.4 | 1 | 287 | -25 | AluJo | Alu | SINE |
| 51172983 | 51173331 | - | 25.6 | 1605 | 2144 | -4681 | L1M4 | L1 | LINE |

  

|  |  |  |  |  |  |  |  |  |  |
| --- | --- | --- | --- | --- | --- | --- | --- | --- | --- |
| chr1:53441276-53446342 | | | 5021 bp | | |  |  |  |  |
| Genome Start | Genome End | Strand | Div | Repeat Start | Repeat End | Repeat Left | Name | Family | Class |
| 53441276 | 53442893 | - | 24.3 | 743 | 2507 | 0 | HAL1 | L1 | LINE |
| 53442894 | 53443262 | + | 14.1 | 1 | 381 | 0 | THE1D | MaLR | LTR |
| 53443265 | 53444160 | + | 12.5 | 3 | 868 | -712 | THE1D-int | MaLR | LTR |
| 53444161 | 53444463 | + | 8.0 | 1 | 302 | -9 | AluY | Alu | SINE |
| 53444464 | 53445180 | + | 12.5 | 869 | 1580 | 0 | THE1D-int | MaLR | LTR |
| 53445181 | 53445546 | + | 12.0 | 1 | 381 | 0 | THE1D | MaLR | LTR |
| 53445547 | 53445715 | - | 29.4 | 487 | 747 | -1760 | HAL1 | L1 | LINE |
| 53445716 | 53446020 | - | 13.2 | 1 | 303 | -6 | AluSc | Alu | SINE |
| 53446021 | 53446078 | - | 25.3 | 419 | 486 | -2021 | HAL1 | L1 | LINE |
| 53446123 | 53446342 | - | 24.2 | 75 | 307 | -2200 | HAL1 | L1 | LINE |

  

|  |  |  |  |  |  |  |  |  |  |
| --- | --- | --- | --- | --- | --- | --- | --- | --- | --- |
| chr1:53659907-53668996 | | | 9120 bp | | |  |  |  |  |
| Genome Start | Genome End | Strand | Div | Repeat Start | Repeat End | Repeat Left | Name | Family | Class |
| 53659907 | 53661080 | + | 13.0 | 3481 | 4706 | -1625 | LTR49-int | ERV1 | LTR |
| 53661081 | 53661381 | + | 12.0 | 1 | 301 | -11 | AluSx | Alu | SINE |
| 53661382 | 53662207 | + | 12.8 | 4707 | 5491 | -840 | LTR49-int | ERV1 | LTR |
| 53662208 | 53662484 | - | 10.5 | 1 | 277 | -32 | AluSc | Alu | SINE |
| 53662485 | 53662594 | + | 12.8 | 5492 | 5598 | -733 | LTR49-int | ERV1 | LTR |
| 53662597 | 53663026 | + | 5.4 | 1 | 462 | -2 | LTR7B | ERV1 | LTR |
| 53663029 | 53665958 | + | 9.0 | 1 | 2987 | -4726 | HERVH | ERV1 | LTR |
| 53665931 | 53666100 | + | 15.4 | 3126 | 3290 | -4423 | HERVH | ERV1 | LTR |
| 53666091 | 53666592 | + | 11.2 | 3492 | 3993 | -3720 | HERVH | ERV1 | LTR |
| 53666595 | 53667256 | + | 14.7 | 4488 | 5149 | -2564 | HERVH | ERV1 | LTR |
| 53667258 | 53667859 | + | 10.6 | 5600 | 7713 | 0 | HERVH | ERV1 | LTR |
| 53667860 | 53668289 | + | 5.1 | 1 | 462 | -2 | LTR7B | ERV1 | LTR |
| 53668291 | 53668996 | + | 10.9 | 5595 | 6331 | 0 | LTR49-int | ERV1 | LTR |

  

|  |  |  |  |  |  |  |  |  |  |
| --- | --- | --- | --- | --- | --- | --- | --- | --- | --- |
| chr1:53994673-54001024 | | | 6199 bp | | |  |  |  |  |
| Genome Start | Genome End | Strand | Div | Repeat Start | Repeat End | Repeat Left | Name | Family | Class |
| 53994673 | 53994788 | - | 23.1 | 2950 | 3069 | -3060 | L1MEd | L1 | LINE |
| 53994789 | 53995081 | - | 14.7 | 1 | 305 | -7 | AluSx | Alu | SINE |
| 53995082 | 53995480 | - | 23.1 | 2535 | 2949 | -3180 | L1MEd | L1 | LINE |
| 53995490 | 53995791 | - | 10.7 | 1 | 299 | -13 | AluSx | Alu | SINE |
| 53995832 | 53996477 | - | 23.3 | 5453 | 6140 | -22 | L1ME3 | L1 | LINE |
| 53996478 | 53996844 | - | 11.2 | 1 | 364 | 0 | THE1B | MaLR | LTR |
| 53996845 | 53998051 | - | 29.6 | 433 | 1735 | 0 | MLT-int | MaLR | LTR |
| 53998052 | 53998354 | + | 14.2 | 1 | 302 | -10 | AluSx | Alu | SINE |
| 53998355 | 53998719 | - | 29.6 | 2 | 432 | -1303 | MLT-int | MaLR | LTR |
| 53998722 | 53999088 | - | 13.5 | 1 | 364 | 0 | THE1B | MaLR | LTR |
| 53999089 | 53999210 | - | 23.3 | 5322 | 5452 | -710 | L1ME3 | L1 | LINE |
| 53999213 | 53999270 | - | 28.8 | 2414 | 2472 | -3651 | L1MEd | L1 | LINE |
| 53999271 | 53999577 | + | 6.9 | 1 | 304 | -7 | AluY | Alu | SINE |
| 53999578 | 53999967 | - | 25.9 | 1379 | 2413 | -3710 | L1MEd | L1 | LINE |
| 53999968 | 54000260 | + | 15.0 | 1 | 297 | -15 | AluJb | Alu | SINE |
| 54000261 | 54000703 | - | 29.9 | 886 | 1378 | -4847 | L1MEd | L1 | LINE |
| 54000804 | 54001024 | - | 33.3 | 596 | 828 | -5291 | L1MEd | L1 | LINE |

  

|  |  |  |  |  |  |  |  |  |  |
| --- | --- | --- | --- | --- | --- | --- | --- | --- | --- |
| chr1:54704823-54708136 | | | 3311 bp | | |  |  |  |  |
| Genome Start | Genome End | Strand | Div | Repeat Start | Repeat End | Repeat Left | Name | Family | Class |
| 54704823 | 54704890 | - | 21.6 | 131 | 193 | 0 | MER53 | DNA | DNA |
| 54704891 | 54705148 | - | 5.4 | 2 | 311 | 0 | AluY | Alu | SINE |
| 54705149 | 54705177 | - | 21.6 | 105 | 130 | -63 | MER53 | DNA | DNA |
| 54705179 | 54705528 | + | 9.7 | 1 | 355 | 0 | THE1A | MaLR | LTR |
| 54705529 | 54705890 | + | 8.6 | 1 | 370 | -1210 | THE1A-int | MaLR | LTR |
| 54705891 | 54706195 | - | 11.2 | 1 | 304 | -8 | AluSx | Alu | SINE |
| 54706196 | 54706241 | + | 8.6 | 371 | 405 | -1175 | THE1A-int | MaLR | LTR |
| 54706242 | 54706530 | + | 6.9 | 1 | 289 | -22 | AluY | Alu | SINE |
| 54706531 | 54707713 | + | 8.6 | 406 | 1580 | 0 | THE1A-int | MaLR | LTR |
| 54707716 | 54708068 | + | 9.4 | 3 | 355 | 0 | THE1A | MaLR | LTR |
| 54708069 | 54708136 | - | 11.8 | 40 | 108 | -85 | MER53 | DNA | DNA |

  

|  |  |  |  |  |  |  |  |  |  |
| --- | --- | --- | --- | --- | --- | --- | --- | --- | --- |
| chr1:55180009-55183220 | | | 3210 bp | | |  |  |  |  |
| Genome Start | Genome End | Strand | Div | Repeat Start | Repeat End | Repeat Left | Name | Family | Class |
| 55180009 | 55180417 | + | 30.0 | 2732 | 3202 | -217 | L2 | L2 | LINE |
| 55180418 | 55180778 | + | 14.0 | 1 | 375 | 0 | THE1C | MaLR | LTR |
| 55180781 | 55182348 | + | 30.9 | 2 | 1735 | 0 | MLT1L-int | MaLR | LTR |
| 55182349 | 55182716 | + | 15.2 | 1 | 375 | 0 | THE1C | MaLR | LTR |
| 55182717 | 55182796 | + | 29.1 | 3203 | 3282 | -137 | L2 | L2 | LINE |
| 55182797 | 55183116 | + | 16.2 | 1 | 299 | -13 | AluJb | Alu | SINE |
| 55183117 | 55183220 | + | 29.1 | 3283 | 3381 | -38 | L2 | L2 | LINE |

  

|  |  |  |  |  |  |  |  |  |  |
| --- | --- | --- | --- | --- | --- | --- | --- | --- | --- |
| chr1:55260582-55271941 | | | 11135 bp | | |  |  |  |  |
| Genome Start | Genome End | Strand | Div | Repeat Start | Repeat End | Repeat Left | Name | Family | Class |
| 55260582 | 55260848 | + | 26.8 | 306 | 625 | -2794 | L2 | L2 | LINE |
| 55260851 | 55260968 | + | 18.6 | 1 | 113 | -489 | MER7C | MER2\_type | DNA |
| 55260969 | 55261418 | + | 6.0 | 1 | 447 | -3 | LTR7 | ERV1 | LTR |
| 55261278 | 55261421 | + | 4.2 | 329 | 472 | 0 | LTR7Y | ERV1 | LTR |
| 55261432 | 55264405 | + | 10.4 | 1 | 3290 | -4423 | HERVH | ERV1 | LTR |
| 55264396 | 55264898 | + | 11.6 | 3492 | 3993 | -3720 | HERVH | ERV1 | LTR |
| 55264901 | 55265557 | + | 14.4 | 4488 | 5149 | -2564 | HERVH | ERV1 | LTR |
| 55265559 | 55266147 | + | 10.1 | 5600 | 7713 | 0 | HERVH | ERV1 | LTR |
| 55266148 | 55266630 | + | 5.8 | 1 | 448 | -2 | LTR7 | ERV1 | LTR |
| 55266644 | 55266905 | + | 13.9 | 110 | 363 | -239 | MER7C | MER2\_type | DNA |
| 55266906 | 55267207 | - | 5.3 | 1 | 302 | -9 | AluY | Alu | SINE |
| 55267208 | 55267390 | + | 13.9 | 364 | 551 | -51 | MER7C | MER2\_type | DNA |
| 55267479 | 55268030 | + | 30.3 | 783 | 1424 | -1995 | L2 | L2 | LINE |
| 55268059 | 55268191 | - | 27.5 | 93 | 232 | -30 | MIR | MIR | SINE |
| 55268250 | 55268816 | + | 28.1 | 1425 | 2114 | -1305 | L2 | L2 | LINE |
| 55268818 | 55268896 | + | 26.6 | 2287 | 2380 | -1039 | L2 | L2 | LINE |
| 55269068 | 55269213 | + | 30.4 | 2671 | 2832 | -587 | L2 | L2 | LINE |
| 55269214 | 55269567 | + | 8.2 | 1 | 355 | 0 | THE1A | MaLR | LTR |
| 55269570 | 55271142 | + | 31.2 | 2 | 1735 | 0 | MLT1I-int | MaLR | LTR |
| 55271143 | 55271492 | + | 10.0 | 1 | 352 | -3 | THE1A | MaLR | LTR |
| 55271493 | 55271941 | + | 29.4 | 2833 | 3401 | -18 | L2 | L2 | LINE |

  

|  |  |  |  |  |  |  |  |  |  |
| --- | --- | --- | --- | --- | --- | --- | --- | --- | --- |
| chr1:56018157-56029349 | | | 10897 bp | | |  |  |  |  |
| Genome Start | Genome End | Strand | Div | Repeat Start | Repeat End | Repeat Left | Name | Family | Class |
| 56018157 | 56021444 | - | 16.4 | 2244 | 5757 | 0 | ERVL-B1 | ERVL | LTR |
| 56021445 | 56022207 | - | 11.5 | 1 | 779 | -1 | LTR17 | ERV1 | LTR |
| 56022212 | 56022498 | - | 7.7 | 8335 | 8621 | -5 | HERV17 | ERV1 | LTR |
| 56022492 | 56024236 | - | 5.0 | 2430 | 4182 | -4444 | HERV17 | ERV1 | LTR |
| 56024222 | 56025450 | - | 7.6 | 779 | 2014 | -6612 | HERV17 | ERV1 | LTR |
| 56025757 | 56026436 | - | 9.8 | 2 | 691 | -7935 | HERV17 | ERV1 | LTR |
| 56026445 | 56027229 | - | 13.0 | 1 | 780 | 0 | LTR17 | ERV1 | LTR |
| 56027230 | 56029349 | - | 18.3 | 1 | 2199 | -3515 | ERVL-B1 | ERVL | LTR |

  

|  |  |  |  |  |  |  |  |  |  |
| --- | --- | --- | --- | --- | --- | --- | --- | --- | --- |
| chr1:56170821-56173657 | | | 2764 bp | | |  |  |  |  |
| Genome Start | Genome End | Strand | Div | Repeat Start | Repeat End | Repeat Left | Name | Family | Class |
| 56170821 | 56171012 | + | 35.9 | 2704 | 2903 | -516 | L2 | L2 | LINE |
| 56171084 | 56171313 | + | 30.9 | 3079 | 3310 | -68 | L2 | L2 | LINE |
| 56171314 | 56171668 | - | 12.2 | 1 | 355 | 0 | THE1A | MaLR | LTR |
| 56171669 | 56173234 | - | 31.3 | 2 | 1735 | 0 | MLT1A0-int | MaLR | LTR |
| 56173237 | 56173592 | - | 10.8 | 1 | 355 | 0 | THE1A | MaLR | LTR |
| 56173593 | 56173657 | + | 30.9 | 3311 | 3378 | 0 | L2 | L2 | LINE |

  

|  |  |  |  |  |  |  |  |  |  |
| --- | --- | --- | --- | --- | --- | --- | --- | --- | --- |
| chr1:57266155-57268758 | | | 2601 bp | | |  |  |  |  |
| Genome Start | Genome End | Strand | Div | Repeat Start | Repeat End | Repeat Left | Name | Family | Class |
| 57266155 | 57266344 | - | 30.8 | 3182 | 3346 | -32 | L2 | L2 | LINE |
| 57266345 | 57266698 | + | 7.3 | 1 | 355 | 0 | THE1A | MaLR | LTR |
| 57266702 | 57268271 | + | 30.1 | 2 | 1735 | 0 | MLT1A-int | MaLR | LTR |
| 57268272 | 57268612 | + | 10.3 | 1 | 355 | 0 | THE1A | MaLR | LTR |
| 57268613 | 57268758 | - | 30.8 | 3036 | 3181 | -197 | L2 | L2 | LINE |

  

|  |  |  |  |  |  |  |  |  |  |
| --- | --- | --- | --- | --- | --- | --- | --- | --- | --- |
| chr1:58078706-58081453 | | | 2752 bp | | |  |  |  |  |
| Genome Start | Genome End | Strand | Div | Repeat Start | Repeat End | Repeat Left | Name | Family | Class |
| 58078706 | 58078752 | - | 12.8 | 2312 | 2358 | 0 | Tigger5 | MER2\_type | DNA |
| 58078756 | 58079120 | - | 11.1 | 1 | 380 | -1 | THE1D | MaLR | LTR |
| 58079121 | 58080726 | - | 12.4 | 3 | 1580 | 0 | THE1D-int | MaLR | LTR |
| 58080729 | 58081101 | - | 13.7 | 1 | 381 | 0 | THE1D | MaLR | LTR |
| 58081102 | 58081223 | - | 14.8 | 2169 | 2291 | -32 | Tigger5 | MER2\_type | DNA |
| 58081215 | 58081453 | - | 18.8 | 4 | 238 | -2085 | Tigger5 | MER2\_type | DNA |

  

|  |  |  |  |  |  |  |  |  |  |
| --- | --- | --- | --- | --- | --- | --- | --- | --- | --- |
| chr1:58404408-58413936 | | | 9520 bp | | |  |  |  |  |
| Genome Start | Genome End | Strand | Div | Repeat Start | Repeat End | Repeat Left | Name | Family | Class |
| 58404408 | 58404996 | - | 23.1 | 5592 | 6184 | 0 | L1MB7 | L1 | LINE |
| 58404997 | 58405307 | - | 15.4 | 1 | 312 | 0 | AluSx | Alu | SINE |
| 58405308 | 58405417 | - | 23.1 | 5505 | 5591 | -593 | L1MB7 | L1 | LINE |
| 58405420 | 58406256 | + | 7.6 | 1 | 826 | 0 | LTR12 | ERV1 | LTR |
| 58406270 | 58407463 | + | 8.8 | 40 | 1244 | -7192 | HERV9 | ERV1 | LTR |
| 58407466 | 58411539 | + | 5.2 | 1087 | 5203 | -3233 | HERV9 | ERV1 | LTR |
| 58411532 | 58412861 | + | 5.0 | 7103 | 8436 | 0 | HERV9 | ERV1 | LTR |
| 58412862 | 58413738 | + | 7.4 | 1 | 826 | 0 | LTR12 | ERV1 | LTR |
| 58413739 | 58413936 | - | 20.0 | 5282 | 5502 | -676 | L1MB7 | L1 | LINE |

  

|  |  |  |  |  |  |  |  |  |  |
| --- | --- | --- | --- | --- | --- | --- | --- | --- | --- |
| chr1:58608137-58611370 | | | 2917 bp | | |  |  |  |  |
| Genome Start | Genome End | Strand | Div | Repeat Start | Repeat End | Repeat Left | Name | Family | Class |
| 58608137 | 58608553 | + | 34.3 | 5461 | 5894 | -346 | L1ME4a | L1 | LINE |
| 58608867 | 58609236 | + | 13.8 | 1 | 381 | 0 | THE1D | MaLR | LTR |
| 58609240 | 58610862 | + | 31.0 | 3 | 1735 | 0 | MLT1J-int | MaLR | LTR |
| 58610863 | 58611233 | + | 16.9 | 1 | 381 | 0 | THE1D | MaLR | LTR |
| 58611235 | 58611370 | + | 30.6 | 5950 | 6090 | -31 | L1ME4a | L1 | LINE |

  

|  |  |  |  |  |  |  |  |  |  |
| --- | --- | --- | --- | --- | --- | --- | --- | --- | --- |
| chr1:59190076-59192695 | | | 2617 bp | | |  |  |  |  |
| Genome Start | Genome End | Strand | Div | Repeat Start | Repeat End | Repeat Left | Name | Family | Class |
| 59190076 | 59190200 | + | 21.6 | 25 | 157 | -62 | MER20 | MER1\_type | DNA |
| 59190202 | 59190338 | + | 7.3 | 1 | 137 | -218 | THE1A | MaLR | LTR |
| 59190350 | 59190504 | + | 18.1 | 418 | 572 | -1008 | THE1-int | MaLR | LTR |
| 59190493 | 59190705 | + | 8.0 | 143 | 355 | 0 | THE1A | MaLR | LTR |
| 59190709 | 59192277 | + | 30.2 | 3 | 1735 | 0 | MLT-int | MaLR | LTR |
| 59192278 | 59192630 | + | 8.8 | 1 | 355 | 0 | THE1A | MaLR | LTR |
| 59192631 | 59192695 | + | 21.5 | 154 | 219 | 0 | MER20 | MER1\_type | DNA |

  

|  |  |  |  |  |  |  |  |  |  |
| --- | --- | --- | --- | --- | --- | --- | --- | --- | --- |
| chr1:60256782-60259738 | | | 2641 bp | | |  |  |  |  |
| Genome Start | Genome End | Strand | Div | Repeat Start | Repeat End | Repeat Left | Name | Family | Class |
| 60256782 | 60256824 | - | 20.9 | 3371 | 3414 | -5 | L2 | L2 | LINE |
| 60257085 | 60257296 | + | 28.9 | 3199 | 3419 | 0 | L2 | L2 | LINE |
| 60257341 | 60257719 | + | 17.5 | 1 | 428 | 0 | MSTA | MaLR | LTR |
| 60257720 | 60258517 | + | 18.8 | 1 | 813 | -838 | MSTA-int | MaLR | LTR |
| 60258530 | 60259119 | + | 14.6 | 993 | 1580 | 0 | MSTA-int | MaLR | LTR |
| 60259120 | 60259516 | + | 13.7 | 1 | 428 | 0 | MSTA | MaLR | LTR |
| 60259517 | 60259738 | - | 31.8 | 3140 | 3378 | -41 | L2 | L2 | LINE |

  

|  |  |  |  |  |  |  |  |  |  |
| --- | --- | --- | --- | --- | --- | --- | --- | --- | --- |
| chr1:60421655-60431713 | | | 10057 bp | | |  |  |  |  |
| Genome Start | Genome End | Strand | Div | Repeat Start | Repeat End | Repeat Left | Name | Family | Class |
| 60421655 | 60422027 | + | 17.1 | 2 | 374 | -6134 | L1MC1 | L1 | LINE |
| 60422028 | 60422405 | - | 11.3 | 1 | 364 | 0 | THE1B | MaLR | LTR |
| 60422406 | 60423973 | - | 30.3 | 2 | 1735 | 0 | MLT-int | MaLR | LTR |
| 60423976 | 60424319 | - | 17.4 | 1 | 364 | 0 | THE1B | MaLR | LTR |
| 60424320 | 60425754 | + | 17.1 | 375 | 1832 | -4676 | L1MC1 | L1 | LINE |
| 60425755 | 60426042 | - | 3.1 | 5867 | 6155 | 0 | L1PA4 | L1 | LINE |
| 60426043 | 60428537 | + | 15.6 | 1833 | 4047 | -2274 | L1MC1 | L1 | LINE |
| 60428538 | 60428953 | + | 13.2 | 1 | 426 | 0 | MSTB | MaLR | LTR |
| 60428954 | 60430156 | + | 15.2 | 4048 | 5275 | -1046 | L1MC1 | L1 | LINE |
| 60430157 | 60430529 | + | 21.6 | 1 | 396 | 0 | MSTD | MaLR | LTR |
| 60430530 | 60431713 | + | 15.5 | 5276 | 6507 | -1 | L1MC1 | L1 | LINE |

  

|  |  |  |  |  |  |  |  |  |  |
| --- | --- | --- | --- | --- | --- | --- | --- | --- | --- |
| chr1:60922349-60925266 | | | 2918 bp | | |  |  |  |  |
| Genome Start | Genome End | Strand | Div | Repeat Start | Repeat End | Repeat Left | Name | Family | Class |
| 60922349 | 60922969 | - | 10.6 | 5676 | 6303 | -1 | L1MA2 | L1 | LINE |
| 60922970 | 60923325 | + | 12.4 | 1 | 364 | 0 | THE1B | MaLR | LTR |
| 60923326 | 60924831 | + | 9.8 | 1 | 1580 | 0 | THE1B-int | MaLR | LTR |
| 60924832 | 60925194 | + | 10.7 | 1 | 364 | 0 | THE1B | MaLR | LTR |
| 60925195 | 60925266 | - | 8.4 | 5607 | 5680 | -624 | L1MA2 | L1 | LINE |

  

|  |  |  |  |  |  |  |  |  |  |
| --- | --- | --- | --- | --- | --- | --- | --- | --- | --- |
| chr1:66212738-66215265 | | | 2528 bp | | |  |  |  |  |
| Genome Start | Genome End | Strand | Div | Repeat Start | Repeat End | Repeat Left | Name | Family | Class |
| 66212738 | 66212839 | - | 30.9 | 73 | 171 | -26 | MER117 | MER1\_type | DNA |
| 66212840 | 66213205 | + | 16.0 | 1 | 428 | 0 | MSTA | MaLR | LTR |
| 66213206 | 66214831 | + | 31.4 | 1 | 1735 | 0 | MLT-int | MaLR | LTR |
| 66214832 | 66215195 | + | 14.2 | 1 | 424 | -4 | MSTA | MaLR | LTR |
| 66215196 | 66215265 | - | 30.9 | 3 | 72 | -125 | MER117 | MER1\_type | DNA |

  

|  |  |  |  |  |  |  |  |  |  |
| --- | --- | --- | --- | --- | --- | --- | --- | --- | --- |
| chr1:67115654-67125751 | | | 10031 bp | | |  |  |  |  |
| Genome Start | Genome End | Strand | Div | Repeat Start | Repeat End | Repeat Left | Name | Family | Class |
| 67115654 | 67115734 | - | 23.5 | 103 | 189 | 0 | MER5A | MER1\_type | DNA |
| 67115747 | 67118144 | - | 19.1 | 5308 | 7775 | -10 | L1MC3 | L1 | LINE |
| 67118145 | 67118506 | + | 13.3 | 1 | 364 | 0 | THE1B | MaLR | LTR |
| 67118509 | 67120075 | + | 30.1 | 2 | 1735 | 0 | MLT1J-int | MaLR | LTR |
| 67120076 | 67120433 | + | 12.9 | 1 | 364 | 0 | THE1B | MaLR | LTR |
| 67120434 | 67121559 | - | 22.7 | 4930 | 6102 | -839 | L1M | L1 | LINE |
| 67121560 | 67121848 | - | 11.8 | 1 | 290 | -23 | AluSq | Alu | SINE |
| 67121849 | 67121992 | - | 22.9 | 4703 | 4929 | -2012 | L1M | L1 | LINE |
| 67121993 | 67122739 | - | 14.9 | 5507 | 6278 | -13 | L1MA7 | L1 | LINE |
| 67122740 | 67123029 | - | 22.9 | 4408 | 4702 | -2239 | L1M | L1 | LINE |
| 67123046 | 67123179 | + | 35.6 | 853 | 986 | -5295 | L1M3b | L1 | LINE |
| 67123188 | 67123536 | - | 15.7 | 2720 | 3069 | -3077 | L1MC | L1 | LINE |
| 67123552 | 67123842 | - | 21.1 | 2112 | 2403 | -4725 | L1M | L1 | LINE |
| 67123843 | 67123988 | + | 17.6 | 1 | 143 | 0 | FLAM\_C | Alu | SINE |
| 67124002 | 67125714 | - | 25.6 | 3 | 2126 | -5002 | L1MCc | L1 | LINE |
| 67125716 | 67125751 | - | 16.7 | 80 | 115 | -74 | MER5A | MER1\_type | DNA |

  

|  |  |  |  |  |  |  |  |  |  |
| --- | --- | --- | --- | --- | --- | --- | --- | --- | --- |
| chr1:67132561-67139737 | | | 7098 bp | | |  |  |  |  |
| Genome Start | Genome End | Strand | Div | Repeat Start | Repeat End | Repeat Left | Name | Family | Class |
| 67132561 | 67133470 | - | 25.5 | 4312 | 5398 | -748 | L1M5 | L1 | LINE |
| 67133471 | 67133747 | + | 21.3 | 5 | 290 | -22 | AluJo | Alu | SINE |
| 67133748 | 67134193 | - | 25.5 | 3769 | 4311 | -1835 | L1M5 | L1 | LINE |
| 67134217 | 67134565 | - | 25.5 | 3378 | 3768 | -2378 | L1M5 | L1 | LINE |
| 67134619 | 67134731 | - | 19.8 | 6009 | 6131 | -53 | L1MB7 | L1 | LINE |
| 67134732 | 67135145 | + | 20.1 | 1 | 426 | -2 | MSTC | MaLR | LTR |
| 67135146 | 67136648 | + | 22.7 | 2 | 1735 | 0 | MSTC-int | MaLR | LTR |
| 67136650 | 67137043 | + | 20.9 | 2 | 428 | 0 | MSTC | MaLR | LTR |
| 67137044 | 67138328 | - | 19.1 | 4657 | 6008 | -176 | L1MB7 | L1 | LINE |
| 67138329 | 67138671 | - | 10.9 | 1 | 312 | 0 | AluSp | Alu | SINE |
| 67138672 | 67139383 | - | 18.5 | 3913 | 4656 | -1490 | L1MB7 | L1 | LINE |
| 67139386 | 67139737 | - | 15.5 | 2879 | 3230 | -2916 | L1M5 | L1 | LINE |

  

|  |  |  |  |  |  |  |  |  |  |
| --- | --- | --- | --- | --- | --- | --- | --- | --- | --- |
| chr1:67303342-67315248 | | | 11529 bp | | |  |  |  |  |
| Genome Start | Genome End | Strand | Div | Repeat Start | Repeat End | Repeat Left | Name | Family | Class |
| 67303342 | 67303841 | + | 24.9 | 2481 | 3006 | -413 | L2 | L2 | LINE |
| 67303911 | 67304010 | - | 24.2 | 216 | 329 | -466 | LTR50 | ERVL | LTR |
| 67304027 | 67304224 | + | 28.9 | 2989 | 3155 | -264 | L2 | L2 | LINE |
| 67304226 | 67304807 | + | 21.7 | 4464 | 5063 | -1083 | L1MD | L1 | LINE |
| 67304855 | 67305232 | + | 10.8 | 1 | 375 | 0 | THE1C | MaLR | LTR |
| 67305233 | 67306764 | + | 11.0 | 1 | 1580 | 0 | THE1C-int | MaLR | LTR |
| 67306765 | 67306952 | + | 10.2 | 1 | 178 | -197 | THE1C | MaLR | LTR |
| 67306953 | 67307263 | - | 8.4 | 1 | 311 | 0 | AluY | Alu | SINE |
| 67307264 | 67307462 | + | 10.2 | 179 | 375 | 0 | THE1C | MaLR | LTR |
| 67307463 | 67307939 | - | 16.5 | 5729 | 6209 | -195 | L1MB1 | L1 | LINE |
| 67307940 | 67308250 | - | 12.6 | 1 | 310 | -1 | AluY | Alu | SINE |
| 67308251 | 67308680 | - | 14.6 | 5268 | 5728 | -676 | L1MB1 | L1 | LINE |
| 67308678 | 67309172 | - | 15.0 | 2371 | 2859 | -3517 | L1MB1 | L1 | LINE |
| 67309173 | 67309424 | + | 15.1 | 29 | 283 | -30 | AluSq | Alu | SINE |
| 67309425 | 67310029 | - | 24.5 | 1683 | 2383 | -4001 | L1M4c | L1 | LINE |
| 67310030 | 67310230 | - | 22.4 | 1 | 48 | -1 | FAM | Alu | SINE |
| 67310231 | 67310460 | - | 24.5 | 1417 | 1682 | -4702 | L1M4c | L1 | LINE |
| 67310461 | 67310897 | - | 12.4 | 1 | 434 | 0 | MER57B | ERV1 | LTR |
| 67310898 | 67311017 | - | 24.5 | 1298 | 1787 | -4970 | L1MB1 | L1 | LINE |
| 67311039 | 67311589 | - | 26.9 | 1107 | 1689 | -4614 | L1MB1 | L1 | LINE |
| 67311722 | 67311903 | - | 34.9 | 932 | 1103 | -5200 | L1MB1 | L1 | LINE |
| 67311966 | 67312600 | - | 32.8 | 416 | 1090 | -5213 | L1MB1 | L1 | LINE |
| 67312604 | 67312983 | + | 19.5 | 5077 | 5462 | -704 | L1M5 | L1 | LINE |
| 67312981 | 67313500 | + | 19.1 | 7291 | 7821 | -174 | L1MD3 | L1 | LINE |
| 67313523 | 67313672 | + | 26.2 | 3152 | 3317 | -102 | L2 | L2 | LINE |
| 67313674 | 67313998 | - | 26.8 | 2269 | 2639 | -1 | MER69B | Tip100 | DNA |
| 67313999 | 67314322 | - | 13.5 | 1 | 311 | -1 | AluSx | Alu | SINE |
| 67314323 | 67315130 | - | 26.8 | 20 | 2268 | -372 | MER69B | Tip100 | DNA |
| 67315141 | 67315248 | + | 29.0 | 3310 | 3419 | 0 | L2 | L2 | LINE |

  

|  |  |  |  |  |  |  |  |  |  |
| --- | --- | --- | --- | --- | --- | --- | --- | --- | --- |
| chr1:67445465-67448059 | | | 2593 bp | | |  |  |  |  |
| Genome Start | Genome End | Strand | Div | Repeat Start | Repeat End | Repeat Left | Name | Family | Class |
| 67445465 | 67445692 | - | 13.2 | 5949 | 6166 | 0 | L1PA16 | L1 | LINE |
| 67445693 | 67446045 | + | 8.5 | 1 | 355 | 0 | THE1A | MaLR | LTR |
| 67446048 | 67447613 | + | 29.1 | 2 | 1735 | 0 | MLT-int | MaLR | LTR |
| 67447614 | 67447966 | + | 9.9 | 1 | 355 | 0 | THE1A | MaLR | LTR |
| 67447967 | 67448059 | - | 13.2 | 5855 | 5948 | -218 | L1PA16 | L1 | LINE |

  

|  |  |  |  |  |  |  |  |  |  |
| --- | --- | --- | --- | --- | --- | --- | --- | --- | --- |
| chr1:68785495-68795513 | | | 10016 bp | | |  |  |  |  |
| Genome Start | Genome End | Strand | Div | Repeat Start | Repeat End | Repeat Left | Name | Family | Class |
| 68785495 | 68791606 | + | 14.5 | 7 | 7035 | -574 | L1PA11 | L1 | LINE |
| 68791607 | 68791962 | + | 17.7 | 1 | 364 | 0 | THE1B | MaLR | LTR |
| 68791963 | 68793247 | + | 10.7 | 1 | 1285 | -295 | THE1B-int | MaLR | LTR |
| 68793248 | 68794277 | + | 5.9 | 5123 | 6154 | 0 | L1PA6 | L1 | LINE |
| 68794281 | 68794592 | + | 10.6 | 1269 | 1580 | 0 | THE1B-int | MaLR | LTR |
| 68794593 | 68794948 | + | 15.7 | 1 | 364 | 0 | THE1B | MaLR | LTR |
| 68794949 | 68795513 | + | 8.2 | 7031 | 7608 | -1 | L1PA11 | L1 | LINE |

  

|  |  |  |  |  |  |  |  |  |  |
| --- | --- | --- | --- | --- | --- | --- | --- | --- | --- |
| chr1:69178331-69182093 | | | 3744 bp | | |  |  |  |  |
| Genome Start | Genome End | Strand | Div | Repeat Start | Repeat End | Repeat Left | Name | Family | Class |
| 69178331 | 69179126 | - | 28.6 | 1702 | 2503 | -4 | HAL1 | L1 | LINE |
| 69179128 | 69179677 | + | 28.6 | 3 | 584 | 0 | MLT1E | MaLR | LTR |
| 69179678 | 69181034 | + | 23.1 | 1 | 1489 | 0 | MLT1E-int | MaLR | LTR |
| 69181037 | 69181559 | + | 25.1 | 3 | 590 | -3 | MLT1E | MaLR | LTR |
| 69181576 | 69182093 | - | 26.3 | 1050 | 1622 | -885 | HAL1 | L1 | LINE |

  

|  |  |  |  |  |  |  |  |  |  |
| --- | --- | --- | --- | --- | --- | --- | --- | --- | --- |
| chr1:69303567-69315290 | | | 11668 bp | | |  |  |  |  |
| Genome Start | Genome End | Strand | Div | Repeat Start | Repeat End | Repeat Left | Name | Family | Class |
| 69303567 | 69303939 | - | 20.4 | 4585 | 6421 | -255 | LOR1-int | ERV1 | LTR |
| 69303941 | 69305460 | - | 9.6 | 4976 | 6491 | 0 | L1PA10 | L1 | LINE |
| 69305461 | 69305768 | - | 14.6 | 1 | 309 | -3 | AluSx | Alu | SINE |
| 69305769 | 69310692 | - | 13.3 | 98 | 4975 | -1486 | L1PA10 | L1 | LINE |
| 69310693 | 69310747 | - | 14.8 | 310 | 364 | 0 | THE1B | MaLR | LTR |
| 69310793 | 69311073 | - | 14.8 | 2 | 309 | -55 | THE1B | MaLR | LTR |
| 69311074 | 69312635 | - | 29.7 | 2 | 1735 | 0 | MLT-int | MaLR | LTR |
| 69312638 | 69312996 | - | 11.9 | 1 | 364 | 0 | THE1B | MaLR | LTR |
| 69312997 | 69313092 | - | 27.0 | 3 | 97 | -6364 | L1PA10 | L1 | LINE |
| 69313096 | 69313703 | - | 16.1 | 4974 | 5593 | -738 | LOR1-int | ERV1 | LTR |
| 69313704 | 69313810 | + | 14.0 | 6045 | 6161 | -5 | L1PA16 | L1 | LINE |
| 69313811 | 69315008 | - | 16.1 | 3766 | 4973 | -1358 | LOR1-int | ERV1 | LTR |
| 69315013 | 69315239 | + | 15.3 | 151 | 381 | 0 | THE1D | MaLR | LTR |
| 69315241 | 69315290 | - | 12.5 | 4164 | 4211 | -3909 | LOR1-int | ERV1 | LTR |

  

|  |  |  |  |  |  |  |  |  |  |
| --- | --- | --- | --- | --- | --- | --- | --- | --- | --- |
| chr1:69406799-69409650 | | | 2830 bp | | |  |  |  |  |
| Genome Start | Genome End | Strand | Div | Repeat Start | Repeat End | Repeat Left | Name | Family | Class |
| 69406799 | 69407346 | + | 14.7 | 5 | 555 | -24 | MER34D | ERV1 | LTR |
| 69407347 | 69407694 | + | 15.7 | 1 | 355 | 0 | THE1A | MaLR | LTR |
| 69407697 | 69409253 | + | 30.9 | 2 | 1735 | 0 | MLT1A0-int | MaLR | LTR |
| 69409254 | 69409469 | + | 10.8 | 1 | 209 | -146 | THE1A | MaLR | LTR |
| 69409490 | 69409626 | + | 10.8 | 210 | 354 | -1 | THE1A | MaLR | LTR |
| 69409627 | 69409650 | + | 14.7 | 556 | 577 | -2 | MER34D | ERV1 | LTR |

  

|  |  |  |  |  |  |  |  |  |  |
| --- | --- | --- | --- | --- | --- | --- | --- | --- | --- |
| chr1:69566798-69573379 | | | 6337 bp | | |  |  |  |  |
| Genome Start | Genome End | Strand | Div | Repeat Start | Repeat End | Repeat Left | Name | Family | Class |
| 69566798 | 69568477 | - | 17.5 | 3886 | 5575 | -1630 | L1M2 | L1 | LINE |
| 69568510 | 69568769 | - | 20.0 | 3365 | 3626 | -3579 | L1M2 | L1 | LINE |
| 69568774 | 69569123 | - | 19.9 | 1 | 381 | 0 | THE1D | MaLR | LTR |
| 69569124 | 69570763 | - | 16.7 | 1 | 1580 | 0 | THE1D-int | MaLR | LTR |
| 69570764 | 69571117 | - | 14.7 | 1 | 381 | 0 | THE1D | MaLR | LTR |
| 69571121 | 69571655 | - | 22.5 | 2831 | 3362 | -3846 | L1M2 | L1 | LINE |
| 69571685 | 69572584 | - | 23.0 | 1881 | 2830 | -4379 | L1M2 | L1 | LINE |
| 69572762 | 69573379 | - | 31.6 | 33 | 641 | -6568 | L1M2 | L1 | LINE |

  

|  |  |  |  |  |  |  |  |  |  |
| --- | --- | --- | --- | --- | --- | --- | --- | --- | --- |
| chr1:71069185-71074598 | | | 5218 bp | | |  |  |  |  |
| Genome Start | Genome End | Strand | Div | Repeat Start | Repeat End | Repeat Left | Name | Family | Class |
| 71069185 | 71070355 | + | 22.0 | 907 | 2113 | -4563 | L1M4 | L1 | LINE |
| 71070396 | 71070626 | + | 28.5 | 2280 | 2508 | -4138 | L1M4 | L1 | LINE |
| 71070633 | 71070985 | + | 12.4 | 1 | 364 | 0 | THE1B | MaLR | LTR |
| 71070988 | 71072624 | + | 9.6 | 3 | 1574 | -6 | THE1B-int | MaLR | LTR |
| 71072630 | 71072984 | + | 11.6 | 1 | 364 | 0 | THE1B | MaLR | LTR |
| 71072995 | 71073225 | + | 20.4 | 2521 | 3179 | -3793 | L1M4 | L1 | LINE |
| 71073272 | 71073524 | + | 32.0 | 4240 | 4524 | -2120 | L1M4 | L1 | LINE |
| 71073529 | 71073652 | - | 29.4 | 3294 | 3418 | -1 | L2 | L2 | LINE |
| 71073736 | 71074598 | + | 28.9 | 4849 | 5790 | -854 | L1M4 | L1 | LINE |

  

|  |  |  |  |  |  |  |  |  |  |
| --- | --- | --- | --- | --- | --- | --- | --- | --- | --- |
| chr1:71082075-71084846 | | | 2770 bp | | |  |  |  |  |
| Genome Start | Genome End | Strand | Div | Repeat Start | Repeat End | Repeat Left | Name | Family | Class |
| 71082075 | 71082181 | - | 29.4 | 3306 | 3419 | 0 | L2 | L2 | LINE |
| 71082182 | 71082537 | + | 14.2 | 1 | 355 | 0 | THE1A | MaLR | LTR |
| 71082540 | 71084120 | + | 32.0 | 2 | 1735 | 0 | MLT-int | MaLR | LTR |
| 71084121 | 71084476 | + | 15.6 | 1 | 355 | 0 | THE1A | MaLR | LTR |
| 71084477 | 71084846 | - | 29.4 | 2970 | 3305 | -114 | L2 | L2 | LINE |

  

|  |  |  |  |  |  |  |  |  |  |
| --- | --- | --- | --- | --- | --- | --- | --- | --- | --- |
| chr1:71381661-71394833 | | | 13152 bp | | |  |  |  |  |
| Genome Start | Genome End | Strand | Div | Repeat Start | Repeat End | Repeat Left | Name | Family | Class |
| 71381661 | 71383972 | - | 12.4 | 3316 | 5687 | -618 | L1M1 | L1 | LINE |
| 71383973 | 71384338 | + | 15.0 | 1 | 381 | 0 | THE1D | MaLR | LTR |
| 71384341 | 71385940 | + | 30.7 | 2 | 1735 | 0 | MLT-int | MaLR | LTR |
| 71385941 | 71386309 | + | 15.8 | 1 | 381 | 0 | THE1D | MaLR | LTR |
| 71386310 | 71387865 | - | 12.3 | 3154 | 4734 | -2831 | L1M1 | L1 | LINE |
| 71387878 | 71387976 | - | 17.2 | 3227 | 3327 | -4238 | L1M1 | L1 | LINE |
| 71387978 | 71390720 | + | 4.9 | 3415 | 6152 | -2 | L1PA5 | L1 | LINE |
| 71390722 | 71391934 | - | 11.4 | 1913 | 3235 | -4330 | L1M1 | L1 | LINE |
| 71391935 | 71392654 | + | 13.4 | 1 | 711 | -23 | MER50 | ERV1 | LTR |
| 71392660 | 71394354 | - | 17.1 | 200 | 1912 | -5653 | L1M1 | L1 | LINE |
| 71394355 | 71394646 | + | 17.5 | 1 | 290 | -22 | AluJb | Alu | SINE |
| 71394647 | 71394833 | - | 17.1 | 16 | 199 | -7366 | L1M1 | L1 | LINE |

  

|  |  |  |  |  |  |  |  |  |  |
| --- | --- | --- | --- | --- | --- | --- | --- | --- | --- |
| chr1:71409516-71412110 | | | 2592 bp | | |  |  |  |  |
| Genome Start | Genome End | Strand | Div | Repeat Start | Repeat End | Repeat Left | Name | Family | Class |
| 71409516 | 71409782 | + | 32.8 | 2920 | 3212 | -207 | L2 | L2 | LINE |
| 71409783 | 71410149 | - | 11.2 | 1 | 375 | 0 | THE1C | MaLR | LTR |
| 71410150 | 71411681 | - | 29.5 | 2 | 1735 | 0 | MLT1I-int | MaLR | LTR |
| 71411685 | 71412053 | - | 11.4 | 1 | 374 | -1 | THE1C | MaLR | LTR |
| 71412054 | 71412110 | + | 32.8 | 3213 | 3271 | -148 | L2 | L2 | LINE |

  

|  |  |  |  |  |  |  |  |  |  |
| --- | --- | --- | --- | --- | --- | --- | --- | --- | --- |
| chr1:71527882-71530914 | | | 3010 bp | | |  |  |  |  |
| Genome Start | Genome End | Strand | Div | Repeat Start | Repeat End | Repeat Left | Name | Family | Class |
| 71527882 | 71527968 | + | 26.4 | 492 | 578 | -894 | MLT1H-int | MaLR | LTR |
| 71527989 | 71528353 | + | 11.9 | 1 | 364 | 0 | THE1B | MaLR | LTR |
| 71528356 | 71529937 | + | 29.6 | 1 | 1735 | 0 | MLT1H-int | MaLR | LTR |
| 71529939 | 71530305 | + | 13.2 | 2 | 364 | 0 | THE1B | MaLR | LTR |
| 71530306 | 71530914 | + | 30.5 | 551 | 1160 | -312 | MLT1H-int | MaLR | LTR |

  

|  |  |  |  |  |  |  |  |  |  |
| --- | --- | --- | --- | --- | --- | --- | --- | --- | --- |
| chr1:71630451-71633516 | | | 3057 bp | | |  |  |  |  |
| Genome Start | Genome End | Strand | Div | Repeat Start | Repeat End | Repeat Left | Name | Family | Class |
| 71630451 | 71630714 | - | 32.3 | 285 | 535 | -7 | MER34 | ERV1 | LTR |
| 71630715 | 71631167 | + | 16.3 | 3 | 422 | -4 | MSTB | MaLR | LTR |
| 71631176 | 71632831 | + | 29.2 | 3 | 1734 | -1 | MLT-int | MaLR | LTR |
| 71632833 | 71633281 | + | 17.5 | 1 | 426 | 0 | MSTB | MaLR | LTR |
| 71633282 | 71633516 | - | 32.3 | 19 | 277 | -265 | MER34 | ERV1 | LTR |

  

|  |  |  |  |  |  |  |  |  |  |
| --- | --- | --- | --- | --- | --- | --- | --- | --- | --- |
| chr1:71952195-71954651 | | | 2453 bp | | |  |  |  |  |
| Genome Start | Genome End | Strand | Div | Repeat Start | Repeat End | Repeat Left | Name | Family | Class |
| 71952195 | 71952268 | + | 30.9 | 89 | 160 | -108 | MIRb | MIR | SINE |
| 71952269 | 71952624 | - | 11.2 | 1 | 381 | 0 | THE1D | MaLR | LTR |
| 71952625 | 71954236 | - | 32.2 | 2 | 1735 | 0 | MLT1K-int | MaLR | LTR |
| 71954241 | 71954597 | - | 16.1 | 1 | 379 | -2 | THE1D | MaLR | LTR |
| 71954598 | 71954651 | + | 30.9 | 161 | 212 | -56 | MIRb | MIR | SINE |

  

|  |  |  |  |  |  |  |  |  |  |
| --- | --- | --- | --- | --- | --- | --- | --- | --- | --- |
| chr1:73427393-73436159 | | | 7956 bp | | |  |  |  |  |
| Genome Start | Genome End | Strand | Div | Repeat Start | Repeat End | Repeat Left | Name | Family | Class |
| 73427393 | 73427541 | + | 35.6 | 265 | 415 | -5985 | L1ME1 | L1 | LINE |
| 73427737 | 73427898 | + | 27.2 | 1018 | 1181 | -5219 | L1ME1 | L1 | LINE |
| 73427978 | 73428606 | + | 27.1 | 1337 | 2038 | -4362 | L1ME1 | L1 | LINE |
| 73428626 | 73428968 | - | 20.2 | 4 | 365 | 0 | MLT1A0 | MaLR | LTR |
| 73428972 | 73429033 | - | 21.7 | 1675 | 1735 | 0 | MLT1A0-int | MaLR | LTR |
| 73429034 | 73429396 | - | 16.8 | 1 | 364 | 0 | THE1B | MaLR | LTR |
| 73429397 | 73429422 | - | 21.7 | 1653 | 1674 | -61 | MLT1A0-int | MaLR | LTR |
| 73429543 | 73429964 | + | 26.1 | 972 | 1456 | -279 | MLT-int | MaLR | LTR |
| 73430124 | 73430383 | + | 15.8 | 1438 | 1706 | -29 | MLT1A0-int | MaLR | LTR |
| 73430371 | 73430635 | + | 22.6 | 97 | 365 | 0 | MLT1A0 | MaLR | LTR |
| 73430637 | 73430937 | - | 23.6 | 2 | 298 | -1437 | MLT1A0-int | MaLR | LTR |
| 73430939 | 73431280 | - | 19.9 | 17 | 365 | 0 | MLT1A0 | MaLR | LTR |
| 73431283 | 73431337 | - | 21.8 | 1 | 55 | -319 | MLT1A0 | MaLR | LTR |
| 73431443 | 73431859 | + | 28.6 | 4261 | 4694 | -1525 | L1ME1 | L1 | LINE |
| 73431855 | 73433278 | + | 2.5 | 1716 | 3139 | -2884 | L1HS | L1 | LINE |
| 73433308 | 73433655 | - | 25.6 | 284 | 654 | 0 | MLT1E | MaLR | LTR |
| 73433752 | 73434067 | - | 11.3 | 1 | 310 | -1 | AluY | Alu | SINE |
| 73434087 | 73434147 | - | 21.7 | 1 | 60 | -502 | MLT1F2 | MaLR | LTR |
| 73434149 | 73434201 | + | 31.7 | 4653 | 4708 | -1438 | L1MD | L1 | LINE |
| 73434202 | 73434510 | + | 13.7 | 1 | 311 | -1 | AluSx | Alu | SINE |
| 73434511 | 73434774 | + | 27.7 | 4782 | 5068 | -1151 | L1ME1 | L1 | LINE |
| 73434775 | 73435069 | + | 20.7 | 12 | 306 | -6 | AluJb | Alu | SINE |
| 73435070 | 73436159 | + | 27.7 | 5069 | 6233 | -6 | L1ME1 | L1 | LINE |

  

|  |  |  |  |  |  |  |  |  |  |
| --- | --- | --- | --- | --- | --- | --- | --- | --- | --- |
| chr1:73580565-73584393 | | | 3691 bp | | |  |  |  |  |
| Genome Start | Genome End | Strand | Div | Repeat Start | Repeat End | Repeat Left | Name | Family | Class |
| 73580565 | 73580671 | - | 20.0 | 2689 | 2825 | -3814 | LOR1-int | ERV1 | LTR |
| 73580796 | 73581380 | - | 22.0 | 1773 | 3527 | -3071 | LOR1-int | ERV1 | LTR |
| 73581381 | 73581721 | + | 14.8 | 1 | 364 | 0 | THE1B | MaLR | LTR |
| 73581726 | 73583293 | + | 29.9 | 4 | 1735 | 0 | MLT-int | MaLR | LTR |
| 73583294 | 73583646 | + | 14.1 | 1 | 364 | 0 | THE1B | MaLR | LTR |
| 73583647 | 73583702 | - | 25.6 | 1720 | 1772 | -6348 | LOR1-int | ERV1 | LTR |
| 73583713 | 73584393 | - | 23.2 | 1173 | 2195 | -4444 | LOR1-int | ERV1 | LTR |

  

|  |  |  |  |  |  |  |  |  |  |
| --- | --- | --- | --- | --- | --- | --- | --- | --- | --- |
| chr1:73685283-73687965 | | | 2681 bp | | |  |  |  |  |
| Genome Start | Genome End | Strand | Div | Repeat Start | Repeat End | Repeat Left | Name | Family | Class |
| 73685283 | 73685353 | - | 32.9 | 5809 | 5885 | -236 | L1M5 | L1 | LINE |
| 73685354 | 73685744 | - | 16.0 | 1 | 428 | 0 | MSTA | MaLR | LTR |
| 73685745 | 73687355 | - | 30.5 | 2 | 1735 | 0 | MLT-int | MaLR | LTR |
| 73687358 | 73687750 | - | 19.0 | 1 | 428 | 0 | MSTA | MaLR | LTR |
| 73687751 | 73687965 | - | 32.9 | 5595 | 5808 | -313 | L1M5 | L1 | LINE |

  

|  |  |  |  |  |  |  |  |  |  |
| --- | --- | --- | --- | --- | --- | --- | --- | --- | --- |
| chr1:73787091-73789906 | | | 2652 bp | | |  |  |  |  |
| Genome Start | Genome End | Strand | Div | Repeat Start | Repeat End | Repeat Left | Name | Family | Class |
| 73787091 | 73787332 | - | 27.6 | 429 | 669 | -4185 | L1MEe | L1 | LINE |
| 73787335 | 73787692 | - | 16.0 | 1 | 362 | -2 | THE1B | MaLR | LTR |
| 73787698 | 73789231 | - | 11.7 | 3 | 1574 | -6 | THE1B-int | MaLR | LTR |
| 73789237 | 73789596 | - | 17.4 | 1 | 360 | -4 | THE1B | MaLR | LTR |
| 73789749 | 73789906 | - | 34.0 | 84 | 243 | -4611 | L1MEe | L1 | LINE |

  

|  |  |  |  |  |  |  |  |  |  |
| --- | --- | --- | --- | --- | --- | --- | --- | --- | --- |
| chr1:73837749-73840660 | | | 2911 bp | | |  |  |  |  |
| Genome Start | Genome End | Strand | Div | Repeat Start | Repeat End | Repeat Left | Name | Family | Class |
| 73837749 | 73838191 | - | 22.6 | 87 | 515 | 0 | MLT2B2 | ERVL | LTR |
| 73838192 | 73838551 | - | 14.5 | 1 | 364 | 0 | THE1B | MaLR | LTR |
| 73838552 | 73840210 | - | 12.4 | 1 | 1580 | 0 | THE1B-int | MaLR | LTR |
| 73840211 | 73840568 | - | 13.8 | 2 | 364 | 0 | THE1B | MaLR | LTR |
| 73840570 | 73840660 | - | 15.4 | 1 | 91 | -463 | MLT2B2 | ERVL | LTR |

  

|  |  |  |  |  |  |  |  |  |  |
| --- | --- | --- | --- | --- | --- | --- | --- | --- | --- |
| chr1:73864360-73873274 | | | 8924 bp | | |  |  |  |  |
| Genome Start | Genome End | Strand | Div | Repeat Start | Repeat End | Repeat Left | Name | Family | Class |
| 73864360 | 73866283 | + | 15.9 | 19 | 1987 | -5578 | L1M1 | L1 | LINE |
| 73866284 | 73866638 | - | 14.4 | 1 | 364 | 0 | THE1B | MaLR | LTR |
| 73866639 | 73868194 | - | 31.1 | 2 | 1735 | 0 | MLT-int | MaLR | LTR |
| 73868197 | 73868263 | - | 16.4 | 309 | 375 | 0 | THE1B | MaLR | LTR |
| 73868253 | 73868430 | - | 12.0 | 1 | 201 | -163 | THE1B | MaLR | LTR |
| 73868431 | 73873274 | + | 15.5 | 2157 | 6687 | -878 | L1M1 | L1 | LINE |

  

|  |  |  |  |  |  |  |  |  |  |
| --- | --- | --- | --- | --- | --- | --- | --- | --- | --- |
| chr1:74220162-74223933 | | | 3772 bp | | |  |  |  |  |
| Genome Start | Genome End | Strand | Div | Repeat Start | Repeat End | Repeat Left | Name | Family | Class |
| 74220162 | 74220702 | - | 10.9 | 4561 | 5103 | -1043 | L1PB | L1 | LINE |
| 74220703 | 74221065 | - | 15.7 | 1 | 364 | 0 | THE1B | MaLR | LTR |
| 74221066 | 74222561 | - | 13.0 | 1 | 1580 | 0 | THE1B-int | MaLR | LTR |
| 74222562 | 74222906 | - | 16.9 | 1 | 364 | 0 | THE1B | MaLR | LTR |
| 74222907 | 74223933 | - | 10.3 | 3505 | 4565 | -1581 | L1PB | L1 | LINE |

  

|  |  |  |  |  |  |  |  |  |  |
| --- | --- | --- | --- | --- | --- | --- | --- | --- | --- |
| chr1:75114068-75126699 | | | 12578 bp | | |  |  |  |  |
| Genome Start | Genome End | Strand | Div | Repeat Start | Repeat End | Repeat Left | Name | Family | Class |
| 75114068 | 75114878 | + | 20.6 | 2689 | 3499 | -2647 | L1P5 | L1 | LINE |
| 75114879 | 75115190 | + | 17.9 | 1 | 312 | 0 | AluSx | Alu | SINE |
| 75115191 | 75115389 | + | 20.6 | 3500 | 3697 | -2449 | L1P5 | L1 | LINE |
| 75115393 | 75121454 | + | 3.5 | 2 | 6047 | 0 | L1PA3 | L1 | LINE |
| 75121451 | 75122302 | + | 16.8 | 3687 | 4583 | -1563 | L1P5 | L1 | LINE |
| 75122303 | 75122656 | + | 14.7 | 1 | 364 | 0 | THE1B | MaLR | LTR |
| 75122659 | 75124228 | + | 30.5 | 2 | 1731 | -4 | MLT-int | MaLR | LTR |
| 75124233 | 75124587 | + | 12.1 | 1 | 364 | 0 | THE1B | MaLR | LTR |
| 75124588 | 75125143 | + | 16.8 | 4584 | 5132 | -1014 | L1P5 | L1 | LINE |
| 75125144 | 75125512 | + | 10.4 | 1 | 372 | -3 | THE1C | MaLR | LTR |
| 75125513 | 75126499 | + | 14.2 | 5133 | 6143 | -13 | L1PB4 | L1 | LINE |
| 75126549 | 75126699 | + | 12.2 | 5390 | 5539 | -617 | L1P5 | L1 | LINE |

  

|  |  |  |  |  |  |  |  |  |  |
| --- | --- | --- | --- | --- | --- | --- | --- | --- | --- |
| chr1:75150668-75159837 | | | 9167 bp | | |  |  |  |  |
| Genome Start | Genome End | Strand | Div | Repeat Start | Repeat End | Repeat Left | Name | Family | Class |
| 75150668 | 75151191 | - | 11.6 | 5627 | 6160 | 0 | L1PREC2 | L1 | LINE |
| 75151192 | 75151550 | - | 14.7 | 1 | 364 | 0 | THE1B | MaLR | LTR |
| 75151551 | 75153163 | - | 30.1 | 2 | 1735 | 0 | MLT-int | MaLR | LTR |
| 75153166 | 75153525 | - | 15.9 | 1 | 364 | 0 | THE1B | MaLR | LTR |
| 75153526 | 75153960 | - | 11.8 | 5191 | 5626 | -534 | L1PREC2 | L1 | LINE |
| 75153964 | 75159296 | - | 7.0 | 829 | 6154 | 0 | L1PA6 | L1 | LINE |
| 75159295 | 75159358 | - | 9.7 | 33 | 94 | -2181 | L1PA6 | L1 | LINE |
| 75159359 | 75159537 | + | 13.4 | 1764 | 1942 | -5771 | HERVH | ERV1 | LTR |
| 75159538 | 75159837 | - | 13.0 | 4899 | 5200 | -946 | L1PREC2 | L1 | LINE |

  

|  |  |  |  |  |  |  |  |  |  |
| --- | --- | --- | --- | --- | --- | --- | --- | --- | --- |
| chr1:75611400-75630144 | | | 18742 bp | | |  |  |  |  |
| Genome Start | Genome End | Strand | Div | Repeat Start | Repeat End | Repeat Left | Name | Family | Class |
| 75611400 | 75614097 | + | 18.5 | 4 | 2730 | -4205 | L1P4d | L1 | LINE |
| 75614100 | 75615358 | + | 3.6 | 4 | 1272 | -4760 | L1HS | L1 | LINE |
| 75615359 | 75616326 | + | 0.9 | 1 | 968 | 0 | LTR5\_Hs | ERVK | LTR |
| 75616327 | 75618870 | + | 1.0 | 1 | 2543 | -4993 | HERVK | ERVK | LTR |
| 75618855 | 75619053 | + | 4.0 | 5336 | 5534 | -2002 | HERVK | ERVK | LTR |
| 75619053 | 75620763 | + | 0.7 | 5826 | 7536 | 0 | HERVK | ERVK | LTR |
| 75620764 | 75621731 | + | 0.9 | 1 | 968 | 0 | LTR5\_Hs | ERVK | LTR |
| 75621732 | 75626519 | + | 2.6 | 1267 | 6043 | 0 | L1PA2 | L1 | LINE |
| 75626538 | 75626777 | + | 14.2 | 2707 | 2904 | -4015 | L1P4d | L1 | LINE |
| 75626778 | 75627074 | + | 7.1 | 7 | 304 | -7 | AluY | Alu | SINE |
| 75627075 | 75630144 | + | 13.7 | 2895 | 5900 | -1015 | L1P4d | L1 | LINE |

  

|  |  |  |  |  |  |  |  |  |  |
| --- | --- | --- | --- | --- | --- | --- | --- | --- | --- |
| chr1:75652682-75656135 | | | 3391 bp | | |  |  |  |  |
| Genome Start | Genome End | Strand | Div | Repeat Start | Repeat End | Repeat Left | Name | Family | Class |
| 75652682 | 75653385 | - | 32.5 | 2657 | 3378 | 0 | L2 | L2 | LINE |
| 75653429 | 75653791 | + | 14.9 | 1 | 364 | 0 | THE1B | MaLR | LTR |
| 75653792 | 75655355 | + | 9.7 | 1 | 1571 | -9 | THE1B-int | MaLR | LTR |
| 75655360 | 75655723 | + | 14.3 | 2 | 364 | 0 | THE1B | MaLR | LTR |
| 75655740 | 75656135 | - | 33.5 | 2174 | 2598 | -821 | L2 | L2 | LINE |

  

|  |  |  |  |  |  |  |  |  |  |
| --- | --- | --- | --- | --- | --- | --- | --- | --- | --- |
| chr1:76929783-76932898 | | | 3114 bp | | |  |  |  |  |
| Genome Start | Genome End | Strand | Div | Repeat Start | Repeat End | Repeat Left | Name | Family | Class |
| 76929783 | 76929923 | - | 30.9 | 534 | 688 | -5 | MER115 | Tip100 | DNA |
| 76929924 | 76930344 | - | 13.9 | 1 | 428 | 0 | MSTA | MaLR | LTR |
| 76930345 | 76931958 | - | 29.4 | 2 | 1735 | 0 | MLT-int | MaLR | LTR |
| 76931961 | 76932380 | - | 13.4 | 1 | 428 | 0 | MSTA | MaLR | LTR |
| 76932381 | 76932898 | - | 30.9 | 2 | 533 | -160 | MER115 | Tip100 | DNA |

  

|  |  |  |  |  |  |  |  |  |  |
| --- | --- | --- | --- | --- | --- | --- | --- | --- | --- |
| chr1:77459044-77462434 | | | 3374 bp | | |  |  |  |  |
| Genome Start | Genome End | Strand | Div | Repeat Start | Repeat End | Repeat Left | Name | Family | Class |
| 77459044 | 77459075 | - | 18.8 | 3388 | 3419 | 0 | L2 | L2 | LINE |
| 77459083 | 77459392 | + | 20.8 | 1 | 305 | -223 | LTR64 | ERV1 | LTR |
| 77459393 | 77459598 | - | 14.7 | 171 | 364 | 0 | THE1B | MaLR | LTR |
| 77459599 | 77459896 | + | 13.2 | 1 | 309 | -3 | AluSx | Alu | SINE |
| 77459897 | 77460078 | - | 14.7 | 1 | 170 | -194 | THE1B | MaLR | LTR |
| 77460079 | 77461577 | - | 10.4 | 1 | 1580 | 0 | THE1B-int | MaLR | LTR |
| 77461578 | 77461946 | - | 12.9 | 1 | 364 | 0 | THE1B | MaLR | LTR |
| 77461947 | 77462154 | + | 20.5 | 306 | 528 | 0 | LTR64 | ERV1 | LTR |
| 77462165 | 77462434 | - | 32.5 | 3067 | 3354 | -65 | L2 | L2 | LINE |

  

|  |  |  |  |  |  |  |  |  |  |
| --- | --- | --- | --- | --- | --- | --- | --- | --- | --- |
| chr1:78531364-78534100 | | | 2735 bp | | |  |  |  |  |
| Genome Start | Genome End | Strand | Div | Repeat Start | Repeat End | Repeat Left | Name | Family | Class |
| 78531364 | 78531641 | + | 12.5 | 1 | 269 | -165 | Tigger2a | MER2\_type | DNA |
| 78531642 | 78531992 | + | 8.3 | 1 | 355 | 0 | THE1A | MaLR | LTR |
| 78531995 | 78533586 | + | 29.6 | 2 | 1735 | 0 | MLT-int | MaLR | LTR |
| 78533587 | 78533929 | + | 7.9 | 1 | 355 | 0 | THE1A | MaLR | LTR |
| 78533930 | 78534100 | + | 12.5 | 270 | 434 | 0 | Tigger2a | MER2\_type | DNA |

  

|  |  |  |  |  |  |  |  |  |  |
| --- | --- | --- | --- | --- | --- | --- | --- | --- | --- |
| chr1:78750545-78753410 | | | 2864 bp | | |  |  |  |  |
| Genome Start | Genome End | Strand | Div | Repeat Start | Repeat End | Repeat Left | Name | Family | Class |
| 78750545 | 78750951 | - | 18.5 | 56 | 424 | -2 | MSTB | MaLR | LTR |
| 78750952 | 78751376 | + | 19.8 | 1 | 426 | 0 | MSTB | MaLR | LTR |
| 78751379 | 78752936 | + | 26.7 | 2 | 1735 | 0 | MLT-int | MaLR | LTR |
| 78752937 | 78753356 | + | 17.6 | 1 | 426 | 0 | MSTB | MaLR | LTR |
| 78753357 | 78753410 | - | 18.5 | 3 | 55 | -371 | MSTB | MaLR | LTR |

  

|  |  |  |  |  |  |  |  |  |  |
| --- | --- | --- | --- | --- | --- | --- | --- | --- | --- |
| chr1:79110395-79119555 | | | 9161 bp | | |  |  |  |  |
| Genome Start | Genome End | Strand | Div | Repeat Start | Repeat End | Repeat Left | Name | Family | Class |
| 79110395 | 79110459 | - | 13.8 | 362 | 426 | 0 | MSTB | MaLR | LTR |
| 79110460 | 79110892 | + | 5.3 | 1 | 464 | 0 | LTR7B | ERV1 | LTR |
| 79110893 | 79118761 | + | 16.9 | 1 | 7713 | 0 | HERVH | ERV1 | LTR |
| 79118762 | 79119188 | + | 6.3 | 1 | 464 | 0 | LTR7B | ERV1 | LTR |
| 79119189 | 79119555 | - | 20.3 | 1 | 366 | -60 | MSTB | MaLR | LTR |

  

|  |  |  |  |  |  |  |  |  |  |
| --- | --- | --- | --- | --- | --- | --- | --- | --- | --- |
| chr1:79929038-79943908 | | | 14876 bp | | |  |  |  |  |
| Genome Start | Genome End | Strand | Div | Repeat Start | Repeat End | Repeat Left | Name | Family | Class |
| 79929038 | 79930118 | - | 17.9 | 6275 | 7332 | -1 | L1MA9 | L1 | LINE |
| 79930124 | 79930354 | - | 18.2 | 204 | 428 | 0 | MSTC | MaLR | LTR |
| 79930355 | 79930670 | - | 17.9 | 1 | 309 | -3 | AluJb | Alu | SINE |
| 79930671 | 79930880 | - | 18.2 | 1 | 203 | -225 | MSTC | MaLR | LTR |
| 79930881 | 79932536 | - | 28.0 | 2 | 1735 | 0 | MSTC-int | MaLR | LTR |
| 79932539 | 79932653 | - | 24.0 | 320 | 428 | 0 | MSTC | MaLR | LTR |
| 79932654 | 79933310 | + | 8.0 | 3 | 660 | 0 | PABL\_A | ERV1 | LTR |
| 79933311 | 79933377 | - | 24.0 | 253 | 319 | -109 | MSTC | MaLR | LTR |
| 79933380 | 79933567 | - | 18.5 | 303 | 531 | 0 | LTR26B | ERV1 | LTR |
| 79933565 | 79933776 | - | 15.7 | 1 | 235 | -193 | MSTC | MaLR | LTR |
| 79933780 | 79934424 | - | 21.2 | 5618 | 6264 | -897 | L1MA9 | L1 | LINE |
| 79934426 | 79934619 | - | 8.2 | 101 | 295 | -16 | AluY | Alu | SINE |
| 79934620 | 79935048 | - | 8.9 | 1 | 467 | -5 | LTR7Y | ERV1 | LTR |
| 79935050 | 79935423 | - | 8.3 | 7322 | 7712 | -1 | HERVH | ERV1 | LTR |
| 79935422 | 79935643 | - | 11.4 | 5597 | 5819 | -1894 | HERVH | ERV1 | LTR |
| 79935641 | 79936325 | - | 15.7 | 4488 | 5149 | -2564 | HERVH | ERV1 | LTR |
| 79936328 | 79936830 | - | 9.8 | 3492 | 3993 | -3720 | HERVH | ERV1 | LTR |
| 79936821 | 79936968 | - | 16.2 | 3140 | 3290 | -4423 | HERVH | ERV1 | LTR |
| 79936963 | 79939903 | - | 9.2 | 1 | 2987 | -4726 | HERVH | ERV1 | LTR |
| 79939904 | 79940335 | - | 9.2 | 1 | 472 | 0 | LTR7Y | ERV1 | LTR |
| 79940336 | 79940446 | - | 7.2 | 1 | 111 | -200 | AluY | Alu | SINE |
| 79940448 | 79940613 | - | 18.3 | 5463 | 5626 | -1535 | L1MA9 | L1 | LINE |
| 79940614 | 79941567 | + | 11.6 | 1 | 968 | 0 | LTR5\_Hs | ERVK | LTR |
| 79941568 | 79942023 | - | 18.3 | 5002 | 5462 | -1699 | L1MA9 | L1 | LINE |
| 79942024 | 79942352 | - | 18.9 | 1 | 331 | 0 | AluSx | Alu | SINE |
| 79942353 | 79943621 | - | 18.3 | 3716 | 5001 | -2160 | L1MA9 | L1 | LINE |
| 79943624 | 79943908 | - | 32.0 | 1587 | 1879 | -5289 | L1MA9 | L1 | LINE |

  

|  |  |  |  |  |  |  |  |  |  |
| --- | --- | --- | --- | --- | --- | --- | --- | --- | --- |
| chr1:80142920-80145514 | | | 2591 bp | | |  |  |  |  |
| Genome Start | Genome End | Strand | Div | Repeat Start | Repeat End | Repeat Left | Name | Family | Class |
| 80142920 | 80142966 | + | 17.0 | 1 | 47 | -255 | AluSx | Alu | SINE |
| 80142967 | 80143342 | - | 16.2 | 1 | 364 | 0 | THE1B | MaLR | LTR |
| 80143343 | 80144871 | - | 29.4 | 2 | 1735 | 0 | MLT1H-int | MaLR | LTR |
| 80144874 | 80145246 | - | 13.6 | 1 | 364 | 0 | THE1B | MaLR | LTR |
| 80145249 | 80145514 | + | 13.2 | 45 | 310 | -2 | AluSx | Alu | SINE |

  

|  |  |  |  |  |  |  |  |  |  |
| --- | --- | --- | --- | --- | --- | --- | --- | --- | --- |
| chr1:80364865-80367929 | | | 3045 bp | | |  |  |  |  |
| Genome Start | Genome End | Strand | Div | Repeat Start | Repeat End | Repeat Left | Name | Family | Class |
| 80364865 | 80364952 | + | 25.5 | 5502 | 5626 | -676 | L1MA10 | L1 | LINE |
| 80364963 | 80365283 | - | 13.1 | 1 | 331 | -33 | THE1B | MaLR | LTR |
| 80365284 | 80366856 | - | 29.2 | 2 | 1735 | 0 | MLT-int | MaLR | LTR |
| 80366859 | 80367211 | - | 13.1 | 1 | 364 | 0 | THE1B | MaLR | LTR |
| 80367220 | 80367929 | + | 17.4 | 5648 | 6332 | -2 | L1MA10 | L1 | LINE |

  

|  |  |  |  |  |  |  |  |  |  |
| --- | --- | --- | --- | --- | --- | --- | --- | --- | --- |
| chr1:80438227-80446554 | | | 8113 bp | | |  |  |  |  |
| Genome Start | Genome End | Strand | Div | Repeat Start | Repeat End | Repeat Left | Name | Family | Class |
| 80438227 | 80439414 | - | 25.0 | 6666 | 7876 | 0 | L1MC4a | L1 | LINE |
| 80439469 | 80440041 | - | 25.0 | 6153 | 6665 | -1211 | L1MC4a | L1 | LINE |
| 80440072 | 80440444 | - | 22.8 | 2 | 365 | 0 | MLT1A0 | MaLR | LTR |
| 80440446 | 80442097 | - | 22.2 | 1 | 1735 | 0 | MLT1A0-int | MaLR | LTR |
| 80442098 | 80442216 | - | 12.7 | 290 | 408 | 0 | MLT1A0 | MaLR | LTR |
| 80442218 | 80442397 | - | 26.3 | 5938 | 6126 | -1750 | L1MC4a | L1 | LINE |
| 80442402 | 80442610 | + | 22.2 | 3 | 230 | -6 | MER106A | MER1\_type | DNA |
| 80442625 | 80444297 | - | 28.2 | 4179 | 5934 | -1942 | L1MC4a | L1 | LINE |
| 80444302 | 80444604 | - | 26.9 | 2151 | 2463 | -3752 | L1MC4a | L1 | LINE |
| 80444635 | 80444928 | - | 6.5 | 1 | 306 | -7 | AluSp | Alu | SINE |
| 80445006 | 80445229 | - | 28.3 | 1081 | 1291 | -4924 | L1MC4a | L1 | LINE |
| 80445230 | 80445516 | - | 13.3 | 12 | 302 | -10 | AluJo | Alu | SINE |
| 80445517 | 80446554 | - | 28.3 | 3 | 1080 | -5135 | L1MC4a | L1 | LINE |

  

|  |  |  |  |  |  |  |  |  |  |
| --- | --- | --- | --- | --- | --- | --- | --- | --- | --- |
| chr1:80829515-80847719 | | | 17736 bp | | |  |  |  |  |
| Genome Start | Genome End | Strand | Div | Repeat Start | Repeat End | Repeat Left | Name | Family | Class |
| 80829515 | 80830375 | - | 26.7 | 4787 | 5702 | -468 | L1M5 | L1 | LINE |
| 80830433 | 80831213 | - | 5.6 | 1 | 780 | 0 | LTR17 | ERV1 | LTR |
| 80831241 | 80831308 | - | 14.7 | 438 | 505 | 0 | MLT1D | MaLR | LTR |
| 80831311 | 80831721 | + | 36.6 | 1 | 420 | -5721 | L1M3c | L1 | LINE |
| 80831842 | 80831895 | + | 13.0 | 3796 | 3850 | -2290 | L1M2 | L1 | LINE |
| 80831904 | 80832952 | + | 15.2 | 2 | 1058 | -5805 | L1PB1 | L1 | LINE |
| 80832958 | 80835438 | + | 10.6 | 1627 | 4177 | -2685 | L1PB1 | L1 | LINE |
| 80835439 | 80835746 | + | 16.6 | 1 | 312 | 0 | AluSx | Alu | SINE |
| 80835747 | 80837979 | + | 10.4 | 4178 | 6398 | -469 | L1PB1 | L1 | LINE |
| 80837980 | 80838059 | + | 8.8 | 1 | 80 | 0 | MADE1 | Mariner | DNA |
| 80838060 | 80838160 | + | 8.7 | 6399 | 6498 | -369 | L1PB1 | L1 | LINE |
| 80838201 | 80838535 | + | 8.7 | 6499 | 6866 | -1 | L1PB1 | L1 | LINE |
| 80838577 | 80839979 | + | 22.7 | 3832 | 5371 | -772 | L1M2 | L1 | LINE |
| 80839980 | 80840298 | + | 10.9 | 1 | 313 | 0 | AluSq | Alu | SINE |
| 80840299 | 80840330 | + | 17.4 | 5369 | 5397 | -743 | L1MB1 | L1 | LINE |
| 80840333 | 80840750 | - | 18.5 | 1 | 425 | -1 | MSTB | MaLR | LTR |
| 80840751 | 80841503 | + | 17.5 | 5405 | 6148 | -20 | L1MB1 | L1 | LINE |
| 80841504 | 80841980 | + | 21.6 | 1 | 499 | -55 | MLT2B3 | ERVL | LTR |
| 80842050 | 80842084 | + | 21.6 | 500 | 553 | -1 | MLT2B3 | ERVL | LTR |
| 80842085 | 80842105 | + | 17.5 | 6149 | 6166 | -2 | L1MB1 | L1 | LINE |
| 80842113 | 80842516 | - | 24.5 | 1 | 448 | -57 | MLT1D | MaLR | LTR |
| 80842565 | 80842659 | - | 26.2 | 1243 | 1325 | -46 | MLT1D-int | MaLR | LTR |
| 80842660 | 80843298 | - | 12.9 | 1 | 635 | 0 | LTR24C | ERV1 | LTR |
| 80843299 | 80843582 | - | 26.2 | 968 | 1242 | -129 | MLT1D-int | MaLR | LTR |
| 80843624 | 80843893 | - | 26.4 | 4234 | 4521 | -1625 | L1M5 | L1 | LINE |
| 80843894 | 80844291 | + | 25.1 | 1 | 396 | 0 | MSTD | MaLR | LTR |
| 80844294 | 80845957 | + | 25.2 | 2 | 1735 | 0 | MSTD-int | MaLR | LTR |
| 80845958 | 80846361 | + | 27.1 | 1 | 396 | 0 | MSTD | MaLR | LTR |
| 80846362 | 80846852 | - | 26.4 | 3689 | 4233 | -1913 | L1M5 | L1 | LINE |
| 80846853 | 80847160 | - | 7.5 | 1 | 310 | -1 | AluY | Alu | SINE |
| 80847161 | 80847719 | - | 26.4 | 3136 | 3688 | -2458 | L1M5 | L1 | LINE |

  

|  |  |  |  |  |  |  |  |  |  |
| --- | --- | --- | --- | --- | --- | --- | --- | --- | --- |
| chr1:83193240-83201170 | | | 7864 bp | | |  |  |  |  |
| Genome Start | Genome End | Strand | Div | Repeat Start | Repeat End | Repeat Left | Name | Family | Class |
| 83193240 | 83193581 | - | 29.3 | 3086 | 3406 | -13 | L2 | L2 | LINE |
| 83193582 | 83193995 | + | 9.9 | 1 | 450 | 0 | LTR7 | ERV1 | LTR |
| 83194001 | 83197159 | + | 10.3 | 6 | 3290 | -4423 | HERVH | ERV1 | LTR |
| 83197151 | 83197645 | + | 11.2 | 3493 | 3993 | -3720 | HERVH | ERV1 | LTR |
| 83197710 | 83198857 | + | 10.8 | 3993 | 5146 | -2567 | HERVH | ERV1 | LTR |
| 83198859 | 83199077 | + | 14.6 | 5597 | 5819 | -1894 | HERVH | ERV1 | LTR |
| 83199076 | 83199455 | + | 11.5 | 7322 | 7713 | 0 | HERVH | ERV1 | LTR |
| 83199456 | 83199865 | + | 9.3 | 1 | 448 | -2 | LTR7 | ERV1 | LTR |
| 83199874 | 83201170 | - | 32.1 | 1635 | 3082 | -337 | L2 | L2 | LINE |

  

|  |  |  |  |  |  |  |  |  |  |
| --- | --- | --- | --- | --- | --- | --- | --- | --- | --- |
| chr1:84266185-84272703 | | | 6517 bp | | |  |  |  |  |
| Genome Start | Genome End | Strand | Div | Repeat Start | Repeat End | Repeat Left | Name | Family | Class |
| 84266185 | 84266478 | + | 16.0 | 2163 | 2459 | -3255 | ERVL-B1 | ERVL | LTR |
| 84266479 | 84266527 | - | 6.1 | 32 | 80 | 0 | MADE1 | Mariner | DNA |
| 84266529 | 84266663 | + | 8.9 | 2 | 136 | -176 | AluSq/x | Alu | SINE |
| 84266664 | 84266967 | + | 9.1 | 1 | 311 | 0 | AluSx | Alu | SINE |
| 84266986 | 84267027 | - | 9.5 | 5 | 46 | -34 | MADE1 | Mariner | DNA |
| 84267032 | 84267570 | + | 15.7 | 2458 | 2993 | -2721 | ERVL-B1 | ERVL | LTR |
| 84267571 | 84268004 | + | 6.7 | 1 | 444 | 0 | MLT2A1 | ERVL | LTR |
| 84268005 | 84268951 | + | 15.7 | 2994 | 3970 | -1744 | ERVL-B1 | ERVL | LTR |
| 84268952 | 84269311 | - | 10.9 | 1 | 364 | 0 | THE1B | MaLR | LTR |
| 84269314 | 84270525 | - | 12.9 | 310 | 1571 | -9 | THE1B-int | MaLR | LTR |
| 84270503 | 84270669 | - | 13.8 | 1 | 170 | -1410 | THE1B-int | MaLR | LTR |
| 84270670 | 84271028 | - | 11.5 | 1 | 364 | 0 | THE1B | MaLR | LTR |
| 84271029 | 84272703 | + | 15.2 | 3966 | 5712 | -2 | ERVL-B1 | ERVL | LTR |

  

|  |  |  |  |  |  |  |  |  |  |
| --- | --- | --- | --- | --- | --- | --- | --- | --- | --- |
| chr1:84570007-84577982 | | | 7970 bp | | |  |  |  |  |
| Genome Start | Genome End | Strand | Div | Repeat Start | Repeat End | Repeat Left | Name | Family | Class |
| 84570007 | 84574567 | - | 13.1 | 2025 | 6778 | -7 | L1PA15 | L1 | LINE |
| 84574568 | 84574934 | - | 10.7 | 1 | 364 | 0 | THE1B | MaLR | LTR |
| 84574939 | 84576496 | - | 29.7 | 2 | 1731 | -4 | MLT-int | MaLR | LTR |
| 84576499 | 84576703 | - | 11.0 | 185 | 364 | 0 | THE1B | MaLR | LTR |
| 84576704 | 84577008 | - | 12.1 | 1 | 305 | -7 | AluSx | Alu | SINE |
| 84577009 | 84577177 | - | 11.0 | 1 | 184 | -180 | THE1B | MaLR | LTR |
| 84577178 | 84577982 | - | 17.4 | 1234 | 2024 | -4761 | L1PA15 | L1 | LINE |

  

|  |  |  |  |  |  |  |  |  |  |
| --- | --- | --- | --- | --- | --- | --- | --- | --- | --- |
| chr1:85240845-85253691 | | | 12718 bp | | |  |  |  |  |
| Genome Start | Genome End | Strand | Div | Repeat Start | Repeat End | Repeat Left | Name | Family | Class |
| 85240845 | 85241047 | - | 17.6 | 5085 | 5274 | -872 | L1MC | L1 | LINE |
| 85241062 | 85241702 | + | 9.4 | 3 | 674 | -6189 | L1PBa | L1 | LINE |
| 85241741 | 85244417 | + | 8.5 | 1301 | 4144 | -2718 | L1PBa | L1 | LINE |
| 85244418 | 85244718 | + | 13.0 | 1 | 301 | -11 | AluSx | Alu | SINE |
| 85244719 | 85245264 | + | 8.7 | 4145 | 4480 | -2382 | L1PBa | L1 | LINE |
| 85245265 | 85245617 | - | 14.2 | 1 | 364 | 0 | THE1B | MaLR | LTR |
| 85245618 | 85247182 | - | 30.7 | 2 | 1735 | 0 | MLT-int | MaLR | LTR |
| 85247185 | 85247260 | - | 13.8 | 300 | 364 | 0 | THE1B | MaLR | LTR |
| 85247261 | 85247566 | + | 12.1 | 1 | 303 | -10 | AluSq | Alu | SINE |
| 85247567 | 85247855 | - | 13.8 | 1 | 299 | -65 | THE1B | MaLR | LTR |
| 85247856 | 85249657 | + | 8.5 | 3765 | 5590 | -561 | L1PB1 | L1 | LINE |
| 85249658 | 85249961 | - | 15.6 | 1 | 307 | -5 | AluSx | Alu | SINE |
| 85249962 | 85250518 | + | 7.2 | 5591 | 6145 | -6 | L1PB1 | L1 | LINE |
| 85250593 | 85250905 | + | 7.2 | 1 | 306 | -5 | AluY | Alu | SINE |
| 85250907 | 85251177 | - | 13.9 | 4820 | 5088 | -1058 | L1MC | L1 | LINE |
| 85251178 | 85251477 | - | 16.1 | 1 | 293 | -19 | AluJb | Alu | SINE |
| 85251478 | 85251548 | - | 13.9 | 4762 | 4819 | -1327 | L1MC | L1 | LINE |
| 85251549 | 85251864 | - | 10.3 | 1 | 302 | -11 | AluSq | Alu | SINE |
| 85251865 | 85253691 | - | 13.9 | 2912 | 4761 | -1385 | L1MC | L1 | LINE |

  

|  |  |  |  |  |  |  |  |  |  |
| --- | --- | --- | --- | --- | --- | --- | --- | --- | --- |
| chr1:85486039-85488553 | | | 2512 bp | | |  |  |  |  |
| Genome Start | Genome End | Strand | Div | Repeat Start | Repeat End | Repeat Left | Name | Family | Class |
| 85486039 | 85486233 | - | 16.5 | 111 | 299 | -13 | AluJo | Alu | SINE |
| 85486234 | 85486601 | - | 14.4 | 1 | 364 | 0 | THE1B | MaLR | LTR |
| 85486602 | 85488168 | - | 29.9 | 3 | 1735 | 0 | MLT1C-int | MaLR | LTR |
| 85488172 | 85488529 | - | 11.4 | 1 | 364 | 0 | THE1B | MaLR | LTR |
| 85488530 | 85488553 | - | 16.5 | 87 | 110 | -202 | AluJo | Alu | SINE |

  

|  |  |  |  |  |  |  |  |  |  |
| --- | --- | --- | --- | --- | --- | --- | --- | --- | --- |
| chr1:87159372-87164916 | | | 5513 bp | | |  |  |  |  |
| Genome Start | Genome End | Strand | Div | Repeat Start | Repeat End | Repeat Left | Name | Family | Class |
| 87159372 | 87159712 | - | 22.7 | 2429 | 2795 | -3424 | L1MEc | L1 | LINE |
| 87159713 | 87160082 | + | 21.5 | 1 | 396 | 0 | MSTD | MaLR | LTR |
| 87160083 | 87160690 | - | 22.2 | 1827 | 2480 | -3920 | L1MEc | L1 | LINE |
| 87160721 | 87161493 | - | 22.2 | 992 | 1826 | -4574 | L1MEc | L1 | LINE |
| 87161494 | 87161669 | - | 12.8 | 178 | 364 | 0 | THE1B | MaLR | LTR |
| 87161670 | 87161965 | - | 8.1 | 2 | 297 | -14 | AluY | Alu | SINE |
| 87161966 | 87162134 | - | 12.8 | 1 | 177 | -187 | THE1B | MaLR | LTR |
| 87162135 | 87163682 | - | 29.0 | 2 | 1735 | 0 | MLT-int | MaLR | LTR |
| 87163685 | 87164028 | - | 13.8 | 1 | 364 | 0 | THE1B | MaLR | LTR |
| 87164029 | 87164916 | - | 22.2 | 40 | 991 | -5409 | L1MEc | L1 | LINE |

  

|  |  |  |  |  |  |  |  |  |  |
| --- | --- | --- | --- | --- | --- | --- | --- | --- | --- |
| chr1:87681849-87684458 | | | 2604 bp | | |  |  |  |  |
| Genome Start | Genome End | Strand | Div | Repeat Start | Repeat End | Repeat Left | Name | Family | Class |
| 87681849 | 87681936 | + | 17.1 | 1 | 89 | -301 | MLT1B | MaLR | LTR |
| 87681939 | 87682309 | - | 14.1 | 5 | 428 | 0 | MSTA | MaLR | LTR |
| 87682314 | 87683784 | - | 19.6 | 1 | 1580 | 0 | MSTA-int | MaLR | LTR |
| 87683785 | 87684163 | - | 13.3 | 1 | 428 | 0 | MSTA | MaLR | LTR |
| 87684164 | 87684458 | + | 16.1 | 88 | 379 | -11 | MLT1B | MaLR | LTR |

  

|  |  |  |  |  |  |  |  |  |  |
| --- | --- | --- | --- | --- | --- | --- | --- | --- | --- |
| chr1:87691655-87702765 | | | 10758 bp | | |  |  |  |  |
| Genome Start | Genome End | Strand | Div | Repeat Start | Repeat End | Repeat Left | Name | Family | Class |
| 87691655 | 87691790 | - | 21.5 | 7543 | 7669 | -207 | L1MC4a | L1 | LINE |
| 87691791 | 87692456 | - | 12.8 | 3 | 697 | 0 | MER50B | ERV1 | LTR |
| 87692457 | 87692682 | - | 21.5 | 7323 | 7542 | -334 | L1MC4a | L1 | LINE |
| 87692683 | 87693409 | + | 12.1 | 1 | 727 | 0 | LTR8A | ERV1 | LTR |
| 87693410 | 87693488 | - | 21.5 | 7241 | 7322 | -554 | L1MC4a | L1 | LINE |
| 87693486 | 87695326 | - | 27.6 | 4074 | 4962 | -1252 | L1MC4a | L1 | LINE |
| 87695327 | 87695779 | - | 11.1 | 1 | 472 | 0 | MER4A1 | ERV1 | LTR |
| 87695780 | 87695921 | - | 29.2 | 3943 | 4073 | -2141 | L1MC4a | L1 | LINE |
| 87695934 | 87696043 | + | 25.2 | 1 | 116 | -389 | MLT1D | MaLR | LTR |
| 87696044 | 87696350 | - | 1.0 | 1 | 307 | -3 | AluYa5 | Alu | SINE |
| 87696351 | 87696593 | + | 25.2 | 117 | 390 | -115 | MLT1D | MaLR | LTR |
| 87696594 | 87696874 | - | 19.4 | 1 | 280 | -32 | AluJb | Alu | SINE |
| 87696875 | 87696990 | + | 25.2 | 391 | 505 | 0 | MLT1D | MaLR | LTR |
| 87696991 | 87698333 | + | 26.6 | 1 | 1453 | -36 | MLT1D-int | MaLR | LTR |
| 87698365 | 87698639 | + | 23.5 | 1 | 268 | -237 | MLT1D | MaLR | LTR |
| 87698640 | 87699070 | - | 21.8 | 3 | 457 | 0 | MSTB2 | MaLR | LTR |
| 87699071 | 87699273 | + | 23.5 | 269 | 504 | -1 | MLT1D | MaLR | LTR |
| 87699333 | 87699938 | - | 29.9 | 3270 | 3898 | -2316 | L1MC4a | L1 | LINE |
| 87699947 | 87701016 | - | 22.5 | 847 | 1971 | -4244 | L1MC4a | L1 | LINE |
| 87701102 | 87701307 | - | 21.9 | 512 | 727 | -5488 | L1MC4a | L1 | LINE |
| 87701302 | 87702195 | + | 20.0 | 5366 | 6274 | -38 | L1MA9 | L1 | LINE |
| 87702218 | 87702310 | - | 22.1 | 432 | 517 | -5698 | L1MC4a | L1 | LINE |
| 87702456 | 87702765 | - | 29.2 | 5 | 322 | -5893 | L1MC4a | L1 | LINE |

  

|  |  |  |  |  |  |  |  |  |  |
| --- | --- | --- | --- | --- | --- | --- | --- | --- | --- |
| chr1:90376393-90379524 | | | 3132 bp | | |  |  |  |  |
| Genome Start | Genome End | Strand | Div | Repeat Start | Repeat End | Repeat Left | Name | Family | Class |
| 90376393 | 90376896 | + | 18.3 | 5289 | 5839 | -344 | L1MB3 | L1 | LINE |
| 90376897 | 90377260 | - | 9.9 | 1 | 364 | 0 | THE1B | MaLR | LTR |
| 90377261 | 90378843 | - | 11.5 | 1 | 1580 | 0 | THE1B-int | MaLR | LTR |
| 90378844 | 90379181 | - | 9.9 | 1 | 364 | 0 | THE1B | MaLR | LTR |
| 90379182 | 90379524 | + | 20.8 | 5836 | 6181 | -2 | L1MB3 | L1 | LINE |

  

|  |  |  |  |  |  |  |  |  |  |
| --- | --- | --- | --- | --- | --- | --- | --- | --- | --- |
| chr1:90460636-90478492 | | | 17673 bp | | |  |  |  |  |
| Genome Start | Genome End | Strand | Div | Repeat Start | Repeat End | Repeat Left | Name | Family | Class |
| 90460636 | 90461034 | + | 23.8 | 2529 | 2958 | -461 | L2 | L2 | LINE |
| 90461042 | 90461887 | - | 20.6 | 15 | 862 | -1 | MER21B | ERV1 | LTR |
| 90461933 | 90462220 | + | 30.1 | 2960 | 3268 | -151 | L2 | L2 | LINE |
| 90462261 | 90462541 | - | 12.9 | 27 | 307 | -5 | AluSx | Alu | SINE |
| 90462585 | 90462952 | + | 12.1 | 3 | 364 | 0 | THE1B | MaLR | LTR |
| 90462953 | 90464421 | + | 10.2 | 1 | 1516 | -64 | THE1B-int | MaLR | LTR |
| 90464422 | 90464586 | + | 10.3 | 125 | 289 | -22 | AluY | Alu | SINE |
| 90464587 | 90464665 | + | 7.6 | 1502 | 1580 | 0 | THE1B-int | MaLR | LTR |
| 90464668 | 90465022 | + | 11.8 | 3 | 364 | 0 | THE1B | MaLR | LTR |
| 90465029 | 90466236 | - | 24.9 | 4948 | 6186 | -369 | L1M5 | L1 | LINE |
| 90466237 | 90466628 | - | 17.8 | 1 | 390 | 0 | MLT1B | MaLR | LTR |
| 90466629 | 90467342 | - | 24.9 | 4236 | 4947 | -1590 | L1M5 | L1 | LINE |
| 90467355 | 90470448 | - | 11.0 | 4619 | 7723 | 0 | L1MA2 | L1 | LINE |
| 90470449 | 90470762 | - | 1.3 | 1 | 314 | -4 | AluYb8 | Alu | SINE |
| 90470763 | 90475195 | - | 14.9 | 9 | 4618 | -2947 | L1MA2 | L1 | LINE |
| 90475199 | 90475503 | - | 12.8 | 1 | 305 | -7 | AluSx | Alu | SINE |
| 90475505 | 90476072 | - | 24.9 | 3653 | 4242 | -2295 | L1M5 | L1 | LINE |
| 90476073 | 90476586 | - | 16.9 | 5814 | 6332 | -12 | L1MC2 | L1 | LINE |
| 90476587 | 90476675 | - | 24.9 | 3557 | 3652 | -2885 | L1M5 | L1 | LINE |
| 90476672 | 90478314 | - | 28.8 | 1527 | 3198 | -3339 | L1M5 | L1 | LINE |
| 90478344 | 90478492 | + | 40.5 | 3218 | 3376 | -2 | L2 | L2 | LINE |

  

|  |  |  |  |  |  |  |  |  |  |
| --- | --- | --- | --- | --- | --- | --- | --- | --- | --- |
| chr1:91098591-91105376 | | | 6735 bp | | |  |  |  |  |
| Genome Start | Genome End | Strand | Div | Repeat Start | Repeat End | Repeat Left | Name | Family | Class |
| 91098591 | 91099000 | + | 24.8 | 2205 | 2660 | -3486 | L1M4 | L1 | LINE |
| 91099001 | 91099353 | - | 6.5 | 1 | 355 | 0 | THE1A | MaLR | LTR |
| 91099354 | 91100923 | - | 30.9 | 2 | 1735 | 0 | MLT-int | MaLR | LTR |
| 91100926 | 91101276 | - | 10.3 | 1 | 355 | 0 | THE1A | MaLR | LTR |
| 91101277 | 91101583 | + | 24.8 | 2661 | 3027 | -3119 | L1M4 | L1 | LINE |
| 91101584 | 91101872 | - | 11.4 | 1 | 289 | -24 | AluSq | Alu | SINE |
| 91101873 | 91101936 | + | 24.8 | 3028 | 3087 | -3059 | L1M4 | L1 | LINE |
| 91101937 | 91102252 | + | 13.3 | 3 | 312 | 0 | AluSx | Alu | SINE |
| 91102253 | 91102681 | + | 24.8 | 3088 | 3390 | -2756 | L1M4 | L1 | LINE |
| 91102731 | 91102840 | - | 20.0 | 10 | 122 | -21 | FLAM\_C | Alu | SINE |
| 91102841 | 91102882 | + | 24.8 | 3391 | 3412 | -2734 | L1M4 | L1 | LINE |
| 91102883 | 91103166 | + | 17.0 | 5 | 286 | -26 | AluJo | Alu | SINE |
| 91103167 | 91103371 | + | 24.8 | 3413 | 3614 | -2532 | L1M4 | L1 | LINE |
| 91103372 | 91103669 | + | 13.4 | 1 | 298 | -14 | AluSx | Alu | SINE |
| 91103670 | 91103976 | + | 24.8 | 3615 | 3923 | -2223 | L1M4 | L1 | LINE |
| 91103977 | 91104270 | - | 13.3 | 1 | 295 | -17 | AluSx | Alu | SINE |
| 91104271 | 91104423 | + | 24.8 | 3924 | 4097 | -2049 | L1M4 | L1 | LINE |
| 91104424 | 91104734 | - | 9.7 | 1 | 310 | 0 | AluSg | Alu | SINE |
| 91104735 | 91105376 | + | 24.8 | 4098 | 4786 | -1360 | L1M4 | L1 | LINE |

  

|  |  |  |  |  |  |  |  |  |  |
| --- | --- | --- | --- | --- | --- | --- | --- | --- | --- |
| chr1:93478746-93483321 | | | 4514 bp | | |  |  |  |  |
| Genome Start | Genome End | Strand | Div | Repeat Start | Repeat End | Repeat Left | Name | Family | Class |
| 93478746 | 93479120 | + | 29.5 | 2229 | 2643 | -776 | L2 | L2 | LINE |
| 93479121 | 93479399 | - | 12.2 | 1 | 291 | -22 | AluSq | Alu | SINE |
| 93479400 | 93479586 | + | 29.5 | 2644 | 2819 | -600 | L2 | L2 | LINE |
| 93479587 | 93479891 | + | 13.5 | 1 | 303 | -7 | AluSg | Alu | SINE |
| 93479892 | 93479964 | + | 29.5 | 2820 | 2891 | -528 | L2 | L2 | LINE |
| 93479968 | 93480321 | + | 17.0 | 5417 | 5781 | -393 | L1MB5 | L1 | LINE |
| 93480322 | 93480660 | - | 12.1 | 1 | 364 | 0 | THE1B | MaLR | LTR |
| 93480661 | 93482164 | - | 29.9 | 2 | 1735 | 0 | MLT-int | MaLR | LTR |
| 93482167 | 93482502 | - | 13.1 | 1 | 364 | 0 | THE1B | MaLR | LTR |
| 93482503 | 93482892 | + | 17.0 | 5782 | 6174 | 0 | L1MB5 | L1 | LINE |
| 93482950 | 93483321 | + | 31.2 | 2989 | 3419 | 0 | L2 | L2 | LINE |

  

|  |  |  |  |  |  |  |  |  |  |
| --- | --- | --- | --- | --- | --- | --- | --- | --- | --- |
| chr1:94067942-94075447 | | | 7454 bp | | |  |  |  |  |
| Genome Start | Genome End | Strand | Div | Repeat Start | Repeat End | Repeat Left | Name | Family | Class |
| 94067942 | 94068324 | + | 11.6 | 1 | 388 | -40 | MSTA | MaLR | LTR |
| 94068325 | 94068455 | + | 18.3 | 1 | 133 | 0 | FLAM\_C | Alu | SINE |
| 94068481 | 94068891 | + | 12.1 | 1 | 450 | 0 | LTR7 | ERV1 | LTR |
| 94068892 | 94071172 | + | 14.1 | 1 | 2558 | -5155 | HERVH | ERV1 | LTR |
| 94071173 | 94072247 | - | 12.4 | 1 | 1232 | -4 | MER11B | ERVK | LTR |
| 94072248 | 94072531 | + | 13.1 | 2559 | 2830 | -4883 | HERVH | ERV1 | LTR |
| 94072532 | 94074385 | + | 12.7 | 3357 | 5250 | -1841 | HERVH | ERV1 | LTR |
| 94074404 | 94074824 | + | 9.4 | 7278 | 7713 | 0 | HERVH | ERV1 | LTR |
| 94074825 | 94075232 | + | 10.3 | 1 | 450 | 0 | LTR7 | ERV1 | LTR |
| 94075239 | 94075388 | + | 12.0 | 160 | 310 | -2 | AluSg/x | Alu | SINE |
| 94075392 | 94075447 | + | 10.7 | 373 | 428 | 0 | MSTA | MaLR | LTR |

  

|  |  |  |  |  |  |  |  |  |  |
| --- | --- | --- | --- | --- | --- | --- | --- | --- | --- |
| chr1:94184670-94195267 | | | 10542 bp | | |  |  |  |  |
| Genome Start | Genome End | Strand | Div | Repeat Start | Repeat End | Repeat Left | Name | Family | Class |
| 94184670 | 94184972 | - | 10.2 | 6679 | 6988 | -17 | L1MA4A | L1 | LINE |
| 94184973 | 94185272 | - | 11.9 | 1 | 295 | -15 | AluSg | Alu | SINE |
| 94185273 | 94187190 | - | 13.7 | 4703 | 6678 | -327 | L1MA4A | L1 | LINE |
| 94187191 | 94187557 | - | 13.6 | 1 | 381 | 0 | THE1D | MaLR | LTR |
| 94187558 | 94189167 | - | 29.4 | 2 | 1735 | 0 | MLT-int | MaLR | LTR |
| 94189170 | 94189536 | - | 11.9 | 1 | 381 | 0 | THE1D | MaLR | LTR |
| 94189537 | 94189824 | - | 15.7 | 4407 | 4702 | -2143 | L1MA4A | L1 | LINE |
| 94189841 | 94190913 | - | 9.6 | 1 | 1058 | -13 | MER11C | ERVK | LTR |
| 94190914 | 94191571 | - | 14.7 | 3745 | 4409 | -2436 | L1MA4A | L1 | LINE |
| 94191572 | 94191878 | + | 11.7 | 1 | 307 | -5 | AluSx | Alu | SINE |
| 94191879 | 94192609 | - | 14.7 | 3012 | 3744 | -3101 | L1MA4A | L1 | LINE |
| 94192648 | 94192777 | - | 13.8 | 1 | 135 | -177 | AluJo | Alu | SINE |
| 94192778 | 94193123 | - | 16.7 | 2654 | 2993 | -3852 | L1MA4A | L1 | LINE |
| 94193124 | 94193430 | - | 8.1 | 1 | 295 | -16 | AluY | Alu | SINE |
| 94193431 | 94195267 | - | 16.1 | 827 | 2653 | -4196 | L1MA4A | L1 | LINE |

  

|  |  |  |  |  |  |  |  |  |  |
| --- | --- | --- | --- | --- | --- | --- | --- | --- | --- |
| chr1:94682036-94689612 | | | 7136 bp | | |  |  |  |  |
| Genome Start | Genome End | Strand | Div | Repeat Start | Repeat End | Repeat Left | Name | Family | Class |
| 94682036 | 94682178 | - | 25.4 | 3117 | 3276 | -2853 | L1MEe | L1 | LINE |
| 94682182 | 94682195 | - | 18.1 | 120 | 133 | -10 | FLAM | Alu | SINE |
| 94682196 | 94682496 | + | 11.3 | 1 | 300 | -9 | AluSc | Alu | SINE |
| 94682497 | 94682608 | - | 18.1 | 2 | 119 | -193 | AluJo/FLAM | Alu | SINE |
| 94682657 | 94682803 | - | 19.8 | 2897 | 3020 | -3109 | L1MEe | L1 | LINE |
| 94682964 | 94683442 | + | 12.0 | 34 | 511 | 0 | MER9 | ERVK | LTR |
| 94683443 | 94683850 | + | 9.1 | 1 | 420 | -5496 | HERVK9 | ERVK | LTR |
| 94683839 | 94686804 | + | 7.7 | 927 | 3919 | -1997 | HERVK9 | ERVK | LTR |
| 94686803 | 94688069 | + | 9.6 | 4369 | 5634 | -282 | HERVK9 | ERVK | LTR |
| 94688095 | 94688447 | + | 10.7 | 5635 | 8048 | 0 | HERVK9 | ERVK | LTR |
| 94688448 | 94688933 | + | 7.9 | 1 | 511 | 0 | MER9 | ERVK | LTR |
| 94688934 | 94689128 | - | 23.8 | 1815 | 2014 | -4135 | L1MEe | L1 | LINE |
| 94689348 | 94689612 | - | 36.4 | 541 | 824 | -5295 | L1MEe | L1 | LINE |

  

|  |  |  |  |  |  |  |  |  |  |
| --- | --- | --- | --- | --- | --- | --- | --- | --- | --- |
| chr1:94804199-94820263 | | | 16064 bp | | |  |  |  |  |
| Genome Start | Genome End | Strand | Div | Repeat Start | Repeat End | Repeat Left | Name | Family | Class |
| 94804199 | 94804610 | - | 22.6 | 1981 | 2475 | -4171 | L1M4c | L1 | LINE |
| 94804622 | 94805008 | - | 9.8 | 1 | 440 | -10 | LTR7 | ERV1 | LTR |
| 94805009 | 94805445 | - | 11.1 | 7277 | 7713 | 0 | HERVH | ERV1 | LTR |
| 94805463 | 94806343 | - | 12.7 | 4222 | 5149 | -2564 | HERVH | ERV1 | LTR |
| 94806343 | 94807199 | - | 12.4 | 3492 | 4290 | -3423 | HERVH | ERV1 | LTR |
| 94807190 | 94807340 | - | 12.7 | 3140 | 3290 | -4423 | HERVH | ERV1 | LTR |
| 94807320 | 94808933 | - | 12.7 | 1434 | 2995 | -4718 | HERVH | ERV1 | LTR |
| 94808934 | 94809233 | - | 9.7 | 1 | 300 | -11 | AluY | Alu | SINE |
| 94809234 | 94810525 | - | 12.8 | 1 | 1433 | -6280 | HERVH | ERV1 | LTR |
| 94810528 | 94810970 | - | 8.7 | 1 | 448 | -2 | LTR7 | ERV1 | LTR |
| 94810971 | 94811632 | - | 21.0 | 1307 | 1969 | -4677 | L1M4c | L1 | LINE |
| 94811636 | 94814492 | - | 9.8 | 4703 | 7588 | -21 | L1PA11 | L1 | LINE |
| 94814493 | 94814799 | - | 6.5 | 1 | 308 | -3 | AluY | Alu | SINE |
| 94814800 | 94815227 | - | 9.9 | 4273 | 4702 | -2879 | L1PA11 | L1 | LINE |
| 94815228 | 94815564 | - | 9.0 | 1 | 313 | 0 | AluSp | Alu | SINE |
| 94815565 | 94818989 | - | 17.3 | 3 | 4272 | -3309 | L1PA11 | L1 | LINE |
| 94818990 | 94820263 | - | 20.0 | 6 | 1341 | -5201 | L1M4c | L1 | LINE |

  

|  |  |  |  |  |  |  |  |  |  |
| --- | --- | --- | --- | --- | --- | --- | --- | --- | --- |
| chr1:95641001-95644003 | | | 2996 bp | | |  |  |  |  |
| Genome Start | Genome End | Strand | Div | Repeat Start | Repeat End | Repeat Left | Name | Family | Class |
| 95641001 | 95641083 | - | 21.7 | 6077 | 6162 | 0 | L1ME3 | L1 | LINE |
| 95641088 | 95641463 | + | 14.7 | 1 | 428 | 0 | MSTA | MaLR | LTR |
| 95641466 | 95643100 | + | 30.3 | 2 | 1735 | 0 | MLT-int | MaLR | LTR |
| 95643101 | 95643473 | + | 14.8 | 1 | 423 | -5 | MSTA | MaLR | LTR |
| 95643475 | 95644003 | - | 28.6 | 5514 | 6074 | -88 | L1ME3 | L1 | LINE |

  

|  |  |  |  |  |  |  |  |  |  |
| --- | --- | --- | --- | --- | --- | --- | --- | --- | --- |
| chr1:96086254-96090374 | | | 4033 bp | | |  |  |  |  |
| Genome Start | Genome End | Strand | Div | Repeat Start | Repeat End | Repeat Left | Name | Family | Class |
| 96086254 | 96086353 | + | 26.5 | 54 | 171 | -350 | LTR33 | ERVL | LTR |
| 96086440 | 96086515 | + | 29.9 | 337 | 414 | -107 | LTR33 | ERVL | LTR |
| 96086516 | 96086875 | + | 15.0 | 1 | 364 | 0 | THE1B | MaLR | LTR |
| 96086878 | 96087319 | + | 29.3 | 2 | 556 | -1179 | MLT1H-int | MaLR | LTR |
| 96087320 | 96087622 | + | 11.5 | 1 | 304 | -8 | AluSx | Alu | SINE |
| 96087623 | 96088747 | + | 29.3 | 557 | 1735 | 0 | MLT1H-int | MaLR | LTR |
| 96088748 | 96089102 | + | 14.8 | 1 | 364 | 0 | THE1B | MaLR | LTR |
| 96089103 | 96089135 | + | 29.9 | 415 | 445 | -76 | LTR33 | ERVL | LTR |
| 96089136 | 96090303 | - | 15.3 | 1 | 1231 | 0 | Tigger3b | MER2\_type | DNA |
| 96090304 | 96090374 | + | 29.9 | 446 | 519 | -2 | LTR33 | ERVL | LTR |

  

|  |  |  |  |  |  |  |  |  |  |
| --- | --- | --- | --- | --- | --- | --- | --- | --- | --- |
| chr1:96428020-96436050 | | | 7637 bp | | |  |  |  |  |
| Genome Start | Genome End | Strand | Div | Repeat Start | Repeat End | Repeat Left | Name | Family | Class |
| 96428020 | 96429646 | + | 28.9 | 10 | 1597 | -4622 | L1MEc | L1 | LINE |
| 96429655 | 96430057 | + | 15.0 | 1 | 426 | -2 | MSTA | MaLR | LTR |
| 96430064 | 96431690 | + | 17.6 | 5 | 1651 | 0 | MSTA-int | MaLR | LTR |
| 96431691 | 96432095 | + | 14.4 | 1 | 426 | -2 | MSTA | MaLR | LTR |
| 96432096 | 96432945 | + | 26.9 | 1722 | 2515 | -3704 | L1MEc | L1 | LINE |
| 96432996 | 96433395 | + | 24.5 | 2516 | 2938 | -3281 | L1MEc | L1 | LINE |
| 96433405 | 96433803 | + | 29.2 | 4184 | 4633 | -1586 | L1MEc | L1 | LINE |
| 96434016 | 96434430 | + | 34.2 | 4719 | 5139 | -1080 | L1MEc | L1 | LINE |
| 96434540 | 96435294 | + | 17.3 | 6918 | 7726 | -59 | L1MC3 | L1 | LINE |
| 96435295 | 96435654 | - | 18.4 | 1 | 365 | 0 | MLT1A0 | MaLR | LTR |
| 96435655 | 96435718 | + | 17.3 | 7727 | 7784 | -1 | L1MC3 | L1 | LINE |
| 96435719 | 96436050 | + | 29.4 | 5269 | 5609 | -704 | L1MEc | L1 | LINE |

  

|  |  |  |  |  |  |  |  |  |  |
| --- | --- | --- | --- | --- | --- | --- | --- | --- | --- |
| chr1:97292986-97296403 | | | 3345 bp | | |  |  |  |  |
| Genome Start | Genome End | Strand | Div | Repeat Start | Repeat End | Repeat Left | Name | Family | Class |
| 97292986 | 97293503 | - | 22.5 | 5618 | 6161 | -5 | L1ME1 | L1 | LINE |
| 97293511 | 97293750 | - | 20.7 | 115 | 365 | 0 | MLT1A0 | MaLR | LTR |
| 97293751 | 97294117 | + | 11.2 | 1 | 375 | 0 | THE1C | MaLR | LTR |
| 97294118 | 97295639 | + | 12.3 | 1 | 1579 | -1 | THE1C-int | MaLR | LTR |
| 97295641 | 97296012 | + | 9.1 | 1 | 375 | 0 | THE1C | MaLR | LTR |
| 97296013 | 97296139 | - | 22.1 | 4 | 131 | -243 | MLT1A0 | MaLR | LTR |
| 97296205 | 97296403 | - | 22.8 | 5164 | 5363 | -783 | L1ME1 | L1 | LINE |

  

|  |  |  |  |  |  |  |  |  |  |
| --- | --- | --- | --- | --- | --- | --- | --- | --- | --- |
| chr1:97746844-97749466 | | | 2577 bp | | |  |  |  |  |
| Genome Start | Genome End | Strand | Div | Repeat Start | Repeat End | Repeat Left | Name | Family | Class |
| 97746844 | 97746904 | + | 22.9 | 10 | 72 | -196 | MIRb | MIR | SINE |
| 97746928 | 97747384 | + | 33.8 | 95 | 548 | -1 | MLT1H | MaLR | LTR |
| 97747401 | 97748761 | + | 28.8 | 24 | 1468 | -4 | MLT1H-int | MaLR | LTR |
| 97748768 | 97749322 | + | 31.3 | 8 | 508 | -47 | MLT1H | MaLR | LTR |
| 97749324 | 97749466 | + | 30.1 | 99 | 260 | -8 | MIRb | MIR | SINE |

  

|  |  |  |  |  |  |  |  |  |  |
| --- | --- | --- | --- | --- | --- | --- | --- | --- | --- |
| chr1:98486591-98490919 | | | 4328 bp | | |  |  |  |  |
| Genome Start | Genome End | Strand | Div | Repeat Start | Repeat End | Repeat Left | Name | Family | Class |
| 98486591 | 98487429 | + | 15.3 | 5296 | 6099 | -201 | L1MA6 | L1 | LINE |
| 98487431 | 98487773 | + | 15.4 | 1 | 364 | 0 | THE1B | MaLR | LTR |
| 98487774 | 98488201 | + | 9.6 | 1 | 429 | -1151 | THE1B-int | MaLR | LTR |
| 98488202 | 98489225 | - | 3.3 | 5119 | 6150 | -5 | L1PA4 | L1 | LINE |
| 98489226 | 98490391 | + | 12.5 | 407 | 1580 | 0 | THE1B-int | MaLR | LTR |
| 98490392 | 98490721 | + | 15.1 | 1 | 364 | 0 | THE1B | MaLR | LTR |
| 98490722 | 98490919 | + | 18.2 | 6096 | 6300 | 0 | L1MA6 | L1 | LINE |

  

|  |  |  |  |  |  |  |  |  |  |
| --- | --- | --- | --- | --- | --- | --- | --- | --- | --- |
| chr1:98799979-98802810 | | | 2816 bp | | |  |  |  |  |
| Genome Start | Genome End | Strand | Div | Repeat Start | Repeat End | Repeat Left | Name | Family | Class |
| 98799979 | 98800177 | + | 34.0 | 413 | 618 | -2801 | L2 | L2 | LINE |
| 98800190 | 98800547 | + | 12.9 | 1 | 364 | 0 | THE1B | MaLR | LTR |
| 98800548 | 98800669 | + | 8.2 | 1 | 129 | -1451 | THE1B-int | MaLR | LTR |
| 98800670 | 98802056 | + | 14.3 | 129 | 1651 | 0 | THE1B-int | MaLR | LTR |
| 98802057 | 98802427 | + | 13.8 | 1 | 363 | -1 | THE1B | MaLR | LTR |
| 98802432 | 98802810 | + | 27.5 | 632 | 1031 | -2388 | L2 | L2 | LINE |

  

|  |  |  |  |  |  |  |  |  |  |
| --- | --- | --- | --- | --- | --- | --- | --- | --- | --- |
| chr1:98915609-98920080 | | | 3973 bp | | |  |  |  |  |
| Genome Start | Genome End | Strand | Div | Repeat Start | Repeat End | Repeat Left | Name | Family | Class |
| 98915609 | 98915738 | + | 20.8 | 6459 | 6591 | -1451 | L1MC4 | L1 | LINE |
| 98915742 | 98916042 | + | 8.3 | 1 | 301 | -9 | AluSg | Alu | SINE |
| 98916169 | 98916581 | - | 17.3 | 1 | 421 | -5 | MSTB | MaLR | LTR |
| 98916619 | 98916927 | + | 19.8 | 6765 | 7075 | -967 | L1MC4 | L1 | LINE |
| 98917114 | 98917159 | + | 17.3 | 6 | 46 | -253 | Alu | Alu | SINE |
| 98917182 | 98917544 | - | 13.4 | 1 | 362 | -2 | THE1B | MaLR | LTR |
| 98917546 | 98919128 | - | 30.3 | 2 | 1734 | -1 | MLT1A1-int | MaLR | LTR |
| 98919131 | 98919496 | - | 13.6 | 1 | 364 | 0 | THE1B | MaLR | LTR |
| 98919498 | 98919575 | + | 19.2 | 44 | 121 | -22 | FLAM | Alu | SINE |
| 98919586 | 98919644 | + | 18.6 | 2064 | 2122 | -385 | HAL1 | L1 | LINE |
| 98919756 | 98920080 | + | 25.7 | 7556 | 7884 | -158 | L1MC4 | L1 | LINE |

  

|  |  |  |  |  |  |  |  |  |  |
| --- | --- | --- | --- | --- | --- | --- | --- | --- | --- |
| chr1:99037481-99045126 | | | 7586 bp | | |  |  |  |  |
| Genome Start | Genome End | Strand | Div | Repeat Start | Repeat End | Repeat Left | Name | Family | Class |
| 99037481 | 99038924 | - | 12.5 | 4703 | 6163 | -3 | L1PA16 | L1 | LINE |
| 99038952 | 99039196 | - | 12.2 | 4451 | 4702 | -1444 | L1PA16 | L1 | LINE |
| 99039199 | 99039642 | - | 7.5 | 1 | 448 | -2 | LTR7 | ERV1 | LTR |
| 99039643 | 99040018 | - | 8.9 | 7324 | 7713 | 0 | HERVH | ERV1 | LTR |
| 99040019 | 99040223 | - | 12.2 | 5614 | 5819 | -1894 | HERVH | ERV1 | LTR |
| 99040272 | 99040882 | - | 12.4 | 4488 | 5146 | -2567 | HERVH | ERV1 | LTR |
| 99040885 | 99041730 | - | 11.3 | 3140 | 3993 | -3720 | HERVH | ERV1 | LTR |
| 99041710 | 99044556 | - | 10.6 | 1 | 2995 | -4718 | HERVH | ERV1 | LTR |
| 99044559 | 99045002 | - | 7.3 | 1 | 448 | -2 | LTR7 | ERV1 | LTR |
| 99045003 | 99045126 | - | 13.7 | 4324 | 4455 | -1691 | L1PA16 | L1 | LINE |

  

|  |  |  |  |  |  |  |  |  |  |
| --- | --- | --- | --- | --- | --- | --- | --- | --- | --- |
| chr1:99045123-99049781 | | | 4618 bp | | |  |  |  |  |
| Genome Start | Genome End | Strand | Div | Repeat Start | Repeat End | Repeat Left | Name | Family | Class |
| 99045123 | 99046451 | + | 12.9 | 2723 | 4009 | -2840 | L1PA15-16 | L1 | LINE |
| 99046456 | 99046823 | - | 10.1 | 3 | 370 | -5 | THE1C | MaLR | LTR |
| 99046827 | 99048363 | - | 10.8 | 10 | 1579 | -1 | THE1C-int | MaLR | LTR |
| 99048398 | 99048755 | - | 11.7 | 3 | 375 | 0 | THE1C | MaLR | LTR |
| 99048756 | 99049781 | + | 11.3 | 4003 | 5016 | -1833 | L1PA15-16 | L1 | LINE |

  

|  |  |  |  |  |  |  |  |  |  |
| --- | --- | --- | --- | --- | --- | --- | --- | --- | --- |
| chr1:99100414-99106299 | | | 5803 bp | | |  |  |  |  |
| Genome Start | Genome End | Strand | Div | Repeat Start | Repeat End | Repeat Left | Name | Family | Class |
| 99100414 | 99101228 | - | 26.9 | 5320 | 6116 | -50 | L1ME1 | L1 | LINE |
| 99101229 | 99101348 | - | 20.8 | 36 | 155 | -21 | FRAM | Alu | SINE |
| 99101390 | 99103120 | - | 16.6 | 4397 | 6155 | -1 | L1PB4 | L1 | LINE |
| 99103147 | 99103642 | - | 18.9 | 3892 | 4396 | -1750 | L1P4 | L1 | LINE |
| 99103646 | 99104005 | - | 14.1 | 1 | 364 | 0 | THE1B | MaLR | LTR |
| 99104006 | 99105567 | - | 12.1 | 14 | 1580 | 0 | THE1B-int | MaLR | LTR |
| 99105569 | 99105927 | - | 13.2 | 1 | 362 | -2 | THE1B | MaLR | LTR |
| 99105933 | 99105993 | - | 19.7 | 3826 | 3887 | -2259 | L1PREC2 | L1 | LINE |
| 99106000 | 99106226 | + | 12.8 | 2169 | 2406 | 0 | Tigger5 | MER2\_type | DNA |
| 99106228 | 99106299 | - | 26.4 | 5169 | 5240 | -906 | L1ME1 | L1 | LINE |

  

|  |  |  |  |  |  |  |  |  |  |
| --- | --- | --- | --- | --- | --- | --- | --- | --- | --- |
| chr1:99270816-99276294 | | | 5409 bp | | |  |  |  |  |
| Genome Start | Genome End | Strand | Div | Repeat Start | Repeat End | Repeat Left | Name | Family | Class |
| 99270816 | 99271175 | + | 30.5 | 348 | 745 | -5210 | L1MA9 | L1 | LINE |
| 99271236 | 99271854 | + | 22.5 | 712 | 1356 | -4609 | L1MA9 | L1 | LINE |
| 99271853 | 99272459 | + | 15.4 | 3632 | 4246 | -1782 | L1MA9 | L1 | LINE |
| 99272470 | 99273857 | + | 16.0 | 4586 | 6018 | -182 | L1MA9 | L1 | LINE |
| 99273858 | 99274220 | - | 16.1 | 1 | 364 | 0 | THE1B | MaLR | LTR |
| 99274221 | 99275778 | - | 29.9 | 2 | 1735 | 0 | MLT-int | MaLR | LTR |
| 99275781 | 99276143 | - | 13.8 | 1 | 364 | 0 | THE1B | MaLR | LTR |
| 99276144 | 99276294 | + | 15.6 | 6019 | 6200 | 0 | L1MA9 | L1 | LINE |

  

|  |  |  |  |  |  |  |  |  |  |
| --- | --- | --- | --- | --- | --- | --- | --- | --- | --- |
| chr1:99325119-99328767 | | | 3481 bp | | |  |  |  |  |
| Genome Start | Genome End | Strand | Div | Repeat Start | Repeat End | Repeat Left | Name | Family | Class |
| 99325119 | 99325551 | - | 31.0 | 2934 | 3386 | -33 | L2 | L2 | LINE |
| 99325560 | 99325948 | + | 13.7 | 1 | 428 | 0 | MSTA | MaLR | LTR |
| 99325949 | 99326055 | + | 16.0 | 3 | 108 | -35 | FLAM\_C | Alu | SINE |
| 99326216 | 99326390 | - | 31.4 | 2750 | 2970 | -449 | L2 | L2 | LINE |
| 99326391 | 99326732 | + | 13.7 | 1 | 362 | -2 | THE1B | MaLR | LTR |
| 99326733 | 99328314 | + | 30.8 | 2 | 1735 | 0 | MLT1B-int | MaLR | LTR |
| 99328315 | 99328667 | + | 14.3 | 1 | 364 | 0 | THE1B | MaLR | LTR |
| 99328668 | 99328767 | - | 31.4 | 2636 | 2749 | -670 | L2 | L2 | LINE |

  

|  |  |  |  |  |  |  |  |  |  |
| --- | --- | --- | --- | --- | --- | --- | --- | --- | --- |
| chr1:101016927-101020637 | | | 3709 bp | | |  |  |  |  |
| Genome Start | Genome End | Strand | Div | Repeat Start | Repeat End | Repeat Left | Name | Family | Class |
| 101016927 | 101017826 | + | 30.4 | 1740 | 2813 | -606 | L2 | L2 | LINE |
| 101017827 | 101018169 | - | 13.0 | 1 | 364 | 0 | THE1B | MaLR | LTR |
| 101018170 | 101019733 | - | 29.0 | 2 | 1735 | 0 | MLT1I-int | MaLR | LTR |
| 101019736 | 101020079 | - | 11.8 | 1 | 364 | 0 | THE1B | MaLR | LTR |
| 101020080 | 101020637 | + | 29.5 | 2814 | 3412 | -7 | L2 | L2 | LINE |

  

|  |  |  |  |  |  |  |  |  |  |
| --- | --- | --- | --- | --- | --- | --- | --- | --- | --- |
| chr1:101332563-101337558 | | | 4985 bp | | |  |  |  |  |
| Genome Start | Genome End | Strand | Div | Repeat Start | Repeat End | Repeat Left | Name | Family | Class |
| 101332563 | 101332916 | - | 15.7 | 1271 | 1632 | -4876 | L1MCa | L1 | LINE |
| 101332921 | 101333286 | - | 22.6 | 1 | 364 | -1 | MLT1A0 | MaLR | LTR |
| 101333287 | 101334941 | - | 23.6 | 2 | 1735 | 0 | MLT1A0-int | MaLR | LTR |
| 101334943 | 101335304 | - | 21.4 | 1 | 365 | 0 | MLT1A0 | MaLR | LTR |
| 101335310 | 101335668 | - | 13.8 | 922 | 1265 | -5243 | L1MCa | L1 | LINE |
| 101335669 | 101335849 | - | 7.8 | 5975 | 6154 | 0 | L1PA7 | L1 | LINE |
| 101335850 | 101335989 | - | 13.8 | 809 | 921 | -5587 | L1MCa | L1 | LINE |
| 101335991 | 101336042 | + | 22.4 | 1 | 50 | -346 | MSTD | MaLR | LTR |
| 101336043 | 101336446 | + | 16.4 | 1 | 457 | 0 | MSTB2 | MaLR | LTR |
| 101336447 | 101336772 | + | 22.4 | 51 | 396 | 0 | MSTD | MaLR | LTR |
| 101336773 | 101337558 | - | 17.1 | 1 | 812 | -5696 | L1MCa | L1 | LINE |

  

|  |  |  |  |  |  |  |  |  |  |
| --- | --- | --- | --- | --- | --- | --- | --- | --- | --- |
| chr1:102830174-102834157 | | | 3540 bp | | |  |  |  |  |
| Genome Start | Genome End | Strand | Div | Repeat Start | Repeat End | Repeat Left | Name | Family | Class |
| 102830174 | 102830331 | + | 27.6 | 373 | 542 | -346 | Kanga2\_a | Tc2 | DNA |
| 102830369 | 102830663 | + | 36.3 | 2250 | 2557 | -862 | L2 | L2 | LINE |
| 102830926 | 102831373 | + | 25.5 | 3 | 467 | 0 | MLT1C | MaLR | LTR |
| 102831376 | 102832084 | + | 28.8 | 353 | 1103 | -268 | MLT1C-int | MaLR | LTR |
| 102832085 | 102832426 | + | 17.0 | 1 | 365 | 0 | MLT1A0 | MaLR | LTR |
| 102832427 | 102832701 | + | 26.2 | 1100 | 1342 | -29 | MLT1C-int | MaLR | LTR |
| 102832779 | 102833237 | + | 22.9 | 2 | 467 | 0 | MLT1C | MaLR | LTR |
| 102833239 | 102833611 | + | 24.4 | 70 | 467 | 0 | MLT1C | MaLR | LTR |
| 102833612 | 102834012 | + | 34.8 | 2925 | 3341 | -78 | L2 | L2 | LINE |
| 102834078 | 102834157 | + | 22.5 | 545 | 625 | -263 | Kanga2\_a | Tc2 | DNA |

  

|  |  |  |  |  |  |  |  |  |  |
| --- | --- | --- | --- | --- | --- | --- | --- | --- | --- |
| chr1:103459493-103464722 | | | 5170 bp | | |  |  |  |  |
| Genome Start | Genome End | Strand | Div | Repeat Start | Repeat End | Repeat Left | Name | Family | Class |
| 103459493 | 103461393 | - | 27.1 | 3599 | 5698 | -464 | L1M5 | L1 | LINE |
| 103461419 | 103461739 | - | 27.7 | 3268 | 3598 | -2548 | L1M5 | L1 | LINE |
| 103461772 | 103462203 | + | 16.2 | 1 | 432 | 0 | MSTB1 | MaLR | LTR |
| 103462204 | 103463772 | + | 29.4 | 3 | 1735 | 0 | MLT-int | MaLR | LTR |
| 103463773 | 103464207 | + | 17.3 | 1 | 430 | -2 | MSTB1 | MaLR | LTR |
| 103464211 | 103464722 | - | 31.3 | 2264 | 2814 | -3332 | L1M5 | L1 | LINE |

  

|  |  |  |  |  |  |  |  |  |  |
| --- | --- | --- | --- | --- | --- | --- | --- | --- | --- |
| chr1:104959710-104962347 | | | 2636 bp | | |  |  |  |  |
| Genome Start | Genome End | Strand | Div | Repeat Start | Repeat End | Repeat Left | Name | Family | Class |
| 104959710 | 104959902 | - | 19.0 | 208 | 428 | 0 | MSTA | MaLR | LTR |
| 104959903 | 104960256 | - | 13.6 | 1 | 364 | 0 | THE1B | MaLR | LTR |
| 104960257 | 104961815 | - | 30.8 | 2 | 1735 | 0 | MLT-int | MaLR | LTR |
| 104961818 | 104962166 | - | 15.5 | 2 | 364 | 0 | THE1B | MaLR | LTR |
| 104962167 | 104962347 | - | 19.0 | 1 | 207 | -221 | MSTA | MaLR | LTR |

  

|  |  |  |  |  |  |  |  |  |  |
| --- | --- | --- | --- | --- | --- | --- | --- | --- | --- |
| chr1:106122117-106126968 | | | 4850 bp | | |  |  |  |  |
| Genome Start | Genome End | Strand | Div | Repeat Start | Repeat End | Repeat Left | Name | Family | Class |
| 106122117 | 106122555 | - | 31.7 | 1703 | 2182 | -325 | HAL1 | L1 | LINE |
| 106122556 | 106122931 | + | 14.2 | 1 | 375 | 0 | THE1C | MaLR | LTR |
| 106122934 | 106124523 | + | 29.9 | 2 | 1735 | 0 | MLT-int | MaLR | LTR |
| 106124524 | 106124885 | + | 13.0 | 1 | 374 | -1 | THE1C | MaLR | LTR |
| 106124886 | 106126404 | - | 30.7 | 136 | 1702 | -805 | HAL1 | L1 | LINE |
| 106126405 | 106126899 | - | 15.3 | 5829 | 6295 | -10 | L1MA3 | L1 | LINE |
| 106126900 | 106126968 | - | 30.7 | 61 | 135 | -2372 | HAL1 | L1 | LINE |

  

|  |  |  |  |  |  |  |  |  |  |
| --- | --- | --- | --- | --- | --- | --- | --- | --- | --- |
| chr1:106492676-106495883 | | | 3084 bp | | |  |  |  |  |
| Genome Start | Genome End | Strand | Div | Repeat Start | Repeat End | Repeat Left | Name | Family | Class |
| 106492676 | 106492750 | + | 26.7 | 6 | 81 | -424 | MLT1D | MaLR | LTR |
| 106492753 | 106492930 | - | 24.0 | 283 | 463 | -4 | MLT1C | MaLR | LTR |
| 106492940 | 106493301 | + | 13.8 | 1 | 364 | 0 | THE1B | MaLR | LTR |
| 106493302 | 106494894 | + | 12.8 | 1 | 1574 | -6 | THE1B-int | MaLR | LTR |
| 106494900 | 106495262 | + | 11.3 | 1 | 364 | 0 | THE1B | MaLR | LTR |
| 106495322 | 106495429 | - | 26.2 | 66 | 172 | -295 | MLT1C | MaLR | LTR |
| 106495479 | 106495883 | + | 23.1 | 66 | 505 | 0 | MLT1D | MaLR | LTR |

  

|  |  |  |  |  |  |  |  |  |  |
| --- | --- | --- | --- | --- | --- | --- | --- | --- | --- |
| chr1:106645758-106650286 | | | 4501 bp | | |  |  |  |  |
| Genome Start | Genome End | Strand | Div | Repeat Start | Repeat End | Repeat Left | Name | Family | Class |
| 106645758 | 106646943 | - | 20.9 | 4970 | 6158 | -186 | L1MC2 | L1 | LINE |
| 106646944 | 106647272 | - | 15.5 | 2 | 428 | 0 | MSTC | MaLR | LTR |
| 106647274 | 106648771 | - | 29.6 | 43 | 1651 | 0 | MSTC-int | MaLR | LTR |
| 106648799 | 106649231 | - | 18.2 | 1 | 428 | 0 | MSTC | MaLR | LTR |
| 106649232 | 106649360 | - | 19.7 | 4849 | 4974 | -1172 | L1MC2 | L1 | LINE |
| 106649361 | 106649778 | - | 21.1 | 1 | 422 | -4 | MSTB | MaLR | LTR |
| 106649779 | 106650286 | - | 19.7 | 4321 | 4848 | -1298 | L1MC2 | L1 | LINE |

  

|  |  |  |  |  |  |  |  |  |  |
| --- | --- | --- | --- | --- | --- | --- | --- | --- | --- |
| chr1:106811218-106827589 | | | 16181 bp | | |  |  |  |  |
| Genome Start | Genome End | Strand | Div | Repeat Start | Repeat End | Repeat Left | Name | Family | Class |
| 106811218 | 106811295 | + | 17.9 | 561 | 640 | -6569 | L1MA4A | L1 | LINE |
| 106811375 | 106812990 | + | 24.9 | 942 | 2521 | -4688 | L1MA4A | L1 | LINE |
| 106812983 | 106816158 | + | 21.9 | 3436 | 6841 | -524 | L1MA4A | L1 | LINE |
| 106816159 | 106816451 | + | 12.0 | 1 | 293 | -16 | AluSc | Alu | SINE |
| 106816471 | 106820387 | + | 20.7 | 3 | 3014 | -3848 | L1PB2 | L1 | LINE |
| 106820388 | 106820696 | + | 14.1 | 1 | 278 | -34 | AluSx | Alu | SINE |
| 106820769 | 106822471 | + | 16.8 | 3015 | 4723 | -2139 | L1PB2 | L1 | LINE |
| 106822472 | 106822831 | + | 17.2 | 1 | 364 | 0 | THE1B | MaLR | LTR |
| 106822832 | 106824399 | + | 11.3 | 1 | 1580 | 0 | THE1B-int | MaLR | LTR |
| 106824400 | 106824757 | + | 13.3 | 1 | 364 | 0 | THE1B | MaLR | LTR |
| 106824758 | 106826874 | + | 14.8 | 4719 | 6864 | -3 | L1PB2 | L1 | LINE |
| 106826882 | 106826999 | + | 18.6 | 6813 | 6931 | -434 | L1MA4A | L1 | LINE |
| 106827000 | 106827079 | - | 25.3 | 222 | 300 | 0 | AluJb | Alu | SINE |
| 106827095 | 106827149 | - | 9.3 | 85 | 138 | -164 | AluJb | Alu | SINE |
| 106827157 | 106827589 | + | 14.7 | 6928 | 7361 | -4 | L1MA4A | L1 | LINE |

  

|  |  |  |  |  |  |  |  |  |  |
| --- | --- | --- | --- | --- | --- | --- | --- | --- | --- |
| chr1:107002723-107013603 | | | 10859 bp | | |  |  |  |  |
| Genome Start | Genome End | Strand | Div | Repeat Start | Repeat End | Repeat Left | Name | Family | Class |
| 107002723 | 107005504 | + | 18.2 | 36 | 2886 | -4679 | L1MA1 | L1 | LINE |
| 107005505 | 107005902 | + | 17.7 | 1 | 428 | 0 | MSTA | MaLR | LTR |
| 107005905 | 107007520 | + | 30.2 | 2 | 1735 | 0 | MLT-int | MaLR | LTR |
| 107007521 | 107007895 | + | 18.1 | 1 | 428 | 0 | MSTA | MaLR | LTR |
| 107007896 | 107008748 | + | 16.6 | 2887 | 3735 | -3830 | L1MA1 | L1 | LINE |
| 107008749 | 107009040 | + | 14.8 | 1 | 296 | -16 | AluSx | Alu | SINE |
| 107008993 | 107009067 | + | 10.4 | 233 | 311 | 0 | Alu | Alu | SINE |
| 107009068 | 107009470 | + | 11.9 | 3734 | 4130 | -3435 | L1MA1 | L1 | LINE |
| 107009505 | 107009647 | - | 16.1 | 137 | 283 | -29 | AluSg/x | Alu | SINE |
| 107009648 | 107010201 | + | 11.5 | 4131 | 4690 | -2875 | L1MA1 | L1 | LINE |
| 107010236 | 107011678 | + | 11.5 | 4691 | 6145 | -1420 | L1MA1 | L1 | LINE |
| 107011679 | 107012040 | - | 16.0 | 1 | 381 | 0 | THE1D | MaLR | LTR |
| 107012041 | 107013603 | + | 11.0 | 6146 | 7714 | -7 | L1MA1 | L1 | LINE |

  

|  |  |  |  |  |  |  |  |  |  |
| --- | --- | --- | --- | --- | --- | --- | --- | --- | --- |
| chr1:107382559-107385426 | | | 2812 bp | | |  |  |  |  |
| Genome Start | Genome End | Strand | Div | Repeat Start | Repeat End | Repeat Left | Name | Family | Class |
| 107382559 | 107382781 | + | 26.4 | 125 | 373 | -2134 | HAL1 | L1 | LINE |
| 107382810 | 107383182 | + | 14.1 | 1 | 362 | -2 | THE1B | MaLR | LTR |
| 107383187 | 107384758 | + | 30.2 | 2 | 1731 | -4 | MLT-int | MaLR | LTR |
| 107384762 | 107385091 | + | 14.1 | 1 | 321 | -43 | THE1B | MaLR | LTR |
| 107385113 | 107385426 | + | 27.4 | 549 | 1063 | -1444 | HAL1 | L1 | LINE |

  

|  |  |  |  |  |  |  |  |  |  |
| --- | --- | --- | --- | --- | --- | --- | --- | --- | --- |
| chr1:107693552-107698310 | | | 4750 bp | | |  |  |  |  |
| Genome Start | Genome End | Strand | Div | Repeat Start | Repeat End | Repeat Left | Name | Family | Class |
| 107693552 | 107694575 | - | 29.1 | 5100 | 6208 | -32 | L1ME3B | L1 | LINE |
| 107694576 | 107695295 | + | 15.0 | 5587 | 6312 | 0 | L1MA9 | L1 | LINE |
| 107695296 | 107695415 | - | 29.0 | 4973 | 5099 | -1047 | L1ME3B | L1 | LINE |
| 107695417 | 107695797 | - | 15.8 | 1 | 428 | 0 | MSTA | MaLR | LTR |
| 107695798 | 107697844 | - | 17.5 | 3 | 1580 | 0 | MSTA-int | MaLR | LTR |
| 107697846 | 107698235 | - | 16.4 | 1 | 428 | 0 | MSTA | MaLR | LTR |
| 107698243 | 107698310 | - | 20.6 | 4900 | 4969 | -1177 | L1ME3B | L1 | LINE |

  

|  |  |  |  |  |  |  |  |  |  |
| --- | --- | --- | --- | --- | --- | --- | --- | --- | --- |
| chr1:107851146-107857262 | | | 6061 bp | | |  |  |  |  |
| Genome Start | Genome End | Strand | Div | Repeat Start | Repeat End | Repeat Left | Name | Family | Class |
| 107851146 | 107851296 | - | 18.4 | 3700 | 3858 | -82 | Arthur1 | Tip100 | DNA |
| 107851299 | 107853063 | + | 13.4 | 3 | 1791 | -627 | Tigger1 | MER2\_type | DNA |
| 107853064 | 107853351 | + | 11.8 | 6009 | 6297 | -6 | L1MA4A | L1 | LINE |
| 107853352 | 107853636 | + | 13.4 | 1792 | 2073 | -345 | Tigger1 | MER2\_type | DNA |
| 107853637 | 107854010 | + | 9.0 | 1 | 375 | 0 | THE1C | MaLR | LTR |
| 107854011 | 107855593 | + | 12.4 | 1 | 1563 | -17 | THE1C-int | MaLR | LTR |
| 107855644 | 107856029 | + | 14.1 | 1 | 373 | -2 | THE1C | MaLR | LTR |
| 107856033 | 107856380 | + | 15.5 | 2070 | 2417 | -1 | Tigger1 | MER2\_type | DNA |
| 107856381 | 107856622 | - | 24.6 | 3453 | 3702 | -238 | Arthur1 | Tip100 | DNA |
| 107856624 | 107857262 | - | 29.1 | 3 | 640 | -586 | Arthur1 | Tip100 | DNA |

  

|  |  |  |  |  |  |  |  |  |  |
| --- | --- | --- | --- | --- | --- | --- | --- | --- | --- |
| chr1:108517240-108519864 | | | 2615 bp | | |  |  |  |  |
| Genome Start | Genome End | Strand | Div | Repeat Start | Repeat End | Repeat Left | Name | Family | Class |
| 108517240 | 108517322 | + | 12.1 | 11 | 93 | -219 | AluJb | Alu | SINE |
| 108517324 | 108517696 | + | 10.8 | 1 | 375 | 0 | THE1C | MaLR | LTR |
| 108517697 | 108519265 | + | 12.1 | 1 | 1574 | -6 | THE1C-int | MaLR | LTR |
| 108519272 | 108519646 | + | 11.1 | 1 | 375 | 0 | THE1C | MaLR | LTR |
| 108519650 | 108519864 | + | 16.8 | 93 | 312 | 0 | AluJb | Alu | SINE |

  

|  |  |  |  |  |  |  |  |  |  |
| --- | --- | --- | --- | --- | --- | --- | --- | --- | --- |
| chr1:108629696-108638004 | | | 7868 bp | | |  |  |  |  |
| Genome Start | Genome End | Strand | Div | Repeat Start | Repeat End | Repeat Left | Name | Family | Class |
| 108629696 | 108630590 | - | 32.7 | 4696 | 5603 | -637 | L1M5 | L1 | LINE |
| 108630621 | 108631868 | - | 7.3 | 4892 | 6170 | 0 | L1PA7 | L1 | LINE |
| 108631867 | 108633682 | + | 8.5 | 3079 | 4896 | -1250 | L1PA7 | L1 | LINE |
| 108633825 | 108634179 | - | 21.1 | 1 | 358 | -7 | MLT1A0 | MaLR | LTR |
| 108634181 | 108635641 | - | 24.4 | 5 | 1370 | -1 | MLT1A0-int | MaLR | LTR |
| 108635646 | 108635974 | - | 23.7 | 1 | 374 | 0 | MLT1A0 | MaLR | LTR |
| 108635975 | 108636569 | - | 33.0 | 3979 | 4589 | -1557 | L1M5 | L1 | LINE |
| 108636588 | 108636795 | - | 23.9 | 5956 | 6165 | -13 | L1MB8 | L1 | LINE |
| 108636883 | 108637038 | + | 22.6 | 2 | 161 | -101 | MIR | MIR | SINE |
| 108637042 | 108637100 | + | 15.2 | 5 | 63 | -17 | MADE2 | Mariner | DNA |
| 108637092 | 108637181 | + | 17.8 | 264 | 354 | -74 | MSTC | MaLR | LTR |
| 108637154 | 108637209 | + | 18.9 | 455 | 514 | -40 | MLT2B3 | ERVL | LTR |
| 108637283 | 108637321 | + | 18.9 | 515 | 553 | -1 | MLT2B3 | ERVL | LTR |
| 108637444 | 108638004 | - | 31.6 | 2405 | 2966 | -3180 | L1M5 | L1 | LINE |

  

|  |  |  |  |  |  |  |  |  |  |
| --- | --- | --- | --- | --- | --- | --- | --- | --- | --- |
| chr1:109127377-109137587 | | | 9857 bp | | |  |  |  |  |
| Genome Start | Genome End | Strand | Div | Repeat Start | Repeat End | Repeat Left | Name | Family | Class |
| 109127377 | 109128379 | - | 21.5 | 5068 | 6233 | -2 | L1ME3 | L1 | LINE |
| 109128380 | 109128722 | - | 13.2 | 1 | 375 | 0 | THE1C | MaLR | LTR |
| 109128723 | 109130232 | - | 30.6 | 5 | 1735 | 0 | MLT-int | MaLR | LTR |
| 109130236 | 109130587 | - | 14.1 | 1 | 375 | 0 | THE1C | MaLR | LTR |
| 109130588 | 109131295 | - | 22.7 | 4317 | 5067 | -1152 | L1ME3 | L1 | LINE |
| 109131296 | 109131413 | - | 22.9 | 1 | 129 | -4 | FLAM\_A | Alu | SINE |
| 109131414 | 109131487 | - | 22.7 | 4249 | 4316 | -1903 | L1ME3 | L1 | LINE |
| 109131488 | 109131679 | + | 5.8 | 1 | 197 | 0 | MER107 | MER1\_type | DNA |
| 109131680 | 109131920 | - | 22.7 | 4025 | 4248 | -1971 | L1ME3 | L1 | LINE |
| 109131921 | 109132236 | - | 16.4 | 1 | 312 | 0 | AluJb | Alu | SINE |
| 109132237 | 109132926 | - | 22.7 | 3309 | 4024 | -2195 | L1ME3 | L1 | LINE |
| 109132927 | 109133226 | + | 11.4 | 3 | 300 | -12 | AluSx | Alu | SINE |
| 109133227 | 109133362 | - | 22.7 | 3172 | 3308 | -2911 | L1ME3 | L1 | LINE |
| 109133363 | 109133577 | + | 9.3 | 2 | 230 | 0 | MER30 | MER1\_type | DNA |
| 109133578 | 109133928 | - | 22.7 | 2810 | 3171 | -3048 | L1ME3 | L1 | LINE |
| 109133929 | 109134155 | + | 14.1 | 1 | 301 | -1 | AluSx | Alu | SINE |
| 109134156 | 109135188 | - | 24.7 | 1756 | 2809 | -3410 | L1ME3 | L1 | LINE |
| 109135197 | 109135789 | - | 32.4 | 1362 | 1972 | -4661 | L1MDa | L1 | LINE |
| 109135805 | 109136244 | - | 18.9 | 5688 | 6156 | -10 | L1ME1 | L1 | LINE |
| 109136305 | 109136505 | - | 28.3 | 813 | 1018 | -5191 | L1ME3 | L1 | LINE |
| 109136521 | 109136752 | - | 14.3 | 6056 | 6295 | -5 | L1MA4 | L1 | LINE |
| 109136757 | 109136945 | - | 26.9 | 701 | 903 | -5497 | L1ME3 | L1 | LINE |
| 109137195 | 109137587 | - | 40.4 | 31 | 417 | -5983 | L1ME3 | L1 | LINE |

  

|  |  |  |  |  |  |  |  |  |  |
| --- | --- | --- | --- | --- | --- | --- | --- | --- | --- |
| chr1:110046237-110053861 | | | 7612 bp | | |  |  |  |  |
| Genome Start | Genome End | Strand | Div | Repeat Start | Repeat End | Repeat Left | Name | Family | Class |
| 110046237 | 110046759 | + | 6.0 | 1 | 521 | -70 | LTR10C | ERV1 | LTR |
| 110046760 | 110047790 | + | 5.3 | 1 | 1266 | 0 | MER11A | ERVK | LTR |
| 110047791 | 110050838 | + | 6.9 | 1 | 3033 | -4920 | HERVK11 | ERVK | LTR |
| 110050849 | 110052667 | + | 5.0 | 5331 | 7203 | -750 | HERVK11 | ERVK | LTR |
| 110052671 | 110052765 | + | 5.3 | 7859 | 7953 | 0 | HERVK11 | ERVK | LTR |
| 110052766 | 110053788 | + | 5.4 | 1 | 1266 | 0 | MER11A | ERVK | LTR |
| 110053789 | 110053861 | + | 7.9 | 519 | 591 | 0 | LTR10C | ERV1 | LTR |

  

|  |  |  |  |  |  |  |  |  |  |
| --- | --- | --- | --- | --- | --- | --- | --- | --- | --- |
| chr1:113163813-113170263 | | | 6432 bp | | |  |  |  |  |
| Genome Start | Genome End | Strand | Div | Repeat Start | Repeat End | Repeat Left | Name | Family | Class |
| 113163813 | 113163905 | - | 14.2 | 7887 | 7984 | -11 | L1MD3 | L1 | LINE |
| 113163906 | 113164215 | - | 5.5 | 1 | 307 | -4 | AluY | Alu | SINE |
| 113164216 | 113164278 | - | 14.2 | 7828 | 7886 | -109 | L1MD3 | L1 | LINE |
| 113164290 | 113164676 | - | 10.0 | 1 | 438 | -12 | LTR7 | ERV1 | LTR |
| 113164677 | 113165071 | - | 8.2 | 7324 | 7713 | 0 | HERVH | ERV1 | LTR |
| 113165072 | 113165286 | - | 12.1 | 5600 | 5819 | -1894 | HERVH | ERV1 | LTR |
| 113165288 | 113165474 | - | 18.2 | 4959 | 5149 | -2564 | HERVH | ERV1 | LTR |
| 113165474 | 113166413 | - | 11.9 | 3995 | 5522 | -2802 | HERVH | ERV1 | LTR |
| 113166409 | 113166774 | - | 13.4 | 3357 | 3735 | -3978 | HERVH | ERV1 | LTR |
| 113166775 | 113167377 | - | 11.2 | 2251 | 2830 | -4883 | HERVH | ERV1 | LTR |
| 113167378 | 113167686 | + | 5.5 | 1 | 309 | -2 | AluY | Alu | SINE |
| 113167687 | 113169803 | - | 12.1 | 1 | 2250 | -5463 | HERVH | ERV1 | LTR |
| 113169806 | 113170199 | - | 9.9 | 1 | 448 | -2 | LTR7 | ERV1 | LTR |
| 113170211 | 113170263 | - | 17.3 | 7744 | 7796 | -199 | L1MD3 | L1 | LINE |

  

|  |  |  |  |  |  |  |  |  |  |
| --- | --- | --- | --- | --- | --- | --- | --- | --- | --- |
| chr1:113866824-113869583 | | | 2758 bp | | |  |  |  |  |
| Genome Start | Genome End | Strand | Div | Repeat Start | Repeat End | Repeat Left | Name | Family | Class |
| 113866824 | 113867075 | - | 26.7 | 7733 | 8024 | -18 | L1MC4 | L1 | LINE |
| 113867076 | 113867420 | + | 12.9 | 1 | 364 | 0 | THE1B | MaLR | LTR |
| 113867423 | 113868980 | + | 30.2 | 2 | 1735 | 0 | MLT-int | MaLR | LTR |
| 113868981 | 113869341 | + | 12.7 | 1 | 364 | 0 | THE1B | MaLR | LTR |
| 113869342 | 113869583 | - | 26.7 | 7524 | 7732 | -310 | L1MC4 | L1 | LINE |

  

|  |  |  |  |  |  |  |  |  |  |
| --- | --- | --- | --- | --- | --- | --- | --- | --- | --- |
| chr1:114057924-114068438 | | | 10469 bp | | |  |  |  |  |
| Genome Start | Genome End | Strand | Div | Repeat Start | Repeat End | Repeat Left | Name | Family | Class |
| 114057924 | 114059517 | + | 12.7 | 569 | 2203 | -5362 | L1M1 | L1 | LINE |
| 114059518 | 114059823 | + | 15.5 | 1 | 312 | 0 | AluJb | Alu | SINE |
| 114059824 | 114060480 | + | 12.7 | 2204 | 2841 | -4724 | L1M1 | L1 | LINE |
| 114060481 | 114060794 | - | 17.9 | 1 | 312 | 0 | AluJo | Alu | SINE |
| 114060795 | 114061710 | + | 12.7 | 2842 | 3749 | -3816 | L1M1 | L1 | LINE |
| 114061711 | 114062067 | - | 5.7 | 1 | 355 | 0 | THE1A | MaLR | LTR |
| 114062068 | 114063133 | - | 30.6 | 599 | 1735 | 0 | MLT-int | MaLR | LTR |
| 114063134 | 114063447 | - | 12.5 | 1 | 312 | 0 | AluSx | Alu | SINE |
| 114063448 | 114063942 | - | 30.6 | 2 | 598 | -1137 | MLT-int | MaLR | LTR |
| 114063945 | 114064292 | - | 7.8 | 1 | 355 | 0 | THE1A | MaLR | LTR |
| 114064293 | 114064737 | + | 13.5 | 2331 | 2787 | -3359 | L1M1 | L1 | LINE |
| 114064744 | 114065073 | - | 10.3 | 833 | 1162 | -418 | THE1-int | MaLR | LTR |
| 114065074 | 114065252 | + | 19.3 | 114 | 285 | -27 | AluSg/x | Alu | SINE |
| 114065283 | 114065840 | + | 11.1 | 3774 | 4318 | -1828 | L1M1 | L1 | LINE |
| 114065841 | 114066138 | - | 7.2 | 1 | 293 | -18 | AluY | Alu | SINE |
| 114066139 | 114066314 | + | 11.1 | 4319 | 4496 | -1650 | L1M1 | L1 | LINE |
| 114066315 | 114066595 | + | 8.5 | 1 | 293 | -18 | AluY | Alu | SINE |
| 114066596 | 114066775 | + | 11.1 | 4497 | 4651 | -1495 | L1M1 | L1 | LINE |
| 114066776 | 114067111 | + | 8.3 | 1 | 329 | 0 | AluSx | Alu | SINE |
| 114067120 | 114067322 | + | 11.1 | 4652 | 4855 | -1291 | L1M1 | L1 | LINE |
| 114067323 | 114067622 | - | 12.7 | 1 | 300 | -12 | AluSx | Alu | SINE |
| 114067623 | 114068438 | + | 9.6 | 4856 | 5668 | -637 | L1M1 | L1 | LINE |

  

|  |  |  |  |  |  |  |  |  |  |
| --- | --- | --- | --- | --- | --- | --- | --- | --- | --- |
| chr1:115462734-115465830 | | | 3095 bp | | |  |  |  |  |
| Genome Start | Genome End | Strand | Div | Repeat Start | Repeat End | Repeat Left | Name | Family | Class |
| 115462734 | 115463278 | - | 26.0 | 73 | 595 | 0 | MLT1G1 | MaLR | LTR |
| 115463279 | 115463685 | - | 15.5 | 1 | 428 | 0 | MSTA | MaLR | LTR |
| 115463686 | 115465318 | - | 28.5 | 2 | 1735 | 0 | MLT1G1-int | MaLR | LTR |
| 115465321 | 115465753 | - | 14.0 | 1 | 428 | 0 | MSTA | MaLR | LTR |
| 115465754 | 115465830 | - | 26.0 | 3 | 72 | -523 | MLT1G1 | MaLR | LTR |

  

|  |  |  |  |  |  |  |  |  |  |
| --- | --- | --- | --- | --- | --- | --- | --- | --- | --- |
| chr1:115853091-115859693 | | | 6600 bp | | |  |  |  |  |
| Genome Start | Genome End | Strand | Div | Repeat Start | Repeat End | Repeat Left | Name | Family | Class |
| 115853091 | 115853284 | - | 16.8 | 99 | 295 | -17 | AluJo | Alu | SINE |
| 115853285 | 115853691 | - | 15.5 | 1 | 450 | 0 | LTR7 | ERV1 | LTR |
| 115853692 | 115854246 | - | 12.5 | 7157 | 7713 | 0 | HERVH | ERV1 | LTR |
| 115854240 | 115855146 | - | 14.2 | 5108 | 6016 | -1697 | HERVH | ERV1 | LTR |
| 115855146 | 115858995 | - | 18.9 | 982 | 4812 | -2287 | HERVH | ERV1 | LTR |
| 115859007 | 115859172 | - | 16.5 | 1 | 175 | -7538 | HERVH | ERV1 | LTR |
| 115859173 | 115859591 | - | 14.9 | 1 | 450 | 0 | LTR7 | ERV1 | LTR |
| 115859592 | 115859693 | - | 18.6 | 2 | 103 | -209 | AluJo | Alu | SINE |

  

|  |  |  |  |  |  |  |  |  |  |
| --- | --- | --- | --- | --- | --- | --- | --- | --- | --- |
| chr1:116906475-116909060 | | | 2584 bp | | |  |  |  |  |
| Genome Start | Genome End | Strand | Div | Repeat Start | Repeat End | Repeat Left | Name | Family | Class |
| 116906475 | 116906652 | - | 16.9 | 112 | 302 | -10 | AluJb | Alu | SINE |
| 116906653 | 116907009 | - | 12.4 | 1 | 364 | 0 | THE1B | MaLR | LTR |
| 116907010 | 116908590 | - | 31.0 | 2 | 1735 | 0 | MLT-int | MaLR | LTR |
| 116908593 | 116908950 | - | 15.0 | 1 | 364 | 0 | THE1B | MaLR | LTR |
| 116908951 | 116909060 | - | 16.9 | 1 | 111 | -201 | AluJb | Alu | SINE |

  

|  |  |  |  |  |  |  |  |  |  |
| --- | --- | --- | --- | --- | --- | --- | --- | --- | --- |
| chr1:117501313-117503741 | | | 2427 bp | | |  |  |  |  |
| Genome Start | Genome End | Strand | Div | Repeat Start | Repeat End | Repeat Left | Name | Family | Class |
| 117501313 | 117501450 | + | 29.7 | 27 | 161 | -107 | MIRb | MIR | SINE |
| 117501451 | 117501803 | - | 9.1 | 1 | 355 | 0 | THE1A | MaLR | LTR |
| 117501804 | 117503346 | - | 29.6 | 2 | 1735 | 0 | MLT-int | MaLR | LTR |
| 117503349 | 117503704 | - | 11.1 | 1 | 355 | 0 | THE1A | MaLR | LTR |
| 117503705 | 117503741 | + | 29.7 | 162 | 198 | -70 | MIRb | MIR | SINE |

  

|  |  |  |  |  |  |  |  |  |  |
| --- | --- | --- | --- | --- | --- | --- | --- | --- | --- |
| chr1:118057805-118076331 | | | 18115 bp | | |  |  |  |  |
| Genome Start | Genome End | Strand | Div | Repeat Start | Repeat End | Repeat Left | Name | Family | Class |
| 118057805 | 118058287 | - | 31.7 | 2881 | 3403 | -16 | L2 | L2 | LINE |
| 118058445 | 118059201 | + | 29.9 | 2456 | 3278 | -2868 | L1M4 | L1 | LINE |
| 118059275 | 118060196 | + | 20.2 | 8 | 905 | -5707 | L1PB1 | L1 | LINE |
| 118060197 | 118060550 | + | 8.5 | 1 | 364 | 0 | THE1B | MaLR | LTR |
| 118060553 | 118062125 | + | 28.2 | 2 | 1735 | 0 | MLT-int | MaLR | LTR |
| 118062128 | 118062482 | + | 11.6 | 3 | 364 | 0 | THE1B | MaLR | LTR |
| 118062483 | 118062600 | + | 20.2 | 906 | 1025 | -5587 | L1PB1 | L1 | LINE |
| 118062601 | 118063082 | - | 6.3 | 5673 | 6154 | 0 | L1PA5 | L1 | LINE |
| 118063083 | 118064823 | + | 10.2 | 1026 | 2859 | -3753 | L1PB1 | L1 | LINE |
| 118064819 | 118070978 | + | 4.7 | 1 | 6154 | -1 | L1PA4 | L1 | LINE |
| 118071000 | 118074749 | + | 9.1 | 2843 | 6617 | 0 | L1PB1 | L1 | LINE |
| 118074756 | 118075732 | + | 29.4 | 3341 | 4334 | -1812 | L1M5 | L1 | LINE |
| 118075889 | 118076331 | - | 30.4 | 1407 | 1876 | -1543 | L2 | L2 | LINE |

  

|  |  |  |  |  |  |  |  |  |  |
| --- | --- | --- | --- | --- | --- | --- | --- | --- | --- |
| chr1:118718525-118722467 | | | 3937 bp | | |  |  |  |  |
| Genome Start | Genome End | Strand | Div | Repeat Start | Repeat End | Repeat Left | Name | Family | Class |
| 118718525 | 118719574 | + | 17.5 | 3 | 1003 | -577 | MSTA-int | MaLR | LTR |
| 118719581 | 118719951 | - | 13.8 | 1 | 364 | 0 | THE1B | MaLR | LTR |
| 118719952 | 118721527 | - | 10.9 | 1 | 1580 | 0 | THE1B-int | MaLR | LTR |
| 118721528 | 118721901 | - | 14.9 | 1 | 364 | 0 | THE1B | MaLR | LTR |
| 118721902 | 118722467 | + | 12.2 | 1014 | 1580 | 0 | MSTA-int | MaLR | LTR |

  

|  |  |  |  |  |  |  |  |  |  |
| --- | --- | --- | --- | --- | --- | --- | --- | --- | --- |
| chr1:118788970-118795125 | | | 6076 bp | | |  |  |  |  |
| Genome Start | Genome End | Strand | Div | Repeat Start | Repeat End | Repeat Left | Name | Family | Class |
| 118788970 | 118790295 | + | 29.2 | 795 | 2305 | -4197 | L1MEb | L1 | LINE |
| 118790296 | 118790603 | + | 18.0 | 1 | 306 | -6 | AluSx | Alu | SINE |
| 118790604 | 118790761 | + | 28.9 | 2306 | 2477 | -4025 | L1MEb | L1 | LINE |
| 118790833 | 118791726 | + | 22.3 | 2551 | 3470 | -2851 | L1MEb | L1 | LINE |
| 118791728 | 118792038 | + | 22.6 | 3713 | 4042 | -2279 | L1MEb | L1 | LINE |
| 118792039 | 118792176 | + | 15.2 | 3 | 166 | -262 | MSTA | MaLR | LTR |
| 118792173 | 118792203 | + | 6.5 | 349 | 379 | -2 | MSTA | MaLR | LTR |
| 118792208 | 118793802 | + | 30.1 | 2 | 1735 | 0 | MLT-int | MaLR | LTR |
| 118793803 | 118794185 | + | 15.3 | 1 | 428 | 0 | MSTA | MaLR | LTR |
| 118794186 | 118794567 | + | 22.1 | 4036 | 4429 | -1892 | L1MEb | L1 | LINE |
| 118794568 | 118794861 | - | 14.6 | 1 | 296 | -16 | AluSx | Alu | SINE |
| 118794862 | 118795041 | + | 22.1 | 4430 | 4612 | -1709 | L1MEb | L1 | LINE |
| 118795050 | 118795125 | + | 19.2 | 4676 | 4754 | -1567 | L1MEb | L1 | LINE |

  

|  |  |  |  |  |  |  |  |  |  |
| --- | --- | --- | --- | --- | --- | --- | --- | --- | --- |
| chr1:142513945-142516481 | | | 2535 bp | | |  |  |  |  |
| Genome Start | Genome End | Strand | Div | Repeat Start | Repeat End | Repeat Left | Name | Family | Class |
| 142513945 | 142514182 | + | 22.1 | 1 | 242 | -70 | AluJo | Alu | SINE |
| 142514183 | 142514524 | + | 13.9 | 1 | 364 | 0 | THE1B | MaLR | LTR |
| 142514527 | 142516099 | + | 29.5 | 2 | 1735 | 0 | MLT-int | MaLR | LTR |
| 142516100 | 142516424 | + | 12.2 | 5 | 364 | 0 | THE1B | MaLR | LTR |
| 142516425 | 142516481 | + | 22.1 | 243 | 299 | -13 | AluJo | Alu | SINE |

  

|  |  |  |  |  |  |  |  |  |  |
| --- | --- | --- | --- | --- | --- | --- | --- | --- | --- |
| chr1:144842685-144845321 | | | 2633 bp | | |  |  |  |  |
| Genome Start | Genome End | Strand | Div | Repeat Start | Repeat End | Repeat Left | Name | Family | Class |
| 144842685 | 144842991 | - | 15.9 | 88 | 390 | 0 | MLT1B | MaLR | LTR |
| 144842992 | 144843372 | + | 13.6 | 1 | 428 | 0 | MSTA | MaLR | LTR |
| 144843373 | 144844852 | + | 17.2 | 1 | 1651 | 0 | MSTA-int | MaLR | LTR |
| 144844857 | 144845230 | + | 14.6 | 5 | 428 | 0 | MSTA | MaLR | LTR |
| 144845231 | 144845321 | - | 15.8 | 5 | 96 | -294 | MLT1B | MaLR | LTR |

  

|  |  |  |  |  |  |  |  |  |  |
| --- | --- | --- | --- | --- | --- | --- | --- | --- | --- |
| chr1:145754368-145757448 | | | 3079 bp | | |  |  |  |  |
| Genome Start | Genome End | Strand | Div | Repeat Start | Repeat End | Repeat Left | Name | Family | Class |
| 145754368 | 145754475 | + | 21.9 | 324 | 457 | -158 | MLT1L | MaLR | LTR |
| 145754476 | 145754902 | - | 15.9 | 1 | 396 | 0 | MSTD | MaLR | LTR |
| 145754903 | 145756106 | - | 22.8 | 596 | 1735 | 0 | MSTD-int | MaLR | LTR |
| 145756107 | 145756413 | + | 7.5 | 2 | 308 | -3 | AluY | Alu | SINE |
| 145756414 | 145757008 | - | 22.8 | 2 | 595 | -1140 | MSTD-int | MaLR | LTR |
| 145757011 | 145757348 | - | 13.9 | 1 | 396 | 0 | MSTD | MaLR | LTR |
| 145757349 | 145757448 | + | 21.9 | 458 | 609 | -6 | MLT1L | MaLR | LTR |

  

|  |  |  |  |  |  |  |  |  |  |
| --- | --- | --- | --- | --- | --- | --- | --- | --- | --- |
| chr1:146139784-146142420 | | | 2633 bp | | |  |  |  |  |
| Genome Start | Genome End | Strand | Div | Repeat Start | Repeat End | Repeat Left | Name | Family | Class |
| 146139784 | 146140090 | - | 15.9 | 88 | 390 | 0 | MLT1B | MaLR | LTR |
| 146140091 | 146140471 | + | 13.6 | 1 | 428 | 0 | MSTA | MaLR | LTR |
| 146140472 | 146141951 | + | 17.2 | 1 | 1651 | 0 | MSTA-int | MaLR | LTR |
| 146141956 | 146142329 | + | 14.6 | 5 | 428 | 0 | MSTA | MaLR | LTR |
| 146142330 | 146142420 | - | 15.8 | 5 | 96 | -294 | MLT1B | MaLR | LTR |

  

|  |  |  |  |  |  |  |  |  |  |
| --- | --- | --- | --- | --- | --- | --- | --- | --- | --- |
| chr1:146376555-146379091 | | | 2535 bp | | |  |  |  |  |
| Genome Start | Genome End | Strand | Div | Repeat Start | Repeat End | Repeat Left | Name | Family | Class |
| 146376555 | 146376792 | + | 22.1 | 1 | 242 | -70 | AluJo | Alu | SINE |
| 146376793 | 146377134 | + | 13.9 | 1 | 364 | 0 | THE1B | MaLR | LTR |
| 146377137 | 146378709 | + | 29.4 | 2 | 1735 | 0 | MLT-int | MaLR | LTR |
| 146378710 | 146379034 | + | 12.2 | 5 | 364 | 0 | THE1B | MaLR | LTR |
| 146379035 | 146379091 | + | 22.1 | 243 | 299 | -13 | AluJo | Alu | SINE |

  

|  |  |  |  |  |  |  |  |  |  |
| --- | --- | --- | --- | --- | --- | --- | --- | --- | --- |
| chr1:152909366-152919116 | | | 9761 bp | | |  |  |  |  |
| Genome Start | Genome End | Strand | Div | Repeat Start | Repeat End | Repeat Left | Name | Family | Class |
| 152909366 | 152909421 | - | 12.7 | 134 | 189 | 0 | MER5A | MER1\_type | DNA |
| 152909426 | 152909667 | + | 22.5 | 1 | 241 | -71 | AluJo | Alu | SINE |
| 152909668 | 152911098 | + | 7.4 | 2 | 1577 | -1 | LTR12C | ERV1 | LTR |
| 152911100 | 152915984 | + | 5.4 | 1 | 4963 | -3473 | HERV9 | ERV1 | LTR |
| 152915972 | 152916285 | + | 17.1 | 741 | 1049 | -4053 | HERV9 | ERV1 | LTR |
| 152916287 | 152916347 | + | 13.1 | 7542 | 7602 | -834 | HERV9 | ERV1 | LTR |
| 152916344 | 152916954 | + | 4.1 | 7827 | 8436 | 0 | HERV9 | ERV1 | LTR |
| 152916955 | 152918412 | + | 7.4 | 2 | 1578 | 0 | LTR12C | ERV1 | LTR |
| 152918413 | 152918470 | + | 5.2 | 238 | 295 | -17 | AluJo | Alu | SINE |
| 152918472 | 152918505 | - | 15.9 | 107 | 135 | -54 | MER5A | MER1\_type | DNA |
| 152918506 | 152919009 | + | 11.8 | 1 | 511 | 0 | MER9 | ERVK | LTR |
| 152919010 | 152919116 | - | 15.9 | 3 | 106 | -83 | MER5A | MER1\_type | DNA |

  

|  |  |  |  |  |  |  |  |  |  |
| --- | --- | --- | --- | --- | --- | --- | --- | --- | --- |
| chr1:153858805-153873842 | | | 14566 bp | | |  |  |  |  |
| Genome Start | Genome End | Strand | Div | Repeat Start | Repeat End | Repeat Left | Name | Family | Class |
| 153858805 | 153859450 | - | 24.1 | 7979 | 8626 | 0 | HERV17 | ERV1 | LTR |
| 153859451 | 153860088 | - | 10.5 | 1 | 608 | 0 | LTR14B | ERVK | LTR |
| 153860089 | 153860198 | - | 24.1 | 7895 | 7978 | -648 | HERV17 | ERV1 | LTR |
| 153860189 | 153860369 | - | 19.8 | 4085 | 4259 | -4367 | HERV17 | ERV1 | LTR |
| 153860370 | 153860881 | + | 10.8 | 1 | 511 | 0 | MER9 | ERVK | LTR |
| 153860882 | 153863079 | - | 19.8 | 1884 | 4084 | -4542 | HERV17 | ERV1 | LTR |
| 153863081 | 153864048 | - | 1.0 | 1 | 968 | 0 | LTR5\_Hs | ERVK | LTR |
| 153864049 | 153865759 | - | 0.5 | 5826 | 7536 | 0 | HERVK | ERVK | LTR |
| 153865759 | 153871292 | - | 0.8 | 1 | 5534 | -2002 | HERVK | ERVK | LTR |
| 153871293 | 153872260 | - | 1.0 | 1 | 968 | 0 | LTR5\_Hs | ERVK | LTR |
| 153872261 | 153872499 | - | 25.3 | 1655 | 1888 | -6738 | HERV17 | ERV1 | LTR |
| 153872982 | 153873406 | - | 18.9 | 125 | 567 | -7690 | HERV17 | ERV1 | LTR |
| 153873407 | 153873705 | - | 9.8 | 1 | 297 | -14 | AluY | Alu | SINE |
| 153873706 | 153873842 | - | 25.7 | 1 | 153 | -6951 | HERV17 | ERV1 | LTR |

  

|  |  |  |  |  |  |  |  |  |  |
| --- | --- | --- | --- | --- | --- | --- | --- | --- | --- |
| chr1:156131701-156135833 | | | 4131 bp | | |  |  |  |  |
| Genome Start | Genome End | Strand | Div | Repeat Start | Repeat End | Repeat Left | Name | Family | Class |
| 156131701 | 156131774 | - | 18.4 | 6172 | 6242 | 0 | L1MD1 | L1 | LINE |
| 156131775 | 156132261 | + | 15.6 | 1 | 486 | 0 | MER66B | ERV1 | LTR |
| 156132262 | 156132612 | - | 18.4 | 5806 | 6171 | -71 | L1MD1 | L1 | LINE |
| 156132613 | 156132966 | - | 10.2 | 1 | 355 | 0 | THE1A | MaLR | LTR |
| 156132967 | 156134536 | - | 29.5 | 2 | 1735 | 0 | MLT-int | MaLR | LTR |
| 156134539 | 156134893 | - | 10.4 | 1 | 355 | 0 | THE1A | MaLR | LTR |
| 156134894 | 156135833 | - | 17.8 | 4844 | 5805 | -437 | L1MD1 | L1 | LINE |

  

|  |  |  |  |  |  |  |  |  |  |
| --- | --- | --- | --- | --- | --- | --- | --- | --- | --- |
| chr1:156135824-156151665 | | | 15337 bp | | |  |  |  |  |
| Genome Start | Genome End | Strand | Div | Repeat Start | Repeat End | Repeat Left | Name | Family | Class |
| 156135824 | 156136192 | + | 27.0 | 16 | 386 | -5998 | L1M4c | L1 | LINE |
| 156136198 | 156137114 | + | 20.4 | 546 | 1467 | -4917 | L1M4c | L1 | LINE |
| 156137155 | 156137246 | - | 16.3 | 6163 | 6255 | -57 | L1MA9 | L1 | LINE |
| 156137243 | 156137582 | - | 14.4 | 5323 | 5665 | -647 | L1MA9 | L1 | LINE |
| 156137590 | 156138282 | + | 18.9 | 1463 | 2264 | -4120 | L1M4c | L1 | LINE |
| 156138299 | 156144401 | + | 6.8 | 5 | 6152 | -2 | L1PA6 | L1 | LINE |
| 156144402 | 156145080 | + | 7.2 | 5476 | 6154 | 0 | L1PA6 | L1 | LINE |
| 156145524 | 156145679 | + | 16.4 | 2246 | 2406 | -3978 | L1M4c | L1 | LINE |
| 156145680 | 156146108 | + | 21.2 | 1 | 457 | 0 | MSTB2 | MaLR | LTR |
| 156146109 | 156147755 | + | 23.8 | 1 | 1650 | -1 | MSTB2-int | MaLR | LTR |
| 156147761 | 156148169 | + | 22.2 | 6 | 457 | 0 | MSTB2 | MaLR | LTR |
| 156148172 | 156149020 | + | 19.3 | 2404 | 3285 | -3097 | L1M4c | L1 | LINE |
| 156149012 | 156149753 | + | 18.3 | 3813 | 4552 | -1824 | L1M4c | L1 | LINE |
| 156149754 | 156150055 | - | 18.5 | 6 | 302 | -10 | AluJo | Alu | SINE |
| 156150056 | 156150341 | + | 18.3 | 4553 | 4842 | -1534 | L1M4c | L1 | LINE |
| 156150342 | 156150642 | + | 19.0 | 1 | 305 | -7 | AluJo | Alu | SINE |
| 156150643 | 156151665 | + | 18.6 | 4843 | 5881 | -538 | L1M4c | L1 | LINE |

  

|  |  |  |  |  |  |  |  |  |  |
| --- | --- | --- | --- | --- | --- | --- | --- | --- | --- |
| chr1:156451142-156461555 | | | 9947 bp | | |  |  |  |  |
| Genome Start | Genome End | Strand | Div | Repeat Start | Repeat End | Repeat Left | Name | Family | Class |
| 156451142 | 156451426 | + | 29.4 | 23 | 327 | -2455 | Charlie2 | MER1\_type | DNA |
| 156451427 | 156451643 | + | 26.6 | 1856 | 2076 | -786 | Charlie2 | MER1\_type | DNA |
| 156451773 | 156451833 | - | 22.9 | 107 | 171 | -4 | MER96 | MER1\_type? | DNA |
| 156451997 | 156452079 | - | 27.7 | 126 | 210 | -58 | MIRb | MIR | SINE |
| 156452214 | 156458361 | - | 5.2 | 1 | 6155 | 0 | L1PA4 | L1 | LINE |
| 156458368 | 156458856 | + | 22.7 | 2068 | 2555 | -307 | Charlie2 | MER1\_type | DNA |
| 156458859 | 156459235 | + | 15.2 | 1 | 364 | 0 | THE1B | MaLR | LTR |
| 156459236 | 156459271 | + | 5.6 | 1 | 36 | -1544 | THE1B-int | MaLR | LTR |
| 156459273 | 156459440 | + | 29.8 | 1988 | 2163 | -699 | Charlie2 | MER1\_type | DNA |
| 156459435 | 156460943 | + | 10.3 | 42 | 1580 | 0 | THE1B-int | MaLR | LTR |
| 156460944 | 156461286 | + | 13.5 | 1 | 364 | 0 | THE1B | MaLR | LTR |
| 156461325 | 156461555 | + | 23.5 | 2598 | 2831 | -31 | Charlie2 | MER1\_type | DNA |

  

|  |  |  |  |  |  |  |  |  |  |
| --- | --- | --- | --- | --- | --- | --- | --- | --- | --- |
| chr1:156736792-156739781 | | | 2958 bp | | |  |  |  |  |
| Genome Start | Genome End | Strand | Div | Repeat Start | Repeat End | Repeat Left | Name | Family | Class |
| 156736792 | 156736842 | - | 11.8 | 6120 | 6172 | -17 | L1MB1 | L1 | LINE |
| 156736864 | 156737258 | - | 16.7 | 1 | 428 | 0 | MSTA | MaLR | LTR |
| 156737265 | 156738305 | - | 18.8 | 605 | 1645 | -6 | MSTA-int | MaLR | LTR |
| 156738306 | 156738616 | - | 8.8 | 1 | 310 | -1 | AluY | Alu | SINE |
| 156738617 | 156739221 | - | 19.3 | 3 | 604 | -1047 | MSTA-int | MaLR | LTR |
| 156739224 | 156739637 | - | 15.6 | 1 | 428 | 0 | MSTA | MaLR | LTR |
| 156739641 | 156739781 | - | 17.9 | 5940 | 6083 | -85 | L1MB1 | L1 | LINE |

  

|  |  |  |  |  |  |  |  |  |  |
| --- | --- | --- | --- | --- | --- | --- | --- | --- | --- |
| chr1:157621648-157624652 | | | 3003 bp | | |  |  |  |  |
| Genome Start | Genome End | Strand | Div | Repeat Start | Repeat End | Repeat Left | Name | Family | Class |
| 157621648 | 157622289 | + | 21.2 | 5394 | 6007 | -164 | L1MB2 | L1 | LINE |
| 157622292 | 157622664 | + | 13.1 | 3 | 364 | 0 | THE1B | MaLR | LTR |
| 157622665 | 157624207 | + | 12.6 | 1 | 1580 | 0 | THE1B-int | MaLR | LTR |
| 157624208 | 157624589 | + | 15.6 | 1 | 364 | 0 | THE1B | MaLR | LTR |
| 157624590 | 157624652 | + | 17.7 | 6003 | 6064 | -107 | L1MB2 | L1 | LINE |

  

|  |  |  |  |  |  |  |  |  |  |
| --- | --- | --- | --- | --- | --- | --- | --- | --- | --- |
| chr1:157650361-157659490 | | | 9126 bp | | |  |  |  |  |
| Genome Start | Genome End | Strand | Div | Repeat Start | Repeat End | Repeat Left | Name | Family | Class |
| 157650361 | 157653745 | - | 16.2 | 3583 | 7005 | 0 | L1MA4A | L1 | LINE |
| 157653746 | 157654084 | + | 15.6 | 1 | 360 | -4 | THE1B | MaLR | LTR |
| 157654089 | 157655656 | + | 30.5 | 2 | 1735 | 0 | MLT-int | MaLR | LTR |
| 157655657 | 157656015 | + | 11.0 | 1 | 364 | 0 | THE1B | MaLR | LTR |
| 157656016 | 157659490 | - | 19.1 | 1 | 3582 | -3263 | L1MA4A | L1 | LINE |

  

|  |  |  |  |  |  |  |  |  |  |
| --- | --- | --- | --- | --- | --- | --- | --- | --- | --- |
| chr1:159046593-159048895 | | | 2302 bp | | |  |  |  |  |
| Genome Start | Genome End | Strand | Div | Repeat Start | Repeat End | Repeat Left | Name | Family | Class |
| 159046593 | 159046663 | - | 25.8 | 5325 | 5389 | -2419 | L1M | L1 | LINE |
| 159046664 | 159047051 | - | 16.4 | 1 | 390 | 0 | MLT1B | MaLR | LTR |
| 159047053 | 159048428 | - | 21.8 | 1 | 1370 | -1 | MLT1B-int | MaLR | LTR |
| 159048429 | 159048829 | - | 16.6 | 1 | 390 | 0 | MLT1B | MaLR | LTR |
| 159048830 | 159048895 | - | 25.8 | 5260 | 5324 | -2484 | L1M | L1 | LINE |

  

|  |  |  |  |  |  |  |  |  |  |
| --- | --- | --- | --- | --- | --- | --- | --- | --- | --- |
| chr1:161367369-161369929 | | | 2557 bp | | |  |  |  |  |
| Genome Start | Genome End | Strand | Div | Repeat Start | Repeat End | Repeat Left | Name | Family | Class |
| 161367369 | 161367439 | + | 31.2 | 57 | 132 | -136 | MIRb | MIR | SINE |
| 161367440 | 161367862 | + | 17.3 | 1 | 426 | 0 | MSTB | MaLR | LTR |
| 161367865 | 161369460 | + | 28.9 | 2 | 1733 | -2 | MLT1J-int | MaLR | LTR |
| 161369463 | 161369884 | + | 16.3 | 1 | 426 | 0 | MSTB | MaLR | LTR |
| 161369885 | 161369929 | + | 31.2 | 133 | 176 | -92 | MIRb | MIR | SINE |

  

|  |  |  |  |  |  |  |  |  |  |
| --- | --- | --- | --- | --- | --- | --- | --- | --- | --- |
| chr1:161463408-161466367 | | | 2958 bp | | |  |  |  |  |
| Genome Start | Genome End | Strand | Div | Repeat Start | Repeat End | Repeat Left | Name | Family | Class |
| 161463408 | 161463633 | + | 29.4 | 2660 | 2881 | -538 | L2 | L2 | LINE |
| 161463634 | 161463989 | - | 14.8 | 1 | 381 | 0 | THE1D | MaLR | LTR |
| 161463990 | 161465535 | - | 30.8 | 2 | 1735 | 0 | MLT1H1-int | MaLR | LTR |
| 161465538 | 161465895 | - | 13.8 | 1 | 381 | 0 | THE1D | MaLR | LTR |
| 161465896 | 161466367 | + | 28.4 | 2882 | 3417 | -2 | L2 | L2 | LINE |

  

|  |  |  |  |  |  |  |  |  |  |
| --- | --- | --- | --- | --- | --- | --- | --- | --- | --- |
| chr1:161826768-161834340 | | | 7494 bp | | |  |  |  |  |
| Genome Start | Genome End | Strand | Div | Repeat Start | Repeat End | Repeat Left | Name | Family | Class |
| 161826768 | 161826901 | - | 18.4 | 6150 | 6302 | -10 | L1MA9 | L1 | LINE |
| 161826928 | 161827722 | - | 18.4 | 5328 | 6149 | -163 | L1MA9 | L1 | LINE |
| 161827723 | 161828119 | + | 9.8 | 1 | 448 | -2 | LTR7 | ERV1 | LTR |
| 161828122 | 161830673 | + | 12.4 | 1 | 2828 | -4885 | HERVH | ERV1 | LTR |
| 161830672 | 161831056 | + | 13.8 | 3354 | 3736 | -3977 | HERVH | ERV1 | LTR |
| 161831067 | 161832096 | + | 10.9 | 3994 | 5011 | -2702 | HERVH | ERV1 | LTR |
| 161832112 | 161832545 | + | 9.3 | 7283 | 7713 | 0 | HERVH | ERV1 | LTR |
| 161832546 | 161832943 | + | 9.3 | 1 | 448 | -2 | LTR7 | ERV1 | LTR |
| 161832967 | 161833012 | - | 8.7 | 1 | 46 | -6712 | MER4E-int | ERV1 | LTR |
| 161833018 | 161833595 | - | 12.3 | 1 | 761 | -5 | MER4E | ERV1 | LTR |
| 161833596 | 161834340 | - | 18.4 | 4344 | 5098 | -1042 | L1MA9 | L1 | LINE |

  

|  |  |  |  |  |  |  |  |  |  |
| --- | --- | --- | --- | --- | --- | --- | --- | --- | --- |
| chr1:162327535-162332673 | | | 5128 bp | | |  |  |  |  |
| Genome Start | Genome End | Strand | Div | Repeat Start | Repeat End | Repeat Left | Name | Family | Class |
| 162327535 | 162327738 | + | 22.8 | 3578 | 3807 | -2339 | L1ME1 | L1 | LINE |
| 162327763 | 162328114 | - | 16.8 | 3 | 362 | -2 | THE1B | MaLR | LTR |
| 162328122 | 162329632 | - | 12.3 | 1 | 1575 | -5 | THE1B-int | MaLR | LTR |
| 162329633 | 162329965 | - | 17.7 | 21 | 364 | 0 | THE1B | MaLR | LTR |
| 162329966 | 162330552 | + | 24.3 | 3805 | 4391 | -1755 | L1ME1 | L1 | LINE |
| 162330553 | 162330997 | + | 7.5 | 5713 | 6154 | 0 | L1PA7 | L1 | LINE |
| 162330998 | 162331190 | + | 24.3 | 4392 | 4604 | -1542 | L1ME1 | L1 | LINE |
| 162331171 | 162331587 | + | 24.6 | 4976 | 5402 | -767 | L1MD | L1 | LINE |
| 162331588 | 162331881 | + | 15.0 | 1 | 310 | 0 | AluSg | Alu | SINE |
| 162331882 | 162332673 | + | 23.9 | 5400 | 6161 | -5 | L1ME1 | L1 | LINE |

  

|  |  |  |  |  |  |  |  |  |  |
| --- | --- | --- | --- | --- | --- | --- | --- | --- | --- |
| chr1:165061312-165069405 | | | 8006 bp | | |  |  |  |  |
| Genome Start | Genome End | Strand | Div | Repeat Start | Repeat End | Repeat Left | Name | Family | Class |
| 165061312 | 165061558 | + | 34.3 | 617 | 870 | -1090 | L4 | RTE | LINE |
| 165061561 | 165061914 | - | 28.0 | 3026 | 3418 | -1 | L2 | L2 | LINE |
| 165061917 | 165062656 | - | 26.0 | 2091 | 2915 | -504 | L2 | L2 | LINE |
| 165062657 | 165063169 | + | 16.0 | 5666 | 6180 | -3 | L1MB3 | L1 | LINE |
| 165063170 | 165063242 | - | 26.0 | 2062 | 2090 | -1329 | L2 | L2 | LINE |
| 165063243 | 165063729 | - | 21.7 | 1 | 480 | -4 | MLT1H2 | MaLR | LTR |
| 165063730 | 165063901 | - | 26.0 | 1890 | 2061 | -1358 | L2 | L2 | LINE |
| 165063902 | 165064201 | + | 14.1 | 1 | 299 | -13 | AluSx | Alu | SINE |
| 165064202 | 165064350 | - | 26.0 | 1751 | 1889 | -1530 | L2 | L2 | LINE |
| 165064354 | 165064540 | - | 17.9 | 263 | 457 | 0 | MSTB2 | MaLR | LTR |
| 165064544 | 165065158 | + | 10.8 | 1 | 617 | -255 | MER4D0 | ERV1 | LTR |
| 165065159 | 165065397 | + | 10.8 | 1 | 235 | -120 | THE1A | MaLR | LTR |
| 165065398 | 165065691 | + | 13.9 | 3 | 299 | -14 | AluSp | Alu | SINE |
| 165065692 | 165065819 | + | 10.8 | 236 | 355 | 0 | THE1A | MaLR | LTR |
| 165065822 | 165067387 | + | 31.1 | 2 | 1735 | 0 | MLT1J2-int | MaLR | LTR |
| 165067388 | 165067738 | + | 14.9 | 1 | 355 | 0 | THE1A | MaLR | LTR |
| 165067739 | 165067996 | + | 10.8 | 618 | 871 | -1 | MER4D0 | ERV1 | LTR |
| 165068023 | 165068101 | - | 19.7 | 92 | 177 | -280 | MSTB2 | MaLR | LTR |
| 165068152 | 165068201 | + | 28.8 | 866 | 919 | -1041 | L4 | RTE | LINE |
| 165068202 | 165068907 | + | 22.1 | 1 | 734 | 0 | MER50 | ERV1 | LTR |
| 165068908 | 165069405 | + | 33.0 | 920 | 1494 | -466 | L4 | RTE | LINE |

  

|  |  |  |  |  |  |  |  |  |  |
| --- | --- | --- | --- | --- | --- | --- | --- | --- | --- |
| chr1:165832947-165835841 | | | 2893 bp | | |  |  |  |  |
| Genome Start | Genome End | Strand | Div | Repeat Start | Repeat End | Repeat Left | Name | Family | Class |
| 165832947 | 165833015 | - | 15.3 | 272 | 335 | -1 | MER2B | MER2\_type | DNA |
| 165833016 | 165833364 | - | 9.2 | 1 | 355 | 0 | THE1A | MaLR | LTR |
| 165833365 | 165834935 | - | 31.1 | 2 | 1735 | 0 | MLT-int | MaLR | LTR |
| 165834938 | 165835289 | - | 9.1 | 1 | 355 | 0 | THE1A | MaLR | LTR |
| 165835290 | 165835319 | - | 15.3 | 249 | 271 | -65 | MER2B | MER2\_type | DNA |
| 165835320 | 165835620 | + | 1.3 | 1 | 302 | -9 | AluYg6 | Alu | SINE |
| 165835621 | 165835841 | - | 15.3 | 17 | 248 | -88 | MER2B | MER2\_type | DNA |

  

|  |  |  |  |  |  |  |  |  |  |
| --- | --- | --- | --- | --- | --- | --- | --- | --- | --- |
| chr1:165842121-165844967 | | | 2831 bp | | |  |  |  |  |
| Genome Start | Genome End | Strand | Div | Repeat Start | Repeat End | Repeat Left | Name | Family | Class |
| 165842121 | 165842171 | - | 13.7 | 143 | 193 | 0 | MER53 | DNA | DNA |
| 165842186 | 165842257 | + | 16.5 | 1 | 74 | -354 | MSTA | MaLR | LTR |
| 165842258 | 165842565 | + | 6.8 | 1 | 306 | -5 | AluY | Alu | SINE |
| 165842566 | 165842914 | + | 16.5 | 75 | 428 | 0 | MSTA | MaLR | LTR |
| 165842915 | 165844460 | + | 17.4 | 1 | 1651 | 0 | MSTA-int | MaLR | LTR |
| 165844461 | 165844869 | + | 16.1 | 1 | 428 | 0 | MSTA | MaLR | LTR |
| 165844872 | 165844967 | - | 24.0 | 1 | 97 | -96 | MER53 | DNA | DNA |

  

|  |  |  |  |  |  |  |  |  |  |
| --- | --- | --- | --- | --- | --- | --- | --- | --- | --- |
| chr1:166570268-166575173 | | | 4801 bp | | |  |  |  |  |
| Genome Start | Genome End | Strand | Div | Repeat Start | Repeat End | Repeat Left | Name | Family | Class |
| 166570268 | 166571921 | + | 21.5 | 891 | 3010 | -2346 | MER93B-int | ERV1 | LTR |
| 166571943 | 166572065 | + | 21.5 | 1890 | 2014 | -4625 | MER93B-int | ERV1 | LTR |
| 166572129 | 166572426 | - | 12.7 | 1 | 293 | -19 | AluSx | Alu | SINE |
| 166572439 | 166572570 | + | 12.3 | 4042 | 4178 | -2153 | LTR49-int | ERV1 | LTR |
| 166572578 | 166572917 | - | 9.5 | 1 | 327 | -28 | THE1A | MaLR | LTR |
| 166572918 | 166574500 | - | 30.1 | 2 | 1735 | 0 | MLT1J-int | MaLR | LTR |
| 166574503 | 166574856 | - | 9.6 | 1 | 355 | 0 | THE1A | MaLR | LTR |
| 166574857 | 166575173 | + | 19.9 | 3356 | 3703 | -1653 | MER93B-int | ERV1 | LTR |

  

|  |  |  |  |  |  |  |  |  |  |
| --- | --- | --- | --- | --- | --- | --- | --- | --- | --- |
| chr1:168834273-168839562 | | | 5196 bp | | |  |  |  |  |
| Genome Start | Genome End | Strand | Div | Repeat Start | Repeat End | Repeat Left | Name | Family | Class |
| 168834273 | 168834499 | - | 18.3 | 1586 | 1891 | -4509 | L1MEc | L1 | LINE |
| 168834500 | 168835101 | + | 20.6 | 1 | 602 | 0 | MER7C | MER2\_type | DNA |
| 168835102 | 168835347 | - | 18.3 | 1311 | 1585 | -4815 | L1MEc | L1 | LINE |
| 168835356 | 168835415 | + | 28.3 | 1 | 61 | -275 | MER2B | MER2\_type | DNA |
| 168835462 | 168835720 | + | 15.4 | 136 | 345 | 0 | MER2B | MER2\_type | DNA |
| 168835721 | 168836353 | - | 19.3 | 674 | 1315 | -5085 | L1MEc | L1 | LINE |
| 168836386 | 168836557 | - | 16.4 | 109 | 281 | -31 | AluSx | Alu | SINE |
| 168836558 | 168836906 | - | 16.4 | 3 | 364 | 0 | THE1B | MaLR | LTR |
| 168836909 | 168838495 | - | 30.0 | 2 | 1735 | 0 | MLT-int | MaLR | LTR |
| 168838498 | 168838850 | - | 14.9 | 5 | 364 | 0 | THE1B | MaLR | LTR |
| 168838851 | 168838952 | - | 16.4 | 2 | 108 | -204 | AluSx | Alu | SINE |
| 168838957 | 168839562 | - | 24.8 | 33 | 722 | -5678 | L1MEc | L1 | LINE |

  

|  |  |  |  |  |  |  |  |  |  |
| --- | --- | --- | --- | --- | --- | --- | --- | --- | --- |
| chr1:169166704-169171064 | | | 4263 bp | | |  |  |  |  |
| Genome Start | Genome End | Strand | Div | Repeat Start | Repeat End | Repeat Left | Name | Family | Class |
| 169166704 | 169166758 | - | 12.7 | 5768 | 5829 | -411 | L1M5 | L1 | LINE |
| 169166830 | 169167127 | - | 12.9 | 1 | 336 | -92 | MSTA | MaLR | LTR |
| 169167129 | 169168669 | - | 29.2 | 2 | 1734 | -1 | MLT1J-int | MaLR | LTR |
| 169168672 | 169168875 | - | 13.4 | 202 | 428 | 0 | MSTA | MaLR | LTR |
| 169168876 | 169168922 | + | 27.6 | 1557 | 1605 | -4779 | L1MB7 | L1 | LINE |
| 169168923 | 169169220 | + | 9.1 | 1 | 298 | -13 | AluY | Alu | SINE |
| 169169221 | 169169283 | + | 27.6 | 1606 | 1664 | -4720 | L1MB7 | L1 | LINE |
| 169169332 | 169169766 | + | 27.1 | 1694 | 2487 | -4223 | L1MB7 | L1 | LINE |
| 169169757 | 169170389 | + | 19.9 | 4933 | 5558 | -824 | L1MB7 | L1 | LINE |
| 169170383 | 169170653 | + | 15.6 | 6127 | 6412 | -8 | L1MB7 | L1 | LINE |
| 169170647 | 169171064 | - | 31.4 | 5302 | 5722 | -518 | L1M5 | L1 | LINE |

  

|  |  |  |  |  |  |  |  |  |  |
| --- | --- | --- | --- | --- | --- | --- | --- | --- | --- |
| chr1:170059902-170065443 | | | 5013 bp | | |  |  |  |  |
| Genome Start | Genome End | Strand | Div | Repeat Start | Repeat End | Repeat Left | Name | Family | Class |
| 170059902 | 170060134 | - | 23.5 | 3207 | 3417 | -2 | L2 | L2 | LINE |
| 170060188 | 170060350 | - | 15.0 | 11 | 176 | 0 | FRAM | Alu | SINE |
| 170060796 | 170061444 | - | 28.7 | 2505 | 3192 | -227 | L2 | L2 | LINE |
| 170061474 | 170061931 | - | 28.0 | 1302 | 1793 | -1626 | L2 | L2 | LINE |
| 170061934 | 170062283 | - | 11.5 | 1 | 379 | -2 | THE1D | MaLR | LTR |
| 170062284 | 170063813 | - | 11.5 | 1 | 1580 | 0 | THE1D-int | MaLR | LTR |
| 170063814 | 170064173 | - | 14.0 | 1 | 381 | 0 | THE1D | MaLR | LTR |
| 170064174 | 170064870 | - | 27.0 | 596 | 1306 | -2113 | L2 | L2 | LINE |
| 170064871 | 170065196 | + | 5.3 | 1 | 318 | 0 | AluY | Alu | SINE |
| 170065197 | 170065443 | - | 27.0 | 333 | 595 | -2824 | L2 | L2 | LINE |

  

|  |  |  |  |  |  |  |  |  |  |
| --- | --- | --- | --- | --- | --- | --- | --- | --- | --- |
| chr1:171048905-171051868 | | | 2920 bp | | |  |  |  |  |
| Genome Start | Genome End | Strand | Div | Repeat Start | Repeat End | Repeat Left | Name | Family | Class |
| 171048905 | 171049044 | - | 20.1 | 3519 | 3660 | -3346 | L1M4c | L1 | LINE |
| 171049076 | 171049461 | - | 12.9 | 1 | 375 | 0 | THE1C | MaLR | LTR |
| 171049462 | 171050985 | - | 13.4 | 1 | 1580 | 0 | THE1C-int | MaLR | LTR |
| 171050986 | 171051287 | - | 11.2 | 75 | 375 | 0 | THE1C | MaLR | LTR |
| 171051288 | 171051355 | - | 16.7 | 1746 | 1851 | -5157 | L1M4c | L1 | LINE |
| 171051369 | 171051868 | - | 23.6 | 1570 | 2083 | -4563 | L1M4c | L1 | LINE |

  

|  |  |  |  |  |  |  |  |  |  |
| --- | --- | --- | --- | --- | --- | --- | --- | --- | --- |
| chr1:172823353-172841934 | | | 18376 bp | | |  |  |  |  |
| Genome Start | Genome End | Strand | Div | Repeat Start | Repeat End | Repeat Left | Name | Family | Class |
| 172823353 | 172823449 | - | 12.5 | 1199 | 1300 | -2 | HSMAR2 | Mariner | DNA |
| 172823465 | 172823617 | - | 11.4 | 7559 | 7711 | -13 | L1MA3 | L1 | LINE |
| 172823618 | 172823926 | - | 14.3 | 1 | 307 | -5 | AluSx | Alu | SINE |
| 172823929 | 172824225 | - | 13.8 | 1 | 298 | -14 | AluSx | Alu | SINE |
| 172824226 | 172825887 | - | 11.8 | 5896 | 7558 | -166 | L1MA3 | L1 | LINE |
| 172825913 | 172825993 | + | 13.6 | 352 | 432 | 0 | MSTB1 | MaLR | LTR |
| 172826114 | 172832140 | - | 0.5 | 1 | 6029 | -3 | L1HS | L1 | LINE |
| 172832139 | 172833809 | - | 15.7 | 2727 | 4490 | -1653 | L1M2 | L1 | LINE |
| 172833810 | 172834108 | + | 10.4 | 1 | 300 | -9 | AluSc | Alu | SINE |
| 172834109 | 172835715 | - | 13.6 | 2523 | 4145 | -3417 | L1MA3 | L1 | LINE |
| 172835719 | 172836071 | - | 6.5 | 2 | 355 | 0 | THE1A | MaLR | LTR |
| 172836073 | 172837164 | - | 9.5 | 503 | 1580 | 0 | THE1A-int | MaLR | LTR |
| 172837165 | 172837418 | + | 6.3 | 5900 | 6153 | -1 | L1PA6 | L1 | LINE |
| 172837419 | 172837930 | - | 9.5 | 1 | 502 | -1078 | THE1A-int | MaLR | LTR |
| 172837933 | 172838322 | - | 10.0 | 1 | 353 | -2 | THE1A | MaLR | LTR |
| 172838323 | 172840397 | - | 19.1 | 12 | 2526 | -5039 | L1MA3 | L1 | LINE |
| 172840399 | 172840622 | - | 13.7 | 981 | 1210 | -92 | HSMAR2 | Mariner | DNA |
| 172840623 | 172840905 | + | 15.6 | 1 | 286 | -26 | AluSx | Alu | SINE |
| 172840945 | 172841934 | - | 13.7 | 2 | 980 | -322 | HSMAR2 | Mariner | DNA |

  

|  |  |  |  |  |  |  |  |  |  |
| --- | --- | --- | --- | --- | --- | --- | --- | --- | --- |
| chr1:175764913-175778730 | | | 13526 bp | | |  |  |  |  |
| Genome Start | Genome End | Strand | Div | Repeat Start | Repeat End | Repeat Left | Name | Family | Class |
| 175764913 | 175765769 | + | 25.4 | 3120 | 3946 | -2200 | L1M5 | L1 | LINE |
| 175765770 | 175766073 | - | 17.8 | 5994 | 6312 | 0 | L1MA9 | L1 | LINE |
| 175766074 | 175766379 | + | 25.4 | 3947 | 4215 | -1931 | L1M5 | L1 | LINE |
| 175766380 | 175766663 | - | 17.6 | 3 | 286 | -26 | AluSx | Alu | SINE |
| 175766664 | 175767722 | + | 25.4 | 4216 | 5302 | -844 | L1M5 | L1 | LINE |
| 175767905 | 175767970 | + | 34.9 | 1184 | 1249 | -5536 | L1PA13 | L1 | LINE |
| 175768024 | 175768516 | + | 14.9 | 1299 | 1806 | -4977 | L1PA13 | L1 | LINE |
| 175768517 | 175768900 | - | 17.8 | 1 | 428 | 0 | MSTA | MaLR | LTR |
| 175768901 | 175770429 | - | 28.9 | 2 | 1735 | 0 | MLT-int | MaLR | LTR |
| 175770432 | 175770811 | - | 14.8 | 1 | 428 | 0 | MSTA | MaLR | LTR |
| 175770812 | 175770921 | + | 12.9 | 1713 | 1817 | -4872 | L1PA15 | L1 | LINE |
| 175770922 | 175771233 | + | 14.8 | 1 | 311 | -1 | AluSx | Alu | SINE |
| 175771234 | 175772693 | + | 13.2 | 1818 | 3306 | -3367 | L1PA15 | L1 | LINE |
| 175772694 | 175773003 | + | 8.4 | 1 | 310 | -1 | AluY | Alu | SINE |
| 175773004 | 175776269 | + | 13.3 | 3307 | 6636 | -56 | L1PA15 | L1 | LINE |
| 175776270 | 175776318 | + | 2.0 | 6110 | 6158 | -2 | L1PREC2 | L1 | LINE |
| 175776327 | 175778345 | + | 10.2 | 4117 | 6163 | 0 | L1PA13 | L1 | LINE |
| 175778393 | 175778730 | + | 24.2 | 5295 | 5667 | -497 | L1M5 | L1 | LINE |

  

|  |  |  |  |  |  |  |  |  |  |
| --- | --- | --- | --- | --- | --- | --- | --- | --- | --- |
| chr1:177704444-177718540 | | | 13985 bp | | |  |  |  |  |
| Genome Start | Genome End | Strand | Div | Repeat Start | Repeat End | Repeat Left | Name | Family | Class |
| 177704444 | 177704690 | - | 18.8 | 6802 | 7031 | -13 | L1MB7 | L1 | LINE |
| 177704691 | 177704987 | - | 10.4 | 1 | 297 | -16 | AluSq | Alu | SINE |
| 177704988 | 177705595 | - | 18.8 | 6205 | 6801 | -243 | L1MB7 | L1 | LINE |
| 177705615 | 177707645 | - | 17.9 | 3818 | 5982 | -1024 | L1MB7 | L1 | LINE |
| 177707646 | 177707950 | - | 9.6 | 3 | 305 | -5 | AluSg | Alu | SINE |
| 177707951 | 177708987 | - | 19.9 | 2772 | 3817 | -3189 | L1MB7 | L1 | LINE |
| 177708988 | 177709268 | + | 9.0 | 17 | 295 | -18 | AluSq | Alu | SINE |
| 177709269 | 177709386 | - | 28.9 | 2658 | 2771 | -4237 | L1MB7 | L1 | LINE |
| 177709387 | 177709699 | + | 13.8 | 1 | 341 | -40 | THE1D | MaLR | LTR |
| 177709700 | 177709990 | + | 11.0 | 1 | 298 | -14 | AluSx | Alu | SINE |
| 177709991 | 177710043 | + | 13.8 | 342 | 381 | 0 | THE1D | MaLR | LTR |
| 177710045 | 177711562 | + | 30.1 | 1 | 1735 | 0 | MLT-int | MaLR | LTR |
| 177711563 | 177711947 | + | 15.7 | 1 | 381 | 0 | THE1D | MaLR | LTR |
| 177711948 | 177713064 | - | 21.8 | 1507 | 2657 | -4351 | L1MB7 | L1 | LINE |
| 177713065 | 177713432 | - | 14.8 | 1 | 364 | 0 | THE1B | MaLR | LTR |
| 177713433 | 177714575 | - | 30.3 | 518 | 1735 | 0 | MLT-int | MaLR | LTR |
| 177714576 | 177714886 | + | 14.3 | 1 | 309 | -3 | AluSx | Alu | SINE |
| 177714887 | 177714952 | - | 30.3 | 433 | 517 | -1218 | MLT-int | MaLR | LTR |
| 177714953 | 177715253 | + | 10.6 | 1 | 303 | -10 | AluSp | Alu | SINE |
| 177715254 | 177715560 | + | 10.3 | 1 | 307 | 0 | AluY | Alu | SINE |
| 177715561 | 177715926 | - | 30.3 | 2 | 432 | -1303 | MLT-int | MaLR | LTR |
| 177715929 | 177716292 | - | 12.5 | 1 | 364 | 0 | THE1B | MaLR | LTR |
| 177716293 | 177716658 | - | 19.7 | 1045 | 1556 | -5452 | L1MB7 | L1 | LINE |
| 177716747 | 177717535 | - | 3.3 | 5362 | 6149 | -5 | L1PA5 | L1 | LINE |
| 177717538 | 177718540 | - | 31.1 | 11 | 1047 | -6287 | L1MB7 | L1 | LINE |

  

|  |  |  |  |  |  |  |  |  |  |
| --- | --- | --- | --- | --- | --- | --- | --- | --- | --- |
| chr1:180182884-180185846 | | | 2961 bp | | |  |  |  |  |
| Genome Start | Genome End | Strand | Div | Repeat Start | Repeat End | Repeat Left | Name | Family | Class |
| 180182884 | 180183195 | - | 28.6 | 5456 | 5793 | -328 | L1M5 | L1 | LINE |
| 180183196 | 180183564 | + | 11.7 | 1 | 375 | 0 | THE1C | MaLR | LTR |
| 180183567 | 180185233 | + | 31.2 | 2 | 1734 | -1 | MLT-int | MaLR | LTR |
| 180185234 | 180185597 | + | 13.6 | 1 | 375 | 0 | THE1C | MaLR | LTR |
| 180185598 | 180185846 | - | 35.1 | 5119 | 5455 | -666 | L1M5 | L1 | LINE |

  

|  |  |  |  |  |  |  |  |  |  |
| --- | --- | --- | --- | --- | --- | --- | --- | --- | --- |
| chr1:180199365-180202582 | | | 3216 bp | | |  |  |  |  |
| Genome Start | Genome End | Strand | Div | Repeat Start | Repeat End | Repeat Left | Name | Family | Class |
| 180199365 | 180200085 | + | 22.3 | 5177 | 5953 | -209 | L1ME3 | L1 | LINE |
| 180200086 | 180200420 | - | 13.6 | 1 | 373 | -2 | THE1C | MaLR | LTR |
| 180200421 | 180202032 | - | 30.0 | 2 | 1735 | 0 | MLT-int | MaLR | LTR |
| 180202035 | 180202390 | - | 12.5 | 1 | 375 | 0 | THE1C | MaLR | LTR |
| 180202391 | 180202582 | + | 22.1 | 5954 | 6149 | -13 | L1ME3 | L1 | LINE |

  

|  |  |  |  |  |  |  |  |  |  |
| --- | --- | --- | --- | --- | --- | --- | --- | --- | --- |
| chr1:184758190-184761259 | | | 3066 bp | | |  |  |  |  |
| Genome Start | Genome End | Strand | Div | Repeat Start | Repeat End | Repeat Left | Name | Family | Class |
| 184758190 | 184758337 | - | 28.0 | 2644 | 2796 | -4230 | L1MEd | L1 | LINE |
| 184758338 | 184758697 | + | 14.8 | 1 | 381 | 0 | THE1D | MaLR | LTR |
| 184758700 | 184760315 | + | 31.7 | 2 | 1734 | -1 | MLT-int | MaLR | LTR |
| 184760317 | 184760693 | + | 16.6 | 1 | 381 | 0 | THE1D | MaLR | LTR |
| 184760694 | 184761031 | - | 28.1 | 2256 | 2643 | -4383 | L1MEd | L1 | LINE |
| 184761033 | 184761259 | - | 30.1 | 1853 | 2090 | -5244 | L1MEd | L1 | LINE |

  

|  |  |  |  |  |  |  |  |  |  |
| --- | --- | --- | --- | --- | --- | --- | --- | --- | --- |
| chr1:184817351-184820825 | | | 3473 bp | | |  |  |  |  |
| Genome Start | Genome End | Strand | Div | Repeat Start | Repeat End | Repeat Left | Name | Family | Class |
| 184817351 | 184818308 | - | 15.3 | 2260 | 3246 | 0 | Ricksha | MuDR | DNA |
| 184818309 | 184818663 | + | 10.2 | 4 | 364 | 0 | THE1B | MaLR | LTR |
| 184818666 | 184820221 | + | 28.7 | 2 | 1735 | 0 | MLT-int | MaLR | LTR |
| 184820222 | 184820584 | + | 10.2 | 1 | 364 | 0 | THE1B | MaLR | LTR |
| 184820585 | 184820825 | - | 15.3 | 2016 | 2259 | -987 | Ricksha | MuDR | DNA |

  

|  |  |  |  |  |  |  |  |  |  |
| --- | --- | --- | --- | --- | --- | --- | --- | --- | --- |
| chr1:185411069-185420819 | | | 9624 bp | | |  |  |  |  |
| Genome Start | Genome End | Strand | Div | Repeat Start | Repeat End | Repeat Left | Name | Family | Class |
| 185411069 | 185412363 | - | 21.6 | 3635 | 4929 | -1812 | L1M4c | L1 | LINE |
| 185412364 | 185412667 | - | 17.4 | 1 | 299 | -13 | AluJo | Alu | SINE |
| 185412668 | 185412860 | - | 21.6 | 3449 | 3634 | -3107 | L1M4c | L1 | LINE |
| 185412864 | 185413269 | - | 14.8 | 1 | 428 | 0 | MSTA | MaLR | LTR |
| 185413270 | 185413421 | - | 16.4 | 1498 | 1651 | 0 | MSTA-int | MaLR | LTR |
| 185413414 | 185413520 | - | 11.5 | 942 | 1045 | -535 | MSTA-int | MaLR | LTR |
| 185413521 | 185413649 | + | 18.6 | 40 | 168 | -8 | FRAM | Alu | SINE |
| 185413719 | 185415233 | - | 15.5 | 3 | 1466 | -114 | MSTA-int | MaLR | LTR |
| 185415240 | 185415614 | - | 17.2 | 1 | 424 | -4 | MSTA | MaLR | LTR |
| 185415653 | 185415945 | + | 23.4 | 1 | 287 | -25 | AluJo | Alu | SINE |
| 185415965 | 185416765 | - | 19.3 | 2560 | 3392 | -3349 | L1M4c | L1 | LINE |
| 185416767 | 185419993 | + | 17.6 | 3507 | 6789 | -773 | L1M1 | L1 | LINE |
| 185419993 | 185420819 | - | 26.6 | 635 | 2158 | -4936 | L1M4c | L1 | LINE |

  

|  |  |  |  |  |  |  |  |  |  |
| --- | --- | --- | --- | --- | --- | --- | --- | --- | --- |
| chr1:186181263-186185543 | | | 4306 bp | | |  |  |  |  |
| Genome Start | Genome End | Strand | Div | Repeat Start | Repeat End | Repeat Left | Name | Family | Class |
| 186181263 | 186181806 | + | 20.6 | 1 | 1822 | -4686 | L1MCa | L1 | LINE |
| 186181781 | 186182255 | + | 17.6 | 2158 | 2634 | -3687 | L1MCa | L1 | LINE |
| 186182256 | 186182595 | + | 14.5 | 1 | 364 | 0 | THE1B | MaLR | LTR |
| 186182596 | 186184138 | + | 9.6 | 1 | 1580 | 0 | THE1B-int | MaLR | LTR |
| 186184140 | 186184494 | + | 15.6 | 1 | 364 | 0 | THE1B | MaLR | LTR |
| 186184495 | 186185543 | + | 18.2 | 2630 | 3697 | -2624 | L1MCa | L1 | LINE |

  

|  |  |  |  |  |  |  |  |  |  |
| --- | --- | --- | --- | --- | --- | --- | --- | --- | --- |
| chr1:187184208-187198023 | | | 13635 bp | | |  |  |  |  |
| Genome Start | Genome End | Strand | Div | Repeat Start | Repeat End | Repeat Left | Name | Family | Class |
| 187184208 | 187185334 | - | 25.2 | 4286 | 5414 | -992 | L1MDb | L1 | LINE |
| 187185422 | 187185585 | - | 24.4 | 4122 | 4294 | -2112 | L1MDb | L1 | LINE |
| 187185619 | 187185822 | + | 23.9 | 17 | 211 | -294 | MLT1D | MaLR | LTR |
| 187185826 | 187187315 | - | 26.6 | 2593 | 4120 | -2286 | L1MDb | L1 | LINE |
| 187187317 | 187187988 | + | 22.3 | 12 | 705 | 0 | MER44D | MER2\_type | DNA |
| 187187989 | 187188665 | - | 26.5 | 1847 | 2600 | -3806 | L1MDb | L1 | LINE |
| 187188661 | 187190946 | + | 22.1 | 5 | 2292 | -4211 | L1PA16 | L1 | LINE |
| 187190969 | 187191656 | + | 16.1 | 4124 | 4751 | -1732 | L1PA16 | L1 | LINE |
| 187191657 | 187192080 | - | 16.5 | 1 | 428 | 0 | MSTA | MaLR | LTR |
| 187192081 | 187193773 | + | 16.4 | 4752 | 6503 | 0 | L1PA16 | L1 | LINE |
| 187193780 | 187193937 | - | 26.0 | 1677 | 1854 | -4648 | L1MDb | L1 | LINE |
| 187193964 | 187194649 | - | 26.8 | 479 | 1151 | -5258 | L1MDb | L1 | LINE |
| 187194654 | 187195115 | + | 10.6 | 1 | 487 | -6 | LTR15 | ERV1 | LTR |
| 187195118 | 187195594 | - | 31.5 | 221 | 651 | -5758 | L1MDb | L1 | LINE |
| 187195595 | 187195949 | + | 17.5 | 1 | 364 | 0 | THE1B | MaLR | LTR |
| 187195952 | 187197520 | + | 30.1 | 2 | 1735 | 0 | MLT-int | MaLR | LTR |
| 187197521 | 187197874 | + | 16.9 | 1 | 364 | 0 | THE1B | MaLR | LTR |
| 187197875 | 187198023 | - | 30.9 | 64 | 220 | -6189 | L1MDb | L1 | LINE |

  

|  |  |  |  |  |  |  |  |  |  |
| --- | --- | --- | --- | --- | --- | --- | --- | --- | --- |
| chr1:187829063-187831876 | | | 2812 bp | | |  |  |  |  |
| Genome Start | Genome End | Strand | Div | Repeat Start | Repeat End | Repeat Left | Name | Family | Class |
| 187829063 | 187829485 | - | 26.7 | 1977 | 2471 | -4555 | L1M4b | L1 | LINE |
| 187829486 | 187829831 | + | 15.8 | 1 | 428 | 0 | MSTA | MaLR | LTR |
| 187829834 | 187831445 | + | 30.4 | 2 | 1735 | 0 | MLT1F2-int | MaLR | LTR |
| 187831446 | 187831805 | + | 15.8 | 1 | 428 | 0 | MSTA | MaLR | LTR |
| 187831806 | 187831876 | - | 28.6 | 1907 | 1976 | -5358 | L1M4b | L1 | LINE |

  

|  |  |  |  |  |  |  |  |  |  |
| --- | --- | --- | --- | --- | --- | --- | --- | --- | --- |
| chr1:188210384-188218542 | | | 8163 bp | | |  |  |  |  |
| Genome Start | Genome End | Strand | Div | Repeat Start | Repeat End | Repeat Left | Name | Family | Class |
| 188210384 | 188212231 | - | 17.5 | 5834 | 7724 | 0 | L1MA3 | L1 | LINE |
| 188212232 | 188212583 | - | 16.0 | 1 | 362 | -2 | THE1B | MaLR | LTR |
| 188212584 | 188214155 | - | 31.2 | 2 | 1735 | 0 | MLT-int | MaLR | LTR |
| 188214158 | 188214509 | - | 13.7 | 1 | 364 | 0 | THE1B | MaLR | LTR |
| 188214510 | 188216180 | - | 17.9 | 4135 | 5833 | -1732 | L1MA3 | L1 | LINE |
| 188216175 | 188218542 | - | 22.6 | 16 | 3094 | -4471 | L1MA3 | L1 | LINE |

  

|  |  |  |  |  |  |  |  |  |  |
| --- | --- | --- | --- | --- | --- | --- | --- | --- | --- |
| chr1:189087902-189099989 | | | 11998 bp | | |  |  |  |  |
| Genome Start | Genome End | Strand | Div | Repeat Start | Repeat End | Repeat Left | Name | Family | Class |
| 189087902 | 189091300 | - | 17.6 | 3068 | 6501 | -2 | L1PA16 | L1 | LINE |
| 189091301 | 189091726 | + | 19.4 | 1 | 428 | 0 | MSTA | MaLR | LTR |
| 189091727 | 189092194 | + | 17.7 | 1 | 471 | -1180 | MSTA-int | MaLR | LTR |
| 189092223 | 189093897 | - | 12.5 | 4469 | 6156 | -7 | L1PA13 | L1 | LINE |
| 189093898 | 189095069 | + | 21.0 | 457 | 1651 | 0 | MSTA-int | MaLR | LTR |
| 189095070 | 189095224 | + | 15.6 | 1 | 154 | -274 | MSTA | MaLR | LTR |
| 189095225 | 189095578 | - | 13.0 | 1 | 364 | 0 | THE1B | MaLR | LTR |
| 189095579 | 189097149 | - | 32.0 | 2 | 1735 | 0 | MLT1A0-int | MaLR | LTR |
| 189097158 | 189097482 | - | 14.8 | 1 | 358 | -6 | THE1B | MaLR | LTR |
| 189097483 | 189097758 | + | 18.8 | 150 | 428 | 0 | MSTA | MaLR | LTR |
| 189097759 | 189099789 | - | 17.8 | 992 | 3072 | -3411 | L1PA16 | L1 | LINE |
| 189099844 | 189099989 | - | 17.8 | 840 | 991 | -5512 | L1PA16 | L1 | LINE |

  

|  |  |  |  |  |  |  |  |  |  |
| --- | --- | --- | --- | --- | --- | --- | --- | --- | --- |
| chr1:189452237-189455263 | | | 3019 bp | | |  |  |  |  |
| Genome Start | Genome End | Strand | Div | Repeat Start | Repeat End | Repeat Left | Name | Family | Class |
| 189452237 | 189452281 | + | 20.0 | 1 | 45 | -470 | MLT2B1 | ERVL | LTR |
| 189452283 | 189452667 | - | 17.2 | 2 | 428 | 0 | MSTA | MaLR | LTR |
| 189452669 | 189454303 | - | 30.5 | 1 | 1735 | 0 | MLT-int | MaLR | LTR |
| 189454305 | 189454525 | - | 19.5 | 192 | 428 | 0 | MSTA | MaLR | LTR |
| 189454527 | 189454738 | - | 19.0 | 87 | 297 | -15 | AluJb | Alu | SINE |
| 189454743 | 189454920 | - | 15.3 | 1 | 202 | -226 | MSTA | MaLR | LTR |
| 189454921 | 189455263 | + | 19.8 | 42 | 497 | -65 | MLT2B1 | ERVL | LTR |

  

|  |  |  |  |  |  |  |  |  |  |
| --- | --- | --- | --- | --- | --- | --- | --- | --- | --- |
| chr1:189631906-189634738 | | | 2831 bp | | |  |  |  |  |
| Genome Start | Genome End | Strand | Div | Repeat Start | Repeat End | Repeat Left | Name | Family | Class |
| 189631906 | 189632346 | - | 20.6 | 2418 | 3786 | -2360 | L1MC | L1 | LINE |
| 189632347 | 189632712 | + | 15.6 | 1 | 364 | 0 | THE1B | MaLR | LTR |
| 189632715 | 189634272 | + | 31.2 | 2 | 1735 | 0 | MLT-int | MaLR | LTR |
| 189634273 | 189634646 | + | 14.1 | 1 | 364 | 0 | THE1B | MaLR | LTR |
| 189634647 | 189634738 | - | 22.3 | 2335 | 2417 | -3729 | L1MC | L1 | LINE |

  

|  |  |  |  |  |  |  |  |  |  |
| --- | --- | --- | --- | --- | --- | --- | --- | --- | --- |
| chr1:190659196-190671283 | | | 11885 bp | | |  |  |  |  |
| Genome Start | Genome End | Strand | Div | Repeat Start | Repeat End | Repeat Left | Name | Family | Class |
| 190659196 | 190659388 | - | 34.2 | 3207 | 3399 | -20 | L2 | L2 | LINE |
| 190659439 | 190659583 | - | 22.4 | 453 | 668 | -30 | MLT1E | MaLR | LTR |
| 190659655 | 190661670 | + | 22.1 | 1 | 2270 | -4939 | L1MA4A | L1 | LINE |
| 190661671 | 190661982 | + | 14.4 | 1 | 312 | 0 | AluSx | Alu | SINE |
| 190661983 | 190665270 | + | 20.8 | 2271 | 5369 | -1839 | L1MA4A | L1 | LINE |
| 190665271 | 190665644 | + | 14.8 | 1 | 381 | 0 | THE1D | MaLR | LTR |
| 190665648 | 190667266 | + | 32.1 | 3 | 1735 | 0 | MLT-int | MaLR | LTR |
| 190667268 | 190667638 | + | 13.4 | 2 | 381 | 0 | THE1D | MaLR | LTR |
| 190667639 | 190669626 | + | 17.1 | 5370 | 7356 | -9 | L1MA4A | L1 | LINE |
| 190669631 | 190670104 | - | 17.2 | 5786 | 6279 | -33 | L1MA9 | L1 | LINE |
| 190670136 | 190670499 | - | 17.2 | 5419 | 5785 | -527 | L1MA9 | L1 | LINE |
| 190670503 | 190670758 | - | 26.2 | 188 | 478 | -176 | MLT1E | MaLR | LTR |
| 190670759 | 190671068 | - | 12.9 | 1 | 310 | -2 | AluSx | Alu | SINE |
| 190671069 | 190671171 | - | 29.0 | 68 | 187 | -406 | MLT1E | MaLR | LTR |
| 190671212 | 190671283 | - | 32.4 | 3088 | 3158 | -261 | L2 | L2 | LINE |

  

|  |  |  |  |  |  |  |  |  |  |
| --- | --- | --- | --- | --- | --- | --- | --- | --- | --- |
| chr1:191609774-191625568 | | | 15305 bp | | |  |  |  |  |
| Genome Start | Genome End | Strand | Div | Repeat Start | Repeat End | Repeat Left | Name | Family | Class |
| 191609774 | 191610040 | - | 20.7 | 5350 | 5618 | -548 | L1M5 | L1 | LINE |
| 191610041 | 191610097 | + | 8.8 | 6248 | 6304 | 0 | L1MA2 | L1 | LINE |
| 191610331 | 191610474 | - | 24.3 | 6176 | 6334 | 0 | L1MA10 | L1 | LINE |
| 191610587 | 191610836 | - | 14.4 | 5407 | 5658 | -675 | L1MC | L1 | LINE |
| 191610840 | 191611388 | - | 18.1 | 1 | 554 | 0 | MLT2A2 | ERVL | LTR |
| 191611389 | 191613048 | - | 9.2 | 3945 | 5654 | 0 | HERVL-A2 | ERVL | LTR |
| 191613051 | 191613177 | + | 8.7 | 248 | 375 | 0 | THE1C | MaLR | LTR |
| 191613178 | 191613243 | + | 18.5 | 1 | 66 | -1514 | THE1C-int | MaLR | LTR |
| 191613240 | 191614532 | + | 13.6 | 242 | 1580 | 0 | THE1C-int | MaLR | LTR |
| 191614533 | 191614895 | + | 13.7 | 1 | 374 | -1 | THE1C | MaLR | LTR |
| 191614903 | 191617125 | - | 10.0 | 1648 | 3918 | -1736 | HERVL-A2 | ERVL | LTR |
| 191617126 | 191617528 | + | 8.8 | 30 | 435 | 0 | AluY | Alu | SINE |
| 191617529 | 191619121 | - | 15.7 | 5 | 1647 | -4007 | HERVL-A2 | ERVL | LTR |
| 191619126 | 191619698 | - | 18.2 | 1 | 554 | 0 | MLT2A2 | ERVL | LTR |
| 191619704 | 191620071 | - | 20.5 | 5036 | 5403 | -743 | L1MC | L1 | LINE |
| 191620072 | 191620371 | - | 12.0 | 1 | 301 | -11 | AluSx | Alu | SINE |
| 191620374 | 191620566 | - | 16.8 | 117 | 307 | -5 | AluJo | Alu | SINE |
| 191620567 | 191621931 | - | 2.2 | 4774 | 6141 | -14 | L1PA3 | L1 | LINE |
| 191621943 | 191622075 | - | 19.7 | 6 | 137 | -6 | FLAM\_C | Alu | SINE |
| 191622076 | 191622662 | - | 14.1 | 4480 | 5004 | -1142 | L1MC | L1 | LINE |
| 191622686 | 191622977 | + | 14.4 | 1 | 292 | -20 | AluSx | Alu | SINE |
| 191623025 | 191623336 | - | 15.8 | 5969 | 6264 | -27 | L1MA7 | L1 | LINE |
| 191623375 | 191623997 | - | 16.8 | 4836 | 5470 | -821 | L1MA7 | L1 | LINE |
| 191623998 | 191624097 | - | 22.0 | 154 | 255 | -198 | MLT2A2 | ERVL | LTR |
| 191624098 | 191624259 | - | 16.8 | 4671 | 4835 | -1456 | L1MA7 | L1 | LINE |
| 191624260 | 191624474 | - | 13.3 | 191 | 428 | 0 | MSTA | MaLR | LTR |
| 191624482 | 191624741 | - | 13.1 | 512 | 787 | -793 | THE1-int | MaLR | LTR |
| 191624742 | 191625033 | - | 14.3 | 3857 | 4137 | -2009 | L1MC | L1 | LINE |
| 191625034 | 191625314 | - | 18.9 | 1 | 283 | -29 | AluJo | Alu | SINE |
| 191625315 | 191625568 | - | 15.6 | 3738 | 4384 | -1762 | L1M5 | L1 | LINE |

  

|  |  |  |  |  |  |  |  |  |  |
| --- | --- | --- | --- | --- | --- | --- | --- | --- | --- |
| chr1:191675159-191679769 | | | 4550 bp | | |  |  |  |  |
| Genome Start | Genome End | Strand | Div | Repeat Start | Repeat End | Repeat Left | Name | Family | Class |
| 191675159 | 191676611 | + | 18.6 | 4 | 1470 | -4244 | ERVL-B2 | ERVL | LTR |
| 191676612 | 191676913 | + | 22.9 | 5 | 328 | -68 | MSTD | MaLR | LTR |
| 191676968 | 191677018 | + | 22.9 | 329 | 396 | 0 | MSTD | MaLR | LTR |
| 191677021 | 191678708 | + | 24.3 | 2 | 1722 | -13 | MSTD-int | MaLR | LTR |
| 191678714 | 191679070 | + | 22.0 | 1 | 396 | 0 | MSTD | MaLR | LTR |
| 191679071 | 191679769 | + | 20.2 | 1470 | 2166 | -3548 | ERVL-B2 | ERVL | LTR |

  

|  |  |  |  |  |  |  |  |  |  |
| --- | --- | --- | --- | --- | --- | --- | --- | --- | --- |
| chr1:192293433-192296715 | | | 3249 bp | | |  |  |  |  |
| Genome Start | Genome End | Strand | Div | Repeat Start | Repeat End | Repeat Left | Name | Family | Class |
| 192293433 | 192293614 | + | 30.6 | 1 | 194 | -270 | LTR16B | ERVL | LTR |
| 192293624 | 192293800 | - | 20.9 | 11 | 193 | 0 | MER53 | DNA | DNA |
| 192293820 | 192294026 | + | 25.5 | 214 | 424 | -40 | LTR16B | ERVL | LTR |
| 192294027 | 192294249 | - | 18.2 | 203 | 428 | 0 | MSTA | MaLR | LTR |
| 192294250 | 192294479 | - | 7.3 | 80 | 298 | -13 | AluY | Alu | SINE |
| 192294480 | 192294657 | - | 18.2 | 1 | 202 | -226 | MSTA | MaLR | LTR |
| 192294662 | 192296301 | - | 31.0 | 2 | 1731 | -4 | MLT-int | MaLR | LTR |
| 192296304 | 192296673 | - | 16.8 | 1 | 428 | 0 | MSTA | MaLR | LTR |
| 192296674 | 192296715 | + | 25.5 | 425 | 462 | -2 | LTR16B | ERVL | LTR |

  

|  |  |  |  |  |  |  |  |  |  |
| --- | --- | --- | --- | --- | --- | --- | --- | --- | --- |
| chr1:192498292-192501139 | | | 2846 bp | | |  |  |  |  |
| Genome Start | Genome End | Strand | Div | Repeat Start | Repeat End | Repeat Left | Name | Family | Class |
| 192498292 | 192498761 | + | 16.9 | 5611 | 6082 | -83 | L1PA15 | L1 | LINE |
| 192498762 | 192499126 | + | 12.2 | 1 | 355 | 0 | THE1A | MaLR | LTR |
| 192499129 | 192500702 | + | 30.7 | 2 | 1735 | 0 | MLT-int | MaLR | LTR |
| 192500703 | 192501056 | + | 10.2 | 1 | 355 | 0 | THE1A | MaLR | LTR |
| 192501057 | 192501139 | + | 16.9 | 6083 | 6163 | -2 | L1PA15 | L1 | LINE |

  

|  |  |  |  |  |  |  |  |  |  |
| --- | --- | --- | --- | --- | --- | --- | --- | --- | --- |
| chr1:192873881-192877936 | | | 4053 bp | | |  |  |  |  |
| Genome Start | Genome End | Strand | Div | Repeat Start | Repeat End | Repeat Left | Name | Family | Class |
| 192873881 | 192874166 | - | 14.9 | 5881 | 6160 | -5 | L1PA15 | L1 | LINE |
| 192874167 | 192874510 | + | 14.3 | 4 | 364 | 0 | THE1B | MaLR | LTR |
| 192874513 | 192876087 | + | 32.4 | 2 | 1735 | 0 | MLT1H-int | MaLR | LTR |
| 192876089 | 192876445 | + | 16.3 | 1 | 363 | -1 | THE1B | MaLR | LTR |
| 192876446 | 192877936 | - | 14.4 | 4354 | 5880 | -285 | L1PA15 | L1 | LINE |

  

|  |  |  |  |  |  |  |  |  |  |
| --- | --- | --- | --- | --- | --- | --- | --- | --- | --- |
| chr1:193248682-193251351 | | | 2682 bp | | |  |  |  |  |
| Genome Start | Genome End | Strand | Div | Repeat Start | Repeat End | Repeat Left | Name | Family | Class |
| 193248682 | 193248811 | - | 27.7 | 435 | 559 | 0 | MER89 | ERV1 | LTR |
| 193248812 | 193249154 | - | 17.3 | 1 | 364 | 0 | THE1B | MaLR | LTR |
| 193249155 | 193250715 | - | 30.8 | 2 | 1735 | 0 | MLT-int | MaLR | LTR |
| 193250718 | 193251062 | - | 15.4 | 1 | 364 | 0 | THE1B | MaLR | LTR |
| 193251063 | 193251292 | - | 27.7 | 197 | 434 | -125 | MER89 | ERV1 | LTR |
| 193251279 | 193251351 | - | 20.6 | 3 | 76 | -483 | MER89 | ERV1 | LTR |

  

|  |  |  |  |  |  |  |  |  |  |
| --- | --- | --- | --- | --- | --- | --- | --- | --- | --- |
| chr1:193442617-193446558 | | | 3939 bp | | |  |  |  |  |
| Genome Start | Genome End | Strand | Div | Repeat Start | Repeat End | Repeat Left | Name | Family | Class |
| 193442617 | 193443026 | - | 20.6 | 5904 | 6327 | -6 | L1MC1 | L1 | LINE |
| 193443027 | 193443385 | + | 16.5 | 1 | 381 | 0 | THE1D | MaLR | LTR |
| 193443386 | 193444910 | + | 15.1 | 1 | 1579 | -1 | THE1D-int | MaLR | LTR |
| 193444912 | 193445285 | + | 15.8 | 1 | 381 | 0 | THE1D | MaLR | LTR |
| 193445288 | 193445607 | - | 18.9 | 5588 | 5906 | -427 | L1MC1 | L1 | LINE |
| 193445608 | 193445918 | + | 14.8 | 1 | 311 | 0 | AluY | Alu | SINE |
| 193445919 | 193446558 | - | 18.3 | 4900 | 5587 | -746 | L1MC1 | L1 | LINE |

  

|  |  |  |  |  |  |  |  |  |  |
| --- | --- | --- | --- | --- | --- | --- | --- | --- | --- |
| chr1:194901985-194907142 | | | 5048 bp | | |  |  |  |  |
| Genome Start | Genome End | Strand | Div | Repeat Start | Repeat End | Repeat Left | Name | Family | Class |
| 194901985 | 194902258 | - | 27.5 | 3370 | 3649 | -2995 | L1M4 | L1 | LINE |
| 194902259 | 194902578 | + | 8.5 | 2 | 302 | -9 | AluY | Alu | SINE |
| 194902579 | 194902917 | - | 27.5 | 2948 | 3369 | -3275 | L1M4 | L1 | LINE |
| 194902919 | 194903126 | - | 29.9 | 6 | 209 | -1 | MER63A | AcHobo | DNA |
| 194903130 | 194904121 | - | 30.4 | 2003 | 2970 | -3694 | L1M4 | L1 | LINE |
| 194904223 | 194904597 | + | 15.4 | 1 | 381 | 0 | THE1D | MaLR | LTR |
| 194904598 | 194906105 | + | 14.8 | 1 | 1580 | 0 | THE1D-int | MaLR | LTR |
| 194906106 | 194906474 | + | 13.9 | 1 | 381 | 0 | THE1D | MaLR | LTR |
| 194906477 | 194906782 | - | 29.1 | 1576 | 1899 | -4926 | L1M4 | L1 | LINE |
| 194906786 | 194907142 | - | 31.2 | 1020 | 1529 | -5118 | L1M4 | L1 | LINE |

  

|  |  |  |  |  |  |  |  |  |  |
| --- | --- | --- | --- | --- | --- | --- | --- | --- | --- |
| chr1:195256710-195260486 | | | 3749 bp | | |  |  |  |  |
| Genome Start | Genome End | Strand | Div | Repeat Start | Repeat End | Repeat Left | Name | Family | Class |
| 195256710 | 195256855 | + | 39.2 | 189 | 336 | -3083 | L2 | L2 | LINE |
| 195256856 | 195257139 | - | 17.6 | 2 | 285 | -27 | AluJb | Alu | SINE |
| 195257140 | 195257496 | + | 39.2 | 337 | 685 | -2734 | L2 | L2 | LINE |
| 195257497 | 195257875 | + | 14.0 | 1 | 381 | 0 | THE1D | MaLR | LTR |
| 195257878 | 195259476 | + | 29.8 | 2 | 1735 | 0 | MLT-int | MaLR | LTR |
| 195259477 | 195259659 | + | 13.2 | 1 | 196 | -185 | THE1D | MaLR | LTR |
| 195259686 | 195259853 | + | 13.2 | 197 | 379 | -2 | THE1D | MaLR | LTR |
| 195259854 | 195260486 | + | 39.2 | 686 | 1332 | -2087 | L2 | L2 | LINE |

  

|  |  |  |  |  |  |  |  |  |  |
| --- | --- | --- | --- | --- | --- | --- | --- | --- | --- |
| chr1:195462236-195464988 | | | 2671 bp | | |  |  |  |  |
| Genome Start | Genome End | Strand | Div | Repeat Start | Repeat End | Repeat Left | Name | Family | Class |
| 195462236 | 195462405 | + | 21.6 | 1 | 162 | -212 | MLT1A | MaLR | LTR |
| 195462480 | 195462822 | - | 13.8 | 1 | 346 | -9 | THE1A | MaLR | LTR |
| 195462823 | 195464488 | - | 30.7 | 4 | 1735 | 0 | MLT-int | MaLR | LTR |
| 195464493 | 195464846 | - | 12.5 | 1 | 355 | 0 | THE1A | MaLR | LTR |
| 195464851 | 195464988 | + | 23.0 | 239 | 374 | 0 | MLT1A | MaLR | LTR |

  

|  |  |  |  |  |  |  |  |  |  |
| --- | --- | --- | --- | --- | --- | --- | --- | --- | --- |
| chr1:196106733-196109361 | | | 2572 bp | | |  |  |  |  |
| Genome Start | Genome End | Strand | Div | Repeat Start | Repeat End | Repeat Left | Name | Family | Class |
| 196106733 | 196106766 | + | 20.1 | 5565 | 5602 | -568 | L1ME3A | L1 | LINE |
| 196106767 | 196107106 | - | 10.7 | 1 | 355 | 0 | THE1A | MaLR | LTR |
| 196107107 | 196108675 | - | 30.0 | 2 | 1735 | 0 | MLT-int | MaLR | LTR |
| 196108678 | 196109035 | - | 11.9 | 1 | 355 | 0 | THE1A | MaLR | LTR |
| 196109036 | 196109207 | + | 20.1 | 5603 | 5770 | -400 | L1ME3A | L1 | LINE |
| 196109263 | 196109361 | + | 21.2 | 6016 | 6114 | -56 | L1ME3A | L1 | LINE |

  

|  |  |  |  |  |  |  |  |  |  |
| --- | --- | --- | --- | --- | --- | --- | --- | --- | --- |
| chr1:196268843-196279351 | | | 10252 bp | | |  |  |  |  |
| Genome Start | Genome End | Strand | Div | Repeat Start | Repeat End | Repeat Left | Name | Family | Class |
| 196268843 | 196269207 | - | 31.9 | 3033 | 3414 | -5 | L2 | L2 | LINE |
| 196269208 | 196269468 | - | 22.5 | 218 | 505 | 0 | MLT1D | MaLR | LTR |
| 196269506 | 196269703 | - | 22.5 | 1 | 217 | -288 | MLT1D | MaLR | LTR |
| 196269704 | 196270403 | - | 27.4 | 2277 | 3032 | -387 | L2 | L2 | LINE |
| 196270404 | 196270809 | + | 9.4 | 1 | 448 | -2 | LTR7 | ERV1 | LTR |
| 196270812 | 196273934 | + | 10.2 | 1 | 2987 | -4726 | HERVH | ERV1 | LTR |
| 196273935 | 196274076 | + | 16.2 | 3146 | 3290 | -4423 | HERVH | ERV1 | LTR |
| 196274067 | 196275740 | + | 8.8 | 3492 | 5146 | -2567 | HERVH | ERV1 | LTR |
| 196275745 | 196275961 | + | 13.0 | 5600 | 5819 | -1894 | HERVH | ERV1 | LTR |
| 196275962 | 196276268 | + | 11.1 | 7324 | 7649 | -64 | HERVH | ERV1 | LTR |
| 196276270 | 196276335 | + | 9.8 | 7653 | 7713 | 0 | HERVH | ERV1 | LTR |
| 196276336 | 196276743 | + | 7.8 | 1 | 448 | -2 | LTR7 | ERV1 | LTR |
| 196276745 | 196277838 | - | 26.9 | 1004 | 2280 | -1139 | L2 | L2 | LINE |
| 196277839 | 196278322 | + | 26.3 | 1 | 547 | -2 | MLT1H | MaLR | LTR |
| 196278323 | 196279063 | - | 26.9 | 219 | 1003 | -2416 | L2 | L2 | LINE |
| 196279286 | 196279351 | - | 25.0 | 25 | 92 | -3327 | L2 | L2 | LINE |

  

|  |  |  |  |  |  |  |  |  |  |
| --- | --- | --- | --- | --- | --- | --- | --- | --- | --- |
| chr1:197266047-197269322 | | | 3040 bp | | |  |  |  |  |
| Genome Start | Genome End | Strand | Div | Repeat Start | Repeat End | Repeat Left | Name | Family | Class |
| 197266047 | 197266293 | + | 34.3 | 3335 | 3604 | -885 | L3 | CR1 | LINE |
| 197266339 | 197266692 | - | 10.8 | 1 | 355 | 0 | THE1A | MaLR | LTR |
| 197266693 | 197268265 | - | 30.3 | 2 | 1735 | 0 | MLT-int | MaLR | LTR |
| 197268268 | 197268622 | - | 12.5 | 1 | 355 | 0 | THE1A | MaLR | LTR |
| 197268693 | 197268824 | - | 30.5 | 32 | 165 | -43 | MIR3 | MIR | SINE |
| 197268944 | 197269322 | + | 33.0 | 3726 | 4129 | -360 | L3 | CR1 | LINE |

  

|  |  |  |  |  |  |  |  |  |  |
| --- | --- | --- | --- | --- | --- | --- | --- | --- | --- |
| chr1:201392335-201395546 | | | 3196 bp | | |  |  |  |  |
| Genome Start | Genome End | Strand | Div | Repeat Start | Repeat End | Repeat Left | Name | Family | Class |
| 201392335 | 201392406 | + | 23.5 | 2517 | 2590 | -829 | L2 | L2 | LINE |
| 201392416 | 201392783 | - | 18.5 | 1 | 396 | 0 | MSTD | MaLR | LTR |
| 201392789 | 201394416 | - | 21.9 | 2 | 1729 | -6 | MSTD-int | MaLR | LTR |
| 201394419 | 201394820 | - | 16.8 | 1 | 396 | 0 | MSTD | MaLR | LTR |
| 201394821 | 201395546 | + | 29.6 | 2596 | 3418 | -1 | L2 | L2 | LINE |

  

|  |  |  |  |  |  |  |  |  |  |
| --- | --- | --- | --- | --- | --- | --- | --- | --- | --- |
| chr1:207416542-207425178 | | | 8449 bp | | |  |  |  |  |
| Genome Start | Genome End | Strand | Div | Repeat Start | Repeat End | Repeat Left | Name | Family | Class |
| 207416542 | 207416736 | + | 19.0 | 3 | 198 | -6665 | L1PB1 | L1 | LINE |
| 207416739 | 207422087 | + | 9.9 | 634 | 6218 | -649 | L1PB1 | L1 | LINE |
| 207422088 | 207422466 | + | 16.1 | 1 | 375 | 0 | THE1C | MaLR | LTR |
| 207422467 | 207423997 | + | 12.4 | 1 | 1580 | 0 | THE1C-int | MaLR | LTR |
| 207423998 | 207424364 | + | 14.0 | 1 | 375 | 0 | THE1C | MaLR | LTR |
| 207424365 | 207424531 | + | 8.4 | 6214 | 6380 | -487 | L1PB1 | L1 | LINE |
| 207424718 | 207425178 | + | 9.8 | 6419 | 6866 | -1 | L1PB1 | L1 | LINE |

  

|  |  |  |  |  |  |  |  |  |  |
| --- | --- | --- | --- | --- | --- | --- | --- | --- | --- |
| chr1:207685302-207694201 | | | 8843 bp | | |  |  |  |  |
| Genome Start | Genome End | Strand | Div | Repeat Start | Repeat End | Repeat Left | Name | Family | Class |
| 207685302 | 207685858 | - | 35.4 | 4522 | 5014 | -20 | HERV16 | ERVL | LTR |
| 207685859 | 207686154 | + | 11.0 | 1 | 295 | -14 | AluSc | Alu | SINE |
| 207686155 | 207686652 | - | 34.6 | 4268 | 4863 | -791 | ERVL-E | ERVL | LTR |
| 207686653 | 207687110 | + | 7.5 | 5710 | 6171 | -3 | L1PA11 | L1 | LINE |
| 207687111 | 207687531 | - | 34.4 | 3830 | 4340 | -1314 | ERVL-E | ERVL | LTR |
| 207687532 | 207687840 | - | 9.7 | 1 | 310 | -2 | AluSx | Alu | SINE |
| 207687841 | 207688503 | - | 34.4 | 3158 | 3829 | -1838 | HERV16 | ERVL | LTR |
| 207688565 | 207688771 | - | 10.4 | 6095 | 6291 | -12 | L1MA4A | L1 | LINE |
| 207688773 | 207689224 | + | 5.4 | 1 | 448 | -2 | LTR7 | ERV1 | LTR |
| 207689227 | 207692290 | + | 10.1 | 1 | 2987 | -4726 | HERVH | ERV1 | LTR |
| 207692285 | 207692432 | + | 12.8 | 3140 | 3290 | -4423 | HERVH | ERV1 | LTR |
| 207692432 | 207692576 | + | 14.5 | 3500 | 3644 | -4069 | HERVH | ERV1 | LTR |
| 207692576 | 207692926 | + | 15.7 | 4740 | 5149 | -2564 | HERVH | ERV1 | LTR |
| 207692928 | 207693141 | + | 12.2 | 5600 | 5819 | -1894 | HERVH | ERV1 | LTR |
| 207693140 | 207693519 | + | 7.5 | 7322 | 7713 | 0 | HERVH | ERV1 | LTR |
| 207693520 | 207693970 | + | 6.2 | 1 | 448 | -2 | LTR7 | ERV1 | LTR |
| 207693972 | 207694152 | - | 15.4 | 5937 | 6099 | -204 | L1MA4A | L1 | LINE |
| 207694154 | 207694201 | - | 14.6 | 1092 | 1139 | -3857 | HERV16 | ERVL | LTR |

  

|  |  |  |  |  |  |  |  |  |  |
| --- | --- | --- | --- | --- | --- | --- | --- | --- | --- |
| chr1:208224459-208229178 | | | 4718 bp | | |  |  |  |  |
| Genome Start | Genome End | Strand | Div | Repeat Start | Repeat End | Repeat Left | Name | Family | Class |
| 208224459 | 208224624 | - | 23.8 | 1731 | 1924 | -4476 | L1MEc | L1 | LINE |
| 208224625 | 208225020 | - | 14.4 | 1 | 428 | 0 | MSTA | MaLR | LTR |
| 208225021 | 208226291 | - | 29.9 | 433 | 1735 | 0 | MLT-int | MaLR | LTR |
| 208226292 | 208226594 | + | 8.8 | 1 | 295 | -16 | AluY | Alu | SINE |
| 208226595 | 208226622 | - | 29.9 | 405 | 432 | -1303 | MLT-int | MaLR | LTR |
| 208226623 | 208226889 | - | 9.7 | 1 | 298 | -12 | AluSg | Alu | SINE |
| 208226890 | 208227234 | - | 29.9 | 2 | 404 | -1331 | MLT-int | MaLR | LTR |
| 208227237 | 208227630 | - | 17.3 | 2 | 428 | 0 | MSTA | MaLR | LTR |
| 208227631 | 208229178 | - | 23.8 | 43 | 1730 | -4670 | L1MEc | L1 | LINE |

  

|  |  |  |  |  |  |  |  |  |  |
| --- | --- | --- | --- | --- | --- | --- | --- | --- | --- |
| chr1:208427054-208435826 | | | 8706 bp | | |  |  |  |  |
| Genome Start | Genome End | Strand | Div | Repeat Start | Repeat End | Repeat Left | Name | Family | Class |
| 208427054 | 208427268 | - | 28.9 | 1083 | 1292 | -180 | MLT1H-int | MaLR | LTR |
| 208427253 | 208427560 | - | 28.5 | 241 | 562 | -910 | MLT1H-int | MaLR | LTR |
| 208427572 | 208427988 | + | 6.2 | 1 | 464 | 0 | LTR7B | ERV1 | LTR |
| 208427989 | 208435268 | + | 14.7 | 1 | 7713 | 0 | HERVH | ERV1 | LTR |
| 208435269 | 208435646 | + | 6.6 | 1 | 464 | 0 | LTR7B | ERV1 | LTR |
| 208435719 | 208435826 | - | 27.8 | 1 | 114 | -1358 | MLT1H-int | MaLR | LTR |

  

|  |  |  |  |  |  |  |  |  |  |
| --- | --- | --- | --- | --- | --- | --- | --- | --- | --- |
| chr1:210433974-210440086 | | | 5736 bp | | |  |  |  |  |
| Genome Start | Genome End | Strand | Div | Repeat Start | Repeat End | Repeat Left | Name | Family | Class |
| 210433974 | 210434110 | + | 20.4 | 7022 | 7159 | -626 | L1MC3 | L1 | LINE |
| 210434162 | 210434568 | + | 15.1 | 1 | 428 | 0 | MSTA | MaLR | LTR |
| 210434571 | 210436214 | + | 27.5 | 2 | 1735 | 0 | MLT-int | MaLR | LTR |
| 210436215 | 210436634 | + | 18.8 | 1 | 428 | 0 | MSTA | MaLR | LTR |
| 210436644 | 210436813 | - | 22.8 | 1 | 197 | -42 | MER8 | MER2\_type | DNA |
| 210436814 | 210436868 | - | 15.8 | 16 | 71 | -2647 | Tigger2 | MER2\_type | DNA |
| 210436869 | 210437179 | - | 7.7 | 2 | 312 | -1 | AluSp | Alu | SINE |
| 210437217 | 210437411 | + | 16.1 | 7164 | 7389 | -396 | L1MC3 | L1 | LINE |
| 210437412 | 210437548 | + | 21.6 | 1 | 136 | -166 | AluSx | Alu | SINE |
| 210437549 | 210437850 | + | 14.6 | 2 | 303 | -9 | AluSx | Alu | SINE |
| 210437851 | 210437938 | + | 21.6 | 137 | 223 | -79 | AluSx | Alu | SINE |
| 210437939 | 210438248 | + | 4.9 | 1 | 307 | -4 | AluY | Alu | SINE |
| 210438249 | 210438305 | + | 21.6 | 224 | 299 | -3 | AluSx | Alu | SINE |
| 210438309 | 210438611 | + | 9.3 | 1 | 301 | -9 | AluSg | Alu | SINE |
| 210438813 | 210439119 | + | 10.4 | 2 | 313 | 0 | AluSq | Alu | SINE |
| 210439126 | 210439217 | + | 9.1 | 7378 | 7465 | -320 | L1MC3 | L1 | LINE |
| 210439258 | 210439382 | + | 15.2 | 176 | 300 | -12 | AluJb | Alu | SINE |
| 210439411 | 210439457 | + | 18.4 | 7462 | 7498 | -287 | L1MC3 | L1 | LINE |
| 210439458 | 210439766 | + | 10.4 | 1 | 310 | -3 | AluSp | Alu | SINE |
| 210439767 | 210440086 | + | 18.4 | 7499 | 7785 | 0 | L1MC3 | L1 | LINE |

  

|  |  |  |  |  |  |  |  |  |  |
| --- | --- | --- | --- | --- | --- | --- | --- | --- | --- |
| chr1:211816101-211821064 | | | 4910 bp | | |  |  |  |  |
| Genome Start | Genome End | Strand | Div | Repeat Start | Repeat End | Repeat Left | Name | Family | Class |
| 211816101 | 211816172 | + | 28.2 | 14 | 86 | -250 | MER2B | MER2\_type | DNA |
| 211816216 | 211816295 | + | 16.2 | 136 | 215 | -130 | MER2B | MER2\_type | DNA |
| 211816305 | 211816999 | + | 13.1 | 24 | 751 | -1667 | Tigger1 | MER2\_type | DNA |
| 211817002 | 211817366 | + | 12.2 | 1 | 364 | 0 | THE1B | MaLR | LTR |
| 211817367 | 211818917 | + | 10.3 | 1 | 1580 | 0 | THE1B-int | MaLR | LTR |
| 211818918 | 211819281 | + | 14.1 | 1 | 364 | 0 | THE1B | MaLR | LTR |
| 211819282 | 211820931 | + | 12.0 | 749 | 2418 | 0 | Tigger1 | MER2\_type | DNA |
| 211820932 | 211821064 | + | 23.7 | 215 | 345 | 0 | MER2B | MER2\_type | DNA |

  

|  |  |  |  |  |  |  |  |  |  |
| --- | --- | --- | --- | --- | --- | --- | --- | --- | --- |
| chr1:213613425-213616300 | | | 2866 bp | | |  |  |  |  |
| Genome Start | Genome End | Strand | Div | Repeat Start | Repeat End | Repeat Left | Name | Family | Class |
| 213613425 | 213613801 | - | 22.8 | 42 | 459 | -8 | MLT1C | MaLR | LTR |
| 213613802 | 213614214 | - | 22.7 | 1 | 396 | 0 | MSTD | MaLR | LTR |
| 213614220 | 213615869 | - | 25.2 | 3 | 1729 | -6 | MSTD-int | MaLR | LTR |
| 213615873 | 213616253 | - | 22.1 | 1 | 396 | 0 | MSTD | MaLR | LTR |
| 213616256 | 213616300 | - | 9.5 | 1 | 44 | -423 | MLT1C | MaLR | LTR |

  

|  |  |  |  |  |  |  |  |  |  |
| --- | --- | --- | --- | --- | --- | --- | --- | --- | --- |
| chr1:213716476-213719904 | | | 3427 bp | | |  |  |  |  |
| Genome Start | Genome End | Strand | Div | Repeat Start | Repeat End | Repeat Left | Name | Family | Class |
| 213716476 | 213716520 | + | 18.2 | 5161 | 5205 | -935 | L1MB2 | L1 | LINE |
| 213716521 | 213716885 | - | 12.4 | 1 | 428 | 0 | MSTA | MaLR | LTR |
| 213716886 | 213718496 | - | 29.0 | 2 | 1735 | 0 | MLT-int | MaLR | LTR |
| 213718499 | 213718913 | - | 17.0 | 1 | 428 | 0 | MSTA | MaLR | LTR |
| 213718914 | 213719904 | + | 18.7 | 5206 | 6161 | -10 | L1MB2 | L1 | LINE |

  

|  |  |  |  |  |  |  |  |  |  |
| --- | --- | --- | --- | --- | --- | --- | --- | --- | --- |
| chr1:214090544-214094432 | | | 3884 bp | | |  |  |  |  |
| Genome Start | Genome End | Strand | Div | Repeat Start | Repeat End | Repeat Left | Name | Family | Class |
| 214090544 | 214092069 | + | 9.3 | 2522 | 4058 | -2088 | L1PB | L1 | LINE |
| 214092070 | 214092428 | - | 9.3 | 1 | 364 | 0 | THE1B | MaLR | LTR |
| 214092434 | 214094009 | - | 9.9 | 1 | 1574 | -6 | THE1B-int | MaLR | LTR |
| 214094010 | 214094365 | - | 10.5 | 1 | 364 | 0 | THE1B | MaLR | LTR |
| 214094366 | 214094432 | + | 10.0 | 4054 | 4119 | -2027 | L1PB | L1 | LINE |

  

|  |  |  |  |  |  |  |  |  |  |
| --- | --- | --- | --- | --- | --- | --- | --- | --- | --- |
| chr1:215439400-215448951 | | | 9514 bp | | |  |  |  |  |
| Genome Start | Genome End | Strand | Div | Repeat Start | Repeat End | Repeat Left | Name | Family | Class |
| 215439400 | 215439585 | - | 25.5 | 6378 | 6555 | 0 | L1ME2 | L1 | LINE |
| 215439586 | 215439702 | - | 23.9 | 5 | 129 | -4 | FLAM\_C | Alu | SINE |
| 215439703 | 215441182 | - | 27.3 | 4770 | 6377 | -178 | L1ME2 | L1 | LINE |
| 215441158 | 215442234 | - | 13.5 | 5200 | 6305 | 0 | L1MA3 | L1 | LINE |
| 215442240 | 215443577 | - | 24.7 | 3356 | 4792 | -1745 | L1ME2 | L1 | LINE |
| 215443597 | 215444849 | - | 19.1 | 4876 | 6164 | -7 | L1MB2 | L1 | LINE |
| 215444852 | 215444960 | - | 30.8 | 3257 | 3367 | -3170 | L1ME2 | L1 | LINE |
| 215444994 | 215445355 | - | 26.7 | 2474 | 2799 | -3919 | L1ME2 | L1 | LINE |
| 215445356 | 215445728 | + | 15.0 | 1 | 364 | 0 | THE1B | MaLR | LTR |
| 215445731 | 215447292 | + | 31.2 | 2 | 1733 | -2 | MLT-int | MaLR | LTR |
| 215447295 | 215447647 | + | 13.4 | 1 | 364 | 0 | THE1B | MaLR | LTR |
| 215447648 | 215447872 | - | 26.6 | 2212 | 2473 | -4245 | L1ME2 | L1 | LINE |
| 215447873 | 215448181 | + | 12.7 | 1 | 308 | -4 | AluSx | Alu | SINE |
| 215448182 | 215448391 | - | 26.6 | 2015 | 2211 | -4507 | L1ME2 | L1 | LINE |
| 215448392 | 215448688 | - | 13.0 | 1 | 293 | -19 | AluSx | Alu | SINE |
| 215448689 | 215448951 | - | 29.5 | 1651 | 2014 | -4704 | L1ME2 | L1 | LINE |

  

|  |  |  |  |  |  |  |  |  |  |
| --- | --- | --- | --- | --- | --- | --- | --- | --- | --- |
| chr1:216656525-216659558 | | | 3031 bp | | |  |  |  |  |
| Genome Start | Genome End | Strand | Div | Repeat Start | Repeat End | Repeat Left | Name | Family | Class |
| 216656525 | 216656673 | + | 30.8 | 2608 | 2764 | -655 | L2 | L2 | LINE |
| 216656674 | 216657009 | + | 13.5 | 1 | 364 | 0 | THE1B | MaLR | LTR |
| 216657013 | 216658587 | + | 29.5 | 3 | 1735 | 0 | MLT1C-int | MaLR | LTR |
| 216658588 | 216658961 | + | 13.0 | 1 | 364 | 0 | THE1B | MaLR | LTR |
| 216658962 | 216659558 | + | 29.3 | 2765 | 3415 | -4 | L2 | L2 | LINE |

  

|  |  |  |  |  |  |  |  |  |  |
| --- | --- | --- | --- | --- | --- | --- | --- | --- | --- |
| chr1:217309726-217312903 | | | 3174 bp | | |  |  |  |  |
| Genome Start | Genome End | Strand | Div | Repeat Start | Repeat End | Repeat Left | Name | Family | Class |
| 217309726 | 217310358 | + | 18.7 | 5219 | 5870 | -314 | L1MB7 | L1 | LINE |
| 217310359 | 217310709 | + | 12.5 | 1 | 363 | -1 | THE1B | MaLR | LTR |
| 217310713 | 217312288 | + | 29.1 | 2 | 1735 | 0 | MLT-int | MaLR | LTR |
| 217312290 | 217312644 | + | 13.9 | 2 | 364 | 0 | THE1B | MaLR | LTR |
| 217312645 | 217312903 | + | 18.0 | 5871 | 6156 | -28 | L1MB7 | L1 | LINE |

  

|  |  |  |  |  |  |  |  |  |  |
| --- | --- | --- | --- | --- | --- | --- | --- | --- | --- |
| chr1:217328225-217340131 | | | 11819 bp | | |  |  |  |  |
| Genome Start | Genome End | Strand | Div | Repeat Start | Repeat End | Repeat Left | Name | Family | Class |
| 217328225 | 217328423 | + | 29.8 | 2725 | 2916 | -3230 | L1ME3B | L1 | LINE |
| 217328416 | 217334728 | + | 16.2 | 11 | 7719 | 0 | L1MA4 | L1 | LINE |
| 217334769 | 217335973 | + | 28.4 | 2896 | 4231 | -1915 | L1ME3B | L1 | LINE |
| 217335992 | 217336352 | + | 13.1 | 1 | 364 | 0 | THE1B | MaLR | LTR |
| 217336353 | 217336765 | + | 12.1 | 1 | 420 | -1160 | THE1B-int | MaLR | LTR |
| 217336768 | 217337746 | + | 13.5 | 558 | 1580 | 0 | THE1B-int | MaLR | LTR |
| 217337747 | 217338107 | + | 14.4 | 1 | 364 | 0 | THE1B | MaLR | LTR |
| 217338108 | 217339080 | + | 27.6 | 4249 | 5331 | -815 | L1ME3B | L1 | LINE |
| 217339117 | 217339612 | + | 28.9 | 5388 | 5917 | -323 | L1ME3B | L1 | LINE |
| 217339613 | 217340055 | + | 19.0 | 1 | 461 | 0 | LOR1b | ERV1 | LTR |
| 217340056 | 217340131 | + | 28.9 | 5918 | 5986 | -254 | L1ME3B | L1 | LINE |

  

|  |  |  |  |  |  |  |  |  |  |
| --- | --- | --- | --- | --- | --- | --- | --- | --- | --- |
| chr1:219664794-219669715 | | | 4918 bp | | |  |  |  |  |
| Genome Start | Genome End | Strand | Div | Repeat Start | Repeat End | Repeat Left | Name | Family | Class |
| 219664794 | 219664897 | - | 16.9 | 3915 | 4016 | -2622 | L1M4c | L1 | LINE |
| 219664898 | 219665256 | - | 11.7 | 1 | 364 | 0 | THE1B | MaLR | LTR |
| 219665257 | 219666817 | - | 29.9 | 2 | 1735 | 0 | MLT-int | MaLR | LTR |
| 219666822 | 219667180 | - | 11.9 | 1 | 362 | -2 | THE1B | MaLR | LTR |
| 219667181 | 219667538 | - | 16.9 | 3541 | 3914 | -2724 | L1M4c | L1 | LINE |
| 219667539 | 219667833 | - | 8.2 | 1 | 299 | -12 | AluY | Alu | SINE |
| 219667834 | 219669715 | - | 23.5 | 1517 | 3540 | -3098 | L1M4c | L1 | LINE |

  

|  |  |  |  |  |  |  |  |  |  |
| --- | --- | --- | --- | --- | --- | --- | --- | --- | --- |
| chr1:220409606-220420827 | | | 11185 bp | | |  |  |  |  |
| Genome Start | Genome End | Strand | Div | Repeat Start | Repeat End | Repeat Left | Name | Family | Class |
| 220409606 | 220413358 | + | 15.8 | 1 | 3659 | -3190 | L1PA16 | L1 | LINE |
| 220413363 | 220413780 | - | 12.1 | 1 | 423 | -5 | MSTA | MaLR | LTR |
| 220413781 | 220414713 | - | 17.1 | 706 | 1651 | 0 | MSTA-int | MaLR | LTR |
| 220414720 | 220414973 | - | 14.2 | 1 | 257 | -1394 | MSTA-int | MaLR | LTR |
| 220414974 | 220415204 | - | 13.4 | 203 | 428 | 0 | MSTA | MaLR | LTR |
| 220415205 | 220415517 | - | 8.5 | 1 | 313 | 0 | AluY | Alu | SINE |
| 220415518 | 220415710 | - | 13.4 | 1 | 202 | -226 | MSTA | MaLR | LTR |
| 220415711 | 220416250 | + | 12.5 | 3655 | 4194 | -2655 | L1PA16 | L1 | LINE |
| 220416276 | 220416723 | + | 12.5 | 4195 | 4693 | -2156 | L1PA16 | L1 | LINE |
| 220416722 | 220417332 | - | 8.5 | 4570 | 5180 | -966 | L1PA8 | L1 | LINE |
| 220417331 | 220418327 | + | 9.3 | 5170 | 6172 | 0 | L1PA8 | L1 | LINE |
| 220418334 | 220418756 | + | 12.2 | 4680 | 5100 | -1749 | L1PA16 | L1 | LINE |
| 220418757 | 220419057 | + | 9.5 | 1 | 311 | 0 | AluSq | Alu | SINE |
| 220419058 | 220420827 | + | 12.3 | 5101 | 6860 | -9 | L1PA16 | L1 | LINE |

  

|  |  |  |  |  |  |  |  |  |  |
| --- | --- | --- | --- | --- | --- | --- | --- | --- | --- |
| chr1:220696797-220699382 | | | 2582 bp | | |  |  |  |  |
| Genome Start | Genome End | Strand | Div | Repeat Start | Repeat End | Repeat Left | Name | Family | Class |
| 220696797 | 220696849 | + | 22.9 | 1 | 49 | -129 | MER5B | MER1\_type | DNA |
| 220696850 | 220697243 | + | 12.5 | 5 | 428 | 0 | MSTA | MaLR | LTR |
| 220697246 | 220698857 | + | 29.4 | 2 | 1735 | 0 | MLT-int | MaLR | LTR |
| 220698860 | 220699260 | + | 12.0 | 3 | 426 | -2 | MSTA | MaLR | LTR |
| 220699261 | 220699382 | + | 22.9 | 50 | 173 | -5 | MER5B | MER1\_type | DNA |

  

|  |  |  |  |  |  |  |  |  |  |
| --- | --- | --- | --- | --- | --- | --- | --- | --- | --- |
| chr1:221263997-221270871 | | | 6887 bp | | |  |  |  |  |
| Genome Start | Genome End | Strand | Div | Repeat Start | Repeat End | Repeat Left | Name | Family | Class |
| 221263997 | 221264055 | - | 16.7 | 669 | 727 | 0 | LTR8A | ERV1 | LTR |
| 221264056 | 221264359 | - | 12.2 | 153 | 472 | 0 | MER4A1 | ERV1 | LTR |
| 221264361 | 221264812 | - | 6.5 | 1 | 448 | -2 | LTR7 | ERV1 | LTR |
| 221264813 | 221265187 | - | 7.2 | 7322 | 7713 | 0 | HERVH | ERV1 | LTR |
| 221265186 | 221265401 | - | 13.4 | 5600 | 5819 | -1894 | HERVH | ERV1 | LTR |
| 221265403 | 221266059 | - | 15.2 | 4488 | 5149 | -2564 | HERVH | ERV1 | LTR |
| 221266062 | 221266566 | - | 12.6 | 3492 | 3993 | -3720 | HERVH | ERV1 | LTR |
| 221266557 | 221266706 | - | 11.3 | 3140 | 3290 | -4423 | HERVH | ERV1 | LTR |
| 221266701 | 221269664 | - | 9.0 | 1 | 2987 | -4726 | HERVH | ERV1 | LTR |
| 221269667 | 221270121 | - | 5.6 | 1 | 448 | -2 | LTR7 | ERV1 | LTR |
| 221270122 | 221270277 | - | 7.7 | 1 | 158 | -314 | MER4A1 | ERV1 | LTR |
| 221270278 | 221270871 | - | 18.3 | 1 | 672 | -55 | LTR8A | ERV1 | LTR |

  

|  |  |  |  |  |  |  |  |  |  |
| --- | --- | --- | --- | --- | --- | --- | --- | --- | --- |
| chr1:221338865-221342512 | | | 3646 bp | | |  |  |  |  |
| Genome Start | Genome End | Strand | Div | Repeat Start | Repeat End | Repeat Left | Name | Family | Class |
| 221338865 | 221339426 | + | 19.7 | 5006 | 5596 | -568 | L1ME2 | L1 | LINE |
| 221339427 | 221339765 | + | 12.0 | 1 | 364 | 0 | THE1B | MaLR | LTR |
| 221339768 | 221341337 | + | 29.1 | 2 | 1735 | 0 | MLT-int | MaLR | LTR |
| 221341338 | 221341616 | + | 13.6 | 1 | 290 | -74 | THE1B | MaLR | LTR |
| 221341617 | 221341927 | - | 10.9 | 1 | 302 | -10 | AluSx | Alu | SINE |
| 221341928 | 221342002 | + | 13.6 | 291 | 364 | 0 | THE1B | MaLR | LTR |
| 221342003 | 221342512 | + | 18.4 | 5597 | 6140 | -24 | L1ME2 | L1 | LINE |

  

|  |  |  |  |  |  |  |  |  |  |
| --- | --- | --- | --- | --- | --- | --- | --- | --- | --- |
| chr1:221926330-221930998 | | | 4470 bp | | |  |  |  |  |
| Genome Start | Genome End | Strand | Div | Repeat Start | Repeat End | Repeat Left | Name | Family | Class |
| 221926330 | 221926601 | + | 13.0 | 1 | 288 | -647 | MER21C | ERV1 | LTR |
| 221926602 | 221926966 | + | 22.3 | 1 | 359 | 0 | AluJo | Alu | SINE |
| 221927020 | 221927128 | + | 13.0 | 289 | 416 | -519 | MER21C | ERV1 | LTR |
| 221927133 | 221927659 | + | 11.4 | 5592 | 6122 | -38 | L1PREC2 | L1 | LINE |
| 221927660 | 221928016 | - | 13.2 | 3 | 428 | 0 | MSTA | MaLR | LTR |
| 221928017 | 221929619 | - | 29.0 | 2 | 1735 | 0 | MLT1F1-int | MaLR | LTR |
| 221929624 | 221929969 | - | 15.7 | 7 | 426 | -2 | MSTA | MaLR | LTR |
| 221929975 | 221930017 | + | 4.7 | 6117 | 6159 | -1 | L1PREC2 | L1 | LINE |
| 221930151 | 221930270 | + | 16.2 | 406 | 523 | -412 | MER21C | ERV1 | LTR |
| 221930271 | 221930612 | - | 11.8 | 1 | 364 | 0 | THE1B | MaLR | LTR |
| 221930613 | 221930998 | + | 16.2 | 524 | 935 | 0 | MER21C | ERV1 | LTR |

  

|  |  |  |  |  |  |  |  |  |  |
| --- | --- | --- | --- | --- | --- | --- | --- | --- | --- |
| chr1:222731787-222738135 | | | 6348 bp | | |  |  |  |  |
| Genome Start | Genome End | Strand | Div | Repeat Start | Repeat End | Repeat Left | Name | Family | Class |
| 222731787 | 222733036 | - | 9.1 | 2066 | 3344 | -4221 | L1M1 | L1 | LINE |
| 222733037 | 222733390 | - | 12.5 | 1 | 364 | 0 | THE1B | MaLR | LTR |
| 222733391 | 222733469 | - | 8.6 | 1516 | 1580 | 0 | THE1B-int | MaLR | LTR |
| 222733470 | 222733777 | - | 7.8 | 2 | 309 | -4 | AluSp | Alu | SINE |
| 222733778 | 222735279 | - | 8.6 | 1 | 1515 | -65 | THE1B-int | MaLR | LTR |
| 222735280 | 222735635 | - | 13.5 | 1 | 364 | 0 | THE1B | MaLR | LTR |
| 222735637 | 222737522 | - | 14.8 | 143 | 2069 | -5496 | L1M1 | L1 | LINE |
| 222737523 | 222737691 | + | 17.8 | 131 | 299 | -13 | AluSg/x | Alu | SINE |
| 222737692 | 222737715 | - | 11.8 | 132 | 153 | -7412 | L1M1 | L1 | LINE |
| 222737716 | 222738022 | - | 11.4 | 1 | 298 | -14 | AluSx | Alu | SINE |
| 222738023 | 222738135 | - | 11.8 | 16 | 131 | -7434 | L1M1 | L1 | LINE |

  

|  |  |  |  |  |  |  |  |  |  |
| --- | --- | --- | --- | --- | --- | --- | --- | --- | --- |
| chr1:223163337-223166310 | | | 2972 bp | | |  |  |  |  |
| Genome Start | Genome End | Strand | Div | Repeat Start | Repeat End | Repeat Left | Name | Family | Class |
| 223163337 | 223163566 | + | 23.1 | 1 | 236 | -209 | LTR56 | ERV1 | LTR |
| 223163567 | 223163764 | - | 11.4 | 183 | 381 | 0 | THE1D | MaLR | LTR |
| 223163765 | 223164057 | - | 10.6 | 1 | 296 | -16 | AluSx | Alu | SINE |
| 223164058 | 223164230 | - | 11.4 | 2 | 182 | -199 | THE1D | MaLR | LTR |
| 223164231 | 223165747 | - | 30.9 | 2 | 1735 | 0 | MLT1H1-int | MaLR | LTR |
| 223165750 | 223166109 | - | 11.0 | 1 | 381 | 0 | THE1D | MaLR | LTR |
| 223166110 | 223166310 | + | 23.1 | 237 | 445 | 0 | LTR56 | ERV1 | LTR |

  

|  |  |  |  |  |  |  |  |  |  |
| --- | --- | --- | --- | --- | --- | --- | --- | --- | --- |
| chr1:223428612-223435146 | | | 6554 bp | | |  |  |  |  |
| Genome Start | Genome End | Strand | Div | Repeat Start | Repeat End | Repeat Left | Name | Family | Class |
| 223428612 | 223430026 | - | 16.1 | 4469 | 5964 | -1598 | L1M2 | L1 | LINE |
| 223430027 | 223430107 | - | 13.6 | 221 | 301 | -10 | AluSg | Alu | SINE |
| 223430115 | 223430165 | + | 15.7 | 136 | 195 | -169 | THE1B | MaLR | LTR |
| 223430166 | 223430378 | - | 9.9 | 1 | 216 | -94 | AluSg | Alu | SINE |
| 223430379 | 223430884 | - | 16.1 | 3945 | 4459 | -3103 | L1M2 | L1 | LINE |
| 223430885 | 223431221 | + | 11.8 | 1 | 364 | 0 | THE1B | MaLR | LTR |
| 223431222 | 223432531 | + | 8.7 | 1 | 1354 | -226 | THE1B-int | MaLR | LTR |
| 223432532 | 223432884 | - | 10.3 | 1 | 355 | 0 | THE1A | MaLR | LTR |
| 223432885 | 223433115 | + | 8.7 | 1355 | 1580 | 0 | THE1B-int | MaLR | LTR |
| 223433116 | 223433464 | + | 13.2 | 1 | 364 | 0 | THE1B | MaLR | LTR |
| 223433468 | 223433512 | - | 4.4 | 3902 | 3946 | -3616 | L1M2 | L1 | LINE |
| 223433484 | 223434655 | - | 21.4 | 2430 | 3619 | -3947 | L1M2 | L1 | LINE |
| 223434656 | 223435065 | - | 18.8 | 1 | 432 | 0 | MSTB1 | MaLR | LTR |
| 223435066 | 223435146 | - | 21.3 | 2340 | 2429 | -5136 | L1M2 | L1 | LINE |

  

|  |  |  |  |  |  |  |  |  |  |
| --- | --- | --- | --- | --- | --- | --- | --- | --- | --- |
| chr1:225087219-225093438 | | | 6051 bp | | |  |  |  |  |
| Genome Start | Genome End | Strand | Div | Repeat Start | Repeat End | Repeat Left | Name | Family | Class |
| 225087219 | 225087429 | - | 20.4 | 240 | 543 | 0 | MER31A | ERV1 | LTR |
| 225087473 | 225087823 | - | 21.9 | 477 | 811 | -52 | MER21B | ERV1 | LTR |
| 225087827 | 225088268 | + | 16.7 | 1 | 450 | 0 | LTR7 | ERV1 | LTR |
| 225088269 | 225088671 | + | 23.3 | 1 | 446 | -7267 | HERVH | ERV1 | LTR |
| 225088759 | 225091862 | + | 18.8 | 792 | 3858 | -3855 | HERVH | ERV1 | LTR |
| 225091861 | 225092293 | + | 10.5 | 7283 | 7713 | 0 | HERVH | ERV1 | LTR |
| 225092294 | 225092728 | + | 14.8 | 1 | 450 | 0 | LTR7 | ERV1 | LTR |
| 225092757 | 225093189 | - | 19.6 | 4 | 436 | -427 | MER21B | ERV1 | LTR |
| 225093200 | 225093438 | - | 16.7 | 1 | 244 | -241 | MER31A | ERV1 | LTR |

  

|  |  |  |  |  |  |  |  |  |  |
| --- | --- | --- | --- | --- | --- | --- | --- | --- | --- |
| chr1:227614343-227621988 | | | 7535 bp | | |  |  |  |  |
| Genome Start | Genome End | Strand | Div | Repeat Start | Repeat End | Repeat Left | Name | Family | Class |
| 227614343 | 227614589 | + | 28.1 | 2827 | 3079 | -340 | L2 | L2 | LINE |
| 227614685 | 227614787 | - | 20.4 | 190 | 292 | -20 | AluSg/x | Alu | SINE |
| 227614788 | 227615185 | + | 5.3 | 1 | 448 | -2 | LTR7 | ERV1 | LTR |
| 227615190 | 227618146 | + | 9.9 | 3 | 2987 | -4726 | HERVH | ERV1 | LTR |
| 227618119 | 227618290 | + | 14.0 | 3126 | 3290 | -4423 | HERVH | ERV1 | LTR |
| 227618281 | 227618783 | + | 9.8 | 3492 | 3993 | -3720 | HERVH | ERV1 | LTR |
| 227618803 | 227620003 | + | 10.2 | 3951 | 5149 | -2287 | HERVH | ERV1 | LTR |
| 227620005 | 227620212 | + | 15.4 | 5600 | 5819 | -1894 | HERVH | ERV1 | LTR |
| 227620211 | 227620590 | + | 7.5 | 7322 | 7713 | 0 | HERVH | ERV1 | LTR |
| 227620591 | 227621035 | + | 5.4 | 1 | 448 | -2 | LTR7 | ERV1 | LTR |
| 227621038 | 227621120 | - | 12.9 | 111 | 193 | -120 | AluSq | Alu | SINE |
| 227621121 | 227621193 | + | 13.7 | 1 | 73 | -226 | AluSg/x | Alu | SINE |
| 227621194 | 227621561 | + | 8.5 | 65 | 464 | 0 | LTR7B | ERV1 | LTR |
| 227621562 | 227621776 | + | 8.4 | 81 | 295 | -17 | AluSg/x | Alu | SINE |
| 227621805 | 227621915 | - | 9.9 | 1 | 111 | -201 | AluSq | Alu | SINE |
| 227621918 | 227621988 | + | 20.0 | 3271 | 3335 | -84 | L2 | L2 | LINE |

  

|  |  |  |  |  |  |  |  |  |  |
| --- | --- | --- | --- | --- | --- | --- | --- | --- | --- |
| chr1:229352385-229355828 | | | 3442 bp | | |  |  |  |  |
| Genome Start | Genome End | Strand | Div | Repeat Start | Repeat End | Repeat Left | Name | Family | Class |
| 229352385 | 229352713 | - | 22.7 | 5836 | 6145 | -21 | L1ME1 | L1 | LINE |
| 229352714 | 229352783 | - | 13.4 | 365 | 428 | 0 | MSTA | MaLR | LTR |
| 229352784 | 229353069 | + | 18.2 | 1 | 294 | -18 | AluJb | Alu | SINE |
| 229353070 | 229353411 | - | 13.4 | 1 | 364 | -64 | MSTA | MaLR | LTR |
| 229353412 | 229355062 | - | 27.9 | 2 | 1735 | 0 | MLT-int | MaLR | LTR |
| 229355065 | 229355554 | - | 16.1 | 1 | 428 | 0 | MSTA | MaLR | LTR |
| 229355555 | 229355828 | - | 22.7 | 5534 | 5835 | -331 | L1ME1 | L1 | LINE |

  

|  |  |  |  |  |  |  |  |  |  |
| --- | --- | --- | --- | --- | --- | --- | --- | --- | --- |
| chr1:230318273-230331588 | | | 13337 bp | | |  |  |  |  |
| Genome Start | Genome End | Strand | Div | Repeat Start | Repeat End | Repeat Left | Name | Family | Class |
| 230318273 | 230318809 | - | 19.5 | 6098 | 6766 | -9 | L1MB5 | L1 | LINE |
| 230318810 | 230319074 | - | 9.1 | 19 | 283 | -30 | AluSp | Alu | SINE |
| 230319075 | 230320333 | - | 21.2 | 4788 | 6097 | -678 | L1MB5 | L1 | LINE |
| 230320339 | 230320483 | + | 15.3 | 6 | 147 | -165 | AluJ | Alu | SINE |
| 230320484 | 230320934 | + | 4.7 | 1 | 448 | -2 | LTR7 | ERV1 | LTR |
| 230320937 | 230323968 | + | 9.3 | 1 | 2995 | -4718 | HERVH | ERV1 | LTR |
| 230323948 | 230324097 | + | 11.3 | 3140 | 3290 | -4423 | HERVH | ERV1 | LTR |
| 230324088 | 230324591 | + | 11.4 | 3492 | 3993 | -3720 | HERVH | ERV1 | LTR |
| 230324594 | 230325255 | + | 14.5 | 4488 | 5149 | -2564 | HERVH | ERV1 | LTR |
| 230325257 | 230325485 | + | 12.0 | 5600 | 5819 | -1894 | HERVH | ERV1 | LTR |
| 230325484 | 230325863 | + | 7.2 | 7322 | 7713 | 0 | HERVH | ERV1 | LTR |
| 230325864 | 230326314 | + | 5.1 | 1 | 448 | -2 | LTR7 | ERV1 | LTR |
| 230326317 | 230326466 | + | 18.7 | 143 | 292 | -20 | AluJ | Alu | SINE |
| 230326467 | 230328870 | - | 19.4 | 2360 | 4801 | -1946 | L1MB5 | L1 | LINE |
| 230328871 | 230329340 | - | 11.9 | 1 | 472 | 0 | MER4A1 | ERV1 | LTR |
| 230329341 | 230331588 | - | 22.1 | 7 | 2359 | -4390 | L1MB5 | L1 | LINE |

  

|  |  |  |  |  |  |  |  |  |  |
| --- | --- | --- | --- | --- | --- | --- | --- | --- | --- |
| chr1:231194949-231197499 | | | 2543 bp | | |  |  |  |  |
| Genome Start | Genome End | Strand | Div | Repeat Start | Repeat End | Repeat Left | Name | Family | Class |
| 231194949 | 231195060 | + | 31.2 | 9 | 121 | -141 | MIR | MIR | SINE |
| 231195063 | 231195402 | + | 11.6 | 18 | 379 | -2 | THE1D | MaLR | LTR |
| 231195408 | 231197008 | + | 30.4 | 3 | 1735 | 0 | MLT1J-int | MaLR | LTR |
| 231197009 | 231197358 | + | 12.0 | 1 | 381 | 0 | THE1D | MaLR | LTR |
| 231197360 | 231197499 | + | 34.6 | 120 | 260 | -2 | MIR | MIR | SINE |

  

|  |  |  |  |  |  |  |  |  |  |
| --- | --- | --- | --- | --- | --- | --- | --- | --- | --- |
| chr1:232092239-232095168 | | | 2923 bp | | |  |  |  |  |
| Genome Start | Genome End | Strand | Div | Repeat Start | Repeat End | Repeat Left | Name | Family | Class |
| 232092239 | 232092712 | - | 35.2 | 2926 | 3417 | -2 | L2 | L2 | LINE |
| 232092716 | 232093092 | + | 9.3 | 1 | 375 | 0 | THE1C | MaLR | LTR |
| 232093093 | 232094624 | + | 11.5 | 1 | 1580 | 0 | THE1C-int | MaLR | LTR |
| 232094626 | 232094996 | + | 11.1 | 2 | 375 | 0 | THE1C | MaLR | LTR |
| 232095000 | 232095168 | - | 28.2 | 2692 | 2880 | -539 | L2 | L2 | LINE |

  

|  |  |  |  |  |  |  |  |  |  |
| --- | --- | --- | --- | --- | --- | --- | --- | --- | --- |
| chr1:236303409-236315808 | | | 12172 bp | | |  |  |  |  |
| Genome Start | Genome End | Strand | Div | Repeat Start | Repeat End | Repeat Left | Name | Family | Class |
| 236303409 | 236303716 | - | 28.3 | 245 | 561 | -2858 | L2 | L2 | LINE |
| 236303720 | 236304288 | - | 19.1 | 5611 | 6176 | -2 | L1MB8 | L1 | LINE |
| 236304332 | 236304694 | - | 14.8 | 1 | 381 | 0 | THE1D | MaLR | LTR |
| 236304695 | 236306296 | - | 13.4 | 3 | 1579 | -1 | THE1D-int | MaLR | LTR |
| 236306298 | 236306664 | - | 13.7 | 1 | 381 | 0 | THE1D | MaLR | LTR |
| 236306673 | 236307134 | + | 27.1 | 47 | 507 | -41 | LTR55 | ERV | LTR |
| 236307183 | 236308208 | - | 19.6 | 4568 | 5617 | -567 | L1MB8 | L1 | LINE |
| 236308209 | 236308281 | - | 21.9 | 1 | 75 | -392 | MLT1C | MaLR | LTR |
| 236308282 | 236308658 | - | 19.6 | 4183 | 4567 | -1617 | L1MB8 | L1 | LINE |
| 236308660 | 236308792 | - | 7.3 | 11 | 133 | -179 | AluSq/x | Alu | SINE |
| 236308836 | 236308987 | - | 23.2 | 4837 | 4988 | -2018 | L1M4c | L1 | LINE |
| 236308986 | 236310633 | - | 20.7 | 2731 | 4440 | -2566 | L1M4c | L1 | LINE |
| 236310670 | 236311987 | - | 8.5 | 6383 | 7721 | 0 | L1MA1 | L1 | LINE |
| 236311965 | 236312677 | - | 8.3 | 3939 | 4677 | -2888 | L1MA1 | L1 | LINE |
| 236312678 | 236312817 | + | 11.5 | 243 | 381 | 0 | THE1D | MaLR | LTR |
| 236312821 | 236313248 | - | 13.3 | 2967 | 3396 | -4169 | L1MA1 | L1 | LINE |
| 236313245 | 236314494 | - | 20.2 | 16 | 1284 | -6281 | L1MA1 | L1 | LINE |
| 236314525 | 236314672 | - | 31.1 | 957 | 1096 | -5808 | L1M4c | L1 | LINE |
| 236314673 | 236314988 | - | 8.4 | 1 | 310 | -1 | AluY | Alu | SINE |
| 236314989 | 236315581 | - | 30.5 | 11 | 956 | -5948 | L1M4c | L1 | LINE |
| 236315588 | 236315655 | - | 35.0 | 186 | 253 | -3166 | L2 | L2 | LINE |
| 236315691 | 236315808 | - | 35.0 | 65 | 185 | -3234 | L2 | L2 | LINE |

  

|  |  |  |  |  |  |  |  |  |  |
| --- | --- | --- | --- | --- | --- | --- | --- | --- | --- |
| chr1:236774871-236779167 | | | 4270 bp | | |  |  |  |  |
| Genome Start | Genome End | Strand | Div | Repeat Start | Repeat End | Repeat Left | Name | Family | Class |
| 236774871 | 236774952 | + | 23.2 | 1 | 83 | -263 | MER7A | MER2\_type | DNA |
| 236774980 | 236775328 | - | 9.5 | 1 | 355 | 0 | THE1A | MaLR | LTR |
| 236775329 | 236776411 | - | 31.4 | 598 | 1735 | 0 | MLT-int | MaLR | LTR |
| 236776412 | 236776699 | - | 9.0 | 15 | 305 | -4 | AluSc | Alu | SINE |
| 236776700 | 236777183 | - | 31.4 | 3 | 597 | -1138 | MLT-int | MaLR | LTR |
| 236777186 | 236777531 | - | 10.4 | 1 | 355 | 0 | THE1A | MaLR | LTR |
| 236777532 | 236778300 | - | 11.4 | 5357 | 6138 | -13 | L1PB1 | L1 | LINE |
| 236778299 | 236778884 | + | 11.1 | 4757 | 5356 | -790 | L1PB1 | L1 | LINE |
| 236778885 | 236779167 | + | 18.8 | 65 | 345 | -1 | MER7A | MER2\_type | DNA |

  

|  |  |  |  |  |  |  |  |  |  |
| --- | --- | --- | --- | --- | --- | --- | --- | --- | --- |
| chr1:240271947-240282990 | | | 11010 bp | | |  |  |  |  |
| Genome Start | Genome End | Strand | Div | Repeat Start | Repeat End | Repeat Left | Name | Family | Class |
| 240271947 | 240272536 | + | 14.8 | 2895 | 3503 | -2643 | L1M1 | L1 | LINE |
| 240272548 | 240273189 | + | 12.1 | 3597 | 4251 | -1895 | L1M1 | L1 | LINE |
| 240273192 | 240273545 | + | 22.0 | 1 | 382 | -46 | MSTA | MaLR | LTR |
| 240273546 | 240273862 | + | 5.7 | 1 | 317 | 0 | AluY | Alu | SINE |
| 240273863 | 240273908 | + | 22.0 | 383 | 428 | 0 | MSTA | MaLR | LTR |
| 240273909 | 240275471 | + | 20.6 | 1 | 1607 | -44 | MSTA-int | MaLR | LTR |
| 240275487 | 240281510 | - | 1.5 | 4 | 6032 | 0 | L1PA2 | L1 | LINE |
| 240281517 | 240281574 | + | 19.0 | 1594 | 1651 | 0 | MSTA-int | MaLR | LTR |
| 240281575 | 240281985 | + | 18.5 | 1 | 428 | 0 | MSTA | MaLR | LTR |
| 240281986 | 240282990 | + | 14.7 | 4249 | 5263 | -883 | L1M1 | L1 | LINE |

  

|  |  |  |  |  |  |  |  |  |  |
| --- | --- | --- | --- | --- | --- | --- | --- | --- | --- |
| chr1:241835666-241838132 | | | 2463 bp | | |  |  |  |  |
| Genome Start | Genome End | Strand | Div | Repeat Start | Repeat End | Repeat Left | Name | Family | Class |
| 241835666 | 241835720 | - | 14.4 | 2978 | 3028 | -1 | Tigger3(Golem) | MER2\_type | DNA |
| 241835721 | 241836095 | + | 11.7 | 2 | 375 | 0 | THE1C | MaLR | LTR |
| 241836100 | 241837656 | + | 28.6 | 4 | 1735 | 0 | MLT-int | MaLR | LTR |
| 241837657 | 241838009 | + | 11.1 | 1 | 375 | 0 | THE1C | MaLR | LTR |
| 241838010 | 241838132 | - | 14.4 | 2851 | 2977 | -52 | Tigger3(Golem) | MER2\_type | DNA |

  

|  |  |  |  |  |  |  |  |  |  |
| --- | --- | --- | --- | --- | --- | --- | --- | --- | --- |
| chr1:246565033-246572986 | | | 7961 bp | | |  |  |  |  |
| Genome Start | Genome End | Strand | Div | Repeat Start | Repeat End | Repeat Left | Name | Family | Class |
| 246565033 | 246565295 | - | 17.1 | 6033 | 6295 | -5 | L1MA5 | L1 | LINE |
| 246565311 | 246565687 | - | 13.6 | 5762 | 6147 | -4 | L1PB2 | L1 | LINE |
| 246565691 | 246566042 | - | 9.9 | 1 | 448 | -2 | LTR7 | ERV1 | LTR |
| 246566043 | 246566419 | - | 10.2 | 7322 | 7713 | 0 | HERVH | ERV1 | LTR |
| 246566418 | 246566633 | - | 15.7 | 5600 | 5819 | -1894 | HERVH | ERV1 | LTR |
| 246566635 | 246568349 | - | 11.8 | 3492 | 5286 | -1805 | HERVH | ERV1 | LTR |
| 246568340 | 246568487 | - | 14.2 | 3140 | 3290 | -4423 | HERVH | ERV1 | LTR |
| 246568482 | 246570169 | - | 10.1 | 1301 | 2987 | -4726 | HERVH | ERV1 | LTR |
| 246570156 | 246570620 | - | 9.9 | 1 | 468 | -7245 | HERVH | ERV1 | LTR |
| 246570624 | 246571031 | - | 7.8 | 1 | 447 | -3 | LTR7 | ERV1 | LTR |
| 246571041 | 246571245 | - | 12.2 | 5542 | 5756 | -395 | L1P5 | L1 | LINE |
| 246571239 | 246571582 | - | 20.4 | 5715 | 6061 | -239 | L1MA5 | L1 | LINE |
| 246571583 | 246571810 | - | 17.6 | 1 | 243 | -67 | AluSg | Alu | SINE |
| 246571812 | 246572986 | - | 14.8 | 4448 | 5606 | -699 | L1MA5 | L1 | LINE |

  

|  |  |  |  |  |  |  |  |  |  |
| --- | --- | --- | --- | --- | --- | --- | --- | --- | --- |
| chr1:246640178-246645688 | | | 4681 bp | | |  |  |  |  |
| Genome Start | Genome End | Strand | Div | Repeat Start | Repeat End | Repeat Left | Name | Family | Class |
| 246640178 | 246640277 | - | 27.7 | 3208 | 3351 | -68 | L2 | L2 | LINE |
| 246640321 | 246640386 | + | 31.8 | 5592 | 5657 | -513 | L1ME3A | L1 | LINE |
| 246640392 | 246640837 | + | 17.9 | 4 | 457 | 0 | MSTB2 | MaLR | LTR |
| 246640840 | 246642488 | + | 27.4 | 2 | 1735 | 0 | MLT-int | MaLR | LTR |
| 246642489 | 246642935 | + | 19.4 | 1 | 449 | -8 | MSTB2 | MaLR | LTR |
| 246643066 | 246643170 | + | 21.9 | 5857 | 5981 | -189 | L1ME3A | L1 | LINE |
| 246643362 | 246644001 | - | 34.8 | 2499 | 3204 | -215 | L2 | L2 | LINE |
| 246644109 | 246644229 | + | 19.0 | 185 | 306 | -6 | AluJb | Alu | SINE |
| 246644466 | 246645008 | + | 3.0 | 5608 | 6154 | -1 | L1PA4 | L1 | LINE |
| 246645125 | 246645688 | - | 34.2 | 1810 | 2473 | -946 | L2 | L2 | LINE |

  

|  |  |  |  |  |  |  |  |  |  |
| --- | --- | --- | --- | --- | --- | --- | --- | --- | --- |
| chr1:247034746-247039347 | | | 4206 bp | | |  |  |  |  |
| Genome Start | Genome End | Strand | Div | Repeat Start | Repeat End | Repeat Left | Name | Family | Class |
| 247034746 | 247034784 | + | 24.5 | 3800 | 3833 | -2843 | MER101-int | ERV1 | LTR |
| 247034785 | 247035073 | - | 11.1 | 1 | 290 | -22 | AluSx | Alu | SINE |
| 247035074 | 247035718 | + | 19.5 | 2812 | 4170 | -2506 | MER101-int | ERV1 | LTR |
| 247035718 | 247035829 | + | 15.3 | 3075 | 3194 | -3445 | MER101-int | ERV1 | LTR |
| 247035868 | 247036075 | - | 15.9 | 88 | 295 | -17 | AluJb | Alu | SINE |
| 247036079 | 247036321 | + | 20.7 | 3281 | 3525 | -3114 | MER101-int | ERV1 | LTR |
| 247036428 | 247036515 | + | 21.3 | 3786 | 3860 | -4260 | MER101-int | ERV1 | LTR |
| 247036555 | 247036693 | + | 28.7 | 3839 | 3967 | -4153 | MER101-int | ERV1 | LTR |
| 247036874 | 247036930 | + | 18.1 | 3950 | 4002 | -4118 | MER101-int | ERV1 | LTR |
| 247036931 | 247037107 | + | 17.0 | 1 | 210 | -171 | THE1D | MaLR | LTR |
| 247037134 | 247037298 | + | 17.0 | 211 | 377 | -4 | THE1D | MaLR | LTR |
| 247037304 | 247038930 | + | 30.3 | 2 | 1735 | 0 | MLT-int | MaLR | LTR |
| 247038931 | 247039293 | + | 12.6 | 1 | 381 | 0 | THE1D | MaLR | LTR |
| 247039294 | 247039347 | + | 18.1 | 4003 | 4054 | -4066 | MER101-int | ERV1 | LTR |

  


|  |  |  |  |  |  |  |  |  |  |
| --- | --- | --- | --- | --- | --- | --- | --- | --- | --- |
| chr2:521674-527455 | | | 5414 bp | | |  |  |  |  |
| Genome Start | Genome End | Strand | Div | Repeat Start | Repeat End | Repeat Left | Name | Family | Class |
| 521674 | 522762 | + | 26.3 | 382 | 1501 | -5041 | L1M3 | L1 | LINE |
| 522763 | 523091 | - | 21.9 | 10 | 362 | -3 | MLT1A0 | MaLR | LTR |
| 523110 | 523491 | - | 24.3 | 1330 | 1727 | -8 | MLT1A0-int | MaLR | LTR |
| 523660 | 523728 | - | 17.4 | 679 | 747 | -988 | MLT1A0-int | MaLR | LTR |
| 523861 | 524526 | - | 19.5 | 2 | 657 | -1078 | MLT1A0-int | MaLR | LTR |
| 524532 | 524882 | - | 20.7 | 13 | 363 | -2 | MLT1A0 | MaLR | LTR |
| 524937 | 526200 | + | 26.7 | 1510 | 2852 | -3792 | L1M3 | L1 | LINE |
| 526192 | 526386 | + | 20.8 | 5169 | 5361 | -1277 | L1M3 | L1 | LINE |
| 526387 | 526824 | + | 8.2 | 1 | 444 | 0 | MLT2A1 | ERVL | LTR |
| 526825 | 527455 | + | 19.5 | 5362 | 5990 | -691 | L1M3 | L1 | LINE |

  

|  |  |  |  |  |  |  |  |  |  |
| --- | --- | --- | --- | --- | --- | --- | --- | --- | --- |
| chr2:880259-884462 | | | 4032 bp | | |  |  |  |  |
| Genome Start | Genome End | Strand | Div | Repeat Start | Repeat End | Repeat Left | Name | Family | Class |
| 880259 | 880532 | + | 37.0 | 2986 | 3268 | -2878 | L1M4 | L1 | LINE |
| 880536 | 880916 | + | 21.3 | 5899 | 6274 | -38 | L1MA9 | L1 | LINE |
| 880957 | 881342 | + | 34.6 | 3329 | 3755 | -2391 | L1M4 | L1 | LINE |
| 881467 | 881887 | + | 15.9 | 1 | 428 | 0 | MSTA | MaLR | LTR |
| 881890 | 883494 | + | 29.8 | 2 | 1735 | 0 | MLT-int | MaLR | LTR |
| 883496 | 883902 | + | 15.2 | 3 | 428 | 0 | MSTA | MaLR | LTR |
| 883904 | 884360 | - | 19.5 | 1 | 531 | -10 | MER66C | ERV1 | LTR |
| 884362 | 884462 | + | 32.0 | 3900 | 4001 | -2145 | L1M4 | L1 | LINE |

  

|  |  |  |  |  |  |  |  |  |  |
| --- | --- | --- | --- | --- | --- | --- | --- | --- | --- |
| chr2:2368934-2373937 | | | 4582 bp | | |  |  |  |  |
| Genome Start | Genome End | Strand | Div | Repeat Start | Repeat End | Repeat Left | Name | Family | Class |
| 2368934 | 2369120 | + | 34.2 | 1981 | 2187 | -1232 | L2 | L2 | LINE |
| 2369196 | 2369498 | - | 9.1 | 1 | 300 | -11 | AluY | Alu | SINE |
| 2369844 | 2370072 | + | 31.1 | 2392 | 2666 | -753 | L2 | L2 | LINE |
| 2370073 | 2370480 | - | 6.6 | 5765 | 6172 | 0 | L1PA8 | L1 | LINE |
| 2370481 | 2370925 | + | 30.9 | 2667 | 3121 | -298 | L2 | L2 | LINE |
| 2370926 | 2371335 | - | 15.7 | 1 | 428 | 0 | MSTA | MaLR | LTR |
| 2371336 | 2372852 | - | 27.9 | 2 | 1735 | 0 | MLT1A-int | MaLR | LTR |
| 2372855 | 2373234 | - | 12.1 | 56 | 428 | 0 | MSTA | MaLR | LTR |
| 2373235 | 2373559 | - | 10.0 | 1 | 310 | 0 | AluSg | Alu | SINE |
| 2373560 | 2373614 | - | 12.1 | 1 | 55 | -373 | MSTA | MaLR | LTR |
| 2373615 | 2373937 | + | 30.6 | 3122 | 3417 | -2 | L2 | L2 | LINE |

  

|  |  |  |  |  |  |  |  |  |  |
| --- | --- | --- | --- | --- | --- | --- | --- | --- | --- |
| chr2:2481191-2483733 | | | 2544 bp | | |  |  |  |  |
| Genome Start | Genome End | Strand | Div | Repeat Start | Repeat End | Repeat Left | Name | Family | Class |
| 2481191 | 2481296 | - | 25.5 | 361 | 467 | 0 | MLT1C | MaLR | LTR |
| 2481297 | 2481649 | - | 11.1 | 1 | 355 | 0 | THE1A | MaLR | LTR |
| 2481650 | 2483221 | - | 29.7 | 2 | 1735 | 0 | MLT1C-int | MaLR | LTR |
| 2483226 | 2483576 | - | 9.4 | 1 | 353 | -2 | THE1A | MaLR | LTR |
| 2483577 | 2483617 | - | 25.5 | 322 | 360 | -107 | MLT1C | MaLR | LTR |
| 2483613 | 2483733 | - | 16.3 | 16 | 138 | -252 | MLT1C | MaLR | LTR |

  

|  |  |  |  |  |  |  |  |  |  |
| --- | --- | --- | --- | --- | --- | --- | --- | --- | --- |
| chr2:2735331-2737782 | | | 2450 bp | | |  |  |  |  |
| Genome Start | Genome End | Strand | Div | Repeat Start | Repeat End | Repeat Left | Name | Family | Class |
| 2735331 | 2735454 | - | 29.6 | 270 | 403 | -47 | LTR16A | ERVL | LTR |
| 2735455 | 2735768 | + | 12.9 | 1 | 375 | 0 | THE1C | MaLR | LTR |
| 2735771 | 2737343 | + | 31.6 | 2 | 1735 | 0 | MLT-int | MaLR | LTR |
| 2737344 | 2737647 | + | 12.3 | 1 | 375 | 0 | THE1C | MaLR | LTR |
| 2737648 | 2737782 | - | 29.3 | 96 | 269 | -181 | LTR16A | ERVL | LTR |

  

|  |  |  |  |  |  |  |  |  |  |
| --- | --- | --- | --- | --- | --- | --- | --- | --- | --- |
| chr2:3230704-3233814 | | | 3109 bp | | |  |  |  |  |
| Genome Start | Genome End | Strand | Div | Repeat Start | Repeat End | Repeat Left | Name | Family | Class |
| 3230704 | 3230748 | - | 21.5 | 1914 | 1959 | -1 | L4 | RTE | LINE |
| 3230749 | 3230945 | + | 16.3 | 2 | 196 | -230 | MSTB | MaLR | LTR |
| 3230946 | 3231089 | + | 13.3 | 3 | 143 | -169 | AluSx | Alu | SINE |
| 3231090 | 3231394 | + | 10.8 | 1 | 305 | -7 | AluSx | Alu | SINE |
| 3231395 | 3231579 | + | 13.3 | 144 | 311 | -1 | AluSx | Alu | SINE |
| 3231580 | 3231810 | + | 16.3 | 197 | 426 | 0 | MSTB | MaLR | LTR |
| 3231813 | 3233380 | + | 28.0 | 2 | 1735 | 0 | MLT-int | MaLR | LTR |
| 3233381 | 3233782 | + | 16.4 | 1 | 424 | -2 | MSTB | MaLR | LTR |
| 3233783 | 3233814 | - | 21.5 | 1882 | 1913 | -47 | L4 | RTE | LINE |

  

|  |  |  |  |  |  |  |  |  |  |
| --- | --- | --- | --- | --- | --- | --- | --- | --- | --- |
| chr2:3834119-3850955 | | | 16767 bp | | |  |  |  |  |
| Genome Start | Genome End | Strand | Div | Repeat Start | Repeat End | Repeat Left | Name | Family | Class |
| 3834119 | 3835083 | - | 25.2 | 5050 | 6052 | -889 | L1MCc | L1 | LINE |
| 3835091 | 3838504 | - | 8.8 | 3006 | 6434 | -12 | L1PA8A | L1 | LINE |
| 3838534 | 3838831 | - | 12.1 | 1 | 301 | -9 | AluSg | Alu | SINE |
| 3838832 | 3839552 | - | 9.8 | 2291 | 3005 | -3456 | L1PA8A | L1 | LINE |
| 3839550 | 3840109 | - | 14.8 | 1337 | 1903 | -4580 | L1PA8A | L1 | LINE |
| 3840103 | 3840958 | - | 10.0 | 3 | 867 | -5008 | L1PA8A | L1 | LINE |
| 3840969 | 3841010 | - | 16.7 | 5012 | 5053 | -1888 | L1MCc | L1 | LINE |
| 3841014 | 3841396 | - | 10.0 | 1 | 450 | 0 | LTR7 | ERV1 | LTR |
| 3841397 | 3841951 | - | 11.5 | 7157 | 7713 | 0 | HERVH | ERV1 | LTR |
| 3841944 | 3842197 | - | 12.2 | 5760 | 6017 | -1696 | HERVH | ERV1 | LTR |
| 3842194 | 3842922 | - | 12.2 | 3995 | 4712 | -3001 | HERVH | ERV1 | LTR |
| 3842936 | 3846414 | - | 14.8 | 1 | 3734 | -3979 | HERVH | ERV1 | LTR |
| 3846415 | 3846805 | - | 10.3 | 1 | 450 | 0 | LTR7 | ERV1 | LTR |
| 3846806 | 3848237 | - | 20.8 | 3800 | 5013 | -1928 | L1MCc | L1 | LINE |
| 3848238 | 3848589 | + | 12.7 | 1 | 364 | 0 | THE1B | MaLR | LTR |
| 3848590 | 3849126 | - | 16.1 | 3220 | 3799 | -3142 | L1MCc | L1 | LINE |
| 3849098 | 3849242 | - | 26.6 | 2192 | 2369 | -4592 | L1MCc | L1 | LINE |
| 3849265 | 3849337 | - | 30.1 | 1469 | 1535 | -4888 | L1MCc | L1 | LINE |
| 3849338 | 3849552 | - | 26.1 | 1 | 224 | 0 | MER58A | MER1\_type | DNA |
| 3849553 | 3849730 | - | 30.1 | 1295 | 1468 | -4955 | L1MCc | L1 | LINE |
| 3849731 | 3849930 | - | 24.5 | 15 | 224 | 0 | MER58A | MER1\_type | DNA |
| 3849931 | 3850361 | - | 22.0 | 807 | 1283 | -5140 | L1MCc | L1 | LINE |
| 3850399 | 3850955 | - | 31.4 | 61 | 668 | -5755 | L1MCc | L1 | LINE |

  

|  |  |  |  |  |  |  |  |  |  |
| --- | --- | --- | --- | --- | --- | --- | --- | --- | --- |
| chr2:4012816-4023213 | | | 10237 bp | | |  |  |  |  |
| Genome Start | Genome End | Strand | Div | Repeat Start | Repeat End | Repeat Left | Name | Family | Class |
| 4012816 | 4014740 | - | 28.7 | 3127 | 5092 | -1127 | L1MEc | L1 | LINE |
| 4014741 | 4014780 | - | 10.4 | 329 | 364 | 0 | THE1B | MaLR | LTR |
| 4014781 | 4015098 | + | 10.4 | 1 | 306 | -5 | AluY | Alu | SINE |
| 4015099 | 4015434 | - | 10.4 | 1 | 328 | -36 | THE1B | MaLR | LTR |
| 4015435 | 4017014 | - | 30.6 | 2 | 1735 | 0 | MLT1K-int | MaLR | LTR |
| 4017017 | 4017381 | - | 12.9 | 1 | 364 | 0 | THE1B | MaLR | LTR |
| 4017382 | 4017821 | - | 28.7 | 2673 | 3126 | -3093 | L1MEc | L1 | LINE |
| 4017947 | 4019378 | - | 20.3 | 4058 | 5579 | -2279 | L1MC | L1 | LINE |
| 4019389 | 4019534 | - | 17.8 | 4143 | 4301 | -1845 | L1M3 | L1 | LINE |
| 4019533 | 4020759 | - | 8.3 | 4364 | 5581 | -587 | L1P4 | L1 | LINE |
| 4020782 | 4021944 | - | 28.0 | 5 | 1167 | -4878 | L1MCb | L1 | LINE |
| 4021949 | 4022244 | - | 24.2 | 2183 | 2476 | -3924 | L1MEc | L1 | LINE |
| 4022245 | 4022531 | + | 9.4 | 9 | 295 | -14 | AluSg1 | Alu | SINE |
| 4022532 | 4023213 | - | 23.9 | 1491 | 2240 | -4160 | L1MEc | L1 | LINE |

  

|  |  |  |  |  |  |  |  |  |  |
| --- | --- | --- | --- | --- | --- | --- | --- | --- | --- |
| chr2:4216464-4219425 | | | 2895 bp | | |  |  |  |  |
| Genome Start | Genome End | Strand | Div | Repeat Start | Repeat End | Repeat Left | Name | Family | Class |
| 4216464 | 4216618 | - | 28.5 | 2333 | 2507 | 0 | HAL1 | L1 | LINE |
| 4216634 | 4216982 | + | 25.4 | 18 | 374 | 0 | MLT1A | MaLR | LTR |
| 4217034 | 4217408 | - | 12.0 | 1 | 375 | 0 | THE1C | MaLR | LTR |
| 4217409 | 4218941 | - | 12.1 | 1 | 1580 | 0 | THE1C-int | MaLR | LTR |
| 4218942 | 4219157 | - | 13.6 | 159 | 375 | 0 | THE1C | MaLR | LTR |
| 4219159 | 4219425 | - | 33.9 | 235 | 512 | -1995 | HAL1 | L1 | LINE |

  

|  |  |  |  |  |  |  |  |  |  |
| --- | --- | --- | --- | --- | --- | --- | --- | --- | --- |
| chr2:4410645-4421637 | | | 10958 bp | | |  |  |  |  |
| Genome Start | Genome End | Strand | Div | Repeat Start | Repeat End | Repeat Left | Name | Family | Class |
| 4410645 | 4410779 | - | 18.1 | 7561 | 7695 | -24 | L1MA4 | L1 | LINE |
| 4410780 | 4410942 | - | 10.4 | 5999 | 6166 | -2 | L1PA10 | L1 | LINE |
| 4410943 | 4411339 | - | 18.1 | 7208 | 7560 | -159 | L1MA4 | L1 | LINE |
| 4411340 | 4411648 | - | 8.1 | 2 | 308 | -3 | AluY | Alu | SINE |
| 4411649 | 4414408 | - | 15.8 | 4424 | 7207 | -512 | L1MA4 | L1 | LINE |
| 4414409 | 4414819 | + | 15.8 | 1 | 381 | 0 | THE1D | MaLR | LTR |
| 4414820 | 4415035 | - | 15.4 | 4204 | 4423 | -3142 | L1MA4 | L1 | LINE |
| 4415037 | 4415299 | - | 18.8 | 130 | 426 | 0 | MSTB | MaLR | LTR |
| 4415300 | 4415640 | - | 10.0 | 1 | 450 | 0 | LTR7 | ERV1 | LTR |
| 4415641 | 4415769 | - | 18.8 | 1 | 129 | -297 | MSTB | MaLR | LTR |
| 4415771 | 4417347 | - | 29.6 | 2 | 1731 | -4 | MLT1A-int | MaLR | LTR |
| 4417350 | 4417745 | - | 19.1 | 1 | 426 | 0 | MSTB | MaLR | LTR |
| 4417746 | 4418923 | - | 17.2 | 3063 | 4207 | -3358 | L1MA4 | L1 | LINE |
| 4418924 | 4419216 | - | 19.2 | 1 | 288 | -24 | AluJb | Alu | SINE |
| 4419217 | 4419414 | - | 20.1 | 2863 | 3062 | -4503 | L1MA4 | L1 | LINE |
| 4419446 | 4421637 | - | 21.5 | 12 | 2862 | -4703 | L1MA4 | L1 | LINE |

  

|  |  |  |  |  |  |  |  |  |  |
| --- | --- | --- | --- | --- | --- | --- | --- | --- | --- |
| chr2:4457447-4463920 | | | 6413 bp | | |  |  |  |  |
| Genome Start | Genome End | Strand | Div | Repeat Start | Repeat End | Repeat Left | Name | Family | Class |
| 4457447 | 4457725 | - | 26.9 | 4348 | 4628 | -2313 | L1MDa | L1 | LINE |
| 4457726 | 4457805 | + | 11.2 | 1 | 80 | 0 | MADE1 | Mariner | DNA |
| 4457806 | 4457969 | - | 26.9 | 4185 | 4347 | -2594 | L1MDa | L1 | LINE |
| 4458031 | 4458249 | + | 19.8 | 1 | 219 | 0 | MER20 | MER1\_type | DNA |
| 4458250 | 4458493 | - | 25.7 | 3854 | 4111 | -2830 | L1MDa | L1 | LINE |
| 4458494 | 4459149 | - | 14.9 | 5621 | 6291 | 0 | L1MA8 | L1 | LINE |
| 4459149 | 4459836 | - | 28.3 | 3178 | 3869 | -3072 | L1MDa | L1 | LINE |
| 4459837 | 4460214 | - | 16.4 | 1 | 381 | 0 | THE1D | MaLR | LTR |
| 4460215 | 4461833 | - | 30.2 | 1 | 1735 | 0 | MLT-int | MaLR | LTR |
| 4461835 | 4462202 | - | 14.2 | 1 | 381 | 0 | THE1D | MaLR | LTR |
| 4462203 | 4463920 | - | 27.0 | 1318 | 3177 | -3764 | L1MDa | L1 | LINE |

  

|  |  |  |  |  |  |  |  |  |  |
| --- | --- | --- | --- | --- | --- | --- | --- | --- | --- |
| chr2:4714556-4717380 | | | 2817 bp | | |  |  |  |  |
| Genome Start | Genome End | Strand | Div | Repeat Start | Repeat End | Repeat Left | Name | Family | Class |
| 4714556 | 4714915 | + | 18.6 | 4 | 385 | -43 | MSTC | MaLR | LTR |
| 4714916 | 4715326 | - | 22.5 | 1 | 426 | 0 | MSTB | MaLR | LTR |
| 4715335 | 4716923 | - | 21.5 | 1 | 1636 | -15 | MSTB-int | MaLR | LTR |
| 4716924 | 4717338 | - | 18.8 | 1 | 426 | 0 | MSTB | MaLR | LTR |
| 4717339 | 4717380 | + | 7.1 | 381 | 422 | -6 | MSTC | MaLR | LTR |

  

|  |  |  |  |  |  |  |  |  |  |
| --- | --- | --- | --- | --- | --- | --- | --- | --- | --- |
| chr2:4812198-4815925 | | | 3723 bp | | |  |  |  |  |
| Genome Start | Genome End | Strand | Div | Repeat Start | Repeat End | Repeat Left | Name | Family | Class |
| 4812198 | 4812285 | - | 25.0 | 1355 | 1442 | -13 | Charlie1a | MER1\_type | DNA |
| 4812287 | 4812657 | - | 13.4 | 1 | 381 | 0 | THE1D | MaLR | LTR |
| 4812658 | 4814252 | - | 14.0 | 3 | 1580 | 0 | THE1D-int | MaLR | LTR |
| 4814255 | 4814608 | - | 17.3 | 1 | 381 | 0 | THE1D | MaLR | LTR |
| 4814611 | 4815925 | - | 24.2 | 13 | 1344 | -111 | Charlie1a | MER1\_type | DNA |

  

|  |  |  |  |  |  |  |  |  |  |
| --- | --- | --- | --- | --- | --- | --- | --- | --- | --- |
| chr2:5239063-5242754 | | | 3256 bp | | |  |  |  |  |
| Genome Start | Genome End | Strand | Div | Repeat Start | Repeat End | Repeat Left | Name | Family | Class |
| 5239063 | 5239321 | + | 29.3 | 581 | 841 | -5278 | L1MEd | L1 | LINE |
| 5239563 | 5239930 | + | 13.5 | 1 | 375 | 0 | THE1C | MaLR | LTR |
| 5239931 | 5240482 | + | 14.8 | 1 | 427 | -1153 | THE1C-int | MaLR | LTR |
| 5240483 | 5240568 | + | 4.7 | 6067 | 6152 | -3 | L1PA4 | L1 | LINE |
| 5240569 | 5241440 | + | 14.8 | 428 | 1277 | -303 | THE1C-int | MaLR | LTR |
| 5241468 | 5241843 | + | 9.9 | 1 | 375 | 0 | THE1C | MaLR | LTR |
| 5241856 | 5242006 | + | 26.5 | 1044 | 1206 | -4913 | L1MEd | L1 | LINE |
| 5242101 | 5242328 | + | 33.0 | 1408 | 1661 | -4488 | L1MEd | L1 | LINE |
| 5242391 | 5242754 | + | 30.3 | 1762 | 2161 | -3988 | L1MEd | L1 | LINE |

  

|  |  |  |  |  |  |  |  |  |  |
| --- | --- | --- | --- | --- | --- | --- | --- | --- | --- |
| chr2:5600560-5603246 | | | 2675 bp | | |  |  |  |  |
| Genome Start | Genome End | Strand | Div | Repeat Start | Repeat End | Repeat Left | Name | Family | Class |
| 5600560 | 5600688 | + | 22.5 | 4 | 140 | -234 | MLT1A | MaLR | LTR |
| 5600689 | 5601050 | + | 12.8 | 1 | 379 | -2 | THE1D | MaLR | LTR |
| 5601052 | 5602650 | + | 31.0 | 1 | 1735 | 0 | MLT1A-int | MaLR | LTR |
| 5602651 | 5602996 | + | 15.2 | 1 | 367 | -14 | THE1D | MaLR | LTR |
| 5603008 | 5603246 | + | 23.1 | 133 | 374 | 0 | MLT1A | MaLR | LTR |

  

|  |  |  |  |  |  |  |  |  |  |
| --- | --- | --- | --- | --- | --- | --- | --- | --- | --- |
| chr2:6011814-6025507 | | | 13653 bp | | |  |  |  |  |
| Genome Start | Genome End | Strand | Div | Repeat Start | Repeat End | Repeat Left | Name | Family | Class |
| 6011814 | 6012945 | - | 17.7 | 5828 | 7001 | -4 | L1MA4A | L1 | LINE |
| 6012953 | 6014710 | - | 20.2 | 3097 | 5060 | -1802 | L1PBa | L1 | LINE |
| 6014711 | 6015090 | - | 16.7 | 1 | 428 | 0 | MSTA | MaLR | LTR |
| 6015091 | 6018583 | - | 22.3 | 480 | 3096 | -3766 | L1PBa | L1 | LINE |
| 6018589 | 6019290 | - | 19.2 | 4190 | 4909 | -1936 | L1MA4A | L1 | LINE |
| 6019291 | 6019658 | + | 10.7 | 1 | 364 | 0 | THE1B | MaLR | LTR |
| 6019659 | 6020363 | - | 19.2 | 3492 | 4189 | -2656 | L1MA4A | L1 | LINE |
| 6020364 | 6020657 | - | 17.8 | 1 | 298 | -14 | AluSx | Alu | SINE |
| 6020658 | 6021528 | - | 20.1 | 2638 | 3491 | -3354 | L1MA4A | L1 | LINE |
| 6021529 | 6021902 | - | 14.9 | 1 | 364 | 0 | THE1B | MaLR | LTR |
| 6021903 | 6023430 | - | 30.1 | 2 | 1735 | 0 | MLT-int | MaLR | LTR |
| 6023433 | 6023797 | - | 12.2 | 1 | 364 | 0 | THE1B | MaLR | LTR |
| 6023798 | 6023938 | - | 23.5 | 2495 | 2637 | -4212 | L1MA4A | L1 | LINE |
| 6023966 | 6025507 | - | 22.1 | 209 | 2173 | -4676 | L1MA4A | L1 | LINE |

  

|  |  |  |  |  |  |  |  |  |  |
| --- | --- | --- | --- | --- | --- | --- | --- | --- | --- |
| chr2:6915505-6918369 | | | 2725 bp | | |  |  |  |  |
| Genome Start | Genome End | Strand | Div | Repeat Start | Repeat End | Repeat Left | Name | Family | Class |
| 6915505 | 6915673 | + | 29.2 | 2803 | 2980 | -439 | L2 | L2 | LINE |
| 6915809 | 6916041 | + | 31.4 | 3115 | 3333 | -86 | L2 | L2 | LINE |
| 6916042 | 6916383 | + | 14.2 | 1 | 364 | 0 | THE1B | MaLR | LTR |
| 6916386 | 6917946 | + | 28.8 | 2 | 1734 | -1 | MLT-int | MaLR | LTR |
| 6917950 | 6918291 | + | 11.9 | 3 | 364 | 0 | THE1B | MaLR | LTR |
| 6918292 | 6918369 | + | 31.4 | 3334 | 3410 | -9 | L2 | L2 | LINE |

  

|  |  |  |  |  |  |  |  |  |  |
| --- | --- | --- | --- | --- | --- | --- | --- | --- | --- |
| chr2:7819869-7822875 | | | 3002 bp | | |  |  |  |  |
| Genome Start | Genome End | Strand | Div | Repeat Start | Repeat End | Repeat Left | Name | Family | Class |
| 7819869 | 7820197 | - | 26.5 | 4098 | 4434 | -1712 | L1M5 | L1 | LINE |
| 7820201 | 7820557 | - | 13.0 | 1 | 364 | 0 | THE1B | MaLR | LTR |
| 7820558 | 7822140 | - | 10.8 | 1 | 1580 | 0 | THE1B-int | MaLR | LTR |
| 7822141 | 7822496 | - | 12.4 | 1 | 364 | 0 | THE1B | MaLR | LTR |
| 7822499 | 7822875 | - | 31.0 | 3685 | 4100 | -2046 | L1M5 | L1 | LINE |

  

|  |  |  |  |  |  |  |  |  |  |
| --- | --- | --- | --- | --- | --- | --- | --- | --- | --- |
| chr2:9914640-9924940 | | | 10287 bp | | |  |  |  |  |
| Genome Start | Genome End | Strand | Div | Repeat Start | Repeat End | Repeat Left | Name | Family | Class |
| 9914640 | 9914757 | - | 29.1 | 3079 | 3208 | -211 | L2 | L2 | LINE |
| 9914760 | 9914865 | + | 19.0 | 58 | 160 | 0 | MER5A1 | MER1\_type | DNA |
| 9914873 | 9915140 | - | 23.1 | 2425 | 2713 | -706 | L2 | L2 | LINE |
| 9915141 | 9915526 | - | 15.2 | 1 | 379 | -2 | THE1D | MaLR | LTR |
| 9915527 | 9916966 | - | 29.2 | 241 | 1735 | 0 | MLT-int | MaLR | LTR |
| 9916967 | 9917265 | - | 16.1 | 1 | 300 | -12 | AluSx | Alu | SINE |
| 9917266 | 9917448 | - | 29.2 | 1 | 240 | -1495 | MLT-int | MaLR | LTR |
| 9917450 | 9917813 | - | 12.3 | 1 | 381 | 0 | THE1D | MaLR | LTR |
| 9917814 | 9917921 | - | 23.1 | 2309 | 2424 | -995 | L2 | L2 | LINE |
| 9917922 | 9918061 | - | 19.4 | 35 | 173 | -7 | MER104 | Tc2 | DNA |
| 9918062 | 9918538 | - | 23.1 | 1797 | 2308 | -1111 | L2 | L2 | LINE |
| 9918543 | 9924673 | + | 4.6 | 2 | 6149 | -6 | L1PA4 | L1 | LINE |
| 9924674 | 9924940 | - | 24.9 | 1530 | 1807 | -1612 | L2 | L2 | LINE |

  

|  |  |  |  |  |  |  |  |  |  |
| --- | --- | --- | --- | --- | --- | --- | --- | --- | --- |
| chr2:12683700-12690334 | | | 6632 bp | | |  |  |  |  |
| Genome Start | Genome End | Strand | Div | Repeat Start | Repeat End | Repeat Left | Name | Family | Class |
| 12683700 | 12683911 | + | 20.9 | 1 | 217 | -510 | LTR8A | ERV1 | LTR |
| 12683912 | 12684362 | + | 6.2 | 1 | 448 | -2 | LTR7 | ERV1 | LTR |
| 12684365 | 12687626 | + | 9.2 | 1 | 3290 | -4423 | HERVH | ERV1 | LTR |
| 12687617 | 12688117 | + | 12.4 | 3492 | 3993 | -3720 | HERVH | ERV1 | LTR |
| 12688120 | 12688780 | + | 15.3 | 4488 | 5149 | -2564 | HERVH | ERV1 | LTR |
| 12688782 | 12688998 | + | 13.4 | 5600 | 5819 | -1894 | HERVH | ERV1 | LTR |
| 12688997 | 12689376 | + | 5.9 | 7322 | 7713 | 0 | HERVH | ERV1 | LTR |
| 12689377 | 12689834 | + | 6.2 | 1 | 448 | -2 | LTR7 | ERV1 | LTR |
| 12689845 | 12690334 | + | 18.8 | 214 | 724 | -3 | LTR8A | ERV1 | LTR |

  

|  |  |  |  |  |  |  |  |  |  |
| --- | --- | --- | --- | --- | --- | --- | --- | --- | --- |
| chr2:12915666-12920010 | | | 4331 bp | | |  |  |  |  |
| Genome Start | Genome End | Strand | Div | Repeat Start | Repeat End | Repeat Left | Name | Family | Class |
| 12915666 | 12916199 | - | 34.9 | 2767 | 3382 | -37 | L2 | L2 | LINE |
| 12916204 | 12916546 | + | 25.2 | 34 | 356 | -206 | MLT1F2 | MaLR | LTR |
| 12916547 | 12916928 | - | 16.2 | 1 | 388 | -2 | MLT1B | MaLR | LTR |
| 12916929 | 12917135 | + | 25.2 | 357 | 560 | -2 | MLT1F2 | MaLR | LTR |
| 12917139 | 12917833 | + | 25.2 | 1 | 694 | -795 | MLT1F2-int | MaLR | LTR |
| 12917834 | 12918319 | - | 14.8 | 1 | 486 | 0 | MER66B | ERV1 | LTR |
| 12918320 | 12919070 | + | 25.2 | 695 | 1487 | -2 | MLT1F2-int | MaLR | LTR |
| 12919077 | 12919546 | + | 25.8 | 5 | 458 | -104 | MLT1F2 | MaLR | LTR |
| 12919547 | 12919859 | - | 8.1 | 1 | 297 | -14 | AluY | Alu | SINE |
| 12919860 | 12919960 | + | 25.8 | 459 | 559 | -3 | MLT1F2 | MaLR | LTR |
| 12919962 | 12920010 | - | 25.0 | 2728 | 2775 | -644 | L2 | L2 | LINE |

  

|  |  |  |  |  |  |  |  |  |  |
| --- | --- | --- | --- | --- | --- | --- | --- | --- | --- |
| chr2:13125406-13133250 | | | 7348 bp | | |  |  |  |  |
| Genome Start | Genome End | Strand | Div | Repeat Start | Repeat End | Repeat Left | Name | Family | Class |
| 13125406 | 13126375 | + | 19.8 | 397 | 1423 | -5253 | MER66C-int | ERV1 | LTR |
| 13126399 | 13126982 | + | 22.1 | 1532 | 2161 | -4515 | MER66C-int | ERV1 | LTR |
| 13126986 | 13129113 | + | 26.4 | 2342 | 4527 | -2149 | MER66C-int | ERV1 | LTR |
| 13129513 | 13129672 | + | 21.1 | 4526 | 4644 | -2032 | MER66C-int | ERV1 | LTR |
| 13129673 | 13129980 | + | 7.8 | 1 | 312 | -1 | AluSp | Alu | SINE |
| 13129981 | 13130113 | + | 21.1 | 4645 | 4769 | -1907 | MER66C-int | ERV1 | LTR |
| 13130122 | 13130495 | - | 15.8 | 1 | 375 | 0 | THE1C | MaLR | LTR |
| 13130496 | 13130714 | - | 8.7 | 1355 | 1580 | 0 | THE1C-int | MaLR | LTR |
| 13130714 | 13130965 | - | 13.7 | 1 | 253 | -122 | THE1C | MaLR | LTR |
| 13130966 | 13132463 | - | 13.7 | 1 | 1580 | 0 | THE1C-int | MaLR | LTR |
| 13132464 | 13132838 | - | 12.0 | 1 | 375 | 0 | THE1C | MaLR | LTR |
| 13132842 | 13132896 | + | 18.2 | 4773 | 4828 | -1848 | MER66C-int | ERV1 | LTR |
| 13132959 | 13133250 | + | 17.8 | 6359 | 6665 | -11 | MER66C-int | ERV1 | LTR |

  

|  |  |  |  |  |  |  |  |  |  |
| --- | --- | --- | --- | --- | --- | --- | --- | --- | --- |
| chr2:13186524-13190218 | | | 3281 bp | | |  |  |  |  |
| Genome Start | Genome End | Strand | Div | Repeat Start | Repeat End | Repeat Left | Name | Family | Class |
| 13186524 | 13186810 | + | 31.0 | 4553 | 4864 | -1282 | L1M5 | L1 | LINE |
| 13186928 | 13187307 | + | 16.0 | 1 | 428 | 0 | MSTA | MaLR | LTR |
| 13187310 | 13188916 | + | 16.2 | 3 | 1576 | -4 | MSTA-int | MaLR | LTR |
| 13188921 | 13189305 | + | 14.8 | 1 | 426 | -2 | MSTA | MaLR | LTR |
| 13189394 | 13189934 | - | 18.3 | 5598 | 6156 | 0 | L1PB4 | L1 | LINE |
| 13190138 | 13190218 | + | 23.5 | 5348 | 5431 | -809 | L1M5 | L1 | LINE |

  

|  |  |  |  |  |  |  |  |  |  |
| --- | --- | --- | --- | --- | --- | --- | --- | --- | --- |
| chr2:13414112-13419324 | | | 5207 bp | | |  |  |  |  |
| Genome Start | Genome End | Strand | Div | Repeat Start | Repeat End | Repeat Left | Name | Family | Class |
| 13414112 | 13416057 | + | 18.7 | 725 | 2698 | -4867 | L1MA2 | L1 | LINE |
| 13416059 | 13416425 | - | 14.9 | 3 | 379 | -2 | THE1D | MaLR | LTR |
| 13416428 | 13417898 | - | 15.6 | 3 | 1580 | 0 | THE1D-int | MaLR | LTR |
| 13417901 | 13418272 | - | 14.5 | 1 | 381 | 0 | THE1D | MaLR | LTR |
| 13418274 | 13419324 | + | 12.2 | 2696 | 3763 | -3802 | L1MA2 | L1 | LINE |

  

|  |  |  |  |  |  |  |  |  |  |
| --- | --- | --- | --- | --- | --- | --- | --- | --- | --- |
| chr2:13494583-13500815 | | | 5425 bp | | |  |  |  |  |
| Genome Start | Genome End | Strand | Div | Repeat Start | Repeat End | Repeat Left | Name | Family | Class |
| 13494583 | 13494791 | - | 30.1 | 5003 | 5203 | -511 | ERVL-B4 | ERVL | LTR |
| 13495009 | 13495363 | - | 15.3 | 1 | 381 | 0 | THE1D | MaLR | LTR |
| 13495365 | 13495796 | - | 14.2 | 1145 | 1579 | -1 | THE1D-int | MaLR | LTR |
| 13495811 | 13496186 | - | 15.6 | 4 | 381 | 0 | THE1D | MaLR | LTR |
| 13496191 | 13496348 | - | 32.2 | 2052 | 2205 | -3509 | ERVL-B4 | ERVL | LTR |
| 13496695 | 13497059 | - | 36.0 | 1204 | 1576 | -3420 | ERVL-B4 | ERVL | LTR |
| 13497091 | 13497232 | - | 36.0 | 1056 | 1203 | -3793 | ERVL-B4 | ERVL | LTR |
| 13497312 | 13498913 | - | 6.2 | 4551 | 6149 | -5 | L1PA5 | L1 | LINE |
| 13498907 | 13499683 | + | 4.1 | 3642 | 4421 | -1725 | L1PA5 | L1 | LINE |
| 13499760 | 13500363 | - | 28.8 | 84 | 663 | -4333 | ERVL-B4 | ERVL | LTR |
| 13500364 | 13500379 | - | 6.0 | 284 | 311 | 0 | AluY | Alu | SINE |
| 13500427 | 13500726 | - | 6.0 | 1 | 283 | -28 | AluY | Alu | SINE |
| 13500727 | 13500815 | - | 28.8 | 1 | 83 | -4913 | ERVL-B4 | ERVL | LTR |

  

|  |  |  |  |  |  |  |  |  |  |
| --- | --- | --- | --- | --- | --- | --- | --- | --- | --- |
| chr2:13724174-13732372 | | | 7747 bp | | |  |  |  |  |
| Genome Start | Genome End | Strand | Div | Repeat Start | Repeat End | Repeat Left | Name | Family | Class |
| 13724174 | 13725433 | + | 31.5 | 17 | 1475 | -4948 | L1MCc | L1 | LINE |
| 13725434 | 13725907 | + | 24.7 | 1 | 467 | 0 | MLT1C | MaLR | LTR |
| 13725908 | 13726185 | + | 30.3 | 1476 | 1914 | -5017 | L1MCc | L1 | LINE |
| 13726235 | 13726263 | + | 30.8 | 1915 | 1948 | -4983 | L1MCc | L1 | LINE |
| 13726495 | 13726644 | + | 23.7 | 2187 | 2512 | -4610 | L1MCc | L1 | LINE |
| 13726642 | 13728498 | - | 18.5 | 4414 | 6163 | -3 | L1PA16 | L1 | LINE |
| 13728525 | 13728706 | - | 13.1 | 4203 | 4416 | -1730 | L1PA16 | L1 | LINE |
| 13728716 | 13729224 | + | 26.4 | 2515 | 3064 | -4058 | L1MCc | L1 | LINE |
| 13729236 | 13729458 | + | 30.5 | 3240 | 3455 | -3486 | L1MCc | L1 | LINE |
| 13729569 | 13729860 | - | 19.4 | 10 | 300 | -12 | AluJb | Alu | SINE |
| 13729861 | 13730225 | - | 14.8 | 3 | 362 | -2 | THE1B | MaLR | LTR |
| 13730228 | 13731657 | - | 11.9 | 1 | 1580 | 0 | THE1B-int | MaLR | LTR |
| 13731658 | 13732031 | - | 14.6 | 1 | 364 | 0 | THE1B | MaLR | LTR |
| 13732049 | 13732372 | + | 27.6 | 3547 | 3886 | -3055 | L1MCc | L1 | LINE |

  

|  |  |  |  |  |  |  |  |  |  |
| --- | --- | --- | --- | --- | --- | --- | --- | --- | --- |
| chr2:14047066-14051047 | | | 3896 bp | | |  |  |  |  |
| Genome Start | Genome End | Strand | Div | Repeat Start | Repeat End | Repeat Left | Name | Family | Class |
| 14047066 | 14047488 | + | 28.4 | 5 | 461 | -5680 | L1M3c | L1 | LINE |
| 14047513 | 14048343 | + | 28.4 | 462 | 1371 | -4770 | L1M3c | L1 | LINE |
| 14048346 | 14048489 | + | 29.4 | 1 | 124 | -42 | FRAM | Alu | SINE |
| 14048519 | 14048628 | + | 25.7 | 1391 | 1499 | -4642 | L1M3c | L1 | LINE |
| 14048631 | 14048984 | - | 13.6 | 6 | 364 | 0 | THE1B | MaLR | LTR |
| 14048990 | 14050487 | - | 12.2 | 1 | 1580 | 0 | THE1B-int | MaLR | LTR |
| 14050490 | 14050844 | - | 12.8 | 1 | 362 | -2 | THE1B | MaLR | LTR |
| 14050867 | 14051047 | + | 25.0 | 1504 | 1670 | -4541 | L1M3c | L1 | LINE |

  

|  |  |  |  |  |  |  |  |  |  |
| --- | --- | --- | --- | --- | --- | --- | --- | --- | --- |
| chr2:15180924-15184196 | | | 3253 bp | | |  |  |  |  |
| Genome Start | Genome End | Strand | Div | Repeat Start | Repeat End | Repeat Left | Name | Family | Class |
| 15180924 | 15180965 | - | 16.7 | 449 | 490 | -1 | LTR16C | ERVL | LTR |
| 15180976 | 15181434 | + | 13.4 | 1 | 426 | 0 | MSTB | MaLR | LTR |
| 15181439 | 15182740 | + | 20.9 | 4 | 1309 | -379 | MSTB-int | MaLR | LTR |
| 15182741 | 15183157 | - | 16.1 | 1 | 428 | 0 | MSTA | MaLR | LTR |
| 15183158 | 15183553 | + | 17.9 | 1273 | 1651 | 0 | MSTB-int | MaLR | LTR |
| 15183554 | 15183975 | + | 17.7 | 1 | 426 | 0 | MSTB | MaLR | LTR |
| 15183982 | 15184196 | - | 22.1 | 190 | 404 | -87 | LTR16C | ERVL | LTR |

  

|  |  |  |  |  |  |  |  |  |  |
| --- | --- | --- | --- | --- | --- | --- | --- | --- | --- |
| chr2:15261297-15269081 | | | 7633 bp | | |  |  |  |  |
| Genome Start | Genome End | Strand | Div | Repeat Start | Repeat End | Repeat Left | Name | Family | Class |
| 15261297 | 15261421 | + | 22.3 | 6138 | 6271 | -1537 | L1MC4a | L1 | LINE |
| 15261506 | 15261551 | + | 22.3 | 6272 | 6310 | -1498 | L1MC4a | L1 | LINE |
| 15261552 | 15261861 | + | 19.2 | 1 | 312 | 0 | AluJo | Alu | SINE |
| 15261862 | 15262094 | + | 22.3 | 6311 | 6578 | -1230 | L1MC4a | L1 | LINE |
| 15262095 | 15262596 | + | 6.6 | 5652 | 6154 | 0 | L1PA5 | L1 | LINE |
| 15262633 | 15263183 | + | 10.0 | 3207 | 3749 | -3816 | L1M1 | L1 | LINE |
| 15263184 | 15263521 | + | 13.5 | 6 | 381 | 0 | THE1D | MaLR | LTR |
| 15263524 | 15265109 | + | 30.6 | 2 | 1735 | 0 | MLT1J1-int | MaLR | LTR |
| 15265112 | 15265463 | + | 13.5 | 3 | 381 | 0 | THE1D | MaLR | LTR |
| 15265473 | 15265649 | + | 10.8 | 2348 | 2521 | -3625 | L1MA2 | L1 | LINE |
| 15265650 | 15266010 | - | 12.7 | 1 | 364 | 0 | THE1B | MaLR | LTR |
| 15266011 | 15267098 | + | 10.8 | 2522 | 3721 | -2425 | L1MA2 | L1 | LINE |
| 15267095 | 15268851 | + | 10.8 | 4536 | 6304 | 0 | L1MA2 | L1 | LINE |
| 15268875 | 15269081 | + | 25.4 | 7559 | 7808 | 0 | L1MC4a | L1 | LINE |

  

|  |  |  |  |  |  |  |  |  |  |
| --- | --- | --- | --- | --- | --- | --- | --- | --- | --- |
| chr2:16119908-16122814 | | | 2905 bp | | |  |  |  |  |
| Genome Start | Genome End | Strand | Div | Repeat Start | Repeat End | Repeat Left | Name | Family | Class |
| 16119908 | 16119936 | + | 20.3 | 14 | 43 | -610 | MER82 | MER2\_type | DNA |
| 16119937 | 16120310 | - | 10.9 | 1 | 373 | -2 | THE1C | MaLR | LTR |
| 16120311 | 16121852 | - | 28.9 | 2 | 1735 | 0 | MLT-int | MaLR | LTR |
| 16121855 | 16122230 | - | 11.3 | 2 | 375 | 0 | THE1C | MaLR | LTR |
| 16122231 | 16122814 | + | 20.3 | 44 | 647 | -6 | MER82 | MER2\_type | DNA |

  

|  |  |  |  |  |  |  |  |  |  |
| --- | --- | --- | --- | --- | --- | --- | --- | --- | --- |
| chr2:16618258-16620937 | | | 2677 bp | | |  |  |  |  |
| Genome Start | Genome End | Strand | Div | Repeat Start | Repeat End | Repeat Left | Name | Family | Class |
| 16618258 | 16618287 | - | 23.5 | 244 | 270 | -6 | MIRm | MIR | SINE |
| 16618288 | 16618643 | + | 10.7 | 1 | 363 | -1 | THE1B | MaLR | LTR |
| 16618647 | 16620153 | + | 30.1 | 2 | 1735 | 0 | MLT1A0-int | MaLR | LTR |
| 16620154 | 16620322 | + | 12.4 | 1 | 184 | -180 | THE1B | MaLR | LTR |
| 16620323 | 16620658 | + | 3.9 | 1 | 335 | 0 | AluY | Alu | SINE |
| 16620659 | 16620858 | + | 12.4 | 185 | 364 | 0 | THE1B | MaLR | LTR |
| 16620859 | 16620937 | - | 23.5 | 164 | 243 | -33 | MIRm | MIR | SINE |

  

|  |  |  |  |  |  |  |  |  |  |
| --- | --- | --- | --- | --- | --- | --- | --- | --- | --- |
| chr2:16848411-16851472 | | | 3056 bp | | |  |  |  |  |
| Genome Start | Genome End | Strand | Div | Repeat Start | Repeat End | Repeat Left | Name | Family | Class |
| 16848411 | 16848532 | + | 12.6 | 5079 | 5194 | -946 | L1M3 | L1 | LINE |
| 16848533 | 16848889 | - | 12.3 | 1 | 363 | -1 | THE1B | MaLR | LTR |
| 16848894 | 16850452 | - | 29.7 | 2 | 1731 | -4 | MLT1D-int | MaLR | LTR |
| 16850455 | 16850788 | - | 13.6 | 3 | 364 | 0 | THE1B | MaLR | LTR |
| 16850789 | 16851472 | + | 13.6 | 5195 | 5802 | -366 | L1M3 | L1 | LINE |

  

|  |  |  |  |  |  |  |  |  |  |
| --- | --- | --- | --- | --- | --- | --- | --- | --- | --- |
| chr2:17383436-17392209 | | | 8615 bp | | |  |  |  |  |
| Genome Start | Genome End | Strand | Div | Repeat Start | Repeat End | Repeat Left | Name | Family | Class |
| 17383436 | 17383583 | - | 36.7 | 4307 | 4445 | -44 | L3 | CR1 | LINE |
| 17383689 | 17384438 | + | 6.0 | 1 | 780 | 0 | LTR17 | ERV1 | LTR |
| 17384439 | 17390370 | + | 4.9 | 1 | 6019 | -2607 | HERV17 | ERV1 | LTR |
| 17390364 | 17390402 | + | 5.1 | 7510 | 7548 | -1078 | HERV17 | ERV1 | LTR |
| 17390393 | 17390684 | + | 5.8 | 8335 | 8626 | 0 | HERV17 | ERV1 | LTR |
| 17390685 | 17391462 | + | 7.7 | 1 | 780 | 0 | LTR17 | ERV1 | LTR |
| 17391534 | 17392209 | - | 31.8 | 3527 | 4270 | -219 | L3 | CR1 | LINE |

  

|  |  |  |  |  |  |  |  |  |  |
| --- | --- | --- | --- | --- | --- | --- | --- | --- | --- |
| chr2:17446191-17454057 | | | 7622 bp | | |  |  |  |  |
| Genome Start | Genome End | Strand | Div | Repeat Start | Repeat End | Repeat Left | Name | Family | Class |
| 17446191 | 17446368 | - | 28.6 | 2210 | 2382 | -2452 | L1MEe | L1 | LINE |
| 17446374 | 17446773 | - | 29.9 | 3055 | 3480 | -2666 | L1M5 | L1 | LINE |
| 17446891 | 17447604 | - | 17.7 | 1540 | 2285 | -2546 | L1MEe | L1 | LINE |
| 17447605 | 17447915 | - | 14.9 | 1 | 309 | -4 | AluSq | Alu | SINE |
| 17447916 | 17448379 | - | 17.7 | 2388 | 2851 | -3292 | L1M2 | L1 | LINE |
| 17448376 | 17451052 | + | 16.2 | 3592 | 6280 | -17 | L1MA5A | L1 | LINE |
| 17451083 | 17451413 | - | 33.5 | 1095 | 1449 | -3385 | L1MEe | L1 | LINE |
| 17451506 | 17451880 | + | 16.2 | 1 | 364 | 0 | THE1B | MaLR | LTR |
| 17451883 | 17453445 | + | 31.6 | 2 | 1735 | 0 | MLT1D-int | MaLR | LTR |
| 17453448 | 17453822 | + | 16.0 | 3 | 364 | 0 | THE1B | MaLR | LTR |
| 17453824 | 17454057 | - | 25.1 | 754 | 987 | -3867 | L1MEe | L1 | LINE |

  

|  |  |  |  |  |  |  |  |  |  |
| --- | --- | --- | --- | --- | --- | --- | --- | --- | --- |
| chr2:17967238-17973425 | | | 6156 bp | | |  |  |  |  |
| Genome Start | Genome End | Strand | Div | Repeat Start | Repeat End | Repeat Left | Name | Family | Class |
| 17967238 | 17969830 | - | 22.4 | 3406 | 6178 | 0 | L1MB8 | L1 | LINE |
| 17969831 | 17970187 | + | 13.3 | 1 | 319 | -291 | MER4B | ERV1 | LTR |
| 17970188 | 17970545 | - | 12.7 | 1 | 375 | 0 | THE1C | MaLR | LTR |
| 17970546 | 17972188 | - | 29.7 | 2 | 1735 | 0 | MLT-int | MaLR | LTR |
| 17972192 | 17972545 | - | 15.6 | 1 | 374 | -1 | THE1C | MaLR | LTR |
| 17972546 | 17972824 | + | 13.3 | 320 | 574 | -36 | MER4B | ERV1 | LTR |
| 17972854 | 17972999 | - | 19.0 | 3263 | 3409 | -2737 | L1MB8 | L1 | LINE |
| 17973000 | 17973282 | - | 15.8 | 33 | 310 | -2 | AluSx | Alu | SINE |
| 17973283 | 17973425 | - | 19.0 | 3115 | 3262 | -2884 | L1MB8 | L1 | LINE |

  

|  |  |  |  |  |  |  |  |  |  |
| --- | --- | --- | --- | --- | --- | --- | --- | --- | --- |
| chr2:18062955-18070018 | | | 7030 bp | | |  |  |  |  |
| Genome Start | Genome End | Strand | Div | Repeat Start | Repeat End | Repeat Left | Name | Family | Class |
| 18062955 | 18064262 | - | 15.4 | 4984 | 6289 | -2 | L1PA16 | L1 | LINE |
| 18064264 | 18067448 | - | 17.0 | 633 | 3821 | -2450 | L1PA16 | L1 | LINE |
| 18067480 | 18067842 | - | 13.5 | 1 | 364 | 0 | THE1B | MaLR | LTR |
| 18067843 | 18069415 | - | 30.4 | 2 | 1735 | 0 | MLT-int | MaLR | LTR |
| 18069418 | 18069601 | - | 12.6 | 178 | 364 | 0 | THE1B | MaLR | LTR |
| 18069602 | 18070018 | - | 24.0 | 1 | 420 | -5871 | L1PA16 | L1 | LINE |

  

|  |  |  |  |  |  |  |  |  |  |
| --- | --- | --- | --- | --- | --- | --- | --- | --- | --- |
| chr2:19209668-19216756 | | | 6648 bp | | |  |  |  |  |
| Genome Start | Genome End | Strand | Div | Repeat Start | Repeat End | Repeat Left | Name | Family | Class |
| 19209668 | 19210436 | + | 28.2 | 2957 | 3728 | -2418 | L1M5 | L1 | LINE |
| 19210657 | 19211592 | + | 31.8 | 10 | 1030 | -2389 | L2 | L2 | LINE |
| 19211708 | 19212149 | + | 29.7 | 3932 | 4429 | -1717 | L1M5 | L1 | LINE |
| 19212257 | 19212454 | + | 29.7 | 4430 | 4635 | -1511 | L1M5 | L1 | LINE |
| 19212474 | 19212600 | - | 14.2 | 6013 | 6168 | 0 | L1MB1 | L1 | LINE |
| 19212601 | 19212829 | - | 12.6 | 443 | 664 | 0 | MER4A | ERV1 | LTR |
| 19212830 | 19213236 | - | 12.9 | 1 | 426 | -2 | MSTA | MaLR | LTR |
| 19213237 | 19214880 | - | 30.6 | 2 | 1735 | 0 | MLT-int | MaLR | LTR |
| 19214883 | 19215291 | - | 16.7 | 1 | 428 | 0 | MSTA | MaLR | LTR |
| 19215292 | 19215444 | - | 17.1 | 176 | 442 | -222 | MER4A | ERV1 | LTR |
| 19215417 | 19215791 | - | 9.6 | 1 | 357 | -307 | MER4A | ERV1 | LTR |
| 19215792 | 19216254 | - | 17.6 | 5559 | 6016 | -152 | L1MB1 | L1 | LINE |
| 19216261 | 19216756 | + | 35.9 | 4671 | 5172 | -974 | L1M5 | L1 | LINE |

  

|  |  |  |  |  |  |  |  |  |  |
| --- | --- | --- | --- | --- | --- | --- | --- | --- | --- |
| chr2:20145436-20151645 | | | 6208 bp | | |  |  |  |  |
| Genome Start | Genome End | Strand | Div | Repeat Start | Repeat End | Repeat Left | Name | Family | Class |
| 20145436 | 20145692 | + | 21.9 | 1 | 339 | -57 | MSTD | MaLR | LTR |
| 20145698 | 20145803 | - | 13.2 | 206 | 311 | -1 | AluSg/x | Alu | SINE |
| 20145804 | 20146238 | + | 11.1 | 1 | 450 | 0 | LTR7 | ERV1 | LTR |
| 20146261 | 20149548 | + | 11.3 | 4 | 3736 | -3977 | HERVH | ERV1 | LTR |
| 20149542 | 20150544 | + | 12.7 | 3962 | 4992 | -2287 | HERVH | ERV1 | LTR |
| 20150524 | 20150933 | + | 8.9 | 7296 | 7713 | 0 | HERVH | ERV1 | LTR |
| 20150934 | 20151365 | + | 11.1 | 1 | 448 | -2 | LTR7 | ERV1 | LTR |
| 20151368 | 20151575 | - | 13.0 | 1 | 209 | -104 | AluSq | Alu | SINE |
| 20151577 | 20151645 | + | 11.6 | 328 | 396 | 0 | MSTD | MaLR | LTR |

  

|  |  |  |  |  |  |  |  |  |  |
| --- | --- | --- | --- | --- | --- | --- | --- | --- | --- |
| chr2:21607356-21613806 | | | 6339 bp | | |  |  |  |  |
| Genome Start | Genome End | Strand | Div | Repeat Start | Repeat End | Repeat Left | Name | Family | Class |
| 21607356 | 21607538 | - | 19.4 | 4865 | 5048 | -1958 | L1M4b | L1 | LINE |
| 21607561 | 21607616 | - | 25.6 | 3945 | 4000 | -2140 | L1M3 | L1 | LINE |
| 21607634 | 21607774 | - | 19.1 | 1 | 140 | -36 | FRAM | Alu | SINE |
| 21607782 | 21609817 | - | 26.0 | 3075 | 4814 | -2192 | L1M4b | L1 | LINE |
| 21609818 | 21610121 | - | 8.2 | 1 | 300 | 0 | AluY | Alu | SINE |
| 21610122 | 21610407 | - | 29.4 | 2934 | 3201 | -4133 | L1M4b | L1 | LINE |
| 21610448 | 21611062 | - | 26.2 | 1772 | 2460 | -4548 | L1M4b | L1 | LINE |
| 21611063 | 21611434 | + | 14.9 | 1 | 428 | 0 | MSTA | MaLR | LTR |
| 21611437 | 21613042 | + | 30.1 | 2 | 1735 | 0 | MLT-int | MaLR | LTR |
| 21613043 | 21613215 | + | 17.6 | 1 | 224 | -204 | MSTA | MaLR | LTR |
| 21613240 | 21613418 | + | 17.6 | 225 | 427 | -1 | MSTA | MaLR | LTR |
| 21613419 | 21613806 | - | 23.2 | 1390 | 1809 | -5199 | L1M4b | L1 | LINE |

  

|  |  |  |  |  |  |  |  |  |  |
| --- | --- | --- | --- | --- | --- | --- | --- | --- | --- |
| chr2:22066991-22070334 | | | 3342 bp | | |  |  |  |  |
| Genome Start | Genome End | Strand | Div | Repeat Start | Repeat End | Repeat Left | Name | Family | Class |
| 22066991 | 22067670 | + | 33.4 | 2227 | 2997 | -422 | L2 | L2 | LINE |
| 22067671 | 22068025 | - | 11.9 | 1 | 375 | 0 | THE1C | MaLR | LTR |
| 22068026 | 22069603 | - | 30.8 | 2 | 1735 | 0 | MLT1J-int | MaLR | LTR |
| 22069606 | 22069971 | - | 13.1 | 1 | 375 | 0 | THE1C | MaLR | LTR |
| 22069972 | 22070334 | + | 31.9 | 2998 | 3418 | -1 | L2 | L2 | LINE |

  

|  |  |  |  |  |  |  |  |  |  |
| --- | --- | --- | --- | --- | --- | --- | --- | --- | --- |
| chr2:22298846-22301369 | | | 2480 bp | | |  |  |  |  |
| Genome Start | Genome End | Strand | Div | Repeat Start | Repeat End | Repeat Left | Name | Family | Class |
| 22298846 | 22298939 | + | 20.2 | 6 | 115 | -352 | MLT1B | MaLR | LTR |
| 22298982 | 22299060 | + | 18.1 | 285 | 359 | -31 | MLT1B | MaLR | LTR |
| 22299061 | 22299424 | + | 14.3 | 1 | 364 | 0 | THE1B | MaLR | LTR |
| 22299427 | 22300978 | + | 30.1 | 2 | 1735 | 0 | MLT-int | MaLR | LTR |
| 22300979 | 22301337 | + | 13.5 | 1 | 364 | 0 | THE1B | MaLR | LTR |
| 22301338 | 22301369 | + | 18.1 | 360 | 390 | 0 | MLT1B | MaLR | LTR |

  

|  |  |  |  |  |  |  |  |  |  |
| --- | --- | --- | --- | --- | --- | --- | --- | --- | --- |
| chr2:22779270-22785725 | | | 6468 bp | | |  |  |  |  |
| Genome Start | Genome End | Strand | Div | Repeat Start | Repeat End | Repeat Left | Name | Family | Class |
| 22779270 | 22781636 | + | 22.7 | 9 | 2237 | -4612 | L1M2 | L1 | LINE |
| 22781637 | 22781830 | - | 1.0 | 5957 | 6150 | -5 | L1PA3 | L1 | LINE |
| 22781831 | 22782239 | + | 22.7 | 2238 | 2505 | -4344 | L1M2 | L1 | LINE |
| 22782228 | 22782534 | + | 5.9 | 2768 | 3072 | -3074 | L1P1 | L1 | LINE |
| 22782524 | 22782634 | + | 18.4 | 2494 | 2602 | -4247 | L1M2 | L1 | LINE |
| 22782638 | 22783011 | - | 15.6 | 3 | 375 | 0 | THE1C | MaLR | LTR |
| 22783014 | 22784599 | - | 29.5 | 2 | 1735 | 0 | MLT-int | MaLR | LTR |
| 22784604 | 22784962 | - | 13.0 | 16 | 373 | -2 | THE1C | MaLR | LTR |
| 22784965 | 22785725 | + | 18.4 | 2601 | 3362 | -3483 | L1M2 | L1 | LINE |

  

|  |  |  |  |  |  |  |  |  |  |
| --- | --- | --- | --- | --- | --- | --- | --- | --- | --- |
| chr2:22796325-22799838 | | | 3224 bp | | |  |  |  |  |
| Genome Start | Genome End | Strand | Div | Repeat Start | Repeat End | Repeat Left | Name | Family | Class |
| 22796325 | 22796523 | + | 31.6 | 4434 | 4643 | -1071 | ERVL-B4 | ERVL | LTR |
| 22796595 | 22796947 | - | 12.5 | 1 | 355 | 0 | THE1A | MaLR | LTR |
| 22796948 | 22798566 | - | 10.5 | 1 | 1580 | 0 | THE1A-int | MaLR | LTR |
| 22798567 | 22798918 | - | 10.5 | 1 | 355 | 0 | THE1A | MaLR | LTR |
| 22798930 | 22799205 | + | 33.3 | 4747 | 5037 | -677 | ERVL-B4 | ERVL | LTR |
| 22799207 | 22799511 | + | 34.5 | 1 | 324 | -85 | MLT1I | MaLR | LTR |
| 22799719 | 22799838 | + | 31.9 | 5078 | 5200 | -467 | ERVL-B4 | ERVL | LTR |

  

|  |  |  |  |  |  |  |  |  |  |
| --- | --- | --- | --- | --- | --- | --- | --- | --- | --- |
| chr2:28043471-28046005 | | | 2534 bp | | |  |  |  |  |
| Genome Start | Genome End | Strand | Div | Repeat Start | Repeat End | Repeat Left | Name | Family | Class |
| 28043471 | 28043539 | - | 26.1 | 421 | 491 | 0 | LTR16C | ERVL | LTR |
| 28043541 | 28043900 | + | 13.0 | 1 | 364 | 0 | THE1B | MaLR | LTR |
| 28043901 | 28045332 | + | 8.5 | 1 | 1580 | 0 | THE1B-int | MaLR | LTR |
| 28045333 | 28045689 | + | 11.5 | 1 | 364 | 0 | THE1B | MaLR | LTR |
| 28045690 | 28046005 | - | 33.9 | 75 | 424 | -67 | LTR16C | ERVL | LTR |

  

|  |  |  |  |  |  |  |  |  |  |
| --- | --- | --- | --- | --- | --- | --- | --- | --- | --- |
| chr2:31595586-31599855 | | | 3825 bp | | |  |  |  |  |
| Genome Start | Genome End | Strand | Div | Repeat Start | Repeat End | Repeat Left | Name | Family | Class |
| 31595586 | 31596276 | - | 25.1 | 964 | 1787 | -5341 | L1MCc | L1 | LINE |
| 31596277 | 31596379 | + | 20.6 | 7 | 109 | -5 | MER81 | AcHobo | DNA |
| 31596385 | 31596478 | - | 25.8 | 256 | 353 | -6070 | L1MCc | L1 | LINE |
| 31596532 | 31596780 | - | 30.5 | 649 | 898 | -5525 | L1MCc | L1 | LINE |
| 31597128 | 31597431 | + | 9.9 | 1 | 355 | 0 | THE1A | MaLR | LTR |
| 31597434 | 31598991 | + | 29.5 | 2 | 1733 | -2 | MLT-int | MaLR | LTR |
| 31598994 | 31599320 | + | 5.2 | 1 | 355 | 0 | THE1A | MaLR | LTR |
| 31599349 | 31599431 | - | 25.6 | 373 | 455 | -5968 | L1MCc | L1 | LINE |
| 31599438 | 31599549 | + | 25.0 | 1 | 112 | -2 | MER81 | AcHobo | DNA |
| 31599552 | 31599855 | - | 25.7 | 18 | 353 | -6070 | L1MCc | L1 | LINE |

  

|  |  |  |  |  |  |  |  |  |  |
| --- | --- | --- | --- | --- | --- | --- | --- | --- | --- |
| chr2:31692603-31696544 | | | 3928 bp | | |  |  |  |  |
| Genome Start | Genome End | Strand | Div | Repeat Start | Repeat End | Repeat Left | Name | Family | Class |
| 31692603 | 31693373 | - | 12.3 | 807 | 1580 | 0 | THE1B-int | MaLR | LTR |
| 31693377 | 31693745 | + | 14.5 | 1 | 364 | 0 | THE1B | MaLR | LTR |
| 31693746 | 31695414 | + | 9.5 | 1 | 1572 | -8 | THE1B-int | MaLR | LTR |
| 31695426 | 31695784 | + | 13.8 | 3 | 364 | 0 | THE1B | MaLR | LTR |
| 31695785 | 31696544 | - | 13.1 | 6 | 808 | -772 | THE1B-int | MaLR | LTR |

  

|  |  |  |  |  |  |  |  |  |  |
| --- | --- | --- | --- | --- | --- | --- | --- | --- | --- |
| chr2:31895965-31901104 | | | 5152 bp | | |  |  |  |  |
| Genome Start | Genome End | Strand | Div | Repeat Start | Repeat End | Repeat Left | Name | Family | Class |
| 31895965 | 31897147 | - | 11.7 | 6313 | 7507 | -216 | L1MA2 | L1 | LINE |
| 31897148 | 31897188 | - | 16.6 | 399 | 428 | 0 | MSTA | MaLR | LTR |
| 31897189 | 31897495 | + | 9.4 | 1 | 307 | -2 | AluSc | Alu | SINE |
| 31897496 | 31897802 | + | 8.8 | 1 | 307 | -4 | AluY | Alu | SINE |
| 31897803 | 31898192 | - | 16.6 | 1 | 398 | -30 | MSTA | MaLR | LTR |
| 31898193 | 31899260 | - | 17.4 | 550 | 1651 | 0 | MSTA-int | MaLR | LTR |
| 31899249 | 31899340 | - | 9.9 | 1 | 92 | -1559 | MSTA-int | MaLR | LTR |
| 31899341 | 31899665 | - | 15.5 | 97 | 428 | 0 | MSTA | MaLR | LTR |
| 31899666 | 31900201 | - | 10.5 | 5064 | 5471 | -2094 | L1MA2 | L1 | LINE |
| 31900202 | 31900575 | - | 11.3 | 5787 | 6174 | 0 | L1PA11 | L1 | LINE |
| 31900576 | 31901104 | - | 10.5 | 4536 | 5063 | -2502 | L1MA2 | L1 | LINE |

  

|  |  |  |  |  |  |  |  |  |  |
| --- | --- | --- | --- | --- | --- | --- | --- | --- | --- |
| chr2:32802799-32806538 | | | 3718 bp | | |  |  |  |  |
| Genome Start | Genome End | Strand | Div | Repeat Start | Repeat End | Repeat Left | Name | Family | Class |
| 32802799 | 32803380 | + | 19.4 | 1 | 611 | -844 | Charlie1a | MER1\_type | DNA |
| 32803381 | 32803736 | - | 12.4 | 1 | 364 | 0 | THE1B | MaLR | LTR |
| 32803737 | 32805316 | - | 30.0 | 2 | 1735 | 0 | MLT-int | MaLR | LTR |
| 32805319 | 32805676 | - | 10.9 | 1 | 364 | 0 | THE1B | MaLR | LTR |
| 32805677 | 32806307 | + | 19.4 | 612 | 1218 | -237 | Charlie1a | MER1\_type | DNA |
| 32806328 | 32806538 | + | 19.4 | 1219 | 1453 | -2 | Charlie1a | MER1\_type | DNA |

  

|  |  |  |  |  |  |  |  |  |  |
| --- | --- | --- | --- | --- | --- | --- | --- | --- | --- |
| chr2:33733136-33745024 | | | 11554 bp | | |  |  |  |  |
| Genome Start | Genome End | Strand | Div | Repeat Start | Repeat End | Repeat Left | Name | Family | Class |
| 33733136 | 33734491 | + | 17.6 | 206 | 1743 | -5462 | LTR28-int | ERV1 | LTR |
| 33734635 | 33734721 | + | 19.8 | 3910 | 3995 | -885 | MER83B-int | ERV1 | LTR |
| 33734722 | 33735019 | + | 16.0 | 990 | 1278 | -4668 | MER83B-int | ERV1 | LTR |
| 33735020 | 33735429 | + | 2.9 | 5740 | 6152 | -3 | L1PA3 | L1 | LINE |
| 33735430 | 33735601 | + | 16.0 | 1279 | 1449 | -4497 | MER83B-int | ERV1 | LTR |
| 33735595 | 33735991 | + | 12.3 | 2218 | 2608 | -4929 | MER83B-int | ERV1 | LTR |
| 33735993 | 33736479 | + | 8.4 | 256 | 773 | -7 | LTR17 | ERV1 | LTR |
| 33736496 | 33737187 | + | 6.8 | 1 | 684 | -7942 | HERV17 | ERV1 | LTR |
| 33737353 | 33738555 | + | 6.9 | 802 | 2007 | -6619 | HERV17 | ERV1 | LTR |
| 33738548 | 33740494 | + | 4.6 | 2430 | 4416 | -4210 | HERV17 | ERV1 | LTR |
| 33740495 | 33740791 | - | 9.8 | 1 | 298 | -13 | AluY | Alu | SINE |
| 33740792 | 33742405 | + | 4.6 | 4417 | 6019 | -2607 | HERV17 | ERV1 | LTR |
| 33742427 | 33742717 | + | 6.9 | 8335 | 8626 | 0 | HERV17 | ERV1 | LTR |
| 33742718 | 33743026 | + | 5.8 | 1 | 326 | -454 | LTR17 | ERV1 | LTR |
| 33743031 | 33745024 | + | 12.7 | 2244 | 7187 | -1 | LTR28-int | ERV1 | LTR |

  

|  |  |  |  |  |  |  |  |  |  |
| --- | --- | --- | --- | --- | --- | --- | --- | --- | --- |
| chr2:33767889-33773305 | | | 5398 bp | | |  |  |  |  |
| Genome Start | Genome End | Strand | Div | Repeat Start | Repeat End | Repeat Left | Name | Family | Class |
| 33767889 | 33768875 | - | 9.6 | 5171 | 6159 | -1 | L1PREC2 | L1 | LINE |
| 33768893 | 33769195 | - | 11.6 | 1 | 307 | -6 | AluSp | Alu | SINE |
| 33769196 | 33770548 | - | 8.9 | 3791 | 5161 | -985 | L1PREC2 | L1 | LINE |
| 33770549 | 33770896 | - | 9.2 | 1 | 355 | 0 | THE1A | MaLR | LTR |
| 33770897 | 33772489 | - | 30.8 | 2 | 1735 | 0 | MLT1D-int | MaLR | LTR |
| 33772492 | 33772844 | - | 9.3 | 2 | 355 | 0 | THE1A | MaLR | LTR |
| 33772845 | 33773305 | - | 8.9 | 3333 | 3790 | -2356 | L1PREC2 | L1 | LINE |

  

|  |  |  |  |  |  |  |  |  |  |
| --- | --- | --- | --- | --- | --- | --- | --- | --- | --- |
| chr2:33929328-33936846 | | | 6930 bp | | |  |  |  |  |
| Genome Start | Genome End | Strand | Div | Repeat Start | Repeat End | Repeat Left | Name | Family | Class |
| 33929328 | 33930410 | + | 25.9 | 2014 | 3056 | -1135 | MER61D-int | ERV1 | LTR |
| 33930416 | 33930908 | - | 17.9 | 1 | 497 | 0 | LOR1a | ERV1 | LTR |
| 33930925 | 33931291 | - | 17.2 | 1 | 428 | 0 | MSTA | MaLR | LTR |
| 33931293 | 33932901 | - | 16.2 | 15 | 1650 | -1 | MSTA-int | MaLR | LTR |
| 33932926 | 33933320 | - | 15.3 | 4 | 428 | 0 | MSTA | MaLR | LTR |
| 33933364 | 33934052 | + | 24.8 | 3079 | 3736 | -455 | MER61D-int | ERV1 | LTR |
| 33934053 | 33934397 | + | 21.2 | 1 | 396 | 0 | MSTD | MaLR | LTR |
| 33934398 | 33934467 | + | 24.8 | 3737 | 3803 | -388 | MER61D-int | ERV1 | LTR |
| 33934470 | 33934701 | - | 12.7 | 277 | 531 | 0 | LTR26B | ERV1 | LTR |
| 33934702 | 33935086 | + | 10.7 | 1 | 364 | 0 | THE1B | MaLR | LTR |
| 33935087 | 33935148 | - | 12.7 | 237 | 276 | -255 | LTR26B | ERV1 | LTR |
| 33935149 | 33935444 | - | 6.8 | 1 | 307 | -4 | AluY | Alu | SINE |
| 33935445 | 33935500 | - | 12.7 | 189 | 236 | -295 | LTR26B | ERV1 | LTR |
| 33935932 | 33936183 | - | 11.6 | 1 | 286 | -336 | LTR26B | ERV1 | LTR |
| 33936243 | 33936547 | + | 10.2 | 1 | 306 | -5 | AluY | Alu | SINE |
| 33936556 | 33936846 | + | 21.6 | 3853 | 4136 | -55 | MER61D-int | ERV1 | LTR |

  

|  |  |  |  |  |  |  |  |  |  |
| --- | --- | --- | --- | --- | --- | --- | --- | --- | --- |
| chr2:34313654-34317328 | | | 3574 bp | | |  |  |  |  |
| Genome Start | Genome End | Strand | Div | Repeat Start | Repeat End | Repeat Left | Name | Family | Class |
| 34313654 | 34313845 | - | 17.3 | 848 | 1038 | -2 | MER45B | Tip100 | DNA |
| 34313945 | 34314387 | - | 21.1 | 305 | 856 | -184 | MER45B | Tip100 | DNA |
| 34314388 | 34314738 | - | 18.5 | 1 | 365 | 0 | MLT1A0 | MaLR | LTR |
| 34314739 | 34314953 | - | 22.7 | 88 | 304 | -736 | MER45B | Tip100 | DNA |
| 34314954 | 34315312 | + | 12.0 | 1 | 355 | 0 | THE1A | MaLR | LTR |
| 34315315 | 34316886 | + | 31.0 | 2 | 1735 | 0 | MLT-int | MaLR | LTR |
| 34316887 | 34317236 | + | 11.8 | 1 | 355 | 0 | THE1A | MaLR | LTR |
| 34317237 | 34317328 | - | 22.7 | 1 | 87 | -953 | MER45B | Tip100 | DNA |

  

|  |  |  |  |  |  |  |  |  |  |
| --- | --- | --- | --- | --- | --- | --- | --- | --- | --- |
| chr2:34527419-34536340 | | | 8927 bp | | |  |  |  |  |
| Genome Start | Genome End | Strand | Div | Repeat Start | Repeat End | Repeat Left | Name | Family | Class |
| 34527419 | 34528057 | - | 11.7 | 7032 | 7674 | -49 | L1MA2 | L1 | LINE |
| 34528058 | 34528418 | + | 12.2 | 3 | 375 | 0 | THE1C | MaLR | LTR |
| 34528419 | 34530806 | - | 14.3 | 4626 | 7031 | -692 | L1MA2 | L1 | LINE |
| 34530807 | 34530898 | - | 14.1 | 222 | 313 | 0 | AluSp/q | Alu | SINE |
| 34530899 | 34531309 | - | 14.3 | 4212 | 4625 | -3098 | L1MA2 | L1 | LINE |
| 34531310 | 34531685 | - | 13.4 | 1 | 364 | 0 | THE1B | MaLR | LTR |
| 34531686 | 34531754 | - | 11.6 | 1512 | 1580 | 0 | THE1B-int | MaLR | LTR |
| 34531750 | 34532034 | - | 10.5 | 1 | 291 | -1289 | THE1B-int | MaLR | LTR |
| 34532035 | 34532289 | - | 16.7 | 112 | 364 | 0 | THE1B | MaLR | LTR |
| 34532290 | 34532597 | - | 12.7 | 1 | 308 | -4 | AluSx | Alu | SINE |
| 34532598 | 34532708 | - | 16.7 | 1 | 111 | -253 | THE1B | MaLR | LTR |
| 34532709 | 34533324 | - | 14.1 | 3603 | 4214 | -3351 | L1MA2 | L1 | LINE |
| 34533325 | 34533626 | - | 12.6 | 1 | 304 | -5 | AluSc | Alu | SINE |
| 34533627 | 34534686 | - | 15.8 | 2561 | 3602 | -3963 | L1MA2 | L1 | LINE |
| 34534687 | 34534735 | - | 10.2 | 243 | 291 | -8 | Alu | Alu | SINE |
| 34534736 | 34535931 | - | 15.8 | 1386 | 2560 | -5005 | L1MA2 | L1 | LINE |
| 34535932 | 34536296 | - | 12.4 | 1 | 364 | 0 | THE1B | MaLR | LTR |
| 34536297 | 34536340 | - | 15.8 | 1336 | 1383 | -6182 | L1MA2 | L1 | LINE |

  

|  |  |  |  |  |  |  |  |  |  |
| --- | --- | --- | --- | --- | --- | --- | --- | --- | --- |
| chr2:35116808-35120746 | | | 3839 bp | | |  |  |  |  |
| Genome Start | Genome End | Strand | Div | Repeat Start | Repeat End | Repeat Left | Name | Family | Class |
| 35116808 | 35117193 | - | 33.6 | 5736 | 6134 | -36 | L1ME3A | L1 | LINE |
| 35117253 | 35117820 | - | 29.1 | 3 | 619 | -7 | MLT1E2 | MaLR | LTR |
| 35117826 | 35117904 | - | 30.4 | 5533 | 5612 | -558 | L1ME3A | L1 | LINE |
| 35117905 | 35118269 | + | 14.7 | 1 | 379 | -2 | THE1D | MaLR | LTR |
| 35118276 | 35119574 | + | 14.3 | 5 | 1580 | 0 | THE1D-int | MaLR | LTR |
| 35119575 | 35119935 | + | 16.2 | 1 | 381 | 0 | THE1D | MaLR | LTR |
| 35119939 | 35120118 | - | 35.0 | 5350 | 5536 | -704 | L1ME3A | L1 | LINE |
| 35120146 | 35120746 | - | 26.8 | 4317 | 4972 | -1174 | L1ME3A | L1 | LINE |

  

|  |  |  |  |  |  |  |  |  |  |
| --- | --- | --- | --- | --- | --- | --- | --- | --- | --- |
| chr2:35275667-35280364 | | | 4666 bp | | |  |  |  |  |
| Genome Start | Genome End | Strand | Div | Repeat Start | Repeat End | Repeat Left | Name | Family | Class |
| 35275667 | 35275873 | - | 19.3 | 6134 | 6333 | -11 | L1MC2 | L1 | LINE |
| 35275876 | 35276043 | - | 25.6 | 5677 | 5845 | -499 | L1MC2 | L1 | LINE |
| 35276045 | 35276093 | + | 8.2 | 314 | 362 | -2 | THE1B | MaLR | LTR |
| 35276096 | 35277606 | + | 11.6 | 1 | 1580 | 0 | THE1B-int | MaLR | LTR |
| 35277607 | 35277965 | + | 10.6 | 1 | 359 | -5 | THE1B | MaLR | LTR |
| 35277966 | 35279109 | - | 18.8 | 4413 | 5598 | -746 | L1MC2 | L1 | LINE |
| 35279110 | 35279405 | - | 6.4 | 1 | 296 | -15 | AluY | Alu | SINE |
| 35279406 | 35279469 | - | 19.4 | 4351 | 4412 | -1734 | L1MC2 | L1 | LINE |
| 35279481 | 35279641 | - | 31.4 | 309 | 467 | 0 | MLT1C | MaLR | LTR |
| 35279658 | 35280364 | - | 21.0 | 2657 | 3375 | -2771 | L1MC2 | L1 | LINE |

  

|  |  |  |  |  |  |  |  |  |  |
| --- | --- | --- | --- | --- | --- | --- | --- | --- | --- |
| chr2:41549247-41553589 | | | 3652 bp | | |  |  |  |  |
| Genome Start | Genome End | Strand | Div | Repeat Start | Repeat End | Repeat Left | Name | Family | Class |
| 41549247 | 41550129 | - | 26.0 | 1348 | 2275 | -3940 | L1MC4 | L1 | LINE |
| 41550278 | 41550458 | - | 27.9 | 970 | 1168 | -5047 | L1MC4 | L1 | LINE |
| 41550479 | 41550721 | - | 27.9 | 725 | 969 | -5246 | L1MC4 | L1 | LINE |
| 41550807 | 41550865 | - | 23.7 | 642 | 701 | -5514 | L1MC4 | L1 | LINE |
| 41550928 | 41551290 | + | 14.2 | 1 | 381 | 0 | THE1D | MaLR | LTR |
| 41551293 | 41552718 | + | 15.0 | 3 | 1392 | -188 | THE1D-int | MaLR | LTR |
| 41552719 | 41552789 | + | 7.5 | 1514 | 1580 | 0 | THE1D-int | MaLR | LTR |
| 41552790 | 41553164 | + | 12.4 | 1 | 375 | -6 | THE1D | MaLR | LTR |
| 41553539 | 41553589 | - | 22.0 | 119 | 168 | -6047 | L1MC4 | L1 | LINE |

  

|  |  |  |  |  |  |  |  |  |  |
| --- | --- | --- | --- | --- | --- | --- | --- | --- | --- |
| chr2:42808453-42818286 | | | 9810 bp | | |  |  |  |  |
| Genome Start | Genome End | Strand | Div | Repeat Start | Repeat End | Repeat Left | Name | Family | Class |
| 42808453 | 42809730 | - | 9.1 | 6448 | 7724 | 0 | L1MA3 | L1 | LINE |
| 42809731 | 42810044 | - | 7.8 | 1 | 309 | -2 | AluY | Alu | SINE |
| 42810045 | 42811972 | - | 11.2 | 4438 | 6447 | -1118 | L1MA3 | L1 | LINE |
| 42811974 | 42812318 | - | 7.8 | 1 | 355 | 0 | THE1A | MaLR | LTR |
| 42812319 | 42813780 | - | 9.0 | 1 | 1580 | 0 | THE1A-int | MaLR | LTR |
| 42813781 | 42814131 | - | 7.7 | 1 | 355 | 0 | THE1A | MaLR | LTR |
| 42814132 | 42814286 | - | 9.0 | 4286 | 4441 | -3124 | L1MA3 | L1 | LINE |
| 42814310 | 42814443 | - | 14.3 | 1 | 134 | -178 | AluJo | Alu | SINE |
| 42814444 | 42818286 | - | 13.5 | 12 | 4299 | -3266 | L1MA3 | L1 | LINE |

  

|  |  |  |  |  |  |  |  |  |  |
| --- | --- | --- | --- | --- | --- | --- | --- | --- | --- |
| chr2:43169513-43173756 | | | 4111 bp | | |  |  |  |  |
| Genome Start | Genome End | Strand | Div | Repeat Start | Repeat End | Repeat Left | Name | Family | Class |
| 43169513 | 43169642 | - | 31.2 | 133 | 257 | -5 | MIR | MIR | SINE |
| 43169643 | 43169966 | - | 28.0 | 231 | 549 | -3 | MLT1H1 | MaLR | LTR |
| 43169967 | 43170264 | - | 9.9 | 1 | 294 | -19 | AluSq | Alu | SINE |
| 43170265 | 43170499 | - | 30.1 | 1 | 230 | -325 | MLT1H1 | MaLR | LTR |
| 43170500 | 43170750 | - | 24.6 | 1244 | 1489 | 0 | MLT1H1-int | MaLR | LTR |
| 43170751 | 43171054 | + | 12.2 | 1 | 304 | -9 | AluSq | Alu | SINE |
| 43171055 | 43171330 | - | 24.6 | 994 | 1243 | -246 | MLT1H1-int | MaLR | LTR |
| 43171331 | 43171680 | + | 10.5 | 1 | 364 | 0 | THE1B | MaLR | LTR |
| 43171681 | 43172032 | - | 24.6 | 627 | 993 | -496 | MLT1H1-int | MaLR | LTR |
| 43172137 | 43172537 | - | 24.6 | 310 | 626 | -863 | MLT1H1-int | MaLR | LTR |
| 43172538 | 43172845 | - | 12.7 | 1 | 308 | -4 | AluSx | Alu | SINE |
| 43172846 | 43173110 | - | 24.6 | 11 | 309 | -1180 | MLT1H1-int | MaLR | LTR |
| 43173128 | 43173623 | - | 26.7 | 25 | 550 | -5 | MLT1H1 | MaLR | LTR |
| 43173636 | 43173756 | - | 29.2 | 2 | 122 | -140 | MIR | MIR | SINE |

  

|  |  |  |  |  |  |  |  |  |  |
| --- | --- | --- | --- | --- | --- | --- | --- | --- | --- |
| chr2:44971668-44974306 | | | 2452 bp | | |  |  |  |  |
| Genome Start | Genome End | Strand | Div | Repeat Start | Repeat End | Repeat Left | Name | Family | Class |
| 44971668 | 44971748 | - | 22.3 | 84 | 162 | -113 | MIRb | MIR | SINE |
| 44971749 | 44971858 | - | 12.8 | 273 | 381 | 0 | THE1C | MaLR | LTR |
| 44971853 | 44972012 | - | 8.8 | 3 | 163 | -212 | THE1C | MaLR | LTR |
| 44972015 | 44973651 | - | 30.6 | 2 | 1735 | 0 | MLT1L-int | MaLR | LTR |
| 44973654 | 44974027 | - | 9.4 | 1 | 375 | 0 | THE1C | MaLR | LTR |
| 44974217 | 44974306 | - | 19.1 | 7 | 96 | -172 | MIRb | MIR | SINE |

  

|  |  |  |  |  |  |  |  |  |  |
| --- | --- | --- | --- | --- | --- | --- | --- | --- | --- |
| chr2:48890961-48895106 | | | 3998 bp | | |  |  |  |  |
| Genome Start | Genome End | Strand | Div | Repeat Start | Repeat End | Repeat Left | Name | Family | Class |
| 48890961 | 48891922 | - | 26.1 | 1098 | 2136 | -371 | HAL1 | L1 | LINE |
| 48891923 | 48892283 | - | 14.8 | 1 | 364 | 0 | THE1B | MaLR | LTR |
| 48892284 | 48893857 | - | 29.9 | 2 | 1735 | 0 | MLT-int | MaLR | LTR |
| 48893860 | 48894209 | - | 14.1 | 1 | 364 | 0 | THE1B | MaLR | LTR |
| 48894210 | 48894738 | - | 26.1 | 535 | 1097 | -1410 | HAL1 | L1 | LINE |
| 48894880 | 48895023 | - | 27.3 | 147 | 308 | -2199 | HAL1 | L1 | LINE |
| 48895029 | 48895106 | - | 33.3 | 1 | 79 | -2428 | HAL1 | L1 | LINE |

  

|  |  |  |  |  |  |  |  |  |  |
| --- | --- | --- | --- | --- | --- | --- | --- | --- | --- |
| chr2:49156021-49161951 | | | 5908 bp | | |  |  |  |  |
| Genome Start | Genome End | Strand | Div | Repeat Start | Repeat End | Repeat Left | Name | Family | Class |
| 49156021 | 49156275 | + | 27.1 | 3083 | 3349 | -2780 | L1ME3B | L1 | LINE |
| 49156276 | 49156574 | - | 19.2 | 9 | 297 | -15 | AluJb | Alu | SINE |
| 49156575 | 49156823 | + | 27.1 | 3350 | 3590 | -2539 | L1ME3B | L1 | LINE |
| 49156824 | 49157178 | - | 11.9 | 1 | 364 | 0 | THE1B | MaLR | LTR |
| 49157179 | 49158723 | - | 30.2 | 2 | 1735 | 0 | MLT-int | MaLR | LTR |
| 49158726 | 49159024 | - | 11.3 | 71 | 364 | 0 | THE1B | MaLR | LTR |
| 49159046 | 49161311 | + | 29.6 | 3588 | 5946 | -277 | L1ME3B | L1 | LINE |
| 49161312 | 49161877 | + | 19.6 | 1 | 558 | 0 | MER74A | ERVL | LTR |
| 49161878 | 49161951 | + | 29.1 | 5947 | 6028 | -195 | L1ME3B | L1 | LINE |

  

|  |  |  |  |  |  |  |  |  |  |
| --- | --- | --- | --- | --- | --- | --- | --- | --- | --- |
| chr2:49442007-49447021 | | | 4970 bp | | |  |  |  |  |
| Genome Start | Genome End | Strand | Div | Repeat Start | Repeat End | Repeat Left | Name | Family | Class |
| 49442007 | 49442561 | + | 36.9 | 187 | 756 | -5644 | L1MEc | L1 | LINE |
| 49442573 | 49442620 | - | 4.2 | 1 | 48 | -307 | THE1A | MaLR | LTR |
| 49442623 | 49444196 | - | 10.4 | 2 | 1578 | -2 | THE1A-int | MaLR | LTR |
| 49444198 | 49444552 | - | 9.6 | 1 | 355 | 0 | THE1A | MaLR | LTR |
| 49444584 | 49447021 | + | 26.2 | 2060 | 4692 | -1527 | L1MEc | L1 | LINE |

  

|  |  |  |  |  |  |  |  |  |  |
| --- | --- | --- | --- | --- | --- | --- | --- | --- | --- |
| chr2:49608126-49631228 | | | 22589 bp | | |  |  |  |  |
| Genome Start | Genome End | Strand | Div | Repeat Start | Repeat End | Repeat Left | Name | Family | Class |
| 49608126 | 49608666 | - | 32.6 | 2267 | 2822 | -2845 | ERVL-E | ERVL | LTR |
| 49608706 | 49609107 | + | 33.8 | 418 | 831 | -5472 | L1M3 | L1 | LINE |
| 49609167 | 49609598 | + | 32.9 | 615 | 1068 | -5235 | L1M3d | L1 | LINE |
| 49609759 | 49610133 | - | 15.2 | 1 | 375 | 0 | THE1C | MaLR | LTR |
| 49610139 | 49611677 | - | 13.7 | 1 | 1574 | -6 | THE1C-int | MaLR | LTR |
| 49611678 | 49612045 | - | 19.3 | 1 | 375 | 0 | THE1C | MaLR | LTR |
| 49612050 | 49615169 | + | 23.2 | 777 | 3976 | -2400 | L1M3 | L1 | LINE |
| 49615172 | 49615500 | - | 15.5 | 4 | 329 | -1 | MER2 | MER2\_type | DNA |
| 49615503 | 49615954 | + | 16.0 | 3975 | 4425 | -1951 | L1M3 | L1 | LINE |
| 49615926 | 49616635 | + | 16.6 | 5209 | 5909 | -495 | L1M3 | L1 | LINE |
| 49616636 | 49616799 | + | 16.9 | 17 | 187 | -7378 | L1MA3 | L1 | LINE |
| 49616800 | 49617108 | + | 9.2 | 1 | 305 | -6 | AluY | Alu | SINE |
| 49617109 | 49617183 | + | 16.9 | 188 | 252 | -7313 | L1MA3 | L1 | LINE |
| 49617184 | 49618606 | + | 15.5 | 1681 | 3129 | -4436 | L1MA3 | L1 | LINE |
| 49618607 | 49619340 | + | 18.5 | 1 | 691 | 0 | LTR8 | ERV1 | LTR |
| 49619341 | 49620952 | + | 13.2 | 3130 | 4757 | -2808 | L1MA3 | L1 | LINE |
| 49620953 | 49621246 | + | 23.5 | 1 | 296 | -16 | AluJb | Alu | SINE |
| 49621247 | 49623251 | + | 12.5 | 4758 | 6755 | -810 | L1MA3 | L1 | LINE |
| 49623252 | 49623702 | - | 9.2 | 2 | 444 | 0 | MLT2A1 | ERVL | LTR |
| 49623705 | 49625633 | - | 7.9 | 3703 | 5654 | 0 | HERVL-A1 | ERVL | LTR |
| 49625635 | 49626524 | - | 7.7 | 2684 | 3576 | -2570 | L1P1 | L1 | LINE |
| 49626497 | 49627745 | + | 6.8 | 4901 | 6155 | 0 | L1PA4 | L1 | LINE |
| 49627749 | 49628291 | - | 8.5 | 3173 | 3716 | -1938 | HERVL-A1 | ERVL | LTR |
| 49628282 | 49629474 | - | 8.1 | 1 | 1205 | -4449 | HERVL-A1 | ERVL | LTR |
| 49629475 | 49629886 | - | 9.5 | 1 | 444 | 0 | MLT2A1 | ERVL | LTR |
| 49629893 | 49630570 | + | 12.8 | 6757 | 7441 | -283 | L1MA3 | L1 | LINE |
| 49630713 | 49630932 | - | 31.5 | 1931 | 2161 | -3506 | ERVL-E | ERVL | LTR |
| 49631089 | 49631228 | - | 29.5 | 1605 | 1745 | -3922 | ERVL-E | ERVL | LTR |

  

|  |  |  |  |  |  |  |  |  |  |
| --- | --- | --- | --- | --- | --- | --- | --- | --- | --- |
| chr2:53343189-53345642 | | | 2419 bp | | |  |  |  |  |
| Genome Start | Genome End | Strand | Div | Repeat Start | Repeat End | Repeat Left | Name | Family | Class |
| 53343189 | 53343267 | + | 17.7 | 1 | 80 | -113 | MER53 | DNA | DNA |
| 53343277 | 53343629 | - | 10.6 | 1 | 355 | 0 | THE1A | MaLR | LTR |
| 53343635 | 53345171 | - | 9.6 | 1 | 1567 | -13 | THE1A-int | MaLR | LTR |
| 53345172 | 53345526 | - | 11.7 | 1 | 355 | 0 | THE1A | MaLR | LTR |
| 53345548 | 53345642 | + | 16.8 | 84 | 186 | -7 | MER53 | DNA | DNA |

  

|  |  |  |  |  |  |  |  |  |  |
| --- | --- | --- | --- | --- | --- | --- | --- | --- | --- |
| chr2:53836425-53842626 | | | 5764 bp | | |  |  |  |  |
| Genome Start | Genome End | Strand | Div | Repeat Start | Repeat End | Repeat Left | Name | Family | Class |
| 53836425 | 53836496 | + | 25.4 | 5211 | 5282 | -864 | L1M5 | L1 | LINE |
| 53836511 | 53836816 | + | 19.0 | 1 | 289 | -23 | AluJo | Alu | SINE |
| 53836881 | 53837036 | + | 29.7 | 437 | 591 | 0 | MLT1K | MaLR | LTR |
| 53837080 | 53837284 | + | 15.1 | 2 | 211 | -304 | MLT2B2 | ERVL | LTR |
| 53837284 | 53837738 | + | 9.6 | 260 | 780 | 0 | LTR17 | ERV1 | LTR |
| 53837739 | 53838450 | + | 5.3 | 1 | 707 | -7919 | HERV17 | ERV1 | LTR |
| 53838640 | 53838908 | + | 7.8 | 784 | 1058 | -7568 | HERV17 | ERV1 | LTR |
| 53839046 | 53839530 | + | 6.7 | 1537 | 2014 | -6612 | HERV17 | ERV1 | LTR |
| 53839516 | 53840835 | + | 6.1 | 2430 | 3754 | -4872 | HERV17 | ERV1 | LTR |
| 53840815 | 53841427 | + | 5.9 | 5405 | 6019 | -2607 | HERV17 | ERV1 | LTR |
| 53841449 | 53841741 | + | 6.2 | 8335 | 8626 | 0 | HERV17 | ERV1 | LTR |
| 53841742 | 53842066 | + | 9.6 | 1 | 324 | -456 | LTR17 | ERV1 | LTR |
| 53842067 | 53842433 | + | 22.0 | 192 | 548 | -6 | MLT2B3 | ERVL | LTR |
| 53842441 | 53842626 | + | 27.6 | 5624 | 5808 | -313 | L1M5 | L1 | LINE |

  

|  |  |  |  |  |  |  |  |  |  |
| --- | --- | --- | --- | --- | --- | --- | --- | --- | --- |
| chr2:57090899-57096545 | | | 5418 bp | | |  |  |  |  |
| Genome Start | Genome End | Strand | Div | Repeat Start | Repeat End | Repeat Left | Name | Family | Class |
| 57090899 | 57090937 | + | 17.2 | 4130 | 4168 | -1978 | L1MC1 | L1 | LINE |
| 57091098 | 57091249 | + | 17.2 | 4169 | 4330 | -1816 | L1MC1 | L1 | LINE |
| 57091263 | 57091296 | - | 28.4 | 517 | 545 | -10 | MLT2B5 | ERVL | LTR |
| 57091344 | 57091882 | - | 26.4 | 163 | 724 | -39 | MLT2B3 | ERVL | LTR |
| 57091883 | 57092958 | + | 17.5 | 4339 | 5444 | -889 | L1MC1 | L1 | LINE |
| 57092959 | 57093339 | + | 17.2 | 1 | 428 | 0 | MSTA | MaLR | LTR |
| 57093340 | 57094958 | + | 20.2 | 1 | 1646 | -5 | MSTA-int | MaLR | LTR |
| 57094968 | 57095365 | + | 13.2 | 1 | 428 | 0 | MSTA | MaLR | LTR |
| 57095366 | 57095756 | + | 17.1 | 5440 | 5822 | -511 | L1MC1 | L1 | LINE |
| 57095757 | 57096062 | + | 13.1 | 1 | 306 | -4 | AluSg | Alu | SINE |
| 57096063 | 57096545 | + | 17.1 | 5823 | 6322 | -11 | L1MC1 | L1 | LINE |

  

|  |  |  |  |  |  |  |  |  |  |
| --- | --- | --- | --- | --- | --- | --- | --- | --- | --- |
| chr2:57103572-57110008 | | | 6435 bp | | |  |  |  |  |
| Genome Start | Genome End | Strand | Div | Repeat Start | Repeat End | Repeat Left | Name | Family | Class |
| 57103572 | 57103597 | - | 16.1 | 3908 | 3925 | -2924 | L1PA16 | L1 | LINE |
| 57103598 | 57103899 | + | 12.6 | 1 | 303 | -6 | AluSc | Alu | SINE |
| 57103900 | 57105399 | - | 17.0 | 2372 | 3907 | -2942 | L1PA16 | L1 | LINE |
| 57105400 | 57105753 | - | 12.8 | 1 | 364 | 0 | THE1B | MaLR | LTR |
| 57105754 | 57107314 | - | 31.4 | 2 | 1735 | 0 | MLT-int | MaLR | LTR |
| 57107317 | 57107669 | - | 12.5 | 1 | 364 | 0 | THE1B | MaLR | LTR |
| 57107670 | 57110008 | - | 18.9 | 1 | 2371 | -4482 | L1PA16 | L1 | LINE |

  

|  |  |  |  |  |  |  |  |  |  |
| --- | --- | --- | --- | --- | --- | --- | --- | --- | --- |
| chr2:57235576-57239281 | | | 3278 bp | | |  |  |  |  |
| Genome Start | Genome End | Strand | Div | Repeat Start | Repeat End | Repeat Left | Name | Family | Class |
| 57235576 | 57235626 | + | 32.0 | 3 | 61 | -6362 | L1MCc | L1 | LINE |
| 57235627 | 57236040 | - | 20.1 | 1 | 426 | 0 | MSTB | MaLR | LTR |
| 57236041 | 57237697 | - | 29.9 | 2 | 1735 | 0 | MLT-int | MaLR | LTR |
| 57237700 | 57238118 | - | 16.4 | 1 | 426 | 0 | MSTB | MaLR | LTR |
| 57238119 | 57238461 | + | 32.0 | 62 | 422 | -6001 | L1MCc | L1 | LINE |
| 57238754 | 57238890 | + | 31.9 | 799 | 945 | -5478 | L1MCc | L1 | LINE |
| 57239003 | 57239154 | + | 24.8 | 1101 | 1254 | -5169 | L1MCc | L1 | LINE |
| 57239177 | 57239281 | + | 24.8 | 1255 | 1390 | -5033 | L1MCc | L1 | LINE |

  

|  |  |  |  |  |  |  |  |  |  |
| --- | --- | --- | --- | --- | --- | --- | --- | --- | --- |
| chr2:57330829-57334583 | | | 3754 bp | | |  |  |  |  |
| Genome Start | Genome End | Strand | Div | Repeat Start | Repeat End | Repeat Left | Name | Family | Class |
| 57330829 | 57331055 | + | 11.9 | 1 | 226 | -1354 | THE1D-int | MaLR | LTR |
| 57331057 | 57331423 | - | 20.6 | 1 | 364 | 0 | THE1B | MaLR | LTR |
| 57331424 | 57332905 | - | 14.4 | 1 | 1580 | 0 | THE1B-int | MaLR | LTR |
| 57332906 | 57333273 | - | 17.7 | 1 | 364 | 0 | THE1B | MaLR | LTR |
| 57333274 | 57334583 | + | 14.6 | 223 | 1580 | 0 | THE1D-int | MaLR | LTR |

  

|  |  |  |  |  |  |  |  |  |  |
| --- | --- | --- | --- | --- | --- | --- | --- | --- | --- |
| chr2:57345885-57361355 | | | 15389 bp | | |  |  |  |  |
| Genome Start | Genome End | Strand | Div | Repeat Start | Repeat End | Repeat Left | Name | Family | Class |
| 57345885 | 57346681 | + | 18.8 | 2963 | 3766 | -3796 | L1MA4 | L1 | LINE |
| 57346683 | 57347055 | - | 19.8 | 1 | 428 | 0 | MSTA | MaLR | LTR |
| 57347056 | 57348595 | - | 17.0 | 4 | 1580 | 0 | MSTA-int | MaLR | LTR |
| 57348599 | 57348952 | - | 18.3 | 1 | 428 | 0 | MSTA | MaLR | LTR |
| 57348953 | 57350921 | + | 19.2 | 3763 | 5784 | -1778 | L1MA4 | L1 | LINE |
| 57350923 | 57351344 | + | 22.4 | 3 | 432 | 0 | MSTB1 | MaLR | LTR |
| 57351345 | 57351480 | + | 17.8 | 1 | 146 | -1505 | MSTB1-int | MaLR | LTR |
| 57351506 | 57357590 | - | 7.2 | 7 | 6148 | -6 | L1PA5 | L1 | LINE |
| 57357604 | 57359049 | + | 24.5 | 138 | 1610 | -41 | MSTB1-int | MaLR | LTR |
| 57359089 | 57359510 | + | 19.0 | 1 | 432 | 0 | MSTB1 | MaLR | LTR |
| 57359511 | 57361355 | + | 18.2 | 5779 | 7646 | -73 | L1MA4 | L1 | LINE |

  

|  |  |  |  |  |  |  |  |  |  |
| --- | --- | --- | --- | --- | --- | --- | --- | --- | --- |
| chr2:57935683-57937739 | | | 2029 bp | | |  |  |  |  |
| Genome Start | Genome End | Strand | Div | Repeat Start | Repeat End | Repeat Left | Name | Family | Class |
| 57935683 | 57935902 | - | 22.0 | 7079 | 7301 | -741 | L1MC4 | L1 | LINE |
| 57935918 | 57936352 | - | 16.2 | 1 | 428 | 0 | THE1D | MaLR | LTR |
| 57936353 | 57936792 | - | 9.8 | 1142 | 1580 | 0 | THE1D-int | MaLR | LTR |
| 57936804 | 57937178 | - | 13.4 | 1 | 381 | 0 | THE1D | MaLR | LTR |
| 57937181 | 57937739 | - | 27.4 | 6468 | 7065 | -977 | L1MC4 | L1 | LINE |

  

|  |  |  |  |  |  |  |  |  |  |
| --- | --- | --- | --- | --- | --- | --- | --- | --- | --- |
| chr2:59587509-59590163 | | | 2649 bp | | |  |  |  |  |
| Genome Start | Genome End | Strand | Div | Repeat Start | Repeat End | Repeat Left | Name | Family | Class |
| 59587509 | 59587620 | + | 15.6 | 6013 | 6119 | -214 | L1MC1 | L1 | LINE |
| 59587621 | 59587969 | + | 8.9 | 1 | 353 | -2 | THE1A | MaLR | LTR |
| 59587976 | 59589537 | + | 30.2 | 4 | 1735 | 0 | MLT-int | MaLR | LTR |
| 59589538 | 59589953 | + | 10.5 | 1 | 355 | 0 | THE1A | MaLR | LTR |
| 59589954 | 59590163 | + | 15.6 | 6123 | 6333 | 0 | L1MC1 | L1 | LINE |

  

|  |  |  |  |  |  |  |  |  |  |
| --- | --- | --- | --- | --- | --- | --- | --- | --- | --- |
| chr2:59685978-59698810 | | | 12740 bp | | |  |  |  |  |
| Genome Start | Genome End | Strand | Div | Repeat Start | Repeat End | Repeat Left | Name | Family | Class |
| 59685978 | 59686756 | - | 20.2 | 5875 | 6682 | 0 | L1MB7 | L1 | LINE |
| 59686790 | 59687093 | - | 18.5 | 4 | 307 | -5 | AluJb | Alu | SINE |
| 59687117 | 59687635 | - | 21.5 | 5265 | 5796 | -848 | L1MB7 | L1 | LINE |
| 59687672 | 59687983 | + | 11.6 | 14 | 381 | 0 | THE1D | MaLR | LTR |
| 59687986 | 59689588 | + | 14.2 | 3 | 1580 | 0 | THE1D-int | MaLR | LTR |
| 59689589 | 59689943 | + | 16.9 | 1 | 381 | 0 | THE1D | MaLR | LTR |
| 59689944 | 59690932 | - | 20.9 | 4234 | 5269 | -1375 | L1MB7 | L1 | LINE |
| 59690939 | 59691424 | + | 19.9 | 3723 | 4228 | -2416 | L1MB7 | L1 | LINE |
| 59691420 | 59691706 | - | 18.9 | 3526 | 3840 | -2804 | L1MB7 | L1 | LINE |
| 59691701 | 59692466 | - | 10.7 | 5396 | 6161 | -2 | L1PA13 | L1 | LINE |
| 59692467 | 59692839 | - | 7.2 | 4902 | 5282 | -864 | L1PREC2 | L1 | LINE |
| 59692842 | 59694507 | - | 25.1 | 1838 | 3530 | -3114 | L1MB7 | L1 | LINE |
| 59694508 | 59694877 | - | 12.4 | 1 | 364 | 0 | THE1B | MaLR | LTR |
| 59694878 | 59696395 | - | 29.8 | 2 | 1735 | 0 | MLT-int | MaLR | LTR |
| 59696398 | 59696761 | - | 13.0 | 1 | 364 | 0 | THE1B | MaLR | LTR |
| 59696762 | 59696772 | - | 26.4 | 1671 | 1679 | -5293 | L1MB7 | L1 | LINE |
| 59696773 | 59697126 | - | 14.6 | 1 | 375 | 0 | THE1C | MaLR | LTR |
| 59697127 | 59698810 | - | 25.2 | 14 | 1827 | -4998 | L1MB7 | L1 | LINE |

  

|  |  |  |  |  |  |  |  |  |  |
| --- | --- | --- | --- | --- | --- | --- | --- | --- | --- |
| chr2:59775514-59790969 | | | 14792 bp | | |  |  |  |  |
| Genome Start | Genome End | Strand | Div | Repeat Start | Repeat End | Repeat Left | Name | Family | Class |
| 59775514 | 59775723 | + | 26.9 | 397 | 594 | -4260 | L1ME3B | L1 | LINE |
| 59775724 | 59775850 | - | 8.9 | 6025 | 6149 | -2 | L1PB1 | L1 | LINE |
| 59775851 | 59776328 | + | 26.9 | 595 | 1101 | -3753 | L1ME3B | L1 | LINE |
| 59776498 | 59776788 | - | 21.4 | 301 | 619 | -5 | MLT1E3 | MaLR | LTR |
| 59776822 | 59776942 | - | 25.6 | 1 | 133 | -521 | MLT1E3 | MaLR | LTR |
| 59776978 | 59777390 | + | 32.0 | 1318 | 1821 | -3013 | L1ME3B | L1 | LINE |
| 59777404 | 59777561 | + | 14.6 | 132 | 289 | -23 | AluSg/x | Alu | SINE |
| 59777664 | 59777937 | + | 26.9 | 2042 | 2338 | -2496 | L1ME3B | L1 | LINE |
| 59777972 | 59779357 | - | 8.5 | 6306 | 7721 | 0 | L1MA1 | L1 | LINE |
| 59779369 | 59780908 | - | 10.2 | 2529 | 4454 | -3111 | L1MA1 | L1 | LINE |
| 59780906 | 59784053 | - | 16.9 | 9 | 3233 | -4332 | L1M1 | L1 | LINE |
| 59784171 | 59784536 | - | 14.1 | 1 | 381 | 0 | THE1D | MaLR | LTR |
| 59784537 | 59786117 | - | 31.0 | 2 | 1735 | 0 | MSTD-int | MaLR | LTR |
| 59786120 | 59786495 | - | 14.1 | 1 | 381 | 0 | THE1D | MaLR | LTR |
| 59786496 | 59786895 | + | 28.3 | 2433 | 2894 | -1940 | L1ME3B | L1 | LINE |
| 59786896 | 59787939 | + | 15.4 | 1 | 1068 | 0 | Tigger2b\_Pri | MER2\_type | DNA |
| 59787940 | 59788449 | + | 28.3 | 2895 | 3457 | -1377 | L1ME3B | L1 | LINE |
| 59788456 | 59788546 | - | 32.0 | 164 | 248 | -14 | MIR | MIR | SINE |
| 59788547 | 59788907 | + | 17.0 | 5 | 365 | 0 | MLT1A0 | MaLR | LTR |
| 59788908 | 59789045 | - | 32.0 | 21 | 163 | -99 | MIR | MIR | SINE |
| 59789072 | 59789280 | + | 33.5 | 3452 | 3685 | -1149 | L1ME3B | L1 | LINE |
| 59789281 | 59789638 | + | 16.4 | 1 | 236 | 0 | MER46B | MER2\_type | DNA |
| 59789639 | 59789983 | + | 33.5 | 3686 | 4046 | -788 | L1ME3B | L1 | LINE |
| 59789986 | 59790175 | + | 33.0 | 4054 | 4253 | -675 | L1ME3B | L1 | LINE |
| 59790320 | 59790400 | + | 17.9 | 1 | 78 | -2640 | Tigger2 | MER2\_type | DNA |
| 59790373 | 59790820 | + | 15.2 | 1530 | 2718 | 0 | Tigger2 | MER2\_type | DNA |
| 59790822 | 59790969 | + | 26.4 | 4596 | 4756 | -172 | L1ME3B | L1 | LINE |

  

|  |  |  |  |  |  |  |  |  |  |
| --- | --- | --- | --- | --- | --- | --- | --- | --- | --- |
| chr2:62357161-62360526 | | | 3418 bp | | |  |  |  |  |
| Genome Start | Genome End | Strand | Div | Repeat Start | Repeat End | Repeat Left | Name | Family | Class |
| 62357161 | 62357300 | + | 17.9 | 1 | 150 | -445 | MLT1G | MaLR | LTR |
| 62357304 | 62357400 | + | 17.0 | 4 | 97 | -96 | MER53 | DNA | DNA |
| 62357315 | 62357401 | - | 14.0 | 111 | 188 | -5 | MER53 | DNA | DNA |
| 62357402 | 62357803 | + | 16.9 | 1 | 428 | 0 | MSTA | MaLR | LTR |
| 62357807 | 62359445 | + | 26.5 | 3 | 1735 | 0 | MLT-int | MaLR | LTR |
| 62359446 | 62359834 | + | 17.7 | 1 | 428 | 0 | MSTA | MaLR | LTR |
| 62359835 | 62359849 | - | 14.0 | 96 | 110 | -83 | MER53 | DNA | DNA |
| 62359878 | 62360110 | + | 25.1 | 105 | 382 | -203 | MLT1G | MaLR | LTR |
| 62360111 | 62360313 | + | 16.8 | 1 | 209 | 0 | MER3 | MER1\_type | DNA |
| 62360314 | 62360526 | + | 19.6 | 383 | 590 | 0 | MLT1G | MaLR | LTR |

  

|  |  |  |  |  |  |  |  |  |  |
| --- | --- | --- | --- | --- | --- | --- | --- | --- | --- |
| chr2:64332785-64339108 | | | 6011 bp | | |  |  |  |  |
| Genome Start | Genome End | Strand | Div | Repeat Start | Repeat End | Repeat Left | Name | Family | Class |
| 64332785 | 64333020 | - | 27.9 | 816 | 1074 | -16 | MER97c | MER1\_type? | DNA |
| 64333052 | 64333533 | + | 5.8 | 1 | 448 | -2 | LTR7 | ERV1 | LTR |
| 64333536 | 64336573 | + | 9.2 | 1 | 2987 | -4726 | HERVH | ERV1 | LTR |
| 64336568 | 64336712 | + | 13.8 | 3140 | 3285 | -4428 | HERVH | ERV1 | LTR |
| 64336712 | 64336812 | + | 14.8 | 3544 | 3644 | -4069 | HERVH | ERV1 | LTR |
| 64336812 | 64337221 | + | 16.6 | 4740 | 5149 | -2564 | HERVH | ERV1 | LTR |
| 64337223 | 64337239 | + | 12.9 | 5600 | 5622 | -2091 | HERVH | ERV1 | LTR |
| 64337264 | 64337457 | + | 12.9 | 5623 | 5819 | -1894 | HERVH | ERV1 | LTR |
| 64337456 | 64337834 | + | 7.8 | 7322 | 7713 | 0 | HERVH | ERV1 | LTR |
| 64337835 | 64338284 | + | 4.0 | 1 | 448 | -2 | LTR7 | ERV1 | LTR |
| 64338550 | 64339108 | - | 19.4 | 1 | 605 | -485 | MER97c | MER1\_type? | DNA |

  

|  |  |  |  |  |  |  |  |  |  |
| --- | --- | --- | --- | --- | --- | --- | --- | --- | --- |
| chr2:67411175-67418556 | | | 7226 bp | | |  |  |  |  |
| Genome Start | Genome End | Strand | Div | Repeat Start | Repeat End | Repeat Left | Name | Family | Class |
| 67411175 | 67411459 | + | 22.7 | 5337 | 5606 | -560 | L1ME1 | L1 | LINE |
| 67411460 | 67411626 | + | 16.2 | 2 | 169 | -7 | FRAM | Alu | SINE |
| 67411627 | 67411980 | + | 22.4 | 5596 | 5998 | -168 | L1ME1 | L1 | LINE |
| 67411981 | 67412152 | + | 15.9 | 1 | 170 | -142 | AluJo | Alu | SINE |
| 67412153 | 67412550 | + | 8.5 | 1 | 448 | -2 | LTR7 | ERV1 | LTR |
| 67412553 | 67416489 | + | 14.5 | 1 | 3993 | -3720 | HERVH | ERV1 | LTR |
| 67416492 | 67417153 | + | 15.1 | 4488 | 5149 | -2564 | HERVH | ERV1 | LTR |
| 67417155 | 67417370 | + | 13.9 | 5600 | 5819 | -1894 | HERVH | ERV1 | LTR |
| 67417371 | 67417438 | + | 11.2 | 7324 | 7393 | -320 | HERVH | ERV1 | LTR |
| 67417439 | 67417483 | - | 2.2 | 6111 | 6155 | 0 | L1PA3 | L1 | LINE |
| 67417484 | 67417772 | + | 11.2 | 7394 | 7699 | -14 | HERVH | ERV1 | LTR |
| 67417839 | 67418237 | + | 7.0 | 1 | 448 | -2 | LTR7 | ERV1 | LTR |
| 67418240 | 67418361 | + | 18.9 | 166 | 288 | -24 | AluJo | Alu | SINE |
| 67418445 | 67418556 | + | 18.9 | 6036 | 6148 | -18 | L1ME1 | L1 | LINE |

  

|  |  |  |  |  |  |  |  |  |  |
| --- | --- | --- | --- | --- | --- | --- | --- | --- | --- |
| chr2:69521825-69530121 | | | 8278 bp | | |  |  |  |  |
| Genome Start | Genome End | Strand | Div | Repeat Start | Repeat End | Repeat Left | Name | Family | Class |
| 69521825 | 69522043 | + | 10.6 | 4263 | 4481 | -1850 | LTR49-int | ERV1 | LTR |
| 69522044 | 69522167 | - | 3.2 | 6032 | 6155 | 0 | L1PA2 | L1 | LINE |
| 69522168 | 69522693 | + | 10.6 | 4482 | 4970 | -1361 | LTR49-int | ERV1 | LTR |
| 69522694 | 69523010 | - | 14.7 | 1 | 312 | 0 | AluJb | Alu | SINE |
| 69523011 | 69523646 | + | 10.6 | 4971 | 5603 | -728 | LTR49-int | ERV1 | LTR |
| 69523647 | 69524043 | - | 9.4 | 1 | 448 | -2 | LTR7 | ERV1 | LTR |
| 69524044 | 69524420 | - | 7.8 | 7324 | 7713 | 0 | HERVH | ERV1 | LTR |
| 69524421 | 69524640 | - | 14.1 | 5600 | 5819 | -1894 | HERVH | ERV1 | LTR |
| 69524646 | 69525544 | - | 12.0 | 4221 | 5145 | -2568 | HERVH | ERV1 | LTR |
| 69525543 | 69525851 | - | 14.1 | 3995 | 4290 | -3423 | HERVH | ERV1 | LTR |
| 69525866 | 69529577 | - | 12.2 | 1 | 3732 | -3981 | HERVH | ERV1 | LTR |
| 69529580 | 69529977 | - | 8.6 | 1 | 448 | -2 | LTR7 | ERV1 | LTR |
| 69529978 | 69530121 | + | 11.1 | 5598 | 5741 | -590 | LTR49-int | ERV1 | LTR |

  

|  |  |  |  |  |  |  |  |  |  |
| --- | --- | --- | --- | --- | --- | --- | --- | --- | --- |
| chr2:70699293-70711155 | | | 11405 bp | | |  |  |  |  |
| Genome Start | Genome End | Strand | Div | Repeat Start | Repeat End | Repeat Left | Name | Family | Class |
| 70699293 | 70699437 | - | 28.3 | 2964 | 3114 | -305 | L2 | L2 | LINE |
| 70699717 | 70699825 | - | 26.9 | 2060 | 2171 | -1248 | L2 | L2 | LINE |
| 70699826 | 70699945 | - | 28.6 | 6 | 133 | -1 | MER94 | AcHobo | DNA |
| 70699946 | 70700506 | - | 25.9 | 1421 | 2056 | -1363 | L2 | L2 | LINE |
| 70700510 | 70701146 | + | 19.5 | 1 | 612 | 0 | LTR9 | ERV1 | LTR |
| 70701190 | 70701537 | + | 6.9 | 1 | 448 | -2 | LTR7 | ERV1 | LTR |
| 70701540 | 70705255 | + | 9.6 | 1 | 3746 | -3967 | HERVH | ERV1 | LTR |
| 70705251 | 70705394 | + | 5.6 | 3850 | 3993 | -3720 | HERVH | ERV1 | LTR |
| 70705397 | 70706057 | + | 14.4 | 4488 | 5149 | -2564 | HERVH | ERV1 | LTR |
| 70706059 | 70706274 | + | 12.5 | 5600 | 5819 | -1894 | HERVH | ERV1 | LTR |
| 70706273 | 70706648 | + | 10.8 | 7322 | 7713 | 0 | HERVH | ERV1 | LTR |
| 70706649 | 70707049 | + | 7.7 | 1 | 448 | -2 | LTR7 | ERV1 | LTR |
| 70707130 | 70707534 | + | 24.4 | 25 | 469 | -5954 | L1MCc | L1 | LINE |
| 70707567 | 70708178 | + | 26.7 | 1865 | 2481 | -4647 | L1MCc | L1 | LINE |
| 70708190 | 70708402 | - | 20.3 | 1 | 222 | -2 | MER58A | MER1\_type | DNA |
| 70708406 | 70708883 | + | 22.6 | 2656 | 3141 | -3902 | L1MCc | L1 | LINE |
| 70708884 | 70709521 | - | 9.7 | 5525 | 6160 | 0 | L1PREC2 | L1 | LINE |
| 70709522 | 70709766 | + | 21.7 | 3040 | 3272 | -3669 | L1MCc | L1 | LINE |
| 70709767 | 70710065 | + | 12.0 | 1 | 299 | -13 | AluSx | Alu | SINE |
| 70710066 | 70710385 | + | 21.7 | 3273 | 3573 | -3368 | L1MCc | L1 | LINE |
| 70710395 | 70711155 | - | 28.8 | 474 | 1312 | -2107 | L2 | L2 | LINE |

  

|  |  |  |  |  |  |  |  |  |  |
| --- | --- | --- | --- | --- | --- | --- | --- | --- | --- |
| chr2:73129290-73133194 | | | 3778 bp | | |  |  |  |  |
| Genome Start | Genome End | Strand | Div | Repeat Start | Repeat End | Repeat Left | Name | Family | Class |
| 73129290 | 73129375 | - | 30.9 | 7264 | 7348 | -460 | L1MC4a | L1 | LINE |
| 73129373 | 73129491 | - | 25.2 | 5593 | 5722 | -399 | L1M5 | L1 | LINE |
| 73129488 | 73129661 | - | 28.6 | 7166 | 7340 | -702 | L1MC4 | L1 | LINE |
| 73129703 | 73129912 | - | 15.4 | 156 | 408 | 0 | MLT1A1 | MaLR | LTR |
| 73129913 | 73130191 | - | 14.1 | 1 | 293 | -19 | AluJb | Alu | SINE |
| 73130192 | 73130342 | - | 15.4 | 1 | 155 | -253 | MLT1A1 | MaLR | LTR |
| 73130348 | 73131025 | - | 23.7 | 901 | 1645 | -6 | MLT1A1-int | MaLR | LTR |
| 73131059 | 73131894 | - | 21.7 | 107 | 958 | -777 | MLT1A1-int | MaLR | LTR |
| 73131895 | 73132192 | - | 8.4 | 1 | 298 | -11 | AluSg1 | Alu | SINE |
| 73132193 | 73132306 | - | 24.3 | 4 | 106 | -1545 | MLT1A1-int | MaLR | LTR |
| 73132308 | 73132638 | - | 16.4 | 2 | 408 | 0 | MLT1A1 | MaLR | LTR |
| 73132640 | 73132976 | - | 24.1 | 6590 | 6977 | -831 | L1MC4a | L1 | LINE |
| 73133030 | 73133194 | - | 24.1 | 6417 | 6589 | -1219 | L1MC4a | L1 | LINE |

  

|  |  |  |  |  |  |  |  |  |  |
| --- | --- | --- | --- | --- | --- | --- | --- | --- | --- |
| chr2:75812987-75816086 | | | 2847 bp | | |  |  |  |  |
| Genome Start | Genome End | Strand | Div | Repeat Start | Repeat End | Repeat Left | Name | Family | Class |
| 75812987 | 75813335 | - | 31.3 | 5295 | 5650 | -2158 | L1M5 | L1 | LINE |
| 75813460 | 75813810 | - | 8.6 | 3 | 354 | -1 | THE1A | MaLR | LTR |
| 75813813 | 75815274 | - | 29.4 | 2 | 1735 | 0 | MLT-int | MaLR | LTR |
| 75815278 | 75815427 | - | 10.7 | 205 | 354 | -1 | THE1A | MaLR | LTR |
| 75815425 | 75815506 | - | 8.5 | 1 | 82 | -273 | THE1A | MaLR | LTR |
| 75815507 | 75815795 | - | 25.4 | 4865 | 5166 | -980 | L1M5 | L1 | LINE |
| 75815923 | 75816086 | - | 21.9 | 4645 | 4819 | -1327 | L1M5 | L1 | LINE |

  

|  |  |  |  |  |  |  |  |  |  |
| --- | --- | --- | --- | --- | --- | --- | --- | --- | --- |
| chr2:76170308-76186220 | | | 15628 bp | | |  |  |  |  |
| Genome Start | Genome End | Strand | Div | Repeat Start | Repeat End | Repeat Left | Name | Family | Class |
| 76170308 | 76170458 | - | 14.0 | 7633 | 7785 | 0 | L1MC3 | L1 | LINE |
| 76170465 | 76170782 | - | 20.4 | 7121 | 7502 | -283 | L1MC3 | L1 | LINE |
| 76170783 | 76170969 | + | 14.5 | 2746 | 2931 | -3215 | L1P4 | L1 | LINE |
| 76170995 | 76171108 | - | 34.2 | 34 | 159 | -109 | MIRb | MIR | SINE |
| 76171283 | 76177419 | - | 4.1 | 2 | 6152 | -3 | L1PA3 | L1 | LINE |
| 76177422 | 76178087 | + | 14.3 | 2920 | 3596 | -2550 | L1P4 | L1 | LINE |
| 76178088 | 76178431 | - | 16.6 | 1 | 364 | 0 | THE1B | MaLR | LTR |
| 76178448 | 76180024 | - | 30.2 | 2 | 1731 | -4 | MLT1F2-int | MaLR | LTR |
| 76180027 | 76180383 | - | 16.7 | 1 | 364 | 0 | THE1B | MaLR | LTR |
| 76180384 | 76182962 | + | 14.2 | 3597 | 6164 | -2 | L1PA16 | L1 | LINE |
| 76183020 | 76184887 | - | 22.3 | 4677 | 6703 | -1500 | L1MC3 | L1 | LINE |
| 76184892 | 76185269 | - | 6.9 | 4595 | 4973 | -1173 | L1P3 | L1 | LINE |
| 76185269 | 76185444 | - | 19.3 | 2755 | 2924 | -3640 | L1MC3 | L1 | LINE |
| 76185445 | 76185883 | + | 10.0 | 1 | 453 | 0 | MLT2A2 | ERVL | LTR |
| 76185884 | 76186220 | - | 17.5 | 2382 | 2754 | -3810 | L1MC3 | L1 | LINE |

  

|  |  |  |  |  |  |  |  |  |  |
| --- | --- | --- | --- | --- | --- | --- | --- | --- | --- |
| chr2:76483743-76492397 | | | 8678 bp | | |  |  |  |  |
| Genome Start | Genome End | Strand | Div | Repeat Start | Repeat End | Repeat Left | Name | Family | Class |
| 76483743 | 76484107 | + | 17.7 | 1 | 370 | -7750 | LOR1b-int | ERV1 | LTR |
| 76484107 | 76484191 | + | 13.5 | 1912 | 2002 | -6118 | LOR1-int | ERV1 | LTR |
| 76484192 | 76484487 | + | 7.1 | 1 | 308 | 0 | AluY | Alu | SINE |
| 76484488 | 76484499 | + | 7.1 | 284 | 293 | -18 | AluY | Alu | SINE |
| 76484500 | 76485071 | + | 13.5 | 2003 | 2576 | -5544 | LOR1-int | ERV1 | LTR |
| 76485054 | 76486294 | + | 10.9 | 2735 | 3975 | -4145 | LOR1-int | ERV1 | LTR |
| 76486295 | 76487532 | + | 23.9 | 1 | 2119 | -4 | MER52A | ERV1 | LTR |
| 76487538 | 76488303 | + | 13.8 | 3984 | 4751 | -3369 | LOR1b-int | ERV1 | LTR |
| 76488305 | 76488668 | + | 17.8 | 1 | 364 | 0 | THE1B | MaLR | LTR |
| 76488670 | 76490103 | + | 11.3 | 2 | 1435 | -145 | THE1B-int | MaLR | LTR |
| 76490077 | 76490762 | - | 14.0 | 1 | 618 | -208 | LTR12 | ERV1 | LTR |
| 76490763 | 76490911 | + | 8.0 | 1432 | 1580 | 0 | THE1B-int | MaLR | LTR |
| 76490912 | 76491269 | + | 14.7 | 1 | 364 | 0 | THE1B | MaLR | LTR |
| 76491288 | 76492051 | + | 10.4 | 4760 | 5524 | -2596 | LOR1b-int | ERV1 | LTR |
| 76492051 | 76492306 | + | 11.7 | 5809 | 6064 | -2056 | LOR1b-int | ERV1 | LTR |
| 76492306 | 76492397 | + | 11.0 | 8030 | 8120 | 0 | LOR1b-int | ERV1 | LTR |

  

|  |  |  |  |  |  |  |  |  |  |
| --- | --- | --- | --- | --- | --- | --- | --- | --- | --- |
| chr2:77102445-77105080 | | | 2633 bp | | |  |  |  |  |
| Genome Start | Genome End | Strand | Div | Repeat Start | Repeat End | Repeat Left | Name | Family | Class |
| 77102445 | 77102634 | - | 20.7 | 5983 | 6270 | -42 | L1MA9 | L1 | LINE |
| 77102638 | 77102990 | - | 10.8 | 1 | 355 | 0 | THE1A | MaLR | LTR |
| 77102991 | 77104538 | - | 9.8 | 1 | 1580 | 0 | THE1A-int | MaLR | LTR |
| 77104539 | 77104891 | - | 14.7 | 1 | 355 | 0 | THE1A | MaLR | LTR |
| 77104892 | 77105080 | - | 12.7 | 5795 | 5984 | -328 | L1MA9 | L1 | LINE |

  

|  |  |  |  |  |  |  |  |  |  |
| --- | --- | --- | --- | --- | --- | --- | --- | --- | --- |
| chr2:77167515-77179970 | | | 12071 bp | | |  |  |  |  |
| Genome Start | Genome End | Strand | Div | Repeat Start | Repeat End | Repeat Left | Name | Family | Class |
| 77167515 | 77168366 | + | 31.7 | 1 | 847 | -5272 | L1ME3B | L1 | LINE |
| 77168542 | 77168722 | - | 27.6 | 477 | 655 | 0 | LTR16D | ERVL | LTR |
| 77168752 | 77168850 | - | 26.0 | 262 | 367 | -288 | LTR16D | ERVL | LTR |
| 77168881 | 77169308 | + | 5.2 | 24 | 448 | -2 | LTR7 | ERV1 | LTR |
| 77169311 | 77172556 | + | 9.6 | 1 | 3290 | -4423 | HERVH | ERV1 | LTR |
| 77172547 | 77173049 | + | 11.6 | 3492 | 3993 | -3720 | HERVH | ERV1 | LTR |
| 77173052 | 77173705 | + | 13.2 | 4488 | 5149 | -2564 | HERVH | ERV1 | LTR |
| 77173707 | 77173922 | + | 13.0 | 5600 | 5819 | -1894 | HERVH | ERV1 | LTR |
| 77173921 | 77174300 | + | 7.8 | 7322 | 7713 | 0 | HERVH | ERV1 | LTR |
| 77174301 | 77174664 | + | 4.4 | 1 | 365 | -85 | LTR7 | ERV1 | LTR |
| 77174665 | 77176129 | + | 18.4 | 4197 | 5736 | -555 | L1M1 | L1 | LINE |
| 77176143 | 77176446 | - | 11.4 | 1 | 297 | -15 | AluSx | Alu | SINE |
| 77176448 | 77176499 | + | 9.6 | 5802 | 5860 | -445 | L1M1 | L1 | LINE |
| 77176526 | 77176795 | + | 20.1 | 6007 | 6287 | -4 | L1MA7 | L1 | LINE |
| 77176859 | 77178758 | + | 31.3 | 1437 | 3434 | -2695 | L1ME3B | L1 | LINE |
| 77178814 | 77179970 | + | 31.1 | 4627 | 5847 | -376 | L1ME3B | L1 | LINE |

  

|  |  |  |  |  |  |  |  |  |  |
| --- | --- | --- | --- | --- | --- | --- | --- | --- | --- |
| chr2:78360376-78364116 | | | 3309 bp | | |  |  |  |  |
| Genome Start | Genome End | Strand | Div | Repeat Start | Repeat End | Repeat Left | Name | Family | Class |
| 78360376 | 78360760 | + | 34.9 | 867 | 1273 | -2146 | L2 | L2 | LINE |
| 78360795 | 78361099 | + | 17.0 | 9 | 311 | -1 | AluJo | Alu | SINE |
| 78361470 | 78361758 | + | 36.0 | 1361 | 1676 | -1743 | L2 | L2 | LINE |
| 78361759 | 78362106 | + | 15.0 | 1 | 363 | -1 | THE1B | MaLR | LTR |
| 78362132 | 78363498 | + | 13.8 | 146 | 1580 | 0 | THE1B-int | MaLR | LTR |
| 78363499 | 78363853 | + | 14.7 | 1 | 364 | 0 | THE1B | MaLR | LTR |
| 78363857 | 78364116 | + | 34.8 | 1675 | 1953 | -1466 | L2 | L2 | LINE |

  

|  |  |  |  |  |  |  |  |  |  |
| --- | --- | --- | --- | --- | --- | --- | --- | --- | --- |
| chr2:79130666-79138629 | | | 7668 bp | | |  |  |  |  |
| Genome Start | Genome End | Strand | Div | Repeat Start | Repeat End | Repeat Left | Name | Family | Class |
| 79130666 | 79131245 | + | 25.9 | 6190 | 6785 | -1023 | L1MC4a | L1 | LINE |
| 79131379 | 79131927 | + | 19.8 | 1 | 544 | -10 | MLT2A2 | ERVL | LTR |
| 79132034 | 79132432 | + | 8.3 | 1 | 448 | -2 | LTR7 | ERV1 | LTR |
| 79132435 | 79135412 | + | 9.2 | 1 | 2987 | -4726 | HERVH | ERV1 | LTR |
| 79135407 | 79135554 | + | 16.2 | 3140 | 3290 | -4423 | HERVH | ERV1 | LTR |
| 79135545 | 79136047 | + | 11.0 | 3492 | 3993 | -3720 | HERVH | ERV1 | LTR |
| 79136050 | 79136712 | + | 13.8 | 4488 | 5149 | -2564 | HERVH | ERV1 | LTR |
| 79136714 | 79136930 | + | 12.5 | 5600 | 5819 | -1894 | HERVH | ERV1 | LTR |
| 79136931 | 79137307 | + | 10.2 | 7324 | 7713 | 0 | HERVH | ERV1 | LTR |
| 79137308 | 79137706 | + | 10.3 | 1 | 448 | -2 | LTR7 | ERV1 | LTR |
| 79137775 | 79138629 | + | 22.0 | 6844 | 7757 | -51 | L1MC4a | L1 | LINE |

  

|  |  |  |  |  |  |  |  |  |  |
| --- | --- | --- | --- | --- | --- | --- | --- | --- | --- |
| chr2:79308145-79319242 | | | 11074 bp | | |  |  |  |  |
| Genome Start | Genome End | Strand | Div | Repeat Start | Repeat End | Repeat Left | Name | Family | Class |
| 79308145 | 79308388 | - | 12.3 | 2167 | 2418 | 0 | Tigger1 | MER2\_type | DNA |
| 79308395 | 79309875 | - | 14.4 | 5196 | 6684 | -9 | L1PA16 | L1 | LINE |
| 79309876 | 79310231 | + | 8.9 | 1 | 355 | 0 | THE1A | MaLR | LTR |
| 79310232 | 79311799 | + | 9.8 | 1 | 1574 | -6 | THE1A-int | MaLR | LTR |
| 79311805 | 79312167 | + | 13.6 | 1 | 355 | 0 | THE1A | MaLR | LTR |
| 79312170 | 79316777 | - | 18.4 | 601 | 5207 | -1466 | L1PA16 | L1 | LINE |
| 79316785 | 79317061 | - | 21.8 | 6 | 282 | -30 | AluJo | Alu | SINE |
| 79317066 | 79319242 | - | 15.5 | 1 | 2181 | -237 | Tigger1 | MER2\_type | DNA |

  

|  |  |  |  |  |  |  |  |  |  |
| --- | --- | --- | --- | --- | --- | --- | --- | --- | --- |
| chr2:79631762-79634665 | | | 2899 bp | | |  |  |  |  |
| Genome Start | Genome End | Strand | Div | Repeat Start | Repeat End | Repeat Left | Name | Family | Class |
| 79631762 | 79632228 | - | 21.8 | 189 | 655 | -9 | MER4A | ERV1 | LTR |
| 79632229 | 79632606 | - | 14.7 | 1 | 428 | 0 | MSTA | MaLR | LTR |
| 79632607 | 79634079 | - | 15.7 | 3 | 1580 | 0 | MSTA-int | MaLR | LTR |
| 79634082 | 79634457 | - | 17.2 | 4 | 428 | 0 | MSTA | MaLR | LTR |
| 79634461 | 79634665 | - | 14.4 | 1 | 193 | -471 | MER4A | ERV1 | LTR |

  

|  |  |  |  |  |  |  |  |  |  |
| --- | --- | --- | --- | --- | --- | --- | --- | --- | --- |
| chr2:81225205-81228013 | | | 2797 bp | | |  |  |  |  |
| Genome Start | Genome End | Strand | Div | Repeat Start | Repeat End | Repeat Left | Name | Family | Class |
| 81225205 | 81225437 | - | 31.4 | 327 | 740 | -5215 | L1M3a | L1 | LINE |
| 81225438 | 81225787 | - | 8.3 | 2 | 355 | 0 | THE1A | MaLR | LTR |
| 81225789 | 81227378 | - | 11.0 | 1 | 1580 | 0 | THE1A-int | MaLR | LTR |
| 81227379 | 81227734 | - | 12.7 | 1 | 355 | 0 | THE1A | MaLR | LTR |
| 81227746 | 81228013 | - | 27.3 | 11 | 317 | -5638 | L1M3a | L1 | LINE |

  

|  |  |  |  |  |  |  |  |  |  |
| --- | --- | --- | --- | --- | --- | --- | --- | --- | --- |
| chr2:82094072-82113559 | | | 19405 bp | | |  |  |  |  |
| Genome Start | Genome End | Strand | Div | Repeat Start | Repeat End | Repeat Left | Name | Family | Class |
| 82094072 | 82094296 | - | 21.9 | 6823 | 7037 | -1 | L1MB8 | L1 | LINE |
| 82094297 | 82094785 | - | 18.5 | 1 | 509 | 0 | LTR22C | ERVK | LTR |
| 82094786 | 82095353 | - | 21.9 | 6247 | 6822 | -216 | L1MB8 | L1 | LINE |
| 82095352 | 82095419 | - | 22.1 | 5676 | 5744 | -1262 | L1MB8 | L1 | LINE |
| 82095420 | 82095713 | - | 9.2 | 1 | 306 | -4 | AluSg | Alu | SINE |
| 82095714 | 82096511 | - | 22.1 | 4827 | 5675 | -1331 | L1MB8 | L1 | LINE |
| 82096526 | 82099482 | - | 21.1 | 1865 | 4748 | -2258 | L1MB8 | L1 | LINE |
| 82099484 | 82099620 | - | 23.4 | 956 | 1108 | -6226 | L1MB8 | L1 | LINE |
| 82099628 | 82100179 | + | 28.0 | 4011 | 4544 | -1602 | L1PB | L1 | LINE |
| 82100180 | 82100478 | + | 17.4 | 1 | 288 | -24 | AluSx | Alu | SINE |
| 82100479 | 82101925 | + | 18.1 | 4530 | 5926 | -374 | L1MA4 | L1 | LINE |
| 82101927 | 82102292 | - | 15.9 | 1 | 364 | 0 | THE1B | MaLR | LTR |
| 82102293 | 82103295 | - | 13.3 | 542 | 1580 | 0 | THE1B-int | MaLR | LTR |
| 82103296 | 82103878 | + | 4.3 | 1 | 587 | 0 | LTR14C | ERVK | LTR |
| 82103880 | 82110701 | + | 5.4 | 2 | 6806 | -628 | HERVK14C | ERVK | LTR |
| 82110729 | 82111332 | + | 5.4 | 6807 | 7432 | -2 | HERVK14C | ERVK | LTR |
| 82111333 | 82111914 | + | 7.0 | 1 | 587 | 0 | LTR14C | ERVK | LTR |
| 82111915 | 82112456 | - | 8.9 | 1 | 545 | -1035 | THE1B-int | MaLR | LTR |
| 82112457 | 82112808 | - | 14.2 | 3 | 364 | 0 | THE1B | MaLR | LTR |
| 82112811 | 82113171 | + | 18.1 | 5925 | 6297 | -3 | L1MA4 | L1 | LINE |
| 82113189 | 82113469 | - | 23.6 | 3 | 285 | -2744 | Tigger3(Golem) | MER2\_type | DNA |
| 82113485 | 82113559 | - | 20.6 | 3 | 81 | -7253 | L1MB8 | L1 | LINE |

  

|  |  |  |  |  |  |  |  |  |  |
| --- | --- | --- | --- | --- | --- | --- | --- | --- | --- |
| chr2:82376643-82380944 | | | 3529 bp | | |  |  |  |  |
| Genome Start | Genome End | Strand | Div | Repeat Start | Repeat End | Repeat Left | Name | Family | Class |
| 82376643 | 82376734 | - | 28.3 | 5790 | 5883 | -1925 | L1MC4a | L1 | LINE |
| 82377045 | 82377226 | - | 23.1 | 5299 | 5500 | -2308 | L1MC4a | L1 | LINE |
| 82377372 | 82377771 | - | 18.1 | 1 | 426 | 0 | MSTB | MaLR | LTR |
| 82377772 | 82379360 | - | 28.7 | 2 | 1735 | 0 | MLT-int | MaLR | LTR |
| 82379363 | 82379773 | - | 23.2 | 1 | 426 | 0 | MSTB | MaLR | LTR |
| 82380066 | 82380156 | - | 32.4 | 4754 | 4846 | -1300 | L1MC4a | L1 | LINE |
| 82380157 | 82380646 | - | 25.3 | 2 | 467 | 0 | MLT1C | MaLR | LTR |
| 82380647 | 82380823 | - | 32.4 | 4572 | 4753 | -1393 | L1MC4a | L1 | LINE |
| 82380848 | 82380944 | - | 32.0 | 4496 | 4592 | -1554 | L1MC4a | L1 | LINE |

  

|  |  |  |  |  |  |  |  |  |  |
| --- | --- | --- | --- | --- | --- | --- | --- | --- | --- |
| chr2:83460118-83464571 | | | 4415 bp | | |  |  |  |  |
| Genome Start | Genome End | Strand | Div | Repeat Start | Repeat End | Repeat Left | Name | Family | Class |
| 83460118 | 83460403 | + | 12.6 | 4058 | 4343 | -1803 | L1PA16 | L1 | LINE |
| 83460405 | 83460778 | + | 18.1 | 1 | 428 | 0 | MSTA | MaLR | LTR |
| 83460814 | 83462419 | + | 17.8 | 24 | 1650 | -1 | MSTA-int | MaLR | LTR |
| 83462423 | 83462799 | + | 17.6 | 3 | 428 | 0 | MSTA | MaLR | LTR |
| 83462800 | 83464571 | + | 14.5 | 4340 | 6157 | -9 | L1PA16 | L1 | LINE |

  

|  |  |  |  |  |  |  |  |  |  |
| --- | --- | --- | --- | --- | --- | --- | --- | --- | --- |
| chr2:84170091-84177218 | | | 6860 bp | | |  |  |  |  |
| Genome Start | Genome End | Strand | Div | Repeat Start | Repeat End | Repeat Left | Name | Family | Class |
| 84170091 | 84170856 | + | 21.9 | 24 | 898 | -5747 | L1MB5 | L1 | LINE |
| 84171121 | 84172395 | + | 25.6 | 933 | 2231 | -4518 | L1MB5 | L1 | LINE |
| 84172396 | 84172750 | - | 13.1 | 1 | 364 | 0 | THE1B | MaLR | LTR |
| 84172751 | 84174325 | - | 30.7 | 2 | 1735 | 0 | MLT-int | MaLR | LTR |
| 84174328 | 84174642 | - | 13.4 | 1 | 364 | 0 | THE1B | MaLR | LTR |
| 84174643 | 84176097 | + | 22.9 | 2232 | 3845 | -2902 | L1MB5 | L1 | LINE |
| 84176100 | 84177218 | + | 19.1 | 5454 | 6564 | -211 | L1MB5 | L1 | LINE |

  

|  |  |  |  |  |  |  |  |  |  |
| --- | --- | --- | --- | --- | --- | --- | --- | --- | --- |
| chr2:84248205-84250731 | | | 2524 bp | | |  |  |  |  |
| Genome Start | Genome End | Strand | Div | Repeat Start | Repeat End | Repeat Left | Name | Family | Class |
| 84248205 | 84248363 | - | 31.3 | 90 | 262 | 0 | MIR | MIR | SINE |
| 84248364 | 84248722 | + | 13.9 | 1 | 364 | 0 | THE1B | MaLR | LTR |
| 84248725 | 84250302 | + | 30.1 | 2 | 1734 | -1 | MLT1E2-int | MaLR | LTR |
| 84250304 | 84250654 | + | 12.8 | 1 | 364 | 0 | THE1B | MaLR | LTR |
| 84250655 | 84250731 | - | 31.3 | 8 | 89 | -173 | MIR | MIR | SINE |

  

|  |  |  |  |  |  |  |  |  |  |
| --- | --- | --- | --- | --- | --- | --- | --- | --- | --- |
| chr2:84826752-84830208 | | | 3108 bp | | |  |  |  |  |
| Genome Start | Genome End | Strand | Div | Repeat Start | Repeat End | Repeat Left | Name | Family | Class |
| 84826752 | 84827090 | - | 35.1 | 2082 | 2426 | -993 | L2 | L2 | LINE |
| 84827329 | 84827749 | - | 17.8 | 1 | 426 | 0 | MSTB | MaLR | LTR |
| 84827750 | 84829399 | - | 28.6 | 2 | 1735 | 0 | MLT1J-int | MaLR | LTR |
| 84829402 | 84829823 | - | 18.0 | 1 | 426 | 0 | MSTB | MaLR | LTR |
| 84829933 | 84830208 | - | 29.4 | 816 | 1113 | -2306 | L2 | L2 | LINE |

  

|  |  |  |  |  |  |  |  |  |  |
| --- | --- | --- | --- | --- | --- | --- | --- | --- | --- |
| chr2:86020774-86025491 | | | 4410 bp | | |  |  |  |  |
| Genome Start | Genome End | Strand | Div | Repeat Start | Repeat End | Repeat Left | Name | Family | Class |
| 86020774 | 86021140 | + | 22.1 | 4838 | 5237 | -909 | L1M5 | L1 | LINE |
| 86021334 | 86021607 | + | 29.8 | 103 | 448 | -9 | LTR16A | ERVL | LTR |
| 86021658 | 86021786 | + | 19.8 | 5262 | 5389 | -757 | L1M5 | L1 | LINE |
| 86021793 | 86022098 | - | 26.4 | 157 | 467 | 0 | MLT1C | MaLR | LTR |
| 86022129 | 86022488 | - | 11.7 | 1 | 364 | 0 | THE1B | MaLR | LTR |
| 86022494 | 86024011 | - | 10.6 | 1 | 1574 | -6 | THE1B-int | MaLR | LTR |
| 86024012 | 86024369 | - | 11.2 | 1 | 364 | 0 | THE1B | MaLR | LTR |
| 86024387 | 86024536 | - | 20.8 | 1 | 156 | -349 | MLT1C | MaLR | LTR |
| 86024543 | 86024772 | + | 24.2 | 5400 | 5650 | -520 | L1M5 | L1 | LINE |
| 86024774 | 86025334 | + | 29.5 | 28 | 590 | 0 | MLT1G | MaLR | LTR |
| 86025335 | 86025491 | + | 19.7 | 5646 | 5803 | -367 | L1M5 | L1 | LINE |

  

|  |  |  |  |  |  |  |  |  |  |
| --- | --- | --- | --- | --- | --- | --- | --- | --- | --- |
| chr2:88323399-88325966 | | | 2565 bp | | |  |  |  |  |
| Genome Start | Genome End | Strand | Div | Repeat Start | Repeat End | Repeat Left | Name | Family | Class |
| 88323399 | 88323461 | + | 17.5 | 1 | 60 | -252 | AluJo | Alu | SINE |
| 88323462 | 88323804 | + | 11.7 | 1 | 354 | -1 | THE1A | MaLR | LTR |
| 88323808 | 88325370 | + | 30.4 | 2 | 1735 | 0 | MLT1L-int | MaLR | LTR |
| 88325371 | 88325702 | + | 14.6 | 1 | 355 | 0 | THE1A | MaLR | LTR |
| 88325703 | 88325966 | + | 17.5 | 61 | 334 | 0 | AluJo | Alu | SINE |

  

|  |  |  |  |  |  |  |  |  |  |
| --- | --- | --- | --- | --- | --- | --- | --- | --- | --- |
| chr2:88839146-88841530 | | | 2383 bp | | |  |  |  |  |
| Genome Start | Genome End | Strand | Div | Repeat Start | Repeat End | Repeat Left | Name | Family | Class |
| 88839146 | 88839167 | + | 23.2 | 2422 | 2439 | -4194 | L1MDa | L1 | LINE |
| 88839168 | 88839522 | + | 13.5 | 1 | 364 | 0 | THE1B | MaLR | LTR |
| 88839525 | 88841088 | + | 30.2 | 2 | 1735 | 0 | MLT-int | MaLR | LTR |
| 88841089 | 88841446 | + | 13.1 | 1 | 364 | 0 | THE1B | MaLR | LTR |
| 88841447 | 88841530 | + | 23.2 | 2440 | 2520 | -4113 | L1MDa | L1 | LINE |

  

|  |  |  |  |  |  |  |  |  |  |
| --- | --- | --- | --- | --- | --- | --- | --- | --- | --- |
| chr2:95165707-95172711 | | | 6904 bp | | |  |  |  |  |
| Genome Start | Genome End | Strand | Div | Repeat Start | Repeat End | Repeat Left | Name | Family | Class |
| 95165707 | 95165736 | + | 26.9 | 3380 | 3409 | -3392 | MER89-int | ERV1 | LTR |
| 95165737 | 95166018 | - | 12.8 | 1 | 292 | -20 | AluSx | Alu | SINE |
| 95166019 | 95167277 | + | 26.9 | 3410 | 4609 | -2192 | MER89-int | ERV1 | LTR |
| 95167281 | 95167662 | + | 15.6 | 5 | 428 | 0 | MSTA | MaLR | LTR |
| 95167665 | 95169254 | + | 27.7 | 2 | 1735 | 0 | MLT-int | MaLR | LTR |
| 95169255 | 95169407 | + | 15.5 | 1 | 176 | -205 | MSTA | MaLR | LTR |
| 95169417 | 95169764 | + | 6.1 | 5806 | 6151 | -3 | L1PA6 | L1 | LINE |
| 95169810 | 95170091 | + | 12.8 | 1 | 289 | -23 | AluSx | Alu | SINE |
| 95170120 | 95170165 | + | 9.8 | 5929 | 5974 | -177 | L1PB1 | L1 | LINE |
| 95170166 | 95170468 | + | 10.6 | 1 | 302 | -8 | AluSg | Alu | SINE |
| 95170469 | 95170653 | + | 9.8 | 5975 | 6137 | -14 | L1PB1 | L1 | LINE |
| 95170654 | 95170697 | + | 11.4 | 385 | 428 | 0 | MSTA | MaLR | LTR |
| 95170698 | 95172509 | + | 23.0 | 4597 | 6436 | -365 | MER89-int | ERV1 | LTR |
| 95172524 | 95172711 | + | 16.5 | 6609 | 6799 | -2 | MER89-int | ERV1 | LTR |

  

|  |  |  |  |  |  |  |  |  |  |
| --- | --- | --- | --- | --- | --- | --- | --- | --- | --- |
| chr2:96246374-96254506 | | | 7920 bp | | |  |  |  |  |
| Genome Start | Genome End | Strand | Div | Repeat Start | Repeat End | Repeat Left | Name | Family | Class |
| 96246374 | 96246596 | - | 19.9 | 2614 | 2852 | -10 | Charlie2 | MER1\_type | DNA |
| 96246609 | 96246933 | - | 10.2 | 1 | 324 | -456 | LTR17 | ERV1 | LTR |
| 96246934 | 96247224 | - | 5.3 | 6831 | 8626 | 0 | HERV17 | ERV1 | LTR |
| 96247202 | 96247240 | - | 7.7 | 7510 | 7548 | -1078 | HERV17 | ERV1 | LTR |
| 96247237 | 96247404 | - | 5.5 | 6985 | 7147 | -1479 | HERV17 | ERV1 | LTR |
| 96247396 | 96248461 | - | 5.3 | 4830 | 5902 | -2724 | HERV17 | ERV1 | LTR |
| 96248462 | 96248774 | - | 8.4 | 1 | 311 | 0 | AluY | Alu | SINE |
| 96248775 | 96249346 | - | 5.3 | 4259 | 4829 | -3797 | HERV17 | ERV1 | LTR |
| 96249347 | 96250490 | - | 4.6 | 2577 | 3727 | -4899 | HERV17 | ERV1 | LTR |
| 96250487 | 96251377 | - | 5.5 | 763 | 1634 | -6992 | HERV17 | ERV1 | LTR |
| 96251563 | 96252283 | - | 6.6 | 1 | 709 | -7917 | HERV17 | ERV1 | LTR |
| 96252284 | 96252790 | - | 9.1 | 256 | 780 | 0 | LTR17 | ERV1 | LTR |
| 96252792 | 96252925 | - | 12.2 | 1 | 131 | -12 | FLAM\_C | Alu | SINE |
| 96252976 | 96253026 | - | 18.5 | 2485 | 2535 | -327 | Charlie2 | MER1\_type | DNA |
| 96253027 | 96253327 | - | 10.0 | 1 | 302 | -11 | AluSp | Alu | SINE |
| 96253333 | 96253558 | - | 12.8 | 5 | 237 | -75 | AluSx | Alu | SINE |
| 96253559 | 96253716 | - | 18.5 | 2327 | 2484 | -378 | Charlie2 | MER1\_type | DNA |
| 96253717 | 96254011 | + | 7.8 | 1 | 293 | -17 | AluSg | Alu | SINE |
| 96254012 | 96254061 | - | 18.5 | 2274 | 2326 | -536 | Charlie2 | MER1\_type | DNA |
| 96254062 | 96254351 | + | 11.4 | 3 | 292 | -19 | AluY | Alu | SINE |
| 96254352 | 96254506 | - | 18.5 | 2109 | 2273 | -589 | Charlie2 | MER1\_type | DNA |

  

|  |  |  |  |  |  |  |  |  |  |
| --- | --- | --- | --- | --- | --- | --- | --- | --- | --- |
| chr2:96466677-96469791 | | | 3113 bp | | |  |  |  |  |
| Genome Start | Genome End | Strand | Div | Repeat Start | Repeat End | Repeat Left | Name | Family | Class |
| 96466677 | 96467030 | - | 13.3 | 230 | 635 | 0 | LTR24C | ERV1 | LTR |
| 96467031 | 96467379 | + | 13.0 | 1 | 381 | 0 | THE1D | MaLR | LTR |
| 96467382 | 96469162 | + | 29.9 | 2 | 1735 | 0 | MLT-int | MaLR | LTR |
| 96469163 | 96469552 | + | 11.4 | 1 | 381 | 0 | THE1D | MaLR | LTR |
| 96469553 | 96469791 | - | 13.3 | 1 | 229 | -406 | LTR24C | ERV1 | LTR |

  

|  |  |  |  |  |  |  |  |  |  |
| --- | --- | --- | --- | --- | --- | --- | --- | --- | --- |
| chr2:101149368-101157003 | | | 7396 bp | | |  |  |  |  |
| Genome Start | Genome End | Strand | Div | Repeat Start | Repeat End | Repeat Left | Name | Family | Class |
| 101149368 | 101149546 | + | 24.7 | 10 | 182 | -2325 | HAL1 | L1 | LINE |
| 101149696 | 101149829 | + | 14.3 | 1 | 133 | 0 | FLAM\_C | Alu | SINE |
| 101149909 | 101150323 | + | 13.0 | 1 | 428 | 0 | MSTA | MaLR | LTR |
| 101150324 | 101151852 | + | 16.3 | 1 | 1651 | 0 | MSTA-int | MaLR | LTR |
| 101151853 | 101152042 | + | 13.2 | 1 | 195 | -233 | MSTA | MaLR | LTR |
| 101152050 | 101152308 | - | 9.7 | 1 | 259 | -53 | AluSx | Alu | SINE |
| 101152309 | 101152651 | + | 11.0 | 26 | 364 | 0 | THE1B | MaLR | LTR |
| 101152654 | 101154227 | + | 30.9 | 2 | 1735 | 0 | MLT1A0-int | MaLR | LTR |
| 101154228 | 101154634 | + | 14.4 | 1 | 364 | 0 | THE1B | MaLR | LTR |
| 101154637 | 101154731 | + | 14.7 | 334 | 428 | 0 | MSTA | MaLR | LTR |
| 101154733 | 101155256 | + | 25.9 | 664 | 1160 | -1347 | HAL1 | L1 | LINE |
| 101155257 | 101155343 | + | 12.6 | 222 | 310 | -1 | AluS | Alu | SINE |
| 101155344 | 101155581 | + | 25.9 | 1161 | 1386 | -1121 | HAL1 | L1 | LINE |
| 101155582 | 101155749 | + | 19.2 | 1 | 144 | -168 | AluJb | Alu | SINE |
| 101155750 | 101156041 | + | 9.6 | 1 | 292 | -17 | AluSc | Alu | SINE |
| 101156042 | 101156203 | + | 19.2 | 145 | 302 | -10 | AluJb | Alu | SINE |
| 101156204 | 101156371 | + | 25.9 | 1389 | 1557 | -950 | HAL1 | L1 | LINE |
| 101156372 | 101156669 | + | 19.2 | 1 | 297 | -15 | AluJo | Alu | SINE |
| 101156670 | 101157003 | + | 25.9 | 1558 | 1901 | -606 | HAL1 | L1 | LINE |

  

|  |  |  |  |  |  |  |  |  |  |
| --- | --- | --- | --- | --- | --- | --- | --- | --- | --- |
| chr2:101888417-101891362 | | | 2946 bp | | |  |  |  |  |
| Genome Start | Genome End | Strand | Div | Repeat Start | Repeat End | Repeat Left | Name | Family | Class |
| 101888417 | 101888840 | + | 11.5 | 1 | 412 | -228 | MER51C | ERV1 | LTR |
| 101888841 | 101889221 | - | 11.3 | 1 | 375 | 0 | THE1C | MaLR | LTR |
| 101889222 | 101890776 | - | 11.7 | 1 | 1580 | 0 | THE1C-int | MaLR | LTR |
| 101890777 | 101891129 | - | 8.0 | 1 | 375 | 0 | THE1C | MaLR | LTR |
| 101891130 | 101891362 | + | 7.7 | 408 | 640 | 0 | MER51C | ERV1 | LTR |

  

|  |  |  |  |  |  |  |  |  |  |
| --- | --- | --- | --- | --- | --- | --- | --- | --- | --- |
| chr2:101951162-101954142 | | | 2979 bp | | |  |  |  |  |
| Genome Start | Genome End | Strand | Div | Repeat Start | Repeat End | Repeat Left | Name | Family | Class |
| 101951162 | 101951405 | - | 18.6 | 312 | 559 | 0 | MER89 | ERV1 | LTR |
| 101951406 | 101951820 | - | 14.5 | 1 | 426 | 0 | MSTB | MaLR | LTR |
| 101951821 | 101953418 | - | 28.7 | 2 | 1735 | 0 | MLT-int | MaLR | LTR |
| 101953421 | 101953840 | - | 12.4 | 1 | 426 | 0 | MSTB | MaLR | LTR |
| 101953841 | 101954142 | - | 20.2 | 1 | 311 | -248 | MER89 | ERV1 | LTR |

  

|  |  |  |  |  |  |  |  |  |  |
| --- | --- | --- | --- | --- | --- | --- | --- | --- | --- |
| chr2:103573471-103583412 | | | 9856 bp | | |  |  |  |  |
| Genome Start | Genome End | Strand | Div | Repeat Start | Repeat End | Repeat Left | Name | Family | Class |
| 103573471 | 103573625 | - | 16.7 | 1398 | 1556 | -2 | Looper | PiggyBac | DNA |
| 103573642 | 103574263 | - | 25.3 | 252 | 960 | -598 | Looper | PiggyBac | DNA |
| 103574313 | 103575441 | + | 7.0 | 1 | 1266 | 0 | MER11A | ERVK | LTR |
| 103575442 | 103576548 | + | 20.5 | 1391 | 2502 | -4286 | L1PA13 | L1 | LINE |
| 103576549 | 103576892 | + | 13.9 | 1 | 364 | 0 | THE1B | MaLR | LTR |
| 103576895 | 103578483 | + | 31.1 | 2 | 1735 | 0 | MLT1F2-int | MaLR | LTR |
| 103578484 | 103578828 | + | 14.2 | 1 | 364 | 0 | THE1B | MaLR | LTR |
| 103578829 | 103583167 | + | 16.3 | 2501 | 6770 | -16 | L1PA16 | L1 | LINE |
| 103583187 | 103583412 | - | 22.9 | 24 | 254 | -1304 | Looper | PiggyBac | DNA |

  

|  |  |  |  |  |  |  |  |  |  |
| --- | --- | --- | --- | --- | --- | --- | --- | --- | --- |
| chr2:103737403-103744796 | | | 7292 bp | | |  |  |  |  |
| Genome Start | Genome End | Strand | Div | Repeat Start | Repeat End | Repeat Left | Name | Family | Class |
| 103737403 | 103739426 | + | 20.5 | 5 | 2348 | -4986 | L1M4b | L1 | LINE |
| 103739451 | 103740366 | + | 20.8 | 2349 | 3330 | -3855 | L1M4b | L1 | LINE |
| 103740382 | 103740661 | - | 26.5 | 222 | 505 | 0 | MLT1D | MaLR | LTR |
| 103740662 | 103740943 | + | 14.5 | 1 | 282 | -30 | AluSx | Alu | SINE |
| 103740944 | 103741206 | - | 26.5 | 3 | 221 | -284 | MLT1D | MaLR | LTR |
| 103741221 | 103741737 | - | 26.7 | 969 | 1468 | -21 | MLT1D-int | MaLR | LTR |
| 103741758 | 103742753 | - | 26.7 | 4 | 968 | -521 | MLT1D-int | MaLR | LTR |
| 103742755 | 103743190 | - | 27.6 | 44 | 505 | 0 | MLT1D | MaLR | LTR |
| 103743219 | 103744796 | + | 20.7 | 3171 | 4708 | -2298 | L1M4b | L1 | LINE |

  

|  |  |  |  |  |  |  |  |  |  |
| --- | --- | --- | --- | --- | --- | --- | --- | --- | --- |
| chr2:105989202-105992555 | | | 3352 bp | | |  |  |  |  |
| Genome Start | Genome End | Strand | Div | Repeat Start | Repeat End | Repeat Left | Name | Family | Class |
| 105989202 | 105989452 | - | 19.0 | 217 | 661 | 0 | MER31B | ERV1 | LTR |
| 105989453 | 105989812 | + | 10.9 | 1 | 364 | 0 | THE1B | MaLR | LTR |
| 105989815 | 105991346 | + | 28.8 | 2 | 1735 | 0 | MLT-int | MaLR | LTR |
| 105991347 | 105991709 | + | 11.3 | 1 | 364 | 0 | THE1B | MaLR | LTR |
| 105991710 | 105991753 | - | 20.0 | 177 | 216 | -250 | MER31B | ERV1 | LTR |
| 105991754 | 105992369 | - | 15.2 | 1 | 634 | 0 | MER51A | ERV1 | LTR |
| 105992370 | 105992555 | - | 20.0 | 1 | 176 | -290 | MER31B | ERV1 | LTR |

  

|  |  |  |  |  |  |  |  |  |  |
| --- | --- | --- | --- | --- | --- | --- | --- | --- | --- |
| chr2:106582091-106586313 | | | 4151 bp | | |  |  |  |  |
| Genome Start | Genome End | Strand | Div | Repeat Start | Repeat End | Repeat Left | Name | Family | Class |
| 106582091 | 106583754 | - | 20.7 | 4551 | 6178 | 0 | L1MB8 | L1 | LINE |
| 106583756 | 106583955 | - | 19.8 | 218 | 425 | -1 | MSTB | MaLR | LTR |
| 106583982 | 106584181 | - | 19.8 | 3 | 217 | -209 | MSTB | MaLR | LTR |
| 106584187 | 106585791 | - | 20.1 | 1 | 1645 | -6 | MSTB-int | MaLR | LTR |
| 106585793 | 106586197 | - | 16.7 | 1 | 425 | -1 | MSTB | MaLR | LTR |
| 106586237 | 106586313 | - | 14.7 | 4433 | 4509 | -1637 | L1MB8 | L1 | LINE |

  

|  |  |  |  |  |  |  |  |  |  |
| --- | --- | --- | --- | --- | --- | --- | --- | --- | --- |
| chr2:106644917-106648172 | | | 3194 bp | | |  |  |  |  |
| Genome Start | Genome End | Strand | Div | Repeat Start | Repeat End | Repeat Left | Name | Family | Class |
| 106644917 | 106645063 | - | 26.1 | 5903 | 6060 | -180 | L1ME3B | L1 | LINE |
| 106645092 | 106645377 | - | 11.6 | 1 | 293 | -17 | AluSg | Alu | SINE |
| 106645378 | 106645667 | - | 26.1 | 5591 | 5902 | -338 | L1ME3B | L1 | LINE |
| 106645677 | 106646042 | + | 16.1 | 1 | 378 | -3 | THE1D | MaLR | LTR |
| 106646048 | 106647536 | + | 11.8 | 3 | 1580 | 0 | THE1D-int | MaLR | LTR |
| 106647537 | 106647904 | + | 14.2 | 1 | 381 | 0 | THE1D | MaLR | LTR |
| 106647925 | 106648172 | - | 29.1 | 3526 | 3791 | -2355 | L1ME3B | L1 | LINE |

  

|  |  |  |  |  |  |  |  |  |  |
| --- | --- | --- | --- | --- | --- | --- | --- | --- | --- |
| chr2:106652460-106656255 | | | 3647 bp | | |  |  |  |  |
| Genome Start | Genome End | Strand | Div | Repeat Start | Repeat End | Repeat Left | Name | Family | Class |
| 106652460 | 106652669 | - | 29.1 | 5859 | 6081 | -159 | L1ME3B | L1 | LINE |
| 106652684 | 106652706 | - | 26.4 | 6257 | 6279 | -24 | L1MA4A | L1 | LINE |
| 106652707 | 106652824 | - | 24.5 | 6014 | 6127 | -29 | L1PB4 | L1 | LINE |
| 106652825 | 106653232 | + | 15.0 | 3 | 424 | -4 | MSTA | MaLR | LTR |
| 106653239 | 106654848 | + | 30.2 | 2 | 1735 | 0 | MLT-int | MaLR | LTR |
| 106654851 | 106655251 | + | 14.2 | 3 | 428 | 0 | MSTA | MaLR | LTR |
| 106655252 | 106655282 | - | 24.5 | 5985 | 6013 | -143 | L1PB4 | L1 | LINE |
| 106655308 | 106655467 | - | 25.9 | 5668 | 5841 | -399 | L1ME3B | L1 | LINE |
| 106655570 | 106656255 | - | 27.7 | 4698 | 5470 | -770 | L1ME3B | L1 | LINE |

  

|  |  |  |  |  |  |  |  |  |  |
| --- | --- | --- | --- | --- | --- | --- | --- | --- | --- |
| chr2:108023737-108029387 | | | 5507 bp | | |  |  |  |  |
| Genome Start | Genome End | Strand | Div | Repeat Start | Repeat End | Repeat Left | Name | Family | Class |
| 108023737 | 108024043 | + | 26.7 | 1700 | 2002 | -4631 | L1MDa | L1 | LINE |
| 108024044 | 108024163 | + | 24.2 | 1 | 132 | -1 | FLAM\_A | Alu | SINE |
| 108024164 | 108024200 | + | 26.7 | 2003 | 2038 | -4595 | L1MDa | L1 | LINE |
| 108024201 | 108024569 | + | 14.2 | 1 | 364 | 0 | THE1B | MaLR | LTR |
| 108024583 | 108025810 | + | 12.0 | 342 | 1580 | 0 | THE1B-int | MaLR | LTR |
| 108025811 | 108026168 | + | 13.8 | 1 | 364 | 0 | THE1B | MaLR | LTR |
| 108026194 | 108026947 | + | 30.5 | 2155 | 2841 | -3696 | L1MDa | L1 | LINE |
| 108026917 | 108027496 | + | 28.2 | 4218 | 4826 | -1711 | L1MDa | L1 | LINE |
| 108027498 | 108028255 | - | 15.1 | 545 | 1302 | 0 | HSMAR2 | Mariner | DNA |
| 108028391 | 108028926 | - | 18.8 | 1 | 545 | -757 | HSMAR2 | Mariner | DNA |
| 108028928 | 108029387 | + | 24.7 | 4827 | 5340 | -1197 | L1MDa | L1 | LINE |

  

|  |  |  |  |  |  |  |  |  |  |
| --- | --- | --- | --- | --- | --- | --- | --- | --- | --- |
| chr2:112513302-112520967 | | | 7493 bp | | |  |  |  |  |
| Genome Start | Genome End | Strand | Div | Repeat Start | Repeat End | Repeat Left | Name | Family | Class |
| 112513302 | 112513363 | - | 15.0 | 1643 | 1702 | -4698 | L1MEc | L1 | LINE |
| 112513394 | 112513718 | - | 6.2 | 1 | 326 | -454 | LTR17 | ERV1 | LTR |
| 112513719 | 112514012 | - | 6.8 | 8335 | 8626 | 0 | HERV17 | ERV1 | LTR |
| 112514003 | 112514041 | - | 5.1 | 7510 | 7548 | -1078 | HERV17 | ERV1 | LTR |
| 112514035 | 112514276 | - | 5.3 | 5778 | 6019 | -2607 | HERV17 | ERV1 | LTR |
| 112514277 | 112514722 | + | 8.6 | 1 | 447 | -3 | LTR7 | ERV1 | LTR |
| 112514723 | 112516414 | - | 5.9 | 2430 | 5777 | -2849 | HERV17 | ERV1 | LTR |
| 112516398 | 112517667 | - | 6.8 | 757 | 2016 | -6610 | HERV17 | ERV1 | LTR |
| 112517826 | 112518524 | - | 5.7 | 1 | 703 | -7923 | HERV17 | ERV1 | LTR |
| 112518540 | 112519037 | - | 9.3 | 256 | 774 | -6 | LTR17 | ERV1 | LTR |
| 112519042 | 112519069 | - | 19.7 | 1568 | 1589 | -4811 | L1MEc | L1 | LINE |
| 112519070 | 112519367 | - | 16.4 | 1 | 298 | -14 | AluSx | Alu | SINE |
| 112519368 | 112520967 | - | 19.7 | 35 | 1567 | -4833 | L1MEc | L1 | LINE |

  

|  |  |  |  |  |  |  |  |  |  |
| --- | --- | --- | --- | --- | --- | --- | --- | --- | --- |
| chr2:114820389-114825619 | | | 5228 bp | | |  |  |  |  |
| Genome Start | Genome End | Strand | Div | Repeat Start | Repeat End | Repeat Left | Name | Family | Class |
| 114820389 | 114820703 | + | 26.8 | 2102 | 2435 | -984 | L2 | L2 | LINE |
| 114820704 | 114821100 | + | 21.2 | 1 | 381 | -182 | MER68 | ERVL | LTR |
| 114821101 | 114821396 | - | 10.9 | 1 | 293 | -17 | AluSg | Alu | SINE |
| 114821397 | 114821588 | + | 21.2 | 382 | 563 | 0 | MER68 | ERVL | LTR |
| 114821589 | 114821791 | + | 26.8 | 2436 | 2570 | -849 | L2 | L2 | LINE |
| 114821792 | 114822175 | - | 20.3 | 1 | 396 | 0 | MSTD | MaLR | LTR |
| 114822177 | 114822428 | - | 23.1 | 1463 | 1734 | -1 | MSTD-int | MaLR | LTR |
| 114822429 | 114822734 | + | 15.4 | 1 | 292 | -20 | AluJb | Alu | SINE |
| 114822735 | 114824153 | - | 23.1 | 2 | 1462 | -273 | MSTD-int | MaLR | LTR |
| 114824156 | 114824502 | - | 19.9 | 1 | 396 | 0 | MSTD | MaLR | LTR |
| 114824503 | 114825071 | + | 27.4 | 2571 | 3259 | -160 | L2 | L2 | LINE |
| 114825072 | 114825449 | + | 17.0 | 4 | 390 | 0 | MLT1B | MaLR | LTR |
| 114825450 | 114825619 | + | 28.9 | 3260 | 3418 | -1 | L2 | L2 | LINE |

  

|  |  |  |  |  |  |  |  |  |  |
| --- | --- | --- | --- | --- | --- | --- | --- | --- | --- |
| chr2:115727402-115731322 | | | 3919 bp | | |  |  |  |  |
| Genome Start | Genome End | Strand | Div | Repeat Start | Repeat End | Repeat Left | Name | Family | Class |
| 115727402 | 115728132 | - | 19.4 | 874 | 1631 | -20 | MSTB-int | MaLR | LTR |
| 115728133 | 115728503 | - | 13.1 | 1 | 375 | 0 | THE1C | MaLR | LTR |
| 115728504 | 115730082 | - | 31.0 | 2 | 1735 | 0 | MLT1H-int | MaLR | LTR |
| 115730085 | 115730459 | - | 10.7 | 1 | 375 | 0 | THE1C | MaLR | LTR |
| 115730460 | 115731322 | - | 19.4 | 1 | 873 | -778 | MSTB-int | MaLR | LTR |

  

|  |  |  |  |  |  |  |  |  |  |
| --- | --- | --- | --- | --- | --- | --- | --- | --- | --- |
| chr2:116463020-116472699 | | | 9645 bp | | |  |  |  |  |
| Genome Start | Genome End | Strand | Div | Repeat Start | Repeat End | Repeat Left | Name | Family | Class |
| 116463020 | 116463902 | - | 12.7 | 6862 | 7724 | 0 | L1MA3 | L1 | LINE |
| 116463903 | 116464183 | - | 14.2 | 9 | 298 | -14 | AluSx | Alu | SINE |
| 116464184 | 116465875 | - | 12.9 | 5173 | 6861 | -863 | L1MA3 | L1 | LINE |
| 116465873 | 116466605 | - | 13.9 | 3747 | 4498 | -3067 | L1MA3 | L1 | LINE |
| 116466606 | 116467005 | + | 19.2 | 1 | 428 | 0 | MSTA | MaLR | LTR |
| 116467006 | 116467353 | + | 19.4 | 1 | 335 | -1316 | MSTA-int | MaLR | LTR |
| 116467354 | 116467830 | + | 3.8 | 5665 | 6141 | -13 | L1PA5 | L1 | LINE |
| 116467831 | 116469010 | + | 19.4 | 336 | 1519 | -132 | MSTA-int | MaLR | LTR |
| 116469011 | 116469684 | - | 7.1 | 5476 | 6154 | 0 | L1PA6 | L1 | LINE |
| 116469685 | 116469700 | + | 19.4 | 1520 | 1530 | -121 | MSTA-int | MaLR | LTR |
| 116469730 | 116469897 | + | 19.4 | 1531 | 1650 | -1 | MSTA-int | MaLR | LTR |
| 116469905 | 116470303 | + | 19.6 | 8 | 428 | 0 | MSTA | MaLR | LTR |
| 116470306 | 116470714 | - | 13.8 | 3346 | 3749 | -3816 | L1MA3 | L1 | LINE |
| 116470715 | 116471085 | - | 8.4 | 5750 | 6127 | -4 | L1PA8A | L1 | LINE |
| 116471086 | 116472699 | - | 13.8 | 1725 | 3345 | -4220 | L1MA3 | L1 | LINE |

  

|  |  |  |  |  |  |  |  |  |  |
| --- | --- | --- | --- | --- | --- | --- | --- | --- | --- |
| chr2:116592734-116600527 | | | 7780 bp | | |  |  |  |  |
| Genome Start | Genome End | Strand | Div | Repeat Start | Repeat End | Repeat Left | Name | Family | Class |
| 116592734 | 116593015 | - | 14.0 | 1302 | 1580 | 0 | MSTA-int | MaLR | LTR |
| 116593016 | 116593366 | + | 12.7 | 1 | 355 | 0 | THE1A | MaLR | LTR |
| 116593369 | 116593741 | + | 8.6 | 3 | 375 | -1205 | THE1A-int | MaLR | LTR |
| 116593754 | 116597610 | - | 2.7 | 2292 | 6155 | 0 | L1PA3 | L1 | LINE |
| 116597611 | 116598828 | + | 11.0 | 357 | 1580 | 0 | THE1A-int | MaLR | LTR |
| 116598829 | 116599183 | + | 14.5 | 1 | 355 | 0 | THE1A | MaLR | LTR |
| 116599184 | 116600527 | - | 18.1 | 3 | 1306 | -274 | MSTA-int | MaLR | LTR |

  

|  |  |  |  |  |  |  |  |  |  |
| --- | --- | --- | --- | --- | --- | --- | --- | --- | --- |
| chr2:116637204-116640202 | | | 2985 bp | | |  |  |  |  |
| Genome Start | Genome End | Strand | Div | Repeat Start | Repeat End | Repeat Left | Name | Family | Class |
| 116637204 | 116637442 | - | 13.5 | 214 | 477 | -118 | LTR49 | ERV1 | LTR |
| 116637444 | 116637792 | + | 10.6 | 1 | 355 | 0 | THE1A | MaLR | LTR |
| 116637795 | 116639050 | + | 30.9 | 2 | 1369 | -366 | MLT-int | MaLR | LTR |
| 116639051 | 116639302 | - | 10.7 | 39 | 292 | -21 | AluSq | Alu | SINE |
| 116639303 | 116639626 | + | 30.9 | 1370 | 1735 | 0 | MLT-int | MaLR | LTR |
| 116639629 | 116639970 | + | 10.1 | 3 | 347 | -8 | THE1A | MaLR | LTR |
| 116639980 | 116640202 | - | 18.4 | 1 | 217 | -378 | LTR49 | ERV1 | LTR |

  

|  |  |  |  |  |  |  |  |  |  |
| --- | --- | --- | --- | --- | --- | --- | --- | --- | --- |
| chr2:116663757-116671142 | | | 7380 bp | | |  |  |  |  |
| Genome Start | Genome End | Strand | Div | Repeat Start | Repeat End | Repeat Left | Name | Family | Class |
| 116663757 | 116664588 | - | 11.6 | 4668 | 5450 | -2115 | L1M1 | L1 | LINE |
| 116664589 | 116664995 | - | 8.1 | 1 | 448 | -2 | LTR7 | ERV1 | LTR |
| 116664996 | 116665124 | - | 11.6 | 4537 | 4667 | -2898 | L1M1 | L1 | LINE |
| 116665126 | 116667308 | - | 15.0 | 1731 | 3973 | -3592 | L1M1 | L1 | LINE |
| 116667309 | 116667675 | - | 15.4 | 3 | 381 | 0 | THE1D | MaLR | LTR |
| 116667679 | 116669187 | - | 32.1 | 2 | 1734 | -1 | MLT1B-int | MaLR | LTR |
| 116669190 | 116669565 | - | 14.1 | 1 | 381 | 0 | THE1D | MaLR | LTR |
| 116669566 | 116671142 | - | 20.7 | 12 | 1730 | -5835 | L1M1 | L1 | LINE |

  

|  |  |  |  |  |  |  |  |  |  |
| --- | --- | --- | --- | --- | --- | --- | --- | --- | --- |
| chr2:116879933-116883249 | | | 3315 bp | | |  |  |  |  |
| Genome Start | Genome End | Strand | Div | Repeat Start | Repeat End | Repeat Left | Name | Family | Class |
| 116879933 | 116880510 | + | 28.9 | 6704 | 7400 | -642 | L1MC4 | L1 | LINE |
| 116880511 | 116880866 | + | 10.4 | 1 | 364 | 0 | THE1B | MaLR | LTR |
| 116880869 | 116882467 | + | 31.4 | 2 | 1735 | 0 | MLT-int | MaLR | LTR |
| 116882468 | 116882820 | + | 13.1 | 1 | 364 | 0 | THE1B | MaLR | LTR |
| 116882821 | 116883249 | + | 28.9 | 7401 | 7830 | -212 | L1MC4 | L1 | LINE |

  

|  |  |  |  |  |  |  |  |  |  |
| --- | --- | --- | --- | --- | --- | --- | --- | --- | --- |
| chr2:116904991-116908755 | | | 3359 bp | | |  |  |  |  |
| Genome Start | Genome End | Strand | Div | Repeat Start | Repeat End | Repeat Left | Name | Family | Class |
| 116904991 | 116905378 | + | 30.9 | 2178 | 2627 | -3519 | L1M5 | L1 | LINE |
| 116905390 | 116905958 | - | 33.9 | 2597 | 3416 | -3 | L2 | L2 | LINE |
| 116906128 | 116906491 | - | 14.4 | 1 | 364 | 0 | THE1B | MaLR | LTR |
| 116906495 | 116908078 | - | 30.9 | 2 | 1731 | -4 | MLT-int | MaLR | LTR |
| 116908081 | 116908429 | - | 18.1 | 1 | 364 | 0 | THE1B | MaLR | LTR |
| 116908651 | 116908755 | + | 23.8 | 2933 | 3054 | -3092 | L1M5 | L1 | LINE |

  

|  |  |  |  |  |  |  |  |  |  |
| --- | --- | --- | --- | --- | --- | --- | --- | --- | --- |
| chr2:116915612-116921124 | | | 5421 bp | | |  |  |  |  |
| Genome Start | Genome End | Strand | Div | Repeat Start | Repeat End | Repeat Left | Name | Family | Class |
| 116915612 | 116915907 | + | 23.6 | 5460 | 5747 | -2038 | L1MC3 | L1 | LINE |
| 116915908 | 116916298 | + | 20.2 | 3 | 398 | -28 | MSTB | MaLR | LTR |
| 116916299 | 116916532 | - | 10.7 | 1 | 234 | -77 | AluY | Alu | SINE |
| 116916533 | 116916572 | + | 20.2 | 399 | 426 | 0 | MSTB | MaLR | LTR |
| 116916573 | 116916637 | + | 23.6 | 5748 | 5807 | -1978 | L1MC3 | L1 | LINE |
| 116916638 | 116916986 | - | 18.1 | 1 | 408 | 0 | MLT1A1 | MaLR | LTR |
| 116916987 | 116918000 | + | 23.6 | 5808 | 6866 | -919 | L1MC3 | L1 | LINE |
| 116918002 | 116918375 | - | 19.4 | 1 | 428 | 0 | MSTA | MaLR | LTR |
| 116918376 | 116919995 | - | 16.0 | 3 | 1580 | 0 | MSTA-int | MaLR | LTR |
| 116919998 | 116920348 | - | 17.8 | 3 | 428 | 0 | MSTA | MaLR | LTR |
| 116920438 | 116921124 | + | 19.9 | 6995 | 7765 | -20 | L1MC3 | L1 | LINE |

  

|  |  |  |  |  |  |  |  |  |  |
| --- | --- | --- | --- | --- | --- | --- | --- | --- | --- |
| chr2:117075424-117079542 | | | 4122 bp | | |  |  |  |  |
| Genome Start | Genome End | Strand | Div | Repeat Start | Repeat End | Repeat Left | Name | Family | Class |
| 117075424 | 117075817 | - | 14.1 | 2015 | 2418 | 0 | Tigger1 | MER2\_type | DNA |
| 117075815 | 117075855 | - | 14.3 | 415 | 455 | -1963 | Tigger1 | MER2\_type | DNA |
| 117075856 | 117076210 | + | 10.8 | 1 | 355 | 0 | THE1A | MaLR | LTR |
| 117076220 | 117076966 | + | 10.6 | 1 | 861 | -719 | THE1A-int | MaLR | LTR |
| 117076967 | 117078238 | + | 7.5 | 4868 | 6151 | -3 | L1PA7 | L1 | LINE |
| 117078239 | 117078383 | + | 11.0 | 846 | 990 | -590 | THE1A-int | MaLR | LTR |
| 117078375 | 117078776 | + | 9.0 | 1174 | 1580 | 0 | THE1A-int | MaLR | LTR |
| 117078777 | 117079127 | + | 13.2 | 1 | 355 | 0 | THE1A | MaLR | LTR |
| 117079128 | 117079542 | - | 15.3 | 1 | 418 | -2000 | Tigger1 | MER2\_type | DNA |

  

|  |  |  |  |  |  |  |  |  |  |
| --- | --- | --- | --- | --- | --- | --- | --- | --- | --- |
| chr2:117126891-117136252 | | | 9282 bp | | |  |  |  |  |
| Genome Start | Genome End | Strand | Div | Repeat Start | Repeat End | Repeat Left | Name | Family | Class |
| 117126891 | 117129006 | + | 19.1 | 11 | 2132 | -5433 | L1M1 | L1 | LINE |
| 117129007 | 117129381 | - | 14.2 | 1 | 364 | 0 | THE1B | MaLR | LTR |
| 117129382 | 117130953 | - | 10.6 | 1 | 1580 | 0 | THE1B-int | MaLR | LTR |
| 117130954 | 117131328 | - | 15.1 | 1 | 364 | 0 | THE1B | MaLR | LTR |
| 117131329 | 117131667 | + | 15.5 | 2128 | 2455 | -5110 | L1M1 | L1 | LINE |
| 117131692 | 117133130 | + | 15.7 | 2456 | 3952 | -3613 | L1M1 | L1 | LINE |
| 117133131 | 117133397 | - | 15.4 | 84 | 428 | 0 | MER61C | ERV1 | LTR |
| 117133411 | 117133658 | + | 14.3 | 3947 | 4194 | -3371 | L1M1 | L1 | LINE |
| 117133659 | 117133966 | + | 13.7 | 1 | 309 | -4 | AluSq | Alu | SINE |
| 117133967 | 117135514 | + | 14.3 | 4195 | 5668 | -1897 | L1M1 | L1 | LINE |
| 117135558 | 117136252 | + | 14.3 | 5669 | 6364 | -1201 | L1M1 | L1 | LINE |

  

|  |  |  |  |  |  |  |  |  |  |
| --- | --- | --- | --- | --- | --- | --- | --- | --- | --- |
| chr2:117195830-117198489 | | | 2658 bp | | |  |  |  |  |
| Genome Start | Genome End | Strand | Div | Repeat Start | Repeat End | Repeat Left | Name | Family | Class |
| 117195830 | 117195941 | + | 19.2 | 1 | 112 | -200 | AluJb | Alu | SINE |
| 117195942 | 117196309 | - | 17.7 | 1 | 428 | 0 | MSTA | MaLR | LTR |
| 117196310 | 117197907 | - | 30.0 | 2 | 1735 | 0 | MLT-int | MaLR | LTR |
| 117197910 | 117198303 | - | 15.9 | 1 | 428 | 0 | MSTA | MaLR | LTR |
| 117198304 | 117198489 | + | 19.2 | 113 | 286 | -26 | AluJb | Alu | SINE |

  

|  |  |  |  |  |  |  |  |  |  |
| --- | --- | --- | --- | --- | --- | --- | --- | --- | --- |
| chr2:117503168-117508548 | | | 5378 bp | | |  |  |  |  |
| Genome Start | Genome End | Strand | Div | Repeat Start | Repeat End | Repeat Left | Name | Family | Class |
| 117503168 | 117505849 | + | 19.7 | 24 | 3575 | -4028 | L1PA12 | L1 | LINE |
| 117505850 | 117506204 | - | 13.6 | 1 | 355 | 0 | THE1A | MaLR | LTR |
| 117506205 | 117507778 | - | 31.6 | 2 | 1735 | 0 | MLT-int | MaLR | LTR |
| 117507782 | 117508134 | - | 15.1 | 1 | 354 | -1 | THE1A | MaLR | LTR |
| 117508135 | 117508548 | + | 12.3 | 3576 | 3979 | -3602 | L1PA12 | L1 | LINE |

  

|  |  |  |  |  |  |  |  |  |  |
| --- | --- | --- | --- | --- | --- | --- | --- | --- | --- |
| chr2:117604247-117610692 | | | 6426 bp | | |  |  |  |  |
| Genome Start | Genome End | Strand | Div | Repeat Start | Repeat End | Repeat Left | Name | Family | Class |
| 117604247 | 117604299 | - | 24.2 | 554 | 622 | 0 | LTR29 | ERV1 | LTR |
| 117604300 | 117604600 | + | 1.0 | 1 | 301 | -10 | AluY | Alu | SINE |
| 117604601 | 117604674 | - | 24.2 | 480 | 553 | -69 | LTR29 | ERV1 | LTR |
| 117604675 | 117605042 | + | 11.5 | 1 | 450 | 0 | LTR7 | ERV1 | LTR |
| 117605043 | 117605478 | + | 19.4 | 1 | 459 | -7254 | HERVH | ERV1 | LTR |
| 117605537 | 117607193 | + | 15.5 | 800 | 2419 | -5294 | HERVH | ERV1 | LTR |
| 117607190 | 117607977 | + | 19.3 | 3608 | 4336 | -2287 | HERVH | ERV1 | LTR |
| 117607966 | 117608154 | + | 13.8 | 6758 | 6948 | -765 | HERVH | ERV1 | LTR |
| 117608132 | 117608204 | + | 11.8 | 7638 | 7713 | 0 | HERVH | ERV1 | LTR |
| 117608206 | 117608758 | + | 11.4 | 7173 | 7713 | 0 | HERVH | ERV1 | LTR |
| 117608759 | 117609124 | + | 11.3 | 1 | 450 | 0 | LTR7 | ERV1 | LTR |
| 117609125 | 117609325 | - | 17.8 | 284 | 483 | -139 | LTR29 | ERV1 | LTR |
| 117609326 | 117609533 | - | 9.6 | 585 | 781 | 0 | MER4E1 | ERV1 | LTR |
| 117609534 | 117609851 | + | 1.6 | 1 | 318 | 0 | AluYb8 | Alu | SINE |
| 117609852 | 117610431 | - | 9.6 | 1 | 584 | -197 | MER4E1 | ERV1 | LTR |
| 117610432 | 117610692 | - | 17.8 | 9 | 283 | -339 | LTR29 | ERV1 | LTR |

  

|  |  |  |  |  |  |  |  |  |  |
| --- | --- | --- | --- | --- | --- | --- | --- | --- | --- |
| chr2:117616942-117622182 | | | 5129 bp | | |  |  |  |  |
| Genome Start | Genome End | Strand | Div | Repeat Start | Repeat End | Repeat Left | Name | Family | Class |
| 117616942 | 117617468 | - | 22.5 | 3802 | 4364 | -2642 | L1M4b | L1 | LINE |
| 117617518 | 117617890 | - | 26.3 | 2846 | 3244 | -4090 | L1M4b | L1 | LINE |
| 117617891 | 117618185 | + | 10.6 | 6 | 299 | -11 | AluSg | Alu | SINE |
| 117618186 | 117618965 | - | 26.5 | 1706 | 2845 | -4489 | L1M4b | L1 | LINE |
| 117618971 | 117619320 | - | 11.8 | 1 | 349 | -15 | THE1B | MaLR | LTR |
| 117619325 | 117620522 | - | 11.8 | 329 | 1576 | -4 | THE1B-int | MaLR | LTR |
| 117620523 | 117620816 | + | 10.5 | 1 | 295 | -18 | AluSp | Alu | SINE |
| 117620817 | 117621137 | - | 11.8 | 2 | 328 | -1252 | THE1B-int | MaLR | LTR |
| 117621138 | 117621350 | - | 11.6 | 154 | 364 | 0 | THE1B | MaLR | LTR |
| 117621351 | 117621663 | + | 8.2 | 1 | 309 | 0 | AluSc | Alu | SINE |
| 117621664 | 117621831 | - | 11.6 | 1 | 153 | -211 | THE1B | MaLR | LTR |
| 117621886 | 117622182 | - | 28.7 | 1357 | 1663 | -5671 | L1M4b | L1 | LINE |

  

|  |  |  |  |  |  |  |  |  |  |
| --- | --- | --- | --- | --- | --- | --- | --- | --- | --- |
| chr2:117751498-117766403 | | | 14832 bp | | |  |  |  |  |
| Genome Start | Genome End | Strand | Div | Repeat Start | Repeat End | Repeat Left | Name | Family | Class |
| 117751498 | 117752362 | + | 29.0 | 340 | 1239 | -4976 | L1MC4 | L1 | LINE |
| 117752448 | 117752793 | + | 29.0 | 1240 | 1641 | -4574 | L1MC4 | L1 | LINE |
| 117752799 | 117753249 | + | 5.1 | 1 | 448 | -2 | LTR7 | ERV1 | LTR |
| 117753252 | 117756200 | + | 8.9 | 1 | 2995 | -4718 | HERVH | ERV1 | LTR |
| 117756180 | 117756326 | + | 13.6 | 3140 | 3290 | -4423 | HERVH | ERV1 | LTR |
| 117756317 | 117756820 | + | 12.0 | 3492 | 3993 | -3720 | HERVH | ERV1 | LTR |
| 117756823 | 117757484 | + | 15.5 | 4488 | 5149 | -2564 | HERVH | ERV1 | LTR |
| 117757486 | 117757701 | + | 12.5 | 5600 | 5819 | -1894 | HERVH | ERV1 | LTR |
| 117757705 | 117758074 | + | 9.2 | 7327 | 7713 | 0 | HERVH | ERV1 | LTR |
| 117758075 | 117758555 | + | 5.4 | 1 | 448 | -2 | LTR7 | ERV1 | LTR |
| 117758589 | 117759059 | + | 25.4 | 1696 | 2183 | -4032 | L1MC4 | L1 | LINE |
| 117759047 | 117760438 | + | 20.5 | 2914 | 4272 | -3290 | L1MA4 | L1 | LINE |
| 117760439 | 117760737 | + | 5.8 | 1 | 294 | -17 | AluY | Alu | SINE |
| 117760738 | 117761564 | + | 18.8 | 4273 | 5108 | -2454 | L1MA4 | L1 | LINE |
| 117761565 | 117761874 | + | 9.4 | 1 | 310 | -2 | AluSx | Alu | SINE |
| 117761875 | 117762425 | + | 18.8 | 5109 | 5666 | -1896 | L1MA4 | L1 | LINE |
| 117762426 | 117762727 | + | 9.0 | 2 | 303 | -8 | AluY | Alu | SINE |
| 117762728 | 117763346 | + | 18.8 | 5667 | 6287 | -1275 | L1MA4 | L1 | LINE |
| 117763347 | 117763759 | - | 16.8 | 1 | 426 | 0 | MSTB | MaLR | LTR |
| 117763760 | 117764070 | + | 18.8 | 6288 | 6599 | -963 | L1MA4 | L1 | LINE |
| 117764078 | 117764300 | + | 22.0 | 5952 | 6170 | -130 | L1MA4 | L1 | LINE |
| 117764289 | 117764417 | + | 18.1 | 6598 | 6724 | -838 | L1MA4 | L1 | LINE |
| 117764418 | 117764626 | + | 18.4 | 1 | 213 | -855 | Tigger2b\_Pri | MER2\_type | DNA |
| 117764628 | 117765223 | + | 17.3 | 464 | 1068 | 0 | Tigger2b\_Pri | MER2\_type | DNA |
| 117765232 | 117766197 | + | 16.9 | 6742 | 7714 | -5 | L1MA4 | L1 | LINE |
| 117766181 | 117766403 | + | 26.2 | 2157 | 2385 | -3830 | L1MC4 | L1 | LINE |

  

|  |  |  |  |  |  |  |  |  |  |
| --- | --- | --- | --- | --- | --- | --- | --- | --- | --- |
| chr2:117833344-117842822 | | | 9453 bp | | |  |  |  |  |
| Genome Start | Genome End | Strand | Div | Repeat Start | Repeat End | Repeat Left | Name | Family | Class |
| 117833344 | 117838018 | + | 20.6 | 599 | 4394 | -2468 | L1PB3 | L1 | LINE |
| 117838041 | 117839394 | + | 17.7 | 4395 | 5759 | -1103 | L1PB3 | L1 | LINE |
| 117839395 | 117839755 | - | 10.4 | 3 | 364 | 0 | THE1B | MaLR | LTR |
| 117839758 | 117841323 | - | 29.9 | 2 | 1735 | 0 | MLT-int | MaLR | LTR |
| 117841326 | 117841692 | - | 13.9 | 1 | 364 | 0 | THE1B | MaLR | LTR |
| 117841693 | 117842822 | + | 15.7 | 5760 | 6865 | -5 | L1PB3 | L1 | LINE |

  

|  |  |  |  |  |  |  |  |  |  |
| --- | --- | --- | --- | --- | --- | --- | --- | --- | --- |
| chr2:118107786-118115008 | | | 6483 bp | | |  |  |  |  |
| Genome Start | Genome End | Strand | Div | Repeat Start | Repeat End | Repeat Left | Name | Family | Class |
| 118107786 | 118108366 | - | 36.3 | 4675 | 5277 | -390 | ERVL-B4 | ERVL | LTR |
| 118108412 | 118108882 | - | 30.8 | 4154 | 4633 | -1081 | ERVL-B4 | ERVL | LTR |
| 118109012 | 118109091 | - | 37.0 | 3827 | 3956 | -1801 | ERVL-B4 | ERVL | LTR |
| 118109172 | 118110353 | - | 37.0 | 2590 | 3826 | -1888 | ERVL-B4 | ERVL | LTR |
| 118110354 | 118110712 | + | 12.7 | 1 | 364 | 0 | THE1B | MaLR | LTR |
| 118110715 | 118112282 | + | 29.7 | 2 | 1735 | 0 | MLT-int | MaLR | LTR |
| 118112285 | 118112639 | + | 11.4 | 5 | 364 | 0 | THE1B | MaLR | LTR |
| 118112640 | 118114421 | - | 33.8 | 644 | 2589 | -3125 | ERVL-B4 | ERVL | LTR |
| 118114904 | 118115008 | - | 30.5 | 1 | 108 | -4888 | ERVL-B4 | ERVL | LTR |

  

|  |  |  |  |  |  |  |  |  |  |
| --- | --- | --- | --- | --- | --- | --- | --- | --- | --- |
| chr2:118354195-118359451 | | | 5191 bp | | |  |  |  |  |
| Genome Start | Genome End | Strand | Div | Repeat Start | Repeat End | Repeat Left | Name | Family | Class |
| 118354195 | 118354214 | - | 17.5 | 6227 | 6246 | -45 | L1MA7 | L1 | LINE |
| 118354248 | 118354685 | - | 17.5 | 5790 | 6226 | -65 | L1MA7 | L1 | LINE |
| 118354710 | 118355750 | - | 17.2 | 4765 | 5789 | -502 | L1MA7 | L1 | LINE |
| 118355751 | 118356112 | + | 12.8 | 1 | 375 | 0 | THE1C | MaLR | LTR |
| 118356113 | 118357685 | + | 11.5 | 1 | 1572 | -8 | THE1C-int | MaLR | LTR |
| 118357695 | 118358046 | + | 13.6 | 2 | 375 | 0 | THE1C | MaLR | LTR |
| 118358047 | 118358581 | - | 13.1 | 4210 | 4769 | -1374 | L1MA7 | L1 | LINE |
| 118358582 | 118358865 | + | 12.7 | 3 | 285 | -24 | AluSc | Alu | SINE |
| 118358866 | 118359451 | - | 13.1 | 3666 | 4209 | -1934 | L1MA7 | L1 | LINE |

  

|  |  |  |  |  |  |  |  |  |  |
| --- | --- | --- | --- | --- | --- | --- | --- | --- | --- |
| chr2:119893118-119896834 | | | 3662 bp | | |  |  |  |  |
| Genome Start | Genome End | Strand | Div | Repeat Start | Repeat End | Repeat Left | Name | Family | Class |
| 119893118 | 119893505 | + | 20.2 | 10 | 374 | -191 | MER34B | ERV1 | LTR |
| 119893506 | 119893540 | - | 18.8 | 400 | 430 | -2 | MSTB1 | MaLR | LTR |
| 119893541 | 119893839 | + | 8.7 | 1 | 298 | -13 | AluY | Alu | SINE |
| 119893840 | 119894235 | - | 18.8 | 1 | 399 | -33 | MSTB1 | MaLR | LTR |
| 119894236 | 119894574 | - | 11.5 | 1306 | 1651 | 0 | MSTB1-int | MaLR | LTR |
| 119894575 | 119894992 | - | 12.5 | 1 | 437 | 0 | LTR23 | ERV1 | LTR |
| 119894993 | 119896246 | - | 20.3 | 3 | 1305 | -346 | MSTB1-int | MaLR | LTR |
| 119896247 | 119896654 | - | 14.0 | 2 | 430 | -2 | MSTB1 | MaLR | LTR |
| 119896710 | 119896834 | + | 15.0 | 445 | 563 | -2 | MER34B | ERV1 | LTR |

  

|  |  |  |  |  |  |  |  |  |  |
| --- | --- | --- | --- | --- | --- | --- | --- | --- | --- |
| chr2:121845727-121849901 | | | 3600 bp | | |  |  |  |  |
| Genome Start | Genome End | Strand | Div | Repeat Start | Repeat End | Repeat Left | Name | Family | Class |
| 121845727 | 121845887 | + | 30.8 | 1587 | 1756 | -1663 | L2 | L2 | LINE |
| 121846225 | 121846722 | + | 32.8 | 2023 | 2606 | -813 | L2 | L2 | LINE |
| 121846725 | 121846938 | + | 32.7 | 2761 | 2987 | -432 | L2 | L2 | LINE |
| 121847084 | 121847469 | + | 13.9 | 1 | 428 | 0 | MSTA | MaLR | LTR |
| 121847472 | 121849072 | + | 30.1 | 2 | 1735 | 0 | MLT1A-int | MaLR | LTR |
| 121849073 | 121849407 | + | 16.0 | 1 | 391 | -37 | MSTA | MaLR | LTR |
| 121849408 | 121849707 | - | 9.0 | 1 | 300 | -9 | AluSc | Alu | SINE |
| 121849708 | 121849745 | + | 16.0 | 392 | 428 | 0 | MSTA | MaLR | LTR |
| 121849835 | 121849901 | + | 32.3 | 3242 | 3307 | -71 | L2 | L2 | LINE |

  

|  |  |  |  |  |  |  |  |  |  |
| --- | --- | --- | --- | --- | --- | --- | --- | --- | --- |
| chr2:122590613-122606277 | | | 15513 bp | | |  |  |  |  |
| Genome Start | Genome End | Strand | Div | Repeat Start | Repeat End | Repeat Left | Name | Family | Class |
| 122590613 | 122592971 | - | 16.7 | 4945 | 7337 | -28 | L1MA4A | L1 | LINE |
| 122593002 | 122595147 | - | 8.4 | 4291 | 6451 | -18 | L1PA7 | L1 | LINE |
| 122595148 | 122595462 | - | 11.6 | 2 | 319 | 0 | AluY | Alu | SINE |
| 122595463 | 122599229 | - | 10.3 | 415 | 4290 | -2171 | L1PA7 | L1 | LINE |
| 122599251 | 122599652 | - | 11.6 | 6 | 414 | -6047 | L1PA7 | L1 | LINE |
| 122599659 | 122600229 | - | 17.7 | 4379 | 4960 | -2245 | L1MA4A | L1 | LINE |
| 122600230 | 122600607 | + | 12.1 | 1 | 428 | 0 | MSTA | MaLR | LTR |
| 122600608 | 122601577 | + | 19.4 | 1 | 1000 | -651 | MSTA-int | MaLR | LTR |
| 122601574 | 122601686 | + | 10.7 | 1539 | 1651 | 0 | MSTA-int | MaLR | LTR |
| 122601687 | 122601759 | + | 15.1 | 1 | 73 | -355 | MSTA | MaLR | LTR |
| 122601757 | 122601917 | + | 16.7 | 259 | 428 | 0 | MSTA | MaLR | LTR |
| 122601918 | 122603066 | - | 19.4 | 3287 | 4386 | -2822 | L1MA4A | L1 | LINE |
| 122603168 | 122603231 | - | 9.4 | 231 | 294 | -17 | Alu | Alu | SINE |
| 122603233 | 122606277 | - | 22.9 | 19 | 3255 | -3954 | L1MA4A | L1 | LINE |

  

|  |  |  |  |  |  |  |  |  |  |
| --- | --- | --- | --- | --- | --- | --- | --- | --- | --- |
| chr2:123604708-123607516 | | | 2726 bp | | |  |  |  |  |
| Genome Start | Genome End | Strand | Div | Repeat Start | Repeat End | Repeat Left | Name | Family | Class |
| 123604708 | 123605138 | + | 26.6 | 21 | 485 | -35 | MLT2B1 | ERVL | LTR |
| 123605216 | 123605583 | + | 11.3 | 1 | 364 | 0 | THE1B | MaLR | LTR |
| 123605584 | 123607101 | + | 13.7 | 1 | 1556 | -24 | THE1B-int | MaLR | LTR |
| 123607108 | 123607472 | + | 12.4 | 1 | 364 | 0 | THE1B | MaLR | LTR |
| 123607473 | 123607516 | + | 11.4 | 517 | 560 | 0 | MLT2B1 | ERVL | LTR |

  

|  |  |  |  |  |  |  |  |  |  |
| --- | --- | --- | --- | --- | --- | --- | --- | --- | --- |
| chr2:125690126-125699553 | | | 9424 bp | | |  |  |  |  |
| Genome Start | Genome End | Strand | Div | Repeat Start | Repeat End | Repeat Left | Name | Family | Class |
| 125690126 | 125695565 | - | 20.6 | 1332 | 6939 | 0 | L1MA5A | L1 | LINE |
| 125695566 | 125695935 | - | 15.9 | 1 | 364 | 0 | THE1B | MaLR | LTR |
| 125695936 | 125697505 | - | 30.4 | 2 | 1735 | 0 | MLT-int | MaLR | LTR |
| 125697510 | 125697865 | - | 14.7 | 1 | 362 | -2 | THE1B | MaLR | LTR |
| 125697866 | 125699553 | - | 27.5 | 4 | 1380 | -5409 | L1MA5A | L1 | LINE |

  

|  |  |  |  |  |  |  |  |  |  |
| --- | --- | --- | --- | --- | --- | --- | --- | --- | --- |
| chr2:125949131-125953898 | | | 4732 bp | | |  |  |  |  |
| Genome Start | Genome End | Strand | Div | Repeat Start | Repeat End | Repeat Left | Name | Family | Class |
| 125949131 | 125949476 | + | 22.3 | 1 | 362 | -192 | MLT2B3 | ERVL | LTR |
| 125949478 | 125949844 | + | 15.9 | 1 | 364 | 0 | THE1B | MaLR | LTR |
| 125949845 | 125951419 | + | 9.8 | 1 | 1579 | -1 | THE1B-int | MaLR | LTR |
| 125951420 | 125951786 | + | 13.3 | 1 | 364 | 0 | THE1B | MaLR | LTR |
| 125951788 | 125951946 | + | 23.9 | 698 | 865 | -1183 | Ricksha | MuDR | DNA |
| 125951981 | 125952259 | + | 22.4 | 25 | 318 | -116 | Tigger2a | MER2\_type | DNA |
| 125952260 | 125953744 | - | 12.3 | 1 | 1578 | 0 | LTR12C | ERV1 | LTR |
| 125953745 | 125953850 | + | 22.4 | 319 | 432 | -2 | Tigger2a | MER2\_type | DNA |
| 125953851 | 125953898 | + | 8.3 | 516 | 562 | 0 | MLT2B3 | ERVL | LTR |

  

|  |  |  |  |  |  |  |  |  |  |
| --- | --- | --- | --- | --- | --- | --- | --- | --- | --- |
| chr2:126076486-126079983 | | | 3410 bp | | |  |  |  |  |
| Genome Start | Genome End | Strand | Div | Repeat Start | Repeat End | Repeat Left | Name | Family | Class |
| 126076486 | 126076703 | - | 18.8 | 124 | 345 | 0 | MER2B | MER2\_type | DNA |
| 126076704 | 126076896 | + | 18.9 | 80 | 268 | -44 | AluJb | Alu | SINE |
| 126076897 | 126077278 | + | 13.3 | 1 | 375 | 0 | THE1C | MaLR | LTR |
| 126077281 | 126078879 | + | 31.7 | 2 | 1734 | -1 | MLT-int | MaLR | LTR |
| 126078881 | 126079216 | + | 11.5 | 1 | 331 | -44 | THE1C | MaLR | LTR |
| 126079217 | 126079468 | + | 9.9 | 47 | 298 | -13 | AluY | Alu | SINE |
| 126079469 | 126079520 | + | 11.5 | 332 | 375 | 0 | THE1C | MaLR | LTR |
| 126079521 | 126079545 | + | 18.9 | 269 | 288 | -24 | AluJb | Alu | SINE |
| 126079586 | 126079876 | + | 15.1 | 1 | 299 | -11 | AluSg | Alu | SINE |
| 126079922 | 126079983 | - | 29.0 | 1 | 62 | -274 | MER2B | MER2\_type | DNA |

  

|  |  |  |  |  |  |  |  |  |  |
| --- | --- | --- | --- | --- | --- | --- | --- | --- | --- |
| chr2:126545534-126553713 | | | 8171 bp | | |  |  |  |  |
| Genome Start | Genome End | Strand | Div | Repeat Start | Repeat End | Repeat Left | Name | Family | Class |
| 126545534 | 126549396 | + | 10.4 | 1 | 3911 | -1743 | HERVL-A2 | ERVL | LTR |
| 126549397 | 126549788 | - | 12.5 | 1 | 375 | 0 | THE1C | MaLR | LTR |
| 126549789 | 126549855 | + | 9.9 | 3912 | 3976 | -1678 | HERVL-A2 | ERVL | LTR |
| 126549856 | 126550211 | + | 14.3 | 1 | 364 | 0 | THE1B | MaLR | LTR |
| 126550212 | 126551731 | + | 14.6 | 1 | 1580 | 0 | THE1B-int | MaLR | LTR |
| 126551732 | 126552038 | + | 12.4 | 1 | 364 | 0 | THE1B | MaLR | LTR |
| 126552048 | 126553713 | + | 13.2 | 3992 | 5654 | 0 | HERVL-A2 | ERVL | LTR |

  

|  |  |  |  |  |  |  |  |  |  |
| --- | --- | --- | --- | --- | --- | --- | --- | --- | --- |
| chr2:126623833-126629740 | | | 5907 bp | | |  |  |  |  |
| Genome Start | Genome End | Strand | Div | Repeat Start | Repeat End | Repeat Left | Name | Family | Class |
| 126623833 | 126624203 | - | 17.6 | 5949 | 6333 | 0 | L1MC1 | L1 | LINE |
| 126624204 | 126624574 | - | 20.2 | 4 | 381 | 0 | THE1D | MaLR | LTR |
| 126624578 | 126626101 | - | 12.4 | 1 | 1580 | 0 | THE1D-int | MaLR | LTR |
| 126626104 | 126626471 | - | 15.5 | 5 | 379 | -2 | THE1D | MaLR | LTR |
| 126626476 | 126626615 | - | 10.7 | 5814 | 5953 | -391 | L1MC1 | L1 | LINE |
| 126626612 | 126628450 | - | 11.6 | 4318 | 6160 | 0 | L1PREC2 | L1 | LINE |
| 126628447 | 126629740 | - | 19.0 | 4559 | 5823 | -510 | L1MC1 | L1 | LINE |

  

|  |  |  |  |  |  |  |  |  |  |
| --- | --- | --- | --- | --- | --- | --- | --- | --- | --- |
| chr2:126858178-126864981 | | | 6201 bp | | |  |  |  |  |
| Genome Start | Genome End | Strand | Div | Repeat Start | Repeat End | Repeat Left | Name | Family | Class |
| 126858178 | 126858305 | - | 20.3 | 6128 | 6264 | -486 | L1MD2 | L1 | LINE |
| 126858337 | 126859126 | - | 20.4 | 5361 | 6127 | -623 | L1MD2 | L1 | LINE |
| 126859128 | 126859677 | - | 24.1 | 3582 | 4148 | -2389 | L1MD2 | L1 | LINE |
| 126859667 | 126860616 | - | 24.8 | 1428 | 2404 | -4229 | L1MD2 | L1 | LINE |
| 126860617 | 126860842 | - | 13.9 | 1 | 230 | 0 | MER30 | MER1\_type | DNA |
| 126860843 | 126860923 | - | 24.8 | 1356 | 1427 | -5206 | L1MD2 | L1 | LINE |
| 126861031 | 126861388 | + | 16.1 | 3 | 381 | 0 | THE1D | MaLR | LTR |
| 126861391 | 126862679 | + | 14.3 | 3 | 1243 | -337 | THE1D-int | MaLR | LTR |
| 126862680 | 126863144 | - | 9.9 | 1 | 468 | -4 | MER4A1 | ERV1 | LTR |
| 126863145 | 126863484 | + | 14.3 | 1244 | 1580 | 0 | THE1D-int | MaLR | LTR |
| 126863486 | 126863859 | + | 12.7 | 2 | 381 | 0 | THE1D | MaLR | LTR |
| 126863889 | 126864162 | - | 20.1 | 974 | 1282 | -5258 | L1MD2 | L1 | LINE |
| 126864606 | 126864981 | - | 26.9 | 23 | 385 | -6248 | L1MD2 | L1 | LINE |

  

|  |  |  |  |  |  |  |  |  |  |
| --- | --- | --- | --- | --- | --- | --- | --- | --- | --- |
| chr2:126996614-127002216 | | | 5597 bp | | |  |  |  |  |
| Genome Start | Genome End | Strand | Div | Repeat Start | Repeat End | Repeat Left | Name | Family | Class |
| 126996614 | 126996921 | - | 17.2 | 5398 | 5692 | -22 | ERVL-B4 | ERVL | LTR |
| 126996922 | 126997506 | + | 10.3 | 5581 | 6171 | -3 | L1PA11 | L1 | LINE |
| 126997507 | 126997614 | - | 17.2 | 5290 | 5397 | -317 | ERVL-B4 | ERVL | LTR |
| 126997615 | 126997944 | - | 16.1 | 157 | 475 | -10 | MER31A | ERV1 | LTR |
| 126997945 | 126998015 | + | 14.9 | 228 | 294 | -18 | AluJ/FRAM | Alu | SINE |
| 126998016 | 126998175 | - | 16.1 | 3 | 156 | -329 | MER31A | ERV1 | LTR |
| 126998176 | 126998271 | - | 16.8 | 5204 | 5297 | -417 | ERVL-B4 | ERVL | LTR |
| 126998272 | 126998633 | - | 21.9 | 1 | 365 | 0 | MLT1A0 | MaLR | LTR |
| 126998634 | 126999381 | - | 16.8 | 4441 | 5203 | -511 | ERVL-B4 | ERVL | LTR |
| 126999387 | 126999469 | - | 10.8 | 4204 | 4288 | -1426 | ERVL-B4 | ERVL | LTR |
| 126999469 | 126999599 | - | 16.8 | 4318 | 4443 | -1271 | ERVL-B4 | ERVL | LTR |
| 126999600 | 126999988 | - | 17.0 | 1 | 432 | 0 | MSTB1 | MaLR | LTR |
| 126999989 | 127001529 | - | 27.5 | 2 | 1735 | 0 | MLT-int | MaLR | LTR |
| 127001532 | 127001957 | - | 17.2 | 1 | 432 | 0 | MSTB1 | MaLR | LTR |
| 127001958 | 127002216 | - | 16.8 | 4059 | 4317 | -1397 | ERVL-B4 | ERVL | LTR |

  

|  |  |  |  |  |  |  |  |  |  |
| --- | --- | --- | --- | --- | --- | --- | --- | --- | --- |
| chr2:127396150-127407159 | | | 11002 bp | | |  |  |  |  |
| Genome Start | Genome End | Strand | Div | Repeat Start | Repeat End | Repeat Left | Name | Family | Class |
| 127396150 | 127396201 | + | 18.5 | 10 | 64 | -310 | MLT1B | MaLR | LTR |
| 127396208 | 127396723 | - | 15.9 | 2502 | 3029 | 0 | Tigger3(Golem) | MER2\_type | DNA |
| 127396724 | 127397489 | - | 13.5 | 6216 | 7005 | 0 | L1MA4A | L1 | LINE |
| 127397490 | 127397514 | - | 15.9 | 2476 | 2501 | -528 | Tigger3(Golem) | MER2\_type | DNA |
| 127397515 | 127398417 | - | 16.0 | 4919 | 5814 | -1031 | L1MA4A | L1 | LINE |
| 127398418 | 127398715 | + | 13.4 | 1 | 303 | -7 | AluSg | Alu | SINE |
| 127398716 | 127399245 | - | 16.0 | 4379 | 4918 | -1927 | L1MA4A | L1 | LINE |
| 127399248 | 127399622 | + | 14.7 | 1 | 381 | 0 | THE1D | MaLR | LTR |
| 127399625 | 127401152 | + | 11.7 | 3 | 1580 | 0 | THE1D-int | MaLR | LTR |
| 127401153 | 127401529 | + | 11.4 | 1 | 381 | 0 | THE1D | MaLR | LTR |
| 127401530 | 127402707 | - | 17.3 | 3216 | 4381 | -2464 | L1MA4A | L1 | LINE |
| 127402708 | 127403018 | - | 16.4 | 1 | 312 | 0 | AluSx | Alu | SINE |
| 127403019 | 127406209 | - | 17.3 | 4 | 3215 | -3630 | L1MA4A | L1 | LINE |
| 127406211 | 127406832 | - | 14.0 | 17 | 701 | -530 | Tigger3b | MER2\_type | DNA |
| 127406830 | 127407159 | + | 23.1 | 77 | 390 | 0 | MLT1B | MaLR | LTR |

  

|  |  |  |  |  |  |  |  |  |  |
| --- | --- | --- | --- | --- | --- | --- | --- | --- | --- |
| chr2:127709449-127720589 | | | 10874 bp | | |  |  |  |  |
| Genome Start | Genome End | Strand | Div | Repeat Start | Repeat End | Repeat Left | Name | Family | Class |
| 127709449 | 127710640 | + | 18.3 | 1 | 1079 | -5429 | L1M | L1 | LINE |
| 127710641 | 127710946 | + | 12.9 | 1 | 303 | -9 | AluSx | Alu | SINE |
| 127710947 | 127711597 | + | 18.3 | 1080 | 1754 | -4754 | L1M | L1 | LINE |
| 127711598 | 127711970 | + | 13.8 | 1 | 381 | 0 | THE1D | MaLR | LTR |
| 127711973 | 127712862 | + | 30.2 | 2 | 979 | -756 | MLT-int | MaLR | LTR |
| 127712863 | 127713160 | + | 13.1 | 1 | 298 | -14 | AluSx | Alu | SINE |
| 127713161 | 127713837 | + | 30.2 | 980 | 1735 | 0 | MLT-int | MaLR | LTR |
| 127713838 | 127714217 | + | 12.4 | 1 | 381 | 0 | THE1D | MaLR | LTR |
| 127714218 | 127714557 | + | 18.3 | 1755 | 2146 | -4362 | L1M | L1 | LINE |
| 127714558 | 127714841 | + | 13.4 | 1 | 292 | -20 | AluSx | Alu | SINE |
| 127714842 | 127714910 | + | 18.3 | 2147 | 2214 | -4294 | L1M | L1 | LINE |
| 127714911 | 127715048 | + | 17.4 | 1 | 132 | -180 | AluJ | Alu | SINE |
| 127715089 | 127715167 | + | 10.8 | 2211 | 2284 | -4224 | L1M | L1 | LINE |
| 127715168 | 127715538 | - | 11.6 | 1 | 375 | 0 | THE1C | MaLR | LTR |
| 127715540 | 127716211 | - | 12.1 | 905 | 1579 | -1 | THE1C-int | MaLR | LTR |
| 127716213 | 127716560 | - | 14.0 | 1 | 359 | -69 | MSTC | MaLR | LTR |
| 127716561 | 127717766 | + | 27.9 | 2958 | 4133 | -2188 | L1M | L1 | LINE |
| 127717776 | 127717900 | - | 16.0 | 12 | 136 | -176 | AluJb | Alu | SINE |
| 127717901 | 127718072 | + | 16.7 | 107 | 301 | -11 | AluJ | Alu | SINE |
| 127718091 | 127718382 | + | 12.7 | 1 | 295 | -17 | AluSx | Alu | SINE |
| 127718416 | 127718705 | - | 19.1 | 1 | 288 | -24 | AluJo | Alu | SINE |
| 127718803 | 127719992 | + | 30.5 | 4212 | 5415 | -906 | L1M | L1 | LINE |
| 127720014 | 127720322 | + | 17.3 | 1 | 306 | 0 | AluJb | Alu | SINE |
| 127720355 | 127720531 | - | 19.6 | 1 | 175 | -10 | FAM | Alu | SINE |
| 127720545 | 127720589 | + | 17.2 | 5482 | 5550 | -2433 | L1M | L1 | LINE |

  

|  |  |  |  |  |  |  |  |  |  |
| --- | --- | --- | --- | --- | --- | --- | --- | --- | --- |
| chr2:128162256-128166484 | | | 4227 bp | | |  |  |  |  |
| Genome Start | Genome End | Strand | Div | Repeat Start | Repeat End | Repeat Left | Name | Family | Class |
| 128162256 | 128162933 | - | 17.6 | 1983 | 2712 | -4622 | L1M4b | L1 | LINE |
| 128162934 | 128163225 | + | 22.1 | 1 | 281 | -31 | AluJo | Alu | SINE |
| 128163226 | 128163348 | - | 17.6 | 1846 | 1982 | -5352 | L1M4b | L1 | LINE |
| 128163349 | 128163583 | - | 12.4 | 194 | 428 | 0 | MSTA | MaLR | LTR |
| 128163584 | 128163880 | - | 12.2 | 1 | 298 | -14 | AluSx | Alu | SINE |
| 128163881 | 128164051 | - | 12.4 | 1 | 193 | -235 | MSTA | MaLR | LTR |
| 128164052 | 128165641 | - | 27.3 | 2 | 1735 | 0 | MLT1A-int | MaLR | LTR |
| 128165644 | 128166055 | - | 12.2 | 1 | 428 | 0 | MSTA | MaLR | LTR |
| 128166056 | 128166236 | - | 17.6 | 1668 | 1845 | -5489 | L1M4b | L1 | LINE |
| 128166237 | 128166367 | - | 15.3 | 1 | 131 | -2 | FLAM\_C | Alu | SINE |
| 128166368 | 128166484 | - | 17.6 | 1555 | 1667 | -5667 | L1M4b | L1 | LINE |

  

|  |  |  |  |  |  |  |  |  |  |
| --- | --- | --- | --- | --- | --- | --- | --- | --- | --- |
| chr2:129175436-129187579 | | | 11608 bp | | |  |  |  |  |
| Genome Start | Genome End | Strand | Div | Repeat Start | Repeat End | Repeat Left | Name | Family | Class |
| 129175436 | 129178593 | - | 19.3 | 3292 | 6507 | -537 | L1M4 | L1 | LINE |
| 129178723 | 129179015 | - | 0.3 | 1 | 293 | -17 | AluYa5 | Alu | SINE |
| 129179118 | 129179538 | + | 23.4 | 145 | 596 | -11 | MER77 | ERVL | LTR |
| 129179626 | 129180343 | - | 18.0 | 2551 | 3369 | -3965 | L1M4 | L1 | LINE |
| 129180360 | 129180721 | - | 20.1 | 2039 | 2468 | -4866 | L1M4 | L1 | LINE |
| 129180723 | 129181130 | - | 13.8 | 1 | 414 | -12 | MSTB | MaLR | LTR |
| 129181136 | 129182711 | - | 19.3 | 1 | 1646 | -5 | MSTB-int | MaLR | LTR |
| 129182712 | 129183114 | - | 16.4 | 1 | 426 | 0 | MSTB | MaLR | LTR |
| 129183126 | 129183435 | - | 8.8 | 1 | 306 | -5 | AluY | Alu | SINE |
| 129183447 | 129183803 | - | 21.7 | 1648 | 2035 | -5299 | L1M4 | L1 | LINE |
| 129183804 | 129184618 | - | 23.4 | 320 | 1169 | -6165 | L1M4 | L1 | LINE |
| 129184619 | 129184957 | - | 14.9 | 3 | 375 | 0 | THE1C | MaLR | LTR |
| 129184958 | 129185188 | - | 23.4 | 84 | 319 | -7015 | L1M4 | L1 | LINE |
| 129185257 | 129185440 | + | 25.5 | 5581 | 5794 | -327 | L1M5 | L1 | LINE |
| 129185513 | 129185555 | - | 15.7 | 6132 | 6175 | -8 | L1MB3 | L1 | LINE |
| 129185556 | 129185847 | - | 9.9 | 1 | 294 | -15 | AluSc | Alu | SINE |
| 129185848 | 129186148 | - | 15.7 | 5823 | 6131 | -52 | L1MB3 | L1 | LINE |
| 129186149 | 129186450 | - | 13.8 | 1 | 292 | -21 | AluSq | Alu | SINE |
| 129186451 | 129186986 | - | 15.7 | 5297 | 5822 | -361 | L1MB3 | L1 | LINE |
| 129187019 | 129187438 | - | 19.4 | 4915 | 5327 | -819 | L1M4 | L1 | LINE |
| 129187441 | 129187579 | - | 15.3 | 3138 | 3279 | -2867 | L1M4 | L1 | LINE |

  

|  |  |  |  |  |  |  |  |  |  |
| --- | --- | --- | --- | --- | --- | --- | --- | --- | --- |
| chr2:129494107-129496463 | | | 2354 bp | | |  |  |  |  |
| Genome Start | Genome End | Strand | Div | Repeat Start | Repeat End | Repeat Left | Name | Family | Class |
| 129494107 | 129494185 | - | 13.7 | 6090 | 6165 | 0 | L1PA15 | L1 | LINE |
| 129494186 | 129494532 | + | 14.2 | 1 | 364 | 0 | THE1B | MaLR | LTR |
| 129494535 | 129496076 | + | 28.8 | 2 | 1735 | 0 | MLT-int | MaLR | LTR |
| 129496078 | 129496420 | + | 14.4 | 2 | 364 | 0 | THE1B | MaLR | LTR |
| 129496421 | 129496463 | - | 13.7 | 6048 | 6089 | -76 | L1PA15 | L1 | LINE |

  

|  |  |  |  |  |  |  |  |  |  |
| --- | --- | --- | --- | --- | --- | --- | --- | --- | --- |
| chr2:130051027-130057943 | | | 6414 bp | | |  |  |  |  |
| Genome Start | Genome End | Strand | Div | Repeat Start | Repeat End | Repeat Left | Name | Family | Class |
| 130051027 | 130052207 | - | 31.0 | 5678 | 6900 | -908 | L1MC4a | L1 | LINE |
| 130052208 | 130052491 | - | 16.6 | 13 | 305 | -7 | AluJb | Alu | SINE |
| 130052492 | 130052995 | - | 31.0 | 5257 | 5677 | -2131 | L1MC4a | L1 | LINE |
| 130053001 | 130053503 | - | 31.4 | 4698 | 5251 | -895 | L1MC4a | L1 | LINE |
| 130053584 | 130053722 | - | 26.8 | 4400 | 4548 | -1598 | L1MC4a | L1 | LINE |
| 130054053 | 130054100 | - | 12.5 | 6097 | 6144 | -12 | L1PB4 | L1 | LINE |
| 130054101 | 130054502 | - | 15.0 | 1 | 426 | 0 | MSTB | MaLR | LTR |
| 130054503 | 130056105 | - | 18.1 | 1 | 1651 | 0 | MSTB-int | MaLR | LTR |
| 130056106 | 130056316 | - | 15.7 | 203 | 426 | 0 | MSTB | MaLR | LTR |
| 130056317 | 130056603 | + | 10.1 | 1 | 287 | -25 | AluSx | Alu | SINE |
| 130056632 | 130056834 | - | 15.7 | 1 | 202 | -224 | MSTB | MaLR | LTR |
| 130056835 | 130057067 | - | 19.6 | 5868 | 6101 | -55 | L1PB4 | L1 | LINE |
| 130057128 | 130057943 | - | 31.0 | 3381 | 4230 | -1910 | L1MC4a | L1 | LINE |

  

|  |  |  |  |  |  |  |  |  |  |
| --- | --- | --- | --- | --- | --- | --- | --- | --- | --- |
| chr2:134511456-134525883 | | | 14400 bp | | |  |  |  |  |
| Genome Start | Genome End | Strand | Div | Repeat Start | Repeat End | Repeat Left | Name | Family | Class |
| 134511456 | 134511767 | - | 23.2 | 166 | 505 | 0 | MLT1D | MaLR | LTR |
| 134511770 | 134512392 | + | 11.3 | 1697 | 2354 | -3967 | L1MCa | L1 | LINE |
| 134512393 | 134512687 | + | 19.7 | 1 | 297 | -15 | AluJo | Alu | SINE |
| 134512688 | 134512771 | + | 11.3 | 2355 | 2430 | -4078 | L1MCa | L1 | LINE |
| 134512778 | 134513153 | - | 15.6 | 1 | 428 | 0 | MSTA | MaLR | LTR |
| 134513154 | 134514822 | - | 29.7 | 1 | 1735 | 0 | MLT1B-int | MaLR | LTR |
| 134514823 | 134515205 | - | 13.7 | 1 | 428 | 0 | MSTA | MaLR | LTR |
| 134515206 | 134516818 | + | 14.3 | 2257 | 3887 | -2259 | L1MC1 | L1 | LINE |
| 134516821 | 134522945 | + | 5.1 | 26 | 6155 | 0 | L1PA4 | L1 | LINE |
| 134522959 | 134523946 | + | 15.0 | 3876 | 4882 | -1264 | L1MC1 | L1 | LINE |
| 134523947 | 134524235 | + | 16.6 | 1 | 297 | -15 | AluJb | Alu | SINE |
| 134524236 | 134525705 | + | 14.8 | 4883 | 6324 | -9 | L1MC1 | L1 | LINE |
| 134525711 | 134525883 | - | 22.9 | 1 | 177 | -328 | MLT1D | MaLR | LTR |

  

|  |  |  |  |  |  |  |  |  |  |
| --- | --- | --- | --- | --- | --- | --- | --- | --- | --- |
| chr2:135722969-135726396 | | | 3426 bp | | |  |  |  |  |
| Genome Start | Genome End | Strand | Div | Repeat Start | Repeat End | Repeat Left | Name | Family | Class |
| 135722969 | 135723570 | + | 8.9 | 5328 | 5929 | -232 | L1PA14 | L1 | LINE |
| 135723571 | 135723927 | + | 9.9 | 1 | 355 | 0 | THE1A | MaLR | LTR |
| 135723930 | 135725491 | + | 29.6 | 2 | 1735 | 0 | MLT1J-int | MaLR | LTR |
| 135725492 | 135725795 | + | 8.0 | 1 | 304 | -51 | THE1A | MaLR | LTR |
| 135725796 | 135726103 | + | 10.4 | 2 | 310 | 0 | AluSg | Alu | SINE |
| 135726104 | 135726165 | + | 8.0 | 305 | 355 | 0 | THE1A | MaLR | LTR |
| 135726166 | 135726396 | + | 8.9 | 5930 | 6158 | -3 | L1PA14 | L1 | LINE |

  

|  |  |  |  |  |  |  |  |  |  |
| --- | --- | --- | --- | --- | --- | --- | --- | --- | --- |
| chr2:137173437-137176867 | | | 3404 bp | | |  |  |  |  |
| Genome Start | Genome End | Strand | Div | Repeat Start | Repeat End | Repeat Left | Name | Family | Class |
| 137173437 | 137173554 | + | 20.8 | 4 | 118 | -144 | MIR | MIR | SINE |
| 137173563 | 137173665 | - | 18.0 | 257 | 357 | -8 | MLT1A1 | MaLR | LTR |
| 137173668 | 137173777 | - | 8.2 | 1 | 114 | -294 | MLT1A1 | MaLR | LTR |
| 137173778 | 137174004 | - | 19.9 | 1496 | 1735 | 0 | MLT1A1-int | MaLR | LTR |
| 137174005 | 137174092 | + | 4.5 | 6059 | 6146 | -8 | L1PA6 | L1 | LINE |
| 137174100 | 137174944 | + | 6.4 | 5295 | 6141 | -13 | L1PA7 | L1 | LINE |
| 137174950 | 137176339 | - | 22.4 | 1 | 1499 | -236 | MLT1A1-int | MaLR | LTR |
| 137176340 | 137176726 | - | 20.9 | 1 | 408 | 0 | MLT1A1 | MaLR | LTR |
| 137176732 | 137176867 | + | 18.9 | 114 | 252 | -10 | MIR | MIR | SINE |

  

|  |  |  |  |  |  |  |  |  |  |
| --- | --- | --- | --- | --- | --- | --- | --- | --- | --- |
| chr2:137386498-137388943 | | | 2444 bp | | |  |  |  |  |
| Genome Start | Genome End | Strand | Div | Repeat Start | Repeat End | Repeat Left | Name | Family | Class |
| 137386498 | 137386580 | + | 28.9 | 80 | 158 | -104 | MIR | MIR | SINE |
| 137386581 | 137386934 | + | 8.5 | 1 | 355 | 0 | THE1A | MaLR | LTR |
| 137386937 | 137388506 | + | 30.4 | 2 | 1735 | 0 | MLT1I-int | MaLR | LTR |
| 137388507 | 137388861 | + | 9.0 | 1 | 355 | 0 | THE1A | MaLR | LTR |
| 137388862 | 137388943 | + | 28.9 | 159 | 251 | -11 | MIR | MIR | SINE |

  

|  |  |  |  |  |  |  |  |  |  |
| --- | --- | --- | --- | --- | --- | --- | --- | --- | --- |
| chr2:138677724-138680628 | | | 2903 bp | | |  |  |  |  |
| Genome Start | Genome End | Strand | Div | Repeat Start | Repeat End | Repeat Left | Name | Family | Class |
| 138677724 | 138677768 | - | 14.9 | 176 | 225 | -11 | MER46B | MER2\_type | DNA |
| 138677769 | 138678046 | + | 6.8 | 24 | 301 | -10 | AluY | Alu | SINE |
| 138678047 | 138678064 | - | 14.9 | 152 | 175 | -61 | MER46B | MER2\_type | DNA |
| 138678065 | 138678422 | - | 15.6 | 1 | 364 | 0 | THE1B | MaLR | LTR |
| 138678423 | 138679989 | - | 31.5 | 2 | 1735 | 0 | MLT-int | MaLR | LTR |
| 138679992 | 138680350 | - | 14.7 | 1 | 364 | 0 | THE1B | MaLR | LTR |
| 138680351 | 138680628 | - | 14.9 | 1 | 151 | -85 | MER46B | MER2\_type | DNA |

  

|  |  |  |  |  |  |  |  |  |  |
| --- | --- | --- | --- | --- | --- | --- | --- | --- | --- |
| chr2:140044725-140049795 | | | 4839 bp | | |  |  |  |  |
| Genome Start | Genome End | Strand | Div | Repeat Start | Repeat End | Repeat Left | Name | Family | Class |
| 140044725 | 140044925 | + | 28.6 | 1937 | 2167 | -2322 | L3 | CR1 | LINE |
| 140044945 | 140045175 | + | 35.8 | 29 | 262 | 0 | MIR | MIR | SINE |
| 140045229 | 140045329 | + | 30.4 | 2233 | 2329 | -2160 | L3 | CR1 | LINE |
| 140045330 | 140045535 | + | 20.8 | 4 | 216 | -210 | MSTB | MaLR | LTR |
| 140045571 | 140045774 | + | 20.8 | 217 | 425 | -1 | MSTB | MaLR | LTR |
| 140045777 | 140047415 | + | 28.0 | 2 | 1735 | 0 | MLT-int | MaLR | LTR |
| 140047416 | 140047832 | + | 19.0 | 1 | 426 | 0 | MSTB | MaLR | LTR |
| 140047833 | 140048330 | + | 32.0 | 2330 | 2841 | -1648 | L3 | CR1 | LINE |
| 140048454 | 140049795 | + | 34.3 | 3053 | 4464 | -25 | L3 | CR1 | LINE |

  

|  |  |  |  |  |  |  |  |  |  |
| --- | --- | --- | --- | --- | --- | --- | --- | --- | --- |
| chr2:140510164-140515096 | | | 4747 bp | | |  |  |  |  |
| Genome Start | Genome End | Strand | Div | Repeat Start | Repeat End | Repeat Left | Name | Family | Class |
| 140510164 | 140511718 | + | 24.0 | 1 | 1375 | -5133 | L1MCa | L1 | LINE |
| 140511739 | 140512093 | - | 15.8 | 1 | 364 | 0 | THE1B | MaLR | LTR |
| 140512094 | 140513771 | - | 11.5 | 1 | 1580 | 0 | THE1B-int | MaLR | LTR |
| 140513774 | 140514124 | - | 13.7 | 1 | 362 | -2 | THE1B | MaLR | LTR |
| 140514125 | 140514377 | + | 20.3 | 1410 | 1643 | -4865 | L1MCa | L1 | LINE |
| 140514378 | 140514498 | + | 29.1 | 1 | 122 | -11 | FLAM\_A | Alu | SINE |
| 140514663 | 140515096 | + | 20.5 | 1634 | 2133 | -4375 | L1MCa | L1 | LINE |

  

|  |  |  |  |  |  |  |  |  |  |
| --- | --- | --- | --- | --- | --- | --- | --- | --- | --- |
| chr2:143422225-143425285 | | | 3059 bp | | |  |  |  |  |
| Genome Start | Genome End | Strand | Div | Repeat Start | Repeat End | Repeat Left | Name | Family | Class |
| 143422225 | 143422441 | - | 14.0 | 659 | 881 | -1537 | Tigger1 | MER2\_type | DNA |
| 143422442 | 143422797 | - | 13.3 | 1 | 364 | 0 | THE1B | MaLR | LTR |
| 143422798 | 143424365 | - | 30.1 | 2 | 1735 | 0 | MLT-int | MaLR | LTR |
| 143424368 | 143424717 | - | 10.6 | 3 | 364 | 0 | THE1B | MaLR | LTR |
| 143424718 | 143425285 | - | 14.0 | 61 | 658 | -1760 | Tigger1 | MER2\_type | DNA |

  

|  |  |  |  |  |  |  |  |  |  |
| --- | --- | --- | --- | --- | --- | --- | --- | --- | --- |
| chr2:144392081-144394993 | | | 2909 bp | | |  |  |  |  |
| Genome Start | Genome End | Strand | Div | Repeat Start | Repeat End | Repeat Left | Name | Family | Class |
| 144392081 | 144392473 | - | 19.5 | 323 | 717 | 0 | MLT2B3 | ERVL | LTR |
| 144392474 | 144392836 | + | 15.9 | 1 | 364 | 0 | THE1B | MaLR | LTR |
| 144392839 | 144394405 | + | 29.5 | 2 | 1733 | -2 | MLT-int | MaLR | LTR |
| 144394408 | 144394779 | + | 13.5 | 1 | 364 | 0 | THE1B | MaLR | LTR |
| 144394780 | 144394993 | - | 13.0 | 1 | 213 | -341 | MLT2B3 | ERVL | LTR |

  

|  |  |  |  |  |  |  |  |  |  |
| --- | --- | --- | --- | --- | --- | --- | --- | --- | --- |
| chr2:145893129-145896544 | | | 3414 bp | | |  |  |  |  |
| Genome Start | Genome End | Strand | Div | Repeat Start | Repeat End | Repeat Left | Name | Family | Class |
| 145893129 | 145893440 | + | 25.1 | 1357 | 1672 | -5824 | L1M3c | L1 | LINE |
| 145893441 | 145893817 | - | 18.1 | 1 | 380 | -1 | THE1D | MaLR | LTR |
| 145893818 | 145895426 | - | 30.1 | 2 | 1735 | 0 | MLT-int | MaLR | LTR |
| 145895429 | 145895811 | - | 15.8 | 1 | 381 | 0 | THE1D | MaLR | LTR |
| 145895812 | 145896544 | + | 25.1 | 1673 | 2454 | -5042 | L1M3c | L1 | LINE |

  

|  |  |  |  |  |  |  |  |  |  |
| --- | --- | --- | --- | --- | --- | --- | --- | --- | --- |
| chr2:147736449-147744416 | | | 7965 bp | | |  |  |  |  |
| Genome Start | Genome End | Strand | Div | Repeat Start | Repeat End | Repeat Left | Name | Family | Class |
| 147736449 | 147741881 | - | 18.9 | 216 | 5560 | -778 | L1M3f | L1 | LINE |
| 147741882 | 147742261 | - | 14.2 | 1 | 364 | 0 | THE1B | MaLR | LTR |
| 147742263 | 147743819 | - | 29.2 | 2 | 1732 | -3 | MLT-int | MaLR | LTR |
| 147743822 | 147744203 | - | 16.8 | 1 | 364 | 0 | THE1B | MaLR | LTR |
| 147744204 | 147744416 | - | 20.1 | 2 | 215 | -5113 | L1M3f | L1 | LINE |

  

|  |  |  |  |  |  |  |  |  |  |
| --- | --- | --- | --- | --- | --- | --- | --- | --- | --- |
| chr2:151141254-151145647 | | | 4181 bp | | |  |  |  |  |
| Genome Start | Genome End | Strand | Div | Repeat Start | Repeat End | Repeat Left | Name | Family | Class |
| 151141254 | 151141696 | + | 28.8 | 2012 | 2455 | -3779 | L1MC4 | L1 | LINE |
| 151141697 | 151141989 | + | 7.5 | 7 | 302 | -9 | AluY | Alu | SINE |
| 151141990 | 151142024 | + | 30.3 | 2456 | 2493 | -3741 | L1MC4 | L1 | LINE |
| 151142209 | 151142607 | - | 15.1 | 1 | 411 | -17 | MSTA | MaLR | LTR |
| 151142608 | 151142705 | - | 10.3 | 1554 | 1651 | 0 | MSTA-int | MaLR | LTR |
| 151142692 | 151142852 | - | 14.4 | 1 | 164 | -264 | MSTA | MaLR | LTR |
| 151142853 | 151144352 | - | 29.1 | 2 | 1735 | 0 | MLT-int | MaLR | LTR |
| 151144355 | 151144756 | - | 14.6 | 1 | 428 | 0 | MSTA | MaLR | LTR |
| 151144772 | 151145071 | - | 5.3 | 1 | 300 | -11 | AluY | Alu | SINE |
| 151145098 | 151145647 | + | 31.1 | 2661 | 3244 | -2970 | L1MC4 | L1 | LINE |

  

|  |  |  |  |  |  |  |  |  |  |
| --- | --- | --- | --- | --- | --- | --- | --- | --- | --- |
| chr2:151427192-151430644 | | | 3453 bp | | |  |  |  |  |
| Genome Start | Genome End | Strand | Div | Repeat Start | Repeat End | Repeat Left | Name | Family | Class |
| 151427192 | 151427573 | - | 22.9 | 200 | 586 | -36 | MER74B | ERVL | LTR |
| 151427574 | 151427902 | - | 14.1 | 1 | 408 | 0 | MLT1A1 | MaLR | LTR |
| 151427903 | 151428927 | - | 21.0 | 790 | 1735 | 0 | MLT1A1-int | MaLR | LTR |
| 151428928 | 151429283 | - | 12.7 | 1 | 364 | 0 | THE1B | MaLR | LTR |
| 151429284 | 151430071 | - | 21.0 | 1 | 789 | -946 | MLT1A1-int | MaLR | LTR |
| 151430072 | 151430449 | - | 21.3 | 1 | 408 | 0 | MLT1A1 | MaLR | LTR |
| 151430450 | 151430644 | - | 22.8 | 8 | 204 | -418 | MER74B | ERVL | LTR |

  

|  |  |  |  |  |  |  |  |  |  |
| --- | --- | --- | --- | --- | --- | --- | --- | --- | --- |
| chr2:151873578-151885785 | | | 12145 bp | | |  |  |  |  |
| Genome Start | Genome End | Strand | Div | Repeat Start | Repeat End | Repeat Left | Name | Family | Class |
| 151873578 | 151873826 | + | 18.3 | 5 | 245 | -6367 | L1PBa1 | L1 | LINE |
| 151873828 | 151874242 | + | 6.5 | 1 | 464 | 0 | LTR7B | ERV1 | LTR |
| 151874243 | 151874708 | + | 13.0 | 1 | 459 | -7254 | HERVH | ERV1 | LTR |
| 151874767 | 151877017 | + | 12.8 | 800 | 2995 | -4718 | HERVH | ERV1 | LTR |
| 151876997 | 151878732 | + | 13.4 | 3140 | 4834 | -2228 | HERVH | ERV1 | LTR |
| 151878761 | 151879029 | + | 17.1 | 5747 | 6016 | -1697 | HERVH | ERV1 | LTR |
| 151879023 | 151879558 | + | 11.2 | 7157 | 7713 | 0 | HERVH | ERV1 | LTR |
| 151879559 | 151879983 | + | 4.7 | 1 | 462 | -2 | LTR7B | ERV1 | LTR |
| 151879988 | 151881367 | + | 13.5 | 242 | 1616 | -4997 | L1PBa1 | L1 | LINE |
| 151881368 | 151881671 | + | 11.7 | 1 | 300 | -13 | AluSq | Alu | SINE |
| 151881672 | 151881684 | + | 11.4 | 1617 | 1620 | -4993 | L1PBa1 | L1 | LINE |
| 151881685 | 151881980 | + | 12.3 | 1 | 293 | -20 | AluSq | Alu | SINE |
| 151881981 | 151885785 | + | 8.7 | 1621 | 5507 | -1105 | L1PBa1 | L1 | LINE |

  

|  |  |  |  |  |  |  |  |  |  |
| --- | --- | --- | --- | --- | --- | --- | --- | --- | --- |
| chr2:152837649-152845947 | | | 8300 bp | | |  |  |  |  |
| Genome Start | Genome End | Strand | Div | Repeat Start | Repeat End | Repeat Left | Name | Family | Class |
| 152837649 | 152837926 | + | 23.9 | 28 | 311 | -6322 | L1MDa | L1 | LINE |
| 152837927 | 152838387 | + | 21.2 | 1 | 458 | -96 | MLT2B3 | ERVL | LTR |
| 152838388 | 152838901 | - | 10.8 | 1 | 511 | 0 | MER9 | ERVK | LTR |
| 152838902 | 152842653 | - | 6.7 | 2137 | 5916 | 0 | HERVK9 | ERVK | LTR |
| 152842653 | 152843181 | - | 8.2 | 804 | 1361 | -4555 | HERVK9 | ERVK | LTR |
| 152843182 | 152843250 | - | 25.4 | 55 | 117 | -61 | MER5B | MER1\_type | DNA |
| 152843251 | 152844009 | - | 8.2 | 3 | 803 | -5113 | HERVK9 | ERVK | LTR |
| 152844010 | 152844522 | - | 10.4 | 1 | 511 | 0 | MER9 | ERVK | LTR |
| 152844523 | 152844634 | + | 12.5 | 456 | 567 | 0 | MLT2B1 | ERVL | LTR |
| 152844635 | 152845947 | + | 23.6 | 307 | 1741 | -4892 | L1MDa | L1 | LINE |

  

|  |  |  |  |  |  |  |  |  |  |
| --- | --- | --- | --- | --- | --- | --- | --- | --- | --- |
| chr2:154102271-154106757 | | | 4482 bp | | |  |  |  |  |
| Genome Start | Genome End | Strand | Div | Repeat Start | Repeat End | Repeat Left | Name | Family | Class |
| 154102271 | 154102331 | + | 14.8 | 1 | 77 | -576 | MER39B | ERV1 | LTR |
| 154102333 | 154102701 | + | 13.8 | 4 | 375 | 0 | THE1C | MaLR | LTR |
| 154102703 | 154103065 | + | 11.6 | 2 | 375 | -1205 | THE1C-int | MaLR | LTR |
| 154103066 | 154104607 | - | 2.0 | 4698 | 6239 | 0 | L1PA2 | L1 | LINE |
| 154104608 | 154105803 | + | 13.2 | 345 | 1580 | 0 | THE1C-int | MaLR | LTR |
| 154105804 | 154106175 | + | 12.2 | 1 | 372 | -3 | THE1C | MaLR | LTR |
| 154106179 | 154106757 | + | 15.1 | 71 | 653 | 0 | MER39B | ERV1 | LTR |

  

|  |  |  |  |  |  |  |  |  |  |
| --- | --- | --- | --- | --- | --- | --- | --- | --- | --- |
| chr2:154391380-154394498 | | | 3117 bp | | |  |  |  |  |
| Genome Start | Genome End | Strand | Div | Repeat Start | Repeat End | Repeat Left | Name | Family | Class |
| 154391380 | 154391749 | - | 30.6 | 2427 | 2851 | -568 | L2 | L2 | LINE |
| 154391750 | 154392100 | - | 15.5 | 33 | 364 | 0 | THE1B | MaLR | LTR |
| 154392101 | 154392402 | + | 9.4 | 1 | 299 | -12 | AluY | Alu | SINE |
| 154392403 | 154392435 | - | 15.5 | 1 | 32 | -332 | THE1B | MaLR | LTR |
| 154392436 | 154394015 | - | 31.7 | 2 | 1735 | 0 | MLT1J-int | MaLR | LTR |
| 154394018 | 154394389 | - | 13.3 | 2 | 364 | 0 | THE1B | MaLR | LTR |
| 154394390 | 154394498 | - | 30.6 | 2313 | 2426 | -993 | L2 | L2 | LINE |

  

|  |  |  |  |  |  |  |  |  |  |
| --- | --- | --- | --- | --- | --- | --- | --- | --- | --- |
| chr2:154407630-154411331 | | | 3420 bp | | |  |  |  |  |
| Genome Start | Genome End | Strand | Div | Repeat Start | Repeat End | Repeat Left | Name | Family | Class |
| 154407630 | 154407865 | + | 29.8 | 927 | 1280 | -5031 | L1MDa | L1 | LINE |
| 154407866 | 154408285 | - | 17.4 | 1 | 426 | 0 | MSTB | MaLR | LTR |
| 154408294 | 154409933 | - | 18.6 | 1 | 1649 | -2 | MSTB-int | MaLR | LTR |
| 154409934 | 154410378 | - | 15.6 | 1 | 426 | 0 | MSTB | MaLR | LTR |
| 154410380 | 154410961 | + | 30.3 | 1237 | 1930 | -4411 | L1MDa | L1 | LINE |
| 154411235 | 154411331 | + | 22.1 | 2341 | 2441 | -4061 | L1MDa | L1 | LINE |

  

|  |  |  |  |  |  |  |  |  |  |
| --- | --- | --- | --- | --- | --- | --- | --- | --- | --- |
| chr2:154852249-154855427 | | | 3045 bp | | |  |  |  |  |
| Genome Start | Genome End | Strand | Div | Repeat Start | Repeat End | Repeat Left | Name | Family | Class |
| 154852249 | 154852756 | - | 21.3 | 1417 | 1942 | -4711 | L1M3de | L1 | LINE |
| 154852757 | 154853115 | - | 11.0 | 3 | 364 | 0 | THE1B | MaLR | LTR |
| 154853118 | 154854076 | - | 12.8 | 625 | 1580 | 0 | THE1B-int | MaLR | LTR |
| 154854088 | 154854450 | - | 11.0 | 1 | 364 | 0 | THE1B | MaLR | LTR |
| 154854453 | 154854932 | - | 29.4 | 935 | 1419 | -5234 | L1M3de | L1 | LINE |
| 154854961 | 154855048 | - | 28.2 | 279 | 366 | -6287 | L1M3de | L1 | LINE |
| 154855093 | 154855153 | - | 28.3 | 278 | 337 | -6316 | L1M3de | L1 | LINE |
| 154855201 | 154855427 | - | 33.2 | 744 | 988 | -5665 | L1M3de | L1 | LINE |

  

|  |  |  |  |  |  |  |  |  |  |
| --- | --- | --- | --- | --- | --- | --- | --- | --- | --- |
| chr2:155466203-155474351 | | | 8136 bp | | |  |  |  |  |
| Genome Start | Genome End | Strand | Div | Repeat Start | Repeat End | Repeat Left | Name | Family | Class |
| 155466203 | 155468225 | - | 20.6 | 3466 | 5507 | -1608 | L1M2 | L1 | LINE |
| 155468229 | 155468580 | - | 12.7 | 1 | 375 | 0 | THE1C | MaLR | LTR |
| 155468581 | 155468632 | - | 11.8 | 1530 | 1580 | 0 | THE1C-int | MaLR | LTR |
| 155468633 | 155468982 | + | 13.5 | 1 | 353 | -11 | THE1B | MaLR | LTR |
| 155468991 | 155470476 | - | 13.3 | 1 | 1533 | -47 | THE1C-int | MaLR | LTR |
| 155470479 | 155470815 | - | 12.9 | 1 | 373 | -2 | THE1C | MaLR | LTR |
| 155470816 | 155474351 | - | 28.5 | 2 | 3464 | -3651 | L1M2 | L1 | LINE |

  

|  |  |  |  |  |  |  |  |  |  |
| --- | --- | --- | --- | --- | --- | --- | --- | --- | --- |
| chr2:155664255-155674072 | | | 9769 bp | | |  |  |  |  |
| Genome Start | Genome End | Strand | Div | Repeat Start | Repeat End | Repeat Left | Name | Family | Class |
| 155664255 | 155666128 | + | 18.4 | 23 | 2231 | -5334 | L1M1 | L1 | LINE |
| 155666129 | 155666485 | - | 17.1 | 1 | 364 | 0 | THE1B | MaLR | LTR |
| 155666486 | 155668095 | - | 31.9 | 2 | 1735 | 0 | MLT-int | MaLR | LTR |
| 155668098 | 155668453 | - | 13.6 | 1 | 364 | 0 | THE1B | MaLR | LTR |
| 155668454 | 155671249 | + | 14.9 | 2232 | 4993 | -2572 | L1M1 | L1 | LINE |
| 155671250 | 155672460 | + | 14.6 | 4919 | 6165 | 0 | L1PA15 | L1 | LINE |
| 155672508 | 155674072 | + | 13.4 | 4981 | 6495 | -1070 | L1M1 | L1 | LINE |

  

|  |  |  |  |  |  |  |  |  |  |
| --- | --- | --- | --- | --- | --- | --- | --- | --- | --- |
| chr2:158849586-158854772 | | | 5066 bp | | |  |  |  |  |
| Genome Start | Genome End | Strand | Div | Repeat Start | Repeat End | Repeat Left | Name | Family | Class |
| 158849586 | 158849763 | + | 32.6 | 796 | 963 | -5156 | L1ME3B | L1 | LINE |
| 158849764 | 158850064 | + | 12.3 | 1 | 301 | -12 | AluSq | Alu | SINE |
| 158850065 | 158850215 | + | 32.6 | 964 | 1116 | -5003 | L1ME3B | L1 | LINE |
| 158850293 | 158850593 | - | 5.1 | 1 | 295 | -16 | AluY | Alu | SINE |
| 158850622 | 158851035 | + | 26.2 | 4043 | 4464 | -1665 | L1ME3B | L1 | LINE |
| 158851036 | 158851090 | + | 7.3 | 398 | 453 | -101 | MER41A | ERV1 | LTR |
| 158851091 | 158851665 | + | 26.2 | 4465 | 5051 | -1078 | L1ME3B | L1 | LINE |
| 158851666 | 158852018 | - | 9.1 | 1 | 355 | 0 | THE1A | MaLR | LTR |
| 158852019 | 158853550 | - | 10.3 | 1 | 1580 | 0 | THE1A-int | MaLR | LTR |
| 158853551 | 158853905 | - | 8.2 | 1 | 355 | 0 | THE1A | MaLR | LTR |
| 158853906 | 158854606 | + | 27.1 | 5049 | 5766 | -457 | L1ME3B | L1 | LINE |
| 158854623 | 158854772 | + | 23.5 | 5846 | 6008 | -215 | L1ME3B | L1 | LINE |

  

|  |  |  |  |  |  |  |  |  |  |
| --- | --- | --- | --- | --- | --- | --- | --- | --- | --- |
| chr2:159578512-159581228 | | | 2715 bp | | |  |  |  |  |
| Genome Start | Genome End | Strand | Div | Repeat Start | Repeat End | Repeat Left | Name | Family | Class |
| 159578512 | 159578884 | - | 16.7 | 47 | 390 | 0 | MLT1B | MaLR | LTR |
| 159578885 | 159579249 | - | 13.6 | 1 | 355 | 0 | THE1A | MaLR | LTR |
| 159579250 | 159580812 | - | 31.2 | 2 | 1735 | 0 | MLT1B-int | MaLR | LTR |
| 159580815 | 159581185 | - | 13.8 | 1 | 355 | 0 | THE1A | MaLR | LTR |
| 159581186 | 159581228 | - | 16.7 | 1 | 46 | -344 | MLT1B | MaLR | LTR |

  

|  |  |  |  |  |  |  |  |  |  |
| --- | --- | --- | --- | --- | --- | --- | --- | --- | --- |
| chr2:162642470-162645003 | | | 2505 bp | | |  |  |  |  |
| Genome Start | Genome End | Strand | Div | Repeat Start | Repeat End | Repeat Left | Name | Family | Class |
| 162642470 | 162642621 | + | 15.9 | 100 | 251 | -139 | MLT1C | MaLR | LTR |
| 162642633 | 162642995 | - | 9.6 | 1 | 364 | 0 | THE1B | MaLR | LTR |
| 162642996 | 162644481 | - | 10.8 | 1 | 1580 | 0 | THE1B-int | MaLR | LTR |
| 162644482 | 162644846 | - | 9.9 | 1 | 364 | 0 | THE1B | MaLR | LTR |
| 162644865 | 162645003 | + | 17.3 | 325 | 464 | -3 | MLT1C | MaLR | LTR |

  

|  |  |  |  |  |  |  |  |  |  |
| --- | --- | --- | --- | --- | --- | --- | --- | --- | --- |
| chr2:164385921-164396513 | | | 10535 bp | | |  |  |  |  |
| Genome Start | Genome End | Strand | Div | Repeat Start | Repeat End | Repeat Left | Name | Family | Class |
| 164385921 | 164386005 | + | 24.1 | 3221 | 3303 | -116 | L2 | L2 | LINE |
| 164386016 | 164386128 | - | 8.0 | 7609 | 7721 | 0 | L1MA1 | L1 | LINE |
| 164386129 | 164388816 | - | 16.6 | 4695 | 7395 | -326 | L1MA1 | L1 | LINE |
| 164388817 | 164389123 | - | 12.4 | 1 | 307 | -2 | AluSc | Alu | SINE |
| 164389124 | 164392576 | - | 18.2 | 1138 | 4694 | -2865 | L1MA1 | L1 | LINE |
| 164392577 | 164392917 | - | 14.6 | 2 | 364 | 0 | THE1B | MaLR | LTR |
| 164392919 | 164394482 | - | 30.1 | 2 | 1735 | 0 | MLT-int | MaLR | LTR |
| 164394485 | 164394829 | - | 13.6 | 1 | 364 | 0 | THE1B | MaLR | LTR |
| 164394830 | 164395958 | - | 17.7 | 11 | 1137 | -6428 | L1MA1 | L1 | LINE |
| 164396000 | 164396410 | - | 22.5 | 6 | 426 | -41 | MLT1C | MaLR | LTR |
| 164396415 | 164396513 | + | 26.3 | 3314 | 3414 | -5 | L2 | L2 | LINE |

  

|  |  |  |  |  |  |  |  |  |  |
| --- | --- | --- | --- | --- | --- | --- | --- | --- | --- |
| chr2:164455771-164458444 | | | 2670 bp | | |  |  |  |  |
| Genome Start | Genome End | Strand | Div | Repeat Start | Repeat End | Repeat Left | Name | Family | Class |
| 164455771 | 164455996 | + | 19.1 | 1 | 225 | -87 | AluJo | Alu | SINE |
| 164456000 | 164456363 | - | 12.7 | 1 | 375 | 0 | THE1C | MaLR | LTR |
| 164456364 | 164457947 | - | 12.6 | 1 | 1580 | 0 | THE1C-int | MaLR | LTR |
| 164457948 | 164458311 | - | 11.1 | 1 | 375 | 0 | THE1C | MaLR | LTR |
| 164458313 | 164458416 | + | 18.0 | 222 | 325 | 0 | AluJ/FRAM | Alu | SINE |
| 164458417 | 164458444 | + | 18.0 | 284 | 310 | -2 | AluJo | Alu | SINE |

  

|  |  |  |  |  |  |  |  |  |  |
| --- | --- | --- | --- | --- | --- | --- | --- | --- | --- |
| chr2:164860309-164872184 | | | 11914 bp | | |  |  |  |  |
| Genome Start | Genome End | Strand | Div | Repeat Start | Repeat End | Repeat Left | Name | Family | Class |
| 164860309 | 164860581 | + | 25.6 | 4235 | 4512 | -1634 | L1MC3 | L1 | LINE |
| 164860582 | 164861014 | + | 7.9 | 1 | 462 | -2 | LTR7B | ERV1 | LTR |
| 164861017 | 164864006 | + | 10.0 | 1 | 2987 | -4726 | HERVH | ERV1 | LTR |
| 164864001 | 164864150 | + | 12.7 | 3140 | 3290 | -4423 | HERVH | ERV1 | LTR |
| 164864141 | 164864659 | + | 12.3 | 3492 | 4006 | -3707 | HERVH | ERV1 | LTR |
| 164864644 | 164865297 | + | 15.3 | 4492 | 5149 | -2564 | HERVH | ERV1 | LTR |
| 164865295 | 164865515 | + | 13.3 | 5597 | 5819 | -1894 | HERVH | ERV1 | LTR |
| 164865516 | 164865893 | + | 7.0 | 7324 | 7713 | 0 | HERVH | ERV1 | LTR |
| 164865894 | 164866322 | + | 5.8 | 1 | 462 | -2 | LTR7B | ERV1 | LTR |
| 164866324 | 164866690 | + | 24.9 | 4507 | 4877 | -1269 | L1MC3 | L1 | LINE |
| 164866691 | 164866995 | + | 10.2 | 1 | 303 | -10 | AluSq | Alu | SINE |
| 164866996 | 164867418 | + | 24.9 | 4878 | 5345 | -801 | L1MC3 | L1 | LINE |
| 164867426 | 164867761 | + | 26.5 | 10 | 353 | -6981 | L1MB5 | L1 | LINE |
| 164867765 | 164868012 | + | 17.5 | 670 | 934 | -6400 | L1MB5 | L1 | LINE |
| 164868027 | 164868826 | + | 24.0 | 994 | 1850 | -5484 | L1MB5 | L1 | LINE |
| 164868806 | 164869051 | + | 18.0 | 4517 | 4764 | -2242 | L1MB5 | L1 | LINE |
| 164869057 | 164869370 | + | 17.7 | 1 | 428 | 0 | MSTC | MaLR | LTR |
| 164869376 | 164870890 | + | 22.0 | 3912 | 5449 | -910 | L1MD | L1 | LINE |
| 164870865 | 164871238 | + | 21.8 | 5781 | 6174 | -4 | L1MB8 | L1 | LINE |
| 164871246 | 164872184 | + | 26.8 | 5328 | 6290 | -1495 | L1MC3 | L1 | LINE |

  

|  |  |  |  |  |  |  |  |  |  |
| --- | --- | --- | --- | --- | --- | --- | --- | --- | --- |
| chr2:167000793-167003253 | | | 2459 bp | | |  |  |  |  |
| Genome Start | Genome End | Strand | Div | Repeat Start | Repeat End | Repeat Left | Name | Family | Class |
| 167000793 | 167000916 | - | 36.7 | 81 | 201 | -67 | MIRb | MIR | SINE |
| 167000917 | 167001289 | + | 14.2 | 1 | 375 | 0 | THE1C | MaLR | LTR |
| 167001292 | 167002822 | + | 28.6 | 2 | 1735 | 0 | MLT1K-int | MaLR | LTR |
| 167002823 | 167003195 | + | 12.4 | 1 | 375 | 0 | THE1C | MaLR | LTR |
| 167003196 | 167003253 | - | 36.7 | 21 | 80 | -188 | MIRb | MIR | SINE |

  

|  |  |  |  |  |  |  |  |  |  |
| --- | --- | --- | --- | --- | --- | --- | --- | --- | --- |
| chr2:167302856-167309522 | | | 6651 bp | | |  |  |  |  |
| Genome Start | Genome End | Strand | Div | Repeat Start | Repeat End | Repeat Left | Name | Family | Class |
| 167302856 | 167302915 | - | 11.7 | 238 | 297 | -12 | AluSc | Alu | SINE |
| 167302920 | 167303794 | - | 8.1 | 1 | 824 | -2 | LTR12 | ERV1 | LTR |
| 167303795 | 167304566 | - | 5.8 | 7664 | 8436 | 0 | HERV9 | ERV1 | LTR |
| 167304565 | 167304991 | - | 6.3 | 1432 | 1864 | -3238 | HERV9 | ERV1 | LTR |
| 167304974 | 167305351 | - | 9.8 | 741 | 1200 | -3902 | HERV9 | ERV1 | LTR |
| 167305335 | 167305527 | - | 5.2 | 4775 | 4970 | -3466 | HERV9 | ERV1 | LTR |
| 167305527 | 167308356 | - | 7.7 | 60 | 2980 | -5456 | HERV9 | ERV1 | LTR |
| 167308407 | 167309283 | - | 8.9 | 1 | 826 | 0 | LTR12 | ERV1 | LTR |
| 167309284 | 167309522 | - | 10.9 | 1 | 239 | -70 | AluSc | Alu | SINE |

  

|  |  |  |  |  |  |  |  |  |  |
| --- | --- | --- | --- | --- | --- | --- | --- | --- | --- |
| chr2:167724948-167742345 | | | 17339 bp | | |  |  |  |  |
| Genome Start | Genome End | Strand | Div | Repeat Start | Repeat End | Repeat Left | Name | Family | Class |
| 167724948 | 167725151 | - | 27.9 | 5896 | 6121 | 0 | L1ME4a | L1 | LINE |
| 167725152 | 167725493 | - | 14.1 | 1 | 362 | -2 | THE1B | MaLR | LTR |
| 167725494 | 167727061 | - | 31.0 | 2 | 1735 | 0 | MLT1D-int | MaLR | LTR |
| 167727064 | 167727407 | - | 11.8 | 1 | 364 | 0 | THE1B | MaLR | LTR |
| 167727459 | 167728914 | - | 24.1 | 4626 | 6155 | -9 | L1ME2 | L1 | LINE |
| 167728915 | 167729229 | + | 5.5 | 1 | 309 | -2 | AluY | Alu | SINE |
| 167729230 | 167729501 | - | 27.3 | 4362 | 4625 | -1521 | L1ME2 | L1 | LINE |
| 167729470 | 167730890 | - | 16.8 | 5020 | 6507 | -1 | L1MC1 | L1 | LINE |
| 167730888 | 167731242 | - | 16.8 | 2800 | 3148 | -3173 | L1MC1 | L1 | LINE |
| 167731243 | 167731617 | - | 15.2 | 1 | 426 | 0 | MSTB | MaLR | LTR |
| 167731618 | 167733358 | - | 15.5 | 995 | 2799 | -3522 | L1MC1 | L1 | LINE |
| 167733359 | 167733636 | - | 6.8 | 34 | 311 | 0 | AluY | Alu | SINE |
| 167733637 | 167734415 | - | 18.7 | 1 | 994 | -5514 | L1MC1 | L1 | LINE |
| 167734418 | 167735643 | - | 29.0 | 3040 | 4397 | -1743 | L1M3 | L1 | LINE |
| 167735644 | 167735955 | - | 10.6 | 1 | 312 | 0 | AluSx | Alu | SINE |
| 167735956 | 167736624 | - | 31.4 | 2330 | 3039 | -3101 | L1M3 | L1 | LINE |
| 167736592 | 167736687 | - | 26.9 | 3554 | 3647 | -3687 | L1MCc | L1 | LINE |
| 167736681 | 167736792 | - | 11.2 | 6204 | 6304 | 0 | L1MA2 | L1 | LINE |
| 167736793 | 167737844 | - | 8.4 | 1 | 1232 | -4 | MER11B | ERVK | LTR |
| 167737845 | 167738666 | - | 11.2 | 5361 | 6203 | -101 | L1MA2 | L1 | LINE |
| 167738683 | 167740093 | - | 9.0 | 2699 | 4154 | -2307 | L1P3 | L1 | LINE |
| 167740077 | 167741220 | - | 9.0 | 1 | 1284 | -5177 | L1P3 | L1 | LINE |
| 167741225 | 167741684 | - | 33.8 | 1043 | 1510 | -4978 | L1MCc | L1 | LINE |
| 167741742 | 167742181 | - | 30.3 | 79 | 518 | -5970 | L1MCc | L1 | LINE |
| 167742201 | 167742345 | - | 23.0 | 5714 | 5861 | -260 | L1ME4a | L1 | LINE |

  

|  |  |  |  |  |  |  |  |  |  |
| --- | --- | --- | --- | --- | --- | --- | --- | --- | --- |
| chr2:167831659-167834583 | | | 2840 bp | | |  |  |  |  |
| Genome Start | Genome End | Strand | Div | Repeat Start | Repeat End | Repeat Left | Name | Family | Class |
| 167831659 | 167832041 | + | 37.4 | 2256 | 2663 | -756 | L2 | L2 | LINE |
| 167832123 | 167832478 | + | 11.5 | 2 | 364 | 0 | THE1B | MaLR | LTR |
| 167832481 | 167834100 | + | 30.3 | 2 | 1735 | 0 | MLT1J2-int | MaLR | LTR |
| 167834101 | 167834452 | + | 18.0 | 1 | 362 | -2 | THE1B | MaLR | LTR |
| 167834455 | 167834583 | + | 35.9 | 3248 | 3378 | 0 | L2 | L2 | LINE |

  

|  |  |  |  |  |  |  |  |  |  |
| --- | --- | --- | --- | --- | --- | --- | --- | --- | --- |
| chr2:167979728-167982343 | | | 2614 bp | | |  |  |  |  |
| Genome Start | Genome End | Strand | Div | Repeat Start | Repeat End | Repeat Left | Name | Family | Class |
| 167979728 | 167979845 | - | 31.6 | 116 | 243 | -25 | MIRb | MIR | SINE |
| 167979846 | 167980238 | + | 15.8 | 1 | 426 | -2 | MSTC | MaLR | LTR |
| 167980239 | 167981863 | + | 25.0 | 2 | 1733 | -2 | MSTC-int | MaLR | LTR |
| 167981866 | 167982287 | + | 15.7 | 1 | 428 | 0 | MSTC | MaLR | LTR |
| 167982288 | 167982343 | - | 31.6 | 62 | 115 | -153 | MIRb | MIR | SINE |

  

|  |  |  |  |  |  |  |  |  |  |
| --- | --- | --- | --- | --- | --- | --- | --- | --- | --- |
| chr2:169169171-169172018 | | | 2676 bp | | |  |  |  |  |
| Genome Start | Genome End | Strand | Div | Repeat Start | Repeat End | Repeat Left | Name | Family | Class |
| 169169171 | 169169248 | + | 26.9 | 1 | 78 | -137 | MER58 | MER1\_type | DNA |
| 169169249 | 169169666 | + | 12.5 | 1 | 428 | 0 | MSTA | MaLR | LTR |
| 169169667 | 169171147 | + | 14.5 | 1 | 1479 | -172 | MSTA-int | MaLR | LTR |
| 169171253 | 169171436 | + | 14.5 | 1480 | 1649 | -2 | MSTA-int | MaLR | LTR |
| 169171439 | 169171852 | + | 10.3 | 1 | 428 | 0 | MSTA | MaLR | LTR |
| 169171918 | 169172018 | + | 20.8 | 2318 | 2420 | 0 | MER58 | MER1\_type | DNA |

  

|  |  |  |  |  |  |  |  |  |  |
| --- | --- | --- | --- | --- | --- | --- | --- | --- | --- |
| chr2:169607506-169615521 | | | 8004 bp | | |  |  |  |  |
| Genome Start | Genome End | Strand | Div | Repeat Start | Repeat End | Repeat Left | Name | Family | Class |
| 169607506 | 169607806 | + | 18.1 | 3518 | 3857 | -2289 | L1MB5 | L1 | LINE |
| 169607807 | 169608159 | + | 9.1 | 1 | 355 | 0 | THE1A | MaLR | LTR |
| 169608162 | 169609741 | + | 30.6 | 2 | 1735 | 0 | MLT-int | MaLR | LTR |
| 169609744 | 169610094 | + | 7.4 | 3 | 355 | 0 | THE1A | MaLR | LTR |
| 169610095 | 169611071 | + | 18.1 | 3858 | 4836 | -1310 | L1MB5 | L1 | LINE |
| 169611073 | 169611671 | + | 14.1 | 15 | 602 | -6963 | L1M1 | L1 | LINE |
| 169611669 | 169614302 | + | 11.2 | 819 | 3519 | -4046 | L1M1 | L1 | LINE |
| 169614301 | 169614750 | + | 14.1 | 4823 | 5274 | -872 | L1MB5 | L1 | LINE |
| 169614763 | 169615521 | + | 18.4 | 5409 | 6164 | -10 | L1MB5 | L1 | LINE |

  

|  |  |  |  |  |  |  |  |  |  |
| --- | --- | --- | --- | --- | --- | --- | --- | --- | --- |
| chr2:170249066-170257890 | | | 8438 bp | | |  |  |  |  |
| Genome Start | Genome End | Strand | Div | Repeat Start | Repeat End | Repeat Left | Name | Family | Class |
| 170249066 | 170249404 | - | 17.0 | 6322 | 6666 | -16 | L1MB7 | L1 | LINE |
| 170249434 | 170249711 | - | 14.2 | 1 | 276 | -36 | AluSx | Alu | SINE |
| 170249712 | 170250763 | - | 17.2 | 5249 | 6321 | -361 | L1MB7 | L1 | LINE |
| 170250764 | 170250939 | - | 21.1 | 1 | 175 | -10 | FAM | Alu | SINE |
| 170250947 | 170251158 | - | 15.4 | 5050 | 5253 | -1391 | L1MB7 | L1 | LINE |
| 170251159 | 170251577 | - | 12.7 | 5 | 428 | 0 | MSTC | MaLR | LTR |
| 170251578 | 170251617 | - | 15.4 | 5010 | 5049 | -1595 | L1MB7 | L1 | LINE |
| 170251618 | 170251971 | - | 9.0 | 1 | 355 | 0 | THE1A | MaLR | LTR |
| 170251972 | 170253658 | - | 28.9 | 2 | 1735 | 0 | MLT-int | MaLR | LTR |
| 170253661 | 170254010 | - | 8.6 | 1 | 355 | 0 | THE1A | MaLR | LTR |
| 170254011 | 170254750 | - | 15.4 | 4237 | 5009 | -1635 | L1MB7 | L1 | LINE |
| 170254766 | 170254913 | - | 15.5 | 11 | 165 | -20 | FAM | Alu | SINE |
| 170254926 | 170255189 | - | 17.1 | 3980 | 4251 | -2393 | L1MB7 | L1 | LINE |
| 170255194 | 170255438 | + | 9.0 | 58 | 302 | -11 | AluSq | Alu | SINE |
| 170255450 | 170256179 | - | 18.5 | 1101 | 1866 | -5106 | L1MB7 | L1 | LINE |
| 170256393 | 170257003 | - | 19.4 | 502 | 1140 | -5402 | L1MB7 | L1 | LINE |
| 170257004 | 170257196 | - | 18.0 | 217 | 389 | -7 | MSTD | MaLR | LTR |
| 170257244 | 170257413 | - | 18.0 | 31 | 216 | -180 | MSTD | MaLR | LTR |
| 170257461 | 170257890 | - | 27.0 | 1 | 432 | -6110 | L1MB7 | L1 | LINE |

  

|  |  |  |  |  |  |  |  |  |  |
| --- | --- | --- | --- | --- | --- | --- | --- | --- | --- |
| chr2:171985220-171991285 | | | 5972 bp | | |  |  |  |  |
| Genome Start | Genome End | Strand | Div | Repeat Start | Repeat End | Repeat Left | Name | Family | Class |
| 171985220 | 171985396 | + | 31.5 | 2 | 170 | -5949 | L1ME3B | L1 | LINE |
| 171985421 | 171986053 | + | 27.2 | 3757 | 4386 | -1765 | L1MC | L1 | LINE |
| 171986054 | 171986215 | + | 16.1 | 5 | 170 | -15 | FAM | Alu | SINE |
| 171986254 | 171986386 | + | 27.8 | 4365 | 4498 | -1631 | L1ME3B | L1 | LINE |
| 171986387 | 171986619 | + | 18.9 | 5 | 238 | -1 | MER8 | MER2\_type | DNA |
| 171986620 | 171987671 | + | 29.9 | 4498 | 5587 | -636 | L1ME3B | L1 | LINE |
| 171987672 | 171988044 | + | 10.8 | 1 | 375 | 0 | THE1C | MaLR | LTR |
| 171988047 | 171989628 | + | 27.8 | 2 | 1735 | 0 | MLT-int | MaLR | LTR |
| 171989629 | 171990005 | + | 6.4 | 1 | 375 | 0 | THE1C | MaLR | LTR |
| 171990006 | 171990062 | + | 29.1 | 5588 | 5644 | -579 | L1ME3B | L1 | LINE |
| 171990066 | 171990556 | + | 23.1 | 1 | 654 | 0 | MLT1E1 | MaLR | LTR |
| 171990566 | 171990632 | + | 24.1 | 5654 | 5715 | -508 | L1ME3B | L1 | LINE |
| 171990633 | 171990938 | + | 12.1 | 1 | 306 | -4 | AluSg | Alu | SINE |
| 171990939 | 171991134 | + | 24.1 | 5716 | 5931 | -292 | L1ME3B | L1 | LINE |
| 171991153 | 171991285 | + | 29.4 | 6037 | 6172 | -51 | L1ME3B | L1 | LINE |

  

|  |  |  |  |  |  |  |  |  |  |
| --- | --- | --- | --- | --- | --- | --- | --- | --- | --- |
| chr2:173320187-173323947 | | | 3759 bp | | |  |  |  |  |
| Genome Start | Genome End | Strand | Div | Repeat Start | Repeat End | Repeat Left | Name | Family | Class |
| 173320187 | 173320447 | - | 17.4 | 6021 | 6281 | -10 | L1MA8 | L1 | LINE |
| 173320448 | 173320736 | - | 16.6 | 1 | 293 | -19 | AluJo | Alu | SINE |
| 173320737 | 173321252 | - | 17.4 | 5550 | 6045 | -246 | L1MA8 | L1 | LINE |
| 173321253 | 173321615 | - | 12.9 | 1 | 364 | 0 | THE1B | MaLR | LTR |
| 173321616 | 173323194 | - | 30.0 | 2 | 1735 | 0 | MLT-int | MaLR | LTR |
| 173323197 | 173323565 | - | 13.9 | 1 | 364 | 0 | THE1B | MaLR | LTR |
| 173323566 | 173323947 | - | 16.6 | 5160 | 5549 | -742 | L1MA8 | L1 | LINE |

  

|  |  |  |  |  |  |  |  |  |  |
| --- | --- | --- | --- | --- | --- | --- | --- | --- | --- |
| chr2:174935439-174941504 | | | 6062 bp | | |  |  |  |  |
| Genome Start | Genome End | Strand | Div | Repeat Start | Repeat End | Repeat Left | Name | Family | Class |
| 174935439 | 174935505 | + | 20.6 | 12 | 79 | -5966 | L1MCb | L1 | LINE |
| 174935506 | 174935689 | + | 6.1 | 131 | 310 | -1 | AluY | Alu | SINE |
| 174935690 | 174936513 | + | 20.6 | 80 | 963 | -5082 | L1MCb | L1 | LINE |
| 174936514 | 174936870 | - | 10.4 | 1 | 364 | 0 | THE1B | MaLR | LTR |
| 174936873 | 174938451 | - | 29.4 | 2 | 1733 | -2 | MLT-int | MaLR | LTR |
| 174938454 | 174938808 | - | 13.6 | 1 | 364 | 0 | THE1B | MaLR | LTR |
| 174938809 | 174940137 | + | 23.3 | 964 | 2603 | -4148 | L1MCb | L1 | LINE |
| 174940138 | 174940275 | + | 16.8 | 1 | 145 | -167 | AluJo | Alu | SINE |
| 174940276 | 174940552 | + | 8.7 | 5 | 279 | -30 | AluSc | Alu | SINE |
| 174940553 | 174940739 | + | 16.8 | 146 | 302 | -10 | AluJo | Alu | SINE |
| 174940740 | 174941504 | + | 15.6 | 2604 | 3355 | -3209 | L1MCb | L1 | LINE |

  

|  |  |  |  |  |  |  |  |  |  |
| --- | --- | --- | --- | --- | --- | --- | --- | --- | --- |
| chr2:174961549-174964176 | | | 2626 bp | | |  |  |  |  |
| Genome Start | Genome End | Strand | Div | Repeat Start | Repeat End | Repeat Left | Name | Family | Class |
| 174961549 | 174961720 | - | 31.4 | 5852 | 6024 | -1784 | L1MC4a | L1 | LINE |
| 174961721 | 174962066 | + | 9.6 | 1 | 375 | 0 | THE1C | MaLR | LTR |
| 174962069 | 174963600 | + | 29.3 | 2 | 1735 | 0 | MLT-int | MaLR | LTR |
| 174963601 | 174963980 | + | 10.5 | 1 | 374 | -1 | THE1C | MaLR | LTR |
| 174963981 | 174964176 | - | 31.4 | 5664 | 5851 | -1957 | L1MC4a | L1 | LINE |

  

|  |  |  |  |  |  |  |  |  |  |
| --- | --- | --- | --- | --- | --- | --- | --- | --- | --- |
| chr2:176368676-176371737 | | | 3059 bp | | |  |  |  |  |
| Genome Start | Genome End | Strand | Div | Repeat Start | Repeat End | Repeat Left | Name | Family | Class |
| 176368676 | 176368778 | + | 23.8 | 1 | 103 | -487 | MLT1G | MaLR | LTR |
| 176368779 | 176369065 | - | 13.2 | 1 | 287 | -23 | AluSg | Alu | SINE |
| 176369066 | 176369235 | + | 23.8 | 104 | 281 | -309 | MLT1G | MaLR | LTR |
| 176369236 | 176369578 | + | 11.8 | 1 | 364 | 0 | THE1B | MaLR | LTR |
| 176369581 | 176371106 | + | 30.0 | 2 | 1735 | 0 | MLT-int | MaLR | LTR |
| 176371108 | 176371447 | + | 11.9 | 2 | 362 | -2 | THE1B | MaLR | LTR |
| 176371448 | 176371737 | + | 23.8 | 282 | 570 | -20 | MLT1G | MaLR | LTR |

  

|  |  |  |  |  |  |  |  |  |  |
| --- | --- | --- | --- | --- | --- | --- | --- | --- | --- |
| chr2:178215576-178218505 | | | 2928 bp | | |  |  |  |  |
| Genome Start | Genome End | Strand | Div | Repeat Start | Repeat End | Repeat Left | Name | Family | Class |
| 178215576 | 178215697 | + | 26.7 | 156 | 265 | -246 | MLT1J | MaLR | LTR |
| 178215698 | 178216094 | + | 12.4 | 1 | 364 | 0 | THE1B | MaLR | LTR |
| 178216097 | 178217673 | + | 30.6 | 2 | 1735 | 0 | MLT-int | MaLR | LTR |
| 178217674 | 178218044 | + | 13.6 | 1 | 364 | 0 | THE1B | MaLR | LTR |
| 178218045 | 178218149 | + | 26.7 | 266 | 348 | -163 | MLT1J | MaLR | LTR |
| 178218150 | 178218469 | - | 15.2 | 1 | 310 | -2 | AluSx | Alu | SINE |
| 178218470 | 178218505 | + | 26.7 | 349 | 388 | -123 | MLT1J | MaLR | LTR |

  

|  |  |  |  |  |  |  |  |  |  |
| --- | --- | --- | --- | --- | --- | --- | --- | --- | --- |
| chr2:178726497-178729097 | | | 2597 bp | | |  |  |  |  |
| Genome Start | Genome End | Strand | Div | Repeat Start | Repeat End | Repeat Left | Name | Family | Class |
| 178726497 | 178726594 | + | 18.4 | 1 | 98 | -214 | AluJo | Alu | SINE |
| 178726595 | 178726951 | - | 15.3 | 1 | 364 | 0 | THE1B | MaLR | LTR |
| 178726952 | 178728533 | - | 30.4 | 2 | 1735 | 0 | MLT1H-int | MaLR | LTR |
| 178728538 | 178728889 | - | 12.0 | 1 | 362 | -2 | THE1B | MaLR | LTR |
| 178728890 | 178729097 | + | 18.4 | 99 | 295 | -17 | AluJo | Alu | SINE |

  

|  |  |  |  |  |  |  |  |  |  |
| --- | --- | --- | --- | --- | --- | --- | --- | --- | --- |
| chr2:179598079-179600966 | | | 2882 bp | | |  |  |  |  |
| Genome Start | Genome End | Strand | Div | Repeat Start | Repeat End | Repeat Left | Name | Family | Class |
| 179598079 | 179598232 | - | 12.3 | 6010 | 6163 | -2 | L1PA15 | L1 | LINE |
| 179598234 | 179598603 | + | 12.0 | 2 | 364 | 0 | THE1B | MaLR | LTR |
| 179598604 | 179600146 | + | 9.5 | 1 | 1574 | -6 | THE1B-int | MaLR | LTR |
| 179600152 | 179600334 | + | 14.4 | 1 | 184 | -180 | THE1B | MaLR | LTR |
| 179600335 | 179600648 | + | 7.7 | 1 | 311 | 0 | AluY | Alu | SINE |
| 179600649 | 179600843 | + | 14.4 | 185 | 364 | 0 | THE1B | MaLR | LTR |
| 179600844 | 179600966 | - | 13.9 | 5891 | 6014 | -151 | L1PA15 | L1 | LINE |

  

|  |  |  |  |  |  |  |  |  |  |
| --- | --- | --- | --- | --- | --- | --- | --- | --- | --- |
| chr2:179654467-179670867 | | | 16368 bp | | |  |  |  |  |
| Genome Start | Genome End | Strand | Div | Repeat Start | Repeat End | Repeat Left | Name | Family | Class |
| 179654467 | 179654665 | - | 15.9 | 7397 | 7591 | -132 | L1MA2 | L1 | LINE |
| 179654666 | 179655040 | - | 9.7 | 1 | 375 | 0 | THE1C | MaLR | LTR |
| 179655041 | 179656579 | - | 10.8 | 1 | 1580 | 0 | THE1C-int | MaLR | LTR |
| 179656580 | 179656953 | - | 9.1 | 1 | 375 | 0 | THE1C | MaLR | LTR |
| 179656954 | 179659128 | - | 10.6 | 5209 | 7401 | -322 | L1MA2 | L1 | LINE |
| 179659129 | 179659433 | - | 7.5 | 1 | 305 | -6 | AluY | Alu | SINE |
| 179659434 | 179660327 | - | 10.6 | 4314 | 5208 | -2357 | L1MA2 | L1 | LINE |
| 179660360 | 179666532 | - | 4.6 | 1 | 6152 | -3 | L1PA4 | L1 | LINE |
| 179666534 | 179670867 | - | 15.3 | 19 | 4339 | -3226 | L1MA2 | L1 | LINE |

  

|  |  |  |  |  |  |  |  |  |  |
| --- | --- | --- | --- | --- | --- | --- | --- | --- | --- |
| chr2:182275325-182278329 | | | 2996 bp | | |  |  |  |  |
| Genome Start | Genome End | Strand | Div | Repeat Start | Repeat End | Repeat Left | Name | Family | Class |
| 182275325 | 182275701 | - | 7.2 | 5792 | 6155 | -8 | L1PA13 | L1 | LINE |
| 182275706 | 182276068 | - | 15.7 | 3 | 375 | 0 | THE1C | MaLR | LTR |
| 182276071 | 182277619 | - | 16.1 | 4 | 1580 | 0 | THE1C-int | MaLR | LTR |
| 182277623 | 182277984 | - | 15.2 | 1 | 375 | 0 | THE1C | MaLR | LTR |
| 182277985 | 182278329 | - | 8.8 | 5306 | 5792 | -371 | L1PA13 | L1 | LINE |

  

|  |  |  |  |  |  |  |  |  |  |
| --- | --- | --- | --- | --- | --- | --- | --- | --- | --- |
| chr2:182283950-182287067 | | | 3116 bp | | |  |  |  |  |
| Genome Start | Genome End | Strand | Div | Repeat Start | Repeat End | Repeat Left | Name | Family | Class |
| 182283950 | 182284035 | - | 28.6 | 3259 | 3344 | -75 | L2 | L2 | LINE |
| 182284036 | 182284389 | + | 11.1 | 1 | 355 | 0 | THE1A | MaLR | LTR |
| 182284392 | 182285953 | + | 28.9 | 2 | 1735 | 0 | MLT-int | MaLR | LTR |
| 182285954 | 182286314 | + | 11.1 | 1 | 355 | 0 | THE1A | MaLR | LTR |
| 182286315 | 182286672 | - | 27.6 | 2912 | 3258 | -161 | L2 | L2 | LINE |
| 182286673 | 182287037 | + | 9.9 | 1 | 364 | 0 | THE1B | MaLR | LTR |
| 182287038 | 182287067 | - | 26.6 | 2882 | 2911 | -508 | L2 | L2 | LINE |

  

|  |  |  |  |  |  |  |  |  |  |
| --- | --- | --- | --- | --- | --- | --- | --- | --- | --- |
| chr2:182947231-182951482 | | | 4250 bp | | |  |  |  |  |
| Genome Start | Genome End | Strand | Div | Repeat Start | Repeat End | Repeat Left | Name | Family | Class |
| 182947231 | 182947750 | + | 26.1 | 4920 | 5451 | -713 | L1ME2 | L1 | LINE |
| 182947751 | 182948124 | - | 12.5 | 1 | 364 | 0 | THE1B | MaLR | LTR |
| 182948125 | 182949665 | - | 30.7 | 2 | 1735 | 0 | MLT-int | MaLR | LTR |
| 182949668 | 182950043 | - | 15.1 | 1 | 364 | 0 | THE1B | MaLR | LTR |
| 182950044 | 182950088 | + | 24.8 | 5452 | 5484 | -680 | L1ME2 | L1 | LINE |
| 182950089 | 182950496 | + | 16.1 | 1 | 428 | 0 | MSTA | MaLR | LTR |
| 182950497 | 182950824 | + | 24.8 | 5485 | 5809 | -355 | L1ME2 | L1 | LINE |
| 182950825 | 182951117 | - | 6.8 | 1 | 294 | -17 | AluY | Alu | SINE |
| 182951118 | 182951482 | + | 24.8 | 5810 | 6163 | -1 | L1ME2 | L1 | LINE |

  

|  |  |  |  |  |  |  |  |  |  |
| --- | --- | --- | --- | --- | --- | --- | --- | --- | --- |
| chr2:183099154-183103068 | | | 3752 bp | | |  |  |  |  |
| Genome Start | Genome End | Strand | Div | Repeat Start | Repeat End | Repeat Left | Name | Family | Class |
| 183099154 | 183099301 | + | 34.0 | 11 | 154 | -5965 | L1MEd | L1 | LINE |
| 183099312 | 183099849 | + | 36.8 | 213 | 763 | -5356 | L1MEd | L1 | LINE |
| 183099926 | 183100156 | + | 29.8 | 786 | 1018 | -5101 | L1MEd | L1 | LINE |
| 183100157 | 183100299 | + | 17.8 | 1 | 125 | -187 | AluJb | Alu | SINE |
| 183100375 | 183100553 | + | 17.8 | 126 | 308 | -4 | AluJb | Alu | SINE |
| 183100554 | 183100656 | + | 29.8 | 1019 | 1120 | -4999 | L1MEd | L1 | LINE |
| 183100657 | 183101008 | + | 13.8 | 1 | 364 | 0 | THE1B | MaLR | LTR |
| 183101011 | 183102588 | + | 32.1 | 2 | 1735 | 0 | MLT-int | MaLR | LTR |
| 183102589 | 183102931 | + | 13.9 | 1 | 364 | 0 | THE1B | MaLR | LTR |
| 183102932 | 183103068 | + | 29.8 | 1121 | 1249 | -4870 | L1MEd | L1 | LINE |

  

|  |  |  |  |  |  |  |  |  |  |
| --- | --- | --- | --- | --- | --- | --- | --- | --- | --- |
| chr2:183616468-183627405 | | | 10439 bp | | |  |  |  |  |
| Genome Start | Genome End | Strand | Div | Repeat Start | Repeat End | Repeat Left | Name | Family | Class |
| 183616468 | 183616567 | - | 31.3 | 2290 | 2395 | -1024 | L2 | L2 | LINE |
| 183616760 | 183616849 | + | 10.4 | 1 | 132 | -318 | LTR7 | ERV1 | LTR |
| 183616850 | 183617158 | + | 7.8 | 1 | 309 | -4 | AluSp | Alu | SINE |
| 183617159 | 183617452 | + | 10.4 | 133 | 447 | -3 | LTR7 | ERV1 | LTR |
| 183617455 | 183620288 | + | 12.8 | 1 | 3004 | -4709 | HERVH | ERV1 | LTR |
| 183620259 | 183620827 | + | 16.9 | 3140 | 3736 | -3977 | HERVH | ERV1 | LTR |
| 183620838 | 183621850 | + | 10.4 | 3995 | 4992 | -2721 | HERVH | ERV1 | LTR |
| 183621830 | 183622249 | + | 7.0 | 7296 | 7713 | 0 | HERVH | ERV1 | LTR |
| 183622250 | 183622623 | + | 7.8 | 1 | 448 | -2 | LTR7 | ERV1 | LTR |
| 183622627 | 183623070 | - | 29.5 | 1670 | 2162 | -1257 | L2 | L2 | LINE |
| 183623142 | 183623244 | - | 28.4 | 159 | 261 | -1 | MIR | MIR | SINE |
| 183623245 | 183623752 | - | 12.4 | 1 | 511 | 0 | MER9 | ERVK | LTR |
| 183623753 | 183624091 | - | 12.4 | 5635 | 7337 | 0 | HERVK9 | ERVK | LTR |
| 183624118 | 183624866 | - | 9.5 | 4893 | 5641 | -275 | HERVK9 | ERVK | LTR |
| 183624867 | 183625656 | - | 6.1 | 1424 | 2213 | -3703 | HERVK9 | ERVK | LTR |
| 183625655 | 183626227 | - | 8.9 | 1 | 568 | -5348 | HERVK9 | ERVK | LTR |
| 183626228 | 183626740 | - | 11.9 | 1 | 511 | 0 | MER9 | ERVK | LTR |
| 183626822 | 183626908 | - | 27.4 | 1496 | 1584 | -1835 | L2 | L2 | LINE |
| 183626987 | 183627170 | + | 24.4 | 14 | 201 | -67 | MIRb | MIR | SINE |
| 183627260 | 183627405 | - | 28.7 | 1150 | 1313 | -2106 | L2 | L2 | LINE |

  

|  |  |  |  |  |  |  |  |  |  |
| --- | --- | --- | --- | --- | --- | --- | --- | --- | --- |
| chr2:185290834-185295099 | | | 4263 bp | | |  |  |  |  |
| Genome Start | Genome End | Strand | Div | Repeat Start | Repeat End | Repeat Left | Name | Family | Class |
| 185290834 | 185290974 | + | 17.0 | 4028 | 4170 | -1973 | L1MA4A | L1 | LINE |
| 185290975 | 185291318 | - | 16.5 | 1 | 364 | 0 | THE1B | MaLR | LTR |
| 185291319 | 185292916 | - | 11.1 | 2 | 1580 | 0 | THE1B-int | MaLR | LTR |
| 185292919 | 185293261 | - | 18.6 | 1 | 363 | -1 | THE1B | MaLR | LTR |
| 185293263 | 185295099 | + | 16.9 | 4167 | 6023 | -280 | L1MA4A | L1 | LINE |

  

|  |  |  |  |  |  |  |  |  |  |
| --- | --- | --- | --- | --- | --- | --- | --- | --- | --- |
| chr2:185453911-185461103 | | | 7193 bp | | |  |  |  |  |
| Genome Start | Genome End | Strand | Div | Repeat Start | Repeat End | Repeat Left | Name | Family | Class |
| 185453911 | 185458533 | + | 15.4 | 1684 | 6393 | -299 | L1PA15 | L1 | LINE |
| 185458534 | 185458886 | - | 12.5 | 1 | 355 | 0 | THE1A | MaLR | LTR |
| 185458887 | 185460458 | - | 11.3 | 1 | 1580 | 0 | THE1A-int | MaLR | LTR |
| 185460459 | 185460812 | - | 10.2 | 1 | 355 | 0 | THE1A | MaLR | LTR |
| 185460813 | 185461103 | + | 15.5 | 6389 | 6679 | -13 | L1PA15 | L1 | LINE |

  

|  |  |  |  |  |  |  |  |  |  |
| --- | --- | --- | --- | --- | --- | --- | --- | --- | --- |
| chr2:185836187-185840486 | | | 3858 bp | | |  |  |  |  |
| Genome Start | Genome End | Strand | Div | Repeat Start | Repeat End | Repeat Left | Name | Family | Class |
| 185836187 | 185836549 | - | 27.0 | 1115 | 1581 | -4982 | L1MEb | L1 | LINE |
| 185836598 | 185836744 | - | 23.9 | 6015 | 6333 | -1 | L1ME1 | L1 | LINE |
| 185836745 | 185837107 | - | 15.9 | 1 | 381 | 0 | THE1D | MaLR | LTR |
| 185837108 | 185838701 | - | 31.0 | 2 | 1735 | 0 | MLT-int | MaLR | LTR |
| 185838704 | 185839067 | - | 16.6 | 1 | 381 | 0 | THE1D | MaLR | LTR |
| 185839068 | 185839613 | - | 28.6 | 5467 | 6014 | -152 | L1ME1 | L1 | LINE |
| 185839610 | 185839789 | - | 27.5 | 1011 | 1196 | -5176 | L1MEb | L1 | LINE |
| 185840186 | 185840486 | - | 30.4 | 255 | 573 | -5990 | L1MEb | L1 | LINE |

  

|  |  |  |  |  |  |  |  |  |  |
| --- | --- | --- | --- | --- | --- | --- | --- | --- | --- |
| chr2:186022585-186026028 | | | 3273 bp | | |  |  |  |  |
| Genome Start | Genome End | Strand | Div | Repeat Start | Repeat End | Repeat Left | Name | Family | Class |
| 186022585 | 186022926 | + | 24.3 | 2975 | 3342 | -3195 | L1MDa | L1 | LINE |
| 186022964 | 186023266 | - | 12.5 | 1 | 305 | -4 | AluSc | Alu | SINE |
| 186023267 | 186023614 | - | 16.2 | 1 | 363 | -1 | THE1B | MaLR | LTR |
| 186023615 | 186025188 | - | 10.4 | 1 | 1580 | 0 | THE1B-int | MaLR | LTR |
| 186025189 | 186025541 | - | 14.9 | 1 | 364 | 0 | THE1B | MaLR | LTR |
| 186025676 | 186026028 | + | 28.0 | 3486 | 3841 | -2696 | L1MDa | L1 | LINE |

  

|  |  |  |  |  |  |  |  |  |  |
| --- | --- | --- | --- | --- | --- | --- | --- | --- | --- |
| chr2:186924218-186927894 | | | 3295 bp | | |  |  |  |  |
| Genome Start | Genome End | Strand | Div | Repeat Start | Repeat End | Repeat Left | Name | Family | Class |
| 186924218 | 186924564 | + | 25.1 | 4473 | 4861 | -1285 | L1ME4a | L1 | LINE |
| 186924565 | 186924961 | + | 31.6 | 65 | 457 | 0 | LTR16A1 | ERVL | LTR |
| 186925225 | 186925594 | + | 13.0 | 1 | 364 | 0 | THE1B | MaLR | LTR |
| 186925595 | 186927162 | + | 10.8 | 1 | 1580 | 0 | THE1B-int | MaLR | LTR |
| 186927163 | 186927496 | + | 12.4 | 1 | 361 | -3 | THE1B | MaLR | LTR |
| 186927616 | 186927894 | + | 26.6 | 5791 | 6116 | -5 | L1ME4a | L1 | LINE |

  

|  |  |  |  |  |  |  |  |  |  |
| --- | --- | --- | --- | --- | --- | --- | --- | --- | --- |
| chr2:187780623-187786489 | | | 5880 bp | | |  |  |  |  |
| Genome Start | Genome End | Strand | Div | Repeat Start | Repeat End | Repeat Left | Name | Family | Class |
| 187780623 | 187782400 | + | 15.9 | 221 | 2013 | -4772 | L1PA13 | L1 | LINE |
| 187782401 | 187782734 | + | 12.6 | 1 | 364 | 0 | THE1B | MaLR | LTR |
| 187782737 | 187784312 | + | 29.3 | 2 | 1735 | 0 | MLT-int | MaLR | LTR |
| 187784313 | 187784657 | + | 14.6 | 1 | 364 | 0 | THE1B | MaLR | LTR |
| 187784658 | 187785722 | + | 14.6 | 2014 | 3087 | -3679 | L1PA13 | L1 | LINE |
| 187785708 | 187786489 | + | 14.1 | 5986 | 6778 | -5 | L1PA13 | L1 | LINE |

  

|  |  |  |  |  |  |  |  |  |  |
| --- | --- | --- | --- | --- | --- | --- | --- | --- | --- |
| chr2:189124085-189135462 | | | 11106 bp | | |  |  |  |  |
| Genome Start | Genome End | Strand | Div | Repeat Start | Repeat End | Repeat Left | Name | Family | Class |
| 189124085 | 189128750 | - | 18.3 | 2189 | 6982 | -23 | L1MA4A | L1 | LINE |
| 189128751 | 189129059 | + | 14.2 | 1 | 310 | 0 | AluSg | Alu | SINE |
| 189129060 | 189129683 | - | 20.3 | 1593 | 2188 | -4661 | L1MA4A | L1 | LINE |
| 189129684 | 189129726 | - | 14.5 | 98 | 133 | -295 | MSTA | MaLR | LTR |
| 189129727 | 189130017 | + | 6.2 | 2 | 303 | -8 | AluY | Alu | SINE |
| 189130018 | 189130119 | - | 14.5 | 2 | 97 | -331 | MSTA | MaLR | LTR |
| 189130121 | 189130720 | - | 16.8 | 1053 | 1651 | 0 | MSTA-int | MaLR | LTR |
| 189130741 | 189131132 | - | 22.4 | 511 | 928 | -723 | MSTA-int | MaLR | LTR |
| 189131133 | 189131485 | + | 10.0 | 1 | 353 | -2 | THE1A | MaLR | LTR |
| 189131488 | 189132527 | + | 8.9 | 1 | 1048 | -532 | THE1A-int | MaLR | LTR |
| 189132526 | 189132704 | + | 6.7 | 1401 | 1580 | 0 | THE1A-int | MaLR | LTR |
| 189132705 | 189133059 | + | 12.5 | 1 | 355 | 0 | THE1A | MaLR | LTR |
| 189133063 | 189133564 | - | 14.3 | 1 | 512 | -1139 | MSTA-int | MaLR | LTR |
| 189133565 | 189134005 | - | 14.6 | 1 | 428 | 0 | MSTA | MaLR | LTR |
| 189134006 | 189134812 | - | 31.8 | 659 | 1488 | -5361 | L1MA4A | L1 | LINE |
| 189135061 | 189135462 | - | 24.0 | 80 | 483 | -6366 | L1MA4A | L1 | LINE |

  

|  |  |  |  |  |  |  |  |  |  |
| --- | --- | --- | --- | --- | --- | --- | --- | --- | --- |
| chr2:190080637-190084198 | | | 3558 bp | | |  |  |  |  |
| Genome Start | Genome End | Strand | Div | Repeat Start | Repeat End | Repeat Left | Name | Family | Class |
| 190080637 | 190080762 | + | 16.7 | 5324 | 5439 | -920 | L1MD2 | L1 | LINE |
| 190080763 | 190081041 | + | 15.8 | 9 | 291 | -21 | AluJb | Alu | SINE |
| 190081042 | 190081650 | + | 16.7 | 5440 | 6088 | -271 | L1MD2 | L1 | LINE |
| 190081651 | 190082019 | + | 12.3 | 1 | 364 | 0 | THE1B | MaLR | LTR |
| 190082024 | 190083603 | + | 29.4 | 4 | 1735 | 0 | MLT-int | MaLR | LTR |
| 190083604 | 190083937 | + | 13.2 | 1 | 364 | 0 | THE1B | MaLR | LTR |
| 190083938 | 190084198 | + | 16.7 | 6089 | 6347 | -12 | L1MD2 | L1 | LINE |

  

|  |  |  |  |  |  |  |  |  |  |
| --- | --- | --- | --- | --- | --- | --- | --- | --- | --- |
| chr2:190510366-190518256 | | | 7884 bp | | |  |  |  |  |
| Genome Start | Genome End | Strand | Div | Repeat Start | Repeat End | Repeat Left | Name | Family | Class |
| 190510366 | 190510527 | + | 15.9 | 11 | 176 | -5152 | L1M3f | L1 | LINE |
| 190510527 | 190511024 | + | 8.5 | 5285 | 5787 | -517 | L1MA2 | L1 | LINE |
| 190511025 | 190511332 | + | 12.7 | 1 | 308 | -4 | AluSx | Alu | SINE |
| 190511333 | 190511830 | + | 8.5 | 5788 | 6304 | 0 | L1MA2 | L1 | LINE |
| 190511836 | 190512834 | + | 15.4 | 161 | 1194 | -4134 | L1M3f | L1 | LINE |
| 190512835 | 190512968 | + | 14.2 | 1 | 133 | -178 | AluY | Alu | SINE |
| 190512969 | 190513267 | + | 9.0 | 1 | 299 | -12 | AluY | Alu | SINE |
| 190513268 | 190513402 | + | 14.2 | 134 | 267 | -44 | AluY | Alu | SINE |
| 190513403 | 190513628 | + | 20.5 | 1195 | 1394 | -4934 | L1M3f | L1 | LINE |
| 190513629 | 190513871 | + | 10.7 | 1 | 291 | -19 | AluYa5 | Alu | SINE |
| 190513872 | 190515063 | + | 21.5 | 1395 | 2599 | -3726 | L1M3f | L1 | LINE |
| 190515064 | 190515457 | + | 18.4 | 1 | 428 | 0 | MSTA | MaLR | LTR |
| 190515460 | 190517102 | + | 28.4 | 2 | 1734 | -1 | MLT-int | MaLR | LTR |
| 190517104 | 190517502 | + | 17.0 | 1 | 428 | 0 | MSTA | MaLR | LTR |
| 190517503 | 190518256 | + | 14.8 | 2600 | 3361 | -2964 | L1M3f | L1 | LINE |

  

|  |  |  |  |  |  |  |  |  |  |
| --- | --- | --- | --- | --- | --- | --- | --- | --- | --- |
| chr2:190583465-190593429 | | | 9613 bp | | |  |  |  |  |
| Genome Start | Genome End | Strand | Div | Repeat Start | Repeat End | Repeat Left | Name | Family | Class |
| 190583465 | 190583550 | + | 22.6 | 2370 | 2453 | -409 | Charlie2 | MER1\_type | DNA |
| 190583722 | 190583837 | - | 16.4 | 6161 | 6280 | -11 | L1MA7 | L1 | LINE |
| 190583844 | 190584021 | - | 17.7 | 6634 | 6757 | -965 | L1M | L1 | LINE |
| 190584022 | 190584330 | - | 14.2 | 2 | 306 | -7 | AluSq | Alu | SINE |
| 190584331 | 190585109 | - | 17.4 | 5863 | 6641 | -924 | L1M | L1 | LINE |
| 190585110 | 190585286 | - | 17.5 | 110 | 292 | -20 | AluJb | Alu | SINE |
| 190585287 | 190586464 | - | 17.4 | 4685 | 5862 | -1703 | L1M | L1 | LINE |
| 190586465 | 190586786 | - | 9.2 | 3 | 321 | 0 | AluY | Alu | SINE |
| 190586787 | 190587151 | - | 27.3 | 3094 | 3467 | -2883 | L1M3b | L1 | LINE |
| 190587160 | 190587534 | - | 16.5 | 1 | 381 | 0 | THE1D | MaLR | LTR |
| 190587535 | 190587612 | - | 15.4 | 1502 | 1580 | 0 | THE1D-int | MaLR | LTR |
| 190587614 | 190587751 | - | 18.1 | 156 | 294 | -18 | AluSg/x | Alu | SINE |
| 190587752 | 190589155 | - | 13.5 | 1 | 1367 | -213 | THE1D-int | MaLR | LTR |
| 190589156 | 190589522 | - | 9.5 | 1 | 381 | 0 | THE1D | MaLR | LTR |
| 190589523 | 190592986 | - | 24.7 | 1 | 3090 | -3257 | L1M3b | L1 | LINE |
| 190593153 | 190593429 | + | 22.8 | 2488 | 2782 | -80 | Charlie2 | MER1\_type | DNA |

  

|  |  |  |  |  |  |  |  |  |  |
| --- | --- | --- | --- | --- | --- | --- | --- | --- | --- |
| chr2:193702385-193707176 | | | 4627 bp | | |  |  |  |  |
| Genome Start | Genome End | Strand | Div | Repeat Start | Repeat End | Repeat Left | Name | Family | Class |
| 193702385 | 193702697 | - | 28.9 | 4426 | 4932 | -1389 | L1MCa | L1 | LINE |
| 193702725 | 193703468 | - | 27.2 | 3656 | 4425 | -1896 | L1MCa | L1 | LINE |
| 193703469 | 193703850 | - | 16.6 | 1 | 428 | 0 | MSTA | MaLR | LTR |
| 193703851 | 193705450 | - | 31.1 | 2 | 1735 | 0 | MLT-int | MaLR | LTR |
| 193705453 | 193705839 | - | 19.1 | 1 | 428 | 0 | MSTA | MaLR | LTR |
| 193705840 | 193706043 | - | 27.2 | 3467 | 3655 | -2666 | L1MCa | L1 | LINE |
| 193706128 | 193706537 | - | 23.3 | 2267 | 2679 | -3642 | L1MCa | L1 | LINE |
| 193706590 | 193707176 | - | 28.8 | 1504 | 2227 | -4275 | L1MCa | L1 | LINE |

  

|  |  |  |  |  |  |  |  |  |  |
| --- | --- | --- | --- | --- | --- | --- | --- | --- | --- |
| chr2:193709404-193714526 | | | 5113 bp | | |  |  |  |  |
| Genome Start | Genome End | Strand | Div | Repeat Start | Repeat End | Repeat Left | Name | Family | Class |
| 193709404 | 193710165 | + | 21.2 | 2719 | 3493 | -3352 | L1MA4A | L1 | LINE |
| 193710172 | 193710328 | + | 18.2 | 4852 | 5006 | -1839 | L1MA4A | L1 | LINE |
| 193710329 | 193710662 | + | 17.1 | 1 | 364 | 0 | THE1B | MaLR | LTR |
| 193710663 | 193712366 | + | 19.1 | 5007 | 6748 | -257 | L1MA4A | L1 | LINE |
| 193712369 | 193712733 | - | 14.0 | 3 | 364 | 0 | THE1B | MaLR | LTR |
| 193712736 | 193714290 | - | 12.7 | 1 | 1580 | 0 | THE1B-int | MaLR | LTR |
| 193714291 | 193714471 | - | 10.7 | 187 | 364 | 0 | THE1B | MaLR | LTR |
| 193714472 | 193714526 | + | 10.9 | 6939 | 6993 | -12 | L1MA4A | L1 | LINE |

  

|  |  |  |  |  |  |  |  |  |  |
| --- | --- | --- | --- | --- | --- | --- | --- | --- | --- |
| chr2:194703570-194709743 | | | 6168 bp | | |  |  |  |  |
| Genome Start | Genome End | Strand | Div | Repeat Start | Repeat End | Repeat Left | Name | Family | Class |
| 194703570 | 194703667 | + | 21.4 | 5804 | 5903 | -263 | L1ME2 | L1 | LINE |
| 194703671 | 194704082 | - | 8.1 | 1 | 448 | -2 | LTR7 | ERV1 | LTR |
| 194704083 | 194704456 | - | 12.5 | 7322 | 7713 | 0 | HERVH | ERV1 | LTR |
| 194704455 | 194704670 | - | 13.4 | 5600 | 5819 | -1894 | HERVH | ERV1 | LTR |
| 194704672 | 194705345 | - | 15.5 | 4478 | 5149 | -2564 | HERVH | ERV1 | LTR |
| 194705337 | 194706188 | - | 8.7 | 3140 | 3993 | -3720 | HERVH | ERV1 | LTR |
| 194706168 | 194709111 | - | 9.9 | 1 | 2995 | -4718 | HERVH | ERV1 | LTR |
| 194709114 | 194709523 | - | 8.6 | 1 | 448 | -2 | LTR7 | ERV1 | LTR |
| 194709556 | 194709743 | + | 25.1 | 5935 | 6130 | -34 | L1ME2 | L1 | LINE |

  

|  |  |  |  |  |  |  |  |  |  |
| --- | --- | --- | --- | --- | --- | --- | --- | --- | --- |
| chr2:195368565-195371747 | | | 2978 bp | | |  |  |  |  |
| Genome Start | Genome End | Strand | Div | Repeat Start | Repeat End | Repeat Left | Name | Family | Class |
| 195368565 | 195368815 | + | 36.0 | 1924 | 2203 | -1216 | L2 | L2 | LINE |
| 195368816 | 195369214 | + | 13.5 | 1 | 364 | 0 | THE1B | MaLR | LTR |
| 195369217 | 195370803 | + | 31.8 | 2 | 1735 | 0 | MLT-int | MaLR | LTR |
| 195370805 | 195371165 | + | 11.7 | 2 | 362 | -2 | THE1B | MaLR | LTR |
| 195371368 | 195371747 | + | 34.3 | 2291 | 2638 | -781 | L2 | L2 | LINE |

  

|  |  |  |  |  |  |  |  |  |  |
| --- | --- | --- | --- | --- | --- | --- | --- | --- | --- |
| chr2:199554352-199560946 | | | 6464 bp | | |  |  |  |  |
| Genome Start | Genome End | Strand | Div | Repeat Start | Repeat End | Repeat Left | Name | Family | Class |
| 199554352 | 199554468 | + | 26.4 | 1 | 107 | -398 | MLT1D | MaLR | LTR |
| 199554542 | 199554728 | + | 15.2 | 2987 | 3178 | -3025 | L1PBb | L1 | LINE |
| 199554729 | 199555103 | + | 15.8 | 1 | 381 | 0 | THE1D | MaLR | LTR |
| 199555104 | 199555155 | + | 15.2 | 3179 | 3236 | -2967 | L1PBb | L1 | LINE |
| 199555156 | 199555277 | + | 17.2 | 266 | 381 | 0 | THE1D | MaLR | LTR |
| 199555278 | 199557413 | + | 9.9 | 3125 | 5278 | -868 | L1PREC2 | L1 | LINE |
| 199557414 | 199557796 | - | 17.2 | 1 | 428 | 0 | MSTA | MaLR | LTR |
| 199557800 | 199559409 | - | 29.5 | 2 | 1733 | -2 | MLT1D-int | MaLR | LTR |
| 199559413 | 199559793 | - | 18.8 | 3 | 428 | 0 | MSTA | MaLR | LTR |
| 199559820 | 199560548 | + | 12.9 | 5410 | 6158 | -2 | L1PREC2 | L1 | LINE |
| 199560575 | 199560946 | + | 23.3 | 118 | 505 | 0 | MLT1D | MaLR | LTR |

  

|  |  |  |  |  |  |  |  |  |  |
| --- | --- | --- | --- | --- | --- | --- | --- | --- | --- |
| chr2:200589672-200591948 | | | 2261 bp | | |  |  |  |  |
| Genome Start | Genome End | Strand | Div | Repeat Start | Repeat End | Repeat Left | Name | Family | Class |
| 200589672 | 200589797 | - | 11.1 | 237 | 365 | 0 | MLT1A0 | MaLR | LTR |
| 200589798 | 200589997 | - | 13.6 | 175 | 364 | 0 | THE1B | MaLR | LTR |
| 200589998 | 200590300 | - | 10.9 | 1 | 302 | -8 | AluSg | Alu | SINE |
| 200590301 | 200590468 | - | 13.6 | 1 | 174 | -190 | THE1B | MaLR | LTR |
| 200590469 | 200590516 | - | 6.2 | 1533 | 1580 | 0 | THE1B-int | MaLR | LTR |
| 200590515 | 200591372 | - | 9.1 | 1 | 885 | -695 | THE1B-int | MaLR | LTR |
| 200591373 | 200591729 | - | 11.5 | 1 | 364 | 0 | THE1B | MaLR | LTR |
| 200591748 | 200591948 | - | 12.5 | 1 | 200 | -165 | MLT1A0 | MaLR | LTR |

  

|  |  |  |  |  |  |  |  |  |  |
| --- | --- | --- | --- | --- | --- | --- | --- | --- | --- |
| chr2:204534738-204541712 | | | 6940 bp | | |  |  |  |  |
| Genome Start | Genome End | Strand | Div | Repeat Start | Repeat End | Repeat Left | Name | Family | Class |
| 204534738 | 204534869 | - | 27.3 | 7734 | 7880 | -162 | L1MC4 | L1 | LINE |
| 204534910 | 204535319 | - | 8.1 | 1 | 448 | -2 | LTR7 | ERV1 | LTR |
| 204535320 | 204535696 | - | 9.9 | 7324 | 7713 | 0 | HERVH | ERV1 | LTR |
| 204535697 | 204535913 | - | 14.8 | 5600 | 5819 | -1894 | HERVH | ERV1 | LTR |
| 204535918 | 204536577 | - | 12.9 | 4488 | 5146 | -2567 | HERVH | ERV1 | LTR |
| 204536581 | 204537081 | - | 9.2 | 3492 | 3992 | -3721 | HERVH | ERV1 | LTR |
| 204537072 | 204537219 | - | 14.9 | 3140 | 3290 | -4423 | HERVH | ERV1 | LTR |
| 204537214 | 204540243 | - | 8.7 | 1 | 2987 | -4726 | HERVH | ERV1 | LTR |
| 204540246 | 204540602 | - | 7.0 | 1 | 448 | -2 | LTR7 | ERV1 | LTR |
| 204540605 | 204541712 | - | 22.3 | 6452 | 7632 | -410 | L1MC4 | L1 | LINE |

  

|  |  |  |  |  |  |  |  |  |  |
| --- | --- | --- | --- | --- | --- | --- | --- | --- | --- |
| chr2:205222495-205226259 | | | 3763 bp | | |  |  |  |  |
| Genome Start | Genome End | Strand | Div | Repeat Start | Repeat End | Repeat Left | Name | Family | Class |
| 205222495 | 205223671 | + | 9.7 | 4768 | 5944 | -216 | L1PREC2 | L1 | LINE |
| 205223672 | 205224056 | - | 18.4 | 1 | 428 | 0 | MSTA | MaLR | LTR |
| 205224057 | 205225645 | - | 29.9 | 2 | 1735 | 0 | MLT1H-int | MaLR | LTR |
| 205225648 | 205226032 | - | 15.7 | 1 | 428 | 0 | MSTA | MaLR | LTR |
| 205226033 | 205226259 | + | 10.1 | 5945 | 6160 | 0 | L1PREC2 | L1 | LINE |

  

|  |  |  |  |  |  |  |  |  |  |
| --- | --- | --- | --- | --- | --- | --- | --- | --- | --- |
| chr2:206919059-206924410 | | | 5353 bp | | |  |  |  |  |
| Genome Start | Genome End | Strand | Div | Repeat Start | Repeat End | Repeat Left | Name | Family | Class |
| 206919059 | 206919213 | - | 14.8 | 3914 | 4073 | -1612 | HERVL74 | ERVL | LTR |
| 206919200 | 206919369 | - | 20.2 | 3488 | 3658 | -2027 | HERVL74 | ERVL | LTR |
| 206919371 | 206919678 | + | 12.7 | 1 | 308 | -4 | AluSx | Alu | SINE |
| 206919682 | 206921292 | - | 19.5 | 1668 | 3340 | -2345 | HERVL74 | ERVL | LTR |
| 206921293 | 206921653 | - | 14.2 | 1 | 381 | 0 | THE1D | MaLR | LTR |
| 206921654 | 206923171 | - | 12.5 | 117 | 1580 | 0 | THE1D-int | MaLR | LTR |
| 206923172 | 206923347 | - | 12.5 | 5998 | 6173 | -1 | L1PA11 | L1 | LINE |
| 206923348 | 206923461 | - | 12.8 | 4 | 116 | -1464 | THE1D-int | MaLR | LTR |
| 206923465 | 206923823 | - | 12.4 | 1 | 381 | 0 | THE1D | MaLR | LTR |
| 206923830 | 206924410 | - | 18.8 | 1050 | 1666 | -4019 | HERVL74 | ERVL | LTR |

  

|  |  |  |  |  |  |  |  |  |  |
| --- | --- | --- | --- | --- | --- | --- | --- | --- | --- |
| chr2:207379598-207385678 | | | 6105 bp | | |  |  |  |  |
| Genome Start | Genome End | Strand | Div | Repeat Start | Repeat End | Repeat Left | Name | Family | Class |
| 207379598 | 207379806 | - | 6.8 | 93 | 298 | -15 | AluSp | Alu | SINE |
| 207379809 | 207380258 | - | 6.5 | 1 | 448 | -2 | LTR7 | ERV1 | LTR |
| 207380259 | 207380636 | - | 7.5 | 7324 | 7713 | 0 | HERVH | ERV1 | LTR |
| 207380637 | 207380861 | - | 13.5 | 5600 | 5819 | -1894 | HERVH | ERV1 | LTR |
| 207380863 | 207381519 | - | 14.3 | 4488 | 5149 | -2564 | HERVH | ERV1 | LTR |
| 207381522 | 207382025 | - | 11.6 | 3492 | 3993 | -3720 | HERVH | ERV1 | LTR |
| 207382016 | 207382165 | - | 12.0 | 3140 | 3290 | -4423 | HERVH | ERV1 | LTR |
| 207382145 | 207385143 | - | 8.7 | 1 | 2995 | -4718 | HERVH | ERV1 | LTR |
| 207385146 | 207385596 | - | 6.0 | 1 | 448 | -2 | LTR7 | ERV1 | LTR |
| 207385597 | 207385678 | - | 6.1 | 16 | 97 | -215 | AluSp | Alu | SINE |

  

|  |  |  |  |  |  |  |  |  |  |
| --- | --- | --- | --- | --- | --- | --- | --- | --- | --- |
| chr2:207589919-207604232 | | | 14297 bp | | |  |  |  |  |
| Genome Start | Genome End | Strand | Div | Repeat Start | Repeat End | Repeat Left | Name | Family | Class |
| 207589919 | 207590186 | + | 21.2 | 1 | 258 | -6951 | L1MA5 | L1 | LINE |
| 207590187 | 207590596 | + | 7.8 | 1 | 448 | -2 | LTR7 | ERV1 | LTR |
| 207590599 | 207594627 | + | 9.9 | 1 | 3993 | -3720 | HERVH | ERV1 | LTR |
| 207594630 | 207595247 | + | 14.6 | 4488 | 5149 | -2564 | HERVH | ERV1 | LTR |
| 207595249 | 207595464 | + | 12.5 | 5600 | 5819 | -1894 | HERVH | ERV1 | LTR |
| 207595465 | 207595841 | + | 9.4 | 7324 | 7713 | 0 | HERVH | ERV1 | LTR |
| 207595842 | 207596253 | + | 7.8 | 1 | 448 | -2 | LTR7 | ERV1 | LTR |
| 207596257 | 207596993 | + | 26.8 | 255 | 1008 | -6201 | L1MA5 | L1 | LINE |
| 207596976 | 207598911 | + | 18.0 | 1722 | 3688 | -3517 | L1MA5 | L1 | LINE |
| 207598912 | 207599211 | + | 1.0 | 1 | 300 | -11 | AluY | Alu | SINE |
| 207599212 | 207600730 | + | 16.4 | 3689 | 5141 | -2064 | L1MA5 | L1 | LINE |
| 207600731 | 207601031 | + | 16.7 | 1 | 300 | -12 | AluJb | Alu | SINE |
| 207601059 | 207601075 | + | 16.7 | 301 | 312 | 0 | AluJb | Alu | SINE |
| 207601076 | 207601541 | + | 16.4 | 5142 | 5595 | -1610 | L1MA5 | L1 | LINE |
| 207601542 | 207601835 | + | 10.9 | 1 | 303 | -10 | AluSp | Alu | SINE |
| 207601836 | 207602144 | + | 16.4 | 5596 | 5907 | -1298 | L1MA5 | L1 | LINE |
| 207602145 | 207602450 | - | 1.3 | 1 | 306 | -12 | AluYb8 | Alu | SINE |
| 207602451 | 207602998 | + | 16.4 | 5908 | 6425 | -780 | L1MA5 | L1 | LINE |
| 207602999 | 207603289 | + | 11.7 | 1 | 291 | -18 | AluSc | Alu | SINE |
| 207603290 | 207604232 | + | 13.1 | 6429 | 7362 | 0 | L1MA5 | L1 | LINE |

  

|  |  |  |  |  |  |  |  |  |  |
| --- | --- | --- | --- | --- | --- | --- | --- | --- | --- |
| chr2:208788111-208795131 | | | 6965 bp | | |  |  |  |  |
| Genome Start | Genome End | Strand | Div | Repeat Start | Repeat End | Repeat Left | Name | Family | Class |
| 208788111 | 208788673 | - | 13.9 | 129 | 711 | -23 | MER50 | ERV1 | LTR |
| 208788722 | 208788890 | - | 16.2 | 93 | 284 | -28 | AluSx | Alu | SINE |
| 208788894 | 208789308 | - | 8.6 | 1 | 448 | -2 | LTR7 | ERV1 | LTR |
| 208789309 | 208789685 | - | 10.2 | 7322 | 7713 | 0 | HERVH | ERV1 | LTR |
| 208789684 | 208789899 | - | 13.4 | 5600 | 5819 | -1894 | HERVH | ERV1 | LTR |
| 208789901 | 208790518 | - | 14.3 | 4488 | 5149 | -2564 | HERVH | ERV1 | LTR |
| 208790521 | 208794497 | - | 10.2 | 1 | 3993 | -3720 | HERVH | ERV1 | LTR |
| 208794500 | 208794904 | - | 9.7 | 6 | 448 | -2 | LTR7 | ERV1 | LTR |
| 208794905 | 208794999 | - | 11.6 | 2 | 96 | -216 | AluSx | Alu | SINE |
| 208795002 | 208795131 | - | 11.5 | 4 | 134 | -600 | MER50 | ERV1 | LTR |

  

|  |  |  |  |  |  |  |  |  |  |
| --- | --- | --- | --- | --- | --- | --- | --- | --- | --- |
| chr2:209086935-209092215 | | | 4767 bp | | |  |  |  |  |
| Genome Start | Genome End | Strand | Div | Repeat Start | Repeat End | Repeat Left | Name | Family | Class |
| 209086935 | 209087086 | - | 26.7 | 5965 | 6143 | -23 | L1ME1 | L1 | LINE |
| 209087123 | 209087191 | - | 20.3 | 5815 | 5884 | -280 | L1ME1 | L1 | LINE |
| 209087192 | 209087602 | - | 14.2 | 1 | 428 | 0 | MSTA | MaLR | LTR |
| 209087603 | 209088972 | - | 17.3 | 1 | 1651 | 0 | MSTA-int | MaLR | LTR |
| 209088973 | 209089393 | - | 12.1 | 1 | 428 | 0 | MSTA | MaLR | LTR |
| 209089396 | 209089730 | - | 24.2 | 5479 | 5813 | -353 | L1ME1 | L1 | LINE |
| 209090207 | 209091266 | - | 22.8 | 4410 | 5449 | -713 | L1ME1 | L1 | LINE |
| 209091267 | 209091563 | - | 12.5 | 1 | 298 | -14 | AluSx | Alu | SINE |
| 209091564 | 209092215 | - | 24.3 | 3763 | 4409 | -1737 | L1ME1 | L1 | LINE |

  

|  |  |  |  |  |  |  |  |  |  |
| --- | --- | --- | --- | --- | --- | --- | --- | --- | --- |
| chr2:209465841-209479004 | | | 12703 bp | | |  |  |  |  |
| Genome Start | Genome End | Strand | Div | Repeat Start | Repeat End | Repeat Left | Name | Family | Class |
| 209465841 | 209466187 | + | 29.3 | 5587 | 5936 | -1872 | L1MC4a | L1 | LINE |
| 209466384 | 209466736 | - | 20.5 | 44 | 390 | 0 | MLT1C | MaLR | LTR |
| 209466737 | 209467136 | + | 8.8 | 1 | 448 | -2 | LTR7 | ERV1 | LTR |
| 209467139 | 209467507 | + | 10.1 | 1 | 369 | -7344 | HERVH | ERV1 | LTR |
| 209467508 | 209467647 | + | 3.6 | 2 | 142 | -5890 | L1HS | L1 | LINE |
| 209467644 | 209473048 | + | 2.5 | 619 | 6032 | 0 | L1PA2 | L1 | LINE |
| 209473084 | 209475614 | + | 11.8 | 362 | 2995 | -4718 | HERVH | ERV1 | LTR |
| 209475584 | 209475743 | + | 12.5 | 3128 | 3290 | -4423 | HERVH | ERV1 | LTR |
| 209475743 | 209477453 | + | 10.6 | 3496 | 5225 | -1866 | HERVH | ERV1 | LTR |
| 209477455 | 209477664 | + | 11.4 | 5600 | 5819 | -1894 | HERVH | ERV1 | LTR |
| 209477665 | 209478041 | + | 8.9 | 7324 | 7713 | 0 | HERVH | ERV1 | LTR |
| 209478042 | 209478436 | + | 8.1 | 1 | 448 | -2 | LTR7 | ERV1 | LTR |
| 209478677 | 209478889 | + | 32.0 | 6391 | 6590 | -1218 | L1MC4a | L1 | LINE |
| 209478913 | 209479004 | + | 32.0 | 6591 | 6676 | -1132 | L1MC4a | L1 | LINE |

  

|  |  |  |  |  |  |  |  |  |  |
| --- | --- | --- | --- | --- | --- | --- | --- | --- | --- |
| chr2:210367474-210370149 | | | 2674 bp | | |  |  |  |  |
| Genome Start | Genome End | Strand | Div | Repeat Start | Repeat End | Repeat Left | Name | Family | Class |
| 210367474 | 210367545 | - | 17.3 | 6114 | 6184 | 0 | L1MB7 | L1 | LINE |
| 210367546 | 210367889 | + | 9.9 | 4 | 355 | 0 | THE1A | MaLR | LTR |
| 210367892 | 210369471 | + | 29.2 | 2 | 1735 | 0 | MLT-int | MaLR | LTR |
| 210369472 | 210369832 | + | 9.6 | 1 | 355 | 0 | THE1A | MaLR | LTR |
| 210369833 | 210370149 | - | 17.3 | 5797 | 6113 | -71 | L1MB7 | L1 | LINE |

  

|  |  |  |  |  |  |  |  |  |  |
| --- | --- | --- | --- | --- | --- | --- | --- | --- | --- |
| chr2:211071632-211075321 | | | 3683 bp | | |  |  |  |  |
| Genome Start | Genome End | Strand | Div | Repeat Start | Repeat End | Repeat Left | Name | Family | Class |
| 211071632 | 211072864 | - | 19.7 | 116 | 1257 | -5596 | L1PA15-16 | L1 | LINE |
| 211072865 | 211073232 | - | 13.5 | 1 | 375 | 0 | THE1C | MaLR | LTR |
| 211073235 | 211074846 | - | 30.2 | 5 | 1733 | -2 | MLT-int | MaLR | LTR |
| 211074852 | 211075208 | - | 14.8 | 1 | 375 | 0 | THE1C | MaLR | LTR |
| 211075209 | 211075321 | - | 19.5 | 3 | 115 | -6738 | L1PA15-16 | L1 | LINE |

  

|  |  |  |  |  |  |  |  |  |  |
| --- | --- | --- | --- | --- | --- | --- | --- | --- | --- |
| chr2:212742325-212745909 | | | 3584 bp | | |  |  |  |  |
| Genome Start | Genome End | Strand | Div | Repeat Start | Repeat End | Repeat Left | Name | Family | Class |
| 212742325 | 212742585 | + | 12.3 | 4835 | 5095 | -1051 | L1PA11 | L1 | LINE |
| 212742587 | 212742959 | + | 14.1 | 1 | 381 | 0 | THE1D | MaLR | LTR |
| 212742960 | 212744479 | + | 13.3 | 1 | 1580 | 0 | THE1D-int | MaLR | LTR |
| 212744480 | 212744840 | + | 16.7 | 1 | 381 | 0 | THE1D | MaLR | LTR |
| 212744841 | 212745909 | + | 11.7 | 5092 | 6150 | -24 | L1PA11 | L1 | LINE |

  

|  |  |  |  |  |  |  |  |  |  |
| --- | --- | --- | --- | --- | --- | --- | --- | --- | --- |
| chr2:213032305-213036363 | | | 4056 bp | | |  |  |  |  |
| Genome Start | Genome End | Strand | Div | Repeat Start | Repeat End | Repeat Left | Name | Family | Class |
| 213032305 | 213032882 | + | 15.8 | 4380 | 4961 | -1182 | L1MA5 | L1 | LINE |
| 213032883 | 213033235 | + | 12.5 | 1 | 364 | 0 | THE1B | MaLR | LTR |
| 213033238 | 213034685 | + | 30.1 | 2 | 1735 | 0 | MLT-int | MaLR | LTR |
| 213034687 | 213035044 | + | 12.7 | 2 | 364 | 0 | THE1B | MaLR | LTR |
| 213035045 | 213036363 | + | 15.1 | 4962 | 6298 | -2 | L1MA5 | L1 | LINE |

  

|  |  |  |  |  |  |  |  |  |  |
| --- | --- | --- | --- | --- | --- | --- | --- | --- | --- |
| chr2:213039179-213041721 | | | 2539 bp | | |  |  |  |  |
| Genome Start | Genome End | Strand | Div | Repeat Start | Repeat End | Repeat Left | Name | Family | Class |
| 213039179 | 213039320 | + | 24.1 | 3 | 143 | -96 | MER8 | MER2\_type | DNA |
| 213039321 | 213039681 | + | 13.0 | 1 | 364 | 0 | THE1B | MaLR | LTR |
| 213039686 | 213041267 | + | 30.9 | 4 | 1735 | 0 | MLT-int | MaLR | LTR |
| 213041268 | 213041621 | + | 14.5 | 1 | 364 | 0 | THE1B | MaLR | LTR |
| 213041622 | 213041721 | + | 24.1 | 144 | 239 | 0 | MER8 | MER2\_type | DNA |

  

|  |  |  |  |  |  |  |  |  |  |
| --- | --- | --- | --- | --- | --- | --- | --- | --- | --- |
| chr2:213849606-213853006 | | | 3399 bp | | |  |  |  |  |
| Genome Start | Genome End | Strand | Div | Repeat Start | Repeat End | Repeat Left | Name | Family | Class |
| 213849606 | 213849915 | - | 18.2 | 7458 | 7783 | -2 | L1MC3 | L1 | LINE |
| 213849916 | 213850279 | + | 12.8 | 1 | 364 | 0 | THE1B | MaLR | LTR |
| 213850282 | 213851855 | + | 30.8 | 2 | 1735 | 0 | MLT-int | MaLR | LTR |
| 213851856 | 213852218 | + | 14.3 | 1 | 364 | 0 | THE1B | MaLR | LTR |
| 213852219 | 213853006 | - | 18.2 | 6632 | 7457 | -328 | L1MC3 | L1 | LINE |

  

|  |  |  |  |  |  |  |  |  |  |
| --- | --- | --- | --- | --- | --- | --- | --- | --- | --- |
| chr2:213901557-213904096 | | | 2535 bp | | |  |  |  |  |
| Genome Start | Genome End | Strand | Div | Repeat Start | Repeat End | Repeat Left | Name | Family | Class |
| 213901557 | 213901780 | - | 32.3 | 700 | 918 | -5505 | L1MCc | L1 | LINE |
| 213901781 | 213902131 | + | 9.5 | 1 | 364 | 0 | THE1B | MaLR | LTR |
| 213902134 | 213903708 | + | 29.8 | 2 | 1731 | -4 | MLT-int | MaLR | LTR |
| 213903712 | 213904048 | + | 11.0 | 3 | 364 | 0 | THE1B | MaLR | LTR |
| 213904049 | 213904096 | - | 32.3 | 656 | 699 | -5724 | L1MCc | L1 | LINE |

  

|  |  |  |  |  |  |  |  |  |  |
| --- | --- | --- | --- | --- | --- | --- | --- | --- | --- |
| chr2:216105314-216108563 | | | 3170 bp | | |  |  |  |  |
| Genome Start | Genome End | Strand | Div | Repeat Start | Repeat End | Repeat Left | Name | Family | Class |
| 216105314 | 216105757 | + | 15.9 | 5231 | 5677 | -506 | L1MB3 | L1 | LINE |
| 216105835 | 216106223 | + | 17.1 | 1 | 428 | 0 | MSTA | MaLR | LTR |
| 216106226 | 216107824 | + | 14.1 | 3 | 1580 | 0 | MSTA-int | MaLR | LTR |
| 216107825 | 216108205 | + | 12.8 | 1 | 421 | -7 | MSTA | MaLR | LTR |
| 216108206 | 216108278 | + | 15.3 | 5709 | 5780 | -403 | L1MB3 | L1 | LINE |
| 216108280 | 216108563 | + | 14.6 | 5876 | 6181 | -2 | L1MB3 | L1 | LINE |

  

|  |  |  |  |  |  |  |  |  |  |
| --- | --- | --- | --- | --- | --- | --- | --- | --- | --- |
| chr2:216495168-216501191 | | | 6037 bp | | |  |  |  |  |
| Genome Start | Genome End | Strand | Div | Repeat Start | Repeat End | Repeat Left | Name | Family | Class |
| 216495168 | 216495267 | - | 29.0 | 155 | 268 | 0 | MIRb | MIR | SINE |
| 216495271 | 216495723 | + | 6.2 | 1 | 448 | -2 | LTR7 | ERV1 | LTR |
| 216495726 | 216498762 | + | 9.3 | 1 | 2995 | -4718 | HERVH | ERV1 | LTR |
| 216498742 | 216498828 | + | 13.8 | 3140 | 3227 | -4486 | HERVH | ERV1 | LTR |
| 216498829 | 216498891 | + | 9.5 | 3228 | 3290 | -4423 | HERVH | ERV1 | LTR |
| 216498882 | 216499386 | + | 10.6 | 3492 | 3993 | -3720 | HERVH | ERV1 | LTR |
| 216499389 | 216500050 | + | 13.8 | 4488 | 5149 | -2564 | HERVH | ERV1 | LTR |
| 216500052 | 216500263 | + | 11.8 | 5600 | 5819 | -1894 | HERVH | ERV1 | LTR |
| 216500266 | 216500641 | + | 8.6 | 7326 | 7713 | 0 | HERVH | ERV1 | LTR |
| 216500642 | 216501094 | + | 5.1 | 1 | 448 | -2 | LTR7 | ERV1 | LTR |
| 216501103 | 216501191 | - | 22.5 | 47 | 154 | -114 | MIRb | MIR | SINE |

  

|  |  |  |  |  |  |  |  |  |  |
| --- | --- | --- | --- | --- | --- | --- | --- | --- | --- |
| chr2:217471923-217478268 | | | 6274 bp | | |  |  |  |  |
| Genome Start | Genome End | Strand | Div | Repeat Start | Repeat End | Repeat Left | Name | Family | Class |
| 217471923 | 217472637 | - | 16.7 | 1835 | 2658 | -4093 | L1MCa | L1 | LINE |
| 217472638 | 217473023 | + | 15.1 | 1 | 347 | -317 | MER4A | ERV1 | LTR |
| 217473064 | 217473461 | - | 18.0 | 5 | 424 | -2 | MSTB | MaLR | LTR |
| 217473463 | 217474312 | - | 17.2 | 782 | 1651 | 0 | MSTB-int | MaLR | LTR |
| 217474304 | 217475124 | + | 19.4 | 506 | 1344 | -307 | MST-int | MaLR | LTR |
| 217475125 | 217475433 | + | 9.2 | 1 | 303 | -6 | AluSc | Alu | SINE |
| 217475434 | 217475745 | + | 19.4 | 1345 | 1651 | 0 | MST-int | MaLR | LTR |
| 217475771 | 217476267 | - | 15.9 | 2 | 504 | -1147 | MSTB-int | MaLR | LTR |
| 217476269 | 217476691 | - | 17.2 | 1 | 426 | 0 | MSTB | MaLR | LTR |
| 217476699 | 217476978 | + | 23.0 | 275 | 695 | -3 | MER4C | ERV1 | LTR |
| 217476987 | 217477465 | - | 18.4 | 1340 | 1831 | -4920 | L1MCa | L1 | LINE |
| 217477465 | 217478268 | - | 26.7 | 1 | 827 | -5218 | L1MCa | L1 | LINE |

  

|  |  |  |  |  |  |  |  |  |  |
| --- | --- | --- | --- | --- | --- | --- | --- | --- | --- |
| chr2:218682229-218694449 | | | 12216 bp | | |  |  |  |  |
| Genome Start | Genome End | Strand | Div | Repeat Start | Repeat End | Repeat Left | Name | Family | Class |
| 218682229 | 218683270 | - | 4.9 | 7401 | 8436 | 0 | HERV9 | ERV1 | LTR |
| 218683271 | 218685223 | - | 5.0 | 4516 | 6479 | -1957 | HERV9 | ERV1 | LTR |
| 218685224 | 218685734 | + | 10.6 | 1 | 511 | 0 | MER9 | ERVK | LTR |
| 218685735 | 218686312 | + | 6.9 | 1 | 568 | -5348 | HERVK9 | ERVK | LTR |
| 218686314 | 218687659 | + | 8.0 | 1113 | 2469 | -3447 | HERVK9 | ERVK | LTR |
| 218687659 | 218687791 | + | 4.5 | 2654 | 2786 | -3130 | HERVK9 | ERVK | LTR |
| 218687792 | 218688021 | + | 3.5 | 3711 | 3941 | -1975 | HERVK9 | ERVK | LTR |
| 218688021 | 218689082 | + | 9.5 | 4582 | 5634 | -282 | HERVK9 | ERVK | LTR |
| 218689089 | 218689461 | + | 13.5 | 5635 | 7337 | 0 | HERVK9 | ERVK | LTR |
| 218689462 | 218689974 | + | 11.1 | 1 | 511 | 0 | MER9 | ERVK | LTR |
| 218689975 | 218694449 | - | 5.3 | 43 | 4521 | -3915 | HERV9 | ERV1 | LTR |

  

|  |  |  |  |  |  |  |  |  |  |
| --- | --- | --- | --- | --- | --- | --- | --- | --- | --- |
| chr2:218746262-218752996 | | | 6656 bp | | |  |  |  |  |
| Genome Start | Genome End | Strand | Div | Repeat Start | Repeat End | Repeat Left | Name | Family | Class |
| 218746262 | 218746393 | + | 21.2 | 16 | 149 | -804 | MER45C | Tip100 | DNA |
| 218746395 | 218746523 | + | 16.4 | 2 | 129 | -4 | FLAM\_C | Alu | SINE |
| 218746535 | 218746823 | + | 9.0 | 2 | 290 | -20 | AluSg | Alu | SINE |
| 218746858 | 218746965 | + | 26.9 | 152 | 266 | -687 | MER45C | Tip100 | DNA |
| 218746988 | 218747416 | - | 5.1 | 1 | 462 | -2 | LTR7B | ERV1 | LTR |
| 218747417 | 218747796 | - | 10.4 | 7322 | 7713 | 0 | HERVH | ERV1 | LTR |
| 218747795 | 218748011 | - | 14.8 | 5600 | 5819 | -1894 | HERVH | ERV1 | LTR |
| 218748013 | 218748673 | - | 13.8 | 4489 | 5149 | -2564 | HERVH | ERV1 | LTR |
| 218748677 | 218749179 | - | 10.8 | 3492 | 3993 | -3720 | HERVH | ERV1 | LTR |
| 218749170 | 218749317 | - | 14.2 | 3140 | 3290 | -4423 | HERVH | ERV1 | LTR |
| 218749312 | 218752423 | - | 10.5 | 1 | 2987 | -4726 | HERVH | ERV1 | LTR |
| 218752426 | 218752854 | - | 5.4 | 1 | 462 | -2 | LTR7B | ERV1 | LTR |
| 218752878 | 218752996 | + | 25.4 | 695 | 811 | -142 | MER45C | Tip100 | DNA |

  

|  |  |  |  |  |  |  |  |  |  |
| --- | --- | --- | --- | --- | --- | --- | --- | --- | --- |
| chr2:220510843-220517178 | | | 6285 bp | | |  |  |  |  |
| Genome Start | Genome End | Strand | Div | Repeat Start | Repeat End | Repeat Left | Name | Family | Class |
| 220510843 | 220511345 | - | 23.2 | 5653 | 6158 | -6 | L1ME2 | L1 | LINE |
| 220511346 | 220511696 | + | 18.7 | 1 | 408 | 0 | MLT1A1 | MaLR | LTR |
| 220511697 | 220513463 | + | 21.9 | 1 | 1735 | 0 | MLT1A1-int | MaLR | LTR |
| 220513464 | 220513819 | + | 19.5 | 1 | 406 | -2 | MLT1A1 | MaLR | LTR |
| 220513820 | 220514331 | - | 23.0 | 5115 | 5652 | -512 | L1ME2 | L1 | LINE |
| 220514358 | 220515002 | - | 18.1 | 3988 | 4445 | -1698 | L1M2 | L1 | LINE |
| 220515003 | 220515066 | - | 6.2 | 6080 | 6143 | -11 | L1PA7 | L1 | LINE |
| 220515067 | 220516326 | - | 18.1 | 2683 | 3987 | -2156 | L1M2 | L1 | LINE |
| 220516334 | 220516412 | - | 19.0 | 4720 | 4798 | -1348 | L1MC | L1 | LINE |
| 220516431 | 220517178 | - | 24.5 | 3870 | 4620 | -1526 | L1ME2 | L1 | LINE |

  

|  |  |  |  |  |  |  |  |  |  |
| --- | --- | --- | --- | --- | --- | --- | --- | --- | --- |
| chr2:220976797-220987936 | | | 11045 bp | | |  |  |  |  |
| Genome Start | Genome End | Strand | Div | Repeat Start | Repeat End | Repeat Left | Name | Family | Class |
| 220976797 | 220976956 | - | 23.9 | 3179 | 3337 | -2882 | L1MEc | L1 | LINE |
| 220976954 | 220977472 | - | 14.0 | 5653 | 6160 | 0 | L1PREC2 | L1 | LINE |
| 220977473 | 220977836 | - | 16.4 | 1 | 375 | 0 | THE1C | MaLR | LTR |
| 220977837 | 220979373 | - | 30.7 | 2 | 1735 | 0 | MLT-int | MaLR | LTR |
| 220979376 | 220979738 | - | 16.1 | 3 | 375 | 0 | THE1C | MaLR | LTR |
| 220979739 | 220979759 | - | 14.0 | 6159 | 6179 | -508 | L1PREC2 | L1 | LINE |
| 220979797 | 220980027 | - | 10.9 | 85 | 308 | -5 | AluSp | Alu | SINE |
| 220980028 | 220980152 | - | 14.0 | 6026 | 6158 | -529 | L1PREC2 | L1 | LINE |
| 220980182 | 220985949 | - | 14.7 | 179 | 6025 | -662 | L1PREC2 | L1 | LINE |
| 220985952 | 220986041 | + | 8.9 | 147 | 236 | -6453 | L1PA14 | L1 | LINE |
| 220986070 | 220987936 | - | 21.0 | 31 | 1934 | -4466 | L1MEc | L1 | LINE |

  

|  |  |  |  |  |  |  |  |  |  |
| --- | --- | --- | --- | --- | --- | --- | --- | --- | --- |
| chr2:221500104-221502738 | | | 2633 bp | | |  |  |  |  |
| Genome Start | Genome End | Strand | Div | Repeat Start | Repeat End | Repeat Left | Name | Family | Class |
| 221500104 | 221500203 | - | 13.8 | 271 | 364 | 0 | THE1B | MaLR | LTR |
| 221500204 | 221500546 | - | 13.9 | 1 | 364 | 0 | THE1B | MaLR | LTR |
| 221500547 | 221502121 | - | 30.3 | 2 | 1735 | 0 | MLT1K-int | MaLR | LTR |
| 221502124 | 221502468 | - | 13.9 | 1 | 364 | 0 | THE1B | MaLR | LTR |
| 221502469 | 221502738 | - | 13.8 | 1 | 270 | -94 | THE1B | MaLR | LTR |

  

|  |  |  |  |  |  |  |  |  |  |
| --- | --- | --- | --- | --- | --- | --- | --- | --- | --- |
| chr2:221643343-221646052 | | | 2707 bp | | |  |  |  |  |
| Genome Start | Genome End | Strand | Div | Repeat Start | Repeat End | Repeat Left | Name | Family | Class |
| 221643343 | 221643670 | + | 29.6 | 105 | 407 | -142 | MLT1H | MaLR | LTR |
| 221643671 | 221644036 | - | 17.0 | 1 | 375 | 0 | THE1C | MaLR | LTR |
| 221644038 | 221645558 | - | 30.4 | 2 | 1734 | -1 | MLT-int | MaLR | LTR |
| 221645561 | 221645938 | - | 13.7 | 1 | 375 | 0 | THE1C | MaLR | LTR |
| 221645939 | 221646052 | + | 29.6 | 408 | 547 | -2 | MLT1H | MaLR | LTR |

  

|  |  |  |  |  |  |  |  |  |  |
| --- | --- | --- | --- | --- | --- | --- | --- | --- | --- |
| chr2:223560794-223563739 | | | 2944 bp | | |  |  |  |  |
| Genome Start | Genome End | Strand | Div | Repeat Start | Repeat End | Repeat Left | Name | Family | Class |
| 223560794 | 223561011 | - | 21.2 | 140 | 358 | -7 | MLT1A0 | MaLR | LTR |
| 223561012 | 223561364 | - | 13.5 | 1 | 364 | 0 | THE1B | MaLR | LTR |
| 223561365 | 223561420 | - | 30.7 | 1681 | 1735 | 0 | MLT1A0-int | MaLR | LTR |
| 223561421 | 223561731 | + | 14.2 | 1 | 310 | 0 | AluSg | Alu | SINE |
| 223561732 | 223563251 | - | 30.7 | 2 | 1680 | -55 | MLT1A0-int | MaLR | LTR |
| 223563254 | 223563609 | - | 11.3 | 3 | 364 | 0 | THE1B | MaLR | LTR |
| 223563610 | 223563739 | - | 21.2 | 2 | 139 | -226 | MLT1A0 | MaLR | LTR |

  

|  |  |  |  |  |  |  |  |  |  |
| --- | --- | --- | --- | --- | --- | --- | --- | --- | --- |
| chr2:225307465-225311725 | | | 3964 bp | | |  |  |  |  |
| Genome Start | Genome End | Strand | Div | Repeat Start | Repeat End | Repeat Left | Name | Family | Class |
| 225307465 | 225307502 | - | 14.9 | 527 | 565 | -2 | MLT2B1 | ERVL | LTR |
| 225307544 | 225307579 | - | 14.9 | 499 | 526 | -41 | MLT2B1 | ERVL | LTR |
| 225307740 | 225307862 | - | 16.7 | 1 | 147 | -217 | THE1B | MaLR | LTR |
| 225307866 | 225307953 | + | 13.0 | 585 | 677 | -40 | MLT2B4 | ERVL | LTR |
| 225308005 | 225308329 | + | 15.8 | 77 | 428 | 0 | MSTA | MaLR | LTR |
| 225308332 | 225308915 | + | 28.2 | 2 | 669 | -1066 | MLT-int | MaLR | LTR |
| 225308916 | 225309213 | - | 6.1 | 2 | 298 | -13 | AluY | Alu | SINE |
| 225309214 | 225310257 | + | 28.2 | 670 | 1735 | 0 | MLT-int | MaLR | LTR |
| 225310261 | 225310623 | + | 15.3 | 4 | 428 | 0 | MSTA | MaLR | LTR |
| 225310661 | 225310703 | - | 19.8 | 315 | 346 | -221 | MLT2B1 | ERVL | LTR |
| 225310704 | 225311455 | - | 15.1 | 1 | 783 | 0 | LTR39 | ERV1 | LTR |
| 225311456 | 225311725 | - | 19.8 | 1 | 267 | -253 | MLT2B1 | ERVL | LTR |

  

|  |  |  |  |  |  |  |  |  |  |
| --- | --- | --- | --- | --- | --- | --- | --- | --- | --- |
| chr2:225874101-225877487 | | | 3351 bp | | |  |  |  |  |
| Genome Start | Genome End | Strand | Div | Repeat Start | Repeat End | Repeat Left | Name | Family | Class |
| 225874101 | 225874517 | + | 24.1 | 5318 | 5767 | -399 | L1ME1 | L1 | LINE |
| 225874543 | 225874908 | + | 10.4 | 1 | 364 | 0 | THE1B | MaLR | LTR |
| 225874909 | 225876311 | + | 9.7 | 1 | 1402 | -178 | THE1B-int | MaLR | LTR |
| 225876312 | 225876615 | - | 12.5 | 1 | 305 | -7 | AluSx | Alu | SINE |
| 225876616 | 225876800 | + | 9.7 | 1403 | 1574 | -6 | THE1B-int | MaLR | LTR |
| 225876801 | 225877166 | + | 13.2 | 6 | 364 | 0 | THE1B | MaLR | LTR |
| 225877178 | 225877487 | + | 24.3 | 5838 | 6161 | -5 | L1ME1 | L1 | LINE |

  

|  |  |  |  |  |  |  |  |  |  |
| --- | --- | --- | --- | --- | --- | --- | --- | --- | --- |
| chr2:226109497-226120856 | | | 11360 bp | | |  |  |  |  |
| Genome Start | Genome End | Strand | Div | Repeat Start | Repeat End | Repeat Left | Name | Family | Class |
| 226109497 | 226109726 | - | 13.1 | 7488 | 7724 | 0 | L1MA3 | L1 | LINE |
| 226109727 | 226110037 | - | 7.8 | 1 | 311 | 0 | AluY | Alu | SINE |
| 226110038 | 226110286 | - | 13.1 | 7226 | 7487 | -237 | L1MA3 | L1 | LINE |
| 226110287 | 226110631 | - | 11.2 | 1 | 364 | 0 | THE1B | MaLR | LTR |
| 226110632 | 226111718 | - | 8.9 | 488 | 1580 | 0 | THE1B-int | MaLR | LTR |
| 226111719 | 226112739 | - | 6.8 | 5222 | 6241 | 0 | L1PA7 | L1 | LINE |
| 226112740 | 226113245 | - | 9.1 | 1 | 506 | -1074 | THE1B-int | MaLR | LTR |
| 226113246 | 226113590 | - | 14.4 | 1 | 364 | 0 | THE1B | MaLR | LTR |
| 226113591 | 226120856 | - | 13.6 | 19 | 7230 | -494 | L1MA3 | L1 | LINE |

  

|  |  |  |  |  |  |  |  |  |  |
| --- | --- | --- | --- | --- | --- | --- | --- | --- | --- |
| chr2:228659057-228664215 | | | 5160 bp | | |  |  |  |  |
| Genome Start | Genome End | Strand | Div | Repeat Start | Repeat End | Repeat Left | Name | Family | Class |
| 228659057 | 228660875 | + | 21.5 | 4 | 1971 | -5363 | L1M4b | L1 | LINE |
| 228660873 | 228661519 | + | 18.4 | 2571 | 3270 | -4064 | L1M4b | L1 | LINE |
| 228661520 | 228661919 | + | 16.0 | 3 | 426 | 0 | MSTB | MaLR | LTR |
| 228661922 | 228663522 | + | 26.6 | 2 | 1735 | 0 | MLT-int | MaLR | LTR |
| 228663523 | 228663942 | + | 19.4 | 1 | 426 | 0 | MSTB | MaLR | LTR |
| 228663943 | 228664215 | + | 19.3 | 3271 | 3659 | -3675 | L1M4b | L1 | LINE |

  

|  |  |  |  |  |  |  |  |  |  |
| --- | --- | --- | --- | --- | --- | --- | --- | --- | --- |
| chr2:228965821-228975165 | | | 9281 bp | | |  |  |  |  |
| Genome Start | Genome End | Strand | Div | Repeat Start | Repeat End | Repeat Left | Name | Family | Class |
| 228965821 | 228966382 | - | 17.7 | 5944 | 6503 | 0 | L1PA16 | L1 | LINE |
| 228966383 | 228967687 | - | 14.7 | 4172 | 5464 | -1019 | L1PA16 | L1 | LINE |
| 228967688 | 228967981 | - | 9.2 | 71 | 364 | 0 | THE1B | MaLR | LTR |
| 228967976 | 228968116 | + | 9.2 | 1337 | 1477 | -103 | THE1-int | MaLR | LTR |
| 228968182 | 228968246 | - | 9.2 | 1 | 65 | -299 | THE1B | MaLR | LTR |
| 228968251 | 228969772 | - | 10.5 | 2 | 1576 | -4 | THE1B-int | MaLR | LTR |
| 228969774 | 228970062 | - | 10.8 | 74 | 364 | 0 | THE1B | MaLR | LTR |
| 228970063 | 228971054 | - | 16.7 | 1 | 1071 | 0 | MER11C | ERVK | LTR |
| 228971055 | 228971129 | - | 10.8 | 1 | 73 | -291 | THE1B | MaLR | LTR |
| 228971130 | 228975165 | - | 18.7 | 116 | 4176 | -2307 | L1PA16 | L1 | LINE |

  

|  |  |  |  |  |  |  |  |  |  |
| --- | --- | --- | --- | --- | --- | --- | --- | --- | --- |
| chr2:229570166-229574489 | | | 4143 bp | | |  |  |  |  |
| Genome Start | Genome End | Strand | Div | Repeat Start | Repeat End | Repeat Left | Name | Family | Class |
| 229570166 | 229571043 | - | 20.0 | 514 | 1446 | -9 | Charlie1a | MER1\_type | DNA |
| 229571044 | 229571419 | + | 12.4 | 1 | 379 | -2 | THE1D | MaLR | LTR |
| 229571425 | 229571774 | + | 16.0 | 4 | 355 | -1225 | THE1D-int | MaLR | LTR |
| 229571775 | 229572133 | + | 13.4 | 3 | 364 | 0 | THE1B | MaLR | LTR |
| 229572134 | 229573407 | + | 16.0 | 356 | 1580 | 0 | THE1D-int | MaLR | LTR |
| 229573408 | 229573767 | + | 15.4 | 1 | 381 | 0 | THE1D | MaLR | LTR |
| 229573827 | 229573976 | - | 23.3 | 275 | 423 | -1032 | Charlie1a | MER1\_type | DNA |
| 229573978 | 229574133 | + | 20.6 | 135 | 289 | -23 | AluJb | Alu | SINE |
| 229574250 | 229574489 | - | 23.0 | 7 | 248 | -1207 | Charlie1a | MER1\_type | DNA |

  

|  |  |  |  |  |  |  |  |  |  |
| --- | --- | --- | --- | --- | --- | --- | --- | --- | --- |
| chr2:229758176-229760898 | | | 2721 bp | | |  |  |  |  |
| Genome Start | Genome End | Strand | Div | Repeat Start | Repeat End | Repeat Left | Name | Family | Class |
| 229758176 | 229758381 | + | 27.7 | 1497 | 1721 | -1698 | L2 | L2 | LINE |
| 229758382 | 229758765 | - | 11.3 | 1 | 364 | 0 | THE1B | MaLR | LTR |
| 229758766 | 229760264 | - | 30.2 | 2 | 1735 | 0 | MLT1H1-int | MaLR | LTR |
| 229760267 | 229760619 | - | 11.6 | 1 | 364 | 0 | THE1B | MaLR | LTR |
| 229760620 | 229760898 | + | 27.7 | 1722 | 1989 | -1430 | L2 | L2 | LINE |

  

|  |  |  |  |  |  |  |  |  |  |
| --- | --- | --- | --- | --- | --- | --- | --- | --- | --- |
| chr2:233787727-233792503 | | | 4747 bp | | |  |  |  |  |
| Genome Start | Genome End | Strand | Div | Repeat Start | Repeat End | Repeat Left | Name | Family | Class |
| 233787727 | 233787861 | - | 16.9 | 354 | 487 | -4 | LTR16C | ERVL | LTR |
| 233787884 | 233788168 | + | 11.9 | 2 | 289 | -21 | AluSg | Alu | SINE |
| 233788185 | 233788480 | - | 10.1 | 146 | 464 | 0 | LTR7B | ERV1 | LTR |
| 233788459 | 233788574 | - | 19.1 | 146 | 264 | -208 | LTR7Y | ERV1 | LTR |
| 233788553 | 233788667 | - | 21.7 | 146 | 264 | -200 | LTR7B | ERV1 | LTR |
| 233788677 | 233788937 | - | 9.6 | 1 | 264 | -200 | LTR7B | ERV1 | LTR |
| 233788938 | 233789482 | - | 10.8 | 7157 | 7713 | 0 | HERVH | ERV1 | LTR |
| 233789476 | 233789742 | - | 19.5 | 5749 | 6016 | -1697 | HERVH | ERV1 | LTR |
| 233789740 | 233791078 | - | 11.7 | 3634 | 4915 | -2287 | HERVH | ERV1 | LTR |
| 233791085 | 233791206 | - | 7.4 | 341 | 464 | 0 | LTR7B | ERV1 | LTR |
| 233791207 | 233791269 | + | 6.3 | 239 | 301 | -9 | Alu | Alu | SINE |
| 233791272 | 233791653 | - | 10.6 | 26 | 437 | -175 | LTR9 | ERV1 | LTR |
| 233791654 | 233791701 | + | 8.3 | 1 | 48 | -416 | LTR7B | ERV1 | LTR |
| 233791706 | 233791767 | - | 8.1 | 173 | 234 | -76 | Alu | Alu | SINE |
| 233791772 | 233791949 | + | 12.6 | 443 | 612 | 0 | LTR9 | ERV1 | LTR |
| 233791971 | 233792055 | - | 24.3 | 241 | 332 | -159 | LTR16C | ERVL | LTR |
| 233792056 | 233792346 | + | 15.8 | 3 | 289 | -23 | AluJo | Alu | SINE |
| 233792347 | 233792503 | - | 24.3 | 84 | 240 | -251 | LTR16C | ERVL | LTR |

  

|  |  |  |  |  |  |  |  |  |  |
| --- | --- | --- | --- | --- | --- | --- | --- | --- | --- |
| chr2:235208753-235211896 | | | 2898 bp | | |  |  |  |  |
| Genome Start | Genome End | Strand | Div | Repeat Start | Repeat End | Repeat Left | Name | Family | Class |
| 235208753 | 235208879 | + | 25.4 | 24 | 145 | -628 | LTR41 | ERVL | LTR |
| 235208938 | 235209287 | + | 13.5 | 1 | 363 | -1 | THE1B | MaLR | LTR |
| 235209291 | 235210803 | + | 29.1 | 2 | 1735 | 0 | MLT1K-int | MaLR | LTR |
| 235210804 | 235211158 | + | 12.9 | 1 | 364 | 0 | THE1B | MaLR | LTR |
| 235211301 | 235211609 | - | 7.4 | 1 | 310 | -1 | AluY | Alu | SINE |
| 235211653 | 235211896 | + | 28.2 | 531 | 751 | -22 | LTR41 | ERVL | LTR |

  

|  |  |  |  |  |  |  |  |  |  |
| --- | --- | --- | --- | --- | --- | --- | --- | --- | --- |
| chr2:237437451-237440147 | | | 2695 bp | | |  |  |  |  |
| Genome Start | Genome End | Strand | Div | Repeat Start | Repeat End | Repeat Left | Name | Family | Class |
| 237437451 | 237437812 | + | 20.5 | 1 | 398 | -117 | MLT2B2 | ERVL | LTR |
| 237437813 | 237438160 | - | 11.3 | 1 | 355 | 0 | THE1A | MaLR | LTR |
| 237438161 | 237439733 | - | 29.9 | 2 | 1735 | 0 | MLT-int | MaLR | LTR |
| 237439736 | 237440032 | - | 11.2 | 1 | 355 | 0 | THE1A | MaLR | LTR |
| 237440033 | 237440147 | + | 20.5 | 446 | 559 | -3 | MLT2B2 | ERVL | LTR |

  

|  |  |  |  |  |  |  |  |  |  |
| --- | --- | --- | --- | --- | --- | --- | --- | --- | --- |
| chr2:237875593-237879921 | | | 4034 bp | | |  |  |  |  |
| Genome Start | Genome End | Strand | Div | Repeat Start | Repeat End | Repeat Left | Name | Family | Class |
| 237875593 | 237875805 | - | 32.9 | 5385 | 5579 | -88 | ERVL-E | ERVL | LTR |
| 237875806 | 237876165 | + | 7.7 | 3 | 355 | 0 | THE1A | MaLR | LTR |
| 237876168 | 237877711 | + | 29.6 | 2 | 1735 | 0 | MLT-int | MaLR | LTR |
| 237877712 | 237878073 | + | 9.3 | 1 | 355 | 0 | THE1A | MaLR | LTR |
| 237878074 | 237878918 | - | 32.9 | 4533 | 5384 | -283 | ERVL-E | ERVL | LTR |
| 237879212 | 237879921 | - | 27.8 | 3742 | 4621 | -1093 | ERVL-E | ERVL | LTR |

  

|  |  |  |  |  |  |  |  |  |  |
| --- | --- | --- | --- | --- | --- | --- | --- | --- | --- |
| chr2:241429813-241435302 | | | 5349 bp | | |  |  |  |  |
| Genome Start | Genome End | Strand | Div | Repeat Start | Repeat End | Repeat Left | Name | Family | Class |
| 241429813 | 241429951 | - | 16.8 | 476 | 653 | 0 | LTR49 | ERV1 | LTR |
| 241429952 | 241430257 | + | 15.3 | 1 | 309 | -3 | AluJo | Alu | SINE |
| 241430258 | 241430282 | - | 23.9 | 481 | 507 | -146 | MER39B | ERV1 | LTR |
| 241430295 | 241430345 | + | 14.0 | 1 | 50 | -850 | MER4D1 | ERV1 | LTR |
| 241430350 | 241430743 | - | 10.7 | 104 | 508 | -3 | LTR10F | ERV1 | LTR |
| 241430744 | 241430794 | - | 11.8 | 107 | 159 | -352 | LTR10F | ERV1 | LTR |
| 241430795 | 241430878 | - | 8.3 | 104 | 197 | -314 | LTR10F | ERV1 | LTR |
| 241430902 | 241430987 | - | 8.1 | 104 | 197 | -314 | LTR10F | ERV1 | LTR |
| 241431011 | 241431096 | - | 8.1 | 104 | 197 | -314 | LTR10F | ERV1 | LTR |
| 241431120 | 241431204 | - | 5.9 | 104 | 197 | -314 | LTR10F | ERV1 | LTR |
| 241431228 | 241431310 | - | 3.6 | 104 | 197 | -314 | LTR10F | ERV1 | LTR |
| 241431311 | 241431361 | - | 11.8 | 107 | 159 | -352 | LTR10F | ERV1 | LTR |
| 241431362 | 241431447 | - | 8.1 | 104 | 197 | -314 | LTR10F | ERV1 | LTR |
| 241431471 | 241431551 | - | 6.2 | 108 | 197 | -314 | LTR10F | ERV1 | LTR |
| 241431553 | 241431738 | - | 8.6 | 1 | 197 | -314 | LTR10F | ERV1 | LTR |
| 241431740 | 241432595 | + | 16.0 | 49 | 895 | -8 | MER4D | ERV1 | LTR |
| 241432602 | 241432761 | - | 16.2 | 205 | 415 | -180 | LTR49 | ERV1 | LTR |
| 241432762 | 241433122 | + | 14.6 | 1 | 373 | -2 | THE1C | MaLR | LTR |
| 241433125 | 241434727 | + | 10.9 | 1 | 1580 | 0 | THE1C-int | MaLR | LTR |
| 241434728 | 241435098 | + | 11.2 | 1 | 375 | 0 | THE1C | MaLR | LTR |
| 241435099 | 241435302 | - | 10.3 | 3 | 207 | -388 | LTR49 | ERV1 | LTR |

  


|  |  |  |  |  |  |  |  |  |  |
| --- | --- | --- | --- | --- | --- | --- | --- | --- | --- |
| chr3:906420-914968 | | | 8115 bp | | |  |  |  |  |
| Genome Start | Genome End | Strand | Div | Repeat Start | Repeat End | Repeat Left | Name | Family | Class |
| 906420 | 906755 | + | 32.9 | 979 | 1319 | -4890 | L1MEd | L1 | LINE |
| 906789 | 907047 | + | 33.0 | 1403 | 1656 | -4583 | L1MEd | L1 | LINE |
| 907157 | 907360 | + | 32.3 | 1629 | 1854 | -4385 | L1MEd | L1 | LINE |
| 907377 | 907788 | - | 8.1 | 1 | 448 | -2 | LTR7 | ERV1 | LTR |
| 907789 | 908167 | - | 10.7 | 7322 | 7713 | 0 | HERVH | ERV1 | LTR |
| 908166 | 908377 | - | 11.3 | 5600 | 5819 | -1894 | HERVH | ERV1 | LTR |
| 908379 | 909039 | - | 13.8 | 4490 | 5149 | -2564 | HERVH | ERV1 | LTR |
| 909044 | 909547 | - | 11.4 | 3492 | 3993 | -3720 | HERVH | ERV1 | LTR |
| 909538 | 912682 | - | 9.9 | 1 | 3290 | -4423 | HERVH | ERV1 | LTR |
| 912685 | 913096 | - | 9.8 | 1 | 448 | -2 | LTR7 | ERV1 | LTR |
| 913378 | 914476 | - | 6.4 | 5070 | 6172 | 0 | L1PA8 | L1 | LINE |
| 914477 | 914968 | + | 28.5 | 2183 | 2656 | -3563 | L1MEd | L1 | LINE |

  

|  |  |  |  |  |  |  |  |  |  |
| --- | --- | --- | --- | --- | --- | --- | --- | --- | --- |
| chr3:2121608-2124395 | | | 2605 bp | | |  |  |  |  |
| Genome Start | Genome End | Strand | Div | Repeat Start | Repeat End | Repeat Left | Name | Family | Class |
| 2121608 | 2121748 | + | 31.9 | 94 | 233 | -358 | MLT1K | MaLR | LTR |
| 2121930 | 2122044 | + | 27.8 | 375 | 489 | -102 | MLT1K | MaLR | LTR |
| 2122045 | 2122404 | - | 14.7 | 1 | 375 | 0 | THE1C | MaLR | LTR |
| 2122405 | 2123940 | - | 29.9 | 2 | 1735 | 0 | MLT-int | MaLR | LTR |
| 2123943 | 2124318 | - | 14.2 | 1 | 375 | 0 | THE1C | MaLR | LTR |
| 2124319 | 2124395 | + | 27.8 | 490 | 564 | -27 | MLT1K | MaLR | LTR |

  

|  |  |  |  |  |  |  |  |  |  |
| --- | --- | --- | --- | --- | --- | --- | --- | --- | --- |
| chr3:4344939-4349075 | | | 4135 bp | | |  |  |  |  |
| Genome Start | Genome End | Strand | Div | Repeat Start | Repeat End | Repeat Left | Name | Family | Class |
| 4344939 | 4345030 | - | 21.3 | 3437 | 4705 | -1382 | MER83B-int | ERV1 | LTR |
| 4345031 | 4345370 | - | 12.7 | 1 | 363 | -1 | THE1B | MaLR | LTR |
| 4345371 | 4346983 | - | 29.3 | 2 | 1735 | 0 | MLT-int | MaLR | LTR |
| 4346986 | 4347340 | - | 12.5 | 1 | 364 | 0 | THE1B | MaLR | LTR |
| 4347341 | 4348229 | - | 21.8 | 2591 | 4612 | -1444 | MER83B-int | ERV1 | LTR |
| 4348230 | 4348537 | + | 12.5 | 1 | 304 | -8 | AluSx | Alu | SINE |
| 4348538 | 4349075 | - | 16.6 | 2302 | 3900 | -2290 | MER83B-int | ERV1 | LTR |

  

|  |  |  |  |  |  |  |  |  |  |
| --- | --- | --- | --- | --- | --- | --- | --- | --- | --- |
| chr3:5997174-5999737 | | | 2562 bp | | |  |  |  |  |
| Genome Start | Genome End | Strand | Div | Repeat Start | Repeat End | Repeat Left | Name | Family | Class |
| 5997174 | 5997327 | - | 20.1 | 209 | 341 | 0 | MER58B | MER1\_type | DNA |
| 5997328 | 5997680 | - | 16.2 | 1 | 364 | 0 | THE1B | MaLR | LTR |
| 5997681 | 5999205 | - | 30.8 | 2 | 1735 | 0 | MLT-int | MaLR | LTR |
| 5999208 | 5999552 | - | 15.7 | 1 | 364 | 0 | THE1B | MaLR | LTR |
| 5999553 | 5999737 | - | 20.1 | 11 | 208 | -133 | MER58B | MER1\_type | DNA |

  

|  |  |  |  |  |  |  |  |  |  |
| --- | --- | --- | --- | --- | --- | --- | --- | --- | --- |
| chr3:6439431-6442709 | | | 3277 bp | | |  |  |  |  |
| Genome Start | Genome End | Strand | Div | Repeat Start | Repeat End | Repeat Left | Name | Family | Class |
| 6439431 | 6439906 | + | 11.1 | 1 | 491 | -169 | PABL\_A | ERV1 | LTR |
| 6439907 | 6440110 | - | 12.3 | 174 | 363 | -1 | THE1B | MaLR | LTR |
| 6440111 | 6440422 | - | 13.1 | 1 | 312 | 0 | AluSx | Alu | SINE |
| 6440423 | 6440601 | - | 12.3 | 1 | 173 | -191 | THE1B | MaLR | LTR |
| 6440602 | 6442165 | - | 29.1 | 2 | 1735 | 0 | MLT1K-int | MaLR | LTR |
| 6442168 | 6442537 | - | 14.1 | 1 | 364 | 0 | THE1B | MaLR | LTR |
| 6442538 | 6442709 | + | 8.8 | 485 | 660 | 0 | PABL\_A | ERV1 | LTR |

  

|  |  |  |  |  |  |  |  |  |  |
| --- | --- | --- | --- | --- | --- | --- | --- | --- | --- |
| chr3:7520952-7523799 | | | 2693 bp | | |  |  |  |  |
| Genome Start | Genome End | Strand | Div | Repeat Start | Repeat End | Repeat Left | Name | Family | Class |
| 7520952 | 7521086 | + | 37.3 | 3004 | 3143 | -276 | L2 | L2 | LINE |
| 7521116 | 7521494 | - | 24.4 | 1 | 396 | 0 | MSTD | MaLR | LTR |
| 7521495 | 7523207 | - | 24.5 | 9 | 1735 | 0 | MSTD-int | MaLR | LTR |
| 7523216 | 7523606 | - | 26.2 | 6 | 396 | 0 | MSTD | MaLR | LTR |
| 7523725 | 7523799 | + | 32.9 | 3344 | 3416 | -3 | L2 | L2 | LINE |

  

|  |  |  |  |  |  |  |  |  |  |
| --- | --- | --- | --- | --- | --- | --- | --- | --- | --- |
| chr3:11422789-11425380 | | | 2587 bp | | |  |  |  |  |
| Genome Start | Genome End | Strand | Div | Repeat Start | Repeat End | Repeat Left | Name | Family | Class |
| 11422789 | 11423039 | - | 24.0 | 43 | 289 | -23 | AluJo | Alu | SINE |
| 11423040 | 11423403 | - | 13.7 | 1 | 364 | 0 | THE1B | MaLR | LTR |
| 11423409 | 11424977 | - | 8.6 | 1 | 1576 | -4 | THE1B-int | MaLR | LTR |
| 11424978 | 11425339 | - | 15.8 | 1 | 364 | 0 | THE1B | MaLR | LTR |
| 11425340 | 11425380 | - | 14.6 | 7 | 47 | -252 | AluJo | Alu | SINE |

  

|  |  |  |  |  |  |  |  |  |  |
| --- | --- | --- | --- | --- | --- | --- | --- | --- | --- |
| chr3:12050017-12055809 | | | 5764 bp | | |  |  |  |  |
| Genome Start | Genome End | Strand | Div | Repeat Start | Repeat End | Repeat Left | Name | Family | Class |
| 12050017 | 12050944 | - | 16.2 | 5255 | 6165 | -19 | L1MB7 | L1 | LINE |
| 12050949 | 12051325 | - | 18.6 | 1 | 396 | 0 | MSTD | MaLR | LTR |
| 12051346 | 12051881 | - | 22.0 | 1160 | 1706 | -29 | MSTD-int | MaLR | LTR |
| 12051882 | 12052658 | - | 18.4 | 1 | 783 | 0 | LTR39 | ERV1 | LTR |
| 12052659 | 12053880 | - | 22.0 | 2 | 1159 | -576 | MSTD-int | MaLR | LTR |
| 12053882 | 12054308 | - | 17.5 | 1 | 396 | 0 | MSTD | MaLR | LTR |
| 12054313 | 12055809 | - | 14.7 | 3741 | 5251 | -895 | L1MB7 | L1 | LINE |

  

|  |  |  |  |  |  |  |  |  |  |
| --- | --- | --- | --- | --- | --- | --- | --- | --- | --- |
| chr3:16003054-16012346 | | | 9259 bp | | |  |  |  |  |
| Genome Start | Genome End | Strand | Div | Repeat Start | Repeat End | Repeat Left | Name | Family | Class |
| 16003054 | 16003606 | - | 14.3 | 5930 | 6489 | -19 | L1MC1 | L1 | LINE |
| 16003607 | 16003967 | - | 13.0 | 1 | 364 | 0 | THE1B | MaLR | LTR |
| 16003970 | 16005538 | - | 7.8 | 1 | 1578 | -2 | THE1B-int | MaLR | LTR |
| 16005539 | 16005902 | - | 11.9 | 1 | 364 | 0 | THE1B | MaLR | LTR |
| 16005905 | 16006332 | - | 13.5 | 5483 | 5932 | -576 | L1MC1 | L1 | LINE |
| 16006333 | 16006644 | - | 17.7 | 1 | 300 | -12 | AluJb | Alu | SINE |
| 16006645 | 16007431 | - | 13.2 | 4610 | 5482 | -839 | L1MC1 | L1 | LINE |
| 16007438 | 16007893 | + | 15.1 | 8 | 2406 | 0 | Tigger5 | MER2\_type | DNA |
| 16007894 | 16008970 | - | 14.3 | 3521 | 4611 | -1710 | L1MC1 | L1 | LINE |
| 16008971 | 16009252 | - | 17.0 | 1 | 307 | -5 | AluJo | Alu | SINE |
| 16009253 | 16010076 | - | 14.3 | 2577 | 3520 | -2801 | L1MC1 | L1 | LINE |
| 16010077 | 16010385 | - | 10.0 | 1 | 299 | -12 | AluY | Alu | SINE |
| 16010386 | 16011375 | - | 15.3 | 1542 | 2576 | -3745 | L1MC1 | L1 | LINE |
| 16011397 | 16011737 | - | 15.7 | 1206 | 1541 | -4967 | L1MC1 | L1 | LINE |
| 16011741 | 16012346 | - | 13.0 | 474 | 1112 | -5396 | L1MC1 | L1 | LINE |

  

|  |  |  |  |  |  |  |  |  |  |
| --- | --- | --- | --- | --- | --- | --- | --- | --- | --- |
| chr3:16631506-16634165 | | | 2658 bp | | |  |  |  |  |
| Genome Start | Genome End | Strand | Div | Repeat Start | Repeat End | Repeat Left | Name | Family | Class |
| 16631506 | 16631709 | - | 19.9 | 108 | 302 | -10 | AluJo | Alu | SINE |
| 16631710 | 16632081 | + | 14.2 | 1 | 381 | 0 | THE1D | MaLR | LTR |
| 16632084 | 16633677 | + | 32.9 | 2 | 1735 | 0 | MLT1I-int | MaLR | LTR |
| 16633678 | 16634054 | + | 12.4 | 1 | 379 | -2 | THE1D | MaLR | LTR |
| 16634055 | 16634165 | - | 19.9 | 1 | 107 | -205 | AluJo | Alu | SINE |

  

|  |  |  |  |  |  |  |  |  |  |
| --- | --- | --- | --- | --- | --- | --- | --- | --- | --- |
| chr3:16759086-16769092 | | | 9905 bp | | |  |  |  |  |
| Genome Start | Genome End | Strand | Div | Repeat Start | Repeat End | Repeat Left | Name | Family | Class |
| 16759086 | 16759251 | - | 30.3 | 5993 | 6163 | -77 | L1ME3B | L1 | LINE |
| 16759249 | 16760142 | - | 16.6 | 5299 | 6178 | 0 | L1MB8 | L1 | LINE |
| 16760159 | 16760241 | - | 9.6 | 6214 | 6296 | -4 | L1MA4 | L1 | LINE |
| 16760244 | 16760690 | - | 27.6 | 5495 | 5937 | -303 | L1ME3B | L1 | LINE |
| 16760697 | 16761191 | + | 4.7 | 1 | 448 | -2 | LTR7 | ERV1 | LTR |
| 16761194 | 16764238 | + | 9.1 | 1 | 2992 | -4721 | HERVH | ERV1 | LTR |
| 16764225 | 16764372 | + | 14.9 | 3140 | 3290 | -4423 | HERVH | ERV1 | LTR |
| 16764363 | 16764865 | + | 12.2 | 3492 | 3993 | -3720 | HERVH | ERV1 | LTR |
| 16764868 | 16765530 | + | 13.8 | 4488 | 5149 | -2564 | HERVH | ERV1 | LTR |
| 16765532 | 16765748 | + | 12.0 | 5600 | 5819 | -1894 | HERVH | ERV1 | LTR |
| 16765747 | 16766129 | + | 8.8 | 7322 | 7713 | 0 | HERVH | ERV1 | LTR |
| 16766130 | 16766575 | + | 4.7 | 1 | 448 | -2 | LTR7 | ERV1 | LTR |
| 16766581 | 16767080 | - | 26.0 | 4967 | 5490 | -750 | L1ME3B | L1 | LINE |
| 16767083 | 16767246 | + | 11.0 | 6131 | 6295 | -7 | L1MA1 | L1 | LINE |
| 16767266 | 16767460 | - | 27.1 | 4417 | 4618 | -1528 | L1ME3B | L1 | LINE |
| 16767461 | 16767590 | - | 20.8 | 1 | 130 | -3 | FLAM\_A | Alu | SINE |
| 16767591 | 16768121 | - | 27.1 | 3865 | 4416 | -1730 | L1ME3B | L1 | LINE |
| 16768122 | 16768196 | + | 10.7 | 210 | 284 | -27 | AluY | Alu | SINE |
| 16768263 | 16768628 | - | 27.1 | 3496 | 3864 | -2282 | L1ME3B | L1 | LINE |
| 16768638 | 16768800 | - | 22.1 | 1 | 163 | -22 | FAM | Alu | SINE |
| 16768802 | 16769092 | - | 24.5 | 3212 | 3510 | -2636 | L1ME3B | L1 | LINE |

  

|  |  |  |  |  |  |  |  |  |  |
| --- | --- | --- | --- | --- | --- | --- | --- | --- | --- |
| chr3:17142009-17146589 | | | 4313 bp | | |  |  |  |  |
| Genome Start | Genome End | Strand | Div | Repeat Start | Repeat End | Repeat Left | Name | Family | Class |
| 17142009 | 17142082 | - | 16.2 | 5567 | 5653 | -517 | L1M5 | L1 | LINE |
| 17142118 | 17142258 | - | 23.4 | 5454 | 5580 | -603 | L1M3 | L1 | LINE |
| 17142363 | 17142770 | + | 22.4 | 187 | 621 | -3410 | Zaphod | Tip100 | DNA |
| 17142771 | 17142984 | - | 21.6 | 1 | 219 | 0 | MER20 | MER1\_type | DNA |
| 17142985 | 17143369 | + | 22.4 | 622 | 1018 | -3013 | Zaphod | Tip100 | DNA |
| 17143343 | 17143679 | + | 17.9 | 3596 | 3964 | -67 | Zaphod | Tip100 | DNA |
| 17143833 | 17144035 | - | 27.1 | 5254 | 5462 | -712 | L1P4 | L1 | LINE |
| 17144036 | 17144388 | + | 8.6 | 1 | 355 | 0 | THE1A | MaLR | LTR |
| 17144392 | 17145955 | + | 30.6 | 3 | 1735 | 0 | MLT-int | MaLR | LTR |
| 17145956 | 17146265 | + | 12.4 | 1 | 355 | 0 | THE1A | MaLR | LTR |
| 17146266 | 17146589 | - | 26.7 | 4940 | 5254 | -892 | L1M5 | L1 | LINE |

  

|  |  |  |  |  |  |  |  |  |  |
| --- | --- | --- | --- | --- | --- | --- | --- | --- | --- |
| chr3:17449330-17452484 | | | 3091 bp | | |  |  |  |  |
| Genome Start | Genome End | Strand | Div | Repeat Start | Repeat End | Repeat Left | Name | Family | Class |
| 17449330 | 17449721 | + | 24.5 | 9 | 428 | -6179 | L1MB4 | L1 | LINE |
| 17449777 | 17450140 | - | 13.5 | 1 | 369 | -6 | THE1C | MaLR | LTR |
| 17450147 | 17451811 | - | 12.3 | 1 | 1568 | -12 | THE1C-int | MaLR | LTR |
| 17451812 | 17452188 | - | 11.0 | 1 | 375 | 0 | THE1C | MaLR | LTR |
| 17452192 | 17452484 | + | 18.9 | 492 | 790 | -5817 | L1MB4 | L1 | LINE |

  

|  |  |  |  |  |  |  |  |  |  |
| --- | --- | --- | --- | --- | --- | --- | --- | --- | --- |
| chr3:17966279-17970466 | | | 3979 bp | | |  |  |  |  |
| Genome Start | Genome End | Strand | Div | Repeat Start | Repeat End | Repeat Left | Name | Family | Class |
| 17966279 | 17966678 | - | 28.6 | 5733 | 6159 | -81 | L1ME3B | L1 | LINE |
| 17966712 | 17966822 | + | 21.3 | 2412 | 2520 | -342 | Charlie2 | MER1\_type | DNA |
| 17966831 | 17967201 | + | 10.8 | 1 | 375 | 0 | THE1C | MaLR | LTR |
| 17967204 | 17968787 | + | 29.0 | 2 | 1735 | 0 | MLT-int | MaLR | LTR |
| 17968788 | 17969164 | + | 9.4 | 1 | 375 | 0 | THE1C | MaLR | LTR |
| 17969221 | 17969474 | - | 16.0 | 105 | 358 | 0 | AluJo | Alu | SINE |
| 17969540 | 17969753 | - | 29.9 | 5336 | 5553 | -687 | L1ME3B | L1 | LINE |
| 17969798 | 17970130 | + | 28.8 | 71 | 407 | -2 | MLT1I | MaLR | LTR |
| 17970132 | 17970466 | - | 24.6 | 4980 | 5319 | -827 | L1ME3B | L1 | LINE |

  

|  |  |  |  |  |  |  |  |  |  |
| --- | --- | --- | --- | --- | --- | --- | --- | --- | --- |
| chr3:19087270-19093161 | | | 5614 bp | | |  |  |  |  |
| Genome Start | Genome End | Strand | Div | Repeat Start | Repeat End | Repeat Left | Name | Family | Class |
| 19087270 | 19087595 | + | 14.3 | 3641 | 3964 | -2182 | L1MC1 | L1 | LINE |
| 19087602 | 19087683 | + | 18.3 | 6207 | 6292 | -5 | L1MA5A | L1 | LINE |
| 19087915 | 19089744 | + | 18.0 | 3945 | 5830 | -514 | L1MC1 | L1 | LINE |
| 19089745 | 19089968 | + | 16.1 | 1 | 229 | -197 | MSTB | MaLR | LTR |
| 19089991 | 19090362 | + | 19.3 | 5 | 426 | 0 | MSTB | MaLR | LTR |
| 19090363 | 19090563 | + | 16.6 | 231 | 426 | 0 | MSTB | MaLR | LTR |
| 19090564 | 19092204 | + | 19.5 | 1 | 1634 | -17 | MSTB-int | MaLR | LTR |
| 19092224 | 19092658 | + | 17.9 | 1 | 426 | 0 | MSTB | MaLR | LTR |
| 19092659 | 19093161 | + | 16.7 | 5826 | 6331 | -2 | L1MC1 | L1 | LINE |

  

|  |  |  |  |  |  |  |  |  |  |
| --- | --- | --- | --- | --- | --- | --- | --- | --- | --- |
| chr3:19668325-19675868 | | | 7339 bp | | |  |  |  |  |
| Genome Start | Genome End | Strand | Div | Repeat Start | Repeat End | Repeat Left | Name | Family | Class |
| 19668325 | 19668415 | + | 14.2 | 5248 | 5341 | -2676 | L1MD2 | L1 | LINE |
| 19668419 | 19670066 | + | 26.1 | 835 | 2456 | -5030 | L1M3d | L1 | LINE |
| 19670071 | 19670421 | - | 11.8 | 1 | 377 | -4 | THE1D | MaLR | LTR |
| 19670422 | 19670628 | - | 14.0 | 1388 | 1580 | 0 | THE1D-int | MaLR | LTR |
| 19670629 | 19670895 | + | 11.0 | 1 | 295 | -7 | AluSx | Alu | SINE |
| 19670896 | 19672318 | - | 14.0 | 4 | 1387 | -193 | THE1D-int | MaLR | LTR |
| 19672322 | 19672687 | - | 13.1 | 1 | 381 | 0 | THE1D | MaLR | LTR |
| 19672688 | 19672934 | + | 12.5 | 2451 | 2685 | -4801 | L1M3d | L1 | LINE |
| 19672927 | 19674580 | + | 20.3 | 2986 | 4669 | -2893 | L1M3d | L1 | LINE |
| 19674582 | 19674858 | + | 17.7 | 1 | 301 | -127 | MSTC | MaLR | LTR |
| 19674859 | 19675217 | + | 11.8 | 5814 | 6174 | 0 | L1PA11 | L1 | LINE |
| 19675420 | 19675868 | + | 19.9 | 5482 | 5929 | -430 | L1MD2 | L1 | LINE |

  

|  |  |  |  |  |  |  |  |  |  |
| --- | --- | --- | --- | --- | --- | --- | --- | --- | --- |
| chr3:20262928-20274902 | | | 11606 bp | | |  |  |  |  |
| Genome Start | Genome End | Strand | Div | Repeat Start | Repeat End | Repeat Left | Name | Family | Class |
| 20262928 | 20263323 | + | 31.1 | 4099 | 4515 | -1631 | L1M5 | L1 | LINE |
| 20263403 | 20263538 | + | 30.6 | 4509 | 4649 | -1541 | L1M5 | L1 | LINE |
| 20263565 | 20263797 | + | 22.0 | 7771 | 7991 | -4 | L1MD3 | L1 | LINE |
| 20263798 | 20264891 | - | 12.7 | 5757 | 6868 | -1 | L1PA16 | L1 | LINE |
| 20264892 | 20265271 | - | 8.8 | 1 | 375 | 0 | THE1C | MaLR | LTR |
| 20265272 | 20265771 | - | 10.8 | 5254 | 5756 | -1093 | L1PA16 | L1 | LINE |
| 20265772 | 20266190 | - | 15.2 | 2 | 426 | 0 | MSTB | MaLR | LTR |
| 20266212 | 20266251 | - | 19.1 | 1602 | 1632 | -19 | MSTB-int | MaLR | LTR |
| 20266252 | 20266557 | - | 9.5 | 1 | 307 | -5 | AluSx | Alu | SINE |
| 20266558 | 20268082 | - | 19.1 | 1 | 1601 | -50 | MSTB-int | MaLR | LTR |
| 20268083 | 20268500 | - | 16.4 | 1 | 426 | 0 | MSTB | MaLR | LTR |
| 20268501 | 20268713 | - | 14.4 | 5054 | 5258 | -1591 | L1PA16 | L1 | LINE |
| 20268701 | 20271639 | - | 10.7 | 2 | 2922 | -3539 | L1P2 | L1 | LINE |
| 20271640 | 20271651 | - | 12.9 | 3789 | 3800 | -3049 | L1PA16 | L1 | LINE |
| 20271682 | 20273511 | - | 14.2 | 1938 | 3788 | -3061 | L1PA16 | L1 | LINE |
| 20273512 | 20273808 | + | 13.7 | 1 | 296 | -16 | AluJb | Alu | SINE |
| 20273809 | 20274502 | - | 15.6 | 1236 | 1937 | -4916 | L1PA16 | L1 | LINE |
| 20274729 | 20274902 | + | 30.8 | 5634 | 5811 | -310 | L1M5 | L1 | LINE |

  

|  |  |  |  |  |  |  |  |  |  |
| --- | --- | --- | --- | --- | --- | --- | --- | --- | --- |
| chr3:20981810-20992296 | | | 10053 bp | | |  |  |  |  |
| Genome Start | Genome End | Strand | Div | Repeat Start | Repeat End | Repeat Left | Name | Family | Class |
| 20981810 | 20983379 | - | 19.8 | 4980 | 6507 | -858 | L1M1 | L1 | LINE |
| 20983395 | 20984031 | - | 25.5 | 2654 | 3286 | -3919 | L1M1 | L1 | LINE |
| 20984037 | 20984447 | - | 9.5 | 1 | 448 | -2 | LTR7 | ERV1 | LTR |
| 20984448 | 20984821 | - | 11.7 | 7322 | 7713 | 0 | HERVH | ERV1 | LTR |
| 20984820 | 20985034 | - | 11.6 | 5600 | 5819 | -1894 | HERVH | ERV1 | LTR |
| 20985036 | 20985698 | - | 14.9 | 4488 | 5149 | -2564 | HERVH | ERV1 | LTR |
| 20985701 | 20986203 | - | 12.4 | 3492 | 3993 | -3720 | HERVH | ERV1 | LTR |
| 20986194 | 20986338 | - | 15.9 | 3140 | 3290 | -4423 | HERVH | ERV1 | LTR |
| 20986333 | 20989313 | - | 10.2 | 1 | 2987 | -4726 | HERVH | ERV1 | LTR |
| 20989316 | 20989727 | - | 9.1 | 1 | 448 | -2 | LTR7 | ERV1 | LTR |
| 20989735 | 20991176 | - | 29.2 | 1276 | 2373 | -4480 | L1PA17 | L1 | LINE |
| 20991597 | 20992296 | - | 35.0 | 1 | 738 | -6471 | L1M1 | L1 | LINE |

  

|  |  |  |  |  |  |  |  |  |  |
| --- | --- | --- | --- | --- | --- | --- | --- | --- | --- |
| chr3:21213161-21222984 | | | 9783 bp | | |  |  |  |  |
| Genome Start | Genome End | Strand | Div | Repeat Start | Repeat End | Repeat Left | Name | Family | Class |
| 21213161 | 21213232 | - | 15.6 | 6930 | 6998 | -7 | L1MA4A | L1 | LINE |
| 21213233 | 21213531 | + | 12.4 | 1 | 303 | -10 | AluSp | Alu | SINE |
| 21213532 | 21218018 | - | 18.4 | 2289 | 6929 | -76 | L1MA4A | L1 | LINE |
| 21218019 | 21218390 | + | 14.1 | 1 | 381 | 0 | THE1D | MaLR | LTR |
| 21218391 | 21219091 | - | 25.6 | 1574 | 2288 | -4561 | L1MA4A | L1 | LINE |
| 21219125 | 21220598 | - | 25.6 | 175 | 1573 | -5276 | L1MA4A | L1 | LINE |
| 21220602 | 21220975 | + | 13.5 | 1 | 364 | 0 | THE1B | MaLR | LTR |
| 21220976 | 21222530 | + | 10.2 | 1 | 1580 | 0 | THE1B-int | MaLR | LTR |
| 21222531 | 21222892 | + | 14.8 | 1 | 364 | 0 | THE1B | MaLR | LTR |
| 21222898 | 21222984 | - | 13.8 | 82 | 171 | -6678 | L1MA4A | L1 | LINE |

  

|  |  |  |  |  |  |  |  |  |  |
| --- | --- | --- | --- | --- | --- | --- | --- | --- | --- |
| chr3:21717419-21720082 | | | 2518 bp | | |  |  |  |  |
| Genome Start | Genome End | Strand | Div | Repeat Start | Repeat End | Repeat Left | Name | Family | Class |
| 21717419 | 21717542 | + | 22.9 | 6 | 140 | -381 | LTR33 | ERVL | LTR |
| 21717657 | 21718007 | - | 8.8 | 1 | 355 | 0 | THE1A | MaLR | LTR |
| 21718008 | 21719578 | - | 9.9 | 1 | 1580 | 0 | THE1A-int | MaLR | LTR |
| 21719579 | 21719932 | - | 8.2 | 1 | 355 | 0 | THE1A | MaLR | LTR |
| 21719965 | 21720082 | + | 29.7 | 385 | 506 | -15 | LTR33 | ERVL | LTR |

  

|  |  |  |  |  |  |  |  |  |  |
| --- | --- | --- | --- | --- | --- | --- | --- | --- | --- |
| chr3:22611419-22618910 | | | 7427 bp | | |  |  |  |  |
| Genome Start | Genome End | Strand | Div | Repeat Start | Repeat End | Repeat Left | Name | Family | Class |
| 22611419 | 22611699 | + | 22.9 | 12 | 288 | -7046 | L1MB8 | L1 | LINE |
| 22611715 | 22612535 | + | 22.1 | 1271 | 2150 | -5184 | L1MB8 | L1 | LINE |
| 22612539 | 22612903 | + | 20.2 | 1 | 428 | 0 | MSTA | MaLR | LTR |
| 22612906 | 22614535 | + | 15.0 | 3 | 1580 | 0 | MSTA-int | MaLR | LTR |
| 22614538 | 22614702 | + | 15.4 | 3 | 223 | -205 | MSTA | MaLR | LTR |
| 22614726 | 22614910 | + | 15.4 | 224 | 426 | -2 | MSTA | MaLR | LTR |
| 22614944 | 22615247 | + | 24.3 | 2733 | 3207 | -4127 | L1MB8 | L1 | LINE |
| 22615235 | 22616375 | + | 19.6 | 3890 | 5012 | -1994 | L1MB8 | L1 | LINE |
| 22616376 | 22616620 | - | 13.9 | 2 | 246 | -67 | AluSq | Alu | SINE |
| 22616621 | 22617667 | + | 19.6 | 5013 | 6070 | -936 | L1MB8 | L1 | LINE |
| 22617668 | 22618042 | + | 17.9 | 2 | 365 | 0 | MLT1A0 | MaLR | LTR |
| 22618043 | 22618910 | + | 20.7 | 6071 | 7038 | 0 | L1MB8 | L1 | LINE |

  

|  |  |  |  |  |  |  |  |  |  |
| --- | --- | --- | --- | --- | --- | --- | --- | --- | --- |
| chr3:22833905-22838202 | | | 4296 bp | | |  |  |  |  |
| Genome Start | Genome End | Strand | Div | Repeat Start | Repeat End | Repeat Left | Name | Family | Class |
| 22833905 | 22834249 | + | 28.3 | 3986 | 4355 | -1791 | L1M5 | L1 | LINE |
| 22834250 | 22834541 | + | 14.0 | 1 | 298 | -14 | AluSx | Alu | SINE |
| 22834542 | 22834576 | + | 28.3 | 4356 | 4382 | -1764 | L1M5 | L1 | LINE |
| 22834577 | 22834996 | - | 15.4 | 1 | 428 | 0 | MSTA | MaLR | LTR |
| 22834997 | 22836551 | - | 28.1 | 2 | 1735 | 0 | MLT-int | MaLR | LTR |
| 22836554 | 22836981 | - | 19.3 | 1 | 428 | 0 | MSTA | MaLR | LTR |
| 22836982 | 22838202 | + | 26.1 | 4383 | 5799 | -441 | L1M5 | L1 | LINE |

  

|  |  |  |  |  |  |  |  |  |  |
| --- | --- | --- | --- | --- | --- | --- | --- | --- | --- |
| chr3:23641608-23644245 | | | 2636 bp | | |  |  |  |  |
| Genome Start | Genome End | Strand | Div | Repeat Start | Repeat End | Repeat Left | Name | Family | Class |
| 23641608 | 23641641 | + | 29.8 | 3266 | 3294 | -125 | L2 | L2 | LINE |
| 23641642 | 23642009 | - | 24.3 | 1 | 393 | -3 | MSTD | MaLR | LTR |
| 23642010 | 23643776 | - | 25.2 | 2 | 1735 | 0 | MSTD-int | MaLR | LTR |
| 23643779 | 23644138 | - | 24.7 | 1 | 396 | 0 | MSTD | MaLR | LTR |
| 23644139 | 23644245 | + | 29.8 | 3295 | 3411 | -8 | L2 | L2 | LINE |

  

|  |  |  |  |  |  |  |  |  |  |
| --- | --- | --- | --- | --- | --- | --- | --- | --- | --- |
| chr3:26297766-26305925 | | | 8103 bp | | |  |  |  |  |
| Genome Start | Genome End | Strand | Div | Repeat Start | Repeat End | Repeat Left | Name | Family | Class |
| 26297766 | 26298125 | - | 31.4 | 2792 | 3219 | -200 | L2 | L2 | LINE |
| 26298142 | 26298755 | - | 17.3 | 1 | 673 | -6 | LTR58 | ERV1 | LTR |
| 26298756 | 26298938 | - | 30.7 | 2598 | 2797 | -622 | L2 | L2 | LINE |
| 26298940 | 26299212 | - | 14.0 | 505 | 781 | 0 | MER4E1 | ERV1 | LTR |
| 26299213 | 26299315 | + | 16.8 | 1 | 103 | -209 | AluSx | Alu | SINE |
| 26299316 | 26299650 | - | 13.0 | 1 | 364 | 0 | THE1B | MaLR | LTR |
| 26299651 | 26301215 | - | 29.9 | 5 | 1735 | 0 | MLT1H-int | MaLR | LTR |
| 26301221 | 26301553 | - | 14.0 | 1 | 364 | 0 | THE1B | MaLR | LTR |
| 26301554 | 26301746 | + | 16.8 | 104 | 291 | -21 | AluSx | Alu | SINE |
| 26301756 | 26302103 | - | 16.5 | 1 | 360 | -250 | MER4B | ERV1 | LTR |
| 26302104 | 26302144 | - | 25.8 | 2560 | 2600 | -819 | L2 | L2 | LINE |
| 26302145 | 26302760 | + | 22.8 | 1 | 644 | 0 | LTR48B | ERV1 | LTR |
| 26302761 | 26302847 | - | 25.8 | 2481 | 2559 | -860 | L2 | L2 | LINE |
| 26302874 | 26303738 | + | 17.6 | 1 | 838 | 0 | MER4D | ERV1 | LTR |
| 26303742 | 26303883 | - | 28.3 | 2350 | 2495 | -924 | L2 | L2 | LINE |
| 26303879 | 26303978 | - | 34.0 | 2102 | 2201 | -1218 | L2 | L2 | LINE |
| 26303981 | 26304387 | - | 14.8 | 155 | 595 | 0 | LTR49 | ERV1 | LTR |
| 26304388 | 26304732 | + | 18.8 | 1 | 308 | -4 | AluJb | Alu | SINE |
| 26304733 | 26304888 | - | 14.8 | 1 | 154 | -441 | LTR49 | ERV1 | LTR |
| 26304889 | 26305002 | - | 26.4 | 1967 | 2101 | -1318 | L2 | L2 | LINE |
| 26305003 | 26305550 | + | 16.1 | 1 | 595 | 0 | LTR49 | ERV1 | LTR |
| 26305551 | 26305925 | - | 26.4 | 1548 | 1966 | -1453 | L2 | L2 | LINE |

  

|  |  |  |  |  |  |  |  |  |  |
| --- | --- | --- | --- | --- | --- | --- | --- | --- | --- |
| chr3:27565132-27570815 | | | 5551 bp | | |  |  |  |  |
| Genome Start | Genome End | Strand | Div | Repeat Start | Repeat End | Repeat Left | Name | Family | Class |
| 27565132 | 27565273 | + | 27.6 | 2372 | 2521 | -898 | L2 | L2 | LINE |
| 27565283 | 27565711 | - | 33.7 | 183 | 586 | -5 | MLT1K | MaLR | LTR |
| 27565841 | 27566065 | - | 16.0 | 70 | 304 | -8 | AluJo | Alu | SINE |
| 27566066 | 27566497 | - | 5.1 | 1 | 464 | 0 | LTR7B | ERV1 | LTR |
| 27566498 | 27566885 | - | 10.4 | 7324 | 7713 | 0 | HERVH | ERV1 | LTR |
| 27566886 | 27567384 | - | 10.3 | 5318 | 5819 | -1894 | HERVH | ERV1 | LTR |
| 27567373 | 27569229 | - | 11.4 | 756 | 2571 | -5142 | HERVH | ERV1 | LTR |
| 27569217 | 27569418 | - | 22.7 | 346 | 620 | -7093 | HERVH | ERV1 | LTR |
| 27569401 | 27569866 | - | 14.1 | 1 | 459 | -7254 | HERVH | ERV1 | LTR |
| 27569867 | 27570298 | - | 4.9 | 1 | 464 | 0 | LTR7B | ERV1 | LTR |
| 27570299 | 27570396 | - | 21.6 | 113 | 209 | -103 | AluSg/x | Alu | SINE |
| 27570435 | 27570815 | + | 33.7 | 2516 | 2951 | -468 | L2 | L2 | LINE |

  

|  |  |  |  |  |  |  |  |  |  |
| --- | --- | --- | --- | --- | --- | --- | --- | --- | --- |
| chr3:27614342-27620127 | | | 5781 bp | | |  |  |  |  |
| Genome Start | Genome End | Strand | Div | Repeat Start | Repeat End | Repeat Left | Name | Family | Class |
| 27614342 | 27614421 | - | 21.2 | 6078 | 6155 | -9 | L1ME2 | L1 | LINE |
| 27614422 | 27614737 | + | 11.6 | 1 | 310 | -2 | AluSx | Alu | SINE |
| 27614738 | 27614963 | - | 21.2 | 5887 | 6077 | -87 | L1ME2 | L1 | LINE |
| 27614964 | 27615252 | - | 12.5 | 1 | 289 | -21 | AluSg | Alu | SINE |
| 27615253 | 27615472 | - | 21.2 | 5646 | 5886 | -278 | L1ME2 | L1 | LINE |
| 27615497 | 27615928 | + | 4.6 | 1 | 464 | 0 | LTR7B | ERV1 | LTR |
| 27615929 | 27616789 | + | 20.0 | 1 | 1182 | -6531 | HERVH | ERV1 | LTR |
| 27616788 | 27617061 | + | 16.1 | 2728 | 2995 | -4718 | HERVH | ERV1 | LTR |
| 27617041 | 27618758 | + | 13.0 | 3140 | 5286 | -1805 | HERVH | ERV1 | LTR |
| 27618762 | 27619194 | + | 8.2 | 7283 | 7713 | 0 | HERVH | ERV1 | LTR |
| 27619195 | 27619658 | + | 5.8 | 1 | 464 | 0 | LTR7B | ERV1 | LTR |
| 27619660 | 27619937 | - | 16.6 | 1 | 278 | -34 | AluJo | Alu | SINE |
| 27619938 | 27620127 | - | 21.2 | 5464 | 5657 | -507 | L1ME2 | L1 | LINE |

  

|  |  |  |  |  |  |  |  |  |  |
| --- | --- | --- | --- | --- | --- | --- | --- | --- | --- |
| chr3:28039725-28043930 | | | 4175 bp | | |  |  |  |  |
| Genome Start | Genome End | Strand | Div | Repeat Start | Repeat End | Repeat Left | Name | Family | Class |
| 28039725 | 28040036 | - | 14.2 | 6013 | 6312 | 0 | L1MA9 | L1 | LINE |
| 28040037 | 28040391 | + | 8.8 | 1 | 355 | 0 | THE1A | MaLR | LTR |
| 28040394 | 28041637 | + | 31.6 | 2 | 1358 | -377 | MLT1B-int | MaLR | LTR |
| 28041638 | 28041919 | - | 14.2 | 1 | 289 | -21 | AluSg | Alu | SINE |
| 28041920 | 28042243 | + | 31.6 | 1359 | 1735 | 0 | MLT1B-int | MaLR | LTR |
| 28042244 | 28042599 | + | 15.0 | 1 | 355 | 0 | THE1A | MaLR | LTR |
| 28042600 | 28042799 | - | 14.2 | 5793 | 6012 | -300 | L1MA9 | L1 | LINE |
| 28042829 | 28043930 | - | 17.4 | 4782 | 5795 | -376 | L1MA9 | L1 | LINE |

  

|  |  |  |  |  |  |  |  |  |  |
| --- | --- | --- | --- | --- | --- | --- | --- | --- | --- |
| chr3:29050793-29059149 | | | 8348 bp | | |  |  |  |  |
| Genome Start | Genome End | Strand | Div | Repeat Start | Repeat End | Repeat Left | Name | Family | Class |
| 29050793 | 29051890 | - | 19.0 | 4635 | 5788 | -749 | L1M | L1 | LINE |
| 29051891 | 29052188 | - | 11.7 | 1 | 298 | -14 | AluSx | Alu | SINE |
| 29052189 | 29055420 | - | 19.8 | 1175 | 4634 | -1903 | L1M | L1 | LINE |
| 29055422 | 29055530 | + | 19.9 | 1 | 108 | -204 | AluJb | Alu | SINE |
| 29055531 | 29055895 | + | 11.8 | 1 | 364 | 0 | THE1B | MaLR | LTR |
| 29055900 | 29057492 | + | 31.5 | 4 | 1735 | 0 | MLT-int | MaLR | LTR |
| 29057495 | 29057878 | + | 9.2 | 3 | 364 | 0 | THE1B | MaLR | LTR |
| 29057879 | 29058082 | + | 19.9 | 109 | 303 | -9 | AluJb | Alu | SINE |
| 29058085 | 29059149 | - | 26.9 | 26 | 1181 | -5452 | L1M | L1 | LINE |

  

|  |  |  |  |  |  |  |  |  |  |
| --- | --- | --- | --- | --- | --- | --- | --- | --- | --- |
| chr3:30814635-30817220 | | | 2441 bp | | |  |  |  |  |
| Genome Start | Genome End | Strand | Div | Repeat Start | Repeat End | Repeat Left | Name | Family | Class |
| 30814635 | 30814734 | + | 29.9 | 2995 | 3088 | -290 | L2 | L2 | LINE |
| 30814735 | 30815092 | - | 11.8 | 1 | 355 | 0 | THE1A | MaLR | LTR |
| 30815093 | 30816629 | - | 28.4 | 2 | 1735 | 0 | MLT1K-int | MaLR | LTR |
| 30816634 | 30816981 | - | 11.1 | 1 | 353 | -2 | THE1A | MaLR | LTR |
| 30816982 | 30817030 | + | 29.9 | 3089 | 3143 | -235 | L2 | L2 | LINE |
| 30817172 | 30817220 | + | 28.6 | 3325 | 3373 | -5 | L2 | L2 | LINE |

  

|  |  |  |  |  |  |  |  |  |  |
| --- | --- | --- | --- | --- | --- | --- | --- | --- | --- |
| chr3:31160848-31169464 | | | 8615 bp | | |  |  |  |  |
| Genome Start | Genome End | Strand | Div | Repeat Start | Repeat End | Repeat Left | Name | Family | Class |
| 31160848 | 31162952 | - | 9.9 | 2811 | 4901 | -2664 | L1M1 | L1 | LINE |
| 31162953 | 31163316 | - | 15.1 | 1 | 364 | 0 | THE1B | MaLR | LTR |
| 31163317 | 31164412 | - | 11.6 | 483 | 1580 | 0 | THE1B-int | MaLR | LTR |
| 31164413 | 31165621 | - | 3.5 | 4940 | 6150 | -5 | L1PA4 | L1 | LINE |
| 31165622 | 31166120 | - | 10.6 | 1 | 499 | -1081 | THE1B-int | MaLR | LTR |
| 31166121 | 31166482 | - | 13.2 | 3 | 364 | 0 | THE1B | MaLR | LTR |
| 31166485 | 31169045 | - | 17.2 | 110 | 2815 | -4750 | L1M1 | L1 | LINE |
| 31169046 | 31169352 | + | 10.2 | 1 | 305 | -4 | AluSc | Alu | SINE |
| 31169353 | 31169464 | - | 21.8 | 1 | 109 | -7456 | L1M1 | L1 | LINE |

  

|  |  |  |  |  |  |  |  |  |  |
| --- | --- | --- | --- | --- | --- | --- | --- | --- | --- |
| chr3:31331047-31334029 | | | 2981 bp | | |  |  |  |  |
| Genome Start | Genome End | Strand | Div | Repeat Start | Repeat End | Repeat Left | Name | Family | Class |
| 31331047 | 31331076 | - | 25.3 | 570 | 595 | 0 | MLT1G1 | MaLR | LTR |
| 31331077 | 31331464 | + | 15.9 | 1 | 428 | 0 | MSTA | MaLR | LTR |
| 31331467 | 31333119 | + | 29.2 | 2 | 1735 | 0 | MLT-int | MaLR | LTR |
| 31333120 | 31333503 | + | 13.7 | 1 | 428 | 0 | MSTA | MaLR | LTR |
| 31333504 | 31334029 | - | 25.3 | 1 | 569 | -26 | MLT1G1 | MaLR | LTR |

  

|  |  |  |  |  |  |  |  |  |  |
| --- | --- | --- | --- | --- | --- | --- | --- | --- | --- |
| chr3:31342673-31345950 | | | 3274 bp | | |  |  |  |  |
| Genome Start | Genome End | Strand | Div | Repeat Start | Repeat End | Repeat Left | Name | Family | Class |
| 31342673 | 31342723 | + | 26.9 | 5 | 46 | -549 | MLT1G1 | MaLR | LTR |
| 31342724 | 31343123 | - | 11.4 | 1 | 428 | 0 | MSTA | MaLR | LTR |
| 31343124 | 31344729 | - | 28.4 | 2 | 1735 | 0 | MLT-int | MaLR | LTR |
| 31344734 | 31344955 | - | 10.9 | 193 | 426 | -2 | MSTA | MaLR | LTR |
| 31344956 | 31345261 | - | 9.3 | 1 | 304 | 0 | AluSg | Alu | SINE |
| 31345262 | 31345443 | - | 10.9 | 1 | 192 | -236 | MSTA | MaLR | LTR |
| 31345444 | 31345950 | + | 26.9 | 47 | 589 | -6 | MLT1G1 | MaLR | LTR |

  

|  |  |  |  |  |  |  |  |  |  |
| --- | --- | --- | --- | --- | --- | --- | --- | --- | --- |
| chr3:32943920-32949925 | | | 5876 bp | | |  |  |  |  |
| Genome Start | Genome End | Strand | Div | Repeat Start | Repeat End | Repeat Left | Name | Family | Class |
| 32943920 | 32944123 | - | 16.3 | 136 | 340 | -5 | MER2 | MER2\_type | DNA |
| 32944171 | 32944212 | - | 7.1 | 6111 | 6152 | -2 | L1PB3 | L1 | LINE |
| 32944211 | 32944248 | - | 7.9 | 6113 | 6150 | -4 | L1PB3 | L1 | LINE |
| 32944249 | 32945248 | - | 11.4 | 5147 | 6147 | -4 | L1PB2 | L1 | LINE |
| 32945249 | 32945396 | + | 11.7 | 1 | 162 | -202 | THE1B | MaLR | LTR |
| 32945397 | 32945703 | - | 11.1 | 2 | 306 | -3 | AluSc | Alu | SINE |
| 32945704 | 32945826 | + | 11.7 | 163 | 299 | -65 | THE1B | MaLR | LTR |
| 32945827 | 32946143 | - | 9.6 | 1 | 302 | -9 | AluY | Alu | SINE |
| 32946144 | 32946215 | + | 11.7 | 300 | 364 | 0 | THE1B | MaLR | LTR |
| 32946218 | 32947788 | + | 29.8 | 2 | 1735 | 0 | MLT1D-int | MaLR | LTR |
| 32947789 | 32948124 | + | 11.0 | 1 | 364 | 0 | THE1B | MaLR | LTR |
| 32948125 | 32948323 | - | 13.1 | 4947 | 5146 | -1000 | L1PB | L1 | LINE |
| 32948324 | 32948625 | - | 13.0 | 1 | 299 | -11 | AluSg | Alu | SINE |
| 32948626 | 32948672 | - | 13.1 | 4925 | 4946 | -1200 | L1PB | L1 | LINE |
| 32948717 | 32948999 | - | 11.1 | 1 | 280 | -30 | AluSg | Alu | SINE |
| 32949000 | 32949743 | - | 13.1 | 4211 | 4924 | -1222 | L1PB | L1 | LINE |
| 32949766 | 32949831 | - | 15.2 | 1 | 66 | -246 | AluJ/FLAM | Alu | SINE |
| 32949849 | 32949925 | - | 32.5 | 1 | 77 | -268 | MER2 | MER2\_type | DNA |

  

|  |  |  |  |  |  |  |  |  |  |
| --- | --- | --- | --- | --- | --- | --- | --- | --- | --- |
| chr3:33276801-33289774 | | | 12923 bp | | |  |  |  |  |
| Genome Start | Genome End | Strand | Div | Repeat Start | Repeat End | Repeat Left | Name | Family | Class |
| 33276801 | 33277188 | - | 21.9 | 1450 | 1833 | -4813 | L1M4 | L1 | LINE |
| 33277206 | 33278947 | - | 10.8 | 5099 | 6867 | 0 | L1PB1 | L1 | LINE |
| 33278948 | 33279258 | + | 9.0 | 1 | 311 | -2 | AluSp | Alu | SINE |
| 33279259 | 33279867 | - | 11.2 | 4511 | 5098 | -1764 | L1PB1 | L1 | LINE |
| 33279868 | 33280175 | + | 11.1 | 1 | 309 | -3 | AluSx | Alu | SINE |
| 33280176 | 33282515 | - | 11.6 | 2139 | 4510 | -2352 | L1PB1 | L1 | LINE |
| 33282516 | 33282870 | - | 12.3 | 1 | 355 | 0 | THE1A | MaLR | LTR |
| 33282871 | 33284427 | - | 29.8 | 5 | 1735 | 0 | MLT1H-int | MaLR | LTR |
| 33284433 | 33284775 | - | 12.7 | 1 | 355 | 0 | THE1A | MaLR | LTR |
| 33284776 | 33286402 | - | 12.6 | 497 | 2138 | -4725 | L1PBa | L1 | LINE |
| 33286403 | 33286694 | + | 11.0 | 3 | 295 | -17 | AluSx | Alu | SINE |
| 33286695 | 33287194 | - | 12.6 | 2 | 496 | -6367 | L1PBa | L1 | LINE |
| 33287195 | 33287509 | - | 22.4 | 1130 | 1461 | -5081 | L1M4 | L1 | LINE |
| 33287539 | 33288135 | - | 12.7 | 1 | 607 | -7 | LTR18B | ERVL | LTR |
| 33288136 | 33288909 | - | 30.1 | 379 | 1135 | -5407 | L1M4 | L1 | LINE |
| 33288910 | 33289434 | + | 14.1 | 1 | 529 | 0 | MER1A | MER1\_type | DNA |
| 33289435 | 33289774 | - | 30.1 | 15 | 378 | -6164 | L1M4 | L1 | LINE |

  

|  |  |  |  |  |  |  |  |  |  |
| --- | --- | --- | --- | --- | --- | --- | --- | --- | --- |
| chr3:34715605-34720142 | | | 4436 bp | | |  |  |  |  |
| Genome Start | Genome End | Strand | Div | Repeat Start | Repeat End | Repeat Left | Name | Family | Class |
| 34715605 | 34716330 | + | 22.2 | 2097 | 2836 | -3546 | L1M4c | L1 | LINE |
| 34716330 | 34716377 | - | 10.4 | 5320 | 5367 | -779 | L1PREC2 | L1 | LINE |
| 34716380 | 34717131 | + | 12.0 | 5374 | 6159 | -1 | L1PREC2 | L1 | LINE |
| 34717164 | 34717221 | + | 8.6 | 2827 | 2884 | -3498 | L1M4c | L1 | LINE |
| 34717222 | 34717588 | - | 16.5 | 1 | 364 | 0 | THE1B | MaLR | LTR |
| 34717589 | 34717641 | - | 7.5 | 1528 | 1580 | 0 | THE1B-int | MaLR | LTR |
| 34717682 | 34718778 | - | 10.8 | 435 | 1541 | -39 | THE1B-int | MaLR | LTR |
| 34718779 | 34719091 | - | 14.4 | 1 | 312 | -1 | AluSp | Alu | SINE |
| 34719092 | 34719532 | - | 10.8 | 1 | 434 | -1146 | THE1B-int | MaLR | LTR |
| 34719535 | 34719901 | - | 17.8 | 1 | 362 | -2 | THE1B | MaLR | LTR |
| 34719902 | 34719950 | + | 12.2 | 2880 | 2928 | -3454 | L1M4c | L1 | LINE |
| 34719978 | 34720142 | + | 15.4 | 2920 | 3078 | -3304 | L1M4c | L1 | LINE |

  

|  |  |  |  |  |  |  |  |  |  |
| --- | --- | --- | --- | --- | --- | --- | --- | --- | --- |
| chr3:35990069-35992750 | | | 2676 bp | | |  |  |  |  |
| Genome Start | Genome End | Strand | Div | Repeat Start | Repeat End | Repeat Left | Name | Family | Class |
| 35990069 | 35990256 | + | 30.3 | 1199 | 1417 | -1090 | HAL1 | L1 | LINE |
| 35990257 | 35990632 | - | 14.4 | 1 | 381 | 0 | THE1D | MaLR | LTR |
| 35990639 | 35992171 | - | 12.4 | 1 | 1573 | -7 | THE1D-int | MaLR | LTR |
| 35992172 | 35992547 | - | 16.0 | 1 | 381 | 0 | THE1D | MaLR | LTR |
| 35992548 | 35992750 | + | 33.7 | 1413 | 1640 | -867 | HAL1 | L1 | LINE |

  

|  |  |  |  |  |  |  |  |  |  |
| --- | --- | --- | --- | --- | --- | --- | --- | --- | --- |
| chr3:46352676-46357153 | | | 4473 bp | | |  |  |  |  |
| Genome Start | Genome End | Strand | Div | Repeat Start | Repeat End | Repeat Left | Name | Family | Class |
| 46352676 | 46353201 | + | 22.0 | 4537 | 5004 | -1142 | L1MB7 | L1 | LINE |
| 46353202 | 46353244 | + | 8.9 | 1 | 32 | -48 | MADE1 | Mariner | DNA |
| 46353245 | 46353539 | - | 11.9 | 1 | 306 | -5 | AluY | Alu | SINE |
| 46353540 | 46353588 | + | 8.9 | 33 | 80 | 0 | MADE1 | Mariner | DNA |
| 46353589 | 46354186 | + | 15.1 | 5005 | 5598 | -586 | L1MB7 | L1 | LINE |
| 46354188 | 46354310 | + | 19.5 | 3 | 125 | -18 | FLAM\_C | Alu | SINE |
| 46354313 | 46354572 | + | 18.1 | 5593 | 5913 | -271 | L1MB7 | L1 | LINE |
| 46354573 | 46354911 | - | 14.4 | 1 | 428 | 0 | MSTA | MaLR | LTR |
| 46354912 | 46356492 | - | 30.3 | 2 | 1735 | 0 | MLT-int | MaLR | LTR |
| 46356495 | 46356878 | - | 13.3 | 1 | 428 | 0 | MSTA | MaLR | LTR |
| 46356879 | 46357153 | + | 18.1 | 5914 | 6172 | -12 | L1MB7 | L1 | LINE |

  

|  |  |  |  |  |  |  |  |  |  |
| --- | --- | --- | --- | --- | --- | --- | --- | --- | --- |
| chr3:46834831-46841848 | | | 6956 bp | | |  |  |  |  |
| Genome Start | Genome End | Strand | Div | Repeat Start | Repeat End | Repeat Left | Name | Family | Class |
| 46834831 | 46835744 | - | 22.7 | 2316 | 3255 | -2964 | L1MEc | L1 | LINE |
| 46835745 | 46836038 | + | 18.1 | 2 | 298 | -14 | AluJb | Alu | SINE |
| 46836039 | 46836242 | - | 22.9 | 2183 | 2417 | -3983 | L1MEc | L1 | LINE |
| 46836270 | 46836386 | - | 23.4 | 2081 | 2198 | -4202 | L1MEc | L1 | LINE |
| 46836387 | 46836749 | + | 12.5 | 1 | 360 | -4 | THE1B | MaLR | LTR |
| 46836750 | 46836780 | - | 23.4 | 2052 | 2080 | -4320 | L1MEc | L1 | LINE |
| 46836793 | 46837077 | - | 4.6 | 1 | 297 | -14 | AluY | Alu | SINE |
| 46837095 | 46837265 | + | 16.5 | 131 | 302 | -10 | AluJb | Alu | SINE |
| 46837270 | 46838411 | - | 27.7 | 641 | 1895 | -4505 | L1MEc | L1 | LINE |
| 46838412 | 46838777 | - | 11.3 | 1 | 381 | 0 | THE1D | MaLR | LTR |
| 46838778 | 46838835 | - | 28.8 | 1683 | 1735 | 0 | MLT-int | MaLR | LTR |
| 46838836 | 46839150 | + | 16.4 | 1 | 310 | -2 | AluSx | Alu | SINE |
| 46839151 | 46840722 | - | 28.8 | 2 | 1682 | -53 | MLT-int | MaLR | LTR |
| 46840725 | 46841093 | - | 11.7 | 1 | 381 | 0 | THE1D | MaLR | LTR |
| 46841094 | 46841375 | - | 27.7 | 363 | 640 | -5760 | L1MEc | L1 | LINE |
| 46841376 | 46841566 | - | 24.7 | 1 | 215 | 0 | MER58C | MER1\_type | DNA |
| 46841567 | 46841848 | - | 27.7 | 76 | 362 | -6038 | L1MEc | L1 | LINE |

  

|  |  |  |  |  |  |  |  |  |  |
| --- | --- | --- | --- | --- | --- | --- | --- | --- | --- |
| chr3:49232087-49246127 | | | 13996 bp | | |  |  |  |  |
| Genome Start | Genome End | Strand | Div | Repeat Start | Repeat End | Repeat Left | Name | Family | Class |
| 49232087 | 49232656 | + | 9.5 | 1 | 568 | -5348 | HERVK9 | ERVK | LTR |
| 49232655 | 49232781 | + | 10.2 | 1424 | 1550 | -4366 | HERVK9 | ERVK | LTR |
| 49232782 | 49233023 | - | 8.8 | 522 | 826 | 0 | LTR12 | ERV1 | LTR |
| 49233024 | 49233320 | + | 10.8 | 1 | 299 | -12 | AluY | Alu | SINE |
| 49233321 | 49233947 | - | 8.8 | 3 | 521 | -305 | LTR12 | ERV1 | LTR |
| 49233948 | 49234682 | - | 6.5 | 7701 | 8436 | 0 | HERV9 | ERV1 | LTR |
| 49234681 | 49235847 | - | 5.2 | 5606 | 6767 | -1669 | HERV9 | ERV1 | LTR |
| 49235848 | 49236139 | + | 6.8 | 1 | 293 | -18 | AluY | Alu | SINE |
| 49236140 | 49237620 | - | 5.2 | 4128 | 5605 | -2831 | HERV9 | ERV1 | LTR |
| 49237621 | 49237917 | + | 9.1 | 1 | 298 | -12 | AluSg | Alu | SINE |
| 49237918 | 49237953 | - | 5.2 | 4092 | 4127 | -4309 | HERV9 | ERV1 | LTR |
| 49237961 | 49240949 | - | 7.3 | 60 | 3149 | -5287 | HERV9 | ERV1 | LTR |
| 49240976 | 49241693 | - | 8.7 | 1 | 826 | 0 | LTR12 | ERV1 | LTR |
| 49241696 | 49245748 | + | 8.9 | 1549 | 5634 | -282 | HERVK9 | ERVK | LTR |
| 49245763 | 49246127 | + | 13.8 | 5635 | 7377 | 0 | HERVK9 | ERVK | LTR |

  

|  |  |  |  |  |  |  |  |  |  |
| --- | --- | --- | --- | --- | --- | --- | --- | --- | --- |
| chr3:54124237-54127358 | | | 3111 bp | | |  |  |  |  |
| Genome Start | Genome End | Strand | Div | Repeat Start | Repeat End | Repeat Left | Name | Family | Class |
| 54124237 | 54124286 | - | 26.5 | 6120 | 6168 | -1640 | L1MC4a | L1 | LINE |
| 54124287 | 54124586 | + | 9.0 | 1 | 299 | -11 | AluSg | Alu | SINE |
| 54124587 | 54125006 | - | 26.5 | 5670 | 6119 | -1689 | L1MC4a | L1 | LINE |
| 54125015 | 54125349 | + | 13.2 | 23 | 381 | 0 | THE1D | MaLR | LTR |
| 54125352 | 54126896 | + | 29.3 | 2 | 1735 | 0 | MLT-int | MaLR | LTR |
| 54126897 | 54127254 | + | 13.8 | 1 | 381 | 0 | THE1D | MaLR | LTR |
| 54127256 | 54127358 | - | 26.2 | 5532 | 5656 | -2152 | L1MC4a | L1 | LINE |

  

|  |  |  |  |  |  |  |  |  |  |
| --- | --- | --- | --- | --- | --- | --- | --- | --- | --- |
| chr3:58852239-58858942 | | | 6698 bp | | |  |  |  |  |
| Genome Start | Genome End | Strand | Div | Repeat Start | Repeat End | Repeat Left | Name | Family | Class |
| 58852239 | 58853809 | - | 9.0 | 4130 | 5675 | -684 | L1P4 | L1 | LINE |
| 58853810 | 58854109 | - | 7.3 | 1 | 299 | -12 | AluY | Alu | SINE |
| 58854110 | 58854565 | - | 9.2 | 3677 | 4129 | -2202 | L1P4 | L1 | LINE |
| 58854572 | 58854924 | - | 11.3 | 1 | 364 | 0 | THE1B | MaLR | LTR |
| 58854925 | 58856465 | - | 8.7 | 1 | 1580 | 0 | THE1B-int | MaLR | LTR |
| 58856466 | 58856820 | - | 11.8 | 1 | 364 | 0 | THE1B | MaLR | LTR |
| 58856821 | 58858942 | - | 12.3 | 1542 | 3675 | -2656 | L1P4 | L1 | LINE |

  

|  |  |  |  |  |  |  |  |  |  |
| --- | --- | --- | --- | --- | --- | --- | --- | --- | --- |
| chr3:66553030-66556802 | | | 3758 bp | | |  |  |  |  |
| Genome Start | Genome End | Strand | Div | Repeat Start | Repeat End | Repeat Left | Name | Family | Class |
| 66553030 | 66553121 | + | 19.8 | 2725 | 2819 | -600 | L2 | L2 | LINE |
| 66553126 | 66553930 | + | 17.4 | 5194 | 6020 | -151 | L1MB2 | L1 | LINE |
| 66553931 | 66554298 | - | 12.3 | 1 | 364 | 0 | THE1B | MaLR | LTR |
| 66554299 | 66555868 | - | 29.2 | 2 | 1735 | 0 | MLT-int | MaLR | LTR |
| 66555871 | 66556241 | - | 13.4 | 1 | 364 | 0 | THE1B | MaLR | LTR |
| 66556242 | 66556393 | + | 17.5 | 6021 | 6169 | -2 | L1MB2 | L1 | LINE |
| 66556403 | 66556708 | + | 9.5 | 1 | 306 | -7 | AluSp | Alu | SINE |
| 66556709 | 66556802 | + | 25.5 | 2813 | 2916 | -503 | L2 | L2 | LINE |

  

|  |  |  |  |  |  |  |  |  |  |
| --- | --- | --- | --- | --- | --- | --- | --- | --- | --- |
| chr3:66691904-66694677 | | | 2772 bp | | |  |  |  |  |
| Genome Start | Genome End | Strand | Div | Repeat Start | Repeat End | Repeat Left | Name | Family | Class |
| 66691904 | 66691977 | - | 29.2 | 161 | 241 | -21 | MIR | MIR | SINE |
| 66691978 | 66692336 | - | 8.2 | 1 | 355 | 0 | THE1A | MaLR | LTR |
| 66692337 | 66693875 | - | 27.9 | 2 | 1735 | 0 | MLT1L-int | MaLR | LTR |
| 66693878 | 66694204 | - | 8.0 | 39 | 355 | 0 | THE1A | MaLR | LTR |
| 66694205 | 66694521 | + | 14.2 | 1 | 304 | -9 | AluSq | Alu | SINE |
| 66694522 | 66694561 | - | 8.0 | 1 | 38 | -317 | THE1A | MaLR | LTR |
| 66694562 | 66694677 | - | 29.2 | 41 | 160 | -102 | MIR | MIR | SINE |

  

|  |  |  |  |  |  |  |  |  |  |
| --- | --- | --- | --- | --- | --- | --- | --- | --- | --- |
| chr3:68441776-68445522 | | | 3743 bp | | |  |  |  |  |
| Genome Start | Genome End | Strand | Div | Repeat Start | Repeat End | Repeat Left | Name | Family | Class |
| 68441776 | 68442749 | - | 25.7 | 2492 | 3523 | -1 | Zaphod2 | Tip100 | DNA |
| 68442750 | 68443122 | - | 11.5 | 1 | 364 | 0 | THE1B | MaLR | LTR |
| 68443123 | 68444661 | - | 30.2 | 2 | 1735 | 0 | MLT-int | MaLR | LTR |
| 68444664 | 68445027 | - | 15.3 | 3 | 364 | 0 | THE1B | MaLR | LTR |
| 68445028 | 68445158 | - | 25.7 | 2364 | 2491 | -1033 | Zaphod2 | Tip100 | DNA |
| 68445159 | 68445324 | - | 28.9 | 149 | 320 | -3204 | Zaphod2 | Tip100 | DNA |
| 68445327 | 68445522 | - | 27.0 | 12 | 208 | -3316 | Zaphod2 | Tip100 | DNA |

  

|  |  |  |  |  |  |  |  |  |  |
| --- | --- | --- | --- | --- | --- | --- | --- | --- | --- |
| chr3:68508088-68511488 | | | 3399 bp | | |  |  |  |  |
| Genome Start | Genome End | Strand | Div | Repeat Start | Repeat End | Repeat Left | Name | Family | Class |
| 68508088 | 68508612 | - | 8.8 | 5638 | 6163 | 0 | L1PA13 | L1 | LINE |
| 68508613 | 68508976 | + | 13.3 | 1 | 381 | 0 | THE1D | MaLR | LTR |
| 68508977 | 68510492 | + | 13.4 | 1 | 1578 | -2 | THE1D-int | MaLR | LTR |
| 68510495 | 68510862 | + | 13.2 | 1 | 381 | 0 | THE1D | MaLR | LTR |
| 68510863 | 68511488 | - | 9.8 | 4999 | 5642 | -526 | L1PA13 | L1 | LINE |

  

|  |  |  |  |  |  |  |  |  |  |
| --- | --- | --- | --- | --- | --- | --- | --- | --- | --- |
| chr3:68568801-68574721 | | | 5475 bp | | |  |  |  |  |
| Genome Start | Genome End | Strand | Div | Repeat Start | Repeat End | Repeat Left | Name | Family | Class |
| 68568801 | 68569061 | - | 37.9 | 1696 | 1956 | -4 | L4 | RTE | LINE |
| 68569212 | 68569635 | - | 17.2 | 1 | 426 | -2 | MSTC | MaLR | LTR |
| 68569636 | 68569917 | - | 25.8 | 1365 | 1735 | 0 | MSTC-int | MaLR | LTR |
| 68569918 | 68570206 | + | 21.1 | 1 | 291 | -21 | AluJo | Alu | SINE |
| 68570207 | 68571199 | - | 25.8 | 378 | 1364 | -371 | MSTC-int | MaLR | LTR |
| 68571200 | 68571239 | - | 9.6 | 522 | 561 | 0 | MLT2A2 | ERVL | LTR |
| 68571286 | 68571672 | - | 9.6 | 2 | 413 | -40 | MLT2A2 | ERVL | LTR |
| 68571673 | 68571862 | - | 7.9 | 5469 | 5654 | 0 | HERVL-A2 | ERVL | LTR |
| 68571863 | 68572224 | + | 17.5 | 1 | 364 | 0 | THE1B | MaLR | LTR |
| 68572225 | 68572811 | - | 7.9 | 4861 | 5468 | -186 | HERVL-A2 | ERVL | LTR |
| 68572805 | 68573843 | + | 7.8 | 2816 | 3854 | -1800 | HERVL | ERVL | LTR |
| 68573884 | 68573984 | - | 18.2 | 2 | 101 | -1550 | MSTC-int | MaLR | LTR |
| 68573990 | 68574376 | - | 14.0 | 1 | 424 | -4 | MSTC | MaLR | LTR |
| 68574589 | 68574721 | - | 43.6 | 1269 | 1403 | -557 | L4 | RTE | LINE |

  

|  |  |  |  |  |  |  |  |  |  |
| --- | --- | --- | --- | --- | --- | --- | --- | --- | --- |
| chr3:68731801-68743028 | | | 10941 bp | | |  |  |  |  |
| Genome Start | Genome End | Strand | Div | Repeat Start | Repeat End | Repeat Left | Name | Family | Class |
| 68731801 | 68732197 | - | 30.0 | 2978 | 3416 | -3 | L2 | L2 | LINE |
| 68732198 | 68732408 | - | 19.5 | 1 | 224 | 0 | MER58A | MER1\_type | DNA |
| 68732409 | 68732445 | - | 30.0 | 2942 | 2977 | -442 | L2 | L2 | LINE |
| 68732481 | 68732778 | - | 19.1 | 5 | 296 | -16 | AluJb | Alu | SINE |
| 68732789 | 68732828 | - | 19.3 | 521 | 556 | 0 | MLT2B4 | ERVL | LTR |
| 68732856 | 68733297 | - | 19.3 | 17 | 520 | -36 | MLT2B4 | ERVL | LTR |
| 68733301 | 68733873 | - | 14.0 | 5733 | 6287 | -4 | L1MA8 | L1 | LINE |
| 68733874 | 68734283 | - | 18.3 | 1 | 426 | 0 | MSTB | MaLR | LTR |
| 68734284 | 68734753 | - | 14.0 | 5257 | 5732 | -559 | L1MA8 | L1 | LINE |
| 68734929 | 68735803 | + | 15.8 | 5 | 884 | -5469 | L1PA10 | L1 | LINE |
| 68735796 | 68737758 | + | 21.3 | 1974 | 4295 | -2036 | L1PA10 | L1 | LINE |
| 68737759 | 68738037 | - | 12.9 | 23 | 301 | -9 | AluSg | Alu | SINE |
| 68738038 | 68739514 | + | 8.9 | 4111 | 5621 | -547 | L1P4 | L1 | LINE |
| 68739515 | 68739879 | - | 11.1 | 1 | 362 | -2 | THE1B | MaLR | LTR |
| 68739880 | 68741458 | - | 30.4 | 2 | 1735 | 0 | MLT-int | MaLR | LTR |
| 68741461 | 68741826 | - | 10.5 | 1 | 364 | 0 | THE1B | MaLR | LTR |
| 68741827 | 68742373 | + | 9.7 | 5622 | 6159 | -9 | L1PA10 | L1 | LINE |
| 68742395 | 68742525 | - | 13.8 | 5125 | 5259 | -884 | L1M2 | L1 | LINE |
| 68742548 | 68743028 | - | 31.8 | 2360 | 2875 | -544 | L2 | L2 | LINE |

  

|  |  |  |  |  |  |  |  |  |  |
| --- | --- | --- | --- | --- | --- | --- | --- | --- | --- |
| chr3:68909868-68915208 | | | 5307 bp | | |  |  |  |  |
| Genome Start | Genome End | Strand | Div | Repeat Start | Repeat End | Repeat Left | Name | Family | Class |
| 68909868 | 68910298 | + | 25.7 | 2203 | 2617 | -3602 | L1MEc | L1 | LINE |
| 68910299 | 68910663 | + | 11.5 | 1 | 364 | 0 | THE1B | MaLR | LTR |
| 68910666 | 68912221 | + | 29.8 | 2 | 1735 | 0 | MLT-int | MaLR | LTR |
| 68912222 | 68912582 | + | 13.9 | 1 | 364 | 0 | THE1B | MaLR | LTR |
| 68912583 | 68913713 | + | 26.2 | 2618 | 3829 | -2390 | L1MEc | L1 | LINE |
| 68913716 | 68914727 | + | 16.4 | 5014 | 6136 | -38 | L1MB5 | L1 | LINE |
| 68914758 | 68915208 | + | 20.1 | 3823 | 4321 | -1898 | L1MEc | L1 | LINE |

  

|  |  |  |  |  |  |  |  |  |  |
| --- | --- | --- | --- | --- | --- | --- | --- | --- | --- |
| chr3:69123514-69126167 | | | 2560 bp | | |  |  |  |  |
| Genome Start | Genome End | Strand | Div | Repeat Start | Repeat End | Repeat Left | Name | Family | Class |
| 69123514 | 69123585 | + | 16.7 | 14 | 85 | -227 | AluJo | Alu | SINE |
| 69123586 | 69123939 | - | 6.2 | 1 | 355 | 0 | THE1A | MaLR | LTR |
| 69123940 | 69125515 | - | 29.8 | 2 | 1735 | 0 | MLT-int | MaLR | LTR |
| 69125518 | 69125869 | - | 6.8 | 1 | 355 | 0 | THE1A | MaLR | LTR |
| 69125870 | 69125938 | + | 16.7 | 86 | 145 | -167 | AluJo | Alu | SINE |
| 69126031 | 69126167 | + | 20.4 | 129 | 265 | -47 | AluJo | Alu | SINE |

  

|  |  |  |  |  |  |  |  |  |  |
| --- | --- | --- | --- | --- | --- | --- | --- | --- | --- |
| chr3:69740456-69743259 | | | 2745 bp | | |  |  |  |  |
| Genome Start | Genome End | Strand | Div | Repeat Start | Repeat End | Repeat Left | Name | Family | Class |
| 69740456 | 69740536 | + | 27.2 | 187 | 267 | -324 | MLT1K | MaLR | LTR |
| 69740582 | 69740683 | + | 28.7 | 1 | 110 | -4 | MER81 | AcHobo | DNA |
| 69740684 | 69741046 | + | 13.1 | 1 | 364 | 0 | THE1B | MaLR | LTR |
| 69741047 | 69742626 | + | 9.5 | 1 | 1580 | 0 | THE1B-int | MaLR | LTR |
| 69742627 | 69742971 | + | 12.6 | 1 | 364 | 0 | THE1B | MaLR | LTR |
| 69742986 | 69743259 | + | 29.4 | 314 | 591 | 0 | MLT1K | MaLR | LTR |

  

|  |  |  |  |  |  |  |  |  |  |
| --- | --- | --- | --- | --- | --- | --- | --- | --- | --- |
| chr3:69883483-69886643 | | | 3159 bp | | |  |  |  |  |
| Genome Start | Genome End | Strand | Div | Repeat Start | Repeat End | Repeat Left | Name | Family | Class |
| 69883483 | 69883605 | + | 30.9 | 19 | 142 | -126 | MIRb | MIR | SINE |
| 69883606 | 69884049 | + | 12.9 | 1 | 428 | 0 | MSTA | MaLR | LTR |
| 69884052 | 69885859 | + | 27.1 | 2 | 1735 | 0 | MLT-int | MaLR | LTR |
| 69885860 | 69886062 | + | 14.3 | 1 | 202 | -226 | MSTA | MaLR | LTR |
| 69886063 | 69886301 | - | 12.6 | 1 | 293 | -17 | AluSg | Alu | SINE |
| 69886302 | 69886541 | + | 14.3 | 203 | 428 | 0 | MSTA | MaLR | LTR |
| 69886542 | 69886643 | + | 30.9 | 143 | 231 | -37 | MIRb | MIR | SINE |

  

|  |  |  |  |  |  |  |  |  |  |
| --- | --- | --- | --- | --- | --- | --- | --- | --- | --- |
| chr3:70564321-70572730 | | | 8328 bp | | |  |  |  |  |
| Genome Start | Genome End | Strand | Div | Repeat Start | Repeat End | Repeat Left | Name | Family | Class |
| 70564321 | 70566509 | - | 17.0 | 4097 | 6335 | -9 | L1MC2 | L1 | LINE |
| 70566513 | 70566924 | - | 8.8 | 1 | 448 | -2 | LTR7 | ERV1 | LTR |
| 70566925 | 70567303 | - | 11.5 | 7322 | 7713 | 0 | HERVH | ERV1 | LTR |
| 70567302 | 70567517 | - | 12.0 | 5600 | 5819 | -1894 | HERVH | ERV1 | LTR |
| 70567519 | 70568181 | - | 15.2 | 4488 | 5149 | -2564 | HERVH | ERV1 | LTR |
| 70568184 | 70569038 | - | 8.3 | 3140 | 3993 | -3720 | HERVH | ERV1 | LTR |
| 70569033 | 70571293 | - | 9.9 | 714 | 2987 | -4726 | HERVH | ERV1 | LTR |
| 70571269 | 70571843 | - | 10.2 | 1 | 572 | -7141 | HERVH | ERV1 | LTR |
| 70571851 | 70572260 | - | 7.9 | 1 | 443 | -7 | LTR7 | ERV1 | LTR |
| 70572261 | 70572407 | - | 15.1 | 3951 | 4100 | -2046 | L1MC2 | L1 | LINE |
| 70572488 | 70572595 | + | 6.2 | 1 | 80 | 0 | MADE1 | Mariner | DNA |
| 70572618 | 70572730 | - | 23.9 | 3835 | 3953 | -2193 | L1MC2 | L1 | LINE |

  

|  |  |  |  |  |  |  |  |  |  |
| --- | --- | --- | --- | --- | --- | --- | --- | --- | --- |
| chr3:70591902-70594929 | | | 3022 bp | | |  |  |  |  |
| Genome Start | Genome End | Strand | Div | Repeat Start | Repeat End | Repeat Left | Name | Family | Class |
| 70591902 | 70592272 | + | 30.6 | 2666 | 3078 | -341 | L2 | L2 | LINE |
| 70592279 | 70592634 | + | 11.4 | 1 | 355 | 0 | THE1A | MaLR | LTR |
| 70592635 | 70594230 | + | 9.8 | 1 | 1580 | 0 | THE1A-int | MaLR | LTR |
| 70594231 | 70594579 | + | 13.5 | 1 | 355 | 0 | THE1A | MaLR | LTR |
| 70594580 | 70594929 | + | 30.8 | 3084 | 3419 | 0 | L2 | L2 | LINE |

  

|  |  |  |  |  |  |  |  |  |  |
| --- | --- | --- | --- | --- | --- | --- | --- | --- | --- |
| chr3:70745513-70749868 | | | 3903 bp | | |  |  |  |  |
| Genome Start | Genome End | Strand | Div | Repeat Start | Repeat End | Repeat Left | Name | Family | Class |
| 70745513 | 70745733 | - | 26.5 | 1327 | 1565 | -4584 | L1MEe | L1 | LINE |
| 70745957 | 70746367 | - | 17.3 | 5 | 426 | 0 | MSTB | MaLR | LTR |
| 70746370 | 70747970 | - | 18.4 | 2 | 1651 | 0 | MSTB-int | MaLR | LTR |
| 70747972 | 70748381 | - | 15.9 | 1 | 426 | 0 | MSTB | MaLR | LTR |
| 70748383 | 70748758 | - | 29.7 | 660 | 1032 | -5087 | L1MEe | L1 | LINE |
| 70748961 | 70749707 | + | 6.7 | 1 | 780 | 0 | LTR17 | ERV1 | LTR |
| 70749732 | 70749868 | - | 32.1 | 2 | 138 | -5981 | L1MEe | L1 | LINE |

  

|  |  |  |  |  |  |  |  |  |  |
| --- | --- | --- | --- | --- | --- | --- | --- | --- | --- |
| chr3:70839536-70846466 | | | 6820 bp | | |  |  |  |  |
| Genome Start | Genome End | Strand | Div | Repeat Start | Repeat End | Repeat Left | Name | Family | Class |
| 70839536 | 70839832 | + | 26.7 | 5268 | 5582 | -658 | L1ME3B | L1 | LINE |
| 70839833 | 70840148 | - | 17.0 | 3 | 324 | 0 | MER33 | MER1\_type | DNA |
| 70840149 | 70840176 | + | 26.7 | 5583 | 5602 | -638 | L1ME3B | L1 | LINE |
| 70840184 | 70840445 | + | 19.9 | 1 | 264 | -2227 | Tigger7 | MER2\_type | DNA |
| 70840467 | 70843120 | - | 14.2 | 50 | 2717 | -1 | Tigger2 | MER2\_type | DNA |
| 70843121 | 70843520 | - | 16.8 | 1 | 428 | 0 | MSTA | MaLR | LTR |
| 70843521 | 70843553 | - | 29.8 | 1713 | 1735 | 0 | MLT-int | MaLR | LTR |
| 70843554 | 70843856 | - | 9.9 | 1 | 304 | -7 | AluY | Alu | SINE |
| 70843857 | 70845439 | - | 29.8 | 2 | 1712 | -23 | MLT-int | MaLR | LTR |
| 70845442 | 70845827 | - | 16.4 | 1 | 428 | 0 | MSTA | MaLR | LTR |
| 70845828 | 70845881 | - | 22.2 | 1 | 54 | -2664 | Tigger2 | MER2\_type | DNA |
| 70845882 | 70845964 | + | 21.7 | 2409 | 2491 | 0 | Tigger7 | MER2\_type | DNA |
| 70846046 | 70846466 | + | 28.0 | 5745 | 6188 | -52 | L1ME3B | L1 | LINE |

  

|  |  |  |  |  |  |  |  |  |  |
| --- | --- | --- | --- | --- | --- | --- | --- | --- | --- |
| chr3:75601245-75608107 | | | 6803 bp | | |  |  |  |  |
| Genome Start | Genome End | Strand | Div | Repeat Start | Repeat End | Repeat Left | Name | Family | Class |
| 75601245 | 75601425 | + | 22.9 | 1 | 185 | -320 | MLT1D | MaLR | LTR |
| 75601436 | 75601734 | + | 10.0 | 3 | 302 | -11 | AluSp | Alu | SINE |
| 75601747 | 75602145 | - | 9.1 | 3 | 448 | -2 | LTR7 | ERV1 | LTR |
| 75602148 | 75602524 | - | 10.8 | 7324 | 7713 | 0 | HERVH | ERV1 | LTR |
| 75602525 | 75602736 | - | 11.8 | 5600 | 5819 | -1894 | HERVH | ERV1 | LTR |
| 75602741 | 75603399 | - | 14.7 | 4488 | 5146 | -2567 | HERVH | ERV1 | LTR |
| 75603402 | 75604251 | - | 10.0 | 3140 | 3993 | -3720 | HERVH | ERV1 | LTR |
| 75604238 | 75607173 | - | 9.9 | 1 | 2992 | -4721 | HERVH | ERV1 | LTR |
| 75607176 | 75607524 | - | 9.5 | 1 | 448 | -2 | LTR7 | ERV1 | LTR |
| 75607567 | 75607738 | + | 22.9 | 206 | 425 | -79 | MLT1D | MaLR | LTR |
| 75607739 | 75608042 | - | 15.2 | 1 | 303 | -9 | AluSx | Alu | SINE |
| 75608043 | 75608107 | + | 23.4 | 389 | 484 | 0 | MLT1D | MaLR | LTR |

  

|  |  |  |  |  |  |  |  |  |  |
| --- | --- | --- | --- | --- | --- | --- | --- | --- | --- |
| chr3:75656576-75668270 | | | 11603 bp | | |  |  |  |  |
| Genome Start | Genome End | Strand | Div | Repeat Start | Repeat End | Repeat Left | Name | Family | Class |
| 75656576 | 75656989 | + | 20.5 | 1056 | 1438 | -5411 | L1MA4 | L1 | LINE |
| 75656990 | 75657316 | + | 10.8 | 3 | 309 | -1 | AluSg | Alu | SINE |
| 75657317 | 75658361 | + | 20.5 | 1439 | 2522 | -4327 | L1MA4 | L1 | LINE |
| 75658362 | 75658520 | + | 19.5 | 1 | 160 | -6 | FRAM | Alu | SINE |
| 75658521 | 75658772 | + | 20.5 | 2523 | 2779 | -4070 | L1MA4 | L1 | LINE |
| 75658773 | 75659130 | - | 13.6 | 1 | 364 | 0 | THE1B | MaLR | LTR |
| 75659131 | 75660702 | - | 30.8 | 2 | 1735 | 0 | MLT-int | MaLR | LTR |
| 75660705 | 75661058 | - | 15.8 | 1 | 364 | 0 | THE1B | MaLR | LTR |
| 75661059 | 75663263 | + | 16.6 | 2780 | 4969 | -1876 | L1MA4 | L1 | LINE |
| 75663264 | 75663562 | + | 13.4 | 1 | 303 | -9 | AluSx | Alu | SINE |
| 75663563 | 75664058 | + | 16.5 | 4970 | 5444 | -1401 | L1MA4 | L1 | LINE |
| 75664059 | 75664355 | + | 8.8 | 1 | 278 | -33 | AluY | Alu | SINE |
| 75664380 | 75664396 | + | 8.8 | 279 | 293 | -18 | AluY | Alu | SINE |
| 75664397 | 75664855 | + | 16.5 | 5445 | 5905 | -940 | L1MA4 | L1 | LINE |
| 75664856 | 75665099 | + | 9.9 | 4958 | 5204 | -942 | L1PREC2 | L1 | LINE |
| 75665095 | 75665673 | + | 13.9 | 6043 | 6629 | -373 | L1MA4 | L1 | LINE |
| 75665733 | 75665942 | + | 15.8 | 6601 | 6827 | -175 | L1MA4 | L1 | LINE |
| 75665948 | 75666630 | - | 10.9 | 5469 | 6151 | 0 | L1PB1 | L1 | LINE |
| 75666631 | 75666940 | - | 11.0 | 1 | 310 | 0 | AluSg | Alu | SINE |
| 75666941 | 75668089 | - | 9.3 | 4294 | 5468 | -683 | L1PB1 | L1 | LINE |
| 75668097 | 75668270 | + | 12.6 | 6812 | 6990 | -12 | L1MA4 | L1 | LINE |

  

|  |  |  |  |  |  |  |  |  |  |
| --- | --- | --- | --- | --- | --- | --- | --- | --- | --- |
| chr3:75823534-75834605 | | | 11070 bp | | |  |  |  |  |
| Genome Start | Genome End | Strand | Div | Repeat Start | Repeat End | Repeat Left | Name | Family | Class |
| 75823534 | 75828451 | + | 21.9 | 17 | 5299 | -2266 | L1MA2 | L1 | LINE |
| 75828452 | 75828758 | + | 13.7 | 1 | 307 | -5 | AluSx | Alu | SINE |
| 75828759 | 75829135 | + | 26.0 | 5300 | 5670 | -1895 | L1MA2 | L1 | LINE |
| 75829136 | 75829554 | + | 20.6 | 1 | 428 | 0 | MSTA | MaLR | LTR |
| 75829557 | 75831193 | + | 31.8 | 2 | 1735 | 0 | MLT-int | MaLR | LTR |
| 75831194 | 75831600 | + | 20.9 | 1 | 428 | 0 | MSTA | MaLR | LTR |
| 75831601 | 75831952 | + | 26.0 | 5671 | 6027 | -1538 | L1MA2 | L1 | LINE |
| 75831953 | 75832246 | + | 10.9 | 1 | 293 | -17 | AluSg | Alu | SINE |
| 75832247 | 75832904 | + | 26.0 | 6028 | 6683 | -882 | L1MA2 | L1 | LINE |
| 75832905 | 75833230 | + | 15.6 | 1 | 326 | -49 | THE1C | MaLR | LTR |
| 75833231 | 75833540 | + | 9.4 | 2 | 289 | -20 | AluSg1 | Alu | SINE |
| 75833541 | 75833595 | + | 15.6 | 327 | 375 | 0 | THE1C | MaLR | LTR |
| 75833596 | 75834605 | + | 14.9 | 6680 | 7721 | -2 | L1MA2 | L1 | LINE |

  

|  |  |  |  |  |  |  |  |  |  |
| --- | --- | --- | --- | --- | --- | --- | --- | --- | --- |
| chr3:76014268-76017061 | | | 2788 bp | | |  |  |  |  |
| Genome Start | Genome End | Strand | Div | Repeat Start | Repeat End | Repeat Left | Name | Family | Class |
| 76014268 | 76014456 | + | 17.8 | 5616 | 5806 | -350 | L1PB4 | L1 | LINE |
| 76014457 | 76014796 | - | 13.1 | 5 | 364 | 0 | THE1B | MaLR | LTR |
| 76014801 | 76016376 | - | 30.2 | 2 | 1735 | 0 | MLT-int | MaLR | LTR |
| 76016379 | 76016720 | - | 14.2 | 1 | 364 | 0 | THE1B | MaLR | LTR |
| 76016721 | 76017061 | + | 17.8 | 5807 | 6155 | -1 | L1PB4 | L1 | LINE |

  

|  |  |  |  |  |  |  |  |  |  |
| --- | --- | --- | --- | --- | --- | --- | --- | --- | --- |
| chr3:78027876-78031589 | | | 3707 bp | | |  |  |  |  |
| Genome Start | Genome End | Strand | Div | Repeat Start | Repeat End | Repeat Left | Name | Family | Class |
| 78027876 | 78027939 | + | 20.3 | 1 | 65 | -309 | MLT1A | MaLR | LTR |
| 78027942 | 78028285 | - | 14.4 | 1 | 364 | 0 | THE1B | MaLR | LTR |
| 78028286 | 78029824 | - | 8.8 | 1 | 1580 | 0 | THE1B-int | MaLR | LTR |
| 78029825 | 78030168 | - | 13.3 | 1 | 364 | 0 | THE1B | MaLR | LTR |
| 78030169 | 78030318 | + | 24.1 | 63 | 214 | -160 | MLT1A | MaLR | LTR |
| 78030324 | 78031463 | - | 15.6 | 1 | 1231 | 0 | Tigger3b | MER2\_type | DNA |
| 78031464 | 78031589 | + | 24.2 | 218 | 342 | -32 | MLT1A | MaLR | LTR |

  

|  |  |  |  |  |  |  |  |  |  |
| --- | --- | --- | --- | --- | --- | --- | --- | --- | --- |
| chr3:79179799-79196730 | | | 16556 bp | | |  |  |  |  |
| Genome Start | Genome End | Strand | Div | Repeat Start | Repeat End | Repeat Left | Name | Family | Class |
| 79179799 | 79180208 | + | 34.3 | 348 | 806 | -2613 | L2 | L2 | LINE |
| 79180256 | 79180353 | + | 18.8 | 368 | 464 | 0 | LTR16B | ERVL | LTR |
| 79180358 | 79180562 | + | 26.2 | 1261 | 1516 | -1903 | L2 | L2 | LINE |
| 79180564 | 79183502 | + | 8.4 | 827 | 3745 | -2401 | L1P2 | L1 | LINE |
| 79183503 | 79184580 | + | 12.8 | 1 | 1232 | -4 | MER11B | ERVK | LTR |
| 79184586 | 79189150 | + | 7.7 | 1 | 4601 | -3352 | HERVK11 | ERVK | LTR |
| 79189149 | 79190372 | + | 5.9 | 5525 | 6767 | -1186 | HERVK11 | ERVK | LTR |
| 79190358 | 79191195 | + | 5.3 | 6957 | 7794 | -159 | HERVK11 | ERVK | LTR |
| 79191196 | 79191307 | + | 29.6 | 4329 | 4453 | -55 | L3b | CR1 | LINE |
| 79191308 | 79191354 | + | 10.6 | 7907 | 7953 | 0 | HERVK11 | ERVK | LTR |
| 79191355 | 79192432 | + | 12.9 | 1 | 1232 | -4 | MER11B | ERVK | LTR |
| 79192433 | 79194834 | + | 7.8 | 3735 | 6154 | 0 | L1PA6 | L1 | LINE |
| 79194872 | 79195075 | - | 27.4 | 4 | 207 | -17 | MER58A | MER1\_type | DNA |
| 79195086 | 79195507 | + | 29.2 | 1651 | 2135 | -1284 | L2 | L2 | LINE |
| 79195508 | 79195590 | + | 25.9 | 1 | 84 | -977 | MER63 | AcHobo | DNA |
| 79195674 | 79196070 | + | 18.9 | 5888 | 6290 | -10 | L1MA6 | L1 | LINE |
| 79196170 | 79196270 | + | 34.6 | 2141 | 2243 | -1176 | L2 | L2 | LINE |
| 79196374 | 79196489 | - | 15.8 | 6 | 126 | -186 | AluJb | Alu | SINE |
| 79196494 | 79196730 | + | 33.3 | 2792 | 3038 | -381 | L2 | L2 | LINE |

  

|  |  |  |  |  |  |  |  |  |  |
| --- | --- | --- | --- | --- | --- | --- | --- | --- | --- |
| chr3:80438666-80450737 | | | 12077 bp | | |  |  |  |  |
| Genome Start | Genome End | Strand | Div | Repeat Start | Repeat End | Repeat Left | Name | Family | Class |
| 80438666 | 80438817 | + | 17.8 | 1598 | 1749 | -4940 | L1PA15 | L1 | LINE |
| 80438818 | 80439516 | + | 7.0 | 1 | 722 | 0 | LTR30 | ERV1 | LTR |
| 80439517 | 80442979 | + | 9.3 | 1 | 3462 | -4795 | HERV30 | ERV1 | LTR |
| 80442973 | 80443873 | + | 12.9 | 7344 | 8257 | 0 | HERV30 | ERV1 | LTR |
| 80443874 | 80444569 | + | 6.3 | 1 | 722 | 0 | LTR30 | ERV1 | LTR |
| 80444572 | 80446160 | + | 16.8 | 1748 | 3361 | -3312 | L1PA15 | L1 | LINE |
| 80446161 | 80447541 | + | 6.9 | 2 | 1578 | 0 | LTR12C | ERV1 | LTR |
| 80447542 | 80450737 | + | 15.7 | 3362 | 6616 | -76 | L1PA15 | L1 | LINE |

  

|  |  |  |  |  |  |  |  |  |  |
| --- | --- | --- | --- | --- | --- | --- | --- | --- | --- |
| chr3:80834947-80838856 | | | 3900 bp | | |  |  |  |  |
| Genome Start | Genome End | Strand | Div | Repeat Start | Repeat End | Repeat Left | Name | Family | Class |
| 80834947 | 80836068 | + | 18.3 | 4558 | 5680 | -476 | L1PB4 | L1 | LINE |
| 80836076 | 80836431 | - | 13.6 | 1 | 362 | -2 | THE1B | MaLR | LTR |
| 80836432 | 80838009 | - | 12.8 | 1 | 1580 | 0 | THE1B-int | MaLR | LTR |
| 80838010 | 80838368 | - | 14.0 | 1 | 364 | 0 | THE1B | MaLR | LTR |
| 80838372 | 80838856 | + | 19.5 | 5683 | 6156 | 0 | L1PB4 | L1 | LINE |

  

|  |  |  |  |  |  |  |  |  |  |
| --- | --- | --- | --- | --- | --- | --- | --- | --- | --- |
| chr3:81112886-81115431 | | | 2544 bp | | |  |  |  |  |
| Genome Start | Genome End | Strand | Div | Repeat Start | Repeat End | Repeat Left | Name | Family | Class |
| 81112886 | 81112938 | + | 37.9 | 3030 | 3079 | -299 | L2 | L2 | LINE |
| 81112939 | 81113343 | + | 18.1 | 1 | 428 | 0 | MSTA | MaLR | LTR |
| 81113346 | 81114926 | + | 29.7 | 2 | 1735 | 0 | MLT-int | MaLR | LTR |
| 81114927 | 81115308 | + | 19.3 | 1 | 428 | 0 | MSTA | MaLR | LTR |
| 81115309 | 81115431 | + | 37.9 | 3080 | 3202 | -176 | L2 | L2 | LINE |

  

|  |  |  |  |  |  |  |  |  |  |
| --- | --- | --- | --- | --- | --- | --- | --- | --- | --- |
| chr3:81910839-81916025 | | | 4901 bp | | |  |  |  |  |
| Genome Start | Genome End | Strand | Div | Repeat Start | Repeat End | Repeat Left | Name | Family | Class |
| 81910839 | 81911184 | + | 34.2 | 1864 | 2229 | -1190 | L2 | L2 | LINE |
| 81911252 | 81911624 | - | 9.9 | 1 | 375 | 0 | THE1C | MaLR | LTR |
| 81911627 | 81911662 | - | 30.3 | 1705 | 1733 | -2 | MLT-int | MaLR | LTR |
| 81911663 | 81911960 | - | 12.8 | 1 | 298 | -14 | AluSx | Alu | SINE |
| 81911961 | 81913643 | - | 30.3 | 2 | 1704 | -31 | MLT-int | MaLR | LTR |
| 81913646 | 81913759 | - | 12.9 | 261 | 375 | 0 | THE1C | MaLR | LTR |
| 81913760 | 81914521 | + | 11.9 | 1 | 780 | 0 | LTR17 | ERV1 | LTR |
| 81914522 | 81914779 | - | 12.9 | 1 | 260 | -115 | THE1C | MaLR | LTR |
| 81914790 | 81915107 | + | 31.3 | 2536 | 2883 | -536 | L2 | L2 | LINE |
| 81915114 | 81915545 | - | 2.1 | 5725 | 6155 | 0 | L1PA4 | L1 | LINE |
| 81915745 | 81916025 | + | 40.4 | 3072 | 3377 | -1 | L2 | L2 | LINE |

  

|  |  |  |  |  |  |  |  |  |  |
| --- | --- | --- | --- | --- | --- | --- | --- | --- | --- |
| chr3:82773177-82782204 | | | 9045 bp | | |  |  |  |  |
| Genome Start | Genome End | Strand | Div | Repeat Start | Repeat End | Repeat Left | Name | Family | Class |
| 82773177 | 82774123 | + | 12.0 | 6 | 1033 | -6058 | MER52C-int | ERV1 | LTR |
| 82774125 | 82775818 | + | 13.3 | 833 | 2561 | -4530 | MER52-int | ERV1 | LTR |
| 82775795 | 82776713 | + | 11.8 | 2727 | 3709 | -3382 | MER52-int | ERV1 | LTR |
| 82776714 | 82776787 | + | 26.1 | 1 | 73 | -105 | MER5B | MER1\_type | DNA |
| 82776788 | 82777281 | + | 11.8 | 3710 | 4237 | -2854 | MER52-int | ERV1 | LTR |
| 82777282 | 82777627 | + | 14.0 | 1 | 364 | 0 | THE1B | MaLR | LTR |
| 82777630 | 82779196 | + | 32.5 | 2 | 1735 | 0 | MLT1A0-int | MaLR | LTR |
| 82779197 | 82779537 | + | 13.1 | 1 | 364 | 0 | THE1B | MaLR | LTR |
| 82779538 | 82781973 | + | 11.8 | 4240 | 6738 | -353 | MER52C-int | ERV1 | LTR |
| 82781978 | 82782204 | + | 9.7 | 6850 | 7089 | -2 | MER52C-int | ERV1 | LTR |

  

|  |  |  |  |  |  |  |  |  |  |
| --- | --- | --- | --- | --- | --- | --- | --- | --- | --- |
| chr3:83191568-83194281 | | | 2712 bp | | |  |  |  |  |
| Genome Start | Genome End | Strand | Div | Repeat Start | Repeat End | Repeat Left | Name | Family | Class |
| 83191568 | 83191961 | + | 18.1 | 1 | 391 | -37 | MSTC | MaLR | LTR |
| 83191962 | 83192319 | - | 12.8 | 1 | 364 | 0 | THE1B | MaLR | LTR |
| 83192320 | 83193884 | - | 32.1 | 2 | 1735 | 0 | MLT-int | MaLR | LTR |
| 83193887 | 83194241 | - | 16.1 | 1 | 364 | 0 | THE1B | MaLR | LTR |
| 83194242 | 83194281 | + | 18.1 | 392 | 428 | 0 | MSTC | MaLR | LTR |

  

|  |  |  |  |  |  |  |  |  |  |
| --- | --- | --- | --- | --- | --- | --- | --- | --- | --- |
| chr3:83475888-83483388 | | | 6808 bp | | |  |  |  |  |
| Genome Start | Genome End | Strand | Div | Repeat Start | Repeat End | Repeat Left | Name | Family | Class |
| 83475888 | 83476424 | - | 17.9 | 2976 | 3521 | -2666 | MER34-int | ERV1 | LTR |
| 83476425 | 83476875 | - | 18.9 | 281 | 716 | -18 | MER50 | ERV1 | LTR |
| 83476875 | 83477267 | - | 17.6 | 1 | 398 | -5 | MER57A | ERV1 | LTR |
| 83477270 | 83477534 | - | 17.4 | 14 | 287 | -447 | MER50 | ERV1 | LTR |
| 83477537 | 83477638 | - | 23.9 | 4018 | 4115 | -2216 | MER34-int | ERV1 | LTR |
| 83477639 | 83477957 | + | 15.5 | 1 | 364 | 0 | THE1B | MaLR | LTR |
| 83477960 | 83479508 | + | 30.9 | 2 | 1735 | 0 | MLT-int | MaLR | LTR |
| 83479511 | 83479870 | + | 14.3 | 3 | 364 | 0 | THE1B | MaLR | LTR |
| 83479871 | 83481655 | - | 24.1 | 2332 | 4017 | -2314 | MER34-int | ERV1 | LTR |
| 83481807 | 83482164 | - | 29.8 | 1911 | 2274 | -4057 | MER34-int | ERV1 | LTR |
| 83482255 | 83482319 | - | 21.0 | 1798 | 1859 | -4472 | MER34-int | ERV1 | LTR |
| 83482326 | 83482478 | - | 18.6 | 2051 | 2201 | -4062 | MER34-int | ERV1 | LTR |
| 83482796 | 83482971 | - | 26.1 | 1091 | 1252 | -7792 | MER34-int | ERV1 | LTR |
| 83482995 | 83483131 | - | 31.9 | 1089 | 1233 | -5098 | MER34-int | ERV1 | LTR |
| 83483231 | 83483388 | - | 22.8 | 918 | 1088 | -7119 | MER34-int | ERV1 | LTR |

  

|  |  |  |  |  |  |  |  |  |  |
| --- | --- | --- | --- | --- | --- | --- | --- | --- | --- |
| chr3:83726720-83729776 | | | 3049 bp | | |  |  |  |  |
| Genome Start | Genome End | Strand | Div | Repeat Start | Repeat End | Repeat Left | Name | Family | Class |
| 83726720 | 83726932 | + | 9.5 | 5438 | 5659 | -509 | L1PA13 | L1 | LINE |
| 83726933 | 83727291 | + | 15.9 | 1 | 363 | -1 | THE1B | MaLR | LTR |
| 83727293 | 83728838 | + | 12.8 | 1 | 1574 | -6 | THE1B-int | MaLR | LTR |
| 83728846 | 83729202 | + | 15.3 | 3 | 364 | 0 | THE1B | MaLR | LTR |
| 83729203 | 83729655 | + | 12.2 | 5655 | 6111 | -52 | L1PA13 | L1 | LINE |
| 83729656 | 83729726 | - | 11.3 | 1 | 80 | 0 | MADE1 | Mariner | DNA |
| 83729727 | 83729776 | + | 12.2 | 6112 | 6160 | -3 | L1PA13 | L1 | LINE |

  

|  |  |  |  |  |  |  |  |  |  |
| --- | --- | --- | --- | --- | --- | --- | --- | --- | --- |
| chr3:83884927-83891427 | | | 6374 bp | | |  |  |  |  |
| Genome Start | Genome End | Strand | Div | Repeat Start | Repeat End | Repeat Left | Name | Family | Class |
| 83884927 | 83885070 | - | 23.9 | 6028 | 6164 | -255 | L1MB3 | L1 | LINE |
| 83885071 | 83885418 | - | 21.0 | 1 | 396 | 0 | MSTD | MaLR | LTR |
| 83885419 | 83886666 | - | 24.1 | 4670 | 6027 | -392 | L1MB3 | L1 | LINE |
| 83886667 | 83887017 | + | 13.2 | 1 | 355 | 0 | THE1A | MaLR | LTR |
| 83887021 | 83888572 | + | 30.4 | 3 | 1735 | 0 | MLT-int | MaLR | LTR |
| 83888573 | 83888928 | + | 13.9 | 1 | 355 | 0 | THE1A | MaLR | LTR |
| 83888929 | 83889735 | - | 24.1 | 3848 | 4669 | -1707 | L1MB3 | L1 | LINE |
| 83889748 | 83890090 | - | 24.4 | 1 | 368 | -6 | MLT1A | MaLR | LTR |
| 83890091 | 83890370 | - | 25.5 | 3567 | 3846 | -2530 | L1MB3 | L1 | LINE |
| 83890502 | 83891248 | - | 26.0 | 772 | 1617 | -4767 | L1MB3 | L1 | LINE |
| 83891230 | 83891427 | - | 26.5 | 130 | 324 | -5918 | L1MB3 | L1 | LINE |

  

|  |  |  |  |  |  |  |  |  |  |
| --- | --- | --- | --- | --- | --- | --- | --- | --- | --- |
| chr3:84344566-84351674 | | | 7090 bp | | |  |  |  |  |
| Genome Start | Genome End | Strand | Div | Repeat Start | Repeat End | Repeat Left | Name | Family | Class |
| 84344566 | 84344960 | + | 23.7 | 404 | 805 | -5888 | L1PA15 | L1 | LINE |
| 84344978 | 84346168 | + | 18.0 | 1941 | 3151 | -3522 | L1PA15 | L1 | LINE |
| 84346169 | 84346521 | - | 11.1 | 1 | 355 | 0 | THE1A | MaLR | LTR |
| 84346522 | 84347708 | - | 32.2 | 475 | 1735 | 0 | MLT-int | MaLR | LTR |
| 84347709 | 84348015 | + | 16.0 | 1 | 307 | -2 | AluSc | Alu | SINE |
| 84348016 | 84348397 | - | 32.2 | 4 | 474 | -1261 | MLT-int | MaLR | LTR |
| 84348400 | 84348750 | - | 10.6 | 1 | 355 | 0 | THE1A | MaLR | LTR |
| 84348751 | 84351237 | + | 17.8 | 3152 | 5637 | -1036 | L1PA15 | L1 | LINE |
| 84351238 | 84351674 | + | 22.0 | 6244 | 6692 | 0 | L1PA15 | L1 | LINE |

  

|  |  |  |  |  |  |  |  |  |  |
| --- | --- | --- | --- | --- | --- | --- | --- | --- | --- |
| chr3:84703384-84711708 | | | 7751 bp | | |  |  |  |  |
| Genome Start | Genome End | Strand | Div | Repeat Start | Repeat End | Repeat Left | Name | Family | Class |
| 84703384 | 84703477 | + | 30.1 | 898 | 991 | -6046 | L1MA8 | L1 | LINE |
| 84703479 | 84703825 | - | 10.7 | 1 | 355 | 0 | THE1A | MaLR | LTR |
| 84703826 | 84705415 | - | 31.4 | 2 | 1735 | 0 | MLT-int | MaLR | LTR |
| 84705422 | 84705768 | - | 9.5 | 1 | 350 | -5 | THE1A | MaLR | LTR |
| 84705992 | 84708091 | + | 26.8 | 1589 | 3414 | -3686 | L1MA8 | L1 | LINE |
| 84708434 | 84709678 | + | 21.1 | 3398 | 4644 | -2456 | L1MA8 | L1 | LINE |
| 84709679 | 84709986 | + | 11.0 | 2 | 310 | -3 | AluSp | Alu | SINE |
| 84709987 | 84710856 | + | 21.1 | 4645 | 5500 | -1600 | L1MA8 | L1 | LINE |
| 84710859 | 84711115 | - | 13.1 | 5212 | 5489 | -679 | L1M3 | L1 | LINE |
| 84711116 | 84711708 | + | 23.9 | 6489 | 7121 | -130 | L1MA8 | L1 | LINE |

  

|  |  |  |  |  |  |  |  |  |  |
| --- | --- | --- | --- | --- | --- | --- | --- | --- | --- |
| chr3:84979957-84982681 | | | 2723 bp | | |  |  |  |  |
| Genome Start | Genome End | Strand | Div | Repeat Start | Repeat End | Repeat Left | Name | Family | Class |
| 84979957 | 84980028 | + | 20.7 | 24 | 117 | -248 | MLT1A0 | MaLR | LTR |
| 84980029 | 84980422 | - | 17.2 | 1 | 428 | 0 | MSTA | MaLR | LTR |
| 84980423 | 84982042 | - | 29.9 | 2 | 1735 | 0 | MLT-int | MaLR | LTR |
| 84982045 | 84982437 | - | 14.7 | 1 | 428 | 0 | MSTA | MaLR | LTR |
| 84982438 | 84982681 | + | 20.7 | 118 | 360 | -5 | MLT1A0 | MaLR | LTR |

  

|  |  |  |  |  |  |  |  |  |  |
| --- | --- | --- | --- | --- | --- | --- | --- | --- | --- |
| chr3:85829919-85833452 | | | 3402 bp | | |  |  |  |  |
| Genome Start | Genome End | Strand | Div | Repeat Start | Repeat End | Repeat Left | Name | Family | Class |
| 85829919 | 85830219 | - | 26.6 | 162 | 524 | -31 | MLT1H1 | MaLR | LTR |
| 85830225 | 85830625 | - | 14.0 | 1 | 428 | 0 | MSTA | MaLR | LTR |
| 85830636 | 85831786 | - | 18.7 | 469 | 1646 | -5 | MSTA-int | MaLR | LTR |
| 85831787 | 85832337 | + | 8.7 | 5604 | 6152 | -2 | L1PA7 | L1 | LINE |
| 85832338 | 85832798 | - | 18.7 | 2 | 468 | -1183 | MSTA-int | MaLR | LTR |
| 85832801 | 85833201 | - | 15.3 | 1 | 428 | 0 | MSTA | MaLR | LTR |
| 85833202 | 85833295 | - | 27.2 | 59 | 155 | -435 | MLT1H1 | MaLR | LTR |
| 85833411 | 85833452 | - | 11.9 | 3 | 45 | -545 | MLT1H1 | MaLR | LTR |

  

|  |  |  |  |  |  |  |  |  |  |
| --- | --- | --- | --- | --- | --- | --- | --- | --- | --- |
| chr3:86659199-86675894 | | | 16329 bp | | |  |  |  |  |
| Genome Start | Genome End | Strand | Div | Repeat Start | Repeat End | Repeat Left | Name | Family | Class |
| 86659199 | 86660401 | + | 18.1 | 2350 | 3444 | -2699 | L1MA9 | L1 | LINE |
| 86660392 | 86660891 | + | 14.1 | 3912 | 4421 | -1725 | L1MA3 | L1 | LINE |
| 86660893 | 86662072 | + | 14.9 | 5108 | 6304 | -1 | L1MA3 | L1 | LINE |
| 86662148 | 86662639 | + | 23.1 | 3 | 472 | 0 | MER70A | ERVL | LTR |
| 86662737 | 86664117 | + | 30.7 | 612 | 1881 | -4470 | L1M3b | L1 | LINE |
| 86664118 | 86664523 | - | 17.1 | 1 | 426 | 0 | MSTB | MaLR | LTR |
| 86664524 | 86666200 | - | 29.9 | 2 | 1735 | 0 | MLT-int | MaLR | LTR |
| 86666203 | 86666605 | - | 19.3 | 5 | 426 | 0 | MSTB | MaLR | LTR |
| 86666606 | 86666893 | + | 31.4 | 1882 | 2176 | -4175 | L1M3b | L1 | LINE |
| 86667009 | 86668122 | + | 19.5 | 4593 | 5727 | -585 | L1MA9 | L1 | LINE |
| 86668123 | 86668426 | + | 22.1 | 1 | 298 | -14 | AluJo | Alu | SINE |
| 86668427 | 86668487 | + | 22.4 | 5728 | 5786 | -526 | L1MA9 | L1 | LINE |
| 86668579 | 86668907 | + | 22.4 | 5787 | 6150 | -162 | L1MA9 | L1 | LINE |
| 86668909 | 86669340 | - | 13.5 | 1 | 448 | -2 | LTR7 | ERV1 | LTR |
| 86669341 | 86669896 | - | 10.0 | 7157 | 7713 | 0 | HERVH | ERV1 | LTR |
| 86669890 | 86670324 | - | 13.9 | 5590 | 6016 | -1697 | HERVH | ERV1 | LTR |
| 86670316 | 86675440 | - | 12.9 | 1 | 5317 | -1774 | HERVH | ERV1 | LTR |
| 86675451 | 86675770 | - | 15.1 | 1 | 337 | -113 | LTR7 | ERV1 | LTR |
| 86675772 | 86675894 | + | 20.3 | 6146 | 6274 | -38 | L1MA9 | L1 | LINE |

  

|  |  |  |  |  |  |  |  |  |  |
| --- | --- | --- | --- | --- | --- | --- | --- | --- | --- |
| chr3:86734276-86736818 | | | 2541 bp | | |  |  |  |  |
| Genome Start | Genome End | Strand | Div | Repeat Start | Repeat End | Repeat Left | Name | Family | Class |
| 86734276 | 86734342 | - | 18.7 | 226 | 285 | -638 | MER49 | ERV1 | LTR |
| 86734343 | 86734709 | + | 13.8 | 3 | 375 | 0 | THE1C | MaLR | LTR |
| 86734712 | 86736246 | + | 29.3 | 2 | 1735 | 0 | MLT-int | MaLR | LTR |
| 86736247 | 86736594 | + | 13.8 | 1 | 375 | 0 | THE1C | MaLR | LTR |
| 86736595 | 86736818 | - | 18.7 | 1 | 225 | -698 | MER49 | ERV1 | LTR |

  

|  |  |  |  |  |  |  |  |  |  |
| --- | --- | --- | --- | --- | --- | --- | --- | --- | --- |
| chr3:86806859-86831031 | | | 23844 bp | | |  |  |  |  |
| Genome Start | Genome End | Strand | Div | Repeat Start | Repeat End | Repeat Left | Name | Family | Class |
| 86806859 | 86807032 | - | 24.0 | 3576 | 3754 | -2375 | L1MEe | L1 | LINE |
| 86807056 | 86807312 | - | 14.8 | 6034 | 6299 | -6 | L1MA3 | L1 | LINE |
| 86807313 | 86807696 | + | 16.4 | 1 | 428 | 0 | MSTA | MaLR | LTR |
| 86807697 | 86807944 | + | 11.3 | 1 | 249 | -1402 | MSTA-int | MaLR | LTR |
| 86807972 | 86808244 | - | 16.4 | 5771 | 6039 | -266 | L1MA3 | L1 | LINE |
| 86808246 | 86809082 | - | 14.9 | 5327 | 6163 | -3 | L1PA16 | L1 | LINE |
| 86809083 | 86809381 | - | 11.7 | 9 | 308 | -2 | AluSg | Alu | SINE |
| 86809382 | 86813359 | - | 16.2 | 1631 | 5663 | -820 | L1P4a | L1 | LINE |
| 86813360 | 86813658 | - | 20.6 | 13 | 308 | -4 | AluJo | Alu | SINE |
| 86813659 | 86815257 | - | 20.0 | 3 | 1630 | -4873 | L1P4a | L1 | LINE |
| 86815279 | 86815721 | - | 29.0 | 3069 | 3538 | -2591 | L1MEe | L1 | LINE |
| 86815786 | 86816467 | - | 25.6 | 5646 | 6342 | -17 | L1MD2 | L1 | LINE |
| 86816468 | 86816810 | + | 25.7 | 4 | 374 | 0 | MLT1A | MaLR | LTR |
| 86816811 | 86816853 | - | 25.6 | 5611 | 5645 | -714 | L1MD2 | L1 | LINE |
| 86816859 | 86817041 | + | 24.9 | 4946 | 5125 | -1021 | L1MD | L1 | LINE |
| 86817042 | 86817352 | + | 9.0 | 1 | 310 | -1 | AluY | Alu | SINE |
| 86817353 | 86817804 | + | 23.0 | 5126 | 5594 | -765 | L1MD | L1 | LINE |
| 86817860 | 86818076 | - | 32.8 | 1535 | 1751 | -4398 | L1MEe | L1 | LINE |
| 86818077 | 86818196 | + | 13.2 | 1 | 120 | -190 | AluSg | Alu | SINE |
| 86818232 | 86818398 | + | 13.2 | 121 | 298 | -12 | AluSg | Alu | SINE |
| 86818399 | 86818446 | - | 32.8 | 1497 | 1534 | -4615 | L1MEe | L1 | LINE |
| 86818515 | 86820680 | + | 15.1 | 808 | 3017 | -4548 | L1MA1 | L1 | LINE |
| 86820668 | 86823391 | + | 17.6 | 3475 | 6134 | -1428 | L1MA1 | L1 | LINE |
| 86823392 | 86824089 | - | 10.2 | 1 | 722 | 0 | LTR30 | ERV1 | LTR |
| 86824090 | 86824554 | - | 10.0 | 7797 | 8257 | 0 | HERV30 | ERV1 | LTR |
| 86824551 | 86828159 | - | 11.3 | 1 | 3621 | -4636 | HERV30 | ERV1 | LTR |
| 86828161 | 86828863 | - | 9.6 | 1 | 721 | -1 | LTR30 | ERV1 | LTR |
| 86828875 | 86830349 | + | 10.3 | 6223 | 7711 | -10 | L1MA1 | L1 | LINE |
| 86830385 | 86830492 | - | 31.3 | 1085 | 1193 | -4926 | L1MEe | L1 | LINE |
| 86830493 | 86830801 | - | 6.5 | 1 | 308 | -3 | AluY | Alu | SINE |
| 86830802 | 86831031 | - | 31.3 | 829 | 1084 | -5035 | L1MEe | L1 | LINE |

  

|  |  |  |  |  |  |  |  |  |  |
| --- | --- | --- | --- | --- | --- | --- | --- | --- | --- |
| chr3:86993171-86996193 | | | 2948 bp | | |  |  |  |  |
| Genome Start | Genome End | Strand | Div | Repeat Start | Repeat End | Repeat Left | Name | Family | Class |
| 86993171 | 86993616 | - | 29.9 | 3034 | 3540 | -4025 | L1M3e | L1 | LINE |
| 86993671 | 86994019 | + | 12.9 | 1 | 352 | -3 | THE1A | MaLR | LTR |
| 86994025 | 86995598 | + | 31.5 | 2 | 1735 | 0 | MLT-int | MaLR | LTR |
| 86995599 | 86995951 | + | 14.8 | 1 | 355 | 0 | THE1A | MaLR | LTR |
| 86995968 | 86996193 | - | 33.2 | 979 | 1765 | -5724 | L1M3e | L1 | LINE |

  

|  |  |  |  |  |  |  |  |  |  |
| --- | --- | --- | --- | --- | --- | --- | --- | --- | --- |
| chr3:87185822-87191781 | | | 5471 bp | | |  |  |  |  |
| Genome Start | Genome End | Strand | Div | Repeat Start | Repeat End | Repeat Left | Name | Family | Class |
| 87185822 | 87185911 | + | 36.7 | 2639 | 2730 | -689 | L2 | L2 | LINE |
| 87185943 | 87186160 | - | 24.7 | 2302 | 2502 | -5 | HAL1 | L1 | LINE |
| 87186177 | 87187717 | - | 29.4 | 433 | 2079 | -428 | HAL1 | L1 | LINE |
| 87187747 | 87187948 | - | 30.5 | 120 | 322 | -2185 | HAL1 | L1 | LINE |
| 87188310 | 87188367 | + | 17.2 | 135 | 193 | 0 | MER53 | DNA | DNA |
| 87188377 | 87188769 | - | 13.0 | 1 | 364 | 0 | THE1B | MaLR | LTR |
| 87188770 | 87190329 | - | 30.5 | 2 | 1735 | 0 | MLT1H-int | MaLR | LTR |
| 87190334 | 87190522 | - | 12.6 | 176 | 364 | 0 | THE1B | MaLR | LTR |
| 87190552 | 87190718 | - | 12.6 | 3 | 175 | -189 | THE1B | MaLR | LTR |
| 87190720 | 87191011 | + | 34.6 | 2990 | 3270 | -149 | L2 | L2 | LINE |
| 87191012 | 87191290 | + | 11.5 | 9 | 289 | -24 | AluSq | Alu | SINE |
| 87191300 | 87191632 | + | 7.1 | 1 | 333 | 0 | AluY | Alu | SINE |
| 87191633 | 87191781 | + | 34.6 | 3274 | 3419 | 0 | L2 | L2 | LINE |

  

|  |  |  |  |  |  |  |  |  |  |
| --- | --- | --- | --- | --- | --- | --- | --- | --- | --- |
| chr3:87237173-87240256 | | | 3082 bp | | |  |  |  |  |
| Genome Start | Genome End | Strand | Div | Repeat Start | Repeat End | Repeat Left | Name | Family | Class |
| 87237173 | 87237826 | + | 25.0 | 5259 | 5918 | -441 | L1MD2 | L1 | LINE |
| 87237827 | 87238187 | + | 14.0 | 1 | 364 | 0 | THE1B | MaLR | LTR |
| 87238190 | 87239744 | + | 29.9 | 2 | 1735 | 0 | MLT-int | MaLR | LTR |
| 87239745 | 87240105 | + | 13.1 | 1 | 364 | 0 | THE1B | MaLR | LTR |
| 87240106 | 87240256 | + | 25.0 | 5919 | 6089 | -270 | L1MD2 | L1 | LINE |

  

|  |  |  |  |  |  |  |  |  |  |
| --- | --- | --- | --- | --- | --- | --- | --- | --- | --- |
| chr3:87655409-87661544 | | | 6134 bp | | |  |  |  |  |
| Genome Start | Genome End | Strand | Div | Repeat Start | Repeat End | Repeat Left | Name | Family | Class |
| 87655409 | 87656222 | - | 18.3 | 5467 | 6289 | -2 | L1MA8 | L1 | LINE |
| 87656223 | 87656608 | + | 13.7 | 1 | 375 | 0 | THE1C | MaLR | LTR |
| 87656609 | 87658145 | + | 12.9 | 1 | 1580 | 0 | THE1C-int | MaLR | LTR |
| 87658147 | 87658330 | + | 13.2 | 2 | 168 | -207 | THE1C | MaLR | LTR |
| 87658331 | 87658614 | - | 14.8 | 3 | 288 | -24 | AluSx | Alu | SINE |
| 87658615 | 87658841 | + | 13.2 | 169 | 375 | 0 | THE1C | MaLR | LTR |
| 87658843 | 87661544 | - | 17.7 | 2713 | 5470 | -821 | L1MA8 | L1 | LINE |

  

|  |  |  |  |  |  |  |  |  |  |
| --- | --- | --- | --- | --- | --- | --- | --- | --- | --- |
| chr3:88675513-88678084 | | | 2570 bp | | |  |  |  |  |
| Genome Start | Genome End | Strand | Div | Repeat Start | Repeat End | Repeat Left | Name | Family | Class |
| 88675513 | 88675657 | + | 21.7 | 3 | 151 | -161 | AluJo | Alu | SINE |
| 88675658 | 88676011 | + | 12.2 | 1 | 364 | 0 | THE1B | MaLR | LTR |
| 88676014 | 88677583 | + | 29.9 | 2 | 1735 | 0 | MLT-int | MaLR | LTR |
| 88677584 | 88677938 | + | 10.7 | 1 | 364 | 0 | THE1B | MaLR | LTR |
| 88677939 | 88678084 | + | 21.7 | 152 | 292 | -20 | AluJo | Alu | SINE |

  

|  |  |  |  |  |  |  |  |  |  |
| --- | --- | --- | --- | --- | --- | --- | --- | --- | --- |
| chr3:88906102-88914829 | | | 8724 bp | | |  |  |  |  |
| Genome Start | Genome End | Strand | Div | Repeat Start | Repeat End | Repeat Left | Name | Family | Class |
| 88906102 | 88910912 | + | 18.4 | 18 | 5639 | -1926 | L1M1 | L1 | LINE |
| 88910913 | 88911222 | - | 11.7 | 1 | 309 | 0 | AluSc | Alu | SINE |
| 88911223 | 88911404 | + | 16.2 | 5640 | 5811 | -1754 | L1M1 | L1 | LINE |
| 88911407 | 88911780 | - | 15.9 | 1 | 377 | -4 | THE1D | MaLR | LTR |
| 88911781 | 88913279 | - | 12.6 | 1 | 1580 | 0 | THE1D-int | MaLR | LTR |
| 88913280 | 88913650 | - | 14.9 | 1 | 381 | 0 | THE1D | MaLR | LTR |
| 88913653 | 88914829 | + | 12.8 | 5807 | 6980 | -744 | L1M1 | L1 | LINE |

  

|  |  |  |  |  |  |  |  |  |  |
| --- | --- | --- | --- | --- | --- | --- | --- | --- | --- |
| chr3:88921301-88931188 | | | 9870 bp | | |  |  |  |  |
| Genome Start | Genome End | Strand | Div | Repeat Start | Repeat End | Repeat Left | Name | Family | Class |
| 88921301 | 88921854 | - | 15.5 | 7174 | 7723 | 0 | L1MA2 | L1 | LINE |
| 88921873 | 88922035 | - | 22.1 | 137 | 299 | -13 | AluJb | Alu | SINE |
| 88922037 | 88925251 | - | 14.3 | 3923 | 7187 | -537 | L1MA2 | L1 | LINE |
| 88925246 | 88926288 | - | 16.6 | 2647 | 3700 | -3865 | L1MA2 | L1 | LINE |
| 88926289 | 88926631 | - | 13.3 | 1 | 377 | -4 | THE1D | MaLR | LTR |
| 88926632 | 88928251 | - | 30.4 | 3 | 1735 | 0 | MLT-int | MaLR | LTR |
| 88928257 | 88928621 | - | 14.4 | 1 | 379 | -2 | THE1D | MaLR | LTR |
| 88928622 | 88931188 | - | 17.8 | 16 | 2646 | -4919 | L1MA2 | L1 | LINE |

  

|  |  |  |  |  |  |  |  |  |  |
| --- | --- | --- | --- | --- | --- | --- | --- | --- | --- |
| chr3:89796847-89807934 | | | 10828 bp | | |  |  |  |  |
| Genome Start | Genome End | Strand | Div | Repeat Start | Repeat End | Repeat Left | Name | Family | Class |
| 89796847 | 89799475 | - | 28.2 | 2634 | 5255 | -964 | L1MEc | L1 | LINE |
| 89799509 | 89799938 | + | 22.1 | 452 | 888 | -5316 | L1PBb | L1 | LINE |
| 89799939 | 89800250 | + | 12.9 | 1 | 311 | -1 | AluSx | Alu | SINE |
| 89800251 | 89801436 | + | 14.8 | 889 | 2176 | -4028 | L1PBb | L1 | LINE |
| 89801437 | 89801800 | + | 16.1 | 1 | 362 | -2 | THE1B | MaLR | LTR |
| 89801805 | 89803382 | + | 31.7 | 2 | 1735 | 0 | MLT-int | MaLR | LTR |
| 89803383 | 89803717 | + | 17.5 | 1 | 364 | 0 | THE1B | MaLR | LTR |
| 89803718 | 89805928 | + | 14.5 | 2174 | 4394 | -1809 | L1PBb | L1 | LINE |
| 89805929 | 89805974 | - | 13.2 | 5943 | 5988 | -163 | L1PB2 | L1 | LINE |
| 89805975 | 89806132 | - | 28.4 | 5770 | 5925 | -264 | L1MB1 | L1 | LINE |
| 89806245 | 89806537 | - | 24.1 | 2147 | 2454 | -3946 | L1MEc | L1 | LINE |
| 89806600 | 89806729 | - | 24.6 | 1464 | 1599 | -4801 | L1MEc | L1 | LINE |
| 89806779 | 89807934 | - | 26.4 | 46 | 1281 | -5119 | L1MEc | L1 | LINE |

  

|  |  |  |  |  |  |  |  |  |  |
| --- | --- | --- | --- | --- | --- | --- | --- | --- | --- |
| chr3:89830673-89834520 | | | 3846 bp | | |  |  |  |  |
| Genome Start | Genome End | Strand | Div | Repeat Start | Repeat End | Repeat Left | Name | Family | Class |
| 89830673 | 89831817 | + | 25.2 | 3 | 1155 | -5708 | L1PBa | L1 | LINE |
| 89831818 | 89832174 | - | 14.2 | 1 | 375 | 0 | THE1C | MaLR | LTR |
| 89832175 | 89833655 | - | 12.8 | 1 | 1580 | 0 | THE1C-int | MaLR | LTR |
| 89833656 | 89834028 | - | 13.0 | 1 | 375 | 0 | THE1C | MaLR | LTR |
| 89834031 | 89834520 | + | 21.9 | 1153 | 1639 | -5224 | L1PBa | L1 | LINE |

  

|  |  |  |  |  |  |  |  |  |  |
| --- | --- | --- | --- | --- | --- | --- | --- | --- | --- |
| chr3:89834492-89841631 | | | 7046 bp | | |  |  |  |  |
| Genome Start | Genome End | Strand | Div | Repeat Start | Repeat End | Repeat Left | Name | Family | Class |
| 89834492 | 89834874 | + | 25.5 | 660 | 1051 | -5812 | L1PB2 | L1 | LINE |
| 89834876 | 89835247 | - | 13.3 | 2 | 364 | 0 | THE1B | MaLR | LTR |
| 89835249 | 89836392 | - | 12.1 | 418 | 1580 | 0 | THE1B-int | MaLR | LTR |
| 89836393 | 89836745 | - | 16.5 | 1 | 355 | 0 | THE1A | MaLR | LTR |
| 89836746 | 89837146 | - | 12.1 | 1 | 417 | -1163 | THE1B-int | MaLR | LTR |
| 89837147 | 89837504 | - | 13.6 | 1 | 364 | 0 | THE1B | MaLR | LTR |
| 89837505 | 89837770 | + | 16.9 | 1051 | 1317 | -5546 | L1PB2 | L1 | LINE |
| 89837771 | 89840087 | + | 14.2 | 2935 | 5355 | -1507 | L1PB2 | L1 | LINE |
| 89840115 | 89840823 | + | 14.2 | 5356 | 6073 | -789 | L1PB2 | L1 | LINE |
| 89840889 | 89841631 | + | 11.4 | 6074 | 6820 | -47 | L1PB2 | L1 | LINE |

  

|  |  |  |  |  |  |  |  |  |  |
| --- | --- | --- | --- | --- | --- | --- | --- | --- | --- |
| chr3:89938688-89941909 | | | 3098 bp | | |  |  |  |  |
| Genome Start | Genome End | Strand | Div | Repeat Start | Repeat End | Repeat Left | Name | Family | Class |
| 89938688 | 89939122 | + | 28.6 | 7 | 449 | -4405 | L1MEe | L1 | LINE |
| 89939217 | 89939365 | + | 26.2 | 616 | 767 | -4087 | L1MEe | L1 | LINE |
| 89939385 | 89939751 | - | 13.2 | 2 | 367 | -8 | THE1C | MaLR | LTR |
| 89939755 | 89941320 | - | 13.9 | 1 | 1575 | -5 | THE1C-int | MaLR | LTR |
| 89941321 | 89941704 | - | 16.0 | 1 | 375 | 0 | THE1C | MaLR | LTR |
| 89941713 | 89941909 | + | 31.8 | 784 | 985 | -3869 | L1MEe | L1 | LINE |

  

|  |  |  |  |  |  |  |  |  |  |
| --- | --- | --- | --- | --- | --- | --- | --- | --- | --- |
| chr3:89943082-89953408 | | | 10326 bp | | |  |  |  |  |
| Genome Start | Genome End | Strand | Div | Repeat Start | Repeat End | Repeat Left | Name | Family | Class |
| 89943082 | 89946188 | + | 18.5 | 416 | 3371 | -3302 | L1PA15 | L1 | LINE |
| 89946190 | 89946525 | - | 18.2 | 1 | 364 | 0 | THE1B | MaLR | LTR |
| 89946526 | 89948092 | - | 11.2 | 1 | 1580 | 0 | THE1B-int | MaLR | LTR |
| 89948093 | 89948439 | - | 17.1 | 1 | 364 | 0 | THE1B | MaLR | LTR |
| 89948440 | 89949594 | + | 17.6 | 3368 | 4537 | -2136 | L1PA15 | L1 | LINE |
| 89949595 | 89951176 | - | 21.1 | 1 | 1755 | 0 | MER52A | ERV1 | LTR |
| 89951177 | 89953408 | + | 16.7 | 4538 | 6687 | -5 | L1PA15 | L1 | LINE |

  

|  |  |  |  |  |  |  |  |  |  |
| --- | --- | --- | --- | --- | --- | --- | --- | --- | --- |
| chr3:90115028-90126245 | | | 11142 bp | | |  |  |  |  |
| Genome Start | Genome End | Strand | Div | Repeat Start | Repeat End | Repeat Left | Name | Family | Class |
| 90115028 | 90115657 | + | 18.4 | 1664 | 2268 | -5297 | L1MA3 | L1 | LINE |
| 90115657 | 90115882 | - | 13.1 | 2110 | 2344 | -3802 | L1M1 | L1 | LINE |
| 90115888 | 90116474 | + | 13.1 | 2262 | 2861 | -4704 | L1MA3 | L1 | LINE |
| 90116498 | 90116567 | + | 13.1 | 2862 | 2927 | -4638 | L1MA3 | L1 | LINE |
| 90116568 | 90116881 | + | 0.3 | 1 | 314 | -4 | AluYb8 | Alu | SINE |
| 90116882 | 90119910 | + | 15.2 | 2928 | 5940 | -1625 | L1MA3 | L1 | LINE |
| 90119911 | 90120184 | + | 17.6 | 1 | 294 | -134 | MSTA | MaLR | LTR |
| 90120185 | 90120533 | + | 16.4 | 1 | 355 | 0 | THE1A | MaLR | LTR |
| 90120534 | 90122108 | + | 10.1 | 1 | 1580 | 0 | THE1A-int | MaLR | LTR |
| 90122111 | 90122459 | + | 15.8 | 3 | 355 | 0 | THE1A | MaLR | LTR |
| 90122479 | 90122579 | + | 14.8 | 328 | 428 | 0 | MSTA | MaLR | LTR |
| 90122580 | 90123102 | + | 18.2 | 14 | 538 | -1113 | MST-int | MaLR | LTR |
| 90123102 | 90123699 | + | 17.0 | 1006 | 1574 | -6 | MSTA-int | MaLR | LTR |
| 90123705 | 90124098 | + | 17.6 | 1 | 428 | 0 | MSTA | MaLR | LTR |
| 90124099 | 90124238 | + | 14.3 | 5933 | 6072 | -1490 | L1MA3 | L1 | LINE |
| 90124263 | 90125766 | + | 13.6 | 6044 | 7522 | -202 | L1MA3 | L1 | LINE |
| 90125767 | 90126051 | - | 15.6 | 23 | 305 | -7 | AluSx | Alu | SINE |
| 90126052 | 90126245 | + | 14.1 | 7523 | 7714 | -10 | L1MA3 | L1 | LINE |

  

|  |  |  |  |  |  |  |  |  |  |
| --- | --- | --- | --- | --- | --- | --- | --- | --- | --- |
| chr3:97539123-97542015 | | | 2891 bp | | |  |  |  |  |
| Genome Start | Genome End | Strand | Div | Repeat Start | Repeat End | Repeat Left | Name | Family | Class |
| 97539123 | 97539305 | + | 32.8 | 4271 | 4453 | -501 | HERV35I | ERV1 | LTR |
| 97539306 | 97539662 | + | 15.8 | 1 | 364 | 0 | THE1B | MaLR | LTR |
| 97539665 | 97541255 | + | 30.3 | 2 | 1735 | 0 | MLT-int | MaLR | LTR |
| 97541256 | 97541622 | + | 17.3 | 1 | 364 | 0 | THE1B | MaLR | LTR |
| 97541623 | 97542015 | + | 29.3 | 4454 | 6918 | 0 | HERV35I | ERV1 | LTR |

  

|  |  |  |  |  |  |  |  |  |  |
| --- | --- | --- | --- | --- | --- | --- | --- | --- | --- |
| chr3:98124178-98133846 | | | 9615 bp | | |  |  |  |  |
| Genome Start | Genome End | Strand | Div | Repeat Start | Repeat End | Repeat Left | Name | Family | Class |
| 98124178 | 98127139 | - | 11.6 | 4624 | 7608 | -1 | L1PA11 | L1 | LINE |
| 98127140 | 98127439 | - | 13.7 | 1 | 300 | -13 | AluSq | Alu | SINE |
| 98127440 | 98127552 | - | 11.6 | 4518 | 4623 | -2958 | L1PA11 | L1 | LINE |
| 98127553 | 98127912 | - | 10.3 | 1 | 381 | 0 | THE1D | MaLR | LTR |
| 98127926 | 98129345 | - | 14.2 | 3 | 1575 | -5 | THE1D-int | MaLR | LTR |
| 98129348 | 98129722 | - | 19.0 | 1 | 381 | 0 | THE1D | MaLR | LTR |
| 98129723 | 98130609 | - | 11.1 | 3627 | 4522 | -3059 | L1PA11 | L1 | LINE |
| 98130646 | 98130721 | - | 21.4 | 704 | 773 | -807 | THE1-int | MaLR | LTR |
| 98130725 | 98132243 | - | 20.0 | 2071 | 3627 | -3976 | L1PA11 | L1 | LINE |
| 98132244 | 98132555 | + | 13.5 | 1 | 312 | 0 | AluSx | Alu | SINE |
| 98132556 | 98133846 | - | 23.7 | 1 | 2070 | -5533 | L1PA11 | L1 | LINE |

  

|  |  |  |  |  |  |  |  |  |  |
| --- | --- | --- | --- | --- | --- | --- | --- | --- | --- |
| chr3:98623919-98629225 | | | 5314 bp | | |  |  |  |  |
| Genome Start | Genome End | Strand | Div | Repeat Start | Repeat End | Repeat Left | Name | Family | Class |
| 98623919 | 98624246 | + | 19.8 | 2417 | 2773 | -3373 | L1PA13 | L1 | LINE |
| 98624235 | 98625508 | + | 15.1 | 3677 | 4963 | -1183 | L1PA13 | L1 | LINE |
| 98625513 | 98625866 | - | 13.1 | 2 | 362 | -2 | THE1B | MaLR | LTR |
| 98625868 | 98626056 | - | 11.9 | 1407 | 1580 | 0 | THE1B-int | MaLR | LTR |
| 98626057 | 98626359 | + | 10.3 | 1 | 302 | -11 | AluSq | Alu | SINE |
| 98626360 | 98627700 | - | 11.9 | 1 | 1406 | -174 | THE1B-int | MaLR | LTR |
| 98627701 | 98628052 | - | 15.5 | 1 | 364 | 0 | THE1B | MaLR | LTR |
| 98628053 | 98629225 | + | 12.8 | 4961 | 6147 | -16 | L1PA13 | L1 | LINE |

  

|  |  |  |  |  |  |  |  |  |  |
| --- | --- | --- | --- | --- | --- | --- | --- | --- | --- |
| chr3:100210101-100211819 | | | 1675 bp | | |  |  |  |  |
| Genome Start | Genome End | Strand | Div | Repeat Start | Repeat End | Repeat Left | Name | Family | Class |
| 100210101 | 100210239 | + | 29.4 | 147 | 287 | -268 | MLT1F | MaLR | LTR |
| 100210285 | 100210612 | - | 16.9 | 1 | 354 | -72 | MSTB | MaLR | LTR |
| 100210613 | 100210668 | - | 12.5 | 1595 | 1651 | 0 | MSTB-int | MaLR | LTR |
| 100210668 | 100211199 | - | 17.4 | 1 | 550 | -1101 | MSTB-int | MaLR | LTR |
| 100211200 | 100211592 | - | 16.6 | 1 | 426 | 0 | MSTB | MaLR | LTR |
| 100211593 | 100211819 | + | 29.8 | 289 | 528 | -14 | MLT1F | MaLR | LTR |

  

|  |  |  |  |  |  |  |  |  |  |
| --- | --- | --- | --- | --- | --- | --- | --- | --- | --- |
| chr3:100353793-100364066 | | | 10073 bp | | |  |  |  |  |
| Genome Start | Genome End | Strand | Div | Repeat Start | Repeat End | Repeat Left | Name | Family | Class |
| 100353793 | 100354343 | + | 27.2 | 685 | 1321 | -4940 | L1MEb | L1 | LINE |
| 100354379 | 100355269 | + | 27.0 | 1322 | 2266 | -3995 | L1MEb | L1 | LINE |
| 100355279 | 100355453 | + | 12.3 | 6113 | 6292 | -8 | L1MA4 | L1 | LINE |
| 100355552 | 100356367 | + | 29.2 | 2188 | 3062 | -3018 | L1MEb | L1 | LINE |
| 100356368 | 100356754 | - | 17.3 | 1 | 426 | -2 | MSTA | MaLR | LTR |
| 100356755 | 100357006 | + | 29.2 | 3063 | 3339 | -2741 | L1MEb | L1 | LINE |
| 100357017 | 100357323 | + | 16.6 | 91 | 381 | 0 | THE1D | MaLR | LTR |
| 100357326 | 100358907 | + | 30.1 | 2 | 1735 | 0 | MLT-int | MaLR | LTR |
| 100358908 | 100359276 | + | 17.0 | 1 | 428 | 0 | MSTA | MaLR | LTR |
| 100359305 | 100359528 | - | 18.6 | 207 | 428 | 0 | MSTA | MaLR | LTR |
| 100359529 | 100359900 | + | 15.2 | 1 | 364 | 0 | THE1B | MaLR | LTR |
| 100359901 | 100360107 | - | 18.6 | 1 | 206 | -222 | MSTA | MaLR | LTR |
| 100360108 | 100361681 | - | 30.7 | 2 | 1735 | 0 | MLT-int | MaLR | LTR |
| 100361684 | 100362102 | - | 15.7 | 4 | 428 | 0 | MSTA | MaLR | LTR |
| 100362112 | 100363370 | + | 24.8 | 3693 | 5008 | -1072 | L1MEb | L1 | LINE |
| 100363376 | 100363813 | + | 29.2 | 2 | 465 | -2 | MLT1D | MaLR | LTR |
| 100363817 | 100364066 | + | 24.4 | 5010 | 5279 | -801 | L1MEb | L1 | LINE |

  

|  |  |  |  |  |  |  |  |  |  |
| --- | --- | --- | --- | --- | --- | --- | --- | --- | --- |
| chr3:100644856-100649392 | | | 4503 bp | | |  |  |  |  |
| Genome Start | Genome End | Strand | Div | Repeat Start | Repeat End | Repeat Left | Name | Family | Class |
| 100644856 | 100645741 | - | 34.4 | 2433 | 3419 | 0 | L2 | L2 | LINE |
| 100645743 | 100646090 | + | 14.8 | 1 | 354 | -10 | THE1B | MaLR | LTR |
| 100646124 | 100647834 | + | 29.8 | 2 | 1735 | 0 | MLT1I-int | MaLR | LTR |
| 100647835 | 100648002 | + | 15.5 | 1 | 169 | -195 | THE1B | MaLR | LTR |
| 100648003 | 100648301 | + | 9.1 | 1 | 298 | -13 | AluY | Alu | SINE |
| 100648302 | 100648506 | + | 15.5 | 170 | 364 | 0 | THE1B | MaLR | LTR |
| 100648507 | 100648849 | - | 30.7 | 2086 | 2436 | -983 | L2 | L2 | LINE |
| 100648850 | 100649113 | + | 9.8 | 23 | 286 | -25 | AluY | Alu | SINE |
| 100649114 | 100649392 | - | 30.7 | 1828 | 2085 | -1334 | L2 | L2 | LINE |

  

|  |  |  |  |  |  |  |  |  |  |
| --- | --- | --- | --- | --- | --- | --- | --- | --- | --- |
| chr3:101195767-101198901 | | | 3132 bp | | |  |  |  |  |
| Genome Start | Genome End | Strand | Div | Repeat Start | Repeat End | Repeat Left | Name | Family | Class |
| 101195767 | 101196173 | + | 16.7 | 2484 | 2893 | -3250 | L1M2 | L1 | LINE |
| 101196174 | 101196513 | - | 9.4 | 1 | 355 | 0 | THE1A | MaLR | LTR |
| 101196514 | 101198077 | - | 29.1 | 3 | 1735 | 0 | MLT-int | MaLR | LTR |
| 101198081 | 101198433 | - | 8.2 | 1 | 355 | 0 | THE1A | MaLR | LTR |
| 101198434 | 101198901 | + | 16.7 | 2894 | 3394 | -2749 | L1M2 | L1 | LINE |

  

|  |  |  |  |  |  |  |  |  |  |
| --- | --- | --- | --- | --- | --- | --- | --- | --- | --- |
| chr3:101667943-101673873 | | | 5788 bp | | |  |  |  |  |
| Genome Start | Genome End | Strand | Div | Repeat Start | Repeat End | Repeat Left | Name | Family | Class |
| 101667943 | 101668001 | + | 16.9 | 434 | 494 | -6694 | LTR25-int | ERV1 | LTR |
| 101668063 | 101669067 | + | 21.3 | 203 | 1456 | -4763 | LTR27-int | ERV1 | LTR |
| 101669139 | 101670467 | + | 14.1 | 609 | 4916 | -301 | MER57-int | ERV1 | LTR |
| 101670468 | 101670821 | - | 14.4 | 1 | 364 | 0 | THE1B | MaLR | LTR |
| 101670824 | 101672396 | - | 10.8 | 1 | 1572 | -8 | THE1B-int | MaLR | LTR |
| 101672397 | 101672748 | - | 13.0 | 1 | 364 | 0 | THE1B | MaLR | LTR |
| 101672758 | 101673873 | + | 16.4 | 4123 | 5206 | -1982 | LTR25-int | ERV1 | LTR |

  

|  |  |  |  |  |  |  |  |  |  |
| --- | --- | --- | --- | --- | --- | --- | --- | --- | --- |
| chr3:102508224-102511698 | | | 3473 bp | | |  |  |  |  |
| Genome Start | Genome End | Strand | Div | Repeat Start | Repeat End | Repeat Left | Name | Family | Class |
| 102508224 | 102508886 | + | 24.5 | 4931 | 5614 | -550 | L1ME2 | L1 | LINE |
| 102508887 | 102509263 | - | 16.9 | 1 | 375 | 0 | THE1C | MaLR | LTR |
| 102509264 | 102510826 | - | 30.4 | 2 | 1735 | 0 | MLT-int | MaLR | LTR |
| 102510829 | 102511224 | - | 15.2 | 1 | 375 | 0 | THE1C | MaLR | LTR |
| 102511225 | 102511698 | + | 21.7 | 5615 | 6151 | -13 | L1ME2 | L1 | LINE |

  

|  |  |  |  |  |  |  |  |  |  |
| --- | --- | --- | --- | --- | --- | --- | --- | --- | --- |
| chr3:102893374-102902795 | | | 9422 bp | | |  |  |  |  |
| Genome Start | Genome End | Strand | Div | Repeat Start | Repeat End | Repeat Left | Name | Family | Class |
| 102893374 | 102893425 | + | 9.6 | 1 | 52 | -250 | AluSx | Alu | SINE |
| 102893427 | 102894395 | + | 4.0 | 1 | 968 | 0 | LTR5\_Hs | ERVK | LTR |
| 102894396 | 102899874 | + | 10.8 | 1 | 5534 | -2002 | HERVK | ERVK | LTR |
| 102899874 | 102901580 | + | 1.9 | 5826 | 7536 | 0 | HERVK | ERVK | LTR |
| 102901581 | 102902549 | + | 3.8 | 1 | 968 | 0 | LTR5\_Hs | ERVK | LTR |
| 102902550 | 102902795 | + | 10.2 | 49 | 299 | -13 | AluSx | Alu | SINE |

  

|  |  |  |  |  |  |  |  |  |  |
| --- | --- | --- | --- | --- | --- | --- | --- | --- | --- |
| chr3:103812256-103814508 | | | 2201 bp | | |  |  |  |  |
| Genome Start | Genome End | Strand | Div | Repeat Start | Repeat End | Repeat Left | Name | Family | Class |
| 103812256 | 103812317 | + | 8.2 | 1 | 61 | -575 | MER92B | ERV1 | LTR |
| 103812328 | 103812587 | - | 19.9 | 443 | 707 | 0 | MER39 | ERV1 | LTR |
| 103812588 | 103812964 | - | 26.5 | 1 | 396 | 0 | MSTD | MaLR | LTR |
| 103812986 | 103813227 | - | 18.7 | 1457 | 1710 | -25 | MSTD-int | MaLR | LTR |
| 103813272 | 103813662 | - | 21.8 | 4 | 396 | 0 | MSTD | MaLR | LTR |
| 103813666 | 103813857 | - | 29.8 | 260 | 444 | -263 | MER39 | ERV1 | LTR |
| 103813831 | 103813932 | - | 25.0 | 1 | 122 | -420 | MER34 | ERV1 | LTR |
| 103813934 | 103814508 | + | 21.1 | 62 | 636 | 0 | MER92B | ERV1 | LTR |

  

|  |  |  |  |  |  |  |  |  |  |
| --- | --- | --- | --- | --- | --- | --- | --- | --- | --- |
| chr3:104055414-104060048 | | | 4217 bp | | |  |  |  |  |
| Genome Start | Genome End | Strand | Div | Repeat Start | Repeat End | Repeat Left | Name | Family | Class |
| 104055414 | 104055620 | - | 29.6 | 4602 | 4817 | -41 | L1ME3A | L1 | LINE |
| 104055621 | 104055967 | - | 8.8 | 1 | 355 | 0 | THE1A | MaLR | LTR |
| 104055968 | 104057542 | - | 11.2 | 18 | 1580 | 0 | THE1A-int | MaLR | LTR |
| 104057543 | 104057887 | - | 8.1 | 5 | 353 | -2 | THE1A | MaLR | LTR |
| 104057888 | 104057900 | - | 28.9 | 4 | 16 | -1473 | MLT1F-int | MaLR | LTR |
| 104057902 | 104059217 | - | 27.0 | 3233 | 4596 | -262 | L1ME3A | L1 | LINE |
| 104059247 | 104059309 | - | 27.0 | 2615 | 2677 | -2157 | L1ME3A | L1 | LINE |
| 104059698 | 104059845 | - | 30.2 | 2 | 132 | -1 | FLAM\_A | Alu | SINE |
| 104059846 | 104060048 | - | 24.1 | 173 | 436 | -4579 | L1ME3A | L1 | LINE |

  

|  |  |  |  |  |  |  |  |  |  |
| --- | --- | --- | --- | --- | --- | --- | --- | --- | --- |
| chr3:104207953-104211054 | | | 3100 bp | | |  |  |  |  |
| Genome Start | Genome End | Strand | Div | Repeat Start | Repeat End | Repeat Left | Name | Family | Class |
| 104207953 | 104208385 | - | 19.1 | 282 | 722 | -12 | MER50 | ERV1 | LTR |
| 104208386 | 104208771 | - | 17.1 | 1 | 428 | 0 | MSTA | MaLR | LTR |
| 104208772 | 104210393 | - | 31.6 | 2 | 1735 | 0 | MLT-int | MaLR | LTR |
| 104210396 | 104210768 | - | 19.8 | 1 | 428 | 0 | MSTA | MaLR | LTR |
| 104210769 | 104211054 | - | 19.1 | 1 | 281 | -453 | MER50 | ERV1 | LTR |

  

|  |  |  |  |  |  |  |  |  |  |
| --- | --- | --- | --- | --- | --- | --- | --- | --- | --- |
| chr3:104831974-104835788 | | | 3340 bp | | |  |  |  |  |
| Genome Start | Genome End | Strand | Div | Repeat Start | Repeat End | Repeat Left | Name | Family | Class |
| 104831974 | 104832180 | - | 22.3 | 3984 | 4186 | -1954 | L1M3 | L1 | LINE |
| 104832635 | 104832988 | + | 11.9 | 1 | 375 | 0 | THE1C | MaLR | LTR |
| 104832989 | 104834626 | + | 15.1 | 1 | 1574 | -6 | THE1C-int | MaLR | LTR |
| 104834631 | 104834997 | + | 11.5 | 3 | 375 | 0 | THE1C | MaLR | LTR |
| 104835004 | 104835140 | + | 23.5 | 3985 | 4121 | -2019 | L1M3 | L1 | LINE |
| 104835152 | 104835788 | - | 23.9 | 3316 | 3965 | -2175 | L1M3 | L1 | LINE |

  

|  |  |  |  |  |  |  |  |  |  |
| --- | --- | --- | --- | --- | --- | --- | --- | --- | --- |
| chr3:105591347-105594562 | | | 3183 bp | | |  |  |  |  |
| Genome Start | Genome End | Strand | Div | Repeat Start | Repeat End | Repeat Left | Name | Family | Class |
| 105591347 | 105591736 | + | 27.4 | 9 | 358 | -147 | MLT1D | MaLR | LTR |
| 105591750 | 105592119 | - | 16.4 | 1 | 379 | -2 | THE1D | MaLR | LTR |
| 105592120 | 105593586 | - | 14.8 | 1 | 1580 | 0 | THE1D-int | MaLR | LTR |
| 105593587 | 105593958 | - | 13.6 | 1 | 381 | 0 | THE1D | MaLR | LTR |
| 105593968 | 105594025 | + | 22.4 | 373 | 433 | -72 | MLT1D | MaLR | LTR |
| 105594027 | 105594502 | - | 25.4 | 11 | 540 | -2 | MER34 | ERV1 | LTR |
| 105594513 | 105594562 | + | 16.0 | 429 | 480 | -25 | MLT1D | MaLR | LTR |

  

|  |  |  |  |  |  |  |  |  |  |
| --- | --- | --- | --- | --- | --- | --- | --- | --- | --- |
| chr3:106222789-106226239 | | | 3417 bp | | |  |  |  |  |
| Genome Start | Genome End | Strand | Div | Repeat Start | Repeat End | Repeat Left | Name | Family | Class |
| 106222789 | 106223358 | + | 33.1 | 4062 | 4667 | -1000 | ERVL-E | ERVL | LTR |
| 106223363 | 106223731 | + | 17.2 | 1 | 365 | 0 | MLT1A0 | MaLR | LTR |
| 106223759 | 106225460 | + | 21.6 | 16 | 1735 | 0 | MLT1A0-int | MaLR | LTR |
| 106225461 | 106225815 | + | 20.8 | 1 | 359 | -6 | MLT1A0 | MaLR | LTR |
| 106225819 | 106226239 | + | 36.2 | 4674 | 5161 | -553 | ERVL-E | ERVL | LTR |

  

|  |  |  |  |  |  |  |  |  |  |
| --- | --- | --- | --- | --- | --- | --- | --- | --- | --- |
| chr3:106274858-106282968 | | | 8040 bp | | |  |  |  |  |
| Genome Start | Genome End | Strand | Div | Repeat Start | Repeat End | Repeat Left | Name | Family | Class |
| 106274858 | 106276348 | + | 26.6 | 2166 | 3627 | -2592 | L1ME1 | L1 | LINE |
| 106276383 | 106277017 | + | 26.7 | 4195 | 4839 | -1380 | L1ME1 | L1 | LINE |
| 106277020 | 106277277 | + | 24.8 | 43 | 308 | -4 | AluJo | Alu | SINE |
| 106277279 | 106277701 | + | 28.7 | 4825 | 5242 | -977 | L1ME1 | L1 | LINE |
| 106277702 | 106277944 | - | 20.6 | 1 | 296 | -6 | AluJo | Alu | SINE |
| 106277945 | 106278464 | + | 24.5 | 5243 | 5779 | -458 | L1ME1 | L1 | LINE |
| 106278467 | 106278851 | - | 25.7 | 4 | 467 | 0 | MLT1A | MaLR | LTR |
| 106278862 | 106280149 | - | 29.4 | 99 | 1363 | -8 | MLT1A-int | MaLR | LTR |
| 106280150 | 106280513 | - | 15.1 | 1 | 381 | 0 | THE1D | MaLR | LTR |
| 106280514 | 106282149 | - | 30.1 | 2 | 1735 | 0 | MLT-int | MaLR | LTR |
| 106282153 | 106282489 | - | 15.4 | 29 | 380 | -1 | THE1D | MaLR | LTR |
| 106282491 | 106282558 | - | 11.8 | 33 | 101 | -1634 | MLT-int | MaLR | LTR |
| 106282577 | 106282968 | + | 26.8 | 5773 | 6174 | -65 | L1ME1 | L1 | LINE |

  

|  |  |  |  |  |  |  |  |  |  |
| --- | --- | --- | --- | --- | --- | --- | --- | --- | --- |
| chr3:106841682-106845067 | | | 2904 bp | | |  |  |  |  |
| Genome Start | Genome End | Strand | Div | Repeat Start | Repeat End | Repeat Left | Name | Family | Class |
| 106841682 | 106841870 | - | 31.7 | 1075 | 1257 | -4862 | L1MEd | L1 | LINE |
| 106842140 | 106842496 | - | 12.7 | 1 | 364 | 0 | THE1B | MaLR | LTR |
| 106842497 | 106844064 | - | 30.2 | 2 | 1735 | 0 | MLT-int | MaLR | LTR |
| 106844067 | 106844422 | - | 15.1 | 1 | 364 | 0 | THE1B | MaLR | LTR |
| 106844634 | 106845067 | - | 34.4 | 36 | 469 | -5650 | L1MEd | L1 | LINE |

  

|  |  |  |  |  |  |  |  |  |  |
| --- | --- | --- | --- | --- | --- | --- | --- | --- | --- |
| chr3:108033568-108040309 | | | 6369 bp | | |  |  |  |  |
| Genome Start | Genome End | Strand | Div | Repeat Start | Repeat End | Repeat Left | Name | Family | Class |
| 108033568 | 108033632 | - | 29.2 | 381 | 445 | -5 | LTR16A | ERVL | LTR |
| 108033640 | 108034143 | - | 14.4 | 1 | 497 | 0 | LOR1a | ERV1 | LTR |
| 108034144 | 108034245 | - | 11.0 | 8021 | 8120 | 0 | LOR1a-int | ERV1 | LTR |
| 108034303 | 108034626 | - | 15.4 | 3777 | 6163 | -595 | LOR1a-int | ERV1 | LTR |
| 108034617 | 108034742 | - | 19.2 | 3357 | 3482 | -3157 | LOR1a-int | ERV1 | LTR |
| 108034934 | 108036181 | - | 11.8 | 2499 | 3778 | -4342 | LOR1a-int | ERV1 | LTR |
| 108036237 | 108036320 | - | 11.8 | 2407 | 2498 | -5622 | LOR1a-int | ERV1 | LTR |
| 108036321 | 108036625 | + | 13.1 | 1 | 305 | -7 | AluSx | Alu | SINE |
| 108036626 | 108036914 | - | 11.8 | 2114 | 2406 | -5714 | LOR1a-int | ERV1 | LTR |
| 108036915 | 108037219 | - | 6.6 | 1 | 305 | -6 | AluY | Alu | SINE |
| 108037220 | 108038802 | - | 11.8 | 528 | 2113 | -6007 | LOR1a-int | ERV1 | LTR |
| 108038849 | 108039369 | - | 11.8 | 7 | 527 | -7593 | LOR1a-int | ERV1 | LTR |
| 108039371 | 108039862 | - | 13.9 | 1 | 497 | 0 | LOR1a | ERV1 | LTR |
| 108039865 | 108040179 | - | 12.1 | 1 | 314 | 0 | AluSg | Alu | SINE |
| 108040204 | 108040309 | - | 31.1 | 230 | 341 | -109 | LTR16A | ERVL | LTR |

  

|  |  |  |  |  |  |  |  |  |  |
| --- | --- | --- | --- | --- | --- | --- | --- | --- | --- |
| chr3:108375827-108383730 | | | 7840 bp | | |  |  |  |  |
| Genome Start | Genome End | Strand | Div | Repeat Start | Repeat End | Repeat Left | Name | Family | Class |
| 108375827 | 108376309 | + | 17.0 | 2188 | 2725 | -3421 | L1MB7 | L1 | LINE |
| 108376320 | 108376548 | - | 17.6 | 658 | 881 | -699 | THE1-int | MaLR | LTR |
| 108376564 | 108376700 | - | 14.2 | 1 | 138 | -243 | THE1D | MaLR | LTR |
| 108376702 | 108376983 | + | 19.5 | 129 | 428 | 0 | MSTA | MaLR | LTR |
| 108376984 | 108378458 | + | 14.2 | 1 | 1580 | 0 | MSTA-int | MaLR | LTR |
| 108378459 | 108378841 | + | 13.5 | 1 | 379 | -2 | THE1D | MaLR | LTR |
| 108378844 | 108378954 | - | 17.3 | 1 | 110 | -318 | MSTA | MaLR | LTR |
| 108378955 | 108380588 | - | 30.9 | 2 | 1735 | 0 | MLT1A0-int | MaLR | LTR |
| 108380591 | 108380979 | - | 15.3 | 1 | 428 | 0 | MSTA | MaLR | LTR |
| 108380990 | 108381114 | + | 17.6 | 3359 | 3487 | -2659 | L1MB7 | L1 | LINE |
| 108381139 | 108383730 | + | 18.1 | 3488 | 6160 | -24 | L1MB7 | L1 | LINE |

  

|  |  |  |  |  |  |  |  |  |  |
| --- | --- | --- | --- | --- | --- | --- | --- | --- | --- |
| chr3:108385501-108393926 | | | 8039 bp | | |  |  |  |  |
| Genome Start | Genome End | Strand | Div | Repeat Start | Repeat End | Repeat Left | Name | Family | Class |
| 108385501 | 108387372 | + | 24.3 | 19 | 1916 | -4586 | L1MD2 | L1 | LINE |
| 108387492 | 108387769 | + | 12.9 | 1 | 291 | -21 | AluSx | Alu | SINE |
| 108388005 | 108388724 | + | 21.8 | 1916 | 2683 | -3723 | L1MD2 | L1 | LINE |
| 108388719 | 108389793 | + | 19.1 | 3176 | 4290 | -2116 | L1MD2 | L1 | LINE |
| 108389809 | 108390016 | + | 18.6 | 4639 | 4841 | -1565 | L1MD2 | L1 | LINE |
| 108390017 | 108390382 | - | 21.4 | 1 | 365 | 0 | MLT1A0 | MaLR | LTR |
| 108390383 | 108391955 | - | 23.6 | 1 | 1651 | 0 | MLT1A0-int | MaLR | LTR |
| 108391959 | 108392303 | - | 16.7 | 11 | 362 | -3 | MLT1A0 | MaLR | LTR |
| 108392314 | 108392633 | + | 21.4 | 4837 | 5223 | -1183 | L1MD2 | L1 | LINE |
| 108392645 | 108393926 | + | 16.8 | 5313 | 6619 | 0 | L1MD2 | L1 | LINE |

  

|  |  |  |  |  |  |  |  |  |  |
| --- | --- | --- | --- | --- | --- | --- | --- | --- | --- |
| chr3:109444726-109451796 | | | 7041 bp | | |  |  |  |  |
| Genome Start | Genome End | Strand | Div | Repeat Start | Repeat End | Repeat Left | Name | Family | Class |
| 109444726 | 109445408 | + | 23.6 | 1188 | 1884 | -4755 | MER101-int | ERV1 | LTR |
| 109445409 | 109445771 | + | 9.4 | 1 | 375 | 0 | THE1C | MaLR | LTR |
| 109445774 | 109447346 | + | 29.6 | 2 | 1735 | 0 | MLT-int | MaLR | LTR |
| 109447347 | 109447720 | + | 9.1 | 1 | 375 | 0 | THE1C | MaLR | LTR |
| 109447721 | 109448737 | + | 20.9 | 1885 | 3730 | -2868 | MER101-int | ERV1 | LTR |
| 109448757 | 109450801 | + | 15.7 | 3302 | 5377 | -1262 | MER101-int | ERV1 | LTR |
| 109450811 | 109451796 | + | 14.9 | 5556 | 6582 | -57 | MER101-int | ERV1 | LTR |

  

|  |  |  |  |  |  |  |  |  |  |
| --- | --- | --- | --- | --- | --- | --- | --- | --- | --- |
| chr3:109474249-109482057 | | | 7090 bp | | |  |  |  |  |
| Genome Start | Genome End | Strand | Div | Repeat Start | Repeat End | Repeat Left | Name | Family | Class |
| 109474249 | 109474475 | - | 35.3 | 2420 | 2657 | -3057 | HERVL | ERVL | LTR |
| 109474476 | 109474679 | - | 10.9 | 516 | 722 | 0 | LTR30 | ERV1 | LTR |
| 109475025 | 109477027 | - | 3.5 | 4120 | 6152 | -3 | L1PA4 | L1 | LINE |
| 109477014 | 109477296 | + | 4.2 | 3823 | 4106 | -2040 | L1PA4 | L1 | LINE |
| 109477297 | 109477825 | - | 7.2 | 1 | 530 | -192 | LTR30 | ERV1 | LTR |
| 109477826 | 109477979 | - | 37.7 | 2269 | 2423 | -3291 | HERVL | ERVL | LTR |
| 109477980 | 109478283 | - | 12.9 | 1 | 304 | -8 | AluSx | Alu | SINE |
| 109478284 | 109478442 | - | 37.7 | 2104 | 2268 | -3446 | HERVL | ERVL | LTR |
| 109478759 | 109478841 | - | 31.3 | 1908 | 1990 | -3664 | HERVL | ERVL | LTR |
| 109478896 | 109479254 | - | 14.0 | 1 | 362 | -2 | THE1B | MaLR | LTR |
| 109479261 | 109480827 | - | 11.3 | 1 | 1574 | -6 | THE1B-int | MaLR | LTR |
| 109480831 | 109481182 | - | 11.4 | 1 | 357 | -7 | THE1B | MaLR | LTR |
| 109481192 | 109482057 | - | 36.8 | 6 | 865 | -4131 | HERVL | ERVL | LTR |

  

|  |  |  |  |  |  |  |  |  |  |
| --- | --- | --- | --- | --- | --- | --- | --- | --- | --- |
| chr3:110669986-110672544 | | | 2500 bp | | |  |  |  |  |
| Genome Start | Genome End | Strand | Div | Repeat Start | Repeat End | Repeat Left | Name | Family | Class |
| 110669986 | 110670148 | - | 17.9 | 138 | 299 | -13 | AluJb | Alu | SINE |
| 110670149 | 110670501 | + | 12.8 | 1 | 357 | -7 | THE1B | MaLR | LTR |
| 110670530 | 110672028 | + | 11.8 | 9 | 1497 | -83 | THE1B-int | MaLR | LTR |
| 110672046 | 110672408 | + | 14.1 | 1 | 364 | 0 | THE1B | MaLR | LTR |
| 110672423 | 110672544 | - | 9.0 | 11 | 133 | -179 | AluJb | Alu | SINE |

  

|  |  |  |  |  |  |  |  |  |  |
| --- | --- | --- | --- | --- | --- | --- | --- | --- | --- |
| chr3:110937434-110941501 | | | 4066 bp | | |  |  |  |  |
| Genome Start | Genome End | Strand | Div | Repeat Start | Repeat End | Repeat Left | Name | Family | Class |
| 110937434 | 110937918 | + | 15.0 | 4476 | 4971 | -1172 | L1MA9 | L1 | LINE |
| 110937919 | 110938288 | - | 14.7 | 3 | 381 | 0 | THE1D | MaLR | LTR |
| 110938291 | 110939840 | - | 11.4 | 1 | 1580 | 0 | THE1D-int | MaLR | LTR |
| 110939841 | 110940211 | - | 14.4 | 1 | 381 | 0 | THE1D | MaLR | LTR |
| 110940212 | 110941501 | + | 16.7 | 4964 | 6312 | 0 | L1MA9 | L1 | LINE |

  

|  |  |  |  |  |  |  |  |  |  |
| --- | --- | --- | --- | --- | --- | --- | --- | --- | --- |
| chr3:111528880-111531814 | | | 2931 bp | | |  |  |  |  |
| Genome Start | Genome End | Strand | Div | Repeat Start | Repeat End | Repeat Left | Name | Family | Class |
| 111528880 | 111529268 | - | 12.2 | 3476 | 3859 | -4296 | L1PREC2 | L1 | LINE |
| 111529269 | 111529652 | + | 16.1 | 1 | 381 | 0 | THE1D | MaLR | LTR |
| 111529655 | 111531235 | + | 31.3 | 2 | 1733 | -2 | MLT-int | MaLR | LTR |
| 111531238 | 111531608 | + | 13.7 | 1 | 381 | 0 | THE1D | MaLR | LTR |
| 111531609 | 111531814 | - | 12.2 | 3249 | 3475 | -4680 | L1PREC2 | L1 | LINE |

  

|  |  |  |  |  |  |  |  |  |  |
| --- | --- | --- | --- | --- | --- | --- | --- | --- | --- |
| chr3:111531812-111535603 | | | 3786 bp | | |  |  |  |  |
| Genome Start | Genome End | Strand | Div | Repeat Start | Repeat End | Repeat Left | Name | Family | Class |
| 111531812 | 111533131 | + | 13.0 | 4610 | 5952 | -209 | L1PA14 | L1 | LINE |
| 111533132 | 111533483 | - | 13.3 | 3 | 364 | 0 | THE1B | MaLR | LTR |
| 111533486 | 111535062 | - | 30.9 | 2 | 1735 | 0 | MLT1I-int | MaLR | LTR |
| 111535067 | 111535392 | - | 12.4 | 1 | 362 | -2 | THE1B | MaLR | LTR |
| 111535393 | 111535603 | + | 14.3 | 5953 | 6161 | 0 | L1PA14 | L1 | LINE |

  

|  |  |  |  |  |  |  |  |  |  |
| --- | --- | --- | --- | --- | --- | --- | --- | --- | --- |
| chr3:111793442-111797380 | | | 3907 bp | | |  |  |  |  |
| Genome Start | Genome End | Strand | Div | Repeat Start | Repeat End | Repeat Left | Name | Family | Class |
| 111793442 | 111794046 | - | 16.3 | 1029 | 1651 | 0 | MSTB-int | MaLR | LTR |
| 111794048 | 111794449 | + | 10.5 | 1 | 375 | 0 | THE1C | MaLR | LTR |
| 111794450 | 111795984 | + | 13.9 | 1 | 1580 | 0 | THE1C-int | MaLR | LTR |
| 111795985 | 111796360 | + | 11.8 | 1 | 375 | 0 | THE1C | MaLR | LTR |
| 111796362 | 111797089 | - | 17.9 | 281 | 1031 | -620 | MSTB-int | MaLR | LTR |
| 111797120 | 111797380 | - | 17.9 | 2 | 280 | -1371 | MSTB-int | MaLR | LTR |

  

|  |  |  |  |  |  |  |  |  |  |
| --- | --- | --- | --- | --- | --- | --- | --- | --- | --- |
| chr3:111901906-111910245 | | | 8222 bp | | |  |  |  |  |
| Genome Start | Genome End | Strand | Div | Repeat Start | Repeat End | Repeat Left | Name | Family | Class |
| 111901906 | 111903232 | - | 11.1 | 6426 | 7724 | 0 | L1MA3 | L1 | LINE |
| 111903233 | 111903548 | - | 12.4 | 1 | 316 | 0 | AluSg | Alu | SINE |
| 111903549 | 111904181 | - | 12.4 | 5789 | 6425 | -1140 | L1MA3 | L1 | LINE |
| 111904182 | 111904492 | - | 21.6 | 1 | 307 | -5 | AluJo | Alu | SINE |
| 111904493 | 111904764 | - | 12.4 | 5516 | 5788 | -1777 | L1MA3 | L1 | LINE |
| 111904765 | 111905121 | - | 11.9 | 1 | 364 | 0 | THE1B | MaLR | LTR |
| 111905122 | 111906647 | - | 11.8 | 1 | 1580 | 0 | THE1B-int | MaLR | LTR |
| 111906648 | 111907011 | - | 12.4 | 1 | 364 | 0 | THE1B | MaLR | LTR |
| 111907013 | 111907419 | - | 15.7 | 5103 | 5514 | -2051 | L1MA3 | L1 | LINE |
| 111907544 | 111910173 | - | 13.2 | 1595 | 5113 | -2452 | L1MA3 | L1 | LINE |
| 111910167 | 111910245 | - | 15.2 | 1095 | 1173 | -6392 | L1MA3 | L1 | LINE |

  

|  |  |  |  |  |  |  |  |  |  |
| --- | --- | --- | --- | --- | --- | --- | --- | --- | --- |
| chr3:112149633-112153481 | | | 3847 bp | | |  |  |  |  |
| Genome Start | Genome End | Strand | Div | Repeat Start | Repeat End | Repeat Left | Name | Family | Class |
| 112149633 | 112149683 | + | 16.9 | 1 | 52 | -1599 | MSTA-int | MaLR | LTR |
| 112149684 | 112150044 | - | 13.6 | 1 | 364 | 0 | THE1B | MaLR | LTR |
| 112150045 | 112151563 | - | 30.5 | 2 | 1735 | 0 | MLT-int | MaLR | LTR |
| 112151566 | 112151928 | - | 14.8 | 1 | 364 | 0 | THE1B | MaLR | LTR |
| 112151929 | 112153481 | + | 16.9 | 53 | 1644 | -7 | MSTA-int | MaLR | LTR |

  

|  |  |  |  |  |  |  |  |  |  |
| --- | --- | --- | --- | --- | --- | --- | --- | --- | --- |
| chr3:112725632-112732082 | | | 6437 bp | | |  |  |  |  |
| Genome Start | Genome End | Strand | Div | Repeat Start | Repeat End | Repeat Left | Name | Family | Class |
| 112725632 | 112725678 | - | 20.0 | 110 | 154 | -114 | MIRb | MIR | SINE |
| 112725682 | 112726696 | + | 8.1 | 1 | 1266 | 0 | MER11A | ERVK | LTR |
| 112726697 | 112727005 | + | 12.1 | 1 | 308 | -7645 | HERVK11 | ERVK | LTR |
| 112727009 | 112728303 | + | 8.4 | 2257 | 3534 | -4419 | HERVK11 | ERVK | LTR |
| 112728294 | 112730986 | + | 7.4 | 5256 | 7953 | 0 | HERVK11 | ERVK | LTR |
| 112730988 | 112732001 | + | 5.5 | 2 | 1266 | 0 | MER11A | ERVK | LTR |
| 112732019 | 112732082 | - | 18.2 | 21 | 92 | -176 | MIRb | MIR | SINE |

  

|  |  |  |  |  |  |  |  |  |  |
| --- | --- | --- | --- | --- | --- | --- | --- | --- | --- |
| chr3:112859461-112866807 | | | 7215 bp | | |  |  |  |  |
| Genome Start | Genome End | Strand | Div | Repeat Start | Repeat End | Repeat Left | Name | Family | Class |
| 112859461 | 112860346 | + | 28.5 | 6658 | 7535 | -507 | L1MC4 | L1 | LINE |
| 112860347 | 112861165 | - | 14.6 | 2857 | 3730 | -2416 | L1P4 | L1 | LINE |
| 112861166 | 112862492 | + | 13.7 | 3742 | 5079 | -1067 | L1P4 | L1 | LINE |
| 112862493 | 112862874 | - | 14.4 | 1 | 426 | -2 | MSTA | MaLR | LTR |
| 112862875 | 112864481 | - | 30.4 | 2 | 1735 | 0 | MLT-int | MaLR | LTR |
| 112864484 | 112864895 | - | 15.6 | 1 | 428 | 0 | MSTA | MaLR | LTR |
| 112864896 | 112865289 | + | 13.9 | 5080 | 5525 | -641 | L1PA16 | L1 | LINE |
| 112865290 | 112865586 | - | 8.8 | 1 | 295 | -16 | AluY | Alu | SINE |
| 112865587 | 112866215 | + | 14.3 | 5526 | 6166 | 0 | L1PA16 | L1 | LINE |
| 112866240 | 112866549 | + | 24.6 | 7546 | 7889 | -153 | L1MC4 | L1 | LINE |
| 112866618 | 112866709 | + | 22.4 | 7902 | 7984 | -58 | L1MC4 | L1 | LINE |
| 112866748 | 112866807 | + | 22.4 | 7985 | 8041 | -1 | L1MC4 | L1 | LINE |

  

|  |  |  |  |  |  |  |  |  |  |
| --- | --- | --- | --- | --- | --- | --- | --- | --- | --- |
| chr3:113016109-113018865 | | | 2753 bp | | |  |  |  |  |
| Genome Start | Genome End | Strand | Div | Repeat Start | Repeat End | Repeat Left | Name | Family | Class |
| 113016109 | 113016403 | - | 20.4 | 171 | 457 | 0 | MSTB2 | MaLR | LTR |
| 113016404 | 113016779 | + | 13.5 | 1 | 364 | 0 | THE1B | MaLR | LTR |
| 113016784 | 113018331 | + | 30.6 | 4 | 1735 | 0 | MLT-int | MaLR | LTR |
| 113018332 | 113018707 | + | 14.3 | 1 | 364 | 0 | THE1B | MaLR | LTR |
| 113018708 | 113018865 | - | 20.4 | 2 | 170 | -287 | MSTB2 | MaLR | LTR |

  

|  |  |  |  |  |  |  |  |  |  |
| --- | --- | --- | --- | --- | --- | --- | --- | --- | --- |
| chr3:113632274-113635038 | | | 2760 bp | | |  |  |  |  |
| Genome Start | Genome End | Strand | Div | Repeat Start | Repeat End | Repeat Left | Name | Family | Class |
| 113632274 | 113632372 | - | 18.5 | 88 | 193 | 0 | MER53 | DNA | DNA |
| 113632373 | 113632712 | + | 12.3 | 1 | 363 | -1 | THE1B | MaLR | LTR |
| 113632716 | 113634293 | + | 30.4 | 2 | 1735 | 0 | MLT-int | MaLR | LTR |
| 113634296 | 113634635 | + | 11.4 | 3 | 364 | 0 | THE1B | MaLR | LTR |
| 113634636 | 113634671 | - | 18.5 | 64 | 87 | -106 | MER53 | DNA | DNA |
| 113634672 | 113634974 | - | 16.9 | 1 | 302 | -10 | AluSx | Alu | SINE |
| 113634975 | 113635038 | - | 18.5 | 3 | 63 | -130 | MER53 | DNA | DNA |

  

|  |  |  |  |  |  |  |  |  |  |
| --- | --- | --- | --- | --- | --- | --- | --- | --- | --- |
| chr3:114511216-114518123 | | | 6882 bp | | |  |  |  |  |
| Genome Start | Genome End | Strand | Div | Repeat Start | Repeat End | Repeat Left | Name | Family | Class |
| 114511216 | 114512154 | + | 21.1 | 71 | 1106 | -5527 | L1MDa | L1 | LINE |
| 114512155 | 114512579 | + | 16.2 | 1 | 428 | 0 | MSTA | MaLR | LTR |
| 114512580 | 114512803 | + | 21.1 | 1107 | 1350 | -5283 | L1MDa | L1 | LINE |
| 114512805 | 114513159 | + | 11.4 | 1 | 355 | 0 | THE1A | MaLR | LTR |
| 114513160 | 114513586 | + | 8.2 | 1 | 428 | -1152 | THE1A-int | MaLR | LTR |
| 114513612 | 114513919 | - | 10.5 | 1 | 298 | -13 | AluY | Alu | SINE |
| 114513920 | 114515088 | + | 8.2 | 429 | 1580 | 0 | THE1A-int | MaLR | LTR |
| 114515089 | 114515443 | + | 11.1 | 1 | 355 | 0 | THE1A | MaLR | LTR |
| 114515444 | 114517165 | + | 16.9 | 1347 | 3074 | -3463 | L1MDa | L1 | LINE |
| 114517166 | 114517527 | + | 17.1 | 1 | 365 | -1 | Tigger5a | MER2\_type | DNA |
| 114517528 | 114518123 | + | 15.8 | 3075 | 3685 | -2852 | L1MDa | L1 | LINE |

  

|  |  |  |  |  |  |  |  |  |  |
| --- | --- | --- | --- | --- | --- | --- | --- | --- | --- |
| chr3:116706019-116708807 | | | 2787 bp | | |  |  |  |  |
| Genome Start | Genome End | Strand | Div | Repeat Start | Repeat End | Repeat Left | Name | Family | Class |
| 116706019 | 116706207 | + | 21.8 | 1 | 195 | -324 | LTR40a | ERVL | LTR |
| 116706208 | 116706562 | + | 12.8 | 1 | 364 | 0 | THE1B | MaLR | LTR |
| 116706565 | 116708160 | + | 29.6 | 2 | 1735 | 0 | MLT-int | MaLR | LTR |
| 116708161 | 116708515 | + | 13.3 | 1 | 364 | 0 | THE1B | MaLR | LTR |
| 116708516 | 116708807 | + | 21.8 | 196 | 519 | 0 | LTR40a | ERVL | LTR |

  

|  |  |  |  |  |  |  |  |  |  |
| --- | --- | --- | --- | --- | --- | --- | --- | --- | --- |
| chr3:117677994-117682152 | | | 3700 bp | | |  |  |  |  |
| Genome Start | Genome End | Strand | Div | Repeat Start | Repeat End | Repeat Left | Name | Family | Class |
| 117677994 | 117678381 | + | 35.1 | 17 | 410 | -6013 | L1MCc | L1 | LINE |
| 117678830 | 117679429 | + | 27.5 | 1252 | 1938 | -4901 | L1MCc | L1 | LINE |
| 117679432 | 117679795 | + | 12.7 | 1 | 364 | 0 | THE1B | MaLR | LTR |
| 117679796 | 117681355 | + | 12.6 | 1 | 1580 | 0 | THE1B-int | MaLR | LTR |
| 117681358 | 117681690 | + | 13.2 | 3 | 336 | -28 | THE1B | MaLR | LTR |
| 117681691 | 117681971 | - | 11.8 | 1 | 282 | -31 | AluSq | Alu | SINE |
| 117681972 | 117682011 | + | 13.2 | 337 | 364 | 0 | THE1B | MaLR | LTR |
| 117682019 | 117682152 | + | 32.8 | 2101 | 2238 | -4884 | L1MCc | L1 | LINE |

  

|  |  |  |  |  |  |  |  |  |  |
| --- | --- | --- | --- | --- | --- | --- | --- | --- | --- |
| chr3:118093227-118095327 | | | 1897 bp | | |  |  |  |  |
| Genome Start | Genome End | Strand | Div | Repeat Start | Repeat End | Repeat Left | Name | Family | Class |
| 118093227 | 118093364 | + | 30.8 | 3844 | 3973 | -516 | L3 | CR1 | LINE |
| 118093372 | 118093535 | + | 29.8 | 11 | 173 | -95 | MIR | MIR | SINE |
| 118093688 | 118094043 | - | 19.2 | 5 | 365 | 0 | MLT1A0 | MaLR | LTR |
| 118094048 | 118094810 | - | 23.2 | 1075 | 1735 | 0 | MLT1A0-int | MaLR | LTR |
| 118094827 | 118095086 | - | 20.5 | 1 | 255 | -1480 | MLT1A0-int | MaLR | LTR |
| 118095087 | 118095231 | - | 20.5 | 239 | 428 | 0 | MLT1A0 | MaLR | LTR |
| 118095257 | 118095327 | + | 35.2 | 4162 | 4232 | -257 | L3 | CR1 | LINE |

  

|  |  |  |  |  |  |  |  |  |  |
| --- | --- | --- | --- | --- | --- | --- | --- | --- | --- |
| chr3:119292329-119297542 | | | 5206 bp | | |  |  |  |  |
| Genome Start | Genome End | Strand | Div | Repeat Start | Repeat End | Repeat Left | Name | Family | Class |
| 119292329 | 119293570 | + | 22.2 | 4066 | 5369 | -777 | L1MD2 | L1 | LINE |
| 119293575 | 119293949 | + | 12.3 | 3 | 375 | 0 | THE1C | MaLR | LTR |
| 119293950 | 119295576 | + | 12.3 | 1 | 1580 | 0 | THE1C-int | MaLR | LTR |
| 119295579 | 119295948 | + | 10.3 | 3 | 371 | -4 | THE1C | MaLR | LTR |
| 119295951 | 119296478 | + | 21.6 | 5365 | 5885 | -474 | L1MD2 | L1 | LINE |
| 119296479 | 119296768 | + | 7.3 | 16 | 302 | -9 | AluY | Alu | SINE |
| 119296769 | 119296831 | + | 21.6 | 5886 | 5946 | -413 | L1MD2 | L1 | LINE |
| 119296832 | 119297238 | - | 5.4 | 5762 | 6168 | -4 | L1PA8 | L1 | LINE |
| 119297239 | 119297542 | + | 21.6 | 5947 | 6267 | -92 | L1MD2 | L1 | LINE |

  

|  |  |  |  |  |  |  |  |  |  |
| --- | --- | --- | --- | --- | --- | --- | --- | --- | --- |
| chr3:119458696-119462419 | | | 3721 bp | | |  |  |  |  |
| Genome Start | Genome End | Strand | Div | Repeat Start | Repeat End | Repeat Left | Name | Family | Class |
| 119458696 | 119459653 | + | 19.1 | 4626 | 5627 | -557 | L1MB7 | L1 | LINE |
| 119459654 | 119460009 | + | 14.1 | 1 | 364 | 0 | THE1B | MaLR | LTR |
| 119460013 | 119461573 | + | 29.7 | 3 | 1735 | 0 | MLT-int | MaLR | LTR |
| 119461574 | 119461934 | + | 13.5 | 1 | 364 | 0 | THE1B | MaLR | LTR |
| 119461935 | 119462419 | + | 20.4 | 5628 | 6167 | -13 | L1MB7 | L1 | LINE |

  

|  |  |  |  |  |  |  |  |  |  |
| --- | --- | --- | --- | --- | --- | --- | --- | --- | --- |
| chr3:119584304-119586796 | | | 2491 bp | | |  |  |  |  |
| Genome Start | Genome End | Strand | Div | Repeat Start | Repeat End | Repeat Left | Name | Family | Class |
| 119584304 | 119584451 | - | 26.1 | 91 | 246 | -16 | MIR | MIR | SINE |
| 119584452 | 119584800 | + | 8.0 | 5 | 355 | 0 | THE1A | MaLR | LTR |
| 119584803 | 119586382 | + | 30.2 | 2 | 1735 | 0 | MSTD-int | MaLR | LTR |
| 119586383 | 119586736 | + | 10.7 | 1 | 355 | 0 | THE1A | MaLR | LTR |
| 119586737 | 119586796 | - | 26.1 | 32 | 90 | -172 | MIR | MIR | SINE |

  

|  |  |  |  |  |  |  |  |  |  |
| --- | --- | --- | --- | --- | --- | --- | --- | --- | --- |
| chr3:120954931-120959322 | | | 4166 bp | | |  |  |  |  |
| Genome Start | Genome End | Strand | Div | Repeat Start | Repeat End | Repeat Left | Name | Family | Class |
| 120954931 | 120955200 | + | 36.7 | 521 | 813 | -5306 | L1MEd | L1 | LINE |
| 120955202 | 120955402 | - | 12.9 | 5950 | 6150 | -1 | L1PB1 | L1 | LINE |
| 120955444 | 120955537 | + | 14.9 | 293 | 386 | -4 | MLT1B | MaLR | LTR |
| 120955542 | 120956045 | + | 21.5 | 1 | 661 | -710 | MLT1F-int | MaLR | LTR |
| 120956065 | 120956226 | + | 26.2 | 679 | 1023 | -466 | MLT1F-int | MaLR | LTR |
| 120956256 | 120956825 | + | 28.2 | 1141 | 1735 | 0 | MLT1F-int | MaLR | LTR |
| 120956829 | 120957251 | + | 21.7 | 3 | 408 | 0 | MLT1B | MaLR | LTR |
| 120957271 | 120957473 | + | 27.9 | 833 | 1067 | -5158 | L1MEd | L1 | LINE |
| 120957584 | 120958898 | + | 25.6 | 1078 | 2584 | -3545 | L1MEd | L1 | LINE |
| 120958899 | 120959202 | + | 14.6 | 1 | 301 | -11 | AluJb | Alu | SINE |
| 120959203 | 120959322 | + | 24.6 | 2585 | 2699 | -3430 | L1MEd | L1 | LINE |

  

|  |  |  |  |  |  |  |  |  |  |
| --- | --- | --- | --- | --- | --- | --- | --- | --- | --- |
| chr3:125047843-125056960 | | | 8314 bp | | |  |  |  |  |
| Genome Start | Genome End | Strand | Div | Repeat Start | Repeat End | Repeat Left | Name | Family | Class |
| 125047843 | 125048060 | - | 28.0 | 5694 | 5941 | -282 | L1ME3B | L1 | LINE |
| 125048224 | 125048530 | - | 29.4 | 4242 | 4573 | -1556 | L1ME3B | L1 | LINE |
| 125048531 | 125048858 | + | 21.8 | 1 | 345 | 0 | MER2 | MER2\_type | DNA |
| 125048859 | 125049664 | - | 29.4 | 3377 | 4241 | -1888 | L1ME3B | L1 | LINE |
| 125049852 | 125049946 | - | 24.2 | 3040 | 3145 | -2984 | L1ME3B | L1 | LINE |
| 125050050 | 125050469 | + | 33.9 | 95 | 492 | -19 | MLT1J | MaLR | LTR |
| 125050532 | 125050810 | - | 17.9 | 1 | 281 | -31 | AluJb | Alu | SINE |
| 125050841 | 125050892 | - | 15.4 | 2975 | 3026 | -3120 | L1M5 | L1 | LINE |
| 125050922 | 125051035 | + | 20.0 | 366 | 549 | 0 | MLT1H | MaLR | LTR |
| 125051052 | 125051254 | + | 28.9 | 18 | 293 | -1179 | MLT1H-int | MaLR | LTR |
| 125051285 | 125054228 | - | 4.8 | 3191 | 6150 | -4 | L1PA5 | L1 | LINE |
| 125054245 | 125055348 | + | 22.9 | 304 | 1472 | 0 | MLT1H-int | MaLR | LTR |
| 125055350 | 125055805 | + | 28.2 | 3 | 511 | -38 | MLT1H | MaLR | LTR |
| 125055932 | 125056111 | - | 29.1 | 1634 | 1826 | -4323 | L1ME3B | L1 | LINE |
| 125056112 | 125056307 | + | 14.1 | 5963 | 6166 | 0 | L1PA17 | L1 | LINE |
| 125056308 | 125056613 | - | 29.1 | 1317 | 1633 | -4516 | L1ME3B | L1 | LINE |
| 125056655 | 125056960 | - | 31.6 | 919 | 1225 | -4894 | L1ME3B | L1 | LINE |

  

|  |  |  |  |  |  |  |  |  |  |
| --- | --- | --- | --- | --- | --- | --- | --- | --- | --- |
| chr3:127003094-127014859 | | | 11666 bp | | |  |  |  |  |
| Genome Start | Genome End | Strand | Div | Repeat Start | Repeat End | Repeat Left | Name | Family | Class |
| 127003094 | 127003270 | - | 12.5 | 6812 | 6995 | -7 | L1MA4 | L1 | LINE |
| 127003278 | 127004421 | + | 9.0 | 4294 | 5484 | -667 | L1PB1 | L1 | LINE |
| 127004422 | 127004708 | + | 9.4 | 1 | 288 | -22 | AluSg | Alu | SINE |
| 127004742 | 127005036 | + | 9.6 | 5485 | 5790 | -361 | L1PB1 | L1 | LINE |
| 127005082 | 127005430 | + | 9.6 | 5791 | 6149 | -2 | L1PB1 | L1 | LINE |
| 127005434 | 127006310 | - | 15.0 | 6029 | 6827 | -175 | L1MA4 | L1 | LINE |
| 127006294 | 127006539 | - | 11.4 | 4958 | 5204 | -942 | L1PREC2 | L1 | LINE |
| 127006540 | 127007084 | - | 16.5 | 5451 | 5905 | -940 | L1MA4 | L1 | LINE |
| 127007085 | 127007385 | - | 5.5 | 1 | 294 | -17 | AluY | Alu | SINE |
| 127007386 | 127007884 | - | 16.5 | 4970 | 5450 | -1395 | L1MA4 | L1 | LINE |
| 127007885 | 127008183 | - | 13.4 | 1 | 302 | -10 | AluSx | Alu | SINE |
| 127008184 | 127009206 | - | 16.5 | 3957 | 4969 | -1876 | L1MA4 | L1 | LINE |
| 127009234 | 127010391 | - | 16.6 | 2775 | 3956 | -2889 | L1MA4 | L1 | LINE |
| 127010392 | 127010745 | + | 14.7 | 1 | 364 | 0 | THE1B | MaLR | LTR |
| 127010748 | 127012313 | + | 31.2 | 2 | 1735 | 0 | MLT-int | MaLR | LTR |
| 127012314 | 127012671 | + | 12.4 | 1 | 364 | 0 | THE1B | MaLR | LTR |
| 127012672 | 127012935 | - | 19.9 | 2525 | 2774 | -4075 | L1MA4 | L1 | LINE |
| 127012936 | 127013093 | - | 19.6 | 1 | 158 | -8 | FRAM | Alu | SINE |
| 127013094 | 127014137 | - | 19.9 | 1443 | 2524 | -4325 | L1MA4 | L1 | LINE |
| 127014138 | 127014448 | - | 11.0 | 1 | 310 | 0 | AluSg | Alu | SINE |
| 127014449 | 127014859 | - | 19.9 | 987 | 1442 | -5407 | L1MA4 | L1 | LINE |

  

|  |  |  |  |  |  |  |  |  |  |
| --- | --- | --- | --- | --- | --- | --- | --- | --- | --- |
| chr3:127064699-127071540 | | | 6776 bp | | |  |  |  |  |
| Genome Start | Genome End | Strand | Div | Repeat Start | Repeat End | Repeat Left | Name | Family | Class |
| 127064699 | 127064763 | - | 22.6 | 389 | 484 | 0 | MLT1D | MaLR | LTR |
| 127064764 | 127065067 | + | 14.9 | 1 | 303 | -9 | AluSx | Alu | SINE |
| 127065068 | 127065240 | - | 22.2 | 206 | 430 | -75 | MLT1D | MaLR | LTR |
| 127065283 | 127065627 | + | 9.6 | 1 | 448 | -2 | LTR7 | ERV1 | LTR |
| 127065630 | 127068526 | + | 9.3 | 1 | 2984 | -4729 | HERVH | ERV1 | LTR |
| 127068523 | 127069378 | + | 10.1 | 3138 | 3993 | -3720 | HERVH | ERV1 | LTR |
| 127069381 | 127070039 | + | 14.3 | 4488 | 5145 | -2568 | HERVH | ERV1 | LTR |
| 127070037 | 127070251 | + | 12.6 | 5593 | 5819 | -1894 | HERVH | ERV1 | LTR |
| 127070252 | 127070627 | + | 11.1 | 7324 | 7713 | 0 | HERVH | ERV1 | LTR |
| 127070629 | 127071030 | + | 7.8 | 2 | 448 | -2 | LTR7 | ERV1 | LTR |
| 127071047 | 127071344 | - | 9.7 | 3 | 301 | -12 | AluSp | Alu | SINE |
| 127071355 | 127071540 | - | 23.3 | 1 | 185 | -320 | MLT1D | MaLR | LTR |

  

|  |  |  |  |  |  |  |  |  |  |
| --- | --- | --- | --- | --- | --- | --- | --- | --- | --- |
| chr3:127292738-127299095 | | | 6320 bp | | |  |  |  |  |
| Genome Start | Genome End | Strand | Div | Repeat Start | Repeat End | Repeat Left | Name | Family | Class |
| 127292738 | 127293566 | - | 11.0 | 41 | 828 | 0 | MER4D1 | ERV1 | LTR |
| 127293567 | 127293626 | - | 16.7 | 638 | 697 | 0 | MER50B | ERV1 | LTR |
| 127293628 | 127294139 | + | 8.1 | 1 | 511 | 0 | MER9 | ERVK | LTR |
| 127294140 | 127294732 | + | 7.3 | 1 | 592 | -5324 | HERVK9 | ERVK | LTR |
| 127294733 | 127296252 | + | 5.8 | 1268 | 2786 | -3130 | HERVK9 | ERVK | LTR |
| 127296253 | 127296472 | + | 5.5 | 3711 | 3940 | -1976 | HERVK9 | ERVK | LTR |
| 127296474 | 127297521 | + | 9.2 | 4583 | 5634 | -282 | HERVK9 | ERVK | LTR |
| 127297558 | 127297902 | + | 11.7 | 5635 | 7339 | 0 | HERVK9 | ERVK | LTR |
| 127297903 | 127298421 | + | 8.5 | 1 | 511 | 0 | MER9 | ERVK | LTR |
| 127298422 | 127299051 | - | 12.6 | 1 | 641 | -56 | MER50B | ERV1 | LTR |
| 127299052 | 127299095 | - | 11.4 | 1 | 44 | -856 | MER4D1 | ERV1 | LTR |

  

|  |  |  |  |  |  |  |  |  |  |
| --- | --- | --- | --- | --- | --- | --- | --- | --- | --- |
| chr3:128694472-128697391 | | | 2912 bp | | |  |  |  |  |
| Genome Start | Genome End | Strand | Div | Repeat Start | Repeat End | Repeat Left | Name | Family | Class |
| 128694472 | 128694582 | - | 25.2 | 382 | 505 | -6 | MLT1J | MaLR | LTR |
| 128694590 | 128694992 | - | 13.9 | 1 | 426 | 0 | MSTB | MaLR | LTR |
| 128694993 | 128696648 | - | 17.9 | 1 | 1651 | 0 | MSTB-int | MaLR | LTR |
| 128696649 | 128697057 | - | 13.9 | 1 | 426 | 0 | MSTB | MaLR | LTR |
| 128697059 | 128697391 | - | 25.4 | 1 | 363 | -148 | MLT1J | MaLR | LTR |

  

|  |  |  |  |  |  |  |  |  |  |
| --- | --- | --- | --- | --- | --- | --- | --- | --- | --- |
| chr3:131102014-131112472 | | | 10236 bp | | |  |  |  |  |
| Genome Start | Genome End | Strand | Div | Repeat Start | Repeat End | Repeat Left | Name | Family | Class |
| 131102014 | 131102073 | + | 24.6 | 1452 | 1524 | -2000 | Zaphod2 | Tip100 | DNA |
| 131102074 | 131102377 | + | 11.2 | 1 | 304 | -8 | AluSx | Alu | SINE |
| 131102413 | 131102475 | + | 24.6 | 1525 | 1587 | -1937 | Zaphod2 | Tip100 | DNA |
| 131102485 | 131102788 | + | 11.2 | 1 | 303 | -7 | AluSg | Alu | SINE |
| 131102795 | 131103281 | + | 11.0 | 1 | 511 | 0 | MER9 | ERVK | LTR |
| 131103282 | 131103945 | + | 8.7 | 1 | 662 | -5254 | HERVK9 | ERVK | LTR |
| 131103946 | 131107963 | + | 8.8 | 1625 | 5634 | -282 | HERVK9 | ERVK | LTR |
| 131107989 | 131108335 | + | 12.9 | 5635 | 8029 | 0 | HERVK9 | ERVK | LTR |
| 131108336 | 131108776 | + | 13.1 | 1 | 443 | -68 | MER9 | ERVK | LTR |
| 131108812 | 131108870 | + | 13.1 | 444 | 511 | 0 | MER9 | ERVK | LTR |
| 131108894 | 131109377 | + | 21.4 | 1622 | 2103 | -1421 | Zaphod2 | Tip100 | DNA |
| 131109433 | 131109521 | - | 22.1 | 134 | 220 | -92 | AluJo | Alu | SINE |
| 131109546 | 131109595 | - | 22.1 | 85 | 133 | -179 | AluJo | Alu | SINE |
| 131109596 | 131109637 | - | 7.5 | 211 | 250 | -52 | AluSg/x | Alu | SINE |
| 131109609 | 131109687 | + | 10.7 | 230 | 296 | -4 | Alu | Alu | SINE |
| 131109683 | 131109811 | + | 19.2 | 2177 | 2301 | -1223 | Zaphod2 | Tip100 | DNA |
| 131109812 | 131110365 | - | 12.9 | 1 | 614 | -4 | LTR35 | ERV1 | LTR |
| 131110366 | 131110627 | + | 19.2 | 2302 | 2553 | -971 | Zaphod2 | Tip100 | DNA |
| 131110666 | 131110751 | - | 17.6 | 2 | 86 | -226 | AluJ/FLAM | Alu | SINE |
| 131110753 | 131110976 | + | 16.7 | 2541 | 2764 | -760 | Zaphod2 | Tip100 | DNA |
| 131110977 | 131111285 | - | 10.0 | 1 | 310 | -2 | AluSx | Alu | SINE |
| 131111286 | 131111369 | + | 16.7 | 2765 | 2829 | -695 | Zaphod2 | Tip100 | DNA |
| 131111370 | 131111527 | + | 21.5 | 1 | 160 | 0 | MER5A1 | MER1\_type | DNA |
| 131111528 | 131111731 | + | 16.7 | 2830 | 3034 | -490 | Zaphod2 | Tip100 | DNA |
| 131111732 | 131112054 | - | 6.5 | 5 | 318 | 0 | AluY | Alu | SINE |
| 131112061 | 131112192 | - | 16.7 | 1 | 132 | -11 | FLAM\_C | Alu | SINE |
| 131112193 | 131112472 | + | 16.7 | 3041 | 3320 | -204 | Zaphod2 | Tip100 | DNA |

  

|  |  |  |  |  |  |  |  |  |  |
| --- | --- | --- | --- | --- | --- | --- | --- | --- | --- |
| chr3:131338681-131354804 | | | 16093 bp | | |  |  |  |  |
| Genome Start | Genome End | Strand | Div | Repeat Start | Repeat End | Repeat Left | Name | Family | Class |
| 131338681 | 131338861 | + | 24.0 | 1 | 185 | -320 | MLT1D | MaLR | LTR |
| 131338872 | 131339161 | + | 10.7 | 3 | 293 | -20 | AluSp | Alu | SINE |
| 131339174 | 131339571 | - | 10.3 | 2 | 448 | -2 | LTR7 | ERV1 | LTR |
| 131339573 | 131339948 | - | 8.9 | 7323 | 7713 | 0 | HERVH | ERV1 | LTR |
| 131339949 | 131340154 | - | 11.7 | 5600 | 5818 | -1895 | HERVH | ERV1 | LTR |
| 131340156 | 131340221 | - | 15.0 | 5090 | 5149 | -2564 | HERVH | ERV1 | LTR |
| 131340222 | 131341289 | - | 4.3 | 1 | 1071 | 0 | MER11C | ERVK | LTR |
| 131341290 | 131341892 | - | 15.0 | 4488 | 5089 | -2624 | HERVH | ERV1 | LTR |
| 131341895 | 131342444 | - | 9.1 | 3442 | 3993 | -3720 | HERVH | ERV1 | LTR |
| 131342445 | 131342941 | - | 8.9 | 1 | 501 | 0 | LTR2C | ERV1 | LTR |
| 131342942 | 131350763 | - | 6.6 | 3 | 7811 | -2 | HERVE | ERV1 | LTR |
| 131350764 | 131351067 | - | 11.4 | 3140 | 3441 | -4272 | HERVH | ERV1 | LTR |
| 131351054 | 131353871 | - | 10.2 | 1 | 2992 | -4721 | HERVH | ERV1 | LTR |
| 131353874 | 131354223 | - | 9.8 | 1 | 448 | -2 | LTR7 | ERV1 | LTR |
| 131354241 | 131354434 | + | 25.1 | 193 | 430 | -74 | MLT1D | MaLR | LTR |
| 131354435 | 131354739 | - | 15.5 | 1 | 303 | -9 | AluSx | Alu | SINE |
| 131354740 | 131354804 | + | 25.2 | 394 | 484 | 0 | MLT1D | MaLR | LTR |

  

|  |  |  |  |  |  |  |  |  |  |
| --- | --- | --- | --- | --- | --- | --- | --- | --- | --- |
| chr3:134210177-134216565 | | | 6343 bp | | |  |  |  |  |
| Genome Start | Genome End | Strand | Div | Repeat Start | Repeat End | Repeat Left | Name | Family | Class |
| 134210177 | 134210248 | + | 29.3 | 5405 | 5481 | -640 | L1MC4a | L1 | LINE |
| 134210249 | 134210526 | + | 8.6 | 1 | 278 | -21 | AluYd8 | Alu | SINE |
| 134210527 | 134210683 | + | 29.3 | 5482 | 5648 | -473 | L1MC4a | L1 | LINE |
| 134210722 | 134211123 | + | 13.5 | 7 | 428 | 0 | MSTA | MaLR | LTR |
| 134211124 | 134211660 | + | 16.7 | 1 | 543 | -1108 | MSTA-int | MaLR | LTR |
| 134211661 | 134211967 | + | 10.4 | 1 | 307 | -4 | AluY | Alu | SINE |
| 134211968 | 134212142 | + | 16.7 | 544 | 726 | -925 | MSTA-int | MaLR | LTR |
| 134212143 | 134212494 | - | 8.5 | 1 | 355 | 0 | THE1A | MaLR | LTR |
| 134212495 | 134214098 | - | 28.8 | 2 | 1735 | 0 | MLT-int | MaLR | LTR |
| 134214103 | 134214456 | - | 9.4 | 1 | 354 | -1 | THE1A | MaLR | LTR |
| 134214457 | 134215094 | + | 16.7 | 727 | 1338 | -313 | MSTA-int | MaLR | LTR |
| 134215095 | 134215372 | + | 9.0 | 4 | 297 | -13 | AluSg | Alu | SINE |
| 134215373 | 134215608 | + | 16.7 | 1339 | 1569 | -82 | MSTA-int | MaLR | LTR |
| 134215609 | 134215829 | + | 11.4 | 26 | 244 | -111 | THE1A | MaLR | LTR |
| 134215829 | 134215911 | + | 8.9 | 1502 | 1580 | 0 | MSTA-int | MaLR | LTR |
| 134215912 | 134216323 | + | 15.9 | 1 | 428 | 0 | MSTA | MaLR | LTR |
| 134216329 | 134216565 | + | 31.7 | 5694 | 5926 | -1882 | L1MC4a | L1 | LINE |

  

|  |  |  |  |  |  |  |  |  |  |
| --- | --- | --- | --- | --- | --- | --- | --- | --- | --- |
| chr3:137284558-137287111 | | | 2552 bp | | |  |  |  |  |
| Genome Start | Genome End | Strand | Div | Repeat Start | Repeat End | Repeat Left | Name | Family | Class |
| 137284558 | 137284739 | - | 16.2 | 110 | 289 | -23 | AluJb | Alu | SINE |
| 137284740 | 137285102 | + | 10.2 | 1 | 364 | 0 | THE1B | MaLR | LTR |
| 137285103 | 137286639 | + | 10.6 | 1 | 1580 | 0 | THE1B-int | MaLR | LTR |
| 137286642 | 137286997 | + | 9.0 | 3 | 364 | 0 | THE1B | MaLR | LTR |
| 137286998 | 137287111 | - | 14.0 | 1 | 114 | -198 | AluJb | Alu | SINE |

  

|  |  |  |  |  |  |  |  |  |  |
| --- | --- | --- | --- | --- | --- | --- | --- | --- | --- |
| chr3:137418231-137425825 | | | 7514 bp | | |  |  |  |  |
| Genome Start | Genome End | Strand | Div | Repeat Start | Repeat End | Repeat Left | Name | Family | Class |
| 137418231 | 137418256 | - | 24.2 | 5995 | 6021 | -100 | L1ME4a | L1 | LINE |
| 137418257 | 137418344 | + | 20.4 | 1 | 93 | -146 | MER8 | MER2\_type | DNA |
| 137418345 | 137418645 | + | 8.0 | 1 | 301 | -10 | AluY | Alu | SINE |
| 137418646 | 137418802 | + | 20.4 | 94 | 239 | 0 | MER8 | MER2\_type | DNA |
| 137418803 | 137418965 | - | 24.2 | 5824 | 5994 | -127 | L1ME4a | L1 | LINE |
| 137418971 | 137419434 | - | 4.7 | 1 | 464 | 0 | LTR7B | ERV1 | LTR |
| 137419435 | 137419865 | - | 14.1 | 7283 | 7713 | 0 | HERVH | ERV1 | LTR |
| 137419878 | 137421591 | - | 13.3 | 3357 | 5251 | -1840 | HERVH | ERV1 | LTR |
| 137421585 | 137422131 | - | 16.5 | 2300 | 3078 | -3576 | HERVH | ERV1 | LTR |
| 137422132 | 137422428 | + | 9.8 | 1 | 297 | -14 | AluY | Alu | SINE |
| 137422429 | 137424334 | - | 20.3 | 801 | 2488 | -4166 | HERVH | ERV1 | LTR |
| 137424389 | 137424816 | - | 18.5 | 1 | 459 | -7254 | HERVH | ERV1 | LTR |
| 137424817 | 137425246 | - | 4.9 | 1 | 464 | 0 | LTR7B | ERV1 | LTR |
| 137425247 | 137425419 | + | 15.0 | 6 | 189 | 0 | MER5A | MER1\_type | DNA |
| 137425433 | 137425730 | - | 7.4 | 1 | 297 | -14 | AluY | Alu | SINE |
| 137425735 | 137425825 | - | 26.7 | 5735 | 5825 | -296 | L1ME4a | L1 | LINE |

  

|  |  |  |  |  |  |  |  |  |  |
| --- | --- | --- | --- | --- | --- | --- | --- | --- | --- |
| chr3:137963535-137966504 | | | 2968 bp | | |  |  |  |  |
| Genome Start | Genome End | Strand | Div | Repeat Start | Repeat End | Repeat Left | Name | Family | Class |
| 137963535 | 137963662 | - | 27.4 | 1743 | 1900 | -607 | HAL1 | L1 | LINE |
| 137963663 | 137964019 | + | 12.0 | 2 | 364 | 0 | THE1B | MaLR | LTR |
| 137964022 | 137965621 | + | 29.4 | 2 | 1735 | 0 | MLT-int | MaLR | LTR |
| 137965622 | 137965789 | + | 9.0 | 1 | 157 | -207 | THE1B | MaLR | LTR |
| 137965790 | 137966101 | + | 6.8 | 1 | 308 | -3 | AluY | Alu | SINE |
| 137966102 | 137966308 | + | 9.0 | 158 | 364 | 0 | THE1B | MaLR | LTR |
| 137966309 | 137966504 | - | 27.4 | 1514 | 1742 | -765 | HAL1 | L1 | LINE |

  

|  |  |  |  |  |  |  |  |  |  |
| --- | --- | --- | --- | --- | --- | --- | --- | --- | --- |
| chr3:138072984-138076081 | | | 3096 bp | | |  |  |  |  |
| Genome Start | Genome End | Strand | Div | Repeat Start | Repeat End | Repeat Left | Name | Family | Class |
| 138072984 | 138073197 | - | 23.0 | 5918 | 6131 | -31 | L1ME3 | L1 | LINE |
| 138073198 | 138073559 | + | 13.0 | 1 | 364 | 0 | THE1B | MaLR | LTR |
| 138073562 | 138075140 | + | 30.9 | 2 | 1735 | 0 | MLT-int | MaLR | LTR |
| 138075141 | 138075484 | + | 14.9 | 1 | 364 | 0 | THE1B | MaLR | LTR |
| 138075485 | 138075699 | - | 23.0 | 5687 | 5917 | -245 | L1ME3 | L1 | LINE |
| 138075700 | 138076011 | - | 10.5 | 1 | 305 | -7 | AluSx | Alu | SINE |
| 138076012 | 138076081 | - | 23.0 | 5628 | 5686 | -476 | L1ME3 | L1 | LINE |

  

|  |  |  |  |  |  |  |  |  |  |
| --- | --- | --- | --- | --- | --- | --- | --- | --- | --- |
| chr3:138645349-138648110 | | | 2713 bp | | |  |  |  |  |
| Genome Start | Genome End | Strand | Div | Repeat Start | Repeat End | Repeat Left | Name | Family | Class |
| 138645349 | 138645550 | - | 25.9 | 197 | 402 | -7 | MLT1I | MaLR | LTR |
| 138645552 | 138645928 | - | 10.2 | 3 | 375 | 0 | THE1C | MaLR | LTR |
| 138645930 | 138647512 | - | 11.7 | 2 | 1580 | 0 | THE1C-int | MaLR | LTR |
| 138647515 | 138647912 | - | 11.0 | 2 | 374 | -1 | THE1C | MaLR | LTR |
| 138647958 | 138648110 | - | 24.5 | 2 | 154 | -255 | MLT1I | MaLR | LTR |

  

|  |  |  |  |  |  |  |  |  |  |
| --- | --- | --- | --- | --- | --- | --- | --- | --- | --- |
| chr3:138786208-138789763 | | | 3554 bp | | |  |  |  |  |
| Genome Start | Genome End | Strand | Div | Repeat Start | Repeat End | Repeat Left | Name | Family | Class |
| 138786208 | 138786314 | + | 24.8 | 6346 | 6453 | -1355 | L1MC4a | L1 | LINE |
| 138786315 | 138786668 | - | 13.4 | 1 | 355 | 0 | THE1A | MaLR | LTR |
| 138786669 | 138788231 | - | 29.6 | 2 | 1735 | 0 | MLT-int | MaLR | LTR |
| 138788234 | 138788582 | - | 10.1 | 1 | 355 | 0 | THE1A | MaLR | LTR |
| 138788583 | 138789763 | + | 24.8 | 6454 | 7714 | -94 | L1MC4a | L1 | LINE |

  

|  |  |  |  |  |  |  |  |  |  |
| --- | --- | --- | --- | --- | --- | --- | --- | --- | --- |
| chr3:141374074-141376773 | | | 2697 bp | | |  |  |  |  |
| Genome Start | Genome End | Strand | Div | Repeat Start | Repeat End | Repeat Left | Name | Family | Class |
| 141374074 | 141374119 | - | 23.0 | 422 | 467 | 0 | MLT1C | MaLR | LTR |
| 141374120 | 141374493 | - | 12.7 | 1 | 355 | 0 | THE1A | MaLR | LTR |
| 141374494 | 141376059 | - | 29.7 | 3 | 1735 | 0 | MLT1C-int | MaLR | LTR |
| 141376063 | 141376387 | - | 14.2 | 1 | 355 | 0 | THE1A | MaLR | LTR |
| 141376388 | 141376773 | - | 23.0 | 3 | 421 | -46 | MLT1C | MaLR | LTR |

  

|  |  |  |  |  |  |  |  |  |  |
| --- | --- | --- | --- | --- | --- | --- | --- | --- | --- |
| chr3:142072440-142081360 | | | 8713 bp | | |  |  |  |  |
| Genome Start | Genome End | Strand | Div | Repeat Start | Repeat End | Repeat Left | Name | Family | Class |
| 142072440 | 142073343 | + | 32.0 | 2341 | 3283 | -2384 | ERVL-E | ERVL | LTR |
| 142073547 | 142074414 | + | 7.6 | 1 | 826 | 0 | LTR12 | ERV1 | LTR |
| 142074431 | 142077453 | + | 7.1 | 43 | 3149 | -5287 | HERV9 | ERV1 | LTR |
| 142077461 | 142078337 | + | 6.5 | 4092 | 4963 | -3473 | HERV9 | ERV1 | LTR |
| 142078339 | 142079134 | + | 10.0 | 840 | 1864 | -3238 | HERV9 | ERV1 | LTR |
| 142079114 | 142079901 | + | 6.1 | 7642 | 8436 | 0 | HERV9 | ERV1 | LTR |
| 142079902 | 142080658 | + | 8.0 | 1 | 826 | 0 | LTR12 | ERV1 | LTR |
| 142080661 | 142081360 | + | 30.9 | 3515 | 4332 | -1322 | ERVL-E | ERVL | LTR |

  

|  |  |  |  |  |  |  |  |  |  |
| --- | --- | --- | --- | --- | --- | --- | --- | --- | --- |
| chr3:142084868-142091942 | | | 6862 bp | | |  |  |  |  |
| Genome Start | Genome End | Strand | Div | Repeat Start | Repeat End | Repeat Left | Name | Family | Class |
| 142084868 | 142084952 | + | 25.7 | 3342 | 3430 | -2716 | L1M5 | L1 | LINE |
| 142084953 | 142085666 | - | 24.2 | 1 | 727 | 0 | LTR8A | ERV1 | LTR |
| 142085667 | 142086088 | + | 25.7 | 3431 | 3891 | -2255 | L1M5 | L1 | LINE |
| 142086165 | 142086320 | + | 11.8 | 1 | 158 | -206 | THE1B | MaLR | LTR |
| 142086321 | 142086630 | - | 14.5 | 1 | 310 | -2 | AluSx | Alu | SINE |
| 142086631 | 142086836 | + | 11.8 | 159 | 364 | 0 | THE1B | MaLR | LTR |
| 142086837 | 142088294 | + | 8.8 | 1 | 1579 | -1 | THE1B-int | MaLR | LTR |
| 142088296 | 142088639 | + | 9.1 | 1 | 364 | 0 | THE1B | MaLR | LTR |
| 142088641 | 142088924 | + | 14.4 | 15 | 299 | -13 | AluJb | Alu | SINE |
| 142088952 | 142089721 | + | 24.4 | 120 | 816 | -119 | MER21C | ERV1 | LTR |
| 142089775 | 142090083 | + | 27.9 | 6049 | 6373 | -1435 | L1MC4a | L1 | LINE |
| 142090139 | 142090421 | + | 28.7 | 6753 | 7039 | -1003 | L1MC4 | L1 | LINE |
| 142090436 | 142090931 | + | 25.1 | 6920 | 7417 | -544 | L1MC5 | L1 | LINE |
| 142090932 | 142091057 | + | 20.3 | 1 | 123 | -10 | FLAM\_A | Alu | SINE |
| 142091058 | 142091570 | + | 25.1 | 7418 | 7929 | -32 | L1MC5 | L1 | LINE |
| 142091557 | 142091942 | + | 23.4 | 3867 | 4271 | -1875 | L1M5 | L1 | LINE |

  

|  |  |  |  |  |  |  |  |  |  |
| --- | --- | --- | --- | --- | --- | --- | --- | --- | --- |
| chr3:142406172-142413422 | | | 7251 bp | | |  |  |  |  |
| Genome Start | Genome End | Strand | Div | Repeat Start | Repeat End | Repeat Left | Name | Family | Class |
| 142406172 | 142406428 | + | 14.6 | 1 | 271 | -163 | MER57B | ERV1 | LTR |
| 142406433 | 142406838 | + | 6.7 | 1 | 448 | -2 | LTR7 | ERV1 | LTR |
| 142406841 | 142410731 | + | 10.9 | 1 | 3992 | -3721 | HERVH | ERV1 | LTR |
| 142410735 | 142410918 | + | 10.9 | 4488 | 4680 | -3033 | HERVH | ERV1 | LTR |
| 142410905 | 142412855 | + | 6.7 | 5769 | 7713 | 0 | HERVH | ERV1 | LTR |
| 142412856 | 142413261 | + | 6.4 | 1 | 448 | -2 | LTR7 | ERV1 | LTR |
| 142413267 | 142413422 | + | 12.3 | 274 | 431 | -3 | MER57B | ERV1 | LTR |

  

|  |  |  |  |  |  |  |  |  |  |
| --- | --- | --- | --- | --- | --- | --- | --- | --- | --- |
| chr3:145220490-145229125 | | | 8607 bp | | |  |  |  |  |
| Genome Start | Genome End | Strand | Div | Repeat Start | Repeat End | Repeat Left | Name | Family | Class |
| 145220490 | 145220626 | - | 18.8 | 137 | 302 | 0 | AluJo | Alu | SINE |
| 145220627 | 145220980 | - | 11.9 | 1 | 355 | 0 | THE1A | MaLR | LTR |
| 145220981 | 145222537 | - | 11.8 | 14 | 1580 | 0 | THE1A-int | MaLR | LTR |
| 145222538 | 145222885 | - | 8.1 | 7 | 355 | 0 | THE1A | MaLR | LTR |
| 145222909 | 145225651 | - | 7.4 | 11 | 2769 | -3377 | L1PA5 | L1 | LINE |
| 145225653 | 145228993 | + | 5.5 | 2786 | 6147 | -7 | L1PA5 | L1 | LINE |
| 145228999 | 145229125 | - | 18.9 | 5 | 133 | -169 | AluJo | Alu | SINE |

  

|  |  |  |  |  |  |  |  |  |  |
| --- | --- | --- | --- | --- | --- | --- | --- | --- | --- |
| chr3:145864779-145868633 | | | 3501 bp | | |  |  |  |  |
| Genome Start | Genome End | Strand | Div | Repeat Start | Repeat End | Repeat Left | Name | Family | Class |
| 145864779 | 145865402 | - | 31.5 | 441 | 1121 | -4925 | L1MCc | L1 | LINE |
| 145865587 | 145866051 | + | 22.6 | 4 | 467 | 0 | MLT1C | MaLR | LTR |
| 145866183 | 145866451 | - | 13.0 | 1 | 364 | 0 | THE1B | MaLR | LTR |
| 145866452 | 145867958 | - | 11.8 | 1 | 1580 | 0 | THE1B-int | MaLR | LTR |
| 145867959 | 145868322 | - | 13.5 | 1 | 364 | 0 | THE1B | MaLR | LTR |
| 145868362 | 145868633 | - | 29.8 | 28 | 294 | -5751 | L1MCc | L1 | LINE |

  

|  |  |  |  |  |  |  |  |  |  |
| --- | --- | --- | --- | --- | --- | --- | --- | --- | --- |
| chr3:145913211-145921252 | | | 8000 bp | | |  |  |  |  |
| Genome Start | Genome End | Strand | Div | Repeat Start | Repeat End | Repeat Left | Name | Family | Class |
| 145913211 | 145913361 | + | 21.2 | 2516 | 2667 | -3476 | L1MA8 | L1 | LINE |
| 145913360 | 145913497 | - | 15.9 | 5781 | 5913 | -387 | L1MA5 | L1 | LINE |
| 145913549 | 145915269 | - | 18.1 | 4116 | 5780 | -520 | L1MA5 | L1 | LINE |
| 145915267 | 145915501 | + | 20.2 | 2668 | 2909 | -3231 | L1MA8 | L1 | LINE |
| 145915502 | 145915807 | + | 7.2 | 1 | 305 | -6 | AluY | Alu | SINE |
| 145915808 | 145915854 | + | 20.2 | 2910 | 2952 | -3188 | L1MA8 | L1 | LINE |
| 145915855 | 145916213 | - | 14.4 | 1 | 355 | 0 | THE1A | MaLR | LTR |
| 145916219 | 145917786 | - | 11.5 | 1 | 1573 | -7 | THE1A-int | MaLR | LTR |
| 145917789 | 145918139 | - | 11.8 | 1 | 353 | -2 | THE1A | MaLR | LTR |
| 145918140 | 145918544 | + | 16.3 | 2948 | 3383 | -2760 | L1MA8 | L1 | LINE |
| 145918534 | 145918677 | + | 20.7 | 4048 | 4191 | -1952 | L1MA8 | L1 | LINE |
| 145918678 | 145918972 | + | 13.6 | 1 | 297 | -15 | AluSx | Alu | SINE |
| 145918973 | 145920779 | + | 19.5 | 4192 | 6130 | -161 | L1MA8 | L1 | LINE |
| 145920780 | 145921087 | + | 9.7 | 5 | 312 | 0 | AluSx | Alu | SINE |
| 145921088 | 145921252 | + | 17.9 | 6131 | 6287 | -4 | L1MA8 | L1 | LINE |

  

|  |  |  |  |  |  |  |  |  |  |
| --- | --- | --- | --- | --- | --- | --- | --- | --- | --- |
| chr3:146992570-146998137 | | | 5254 bp | | |  |  |  |  |
| Genome Start | Genome End | Strand | Div | Repeat Start | Repeat End | Repeat Left | Name | Family | Class |
| 146992570 | 146993109 | + | 18.3 | 1 | 555 | -156 | MER67C | ERV1 | LTR |
| 146993110 | 146993923 | - | 14.2 | 4566 | 5352 | -794 | L1P4 | L1 | LINE |
| 146993924 | 146994139 | - | 15.0 | 156 | 364 | 0 | THE1B | MaLR | LTR |
| 146994140 | 146994427 | + | 13.4 | 1 | 297 | -13 | AluSg | Alu | SINE |
| 146994428 | 146994598 | - | 15.0 | 1 | 155 | -209 | THE1B | MaLR | LTR |
| 146994599 | 146996176 | - | 32.3 | 2 | 1735 | 0 | MLT1A-int | MaLR | LTR |
| 146996179 | 146996547 | - | 13.3 | 1 | 364 | 0 | THE1B | MaLR | LTR |
| 146996548 | 146996792 | - | 14.2 | 4317 | 4565 | -1581 | L1P4 | L1 | LINE |
| 146996793 | 146997529 | + | 14.9 | 5373 | 6157 | -9 | L1PA16 | L1 | LINE |
| 146997680 | 146997800 | - | 18.2 | 4675 | 4798 | -1348 | L1P4 | L1 | LINE |
| 146997963 | 146998137 | + | 17.4 | 537 | 711 | 0 | MER67C | ERV1 | LTR |

  

|  |  |  |  |  |  |  |  |  |  |
| --- | --- | --- | --- | --- | --- | --- | --- | --- | --- |
| chr3:147238519-147247712 | | | 8976 bp | | |  |  |  |  |
| Genome Start | Genome End | Strand | Div | Repeat Start | Repeat End | Repeat Left | Name | Family | Class |
| 147238519 | 147239078 | + | 32.3 | 2283 | 2901 | -518 | L2 | L2 | LINE |
| 147239153 | 147239244 | + | 28.4 | 3075 | 3168 | -210 | L2 | L2 | LINE |
| 147239309 | 147240147 | - | 30.1 | 2516 | 3418 | -1 | L2 | L2 | LINE |
| 147240149 | 147242131 | - | 14.3 | 467 | 2490 | -1 | Tigger7 | MER2\_type | DNA |
| 147242132 | 147242492 | - | 13.8 | 1 | 364 | 0 | THE1B | MaLR | LTR |
| 147242493 | 147244080 | - | 30.4 | 2 | 1735 | 0 | MLT-int | MaLR | LTR |
| 147244083 | 147244443 | - | 13.3 | 1 | 364 | 0 | THE1B | MaLR | LTR |
| 147244445 | 147244919 | - | 22.1 | 1 | 470 | -2021 | Tigger7 | MER2\_type | DNA |
| 147244925 | 147245063 | - | 29.0 | 2374 | 2513 | -906 | L2 | L2 | LINE |
| 147245083 | 147245466 | - | 17.2 | 1 | 428 | 0 | MSTA | MaLR | LTR |
| 147245467 | 147246859 | - | 15.4 | 3 | 1580 | 0 | MSTA-int | MaLR | LTR |
| 147246862 | 147247070 | - | 13.5 | 191 | 428 | 0 | MSTA | MaLR | LTR |
| 147247071 | 147247392 | - | 9.6 | 2 | 324 | 0 | AluSp | Alu | SINE |
| 147247393 | 147247568 | - | 13.5 | 1 | 190 | -238 | MSTA | MaLR | LTR |
| 147247619 | 147247712 | + | 21.6 | 3327 | 3416 | -3 | L2 | L2 | LINE |

  

|  |  |  |  |  |  |  |  |  |  |
| --- | --- | --- | --- | --- | --- | --- | --- | --- | --- |
| chr3:147618054-147620715 | | | 2575 bp | | |  |  |  |  |
| Genome Start | Genome End | Strand | Div | Repeat Start | Repeat End | Repeat Left | Name | Family | Class |
| 147618054 | 147618129 | - | 15.8 | 5549 | 5625 | -559 | L1M4 | L1 | LINE |
| 147618130 | 147618364 | + | 15.9 | 6066 | 6300 | -3 | L1MA4A | L1 | LINE |
| 147618366 | 147618481 | - | 23.5 | 310 | 449 | -56 | MLT1D | MaLR | LTR |
| 147618501 | 147618689 | - | 28.6 | 5 | 184 | -321 | MLT1D | MaLR | LTR |
| 147618726 | 147619974 | - | 23.9 | 1 | 1337 | -34 | MLT1D-int | MaLR | LTR |
| 147619975 | 147620456 | - | 24.8 | 1 | 505 | 0 | MLT1D | MaLR | LTR |
| 147620488 | 147620715 | - | 22.0 | 23 | 252 | -6290 | L1M4 | L1 | LINE |

  

|  |  |  |  |  |  |  |  |  |  |
| --- | --- | --- | --- | --- | --- | --- | --- | --- | --- |
| chr3:149052636-149066578 | | | 13823 bp | | |  |  |  |  |
| Genome Start | Genome End | Strand | Div | Repeat Start | Repeat End | Repeat Left | Name | Family | Class |
| 149052636 | 149054188 | + | 23.9 | 39 | 1629 | -4771 | L1ME1 | L1 | LINE |
| 149054189 | 149054774 | + | 16.3 | 5577 | 6179 | -1 | L1MB4 | L1 | LINE |
| 149054775 | 149055001 | + | 23.9 | 1630 | 1830 | -4570 | L1ME1 | L1 | LINE |
| 149055002 | 149055123 | + | 18.0 | 1 | 121 | -181 | AluJo | Alu | SINE |
| 149055150 | 149055282 | + | 18.0 | 122 | 292 | -10 | AluJo | Alu | SINE |
| 149055283 | 149055394 | + | 23.9 | 1831 | 1928 | -4472 | L1ME1 | L1 | LINE |
| 149055395 | 149055685 | + | 15.1 | 1 | 293 | -19 | AluJb | Alu | SINE |
| 149055686 | 149057725 | + | 27.2 | 1929 | 4046 | -2167 | L1ME1 | L1 | LINE |
| 149057732 | 149058136 | - | 19.6 | 4 | 421 | -5 | MSTB | MaLR | LTR |
| 149058140 | 149059775 | - | 28.7 | 2 | 1735 | 0 | MLT-int | MaLR | LTR |
| 149059778 | 149060209 | - | 20.8 | 1 | 426 | 0 | MSTB | MaLR | LTR |
| 149060210 | 149060643 | + | 27.4 | 4049 | 4483 | -1736 | L1ME1 | L1 | LINE |
| 149060644 | 149060937 | + | 7.1 | 3 | 296 | -15 | AluY | Alu | SINE |
| 149060938 | 149061559 | + | 27.4 | 4484 | 5133 | -1086 | L1ME1 | L1 | LINE |
| 149061560 | 149061713 | - | 15.9 | 97 | 236 | 0 | MER46B | MER2\_type | DNA |
| 149061714 | 149061969 | - | 20.2 | 1 | 295 | -7 | AluJo | Alu | SINE |
| 149061970 | 149062112 | - | 15.9 | 66 | 96 | -140 | MER46B | MER2\_type | DNA |
| 149062136 | 149062448 | + | 28.6 | 5522 | 5833 | -406 | L1ME1 | L1 | LINE |
| 149062479 | 149062603 | + | 22.4 | 33 | 157 | -19 | FRAM | Alu | SINE |
| 149062614 | 149062737 | + | 21.4 | 5808 | 5939 | -300 | L1ME1 | L1 | LINE |
| 149062763 | 149063024 | + | 15.9 | 1 | 262 | -116 | MER83B | ERV1 | LTR |
| 149063025 | 149063393 | - | 10.4 | 1 | 381 | 0 | THE1D | MaLR | LTR |
| 149063394 | 149063509 | + | 15.9 | 263 | 378 | 0 | MER83B | ERV1 | LTR |
| 149063525 | 149064277 | + | 23.5 | 47 | 822 | -4058 | MER83B-int | ERV1 | LTR |
| 149064277 | 149064704 | + | 15.8 | 1069 | 1501 | -3379 | MER83B-int | ERV1 | LTR |
| 149064682 | 149065997 | + | 12.1 | 3547 | 4867 | -13 | MER83B-int | ERV1 | LTR |
| 149066001 | 149066366 | + | 16.7 | 1 | 378 | 0 | MER83B | ERV1 | LTR |
| 149066368 | 149066578 | + | 28.7 | 5985 | 6181 | -58 | L1ME1 | L1 | LINE |

  

|  |  |  |  |  |  |  |  |  |  |
| --- | --- | --- | --- | --- | --- | --- | --- | --- | --- |
| chr3:150011071-150013386 | | | 2210 bp | | |  |  |  |  |
| Genome Start | Genome End | Strand | Div | Repeat Start | Repeat End | Repeat Left | Name | Family | Class |
| 150011071 | 150011150 | + | 18.4 | 1 | 77 | -142 | MER20 | MER1\_type | DNA |
| 150011227 | 150011304 | + | 14.1 | 276 | 353 | -2 | THE1A | MaLR | LTR |
| 150011309 | 150012878 | + | 29.7 | 2 | 1735 | 0 | MLT-int | MaLR | LTR |
| 150012879 | 150013233 | + | 8.7 | 1 | 355 | 0 | THE1A | MaLR | LTR |
| 150013260 | 150013386 | + | 17.3 | 93 | 219 | 0 | MER20 | MER1\_type | DNA |

  

|  |  |  |  |  |  |  |  |  |  |
| --- | --- | --- | --- | --- | --- | --- | --- | --- | --- |
| chr3:152054469-152062068 | | | 7607 bp | | |  |  |  |  |
| Genome Start | Genome End | Strand | Div | Repeat Start | Repeat End | Repeat Left | Name | Family | Class |
| 152054469 | 152054631 | + | 18.0 | 5126 | 5308 | -838 | L1M5 | L1 | LINE |
| 152054632 | 152054725 | + | 16.9 | 42 | 130 | -12 | FLAM\_A | Alu | SINE |
| 152054733 | 152055254 | + | 7.3 | 256 | 780 | 0 | LTR17 | ERV1 | LTR |
| 152055255 | 152061174 | + | 5.1 | 1 | 6019 | -2607 | HERV17 | ERV1 | LTR |
| 152061168 | 152061206 | + | 7.7 | 7510 | 7548 | -1078 | HERV17 | ERV1 | LTR |
| 152061197 | 152061488 | + | 5.1 | 8335 | 8626 | 0 | HERV17 | ERV1 | LTR |
| 152061489 | 152061814 | + | 4.3 | 1 | 326 | -454 | LTR17 | ERV1 | LTR |
| 152061818 | 152062068 | + | 25.3 | 5299 | 5562 | -678 | L1M5 | L1 | LINE |

  

|  |  |  |  |  |  |  |  |  |  |
| --- | --- | --- | --- | --- | --- | --- | --- | --- | --- |
| chr3:152420855-152423243 | | | 2387 bp | | |  |  |  |  |
| Genome Start | Genome End | Strand | Div | Repeat Start | Repeat End | Repeat Left | Name | Family | Class |
| 152420855 | 152420942 | - | 20.1 | 73 | 160 | 0 | MER5A1 | MER1\_type | DNA |
| 152420943 | 152421295 | + | 13.9 | 1 | 364 | 0 | THE1B | MaLR | LTR |
| 152421298 | 152422854 | + | 31.0 | 2 | 1735 | 0 | MLT-int | MaLR | LTR |
| 152422855 | 152423198 | + | 14.2 | 1 | 362 | -2 | THE1B | MaLR | LTR |
| 152423199 | 152423243 | - | 20.1 | 9 | 72 | -88 | MER5A1 | MER1\_type | DNA |

  

|  |  |  |  |  |  |  |  |  |  |
| --- | --- | --- | --- | --- | --- | --- | --- | --- | --- |
| chr3:153899240-153903907 | | | 4661 bp | | |  |  |  |  |
| Genome Start | Genome End | Strand | Div | Repeat Start | Repeat End | Repeat Left | Name | Family | Class |
| 153899240 | 153900214 | - | 12.2 | 1406 | 2418 | 0 | Tigger1 | MER2\_type | DNA |
| 153900215 | 153900567 | - | 10.5 | 1 | 355 | 0 | THE1A | MaLR | LTR |
| 153900573 | 153902132 | - | 29.5 | 2 | 1735 | 0 | MLT-int | MaLR | LTR |
| 153902135 | 153902487 | - | 8.8 | 1 | 355 | 0 | THE1A | MaLR | LTR |
| 153902488 | 153903907 | - | 14.2 | 1 | 1410 | -1008 | Tigger1 | MER2\_type | DNA |

  

|  |  |  |  |  |  |  |  |  |  |
| --- | --- | --- | --- | --- | --- | --- | --- | --- | --- |
| chr3:154046021-154048873 | | | 2849 bp | | |  |  |  |  |
| Genome Start | Genome End | Strand | Div | Repeat Start | Repeat End | Repeat Left | Name | Family | Class |
| 154046021 | 154046191 | - | 12.5 | 5984 | 6157 | -9 | L1PA16 | L1 | LINE |
| 154046192 | 154046532 | + | 13.4 | 1 | 362 | -2 | THE1B | MaLR | LTR |
| 154046537 | 154048120 | + | 28.5 | 2 | 1735 | 0 | MLT1G-int | MaLR | LTR |
| 154048121 | 154048473 | + | 10.7 | 1 | 364 | 0 | THE1B | MaLR | LTR |
| 154048474 | 154048873 | - | 12.5 | 5586 | 5983 | -183 | L1PA16 | L1 | LINE |

  

|  |  |  |  |  |  |  |  |  |  |
| --- | --- | --- | --- | --- | --- | --- | --- | --- | --- |
| chr3:154426329-154433117 | | | 6778 bp | | |  |  |  |  |
| Genome Start | Genome End | Strand | Div | Repeat Start | Repeat End | Repeat Left | Name | Family | Class |
| 154426329 | 154426612 | - | 14.8 | 175 | 457 | 0 | MSTB2 | MaLR | LTR |
| 154426629 | 154427041 | - | 5.8 | 1 | 448 | -2 | LTR7 | ERV1 | LTR |
| 154427042 | 154427417 | - | 9.4 | 7322 | 7713 | 0 | HERVH | ERV1 | LTR |
| 154427416 | 154427631 | - | 14.8 | 5600 | 5819 | -1894 | HERVH | ERV1 | LTR |
| 154427633 | 154429362 | - | 11.4 | 3492 | 5273 | -1818 | HERVH | ERV1 | LTR |
| 154429353 | 154429502 | - | 12.7 | 3140 | 3290 | -4423 | HERVH | ERV1 | LTR |
| 154429497 | 154432587 | - | 9.7 | 1 | 2987 | -4726 | HERVH | ERV1 | LTR |
| 154432590 | 154433002 | - | 6.1 | 1 | 448 | -2 | LTR7 | ERV1 | LTR |
| 154433013 | 154433117 | - | 16.7 | 1 | 102 | -326 | MSTB2 | MaLR | LTR |

  

|  |  |  |  |  |  |  |  |  |  |
| --- | --- | --- | --- | --- | --- | --- | --- | --- | --- |
| chr3:154763413-154767028 | | | 3445 bp | | |  |  |  |  |
| Genome Start | Genome End | Strand | Div | Repeat Start | Repeat End | Repeat Left | Name | Family | Class |
| 154763413 | 154763509 | + | 25.3 | 3558 | 3656 | -833 | L3 | CR1 | LINE |
| 154763539 | 154763928 | + | 16.2 | 1 | 428 | 0 | MSTA | MaLR | LTR |
| 154763931 | 154765551 | + | 30.3 | 2 | 1735 | 0 | MLT-int | MaLR | LTR |
| 154765552 | 154765900 | + | 12.7 | 1 | 401 | -27 | MSTA | MaLR | LTR |
| 154765901 | 154766216 | - | 11.3 | 1 | 310 | -1 | AluY | Alu | SINE |
| 154766217 | 154766256 | + | 12.7 | 402 | 428 | 0 | MSTA | MaLR | LTR |
| 154766258 | 154766413 | + | 28.8 | 61 | 236 | -32 | MIRb | MIR | SINE |
| 154766553 | 154767028 | + | 37.3 | 3694 | 4214 | -275 | L3 | CR1 | LINE |

  

|  |  |  |  |  |  |  |  |  |  |
| --- | --- | --- | --- | --- | --- | --- | --- | --- | --- |
| chr3:155563485-155573798 | | | 10108 bp | | |  |  |  |  |
| Genome Start | Genome End | Strand | Div | Repeat Start | Repeat End | Repeat Left | Name | Family | Class |
| 155563485 | 155563761 | - | 34.8 | 2764 | 3078 | -341 | L2 | L2 | LINE |
| 155563763 | 155564039 | - | 21.8 | 129 | 408 | 0 | MLT1A1 | MaLR | LTR |
| 155564040 | 155564480 | + | 21.0 | 1 | 1227 | -15 | LTR27B | ERV1 | LTR |
| 155564674 | 155564799 | - | 18.5 | 1 | 125 | -303 | MLT1A1 | MaLR | LTR |
| 155564800 | 155566551 | - | 22.0 | 4 | 1735 | 0 | MLT1A1-int | MaLR | LTR |
| 155566553 | 155567435 | + | 16.3 | 1 | 903 | 0 | MER4D | ERV1 | LTR |
| 155567444 | 155567586 | - | 13.3 | 256 | 406 | -2 | MLT1A1 | MaLR | LTR |
| 155567587 | 155568283 | + | 7.0 | 1 | 722 | 0 | LTR30 | ERV1 | LTR |
| 155568284 | 155571737 | + | 9.0 | 1 | 3462 | -4795 | HERV30 | ERV1 | LTR |
| 155571731 | 155572625 | + | 11.1 | 7344 | 8257 | 0 | HERV30 | ERV1 | LTR |
| 155572626 | 155573015 | + | 5.1 | 1 | 401 | -321 | LTR30 | ERV1 | LTR |
| 155573026 | 155573116 | - | 15.7 | 1 | 97 | -311 | MLT1A1 | MaLR | LTR |
| 155573117 | 155573798 | - | 29.6 | 2025 | 2767 | -652 | L2 | L2 | LINE |

  

|  |  |  |  |  |  |  |  |  |  |
| --- | --- | --- | --- | --- | --- | --- | --- | --- | --- |
| chr3:155800431-155803632 | | | 3197 bp | | |  |  |  |  |
| Genome Start | Genome End | Strand | Div | Repeat Start | Repeat End | Repeat Left | Name | Family | Class |
| 155800431 | 155800741 | + | 28.6 | 2683 | 2988 | -431 | L2 | L2 | LINE |
| 155800742 | 155801162 | - | 15.6 | 1 | 428 | 0 | MSTA | MaLR | LTR |
| 155801163 | 155802775 | - | 30.2 | 2 | 1735 | 0 | MLT1L-int | MaLR | LTR |
| 155802781 | 155803214 | - | 13.1 | 1 | 426 | -2 | MSTA | MaLR | LTR |
| 155803215 | 155803632 | + | 32.2 | 2992 | 3414 | -5 | L2 | L2 | LINE |

  

|  |  |  |  |  |  |  |  |  |  |
| --- | --- | --- | --- | --- | --- | --- | --- | --- | --- |
| chr3:156120454-156124801 | | | 4223 bp | | |  |  |  |  |
| Genome Start | Genome End | Strand | Div | Repeat Start | Repeat End | Repeat Left | Name | Family | Class |
| 156120454 | 156120670 | + | 17.1 | 1644 | 1869 | -4531 | L1MEc | L1 | LINE |
| 156120686 | 156121075 | - | 12.2 | 1 | 428 | 0 | MSTA | MaLR | LTR |
| 156121091 | 156122693 | - | 17.5 | 1 | 1635 | -16 | MSTA-int | MaLR | LTR |
| 156122694 | 156123082 | - | 14.8 | 1 | 428 | 0 | MSTA | MaLR | LTR |
| 156123085 | 156124416 | + | 24.2 | 1892 | 3192 | -3027 | L1MEc | L1 | LINE |
| 156124510 | 156124801 | + | 25.1 | 3529 | 3825 | -2394 | L1MEc | L1 | LINE |

  

|  |  |  |  |  |  |  |  |  |  |
| --- | --- | --- | --- | --- | --- | --- | --- | --- | --- |
| chr3:156249629-156254629 | | | 4894 bp | | |  |  |  |  |
| Genome Start | Genome End | Strand | Div | Repeat Start | Repeat End | Repeat Left | Name | Family | Class |
| 156249629 | 156249926 | + | 23.4 | 6 | 318 | -149 | MLT1C | MaLR | LTR |
| 156249927 | 156250110 | + | 18.4 | 52 | 229 | -1142 | MLT1C-int | MaLR | LTR |
| 156250162 | 156250288 | + | 18.4 | 230 | 369 | -1002 | MLT1C-int | MaLR | LTR |
| 156250311 | 156251393 | - | 13.1 | 5074 | 6161 | -4 | L1PA15 | L1 | LINE |
| 156251394 | 156251857 | + | 22.9 | 354 | 837 | -534 | MLT1C-int | MaLR | LTR |
| 156251859 | 156252233 | - | 14.5 | 1 | 375 | 0 | THE1C | MaLR | LTR |
| 156252234 | 156253754 | - | 13.6 | 1 | 1580 | 0 | THE1C-int | MaLR | LTR |
| 156253755 | 156254122 | - | 15.2 | 1 | 375 | 0 | THE1C | MaLR | LTR |
| 156254123 | 156254487 | + | 27.2 | 876 | 1223 | -247 | MLT1B-int | MaLR | LTR |
| 156254521 | 156254629 | + | 15.5 | 381 | 567 | 0 | MLT1C | MaLR | LTR |

  

|  |  |  |  |  |  |  |  |  |  |
| --- | --- | --- | --- | --- | --- | --- | --- | --- | --- |
| chr3:156471072-156483259 | | | 12158 bp | | |  |  |  |  |
| Genome Start | Genome End | Strand | Div | Repeat Start | Repeat End | Repeat Left | Name | Family | Class |
| 156471072 | 156471269 | + | 15.2 | 2 | 200 | -6663 | L1PBa | L1 | LINE |
| 156471272 | 156473498 | + | 10.4 | 636 | 3105 | -3757 | L1PBa | L1 | LINE |
| 156473499 | 156473794 | + | 12.5 | 1 | 305 | -8 | AluSq | Alu | SINE |
| 156473795 | 156474917 | + | 9.6 | 3106 | 4185 | -2677 | L1PBa | L1 | LINE |
| 156474920 | 156475324 | - | 7.8 | 1 | 448 | -2 | LTR7 | ERV1 | LTR |
| 156475325 | 156475701 | - | 8.9 | 7324 | 7713 | 0 | HERVH | ERV1 | LTR |
| 156475702 | 156475921 | - | 13.6 | 5600 | 5819 | -1894 | HERVH | ERV1 | LTR |
| 156475923 | 156480875 | - | 15.9 | 1 | 5149 | -2564 | HERVH | ERV1 | LTR |
| 156480878 | 156481281 | - | 9.1 | 1 | 448 | -2 | LTR7 | ERV1 | LTR |
| 156481282 | 156481482 | + | 9.5 | 4181 | 4396 | -2466 | L1PBa | L1 | LINE |
| 156481514 | 156482464 | + | 9.5 | 4397 | 5324 | -1538 | L1PBa | L1 | LINE |
| 156482465 | 156482671 | + | 11.4 | 1 | 162 | -215 | MER51A | ERV1 | LTR |
| 156482664 | 156482970 | + | 12.5 | 63 | 377 | 0 | MER51B | ERV1 | LTR |
| 156482971 | 156483259 | + | 9.6 | 5322 | 5604 | -1258 | L1PBa | L1 | LINE |

  

|  |  |  |  |  |  |  |  |  |  |
| --- | --- | --- | --- | --- | --- | --- | --- | --- | --- |
| chr3:156729904-156742375 | | | 12449 bp | | |  |  |  |  |
| Genome Start | Genome End | Strand | Div | Repeat Start | Repeat End | Repeat Left | Name | Family | Class |
| 156729904 | 156730242 | - | 12.3 | 2088 | 2418 | 0 | Tigger1 | MER2\_type | DNA |
| 156730243 | 156730534 | - | 9.8 | 1 | 307 | 0 | AluSq | Alu | SINE |
| 156730535 | 156730809 | - | 12.3 | 1739 | 2087 | -331 | Tigger1 | MER2\_type | DNA |
| 156730810 | 156731063 | - | 6.7 | 37 | 298 | -13 | AluY | Alu | SINE |
| 156731064 | 156731825 | - | 12.3 | 972 | 1738 | -680 | Tigger1 | MER2\_type | DNA |
| 156731826 | 156732123 | + | 11.4 | 1 | 298 | -14 | AluSx | Alu | SINE |
| 156732124 | 156732633 | - | 12.3 | 458 | 971 | -1447 | Tigger1 | MER2\_type | DNA |
| 156732645 | 156732919 | + | 14.9 | 1 | 285 | -6578 | L1PB1 | L1 | LINE |
| 156732920 | 156733310 | + | 13.2 | 1 | 381 | 0 | THE1D | MaLR | LTR |
| 156733311 | 156733747 | + | 12.0 | 1 | 496 | -1084 | THE1D-int | MaLR | LTR |
| 156733748 | 156734055 | + | 11.0 | 1 | 309 | -4 | AluSq | Alu | SINE |
| 156734056 | 156735122 | + | 12.0 | 497 | 1580 | 0 | THE1D-int | MaLR | LTR |
| 156735123 | 156735505 | + | 11.7 | 1 | 379 | -2 | THE1D | MaLR | LTR |
| 156735512 | 156736337 | + | 11.9 | 285 | 1159 | -5704 | L1PB1 | L1 | LINE |
| 156736340 | 156741876 | + | 10.0 | 1290 | 6867 | 0 | L1PB1 | L1 | LINE |
| 156741881 | 156742375 | - | 17.4 | 4 | 483 | -1935 | Tigger1 | MER2\_type | DNA |

  

|  |  |  |  |  |  |  |  |  |  |
| --- | --- | --- | --- | --- | --- | --- | --- | --- | --- |
| chr3:156915252-156919463 | | | 4208 bp | | |  |  |  |  |
| Genome Start | Genome End | Strand | Div | Repeat Start | Repeat End | Repeat Left | Name | Family | Class |
| 156915252 | 156915789 | - | 27.1 | 3113 | 3778 | -2368 | L1M5 | L1 | LINE |
| 156915790 | 156916087 | - | 17.3 | 1 | 295 | -17 | AluJb | Alu | SINE |
| 156916090 | 156916386 | - | 16.9 | 1 | 304 | -8 | AluJo | Alu | SINE |
| 156916387 | 156916861 | - | 27.1 | 2606 | 3112 | -3034 | L1M5 | L1 | LINE |
| 156916862 | 156917264 | - | 15.5 | 1 | 428 | 0 | MSTA | MaLR | LTR |
| 156917265 | 156918920 | - | 28.7 | 2 | 1735 | 0 | MLT-int | MaLR | LTR |
| 156918923 | 156919310 | - | 18.0 | 1 | 428 | 0 | MSTA | MaLR | LTR |
| 156919311 | 156919463 | - | 27.1 | 2440 | 2605 | -3541 | L1M5 | L1 | LINE |

  

|  |  |  |  |  |  |  |  |  |  |
| --- | --- | --- | --- | --- | --- | --- | --- | --- | --- |
| chr3:157767409-157770120 | | | 2709 bp | | |  |  |  |  |
| Genome Start | Genome End | Strand | Div | Repeat Start | Repeat End | Repeat Left | Name | Family | Class |
| 157767409 | 157767559 | - | 30.4 | 376 | 532 | -59 | MLT1K | MaLR | LTR |
| 157767560 | 157767913 | + | 10.7 | 1 | 355 | 0 | THE1A | MaLR | LTR |
| 157767916 | 157769492 | + | 31.7 | 2 | 1735 | 0 | MLT-int | MaLR | LTR |
| 157769494 | 157769847 | + | 7.7 | 2 | 355 | 0 | THE1A | MaLR | LTR |
| 157769848 | 157770120 | - | 30.4 | 76 | 375 | -216 | MLT1K | MaLR | LTR |

  

|  |  |  |  |  |  |  |  |  |  |
| --- | --- | --- | --- | --- | --- | --- | --- | --- | --- |
| chr3:158030246-158040784 | | | 10448 bp | | |  |  |  |  |
| Genome Start | Genome End | Strand | Div | Repeat Start | Repeat End | Repeat Left | Name | Family | Class |
| 158030246 | 158031421 | + | 15.2 | 1 | 419 | -1562 | Tigger2 | MER2\_type | DNA |
| 158031472 | 158031968 | - | 15.5 | 111 | 674 | -53 | LTR8A | ERV1 | LTR |
| 158031969 | 158032321 | - | 10.8 | 1 | 364 | 0 | THE1B | MaLR | LTR |
| 158032322 | 158032414 | - | 15.9 | 1 | 110 | -617 | LTR8A | ERV1 | LTR |
| 158032415 | 158036346 | - | 17.8 | 2780 | 6263 | 0 | HERV35I | ERV1 | LTR |
| 158036347 | 158036655 | - | 13.3 | 1 | 311 | -1 | AluSx | Alu | SINE |
| 158036656 | 158039045 | - | 18.1 | 393 | 2779 | -4139 | HERV35I | ERV1 | LTR |
| 158039097 | 158039322 | - | 30.4 | 3 | 219 | -6044 | HERV35I | ERV1 | LTR |
| 158039325 | 158039466 | - | 17.1 | 595 | 727 | 0 | LTR8A | ERV1 | LTR |
| 158039461 | 158039792 | + | 13.6 | 422 | 760 | -1221 | Tigger2 | MER2\_type | DNA |
| 158039787 | 158040784 | + | 13.3 | 930 | 2717 | -1 | Tigger2 | MER2\_type | DNA |

  

|  |  |  |  |  |  |  |  |  |  |
| --- | --- | --- | --- | --- | --- | --- | --- | --- | --- |
| chr3:158075025-158087512 | | | 12447 bp | | |  |  |  |  |
| Genome Start | Genome End | Strand | Div | Repeat Start | Repeat End | Repeat Left | Name | Family | Class |
| 158075025 | 158076196 | - | 13.1 | 5681 | 6867 | 0 | L1PB1 | L1 | LINE |
| 158076197 | 158076503 | + | 8.1 | 1 | 309 | -2 | AluY | Alu | SINE |
| 158076504 | 158078242 | - | 13.3 | 3908 | 5680 | -1182 | L1PB1 | L1 | LINE |
| 158078243 | 158078259 | - | 9.7 | 283 | 310 | 0 | AluSg | Alu | SINE |
| 158078283 | 158078566 | - | 9.7 | 2 | 282 | -28 | AluSg | Alu | SINE |
| 158078567 | 158079187 | - | 13.3 | 3289 | 3907 | -2955 | L1PB1 | L1 | LINE |
| 158079188 | 158079523 | + | 15.0 | 1 | 364 | 0 | THE1B | MaLR | LTR |
| 158079524 | 158080793 | + | 10.7 | 1 | 1281 | -299 | THE1B-int | MaLR | LTR |
| 158080794 | 158083647 | + | 2.5 | 3300 | 6154 | -1 | L1PA3 | L1 | LINE |
| 158083665 | 158083977 | + | 6.4 | 1269 | 1580 | 0 | THE1B-int | MaLR | LTR |
| 158083978 | 158084333 | + | 12.9 | 1 | 363 | -1 | THE1B | MaLR | LTR |
| 158084335 | 158087512 | - | 15.4 | 3 | 3291 | -3571 | L1PB1 | L1 | LINE |

  

|  |  |  |  |  |  |  |  |  |  |
| --- | --- | --- | --- | --- | --- | --- | --- | --- | --- |
| chr3:158762255-158766131 | | | 3855 bp | | |  |  |  |  |
| Genome Start | Genome End | Strand | Div | Repeat Start | Repeat End | Repeat Left | Name | Family | Class |
| 158762255 | 158762876 | - | 26.6 | 2327 | 2919 | -3300 | L1MEc | L1 | LINE |
| 158762892 | 158763243 | - | 12.3 | 3 | 355 | 0 | THE1A | MaLR | LTR |
| 158763246 | 158764318 | - | 9.5 | 497 | 1580 | 0 | THE1A-int | MaLR | LTR |
| 158764319 | 158764633 | - | 9.7 | 1 | 309 | -4 | AluSq | Alu | SINE |
| 158764634 | 158765128 | - | 9.5 | 3 | 496 | -1084 | THE1A-int | MaLR | LTR |
| 158765131 | 158765485 | - | 11.4 | 1 | 355 | 0 | THE1A | MaLR | LTR |
| 158765489 | 158766131 | - | 29.9 | 336 | 1008 | -5392 | L1MEc | L1 | LINE |

  

|  |  |  |  |  |  |  |  |  |  |
| --- | --- | --- | --- | --- | --- | --- | --- | --- | --- |
| chr3:158779544-158788866 | | | 9259 bp | | |  |  |  |  |
| Genome Start | Genome End | Strand | Div | Repeat Start | Repeat End | Repeat Left | Name | Family | Class |
| 158779544 | 158779710 | + | 6.4 | 1 | 180 | -264 | MLT2A1 | ERVL | LTR |
| 158779711 | 158780165 | - | 5.7 | 8 | 463 | 0 | LTR2 | ERV1 | LTR |
| 158780170 | 158788030 | - | 19.2 | 1 | 7812 | -1 | HERVE | ERV1 | LTR |
| 158788031 | 158788489 | - | 6.1 | 1 | 463 | 0 | LTR2 | ERV1 | LTR |
| 158788493 | 158788730 | + | 10.8 | 183 | 503 | -44 | MLT2A1 | ERVL | LTR |
| 158788788 | 158788866 | + | 6.7 | 474 | 555 | 0 | MLT2A1 | ERVL | LTR |

  

|  |  |  |  |  |  |  |  |  |  |
| --- | --- | --- | --- | --- | --- | --- | --- | --- | --- |
| chr3:163258047-163261416 | | | 2994 bp | | |  |  |  |  |
| Genome Start | Genome End | Strand | Div | Repeat Start | Repeat End | Repeat Left | Name | Family | Class |
| 163258047 | 163258181 | - | 40.6 | 3233 | 3378 | 0 | L2 | L2 | LINE |
| 163258555 | 163258990 | - | 38.1 | 2387 | 2829 | -590 | L2 | L2 | LINE |
| 163258991 | 163259358 | - | 14.2 | 1 | 381 | 0 | THE1D | MaLR | LTR |
| 163259359 | 163261001 | - | 30.2 | 3 | 1735 | 0 | MLT1G1-int | MaLR | LTR |
| 163261005 | 163261347 | - | 12.7 | 1 | 381 | 0 | THE1D | MaLR | LTR |
| 163261348 | 163261416 | - | 38.1 | 2318 | 2386 | -1033 | L2 | L2 | LINE |

  

|  |  |  |  |  |  |  |  |  |  |
| --- | --- | --- | --- | --- | --- | --- | --- | --- | --- |
| chr3:164306836-164309344 | | | 2502 bp | | |  |  |  |  |
| Genome Start | Genome End | Strand | Div | Repeat Start | Repeat End | Repeat Left | Name | Family | Class |
| 164306836 | 164306895 | - | 16.7 | 245 | 314 | -917 | Tigger3b | MER2\_type | DNA |
| 164306896 | 164307260 | - | 18.7 | 3 | 375 | 0 | THE1C | MaLR | LTR |
| 164307268 | 164308750 | - | 14.7 | 1 | 1574 | -6 | THE1C-int | MaLR | LTR |
| 164308751 | 164309100 | - | 14.6 | 1 | 371 | -4 | THE1C | MaLR | LTR |
| 164309101 | 164309344 | - | 22.4 | 7 | 249 | -982 | Tigger3b | MER2\_type | DNA |

  

|  |  |  |  |  |  |  |  |  |  |
| --- | --- | --- | --- | --- | --- | --- | --- | --- | --- |
| chr3:164490976-164496404 | | | 5356 bp | | |  |  |  |  |
| Genome Start | Genome End | Strand | Div | Repeat Start | Repeat End | Repeat Left | Name | Family | Class |
| 164490976 | 164491168 | + | 17.6 | 2897 | 3093 | -3047 | L1MB1 | L1 | LINE |
| 164491169 | 164491524 | - | 13.0 | 1 | 364 | 0 | THE1B | MaLR | LTR |
| 164491525 | 164493098 | - | 14.3 | 3 | 1580 | 0 | THE1B-int | MaLR | LTR |
| 164493103 | 164493462 | - | 13.3 | 1 | 424 | -2 | THE1B | MaLR | LTR |
| 164493463 | 164494889 | + | 18.0 | 3089 | 4631 | -1509 | L1MB1 | L1 | LINE |
| 164494958 | 164496299 | + | 18.2 | 4632 | 6021 | -147 | L1MB1 | L1 | LINE |
| 164496301 | 164496404 | + | 19.2 | 6065 | 6168 | 0 | L1MB1 | L1 | LINE |

  

|  |  |  |  |  |  |  |  |  |  |
| --- | --- | --- | --- | --- | --- | --- | --- | --- | --- |
| chr3:164771503-164775074 | | | 3561 bp | | |  |  |  |  |
| Genome Start | Genome End | Strand | Div | Repeat Start | Repeat End | Repeat Left | Name | Family | Class |
| 164771503 | 164771607 | + | 19.1 | 16 | 124 | -7441 | L1M1 | L1 | LINE |
| 164771615 | 164771687 | + | 16.0 | 1100 | 1173 | -6392 | L1M1 | L1 | LINE |
| 164771688 | 164772042 | + | 9.9 | 1 | 353 | -2 | THE1A | MaLR | LTR |
| 164772047 | 164773590 | + | 32.0 | 2 | 1735 | 0 | MLT-int | MaLR | LTR |
| 164773591 | 164773938 | + | 11.8 | 1 | 351 | -4 | THE1A | MaLR | LTR |
| 164773939 | 164775074 | + | 16.0 | 1174 | 2343 | -5222 | L1M1 | L1 | LINE |

  

|  |  |  |  |  |  |  |  |  |  |
| --- | --- | --- | --- | --- | --- | --- | --- | --- | --- |
| chr3:164937928-164947388 | | | 8884 bp | | |  |  |  |  |
| Genome Start | Genome End | Strand | Div | Repeat Start | Repeat End | Repeat Left | Name | Family | Class |
| 164937928 | 164938631 | + | 28.3 | 1217 | 1941 | -5555 | L1M3b | L1 | LINE |
| 164938913 | 164940539 | + | 27.4 | 2259 | 3863 | -3699 | L1M3b | L1 | LINE |
| 164940547 | 164940751 | - | 24.5 | 136 | 344 | -1 | MER2 | MER2\_type | DNA |
| 164940859 | 164941256 | + | 23.4 | 3877 | 4273 | -3289 | L1M3b | L1 | LINE |
| 164941258 | 164941430 | - | 24.3 | 2847 | 3027 | -2 | Tigger3(Golem) | MER2\_type | DNA |
| 164941431 | 164941648 | + | 17.4 | 4291 | 4532 | -3030 | L1M3b | L1 | LINE |
| 164941646 | 164942216 | + | 19.2 | 5138 | 5724 | -1838 | L1M3b | L1 | LINE |
| 164942217 | 164942866 | + | 3.7 | 5505 | 6155 | 0 | L1PA4 | L1 | LINE |
| 164942867 | 164943080 | + | 15.1 | 189 | 428 | 0 | MSTA | MaLR | LTR |
| 164943081 | 164943822 | + | 18.9 | 5357 | 6129 | -171 | L1MA4 | L1 | LINE |
| 164943851 | 164944205 | - | 22.7 | 1 | 426 | 0 | MSTB | MaLR | LTR |
| 164944206 | 164945296 | - | 20.2 | 540 | 1651 | 0 | MSTB-int | MaLR | LTR |
| 164945297 | 164945598 | + | 13.2 | 1 | 302 | -7 | AluSc | Alu | SINE |
| 164945599 | 164946138 | - | 20.2 | 1 | 539 | -1112 | MSTB-int | MaLR | LTR |
| 164946139 | 164946525 | - | 19.0 | 1 | 426 | 0 | MSTB | MaLR | LTR |
| 164946526 | 164946660 | + | 14.1 | 6158 | 6296 | -29 | L1MA3 | L1 | LINE |
| 164946817 | 164946864 | + | 15.4 | 5701 | 5748 | -1817 | L1M3b | L1 | LINE |
| 164946865 | 164947167 | - | 1.3 | 1 | 303 | -8 | AluY | Alu | SINE |
| 164947168 | 164947388 | + | 15.4 | 5749 | 5967 | -1598 | L1M3b | L1 | LINE |

  

|  |  |  |  |  |  |  |  |  |  |
| --- | --- | --- | --- | --- | --- | --- | --- | --- | --- |
| chr3:165159507-165170972 | | | 11302 bp | | |  |  |  |  |
| Genome Start | Genome End | Strand | Div | Repeat Start | Repeat End | Repeat Left | Name | Family | Class |
| 165159507 | 165161131 | + | 25.6 | 1 | 1690 | -5159 | L1MA4A | L1 | LINE |
| 165161152 | 165161268 | + | 21.0 | 1783 | 1901 | -4948 | L1MA4A | L1 | LINE |
| 165161269 | 165161541 | + | 18.3 | 25 | 307 | -5 | AluSx | Alu | SINE |
| 165161542 | 165163254 | + | 20.7 | 1902 | 3612 | -3233 | L1MA4A | L1 | LINE |
| 165163258 | 165163451 | + | 16.2 | 1 | 216 | -212 | MSTA | MaLR | LTR |
| 165163475 | 165163680 | + | 16.2 | 217 | 427 | -1 | MSTA | MaLR | LTR |
| 165163682 | 165165272 | + | 20.4 | 2 | 1651 | 0 | MSTA-int | MaLR | LTR |
| 165165273 | 165165693 | + | 15.8 | 1 | 428 | 0 | MSTA | MaLR | LTR |
| 165165706 | 165166074 | + | 20.8 | 3624 | 4009 | -2839 | L1MA4A | L1 | LINE |
| 165166070 | 165166206 | + | 15.3 | 5476 | 5602 | -564 | L1PA16 | L1 | LINE |
| 165166234 | 165166253 | + | 15.3 | 5603 | 5603 | -563 | L1PA16 | L1 | LINE |
| 165166254 | 165166520 | - | 10.9 | 11 | 288 | -25 | AluSp | Alu | SINE |
| 165166521 | 165167064 | + | 15.3 | 5604 | 6145 | -21 | L1PA16 | L1 | LINE |
| 165167084 | 165167291 | + | 18.6 | 3998 | 4196 | -2649 | L1MA4A | L1 | LINE |
| 165167292 | 165167578 | + | 20.6 | 1 | 296 | -16 | AluJb | Alu | SINE |
| 165167579 | 165168611 | + | 18.6 | 4197 | 5242 | -1603 | L1MA4A | L1 | LINE |
| 165168615 | 165169367 | + | 22.3 | 1 | 813 | -5478 | L1P4e | L1 | LINE |
| 165169377 | 165169623 | + | 16.3 | 5910 | 6161 | -4 | L1PA15 | L1 | LINE |
| 165169676 | 165170972 | + | 18.2 | 5231 | 6538 | -467 | L1MA4A | L1 | LINE |

  

|  |  |  |  |  |  |  |  |  |  |
| --- | --- | --- | --- | --- | --- | --- | --- | --- | --- |
| chr3:165257284-165268105 | | | 10418 bp | | |  |  |  |  |
| Genome Start | Genome End | Strand | Div | Repeat Start | Repeat End | Repeat Left | Name | Family | Class |
| 165257284 | 165257628 | - | 9.6 | 3890 | 4232 | -2150 | L1M4c | L1 | LINE |
| 165257644 | 165258554 | - | 21.7 | 2137 | 3106 | -3270 | L1M4c | L1 | LINE |
| 165258561 | 165259118 | - | 20.2 | 1386 | 2078 | -4306 | L1M4c | L1 | LINE |
| 165259120 | 165260878 | - | 18.5 | 2 | 1784 | -947 | Tigger4(Zombi) | MER2\_type | DNA |
| 165260880 | 165261327 | + | 22.3 | 3338 | 3778 | -2365 | L1MA7 | L1 | LINE |
| 165261338 | 165261596 | - | 23.3 | 3936 | 4203 | -1937 | L1M3 | L1 | LINE |
| 165261615 | 165261820 | + | 17.3 | 4998 | 5194 | -949 | L1MA7 | L1 | LINE |
| 165261821 | 165262224 | - | 20.6 | 1 | 426 | 0 | MSTB | MaLR | LTR |
| 165262225 | 165263309 | + | 20.3 | 5195 | 6288 | -3 | L1MA7 | L1 | LINE |
| 165263452 | 165263526 | - | 9.3 | 6080 | 6154 | 0 | L1PA7 | L1 | LINE |
| 165263551 | 165264001 | - | 19.9 | 425 | 926 | -5458 | L1M4c | L1 | LINE |
| 165264018 | 165264392 | + | 11.5 | 1 | 375 | 0 | THE1C | MaLR | LTR |
| 165264394 | 165265886 | + | 14.4 | 2 | 1580 | 0 | THE1C-int | MaLR | LTR |
| 165265887 | 165266253 | + | 12.3 | 1 | 375 | 0 | THE1C | MaLR | LTR |
| 165266254 | 165266568 | - | 22.9 | 13 | 358 | -7 | MLT1A0 | MaLR | LTR |
| 165266575 | 165267188 | - | 26.8 | 1128 | 1735 | 0 | MLT1A0-int | MaLR | LTR |
| 165267349 | 165267704 | - | 23.1 | 1 | 363 | -2 | MLT1A0 | MaLR | LTR |
| 165267709 | 165268105 | - | 19.8 | 2 | 400 | -5984 | L1M4c | L1 | LINE |

  

|  |  |  |  |  |  |  |  |  |  |
| --- | --- | --- | --- | --- | --- | --- | --- | --- | --- |
| chr3:165348862-165351888 | | | 3005 bp | | |  |  |  |  |
| Genome Start | Genome End | Strand | Div | Repeat Start | Repeat End | Repeat Left | Name | Family | Class |
| 165348862 | 165349100 | + | 19.2 | 1 | 249 | -485 | MER50 | ERV1 | LTR |
| 165349101 | 165349461 | - | 15.9 | 3 | 428 | 0 | MSTA | MaLR | LTR |
| 165349464 | 165351036 | - | 21.7 | 93 | 1651 | 0 | MSTA-int | MaLR | LTR |
| 165351057 | 165351426 | - | 18.5 | 1 | 428 | 0 | MSTA | MaLR | LTR |
| 165351427 | 165351888 | + | 19.2 | 249 | 701 | -33 | MER50 | ERV1 | LTR |

  

|  |  |  |  |  |  |  |  |  |  |
| --- | --- | --- | --- | --- | --- | --- | --- | --- | --- |
| chr3:165506015-165514347 | | | 8324 bp | | |  |  |  |  |
| Genome Start | Genome End | Strand | Div | Repeat Start | Repeat End | Repeat Left | Name | Family | Class |
| 165506015 | 165507615 | + | 13.8 | 13 | 2219 | -4644 | L1PBa | L1 | LINE |
| 165507604 | 165509063 | + | 13.8 | 2503 | 4031 | -2831 | L1PBa | L1 | LINE |
| 165509064 | 165509427 | - | 16.3 | 1 | 364 | 0 | THE1B | MaLR | LTR |
| 165509433 | 165510926 | - | 14.7 | 1 | 1574 | -6 | THE1B-int | MaLR | LTR |
| 165510929 | 165511290 | - | 15.6 | 1 | 362 | -2 | THE1B | MaLR | LTR |
| 165511301 | 165511883 | - | 8.4 | 1 | 688 | 0 | LTR12\_ | ERV1 | LTR |
| 165511888 | 165514347 | + | 10.8 | 4037 | 6495 | -372 | L1PBa | L1 | LINE |

  

|  |  |  |  |  |  |  |  |  |  |
| --- | --- | --- | --- | --- | --- | --- | --- | --- | --- |
| chr3:165527543-165549342 | | | 21330 bp | | |  |  |  |  |
| Genome Start | Genome End | Strand | Div | Repeat Start | Repeat End | Repeat Left | Name | Family | Class |
| 165527543 | 165527862 | - | 22.2 | 6267 | 6593 | -5 | MER4C-int | ERV1 | LTR |
| 165527897 | 165528215 | - | 22.2 | 5938 | 6266 | -332 | MER4C-int | ERV1 | LTR |
| 165528216 | 165528295 | - | 12.8 | 409 | 486 | 0 | LTR19A | ERV1 | LTR |
| 165528296 | 165529118 | + | 6.3 | 5286 | 6142 | -12 | L1PA7 | L1 | LINE |
| 165529123 | 165529617 | - | 15.1 | 1 | 518 | -62 | LTR19B | ERV1 | LTR |
| 165529618 | 165530054 | - | 18.3 | 5525 | 5942 | -656 | MER4C-int | ERV1 | LTR |
| 165530055 | 165530349 | - | 20.0 | 1 | 298 | -14 | AluSx | Alu | SINE |
| 165530350 | 165532115 | - | 18.3 | 3708 | 5524 | -1074 | MER4C-int | ERV1 | LTR |
| 165532116 | 165532403 | - | 16.3 | 1 | 292 | -20 | AluSx | Alu | SINE |
| 165532404 | 165532588 | - | 18.3 | 3526 | 3707 | -2891 | MER4C-int | ERV1 | LTR |
| 165532624 | 165532920 | - | 18.4 | 2467 | 6811 | -280 | HUERS-P2 | ERV1 | LTR |
| 165532906 | 165533672 | - | 23.3 | 631 | 1398 | -1690 | HUERS-P2 | ERV1 | LTR |
| 165533657 | 165533800 | - | 25.4 | 2 | 136 | -2952 | HUERS-P2 | ERV1 | LTR |
| 165533802 | 165534315 | - | 15.9 | 17 | 656 | 0 | LTR1C | ERV1 | LTR |
| 165534318 | 165534427 | - | 20.8 | 2613 | 2722 | -4815 | MER57-int | ERV1 | LTR |
| 165534491 | 165534974 | - | 7.6 | 1 | 530 | -28 | LTR6B | ERV1 | LTR |
| 165534975 | 165537741 | - | 11.3 | 6070 | 8909 | -69 | HERVS71 | ERV1 | LTR |
| 165537741 | 165538612 | - | 12.8 | 4803 | 5673 | -3305 | HERVS71 | ERV1 | LTR |
| 165538596 | 165539952 | - | 10.6 | 1857 | 3235 | -5743 | HERVS71 | ERV1 | LTR |
| 165539979 | 165541389 | - | 10.6 | 480 | 1856 | -7122 | HERVS71 | ERV1 | LTR |
| 165541411 | 165541865 | - | 10.6 | 6 | 479 | -8499 | HERVS71 | ERV1 | LTR |
| 165541869 | 165542379 | - | 7.4 | 1 | 558 | 0 | LTR6B | ERV1 | LTR |
| 165542663 | 165543071 | - | 8.2 | 1 | 448 | -2 | LTR7 | ERV1 | LTR |
| 165543072 | 165543428 | - | 8.5 | 7322 | 7713 | 0 | HERVH | ERV1 | LTR |
| 165543427 | 165543647 | - | 13.3 | 5597 | 5819 | -1894 | HERVH | ERV1 | LTR |
| 165543645 | 165544304 | - | 13.7 | 4488 | 5149 | -2564 | HERVH | ERV1 | LTR |
| 165544307 | 165544808 | - | 12.2 | 3492 | 3993 | -3720 | HERVH | ERV1 | LTR |
| 165544799 | 165544946 | - | 13.5 | 3140 | 3290 | -4423 | HERVH | ERV1 | LTR |
| 165544941 | 165547920 | - | 9.7 | 1 | 2987 | -4726 | HERVH | ERV1 | LTR |
| 165547923 | 165548331 | - | 8.1 | 1 | 448 | -2 | LTR7 | ERV1 | LTR |
| 165548353 | 165548574 | - | 17.7 | 865 | 1096 | -5502 | MER4C-int | ERV1 | LTR |
| 165548575 | 165548625 | - | 18.0 | 264 | 313 | -282 | LTR49 | ERV1 | LTR |
| 165548669 | 165549342 | - | 15.7 | 1 | 812 | -5786 | MER4C-int | ERV1 | LTR |

  

|  |  |  |  |  |  |  |  |  |  |
| --- | --- | --- | --- | --- | --- | --- | --- | --- | --- |
| chr3:165848276-165852066 | | | 3757 bp | | |  |  |  |  |
| Genome Start | Genome End | Strand | Div | Repeat Start | Repeat End | Repeat Left | Name | Family | Class |
| 165848276 | 165848320 | - | 6.7 | 365 | 409 | -19 | MSTA | MaLR | LTR |
| 165848350 | 165848406 | + | 10.5 | 1 | 57 | -324 | THE1D | MaLR | LTR |
| 165848409 | 165848755 | - | 12.5 | 80 | 445 | -8 | MLT2A2 | ERVL | LTR |
| 165848756 | 165848986 | - | 16.2 | 506 | 734 | -4920 | HERVL | ERVL | LTR |
| 165848987 | 165849344 | + | 14.1 | 1 | 364 | 0 | THE1B | MaLR | LTR |
| 165849348 | 165850923 | + | 30.6 | 3 | 1735 | 0 | MLT1F1-int | MaLR | LTR |
| 165850924 | 165851285 | + | 14.4 | 1 | 364 | 0 | THE1B | MaLR | LTR |
| 165851286 | 165851417 | - | 9.8 | 1 | 132 | -321 | MLT2A2 | ERVL | LTR |
| 165851418 | 165851744 | + | 16.7 | 51 | 381 | 0 | THE1D | MaLR | LTR |
| 165851745 | 165852066 | - | 18.3 | 1 | 355 | -73 | MSTA | MaLR | LTR |

  

|  |  |  |  |  |  |  |  |  |  |
| --- | --- | --- | --- | --- | --- | --- | --- | --- | --- |
| chr3:166711076-166717812 | | | 6123 bp | | |  |  |  |  |
| Genome Start | Genome End | Strand | Div | Repeat Start | Repeat End | Repeat Left | Name | Family | Class |
| 166711076 | 166711255 | + | 31.5 | 1687 | 1889 | -1530 | L2 | L2 | LINE |
| 166711265 | 166711385 | - | 9.1 | 5059 | 5179 | -967 | L1P1 | L1 | LINE |
| 166711386 | 166711663 | + | 9.3 | 5884 | 6152 | -2 | L1PA7 | L1 | LINE |
| 166711875 | 166712453 | + | 31.6 | 1 | 595 | 0 | MLT1G | MaLR | LTR |
| 166712459 | 166713220 | + | 35.5 | 2118 | 2869 | -550 | L2 | L2 | LINE |
| 166713224 | 166713354 | + | 26.1 | 5 | 139 | -456 | MLT1G1 | MaLR | LTR |
| 166713410 | 166713705 | + | 27.5 | 230 | 589 | -6 | MLT1G1 | MaLR | LTR |
| 166713713 | 166715067 | + | 30.3 | 2 | 1489 | 0 | MLT1G1-int | MaLR | LTR |
| 166715068 | 166715130 | + | 30.4 | 1 | 50 | -545 | MLT1G1 | MaLR | LTR |
| 166715131 | 166715157 | - | 21.2 | 282 | 308 | -4 | AluJo | Alu | SINE |
| 166715249 | 166715527 | - | 21.2 | 2 | 281 | -31 | AluJo | Alu | SINE |
| 166715528 | 166715943 | + | 30.4 | 51 | 529 | -66 | MLT1G1 | MaLR | LTR |
| 166715944 | 166716216 | - | 21.3 | 1 | 288 | -24 | AluJo | Alu | SINE |
| 166716217 | 166716277 | + | 30.4 | 530 | 588 | -7 | MLT1G1 | MaLR | LTR |
| 166716294 | 166716473 | + | 38.1 | 2871 | 3063 | -356 | L2 | L2 | LINE |
| 166716683 | 166717064 | - | 6.8 | 4786 | 5168 | -978 | L1P1 | L1 | LINE |
| 166717065 | 166717701 | + | 3.9 | 5518 | 6155 | 0 | L1PA4 | L1 | LINE |
| 166717710 | 166717812 | + | 31.1 | 3316 | 3418 | -1 | L2 | L2 | LINE |

  

|  |  |  |  |  |  |  |  |  |  |
| --- | --- | --- | --- | --- | --- | --- | --- | --- | --- |
| chr3:167270042-167274737 | | | 4682 bp | | |  |  |  |  |
| Genome Start | Genome End | Strand | Div | Repeat Start | Repeat End | Repeat Left | Name | Family | Class |
| 167270042 | 167270505 | + | 18.0 | 5496 | 5961 | -207 | L1MB1 | L1 | LINE |
| 167270506 | 167270816 | + | 8.4 | 1 | 312 | -1 | AluSp | Alu | SINE |
| 167270817 | 167270884 | + | 18.0 | 5962 | 6007 | -161 | L1MB1 | L1 | LINE |
| 167270886 | 167271309 | - | 20.2 | 1 | 426 | 0 | MSTB | MaLR | LTR |
| 167271310 | 167272184 | - | 20.1 | 770 | 1651 | 0 | MSTB-int | MaLR | LTR |
| 167272189 | 167272237 | + | 20.4 | 1034 | 1082 | -498 | THE1-int | MaLR | LTR |
| 167272244 | 167273481 | + | 20.8 | 364 | 1651 | 0 | MSTA-int | MaLR | LTR |
| 167273482 | 167273896 | + | 17.4 | 1 | 428 | 0 | MSTA | MaLR | LTR |
| 167273897 | 167274112 | - | 14.8 | 1 | 216 | -1435 | MSTB-int | MaLR | LTR |
| 167274113 | 167274567 | - | 19.6 | 1 | 426 | 0 | MSTB | MaLR | LTR |
| 167274571 | 167274737 | + | 15.5 | 6006 | 6168 | 0 | L1MB1 | L1 | LINE |

  

|  |  |  |  |  |  |  |  |  |  |
| --- | --- | --- | --- | --- | --- | --- | --- | --- | --- |
| chr3:167448338-167450923 | | | 2556 bp | | |  |  |  |  |
| Genome Start | Genome End | Strand | Div | Repeat Start | Repeat End | Repeat Left | Name | Family | Class |
| 167448338 | 167448434 | - | 33.7 | 250 | 336 | -73 | MLT1I | MaLR | LTR |
| 167448463 | 167448492 | - | 33.7 | 222 | 249 | -160 | MLT1I | MaLR | LTR |
| 167448493 | 167448850 | + | 14.7 | 1 | 364 | 0 | THE1B | MaLR | LTR |
| 167448853 | 167450399 | + | 31.2 | 2 | 1735 | 0 | MLT1I-int | MaLR | LTR |
| 167450400 | 167450749 | + | 16.5 | 1 | 364 | 0 | THE1B | MaLR | LTR |
| 167450750 | 167450923 | - | 33.7 | 56 | 221 | -188 | MLT1I | MaLR | LTR |

  

|  |  |  |  |  |  |  |  |  |  |
| --- | --- | --- | --- | --- | --- | --- | --- | --- | --- |
| chr3:168258533-168261386 | | | 2852 bp | | |  |  |  |  |
| Genome Start | Genome End | Strand | Div | Repeat Start | Repeat End | Repeat Left | Name | Family | Class |
| 168258533 | 168258729 | + | 30.8 | 1 | 207 | -408 | MLT1L | MaLR | LTR |
| 168258730 | 168259092 | - | 12.2 | 1 | 373 | -2 | THE1C | MaLR | LTR |
| 168259093 | 168260628 | - | 30.9 | 2 | 1735 | 0 | MLT1B-int | MaLR | LTR |
| 168260631 | 168260996 | - | 14.6 | 1 | 375 | 0 | THE1C | MaLR | LTR |
| 168260997 | 168261386 | + | 30.8 | 208 | 615 | 0 | MLT1L | MaLR | LTR |

  

|  |  |  |  |  |  |  |  |  |  |
| --- | --- | --- | --- | --- | --- | --- | --- | --- | --- |
| chr3:169350856-169356372 | | | 5515 bp | | |  |  |  |  |
| Genome Start | Genome End | Strand | Div | Repeat Start | Repeat End | Repeat Left | Name | Family | Class |
| 169350856 | 169352022 | - | 31.0 | 1771 | 2920 | -3933 | L1PA15-16 | L1 | LINE |
| 169352023 | 169352399 | + | 16.5 | 1 | 428 | 0 | MSTA | MaLR | LTR |
| 169352402 | 169354022 | + | 29.1 | 2 | 1735 | 0 | MLT-int | MaLR | LTR |
| 169354023 | 169354405 | + | 15.0 | 1 | 428 | 0 | MSTA | MaLR | LTR |
| 169354406 | 169356372 | - | 31.0 | 72 | 1770 | -5083 | L1PA15-16 | L1 | LINE |

  

|  |  |  |  |  |  |  |  |  |  |
| --- | --- | --- | --- | --- | --- | --- | --- | --- | --- |
| chr3:169373783-169377597 | | | 3815 bp | | |  |  |  |  |
| Genome Start | Genome End | Strand | Div | Repeat Start | Repeat End | Repeat Left | Name | Family | Class |
| 169373783 | 169373866 | - | 24.5 | 5114 | 5193 | -953 | L1M4 | L1 | LINE |
| 169373867 | 169374276 | - | 13.8 | 3 | 428 | 0 | MSTA | MaLR | LTR |
| 169374277 | 169375889 | - | 29.0 | 1 | 1735 | 0 | MLT-int | MaLR | LTR |
| 169375890 | 169376302 | - | 13.1 | 1 | 428 | 0 | MSTA | MaLR | LTR |
| 169376303 | 169377597 | - | 24.0 | 3510 | 5113 | -1033 | L1M4 | L1 | LINE |

  

|  |  |  |  |  |  |  |  |  |  |
| --- | --- | --- | --- | --- | --- | --- | --- | --- | --- |
| chr3:169455177-169457764 | | | 2584 bp | | |  |  |  |  |
| Genome Start | Genome End | Strand | Div | Repeat Start | Repeat End | Repeat Left | Name | Family | Class |
| 169455177 | 169455304 | - | 29.6 | 113 | 243 | -19 | MIR | MIR | SINE |
| 169455305 | 169455674 | - | 13.7 | 3 | 381 | 0 | THE1D | MaLR | LTR |
| 169455677 | 169457296 | - | 29.4 | 2 | 1735 | 0 | MLT-int | MaLR | LTR |
| 169457299 | 169457661 | - | 11.5 | 1 | 381 | 0 | THE1D | MaLR | LTR |
| 169457662 | 169457764 | - | 29.6 | 8 | 112 | -150 | MIR | MIR | SINE |

  

|  |  |  |  |  |  |  |  |  |  |
| --- | --- | --- | --- | --- | --- | --- | --- | --- | --- |
| chr3:170131289-170134041 | | | 2701 bp | | |  |  |  |  |
| Genome Start | Genome End | Strand | Div | Repeat Start | Repeat End | Repeat Left | Name | Family | Class |
| 170131289 | 170131401 | - | 7.1 | 241 | 354 | -1 | THE1A | MaLR | LTR |
| 170131402 | 170131762 | - | 8.6 | 1 | 450 | 0 | LTR7 | ERV1 | LTR |
| 170131763 | 170132300 | - | 11.3 | 7173 | 7713 | 0 | HERVH | ERV1 | LTR |
| 170132355 | 170132547 | - | 15.0 | 6756 | 6948 | -765 | HERVH | ERV1 | LTR |
| 170132529 | 170133251 | - | 15.3 | 5108 | 5840 | -1873 | HERVH | ERV1 | LTR |
| 170133251 | 170133420 | - | 14.7 | 4643 | 4812 | -2901 | HERVH | ERV1 | LTR |
| 170133437 | 170133797 | - | 8.6 | 1 | 450 | 0 | LTR7 | ERV1 | LTR |
| 170133800 | 170134041 | - | 11.7 | 1 | 243 | -112 | THE1A | MaLR | LTR |

  

|  |  |  |  |  |  |  |  |  |  |
| --- | --- | --- | --- | --- | --- | --- | --- | --- | --- |
| chr3:171882109-171889955 | | | 7840 bp | | |  |  |  |  |
| Genome Start | Genome End | Strand | Div | Repeat Start | Repeat End | Repeat Left | Name | Family | Class |
| 171882109 | 171882239 | - | 12.2 | 162 | 292 | -20 | AluJo | Alu | SINE |
| 171882241 | 171882740 | - | 5.6 | 1 | 448 | -2 | LTR7 | ERV1 | LTR |
| 171882741 | 171884842 | - | 6.2 | 5600 | 7713 | 0 | HERVH | ERV1 | LTR |
| 171884844 | 171885506 | - | 13.9 | 4488 | 5149 | -2564 | HERVH | ERV1 | LTR |
| 171885509 | 171886011 | - | 12.4 | 3492 | 3993 | -3720 | HERVH | ERV1 | LTR |
| 171886002 | 171886147 | - | 13.0 | 3140 | 3290 | -4423 | HERVH | ERV1 | LTR |
| 171886142 | 171889287 | - | 9.6 | 1 | 2987 | -4726 | HERVH | ERV1 | LTR |
| 171889290 | 171889789 | - | 5.8 | 1 | 448 | -2 | LTR7 | ERV1 | LTR |
| 171889807 | 171889955 | - | 17.4 | 3 | 155 | -157 | AluJo | Alu | SINE |

  

|  |  |  |  |  |  |  |  |  |  |
| --- | --- | --- | --- | --- | --- | --- | --- | --- | --- |
| chr3:172118138-172123447 | | | 5303 bp | | |  |  |  |  |
| Genome Start | Genome End | Strand | Div | Repeat Start | Repeat End | Repeat Left | Name | Family | Class |
| 172118138 | 172118413 | + | 9.3 | 3868 | 4139 | -4016 | L1PREC2 | L1 | LINE |
| 172118414 | 172118767 | + | 11.9 | 1 | 363 | -1 | THE1B | MaLR | LTR |
| 172118769 | 172120295 | + | 9.9 | 1 | 1579 | -1 | THE1B-int | MaLR | LTR |
| 172120297 | 172120655 | + | 12.4 | 1 | 358 | -6 | THE1B | MaLR | LTR |
| 172120661 | 172123447 | + | 9.2 | 4134 | 6986 | -1155 | L1PREC2 | L1 | LINE |

  

|  |  |  |  |  |  |  |  |  |  |
| --- | --- | --- | --- | --- | --- | --- | --- | --- | --- |
| chr3:173441719-173444377 | | | 2652 bp | | |  |  |  |  |
| Genome Start | Genome End | Strand | Div | Repeat Start | Repeat End | Repeat Left | Name | Family | Class |
| 173441719 | 173441885 | - | 12.9 | 228 | 390 | 0 | MLT1B | MaLR | LTR |
| 173441886 | 173442241 | - | 14.4 | 1 | 364 | 0 | THE1B | MaLR | LTR |
| 173442249 | 173443798 | - | 9.1 | 1 | 1573 | -7 | THE1B-int | MaLR | LTR |
| 173443799 | 173444154 | - | 11.8 | 1 | 364 | 0 | THE1B | MaLR | LTR |
| 173444155 | 173444377 | - | 20.6 | 1 | 232 | -158 | MLT1B | MaLR | LTR |

  

|  |  |  |  |  |  |  |  |  |  |
| --- | --- | --- | --- | --- | --- | --- | --- | --- | --- |
| chr3:173777545-173781092 | | | 3540 bp | | |  |  |  |  |
| Genome Start | Genome End | Strand | Div | Repeat Start | Repeat End | Repeat Left | Name | Family | Class |
| 173777545 | 173777720 | - | 18.3 | 822 | 996 | -5388 | L1M4c | L1 | LINE |
| 173777721 | 173778087 | + | 16.2 | 1 | 364 | 0 | THE1B | MaLR | LTR |
| 173778088 | 173778688 | - | 18.3 | 171 | 821 | -5563 | L1M4c | L1 | LINE |
| 173778693 | 173779072 | + | 11.5 | 1 | 381 | 0 | THE1D | MaLR | LTR |
| 173779073 | 173780565 | + | 11.6 | 1 | 1580 | 0 | THE1D-int | MaLR | LTR |
| 173780566 | 173780948 | + | 11.3 | 1 | 381 | 0 | THE1D | MaLR | LTR |
| 173780953 | 173781092 | - | 15.7 | 23 | 167 | -6217 | L1M4c | L1 | LINE |

  

|  |  |  |  |  |  |  |  |  |  |
| --- | --- | --- | --- | --- | --- | --- | --- | --- | --- |
| chr3:174272279-174275649 | | | 3369 bp | | |  |  |  |  |
| Genome Start | Genome End | Strand | Div | Repeat Start | Repeat End | Repeat Left | Name | Family | Class |
| 174272279 | 174272871 | + | 13.8 | 5370 | 5962 | -204 | L1PA16 | L1 | LINE |
| 174272872 | 174273234 | + | 15.3 | 1 | 364 | 0 | THE1B | MaLR | LTR |
| 174273237 | 174274801 | + | 29.5 | 2 | 1735 | 0 | MLT-int | MaLR | LTR |
| 174274802 | 174275167 | + | 11.7 | 1 | 364 | 0 | THE1B | MaLR | LTR |
| 174275168 | 174275240 | + | 13.8 | 5963 | 6032 | -134 | L1PA16 | L1 | LINE |
| 174275241 | 174275506 | + | 13.5 | 42 | 312 | 0 | AluSx | Alu | SINE |
| 174275507 | 174275649 | + | 13.8 | 6033 | 6166 | 0 | L1PA16 | L1 | LINE |

  

|  |  |  |  |  |  |  |  |  |  |
| --- | --- | --- | --- | --- | --- | --- | --- | --- | --- |
| chr3:174343041-174362375 | | | 18864 bp | | |  |  |  |  |
| Genome Start | Genome End | Strand | Div | Repeat Start | Repeat End | Repeat Left | Name | Family | Class |
| 174343041 | 174344608 | - | 22.7 | 4496 | 6117 | -32 | L1ME1 | L1 | LINE |
| 174344610 | 174346522 | - | 13.4 | 457 | 2418 | 0 | Tigger1 | MER2\_type | DNA |
| 174346526 | 174347410 | + | 21.4 | 19 | 929 | -6636 | L1M1 | L1 | LINE |
| 174347406 | 174347790 | - | 19.2 | 983 | 1400 | -6165 | L1M1 | L1 | LINE |
| 174347788 | 174352421 | + | 8.8 | 2978 | 7721 | 0 | L1MA1 | L1 | LINE |
| 174352439 | 174352901 | - | 20.1 | 1 | 475 | -1943 | Tigger1 | MER2\_type | DNA |
| 174352930 | 174353591 | - | 23.2 | 3803 | 4468 | -1678 | L1MD | L1 | LINE |
| 174353602 | 174354130 | - | 24.4 | 2783 | 3447 | -2682 | L1ME1 | L1 | LINE |
| 174354141 | 174354188 | + | 25.1 | 1 | 48 | -141 | MER5A | MER1\_type | DNA |
| 174354189 | 174354431 | + | 20.7 | 1 | 243 | -224 | MLT1C | MaLR | LTR |
| 174354432 | 174354727 | - | 20.6 | 1 | 301 | -11 | AluJb | Alu | SINE |
| 174354728 | 174354964 | + | 20.7 | 244 | 467 | 0 | MLT1C | MaLR | LTR |
| 174354965 | 174355107 | + | 25.1 | 49 | 188 | -1 | MER5A | MER1\_type | DNA |
| 174355109 | 174355479 | - | 28.7 | 2409 | 2779 | -3350 | L1ME1 | L1 | LINE |
| 174355702 | 174356115 | - | 9.3 | 1 | 448 | -2 | LTR7 | ERV1 | LTR |
| 174356116 | 174356492 | - | 8.3 | 7324 | 7713 | 0 | HERVH | ERV1 | LTR |
| 174356493 | 174356712 | - | 12.3 | 5600 | 5819 | -1894 | HERVH | ERV1 | LTR |
| 174356714 | 174357941 | - | 12.3 | 3953 | 5149 | -2564 | HERVH | ERV1 | LTR |
| 174357931 | 174358777 | - | 10.3 | 3140 | 3993 | -3720 | HERVH | ERV1 | LTR |
| 174358774 | 174361608 | - | 12.3 | 1 | 2985 | -4728 | HERVH | ERV1 | LTR |
| 174361611 | 174362028 | - | 10.0 | 1 | 448 | -2 | LTR7 | ERV1 | LTR |
| 174362228 | 174362375 | - | 27.8 | 41 | 186 | -5933 | L1ME1 | L1 | LINE |

  

|  |  |  |  |  |  |  |  |  |  |
| --- | --- | --- | --- | --- | --- | --- | --- | --- | --- |
| chr3:174397238-174402420 | | | 4947 bp | | |  |  |  |  |
| Genome Start | Genome End | Strand | Div | Repeat Start | Repeat End | Repeat Left | Name | Family | Class |
| 174397238 | 174397348 | - | 24.3 | 4359 | 4469 | -467 | MER39-int | ERV1 | LTR |
| 174397405 | 174397491 | - | 23.5 | 4 | 100 | -326 | LTR37A | ERV1 | LTR |
| 174397515 | 174397630 | - | 21.0 | 4093 | 4209 | -727 | MER39-int | ERV1 | LTR |
| 174397631 | 174398027 | - | 13.3 | 1 | 428 | 0 | MSTA | MaLR | LTR |
| 174398028 | 174398660 | - | 21.0 | 3491 | 4092 | -844 | MER39-int | ERV1 | LTR |
| 174398669 | 174398720 | - | 19.2 | 381 | 432 | 0 | MSTB1 | MaLR | LTR |
| 174398712 | 174398810 | - | 15.2 | 2 | 107 | -1544 | MSTB-int | MaLR | LTR |
| 174398812 | 174399223 | - | 19.7 | 1 | 426 | 0 | MSTB | MaLR | LTR |
| 174399224 | 174400901 | - | 19.0 | 1 | 1611 | -40 | MSTB-int | MaLR | LTR |
| 174400902 | 174401312 | - | 18.4 | 1 | 426 | 0 | MSTB | MaLR | LTR |
| 174401325 | 174402016 | - | 21.5 | 331 | 1007 | -3929 | MER39-int | ERV1 | LTR |
| 174402098 | 174402199 | - | 27.4 | 200 | 306 | -4630 | MER39-int | ERV1 | LTR |
| 174402264 | 174402420 | - | 23.4 | 20 | 173 | -4763 | MER39-int | ERV1 | LTR |

  

|  |  |  |  |  |  |  |  |  |  |
| --- | --- | --- | --- | --- | --- | --- | --- | --- | --- |
| chr3:174452191-174456607 | | | 4340 bp | | |  |  |  |  |
| Genome Start | Genome End | Strand | Div | Repeat Start | Repeat End | Repeat Left | Name | Family | Class |
| 174452191 | 174452432 | - | 24.1 | 265 | 519 | -2 | MER113 | MER1\_type | DNA |
| 174452433 | 174452847 | - | 15.3 | 3 | 428 | 0 | MSTA | MaLR | LTR |
| 174452850 | 174453678 | - | 20.4 | 820 | 1651 | 0 | MSTA-int | MaLR | LTR |
| 174453679 | 174453979 | - | 9.4 | 1 | 301 | -10 | AluY | Alu | SINE |
| 174453980 | 174454341 | - | 20.4 | 470 | 819 | -832 | MSTA-int | MaLR | LTR |
| 174454375 | 174455550 | - | 2.6 | 4970 | 6145 | -10 | L1PA3 | L1 | LINE |
| 174455551 | 174456013 | - | 13.6 | 1 | 465 | -1186 | MSTA-int | MaLR | LTR |
| 174456014 | 174456422 | - | 14.7 | 1 | 428 | 0 | MSTA | MaLR | LTR |
| 174456465 | 174456607 | - | 26.8 | 66 | 218 | -303 | MER113 | MER1\_type | DNA |

  

|  |  |  |  |  |  |  |  |  |  |
| --- | --- | --- | --- | --- | --- | --- | --- | --- | --- |
| chr3:174621773-174633623 | | | 11416 bp | | |  |  |  |  |
| Genome Start | Genome End | Strand | Div | Repeat Start | Repeat End | Repeat Left | Name | Family | Class |
| 174621773 | 174621995 | - | 27.3 | 7665 | 7896 | -146 | L1MC4 | L1 | LINE |
| 174621996 | 174622304 | - | 16.4 | 1 | 297 | -15 | AluJb | Alu | SINE |
| 174622305 | 174622402 | - | 27.3 | 7565 | 7664 | -378 | L1MC4 | L1 | LINE |
| 174622404 | 174622532 | - | 18.8 | 6167 | 6295 | -5 | L1MA5 | L1 | LINE |
| 174622613 | 174622630 | + | 14.4 | 5776 | 5792 | -508 | L1MA4 | L1 | LINE |
| 174622656 | 174622852 | + | 14.4 | 5793 | 5991 | -309 | L1MA4 | L1 | LINE |
| 174622882 | 174623380 | - | 16.7 | 5397 | 5920 | -562 | L1M1 | L1 | LINE |
| 174623381 | 174623768 | - | 13.0 | 1 | 381 | 0 | THE1D | MaLR | LTR |
| 174623769 | 174625407 | - | 30.3 | 3 | 1735 | 0 | MLT-int | MaLR | LTR |
| 174625411 | 174625776 | - | 13.8 | 1 | 381 | 0 | THE1D | MaLR | LTR |
| 174625777 | 174627107 | - | 17.3 | 4032 | 5396 | -929 | L1M1 | L1 | LINE |
| 174627131 | 174630851 | - | 20.4 | 15 | 4031 | -2294 | L1M1 | L1 | LINE |
| 174630865 | 174631063 | - | 22.3 | 7245 | 7490 | -318 | L1MC4a | L1 | LINE |
| 174631064 | 174631463 | + | 20.0 | 1 | 432 | 0 | MSTB1 | MaLR | LTR |
| 174631464 | 174631485 | - | 22.3 | 7224 | 7244 | -564 | L1MC4a | L1 | LINE |
| 174631509 | 174631788 | - | 25.7 | 6806 | 7089 | -953 | L1MC4 | L1 | LINE |
| 174632044 | 174633324 | - | 18.1 | 5029 | 6322 | -11 | L1MC1 | L1 | LINE |
| 174633308 | 174633623 | - | 24.4 | 6467 | 6830 | -1212 | L1MC4 | L1 | LINE |

  

|  |  |  |  |  |  |  |  |  |  |
| --- | --- | --- | --- | --- | --- | --- | --- | --- | --- |
| chr3:175586024-175591810 | | | 5406 bp | | |  |  |  |  |
| Genome Start | Genome End | Strand | Div | Repeat Start | Repeat End | Repeat Left | Name | Family | Class |
| 175586024 | 175586280 | - | 30.8 | 4746 | 5005 | -1214 | L1ME1 | L1 | LINE |
| 175586501 | 175586708 | - | 20.4 | 163 | 365 | 0 | MLT1A0 | MaLR | LTR |
| 175586709 | 175587052 | - | 12.4 | 1 | 364 | 0 | THE1B | MaLR | LTR |
| 175587053 | 175587218 | - | 20.4 | 1 | 162 | -203 | MLT1A0 | MaLR | LTR |
| 175587276 | 175588520 | - | 25.6 | 1655 | 2902 | -3317 | L1ME1 | L1 | LINE |
| 175588610 | 175589022 | - | 16.1 | 5 | 426 | -2 | MSTA | MaLR | LTR |
| 175589028 | 175589223 | - | 17.6 | 1456 | 1646 | -5 | MSTA-int | MaLR | LTR |
| 175589224 | 175589607 | - | 12.7 | 1 | 381 | 0 | THE1D | MaLR | LTR |
| 175589608 | 175591028 | - | 17.6 | 1 | 1455 | -196 | MSTA-int | MaLR | LTR |
| 175591035 | 175591449 | - | 12.5 | 1 | 418 | -10 | MSTA | MaLR | LTR |
| 175591458 | 175591693 | + | 32.5 | 159 | 414 | -5986 | L1MEc | L1 | LINE |
| 175591690 | 175591810 | - | 25.2 | 176 | 305 | -5904 | L1ME1 | L1 | LINE |

  

|  |  |  |  |  |  |  |  |  |  |
| --- | --- | --- | --- | --- | --- | --- | --- | --- | --- |
| chr3:175995900-176002282 | | | 6249 bp | | |  |  |  |  |
| Genome Start | Genome End | Strand | Div | Repeat Start | Repeat End | Repeat Left | Name | Family | Class |
| 175995900 | 175996410 | - | 21.5 | 5256 | 5779 | -405 | L1M4 | L1 | LINE |
| 175996418 | 175996696 | + | 20.5 | 1 | 272 | -118 | MLT1B | MaLR | LTR |
| 175996721 | 175997210 | - | 16.7 | 5649 | 6143 | -13 | L1PB4 | L1 | LINE |
| 175997223 | 175997783 | - | 18.2 | 4815 | 5398 | -748 | L1P4 | L1 | LINE |
| 175997784 | 175998007 | - | 9.6 | 94 | 312 | 0 | AluSg/x | Alu | SINE |
| 175998008 | 175998193 | - | 18.2 | 4636 | 4814 | -1332 | L1P4 | L1 | LINE |
| 175998195 | 175998253 | + | 15.2 | 285 | 344 | -46 | MLT1B | MaLR | LTR |
| 175998259 | 175998744 | - | 21.3 | 4742 | 5248 | -898 | L1M4 | L1 | LINE |
| 175998828 | 175999371 | - | 18.6 | 4191 | 4737 | -1409 | L1M4 | L1 | LINE |
| 175999372 | 175999669 | + | 11.7 | 1 | 298 | -14 | AluSx | Alu | SINE |
| 175999670 | 175999775 | - | 18.6 | 4088 | 4190 | -1956 | L1M4 | L1 | LINE |
| 175999776 | 176000129 | + | 13.6 | 1 | 364 | 0 | THE1B | MaLR | LTR |
| 176000130 | 176001661 | + | 10.6 | 1 | 1580 | 0 | THE1B-int | MaLR | LTR |
| 176001664 | 176002013 | + | 11.7 | 3 | 362 | -2 | THE1B | MaLR | LTR |
| 176002014 | 176002282 | - | 16.6 | 3846 | 4094 | -2052 | L1M4 | L1 | LINE |

  

|  |  |  |  |  |  |  |  |  |  |
| --- | --- | --- | --- | --- | --- | --- | --- | --- | --- |
| chr3:177133205-177149871 | | | 16182 bp | | |  |  |  |  |
| Genome Start | Genome End | Strand | Div | Repeat Start | Repeat End | Repeat Left | Name | Family | Class |
| 177133205 | 177133349 | + | 29.2 | 3 | 157 | -105 | MIR | MIR | SINE |
| 177133385 | 177135171 | + | 23.6 | 1 | 1911 | -4840 | L1MCb | L1 | LINE |
| 177135195 | 177135405 | + | 23.1 | 1912 | 2126 | -4625 | L1MCb | L1 | LINE |
| 177135514 | 177135914 | + | 12.8 | 1 | 428 | 0 | MSTA | MaLR | LTR |
| 177135917 | 177137487 | + | 27.8 | 2 | 1735 | 0 | MLT-int | MaLR | LTR |
| 177137488 | 177137674 | + | 16.2 | 1 | 199 | -229 | MSTA | MaLR | LTR |
| 177137675 | 177139160 | + | 8.3 | 2 | 1578 | 0 | LTR12C | ERV1 | LTR |
| 177139161 | 177139381 | + | 16.2 | 200 | 428 | 0 | MSTA | MaLR | LTR |
| 177139480 | 177140457 | + | 22.0 | 2380 | 3380 | -3184 | L1MCb | L1 | LINE |
| 177140468 | 177141900 | + | 35.0 | 1 | 1386 | -5464 | L1PA15-16 | L1 | LINE |
| 177141891 | 177146267 | + | 23.1 | 1275 | 5553 | -1296 | L1PA15-16 | L1 | LINE |
| 177146277 | 177147003 | + | 19.6 | 6875 | 7650 | -135 | L1MC3 | L1 | LINE |
| 177147004 | 177147366 | - | 11.8 | 1 | 381 | 0 | THE1D | MaLR | LTR |
| 177147367 | 177147384 | + | 19.6 | 7651 | 7663 | -122 | L1MC3 | L1 | LINE |
| 177147385 | 177147882 | + | 23.9 | 1 | 478 | -42 | MLT2B1 | ERVL | LTR |
| 177148033 | 177149618 | + | 13.9 | 4573 | 6158 | -7 | L1PA15 | L1 | LINE |
| 177149652 | 177149744 | + | 17.6 | 7695 | 7785 | 0 | L1MC3 | L1 | LINE |
| 177149772 | 177149871 | + | 27.3 | 162 | 262 | 0 | MIR | MIR | SINE |

  

|  |  |  |  |  |  |  |  |  |  |
| --- | --- | --- | --- | --- | --- | --- | --- | --- | --- |
| chr3:177424394-177433767 | | | 9349 bp | | |  |  |  |  |
| Genome Start | Genome End | Strand | Div | Repeat Start | Repeat End | Repeat Left | Name | Family | Class |
| 177424394 | 177427415 | + | 24.5 | 2351 | 5429 | -2130 | L1MA9 | L1 | LINE |
| 177427421 | 177427833 | + | 18.1 | 1 | 428 | 0 | MSTA | MaLR | LTR |
| 177427838 | 177429470 | + | 18.0 | 11 | 1651 | 0 | MSTA-int | MaLR | LTR |
| 177429473 | 177429880 | + | 13.9 | 3 | 426 | -2 | MSTA | MaLR | LTR |
| 177429884 | 177430022 | + | 21.7 | 5432 | 5571 | -1988 | L1MA9 | L1 | LINE |
| 177430032 | 177430788 | - | 16.6 | 1588 | 2346 | -72 | Tigger1 | MER2\_type | DNA |
| 177430789 | 177431086 | - | 13.4 | 1 | 298 | -12 | AluSg | Alu | SINE |
| 177431087 | 177432688 | - | 16.6 | 5 | 1587 | -831 | Tigger1 | MER2\_type | DNA |
| 177432691 | 177433767 | + | 20.7 | 6651 | 7693 | -38 | L1MA9 | L1 | LINE |

  

|  |  |  |  |  |  |  |  |  |  |
| --- | --- | --- | --- | --- | --- | --- | --- | --- | --- |
| chr3:178089435-178095669 | | | 6228 bp | | |  |  |  |  |
| Genome Start | Genome End | Strand | Div | Repeat Start | Repeat End | Repeat Left | Name | Family | Class |
| 178089435 | 178091351 | + | 11.0 | 2509 | 4450 | -1696 | L1PB1 | L1 | LINE |
| 178091352 | 178091653 | + | 11.7 | 1 | 299 | -65 | THE1B | MaLR | LTR |
| 178091654 | 178091962 | - | 6.8 | 1 | 309 | -2 | AluY | Alu | SINE |
| 178091963 | 178092039 | + | 11.7 | 300 | 364 | 0 | THE1B | MaLR | LTR |
| 178092040 | 178093609 | + | 10.1 | 1 | 1567 | -13 | THE1B-int | MaLR | LTR |
| 178093616 | 178093980 | + | 12.6 | 1 | 362 | -2 | THE1B | MaLR | LTR |
| 178093982 | 178095669 | + | 7.2 | 4445 | 6150 | -1 | L1PB1 | L1 | LINE |

  

|  |  |  |  |  |  |  |  |  |  |
| --- | --- | --- | --- | --- | --- | --- | --- | --- | --- |
| chr3:178956944-178960565 | | | 3257 bp | | |  |  |  |  |
| Genome Start | Genome End | Strand | Div | Repeat Start | Repeat End | Repeat Left | Name | Family | Class |
| 178956944 | 178957025 | - | 37.8 | 179 | 261 | -7 | MIRb | MIR | SINE |
| 178957323 | 178957720 | - | 13.0 | 1 | 428 | 0 | MSTA | MaLR | LTR |
| 178957721 | 178959352 | - | 18.5 | 6 | 1651 | 0 | MSTA-int | MaLR | LTR |
| 178959357 | 178959574 | - | 12.6 | 212 | 428 | 0 | MSTA | MaLR | LTR |
| 178959575 | 178960204 | + | 11.1 | 3 | 632 | -2 | MER41B | ERV1 | LTR |
| 178960205 | 178960394 | - | 12.6 | 2 | 211 | -217 | MSTA | MaLR | LTR |
| 178960459 | 178960565 | - | 32.7 | 82 | 187 | -81 | MIRb | MIR | SINE |

  

|  |  |  |  |  |  |  |  |  |  |
| --- | --- | --- | --- | --- | --- | --- | --- | --- | --- |
| chr3:179891733-179900494 | | | 8760 bp | | |  |  |  |  |
| Genome Start | Genome End | Strand | Div | Repeat Start | Repeat End | Repeat Left | Name | Family | Class |
| 179891733 | 179894480 | - | 11.6 | 3418 | 6160 | -3 | L1PA13 | L1 | LINE |
| 179894481 | 179894839 | - | 15.9 | 1 | 375 | 0 | THE1C | MaLR | LTR |
| 179894840 | 179896431 | - | 30.8 | 2 | 1735 | 0 | MSTD-int | MaLR | LTR |
| 179896434 | 179896809 | - | 12.7 | 1 | 375 | 0 | THE1C | MaLR | LTR |
| 179896810 | 179900494 | - | 15.6 | 164 | 4037 | -2729 | L1PA13 | L1 | LINE |

  

|  |  |  |  |  |  |  |  |  |  |
| --- | --- | --- | --- | --- | --- | --- | --- | --- | --- |
| chr3:180715649-180726899 | | | 11412 bp | | |  |  |  |  |
| Genome Start | Genome End | Strand | Div | Repeat Start | Repeat End | Repeat Left | Name | Family | Class |
| 180715649 | 180715786 | - | 20.3 | 123 | 266 | -2 | MIR | MIR | SINE |
| 180715787 | 180716174 | - | 11.4 | 1 | 450 | 0 | LTR7 | ERV1 | LTR |
| 180716175 | 180716609 | - | 9.8 | 7282 | 7713 | 0 | HERVH | ERV1 | LTR |
| 180716613 | 180717550 | - | 11.5 | 4070 | 5020 | -2693 | HERVH | ERV1 | LTR |
| 180717551 | 180717961 | + | 7.8 | 1 | 448 | -2 | LTR7 | ERV1 | LTR |
| 180717849 | 180717963 | + | 6.1 | 337 | 450 | 0 | LTR7A | ERV1 | LTR |
| 180717965 | 180720749 | + | 11.8 | 1 | 2992 | -4721 | HERVH | ERV1 | LTR |
| 180720737 | 180720885 | + | 14.1 | 3141 | 3290 | -4423 | HERVH | ERV1 | LTR |
| 180720876 | 180721380 | + | 11.6 | 3492 | 3993 | -3720 | HERVH | ERV1 | LTR |
| 180721383 | 180721965 | + | 11.9 | 4488 | 5149 | -2564 | HERVH | ERV1 | LTR |
| 180721967 | 180722562 | + | 10.8 | 5600 | 7713 | 0 | HERVH | ERV1 | LTR |
| 180722563 | 180722964 | + | 8.8 | 1 | 448 | -2 | LTR7 | ERV1 | LTR |
| 180722968 | 180723125 | - | 17.1 | 2947 | 4073 | -998 | HERVH | ERV1 | LTR |
| 180723112 | 180723298 | - | 8.6 | 3806 | 3993 | -3720 | HERVH | ERV1 | LTR |
| 180723293 | 180723737 | - | 16.4 | 3140 | 3587 | -4126 | HERVH | ERV1 | LTR |
| 180723717 | 180726440 | - | 15.8 | 1 | 2995 | -4718 | HERVH | ERV1 | LTR |
| 180726441 | 180726830 | - | 8.8 | 1 | 450 | 0 | LTR7 | ERV1 | LTR |
| 180726837 | 180726899 | - | 28.6 | 57 | 120 | -142 | MIR | MIR | SINE |

  

|  |  |  |  |  |  |  |  |  |  |
| --- | --- | --- | --- | --- | --- | --- | --- | --- | --- |
| chr3:181277863-181287767 | | | 9860 bp | | |  |  |  |  |
| Genome Start | Genome End | Strand | Div | Repeat Start | Repeat End | Repeat Left | Name | Family | Class |
| 181277863 | 181277956 | + | 23.5 | 338 | 430 | -5372 | L1MCb | L1 | LINE |
| 181277957 | 181278378 | - | 13.3 | 1 | 428 | 0 | MSTA | MaLR | LTR |
| 181278379 | 181279962 | - | 29.2 | 2 | 1735 | 0 | MLT-int | MaLR | LTR |
| 181279965 | 181280340 | - | 15.7 | 1 | 428 | 0 | MSTA | MaLR | LTR |
| 181280341 | 181280982 | + | 21.6 | 431 | 1156 | -5352 | L1MCb | L1 | LINE |
| 181281011 | 181281992 | + | 16.9 | 3957 | 4966 | -1180 | L1MC1 | L1 | LINE |
| 181281993 | 181282393 | - | 17.8 | 1 | 430 | -2 | MSTB1 | MaLR | LTR |
| 181282394 | 181283746 | + | 17.1 | 4967 | 6332 | -1 | L1MC1 | L1 | LINE |
| 181283742 | 181284351 | + | 21.2 | 1172 | 1813 | -4695 | L1MCb | L1 | LINE |
| 181284356 | 181284640 | + | 5.6 | 1 | 297 | -14 | AluY | Alu | SINE |
| 181284644 | 181284977 | + | 24.1 | 1 | 408 | 0 | MLT1A1 | MaLR | LTR |
| 181284978 | 181285947 | + | 23.4 | 1 | 965 | -770 | MLT1A1-int | MaLR | LTR |
| 181285948 | 181286303 | + | 14.6 | 1 | 364 | 0 | THE1B | MaLR | LTR |
| 181286304 | 181287033 | + | 21.8 | 963 | 1735 | 0 | MLT1A1-int | MaLR | LTR |
| 181287038 | 181287387 | + | 18.1 | 5 | 408 | 0 | MLT1A1 | MaLR | LTR |
| 181287397 | 181287767 | + | 24.9 | 1938 | 2339 | -4257 | L1MCb | L1 | LINE |

  

|  |  |  |  |  |  |  |  |  |  |
| --- | --- | --- | --- | --- | --- | --- | --- | --- | --- |
| chr3:181428783-181435991 | | | 7213 bp | | |  |  |  |  |
| Genome Start | Genome End | Strand | Div | Repeat Start | Repeat End | Repeat Left | Name | Family | Class |
| 181428783 | 181429014 | + | 20.6 | 226 | 454 | -4763 | MER61C-int | ERV1 | LTR |
| 181429034 | 181429455 | + | 11.8 | 422 | 841 | -4376 | MER61C-int | ERV1 | LTR |
| 181429456 | 181430132 | + | 12.4 | 959 | 1640 | -3577 | MER61C-int | ERV1 | LTR |
| 181430112 | 181431699 | + | 11.0 | 1972 | 3572 | -1645 | MER61C-int | ERV1 | LTR |
| 181431694 | 181431934 | + | 11.4 | 3446 | 3685 | -1532 | MER61-int | ERV1 | LTR |
| 181431935 | 181432229 | + | 10.7 | 1 | 295 | -18 | AluSp | Alu | SINE |
| 181432230 | 181433301 | + | 11.4 | 3686 | 4742 | -475 | MER61-int | ERV1 | LTR |
| 181433302 | 181433645 | - | 10.9 | 1 | 364 | 0 | THE1B | MaLR | LTR |
| 181433646 | 181435213 | - | 30.8 | 2 | 1735 | 0 | MLT1J-int | MaLR | LTR |
| 181435218 | 181435563 | - | 10.5 | 1 | 362 | -2 | THE1B | MaLR | LTR |
| 181435564 | 181435991 | + | 11.4 | 4743 | 5217 | 0 | MER61C-int | ERV1 | LTR |

  

|  |  |  |  |  |  |  |  |  |  |
| --- | --- | --- | --- | --- | --- | --- | --- | --- | --- |
| chr3:184366523-184372937 | | | 6004 bp | | |  |  |  |  |
| Genome Start | Genome End | Strand | Div | Repeat Start | Repeat End | Repeat Left | Name | Family | Class |
| 184366523 | 184366907 | - | 25.6 | 3866 | 4193 | -2748 | L1MCc | L1 | LINE |
| 184366908 | 184367188 | + | 7.8 | 28 | 308 | -5 | AluSq | Alu | SINE |
| 184367189 | 184367515 | - | 26.4 | 3518 | 3865 | -3076 | L1MCc | L1 | LINE |
| 184367523 | 184367642 | - | 15.3 | 267 | 387 | -3 | MLT1B | MaLR | LTR |
| 184367643 | 184367954 | - | 11.9 | 1 | 312 | 0 | AluSx | Alu | SINE |
| 184367955 | 184368168 | - | 15.3 | 43 | 266 | -124 | MLT1B | MaLR | LTR |
| 184368169 | 184368527 | - | 8.9 | 1 | 355 | 0 | THE1A | MaLR | LTR |
| 184368528 | 184369955 | - | 8.1 | 1 | 1580 | 0 | THE1A-int | MaLR | LTR |
| 184369956 | 184370309 | - | 4.8 | 1 | 355 | 0 | THE1A | MaLR | LTR |
| 184370311 | 184370356 | - | 10.9 | 1 | 46 | -421 | MLT1B | MaLR | LTR |
| 184370358 | 184370462 | - | 21.4 | 3423 | 3521 | -3420 | L1MCc | L1 | LINE |
| 184370463 | 184371489 | + | 5.3 | 1 | 1033 | 0 | LTR5A | ERVK | LTR |
| 184371490 | 184371508 | - | 21.4 | 3405 | 3422 | -3519 | L1MCc | L1 | LINE |
| 184371521 | 184371721 | + | 19.4 | 12 | 220 | -2103 | Tigger5 | MER2\_type | DNA |
| 184371718 | 184371933 | + | 15.3 | 2141 | 2357 | 0 | Tigger5 | MER2\_type | DNA |
| 184371934 | 184371978 | - | 21.1 | 132 | 176 | -123 | AluSc | Alu | SINE |
| 184371979 | 184372100 | - | 22.1 | 1 | 125 | -177 | AluJo/FLAM | Alu | SINE |
| 184372102 | 184372185 | - | 20.7 | 2636 | 2721 | -4401 | L1MCc | L1 | LINE |
| 184372579 | 184372645 | - | 19.4 | 2547 | 2614 | -4508 | L1MCc | L1 | LINE |
| 184372646 | 184372937 | - | 28.7 | 854 | 1179 | -5244 | L1MCc | L1 | LINE |

  

|  |  |  |  |  |  |  |  |  |  |
| --- | --- | --- | --- | --- | --- | --- | --- | --- | --- |
| chr3:184613084-184623399 | | | 10313 bp | | |  |  |  |  |
| Genome Start | Genome End | Strand | Div | Repeat Start | Repeat End | Repeat Left | Name | Family | Class |
| 184613084 | 184613634 | - | 12.5 | 4581 | 5128 | -1878 | L1M4b | L1 | LINE |
| 184613635 | 184613873 | - | 12.9 | 2463 | 2707 | -1 | Tigger2 | MER2\_type | DNA |
| 184613874 | 184614278 | + | 11.6 | 1 | 437 | 0 | LTR21B | ERV1 | LTR |
| 184614279 | 184614487 | - | 12.9 | 1 | 1725 | 0 | Tigger2a | MER2\_type | DNA |
| 184614488 | 184615671 | - | 16.7 | 3389 | 4583 | -2423 | L1M4 | L1 | LINE |
| 184615672 | 184615968 | - | 9.2 | 1 | 295 | -17 | AluSx | Alu | SINE |
| 184615969 | 184617060 | - | 17.2 | 2629 | 3704 | -3618 | L1M4b | L1 | LINE |
| 184617061 | 184617129 | + | 20.5 | 1 | 68 | -2255 | Tigger5 | MER2\_type | DNA |
| 184617130 | 184617309 | + | 19.4 | 2 | 207 | -138 | MER2 | MER2\_type | DNA |
| 184617310 | 184617627 | + | 9.7 | 1 | 309 | 0 | AluSc | Alu | SINE |
| 184617628 | 184617778 | + | 19.4 | 208 | 345 | 0 | MER2 | MER2\_type | DNA |
| 184617779 | 184617800 | + | 20.5 | 69 | 88 | -2235 | Tigger5 | MER2\_type | DNA |
| 184617801 | 184617818 | - | 17.4 | 2629 | 2628 | -4706 | L1M4b | L1 | LINE |
| 184617819 | 184617914 | - | 20.8 | 1 | 96 | -1 | Tigger5c | MER2\_type | DNA |
| 184617915 | 184618176 | - | 17.4 | 2309 | 2628 | -4706 | L1M4b | L1 | LINE |
| 184618177 | 184618407 | - | 13.5 | 165 | 381 | 0 | THE1D | MaLR | LTR |
| 184618408 | 184618692 | + | 12.4 | 21 | 303 | -9 | AluSx | Alu | SINE |
| 184618693 | 184618850 | - | 13.5 | 1 | 164 | -217 | THE1D | MaLR | LTR |
| 184618851 | 184620072 | - | 28.4 | 443 | 1735 | 0 | MLT-int | MaLR | LTR |
| 184620073 | 184620373 | + | 12.6 | 1 | 301 | -11 | AluSx | Alu | SINE |
| 184620374 | 184620758 | - | 28.4 | 3 | 442 | -1293 | MLT-int | MaLR | LTR |
| 184620762 | 184620801 | - | 14.3 | 347 | 381 | 0 | THE1D | MaLR | LTR |
| 184620802 | 184621094 | + | 10.3 | 1 | 294 | -16 | AluSg | Alu | SINE |
| 184621095 | 184621434 | - | 14.3 | 1 | 346 | -35 | THE1D | MaLR | LTR |
| 184621435 | 184621941 | - | 17.9 | 1675 | 2308 | -5026 | L1M4b | L1 | LINE |
| 184621942 | 184622064 | - | 15.4 | 2 | 137 | -175 | AluJo | Alu | SINE |
| 184622065 | 184623399 | - | 17.9 | 5 | 1674 | -5660 | L1M4b | L1 | LINE |

  

|  |  |  |  |  |  |  |  |  |  |
| --- | --- | --- | --- | --- | --- | --- | --- | --- | --- |
| chr3:188406212-188408800 | | | 2485 bp | | |  |  |  |  |
| Genome Start | Genome End | Strand | Div | Repeat Start | Repeat End | Repeat Left | Name | Family | Class |
| 188406212 | 188406403 | - | 16.8 | 5935 | 6154 | -29 | L1MB3 | L1 | LINE |
| 188406497 | 188406644 | - | 15.5 | 5761 | 5909 | -271 | L1MB3 | L1 | LINE |
| 188406646 | 188407068 | - | 13.5 | 1 | 428 | 0 | MSTC | MaLR | LTR |
| 188407075 | 188408630 | - | 25.0 | 2 | 1728 | -7 | MSTC-int | MaLR | LTR |
| 188408635 | 188408677 | - | 13.9 | 384 | 426 | -2 | MSTC | MaLR | LTR |
| 188408678 | 188408800 | - | 17.8 | 5647 | 5765 | -415 | L1MB3 | L1 | LINE |

  

|  |  |  |  |  |  |  |  |  |  |
| --- | --- | --- | --- | --- | --- | --- | --- | --- | --- |
| chr3:188687039-188697464 | | | 10408 bp | | |  |  |  |  |
| Genome Start | Genome End | Strand | Div | Repeat Start | Repeat End | Repeat Left | Name | Family | Class |
| 188687039 | 188687280 | + | 22.9 | 3086 | 3361 | -2785 | L1ME1 | L1 | LINE |
| 188687281 | 188687634 | + | 9.6 | 1 | 355 | 0 | THE1A | MaLR | LTR |
| 188687635 | 188688201 | + | 6.9 | 1 | 572 | -1008 | THE1A-int | MaLR | LTR |
| 188688210 | 188688423 | + | 10.8 | 1365 | 1580 | 0 | THE1A-int | MaLR | LTR |
| 188688424 | 188688778 | + | 10.1 | 1 | 355 | 0 | THE1A | MaLR | LTR |
| 188688780 | 188689987 | + | 27.5 | 3358 | 4613 | -1533 | L1ME1 | L1 | LINE |
| 188689988 | 188690297 | - | 13.2 | 1 | 306 | -6 | AluSx | Alu | SINE |
| 188690298 | 188690910 | + | 27.5 | 4614 | 5199 | -947 | L1ME1 | L1 | LINE |
| 188690914 | 188694067 | + | 16.7 | 1 | 2236 | -4627 | L1PBa | L1 | LINE |
| 188694069 | 188694114 | + | 4.3 | 2570 | 2615 | -4248 | L1PBa | L1 | LINE |
| 188694138 | 188694206 | + | 2.9 | 3246 | 3314 | -3548 | L1PBa | L1 | LINE |
| 188694188 | 188695782 | + | 12.4 | 3327 | 4906 | -1240 | L1PB2 | L1 | LINE |
| 188695771 | 188696515 | + | 10.8 | 5397 | 6151 | 0 | L1PB2 | L1 | LINE |
| 188696529 | 188697464 | + | 25.3 | 5204 | 6162 | -4 | L1ME1 | L1 | LINE |

  

|  |  |  |  |  |  |  |  |  |  |
| --- | --- | --- | --- | --- | --- | --- | --- | --- | --- |
| chr3:190959873-190962922 | | | 3048 bp | | |  |  |  |  |
| Genome Start | Genome End | Strand | Div | Repeat Start | Repeat End | Repeat Left | Name | Family | Class |
| 190959873 | 190960266 | - | 18.8 | 378 | 781 | 0 | MER4E1 | ERV1 | LTR |
| 190960267 | 190960632 | - | 14.0 | 1 | 364 | 0 | THE1B | MaLR | LTR |
| 190960633 | 190962196 | - | 31.6 | 2 | 1735 | 0 | MLT-int | MaLR | LTR |
| 190962199 | 190962560 | - | 12.1 | 1 | 364 | 0 | THE1B | MaLR | LTR |
| 190962561 | 190962922 | - | 18.8 | 1 | 377 | -404 | MER4E1 | ERV1 | LTR |

  

|  |  |  |  |  |  |  |  |  |  |
| --- | --- | --- | --- | --- | --- | --- | --- | --- | --- |
| chr3:191402702-191406218 | | | 3510 bp | | |  |  |  |  |
| Genome Start | Genome End | Strand | Div | Repeat Start | Repeat End | Repeat Left | Name | Family | Class |
| 191402702 | 191403318 | + | 35.0 | 4399 | 5031 | -1115 | L1M5 | L1 | LINE |
| 191403319 | 191403682 | + | 13.4 | 1 | 364 | 0 | THE1B | MaLR | LTR |
| 191403685 | 191405241 | + | 31.1 | 2 | 1735 | 0 | MLT1H-int | MaLR | LTR |
| 191405242 | 191405604 | + | 16.4 | 1 | 364 | 0 | THE1B | MaLR | LTR |
| 191405605 | 191405781 | + | 35.0 | 5032 | 5219 | -927 | L1M5 | L1 | LINE |
| 191405787 | 191406218 | + | 26.2 | 5259 | 5736 | -385 | L1M5 | L1 | LINE |

  

|  |  |  |  |  |  |  |  |  |  |
| --- | --- | --- | --- | --- | --- | --- | --- | --- | --- |
| chr3:191491293-191494498 | | | 3139 bp | | |  |  |  |  |
| Genome Start | Genome End | Strand | Div | Repeat Start | Repeat End | Repeat Left | Name | Family | Class |
| 191491293 | 191491733 | - | 30.7 | 2920 | 3401 | -18 | L2 | L2 | LINE |
| 191491734 | 191492098 | - | 17.9 | 1 | 375 | 0 | THE1C | MaLR | LTR |
| 191492132 | 191492192 | - | 13.5 | 1415 | 1473 | -107 | THE1C-int | MaLR | LTR |
| 191492216 | 191493513 | - | 13.5 | 3 | 1414 | -166 | THE1C-int | MaLR | LTR |
| 191493514 | 191493865 | - | 16.5 | 1 | 375 | 0 | THE1C | MaLR | LTR |
| 191493877 | 191494498 | - | 32.8 | 2337 | 2915 | -504 | L2 | L2 | LINE |

  

|  |  |  |  |  |  |  |  |  |  |
| --- | --- | --- | --- | --- | --- | --- | --- | --- | --- |
| chr3:191540346-191544578 | | | 4237 bp | | |  |  |  |  |
| Genome Start | Genome End | Strand | Div | Repeat Start | Repeat End | Repeat Left | Name | Family | Class |
| 191540346 | 191540750 | + | 21.8 | 3685 | 4093 | -2050 | L1MA9 | L1 | LINE |
| 191540751 | 191541109 | - | 16.6 | 1 | 364 | 0 | THE1B | MaLR | LTR |
| 191541110 | 191542686 | - | 30.4 | 2 | 1735 | 0 | MLT-int | MaLR | LTR |
| 191542689 | 191542840 | - | 15.1 | 203 | 364 | 0 | THE1B | MaLR | LTR |
| 191542841 | 191543057 | + | 16.6 | 4277 | 4496 | -1647 | L1MA9 | L1 | LINE |
| 191543052 | 191544578 | + | 21.0 | 4738 | 6274 | -38 | L1MA9 | L1 | LINE |

  

|  |  |  |  |  |  |  |  |  |  |
| --- | --- | --- | --- | --- | --- | --- | --- | --- | --- |
| chr3:191615748-191618988 | | | 3231 bp | | |  |  |  |  |
| Genome Start | Genome End | Strand | Div | Repeat Start | Repeat End | Repeat Left | Name | Family | Class |
| 191615748 | 191616107 | - | 26.7 | 5616 | 5985 | -185 | L1ME3A | L1 | LINE |
| 191616108 | 191616488 | - | 14.8 | 1 | 381 | 0 | THE1D | MaLR | LTR |
| 191616493 | 191618006 | - | 31.0 | 3 | 1731 | -4 | MLT-int | MaLR | LTR |
| 191618013 | 191618394 | - | 14.6 | 3 | 378 | -3 | THE1D | MaLR | LTR |
| 191618395 | 191618988 | - | 25.7 | 5022 | 5615 | -555 | L1ME3A | L1 | LINE |

  

|  |  |  |  |  |  |  |  |  |  |
| --- | --- | --- | --- | --- | --- | --- | --- | --- | --- |
| chr3:191996248-192003484 | | | 7260 bp | | |  |  |  |  |
| Genome Start | Genome End | Strand | Div | Repeat Start | Repeat End | Repeat Left | Name | Family | Class |
| 191996248 | 191999991 | + | 16.8 | 13 | 3816 | -3749 | L1M1 | L1 | LINE |
| 191999992 | 192000359 | + | 17.3 | 1 | 428 | 0 | MSTA | MaLR | LTR |
| 192000360 | 192000680 | + | 11.8 | 3817 | 4136 | -3429 | L1M1 | L1 | LINE |
| 192000681 | 192001020 | + | 7.7 | 1 | 355 | 0 | THE1A | MaLR | LTR |
| 192001021 | 192001112 | + | 5.4 | 1 | 92 | -1488 | THE1A-int | MaLR | LTR |
| 192001098 | 192001224 | - | 9.0 | 76 | 197 | -158 | THE1A | MaLR | LTR |
| 192001215 | 192001897 | + | 8.7 | 899 | 1580 | 0 | THE1A-int | MaLR | LTR |
| 192001898 | 192002134 | + | 9.9 | 1 | 228 | -127 | THE1A | MaLR | LTR |
| 192002135 | 192002444 | + | 13.7 | 1 | 306 | -6 | AluSx | Alu | SINE |
| 192002445 | 192002572 | + | 9.9 | 229 | 355 | 0 | THE1A | MaLR | LTR |
| 192002575 | 192003484 | + | 9.0 | 4134 | 5050 | -2515 | L1M1 | L1 | LINE |

  

|  |  |  |  |  |  |  |  |  |  |
| --- | --- | --- | --- | --- | --- | --- | --- | --- | --- |
| chr3:192115907-192127405 | | | 11529 bp | | |  |  |  |  |
| Genome Start | Genome End | Strand | Div | Repeat Start | Repeat End | Repeat Left | Name | Family | Class |
| 192115907 | 192120274 | - | 17.3 | 2346 | 6785 | 0 | L1PA15 | L1 | LINE |
| 192120275 | 192120606 | - | 14.8 | 1 | 310 | -2 | AluSx | Alu | SINE |
| 192120607 | 192120646 | - | 16.7 | 2305 | 2345 | -4437 | L1PA15 | L1 | LINE |
| 192120647 | 192121093 | + | 7.0 | 1 | 448 | -2 | LTR7 | ERV1 | LTR |
| 192121096 | 192124010 | + | 8.7 | 1 | 2995 | -4718 | HERVH | ERV1 | LTR |
| 192123990 | 192124139 | + | 13.3 | 3140 | 3290 | -4423 | HERVH | ERV1 | LTR |
| 192124130 | 192124632 | + | 11.0 | 3492 | 3993 | -3720 | HERVH | ERV1 | LTR |
| 192124635 | 192125297 | + | 14.3 | 4488 | 5149 | -2564 | HERVH | ERV1 | LTR |
| 192125295 | 192125515 | + | 12.4 | 5597 | 5819 | -1894 | HERVH | ERV1 | LTR |
| 192125514 | 192125893 | + | 8.5 | 7322 | 7713 | 0 | HERVH | ERV1 | LTR |
| 192125894 | 192126343 | + | 6.3 | 1 | 448 | -2 | LTR7 | ERV1 | LTR |
| 192126346 | 192127405 | - | 24.3 | 1246 | 2310 | -4475 | L1PA15 | L1 | LINE |

  

|  |  |  |  |  |  |  |  |  |  |
| --- | --- | --- | --- | --- | --- | --- | --- | --- | --- |
| chr3:192603488-192611817 | | | 8321 bp | | |  |  |  |  |
| Genome Start | Genome End | Strand | Div | Repeat Start | Repeat End | Repeat Left | Name | Family | Class |
| 192603488 | 192604159 | - | 19.8 | 6046 | 6781 | 0 | L1MB4 | L1 | LINE |
| 192604149 | 192604938 | - | 20.1 | 4772 | 5573 | -1174 | L1MB4 | L1 | LINE |
| 192604939 | 192605338 | - | 20.0 | 3 | 426 | 0 | MSTB | MaLR | LTR |
| 192605341 | 192606963 | - | 28.8 | 2 | 1735 | 0 | MLT-int | MaLR | LTR |
| 192606969 | 192607363 | - | 17.5 | 1 | 422 | -4 | MSTB | MaLR | LTR |
| 192607364 | 192607445 | - | 20.1 | 4690 | 4771 | -1976 | L1MB4 | L1 | LINE |
| 192607449 | 192607603 | - | 19.5 | 4108 | 4257 | -2490 | L1MB4 | L1 | LINE |
| 192607604 | 192607892 | - | 10.0 | 5 | 293 | -18 | AluY | Alu | SINE |
| 192607893 | 192608426 | - | 19.5 | 3559 | 4107 | -2640 | L1MB4 | L1 | LINE |
| 192608456 | 192609511 | - | 20.0 | 2455 | 3558 | -3189 | L1MB4 | L1 | LINE |
| 192609512 | 192609715 | + | 6.4 | 5947 | 6151 | -3 | L1PA5 | L1 | LINE |
| 192609716 | 192609826 | - | 21.4 | 2317 | 2454 | -4295 | L1MB4 | L1 | LINE |
| 192609827 | 192610147 | + | 14.0 | 1 | 339 | 0 | MER1B | MER1\_type | DNA |
| 192610148 | 192610671 | - | 21.4 | 1702 | 2316 | -4433 | L1MB4 | L1 | LINE |
| 192610653 | 192611817 | - | 25.0 | 10 | 1309 | -5336 | L1MB4 | L1 | LINE |

  

|  |  |  |  |  |  |  |  |  |  |
| --- | --- | --- | --- | --- | --- | --- | --- | --- | --- |
| chr3:192681265-192689717 | | | 8453 bp | | |  |  |  |  |
| Genome Start | Genome End | Strand | Div | Repeat Start | Repeat End | Repeat Left | Name | Family | Class |
| 192681265 | 192682558 | + | 17.9 | 16 | 1315 | -6250 | L1MA3 | L1 | LINE |
| 192682559 | 192682903 | + | 11.6 | 1 | 355 | 0 | THE1A | MaLR | LTR |
| 192682906 | 192684479 | + | 30.0 | 2 | 1735 | 0 | MLT-int | MaLR | LTR |
| 192684480 | 192684831 | + | 11.2 | 1 | 355 | 0 | THE1A | MaLR | LTR |
| 192684832 | 192685354 | + | 17.9 | 1316 | 1831 | -5734 | L1MA3 | L1 | LINE |
| 192685353 | 192689717 | + | 13.2 | 3300 | 7723 | -1 | L1MA3 | L1 | LINE |

  

|  |  |  |  |  |  |  |  |  |  |
| --- | --- | --- | --- | --- | --- | --- | --- | --- | --- |
| chr3:193728158-193730608 | | | 2449 bp | | |  |  |  |  |
| Genome Start | Genome End | Strand | Div | Repeat Start | Repeat End | Repeat Left | Name | Family | Class |
| 193728158 | 193728177 | - | 23.2 | 6262 | 6280 | -23 | L1MA4A | L1 | LINE |
| 193728178 | 193728522 | - | 11.2 | 1 | 364 | 0 | THE1B | MaLR | LTR |
| 193728523 | 193730062 | - | 29.7 | 2 | 1735 | 0 | MLT-int | MaLR | LTR |
| 193730065 | 193730405 | - | 13.4 | 1 | 364 | 0 | THE1B | MaLR | LTR |
| 193730406 | 193730608 | - | 22.4 | 6001 | 6261 | -42 | L1MA4A | L1 | LINE |

  

|  |  |  |  |  |  |  |  |  |  |
| --- | --- | --- | --- | --- | --- | --- | --- | --- | --- |
| chr3:194194554-194209690 | | | 15540 bp | | |  |  |  |  |
| Genome Start | Genome End | Strand | Div | Repeat Start | Repeat End | Repeat Left | Name | Family | Class |
| 194194554 | 194194757 | + | 29.8 | 765 | 1001 | -2418 | L2 | L2 | LINE |
| 194194758 | 194195059 | - | 13.0 | 1 | 301 | -11 | AluSx | Alu | SINE |
| 194195060 | 194195386 | + | 29.8 | 1002 | 1323 | -2096 | L2 | L2 | LINE |
| 194195404 | 194196127 | - | 13.8 | 5428 | 6157 | -9 | L1PA16 | L1 | LINE |
| 194196128 | 194196539 | + | 8.6 | 1 | 448 | -2 | LTR7 | ERV1 | LTR |
| 194196542 | 194199572 | + | 9.8 | 1 | 2987 | -4726 | HERVH | ERV1 | LTR |
| 194199567 | 194199714 | + | 12.8 | 3140 | 3290 | -4423 | HERVH | ERV1 | LTR |
| 194199705 | 194200207 | + | 11.4 | 3492 | 3993 | -3720 | HERVH | ERV1 | LTR |
| 194200210 | 194200872 | + | 13.8 | 4488 | 5149 | -2564 | HERVH | ERV1 | LTR |
| 194200874 | 194201089 | + | 13.9 | 5600 | 5819 | -1894 | HERVH | ERV1 | LTR |
| 194201090 | 194201465 | + | 10.0 | 7324 | 7712 | -1 | HERVH | ERV1 | LTR |
| 194201467 | 194201871 | + | 7.2 | 1 | 448 | -2 | LTR7 | ERV1 | LTR |
| 194201874 | 194202914 | - | 11.6 | 4373 | 5432 | -734 | L1PA16 | L1 | LINE |
| 194203004 | 194203051 | - | 11.0 | 6104 | 6143 | -8 | L1PB1 | L1 | LINE |
| 194203052 | 194203348 | - | 12.1 | 1 | 297 | -12 | AluSc | Alu | SINE |
| 194203349 | 194203892 | - | 11.0 | 5563 | 6103 | -48 | L1PB1 | L1 | LINE |
| 194203393 | 194207310 | - | 9.4 | 2778 | 6783 | -99 | L1PA16 | L1 | LINE |
| 194207278 | 194207915 | - | 9.3 | 2099 | 2750 | -4113 | L1PBa | L1 | LINE |
| 194207918 | 194208168 | - | 10.4 | 4128 | 4384 | -1762 | L1P4 | L1 | LINE |
| 194208172 | 194208822 | + | 27.2 | 1311 | 1982 | -1437 | L2 | L2 | LINE |
| 194208843 | 194208924 | + | 27.2 | 1983 | 2056 | -1363 | L2 | L2 | LINE |
| 194208932 | 194209690 | + | 28.2 | 2593 | 3416 | -3 | L2 | L2 | LINE |

  

|  |  |  |  |  |  |  |  |  |  |
| --- | --- | --- | --- | --- | --- | --- | --- | --- | --- |
| chr3:196087962-196090539 | | | 2575 bp | | |  |  |  |  |
| Genome Start | Genome End | Strand | Div | Repeat Start | Repeat End | Repeat Left | Name | Family | Class |
| 196087962 | 196088136 | + | 15.4 | 1 | 169 | -143 | AluJb | Alu | SINE |
| 196088137 | 196088494 | - | 18.9 | 2 | 364 | 0 | THE1B | MaLR | LTR |
| 196088496 | 196090060 | - | 32.6 | 2 | 1735 | 0 | MLT1L-int | MaLR | LTR |
| 196090063 | 196090423 | - | 11.7 | 1 | 364 | 0 | THE1B | MaLR | LTR |
| 196090424 | 196090539 | + | 15.4 | 170 | 280 | -32 | AluJb | Alu | SINE |

  

|  |  |  |  |  |  |  |  |  |  |
| --- | --- | --- | --- | --- | --- | --- | --- | --- | --- |
| chr3:198570137-198572894 | | | 2756 bp | | |  |  |  |  |
| Genome Start | Genome End | Strand | Div | Repeat Start | Repeat End | Repeat Left | Name | Family | Class |
| 198570137 | 198570586 | + | 20.5 | 1 | 479 | -63 | MLT1F | MaLR | LTR |
| 198570587 | 198570915 | + | 10.3 | 1 | 364 | 0 | THE1B | MaLR | LTR |
| 198570916 | 198572453 | + | 8.9 | 1 | 1580 | 0 | THE1B-int | MaLR | LTR |
| 198572454 | 198572825 | + | 13.1 | 1 | 364 | 0 | THE1B | MaLR | LTR |
| 198572828 | 198572894 | + | 12.1 | 497 | 562 | 0 | MLT1F | MaLR | LTR |

  


|  |  |  |  |  |  |  |  |  |  |
| --- | --- | --- | --- | --- | --- | --- | --- | --- | --- |
| chr4:2351531-2355203 | | | 3631 bp | | |  |  |  |  |
| Genome Start | Genome End | Strand | Div | Repeat Start | Repeat End | Repeat Left | Name | Family | Class |
| 2351531 | 2352062 | - | 29.4 | 1886 | 2419 | -443 | Charlie2a | MER1\_type | DNA |
| 2352101 | 2352450 | + | 9.7 | 1 | 355 | 0 | THE1A | MaLR | LTR |
| 2352451 | 2352926 | + | 7.7 | 1 | 475 | -1105 | THE1A-int | MaLR | LTR |
| 2352927 | 2353231 | - | 13.8 | 1 | 305 | -7 | AluSx | Alu | SINE |
| 2353232 | 2354272 | + | 7.7 | 476 | 1514 | -66 | THE1A-int | MaLR | LTR |
| 2354273 | 2354626 | - | 9.3 | 1 | 355 | 0 | THE1A | MaLR | LTR |
| 2354627 | 2354696 | + | 7.7 | 1515 | 1580 | 0 | THE1A-int | MaLR | LTR |
| 2354699 | 2355055 | + | 11.5 | 3 | 354 | -1 | THE1A | MaLR | LTR |
| 2355058 | 2355203 | - | 30.8 | 1628 | 1786 | -1075 | Charlie2a | MER1\_type | DNA |

  

|  |  |  |  |  |  |  |  |  |  |
| --- | --- | --- | --- | --- | --- | --- | --- | --- | --- |
| chr4:4052210-4058947 | | | 6685 bp | | |  |  |  |  |
| Genome Start | Genome End | Strand | Div | Repeat Start | Repeat End | Repeat Left | Name | Family | Class |
| 4052210 | 4052395 | + | 23.3 | 1 | 185 | -320 | MLT1D | MaLR | LTR |
| 4052408 | 4052705 | + | 10.7 | 5 | 303 | -10 | AluSp | Alu | SINE |
| 4052715 | 4053113 | - | 10.4 | 2 | 448 | -2 | LTR7 | ERV1 | LTR |
| 4053115 | 4053491 | - | 8.9 | 7324 | 7713 | 0 | HERVH | ERV1 | LTR |
| 4053492 | 4053701 | - | 12.4 | 5600 | 5819 | -1894 | HERVH | ERV1 | LTR |
| 4053703 | 4054364 | - | 15.4 | 4488 | 5149 | -2564 | HERVH | ERV1 | LTR |
| 4054367 | 4055221 | - | 10.3 | 3140 | 3993 | -3720 | HERVH | ERV1 | LTR |
| 4055207 | 4058038 | - | 10.0 | 1 | 2993 | -4720 | HERVH | ERV1 | LTR |
| 4058044 | 4058389 | - | 9.9 | 1 | 447 | -3 | LTR7 | ERV1 | LTR |
| 4058428 | 4058600 | + | 23.0 | 206 | 435 | -69 | MLT1D | MaLR | LTR |
| 4058601 | 4058903 | - | 14.6 | 1 | 302 | -10 | AluSx | Alu | SINE |
| 4058904 | 4058947 | + | 22.2 | 436 | 483 | -22 | MLT1D | MaLR | LTR |

  

|  |  |  |  |  |  |  |  |  |  |
| --- | --- | --- | --- | --- | --- | --- | --- | --- | --- |
| chr4:4091966-4101848 | | | 9849 bp | | |  |  |  |  |
| Genome Start | Genome End | Strand | Div | Repeat Start | Repeat End | Repeat Left | Name | Family | Class |
| 4091966 | 4092136 | + | 35.5 | 2233 | 2401 | -1018 | L2 | L2 | LINE |
| 4092137 | 4092454 | - | 1.3 | 1 | 318 | 0 | AluYb9 | Alu | SINE |
| 4092455 | 4092535 | + | 35.5 | 2402 | 2468 | -951 | L2 | L2 | LINE |
| 4092545 | 4092864 | + | 18.2 | 11 | 290 | -22 | AluJo | Alu | SINE |
| 4092866 | 4093554 | + | 30.5 | 2452 | 3207 | -212 | L2 | L2 | LINE |
| 4093564 | 4094638 | + | 6.9 | 1 | 1266 | 0 | MER11A | ERVK | LTR |
| 4094671 | 4094750 | + | 30.8 | 3249 | 3327 | -51 | L2 | L2 | LINE |
| 4094751 | 4095817 | - | 5.8 | 1 | 1071 | 0 | MER11C | ERVK | LTR |
| 4095818 | 4096816 | - | 7.5 | 6954 | 7953 | 0 | HERVK11 | ERVK | LTR |
| 4096801 | 4098032 | - | 8.0 | 5525 | 6765 | -1188 | HERVK11 | ERVK | LTR |
| 4098031 | 4099943 | - | 7.3 | 1165 | 4601 | -3352 | HERVK11 | ERVK | LTR |
| 4099944 | 4100729 | - | 15.7 | 1 | 851 | -7102 | HERVK11 | ERVK | LTR |
| 4100730 | 4101788 | - | 4.9 | 1 | 1071 | 0 | MER11C | ERVK | LTR |
| 4101790 | 4101848 | + | 21.4 | 3364 | 3419 | 0 | L2 | L2 | LINE |

  

|  |  |  |  |  |  |  |  |  |  |
| --- | --- | --- | --- | --- | --- | --- | --- | --- | --- |
| chr4:5458037-5460779 | | | 2736 bp | | |  |  |  |  |
| Genome Start | Genome End | Strand | Div | Repeat Start | Repeat End | Repeat Left | Name | Family | Class |
| 5458037 | 5458120 | + | 8.4 | 1 | 83 | -282 | MLT1A0 | MaLR | LTR |
| 5458121 | 5458495 | - | 15.1 | 1 | 428 | 0 | MSTA | MaLR | LTR |
| 5458503 | 5460088 | - | 17.9 | 1 | 1651 | 0 | MSTA-int | MaLR | LTR |
| 5460089 | 5460502 | - | 15.1 | 1 | 428 | 0 | MSTA | MaLR | LTR |
| 5460503 | 5460779 | + | 16.2 | 79 | 363 | -2 | MLT1A0 | MaLR | LTR |

  

|  |  |  |  |  |  |  |  |  |  |
| --- | --- | --- | --- | --- | --- | --- | --- | --- | --- |
| chr4:8531945-8547597 | | | 15567 bp | | |  |  |  |  |
| Genome Start | Genome End | Strand | Div | Repeat Start | Repeat End | Repeat Left | Name | Family | Class |
| 8531945 | 8532495 | - | 20.1 | 2425 | 3028 | -1 | Tigger3(Golem) | MER2\_type | DNA |
| 8532496 | 8532710 | - | 14.7 | 53 | 309 | -3 | AluJb | Alu | SINE |
| 8532716 | 8533338 | - | 21.3 | 87 | 727 | -7 | MER50 | ERV1 | LTR |
| 8533339 | 8534125 | + | 7.5 | 3 | 781 | 0 | MER4E1 | ERV1 | LTR |
| 8534126 | 8534207 | - | 21.3 | 7 | 86 | -648 | MER50 | ERV1 | LTR |
| 8534214 | 8534251 | - | 10.5 | 21 | 58 | -244 | AluJb | Alu | SINE |
| 8534254 | 8534999 | - | 15.7 | 1646 | 2413 | -616 | Tigger3(Golem) | MER2\_type | DNA |
| 8535026 | 8535469 | - | 5.4 | 1 | 448 | -2 | LTR7 | ERV1 | LTR |
| 8535470 | 8535845 | - | 9.9 | 7322 | 7713 | 0 | HERVH | ERV1 | LTR |
| 8535844 | 8536047 | - | 12.8 | 5612 | 5819 | -1894 | HERVH | ERV1 | LTR |
| 8536067 | 8536724 | - | 14.6 | 4488 | 5149 | -2564 | HERVH | ERV1 | LTR |
| 8536727 | 8540886 | - | 9.9 | 1 | 3993 | -3720 | HERVH | ERV1 | LTR |
| 8540889 | 8541336 | - | 6.8 | 1 | 448 | -2 | LTR7 | ERV1 | LTR |
| 8541337 | 8541938 | - | 20.5 | 1079 | 1638 | -1391 | Tigger3(Golem) | MER2\_type | DNA |
| 8541939 | 8542076 | + | 17.0 | 1 | 145 | -582 | LTR8A | ERV1 | LTR |
| 8542077 | 8542404 | - | 8.2 | 1 | 327 | 0 | AluSx | Alu | SINE |
| 8542405 | 8542871 | + | 17.0 | 146 | 618 | -109 | LTR8A | ERV1 | LTR |
| 8542872 | 8544600 | - | 7.2 | 2 | 1578 | 0 | LTR12C | ERV1 | LTR |
| 8544601 | 8544713 | + | 17.0 | 619 | 727 | 0 | LTR8A | ERV1 | LTR |
| 8544714 | 8545000 | - | 15.1 | 785 | 1082 | -1947 | Tigger3(Golem) | MER2\_type | DNA |
| 8545001 | 8545151 | + | 16.8 | 1 | 142 | -549 | LTR8 | ERV1 | LTR |
| 8545152 | 8545679 | + | 10.4 | 1 | 600 | 0 | MER4A1\_ | ERV1 | LTR |
| 8545680 | 8546244 | + | 16.8 | 143 | 691 | 0 | LTR8 | ERV1 | LTR |
| 8546245 | 8546703 | - | 21.6 | 369 | 788 | -2241 | Tigger3(Golem) | MER2\_type | DNA |
| 8546722 | 8547402 | + | 10.5 | 1 | 662 | -2 | MER4A | ERV1 | LTR |
| 8547411 | 8547535 | - | 9.7 | 29 | 142 | -522 | MER4A | ERV1 | LTR |
| 8547536 | 8547597 | - | 24.2 | 5 | 66 | -2963 | Tigger3(Golem) | MER2\_type | DNA |

  

|  |  |  |  |  |  |  |  |  |  |
| --- | --- | --- | --- | --- | --- | --- | --- | --- | --- |
| chr4:9182914-9191914 | | | 8924 bp | | |  |  |  |  |
| Genome Start | Genome End | Strand | Div | Repeat Start | Repeat End | Repeat Left | Name | Family | Class |
| 9182914 | 9183498 | - | 13.3 | 6037 | 6629 | -376 | L1MA4A | L1 | LINE |
| 9183488 | 9183733 | - | 11.8 | 4958 | 5204 | -942 | L1PREC2 | L1 | LINE |
| 9183734 | 9184180 | - | 16.9 | 5451 | 5905 | -940 | L1MA4A | L1 | LINE |
| 9184208 | 9184522 | - | 9.7 | 1 | 300 | -11 | AluY | Alu | SINE |
| 9184523 | 9185018 | - | 16.9 | 4966 | 5450 | -1395 | L1MA4A | L1 | LINE |
| 9185019 | 9185246 | - | 13.2 | 1 | 302 | 0 | AluSx | Alu | SINE |
| 9185247 | 9187438 | - | 16.9 | 2778 | 4965 | -1880 | L1MA4A | L1 | LINE |
| 9187439 | 9187791 | + | 14.2 | 1 | 364 | 0 | THE1B | MaLR | LTR |
| 9187794 | 9189363 | + | 30.6 | 2 | 1735 | 0 | MLT-int | MaLR | LTR |
| 9189364 | 9189721 | + | 13.6 | 1 | 364 | 0 | THE1B | MaLR | LTR |
| 9189722 | 9189971 | - | 15.7 | 2521 | 2777 | -4072 | L1MA4A | L1 | LINE |
| 9190000 | 9190142 | - | 18.9 | 1 | 143 | -33 | FRAM | Alu | SINE |
| 9190143 | 9191193 | - | 21.4 | 1440 | 2522 | -4327 | L1MA4A | L1 | LINE |
| 9191225 | 9191502 | - | 12.6 | 1 | 279 | -31 | AluSg | Alu | SINE |
| 9191503 | 9191914 | - | 22.7 | 805 | 1439 | -5410 | L1MA4A | L1 | LINE |

  

|  |  |  |  |  |  |  |  |  |  |
| --- | --- | --- | --- | --- | --- | --- | --- | --- | --- |
| chr4:9249116-9255979 | | | 6809 bp | | |  |  |  |  |
| Genome Start | Genome End | Strand | Div | Repeat Start | Repeat End | Repeat Left | Name | Family | Class |
| 9249116 | 9249180 | - | 23.0 | 389 | 484 | 0 | MLT1D | MaLR | LTR |
| 9249181 | 9249482 | + | 14.7 | 1 | 301 | -11 | AluSx | Alu | SINE |
| 9249483 | 9249655 | - | 22.5 | 206 | 430 | -75 | MLT1D | MaLR | LTR |
| 9249695 | 9250041 | + | 9.3 | 1 | 447 | -3 | LTR7 | ERV1 | LTR |
| 9250047 | 9252988 | + | 9.5 | 1 | 2993 | -4720 | HERVH | ERV1 | LTR |
| 9252974 | 9253825 | + | 10.1 | 3140 | 3993 | -3720 | HERVH | ERV1 | LTR |
| 9253828 | 9254490 | + | 15.9 | 4488 | 5149 | -2564 | HERVH | ERV1 | LTR |
| 9254492 | 9254702 | + | 13.3 | 5600 | 5819 | -1894 | HERVH | ERV1 | LTR |
| 9254703 | 9255079 | + | 9.9 | 7324 | 7713 | 0 | HERVH | ERV1 | LTR |
| 9255081 | 9255475 | + | 10.4 | 2 | 448 | -2 | LTR7 | ERV1 | LTR |
| 9255486 | 9255781 | - | 9.8 | 5 | 301 | -12 | AluSp | Alu | SINE |
| 9255794 | 9255979 | - | 23.9 | 1 | 185 | -320 | MLT1D | MaLR | LTR |

  

|  |  |  |  |  |  |  |  |  |  |
| --- | --- | --- | --- | --- | --- | --- | --- | --- | --- |
| chr4:10933169-10936403 | | | 3202 bp | | |  |  |  |  |
| Genome Start | Genome End | Strand | Div | Repeat Start | Repeat End | Repeat Left | Name | Family | Class |
| 10933169 | 10933420 | + | 17.4 | 5819 | 6053 | -259 | L1MA9 | L1 | LINE |
| 10933422 | 10933624 | - | 17.3 | 212 | 426 | 0 | MSTB | MaLR | LTR |
| 10933630 | 10933725 | + | 7.4 | 1 | 95 | -333 | MSTA | MaLR | LTR |
| 10933749 | 10934031 | + | 18.7 | 130 | 428 | 0 | MSTA | MaLR | LTR |
| 10934034 | 10935568 | + | 28.6 | 2 | 1735 | 0 | MLT1F2-int | MaLR | LTR |
| 10935569 | 10935966 | + | 18.5 | 1 | 428 | 0 | MSTA | MaLR | LTR |
| 10935967 | 10936143 | - | 15.2 | 3 | 200 | -226 | MSTB | MaLR | LTR |
| 10936146 | 10936403 | + | 17.6 | 6050 | 6312 | 0 | L1MA9 | L1 | LINE |

  

|  |  |  |  |  |  |  |  |  |  |
| --- | --- | --- | --- | --- | --- | --- | --- | --- | --- |
| chr4:10995647-10999177 | | | 3432 bp | | |  |  |  |  |
| Genome Start | Genome End | Strand | Div | Repeat Start | Repeat End | Repeat Left | Name | Family | Class |
| 10995647 | 10995985 | - | 21.6 | 568 | 914 | -21 | MER21C | ERV1 | LTR |
| 10996016 | 10996387 | + | 19.9 | 1 | 396 | 0 | MSTD | MaLR | LTR |
| 10996390 | 10998130 | + | 26.0 | 2 | 1735 | 0 | MSTD-int | MaLR | LTR |
| 10998131 | 10998394 | + | 17.2 | 1 | 264 | -132 | MSTD | MaLR | LTR |
| 10998390 | 10998713 | - | 24.0 | 206 | 512 | -423 | MER21C | ERV1 | LTR |
| 10998715 | 10999031 | + | 0.6 | 1 | 317 | -1 | AluYb8 | Alu | SINE |
| 10999103 | 10999177 | - | 18.7 | 25 | 189 | -746 | MER21C | ERV1 | LTR |

  

|  |  |  |  |  |  |  |  |  |  |
| --- | --- | --- | --- | --- | --- | --- | --- | --- | --- |
| chr4:11482138-11489135 | | | 6746 bp | | |  |  |  |  |
| Genome Start | Genome End | Strand | Div | Repeat Start | Repeat End | Repeat Left | Name | Family | Class |
| 11482138 | 11482272 | - | 22.5 | 2795 | 2939 | -5 | BLACKJACK | AcHobo | DNA |
| 11482273 | 11482558 | - | 12.9 | 1 | 292 | -20 | AluSx | Alu | SINE |
| 11482559 | 11484068 | - | 22.5 | 1227 | 2794 | -150 | BLACKJACK | AcHobo | DNA |
| 11484214 | 11484938 | - | 27.1 | 134 | 926 | -2018 | BLACKJACK | AcHobo | DNA |
| 11484952 | 11485465 | - | 19.5 | 5817 | 6332 | -1 | L1MC1 | L1 | LINE |
| 11485545 | 11485683 | - | 15.1 | 6014 | 6157 | -9 | L1PA16 | L1 | LINE |
| 11485688 | 11486087 | - | 14.4 | 1 | 428 | 0 | THE1D | MaLR | LTR |
| 11486088 | 11487702 | - | 13.4 | 3 | 1580 | 0 | THE1D-int | MaLR | LTR |
| 11487709 | 11488077 | - | 16.0 | 1 | 377 | -4 | THE1D | MaLR | LTR |
| 11488078 | 11488405 | - | 14.7 | 5680 | 6011 | -155 | L1PA16 | L1 | LINE |
| 11488406 | 11488730 | - | 19.8 | 3 | 308 | -4 | AluJo | Alu | SINE |
| 11488732 | 11489026 | - | 18.0 | 5500 | 5806 | -527 | L1MC1 | L1 | LINE |
| 11489031 | 11489135 | - | 25.7 | 37 | 146 | -2798 | BLACKJACK | AcHobo | DNA |

  

|  |  |  |  |  |  |  |  |  |  |
| --- | --- | --- | --- | --- | --- | --- | --- | --- | --- |
| chr4:12032935-12042610 | | | 9671 bp | | |  |  |  |  |
| Genome Start | Genome End | Strand | Div | Repeat Start | Repeat End | Repeat Left | Name | Family | Class |
| 12032935 | 12034588 | + | 14.2 | 1477 | 4184 | -2414 | MER4A-int | ERV1 | LTR |
| 12034586 | 12036424 | + | 14.8 | 4481 | 6357 | -241 | MER4A-int | ERV1 | LTR |
| 12036425 | 12036982 | - | 8.2 | 7 | 511 | 0 | LTR10F | ERV1 | LTR |
| 12036989 | 12037376 | - | 4.9 | 4716 | 5102 | 0 | HERVIP10FH | ERV1 | LTR |
| 12037379 | 12039171 | - | 9.1 | 2145 | 3948 | -1154 | HERVIP10FH | ERV1 | LTR |
| 12039172 | 12039471 | - | 6.0 | 1 | 300 | -11 | AluY | Alu | SINE |
| 12039472 | 12041829 | - | 9.1 | 1 | 2144 | -2958 | HERVIP10FH | ERV1 | LTR |
| 12041830 | 12042374 | - | 7.9 | 1 | 511 | 0 | LTR10F | ERV1 | LTR |
| 12042375 | 12042610 | + | 15.7 | 6354 | 6596 | -2 | MER4A-int | ERV1 | LTR |

  

|  |  |  |  |  |  |  |  |  |  |
| --- | --- | --- | --- | --- | --- | --- | --- | --- | --- |
| chr4:12441134-12444808 | | | 3673 bp | | |  |  |  |  |
| Genome Start | Genome End | Strand | Div | Repeat Start | Repeat End | Repeat Left | Name | Family | Class |
| 12441134 | 12442003 | + | 33.5 | 1849 | 2800 | -619 | L2 | L2 | LINE |
| 12442004 | 12442346 | - | 12.1 | 1 | 364 | 0 | THE1B | MaLR | LTR |
| 12442348 | 12443913 | - | 31.2 | 1 | 1734 | -1 | MLT1A0-int | MaLR | LTR |
| 12443915 | 12444256 | - | 12.6 | 1 | 364 | 0 | THE1B | MaLR | LTR |
| 12444257 | 12444808 | + | 32.9 | 2801 | 3416 | -3 | L2 | L2 | LINE |

  

|  |  |  |  |  |  |  |  |  |  |
| --- | --- | --- | --- | --- | --- | --- | --- | --- | --- |
| chr4:12716525-12725985 | | | 8957 bp | | |  |  |  |  |
| Genome Start | Genome End | Strand | Div | Repeat Start | Repeat End | Repeat Left | Name | Family | Class |
| 12716525 | 12716610 | + | 25.6 | 13 | 104 | -2403 | HAL1 | L1 | LINE |
| 12716612 | 12716713 | - | 20.7 | 221 | 318 | -78 | MSTD | MaLR | LTR |
| 12716714 | 12717039 | + | 16.0 | 1 | 312 | 0 | AluSx | Alu | SINE |
| 12717040 | 12717229 | - | 20.7 | 4 | 220 | -176 | MSTD | MaLR | LTR |
| 12717232 | 12718878 | - | 25.5 | 2 | 1735 | 0 | MSTD-int | MaLR | LTR |
| 12718881 | 12719270 | - | 21.5 | 1 | 396 | 0 | MSTD | MaLR | LTR |
| 12719277 | 12719863 | + | 35.7 | 110 | 801 | -1706 | HAL1 | L1 | LINE |
| 12719874 | 12719992 | + | 27.4 | 1495 | 1594 | -913 | HAL1 | L1 | LINE |
| 12719993 | 12720526 | + | 19.5 | 2 | 556 | 0 | MLT2B4 | ERVL | LTR |
| 12720527 | 12720878 | + | 27.4 | 1595 | 1982 | -525 | HAL1 | L1 | LINE |
| 12720916 | 12721024 | + | 26.6 | 2725 | 2845 | -4008 | L1PA15-16 | L1 | LINE |
| 12721096 | 12722932 | + | 19.4 | 2146 | 3913 | -2243 | L1PB4 | L1 | LINE |
| 12722933 | 12722996 | + | 9.8 | 3 | 63 | -17 | MADE1 | Mariner | DNA |
| 12722997 | 12723171 | + | 19.4 | 3914 | 4081 | -2075 | L1PB4 | L1 | LINE |
| 12723172 | 12723224 | + | 5.7 | 28 | 80 | 0 | MADE1 | Mariner | DNA |
| 12723225 | 12724995 | + | 19.4 | 4082 | 5786 | -370 | L1PB4 | L1 | LINE |
| 12725137 | 12725489 | + | 13.0 | 5793 | 6149 | -7 | L1PB4 | L1 | LINE |
| 12725671 | 12725737 | + | 17.9 | 14 | 80 | 0 | MADE2 | Mariner | DNA |
| 12725791 | 12725985 | + | 27.4 | 2233 | 2429 | -78 | HAL1 | L1 | LINE |

  

|  |  |  |  |  |  |  |  |  |  |
| --- | --- | --- | --- | --- | --- | --- | --- | --- | --- |
| chr4:13379653-13388688 | | | 8726 bp | | |  |  |  |  |
| Genome Start | Genome End | Strand | Div | Repeat Start | Repeat End | Repeat Left | Name | Family | Class |
| 13379653 | 13379981 | + | 27.6 | 6611 | 7005 | -1037 | L1MC5 | L1 | LINE |
| 13379981 | 13380250 | - | 11.2 | 6510 | 6779 | -88 | L1PB1 | L1 | LINE |
| 13380286 | 13380582 | - | 11.2 | 6204 | 6509 | -358 | L1PB1 | L1 | LINE |
| 13380606 | 13383708 | - | 8.1 | 3093 | 6203 | -664 | L1PB1 | L1 | LINE |
| 13383709 | 13384006 | - | 8.8 | 1 | 296 | -15 | AluY | Alu | SINE |
| 13384007 | 13385391 | - | 12.3 | 1680 | 3092 | -3770 | L1PB1 | L1 | LINE |
| 13385421 | 13385657 | + | 11.5 | 2168 | 2406 | 0 | Tigger5 | MER2\_type | DNA |
| 13385805 | 13386038 | + | 23.1 | 7305 | 7534 | -427 | L1MC5 | L1 | LINE |
| 13386101 | 13386464 | - | 8.8 | 1 | 364 | 0 | THE1B | MaLR | LTR |
| 13386470 | 13388047 | - | 12.7 | 1 | 1574 | -6 | THE1B-int | MaLR | LTR |
| 13388048 | 13388411 | - | 14.8 | 1 | 364 | 0 | THE1B | MaLR | LTR |
| 13388422 | 13388688 | + | 27.2 | 7660 | 7935 | -26 | L1MC5 | L1 | LINE |

  

|  |  |  |  |  |  |  |  |  |  |
| --- | --- | --- | --- | --- | --- | --- | --- | --- | --- |
| chr4:13434635-13437508 | | | 2749 bp | | |  |  |  |  |
| Genome Start | Genome End | Strand | Div | Repeat Start | Repeat End | Repeat Left | Name | Family | Class |
| 13434635 | 13434853 | + | 36.3 | 2825 | 3046 | -373 | L2 | L2 | LINE |
| 13434928 | 13435282 | + | 12.2 | 1 | 355 | 0 | THE1A | MaLR | LTR |
| 13435285 | 13436855 | + | 30.1 | 2 | 1735 | 0 | MSTD-int | MaLR | LTR |
| 13436858 | 13437210 | + | 10.0 | 3 | 355 | 0 | THE1A | MaLR | LTR |
| 13437258 | 13437508 | + | 29.7 | 3181 | 3418 | -1 | L2 | L2 | LINE |

  

|  |  |  |  |  |  |  |  |  |  |
| --- | --- | --- | --- | --- | --- | --- | --- | --- | --- |
| chr4:14343050-14350985 | | | 7883 bp | | |  |  |  |  |
| Genome Start | Genome End | Strand | Div | Repeat Start | Repeat End | Repeat Left | Name | Family | Class |
| 14343050 | 14343399 | + | 33.9 | 366 | 785 | -2634 | L2 | L2 | LINE |
| 14343402 | 14343769 | - | 32.7 | 3073 | 3414 | -5 | L2 | L2 | LINE |
| 14343771 | 14344109 | - | 10.4 | 1 | 355 | 0 | THE1A | MaLR | LTR |
| 14344110 | 14345683 | - | 30.2 | 2 | 1735 | 0 | MLT1B-int | MaLR | LTR |
| 14345686 | 14346035 | - | 10.7 | 7 | 355 | 0 | THE1A | MaLR | LTR |
| 14346041 | 14346478 | - | 29.6 | 2581 | 3077 | -342 | L2 | L2 | LINE |
| 14346492 | 14346602 | - | 33.6 | 2513 | 2629 | -790 | L2 | L2 | LINE |
| 14346612 | 14346645 | - | 8.8 | 1106 | 1139 | -441 | THE1-int | MaLR | LTR |
| 14346646 | 14346706 | - | 22.9 | 2451 | 2512 | -907 | L2 | L2 | LINE |
| 14346713 | 14347264 | + | 34.4 | 781 | 1330 | -2089 | L2 | L2 | LINE |
| 14347266 | 14348087 | + | 10.4 | 4190 | 5014 | -1132 | L1PREC2 | L1 | LINE |
| 14348090 | 14348255 | + | 13.2 | 1 | 167 | -145 | AluSx | Alu | SINE |
| 14348256 | 14348330 | + | 4.1 | 3645 | 3717 | -2429 | L1PREC2 | L1 | LINE |
| 14348331 | 14348406 | + | 12.2 | 6084 | 6157 | -6 | L1PA13 | L1 | LINE |
| 14348414 | 14349381 | + | 32.1 | 1342 | 2324 | -1095 | L2 | L2 | LINE |
| 14349382 | 14349702 | - | 14.2 | 1 | 338 | -1 | MER44A | MER2\_type | DNA |
| 14349703 | 14349921 | + | 32.1 | 2325 | 2543 | -876 | L2 | L2 | LINE |
| 14349922 | 14350333 | - | 18.9 | 1 | 388 | -2 | MLT1B | MaLR | LTR |
| 14350334 | 14350778 | + | 32.1 | 2544 | 3081 | -338 | L2 | L2 | LINE |
| 14350784 | 14350985 | + | 32.5 | 3183 | 3397 | -22 | L2 | L2 | LINE |

  

|  |  |  |  |  |  |  |  |  |  |
| --- | --- | --- | --- | --- | --- | --- | --- | --- | --- |
| chr4:14695210-14701430 | | | 6219 bp | | |  |  |  |  |
| Genome Start | Genome End | Strand | Div | Repeat Start | Repeat End | Repeat Left | Name | Family | Class |
| 14695210 | 14695959 | - | 17.9 | 5484 | 6242 | 0 | L1MD1 | L1 | LINE |
| 14695960 | 14696268 | - | 9.7 | 1 | 355 | 0 | THE1A | MaLR | LTR |
| 14696269 | 14697819 | - | 30.7 | 2 | 1735 | 0 | MLT-int | MaLR | LTR |
| 14697822 | 14698174 | - | 10.2 | 1 | 355 | 0 | THE1A | MaLR | LTR |
| 14698175 | 14699280 | - | 16.6 | 4461 | 5483 | -759 | L1MD1 | L1 | LINE |
| 14699281 | 14699337 | - | 3.8 | 1 | 53 | -27 | MADE1 | Mariner | DNA |
| 14699338 | 14700032 | - | 16.6 | 3818 | 4460 | -1782 | L1MD1 | L1 | LINE |
| 14700033 | 14700333 | - | 8.7 | 1 | 301 | -8 | AluSc | Alu | SINE |
| 14700334 | 14701430 | - | 16.6 | 2693 | 3815 | -2331 | L1MD1 | L1 | LINE |

  

|  |  |  |  |  |  |  |  |  |  |
| --- | --- | --- | --- | --- | --- | --- | --- | --- | --- |
| chr4:14745493-14747995 | | | 2501 bp | | |  |  |  |  |
| Genome Start | Genome End | Strand | Div | Repeat Start | Repeat End | Repeat Left | Name | Family | Class |
| 14745493 | 14745594 | + | 29.4 | 58 | 161 | -101 | MIR | MIR | SINE |
| 14745595 | 14745955 | - | 12.8 | 1 | 362 | -2 | THE1B | MaLR | LTR |
| 14745956 | 14747546 | - | 29.8 | 2 | 1735 | 0 | MLT-int | MaLR | LTR |
| 14747549 | 14747909 | - | 12.2 | 1 | 364 | 0 | THE1B | MaLR | LTR |
| 14747910 | 14747995 | + | 29.4 | 162 | 245 | -17 | MIR | MIR | SINE |

  

|  |  |  |  |  |  |  |  |  |  |
| --- | --- | --- | --- | --- | --- | --- | --- | --- | --- |
| chr4:14816420-14819054 | | | 2282 bp | | |  |  |  |  |
| Genome Start | Genome End | Strand | Div | Repeat Start | Repeat End | Repeat Left | Name | Family | Class |
| 14816420 | 14817066 | + | 30.9 | 1754 | 2411 | -1008 | L2 | L2 | LINE |
| 14817070 | 14817431 | + | 15.6 | 1 | 381 | 0 | THE1D | MaLR | LTR |
| 14817432 | 14817895 | + | 11.9 | 1 | 397 | -1183 | THE1D-int | MaLR | LTR |
| 14817910 | 14818237 | + | 10.2 | 1251 | 1580 | 0 | THE1D-int | MaLR | LTR |
| 14818238 | 14818589 | + | 15.4 | 1 | 381 | 0 | THE1D | MaLR | LTR |
| 14818926 | 14819054 | + | 25.6 | 3245 | 3379 | -40 | L2 | L2 | LINE |

  

|  |  |  |  |  |  |  |  |  |  |
| --- | --- | --- | --- | --- | --- | --- | --- | --- | --- |
| chr4:15893631-15896203 | | | 2570 bp | | |  |  |  |  |
| Genome Start | Genome End | Strand | Div | Repeat Start | Repeat End | Repeat Left | Name | Family | Class |
| 15893631 | 15893885 | + | 13.7 | 26 | 281 | -186 | MLT1C | MaLR | LTR |
| 15893887 | 15894230 | + | 13.9 | 1 | 363 | -1 | THE1B | MaLR | LTR |
| 15894233 | 15895681 | + | 9.7 | 2 | 1580 | 0 | THE1B-int | MaLR | LTR |
| 15895682 | 15896029 | + | 14.3 | 1 | 364 | 0 | THE1B | MaLR | LTR |
| 15896030 | 15896203 | + | 16.7 | 278 | 459 | -8 | MLT1C | MaLR | LTR |

  

|  |  |  |  |  |  |  |  |  |  |
| --- | --- | --- | --- | --- | --- | --- | --- | --- | --- |
| chr4:18425073-18428373 | | | 3264 bp | | |  |  |  |  |
| Genome Start | Genome End | Strand | Div | Repeat Start | Repeat End | Repeat Left | Name | Family | Class |
| 18425073 | 18425300 | + | 14.5 | 5149 | 5378 | -762 | L1M3 | L1 | LINE |
| 18425311 | 18425662 | + | 18.2 | 5 | 430 | -2 | MSTB1 | MaLR | LTR |
| 18425666 | 18427315 | + | 22.5 | 2 | 1637 | -14 | MSTB1-int | MaLR | LTR |
| 18427328 | 18427492 | + | 17.3 | 1 | 197 | -235 | MSTB1 | MaLR | LTR |
| 18427493 | 18427789 | + | 10.1 | 2 | 300 | -13 | AluSp | Alu | SINE |
| 18427790 | 18428006 | + | 17.3 | 198 | 419 | -13 | MSTB1 | MaLR | LTR |
| 18428019 | 18428373 | + | 12.9 | 5410 | 5752 | -416 | L1M3 | L1 | LINE |

  

|  |  |  |  |  |  |  |  |  |  |
| --- | --- | --- | --- | --- | --- | --- | --- | --- | --- |
| chr4:18429377-18438983 | | | 9549 bp | | |  |  |  |  |
| Genome Start | Genome End | Strand | Div | Repeat Start | Repeat End | Repeat Left | Name | Family | Class |
| 18429377 | 18430755 | + | 27.3 | 52 | 1599 | -1345 | BLACKJACK | AcHobo | DNA |
| 18430756 | 18430833 | + | 19.2 | 1315 | 1392 | -5215 | L1MB4 | L1 | LINE |
| 18430838 | 18431247 | + | 20.7 | 3248 | 3655 | -3086 | L1MB4 | L1 | LINE |
| 18431248 | 18431658 | - | 12.7 | 1 | 428 | 0 | MSTA | MaLR | LTR |
| 18431659 | 18432249 | + | 20.7 | 3656 | 4313 | -2428 | L1MB4 | L1 | LINE |
| 18432256 | 18432653 | + | 18.5 | 1 | 426 | 0 | MSTB | MaLR | LTR |
| 18432654 | 18433000 | + | 18.2 | 3 | 351 | -1300 | MSTB-int | MaLR | LTR |
| 18433001 | 18433280 | + | 8.2 | 1 | 281 | -32 | AluSp | Alu | SINE |
| 18433281 | 18433578 | + | 25.3 | 410 | 675 | -1060 | MLT-int | MaLR | LTR |
| 18433570 | 18433738 | + | 21.9 | 763 | 949 | -702 | MSTB-int | MaLR | LTR |
| 18433741 | 18434377 | + | 17.9 | 951 | 1644 | -7 | MSTB-int | MaLR | LTR |
| 18434384 | 18434773 | + | 16.9 | 1 | 426 | 0 | MSTB | MaLR | LTR |
| 18434776 | 18436579 | + | 23.3 | 3714 | 5460 | -723 | L1MB2 | L1 | LINE |
| 18436580 | 18436946 | + | 8.2 | 5819 | 6182 | 0 | L1PA7 | L1 | LINE |
| 18436947 | 18436979 | + | 17.5 | 5461 | 5490 | -693 | L1MB2 | L1 | LINE |
| 18436984 | 18437075 | + | 6.5 | 6210 | 6302 | 0 | L1MA1 | L1 | LINE |
| 18437093 | 18437202 | + | 17.2 | 5477 | 5585 | -586 | L1MB2 | L1 | LINE |
| 18437203 | 18437568 | - | 12.6 | 1 | 364 | 0 | THE1B | MaLR | LTR |
| 18437569 | 18438156 | + | 17.2 | 5586 | 6167 | -4 | L1MB2 | L1 | LINE |
| 18438183 | 18438983 | + | 24.2 | 1698 | 2519 | -425 | BLACKJACK | AcHobo | DNA |

  

|  |  |  |  |  |  |  |  |  |  |
| --- | --- | --- | --- | --- | --- | --- | --- | --- | --- |
| chr4:18604374-18609429 | | | 4988 bp | | |  |  |  |  |
| Genome Start | Genome End | Strand | Div | Repeat Start | Repeat End | Repeat Left | Name | Family | Class |
| 18604374 | 18604505 | - | 18.4 | 4719 | 4847 | -1299 | L1MC | L1 | LINE |
| 18604528 | 18604904 | + | 24.5 | 14 | 396 | 0 | MSTD | MaLR | LTR |
| 18604907 | 18606606 | + | 27.2 | 2 | 1735 | 0 | MSTD-int | MaLR | LTR |
| 18606607 | 18606967 | + | 23.8 | 1 | 364 | -32 | MSTD | MaLR | LTR |
| 18606968 | 18607323 | - | 12.1 | 1 | 364 | 0 | THE1B | MaLR | LTR |
| 18607328 | 18608956 | - | 12.9 | 14 | 1574 | -6 | THE1B-int | MaLR | LTR |
| 18608959 | 18609309 | - | 15.4 | 3 | 361 | -3 | THE1B | MaLR | LTR |
| 18609348 | 18609429 | - | 19.2 | 4233 | 4307 | -1839 | L1MC | L1 | LINE |

  

|  |  |  |  |  |  |  |  |  |  |
| --- | --- | --- | --- | --- | --- | --- | --- | --- | --- |
| chr4:18962692-18965492 | | | 2769 bp | | |  |  |  |  |
| Genome Start | Genome End | Strand | Div | Repeat Start | Repeat End | Repeat Left | Name | Family | Class |
| 18962692 | 18962773 | + | 27.2 | 7325 | 7407 | -635 | L1MC4 | L1 | LINE |
| 18962774 | 18963134 | - | 15.2 | 1 | 364 | 0 | THE1B | MaLR | LTR |
| 18963135 | 18964601 | - | 11.9 | 1 | 1580 | 0 | THE1B-int | MaLR | LTR |
| 18964602 | 18964946 | - | 11.6 | 1 | 364 | 0 | THE1B | MaLR | LTR |
| 18964947 | 18965270 | + | 26.6 | 7403 | 7690 | -352 | L1MC4 | L1 | LINE |
| 18965303 | 18965492 | + | 26.6 | 7691 | 7886 | -156 | L1MC4 | L1 | LINE |

  

|  |  |  |  |  |  |  |  |  |  |
| --- | --- | --- | --- | --- | --- | --- | --- | --- | --- |
| chr4:20237906-20248337 | | | 10363 bp | | |  |  |  |  |
| Genome Start | Genome End | Strand | Div | Repeat Start | Repeat End | Repeat Left | Name | Family | Class |
| 20237906 | 20239028 | - | 11.0 | 5432 | 6593 | -5 | MER4A1-int | ERV1 | LTR |
| 20239029 | 20239147 | + | 16.0 | 123 | 257 | -434 | LTR8 | ERV1 | LTR |
| 20239148 | 20239844 | - | 7.0 | 1764 | 2495 | -4103 | MER4-int | ERV1 | LTR |
| 20239925 | 20240490 | - | 12.0 | 531 | 1150 | -5608 | MER4-int | ERV1 | LTR |
| 20240492 | 20240900 | - | 9.1 | 1 | 448 | -2 | LTR7 | ERV1 | LTR |
| 20240901 | 20241278 | - | 9.1 | 7322 | 7713 | 0 | HERVH | ERV1 | LTR |
| 20241277 | 20241488 | - | 12.7 | 5600 | 5819 | -1894 | HERVH | ERV1 | LTR |
| 20241490 | 20243211 | - | 13.2 | 3492 | 5149 | -2287 | HERVH | ERV1 | LTR |
| 20243202 | 20243349 | - | 16.2 | 3140 | 3290 | -4423 | HERVH | ERV1 | LTR |
| 20243344 | 20246287 | - | 10.2 | 1 | 2987 | -4726 | HERVH | ERV1 | LTR |
| 20246290 | 20246699 | - | 8.1 | 1 | 448 | -2 | LTR7 | ERV1 | LTR |
| 20246700 | 20247045 | - | 12.8 | 189 | 536 | -6222 | MER4B-int | ERV1 | LTR |
| 20247045 | 20247286 | - | 26.9 | 1948 | 2224 | -4107 | LTR49-int | ERV1 | LTR |
| 20247287 | 20247575 | + | 9.0 | 1 | 292 | -19 | AluY | Alu | SINE |
| 20247576 | 20247685 | - | 26.9 | 1848 | 1947 | -4384 | MER4A1-int | ERV1 | LTR |
| 20247715 | 20247886 | - | 20.0 | 620 | 1013 | -5745 | MER4A1-int | ERV1 | LTR |
| 20247862 | 20248337 | - | 11.1 | 3 | 500 | -6151 | MER4A1-int | ERV1 | LTR |

  

|  |  |  |  |  |  |  |  |  |  |
| --- | --- | --- | --- | --- | --- | --- | --- | --- | --- |
| chr4:21666732-21669754 | | | 3021 bp | | |  |  |  |  |
| Genome Start | Genome End | Strand | Div | Repeat Start | Repeat End | Repeat Left | Name | Family | Class |
| 21666732 | 21666778 | - | 22.1 | 891 | 933 | -2 | MER21C | ERV1 | LTR |
| 21666779 | 21667113 | + | 16.4 | 1 | 364 | 0 | THE1B | MaLR | LTR |
| 21667116 | 21668690 | + | 31.1 | 2 | 1735 | 0 | MLT-int | MaLR | LTR |
| 21668691 | 21669050 | + | 14.4 | 1 | 364 | 0 | THE1B | MaLR | LTR |
| 21669051 | 21669754 | - | 22.1 | 148 | 890 | -45 | MER21C | ERV1 | LTR |

  

|  |  |  |  |  |  |  |  |  |  |
| --- | --- | --- | --- | --- | --- | --- | --- | --- | --- |
| chr4:23200698-23203789 | | | 3080 bp | | |  |  |  |  |
| Genome Start | Genome End | Strand | Div | Repeat Start | Repeat End | Repeat Left | Name | Family | Class |
| 23200698 | 23201372 | - | 24.4 | 104 | 887 | -484 | MLT1-int | MaLR | LTR |
| 23201373 | 23201743 | + | 11.1 | 1 | 375 | 0 | THE1C | MaLR | LTR |
| 23201744 | 23203315 | + | 12.3 | 1 | 1579 | -1 | THE1C-int | MaLR | LTR |
| 23203318 | 23203691 | + | 11.9 | 3 | 375 | 0 | THE1C | MaLR | LTR |
| 23203702 | 23203789 | - | 15.9 | 2 | 97 | -1638 | MLT1-int | MaLR | LTR |

  

|  |  |  |  |  |  |  |  |  |  |
| --- | --- | --- | --- | --- | --- | --- | --- | --- | --- |
| chr4:23272383-23277098 | | | 4646 bp | | |  |  |  |  |
| Genome Start | Genome End | Strand | Div | Repeat Start | Repeat End | Repeat Left | Name | Family | Class |
| 23272383 | 23272965 | + | 32.3 | 2102 | 2692 | -727 | L2 | L2 | LINE |
| 23273000 | 23273409 | + | 21.2 | 4 | 396 | 0 | MSTD | MaLR | LTR |
| 23273410 | 23274097 | + | 22.7 | 2 | 651 | -1084 | MSTD-int | MaLR | LTR |
| 23274098 | 23274427 | - | 21.2 | 554 | 903 | 0 | MER4D | ERV1 | LTR |
| 23274458 | 23274725 | - | 20.2 | 1 | 285 | -638 | MER49 | ERV1 | LTR |
| 23274726 | 23275730 | + | 25.1 | 650 | 1731 | -4 | MSTD-int | MaLR | LTR |
| 23275736 | 23275835 | + | 18.4 | 2 | 104 | -304 | MSTD | MaLR | LTR |
| 23275835 | 23275937 | + | 18.7 | 210 | 314 | -82 | MSTD | MaLR | LTR |
| 23275938 | 23276356 | + | 18.4 | 1 | 426 | 0 | MSTB | MaLR | LTR |
| 23276357 | 23276440 | + | 18.7 | 315 | 395 | -1 | MSTD | MaLR | LTR |
| 23276443 | 23277098 | + | 35.5 | 2738 | 3419 | 0 | L2 | L2 | LINE |

  

|  |  |  |  |  |  |  |  |  |  |
| --- | --- | --- | --- | --- | --- | --- | --- | --- | --- |
| chr4:23333439-23338740 | | | 5333 bp | | |  |  |  |  |
| Genome Start | Genome End | Strand | Div | Repeat Start | Repeat End | Repeat Left | Name | Family | Class |
| 23333439 | 23333591 | + | 6.5 | 1 | 153 | -158 | AluY | Alu | SINE |
| 23333594 | 23334042 | - | 5.1 | 1 | 448 | -2 | LTR7 | ERV1 | LTR |
| 23334043 | 23334211 | - | 6.1 | 7550 | 7713 | 0 | HERVH | ERV1 | LTR |
| 23334204 | 23334432 | - | 11.4 | 4488 | 4719 | -2994 | HERVH | ERV1 | LTR |
| 23334435 | 23334939 | - | 11.2 | 3492 | 3993 | -3720 | HERVH | ERV1 | LTR |
| 23334930 | 23335079 | - | 11.3 | 3140 | 3290 | -4423 | HERVH | ERV1 | LTR |
| 23335059 | 23338138 | - | 9.0 | 1 | 2995 | -4718 | HERVH | ERV1 | LTR |
| 23338141 | 23338587 | - | 5.2 | 3 | 448 | -2 | LTR7 | ERV1 | LTR |
| 23338590 | 23338740 | + | 7.3 | 149 | 302 | -9 | AluY | Alu | SINE |

  

|  |  |  |  |  |  |  |  |  |  |
| --- | --- | --- | --- | --- | --- | --- | --- | --- | --- |
| chr4:24111236-24117796 | | | 6480 bp | | |  |  |  |  |
| Genome Start | Genome End | Strand | Div | Repeat Start | Repeat End | Repeat Left | Name | Family | Class |
| 24111236 | 24111409 | + | 30.1 | 57 | 238 | -353 | MLT1K | MaLR | LTR |
| 24111506 | 24111591 | + | 25.6 | 377 | 463 | -128 | MLT1K | MaLR | LTR |
| 24111595 | 24111695 | - | 7.9 | 205 | 305 | -7 | AluSg | Alu | SINE |
| 24111696 | 24112146 | + | 6.0 | 1 | 448 | -2 | LTR7 | ERV1 | LTR |
| 24112149 | 24115150 | + | 9.8 | 1 | 2995 | -4718 | HERVH | ERV1 | LTR |
| 24115130 | 24115276 | + | 13.6 | 3140 | 3290 | -4423 | HERVH | ERV1 | LTR |
| 24115267 | 24115765 | + | 12.2 | 3492 | 3993 | -3720 | HERVH | ERV1 | LTR |
| 24115768 | 24116430 | + | 15.0 | 4488 | 5149 | -2564 | HERVH | ERV1 | LTR |
| 24116432 | 24116644 | + | 12.7 | 5600 | 5816 | -1897 | HERVH | ERV1 | LTR |
| 24116646 | 24117020 | + | 7.2 | 7322 | 7713 | 0 | HERVH | ERV1 | LTR |
| 24117021 | 24117471 | + | 6.7 | 1 | 448 | -2 | LTR7 | ERV1 | LTR |
| 24117474 | 24117680 | - | 9.2 | 1 | 207 | -103 | AluSg | Alu | SINE |
| 24117686 | 24117796 | + | 25.9 | 466 | 573 | -18 | MLT1K | MaLR | LTR |

  

|  |  |  |  |  |  |  |  |  |  |
| --- | --- | --- | --- | --- | --- | --- | --- | --- | --- |
| chr4:24824894-24828251 | | | 3355 bp | | |  |  |  |  |
| Genome Start | Genome End | Strand | Div | Repeat Start | Repeat End | Repeat Left | Name | Family | Class |
| 24824894 | 24825178 | + | 19.6 | 5 | 309 | -158 | MLT1C | MaLR | LTR |
| 24825179 | 24825535 | - | 13.0 | 1 | 381 | 0 | THE1D | MaLR | LTR |
| 24825537 | 24826615 | - | 29.7 | 599 | 1734 | -1 | MLT-int | MaLR | LTR |
| 24826616 | 24826874 | - | 10.1 | 54 | 312 | -1 | AluSp | Alu | SINE |
| 24826875 | 24827441 | - | 29.7 | 2 | 598 | -1137 | MLT-int | MaLR | LTR |
| 24827444 | 24827513 | - | 12.6 | 325 | 381 | 0 | THE1D | MaLR | LTR |
| 24827514 | 24827811 | - | 12.7 | 1 | 294 | -18 | AluSx | Alu | SINE |
| 24827812 | 24828105 | - | 12.6 | 1 | 324 | -57 | THE1D | MaLR | LTR |
| 24828106 | 24828251 | + | 19.6 | 310 | 466 | -1 | MLT1C | MaLR | LTR |

  

|  |  |  |  |  |  |  |  |  |  |
| --- | --- | --- | --- | --- | --- | --- | --- | --- | --- |
| chr4:26910032-26913092 | | | 3059 bp | | |  |  |  |  |
| Genome Start | Genome End | Strand | Div | Repeat Start | Repeat End | Repeat Left | Name | Family | Class |
| 26910032 | 26910301 | - | 34.3 | 136 | 423 | -61 | MLT1H2 | MaLR | LTR |
| 26910302 | 26910678 | - | 12.3 | 1 | 375 | 0 | THE1C | MaLR | LTR |
| 26910679 | 26911705 | - | 31.1 | 660 | 1735 | 0 | MLT1H2-int | MaLR | LTR |
| 26911706 | 26912017 | - | 11.6 | 1 | 310 | 0 | AluSg | Alu | SINE |
| 26912018 | 26912597 | - | 31.1 | 2 | 659 | -1076 | MLT1H2-int | MaLR | LTR |
| 26912600 | 26912970 | - | 11.3 | 1 | 375 | 0 | THE1C | MaLR | LTR |
| 26912971 | 26913092 | - | 34.3 | 17 | 135 | -349 | MLT1H2 | MaLR | LTR |

  

|  |  |  |  |  |  |  |  |  |  |
| --- | --- | --- | --- | --- | --- | --- | --- | --- | --- |
| chr4:27641879-27652149 | | | 10202 bp | | |  |  |  |  |
| Genome Start | Genome End | Strand | Div | Repeat Start | Repeat End | Repeat Left | Name | Family | Class |
| 27641879 | 27641995 | - | 36.8 | 3303 | 3419 | 0 | L2 | L2 | LINE |
| 27641996 | 27643164 | - | 17.7 | 2484 | 3666 | -2480 | L1PA15 | L1 | LINE |
| 27643171 | 27643295 | + | 14.5 | 3658 | 3785 | -2361 | L1PA15 | L1 | LINE |
| 27643301 | 27644180 | + | 15.5 | 2958 | 3856 | -2290 | L1MA2 | L1 | LINE |
| 27644179 | 27645693 | + | 14.2 | 4712 | 6297 | -7 | L1MA2 | L1 | LINE |
| 27645703 | 27646042 | + | 17.4 | 3775 | 4131 | -2015 | L1PA15 | L1 | LINE |
| 27646043 | 27646454 | - | 18.4 | 5 | 428 | 0 | MSTA | MaLR | LTR |
| 27646457 | 27646540 | - | 18.7 | 1570 | 1651 | 0 | MSTA-int | MaLR | LTR |
| 27646541 | 27646711 | - | 11.9 | 130 | 296 | -17 | AluSp | Alu | SINE |
| 27646732 | 27646861 | - | 11.9 | 3 | 129 | -184 | AluSp | Alu | SINE |
| 27646862 | 27648385 | - | 18.7 | 1 | 1569 | -82 | MSTA-int | MaLR | LTR |
| 27648386 | 27648785 | - | 16.9 | 3 | 428 | 0 | MSTA | MaLR | LTR |
| 27648788 | 27648883 | + | 15.5 | 4127 | 4211 | -1935 | L1PA15 | L1 | LINE |
| 27648884 | 27649165 | - | 13.5 | 12 | 295 | -17 | AluSx | Alu | SINE |
| 27649166 | 27649485 | + | 15.5 | 4212 | 4511 | -1635 | L1PA15 | L1 | LINE |
| 27649486 | 27649775 | + | 15.9 | 3 | 299 | -13 | AluSx | Alu | SINE |
| 27649776 | 27650749 | + | 15.6 | 4512 | 5492 | -673 | L1PA15 | L1 | LINE |
| 27650750 | 27651060 | + | 8.8 | 1 | 307 | -4 | AluY | Alu | SINE |
| 27651061 | 27651731 | + | 16.9 | 5493 | 6159 | -6 | L1PA15 | L1 | LINE |
| 27651759 | 27652149 | - | 32.2 | 2838 | 3284 | -135 | L2 | L2 | LINE |

  

|  |  |  |  |  |  |  |  |  |  |
| --- | --- | --- | --- | --- | --- | --- | --- | --- | --- |
| chr4:27693071-27697362 | | | 4291 bp | | |  |  |  |  |
| Genome Start | Genome End | Strand | Div | Repeat Start | Repeat End | Repeat Left | Name | Family | Class |
| 27693071 | 27693827 | + | 27.7 | 113 | 890 | -6444 | L1M4b | L1 | LINE |
| 27693828 | 27694071 | - | 19.2 | 154 | 403 | 0 | MER57A | ERV1 | LTR |
| 27694072 | 27694447 | - | 14.8 | 1 | 375 | 0 | THE1C | MaLR | LTR |
| 27694448 | 27695965 | - | 32.0 | 2 | 1735 | 0 | MLT-int | MaLR | LTR |
| 27695966 | 27696338 | - | 11.9 | 1 | 371 | -4 | THE1C | MaLR | LTR |
| 27696339 | 27696468 | - | 19.2 | 28 | 153 | -250 | MER57A | ERV1 | LTR |
| 27696470 | 27697362 | + | 20.9 | 922 | 1849 | -5485 | L1M4b | L1 | LINE |

  

|  |  |  |  |  |  |  |  |  |  |
| --- | --- | --- | --- | --- | --- | --- | --- | --- | --- |
| chr4:28082468-28085351 | | | 2878 bp | | |  |  |  |  |
| Genome Start | Genome End | Strand | Div | Repeat Start | Repeat End | Repeat Left | Name | Family | Class |
| 28082468 | 28082738 | - | 33.0 | 2776 | 3061 | -358 | L2 | L2 | LINE |
| 28082741 | 28083115 | + | 14.0 | 1 | 381 | 0 | THE1D | MaLR | LTR |
| 28083116 | 28084712 | + | 14.1 | 1 | 1580 | 0 | THE1D-int | MaLR | LTR |
| 28084713 | 28085083 | + | 14.2 | 1 | 381 | 0 | THE1D | MaLR | LTR |
| 28085088 | 28085351 | - | 27.0 | 2481 | 2773 | -646 | L2 | L2 | LINE |

  

|  |  |  |  |  |  |  |  |  |  |
| --- | --- | --- | --- | --- | --- | --- | --- | --- | --- |
| chr4:28177696-28181255 | | | 3542 bp | | |  |  |  |  |
| Genome Start | Genome End | Strand | Div | Repeat Start | Repeat End | Repeat Left | Name | Family | Class |
| 28177696 | 28178126 | + | 23.6 | 1 | 430 | -5861 | L1P4e | L1 | LINE |
| 28178127 | 28178483 | - | 14.2 | 1 | 381 | 0 | THE1D | MaLR | LTR |
| 28178484 | 28180113 | - | 32.2 | 3 | 1735 | 0 | MLT-int | MaLR | LTR |
| 28180117 | 28180462 | - | 15.1 | 1 | 381 | 0 | THE1D | MaLR | LTR |
| 28180463 | 28180857 | + | 23.6 | 431 | 823 | -5468 | L1P4e | L1 | LINE |
| 28180873 | 28181255 | + | 13.2 | 1007 | 1387 | -4904 | L1P4e | L1 | LINE |

  

|  |  |  |  |  |  |  |  |  |  |
| --- | --- | --- | --- | --- | --- | --- | --- | --- | --- |
| chr4:28603693-28610796 | | | 6179 bp | | |  |  |  |  |
| Genome Start | Genome End | Strand | Div | Repeat Start | Repeat End | Repeat Left | Name | Family | Class |
| 28603693 | 28603971 | + | 39.0 | 1 | 290 | -4706 | HERV16 | ERVL | LTR |
| 28604072 | 28604465 | - | 24.9 | 3 | 421 | 0 | LTR52 | ERVL | LTR |
| 28604522 | 28605289 | + | 37.8 | 477 | 1233 | -3763 | HERV16 | ERVL | LTR |
| 28605290 | 28605507 | - | 24.9 | 1 | 218 | -1 | MER20 | MER1\_type | DNA |
| 28605508 | 28605737 | + | 37.8 | 1234 | 1484 | -3512 | HERV16 | ERVL | LTR |
| 28605744 | 28606244 | + | 32.9 | 1589 | 2091 | -2905 | HERV16 | ERVL | LTR |
| 28606307 | 28606480 | + | 33.0 | 2225 | 2407 | -3350 | HERV16 | ERVL | LTR |
| 28606649 | 28606979 | + | 35.1 | 2384 | 2723 | -2944 | HERV16 | ERVL | LTR |
| 28607178 | 28607628 | + | 36.9 | 2949 | 3420 | -2247 | HERV16 | ERVL | LTR |
| 28607825 | 28608037 | - | 16.9 | 81 | 295 | -17 | AluSg/x | Alu | SINE |
| 28608137 | 28608497 | + | 11.1 | 1 | 364 | 0 | THE1B | MaLR | LTR |
| 28608501 | 28610080 | + | 30.7 | 3 | 1735 | 0 | MLT-int | MaLR | LTR |
| 28610081 | 28610445 | + | 12.2 | 1 | 362 | -2 | THE1B | MaLR | LTR |
| 28610478 | 28610653 | - | 22.2 | 125 | 296 | -16 | AluJo | Alu | SINE |
| 28610659 | 28610796 | + | 26.8 | 3772 | 3911 | -1756 | HERV16 | ERVL | LTR |

  

|  |  |  |  |  |  |  |  |  |  |
| --- | --- | --- | --- | --- | --- | --- | --- | --- | --- |
| chr4:29155181-29159224 | | | 3627 bp | | |  |  |  |  |
| Genome Start | Genome End | Strand | Div | Repeat Start | Repeat End | Repeat Left | Name | Family | Class |
| 29155181 | 29155467 | + | 29.9 | 1526 | 1821 | -3013 | L1MEe | L1 | LINE |
| 29155525 | 29155830 | + | 20.6 | 1 | 354 | -11 | MLT1A0 | MaLR | LTR |
| 29155875 | 29156035 | + | 25.2 | 1918 | 2069 | -2765 | L1MEe | L1 | LINE |
| 29156308 | 29156352 | + | 25.0 | 1 | 44 | -610 | MLT1E2 | MaLR | LTR |
| 29156357 | 29156725 | - | 14.8 | 3 | 364 | 0 | THE1B | MaLR | LTR |
| 29156728 | 29158199 | - | 11.4 | 2 | 1580 | 0 | THE1B-int | MaLR | LTR |
| 29158201 | 29158565 | - | 13.6 | 1 | 364 | 0 | THE1B | MaLR | LTR |
| 29158575 | 29158651 | + | 18.9 | 87 | 167 | -457 | MLT1E2 | MaLR | LTR |
| 29158680 | 29159063 | + | 27.7 | 233 | 626 | 0 | MLT1E2 | MaLR | LTR |
| 29159064 | 29159224 | + | 27.7 | 2346 | 2514 | -2320 | L1MEe | L1 | LINE |

  

|  |  |  |  |  |  |  |  |  |  |
| --- | --- | --- | --- | --- | --- | --- | --- | --- | --- |
| chr4:29848843-29858092 | | | 9213 bp | | |  |  |  |  |
| Genome Start | Genome End | Strand | Div | Repeat Start | Repeat End | Repeat Left | Name | Family | Class |
| 29848843 | 29850022 | - | 13.7 | 5051 | 6206 | -2 | L1PB2 | L1 | LINE |
| 29850055 | 29850358 | - | 11.9 | 1 | 285 | -24 | AluSc | Alu | SINE |
| 29850359 | 29851480 | - | 15.7 | 3946 | 5050 | -1153 | L1PB2 | L1 | LINE |
| 29851481 | 29851788 | - | 7.8 | 3 | 311 | 0 | AluY | Alu | SINE |
| 29851789 | 29854054 | - | 15.7 | 1596 | 3945 | -2258 | L1PB2 | L1 | LINE |
| 29854055 | 29854434 | - | 17.0 | 1 | 428 | 0 | MSTA | MaLR | LTR |
| 29854435 | 29856043 | - | 30.9 | 3 | 1735 | 0 | MLT-int | MaLR | LTR |
| 29856047 | 29856430 | - | 18.9 | 1 | 428 | 0 | MSTA | MaLR | LTR |
| 29856431 | 29856964 | - | 15.6 | 1060 | 1595 | -4609 | L1PB2 | L1 | LINE |
| 29856966 | 29857332 | - | 16.7 | 673 | 1058 | -5146 | L1PB2 | L1 | LINE |
| 29857353 | 29857420 | - | 16.7 | 607 | 672 | -5532 | L1PB2 | L1 | LINE |
| 29857401 | 29857469 | + | 20.3 | 34 | 105 | -6099 | L1PBb | L1 | LINE |
| 29857471 | 29858092 | - | 20.4 | 8 | 618 | -5586 | L1PB2 | L1 | LINE |

  

|  |  |  |  |  |  |  |  |  |  |
| --- | --- | --- | --- | --- | --- | --- | --- | --- | --- |
| chr4:30023448-30032089 | | | 8471 bp | | |  |  |  |  |
| Genome Start | Genome End | Strand | Div | Repeat Start | Repeat End | Repeat Left | Name | Family | Class |
| 30023448 | 30023786 | + | 30.0 | 31 | 401 | -5983 | L1MB5 | L1 | LINE |
| 30023881 | 30026046 | + | 25.3 | 566 | 2867 | -3515 | L1MB5 | L1 | LINE |
| 30026064 | 30026503 | + | 28.2 | 3695 | 4091 | -2336 | L1MB5 | L1 | LINE |
| 30026506 | 30026812 | + | 14.2 | 51 | 364 | 0 | THE1B | MaLR | LTR |
| 30026815 | 30028366 | + | 29.7 | 2 | 1731 | -4 | MLT-int | MaLR | LTR |
| 30028371 | 30028721 | + | 14.8 | 1 | 364 | 0 | THE1B | MaLR | LTR |
| 30028726 | 30029368 | + | 24.5 | 4100 | 4790 | -1592 | L1MB5 | L1 | LINE |
| 30029369 | 30029776 | + | 26.0 | 1 | 436 | -118 | MLT2B3 | ERVL | LTR |
| 30029825 | 30030221 | + | 22.8 | 4794 | 5206 | -1176 | L1MB5 | L1 | LINE |
| 30030222 | 30030583 | - | 24.2 | 1 | 365 | 0 | MLT1A0 | MaLR | LTR |
| 30030584 | 30031462 | + | 23.5 | 5207 | 6104 | -306 | L1MB5 | L1 | LINE |
| 30031463 | 30031772 | - | 6.2 | 1 | 309 | -2 | AluY | Alu | SINE |
| 30031773 | 30032089 | + | 24.1 | 6105 | 6410 | 0 | L1MB5 | L1 | LINE |

  

|  |  |  |  |  |  |  |  |  |  |
| --- | --- | --- | --- | --- | --- | --- | --- | --- | --- |
| chr4:30173670-30178805 | | | 5132 bp | | |  |  |  |  |
| Genome Start | Genome End | Strand | Div | Repeat Start | Repeat End | Repeat Left | Name | Family | Class |
| 30173670 | 30175946 | + | 17.4 | 3509 | 5811 | -489 | L1MA6 | L1 | LINE |
| 30175947 | 30176327 | + | 11.3 | 1 | 375 | 0 | THE1C | MaLR | LTR |
| 30176330 | 30177887 | + | 29.6 | 2 | 1735 | 0 | MLT-int | MaLR | LTR |
| 30177890 | 30178253 | + | 13.4 | 3 | 375 | 0 | THE1C | MaLR | LTR |
| 30178254 | 30178805 | + | 19.3 | 5806 | 6288 | -3 | L1MA6 | L1 | LINE |

  

|  |  |  |  |  |  |  |  |  |  |
| --- | --- | --- | --- | --- | --- | --- | --- | --- | --- |
| chr4:30287772-30290454 | | | 2681 bp | | |  |  |  |  |
| Genome Start | Genome End | Strand | Div | Repeat Start | Repeat End | Repeat Left | Name | Family | Class |
| 30287772 | 30287883 | - | 30.2 | 382 | 487 | -4 | LTR16C | ERVL | LTR |
| 30287884 | 30288239 | + | 13.8 | 1 | 364 | 0 | THE1B | MaLR | LTR |
| 30288242 | 30289816 | + | 31.6 | 2 | 1735 | 0 | MLT-int | MaLR | LTR |
| 30289817 | 30290170 | + | 14.2 | 1 | 364 | 0 | THE1B | MaLR | LTR |
| 30290171 | 30290454 | - | 30.2 | 100 | 381 | -110 | LTR16C | ERVL | LTR |

  

|  |  |  |  |  |  |  |  |  |  |
| --- | --- | --- | --- | --- | --- | --- | --- | --- | --- |
| chr4:30809048-30813565 | | | 4516 bp | | |  |  |  |  |
| Genome Start | Genome End | Strand | Div | Repeat Start | Repeat End | Repeat Left | Name | Family | Class |
| 30809048 | 30809071 | - | 23.6 | 5393 | 5414 | -756 | L1M | L1 | LINE |
| 30809072 | 30809136 | - | 10.2 | 301 | 355 | 0 | THE1A | MaLR | LTR |
| 30809137 | 30809407 | + | 10.7 | 41 | 313 | 0 | AluSq | Alu | SINE |
| 30809408 | 30809686 | - | 10.2 | 1 | 300 | -55 | THE1A | MaLR | LTR |
| 30809687 | 30811212 | - | 29.3 | 2 | 1735 | 0 | MLT1C-int | MaLR | LTR |
| 30811215 | 30811562 | - | 10.7 | 1 | 355 | 0 | THE1A | MaLR | LTR |
| 30811563 | 30812415 | - | 29.5 | 4478 | 5392 | -778 | L1M | L1 | LINE |
| 30812416 | 30813115 | + | 7.9 | 1 | 688 | 0 | LTR12\_ | ERV1 | LTR |
| 30813116 | 30813565 | - | 30.5 | 4021 | 4477 | -1669 | L1M | L1 | LINE |

  

|  |  |  |  |  |  |  |  |  |  |
| --- | --- | --- | --- | --- | --- | --- | --- | --- | --- |
| chr4:31492167-31500661 | | | 8727 bp | | |  |  |  |  |
| Genome Start | Genome End | Strand | Div | Repeat Start | Repeat End | Repeat Left | Name | Family | Class |
| 31492167 | 31492734 | + | 33.2 | 186 | 765 | -5777 | L1MB5 | L1 | LINE |
| 31492736 | 31493040 | + | 24.7 | 882 | 1205 | -5337 | L1MB5 | L1 | LINE |
| 31493041 | 31493781 | + | 16.1 | 1 | 697 | 0 | MER50B | ERV1 | LTR |
| 31493782 | 31493888 | + | 24.7 | 1206 | 1282 | -5260 | L1MB5 | L1 | LINE |
| 31493941 | 31494071 | + | 18.3 | 21 | 151 | -25 | FRAM | Alu | SINE |
| 31494106 | 31494776 | + | 24.0 | 3141 | 3834 | -2810 | L1MB5 | L1 | LINE |
| 31494806 | 31495081 | - | 17.5 | 1 | 280 | -30 | AluSg | Alu | SINE |
| 31495082 | 31495183 | + | 24.0 | 3835 | 3923 | -2721 | L1MB5 | L1 | LINE |
| 31495187 | 31495554 | + | 25.0 | 4 | 367 | -7 | MLT1A | MaLR | LTR |
| 31495555 | 31495938 | + | 15.0 | 1 | 381 | 0 | THE1D | MaLR | LTR |
| 31495939 | 31497455 | + | 15.8 | 1 | 1579 | -1 | THE1D-int | MaLR | LTR |
| 31497456 | 31497798 | + | 16.5 | 9 | 381 | 0 | THE1D | MaLR | LTR |
| 31497821 | 31499053 | + | 24.3 | 3929 | 5198 | -1446 | L1MB5 | L1 | LINE |
| 31499169 | 31499399 | + | 24.0 | 1 | 232 | -235 | MLT1C | MaLR | LTR |
| 31499400 | 31499657 | + | 9.4 | 6039 | 6302 | 0 | L1MA1 | L1 | LINE |
| 31499658 | 31499890 | + | 24.0 | 233 | 467 | 0 | MLT1C | MaLR | LTR |
| 31499901 | 31500549 | + | 27.7 | 5326 | 6026 | -265 | L1MA8 | L1 | LINE |
| 31500052 | 31500661 | + | 21.2 | 5510 | 6138 | -36 | L1MB5 | L1 | LINE |

  

|  |  |  |  |  |  |  |  |  |  |
| --- | --- | --- | --- | --- | --- | --- | --- | --- | --- |
| chr4:31560619-31563216 | | | 2586 bp | | |  |  |  |  |
| Genome Start | Genome End | Strand | Div | Repeat Start | Repeat End | Repeat Left | Name | Family | Class |
| 31560619 | 31560730 | - | 20.0 | 402 | 519 | 0 | LTR40a | ERVL | LTR |
| 31560735 | 31561105 | + | 11.8 | 3 | 375 | 0 | THE1C | MaLR | LTR |
| 31561106 | 31562637 | + | 14.4 | 1 | 1580 | 0 | THE1C-int | MaLR | LTR |
| 31562640 | 31563015 | + | 15.4 | 3 | 375 | 0 | THE1C | MaLR | LTR |
| 31563022 | 31563216 | - | 28.2 | 199 | 400 | -119 | LTR40a | ERVL | LTR |

  

|  |  |  |  |  |  |  |  |  |  |
| --- | --- | --- | --- | --- | --- | --- | --- | --- | --- |
| chr4:31721921-31729896 | | | 7947 bp | | |  |  |  |  |
| Genome Start | Genome End | Strand | Div | Repeat Start | Repeat End | Repeat Left | Name | Family | Class |
| 31721921 | 31722288 | - | 15.7 | 838 | 1230 | -1 | Tigger3b | MER2\_type | DNA |
| 31722289 | 31722579 | + | 12.4 | 1 | 294 | -15 | AluSc | Alu | SINE |
| 31722580 | 31723387 | - | 15.7 | 68 | 837 | -394 | Tigger3b | MER2\_type | DNA |
| 31723407 | 31723764 | - | 16.1 | 5799 | 6154 | 0 | L1PB3 | L1 | LINE |
| 31723767 | 31724153 | - | 14.7 | 1 | 381 | 0 | THE1D | MaLR | LTR |
| 31724154 | 31725581 | - | 14.9 | 4 | 1580 | 0 | THE1D-int | MaLR | LTR |
| 31725585 | 31725964 | - | 14.4 | 1 | 381 | 0 | THE1D | MaLR | LTR |
| 31725965 | 31728781 | - | 18.3 | 2920 | 5801 | -353 | L1PB3 | L1 | LINE |
| 31728782 | 31729080 | - | 15.2 | 1 | 307 | -5 | AluSx | Alu | SINE |
| 31729081 | 31729815 | - | 19.4 | 2175 | 2919 | -3227 | L1PB3 | L1 | LINE |
| 31729821 | 31729896 | - | 22.4 | 2 | 83 | -1148 | Tigger3b | MER2\_type | DNA |

  

|  |  |  |  |  |  |  |  |  |  |
| --- | --- | --- | --- | --- | --- | --- | --- | --- | --- |
| chr4:31942194-31948229 | | | 5665 bp | | |  |  |  |  |
| Genome Start | Genome End | Strand | Div | Repeat Start | Repeat End | Repeat Left | Name | Family | Class |
| 31942194 | 31942293 | + | 30.3 | 940 | 1050 | -5069 | L1ME3 | L1 | LINE |
| 31942294 | 31942594 | + | 14.2 | 1 | 296 | -16 | AluSx | Alu | SINE |
| 31942595 | 31942651 | + | 30.3 | 1051 | 1110 | -5009 | L1ME3 | L1 | LINE |
| 31942815 | 31943066 | + | 29.0 | 1328 | 1586 | -4563 | L1ME3 | L1 | LINE |
| 31943108 | 31943456 | - | 18.0 | 1 | 364 | 0 | THE1B | MaLR | LTR |
| 31943490 | 31943795 | + | 15.0 | 1 | 307 | -5 | AluSx | Alu | SINE |
| 31943886 | 31944166 | + | 33.0 | 1812 | 2093 | -4056 | L1ME3 | L1 | LINE |
| 31944169 | 31944517 | + | 28.6 | 4654 | 5019 | -1110 | L1ME3 | L1 | LINE |
| 31944518 | 31944814 | - | 24.2 | 1 | 345 | 0 | MER2 | MER2\_type | DNA |
| 31944815 | 31945315 | + | 27.0 | 5020 | 5579 | -574 | L1ME3 | L1 | LINE |
| 31945354 | 31945715 | + | 14.4 | 1 | 362 | -2 | THE1B | MaLR | LTR |
| 31945720 | 31947277 | + | 29.9 | 2 | 1735 | 0 | MLT-int | MaLR | LTR |
| 31947278 | 31947637 | + | 15.2 | 1 | 353 | -11 | THE1B | MaLR | LTR |
| 31947638 | 31947902 | + | 28.5 | 5786 | 6081 | -64 | L1ME3 | L1 | LINE |
| 31947903 | 31948171 | + | 21.9 | 1 | 300 | -2 | AluJb | Alu | SINE |
| 31948172 | 31948229 | + | 28.5 | 6082 | 6138 | -7 | L1ME3 | L1 | LINE |

  

|  |  |  |  |  |  |  |  |  |  |
| --- | --- | --- | --- | --- | --- | --- | --- | --- | --- |
| chr4:32875534-32879401 | | | 3826 bp | | |  |  |  |  |
| Genome Start | Genome End | Strand | Div | Repeat Start | Repeat End | Repeat Left | Name | Family | Class |
| 32875534 | 32875708 | - | 28.9 | 3256 | 3411 | -8 | L2 | L2 | LINE |
| 32875709 | 32875929 | - | 19.4 | 272 | 554 | -2 | MLT2B4 | ERVL | LTR |
| 32875956 | 32876204 | - | 19.4 | 3 | 271 | -285 | MLT2B4 | ERVL | LTR |
| 32876205 | 32876751 | - | 28.9 | 2726 | 3255 | -164 | L2 | L2 | LINE |
| 32876758 | 32877112 | + | 14.7 | 1 | 364 | 0 | THE1B | MaLR | LTR |
| 32877118 | 32878707 | + | 31.1 | 5 | 1735 | 0 | MLT1F2-int | MaLR | LTR |
| 32878713 | 32879060 | + | 14.7 | 6 | 364 | 0 | THE1B | MaLR | LTR |
| 32879061 | 32879401 | - | 29.5 | 2349 | 2717 | -702 | L2 | L2 | LINE |

  

|  |  |  |  |  |  |  |  |  |  |
| --- | --- | --- | --- | --- | --- | --- | --- | --- | --- |
| chr4:33183635-33190398 | | | 6755 bp | | |  |  |  |  |
| Genome Start | Genome End | Strand | Div | Repeat Start | Repeat End | Repeat Left | Name | Family | Class |
| 33183635 | 33184469 | + | 19.3 | 1666 | 2510 | -3677 | MER57A-int | ERV1 | LTR |
| 33184470 | 33184842 | + | 13.5 | 3 | 375 | 0 | THE1C | MaLR | LTR |
| 33184843 | 33186354 | + | 15.1 | 1 | 1580 | 0 | THE1C-int | MaLR | LTR |
| 33186358 | 33186723 | + | 12.4 | 5 | 375 | 0 | THE1C | MaLR | LTR |
| 33186730 | 33190398 | + | 19.2 | 2507 | 6158 | -29 | MER57A-int | ERV1 | LTR |

  

|  |  |  |  |  |  |  |  |  |  |
| --- | --- | --- | --- | --- | --- | --- | --- | --- | --- |
| chr4:33866912-33869677 | | | 2763 bp | | |  |  |  |  |
| Genome Start | Genome End | Strand | Div | Repeat Start | Repeat End | Repeat Left | Name | Family | Class |
| 33866912 | 33867065 | - | 18.1 | 158 | 306 | -6 | AluJ | Alu | SINE |
| 33867066 | 33867477 | + | 15.6 | 1 | 428 | 0 | MSTA | MaLR | LTR |
| 33867481 | 33869098 | + | 31.2 | 2 | 1735 | 0 | MLT-int | MaLR | LTR |
| 33869099 | 33869512 | + | 15.8 | 1 | 428 | 0 | MSTA | MaLR | LTR |
| 33869513 | 33869535 | - | 18.1 | 135 | 157 | -155 | AluJ | Alu | SINE |
| 33869536 | 33869677 | - | 18.8 | 1 | 133 | -169 | AluJ | Alu | SINE |

  

|  |  |  |  |  |  |  |  |  |  |
| --- | --- | --- | --- | --- | --- | --- | --- | --- | --- |
| chr4:33901556-33909122 | | | 7523 bp | | |  |  |  |  |
| Genome Start | Genome End | Strand | Div | Repeat Start | Repeat End | Repeat Left | Name | Family | Class |
| 33901556 | 33902982 | - | 13.8 | 6291 | 7724 | 0 | L1MA3 | L1 | LINE |
| 33902983 | 33903333 | + | 12.4 | 1 | 355 | 0 | THE1A | MaLR | LTR |
| 33903336 | 33903707 | + | 30.3 | 2 | 436 | -1299 | MLT-int | MaLR | LTR |
| 33903708 | 33904015 | - | 7.5 | 1 | 309 | -2 | AluY | Alu | SINE |
| 33904016 | 33905226 | + | 30.3 | 437 | 1734 | -1 | MLT-int | MaLR | LTR |
| 33905228 | 33905582 | + | 17.3 | 1 | 355 | 0 | THE1A | MaLR | LTR |
| 33905583 | 33905942 | - | 15.6 | 5897 | 6290 | -1275 | L1MA3 | L1 | LINE |
| 33905984 | 33907395 | - | 15.4 | 4471 | 5896 | -1669 | L1MA3 | L1 | LINE |
| 33907396 | 33907500 | - | 22.9 | 670 | 776 | -264 | MER45B | Tip100 | DNA |
| 33907501 | 33909122 | - | 15.4 | 2832 | 4470 | -3095 | L1MA3 | L1 | LINE |

  

|  |  |  |  |  |  |  |  |  |  |
| --- | --- | --- | --- | --- | --- | --- | --- | --- | --- |
| chr4:33939299-33943972 | | | 4657 bp | | |  |  |  |  |
| Genome Start | Genome End | Strand | Div | Repeat Start | Repeat End | Repeat Left | Name | Family | Class |
| 33939299 | 33940459 | + | 27.9 | 1 | 1945 | -5264 | L1M2b | L1 | LINE |
| 33940461 | 33940818 | - | 13.6 | 2 | 373 | -2 | THE1C | MaLR | LTR |
| 33940828 | 33942418 | - | 11.9 | 1 | 1573 | -7 | THE1C-int | MaLR | LTR |
| 33942421 | 33942786 | - | 14.5 | 4 | 373 | -2 | THE1C | MaLR | LTR |
| 33942792 | 33943972 | + | 24.6 | 1952 | 3147 | -4062 | L1M2b | L1 | LINE |

  

|  |  |  |  |  |  |  |  |  |  |
| --- | --- | --- | --- | --- | --- | --- | --- | --- | --- |
| chr4:34586926-34590398 | | | 3199 bp | | |  |  |  |  |
| Genome Start | Genome End | Strand | Div | Repeat Start | Repeat End | Repeat Left | Name | Family | Class |
| 34586926 | 34586966 | + | 33.0 | 2 | 33 | -6820 | L1PA15-16 | L1 | LINE |
| 34586967 | 34587312 | + | 16.6 | 1 | 364 | 0 | THE1B | MaLR | LTR |
| 34587317 | 34588873 | + | 31.1 | 4 | 1735 | 0 | MLT-int | MaLR | LTR |
| 34588874 | 34589233 | + | 15.8 | 1 | 364 | 0 | THE1B | MaLR | LTR |
| 34589234 | 34590008 | + | 33.6 | 34 | 1028 | -5841 | L1PA15-16 | L1 | LINE |
| 34590279 | 34590398 | + | 23.9 | 1408 | 1525 | -5325 | L1PA15-16 | L1 | LINE |

  

|  |  |  |  |  |  |  |  |  |  |
| --- | --- | --- | --- | --- | --- | --- | --- | --- | --- |
| chr4:34849370-34852607 | | | 3236 bp | | |  |  |  |  |
| Genome Start | Genome End | Strand | Div | Repeat Start | Repeat End | Repeat Left | Name | Family | Class |
| 34849370 | 34849455 | + | 24.4 | 1 | 95 | -410 | MLT1D | MaLR | LTR |
| 34849461 | 34849836 | + | 21.4 | 4 | 396 | 0 | MSTD | MaLR | LTR |
| 34849836 | 34850111 | + | 26.4 | 3 | 278 | -1457 | MSTD-int | MaLR | LTR |
| 34850112 | 34850535 | + | 13.5 | 1 | 428 | 0 | MSTA | MaLR | LTR |
| 34850536 | 34851904 | + | 26.4 | 279 | 1729 | -6 | MSTD-int | MaLR | LTR |
| 34851910 | 34852271 | + | 24.8 | 1 | 396 | 0 | MSTD | MaLR | LTR |
| 34852272 | 34852548 | + | 23.9 | 93 | 397 | -108 | MLT1D | MaLR | LTR |
| 34852542 | 34852607 | + | 17.2 | 558 | 621 | -3 | MLT1D | MaLR | LTR |

  

|  |  |  |  |  |  |  |  |  |  |
| --- | --- | --- | --- | --- | --- | --- | --- | --- | --- |
| chr4:34918398-34921061 | | | 2592 bp | | |  |  |  |  |
| Genome Start | Genome End | Strand | Div | Repeat Start | Repeat End | Repeat Left | Name | Family | Class |
| 34918398 | 34918470 | + | 21.1 | 3101 | 3171 | -2514 | HERVL74 | ERVL | LTR |
| 34918541 | 34918902 | + | 16.8 | 1 | 364 | 0 | THE1B | MaLR | LTR |
| 34918903 | 34920627 | + | 12.1 | 1 | 1580 | 0 | THE1B-int | MaLR | LTR |
| 34920630 | 34920989 | + | 16.1 | 3 | 364 | 0 | THE1B | MaLR | LTR |
| 34920990 | 34921061 | + | 22.2 | 3477 | 3549 | -7 | HERVL74 | ERVL | LTR |

  

|  |  |  |  |  |  |  |  |  |  |
| --- | --- | --- | --- | --- | --- | --- | --- | --- | --- |
| chr4:35108209-35111198 | | | 2682 bp | | |  |  |  |  |
| Genome Start | Genome End | Strand | Div | Repeat Start | Repeat End | Repeat Left | Name | Family | Class |
| 35108209 | 35108400 | + | 25.1 | 1 | 176 | -329 | MLT1D | MaLR | LTR |
| 35108401 | 35108760 | + | 15.0 | 1 | 381 | 0 | THE1D | MaLR | LTR |
| 35108763 | 35110044 | + | 15.4 | 3 | 1304 | -276 | THE1D-int | MaLR | LTR |
| 35110328 | 35110524 | + | 12.9 | 1379 | 1580 | 0 | THE1D-int | MaLR | LTR |
| 35110525 | 35110900 | + | 15.5 | 1 | 381 | 0 | THE1D | MaLR | LTR |
| 35110924 | 35111198 | + | 27.1 | 224 | 505 | 0 | MLT1D | MaLR | LTR |

  

|  |  |  |  |  |  |  |  |  |  |
| --- | --- | --- | --- | --- | --- | --- | --- | --- | --- |
| chr4:36626226-36632929 | | | 6674 bp | | |  |  |  |  |
| Genome Start | Genome End | Strand | Div | Repeat Start | Repeat End | Repeat Left | Name | Family | Class |
| 36626226 | 36626347 | + | 15.6 | 136 | 262 | -83 | MER2B | MER2\_type | DNA |
| 36626352 | 36627042 | - | 11.6 | 5475 | 6167 | -7 | L1PA11 | L1 | LINE |
| 36627067 | 36627171 | - | 8.4 | 6066 | 6168 | 0 | L1PA10 | L1 | LINE |
| 36627172 | 36627531 | - | 11.5 | 1 | 375 | 0 | THE1C | MaLR | LTR |
| 36627532 | 36629052 | - | 29.4 | 2 | 1735 | 0 | MLT-int | MaLR | LTR |
| 36629055 | 36629433 | - | 12.4 | 1 | 375 | 0 | THE1C | MaLR | LTR |
| 36629434 | 36632817 | - | 10.1 | 2717 | 6065 | -103 | L1PA10 | L1 | LINE |
| 36632818 | 36632929 | + | 26.8 | 224 | 336 | 0 | MER2B | MER2\_type | DNA |

  

|  |  |  |  |  |  |  |  |  |  |
| --- | --- | --- | --- | --- | --- | --- | --- | --- | --- |
| chr4:36820116-36829537 | | | 9412 bp | | |  |  |  |  |
| Genome Start | Genome End | Strand | Div | Repeat Start | Repeat End | Repeat Left | Name | Family | Class |
| 36820116 | 36823464 | - | 10.0 | 4313 | 7692 | -29 | L1MA1 | L1 | LINE |
| 36823466 | 36823818 | + | 13.4 | 1 | 375 | 0 | THE1C | MaLR | LTR |
| 36823819 | 36825336 | + | 14.2 | 1 | 1569 | -11 | THE1C-int | MaLR | LTR |
| 36825346 | 36825710 | + | 14.4 | 1 | 375 | 0 | THE1C | MaLR | LTR |
| 36825711 | 36829537 | - | 20.4 | 15 | 4316 | -3249 | L1MA1 | L1 | LINE |

  

|  |  |  |  |  |  |  |  |  |  |
| --- | --- | --- | --- | --- | --- | --- | --- | --- | --- |
| chr4:37159518-37161412 | | | 1895 bp | | |  |  |  |  |
| Genome Start | Genome End | Strand | Div | Repeat Start | Repeat End | Repeat Left | Name | Family | Class |
| 37159518 | 37159777 | - | 20.2 | 43 | 299 | -91 | MLT1B | MaLR | LTR |
| 37159779 | 37160154 | + | 11.6 | 1 | 375 | 0 | THE1C | MaLR | LTR |
| 37160155 | 37160898 | + | 12.8 | 1 | 781 | -799 | THE1C-int | MaLR | LTR |
| 37160898 | 37160992 | + | 6.4 | 1487 | 1580 | 0 | THE1C-int | MaLR | LTR |
| 37160993 | 37161365 | + | 10.3 | 1 | 373 | -2 | THE1C | MaLR | LTR |
| 37161366 | 37161412 | - | 17.0 | 2 | 48 | -342 | MLT1B | MaLR | LTR |

  

|  |  |  |  |  |  |  |  |  |  |
| --- | --- | --- | --- | --- | --- | --- | --- | --- | --- |
| chr4:37394949-37398373 | | | 3402 bp | | |  |  |  |  |
| Genome Start | Genome End | Strand | Div | Repeat Start | Repeat End | Repeat Left | Name | Family | Class |
| 37394949 | 37395090 | + | 26.1 | 5065 | 5208 | -938 | L1M | L1 | LINE |
| 37395091 | 37395188 | + | 28.2 | 3 | 91 | -504 | MLT1G1 | MaLR | LTR |
| 37395189 | 37395497 | + | 6.8 | 1 | 309 | -2 | AluY | Alu | SINE |
| 37395498 | 37395972 | + | 28.2 | 92 | 588 | -7 | MLT1G1 | MaLR | LTR |
| 37395980 | 37397401 | + | 24.7 | 4 | 1489 | 0 | MLT1G1-int | MaLR | LTR |
| 37397402 | 37397820 | + | 26.5 | 1 | 356 | -199 | MLT1G1 | MaLR | LTR |
| 37397821 | 37398148 | + | 7.5 | 1 | 308 | -3 | AluY | Alu | SINE |
| 37398149 | 37398316 | + | 27.3 | 357 | 551 | -4 | MLT1G1 | MaLR | LTR |
| 37398333 | 37398373 | + | 25.4 | 5280 | 5318 | -844 | L1M | L1 | LINE |

  

|  |  |  |  |  |  |  |  |  |  |
| --- | --- | --- | --- | --- | --- | --- | --- | --- | --- |
| chr4:38096159-38098741 | | | 2540 bp | | |  |  |  |  |
| Genome Start | Genome End | Strand | Div | Repeat Start | Repeat End | Repeat Left | Name | Family | Class |
| 38096159 | 38096327 | - | 23.9 | 84 | 258 | -3 | MER112 | MER1\_type | DNA |
| 38096371 | 38096739 | - | 14.0 | 1 | 364 | 0 | THE1B | MaLR | LTR |
| 38096740 | 38098304 | - | 8.8 | 1 | 1580 | 0 | THE1B-int | MaLR | LTR |
| 38098305 | 38098673 | - | 10.9 | 1 | 364 | 0 | THE1B | MaLR | LTR |
| 38098674 | 38098741 | - | 19.7 | 1 | 72 | -188 | MER112 | MER1\_type | DNA |

  

|  |  |  |  |  |  |  |  |  |  |
| --- | --- | --- | --- | --- | --- | --- | --- | --- | --- |
| chr4:41668536-41671228 | | | 2676 bp | | |  |  |  |  |
| Genome Start | Genome End | Strand | Div | Repeat Start | Repeat End | Repeat Left | Name | Family | Class |
| 41668536 | 41668808 | - | 21.3 | 135 | 467 | 0 | MLT1B | MaLR | LTR |
| 41668824 | 41669173 | + | 13.7 | 15 | 364 | 0 | THE1B | MaLR | LTR |
| 41669174 | 41670742 | + | 8.4 | 1 | 1580 | 0 | THE1B-int | MaLR | LTR |
| 41670743 | 41671107 | + | 11.6 | 1 | 364 | 0 | THE1B | MaLR | LTR |
| 41671110 | 41671228 | - | 22.7 | 1 | 123 | -251 | MLT1B | MaLR | LTR |

  

|  |  |  |  |  |  |  |  |  |  |
| --- | --- | --- | --- | --- | --- | --- | --- | --- | --- |
| chr4:42497276-42501728 | | | 4162 bp | | |  |  |  |  |
| Genome Start | Genome End | Strand | Div | Repeat Start | Repeat End | Repeat Left | Name | Family | Class |
| 42497276 | 42497674 | - | 21.7 | 53 | 466 | -1 | MLT1C | MaLR | LTR |
| 42497823 | 42497958 | - | 13.6 | 1 | 132 | -1 | FLAM\_C | Alu | SINE |
| 42497960 | 42498399 | - | 14.0 | 5030 | 5474 | -859 | L1MC | L1 | LINE |
| 42498400 | 42498812 | - | 13.4 | 1 | 428 | 0 | MSTA | MaLR | LTR |
| 42498813 | 42500373 | - | 27.9 | 2 | 1735 | 0 | MLT-int | MaLR | LTR |
| 42500376 | 42500787 | - | 14.6 | 1 | 428 | 0 | MSTA | MaLR | LTR |
| 42500788 | 42501413 | - | 15.0 | 4464 | 5029 | -1117 | L1MC | L1 | LINE |
| 42501424 | 42501511 | - | 27.3 | 1443 | 1532 | -975 | HAL1 | L1 | LINE |
| 42501642 | 42501728 | - | 16.2 | 1 | 80 | -285 | MLT1C | MaLR | LTR |

  

|  |  |  |  |  |  |  |  |  |  |
| --- | --- | --- | --- | --- | --- | --- | --- | --- | --- |
| chr4:42528373-42530878 | | | 2504 bp | | |  |  |  |  |
| Genome Start | Genome End | Strand | Div | Repeat Start | Repeat End | Repeat Left | Name | Family | Class |
| 42528373 | 42528420 | - | 26.3 | 198 | 246 | -22 | MIRb | MIR | SINE |
| 42528421 | 42528782 | + | 12.4 | 1 | 364 | 0 | THE1B | MaLR | LTR |
| 42528785 | 42530390 | + | 31.4 | 2 | 1735 | 0 | MLT1H1-int | MaLR | LTR |
| 42530391 | 42530754 | + | 11.0 | 1 | 364 | 0 | THE1B | MaLR | LTR |
| 42530755 | 42530878 | - | 26.4 | 20 | 197 | -71 | MIRb | MIR | SINE |

  

|  |  |  |  |  |  |  |  |  |  |
| --- | --- | --- | --- | --- | --- | --- | --- | --- | --- |
| chr4:42809472-42821072 | | | 11520 bp | | |  |  |  |  |
| Genome Start | Genome End | Strand | Div | Repeat Start | Repeat End | Repeat Left | Name | Family | Class |
| 42809472 | 42809830 | - | 21.9 | 1392 | 1753 | -1108 | Charlie2a | MER1\_type | DNA |
| 42809832 | 42811546 | + | 16.4 | 17 | 1793 | -5772 | L1MA1 | L1 | LINE |
| 42811547 | 42811647 | + | 7.9 | 211 | 311 | 0 | AluY | Alu | SINE |
| 42811648 | 42812699 | + | 16.4 | 1794 | 2883 | -4682 | L1MA1 | L1 | LINE |
| 42812741 | 42815177 | + | 12.2 | 2886 | 5371 | -2194 | L1MA1 | L1 | LINE |
| 42815178 | 42815359 | + | 15.9 | 131 | 312 | 0 | AluSg/x | Alu | SINE |
| 42815360 | 42816066 | + | 12.2 | 5372 | 6092 | -1473 | L1MA1 | L1 | LINE |
| 42816054 | 42816127 | + | 11.0 | 6715 | 6788 | -799 | L1P | L1 | LINE |
| 42816180 | 42816534 | - | 10.5 | 1 | 344 | -11 | THE1A | MaLR | LTR |
| 42816535 | 42818125 | - | 10.4 | 1 | 1580 | 0 | THE1A-int | MaLR | LTR |
| 42818126 | 42818473 | - | 6.6 | 1 | 355 | 0 | THE1A | MaLR | LTR |
| 42818474 | 42819411 | + | 9.8 | 6779 | 7714 | -7 | L1MA1 | L1 | LINE |
| 42819412 | 42819617 | - | 29.4 | 1219 | 1452 | -1409 | Charlie2a | MER1\_type | DNA |
| 42819618 | 42820056 | - | 22.0 | 1 | 467 | 0 | MLT1C | MaLR | LTR |
| 42820057 | 42821072 | - | 28.8 | 65 | 1218 | -1643 | Charlie2a | MER1\_type | DNA |

  

|  |  |  |  |  |  |  |  |  |  |
| --- | --- | --- | --- | --- | --- | --- | --- | --- | --- |
| chr4:43008528-43011334 | | | 2627 bp | | |  |  |  |  |
| Genome Start | Genome End | Strand | Div | Repeat Start | Repeat End | Repeat Left | Name | Family | Class |
| 43008528 | 43008623 | + | 30.2 | 21 | 118 | -403 | LTR33 | ERVL | LTR |
| 43008648 | 43009006 | + | 13.7 | 1 | 364 | 0 | THE1B | MaLR | LTR |
| 43009007 | 43010616 | + | 12.0 | 1 | 1571 | -9 | THE1B-int | MaLR | LTR |
| 43010625 | 43010984 | + | 11.7 | 1 | 364 | 0 | THE1B | MaLR | LTR |
| 43011133 | 43011334 | + | 28.1 | 328 | 518 | -3 | LTR33 | ERVL | LTR |

  

|  |  |  |  |  |  |  |  |  |  |
| --- | --- | --- | --- | --- | --- | --- | --- | --- | --- |
| chr4:43248627-43254273 | | | 5632 bp | | |  |  |  |  |
| Genome Start | Genome End | Strand | Div | Repeat Start | Repeat End | Repeat Left | Name | Family | Class |
| 43248627 | 43249153 | - | 16.0 | 3171 | 3733 | -2592 | L1M3f | L1 | LINE |
| 43249157 | 43249532 | + | 11.9 | 1 | 375 | 0 | THE1C | MaLR | LTR |
| 43249533 | 43251065 | + | 12.9 | 1 | 1580 | 0 | THE1C-int | MaLR | LTR |
| 43251067 | 43251431 | + | 14.6 | 2 | 368 | -7 | THE1C | MaLR | LTR |
| 43251435 | 43251501 | - | 17.7 | 3107 | 3176 | -3149 | L1M3f | L1 | LINE |
| 43251502 | 43251809 | - | 13.0 | 1 | 309 | 0 | AluSc | Alu | SINE |
| 43251810 | 43253030 | - | 20.7 | 1856 | 3106 | -3219 | L1M3f | L1 | LINE |
| 43253039 | 43254273 | - | 27.1 | 556 | 1787 | -4541 | L1M3f | L1 | LINE |

  

|  |  |  |  |  |  |  |  |  |  |
| --- | --- | --- | --- | --- | --- | --- | --- | --- | --- |
| chr4:44492933-44497924 | | | 4989 bp | | |  |  |  |  |
| Genome Start | Genome End | Strand | Div | Repeat Start | Repeat End | Repeat Left | Name | Family | Class |
| 44492933 | 44493420 | - | 25.2 | 2371 | 2872 | -4100 | L1M4b | L1 | LINE |
| 44493422 | 44493889 | - | 21.4 | 1399 | 1956 | -4690 | L1M4b | L1 | LINE |
| 44493890 | 44494080 | + | 15.1 | 3 | 199 | -229 | MSTA | MaLR | LTR |
| 44494081 | 44494403 | + | 14.8 | 1 | 318 | 0 | AluSc | Alu | SINE |
| 44494404 | 44494631 | + | 15.1 | 200 | 428 | 0 | MSTA | MaLR | LTR |
| 44494634 | 44496233 | + | 31.1 | 2 | 1735 | 0 | MLT-int | MaLR | LTR |
| 44496234 | 44496638 | + | 11.9 | 1 | 428 | 0 | MSTA | MaLR | LTR |
| 44496639 | 44497924 | - | 22.8 | 51 | 1442 | -5100 | L1M4b | L1 | LINE |

  

|  |  |  |  |  |  |  |  |  |  |
| --- | --- | --- | --- | --- | --- | --- | --- | --- | --- |
| chr4:44547705-44551297 | | | 3591 bp | | |  |  |  |  |
| Genome Start | Genome End | Strand | Div | Repeat Start | Repeat End | Repeat Left | Name | Family | Class |
| 44547705 | 44547822 | - | 16.7 | 5139 | 5256 | -887 | L1M2 | L1 | LINE |
| 44547823 | 44548196 | + | 12.5 | 1 | 364 | 0 | THE1B | MaLR | LTR |
| 44548199 | 44549774 | + | 30.4 | 2 | 1735 | 0 | MLT-int | MaLR | LTR |
| 44549775 | 44550149 | + | 11.3 | 1 | 364 | 0 | THE1B | MaLR | LTR |
| 44550150 | 44551297 | - | 16.7 | 3995 | 5138 | -1005 | L1M2 | L1 | LINE |

  

|  |  |  |  |  |  |  |  |  |  |
| --- | --- | --- | --- | --- | --- | --- | --- | --- | --- |
| chr4:44788347-44792571 | | | 4201 bp | | |  |  |  |  |
| Genome Start | Genome End | Strand | Div | Repeat Start | Repeat End | Repeat Left | Name | Family | Class |
| 44788347 | 44789222 | + | 13.2 | 4794 | 5651 | -514 | L1PA15 | L1 | LINE |
| 44789223 | 44789638 | - | 16.5 | 2 | 426 | 0 | MSTB | MaLR | LTR |
| 44789651 | 44790614 | - | 19.4 | 679 | 1641 | -10 | MSTB-int | MaLR | LTR |
| 44790620 | 44790986 | + | 11.3 | 6 | 375 | 0 | THE1C | MaLR | LTR |
| 44790988 | 44791660 | - | 19.4 | 1 | 682 | -969 | MSTB-int | MaLR | LTR |
| 44791661 | 44792062 | - | 17.2 | 1 | 426 | 0 | MSTB | MaLR | LTR |
| 44792069 | 44792571 | + | 13.7 | 5654 | 6158 | -7 | L1PA15 | L1 | LINE |

  

|  |  |  |  |  |  |  |  |  |  |
| --- | --- | --- | --- | --- | --- | --- | --- | --- | --- |
| chr4:45052207-45060025 | | | 7165 bp | | |  |  |  |  |
| Genome Start | Genome End | Strand | Div | Repeat Start | Repeat End | Repeat Left | Name | Family | Class |
| 45052207 | 45052261 | + | 16.4 | 1573 | 1628 | -4587 | L1MC | L1 | LINE |
| 45052310 | 45053104 | + | 25.2 | 1733 | 2555 | -3660 | L1MC | L1 | LINE |
| 45053271 | 45053730 | + | 29.6 | 2744 | 3244 | -2970 | L1MC | L1 | LINE |
| 45053721 | 45054042 | + | 30.2 | 4146 | 4477 | -1737 | L1MC | L1 | LINE |
| 45054059 | 45054578 | - | 27.0 | 33 | 623 | -3 | MLT1E2 | MaLR | LTR |
| 45054905 | 45055282 | + | 12.8 | 2 | 428 | 0 | MSTA | MaLR | LTR |
| 45055284 | 45056194 | + | 17.8 | 2 | 933 | -718 | MSTA-int | MaLR | LTR |
| 45056195 | 45056550 | + | 18.3 | 1 | 364 | -11 | THE1C | MaLR | LTR |
| 45056562 | 45058171 | + | 15.2 | 1 | 1571 | -9 | THE1C-int | MaLR | LTR |
| 45058181 | 45058510 | + | 16.0 | 1 | 375 | 0 | THE1C | MaLR | LTR |
| 45058511 | 45059225 | + | 15.7 | 861 | 1580 | 0 | MSTA-int | MaLR | LTR |
| 45059229 | 45059601 | + | 18.1 | 4 | 426 | -2 | MSTA | MaLR | LTR |
| 45059686 | 45060025 | + | 36.0 | 4950 | 5308 | -906 | L1MC | L1 | LINE |

  

|  |  |  |  |  |  |  |  |  |  |
| --- | --- | --- | --- | --- | --- | --- | --- | --- | --- |
| chr4:45236897-45242954 | | | 6054 bp | | |  |  |  |  |
| Genome Start | Genome End | Strand | Div | Repeat Start | Repeat End | Repeat Left | Name | Family | Class |
| 45236897 | 45237041 | + | 13.8 | 7 | 179 | -7976 | L1PREC2 | L1 | LINE |
| 45237044 | 45237428 | + | 15.8 | 11 | 428 | 0 | MSTA | MaLR | LTR |
| 45237431 | 45239015 | + | 30.7 | 2 | 1735 | 0 | MLT-int | MaLR | LTR |
| 45239016 | 45239206 | + | 11.2 | 1 | 196 | -232 | MSTA | MaLR | LTR |
| 45239207 | 45239691 | + | 9.7 | 1 | 484 | -204 | LTR12\_ | ERV1 | LTR |
| 45239692 | 45242954 | + | 20.6 | 2685 | 6159 | -1 | L1PREC2 | L1 | LINE |

  

|  |  |  |  |  |  |  |  |  |  |
| --- | --- | --- | --- | --- | --- | --- | --- | --- | --- |
| chr4:45656326-45668237 | | | 11413 bp | | |  |  |  |  |
| Genome Start | Genome End | Strand | Div | Repeat Start | Repeat End | Repeat Left | Name | Family | Class |
| 45656326 | 45656654 | - | 16.5 | 5190 | 5520 | -2042 | L1M1 | L1 | LINE |
| 45656732 | 45657771 | - | 11.0 | 5105 | 6159 | -4 | L1PA13 | L1 | LINE |
| 45657772 | 45658124 | - | 15.0 | 1 | 355 | 0 | THE1A | MaLR | LTR |
| 45658125 | 45659685 | - | 30.4 | 2 | 1735 | 0 | MLT1K-int | MaLR | LTR |
| 45659688 | 45660042 | - | 13.1 | 1 | 355 | 0 | THE1A | MaLR | LTR |
| 45660043 | 45661621 | - | 12.9 | 3511 | 5104 | -1042 | L1PREC2 | L1 | LINE |
| 45661645 | 45662588 | - | 12.9 | 2571 | 3510 | -2636 | L1PREC2 | L1 | LINE |
| 45662585 | 45662957 | - | 22.0 | 4 | 382 | -6403 | L1PA13 | L1 | LINE |
| 45662964 | 45663268 | - | 20.4 | 2 | 305 | -7 | AluJo | Alu | SINE |
| 45663270 | 45664124 | - | 18.8 | 4351 | 5205 | -2357 | L1M1 | L1 | LINE |
| 45664125 | 45664500 | - | 17.7 | 5794 | 6166 | 0 | L1PA16 | L1 | LINE |
| 45664501 | 45665137 | - | 18.8 | 3706 | 4350 | -3212 | L1M1 | L1 | LINE |
| 45665138 | 45665556 | + | 19.5 | 1 | 426 | 0 | MSTB | MaLR | LTR |
| 45665557 | 45665844 | - | 19.1 | 3299 | 3705 | -3857 | L1M1 | L1 | LINE |
| 45666216 | 45666250 | + | 5.7 | 1979 | 2013 | -5155 | L1M2a | L1 | LINE |
| 45666254 | 45666360 | + | 20.6 | 1986 | 2096 | -5072 | L1M2a | L1 | LINE |
| 45666358 | 45666789 | + | 24.6 | 2522 | 2945 | -4223 | L1M2a | L1 | LINE |
| 45666813 | 45668237 | - | 33.5 | 783 | 2401 | -5165 | L1M1 | L1 | LINE |

  

|  |  |  |  |  |  |  |  |  |  |
| --- | --- | --- | --- | --- | --- | --- | --- | --- | --- |
| chr4:47332786-47335888 | | | 3101 bp | | |  |  |  |  |
| Genome Start | Genome End | Strand | Div | Repeat Start | Repeat End | Repeat Left | Name | Family | Class |
| 47332786 | 47333239 | - | 39.5 | 1157 | 1640 | -320 | L4 | RTE | LINE |
| 47333240 | 47333604 | + | 14.1 | 1 | 364 | 0 | THE1B | MaLR | LTR |
| 47333607 | 47335168 | + | 28.8 | 2 | 1735 | 0 | MLT-int | MaLR | LTR |
| 47335169 | 47335532 | + | 13.8 | 1 | 364 | 0 | THE1B | MaLR | LTR |
| 47335533 | 47335888 | - | 39.5 | 793 | 1156 | -804 | L4 | RTE | LINE |

  

|  |  |  |  |  |  |  |  |  |  |
| --- | --- | --- | --- | --- | --- | --- | --- | --- | --- |
| chr4:47351029-47358254 | | | 7222 bp | | |  |  |  |  |
| Genome Start | Genome End | Strand | Div | Repeat Start | Repeat End | Repeat Left | Name | Family | Class |
| 47351029 | 47352428 | + | 13.6 | 13 | 1423 | -6142 | L1MA3 | L1 | LINE |
| 47352429 | 47352926 | + | 13.4 | 4736 | 5223 | -2342 | L1MA3 | L1 | LINE |
| 47352927 | 47353227 | - | 12.0 | 3 | 303 | -10 | AluSq | Alu | SINE |
| 47353228 | 47353978 | + | 13.4 | 5224 | 5981 | -1584 | L1MA3 | L1 | LINE |
| 47353981 | 47354025 | - | 13.9 | 383 | 426 | 0 | MSTB | MaLR | LTR |
| 47354026 | 47354337 | + | 8.7 | 1 | 310 | -1 | AluY | Alu | SINE |
| 47354338 | 47354691 | - | 13.9 | 1 | 382 | -44 | MSTB | MaLR | LTR |
| 47354692 | 47356126 | - | 18.3 | 3 | 1580 | 0 | MSTB-int | MaLR | LTR |
| 47356129 | 47356553 | - | 16.5 | 1 | 426 | 0 | MSTB | MaLR | LTR |
| 47356554 | 47358254 | + | 11.2 | 5980 | 7722 | -2 | L1MA3 | L1 | LINE |

  

|  |  |  |  |  |  |  |  |  |  |
| --- | --- | --- | --- | --- | --- | --- | --- | --- | --- |
| chr4:52505952-52517353 | | | 11108 bp | | |  |  |  |  |
| Genome Start | Genome End | Strand | Div | Repeat Start | Repeat End | Repeat Left | Name | Family | Class |
| 52505952 | 52506076 | - | 16.0 | 1610 | 1735 | 0 | MLT1A0-int | MaLR | LTR |
| 52506090 | 52506490 | + | 16.0 | 1 | 428 | 0 | MSTA | MaLR | LTR |
| 52506505 | 52508060 | + | 18.6 | 45 | 1651 | 0 | MSTA-int | MaLR | LTR |
| 52508061 | 52508432 | + | 17.2 | 1 | 401 | -27 | MSTA | MaLR | LTR |
| 52508427 | 52514289 | - | 7.8 | 11 | 6031 | 0 | L1PA5 | L1 | LINE |
| 52514343 | 52514986 | - | 27.5 | 893 | 1583 | -152 | MLT1A0-int | MaLR | LTR |
| 52515043 | 52515424 | + | 14.2 | 1 | 414 | -14 | MSTA | MaLR | LTR |
| 52515455 | 52515791 | - | 20.9 | 451 | 790 | -945 | MLT1A0-int | MaLR | LTR |
| 52515893 | 52516482 | - | 17.8 | 19 | 635 | 0 | LTR24C | ERV1 | LTR |
| 52516502 | 52516604 | - | 17.9 | 27 | 122 | -11 | FLAM\_C | Alu | SINE |
| 52516619 | 52516993 | - | 19.8 | 82 | 451 | -1284 | MLT1A0-int | MaLR | LTR |
| 52516994 | 52517271 | + | 10.1 | 1 | 281 | -30 | AluY | Alu | SINE |
| 52517272 | 52517353 | - | 19.8 | 10 | 81 | -1654 | MLT1A0-int | MaLR | LTR |

  

|  |  |  |  |  |  |  |  |  |  |
| --- | --- | --- | --- | --- | --- | --- | --- | --- | --- |
| chr4:57982111-57984814 | | | 2451 bp | | |  |  |  |  |
| Genome Start | Genome End | Strand | Div | Repeat Start | Repeat End | Repeat Left | Name | Family | Class |
| 57982111 | 57982155 | + | 28.9 | 4084 | 4128 | -361 | L3 | CR1 | LINE |
| 57982166 | 57982543 | + | 13.8 | 1 | 381 | 0 | THE1D | MaLR | LTR |
| 57982544 | 57984051 | + | 10.5 | 1 | 1580 | 0 | THE1D-int | MaLR | LTR |
| 57984052 | 57984355 | + | 18.5 | 1 | 381 | 0 | THE1D | MaLR | LTR |
| 57984599 | 57984814 | + | 32.4 | 4222 | 4453 | -36 | L3 | CR1 | LINE |

  

|  |  |  |  |  |  |  |  |  |  |
| --- | --- | --- | --- | --- | --- | --- | --- | --- | --- |
| chr4:58603451-58606067 | | | 2301 bp | | |  |  |  |  |
| Genome Start | Genome End | Strand | Div | Repeat Start | Repeat End | Repeat Left | Name | Family | Class |
| 58603451 | 58603993 | + | 40.8 | 352 | 901 | -2518 | L2 | L2 | LINE |
| 58604055 | 58604411 | + | 16.1 | 1 | 364 | 0 | THE1B | MaLR | LTR |
| 58604412 | 58604462 | + | 17.6 | 1 | 53 | -1527 | THE1B-int | MaLR | LTR |
| 58604463 | 58604561 | + | 17.2 | 1 | 99 | -258 | LTR18A | ERVL | LTR |
| 58604503 | 58605291 | + | 13.8 | 803 | 1580 | 0 | THE1B-int | MaLR | LTR |
| 58605292 | 58605647 | + | 16.9 | 1 | 362 | -2 | THE1B | MaLR | LTR |
| 58605962 | 58606067 | + | 37.9 | 1338 | 1442 | -1977 | L2 | L2 | LINE |

  

|  |  |  |  |  |  |  |  |  |  |
| --- | --- | --- | --- | --- | --- | --- | --- | --- | --- |
| chr4:58841121-58844481 | | | 3258 bp | | |  |  |  |  |
| Genome Start | Genome End | Strand | Div | Repeat Start | Repeat End | Repeat Left | Name | Family | Class |
| 58841121 | 58841249 | + | 22.6 | 4 | 129 | -337 | MER31B | ERV1 | LTR |
| 58841250 | 58841605 | + | 14.7 | 1 | 364 | 0 | THE1B | MaLR | LTR |
| 58841608 | 58843212 | + | 32.5 | 2 | 1735 | 0 | MLT-int | MaLR | LTR |
| 58843213 | 58843586 | + | 17.2 | 1 | 364 | 0 | THE1B | MaLR | LTR |
| 58843587 | 58843816 | + | 22.6 | 130 | 356 | -110 | MER31B | ERV1 | LTR |
| 58843930 | 58844077 | - | 27.9 | 233 | 379 | -95 | MER101 | ERV1 | LTR |
| 58844066 | 58844372 | - | 18.8 | 1 | 298 | -272 | MER101B | ERV1 | LTR |
| 58844373 | 58844481 | + | 23.9 | 353 | 466 | 0 | MER31B | ERV1 | LTR |

  

|  |  |  |  |  |  |  |  |  |  |
| --- | --- | --- | --- | --- | --- | --- | --- | --- | --- |
| chr4:58903448-58907292 | | | 3457 bp | | |  |  |  |  |
| Genome Start | Genome End | Strand | Div | Repeat Start | Repeat End | Repeat Left | Name | Family | Class |
| 58903448 | 58903703 | - | 31.0 | 1815 | 2125 | -1294 | L2 | L2 | LINE |
| 58903737 | 58903856 | + | 32.2 | 7 | 133 | -45 | MER5B | MER1\_type | DNA |
| 58903911 | 58904550 | - | 38.4 | 1099 | 1778 | -1641 | L2 | L2 | LINE |
| 58904727 | 58905096 | + | 10.8 | 1 | 381 | 0 | THE1D | MaLR | LTR |
| 58905101 | 58906597 | + | 14.0 | 5 | 1580 | 0 | THE1D-int | MaLR | LTR |
| 58906598 | 58906968 | + | 14.1 | 1 | 380 | -1 | THE1D | MaLR | LTR |
| 58907090 | 58907292 | - | 43.4 | 524 | 733 | -2686 | L2 | L2 | LINE |

  

|  |  |  |  |  |  |  |  |  |  |
| --- | --- | --- | --- | --- | --- | --- | --- | --- | --- |
| chr4:58917810-58924551 | | | 6101 bp | | |  |  |  |  |
| Genome Start | Genome End | Strand | Div | Repeat Start | Repeat End | Repeat Left | Name | Family | Class |
| 58917810 | 58917950 | + | 24.3 | 73 | 219 | -5448 | ERVL-F | ERVL | LTR |
| 58917958 | 58918313 | + | 14.1 | 1 | 364 | 0 | THE1B | MaLR | LTR |
| 58918314 | 58918593 | + | 9.2 | 1 | 227 | -1353 | THE1B-int | MaLR | LTR |
| 58918594 | 58918947 | - | 9.9 | 1 | 355 | 0 | THE1A | MaLR | LTR |
| 58918948 | 58919053 | + | 9.2 | 228 | 334 | -1246 | THE1B-int | MaLR | LTR |
| 58919053 | 58920007 | + | 15.5 | 626 | 1574 | -6 | THE1B-int | MaLR | LTR |
| 58920013 | 58920406 | + | 15.0 | 1 | 364 | 0 | THE1B | MaLR | LTR |
| 58920467 | 58921996 | - | 19.9 | 1 | 1726 | -29 | MER52A | ERV1 | LTR |
| 58922000 | 58922877 | + | 28.9 | 273 | 1213 | -4454 | ERVL-F | ERVL | LTR |
| 58923165 | 58923435 | - | 15.9 | 1 | 279 | -33 | AluSx | Alu | SINE |
| 58923614 | 58924358 | + | 28.8 | 1276 | 2047 | -3667 | ERVL-F | ERVL | LTR |
| 58924461 | 58924551 | + | 30.8 | 2172 | 2263 | -3451 | ERVL-F | ERVL | LTR |

  

|  |  |  |  |  |  |  |  |  |  |
| --- | --- | --- | --- | --- | --- | --- | --- | --- | --- |
| chr4:63589671-63600478 | | | 10618 bp | | |  |  |  |  |
| Genome Start | Genome End | Strand | Div | Repeat Start | Repeat End | Repeat Left | Name | Family | Class |
| 63589671 | 63590455 | - | 22.7 | 3341 | 4126 | -2195 | L1MCa | L1 | LINE |
| 63590456 | 63590815 | - | 17.3 | 1 | 381 | 0 | THE1D | MaLR | LTR |
| 63590816 | 63592425 | - | 30.0 | 2 | 1735 | 0 | MLT-int | MaLR | LTR |
| 63592428 | 63592790 | - | 15.8 | 1 | 381 | 0 | THE1D | MaLR | LTR |
| 63592791 | 63593417 | - | 22.7 | 2654 | 3340 | -2981 | L1MCa | L1 | LINE |
| 63593418 | 63593694 | - | 19.1 | 15 | 291 | -21 | AluSx | Alu | SINE |
| 63593695 | 63593979 | - | 22.7 | 2390 | 2653 | -3668 | L1MCa | L1 | LINE |
| 63593980 | 63594265 | + | 15.7 | 1 | 294 | -18 | AluSx | Alu | SINE |
| 63594266 | 63594713 | - | 24.7 | 1952 | 2389 | -4119 | L1MCa | L1 | LINE |
| 63594716 | 63594861 | - | 22.6 | 529 | 691 | 0 | MER76 | ERVL | LTR |
| 63594862 | 63595170 | - | 8.7 | 1 | 309 | -3 | AluSx | Alu | SINE |
| 63595171 | 63595202 | - | 22.6 | 501 | 528 | -163 | MER76 | ERVL | LTR |
| 63595237 | 63595608 | - | 19.6 | 32 | 408 | 0 | MLT1A1 | MaLR | LTR |
| 63595639 | 63596443 | - | 24.8 | 858 | 1716 | -19 | MLT1A1-int | MaLR | LTR |
| 63596463 | 63598567 | - | 17.7 | 2 | 2205 | -4303 | L1MCa | L1 | LINE |
| 63598675 | 63599072 | - | 20.8 | 323 | 727 | 0 | LTR8A | ERV1 | LTR |
| 63599068 | 63599129 | - | 11.7 | 1 | 60 | -667 | LTR8A | ERV1 | LTR |
| 63599131 | 63599471 | - | 27.5 | 775 | 1120 | -5388 | L1MCa | L1 | LINE |
| 63599472 | 63599781 | + | 13.6 | 1 | 296 | -17 | AluSq | Alu | SINE |
| 63599782 | 63600478 | - | 27.5 | 1 | 774 | -5734 | L1MCa | L1 | LINE |

  

|  |  |  |  |  |  |  |  |  |  |
| --- | --- | --- | --- | --- | --- | --- | --- | --- | --- |
| chr4:64235352-64241560 | | | 6105 bp | | |  |  |  |  |
| Genome Start | Genome End | Strand | Div | Repeat Start | Repeat End | Repeat Left | Name | Family | Class |
| 64235352 | 64236148 | + | 26.7 | 2 | 799 | -5246 | L1MCb | L1 | LINE |
| 64236149 | 64236341 | + | 14.9 | 1 | 233 | -195 | MSTA | MaLR | LTR |
| 64236373 | 64236543 | + | 14.9 | 234 | 423 | -5 | MSTA | MaLR | LTR |
| 64236549 | 64238169 | + | 30.5 | 2 | 1735 | 0 | MLT-int | MaLR | LTR |
| 64238170 | 64238570 | + | 14.9 | 1 | 428 | 0 | MSTA | MaLR | LTR |
| 64238571 | 64238670 | + | 26.7 | 803 | 875 | -5170 | L1MCb | L1 | LINE |
| 64238713 | 64239442 | + | 24.4 | 876 | 1715 | -5036 | L1MCb | L1 | LINE |
| 64239443 | 64239812 | - | 15.8 | 1 | 364 | 0 | THE1B | MaLR | LTR |
| 64239813 | 64240202 | + | 24.4 | 1716 | 2136 | -4615 | L1MCb | L1 | LINE |
| 64240229 | 64241560 | + | 23.7 | 2237 | 3723 | -2841 | L1MCb | L1 | LINE |

  

|  |  |  |  |  |  |  |  |  |  |
| --- | --- | --- | --- | --- | --- | --- | --- | --- | --- |
| chr4:64812622-64816172 | | | 3563 bp | | |  |  |  |  |
| Genome Start | Genome End | Strand | Div | Repeat Start | Repeat End | Repeat Left | Name | Family | Class |
| 64812622 | 64813248 | - | 16.1 | 1703 | 2399 | -5138 | MER41A-int | ERV1 | LTR |
| 64813235 | 64813838 | - | 18.1 | 42 | 640 | -5306 | MER41A-int | ERV1 | LTR |
| 64813839 | 64814201 | - | 16.2 | 1 | 364 | 0 | THE1B | MaLR | LTR |
| 64814202 | 64815771 | - | 31.4 | 2 | 1735 | 0 | MLT-int | MaLR | LTR |
| 64815774 | 64816128 | - | 16.8 | 1 | 364 | 0 | THE1B | MaLR | LTR |
| 64816129 | 64816172 | - | 18.1 | 1 | 41 | -5905 | MER41A-int | ERV1 | LTR |

  

|  |  |  |  |  |  |  |  |  |  |
| --- | --- | --- | --- | --- | --- | --- | --- | --- | --- |
| chr4:64895656-64897118 | | | 1431 bp | | |  |  |  |  |
| Genome Start | Genome End | Strand | Div | Repeat Start | Repeat End | Repeat Left | Name | Family | Class |
| 64895656 | 64895750 | + | 30.1 | 2387 | 2492 | -927 | L2 | L2 | LINE |
| 64895762 | 64896147 | - | 14.3 | 1 | 381 | 0 | THE1D | MaLR | LTR |
| 64896153 | 64896219 | - | 10.8 | 1509 | 1575 | -5 | THE1D-int | MaLR | LTR |
| 64896222 | 64896398 | - | 15.9 | 1 | 179 | -1401 | THE1D-int | MaLR | LTR |
| 64896400 | 64896762 | - | 16.9 | 3 | 380 | -1 | THE1D | MaLR | LTR |
| 64896776 | 64897118 | + | 34.2 | 2527 | 2860 | -559 | L2 | L2 | LINE |

  

|  |  |  |  |  |  |  |  |  |  |
| --- | --- | --- | --- | --- | --- | --- | --- | --- | --- |
| chr4:64999252-65004846 | | | 5381 bp | | |  |  |  |  |
| Genome Start | Genome End | Strand | Div | Repeat Start | Repeat End | Repeat Left | Name | Family | Class |
| 64999252 | 64999489 | + | 32.6 | 332 | 580 | -2839 | L2 | L2 | LINE |
| 64999504 | 65000413 | + | 34.1 | 727 | 1674 | -1745 | L2 | L2 | LINE |
| 65000414 | 65000846 | - | 18.5 | 1 | 428 | 0 | MSTA | MaLR | LTR |
| 65000847 | 65000897 | + | 34.1 | 1675 | 1723 | -1696 | L2 | L2 | LINE |
| 65000912 | 65001282 | - | 14.6 | 1 | 371 | -4 | THE1C | MaLR | LTR |
| 65001283 | 65002742 | - | 14.9 | 49 | 1580 | 0 | THE1C-int | MaLR | LTR |
| 65002743 | 65003013 | - | 2.2 | 28 | 298 | -1 | AluYd8 | Alu | SINE |
| 65003014 | 65003062 | - | 14.9 | 1 | 48 | -1532 | THE1C-int | MaLR | LTR |
| 65003067 | 65003432 | - | 14.2 | 1 | 371 | -4 | THE1C | MaLR | LTR |
| 65003557 | 65004220 | + | 32.8 | 1844 | 2530 | -889 | L2 | L2 | LINE |
| 65004279 | 65004846 | + | 37.2 | 2807 | 3419 | 0 | L2 | L2 | LINE |

  

|  |  |  |  |  |  |  |  |  |  |
| --- | --- | --- | --- | --- | --- | --- | --- | --- | --- |
| chr4:66444110-66452354 | | | 8214 bp | | |  |  |  |  |
| Genome Start | Genome End | Strand | Div | Repeat Start | Repeat End | Repeat Left | Name | Family | Class |
| 66444110 | 66444169 | - | 22.4 | 5100 | 5144 | -1718 | L1PBa | L1 | LINE |
| 66444170 | 66444485 | - | 6.4 | 5856 | 6169 | -3 | L1PA8 | L1 | LINE |
| 66444486 | 66444642 | - | 22.4 | 4938 | 5099 | -1763 | L1PBa | L1 | LINE |
| 66444643 | 66445046 | - | 18.4 | 1 | 428 | 0 | MSTA | MaLR | LTR |
| 66445047 | 66446676 | - | 30.7 | 2 | 1735 | 0 | MLT-int | MaLR | LTR |
| 66446691 | 66447098 | - | 16.5 | 1 | 408 | -20 | MSTA | MaLR | LTR |
| 66447101 | 66447314 | - | 22.6 | 4724 | 4940 | -1922 | L1PBa | L1 | LINE |
| 66447315 | 66447669 | + | 14.6 | 1 | 364 | 0 | THE1B | MaLR | LTR |
| 66447672 | 66449246 | + | 31.9 | 2 | 1735 | 0 | MLT-int | MaLR | LTR |
| 66449248 | 66449606 | + | 16.9 | 2 | 364 | 0 | THE1B | MaLR | LTR |
| 66449607 | 66450505 | - | 22.6 | 3847 | 4723 | -2139 | L1PBa | L1 | LINE |
| 66450506 | 66450825 | - | 10.0 | 1 | 311 | 0 | AluY | Alu | SINE |
| 66450826 | 66452210 | - | 23.2 | 2384 | 3846 | -3016 | L1PBa | L1 | LINE |
| 66452223 | 66452354 | - | 27.4 | 926 | 1064 | -5799 | L1PBa | L1 | LINE |

  

|  |  |  |  |  |  |  |  |  |  |
| --- | --- | --- | --- | --- | --- | --- | --- | --- | --- |
| chr4:66728378-66737465 | | | 8541 bp | | |  |  |  |  |
| Genome Start | Genome End | Strand | Div | Repeat Start | Repeat End | Repeat Left | Name | Family | Class |
| 66728378 | 66728692 | - | 33.4 | 3044 | 3399 | -20 | L2 | L2 | LINE |
| 66728693 | 66729003 | + | 10.0 | 1 | 311 | 0 | AluY | Alu | SINE |
| 66729045 | 66729245 | + | 23.5 | 10 | 209 | -15 | MER58A | MER1\_type | DNA |
| 66729316 | 66730375 | - | 34.4 | 1805 | 2877 | -542 | L2 | L2 | LINE |
| 66730395 | 66730537 | - | 17.4 | 7124 | 7267 | -5 | L1MA9 | L1 | LINE |
| 66730583 | 66730970 | - | 17.4 | 6723 | 7123 | -149 | L1MA9 | L1 | LINE |
| 66730971 | 66731052 | + | 17.8 | 1 | 74 | -291 | MLT1A0 | MaLR | LTR |
| 66731055 | 66731317 | - | 17.5 | 4810 | 5073 | -2027 | L1MA9 | L1 | LINE |
| 66731320 | 66731376 | - | 20.5 | 304 | 358 | -7 | MLT1A0 | MaLR | LTR |
| 66731377 | 66731705 | + | 7.5 | 1 | 328 | 0 | AluY | Alu | SINE |
| 66731706 | 66732003 | - | 20.5 | 1 | 303 | -62 | MLT1A0 | MaLR | LTR |
| 66732004 | 66733675 | - | 25.0 | 2 | 1735 | 0 | MLT1A0-int | MaLR | LTR |
| 66733682 | 66734039 | - | 26.1 | 1 | 363 | -2 | MLT1A0 | MaLR | LTR |
| 66734040 | 66735188 | - | 21.0 | 2976 | 4148 | -2955 | L1MA9 | L1 | LINE |
| 66735196 | 66735927 | - | 28.2 | 162 | 1876 | -5154 | L1MA9 | L1 | LINE |
| 66735976 | 66736116 | - | 28.1 | 1 | 161 | -6869 | L1MA9 | L1 | LINE |
| 66736144 | 66736712 | - | 36.8 | 1208 | 1811 | -1608 | L2 | L2 | LINE |
| 66736755 | 66736908 | + | 28.8 | 38 | 192 | -217 | MLT1I | MaLR | LTR |
| 66737147 | 66737465 | - | 32.8 | 820 | 1158 | -2261 | L2 | L2 | LINE |

  

|  |  |  |  |  |  |  |  |  |  |
| --- | --- | --- | --- | --- | --- | --- | --- | --- | --- |
| chr4:67072958-67078295 | | | 5098 bp | | |  |  |  |  |
| Genome Start | Genome End | Strand | Div | Repeat Start | Repeat End | Repeat Left | Name | Family | Class |
| 67072958 | 67073688 | - | 36.1 | 2686 | 3417 | -2 | L2 | L2 | LINE |
| 67073710 | 67073760 | - | 15.7 | 3689 | 3740 | -2406 | L1MC | L1 | LINE |
| 67073937 | 67075732 | - | 30.3 | 34 | 1997 | -4403 | L1MEc | L1 | LINE |
| 67075733 | 67075880 | - | 30.4 | 2481 | 2646 | -773 | L2 | L2 | LINE |
| 67075927 | 67076287 | + | 15.0 | 1 | 364 | 0 | THE1B | MaLR | LTR |
| 67076291 | 67077949 | + | 32.6 | 3 | 1734 | -1 | MLT1J-int | MaLR | LTR |
| 67077951 | 67078059 | + | 15.6 | 1 | 117 | -247 | THE1B | MaLR | LTR |
| 67078048 | 67078087 | + | 12.5 | 325 | 364 | 0 | THE1B | MaLR | LTR |
| 67078093 | 67078295 | - | 34.7 | 2255 | 2455 | -964 | L2 | L2 | LINE |

  

|  |  |  |  |  |  |  |  |  |  |
| --- | --- | --- | --- | --- | --- | --- | --- | --- | --- |
| chr4:67313378-67317206 | | | 3593 bp | | |  |  |  |  |
| Genome Start | Genome End | Strand | Div | Repeat Start | Repeat End | Repeat Left | Name | Family | Class |
| 67313378 | 67313684 | - | 30.8 | 2145 | 2465 | -2369 | L1MEe | L1 | LINE |
| 67313877 | 67314057 | - | 30.4 | 1784 | 1986 | -2848 | L1MEe | L1 | LINE |
| 67314058 | 67314405 | + | 12.3 | 3 | 364 | 0 | THE1B | MaLR | LTR |
| 67314408 | 67315918 | + | 28.9 | 2 | 1735 | 0 | MLT-int | MaLR | LTR |
| 67315919 | 67316269 | + | 13.3 | 1 | 364 | 0 | THE1B | MaLR | LTR |
| 67316270 | 67316481 | - | 30.4 | 1568 | 1783 | -3051 | L1MEe | L1 | LINE |
| 67316524 | 67317206 | - | 27.2 | 613 | 1355 | -3479 | L1MEe | L1 | LINE |

  

|  |  |  |  |  |  |  |  |  |  |
| --- | --- | --- | --- | --- | --- | --- | --- | --- | --- |
| chr4:67869652-67872889 | | | 3237 bp | | |  |  |  |  |
| Genome Start | Genome End | Strand | Div | Repeat Start | Repeat End | Repeat Left | Name | Family | Class |
| 67869652 | 67869931 | + | 13.8 | 5584 | 5866 | -467 | L1MC1 | L1 | LINE |
| 67869932 | 67870228 | - | 14.5 | 1 | 297 | -15 | AluSx | Alu | SINE |
| 67870229 | 67870533 | + | 13.8 | 5867 | 6173 | -160 | L1MC1 | L1 | LINE |
| 67870534 | 67870913 | + | 11.6 | 1 | 375 | 0 | THE1C | MaLR | LTR |
| 67870914 | 67872371 | + | 10.8 | 1 | 1580 | 0 | THE1C-int | MaLR | LTR |
| 67872372 | 67872741 | + | 7.9 | 1 | 375 | 0 | THE1C | MaLR | LTR |
| 67872743 | 67872889 | + | 15.0 | 6170 | 6316 | -17 | L1MC1 | L1 | LINE |

  

|  |  |  |  |  |  |  |  |  |  |
| --- | --- | --- | --- | --- | --- | --- | --- | --- | --- |
| chr4:67898711-67901624 | | | 2912 bp | | |  |  |  |  |
| Genome Start | Genome End | Strand | Div | Repeat Start | Repeat End | Repeat Left | Name | Family | Class |
| 67898711 | 67899090 | + | 34.0 | 25 | 450 | -243 | MER115 | Tip100 | DNA |
| 67899091 | 67899451 | - | 14.9 | 1 | 364 | 0 | THE1B | MaLR | LTR |
| 67899452 | 67901022 | - | 30.2 | 2 | 1735 | 0 | MLT-int | MaLR | LTR |
| 67901025 | 67901389 | - | 16.9 | 3 | 364 | 0 | THE1B | MaLR | LTR |
| 67901390 | 67901624 | + | 34.0 | 451 | 682 | -11 | MER115 | Tip100 | DNA |

  

|  |  |  |  |  |  |  |  |  |  |
| --- | --- | --- | --- | --- | --- | --- | --- | --- | --- |
| chr4:69027819-69039817 | | | 11884 bp | | |  |  |  |  |
| Genome Start | Genome End | Strand | Div | Repeat Start | Repeat End | Repeat Left | Name | Family | Class |
| 69027819 | 69029045 | - | 20.4 | 1040 | 2332 | -4301 | L1MDa | L1 | LINE |
| 69029109 | 69029940 | - | 13.5 | 5457 | 6289 | -1 | L1PA15 | L1 | LINE |
| 69029941 | 69029980 | - | 18.4 | 389 | 428 | 0 | MSTA | MaLR | LTR |
| 69029981 | 69030270 | + | 17.3 | 1 | 300 | -12 | AluJb | Alu | SINE |
| 69030271 | 69030665 | - | 18.4 | 1 | 388 | -40 | MSTA | MaLR | LTR |
| 69030666 | 69030784 | - | 12.1 | 1543 | 1651 | 0 | MSTA-int | MaLR | LTR |
| 69030785 | 69031099 | + | 21.9 | 1 | 297 | -15 | AluJb | Alu | SINE |
| 69031100 | 69031157 | - | 12.1 | 1486 | 1542 | -109 | MSTA-int | MaLR | LTR |
| 69031144 | 69031353 | - | 18.1 | 1 | 217 | -1434 | MSTA-int | MaLR | LTR |
| 69031354 | 69031758 | - | 19.2 | 2 | 428 | 0 | MSTA | MaLR | LTR |
| 69031765 | 69032618 | - | 15.4 | 4615 | 5458 | -813 | L1PA15 | L1 | LINE |
| 69032619 | 69032920 | - | 17.3 | 1 | 301 | -11 | AluJo | Alu | SINE |
| 69032921 | 69033455 | - | 15.4 | 4066 | 4614 | -1657 | L1PA15 | L1 | LINE |
| 69033456 | 69033759 | - | 11.5 | 1 | 304 | -8 | AluSx | Alu | SINE |
| 69033760 | 69035567 | - | 15.5 | 2145 | 4065 | -2206 | L1PA15 | L1 | LINE |
| 69035568 | 69035864 | - | 14.5 | 1 | 298 | -15 | AluSq | Alu | SINE |
| 69035865 | 69036054 | - | 17.6 | 1957 | 2144 | -4143 | L1PA15 | L1 | LINE |
| 69036055 | 69036281 | + | 12.8 | 84 | 310 | -3 | AluSq | Alu | SINE |
| 69036282 | 69036843 | - | 17.6 | 1416 | 1956 | -4331 | L1PA15 | L1 | LINE |
| 69036844 | 69037152 | - | 9.4 | 1 | 311 | 0 | AluY | Alu | SINE |
| 69037153 | 69038633 | - | 21.5 | 5 | 1415 | -4872 | L1PA15 | L1 | LINE |
| 69038684 | 69038765 | - | 30.5 | 968 | 1050 | -5583 | L1MDa | L1 | LINE |
| 69038776 | 69039061 | - | 29.5 | 523 | 857 | -5776 | L1MDa | L1 | LINE |
| 69039062 | 69039369 | - | 14.3 | 3 | 311 | -1 | AluSx | Alu | SINE |
| 69039370 | 69039817 | - | 29.5 | 34 | 522 | -6111 | L1MDa | L1 | LINE |

  

|  |  |  |  |  |  |  |  |  |  |
| --- | --- | --- | --- | --- | --- | --- | --- | --- | --- |
| chr4:69497056-69509159 | | | 11989 bp | | |  |  |  |  |
| Genome Start | Genome End | Strand | Div | Repeat Start | Repeat End | Repeat Left | Name | Family | Class |
| 69497056 | 69498392 | - | 20.6 | 1040 | 2415 | -4218 | L1MDa | L1 | LINE |
| 69498456 | 69499287 | - | 13.5 | 5457 | 6289 | -1 | L1PA15 | L1 | LINE |
| 69499288 | 69499327 | - | 18.9 | 389 | 428 | 0 | MSTA | MaLR | LTR |
| 69499328 | 69499617 | + | 16.6 | 1 | 300 | -12 | AluJb | Alu | SINE |
| 69499618 | 69500023 | - | 18.9 | 1 | 388 | -40 | MSTA | MaLR | LTR |
| 69500024 | 69500142 | - | 12.1 | 1543 | 1651 | 0 | MSTA-int | MaLR | LTR |
| 69500143 | 69500434 | + | 21.9 | 1 | 297 | -15 | AluJb | Alu | SINE |
| 69500435 | 69500492 | - | 12.1 | 1486 | 1542 | -109 | MSTA-int | MaLR | LTR |
| 69500479 | 69500688 | - | 18.1 | 1 | 217 | -1434 | MSTA-int | MaLR | LTR |
| 69500689 | 69501091 | - | 18.7 | 2 | 428 | 0 | MSTA | MaLR | LTR |
| 69501098 | 69501951 | - | 15.6 | 4615 | 5458 | -813 | L1PA15 | L1 | LINE |
| 69501952 | 69502253 | - | 17.3 | 1 | 301 | -11 | AluJo | Alu | SINE |
| 69502254 | 69502788 | - | 15.6 | 4066 | 4614 | -1657 | L1PA15 | L1 | LINE |
| 69502789 | 69503090 | - | 11.6 | 3 | 304 | -8 | AluSx | Alu | SINE |
| 69503091 | 69504900 | - | 15.7 | 2145 | 4065 | -2206 | L1PA15 | L1 | LINE |
| 69504901 | 69505197 | - | 14.1 | 1 | 298 | -15 | AluSq | Alu | SINE |
| 69505198 | 69505387 | - | 17.4 | 1957 | 2144 | -4143 | L1PA15 | L1 | LINE |
| 69505388 | 69505615 | + | 12.7 | 84 | 311 | -2 | AluSq | Alu | SINE |
| 69505616 | 69506177 | - | 17.4 | 1416 | 1956 | -4331 | L1PA15 | L1 | LINE |
| 69506178 | 69506489 | - | 9.4 | 1 | 311 | 0 | AluY | Alu | SINE |
| 69506490 | 69507970 | - | 21.4 | 5 | 1415 | -4872 | L1PA15 | L1 | LINE |
| 69508021 | 69508102 | - | 30.5 | 968 | 1050 | -5583 | L1MDa | L1 | LINE |
| 69508113 | 69508401 | - | 29.5 | 523 | 857 | -5776 | L1MDa | L1 | LINE |
| 69508402 | 69508708 | - | 14.0 | 3 | 310 | -2 | AluSx | Alu | SINE |
| 69508709 | 69509159 | - | 29.5 | 34 | 522 | -6111 | L1MDa | L1 | LINE |

  

|  |  |  |  |  |  |  |  |  |  |
| --- | --- | --- | --- | --- | --- | --- | --- | --- | --- |
| chr4:69629412-69639452 | | | 9879 bp | | |  |  |  |  |
| Genome Start | Genome End | Strand | Div | Repeat Start | Repeat End | Repeat Left | Name | Family | Class |
| 69629412 | 69630001 | - | 26.6 | 3901 | 4473 | -1852 | L1M3f | L1 | LINE |
| 69630006 | 69630358 | - | 14.6 | 3 | 355 | 0 | THE1A | MaLR | LTR |
| 69630361 | 69631935 | - | 31.5 | 2 | 1735 | 0 | MLT-int | MaLR | LTR |
| 69631940 | 69632001 | - | 14.3 | 299 | 353 | -2 | THE1A | MaLR | LTR |
| 69632002 | 69632312 | + | 10.3 | 1 | 311 | 0 | AluY | Alu | SINE |
| 69632313 | 69632611 | - | 14.3 | 1 | 298 | -57 | THE1A | MaLR | LTR |
| 69632612 | 69633137 | - | 24.4 | 3395 | 3901 | -2424 | L1M3f | L1 | LINE |
| 69633159 | 69633390 | - | 20.7 | 399 | 639 | -37 | MER61F | ERV1 | LTR |
| 69633396 | 69633869 | - | 16.1 | 1 | 449 | -227 | MER61F | ERV1 | LTR |
| 69633870 | 69635473 | - | 24.2 | 1720 | 3399 | -2926 | L1M3f | L1 | LINE |
| 69635480 | 69635772 | - | 16.9 | 2 | 338 | -5 | MER61A | ERV1 | LTR |
| 69635774 | 69636751 | - | 13.3 | 4191 | 5217 | 0 | MER61A-int | ERV1 | LTR |
| 69636856 | 69637095 | + | 13.0 | 1411 | 1659 | -3558 | MER61-int | ERV1 | LTR |
| 69637096 | 69637408 | - | 21.0 | 1 | 311 | -32 | MER61A | ERV1 | LTR |
| 69637424 | 69638767 | - | 25.2 | 277 | 1704 | -4624 | L1M3f | L1 | LINE |
| 69638768 | 69639172 | - | 17.9 | 3 | 426 | -2 | MSTA | MaLR | LTR |
| 69639173 | 69639452 | - | 23.6 | 2 | 276 | -5052 | L1M3f | L1 | LINE |

  

|  |  |  |  |  |  |  |  |  |  |
| --- | --- | --- | --- | --- | --- | --- | --- | --- | --- |
| chr4:69657318-69666880 | | | 8527 bp | | |  |  |  |  |
| Genome Start | Genome End | Strand | Div | Repeat Start | Repeat End | Repeat Left | Name | Family | Class |
| 69657318 | 69657491 | + | 34.7 | 5 | 181 | -4815 | HERV16 | ERVL | LTR |
| 69657691 | 69658156 | - | 20.1 | 2 | 557 | 0 | MER41D | ERV1 | LTR |
| 69658369 | 69658492 | + | 27.4 | 589 | 720 | -4276 | HERV16 | ERVL | LTR |
| 69658514 | 69659569 | - | 20.4 | 2073 | 3124 | -3359 | L1P4a | L1 | LINE |
| 69659568 | 69659751 | + | 12.0 | 1727 | 1912 | -4591 | L1P4a | L1 | LINE |
| 69659761 | 69660695 | - | 31.0 | 9 | 967 | -5536 | L1P4a | L1 | LINE |
| 69660708 | 69660976 | - | 19.7 | 5018 | 5366 | -780 | L1P4 | L1 | LINE |
| 69660977 | 69661340 | - | 15.4 | 1 | 364 | 0 | THE1B | MaLR | LTR |
| 69661341 | 69662991 | - | 31.4 | 2 | 1735 | 0 | MLT-int | MaLR | LTR |
| 69662994 | 69663353 | - | 14.4 | 1 | 364 | 0 | THE1B | MaLR | LTR |
| 69663354 | 69664410 | - | 19.7 | 3943 | 5017 | -1129 | L1P4 | L1 | LINE |
| 69664405 | 69665192 | + | 22.3 | 3151 | 3944 | -2202 | L1P4 | L1 | LINE |
| 69665199 | 69665413 | + | 37.7 | 976 | 1190 | -3806 | HERV16 | ERVL | LTR |
| 69665662 | 69665857 | + | 34.0 | 1608 | 1808 | -3188 | HERV16 | ERVL | LTR |
| 69666102 | 69666290 | - | 33.3 | 226 | 418 | -49 | MLT1C | MaLR | LTR |
| 69666342 | 69666417 | - | 20.0 | 50 | 117 | -16 | FLAM | Alu | SINE |
| 69666405 | 69666597 | - | 27.7 | 6 | 199 | -285 | MLT1C | MaLR | LTR |
| 69666651 | 69666880 | + | 27.8 | 2255 | 2495 | -2501 | HERV16 | ERVL | LTR |

  

|  |  |  |  |  |  |  |  |  |  |
| --- | --- | --- | --- | --- | --- | --- | --- | --- | --- |
| chr4:69705989-69709305 | | | 3315 bp | | |  |  |  |  |
| Genome Start | Genome End | Strand | Div | Repeat Start | Repeat End | Repeat Left | Name | Family | Class |
| 69705989 | 69706679 | + | 19.7 | 4315 | 5017 | -1129 | L1P4 | L1 | LINE |
| 69706680 | 69707043 | + | 15.1 | 1 | 364 | 0 | THE1B | MaLR | LTR |
| 69707046 | 69708673 | + | 31.0 | 2 | 1735 | 0 | MLT-int | MaLR | LTR |
| 69708674 | 69709037 | + | 15.9 | 1 | 364 | 0 | THE1B | MaLR | LTR |
| 69709038 | 69709305 | + | 19.7 | 5018 | 5366 | -780 | L1P4 | L1 | LINE |

  

|  |  |  |  |  |  |  |  |  |  |
| --- | --- | --- | --- | --- | --- | --- | --- | --- | --- |
| chr4:70090958-70099966 | | | 8911 bp | | |  |  |  |  |
| Genome Start | Genome End | Strand | Div | Repeat Start | Repeat End | Repeat Left | Name | Family | Class |
| 70090958 | 70092098 | + | 16.8 | 4668 | 5791 | -360 | L1PB1 | L1 | LINE |
| 70092115 | 70092204 | + | 7.8 | 1 | 91 | -1175 | MER11A | ERVK | LTR |
| 70092202 | 70092648 | + | 7.1 | 861 | 1266 | 0 | MER11A | ERVK | LTR |
| 70092656 | 70095743 | + | 9.9 | 6 | 3066 | -4887 | HERVK11 | ERVK | LTR |
| 70095745 | 70096476 | + | 11.4 | 5331 | 6075 | -1878 | HERVK11 | ERVK | LTR |
| 70096496 | 70096651 | - | 9.6 | 1111 | 1266 | 0 | MER11A | ERVK | LTR |
| 70096655 | 70098517 | + | 8.6 | 6082 | 7953 | 0 | HERVK11 | ERVK | LTR |
| 70098518 | 70099541 | + | 8.3 | 1 | 1266 | 0 | MER11A | ERVK | LTR |
| 70099597 | 70099966 | + | 19.8 | 5782 | 6151 | 0 | L1PB1 | L1 | LINE |

  

|  |  |  |  |  |  |  |  |  |  |
| --- | --- | --- | --- | --- | --- | --- | --- | --- | --- |
| chr4:70164339-70177295 | | | 12358 bp | | |  |  |  |  |
| Genome Start | Genome End | Strand | Div | Repeat Start | Repeat End | Repeat Left | Name | Family | Class |
| 70164339 | 70164516 | - | 32.8 | 2315 | 2499 | -2497 | HERV16 | ERVL | LTR |
| 70164626 | 70164806 | + | 26.4 | 6 | 182 | -285 | MLT1C | MaLR | LTR |
| 70164806 | 70164886 | + | 19.8 | 50 | 133 | 0 | FLAM | Alu | SINE |
| 70164957 | 70165156 | + | 29.8 | 219 | 425 | -42 | MLT1C | MaLR | LTR |
| 70165319 | 70165580 | - | 39.7 | 1608 | 1872 | -3124 | HERV16 | ERVL | LTR |
| 70165816 | 70166047 | - | 38.9 | 972 | 1202 | -3794 | HERV16 | ERVL | LTR |
| 70166051 | 70166774 | - | 22.1 | 3216 | 3944 | -2202 | L1PREC2 | L1 | LINE |
| 70166785 | 70166885 | - | 26.0 | 3150 | 3250 | -2896 | L1P4 | L1 | LINE |
| 70166888 | 70167246 | + | 21.2 | 3951 | 4318 | -1828 | L1P4 | L1 | LINE |
| 70167247 | 70167946 | + | 7.5 | 1 | 719 | -3 | LTR30 | ERV1 | LTR |
| 70167947 | 70171376 | + | 9.5 | 2 | 3462 | -4795 | HERV30 | ERV1 | LTR |
| 70171370 | 70172270 | + | 11.9 | 7344 | 8257 | 0 | HERV30 | ERV1 | LTR |
| 70172272 | 70172972 | + | 7.3 | 4 | 722 | 0 | LTR30 | ERV1 | LTR |
| 70172973 | 70173666 | + | 19.0 | 4315 | 5017 | -1129 | L1P4 | L1 | LINE |
| 70173667 | 70174028 | + | 15.8 | 1 | 364 | 0 | THE1B | MaLR | LTR |
| 70174031 | 70175671 | + | 33.1 | 2 | 1735 | 0 | MLT1A0-int | MaLR | LTR |
| 70175672 | 70176031 | + | 18.3 | 1 | 364 | 0 | THE1B | MaLR | LTR |
| 70176032 | 70176299 | + | 23.4 | 5015 | 5363 | -780 | L1M2 | L1 | LINE |
| 70176312 | 70176704 | + | 30.6 | 9 | 406 | -6097 | L1P4a | L1 | LINE |
| 70176706 | 70176921 | + | 18.5 | 342 | 557 | 0 | MER41D | ERV1 | LTR |
| 70176922 | 70177295 | - | 35.5 | 5 | 387 | -4609 | HERV16 | ERVL | LTR |

  

|  |  |  |  |  |  |  |  |  |  |
| --- | --- | --- | --- | --- | --- | --- | --- | --- | --- |
| chr4:70272941-70286282 | | | 12613 bp | | |  |  |  |  |
| Genome Start | Genome End | Strand | Div | Repeat Start | Repeat End | Repeat Left | Name | Family | Class |
| 70272941 | 70273170 | - | 31.4 | 2254 | 2494 | -2502 | HERV16 | ERVL | LTR |
| 70273264 | 70273402 | + | 27.3 | 48 | 181 | -286 | MLT1C | MaLR | LTR |
| 70273403 | 70273485 | + | 19.3 | 50 | 133 | -10 | FLAM | Alu | SINE |
| 70273548 | 70273747 | + | 29.3 | 219 | 425 | -42 | MLT1C | MaLR | LTR |
| 70273768 | 70273888 | - | 30.8 | 2104 | 2226 | -3428 | HERV16 | ERVL | LTR |
| 70273961 | 70274172 | - | 38.6 | 1608 | 1824 | -3172 | HERV16 | ERVL | LTR |
| 70274425 | 70274831 | + | 5.9 | 5745 | 6152 | -3 | L1PA4 | L1 | LINE |
| 70274843 | 70275060 | - | 39.4 | 972 | 1188 | -3808 | HERV16 | ERVL | LTR |
| 70275064 | 70275850 | - | 22.5 | 3150 | 3944 | -2202 | L1P4 | L1 | LINE |
| 70275853 | 70276208 | + | 19.4 | 3951 | 4318 | -1828 | L1P4 | L1 | LINE |
| 70276209 | 70276914 | + | 8.3 | 1 | 722 | 0 | LTR30 | ERV1 | LTR |
| 70276916 | 70280356 | + | 9.3 | 1 | 3462 | -4795 | HERV30 | ERV1 | LTR |
| 70280350 | 70281250 | + | 10.8 | 7344 | 8257 | 0 | HERV30 | ERV1 | LTR |
| 70281257 | 70281948 | + | 8.6 | 9 | 720 | -2 | LTR30 | ERV1 | LTR |
| 70281950 | 70282647 | + | 19.2 | 4314 | 5015 | -1131 | L1P4 | L1 | LINE |
| 70282648 | 70283008 | + | 17.8 | 1 | 364 | 0 | THE1B | MaLR | LTR |
| 70283011 | 70284658 | + | 32.8 | 2 | 1735 | 0 | MLT1A0-int | MaLR | LTR |
| 70284659 | 70285021 | + | 16.0 | 1 | 364 | 0 | THE1B | MaLR | LTR |
| 70285022 | 70285288 | + | 23.6 | 5013 | 5363 | -780 | L1M2 | L1 | LINE |
| 70285301 | 70285699 | + | 30.1 | 9 | 406 | -6097 | L1P4a | L1 | LINE |
| 70285701 | 70285910 | + | 21.5 | 342 | 557 | 0 | MER41D | ERV1 | LTR |
| 70286109 | 70286282 | - | 28.4 | 5 | 181 | -4815 | HERV16 | ERVL | LTR |

  

|  |  |  |  |  |  |  |  |  |  |
| --- | --- | --- | --- | --- | --- | --- | --- | --- | --- |
| chr4:70635525-70649730 | | | 13781 bp | | |  |  |  |  |
| Genome Start | Genome End | Strand | Div | Repeat Start | Repeat End | Repeat Left | Name | Family | Class |
| 70635525 | 70635695 | + | 25.8 | 185 | 345 | -6288 | L1MDa | L1 | LINE |
| 70636010 | 70636106 | + | 17.5 | 1150 | 1248 | -5292 | L1MDa | L1 | LINE |
| 70636115 | 70636859 | + | 19.3 | 50 | 803 | -6762 | L1M1 | L1 | LINE |
| 70636920 | 70638158 | + | 25.6 | 1265 | 2532 | -4101 | L1MDa | L1 | LINE |
| 70638162 | 70638579 | - | 18.6 | 5745 | 6165 | 0 | L1PA15 | L1 | LINE |
| 70638580 | 70638951 | - | 14.3 | 1 | 381 | 0 | THE1D | MaLR | LTR |
| 70638957 | 70640515 | - | 12.7 | 1 | 1574 | -6 | THE1D-int | MaLR | LTR |
| 70640516 | 70640887 | - | 16.3 | 1 | 381 | 0 | THE1D | MaLR | LTR |
| 70640888 | 70641032 | - | 16.6 | 5600 | 5749 | -417 | L1PA15 | L1 | LINE |
| 70641034 | 70642872 | - | 16.5 | 3792 | 5699 | -784 | L1P4a | L1 | LINE |
| 70642904 | 70644832 | - | 19.6 | 28 | 1755 | 0 | MER52D | ERV1 | LTR |
| 70644834 | 70648342 | - | 22.9 | 1 | 3703 | -2780 | L1P4a | L1 | LINE |
| 70648345 | 70649730 | + | 25.8 | 2528 | 3966 | -2571 | L1MDa | L1 | LINE |

  

|  |  |  |  |  |  |  |  |  |  |
| --- | --- | --- | --- | --- | --- | --- | --- | --- | --- |
| chr4:70706080-70709712 | | | 3542 bp | | |  |  |  |  |
| Genome Start | Genome End | Strand | Div | Repeat Start | Repeat End | Repeat Left | Name | Family | Class |
| 70706080 | 70706819 | + | 26.6 | 5008 | 5762 | -408 | L1ME3A | L1 | LINE |
| 70706820 | 70707184 | + | 13.3 | 1 | 364 | 0 | THE1B | MaLR | LTR |
| 70707187 | 70708765 | + | 31.5 | 2 | 1735 | 0 | MLT-int | MaLR | LTR |
| 70708766 | 70709139 | + | 13.3 | 1 | 364 | 0 | THE1B | MaLR | LTR |
| 70709140 | 70709184 | + | 27.4 | 5763 | 5800 | -370 | L1ME3A | L1 | LINE |
| 70709202 | 70709331 | - | 26.9 | 55 | 185 | -4 | MER5A | MER1\_type | DNA |
| 70709404 | 70709712 | + | 25.7 | 5842 | 6162 | -8 | L1ME3A | L1 | LINE |

  

|  |  |  |  |  |  |  |  |  |  |
| --- | --- | --- | --- | --- | --- | --- | --- | --- | --- |
| chr4:72698934-72711854 | | | 12508 bp | | |  |  |  |  |
| Genome Start | Genome End | Strand | Div | Repeat Start | Repeat End | Repeat Left | Name | Family | Class |
| 72698934 | 72699130 | + | 13.2 | 3 | 200 | -6663 | L1PBa | L1 | LINE |
| 72699131 | 72700366 | + | 11.5 | 634 | 2029 | -4834 | L1PBa | L1 | LINE |
| 72700376 | 72706523 | + | 8.4 | 5 | 6154 | 0 | L1PA6 | L1 | LINE |
| 72706587 | 72706896 | - | 13.2 | 1 | 310 | 0 | AluSg | Alu | SINE |
| 72707231 | 72707290 | + | 8.7 | 2013 | 2064 | -4799 | L1PBa | L1 | LINE |
| 72707291 | 72707646 | - | 15.3 | 4 | 364 | 0 | THE1B | MaLR | LTR |
| 72707650 | 72707957 | - | 29.8 | 1384 | 1735 | 0 | MLT-int | MaLR | LTR |
| 72707958 | 72708253 | - | 11.7 | 1 | 294 | -16 | AluSg | Alu | SINE |
| 72708254 | 72709526 | - | 29.8 | 2 | 1383 | -352 | MLT-int | MaLR | LTR |
| 72709531 | 72709889 | - | 17.3 | 1 | 362 | -2 | THE1B | MaLR | LTR |
| 72709890 | 72711854 | + | 10.8 | 2065 | 4098 | -2764 | L1PBa | L1 | LINE |

  

|  |  |  |  |  |  |  |  |  |  |
| --- | --- | --- | --- | --- | --- | --- | --- | --- | --- |
| chr4:73125580-73129108 | | | 3533 bp | | |  |  |  |  |
| Genome Start | Genome End | Strand | Div | Repeat Start | Repeat End | Repeat Left | Name | Family | Class |
| 73125580 | 73126644 | - | 12.7 | 5072 | 6158 | -8 | L1PA16 | L1 | LINE |
| 73126645 | 73126962 | + | 9.5 | 1 | 373 | -2 | THE1C | MaLR | LTR |
| 73126965 | 73128482 | + | 12.9 | 1 | 1580 | 0 | THE1C-int | MaLR | LTR |
| 73128483 | 73128857 | + | 11.2 | 1 | 375 | 0 | THE1C | MaLR | LTR |
| 73128858 | 73129025 | - | 16.1 | 4909 | 5076 | -1070 | L1PA16 | L1 | LINE |
| 73129020 | 73129108 | - | 16.9 | 4467 | 4555 | -1591 | L1PA16 | L1 | LINE |

  

|  |  |  |  |  |  |  |  |  |  |
| --- | --- | --- | --- | --- | --- | --- | --- | --- | --- |
| chr4:73337910-73341423 | | | 3473 bp | | |  |  |  |  |
| Genome Start | Genome End | Strand | Div | Repeat Start | Repeat End | Repeat Left | Name | Family | Class |
| 73337910 | 73338282 | - | 35.0 | 5568 | 5939 | -1869 | L1MC4a | L1 | LINE |
| 73338311 | 73338626 | + | 15.8 | 1 | 312 | -43 | THE1A | MaLR | LTR |
| 73338627 | 73339064 | + | 8.7 | 1 | 444 | 0 | MLT2A1 | ERVL | LTR |
| 73339065 | 73339083 | + | 15.8 | 313 | 316 | -39 | THE1A | MaLR | LTR |
| 73339084 | 73339390 | - | 12.1 | 1 | 306 | -6 | AluSx | Alu | SINE |
| 73339391 | 73339431 | + | 15.8 | 317 | 355 | 0 | THE1A | MaLR | LTR |
| 73339436 | 73340978 | + | 29.9 | 4 | 1735 | 0 | MLT1C-int | MaLR | LTR |
| 73340981 | 73341290 | + | 15.9 | 3 | 355 | 0 | THE1A | MaLR | LTR |
| 73341298 | 73341423 | - | 30.2 | 5384 | 5518 | -644 | L1MC4a | L1 | LINE |

  

|  |  |  |  |  |  |  |  |  |  |
| --- | --- | --- | --- | --- | --- | --- | --- | --- | --- |
| chr4:74005083-74018774 | | | 13706 bp | | |  |  |  |  |
| Genome Start | Genome End | Strand | Div | Repeat Start | Repeat End | Repeat Left | Name | Family | Class |
| 74005083 | 74008899 | - | 13.0 | 2040 | 5909 | -1656 | L1M1 | L1 | LINE |
| 74008900 | 74009196 | + | 12.1 | 1 | 298 | -15 | AluSq | Alu | SINE |
| 74009197 | 74010156 | - | 14.3 | 1031 | 2039 | -5526 | L1M1 | L1 | LINE |
| 74010157 | 74011004 | - | 6.5 | 1 | 780 | 0 | LTR17 | ERV1 | LTR |
| 74011005 | 74012726 | - | 6.8 | 6902 | 8626 | 0 | HERV17 | ERV1 | LTR |
| 74012718 | 74016107 | - | 4.8 | 1679 | 5067 | -3559 | HERV17 | ERV1 | LTR |
| 74016105 | 74016829 | - | 4.9 | 1147 | 1864 | -6762 | HERV17 | ERV1 | LTR |
| 74016828 | 74017007 | - | 7.8 | 1 | 180 | -8446 | HERV17 | ERV1 | LTR |
| 74017008 | 74017767 | - | 7.4 | 1 | 780 | 0 | LTR17 | ERV1 | LTR |
| 74017768 | 74018774 | - | 17.7 | 6 | 1034 | -6531 | L1M1 | L1 | LINE |

  

|  |  |  |  |  |  |  |  |  |  |
| --- | --- | --- | --- | --- | --- | --- | --- | --- | --- |
| chr4:74439581-74455400 | | | 15587 bp | | |  |  |  |  |
| Genome Start | Genome End | Strand | Div | Repeat Start | Repeat End | Repeat Left | Name | Family | Class |
| 74439581 | 74439975 | + | 29.0 | 1455 | 1886 | -1533 | L2 | L2 | LINE |
| 74439976 | 74440127 | - | 20.9 | 8 | 158 | 0 | FLAM\_A | Alu | SINE |
| 74440016 | 74440988 | + | 29.0 | 1887 | 2803 | -616 | L2 | L2 | LINE |
| 74440983 | 74441147 | - | 27.9 | 20 | 189 | -79 | MIRb | MIR | SINE |
| 74441189 | 74441254 | + | 30.3 | 3350 | 3419 | 0 | L2 | L2 | LINE |
| 74441389 | 74441698 | - | 13.6 | 1 | 311 | -1 | AluSx | Alu | SINE |
| 74441727 | 74442234 | + | 12.3 | 2 | 511 | 0 | MER9 | ERVK | LTR |
| 74442235 | 74444730 | + | 8.1 | 1 | 2500 | -3416 | HERVK9 | ERVK | LTR |
| 74444737 | 74450827 | + | 6.9 | 11 | 6154 | 0 | L1PA5 | L1 | LINE |
| 74450834 | 74453970 | + | 9.4 | 2484 | 5634 | -282 | HERVK9 | ERVK | LTR |
| 74454067 | 74454343 | + | 6.1 | 5635 | 5916 | 0 | HERVK9 | ERVK | LTR |
| 74454344 | 74454853 | + | 13.0 | 1 | 511 | 0 | MER9 | ERVK | LTR |
| 74454894 | 74455400 | + | 30.8 | 2825 | 3338 | -81 | L2 | L2 | LINE |

  

|  |  |  |  |  |  |  |  |  |  |
| --- | --- | --- | --- | --- | --- | --- | --- | --- | --- |
| chr4:74553247-74562565 | | | 9315 bp | | |  |  |  |  |
| Genome Start | Genome End | Strand | Div | Repeat Start | Repeat End | Repeat Left | Name | Family | Class |
| 74553247 | 74558579 | - | 11.6 | 1345 | 6775 | -8 | L1PA13 | L1 | LINE |
| 74558580 | 74558954 | - | 15.9 | 3 | 381 | 0 | THE1D | MaLR | LTR |
| 74558957 | 74560518 | - | 29.3 | 2 | 1735 | 0 | MLT-int | MaLR | LTR |
| 74560521 | 74560900 | - | 11.5 | 1 | 381 | 0 | THE1D | MaLR | LTR |
| 74560901 | 74561015 | - | 17.3 | 1255 | 1344 | -5441 | L1PA13 | L1 | LINE |
| 74561016 | 74561317 | - | 11.9 | 1 | 310 | -2 | AluSx | Alu | SINE |
| 74561318 | 74562565 | - | 17.3 | 6 | 1254 | -5531 | L1PA13 | L1 | LINE |

  

|  |  |  |  |  |  |  |  |  |  |
| --- | --- | --- | --- | --- | --- | --- | --- | --- | --- |
| chr4:77554558-77558382 | | | 3708 bp | | |  |  |  |  |
| Genome Start | Genome End | Strand | Div | Repeat Start | Repeat End | Repeat Left | Name | Family | Class |
| 77554558 | 77554705 | - | 27.6 | 3201 | 3339 | -80 | L2 | L2 | LINE |
| 77554718 | 77555062 | + | 7.8 | 1 | 355 | 0 | THE1A | MaLR | LTR |
| 77555063 | 77555632 | + | 9.6 | 1 | 554 | -1026 | THE1A-int | MaLR | LTR |
| 77555633 | 77556310 | + | 4.5 | 1 | 684 | -323 | LTR13 | ERVK | LTR |
| 77556337 | 77556626 | + | 4.5 | 685 | 1006 | -1 | LTR13 | ERVK | LTR |
| 77556627 | 77557652 | + | 10.1 | 555 | 1578 | -2 | THE1A-int | MaLR | LTR |
| 77557653 | 77557748 | + | 7.3 | 1 | 96 | -259 | THE1A | MaLR | LTR |
| 77557747 | 77557897 | + | 6.0 | 201 | 355 | 0 | THE1A | MaLR | LTR |
| 77557903 | 77558107 | - | 32.7 | 2957 | 3174 | -245 | L2 | L2 | LINE |
| 77558184 | 77558382 | - | 26.3 | 2713 | 2950 | -469 | L2 | L2 | LINE |

  

|  |  |  |  |  |  |  |  |  |  |
| --- | --- | --- | --- | --- | --- | --- | --- | --- | --- |
| chr4:79235630-79238273 | | | 2642 bp | | |  |  |  |  |
| Genome Start | Genome End | Strand | Div | Repeat Start | Repeat End | Repeat Left | Name | Family | Class |
| 79235630 | 79235682 | + | 13.0 | 1 | 47 | -343 | MLT1B | MaLR | LTR |
| 79235683 | 79236044 | - | 12.6 | 1 | 364 | 0 | THE1B | MaLR | LTR |
| 79236045 | 79237600 | - | 29.9 | 2 | 1735 | 0 | MLT1H-int | MaLR | LTR |
| 79237603 | 79237964 | - | 12.4 | 4 | 364 | 0 | THE1B | MaLR | LTR |
| 79237965 | 79238273 | + | 13.0 | 48 | 390 | 0 | MLT1B | MaLR | LTR |

  

|  |  |  |  |  |  |  |  |  |  |
| --- | --- | --- | --- | --- | --- | --- | --- | --- | --- |
| chr4:79853233-79860945 | | | 7459 bp | | |  |  |  |  |
| Genome Start | Genome End | Strand | Div | Repeat Start | Repeat End | Repeat Left | Name | Family | Class |
| 79853233 | 79853405 | + | 24.7 | 7471 | 7678 | -364 | L1MC4 | L1 | LINE |
| 79853411 | 79854019 | + | 14.5 | 1 | 637 | -16 | MER39B | ERV1 | LTR |
| 79854020 | 79854471 | - | 6.5 | 1 | 448 | -2 | LTR7 | ERV1 | LTR |
| 79854477 | 79854851 | - | 8.1 | 7322 | 7708 | -5 | HERVH | ERV1 | LTR |
| 79854850 | 79855065 | - | 13.0 | 5600 | 5819 | -1894 | HERVH | ERV1 | LTR |
| 79855067 | 79855727 | - | 15.2 | 4489 | 5149 | -2564 | HERVH | ERV1 | LTR |
| 79855731 | 79856232 | - | 11.0 | 3492 | 3993 | -3720 | HERVH | ERV1 | LTR |
| 79856223 | 79859447 | - | 9.2 | 1 | 3290 | -4423 | HERVH | ERV1 | LTR |
| 79859450 | 79859899 | - | 6.0 | 1 | 448 | -2 | LTR7 | ERV1 | LTR |
| 79860150 | 79860226 | + | 21.3 | 7918 | 7998 | -44 | L1MC4 | L1 | LINE |
| 79860227 | 79860924 | - | 14.7 | 1 | 727 | 0 | LTR8A | ERV1 | LTR |
| 79860925 | 79860945 | + | 21.3 | 7999 | 8015 | -27 | L1MC4 | L1 | LINE |

  

|  |  |  |  |  |  |  |  |  |  |
| --- | --- | --- | --- | --- | --- | --- | --- | --- | --- |
| chr4:80480338-80492420 | | | 12033 bp | | |  |  |  |  |
| Genome Start | Genome End | Strand | Div | Repeat Start | Repeat End | Repeat Left | Name | Family | Class |
| 80480338 | 80481755 | + | 20.4 | 442 | 1805 | -5044 | L1MA4A | L1 | LINE |
| 80481756 | 80482066 | + | 9.4 | 1 | 310 | -1 | AluY | Alu | SINE |
| 80482067 | 80482132 | + | 20.4 | 1806 | 1861 | -4988 | L1MA4A | L1 | LINE |
| 80482133 | 80482496 | - | 14.6 | 1 | 364 | 0 | THE1B | MaLR | LTR |
| 80482497 | 80483597 | + | 20.5 | 1862 | 2919 | -3929 | L1MA4A | L1 | LINE |
| 80483598 | 80483903 | + | 14.7 | 1 | 314 | 0 | AluY | Alu | SINE |
| 80483904 | 80483945 | + | 26.2 | 2920 | 2958 | -3890 | L1MA4A | L1 | LINE |
| 80483996 | 80486240 | - | 10.8 | 4107 | 6302 | -2 | L1MA2 | L1 | LINE |
| 80486241 | 80486596 | + | 12.8 | 1 | 364 | 0 | THE1B | MaLR | LTR |
| 80486599 | 80488152 | + | 30.1 | 2 | 1735 | 0 | MLT1H-int | MaLR | LTR |
| 80488153 | 80488509 | + | 12.5 | 1 | 364 | 0 | THE1B | MaLR | LTR |
| 80488510 | 80489598 | - | 10.7 | 4446 | 5525 | -2040 | L1M1 | L1 | LINE |
| 80489599 | 80489909 | - | 10.3 | 1 | 311 | 0 | AluY | Alu | SINE |
| 80489910 | 80490349 | - | 10.7 | 4016 | 4445 | -3120 | L1M1 | L1 | LINE |
| 80490342 | 80490564 | - | 9.1 | 3432 | 3660 | -3905 | L1M1 | L1 | LINE |
| 80490563 | 80490854 | - | 23.1 | 15 | 297 | -7268 | L1M1 | L1 | LINE |
| 80490852 | 80491214 | + | 20.4 | 2952 | 3310 | -3535 | L1MA4A | L1 | LINE |
| 80491203 | 80491294 | + | 17.9 | 6073 | 6157 | -9 | L1PA17 | L1 | LINE |
| 80491300 | 80491530 | + | 20.9 | 3294 | 3518 | -3327 | L1MA4A | L1 | LINE |
| 80491549 | 80492069 | + | 18.1 | 3913 | 4427 | -2418 | L1MA4A | L1 | LINE |
| 80492070 | 80492379 | + | 10.1 | 1 | 309 | -4 | AluSp | Alu | SINE |
| 80492380 | 80492420 | + | 18.1 | 4428 | 4472 | -2373 | L1MA4A | L1 | LINE |

  

|  |  |  |  |  |  |  |  |  |  |
| --- | --- | --- | --- | --- | --- | --- | --- | --- | --- |
| chr4:80688999-80701704 | | | 12461 bp | | |  |  |  |  |
| Genome Start | Genome End | Strand | Div | Repeat Start | Repeat End | Repeat Left | Name | Family | Class |
| 80688999 | 80689177 | - | 24.3 | 3244 | 3419 | 0 | L2 | L2 | LINE |
| 80689179 | 80691584 | + | 19.4 | 2174 | 4601 | -1545 | L1PA17 | L1 | LINE |
| 80691593 | 80692275 | + | 19.2 | 5465 | 6150 | -16 | L1PA17 | L1 | LINE |
| 80692293 | 80692455 | - | 27.2 | 3105 | 3291 | -128 | L2 | L2 | LINE |
| 80692488 | 80692931 | + | 21.6 | 1 | 467 | -47 | MER67D | ERV1 | LTR |
| 80692932 | 80693195 | - | 20.3 | 29 | 301 | -11 | AluJb | Alu | SINE |
| 80693253 | 80694004 | - | 30.9 | 1812 | 2596 | -823 | L2 | L2 | LINE |
| 80694148 | 80694594 | - | 4.9 | 1 | 448 | -2 | LTR7 | ERV1 | LTR |
| 80694595 | 80694974 | - | 7.7 | 7322 | 7713 | 0 | HERVH | ERV1 | LTR |
| 80694973 | 80695183 | - | 12.3 | 5600 | 5819 | -1894 | HERVH | ERV1 | LTR |
| 80695185 | 80695843 | - | 15.0 | 4489 | 5149 | -2564 | HERVH | ERV1 | LTR |
| 80695847 | 80696349 | - | 12.2 | 3492 | 3993 | -3720 | HERVH | ERV1 | LTR |
| 80696340 | 80696489 | - | 12.0 | 3140 | 3290 | -4423 | HERVH | ERV1 | LTR |
| 80696476 | 80699435 | - | 8.9 | 1 | 2992 | -4721 | HERVH | ERV1 | LTR |
| 80699438 | 80699887 | - | 6.0 | 2 | 448 | -2 | LTR7 | ERV1 | LTR |
| 80699896 | 80700016 | - | 34.5 | 1652 | 1799 | -1620 | L2 | L2 | LINE |
| 80700017 | 80700308 | + | 11.7 | 1 | 294 | -19 | AluSq | Alu | SINE |
| 80700309 | 80700528 | - | 34.5 | 1415 | 1651 | -1768 | L2 | L2 | LINE |
| 80700529 | 80700980 | - | 31.7 | 819 | 1281 | -2138 | L2 | L2 | LINE |
| 80700987 | 80701316 | + | 29.2 | 3111 | 3418 | -1 | L2 | L2 | LINE |
| 80701310 | 80701704 | - | 33.0 | 421 | 838 | -2581 | L2 | L2 | LINE |

  

|  |  |  |  |  |  |  |  |  |  |
| --- | --- | --- | --- | --- | --- | --- | --- | --- | --- |
| chr4:81509541-81515718 | | | 5765 bp | | |  |  |  |  |
| Genome Start | Genome End | Strand | Div | Repeat Start | Repeat End | Repeat Left | Name | Family | Class |
| 81509541 | 81509940 | - | 32.2 | 4819 | 5231 | -436 | HERV16 | ERVL | LTR |
| 81509974 | 81510121 | - | 35.3 | 4433 | 4579 | -1088 | HERV16 | ERVL | LTR |
| 81510122 | 81510517 | - | 13.2 | 1 | 428 | 0 | MSTA | MaLR | LTR |
| 81510518 | 81512155 | - | 29.9 | 2 | 1735 | 0 | MLT-int | MaLR | LTR |
| 81512158 | 81512549 | - | 15.1 | 4 | 428 | 0 | MSTA | MaLR | LTR |
| 81512550 | 81512646 | - | 35.3 | 4338 | 4432 | -1235 | HERV16 | ERVL | LTR |
| 81512662 | 81512751 | + | 18.9 | 299 | 390 | 0 | MLT1B | MaLR | LTR |
| 81512786 | 81513219 | - | 31.4 | 2399 | 2857 | -2139 | HERV16 | ERVL | LTR |
| 81513213 | 81513570 | - | 31.0 | 1905 | 2172 | -2824 | HERV16 | ERVL | LTR |
| 81513626 | 81514963 | - | 34.0 | 366 | 1783 | -3213 | HERV16 | ERVL | LTR |
| 81515202 | 81515424 | + | 30.9 | 1041 | 1264 | -3732 | HERV16 | ERVL | LTR |
| 81515468 | 81515718 | - | 30.9 | 39 | 280 | -4716 | HERV16 | ERVL | LTR |

  

|  |  |  |  |  |  |  |  |  |  |
| --- | --- | --- | --- | --- | --- | --- | --- | --- | --- |
| chr4:81540403-81542902 | | | 2496 bp | | |  |  |  |  |
| Genome Start | Genome End | Strand | Div | Repeat Start | Repeat End | Repeat Left | Name | Family | Class |
| 81540403 | 81540526 | - | 31.0 | 119 | 260 | -8 | MIR | MIR | SINE |
| 81540527 | 81540875 | + | 9.8 | 1 | 355 | 0 | THE1A | MaLR | LTR |
| 81540878 | 81542455 | + | 30.4 | 2 | 1735 | 0 | MLT1J-int | MaLR | LTR |
| 81542458 | 81542807 | + | 8.3 | 3 | 355 | 0 | THE1A | MaLR | LTR |
| 81542808 | 81542902 | - | 31.0 | 27 | 125 | -143 | MIR | MIR | SINE |

  

|  |  |  |  |  |  |  |  |  |  |
| --- | --- | --- | --- | --- | --- | --- | --- | --- | --- |
| chr4:82148100-82150677 | | | 2574 bp | | |  |  |  |  |
| Genome Start | Genome End | Strand | Div | Repeat Start | Repeat End | Repeat Left | Name | Family | Class |
| 82148100 | 82148211 | + | 34.6 | 1 | 113 | -149 | MIR | MIR | SINE |
| 82148212 | 82148584 | + | 12.5 | 1 | 373 | -2 | THE1C | MaLR | LTR |
| 82148589 | 82150156 | + | 29.4 | 2 | 1735 | 0 | MLT-int | MaLR | LTR |
| 82150157 | 82150530 | + | 15.9 | 1 | 375 | 0 | THE1C | MaLR | LTR |
| 82150531 | 82150677 | + | 34.6 | 114 | 254 | -8 | MIR | MIR | SINE |

  

|  |  |  |  |  |  |  |  |  |  |
| --- | --- | --- | --- | --- | --- | --- | --- | --- | --- |
| chr4:82150982-82154063 | | | 3077 bp | | |  |  |  |  |
| Genome Start | Genome End | Strand | Div | Repeat Start | Repeat End | Repeat Left | Name | Family | Class |
| 82150982 | 82151389 | + | 19.8 | 5055 | 5469 | -831 | L1M1 | L1 | LINE |
| 82151391 | 82151764 | + | 13.2 | 1 | 362 | -2 | THE1B | MaLR | LTR |
| 82151767 | 82153341 | + | 10.3 | 1 | 1580 | 0 | THE1B-int | MaLR | LTR |
| 82153344 | 82153714 | + | 15.8 | 3 | 364 | 0 | THE1B | MaLR | LTR |
| 82153715 | 82154063 | + | 18.6 | 5466 | 5812 | -491 | L1M1 | L1 | LINE |

  

|  |  |  |  |  |  |  |  |  |  |
| --- | --- | --- | --- | --- | --- | --- | --- | --- | --- |
| chr4:82436938-82439875 | | | 2936 bp | | |  |  |  |  |
| Genome Start | Genome End | Strand | Div | Repeat Start | Repeat End | Repeat Left | Name | Family | Class |
| 82436938 | 82436967 | + | 23.3 | 11 | 39 | -551 | MLT1G | MaLR | LTR |
| 82436968 | 82437348 | + | 13.6 | 1 | 380 | -1 | THE1D | MaLR | LTR |
| 82437351 | 82438967 | + | 31.4 | 2 | 1735 | 0 | MLT1G-int | MaLR | LTR |
| 82438968 | 82439347 | + | 12.5 | 1 | 379 | -2 | THE1D | MaLR | LTR |
| 82439348 | 82439875 | + | 23.3 | 40 | 590 | 0 | MLT1G | MaLR | LTR |

  

|  |  |  |  |  |  |  |  |  |  |
| --- | --- | --- | --- | --- | --- | --- | --- | --- | --- |
| chr4:83612743-83621540 | | | 8747 bp | | |  |  |  |  |
| Genome Start | Genome End | Strand | Div | Repeat Start | Repeat End | Repeat Left | Name | Family | Class |
| 83612743 | 83612898 | - | 28.1 | 3249 | 3417 | -2 | L2 | L2 | LINE |
| 83612954 | 83613036 | + | 12.1 | 69 | 155 | -21 | FRAM | Alu | SINE |
| 83613037 | 83613222 | - | 24.3 | 3037 | 3237 | -182 | L2 | L2 | LINE |
| 83613223 | 83613492 | - | 10.8 | 103 | 375 | 0 | THE1C | MaLR | LTR |
| 83613494 | 83614207 | + | 8.6 | 1 | 780 | 0 | LTR17 | ERV1 | LTR |
| 83614210 | 83614545 | + | 8.3 | 3 | 346 | -8280 | HERV17 | ERV1 | LTR |
| 83614546 | 83614856 | - | 1.0 | 1 | 311 | 0 | AluY | Alu | SINE |
| 83614857 | 83618494 | + | 8.3 | 347 | 3947 | -4679 | HERV17 | ERV1 | LTR |
| 83618488 | 83619514 | + | 7.1 | 7599 | 8626 | 0 | HERV17 | ERV1 | LTR |
| 83619515 | 83620230 | + | 8.8 | 1 | 780 | 0 | LTR17 | ERV1 | LTR |
| 83620231 | 83620333 | - | 8.7 | 3 | 105 | -270 | THE1C | MaLR | LTR |
| 83620334 | 83620542 | - | 26.8 | 2821 | 3043 | -376 | L2 | L2 | LINE |
| 83620543 | 83620827 | - | 11.7 | 12 | 302 | -10 | AluJb | Alu | SINE |
| 83620828 | 83621540 | - | 26.8 | 2036 | 2820 | -599 | L2 | L2 | LINE |

  

|  |  |  |  |  |  |  |  |  |  |
| --- | --- | --- | --- | --- | --- | --- | --- | --- | --- |
| chr4:84428564-84431563 | | | 2998 bp | | |  |  |  |  |
| Genome Start | Genome End | Strand | Div | Repeat Start | Repeat End | Repeat Left | Name | Family | Class |
| 84428564 | 84428597 | + | 19.1 | 56 | 89 | -325 | MLT2D | ERVL | LTR |
| 84428598 | 84428959 | + | 9.7 | 1 | 364 | 0 | THE1B | MaLR | LTR |
| 84428962 | 84430552 | + | 30.1 | 2 | 1735 | 0 | MLT-int | MaLR | LTR |
| 84430553 | 84430916 | + | 10.4 | 1 | 364 | 0 | THE1B | MaLR | LTR |
| 84430917 | 84431202 | + | 19.1 | 90 | 359 | -55 | MLT2D | ERVL | LTR |
| 84431203 | 84431504 | + | 7.0 | 1 | 301 | -10 | AluY | Alu | SINE |
| 84431505 | 84431563 | + | 19.1 | 506 | 552 | -8 | MLT2D | ERVL | LTR |

  

|  |  |  |  |  |  |  |  |  |  |
| --- | --- | --- | --- | --- | --- | --- | --- | --- | --- |
| chr4:84485828-84490710 | | | 4881 bp | | |  |  |  |  |
| Genome Start | Genome End | Strand | Div | Repeat Start | Repeat End | Repeat Left | Name | Family | Class |
| 84485828 | 84485889 | + | 17.7 | 5728 | 5789 | -379 | L1MB2 | L1 | LINE |
| 84485890 | 84486054 | + | 12.4 | 3 | 169 | -1133 | HSMAR2 | Mariner | DNA |
| 84486055 | 84486422 | - | 14.3 | 1 | 375 | 0 | THE1C | MaLR | LTR |
| 84486423 | 84488020 | - | 30.4 | 2 | 1735 | 0 | MLT-int | MaLR | LTR |
| 84488023 | 84488399 | - | 15.7 | 1 | 375 | 0 | THE1C | MaLR | LTR |
| 84488400 | 84488464 | + | 12.4 | 170 | 222 | -1080 | HSMAR2 | Mariner | DNA |
| 84488465 | 84488760 | + | 15.5 | 4 | 299 | -13 | AluSx | Alu | SINE |
| 84488761 | 84489742 | + | 12.4 | 223 | 1236 | -66 | HSMAR2 | Mariner | DNA |
| 84489743 | 84490054 | - | 12.6 | 1 | 312 | 0 | AluSx | Alu | SINE |
| 84490055 | 84490092 | + | 12.4 | 1237 | 1276 | -26 | HSMAR2 | Mariner | DNA |
| 84490093 | 84490323 | - | 8.7 | 1 | 236 | -76 | AluSx | Alu | SINE |
| 84490324 | 84490346 | + | 12.4 | 1277 | 1299 | -3 | HSMAR2 | Mariner | DNA |
| 84490347 | 84490710 | + | 16.2 | 5784 | 6164 | -7 | L1MB2 | L1 | LINE |

  

|  |  |  |  |  |  |  |  |  |  |
| --- | --- | --- | --- | --- | --- | --- | --- | --- | --- |
| chr4:84514487-84517426 | | | 2936 bp | | |  |  |  |  |
| Genome Start | Genome End | Strand | Div | Repeat Start | Repeat End | Repeat Left | Name | Family | Class |
| 84514487 | 84514614 | + | 14.7 | 1 | 128 | -594 | MER72 | ERV1 | LTR |
| 84514615 | 84514969 | - | 8.7 | 1 | 355 | 0 | THE1A | MaLR | LTR |
| 84514972 | 84516545 | - | 29.7 | 2 | 1733 | -2 | MLT1D-int | MaLR | LTR |
| 84516548 | 84516723 | - | 8.7 | 181 | 355 | 0 | THE1A | MaLR | LTR |
| 84516724 | 84517031 | + | 10.1 | 1 | 308 | -2 | AluSg | Alu | SINE |
| 84517032 | 84517225 | - | 8.7 | 1 | 180 | -175 | THE1A | MaLR | LTR |
| 84517226 | 84517426 | + | 14.6 | 124 | 321 | -401 | MER72 | ERV1 | LTR |

  

|  |  |  |  |  |  |  |  |  |  |
| --- | --- | --- | --- | --- | --- | --- | --- | --- | --- |
| chr4:85796391-85799611 | | | 3219 bp | | |  |  |  |  |
| Genome Start | Genome End | Strand | Div | Repeat Start | Repeat End | Repeat Left | Name | Family | Class |
| 85796391 | 85796926 | + | 19.9 | 5530 | 6080 | -104 | L1MB7 | L1 | LINE |
| 85796927 | 85797282 | - | 7.1 | 1 | 355 | 0 | THE1A | MaLR | LTR |
| 85797283 | 85797588 | - | 29.6 | 1384 | 1735 | 0 | MLT-int | MaLR | LTR |
| 85797589 | 85797895 | - | 12.1 | 1 | 306 | -7 | AluSq | Alu | SINE |
| 85797896 | 85799171 | - | 29.6 | 2 | 1383 | -352 | MLT-int | MaLR | LTR |
| 85799174 | 85799524 | - | 8.0 | 1 | 355 | 0 | THE1A | MaLR | LTR |
| 85799525 | 85799611 | + | 19.9 | 6081 | 6175 | -9 | L1MB7 | L1 | LINE |

  

|  |  |  |  |  |  |  |  |  |  |
| --- | --- | --- | --- | --- | --- | --- | --- | --- | --- |
| chr4:86227911-86232027 | | | 3910 bp | | |  |  |  |  |
| Genome Start | Genome End | Strand | Div | Repeat Start | Repeat End | Repeat Left | Name | Family | Class |
| 86227911 | 86228123 | - | 31.7 | 2030 | 2237 | -3912 | L1MEe | L1 | LINE |
| 86228167 | 86228541 | + | 10.2 | 1 | 373 | -2 | THE1C | MaLR | LTR |
| 86228546 | 86230075 | + | 30.0 | 2 | 1735 | 0 | MLT-int | MaLR | LTR |
| 86230078 | 86230452 | + | 10.2 | 3 | 375 | 0 | THE1C | MaLR | LTR |
| 86230453 | 86230692 | - | 25.9 | 5352 | 5593 | -647 | L1M5 | L1 | LINE |
| 86230712 | 86231103 | + | 15.1 | 5874 | 6266 | -25 | L1MA7 | L1 | LINE |
| 86231122 | 86231763 | - | 28.0 | 4725 | 5341 | -805 | L1M5 | L1 | LINE |
| 86231885 | 86232027 | - | 26.2 | 13 | 160 | -5959 | L1MEe | L1 | LINE |

  

|  |  |  |  |  |  |  |  |  |  |
| --- | --- | --- | --- | --- | --- | --- | --- | --- | --- |
| chr4:86256063-86260367 | | | 4250 bp | | |  |  |  |  |
| Genome Start | Genome End | Strand | Div | Repeat Start | Repeat End | Repeat Left | Name | Family | Class |
| 86256063 | 86257031 | + | 32.6 | 200 | 1255 | -2164 | L2 | L2 | LINE |
| 86257032 | 86257380 | + | 15.4 | 1 | 364 | 0 | THE1B | MaLR | LTR |
| 86257383 | 86258948 | + | 30.8 | 2 | 1733 | -2 | MLT1I-int | MaLR | LTR |
| 86258951 | 86259315 | + | 13.0 | 1 | 364 | 0 | THE1B | MaLR | LTR |
| 86259316 | 86259644 | + | 32.6 | 1256 | 1607 | -1812 | L2 | L2 | LINE |
| 86259719 | 86260200 | + | 34.6 | 2213 | 2721 | -698 | L2 | L2 | LINE |
| 86260178 | 86260367 | + | 25.0 | 3216 | 3417 | -2 | L2 | L2 | LINE |

  

|  |  |  |  |  |  |  |  |  |  |
| --- | --- | --- | --- | --- | --- | --- | --- | --- | --- |
| chr4:86489581-86498548 | | | 8954 bp | | |  |  |  |  |
| Genome Start | Genome End | Strand | Div | Repeat Start | Repeat End | Repeat Left | Name | Family | Class |
| 86489581 | 86492233 | - | 8.9 | 4222 | 6856 | -11 | L1PB1 | L1 | LINE |
| 86492303 | 86492630 | - | 6.1 | 1 | 327 | -453 | LTR17 | ERV1 | LTR |
| 86492631 | 86492922 | - | 6.5 | 8335 | 8626 | 0 | HERV17 | ERV1 | LTR |
| 86492913 | 86492951 | - | 7.7 | 7510 | 7548 | -1078 | HERV17 | ERV1 | LTR |
| 86492940 | 86494391 | - | 5.2 | 4571 | 6024 | -2602 | HERV17 | ERV1 | LTR |
| 86494384 | 86495023 | - | 4.1 | 2430 | 3071 | -5555 | HERV17 | ERV1 | LTR |
| 86495009 | 86495071 | - | 7.9 | 1952 | 2014 | -6612 | HERV17 | ERV1 | LTR |
| 86495059 | 86495101 | - | 13.9 | 1 | 43 | -8583 | HERV17 | ERV1 | LTR |
| 86495102 | 86495571 | - | 10.5 | 247 | 780 | 0 | LTR17 | ERV1 | LTR |
| 86495575 | 86498548 | - | 10.4 | 998 | 4217 | -2645 | L1PB1 | L1 | LINE |

  

|  |  |  |  |  |  |  |  |  |  |
| --- | --- | --- | --- | --- | --- | --- | --- | --- | --- |
| chr4:86953839-86957664 | | | 3649 bp | | |  |  |  |  |
| Genome Start | Genome End | Strand | Div | Repeat Start | Repeat End | Repeat Left | Name | Family | Class |
| 86953839 | 86953970 | - | 13.0 | 6177 | 6307 | -5 | L1MA9 | L1 | LINE |
| 86954021 | 86954034 | - | 13.0 | 6148 | 6176 | -136 | L1MA9 | L1 | LINE |
| 86954035 | 86954397 | - | 15.4 | 1 | 364 | 0 | THE1B | MaLR | LTR |
| 86954398 | 86955977 | - | 30.0 | 2 | 1735 | 0 | MLT1C-int | MaLR | LTR |
| 86955980 | 86956339 | - | 14.8 | 1 | 364 | 0 | THE1B | MaLR | LTR |
| 86956340 | 86956690 | - | 13.0 | 5781 | 6147 | -165 | L1MA9 | L1 | LINE |
| 86956816 | 86957664 | - | 15.4 | 4916 | 5791 | -521 | L1MA9 | L1 | LINE |

  

|  |  |  |  |  |  |  |  |  |  |
| --- | --- | --- | --- | --- | --- | --- | --- | --- | --- |
| chr4:88830812-88834743 | | | 3921 bp | | |  |  |  |  |
| Genome Start | Genome End | Strand | Div | Repeat Start | Repeat End | Repeat Left | Name | Family | Class |
| 88830812 | 88830954 | - | 31.5 | 3320 | 3463 | -2251 | ERVL-B4 | ERVL | LTR |
| 88830959 | 88831320 | + | 15.0 | 1 | 364 | 0 | THE1B | MaLR | LTR |
| 88831323 | 88831836 | + | 29.5 | 2 | 490 | -1245 | MLT-int | MaLR | LTR |
| 88831837 | 88832147 | + | 11.9 | 1 | 313 | 0 | AluSq | Alu | SINE |
| 88832148 | 88833356 | + | 29.5 | 491 | 1735 | 0 | MLT-int | MaLR | LTR |
| 88833357 | 88833717 | + | 13.0 | 1 | 364 | 0 | THE1B | MaLR | LTR |
| 88833718 | 88833742 | - | 33.3 | 3235 | 3257 | -2410 | ERVL-B4 | ERVL | LTR |
| 88833748 | 88834743 | - | 36.3 | 282 | 1316 | -4351 | ERVL-B4 | ERVL | LTR |

  

|  |  |  |  |  |  |  |  |  |  |
| --- | --- | --- | --- | --- | --- | --- | --- | --- | --- |
| chr4:89359423-89365684 | | | 5834 bp | | |  |  |  |  |
| Genome Start | Genome End | Strand | Div | Repeat Start | Repeat End | Repeat Left | Name | Family | Class |
| 89359423 | 89359693 | - | 32.6 | 3073 | 3410 | -9 | L2 | L2 | LINE |
| 89359703 | 89359752 | - | 13.9 | 6139 | 6183 | 0 | L1MB3 | L1 | LINE |
| 89359753 | 89360044 | - | 6.7 | 21 | 304 | -7 | AluY | Alu | SINE |
| 89360045 | 89360661 | - | 13.9 | 5480 | 6138 | -45 | L1MB3 | L1 | LINE |
| 89360827 | 89361162 | + | 10.3 | 1 | 335 | -40 | THE1C | MaLR | LTR |
| 89361163 | 89361469 | + | 15.3 | 1 | 310 | -3 | AluSq | Alu | SINE |
| 89361470 | 89361524 | + | 10.3 | 336 | 375 | 0 | THE1C | MaLR | LTR |
| 89361525 | 89362506 | + | 11.1 | 1 | 964 | -616 | THE1C-int | MaLR | LTR |
| 89362507 | 89362849 | + | 11.2 | 1 | 364 | 0 | THE1B | MaLR | LTR |
| 89362850 | 89363471 | + | 11.1 | 965 | 1580 | 0 | THE1C-int | MaLR | LTR |
| 89363474 | 89363839 | + | 9.6 | 3 | 375 | 0 | THE1C | MaLR | LTR |
| 89364048 | 89364341 | - | 14.0 | 5179 | 5490 | -690 | L1MB3 | L1 | LINE |
| 89364344 | 89364632 | + | 16.4 | 1 | 300 | -12 | AluJb | Alu | SINE |
| 89364666 | 89364834 | - | 14.2 | 134 | 302 | -10 | AluJb | Alu | SINE |
| 89364843 | 89365286 | - | 14.7 | 4732 | 5180 | -966 | L1M4 | L1 | LINE |
| 89365288 | 89365318 | - | 28.8 | 3033 | 3063 | -356 | L2 | L2 | LINE |
| 89365319 | 89365543 | - | 20.6 | 2 | 224 | 0 | MER58A | MER1\_type | DNA |
| 89365544 | 89365684 | - | 28.8 | 2884 | 3032 | -387 | L2 | L2 | LINE |

  

|  |  |  |  |  |  |  |  |  |  |
| --- | --- | --- | --- | --- | --- | --- | --- | --- | --- |
| chr4:90096735-90100009 | | | 3273 bp | | |  |  |  |  |
| Genome Start | Genome End | Strand | Div | Repeat Start | Repeat End | Repeat Left | Name | Family | Class |
| 90096735 | 90097027 | - | 18.6 | 153 | 505 | 0 | MLT1D | MaLR | LTR |
| 90097028 | 90097217 | + | 17.8 | 1 | 195 | -231 | MSTB | MaLR | LTR |
| 90097218 | 90097513 | + | 14.0 | 1 | 293 | -19 | AluSx | Alu | SINE |
| 90097514 | 90097722 | + | 17.8 | 196 | 426 | 0 | MSTB | MaLR | LTR |
| 90097725 | 90099443 | + | 26.6 | 2 | 1735 | 0 | MLT1F1-int | MaLR | LTR |
| 90099444 | 90099874 | + | 16.4 | 1 | 424 | -2 | MSTB | MaLR | LTR |
| 90099875 | 90100009 | - | 16.9 | 22 | 152 | -353 | MLT1D | MaLR | LTR |

  

|  |  |  |  |  |  |  |  |  |  |
| --- | --- | --- | --- | --- | --- | --- | --- | --- | --- |
| chr4:90989149-90992079 | | | 2906 bp | | |  |  |  |  |
| Genome Start | Genome End | Strand | Div | Repeat Start | Repeat End | Repeat Left | Name | Family | Class |
| 90989149 | 90989444 | + | 28.9 | 1 | 383 | -412 | LTR50 | ERVL | LTR |
| 90989445 | 90989804 | - | 12.2 | 1 | 364 | 0 | THE1B | MaLR | LTR |
| 90989805 | 90991379 | - | 30.2 | 4 | 1735 | 0 | MLT-int | MaLR | LTR |
| 90991384 | 90991749 | - | 9.9 | 3 | 364 | 0 | THE1B | MaLR | LTR |
| 90991750 | 90991917 | + | 28.9 | 384 | 546 | -249 | LTR50 | ERVL | LTR |
| 90991939 | 90992079 | + | 32.6 | 502 | 647 | -148 | LTR50 | ERVL | LTR |

  

|  |  |  |  |  |  |  |  |  |  |
| --- | --- | --- | --- | --- | --- | --- | --- | --- | --- |
| chr4:92314997-92327460 | | | 12462 bp | | |  |  |  |  |
| Genome Start | Genome End | Strand | Div | Repeat Start | Repeat End | Repeat Left | Name | Family | Class |
| 92314997 | 92320856 | - | 7.6 | 260 | 6154 | 0 | L1PA5 | L1 | LINE |
| 92320857 | 92321368 | - | 13.3 | 1 | 511 | 0 | MER9 | ERVK | LTR |
| 92321369 | 92326123 | - | 6.5 | 1114 | 5916 | 0 | HERVK9 | ERVK | LTR |
| 92326126 | 92326687 | - | 7.1 | 1 | 568 | -5348 | HERVK9 | ERVK | LTR |
| 92326688 | 92327200 | - | 11.2 | 1 | 511 | 0 | MER9 | ERVK | LTR |
| 92327201 | 92327460 | - | 13.1 | 4 | 265 | -2010 | L1PA5 | L1 | LINE |

  

|  |  |  |  |  |  |  |  |  |  |
| --- | --- | --- | --- | --- | --- | --- | --- | --- | --- |
| chr4:92792180-92807170 | | | 14824 bp | | |  |  |  |  |
| Genome Start | Genome End | Strand | Div | Repeat Start | Repeat End | Repeat Left | Name | Family | Class |
| 92792180 | 92792476 | - | 28.3 | 4267 | 4574 | -1572 | L1M5 | L1 | LINE |
| 92792556 | 92793545 | - | 17.5 | 4759 | 5789 | -514 | L1M1 | L1 | LINE |
| 92793546 | 92794117 | + | 22.1 | 1 | 550 | -4 | MLT2B3 | ERVL | LTR |
| 92794118 | 92794562 | - | 19.7 | 4303 | 4758 | -1385 | L1M1 | L1 | LINE |
| 92794563 | 92794773 | - | 23.2 | 5946 | 6156 | 0 | L1PB4 | L1 | LINE |
| 92794776 | 92795227 | - | 5.4 | 1 | 448 | -2 | LTR7 | ERV1 | LTR |
| 92795228 | 92795607 | - | 9.9 | 7322 | 7713 | 0 | HERVH | ERV1 | LTR |
| 92795606 | 92795821 | - | 13.0 | 5600 | 5819 | -1894 | HERVH | ERV1 | LTR |
| 92795823 | 92796484 | - | 14.7 | 4488 | 5149 | -2564 | HERVH | ERV1 | LTR |
| 92796486 | 92796976 | - | 11.1 | 3506 | 3994 | -3719 | HERVH | ERV1 | LTR |
| 92796982 | 92800289 | - | 10.0 | 1 | 3290 | -4423 | HERVH | ERV1 | LTR |
| 92800292 | 92800742 | - | 5.4 | 1 | 448 | -2 | LTR7 | ERV1 | LTR |
| 92800743 | 92802599 | - | 18.6 | 4123 | 5950 | -206 | L1PB4 | L1 | LINE |
| 92802610 | 92804512 | - | 25.3 | 27 | 2775 | -4434 | L1M2 | L1 | LINE |
| 92804596 | 92806721 | - | 21.6 | 3929 | 5929 | -255 | L1MB7 | L1 | LINE |
| 92806722 | 92806855 | + | 10.4 | 6030 | 6163 | 0 | L1PA13 | L1 | LINE |
| 92806856 | 92806968 | - | 28.4 | 3812 | 3928 | -2218 | L1M5 | L1 | LINE |
| 92806955 | 92807170 | - | 27.3 | 2249 | 2511 | -3635 | L1M5 | L1 | LINE |

  

|  |  |  |  |  |  |  |  |  |  |
| --- | --- | --- | --- | --- | --- | --- | --- | --- | --- |
| chr4:92833172-92835934 | | | 2762 bp | | |  |  |  |  |
| Genome Start | Genome End | Strand | Div | Repeat Start | Repeat End | Repeat Left | Name | Family | Class |
| 92833172 | 92834222 | - | 13.9 | 495 | 1580 | 0 | THE1D-int | MaLR | LTR |
| 92834223 | 92834595 | + | 17.9 | 1 | 371 | -4 | THE1C | MaLR | LTR |
| 92834600 | 92834959 | + | 11.2 | 1 | 365 | -1215 | THE1C-int | MaLR | LTR |
| 92834957 | 92835037 | + | 12.5 | 1501 | 1580 | 0 | THE1C-int | MaLR | LTR |
| 92835038 | 92835394 | + | 16.8 | 1 | 375 | 0 | THE1C | MaLR | LTR |
| 92835395 | 92835934 | - | 14.1 | 2 | 500 | -1080 | THE1D-int | MaLR | LTR |

  

|  |  |  |  |  |  |  |  |  |  |
| --- | --- | --- | --- | --- | --- | --- | --- | --- | --- |
| chr4:92910802-92916278 | | | 5102 bp | | |  |  |  |  |
| Genome Start | Genome End | Strand | Div | Repeat Start | Repeat End | Repeat Left | Name | Family | Class |
| 92910802 | 92911390 | - | 33.1 | 3318 | 3903 | -1051 | MER83C-int | ERV1 | LTR |
| 92911391 | 92911738 | - | 14.1 | 4 | 353 | -2 | THE1A | MaLR | LTR |
| 92911742 | 92912757 | - | 30.5 | 668 | 1735 | 0 | MLT-int | MaLR | LTR |
| 92912758 | 92913060 | - | 10.6 | 1 | 304 | -7 | AluY | Alu | SINE |
| 92913061 | 92913605 | - | 30.5 | 2 | 667 | -1068 | MLT-int | MaLR | LTR |
| 92913608 | 92913963 | - | 14.2 | 1 | 355 | 0 | THE1A | MaLR | LTR |
| 92913964 | 92914975 | - | 33.1 | 2318 | 3317 | -1637 | MER83C-int | ERV1 | LTR |
| 92915118 | 92915209 | - | 30.4 | 2088 | 2179 | -2775 | MER83C-int | ERV1 | LTR |
| 92915438 | 92916278 | - | 31.4 | 1067 | 2128 | -3505 | MER83C-int | ERV1 | LTR |

  

|  |  |  |  |  |  |  |  |  |  |
| --- | --- | --- | --- | --- | --- | --- | --- | --- | --- |
| chr4:93163408-93187338 | | | 23539 bp | | |  |  |  |  |
| Genome Start | Genome End | Strand | Div | Repeat Start | Repeat End | Repeat Left | Name | Family | Class |
| 93163408 | 93163569 | - | 26.3 | 2200 | 2373 | -134 | HAL1 | L1 | LINE |
| 93163579 | 93163941 | - | 14.1 | 1 | 364 | 0 | THE1B | MaLR | LTR |
| 93163942 | 93165492 | - | 30.7 | 2 | 1735 | 0 | MLT-int | MaLR | LTR |
| 93165495 | 93165534 | - | 15.0 | 325 | 364 | 0 | THE1B | MaLR | LTR |
| 93165536 | 93165945 | - | 10.7 | 24 | 472 | 0 | MER4A1 | ERV1 | LTR |
| 93165946 | 93166416 | + | 3.8 | 1 | 472 | 0 | LTR7Y | ERV1 | LTR |
| 93166417 | 93168148 | + | 15.2 | 1 | 1514 | -5242 | HERVH | ERV1 | LTR |
| 93168183 | 93170866 | + | 7.1 | 1945 | 7713 | 0 | HERVH | ERV1 | LTR |
| 93170867 | 93171303 | + | 2.1 | 1 | 439 | -33 | LTR7Y | ERV1 | LTR |
| 93171304 | 93171336 | + | 6.1 | 440 | 472 | 0 | LTR7Y | ERV1 | LTR |
| 93171368 | 93174054 | - | 11.4 | 3837 | 6596 | -2 | MER4B-int | ERV1 | LTR |
| 93174108 | 93174664 | - | 14.6 | 2953 | 3533 | -3065 | MER4B-int | ERV1 | LTR |
| 93174665 | 93174956 | - | 10.6 | 3 | 294 | -19 | AluSq | Alu | SINE |
| 93174957 | 93175190 | - | 14.6 | 2724 | 2952 | -3646 | MER4B-int | ERV1 | LTR |
| 93175196 | 93175548 | - | 11.1 | 1756 | 2105 | -4493 | MER4B-int | ERV1 | LTR |
| 93175557 | 93175569 | - | 21.6 | 1357 | 1369 | -417 | MER4B-int | ERV1 | LTR |
| 93175605 | 93176543 | - | 14.5 | 170 | 1356 | -430 | MER4B-int | ERV1 | LTR |
| 93176545 | 93176903 | - | 25.2 | 1844 | 2929 | -3989 | LTR49-int | ERV1 | LTR |
| 93176927 | 93177620 | - | 9.4 | 1 | 1039 | -5745 | MER4A1-int | ERV1 | LTR |
| 93177628 | 93178068 | - | 10.4 | 1 | 468 | -4 | MER4A1 | ERV1 | LTR |
| 93178069 | 93178390 | - | 13.0 | 1 | 327 | -37 | THE1B | MaLR | LTR |
| 93178541 | 93178602 | - | 17.7 | 6212 | 6274 | -26 | L1MA4 | L1 | LINE |
| 93178604 | 93178833 | - | 7.5 | 233 | 463 | 0 | LTR2 | ERV1 | LTR |
| 93178862 | 93179070 | - | 7.5 | 2 | 232 | -231 | LTR2 | ERV1 | LTR |
| 93179071 | 93185463 | - | 5.9 | 476 | 6896 | 0 | Harlequin | ERV1 | LTR |
| 93185464 | 93185767 | - | 7.6 | 1 | 304 | -7 | AluY | Alu | SINE |
| 93185768 | 93186233 | - | 5.9 | 1 | 475 | -6421 | Harlequin | ERV1 | LTR |
| 93186234 | 93186695 | - | 7.6 | 1 | 463 | 0 | LTR2 | ERV1 | LTR |
| 93186696 | 93186879 | - | 20.6 | 6030 | 6214 | -86 | L1MA4 | L1 | LINE |
| 93186884 | 93187338 | - | 28.2 | 1603 | 2090 | -417 | HAL1 | L1 | LINE |

  

|  |  |  |  |  |  |  |  |  |  |
| --- | --- | --- | --- | --- | --- | --- | --- | --- | --- |
| chr4:93205289-93214831 | | | 9369 bp | | |  |  |  |  |
| Genome Start | Genome End | Strand | Div | Repeat Start | Repeat End | Repeat Left | Name | Family | Class |
| 93205289 | 93205424 | + | 36.8 | 1231 | 1366 | -5343 | L1MA9 | L1 | LINE |
| 93205424 | 93205660 | + | 20.3 | 1788 | 2020 | -4769 | L1MA9 | L1 | LINE |
| 93205664 | 93206048 | - | 10.4 | 1 | 381 | 0 | THE1D | MaLR | LTR |
| 93206049 | 93206740 | - | 21.2 | 2146 | 2848 | -4320 | L1M2 | L1 | LINE |
| 93206740 | 93206842 | - | 36.9 | 1643 | 1745 | -5343 | L1M2 | L1 | LINE |
| 93206933 | 93206992 | + | 10.0 | 258 | 317 | -1263 | THE1-int | MaLR | LTR |
| 93206999 | 93209197 | + | 20.9 | 3796 | 6081 | -852 | L1MA9 | L1 | LINE |
| 93209200 | 93209628 | + | 17.8 | 1 | 428 | 0 | MSTA | MaLR | LTR |
| 93209629 | 93210735 | + | 20.3 | 1 | 1154 | -497 | MSTA-int | MaLR | LTR |
| 93210751 | 93211074 | - | 12.5 | 2 | 345 | -36 | THE1D | MaLR | LTR |
| 93211076 | 93212599 | - | 14.1 | 2 | 1580 | 0 | THE1D-int | MaLR | LTR |
| 93212601 | 93212962 | - | 16.4 | 1 | 381 | 0 | THE1D | MaLR | LTR |
| 93212963 | 93213215 | + | 20.4 | 1158 | 1411 | -240 | MSTA-int | MaLR | LTR |
| 93213239 | 93213477 | + | 20.4 | 1412 | 1651 | 0 | MSTA-int | MaLR | LTR |
| 93213478 | 93213908 | + | 16.4 | 1 | 428 | 0 | MSTA | MaLR | LTR |
| 93213915 | 93214256 | + | 18.4 | 6085 | 6419 | -535 | L1MA9 | L1 | LINE |
| 93214286 | 93214831 | + | 18.4 | 6420 | 6954 | 0 | L1MA9 | L1 | LINE |

  

|  |  |  |  |  |  |  |  |  |  |
| --- | --- | --- | --- | --- | --- | --- | --- | --- | --- |
| chr4:93426028-93431762 | | | 5730 bp | | |  |  |  |  |
| Genome Start | Genome End | Strand | Div | Repeat Start | Repeat End | Repeat Left | Name | Family | Class |
| 93426028 | 93427433 | - | 15.2 | 5588 | 7000 | -2 | L1MA5 | L1 | LINE |
| 93427434 | 93427800 | - | 14.0 | 1 | 363 | -1 | THE1B | MaLR | LTR |
| 93427801 | 93429360 | - | 31.2 | 2 | 1735 | 0 | MLT-int | MaLR | LTR |
| 93429363 | 93429712 | - | 17.3 | 1 | 364 | 0 | THE1B | MaLR | LTR |
| 93429713 | 93430082 | - | 17.9 | 5192 | 5587 | -1258 | L1MA5 | L1 | LINE |
| 93430093 | 93430989 | - | 17.0 | 3602 | 4512 | -2333 | L1MA5 | L1 | LINE |
| 93430983 | 93431151 | - | 23.3 | 1576 | 1750 | -5099 | L1MA5 | L1 | LINE |
| 93431152 | 93431532 | + | 13.6 | 1 | 381 | 0 | THE1D | MaLR | LTR |
| 93431533 | 93431762 | - | 23.3 | 1320 | 1575 | -5274 | L1MA5 | L1 | LINE |

  

|  |  |  |  |  |  |  |  |  |  |
| --- | --- | --- | --- | --- | --- | --- | --- | --- | --- |
| chr4:95217498-95238978 | | | 21438 bp | | |  |  |  |  |
| Genome Start | Genome End | Strand | Div | Repeat Start | Repeat End | Repeat Left | Name | Family | Class |
| 95217498 | 95218415 | - | 18.9 | 1403 | 2339 | -4061 | L1MEc | L1 | LINE |
| 95218416 | 95218732 | - | 5.5 | 1 | 311 | 0 | AluY | Alu | SINE |
| 95218733 | 95218974 | - | 18.9 | 1149 | 1402 | -4998 | L1MEc | L1 | LINE |
| 95218985 | 95220803 | - | 15.2 | 4488 | 6300 | 0 | L1MA6 | L1 | LINE |
| 95220838 | 95222355 | - | 11.2 | 16 | 1580 | 0 | THE1B-int | MaLR | LTR |
| 95222378 | 95222740 | - | 12.7 | 2 | 364 | 0 | THE1B | MaLR | LTR |
| 95222742 | 95223005 | + | 15.2 | 3895 | 4173 | -1973 | L1P4 | L1 | LINE |
| 95223006 | 95223171 | - | 13.2 | 200 | 364 | 0 | THE1B | MaLR | LTR |
| 95223172 | 95223389 | + | 13.8 | 85 | 302 | -10 | AluSg/x | Alu | SINE |
| 95223390 | 95223580 | - | 13.2 | 3 | 199 | -165 | THE1B | MaLR | LTR |
| 95223583 | 95224775 | - | 10.4 | 383 | 1580 | 0 | THE1B-int | MaLR | LTR |
| 95224821 | 95225237 | - | 7.3 | 1 | 410 | -40 | LTR7 | ERV1 | LTR |
| 95225238 | 95225614 | - | 8.1 | 7324 | 7713 | 0 | HERVH | ERV1 | LTR |
| 95225615 | 95225831 | - | 11.2 | 5600 | 5819 | -1894 | HERVH | ERV1 | LTR |
| 95225833 | 95226493 | - | 14.7 | 4488 | 5149 | -2564 | HERVH | ERV1 | LTR |
| 95226496 | 95227000 | - | 11.6 | 3492 | 3993 | -3720 | HERVH | ERV1 | LTR |
| 95226991 | 95227140 | - | 13.3 | 3140 | 3290 | -4423 | HERVH | ERV1 | LTR |
| 95227120 | 95229958 | - | 8.8 | 1 | 2995 | -4718 | HERVH | ERV1 | LTR |
| 95229959 | 95230065 | - | 9.3 | 366 | 472 | 0 | LTR7Y | ERV1 | LTR |
| 95230066 | 95230456 | - | 8.7 | 1 | 392 | -1188 | THE1B-int | MaLR | LTR |
| 95230457 | 95230796 | - | 12.0 | 1 | 364 | 0 | THE1B | MaLR | LTR |
| 95230797 | 95232641 | + | 15.1 | 4169 | 5937 | -366 | L1MA4A | L1 | LINE |
| 95232460 | 95232835 | + | 13.3 | 5767 | 6147 | -7 | L1PB3 | L1 | LINE |
| 95232855 | 95234182 | - | 14.2 | 4336 | 5750 | -1812 | L1M1 | L1 | LINE |
| 95234234 | 95236273 | - | 9.6 | 4102 | 6159 | -4 | L1PA13 | L1 | LINE |
| 95236276 | 95236502 | - | 17.4 | 4052 | 4262 | -3297 | L1M1 | L1 | LINE |
| 95236503 | 95236609 | - | 12.2 | 187 | 295 | -17 | AluSg/x | Alu | SINE |
| 95236610 | 95236802 | - | 17.4 | 3872 | 4051 | -3508 | L1M1 | L1 | LINE |
| 95236803 | 95237115 | - | 18.1 | 2 | 312 | 0 | AluJb | Alu | SINE |
| 95237116 | 95237969 | - | 24.8 | 2999 | 3869 | -3690 | L1M1 | L1 | LINE |
| 95238037 | 95238978 | - | 22.8 | 44 | 1082 | -5318 | L1MEc | L1 | LINE |

  

|  |  |  |  |  |  |  |  |  |  |
| --- | --- | --- | --- | --- | --- | --- | --- | --- | --- |
| chr4:95521614-95530859 | | | 9142 bp | | |  |  |  |  |
| Genome Start | Genome End | Strand | Div | Repeat Start | Repeat End | Repeat Left | Name | Family | Class |
| 95521614 | 95521732 | - | 13.6 | 2297 | 2417 | -1 | Tigger1 | MER2\_type | DNA |
| 95521755 | 95521947 | + | 4.7 | 1721 | 1917 | -501 | Tigger1 | MER2\_type | DNA |
| 95521993 | 95523098 | - | 10.5 | 1170 | 2290 | -128 | Tigger1 | MER2\_type | DNA |
| 95523099 | 95523494 | - | 9.1 | 1 | 448 | -2 | LTR7 | ERV1 | LTR |
| 95523495 | 95523899 | - | 5.9 | 7324 | 7713 | 0 | HERVH | ERV1 | LTR |
| 95523900 | 95524126 | - | 16.0 | 5591 | 5819 | -1894 | HERVH | ERV1 | LTR |
| 95524117 | 95525283 | - | 10.7 | 3994 | 5149 | -2564 | HERVH | ERV1 | LTR |
| 95525338 | 95525841 | - | 10.8 | 3492 | 3992 | -3721 | HERVH | ERV1 | LTR |
| 95525832 | 95529039 | - | 10.9 | 1 | 3290 | -4423 | HERVH | ERV1 | LTR |
| 95529042 | 95529434 | - | 10.0 | 1 | 448 | -2 | LTR7 | ERV1 | LTR |
| 95529436 | 95530113 | - | 13.0 | 466 | 1166 | -1252 | Tigger1 | MER2\_type | DNA |
| 95530114 | 95530424 | + | 20.8 | 2 | 309 | -3 | AluJo | Alu | SINE |
| 95530425 | 95530859 | - | 13.0 | 5 | 465 | -1953 | Tigger1 | MER2\_type | DNA |

  

|  |  |  |  |  |  |  |  |  |  |
| --- | --- | --- | --- | --- | --- | --- | --- | --- | --- |
| chr4:97886239-97890813 | | | 4573 bp | | |  |  |  |  |
| Genome Start | Genome End | Strand | Div | Repeat Start | Repeat End | Repeat Left | Name | Family | Class |
| 97886239 | 97888471 | + | 13.9 | 3060 | 5336 | -810 | L1PB | L1 | LINE |
| 97888472 | 97888823 | - | 15.7 | 1 | 364 | 0 | THE1B | MaLR | LTR |
| 97888824 | 97890395 | - | 31.2 | 2 | 1735 | 0 | MLT-int | MaLR | LTR |
| 97890398 | 97890744 | - | 11.5 | 1 | 364 | 0 | THE1B | MaLR | LTR |
| 97890745 | 97890813 | + | 13.9 | 5337 | 5403 | -743 | L1PB | L1 | LINE |

  

|  |  |  |  |  |  |  |  |  |  |
| --- | --- | --- | --- | --- | --- | --- | --- | --- | --- |
| chr4:99112714-99115472 | | | 2757 bp | | |  |  |  |  |
| Genome Start | Genome End | Strand | Div | Repeat Start | Repeat End | Repeat Left | Name | Family | Class |
| 99112714 | 99112802 | + | 20.3 | 5520 | 5611 | -555 | L1ME1 | L1 | LINE |
| 99112803 | 99113166 | + | 12.8 | 1 | 364 | 0 | THE1B | MaLR | LTR |
| 99113169 | 99114655 | + | 30.5 | 2 | 1735 | 0 | MLT-int | MaLR | LTR |
| 99114656 | 99115014 | + | 12.7 | 1 | 364 | 0 | THE1B | MaLR | LTR |
| 99115015 | 99115472 | + | 26.2 | 5612 | 6159 | -7 | L1ME1 | L1 | LINE |

  

|  |  |  |  |  |  |  |  |  |  |
| --- | --- | --- | --- | --- | --- | --- | --- | --- | --- |
| chr4:99843527-99846234 | | | 2706 bp | | |  |  |  |  |
| Genome Start | Genome End | Strand | Div | Repeat Start | Repeat End | Repeat Left | Name | Family | Class |
| 99843527 | 99843802 | - | 11.5 | 107 | 375 | 0 | THE1C | MaLR | LTR |
| 99843803 | 99844172 | + | 15.8 | 1 | 364 | 0 | THE1B | MaLR | LTR |
| 99844175 | 99845754 | + | 29.7 | 2 | 1735 | 0 | MSTD-int | MaLR | LTR |
| 99845755 | 99846131 | + | 14.3 | 1 | 364 | 0 | THE1B | MaLR | LTR |
| 99846132 | 99846234 | - | 11.5 | 1 | 106 | -269 | THE1C | MaLR | LTR |

  

|  |  |  |  |  |  |  |  |  |  |
| --- | --- | --- | --- | --- | --- | --- | --- | --- | --- |
| chr4:99980733-99990667 | | | 9931 bp | | |  |  |  |  |
| Genome Start | Genome End | Strand | Div | Repeat Start | Repeat End | Repeat Left | Name | Family | Class |
| 99980733 | 99981148 | - | 24.0 | 5004 | 5473 | -767 | L1M5 | L1 | LINE |
| 99981149 | 99981500 | + | 6.5 | 1 | 355 | 0 | THE1A | MaLR | LTR |
| 99981503 | 99983055 | + | 31.8 | 2 | 1735 | 0 | MLT-int | MaLR | LTR |
| 99983056 | 99983414 | + | 7.4 | 1 | 355 | 0 | THE1A | MaLR | LTR |
| 99983415 | 99984572 | - | 23.6 | 3774 | 5003 | -1143 | L1M5 | L1 | LINE |
| 99984575 | 99985121 | - | 11.5 | 1 | 546 | -88 | MER41B | ERV1 | LTR |
| 99985124 | 99987981 | - | 10.1 | 3051 | 5943 | -3 | MER41B-int | ERV1 | LTR |
| 99987982 | 99988044 | + | 17.5 | 1 | 63 | -236 | Alu | Alu | SINE |
| 99988045 | 99988187 | - | 12.8 | 2781 | 2915 | -4622 | MER41B-int | ERV1 | LTR |
| 99988187 | 99988355 | - | 10.1 | 1944 | 2104 | -4159 | MER41B-int | ERV1 | LTR |
| 99988351 | 99988688 | - | 15.1 | 2006 | 2348 | -5189 | MER41B-int | ERV1 | LTR |
| 99988691 | 99989378 | - | 11.0 | 1 | 690 | -5256 | MER41B-int | ERV1 | LTR |
| 99989379 | 99989529 | - | 10.0 | 481 | 634 | 0 | MER41B | ERV1 | LTR |
| 99989532 | 99989588 | - | 23.8 | 3747 | 3795 | -2351 | L1M5 | L1 | LINE |
| 99989589 | 99989889 | + | 6.3 | 1 | 301 | -12 | AluSp | Alu | SINE |
| 99989890 | 99990667 | - | 23.8 | 2965 | 3746 | -2400 | L1M5 | L1 | LINE |

  

|  |  |  |  |  |  |  |  |  |  |
| --- | --- | --- | --- | --- | --- | --- | --- | --- | --- |
| chr4:100503970-100515607 | | | 11640 bp | | |  |  |  |  |
| Genome Start | Genome End | Strand | Div | Repeat Start | Repeat End | Repeat Left | Name | Family | Class |
| 100503970 | 100504800 | - | 11.3 | 6879 | 7723 | 0 | L1MA2 | L1 | LINE |
| 100504801 | 100505093 | + | 15.1 | 1 | 293 | -16 | AluSc | Alu | SINE |
| 100505094 | 100507234 | - | 12.4 | 4725 | 6878 | -845 | L1MA2 | L1 | LINE |
| 100507235 | 100507742 | + | 11.9 | 1 | 511 | 0 | MER9 | ERVK | LTR |
| 100507743 | 100508313 | + | 10.0 | 1 | 568 | -5348 | HERVK9 | ERVK | LTR |
| 100508312 | 100512802 | + | 9.2 | 1424 | 5916 | 0 | HERVK9 | ERVK | LTR |
| 100512803 | 100513308 | + | 12.8 | 1 | 511 | 0 | MER9 | ERVK | LTR |
| 100513309 | 100514615 | - | 10.6 | 3349 | 4730 | -2835 | L1MA2 | L1 | LINE |
| 100514616 | 100514905 | - | 13.1 | 1 | 290 | -20 | AluSg | Alu | SINE |
| 100514906 | 100515607 | - | 11.4 | 2623 | 3348 | -4217 | L1MA2 | L1 | LINE |

  

|  |  |  |  |  |  |  |  |  |  |
| --- | --- | --- | --- | --- | --- | --- | --- | --- | --- |
| chr4:101190686-101193768 | | | 2797 bp | | |  |  |  |  |
| Genome Start | Genome End | Strand | Div | Repeat Start | Repeat End | Repeat Left | Name | Family | Class |
| 101190686 | 101190867 | + | 36.3 | 1323 | 1520 | -440 | L4 | RTE | LINE |
| 101190903 | 101191052 | + | 22.7 | 1 | 156 | -4 | MER5A1 | MER1\_type | DNA |
| 101191203 | 101191552 | - | 14.6 | 1 | 355 | 0 | THE1A | MaLR | LTR |
| 101191557 | 101193115 | - | 30.6 | 2 | 1731 | -4 | MLT-int | MaLR | LTR |
| 101193118 | 101193469 | - | 11.5 | 1 | 355 | 0 | THE1A | MaLR | LTR |
| 101193470 | 101193644 | + | 27.8 | 91 | 268 | 0 | MIRb | MIR | SINE |
| 101193740 | 101193768 | + | 13.8 | 1730 | 1758 | -202 | L4 | RTE | LINE |

  

|  |  |  |  |  |  |  |  |  |  |
| --- | --- | --- | --- | --- | --- | --- | --- | --- | --- |
| chr4:102131728-102139072 | | | 6960 bp | | |  |  |  |  |
| Genome Start | Genome End | Strand | Div | Repeat Start | Repeat End | Repeat Left | Name | Family | Class |
| 102131728 | 102131872 | + | 14.5 | 5056 | 5202 | -938 | L1MA8 | L1 | LINE |
| 102131873 | 102132271 | - | 10.6 | 1 | 448 | -2 | LTR7 | ERV1 | LTR |
| 102132272 | 102132648 | - | 9.4 | 7324 | 7713 | 0 | HERVH | ERV1 | LTR |
| 102132649 | 102132857 | - | 12.0 | 5607 | 5819 | -1894 | HERVH | ERV1 | LTR |
| 102132866 | 102133054 | - | 16.4 | 4961 | 5149 | -2564 | HERVH | ERV1 | LTR |
| 102133054 | 102133691 | - | 12.0 | 3994 | 4621 | -3092 | HERVH | ERV1 | LTR |
| 102133702 | 102134287 | - | 15.5 | 3140 | 3736 | -3977 | HERVH | ERV1 | LTR |
| 102134267 | 102137256 | - | 13.1 | 1 | 2995 | -4718 | HERVH | ERV1 | LTR |
| 102137259 | 102137655 | - | 10.5 | 1 | 448 | -2 | LTR7 | ERV1 | LTR |
| 102137657 | 102138240 | + | 16.9 | 5200 | 5793 | -498 | L1MA8 | L1 | LINE |
| 102138627 | 102139072 | + | 16.2 | 5816 | 6291 | 0 | L1MA8 | L1 | LINE |

  

|  |  |  |  |  |  |  |  |  |  |
| --- | --- | --- | --- | --- | --- | --- | --- | --- | --- |
| chr4:102142400-102147777 | | | 5330 bp | | |  |  |  |  |
| Genome Start | Genome End | Strand | Div | Repeat Start | Repeat End | Repeat Left | Name | Family | Class |
| 102142400 | 102142969 | - | 23.7 | 1860 | 2466 | -3934 | L1MEc | L1 | LINE |
| 102142971 | 102143483 | + | 30.7 | 3 | 512 | -7 | LTR40a | ERVL | LTR |
| 102143492 | 102144259 | - | 24.1 | 972 | 1766 | -4549 | L1MEc | L1 | LINE |
| 102144302 | 102144397 | - | 26.4 | 80 | 172 | -3 | MER96 | MER1\_type? | DNA |
| 102144404 | 102144760 | - | 22.6 | 615 | 975 | -5425 | L1MEc | L1 | LINE |
| 102144761 | 102145143 | + | 16.3 | 1 | 428 | 0 | MSTA | MaLR | LTR |
| 102145146 | 102146777 | + | 30.3 | 2 | 1735 | 0 | MLT-int | MaLR | LTR |
| 102146778 | 102147199 | + | 16.4 | 1 | 428 | 0 | MSTA | MaLR | LTR |
| 102147200 | 102147244 | - | 22.6 | 569 | 614 | -5786 | L1MEc | L1 | LINE |
| 102147234 | 102147373 | - | 29.4 | 122 | 267 | -6133 | L1MEc | L1 | LINE |
| 102147374 | 102147684 | + | 11.4 | 1 | 311 | 0 | AluSg | Alu | SINE |
| 102147685 | 102147777 | - | 29.4 | 39 | 121 | -6279 | L1MEc | L1 | LINE |

  

|  |  |  |  |  |  |  |  |  |  |
| --- | --- | --- | --- | --- | --- | --- | --- | --- | --- |
| chr4:103302622-103307429 | | | 4702 bp | | |  |  |  |  |
| Genome Start | Genome End | Strand | Div | Repeat Start | Repeat End | Repeat Left | Name | Family | Class |
| 103302622 | 103302837 | - | 21.6 | 227 | 496 | -15 | MLT1J | MaLR | LTR |
| 103302841 | 103303166 | + | 7.4 | 1 | 325 | 0 | AluY | Alu | SINE |
| 103303167 | 103303179 | + | 7.4 | 283 | 295 | -16 | AluY | Alu | SINE |
| 103303210 | 103303321 | + | 20.6 | 1 | 92 | -304 | MSTD | MaLR | LTR |
| 103303322 | 103303949 | - | 12.1 | 1 | 667 | 0 | PABL\_B | ERV1 | LTR |
| 103303950 | 103304047 | + | 20.6 | 93 | 192 | -204 | MSTD | MaLR | LTR |
| 103304067 | 103304152 | - | 5.3 | 173 | 253 | -414 | PABL\_B | ERV1 | LTR |
| 103304137 | 103304190 | - | 10.0 | 1 | 51 | -616 | PABL\_B | ERV1 | LTR |
| 103304193 | 103304282 | + | 25.9 | 95 | 183 | -213 | MSTD | MaLR | LTR |
| 103304308 | 103304429 | - | 10.8 | 136 | 253 | -414 | PABL\_B | ERV1 | LTR |
| 103304461 | 103304616 | + | 18.6 | 228 | 396 | 0 | MSTD | MaLR | LTR |
| 103304621 | 103304771 | + | 24.6 | 4 | 148 | -1587 | MSTD-int | MaLR | LTR |
| 103304772 | 103305078 | - | 10.9 | 1 | 307 | 0 | AluY | Alu | SINE |
| 103305084 | 103305325 | - | 9.1 | 1 | 242 | -69 | AluY | Alu | SINE |
| 103305326 | 103306896 | + | 24.6 | 149 | 1735 | 0 | MSTD-int | MaLR | LTR |
| 103306897 | 103307275 | + | 20.1 | 1 | 396 | 0 | MSTD | MaLR | LTR |
| 103307279 | 103307429 | - | 31.1 | 38 | 205 | -306 | MLT1J | MaLR | LTR |

  

|  |  |  |  |  |  |  |  |  |  |
| --- | --- | --- | --- | --- | --- | --- | --- | --- | --- |
| chr4:105081802-105088037 | | | 6237 bp | | |  |  |  |  |
| Genome Start | Genome End | Strand | Div | Repeat Start | Repeat End | Repeat Left | Name | Family | Class |
| 105081802 | 105081873 | + | 13.9 | 1 | 81 | -391 | MER4A1 | ERV1 | LTR |
| 105081874 | 105082322 | + | 9.3 | 1 | 442 | -8 | LTR7 | ERV1 | LTR |
| 105082332 | 105085160 | + | 11.7 | 1 | 2995 | -4718 | HERVH | ERV1 | LTR |
| 105085140 | 105085991 | + | 10.9 | 3140 | 3993 | -3720 | HERVH | ERV1 | LTR |
| 105085994 | 105086604 | + | 12.9 | 4488 | 5149 | -2564 | HERVH | ERV1 | LTR |
| 105086606 | 105086821 | + | 11.6 | 5600 | 5819 | -1894 | HERVH | ERV1 | LTR |
| 105086822 | 105087198 | + | 9.5 | 7324 | 7708 | -5 | HERVH | ERV1 | LTR |
| 105087204 | 105087635 | + | 6.6 | 1 | 442 | -8 | LTR7 | ERV1 | LTR |
| 105087639 | 105088037 | + | 10.3 | 72 | 472 | 0 | MER4A1 | ERV1 | LTR |

  

|  |  |  |  |  |  |  |  |  |  |
| --- | --- | --- | --- | --- | --- | --- | --- | --- | --- |
| chr4:105244390-105250109 | | | 5695 bp | | |  |  |  |  |
| Genome Start | Genome End | Strand | Div | Repeat Start | Repeat End | Repeat Left | Name | Family | Class |
| 105244390 | 105245288 | + | 18.0 | 2761 | 3680 | -2466 | L1PB3 | L1 | LINE |
| 105245312 | 105246196 | + | 18.0 | 3681 | 4565 | -1581 | L1PB3 | L1 | LINE |
| 105246197 | 105246573 | + | 16.3 | 2 | 428 | 0 | MSTA | MaLR | LTR |
| 105246576 | 105248157 | + | 30.5 | 2 | 1735 | 0 | MLT-int | MaLR | LTR |
| 105248158 | 105248533 | + | 18.5 | 1 | 428 | 0 | MSTA | MaLR | LTR |
| 105248534 | 105250109 | + | 15.9 | 4561 | 6154 | 0 | L1PB3 | L1 | LINE |

  

|  |  |  |  |  |  |  |  |  |  |
| --- | --- | --- | --- | --- | --- | --- | --- | --- | --- |
| chr4:105760416-105764976 | | | 4396 bp | | |  |  |  |  |
| Genome Start | Genome End | Strand | Div | Repeat Start | Repeat End | Repeat Left | Name | Family | Class |
| 105760416 | 105760663 | + | 35.6 | 1251 | 1498 | -3498 | HERV16 | ERVL | LTR |
| 105760721 | 105760940 | + | 35.6 | 1598 | 1825 | -3171 | HERV16 | ERVL | LTR |
| 105760942 | 105761277 | - | 11.2 | 1 | 364 | 0 | THE1B | MaLR | LTR |
| 105761278 | 105762849 | - | 29.0 | 2 | 1735 | 0 | MLT-int | MaLR | LTR |
| 105762852 | 105763187 | - | 12.2 | 1 | 364 | 0 | THE1B | MaLR | LTR |
| 105763293 | 105764205 | + | 35.8 | 3571 | 4502 | -1165 | HERV16 | ERVL | LTR |
| 105764206 | 105764428 | - | 11.7 | 1 | 230 | 0 | MER30 | MER1\_type | DNA |
| 105764429 | 105764976 | + | 35.8 | 4503 | 5068 | -599 | HERV16 | ERVL | LTR |

  

|  |  |  |  |  |  |  |  |  |  |
| --- | --- | --- | --- | --- | --- | --- | --- | --- | --- |
| chr4:105806723-105810650 | | | 3694 bp | | |  |  |  |  |
| Genome Start | Genome End | Strand | Div | Repeat Start | Repeat End | Repeat Left | Name | Family | Class |
| 105806723 | 105807115 | + | 33.2 | 193 | 611 | -2808 | L2 | L2 | LINE |
| 105807205 | 105807323 | - | 15.3 | 114 | 235 | -1 | MER46A | MER2\_type | DNA |
| 105807367 | 105807552 | - | 31.2 | 2 | 192 | -70 | MIR | MIR | SINE |
| 105807600 | 105807931 | - | 9.8 | 1 | 364 | 0 | THE1B | MaLR | LTR |
| 105807932 | 105809503 | - | 29.6 | 2 | 1735 | 0 | MLT1K-int | MaLR | LTR |
| 105809508 | 105809560 | - | 9.9 | 310 | 362 | -2 | THE1B | MaLR | LTR |
| 105809612 | 105809886 | - | 9.9 | 2 | 309 | -55 | THE1B | MaLR | LTR |
| 105809887 | 105810650 | + | 33.5 | 737 | 1647 | -1772 | L2 | L2 | LINE |

  

|  |  |  |  |  |  |  |  |  |  |
| --- | --- | --- | --- | --- | --- | --- | --- | --- | --- |
| chr4:105980899-105983492 | | | 2592 bp | | |  |  |  |  |
| Genome Start | Genome End | Strand | Div | Repeat Start | Repeat End | Repeat Left | Name | Family | Class |
| 105980899 | 105980943 | + | 30.4 | 3106 | 3153 | -266 | L2 | L2 | LINE |
| 105980944 | 105981299 | - | 10.2 | 1 | 355 | 0 | THE1A | MaLR | LTR |
| 105981300 | 105982872 | - | 30.3 | 2 | 1735 | 0 | MLT-int | MaLR | LTR |
| 105982875 | 105983232 | - | 11.8 | 1 | 355 | 0 | THE1A | MaLR | LTR |
| 105983233 | 105983492 | + | 30.4 | 3154 | 3419 | 0 | L2 | L2 | LINE |

  

|  |  |  |  |  |  |  |  |  |  |
| --- | --- | --- | --- | --- | --- | --- | --- | --- | --- |
| chr4:106505026-106507976 | | | 2921 bp | | |  |  |  |  |
| Genome Start | Genome End | Strand | Div | Repeat Start | Repeat End | Repeat Left | Name | Family | Class |
| 106505026 | 106505309 | - | 16.2 | 150 | 426 | 0 | MSTB | MaLR | LTR |
| 106505310 | 106505654 | + | 15.1 | 1 | 342 | -33 | THE1C | MaLR | LTR |
| 106505655 | 106505962 | - | 7.2 | 1 | 310 | -1 | AluY | Alu | SINE |
| 106505963 | 106506000 | + | 15.1 | 343 | 375 | 0 | THE1C | MaLR | LTR |
| 106506008 | 106507529 | + | 11.3 | 18 | 1580 | 0 | THE1C-int | MaLR | LTR |
| 106507530 | 106507899 | + | 18.4 | 1 | 375 | 0 | THE1C | MaLR | LTR |
| 106507923 | 106507976 | - | 9.4 | 1 | 53 | -373 | MSTB | MaLR | LTR |

  

|  |  |  |  |  |  |  |  |  |  |
| --- | --- | --- | --- | --- | --- | --- | --- | --- | --- |
| chr4:106603065-106606630 | | | 3059 bp | | |  |  |  |  |
| Genome Start | Genome End | Strand | Div | Repeat Start | Repeat End | Repeat Left | Name | Family | Class |
| 106603065 | 106603205 | - | 23.6 | 2245 | 2398 | -109 | HAL1 | L1 | LINE |
| 106603271 | 106603603 | + | 14.7 | 1 | 364 | 0 | THE1B | MaLR | LTR |
| 106603606 | 106605158 | + | 29.5 | 2 | 1735 | 0 | MLT-int | MaLR | LTR |
| 106605159 | 106605489 | + | 11.8 | 1 | 364 | 0 | THE1B | MaLR | LTR |
| 106605671 | 106605814 | + | 33.3 | 46 | 189 | 0 | MER5A | MER1\_type | DNA |
| 106605997 | 106606335 | - | 24.5 | 1514 | 1900 | -607 | HAL1 | L1 | LINE |
| 106606413 | 106606630 | - | 25.2 | 1103 | 1334 | -1173 | HAL1 | L1 | LINE |

  

|  |  |  |  |  |  |  |  |  |  |
| --- | --- | --- | --- | --- | --- | --- | --- | --- | --- |
| chr4:107165214-107171303 | | | 6016 bp | | |  |  |  |  |
| Genome Start | Genome End | Strand | Div | Repeat Start | Repeat End | Repeat Left | Name | Family | Class |
| 107165214 | 107165563 | - | 9.9 | 5793 | 6144 | -7 | L1PB1 | L1 | LINE |
| 107165608 | 107166976 | - | 10.3 | 4398 | 5792 | -359 | L1PB1 | L1 | LINE |
| 107167007 | 107168074 | - | 10.4 | 3348 | 4397 | -1749 | L1PB1 | L1 | LINE |
| 107168075 | 107168409 | + | 15.6 | 1 | 334 | -30 | THE1B | MaLR | LTR |
| 107168410 | 107168706 | - | 13.5 | 1 | 297 | -15 | AluSx | Alu | SINE |
| 107168707 | 107168748 | + | 15.6 | 335 | 364 | 0 | THE1B | MaLR | LTR |
| 107168749 | 107170323 | + | 10.6 | 1 | 1580 | 0 | THE1B-int | MaLR | LTR |
| 107170324 | 107170676 | + | 15.0 | 1 | 364 | 0 | THE1B | MaLR | LTR |
| 107170677 | 107171303 | - | 11.0 | 2835 | 3351 | -2795 | L1PB1 | L1 | LINE |

  

|  |  |  |  |  |  |  |  |  |  |
| --- | --- | --- | --- | --- | --- | --- | --- | --- | --- |
| chr4:107912649-107915928 | | | 3113 bp | | |  |  |  |  |
| Genome Start | Genome End | Strand | Div | Repeat Start | Repeat End | Repeat Left | Name | Family | Class |
| 107912649 | 107913075 | - | 25.0 | 5741 | 6170 | 0 | L1ME3A | L1 | LINE |
| 107913076 | 107913449 | + | 14.7 | 1 | 375 | 0 | THE1C | MaLR | LTR |
| 107913452 | 107915039 | + | 30.3 | 2 | 1735 | 0 | MLT-int | MaLR | LTR |
| 107915042 | 107915414 | + | 10.9 | 3 | 375 | 0 | THE1C | MaLR | LTR |
| 107915415 | 107915493 | - | 25.0 | 5664 | 5740 | -430 | L1ME3A | L1 | LINE |
| 107915657 | 107915928 | - | 28.1 | 5055 | 5689 | -485 | L1ME3A | L1 | LINE |

  

|  |  |  |  |  |  |  |  |  |  |
| --- | --- | --- | --- | --- | --- | --- | --- | --- | --- |
| chr4:108895667-108898270 | | | 2602 bp | | |  |  |  |  |
| Genome Start | Genome End | Strand | Div | Repeat Start | Repeat End | Repeat Left | Name | Family | Class |
| 108895667 | 108895842 | + | 24.0 | 16 | 227 | -35 | MIR | MIR | SINE |
| 108895843 | 108896237 | + | 11.3 | 1 | 428 | 0 | MSTA | MaLR | LTR |
| 108896240 | 108897846 | + | 29.3 | 2 | 1735 | 0 | MLT1C-int | MaLR | LTR |
| 108897847 | 108898230 | + | 14.8 | 1 | 428 | 0 | MSTA | MaLR | LTR |
| 108898231 | 108898270 | + | 24.0 | 228 | 262 | 0 | MIR | MIR | SINE |

  

|  |  |  |  |  |  |  |  |  |  |
| --- | --- | --- | --- | --- | --- | --- | --- | --- | --- |
| chr4:111469049-111472631 | | | 3576 bp | | |  |  |  |  |
| Genome Start | Genome End | Strand | Div | Repeat Start | Repeat End | Repeat Left | Name | Family | Class |
| 111469049 | 111469203 | + | 29.6 | 3058 | 3232 | -187 | L2 | L2 | LINE |
| 111469207 | 111469240 | - | 14.7 | 574 | 602 | 0 | LTR43 | ERV1 | LTR |
| 111469241 | 111469623 | + | 13.7 | 1 | 428 | 0 | MSTA | MaLR | LTR |
| 111469626 | 111471232 | + | 29.6 | 2 | 1735 | 0 | MLT-int | MaLR | LTR |
| 111471233 | 111471594 | + | 13.6 | 1 | 401 | -27 | MSTA | MaLR | LTR |
| 111471595 | 111471904 | - | 9.7 | 1 | 312 | -1 | AluSp | Alu | SINE |
| 111471905 | 111471945 | + | 13.6 | 402 | 428 | 0 | MSTA | MaLR | LTR |
| 111471946 | 111472452 | - | 17.0 | 1 | 573 | -29 | LTR43 | ERV1 | LTR |
| 111472455 | 111472631 | + | 27.4 | 3234 | 3419 | 0 | L2 | L2 | LINE |

  

|  |  |  |  |  |  |  |  |  |  |
| --- | --- | --- | --- | --- | --- | --- | --- | --- | --- |
| chr4:111735356-111739069 | | | 3615 bp | | |  |  |  |  |
| Genome Start | Genome End | Strand | Div | Repeat Start | Repeat End | Repeat Left | Name | Family | Class |
| 111735356 | 111735896 | - | 34.8 | 3841 | 4427 | -62 | L3 | CR1 | LINE |
| 111735924 | 111736097 | + | 18.4 | 1 | 174 | -11 | FAM | Alu | SINE |
| 111736106 | 111736600 | - | 20.8 | 8 | 504 | -38 | MLT1F | MaLR | LTR |
| 111736607 | 111736693 | - | 28.7 | 1402 | 1489 | 0 | MLT1F-int | MaLR | LTR |
| 111736694 | 111736940 | + | 4.7 | 5938 | 6184 | 0 | L1PA3 | L1 | LINE |
| 111736962 | 111738287 | - | 23.4 | 13 | 1415 | -74 | MLT1F-int | MaLR | LTR |
| 111738290 | 111738821 | - | 22.8 | 1 | 542 | 0 | MLT1F | MaLR | LTR |
| 111738857 | 111739069 | - | 26.2 | 3553 | 3784 | -705 | L3 | CR1 | LINE |

  

|  |  |  |  |  |  |  |  |  |  |
| --- | --- | --- | --- | --- | --- | --- | --- | --- | --- |
| chr4:112723575-112726669 | | | 3072 bp | | |  |  |  |  |
| Genome Start | Genome End | Strand | Div | Repeat Start | Repeat End | Repeat Left | Name | Family | Class |
| 112723575 | 112723976 | - | 30.6 | 1837 | 2304 | -203 | HAL1 | L1 | LINE |
| 112723998 | 112724026 | - | 36.0 | 315 | 338 | -2169 | HAL1 | L1 | LINE |
| 112724027 | 112724398 | - | 12.9 | 1 | 381 | 0 | THE1D | MaLR | LTR |
| 112724399 | 112726002 | - | 31.5 | 2 | 1735 | 0 | MLT-int | MaLR | LTR |
| 112726005 | 112726377 | - | 16.0 | 1 | 381 | 0 | THE1D | MaLR | LTR |
| 112726378 | 112726669 | - | 36.0 | 22 | 314 | -2193 | HAL1 | L1 | LINE |

  

|  |  |  |  |  |  |  |  |  |  |
| --- | --- | --- | --- | --- | --- | --- | --- | --- | --- |
| chr4:116096058-116104381 | | | 8316 bp | | |  |  |  |  |
| Genome Start | Genome End | Strand | Div | Repeat Start | Repeat End | Repeat Left | Name | Family | Class |
| 116096058 | 116096211 | - | 33.1 | 2695 | 2851 | -568 | L2 | L2 | LINE |
| 116096221 | 116097151 | + | 28.9 | 13 | 948 | -6386 | L1MB8 | L1 | LINE |
| 116097167 | 116097773 | + | 23.4 | 1031 | 1682 | -5652 | L1MB8 | L1 | LINE |
| 116097777 | 116098135 | - | 14.8 | 1 | 364 | 0 | THE1B | MaLR | LTR |
| 116098138 | 116098860 | - | 10.7 | 843 | 1573 | -7 | THE1B-int | MaLR | LTR |
| 116098860 | 116099012 | - | 9.2 | 206 | 361 | -1219 | THE1B-int | MaLR | LTR |
| 116099003 | 116099082 | - | 13.8 | 215 | 295 | -1285 | THE1B-int | MaLR | LTR |
| 116099096 | 116099939 | - | 14.7 | 1 | 822 | -758 | THE1B-int | MaLR | LTR |
| 116099940 | 116100264 | - | 16.1 | 1 | 364 | 0 | THE1B | MaLR | LTR |
| 116100265 | 116100930 | + | 30.3 | 1721 | 2420 | -4588 | L1MB8 | L1 | LINE |
| 116100931 | 116101153 | + | 9.0 | 1 | 225 | -88 | AluSq | Alu | SINE |
| 116101154 | 116101721 | + | 21.8 | 2473 | 3118 | -4216 | L1MB8 | L1 | LINE |
| 116101700 | 116103478 | + | 20.9 | 3734 | 5564 | -1442 | L1MB8 | L1 | LINE |
| 116103477 | 116104213 | + | 18.3 | 6304 | 7036 | -2 | L1MB8 | L1 | LINE |
| 116104215 | 116104381 | - | 24.4 | 2514 | 2707 | -712 | L2 | L2 | LINE |

  

|  |  |  |  |  |  |  |  |  |  |
| --- | --- | --- | --- | --- | --- | --- | --- | --- | --- |
| chr4:116606973-116610093 | | | 3100 bp | | |  |  |  |  |
| Genome Start | Genome End | Strand | Div | Repeat Start | Repeat End | Repeat Left | Name | Family | Class |
| 116606973 | 116607047 | + | 13.3 | 1 | 81 | -284 | MLT1A0 | MaLR | LTR |
| 116607054 | 116607348 | + | 19.3 | 1 | 300 | -12 | AluJb | Alu | SINE |
| 116607361 | 116607791 | + | 17.6 | 1 | 426 | -2 | MSTC | MaLR | LTR |
| 116607795 | 116609429 | + | 27.9 | 1 | 1735 | 0 | MSTC-int | MaLR | LTR |
| 116609430 | 116609857 | + | 18.8 | 1 | 428 | 0 | MSTC | MaLR | LTR |
| 116609858 | 116610093 | + | 31.3 | 131 | 363 | -2 | MLT1A0 | MaLR | LTR |

  

|  |  |  |  |  |  |  |  |  |  |
| --- | --- | --- | --- | --- | --- | --- | --- | --- | --- |
| chr4:117049146-117052572 | | | 3305 bp | | |  |  |  |  |
| Genome Start | Genome End | Strand | Div | Repeat Start | Repeat End | Repeat Left | Name | Family | Class |
| 117049146 | 117049352 | + | 13.8 | 5577 | 5792 | -364 | L1PB4 | L1 | LINE |
| 117049472 | 117049501 | + | 13.8 | 5793 | 5832 | -324 | L1PB4 | L1 | LINE |
| 117049502 | 117049902 | - | 17.4 | 2 | 428 | 0 | MSTA | MaLR | LTR |
| 117049903 | 117049933 | + | 13.8 | 5833 | 5857 | -299 | L1PB4 | L1 | LINE |
| 117049934 | 117050298 | - | 14.8 | 1 | 381 | 0 | THE1D | MaLR | LTR |
| 117050299 | 117051898 | - | 14.8 | 3 | 1580 | 0 | THE1D-int | MaLR | LTR |
| 117051902 | 117052269 | - | 14.4 | 1 | 380 | -1 | THE1D | MaLR | LTR |
| 117052270 | 117052572 | + | 23.9 | 5854 | 6155 | -1 | L1PB4 | L1 | LINE |

  

|  |  |  |  |  |  |  |  |  |  |
| --- | --- | --- | --- | --- | --- | --- | --- | --- | --- |
| chr4:117654931-117660690 | | | 5766 bp | | |  |  |  |  |
| Genome Start | Genome End | Strand | Div | Repeat Start | Repeat End | Repeat Left | Name | Family | Class |
| 117654931 | 117655058 | - | 11.7 | 6840 | 6967 | -238 | MER50-int | ERV1 | LTR |
| 117655062 | 117655323 | - | 15.4 | 4184 | 4445 | -1501 | MER50-int | ERV1 | LTR |
| 117655326 | 117657049 | - | 21.4 | 2788 | 4538 | -2667 | MER50-int | ERV1 | LTR |
| 117657046 | 117657924 | - | 17.2 | 821 | 1850 | -5355 | MER50-int | ERV1 | LTR |
| 117657925 | 117658286 | - | 15.1 | 1 | 364 | 0 | THE1B | MaLR | LTR |
| 117658287 | 117658509 | - | 8.6 | 1283 | 1580 | 0 | THE1B-int | MaLR | LTR |
| 117658503 | 117659520 | - | 11.7 | 1 | 1115 | -465 | THE1B-int | MaLR | LTR |
| 117659521 | 117659880 | - | 16.0 | 1 | 364 | 0 | THE1B | MaLR | LTR |
| 117659881 | 117660690 | - | 16.6 | 33 | 825 | -6380 | MER50-int | ERV1 | LTR |

  

|  |  |  |  |  |  |  |  |  |  |
| --- | --- | --- | --- | --- | --- | --- | --- | --- | --- |
| chr4:117712233-117717221 | | | 4889 bp | | |  |  |  |  |
| Genome Start | Genome End | Strand | Div | Repeat Start | Repeat End | Repeat Left | Name | Family | Class |
| 117712233 | 117713175 | - | 30.0 | 2606 | 3587 | -2080 | ERVL-E | ERVL | LTR |
| 117713176 | 117713524 | - | 13.2 | 5 | 364 | 0 | THE1B | MaLR | LTR |
| 117713529 | 117715094 | - | 32.1 | 2 | 1735 | 0 | MLT-int | MaLR | LTR |
| 117715097 | 117715446 | - | 14.5 | 1 | 364 | 0 | THE1B | MaLR | LTR |
| 117715447 | 117715839 | - | 30.0 | 2178 | 2605 | -3062 | ERVL-E | ERVL | LTR |
| 117715828 | 117716548 | - | 30.6 | 1263 | 2006 | -3661 | ERVL-E | ERVL | LTR |
| 117716588 | 117716817 | + | 18.7 | 1 | 214 | -160 | MLT1A | MaLR | LTR |
| 117716847 | 117716931 | + | 18.7 | 215 | 287 | -87 | MLT1A | MaLR | LTR |
| 117716970 | 117717221 | - | 25.6 | 745 | 1018 | -4649 | ERVL-E | ERVL | LTR |

  

|  |  |  |  |  |  |  |  |  |  |
| --- | --- | --- | --- | --- | --- | --- | --- | --- | --- |
| chr4:118057543-118064128 | | | 6582 bp | | |  |  |  |  |
| Genome Start | Genome End | Strand | Div | Repeat Start | Repeat End | Repeat Left | Name | Family | Class |
| 118057543 | 118059486 | - | 14.1 | 4274 | 6426 | -1139 | L1M1 | L1 | LINE |
| 118059487 | 118059866 | + | 15.2 | 1 | 428 | 0 | MSTA | MaLR | LTR |
| 118059867 | 118060784 | + | 20.4 | 1 | 932 | -719 | MSTA-int | MaLR | LTR |
| 118060786 | 118060844 | - | 15.2 | 370 | 428 | 0 | MSTA | MaLR | LTR |
| 118060845 | 118061231 | - | 16.2 | 3 | 416 | -12 | MSTA | MaLR | LTR |
| 118061234 | 118061950 | + | 15.3 | 861 | 1580 | 0 | MSTA-int | MaLR | LTR |
| 118061951 | 118062330 | + | 14.9 | 1 | 428 | 0 | MSTA | MaLR | LTR |
| 118062332 | 118064128 | - | 15.6 | 2475 | 4277 | -3288 | L1M1 | L1 | LINE |

  

|  |  |  |  |  |  |  |  |  |  |
| --- | --- | --- | --- | --- | --- | --- | --- | --- | --- |
| chr4:118290019-118293557 | | | 3537 bp | | |  |  |  |  |
| Genome Start | Genome End | Strand | Div | Repeat Start | Repeat End | Repeat Left | Name | Family | Class |
| 118290019 | 118290774 | - | 14.9 | 4566 | 5321 | -825 | L1P4 | L1 | LINE |
| 118290775 | 118291150 | - | 13.1 | 1 | 364 | 0 | THE1B | MaLR | LTR |
| 118291151 | 118292804 | - | 32.1 | 2 | 1735 | 0 | MLT1B-int | MaLR | LTR |
| 118292807 | 118293165 | - | 12.8 | 1 | 364 | 0 | THE1B | MaLR | LTR |
| 118293166 | 118293557 | - | 14.9 | 4169 | 4565 | -1581 | L1P4 | L1 | LINE |

  

|  |  |  |  |  |  |  |  |  |  |
| --- | --- | --- | --- | --- | --- | --- | --- | --- | --- |
| chr4:118391195-118398458 | | | 7277 bp | | |  |  |  |  |
| Genome Start | Genome End | Strand | Div | Repeat Start | Repeat End | Repeat Left | Name | Family | Class |
| 118391195 | 118392710 | + | 12.7 | 1 | 1521 | -59 | THE1B-int | MaLR | LTR |
| 118392711 | 118393112 | + | 9.0 | 1 | 448 | -2 | LTR7 | ERV1 | LTR |
| 118393115 | 118396099 | + | 10.6 | 1 | 2987 | -4726 | HERVH | ERV1 | LTR |
| 118396094 | 118396238 | + | 16.6 | 3140 | 3290 | -4423 | HERVH | ERV1 | LTR |
| 118396229 | 118396731 | + | 11.9 | 3492 | 3993 | -3720 | HERVH | ERV1 | LTR |
| 118396734 | 118397396 | + | 13.6 | 4488 | 5150 | -2563 | HERVH | ERV1 | LTR |
| 118397395 | 118397613 | + | 12.9 | 5598 | 5819 | -1894 | HERVH | ERV1 | LTR |
| 118397612 | 118397990 | + | 11.5 | 7322 | 7713 | 0 | HERVH | ERV1 | LTR |
| 118397991 | 118398392 | + | 9.5 | 1 | 448 | -2 | LTR7 | ERV1 | LTR |
| 118398396 | 118398458 | + | 7.9 | 1518 | 1580 | 0 | THE1B-int | MaLR | LTR |

  

|  |  |  |  |  |  |  |  |  |  |
| --- | --- | --- | --- | --- | --- | --- | --- | --- | --- |
| chr4:118892476-118895511 | | | 3003 bp | | |  |  |  |  |
| Genome Start | Genome End | Strand | Div | Repeat Start | Repeat End | Repeat Left | Name | Family | Class |
| 118892476 | 118892618 | + | 24.1 | 3 | 175 | -228 | MER57A | ERV1 | LTR |
| 118892620 | 118892981 | + | 17.6 | 1 | 364 | 0 | THE1B | MaLR | LTR |
| 118892982 | 118894545 | + | 11.4 | 1 | 1574 | -6 | THE1B-int | MaLR | LTR |
| 118894551 | 118894911 | + | 13.7 | 1 | 364 | 0 | THE1B | MaLR | LTR |
| 118894939 | 118895135 | + | 22.2 | 165 | 359 | -44 | MER57A | ERV1 | LTR |
| 118895136 | 118895456 | + | 16.2 | 4 | 325 | 0 | AluSx | Alu | SINE |
| 118895457 | 118895511 | + | 22.2 | 360 | 403 | 0 | MER57A | ERV1 | LTR |

  

|  |  |  |  |  |  |  |  |  |  |
| --- | --- | --- | --- | --- | --- | --- | --- | --- | --- |
| chr4:118897119-118905551 | | | 8132 bp | | |  |  |  |  |
| Genome Start | Genome End | Strand | Div | Repeat Start | Repeat End | Repeat Left | Name | Family | Class |
| 118897119 | 118897256 | + | 25.0 | 305 | 429 | -4507 | MER57A-int | ERV1 | LTR |
| 118897320 | 118897723 | + | 15.3 | 1 | 360 | -4 | THE1B | MaLR | LTR |
| 118897730 | 118899303 | + | 29.1 | 2 | 1735 | 0 | MLT-int | MaLR | LTR |
| 118899304 | 118899671 | + | 12.6 | 1 | 364 | 0 | THE1B | MaLR | LTR |
| 118899676 | 118899976 | - | 26.9 | 1845 | 2160 | -4171 | MER4B-int | ERV1 | LTR |
| 118900019 | 118900134 | - | 20.4 | 751 | 1039 | -5753 | MER4B-int | ERV1 | LTR |
| 118900205 | 118900714 | - | 15.4 | 2 | 474 | -6124 | MER4B-int | ERV1 | LTR |
| 118900719 | 118901298 | - | 14.7 | 1 | 610 | 0 | MER4B | ERV1 | LTR |
| 118901300 | 118901799 | + | 26.4 | 1488 | 1997 | -2939 | MER57A-int | ERV1 | LTR |
| 118901797 | 118902766 | + | 23.8 | 2160 | 3182 | -1916 | MER57A-int | ERV1 | LTR |
| 118902809 | 118903958 | + | 22.7 | 3507 | 4610 | -1577 | MER57A-int | ERV1 | LTR |
| 118904003 | 118904061 | - | 13.6 | 6098 | 6157 | -9 | L1PA16 | L1 | LINE |
| 118904063 | 118905384 | - | 14.5 | 4707 | 6061 | -105 | L1PA16 | L1 | LINE |
| 118905412 | 118905551 | + | 21.8 | 4679 | 6103 | -1430 | MER57A-int | ERV1 | LTR |

  

|  |  |  |  |  |  |  |  |  |  |
| --- | --- | --- | --- | --- | --- | --- | --- | --- | --- |
| chr4:121711454-121718198 | | | 6744 bp | | |  |  |  |  |
| Genome Start | Genome End | Strand | Div | Repeat Start | Repeat End | Repeat Left | Name | Family | Class |
| 121711454 | 121711777 | + | 17.7 | 2790 | 3099 | -3750 | L1PA17 | L1 | LINE |
| 121711779 | 121712028 | - | 16.1 | 139 | 364 | 0 | THE1B | MaLR | LTR |
| 121712029 | 121712325 | - | 13.8 | 1 | 291 | -19 | AluSg | Alu | SINE |
| 121712326 | 121712472 | - | 16.1 | 1 | 138 | -226 | THE1B | MaLR | LTR |
| 121712473 | 121714041 | - | 9.9 | 1 | 1580 | 0 | THE1B-int | MaLR | LTR |
| 121714042 | 121714406 | - | 15.7 | 1 | 364 | 0 | THE1B | MaLR | LTR |
| 121714407 | 121718198 | + | 16.5 | 3096 | 6853 | -16 | L1PA17 | L1 | LINE |

  

|  |  |  |  |  |  |  |  |  |  |
| --- | --- | --- | --- | --- | --- | --- | --- | --- | --- |
| chr4:122571950-122580691 | | | 8662 bp | | |  |  |  |  |
| Genome Start | Genome End | Strand | Div | Repeat Start | Repeat End | Repeat Left | Name | Family | Class |
| 122571950 | 122573890 | - | 14.7 | 5017 | 6998 | -7 | L1MA4A | L1 | LINE |
| 122573897 | 122574001 | - | 12.1 | 6201 | 6305 | 0 | L1MA3 | L1 | LINE |
| 122574002 | 122574348 | - | 15.7 | 2 | 364 | 0 | THE1B | MaLR | LTR |
| 122574350 | 122575924 | - | 32.1 | 2 | 1735 | 0 | MLT-int | MaLR | LTR |
| 122575927 | 122576273 | - | 16.0 | 1 | 363 | -1 | THE1B | MaLR | LTR |
| 122576274 | 122577219 | - | 12.2 | 5229 | 6200 | -105 | L1MA3 | L1 | LINE |
| 122577220 | 122577810 | + | 9.9 | 4045 | 4638 | -579 | MER61-int | ERV1 | LTR |
| 122577811 | 122577850 | - | 20.5 | 2477 | 2515 | -3625 | L1M3 | L1 | LINE |
| 122577922 | 122578228 | - | 20.5 | 2190 | 2476 | -3664 | L1M3 | L1 | LINE |
| 122578229 | 122578544 | - | 15.7 | 1 | 295 | -17 | AluSx | Alu | SINE |
| 122578545 | 122580691 | - | 17.6 | 554 | 2891 | -3958 | L1MA4A | L1 | LINE |

  

|  |  |  |  |  |  |  |  |  |  |
| --- | --- | --- | --- | --- | --- | --- | --- | --- | --- |
| chr4:125579916-125589257 | | | 9269 bp | | |  |  |  |  |
| Genome Start | Genome End | Strand | Div | Repeat Start | Repeat End | Repeat Left | Name | Family | Class |
| 125579916 | 125581754 | - | 14.8 | 5473 | 7360 | -5 | L1MA4A | L1 | LINE |
| 125581823 | 125586339 | - | 20.0 | 563 | 5472 | -1733 | L1MA4A | L1 | LINE |
| 125586340 | 125586702 | - | 9.9 | 1 | 364 | 0 | THE1B | MaLR | LTR |
| 125586707 | 125588310 | - | 11.1 | 2 | 1576 | -4 | THE1B-int | MaLR | LTR |
| 125588312 | 125588685 | - | 13.7 | 1 | 364 | 0 | THE1B | MaLR | LTR |
| 125588686 | 125589257 | - | 21.1 | 1 | 567 | -6642 | L1MA4A | L1 | LINE |

  

|  |  |  |  |  |  |  |  |  |  |
| --- | --- | --- | --- | --- | --- | --- | --- | --- | --- |
| chr4:126701849-126715457 | | | 13468 bp | | |  |  |  |  |
| Genome Start | Genome End | Strand | Div | Repeat Start | Repeat End | Repeat Left | Name | Family | Class |
| 126701849 | 126702222 | - | 14.5 | 6253 | 6638 | -927 | L1M1 | L1 | LINE |
| 126702225 | 126702675 | - | 5.4 | 1 | 448 | -2 | LTR7 | ERV1 | LTR |
| 126702676 | 126703055 | - | 8.3 | 7322 | 7713 | 0 | HERVH | ERV1 | LTR |
| 126703054 | 126703265 | - | 10.8 | 5600 | 5819 | -1894 | HERVH | ERV1 | LTR |
| 126703267 | 126703928 | - | 14.2 | 4488 | 5149 | -2564 | HERVH | ERV1 | LTR |
| 126703931 | 126704435 | - | 10.8 | 3492 | 3993 | -3720 | HERVH | ERV1 | LTR |
| 126704414 | 126707610 | - | 9.4 | 1 | 3308 | -4405 | HERVH | ERV1 | LTR |
| 126707613 | 126708061 | - | 3.4 | 1 | 448 | -2 | LTR7 | ERV1 | LTR |
| 126708062 | 126708112 | - | 8.2 | 6216 | 6257 | -1308 | L1M1 | L1 | LINE |
| 126708113 | 126708400 | - | 9.7 | 1 | 288 | -22 | AluSg | Alu | SINE |
| 126708401 | 126708457 | - | 8.2 | 6158 | 6215 | -1350 | L1M1 | L1 | LINE |
| 126708530 | 126710236 | - | 11.9 | 4523 | 6171 | -1394 | L1M1 | L1 | LINE |
| 126710237 | 126710542 | + | 19.2 | 1 | 299 | -13 | AluJo | Alu | SINE |
| 126710543 | 126710595 | - | 11.9 | 4473 | 4522 | -3043 | L1M1 | L1 | LINE |
| 126710581 | 126711661 | - | 7.5 | 3721 | 4815 | -1331 | L1PA5 | L1 | LINE |
| 126711659 | 126712985 | + | 6.3 | 4830 | 6153 | -1 | L1PA5 | L1 | LINE |
| 126712984 | 126713385 | - | 14.7 | 4079 | 4491 | -3074 | L1M1 | L1 | LINE |
| 126713382 | 126713757 | - | 18.6 | 1363 | 1742 | -5823 | L1M1 | L1 | LINE |
| 126713758 | 126714049 | - | 19.7 | 118 | 437 | 0 | LTR21B | ERV1 | LTR |
| 126714160 | 126715457 | - | 20.9 | 5 | 1367 | -6198 | L1M1 | L1 | LINE |

  

|  |  |  |  |  |  |  |  |  |  |
| --- | --- | --- | --- | --- | --- | --- | --- | --- | --- |
| chr4:126750531-126753022 | | | 2490 bp | | |  |  |  |  |
| Genome Start | Genome End | Strand | Div | Repeat Start | Repeat End | Repeat Left | Name | Family | Class |
| 126750531 | 126750582 | + | 30.1 | 830 | 880 | -2539 | L2 | L2 | LINE |
| 126750583 | 126750937 | + | 13.1 | 1 | 355 | 0 | THE1A | MaLR | LTR |
| 126750940 | 126752494 | + | 30.2 | 2 | 1735 | 0 | MSTC-int | MaLR | LTR |
| 126752495 | 126752842 | + | 10.7 | 1 | 355 | 0 | THE1A | MaLR | LTR |
| 126752843 | 126753022 | + | 30.1 | 881 | 1090 | -2329 | L2 | L2 | LINE |

  

|  |  |  |  |  |  |  |  |  |  |
| --- | --- | --- | --- | --- | --- | --- | --- | --- | --- |
| chr4:126776599-126780405 | | | 3566 bp | | |  |  |  |  |
| Genome Start | Genome End | Strand | Div | Repeat Start | Repeat End | Repeat Left | Name | Family | Class |
| 126776599 | 126776678 | + | 20.7 | 3 | 96 | -419 | MLT2B2 | ERVL | LTR |
| 126776679 | 126776741 | + | 22.9 | 1 | 64 | -530 | LTR27B | ERV1 | LTR |
| 126776742 | 126777104 | - | 12.6 | 1 | 364 | 0 | THE1B | MaLR | LTR |
| 126777105 | 126778669 | - | 30.2 | 2 | 1735 | 0 | MLT-int | MaLR | LTR |
| 126778674 | 126779032 | - | 13.8 | 1 | 362 | -2 | THE1B | MaLR | LTR |
| 126779033 | 126779422 | + | 23.1 | 65 | 1232 | -147 | LTR27B | ERV1 | LTR |
| 126779660 | 126779998 | + | 28.2 | 1307 | 1755 | 0 | MER52A | ERV1 | LTR |
| 126779999 | 126780405 | + | 22.3 | 94 | 501 | -14 | MLT2B2 | ERVL | LTR |

  

|  |  |  |  |  |  |  |  |  |  |
| --- | --- | --- | --- | --- | --- | --- | --- | --- | --- |
| chr4:127662712-127667553 | | | 4761 bp | | |  |  |  |  |
| Genome Start | Genome End | Strand | Div | Repeat Start | Repeat End | Repeat Left | Name | Family | Class |
| 127662712 | 127663249 | + | 28.5 | 5 | 608 | -881 | MLT1H-int | MaLR | LTR |
| 127663282 | 127664286 | - | 15.3 | 164 | 1231 | 0 | Tigger3b | MER2\_type | DNA |
| 127664287 | 127664639 | - | 9.1 | 1 | 355 | 0 | THE1A | MaLR | LTR |
| 127664640 | 127666217 | - | 31.6 | 2 | 1735 | 0 | MLT-int | MaLR | LTR |
| 127666220 | 127666575 | - | 9.6 | 1 | 355 | 0 | THE1A | MaLR | LTR |
| 127666576 | 127666733 | - | 15.3 | 3 | 163 | -1068 | Tigger3b | MER2\_type | DNA |
| 127666781 | 127667553 | + | 28.8 | 693 | 1488 | -1 | MLT1H-int | MaLR | LTR |

  

|  |  |  |  |  |  |  |  |  |  |
| --- | --- | --- | --- | --- | --- | --- | --- | --- | --- |
| chr4:128225706-128235589 | | | 9880 bp | | |  |  |  |  |
| Genome Start | Genome End | Strand | Div | Repeat Start | Repeat End | Repeat Left | Name | Family | Class |
| 128225706 | 128226058 | + | 26.9 | 4408 | 4766 | -1380 | L1ME1 | L1 | LINE |
| 128226059 | 128226429 | + | 11.4 | 1 | 375 | 0 | THE1C | MaLR | LTR |
| 128226432 | 128227987 | + | 30.4 | 2 | 1735 | 0 | MLT-int | MaLR | LTR |
| 128227988 | 128228359 | + | 15.8 | 1 | 375 | 0 | THE1C | MaLR | LTR |
| 128228360 | 128228422 | + | 26.9 | 4767 | 4811 | -1335 | L1ME1 | L1 | LINE |
| 128228423 | 128228713 | + | 21.2 | 3 | 291 | -21 | AluJb | Alu | SINE |
| 128228714 | 128229213 | + | 26.9 | 4812 | 5394 | -752 | L1ME1 | L1 | LINE |
| 128229210 | 128234906 | + | 12.2 | 549 | 6358 | -1 | L1PA11 | L1 | LINE |
| 128234913 | 128235589 | + | 24.7 | 5383 | 6161 | -5 | L1ME1 | L1 | LINE |

  

|  |  |  |  |  |  |  |  |  |  |
| --- | --- | --- | --- | --- | --- | --- | --- | --- | --- |
| chr4:128322399-128330955 | | | 8275 bp | | |  |  |  |  |
| Genome Start | Genome End | Strand | Div | Repeat Start | Repeat End | Repeat Left | Name | Family | Class |
| 128322399 | 128323413 | + | 29.9 | 2766 | 3779 | -1888 | ERVL-E | ERVL | LTR |
| 128323416 | 128324127 | + | 11.9 | 2632 | 3352 | -2794 | L1M1 | L1 | LINE |
| 128324128 | 128324471 | + | 12.1 | 1 | 364 | 0 | THE1B | MaLR | LTR |
| 128324472 | 128326036 | + | 9.1 | 1 | 1580 | 0 | THE1B-int | MaLR | LTR |
| 128326037 | 128326379 | + | 14.2 | 1 | 364 | 0 | THE1B | MaLR | LTR |
| 128326380 | 128328673 | + | 11.7 | 3348 | 5659 | -646 | L1M1 | L1 | LINE |
| 128328674 | 128329396 | + | 12.6 | 5571 | 6304 | 0 | L1MA2 | L1 | LINE |
| 128329450 | 128329593 | + | 29.1 | 3902 | 4046 | -1621 | ERVL-E | ERVL | LTR |
| 128329605 | 128330603 | + | 27.9 | 4148 | 5302 | -365 | ERVL-E | ERVL | LTR |
| 128330820 | 128330955 | + | 21.7 | 5525 | 5667 | 0 | ERVL-E | ERVL | LTR |

  

|  |  |  |  |  |  |  |  |  |  |
| --- | --- | --- | --- | --- | --- | --- | --- | --- | --- |
| chr4:128434484-128443137 | | | 8596 bp | | |  |  |  |  |
| Genome Start | Genome End | Strand | Div | Repeat Start | Repeat End | Repeat Left | Name | Family | Class |
| 128434484 | 128435589 | + | 16.3 | 1 | 1162 | -5701 | L1PB1 | L1 | LINE |
| 128435591 | 128435633 | + | 11.6 | 1313 | 1355 | -5508 | L1PB1 | L1 | LINE |
| 128435635 | 128436012 | - | 13.1 | 1 | 375 | 0 | THE1C | MaLR | LTR |
| 128436034 | 128437412 | - | 12.3 | 1 | 1359 | -221 | THE1C-int | MaLR | LTR |
| 128437413 | 128437781 | - | 11.2 | 3 | 375 | 0 | THE1C | MaLR | LTR |
| 128437784 | 128439443 | + | 10.1 | 1352 | 3174 | -3688 | L1PB1 | L1 | LINE |
| 128439477 | 128443137 | + | 10.2 | 3175 | 6865 | -2 | L1PB1 | L1 | LINE |

  

|  |  |  |  |  |  |  |  |  |  |
| --- | --- | --- | --- | --- | --- | --- | --- | --- | --- |
| chr4:128651347-128657875 | | | 6534 bp | | |  |  |  |  |
| Genome Start | Genome End | Strand | Div | Repeat Start | Repeat End | Repeat Left | Name | Family | Class |
| 128651347 | 128651634 | + | 17.7 | 1 | 332 | -191 | Charlie1b | MER1\_type | DNA |
| 128651643 | 128651991 | - | 8.0 | 1 | 448 | -2 | LTR7 | ERV1 | LTR |
| 128651992 | 128652369 | - | 9.9 | 7322 | 7713 | 0 | HERVH | ERV1 | LTR |
| 128652368 | 128652582 | - | 12.1 | 5600 | 5819 | -1894 | HERVH | ERV1 | LTR |
| 128652584 | 128654275 | - | 15.9 | 3492 | 5149 | -2564 | HERVH | ERV1 | LTR |
| 128654268 | 128654415 | - | 11.5 | 3140 | 3288 | -4425 | HERVH | ERV1 | LTR |
| 128654410 | 128657356 | - | 9.4 | 1 | 2987 | -4726 | HERVH | ERV1 | LTR |
| 128657359 | 128657707 | - | 7.7 | 1 | 448 | -2 | LTR7 | ERV1 | LTR |
| 128657708 | 128657875 | + | 17.7 | 336 | 518 | -5 | Charlie1b | MER1\_type | DNA |

  

|  |  |  |  |  |  |  |  |  |  |
| --- | --- | --- | --- | --- | --- | --- | --- | --- | --- |
| chr4:129516714-129519062 | | | 2346 bp | | |  |  |  |  |
| Genome Start | Genome End | Strand | Div | Repeat Start | Repeat End | Repeat Left | Name | Family | Class |
| 129516714 | 129516793 | - | 13.0 | 6207 | 6277 | -23 | L1MA5 | L1 | LINE |
| 129516794 | 129517180 | - | 9.6 | 1 | 381 | 0 | THE1D | MaLR | LTR |
| 129517181 | 129518666 | - | 11.1 | 3 | 1580 | 0 | THE1D-int | MaLR | LTR |
| 129518669 | 129518993 | - | 10.9 | 1 | 381 | 0 | THE1D | MaLR | LTR |
| 129518995 | 129519062 | - | 19.1 | 6133 | 6201 | -99 | L1MA5 | L1 | LINE |

  

|  |  |  |  |  |  |  |  |  |  |
| --- | --- | --- | --- | --- | --- | --- | --- | --- | --- |
| chr4:130391214-130394187 | | | 2959 bp | | |  |  |  |  |
| Genome Start | Genome End | Strand | Div | Repeat Start | Repeat End | Repeat Left | Name | Family | Class |
| 130391214 | 130391716 | + | 24.8 | 12 | 513 | -94 | MER77 | ERVL | LTR |
| 130391723 | 130392099 | + | 13.4 | 1 | 428 | 0 | MSTA | MaLR | LTR |
| 130392102 | 130393703 | + | 13.6 | 3 | 1580 | 0 | MSTA-int | MaLR | LTR |
| 130393704 | 130394090 | + | 16.5 | 1 | 428 | 0 | MSTA | MaLR | LTR |
| 130394098 | 130394187 | + | 17.5 | 525 | 606 | -1 | MER77 | ERVL | LTR |

  

|  |  |  |  |  |  |  |  |  |  |
| --- | --- | --- | --- | --- | --- | --- | --- | --- | --- |
| chr4:131404826-131412125 | | | 7300 bp | | |  |  |  |  |
| Genome Start | Genome End | Strand | Div | Repeat Start | Repeat End | Repeat Left | Name | Family | Class |
| 131404826 | 131405471 | - | 19.3 | 5847 | 6478 | -4 | L1MA4 | L1 | LINE |
| 131405472 | 131405767 | - | 14.9 | 2 | 296 | -14 | AluSg | Alu | SINE |
| 131405768 | 131406711 | - | 18.6 | 4902 | 5846 | -636 | L1MA4 | L1 | LINE |
| 131406712 | 131407072 | + | 12.4 | 1 | 381 | 0 | THE1D | MaLR | LTR |
| 131407075 | 131408542 | + | 17.5 | 3 | 1580 | 0 | THE1D-int | MaLR | LTR |
| 131408543 | 131408905 | + | 14.5 | 1 | 381 | 0 | THE1D | MaLR | LTR |
| 131408906 | 131410152 | - | 19.8 | 3668 | 4906 | -1419 | L1MA4 | L1 | LINE |
| 131410151 | 131410237 | - | 24.7 | 1632 | 1702 | -4626 | L1MA4 | L1 | LINE |
| 131410238 | 131410548 | - | 9.7 | 1 | 311 | 0 | AluY | Alu | SINE |
| 131410549 | 131412125 | - | 20.0 | 11 | 1631 | -4697 | L1MA4 | L1 | LINE |

  

|  |  |  |  |  |  |  |  |  |  |
| --- | --- | --- | --- | --- | --- | --- | --- | --- | --- |
| chr4:131907788-131915687 | | | 7771 bp | | |  |  |  |  |
| Genome Start | Genome End | Strand | Div | Repeat Start | Repeat End | Repeat Left | Name | Family | Class |
| 131907788 | 131908009 | + | 24.8 | 5539 | 5782 | -2026 | L1MC4a | L1 | LINE |
| 131908014 | 131908415 | + | 9.0 | 1 | 448 | -2 | LTR7 | ERV1 | LTR |
| 131908418 | 131911378 | + | 9.2 | 1 | 2992 | -4721 | HERVH | ERV1 | LTR |
| 131911365 | 131912217 | + | 10.1 | 3140 | 3993 | -3720 | HERVH | ERV1 | LTR |
| 131912220 | 131912882 | + | 16.2 | 4488 | 5149 | -2564 | HERVH | ERV1 | LTR |
| 131912884 | 131913097 | + | 13.1 | 5600 | 5819 | -1894 | HERVH | ERV1 | LTR |
| 131913098 | 131913474 | + | 11.3 | 7324 | 7713 | 0 | HERVH | ERV1 | LTR |
| 131913475 | 131913873 | + | 7.6 | 1 | 448 | -2 | LTR7 | ERV1 | LTR |
| 131913881 | 131914287 | + | 25.5 | 5781 | 6188 | -1620 | L1MC4a | L1 | LINE |
| 131914288 | 131914530 | + | 1.7 | 71 | 310 | -8 | AluYb8 | Alu | SINE |
| 131914531 | 131915091 | + | 25.5 | 6189 | 6756 | -1052 | L1MC4a | L1 | LINE |
| 131915099 | 131915408 | + | 22.2 | 7154 | 7470 | -338 | L1MC4a | L1 | LINE |
| 131915529 | 131915687 | + | 31.2 | 7651 | 7807 | -1 | L1MC4a | L1 | LINE |

  

|  |  |  |  |  |  |  |  |  |  |
| --- | --- | --- | --- | --- | --- | --- | --- | --- | --- |
| chr4:132104112-132108164 | | | 4046 bp | | |  |  |  |  |
| Genome Start | Genome End | Strand | Div | Repeat Start | Repeat End | Repeat Left | Name | Family | Class |
| 132104112 | 132104724 | + | 14.9 | 1 | 622 | -1796 | Tigger1 | MER2\_type | DNA |
| 132104725 | 132105075 | + | 17.3 | 1 | 364 | 0 | THE1B | MaLR | LTR |
| 132105078 | 132106667 | + | 29.9 | 2 | 1735 | 0 | MLT-int | MaLR | LTR |
| 132106668 | 132107003 | + | 19.4 | 1 | 364 | 0 | THE1B | MaLR | LTR |
| 132107005 | 132107545 | + | 15.6 | 620 | 1164 | -1254 | Tigger1 | MER2\_type | DNA |
| 132107546 | 132107780 | + | 16.5 | 76 | 311 | -1 | AluSg/x | Alu | SINE |
| 132107785 | 132108164 | + | 14.0 | 1149 | 1533 | -885 | Tigger1 | MER2\_type | DNA |

  

|  |  |  |  |  |  |  |  |  |  |
| --- | --- | --- | --- | --- | --- | --- | --- | --- | --- |
| chr4:132239593-132243488 | | | 3894 bp | | |  |  |  |  |
| Genome Start | Genome End | Strand | Div | Repeat Start | Repeat End | Repeat Left | Name | Family | Class |
| 132239593 | 132240392 | - | 21.4 | 918 | 1755 | 0 | MER52A | ERV1 | LTR |
| 132240393 | 132240748 | + | 15.4 | 1 | 364 | 0 | THE1B | MaLR | LTR |
| 132240751 | 132242419 | + | 30.1 | 2 | 1735 | 0 | MLT-int | MaLR | LTR |
| 132242420 | 132242775 | + | 16.5 | 1 | 364 | 0 | THE1B | MaLR | LTR |
| 132242776 | 132243488 | - | 21.4 | 11 | 917 | -838 | MER52A | ERV1 | LTR |

  

|  |  |  |  |  |  |  |  |  |  |
| --- | --- | --- | --- | --- | --- | --- | --- | --- | --- |
| chr4:132461268-132466397 | | | 5103 bp | | |  |  |  |  |
| Genome Start | Genome End | Strand | Div | Repeat Start | Repeat End | Repeat Left | Name | Family | Class |
| 132461268 | 132462166 | + | 18.5 | 3395 | 4329 | -1814 | L1MA8 | L1 | LINE |
| 132462192 | 132463481 | + | 18.9 | 4330 | 5651 | -640 | L1MA8 | L1 | LINE |
| 132463482 | 132463842 | + | 15.5 | 1 | 364 | 0 | THE1B | MaLR | LTR |
| 132463845 | 132465404 | + | 30.9 | 2 | 1735 | 0 | MLT-int | MaLR | LTR |
| 132465405 | 132465768 | + | 18.7 | 1 | 364 | 0 | THE1B | MaLR | LTR |
| 132465769 | 132466397 | + | 20.3 | 5652 | 6288 | -3 | L1MA8 | L1 | LINE |

  

|  |  |  |  |  |  |  |  |  |  |
| --- | --- | --- | --- | --- | --- | --- | --- | --- | --- |
| chr4:132771430-132775295 | | | 3842 bp | | |  |  |  |  |
| Genome Start | Genome End | Strand | Div | Repeat Start | Repeat End | Repeat Left | Name | Family | Class |
| 132771430 | 132772148 | - | 21.5 | 4074 | 4809 | -1331 | L1M3 | L1 | LINE |
| 132772150 | 132772310 | - | 9.3 | 146 | 306 | -5 | AluY | Alu | SINE |
| 132772311 | 132772580 | - | 21.6 | 3817 | 4083 | -2057 | L1M3 | L1 | LINE |
| 132772604 | 132772952 | + | 19.3 | 1 | 375 | 0 | THE1C | MaLR | LTR |
| 132772953 | 132774175 | + | 15.6 | 1 | 1261 | -319 | THE1C-int | MaLR | LTR |
| 132774176 | 132774477 | - | 13.6 | 1 | 302 | -10 | AluSx | Alu | SINE |
| 132774478 | 132774795 | + | 15.6 | 1262 | 1580 | 0 | THE1C-int | MaLR | LTR |
| 132774796 | 132775150 | + | 15.2 | 1 | 375 | 0 | THE1C | MaLR | LTR |
| 132775151 | 132775295 | - | 22.1 | 3639 | 3789 | -2351 | L1M3 | L1 | LINE |

  

|  |  |  |  |  |  |  |  |  |  |
| --- | --- | --- | --- | --- | --- | --- | --- | --- | --- |
| chr4:132796951-132812065 | | | 14941 bp | | |  |  |  |  |
| Genome Start | Genome End | Strand | Div | Repeat Start | Repeat End | Repeat Left | Name | Family | Class |
| 132796951 | 132797182 | - | 25.1 | 6382 | 6599 | -20 | L1MD2 | L1 | LINE |
| 132797168 | 132797451 | - | 24.1 | 5855 | 6150 | -469 | L1MD2 | L1 | LINE |
| 132797515 | 132797599 | + | 22.4 | 5987 | 6071 | -233 | L1MA2 | L1 | LINE |
| 132797600 | 132797949 | - | 22.7 | 5523 | 5867 | -635 | L1MD2 | L1 | LINE |
| 132797927 | 132798580 | - | 23.1 | 2904 | 3537 | -2869 | L1MD2 | L1 | LINE |
| 132798609 | 132798845 | - | 23.1 | 2662 | 2903 | -3503 | L1MD2 | L1 | LINE |
| 132798846 | 132799439 | - | 13.7 | 5 | 622 | 0 | LTR77 | ERV1 | LTR |
| 132799440 | 132800489 | - | 26.2 | 1589 | 2661 | -3745 | L1MD2 | L1 | LINE |
| 132800489 | 132800659 | + | 15.8 | 4866 | 5037 | -1106 | L1MA5 | L1 | LINE |
| 132800659 | 132801449 | + | 16.5 | 5530 | 6300 | 0 | L1MA5 | L1 | LINE |
| 132801472 | 132801928 | - | 40.6 | 2795 | 3292 | -1197 | L3 | CR1 | LINE |
| 132801955 | 132802455 | + | 24.4 | 1 | 465 | -40 | MLT1D | MaLR | LTR |
| 132802466 | 132803207 | - | 24.1 | 845 | 1601 | -4901 | L1MD2 | L1 | LINE |
| 132803209 | 132803711 | - | 24.1 | 1 | 472 | 0 | MER70A | ERVL | LTR |
| 132803720 | 132804149 | - | 18.6 | 4593 | 5022 | -1 | MER70A-int | ERVL | LTR |
| 132804150 | 132804454 | + | 13.7 | 1 | 294 | -19 | AluSp | Alu | SINE |
| 132804455 | 132806597 | - | 18.6 | 2380 | 4592 | -431 | MER70A-int | ERVL | LTR |
| 132806598 | 132806988 | + | 11.1 | 1 | 450 | 0 | LTR7 | ERV1 | LTR |
| 132806996 | 132807112 | + | 10.6 | 2613 | 2725 | -4988 | HERVH | ERV1 | LTR |
| 132807115 | 132808604 | + | 11.4 | 3582 | 5286 | -1805 | HERVH | ERV1 | LTR |
| 132808624 | 132809056 | + | 10.3 | 7283 | 7713 | 0 | HERVH | ERV1 | LTR |
| 132809057 | 132809447 | + | 11.4 | 1 | 450 | 0 | LTR7 | ERV1 | LTR |
| 132809448 | 132809531 | - | 19.1 | 2300 | 2384 | -2639 | MER70A-int | ERVL | LTR |
| 132809551 | 132810542 | - | 20.3 | 10 | 1076 | -3947 | MER70A-int | ERVL | LTR |
| 132810552 | 132810783 | - | 23.4 | 246 | 472 | 0 | MER70A | ERVL | LTR |
| 132810784 | 132811077 | - | 7.6 | 1 | 296 | -17 | AluSq | Alu | SINE |
| 132811078 | 132811322 | - | 23.4 | 1 | 245 | -227 | MER70A | ERVL | LTR |
| 132811323 | 132812065 | - | 31.9 | 9 | 848 | -5561 | L1MD2 | L1 | LINE |

  

|  |  |  |  |  |  |  |  |  |  |
| --- | --- | --- | --- | --- | --- | --- | --- | --- | --- |
| chr4:133225344-133234383 | | | 9032 bp | | |  |  |  |  |
| Genome Start | Genome End | Strand | Div | Repeat Start | Repeat End | Repeat Left | Name | Family | Class |
| 133225344 | 133227799 | - | 10.9 | 3154 | 5654 | 0 | HERVL-A2 | ERVL | LTR |
| 133227800 | 133228166 | - | 12.8 | 1 | 375 | 0 | THE1C | MaLR | LTR |
| 133228167 | 133228576 | - | 10.9 | 2730 | 3153 | -2501 | HERVL-A2 | ERVL | LTR |
| 133228577 | 133228946 | + | 11.7 | 2 | 375 | 0 | THE1C | MaLR | LTR |
| 133228947 | 133230530 | + | 13.5 | 1 | 1574 | -6 | THE1C-int | MaLR | LTR |
| 133230538 | 133230900 | + | 10.8 | 1 | 373 | -2 | THE1C | MaLR | LTR |
| 133230902 | 133231013 | - | 11.8 | 2629 | 2736 | -2918 | HERVL-A2 | ERVL | LTR |
| 133231014 | 133231769 | - | 6.8 | 1 | 780 | 0 | LTR17 | ERV1 | LTR |
| 133231770 | 133234383 | - | 11.8 | 1 | 2628 | -3026 | HERVL-A2 | ERVL | LTR |

  

|  |  |  |  |  |  |  |  |  |  |
| --- | --- | --- | --- | --- | --- | --- | --- | --- | --- |
| chr4:133291840-133300626 | | | 8755 bp | | |  |  |  |  |
| Genome Start | Genome End | Strand | Div | Repeat Start | Repeat End | Repeat Left | Name | Family | Class |
| 133291840 | 133293457 | - | 11.7 | 5252 | 6860 | -7 | L1PB1 | L1 | LINE |
| 133293458 | 133293815 | + | 14.4 | 1 | 364 | 0 | THE1B | MaLR | LTR |
| 133293816 | 133295509 | - | 12.2 | 3552 | 5251 | -1611 | L1PB1 | L1 | LINE |
| 133295511 | 133295863 | - | 16.4 | 3 | 364 | 0 | THE1B | MaLR | LTR |
| 133295872 | 133297418 | - | 13.7 | 1 | 1573 | -7 | THE1B-int | MaLR | LTR |
| 133297419 | 133297777 | - | 14.7 | 1 | 364 | 0 | THE1B | MaLR | LTR |
| 133297778 | 133298633 | - | 13.0 | 2642 | 3555 | -3307 | L1PB1 | L1 | LINE |
| 133298657 | 133300498 | - | 13.6 | 762 | 2641 | -4222 | L1PB1 | L1 | LINE |
| 133300499 | 133300626 | - | 14.1 | 1 | 132 | -6731 | L1PB1 | L1 | LINE |

  

|  |  |  |  |  |  |  |  |  |  |
| --- | --- | --- | --- | --- | --- | --- | --- | --- | --- |
| chr4:136307765-136317074 | | | 8904 bp | | |  |  |  |  |
| Genome Start | Genome End | Strand | Div | Repeat Start | Repeat End | Repeat Left | Name | Family | Class |
| 136307765 | 136307895 | + | 21.1 | 109 | 243 | -6355 | MER4C-int | ERV1 | LTR |
| 136308040 | 136308471 | - | 9.0 | 1 | 448 | -2 | LTR7 | ERV1 | LTR |
| 136308472 | 136308851 | - | 7.5 | 7322 | 7713 | 0 | HERVH | ERV1 | LTR |
| 136308850 | 136309014 | - | 14.2 | 5654 | 5819 | -1894 | HERVH | ERV1 | LTR |
| 136309042 | 136309599 | - | 13.8 | 4488 | 5104 | -2609 | HERVH | ERV1 | LTR |
| 136309602 | 136310104 | - | 10.2 | 3492 | 3993 | -3720 | HERVH | ERV1 | LTR |
| 136310095 | 136310239 | - | 13.1 | 3140 | 3290 | -4423 | HERVH | ERV1 | LTR |
| 136310234 | 136313291 | - | 9.6 | 1 | 2987 | -4726 | HERVH | ERV1 | LTR |
| 136313294 | 136313723 | - | 10.2 | 1 | 448 | -2 | LTR7 | ERV1 | LTR |
| 136313963 | 136314220 | + | 21.1 | 994 | 1267 | -5064 | MER4C-int | ERV1 | LTR |
| 136314221 | 136314316 | + | 15.6 | 2985 | 3080 | -3183 | MER4C-int | ERV1 | LTR |
| 136314316 | 136314849 | + | 27.4 | 3798 | 4301 | -4743 | MER4C-int | ERV1 | LTR |
| 136314863 | 136315012 | + | 25.6 | 1580 | 3151 | -3767 | MER4C-int | ERV1 | LTR |
| 136315013 | 136315531 | - | 9.2 | 1 | 521 | 0 | MER41C | ERV1 | LTR |
| 136315532 | 136316458 | + | 21.9 | 1667 | 5109 | -2881 | MER4C-int | ERV1 | LTR |
| 136316459 | 136316754 | + | 20.1 | 9 | 309 | 0 | AluJb | Alu | SINE |
| 136316755 | 136316873 | + | 22.8 | 5110 | 5231 | -3813 | MER4C-int | ERV1 | LTR |
| 136316872 | 136317074 | + | 15.2 | 4248 | 4451 | -3669 | MER4C-int | ERV1 | LTR |

  

|  |  |  |  |  |  |  |  |  |  |
| --- | --- | --- | --- | --- | --- | --- | --- | --- | --- |
| chr4:136334013-136337265 | | | 3166 bp | | |  |  |  |  |
| Genome Start | Genome End | Strand | Div | Repeat Start | Repeat End | Repeat Left | Name | Family | Class |
| 136334013 | 136334231 | - | 30.3 | 3213 | 3419 | 0 | L2 | L2 | LINE |
| 136334232 | 136334584 | + | 18.7 | 1 | 417 | -9 | MSTB | MaLR | LTR |
| 136334626 | 136335756 | + | 18.9 | 443 | 1651 | 0 | MSTB-int | MaLR | LTR |
| 136335757 | 136336147 | + | 15.9 | 1 | 426 | 0 | MSTB | MaLR | LTR |
| 136336150 | 136336337 | - | 37.3 | 3018 | 3215 | -204 | L2 | L2 | LINE |
| 136336369 | 136336764 | - | 27.6 | 1 | 453 | -14 | MLT1C | MaLR | LTR |
| 136336778 | 136337265 | - | 33.8 | 2461 | 2994 | -425 | L2 | L2 | LINE |

  

|  |  |  |  |  |  |  |  |  |  |
| --- | --- | --- | --- | --- | --- | --- | --- | --- | --- |
| chr4:136905425-136910503 | | | 5075 bp | | |  |  |  |  |
| Genome Start | Genome End | Strand | Div | Repeat Start | Repeat End | Repeat Left | Name | Family | Class |
| 136905425 | 136905489 | - | 16.9 | 2200 | 2269 | -149 | Tigger1 | MER2\_type | DNA |
| 136905491 | 136905862 | - | 12.2 | 1 | 375 | 0 | THE1C | MaLR | LTR |
| 136905864 | 136907492 | - | 29.9 | 2 | 1734 | -1 | MLT-int | MaLR | LTR |
| 136907495 | 136907870 | - | 14.4 | 33 | 375 | 0 | THE1C | MaLR | LTR |
| 136907871 | 136908291 | + | 8.8 | 5746 | 6166 | -6 | L1PA8 | L1 | LINE |
| 136908292 | 136908330 | - | 14.4 | 1 | 32 | -343 | THE1C | MaLR | LTR |
| 136908331 | 136910503 | - | 18.0 | 20 | 2203 | -215 | Tigger1 | MER2\_type | DNA |

  

|  |  |  |  |  |  |  |  |  |  |
| --- | --- | --- | --- | --- | --- | --- | --- | --- | --- |
| chr4:137163152-137169545 | | | 6237 bp | | |  |  |  |  |
| Genome Start | Genome End | Strand | Div | Repeat Start | Repeat End | Repeat Left | Name | Family | Class |
| 137163152 | 137165326 | + | 14.8 | 2506 | 4727 | -1419 | L1PB | L1 | LINE |
| 137165327 | 137165696 | + | 20.2 | 1 | 428 | 0 | MSTA | MaLR | LTR |
| 137165699 | 137167323 | + | 31.2 | 2 | 1735 | 0 | MLT-int | MaLR | LTR |
| 137167324 | 137167626 | + | 15.7 | 1 | 380 | -1 | MSTA | MaLR | LTR |
| 137167634 | 137169046 | + | 12.9 | 4729 | 6148 | -3 | L1PB1 | L1 | LINE |
| 137169191 | 137169319 | - | 16.3 | 162 | 291 | -21 | AluSx | Alu | SINE |
| 137169320 | 137169375 | + | 16.1 | 318 | 374 | -1 | THE1C | MaLR | LTR |
| 137169380 | 137169545 | + | 19.3 | 4726 | 4891 | -1255 | L1PB | L1 | LINE |

  

|  |  |  |  |  |  |  |  |  |  |
| --- | --- | --- | --- | --- | --- | --- | --- | --- | --- |
| chr4:137658405-137663412 | | | 4740 bp | | |  |  |  |  |
| Genome Start | Genome End | Strand | Div | Repeat Start | Repeat End | Repeat Left | Name | Family | Class |
| 137658405 | 137659266 | + | 27.0 | 1149 | 2101 | -4138 | L1MEc | L1 | LINE |
| 137659478 | 137659583 | + | 28.6 | 2331 | 2436 | -3783 | L1MEc | L1 | LINE |
| 137659603 | 137659953 | - | 14.1 | 3 | 379 | -2 | THE1D | MaLR | LTR |
| 137659957 | 137661603 | - | 16.1 | 3 | 1579 | -1 | THE1D-int | MaLR | LTR |
| 137661606 | 137661969 | - | 15.0 | 1 | 381 | 0 | THE1D | MaLR | LTR |
| 137661994 | 137662476 | + | 27.8 | 2496 | 3002 | -3217 | L1MEc | L1 | LINE |
| 137662477 | 137662777 | + | 12.7 | 1 | 300 | -13 | AluSq | Alu | SINE |
| 137662778 | 137663307 | + | 27.8 | 3003 | 3525 | -2694 | L1MEc | L1 | LINE |
| 137663317 | 137663412 | + | 22.8 | 3489 | 3583 | -2636 | L1MEc | L1 | LINE |

  

|  |  |  |  |  |  |  |  |  |  |
| --- | --- | --- | --- | --- | --- | --- | --- | --- | --- |
| chr4:138409867-138414781 | | | 4483 bp | | |  |  |  |  |
| Genome Start | Genome End | Strand | Div | Repeat Start | Repeat End | Repeat Left | Name | Family | Class |
| 138409867 | 138410560 | - | 32.2 | 5330 | 6058 | -1750 | L1MC4a | L1 | LINE |
| 138410591 | 138410991 | - | 20.7 | 1 | 408 | 0 | MLT1A1 | MaLR | LTR |
| 138410992 | 138411741 | - | 25.5 | 901 | 1735 | 0 | MLT1A1-int | MaLR | LTR |
| 138411782 | 138413649 | + | 17.6 | 4370 | 6283 | -8 | L1MA7 | L1 | LINE |
| 138413650 | 138413745 | - | 27.4 | 3 | 97 | -1554 | MLT1A1-int | MaLR | LTR |
| 138413747 | 138414122 | - | 18.9 | 29 | 408 | 0 | MLT1A1 | MaLR | LTR |
| 138414484 | 138414781 | - | 38.9 | 4721 | 5045 | -1101 | L1MC4a | L1 | LINE |

  

|  |  |  |  |  |  |  |  |  |  |
| --- | --- | --- | --- | --- | --- | --- | --- | --- | --- |
| chr4:139264871-139270956 | | | 6086 bp | | |  |  |  |  |
| Genome Start | Genome End | Strand | Div | Repeat Start | Repeat End | Repeat Left | Name | Family | Class |
| 139264871 | 139265405 | - | 23.2 | 4586 | 5141 | -544 | ERVL-E | ERVL | LTR |
| 139265394 | 139265550 | - | 20.1 | 3918 | 4073 | -1612 | ERVL-E | ERVL | LTR |
| 139265554 | 139265701 | - | 23.6 | 3494 | 3642 | -2043 | ERVL-E | ERVL | LTR |
| 139265711 | 139266229 | - | 25.2 | 2832 | 3329 | -2356 | ERVL-E | ERVL | LTR |
| 139266230 | 139266651 | + | 15.0 | 1 | 426 | 0 | MSTB | MaLR | LTR |
| 139266652 | 139268217 | + | 19.1 | 1 | 1651 | 0 | MSTB-int | MaLR | LTR |
| 139268218 | 139268622 | + | 14.4 | 1 | 426 | 0 | MSTB | MaLR | LTR |
| 139268623 | 139269036 | - | 22.1 | 2428 | 2836 | -2849 | ERVL-E | ERVL | LTR |
| 139269037 | 139269345 | - | 6.5 | 1 | 309 | 0 | AluSc | Alu | SINE |
| 139269346 | 139269461 | - | 22.1 | 2313 | 2427 | -3258 | ERVL-E | ERVL | LTR |
| 139269462 | 139269769 | - | 14.3 | 1 | 308 | -4 | AluSx | Alu | SINE |
| 139269770 | 139270956 | - | 19.2 | 1041 | 2312 | -3373 | ERVL-E | ERVL | LTR |

  

|  |  |  |  |  |  |  |  |  |  |
| --- | --- | --- | --- | --- | --- | --- | --- | --- | --- |
| chr4:140772873-140780047 | | | 7173 bp | | |  |  |  |  |
| Genome Start | Genome End | Strand | Div | Repeat Start | Repeat End | Repeat Left | Name | Family | Class |
| 140772873 | 140772968 | - | 21.9 | 428 | 523 | 0 | LTR33A | ERVL | LTR |
| 140772970 | 140773374 | + | 5.7 | 1 | 415 | -851 | MER11A | ERVK | LTR |
| 140773369 | 140773820 | + | 6.9 | 828 | 1266 | 0 | MER11A | ERVK | LTR |
| 140773821 | 140775597 | + | 8.4 | 1 | 1748 | -6205 | HERVK11 | ERVK | LTR |
| 140775596 | 140777729 | + | 6.7 | 5357 | 7495 | -458 | HERVK11 | ERVK | LTR |
| 140777730 | 140778030 | + | 8.3 | 1 | 301 | -10 | AluY | Alu | SINE |
| 140778031 | 140778496 | + | 6.7 | 7496 | 7953 | 0 | HERVK11 | ERVK | LTR |
| 140778497 | 140779649 | + | 7.6 | 1 | 1266 | 0 | MER11A | ERVK | LTR |
| 140779659 | 140780047 | - | 28.1 | 2 | 423 | -100 | LTR33A | ERVL | LTR |

  

|  |  |  |  |  |  |  |  |  |  |
| --- | --- | --- | --- | --- | --- | --- | --- | --- | --- |
| chr4:142829193-142834099 | | | 4819 bp | | |  |  |  |  |
| Genome Start | Genome End | Strand | Div | Repeat Start | Repeat End | Repeat Left | Name | Family | Class |
| 142829193 | 142829443 | + | 36.4 | 2253 | 2527 | -892 | L2 | L2 | LINE |
| 142829444 | 142831026 | - | 5.9 | 1 | 1578 | 0 | LTR12C | ERV1 | LTR |
| 142831027 | 142831564 | + | 36.4 | 2528 | 3118 | -260 | L2 | L2 | LINE |
| 142831621 | 142831937 | - | 13.5 | 1 | 381 | 0 | THE1D | MaLR | LTR |
| 142831938 | 142833518 | - | 15.3 | 15 | 1580 | 0 | THE1D-int | MaLR | LTR |
| 142833527 | 142833568 | - | 9.8 | 341 | 381 | 0 | THE1D | MaLR | LTR |
| 142833569 | 142833645 | + | 5.6 | 237 | 307 | -4 | Alu | Alu | SINE |
| 142833651 | 142833977 | - | 12.3 | 1 | 347 | -34 | THE1D | MaLR | LTR |
| 142833997 | 142834099 | + | 29.7 | 3261 | 3378 | 0 | L2 | L2 | LINE |

  

|  |  |  |  |  |  |  |  |  |  |
| --- | --- | --- | --- | --- | --- | --- | --- | --- | --- |
| chr4:143011690-143014779 | | | 3088 bp | | |  |  |  |  |
| Genome Start | Genome End | Strand | Div | Repeat Start | Repeat End | Repeat Left | Name | Family | Class |
| 143011690 | 143011936 | - | 19.5 | 459 | 711 | -23 | MER50 | ERV1 | LTR |
| 143011937 | 143012299 | - | 16.9 | 1 | 381 | 0 | THE1D | MaLR | LTR |
| 143012300 | 143013920 | - | 30.8 | 2 | 1735 | 0 | MLT-int | MaLR | LTR |
| 143013923 | 143014288 | - | 16.9 | 1 | 381 | 0 | THE1D | MaLR | LTR |
| 143014289 | 143014779 | - | 14.7 | 1 | 463 | -271 | MER50 | ERV1 | LTR |

  

|  |  |  |  |  |  |  |  |  |  |
| --- | --- | --- | --- | --- | --- | --- | --- | --- | --- |
| chr4:144030327-144043706 | | | 13305 bp | | |  |  |  |  |
| Genome Start | Genome End | Strand | Div | Repeat Start | Repeat End | Repeat Left | Name | Family | Class |
| 144030327 | 144030413 | - | 21.4 | 5585 | 5672 | -498 | L1M5 | L1 | LINE |
| 144030414 | 144030822 | - | 20.1 | 1 | 428 | 0 | MSTC | MaLR | LTR |
| 144030823 | 144031579 | - | 30.0 | 4671 | 5584 | -600 | L1M5 | L1 | LINE |
| 144031647 | 144031810 | + | 9.8 | 133 | 296 | -15 | AluY | Alu | SINE |
| 144031812 | 144032929 | - | 13.3 | 5049 | 6148 | -17 | L1PA15 | L1 | LINE |
| 144032930 | 144033314 | + | 12.9 | 1 | 428 | 0 | MSTA | MaLR | LTR |
| 144033317 | 144034786 | + | 17.2 | 3 | 1580 | 0 | MSTA-int | MaLR | LTR |
| 144034787 | 144035166 | + | 13.3 | 1 | 428 | 0 | MSTA | MaLR | LTR |
| 144035167 | 144037923 | - | 14.9 | 2888 | 5673 | -1093 | L1PA13 | L1 | LINE |
| 144037924 | 144039349 | + | 7.1 | 2 | 1578 | 0 | LTR12C | ERV1 | LTR |
| 144039350 | 144042132 | - | 20.2 | 106 | 2887 | -3879 | L1PA13 | L1 | LINE |
| 144042138 | 144042381 | - | 31.6 | 4428 | 4665 | -1481 | L1M5 | L1 | LINE |
| 144042382 | 144042687 | - | 20.3 | 1 | 306 | -6 | AluJb | Alu | SINE |
| 144042688 | 144043706 | - | 31.6 | 3504 | 4427 | -1719 | L1M5 | L1 | LINE |

  

|  |  |  |  |  |  |  |  |  |  |
| --- | --- | --- | --- | --- | --- | --- | --- | --- | --- |
| chr4:144105546-144107958 | | | 2364 bp | | |  |  |  |  |
| Genome Start | Genome End | Strand | Div | Repeat Start | Repeat End | Repeat Left | Name | Family | Class |
| 144105546 | 144105713 | + | 19.1 | 6 | 194 | -273 | MLT1C | MaLR | LTR |
| 144105791 | 144106132 | - | 8.2 | 1 | 355 | 0 | THE1A | MaLR | LTR |
| 144106133 | 144107684 | - | 28.9 | 2 | 1735 | 0 | MLT-int | MaLR | LTR |
| 144107687 | 144107823 | - | 10.9 | 213 | 355 | 0 | THE1A | MaLR | LTR |
| 144107794 | 144107958 | + | 21.9 | 303 | 467 | 0 | MLT1C | MaLR | LTR |

  

|  |  |  |  |  |  |  |  |  |  |
| --- | --- | --- | --- | --- | --- | --- | --- | --- | --- |
| chr4:144786972-144789666 | | | 2688 bp | | |  |  |  |  |
| Genome Start | Genome End | Strand | Div | Repeat Start | Repeat End | Repeat Left | Name | Family | Class |
| 144786972 | 144786997 | - | 19.2 | 272 | 297 | -15 | AluJo | Alu | SINE |
| 144786998 | 144787368 | + | 14.3 | 1 | 375 | 0 | THE1C | MaLR | LTR |
| 144787375 | 144789027 | + | 31.4 | 5 | 1734 | -1 | MLT1A0-int | MaLR | LTR |
| 144789029 | 144789389 | + | 14.4 | 1 | 375 | 0 | THE1C | MaLR | LTR |
| 144789390 | 144789666 | - | 19.2 | 1 | 271 | -41 | AluJo | Alu | SINE |

  

|  |  |  |  |  |  |  |  |  |  |
| --- | --- | --- | --- | --- | --- | --- | --- | --- | --- |
| chr4:145336321-145342076 | | | 5688 bp | | |  |  |  |  |
| Genome Start | Genome End | Strand | Div | Repeat Start | Repeat End | Repeat Left | Name | Family | Class |
| 145336321 | 145336419 | - | 16.2 | 2320 | 2418 | 0 | Tigger1 | MER2\_type | DNA |
| 145336421 | 145336795 | + | 14.2 | 2 | 428 | 0 | MSTA | MaLR | LTR |
| 145336796 | 145337077 | + | 14.9 | 1 | 284 | -1367 | MSTA-int | MaLR | LTR |
| 145337103 | 145338247 | + | 16.1 | 427 | 1580 | 0 | MSTA-int | MaLR | LTR |
| 145338248 | 145338622 | + | 14.8 | 1 | 428 | 0 | MSTA | MaLR | LTR |
| 145338623 | 145339858 | - | 13.2 | 973 | 2324 | -94 | Tigger1 | MER2\_type | DNA |
| 145339859 | 145340067 | + | 8.7 | 4954 | 5173 | -973 | L1PA8 | L1 | LINE |
| 145340110 | 145341078 | + | 7.9 | 5174 | 6159 | -13 | L1PA8 | L1 | LINE |
| 145341079 | 145342076 | - | 14.9 | 1 | 1008 | -1410 | Tigger1 | MER2\_type | DNA |

  

|  |  |  |  |  |  |  |  |  |  |
| --- | --- | --- | --- | --- | --- | --- | --- | --- | --- |
| chr4:146837309-146856318 | | | 18824 bp | | |  |  |  |  |
| Genome Start | Genome End | Strand | Div | Repeat Start | Repeat End | Repeat Left | Name | Family | Class |
| 146837309 | 146837352 | - | 15.9 | 6333 | 6376 | 0 | L1MA9 | L1 | LINE |
| 146837353 | 146837651 | + | 12.2 | 1 | 298 | -14 | AluSx | Alu | SINE |
| 146837703 | 146837999 | - | 13.1 | 1 | 297 | -13 | AluSg | Alu | SINE |
| 146838000 | 146838799 | - | 15.1 | 5340 | 6193 | -183 | L1MA9 | L1 | LINE |
| 146838806 | 146839393 | - | 12.2 | 1 | 603 | -7 | MER4B | ERV1 | LTR |
| 146839425 | 146839874 | + | 5.6 | 1 | 448 | -2 | LTR7 | ERV1 | LTR |
| 146839877 | 146843282 | + | 10.0 | 1 | 5819 | -1894 | HERVH | ERV1 | LTR |
| 146843280 | 146843657 | + | 7.2 | 7321 | 7713 | 0 | HERVH | ERV1 | LTR |
| 146843658 | 146844105 | + | 4.2 | 1 | 448 | -2 | LTR7 | ERV1 | LTR |
| 146844106 | 146844460 | - | 17.9 | 4957 | 5313 | -891 | L1MA9 | L1 | LINE |
| 146844461 | 146845079 | - | 18.9 | 1 | 595 | 0 | LTR49 | ERV1 | LTR |
| 146845080 | 146845253 | - | 17.9 | 4783 | 4956 | -1248 | L1MA9 | L1 | LINE |
| 146845255 | 146845308 | - | 13.0 | 1663 | 1721 | -4490 | L1MA9 | L1 | LINE |
| 146845310 | 146846065 | - | 14.4 | 1 | 711 | -23 | MER50 | ERV1 | LTR |
| 146846066 | 146847247 | - | 10.7 | 5365 | 7197 | -8 | MER50-int | ERV1 | LTR |
| 146847248 | 146847410 | - | 8.2 | 134 | 294 | -17 | AluY | Alu | SINE |
| 146847411 | 146847706 | - | 8.6 | 13 | 303 | -10 | AluSq | Alu | SINE |
| 146847707 | 146847840 | - | 8.2 | 1 | 133 | -178 | AluY | Alu | SINE |
| 146847841 | 146848905 | - | 16.7 | 2788 | 4075 | -805 | MER50-int | ERV1 | LTR |
| 146848913 | 146850661 | - | 13.7 | 1 | 1837 | -5368 | MER50-int | ERV1 | LTR |
| 146850736 | 146851465 | - | 14.7 | 1 | 722 | -12 | MER50 | ERV1 | LTR |
| 146851466 | 146851487 | - | 26.0 | 1649 | 1666 | -4545 | L1MA9 | L1 | LINE |
| 146851488 | 146851975 | + | 10.0 | 1 | 507 | 0 | MER84 | ERV1 | LTR |
| 146851976 | 146852363 | - | 19.9 | 1269 | 1648 | -4563 | L1MA9 | L1 | LINE |
| 146852393 | 146854352 | + | 13.5 | 1 | 1976 | -442 | Tigger1 | MER2\_type | DNA |
| 146854341 | 146854373 | + | 15.2 | 2221 | 2252 | -166 | Tigger1 | MER2\_type | DNA |
| 146854374 | 146854684 | - | 12.2 | 1 | 311 | -1 | AluSx | Alu | SINE |
| 146854685 | 146854856 | + | 15.2 | 2253 | 2418 | 0 | Tigger1 | MER2\_type | DNA |
| 146854860 | 146855105 | - | 23.5 | 6005 | 6254 | -58 | L1MA9 | L1 | LINE |
| 146855102 | 146856318 | - | 22.7 | 1 | 1294 | -4847 | L1MA9 | L1 | LINE |

  

|  |  |  |  |  |  |  |  |  |  |
| --- | --- | --- | --- | --- | --- | --- | --- | --- | --- |
| chr4:148691915-148694701 | | | 2785 bp | | |  |  |  |  |
| Genome Start | Genome End | Strand | Div | Repeat Start | Repeat End | Repeat Left | Name | Family | Class |
| 148691915 | 148692282 | - | 16.1 | 59 | 428 | 0 | MSTA | MaLR | LTR |
| 148692283 | 148692646 | + | 11.0 | 1 | 364 | 0 | THE1B | MaLR | LTR |
| 148692649 | 148694284 | + | 30.7 | 2 | 1735 | 0 | MLT-int | MaLR | LTR |
| 148694285 | 148694643 | + | 12.8 | 1 | 364 | 0 | THE1B | MaLR | LTR |
| 148694644 | 148694701 | - | 16.1 | 1 | 58 | -370 | MSTA | MaLR | LTR |

  

|  |  |  |  |  |  |  |  |  |  |
| --- | --- | --- | --- | --- | --- | --- | --- | --- | --- |
| chr4:151659823-151664709 | | | 4684 bp | | |  |  |  |  |
| Genome Start | Genome End | Strand | Div | Repeat Start | Repeat End | Repeat Left | Name | Family | Class |
| 151659823 | 151660741 | - | 24.2 | 5258 | 6228 | -9 | L1ME2 | L1 | LINE |
| 151660819 | 151661121 | - | 21.6 | 4422 | 4740 | -1479 | L1ME2 | L1 | LINE |
| 151661130 | 151661437 | + | 13.2 | 5732 | 6049 | -116 | L1PA15 | L1 | LINE |
| 151661457 | 151661508 | + | 15.4 | 6030 | 6083 | -82 | L1PA15 | L1 | LINE |
| 151661519 | 151662029 | - | 25.1 | 2940 | 3502 | -2644 | L1MD | L1 | LINE |
| 151662032 | 151662156 | - | 20.0 | 1 | 125 | -187 | AluJb | Alu | SINE |
| 151662159 | 151662610 | - | 28.2 | 2529 | 3026 | -3193 | L1ME2 | L1 | LINE |
| 151662612 | 151662760 | - | 19.5 | 340 | 505 | 0 | MLT1D | MaLR | LTR |
| 151662763 | 151662927 | + | 21.5 | 244 | 406 | -2 | MLT1A1 | MaLR | LTR |
| 151662930 | 151663004 | + | 24.0 | 1 | 77 | -1658 | MLT1A1-int | MaLR | LTR |
| 151663012 | 151663576 | + | 25.8 | 591 | 1150 | -545 | MLT1A1-int | MaLR | LTR |
| 151663577 | 151663810 | + | 20.6 | 1479 | 1721 | -14 | MLT1A1-int | MaLR | LTR |
| 151663817 | 151664166 | + | 16.6 | 1 | 408 | 0 | MLT1A1 | MaLR | LTR |
| 151664172 | 151664407 | - | 25.6 | 7316 | 7541 | -454 | L1MD3 | L1 | LINE |
| 151664470 | 151664709 | - | 22.1 | 672 | 921 | -5479 | L1ME2 | L1 | LINE |

  

|  |  |  |  |  |  |  |  |  |  |
| --- | --- | --- | --- | --- | --- | --- | --- | --- | --- |
| chr4:152097640-152101027 | | | 3160 bp | | |  |  |  |  |
| Genome Start | Genome End | Strand | Div | Repeat Start | Repeat End | Repeat Left | Name | Family | Class |
| 152097640 | 152097766 | + | 22.1 | 6623 | 6758 | -1203 | L1MC5 | L1 | LINE |
| 152097767 | 152098130 | - | 6.5 | 1 | 364 | 0 | THE1B | MaLR | LTR |
| 152098131 | 152099651 | - | 11.3 | 1 | 1580 | 0 | THE1B-int | MaLR | LTR |
| 152099658 | 152099994 | - | 9.2 | 5 | 358 | -6 | THE1B | MaLR | LTR |
| 152099999 | 152100308 | + | 23.6 | 6753 | 7041 | -920 | L1MC5 | L1 | LINE |
| 152100523 | 152100653 | - | 20.6 | 1 | 131 | -2 | FLAM\_A | Alu | SINE |
| 152100658 | 152101027 | + | 24.5 | 7064 | 7443 | -518 | L1MC5 | L1 | LINE |

  

|  |  |  |  |  |  |  |  |  |  |
| --- | --- | --- | --- | --- | --- | --- | --- | --- | --- |
| chr4:154150629-154153339 | | | 2709 bp | | |  |  |  |  |
| Genome Start | Genome End | Strand | Div | Repeat Start | Repeat End | Repeat Left | Name | Family | Class |
| 154150629 | 154150779 | - | 22.1 | 168 | 2415 | -5 | MER58 | MER1\_type | DNA |
| 154150782 | 154151177 | - | 14.2 | 1 | 428 | 0 | MSTA | MaLR | LTR |
| 154151178 | 154152785 | - | 15.8 | 1 | 1651 | 0 | MSTA-int | MaLR | LTR |
| 154152786 | 154153166 | - | 16.0 | 1 | 428 | 0 | MSTA | MaLR | LTR |
| 154153167 | 154153339 | - | 21.0 | 1 | 170 | -171 | MER58 | MER1\_type | DNA |

  

|  |  |  |  |  |  |  |  |  |  |
| --- | --- | --- | --- | --- | --- | --- | --- | --- | --- |
| chr4:154804013-154809063 | | | 4901 bp | | |  |  |  |  |
| Genome Start | Genome End | Strand | Div | Repeat Start | Repeat End | Repeat Left | Name | Family | Class |
| 154804013 | 154804302 | - | 35.9 | 2789 | 3079 | -340 | L2 | L2 | LINE |
| 154804310 | 154804513 | + | 16.6 | 8 | 235 | -89 | MER33 | MER1\_type | DNA |
| 154804527 | 154804821 | + | 16.0 | 1 | 296 | -16 | AluJo | Alu | SINE |
| 154804843 | 154804914 | + | 22.4 | 223 | 285 | -39 | MER33 | MER1\_type | DNA |
| 154804915 | 154805211 | + | 11.2 | 11 | 305 | -7 | AluSx | Alu | SINE |
| 154805212 | 154805252 | + | 22.4 | 286 | 323 | -1 | MER33 | MER1\_type | DNA |
| 154805253 | 154805406 | - | 37.5 | 2641 | 2797 | -622 | L2 | L2 | LINE |
| 154805497 | 154805856 | + | 9.7 | 1 | 355 | 0 | THE1A | MaLR | LTR |
| 154805859 | 154807399 | + | 28.4 | 2 | 1735 | 0 | MLT-int | MaLR | LTR |
| 154807400 | 154807754 | + | 11.1 | 1 | 355 | 0 | THE1A | MaLR | LTR |
| 154807772 | 154808077 | - | 31.1 | 2094 | 2465 | -954 | L2 | L2 | LINE |
| 154808078 | 154808392 | + | 11.1 | 1 | 309 | -3 | AluSx | Alu | SINE |
| 154808393 | 154809063 | - | 31.1 | 1374 | 2093 | -1326 | L2 | L2 | LINE |

  

|  |  |  |  |  |  |  |  |  |  |
| --- | --- | --- | --- | --- | --- | --- | --- | --- | --- |
| chr4:155019749-155024127 | | | 4399 bp | | |  |  |  |  |
| Genome Start | Genome End | Strand | Div | Repeat Start | Repeat End | Repeat Left | Name | Family | Class |
| 155019749 | 155020444 | + | 24.4 | 3564 | 4308 | -1359 | ERVL-F | ERVL | LTR |
| 155020445 | 155020818 | + | 15.4 | 1 | 381 | 0 | THE1D | MaLR | LTR |
| 155020821 | 155022433 | + | 30.1 | 2 | 1735 | 0 | MLT-int | MaLR | LTR |
| 155022434 | 155022810 | + | 14.0 | 1 | 381 | 0 | THE1D | MaLR | LTR |
| 155022811 | 155023487 | + | 25.3 | 4309 | 5032 | -635 | ERVL-F | ERVL | LTR |
| 155023466 | 155023674 | + | 23.9 | 5168 | 5401 | -266 | ERVL-F | ERVL | LTR |
| 155023675 | 155023907 | - | 16.7 | 1 | 289 | -13 | AluJb | Alu | SINE |
| 155023908 | 155024127 | + | 23.9 | 5402 | 5634 | -33 | ERVL-F | ERVL | LTR |

  

|  |  |  |  |  |  |  |  |  |  |
| --- | --- | --- | --- | --- | --- | --- | --- | --- | --- |
| chr4:155113856-155125734 | | | 11722 bp | | |  |  |  |  |
| Genome Start | Genome End | Strand | Div | Repeat Start | Repeat End | Repeat Left | Name | Family | Class |
| 155113856 | 155114073 | - | 26.4 | 2249 | 2494 | -13 | HAL1 | L1 | LINE |
| 155114092 | 155114468 | + | 14.8 | 1 | 428 | 0 | MSTA | MaLR | LTR |
| 155114471 | 155116124 | + | 28.2 | 2 | 1735 | 0 | MLT-int | MaLR | LTR |
| 155116125 | 155116386 | + | 14.8 | 1 | 279 | -149 | MSTA | MaLR | LTR |
| 155116387 | 155116730 | + | 15.9 | 1 | 364 | 0 | THE1B | MaLR | LTR |
| 155116731 | 155116871 | + | 14.8 | 280 | 428 | 0 | MSTA | MaLR | LTR |
| 155116880 | 155118212 | - | 25.8 | 814 | 2221 | -286 | HAL1 | L1 | LINE |
| 155118326 | 155118636 | - | 17.0 | 495 | 833 | -61 | MER97a | MER1\_type? | DNA |
| 155118638 | 155124825 | - | 5.0 | 2 | 6197 | 0 | L1PA4 | L1 | LINE |
| 155124828 | 155125299 | - | 25.9 | 3 | 503 | -587 | MER97c | MER1\_type? | DNA |
| 155125313 | 155125734 | - | 29.4 | 279 | 738 | -1769 | HAL1 | L1 | LINE |

  

|  |  |  |  |  |  |  |  |  |  |
| --- | --- | --- | --- | --- | --- | --- | --- | --- | --- |
| chr4:155987152-155991519 | | | 3570 bp | | |  |  |  |  |
| Genome Start | Genome End | Strand | Div | Repeat Start | Repeat End | Repeat Left | Name | Family | Class |
| 155987152 | 155987279 | - | 37.0 | 3228 | 3375 | -3 | L2 | L2 | LINE |
| 155987292 | 155987613 | + | 15.8 | 1 | 364 | 0 | THE1B | MaLR | LTR |
| 155987615 | 155988697 | + | 9.9 | 2 | 1093 | -487 | THE1B-int | MaLR | LTR |
| 155988698 | 155989260 | - | 7.6 | 1 | 580 | 0 | LTR19B | ERV1 | LTR |
| 155989261 | 155989742 | + | 9.9 | 1094 | 1580 | 0 | THE1B-int | MaLR | LTR |
| 155989743 | 155990085 | + | 11.8 | 1 | 364 | 0 | THE1B | MaLR | LTR |
| 155990471 | 155990577 | - | 36.5 | 3264 | 3378 | 0 | L2 | L2 | LINE |
| 155990978 | 155991519 | - | 36.6 | 2573 | 3116 | -262 | L2 | L2 | LINE |

  

|  |  |  |  |  |  |  |  |  |  |
| --- | --- | --- | --- | --- | --- | --- | --- | --- | --- |
| chr4:156293429-156301685 | | | 7989 bp | | |  |  |  |  |
| Genome Start | Genome End | Strand | Div | Repeat Start | Repeat End | Repeat Left | Name | Family | Class |
| 156293429 | 156293642 | + | 25.1 | 115 | 321 | -4550 | MER65A-int | ERV1 | LTR |
| 156293688 | 156294299 | + | 25.6 | 434 | 1075 | -3796 | MER65A-int | ERV1 | LTR |
| 156294303 | 156295868 | + | 19.1 | 1035 | 2829 | -3673 | MER65A-int | ERV1 | LTR |
| 156296057 | 156296655 | + | 19.9 | 2774 | 3377 | -3262 | MER65A-int | ERV1 | LTR |
| 156296656 | 156297076 | + | 14.8 | 1 | 426 | 0 | MSTB | MaLR | LTR |
| 156297079 | 156298716 | + | 29.1 | 2 | 1735 | 0 | MLT-int | MaLR | LTR |
| 156298717 | 156299131 | + | 17.4 | 1 | 426 | 0 | MSTB | MaLR | LTR |
| 156299132 | 156299526 | + | 19.9 | 3378 | 3785 | -2854 | MER65A-int | ERV1 | LTR |
| 156299523 | 156300207 | + | 21.8 | 3075 | 5018 | -1219 | MER65A-int | ERV1 | LTR |
| 156300225 | 156300342 | + | 19.0 | 261 | 376 | -56 | MSTB1 | MaLR | LTR |
| 156300345 | 156300450 | + | 17.1 | 4224 | 4340 | -2299 | MER65A-int | ERV1 | LTR |
| 156300484 | 156300873 | + | 19.4 | 3603 | 4615 | -977 | MER65A-int | ERV1 | LTR |
| 156300856 | 156301685 | + | 23.4 | 3907 | 4871 | 0 | MER65A-int | ERV1 | LTR |

  

|  |  |  |  |  |  |  |  |  |  |
| --- | --- | --- | --- | --- | --- | --- | --- | --- | --- |
| chr4:157073432-157076399 | | | 2946 bp | | |  |  |  |  |
| Genome Start | Genome End | Strand | Div | Repeat Start | Repeat End | Repeat Left | Name | Family | Class |
| 157073432 | 157073679 | + | 31.5 | 2456 | 2698 | -721 | L2 | L2 | LINE |
| 157073691 | 157074040 | + | 6.6 | 1 | 353 | -2 | THE1A | MaLR | LTR |
| 157074043 | 157075582 | + | 29.6 | 2 | 1735 | 0 | MLT-int | MaLR | LTR |
| 157075583 | 157075935 | + | 7.1 | 1 | 355 | 0 | THE1A | MaLR | LTR |
| 157075945 | 157076399 | + | 28.3 | 2939 | 3419 | 0 | L2 | L2 | LINE |

  

|  |  |  |  |  |  |  |  |  |  |
| --- | --- | --- | --- | --- | --- | --- | --- | --- | --- |
| chr4:158675026-158686339 | | | 11174 bp | | |  |  |  |  |
| Genome Start | Genome End | Strand | Div | Repeat Start | Repeat End | Repeat Left | Name | Family | Class |
| 158675026 | 158676173 | - | 19.8 | 6546 | 7715 | -4 | L1MA4 | L1 | LINE |
| 158676174 | 158676597 | + | 15.4 | 1 | 428 | 0 | MSTA | MaLR | LTR |
| 158676600 | 158676952 | + | 17.2 | 3 | 348 | -1232 | MSTA-int | MaLR | LTR |
| 158676953 | 158677097 | - | 15.2 | 93 | 229 | -1 | MER30 | MER1\_type | DNA |
| 158677098 | 158677386 | - | 13.2 | 1 | 289 | -20 | AluSc | Alu | SINE |
| 158677387 | 158677479 | - | 15.2 | 1 | 92 | -138 | MER30 | MER1\_type | DNA |
| 158677480 | 158677612 | + | 17.2 | 349 | 440 | -1140 | MSTA-int | MaLR | LTR |
| 158677613 | 158677966 | - | 13.9 | 1 | 364 | 0 | THE1B | MaLR | LTR |
| 158677967 | 158678783 | + | 17.2 | 441 | 1277 | -303 | MSTA-int | MaLR | LTR |
| 158678791 | 158679145 | - | 15.6 | 1 | 355 | 0 | THE1A | MaLR | LTR |
| 158679148 | 158680714 | - | 11.0 | 2 | 1575 | -5 | THE1A-int | MaLR | LTR |
| 158680716 | 158681035 | - | 15.6 | 38 | 355 | 0 | THE1A | MaLR | LTR |
| 158681037 | 158681139 | - | 15.0 | 1 | 101 | -263 | THE1B | MaLR | LTR |
| 158681144 | 158681422 | + | 15.5 | 1282 | 1561 | -19 | MSTA-int | MaLR | LTR |
| 158681452 | 158681784 | + | 15.9 | 56 | 428 | 0 | MSTA | MaLR | LTR |
| 158681785 | 158682757 | - | 17.7 | 5591 | 6550 | -1012 | L1MA4 | L1 | LINE |
| 158682758 | 158683065 | - | 14.3 | 1 | 312 | -1 | AluSq | Alu | SINE |
| 158683066 | 158684063 | - | 17.7 | 4565 | 5590 | -1972 | L1MA4 | L1 | LINE |
| 158684077 | 158684468 | - | 17.4 | 5781 | 6152 | -4 | L1PB4 | L1 | LINE |
| 158684545 | 158684835 | - | 12.9 | 5504 | 5792 | -364 | L1PB4 | L1 | LINE |
| 158684841 | 158686339 | - | 21.1 | 3075 | 4576 | -2986 | L1MA4 | L1 | LINE |

  

|  |  |  |  |  |  |  |  |  |  |
| --- | --- | --- | --- | --- | --- | --- | --- | --- | --- |
| chr4:158817750-158820391 | | | 2639 bp | | |  |  |  |  |
| Genome Start | Genome End | Strand | Div | Repeat Start | Repeat End | Repeat Left | Name | Family | Class |
| 158817750 | 158818080 | - | 14.1 | 73 | 426 | -2 | MSTA | MaLR | LTR |
| 158818081 | 158818417 | - | 13.3 | 1 | 364 | 0 | THE1B | MaLR | LTR |
| 158818418 | 158819963 | - | 28.9 | 3 | 1735 | 0 | MLT1J2-int | MaLR | LTR |
| 158819967 | 158820317 | - | 11.9 | 1 | 364 | 0 | THE1B | MaLR | LTR |
| 158820318 | 158820391 | - | 14.1 | 1 | 72 | -356 | MSTA | MaLR | LTR |

  

|  |  |  |  |  |  |  |  |  |  |
| --- | --- | --- | --- | --- | --- | --- | --- | --- | --- |
| chr4:158863193-158865977 | | | 2782 bp | | |  |  |  |  |
| Genome Start | Genome End | Strand | Div | Repeat Start | Repeat End | Repeat Left | Name | Family | Class |
| 158863193 | 158863485 | + | 18.8 | 1 | 305 | -251 | MLT2B4 | ERVL | LTR |
| 158863487 | 158863848 | + | 13.3 | 2 | 364 | 0 | THE1B | MaLR | LTR |
| 158863849 | 158865429 | + | 12.0 | 1 | 1580 | 0 | THE1B-int | MaLR | LTR |
| 158865432 | 158865787 | + | 14.4 | 3 | 364 | 0 | THE1B | MaLR | LTR |
| 158865788 | 158865977 | + | 21.8 | 301 | 490 | -66 | MLT2B4 | ERVL | LTR |

  

|  |  |  |  |  |  |  |  |  |  |
| --- | --- | --- | --- | --- | --- | --- | --- | --- | --- |
| chr4:160799285-160807506 | | | 8205 bp | | |  |  |  |  |
| Genome Start | Genome End | Strand | Div | Repeat Start | Repeat End | Repeat Left | Name | Family | Class |
| 160799285 | 160799437 | - | 19.8 | 2957 | 3126 | -3411 | L1MDa | L1 | LINE |
| 160799438 | 160800018 | + | 17.7 | 1 | 554 | 0 | MLT2B3 | ERVL | LTR |
| 160800019 | 160800109 | - | 19.8 | 2866 | 2956 | -3581 | L1MDa | L1 | LINE |
| 160800110 | 160800825 | - | 14.8 | 5547 | 6288 | -3 | L1MA8 | L1 | LINE |
| 160800826 | 160801118 | - | 19.8 | 2640 | 2865 | -3672 | L1MDa | L1 | LINE |
| 160801119 | 160801716 | - | 13.8 | 5551 | 6143 | -17 | L1PREC2 | L1 | LINE |
| 160801717 | 160801838 | - | 19.8 | 2513 | 2639 | -3898 | L1MDa | L1 | LINE |
| 160801839 | 160801864 | + | 13.4 | 1 | 26 | -349 | THE1C | MaLR | LTR |
| 160801865 | 160802216 | - | 13.9 | 2 | 364 | 0 | THE1B | MaLR | LTR |
| 160802217 | 160802573 | + | 13.4 | 27 | 375 | 0 | THE1C | MaLR | LTR |
| 160802574 | 160803318 | - | 19.6 | 1756 | 2516 | -4117 | L1MDa | L1 | LINE |
| 160803324 | 160803687 | - | 13.9 | 1 | 374 | -7 | THE1D | MaLR | LTR |
| 160803688 | 160804528 | - | 30.4 | 878 | 1735 | 0 | MLT-int | MaLR | LTR |
| 160804529 | 160804828 | - | 13.0 | 3 | 301 | -9 | AluSg | Alu | SINE |
| 160804829 | 160805631 | - | 30.4 | 2 | 877 | -858 | MLT-int | MaLR | LTR |
| 160805634 | 160806010 | - | 13.2 | 1 | 381 | 0 | THE1D | MaLR | LTR |
| 160806021 | 160807506 | - | 25.0 | 3 | 1753 | -4880 | L1MDa | L1 | LINE |

  

|  |  |  |  |  |  |  |  |  |  |
| --- | --- | --- | --- | --- | --- | --- | --- | --- | --- |
| chr4:161182401-161191426 | | | 8689 bp | | |  |  |  |  |
| Genome Start | Genome End | Strand | Div | Repeat Start | Repeat End | Repeat Left | Name | Family | Class |
| 161182401 | 161182596 | + | 15.3 | 3 | 200 | -6663 | L1PB1 | L1 | LINE |
| 161182577 | 161182782 | + | 20.2 | 620 | 812 | -6051 | L1PB1 | L1 | LINE |
| 161182783 | 161183122 | - | 7.7 | 1 | 355 | 0 | THE1A | MaLR | LTR |
| 161183123 | 161184696 | - | 10.2 | 1 | 1580 | 0 | THE1A-int | MaLR | LTR |
| 161184697 | 161185045 | - | 9.7 | 1 | 355 | 0 | THE1A | MaLR | LTR |
| 161185046 | 161187816 | + | 12.4 | 808 | 3738 | -3124 | L1PB1 | L1 | LINE |
| 161187817 | 161188032 | + | 14.3 | 86 | 306 | -6 | AluSg/x | Alu | SINE |
| 161188033 | 161188652 | + | 12.4 | 3739 | 4393 | -2469 | L1PB1 | L1 | LINE |
| 161188679 | 161190753 | + | 10.0 | 4397 | 6507 | -360 | L1PB1 | L1 | LINE |
| 161191085 | 161191426 | + | 7.9 | 6523 | 6867 | 0 | L1PB1 | L1 | LINE |

  

|  |  |  |  |  |  |  |  |  |  |
| --- | --- | --- | --- | --- | --- | --- | --- | --- | --- |
| chr4:161224254-161231395 | | | 7066 bp | | |  |  |  |  |
| Genome Start | Genome End | Strand | Div | Repeat Start | Repeat End | Repeat Left | Name | Family | Class |
| 161224254 | 161224848 | - | 31.9 | 2693 | 3419 | 0 | L2 | L2 | LINE |
| 161224858 | 161225091 | - | 20.1 | 6082 | 6306 | -6 | L1MA9 | L1 | LINE |
| 161225092 | 161225455 | - | 21.1 | 5 | 404 | -4 | MLT1A1 | MaLR | LTR |
| 161225456 | 161225657 | - | 18.9 | 5794 | 6081 | -231 | L1MA9 | L1 | LINE |
| 161225697 | 161225893 | - | 16.5 | 5588 | 5793 | -519 | L1MA9 | L1 | LINE |
| 161225894 | 161226266 | - | 15.5 | 1 | 364 | 0 | THE1B | MaLR | LTR |
| 161226267 | 161227861 | - | 30.9 | 2 | 1735 | 0 | MLT-int | MaLR | LTR |
| 161227864 | 161228235 | - | 15.7 | 1 | 364 | 0 | THE1B | MaLR | LTR |
| 161228236 | 161229117 | - | 17.4 | 4668 | 5587 | -725 | L1MA9 | L1 | LINE |
| 161229119 | 161229339 | + | 16.3 | 2120 | 2329 | -3811 | L1M3 | L1 | LINE |
| 161229338 | 161229677 | + | 20.9 | 2643 | 2986 | -3154 | L1M3 | L1 | LINE |
| 161229681 | 161230196 | - | 29.2 | 2178 | 2701 | -718 | L2 | L2 | LINE |
| 161230197 | 161230569 | + | 16.7 | 1 | 364 | 0 | THE1B | MaLR | LTR |
| 161230570 | 161230640 | - | 29.2 | 2105 | 2177 | -1242 | L2 | L2 | LINE |
| 161230641 | 161230943 | + | 12.2 | 1 | 308 | -4 | AluSx | Alu | SINE |
| 161230944 | 161231278 | - | 29.2 | 1766 | 2104 | -1315 | L2 | L2 | LINE |
| 161231303 | 161231395 | - | 27.0 | 1427 | 1515 | -1904 | L2 | L2 | LINE |

  

|  |  |  |  |  |  |  |  |  |  |
| --- | --- | --- | --- | --- | --- | --- | --- | --- | --- |
| chr4:162444045-162447425 | | | 3166 bp | | |  |  |  |  |
| Genome Start | Genome End | Strand | Div | Repeat Start | Repeat End | Repeat Left | Name | Family | Class |
| 162444045 | 162444196 | + | 14.0 | 1 | 165 | -457 | LTR29 | ERV1 | LTR |
| 162444197 | 162444548 | + | 12.5 | 1 | 364 | 0 | THE1B | MaLR | LTR |
| 162444553 | 162446127 | + | 29.8 | 4 | 1735 | 0 | MLT1J1-int | MaLR | LTR |
| 162446128 | 162446486 | + | 11.0 | 1 | 364 | 0 | THE1B | MaLR | LTR |
| 162446487 | 162446630 | + | 14.0 | 166 | 311 | -311 | LTR29 | ERV1 | LTR |
| 162446842 | 162447175 | - | 12.8 | 1 | 381 | 0 | THE1D | MaLR | LTR |
| 162447176 | 162447425 | + | 17.5 | 310 | 556 | -66 | LTR29 | ERV1 | LTR |

  

|  |  |  |  |  |  |  |  |  |  |
| --- | --- | --- | --- | --- | --- | --- | --- | --- | --- |
| chr4:162803022-162807049 | | | 3797 bp | | |  |  |  |  |
| Genome Start | Genome End | Strand | Div | Repeat Start | Repeat End | Repeat Left | Name | Family | Class |
| 162803022 | 162803211 | + | 31.9 | 1589 | 1905 | -5104 | L1M5 | L1 | LINE |
| 162803244 | 162803410 | + | 30.0 | 1890 | 2050 | -4958 | L1M5 | L1 | LINE |
| 162803411 | 162803773 | - | 9.6 | 1 | 355 | 0 | THE1A | MaLR | LTR |
| 162803774 | 162805339 | - | 30.4 | 2 | 1735 | 0 | MLT-int | MaLR | LTR |
| 162805342 | 162805694 | - | 9.1 | 1 | 355 | 0 | THE1A | MaLR | LTR |
| 162805728 | 162805787 | + | 10.0 | 6093 | 6152 | -13 | L1PA15 | L1 | LINE |
| 162805816 | 162805840 | + | 31.9 | 2227 | 2249 | -4777 | L1M5 | L1 | LINE |
| 162805841 | 162806134 | - | 7.1 | 1 | 302 | -9 | AluY | Alu | SINE |
| 162806135 | 162806469 | + | 31.9 | 2250 | 2589 | -4437 | L1M5 | L1 | LINE |
| 162806560 | 162806903 | + | 29.5 | 5903 | 6249 | -757 | L1M5 | L1 | LINE |
| 162806950 | 162807049 | + | 38.0 | 6314 | 6413 | -687 | L1M5 | L1 | LINE |

  

|  |  |  |  |  |  |  |  |  |  |
| --- | --- | --- | --- | --- | --- | --- | --- | --- | --- |
| chr4:163371423-163375278 | | | 3606 bp | | |  |  |  |  |
| Genome Start | Genome End | Strand | Div | Repeat Start | Repeat End | Repeat Left | Name | Family | Class |
| 163371423 | 163371863 | + | 33.2 | 711 | 1170 | -3826 | ERVL-E | ERVL | LTR |
| 163371968 | 163372247 | - | 14.6 | 1 | 381 | 0 | THE1D | MaLR | LTR |
| 163372248 | 163373883 | - | 30.8 | 2 | 1735 | 0 | MLT-int | MaLR | LTR |
| 163373886 | 163374237 | - | 15.4 | 7 | 381 | 0 | THE1D | MaLR | LTR |
| 163374397 | 163375025 | + | 38.6 | 4056 | 4695 | -972 | ERVL-E | ERVL | LTR |
| 163375011 | 163375278 | + | 38.7 | 4798 | 5066 | -691 | ERVL-E | ERVL | LTR |

  

|  |  |  |  |  |  |  |  |  |  |
| --- | --- | --- | --- | --- | --- | --- | --- | --- | --- |
| chr4:163629567-163636443 | | | 6584 bp | | |  |  |  |  |
| Genome Start | Genome End | Strand | Div | Repeat Start | Repeat End | Repeat Left | Name | Family | Class |
| 163629567 | 163629790 | + | 20.0 | 4950 | 5173 | -967 | L1MA9 | L1 | LINE |
| 163629791 | 163630161 | + | 12.1 | 1 | 375 | 0 | THE1C | MaLR | LTR |
| 163630164 | 163631769 | + | 29.7 | 2 | 1735 | 0 | MLT-int | MaLR | LTR |
| 163631770 | 163632145 | + | 10.1 | 1 | 375 | 0 | THE1C | MaLR | LTR |
| 163632146 | 163632763 | + | 18.0 | 5174 | 5775 | -537 | L1MA9 | L1 | LINE |
| 163632796 | 163633150 | + | 11.8 | 1 | 364 | 0 | THE1B | MaLR | LTR |
| 163633151 | 163634571 | + | 13.9 | 1 | 1414 | -166 | THE1B-int | MaLR | LTR |
| 163634596 | 163634751 | + | 13.9 | 1415 | 1570 | -10 | THE1B-int | MaLR | LTR |
| 163634761 | 163634982 | + | 12.8 | 1 | 226 | -138 | THE1B | MaLR | LTR |
| 163634983 | 163635289 | + | 10.7 | 1 | 299 | -12 | AluY | Alu | SINE |
| 163635290 | 163635429 | + | 12.8 | 227 | 364 | 0 | THE1B | MaLR | LTR |
| 163635556 | 163635926 | + | 19.5 | 5776 | 6151 | -161 | L1MA9 | L1 | LINE |
| 163635982 | 163636036 | + | 19.5 | 6152 | 6219 | -93 | L1MA9 | L1 | LINE |
| 163636082 | 163636371 | - | 18.6 | 1 | 293 | -19 | AluJb | Alu | SINE |
| 163636372 | 163636443 | + | 18.2 | 6210 | 6281 | -31 | L1MA9 | L1 | LINE |

  

|  |  |  |  |  |  |  |  |  |  |
| --- | --- | --- | --- | --- | --- | --- | --- | --- | --- |
| chr4:163746693-163750397 | | | 3631 bp | | |  |  |  |  |
| Genome Start | Genome End | Strand | Div | Repeat Start | Repeat End | Repeat Left | Name | Family | Class |
| 163746693 | 163747217 | + | 18.3 | 1 | 534 | -369 | MER4D | ERV1 | LTR |
| 163747228 | 163747648 | + | 12.4 | 7 | 428 | 0 | MSTA | MaLR | LTR |
| 163747649 | 163749271 | + | 19.0 | 1 | 1651 | 0 | MSTA-int | MaLR | LTR |
| 163749272 | 163749643 | + | 16.9 | 1 | 398 | -30 | MSTA | MaLR | LTR |
| 163749708 | 163750010 | - | 15.2 | 1 | 303 | -9 | AluSx | Alu | SINE |
| 163750011 | 163750049 | + | 12.8 | 390 | 428 | 0 | MSTA | MaLR | LTR |
| 163750050 | 163750397 | + | 19.0 | 535 | 895 | -8 | MER4D | ERV1 | LTR |

  

|  |  |  |  |  |  |  |  |  |  |
| --- | --- | --- | --- | --- | --- | --- | --- | --- | --- |
| chr4:164453269-164460942 | | | 7677 bp | | |  |  |  |  |
| Genome Start | Genome End | Strand | Div | Repeat Start | Repeat End | Repeat Left | Name | Family | Class |
| 164453269 | 164454424 | - | 18.0 | 5402 | 6504 | -15 | L1MC2 | L1 | LINE |
| 164454428 | 164454973 | - | 8.6 | 5608 | 6156 | -12 | L1PA10 | L1 | LINE |
| 164454974 | 164455276 | + | 9.6 | 5319 | 5621 | -510 | L1PA10 | L1 | LINE |
| 164455265 | 164455325 | - | 18.5 | 4140 | 4196 | -2125 | L1MC2 | L1 | LINE |
| 164455326 | 164455605 | - | 16.8 | 5874 | 6158 | -8 | L1PA16 | L1 | LINE |
| 164455606 | 164456521 | - | 18.1 | 2053 | 4139 | -2182 | L1MC2 | L1 | LINE |
| 164456522 | 164456932 | - | 15.3 | 5 | 428 | 0 | MSTA | MaLR | LTR |
| 164456937 | 164458554 | - | 27.9 | 2 | 1735 | 0 | MLT-int | MaLR | LTR |
| 164458557 | 164458975 | - | 16.6 | 1 | 428 | 0 | MSTA | MaLR | LTR |
| 164458976 | 164460942 | - | 17.6 | 4 | 2052 | -4456 | L1MC2 | L1 | LINE |

  

|  |  |  |  |  |  |  |  |  |  |
| --- | --- | --- | --- | --- | --- | --- | --- | --- | --- |
| chr4:165590918-165602413 | | | 11424 bp | | |  |  |  |  |
| Genome Start | Genome End | Strand | Div | Repeat Start | Repeat End | Repeat Left | Name | Family | Class |
| 165590918 | 165591190 | + | 23.2 | 5011 | 5288 | -858 | L1ME3A | L1 | LINE |
| 165591191 | 165591538 | + | 21.9 | 1 | 365 | 0 | MLT1A0 | MaLR | LTR |
| 165591539 | 165591724 | + | 23.9 | 5288 | 5485 | -755 | L1ME3A | L1 | LINE |
| 165591726 | 165592036 | + | 15.7 | 1 | 305 | -7 | AluJb | Alu | SINE |
| 165592076 | 165598870 | + | 19.3 | 11 | 7002 | 0 | L1MA5 | L1 | LINE |
| 165598898 | 165599262 | + | 12.4 | 1 | 364 | 0 | THE1B | MaLR | LTR |
| 165599263 | 165599458 | + | 10.2 | 1 | 212 | -1368 | THE1B-int | MaLR | LTR |
| 165599459 | 165599514 | + | 10.7 | 153 | 208 | -1372 | THE1B-int | MaLR | LTR |
| 165599517 | 165599576 | + | 8.3 | 151 | 210 | -1370 | THE1B-int | MaLR | LTR |
| 165599577 | 165601105 | + | 12.6 | 151 | 1580 | 0 | THE1B-int | MaLR | LTR |
| 165601108 | 165601478 | + | 10.3 | 3 | 364 | 0 | THE1B | MaLR | LTR |
| 165601480 | 165601696 | + | 25.6 | 5494 | 5721 | -449 | L1ME3A | L1 | LINE |
| 165601697 | 165602028 | + | 13.2 | 2 | 236 | 0 | MER46B | MER2\_type | DNA |
| 165602029 | 165602413 | + | 27.2 | 5722 | 6126 | -44 | L1ME3A | L1 | LINE |

  

|  |  |  |  |  |  |  |  |  |  |
| --- | --- | --- | --- | --- | --- | --- | --- | --- | --- |
| chr4:166423053-166425859 | | | 2805 bp | | |  |  |  |  |
| Genome Start | Genome End | Strand | Div | Repeat Start | Repeat End | Repeat Left | Name | Family | Class |
| 166423053 | 166423425 | + | 36.3 | 3920 | 4309 | -180 | L3 | CR1 | LINE |
| 166423426 | 166423789 | - | 12.6 | 1 | 364 | 0 | THE1B | MaLR | LTR |
| 166423790 | 166425334 | - | 30.7 | 2 | 1735 | 0 | MLT1H1-int | MaLR | LTR |
| 166425337 | 166425699 | - | 13.0 | 1 | 364 | 0 | THE1B | MaLR | LTR |
| 166425700 | 166425859 | + | 36.3 | 4310 | 4486 | -3 | L3 | CR1 | LINE |

  

|  |  |  |  |  |  |  |  |  |  |
| --- | --- | --- | --- | --- | --- | --- | --- | --- | --- |
| chr4:167661183-167667227 | | | 6053 bp | | |  |  |  |  |
| Genome Start | Genome End | Strand | Div | Repeat Start | Repeat End | Repeat Left | Name | Family | Class |
| 167661183 | 167661301 | + | 8.4 | 1 | 120 | -192 | AluSq | Alu | SINE |
| 167661302 | 167661693 | + | 9.0 | 1 | 441 | -9 | LTR7 | ERV1 | LTR |
| 167661703 | 167664544 | + | 9.7 | 1 | 2995 | -4718 | HERVH | ERV1 | LTR |
| 167664524 | 167665374 | + | 7.7 | 3140 | 3992 | -3721 | HERVH | ERV1 | LTR |
| 167665378 | 167666040 | + | 14.2 | 4488 | 5149 | -2564 | HERVH | ERV1 | LTR |
| 167666042 | 167666255 | + | 13.1 | 5600 | 5819 | -1894 | HERVH | ERV1 | LTR |
| 167666254 | 167666631 | + | 10.5 | 7322 | 7713 | 0 | HERVH | ERV1 | LTR |
| 167666632 | 167667032 | + | 8.0 | 1 | 448 | -2 | LTR7 | ERV1 | LTR |
| 167667035 | 167667227 | + | 14.0 | 115 | 308 | -5 | AluSq | Alu | SINE |

  

|  |  |  |  |  |  |  |  |  |  |
| --- | --- | --- | --- | --- | --- | --- | --- | --- | --- |
| chr4:168657070-168665495 | | | 8427 bp | | |  |  |  |  |
| Genome Start | Genome End | Strand | Div | Repeat Start | Repeat End | Repeat Left | Name | Family | Class |
| 168657070 | 168657472 | - | 11.0 | 5750 | 6159 | -4 | L1PA13 | L1 | LINE |
| 168657473 | 168657826 | - | 13.1 | 1 | 355 | 0 | THE1A | MaLR | LTR |
| 168657827 | 168659389 | - | 31.5 | 2 | 1735 | 0 | MLT1E2-int | MaLR | LTR |
| 168659392 | 168659739 | - | 12.5 | 1 | 355 | 0 | THE1A | MaLR | LTR |
| 168659740 | 168660838 | - | 12.3 | 4621 | 5749 | -414 | L1P4 | L1 | LINE |
| 168660836 | 168661660 | - | 13.7 | 3780 | 4604 | -2162 | L1PA13 | L1 | LINE |
| 168661661 | 168662009 | + | 13.8 | 2 | 362 | -2 | THE1B | MaLR | LTR |
| 168662010 | 168665495 | - | 17.8 | 5 | 3779 | -2987 | L1PA13 | L1 | LINE |

  

|  |  |  |  |  |  |  |  |  |  |
| --- | --- | --- | --- | --- | --- | --- | --- | --- | --- |
| chr4:169031869-169039923 | | | 8064 bp | | |  |  |  |  |
| Genome Start | Genome End | Strand | Div | Repeat Start | Repeat End | Repeat Left | Name | Family | Class |
| 169031869 | 169034036 | - | 21.5 | 195 | 2414 | -6 | Cheshire | MER1\_type | DNA |
| 169034038 | 169034435 | + | 9.6 | 1 | 448 | -2 | LTR7 | ERV1 | LTR |
| 169034438 | 169037253 | + | 9.5 | 1 | 2995 | -4718 | HERVH | ERV1 | LTR |
| 169037233 | 169038086 | + | 8.6 | 3140 | 3992 | -3721 | HERVH | ERV1 | LTR |
| 169038090 | 169038756 | + | 14.2 | 4488 | 5149 | -2564 | HERVH | ERV1 | LTR |
| 169038758 | 169038972 | + | 12.6 | 5600 | 5819 | -1894 | HERVH | ERV1 | LTR |
| 169038973 | 169039349 | + | 10.5 | 7324 | 7713 | 0 | HERVH | ERV1 | LTR |
| 169039352 | 169039747 | + | 7.8 | 3 | 448 | -2 | LTR7 | ERV1 | LTR |
| 169039751 | 169039923 | - | 21.5 | 5 | 197 | -2223 | Cheshire | MER1\_type | DNA |

  

|  |  |  |  |  |  |  |  |  |  |
| --- | --- | --- | --- | --- | --- | --- | --- | --- | --- |
| chr4:169109021-169112223 | | | 3201 bp | | |  |  |  |  |
| Genome Start | Genome End | Strand | Div | Repeat Start | Repeat End | Repeat Left | Name | Family | Class |
| 169109021 | 169109122 | + | 22.8 | 6 | 100 | -553 | MER82 | MER2\_type | DNA |
| 169109123 | 169109680 | - | 14.7 | 5608 | 6163 | -2 | L1PA15 | L1 | LINE |
| 169109681 | 169110044 | + | 14.1 | 1 | 364 | 0 | THE1B | MaLR | LTR |
| 169110047 | 169111659 | + | 30.1 | 2 | 1735 | 0 | MLT-int | MaLR | LTR |
| 169111660 | 169112022 | + | 14.3 | 1 | 364 | 0 | THE1B | MaLR | LTR |
| 169112023 | 169112119 | - | 14.7 | 5523 | 5607 | -558 | L1P4 | L1 | LINE |
| 169112120 | 169112223 | + | 22.8 | 101 | 197 | -456 | MER82 | MER2\_type | DNA |

  

|  |  |  |  |  |  |  |  |  |  |
| --- | --- | --- | --- | --- | --- | --- | --- | --- | --- |
| chr4:169204060-169210113 | | | 5964 bp | | |  |  |  |  |
| Genome Start | Genome End | Strand | Div | Repeat Start | Repeat End | Repeat Left | Name | Family | Class |
| 169204060 | 169204263 | - | 23.1 | 5940 | 6127 | -57 | L1MB7 | L1 | LINE |
| 169204264 | 169205202 | - | 16.7 | 1 | 983 | 0 | LTR1D | ERV1 | LTR |
| 169205203 | 169205221 | - | 23.1 | 5915 | 5939 | -245 | L1MB7 | L1 | LINE |
| 169205222 | 169205576 | + | 6.5 | 1 | 355 | 0 | THE1A | MaLR | LTR |
| 169205579 | 169205849 | + | 29.2 | 2 | 337 | -1398 | MLT-int | MaLR | LTR |
| 169205850 | 169206147 | - | 10.4 | 1 | 298 | -13 | AluY | Alu | SINE |
| 169206148 | 169207445 | + | 29.2 | 338 | 1735 | 0 | MLT-int | MaLR | LTR |
| 169207446 | 169207801 | + | 8.8 | 1 | 355 | 0 | THE1A | MaLR | LTR |
| 169207802 | 169207995 | - | 23.1 | 5706 | 5914 | -270 | L1MB7 | L1 | LINE |
| 169208084 | 169210113 | - | 26.2 | 3546 | 5702 | -482 | L1MB7 | L1 | LINE |

  

|  |  |  |  |  |  |  |  |  |  |
| --- | --- | --- | --- | --- | --- | --- | --- | --- | --- |
| chr4:171910458-171915023 | | | 4566 bp | | |  |  |  |  |
| Genome Start | Genome End | Strand | Div | Repeat Start | Repeat End | Repeat Left | Name | Family | Class |
| 171910458 | 171911059 | - | 12.3 | 2747 | 3346 | -3503 | L1PA15-16 | L1 | LINE |
| 171911060 | 171911434 | + | 10.6 | 1 | 375 | 0 | THE1C | MaLR | LTR |
| 171911437 | 171912986 | + | 30.6 | 2 | 1735 | 0 | MLT1C-int | MaLR | LTR |
| 171912987 | 171913363 | + | 14.8 | 1 | 375 | 0 | THE1C | MaLR | LTR |
| 171913364 | 171914615 | - | 13.4 | 1462 | 2746 | -4107 | L1PA15-16 | L1 | LINE |
| 171914614 | 171915023 | - | 16.4 | 1 | 460 | -6393 | L1PA15-16 | L1 | LINE |

  

|  |  |  |  |  |  |  |  |  |  |
| --- | --- | --- | --- | --- | --- | --- | --- | --- | --- |
| chr4:171972648-171975476 | | | 2757 bp | | |  |  |  |  |
| Genome Start | Genome End | Strand | Div | Repeat Start | Repeat End | Repeat Left | Name | Family | Class |
| 171972648 | 171972718 | + | 27.5 | 1 | 69 | -585 | MLT1E3 | MaLR | LTR |
| 171972766 | 171973118 | + | 12.5 | 1 | 364 | 0 | THE1B | MaLR | LTR |
| 171973119 | 171974653 | + | 12.0 | 1 | 1580 | 0 | THE1B-int | MaLR | LTR |
| 171974654 | 171975011 | + | 12.7 | 1 | 364 | 0 | THE1B | MaLR | LTR |
| 171975037 | 171975476 | + | 24.3 | 139 | 565 | -59 | MLT1E3 | MaLR | LTR |

  

|  |  |  |  |  |  |  |  |  |  |
| --- | --- | --- | --- | --- | --- | --- | --- | --- | --- |
| chr4:172050068-172052883 | | | 2813 bp | | |  |  |  |  |
| Genome Start | Genome End | Strand | Div | Repeat Start | Repeat End | Repeat Left | Name | Family | Class |
| 172050068 | 172050222 | + | 28.3 | 3 | 156 | -1744 | Kanga1 | Tc2 | DNA |
| 172050223 | 172050565 | - | 18.0 | 1 | 364 | 0 | THE1B | MaLR | LTR |
| 172050566 | 172052145 | - | 29.9 | 2 | 1735 | 0 | MLT1H1-int | MaLR | LTR |
| 172052149 | 172052498 | - | 14.9 | 1 | 363 | -1 | THE1B | MaLR | LTR |
| 172052499 | 172052883 | + | 28.0 | 1158 | 1567 | -178 | Kanga1 | Tc2 | DNA |

  

|  |  |  |  |  |  |  |  |  |  |
| --- | --- | --- | --- | --- | --- | --- | --- | --- | --- |
| chr4:173886010-173896195 | | | 10173 bp | | |  |  |  |  |
| Genome Start | Genome End | Strand | Div | Repeat Start | Repeat End | Repeat Left | Name | Family | Class |
| 173886010 | 173888524 | - | 17.4 | 3773 | 6175 | -298 | L1MA8 | L1 | LINE |
| 173888525 | 173888822 | + | 10.1 | 1 | 297 | -13 | AluSg | Alu | SINE |
| 173888823 | 173891126 | - | 19.7 | 1435 | 3772 | -2553 | L1MA8 | L1 | LINE |
| 173891129 | 173891193 | + | 10.8 | 1 | 65 | -310 | THE1C | MaLR | LTR |
| 173891202 | 173891565 | + | 12.1 | 1 | 364 | 0 | THE1B | MaLR | LTR |
| 173891568 | 173893372 | + | 15.2 | 3 | 1651 | 0 | THE1B-int | MaLR | LTR |
| 173893373 | 173893484 | + | 14.0 | 1 | 113 | -251 | THE1B | MaLR | LTR |
| 173893485 | 173894558 | - | 12.4 | 1 | 1236 | 0 | MER11B | ERVK | LTR |
| 173894559 | 173894810 | + | 14.0 | 114 | 363 | -1 | THE1B | MaLR | LTR |
| 173894812 | 173896195 | - | 19.5 | 13 | 1437 | -4891 | L1MA8 | L1 | LINE |

  

|  |  |  |  |  |  |  |  |  |  |
| --- | --- | --- | --- | --- | --- | --- | --- | --- | --- |
| chr4:174145243-174150351 | | | 5109 bp | | |  |  |  |  |
| Genome Start | Genome End | Strand | Div | Repeat Start | Repeat End | Repeat Left | Name | Family | Class |
| 174145243 | 174145306 | - | 15.2 | 5980 | 6043 | -875 | HERV35I | ERV1 | LTR |
| 174145307 | 174145722 | + | 13.7 | 1 | 428 | 0 | MSTA | MaLR | LTR |
| 174145723 | 174147199 | + | 18.6 | 1 | 1651 | 0 | MSTA-int | MaLR | LTR |
| 174147200 | 174147583 | + | 13.6 | 1 | 428 | 0 | MSTA | MaLR | LTR |
| 174147584 | 174149114 | - | 15.2 | 4498 | 5984 | -934 | HERV35I | ERV1 | LTR |
| 174149115 | 174149433 | - | 14.2 | 1 | 310 | 0 | AluSx | Alu | SINE |
| 174149434 | 174150351 | - | 15.2 | 3562 | 4497 | -2421 | HERV35I | ERV1 | LTR |

  

|  |  |  |  |  |  |  |  |  |  |
| --- | --- | --- | --- | --- | --- | --- | --- | --- | --- |
| chr4:175215041-175220425 | | | 5350 bp | | |  |  |  |  |
| Genome Start | Genome End | Strand | Div | Repeat Start | Repeat End | Repeat Left | Name | Family | Class |
| 175215041 | 175215587 | + | 20.3 | 2233 | 2833 | -3313 | L1MB7 | L1 | LINE |
| 175215583 | 175217243 | + | 18.6 | 4074 | 5955 | -223 | L1MB7 | L1 | LINE |
| 175217246 | 175217335 | + | 16.8 | 1 | 92 | -423 | MLT2B3 | ERVL | LTR |
| 175217336 | 175217762 | - | 18.9 | 1 | 426 | 0 | MSTB | MaLR | LTR |
| 175217763 | 175219382 | - | 17.5 | 15 | 1651 | 0 | MSTB-int | MaLR | LTR |
| 175219414 | 175219837 | - | 17.1 | 1 | 426 | 0 | MSTB | MaLR | LTR |
| 175219839 | 175220291 | + | 24.4 | 90 | 554 | 0 | MLT2B3 | ERVL | LTR |
| 175220298 | 175220425 | + | 21.4 | 5961 | 6086 | -98 | L1MB7 | L1 | LINE |

  

|  |  |  |  |  |  |  |  |  |  |
| --- | --- | --- | --- | --- | --- | --- | --- | --- | --- |
| chr4:176837118-176843814 | | | 6399 bp | | |  |  |  |  |
| Genome Start | Genome End | Strand | Div | Repeat Start | Repeat End | Repeat Left | Name | Family | Class |
| 176837118 | 176839650 | - | 28.4 | 5240 | 7717 | -91 | L1MC4a | L1 | LINE |
| 176839651 | 176840008 | + | 17.0 | 5 | 424 | -4 | MSTA | MaLR | LTR |
| 176840015 | 176841648 | + | 29.0 | 2 | 1734 | -1 | MLT-int | MaLR | LTR |
| 176841650 | 176841980 | + | 18.3 | 1 | 365 | -63 | MSTA | MaLR | LTR |
| 176841981 | 176842273 | - | 11.3 | 1 | 293 | -17 | AluSg | Alu | SINE |
| 176842274 | 176842347 | + | 18.3 | 366 | 428 | 0 | MSTA | MaLR | LTR |
| 176842348 | 176842425 | - | 27.9 | 5169 | 5239 | -907 | L1PREC2 | L1 | LINE |
| 176842717 | 176843021 | - | 31.9 | 4662 | 4958 | -1188 | L1MC4a | L1 | LINE |
| 176843022 | 176843333 | - | 6.8 | 1 | 311 | 0 | AluY | Alu | SINE |
| 176843334 | 176843814 | - | 31.9 | 4160 | 4661 | -1485 | L1MC4a | L1 | LINE |

  

|  |  |  |  |  |  |  |  |  |  |
| --- | --- | --- | --- | --- | --- | --- | --- | --- | --- |
| chr4:177500130-177517942 | | | 17510 bp | | |  |  |  |  |
| Genome Start | Genome End | Strand | Div | Repeat Start | Repeat End | Repeat Left | Name | Family | Class |
| 177500130 | 177500210 | - | 19.2 | 2182 | 2263 | -3886 | L1MEe | L1 | LINE |
| 177500455 | 177500495 | + | 17.1 | 141 | 185 | -91 | MIRm | MIR | SINE |
| 177500467 | 177500524 | + | 14.0 | 3299 | 3358 | -61 | L2 | L2 | LINE |
| 177500526 | 177501884 | - | 17.9 | 349 | 1754 | -1 | MER52A | ERV1 | LTR |
| 177501885 | 177502391 | + | 12.1 | 1 | 511 | 0 | MER9 | ERVK | LTR |
| 177502392 | 177503167 | + | 8.6 | 1 | 783 | -5133 | HERVK9 | ERVK | LTR |
| 177503168 | 177503222 | + | 24.0 | 60 | 109 | -69 | MER5B | MER1\_type | DNA |
| 177503223 | 177508027 | + | 8.6 | 784 | 5632 | -284 | HERVK9 | ERVK | LTR |
| 177508053 | 177508402 | + | 10.7 | 5635 | 8032 | 0 | HERVK9 | ERVK | LTR |
| 177508403 | 177508914 | + | 10.0 | 1 | 511 | 0 | MER9 | ERVK | LTR |
| 177508915 | 177509126 | - | 20.3 | 1 | 197 | -1558 | MER52A | ERV1 | LTR |
| 177509142 | 177509260 | - | 23.9 | 1765 | 1888 | -4261 | L1MEe | L1 | LINE |
| 177509266 | 177509736 | - | 12.1 | 1 | 472 | 0 | MER4A1 | ERV1 | LTR |
| 177509741 | 177512507 | - | 11.5 | 3815 | 6596 | -2 | MER4A1-int | ERV1 | LTR |
| 177512506 | 177512768 | - | 12.6 | 3433 | 3676 | -2922 | MER4A1-int | ERV1 | LTR |
| 177512769 | 177513071 | + | 15.2 | 1 | 303 | -9 | AluSx | Alu | SINE |
| 177513072 | 177515436 | - | 12.6 | 983 | 3432 | -3166 | MER4A1-int | ERV1 | LTR |
| 177515438 | 177515959 | - | 13.1 | 1 | 547 | -7 | MER41A | ERV1 | LTR |
| 177515961 | 177516907 | - | 14.3 | 1 | 992 | -5606 | MER4A1-int | ERV1 | LTR |
| 177516911 | 177517385 | - | 10.0 | 1 | 472 | 0 | MER4A1 | ERV1 | LTR |
| 177517410 | 177517550 | - | 22.5 | 1581 | 1745 | -4404 | L1MEe | L1 | LINE |
| 177517562 | 177517942 | - | 33.3 | 557 | 939 | -5180 | L1MEe | L1 | LINE |

  

|  |  |  |  |  |  |  |  |  |  |
| --- | --- | --- | --- | --- | --- | --- | --- | --- | --- |
| chr4:179316472-179322309 | | | 5517 bp | | |  |  |  |  |
| Genome Start | Genome End | Strand | Div | Repeat Start | Repeat End | Repeat Left | Name | Family | Class |
| 179316472 | 179316803 | + | 34.4 | 656 | 986 | -5084 | L1MEb | L1 | LINE |
| 179316845 | 179317484 | + | 30.5 | 1065 | 1871 | -4390 | L1MEb | L1 | LINE |
| 179317680 | 179318507 | + | 27.2 | 4795 | 5611 | -553 | L1M5 | L1 | LINE |
| 179318520 | 179318883 | - | 12.4 | 1 | 363 | -1 | THE1B | MaLR | LTR |
| 179318884 | 179320323 | - | 12.7 | 2 | 1580 | 0 | THE1B-int | MaLR | LTR |
| 179320325 | 179320654 | - | 16.7 | 5 | 364 | 0 | THE1B | MaLR | LTR |
| 179320655 | 179320766 | + | 24.1 | 5620 | 5733 | -433 | L1M5 | L1 | LINE |
| 179320764 | 179321559 | + | 30.2 | 2252 | 3000 | -3080 | L1MEb | L1 | LINE |
| 179321579 | 179321701 | + | 31.1 | 2977 | 3100 | -2980 | L1MEb | L1 | LINE |
| 179321758 | 179322309 | + | 29.6 | 4010 | 4609 | -1471 | L1MEb | L1 | LINE |

  

|  |  |  |  |  |  |  |  |  |  |
| --- | --- | --- | --- | --- | --- | --- | --- | --- | --- |
| chr4:179663173-179667716 | | | 4306 bp | | |  |  |  |  |
| Genome Start | Genome End | Strand | Div | Repeat Start | Repeat End | Repeat Left | Name | Family | Class |
| 179663173 | 179663361 | + | 24.3 | 2335 | 2546 | -3600 | L1MB8 | L1 | LINE |
| 179663363 | 179663415 | - | 19.2 | 414 | 467 | 0 | MLT1C | MaLR | LTR |
| 179663464 | 179663677 | - | 21.4 | 9 | 240 | -227 | MLT1C | MaLR | LTR |
| 179663694 | 179665109 | - | 24.8 | 1 | 1734 | -1 | MLT1C-int | MaLR | LTR |
| 179665114 | 179665416 | - | 25.3 | 162 | 463 | -4 | MLT1C | MaLR | LTR |
| 179665425 | 179666090 | + | 20.4 | 3316 | 4167 | -1979 | L1MB8 | L1 | LINE |
| 179666149 | 179666632 | + | 28.1 | 41 | 560 | -47 | MER77 | ERVL | LTR |
| 179666730 | 179667089 | + | 23.0 | 5025 | 5381 | -765 | L1MB8 | L1 | LINE |
| 179667096 | 179667716 | + | 20.2 | 5554 | 6172 | -6 | L1MB8 | L1 | LINE |

  

|  |  |  |  |  |  |  |  |  |  |
| --- | --- | --- | --- | --- | --- | --- | --- | --- | --- |
| chr4:179810335-179813180 | | | 2844 bp | | |  |  |  |  |
| Genome Start | Genome End | Strand | Div | Repeat Start | Repeat End | Repeat Left | Name | Family | Class |
| 179810335 | 179810477 | + | 15.7 | 1014 | 1149 | -431 | MSTA-int | MaLR | LTR |
| 179810478 | 179810831 | + | 14.8 | 1 | 355 | 0 | THE1A | MaLR | LTR |
| 179810834 | 179812421 | + | 30.7 | 2 | 1735 | 0 | MLT-int | MaLR | LTR |
| 179812422 | 179812776 | + | 14.1 | 1 | 353 | -2 | THE1A | MaLR | LTR |
| 179812777 | 179813180 | + | 15.7 | 1150 | 1548 | -32 | MSTA-int | MaLR | LTR |

  

|  |  |  |  |  |  |  |  |  |  |
| --- | --- | --- | --- | --- | --- | --- | --- | --- | --- |
| chr4:180613904-180618690 | | | 4319 bp | | |  |  |  |  |
| Genome Start | Genome End | Strand | Div | Repeat Start | Repeat End | Repeat Left | Name | Family | Class |
| 180613904 | 180614087 | + | 28.6 | 2217 | 2416 | -3967 | L1M4c | L1 | LINE |
| 180614465 | 180615885 | + | 3.2 | 4779 | 6199 | 0 | L1PA3 | L1 | LINE |
| 180615895 | 180616099 | + | 28.1 | 3965 | 4175 | -2216 | L1M4c | L1 | LINE |
| 180616100 | 180616442 | + | 13.3 | 1 | 363 | -1 | THE1B | MaLR | LTR |
| 180616444 | 180618010 | + | 9.7 | 1 | 1580 | 0 | THE1B-int | MaLR | LTR |
| 180618011 | 180618353 | + | 11.5 | 1 | 364 | 0 | THE1B | MaLR | LTR |
| 180618435 | 180618690 | + | 31.8 | 4275 | 4555 | -1827 | L1M4c | L1 | LINE |

  

|  |  |  |  |  |  |  |  |  |  |
| --- | --- | --- | --- | --- | --- | --- | --- | --- | --- |
| chr4:180878249-180885189 | | | 6937 bp | | |  |  |  |  |
| Genome Start | Genome End | Strand | Div | Repeat Start | Repeat End | Repeat Left | Name | Family | Class |
| 180878249 | 180878671 | - | 13.0 | 167 | 612 | 0 | LTR9 | ERV1 | LTR |
| 180878672 | 180879080 | + | 11.1 | 1 | 448 | -2 | LTR7 | ERV1 | LTR |
| 180879083 | 180884044 | + | 13.9 | 1 | 5149 | -2564 | HERVH | ERV1 | LTR |
| 180884046 | 180884258 | + | 11.4 | 5600 | 5819 | -1894 | HERVH | ERV1 | LTR |
| 180884259 | 180884632 | + | 11.1 | 7324 | 7713 | 0 | HERVH | ERV1 | LTR |
| 180884633 | 180885043 | + | 7.8 | 1 | 448 | -2 | LTR7 | ERV1 | LTR |
| 180885045 | 180885189 | - | 5.5 | 1 | 172 | -440 | LTR9 | ERV1 | LTR |

  

|  |  |  |  |  |  |  |  |  |  |
| --- | --- | --- | --- | --- | --- | --- | --- | --- | --- |
| chr4:185736842-185740476 | | | 3633 bp | | |  |  |  |  |
| Genome Start | Genome End | Strand | Div | Repeat Start | Repeat End | Repeat Left | Name | Family | Class |
| 185736842 | 185737320 | + | 26.7 | 4655 | 5191 | -955 | L1M5 | L1 | LINE |
| 185737321 | 185737627 | + | 9.7 | 1 | 311 | -1 | AluSx | Alu | SINE |
| 185737628 | 185737909 | + | 26.1 | 5192 | 5496 | -744 | L1M5 | L1 | LINE |
| 185737910 | 185738261 | - | 9.1 | 1 | 355 | 0 | THE1A | MaLR | LTR |
| 185738262 | 185739830 | - | 29.6 | 2 | 1735 | 0 | MLT-int | MaLR | LTR |
| 185739833 | 185740186 | - | 8.2 | 1 | 355 | 0 | THE1A | MaLR | LTR |
| 185740187 | 185740476 | + | 25.7 | 5497 | 5776 | -464 | L1M5 | L1 | LINE |

  

|  |  |  |  |  |  |  |  |  |  |
| --- | --- | --- | --- | --- | --- | --- | --- | --- | --- |
| chr4:187202105-187208062 | | | 5970 bp | | |  |  |  |  |
| Genome Start | Genome End | Strand | Div | Repeat Start | Repeat End | Repeat Left | Name | Family | Class |
| 187202105 | 187202167 | + | 6.3 | 1 | 63 | -255 | AluSg | Alu | SINE |
| 187202171 | 187202572 | - | 7.5 | 1 | 448 | -2 | LTR7 | ERV1 | LTR |
| 187202573 | 187202948 | - | 10.0 | 7324 | 7713 | 0 | HERVH | ERV1 | LTR |
| 187202949 | 187203164 | - | 13.9 | 5600 | 5819 | -1894 | HERVH | ERV1 | LTR |
| 187203166 | 187203828 | - | 12.9 | 4488 | 5149 | -2564 | HERVH | ERV1 | LTR |
| 187203831 | 187204685 | - | 12.0 | 3140 | 3993 | -3720 | HERVH | ERV1 | LTR |
| 187204665 | 187207420 | - | 10.7 | 1 | 2995 | -4718 | HERVH | ERV1 | LTR |
| 187207423 | 187207820 | - | 8.8 | 1 | 448 | -2 | LTR7 | ERV1 | LTR |
| 187207822 | 187208062 | + | 10.0 | 61 | 301 | -9 | AluSg | Alu | SINE |

  

|  |  |  |  |  |  |  |  |  |  |
| --- | --- | --- | --- | --- | --- | --- | --- | --- | --- |
| chr4:187378707-187385523 | | | 6812 bp | | |  |  |  |  |
| Genome Start | Genome End | Strand | Div | Repeat Start | Repeat End | Repeat Left | Name | Family | Class |
| 187378707 | 187381017 | - | 18.7 | 2933 | 5325 | -2234 | L1M3c | L1 | LINE |
| 187381018 | 187381386 | + | 13.8 | 1 | 381 | 0 | THE1D | MaLR | LTR |
| 187381390 | 187382976 | + | 30.4 | 3 | 1735 | 0 | MLT-int | MaLR | LTR |
| 187382977 | 187383335 | + | 13.7 | 1 | 381 | 0 | THE1D | MaLR | LTR |
| 187383336 | 187383567 | - | 27.0 | 2692 | 2932 | -4633 | L1M3c | L1 | LINE |
| 187383568 | 187383881 | - | 9.5 | 1 | 304 | -7 | AluY | Alu | SINE |
| 187383882 | 187384717 | - | 25.5 | 2025 | 2691 | -4874 | L1M3c | L1 | LINE |
| 187384720 | 187385523 | - | 26.2 | 473 | 1600 | -6234 | L1M3c | L1 | LINE |

  

|  |  |  |  |  |  |  |  |  |  |
| --- | --- | --- | --- | --- | --- | --- | --- | --- | --- |
| chr4:187400023-187405819 | | | 5785 bp | | |  |  |  |  |
| Genome Start | Genome End | Strand | Div | Repeat Start | Repeat End | Repeat Left | Name | Family | Class |
| 187400023 | 187400086 | + | 35.9 | 3636 | 3700 | -789 | L3 | CR1 | LINE |
| 187400104 | 187400470 | - | 7.7 | 1 | 450 | 0 | LTR7 | ERV1 | LTR |
| 187400471 | 187400903 | - | 10.5 | 7283 | 7713 | 0 | HERVH | ERV1 | LTR |
| 187400908 | 187402148 | - | 13.6 | 3809 | 5020 | -2287 | HERVH | ERV1 | LTR |
| 187402140 | 187405364 | - | 12.4 | 1 | 3601 | -4112 | HERVH | ERV1 | LTR |
| 187405365 | 187405732 | - | 8.5 | 1 | 450 | 0 | LTR7 | ERV1 | LTR |
| 187405733 | 187405819 | + | 23.0 | 3716 | 3827 | -662 | L3 | CR1 | LINE |

  

|  |  |  |  |  |  |  |  |  |  |
| --- | --- | --- | --- | --- | --- | --- | --- | --- | --- |
| chr4:188143618-188148856 | | | 4932 bp | | |  |  |  |  |
| Genome Start | Genome End | Strand | Div | Repeat Start | Repeat End | Repeat Left | Name | Family | Class |
| 188143618 | 188144139 | - | 30.1 | 5316 | 5835 | -1973 | L1MC4a | L1 | LINE |
| 188144279 | 188144323 | - | 13.3 | 6288 | 6333 | -1 | L1MA10 | L1 | LINE |
| 188144324 | 188144544 | - | 22.6 | 3 | 227 | -85 | AluJo | Alu | SINE |
| 188144548 | 188144636 | - | 20.3 | 6205 | 6292 | -42 | L1MA10 | L1 | LINE |
| 188144637 | 188144988 | - | 10.5 | 5 | 364 | 0 | THE1B | MaLR | LTR |
| 188144993 | 188145527 | - | 29.9 | 1154 | 1735 | 0 | MLT-int | MaLR | LTR |
| 188145619 | 188145703 | - | 29.9 | 1154 | 1153 | -582 | MLT-int | MaLR | LTR |
| 188145704 | 188146010 | - | 14.7 | 1 | 306 | -7 | AluSq | Alu | SINE |
| 188146011 | 188147060 | - | 29.9 | 2 | 1153 | -582 | MLT-int | MaLR | LTR |
| 188147063 | 188147416 | - | 13.6 | 1 | 364 | 0 | THE1B | MaLR | LTR |
| 188147417 | 188147873 | - | 20.3 | 5749 | 6204 | -130 | L1MA10 | L1 | LINE |
| 188147942 | 188148856 | - | 33.9 | 4143 | 5114 | -1032 | L1MC4a | L1 | LINE |

  

|  |  |  |  |  |  |  |  |  |  |
| --- | --- | --- | --- | --- | --- | --- | --- | --- | --- |
| chr4:190470260-190476177 | | | 5349 bp | | |  |  |  |  |
| Genome Start | Genome End | Strand | Div | Repeat Start | Repeat End | Repeat Left | Name | Family | Class |
| 190470260 | 190471213 | + | 20.6 | 4020 | 4965 | -1181 | L1MB8 | L1 | LINE |
| 190471214 | 190471619 | - | 17.4 | 1 | 426 | 0 | MSTB | MaLR | LTR |
| 190471620 | 190472557 | - | 22.1 | 710 | 1651 | 0 | MSTB-int | MaLR | LTR |
| 190473031 | 190473613 | - | 18.3 | 160 | 764 | -887 | MSTB-int | MaLR | LTR |
| 190473614 | 190474123 | + | 13.3 | 1 | 512 | -2 | MER75 | PiggyBac | DNA |
| 190474124 | 190474303 | - | 18.3 | 1 | 159 | -1492 | MSTB-int | MaLR | LTR |
| 190474304 | 190474735 | - | 19.3 | 1 | 426 | 0 | MSTB | MaLR | LTR |
| 190474736 | 190475400 | + | 21.6 | 4961 | 5644 | -540 | L1MB8 | L1 | LINE |
| 190475402 | 190475460 | - | 22.0 | 2347 | 2405 | 0 | Tigger5 | MER2\_type | DNA |
| 190475555 | 190475644 | - | 26.7 | 2 | 92 | -5 | Tigger5c | MER2\_type | DNA |
| 190475646 | 190476177 | + | 22.9 | 5643 | 6167 | -11 | L1MB8 | L1 | LINE |

  

|  |  |  |  |  |  |  |  |  |  |
| --- | --- | --- | --- | --- | --- | --- | --- | --- | --- |
| chr4:190680006-190689823 | | | 9813 bp | | |  |  |  |  |
| Genome Start | Genome End | Strand | Div | Repeat Start | Repeat End | Repeat Left | Name | Family | Class |
| 190680006 | 190682603 | - | 16.6 | 4246 | 6863 | -7 | L1PB3 | L1 | LINE |
| 190682604 | 190682974 | + | 12.4 | 1 | 375 | 0 | THE1C | MaLR | LTR |
| 190682977 | 190684557 | + | 29.1 | 2 | 1735 | 0 | MLT-int | MaLR | LTR |
| 190684561 | 190684908 | + | 18.1 | 4 | 375 | 0 | THE1C | MaLR | LTR |
| 190684909 | 190685906 | - | 17.4 | 3257 | 4245 | -2617 | L1PB3 | L1 | LINE |
| 190685907 | 190686274 | - | 15.6 | 1 | 381 | 0 | THE1D | MaLR | LTR |
| 190686275 | 190689823 | - | 21.2 | 547 | 3256 | -3606 | L1PB3 | L1 | LINE |

  

|  |  |  |  |  |  |  |  |  |  |
| --- | --- | --- | --- | --- | --- | --- | --- | --- | --- |
| chr4:190910399-190912878 | | | 2478 bp | | |  |  |  |  |
| Genome Start | Genome End | Strand | Div | Repeat Start | Repeat End | Repeat Left | Name | Family | Class |
| 190910399 | 190910428 | + | 21.6 | 2 | 32 | -187 | MER20 | MER1\_type | DNA |
| 190910429 | 190910779 | + | 18.8 | 2 | 355 | 0 | THE1A | MaLR | LTR |
| 190910782 | 190912350 | + | 30.6 | 2 | 1735 | 0 | MLT-int | MaLR | LTR |
| 190912351 | 190912697 | + | 15.8 | 1 | 353 | -2 | THE1A | MaLR | LTR |
| 190912698 | 190912878 | + | 21.6 | 33 | 214 | -5 | MER20 | MER1\_type | DNA |

  

|  |  |  |  |  |  |  |  |  |  |
| --- | --- | --- | --- | --- | --- | --- | --- | --- | --- |
| chr4:191045369-191050546 | | | 5151 bp | | |  |  |  |  |
| Genome Start | Genome End | Strand | Div | Repeat Start | Repeat End | Repeat Left | Name | Family | Class |
| 191045369 | 191045456 | + | 14.8 | 7 | 94 | -1954 | Ricksha | MuDR | DNA |
| 191045480 | 191045840 | - | 14.6 | 1 | 375 | 0 | THE1C | MaLR | LTR |
| 191045841 | 191047360 | - | 13.1 | 1 | 1580 | 0 | THE1C-int | MaLR | LTR |
| 191047361 | 191047737 | - | 7.6 | 1 | 375 | 0 | THE1C | MaLR | LTR |
| 191047739 | 191047847 | - | 19.3 | 3 | 111 | -32 | FLAM\_C | Alu | SINE |
| 191047849 | 191048175 | + | 14.4 | 79 | 1688 | -1547 | Ricksha | MuDR | DNA |
| 191048178 | 191050069 | + | 14.4 | 1190 | 3123 | -123 | Ricksha | MuDR | DNA |
| 191050070 | 191050364 | - | 13.2 | 1 | 295 | -16 | AluY | Alu | SINE |
| 191050365 | 191050546 | + | 14.4 | 3124 | 3246 | 0 | Ricksha | MuDR | DNA |

  


|  |  |  |  |  |  |  |  |  |  |
| --- | --- | --- | --- | --- | --- | --- | --- | --- | --- |
| chr5:755370-763041 | | | 7674 bp | | |  |  |  |  |
| Genome Start | Genome End | Strand | Div | Repeat Start | Repeat End | Repeat Left | Name | Family | Class |
| 755370 | 755585 | + | 15.7 | 1 | 220 | -1431 | MSTB-int | MaLR | LTR |
| 755586 | 756014 | + | 4.7 | 1 | 462 | -2 | LTR7B | ERV1 | LTR |
| 756017 | 759303 | + | 11.4 | 1 | 2987 | -4726 | HERVH | ERV1 | LTR |
| 759298 | 759445 | + | 18.2 | 3140 | 3290 | -4423 | HERVH | ERV1 | LTR |
| 759445 | 759936 | + | 11.6 | 3500 | 3993 | -3720 | HERVH | ERV1 | LTR |
| 759939 | 760593 | + | 14.0 | 4488 | 5149 | -2564 | HERVH | ERV1 | LTR |
| 760595 | 761189 | + | 10.4 | 5600 | 7713 | 0 | HERVH | ERV1 | LTR |
| 761190 | 761616 | + | 5.4 | 1 | 462 | -2 | LTR7B | ERV1 | LTR |
| 761617 | 763041 | + | 18.3 | 214 | 1651 | 0 | MSTB-int | MaLR | LTR |

  

|  |  |  |  |  |  |  |  |  |  |
| --- | --- | --- | --- | --- | --- | --- | --- | --- | --- |
| chr5:2124847-2127437 | | | 2585 bp | | |  |  |  |  |
| Genome Start | Genome End | Strand | Div | Repeat Start | Repeat End | Repeat Left | Name | Family | Class |
| 2124847 | 2124895 | + | 25.1 | 65 | 113 | -149 | MIR | MIR | SINE |
| 2124896 | 2125327 | + | 16.6 | 1 | 426 | 0 | MSTB | MaLR | LTR |
| 2125330 | 2126936 | + | 26.9 | 2 | 1731 | -4 | MLT1H-int | MaLR | LTR |
| 2126941 | 2127314 | + | 13.4 | 1 | 426 | 0 | MSTB | MaLR | LTR |
| 2127315 | 2127437 | + | 25.1 | 114 | 243 | -19 | MIR | MIR | SINE |

  

|  |  |  |  |  |  |  |  |  |  |
| --- | --- | --- | --- | --- | --- | --- | --- | --- | --- |
| chr5:4446405-4449739 | | | 3326 bp | | |  |  |  |  |
| Genome Start | Genome End | Strand | Div | Repeat Start | Repeat End | Repeat Left | Name | Family | Class |
| 4446405 | 4447114 | + | 18.5 | 5449 | 6160 | -145 | L1MA3 | L1 | LINE |
| 4447117 | 4447144 | + | 16.5 | 1 | 27 | -401 | MSTA | MaLR | LTR |
| 4447145 | 4447495 | - | 14.4 | 1 | 364 | 0 | THE1B | MaLR | LTR |
| 4447496 | 4449075 | - | 31.4 | 3 | 1735 | 0 | MLT-int | MaLR | LTR |
| 4449079 | 4449431 | - | 13.4 | 4 | 364 | 0 | THE1B | MaLR | LTR |
| 4449432 | 4449587 | + | 16.5 | 28 | 200 | -228 | MSTA | MaLR | LTR |
| 4449592 | 4449739 | + | 20.9 | 6158 | 6305 | 0 | L1MA3 | L1 | LINE |

  

|  |  |  |  |  |  |  |  |  |  |
| --- | --- | --- | --- | --- | --- | --- | --- | --- | --- |
| chr5:4801194-4811689 | | | 9842 bp | | |  |  |  |  |
| Genome Start | Genome End | Strand | Div | Repeat Start | Repeat End | Repeat Left | Name | Family | Class |
| 4801194 | 4801368 | - | 28.8 | 2385 | 2557 | -3662 | L1MEc | L1 | LINE |
| 4801369 | 4801649 | + | 10.0 | 1 | 298 | -15 | AluSq | Alu | SINE |
| 4801650 | 4801710 | - | 24.8 | 2327 | 2465 | -3935 | L1MEc | L1 | LINE |
| 4801711 | 4801820 | - | 16.0 | 6202 | 6308 | -4 | L1MA9 | L1 | LINE |
| 4801821 | 4802456 | - | 23.0 | 1765 | 2428 | -3972 | L1MEc | L1 | LINE |
| 4802539 | 4802652 | - | 10.5 | 1 | 114 | -241 | THE1A | MaLR | LTR |
| 4802694 | 4803021 | - | 8.5 | 1 | 331 | -24 | THE1A | MaLR | LTR |
| 4803022 | 4804105 | - | 29.1 | 600 | 1735 | 0 | MLT-int | MaLR | LTR |
| 4804131 | 4804609 | - | 29.1 | 2 | 599 | -1136 | MLT-int | MaLR | LTR |
| 4804612 | 4804963 | - | 9.9 | 1 | 355 | 0 | THE1A | MaLR | LTR |
| 4805018 | 4805599 | - | 23.5 | 1005 | 1593 | -4807 | L1MEc | L1 | LINE |
| 4805630 | 4806070 | - | 23.8 | 7205 | 7738 | -47 | L1MC3 | L1 | LINE |
| 4806101 | 4806250 | - | 20.7 | 6945 | 7104 | -681 | L1MC3 | L1 | LINE |
| 4806254 | 4806300 | + | 10.6 | 6264 | 6311 | -1 | L1MA9 | L1 | LINE |
| 4806324 | 4806393 | - | 15.9 | 6877 | 6939 | -846 | L1MC3 | L1 | LINE |
| 4806439 | 4806906 | + | 23.4 | 17 | 505 | 0 | MLT1D | MaLR | LTR |
| 4806908 | 4807309 | - | 22.4 | 6414 | 6814 | -971 | L1MC3 | L1 | LINE |
| 4807353 | 4808897 | - | 22.3 | 4807 | 6413 | -1372 | L1MC3 | L1 | LINE |
| 4809066 | 4810614 | - | 24.1 | 2378 | 4186 | -2033 | L1MEc | L1 | LINE |
| 4810695 | 4811076 | - | 26.8 | 12 | 429 | -5616 | L1MCb | L1 | LINE |
| 4811104 | 4811689 | - | 27.3 | 357 | 967 | -5433 | L1MEc | L1 | LINE |

  

|  |  |  |  |  |  |  |  |  |  |
| --- | --- | --- | --- | --- | --- | --- | --- | --- | --- |
| chr5:5674784-5678310 | | | 3129 bp | | |  |  |  |  |
| Genome Start | Genome End | Strand | Div | Repeat Start | Repeat End | Repeat Left | Name | Family | Class |
| 5674784 | 5675203 | - | 33.8 | 993 | 1441 | -31 | MLT1H-int | MaLR | LTR |
| 5675353 | 5675774 | + | 19.3 | 2 | 423 | -3 | MSTB | MaLR | LTR |
| 5675781 | 5677407 | + | 28.9 | 2 | 1735 | 0 | MLT1G-int | MaLR | LTR |
| 5677410 | 5677597 | + | 16.0 | 3 | 191 | -235 | MSTB | MaLR | LTR |
| 5677701 | 5678048 | - | 18.5 | 6 | 505 | -9 | MER31B | ERV1 | LTR |
| 5678187 | 5678310 | - | 23.0 | 388 | 510 | -962 | MLT1H-int | MaLR | LTR |

  

|  |  |  |  |  |  |  |  |  |  |
| --- | --- | --- | --- | --- | --- | --- | --- | --- | --- |
| chr5:7315918-7322683 | | | 6734 bp | | |  |  |  |  |
| Genome Start | Genome End | Strand | Div | Repeat Start | Repeat End | Repeat Left | Name | Family | Class |
| 7315918 | 7316692 | + | 25.6 | 2315 | 3119 | -3091 | L1M3c | L1 | LINE |
| 7316693 | 7317141 | + | 6.9 | 1 | 448 | -2 | LTR7 | ERV1 | LTR |
| 7317144 | 7320101 | + | 8.9 | 1 | 2987 | -4726 | HERVH | ERV1 | LTR |
| 7320096 | 7320242 | + | 12.9 | 3140 | 3290 | -4423 | HERVH | ERV1 | LTR |
| 7320233 | 7320736 | + | 11.4 | 3492 | 3993 | -3720 | HERVH | ERV1 | LTR |
| 7320739 | 7321401 | + | 14.8 | 4488 | 5149 | -2564 | HERVH | ERV1 | LTR |
| 7321403 | 7321618 | + | 14.8 | 5600 | 5819 | -1894 | HERVH | ERV1 | LTR |
| 7321619 | 7321996 | + | 8.3 | 7324 | 7713 | 0 | HERVH | ERV1 | LTR |
| 7321997 | 7322444 | + | 4.0 | 1 | 448 | -2 | LTR7 | ERV1 | LTR |
| 7322488 | 7322683 | + | 28.0 | 3129 | 3332 | -2878 | L1M3c | L1 | LINE |

  

|  |  |  |  |  |  |  |  |  |  |
| --- | --- | --- | --- | --- | --- | --- | --- | --- | --- |
| chr5:8393099-8401979 | | | 8876 bp | | |  |  |  |  |
| Genome Start | Genome End | Strand | Div | Repeat Start | Repeat End | Repeat Left | Name | Family | Class |
| 8393099 | 8396599 | + | 20.9 | 6 | 3625 | -2646 | L1PA16 | L1 | LINE |
| 8396603 | 8396970 | - | 16.7 | 1 | 381 | 0 | THE1D | MaLR | LTR |
| 8396971 | 8398579 | - | 16.5 | 3 | 1580 | 0 | THE1D-int | MaLR | LTR |
| 8398582 | 8398952 | - | 16.6 | 1 | 381 | 0 | THE1D | MaLR | LTR |
| 8398953 | 8399469 | + | 14.4 | 3626 | 4114 | -2157 | L1PA16 | L1 | LINE |
| 8399470 | 8399772 | + | 8.9 | 1 | 303 | -8 | AluY | Alu | SINE |
| 8399773 | 8401979 | + | 15.4 | 4115 | 6285 | -6 | L1PA16 | L1 | LINE |

  

|  |  |  |  |  |  |  |  |  |  |
| --- | --- | --- | --- | --- | --- | --- | --- | --- | --- |
| chr5:8521626-8526110 | | | 4326 bp | | |  |  |  |  |
| Genome Start | Genome End | Strand | Div | Repeat Start | Repeat End | Repeat Left | Name | Family | Class |
| 8521626 | 8522053 | - | 31.6 | 5404 | 5858 | -382 | L1ME3B | L1 | LINE |
| 8522107 | 8522759 | - | 32.6 | 4063 | 4705 | -1441 | L1ME3B | L1 | LINE |
| 8522782 | 8523150 | - | 12.8 | 1 | 375 | 0 | THE1C | MaLR | LTR |
| 8523151 | 8524734 | - | 12.8 | 1 | 1580 | 0 | THE1C-int | MaLR | LTR |
| 8524735 | 8525089 | - | 15.6 | 19 | 375 | 0 | THE1C | MaLR | LTR |
| 8525156 | 8525353 | - | 31.5 | 3503 | 3714 | -2432 | L1ME3B | L1 | LINE |
| 8525368 | 8525643 | + | 20.5 | 291 | 563 | 0 | MER68 | ERVL | LTR |
| 8525648 | 8526110 | - | 28.4 | 2932 | 3416 | -2730 | L1ME3B | L1 | LINE |

  

|  |  |  |  |  |  |  |  |  |  |
| --- | --- | --- | --- | --- | --- | --- | --- | --- | --- |
| chr5:8811843-8815693 | | | 3849 bp | | |  |  |  |  |
| Genome Start | Genome End | Strand | Div | Repeat Start | Repeat End | Repeat Left | Name | Family | Class |
| 8811843 | 8812413 | + | 14.4 | 1 | 620 | -960 | THE1D-int | MaLR | LTR |
| 8812414 | 8812792 | + | 13.3 | 1 | 364 | 0 | THE1B | MaLR | LTR |
| 8812795 | 8814357 | + | 30.6 | 2 | 1735 | 0 | MLT-int | MaLR | LTR |
| 8814358 | 8814729 | + | 16.1 | 1 | 364 | 0 | THE1B | MaLR | LTR |
| 8814730 | 8815693 | + | 14.4 | 621 | 1578 | -2 | THE1D-int | MaLR | LTR |

  

|  |  |  |  |  |  |  |  |  |  |
| --- | --- | --- | --- | --- | --- | --- | --- | --- | --- |
| chr5:9046474-9049872 | | | 3356 bp | | |  |  |  |  |
| Genome Start | Genome End | Strand | Div | Repeat Start | Repeat End | Repeat Left | Name | Family | Class |
| 9046474 | 9047378 | - | 26.1 | 5249 | 6198 | -41 | L1ME1 | L1 | LINE |
| 9047382 | 9047742 | + | 14.7 | 3 | 364 | 0 | THE1B | MaLR | LTR |
| 9047743 | 9048294 | + | 11.2 | 1 | 536 | -1044 | THE1B-int | MaLR | LTR |
| 9048291 | 9048406 | + | 6.1 | 1461 | 1574 | -6 | THE1B-int | MaLR | LTR |
| 9048412 | 9048771 | + | 15.0 | 1 | 364 | 0 | THE1B | MaLR | LTR |
| 9048785 | 9048963 | - | 24.2 | 5036 | 5240 | -979 | L1ME1 | L1 | LINE |
| 9048990 | 9049122 | - | 27.5 | 1277 | 1400 | -5000 | L1ME1 | L1 | LINE |
| 9049123 | 9049433 | - | 13.5 | 1 | 312 | 0 | AluSx | Alu | SINE |
| 9049434 | 9049872 | - | 27.5 | 803 | 1276 | -5124 | L1ME1 | L1 | LINE |

  

|  |  |  |  |  |  |  |  |  |  |
| --- | --- | --- | --- | --- | --- | --- | --- | --- | --- |
| chr5:9073705-9081793 | | | 7914 bp | | |  |  |  |  |
| Genome Start | Genome End | Strand | Div | Repeat Start | Repeat End | Repeat Left | Name | Family | Class |
| 9073705 | 9075212 | - | 12.8 | 5292 | 6829 | -40 | L1PA16 | L1 | LINE |
| 9075214 | 9076400 | - | 14.4 | 2532 | 3743 | -3106 | L1PA16 | L1 | LINE |
| 9076396 | 9076588 | + | 12.9 | 1740 | 1938 | -4915 | L1PA15-16 | L1 | LINE |
| 9076589 | 9077167 | - | 15.4 | 1980 | 2583 | -4270 | L1PA16 | L1 | LINE |
| 9077171 | 9077477 | - | 16.8 | 5820 | 6174 | 0 | L1PA11 | L1 | LINE |
| 9077648 | 9079391 | - | 20.9 | 119 | 1979 | -4874 | L1PA16 | L1 | LINE |
| 9079392 | 9079761 | + | 14.0 | 1 | 375 | 0 | THE1C | MaLR | LTR |
| 9079762 | 9081301 | + | 13.8 | 1 | 1574 | -6 | THE1C-int | MaLR | LTR |
| 9081307 | 9081674 | + | 14.6 | 1 | 375 | 0 | THE1C | MaLR | LTR |
| 9081676 | 9081793 | - | 9.3 | 2 | 122 | -6731 | L1PA16 | L1 | LINE |

  

|  |  |  |  |  |  |  |  |  |  |
| --- | --- | --- | --- | --- | --- | --- | --- | --- | --- |
| chr5:9323970-9333141 | | | 9082 bp | | |  |  |  |  |
| Genome Start | Genome End | Strand | Div | Repeat Start | Repeat End | Repeat Left | Name | Family | Class |
| 9323970 | 9324069 | + | 28.0 | 10 | 110 | -231 | MER58 | MER1\_type | DNA |
| 9324109 | 9324491 | + | 15.1 | 1 | 426 | -2 | MSTA | MaLR | LTR |
| 9324494 | 9324717 | + | 17.2 | 1 | 223 | -1378 | MSTA-int | MaLR | LTR |
| 9324725 | 9331082 | + | 9.1 | 39 | 6468 | -1 | L1PA7 | L1 | LINE |
| 9331084 | 9331230 | - | 25.7 | 4723 | 4867 | -4177 | LTR39-int | ERV1 | LTR |
| 9331242 | 9332664 | + | 15.4 | 200 | 1580 | 0 | MSTA-int | MaLR | LTR |
| 9332665 | 9333060 | + | 16.1 | 1 | 426 | -2 | MSTA | MaLR | LTR |
| 9333091 | 9333141 | + | 20.0 | 2371 | 2420 | 0 | MER58 | MER1\_type | DNA |

  

|  |  |  |  |  |  |  |  |  |  |
| --- | --- | --- | --- | --- | --- | --- | --- | --- | --- |
| chr5:9920204-9922998 | | | 2791 bp | | |  |  |  |  |
| Genome Start | Genome End | Strand | Div | Repeat Start | Repeat End | Repeat Left | Name | Family | Class |
| 9920204 | 9920386 | + | 21.9 | 54 | 237 | -326 | MER68 | ERVL | LTR |
| 9920387 | 9920746 | - | 13.1 | 3 | 364 | 0 | THE1B | MaLR | LTR |
| 9920749 | 9922323 | - | 28.6 | 2 | 1735 | 0 | MLT-int | MaLR | LTR |
| 9922326 | 9922683 | - | 11.0 | 1 | 364 | 0 | THE1B | MaLR | LTR |
| 9922684 | 9922998 | + | 21.9 | 238 | 552 | -11 | MER68 | ERVL | LTR |

  

|  |  |  |  |  |  |  |  |  |  |
| --- | --- | --- | --- | --- | --- | --- | --- | --- | --- |
| chr5:10208558-10212208 | | | 3576 bp | | |  |  |  |  |
| Genome Start | Genome End | Strand | Div | Repeat Start | Repeat End | Repeat Left | Name | Family | Class |
| 10208558 | 10208660 | - | 14.8 | 680 | 783 | 0 | MER20B | MER1\_type | DNA |
| 10208666 | 10209345 | + | 16.0 | 5452 | 6128 | -43 | L1MB2 | L1 | LINE |
| 10209383 | 10209454 | - | 16.7 | 314 | 392 | -4 | MSTD | MaLR | LTR |
| 10209452 | 10209559 | - | 11.2 | 1 | 112 | -284 | MSTD | MaLR | LTR |
| 10209560 | 10211278 | - | 24.0 | 2 | 1735 | 0 | MSTD-int | MaLR | LTR |
| 10211283 | 10211660 | - | 19.7 | 1 | 394 | -2 | MSTD | MaLR | LTR |
| 10211665 | 10211937 | - | 30.5 | 427 | 702 | -81 | MER20B | MER1\_type | DNA |
| 10211966 | 10212208 | - | 25.9 | 28 | 347 | -436 | MER20B | MER1\_type | DNA |

  

|  |  |  |  |  |  |  |  |  |  |
| --- | --- | --- | --- | --- | --- | --- | --- | --- | --- |
| chr5:11877608-11885770 | | | 8088 bp | | |  |  |  |  |
| Genome Start | Genome End | Strand | Div | Repeat Start | Repeat End | Repeat Left | Name | Family | Class |
| 11877608 | 11878207 | + | 30.1 | 649 | 1299 | -5124 | L1MCc | L1 | LINE |
| 11878255 | 11881063 | + | 27.6 | 1481 | 4468 | -2473 | L1MCc | L1 | LINE |
| 11881109 | 11881189 | + | 23.2 | 1 | 79 | -6212 | L1P4e | L1 | LINE |
| 11881190 | 11881359 | + | 13.5 | 1 | 185 | -179 | THE1B | MaLR | LTR |
| 11881360 | 11881666 | + | 11.7 | 1 | 307 | -6 | AluSq | Alu | SINE |
| 11881667 | 11881859 | + | 13.5 | 186 | 364 | 0 | THE1B | MaLR | LTR |
| 11881862 | 11883424 | + | 29.6 | 2 | 1735 | 0 | MLT1C-int | MaLR | LTR |
| 11883425 | 11883778 | + | 13.6 | 1 | 363 | -1 | THE1B | MaLR | LTR |
| 11883779 | 11883996 | + | 23.2 | 80 | 301 | -5990 | L1P4e | L1 | LINE |
| 11883978 | 11884499 | + | 27.1 | 5102 | 5613 | -1328 | L1MCc | L1 | LINE |
| 11884500 | 11884794 | + | 6.1 | 2 | 297 | -14 | AluY | Alu | SINE |
| 11884795 | 11885113 | + | 27.1 | 5614 | 5961 | -980 | L1MCc | L1 | LINE |
| 11885114 | 11885419 | + | 9.2 | 1 | 306 | -5 | AluY | Alu | SINE |
| 11885420 | 11885770 | + | 25.2 | 5962 | 6274 | -687 | L1MCc | L1 | LINE |

  

|  |  |  |  |  |  |  |  |  |  |
| --- | --- | --- | --- | --- | --- | --- | --- | --- | --- |
| chr5:12058960-12063243 | | | 3467 bp | | |  |  |  |  |
| Genome Start | Genome End | Strand | Div | Repeat Start | Repeat End | Repeat Left | Name | Family | Class |
| 12058960 | 12059131 | - | 26.4 | 5702 | 5877 | -363 | L1M5 | L1 | LINE |
| 12059132 | 12059441 | - | 12.3 | 1 | 310 | -2 | AluSx | Alu | SINE |
| 12059442 | 12059477 | - | 26.4 | 5666 | 5701 | -539 | L1M5 | L1 | LINE |
| 12059896 | 12060313 | - | 27.3 | 4905 | 5334 | -812 | L1M5 | L1 | LINE |
| 12060396 | 12060765 | - | 11.7 | 1 | 364 | 0 | THE1B | MaLR | LTR |
| 12060766 | 12062340 | - | 10.3 | 1 | 1580 | 0 | THE1B-int | MaLR | LTR |
| 12062341 | 12062684 | - | 15.8 | 1 | 364 | 0 | THE1B | MaLR | LTR |
| 12063002 | 12063243 | - | 24.2 | 4152 | 4426 | -1720 | L1M5 | L1 | LINE |

  

|  |  |  |  |  |  |  |  |  |  |
| --- | --- | --- | --- | --- | --- | --- | --- | --- | --- |
| chr5:13121867-13125473 | | | 3551 bp | | |  |  |  |  |
| Genome Start | Genome End | Strand | Div | Repeat Start | Repeat End | Repeat Left | Name | Family | Class |
| 13121867 | 13122928 | + | 25.9 | 1428 | 2601 | -160 | Charlie1 | MER1\_type | DNA |
| 13122945 | 13123322 | - | 13.1 | 3 | 428 | 0 | MSTA | MaLR | LTR |
| 13123330 | 13124923 | - | 20.9 | 1 | 1645 | -6 | MSTA-int | MaLR | LTR |
| 13124924 | 13125304 | - | 16.1 | 1 | 428 | 0 | MSTA | MaLR | LTR |
| 13125338 | 13125473 | + | 26.1 | 2618 | 2761 | 0 | Charlie1 | MER1\_type | DNA |

  

|  |  |  |  |  |  |  |  |  |  |
| --- | --- | --- | --- | --- | --- | --- | --- | --- | --- |
| chr5:13439732-13443780 | | | 4047 bp | | |  |  |  |  |
| Genome Start | Genome End | Strand | Div | Repeat Start | Repeat End | Repeat Left | Name | Family | Class |
| 13439732 | 13439875 | + | 16.7 | 5333 | 5478 | -1836 | L1PA16 | L1 | LINE |
| 13439876 | 13440229 | + | 14.4 | 1 | 381 | 0 | THE1D | MaLR | LTR |
| 13440232 | 13441844 | + | 13.8 | 3 | 1580 | 0 | THE1D-int | MaLR | LTR |
| 13441845 | 13442057 | + | 17.2 | 1 | 229 | -152 | THE1D | MaLR | LTR |
| 13442058 | 13442465 | + | 11.7 | 5996 | 6398 | -916 | L1PA16 | L1 | LINE |
| 13442466 | 13442871 | - | 14.3 | 1 | 428 | 0 | MSTA | MaLR | LTR |
| 13442872 | 13443780 | + | 14.5 | 6399 | 7334 | 0 | L1PA16 | L1 | LINE |

  

|  |  |  |  |  |  |  |  |  |  |
| --- | --- | --- | --- | --- | --- | --- | --- | --- | --- |
| chr5:16190072-16193844 | | | 3294 bp | | |  |  |  |  |
| Genome Start | Genome End | Strand | Div | Repeat Start | Repeat End | Repeat Left | Name | Family | Class |
| 16190072 | 16190283 | + | 29.1 | 2071 | 2289 | -3926 | L1MC4 | L1 | LINE |
| 16190284 | 16190643 | + | 13.9 | 1 | 364 | 0 | THE1B | MaLR | LTR |
| 16190644 | 16192224 | + | 28.7 | 1 | 1735 | 0 | MLT-int | MaLR | LTR |
| 16192225 | 16192590 | + | 12.1 | 1 | 364 | 0 | THE1B | MaLR | LTR |
| 16192591 | 16192808 | + | 29.1 | 2290 | 2513 | -3702 | L1M5 | L1 | LINE |
| 16193033 | 16193363 | + | 31.3 | 2808 | 3145 | -3069 | L1MC4 | L1 | LINE |
| 16193619 | 16193844 | + | 27.7 | 3605 | 3838 | -2376 | L1MC4 | L1 | LINE |

  

|  |  |  |  |  |  |  |  |  |  |
| --- | --- | --- | --- | --- | --- | --- | --- | --- | --- |
| chr5:17898397-17908438 | | | 9823 bp | | |  |  |  |  |
| Genome Start | Genome End | Strand | Div | Repeat Start | Repeat End | Repeat Left | Name | Family | Class |
| 17898397 | 17898823 | + | 35.0 | 1358 | 1786 | -5710 | L1M3c | L1 | LINE |
| 17898974 | 17900065 | + | 23.3 | 1876 | 2980 | -4993 | L1M3c | L1 | LINE |
| 17900066 | 17900423 | + | 13.3 | 1 | 364 | 0 | THE1B | MaLR | LTR |
| 17900426 | 17901992 | + | 30.3 | 2 | 1735 | 0 | MLT-int | MaLR | LTR |
| 17901993 | 17902346 | + | 11.0 | 1 | 364 | 0 | THE1B | MaLR | LTR |
| 17902347 | 17902366 | + | 26.6 | 1392 | 1422 | -4962 | L1M4c | L1 | LINE |
| 17902409 | 17902462 | + | 26.6 | 1423 | 1486 | -4898 | L1M4c | L1 | LINE |
| 17902468 | 17904100 | + | 24.0 | 2881 | 4612 | -2947 | L1M3c | L1 | LINE |
| 17904121 | 17906665 | - | 12.1 | 3729 | 6304 | 0 | L1MA2 | L1 | LINE |
| 17906666 | 17907122 | + | 19.3 | 4614 | 5056 | -2503 | L1M3c | L1 | LINE |
| 17907123 | 17907167 | + | 6.7 | 1 | 45 | -35 | MADE1 | Mariner | DNA |
| 17907168 | 17908438 | + | 19.3 | 5057 | 6289 | -1270 | L1M3c | L1 | LINE |

  

|  |  |  |  |  |  |  |  |  |  |
| --- | --- | --- | --- | --- | --- | --- | --- | --- | --- |
| chr5:17916301-17923396 | | | 6730 bp | | |  |  |  |  |
| Genome Start | Genome End | Strand | Div | Repeat Start | Repeat End | Repeat Left | Name | Family | Class |
| 17916301 | 17916662 | + | 30.0 | 5040 | 5401 | -745 | L1ME3B | L1 | LINE |
| 17916894 | 17917003 | + | 31.5 | 5574 | 5685 | -555 | L1ME3B | L1 | LINE |
| 17917035 | 17917412 | - | 15.7 | 5781 | 6151 | 0 | L1PB1 | L1 | LINE |
| 17917515 | 17918478 | - | 9.3 | 4810 | 5789 | -362 | L1PB1 | L1 | LINE |
| 17918466 | 17919831 | - | 14.7 | 361 | 1574 | -5289 | L1PBa | L1 | LINE |
| 17919832 | 17920140 | - | 12.4 | 1 | 312 | 0 | AluSx | Alu | SINE |
| 17920141 | 17920400 | - | 14.7 | 94 | 360 | -6503 | L1PBa | L1 | LINE |
| 17920401 | 17920759 | + | 10.4 | 1 | 364 | 0 | THE1B | MaLR | LTR |
| 17920760 | 17922039 | + | 9.4 | 1 | 1279 | -301 | THE1B-int | MaLR | LTR |
| 17922040 | 17922356 | + | 9.7 | 2 | 309 | -2 | AluY | Alu | SINE |
| 17922357 | 17922670 | + | 9.4 | 1280 | 1580 | 0 | THE1B-int | MaLR | LTR |
| 17922673 | 17923028 | + | 16.3 | 3 | 364 | 0 | THE1B | MaLR | LTR |
| 17923029 | 17923122 | - | 9.6 | 2 | 98 | -6765 | L1PBa | L1 | LINE |
| 17923136 | 17923396 | + | 34.1 | 5691 | 5969 | -271 | L1ME3B | L1 | LINE |

  

|  |  |  |  |  |  |  |  |  |  |
| --- | --- | --- | --- | --- | --- | --- | --- | --- | --- |
| chr5:18704262-18710673 | | | 6393 bp | | |  |  |  |  |
| Genome Start | Genome End | Strand | Div | Repeat Start | Repeat End | Repeat Left | Name | Family | Class |
| 18704262 | 18704703 | - | 33.7 | 2918 | 3419 | 0 | L2 | L2 | LINE |
| 18704709 | 18705158 | - | 7.8 | 1 | 448 | -2 | LTR7 | ERV1 | LTR |
| 18705159 | 18705546 | - | 7.6 | 7312 | 7713 | 0 | HERVH | ERV1 | LTR |
| 18705537 | 18705751 | - | 13.9 | 5600 | 5819 | -1894 | HERVH | ERV1 | LTR |
| 18705753 | 18706414 | - | 14.4 | 4488 | 5149 | -2564 | HERVH | ERV1 | LTR |
| 18706418 | 18706918 | - | 11.4 | 3492 | 3992 | -3721 | HERVH | ERV1 | LTR |
| 18706909 | 18707058 | - | 14.0 | 3140 | 3290 | -4423 | HERVH | ERV1 | LTR |
| 18707053 | 18710016 | - | 9.7 | 1 | 2987 | -4726 | HERVH | ERV1 | LTR |
| 18710019 | 18710464 | - | 7.5 | 1 | 448 | -2 | LTR7 | ERV1 | LTR |
| 18710499 | 18710673 | - | 34.9 | 2707 | 2885 | -534 | L2 | L2 | LINE |

  

|  |  |  |  |  |  |  |  |  |  |
| --- | --- | --- | --- | --- | --- | --- | --- | --- | --- |
| chr5:20119240-20123725 | | | 4491 bp | | |  |  |  |  |
| Genome Start | Genome End | Strand | Div | Repeat Start | Repeat End | Repeat Left | Name | Family | Class |
| 20119240 | 20119351 | + | 17.6 | 14 | 133 | -2285 | Tigger1 | MER2\_type | DNA |
| 20119345 | 20119476 | + | 20.0 | 270 | 400 | -2018 | Tigger1 | MER2\_type | DNA |
| 20119477 | 20119829 | - | 10.8 | 1 | 361 | -3 | THE1B | MaLR | LTR |
| 20119830 | 20121408 | - | 31.4 | 2 | 1735 | 0 | MLT-int | MaLR | LTR |
| 20121411 | 20121743 | - | 11.6 | 4 | 364 | 0 | THE1B | MaLR | LTR |
| 20121744 | 20123725 | + | 16.9 | 401 | 2418 | 0 | Tigger1 | MER2\_type | DNA |

  

|  |  |  |  |  |  |  |  |  |  |
| --- | --- | --- | --- | --- | --- | --- | --- | --- | --- |
| chr5:20767751-20770907 | | | 3153 bp | | |  |  |  |  |
| Genome Start | Genome End | Strand | Div | Repeat Start | Repeat End | Repeat Left | Name | Family | Class |
| 20767751 | 20768054 | - | 19.5 | 5866 | 6280 | -11 | L1MA7 | L1 | LINE |
| 20768055 | 20768405 | - | 13.4 | 3 | 381 | 0 | THE1D | MaLR | LTR |
| 20768408 | 20770178 | - | 30.6 | 2 | 1735 | 0 | MLT-int | MaLR | LTR |
| 20770181 | 20770534 | - | 16.9 | 3 | 381 | 0 | THE1D | MaLR | LTR |
| 20770535 | 20770907 | - | 17.7 | 5487 | 5865 | -426 | L1MA7 | L1 | LINE |

  

|  |  |  |  |  |  |  |  |  |  |
| --- | --- | --- | --- | --- | --- | --- | --- | --- | --- |
| chr5:20942144-20945025 | | | 2843 bp | | |  |  |  |  |
| Genome Start | Genome End | Strand | Div | Repeat Start | Repeat End | Repeat Left | Name | Family | Class |
| 20942144 | 20942440 | - | 19.9 | 133 | 428 | 0 | MSTA | MaLR | LTR |
| 20942458 | 20942819 | + | 14.5 | 1 | 364 | 0 | THE1B | MaLR | LTR |
| 20942822 | 20943519 | + | 9.6 | 3 | 670 | -910 | THE1B-int | MaLR | LTR |
| 20943520 | 20944256 | + | 10.6 | 847 | 1580 | 0 | THE1B-int | MaLR | LTR |
| 20944257 | 20944614 | + | 14.5 | 1 | 359 | -5 | THE1B | MaLR | LTR |
| 20944623 | 20944914 | + | 7.9 | 6 | 309 | -2 | AluY | Alu | SINE |
| 20944927 | 20945025 | - | 13.3 | 1 | 98 | -328 | MSTA | MaLR | LTR |

  

|  |  |  |  |  |  |  |  |  |  |
| --- | --- | --- | --- | --- | --- | --- | --- | --- | --- |
| chr5:21093993-21100976 | | | 6972 bp | | |  |  |  |  |
| Genome Start | Genome End | Strand | Div | Repeat Start | Repeat End | Repeat Left | Name | Family | Class |
| 21093993 | 21098200 | - | 12.9 | 1309 | 5643 | -1938 | L1PA12 | L1 | LINE |
| 21098201 | 21098577 | + | 14.1 | 1 | 381 | 0 | THE1D | MaLR | LTR |
| 21098580 | 21100115 | + | 20.7 | 3 | 1646 | -5 | MST-int | MaLR | LTR |
| 21100123 | 21100467 | + | 12.9 | 5 | 378 | -3 | THE1D | MaLR | LTR |
| 21100471 | 21100976 | - | 20.9 | 800 | 1307 | -5674 | L1PA12 | L1 | LINE |

  

|  |  |  |  |  |  |  |  |  |  |
| --- | --- | --- | --- | --- | --- | --- | --- | --- | --- |
| chr5:21697959-21701852 | | | 3894 bp | | |  |  |  |  |
| Genome Start | Genome End | Strand | Div | Repeat Start | Repeat End | Repeat Left | Name | Family | Class |
| 21697959 | 21698284 | - | 21.1 | 3862 | 4198 | -2808 | L1M4b | L1 | LINE |
| 21698283 | 21698369 | - | 20.4 | 2819 | 2895 | -4439 | L1M4b | L1 | LINE |
| 21698370 | 21698731 | - | 15.4 | 1 | 364 | 0 | THE1B | MaLR | LTR |
| 21698732 | 21700293 | - | 29.5 | 2 | 1735 | 0 | MLT-int | MaLR | LTR |
| 21700296 | 21700647 | - | 17.3 | 1 | 364 | 0 | THE1B | MaLR | LTR |
| 21700648 | 21700662 | - | 20.4 | 2810 | 2818 | -4516 | L1M4b | L1 | LINE |
| 21700663 | 21701075 | + | 21.3 | 2 | 432 | 0 | MSTB1 | MaLR | LTR |
| 21701076 | 21701852 | - | 27.1 | 7 | 2809 | -4525 | L1M4b | L1 | LINE |

  

|  |  |  |  |  |  |  |  |  |  |
| --- | --- | --- | --- | --- | --- | --- | --- | --- | --- |
| chr5:21980880-21986563 | | | 5581 bp | | |  |  |  |  |
| Genome Start | Genome End | Strand | Div | Repeat Start | Repeat End | Repeat Left | Name | Family | Class |
| 21980880 | 21981008 | + | 27.4 | 1032 | 1167 | -5466 | L1MDa | L1 | LINE |
| 21981009 | 21981319 | + | 12.3 | 1 | 312 | 0 | AluSx | Alu | SINE |
| 21981320 | 21982468 | + | 27.4 | 1168 | 2359 | -4274 | L1MDa | L1 | LINE |
| 21982486 | 21982712 | + | 19.4 | 1 | 229 | -1002 | Tigger3b | MER2\_type | DNA |
| 21982713 | 21983083 | + | 15.2 | 1 | 379 | -2 | THE1D | MaLR | LTR |
| 21983086 | 21984584 | + | 14.4 | 1 | 1580 | 0 | THE1D-int | MaLR | LTR |
| 21984585 | 21984963 | + | 14.5 | 1 | 381 | 0 | THE1D | MaLR | LTR |
| 21984964 | 21985080 | + | 16.8 | 225 | 339 | -892 | Tigger3b | MER2\_type | DNA |
| 21985081 | 21985337 | + | 5.8 | 47 | 303 | -8 | AluY | Alu | SINE |
| 21985338 | 21986197 | + | 16.8 | 340 | 1225 | -6 | Tigger3b | MER2\_type | DNA |
| 21986205 | 21986419 | + | 20.1 | 2379 | 2601 | -4032 | L1MDa | L1 | LINE |
| 21986497 | 21986563 | + | 22.4 | 2614 | 2683 | -3854 | L1MDa | L1 | LINE |

  

|  |  |  |  |  |  |  |  |  |  |
| --- | --- | --- | --- | --- | --- | --- | --- | --- | --- |
| chr5:23069202-23071828 | | | 2625 bp | | |  |  |  |  |
| Genome Start | Genome End | Strand | Div | Repeat Start | Repeat End | Repeat Left | Name | Family | Class |
| 23069202 | 23069532 | + | 20.4 | 1 | 348 | -17 | MLT1A0 | MaLR | LTR |
| 23069533 | 23069865 | - | 11.1 | 1 | 364 | 0 | THE1B | MaLR | LTR |
| 23069866 | 23071466 | - | 32.1 | 2 | 1735 | 0 | MLT-int | MaLR | LTR |
| 23071469 | 23071807 | - | 14.8 | 1 | 364 | 0 | THE1B | MaLR | LTR |
| 23071808 | 23071828 | + | 20.4 | 349 | 365 | 0 | MLT1A0 | MaLR | LTR |

  

|  |  |  |  |  |  |  |  |  |  |
| --- | --- | --- | --- | --- | --- | --- | --- | --- | --- |
| chr5:23432163-23438314 | | | 6024 bp | | |  |  |  |  |
| Genome Start | Genome End | Strand | Div | Repeat Start | Repeat End | Repeat Left | Name | Family | Class |
| 23432163 | 23432352 | - | 27.2 | 3219 | 3412 | -7 | L2 | L2 | LINE |
| 23432359 | 23432820 | + | 20.9 | 1 | 485 | 0 | MER31A | ERV1 | LTR |
| 23432867 | 23433157 | + | 24.2 | 14 | 282 | -4654 | MER31A-int | ERV1 | LTR |
| 23433176 | 23433319 | + | 25.0 | 215 | 359 | -4577 | MER31A-int | ERV1 | LTR |
| 23433320 | 23433606 | - | 7.0 | 23 | 306 | -5 | AluY | Alu | SINE |
| 23433607 | 23433663 | + | 25.0 | 360 | 408 | -4528 | MER31A-int | ERV1 | LTR |
| 23433651 | 23433787 | + | 32.8 | 611 | 754 | -4182 | MER31A-int | ERV1 | LTR |
| 23433804 | 23434483 | + | 22.9 | 883 | 1526 | -3410 | MER31A-int | ERV1 | LTR |
| 23434537 | 23434660 | + | 17.7 | 2583 | 5682 | -2418 | MER31A-int | ERV1 | LTR |
| 23434644 | 23434720 | + | 29.9 | 2496 | 2573 | -2550 | MER31A-int | ERV1 | LTR |
| 23434695 | 23436027 | + | 22.3 | 2459 | 3882 | -1054 | MER31A-int | ERV1 | LTR |
| 23436041 | 23436345 | - | 12.3 | 1 | 312 | -52 | THE1B | MaLR | LTR |
| 23436347 | 23436916 | + | 22.4 | 3924 | 4518 | -418 | MER31A-int | ERV1 | LTR |
| 23436920 | 23437368 | + | 20.4 | 4466 | 4909 | -27 | MER31A-int | ERV1 | LTR |
| 23437373 | 23437602 | + | 15.4 | 1 | 231 | -254 | MER31A | ERV1 | LTR |
| 23437604 | 23437671 | + | 22.1 | 413 | 481 | -4 | MER31A | ERV1 | LTR |
| 23437704 | 23437969 | - | 39.7 | 2925 | 3192 | -227 | L2 | L2 | LINE |
| 23437961 | 23438314 | - | 41.5 | 2049 | 2416 | -1003 | L2 | L2 | LINE |

  

|  |  |  |  |  |  |  |  |  |  |
| --- | --- | --- | --- | --- | --- | --- | --- | --- | --- |
| chr5:23764968-23767369 | | | 2286 bp | | |  |  |  |  |
| Genome Start | Genome End | Strand | Div | Repeat Start | Repeat End | Repeat Left | Name | Family | Class |
| 23764968 | 23765009 | - | 23.9 | 594 | 649 | -5 | MLT1E1 | MaLR | LTR |
| 23765034 | 23765340 | - | 23.9 | 181 | 593 | -61 | MLT1E1 | MaLR | LTR |
| 23765368 | 23765452 | - | 23.5 | 5 | 97 | -557 | MLT1E1 | MaLR | LTR |
| 23765457 | 23766800 | - | 28.0 | 1 | 1489 | 0 | MLT1E1-int | MaLR | LTR |
| 23766812 | 23767074 | - | 32.8 | 383 | 646 | -8 | MLT1E1 | MaLR | LTR |
| 23767125 | 23767369 | - | 28.8 | 1 | 261 | -393 | MLT1E1 | MaLR | LTR |

  

|  |  |  |  |  |  |  |  |  |  |
| --- | --- | --- | --- | --- | --- | --- | --- | --- | --- |
| chr5:23972717-23984944 | | | 11979 bp | | |  |  |  |  |
| Genome Start | Genome End | Strand | Div | Repeat Start | Repeat End | Repeat Left | Name | Family | Class |
| 23972717 | 23973048 | - | 24.1 | 6257 | 6596 | -1607 | L1MC3 | L1 | LINE |
| 23973067 | 23974986 | - | 19.3 | 4333 | 6303 | -2 | L1MA3 | L1 | LINE |
| 23974987 | 23975149 | - | 20.8 | 1 | 159 | -7 | FRAM | Alu | SINE |
| 23975150 | 23977230 | - | 18.3 | 2224 | 4332 | -1814 | L1MA3 | L1 | LINE |
| 23977245 | 23977756 | - | 23.3 | 5683 | 6254 | -1949 | L1MC3 | L1 | LINE |
| 23977904 | 23978257 | - | 11.9 | 1 | 355 | 0 | THE1A | MaLR | LTR |
| 23978258 | 23979832 | - | 11.6 | 1 | 1580 | 0 | THE1A-int | MaLR | LTR |
| 23979833 | 23980188 | - | 12.7 | 1 | 355 | 0 | THE1A | MaLR | LTR |
| 23980193 | 23980389 | - | 26.0 | 5319 | 5505 | -1059 | L1MC3 | L1 | LINE |
| 23980392 | 23981217 | - | 25.1 | 4306 | 5184 | -1380 | L1MC3 | L1 | LINE |
| 23981257 | 23981377 | + | 15.4 | 2646 | 2762 | -267 | Tigger3(Golem) | MER2\_type | DNA |
| 23981391 | 23981556 | + | 19.8 | 2943 | 3111 | -3035 | L1MC | L1 | LINE |
| 23981557 | 23981816 | - | 26.0 | 2763 | 3028 | -1 | Tigger3(Golem) | MER2\_type | DNA |
| 23981823 | 23983085 | + | 16.1 | 4 | 1301 | -1 | HSMAR2 | Mariner | DNA |
| 23983095 | 23984128 | - | 29.0 | 2206 | 3334 | -3230 | L1MC3 | L1 | LINE |
| 23984126 | 23984944 | - | 29.1 | 572 | 1492 | -5259 | L1MC3 | L1 | LINE |

  

|  |  |  |  |  |  |  |  |  |  |
| --- | --- | --- | --- | --- | --- | --- | --- | --- | --- |
| chr5:24926150-24933831 | | | 7665 bp | | |  |  |  |  |
| Genome Start | Genome End | Strand | Div | Repeat Start | Repeat End | Repeat Left | Name | Family | Class |
| 24926150 | 24927955 | + | 20.0 | 104 | 1991 | -3723 | ERVL-B4 | ERVL | LTR |
| 24927956 | 24928174 | - | 16.4 | 191 | 428 | 0 | MSTA | MaLR | LTR |
| 24928175 | 24928482 | - | 13.0 | 1 | 308 | -4 | AluSx | Alu | SINE |
| 24928483 | 24928669 | - | 16.4 | 1 | 190 | -238 | MSTA | MaLR | LTR |
| 24928670 | 24928840 | - | 18.3 | 1418 | 1580 | 0 | MSTA-int | MaLR | LTR |
| 24928861 | 24929781 | - | 18.3 | 477 | 1417 | -163 | MSTA-int | MaLR | LTR |
| 24929782 | 24930026 | - | 13.1 | 61 | 312 | 0 | AluSx | Alu | SINE |
| 24930027 | 24930526 | - | 18.3 | 3 | 476 | -1104 | MSTA-int | MaLR | LTR |
| 24930527 | 24930912 | - | 18.2 | 1 | 428 | 0 | MSTA | MaLR | LTR |
| 24930913 | 24932843 | + | 21.1 | 1987 | 3929 | -1785 | ERVL-B4 | ERVL | LTR |
| 24932841 | 24933339 | + | 17.6 | 4100 | 4622 | -1092 | ERVL-B4 | ERVL | LTR |
| 24933340 | 24933719 | + | 14.6 | 1 | 426 | -2 | MSTA | MaLR | LTR |
| 24933720 | 24933831 | + | 17.6 | 4623 | 4729 | -985 | ERVL-B4 | ERVL | LTR |

  

|  |  |  |  |  |  |  |  |  |  |
| --- | --- | --- | --- | --- | --- | --- | --- | --- | --- |
| chr5:25564460-25569612 | | | 4837 bp | | |  |  |  |  |
| Genome Start | Genome End | Strand | Div | Repeat Start | Repeat End | Repeat Left | Name | Family | Class |
| 25564460 | 25565182 | - | 28.6 | 4408 | 5121 | -1025 | L1M5 | L1 | LINE |
| 25565184 | 25565554 | + | 17.3 | 3 | 364 | 0 | THE1B | MaLR | LTR |
| 25565555 | 25567113 | + | 12.2 | 1 | 1580 | 0 | THE1B-int | MaLR | LTR |
| 25567114 | 25567482 | + | 17.1 | 1 | 364 | 0 | THE1B | MaLR | LTR |
| 25567497 | 25568348 | - | 28.0 | 3477 | 4362 | -1784 | L1M5 | L1 | LINE |
| 25568440 | 25568639 | - | 27.3 | 3858 | 4080 | -2457 | L1MDa | L1 | LINE |
| 25568795 | 25569091 | - | 28.9 | 2589 | 2904 | -3242 | L1M5 | L1 | LINE |
| 25569147 | 25569612 | - | 28.9 | 2130 | 2588 | -3558 | L1M5 | L1 | LINE |

  

|  |  |  |  |  |  |  |  |  |  |
| --- | --- | --- | --- | --- | --- | --- | --- | --- | --- |
| chr5:25894196-25898609 | | | 4312 bp | | |  |  |  |  |
| Genome Start | Genome End | Strand | Div | Repeat Start | Repeat End | Repeat Left | Name | Family | Class |
| 25894196 | 25894771 | + | 27.0 | 2991 | 3605 | -1266 | MER65A-int | ERV1 | LTR |
| 25894772 | 25895084 | - | 7.4 | 1 | 311 | 0 | AluY | Alu | SINE |
| 25895085 | 25895684 | + | 27.0 | 3606 | 4172 | -699 | MER65A-int | ERV1 | LTR |
| 25895783 | 25896094 | + | 27.2 | 4345 | 4668 | -203 | MER65A-int | ERV1 | LTR |
| 25896095 | 25896438 | - | 11.9 | 3 | 364 | 0 | THE1B | MaLR | LTR |
| 25896441 | 25898041 | - | 30.5 | 2 | 1735 | 0 | MLT-int | MaLR | LTR |
| 25898044 | 25898409 | - | 11.8 | 1 | 364 | 0 | THE1B | MaLR | LTR |
| 25898410 | 25898609 | + | 27.2 | 4669 | 4870 | -1 | MER65A-int | ERV1 | LTR |

  

|  |  |  |  |  |  |  |  |  |  |
| --- | --- | --- | --- | --- | --- | --- | --- | --- | --- |
| chr5:26318875-26322434 | | | 3558 bp | | |  |  |  |  |
| Genome Start | Genome End | Strand | Div | Repeat Start | Repeat End | Repeat Left | Name | Family | Class |
| 26318875 | 26320052 | + | 18.0 | 4936 | 6068 | -100 | L1MB1 | L1 | LINE |
| 26320053 | 26320408 | - | 14.2 | 1 | 364 | 0 | THE1B | MaLR | LTR |
| 26320409 | 26321983 | - | 31.4 | 2 | 1735 | 0 | MLT-int | MaLR | LTR |
| 26321986 | 26322336 | - | 14.0 | 1 | 364 | 0 | THE1B | MaLR | LTR |
| 26322337 | 26322434 | + | 19.8 | 6069 | 6166 | -2 | L1MB1 | L1 | LINE |

  

|  |  |  |  |  |  |  |  |  |  |
| --- | --- | --- | --- | --- | --- | --- | --- | --- | --- |
| chr5:26373052-26375881 | | | 2830 bp | | |  |  |  |  |
| Genome Start | Genome End | Strand | Div | Repeat Start | Repeat End | Repeat Left | Name | Family | Class |
| 26373052 | 26373485 | - | 16.2 | 5868 | 6273 | -18 | L1MA7 | L1 | LINE |
| 26373486 | 26373857 | - | 17.1 | 1 | 428 | 0 | MSTA | MaLR | LTR |
| 26373858 | 26375463 | - | 30.9 | 1 | 1735 | 0 | MLT-int | MaLR | LTR |
| 26375464 | 26375845 | - | 15.9 | 1 | 428 | 0 | MSTA | MaLR | LTR |
| 26375846 | 26375881 | - | 16.2 | 5833 | 5867 | -424 | L1MA7 | L1 | LINE |

  

|  |  |  |  |  |  |  |  |  |  |
| --- | --- | --- | --- | --- | --- | --- | --- | --- | --- |
| chr5:26399066-26404339 | | | 5243 bp | | |  |  |  |  |
| Genome Start | Genome End | Strand | Div | Repeat Start | Repeat End | Repeat Left | Name | Family | Class |
| 26399066 | 26399152 | - | 18.9 | 3192 | 3279 | -3929 | L1M2 | L1 | LINE |
| 26399153 | 26399518 | + | 14.5 | 1 | 381 | 0 | THE1D | MaLR | LTR |
| 26399521 | 26400480 | + | 16.5 | 3 | 982 | -598 | THE1D-int | MaLR | LTR |
| 26400481 | 26400607 | + | 9.7 | 1203 | 1327 | -253 | THE1D-int | MaLR | LTR |
| 26400633 | 26400922 | - | 13.5 | 2 | 290 | -19 | AluSc | Alu | SINE |
| 26400925 | 26401192 | + | 12.2 | 1312 | 1580 | 0 | THE1D-int | MaLR | LTR |
| 26401193 | 26401542 | + | 15.6 | 1 | 379 | -2 | THE1D | MaLR | LTR |
| 26401545 | 26404339 | - | 25.9 | 174 | 3196 | -4013 | L1M2 | L1 | LINE |

  

|  |  |  |  |  |  |  |  |  |  |
| --- | --- | --- | --- | --- | --- | --- | --- | --- | --- |
| chr5:27108176-27117913 | | | 9729 bp | | |  |  |  |  |
| Genome Start | Genome End | Strand | Div | Repeat Start | Repeat End | Repeat Left | Name | Family | Class |
| 27108176 | 27114398 | + | 19.1 | 61 | 6880 | -716 | L1PA14 | L1 | LINE |
| 27114399 | 27114444 | - | 10.2 | 312 | 355 | 0 | THE1A | MaLR | LTR |
| 27114445 | 27114748 | + | 8.2 | 1 | 303 | -8 | AluY | Alu | SINE |
| 27114749 | 27115071 | - | 10.2 | 1 | 311 | -44 | THE1A | MaLR | LTR |
| 27115072 | 27115585 | - | 10.1 | 1063 | 1580 | 0 | THE1A-int | MaLR | LTR |
| 27115587 | 27115804 | + | 7.8 | 5069 | 5289 | -857 | L1PREC2 | L1 | LINE |
| 27115812 | 27116859 | - | 9.9 | 1 | 1061 | -519 | THE1A-int | MaLR | LTR |
| 27116860 | 27117204 | - | 11.6 | 1 | 355 | 0 | THE1A | MaLR | LTR |
| 27117206 | 27117913 | + | 10.6 | 6877 | 7587 | -9 | L1PA14 | L1 | LINE |

  

|  |  |  |  |  |  |  |  |  |  |
| --- | --- | --- | --- | --- | --- | --- | --- | --- | --- |
| chr5:27191929-27195830 | | | 3900 bp | | |  |  |  |  |
| Genome Start | Genome End | Strand | Div | Repeat Start | Repeat End | Repeat Left | Name | Family | Class |
| 27191929 | 27192665 | - | 26.7 | 2141 | 2917 | -4648 | L1M1 | L1 | LINE |
| 27192666 | 27193036 | + | 18.4 | 1 | 381 | 0 | THE1D | MaLR | LTR |
| 27193039 | 27194655 | + | 31.3 | 2 | 1735 | 0 | MLT-int | MaLR | LTR |
| 27194656 | 27195034 | + | 18.6 | 1 | 381 | 0 | THE1D | MaLR | LTR |
| 27195035 | 27195830 | - | 26.7 | 1350 | 2140 | -5425 | L1M1 | L1 | LINE |

  

|  |  |  |  |  |  |  |  |  |  |
| --- | --- | --- | --- | --- | --- | --- | --- | --- | --- |
| chr5:27360960-27368500 | | | 7228 bp | | |  |  |  |  |
| Genome Start | Genome End | Strand | Div | Repeat Start | Repeat End | Repeat Left | Name | Family | Class |
| 27360960 | 27361260 | + | 31.1 | 2642 | 2971 | -3175 | L1ME3A | L1 | LINE |
| 27361343 | 27361404 | + | 14.8 | 1946 | 2012 | -4996 | L1M4b | L1 | LINE |
| 27361400 | 27362293 | + | 22.3 | 5276 | 6166 | -8 | L1MB5 | L1 | LINE |
| 27362288 | 27362727 | + | 28.7 | 3434 | 3955 | -2191 | L1ME3A | L1 | LINE |
| 27362728 | 27363023 | + | 9.1 | 5845 | 6144 | -19 | L1PA13 | L1 | LINE |
| 27363024 | 27363645 | + | 28.7 | 3956 | 4621 | -1525 | L1ME3A | L1 | LINE |
| 27363683 | 27363915 | + | 28.7 | 4622 | 4839 | -1307 | L1ME3A | L1 | LINE |
| 27363916 | 27364215 | + | 8.4 | 1 | 296 | -15 | AluY | Alu | SINE |
| 27364216 | 27364354 | + | 28.7 | 4840 | 4971 | -1175 | L1ME3A | L1 | LINE |
| 27364355 | 27364867 | + | 22.1 | 1 | 522 | -1969 | Tigger7 | MER2\_type | DNA |
| 27364870 | 27364957 | + | 25.0 | 2404 | 2491 | 0 | Tigger7 | MER2\_type | DNA |
| 27364977 | 27365277 | + | 9.0 | 13 | 311 | 0 | AluY | Alu | SINE |
| 27365307 | 27365652 | + | 29.0 | 5006 | 5353 | -793 | L1ME3A | L1 | LINE |
| 27365701 | 27366069 | + | 13.7 | 1 | 381 | 0 | THE1D | MaLR | LTR |
| 27366072 | 27367658 | + | 31.9 | 2 | 1735 | 0 | MLT-int | MaLR | LTR |
| 27367659 | 27367996 | + | 16.7 | 1 | 357 | -24 | THE1D | MaLR | LTR |
| 27368065 | 27368185 | + | 26.7 | 5455 | 5580 | -590 | L1ME3A | L1 | LINE |
| 27368223 | 27368500 | + | 30.6 | 5885 | 6165 | -5 | L1ME3A | L1 | LINE |

  

|  |  |  |  |  |  |  |  |  |  |
| --- | --- | --- | --- | --- | --- | --- | --- | --- | --- |
| chr5:27399207-27402200 | | | 2992 bp | | |  |  |  |  |
| Genome Start | Genome End | Strand | Div | Repeat Start | Repeat End | Repeat Left | Name | Family | Class |
| 27399207 | 27399357 | + | 23.1 | 5413 | 5566 | -778 | L1MC2 | L1 | LINE |
| 27399358 | 27399716 | + | 17.1 | 1 | 364 | 0 | THE1B | MaLR | LTR |
| 27399719 | 27401285 | + | 29.1 | 2 | 1735 | 0 | MLT-int | MaLR | LTR |
| 27401286 | 27401642 | + | 15.2 | 1 | 364 | 0 | THE1B | MaLR | LTR |
| 27401643 | 27402200 | + | 23.1 | 5567 | 6149 | -195 | L1MC2 | L1 | LINE |

  

|  |  |  |  |  |  |  |  |  |  |
| --- | --- | --- | --- | --- | --- | --- | --- | --- | --- |
| chr5:28046127-28052790 | | | 6312 bp | | |  |  |  |  |
| Genome Start | Genome End | Strand | Div | Repeat Start | Repeat End | Repeat Left | Name | Family | Class |
| 28046127 | 28046362 | - | 30.9 | 5658 | 5910 | -260 | L1ME3B | L1 | LINE |
| 28046375 | 28046733 | + | 15.3 | 1 | 364 | 0 | THE1B | MaLR | LTR |
| 28046734 | 28047502 | + | 13.2 | 1 | 778 | -802 | THE1B-int | MaLR | LTR |
| 28047503 | 28048142 | + | 28.7 | 1 | 612 | 0 | LTR9 | ERV1 | LTR |
| 28048177 | 28048244 | + | 20.6 | 25 | 96 | -7322 | HUERS-P3b | ERV1 | LTR |
| 28048238 | 28048531 | + | 23.4 | 244 | 534 | -6884 | HUERS-P3b | ERV1 | LTR |
| 28048550 | 28049380 | + | 20.7 | 749 | 1599 | -5819 | HUERS-P3b | ERV1 | LTR |
| 28049421 | 28050471 | + | 16.4 | 1600 | 2995 | -5315 | HUERS-P3b | ERV1 | LTR |
| 28050470 | 28050667 | + | 19.6 | 2388 | 4320 | -560 | MER83B-int | ERV1 | LTR |
| 28050925 | 28051270 | + | 25.6 | 273 | 612 | 0 | LTR9 | ERV1 | LTR |
| 28051271 | 28052070 | + | 13.0 | 775 | 1580 | 0 | THE1B-int | MaLR | LTR |
| 28052071 | 28052436 | + | 16.9 | 1 | 364 | 0 | THE1B | MaLR | LTR |
| 28052437 | 28052790 | - | 26.5 | 5300 | 5650 | -520 | L1ME3B | L1 | LINE |

  

|  |  |  |  |  |  |  |  |  |  |
| --- | --- | --- | --- | --- | --- | --- | --- | --- | --- |
| chr5:28089451-28092136 | | | 2682 bp | | |  |  |  |  |
| Genome Start | Genome End | Strand | Div | Repeat Start | Repeat End | Repeat Left | Name | Family | Class |
| 28089451 | 28089507 | - | 27.9 | 359 | 412 | 0 | MER92A | ERV1 | LTR |
| 28089508 | 28089861 | - | 15.6 | 1 | 364 | 0 | THE1B | MaLR | LTR |
| 28089862 | 28091432 | - | 31.4 | 2 | 1735 | 0 | MLT-int | MaLR | LTR |
| 28091437 | 28091788 | - | 14.8 | 1 | 362 | -2 | THE1B | MaLR | LTR |
| 28091789 | 28092136 | - | 27.9 | 4 | 358 | -54 | MER92A | ERV1 | LTR |

  

|  |  |  |  |  |  |  |  |  |  |
| --- | --- | --- | --- | --- | --- | --- | --- | --- | --- |
| chr5:28460594-28463455 | | | 2862 bp | | |  |  |  |  |
| Genome Start | Genome End | Strand | Div | Repeat Start | Repeat End | Repeat Left | Name | Family | Class |
| 28460594 | 28461070 | + | 27.8 | 111 | 599 | -27 | MLT1E2 | MaLR | LTR |
| 28461071 | 28461478 | + | 13.2 | 1 | 381 | 0 | THE1D | MaLR | LTR |
| 28461479 | 28463090 | + | 30.0 | 1 | 1735 | 0 | MLT-int | MaLR | LTR |
| 28463091 | 28463428 | + | 19.4 | 1 | 381 | 0 | THE1D | MaLR | LTR |
| 28463429 | 28463455 | + | 27.8 | 600 | 626 | 0 | MLT1E2 | MaLR | LTR |

  

|  |  |  |  |  |  |  |  |  |  |
| --- | --- | --- | --- | --- | --- | --- | --- | --- | --- |
| chr5:28917575-28928585 | | | 10863 bp | | |  |  |  |  |
| Genome Start | Genome End | Strand | Div | Repeat Start | Repeat End | Repeat Left | Name | Family | Class |
| 28917575 | 28921397 | + | 24.1 | 3 | 3048 | -3814 | L1PB3 | L1 | LINE |
| 28921398 | 28921783 | + | 16.9 | 1 | 428 | 0 | MSTA | MaLR | LTR |
| 28921786 | 28923416 | + | 31.7 | 2 | 1735 | 0 | MLT-int | MaLR | LTR |
| 28923417 | 28923788 | + | 19.5 | 1 | 416 | -12 | MSTA | MaLR | LTR |
| 28923791 | 28924674 | + | 18.6 | 3031 | 3903 | -2959 | L1PB3 | L1 | LINE |
| 28924675 | 28925031 | + | 14.5 | 1 | 379 | -2 | THE1D | MaLR | LTR |
| 28925032 | 28926071 | + | 18.6 | 3904 | 4942 | -1920 | L1PB3 | L1 | LINE |
| 28926072 | 28926143 | - | 13.5 | 478 | 552 | 0 | MLT2A2 | ERVL | LTR |
| 28926158 | 28926325 | + | 16.8 | 322 | 505 | -50 | MLT2A2 | ERVL | LTR |
| 28926390 | 28926522 | + | 20.6 | 5993 | 6135 | -29 | L1ME2 | L1 | LINE |
| 28926569 | 28927048 | - | 17.4 | 1 | 500 | -54 | MLT2A2 | ERVL | LTR |
| 28927069 | 28928585 | + | 17.7 | 4948 | 6866 | -4 | L1PB3 | L1 | LINE |

  

|  |  |  |  |  |  |  |  |  |  |
| --- | --- | --- | --- | --- | --- | --- | --- | --- | --- |
| chr5:29678544-29681215 | | | 2645 bp | | |  |  |  |  |
| Genome Start | Genome End | Strand | Div | Repeat Start | Repeat End | Repeat Left | Name | Family | Class |
| 29678544 | 29678622 | + | 17.7 | 6511 | 6595 | -964 | L1M2 | L1 | LINE |
| 29678623 | 29678984 | + | 13.5 | 1 | 364 | 0 | THE1B | MaLR | LTR |
| 29679012 | 29680560 | + | 11.1 | 15 | 1580 | 0 | THE1B-int | MaLR | LTR |
| 29680561 | 29680945 | + | 14.4 | 1 | 364 | 0 | THE1B | MaLR | LTR |
| 29680946 | 29681215 | + | 18.2 | 6594 | 6861 | -858 | L1M2 | L1 | LINE |

  

|  |  |  |  |  |  |  |  |  |  |
| --- | --- | --- | --- | --- | --- | --- | --- | --- | --- |
| chr5:30069076-30074161 | | | 5062 bp | | |  |  |  |  |
| Genome Start | Genome End | Strand | Div | Repeat Start | Repeat End | Repeat Left | Name | Family | Class |
| 30069076 | 30070411 | + | 21.4 | 3423 | 4815 | -1325 | L1MA9 | L1 | LINE |
| 30070430 | 30070738 | + | 16.8 | 54 | 375 | 0 | THE1C | MaLR | LTR |
| 30070741 | 30072311 | + | 30.0 | 2 | 1731 | -4 | MLT-int | MaLR | LTR |
| 30072316 | 30072674 | + | 16.8 | 1 | 374 | -1 | THE1C | MaLR | LTR |
| 30072675 | 30074161 | + | 21.0 | 4810 | 6272 | -40 | L1MA9 | L1 | LINE |

  

|  |  |  |  |  |  |  |  |  |  |
| --- | --- | --- | --- | --- | --- | --- | --- | --- | --- |
| chr5:30175952-30182939 | | | 6974 bp | | |  |  |  |  |
| Genome Start | Genome End | Strand | Div | Repeat Start | Repeat End | Repeat Left | Name | Family | Class |
| 30175952 | 30177629 | - | 13.2 | 5993 | 7721 | 0 | L1MA1 | L1 | LINE |
| 30177633 | 30178009 | - | 14.1 | 1 | 375 | 0 | THE1C | MaLR | LTR |
| 30178010 | 30179589 | - | 13.9 | 3 | 1580 | 0 | THE1C-int | MaLR | LTR |
| 30179592 | 30179943 | - | 12.4 | 26 | 375 | 0 | THE1C | MaLR | LTR |
| 30179953 | 30180094 | - | 12.1 | 5837 | 5974 | -1591 | L1MA1 | L1 | LINE |
| 30180095 | 30180435 | - | 14.3 | 1 | 340 | 0 | AluSg | Alu | SINE |
| 30180436 | 30182939 | - | 12.5 | 3354 | 5836 | -1729 | L1MA1 | L1 | LINE |

  

|  |  |  |  |  |  |  |  |  |  |
| --- | --- | --- | --- | --- | --- | --- | --- | --- | --- |
| chr5:30344960-30350813 | | | 5417 bp | | |  |  |  |  |
| Genome Start | Genome End | Strand | Div | Repeat Start | Repeat End | Repeat Left | Name | Family | Class |
| 30344960 | 30345019 | - | 25.0 | 5280 | 5340 | -756 | L1M | L1 | LINE |
| 30345038 | 30345443 | + | 17.9 | 1 | 428 | 0 | MSTA | MaLR | LTR |
| 30345446 | 30346998 | + | 29.8 | 2 | 1735 | 0 | MLT-int | MaLR | LTR |
| 30346999 | 30347392 | + | 18.2 | 1 | 428 | 0 | MSTA | MaLR | LTR |
| 30347396 | 30347883 | - | 29.3 | 4611 | 5231 | -849 | L1M | L1 | LINE |
| 30347978 | 30348090 | - | 25.7 | 4018 | 4142 | -1938 | L1M | L1 | LINE |
| 30348082 | 30348703 | - | 29.4 | 3130 | 3705 | -2375 | L1M | L1 | LINE |
| 30348864 | 30348932 | - | 23.8 | 6225 | 6290 | -1 | L1MA8 | L1 | LINE |
| 30348933 | 30349326 | - | 17.3 | 1 | 403 | 0 | MER57A | ERV1 | LTR |
| 30349327 | 30349588 | - | 23.9 | 5958 | 6224 | -67 | L1MA8 | L1 | LINE |
| 30349609 | 30349811 | - | 25.5 | 2755 | 2969 | -3111 | L1M | L1 | LINE |
| 30349812 | 30350129 | - | 14.2 | 1 | 312 | 0 | AluSx | Alu | SINE |
| 30350130 | 30350225 | - | 25.5 | 2657 | 2754 | -3326 | L1M | L1 | LINE |
| 30350352 | 30350679 | - | 34.1 | 119 | 453 | -5617 | L1M | L1 | LINE |
| 30350703 | 30350813 | - | 33.3 | 6 | 117 | -5953 | L1M | L1 | LINE |

  

|  |  |  |  |  |  |  |  |  |  |
| --- | --- | --- | --- | --- | --- | --- | --- | --- | --- |
| chr5:30511604-30514268 | | | 2663 bp | | |  |  |  |  |
| Genome Start | Genome End | Strand | Div | Repeat Start | Repeat End | Repeat Left | Name | Family | Class |
| 30511604 | 30511948 | - | 16.2 | 48 | 381 | 0 | THE1D | MaLR | LTR |
| 30511949 | 30512296 | - | 9.2 | 1 | 355 | 0 | THE1A | MaLR | LTR |
| 30512297 | 30513870 | - | 29.3 | 2 | 1735 | 0 | MLT-int | MaLR | LTR |
| 30513873 | 30514225 | - | 12.5 | 1 | 355 | 0 | THE1A | MaLR | LTR |
| 30514226 | 30514268 | - | 16.2 | 4 | 47 | -334 | THE1D | MaLR | LTR |

  

|  |  |  |  |  |  |  |  |  |  |
| --- | --- | --- | --- | --- | --- | --- | --- | --- | --- |
| chr5:30520248-30535842 | | | 15595 bp | | |  |  |  |  |
| Genome Start | Genome End | Strand | Div | Repeat Start | Repeat End | Repeat Left | Name | Family | Class |
| 30520248 | 30522516 | - | 7.4 | 3870 | 6149 | -5 | L1PA6 | L1 | LINE |
| 30522517 | 30523476 | - | 2.5 | 1 | 968 | 0 | LTR5\_Hs | ERVK | LTR |
| 30523477 | 30531011 | - | 1.6 | 1 | 7536 | 0 | HERVK | ERVK | LTR |
| 30531012 | 30531962 | - | 2.7 | 1 | 968 | 0 | LTR5\_Hs | ERVK | LTR |
| 30531963 | 30535842 | - | 8.5 | 5 | 3875 | -2271 | L1PA6 | L1 | LINE |

  

|  |  |  |  |  |  |  |  |  |  |
| --- | --- | --- | --- | --- | --- | --- | --- | --- | --- |
| chr5:30732946-30742823 | | | 9627 bp | | |  |  |  |  |
| Genome Start | Genome End | Strand | Div | Repeat Start | Repeat End | Repeat Left | Name | Family | Class |
| 30732946 | 30733285 | + | 18.7 | 3144 | 3508 | -2638 | L1MA3 | L1 | LINE |
| 30733286 | 30733651 | + | 15.1 | 1 | 375 | 0 | THE1C | MaLR | LTR |
| 30733654 | 30735240 | + | 32.0 | 2 | 1735 | 0 | MLT-int | MaLR | LTR |
| 30735241 | 30735600 | + | 15.9 | 1 | 375 | 0 | THE1C | MaLR | LTR |
| 30735601 | 30735715 | + | 18.7 | 3509 | 3618 | -2528 | L1MA3 | L1 | LINE |
| 30735716 | 30736111 | + | 15.9 | 1 | 375 | 0 | THE1C | MaLR | LTR |
| 30736117 | 30737705 | + | 30.3 | 5 | 1735 | 0 | MLT-int | MaLR | LTR |
| 30737706 | 30738070 | + | 15.0 | 1 | 349 | -26 | THE1C | MaLR | LTR |
| 30738074 | 30738454 | + | 3.7 | 5774 | 6155 | 0 | L1PA4 | L1 | LINE |
| 30738603 | 30738721 | + | 21.2 | 5715 | 5855 | -266 | L1M5 | L1 | LINE |
| 30738810 | 30739735 | + | 16.0 | 3614 | 4565 | -1581 | L1MA3 | L1 | LINE |
| 30739736 | 30740062 | - | 16.5 | 51 | 381 | 0 | THE1D | MaLR | LTR |
| 30740063 | 30741074 | - | 11.4 | 1 | 1265 | -1 | MER11A | ERVK | LTR |
| 30741075 | 30741141 | - | 18.0 | 1 | 59 | -322 | THE1D | MaLR | LTR |
| 30741147 | 30742247 | + | 15.1 | 4569 | 5692 | -613 | L1MA3 | L1 | LINE |
| 30742248 | 30742633 | + | 12.8 | 5886 | 6305 | 0 | L1MA3 | L1 | LINE |
| 30742634 | 30742823 | + | 19.5 | 5693 | 5927 | -239 | L1MA3 | L1 | LINE |

  

|  |  |  |  |  |  |  |  |  |  |
| --- | --- | --- | --- | --- | --- | --- | --- | --- | --- |
| chr5:31598980-31603037 | | | 3955 bp | | |  |  |  |  |
| Genome Start | Genome End | Strand | Div | Repeat Start | Repeat End | Repeat Left | Name | Family | Class |
| 31598980 | 31599151 | + | 32.3 | 5494 | 5656 | -2152 | L1MC4a | L1 | LINE |
| 31599165 | 31599548 | + | 20.8 | 1 | 361 | -366 | LTR8A | ERV1 | LTR |
| 31599549 | 31599849 | - | 12.6 | 1 | 302 | -10 | AluSx | Alu | SINE |
| 31599850 | 31600110 | + | 20.8 | 362 | 618 | -109 | LTR8A | ERV1 | LTR |
| 31600128 | 31600502 | - | 10.6 | 1 | 375 | 0 | THE1C | MaLR | LTR |
| 31600503 | 31600559 | - | 10.6 | 1532 | 1580 | 0 | THE1C-int | MaLR | LTR |
| 31600560 | 31600842 | + | 11.7 | 1 | 284 | -28 | AluSx | Alu | SINE |
| 31600886 | 31602356 | - | 10.6 | 17 | 1531 | -49 | THE1C-int | MaLR | LTR |
| 31602361 | 31602736 | - | 10.3 | 1 | 375 | 0 | THE1C | MaLR | LTR |
| 31602740 | 31602833 | + | 13.6 | 641 | 727 | 0 | LTR8A | ERV1 | LTR |
| 31602857 | 31603037 | + | 31.1 | 5712 | 5915 | -1893 | L1MC4a | L1 | LINE |

  

|  |  |  |  |  |  |  |  |  |  |
| --- | --- | --- | --- | --- | --- | --- | --- | --- | --- |
| chr5:33126450-33148079 | | | 21356 bp | | |  |  |  |  |
| Genome Start | Genome End | Strand | Div | Repeat Start | Repeat End | Repeat Left | Name | Family | Class |
| 33126450 | 33127501 | - | 14.9 | 3972 | 5022 | -1826 | L1M2 | L1 | LINE |
| 33127499 | 33129536 | - | 19.3 | 1 | 1890 | -4400 | L1P4e | L1 | LINE |
| 33129550 | 33130308 | - | 14.2 | 4683 | 5447 | -1402 | L1PA15-16 | L1 | LINE |
| 33130309 | 33130600 | - | 13.7 | 1 | 293 | -19 | AluSx | Alu | SINE |
| 33130601 | 33130884 | - | 14.2 | 4402 | 4682 | -2167 | L1PA15-16 | L1 | LINE |
| 33130885 | 33131142 | - | 21.3 | 43 | 300 | -12 | AluJo | Alu | SINE |
| 33131143 | 33131762 | - | 14.2 | 3788 | 4401 | -2448 | L1PA15-16 | L1 | LINE |
| 33131767 | 33137910 | - | 4.6 | 2 | 6155 | 0 | L1PA4 | L1 | LINE |
| 33137913 | 33138174 | - | 13.5 | 3525 | 3800 | -3049 | L1PA15-16 | L1 | LINE |
| 33138175 | 33138484 | - | 9.9 | 1 | 309 | 0 | AluSp | Alu | SINE |
| 33138485 | 33141946 | - | 14.7 | 4 | 3524 | -3325 | L1PA15-16 | L1 | LINE |
| 33141946 | 33142207 | - | 17.8 | 3615 | 3878 | -2967 | L1M2 | L1 | LINE |
| 33142208 | 33142593 | - | 15.3 | 1 | 428 | 0 | MSTA | MaLR | LTR |
| 33142594 | 33143031 | - | 17.8 | 3177 | 3614 | -3231 | L1M2 | L1 | LINE |
| 33143032 | 33143451 | + | 13.0 | 1 | 428 | 0 | MSTA | MaLR | LTR |
| 33143454 | 33145068 | + | 30.3 | 2 | 1733 | -2 | MLT-int | MaLR | LTR |
| 33145073 | 33145491 | + | 13.0 | 3 | 428 | 0 | MSTA | MaLR | LTR |
| 33145492 | 33145955 | - | 18.6 | 2701 | 3176 | -3669 | L1M2 | L1 | LINE |
| 33145974 | 33147235 | - | 9.6 | 4899 | 6168 | 0 | L1PA10 | L1 | LINE |
| 33147471 | 33148079 | - | 32.7 | 61 | 693 | -6156 | L1M2 | L1 | LINE |

  

|  |  |  |  |  |  |  |  |  |  |
| --- | --- | --- | --- | --- | --- | --- | --- | --- | --- |
| chr5:33166350-33174751 | | | 8216 bp | | |  |  |  |  |
| Genome Start | Genome End | Strand | Div | Repeat Start | Repeat End | Repeat Left | Name | Family | Class |
| 33166350 | 33166550 | - | 16.1 | 4839 | 5042 | -1305 | L1M3b | L1 | LINE |
| 33166551 | 33166879 | + | 13.3 | 1 | 364 | 0 | THE1B | MaLR | LTR |
| 33166882 | 33168445 | + | 30.1 | 2 | 1735 | 0 | MLT-int | MaLR | LTR |
| 33168446 | 33168789 | + | 15.3 | 1 | 364 | 0 | THE1B | MaLR | LTR |
| 33168790 | 33168935 | - | 16.1 | 4692 | 4838 | -1509 | L1M3b | L1 | LINE |
| 33169070 | 33169351 | - | 14.9 | 2 | 283 | -29 | AluSx | Alu | SINE |
| 33169352 | 33170657 | - | 21.7 | 3482 | 4691 | -1656 | L1M3b | L1 | LINE |
| 33170708 | 33174259 | - | 21.7 | 191 | 3481 | -2866 | L1M3b | L1 | LINE |
| 33174260 | 33174567 | - | 7.5 | 1 | 308 | 0 | AluY | Alu | SINE |
| 33174568 | 33174751 | - | 25.9 | 8 | 188 | -6093 | L1M3b | L1 | LINE |

  

|  |  |  |  |  |  |  |  |  |  |
| --- | --- | --- | --- | --- | --- | --- | --- | --- | --- |
| chr5:33418313-33422469 | | | 4015 bp | | |  |  |  |  |
| Genome Start | Genome End | Strand | Div | Repeat Start | Repeat End | Repeat Left | Name | Family | Class |
| 33418313 | 33418400 | - | 22.4 | 3320 | 3415 | -4 | L2 | L2 | LINE |
| 33418406 | 33418895 | - | 25.9 | 1968 | 2530 | -889 | L2 | L2 | LINE |
| 33419017 | 33419398 | - | 21.2 | 1 | 396 | 0 | MSTD | MaLR | LTR |
| 33419405 | 33419834 | + | 23.8 | 262 | 703 | -4 | MER39 | ERV1 | LTR |
| 33419843 | 33420368 | - | 29.2 | 1403 | 1961 | -1458 | L2 | L2 | LINE |
| 33420370 | 33420728 | - | 13.8 | 1 | 428 | 0 | MSTA | MaLR | LTR |
| 33420729 | 33421441 | - | 18.1 | 920 | 1651 | 0 | MSTA-int | MaLR | LTR |
| 33421442 | 33421883 | - | 15.2 | 1 | 446 | -1205 | MSTA-int | MaLR | LTR |
| 33421884 | 33422293 | - | 11.3 | 1 | 428 | 0 | MSTA | MaLR | LTR |
| 33422295 | 33422469 | - | 36.0 | 1229 | 1405 | -2014 | L2 | L2 | LINE |

  

|  |  |  |  |  |  |  |  |  |  |
| --- | --- | --- | --- | --- | --- | --- | --- | --- | --- |
| chr5:33893579-33912557 | | | 18852 bp | | |  |  |  |  |
| Genome Start | Genome End | Strand | Div | Repeat Start | Repeat End | Repeat Left | Name | Family | Class |
| 33893579 | 33894238 | + | 15.2 | 2349 | 3043 | -3103 | L1MD | L1 | LINE |
| 33894239 | 33894548 | + | 18.8 | 3 | 311 | -1 | AluJo | Alu | SINE |
| 33894576 | 33899997 | + | 9.6 | 6 | 5386 | -1075 | L1PA7 | L1 | LINE |
| 33899995 | 33900862 | + | 5.3 | 5598 | 6469 | 0 | L1PA7 | L1 | LINE |
| 33900855 | 33903480 | + | 14.3 | 3510 | 6152 | -2 | L1PB3 | L1 | LINE |
| 33903540 | 33903656 | - | 20.9 | 2564 | 2680 | -3466 | L1M5 | L1 | LINE |
| 33903683 | 33904063 | + | 13.8 | 1 | 428 | 0 | MSTA | MaLR | LTR |
| 33904064 | 33904556 | + | 18.6 | 1 | 475 | -1176 | MSTA-int | MaLR | LTR |
| 33904557 | 33904755 | + | 13.6 | 1 | 229 | -1 | MER30 | MER1\_type | DNA |
| 33904756 | 33905377 | + | 18.6 | 476 | 1111 | -540 | MSTA-int | MaLR | LTR |
| 33905372 | 33906372 | - | 11.9 | 1 | 1148 | -88 | MER11B | ERVK | LTR |
| 33906381 | 33906494 | + | 14.2 | 1538 | 1651 | 0 | MSTA-int | MaLR | LTR |
| 33906495 | 33906878 | + | 15.4 | 1 | 426 | -2 | MSTA | MaLR | LTR |
| 33906906 | 33907676 | + | 19.7 | 3066 | 3637 | -2745 | L1M5 | L1 | LINE |
| 33907677 | 33907727 | + | 19.6 | 371 | 421 | -7 | MSTA | MaLR | LTR |
| 33907728 | 33908125 | + | 19.7 | 3638 | 3932 | -2450 | L1M5 | L1 | LINE |
| 33908126 | 33908438 | + | 10.0 | 1 | 310 | 0 | AluSg | Alu | SINE |
| 33908439 | 33908526 | + | 16.2 | 3698 | 3785 | -2548 | L1MC1 | L1 | LINE |
| 33908527 | 33908593 | + | 23.9 | 769 | 835 | -816 | MST-int | MaLR | LTR |
| 33908594 | 33910649 | + | 16.2 | 3786 | 5862 | -471 | L1MC1 | L1 | LINE |
| 33910650 | 33910920 | + | 13.3 | 1 | 271 | -38 | AluSc | Alu | SINE |
| 33910921 | 33911377 | + | 14.7 | 5865 | 6332 | -1 | L1MC1 | L1 | LINE |
| 33911375 | 33912557 | + | 16.6 | 3840 | 5025 | -1121 | L1MD | L1 | LINE |

  

|  |  |  |  |  |  |  |  |  |  |
| --- | --- | --- | --- | --- | --- | --- | --- | --- | --- |
| chr5:33949701-33956584 | | | 6837 bp | | |  |  |  |  |
| Genome Start | Genome End | Strand | Div | Repeat Start | Repeat End | Repeat Left | Name | Family | Class |
| 33949701 | 33950313 | - | 9.9 | 6252 | 6867 | 0 | L1PB1 | L1 | LINE |
| 33950347 | 33950570 | - | 12.0 | 4113 | 4269 | -2593 | L1PB1 | L1 | LINE |
| 33950571 | 33951252 | - | 7.8 | 5451 | 6152 | -2 | L1PA6 | L1 | LINE |
| 33951253 | 33951656 | - | 12.0 | 3701 | 4112 | -2750 | L1PB1 | L1 | LINE |
| 33951657 | 33952017 | + | 12.8 | 1 | 364 | 0 | THE1B | MaLR | LTR |
| 33952018 | 33952290 | + | 7.8 | 1 | 272 | -1308 | THE1B-int | MaLR | LTR |
| 33952303 | 33952581 | + | 6.8 | 1302 | 1580 | 0 | THE1B-int | MaLR | LTR |
| 33952584 | 33952943 | + | 10.1 | 3 | 364 | 0 | THE1B | MaLR | LTR |
| 33952944 | 33956584 | - | 11.5 | 1 | 3705 | -3157 | L1PB1 | L1 | LINE |

  

|  |  |  |  |  |  |  |  |  |  |
| --- | --- | --- | --- | --- | --- | --- | --- | --- | --- |
| chr5:34328920-34331793 | | | 2840 bp | | |  |  |  |  |
| Genome Start | Genome End | Strand | Div | Repeat Start | Repeat End | Repeat Left | Name | Family | Class |
| 34328920 | 34329018 | + | 15.3 | 1 | 98 | -328 | MSTA | MaLR | LTR |
| 34329027 | 34329316 | - | 7.6 | 6 | 306 | -5 | AluY | Alu | SINE |
| 34329325 | 34329682 | - | 13.9 | 1 | 359 | -5 | THE1B | MaLR | LTR |
| 34329683 | 34331115 | - | 10.5 | 3 | 1580 | 0 | THE1B-int | MaLR | LTR |
| 34331118 | 34331479 | - | 13.6 | 1 | 364 | 0 | THE1B | MaLR | LTR |
| 34331496 | 34331793 | + | 20.1 | 132 | 428 | 0 | MSTA | MaLR | LTR |

  

|  |  |  |  |  |  |  |  |  |  |
| --- | --- | --- | --- | --- | --- | --- | --- | --- | --- |
| chr5:34761957-34764781 | | | 2803 bp | | |  |  |  |  |
| Genome Start | Genome End | Strand | Div | Repeat Start | Repeat End | Repeat Left | Name | Family | Class |
| 34761957 | 34762069 | + | 14.7 | 2 | 110 | -202 | AluJo | Alu | SINE |
| 34762073 | 34762450 | - | 14.4 | 1 | 428 | 0 | MSTA | MaLR | LTR |
| 34762467 | 34762788 | + | 12.5 | 39 | 364 | 0 | THE1B | MaLR | LTR |
| 34762791 | 34764246 | + | 29.5 | 2 | 1734 | -1 | MLT-int | MaLR | LTR |
| 34764248 | 34764605 | + | 11.3 | 1 | 364 | 0 | THE1B | MaLR | LTR |
| 34764606 | 34764781 | + | 13.9 | 108 | 286 | -26 | AluJo | Alu | SINE |

  

|  |  |  |  |  |  |  |  |  |  |
| --- | --- | --- | --- | --- | --- | --- | --- | --- | --- |
| chr5:36047636-36053370 | | | 5730 bp | | |  |  |  |  |
| Genome Start | Genome End | Strand | Div | Repeat Start | Repeat End | Repeat Left | Name | Family | Class |
| 36047636 | 36047705 | + | 26.1 | 5 | 73 | -581 | MLT1E1 | MaLR | LTR |
| 36047706 | 36048122 | + | 11.0 | 1 | 428 | 0 | MSTA | MaLR | LTR |
| 36048123 | 36048250 | + | 16.4 | 1 | 142 | -1509 | MSTA-int | MaLR | LTR |
| 36048251 | 36048636 | + | 14.2 | 1 | 381 | 0 | THE1D | MaLR | LTR |
| 36048639 | 36050171 | + | 31.6 | 2 | 1735 | 0 | MLT1E1-int | MaLR | LTR |
| 36050172 | 36050557 | + | 15.1 | 1 | 388 | -27 | THE1D | MaLR | LTR |
| 36050558 | 36050872 | - | 12.9 | 1 | 312 | 0 | AluSx | Alu | SINE |
| 36050873 | 36050911 | + | 24.1 | 389 | 428 | 0 | MSTA | MaLR | LTR |
| 36050912 | 36052385 | + | 19.8 | 138 | 1651 | 0 | MSTA-int | MaLR | LTR |
| 36052386 | 36052804 | + | 17.8 | 1 | 426 | -2 | MSTA | MaLR | LTR |
| 36052808 | 36053370 | + | 23.8 | 70 | 654 | 0 | MLT1E1 | MaLR | LTR |

  

|  |  |  |  |  |  |  |  |  |  |
| --- | --- | --- | --- | --- | --- | --- | --- | --- | --- |
| chr5:37168106-37174252 | | | 6085 bp | | |  |  |  |  |
| Genome Start | Genome End | Strand | Div | Repeat Start | Repeat End | Repeat Left | Name | Family | Class |
| 37168106 | 37168196 | - | 12.0 | 5289 | 5379 | -776 | L1P | L1 | LINE |
| 37168197 | 37168505 | - | 4.9 | 1 | 306 | -5 | AluY | Alu | SINE |
| 37168506 | 37169356 | - | 10.4 | 4391 | 5288 | -858 | L1P | L1 | LINE |
| 37169397 | 37170653 | - | 10.4 | 2999 | 4390 | -1756 | L1P | L1 | LINE |
| 37170654 | 37170951 | - | 9.4 | 1 | 301 | -12 | AluSp | Alu | SINE |
| 37170952 | 37171186 | - | 10.4 | 2776 | 2998 | -3148 | L1P | L1 | LINE |
| 37171187 | 37171411 | - | 12.3 | 161 | 375 | 0 | THE1C | MaLR | LTR |
| 37171412 | 37171710 | + | 11.7 | 1 | 301 | -8 | AluSc | Alu | SINE |
| 37171711 | 37171863 | - | 12.3 | 1 | 160 | -215 | THE1C | MaLR | LTR |
| 37171864 | 37173383 | - | 10.4 | 1 | 1580 | 0 | THE1C-int | MaLR | LTR |
| 37173384 | 37173761 | - | 11.8 | 1 | 375 | 0 | THE1C | MaLR | LTR |
| 37173784 | 37174252 | - | 11.5 | 2292 | 2767 | -3379 | L1P | L1 | LINE |

  

|  |  |  |  |  |  |  |  |  |  |
| --- | --- | --- | --- | --- | --- | --- | --- | --- | --- |
| chr5:39385346-39389735 | | | 3936 bp | | |  |  |  |  |
| Genome Start | Genome End | Strand | Div | Repeat Start | Repeat End | Repeat Left | Name | Family | Class |
| 39385346 | 39385836 | + | 36.0 | 1789 | 2336 | -1083 | L2 | L2 | LINE |
| 39385839 | 39386214 | - | 17.8 | 1 | 428 | 0 | MSTA | MaLR | LTR |
| 39386215 | 39387790 | - | 15.1 | 3 | 1580 | 0 | MSTA-int | MaLR | LTR |
| 39387793 | 39388168 | - | 15.6 | 1 | 428 | 0 | MSTA | MaLR | LTR |
| 39388169 | 39388698 | + | 35.0 | 2334 | 2891 | -528 | L2 | L2 | LINE |
| 39388722 | 39389211 | + | 20.2 | 1 | 501 | -4 | MLT1D | MaLR | LTR |
| 39389639 | 39389735 | + | 25.8 | 3311 | 3417 | -2 | L2 | L2 | LINE |

  

|  |  |  |  |  |  |  |  |  |  |
| --- | --- | --- | --- | --- | --- | --- | --- | --- | --- |
| chr5:41425705-41438984 | | | 13195 bp | | |  |  |  |  |
| Genome Start | Genome End | Strand | Div | Repeat Start | Repeat End | Repeat Left | Name | Family | Class |
| 41425705 | 41426480 | - | 23.6 | 5457 | 6233 | -6 | L1ME1 | L1 | LINE |
| 41426501 | 41429496 | + | 17.6 | 23 | 3064 | -4501 | L1M1 | L1 | LINE |
| 41429497 | 41429808 | + | 9.6 | 1 | 303 | -8 | AluY | Alu | SINE |
| 41429809 | 41431997 | + | 12.2 | 3065 | 5209 | -2356 | L1M1 | L1 | LINE |
| 41431998 | 41432386 | + | 14.7 | 1 | 428 | 0 | MSTA | MaLR | LTR |
| 41432389 | 41434008 | + | 29.1 | 2 | 1733 | -2 | MLT1F2-int | MaLR | LTR |
| 41434011 | 41434388 | + | 16.9 | 1 | 428 | 0 | MSTA | MaLR | LTR |
| 41434389 | 41436852 | + | 10.8 | 3791 | 6298 | -4 | L1MA1 | L1 | LINE |
| 41436888 | 41437441 | - | 27.0 | 4830 | 5456 | -763 | L1ME1 | L1 | LINE |
| 41437443 | 41437494 | + | 23.1 | 1 | 53 | -509 | MLT1F2 | MaLR | LTR |
| 41437520 | 41438984 | - | 26.8 | 2184 | 3678 | -2541 | L1ME1 | L1 | LINE |

  

|  |  |  |  |  |  |  |  |  |  |
| --- | --- | --- | --- | --- | --- | --- | --- | --- | --- |
| chr5:42135001-42141054 | | | 6048 bp | | |  |  |  |  |
| Genome Start | Genome End | Strand | Div | Repeat Start | Repeat End | Repeat Left | Name | Family | Class |
| 42135001 | 42135606 | - | 17.6 | 5047 | 5635 | -531 | L1P4 | L1 | LINE |
| 42135607 | 42136236 | + | 10.5 | 3 | 608 | 0 | LTR14B | ERVK | LTR |
| 42136237 | 42136681 | - | 17.6 | 4596 | 5046 | -1100 | L1P4 | L1 | LINE |
| 42136682 | 42137049 | + | 13.6 | 1 | 371 | -4 | THE1C | MaLR | LTR |
| 42137056 | 42138637 | + | 29.9 | 2 | 1735 | 0 | MLT-int | MaLR | LTR |
| 42138638 | 42138981 | + | 14.1 | 1 | 375 | 0 | THE1C | MaLR | LTR |
| 42138982 | 42141054 | - | 17.6 | 2451 | 4595 | -1551 | L1P4 | L1 | LINE |

  

|  |  |  |  |  |  |  |  |  |  |
| --- | --- | --- | --- | --- | --- | --- | --- | --- | --- |
| chr5:42372281-42380930 | | | 8578 bp | | |  |  |  |  |
| Genome Start | Genome End | Strand | Div | Repeat Start | Repeat End | Repeat Left | Name | Family | Class |
| 42372281 | 42374701 | + | 22.0 | 1 | 2487 | -4362 | L1MA4A | L1 | LINE |
| 42374702 | 42375069 | - | 13.0 | 1 | 381 | 0 | THE1D | MaLR | LTR |
| 42375070 | 42376702 | - | 30.3 | 2 | 1735 | 0 | MLT-int | MaLR | LTR |
| 42376705 | 42376994 | - | 13.2 | 1 | 381 | 0 | THE1D | MaLR | LTR |
| 42376995 | 42377589 | + | 22.2 | 2488 | 3074 | -3771 | L1MA4A | L1 | LINE |
| 42377591 | 42380104 | + | 18.2 | 3699 | 6246 | -759 | L1MA4A | L1 | LINE |
| 42380174 | 42380930 | + | 14.9 | 6244 | 7003 | -2 | L1MA4A | L1 | LINE |

  

|  |  |  |  |  |  |  |  |  |  |
| --- | --- | --- | --- | --- | --- | --- | --- | --- | --- |
| chr5:42462712-42465401 | | | 2550 bp | | |  |  |  |  |
| Genome Start | Genome End | Strand | Div | Repeat Start | Repeat End | Repeat Left | Name | Family | Class |
| 42462712 | 42462968 | - | 34.2 | 4130 | 4428 | -61 | L3 | CR1 | LINE |
| 42463055 | 42463419 | - | 10.2 | 1 | 364 | 0 | THE1B | MaLR | LTR |
| 42463420 | 42464906 | - | 27.9 | 2 | 1735 | 0 | MLT-int | MaLR | LTR |
| 42464909 | 42465270 | - | 13.0 | 1 | 364 | 0 | THE1B | MaLR | LTR |
| 42465323 | 42465401 | - | 35.4 | 3851 | 3930 | -559 | L3 | CR1 | LINE |

  

|  |  |  |  |  |  |  |  |  |  |
| --- | --- | --- | --- | --- | --- | --- | --- | --- | --- |
| chr5:42559135-42565285 | | | 6146 bp | | |  |  |  |  |
| Genome Start | Genome End | Strand | Div | Repeat Start | Repeat End | Repeat Left | Name | Family | Class |
| 42559135 | 42559573 | + | 13.8 | 1 | 440 | -1978 | Tigger1 | MER2\_type | DNA |
| 42559574 | 42559928 | + | 10.4 | 1 | 355 | 0 | THE1A | MaLR | LTR |
| 42559931 | 42561499 | + | 31.1 | 2 | 1735 | 0 | MLT-int | MaLR | LTR |
| 42561500 | 42561853 | + | 9.3 | 1 | 355 | 0 | THE1A | MaLR | LTR |
| 42561854 | 42562538 | + | 13.8 | 441 | 1129 | -1289 | Tigger1 | MER2\_type | DNA |
| 42562542 | 42563000 | + | 8.5 | 3581 | 4044 | -4111 | L1PREC2 | L1 | LINE |
| 42563001 | 42563704 | + | 7.0 | 4045 | 4771 | -3370 | L1PREC2 | L1 | LINE |
| 42563705 | 42564872 | + | 13.0 | 1139 | 2302 | -116 | Tigger1 | MER2\_type | DNA |
| 42564873 | 42565169 | - | 9.8 | 1 | 297 | -14 | AluY | Alu | SINE |
| 42565170 | 42565285 | + | 13.0 | 2303 | 2418 | 0 | Tigger1 | MER2\_type | DNA |

  

|  |  |  |  |  |  |  |  |  |  |
| --- | --- | --- | --- | --- | --- | --- | --- | --- | --- |
| chr5:43731173-43734862 | | | 3363 bp | | |  |  |  |  |
| Genome Start | Genome End | Strand | Div | Repeat Start | Repeat End | Repeat Left | Name | Family | Class |
| 43731173 | 43731512 | + | 31.7 | 3776 | 4148 | -341 | L3 | CR1 | LINE |
| 43731513 | 43731859 | - | 14.8 | 1 | 364 | 0 | THE1B | MaLR | LTR |
| 43731862 | 43733420 | - | 30.8 | 2 | 1733 | -2 | MLT-int | MaLR | LTR |
| 43733423 | 43733825 | - | 12.4 | 1 | 364 | 0 | THE1B | MaLR | LTR |
| 43733826 | 43733896 | + | 31.7 | 4149 | 4233 | -256 | L3 | CR1 | LINE |
| 43734080 | 43734637 | + | 13.5 | 3 | 605 | 0 | MER6A | MER2\_type | DNA |
| 43734778 | 43734862 | + | 28.6 | 4310 | 4409 | -80 | L3 | CR1 | LINE |

  

|  |  |  |  |  |  |  |  |  |  |
| --- | --- | --- | --- | --- | --- | --- | --- | --- | --- |
| chr5:44146720-44155600 | | | 8883 bp | | |  |  |  |  |
| Genome Start | Genome End | Strand | Div | Repeat Start | Repeat End | Repeat Left | Name | Family | Class |
| 44146720 | 44146946 | - | 10.2 | 7481 | 7713 | 0 | HERVH | ERV1 | LTR |
| 44146947 | 44147727 | + | 7.5 | 1 | 780 | 0 | LTR17 | ERV1 | LTR |
| 44147728 | 44149014 | + | 7.8 | 1 | 1291 | -7335 | HERV17 | ERV1 | LTR |
| 44149027 | 44150155 | + | 6.6 | 7498 | 8626 | 0 | HERV17 | ERV1 | LTR |
| 44150156 | 44150936 | + | 7.0 | 1 | 780 | 0 | LTR17 | ERV1 | LTR |
| 44150937 | 44151268 | - | 9.6 | 7173 | 7484 | -229 | HERVH | ERV1 | LTR |
| 44151270 | 44151324 | - | 10.0 | 7664 | 7713 | 0 | HERVH | ERV1 | LTR |
| 44151322 | 44151513 | - | 12.4 | 6758 | 6943 | -770 | HERVH | ERV1 | LTR |
| 44151502 | 44155600 | - | 14.8 | 1 | 4336 | -3377 | HERVH | ERV1 | LTR |

  

|  |  |  |  |  |  |  |  |  |  |
| --- | --- | --- | --- | --- | --- | --- | --- | --- | --- |
| chr5:44246171-44251724 | | | 5511 bp | | |  |  |  |  |
| Genome Start | Genome End | Strand | Div | Repeat Start | Repeat End | Repeat Left | Name | Family | Class |
| 44246171 | 44247216 | - | 12.1 | 5114 | 6160 | 0 | L1PREC2 | L1 | LINE |
| 44247217 | 44247462 | + | 13.5 | 127 | 381 | 0 | THE1D | MaLR | LTR |
| 44247465 | 44249087 | + | 32.5 | 2 | 1735 | 0 | MLT1A-int | MaLR | LTR |
| 44249088 | 44249467 | + | 14.0 | 1 | 381 | 0 | THE1D | MaLR | LTR |
| 44249468 | 44251586 | - | 12.5 | 2822 | 4999 | -1147 | L1PREC2 | L1 | LINE |
| 44251628 | 44251724 | - | 12.5 | 2726 | 2821 | -3325 | L1PREC2 | L1 | LINE |

  

|  |  |  |  |  |  |  |  |  |  |
| --- | --- | --- | --- | --- | --- | --- | --- | --- | --- |
| chr5:44820667-44830608 | | | 9853 bp | | |  |  |  |  |
| Genome Start | Genome End | Strand | Div | Repeat Start | Repeat End | Repeat Left | Name | Family | Class |
| 44820667 | 44820990 | + | 10.8 | 1 | 350 | -122 | MER4A1 | ERV1 | LTR |
| 44820992 | 44821527 | - | 11.9 | 1 | 520 | -1 | MER41C | ERV1 | LTR |
| 44821528 | 44823763 | - | 11.3 | 2856 | 5946 | 0 | MER41C-int | ERV1 | LTR |
| 44823751 | 44824332 | - | 14.7 | 1081 | 3759 | -4057 | MER41-int | ERV1 | LTR |
| 44824324 | 44824436 | - | 13.4 | 500 | 625 | -5321 | MER41-int | ERV1 | LTR |
| 44824437 | 44824948 | + | 11.5 | 1 | 511 | 0 | MER9 | ERVK | LTR |
| 44824949 | 44825527 | + | 7.8 | 1 | 568 | -5348 | HERVK9 | ERVK | LTR |
| 44825529 | 44827203 | + | 6.2 | 1113 | 2786 | -3130 | HERVK9 | ERVK | LTR |
| 44827204 | 44827433 | + | 6.1 | 3711 | 3941 | -1975 | HERVK9 | ERVK | LTR |
| 44827421 | 44828498 | + | 10.0 | 4571 | 5634 | -282 | HERVK9 | ERVK | LTR |
| 44828535 | 44828876 | + | 12.2 | 5635 | 7369 | 0 | HERVK9 | ERVK | LTR |
| 44828877 | 44829388 | + | 9.4 | 1 | 511 | 0 | MER9 | ERVK | LTR |
| 44829389 | 44829871 | - | 12.9 | 2 | 505 | -5441 | MER41C-int | ERV1 | LTR |
| 44829958 | 44830482 | - | 9.9 | 1 | 521 | 0 | MER41C | ERV1 | LTR |
| 44830483 | 44830608 | + | 12.7 | 347 | 472 | 0 | MER4A1 | ERV1 | LTR |

  

|  |  |  |  |  |  |  |  |  |  |
| --- | --- | --- | --- | --- | --- | --- | --- | --- | --- |
| chr5:49697230-49713576 | | | 16034 bp | | |  |  |  |  |
| Genome Start | Genome End | Strand | Div | Repeat Start | Repeat End | Repeat Left | Name | Family | Class |
| 49697230 | 49697651 | + | 34.4 | 1 | 433 | -5708 | L1M3c | L1 | LINE |
| 49697915 | 49698370 | + | 25.7 | 931 | 1389 | -4752 | L1M3c | L1 | LINE |
| 49698375 | 49700845 | + | 8.0 | 2 | 2495 | -3651 | L1P1 | L1 | LINE |
| 49700848 | 49702345 | + | 8.0 | 2 | 1578 | 0 | LTR12C | ERV1 | LTR |
| 49702384 | 49707327 | + | 6.7 | 13 | 4963 | -3473 | HERV9 | ERV1 | LTR |
| 49707329 | 49707575 | + | 13.1 | 824 | 1141 | -4051 | HERV9 | ERV1 | LTR |
| 49707574 | 49707635 | + | 11.3 | 7541 | 7602 | -834 | HERV9 | ERV1 | LTR |
| 49707632 | 49708241 | + | 7.9 | 7827 | 8436 | 0 | HERV9 | ERV1 | LTR |
| 49708242 | 49709574 | + | 7.9 | 2 | 1574 | -4 | LTR12C | ERV1 | LTR |
| 49709586 | 49713238 | + | 5.0 | 2501 | 6151 | -4 | L1PA4 | L1 | LINE |
| 49713236 | 49713347 | + | 20.9 | 2979 | 3088 | -3122 | L1M3c | L1 | LINE |
| 49713351 | 49713576 | + | 16.8 | 3773 | 4010 | -2200 | L1M3c | L1 | LINE |

  

|  |  |  |  |  |  |  |  |  |  |
| --- | --- | --- | --- | --- | --- | --- | --- | --- | --- |
| chr5:50268702-50278346 | | | 9587 bp | | |  |  |  |  |
| Genome Start | Genome End | Strand | Div | Repeat Start | Repeat End | Repeat Left | Name | Family | Class |
| 50268702 | 50269017 | - | 17.2 | 40 | 396 | 0 | MSTD | MaLR | LTR |
| 50269018 | 50269439 | + | 15.5 | 1 | 422 | -4 | MSTB | MaLR | LTR |
| 50269444 | 50269638 | + | 18.5 | 1 | 195 | -1456 | MSTB-int | MaLR | LTR |
| 50269656 | 50271943 | + | 20.1 | 3 | 1644 | -5540 | L1PB2 | L1 | LINE |
| 50271948 | 50273529 | + | 12.6 | 3095 | 4724 | -2138 | L1PB2 | L1 | LINE |
| 50273530 | 50273898 | + | 12.1 | 1 | 380 | -1 | THE1D | MaLR | LTR |
| 50273899 | 50274800 | + | 12.6 | 4725 | 5615 | -1247 | L1PB2 | L1 | LINE |
| 50274801 | 50275109 | + | 12.8 | 1 | 306 | 0 | AluSq | Alu | SINE |
| 50275110 | 50276365 | + | 11.1 | 5616 | 6864 | -3 | L1PB2 | L1 | LINE |
| 50276384 | 50277873 | + | 18.2 | 187 | 1638 | -13 | MSTB-int | MaLR | LTR |
| 50277889 | 50278302 | + | 17.4 | 6 | 426 | 0 | MSTB | MaLR | LTR |
| 50278303 | 50278346 | - | 20.0 | 1 | 44 | -352 | MSTD | MaLR | LTR |

  

|  |  |  |  |  |  |  |  |  |  |
| --- | --- | --- | --- | --- | --- | --- | --- | --- | --- |
| chr5:51203115-51207564 | | | 4447 bp | | |  |  |  |  |
| Genome Start | Genome End | Strand | Div | Repeat Start | Repeat End | Repeat Left | Name | Family | Class |
| 51203115 | 51203535 | - | 15.1 | 4344 | 4782 | -1364 | L1MC | L1 | LINE |
| 51203536 | 51203901 | + | 15.5 | 1 | 364 | 0 | THE1B | MaLR | LTR |
| 51203905 | 51205462 | + | 30.4 | 3 | 1735 | 0 | MLT-int | MaLR | LTR |
| 51205463 | 51205820 | + | 17.1 | 1 | 364 | 0 | THE1B | MaLR | LTR |
| 51205821 | 51206192 | - | 15.1 | 3982 | 4343 | -1803 | L1MC | L1 | LINE |
| 51206193 | 51206509 | - | 9.4 | 2 | 309 | -1 | AluSg | Alu | SINE |
| 51206510 | 51207564 | - | 15.1 | 2856 | 3981 | -2165 | L1MC | L1 | LINE |

  

|  |  |  |  |  |  |  |  |  |  |
| --- | --- | --- | --- | --- | --- | --- | --- | --- | --- |
| chr5:51273295-51280947 | | | 7641 bp | | |  |  |  |  |
| Genome Start | Genome End | Strand | Div | Repeat Start | Repeat End | Repeat Left | Name | Family | Class |
| 51273295 | 51275261 | + | 23.0 | 1190 | 3341 | -3041 | L1MB7 | L1 | LINE |
| 51275263 | 51275648 | + | 11.4 | 1 | 381 | 0 | THE1D | MaLR | LTR |
| 51275651 | 51277187 | + | 12.6 | 3 | 1580 | 0 | THE1D-int | MaLR | LTR |
| 51277188 | 51277573 | + | 16.7 | 1 | 381 | 0 | THE1D | MaLR | LTR |
| 51277583 | 51280083 | + | 20.9 | 3347 | 5828 | -592 | L1MB7 | L1 | LINE |
| 51280084 | 51280418 | - | 2.1 | 5801 | 6152 | -3 | L1PA3 | L1 | LINE |
| 51280419 | 51280947 | + | 18.7 | 5829 | 6400 | -20 | L1MB7 | L1 | LINE |

  

|  |  |  |  |  |  |  |  |  |  |
| --- | --- | --- | --- | --- | --- | --- | --- | --- | --- |
| chr5:53829623-53837182 | | | 7552 bp | | |  |  |  |  |
| Genome Start | Genome End | Strand | Div | Repeat Start | Repeat End | Repeat Left | Name | Family | Class |
| 53829623 | 53829805 | + | 11.5 | 1 | 189 | -502 | LTR8 | ERV1 | LTR |
| 53829806 | 53830295 | - | 9.6 | 1 | 511 | 0 | MER9 | ERVK | LTR |
| 53830296 | 53833905 | - | 9.3 | 2348 | 8032 | 0 | HERVK9 | ERVK | LTR |
| 53833906 | 53834154 | + | 8.5 | 50 | 297 | -14 | AluY | Alu | SINE |
| 53834155 | 53835681 | - | 7.0 | 784 | 2347 | -3569 | HERVK9 | ERVK | LTR |
| 53835682 | 53835741 | - | 23.6 | 55 | 109 | -69 | MER5B | MER1\_type | DNA |
| 53835742 | 53836503 | - | 7.0 | 4 | 783 | -5133 | HERVK9 | ERVK | LTR |
| 53836504 | 53836977 | - | 10.0 | 40 | 511 | 0 | MER9 | ERVK | LTR |
| 53836986 | 53837182 | + | 13.7 | 176 | 343 | -348 | LTR8 | ERV1 | LTR |

  

|  |  |  |  |  |  |  |  |  |  |
| --- | --- | --- | --- | --- | --- | --- | --- | --- | --- |
| chr5:55045907-55052235 | | | 6191 bp | | |  |  |  |  |
| Genome Start | Genome End | Strand | Div | Repeat Start | Repeat End | Repeat Left | Name | Family | Class |
| 55045907 | 55046614 | + | 27.4 | 61 | 814 | -5728 | L1M4 | L1 | LINE |
| 55046668 | 55047868 | + | 21.8 | 957 | 2322 | -4324 | L1M4 | L1 | LINE |
| 55047863 | 55048022 | + | 17.3 | 3014 | 3178 | -3466 | L1M4 | L1 | LINE |
| 55048026 | 55048398 | + | 13.3 | 1 | 364 | 0 | THE1B | MaLR | LTR |
| 55048399 | 55049859 | + | 7.3 | 1 | 1449 | -131 | THE1B-int | MaLR | LTR |
| 55049860 | 55050157 | - | 14.2 | 1 | 295 | -17 | AluSx | Alu | SINE |
| 55050158 | 55050284 | + | 7.3 | 1450 | 1576 | -4 | THE1B-int | MaLR | LTR |
| 55050289 | 55050450 | + | 13.2 | 1 | 164 | -200 | THE1B | MaLR | LTR |
| 55050451 | 55050758 | - | 10.4 | 1 | 309 | -4 | AluSp | Alu | SINE |
| 55050759 | 55050984 | + | 13.2 | 165 | 364 | 0 | THE1B | MaLR | LTR |
| 55050992 | 55051065 | + | 14.9 | 3184 | 3257 | -3387 | L1M4 | L1 | LINE |
| 55051074 | 55051276 | - | 18.6 | 111 | 310 | -2 | AluJb | Alu | SINE |
| 55051329 | 55051632 | + | 14.5 | 5 | 308 | -4 | AluJb | Alu | SINE |
| 55051634 | 55051752 | + | 16.0 | 3535 | 3653 | -2991 | L1M4 | L1 | LINE |
| 55051769 | 55051985 | + | 16.7 | 87 | 299 | -13 | AluJo | Alu | SINE |
| 55051986 | 55052235 | + | 13.2 | 3652 | 3910 | -2734 | L1M4 | L1 | LINE |

  

|  |  |  |  |  |  |  |  |  |  |
| --- | --- | --- | --- | --- | --- | --- | --- | --- | --- |
| chr5:55665067-55668147 | | | 2891 bp | | |  |  |  |  |
| Genome Start | Genome End | Strand | Div | Repeat Start | Repeat End | Repeat Left | Name | Family | Class |
| 55665067 | 55665296 | - | 31.4 | 5331 | 5561 | -679 | L1M5 | L1 | LINE |
| 55665495 | 55665645 | - | 31.6 | 4987 | 5140 | -1006 | L1M5 | L1 | LINE |
| 55665647 | 55666031 | - | 13.6 | 1 | 428 | 0 | MSTA | MaLR | LTR |
| 55666032 | 55666150 | - | 14.9 | 1469 | 1580 | 0 | MSTA-int | MaLR | LTR |
| 55666151 | 55666461 | + | 13.9 | 1 | 310 | -2 | AluSx | Alu | SINE |
| 55666462 | 55667048 | - | 14.9 | 885 | 1468 | -112 | MSTA-int | MaLR | LTR |
| 55667038 | 55667363 | - | 13.0 | 1 | 325 | -1326 | MSTA-int | MaLR | LTR |
| 55667364 | 55667740 | - | 12.8 | 3 | 428 | 0 | MSTA | MaLR | LTR |
| 55667743 | 55668147 | - | 28.1 | 4533 | 4990 | -1156 | L1M5 | L1 | LINE |

  

|  |  |  |  |  |  |  |  |  |  |
| --- | --- | --- | --- | --- | --- | --- | --- | --- | --- |
| chr5:55903647-55909397 | | | 5654 bp | | |  |  |  |  |
| Genome Start | Genome End | Strand | Div | Repeat Start | Repeat End | Repeat Left | Name | Family | Class |
| 55903647 | 55903746 | + | 25.3 | 4545 | 4635 | -1511 | L1ME3 | L1 | LINE |
| 55903747 | 55904139 | - | 15.2 | 1 | 428 | 0 | MSTA | MaLR | LTR |
| 55904140 | 55905769 | - | 28.9 | 2 | 1735 | 0 | MLT-int | MaLR | LTR |
| 55905772 | 55906180 | - | 15.3 | 1 | 428 | 0 | MSTA | MaLR | LTR |
| 55906181 | 55906396 | + | 25.3 | 4636 | 4841 | -1305 | L1ME3 | L1 | LINE |
| 55906397 | 55906590 | + | 11.1 | 1 | 211 | -1020 | Tigger3b | MER2\_type | DNA |
| 55906591 | 55906887 | + | 20.3 | 1 | 298 | -14 | AluJo | Alu | SINE |
| 55906888 | 55907573 | + | 11.1 | 212 | 852 | -379 | Tigger3b | MER2\_type | DNA |
| 55907608 | 55907928 | + | 11.1 | 853 | 1230 | -1 | Tigger3b | MER2\_type | DNA |
| 55907929 | 55907993 | + | 25.3 | 4842 | 4906 | -1240 | L1ME3 | L1 | LINE |
| 55907994 | 55908073 | - | 10.0 | 1 | 80 | 0 | MADE1 | Mariner | DNA |
| 55908074 | 55908385 | + | 25.3 | 4907 | 5247 | -899 | L1ME3 | L1 | LINE |
| 55908447 | 55909397 | + | 25.1 | 5233 | 6158 | -4 | L1ME3 | L1 | LINE |

  

|  |  |  |  |  |  |  |  |  |  |
| --- | --- | --- | --- | --- | --- | --- | --- | --- | --- |
| chr5:55957589-55962717 | | | 5108 bp | | |  |  |  |  |
| Genome Start | Genome End | Strand | Div | Repeat Start | Repeat End | Repeat Left | Name | Family | Class |
| 55957589 | 55957754 | - | 20.4 | 5960 | 6118 | -3 | L1ME4a | L1 | LINE |
| 55957799 | 55959390 | - | 8.9 | 4532 | 6159 | -2 | L1PA14 | L1 | LINE |
| 55959391 | 55959767 | + | 13.2 | 1 | 364 | 0 | THE1B | MaLR | LTR |
| 55959770 | 55961341 | + | 30.1 | 2 | 1735 | 0 | MLT1J1-int | MaLR | LTR |
| 55961342 | 55961718 | + | 13.5 | 1 | 364 | 0 | THE1B | MaLR | LTR |
| 55961719 | 55961850 | - | 9.0 | 4400 | 4531 | -1615 | L1PREC2 | L1 | LINE |
| 55961845 | 55962054 | - | 4.8 | 5609 | 5819 | -335 | L1P3 | L1 | LINE |
| 55962056 | 55962569 | - | 11.6 | 2676 | 3184 | -2962 | L1PREC2 | L1 | LINE |
| 55962550 | 55962717 | - | 23.4 | 5713 | 5919 | -202 | L1ME4a | L1 | LINE |

  

|  |  |  |  |  |  |  |  |  |  |
| --- | --- | --- | --- | --- | --- | --- | --- | --- | --- |
| chr5:56344722-56354597 | | | 9748 bp | | |  |  |  |  |
| Genome Start | Genome End | Strand | Div | Repeat Start | Repeat End | Repeat Left | Name | Family | Class |
| 56344722 | 56344864 | + | 22.4 | 2225 | 2371 | -4029 | L1MEc | L1 | LINE |
| 56344855 | 56344886 | + | 15.6 | 6157 | 6188 | -156 | L1MC2 | L1 | LINE |
| 56344887 | 56345079 | + | 21.0 | 1 | 188 | -186 | MLT1A | MaLR | LTR |
| 56345106 | 56345275 | + | 21.0 | 189 | 374 | 0 | MLT1A | MaLR | LTR |
| 56345276 | 56345431 | + | 15.6 | 6189 | 6338 | -6 | L1MC2 | L1 | LINE |
| 56345432 | 56346235 | + | 26.1 | 2178 | 2978 | -3168 | L1M5 | L1 | LINE |
| 56346239 | 56346448 | + | 18.1 | 1 | 222 | -283 | MLT1D | MaLR | LTR |
| 56346449 | 56347217 | - | 11.1 | 1 | 780 | 0 | LTR17 | ERV1 | LTR |
| 56347218 | 56347254 | + | 18.1 | 223 | 254 | -251 | MLT1D | MaLR | LTR |
| 56347266 | 56347378 | + | 16.1 | 384 | 505 | 0 | MLT1D | MaLR | LTR |
| 56347426 | 56348033 | + | 22.5 | 3134 | 3762 | -2457 | L1MEc | L1 | LINE |
| 56348036 | 56348491 | - | 8.1 | 1 | 464 | 0 | LTR7B | ERV1 | LTR |
| 56348492 | 56349023 | - | 9.6 | 7157 | 7713 | 0 | HERVH | ERV1 | LTR |
| 56349017 | 56350037 | - | 13.3 | 5011 | 6016 | -1697 | HERVH | ERV1 | LTR |
| 56350036 | 56351066 | - | 14.4 | 3357 | 4319 | -2749 | HERVH | ERV1 | LTR |
| 56351067 | 56353123 | - | 14.1 | 800 | 2830 | -4883 | HERVH | ERV1 | LTR |
| 56353182 | 56353651 | - | 20.9 | 1 | 459 | -7254 | HERVH | ERV1 | LTR |
| 56353652 | 56354038 | - | 6.2 | 1 | 464 | 0 | LTR7B | ERV1 | LTR |
| 56354039 | 56354183 | + | 23.4 | 3765 | 3891 | -2328 | L1MEc | L1 | LINE |
| 56354184 | 56354482 | + | 12.5 | 10 | 305 | -7 | AluSx | Alu | SINE |
| 56354483 | 56354597 | + | 23.4 | 3892 | 3996 | -2223 | L1MEc | L1 | LINE |

  

|  |  |  |  |  |  |  |  |  |  |
| --- | --- | --- | --- | --- | --- | --- | --- | --- | --- |
| chr5:57805230-57808312 | | | 2736 bp | | |  |  |  |  |
| Genome Start | Genome End | Strand | Div | Repeat Start | Repeat End | Repeat Left | Name | Family | Class |
| 57805230 | 57805449 | + | 25.2 | 8 | 241 | -2620 | Charlie2 | MER1\_type | DNA |
| 57805753 | 57805912 | + | 18.5 | 1471 | 1662 | -1199 | Charlie2a | MER1\_type | DNA |
| 57805951 | 57806310 | - | 8.9 | 5 | 364 | 0 | THE1B | MaLR | LTR |
| 57806315 | 57807867 | - | 28.3 | 2 | 1735 | 0 | MLT1L-int | MaLR | LTR |
| 57807870 | 57808226 | - | 11.8 | 1 | 364 | 0 | THE1B | MaLR | LTR |
| 57808227 | 57808312 | + | 19.0 | 2747 | 2833 | -29 | Charlie2 | MER1\_type | DNA |

  

|  |  |  |  |  |  |  |  |  |  |
| --- | --- | --- | --- | --- | --- | --- | --- | --- | --- |
| chr5:58734689-58742409 | | | 7613 bp | | |  |  |  |  |
| Genome Start | Genome End | Strand | Div | Repeat Start | Repeat End | Repeat Left | Name | Family | Class |
| 58734689 | 58734725 | - | 11.8 | 6271 | 6305 | 0 | L1MA3 | L1 | LINE |
| 58734773 | 58735959 | - | 11.4 | 5088 | 6270 | -35 | L1MA3 | L1 | LINE |
| 58735960 | 58736264 | + | 14.1 | 1 | 298 | -14 | AluSx | Alu | SINE |
| 58736265 | 58737973 | - | 10.5 | 3283 | 5087 | -1059 | L1MA3 | L1 | LINE |
| 58737998 | 58738269 | - | 10.7 | 2 | 274 | -36 | AluSg | Alu | SINE |
| 58738270 | 58738324 | - | 16.4 | 3252 | 3306 | -2837 | L1M2 | L1 | LINE |
| 58738308 | 58738650 | - | 31.0 | 743 | 1090 | -1417 | HAL1 | L1 | LINE |
| 58738661 | 58739034 | + | 18.7 | 19 | 426 | 0 | MSTB | MaLR | LTR |
| 58739035 | 58740447 | + | 17.9 | 1 | 1427 | -224 | MSTB-int | MaLR | LTR |
| 58740448 | 58740748 | + | 12.7 | 1 | 300 | -12 | AluSx | Alu | SINE |
| 58740749 | 58740987 | + | 17.9 | 1428 | 1651 | 0 | MSTB-int | MaLR | LTR |
| 58740988 | 58741377 | + | 17.8 | 1 | 426 | 0 | MSTB | MaLR | LTR |
| 58741402 | 58741491 | - | 17.1 | 651 | 738 | -1769 | HAL1 | L1 | LINE |
| 58741512 | 58741827 | - | 12.5 | 5943 | 6304 | -1 | L1MA3 | L1 | LINE |
| 58741828 | 58742128 | - | 15.6 | 2 | 309 | -3 | AluSx | Alu | SINE |
| 58742129 | 58742409 | - | 12.5 | 5676 | 5942 | -363 | L1MA3 | L1 | LINE |

  

|  |  |  |  |  |  |  |  |  |  |
| --- | --- | --- | --- | --- | --- | --- | --- | --- | --- |
| chr5:60537029-60539462 | | | 2432 bp | | |  |  |  |  |
| Genome Start | Genome End | Strand | Div | Repeat Start | Repeat End | Repeat Left | Name | Family | Class |
| 60537029 | 60537119 | - | 28.2 | 389 | 483 | -8 | LTR16C | ERVL | LTR |
| 60537120 | 60537481 | - | 13.5 | 1 | 364 | 0 | THE1B | MaLR | LTR |
| 60537482 | 60539038 | - | 30.3 | 2 | 1735 | 0 | MLT-int | MaLR | LTR |
| 60539041 | 60539403 | - | 13.8 | 1 | 364 | 0 | THE1B | MaLR | LTR |
| 60539404 | 60539462 | - | 28.2 | 326 | 388 | -103 | LTR16C | ERVL | LTR |

  

|  |  |  |  |  |  |  |  |  |  |
| --- | --- | --- | --- | --- | --- | --- | --- | --- | --- |
| chr5:61175244-61184769 | | | 9520 bp | | |  |  |  |  |
| Genome Start | Genome End | Strand | Div | Repeat Start | Repeat End | Repeat Left | Name | Family | Class |
| 61175244 | 61176118 | + | 15.4 | 76 | 936 | -5572 | L1MC1 | L1 | LINE |
| 61176119 | 61176507 | + | 21.8 | 1 | 396 | 0 | MSTD | MaLR | LTR |
| 61176510 | 61178307 | + | 25.0 | 2 | 1731 | -4 | MSTD-int | MaLR | LTR |
| 61178312 | 61178705 | + | 23.5 | 1 | 396 | 0 | MSTD | MaLR | LTR |
| 61178706 | 61181920 | + | 15.0 | 937 | 4272 | -2049 | L1MC1 | L1 | LINE |
| 61181921 | 61182351 | + | 13.9 | 1 | 432 | 0 | MSTB1 | MaLR | LTR |
| 61182352 | 61184769 | + | 15.5 | 4273 | 6508 | 0 | L1MC1 | L1 | LINE |

  

|  |  |  |  |  |  |  |  |  |  |
| --- | --- | --- | --- | --- | --- | --- | --- | --- | --- |
| chr5:61521178-61526133 | | | 4932 bp | | |  |  |  |  |
| Genome Start | Genome End | Strand | Div | Repeat Start | Repeat End | Repeat Left | Name | Family | Class |
| 61521178 | 61521766 | - | 14.2 | 5750 | 6312 | 0 | L1MA9 | L1 | LINE |
| 61521767 | 61522155 | + | 15.3 | 1 | 428 | 0 | MSTA | MaLR | LTR |
| 61522156 | 61522656 | - | 14.2 | 5230 | 5749 | -563 | L1MA9 | L1 | LINE |
| 61522657 | 61523256 | + | 16.5 | 1 | 554 | 0 | MLT2B3 | ERVL | LTR |
| 61523257 | 61523594 | - | 14.7 | 4901 | 5229 | -911 | L1MA9 | L1 | LINE |
| 61523597 | 61524020 | + | 16.3 | 4 | 426 | 0 | MSTB | MaLR | LTR |
| 61524021 | 61525600 | + | 16.3 | 1 | 1651 | 0 | MSTB-int | MaLR | LTR |
| 61525601 | 61526028 | + | 14.2 | 1 | 426 | 0 | MSTB | MaLR | LTR |
| 61526051 | 61526133 | - | 19.3 | 4824 | 4908 | -1232 | L1MA9 | L1 | LINE |

  

|  |  |  |  |  |  |  |  |  |  |
| --- | --- | --- | --- | --- | --- | --- | --- | --- | --- |
| chr5:62316531-62327747 | | | 11176 bp | | |  |  |  |  |
| Genome Start | Genome End | Strand | Div | Repeat Start | Repeat End | Repeat Left | Name | Family | Class |
| 62316531 | 62316678 | - | 22.6 | 5316 | 5462 | -920 | L1M4c | L1 | LINE |
| 62316679 | 62321234 | + | 14.4 | 1396 | 5988 | -515 | L1PA16 | L1 | LINE |
| 62321268 | 62321618 | - | 16.8 | 1 | 417 | -11 | MSTA | MaLR | LTR |
| 62321621 | 62322142 | + | 15.0 | 5985 | 6503 | 0 | L1PA16 | L1 | LINE |
| 62322146 | 62324327 | - | 16.2 | 3124 | 5328 | -1054 | L1M4c | L1 | LINE |
| 62324329 | 62324703 | - | 17.6 | 49 | 432 | 0 | MSTB1 | MaLR | LTR |
| 62324705 | 62325066 | - | 15.9 | 1 | 362 | -2 | THE1B | MaLR | LTR |
| 62325067 | 62326630 | - | 11.0 | 1 | 1580 | 0 | THE1B-int | MaLR | LTR |
| 62326631 | 62326994 | - | 11.9 | 1 | 364 | 0 | THE1B | MaLR | LTR |
| 62326995 | 62327048 | - | 7.5 | 1 | 53 | -379 | MSTB1 | MaLR | LTR |
| 62327050 | 62327747 | - | 18.6 | 2096 | 3126 | -3256 | L1M4c | L1 | LINE |

  

|  |  |  |  |  |  |  |  |  |  |
| --- | --- | --- | --- | --- | --- | --- | --- | --- | --- |
| chr5:63368750-63371505 | | | 2756 bp | | |  |  |  |  |
| Genome Start | Genome End | Strand | Div | Repeat Start | Repeat End | Repeat Left | Name | Family | Class |
| 63368750 | 63368967 | - | 33.1 | 3181 | 3400 | -19 | L2 | L2 | LINE |
| 63368968 | 63369362 | + | 13.4 | 1 | 428 | 0 | MSTA | MaLR | LTR |
| 63369363 | 63370970 | + | 30.4 | 1 | 1735 | 0 | MLT1C-int | MaLR | LTR |
| 63370971 | 63371385 | + | 14.8 | 1 | 428 | 0 | MSTA | MaLR | LTR |
| 63371386 | 63371505 | - | 33.1 | 3061 | 3180 | -239 | L2 | L2 | LINE |

  

|  |  |  |  |  |  |  |  |  |  |
| --- | --- | --- | --- | --- | --- | --- | --- | --- | --- |
| chr5:64929836-64934906 | | | 5007 bp | | |  |  |  |  |
| Genome Start | Genome End | Strand | Div | Repeat Start | Repeat End | Repeat Left | Name | Family | Class |
| 64929836 | 64929984 | + | 26.1 | 92 | 242 | -6365 | L1MB2 | L1 | LINE |
| 64930012 | 64930047 | + | 17.6 | 4335 | 4370 | -2371 | L1MB2 | L1 | LINE |
| 64930048 | 64930345 | + | 7.5 | 1 | 297 | -16 | AluSp | Alu | SINE |
| 64930346 | 64930899 | + | 17.6 | 4371 | 4962 | -1779 | L1MB2 | L1 | LINE |
| 64930966 | 64931026 | + | 17.6 | 4963 | 5027 | -1714 | L1MB2 | L1 | LINE |
| 64931027 | 64931105 | + | 8.9 | 2 | 80 | 0 | MADE1 | Mariner | DNA |
| 64931106 | 64931434 | + | 17.6 | 5030 | 5348 | -1393 | L1MB2 | L1 | LINE |
| 64931435 | 64931858 | - | 13.0 | 1 | 428 | 0 | MSTA | MaLR | LTR |
| 64931859 | 64932872 | + | 17.1 | 5349 | 6391 | -381 | L1MB2 | L1 | LINE |
| 64932873 | 64933218 | - | 6.7 | 9 | 355 | 0 | THE1A | MaLR | LTR |
| 64933189 | 64934184 | - | 12.0 | 1 | 1011 | -569 | THE1A-int | MaLR | LTR |
| 64934185 | 64934537 | - | 6.8 | 1 | 355 | 0 | THE1A | MaLR | LTR |
| 64934539 | 64934906 | + | 14.7 | 6388 | 6771 | -1 | L1MB2 | L1 | LINE |

  

|  |  |  |  |  |  |  |  |  |  |
| --- | --- | --- | --- | --- | --- | --- | --- | --- | --- |
| chr5:65097566-65100837 | | | 3206 bp | | |  |  |  |  |
| Genome Start | Genome End | Strand | Div | Repeat Start | Repeat End | Repeat Left | Name | Family | Class |
| 65097566 | 65097739 | - | 24.4 | 392 | 561 | -6 | MLT1G | MaLR | LTR |
| 65097792 | 65098153 | - | 9.5 | 8 | 381 | 0 | THE1D | MaLR | LTR |
| 65098163 | 65099766 | - | 28.6 | 3 | 1731 | -4 | MLT1B-int | MaLR | LTR |
| 65099770 | 65100133 | - | 10.9 | 1 | 381 | 0 | THE1D | MaLR | LTR |
| 65100135 | 65100552 | - | 23.6 | 2 | 390 | 0 | MLT1B | MaLR | LTR |
| 65100554 | 65100837 | - | 24.6 | 10 | 304 | -286 | MLT1G | MaLR | LTR |

  

|  |  |  |  |  |  |  |  |  |  |
| --- | --- | --- | --- | --- | --- | --- | --- | --- | --- |
| chr5:66999024-67002215 | | | 3171 bp | | |  |  |  |  |
| Genome Start | Genome End | Strand | Div | Repeat Start | Repeat End | Repeat Left | Name | Family | Class |
| 66999024 | 66999537 | - | 15.8 | 5643 | 6178 | 0 | L1MB8 | L1 | LINE |
| 66999539 | 66999909 | + | 13.8 | 1 | 381 | 0 | THE1D | MaLR | LTR |
| 66999913 | 67000281 | + | 13.5 | 4 | 369 | -1211 | THE1D-int | MaLR | LTR |
| 67000282 | 67000401 | - | 3.4 | 6026 | 6151 | -3 | L1PA7 | L1 | LINE |
| 67000402 | 67001626 | + | 13.5 | 370 | 1578 | -2 | THE1D-int | MaLR | LTR |
| 67001631 | 67001989 | + | 14.0 | 3 | 381 | 0 | THE1D | MaLR | LTR |
| 67002003 | 67002215 | - | 22.6 | 5403 | 5615 | -569 | L1MB8 | L1 | LINE |

  

|  |  |  |  |  |  |  |  |  |  |
| --- | --- | --- | --- | --- | --- | --- | --- | --- | --- |
| chr5:68639829-68642564 | | | 2731 bp | | |  |  |  |  |
| Genome Start | Genome End | Strand | Div | Repeat Start | Repeat End | Repeat Left | Name | Family | Class |
| 68639829 | 68639887 | + | 18.2 | 5672 | 5739 | -445 | L1MB7 | L1 | LINE |
| 68639888 | 68640240 | + | 10.8 | 1 | 355 | 0 | THE1A | MaLR | LTR |
| 68640246 | 68641808 | + | 29.6 | 5 | 1735 | 0 | MLT-int | MaLR | LTR |
| 68641809 | 68642161 | + | 10.6 | 1 | 355 | 0 | THE1A | MaLR | LTR |
| 68642162 | 68642564 | + | 18.2 | 5740 | 6158 | -26 | L1MB7 | L1 | LINE |

  

|  |  |  |  |  |  |  |  |  |  |
| --- | --- | --- | --- | --- | --- | --- | --- | --- | --- |
| chr5:69286741-69292411 | | | 5564 bp | | |  |  |  |  |
| Genome Start | Genome End | Strand | Div | Repeat Start | Repeat End | Repeat Left | Name | Family | Class |
| 69286741 | 69286865 | + | 26.1 | 1036 | 1160 | -5473 | L1MDa | L1 | LINE |
| 69286866 | 69287176 | + | 11.9 | 1 | 312 | 0 | AluSx | Alu | SINE |
| 69287177 | 69288324 | + | 26.8 | 1157 | 2359 | -4274 | L1MDa | L1 | LINE |
| 69288342 | 69288568 | + | 19.4 | 1 | 229 | -1002 | Tigger3b | MER2\_type | DNA |
| 69288569 | 69288939 | + | 15.4 | 1 | 379 | -2 | THE1D | MaLR | LTR |
| 69288942 | 69290438 | + | 14.6 | 1 | 1580 | 0 | THE1D-int | MaLR | LTR |
| 69290439 | 69290817 | + | 15.6 | 1 | 381 | 0 | THE1D | MaLR | LTR |
| 69290818 | 69290934 | + | 16.9 | 225 | 338 | -893 | Tigger3b | MER2\_type | DNA |
| 69290935 | 69291189 | + | 5.9 | 47 | 301 | -10 | AluY | Alu | SINE |
| 69291190 | 69292050 | + | 16.9 | 339 | 1225 | -6 | Tigger3b | MER2\_type | DNA |
| 69292062 | 69292271 | + | 19.1 | 2384 | 2601 | -4032 | L1MDa | L1 | LINE |
| 69292349 | 69292411 | + | 22.2 | 2614 | 2679 | -3858 | L1MDa | L1 | LINE |

  

|  |  |  |  |  |  |  |  |  |  |
| --- | --- | --- | --- | --- | --- | --- | --- | --- | --- |
| chr5:69418108-69422311 | | | 3968 bp | | |  |  |  |  |
| Genome Start | Genome End | Strand | Div | Repeat Start | Repeat End | Repeat Left | Name | Family | Class |
| 69418108 | 69418713 | - | 26.5 | 2408 | 3081 | -338 | L2 | L2 | LINE |
| 69418714 | 69418848 | + | 15.7 | 1 | 134 | -178 | AluJo | Alu | SINE |
| 69418849 | 69419324 | - | 26.5 | 1879 | 2407 | -1012 | L2 | L2 | LINE |
| 69419520 | 69419645 | - | 23.8 | 315 | 460 | -2959 | L2 | L2 | LINE |
| 69419651 | 69419866 | + | 12.2 | 210 | 428 | 0 | MSTA | MaLR | LTR |
| 69419869 | 69420008 | + | 28.5 | 2 | 202 | -1533 | MLT-int | MaLR | LTR |
| 69420009 | 69420320 | - | 8.1 | 2 | 312 | 0 | AluSx | Alu | SINE |
| 69420321 | 69421789 | + | 28.5 | 203 | 1735 | 0 | MLT-int | MaLR | LTR |
| 69421790 | 69422142 | + | 14.9 | 1 | 377 | -51 | MSTA | MaLR | LTR |
| 69422177 | 69422311 | - | 26.9 | 206 | 355 | -3064 | L2 | L2 | LINE |

  

|  |  |  |  |  |  |  |  |  |  |
| --- | --- | --- | --- | --- | --- | --- | --- | --- | --- |
| chr5:69489075-69494582 | | | 5478 bp | | |  |  |  |  |
| Genome Start | Genome End | Strand | Div | Repeat Start | Repeat End | Repeat Left | Name | Family | Class |
| 69489075 | 69489272 | - | 19.2 | 2384 | 2601 | -4032 | L1MDa | L1 | LINE |
| 69489284 | 69490145 | - | 17.0 | 339 | 1225 | -6 | Tigger3b | MER2\_type | DNA |
| 69490146 | 69490400 | - | 5.1 | 47 | 301 | -10 | AluY | Alu | SINE |
| 69490401 | 69490517 | - | 17.0 | 225 | 338 | -893 | Tigger3b | MER2\_type | DNA |
| 69490518 | 69490896 | - | 14.7 | 1 | 381 | 0 | THE1D | MaLR | LTR |
| 69490897 | 69492393 | - | 14.4 | 1 | 1580 | 0 | THE1D-int | MaLR | LTR |
| 69492396 | 69492766 | - | 15.4 | 1 | 379 | -2 | THE1D | MaLR | LTR |
| 69492767 | 69492993 | - | 18.9 | 1 | 229 | -1002 | Tigger3b | MER2\_type | DNA |
| 69493011 | 69494154 | - | 27.0 | 1161 | 2359 | -4274 | L1MDa | L1 | LINE |
| 69494155 | 69494457 | - | 12.2 | 1 | 304 | -8 | AluSx | Alu | SINE |
| 69494458 | 69494582 | - | 27.0 | 1036 | 1160 | -5473 | L1MDa | L1 | LINE |

  

|  |  |  |  |  |  |  |  |  |  |
| --- | --- | --- | --- | --- | --- | --- | --- | --- | --- |
| chr5:70161811-70167483 | | | 5566 bp | | |  |  |  |  |
| Genome Start | Genome End | Strand | Div | Repeat Start | Repeat End | Repeat Left | Name | Family | Class |
| 70161811 | 70161935 | + | 27.4 | 1036 | 1160 | -5473 | L1MDa | L1 | LINE |
| 70161936 | 70162246 | + | 11.9 | 1 | 312 | 0 | AluSx | Alu | SINE |
| 70162247 | 70163396 | + | 27.4 | 1161 | 2359 | -4274 | L1MDa | L1 | LINE |
| 70163414 | 70163640 | + | 19.4 | 1 | 229 | -1002 | Tigger3b | MER2\_type | DNA |
| 70163641 | 70164011 | + | 15.4 | 1 | 379 | -2 | THE1D | MaLR | LTR |
| 70164014 | 70165510 | + | 14.6 | 1 | 1580 | 0 | THE1D-int | MaLR | LTR |
| 70165511 | 70165889 | + | 15.6 | 1 | 381 | 0 | THE1D | MaLR | LTR |
| 70165890 | 70166006 | + | 16.9 | 225 | 338 | -893 | Tigger3b | MER2\_type | DNA |
| 70166007 | 70166261 | + | 5.9 | 47 | 301 | -10 | AluY | Alu | SINE |
| 70166262 | 70167122 | + | 16.9 | 339 | 1225 | -6 | Tigger3b | MER2\_type | DNA |
| 70167134 | 70167343 | + | 19.1 | 2384 | 2601 | -4032 | L1MDa | L1 | LINE |
| 70167421 | 70167483 | + | 22.2 | 2614 | 2679 | -3858 | L1MDa | L1 | LINE |

  

|  |  |  |  |  |  |  |  |  |  |
| --- | --- | --- | --- | --- | --- | --- | --- | --- | --- |
| chr5:70426340-70430536 | | | 3978 bp | | |  |  |  |  |
| Genome Start | Genome End | Strand | Div | Repeat Start | Repeat End | Repeat Left | Name | Family | Class |
| 70426340 | 70426945 | - | 25.8 | 2379 | 3081 | -338 | L2 | L2 | LINE |
| 70426946 | 70427079 | + | 15.2 | 1 | 132 | -1 | FLAM\_C | Alu | SINE |
| 70427080 | 70427556 | - | 25.8 | 1876 | 2378 | -1041 | L2 | L2 | LINE |
| 70427736 | 70427872 | - | 25.6 | 315 | 475 | -2944 | L2 | L2 | LINE |
| 70427878 | 70428093 | + | 11.7 | 210 | 428 | 0 | MSTA | MaLR | LTR |
| 70428095 | 70428234 | + | 28.4 | 1 | 189 | -1546 | MLT-int | MaLR | LTR |
| 70428235 | 70428545 | - | 8.7 | 2 | 311 | -1 | AluSx | Alu | SINE |
| 70428546 | 70430014 | + | 28.4 | 190 | 1734 | -1 | MLT-int | MaLR | LTR |
| 70430015 | 70430367 | + | 14.9 | 1 | 377 | -51 | MSTA | MaLR | LTR |
| 70430402 | 70430536 | - | 26.9 | 206 | 355 | -3064 | L2 | L2 | LINE |

  

|  |  |  |  |  |  |  |  |  |  |
| --- | --- | --- | --- | --- | --- | --- | --- | --- | --- |
| chr5:70489404-70494918 | | | 5485 bp | | |  |  |  |  |
| Genome Start | Genome End | Strand | Div | Repeat Start | Repeat End | Repeat Left | Name | Family | Class |
| 70489404 | 70489603 | - | 19.5 | 2384 | 2601 | -4032 | L1MDa | L1 | LINE |
| 70489615 | 70490472 | - | 16.9 | 339 | 1225 | -6 | Tigger3b | MER2\_type | DNA |
| 70490473 | 70490727 | - | 5.9 | 47 | 301 | -10 | AluY | Alu | SINE |
| 70490728 | 70490844 | - | 16.9 | 225 | 338 | -893 | Tigger3b | MER2\_type | DNA |
| 70490845 | 70491223 | - | 14.7 | 1 | 381 | 0 | THE1D | MaLR | LTR |
| 70491224 | 70492720 | - | 14.3 | 1 | 1580 | 0 | THE1D-int | MaLR | LTR |
| 70492723 | 70493093 | - | 15.4 | 1 | 379 | -2 | THE1D | MaLR | LTR |
| 70493094 | 70493320 | - | 18.5 | 1 | 229 | -1002 | Tigger3b | MER2\_type | DNA |
| 70493338 | 70494482 | - | 27.1 | 1161 | 2359 | -4274 | L1MDa | L1 | LINE |
| 70494483 | 70494793 | - | 11.9 | 1 | 312 | 0 | AluSx | Alu | SINE |
| 70494794 | 70494918 | - | 27.1 | 1036 | 1160 | -5473 | L1MDa | L1 | LINE |

  

|  |  |  |  |  |  |  |  |  |  |
| --- | --- | --- | --- | --- | --- | --- | --- | --- | --- |
| chr5:75685713-75694847 | | | 8666 bp | | |  |  |  |  |
| Genome Start | Genome End | Strand | Div | Repeat Start | Repeat End | Repeat Left | Name | Family | Class |
| 75685713 | 75685846 | + | 8.2 | 7 | 140 | -170 | AluSg | Alu | SINE |
| 75685915 | 75686199 | - | 10.9 | 1 | 285 | -25 | AluSg | Alu | SINE |
| 75686226 | 75686578 | + | 13.9 | 1 | 355 | 0 | AluSx | Alu | SINE |
| 75686729 | 75687068 | + | 25.2 | 2 | 318 | -2099 | Charlie8 | MER1\_type | DNA |
| 75687069 | 75687381 | - | 13.8 | 1 | 312 | 0 | AluSx | Alu | SINE |
| 75687442 | 75687512 | + | 21.6 | 1377 | 2427 | 0 | Charlie8 | MER1\_type | DNA |
| 75687541 | 75687591 | - | 25.5 | 88 | 138 | -130 | MIRb | MIR | SINE |
| 75687619 | 75687690 | + | 18.2 | 7 | 79 | -787 | MER21C | ERV1 | LTR |
| 75687692 | 75688000 | + | 10.4 | 1 | 309 | -4 | AluSp | Alu | SINE |
| 75688003 | 75688307 | + | 10.6 | 1 | 301 | -12 | AluSq | Alu | SINE |
| 75688309 | 75688481 | + | 11.6 | 134 | 306 | -6 | AluSg/x | Alu | SINE |
| 75688558 | 75688691 | + | 18.9 | 102 | 240 | -623 | MER21B | ERV1 | LTR |
| 75688727 | 75688836 | + | 9.1 | 1 | 111 | -199 | AluSg | Alu | SINE |
| 75688837 | 75689286 | + | 5.6 | 1 | 448 | -2 | LTR7 | ERV1 | LTR |
| 75689289 | 75692480 | + | 8.8 | 1 | 3290 | -4423 | HERVH | ERV1 | LTR |
| 75692471 | 75692952 | + | 10.8 | 3492 | 3993 | -3720 | HERVH | ERV1 | LTR |
| 75692955 | 75693611 | + | 14.8 | 4488 | 5149 | -2564 | HERVH | ERV1 | LTR |
| 75693613 | 75693828 | + | 13.0 | 5600 | 5819 | -1894 | HERVH | ERV1 | LTR |
| 75693827 | 75694206 | + | 8.8 | 7322 | 7713 | 0 | HERVH | ERV1 | LTR |
| 75694207 | 75694652 | + | 6.1 | 1 | 448 | -2 | LTR7 | ERV1 | LTR |
| 75694655 | 75694847 | + | 9.9 | 109 | 300 | -12 | AluSg | Alu | SINE |

  

|  |  |  |  |  |  |  |  |  |  |
| --- | --- | --- | --- | --- | --- | --- | --- | --- | --- |
| chr5:80178932-80182421 | | | 3488 bp | | |  |  |  |  |
| Genome Start | Genome End | Strand | Div | Repeat Start | Repeat End | Repeat Left | Name | Family | Class |
| 80178932 | 80179524 | - | 32.7 | 1110 | 1729 | -778 | HAL1 | L1 | LINE |
| 80179525 | 80179827 | + | 15.3 | 1 | 305 | -7 | AluJb | Alu | SINE |
| 80179828 | 80180025 | - | 32.7 | 942 | 1109 | -1398 | HAL1 | L1 | LINE |
| 80180026 | 80180385 | - | 14.4 | 1 | 362 | -2 | THE1B | MaLR | LTR |
| 80180386 | 80181958 | - | 31.1 | 2 | 1735 | 0 | MLT-int | MaLR | LTR |
| 80181961 | 80182326 | - | 12.9 | 1 | 364 | 0 | THE1B | MaLR | LTR |
| 80182327 | 80182421 | - | 32.7 | 855 | 941 | -1566 | HAL1 | L1 | LINE |

  

|  |  |  |  |  |  |  |  |  |  |
| --- | --- | --- | --- | --- | --- | --- | --- | --- | --- |
| chr5:81284643-81290855 | | | 6239 bp | | |  |  |  |  |
| Genome Start | Genome End | Strand | Div | Repeat Start | Repeat End | Repeat Left | Name | Family | Class |
| 81284643 | 81284878 | - | 12.3 | 47 | 284 | -28 | AluSx | Alu | SINE |
| 81284879 | 81285236 | + | 7.6 | 1 | 448 | -2 | LTR7 | ERV1 | LTR |
| 81285239 | 81288239 | + | 8.9 | 1 | 2994 | -4719 | HERVH | ERV1 | LTR |
| 81288219 | 81288990 | + | 8.8 | 3104 | 3873 | -3840 | HERVH | ERV1 | LTR |
| 81288976 | 81289142 | + | 9.6 | 3827 | 3993 | -3720 | HERVH | ERV1 | LTR |
| 81289147 | 81289803 | + | 14.3 | 4490 | 5146 | -2567 | HERVH | ERV1 | LTR |
| 81289808 | 81290024 | + | 12.0 | 5600 | 5819 | -1894 | HERVH | ERV1 | LTR |
| 81290023 | 81290399 | + | 10.8 | 7322 | 7713 | 0 | HERVH | ERV1 | LTR |
| 81290400 | 81290810 | + | 7.8 | 1 | 448 | -2 | LTR7 | ERV1 | LTR |
| 81290813 | 81290855 | - | 13.9 | 9 | 51 | -251 | AluSx | Alu | SINE |

  

|  |  |  |  |  |  |  |  |  |  |
| --- | --- | --- | --- | --- | --- | --- | --- | --- | --- |
| chr5:81373308-81376806 | | | 3492 bp | | |  |  |  |  |
| Genome Start | Genome End | Strand | Div | Repeat Start | Repeat End | Repeat Left | Name | Family | Class |
| 81373308 | 81373633 | - | 18.5 | 939 | 1266 | -5134 | L1MEc | L1 | LINE |
| 81373634 | 81374013 | - | 13.9 | 1 | 428 | 0 | MSTA | MaLR | LTR |
| 81374014 | 81375606 | - | 12.6 | 3 | 1580 | 0 | MSTA-int | MaLR | LTR |
| 81375609 | 81375978 | - | 13.1 | 1 | 428 | 0 | MSTA | MaLR | LTR |
| 81375984 | 81376806 | - | 27.9 | 47 | 931 | -5469 | L1MEc | L1 | LINE |

  

|  |  |  |  |  |  |  |  |  |  |
| --- | --- | --- | --- | --- | --- | --- | --- | --- | --- |
| chr5:82097919-82102694 | | | 4668 bp | | |  |  |  |  |
| Genome Start | Genome End | Strand | Div | Repeat Start | Repeat End | Repeat Left | Name | Family | Class |
| 82097919 | 82098767 | - | 22.7 | 1012 | 1925 | -4475 | L1MEc | L1 | LINE |
| 82098871 | 82098915 | - | 22.7 | 980 | 1011 | -5389 | L1MEc | L1 | LINE |
| 82098916 | 82099279 | + | 12.0 | 1 | 364 | 0 | THE1B | MaLR | LTR |
| 82099280 | 82099733 | - | 22.7 | 443 | 979 | -5421 | L1MEc | L1 | LINE |
| 82099734 | 82100106 | + | 14.9 | 1 | 379 | -2 | THE1D | MaLR | LTR |
| 82100111 | 82101624 | + | 29.0 | 2 | 1735 | 0 | MLT-int | MaLR | LTR |
| 82101626 | 82101967 | + | 16.1 | 2 | 343 | -38 | THE1D | MaLR | LTR |
| 82101968 | 82102259 | + | 7.5 | 1 | 292 | -19 | AluY | Alu | SINE |
| 82102260 | 82102312 | + | 16.1 | 344 | 381 | 0 | THE1D | MaLR | LTR |
| 82102313 | 82102694 | - | 22.7 | 57 | 442 | -5958 | L1MEc | L1 | LINE |

  

|  |  |  |  |  |  |  |  |  |  |
| --- | --- | --- | --- | --- | --- | --- | --- | --- | --- |
| chr5:82942305-82945108 | | | 2745 bp | | |  |  |  |  |
| Genome Start | Genome End | Strand | Div | Repeat Start | Repeat End | Repeat Left | Name | Family | Class |
| 82942305 | 82942518 | + | 27.6 | 69 | 289 | -161 | LTR16A | ERVL | LTR |
| 82942521 | 82942875 | + | 19.4 | 1 | 363 | -2 | MLT1A0 | MaLR | LTR |
| 82942884 | 82944605 | + | 22.1 | 7 | 1735 | 0 | MLT1A0-int | MaLR | LTR |
| 82944612 | 82944964 | + | 18.3 | 7 | 365 | 0 | MLT1A0 | MaLR | LTR |
| 82945008 | 82945108 | + | 27.7 | 336 | 447 | -3 | LTR16A | ERVL | LTR |

  

|  |  |  |  |  |  |  |  |  |  |
| --- | --- | --- | --- | --- | --- | --- | --- | --- | --- |
| chr5:84000346-84005966 | | | 5598 bp | | |  |  |  |  |
| Genome Start | Genome End | Strand | Div | Repeat Start | Repeat End | Repeat Left | Name | Family | Class |
| 84000346 | 84000835 | + | 13.3 | 4 | 498 | -3693 | MER21A-int | ERV1 | LTR |
| 84000837 | 84001212 | + | 13.6 | 4 | 428 | 0 | MSTA | MaLR | LTR |
| 84001217 | 84002820 | + | 18.0 | 5 | 1572 | -8 | MSTA-int | MaLR | LTR |
| 84002823 | 84003203 | + | 17.0 | 1 | 428 | 0 | MSTA | MaLR | LTR |
| 84003222 | 84003456 | + | 15.9 | 498 | 732 | -3459 | MER21A-int | ERV1 | LTR |
| 84003455 | 84004616 | + | 13.8 | 1919 | 3116 | -1075 | MER21A-int | ERV1 | LTR |
| 84004617 | 84004920 | - | 11.8 | 1 | 304 | -8 | AluSx | Alu | SINE |
| 84004921 | 84005966 | + | 13.8 | 3117 | 4191 | 0 | MER21A-int | ERV1 | LTR |

  

|  |  |  |  |  |  |  |  |  |  |
| --- | --- | --- | --- | --- | --- | --- | --- | --- | --- |
| chr5:84112597-84115249 | | | 2643 bp | | |  |  |  |  |
| Genome Start | Genome End | Strand | Div | Repeat Start | Repeat End | Repeat Left | Name | Family | Class |
| 84112597 | 84112639 | + | 16.4 | 6035 | 6081 | -219 | L1MA6 | L1 | LINE |
| 84112640 | 84113051 | - | 19.7 | 3 | 426 | 0 | MSTB | MaLR | LTR |
| 84113054 | 84114582 | - | 30.2 | 4 | 1735 | 0 | MLT-int | MaLR | LTR |
| 84114591 | 84115019 | - | 19.8 | 1 | 422 | -4 | MSTB | MaLR | LTR |
| 84115020 | 84115249 | + | 16.4 | 6085 | 6300 | 0 | L1MA6 | L1 | LINE |

  

|  |  |  |  |  |  |  |  |  |  |
| --- | --- | --- | --- | --- | --- | --- | --- | --- | --- |
| chr5:84248832-84255025 | | | 6196 bp | | |  |  |  |  |
| Genome Start | Genome End | Strand | Div | Repeat Start | Repeat End | Repeat Left | Name | Family | Class |
| 84248832 | 84249106 | + | 14.6 | 3706 | 3982 | -2164 | L1PA16 | L1 | LINE |
| 84249105 | 84249349 | + | 15.1 | 4453 | 4700 | -1446 | L1PREC2 | L1 | LINE |
| 84249346 | 84250661 | + | 11.1 | 4830 | 6163 | 0 | L1PA13 | L1 | LINE |
| 84250665 | 84251866 | + | 15.4 | 3983 | 5202 | -944 | L1PA16 | L1 | LINE |
| 84251867 | 84252235 | + | 12.5 | 1 | 364 | 0 | THE1B | MaLR | LTR |
| 84252236 | 84253715 | + | 12.7 | 1 | 1580 | 0 | THE1B-int | MaLR | LTR |
| 84253717 | 84254075 | + | 11.1 | 2 | 364 | 0 | THE1B | MaLR | LTR |
| 84254076 | 84255025 | + | 14.7 | 5198 | 6166 | 0 | L1PA16 | L1 | LINE |

  

|  |  |  |  |  |  |  |  |  |  |
| --- | --- | --- | --- | --- | --- | --- | --- | --- | --- |
| chr5:84882222-84887304 | | | 5068 bp | | |  |  |  |  |
| Genome Start | Genome End | Strand | Div | Repeat Start | Repeat End | Repeat Left | Name | Family | Class |
| 84882222 | 84882724 | + | 23.9 | 2417 | 2936 | -4629 | L1M1 | L1 | LINE |
| 84882737 | 84884175 | + | 18.4 | 4910 | 6388 | -1174 | L1M1 | L1 | LINE |
| 84884176 | 84884560 | - | 18.2 | 1 | 428 | 0 | MSTA | MaLR | LTR |
| 84884562 | 84886171 | - | 31.1 | 2 | 1734 | -1 | MLT-int | MaLR | LTR |
| 84886174 | 84886552 | - | 18.0 | 1 | 428 | 0 | MSTA | MaLR | LTR |
| 84886553 | 84887304 | + | 17.9 | 6389 | 7214 | -505 | L1M1 | L1 | LINE |

  

|  |  |  |  |  |  |  |  |  |  |
| --- | --- | --- | --- | --- | --- | --- | --- | --- | --- |
| chr5:85180716-85183589 | | | 2772 bp | | |  |  |  |  |
| Genome Start | Genome End | Strand | Div | Repeat Start | Repeat End | Repeat Left | Name | Family | Class |
| 85180716 | 85180807 | - | 29.4 | 454 | 549 | 0 | MLT1H | MaLR | LTR |
| 85180812 | 85181184 | - | 18.9 | 1 | 423 | -5 | MSTA | MaLR | LTR |
| 85181185 | 85182802 | - | 31.3 | 3 | 1735 | 0 | MLT1H-int | MaLR | LTR |
| 85182806 | 85183198 | - | 15.5 | 1 | 428 | 0 | MSTA | MaLR | LTR |
| 85183294 | 85183589 | - | 29.5 | 1 | 293 | -116 | MLT1H | MaLR | LTR |

  

|  |  |  |  |  |  |  |  |  |  |
| --- | --- | --- | --- | --- | --- | --- | --- | --- | --- |
| chr5:86837437-86852473 | | | 14976 bp | | |  |  |  |  |
| Genome Start | Genome End | Strand | Div | Repeat Start | Repeat End | Repeat Left | Name | Family | Class |
| 86837437 | 86837498 | + | 16.7 | 3751 | 3811 | -3037 | L1M2 | L1 | LINE |
| 86837471 | 86837943 | + | 24.1 | 4653 | 5136 | -1712 | L1M2 | L1 | LINE |
| 86837944 | 86838458 | + | 16.7 | 1 | 520 | 0 | MLT2B1 | ERVL | LTR |
| 86838459 | 86838769 | + | 24.1 | 5137 | 5449 | -1399 | L1M2 | L1 | LINE |
| 86838850 | 86841545 | + | 12.8 | 2617 | 5327 | -1439 | L1PA13 | L1 | LINE |
| 86841546 | 86841849 | + | 13.2 | 1 | 304 | -5 | AluSc | Alu | SINE |
| 86841850 | 86841903 | + | 13.1 | 5328 | 5381 | -1385 | L1PA13 | L1 | LINE |
| 86841904 | 86842416 | + | 11.6 | 1 | 511 | 0 | MER9 | ERVK | LTR |
| 86842417 | 86847699 | + | 6.8 | 1 | 5284 | -632 | HERVK9 | ERVK | LTR |
| 86847700 | 86850659 | + | 6.4 | 3199 | 6153 | -1 | L1PA5 | L1 | LINE |
| 86850661 | 86850813 | + | 3.9 | 5271 | 5423 | -493 | HERVK9 | ERVK | LTR |
| 86850814 | 86850983 | + | 6.5 | 295 | 464 | -47 | MER9 | ERVK | LTR |
| 86850989 | 86851439 | + | 8.2 | 5438 | 5916 | 0 | HERVK9 | ERVK | LTR |
| 86851440 | 86851948 | + | 10.3 | 1 | 509 | -2 | MER9 | ERVK | LTR |
| 86851952 | 86852473 | + | 21.4 | 5459 | 5950 | -898 | L1M2 | L1 | LINE |

  

|  |  |  |  |  |  |  |  |  |  |
| --- | --- | --- | --- | --- | --- | --- | --- | --- | --- |
| chr5:86884778-86887964 | | | 3131 bp | | |  |  |  |  |
| Genome Start | Genome End | Strand | Div | Repeat Start | Repeat End | Repeat Left | Name | Family | Class |
| 86884778 | 86885225 | - | 24.8 | 4255 | 4718 | -949 | ERVL-E | ERVL | LTR |
| 86885227 | 86885592 | - | 11.2 | 1 | 422 | -10 | THE1C | MaLR | LTR |
| 86885593 | 86887109 | - | 12.7 | 1 | 1580 | 0 | THE1C-int | MaLR | LTR |
| 86887110 | 86887483 | - | 13.3 | 1 | 375 | 0 | THE1C | MaLR | LTR |
| 86887532 | 86887746 | - | 23.7 | 3970 | 4190 | -1524 | ERVL-E | ERVL | LTR |
| 86887754 | 86887964 | - | 25.7 | 3552 | 3773 | -1894 | ERVL-E | ERVL | LTR |

  

|  |  |  |  |  |  |  |  |  |  |
| --- | --- | --- | --- | --- | --- | --- | --- | --- | --- |
| chr5:86917459-86937926 | | | 20257 bp | | |  |  |  |  |
| Genome Start | Genome End | Strand | Div | Repeat Start | Repeat End | Repeat Left | Name | Family | Class |
| 86917459 | 86917635 | - | 20.2 | 6128 | 6353 | 0 | L1MA4 | L1 | LINE |
| 86917636 | 86917942 | - | 8.5 | 1 | 307 | -2 | AluSc | Alu | SINE |
| 86917943 | 86918821 | - | 16.0 | 5286 | 6134 | -348 | L1MA4 | L1 | LINE |
| 86918822 | 86919126 | - | 9.2 | 1 | 305 | -8 | AluSp | Alu | SINE |
| 86919127 | 86920553 | - | 17.8 | 3822 | 5285 | -1040 | L1MA4 | L1 | LINE |
| 86920647 | 86921033 | - | 11.4 | 1 | 404 | -40 | MLT2A1 | ERVL | LTR |
| 86921034 | 86921462 | - | 7.8 | 5221 | 5654 | 0 | HERVL-A2 | ERVL | LTR |
| 86921487 | 86926684 | - | 7.8 | 2 | 5220 | -434 | HERVL-A2 | ERVL | LTR |
| 86926685 | 86927126 | - | 10.8 | 1 | 453 | 0 | MLT2A2 | ERVL | LTR |
| 86927127 | 86927483 | - | 14.0 | 1 | 361 | -3 | THE1B | MaLR | LTR |
| 86927484 | 86929048 | - | 29.8 | 2 | 1735 | 0 | MLT-int | MaLR | LTR |
| 86929051 | 86929419 | - | 11.6 | 1 | 364 | 0 | THE1B | MaLR | LTR |
| 86929496 | 86930443 | - | 11.4 | 5231 | 6194 | 0 | L1PREC2 | L1 | LINE |
| 86930447 | 86934306 | - | 14.6 | 11 | 4254 | -3901 | L1PREC2 | L1 | LINE |
| 86934320 | 86937926 | - | 20.7 | 2 | 3751 | -2577 | L1MA4 | L1 | LINE |

  

|  |  |  |  |  |  |  |  |  |  |
| --- | --- | --- | --- | --- | --- | --- | --- | --- | --- |
| chr5:87302350-87314005 | | | 11586 bp | | |  |  |  |  |
| Genome Start | Genome End | Strand | Div | Repeat Start | Repeat End | Repeat Left | Name | Family | Class |
| 87302350 | 87303859 | - | 25.5 | 4397 | 6070 | -83 | L1ME3A | L1 | LINE |
| 87303860 | 87304193 | - | 11.8 | 1 | 364 | 0 | THE1B | MaLR | LTR |
| 87304195 | 87305772 | - | 30.0 | 2 | 1734 | -1 | MLT-int | MaLR | LTR |
| 87305775 | 87306130 | - | 12.1 | 1 | 364 | 0 | THE1B | MaLR | LTR |
| 87306131 | 87306363 | - | 24.6 | 4138 | 4396 | -1733 | L1ME3A | L1 | LINE |
| 87306378 | 87306519 | - | 14.2 | 6017 | 6157 | -9 | L1PA16 | L1 | LINE |
| 87306520 | 87309018 | - | 14.0 | 1831 | 4383 | -2466 | L1PA15-16 | L1 | LINE |
| 87309019 | 87309404 | + | 14.3 | 1 | 428 | 0 | MSTA | MaLR | LTR |
| 87309407 | 87311043 | + | 31.0 | 2 | 1735 | 0 | MLT-int | MaLR | LTR |
| 87311044 | 87311442 | + | 14.0 | 1 | 428 | 0 | MSTA | MaLR | LTR |
| 87311443 | 87313435 | - | 15.1 | 56 | 1830 | -5023 | L1PA15-16 | L1 | LINE |
| 87313487 | 87314005 | - | 22.1 | 3553 | 4104 | -2025 | L1ME3A | L1 | LINE |

  

|  |  |  |  |  |  |  |  |  |  |
| --- | --- | --- | --- | --- | --- | --- | --- | --- | --- |
| chr5:87507162-87509932 | | | 2664 bp | | |  |  |  |  |
| Genome Start | Genome End | Strand | Div | Repeat Start | Repeat End | Repeat Left | Name | Family | Class |
| 87507162 | 87507471 | - | 16.5 | 5609 | 5918 | -1867 | L1MC4a | L1 | LINE |
| 87507480 | 87508000 | - | 10.8 | 5661 | 6174 | 0 | L1PA11 | L1 | LINE |
| 87508001 | 87508285 | + | 20.1 | 1 | 284 | -28 | AluJb | Alu | SINE |
| 87508344 | 87508658 | - | 3.9 | 1 | 326 | -454 | LTR17 | ERV1 | LTR |
| 87508659 | 87508910 | - | 6.8 | 8375 | 8626 | 0 | HERV17 | ERV1 | LTR |
| 87508941 | 87509456 | - | 8.1 | 254 | 780 | 0 | LTR17 | ERV1 | LTR |
| 87509457 | 87509751 | - | 6.8 | 5379 | 5674 | -500 | L1P4 | L1 | LINE |
| 87509763 | 87509932 | - | 18.4 | 5427 | 5598 | -2187 | L1MC4a | L1 | LINE |

  

|  |  |  |  |  |  |  |  |  |  |
| --- | --- | --- | --- | --- | --- | --- | --- | --- | --- |
| chr5:88748081-88750752 | | | 2638 bp | | |  |  |  |  |
| Genome Start | Genome End | Strand | Div | Repeat Start | Repeat End | Repeat Left | Name | Family | Class |
| 88748081 | 88748246 | - | 24.2 | 7364 | 7540 | -421 | L1MC5 | L1 | LINE |
| 88748276 | 88748640 | + | 10.7 | 1 | 375 | 0 | THE1C | MaLR | LTR |
| 88748641 | 88750179 | + | 12.1 | 1 | 1575 | -5 | THE1C-int | MaLR | LTR |
| 88750185 | 88750544 | + | 13.9 | 1 | 373 | -2 | THE1C | MaLR | LTR |
| 88750545 | 88750752 | - | 24.3 | 7132 | 7330 | -631 | L1MC5 | L1 | LINE |

  

|  |  |  |  |  |  |  |  |  |  |
| --- | --- | --- | --- | --- | --- | --- | --- | --- | --- |
| chr5:88916469-88920126 | | | 3514 bp | | |  |  |  |  |
| Genome Start | Genome End | Strand | Div | Repeat Start | Repeat End | Repeat Left | Name | Family | Class |
| 88916469 | 88917299 | - | 35.3 | 2485 | 3375 | -3 | L2 | L2 | LINE |
| 88917382 | 88917728 | - | 22.1 | 4 | 352 | -13 | MLT1A0 | MaLR | LTR |
| 88917738 | 88919462 | - | 23.2 | 2 | 1729 | -6 | MLT1A0-int | MaLR | LTR |
| 88919465 | 88919819 | - | 20.9 | 1 | 365 | 0 | MLT1A0 | MaLR | LTR |
| 88919823 | 88919891 | - | 24.6 | 3254 | 3333 | -45 | L2 | L2 | LINE |
| 88919940 | 88920126 | - | 37.1 | 2139 | 2346 | -1073 | L2 | L2 | LINE |

  

|  |  |  |  |  |  |  |  |  |  |
| --- | --- | --- | --- | --- | --- | --- | --- | --- | --- |
| chr5:90560617-90563568 | | | 2924 bp | | |  |  |  |  |
| Genome Start | Genome End | Strand | Div | Repeat Start | Repeat End | Repeat Left | Name | Family | Class |
| 90560617 | 90560856 | + | 18.9 | 1 | 231 | -474 | Tigger7 | MER2\_type | DNA |
| 90560885 | 90561245 | - | 11.7 | 1 | 375 | 0 | THE1C | MaLR | LTR |
| 90561246 | 90562779 | - | 11.2 | 1 | 1580 | 0 | THE1C-int | MaLR | LTR |
| 90562780 | 90563002 | - | 12.1 | 171 | 375 | 0 | THE1C | MaLR | LTR |
| 90563003 | 90563316 | + | 13.5 | 1 | 311 | -1 | AluSx | Alu | SINE |
| 90563317 | 90563484 | - | 12.1 | 1 | 170 | -205 | THE1C | MaLR | LTR |
| 90563485 | 90563568 | + | 11.9 | 2404 | 2490 | -1 | Tigger7 | MER2\_type | DNA |

  

|  |  |  |  |  |  |  |  |  |  |
| --- | --- | --- | --- | --- | --- | --- | --- | --- | --- |
| chr5:90677838-90680848 | | | 3001 bp | | |  |  |  |  |
| Genome Start | Genome End | Strand | Div | Repeat Start | Repeat End | Repeat Left | Name | Family | Class |
| 90677838 | 90677936 | - | 23.9 | 146 | 240 | -22 | MIR | MIR | SINE |
| 90677939 | 90678035 | - | 20.0 | 74 | 170 | -15 | FAM | Alu | SINE |
| 90678036 | 90678108 | - | 14.1 | 298 | 364 | 0 | THE1B | MaLR | LTR |
| 90678109 | 90678409 | - | 9.4 | 1 | 299 | -10 | AluSc | Alu | SINE |
| 90678410 | 90678705 | - | 14.1 | 1 | 297 | -67 | THE1B | MaLR | LTR |
| 90678706 | 90680276 | - | 30.4 | 2 | 1735 | 0 | MLT1K-int | MaLR | LTR |
| 90680279 | 90680631 | - | 12.8 | 1 | 364 | 0 | THE1B | MaLR | LTR |
| 90680632 | 90680704 | - | 20.0 | 5 | 73 | -112 | FAM | Alu | SINE |
| 90680711 | 90680848 | - | 28.3 | 16 | 154 | -108 | MIR | MIR | SINE |

  

|  |  |  |  |  |  |  |  |  |  |
| --- | --- | --- | --- | --- | --- | --- | --- | --- | --- |
| chr5:92863949-92870732 | | | 6322 bp | | |  |  |  |  |
| Genome Start | Genome End | Strand | Div | Repeat Start | Repeat End | Repeat Left | Name | Family | Class |
| 92863949 | 92864226 | + | 31.3 | 242 | 510 | -5609 | L1ME1 | L1 | LINE |
| 92864270 | 92864760 | + | 29.7 | 5821 | 6370 | -1438 | L1MC4a | L1 | LINE |
| 92864956 | 92865449 | + | 16.8 | 44 | 563 | 0 | MER68 | ERVL | LTR |
| 92865450 | 92865542 | + | 18.1 | 7422 | 7519 | -442 | L1MC5 | L1 | LINE |
| 92865761 | 92865974 | + | 31.6 | 3406 | 3630 | -2499 | L1ME1 | L1 | LINE |
| 92865976 | 92866331 | - | 7.1 | 1 | 354 | -1 | THE1A | MaLR | LTR |
| 92866334 | 92867910 | - | 30.1 | 3 | 1733 | -2 | MLT-int | MaLR | LTR |
| 92867914 | 92868283 | - | 10.5 | 1 | 355 | 0 | THE1A | MaLR | LTR |
| 92868284 | 92868787 | + | 28.2 | 3631 | 4136 | -1993 | L1ME1 | L1 | LINE |
| 92868788 | 92869119 | - | 9.9 | 1 | 332 | 0 | AluSq | Alu | SINE |
| 92869120 | 92870732 | + | 28.3 | 4137 | 5926 | -223 | L1ME1 | L1 | LINE |

  

|  |  |  |  |  |  |  |  |  |  |
| --- | --- | --- | --- | --- | --- | --- | --- | --- | --- |
| chr5:95661389-95673644 | | | 12260 bp | | |  |  |  |  |
| Genome Start | Genome End | Strand | Div | Repeat Start | Repeat End | Repeat Left | Name | Family | Class |
| 95661389 | 95662447 | - | 19.0 | 4500 | 5570 | -809 | L1M1 | L1 | LINE |
| 95662448 | 95662830 | - | 14.2 | 1 | 428 | 0 | MSTA | MaLR | LTR |
| 95662833 | 95664441 | - | 30.9 | 2 | 1733 | -2 | MLT-int | MaLR | LTR |
| 95664444 | 95664814 | - | 16.0 | 3 | 428 | 0 | MSTA | MaLR | LTR |
| 95664815 | 95664931 | - | 19.0 | 4389 | 4499 | -1880 | L1M1 | L1 | LINE |
| 95664925 | 95671366 | - | 9.3 | 11 | 6466 | -3 | L1PA7 | L1 | LINE |
| 95671366 | 95673644 | - | 17.2 | 2043 | 4410 | -1969 | L1M1 | L1 | LINE |

  

|  |  |  |  |  |  |  |  |  |  |
| --- | --- | --- | --- | --- | --- | --- | --- | --- | --- |
| chr5:96699914-96706766 | | | 6851 bp | | |  |  |  |  |
| Genome Start | Genome End | Strand | Div | Repeat Start | Repeat End | Repeat Left | Name | Family | Class |
| 96699914 | 96703243 | + | 15.9 | 5 | 3373 | -4192 | L1M1 | L1 | LINE |
| 96703244 | 96703589 | - | 13.0 | 1 | 364 | 0 | THE1B | MaLR | LTR |
| 96703590 | 96705162 | - | 30.9 | 2 | 1735 | 0 | MLT-int | MaLR | LTR |
| 96705165 | 96705508 | - | 14.5 | 1 | 364 | 0 | THE1B | MaLR | LTR |
| 96705509 | 96706766 | + | 12.7 | 3374 | 4621 | -2944 | L1M1 | L1 | LINE |

  

|  |  |  |  |  |  |  |  |  |  |
| --- | --- | --- | --- | --- | --- | --- | --- | --- | --- |
| chr5:96925337-96933012 | | | 7655 bp | | |  |  |  |  |
| Genome Start | Genome End | Strand | Div | Repeat Start | Repeat End | Repeat Left | Name | Family | Class |
| 96925337 | 96925437 | + | 27.2 | 37 | 136 | -6264 | L1MEc | L1 | LINE |
| 96925438 | 96925746 | + | 12.3 | 1 | 309 | -3 | AluSx | Alu | SINE |
| 96925747 | 96927285 | + | 27.2 | 137 | 1602 | -4798 | L1MEc | L1 | LINE |
| 96927286 | 96927706 | - | 15.8 | 1 | 428 | 0 | MSTA | MaLR | LTR |
| 96927707 | 96930111 | + | 14.8 | 2 | 2418 | 0 | Tigger1 | MER2\_type | DNA |
| 96930112 | 96930608 | + | 27.2 | 1603 | 2175 | -4225 | L1MEc | L1 | LINE |
| 96930614 | 96930998 | - | 14.6 | 1 | 381 | 0 | THE1D | MaLR | LTR |
| 96931003 | 96932487 | - | 13.3 | 1 | 1576 | -4 | THE1D-int | MaLR | LTR |
| 96932490 | 96932873 | - | 16.6 | 1 | 379 | -2 | THE1D | MaLR | LTR |
| 96932884 | 96933012 | + | 18.9 | 2210 | 2339 | -4061 | L1MEc | L1 | LINE |

  

|  |  |  |  |  |  |  |  |  |  |
| --- | --- | --- | --- | --- | --- | --- | --- | --- | --- |
| chr5:97963181-97968000 | | | 4431 bp | | |  |  |  |  |
| Genome Start | Genome End | Strand | Div | Repeat Start | Repeat End | Repeat Left | Name | Family | Class |
| 97963181 | 97963403 | + | 30.7 | 7140 | 7364 | -678 | L1MC4 | L1 | LINE |
| 97963404 | 97963756 | + | 11.3 | 3 | 364 | 0 | THE1B | MaLR | LTR |
| 97963759 | 97965275 | + | 29.7 | 2 | 1735 | 0 | MLT-int | MaLR | LTR |
| 97965276 | 97965628 | + | 14.7 | 1 | 364 | 0 | THE1B | MaLR | LTR |
| 97965629 | 97965814 | + | 30.7 | 7365 | 7563 | -479 | L1MC4 | L1 | LINE |
| 97965824 | 97966815 | + | 13.6 | 4788 | 5791 | -365 | L1PB4 | L1 | LINE |
| 97967098 | 97967454 | + | 14.0 | 5792 | 6153 | -3 | L1PB4 | L1 | LINE |
| 97967524 | 97967861 | + | 25.9 | 7608 | 7928 | -114 | L1MC4 | L1 | LINE |
| 97967889 | 97968000 | + | 25.9 | 7929 | 8040 | -2 | L1MC4 | L1 | LINE |

  

|  |  |  |  |  |  |  |  |  |  |
| --- | --- | --- | --- | --- | --- | --- | --- | --- | --- |
| chr5:98371574-98381893 | | | 10140 bp | | |  |  |  |  |
| Genome Start | Genome End | Strand | Div | Repeat Start | Repeat End | Repeat Left | Name | Family | Class |
| 98371574 | 98371668 | + | 23.4 | 5288 | 5388 | -758 | L1ME4a | L1 | LINE |
| 98371701 | 98372048 | + | 11.7 | 1 | 364 | 0 | THE1B | MaLR | LTR |
| 98372100 | 98372390 | + | 10.7 | 1 | 292 | -20 | AluSx | Alu | SINE |
| 98372428 | 98372583 | + | 28.3 | 5518 | 5664 | -457 | L1ME4a | L1 | LINE |
| 98372592 | 98372831 | - | 18.3 | 177 | 426 | 0 | MSTB | MaLR | LTR |
| 98372832 | 98373001 | + | 15.9 | 121 | 300 | -9 | AluSc | Alu | SINE |
| 98373006 | 98373167 | - | 20.4 | 1 | 183 | -245 | MSTB | MaLR | LTR |
| 98373173 | 98374427 | - | 19.4 | 367 | 1645 | -6 | MSTA-int | MaLR | LTR |
| 98374428 | 98374858 | - | 4.4 | 1 | 464 | 0 | LTR7B | ERV1 | LTR |
| 98374863 | 98375427 | - | 11.8 | 7157 | 7709 | -4 | HERVH | ERV1 | LTR |
| 98375421 | 98375685 | - | 15.5 | 5747 | 6016 | -1697 | HERVH | ERV1 | LTR |
| 98375686 | 98376620 | - | 10.2 | 3962 | 4910 | -2297 | HERVH | ERV1 | LTR |
| 98376657 | 98376809 | - | 10.5 | 3841 | 3993 | -3720 | HERVH | ERV1 | LTR |
| 98376815 | 98377261 | - | 15.2 | 3140 | 3587 | -4126 | HERVH | ERV1 | LTR |
| 98377256 | 98380125 | - | 14.7 | 2 | 2987 | -4726 | HERVH | ERV1 | LTR |
| 98380127 | 98380558 | - | 4.4 | 1 | 464 | 0 | LTR7B | ERV1 | LTR |
| 98380559 | 98380896 | - | 17.4 | 23 | 371 | -1280 | MSTA-int | MaLR | LTR |
| 98380897 | 98381203 | - | 14.1 | 1 | 305 | -7 | AluSx | Alu | SINE |
| 98381204 | 98381230 | - | 17.4 | 1 | 22 | -1629 | MSTA-int | MaLR | LTR |
| 98381233 | 98381626 | - | 16.0 | 1 | 426 | -2 | MSTA | MaLR | LTR |
| 98381635 | 98381893 | + | 29.6 | 5676 | 5952 | -169 | L1ME4a | L1 | LINE |

  

|  |  |  |  |  |  |  |  |  |  |
| --- | --- | --- | --- | --- | --- | --- | --- | --- | --- |
| chr5:99162872-99166624 | | | 3751 bp | | |  |  |  |  |
| Genome Start | Genome End | Strand | Div | Repeat Start | Repeat End | Repeat Left | Name | Family | Class |
| 99162872 | 99163138 | - | 18.6 | 6030 | 6293 | -7 | L1MA4 | L1 | LINE |
| 99163141 | 99163476 | - | 15.2 | 1 | 360 | -4 | THE1B | MaLR | LTR |
| 99163477 | 99165025 | - | 9.8 | 1 | 1580 | 0 | THE1B-int | MaLR | LTR |
| 99165026 | 99165366 | - | 16.8 | 1 | 364 | 0 | THE1B | MaLR | LTR |
| 99165367 | 99165695 | - | 17.7 | 5706 | 6037 | -263 | L1MA4 | L1 | LINE |
| 99165696 | 99166003 | - | 9.4 | 1 | 308 | -3 | AluY | Alu | SINE |
| 99166004 | 99166624 | - | 18.5 | 5017 | 5705 | -595 | L1MA4 | L1 | LINE |

  

|  |  |  |  |  |  |  |  |  |  |
| --- | --- | --- | --- | --- | --- | --- | --- | --- | --- |
| chr5:99471041-99473600 | | | 2555 bp | | |  |  |  |  |
| Genome Start | Genome End | Strand | Div | Repeat Start | Repeat End | Repeat Left | Name | Family | Class |
| 99471041 | 99471189 | + | 30.4 | 3 | 152 | -298 | MLT1J2 | MaLR | LTR |
| 99471190 | 99471567 | + | 14.0 | 1 | 374 | -1 | THE1C | MaLR | LTR |
| 99471573 | 99473162 | + | 32.4 | 4 | 1735 | 0 | MLT1A-int | MaLR | LTR |
| 99473163 | 99473539 | + | 15.2 | 1 | 375 | 0 | THE1C | MaLR | LTR |
| 99473540 | 99473600 | + | 30.4 | 154 | 213 | -237 | MLT1J2 | MaLR | LTR |

  

|  |  |  |  |  |  |  |  |  |  |
| --- | --- | --- | --- | --- | --- | --- | --- | --- | --- |
| chr5:99850445-99854857 | | | 4411 bp | | |  |  |  |  |
| Genome Start | Genome End | Strand | Div | Repeat Start | Repeat End | Repeat Left | Name | Family | Class |
| 99850445 | 99851219 | + | 20.6 | 3783 | 4558 | -1582 | L1MA9 | L1 | LINE |
| 99851220 | 99851574 | - | 15.6 | 1 | 364 | 0 | THE1B | MaLR | LTR |
| 99851575 | 99853146 | - | 30.8 | 2 | 1735 | 0 | MLT-int | MaLR | LTR |
| 99853149 | 99853511 | - | 13.6 | 1 | 364 | 0 | THE1B | MaLR | LTR |
| 99853512 | 99854857 | + | 19.6 | 4559 | 5932 | -380 | L1MA9 | L1 | LINE |

  

|  |  |  |  |  |  |  |  |  |  |
| --- | --- | --- | --- | --- | --- | --- | --- | --- | --- |
| chr5:100343912-100355791 | | | 11580 bp | | |  |  |  |  |
| Genome Start | Genome End | Strand | Div | Repeat Start | Repeat End | Repeat Left | Name | Family | Class |
| 100343912 | 100344505 | - | 25.4 | 1682 | 2283 | -4117 | L1MEc | L1 | LINE |
| 100344518 | 100344823 | - | 24.6 | 1262 | 1571 | -4829 | L1MEc | L1 | LINE |
| 100344813 | 100345068 | - | 31.5 | 182 | 468 | -5932 | L1MEc | L1 | LINE |
| 100345099 | 100345206 | - | 31.5 | 61 | 181 | -6219 | L1MEc | L1 | LINE |
| 100345207 | 100346008 | + | 19.4 | 1 | 784 | -1 | LTR1 | ERV1 | LTR |
| 100346009 | 100346030 | - | 31.5 | 45 | 60 | -6340 | L1MEc | L1 | LINE |
| 100346035 | 100346208 | - | 25.8 | 2125 | 2295 | -4105 | L1MEc | L1 | LINE |
| 100346221 | 100346651 | - | 10.5 | 113 | 553 | -1 | MER41A | ERV1 | LTR |
| 100346652 | 100347623 | - | 8.9 | 1 | 968 | 0 | LTR5\_Hs | ERVK | LTR |
| 100347624 | 100347732 | - | 10.5 | 1 | 112 | -442 | MER41A | ERV1 | LTR |
| 100347895 | 100348326 | - | 9.7 | 1 | 448 | -2 | LTR7 | ERV1 | LTR |
| 100348327 | 100348706 | - | 8.5 | 7322 | 7713 | 0 | HERVH | ERV1 | LTR |
| 100348705 | 100348925 | - | 11.5 | 5597 | 5819 | -1894 | HERVH | ERV1 | LTR |
| 100348923 | 100349552 | - | 13.1 | 4488 | 5149 | -2564 | HERVH | ERV1 | LTR |
| 100349555 | 100350057 | - | 10.6 | 3492 | 3993 | -3720 | HERVH | ERV1 | LTR |
| 100350048 | 100350195 | - | 13.5 | 3140 | 3290 | -4423 | HERVH | ERV1 | LTR |
| 100350190 | 100353393 | - | 10.9 | 1 | 2987 | -4726 | HERVH | ERV1 | LTR |
| 100353396 | 100353827 | - | 9.7 | 1 | 448 | -2 | LTR7 | ERV1 | LTR |
| 100353828 | 100353992 | - | 26.4 | 1702 | 1864 | -4536 | L1MEc | L1 | LINE |
| 100354054 | 100354414 | - | 29.9 | 1204 | 1584 | -4816 | L1MEc | L1 | LINE |
| 100354423 | 100354718 | - | 8.8 | 1 | 297 | -14 | AluY | Alu | SINE |
| 100354745 | 100355563 | - | 33.3 | 289 | 1144 | -5256 | L1MEc | L1 | LINE |
| 100355577 | 100355791 | - | 36.8 | 55 | 275 | -6125 | L1MEc | L1 | LINE |

  

|  |  |  |  |  |  |  |  |  |  |
| --- | --- | --- | --- | --- | --- | --- | --- | --- | --- |
| chr5:100483750-100486484 | | | 2731 bp | | |  |  |  |  |
| Genome Start | Genome End | Strand | Div | Repeat Start | Repeat End | Repeat Left | Name | Family | Class |
| 100483750 | 100484077 | - | 23.6 | 5975 | 6286 | -5 | L1MA7 | L1 | LINE |
| 100484078 | 100484445 | - | 14.3 | 1 | 364 | 0 | THE1B | MaLR | LTR |
| 100484446 | 100485947 | - | 30.6 | 2 | 1735 | 0 | MLT-int | MaLR | LTR |
| 100485952 | 100486317 | - | 14.4 | 1 | 362 | -2 | THE1B | MaLR | LTR |
| 100486318 | 100486484 | - | 23.6 | 5804 | 5974 | -317 | L1MA7 | L1 | LINE |

  

|  |  |  |  |  |  |  |  |  |  |
| --- | --- | --- | --- | --- | --- | --- | --- | --- | --- |
| chr5:100570901-100574083 | | | 3520 bp | | |  |  |  |  |
| Genome Start | Genome End | Strand | Div | Repeat Start | Repeat End | Repeat Left | Name | Family | Class |
| 100570901 | 100571010 | + | 27.5 | 5330 | 5438 | -728 | L1MC3 | L1 | LINE |
| 100571011 | 100571367 | + | 14.7 | 1 | 381 | 0 | THE1D | MaLR | LTR |
| 100571371 | 100573003 | + | 15.4 | 4 | 1580 | 0 | THE1D-int | MaLR | LTR |
| 100573004 | 100573366 | + | 13.2 | 1 | 381 | 0 | THE1D | MaLR | LTR |
| 100573369 | 100574001 | + | 26.0 | 5439 | 6079 | -163 | L1MD1 | L1 | LINE |
| 100573660 | 100574083 | + | 27.4 | 5661 | 6143 | -1642 | L1MC3 | L1 | LINE |

  

|  |  |  |  |  |  |  |  |  |  |
| --- | --- | --- | --- | --- | --- | --- | --- | --- | --- |
| chr5:100864328-100872801 | | | 7982 bp | | |  |  |  |  |
| Genome Start | Genome End | Strand | Div | Repeat Start | Repeat End | Repeat Left | Name | Family | Class |
| 100864328 | 100865039 | + | 17.4 | 1 | 661 | -2057 | Tigger2 | MER2\_type | DNA |
| 100865040 | 100865350 | + | 14.8 | 1 | 312 | 0 | AluSx | Alu | SINE |
| 100865801 | 100866103 | + | 9.9 | 1 | 303 | -8 | AluY | Alu | SINE |
| 100866112 | 100866449 | + | 14.2 | 1 | 312 | 0 | AluSx | Alu | SINE |
| 100866450 | 100867260 | + | 17.4 | 412 | 1241 | -740 | Tigger2 | MER2\_type | DNA |
| 100867262 | 100867682 | + | 18.9 | 1 | 402 | 0 | MSTB | MaLR | LTR |
| 100867683 | 100868091 | + | 19.5 | 1 | 402 | -1249 | MSTB-int | MaLR | LTR |
| 100868092 | 100868399 | + | 9.1 | 1 | 308 | -5 | AluSp | Alu | SINE |
| 100868400 | 100869455 | + | 19.5 | 403 | 1530 | -121 | MSTB-int | MaLR | LTR |
| 100869457 | 100870902 | + | 13.6 | 1 | 1433 | -145 | LTR12C | ERV1 | LTR |
| 100870903 | 100871026 | + | 17.4 | 1527 | 1649 | -2 | MSTB-int | MaLR | LTR |
| 100871029 | 100871416 | + | 19.5 | 1 | 392 | -34 | MSTB | MaLR | LTR |
| 100871417 | 100871714 | - | 8.8 | 1 | 295 | -16 | AluY | Alu | SINE |
| 100871715 | 100871758 | + | 19.5 | 393 | 425 | -1 | MSTB | MaLR | LTR |
| 100871759 | 100872423 | + | 15.7 | 1242 | 2650 | -68 | Tigger2 | MER2\_type | DNA |
| 100872424 | 100872702 | + | 20.8 | 1 | 282 | -30 | AluJo | Alu | SINE |
| 100872733 | 100872801 | + | 15.7 | 2651 | 2716 | -2 | Tigger2 | MER2\_type | DNA |

  

|  |  |  |  |  |  |  |  |  |  |
| --- | --- | --- | --- | --- | --- | --- | --- | --- | --- |
| chr5:101131273-101134050 | | | 2774 bp | | |  |  |  |  |
| Genome Start | Genome End | Strand | Div | Repeat Start | Repeat End | Repeat Left | Name | Family | Class |
| 101131273 | 101131553 | - | 19.4 | 83 | 236 | 0 | MER46B | MER2\_type | DNA |
| 101131554 | 101131906 | - | 11.1 | 3 | 364 | 0 | THE1B | MaLR | LTR |
| 101131909 | 101133616 | - | 29.4 | 2 | 1735 | 0 | MLT-int | MaLR | LTR |
| 101133619 | 101133967 | - | 13.2 | 3 | 364 | 0 | THE1B | MaLR | LTR |
| 101133968 | 101134050 | - | 19.4 | 1 | 82 | -279 | MER46B | MER2\_type | DNA |

  

|  |  |  |  |  |  |  |  |  |  |
| --- | --- | --- | --- | --- | --- | --- | --- | --- | --- |
| chr5:101725767-101728890 | | | 3122 bp | | |  |  |  |  |
| Genome Start | Genome End | Strand | Div | Repeat Start | Repeat End | Repeat Left | Name | Family | Class |
| 101725767 | 101725947 | - | 17.9 | 756 | 922 | -9 | MER21A | ERV1 | LTR |
| 101725948 | 101726302 | - | 13.2 | 1 | 355 | 0 | THE1A | MaLR | LTR |
| 101726303 | 101727864 | - | 30.6 | 2 | 1735 | 0 | MLT-int | MaLR | LTR |
| 101727867 | 101728218 | - | 12.3 | 1 | 355 | 0 | THE1A | MaLR | LTR |
| 101728219 | 101728890 | - | 18.2 | 9 | 755 | -176 | MER21A | ERV1 | LTR |

  

|  |  |  |  |  |  |  |  |  |  |
| --- | --- | --- | --- | --- | --- | --- | --- | --- | --- |
| chr5:102434087-102448389 | | | 14274 bp | | |  |  |  |  |
| Genome Start | Genome End | Strand | Div | Repeat Start | Repeat End | Repeat Left | Name | Family | Class |
| 102434087 | 102434468 | - | 14.8 | 6156 | 6547 | -1 | L1MA9 | L1 | LINE |
| 102434469 | 102435487 | - | 17.8 | 4930 | 6046 | -502 | L1MA9 | L1 | LINE |
| 102435497 | 102437117 | - | 11.7 | 4624 | 6349 | -8 | L1PA8 | L1 | LINE |
| 102437122 | 102437484 | + | 12.5 | 1 | 361 | -3 | THE1B | MaLR | LTR |
| 102437488 | 102439042 | + | 11.1 | 1 | 1567 | -13 | THE1B-int | MaLR | LTR |
| 102439045 | 102439404 | + | 13.2 | 1 | 364 | 0 | THE1B | MaLR | LTR |
| 102439409 | 102444043 | - | 12.0 | 9 | 4620 | -1711 | L1PA8 | L1 | LINE |
| 102444051 | 102444590 | - | 21.2 | 4418 | 4945 | -1434 | L1MA9 | L1 | LINE |
| 102444591 | 102444745 | - | 20.1 | 178 | 336 | 0 | MER2B | MER2\_type | DNA |
| 102444746 | 102444788 | - | 21.2 | 4376 | 4417 | -1962 | L1MA9 | L1 | LINE |
| 102444789 | 102444854 | - | 25.8 | 10 | 75 | -261 | MER2B | MER2\_type | DNA |
| 102444855 | 102448389 | - | 21.2 | 914 | 4375 | -2004 | L1MA9 | L1 | LINE |

  

|  |  |  |  |  |  |  |  |  |  |
| --- | --- | --- | --- | --- | --- | --- | --- | --- | --- |
| chr5:103151846-103162224 | | | 9909 bp | | |  |  |  |  |
| Genome Start | Genome End | Strand | Div | Repeat Start | Repeat End | Repeat Left | Name | Family | Class |
| 103151846 | 103152413 | + | 25.3 | 202 | 1631 | -4700 | MER39-int | ERV1 | LTR |
| 103152416 | 103152463 | - | 19.2 | 391 | 430 | -2 | MSTB1 | MaLR | LTR |
| 103152464 | 103152759 | - | 13.2 | 1 | 295 | -18 | AluSq | Alu | SINE |
| 103152760 | 103152987 | - | 19.2 | 175 | 390 | -42 | MSTB1 | MaLR | LTR |
| 103152988 | 103153349 | - | 17.3 | 1 | 364 | 0 | THE1B | MaLR | LTR |
| 103153350 | 103154916 | - | 30.6 | 2 | 1735 | 0 | MLT-int | MaLR | LTR |
| 103154919 | 103155278 | - | 12.1 | 1 | 364 | 0 | THE1B | MaLR | LTR |
| 103155279 | 103155430 | - | 19.2 | 17 | 174 | -258 | MSTB1 | MaLR | LTR |
| 103155437 | 103155730 | - | 10.9 | 1 | 299 | -14 | AluSp | Alu | SINE |
| 103155762 | 103155824 | + | 15.9 | 1988 | 2058 | -3888 | MER39-int | ERV1 | LTR |
| 103155846 | 103156223 | + | 26.0 | 1908 | 2274 | -4057 | MER39-int | ERV1 | LTR |
| 103156222 | 103156326 | + | 18.1 | 293 | 484 | -5703 | MER39-int | ERV1 | LTR |
| 103156406 | 103156525 | + | 32.5 | 3020 | 3137 | -3781 | MER39-int | ERV1 | LTR |
| 103156549 | 103156813 | + | 26.3 | 753 | 2650 | -3681 | MER39-int | ERV1 | LTR |
| 103156783 | 103156849 | + | 16.4 | 1005 | 1074 | -5565 | MER39-int | ERV1 | LTR |
| 103156850 | 103156898 | + | 20.8 | 1 | 48 | -745 | MER54B | ERVL | LTR |
| 103156898 | 103156982 | + | 20.0 | 562 | 646 | -255 | MER54A | ERVL | LTR |
| 103156983 | 103157059 | + | 24.7 | 825 | 901 | 0 | MER54A | ERVL | LTR |
| 103157120 | 103157193 | + | 25.7 | 1036 | 1112 | -4244 | MER39-int | ERV1 | LTR |
| 103157204 | 103157418 | + | 26.8 | 1908 | 2121 | -2750 | MER39-int | ERV1 | LTR |
| 103157578 | 103157720 | + | 33.6 | 1667 | 1803 | -4384 | MER39-int | ERV1 | LTR |
| 103157794 | 103157904 | + | 19.3 | 1725 | 1840 | -3283 | MER39-int | ERV1 | LTR |
| 103157905 | 103158194 | - | 12.8 | 1 | 290 | -21 | AluY | Alu | SINE |
| 103158195 | 103158492 | + | 17.2 | 1841 | 2302 | -3194 | MER39-int | ERV1 | LTR |
| 103158480 | 103159306 | + | 18.7 | 3508 | 4279 | -2052 | MER39-int | ERV1 | LTR |
| 103159307 | 103159617 | + | 11.0 | 1 | 309 | -2 | AluY | Alu | SINE |
| 103159618 | 103160287 | + | 18.7 | 4280 | 5026 | -1305 | MER39-int | ERV1 | LTR |
| 103160292 | 103160589 | + | 15.4 | 2 | 301 | -11 | AluSx | Alu | SINE |
| 103160636 | 103161036 | - | 16.1 | 1 | 438 | -388 | LTR12 | ERV1 | LTR |
| 103161037 | 103161206 | + | 15.1 | 5061 | 5214 | -1117 | MER39-int | ERV1 | LTR |
| 103161207 | 103161515 | + | 14.2 | 1 | 310 | -1 | AluY | Alu | SINE |
| 103161516 | 103161899 | + | 15.1 | 5215 | 5593 | -738 | MER39-int | ERV1 | LTR |
| 103161901 | 103162224 | + | 20.4 | 4898 | 5181 | -175 | MER39-int | ERV1 | LTR |

  

|  |  |  |  |  |  |  |  |  |  |
| --- | --- | --- | --- | --- | --- | --- | --- | --- | --- |
| chr5:103394594-103401022 | | | 6434 bp | | |  |  |  |  |
| Genome Start | Genome End | Strand | Div | Repeat Start | Repeat End | Repeat Left | Name | Family | Class |
| 103394594 | 103395309 | + | 18.5 | 1 | 758 | -225 | LTR1D | ERV1 | LTR |
| 103395310 | 103395718 | + | 9.8 | 1 | 448 | -2 | LTR7 | ERV1 | LTR |
| 103395721 | 103398612 | + | 10.4 | 1 | 2987 | -4726 | HERVH | ERV1 | LTR |
| 103398607 | 103398754 | + | 14.9 | 3140 | 3290 | -4423 | HERVH | ERV1 | LTR |
| 103398745 | 103399247 | + | 10.2 | 3492 | 3993 | -3720 | HERVH | ERV1 | LTR |
| 103399250 | 103399909 | + | 13.7 | 4488 | 5146 | -2567 | HERVH | ERV1 | LTR |
| 103399914 | 103400130 | + | 13.0 | 5600 | 5819 | -1894 | HERVH | ERV1 | LTR |
| 103400131 | 103400504 | + | 10.3 | 7324 | 7713 | 0 | HERVH | ERV1 | LTR |
| 103400505 | 103400913 | + | 7.9 | 1 | 448 | -2 | LTR7 | ERV1 | LTR |
| 103400917 | 103401022 | + | 21.4 | 879 | 982 | -1 | LTR1D | ERV1 | LTR |

  

|  |  |  |  |  |  |  |  |  |  |
| --- | --- | --- | --- | --- | --- | --- | --- | --- | --- |
| chr5:105124092-105128117 | | | 3711 bp | | |  |  |  |  |
| Genome Start | Genome End | Strand | Div | Repeat Start | Repeat End | Repeat Left | Name | Family | Class |
| 105124092 | 105124863 | + | 20.3 | 197 | 1090 | -5586 | MER66B-int | ERV1 | LTR |
| 105124942 | 105125091 | + | 29.3 | 2941 | 3102 | -3574 | MER66B-int | ERV1 | LTR |
| 105125294 | 105125649 | - | 13.5 | 1 | 364 | 0 | THE1B | MaLR | LTR |
| 105125650 | 105127192 | - | 11.8 | 13 | 1580 | 0 | THE1B-int | MaLR | LTR |
| 105127221 | 105127576 | - | 15.3 | 6 | 364 | 0 | THE1B | MaLR | LTR |
| 105127584 | 105128117 | + | 15.0 | 6148 | 6676 | 0 | MER66B-int | ERV1 | LTR |

  

|  |  |  |  |  |  |  |  |  |  |
| --- | --- | --- | --- | --- | --- | --- | --- | --- | --- |
| chr5:107115837-107119652 | | | 3813 bp | | |  |  |  |  |
| Genome Start | Genome End | Strand | Div | Repeat Start | Repeat End | Repeat Left | Name | Family | Class |
| 107115837 | 107116194 | + | 25.2 | 4608 | 4965 | -1181 | L1ME2 | L1 | LINE |
| 107116195 | 107116584 | + | 12.1 | 1 | 375 | 0 | THE1C | MaLR | LTR |
| 107116587 | 107118133 | + | 29.8 | 2 | 1735 | 0 | MLT-int | MaLR | LTR |
| 107118135 | 107118529 | + | 11.8 | 2 | 375 | 0 | THE1C | MaLR | LTR |
| 107118530 | 107119652 | + | 24.0 | 4966 | 6153 | -11 | L1ME2 | L1 | LINE |

  

|  |  |  |  |  |  |  |  |  |  |
| --- | --- | --- | --- | --- | --- | --- | --- | --- | --- |
| chr5:107815893-107821567 | | | 5655 bp | | |  |  |  |  |
| Genome Start | Genome End | Strand | Div | Repeat Start | Repeat End | Repeat Left | Name | Family | Class |
| 107815893 | 107816345 | + | 28.0 | 503 | 942 | -1565 | HAL1 | L1 | LINE |
| 107816346 | 107816711 | + | 12.1 | 1 | 364 | 0 | THE1B | MaLR | LTR |
| 107816714 | 107818289 | + | 30.7 | 2 | 1735 | 0 | MLT-int | MaLR | LTR |
| 107818290 | 107818646 | + | 15.6 | 1 | 364 | 0 | THE1B | MaLR | LTR |
| 107818647 | 107818738 | + | 28.0 | 943 | 1030 | -1477 | HAL1 | L1 | LINE |
| 107818744 | 107821337 | - | 8.4 | 3552 | 6150 | -4 | L1PA7 | L1 | LINE |
| 107821351 | 107821567 | + | 23.9 | 1043 | 1260 | -1247 | HAL1 | L1 | LINE |

  

|  |  |  |  |  |  |  |  |  |  |
| --- | --- | --- | --- | --- | --- | --- | --- | --- | --- |
| chr5:107861873-107866395 | | | 4519 bp | | |  |  |  |  |
| Genome Start | Genome End | Strand | Div | Repeat Start | Repeat End | Repeat Left | Name | Family | Class |
| 107861873 | 107862893 | - | 12.2 | 5100 | 6163 | -3 | L1PA17 | L1 | LINE |
| 107862894 | 107863307 | + | 16.5 | 1 | 428 | 0 | MSTA | MaLR | LTR |
| 107863308 | 107864933 | + | 16.2 | 1 | 1647 | -4 | MSTA-int | MaLR | LTR |
| 107864938 | 107865340 | + | 15.6 | 1 | 428 | 0 | MSTA | MaLR | LTR |
| 107865341 | 107866395 | - | 12.4 | 4015 | 5104 | -1042 | L1PA17 | L1 | LINE |

  

|  |  |  |  |  |  |  |  |  |  |
| --- | --- | --- | --- | --- | --- | --- | --- | --- | --- |
| chr5:108009450-108012650 | | | 3191 bp | | |  |  |  |  |
| Genome Start | Genome End | Strand | Div | Repeat Start | Repeat End | Repeat Left | Name | Family | Class |
| 108009450 | 108010060 | - | 16.6 | 5693 | 6333 | 0 | L1MC1 | L1 | LINE |
| 108010064 | 108010466 | + | 15.0 | 17 | 428 | 0 | MSTA | MaLR | LTR |
| 108010472 | 108012021 | + | 16.3 | 62 | 1651 | 0 | MSTA-int | MaLR | LTR |
| 108012022 | 108012405 | + | 16.9 | 1 | 428 | 0 | MSTA | MaLR | LTR |
| 108012408 | 108012650 | - | 16.9 | 5448 | 5695 | -638 | L1MC1 | L1 | LINE |

  

|  |  |  |  |  |  |  |  |  |  |
| --- | --- | --- | --- | --- | --- | --- | --- | --- | --- |
| chr5:108776449-108780547 | | | 4104 bp | | |  |  |  |  |
| Genome Start | Genome End | Strand | Div | Repeat Start | Repeat End | Repeat Left | Name | Family | Class |
| 108776449 | 108776977 | + | 21.9 | 2 | 553 | -5989 | L1M4 | L1 | LINE |
| 108776979 | 108777526 | - | 18.8 | 1 | 541 | -14 | MER66C | ERV1 | LTR |
| 108777527 | 108777573 | + | 19.3 | 551 | 594 | -5948 | L1M4 | L1 | LINE |
| 108777574 | 108777916 | + | 14.8 | 1 | 364 | 0 | THE1B | MaLR | LTR |
| 108777920 | 108779450 | + | 30.1 | 3 | 1735 | 0 | MLT-int | MaLR | LTR |
| 108779451 | 108779792 | + | 14.8 | 1 | 363 | -1 | THE1B | MaLR | LTR |
| 108779793 | 108780281 | + | 19.3 | 595 | 1062 | -5480 | L1M4 | L1 | LINE |
| 108780273 | 108780547 | + | 17.5 | 3185 | 3457 | -2689 | L1M4 | L1 | LINE |

  

|  |  |  |  |  |  |  |  |  |  |
| --- | --- | --- | --- | --- | --- | --- | --- | --- | --- |
| chr5:108911700-108917527 | | | 5772 bp | | |  |  |  |  |
| Genome Start | Genome End | Strand | Div | Repeat Start | Repeat End | Repeat Left | Name | Family | Class |
| 108911700 | 108912541 | + | 25.5 | 2607 | 3446 | -2875 | L1MCa | L1 | LINE |
| 108912542 | 108912824 | - | 18.4 | 14 | 295 | -17 | AluJb | Alu | SINE |
| 108912825 | 108913051 | + | 25.5 | 3447 | 3647 | -2674 | L1MCa | L1 | LINE |
| 108913064 | 108913534 | - | 19.1 | 1 | 550 | -6 | MLT2B4 | ERVL | LTR |
| 108913548 | 108914036 | + | 23.3 | 3660 | 4176 | -2145 | L1MCa | L1 | LINE |
| 108914037 | 108914388 | + | 15.4 | 1 | 355 | 0 | THE1A | MaLR | LTR |
| 108914391 | 108915956 | + | 30.6 | 2 | 1735 | 0 | MLT-int | MaLR | LTR |
| 108915957 | 108916308 | + | 14.3 | 1 | 355 | 0 | THE1A | MaLR | LTR |
| 108916309 | 108916791 | + | 23.3 | 4177 | 4624 | -1697 | L1MCa | L1 | LINE |
| 108916821 | 108917527 | + | 23.3 | 4625 | 5389 | -932 | L1MCa | L1 | LINE |

  

|  |  |  |  |  |  |  |  |  |  |
| --- | --- | --- | --- | --- | --- | --- | --- | --- | --- |
| chr5:109893953-109899182 | | | 5222 bp | | |  |  |  |  |
| Genome Start | Genome End | Strand | Div | Repeat Start | Repeat End | Repeat Left | Name | Family | Class |
| 109893953 | 109894535 | + | 13.4 | 5615 | 6226 | -65 | L1MA8 | L1 | LINE |
| 109894536 | 109894931 | + | 8.1 | 1 | 448 | -2 | LTR7 | ERV1 | LTR |
| 109894934 | 109895228 | + | 8.1 | 1 | 300 | -7413 | HERVH | ERV1 | LTR |
| 109895238 | 109896842 | + | 9.8 | 1382 | 2987 | -4726 | HERVH | ERV1 | LTR |
| 109896837 | 109896982 | + | 15.1 | 3140 | 3288 | -4425 | HERVH | ERV1 | LTR |
| 109896985 | 109897472 | + | 9.9 | 3501 | 3993 | -3720 | HERVH | ERV1 | LTR |
| 109897475 | 109898135 | + | 15.9 | 4488 | 5149 | -2564 | HERVH | ERV1 | LTR |
| 109898137 | 109898353 | + | 11.6 | 5600 | 5819 | -1894 | HERVH | ERV1 | LTR |
| 109898347 | 109898717 | + | 11.3 | 7315 | 7713 | 0 | HERVH | ERV1 | LTR |
| 109898718 | 109899113 | + | 8.6 | 1 | 448 | -2 | LTR7 | ERV1 | LTR |
| 109899119 | 109899182 | + | 18.8 | 6225 | 6289 | -2 | L1MA8 | L1 | LINE |

  

|  |  |  |  |  |  |  |  |  |  |
| --- | --- | --- | --- | --- | --- | --- | --- | --- | --- |
| chr5:110989287-110994076 | | | 4666 bp | | |  |  |  |  |
| Genome Start | Genome End | Strand | Div | Repeat Start | Repeat End | Repeat Left | Name | Family | Class |
| 110989287 | 110989593 | + | 22.7 | 45 | 356 | -6044 | L1ME2 | L1 | LINE |
| 110989594 | 110989957 | + | 9.9 | 1 | 364 | 0 | THE1B | MaLR | LTR |
| 110989963 | 110991484 | + | 29.2 | 5 | 1735 | 0 | MLT-int | MaLR | LTR |
| 110991489 | 110991855 | + | 14.6 | 1 | 364 | 0 | THE1B | MaLR | LTR |
| 110991856 | 110993238 | + | 22.5 | 360 | 1865 | -4535 | L1ME2 | L1 | LINE |
| 110993354 | 110994076 | + | 26.0 | 5501 | 6235 | -2 | L1ME2 | L1 | LINE |

  

|  |  |  |  |  |  |  |  |  |  |
| --- | --- | --- | --- | --- | --- | --- | --- | --- | --- |
| chr5:111015761-111018391 | | | 2626 bp | | |  |  |  |  |
| Genome Start | Genome End | Strand | Div | Repeat Start | Repeat End | Repeat Left | Name | Family | Class |
| 111015761 | 111015979 | - | 18.1 | 132 | 346 | 0 | MER7A | MER2\_type | DNA |
| 111015980 | 111016342 | - | 12.2 | 1 | 355 | 0 | THE1A | MaLR | LTR |
| 111016343 | 111017871 | - | 29.6 | 5 | 1735 | 0 | MLT-int | MaLR | LTR |
| 111017877 | 111018239 | - | 11.9 | 1 | 355 | 0 | THE1A | MaLR | LTR |
| 111018240 | 111018391 | - | 18.1 | 1 | 131 | -215 | MER7A | MER2\_type | DNA |

  

|  |  |  |  |  |  |  |  |  |  |
| --- | --- | --- | --- | --- | --- | --- | --- | --- | --- |
| chr5:112723865-112730036 | | | 5644 bp | | |  |  |  |  |
| Genome Start | Genome End | Strand | Div | Repeat Start | Repeat End | Repeat Left | Name | Family | Class |
| 112723865 | 112724093 | - | 24.4 | 3631 | 3871 | -2258 | L1MEd | L1 | LINE |
| 112724111 | 112724388 | - | 19.9 | 3263 | 3546 | -2583 | L1MEd | L1 | LINE |
| 112724767 | 112725159 | - | 21.1 | 2854 | 3289 | -2840 | L1MEd | L1 | LINE |
| 112725160 | 112725722 | + | 16.1 | 1 | 554 | 0 | MLT2B3 | ERVL | LTR |
| 112725723 | 112725750 | - | 21.1 | 2829 | 2853 | -3276 | L1MEd | L1 | LINE |
| 112725757 | 112725813 | - | 17.5 | 230 | 287 | -12 | Alu | Alu | SINE |
| 112725814 | 112725922 | - | 9.2 | 1 | 109 | -203 | AluSq/x | Alu | SINE |
| 112725924 | 112726288 | - | 26.5 | 2409 | 2765 | -3364 | L1MEd | L1 | LINE |
| 112726342 | 112726481 | + | 29.7 | 1 | 142 | -721 | MER21B | ERV1 | LTR |
| 112726554 | 112726593 | - | 14.6 | 393 | 428 | 0 | MSTA | MaLR | LTR |
| 112726594 | 112726901 | + | 11.0 | 1 | 310 | 0 | AluSg | Alu | SINE |
| 112726902 | 112727281 | - | 14.6 | 2 | 392 | -36 | MSTA | MaLR | LTR |
| 112727283 | 112727547 | - | 17.7 | 1381 | 1651 | 0 | MSTA-int | MaLR | LTR |
| 112727548 | 112727856 | + | 12.3 | 1 | 310 | 0 | AluSg | Alu | SINE |
| 112727857 | 112729217 | - | 17.8 | 1 | 1380 | -271 | MSTA-int | MaLR | LTR |
| 112729218 | 112729630 | - | 11.0 | 1 | 428 | 0 | MSTA | MaLR | LTR |
| 112729631 | 112730036 | - | 26.8 | 645 | 1080 | -5039 | L1MEd | L1 | LINE |

  

|  |  |  |  |  |  |  |  |  |  |
| --- | --- | --- | --- | --- | --- | --- | --- | --- | --- |
| chr5:113067907-113071363 | | | 3419 bp | | |  |  |  |  |
| Genome Start | Genome End | Strand | Div | Repeat Start | Repeat End | Repeat Left | Name | Family | Class |
| 113067907 | 113068180 | + | 31.2 | 1 | 289 | -4707 | HERV16 | ERVL | LTR |
| 113068213 | 113068641 | - | 15.9 | 1 | 428 | 0 | MSTA | MaLR | LTR |
| 113068648 | 113070187 | - | 17.4 | 1 | 1646 | -5 | MSTA-int | MaLR | LTR |
| 113070188 | 113070595 | - | 16.2 | 1 | 428 | 0 | MSTA | MaLR | LTR |
| 113070596 | 113071363 | + | 32.7 | 340 | 1131 | -3865 | HERV16 | ERVL | LTR |

  

|  |  |  |  |  |  |  |  |  |  |
| --- | --- | --- | --- | --- | --- | --- | --- | --- | --- |
| chr5:113129883-113132700 | | | 2816 bp | | |  |  |  |  |
| Genome Start | Genome End | Strand | Div | Repeat Start | Repeat End | Repeat Left | Name | Family | Class |
| 113129883 | 113130213 | - | 25.1 | 364 | 698 | -9 | MER39 | ERV1 | LTR |
| 113130214 | 113130586 | + | 14.0 | 1 | 364 | 0 | THE1B | MaLR | LTR |
| 113130589 | 113132147 | + | 28.8 | 2 | 1735 | 0 | MLT-int | MaLR | LTR |
| 113132148 | 113132519 | + | 13.8 | 1 | 364 | 0 | THE1B | MaLR | LTR |
| 113132520 | 113132700 | - | 26.1 | 1 | 363 | -344 | MER39 | ERV1 | LTR |

  

|  |  |  |  |  |  |  |  |  |  |
| --- | --- | --- | --- | --- | --- | --- | --- | --- | --- |
| chr5:113489130-113492211 | | | 3082 bp | | |  |  |  |  |
| Genome Start | Genome End | Strand | Div | Repeat Start | Repeat End | Repeat Left | Name | Family | Class |
| 113489130 | 113489218 | - | 21.5 | 324 | 411 | -56 | MLT1C | MaLR | LTR |
| 113489219 | 113489580 | - | 13.0 | 1 | 390 | 0 | MLT1B | MaLR | LTR |
| 113489581 | 113489842 | - | 21.5 | 69 | 323 | -144 | MLT1C | MaLR | LTR |
| 113489843 | 113490219 | - | 10.5 | 1 | 375 | 0 | THE1C | MaLR | LTR |
| 113490220 | 113491764 | - | 11.9 | 1 | 1580 | 0 | THE1C-int | MaLR | LTR |
| 113491765 | 113492142 | - | 14.2 | 1 | 375 | 0 | THE1C | MaLR | LTR |
| 113492143 | 113492211 | - | 20.3 | 1 | 73 | -394 | MLT1C | MaLR | LTR |

  

|  |  |  |  |  |  |  |  |  |  |
| --- | --- | --- | --- | --- | --- | --- | --- | --- | --- |
| chr5:114317350-114320195 | | | 2842 bp | | |  |  |  |  |
| Genome Start | Genome End | Strand | Div | Repeat Start | Repeat End | Repeat Left | Name | Family | Class |
| 114317350 | 114317631 | + | 22.5 | 1 | 246 | -259 | MLT1D | MaLR | LTR |
| 114317632 | 114317979 | + | 11.1 | 4 | 373 | -2 | THE1C | MaLR | LTR |
| 114317982 | 114319574 | + | 29.1 | 2 | 1733 | -2 | MLT-int | MaLR | LTR |
| 114319577 | 114319927 | + | 10.6 | 1 | 375 | 0 | THE1C | MaLR | LTR |
| 114319928 | 114320195 | + | 22.5 | 247 | 497 | -8 | MLT1D | MaLR | LTR |

  

|  |  |  |  |  |  |  |  |  |  |
| --- | --- | --- | --- | --- | --- | --- | --- | --- | --- |
| chr5:114324207-114338918 | | | 14310 bp | | |  |  |  |  |
| Genome Start | Genome End | Strand | Div | Repeat Start | Repeat End | Repeat Left | Name | Family | Class |
| 114324207 | 114324590 | - | 23.6 | 5751 | 6220 | -1656 | L1MC4a | L1 | LINE |
| 114324604 | 114324915 | - | 10.4 | 1 | 315 | -49 | THE1B | MaLR | LTR |
| 114324916 | 114326444 | - | 12.2 | 1 | 1580 | 0 | THE1B-int | MaLR | LTR |
| 114326445 | 114326803 | - | 15.2 | 2 | 364 | 0 | THE1B | MaLR | LTR |
| 114326809 | 114326869 | - | 21.3 | 5663 | 5725 | -464 | L1MC4a | L1 | LINE |
| 114326880 | 114327057 | + | 20.2 | 2750 | 2933 | -3210 | L1MA9 | L1 | LINE |
| 114327059 | 114327342 | + | 11.4 | 1 | 282 | -30 | AluSx | Alu | SINE |
| 114327391 | 114328658 | - | 13.7 | 4108 | 5398 | -748 | L1PREC2 | L1 | LINE |
| 114328670 | 114329024 | + | 12.5 | 1 | 362 | -2 | THE1B | MaLR | LTR |
| 114329063 | 114329358 | - | 9.8 | 1 | 296 | -14 | AluSg | Alu | SINE |
| 114329361 | 114332043 | + | 22.0 | 3199 | 6032 | -280 | L1MA9 | L1 | LINE |
| 114332069 | 114332171 | + | 22.3 | 22 | 124 | -18 | FLAM\_A | Alu | SINE |
| 114332257 | 114332409 | + | 26.5 | 6039 | 6193 | -119 | L1MA9 | L1 | LINE |
| 114332411 | 114332528 | - | 14.8 | 497 | 604 | -130 | MER50 | ERV1 | LTR |
| 114332648 | 114333694 | - | 32.1 | 4649 | 5686 | -2190 | L1MC4a | L1 | LINE |
| 114333695 | 114334005 | + | 11.7 | 1 | 309 | 0 | AluSc | Alu | SINE |
| 114334006 | 114334478 | - | 36.7 | 4154 | 4648 | -1566 | L1MC4a | L1 | LINE |
| 114334472 | 114334505 | - | 22.5 | 1648 | 1680 | -4535 | L1MC4a | L1 | LINE |
| 114334506 | 114334804 | + | 8.4 | 1 | 301 | -8 | AluSc | Alu | SINE |
| 114334805 | 114335648 | - | 22.5 | 743 | 1647 | -4568 | L1MC4a | L1 | LINE |
| 114335650 | 114336828 | + | 12.9 | 5108 | 6300 | 0 | L1MA6 | L1 | LINE |
| 114336845 | 114336955 | - | 27.1 | 642 | 750 | -5465 | L1MC4a | L1 | LINE |
| 114336983 | 114337572 | - | 21.4 | 7135 | 7771 | -14 | L1MC3 | L1 | LINE |
| 114337573 | 114337792 | + | 11.9 | 1 | 219 | -94 | AluSq | Alu | SINE |
| 114337793 | 114338101 | - | 21.4 | 6872 | 7134 | -651 | L1MC3 | L1 | LINE |
| 114338109 | 114338245 | - | 27.7 | 470 | 629 | -5586 | L1MC4a | L1 | LINE |
| 114338246 | 114338518 | + | 5.0 | 5917 | 6168 | 0 | L1PA2 | L1 | LINE |
| 114338519 | 114338918 | - | 27.7 | 35 | 469 | -5746 | L1MC4a | L1 | LINE |

  

|  |  |  |  |  |  |  |  |  |  |
| --- | --- | --- | --- | --- | --- | --- | --- | --- | --- |
| chr5:114399460-114408686 | | | 9189 bp | | |  |  |  |  |
| Genome Start | Genome End | Strand | Div | Repeat Start | Repeat End | Repeat Left | Name | Family | Class |
| 114399460 | 114399690 | + | 10.0 | 3095 | 3325 | -2821 | L1PA7 | L1 | LINE |
| 114399691 | 114400171 | - | 12.7 | 1 | 511 | 0 | MER9 | ERVK | LTR |
| 114400172 | 114404803 | - | 9.5 | 1348 | 5916 | 0 | HERVK9 | ERVK | LTR |
| 114404805 | 114405356 | - | 10.7 | 62 | 611 | -5305 | HERVK9 | ERVK | LTR |
| 114405394 | 114405874 | - | 11.5 | 1 | 511 | 0 | MER9 | ERVK | LTR |
| 114405875 | 114408686 | + | 9.3 | 3320 | 6154 | 0 | L1PA7 | L1 | LINE |

  

|  |  |  |  |  |  |  |  |  |  |
| --- | --- | --- | --- | --- | --- | --- | --- | --- | --- |
| chr5:115123135-115129450 | | | 6303 bp | | |  |  |  |  |
| Genome Start | Genome End | Strand | Div | Repeat Start | Repeat End | Repeat Left | Name | Family | Class |
| 115123135 | 115123468 | + | 29.8 | 33 | 356 | -6277 | L1MDa | L1 | LINE |
| 115123469 | 115123749 | - | 13.9 | 5878 | 6157 | -9 | L1PA16 | L1 | LINE |
| 115123750 | 115124126 | + | 12.6 | 1 | 381 | 0 | THE1D | MaLR | LTR |
| 115124127 | 115124424 | - | 13.9 | 5575 | 5877 | -289 | L1PA16 | L1 | LINE |
| 115124425 | 115124817 | + | 26.6 | 357 | 1141 | -5492 | L1MDa | L1 | LINE |
| 115124818 | 115125163 | - | 16.8 | 69 | 396 | 0 | MSTD | MaLR | LTR |
| 115125164 | 115125456 | + | 9.6 | 1 | 291 | -20 | AluY | Alu | SINE |
| 115125466 | 115125826 | - | 13.0 | 1 | 381 | 0 | THE1D | MaLR | LTR |
| 115125827 | 115127452 | - | 31.7 | 2 | 1735 | 0 | MLT-int | MaLR | LTR |
| 115127455 | 115127495 | - | 11.8 | 346 | 381 | 0 | THE1D | MaLR | LTR |
| 115127496 | 115127792 | + | 12.5 | 1 | 296 | -16 | AluSx | Alu | SINE |
| 115127793 | 115128119 | - | 11.8 | 3 | 345 | -36 | THE1D | MaLR | LTR |
| 115128120 | 115128195 | - | 16.8 | 1 | 68 | -328 | MSTD | MaLR | LTR |
| 115128196 | 115128755 | + | 19.9 | 1137 | 1736 | -4897 | L1MDa | L1 | LINE |
| 115128758 | 115129085 | - | 21.6 | 1 | 309 | -94 | MER57A | ERV1 | LTR |
| 115129086 | 115129450 | + | 20.3 | 2179 | 2543 | -4090 | L1MDa | L1 | LINE |

  

|  |  |  |  |  |  |  |  |  |  |
| --- | --- | --- | --- | --- | --- | --- | --- | --- | --- |
| chr5:115691906-115694633 | | | 2726 bp | | |  |  |  |  |
| Genome Start | Genome End | Strand | Div | Repeat Start | Repeat End | Repeat Left | Name | Family | Class |
| 115691906 | 115692034 | + | 30.3 | 2379 | 2504 | -3817 | L1MCa | L1 | LINE |
| 115692035 | 115692397 | - | 11.6 | 3 | 364 | 0 | THE1B | MaLR | LTR |
| 115692398 | 115693968 | - | 30.6 | 2 | 1735 | 0 | MLT-int | MaLR | LTR |
| 115693971 | 115694339 | - | 12.6 | 1 | 364 | 0 | THE1B | MaLR | LTR |
| 115694340 | 115694633 | + | 30.3 | 2505 | 2811 | -3510 | L1MCa | L1 | LINE |

  

|  |  |  |  |  |  |  |  |  |  |
| --- | --- | --- | --- | --- | --- | --- | --- | --- | --- |
| chr5:116117335-116121895 | | | 4557 bp | | |  |  |  |  |
| Genome Start | Genome End | Strand | Div | Repeat Start | Repeat End | Repeat Left | Name | Family | Class |
| 116117335 | 116117459 | - | 19.9 | 7657 | 7785 | 0 | L1MC3 | L1 | LINE |
| 116117460 | 116117676 | - | 19.8 | 1 | 236 | 0 | MER46A | MER2\_type | DNA |
| 116117677 | 116117991 | - | 19.9 | 7340 | 7656 | -129 | L1MC3 | L1 | LINE |
| 116117994 | 116118421 | - | 20.2 | 15 | 471 | 0 | LTR32 | ERVL | LTR |
| 116118422 | 116118617 | - | 15.7 | 7135 | 7342 | -443 | L1MC3 | L1 | LINE |
| 116118618 | 116118987 | + | 13.3 | 1 | 381 | 0 | THE1D | MaLR | LTR |
| 116118990 | 116119749 | + | 29.9 | 2 | 875 | -860 | MLT-int | MaLR | LTR |
| 116119750 | 116120055 | + | 8.5 | 1 | 306 | -3 | AluSc | Alu | SINE |
| 116120056 | 116120882 | + | 29.9 | 876 | 1735 | 0 | MLT-int | MaLR | LTR |
| 116120883 | 116121212 | + | 11.4 | 1 | 342 | -39 | THE1D | MaLR | LTR |
| 116121213 | 116121498 | - | 16.1 | 1 | 303 | -9 | AluSx | Alu | SINE |
| 116121499 | 116121543 | + | 11.4 | 343 | 381 | 0 | THE1D | MaLR | LTR |
| 116121544 | 116121895 | - | 15.7 | 6775 | 7134 | -651 | L1MC3 | L1 | LINE |

  

|  |  |  |  |  |  |  |  |  |  |
| --- | --- | --- | --- | --- | --- | --- | --- | --- | --- |
| chr5:117336223-117343184 | | | 6952 bp | | |  |  |  |  |
| Genome Start | Genome End | Strand | Div | Repeat Start | Repeat End | Repeat Left | Name | Family | Class |
| 117336223 | 117336285 | - | 18.0 | 7615 | 7672 | -52 | L1MA3 | L1 | LINE |
| 117336286 | 117336688 | - | 21.1 | 1 | 426 | -2 | MSTA | MaLR | LTR |
| 117336689 | 117338265 | - | 27.1 | 2 | 1735 | 0 | MLT-int | MaLR | LTR |
| 117338268 | 117338663 | - | 18.0 | 1 | 428 | 0 | MSTA | MaLR | LTR |
| 117338664 | 117339510 | - | 18.0 | 6736 | 7614 | -110 | L1MA3 | L1 | LINE |
| 117339516 | 117339716 | - | 20.0 | 85 | 294 | -18 | AluJo | Alu | SINE |
| 117339720 | 117343184 | - | 16.4 | 3258 | 6744 | -821 | L1MA3 | L1 | LINE |

  

|  |  |  |  |  |  |  |  |  |  |
| --- | --- | --- | --- | --- | --- | --- | --- | --- | --- |
| chr5:117841758-117849489 | | | 7510 bp | | |  |  |  |  |
| Genome Start | Genome End | Strand | Div | Repeat Start | Repeat End | Repeat Left | Name | Family | Class |
| 117841758 | 117841875 | + | 28.2 | 175 | 301 | -290 | MLT1K | MaLR | LTR |
| 117842037 | 117844418 | + | 12.3 | 112 | 2508 | -4105 | L1PBa1 | L1 | LINE |
| 117844426 | 117846189 | + | 9.1 | 2576 | 4365 | -2247 | L1PBa1 | L1 | LINE |
| 117846190 | 117846542 | - | 9.6 | 1 | 355 | 0 | THE1A | MaLR | LTR |
| 117846543 | 117848115 | - | 30.7 | 2 | 1735 | 0 | MLT-int | MaLR | LTR |
| 117848118 | 117848481 | - | 7.7 | 1 | 355 | 0 | THE1A | MaLR | LTR |
| 117848482 | 117849102 | + | 9.1 | 3900 | 4513 | -1633 | L1PB | L1 | LINE |
| 117849103 | 117849354 | + | 11.9 | 5891 | 6143 | -8 | L1PB1 | L1 | LINE |
| 117849407 | 117849489 | + | 27.7 | 456 | 543 | -48 | MLT1K | MaLR | LTR |

  

|  |  |  |  |  |  |  |  |  |  |
| --- | --- | --- | --- | --- | --- | --- | --- | --- | --- |
| chr5:118017786-118040008 | | | 21987 bp | | |  |  |  |  |
| Genome Start | Genome End | Strand | Div | Repeat Start | Repeat End | Repeat Left | Name | Family | Class |
| 118017786 | 118017870 | - | 30.6 | 3277 | 3366 | -12 | L2 | L2 | LINE |
| 118018012 | 118019393 | - | 14.5 | 5078 | 6507 | -1 | L1MC1 | L1 | LINE |
| 118019399 | 118020110 | - | 17.1 | 5562 | 6281 | -10 | L1MA7 | L1 | LINE |
| 118020111 | 118020486 | - | 12.6 | 1 | 428 | 0 | MSTA | MaLR | LTR |
| 118020487 | 118022087 | - | 30.3 | 2 | 1735 | 0 | MLT-int | MaLR | LTR |
| 118022090 | 118022459 | - | 15.0 | 1 | 428 | 0 | MSTA | MaLR | LTR |
| 118022460 | 118025274 | - | 17.6 | 2686 | 5561 | -730 | L1MA7 | L1 | LINE |
| 118025275 | 118025729 | - | 16.6 | 4689 | 5094 | -1227 | L1MC1 | L1 | LINE |
| 118025767 | 118028000 | - | 16.6 | 2413 | 4688 | -1633 | L1MC1 | L1 | LINE |
| 118028000 | 118028471 | + | 11.3 | 3937 | 4394 | -1752 | L1PA16 | L1 | LINE |
| 118028502 | 118028803 | + | 11.3 | 4395 | 4706 | -1440 | L1PA16 | L1 | LINE |
| 118028804 | 118029088 | + | 12.3 | 21 | 306 | -6 | AluSx | Alu | SINE |
| 118029089 | 118030534 | + | 12.6 | 4707 | 6155 | -11 | L1PA16 | L1 | LINE |
| 118030538 | 118031011 | - | 18.3 | 1922 | 2425 | -4083 | L1MC1 | L1 | LINE |
| 118031012 | 118031326 | + | 8.2 | 1 | 315 | 0 | AluY | Alu | SINE |
| 118031327 | 118031879 | - | 18.3 | 1379 | 1921 | -4587 | L1MC1 | L1 | LINE |
| 118031880 | 118031934 | - | 9.1 | 6250 | 6304 | 0 | L1MA2 | L1 | LINE |
| 118031935 | 118032238 | - | 18.3 | 1080 | 1378 | -5130 | L1MC1 | L1 | LINE |
| 118032242 | 118032871 | - | 17.8 | 5034 | 5698 | -470 | L1M3 | L1 | LINE |
| 118032880 | 118037217 | - | 17.9 | 3365 | 7710 | 0 | L1MA7 | L1 | LINE |
| 118037223 | 118037442 | + | 27.6 | 1022 | 1526 | -4146 | L1M2c | L1 | LINE |
| 118037421 | 118037772 | - | 22.7 | 11 | 361 | -6669 | L1M3e | L1 | LINE |
| 118037792 | 118038739 | - | 15.4 | 4075 | 5044 | -1096 | L1M3 | L1 | LINE |
| 118038746 | 118039922 | - | 25.5 | 1 | 1129 | -4916 | L1MCb | L1 | LINE |
| 118039923 | 118040008 | - | 20.2 | 2792 | 2870 | -549 | L2 | L2 | LINE |

  

|  |  |  |  |  |  |  |  |  |  |
| --- | --- | --- | --- | --- | --- | --- | --- | --- | --- |
| chr5:118096046-118099025 | | | 2887 bp | | |  |  |  |  |
| Genome Start | Genome End | Strand | Div | Repeat Start | Repeat End | Repeat Left | Name | Family | Class |
| 118096046 | 118096209 | + | 27.3 | 5570 | 5779 | -342 | L1ME4a | L1 | LINE |
| 118096210 | 118096553 | + | 14.2 | 1 | 364 | 0 | THE1B | MaLR | LTR |
| 118096556 | 118098107 | + | 31.4 | 2 | 1735 | 0 | MLT-int | MaLR | LTR |
| 118098108 | 118098452 | + | 13.3 | 1 | 364 | 0 | THE1B | MaLR | LTR |
| 118098453 | 118098577 | + | 31.4 | 5780 | 5911 | -210 | L1ME4a | L1 | LINE |
| 118098617 | 118098852 | - | 28.2 | 8 | 262 | 0 | MIR | MIR | SINE |
| 118098905 | 118099025 | + | 25.6 | 5973 | 6097 | -24 | L1ME4a | L1 | LINE |

  

|  |  |  |  |  |  |  |  |  |  |
| --- | --- | --- | --- | --- | --- | --- | --- | --- | --- |
| chr5:118498774-118507028 | | | 8107 bp | | |  |  |  |  |
| Genome Start | Genome End | Strand | Div | Repeat Start | Repeat End | Repeat Left | Name | Family | Class |
| 118498774 | 118499119 | - | 32.7 | 3016 | 3393 | -26 | L2 | L2 | LINE |
| 118499120 | 118499474 | + | 11.7 | 1 | 381 | 0 | THE1D | MaLR | LTR |
| 118499478 | 118501091 | + | 30.0 | 3 | 1734 | -1 | MLT-int | MaLR | LTR |
| 118501093 | 118501440 | + | 14.2 | 1 | 381 | 0 | THE1D | MaLR | LTR |
| 118501441 | 118502145 | - | 27.4 | 2256 | 3015 | -404 | L2 | L2 | LINE |
| 118502151 | 118502436 | + | 15.1 | 1 | 287 | -25 | AluSx | Alu | SINE |
| 118502560 | 118506821 | + | 10.9 | 3803 | 8155 | 0 | L1PREC2 | L1 | LINE |
| 118506838 | 118507028 | - | 25.7 | 1983 | 2174 | -1245 | L2 | L2 | LINE |

  

|  |  |  |  |  |  |  |  |  |  |
| --- | --- | --- | --- | --- | --- | --- | --- | --- | --- |
| chr5:118782335-118785617 | | | 3206 bp | | |  |  |  |  |
| Genome Start | Genome End | Strand | Div | Repeat Start | Repeat End | Repeat Left | Name | Family | Class |
| 118782335 | 118782647 | + | 34.4 | 2810 | 3191 | -228 | L2 | L2 | LINE |
| 118782702 | 118782871 | + | 26.0 | 53 | 240 | -26 | MIR | MIR | SINE |
| 118782891 | 118783037 | + | 23.9 | 3221 | 3387 | -32 | L2 | L2 | LINE |
| 118783038 | 118783399 | + | 11.9 | 1 | 362 | -2 | THE1B | MaLR | LTR |
| 118783404 | 118784867 | + | 30.4 | 2 | 1684 | -51 | MLT-int | MaLR | LTR |
| 118784868 | 118785163 | - | 16.6 | 1 | 296 | -16 | AluSx | Alu | SINE |
| 118785164 | 118785220 | + | 30.4 | 1685 | 1735 | 0 | MLT-int | MaLR | LTR |
| 118785221 | 118785584 | + | 11.1 | 1 | 364 | 0 | THE1B | MaLR | LTR |
| 118785585 | 118785617 | + | 23.9 | 3388 | 3418 | -1 | L2 | L2 | LINE |

  

|  |  |  |  |  |  |  |  |  |  |
| --- | --- | --- | --- | --- | --- | --- | --- | --- | --- |
| chr5:119087003-119092976 | | | 5927 bp | | |  |  |  |  |
| Genome Start | Genome End | Strand | Div | Repeat Start | Repeat End | Repeat Left | Name | Family | Class |
| 119087003 | 119087077 | + | 12.5 | 4004 | 4078 | -2065 | L1MA5A | L1 | LINE |
| 119087078 | 119087395 | + | 22.6 | 1 | 312 | 0 | AluJo | Alu | SINE |
| 119087440 | 119088006 | + | 17.8 | 4070 | 4646 | -1497 | L1MA5A | L1 | LINE |
| 119088007 | 119088310 | + | 18.8 | 1 | 303 | -9 | AluJb | Alu | SINE |
| 119088311 | 119089152 | + | 16.6 | 4647 | 5487 | -810 | L1MA5A | L1 | LINE |
| 119089153 | 119089457 | + | 13.1 | 5 | 310 | -2 | AluSx | Alu | SINE |
| 119089458 | 119089942 | + | 13.4 | 5488 | 6012 | -285 | L1MA5A | L1 | LINE |
| 119089943 | 119090308 | + | 15.5 | 1 | 381 | 0 | THE1D | MaLR | LTR |
| 119090312 | 119091920 | + | 30.4 | 3 | 1735 | 0 | MLT1K-int | MaLR | LTR |
| 119091921 | 119092281 | + | 14.9 | 1 | 381 | 0 | THE1D | MaLR | LTR |
| 119092282 | 119092397 | + | 13.4 | 6013 | 6121 | -176 | L1MA5A | L1 | LINE |
| 119092398 | 119092810 | + | 16.2 | 1 | 428 | 0 | MSTA | MaLR | LTR |
| 119092811 | 119092976 | + | 13.4 | 6122 | 6285 | -12 | L1MA5A | L1 | LINE |

  

|  |  |  |  |  |  |  |  |  |  |
| --- | --- | --- | --- | --- | --- | --- | --- | --- | --- |
| chr5:120312118-120325258 | | | 13123 bp | | |  |  |  |  |
| Genome Start | Genome End | Strand | Div | Repeat Start | Repeat End | Repeat Left | Name | Family | Class |
| 120312118 | 120312265 | - | 16.6 | 6847 | 6996 | -9 | L1MA4A | L1 | LINE |
| 120312266 | 120312627 | + | 16.5 | 1 | 364 | 0 | THE1B | MaLR | LTR |
| 120312630 | 120314202 | + | 30.1 | 2 | 1735 | 0 | MLT-int | MaLR | LTR |
| 120314203 | 120314552 | + | 14.5 | 3 | 364 | 0 | THE1B | MaLR | LTR |
| 120314553 | 120316192 | - | 17.7 | 5190 | 6846 | -159 | L1MA4A | L1 | LINE |
| 120316193 | 120316545 | + | 9.9 | 3 | 355 | 0 | THE1A | MaLR | LTR |
| 120316548 | 120318154 | + | 30.1 | 2 | 1735 | 0 | MLT-int | MaLR | LTR |
| 120318158 | 120318507 | + | 10.0 | 4 | 355 | 0 | THE1A | MaLR | LTR |
| 120318508 | 120319190 | - | 18.9 | 4515 | 5189 | -1656 | L1MA4A | L1 | LINE |
| 120319206 | 120320082 | - | 14.9 | 2 | 894 | -9 | MER4D | ERV1 | LTR |
| 120320085 | 120321885 | - | 19.1 | 2764 | 4517 | -2328 | L1MA4A | L1 | LINE |
| 120321886 | 120322198 | - | 19.6 | 1 | 312 | 0 | AluJo | Alu | SINE |
| 120322199 | 120322357 | - | 16.9 | 2607 | 2763 | -4086 | L1MA4A | L1 | LINE |
| 120322358 | 120322934 | + | 23.9 | 1 | 554 | 0 | MLT2B3 | ERVL | LTR |
| 120322935 | 120322961 | - | 16.9 | 2580 | 2606 | -4243 | L1MA4A | L1 | LINE |
| 120322956 | 120325258 | - | 22.4 | 13 | 2360 | -4489 | L1MA4A | L1 | LINE |

  

|  |  |  |  |  |  |  |  |  |  |
| --- | --- | --- | --- | --- | --- | --- | --- | --- | --- |
| chr5:120450410-120452605 | | | 2193 bp | | |  |  |  |  |
| Genome Start | Genome End | Strand | Div | Repeat Start | Repeat End | Repeat Left | Name | Family | Class |
| 120450410 | 120450503 | - | 26.1 | 347 | 440 | -10 | LTR16A | ERVL | LTR |
| 120450508 | 120450895 | - | 16.3 | 1 | 426 | -2 | MSTA | MaLR | LTR |
| 120450896 | 120451776 | - | 15.6 | 721 | 1580 | 0 | MSTA-int | MaLR | LTR |
| 120451775 | 120451907 | - | 10.6 | 2 | 134 | -1517 | MSTA-int | MaLR | LTR |
| 120451909 | 120452309 | - | 16.0 | 1 | 428 | 0 | MSTA | MaLR | LTR |
| 120452310 | 120452605 | - | 30.5 | 27 | 348 | -102 | LTR16A | ERVL | LTR |

  

|  |  |  |  |  |  |  |  |  |  |
| --- | --- | --- | --- | --- | --- | --- | --- | --- | --- |
| chr5:121980173-121988150 | | | 7987 bp | | |  |  |  |  |
| Genome Start | Genome End | Strand | Div | Repeat Start | Repeat End | Repeat Left | Name | Family | Class |
| 121980173 | 121980481 | + | 15.2 | 1 | 305 | -180 | MER31A | ERV1 | LTR |
| 121980487 | 121980935 | - | 7.4 | 1 | 448 | -2 | LTR7 | ERV1 | LTR |
| 121980936 | 121984169 | - | 7.7 | 4488 | 7713 | 0 | HERVH | ERV1 | LTR |
| 121984172 | 121984658 | - | 11.6 | 3492 | 3993 | -3720 | HERVH | ERV1 | LTR |
| 121984649 | 121984798 | - | 14.7 | 3140 | 3290 | -4423 | HERVH | ERV1 | LTR |
| 121984778 | 121987514 | - | 10.1 | 1 | 2995 | -4718 | HERVH | ERV1 | LTR |
| 121987522 | 121987968 | - | 6.1 | 1 | 443 | -7 | LTR7 | ERV1 | LTR |
| 121987977 | 121988150 | + | 14.4 | 312 | 485 | 0 | MER31A | ERV1 | LTR |

  

|  |  |  |  |  |  |  |  |  |  |
| --- | --- | --- | --- | --- | --- | --- | --- | --- | --- |
| chr5:123183795-123196903 | | | 12867 bp | | |  |  |  |  |
| Genome Start | Genome End | Strand | Div | Repeat Start | Repeat End | Repeat Left | Name | Family | Class |
| 123183795 | 123183905 | + | 28.8 | 779 | 878 | -2541 | L2 | L2 | LINE |
| 123183906 | 123184309 | + | 18.9 | 3 | 408 | 0 | MLT1A1 | MaLR | LTR |
| 123184310 | 123185610 | + | 28.8 | 879 | 2297 | -1122 | L2 | L2 | LINE |
| 123185656 | 123186849 | - | 18.6 | 5074 | 6320 | -24 | L1MC2 | L1 | LINE |
| 123186846 | 123188211 | + | 21.9 | 912 | 2308 | -4541 | L1MA4A | L1 | LINE |
| 123188212 | 123188583 | + | 13.0 | 1 | 375 | 0 | THE1C | MaLR | LTR |
| 123188584 | 123190101 | + | 12.9 | 1 | 1580 | 0 | THE1C-int | MaLR | LTR |
| 123190102 | 123190479 | + | 15.3 | 1 | 375 | 0 | THE1C | MaLR | LTR |
| 123190480 | 123192808 | + | 18.0 | 2304 | 4807 | -2038 | L1MA4A | L1 | LINE |
| 123192815 | 123193263 | - | 25.4 | 118 | 554 | 0 | MLT2B3 | ERVL | LTR |
| 123193268 | 123195148 | + | 17.5 | 4813 | 6748 | -257 | L1MA4A | L1 | LINE |
| 123195149 | 123195447 | + | 8.4 | 1 | 299 | -12 | AluY | Alu | SINE |
| 123195448 | 123195532 | + | 15.8 | 6749 | 6824 | -181 | L1MA4A | L1 | LINE |
| 123195699 | 123195965 | - | 13.5 | 276 | 545 | -686 | Tigger3b | MER2\_type | DNA |
| 123195967 | 123196067 | - | 24.8 | 1 | 101 | -1130 | Tigger3b | MER2\_type | DNA |
| 123196075 | 123196670 | - | 16.9 | 3816 | 4406 | -1740 | L1MC | L1 | LINE |
| 123196688 | 123196903 | + | 25.7 | 2307 | 2579 | -840 | L2 | L2 | LINE |

  

|  |  |  |  |  |  |  |  |  |  |
| --- | --- | --- | --- | --- | --- | --- | --- | --- | --- |
| chr5:123666455-123673247 | | | 6823 bp | | |  |  |  |  |
| Genome Start | Genome End | Strand | Div | Repeat Start | Repeat End | Repeat Left | Name | Family | Class |
| 123666455 | 123667344 | + | 25.4 | 6640 | 7593 | -449 | L1MC4 | L1 | LINE |
| 123667350 | 123667805 | + | 8.8 | 3 | 448 | -2 | LTR7 | ERV1 | LTR |
| 123667824 | 123670796 | + | 10.9 | 60 | 2995 | -4718 | HERVH | ERV1 | LTR |
| 123670768 | 123670925 | + | 13.5 | 3134 | 3290 | -4423 | HERVH | ERV1 | LTR |
| 123670916 | 123671420 | + | 9.8 | 3492 | 3993 | -3720 | HERVH | ERV1 | LTR |
| 123671423 | 123672074 | + | 15.2 | 4488 | 5149 | -2564 | HERVH | ERV1 | LTR |
| 123672055 | 123672654 | + | 12.3 | 5588 | 7713 | 0 | HERVH | ERV1 | LTR |
| 123672655 | 123673068 | + | 8.5 | 1 | 448 | -2 | LTR7 | ERV1 | LTR |
| 123673074 | 123673193 | + | 25.4 | 7597 | 7736 | -306 | L1MC4 | L1 | LINE |
| 123673193 | 123673247 | + | 21.8 | 7963 | 8017 | -25 | L1MC4 | L1 | LINE |

  

|  |  |  |  |  |  |  |  |  |  |
| --- | --- | --- | --- | --- | --- | --- | --- | --- | --- |
| chr5:123700805-123703948 | | | 3008 bp | | |  |  |  |  |
| Genome Start | Genome End | Strand | Div | Repeat Start | Repeat End | Repeat Left | Name | Family | Class |
| 123700805 | 123701164 | - | 32.5 | 3010 | 3419 | 0 | L2 | L2 | LINE |
| 123701257 | 123701633 | - | 25.6 | 1 | 396 | 0 | MSTD | MaLR | LTR |
| 123701639 | 123701962 | - | 17.4 | 1367 | 1729 | -6 | MSTD-int | MaLR | LTR |
| 123701999 | 123702355 | - | 21.4 | 1 | 396 | 0 | MSTD | MaLR | LTR |
| 123702359 | 123703948 | - | 35.7 | 1220 | 2986 | -433 | L2 | L2 | LINE |

  

|  |  |  |  |  |  |  |  |  |  |
| --- | --- | --- | --- | --- | --- | --- | --- | --- | --- |
| chr5:124819347-124823797 | | | 4400 bp | | |  |  |  |  |
| Genome Start | Genome End | Strand | Div | Repeat Start | Repeat End | Repeat Left | Name | Family | Class |
| 124819347 | 124819494 | + | 33.1 | 2322 | 2475 | -944 | L2 | L2 | LINE |
| 124819542 | 124820398 | - | 8.2 | 5235 | 6095 | -36 | L1PA8A | L1 | LINE |
| 124820399 | 124820741 | + | 9.7 | 1 | 355 | 0 | THE1A | MaLR | LTR |
| 124820742 | 124822302 | + | 9.8 | 1 | 1580 | 0 | THE1A-int | MaLR | LTR |
| 124822303 | 124822647 | + | 10.0 | 1 | 355 | 0 | THE1A | MaLR | LTR |
| 124822648 | 124823011 | - | 9.1 | 4868 | 5239 | -907 | L1PA8A | L1 | LINE |
| 124823016 | 124823797 | + | 33.3 | 2481 | 3373 | -5 | L2 | L2 | LINE |

  

|  |  |  |  |  |  |  |  |  |  |
| --- | --- | --- | --- | --- | --- | --- | --- | --- | --- |
| chr5:125054176-125056773 | | | 2595 bp | | |  |  |  |  |
| Genome Start | Genome End | Strand | Div | Repeat Start | Repeat End | Repeat Left | Name | Family | Class |
| 125054176 | 125054495 | + | 14.9 | 1 | 315 | -49 | THE1B | MaLR | LTR |
| 125054496 | 125054851 | + | 17.4 | 1 | 363 | -1 | THE1B | MaLR | LTR |
| 125054855 | 125056396 | + | 29.9 | 2 | 1735 | 0 | MLT-int | MaLR | LTR |
| 125056397 | 125056725 | + | 19.6 | 1 | 364 | 0 | THE1B | MaLR | LTR |
| 125056726 | 125056773 | + | 14.9 | 316 | 364 | 0 | THE1B | MaLR | LTR |

  

|  |  |  |  |  |  |  |  |  |  |
| --- | --- | --- | --- | --- | --- | --- | --- | --- | --- |
| chr5:125090933-125108683 | | | 17524 bp | | |  |  |  |  |
| Genome Start | Genome End | Strand | Div | Repeat Start | Repeat End | Repeat Left | Name | Family | Class |
| 125090933 | 125091153 | - | 28.9 | 5610 | 5818 | -1990 | L1MC | L1 | LINE |
| 125091213 | 125091478 | - | 18.9 | 3 | 268 | -44 | AluJb | Alu | SINE |
| 125091647 | 125092189 | - | 33.6 | 2858 | 3419 | 0 | L2 | L2 | LINE |
| 125092201 | 125092585 | + | 19.8 | 1 | 396 | 0 | MSTD | MaLR | LTR |
| 125092588 | 125092988 | + | 22.2 | 2 | 410 | -1325 | MSTD-int | MaLR | LTR |
| 125092993 | 125093380 | - | 7.6 | 4539 | 4916 | -1230 | L1PB | L1 | LINE |
| 125093379 | 125093426 | + | 8.3 | 4939 | 4991 | -1155 | L1PB | L1 | LINE |
| 125093431 | 125093981 | + | 21.2 | 5 | 578 | -5297 | L1P3b | L1 | LINE |
| 125093969 | 125100044 | + | 11.1 | 383 | 6483 | 0 | L1PA10 | L1 | LINE |
| 125100035 | 125100651 | + | 9.9 | 4974 | 5607 | -544 | L1PB1 | L1 | LINE |
| 125100648 | 125104145 | - | 7.1 | 445 | 3950 | -2196 | L1PA5 | L1 | LINE |
| 125104149 | 125106361 | + | 6.5 | 3935 | 6153 | -1 | L1PA5 | L1 | LINE |
| 125106362 | 125106914 | + | 9.4 | 5593 | 6151 | 0 | L1PB1 | L1 | LINE |
| 125106920 | 125108176 | + | 25.9 | 402 | 1735 | 0 | MSTD-int | MaLR | LTR |
| 125108177 | 125108578 | + | 17.5 | 1 | 408 | 0 | MSTD | MaLR | LTR |
| 125108579 | 125108683 | - | 25.0 | 5370 | 5483 | -2325 | L1MC | L1 | LINE |

  

|  |  |  |  |  |  |  |  |  |  |
| --- | --- | --- | --- | --- | --- | --- | --- | --- | --- |
| chr5:125123126-125126505 | | | 3378 bp | | |  |  |  |  |
| Genome Start | Genome End | Strand | Div | Repeat Start | Repeat End | Repeat Left | Name | Family | Class |
| 125123126 | 125124165 | - | 13.9 | 4562 | 5645 | -521 | L1P4 | L1 | LINE |
| 125124166 | 125124520 | + | 12.5 | 1 | 364 | 0 | THE1B | MaLR | LTR |
| 125124523 | 125126101 | + | 30.9 | 2 | 1735 | 0 | MLT-int | MaLR | LTR |
| 125126102 | 125126455 | + | 12.8 | 1 | 364 | 0 | THE1B | MaLR | LTR |
| 125126456 | 125126505 | - | 18.0 | 4517 | 4566 | -1580 | L1P4 | L1 | LINE |

  

|  |  |  |  |  |  |  |  |  |  |
| --- | --- | --- | --- | --- | --- | --- | --- | --- | --- |
| chr5:126478922-126486302 | | | 7327 bp | | |  |  |  |  |
| Genome Start | Genome End | Strand | Div | Repeat Start | Repeat End | Repeat Left | Name | Family | Class |
| 126478922 | 126480415 | + | 33.1 | 3 | 1574 | -3422 | HERV16 | ERVL | LTR |
| 126480467 | 126480509 | - | 11.9 | 1129 | 1171 | -3318 | L3 | CR1 | LINE |
| 126480511 | 126481972 | + | 32.6 | 1744 | 3345 | -2412 | HERV16 | ERVL | LTR |
| 126481973 | 126482353 | + | 18.0 | 3 | 396 | 0 | MSTD | MaLR | LTR |
| 126482354 | 126483704 | + | 34.7 | 3139 | 4690 | -709 | HERV16 | ERVL | LTR |
| 126483705 | 126484068 | - | 11.0 | 1 | 364 | 0 | THE1B | MaLR | LTR |
| 126484069 | 126485647 | - | 29.4 | 2 | 1735 | 0 | MLT-int | MaLR | LTR |
| 126485650 | 126486020 | - | 12.2 | 1 | 364 | 0 | THE1B | MaLR | LTR |
| 126486021 | 126486302 | + | 34.5 | 4691 | 4972 | -695 | HERV16 | ERVL | LTR |

  

|  |  |  |  |  |  |  |  |  |  |
| --- | --- | --- | --- | --- | --- | --- | --- | --- | --- |
| chr5:127082344-127087107 | | | 4581 bp | | |  |  |  |  |
| Genome Start | Genome End | Strand | Div | Repeat Start | Repeat End | Repeat Left | Name | Family | Class |
| 127082344 | 127082994 | - | 12.1 | 5644 | 6305 | 0 | L1MA3 | L1 | LINE |
| 127083173 | 127083226 | - | 12.1 | 5583 | 5643 | -662 | L1MA3 | L1 | LINE |
| 127083227 | 127083606 | - | 13.6 | 1 | 428 | 0 | MSTA | MaLR | LTR |
| 127083612 | 127085240 | - | 17.6 | 1 | 1645 | -6 | MSTA-int | MaLR | LTR |
| 127085241 | 127085623 | - | 13.1 | 1 | 428 | 0 | MSTA | MaLR | LTR |
| 127085624 | 127087107 | - | 11.8 | 4093 | 5586 | -719 | L1MA3 | L1 | LINE |

  

|  |  |  |  |  |  |  |  |  |  |
| --- | --- | --- | --- | --- | --- | --- | --- | --- | --- |
| chr5:127136859-127142822 | | | 5914 bp | | |  |  |  |  |
| Genome Start | Genome End | Strand | Div | Repeat Start | Repeat End | Repeat Left | Name | Family | Class |
| 127136859 | 127138652 | - | 20.0 | 5204 | 7034 | 0 | L1MB5 | L1 | LINE |
| 127138653 | 127139211 | + | 18.9 | 1 | 558 | 0 | MER74A | ERVL | LTR |
| 127139212 | 127139253 | - | 19.8 | 5169 | 5203 | -1803 | L1MB5 | L1 | LINE |
| 127139254 | 127139618 | + | 11.3 | 1 | 364 | 0 | THE1B | MaLR | LTR |
| 127139621 | 127141187 | + | 31.2 | 2 | 1735 | 0 | MLT-int | MaLR | LTR |
| 127141188 | 127141556 | + | 15.3 | 1 | 362 | -2 | THE1B | MaLR | LTR |
| 127141557 | 127141598 | - | 19.8 | 5128 | 5168 | -1838 | L1MB5 | L1 | LINE |
| 127141620 | 127142338 | - | 19.8 | 4356 | 5127 | -1879 | L1MB5 | L1 | LINE |
| 127142366 | 127142822 | - | 19.8 | 3824 | 4355 | -2651 | L1MB5 | L1 | LINE |

  

|  |  |  |  |  |  |  |  |  |  |
| --- | --- | --- | --- | --- | --- | --- | --- | --- | --- |
| chr5:128681104-128687502 | | | 6371 bp | | |  |  |  |  |
| Genome Start | Genome End | Strand | Div | Repeat Start | Repeat End | Repeat Left | Name | Family | Class |
| 128681104 | 128682039 | - | 19.6 | 5561 | 6507 | -858 | L1M1 | L1 | LINE |
| 128682040 | 128682235 | - | 14.3 | 105 | 300 | -12 | AluSx | Alu | SINE |
| 128682236 | 128682493 | - | 19.6 | 5300 | 5560 | -1805 | L1M1 | L1 | LINE |
| 128682494 | 128682786 | - | 10.9 | 1 | 293 | -18 | AluY | Alu | SINE |
| 128682787 | 128683756 | - | 19.9 | 2982 | 4235 | -1905 | L1M3 | L1 | LINE |
| 128683757 | 128684090 | - | 11.4 | 1 | 345 | -19 | THE1B | MaLR | LTR |
| 128684091 | 128685662 | - | 29.3 | 2 | 1735 | 0 | MLT-int | MaLR | LTR |
| 128685665 | 128686021 | - | 11.9 | 1 | 364 | 0 | THE1B | MaLR | LTR |
| 128686048 | 128687502 | - | 23.8 | 1 | 1774 | -5435 | L1M1 | L1 | LINE |

  

|  |  |  |  |  |  |  |  |  |  |
| --- | --- | --- | --- | --- | --- | --- | --- | --- | --- |
| chr5:129565202-129568252 | | | 3038 bp | | |  |  |  |  |
| Genome Start | Genome End | Strand | Div | Repeat Start | Repeat End | Repeat Left | Name | Family | Class |
| 129565202 | 129565362 | - | 23.1 | 5995 | 6162 | 0 | L1ME3 | L1 | LINE |
| 129565363 | 129565730 | - | 11.8 | 1 | 364 | 0 | THE1B | MaLR | LTR |
| 129565731 | 129567351 | - | 11.8 | 1 | 1580 | 0 | THE1B-int | MaLR | LTR |
| 129567352 | 129567706 | - | 11.9 | 1 | 364 | 0 | THE1B | MaLR | LTR |
| 129567720 | 129568252 | - | 24.2 | 5411 | 5990 | -172 | L1ME3 | L1 | LINE |

  

|  |  |  |  |  |  |  |  |  |  |
| --- | --- | --- | --- | --- | --- | --- | --- | --- | --- |
| chr5:129691621-129695511 | | | 3869 bp | | |  |  |  |  |
| Genome Start | Genome End | Strand | Div | Repeat Start | Repeat End | Repeat Left | Name | Family | Class |
| 129691621 | 129692607 | - | 18.1 | 5254 | 6278 | -22 | L1MA4 | L1 | LINE |
| 129692623 | 129693012 | + | 14.2 | 1 | 428 | 0 | MSTA | MaLR | LTR |
| 129693015 | 129694546 | + | 15.8 | 3 | 1502 | -78 | MSTA-int | MaLR | LTR |
| 129694547 | 129694844 | + | 9.7 | 1 | 298 | -13 | AluY | Alu | SINE |
| 129694845 | 129694921 | + | 15.8 | 1503 | 1574 | -6 | MSTA-int | MaLR | LTR |
| 129694927 | 129695311 | + | 12.3 | 1 | 428 | 0 | MSTA | MaLR | LTR |
| 129695312 | 129695511 | - | 13.5 | 5044 | 5243 | -903 | L1MA4 | L1 | LINE |

  

|  |  |  |  |  |  |  |  |  |  |
| --- | --- | --- | --- | --- | --- | --- | --- | --- | --- |
| chr5:130240852-130243350 | | | 2497 bp | | |  |  |  |  |
| Genome Start | Genome End | Strand | Div | Repeat Start | Repeat End | Repeat Left | Name | Family | Class |
| 130240852 | 130240946 | - | 31.5 | 3162 | 3261 | -158 | L2 | L2 | LINE |
| 130240947 | 130241301 | + | 12.7 | 1 | 364 | 0 | THE1B | MaLR | LTR |
| 130241304 | 130242883 | + | 29.6 | 2 | 1735 | 0 | MLT-int | MaLR | LTR |
| 130242884 | 130243241 | + | 13.6 | 1 | 364 | 0 | THE1B | MaLR | LTR |
| 130243242 | 130243350 | - | 31.5 | 3054 | 3161 | -258 | L2 | L2 | LINE |

  

|  |  |  |  |  |  |  |  |  |  |
| --- | --- | --- | --- | --- | --- | --- | --- | --- | --- |
| chr5:130406280-130416764 | | | 10391 bp | | |  |  |  |  |
| Genome Start | Genome End | Strand | Div | Repeat Start | Repeat End | Repeat Left | Name | Family | Class |
| 130406280 | 130406354 | - | 16.0 | 2774 | 2859 | -85 | BLACKJACK | AcHobo | DNA |
| 130406355 | 130406908 | + | 16.0 | 5193 | 5746 | -432 | L1M4 | L1 | LINE |
| 130406904 | 130408631 | + | 8.1 | 2808 | 4535 | -1611 | L1P3 | L1 | LINE |
| 130408632 | 130408740 | - | 7.3 | 247 | 355 | 0 | THE1A | MaLR | LTR |
| 130408741 | 130408769 | + | 8.1 | 4536 | 4564 | -1582 | L1P3 | L1 | LINE |
| 130408770 | 130408815 | - | 6.5 | 1 | 46 | -309 | THE1A | MaLR | LTR |
| 130408816 | 130410376 | - | 29.7 | 2 | 1735 | 0 | MLT1A0-int | MaLR | LTR |
| 130410379 | 130410735 | - | 9.6 | 1 | 355 | 0 | THE1A | MaLR | LTR |
| 130410736 | 130411842 | + | 8.6 | 4566 | 5689 | -483 | L1PA8 | L1 | LINE |
| 130411843 | 130412139 | + | 14.2 | 1 | 297 | -15 | AluSx | Alu | SINE |
| 130412140 | 130412606 | + | 10.0 | 5690 | 6163 | -9 | L1PA8 | L1 | LINE |
| 130412610 | 130412747 | - | 16.9 | 2597 | 2727 | -217 | BLACKJACK | AcHobo | DNA |
| 130412745 | 130413207 | + | 23.1 | 8 | 467 | 0 | MLT1C | MaLR | LTR |
| 130413286 | 130416006 | + | 5.6 | 3458 | 6155 | 0 | L1PA4 | L1 | LINE |
| 130416026 | 130416103 | - | 21.6 | 2472 | 2546 | -398 | BLACKJACK | AcHobo | DNA |
| 130416104 | 130416390 | + | 19.6 | 1 | 281 | -31 | AluJo | Alu | SINE |
| 130416391 | 130416764 | - | 21.6 | 2108 | 2471 | -473 | BLACKJACK | AcHobo | DNA |

  

|  |  |  |  |  |  |  |  |  |  |
| --- | --- | --- | --- | --- | --- | --- | --- | --- | --- |
| chr5:134878231-134889765 | | | 11219 bp | | |  |  |  |  |
| Genome Start | Genome End | Strand | Div | Repeat Start | Repeat End | Repeat Left | Name | Family | Class |
| 134878231 | 134878414 | - | 21.8 | 257 | 506 | -5 | MLT1J2 | MaLR | LTR |
| 134878415 | 134878679 | - | 19.3 | 27 | 324 | 0 | MER5C | MER1\_type | DNA |
| 134878812 | 134879780 | - | 22.0 | 3820 | 4848 | -6 | L1ME1 | L1 | LINE |
| 134879781 | 134879988 | - | 16.8 | 116 | 310 | -2 | AluJo | Alu | SINE |
| 134880011 | 134880038 | - | 16.8 | 90 | 115 | -197 | AluJo | Alu | SINE |
| 134880039 | 134880422 | - | 24.5 | 3363 | 3798 | -1036 | L1ME1 | L1 | LINE |
| 134880431 | 134881085 | - | 25.6 | 2241 | 2908 | -1926 | L1ME1 | L1 | LINE |
| 134881086 | 134881503 | + | 23.5 | 1 | 374 | 0 | MLT1A | MaLR | LTR |
| 134881504 | 134882451 | - | 25.6 | 1267 | 2240 | -2594 | L1ME1 | L1 | LINE |
| 134882452 | 134882754 | - | 8.9 | 1 | 302 | -9 | AluY | Alu | SINE |
| 134882755 | 134883667 | - | 24.8 | 421 | 1267 | -3567 | L1ME1 | L1 | LINE |
| 134883741 | 134884515 | - | 17.0 | 1 | 797 | -66 | MER21B | ERV1 | LTR |
| 134884543 | 134884866 | - | 25.3 | 31 | 340 | -4514 | L1ME1 | L1 | LINE |
| 134884867 | 134885020 | - | 15.6 | 11 | 168 | -8 | FRAM | Alu | SINE |
| 134885021 | 134885415 | - | 15.0 | 5 | 426 | 0 | MSTB | MaLR | LTR |
| 134885434 | 134886227 | - | 26.7 | 1150 | 2018 | -5110 | L1MCc | L1 | LINE |
| 134886228 | 134886590 | - | 15.2 | 1 | 372 | -3 | THE1C | MaLR | LTR |
| 134886591 | 134888111 | - | 29.0 | 2 | 1735 | 0 | MLT-int | MaLR | LTR |
| 134888114 | 134888488 | - | 15.1 | 1 | 375 | 0 | THE1C | MaLR | LTR |
| 134888489 | 134889584 | - | 26.3 | 23 | 1149 | -5274 | L1MCc | L1 | LINE |
| 134889619 | 134889765 | - | 28.3 | 8 | 157 | -293 | MLT1J2 | MaLR | LTR |

  

|  |  |  |  |  |  |  |  |  |  |
| --- | --- | --- | --- | --- | --- | --- | --- | --- | --- |
| chr5:135927548-135937367 | | | 9821 bp | | |  |  |  |  |
| Genome Start | Genome End | Strand | Div | Repeat Start | Repeat End | Repeat Left | Name | Family | Class |
| 135927548 | 135927969 | - | 16.3 | 5730 | 6150 | -16 | L1PA16 | L1 | LINE |
| 135927970 | 135928508 | + | 16.2 | 1 | 601 | -225 | LTR12 | ERV1 | LTR |
| 135928513 | 135928927 | - | 12.1 | 5304 | 5721 | -445 | L1PA16 | L1 | LINE |
| 135928928 | 135929378 | - | 7.6 | 1 | 448 | -2 | LTR7 | ERV1 | LTR |
| 135929379 | 135929745 | - | 8.7 | 7324 | 7713 | 0 | HERVH | ERV1 | LTR |
| 135929746 | 135929963 | - | 12.1 | 5600 | 5819 | -1894 | HERVH | ERV1 | LTR |
| 135929965 | 135930626 | - | 14.5 | 4488 | 5149 | -2564 | HERVH | ERV1 | LTR |
| 135930629 | 135931133 | - | 11.8 | 3492 | 3993 | -3720 | HERVH | ERV1 | LTR |
| 135931124 | 135934382 | - | 9.3 | 1 | 3290 | -4423 | HERVH | ERV1 | LTR |
| 135934385 | 135934833 | - | 6.3 | 1 | 448 | -2 | LTR7 | ERV1 | LTR |
| 135934834 | 135937039 | - | 13.3 | 3039 | 5310 | -836 | L1PA16 | L1 | LINE |
| 135937040 | 135937346 | - | 15.4 | 1 | 305 | -7 | AluSx | Alu | SINE |
| 135937347 | 135937367 | - | 13.3 | 3018 | 3038 | -3108 | L1PA16 | L1 | LINE |

  

|  |  |  |  |  |  |  |  |  |  |
| --- | --- | --- | --- | --- | --- | --- | --- | --- | --- |
| chr5:138861784-138872976 | | | 10932 bp | | |  |  |  |  |
| Genome Start | Genome End | Strand | Div | Repeat Start | Repeat End | Repeat Left | Name | Family | Class |
| 138861784 | 138861946 | + | 32.4 | 3704 | 3871 | -618 | L3 | CR1 | LINE |
| 138861947 | 138862247 | + | 12.0 | 1 | 302 | -11 | AluSp | Alu | SINE |
| 138862257 | 138862550 | + | 12.2 | 1 | 294 | -18 | AluSx | Alu | SINE |
| 138862551 | 138862611 | + | 32.4 | 3876 | 3941 | -548 | L3 | CR1 | LINE |
| 138862712 | 138863542 | + | 7.2 | 1 | 826 | 0 | LTR12 | ERV1 | LTR |
| 138863556 | 138870003 | + | 4.9 | 40 | 6479 | -1957 | HERV9 | ERV1 | LTR |
| 138870004 | 138871035 | + | 4.5 | 7401 | 8436 | 0 | HERV9 | ERV1 | LTR |
| 138871036 | 138871145 | + | 7.5 | 1 | 111 | -715 | LTR12 | ERV1 | LTR |
| 138871146 | 138871446 | - | 9.0 | 1 | 301 | -10 | AluY | Alu | SINE |
| 138871447 | 138872171 | + | 7.5 | 112 | 825 | -1 | LTR12 | ERV1 | LTR |
| 138872241 | 138872410 | + | 25.5 | 289 | 460 | -2959 | L2 | L2 | LINE |
| 138872424 | 138872660 | + | 31.1 | 3140 | 3418 | -1 | L2 | L2 | LINE |
| 138872718 | 138872976 | + | 31.8 | 4162 | 4460 | -29 | L3 | CR1 | LINE |

  

|  |  |  |  |  |  |  |  |  |  |
| --- | --- | --- | --- | --- | --- | --- | --- | --- | --- |
| chr5:140307016-140313457 | | | 6335 bp | | |  |  |  |  |
| Genome Start | Genome End | Strand | Div | Repeat Start | Repeat End | Repeat Left | Name | Family | Class |
| 140307016 | 140310545 | - | 24.7 | 1107 | 4855 | -1225 | L1MEc | L1 | LINE |
| 140310633 | 140311104 | - | 26.6 | 576 | 1053 | -5017 | L1MEc | L1 | LINE |
| 140311105 | 140311497 | - | 14.1 | 1 | 428 | 0 | MSTA | MaLR | LTR |
| 140311518 | 140312063 | - | 15.9 | 1100 | 1638 | -13 | MSTA-int | MaLR | LTR |
| 140312064 | 140312135 | + | 8.3 | 240 | 311 | -2 | AluSp/q | Alu | SINE |
| 140312136 | 140312488 | - | 16.8 | 1 | 369 | -1282 | MSTA-int | MaLR | LTR |
| 140312489 | 140312901 | - | 10.9 | 1 | 428 | 0 | MSTA | MaLR | LTR |
| 140312902 | 140313457 | - | 29.7 | 27 | 580 | -5490 | L1MEc | L1 | LINE |

  

|  |  |  |  |  |  |  |  |  |  |
| --- | --- | --- | --- | --- | --- | --- | --- | --- | --- |
| chr5:141083158-141087398 | | | 4150 bp | | |  |  |  |  |
| Genome Start | Genome End | Strand | Div | Repeat Start | Repeat End | Repeat Left | Name | Family | Class |
| 141083158 | 141083379 | - | 15.4 | 144 | 365 | 0 | MLT1A0 | MaLR | LTR |
| 141083420 | 141083840 | - | 11.2 | 3 | 428 | 0 | MSTA | MaLR | LTR |
| 141083843 | 141084851 | - | 27.8 | 670 | 1735 | 0 | MLT1A0-int | MaLR | LTR |
| 141084896 | 141085155 | - | 27.8 | 430 | 669 | -1066 | MLT1A0-int | MaLR | LTR |
| 141085156 | 141085462 | + | 10.4 | 1 | 312 | -1 | AluSp | Alu | SINE |
| 141085463 | 141085520 | - | 27.8 | 408 | 429 | -1306 | MLT1A0-int | MaLR | LTR |
| 141085521 | 141085806 | - | 19.2 | 1 | 287 | -25 | AluJb | Alu | SINE |
| 141085807 | 141086110 | - | 27.8 | 58 | 407 | -1328 | MLT1A0-int | MaLR | LTR |
| 141086111 | 141086382 | - | 14.0 | 21 | 297 | -15 | AluSx | Alu | SINE |
| 141086383 | 141086438 | - | 27.8 | 3 | 57 | -1678 | MLT1A0-int | MaLR | LTR |
| 141086441 | 141086491 | - | 11.1 | 390 | 428 | 0 | MSTA | MaLR | LTR |
| 141086492 | 141086760 | - | 10.6 | 60 | 313 | 0 | AluSq | Alu | SINE |
| 141086761 | 141087150 | - | 11.1 | 1 | 389 | -39 | MSTA | MaLR | LTR |
| 141087152 | 141087262 | - | 11.7 | 1 | 111 | -32 | FLAM\_C | Alu | SINE |
| 141087265 | 141087398 | - | 11.3 | 1 | 155 | -210 | MLT1A0 | MaLR | LTR |

  

|  |  |  |  |  |  |  |  |  |  |
| --- | --- | --- | --- | --- | --- | --- | --- | --- | --- |
| chr5:142682734-142685559 | | | 2826 bp | | |  |  |  |  |
| Genome Start | Genome End | Strand | Div | Repeat Start | Repeat End | Repeat Left | Name | Family | Class |
| 142682734 | 142682958 | + | 17.8 | 1 | 251 | -299 | MER44B | MER2\_type | DNA |
| 142682959 | 142683322 | - | 7.6 | 1 | 355 | 0 | THE1A | MaLR | LTR |
| 142683323 | 142684893 | - | 9.4 | 1 | 1580 | 0 | THE1A-int | MaLR | LTR |
| 142684894 | 142685248 | - | 9.9 | 1 | 355 | 0 | THE1A | MaLR | LTR |
| 142685249 | 142685559 | + | 16.7 | 246 | 549 | -1 | MER44B | MER2\_type | DNA |

  

|  |  |  |  |  |  |  |  |  |  |
| --- | --- | --- | --- | --- | --- | --- | --- | --- | --- |
| chr5:143784187-143786812 | | | 2624 bp | | |  |  |  |  |
| Genome Start | Genome End | Strand | Div | Repeat Start | Repeat End | Repeat Left | Name | Family | Class |
| 143784187 | 143784298 | + | 36.9 | 2840 | 2959 | -460 | L2 | L2 | LINE |
| 143784299 | 143784671 | - | 24.4 | 1 | 396 | 0 | MSTD | MaLR | LTR |
| 143784672 | 143786326 | - | 24.4 | 2 | 1735 | 0 | MSTD-int | MaLR | LTR |
| 143786329 | 143786712 | - | 22.6 | 4 | 396 | 0 | MSTD | MaLR | LTR |
| 143786713 | 143786812 | + | 36.9 | 2960 | 3073 | -346 | L2 | L2 | LINE |

  

|  |  |  |  |  |  |  |  |  |  |
| --- | --- | --- | --- | --- | --- | --- | --- | --- | --- |
| chr5:144614535-144620292 | | | 5501 bp | | |  |  |  |  |
| Genome Start | Genome End | Strand | Div | Repeat Start | Repeat End | Repeat Left | Name | Family | Class |
| 144614535 | 144615180 | + | 32.0 | 964 | 1621 | -5218 | L1MCc | L1 | LINE |
| 144615248 | 144615541 | + | 21.3 | 1 | 287 | -25 | AluJo | Alu | SINE |
| 144615582 | 144615739 | + | 25.5 | 1765 | 1916 | -4923 | L1MCc | L1 | LINE |
| 144615742 | 144616042 | - | 23.2 | 179 | 502 | -3 | MLT1D | MaLR | LTR |
| 144616178 | 144616364 | - | 15.8 | 2 | 166 | -460 | MLT1D | MaLR | LTR |
| 144616376 | 144617559 | + | 28.7 | 2079 | 3248 | -3693 | L1MCc | L1 | LINE |
| 144617560 | 144617978 | + | 15.5 | 1 | 428 | 0 | MSTA | MaLR | LTR |
| 144617981 | 144619618 | + | 29.1 | 2 | 1735 | 0 | MLT-int | MaLR | LTR |
| 144619619 | 144620042 | + | 14.2 | 1 | 428 | 0 | MSTA | MaLR | LTR |
| 144620043 | 144620292 | + | 30.1 | 3249 | 3506 | -3435 | L1MCc | L1 | LINE |

  

|  |  |  |  |  |  |  |  |  |  |
| --- | --- | --- | --- | --- | --- | --- | --- | --- | --- |
| chr5:144764919-144773842 | | | 8737 bp | | |  |  |  |  |
| Genome Start | Genome End | Strand | Div | Repeat Start | Repeat End | Repeat Left | Name | Family | Class |
| 144764919 | 144765106 | - | 18.0 | 384 | 705 | -6 | MER67C | ERV1 | LTR |
| 144765108 | 144765205 | - | 13.3 | 329 | 426 | 0 | MSTA | MaLR | LTR |
| 144765208 | 144765301 | - | 9.6 | 5845 | 5949 | -342 | L1MA8 | L1 | LINE |
| 144765305 | 144765458 | - | 15.3 | 201 | 341 | -87 | MSTA | MaLR | LTR |
| 144765459 | 144765760 | - | 11.6 | 1 | 304 | -7 | AluY | Alu | SINE |
| 144765761 | 144765960 | - | 15.3 | 1 | 200 | -228 | MSTA | MaLR | LTR |
| 144765961 | 144766226 | - | 20.2 | 115 | 387 | -324 | MER67C | ERV1 | LTR |
| 144766250 | 144766318 | - | 12.3 | 6206 | 6273 | -18 | L1MA8 | L1 | LINE |
| 144766309 | 144768151 | + | 13.4 | 1 | 1860 | -5003 | L1PBa | L1 | LINE |
| 144768243 | 144769387 | + | 12.9 | 1861 | 3109 | -3753 | L1PBa | L1 | LINE |
| 144769388 | 144769519 | + | 11.4 | 1 | 132 | -186 | Alu | Alu | SINE |
| 144769520 | 144769579 | + | 13.3 | 3096 | 3155 | -3707 | L1PBa | L1 | LINE |
| 144769575 | 144770167 | + | 10.7 | 4649 | 5238 | -908 | L1PB | L1 | LINE |
| 144770169 | 144770523 | - | 14.7 | 1 | 381 | 0 | THE1D | MaLR | LTR |
| 144770524 | 144770828 | - | 14.4 | 1276 | 1580 | 0 | THE1D-int | MaLR | LTR |
| 144770829 | 144771180 | - | 10.8 | 2 | 364 | 0 | THE1B | MaLR | LTR |
| 144771181 | 144772475 | - | 14.4 | 1 | 1275 | -305 | THE1D-int | MaLR | LTR |
| 144772476 | 144772837 | - | 14.8 | 1 | 381 | 0 | THE1D | MaLR | LTR |
| 144772838 | 144773367 | + | 10.3 | 5235 | 5777 | -374 | L1P5 | L1 | LINE |
| 144773449 | 144773730 | - | 17.1 | 5713 | 6012 | -279 | L1MA8 | L1 | LINE |
| 144773731 | 144773842 | - | 14.6 | 2 | 119 | -592 | MER67C | ERV1 | LTR |

  

|  |  |  |  |  |  |  |  |  |  |
| --- | --- | --- | --- | --- | --- | --- | --- | --- | --- |
| chr5:147229346-147234570 | | | 5254 bp | | |  |  |  |  |
| Genome Start | Genome End | Strand | Div | Repeat Start | Repeat End | Repeat Left | Name | Family | Class |
| 147229346 | 147229589 | + | 11.5 | 189 | 405 | -286 | LTR8 | ERV1 | LTR |
| 147229592 | 147230042 | - | 6.5 | 1 | 448 | -2 | LTR7 | ERV1 | LTR |
| 147230043 | 147230210 | - | 7.4 | 7550 | 7713 | 0 | HERVH | ERV1 | LTR |
| 147230203 | 147230431 | - | 11.0 | 4488 | 4719 | -2994 | HERVH | ERV1 | LTR |
| 147230434 | 147230937 | - | 12.0 | 3492 | 3993 | -3720 | HERVH | ERV1 | LTR |
| 147230928 | 147231075 | - | 13.5 | 3140 | 3290 | -4423 | HERVH | ERV1 | LTR |
| 147231055 | 147233831 | - | 10.4 | 1 | 2995 | -4718 | HERVH | ERV1 | LTR |
| 147233834 | 147234282 | - | 6.3 | 1 | 448 | -2 | LTR7 | ERV1 | LTR |
| 147234287 | 147234570 | + | 12.5 | 406 | 691 | 0 | LTR8 | ERV1 | LTR |

  

|  |  |  |  |  |  |  |  |  |  |
| --- | --- | --- | --- | --- | --- | --- | --- | --- | --- |
| chr5:147414368-147417166 | | | 2797 bp | | |  |  |  |  |
| Genome Start | Genome End | Strand | Div | Repeat Start | Repeat End | Repeat Left | Name | Family | Class |
| 147414368 | 147414761 | + | 25.7 | 1 | 384 | -159 | MER67A | ERV1 | LTR |
| 147414762 | 147415120 | + | 11.9 | 1 | 364 | 0 | THE1B | MaLR | LTR |
| 147415123 | 147416660 | + | 32.7 | 2 | 1735 | 0 | MLT-int | MaLR | LTR |
| 147416661 | 147417016 | + | 14.4 | 1 | 364 | 0 | THE1B | MaLR | LTR |
| 147417017 | 147417166 | + | 25.7 | 385 | 543 | 0 | MER67A | ERV1 | LTR |

  

|  |  |  |  |  |  |  |  |  |  |
| --- | --- | --- | --- | --- | --- | --- | --- | --- | --- |
| chr5:148449113-148452403 | | | 3289 bp | | |  |  |  |  |
| Genome Start | Genome End | Strand | Div | Repeat Start | Repeat End | Repeat Left | Name | Family | Class |
| 148449113 | 148449589 | + | 16.9 | 5142 | 5596 | -582 | L1MB8 | L1 | LINE |
| 148449590 | 148449943 | + | 12.5 | 1 | 355 | 0 | THE1A | MaLR | LTR |
| 148449946 | 148451500 | + | 29.6 | 2 | 1735 | 0 | MLT-int | MaLR | LTR |
| 148451501 | 148451858 | + | 12.8 | 1 | 355 | 0 | THE1A | MaLR | LTR |
| 148451859 | 148452403 | + | 18.2 | 5597 | 6138 | -40 | L1MB8 | L1 | LINE |

  

|  |  |  |  |  |  |  |  |  |  |
| --- | --- | --- | --- | --- | --- | --- | --- | --- | --- |
| chr5:155437755-155445893 | | | 7781 bp | | |  |  |  |  |
| Genome Start | Genome End | Strand | Div | Repeat Start | Repeat End | Repeat Left | Name | Family | Class |
| 155437755 | 155437818 | - | 12.5 | 6552 | 6619 | 0 | L1MD2 | L1 | LINE |
| 155437821 | 155438109 | + | 12.8 | 2 | 293 | -19 | AluSx | Alu | SINE |
| 155438292 | 155438550 | - | 20.3 | 6297 | 6556 | -63 | L1MD2 | L1 | LINE |
| 155438551 | 155438937 | - | 19.1 | 1 | 426 | 0 | MSTB | MaLR | LTR |
| 155438938 | 155440550 | - | 29.0 | 2 | 1735 | 0 | MLT-int | MaLR | LTR |
| 155440553 | 155440985 | - | 19.2 | 1 | 426 | 0 | MSTB | MaLR | LTR |
| 155440987 | 155441281 | - | 15.9 | 1 | 295 | -17 | AluJo | Alu | SINE |
| 155441282 | 155441318 | - | 20.3 | 6266 | 6296 | -323 | L1MD2 | L1 | LINE |
| 155441319 | 155441969 | - | 21.4 | 4 | 644 | 0 | LTR48B | ERV1 | LTR |
| 155441970 | 155442875 | - | 20.5 | 5393 | 6265 | -354 | L1MD2 | L1 | LINE |
| 155442876 | 155443182 | - | 10.4 | 1 | 312 | -1 | AluSp | Alu | SINE |
| 155443183 | 155443379 | - | 21.8 | 5211 | 5392 | -1014 | L1MD2 | L1 | LINE |
| 155443410 | 155444033 | - | 21.8 | 4560 | 5210 | -1196 | L1MD2 | L1 | LINE |
| 155444125 | 155444752 | - | 20.1 | 3942 | 4573 | -1833 | L1MD2 | L1 | LINE |
| 155444776 | 155444814 | - | 20.1 | 3908 | 3941 | -2465 | L1MD2 | L1 | LINE |
| 155444815 | 155445219 | + | 14.8 | 1 | 426 | 0 | MSTB | MaLR | LTR |
| 155445220 | 155445353 | - | 20.1 | 3765 | 3907 | -2499 | L1MD2 | L1 | LINE |
| 155445366 | 155445501 | + | 24.6 | 6004 | 6130 | -48 | L1MB8 | L1 | LINE |
| 155445517 | 155445893 | - | 29.0 | 1922 | 2346 | -4156 | L1MD2 | L1 | LINE |

  

|  |  |  |  |  |  |  |  |  |  |
| --- | --- | --- | --- | --- | --- | --- | --- | --- | --- |
| chr5:155652828-155670292 | | | 17331 bp | | |  |  |  |  |
| Genome Start | Genome End | Strand | Div | Repeat Start | Repeat End | Repeat Left | Name | Family | Class |
| 155652828 | 155653903 | - | 20.5 | 4416 | 5449 | -933 | L1M4c | L1 | LINE |
| 155653904 | 155654035 | + | 20.0 | 2 | 133 | 0 | FLAM\_C | Alu | SINE |
| 155654036 | 155655132 | - | 20.5 | 3302 | 4415 | -1967 | L1M4c | L1 | LINE |
| 155655133 | 155655998 | - | 20.9 | 1 | 898 | -3 | MER54A | ERVL | LTR |
| 155655999 | 155656481 | - | 20.5 | 2714 | 3301 | -3081 | L1M4c | L1 | LINE |
| 155656589 | 155656786 | - | 12.5 | 7532 | 7724 | 0 | L1MA3 | L1 | LINE |
| 155656788 | 155657415 | + | 2.2 | 5554 | 6182 | 0 | L1PA2 | L1 | LINE |
| 155657416 | 155658158 | - | 13.9 | 6771 | 7529 | -195 | L1MA3 | L1 | LINE |
| 155658160 | 155658318 | - | 14.8 | 1 | 142 | -1 | FLAM\_C | Alu | SINE |
| 155658335 | 155659776 | - | 12.7 | 5314 | 6773 | -792 | L1MA3 | L1 | LINE |
| 155659776 | 155663828 | - | 15.1 | 334 | 4494 | -3071 | L1MA3 | L1 | LINE |
| 155663829 | 155664182 | + | 13.2 | 1 | 364 | 0 | THE1B | MaLR | LTR |
| 155664185 | 155665757 | + | 30.2 | 2 | 1735 | 0 | MLT-int | MaLR | LTR |
| 155665759 | 155666111 | + | 13.9 | 2 | 364 | 0 | THE1B | MaLR | LTR |
| 155666112 | 155666425 | - | 18.2 | 14 | 333 | -7232 | L1MA3 | L1 | LINE |
| 155666433 | 155666624 | - | 24.3 | 2529 | 2722 | -3660 | L1M4c | L1 | LINE |
| 155666625 | 155666928 | - | 18.1 | 2 | 305 | -7 | AluJb | Alu | SINE |
| 155666929 | 155667686 | - | 18.0 | 1780 | 2528 | -3854 | L1M4c | L1 | LINE |
| 155667687 | 155667888 | - | 13.4 | 175 | 381 | 0 | THE1D | MaLR | LTR |
| 155667889 | 155668185 | - | 7.7 | 1 | 297 | -14 | AluY | Alu | SINE |
| 155668186 | 155668361 | - | 13.4 | 3 | 174 | -207 | THE1D | MaLR | LTR |
| 155668362 | 155669189 | - | 17.7 | 943 | 1779 | -4605 | L1M4c | L1 | LINE |
| 155669190 | 155669262 | - | 9.6 | 226 | 298 | -13 | Alu | Alu | SINE |
| 155669263 | 155669369 | - | 9.3 | 5 | 111 | -199 | AluSg | Alu | SINE |
| 155669370 | 155670292 | - | 17.7 | 10 | 942 | -5442 | L1M4c | L1 | LINE |

  

|  |  |  |  |  |  |  |  |  |  |
| --- | --- | --- | --- | --- | --- | --- | --- | --- | --- |
| chr5:156017068-156026569 | | | 9503 bp | | |  |  |  |  |
| Genome Start | Genome End | Strand | Div | Repeat Start | Repeat End | Repeat Left | Name | Family | Class |
| 156017068 | 156017294 | + | 11.1 | 1 | 226 | -86 | AluSx | Alu | SINE |
| 156017295 | 156018262 | - | 0.9 | 1 | 968 | 0 | LTR5\_Hs | ERVK | LTR |
| 156018263 | 156019973 | - | 0.4 | 5826 | 7536 | 0 | HERVK | ERVK | LTR |
| 156019973 | 156025506 | - | 0.8 | 1 | 5534 | -2002 | HERVK | ERVK | LTR |
| 156025507 | 156026474 | - | 0.9 | 1 | 968 | 0 | LTR5\_Hs | ERVK | LTR |
| 156026475 | 156026569 | + | 12.4 | 221 | 311 | 0 | AluSx | Alu | SINE |

  

|  |  |  |  |  |  |  |  |  |  |
| --- | --- | --- | --- | --- | --- | --- | --- | --- | --- |
| chr5:156362475-156369208 | | | 5904 bp | | |  |  |  |  |
| Genome Start | Genome End | Strand | Div | Repeat Start | Repeat End | Repeat Left | Name | Family | Class |
| 156362475 | 156362634 | - | 30.8 | 3133 | 3322 | -97 | L2 | L2 | LINE |
| 156362635 | 156363076 | + | 20.8 | 1 | 469 | -51 | MLT2B1 | ERVL | LTR |
| 156363083 | 156363237 | - | 35.2 | 2953 | 3108 | -270 | L2 | L2 | LINE |
| 156363449 | 156363548 | + | 16.0 | 2 | 126 | -17 | FLAM\_C | Alu | SINE |
| 156363605 | 156363983 | - | 26.9 | 172 | 553 | -2 | MLT1H1 | MaLR | LTR |
| 156363984 | 156364275 | - | 11.3 | 1 | 292 | -21 | AluSq | Alu | SINE |
| 156364276 | 156364418 | - | 26.9 | 39 | 171 | -384 | MLT1H1 | MaLR | LTR |
| 156364419 | 156364974 | - | 23.9 | 1 | 593 | 0 | MLT1E | MaLR | LTR |
| 156364975 | 156365012 | - | 26.9 | 1 | 38 | -517 | MLT1H1 | MaLR | LTR |
| 156365051 | 156365188 | - | 25.6 | 3240 | 3378 | 0 | L2 | L2 | LINE |
| 156365239 | 156365368 | + | 13.1 | 1 | 130 | -3 | FLAM\_C | Alu | SINE |
| 156365694 | 156366183 | - | 33.8 | 2329 | 2848 | -571 | L2 | L2 | LINE |
| 156366302 | 156366707 | - | 16.8 | 1 | 426 | 0 | MSTB | MaLR | LTR |
| 156366708 | 156367656 | - | 21.8 | 491 | 1580 | 0 | MSTB-int | MaLR | LTR |
| 156367657 | 156367959 | + | 11.0 | 1 | 301 | -12 | AluSq | Alu | SINE |
| 156367960 | 156368487 | - | 21.8 | 3 | 490 | -1090 | MSTB-int | MaLR | LTR |
| 156368490 | 156368897 | - | 18.4 | 8 | 426 | 0 | MSTB | MaLR | LTR |
| 156368901 | 156369037 | + | 9.1 | 1 | 135 | -177 | AluJo | Alu | SINE |
| 156369059 | 156369208 | - | 29.9 | 2021 | 2165 | -1254 | L2 | L2 | LINE |

  

|  |  |  |  |  |  |  |  |  |  |
| --- | --- | --- | --- | --- | --- | --- | --- | --- | --- |
| chr5:159035747-159052764 | | | 16999 bp | | |  |  |  |  |
| Genome Start | Genome End | Strand | Div | Repeat Start | Repeat End | Repeat Left | Name | Family | Class |
| 159035747 | 159037219 | + | 21.0 | 130 | 1556 | -6009 | L1MA1 | L1 | LINE |
| 159037250 | 159037576 | + | 21.0 | 1557 | 1854 | -5711 | L1MA1 | L1 | LINE |
| 159037578 | 159037994 | - | 10.9 | 1 | 428 | 0 | MSTA | MaLR | LTR |
| 159037995 | 159038601 | - | 17.7 | 1032 | 1651 | 0 | MSTA-int | MaLR | LTR |
| 159038602 | 159038962 | + | 14.5 | 1 | 375 | 0 | THE1C | MaLR | LTR |
| 159038965 | 159040526 | + | 30.8 | 2 | 1734 | -1 | MLT1K-int | MaLR | LTR |
| 159040528 | 159040907 | + | 16.2 | 1 | 375 | 0 | THE1C | MaLR | LTR |
| 159040908 | 159041930 | - | 17.7 | 1 | 1031 | -620 | MSTA-int | MaLR | LTR |
| 159041931 | 159042358 | - | 14.9 | 1 | 428 | 0 | MSTA | MaLR | LTR |
| 159042359 | 159042846 | + | 15.5 | 1851 | 2357 | -5208 | L1MA1 | L1 | LINE |
| 159042861 | 159043660 | - | 2.6 | 5352 | 6152 | -3 | L1PA4 | L1 | LINE |
| 159043672 | 159047860 | - | 4.4 | 1961 | 6154 | -1 | L1PA4 | L1 | LINE |
| 159047837 | 159048738 | + | 11.9 | 2345 | 3252 | -4313 | L1MA1 | L1 | LINE |
| 159048739 | 159049029 | + | 12.7 | 2 | 302 | -8 | AluSg | Alu | SINE |
| 159049030 | 159050118 | + | 10.0 | 3253 | 4341 | -3224 | L1MA1 | L1 | LINE |
| 159050119 | 159050427 | + | 16.6 | 5 | 312 | 0 | AluJo | Alu | SINE |
| 159050428 | 159050567 | + | 9.3 | 4342 | 4468 | -3097 | L1MA1 | L1 | LINE |
| 159050538 | 159050703 | + | 11.4 | 4724 | 4889 | -2676 | L1MA1 | L1 | LINE |
| 159050719 | 159051014 | - | 9.4 | 4704 | 4980 | -1166 | L1M1 | L1 | LINE |
| 159051014 | 159052764 | + | 10.2 | 5899 | 7675 | -46 | L1MA1 | L1 | LINE |

  

|  |  |  |  |  |  |  |  |  |  |
| --- | --- | --- | --- | --- | --- | --- | --- | --- | --- |
| chr5:160607648-160614860 | | | 7197 bp | | |  |  |  |  |
| Genome Start | Genome End | Strand | Div | Repeat Start | Repeat End | Repeat Left | Name | Family | Class |
| 160607648 | 160607989 | - | 36.5 | 2893 | 3404 | -15 | L2 | L2 | LINE |
| 160607994 | 160608404 | + | 11.1 | 3 | 448 | -2 | LTR7 | ERV1 | LTR |
| 160608407 | 160613589 | + | 10.9 | 1 | 5145 | -2568 | HERVH | ERV1 | LTR |
| 160613587 | 160613814 | + | 13.0 | 5593 | 5819 | -1894 | HERVH | ERV1 | LTR |
| 160613815 | 160614191 | + | 11.0 | 7324 | 7713 | 0 | HERVH | ERV1 | LTR |
| 160614192 | 160614598 | + | 8.9 | 1 | 442 | -8 | LTR7 | ERV1 | LTR |
| 160614612 | 160614860 | - | 32.2 | 2624 | 2892 | -527 | L2 | L2 | LINE |

  

|  |  |  |  |  |  |  |  |  |  |
| --- | --- | --- | --- | --- | --- | --- | --- | --- | --- |
| chr5:161675633-161680141 | | | 4371 bp | | |  |  |  |  |
| Genome Start | Genome End | Strand | Div | Repeat Start | Repeat End | Repeat Left | Name | Family | Class |
| 161675633 | 161675701 | - | 7.2 | 1512 | 1585 | -4923 | L1MCa | L1 | LINE |
| 161675703 | 161675992 | + | 24.2 | 4 | 324 | -21 | MER2 | MER2\_type | DNA |
| 161676017 | 161676237 | - | 20.8 | 1276 | 1513 | -4995 | L1MCa | L1 | LINE |
| 161676319 | 161677153 | - | 20.8 | 398 | 1275 | -5233 | L1MCa | L1 | LINE |
| 161677181 | 161677373 | - | 17.2 | 103 | 291 | -21 | AluSx | Alu | SINE |
| 161677374 | 161677720 | + | 11.9 | 1 | 364 | 0 | THE1B | MaLR | LTR |
| 161677723 | 161679299 | + | 30.1 | 2 | 1733 | -2 | MLT1B-int | MaLR | LTR |
| 161679302 | 161679645 | + | 15.4 | 1 | 364 | 0 | THE1B | MaLR | LTR |
| 161679646 | 161679747 | - | 17.2 | 1 | 102 | -210 | AluSx | Alu | SINE |
| 161679749 | 161680141 | - | 18.7 | 1 | 403 | -6105 | L1MCa | L1 | LINE |

  

|  |  |  |  |  |  |  |  |  |  |
| --- | --- | --- | --- | --- | --- | --- | --- | --- | --- |
| chr5:162392784-162396359 | | | 3572 bp | | |  |  |  |  |
| Genome Start | Genome End | Strand | Div | Repeat Start | Repeat End | Repeat Left | Name | Family | Class |
| 162392784 | 162392835 | - | 24.1 | 499 | 547 | -20 | MLT1F1 | MaLR | LTR |
| 162392836 | 162393240 | + | 16.1 | 1 | 428 | 0 | MSTC | MaLR | LTR |
| 162393241 | 162393656 | - | 24.1 | 63 | 498 | -69 | MLT1F1 | MaLR | LTR |
| 162393657 | 162394014 | + | 13.8 | 1 | 381 | 0 | THE1D | MaLR | LTR |
| 162394017 | 162394904 | + | 31.5 | 2 | 1027 | -708 | MSTC-int | MaLR | LTR |
| 162394905 | 162395218 | + | 13.6 | 1 | 309 | -3 | AluSx | Alu | SINE |
| 162395219 | 162395934 | + | 31.5 | 1028 | 1735 | 0 | MSTC-int | MaLR | LTR |
| 162395937 | 162396299 | + | 14.8 | 3 | 380 | -1 | THE1D | MaLR | LTR |
| 162396300 | 162396359 | - | 24.1 | 4 | 62 | -505 | MLT1F1 | MaLR | LTR |

  

|  |  |  |  |  |  |  |  |  |  |
| --- | --- | --- | --- | --- | --- | --- | --- | --- | --- |
| chr5:163144176-163146835 | | | 2658 bp | | |  |  |  |  |
| Genome Start | Genome End | Strand | Div | Repeat Start | Repeat End | Repeat Left | Name | Family | Class |
| 163144176 | 163144285 | + | 20.0 | 1 | 114 | -260 | MLT1A | MaLR | LTR |
| 163144288 | 163144642 | - | 11.4 | 1 | 355 | 0 | THE1A | MaLR | LTR |
| 163144643 | 163146219 | - | 10.7 | 1 | 1579 | -1 | THE1A-int | MaLR | LTR |
| 163146220 | 163146574 | - | 9.9 | 1 | 355 | 0 | THE1A | MaLR | LTR |
| 163146575 | 163146835 | + | 23.5 | 112 | 374 | 0 | MLT1A | MaLR | LTR |

  

|  |  |  |  |  |  |  |  |  |  |
| --- | --- | --- | --- | --- | --- | --- | --- | --- | --- |
| chr5:163265153-163269769 | | | 4382 bp | | |  |  |  |  |
| Genome Start | Genome End | Strand | Div | Repeat Start | Repeat End | Repeat Left | Name | Family | Class |
| 163265153 | 163265418 | + | 38.0 | 2868 | 3151 | -268 | L2 | L2 | LINE |
| 163265419 | 163265811 | + | 22.8 | 1 | 374 | 0 | MLT1A | MaLR | LTR |
| 163265812 | 163265846 | + | 37.5 | 3152 | 3184 | -235 | L2 | L2 | LINE |
| 163265853 | 163266233 | - | 27.9 | 175 | 541 | -8 | MLT1H | MaLR | LTR |
| 163266234 | 163266597 | - | 11.0 | 1 | 364 | 0 | THE1B | MaLR | LTR |
| 163266598 | 163266743 | - | 27.9 | 2 | 174 | -375 | MLT1H | MaLR | LTR |
| 163266743 | 163266840 | - | 24.7 | 1370 | 1472 | 0 | MLT1H-int | MaLR | LTR |
| 163266844 | 163267746 | - | 13.5 | 5254 | 6159 | -1 | L1PREC2 | L1 | LINE |
| 163267760 | 163268708 | - | 26.6 | 312 | 1380 | -92 | MLT1H-int | MaLR | LTR |
| 163268748 | 163268905 | - | 34.0 | 1 | 161 | -1311 | MLT1H-int | MaLR | LTR |
| 163268906 | 163269388 | - | 32.1 | 80 | 549 | 0 | MLT1H | MaLR | LTR |
| 163269441 | 163269576 | - | 26.7 | 65 | 207 | -8 | MER58C | MER1\_type | DNA |
| 163269700 | 163269769 | + | 20.3 | 3300 | 3376 | -2 | L2 | L2 | LINE |

  

|  |  |  |  |  |  |  |  |  |  |
| --- | --- | --- | --- | --- | --- | --- | --- | --- | --- |
| chr5:163278283-163281766 | | | 3271 bp | | |  |  |  |  |
| Genome Start | Genome End | Strand | Div | Repeat Start | Repeat End | Repeat Left | Name | Family | Class |
| 163278283 | 163278377 | - | 28.4 | 160 | 259 | -9 | MIRb | MIR | SINE |
| 163278381 | 163278593 | + | 28.4 | 2 | 217 | -294 | MLT1J | MaLR | LTR |
| 163278622 | 163278745 | - | 29.5 | 404 | 529 | -20 | MLT1H | MaLR | LTR |
| 163278746 | 163279124 | + | 13.1 | 1 | 375 | 0 | THE1C | MaLR | LTR |
| 163279127 | 163280686 | + | 29.8 | 2 | 1735 | 0 | MLT-int | MaLR | LTR |
| 163280687 | 163281062 | + | 10.7 | 1 | 375 | 0 | THE1C | MaLR | LTR |
| 163281063 | 163281382 | - | 29.5 | 13 | 403 | -146 | MLT1H | MaLR | LTR |
| 163281551 | 163281590 | + | 10.0 | 428 | 467 | -44 | MLT1J | MaLR | LTR |
| 163281603 | 163281766 | - | 26.7 | 18 | 178 | -84 | MIRb | MIR | SINE |

  

|  |  |  |  |  |  |  |  |  |  |
| --- | --- | --- | --- | --- | --- | --- | --- | --- | --- |
| chr5:164327860-164337939 | | | 10028 bp | | |  |  |  |  |
| Genome Start | Genome End | Strand | Div | Repeat Start | Repeat End | Repeat Left | Name | Family | Class |
| 164327860 | 164327920 | - | 16.4 | 6100 | 6160 | -6 | L1PA16 | L1 | LINE |
| 164327926 | 164328396 | - | 11.8 | 2 | 501 | 0 | MER61D | ERV1 | LTR |
| 164328399 | 164328542 | - | 17.1 | 7052 | 7187 | -1 | MER61D-int | ERV1 | LTR |
| 164328543 | 164328833 | + | 3.4 | 1 | 292 | -19 | AluY | Alu | SINE |
| 164328834 | 164333201 | - | 17.0 | 609 | 7051 | -137 | MER61D-int | ERV1 | LTR |
| 164333202 | 164333557 | + | 15.1 | 1 | 364 | 0 | THE1B | MaLR | LTR |
| 164333558 | 164335123 | + | 11.2 | 1 | 1580 | 0 | THE1B-int | MaLR | LTR |
| 164335124 | 164335483 | + | 14.5 | 1 | 364 | 0 | THE1B | MaLR | LTR |
| 164335489 | 164335917 | - | 14.7 | 1 | 483 | -18 | MER61D | ERV1 | LTR |
| 164335921 | 164336539 | - | 16.0 | 5459 | 6098 | -68 | L1PA16 | L1 | LINE |
| 164336540 | 164336659 | - | 15.8 | 176 | 295 | -17 | AluSg/x | Alu | SINE |
| 164336660 | 164337690 | - | 16.0 | 4392 | 5458 | -708 | L1PA16 | L1 | LINE |
| 164337728 | 164337939 | - | 13.3 | 4170 | 4389 | -1757 | L1PA16 | L1 | LINE |

  

|  |  |  |  |  |  |  |  |  |  |
| --- | --- | --- | --- | --- | --- | --- | --- | --- | --- |
| chr5:168884571-168887527 | | | 2953 bp | | |  |  |  |  |
| Genome Start | Genome End | Strand | Div | Repeat Start | Repeat End | Repeat Left | Name | Family | Class |
| 168884571 | 168884661 | + | 25.3 | 5466 | 5566 | -674 | L1M5 | L1 | LINE |
| 168884662 | 168884723 | + | 11.3 | 1 | 68 | -389 | MSTB2 | MaLR | LTR |
| 168884726 | 168885078 | - | 7.9 | 1 | 355 | 0 | THE1A | MaLR | LTR |
| 168885079 | 168886665 | - | 9.0 | 1 | 1580 | 0 | THE1A-int | MaLR | LTR |
| 168886666 | 168886991 | - | 9.0 | 1 | 355 | 0 | THE1A | MaLR | LTR |
| 168886992 | 168887366 | + | 21.2 | 66 | 457 | 0 | MSTB2 | MaLR | LTR |
| 168887369 | 168887527 | + | 27.7 | 5564 | 5723 | -517 | L1M5 | L1 | LINE |

  

|  |  |  |  |  |  |  |  |  |  |
| --- | --- | --- | --- | --- | --- | --- | --- | --- | --- |
| chr5:170338955-170343337 | | | 4379 bp | | |  |  |  |  |
| Genome Start | Genome End | Strand | Div | Repeat Start | Repeat End | Repeat Left | Name | Family | Class |
| 170338955 | 170339068 | + | 27.7 | 5 | 123 | -2384 | HAL1 | L1 | LINE |
| 170339069 | 170339422 | + | 12.5 | 1 | 362 | -2 | THE1B | MaLR | LTR |
| 170339427 | 170340975 | + | 29.7 | 2 | 1735 | 0 | MLT-int | MaLR | LTR |
| 170340976 | 170341343 | + | 10.3 | 1 | 364 | 0 | THE1B | MaLR | LTR |
| 170341344 | 170343337 | + | 27.9 | 124 | 2507 | 0 | HAL1 | L1 | LINE |

  

|  |  |  |  |  |  |  |  |  |  |
| --- | --- | --- | --- | --- | --- | --- | --- | --- | --- |
| chr5:170443130-170451653 | | | 8519 bp | | |  |  |  |  |
| Genome Start | Genome End | Strand | Div | Repeat Start | Repeat End | Repeat Left | Name | Family | Class |
| 170443130 | 170443259 | + | 24.0 | 1 | 128 | -111 | MER8 | MER2\_type | DNA |
| 170443260 | 170443572 | + | 13.6 | 1 | 336 | -28 | THE1B | MaLR | LTR |
| 170443573 | 170449582 | - | 2.2 | 13 | 6029 | -3 | L1PA2 | L1 | LINE |
| 170449586 | 170449625 | + | 17.5 | 316 | 355 | 0 | THE1B | MaLR | LTR |
| 170449628 | 170451196 | + | 30.5 | 2 | 1735 | 0 | MLT-int | MaLR | LTR |
| 170451197 | 170451536 | + | 12.2 | 1 | 364 | 0 | THE1B | MaLR | LTR |
| 170451537 | 170451653 | + | 17.2 | 124 | 239 | 0 | MER8 | MER2\_type | DNA |

  

|  |  |  |  |  |  |  |  |  |  |
| --- | --- | --- | --- | --- | --- | --- | --- | --- | --- |
| chr5:173856626-173861390 | | | 4355 bp | | |  |  |  |  |
| Genome Start | Genome End | Strand | Div | Repeat Start | Repeat End | Repeat Left | Name | Family | Class |
| 173856626 | 173857398 | - | 14.9 | 5385 | 6174 | -6 | L1MB4 | L1 | LINE |
| 173857399 | 173857750 | + | 14.2 | 1 | 355 | 0 | THE1A | MaLR | LTR |
| 173857753 | 173859325 | + | 29.0 | 2 | 1735 | 0 | MLT-int | MaLR | LTR |
| 173859327 | 173859677 | + | 15.8 | 2 | 355 | 0 | THE1A | MaLR | LTR |
| 173859678 | 173859804 | - | 14.9 | 5258 | 5384 | -796 | L1M | L1 | LINE |
| 173859905 | 173860293 | - | 17.8 | 4845 | 5245 | -901 | L1M | L1 | LINE |
| 173860452 | 173860672 | - | 23.7 | 1 | 262 | 0 | MIR | MIR | SINE |
| 173860777 | 173860977 | - | 18.9 | 723 | 953 | 0 | MER45C | Tip100 | DNA |
| 173860968 | 173861290 | - | 27.3 | 13 | 401 | -552 | MER45C | Tip100 | DNA |
| 173861346 | 173861390 | - | 15.6 | 5127 | 5171 | -975 | L1MB4 | L1 | LINE |

  

|  |  |  |  |  |  |  |  |  |  |
| --- | --- | --- | --- | --- | --- | --- | --- | --- | --- |
| chr5:175114971-175129217 | | | 14045 bp | | |  |  |  |  |
| Genome Start | Genome End | Strand | Div | Repeat Start | Repeat End | Repeat Left | Name | Family | Class |
| 175114971 | 175115282 | + | 29.3 | 5362 | 5695 | -545 | L1M5 | L1 | LINE |
| 175115297 | 175115570 | + | 13.1 | 5 | 285 | -27 | AluSx | Alu | SINE |
| 175115634 | 175115891 | + | 20.5 | 32 | 292 | -6341 | L1MDa | L1 | LINE |
| 175115892 | 175116029 | + | 13.0 | 6163 | 6305 | 0 | L1MA3 | L1 | LINE |
| 175116030 | 175116684 | + | 20.5 | 293 | 1028 | -5605 | L1MDa | L1 | LINE |
| 175116685 | 175116983 | + | 11.5 | 1 | 330 | 0 | AluSx | Alu | SINE |
| 175116984 | 175117303 | + | 20.5 | 1029 | 1349 | -5284 | L1MDa | L1 | LINE |
| 175117304 | 175117611 | + | 9.1 | 1 | 309 | -2 | AluY | Alu | SINE |
| 175117612 | 175119626 | + | 18.1 | 1350 | 3377 | -3160 | L1MDa | L1 | LINE |
| 175119627 | 175119997 | + | 9.5 | 1 | 375 | 0 | THE1C | MaLR | LTR |
| 175120000 | 175121500 | + | 28.7 | 2 | 1624 | -111 | MLT-int | MaLR | LTR |
| 175121501 | 175121805 | - | 11.8 | 1 | 305 | -4 | AluSc | Alu | SINE |
| 175121806 | 175121923 | + | 28.7 | 1625 | 1735 | 0 | MLT-int | MaLR | LTR |
| 175121924 | 175122295 | + | 10.3 | 1 | 375 | 0 | THE1C | MaLR | LTR |
| 175122296 | 175123020 | + | 15.1 | 3378 | 4111 | -2426 | L1MDa | L1 | LINE |
| 175123021 | 175123332 | + | 17.0 | 3723 | 4049 | -2097 | L1MD1 | L1 | LINE |
| 175123333 | 175123638 | + | 14.7 | 5 | 310 | -2 | AluSx | Alu | SINE |
| 175123639 | 175123860 | + | 17.0 | 4050 | 4261 | -1885 | L1MD1 | L1 | LINE |
| 175123877 | 175124014 | - | 19.3 | 3949 | 4084 | -2062 | L1MD | L1 | LINE |
| 175124011 | 175124996 | + | 15.4 | 4271 | 5266 | -880 | L1MD1 | L1 | LINE |
| 175125073 | 175125377 | + | 22.8 | 5852 | 6161 | -5 | L1ME1 | L1 | LINE |
| 175125390 | 175125453 | + | 21.9 | 5273 | 5336 | -810 | L1MD1 | L1 | LINE |
| 175125459 | 175125846 | - | 12.7 | 1 | 419 | -9 | MSTA | MaLR | LTR |
| 175125847 | 175126927 | - | 28.4 | 599 | 1735 | 0 | MLT-int | MaLR | LTR |
| 175126928 | 175127235 | - | 14.4 | 3 | 307 | -5 | AluSx | Alu | SINE |
| 175127236 | 175127775 | - | 28.4 | 2 | 598 | -1137 | MLT1K-int | MaLR | LTR |
| 175127778 | 175128180 | - | 13.5 | 1 | 428 | 0 | MSTA | MaLR | LTR |
| 175128181 | 175129076 | + | 17.4 | 5332 | 6241 | -1 | L1MD1 | L1 | LINE |
| 175129093 | 175129217 | + | 24.8 | 5701 | 5818 | -422 | L1M5 | L1 | LINE |

  

|  |  |  |  |  |  |  |  |  |  |
| --- | --- | --- | --- | --- | --- | --- | --- | --- | --- |
| chr5:180712516-180715330 | | | 2811 bp | | |  |  |  |  |
| Genome Start | Genome End | Strand | Div | Repeat Start | Repeat End | Repeat Left | Name | Family | Class |
| 180712516 | 180712809 | - | 29.1 | 252 | 542 | -13 | MLT1H1 | MaLR | LTR |
| 180712810 | 180713169 | + | 15.0 | 3 | 364 | 0 | THE1B | MaLR | LTR |
| 180713174 | 180714731 | + | 31.0 | 4 | 1735 | 0 | MLT-int | MaLR | LTR |
| 180714732 | 180715089 | + | 18.3 | 1 | 364 | 0 | THE1B | MaLR | LTR |
| 180715090 | 180715330 | - | 29.1 | 3 | 251 | -304 | MLT1H1 | MaLR | LTR |

  


|  |  |  |  |  |  |  |  |  |  |
| --- | --- | --- | --- | --- | --- | --- | --- | --- | --- |
| chr6:63403-66219 | | | 2813 bp | | |  |  |  |  |
| Genome Start | Genome End | Strand | Div | Repeat Start | Repeat End | Repeat Left | Name | Family | Class |
| 63403 | 63643 | + | 28.7 | 1 | 249 | -306 | MLT1H1 | MaLR | LTR |
| 63644 | 64001 | - | 18.1 | 1 | 364 | 0 | THE1B | MaLR | LTR |
| 64002 | 65561 | - | 30.7 | 4 | 1735 | 0 | MLT-int | MaLR | LTR |
| 65566 | 65925 | - | 15.8 | 3 | 364 | 0 | THE1B | MaLR | LTR |
| 65926 | 66219 | + | 28.7 | 250 | 540 | -15 | MLT1H1 | MaLR | LTR |

  

|  |  |  |  |  |  |  |  |  |  |
| --- | --- | --- | --- | --- | --- | --- | --- | --- | --- |
| chr6:1397897-1409308 | | | 10945 bp | | |  |  |  |  |
| Genome Start | Genome End | Strand | Div | Repeat Start | Repeat End | Repeat Left | Name | Family | Class |
| 1397897 | 1398142 | - | 14.6 | 140 | 374 | 0 | MLT1B | MaLR | LTR |
| 1398145 | 1398730 | + | 18.9 | 1 | 595 | 0 | LTR49 | ERV1 | LTR |
| 1398735 | 1399978 | + | 18.0 | 196 | 1635 | -5200 | LTR49-int | ERV1 | LTR |
| 1399980 | 1400106 | + | 16.0 | 1 | 128 | -174 | AluJ | Alu | SINE |
| 1400153 | 1400441 | - | 9.7 | 1 | 288 | -24 | AluSx | Alu | SINE |
| 1400593 | 1401483 | + | 20.0 | 1563 | 2437 | -3894 | LTR49-int | ERV1 | LTR |
| 1401484 | 1402766 | - | 6.2 | 2 | 1578 | 0 | LTR12C | ERV1 | LTR |
| 1402767 | 1402975 | + | 20.0 | 2438 | 2673 | -3658 | LTR49-int | ERV1 | LTR |
| 1402989 | 1403114 | + | 20.6 | 2 | 127 | -185 | AluJ | Alu | SINE |
| 1403360 | 1403525 | + | 18.7 | 131 | 300 | -12 | AluJ | Alu | SINE |
| 1403527 | 1404962 | + | 18.1 | 2666 | 4667 | -2251 | HERV35I | ERV1 | LTR |
| 1404963 | 1405269 | - | 12.3 | 5854 | 6164 | -1 | L1PA15 | L1 | LINE |
| 1405270 | 1406179 | + | 12.7 | 4210 | 5656 | -1242 | LTR49-int | ERV1 | LTR |
| 1406180 | 1406477 | - | 12.1 | 1 | 299 | -13 | AluSx | Alu | SINE |
| 1406478 | 1406956 | + | 12.3 | 5090 | 5583 | -748 | LTR49-int | ERV1 | LTR |
| 1406957 | 1407390 | + | 13.9 | 1 | 434 | 0 | MER57B | ERV1 | LTR |
| 1407391 | 1407979 | + | 12.3 | 5584 | 6329 | -172 | LTR49-int | ERV1 | LTR |
| 1407980 | 1408277 | + | 11.1 | 1 | 297 | -16 | AluSq | Alu | SINE |
| 1408278 | 1408439 | + | 12.3 | 6160 | 6331 | 0 | LTR49-int | ERV1 | LTR |
| 1408440 | 1408847 | + | 13.8 | 1 | 425 | -170 | LTR49 | ERV1 | LTR |
| 1408852 | 1408997 | + | 12.3 | 162 | 307 | -5 | AluSg/x | Alu | SINE |
| 1408998 | 1409158 | + | 17.6 | 419 | 595 | 0 | LTR49 | ERV1 | LTR |
| 1409159 | 1409308 | - | 16.3 | 1 | 141 | -249 | MLT1B | MaLR | LTR |

  

|  |  |  |  |  |  |  |  |  |  |
| --- | --- | --- | --- | --- | --- | --- | --- | --- | --- |
| chr6:2266502-2273458 | | | 6744 bp | | |  |  |  |  |
| Genome Start | Genome End | Strand | Div | Repeat Start | Repeat End | Repeat Left | Name | Family | Class |
| 2266502 | 2268222 | - | 16.6 | 3494 | 5208 | -1168 | L1M4c | L1 | LINE |
| 2268223 | 2268533 | - | 11.9 | 1 | 312 | 0 | AluSx | Alu | SINE |
| 2268534 | 2269164 | - | 16.6 | 2861 | 3493 | -2883 | L1M4c | L1 | LINE |
| 2269165 | 2269546 | + | 15.0 | 1 | 428 | 0 | MSTA | MaLR | LTR |
| 2269549 | 2271151 | + | 28.7 | 2 | 1735 | 0 | MLT-int | MaLR | LTR |
| 2271152 | 2271533 | + | 13.2 | 1 | 426 | -2 | MSTA | MaLR | LTR |
| 2271534 | 2272166 | - | 18.0 | 2208 | 2860 | -3516 | L1M4c | L1 | LINE |
| 2272234 | 2272531 | - | 10.2 | 1 | 296 | -17 | AluSp | Alu | SINE |
| 2272532 | 2272645 | - | 24.2 | 2091 | 2207 | -4177 | L1M4c | L1 | LINE |
| 2272790 | 2273458 | - | 25.3 | 1076 | 1945 | -4618 | L1M4c | L1 | LINE |

  

|  |  |  |  |  |  |  |  |  |  |
| --- | --- | --- | --- | --- | --- | --- | --- | --- | --- |
| chr6:4187640-4191120 | | | 3406 bp | | |  |  |  |  |
| Genome Start | Genome End | Strand | Div | Repeat Start | Repeat End | Repeat Left | Name | Family | Class |
| 4187640 | 4187742 | - | 18.6 | 6173 | 6279 | -33 | L1MA9 | L1 | LINE |
| 4187744 | 4188106 | - | 15.4 | 1 | 381 | 0 | THE1D | MaLR | LTR |
| 4188107 | 4189573 | - | 11.0 | 4 | 1580 | 0 | THE1D-int | MaLR | LTR |
| 4189577 | 4189937 | - | 13.0 | 1 | 381 | 0 | THE1D | MaLR | LTR |
| 4189968 | 4190291 | - | 19.3 | 5831 | 6151 | -161 | L1MA9 | L1 | LINE |
| 4190292 | 4190601 | - | 10.5 | 1 | 305 | -6 | AluY | Alu | SINE |
| 4190602 | 4190634 | - | 19.3 | 5793 | 5830 | -482 | L1MA9 | L1 | LINE |
| 4190676 | 4191120 | - | 19.3 | 5341 | 5792 | -520 | L1MA9 | L1 | LINE |

  

|  |  |  |  |  |  |  |  |  |  |
| --- | --- | --- | --- | --- | --- | --- | --- | --- | --- |
| chr6:4450041-4454836 | | | 4736 bp | | |  |  |  |  |
| Genome Start | Genome End | Strand | Div | Repeat Start | Repeat End | Repeat Left | Name | Family | Class |
| 4450041 | 4450166 | + | 28.7 | 1626 | 1752 | -1667 | L2 | L2 | LINE |
| 4450167 | 4450384 | + | 19.5 | 1 | 217 | -2 | MER20 | MER1\_type | DNA |
| 4450385 | 4450431 | + | 28.7 | 1753 | 1792 | -1627 | L2 | L2 | LINE |
| 4450432 | 4450603 | + | 19.4 | 1 | 208 | -182 | MLT1B | MaLR | LTR |
| 4450625 | 4450809 | + | 19.4 | 209 | 390 | 0 | MLT1B | MaLR | LTR |
| 4450810 | 4450826 | + | 28.7 | 1793 | 1807 | -1612 | L2 | L2 | LINE |
| 4450837 | 4450892 | + | 26.4 | 5742 | 5797 | -369 | L1ME1 | L1 | LINE |
| 4450893 | 4451250 | - | 10.7 | 3 | 381 | 0 | THE1D | MaLR | LTR |
| 4451253 | 4452849 | - | 29.8 | 1 | 1735 | 0 | MLT-int | MaLR | LTR |
| 4452851 | 4453199 | - | 11.4 | 1 | 381 | 0 | THE1D | MaLR | LTR |
| 4453200 | 4453556 | + | 26.4 | 5798 | 6161 | -5 | L1ME1 | L1 | LINE |
| 4453583 | 4454438 | + | 28.9 | 1800 | 2790 | -629 | L2 | L2 | LINE |
| 4454439 | 4454804 | + | 18.8 | 1 | 396 | 0 | MSTD | MaLR | LTR |
| 4454805 | 4454836 | + | 28.9 | 2791 | 2817 | -602 | L2 | L2 | LINE |

  

|  |  |  |  |  |  |  |  |  |  |
| --- | --- | --- | --- | --- | --- | --- | --- | --- | --- |
| chr6:5739415-5743001 | | | 3446 bp | | |  |  |  |  |
| Genome Start | Genome End | Strand | Div | Repeat Start | Repeat End | Repeat Left | Name | Family | Class |
| 5739415 | 5740129 | - | 28.6 | 2649 | 3413 | -6 | L2 | L2 | LINE |
| 5740249 | 5740457 | - | 22.6 | 244 | 466 | 0 | MER31B | ERV1 | LTR |
| 5740456 | 5740537 | - | 24.4 | 12 | 106 | -360 | MER31B | ERV1 | LTR |
| 5740539 | 5740892 | - | 6.5 | 1 | 354 | -1 | THE1A | MaLR | LTR |
| 5740893 | 5742465 | - | 29.6 | 2 | 1735 | 0 | MLT1A-int | MaLR | LTR |
| 5742468 | 5742818 | - | 8.0 | 1 | 355 | 0 | THE1A | MaLR | LTR |
| 5742840 | 5743001 | - | 25.5 | 2303 | 2479 | -940 | L2 | L2 | LINE |

  

|  |  |  |  |  |  |  |  |  |  |
| --- | --- | --- | --- | --- | --- | --- | --- | --- | --- |
| chr6:5750035-5753377 | | | 3324 bp | | |  |  |  |  |
| Genome Start | Genome End | Strand | Div | Repeat Start | Repeat End | Repeat Left | Name | Family | Class |
| 5750035 | 5750107 | + | 28.8 | 3752 | 3824 | -2322 | L1ME3B | L1 | LINE |
| 5750115 | 5750384 | + | 27.5 | 5691 | 5959 | -281 | L1ME3B | L1 | LINE |
| 5750388 | 5750434 | + | 19.7 | 1 | 45 | -383 | MSTA | MaLR | LTR |
| 5750435 | 5750812 | + | 14.0 | 1 | 380 | -1 | THE1D | MaLR | LTR |
| 5750813 | 5751141 | + | 19.7 | 46 | 428 | 0 | MSTA | MaLR | LTR |
| 5751142 | 5752756 | + | 16.4 | 1 | 1628 | -6 | MSTA-int | MaLR | LTR |
| 5752762 | 5753132 | + | 17.0 | 1 | 428 | 0 | MSTA | MaLR | LTR |
| 5753137 | 5753377 | + | 26.9 | 5955 | 6225 | -15 | L1ME3B | L1 | LINE |

  

|  |  |  |  |  |  |  |  |  |  |
| --- | --- | --- | --- | --- | --- | --- | --- | --- | --- |
| chr6:6065662-6071120 | | | 5407 bp | | |  |  |  |  |
| Genome Start | Genome End | Strand | Div | Repeat Start | Repeat End | Repeat Left | Name | Family | Class |
| 6065662 | 6066217 | - | 32.0 | 818 | 1242 | -5874 | L1M2 | L1 | LINE |
| 6066218 | 6066563 | + | 18.8 | 1 | 358 | -68 | MSTB | MaLR | LTR |
| 6066614 | 6066674 | + | 16.4 | 318 | 379 | -2 | MSTB | MaLR | LTR |
| 6066677 | 6066735 | + | 17.4 | 1 | 56 | -1595 | MSTB-int | MaLR | LTR |
| 6066736 | 6068420 | + | 20.4 | 1 | 1754 | -1 | MER52A | ERV1 | LTR |
| 6068421 | 6069941 | + | 17.4 | 57 | 1651 | 0 | MSTB-int | MaLR | LTR |
| 6069942 | 6070246 | + | 18.4 | 1 | 307 | -119 | MSTB | MaLR | LTR |
| 6070246 | 6070533 | + | 20.2 | 136 | 426 | 0 | MSTB | MaLR | LTR |
| 6070535 | 6071120 | - | 24.1 | 2 | 748 | -6368 | L1M2 | L1 | LINE |

  

|  |  |  |  |  |  |  |  |  |  |
| --- | --- | --- | --- | --- | --- | --- | --- | --- | --- |
| chr6:6505001-6510021 | | | 5019 bp | | |  |  |  |  |
| Genome Start | Genome End | Strand | Div | Repeat Start | Repeat End | Repeat Left | Name | Family | Class |
| 6505001 | 6505248 | - | 19.1 | 3542 | 3796 | -2350 | L1MD | L1 | LINE |
| 6505249 | 6505823 | + | 28.0 | 2 | 561 | -2 | MER68 | ERVL | LTR |
| 6505824 | 6505927 | - | 19.1 | 3446 | 3541 | -2605 | L1MD | L1 | LINE |
| 6505928 | 6506517 | - | 19.9 | 1 | 2487 | 0 | Tigger7 | MER2\_type | DNA |
| 6506518 | 6506730 | - | 20.2 | 3241 | 3447 | -2699 | L1MD | L1 | LINE |
| 6506731 | 6506967 | - | 9.3 | 75 | 311 | 0 | AluY | Alu | SINE |
| 6506968 | 6507486 | - | 20.2 | 2725 | 3240 | -2906 | L1MD | L1 | LINE |
| 6507487 | 6507860 | - | 13.8 | 1 | 381 | 0 | THE1D | MaLR | LTR |
| 6507861 | 6509443 | - | 29.4 | 2 | 1735 | 0 | MLT-int | MaLR | LTR |
| 6509446 | 6509826 | - | 13.0 | 1 | 381 | 0 | THE1D | MaLR | LTR |
| 6509827 | 6510021 | - | 20.2 | 2530 | 2724 | -3422 | L1MD | L1 | LINE |

  

|  |  |  |  |  |  |  |  |  |  |
| --- | --- | --- | --- | --- | --- | --- | --- | --- | --- |
| chr6:7988392-7991682 | | | 3287 bp | | |  |  |  |  |
| Genome Start | Genome End | Strand | Div | Repeat Start | Repeat End | Repeat Left | Name | Family | Class |
| 7988392 | 7989004 | + | 20.0 | 7320 | 7913 | -82 | L1MD3 | L1 | LINE |
| 7989005 | 7989233 | + | 5.5 | 1 | 236 | -119 | THE1A | MaLR | LTR |
| 7989234 | 7989539 | + | 9.5 | 1 | 304 | -6 | AluSg | Alu | SINE |
| 7989540 | 7989671 | + | 5.5 | 237 | 355 | 0 | THE1A | MaLR | LTR |
| 7989674 | 7991249 | + | 30.7 | 2 | 1735 | 0 | MSTD-int | MaLR | LTR |
| 7991252 | 7991604 | + | 8.0 | 3 | 355 | 0 | THE1A | MaLR | LTR |
| 7991605 | 7991682 | + | 20.0 | 7914 | 7995 | 0 | L1MD3 | L1 | LINE |

  

|  |  |  |  |  |  |  |  |  |  |
| --- | --- | --- | --- | --- | --- | --- | --- | --- | --- |
| chr6:9367656-9372819 | | | 5158 bp | | |  |  |  |  |
| Genome Start | Genome End | Strand | Div | Repeat Start | Repeat End | Repeat Left | Name | Family | Class |
| 9367656 | 9368537 | + | 17.7 | 3683 | 4568 | -1572 | L1MA9 | L1 | LINE |
| 9368544 | 9368913 | - | 11.4 | 1 | 373 | -2 | THE1C | MaLR | LTR |
| 9368914 | 9370468 | - | 12.7 | 1 | 1580 | 0 | THE1C-int | MaLR | LTR |
| 9370469 | 9370843 | - | 11.7 | 1 | 375 | 0 | THE1C | MaLR | LTR |
| 9370844 | 9371717 | + | 18.7 | 4567 | 5470 | -842 | L1MA9 | L1 | LINE |
| 9371718 | 9371971 | - | 0.8 | 56 | 308 | -2 | AluYa5 | Alu | SINE |
| 9371972 | 9372819 | + | 17.3 | 5471 | 6312 | 0 | L1MA9 | L1 | LINE |

  

|  |  |  |  |  |  |  |  |  |  |
| --- | --- | --- | --- | --- | --- | --- | --- | --- | --- |
| chr6:9829372-9837495 | | | 8063 bp | | |  |  |  |  |
| Genome Start | Genome End | Strand | Div | Repeat Start | Repeat End | Repeat Left | Name | Family | Class |
| 9829372 | 9829793 | - | 34.9 | 4748 | 5179 | -488 | ERVL-E | ERVL | LTR |
| 9829802 | 9830098 | - | 30.5 | 4514 | 4842 | -915 | ERVL-E | ERVL | LTR |
| 9830180 | 9830907 | + | 6.7 | 1 | 826 | 0 | LTR12 | ERV1 | LTR |
| 9830908 | 9833941 | + | 7.3 | 1 | 2980 | -5456 | HERV9 | ERV1 | LTR |
| 9833941 | 9834137 | + | 7.1 | 4775 | 4973 | -3463 | HERV9 | ERV1 | LTR |
| 9834117 | 9834620 | + | 10.2 | 741 | 1195 | -3907 | HERV9 | ERV1 | LTR |
| 9834608 | 9835042 | + | 6.5 | 1432 | 1864 | -3238 | HERV9 | ERV1 | LTR |
| 9835041 | 9835803 | + | 4.7 | 7664 | 8436 | 0 | HERV9 | ERV1 | LTR |
| 9835804 | 9836630 | + | 8.4 | 1 | 826 | 0 | LTR12 | ERV1 | LTR |
| 9836640 | 9837495 | - | 34.7 | 3437 | 4387 | -1267 | ERVL-E | ERVL | LTR |

  

|  |  |  |  |  |  |  |  |  |  |
| --- | --- | --- | --- | --- | --- | --- | --- | --- | --- |
| chr6:9967719-9972423 | | | 4436 bp | | |  |  |  |  |
| Genome Start | Genome End | Strand | Div | Repeat Start | Repeat End | Repeat Left | Name | Family | Class |
| 9967719 | 9968043 | + | 33.2 | 19 | 361 | -6062 | L1MCc | L1 | LINE |
| 9968228 | 9968529 | + | 10.6 | 1 | 312 | -1 | AluSq | Alu | SINE |
| 9968595 | 9969355 | + | 29.9 | 360 | 1625 | -5503 | L1MCc | L1 | LINE |
| 9969360 | 9969771 | + | 29.3 | 3489 | 3914 | -3027 | L1MCc | L1 | LINE |
| 9969777 | 9970008 | + | 25.1 | 1 | 232 | -189 | LTR52 | ERVL | LTR |
| 9970009 | 9970395 | - | 13.6 | 1 | 428 | 0 | MSTA | MaLR | LTR |
| 9970405 | 9971798 | - | 17.9 | 3 | 1580 | 0 | MSTA-int | MaLR | LTR |
| 9971801 | 9972168 | - | 13.7 | 1 | 428 | 0 | MSTA | MaLR | LTR |
| 9972169 | 9972360 | + | 20.3 | 228 | 421 | 0 | LTR52 | ERVL | LTR |
| 9972361 | 9972423 | + | 19.1 | 3921 | 3983 | -2958 | L1MCc | L1 | LINE |

  

|  |  |  |  |  |  |  |  |  |  |
| --- | --- | --- | --- | --- | --- | --- | --- | --- | --- |
| chr6:11483379-11486883 | | | 3496 bp | | |  |  |  |  |
| Genome Start | Genome End | Strand | Div | Repeat Start | Repeat End | Repeat Left | Name | Family | Class |
| 11483379 | 11483687 | + | 27.7 | 5771 | 6052 | -188 | L1ME3B | L1 | LINE |
| 11483688 | 11484035 | + | 16.2 | 1 | 357 | -69 | MSTB | MaLR | LTR |
| 11484036 | 11484342 | - | 6.8 | 1 | 307 | -4 | AluY | Alu | SINE |
| 11484343 | 11484413 | + | 16.2 | 358 | 426 | 0 | MSTB | MaLR | LTR |
| 11484423 | 11485995 | + | 27.4 | 2 | 1735 | 0 | MLT-int | MaLR | LTR |
| 11485996 | 11486179 | + | 15.6 | 1 | 185 | -241 | MSTB | MaLR | LTR |
| 11486180 | 11486469 | + | 13.1 | 1 | 290 | -22 | AluSx | Alu | SINE |
| 11486470 | 11486709 | + | 15.6 | 186 | 426 | 0 | MSTB | MaLR | LTR |
| 11486710 | 11486883 | + | 27.7 | 6054 | 6229 | -11 | L1ME3B | L1 | LINE |

  

|  |  |  |  |  |  |  |  |  |  |
| --- | --- | --- | --- | --- | --- | --- | --- | --- | --- |
| chr6:11548011-11555969 | | | 7862 bp | | |  |  |  |  |
| Genome Start | Genome End | Strand | Div | Repeat Start | Repeat End | Repeat Left | Name | Family | Class |
| 11548011 | 11549369 | - | 25.9 | 3397 | 4786 | -1433 | L1MEc | L1 | LINE |
| 11549370 | 11549685 | + | 15.6 | 3 | 323 | -41 | THE1B | MaLR | LTR |
| 11549686 | 11550004 | + | 11.1 | 1 | 323 | 0 | AluSq | Alu | SINE |
| 11550005 | 11550055 | + | 15.6 | 324 | 364 | 0 | THE1B | MaLR | LTR |
| 11550058 | 11551609 | + | 29.6 | 2 | 1735 | 0 | MLT-int | MaLR | LTR |
| 11551610 | 11551970 | + | 13.7 | 1 | 364 | 0 | THE1B | MaLR | LTR |
| 11551971 | 11552106 | - | 25.9 | 3261 | 3396 | -2823 | L1MEc | L1 | LINE |
| 11552107 | 11552335 | - | 14.6 | 199 | 428 | 0 | MSTA | MaLR | LTR |
| 11552336 | 11552637 | - | 0.7 | 1 | 302 | -8 | AluYa5 | Alu | SINE |
| 11552638 | 11552831 | - | 14.6 | 2 | 198 | -230 | MSTA | MaLR | LTR |
| 11552832 | 11555055 | - | 24.5 | 970 | 3260 | -2959 | L1MEc | L1 | LINE |
| 11555079 | 11555435 | - | 25.1 | 20 | 383 | -6017 | L1MEc | L1 | LINE |
| 11555507 | 11555628 | + | 24.0 | 988 | 1119 | -5000 | L1MEd | L1 | LINE |
| 11555630 | 11555969 | - | 30.1 | 53 | 398 | -4456 | L1MEc | L1 | LINE |

  

|  |  |  |  |  |  |  |  |  |  |
| --- | --- | --- | --- | --- | --- | --- | --- | --- | --- |
| chr6:12458511-12461778 | | | 3266 bp | | |  |  |  |  |
| Genome Start | Genome End | Strand | Div | Repeat Start | Repeat End | Repeat Left | Name | Family | Class |
| 12458511 | 12458721 | - | 12.4 | 5950 | 6156 | -4 | L1PREC2 | L1 | LINE |
| 12458722 | 12459105 | - | 12.6 | 1 | 428 | 0 | MSTA | MaLR | LTR |
| 12459106 | 12460742 | - | 27.9 | 2 | 1735 | 0 | MLT1L-int | MaLR | LTR |
| 12460745 | 12461134 | - | 12.3 | 1 | 428 | 0 | MSTA | MaLR | LTR |
| 12461135 | 12461778 | - | 12.6 | 5310 | 5949 | -211 | L1PREC2 | L1 | LINE |

  

|  |  |  |  |  |  |  |  |  |  |
| --- | --- | --- | --- | --- | --- | --- | --- | --- | --- |
| chr6:14405007-14412206 | | | 7207 bp | | |  |  |  |  |
| Genome Start | Genome End | Strand | Div | Repeat Start | Repeat End | Repeat Left | Name | Family | Class |
| 14405007 | 14405149 | - | 14.9 | 6184 | 6324 | -9 | L1MC1 | L1 | LINE |
| 14405152 | 14405600 | - | 5.1 | 1 | 448 | -2 | LTR7 | ERV1 | LTR |
| 14405601 | 14405980 | - | 7.5 | 7322 | 7713 | 0 | HERVH | ERV1 | LTR |
| 14405979 | 14406195 | - | 11.6 | 5600 | 5819 | -1894 | HERVH | ERV1 | LTR |
| 14406197 | 14406858 | - | 13.6 | 4488 | 5149 | -2564 | HERVH | ERV1 | LTR |
| 14406861 | 14407362 | - | 11.6 | 3492 | 3993 | -3720 | HERVH | ERV1 | LTR |
| 14407353 | 14410585 | - | 8.3 | 1 | 3290 | -4423 | HERVH | ERV1 | LTR |
| 14410586 | 14411033 | - | 4.0 | 1 | 450 | 0 | LTR7 | ERV1 | LTR |
| 14411034 | 14412206 | - | 14.3 | 4992 | 6188 | -145 | L1MC1 | L1 | LINE |

  

|  |  |  |  |  |  |  |  |  |  |
| --- | --- | --- | --- | --- | --- | --- | --- | --- | --- |
| chr6:16894823-16899498 | | | 4304 bp | | |  |  |  |  |
| Genome Start | Genome End | Strand | Div | Repeat Start | Repeat End | Repeat Left | Name | Family | Class |
| 16894823 | 16894925 | - | 32.7 | 3096 | 3199 | -179 | L2 | L2 | LINE |
| 16895099 | 16895271 | - | 37.0 | 2698 | 2878 | -541 | L2 | L2 | LINE |
| 16895314 | 16895696 | + | 19.5 | 4 | 374 | 0 | MLT1A | MaLR | LTR |
| 16895832 | 16896367 | - | 33.3 | 1598 | 2203 | -1216 | L2 | L2 | LINE |
| 16896377 | 16896802 | - | 13.2 | 1 | 428 | 0 | MSTA | MaLR | LTR |
| 16896809 | 16898400 | - | 15.1 | 1 | 1644 | -7 | MSTA-int | MaLR | LTR |
| 16898401 | 16898820 | - | 12.6 | 1 | 428 | 0 | MSTA | MaLR | LTR |
| 16898828 | 16899498 | - | 36.4 | 835 | 1564 | -1855 | L2 | L2 | LINE |

  

|  |  |  |  |  |  |  |  |  |  |
| --- | --- | --- | --- | --- | --- | --- | --- | --- | --- |
| chr6:16983725-17005886 | | | 21937 bp | | |  |  |  |  |
| Genome Start | Genome End | Strand | Div | Repeat Start | Repeat End | Repeat Left | Name | Family | Class |
| 16983725 | 16983925 | + | 32.8 | 2935 | 3164 | -214 | L2 | L2 | LINE |
| 16983978 | 16984946 | - | 18.7 | 5103 | 6119 | -59 | L1MB8 | L1 | LINE |
| 16984922 | 16986833 | - | 9.6 | 4238 | 6161 | 0 | L1PA14 | L1 | LINE |
| 16986836 | 16988018 | - | 17.0 | 3951 | 5129 | -1017 | L1MB8 | L1 | LINE |
| 16987997 | 16988416 | - | 15.1 | 2617 | 3036 | -3285 | L1MCa | L1 | LINE |
| 16988417 | 16988734 | + | 8.4 | 1 | 311 | 0 | AluY | Alu | SINE |
| 16988735 | 16990993 | - | 15.5 | 310 | 2616 | -3705 | L1MCa | L1 | LINE |
| 16990994 | 16991292 | - | 12.7 | 3 | 302 | -11 | AluSp | Alu | SINE |
| 16991293 | 16991600 | - | 15.6 | 1 | 309 | -6199 | L1MCa | L1 | LINE |
| 16991602 | 16992581 | - | 18.3 | 2976 | 3964 | -2182 | L1MB8 | L1 | LINE |
| 16992634 | 16992695 | - | 11.0 | 2910 | 2982 | -3161 | L1MB8 | L1 | LINE |
| 16992707 | 16993216 | - | 16.3 | 1 | 523 | -72 | LTR49 | ERV1 | LTR |
| 16993217 | 16993499 | - | 17.1 | 6041 | 6331 | 0 | LTR49-int | ERV1 | LTR |
| 16993509 | 16993687 | - | 17.3 | 2745 | 2926 | -3214 | L1M2 | L1 | LINE |
| 16993689 | 16994194 | + | 11.2 | 1 | 542 | -358 | MER4D1 | ERV1 | LTR |
| 16994195 | 16994594 | + | 9.3 | 1 | 448 | -2 | LTR7 | ERV1 | LTR |
| 16994597 | 16997558 | + | 9.0 | 1 | 2987 | -4726 | HERVH | ERV1 | LTR |
| 16997553 | 16997700 | + | 15.5 | 3140 | 3290 | -4423 | HERVH | ERV1 | LTR |
| 16997700 | 16998193 | + | 10.6 | 3500 | 3993 | -3720 | HERVH | ERV1 | LTR |
| 16998196 | 16998858 | + | 13.1 | 4488 | 5149 | -2564 | HERVH | ERV1 | LTR |
| 16998860 | 16999077 | + | 11.6 | 5600 | 5819 | -1894 | HERVH | ERV1 | LTR |
| 16999078 | 16999455 | + | 11.0 | 7324 | 7713 | 0 | HERVH | ERV1 | LTR |
| 16999456 | 16999857 | + | 8.5 | 1 | 448 | -2 | LTR7 | ERV1 | LTR |
| 16999860 | 17000203 | + | 11.4 | 538 | 895 | -5 | MER4D1 | ERV1 | LTR |
| 17000209 | 17000795 | - | 20.5 | 2149 | 2747 | -3399 | L1MB8 | L1 | LINE |
| 17000802 | 17002210 | - | 16.8 | 1697 | 3280 | -4054 | L1M4b | L1 | LINE |
| 17002231 | 17002966 | + | 14.4 | 47 | 54 | -1927 | Tigger2 | MER2\_type | DNA |
| 17002967 | 17003274 | + | 12.0 | 1 | 309 | -3 | AluSx | Alu | SINE |
| 17003275 | 17003812 | + | 14.4 | 55 | 593 | -1388 | Tigger2 | MER2\_type | DNA |
| 17003879 | 17003947 | - | 10.1 | 681 | 12 | -1969 | Tigger2 | MER2\_type | DNA |
| 17003952 | 17004023 | + | 21.1 | 2645 | 2718 | 0 | Tigger2 | MER2\_type | DNA |
| 17004024 | 17004540 | - | 19.6 | 1122 | 1657 | -5677 | L1M4b | L1 | LINE |
| 17004584 | 17005446 | - | 26.1 | 77 | 926 | -6408 | L1M4b | L1 | LINE |
| 17005447 | 17005725 | + | 18.8 | 2 | 295 | -17 | AluJo | Alu | SINE |
| 17005726 | 17005798 | - | 26.1 | 7 | 76 | -7258 | L1M4b | L1 | LINE |
| 17005799 | 17005886 | + | 36.8 | 3290 | 3378 | 0 | L2 | L2 | LINE |

  

|  |  |  |  |  |  |  |  |  |  |
| --- | --- | --- | --- | --- | --- | --- | --- | --- | --- |
| chr6:17130067-17135167 | | | 5085 bp | | |  |  |  |  |
| Genome Start | Genome End | Strand | Div | Repeat Start | Repeat End | Repeat Left | Name | Family | Class |
| 17130067 | 17130264 | - | 12.5 | 6118 | 6324 | -9 | L1MC1 | L1 | LINE |
| 17130265 | 17130566 | - | 11.0 | 1 | 291 | -22 | AluSq | Alu | SINE |
| 17130567 | 17132332 | - | 13.6 | 4328 | 6117 | -216 | L1MC1 | L1 | LINE |
| 17132333 | 17132741 | + | 13.8 | 1 | 426 | 0 | MSTB | MaLR | LTR |
| 17132742 | 17134379 | + | 18.6 | 1 | 1637 | -14 | MSTB-int | MaLR | LTR |
| 17134393 | 17134805 | + | 16.4 | 1 | 423 | -3 | MSTB | MaLR | LTR |
| 17134809 | 17135167 | - | 13.9 | 3966 | 4332 | -1814 | L1MC1 | L1 | LINE |

  

|  |  |  |  |  |  |  |  |  |  |
| --- | --- | --- | --- | --- | --- | --- | --- | --- | --- |
| chr6:17176420-17188453 | | | 11957 bp | | |  |  |  |  |
| Genome Start | Genome End | Strand | Div | Repeat Start | Repeat End | Repeat Left | Name | Family | Class |
| 17176420 | 17176564 | - | 25.6 | 108 | 241 | -21 | MIR | MIR | SINE |
| 17176568 | 17176633 | + | 14.0 | 1 | 57 | -317 | MLT1A | MaLR | LTR |
| 17176648 | 17177007 | - | 9.1 | 5794 | 6146 | -5 | L1PB1 | L1 | LINE |
| 17177008 | 17177360 | + | 11.1 | 1 | 355 | 0 | THE1A | MaLR | LTR |
| 17177363 | 17178929 | + | 30.6 | 2 | 1735 | 0 | MLT1A-int | MaLR | LTR |
| 17178930 | 17179282 | + | 11.4 | 1 | 355 | 0 | THE1A | MaLR | LTR |
| 17179283 | 17181433 | - | 6.7 | 3651 | 5793 | -358 | L1P5 | L1 | LINE |
| 17181434 | 17181551 | + | 14.9 | 39 | 152 | -213 | MLT1A | MaLR | LTR |
| 17181565 | 17184492 | + | 12.4 | 10 | 2838 | -3308 | L1PA7 | L1 | LINE |
| 17184493 | 17184809 | + | 14.2 | 1 | 309 | 0 | AluSc | Alu | SINE |
| 17184810 | 17188127 | + | 7.2 | 2839 | 6153 | -1 | L1PA7 | L1 | LINE |
| 17188130 | 17188312 | + | 23.3 | 155 | 394 | -34 | MLT1A | MaLR | LTR |
| 17188356 | 17188453 | - | 27.8 | 5 | 105 | -157 | MIR | MIR | SINE |

  

|  |  |  |  |  |  |  |  |  |  |
| --- | --- | --- | --- | --- | --- | --- | --- | --- | --- |
| chr6:19304004-19316081 | | | 11893 bp | | |  |  |  |  |
| Genome Start | Genome End | Strand | Div | Repeat Start | Repeat End | Repeat Left | Name | Family | Class |
| 19304004 | 19304215 | + | 31.1 | 557 | 785 | -1224 | HAL1b | L1 | LINE |
| 19304216 | 19304524 | + | 16.5 | 1 | 309 | -3 | AluSx | Alu | SINE |
| 19304525 | 19305491 | + | 31.1 | 786 | 1762 | -247 | HAL1b | L1 | LINE |
| 19305514 | 19306206 | - | 9.4 | 5471 | 6163 | 0 | L1PA13 | L1 | LINE |
| 19306207 | 19306515 | + | 10.4 | 1 | 311 | -2 | AluSq | Alu | SINE |
| 19306516 | 19308924 | - | 22.1 | 2859 | 5470 | -693 | L1PA13 | L1 | LINE |
| 19308925 | 19309286 | - | 12.3 | 1 | 364 | 0 | THE1B | MaLR | LTR |
| 19309287 | 19310710 | - | 11.3 | 1 | 1580 | 0 | THE1B-int | MaLR | LTR |
| 19310711 | 19311070 | - | 12.4 | 3 | 364 | 0 | THE1B | MaLR | LTR |
| 19311073 | 19314346 | - | 16.1 | 92 | 3483 | -3283 | L1PA13 | L1 | LINE |
| 19314402 | 19315082 | - | 24.2 | 5471 | 6166 | 0 | L1ME1 | L1 | LINE |
| 19315107 | 19315417 | + | 19.4 | 3 | 312 | 0 | AluJb | Alu | SINE |
| 19315434 | 19315846 | + | 16.2 | 5746 | 6157 | -9 | L1PA16 | L1 | LINE |
| 19315887 | 19315900 | + | 27.3 | 1827 | 1841 | -168 | HAL1b | L1 | LINE |
| 19315927 | 19316081 | + | 27.3 | 1842 | 2007 | -2 | HAL1b | L1 | LINE |

  

|  |  |  |  |  |  |  |  |  |  |
| --- | --- | --- | --- | --- | --- | --- | --- | --- | --- |
| chr6:19623129-19625977 | | | 2847 bp | | |  |  |  |  |
| Genome Start | Genome End | Strand | Div | Repeat Start | Repeat End | Repeat Left | Name | Family | Class |
| 19623129 | 19623260 | + | 12.7 | 1 | 130 | -397 | MER1A | MER1\_type | DNA |
| 19623261 | 19623579 | + | 13.5 | 3 | 364 | 0 | THE1B | MaLR | LTR |
| 19623582 | 19625244 | + | 29.5 | 2 | 1735 | 0 | MLT-int | MaLR | LTR |
| 19625245 | 19625576 | + | 14.8 | 1 | 364 | 0 | THE1B | MaLR | LTR |
| 19625577 | 19625977 | + | 12.7 | 131 | 529 | 0 | MER1A | MER1\_type | DNA |

  

|  |  |  |  |  |  |  |  |  |  |
| --- | --- | --- | --- | --- | --- | --- | --- | --- | --- |
| chr6:22884146-22888885 | | | 4755 bp | | |  |  |  |  |
| Genome Start | Genome End | Strand | Div | Repeat Start | Repeat End | Repeat Left | Name | Family | Class |
| 22884146 | 22884313 | - | 30.3 | 4412 | 4577 | -953 | HERVL40 | ERVL | LTR |
| 22884294 | 22885167 | - | 26.4 | 3377 | 4293 | -1237 | HERVL40 | ERVL | LTR |
| 22885160 | 22886055 | - | 27.1 | 2051 | 2998 | -2532 | HERVL40 | ERVL | LTR |
| 22886067 | 22886444 | - | 28.5 | 1228 | 1606 | -3924 | HERVL40 | ERVL | LTR |
| 22886445 | 22886808 | + | 14.7 | 1 | 364 | 0 | THE1B | MaLR | LTR |
| 22886811 | 22888363 | + | 31.2 | 2 | 1735 | 0 | MLT-int | MaLR | LTR |
| 22888364 | 22888723 | + | 16.6 | 1 | 364 | 0 | THE1B | MaLR | LTR |
| 22888724 | 22888885 | - | 28.5 | 1039 | 1227 | -4303 | HERVL40 | ERVL | LTR |

  

|  |  |  |  |  |  |  |  |  |  |
| --- | --- | --- | --- | --- | --- | --- | --- | --- | --- |
| chr6:23661688-23664225 | | | 2536 bp | | |  |  |  |  |
| Genome Start | Genome End | Strand | Div | Repeat Start | Repeat End | Repeat Left | Name | Family | Class |
| 23661688 | 23661769 | + | 24.5 | 7298 | 7363 | -445 | L1MC4a | L1 | LINE |
| 23661770 | 23662148 | - | 12.3 | 1 | 381 | 0 | THE1D | MaLR | LTR |
| 23662149 | 23663657 | - | 30.8 | 2 | 1735 | 0 | MLT-int | MaLR | LTR |
| 23663660 | 23664025 | - | 18.0 | 1 | 381 | 0 | THE1D | MaLR | LTR |
| 23664026 | 23664225 | + | 24.5 | 7364 | 7557 | -251 | L1MC4a | L1 | LINE |

  

|  |  |  |  |  |  |  |  |  |  |
| --- | --- | --- | --- | --- | --- | --- | --- | --- | --- |
| chr6:23775041-23783160 | | | 8082 bp | | |  |  |  |  |
| Genome Start | Genome End | Strand | Div | Repeat Start | Repeat End | Repeat Left | Name | Family | Class |
| 23775041 | 23776818 | - | 16.0 | 3726 | 5546 | -775 | L1MCa | L1 | LINE |
| 23776819 | 23776992 | - | 16.3 | 38 | 210 | 0 | MER6B | MER2\_type | DNA |
| 23776993 | 23778743 | - | 16.0 | 1933 | 3725 | -2596 | L1MCa | L1 | LINE |
| 23778756 | 23779166 | - | 17.9 | 1 | 394 | -32 | MSTB | MaLR | LTR |
| 23779167 | 23780828 | - | 27.8 | 2 | 1735 | 0 | MLT-int | MaLR | LTR |
| 23780831 | 23781249 | - | 17.5 | 1 | 426 | 0 | MSTB | MaLR | LTR |
| 23781251 | 23782609 | - | 19.6 | 528 | 1922 | -4586 | L1MCa | L1 | LINE |
| 23782633 | 23783160 | - | 19.6 | 1 | 527 | -5981 | L1MCa | L1 | LINE |

  

|  |  |  |  |  |  |  |  |  |  |
| --- | --- | --- | --- | --- | --- | --- | --- | --- | --- |
| chr6:24171489-24177381 | | | 5341 bp | | |  |  |  |  |
| Genome Start | Genome End | Strand | Div | Repeat Start | Repeat End | Repeat Left | Name | Family | Class |
| 24171489 | 24171629 | + | 30.7 | 2771 | 2922 | -1567 | L3 | CR1 | LINE |
| 24171739 | 24172665 | - | 36.5 | 2360 | 3361 | -58 | L2 | L2 | LINE |
| 24172771 | 24172849 | + | 35.0 | 2974 | 3056 | -1433 | L3 | CR1 | LINE |
| 24172850 | 24173153 | + | 10.9 | 1 | 306 | -3 | AluSc | Alu | SINE |
| 24173154 | 24173530 | + | 35.0 | 3057 | 3470 | -1019 | L3 | CR1 | LINE |
| 24173577 | 24173786 | + | 21.6 | 43 | 214 | -5 | MER20 | MER1\_type | DNA |
| 24174022 | 24174084 | + | 33.3 | 101 | 163 | -45 | MIR3 | MIR | SINE |
| 24174088 | 24174547 | + | 24.6 | 1 | 505 | 0 | MLT1D | MaLR | LTR |
| 24174548 | 24174650 | + | 27.8 | 1 | 104 | -1267 | MLT1D-int | MaLR | LTR |
| 24174651 | 24175094 | + | 24.9 | 1 | 504 | -1 | MLT1D | MaLR | LTR |
| 24175095 | 24176394 | + | 23.4 | 1 | 1217 | -154 | MLT1D-int | MaLR | LTR |
| 24176401 | 24176706 | + | 20.8 | 135 | 465 | -2 | MLT1C | MaLR | LTR |
| 24176755 | 24177381 | + | 30.7 | 3799 | 4487 | -2 | L3 | CR1 | LINE |

  

|  |  |  |  |  |  |  |  |  |  |
| --- | --- | --- | --- | --- | --- | --- | --- | --- | --- |
| chr6:27518899-27524523 | | | 5532 bp | | |  |  |  |  |
| Genome Start | Genome End | Strand | Div | Repeat Start | Repeat End | Repeat Left | Name | Family | Class |
| 27518899 | 27520610 | + | 27.5 | 421 | 2218 | -1201 | L2 | L2 | LINE |
| 27520618 | 27520682 | + | 18.5 | 2375 | 2442 | -977 | L2 | L2 | LINE |
| 27520705 | 27521120 | + | 22.9 | 5486 | 5915 | -255 | L1ME3B | L1 | LINE |
| 27521121 | 27521500 | + | 20.8 | 1 | 396 | 0 | MSTD | MaLR | LTR |
| 27521508 | 27522620 | + | 24.9 | 255 | 1385 | -350 | MSTD-int | MaLR | LTR |
| 27522621 | 27522814 | + | 10.3 | 1 | 194 | -119 | AluSq | Alu | SINE |
| 27522815 | 27523092 | + | 24.9 | 1386 | 1734 | -1 | MSTD-int | MaLR | LTR |
| 27523095 | 27523188 | + | 21.0 | 3 | 100 | -296 | MSTD | MaLR | LTR |
| 27523189 | 27523499 | + | 10.0 | 1 | 311 | -2 | AluSq | Alu | SINE |
| 27523500 | 27523673 | + | 21.0 | 101 | 276 | -120 | MSTD | MaLR | LTR |
| 27523694 | 27523821 | + | 31.8 | 6025 | 6158 | -82 | L1ME3B | L1 | LINE |
| 27523857 | 27524523 | + | 29.1 | 2440 | 3158 | -261 | L2 | L2 | LINE |

  

|  |  |  |  |  |  |  |  |  |  |
| --- | --- | --- | --- | --- | --- | --- | --- | --- | --- |
| chr6:29320937-29331027 | | | 10022 bp | | |  |  |  |  |
| Genome Start | Genome End | Strand | Div | Repeat Start | Repeat End | Repeat Left | Name | Family | Class |
| 29320937 | 29321231 | + | 14.7 | 3333 | 3625 | -2521 | L1P4 | L1 | LINE |
| 29321233 | 29321671 | - | 13.0 | 1 | 450 | 0 | LTR7 | ERV1 | LTR |
| 29321672 | 29322395 | - | 11.6 | 6996 | 7713 | 0 | HERVH | ERV1 | LTR |
| 29322390 | 29327529 | - | 18.9 | 791 | 5835 | -1878 | HERVH | ERV1 | LTR |
| 29327585 | 29328122 | - | 21.6 | 1 | 459 | -7254 | HERVH | ERV1 | LTR |
| 29328123 | 29328564 | - | 16.4 | 1 | 450 | 0 | LTR7 | ERV1 | LTR |
| 29328565 | 29329547 | + | 13.9 | 3622 | 4594 | -1552 | L1P4 | L1 | LINE |
| 29329548 | 29330005 | + | 10.0 | 1 | 472 | 0 | MER4A1 | ERV1 | LTR |
| 29330006 | 29330145 | + | 13.9 | 4595 | 4700 | -1446 | L1P4 | L1 | LINE |
| 29330173 | 29330766 | + | 13.9 | 4701 | 5302 | -844 | L1P4 | L1 | LINE |
| 29330759 | 29331027 | + | 13.0 | 5510 | 5782 | -383 | L1P4 | L1 | LINE |

  

|  |  |  |  |  |  |  |  |  |  |
| --- | --- | --- | --- | --- | --- | --- | --- | --- | --- |
| chr6:29345242-29348366 | | | 3065 bp | | |  |  |  |  |
| Genome Start | Genome End | Strand | Div | Repeat Start | Repeat End | Repeat Left | Name | Family | Class |
| 29345242 | 29345788 | - | 27.5 | 1434 | 1996 | -5132 | L1MCa | L1 | LINE |
| 29345803 | 29345961 | - | 10.7 | 150 | 310 | -2 | AluSg/x | Alu | SINE |
| 29346006 | 29346030 | - | 31.6 | 1300 | 1324 | -5099 | L1MCa | L1 | LINE |
| 29346031 | 29346416 | - | 18.5 | 1 | 374 | 0 | MLT1A | MaLR | LTR |
| 29346418 | 29347835 | - | 25.6 | 2 | 1370 | -1 | MLT1A-int | MaLR | LTR |
| 29347837 | 29348191 | - | 19.9 | 1 | 374 | 0 | MLT1A | MaLR | LTR |
| 29348192 | 29348366 | - | 31.6 | 1131 | 1299 | -5124 | L1MCa | L1 | LINE |

  

|  |  |  |  |  |  |  |  |  |  |
| --- | --- | --- | --- | --- | --- | --- | --- | --- | --- |
| chr6:33540137-33546855 | | | 6689 bp | | |  |  |  |  |
| Genome Start | Genome End | Strand | Div | Repeat Start | Repeat End | Repeat Left | Name | Family | Class |
| 33540137 | 33540270 | - | 19.4 | 2275 | 2418 | -2 | Cheshire | MER1\_type | DNA |
| 33540273 | 33540425 | - | 11.8 | 222 | 377 | -51 | MSTC | MaLR | LTR |
| 33540430 | 33540545 | - | 13.5 | 2127 | 2274 | -146 | Cheshire | MER1\_type | DNA |
| 33540546 | 33540845 | - | 11.3 | 1 | 300 | -12 | AluSx | Alu | SINE |
| 33540846 | 33541017 | - | 13.5 | 1962 | 2126 | -294 | Cheshire | MER1\_type | DNA |
| 33541018 | 33541369 | + | 8.2 | 3 | 355 | 0 | THE1A | MaLR | LTR |
| 33541372 | 33542942 | + | 30.9 | 2 | 1735 | 0 | MLT-int | MaLR | LTR |
| 33542943 | 33543299 | + | 9.4 | 1 | 355 | 0 | THE1A | MaLR | LTR |
| 33543300 | 33543411 | - | 13.5 | 1860 | 1961 | -459 | Cheshire | MER1\_type | DNA |
| 33543412 | 33543700 | - | 14.9 | 11 | 295 | -17 | AluJb | Alu | SINE |
| 33543701 | 33543968 | - | 13.5 | 1585 | 1859 | -561 | Cheshire | MER1\_type | DNA |
| 33543969 | 33544268 | + | 16.2 | 1 | 298 | -14 | AluSx | Alu | SINE |
| 33544275 | 33544600 | + | 10.3 | 5838 | 6160 | -5 | L1PA15 | L1 | LINE |
| 33544601 | 33544942 | - | 17.9 | 1 | 340 | -57 | MLT2C1 | ERVL | LTR |
| 33544959 | 33545085 | - | 20.5 | 1376 | 1501 | -919 | Cheshire | MER1\_type | DNA |
| 33545086 | 33545396 | - | 18.7 | 1 | 365 | 0 | MLT1A0 | MaLR | LTR |
| 33545397 | 33546179 | - | 18.1 | 565 | 1367 | -1053 | Cheshire | MER1\_type | DNA |
| 33546180 | 33546475 | - | 8.1 | 1 | 295 | -16 | AluY | Alu | SINE |
| 33546476 | 33546516 | - | 18.1 | 543 | 564 | -1856 | Cheshire | MER1\_type | DNA |
| 33546517 | 33546829 | - | 10.8 | 1 | 307 | -5 | AluSx | Alu | SINE |
| 33546830 | 33546855 | - | 18.1 | 518 | 542 | -1878 | Cheshire | MER1\_type | DNA |

  

|  |  |  |  |  |  |  |  |  |  |
| --- | --- | --- | --- | --- | --- | --- | --- | --- | --- |
| chr6:33678119-33682316 | | | 4187 bp | | |  |  |  |  |
| Genome Start | Genome End | Strand | Div | Repeat Start | Repeat End | Repeat Left | Name | Family | Class |
| 33678119 | 33678195 | - | 29.6 | 173 | 244 | -18 | MIR | MIR | SINE |
| 33678196 | 33678433 | - | 13.7 | 189 | 426 | 0 | MSTB | MaLR | LTR |
| 33678434 | 33678728 | - | 10.2 | 1 | 293 | -19 | AluSx | Alu | SINE |
| 33678729 | 33678927 | - | 13.7 | 1 | 188 | -238 | MSTB | MaLR | LTR |
| 33678931 | 33679260 | - | 26.8 | 1385 | 1732 | -3 | MLT-int | MaLR | LTR |
| 33679261 | 33679571 | - | 11.9 | 1 | 312 | 0 | AluSx | Alu | SINE |
| 33679572 | 33680332 | - | 26.8 | 597 | 1384 | -351 | MLT-int | MaLR | LTR |
| 33680333 | 33680627 | + | 10.9 | 1 | 300 | -13 | AluSq | Alu | SINE |
| 33680628 | 33681164 | - | 26.8 | 2 | 596 | -1139 | MLT-int | MaLR | LTR |
| 33681173 | 33681548 | - | 15.1 | 53 | 424 | -2 | MSTB | MaLR | LTR |
| 33681549 | 33681849 | - | 8.3 | 3 | 303 | -7 | AluSg | Alu | SINE |
| 33681850 | 33681888 | - | 15.1 | 2 | 52 | -374 | MSTB | MaLR | LTR |
| 33681889 | 33681981 | - | 29.6 | 78 | 168 | -94 | MIR | MIR | SINE |
| 33681982 | 33682280 | + | 9.7 | 1 | 299 | -11 | AluSg | Alu | SINE |
| 33682281 | 33682316 | - | 29.6 | 50 | 77 | -185 | MIR | MIR | SINE |

  

|  |  |  |  |  |  |  |  |  |  |
| --- | --- | --- | --- | --- | --- | --- | --- | --- | --- |
| chr6:39142806-39148209 | | | 5404 bp | | |  |  |  |  |
| Genome Start | Genome End | Strand | Div | Repeat Start | Repeat End | Repeat Left | Name | Family | Class |
| 39142806 | 39143036 | - | 29.6 | 5906 | 6150 | -90 | L1ME3B | L1 | LINE |
| 39143037 | 39143409 | - | 16.3 | 1 | 408 | 0 | MLT1A1 | MaLR | LTR |
| 39143410 | 39145105 | - | 19.4 | 192 | 1735 | 0 | MLT1A1-int | MaLR | LTR |
| 39145106 | 39145406 | + | 6.1 | 1 | 296 | -15 | AluY | Alu | SINE |
| 39145407 | 39145577 | - | 19.4 | 1 | 191 | -1544 | MLT1A1-int | MaLR | LTR |
| 39145578 | 39145987 | - | 17.4 | 3 | 408 | 0 | MLT1A1 | MaLR | LTR |
| 39145988 | 39146818 | - | 29.6 | 5094 | 5905 | -335 | L1ME3B | L1 | LINE |
| 39146819 | 39147129 | + | 11.2 | 1 | 311 | 0 | AluY | Alu | SINE |
| 39147130 | 39148209 | - | 29.8 | 3914 | 5093 | -1053 | L1ME3B | L1 | LINE |

  

|  |  |  |  |  |  |  |  |  |  |
| --- | --- | --- | --- | --- | --- | --- | --- | --- | --- |
| chr6:44938897-44941569 | | | 2609 bp | | |  |  |  |  |
| Genome Start | Genome End | Strand | Div | Repeat Start | Repeat End | Repeat Left | Name | Family | Class |
| 44938897 | 44939398 | - | 32.4 | 2910 | 3391 | -28 | L2 | L2 | LINE |
| 44939404 | 44939757 | - | 12.6 | 1 | 354 | -10 | THE1B | MaLR | LTR |
| 44939758 | 44941120 | - | 8.8 | 1 | 1580 | 0 | THE1B-int | MaLR | LTR |
| 44941121 | 44941482 | - | 15.8 | 1 | 364 | 0 | THE1B | MaLR | LTR |
| 44941542 | 44941569 | - | 17.9 | 1865 | 1892 | -1527 | L2 | L2 | LINE |

  

|  |  |  |  |  |  |  |  |  |  |
| --- | --- | --- | --- | --- | --- | --- | --- | --- | --- |
| chr6:45046906-45050394 | | | 2995 bp | | |  |  |  |  |
| Genome Start | Genome End | Strand | Div | Repeat Start | Repeat End | Repeat Left | Name | Family | Class |
| 45046906 | 45047025 | + | 22.7 | 2610 | 2721 | -698 | L2 | L2 | LINE |
| 45047027 | 45047403 | - | 9.9 | 1 | 373 | -2 | THE1C | MaLR | LTR |
| 45047405 | 45047492 | - | 12.1 | 1515 | 1580 | 0 | THE1C-int | MaLR | LTR |
| 45047493 | 45047788 | - | 6.8 | 1 | 294 | -17 | AluY | Alu | SINE |
| 45047789 | 45049364 | - | 12.1 | 3 | 1514 | -66 | THE1C-int | MaLR | LTR |
| 45049367 | 45049723 | - | 9.0 | 1 | 375 | 0 | THE1C | MaLR | LTR |
| 45049757 | 45049863 | - | 33.6 | 17 | 130 | -138 | MIRb | MIR | SINE |
| 45050321 | 45050394 | + | 29.7 | 2774 | 2850 | -569 | L2 | L2 | LINE |

  

|  |  |  |  |  |  |  |  |  |  |
| --- | --- | --- | --- | --- | --- | --- | --- | --- | --- |
| chr6:45345476-45350909 | | | 5428 bp | | |  |  |  |  |
| Genome Start | Genome End | Strand | Div | Repeat Start | Repeat End | Repeat Left | Name | Family | Class |
| 45345476 | 45345800 | - | 16.3 | 5851 | 6175 | -9 | L1MB7 | L1 | LINE |
| 45345803 | 45346033 | - | 12.1 | 1090 | 1301 | -1 | HSMAR2 | Mariner | DNA |
| 45346034 | 45346393 | + | 15.0 | 2 | 364 | 0 | THE1B | MaLR | LTR |
| 45346396 | 45347965 | + | 30.2 | 2 | 1735 | 0 | MLT-int | MaLR | LTR |
| 45347966 | 45348284 | + | 14.8 | 1 | 364 | 0 | THE1B | MaLR | LTR |
| 45348285 | 45349345 | - | 12.1 | 1 | 1089 | -213 | HSMAR2 | Mariner | DNA |
| 45349346 | 45350311 | - | 18.5 | 4848 | 5837 | -341 | L1MB7 | L1 | LINE |
| 45350314 | 45350606 | + | 18.3 | 1 | 298 | -14 | AluJb | Alu | SINE |
| 45350607 | 45350909 | - | 23.1 | 3620 | 3940 | -2206 | L1MB7 | L1 | LINE |

  

|  |  |  |  |  |  |  |  |  |  |
| --- | --- | --- | --- | --- | --- | --- | --- | --- | --- |
| chr6:46169247-46172782 | | | 3342 bp | | |  |  |  |  |
| Genome Start | Genome End | Strand | Div | Repeat Start | Repeat End | Repeat Left | Name | Family | Class |
| 46169247 | 46169532 | + | 20.9 | 5417 | 5699 | -465 | L1ME2 | L1 | LINE |
| 46169546 | 46169941 | - | 17.2 | 3 | 428 | 0 | MSTA | MaLR | LTR |
| 46169944 | 46171547 | - | 16.8 | 1 | 1651 | 0 | MSTA-int | MaLR | LTR |
| 46171618 | 46172014 | - | 16.9 | 1 | 428 | 0 | MSTA | MaLR | LTR |
| 46172016 | 46172097 | + | 26.8 | 5719 | 5800 | -366 | L1ME2 | L1 | LINE |
| 46172124 | 46172339 | - | 21.8 | 177 | 419 | -123 | MLT1F | MaLR | LTR |
| 46172422 | 46172782 | + | 22.4 | 5811 | 6154 | -10 | L1ME2 | L1 | LINE |

  

|  |  |  |  |  |  |  |  |  |  |
| --- | --- | --- | --- | --- | --- | --- | --- | --- | --- |
| chr6:46271983-46274895 | | | 2883 bp | | |  |  |  |  |
| Genome Start | Genome End | Strand | Div | Repeat Start | Repeat End | Repeat Left | Name | Family | Class |
| 46271983 | 46272029 | + | 15.6 | 6 | 51 | -454 | MLT1D | MaLR | LTR |
| 46272030 | 46272382 | - | 11.5 | 1 | 364 | 0 | THE1B | MaLR | LTR |
| 46272383 | 46273949 | - | 29.5 | 2 | 1735 | 0 | MLT-int | MaLR | LTR |
| 46273952 | 46274293 | - | 13.1 | 1 | 364 | 0 | THE1B | MaLR | LTR |
| 46274294 | 46274413 | + | 15.6 | 52 | 166 | -339 | MLT1D | MaLR | LTR |
| 46274431 | 46274590 | - | 14.4 | 130 | 289 | -20 | AluSc | Alu | SINE |
| 46274602 | 46274895 | + | 24.8 | 161 | 501 | -4 | MLT1D | MaLR | LTR |

  

|  |  |  |  |  |  |  |  |  |  |
| --- | --- | --- | --- | --- | --- | --- | --- | --- | --- |
| chr6:47500003-47508423 | | | 8031 bp | | |  |  |  |  |
| Genome Start | Genome End | Strand | Div | Repeat Start | Repeat End | Repeat Left | Name | Family | Class |
| 47500003 | 47500092 | - | 14.6 | 154 | 245 | -100 | MER2 | MER2\_type | DNA |
| 47500094 | 47500809 | + | 15.5 | 9 | 722 | 0 | MER72 | ERV1 | LTR |
| 47501041 | 47501264 | + | 32.9 | 285 | 495 | -6144 | MER72-int | ERV1 | LTR |
| 47501283 | 47501904 | - | 6.6 | 5543 | 6169 | -3 | L1PA8 | L1 | LINE |
| 47502001 | 47502394 | + | 17.5 | 968 | 1382 | -4949 | MER72-int | ERV1 | LTR |
| 47502395 | 47503155 | + | 21.1 | 1 | 727 | 0 | LTR8A | ERV1 | LTR |
| 47503156 | 47503188 | + | 22.2 | 1180 | 1207 | -7837 | MER72-int | ERV1 | LTR |
| 47503189 | 47503617 | - | 14.9 | 1 | 428 | 0 | MSTA | MaLR | LTR |
| 47503618 | 47505350 | - | 27.8 | 2 | 1735 | 0 | MLT-int | MaLR | LTR |
| 47505354 | 47505835 | - | 14.3 | 1 | 427 | -1 | MSTA | MaLR | LTR |
| 47505836 | 47506080 | + | 13.5 | 1208 | 1651 | -4680 | MER72-int | ERV1 | LTR |
| 47506081 | 47506201 | + | 14.9 | 46 | 168 | -8 | FRAM | Alu | SINE |
| 47506210 | 47506286 | + | 19.5 | 1856 | 1935 | -4396 | MER72-int | ERV1 | LTR |
| 47506289 | 47506466 | - | 13.7 | 244 | 428 | 0 | MSTA | MaLR | LTR |
| 47506467 | 47506946 | + | 8.4 | 1 | 511 | 0 | LTR10F | ERV1 | LTR |
| 47506947 | 47507180 | - | 13.7 | 1 | 243 | -185 | MSTA | MaLR | LTR |
| 47507181 | 47507485 | + | 27.4 | 1933 | 2247 | -4084 | MER72-int | ERV1 | LTR |
| 47507508 | 47507600 | + | 28.1 | 3218 | 3315 | -3603 | MER72-int | ERV1 | LTR |
| 47507601 | 47507908 | + | 14.0 | 1 | 307 | -5 | AluSx | Alu | SINE |
| 47507909 | 47508349 | + | 20.2 | 1181 | 3752 | -3166 | MER72-int | ERV1 | LTR |
| 47508359 | 47508423 | - | 23.1 | 2 | 66 | -279 | MER2 | MER2\_type | DNA |

  

|  |  |  |  |  |  |  |  |  |  |
| --- | --- | --- | --- | --- | --- | --- | --- | --- | --- |
| chr6:48926696-48929624 | | | 2787 bp | | |  |  |  |  |
| Genome Start | Genome End | Strand | Div | Repeat Start | Repeat End | Repeat Left | Name | Family | Class |
| 48926696 | 48926773 | + | 10.3 | 4969 | 5049 | -1282 | LTR49-int | ERV1 | LTR |
| 48926781 | 48927129 | - | 13.9 | 3 | 353 | -11 | THE1B | MaLR | LTR |
| 48927132 | 48928737 | - | 32.2 | 2 | 1735 | 0 | MLT1H1-int | MaLR | LTR |
| 48928740 | 48929103 | - | 13.5 | 1 | 364 | 0 | THE1B | MaLR | LTR |
| 48929104 | 48929216 | + | 15.9 | 5045 | 5158 | -1173 | LTR49-int | ERV1 | LTR |
| 48929220 | 48929375 | + | 21.4 | 1 | 153 | -23 | FRAM | Alu | SINE |
| 48929504 | 48929624 | + | 13.2 | 5313 | 5436 | -895 | LTR49-int | ERV1 | LTR |

  

|  |  |  |  |  |  |  |  |  |  |
| --- | --- | --- | --- | --- | --- | --- | --- | --- | --- |
| chr6:49204331-49206813 | | | 2481 bp | | |  |  |  |  |
| Genome Start | Genome End | Strand | Div | Repeat Start | Repeat End | Repeat Left | Name | Family | Class |
| 49204331 | 49204454 | + | 22.9 | 1 | 135 | 0 | MER58A | MER1\_type | DNA |
| 49204455 | 49204801 | + | 16.6 | 1 | 364 | 0 | THE1B | MaLR | LTR |
| 49204804 | 49206371 | + | 30.6 | 2 | 1735 | 0 | MLT-int | MaLR | LTR |
| 49206372 | 49206731 | + | 14.6 | 1 | 364 | 0 | THE1B | MaLR | LTR |
| 49206732 | 49206813 | + | 22.9 | 136 | 219 | -5 | MER58A | MER1\_type | DNA |

  

|  |  |  |  |  |  |  |  |  |  |
| --- | --- | --- | --- | --- | --- | --- | --- | --- | --- |
| chr6:49441043-49443554 | | | 2418 bp | | |  |  |  |  |
| Genome Start | Genome End | Strand | Div | Repeat Start | Repeat End | Repeat Left | Name | Family | Class |
| 49441043 | 49441092 | + | 16.0 | 88 | 144 | -367 | MLT1J | MaLR | LTR |
| 49441093 | 49441466 | - | 16.3 | 1 | 381 | 0 | THE1D | MaLR | LTR |
| 49441467 | 49442619 | - | 13.9 | 427 | 1580 | 0 | THE1D-int | MaLR | LTR |
| 49442623 | 49442857 | - | 9.0 | 4 | 240 | -1340 | THE1D-int | MaLR | LTR |
| 49442861 | 49443239 | - | 13.2 | 1 | 381 | 0 | THE1D | MaLR | LTR |
| 49443328 | 49443554 | + | 35.3 | 222 | 461 | -50 | MLT1J | MaLR | LTR |

  

|  |  |  |  |  |  |  |  |  |  |
| --- | --- | --- | --- | --- | --- | --- | --- | --- | --- |
| chr6:51558574-51561144 | | | 2558 bp | | |  |  |  |  |
| Genome Start | Genome End | Strand | Div | Repeat Start | Repeat End | Repeat Left | Name | Family | Class |
| 51558574 | 51559113 | - | 25.6 | 5579 | 6135 | -31 | L1ME1 | L1 | LINE |
| 51559114 | 51559492 | - | 13.5 | 5 | 428 | 0 | MSTA | MaLR | LTR |
| 51559502 | 51559626 | - | 9.8 | 1458 | 1580 | 0 | MSTA-int | MaLR | LTR |
| 51559626 | 51560023 | - | 12.6 | 6 | 405 | -1175 | MSTA-int | MaLR | LTR |
| 51560029 | 51560435 | - | 17.9 | 1 | 428 | 0 | MSTA | MaLR | LTR |
| 51560436 | 51561144 | - | 26.4 | 4891 | 5583 | -583 | L1ME1 | L1 | LINE |

  

|  |  |  |  |  |  |  |  |  |  |
| --- | --- | --- | --- | --- | --- | --- | --- | --- | --- |
| chr6:51910647-51917577 | | | 6904 bp | | |  |  |  |  |
| Genome Start | Genome End | Strand | Div | Repeat Start | Repeat End | Repeat Left | Name | Family | Class |
| 51910647 | 51910997 | + | 18.8 | 1 | 351 | -376 | LTR8A | ERV1 | LTR |
| 51910998 | 51911438 | + | 8.1 | 1 | 442 | -8 | LTR7 | ERV1 | LTR |
| 51911448 | 51913993 | + | 10.7 | 1 | 2643 | -5070 | HERVH | ERV1 | LTR |
| 51913994 | 51914311 | - | 0.9 | 1 | 318 | 0 | AluYb8 | Alu | SINE |
| 51914312 | 51914684 | + | 10.4 | 2644 | 2995 | -4718 | HERVH | ERV1 | LTR |
| 51914664 | 51915509 | + | 11.0 | 3140 | 3993 | -3720 | HERVH | ERV1 | LTR |
| 51915512 | 51916127 | + | 12.2 | 4488 | 5149 | -2564 | HERVH | ERV1 | LTR |
| 51916159 | 51916363 | + | 11.2 | 5615 | 5819 | -1894 | HERVH | ERV1 | LTR |
| 51916364 | 51916739 | + | 10.5 | 7324 | 7713 | 0 | HERVH | ERV1 | LTR |
| 51916740 | 51917183 | + | 7.8 | 1 | 448 | -2 | LTR7 | ERV1 | LTR |
| 51917190 | 51917577 | + | 20.3 | 351 | 727 | 0 | LTR8A | ERV1 | LTR |

  

|  |  |  |  |  |  |  |  |  |  |
| --- | --- | --- | --- | --- | --- | --- | --- | --- | --- |
| chr6:53523505-53527020 | | | 3514 bp | | |  |  |  |  |
| Genome Start | Genome End | Strand | Div | Repeat Start | Repeat End | Repeat Left | Name | Family | Class |
| 53523505 | 53523588 | + | 10.5 | 2 | 85 | -815 | MER4D1 | ERV1 | LTR |
| 53523589 | 53523898 | + | 11.7 | 1 | 309 | -3 | AluSx | Alu | SINE |
| 53523899 | 53524469 | + | 10.5 | 86 | 668 | -232 | MER4D1 | ERV1 | LTR |
| 53524470 | 53524824 | + | 9.9 | 1 | 355 | 0 | THE1A | MaLR | LTR |
| 53524827 | 53526405 | + | 30.1 | 2 | 1735 | 0 | MLT1A0-int | MaLR | LTR |
| 53526406 | 53526759 | + | 9.0 | 1 | 355 | 0 | THE1A | MaLR | LTR |
| 53526760 | 53527020 | + | 10.5 | 669 | 900 | 0 | MER4D1 | ERV1 | LTR |

  

|  |  |  |  |  |  |  |  |  |  |
| --- | --- | --- | --- | --- | --- | --- | --- | --- | --- |
| chr6:53848727-53851482 | | | 2750 bp | | |  |  |  |  |
| Genome Start | Genome End | Strand | Div | Repeat Start | Repeat End | Repeat Left | Name | Family | Class |
| 53848727 | 53848778 | - | 11.6 | 483 | 529 | 0 | MER1A | MER1\_type | DNA |
| 53848779 | 53849131 | - | 12.4 | 1 | 364 | 0 | THE1B | MaLR | LTR |
| 53849132 | 53850687 | - | 28.5 | 2 | 1735 | 0 | MLT-int | MaLR | LTR |
| 53850694 | 53851017 | - | 9.4 | 3 | 360 | -4 | THE1B | MaLR | LTR |
| 53851018 | 53851482 | - | 11.6 | 1 | 482 | -47 | MER1A | MER1\_type | DNA |

  

|  |  |  |  |  |  |  |  |  |  |
| --- | --- | --- | --- | --- | --- | --- | --- | --- | --- |
| chr6:54768662-54771322 | | | 2657 bp | | |  |  |  |  |
| Genome Start | Genome End | Strand | Div | Repeat Start | Repeat End | Repeat Left | Name | Family | Class |
| 54768662 | 54768862 | - | 12.4 | 177 | 364 | 0 | THE1B | MaLR | LTR |
| 54768863 | 54769215 | + | 11.1 | 1 | 355 | 0 | THE1A | MaLR | LTR |
| 54769218 | 54770792 | + | 30.1 | 2 | 1735 | 0 | MLT1J-int | MaLR | LTR |
| 54770795 | 54771146 | + | 10.5 | 3 | 355 | 0 | THE1A | MaLR | LTR |
| 54771147 | 54771322 | - | 12.4 | 1 | 176 | -188 | THE1B | MaLR | LTR |

  

|  |  |  |  |  |  |  |  |  |  |
| --- | --- | --- | --- | --- | --- | --- | --- | --- | --- |
| chr6:55601319-55604145 | | | 2827 bp | | |  |  |  |  |
| Genome Start | Genome End | Strand | Div | Repeat Start | Repeat End | Repeat Left | Name | Family | Class |
| 55601319 | 55601403 | - | 12.6 | 2139 | 2292 | -5112 | L1M1 | L1 | LINE |
| 55601404 | 55601755 | + | 13.0 | 1 | 364 | 0 | THE1B | MaLR | LTR |
| 55601758 | 55603329 | + | 31.6 | 2 | 1735 | 0 | MLT1J2-int | MaLR | LTR |
| 55603330 | 55603686 | + | 12.8 | 1 | 360 | -4 | THE1B | MaLR | LTR |
| 55603687 | 55604051 | - | 12.3 | 2459 | 2772 | -4793 | L1M1 | L1 | LINE |
| 55604050 | 55604145 | - | 23.7 | 27 | 119 | -7446 | L1M1 | L1 | LINE |

  

|  |  |  |  |  |  |  |  |  |  |
| --- | --- | --- | --- | --- | --- | --- | --- | --- | --- |
| chr6:57661871-57668161 | | | 6275 bp | | |  |  |  |  |
| Genome Start | Genome End | Strand | Div | Repeat Start | Repeat End | Repeat Left | Name | Family | Class |
| 57661871 | 57662022 | + | 18.9 | 4837 | 4975 | -1168 | L1MA8 | L1 | LINE |
| 57662025 | 57662397 | + | 18.2 | 3 | 420 | -6433 | L1PA15-16 | L1 | LINE |
| 57662398 | 57662711 | - | 14.7 | 1 | 312 | 0 | AluSx | Alu | SINE |
| 57662712 | 57663054 | + | 18.2 | 421 | 755 | -6098 | L1PA15-16 | L1 | LINE |
| 57663055 | 57663448 | - | 17.2 | 1 | 428 | 0 | MSTA | MaLR | LTR |
| 57663449 | 57664993 | - | 16.7 | 1 | 1651 | 0 | MSTA-int | MaLR | LTR |
| 57664994 | 57665380 | - | 17.1 | 1 | 428 | 0 | MSTA | MaLR | LTR |
| 57665392 | 57665712 | + | 25.6 | 761 | 1076 | -5777 | L1PA15-16 | L1 | LINE |
| 57665704 | 57666895 | + | 12.0 | 5641 | 6867 | -1 | L1PA16 | L1 | LINE |
| 57666908 | 57668161 | + | 15.9 | 4977 | 6287 | -4 | L1MA8 | L1 | LINE |

  

|  |  |  |  |  |  |  |  |  |  |
| --- | --- | --- | --- | --- | --- | --- | --- | --- | --- |
| chr6:58145496-58155361 | | | 9836 bp | | |  |  |  |  |
| Genome Start | Genome End | Strand | Div | Repeat Start | Repeat End | Repeat Left | Name | Family | Class |
| 58145496 | 58146230 | - | 16.8 | 6656 | 7395 | -315 | L1MA8 | L1 | LINE |
| 58146231 | 58146653 | + | 17.3 | 1 | 432 | 0 | MSTB1 | MaLR | LTR |
| 58146657 | 58148182 | + | 27.0 | 2 | 1735 | 0 | MLT-int | MaLR | LTR |
| 58148187 | 58148609 | + | 18.7 | 5 | 432 | 0 | MSTB1 | MaLR | LTR |
| 58148610 | 58149096 | - | 17.0 | 6157 | 6655 | -907 | L1MA8 | L1 | LINE |
| 58149097 | 58149529 | + | 15.7 | 1 | 434 | 0 | Tigger2a | MER2\_type | DNA |
| 58149530 | 58150435 | - | 17.0 | 5274 | 6156 | -1406 | L1MA8 | L1 | LINE |
| 58150436 | 58150797 | + | 17.9 | 1 | 364 | 0 | THE1B | MaLR | LTR |
| 58150798 | 58150958 | - | 17.0 | 5123 | 5273 | -2289 | L1MA8 | L1 | LINE |
| 58150959 | 58151249 | - | 18.1 | 1 | 299 | -13 | AluJb | Alu | SINE |
| 58151250 | 58152567 | - | 17.0 | 3924 | 5122 | -2440 | L1MA8 | L1 | LINE |
| 58152568 | 58152875 | - | 10.1 | 1 | 309 | -2 | AluY | Alu | SINE |
| 58152876 | 58154770 | - | 24.6 | 2008 | 3923 | -3639 | L1MA8 | L1 | LINE |
| 58154794 | 58155361 | - | 25.1 | 466 | 1050 | -6439 | L1MA8 | L1 | LINE |

  

|  |  |  |  |  |  |  |  |  |  |
| --- | --- | --- | --- | --- | --- | --- | --- | --- | --- |
| chr6:58715350-58720065 | | | 4708 bp | | |  |  |  |  |
| Genome Start | Genome End | Strand | Div | Repeat Start | Repeat End | Repeat Left | Name | Family | Class |
| 58715350 | 58715941 | + | 17.4 | 2477 | 3095 | -3045 | L1M3 | L1 | LINE |
| 58715942 | 58716320 | + | 15.6 | 1 | 375 | 0 | THE1C | MaLR | LTR |
| 58716321 | 58717591 | + | 13.2 | 1 | 1312 | -268 | THE1C-int | MaLR | LTR |
| 58717592 | 58718065 | - | 13.7 | 1 | 481 | 0 | LTR21A | ERV1 | LTR |
| 58718066 | 58718357 | + | 13.2 | 1313 | 1580 | 0 | THE1C-int | MaLR | LTR |
| 58718358 | 58718734 | + | 14.2 | 1 | 375 | 0 | THE1C | MaLR | LTR |
| 58718743 | 58720065 | + | 18.8 | 3103 | 4488 | -1652 | L1M3 | L1 | LINE |

  

|  |  |  |  |  |  |  |  |  |  |
| --- | --- | --- | --- | --- | --- | --- | --- | --- | --- |
| chr6:58801726-58811597 | | | 9842 bp | | |  |  |  |  |
| Genome Start | Genome End | Strand | Div | Repeat Start | Repeat End | Repeat Left | Name | Family | Class |
| 58801726 | 58802293 | + | 26.3 | 466 | 1050 | -6439 | L1MA8 | L1 | LINE |
| 58802317 | 58804208 | + | 24.7 | 2008 | 3923 | -3639 | L1MA8 | L1 | LINE |
| 58804209 | 58804518 | + | 13.2 | 1 | 311 | 0 | AluY | Alu | SINE |
| 58804519 | 58805832 | + | 17.1 | 3924 | 5122 | -2440 | L1MA8 | L1 | LINE |
| 58805833 | 58806123 | + | 18.8 | 1 | 299 | -13 | AluJb | Alu | SINE |
| 58806124 | 58806284 | + | 17.1 | 5123 | 5273 | -2289 | L1MA8 | L1 | LINE |
| 58806285 | 58806646 | - | 17.3 | 1 | 364 | 0 | THE1B | MaLR | LTR |
| 58806647 | 58807551 | + | 17.1 | 5274 | 6156 | -1406 | L1MA8 | L1 | LINE |
| 58807552 | 58807983 | - | 16.4 | 1 | 434 | 0 | Tigger2a | MER2\_type | DNA |
| 58807984 | 58808478 | + | 17.1 | 6157 | 6655 | -907 | L1MA8 | L1 | LINE |
| 58808479 | 58808900 | - | 18.5 | 5 | 432 | 0 | MSTB1 | MaLR | LTR |
| 58808905 | 58810437 | - | 27.2 | 2 | 1735 | 0 | MLT-int | MaLR | LTR |
| 58810441 | 58810863 | - | 18.3 | 1 | 432 | 0 | MSTB1 | MaLR | LTR |
| 58810864 | 58811597 | + | 16.8 | 6656 | 7395 | -315 | L1MA8 | L1 | LINE |

  

|  |  |  |  |  |  |  |  |  |  |
| --- | --- | --- | --- | --- | --- | --- | --- | --- | --- |
| chr6:62367202-62374243 | | | 7038 bp | | |  |  |  |  |
| Genome Start | Genome End | Strand | Div | Repeat Start | Repeat End | Repeat Left | Name | Family | Class |
| 62367202 | 62367974 | - | 23.5 | 157 | 915 | -5416 | LTR49-int | ERV1 | LTR |
| 62367995 | 62368049 | - | 23.5 | 101 | 156 | -6175 | LTR49-int | ERV1 | LTR |
| 62368050 | 62368498 | + | 9.4 | 1 | 448 | -2 | LTR7 | ERV1 | LTR |
| 62368501 | 62369096 | + | 18.7 | 1 | 560 | -7153 | HERVH | ERV1 | LTR |
| 62369085 | 62371365 | + | 11.5 | 756 | 2985 | -4728 | HERVH | ERV1 | LTR |
| 62371363 | 62373080 | + | 12.5 | 3504 | 5225 | -1866 | HERVH | ERV1 | LTR |
| 62373082 | 62373317 | + | 14.9 | 5600 | 5824 | -1889 | HERVH | ERV1 | LTR |
| 62373312 | 62373687 | + | 11.1 | 7324 | 7713 | 0 | HERVH | ERV1 | LTR |
| 62373688 | 62374136 | + | 9.2 | 1 | 448 | -2 | LTR7 | ERV1 | LTR |
| 62374139 | 62374243 | - | 12.4 | 1 | 105 | -6226 | LTR49-int | ERV1 | LTR |

  

|  |  |  |  |  |  |  |  |  |  |
| --- | --- | --- | --- | --- | --- | --- | --- | --- | --- |
| chr6:62627992-62641914 | | | 13709 bp | | |  |  |  |  |
| Genome Start | Genome End | Strand | Div | Repeat Start | Repeat End | Repeat Left | Name | Family | Class |
| 62627992 | 62628130 | + | 28.9 | 1 | 148 | -4706 | L1ME3B | L1 | LINE |
| 62628141 | 62633128 | - | 13.4 | 2591 | 7534 | -69 | L1PA12 | L1 | LINE |
| 62633129 | 62633478 | - | 13.6 | 1 | 364 | 0 | THE1B | MaLR | LTR |
| 62633480 | 62634968 | - | 28.7 | 2 | 1734 | -1 | MLT-int | MaLR | LTR |
| 62634971 | 62635306 | - | 14.1 | 1 | 364 | 0 | THE1B | MaLR | LTR |
| 62635307 | 62637134 | - | 20.3 | 24 | 2590 | -5013 | L1PA12 | L1 | LINE |
| 62637158 | 62637426 | + | 26.4 | 139 | 427 | -4427 | L1ME3B | L1 | LINE |
| 62637427 | 62639716 | + | 10.5 | 1 | 2315 | -275 | L1P3 | L1 | LINE |
| 62639717 | 62639968 | + | 10.7 | 5902 | 6153 | -1 | L1PA7 | L1 | LINE |
| 62639969 | 62640197 | + | 29.1 | 414 | 658 | -4196 | L1ME3B | L1 | LINE |
| 62640268 | 62640563 | + | 26.8 | 2961 | 3272 | -1562 | L1ME3B | L1 | LINE |
| 62640568 | 62641341 | + | 29.2 | 3336 | 4160 | -768 | L1ME3B | L1 | LINE |
| 62641446 | 62641914 | + | 27.7 | 4361 | 4911 | -17 | L1ME3B | L1 | LINE |

  

|  |  |  |  |  |  |  |  |  |  |
| --- | --- | --- | --- | --- | --- | --- | --- | --- | --- |
| chr6:62900817-62904150 | | | 3292 bp | | |  |  |  |  |
| Genome Start | Genome End | Strand | Div | Repeat Start | Repeat End | Repeat Left | Name | Family | Class |
| 62900817 | 62900936 | - | 17.6 | 85 | 209 | -15 | MER58A | MER1\_type | DNA |
| 62900951 | 62901046 | + | 20.3 | 5461 | 5557 | -755 | L1MA9 | L1 | LINE |
| 62901047 | 62901404 | + | 13.6 | 1 | 364 | 0 | THE1B | MaLR | LTR |
| 62901407 | 62902945 | + | 29.7 | 2 | 1735 | 0 | MLT-int | MaLR | LTR |
| 62902946 | 62903301 | + | 11.9 | 1 | 364 | 0 | THE1B | MaLR | LTR |
| 62903302 | 62904060 | + | 20.3 | 5558 | 6274 | -38 | L1MA9 | L1 | LINE |
| 62904087 | 62904150 | - | 29.7 | 1 | 68 | -39 | MER58A | MER1\_type | DNA |

  

|  |  |  |  |  |  |  |  |  |  |
| --- | --- | --- | --- | --- | --- | --- | --- | --- | --- |
| chr6:63379180-63383392 | | | 3921 bp | | |  |  |  |  |
| Genome Start | Genome End | Strand | Div | Repeat Start | Repeat End | Repeat Left | Name | Family | Class |
| 63379180 | 63379329 | - | 25.4 | 1634 | 1792 | -5413 | MER50-int | ERV1 | LTR |
| 63379416 | 63379477 | - | 18.0 | 1555 | 1618 | -5587 | MER50-int | ERV1 | LTR |
| 63379682 | 63381034 | - | 28.9 | 124 | 1450 | -5755 | MER50-int | ERV1 | LTR |
| 63381035 | 63381355 | + | 15.3 | 1 | 364 | 0 | THE1B | MaLR | LTR |
| 63381358 | 63382929 | + | 30.2 | 2 | 1735 | 0 | MLT-int | MaLR | LTR |
| 63382930 | 63383273 | + | 16.6 | 1 | 364 | 0 | THE1B | MaLR | LTR |
| 63383274 | 63383392 | - | 28.9 | 3 | 123 | -7082 | MER50-int | ERV1 | LTR |

  

|  |  |  |  |  |  |  |  |  |  |
| --- | --- | --- | --- | --- | --- | --- | --- | --- | --- |
| chr6:63412715-63415653 | | | 2936 bp | | |  |  |  |  |
| Genome Start | Genome End | Strand | Div | Repeat Start | Repeat End | Repeat Left | Name | Family | Class |
| 63412715 | 63412970 | + | 19.0 | 34 | 296 | -431 | LTR8A | ERV1 | LTR |
| 63412971 | 63413326 | + | 15.0 | 1 | 364 | 0 | THE1B | MaLR | LTR |
| 63413330 | 63414886 | + | 31.2 | 3 | 1735 | 0 | MLT-int | MaLR | LTR |
| 63414887 | 63415244 | + | 13.0 | 1 | 364 | 0 | THE1B | MaLR | LTR |
| 63415245 | 63415653 | + | 19.0 | 297 | 720 | -7 | LTR8A | ERV1 | LTR |

  

|  |  |  |  |  |  |  |  |  |  |
| --- | --- | --- | --- | --- | --- | --- | --- | --- | --- |
| chr6:63559226-63571815 | | | 12578 bp | | |  |  |  |  |
| Genome Start | Genome End | Strand | Div | Repeat Start | Repeat End | Repeat Left | Name | Family | Class |
| 63559226 | 63559534 | - | 13.4 | 6585 | 6890 | 0 | L1PB1 | L1 | LINE |
| 63559535 | 63559889 | + | 9.0 | 1 | 448 | -2 | LTR7 | ERV1 | LTR |
| 63559892 | 63562787 | + | 12.2 | 1 | 2995 | -4718 | HERVH | ERV1 | LTR |
| 63562767 | 63562916 | + | 16.7 | 3140 | 3290 | -4423 | HERVH | ERV1 | LTR |
| 63562907 | 63563148 | + | 14.1 | 3492 | 3734 | -3979 | HERVH | ERV1 | LTR |
| 63563161 | 63564313 | + | 11.2 | 3994 | 5149 | -2564 | HERVH | ERV1 | LTR |
| 63564315 | 63564530 | + | 12.5 | 5600 | 5819 | -1894 | HERVH | ERV1 | LTR |
| 63564529 | 63564905 | + | 12.9 | 7322 | 7713 | 0 | HERVH | ERV1 | LTR |
| 63564906 | 63565314 | + | 9.1 | 1 | 448 | -2 | LTR7 | ERV1 | LTR |
| 63565316 | 63565391 | - | 11.8 | 6514 | 6589 | -278 | L1PB1 | L1 | LINE |
| 63565385 | 63566472 | - | 12.5 | 5129 | 6210 | -657 | L1PB1 | L1 | LINE |
| 63566509 | 63567988 | - | 13.2 | 3626 | 5128 | -1734 | L1PB1 | L1 | LINE |
| 63567989 | 63568290 | - | 15.0 | 1 | 301 | -11 | AluSx | Alu | SINE |
| 63568291 | 63571815 | - | 13.6 | 1 | 3625 | -3237 | L1PB1 | L1 | LINE |

  

|  |  |  |  |  |  |  |  |  |  |
| --- | --- | --- | --- | --- | --- | --- | --- | --- | --- |
| chr6:63756486-63760767 | | | 4205 bp | | |  |  |  |  |
| Genome Start | Genome End | Strand | Div | Repeat Start | Repeat End | Repeat Left | Name | Family | Class |
| 63756486 | 63756975 | - | 26.5 | 2426 | 2804 | -3733 | L1MDa | L1 | LINE |
| 63756994 | 63757377 | - | 9.6 | 5786 | 6149 | -2 | L1PB1 | L1 | LINE |
| 63757415 | 63757796 | - | 15.8 | 1 | 375 | 0 | THE1C | MaLR | LTR |
| 63757803 | 63758105 | - | 13.8 | 1275 | 1574 | -6 | THE1C-int | MaLR | LTR |
| 63758106 | 63758396 | - | 15.0 | 15 | 294 | -16 | AluSg | Alu | SINE |
| 63758397 | 63759613 | - | 13.8 | 1 | 1274 | -306 | THE1C-int | MaLR | LTR |
| 63759614 | 63759988 | - | 13.4 | 1 | 375 | 0 | THE1C | MaLR | LTR |
| 63759991 | 63760293 | - | 17.5 | 9 | 311 | -1 | AluSx | Alu | SINE |
| 63760308 | 63760767 | - | 28.0 | 1827 | 2398 | -4320 | L1MDa | L1 | LINE |

  

|  |  |  |  |  |  |  |  |  |  |
| --- | --- | --- | --- | --- | --- | --- | --- | --- | --- |
| chr6:63907162-63916353 | | | 9186 bp | | |  |  |  |  |
| Genome Start | Genome End | Strand | Div | Repeat Start | Repeat End | Repeat Left | Name | Family | Class |
| 63907162 | 63909686 | + | 18.6 | 17 | 2585 | -4980 | L1MA2 | L1 | LINE |
| 63909687 | 63909989 | + | 10.6 | 1 | 311 | 0 | AluY | Alu | SINE |
| 63909990 | 63912469 | + | 15.1 | 2586 | 5000 | -2565 | L1MA2 | L1 | LINE |
| 63912470 | 63912842 | + | 16.6 | 1 | 381 | 0 | THE1D | MaLR | LTR |
| 63912843 | 63914358 | + | 13.7 | 1 | 1574 | -6 | THE1D-int | MaLR | LTR |
| 63914364 | 63914739 | + | 12.7 | 1 | 379 | -2 | THE1D | MaLR | LTR |
| 63914741 | 63916353 | + | 10.9 | 5988 | 7671 | -52 | L1MA2 | L1 | LINE |

  

|  |  |  |  |  |  |  |  |  |  |
| --- | --- | --- | --- | --- | --- | --- | --- | --- | --- |
| chr6:63970199-63973541 | | | 3293 bp | | |  |  |  |  |
| Genome Start | Genome End | Strand | Div | Repeat Start | Repeat End | Repeat Left | Name | Family | Class |
| 63970199 | 63970613 | + | 31.0 | 2115 | 2542 | -877 | L2 | L2 | LINE |
| 63970614 | 63970956 | - | 15.1 | 1 | 364 | 0 | THE1B | MaLR | LTR |
| 63970957 | 63972502 | - | 10.2 | 31 | 1580 | 0 | THE1B-int | MaLR | LTR |
| 63972553 | 63972940 | - | 19.0 | 1 | 363 | -1 | THE1B | MaLR | LTR |
| 63972941 | 63973541 | + | 25.4 | 2538 | 3329 | -90 | L2 | L2 | LINE |

  

|  |  |  |  |  |  |  |  |  |  |
| --- | --- | --- | --- | --- | --- | --- | --- | --- | --- |
| chr6:64790240-64800261 | | | 10024 bp | | |  |  |  |  |
| Genome Start | Genome End | Strand | Div | Repeat Start | Repeat End | Repeat Left | Name | Family | Class |
| 64790240 | 64790389 | - | 19.9 | 6025 | 6179 | -1 | L1MB4 | L1 | LINE |
| 64790390 | 64790837 | + | 15.7 | 1 | 461 | 0 | MER65C | ERV1 | LTR |
| 64790838 | 64791460 | - | 20.8 | 5442 | 6025 | -156 | L1MB4 | L1 | LINE |
| 64791461 | 64792795 | + | 7.2 | 2 | 1578 | 0 | LTR12C | ERV1 | LTR |
| 64792796 | 64792849 | + | 14.8 | 1 | 54 | -8382 | HERV9 | ERV1 | LTR |
| 64792857 | 64797882 | + | 6.2 | 17 | 4963 | -3473 | HERV9 | ERV1 | LTR |
| 64797870 | 64798179 | + | 16.2 | 741 | 1051 | -4051 | HERV9 | ERV1 | LTR |
| 64798178 | 64798239 | + | 9.7 | 7541 | 7602 | -834 | HERV9 | ERV1 | LTR |
| 64798236 | 64798833 | + | 5.2 | 7827 | 8426 | -10 | HERV9 | ERV1 | LTR |
| 64798844 | 64800183 | + | 8.0 | 2 | 1578 | 0 | LTR12C | ERV1 | LTR |
| 64800184 | 64800261 | - | 14.1 | 5367 | 5445 | -733 | L1MB4 | L1 | LINE |

  

|  |  |  |  |  |  |  |  |  |  |
| --- | --- | --- | --- | --- | --- | --- | --- | --- | --- |
| chr6:65207452-65211017 | | | 3564 bp | | |  |  |  |  |
| Genome Start | Genome End | Strand | Div | Repeat Start | Repeat End | Repeat Left | Name | Family | Class |
| 65207452 | 65208189 | + | 14.7 | 4 | 772 | -530 | HSMAR2 | Mariner | DNA |
| 65208190 | 65208542 | + | 12.7 | 1 | 364 | 0 | THE1B | MaLR | LTR |
| 65208545 | 65210123 | + | 31.0 | 2 | 1735 | 0 | MLT-int | MaLR | LTR |
| 65210124 | 65210512 | + | 16.6 | 1 | 362 | -2 | THE1B | MaLR | LTR |
| 65210513 | 65211017 | + | 14.7 | 773 | 1301 | -1 | HSMAR2 | Mariner | DNA |

  

|  |  |  |  |  |  |  |  |  |  |
| --- | --- | --- | --- | --- | --- | --- | --- | --- | --- |
| chr6:65689568-65693409 | | | 3578 bp | | |  |  |  |  |
| Genome Start | Genome End | Strand | Div | Repeat Start | Repeat End | Repeat Left | Name | Family | Class |
| 65689568 | 65689715 | - | 26.5 | 1801 | 1974 | -5175 | L1PA17 | L1 | LINE |
| 65689717 | 65690069 | - | 11.1 | 1 | 355 | 0 | THE1A | MaLR | LTR |
| 65690070 | 65691636 | - | 8.9 | 1 | 1580 | 0 | THE1A-int | MaLR | LTR |
| 65691637 | 65691994 | - | 10.5 | 1 | 355 | 0 | THE1A | MaLR | LTR |
| 65692002 | 65692530 | - | 31.5 | 818 | 1502 | -5347 | L1M2 | L1 | LINE |
| 65692787 | 65693409 | - | 31.9 | 2 | 615 | -6238 | L1PA17 | L1 | LINE |

  

|  |  |  |  |  |  |  |  |  |  |
| --- | --- | --- | --- | --- | --- | --- | --- | --- | --- |
| chr6:65796070-65804391 | | | 8252 bp | | |  |  |  |  |
| Genome Start | Genome End | Strand | Div | Repeat Start | Repeat End | Repeat Left | Name | Family | Class |
| 65796070 | 65796134 | + | 23.1 | 4 | 69 | -2649 | Tigger2 | MER2\_type | DNA |
| 65796122 | 65796287 | + | 14.0 | 1533 | 1696 | -285 | Tigger2 | MER2\_type | DNA |
| 65796300 | 65797085 | + | 25.8 | 496 | 1294 | -5492 | L1PA15 | L1 | LINE |
| 65797092 | 65797344 | + | 31.9 | 1200 | 1460 | -5325 | L1PA15 | L1 | LINE |
| 65797360 | 65797772 | + | 15.7 | 2696 | 3078 | -3688 | L1PA15 | L1 | LINE |
| 65797817 | 65798937 | + | 17.4 | 3079 | 4193 | -2573 | L1PA15 | L1 | LINE |
| 65798938 | 65799354 | + | 20.2 | 1 | 424 | -4 | MSTA | MaLR | LTR |
| 65799361 | 65800990 | + | 28.2 | 4 | 1735 | 0 | MLT-int | MaLR | LTR |
| 65800991 | 65801403 | + | 20.6 | 1 | 427 | -1 | MSTA | MaLR | LTR |
| 65801404 | 65804071 | + | 16.8 | 4194 | 6756 | -29 | L1PA15 | L1 | LINE |
| 65804072 | 65804391 | + | 19.4 | 1652 | 2707 | -1 | Tigger2 | MER2\_type | DNA |

  

|  |  |  |  |  |  |  |  |  |  |
| --- | --- | --- | --- | --- | --- | --- | --- | --- | --- |
| chr6:65853921-65857224 | | | 2941 bp | | |  |  |  |  |
| Genome Start | Genome End | Strand | Div | Repeat Start | Repeat End | Repeat Left | Name | Family | Class |
| 65853921 | 65854188 | + | 30.4 | 1964 | 2245 | -1174 | L2 | L2 | LINE |
| 65854430 | 65854806 | + | 19.2 | 1 | 381 | 0 | THE1D | MaLR | LTR |
| 65854807 | 65856282 | + | 13.7 | 1 | 1507 | -73 | THE1D-int | MaLR | LTR |
| 65856283 | 65856574 | + | 16.1 | 1 | 299 | -13 | AluSx | Alu | SINE |
| 65856637 | 65856721 | + | 13.7 | 1508 | 1580 | 0 | THE1D-int | MaLR | LTR |
| 65856722 | 65857097 | + | 14.8 | 1 | 381 | 0 | THE1D | MaLR | LTR |
| 65857158 | 65857224 | + | 32.8 | 3352 | 3418 | -1 | L2 | L2 | LINE |

  

|  |  |  |  |  |  |  |  |  |  |
| --- | --- | --- | --- | --- | --- | --- | --- | --- | --- |
| chr6:67013787-67023803 | | | 9760 bp | | |  |  |  |  |
| Genome Start | Genome End | Strand | Div | Repeat Start | Repeat End | Repeat Left | Name | Family | Class |
| 67013787 | 67014704 | + | 14.2 | 2875 | 3801 | -3764 | L1MA3 | L1 | LINE |
| 67014699 | 67017608 | + | 7.6 | 3212 | 6154 | 0 | L1PA6 | L1 | LINE |
| 67017603 | 67018849 | + | 16.5 | 3795 | 5064 | -2501 | L1MA3 | L1 | LINE |
| 67018850 | 67019211 | - | 16.3 | 6 | 364 | 0 | THE1B | MaLR | LTR |
| 67019210 | 67020199 | - | 12.7 | 521 | 1508 | -72 | THE1B-int | MaLR | LTR |
| 67020197 | 67020557 | - | 13.3 | 2 | 364 | -1216 | THE1B-int | MaLR | LTR |
| 67020562 | 67020925 | - | 16.8 | 1 | 361 | -3 | THE1B | MaLR | LTR |
| 67020929 | 67022104 | + | 15.6 | 5063 | 6249 | -1316 | L1MA3 | L1 | LINE |
| 67022372 | 67023803 | + | 14.2 | 6251 | 7719 | -5 | L1MA3 | L1 | LINE |

  

|  |  |  |  |  |  |  |  |  |  |
| --- | --- | --- | --- | --- | --- | --- | --- | --- | --- |
| chr6:67024429-67032680 | | | 8125 bp | | |  |  |  |  |
| Genome Start | Genome End | Strand | Div | Repeat Start | Repeat End | Repeat Left | Name | Family | Class |
| 67024429 | 67024515 | - | 27.6 | 4763 | 4851 | -1295 | L1MEc | L1 | LINE |
| 67024527 | 67025039 | - | 27.2 | 4019 | 4559 | -1587 | L1MEc | L1 | LINE |
| 67025073 | 67025246 | - | 16.1 | 113 | 286 | -26 | AluSg/x | Alu | SINE |
| 67025259 | 67025796 | + | 10.7 | 1 | 511 | 0 | LTR10F | ERV1 | LTR |
| 67025797 | 67026048 | + | 11.9 | 1 | 271 | -7466 | HERVIP10F | ERV1 | LTR |
| 67026118 | 67030971 | + | 8.1 | 288 | 5102 | 0 | HERVIP10F | ERV1 | LTR |
| 67030972 | 67031471 | + | 10.5 | 1 | 509 | -2 | LTR10F | ERV1 | LTR |
| 67031474 | 67032680 | - | 26.0 | 2110 | 3343 | -2803 | L1MEc | L1 | LINE |

  

|  |  |  |  |  |  |  |  |  |  |
| --- | --- | --- | --- | --- | --- | --- | --- | --- | --- |
| chr6:67049732-67053473 | | | 3726 bp | | |  |  |  |  |
| Genome Start | Genome End | Strand | Div | Repeat Start | Repeat End | Repeat Left | Name | Family | Class |
| 67049732 | 67050277 | - | 21.9 | 7220 | 7756 | -29 | L1MC3 | L1 | LINE |
| 67050278 | 67050592 | + | 13.5 | 1 | 316 | 0 | AluSx | Alu | SINE |
| 67050593 | 67050860 | - | 21.9 | 6919 | 7219 | -566 | L1MC3 | L1 | LINE |
| 67050861 | 67051234 | + | 14.6 | 1 | 375 | 0 | THE1C | MaLR | LTR |
| 67051235 | 67052724 | + | 14.2 | 1 | 1579 | -1 | THE1C-int | MaLR | LTR |
| 67052726 | 67053097 | + | 12.7 | 1 | 371 | -4 | THE1C | MaLR | LTR |
| 67053113 | 67053473 | - | 24.7 | 6577 | 6929 | -856 | L1MC3 | L1 | LINE |

  

|  |  |  |  |  |  |  |  |  |  |
| --- | --- | --- | --- | --- | --- | --- | --- | --- | --- |
| chr6:67834231-67839751 | | | 4885 bp | | |  |  |  |  |
| Genome Start | Genome End | Strand | Div | Repeat Start | Repeat End | Repeat Left | Name | Family | Class |
| 67834231 | 67834325 | - | 21.1 | 4235 | 4333 | -2673 | L1M4c | L1 | LINE |
| 67834721 | 67835071 | + | 15.4 | 7 | 364 | 0 | THE1B | MaLR | LTR |
| 67835074 | 67836619 | + | 30.9 | 2 | 1735 | 0 | MLT-int | MaLR | LTR |
| 67836620 | 67836974 | + | 17.9 | 1 | 352 | -12 | THE1B | MaLR | LTR |
| 67837046 | 67838428 | - | 24.9 | 2660 | 4188 | -2818 | L1M4c | L1 | LINE |
| 67838560 | 67838884 | - | 25.7 | 2112 | 2473 | -4861 | L1M4c | L1 | LINE |
| 67838887 | 67839188 | - | 12.9 | 1 | 303 | -10 | AluSq | Alu | SINE |
| 67839210 | 67839435 | - | 22.2 | 6050 | 6278 | -22 | L1MA6 | L1 | LINE |
| 67839450 | 67839751 | - | 30.2 | 368 | 673 | -6231 | L1M4c | L1 | LINE |

  

|  |  |  |  |  |  |  |  |  |  |
| --- | --- | --- | --- | --- | --- | --- | --- | --- | --- |
| chr6:68070856-68079926 | | | 9065 bp | | |  |  |  |  |
| Genome Start | Genome End | Strand | Div | Repeat Start | Repeat End | Repeat Left | Name | Family | Class |
| 68070856 | 68073657 | - | 23.8 | 5579 | 8392 | -109 | L1PB3 | L1 | LINE |
| 68073658 | 68074020 | + | 16.6 | 1 | 381 | 0 | THE1D | MaLR | LTR |
| 68074027 | 68074537 | + | 16.6 | 7 | 474 | -1106 | THE1D-int | MaLR | LTR |
| 68074538 | 68074882 | + | 6.7 | 5809 | 6154 | 0 | L1PA7 | L1 | LINE |
| 68074883 | 68075977 | + | 16.6 | 475 | 1580 | 0 | THE1D-int | MaLR | LTR |
| 68075978 | 68076360 | + | 14.8 | 1 | 381 | 0 | THE1D | MaLR | LTR |
| 68076361 | 68079926 | - | 20.4 | 1931 | 5583 | -1279 | L1PB3 | L1 | LINE |

  

|  |  |  |  |  |  |  |  |  |  |
| --- | --- | --- | --- | --- | --- | --- | --- | --- | --- |
| chr6:68399497-68402986 | | | 3384 bp | | |  |  |  |  |
| Genome Start | Genome End | Strand | Div | Repeat Start | Repeat End | Repeat Left | Name | Family | Class |
| 68399497 | 68399613 | + | 23.1 | 5203 | 5338 | -808 | L1MD2 | L1 | LINE |
| 68399690 | 68399871 | + | 18.4 | 1 | 210 | -218 | MSTA | MaLR | LTR |
| 68399895 | 68400081 | + | 18.4 | 211 | 427 | -1 | MSTA | MaLR | LTR |
| 68400084 | 68401738 | + | 18.6 | 3 | 1567 | -6 | MSTA-int | MaLR | LTR |
| 68401744 | 68402123 | + | 17.0 | 1 | 428 | 0 | MSTA | MaLR | LTR |
| 68402124 | 68402986 | + | 22.4 | 5447 | 6358 | -1 | L1MD2 | L1 | LINE |

  

|  |  |  |  |  |  |  |  |  |  |
| --- | --- | --- | --- | --- | --- | --- | --- | --- | --- |
| chr6:69353096-69365927 | | | 12442 bp | | |  |  |  |  |
| Genome Start | Genome End | Strand | Div | Repeat Start | Repeat End | Repeat Left | Name | Family | Class |
| 69353096 | 69354223 | + | 17.4 | 3210 | 4423 | -1720 | L1MA7 | L1 | LINE |
| 69354225 | 69354720 | + | 18.2 | 5148 | 5646 | -645 | L1MA7 | L1 | LINE |
| 69354721 | 69355002 | - | 18.9 | 58 | 345 | 0 | MER2 | MER2\_type | DNA |
| 69355003 | 69356267 | - | 15.8 | 4894 | 6161 | -4 | L1PA15 | L1 | LINE |
| 69356268 | 69356619 | - | 13.7 | 6 | 375 | 0 | THE1C | MaLR | LTR |
| 69356625 | 69358199 | - | 30.4 | 2 | 1735 | 0 | MLT-int | MaLR | LTR |
| 69358203 | 69358573 | - | 15.1 | 1 | 374 | -1 | THE1C | MaLR | LTR |
| 69358574 | 69361253 | - | 16.4 | 2423 | 4898 | -1248 | L1PA15 | L1 | LINE |
| 69361249 | 69361920 | - | 6.5 | 5490 | 6154 | 0 | L1PA7 | L1 | LINE |
| 69361921 | 69362214 | - | 8.2 | 1 | 305 | -6 | AluY | Alu | SINE |
| 69362215 | 69363040 | - | 9.0 | 4665 | 5489 | -665 | L1PA7 | L1 | LINE |
| 69363041 | 69363106 | + | 3.0 | 4590 | 4655 | -1491 | L1PA7 | L1 | LINE |
| 69363106 | 69364804 | - | 18.2 | 1340 | 3058 | -3708 | L1PA13 | L1 | LINE |
| 69365190 | 69365239 | - | 20.0 | 20 | 69 | -276 | MER2 | MER2\_type | DNA |
| 69365239 | 69365295 | - | 28.1 | 2 | 58 | -287 | MER2 | MER2\_type | DNA |
| 69365299 | 69365927 | + | 21.2 | 5650 | 6285 | -6 | L1MA7 | L1 | LINE |

  

|  |  |  |  |  |  |  |  |  |  |
| --- | --- | --- | --- | --- | --- | --- | --- | --- | --- |
| chr6:69622354-69625224 | | | 2846 bp | | |  |  |  |  |
| Genome Start | Genome End | Strand | Div | Repeat Start | Repeat End | Repeat Left | Name | Family | Class |
| 69622354 | 69622475 | + | 15.8 | 5 | 124 | -188 | AluJ | Alu | SINE |
| 69622497 | 69622850 | + | 13.7 | 1 | 364 | 0 | THE1B | MaLR | LTR |
| 69622851 | 69624401 | + | 11.1 | 1 | 1578 | -2 | THE1B-int | MaLR | LTR |
| 69624406 | 69624764 | + | 14.5 | 3 | 364 | 0 | THE1B | MaLR | LTR |
| 69624765 | 69624835 | + | 22.5 | 132 | 201 | -111 | AluJ | Alu | SINE |
| 69624836 | 69625127 | + | 13.1 | 1 | 296 | -14 | AluSg | Alu | SINE |
| 69625128 | 69625224 | + | 22.5 | 202 | 296 | -16 | AluJ | Alu | SINE |

  

|  |  |  |  |  |  |  |  |  |  |
| --- | --- | --- | --- | --- | --- | --- | --- | --- | --- |
| chr6:71106825-71115042 | | | 8207 bp | | |  |  |  |  |
| Genome Start | Genome End | Strand | Div | Repeat Start | Repeat End | Repeat Left | Name | Family | Class |
| 71106825 | 71106990 | - | 11.7 | 6522 | 6686 | -1455 | L1PREC2 | L1 | LINE |
| 71106991 | 71107366 | - | 13.9 | 1 | 375 | 0 | THE1C | MaLR | LTR |
| 71107374 | 71108887 | - | 11.6 | 1 | 1573 | -7 | THE1C-int | MaLR | LTR |
| 71108888 | 71109266 | - | 13.7 | 1 | 375 | 0 | THE1C | MaLR | LTR |
| 71109267 | 71114167 | - | 14.9 | 1565 | 6526 | -1615 | L1PREC2 | L1 | LINE |
| 71114172 | 71114627 | + | 7.4 | 1 | 444 | -223 | LTR12B | ERV1 | LTR |
| 71114628 | 71115042 | - | 26.3 | 1143 | 1553 | -6602 | L1PREC2 | L1 | LINE |

  

|  |  |  |  |  |  |  |  |  |  |
| --- | --- | --- | --- | --- | --- | --- | --- | --- | --- |
| chr6:71485867-71488517 | | | 2649 bp | | |  |  |  |  |
| Genome Start | Genome End | Strand | Div | Repeat Start | Repeat End | Repeat Left | Name | Family | Class |
| 71485867 | 71486003 | - | 22.1 | 7830 | 7970 | -72 | L1MC4 | L1 | LINE |
| 71486004 | 71486356 | - | 9.9 | 1 | 355 | 0 | THE1A | MaLR | LTR |
| 71486357 | 71488046 | - | 29.6 | 2 | 1735 | 0 | MLT-int | MaLR | LTR |
| 71488049 | 71488410 | - | 9.0 | 1 | 355 | 0 | THE1A | MaLR | LTR |
| 71488411 | 71488517 | - | 22.1 | 7698 | 7829 | -213 | L1MC4 | L1 | LINE |

  

|  |  |  |  |  |  |  |  |  |  |
| --- | --- | --- | --- | --- | --- | --- | --- | --- | --- |
| chr6:72318713-72327355 | | | 8425 bp | | |  |  |  |  |
| Genome Start | Genome End | Strand | Div | Repeat Start | Repeat End | Repeat Left | Name | Family | Class |
| 72318713 | 72319256 | - | 15.5 | 3045 | 3610 | -3595 | L1M2c | L1 | LINE |
| 72319246 | 72320335 | - | 13.4 | 3492 | 4572 | -2290 | L1PBa | L1 | LINE |
| 72320336 | 72320652 | - | 10.9 | 1 | 305 | -7 | AluSx | Alu | SINE |
| 72320653 | 72321439 | - | 13.5 | 2688 | 3491 | -3371 | L1PBa | L1 | LINE |
| 72321441 | 72321796 | + | 16.2 | 1 | 375 | 0 | THE1C | MaLR | LTR |
| 72321798 | 72323470 | + | 13.4 | 2 | 1734 | -1 | THE1C-int | MaLR | LTR |
| 72323472 | 72323822 | + | 14.1 | 1 | 375 | 0 | THE1C | MaLR | LTR |
| 72323823 | 72323966 | - | 13.4 | 2546 | 2691 | -4172 | L1PBa | L1 | LINE |
| 72323963 | 72324801 | - | 12.9 | 1259 | 2110 | -4753 | L1PBa | L1 | LINE |
| 72324802 | 72325097 | + | 13.5 | 1 | 298 | -14 | AluSx | Alu | SINE |
| 72325098 | 72325246 | - | 12.9 | 1110 | 1258 | -5605 | L1PBa | L1 | LINE |
| 72325288 | 72325361 | - | 20.3 | 389 | 463 | -95 | MER74A | ERVL | LTR |
| 72325533 | 72326614 | - | 21.9 | 1748 | 2750 | -5101 | L1M2c | L1 | LINE |
| 72326633 | 72327355 | - | 30.7 | 1 | 638 | -6571 | L1M2c | L1 | LINE |

  

|  |  |  |  |  |  |  |  |  |  |
| --- | --- | --- | --- | --- | --- | --- | --- | --- | --- |
| chr6:72390480-72393646 | | | 3112 bp | | |  |  |  |  |
| Genome Start | Genome End | Strand | Div | Repeat Start | Repeat End | Repeat Left | Name | Family | Class |
| 72390480 | 72390580 | + | 25.0 | 1943 | 2044 | -1375 | L2 | L2 | LINE |
| 72390601 | 72390661 | + | 36.2 | 2331 | 2397 | -1022 | L2 | L2 | LINE |
| 72390662 | 72391037 | + | 9.6 | 1 | 375 | 0 | THE1C | MaLR | LTR |
| 72391040 | 72392637 | + | 29.6 | 2 | 1735 | 0 | MLT-int | MaLR | LTR |
| 72392638 | 72393006 | + | 12.3 | 1 | 375 | 0 | THE1C | MaLR | LTR |
| 72393007 | 72393485 | + | 36.2 | 2398 | 2961 | -458 | L2 | L2 | LINE |
| 72393519 | 72393646 | + | 33.3 | 2999 | 3152 | -226 | L2 | L2 | LINE |

  

|  |  |  |  |  |  |  |  |  |  |
| --- | --- | --- | --- | --- | --- | --- | --- | --- | --- |
| chr6:72410065-72414957 | | | 4458 bp | | |  |  |  |  |
| Genome Start | Genome End | Strand | Div | Repeat Start | Repeat End | Repeat Left | Name | Family | Class |
| 72410065 | 72410268 | - | 22.5 | 4587 | 4807 | -1837 | L1MEb | L1 | LINE |
| 72410260 | 72410568 | - | 28.4 | 2973 | 3291 | -3353 | L1MEb | L1 | LINE |
| 72410579 | 72410621 | - | 4.7 | 6246 | 6291 | 0 | L1MA7 | L1 | LINE |
| 72410645 | 72410957 | - | 14.6 | 5966 | 6264 | -27 | L1MA7 | L1 | LINE |
| 72410958 | 72411267 | - | 5.9 | 1 | 306 | -5 | AluY | Alu | SINE |
| 72411268 | 72411626 | - | 14.6 | 5647 | 5965 | -326 | L1MA7 | L1 | LINE |
| 72411628 | 72411828 | - | 18.9 | 194 | 396 | 0 | MSTD | MaLR | LTR |
| 72412178 | 72412259 | - | 27.2 | 1 | 81 | -315 | MSTD | MaLR | LTR |
| 72412260 | 72413335 | - | 23.9 | 646 | 1734 | -1 | MLT-int | MaLR | LTR |
| 72413336 | 72413463 | - | 17.2 | 265 | 394 | -2 | MSTD | MaLR | LTR |
| 72413474 | 72414393 | + | 14.9 | 5214 | 6156 | 0 | L1PB4 | L1 | LINE |
| 72414407 | 72414697 | - | 33.7 | 1714 | 2009 | -4731 | L1MEb | L1 | LINE |
| 72414736 | 72414957 | - | 29.8 | 1389 | 1614 | -4928 | L1MEb | L1 | LINE |

  

|  |  |  |  |  |  |  |  |  |  |
| --- | --- | --- | --- | --- | --- | --- | --- | --- | --- |
| chr6:74655366-74661507 | | | 6074 bp | | |  |  |  |  |
| Genome Start | Genome End | Strand | Div | Repeat Start | Repeat End | Repeat Left | Name | Family | Class |
| 74655366 | 74656906 | - | 15.5 | 6144 | 7715 | -4 | L1MA6 | L1 | LINE |
| 74656912 | 74657292 | + | 17.2 | 1 | 428 | 0 | MSTA | MaLR | LTR |
| 74657321 | 74658935 | + | 14.3 | 19 | 1580 | 0 | MSTA-int | MaLR | LTR |
| 74658936 | 74659309 | + | 16.2 | 1 | 428 | 0 | MSTA | MaLR | LTR |
| 74659310 | 74660167 | - | 14.5 | 5282 | 6143 | -1419 | L1MA6 | L1 | LINE |
| 74660155 | 74660459 | - | 17.0 | 4037 | 4340 | -3222 | L1MA6 | L1 | LINE |
| 74660460 | 74660759 | + | 12.5 | 1 | 284 | -29 | AluSq | Alu | SINE |
| 74660808 | 74660822 | + | 12.5 | 285 | 296 | -17 | AluSq | Alu | SINE |
| 74660823 | 74661507 | - | 17.3 | 3350 | 4036 | -3526 | L1MA6 | L1 | LINE |

  

|  |  |  |  |  |  |  |  |  |  |
| --- | --- | --- | --- | --- | --- | --- | --- | --- | --- |
| chr6:75565151-75568927 | | | 3765 bp | | |  |  |  |  |
| Genome Start | Genome End | Strand | Div | Repeat Start | Repeat End | Repeat Left | Name | Family | Class |
| 75565151 | 75565220 | + | 23.9 | 2857 | 2925 | -494 | L2 | L2 | LINE |
| 75565221 | 75565395 | + | 17.6 | 1 | 189 | -192 | THE1D | MaLR | LTR |
| 75565396 | 75565536 | + | 5.8 | 1 | 140 | -171 | AluY | Alu | SINE |
| 75565537 | 75565841 | + | 2.3 | 1 | 305 | -5 | AluYa5 | Alu | SINE |
| 75565842 | 75566004 | + | 5.8 | 141 | 297 | -14 | AluY | Alu | SINE |
| 75566005 | 75566201 | + | 17.6 | 190 | 381 | 0 | THE1D | MaLR | LTR |
| 75566205 | 75567139 | + | 13.3 | 4 | 906 | -674 | THE1D-int | MaLR | LTR |
| 75567140 | 75567440 | + | 13.0 | 1 | 304 | -8 | AluSx | Alu | SINE |
| 75567441 | 75568112 | + | 13.3 | 907 | 1573 | -7 | THE1D-int | MaLR | LTR |
| 75568118 | 75568484 | + | 15.1 | 1 | 381 | 0 | THE1D | MaLR | LTR |
| 75568489 | 75568927 | + | 36.7 | 2954 | 3415 | -4 | L2 | L2 | LINE |

  

|  |  |  |  |  |  |  |  |  |  |
| --- | --- | --- | --- | --- | --- | --- | --- | --- | --- |
| chr6:75824160-75826792 | | | 2630 bp | | |  |  |  |  |
| Genome Start | Genome End | Strand | Div | Repeat Start | Repeat End | Repeat Left | Name | Family | Class |
| 75824160 | 75824441 | + | 22.1 | 6902 | 7180 | -862 | L1MC4 | L1 | LINE |
| 75824442 | 75824783 | - | 10.4 | 1 | 377 | -4 | THE1D | MaLR | LTR |
| 75824785 | 75826364 | - | 29.8 | 2 | 1734 | -1 | MLT-int | MaLR | LTR |
| 75826367 | 75826730 | - | 13.0 | 1 | 381 | 0 | THE1D | MaLR | LTR |
| 75826731 | 75826792 | + | 22.1 | 7181 | 7236 | -806 | L1MC4 | L1 | LINE |

  

|  |  |  |  |  |  |  |  |  |  |
| --- | --- | --- | --- | --- | --- | --- | --- | --- | --- |
| chr6:76847630-76851091 | | | 3349 bp | | |  |  |  |  |
| Genome Start | Genome End | Strand | Div | Repeat Start | Repeat End | Repeat Left | Name | Family | Class |
| 76847630 | 76847799 | - | 26.6 | 5749 | 5951 | -289 | L1ME3B | L1 | LINE |
| 76847803 | 76847947 | - | 24.3 | 5415 | 5562 | -678 | L1ME3B | L1 | LINE |
| 76847951 | 76848022 | - | 17.3 | 316 | 374 | -1 | THE1C | MaLR | LTR |
| 76848023 | 76848323 | - | 12.6 | 2 | 302 | -8 | AluSg | Alu | SINE |
| 76848324 | 76848638 | - | 17.3 | 1 | 315 | -60 | THE1C | MaLR | LTR |
| 76848641 | 76850174 | - | 13.7 | 1 | 1568 | -12 | THE1C-int | MaLR | LTR |
| 76850175 | 76850562 | - | 12.5 | 1 | 375 | 0 | THE1C | MaLR | LTR |
| 76850563 | 76850658 | - | 28.1 | 5320 | 5416 | -735 | L1P | L1 | LINE |
| 76850764 | 76851091 | - | 28.8 | 4473 | 4843 | -1303 | L1ME3B | L1 | LINE |

  

|  |  |  |  |  |  |  |  |  |  |
| --- | --- | --- | --- | --- | --- | --- | --- | --- | --- |
| chr6:77241878-77265059 | | | 23061 bp | | |  |  |  |  |
| Genome Start | Genome End | Strand | Div | Repeat Start | Repeat End | Repeat Left | Name | Family | Class |
| 77241878 | 77242405 | + | 27.4 | 414 | 950 | -1994 | BLACKJACK | AcHobo | DNA |
| 77242411 | 77245982 | + | 26.5 | 1 | 3552 | -3296 | L1M2 | L1 | LINE |
| 77245983 | 77246292 | + | 1.9 | 1 | 310 | -1 | AluY | Alu | SINE |
| 77246293 | 77247486 | + | 18.4 | 3553 | 4718 | -2130 | L1M2 | L1 | LINE |
| 77247487 | 77248039 | - | 12.4 | 5 | 489 | -3 | LTR22B | ERVK | LTR |
| 77248040 | 77248768 | + | 17.2 | 4719 | 5510 | -1343 | L1PB2 | L1 | LINE |
| 77248769 | 77248840 | - | 11.3 | 1 | 67 | -37 | MADE1 | Mariner | DNA |
| 77248841 | 77249842 | + | 17.2 | 5511 | 6599 | -254 | L1PB2 | L1 | LINE |
| 77249835 | 77251007 | + | 11.5 | 4976 | 6158 | -2 | L1PREC2 | L1 | LINE |
| 77251013 | 77251272 | + | 21.2 | 5891 | 6153 | -3 | L1PB4 | L1 | LINE |
| 77251275 | 77251630 | + | 23.2 | 941 | 1295 | -1649 | BLACKJACK | AcHobo | DNA |
| 77251652 | 77251811 | - | 25.7 | 306 | 517 | -14 | LTR45B | ERV1 | LTR |
| 77251815 | 77251904 | - | 11.4 | 1179 | 1266 | 0 | MER11A | ERVK | LTR |
| 77251905 | 77253776 | - | 4.6 | 3580 | 5456 | -699 | L1P2 | L1 | LINE |
| 77253777 | 77254346 | - | 11.5 | 1 | 581 | -655 | MER11B | ERVK | LTR |
| 77254347 | 77255342 | - | 7.2 | 6954 | 7953 | 0 | HERVK11 | ERVK | LTR |
| 77255325 | 77258472 | - | 7.9 | 3568 | 6764 | -1189 | HERVK11 | ERVK | LTR |
| 77258473 | 77258538 | - | 28.8 | 69 | 138 | -130 | MIRb | MIR | SINE |
| 77258539 | 77262049 | - | 7.9 | 3 | 3567 | -4386 | HERVK11 | ERVK | LTR |
| 77262055 | 77263134 | - | 11.2 | 1 | 1232 | -4 | MER11B | ERVK | LTR |
| 77263135 | 77263432 | - | 18.7 | 3 | 307 | -182 | LTR45B | ERV1 | LTR |
| 77263482 | 77264841 | + | 23.1 | 1313 | 2806 | -138 | BLACKJACK | AcHobo | DNA |
| 77264899 | 77265059 | + | 28.0 | 2787 | 2940 | -4 | BLACKJACK | AcHobo | DNA |

  

|  |  |  |  |  |  |  |  |  |  |
| --- | --- | --- | --- | --- | --- | --- | --- | --- | --- |
| chr6:77526183-77529022 | | | 2592 bp | | |  |  |  |  |
| Genome Start | Genome End | Strand | Div | Repeat Start | Repeat End | Repeat Left | Name | Family | Class |
| 77526183 | 77526360 | + | 27.5 | 1433 | 1624 | -883 | HAL1 | L1 | LINE |
| 77526361 | 77526739 | - | 17.1 | 11 | 396 | 0 | MSTD | MaLR | LTR |
| 77526756 | 77527531 | - | 19.0 | 3 | 812 | -51 | MER21B | ERV1 | LTR |
| 77527752 | 77528130 | - | 12.7 | 1 | 375 | 0 | THE1C | MaLR | LTR |
| 77528131 | 77528570 | - | 7.8 | 1142 | 1580 | 0 | THE1C-int | MaLR | LTR |
| 77528582 | 77528950 | - | 14.3 | 1 | 375 | 0 | THE1C | MaLR | LTR |
| 77528952 | 77529022 | + | 17.1 | 1846 | 1917 | -590 | HAL1 | L1 | LINE |

  

|  |  |  |  |  |  |  |  |  |  |
| --- | --- | --- | --- | --- | --- | --- | --- | --- | --- |
| chr6:77724867-77736548 | | | 11647 bp | | |  |  |  |  |
| Genome Start | Genome End | Strand | Div | Repeat Start | Repeat End | Repeat Left | Name | Family | Class |
| 77724867 | 77725400 | + | 19.0 | 2204 | 2744 | -3945 | L1PA14 | L1 | LINE |
| 77725402 | 77731461 | + | 3.2 | 5 | 6058 | 0 | L1PA3 | L1 | LINE |
| 77731462 | 77732009 | + | 19.1 | 2729 | 3280 | -3393 | L1PA14 | L1 | LINE |
| 77732042 | 77732391 | + | 12.0 | 7 | 364 | 0 | THE1B | MaLR | LTR |
| 77732394 | 77733974 | + | 30.1 | 2 | 1735 | 0 | MLT-int | MaLR | LTR |
| 77733975 | 77734330 | + | 13.5 | 1 | 364 | 0 | THE1B | MaLR | LTR |
| 77734331 | 77736548 | + | 15.8 | 3302 | 5420 | -1253 | L1PA14 | L1 | LINE |

  

|  |  |  |  |  |  |  |  |  |  |
| --- | --- | --- | --- | --- | --- | --- | --- | --- | --- |
| chr6:77834829-77838987 | | | 4155 bp | | |  |  |  |  |
| Genome Start | Genome End | Strand | Div | Repeat Start | Repeat End | Repeat Left | Name | Family | Class |
| 77834829 | 77835051 | - | 23.1 | 5237 | 5473 | -886 | L1MD | L1 | LINE |
| 77835052 | 77835419 | - | 11.9 | 1 | 381 | 0 | THE1D | MaLR | LTR |
| 77835420 | 77836918 | - | 16.8 | 3 | 1580 | 0 | THE1D-int | MaLR | LTR |
| 77836921 | 77837293 | - | 13.3 | 1 | 381 | 0 | THE1D | MaLR | LTR |
| 77837296 | 77837504 | - | 26.8 | 5058 | 5239 | -907 | L1MD | L1 | LINE |
| 77837505 | 77837791 | - | 12.3 | 1 | 285 | -24 | AluSc | Alu | SINE |
| 77837792 | 77838076 | - | 26.8 | 4785 | 5057 | -1089 | L1MD | L1 | LINE |
| 77838077 | 77838197 | - | 24.8 | 1 | 122 | -11 | FLAM\_A | Alu | SINE |
| 77838198 | 77838987 | - | 26.8 | 3989 | 4784 | -1362 | L1MD | L1 | LINE |

  

|  |  |  |  |  |  |  |  |  |  |
| --- | --- | --- | --- | --- | --- | --- | --- | --- | --- |
| chr6:78472390-78475491 | | | 3098 bp | | |  |  |  |  |
| Genome Start | Genome End | Strand | Div | Repeat Start | Repeat End | Repeat Left | Name | Family | Class |
| 78472390 | 78473001 | + | 16.1 | 6 | 635 | -72 | MER39 | ERV1 | LTR |
| 78473002 | 78473409 | + | 17.9 | 1 | 426 | 0 | MSTB | MaLR | LTR |
| 78473412 | 78475001 | + | 27.7 | 2 | 1735 | 0 | MLT-int | MaLR | LTR |
| 78475004 | 78475414 | + | 19.3 | 3 | 426 | 0 | MSTB | MaLR | LTR |
| 78475415 | 78475491 | + | 16.1 | 636 | 705 | -2 | MER39 | ERV1 | LTR |

  

|  |  |  |  |  |  |  |  |  |  |
| --- | --- | --- | --- | --- | --- | --- | --- | --- | --- |
| chr6:78726652-78731416 | | | 4763 bp | | |  |  |  |  |
| Genome Start | Genome End | Strand | Div | Repeat Start | Repeat End | Repeat Left | Name | Family | Class |
| 78726652 | 78728327 | - | 17.1 | 4413 | 6123 | -43 | L1PA16 | L1 | LINE |
| 78728328 | 78728678 | + | 12.9 | 1 | 353 | -2 | THE1A | MaLR | LTR |
| 78728681 | 78730250 | + | 10.6 | 1 | 1580 | 0 | THE1A-int | MaLR | LTR |
| 78730251 | 78730602 | + | 14.0 | 1 | 355 | 0 | THE1A | MaLR | LTR |
| 78730603 | 78731416 | - | 16.1 | 3559 | 4417 | -1729 | L1PA16 | L1 | LINE |

  

|  |  |  |  |  |  |  |  |  |  |
| --- | --- | --- | --- | --- | --- | --- | --- | --- | --- |
| chr6:78810427-78816948 | | | 6471 bp | | |  |  |  |  |
| Genome Start | Genome End | Strand | Div | Repeat Start | Repeat End | Repeat Left | Name | Family | Class |
| 78810427 | 78811372 | - | 26.6 | 4081 | 5047 | -1099 | L1MD | L1 | LINE |
| 78811375 | 78811756 | - | 13.1 | 1 | 381 | 0 | THE1D | MaLR | LTR |
| 78811757 | 78811878 | - | 17.2 | 1452 | 1580 | 0 | THE1D-int | MaLR | LTR |
| 78811920 | 78812265 | - | 11.0 | 1 | 349 | -1231 | THE1D-int | MaLR | LTR |
| 78812268 | 78812636 | - | 12.6 | 1 | 379 | -2 | THE1D | MaLR | LTR |
| 78812638 | 78812861 | - | 27.4 | 3841 | 4082 | -2064 | L1MC | L1 | LINE |
| 78812868 | 78815661 | - | 16.6 | 3337 | 6153 | -3 | L1PB4 | L1 | LINE |
| 78815661 | 78816948 | - | 26.5 | 2496 | 3855 | -2291 | L1MD | L1 | LINE |

  

|  |  |  |  |  |  |  |  |  |  |
| --- | --- | --- | --- | --- | --- | --- | --- | --- | --- |
| chr6:79218866-79225931 | | | 6685 bp | | |  |  |  |  |
| Genome Start | Genome End | Strand | Div | Repeat Start | Repeat End | Repeat Left | Name | Family | Class |
| 79218866 | 79219171 | + | 28.9 | 312 | 671 | -2748 | L2 | L2 | LINE |
| 79219186 | 79219302 | + | 15.5 | 23 | 144 | -361 | MLT1D | MaLR | LTR |
| 79219339 | 79219492 | + | 20.5 | 319 | 505 | 0 | MLT1D | MaLR | LTR |
| 79219504 | 79219628 | + | 30.8 | 683 | 803 | -2616 | L2 | L2 | LINE |
| 79219676 | 79219773 | + | 14.3 | 184 | 282 | -93 | THE1C | MaLR | LTR |
| 79219779 | 79220298 | + | 32.5 | 862 | 1407 | -2012 | L2 | L2 | LINE |
| 79220328 | 79220404 | + | 39.0 | 3275 | 3351 | -27 | L2 | L2 | LINE |
| 79220441 | 79220534 | + | 33.3 | 1434 | 1530 | -1889 | L2 | L2 | LINE |
| 79220546 | 79220653 | + | 24.2 | 4820 | 4930 | -1216 | L1MD | L1 | LINE |
| 79220662 | 79220935 | + | 25.3 | 5011 | 5301 | -845 | L1MD | L1 | LINE |
| 79220979 | 79221351 | - | 10.5 | 1 | 371 | -4 | THE1C | MaLR | LTR |
| 79221352 | 79222926 | - | 30.6 | 2 | 1735 | 0 | MLT-int | MaLR | LTR |
| 79222929 | 79223303 | - | 12.1 | 1 | 375 | 0 | THE1C | MaLR | LTR |
| 79223304 | 79223394 | - | 22.8 | 1 | 115 | -18 | FLAM\_C | Alu | SINE |
| 79223423 | 79223833 | + | 26.2 | 5350 | 5790 | -376 | L1M5 | L1 | LINE |
| 79223843 | 79223999 | - | 36.4 | 7 | 165 | -103 | MIRb | MIR | SINE |
| 79224076 | 79225079 | - | 29.1 | 2272 | 3419 | 0 | L2 | L2 | LINE |
| 79225082 | 79225442 | + | 14.4 | 5911 | 6280 | -11 | L1MA8 | L1 | LINE |
| 79225467 | 79225931 | + | 29.8 | 2826 | 3411 | -8 | L2 | L2 | LINE |

  

|  |  |  |  |  |  |  |  |  |  |
| --- | --- | --- | --- | --- | --- | --- | --- | --- | --- |
| chr6:79321559-79324845 | | | 3282 bp | | |  |  |  |  |
| Genome Start | Genome End | Strand | Div | Repeat Start | Repeat End | Repeat Left | Name | Family | Class |
| 79321559 | 79321693 | - | 16.9 | 6021 | 6150 | -6 | L1PB4 | L1 | LINE |
| 79321694 | 79322058 | - | 15.2 | 1 | 428 | 0 | MSTA | MaLR | LTR |
| 79322059 | 79323664 | - | 14.9 | 3 | 1580 | 0 | MSTA-int | MaLR | LTR |
| 79323670 | 79324050 | - | 16.5 | 1 | 426 | -2 | MSTA | MaLR | LTR |
| 79324051 | 79324845 | - | 15.0 | 5196 | 6025 | -131 | L1PB4 | L1 | LINE |

  

|  |  |  |  |  |  |  |  |  |  |
| --- | --- | --- | --- | --- | --- | --- | --- | --- | --- |
| chr6:79519076-79536877 | | | 17780 bp | | |  |  |  |  |
| Genome Start | Genome End | Strand | Div | Repeat Start | Repeat End | Repeat Left | Name | Family | Class |
| 79519076 | 79521974 | + | 16.8 | 16 | 3029 | -4536 | L1MA2 | L1 | LINE |
| 79521975 | 79522353 | + | 15.9 | 4 | 381 | 0 | THE1D | MaLR | LTR |
| 79522354 | 79522554 | + | 16.8 | 3030 | 3234 | -4331 | L1MA2 | L1 | LINE |
| 79522565 | 79524590 | + | 10.7 | 5 | 2035 | -240 | L1P2 | L1 | LINE |
| 79524590 | 79530578 | + | 6.4 | 22 | 6144 | -11 | L1PA3 | L1 | LINE |
| 79530584 | 79530682 | - | 13.1 | 33 | 131 | -181 | AluSq/x | Alu | SINE |
| 79530683 | 79533005 | + | 11.8 | 4069 | 6378 | -1187 | L1MA2 | L1 | LINE |
| 79533009 | 79533387 | + | 15.7 | 1 | 428 | 0 | MSTA | MaLR | LTR |
| 79533388 | 79534976 | + | 16.5 | 1 | 1645 | -6 | MSTA-int | MaLR | LTR |
| 79534982 | 79535361 | + | 13.2 | 1 | 428 | 0 | MSTA | MaLR | LTR |
| 79535362 | 79535660 | + | 11.0 | 6377 | 6677 | -888 | L1MA2 | L1 | LINE |
| 79535661 | 79536274 | + | 9.6 | 6847 | 7468 | -255 | L1MA2 | L1 | LINE |
| 79536275 | 79536614 | + | 15.5 | 1 | 339 | 0 | MER1B | MER1\_type | DNA |
| 79536615 | 79536877 | + | 9.6 | 7469 | 7723 | 0 | L1MA2 | L1 | LINE |

  

|  |  |  |  |  |  |  |  |  |  |
| --- | --- | --- | --- | --- | --- | --- | --- | --- | --- |
| chr6:80207377-80211294 | | | 3918 bp | | |  |  |  |  |
| Genome Start | Genome End | Strand | Div | Repeat Start | Repeat End | Repeat Left | Name | Family | Class |
| 80207377 | 80208515 | - | 27.2 | 371 | 1472 | 0 | MLT1H-int | MaLR | LTR |
| 80208516 | 80208876 | - | 10.8 | 1 | 364 | 0 | THE1B | MaLR | LTR |
| 80208877 | 80210388 | - | 28.9 | 3 | 1735 | 0 | MLT-int | MaLR | LTR |
| 80210389 | 80210749 | - | 12.8 | 1 | 361 | -3 | THE1B | MaLR | LTR |
| 80210750 | 80210782 | - | 27.2 | 346 | 370 | -1102 | MLT1H-int | MaLR | LTR |
| 80210783 | 80211091 | + | 7.7 | 1 | 300 | -11 | AluY | Alu | SINE |
| 80211092 | 80211294 | - | 27.2 | 138 | 345 | -1127 | MLT1H-int | MaLR | LTR |

  

|  |  |  |  |  |  |  |  |  |  |
| --- | --- | --- | --- | --- | --- | --- | --- | --- | --- |
| chr6:81186226-81189912 | | | 3600 bp | | |  |  |  |  |
| Genome Start | Genome End | Strand | Div | Repeat Start | Repeat End | Repeat Left | Name | Family | Class |
| 81186226 | 81186989 | - | 20.1 | 664 | 1479 | -4662 | L1M3c | L1 | LINE |
| 81187074 | 81187469 | - | 28.8 | 102 | 503 | -5638 | L1M3c | L1 | LINE |
| 81187470 | 81187832 | + | 12.7 | 1 | 381 | 0 | THE1D | MaLR | LTR |
| 81187836 | 81189440 | + | 31.2 | 3 | 1735 | 0 | MLT-int | MaLR | LTR |
| 81189441 | 81189808 | + | 13.6 | 1 | 381 | 0 | THE1D | MaLR | LTR |
| 81189809 | 81189912 | - | 28.8 | 5 | 101 | -6040 | L1M3c | L1 | LINE |

  

|  |  |  |  |  |  |  |  |  |  |
| --- | --- | --- | --- | --- | --- | --- | --- | --- | --- |
| chr6:81663506-81668066 | | | 4551 bp | | |  |  |  |  |
| Genome Start | Genome End | Strand | Div | Repeat Start | Repeat End | Repeat Left | Name | Family | Class |
| 81663506 | 81665402 | - | 31.9 | 458 | 2427 | -992 | L2 | L2 | LINE |
| 81665407 | 81665540 | - | 16.7 | 1 | 132 | -180 | AluSq/x | Alu | SINE |
| 81665543 | 81665684 | - | 32.3 | 330 | 474 | -2945 | L2 | L2 | LINE |
| 81665685 | 81666072 | - | 17.2 | 3 | 428 | 0 | MSTA | MaLR | LTR |
| 81666075 | 81667664 | - | 31.2 | 2 | 1735 | 0 | MLT-int | MaLR | LTR |
| 81667667 | 81668051 | - | 17.1 | 1 | 428 | 0 | MSTA | MaLR | LTR |
| 81668052 | 81668066 | - | 32.3 | 315 | 329 | -3090 | L2 | L2 | LINE |

  

|  |  |  |  |  |  |  |  |  |  |
| --- | --- | --- | --- | --- | --- | --- | --- | --- | --- |
| chr6:81739658-81742622 | | | 2963 bp | | |  |  |  |  |
| Genome Start | Genome End | Strand | Div | Repeat Start | Repeat End | Repeat Left | Name | Family | Class |
| 81739658 | 81740120 | - | 22.0 | 80 | 499 | -6 | MLT1D | MaLR | LTR |
| 81740121 | 81740541 | + | 16.4 | 1 | 428 | 0 | MSTA | MaLR | LTR |
| 81740544 | 81742158 | + | 30.3 | 2 | 1735 | 0 | MLT-int | MaLR | LTR |
| 81742159 | 81742541 | + | 16.0 | 1 | 428 | 0 | MSTA | MaLR | LTR |
| 81742542 | 81742622 | - | 22.0 | 1 | 79 | -426 | MLT1D | MaLR | LTR |

  

|  |  |  |  |  |  |  |  |  |  |
| --- | --- | --- | --- | --- | --- | --- | --- | --- | --- |
| chr6:81982389-81985885 | | | 3392 bp | | |  |  |  |  |
| Genome Start | Genome End | Strand | Div | Repeat Start | Repeat End | Repeat Left | Name | Family | Class |
| 81982389 | 81982838 | + | 22.2 | 6502 | 6936 | -849 | L1MC3 | L1 | LINE |
| 81982879 | 81982981 | + | 11.8 | 263 | 364 | 0 | THE1B | MaLR | LTR |
| 81982982 | 81983086 | + | 10.5 | 1 | 105 | -1475 | THE1B-int | MaLR | LTR |
| 81983125 | 81983375 | + | 24.9 | 24 | 276 | -36 | AluJo | Alu | SINE |
| 81983376 | 81983740 | + | 14.1 | 1 | 364 | 0 | THE1B | MaLR | LTR |
| 81983743 | 81985318 | + | 30.3 | 2 | 1735 | 0 | MLT-int | MaLR | LTR |
| 81985319 | 81985680 | + | 17.4 | 1 | 364 | 0 | THE1B | MaLR | LTR |
| 81985706 | 81985885 | + | 19.4 | 6985 | 7166 | -619 | L1MC3 | L1 | LINE |

  

|  |  |  |  |  |  |  |  |  |  |
| --- | --- | --- | --- | --- | --- | --- | --- | --- | --- |
| chr6:82187592-82190419 | | | 2824 bp | | |  |  |  |  |
| Genome Start | Genome End | Strand | Div | Repeat Start | Repeat End | Repeat Left | Name | Family | Class |
| 82187592 | 82188023 | + | 22.7 | 5379 | 5850 | -314 | L1ME2 | L1 | LINE |
| 82188024 | 82188383 | + | 15.8 | 1 | 375 | 0 | THE1C | MaLR | LTR |
| 82188386 | 82189953 | + | 29.1 | 2 | 1735 | 0 | MLT-int | MaLR | LTR |
| 82189956 | 82190318 | + | 13.0 | 5 | 375 | 0 | THE1C | MaLR | LTR |
| 82190319 | 82190419 | + | 22.7 | 5851 | 5966 | -198 | L1ME2 | L1 | LINE |

  

|  |  |  |  |  |  |  |  |  |  |
| --- | --- | --- | --- | --- | --- | --- | --- | --- | --- |
| chr6:82266193-82270478 | | | 4280 bp | | |  |  |  |  |
| Genome Start | Genome End | Strand | Div | Repeat Start | Repeat End | Repeat Left | Name | Family | Class |
| 82266193 | 82266686 | + | 15.1 | 4468 | 4960 | -1186 | L1PA16 | L1 | LINE |
| 82266687 | 82267043 | + | 11.1 | 1 | 375 | 0 | THE1C | MaLR | LTR |
| 82267048 | 82268582 | + | 29.8 | 4 | 1735 | 0 | MLT-int | MaLR | LTR |
| 82268585 | 82268961 | + | 13.6 | 3 | 375 | 0 | THE1C | MaLR | LTR |
| 82268962 | 82269356 | + | 15.0 | 4961 | 5364 | -802 | L1PA16 | L1 | LINE |
| 82269357 | 82269686 | + | 11.7 | 1 | 308 | -5 | AluSp | Alu | SINE |
| 82269687 | 82270478 | + | 14.6 | 5365 | 6164 | -2 | L1PA16 | L1 | LINE |

  

|  |  |  |  |  |  |  |  |  |  |
| --- | --- | --- | --- | --- | --- | --- | --- | --- | --- |
| chr6:82437772-82442067 | | | 4293 bp | | |  |  |  |  |
| Genome Start | Genome End | Strand | Div | Repeat Start | Repeat End | Repeat Left | Name | Family | Class |
| 82437772 | 82437949 | - | 15.5 | 2239 | 2413 | -5 | Tigger1 | MER2\_type | DNA |
| 82437952 | 82438576 | + | 11.4 | 4771 | 5402 | -903 | L1M1 | L1 | LINE |
| 82438577 | 82438933 | - | 12.7 | 1 | 364 | 0 | THE1B | MaLR | LTR |
| 82438934 | 82440465 | - | 30.2 | 2 | 1735 | 0 | MLT-int | MaLR | LTR |
| 82440468 | 82440828 | - | 13.6 | 1 | 364 | 0 | THE1B | MaLR | LTR |
| 82440829 | 82441702 | + | 11.1 | 5403 | 6305 | 0 | L1MA3 | L1 | LINE |
| 82441705 | 82441801 | - | 12.4 | 2142 | 2255 | -163 | Tigger1 | MER2\_type | DNA |
| 82441799 | 82442067 | - | 20.4 | 4 | 277 | -2141 | Tigger1 | MER2\_type | DNA |

  

|  |  |  |  |  |  |  |  |  |  |
| --- | --- | --- | --- | --- | --- | --- | --- | --- | --- |
| chr6:83140615-83145128 | | | 4457 bp | | |  |  |  |  |
| Genome Start | Genome End | Strand | Div | Repeat Start | Repeat End | Repeat Left | Name | Family | Class |
| 83140615 | 83141802 | - | 18.8 | 2354 | 3556 | 0 | LTR52-int | ERVL | LTR |
| 83141803 | 83142024 | - | 8.4 | 162 | 375 | 0 | THE1C | MaLR | LTR |
| 83142025 | 83142225 | + | 13.4 | 85 | 285 | -27 | AluSg/x | Alu | SINE |
| 83142247 | 83142365 | - | 8.4 | 1 | 161 | -214 | THE1C | MaLR | LTR |
| 83142366 | 83143931 | - | 27.7 | 2 | 1735 | 0 | MLT1J1-int | MaLR | LTR |
| 83143934 | 83144311 | - | 8.6 | 1 | 375 | 0 | THE1C | MaLR | LTR |
| 83144312 | 83144439 | - | 18.8 | 2234 | 2353 | -1203 | LTR52-int | ERVL | LTR |
| 83144474 | 83145128 | - | 20.9 | 1460 | 2137 | -1419 | LTR52-int | ERVL | LTR |

  

|  |  |  |  |  |  |  |  |  |  |
| --- | --- | --- | --- | --- | --- | --- | --- | --- | --- |
| chr6:83695837-83699277 | | | 3300 bp | | |  |  |  |  |
| Genome Start | Genome End | Strand | Div | Repeat Start | Repeat End | Repeat Left | Name | Family | Class |
| 83695837 | 83696173 | - | 24.4 | 457 | 793 | -5422 | L1MC4 | L1 | LINE |
| 83696174 | 83696409 | - | 15.2 | 6081 | 6328 | -5 | L1MC1 | L1 | LINE |
| 83696412 | 83696779 | + | 14.3 | 1 | 375 | 0 | THE1C | MaLR | LTR |
| 83696787 | 83698297 | + | 12.7 | 8 | 1580 | 0 | THE1C-int | MaLR | LTR |
| 83698299 | 83698668 | + | 10.6 | 2 | 375 | 0 | THE1C | MaLR | LTR |
| 83698669 | 83698718 | - | 12.0 | 6034 | 6083 | -250 | L1MC1 | L1 | LINE |
| 83698850 | 83699277 | - | 29.4 | 9 | 454 | -5761 | L1MC4 | L1 | LINE |

  

|  |  |  |  |  |  |  |  |  |  |
| --- | --- | --- | --- | --- | --- | --- | --- | --- | --- |
| chr6:83796604-83799830 | | | 3225 bp | | |  |  |  |  |
| Genome Start | Genome End | Strand | Div | Repeat Start | Repeat End | Repeat Left | Name | Family | Class |
| 83796604 | 83796665 | - | 19.8 | 360 | 415 | -11 | MSTB | MaLR | LTR |
| 83796666 | 83797032 | + | 11.0 | 1 | 364 | 0 | THE1B | MaLR | LTR |
| 83797035 | 83797446 | + | 30.4 | 2 | 423 | -1312 | MLT-int | MaLR | LTR |
| 83797447 | 83797746 | - | 15.7 | 1 | 299 | -13 | AluSx | Alu | SINE |
| 83797747 | 83799095 | + | 30.4 | 424 | 1735 | 0 | MLT-int | MaLR | LTR |
| 83799096 | 83799456 | + | 11.4 | 1 | 364 | 0 | THE1B | MaLR | LTR |
| 83799457 | 83799830 | - | 19.7 | 1 | 359 | -67 | MSTB | MaLR | LTR |

  

|  |  |  |  |  |  |  |  |  |  |
| --- | --- | --- | --- | --- | --- | --- | --- | --- | --- |
| chr6:85237558-85257343 | | | 19736 bp | | |  |  |  |  |
| Genome Start | Genome End | Strand | Div | Repeat Start | Repeat End | Repeat Left | Name | Family | Class |
| 85237558 | 85237900 | + | 24.3 | 8 | 325 | -6217 | L1MB7 | L1 | LINE |
| 85237901 | 85238268 | + | 14.1 | 1 | 364 | 0 | THE1B | MaLR | LTR |
| 85238269 | 85241127 | + | 22.9 | 326 | 3374 | -3270 | L1MB7 | L1 | LINE |
| 85241133 | 85241310 | - | 21.9 | 272 | 452 | -53 | MLT1D | MaLR | LTR |
| 85241370 | 85241553 | + | 16.7 | 3363 | 3543 | -3101 | L1MB7 | L1 | LINE |
| 85241549 | 85242237 | - | 3.3 | 3777 | 4469 | -1677 | L1PA4 | L1 | LINE |
| 85242234 | 85243937 | + | 5.2 | 4446 | 6150 | -5 | L1PA4 | L1 | LINE |
| 85243932 | 85245077 | + | 20.3 | 3526 | 4686 | -1958 | L1MB7 | L1 | LINE |
| 85245080 | 85251647 | + | 11.1 | 5 | 6620 | 0 | L1PB3 | L1 | LINE |
| 85251641 | 85253087 | + | 20.0 | 4649 | 6103 | -573 | L1MB7 | L1 | LINE |
| 85253090 | 85254571 | + | 15.1 | 4819 | 6333 | 0 | L1MC1 | L1 | LINE |
| 85254572 | 85254993 | + | 19.5 | 6091 | 6532 | -150 | L1MB7 | L1 | LINE |
| 85254994 | 85255377 | - | 13.7 | 1 | 375 | 0 | THE1C | MaLR | LTR |
| 85255380 | 85256859 | - | 12.2 | 4 | 1578 | -2 | THE1C-int | MaLR | LTR |
| 85256862 | 85257240 | - | 14.7 | 1 | 375 | 0 | THE1C | MaLR | LTR |
| 85257241 | 85257343 | + | 16.5 | 6528 | 6631 | -51 | L1MB7 | L1 | LINE |

  

|  |  |  |  |  |  |  |  |  |  |
| --- | --- | --- | --- | --- | --- | --- | --- | --- | --- |
| chr6:87133749-87139681 | | | 5913 bp | | |  |  |  |  |
| Genome Start | Genome End | Strand | Div | Repeat Start | Repeat End | Repeat Left | Name | Family | Class |
| 87133749 | 87133899 | - | 19.5 | 6030 | 6181 | -2 | L1MB3 | L1 | LINE |
| 87133915 | 87133954 | - | 15.0 | 363 | 402 | -6 | MLT1A1 | MaLR | LTR |
| 87133955 | 87134355 | + | 19.0 | 1 | 426 | 0 | MSTB | MaLR | LTR |
| 87134356 | 87135873 | + | 18.5 | 1 | 1651 | 0 | MSTB-int | MaLR | LTR |
| 87135874 | 87136081 | + | 20.9 | 1 | 223 | -203 | MSTB | MaLR | LTR |
| 87136082 | 87136127 | - | 16.4 | 334 | 364 | 0 | THE1B | MaLR | LTR |
| 87136128 | 87136434 | + | 12.4 | 1 | 308 | -1 | AluSg1 | Alu | SINE |
| 87136435 | 87136768 | - | 16.4 | 1 | 333 | -31 | THE1B | MaLR | LTR |
| 87136769 | 87138325 | - | 30.9 | 2 | 1735 | 0 | MLT1A1-int | MaLR | LTR |
| 87138328 | 87138696 | - | 21.0 | 1 | 364 | 0 | THE1B | MaLR | LTR |
| 87138697 | 87138883 | + | 20.9 | 224 | 425 | -1 | MSTB | MaLR | LTR |
| 87138886 | 87139159 | - | 19.3 | 4 | 313 | -95 | MLT1A1 | MaLR | LTR |
| 87139161 | 87139681 | - | 18.4 | 5492 | 6026 | -157 | L1MB3 | L1 | LINE |

  

|  |  |  |  |  |  |  |  |  |  |
| --- | --- | --- | --- | --- | --- | --- | --- | --- | --- |
| chr6:87158258-87170633 | | | 12455 bp | | |  |  |  |  |
| Genome Start | Genome End | Strand | Div | Repeat Start | Repeat End | Repeat Left | Name | Family | Class |
| 87158258 | 87158488 | + | 28.3 | 30 | 262 | -778 | MER45B | Tip100 | DNA |
| 87158560 | 87158651 | + | 31.6 | 249 | 340 | -700 | MER45B | Tip100 | DNA |
| 87158652 | 87158692 | - | 13.9 | 393 | 425 | -3 | MSTA | MaLR | LTR |
| 87158693 | 87158956 | + | 15.5 | 2 | 283 | -30 | AluSq | Alu | SINE |
| 87158773 | 87158992 | + | 14.7 | 82 | 319 | 0 | AluSq | Alu | SINE |
| 87158993 | 87159031 | - | 13.9 | 354 | 392 | -36 | MSTA | MaLR | LTR |
| 87159041 | 87165167 | + | 7.6 | 4 | 6143 | -11 | L1PA5 | L1 | LINE |
| 87165169 | 87165555 | - | 17.0 | 1 | 371 | -57 | MSTA | MaLR | LTR |
| 87165556 | 87167164 | - | 30.6 | 2 | 1735 | 0 | MLT-int | MaLR | LTR |
| 87167167 | 87167348 | - | 18.2 | 264 | 432 | 0 | MSTA | MaLR | LTR |
| 87167349 | 87167715 | - | 14.9 | 1 | 364 | 0 | THE1B | MaLR | LTR |
| 87167718 | 87169266 | - | 30.0 | 2 | 1733 | -2 | MLT-int | MaLR | LTR |
| 87169269 | 87169594 | - | 12.7 | 1 | 364 | 0 | THE1B | MaLR | LTR |
| 87169595 | 87169880 | - | 19.5 | 1 | 278 | -150 | MSTA | MaLR | LTR |
| 87169899 | 87170119 | + | 21.7 | 355 | 578 | -462 | MER45B | Tip100 | DNA |
| 87170120 | 87170633 | + | 19.9 | 515 | 1040 | 0 | MER45B | Tip100 | DNA |

  

|  |  |  |  |  |  |  |  |  |  |
| --- | --- | --- | --- | --- | --- | --- | --- | --- | --- |
| chr6:87426048-87449523 | | | 23453 bp | | |  |  |  |  |
| Genome Start | Genome End | Strand | Div | Repeat Start | Repeat End | Repeat Left | Name | Family | Class |
| 87426048 | 87430102 | - | 9.8 | 2924 | 6866 | -1 | L1PB1 | L1 | LINE |
| 87430103 | 87430934 | + | 8.2 | 1 | 826 | 0 | LTR12 | ERV1 | LTR |
| 87430935 | 87431648 | - | 8.4 | 2140 | 2923 | -3940 | L1PB1 | L1 | LINE |
| 87431646 | 87437675 | - | 3.3 | 1 | 6031 | -1 | L1PA2 | L1 | LINE |
| 87437676 | 87438135 | - | 10.7 | 1691 | 2152 | -4711 | L1PB1 | L1 | LINE |
| 87438136 | 87438968 | - | 8.6 | 1 | 826 | 0 | LTR12 | ERV1 | LTR |
| 87438969 | 87447234 | - | 5.9 | 60 | 8436 | 0 | HERV9 | ERV1 | LTR |
| 87447261 | 87447987 | - | 9.1 | 3 | 826 | 0 | LTR12 | ERV1 | LTR |
| 87447988 | 87448673 | - | 13.1 | 908 | 1694 | -5169 | L1PB1 | L1 | LINE |
| 87448674 | 87448737 | + | 14.1 | 343 | 406 | -2 | MLT1A1 | MaLR | LTR |
| 87448738 | 87449523 | - | 13.1 | 6 | 907 | -5956 | L1PB1 | L1 | LINE |

  

|  |  |  |  |  |  |  |  |  |  |
| --- | --- | --- | --- | --- | --- | --- | --- | --- | --- |
| chr6:88134448-88137028 | | | 2574 bp | | |  |  |  |  |
| Genome Start | Genome End | Strand | Div | Repeat Start | Repeat End | Repeat Left | Name | Family | Class |
| 88134448 | 88134711 | + | 14.8 | 1 | 264 | -48 | AluJb | Alu | SINE |
| 88134712 | 88135073 | - | 11.5 | 1 | 364 | 0 | THE1B | MaLR | LTR |
| 88135074 | 88136629 | - | 30.1 | 4 | 1735 | 0 | MLT-int | MaLR | LTR |
| 88136634 | 88136985 | - | 13.9 | 4 | 364 | 0 | THE1B | MaLR | LTR |
| 88136989 | 88137028 | + | 12.5 | 260 | 299 | -3 | AluJb | Alu | SINE |

  

|  |  |  |  |  |  |  |  |  |  |
| --- | --- | --- | --- | --- | --- | --- | --- | --- | --- |
| chr6:88793680-88796457 | | | 2776 bp | | |  |  |  |  |
| Genome Start | Genome End | Strand | Div | Repeat Start | Repeat End | Repeat Left | Name | Family | Class |
| 88793680 | 88793714 | + | 14.7 | 6024 | 6058 | -275 | L1MC1 | L1 | LINE |
| 88793715 | 88794114 | - | 17.8 | 1 | 428 | 0 | MSTA | MaLR | LTR |
| 88794115 | 88795756 | - | 28.6 | 2 | 1735 | 0 | MLT-int | MaLR | LTR |
| 88795759 | 88796174 | - | 19.6 | 1 | 428 | 0 | MSTA | MaLR | LTR |
| 88796175 | 88796457 | + | 14.7 | 6059 | 6333 | 0 | L1MC1 | L1 | LINE |

  

|  |  |  |  |  |  |  |  |  |  |
| --- | --- | --- | --- | --- | --- | --- | --- | --- | --- |
| chr6:89233517-89237303 | | | 3690 bp | | |  |  |  |  |
| Genome Start | Genome End | Strand | Div | Repeat Start | Repeat End | Repeat Left | Name | Family | Class |
| 89233517 | 89233705 | + | 20.2 | 5 | 192 | -275 | MLT1C | MaLR | LTR |
| 89233753 | 89233938 | + | 19.0 | 6 | 312 | -623 | MER21C | ERV1 | LTR |
| 89233941 | 89234353 | + | 14.5 | 1 | 428 | 0 | MSTA | MaLR | LTR |
| 89234356 | 89236093 | + | 27.1 | 2 | 1735 | 0 | MLT-int | MaLR | LTR |
| 89236094 | 89236517 | + | 15.5 | 1 | 428 | 0 | MSTA | MaLR | LTR |
| 89236555 | 89237111 | + | 21.7 | 270 | 852 | -11 | MER21B | ERV1 | LTR |
| 89237121 | 89237303 | + | 24.3 | 243 | 437 | -30 | MLT1C | MaLR | LTR |

  

|  |  |  |  |  |  |  |  |  |  |
| --- | --- | --- | --- | --- | --- | --- | --- | --- | --- |
| chr6:89488760-89502836 | | | 14225 bp | | |  |  |  |  |
| Genome Start | Genome End | Strand | Div | Repeat Start | Repeat End | Repeat Left | Name | Family | Class |
| 89488760 | 89489182 | + | 27.8 | 3 | 415 | -5630 | L1MCb | L1 | LINE |
| 89489183 | 89489702 | + | 14.1 | 2 | 539 | -692 | Tigger3b | MER2\_type | DNA |
| 89489703 | 89490025 | + | 12.2 | 1 | 378 | 0 | MER83B | ERV1 | LTR |
| 89490026 | 89490162 | + | 14.1 | 540 | 658 | -573 | Tigger3b | MER2\_type | DNA |
| 89490163 | 89490462 | - | 9.7 | 1 | 300 | -12 | AluSx | Alu | SINE |
| 89490463 | 89490933 | + | 14.1 | 659 | 1134 | -97 | Tigger3b | MER2\_type | DNA |
| 89490935 | 89491113 | - | 14.5 | 143 | 339 | 0 | MER1B | MER1\_type | DNA |
| 89491114 | 89491559 | + | 9.6 | 1 | 511 | 0 | LTR10F | ERV1 | LTR |
| 89491560 | 89496573 | + | 5.4 | 1 | 5102 | 0 | HERVIP10FH | ERV1 | LTR |
| 89496574 | 89497020 | + | 9.9 | 1 | 511 | 0 | LTR10F | ERV1 | LTR |
| 89497021 | 89497151 | - | 11.4 | 2 | 147 | -150 | MER1B | MER1\_type | DNA |
| 89497157 | 89497200 | + | 13.6 | 1143 | 1187 | -44 | Tigger3b | MER2\_type | DNA |
| 89497226 | 89498018 | + | 21.6 | 420 | 1179 | -4866 | L1MCb | L1 | LINE |
| 89498023 | 89498169 | + | 19.1 | 5111 | 5262 | -878 | L1M | L1 | LINE |
| 89498170 | 89498598 | - | 12.3 | 1 | 434 | 0 | Tigger2a | MER2\_type | DNA |
| 89498599 | 89499377 | + | 16.3 | 5269 | 6137 | -47 | L1MB3 | L1 | LINE |
| 89499188 | 89499422 | + | 17.8 | 5943 | 6183 | 0 | L1MB3 | L1 | LINE |
| 89499430 | 89499514 | + | 20.0 | 1257 | 1351 | -5400 | L1MCb | L1 | LINE |
| 89499515 | 89499795 | + | 17.4 | 1 | 281 | -31 | AluJb | Alu | SINE |
| 89499796 | 89501105 | + | 19.8 | 1352 | 2749 | -3815 | L1MCb | L1 | LINE |
| 89501106 | 89501468 | + | 17.6 | 1 | 366 | 0 | Tigger5a | MER2\_type | DNA |
| 89501469 | 89502836 | + | 18.6 | 2750 | 4140 | -2424 | L1MCb | L1 | LINE |

  

|  |  |  |  |  |  |  |  |  |  |
| --- | --- | --- | --- | --- | --- | --- | --- | --- | --- |
| chr6:91099232-91101696 | | | 2441 bp | | |  |  |  |  |
| Genome Start | Genome End | Strand | Div | Repeat Start | Repeat End | Repeat Left | Name | Family | Class |
| 91099232 | 91099295 | - | 28.6 | 125 | 188 | -1 | MER5A | MER1\_type | DNA |
| 91099299 | 91099673 | - | 13.6 | 4 | 375 | 0 | THE1C | MaLR | LTR |
| 91099677 | 91101252 | - | 28.9 | 2 | 1735 | 0 | MLT-int | MaLR | LTR |
| 91101255 | 91101605 | - | 12.2 | 5 | 375 | 0 | THE1C | MaLR | LTR |
| 91101622 | 91101696 | - | 27.0 | 48 | 122 | -67 | MER5A | MER1\_type | DNA |

  

|  |  |  |  |  |  |  |  |  |  |
| --- | --- | --- | --- | --- | --- | --- | --- | --- | --- |
| chr6:92020981-92023804 | | | 2821 bp | | |  |  |  |  |
| Genome Start | Genome End | Strand | Div | Repeat Start | Repeat End | Repeat Left | Name | Family | Class |
| 92020981 | 92021028 | - | 26.1 | 208 | 252 | -5104 | MER39-int | ERV1 | LTR |
| 92021029 | 92021401 | + | 12.5 | 1 | 363 | -1 | THE1B | MaLR | LTR |
| 92021405 | 92022991 | + | 30.5 | 2 | 1735 | 0 | MLT-int | MaLR | LTR |
| 92022992 | 92023364 | + | 11.1 | 1 | 364 | 0 | THE1B | MaLR | LTR |
| 92023365 | 92023385 | - | 26.1 | 197 | 207 | -5149 | MER39-int | ERV1 | LTR |
| 92023386 | 92023665 | - | 11.4 | 17 | 298 | -15 | AluSp | Alu | SINE |
| 92023666 | 92023804 | - | 26.1 | 39 | 196 | -5160 | MER39-int | ERV1 | LTR |

  

|  |  |  |  |  |  |  |  |  |  |
| --- | --- | --- | --- | --- | --- | --- | --- | --- | --- |
| chr6:92184665-92189137 | | | 4440 bp | | |  |  |  |  |
| Genome Start | Genome End | Strand | Div | Repeat Start | Repeat End | Repeat Left | Name | Family | Class |
| 92184665 | 92185069 | + | 11.9 | 3985 | 4393 | -1753 | L1PA16 | L1 | LINE |
| 92185099 | 92186724 | + | 13.0 | 4394 | 6042 | -124 | L1PA16 | L1 | LINE |
| 92186725 | 92187081 | + | 17.8 | 1 | 362 | -2 | THE1B | MaLR | LTR |
| 92187086 | 92188651 | + | 29.8 | 2 | 1735 | 0 | MLT1E1-int | MaLR | LTR |
| 92188652 | 92189012 | + | 15.3 | 1 | 364 | 0 | THE1B | MaLR | LTR |
| 92189013 | 92189137 | + | 14.6 | 6043 | 6163 | -3 | L1PA16 | L1 | LINE |

  

|  |  |  |  |  |  |  |  |  |  |
| --- | --- | --- | --- | --- | --- | --- | --- | --- | --- |
| chr6:92366476-92371686 | | | 5162 bp | | |  |  |  |  |
| Genome Start | Genome End | Strand | Div | Repeat Start | Repeat End | Repeat Left | Name | Family | Class |
| 92366476 | 92366768 | - | 14.2 | 2724 | 3028 | -1 | Tigger3(Golem) | MER2\_type | DNA |
| 92366769 | 92366985 | + | 20.6 | 1 | 206 | -657 | MER21B | ERV1 | LTR |
| 92366986 | 92367354 | + | 16.4 | 1 | 428 | 0 | MSTA | MaLR | LTR |
| 92367355 | 92367986 | + | 20.6 | 207 | 861 | -2 | MER21B | ERV1 | LTR |
| 92367996 | 92368411 | - | 15.4 | 2303 | 2725 | -304 | Tigger3(Golem) | MER2\_type | DNA |
| 92368412 | 92368721 | + | 22.3 | 1 | 292 | -435 | LTR8 | ERV1 | LTR |
| 92368749 | 92369212 | + | 21.3 | 287 | 723 | -4 | LTR8A | ERV1 | LTR |
| 92369220 | 92369637 | - | 17.5 | 1 | 428 | 0 | MSTA | MaLR | LTR |
| 92369638 | 92371247 | - | 18.9 | 5 | 1651 | 0 | MSTA-int | MaLR | LTR |
| 92371252 | 92371657 | - | 15.8 | 1 | 428 | 0 | MSTA | MaLR | LTR |
| 92371660 | 92371686 | - | 3.7 | 2279 | 2305 | -724 | Tigger3(Golem) | MER2\_type | DNA |

  

|  |  |  |  |  |  |  |  |  |  |
| --- | --- | --- | --- | --- | --- | --- | --- | --- | --- |
| chr6:93273967-93282665 | | | 8690 bp | | |  |  |  |  |
| Genome Start | Genome End | Strand | Div | Repeat Start | Repeat End | Repeat Left | Name | Family | Class |
| 93273967 | 93276900 | + | 18.4 | 1 | 3765 | -3816 | L1PA11 | L1 | LINE |
| 93276905 | 93277259 | + | 18.3 | 8 | 373 | -2 | THE1C | MaLR | LTR |
| 93277265 | 93278771 | + | 16.2 | 3 | 1580 | 0 | THE1C-int | MaLR | LTR |
| 93278772 | 93279141 | + | 14.7 | 1 | 375 | 0 | THE1C | MaLR | LTR |
| 93279142 | 93282665 | + | 11.2 | 3763 | 7365 | -244 | L1PA11 | L1 | LINE |

  

|  |  |  |  |  |  |  |  |  |  |
| --- | --- | --- | --- | --- | --- | --- | --- | --- | --- |
| chr6:93501399-93506362 | | | 4962 bp | | |  |  |  |  |
| Genome Start | Genome End | Strand | Div | Repeat Start | Repeat End | Repeat Left | Name | Family | Class |
| 93501399 | 93501766 | - | 30.2 | 5810 | 6136 | -87 | L1ME3B | L1 | LINE |
| 93501767 | 93502048 | - | 13.8 | 1 | 283 | -30 | AluSq | Alu | SINE |
| 93502049 | 93502608 | - | 30.2 | 5241 | 5809 | -414 | L1ME3B | L1 | LINE |
| 93502609 | 93502867 | - | 19.3 | 1 | 260 | -53 | AluSq | Alu | SINE |
| 93502868 | 93503261 | - | 30.0 | 4863 | 5240 | -889 | L1ME3B | L1 | LINE |
| 93503262 | 93503634 | + | 13.5 | 1 | 375 | 0 | THE1C | MaLR | LTR |
| 93503637 | 93505179 | + | 29.9 | 2 | 1735 | 0 | MLT-int | MaLR | LTR |
| 93505180 | 93505551 | + | 16.5 | 1 | 375 | 0 | THE1C | MaLR | LTR |
| 93505552 | 93505889 | - | 30.0 | 4498 | 4862 | -1267 | L1ME3B | L1 | LINE |
| 93505890 | 93506273 | - | 17.0 | 1 | 378 | -3 | THE1D | MaLR | LTR |
| 93506274 | 93506362 | - | 29.5 | 4411 | 4497 | -1632 | L1ME3B | L1 | LINE |

  

|  |  |  |  |  |  |  |  |  |  |
| --- | --- | --- | --- | --- | --- | --- | --- | --- | --- |
| chr6:93534876-93541167 | | | 5740 bp | | |  |  |  |  |
| Genome Start | Genome End | Strand | Div | Repeat Start | Repeat End | Repeat Left | Name | Family | Class |
| 93534876 | 93535214 | + | 33.2 | 26 | 388 | -5731 | L1MEd | L1 | LINE |
| 93535215 | 93535604 | + | 19.5 | 1 | 396 | 0 | MSTD | MaLR | LTR |
| 93535607 | 93537276 | + | 23.8 | 2 | 1735 | 0 | MSTD-int | MaLR | LTR |
| 93537277 | 93537669 | + | 20.0 | 1 | 396 | 0 | MSTD | MaLR | LTR |
| 93537670 | 93537709 | + | 33.2 | 389 | 437 | -5682 | L1MEd | L1 | LINE |
| 93537742 | 93538124 | + | 33.2 | 438 | 840 | -5279 | L1MEd | L1 | LINE |
| 93538159 | 93538435 | - | 11.9 | 94 | 375 | 0 | THE1C | MaLR | LTR |
| 93538436 | 93539112 | - | 14.4 | 1702 | 2338 | -80 | Tigger1 | MER2\_type | DNA |
| 93539111 | 93539590 | - | 19.6 | 2 | 484 | -1934 | Tigger1 | MER2\_type | DNA |
| 93539934 | 93540224 | - | 20.9 | 1 | 282 | -30 | AluJb | Alu | SINE |
| 93540227 | 93540717 | + | 27.4 | 1633 | 2154 | -3995 | L1MEd | L1 | LINE |
| 93540859 | 93541167 | + | 28.9 | 2381 | 2707 | -3422 | L1MEd | L1 | LINE |

  

|  |  |  |  |  |  |  |  |  |  |
| --- | --- | --- | --- | --- | --- | --- | --- | --- | --- |
| chr6:94472477-94475327 | | | 2849 bp | | |  |  |  |  |
| Genome Start | Genome End | Strand | Div | Repeat Start | Repeat End | Repeat Left | Name | Family | Class |
| 94472477 | 94472978 | - | 34.5 | 4816 | 5331 | -815 | L1M5 | L1 | LINE |
| 94472979 | 94473319 | + | 14.0 | 2 | 364 | 0 | THE1B | MaLR | LTR |
| 94473322 | 94474903 | + | 32.4 | 2 | 1735 | 0 | MLT-int | MaLR | LTR |
| 94474904 | 94475248 | + | 14.6 | 1 | 364 | 0 | THE1B | MaLR | LTR |
| 94475249 | 94475327 | - | 34.5 | 4741 | 4815 | -1331 | L1M5 | L1 | LINE |

  

|  |  |  |  |  |  |  |  |  |  |
| --- | --- | --- | --- | --- | --- | --- | --- | --- | --- |
| chr6:94595370-94602806 | | | 7440 bp | | |  |  |  |  |
| Genome Start | Genome End | Strand | Div | Repeat Start | Repeat End | Repeat Left | Name | Family | Class |
| 94595370 | 94596070 | - | 14.5 | 3688 | 4407 | -1739 | L1P3 | L1 | LINE |
| 94596098 | 94596262 | - | 14.5 | 3502 | 3687 | -2459 | L1P3 | L1 | LINE |
| 94596263 | 94596596 | + | 11.2 | 1 | 331 | -44 | THE1C | MaLR | LTR |
| 94596595 | 94597000 | - | 6.2 | 1 | 404 | -46 | LTR7 | ERV1 | LTR |
| 94597001 | 94597326 | - | 8.0 | 7322 | 7713 | 0 | HERVH | ERV1 | LTR |
| 94597325 | 94597540 | - | 13.4 | 5600 | 5819 | -1894 | HERVH | ERV1 | LTR |
| 94597542 | 94598203 | - | 14.4 | 4488 | 5149 | -2564 | HERVH | ERV1 | LTR |
| 94598206 | 94598711 | - | 11.2 | 3492 | 3993 | -3720 | HERVH | ERV1 | LTR |
| 94598702 | 94598851 | - | 14.0 | 3140 | 3290 | -4423 | HERVH | ERV1 | LTR |
| 94598831 | 94601735 | - | 8.9 | 1 | 2995 | -4718 | HERVH | ERV1 | LTR |
| 94601738 | 94602188 | - | 5.6 | 1 | 448 | -2 | LTR7 | ERV1 | LTR |
| 94602189 | 94602806 | - | 13.9 | 2369 | 3017 | -3129 | L1P3 | L1 | LINE |

  

|  |  |  |  |  |  |  |  |  |  |
| --- | --- | --- | --- | --- | --- | --- | --- | --- | --- |
| chr6:94801564-94809348 | | | 7497 bp | | |  |  |  |  |
| Genome Start | Genome End | Strand | Div | Repeat Start | Repeat End | Repeat Left | Name | Family | Class |
| 94801564 | 94802193 | - | 22.6 | 3531 | 4192 | -2452 | L1M4b | L1 | LINE |
| 94802213 | 94802278 | - | 19.7 | 274 | 339 | 0 | MER1B | MER1\_type | DNA |
| 94802279 | 94802634 | + | 17.1 | 1 | 375 | 0 | THE1C | MaLR | LTR |
| 94802637 | 94804221 | + | 14.8 | 3 | 1580 | 0 | THE1C-int | MaLR | LTR |
| 94804232 | 94804544 | + | 14.8 | 3 | 367 | -8 | THE1C | MaLR | LTR |
| 94804548 | 94804806 | - | 22.4 | 1 | 266 | -73 | MER1B | MER1\_type | DNA |
| 94804810 | 94805136 | - | 24.2 | 3184 | 3513 | -3131 | L1M4b | L1 | LINE |
| 94805152 | 94805219 | - | 19.1 | 2771 | 2841 | -3803 | L1M4b | L1 | LINE |
| 94805221 | 94805924 | - | 21.7 | 2 | 787 | 0 | LTR48 | ERV1 | LTR |
| 94805938 | 94807098 | - | 28.8 | 1207 | 2780 | -4192 | L1M4b | L1 | LINE |
| 94807153 | 94807223 | - | 23.9 | 1128 | 1198 | -5344 | L1M4b | L1 | LINE |
| 94807359 | 94808236 | - | 13.6 | 5255 | 6147 | -7 | L1PB3 | L1 | LINE |
| 94808270 | 94809348 | - | 25.3 | 20 | 1105 | -5437 | L1M4b | L1 | LINE |

  

|  |  |  |  |  |  |  |  |  |  |
| --- | --- | --- | --- | --- | --- | --- | --- | --- | --- |
| chr6:95369750-95373586 | | | 3835 bp | | |  |  |  |  |
| Genome Start | Genome End | Strand | Div | Repeat Start | Repeat End | Repeat Left | Name | Family | Class |
| 95369750 | 95370082 | + | 23.4 | 345 | 693 | -5707 | L1MEc | L1 | LINE |
| 95370083 | 95370438 | - | 17.3 | 1 | 364 | 0 | THE1B | MaLR | LTR |
| 95370439 | 95371972 | - | 30.6 | 2 | 1735 | 0 | MLT-int | MaLR | LTR |
| 95371975 | 95372330 | - | 15.3 | 1 | 364 | 0 | THE1B | MaLR | LTR |
| 95372331 | 95372532 | + | 23.4 | 694 | 887 | -5513 | L1MEc | L1 | LINE |
| 95372533 | 95372813 | + | 10.4 | 5874 | 6151 | 0 | L1PB1 | L1 | LINE |
| 95372814 | 95373586 | + | 23.4 | 888 | 1808 | -4592 | L1MEc | L1 | LINE |

  

|  |  |  |  |  |  |  |  |  |  |
| --- | --- | --- | --- | --- | --- | --- | --- | --- | --- |
| chr6:95434309-95437657 | | | 3340 bp | | |  |  |  |  |
| Genome Start | Genome End | Strand | Div | Repeat Start | Repeat End | Repeat Left | Name | Family | Class |
| 95434309 | 95434539 | - | 20.4 | 339 | 558 | 0 | MER74A | ERVL | LTR |
| 95434540 | 95434906 | + | 12.6 | 1 | 362 | -2 | THE1B | MaLR | LTR |
| 95434914 | 95436457 | + | 30.1 | 5 | 1735 | 0 | MLT-int | MaLR | LTR |
| 95436458 | 95436814 | + | 19.4 | 1 | 364 | 0 | THE1B | MaLR | LTR |
| 95436815 | 95436960 | + | 5.7 | 1439 | 1579 | -1 | THE1B-int | MaLR | LTR |
| 95436963 | 95437318 | + | 12.2 | 3 | 364 | 0 | THE1B | MaLR | LTR |
| 95437319 | 95437657 | - | 23.1 | 1 | 343 | -215 | MER74A | ERVL | LTR |

  

|  |  |  |  |  |  |  |  |  |  |
| --- | --- | --- | --- | --- | --- | --- | --- | --- | --- |
| chr6:95492833-95495440 | | | 2605 bp | | |  |  |  |  |
| Genome Start | Genome End | Strand | Div | Repeat Start | Repeat End | Repeat Left | Name | Family | Class |
| 95492833 | 95493060 | - | 24.2 | 149 | 374 | 0 | MLT1A | MaLR | LTR |
| 95493061 | 95493418 | - | 13.9 | 2 | 375 | 0 | THE1C | MaLR | LTR |
| 95493420 | 95495014 | - | 29.6 | 2 | 1735 | 0 | MLT1A-int | MaLR | LTR |
| 95495017 | 95495382 | - | 15.6 | 2 | 375 | 0 | THE1C | MaLR | LTR |
| 95495383 | 95495440 | - | 24.2 | 93 | 148 | -226 | MLT1A | MaLR | LTR |

  

|  |  |  |  |  |  |  |  |  |  |
| --- | --- | --- | --- | --- | --- | --- | --- | --- | --- |
| chr6:96442226-96452728 | | | 10262 bp | | |  |  |  |  |
| Genome Start | Genome End | Strand | Div | Repeat Start | Repeat End | Repeat Left | Name | Family | Class |
| 96442226 | 96442288 | - | 17.5 | 7744 | 7806 | -47 | L1MC3 | L1 | LINE |
| 96442289 | 96442651 | - | 15.3 | 1 | 364 | 0 | THE1B | MaLR | LTR |
| 96442652 | 96443838 | - | 9.7 | 396 | 1580 | 0 | THE1B-int | MaLR | LTR |
| 96443839 | 96443981 | - | 9.1 | 1 | 144 | -1436 | THE1B-int | MaLR | LTR |
| 96443982 | 96444340 | - | 13.8 | 1 | 364 | 0 | THE1B | MaLR | LTR |
| 96444341 | 96444530 | - | 22.5 | 7556 | 7748 | -105 | L1MC3 | L1 | LINE |
| 96444510 | 96444832 | - | 23.3 | 6408 | 6747 | -1106 | L1MC3 | L1 | LINE |
| 96444833 | 96445116 | + | 18.2 | 3 | 296 | -16 | AluJo | Alu | SINE |
| 96445117 | 96445295 | - | 23.3 | 6224 | 6407 | -1446 | L1MC3 | L1 | LINE |
| 96445296 | 96445598 | - | 22.1 | 1 | 306 | -6 | AluJo | Alu | SINE |
| 96445599 | 96446310 | - | 23.3 | 5510 | 6223 | -1630 | L1MC3 | L1 | LINE |
| 96446338 | 96446371 | - | 20.7 | 434 | 465 | -2 | MLT1C | MaLR | LTR |
| 96446372 | 96446672 | + | 5.7 | 1 | 302 | -9 | AluY | Alu | SINE |
| 96446673 | 96447036 | - | 20.7 | 33 | 433 | -34 | MLT1C | MaLR | LTR |
| 96447045 | 96448167 | - | 22.2 | 2189 | 3388 | -3618 | L1M4b | L1 | LINE |
| 96448191 | 96448625 | - | 31.5 | 4592 | 5028 | -1186 | L1MC3 | L1 | LINE |
| 96448654 | 96448770 | - | 2.6 | 6021 | 6137 | -18 | L1PA4 | L1 | LINE |
| 96448771 | 96449044 | - | 7.3 | 1 | 274 | -37 | AluY | Alu | SINE |
| 96449045 | 96449805 | - | 31.5 | 3756 | 4591 | -1623 | L1MC3 | L1 | LINE |
| 96449806 | 96450201 | - | 11.1 | 5767 | 6163 | 0 | L1PA13 | L1 | LINE |
| 96450202 | 96450852 | - | 31.5 | 3018 | 3755 | -2459 | L1MC3 | L1 | LINE |
| 96450850 | 96451584 | - | 23.0 | 1718 | 2551 | -3664 | L1MC3 | L1 | LINE |
| 96451585 | 96451994 | + | 17.2 | 54 | 515 | 0 | MLT2B2 | ERVL | LTR |
| 96451999 | 96452080 | - | 23.2 | 47 | 128 | 0 | MER58A | MER1\_type | DNA |
| 96452093 | 96452197 | - | 26.2 | 1609 | 1716 | -4499 | L1MC3 | L1 | LINE |
| 96452361 | 96452728 | - | 26.0 | 1204 | 1615 | -4600 | L1MC3 | L1 | LINE |

  

|  |  |  |  |  |  |  |  |  |  |
| --- | --- | --- | --- | --- | --- | --- | --- | --- | --- |
| chr6:96885676-96892785 | | | 7069 bp | | |  |  |  |  |
| Genome Start | Genome End | Strand | Div | Repeat Start | Repeat End | Repeat Left | Name | Family | Class |
| 96885676 | 96885750 | - | 23.3 | 7915 | 7987 | -8 | L1MD3 | L1 | LINE |
| 96885751 | 96885974 | - | 21.8 | 2 | 224 | 0 | MER58A | MER1\_type | DNA |
| 96885978 | 96886726 | - | 24.9 | 7043 | 7823 | -172 | L1MD3 | L1 | LINE |
| 96886755 | 96887189 | - | 24.9 | 6638 | 7042 | -953 | L1MD3 | L1 | LINE |
| 96887190 | 96887324 | - | 17.4 | 72 | 193 | 0 | MER53 | DNA | DNA |
| 96887325 | 96887620 | + | 9.1 | 1 | 296 | -15 | AluY | Alu | SINE |
| 96887621 | 96887692 | - | 17.4 | 5 | 71 | -122 | MER53 | DNA | DNA |
| 96887693 | 96889138 | - | 24.8 | 5116 | 6637 | -1358 | L1MD3 | L1 | LINE |
| 96889139 | 96889499 | + | 15.4 | 1 | 364 | 0 | THE1B | MaLR | LTR |
| 96889502 | 96891049 | + | 29.9 | 2 | 1735 | 0 | MLT-int | MaLR | LTR |
| 96891052 | 96891406 | + | 16.7 | 3 | 364 | 0 | THE1B | MaLR | LTR |
| 96891407 | 96892215 | - | 24.0 | 4286 | 5115 | -1031 | L1MC | L1 | LINE |
| 96892222 | 96892785 | - | 26.2 | 2157 | 2725 | -3421 | L1MD3 | L1 | LINE |

  

|  |  |  |  |  |  |  |  |  |  |
| --- | --- | --- | --- | --- | --- | --- | --- | --- | --- |
| chr6:96893486-96898113 | | | 4401 bp | | |  |  |  |  |
| Genome Start | Genome End | Strand | Div | Repeat Start | Repeat End | Repeat Left | Name | Family | Class |
| 96893486 | 96893906 | - | 24.8 | 2375 | 2982 | -4140 | L1MCc | L1 | LINE |
| 96893899 | 96894438 | - | 25.9 | 1563 | 2155 | -4973 | L1MCc | L1 | LINE |
| 96894439 | 96894642 | - | 27.6 | 1 | 219 | 0 | MER20 | MER1\_type | DNA |
| 96894643 | 96895251 | - | 28.7 | 816 | 1562 | -5566 | L1MCc | L1 | LINE |
| 96895483 | 96895587 | - | 30.0 | 323 | 421 | -6002 | L1MCc | L1 | LINE |
| 96895588 | 96895941 | - | 14.0 | 1 | 355 | 0 | THE1A | MaLR | LTR |
| 96895942 | 96897508 | - | 30.4 | 4 | 1735 | 0 | MLT-int | MaLR | LTR |
| 96897513 | 96897867 | - | 12.2 | 1 | 355 | 0 | THE1A | MaLR | LTR |
| 96897868 | 96898113 | - | 30.0 | 37 | 322 | -6101 | L1MCc | L1 | LINE |

  

|  |  |  |  |  |  |  |  |  |  |
| --- | --- | --- | --- | --- | --- | --- | --- | --- | --- |
| chr6:97413043-97416290 | | | 3092 bp | | |  |  |  |  |
| Genome Start | Genome End | Strand | Div | Repeat Start | Repeat End | Repeat Left | Name | Family | Class |
| 97413043 | 97413151 | + | 21.1 | 5311 | 5431 | -690 | L1ME4a | L1 | LINE |
| 97413311 | 97413479 | + | 12.1 | 1 | 183 | -181 | THE1B | MaLR | LTR |
| 97413480 | 97413770 | + | 12.0 | 1 | 291 | -18 | AluSc | Alu | SINE |
| 97413771 | 97413956 | + | 12.1 | 184 | 364 | 0 | THE1B | MaLR | LTR |
| 97413959 | 97415533 | + | 28.9 | 2 | 1735 | 0 | MLT-int | MaLR | LTR |
| 97415534 | 97415611 | + | 10.3 | 1 | 93 | -282 | THE1B | MaLR | LTR |
| 97415608 | 97415736 | - | 12.4 | 1059 | 1187 | -393 | THE1-int | MaLR | LTR |
| 97415736 | 97415999 | + | 14.7 | 96 | 364 | 0 | THE1B | MaLR | LTR |
| 97416000 | 97416290 | + | 24.7 | 5806 | 6120 | -1 | L1ME4a | L1 | LINE |

  

|  |  |  |  |  |  |  |  |  |  |
| --- | --- | --- | --- | --- | --- | --- | --- | --- | --- |
| chr6:98696242-98699071 | | | 2826 bp | | |  |  |  |  |
| Genome Start | Genome End | Strand | Div | Repeat Start | Repeat End | Repeat Left | Name | Family | Class |
| 98696242 | 98696642 | - | 24.0 | 75 | 492 | -13 | MLT1D | MaLR | LTR |
| 98696643 | 98697005 | - | 17.3 | 1 | 381 | 0 | THE1D | MaLR | LTR |
| 98697006 | 98698643 | - | 14.7 | 4 | 1580 | 0 | THE1D-int | MaLR | LTR |
| 98698647 | 98699013 | - | 15.3 | 1 | 381 | 0 | THE1D | MaLR | LTR |
| 98699015 | 98699071 | - | 24.6 | 19 | 75 | -551 | MLT1D | MaLR | LTR |

  

|  |  |  |  |  |  |  |  |  |  |
| --- | --- | --- | --- | --- | --- | --- | --- | --- | --- |
| chr6:100190594-100193737 | | | 3142 bp | | |  |  |  |  |
| Genome Start | Genome End | Strand | Div | Repeat Start | Repeat End | Repeat Left | Name | Family | Class |
| 100190594 | 100190971 | + | 27.1 | 7348 | 7710 | -332 | L1MC4 | L1 | LINE |
| 100190972 | 100191335 | - | 11.3 | 1 | 364 | 0 | THE1B | MaLR | LTR |
| 100191336 | 100193158 | - | 29.2 | 2 | 1735 | 0 | MLT-int | MaLR | LTR |
| 100193161 | 100193517 | - | 10.7 | 1 | 364 | 0 | THE1B | MaLR | LTR |
| 100193518 | 100193737 | + | 27.1 | 7711 | 7911 | -131 | L1MC4 | L1 | LINE |

  

|  |  |  |  |  |  |  |  |  |  |
| --- | --- | --- | --- | --- | --- | --- | --- | --- | --- |
| chr6:100269511-100280886 | | | 11337 bp | | |  |  |  |  |
| Genome Start | Genome End | Strand | Div | Repeat Start | Repeat End | Repeat Left | Name | Family | Class |
| 100269511 | 100269754 | - | 11.3 | 7492 | 7721 | 0 | L1MA1 | L1 | LINE |
| 100269755 | 100270056 | - | 10.9 | 1 | 309 | -4 | AluSq | Alu | SINE |
| 100270057 | 100274278 | - | 10.2 | 3217 | 7491 | -230 | L1MA1 | L1 | LINE |
| 100274335 | 100275661 | - | 10.0 | 4807 | 6153 | -7 | L1PREC2 | L1 | LINE |
| 100275635 | 100275998 | - | 13.1 | 2867 | 3230 | -4335 | L1MA1 | L1 | LINE |
| 100275999 | 100276368 | + | 11.6 | 1 | 381 | 0 | THE1D | MaLR | LTR |
| 100276372 | 100276606 | + | 9.4 | 4 | 238 | -1342 | THE1D-int | MaLR | LTR |
| 100276612 | 100277733 | + | 12.2 | 427 | 1580 | 0 | THE1D-int | MaLR | LTR |
| 100277736 | 100278102 | + | 14.9 | 3 | 381 | 0 | THE1D | MaLR | LTR |
| 100278103 | 100280886 | - | 16.9 | 22 | 2871 | -4694 | L1MA1 | L1 | LINE |

  

|  |  |  |  |  |  |  |  |  |  |
| --- | --- | --- | --- | --- | --- | --- | --- | --- | --- |
| chr6:101253514-101257328 | | | 3434 bp | | |  |  |  |  |
| Genome Start | Genome End | Strand | Div | Repeat Start | Repeat End | Repeat Left | Name | Family | Class |
| 101253514 | 101253582 | - | 26.1 | 3309 | 3378 | 0 | L2 | L2 | LINE |
| 101253585 | 101253683 | + | 20.2 | 3 | 105 | -216 | MER7D | MER2\_type | DNA |
| 101253652 | 101253741 | + | 10.9 | 212 | 266 | -55 | MER7D | MER2\_type | DNA |
| 101253747 | 101254117 | + | 13.4 | 3 | 364 | 0 | THE1B | MaLR | LTR |
| 101254118 | 101255610 | + | 9.7 | 1 | 1580 | 0 | THE1B-int | MaLR | LTR |
| 101255611 | 101255707 | + | 9.3 | 1 | 97 | -267 | THE1B | MaLR | LTR |
| 101255863 | 101256132 | + | 12.6 | 100 | 364 | 0 | THE1B | MaLR | LTR |
| 101256135 | 101256185 | + | 14.0 | 268 | 317 | -4 | MER7D | MER2\_type | DNA |
| 101256435 | 101257328 | - | 34.8 | 2145 | 3077 | -342 | L2 | L2 | LINE |

  

|  |  |  |  |  |  |  |  |  |  |
| --- | --- | --- | --- | --- | --- | --- | --- | --- | --- |
| chr6:101846726-101849472 | | | 2726 bp | | |  |  |  |  |
| Genome Start | Genome End | Strand | Div | Repeat Start | Repeat End | Repeat Left | Name | Family | Class |
| 101846726 | 101846950 | - | 37.2 | 2616 | 2865 | -554 | L2 | L2 | LINE |
| 101846956 | 101847317 | - | 15.6 | 1 | 381 | 0 | THE1D | MaLR | LTR |
| 101847318 | 101848922 | - | 14.2 | 4 | 1580 | 0 | THE1D-int | MaLR | LTR |
| 101848926 | 101849291 | - | 12.5 | 1 | 381 | 0 | THE1D | MaLR | LTR |
| 101849305 | 101849472 | - | 34.6 | 2415 | 2586 | -833 | L2 | L2 | LINE |

  

|  |  |  |  |  |  |  |  |  |  |
| --- | --- | --- | --- | --- | --- | --- | --- | --- | --- |
| chr6:102336586-102350444 | | | 13847 bp | | |  |  |  |  |
| Genome Start | Genome End | Strand | Div | Repeat Start | Repeat End | Repeat Left | Name | Family | Class |
| 102336586 | 102337388 | - | 14.2 | 6244 | 7003 | -2 | L1MA4A | L1 | LINE |
| 102337390 | 102337425 | + | 13.8 | 1 | 30 | -351 | THE1D | MaLR | LTR |
| 102337426 | 102337725 | - | 6.4 | 1 | 300 | -11 | AluY | Alu | SINE |
| 102337726 | 102338069 | + | 13.8 | 31 | 379 | -2 | THE1D | MaLR | LTR |
| 102338075 | 102338897 | + | 14.2 | 4 | 777 | -803 | THE1D-int | MaLR | LTR |
| 102338904 | 102339129 | + | 9.4 | 1357 | 1580 | 0 | THE1D-int | MaLR | LTR |
| 102339130 | 102339479 | + | 11.6 | 1 | 381 | 0 | THE1D | MaLR | LTR |
| 102339480 | 102342775 | - | 17.8 | 2936 | 6247 | -758 | L1MA4A | L1 | LINE |
| 102342778 | 102348929 | - | 4.2 | 2 | 6154 | -1 | L1PA3 | L1 | LINE |
| 102348930 | 102349131 | - | 13.7 | 2717 | 2949 | -3896 | L1MA4A | L1 | LINE |
| 102349130 | 102349434 | - | 22.4 | 1616 | 1923 | -4926 | L1MA4A | L1 | LINE |
| 102349435 | 102349734 | - | 14.1 | 1 | 299 | -13 | AluSx | Alu | SINE |
| 102349735 | 102350444 | - | 22.4 | 817 | 1615 | -5234 | L1MA4A | L1 | LINE |

  

|  |  |  |  |  |  |  |  |  |  |
| --- | --- | --- | --- | --- | --- | --- | --- | --- | --- |
| chr6:102861273-102864197 | | | 2921 bp | | |  |  |  |  |
| Genome Start | Genome End | Strand | Div | Repeat Start | Repeat End | Repeat Left | Name | Family | Class |
| 102861273 | 102861700 | + | 15.9 | 5666 | 6098 | -205 | L1MA4A | L1 | LINE |
| 102861701 | 102862045 | - | 16.1 | 3 | 364 | 0 | THE1B | MaLR | LTR |
| 102862048 | 102863623 | - | 31.5 | 2 | 1735 | 0 | MLT-int | MaLR | LTR |
| 102863626 | 102863994 | - | 15.8 | 1 | 364 | 0 | THE1B | MaLR | LTR |
| 102863995 | 102864197 | + | 15.9 | 6099 | 6298 | -5 | L1MA4A | L1 | LINE |

  

|  |  |  |  |  |  |  |  |  |  |
| --- | --- | --- | --- | --- | --- | --- | --- | --- | --- |
| chr6:103793544-103796475 | | | 2868 bp | | |  |  |  |  |
| Genome Start | Genome End | Strand | Div | Repeat Start | Repeat End | Repeat Left | Name | Family | Class |
| 103793544 | 103793815 | + | 16.7 | 5479 | 5748 | -408 | L1PB4 | L1 | LINE |
| 103793820 | 103794180 | + | 16.3 | 1 | 364 | 0 | THE1B | MaLR | LTR |
| 103794181 | 103795632 | + | 12.7 | 1 | 1467 | -113 | THE1B-int | MaLR | LTR |
| 103795651 | 103796007 | + | 20.2 | 1 | 364 | 0 | THE1B | MaLR | LTR |
| 103796014 | 103796085 | + | 10.2 | 5754 | 5812 | -344 | L1PB4 | L1 | LINE |
| 103796122 | 103796475 | + | 15.6 | 5785 | 6154 | -2 | L1PB4 | L1 | LINE |

  

|  |  |  |  |  |  |  |  |  |  |
| --- | --- | --- | --- | --- | --- | --- | --- | --- | --- |
| chr6:104211878-104214635 | | | 2747 bp | | |  |  |  |  |
| Genome Start | Genome End | Strand | Div | Repeat Start | Repeat End | Repeat Left | Name | Family | Class |
| 104211878 | 104211940 | + | 25.4 | 7 | 70 | -492 | MLT1F2 | MaLR | LTR |
| 104211944 | 104212303 | - | 16.3 | 1 | 364 | 0 | THE1B | MaLR | LTR |
| 104212304 | 104213798 | - | 12.0 | 1 | 1580 | 0 | THE1B-int | MaLR | LTR |
| 104213799 | 104214152 | - | 14.1 | 1 | 364 | 0 | THE1B | MaLR | LTR |
| 104214161 | 104214635 | + | 30.3 | 77 | 562 | 0 | MLT1F2 | MaLR | LTR |

  

|  |  |  |  |  |  |  |  |  |  |
| --- | --- | --- | --- | --- | --- | --- | --- | --- | --- |
| chr6:104703501-104707622 | | | 4116 bp | | |  |  |  |  |
| Genome Start | Genome End | Strand | Div | Repeat Start | Repeat End | Repeat Left | Name | Family | Class |
| 104703501 | 104705147 | - | 10.9 | 4483 | 6147 | -4 | L1PB2 | L1 | LINE |
| 104705148 | 104705495 | - | 10.4 | 7 | 355 | 0 | THE1A | MaLR | LTR |
| 104705498 | 104707066 | - | 9.4 | 1 | 1574 | -6 | THE1A-int | MaLR | LTR |
| 104707067 | 104707422 | - | 9.1 | 1 | 355 | 0 | THE1A | MaLR | LTR |
| 104707427 | 104707622 | - | 14.3 | 4281 | 4481 | -1665 | L1PB2 | L1 | LINE |

  

|  |  |  |  |  |  |  |  |  |  |
| --- | --- | --- | --- | --- | --- | --- | --- | --- | --- |
| chr6:104754744-104757394 | | | 2643 bp | | |  |  |  |  |
| Genome Start | Genome End | Strand | Div | Repeat Start | Repeat End | Repeat Left | Name | Family | Class |
| 104754744 | 104754949 | - | 18.3 | 715 | 927 | -8 | MER21C | ERV1 | LTR |
| 104754950 | 104755316 | + | 14.7 | 1 | 364 | 0 | THE1B | MaLR | LTR |
| 104755319 | 104756877 | + | 31.2 | 2 | 1735 | 0 | MLT-int | MaLR | LTR |
| 104756878 | 104757222 | + | 14.6 | 1 | 352 | -12 | THE1B | MaLR | LTR |
| 104757229 | 104757394 | - | 16.0 | 442 | 650 | -251 | MER21C | ERV1 | LTR |

  

|  |  |  |  |  |  |  |  |  |  |
| --- | --- | --- | --- | --- | --- | --- | --- | --- | --- |
| chr6:106705885-106707606 | | | 1720 bp | | |  |  |  |  |
| Genome Start | Genome End | Strand | Div | Repeat Start | Repeat End | Repeat Left | Name | Family | Class |
| 106705885 | 106706261 | - | 19.9 | 5738 | 6100 | -80 | L1MB4 | L1 | LINE |
| 106706263 | 106706625 | + | 14.2 | 1 | 364 | 0 | THE1B | MaLR | LTR |
| 106706626 | 106706711 | + | 15.1 | 1 | 86 | -1494 | THE1B-int | MaLR | LTR |
| 106706711 | 106707078 | + | 6.3 | 1212 | 1580 | 0 | THE1B-int | MaLR | LTR |
| 106707079 | 106707435 | + | 14.0 | 1 | 354 | -10 | THE1B | MaLR | LTR |
| 106707438 | 106707606 | - | 18.3 | 5573 | 5741 | -439 | L1MB4 | L1 | LINE |

  

|  |  |  |  |  |  |  |  |  |  |
| --- | --- | --- | --- | --- | --- | --- | --- | --- | --- |
| chr6:109617887-109623280 | | | 4963 bp | | |  |  |  |  |
| Genome Start | Genome End | Strand | Div | Repeat Start | Repeat End | Repeat Left | Name | Family | Class |
| 109617887 | 109618118 | - | 22.7 | 1746 | 2146 | -4572 | L1MEc | L1 | LINE |
| 109618397 | 109618755 | + | 9.5 | 1 | 364 | 0 | THE1B | MaLR | LTR |
| 109618756 | 109620368 | + | 9.3 | 1 | 1580 | 0 | THE1B-int | MaLR | LTR |
| 109620369 | 109620732 | + | 10.5 | 1 | 363 | -1 | THE1B | MaLR | LTR |
| 109620872 | 109621237 | - | 10.5 | 5794 | 6157 | -9 | L1PA16 | L1 | LINE |
| 109621252 | 109621731 | - | 27.5 | 1311 | 1809 | -4909 | L1MEc | L1 | LINE |
| 109621732 | 109622047 | - | 14.9 | 1 | 311 | -1 | AluSx | Alu | SINE |
| 109622048 | 109622121 | - | 27.5 | 1266 | 1310 | -5408 | L1MEc | L1 | LINE |
| 109622122 | 109622425 | + | 5.3 | 1 | 304 | -7 | AluY | Alu | SINE |
| 109622426 | 109623280 | - | 27.5 | 365 | 1265 | -5453 | L1MEc | L1 | LINE |

  

|  |  |  |  |  |  |  |  |  |  |
| --- | --- | --- | --- | --- | --- | --- | --- | --- | --- |
| chr6:114439027-114446308 | | | 7217 bp | | |  |  |  |  |
| Genome Start | Genome End | Strand | Div | Repeat Start | Repeat End | Repeat Left | Name | Family | Class |
| 114439027 | 114439293 | - | 8.6 | 86 | 376 | -1 | MER51B | ERV1 | LTR |
| 114439294 | 114439631 | + | 9.8 | 1 | 330 | -218 | LTR14 | ERVK | LTR |
| 114439632 | 114440049 | - | 2.6 | 45 | 464 | 0 | LTR7B | ERV1 | LTR |
| 114440104 | 114440142 | - | 2.6 | 2 | 44 | -420 | LTR7B | ERV1 | LTR |
| 114440143 | 114440572 | - | 12.6 | 7283 | 7713 | 0 | HERVH | ERV1 | LTR |
| 114440577 | 114445569 | - | 20.4 | 1 | 5020 | -2693 | HERVH | ERV1 | LTR |
| 114445570 | 114446002 | - | 4.2 | 1 | 464 | 0 | LTR7B | ERV1 | LTR |
| 114446006 | 114446222 | + | 8.8 | 329 | 548 | 0 | LTR14 | ERVK | LTR |
| 114446227 | 114446308 | - | 2.5 | 1 | 81 | -296 | MER51B | ERV1 | LTR |

  

|  |  |  |  |  |  |  |  |  |  |
| --- | --- | --- | --- | --- | --- | --- | --- | --- | --- |
| chr6:115456312-115468893 | | | 11698 bp | | |  |  |  |  |
| Genome Start | Genome End | Strand | Div | Repeat Start | Repeat End | Repeat Left | Name | Family | Class |
| 115456312 | 115456463 | + | 34.4 | 27 | 181 | -6366 | L1MD2 | L1 | LINE |
| 115456897 | 115457389 | + | 28.2 | 755 | 1283 | -5264 | L1MD2 | L1 | LINE |
| 115457417 | 115458286 | + | 27.4 | 1284 | 2175 | -4465 | L1MD2 | L1 | LINE |
| 115458291 | 115458357 | + | 11.9 | 3 | 69 | -2649 | Tigger2 | MER2\_type | DNA |
| 115458342 | 115458752 | + | 16.6 | 1530 | 2680 | -38 | Tigger2 | MER2\_type | DNA |
| 115459161 | 115459464 | + | 29.4 | 2360 | 2678 | -3866 | L1MD2 | L1 | LINE |
| 115459445 | 115459648 | + | 11.2 | 4671 | 4871 | -1275 | L1PA7 | L1 | LINE |
| 115459650 | 115460100 | + | 6.2 | 1 | 448 | -2 | LTR7 | ERV1 | LTR |
| 115460103 | 115463280 | + | 9.7 | 1 | 3290 | -4423 | HERVH | ERV1 | LTR |
| 115463280 | 115463775 | + | 11.3 | 3500 | 3993 | -3720 | HERVH | ERV1 | LTR |
| 115463778 | 115464431 | + | 13.6 | 4488 | 5149 | -2564 | HERVH | ERV1 | LTR |
| 115464433 | 115464648 | + | 13.4 | 5600 | 5819 | -1894 | HERVH | ERV1 | LTR |
| 115464647 | 115465026 | + | 7.2 | 7322 | 7713 | 0 | HERVH | ERV1 | LTR |
| 115465027 | 115465476 | + | 6.7 | 1 | 448 | -2 | LTR7 | ERV1 | LTR |
| 115465480 | 115466743 | + | 7.6 | 4869 | 6151 | -3 | L1PA7 | L1 | LINE |
| 115466759 | 115467154 | + | 28.0 | 2670 | 3089 | -3455 | L1MD2 | L1 | LINE |
| 115467155 | 115467340 | - | 10.3 | 5975 | 6159 | -4 | L1PA13 | L1 | LINE |
| 115467341 | 115468252 | + | 28.0 | 3090 | 3929 | -2615 | L1MD2 | L1 | LINE |
| 115468249 | 115468665 | + | 25.3 | 4143 | 4646 | -1898 | L1MD2 | L1 | LINE |
| 115468697 | 115468893 | + | 25.3 | 4647 | 4849 | -1695 | L1MD2 | L1 | LINE |

  

|  |  |  |  |  |  |  |  |  |  |
| --- | --- | --- | --- | --- | --- | --- | --- | --- | --- |
| chr6:115625107-115628594 | | | 3086 bp | | |  |  |  |  |
| Genome Start | Genome End | Strand | Div | Repeat Start | Repeat End | Repeat Left | Name | Family | Class |
| 115625107 | 115625254 | - | 27.1 | 3260 | 3410 | -9 | L2 | L2 | LINE |
| 115625311 | 115625675 | - | 15.2 | 1 | 381 | 0 | THE1D | MaLR | LTR |
| 115625678 | 115627286 | - | 29.6 | 2 | 1733 | -2 | MLT-int | MaLR | LTR |
| 115627289 | 115627653 | - | 13.8 | 1 | 381 | 0 | THE1D | MaLR | LTR |
| 115627781 | 115628198 | - | 17.2 | 1 | 426 | 0 | MSTB | MaLR | LTR |
| 115628414 | 115628594 | - | 33.7 | 2604 | 2800 | -619 | L2 | L2 | LINE |

  

|  |  |  |  |  |  |  |  |  |  |
| --- | --- | --- | --- | --- | --- | --- | --- | --- | --- |
| chr6:117276507-117287449 | | | 10915 bp | | |  |  |  |  |
| Genome Start | Genome End | Strand | Div | Repeat Start | Repeat End | Repeat Left | Name | Family | Class |
| 117276507 | 117276845 | - | 19.8 | 3605 | 3969 | -2160 | L1ME3 | L1 | LINE |
| 117276846 | 117277203 | + | 16.7 | 1 | 428 | 0 | MSTA | MaLR | LTR |
| 117277206 | 117277559 | + | 14.5 | 3 | 362 | -1218 | MSTA-int | MaLR | LTR |
| 117277560 | 117278111 | + | 21.8 | 1 | 554 | 0 | MLT2B3 | ERVL | LTR |
| 117278112 | 117279384 | + | 14.5 | 363 | 1580 | 0 | MSTA-int | MaLR | LTR |
| 117279385 | 117279608 | + | 14.8 | 1 | 239 | -142 | MSTA | MaLR | LTR |
| 117279611 | 117280891 | + | 22.5 | 2712 | 4360 | -2406 | L1PA11 | L1 | LINE |
| 117280892 | 117281176 | + | 14.8 | 5 | 291 | -21 | AluSx | Alu | SINE |
| 117281177 | 117283580 | + | 9.2 | 4361 | 6782 | -12 | L1PA11 | L1 | LINE |
| 117283582 | 117283891 | + | 8.5 | 1 | 308 | -3 | AluY | Alu | SINE |
| 117283892 | 117284056 | + | 14.6 | 239 | 428 | 0 | MSTA | MaLR | LTR |
| 117284076 | 117284584 | - | 24.5 | 3056 | 3587 | -2542 | L1ME3 | L1 | LINE |
| 117284579 | 117285765 | - | 6.7 | 4979 | 6154 | 0 | L1PA7 | L1 | LINE |
| 117285761 | 117286532 | - | 25.0 | 2271 | 3070 | -3059 | L1ME3 | L1 | LINE |
| 117286548 | 117286702 | - | 29.3 | 1085 | 1250 | -4869 | L1ME3 | L1 | LINE |
| 117286703 | 117287234 | + | 19.8 | 1 | 552 | -4 | MLT2B4 | ERVL | LTR |
| 117287235 | 117287449 | - | 29.3 | 895 | 1084 | -5035 | L1ME3 | L1 | LINE |

  

|  |  |  |  |  |  |  |  |  |  |
| --- | --- | --- | --- | --- | --- | --- | --- | --- | --- |
| chr6:117572346-117581287 | | | 8705 bp | | |  |  |  |  |
| Genome Start | Genome End | Strand | Div | Repeat Start | Repeat End | Repeat Left | Name | Family | Class |
| 117572346 | 117572522 | + | 28.2 | 1 | 179 | -2433 | Charlie7 | MER1\_type | DNA |
| 117572556 | 117572878 | - | 14.2 | 52 | 375 | 0 | THE1C | MaLR | LTR |
| 117572877 | 117573027 | - | 10.6 | 788 | 938 | -642 | THE1-int | MaLR | LTR |
| 117573026 | 117573079 | - | 3.7 | 1 | 54 | -321 | THE1C | MaLR | LTR |
| 117573080 | 117574650 | - | 30.8 | 2 | 1735 | 0 | MLT-int | MaLR | LTR |
| 117574653 | 117575029 | - | 13.2 | 1 | 375 | 0 | THE1C | MaLR | LTR |
| 117575040 | 117575102 | + | 30.2 | 241 | 303 | -2309 | Charlie7 | MER1\_type | DNA |
| 117575209 | 117576643 | + | 24.6 | 531 | 2137 | -475 | Charlie7 | MER1\_type | DNA |
| 117576645 | 117577501 | + | 11.2 | 4927 | 5795 | -371 | L1P4 | L1 | LINE |
| 117577502 | 117577874 | - | 13.4 | 1 | 364 | 0 | THE1B | MaLR | LTR |
| 117577875 | 117579442 | - | 30.8 | 2 | 1735 | 0 | MLT1C-int | MaLR | LTR |
| 117579445 | 117579814 | - | 14.1 | 1 | 364 | 0 | THE1B | MaLR | LTR |
| 117579815 | 117580184 | + | 12.5 | 5796 | 6164 | -2 | L1PA16 | L1 | LINE |
| 117580207 | 117580380 | + | 24.7 | 2128 | 2335 | -277 | Charlie7 | MER1\_type | DNA |
| 117580435 | 117580527 | + | 20.7 | 1 | 98 | -214 | AluJb | Alu | SINE |
| 117580528 | 117580911 | - | 13.8 | 1 | 428 | 0 | MSTA | MaLR | LTR |
| 117580912 | 117581118 | + | 20.7 | 99 | 306 | -6 | AluJb | Alu | SINE |
| 117581130 | 117581287 | + | 21.5 | 2382 | 2569 | -43 | Charlie7 | MER1\_type | DNA |

  

|  |  |  |  |  |  |  |  |  |  |
| --- | --- | --- | --- | --- | --- | --- | --- | --- | --- |
| chr6:118518115-118523103 | | | 4898 bp | | |  |  |  |  |
| Genome Start | Genome End | Strand | Div | Repeat Start | Repeat End | Repeat Left | Name | Family | Class |
| 118518115 | 118519088 | - | 30.7 | 2391 | 3417 | -2 | L2 | L2 | LINE |
| 118519111 | 118519482 | + | 14.0 | 1 | 364 | 0 | THE1B | MaLR | LTR |
| 118519483 | 118520891 | + | 11.9 | 1 | 1550 | -30 | THE1B-int | MaLR | LTR |
| 118520892 | 118521182 | + | 11.3 | 1 | 291 | -19 | AluSg | Alu | SINE |
| 118521183 | 118521226 | + | 11.9 | 1551 | 1579 | -1 | THE1B-int | MaLR | LTR |
| 118521227 | 118521582 | + | 12.4 | 3 | 360 | -4 | THE1B | MaLR | LTR |
| 118521587 | 118522332 | - | 27.6 | 1403 | 2289 | -1130 | L2 | L2 | LINE |
| 118522358 | 118522510 | - | 28.1 | 2106 | 2294 | -1125 | L2 | L2 | LINE |
| 118522550 | 118522838 | + | 9.7 | 9 | 297 | -15 | AluSx | Alu | SINE |
| 118522840 | 118523002 | + | 17.8 | 135 | 297 | -15 | AluSg/x | Alu | SINE |
| 118523003 | 118523103 | - | 28.0 | 1942 | 2045 | -1374 | L2 | L2 | LINE |

  

|  |  |  |  |  |  |  |  |  |  |
| --- | --- | --- | --- | --- | --- | --- | --- | --- | --- |
| chr6:119038137-119055184 | | | 16677 bp | | |  |  |  |  |
| Genome Start | Genome End | Strand | Div | Repeat Start | Repeat End | Repeat Left | Name | Family | Class |
| 119038137 | 119038563 | + | 31.8 | 584 | 1009 | -5624 | L1MDa | L1 | LINE |
| 119038543 | 119040231 | + | 17.2 | 1676 | 3424 | -3113 | L1MDa | L1 | LINE |
| 119040232 | 119040530 | + | 8.9 | 1 | 295 | -16 | AluY | Alu | SINE |
| 119040531 | 119040792 | + | 17.5 | 3425 | 3677 | -2860 | L1MDa | L1 | LINE |
| 119040793 | 119041085 | + | 7.8 | 1 | 301 | -12 | AluSq | Alu | SINE |
| 119041086 | 119041330 | + | 17.5 | 3678 | 3913 | -2624 | L1MDa | L1 | LINE |
| 119041331 | 119041629 | + | 12.0 | 1 | 299 | -14 | AluSq | Alu | SINE |
| 119041630 | 119042417 | + | 17.5 | 3914 | 4715 | -1822 | L1MDa | L1 | LINE |
| 119042418 | 119042695 | + | 9.3 | 1 | 281 | -32 | AluSp | Alu | SINE |
| 119042766 | 119043085 | + | 17.5 | 4716 | 5014 | -1523 | L1MDa | L1 | LINE |
| 119043195 | 119043257 | + | 14.3 | 5029 | 5093 | -1444 | L1MDa | L1 | LINE |
| 119043292 | 119043325 | + | 19.6 | 5330 | 5363 | -1174 | L1MDa | L1 | LINE |
| 119043326 | 119043912 | + | 21.6 | 1 | 607 | 0 | MER77 | ERVL | LTR |
| 119043913 | 119044099 | + | 19.6 | 5364 | 5567 | -970 | L1MDa | L1 | LINE |
| 119044100 | 119044407 | + | 19.3 | 1 | 303 | -9 | AluJo | Alu | SINE |
| 119044457 | 119044520 | + | 15.6 | 238 | 301 | -1 | Alu | Alu | SINE |
| 119044523 | 119044813 | + | 8.9 | 3 | 293 | -17 | AluSg | Alu | SINE |
| 119044814 | 119044909 | + | 12.8 | 44 | 137 | -5 | FLAM\_A | Alu | SINE |
| 119044958 | 119045654 | + | 14.0 | 1 | 722 | 0 | LTR30 | ERV1 | LTR |
| 119045657 | 119052134 | + | 10.6 | 3 | 6502 | -1755 | HERV30 | ERV1 | LTR |
| 119052213 | 119053960 | + | 19.1 | 6509 | 8257 | 0 | HERV30 | ERV1 | LTR |
| 119053961 | 119054658 | + | 15.6 | 1 | 722 | 0 | LTR30 | ERV1 | LTR |
| 119054659 | 119054775 | + | 16.6 | 5580 | 5698 | -839 | L1MDa | L1 | LINE |
| 119054776 | 119055079 | + | 12.8 | 3 | 307 | -5 | AluSx | Alu | SINE |
| 119055080 | 119055184 | + | 16.6 | 5699 | 5794 | -743 | L1MDa | L1 | LINE |

  

|  |  |  |  |  |  |  |  |  |  |
| --- | --- | --- | --- | --- | --- | --- | --- | --- | --- |
| chr6:120589433-120595810 | | | 6375 bp | | |  |  |  |  |
| Genome Start | Genome End | Strand | Div | Repeat Start | Repeat End | Repeat Left | Name | Family | Class |
| 120589433 | 120590031 | + | 19.9 | 2 | 605 | -6244 | L1M1 | L1 | LINE |
| 120590034 | 120590419 | - | 13.8 | 1 | 381 | 0 | THE1D | MaLR | LTR |
| 120590420 | 120591916 | - | 13.9 | 3 | 1580 | 0 | THE1D-int | MaLR | LTR |
| 120591919 | 120592305 | - | 18.2 | 1 | 381 | 0 | THE1D | MaLR | LTR |
| 120592306 | 120593812 | + | 20.4 | 603 | 2100 | -4749 | L1M1 | L1 | LINE |
| 120593813 | 120594170 | + | 11.3 | 1 | 381 | 0 | THE1D | MaLR | LTR |
| 120594171 | 120594892 | + | 20.4 | 2101 | 2851 | -3998 | L1M1 | L1 | LINE |
| 120594892 | 120595810 | + | 18.8 | 5448 | 6389 | -616 | L1M1 | L1 | LINE |

  

|  |  |  |  |  |  |  |  |  |  |
| --- | --- | --- | --- | --- | --- | --- | --- | --- | --- |
| chr6:122669213-122672506 | | | 3200 bp | | |  |  |  |  |
| Genome Start | Genome End | Strand | Div | Repeat Start | Repeat End | Repeat Left | Name | Family | Class |
| 122669213 | 122669509 | + | 24.8 | 1026 | 1362 | -5038 | L1MEb | L1 | LINE |
| 122669524 | 122669947 | + | 18.6 | 1 | 424 | -2 | MSTB | MaLR | LTR |
| 122669961 | 122671535 | + | 19.5 | 11 | 1651 | 0 | MSTB-int | MaLR | LTR |
| 122671538 | 122671939 | + | 18.7 | 3 | 426 | 0 | MSTB | MaLR | LTR |
| 122672005 | 122672506 | + | 26.7 | 1402 | 1955 | -4445 | L1MEb | L1 | LINE |

  

|  |  |  |  |  |  |  |  |  |  |
| --- | --- | --- | --- | --- | --- | --- | --- | --- | --- |
| chr6:122799946-122805343 | | | 5394 bp | | |  |  |  |  |
| Genome Start | Genome End | Strand | Div | Repeat Start | Repeat End | Repeat Left | Name | Family | Class |
| 122799946 | 122800019 | + | 23.9 | 1838 | 1917 | -4591 | L1MCa | L1 | LINE |
| 122800047 | 122800760 | + | 19.0 | 1918 | 2689 | -3632 | L1MCa | L1 | LINE |
| 122800761 | 122801165 | + | 19.9 | 1 | 422 | -6 | MSTA | MaLR | LTR |
| 122801172 | 122801219 | + | 8.3 | 1 | 48 | -1603 | MSTA-int | MaLR | LTR |
| 122801220 | 122801825 | + | 10.8 | 1595 | 2270 | -4419 | L1PA14 | L1 | LINE |
| 122801826 | 122802662 | + | 17.8 | 745 | 1651 | 0 | MSTA-int | MaLR | LTR |
| 122802663 | 122803080 | + | 17.7 | 1 | 428 | 0 | MSTA | MaLR | LTR |
| 122803081 | 122803166 | + | 10.5 | 2687 | 2773 | -3548 | L1MCa | L1 | LINE |
| 122803138 | 122803893 | + | 15.8 | 3319 | 4144 | -2177 | L1MCa | L1 | LINE |
| 122803894 | 122804192 | + | 9.7 | 1 | 303 | -9 | AluSx | Alu | SINE |
| 122804193 | 122805343 | + | 15.8 | 4145 | 5297 | -1024 | L1MCa | L1 | LINE |

  

|  |  |  |  |  |  |  |  |  |  |
| --- | --- | --- | --- | --- | --- | --- | --- | --- | --- |
| chr6:123273383-123281521 | | | 8162 bp | | |  |  |  |  |
| Genome Start | Genome End | Strand | Div | Repeat Start | Repeat End | Repeat Left | Name | Family | Class |
| 123273383 | 123273878 | - | 17.1 | 6352 | 6875 | -2 | L1MA7 | L1 | LINE |
| 123273854 | 123275544 | - | 18.6 | 3171 | 4879 | -1850 | L1MA7 | L1 | LINE |
| 123275545 | 123275908 | + | 14.5 | 8 | 375 | 0 | THE1C | MaLR | LTR |
| 123275911 | 123277502 | + | 29.1 | 2 | 1735 | 0 | MLT-int | MaLR | LTR |
| 123277503 | 123277875 | + | 14.3 | 1 | 374 | -1 | THE1C | MaLR | LTR |
| 123277876 | 123281521 | - | 24.2 | 11 | 3160 | -3569 | L1MA7 | L1 | LINE |

  

|  |  |  |  |  |  |  |  |  |  |
| --- | --- | --- | --- | --- | --- | --- | --- | --- | --- |
| chr6:123443685-123446341 | | | 2655 bp | | |  |  |  |  |
| Genome Start | Genome End | Strand | Div | Repeat Start | Repeat End | Repeat Left | Name | Family | Class |
| 123443685 | 123443974 | - | 18.3 | 85 | 390 | 0 | MLT1B | MaLR | LTR |
| 123443975 | 123444330 | - | 12.4 | 1 | 355 | 0 | THE1A | MaLR | LTR |
| 123444331 | 123445902 | - | 29.6 | 2 | 1735 | 0 | MLT1B-int | MaLR | LTR |
| 123445905 | 123446258 | - | 10.4 | 1 | 355 | 0 | THE1A | MaLR | LTR |
| 123446259 | 123446341 | - | 18.3 | 1 | 84 | -306 | MLT1B | MaLR | LTR |

  

|  |  |  |  |  |  |  |  |  |  |
| --- | --- | --- | --- | --- | --- | --- | --- | --- | --- |
| chr6:123919623-123925487 | | | 5823 bp | | |  |  |  |  |
| Genome Start | Genome End | Strand | Div | Repeat Start | Repeat End | Repeat Left | Name | Family | Class |
| 123919623 | 123919687 | + | 23.1 | 3 | 67 | -298 | MLT1A0 | MaLR | LTR |
| 123919688 | 123920099 | + | 8.3 | 1 | 448 | -2 | LTR7 | ERV1 | LTR |
| 123920109 | 123922868 | + | 10.3 | 1 | 2984 | -4729 | HERVH | ERV1 | LTR |
| 123922863 | 123923098 | + | 13.1 | 3500 | 3735 | -3978 | HERVH | ERV1 | LTR |
| 123923109 | 123923416 | + | 16.2 | 3995 | 4290 | -3423 | HERVH | ERV1 | LTR |
| 123923415 | 123924346 | + | 12.1 | 4221 | 5149 | -2564 | HERVH | ERV1 | LTR |
| 123924364 | 123924781 | + | 9.6 | 7277 | 7713 | 0 | HERVH | ERV1 | LTR |
| 123924782 | 123925188 | + | 9.6 | 1 | 443 | -7 | LTR7 | ERV1 | LTR |
| 123925203 | 123925487 | + | 17.6 | 63 | 363 | -2 | MLT1A0 | MaLR | LTR |

  

|  |  |  |  |  |  |  |  |  |  |
| --- | --- | --- | --- | --- | --- | --- | --- | --- | --- |
| chr6:123944790-123951386 | | | 6625 bp | | |  |  |  |  |
| Genome Start | Genome End | Strand | Div | Repeat Start | Repeat End | Repeat Left | Name | Family | Class |
| 123944790 | 123945177 | - | 20.4 | 538 | 897 | -6 | MER4D | ERV1 | LTR |
| 123945180 | 123945578 | - | 8.8 | 1 | 448 | -2 | LTR7 | ERV1 | LTR |
| 123945579 | 123945956 | - | 11.8 | 7322 | 7713 | 0 | HERVH | ERV1 | LTR |
| 123945955 | 123946187 | - | 12.1 | 5588 | 5819 | -1894 | HERVH | ERV1 | LTR |
| 123946171 | 123946830 | - | 15.3 | 4488 | 5146 | -2567 | HERVH | ERV1 | LTR |
| 123946833 | 123947335 | - | 9.8 | 3492 | 3993 | -3720 | HERVH | ERV1 | LTR |
| 123947326 | 123947472 | - | 14.3 | 3140 | 3290 | -4423 | HERVH | ERV1 | LTR |
| 123947467 | 123950448 | - | 9.0 | 1 | 2987 | -4726 | HERVH | ERV1 | LTR |
| 123950451 | 123950851 | - | 8.3 | 1 | 448 | -2 | LTR7 | ERV1 | LTR |
| 123950853 | 123951386 | - | 13.8 | 1 | 541 | -362 | MER4D | ERV1 | LTR |

  

|  |  |  |  |  |  |  |  |  |  |
| --- | --- | --- | --- | --- | --- | --- | --- | --- | --- |
| chr6:125011663-125016834 | | | 5116 bp | | |  |  |  |  |
| Genome Start | Genome End | Strand | Div | Repeat Start | Repeat End | Repeat Left | Name | Family | Class |
| 125011663 | 125013276 | + | 17.0 | 2814 | 4425 | -1721 | L1M | L1 | LINE |
| 125013278 | 125013620 | - | 23.2 | 1 | 396 | 0 | MSTD | MaLR | LTR |
| 125013621 | 125015381 | - | 26.3 | 1 | 1730 | -5 | MSTD-int | MaLR | LTR |
| 125015382 | 125015793 | - | 22.9 | 1 | 396 | 0 | MSTD | MaLR | LTR |
| 125015794 | 125016612 | + | 15.2 | 4422 | 5255 | -891 | L1M | L1 | LINE |
| 125016668 | 125016834 | + | 15.0 | 5260 | 5427 | -906 | L1M | L1 | LINE |

  

|  |  |  |  |  |  |  |  |  |  |
| --- | --- | --- | --- | --- | --- | --- | --- | --- | --- |
| chr6:125210069-125214388 | | | 4289 bp | | |  |  |  |  |
| Genome Start | Genome End | Strand | Div | Repeat Start | Repeat End | Repeat Left | Name | Family | Class |
| 125210069 | 125210775 | + | 29.7 | 1705 | 2486 | -933 | L2 | L2 | LINE |
| 125210776 | 125211140 | + | 13.3 | 1 | 364 | 0 | THE1B | MaLR | LTR |
| 125211143 | 125212719 | + | 30.0 | 2 | 1735 | 0 | MLT1G3-int | MaLR | LTR |
| 125212720 | 125213090 | + | 14.7 | 1 | 364 | 0 | THE1B | MaLR | LTR |
| 125213091 | 125213329 | + | 29.7 | 2487 | 2715 | -704 | L2 | L2 | LINE |
| 125213356 | 125213765 | + | 28.6 | 27 | 450 | 0 | LTR16A | ERVL | LTR |
| 125213769 | 125214388 | + | 31.0 | 2714 | 3419 | 0 | L2 | L2 | LINE |

  

|  |  |  |  |  |  |  |  |  |  |
| --- | --- | --- | --- | --- | --- | --- | --- | --- | --- |
| chr6:125820179-125826520 | | | 6221 bp | | |  |  |  |  |
| Genome Start | Genome End | Strand | Div | Repeat Start | Repeat End | Repeat Left | Name | Family | Class |
| 125820179 | 125820393 | - | 14.9 | 5775 | 5992 | -516 | L1MC | L1 | LINE |
| 125820403 | 125820751 | - | 9.7 | 1 | 353 | -2 | THE1A | MaLR | LTR |
| 125820752 | 125822189 | - | 9.1 | 1 | 1580 | 0 | THE1A-int | MaLR | LTR |
| 125822192 | 125822547 | - | 7.4 | 1 | 353 | -2 | THE1A | MaLR | LTR |
| 125822548 | 125822652 | - | 17.4 | 5662 | 5762 | -746 | L1MC | L1 | LINE |
| 125822653 | 125823069 | - | 11.9 | 1 | 428 | 0 | MSTA | MaLR | LTR |
| 125823070 | 125823314 | - | 17.1 | 5395 | 5661 | -847 | L1MC | L1 | LINE |
| 125823357 | 125823416 | + | 16.7 | 330 | 394 | -2 | MSTD | MaLR | LTR |
| 125823456 | 125824025 | - | 14.5 | 4834 | 5387 | -934 | L1MC | L1 | LINE |
| 125824035 | 125824796 | - | 16.7 | 1331 | 2143 | -4365 | L1MC | L1 | LINE |
| 125824797 | 125825191 | - | 25.0 | 1 | 406 | -2 | MLT1A1 | MaLR | LTR |
| 125825192 | 125825900 | - | 16.7 | 622 | 1330 | -5178 | L1MC | L1 | LINE |
| 125825921 | 125826520 | - | 16.7 | 1 | 621 | -5887 | L1MC | L1 | LINE |

  

|  |  |  |  |  |  |  |  |  |  |
| --- | --- | --- | --- | --- | --- | --- | --- | --- | --- |
| chr6:125827225-125831023 | | | 3785 bp | | |  |  |  |  |
| Genome Start | Genome End | Strand | Div | Repeat Start | Repeat End | Repeat Left | Name | Family | Class |
| 125827225 | 125827336 | - | 17.1 | 7666 | 7784 | -1 | L1MC3 | L1 | LINE |
| 125827346 | 125827689 | + | 13.3 | 1 | 364 | 0 | THE1B | MaLR | LTR |
| 125827690 | 125829264 | + | 9.7 | 1 | 1580 | 0 | THE1B-int | MaLR | LTR |
| 125829265 | 125829610 | + | 13.6 | 1 | 364 | 0 | THE1B | MaLR | LTR |
| 125829616 | 125830351 | - | 18.8 | 6876 | 7653 | -132 | L1MC3 | L1 | LINE |
| 125830352 | 125830685 | + | 8.7 | 1 | 354 | -1 | THE1A | MaLR | LTR |
| 125830686 | 125831023 | - | 18.8 | 6503 | 6875 | -910 | L1MC3 | L1 | LINE |

  

|  |  |  |  |  |  |  |  |  |  |
| --- | --- | --- | --- | --- | --- | --- | --- | --- | --- |
| chr6:128737006-128740393 | | | 3305 bp | | |  |  |  |  |
| Genome Start | Genome End | Strand | Div | Repeat Start | Repeat End | Repeat Left | Name | Family | Class |
| 128737006 | 128737864 | + | 26.5 | 2150 | 3122 | -297 | L2 | L2 | LINE |
| 128737946 | 128738045 | + | 28.4 | 3288 | 3383 | -36 | L2 | L2 | LINE |
| 128738046 | 128738412 | - | 13.2 | 1 | 364 | 0 | THE1B | MaLR | LTR |
| 128738413 | 128739987 | - | 30.1 | 2 | 1735 | 0 | MLT-int | MaLR | LTR |
| 128739990 | 128740361 | - | 13.4 | 1 | 364 | 0 | THE1B | MaLR | LTR |
| 128740362 | 128740393 | + | 28.4 | 3384 | 3415 | -4 | L2 | L2 | LINE |

  

|  |  |  |  |  |  |  |  |  |  |
| --- | --- | --- | --- | --- | --- | --- | --- | --- | --- |
| chr6:129064831-129068405 | | | 3237 bp | | |  |  |  |  |
| Genome Start | Genome End | Strand | Div | Repeat Start | Repeat End | Repeat Left | Name | Family | Class |
| 129064831 | 129065346 | + | 28.1 | 6729 | 7280 | -762 | L1MC4 | L1 | LINE |
| 129065347 | 129065699 | + | 13.9 | 1 | 381 | 0 | THE1D | MaLR | LTR |
| 129065702 | 129067278 | + | 28.8 | 2 | 1735 | 0 | MLT-int | MaLR | LTR |
| 129067279 | 129067624 | + | 12.8 | 1 | 381 | 0 | THE1D | MaLR | LTR |
| 129067625 | 129067821 | + | 28.1 | 7281 | 7488 | -554 | L1MC4 | L1 | LINE |
| 129067992 | 129068113 | + | 17.4 | 1 | 121 | -12 | FLAM\_C | Alu | SINE |
| 129068280 | 129068405 | + | 29.0 | 7709 | 7840 | -202 | L1MC4 | L1 | LINE |

  

|  |  |  |  |  |  |  |  |  |  |
| --- | --- | --- | --- | --- | --- | --- | --- | --- | --- |
| chr6:131368511-131372341 | | | 3479 bp | | |  |  |  |  |
| Genome Start | Genome End | Strand | Div | Repeat Start | Repeat End | Repeat Left | Name | Family | Class |
| 131368511 | 131368569 | + | 28.1 | 857 | 913 | -2506 | L2 | L2 | LINE |
| 131368572 | 131368652 | - | 21.0 | 2668 | 2754 | 0 | Charlie9 | MER1\_type | DNA |
| 131368883 | 131369188 | - | 11.2 | 2 | 307 | -5 | AluSx | Alu | SINE |
| 131369213 | 131369514 | + | 12.2 | 1 | 296 | -16 | AluSx | Alu | SINE |
| 131369600 | 131369952 | + | 26.4 | 902 | 1351 | -2068 | L2 | L2 | LINE |
| 131369954 | 131370284 | + | 15.0 | 2 | 375 | 0 | THE1C | MaLR | LTR |
| 131370285 | 131371898 | + | 10.8 | 1 | 1573 | -7 | THE1C-int | MaLR | LTR |
| 131371907 | 131372256 | + | 11.9 | 3 | 375 | 0 | THE1C | MaLR | LTR |
| 131372259 | 131372341 | + | 32.5 | 1349 | 1431 | -1988 | L2 | L2 | LINE |

  

|  |  |  |  |  |  |  |  |  |  |
| --- | --- | --- | --- | --- | --- | --- | --- | --- | --- |
| chr6:131657992-131664184 | | | 6207 bp | | |  |  |  |  |
| Genome Start | Genome End | Strand | Div | Repeat Start | Repeat End | Repeat Left | Name | Family | Class |
| 131657992 | 131658189 | + | 15.3 | 1 | 193 | -150 | MER61A | ERV1 | LTR |
| 131658190 | 131658639 | + | 6.7 | 1 | 448 | -2 | LTR7 | ERV1 | LTR |
| 131658642 | 131661699 | + | 9.9 | 1 | 2995 | -4718 | HERVH | ERV1 | LTR |
| 131661679 | 131661828 | + | 13.3 | 3140 | 3290 | -4423 | HERVH | ERV1 | LTR |
| 131661831 | 131662320 | + | 11.9 | 3503 | 3993 | -3720 | HERVH | ERV1 | LTR |
| 131662323 | 131662983 | + | 13.5 | 4488 | 5149 | -2564 | HERVH | ERV1 | LTR |
| 131662985 | 131663201 | + | 13.0 | 5600 | 5819 | -1894 | HERVH | ERV1 | LTR |
| 131663200 | 131663579 | + | 8.8 | 7322 | 7713 | 0 | HERVH | ERV1 | LTR |
| 131663580 | 131664029 | + | 6.5 | 1 | 448 | -2 | LTR7 | ERV1 | LTR |
| 131664032 | 131664184 | + | 14.5 | 189 | 343 | 0 | MER61A | ERV1 | LTR |

  

|  |  |  |  |  |  |  |  |  |  |
| --- | --- | --- | --- | --- | --- | --- | --- | --- | --- |
| chr6:132536252-132540319 | | | 3684 bp | | |  |  |  |  |
| Genome Start | Genome End | Strand | Div | Repeat Start | Repeat End | Repeat Left | Name | Family | Class |
| 132536252 | 132536652 | + | 28.6 | 1552 | 2009 | -4987 | L1ME3B | L1 | LINE |
| 132536653 | 132537040 | - | 14.4 | 1 | 428 | 0 | MSTA | MaLR | LTR |
| 132537042 | 132538657 | - | 28.8 | 2 | 1734 | -1 | MLT-int | MaLR | LTR |
| 132538660 | 132538881 | - | 14.9 | 196 | 428 | 0 | MSTA | MaLR | LTR |
| 132538882 | 132539180 | - | 13.0 | 1 | 299 | -10 | AluSc | Alu | SINE |
| 132539181 | 132539356 | - | 14.9 | 1 | 195 | -233 | MSTA | MaLR | LTR |
| 132539357 | 132539860 | + | 29.7 | 1893 | 2600 | -4426 | L1ME3B | L1 | LINE |
| 132540242 | 132540319 | + | 22.4 | 4453 | 4529 | -2477 | L1ME3B | L1 | LINE |

  

|  |  |  |  |  |  |  |  |  |  |
| --- | --- | --- | --- | --- | --- | --- | --- | --- | --- |
| chr6:134513083-134515710 | | | 2591 bp | | |  |  |  |  |
| Genome Start | Genome End | Strand | Div | Repeat Start | Repeat End | Repeat Left | Name | Family | Class |
| 134513083 | 134513300 | - | 26.1 | 3165 | 3419 | 0 | L2 | L2 | LINE |
| 134513301 | 134513647 | + | 8.9 | 1 | 355 | 0 | THE1A | MaLR | LTR |
| 134513648 | 134515228 | + | 9.7 | 1 | 1580 | 0 | THE1A-int | MaLR | LTR |
| 134515229 | 134515582 | + | 7.9 | 1 | 355 | 0 | THE1A | MaLR | LTR |
| 134515620 | 134515710 | - | 27.8 | 3002 | 3086 | -292 | L2 | L2 | LINE |

  

|  |  |  |  |  |  |  |  |  |  |
| --- | --- | --- | --- | --- | --- | --- | --- | --- | --- |
| chr6:135840171-135845723 | | | 5433 bp | | |  |  |  |  |
| Genome Start | Genome End | Strand | Div | Repeat Start | Repeat End | Repeat Left | Name | Family | Class |
| 135840171 | 135840309 | - | 26.3 | 2093 | 2234 | -4166 | L1MEc | L1 | LINE |
| 135840310 | 135840652 | + | 13.0 | 1 | 383 | -45 | MSTA | MaLR | LTR |
| 135840653 | 135840931 | - | 12.0 | 1 | 275 | -37 | AluSx | Alu | SINE |
| 135840932 | 135840970 | + | 13.0 | 384 | 425 | -3 | MSTA | MaLR | LTR |
| 135840974 | 135842594 | + | 29.4 | 2 | 1735 | 0 | MSTC-int | MaLR | LTR |
| 135842595 | 135842960 | + | 17.1 | 1 | 428 | 0 | MSTA | MaLR | LTR |
| 135842961 | 135843008 | - | 26.3 | 2046 | 2092 | -4308 | L1MEc | L1 | LINE |
| 135843009 | 135843311 | + | 17.2 | 1 | 305 | -7 | AluJb | Alu | SINE |
| 135843312 | 135843357 | - | 26.3 | 2002 | 2045 | -4355 | L1MEc | L1 | LINE |
| 135843367 | 135843543 | + | 17.2 | 4 | 169 | -259 | MSTC | MaLR | LTR |
| 135843544 | 135843883 | + | 16.1 | 1 | 339 | 0 | MER1B | MER1\_type | DNA |
| 135843884 | 135844134 | + | 17.2 | 170 | 428 | 0 | MSTC | MaLR | LTR |
| 135844164 | 135844375 | - | 16.5 | 4 | 235 | -1 | MER46A | MER2\_type | DNA |
| 135844379 | 135845058 | - | 23.2 | 1165 | 1974 | -4426 | L1MEc | L1 | LINE |
| 135845138 | 135845217 | - | 24.1 | 937 | 1018 | -44 | MER63 | AcHobo | DNA |
| 135845209 | 135845300 | - | 26.1 | 1 | 98 | -963 | MER63 | AcHobo | DNA |
| 135845302 | 135845513 | - | 31.9 | 927 | 1146 | -5169 | L1MEc | L1 | LINE |
| 135845519 | 135845723 | - | 26.5 | 695 | 902 | -5498 | L1MEc | L1 | LINE |

  

|  |  |  |  |  |  |  |  |  |  |
| --- | --- | --- | --- | --- | --- | --- | --- | --- | --- |
| chr6:135950838-135953908 | | | 3064 bp | | |  |  |  |  |
| Genome Start | Genome End | Strand | Div | Repeat Start | Repeat End | Repeat Left | Name | Family | Class |
| 135950838 | 135950965 | - | 19.5 | 31 | 159 | -1 | MER5A1 | MER1\_type | DNA |
| 135950968 | 135951375 | - | 13.7 | 2 | 425 | -3 | MSTA | MaLR | LTR |
| 135951379 | 135953119 | - | 16.0 | 1 | 1645 | -6 | MSTA-int | MaLR | LTR |
| 135953122 | 135953549 | - | 17.4 | 1 | 426 | -2 | MSTA | MaLR | LTR |
| 135953550 | 135953873 | - | 18.7 | 1 | 341 | -87 | MSTA | MaLR | LTR |
| 135953874 | 135953908 | - | 28.6 | 1 | 35 | -125 | MER5A1 | MER1\_type | DNA |

  

|  |  |  |  |  |  |  |  |  |  |
| --- | --- | --- | --- | --- | --- | --- | --- | --- | --- |
| chr6:136008794-136014959 | | | 6162 bp | | |  |  |  |  |
| Genome Start | Genome End | Strand | Div | Repeat Start | Repeat End | Repeat Left | Name | Family | Class |
| 136008794 | 136010363 | + | 18.2 | 5 | 1575 | -4933 | L1MCa | L1 | LINE |
| 136010364 | 136010722 | - | 15.8 | 3 | 364 | 0 | THE1B | MaLR | LTR |
| 136010725 | 136011922 | - | 12.4 | 1 | 1580 | 0 | THE1B-int | MaLR | LTR |
| 136011923 | 136012275 | - | 11.0 | 1 | 364 | 0 | THE1B | MaLR | LTR |
| 136012279 | 136013322 | - | 14.6 | 1574 | 2624 | -3697 | L1MCa | L1 | LINE |
| 136013326 | 136013377 | + | 5.8 | 1 | 52 | -312 | THE1B | MaLR | LTR |
| 136013373 | 136014028 | + | 8.7 | 922 | 1580 | 0 | THE1B-int | MaLR | LTR |
| 136014029 | 136014384 | + | 10.1 | 1 | 363 | -1 | THE1B | MaLR | LTR |
| 136014386 | 136014959 | + | 11.6 | 2629 | 3182 | -3139 | L1MCa | L1 | LINE |

  

|  |  |  |  |  |  |  |  |  |  |
| --- | --- | --- | --- | --- | --- | --- | --- | --- | --- |
| chr6:136123952-136128303 | | | 4322 bp | | |  |  |  |  |
| Genome Start | Genome End | Strand | Div | Repeat Start | Repeat End | Repeat Left | Name | Family | Class |
| 136123952 | 136124117 | - | 26.7 | 5531 | 5662 | -516 | L1M4 | L1 | LINE |
| 136124136 | 136124488 | - | 10.6 | 1 | 353 | -2 | THE1A | MaLR | LTR |
| 136124489 | 136126058 | - | 29.8 | 2 | 1735 | 0 | MLT-int | MaLR | LTR |
| 136126061 | 136126413 | - | 10.5 | 1 | 355 | 0 | THE1A | MaLR | LTR |
| 136126426 | 136126977 | - | 21.4 | 4952 | 5492 | -674 | L1M4 | L1 | LINE |
| 136126978 | 136127333 | - | 12.4 | 1 | 311 | 0 | AluY | Alu | SINE |
| 136127334 | 136127808 | - | 26.4 | 4453 | 4951 | -1195 | L1M4 | L1 | LINE |
| 136127807 | 136128303 | - | 26.9 | 3392 | 3920 | -2226 | L1M4 | L1 | LINE |

  

|  |  |  |  |  |  |  |  |  |  |
| --- | --- | --- | --- | --- | --- | --- | --- | --- | --- |
| chr6:136524738-136527913 | | | 3072 bp | | |  |  |  |  |
| Genome Start | Genome End | Strand | Div | Repeat Start | Repeat End | Repeat Left | Name | Family | Class |
| 136524738 | 136524954 | + | 23.4 | 6 | 241 | -2541 | Charlie2 | MER1\_type | DNA |
| 136525000 | 136525370 | - | 11.7 | 1 | 375 | 0 | THE1C | MaLR | LTR |
| 136525371 | 136526892 | - | 11.1 | 17 | 1580 | 0 | THE1C-int | MaLR | LTR |
| 136526899 | 136527270 | - | 10.2 | 1 | 375 | 0 | THE1C | MaLR | LTR |
| 136527324 | 136527913 | + | 23.2 | 2230 | 2822 | -40 | Charlie2 | MER1\_type | DNA |

  

|  |  |  |  |  |  |  |  |  |  |
| --- | --- | --- | --- | --- | --- | --- | --- | --- | --- |
| chr6:137422193-137431309 | | | 9071 bp | | |  |  |  |  |
| Genome Start | Genome End | Strand | Div | Repeat Start | Repeat End | Repeat Left | Name | Family | Class |
| 137422193 | 137422233 | + | 18.1 | 4665 | 4702 | -1444 | L1ME3 | L1 | LINE |
| 137422234 | 137422469 | + | 14.0 | 76 | 312 | 0 | AluSg/x | Alu | SINE |
| 137422470 | 137422920 | + | 22.0 | 4703 | 5565 | -597 | L1ME3 | L1 | LINE |
| 137422944 | 137423079 | + | 24.3 | 3 | 139 | -901 | MER45B | Tip100 | DNA |
| 137423098 | 137423526 | - | 7.7 | 1 | 472 | 0 | LTR7 | ERV1 | LTR |
| 137423527 | 137423901 | - | 11.1 | 7322 | 7713 | 0 | HERVH | ERV1 | LTR |
| 137423900 | 137423937 | - | 12.3 | 5784 | 5819 | -1894 | HERVH | ERV1 | LTR |
| 137423938 | 137424252 | + | 1.6 | 1 | 318 | 0 | AluYb8 | Alu | SINE |
| 137424253 | 137424439 | - | 12.3 | 5600 | 5783 | -1930 | HERVH | ERV1 | LTR |
| 137424441 | 137429537 | - | 10.6 | 1 | 5149 | -2564 | HERVH | ERV1 | LTR |
| 137429540 | 137429951 | - | 6.8 | 1 | 448 | -2 | LTR7 | ERV1 | LTR |
| 137429952 | 137430794 | + | 20.2 | 159 | 1038 | -2 | MER45B | Tip100 | DNA |
| 137430799 | 137431309 | + | 24.4 | 5617 | 6147 | -15 | L1ME3 | L1 | LINE |

  

|  |  |  |  |  |  |  |  |  |  |
| --- | --- | --- | --- | --- | --- | --- | --- | --- | --- |
| chr6:137951135-137959898 | | | 8762 bp | | |  |  |  |  |
| Genome Start | Genome End | Strand | Div | Repeat Start | Repeat End | Repeat Left | Name | Family | Class |
| 137951135 | 137955388 | + | 23.1 | 190 | 4342 | -2573 | L1P4d | L1 | LINE |
| 137955389 | 137955749 | - | 11.2 | 1 | 364 | 0 | THE1B | MaLR | LTR |
| 137955750 | 137957357 | - | 11.8 | 13 | 1580 | 0 | THE1B-int | MaLR | LTR |
| 137957358 | 137957715 | - | 10.2 | 1 | 363 | -1 | THE1B | MaLR | LTR |
| 137957718 | 137959898 | + | 15.5 | 4340 | 6551 | -374 | L1P4d | L1 | LINE |

  

|  |  |  |  |  |  |  |  |  |  |
| --- | --- | --- | --- | --- | --- | --- | --- | --- | --- |
| chr6:138408334-138411763 | | | 3426 bp | | |  |  |  |  |
| Genome Start | Genome End | Strand | Div | Repeat Start | Repeat End | Repeat Left | Name | Family | Class |
| 138408334 | 138409139 | + | 31.2 | 2108 | 3017 | -402 | L2 | L2 | LINE |
| 138409140 | 138409493 | - | 13.0 | 1 | 364 | 0 | THE1B | MaLR | LTR |
| 138409494 | 138411061 | - | 30.7 | 4 | 1735 | 0 | MLT1C-int | MaLR | LTR |
| 138411066 | 138411419 | - | 12.7 | 1 | 364 | 0 | THE1B | MaLR | LTR |
| 138411420 | 138411763 | + | 32.8 | 3018 | 3397 | -22 | L2 | L2 | LINE |

  

|  |  |  |  |  |  |  |  |  |  |
| --- | --- | --- | --- | --- | --- | --- | --- | --- | --- |
| chr6:139596280-139599741 | | | 3458 bp | | |  |  |  |  |
| Genome Start | Genome End | Strand | Div | Repeat Start | Repeat End | Repeat Left | Name | Family | Class |
| 139596280 | 139596364 | - | 17.6 | 203 | 290 | -22 | AluJb | Alu | SINE |
| 139596367 | 139596608 | + | 11.4 | 141 | 381 | 0 | THE1D | MaLR | LTR |
| 139596611 | 139598322 | + | 29.0 | 2 | 1735 | 0 | MLT-int | MaLR | LTR |
| 139598323 | 139598460 | + | 12.6 | 1 | 137 | -244 | THE1D | MaLR | LTR |
| 139598461 | 139599286 | + | 17.6 | 1 | 785 | 0 | LTR1 | ERV1 | LTR |
| 139599287 | 139599526 | + | 12.6 | 138 | 381 | 0 | THE1D | MaLR | LTR |
| 139599527 | 139599741 | - | 15.5 | 1 | 208 | -104 | AluJb | Alu | SINE |

  

|  |  |  |  |  |  |  |  |  |  |
| --- | --- | --- | --- | --- | --- | --- | --- | --- | --- |
| chr6:140531248-140537137 | | | 5365 bp | | |  |  |  |  |
| Genome Start | Genome End | Strand | Div | Repeat Start | Repeat End | Repeat Left | Name | Family | Class |
| 140531248 | 140532121 | - | 30.1 | 2111 | 3032 | -3097 | L1MEe | L1 | LINE |
| 140532403 | 140532876 | - | 28.6 | 1356 | 1798 | -4351 | L1MEe | L1 | LINE |
| 140532918 | 140533047 | - | 29.3 | 1121 | 1255 | -4864 | L1MEe | L1 | LINE |
| 140533048 | 140533387 | - | 15.4 | 1 | 364 | 0 | THE1B | MaLR | LTR |
| 140533392 | 140534968 | - | 31.1 | 2 | 1731 | -4 | MLT-int | MaLR | LTR |
| 140534971 | 140535160 | - | 15.0 | 178 | 364 | 0 | THE1B | MaLR | LTR |
| 140535161 | 140535456 | - | 12.6 | 1 | 301 | -11 | AluSx | Alu | SINE |
| 140535457 | 140535479 | - | 15.0 | 165 | 177 | -187 | THE1B | MaLR | LTR |
| 140535480 | 140535791 | + | 8.2 | 1 | 303 | -8 | AluY | Alu | SINE |
| 140535792 | 140535935 | - | 15.0 | 1 | 164 | -200 | THE1B | MaLR | LTR |
| 140535936 | 140536076 | - | 29.3 | 968 | 1120 | -4999 | L1MEe | L1 | LINE |
| 140536200 | 140536370 | + | 26.4 | 5777 | 5959 | -281 | L1ME3B | L1 | LINE |
| 140536374 | 140536533 | + | 30.4 | 5985 | 6151 | -89 | L1ME3B | L1 | LINE |
| 140536605 | 140537137 | - | 33.2 | 324 | 896 | -5223 | L1MEe | L1 | LINE |

  

|  |  |  |  |  |  |  |  |  |  |
| --- | --- | --- | --- | --- | --- | --- | --- | --- | --- |
| chr6:140547519-140559913 | | | 12431 bp | | |  |  |  |  |
| Genome Start | Genome End | Strand | Div | Repeat Start | Repeat End | Repeat Left | Name | Family | Class |
| 140547519 | 140550179 | + | 18.4 | 718 | 3405 | -2866 | L1PA16 | L1 | LINE |
| 140550180 | 140550527 | + | 12.7 | 1 | 364 | 0 | THE1B | MaLR | LTR |
| 140550530 | 140552066 | + | 30.9 | 2 | 1733 | -2 | MLT-int | MaLR | LTR |
| 140552079 | 140552420 | + | 12.1 | 1 | 364 | 0 | THE1B | MaLR | LTR |
| 140552421 | 140553620 | + | 17.1 | 3405 | 4618 | -1861 | L1PA16 | L1 | LINE |
| 140553621 | 140554165 | - | 14.0 | 1080 | 1633 | -4313 | MER41-int | ERV1 | LTR |
| 140554155 | 140554387 | - | 15.2 | 403 | 635 | -5311 | MER41-int | ERV1 | LTR |
| 140554362 | 140554483 | + | 14.2 | 720 | 840 | -4377 | MER41-int | ERV1 | LTR |
| 140554487 | 140554595 | + | 21.3 | 1902 | 2017 | -3929 | MER41-int | ERV1 | LTR |
| 140554588 | 140555304 | + | 14.2 | 2163 | 2946 | -3000 | MER41-int | ERV1 | LTR |
| 140555292 | 140556455 | + | 12.2 | 2856 | 4837 | -992 | MER41-int | ERV1 | LTR |
| 140556456 | 140556711 | - | 14.8 | 52 | 307 | -3 | AluSg | Alu | SINE |
| 140556712 | 140557785 | + | 10.9 | 4024 | 5944 | -2 | MER41-int | ERV1 | LTR |
| 140557786 | 140558247 | + | 11.5 | 1 | 466 | -88 | MER41A | ERV1 | LTR |
| 140558253 | 140559913 | + | 15.2 | 4602 | 6291 | 0 | L1PA16 | L1 | LINE |

  

|  |  |  |  |  |  |  |  |  |  |
| --- | --- | --- | --- | --- | --- | --- | --- | --- | --- |
| chr6:140724950-140729425 | | | 4398 bp | | |  |  |  |  |
| Genome Start | Genome End | Strand | Div | Repeat Start | Repeat End | Repeat Left | Name | Family | Class |
| 140724950 | 140725576 | - | 22.7 | 5275 | 5943 | -223 | L1ME1 | L1 | LINE |
| 140725644 | 140725841 | - | 24.7 | 5074 | 5274 | -872 | L1MC | L1 | LINE |
| 140725842 | 140726137 | + | 12.2 | 1 | 364 | 0 | THE1B | MaLR | LTR |
| 140726138 | 140727735 | + | 30.8 | 1 | 1735 | 0 | MLT-int | MaLR | LTR |
| 140727736 | 140728087 | + | 15.4 | 1 | 364 | 0 | THE1B | MaLR | LTR |
| 140728088 | 140728622 | - | 24.7 | 4522 | 5073 | -1073 | L1MC | L1 | LINE |
| 140728620 | 140729114 | - | 23.4 | 955 | 1431 | -4969 | L1MEc | L1 | LINE |
| 140729129 | 140729425 | - | 32.4 | 4419 | 4743 | -1403 | L1ME1 | L1 | LINE |

  

|  |  |  |  |  |  |  |  |  |  |
| --- | --- | --- | --- | --- | --- | --- | --- | --- | --- |
| chr6:140791611-140799683 | | | 8071 bp | | |  |  |  |  |
| Genome Start | Genome End | Strand | Div | Repeat Start | Repeat End | Repeat Left | Name | Family | Class |
| 140791611 | 140793120 | - | 28.0 | 4183 | 5632 | -932 | L1MCb | L1 | LINE |
| 140793121 | 140793220 | - | 17.0 | 1 | 100 | -212 | AluJ/FLAM | Alu | SINE |
| 140793221 | 140794516 | - | 28.0 | 2938 | 4182 | -2382 | L1MCb | L1 | LINE |
| 140794517 | 140794766 | - | 12.4 | 1 | 251 | -62 | AluSq | Alu | SINE |
| 140794767 | 140795681 | - | 25.7 | 1945 | 2935 | -3629 | L1MCb | L1 | LINE |
| 140795682 | 140796057 | + | 17.2 | 1 | 428 | 0 | MSTA | MaLR | LTR |
| 140796060 | 140797664 | + | 30.1 | 2 | 1735 | 0 | MLT-int | MaLR | LTR |
| 140797665 | 140798028 | + | 16.5 | 1 | 428 | 0 | MSTA | MaLR | LTR |
| 140798029 | 140799683 | - | 24.0 | 250 | 1944 | -4807 | L1MCb | L1 | LINE |

  

|  |  |  |  |  |  |  |  |  |  |
| --- | --- | --- | --- | --- | --- | --- | --- | --- | --- |
| chr6:140987530-140991104 | | | 3530 bp | | |  |  |  |  |
| Genome Start | Genome End | Strand | Div | Repeat Start | Repeat End | Repeat Left | Name | Family | Class |
| 140987530 | 140987876 | + | 27.3 | 5335 | 5712 | -452 | L1ME3 | L1 | LINE |
| 140987877 | 140988168 | + | 7.5 | 1 | 292 | -19 | AluY | Alu | SINE |
| 140988169 | 140988432 | + | 27.3 | 5713 | 5998 | -166 | L1ME3 | L1 | LINE |
| 140988477 | 140988830 | - | 17.2 | 1 | 364 | 0 | THE1B | MaLR | LTR |
| 140988831 | 140989013 | - | 12.4 | 1400 | 1580 | 0 | THE1B-int | MaLR | LTR |
| 140989014 | 140989310 | + | 14.8 | 1 | 298 | -14 | AluSx | Alu | SINE |
| 140989311 | 140990666 | - | 12.4 | 1 | 1399 | -181 | THE1B-int | MaLR | LTR |
| 140990667 | 140991015 | - | 13.3 | 1 | 364 | 0 | THE1B | MaLR | LTR |
| 140991017 | 140991104 | + | 19.2 | 6080 | 6159 | -3 | L1ME3 | L1 | LINE |

  

|  |  |  |  |  |  |  |  |  |  |
| --- | --- | --- | --- | --- | --- | --- | --- | --- | --- |
| chr6:141106441-141113547 | | | 7071 bp | | |  |  |  |  |
| Genome Start | Genome End | Strand | Div | Repeat Start | Repeat End | Repeat Left | Name | Family | Class |
| 141106441 | 141106526 | + | 15.9 | 2 | 84 | -311 | MLT1A0 | MaLR | LTR |
| 141106530 | 141107402 | + | 20.5 | 40 | 964 | -6601 | L1M1 | L1 | LINE |
| 141107403 | 141107950 | - | 24.6 | 1 | 554 | 0 | MLT2B3 | ERVL | LTR |
| 141107951 | 141108433 | + | 20.5 | 965 | 1433 | -6132 | L1M1 | L1 | LINE |
| 141108435 | 141108457 | + | 13.7 | 2 | 19 | -1561 | THE1-int | MaLR | LTR |
| 141108458 | 141108826 | + | 12.9 | 1 | 364 | 0 | THE1B | MaLR | LTR |
| 141108827 | 141109403 | + | 13.7 | 20 | 497 | -1083 | THE1B-int | MaLR | LTR |
| 141109404 | 141109667 | + | 12.5 | 1 | 317 | -1 | AluYb9 | Alu | SINE |
| 141109668 | 141109946 | + | 12.0 | 1 | 276 | -33 | AluSg1 | Alu | SINE |
| 141109969 | 141111030 | + | 13.7 | 498 | 1580 | 0 | THE1B-int | MaLR | LTR |
| 141111031 | 141111386 | + | 11.2 | 1 | 364 | 0 | THE1B | MaLR | LTR |
| 141111389 | 141112990 | + | 15.9 | 1458 | 3117 | -4448 | L1M1 | L1 | LINE |
| 141112993 | 141113285 | - | 10.6 | 1 | 293 | -17 | AluSg | Alu | SINE |
| 141113292 | 141113547 | + | 19.5 | 131 | 408 | 0 | MLT1A0 | MaLR | LTR |

  

|  |  |  |  |  |  |  |  |  |  |
| --- | --- | --- | --- | --- | --- | --- | --- | --- | --- |
| chr6:141411495-141417421 | | | 5334 bp | | |  |  |  |  |
| Genome Start | Genome End | Strand | Div | Repeat Start | Repeat End | Repeat Left | Name | Family | Class |
| 141411495 | 141411559 | + | 27.7 | 327 | 391 | -4605 | HERVL | ERVL | LTR |
| 141411560 | 141412116 | - | 28.3 | 1 | 590 | 0 | MLT1G | MaLR | LTR |
| 141412130 | 141413344 | - | 30.3 | 153 | 1472 | -17 | MLT1G-int | MaLR | LTR |
| 141413427 | 141413904 | - | 29.2 | 33 | 590 | 0 | MLT1G | MaLR | LTR |
| 141413916 | 141414199 | - | 19.7 | 3 | 289 | -23 | AluJo | Alu | SINE |
| 141414228 | 141414279 | + | 23.1 | 385 | 436 | -4560 | HERVL | ERVL | LTR |
| 141414528 | 141415134 | + | 31.4 | 701 | 1343 | -3653 | HERVL | ERVL | LTR |
| 141415185 | 141416889 | + | 32.6 | 1427 | 3201 | -1905 | HERVL | ERVL | LTR |
| 141417051 | 141417421 | + | 39.3 | 4509 | 4898 | -756 | HERVL | ERVL | LTR |

  

|  |  |  |  |  |  |  |  |  |  |
| --- | --- | --- | --- | --- | --- | --- | --- | --- | --- |
| chr6:141580036-141585869 | | | 5830 bp | | |  |  |  |  |
| Genome Start | Genome End | Strand | Div | Repeat Start | Repeat End | Repeat Left | Name | Family | Class |
| 141580036 | 141580446 | + | 19.0 | 22 | 429 | -5955 | L1M4c | L1 | LINE |
| 141580447 | 141580955 | - | 26.9 | 5 | 542 | 0 | MER34 | ERV1 | LTR |
| 141580956 | 141582870 | + | 19.0 | 430 | 2333 | -4051 | L1M4c | L1 | LINE |
| 141582873 | 141582982 | + | 19.9 | 3777 | 3881 | -2495 | L1M4c | L1 | LINE |
| 141582983 | 141583351 | - | 17.2 | 3 | 381 | 0 | THE1D | MaLR | LTR |
| 141583354 | 141584956 | - | 32.2 | 1 | 1735 | 0 | MLT-int | MaLR | LTR |
| 141584957 | 141585322 | - | 11.7 | 1 | 381 | 0 | THE1D | MaLR | LTR |
| 141585323 | 141585869 | + | 19.9 | 3882 | 4446 | -1930 | L1M4c | L1 | LINE |

  

|  |  |  |  |  |  |  |  |  |  |
| --- | --- | --- | --- | --- | --- | --- | --- | --- | --- |
| chr6:141791951-141795037 | | | 3068 bp | | |  |  |  |  |
| Genome Start | Genome End | Strand | Div | Repeat Start | Repeat End | Repeat Left | Name | Family | Class |
| 141791951 | 141792037 | + | 18.4 | 6896 | 6993 | -792 | L1MC3 | L1 | LINE |
| 141792053 | 141792405 | - | 12.2 | 1 | 355 | 0 | THE1A | MaLR | LTR |
| 141792406 | 141793973 | - | 29.6 | 2 | 1735 | 0 | MLT-int | MaLR | LTR |
| 141793976 | 141794317 | - | 9.9 | 13 | 355 | 0 | THE1A | MaLR | LTR |
| 141794320 | 141795037 | + | 21.8 | 7021 | 7784 | -1 | L1MC3 | L1 | LINE |

  

|  |  |  |  |  |  |  |  |  |  |
| --- | --- | --- | --- | --- | --- | --- | --- | --- | --- |
| chr6:141815001-141823685 | | | 8645 bp | | |  |  |  |  |
| Genome Start | Genome End | Strand | Div | Repeat Start | Repeat End | Repeat Left | Name | Family | Class |
| 141815001 | 141815218 | + | 25.2 | 7617 | 7842 | -153 | L1MD3 | L1 | LINE |
| 141815219 | 141815652 | - | 17.9 | 32 | 486 | 0 | MER66B | ERV1 | LTR |
| 141815655 | 141816054 | + | 8.0 | 1 | 448 | -2 | LTR7 | ERV1 | LTR |
| 141816057 | 141819892 | + | 9.8 | 1 | 3991 | -3722 | HERVH | ERV1 | LTR |
| 141819892 | 141823120 | + | 5.6 | 4481 | 7713 | 0 | HERVH | ERV1 | LTR |
| 141823121 | 141823510 | + | 8.7 | 1 | 448 | -2 | LTR7 | ERV1 | LTR |
| 141823548 | 141823685 | + | 17.9 | 7840 | 7990 | -5 | L1MD3 | L1 | LINE |

  

|  |  |  |  |  |  |  |  |  |  |
| --- | --- | --- | --- | --- | --- | --- | --- | --- | --- |
| chr6:141866447-141870126 | | | 3677 bp | | |  |  |  |  |
| Genome Start | Genome End | Strand | Div | Repeat Start | Repeat End | Repeat Left | Name | Family | Class |
| 141866447 | 141867208 | - | 29.6 | 709 | 1489 | 0 | MLT1G-int | MaLR | LTR |
| 141867209 | 141867553 | - | 11.6 | 1 | 364 | 0 | THE1B | MaLR | LTR |
| 141867555 | 141869125 | - | 30.6 | 2 | 1734 | -1 | MLT1G-int | MaLR | LTR |
| 141869128 | 141869473 | - | 12.8 | 1 | 364 | 0 | THE1B | MaLR | LTR |
| 141869474 | 141870126 | - | 29.6 | 6 | 708 | -781 | MLT1G-int | MaLR | LTR |

  

|  |  |  |  |  |  |  |  |  |  |
| --- | --- | --- | --- | --- | --- | --- | --- | --- | --- |
| chr6:142007299-142011000 | | | 3510 bp | | |  |  |  |  |
| Genome Start | Genome End | Strand | Div | Repeat Start | Repeat End | Repeat Left | Name | Family | Class |
| 142007299 | 142007582 | + | 20.5 | 4740 | 5031 | -1115 | L1MB8 | L1 | LINE |
| 142007689 | 142007957 | - | 21.6 | 9 | 267 | -45 | AluJo | Alu | SINE |
| 142007960 | 142008414 | + | 18.1 | 5024 | 5504 | -674 | L1MB8 | L1 | LINE |
| 142008417 | 142008790 | + | 12.6 | 1 | 375 | 0 | THE1C | MaLR | LTR |
| 142008833 | 142010203 | + | 13.2 | 26 | 1580 | 0 | THE1C-int | MaLR | LTR |
| 142010204 | 142010557 | + | 10.5 | 1 | 375 | 0 | THE1C | MaLR | LTR |
| 142010598 | 142011000 | + | 21.4 | 5526 | 5939 | -239 | L1MB8 | L1 | LINE |

  

|  |  |  |  |  |  |  |  |  |  |
| --- | --- | --- | --- | --- | --- | --- | --- | --- | --- |
| chr6:142160854-142165285 | | | 4427 bp | | |  |  |  |  |
| Genome Start | Genome End | Strand | Div | Repeat Start | Repeat End | Repeat Left | Name | Family | Class |
| 142160854 | 142162233 | + | 18.5 | 3523 | 4916 | -1230 | L1MC1 | L1 | LINE |
| 142162235 | 142162593 | + | 13.3 | 1 | 364 | 0 | THE1B | MaLR | LTR |
| 142162594 | 142163064 | + | 9.6 | 1 | 478 | -1102 | THE1B-int | MaLR | LTR |
| 142163064 | 142163781 | + | 11.2 | 861 | 1580 | 0 | THE1B-int | MaLR | LTR |
| 142163782 | 142164132 | + | 12.5 | 1 | 361 | -3 | THE1B | MaLR | LTR |
| 142164138 | 142164295 | + | 18.1 | 4931 | 5079 | -1067 | L1MC1 | L1 | LINE |
| 142164296 | 142164465 | + | 18.4 | 5662 | 5827 | -506 | L1MC1 | L1 | LINE |
| 142164466 | 142164775 | + | 7.6 | 1 | 303 | -10 | AluSq | Alu | SINE |
| 142164776 | 142165285 | + | 18.4 | 5828 | 6333 | 0 | L1MC1 | L1 | LINE |

  

|  |  |  |  |  |  |  |  |  |  |
| --- | --- | --- | --- | --- | --- | --- | --- | --- | --- |
| chr6:144230625-144242216 | | | 11403 bp | | |  |  |  |  |
| Genome Start | Genome End | Strand | Div | Repeat Start | Repeat End | Repeat Left | Name | Family | Class |
| 144230625 | 144231026 | - | 24.1 | 1405 | 1876 | -631 | HAL1 | L1 | LINE |
| 144231056 | 144231500 | - | 8.5 | 1 | 444 | 0 | MLT2A1 | ERVL | LTR |
| 144231501 | 144232118 | - | 5.3 | 5036 | 5654 | 0 | HERVL-A1 | ERVL | LTR |
| 144232116 | 144235383 | - | 7.1 | 1464 | 4690 | -964 | HERVL-A1 | ERVL | LTR |
| 144235384 | 144235734 | - | 10.3 | 1 | 355 | 0 | THE1A | MaLR | LTR |
| 144235735 | 144237287 | - | 29.6 | 2 | 1735 | 0 | MLT-int | MaLR | LTR |
| 144237290 | 144237643 | - | 8.8 | 1 | 355 | 0 | THE1A | MaLR | LTR |
| 144237653 | 144237965 | - | 17.1 | 1 | 310 | -2 | AluSx | Alu | SINE |
| 144237966 | 144239401 | - | 7.1 | 5 | 1463 | -4191 | HERVL-A1 | ERVL | LTR |
| 144239406 | 144239865 | - | 7.7 | 1 | 444 | 0 | MLT2A1 | ERVL | LTR |
| 144239866 | 144240099 | - | 26.9 | 1076 | 1336 | -1171 | HAL1 | L1 | LINE |
| 144240100 | 144240411 | - | 9.3 | 2 | 313 | 0 | AluSp | Alu | SINE |
| 144240412 | 144240782 | - | 26.9 | 567 | 1075 | -1432 | HAL1 | L1 | LINE |
| 144240859 | 144241155 | - | 22.3 | 353 | 652 | -1 | MER82 | MER2\_type | DNA |
| 144241159 | 144241310 | + | 15.1 | 134 | 285 | -27 | AluSg/x | Alu | SINE |
| 144241335 | 144241674 | - | 14.1 | 1 | 352 | -301 | MER82 | MER2\_type | DNA |
| 144241720 | 144242216 | - | 28.5 | 35 | 515 | -1992 | HAL1 | L1 | LINE |

  

|  |  |  |  |  |  |  |  |  |  |
| --- | --- | --- | --- | --- | --- | --- | --- | --- | --- |
| chr6:144403966-144412695 | | | 8725 bp | | |  |  |  |  |
| Genome Start | Genome End | Strand | Div | Repeat Start | Repeat End | Repeat Left | Name | Family | Class |
| 144403966 | 144404026 | - | 26.2 | 196 | 262 | 0 | MIR | MIR | SINE |
| 144404028 | 144410165 | - | 6.5 | 1 | 6139 | -15 | L1PA6 | L1 | LINE |
| 144410167 | 144410233 | - | 26.6 | 156 | 221 | -41 | MIR | MIR | SINE |
| 144410234 | 144410604 | - | 12.5 | 1 | 379 | -2 | THE1D | MaLR | LTR |
| 144410605 | 144412215 | - | 31.8 | 3 | 1735 | 0 | MLT-int | MaLR | LTR |
| 144412219 | 144412598 | - | 13.0 | 1 | 381 | 0 | THE1D | MaLR | LTR |
| 144412599 | 144412695 | - | 26.6 | 57 | 155 | -107 | MIR | MIR | SINE |

  

|  |  |  |  |  |  |  |  |  |  |
| --- | --- | --- | --- | --- | --- | --- | --- | --- | --- |
| chr6:144476022-144486196 | | | 10024 bp | | |  |  |  |  |
| Genome Start | Genome End | Strand | Div | Repeat Start | Repeat End | Repeat Left | Name | Family | Class |
| 144476022 | 144476140 | - | 32.8 | 4146 | 4264 | -1450 | ERVL-B4 | ERVL | LTR |
| 144476132 | 144476283 | - | 31.3 | 2918 | 3070 | -2597 | ERVL-B4 | ERVL | LTR |
| 144476294 | 144477617 | - | 30.6 | 1592 | 2943 | -2053 | ERVL-B4 | ERVL | LTR |
| 144477632 | 144477757 | + | 22.2 | 5914 | 6044 | -120 | L1ME2 | L1 | LINE |
| 144477771 | 144478276 | - | 14.3 | 5783 | 6291 | 0 | L1MA8 | L1 | LINE |
| 144478277 | 144478307 | + | 22.6 | 6055 | 6088 | -74 | L1ME3 | L1 | LINE |
| 144478332 | 144479143 | - | 11.5 | 5476 | 6300 | 0 | L1MA6 | L1 | LINE |
| 144479155 | 144479480 | - | 5.5 | 1 | 326 | -454 | LTR17 | ERV1 | LTR |
| 144479481 | 144479773 | - | 6.5 | 8335 | 8626 | 0 | HERV17 | ERV1 | LTR |
| 144479796 | 144483860 | - | 4.5 | 1953 | 6019 | -2607 | HERV17 | ERV1 | LTR |
| 144483853 | 144483891 | - | 15.4 | 1 | 39 | -8587 | HERV17 | ERV1 | LTR |
| 144483893 | 144484391 | - | 8.2 | 260 | 779 | -1 | LTR17 | ERV1 | LTR |
| 144484397 | 144485773 | - | 14.4 | 4119 | 5491 | -800 | L1MA6 | L1 | LINE |
| 144485842 | 144486196 | - | 31.0 | 1052 | 1427 | -3569 | ERVL-B4 | ERVL | LTR |

  

|  |  |  |  |  |  |  |  |  |  |
| --- | --- | --- | --- | --- | --- | --- | --- | --- | --- |
| chr6:146152710-146155432 | | | 2721 bp | | |  |  |  |  |
| Genome Start | Genome End | Strand | Div | Repeat Start | Repeat End | Repeat Left | Name | Family | Class |
| 146152710 | 146152735 | - | 41.5 | 3348 | 3368 | -10 | L2 | L2 | LINE |
| 146152736 | 146153097 | - | 14.7 | 1 | 364 | 0 | THE1B | MaLR | LTR |
| 146153098 | 146154670 | - | 29.4 | 2 | 1735 | 0 | MLT1A1-int | MaLR | LTR |
| 146154673 | 146155025 | - | 14.5 | 1 | 364 | 0 | THE1B | MaLR | LTR |
| 146155026 | 146155432 | - | 41.5 | 2945 | 3347 | -31 | L2 | L2 | LINE |

  

|  |  |  |  |  |  |  |  |  |  |
| --- | --- | --- | --- | --- | --- | --- | --- | --- | --- |
| chr6:146361368-146373055 | | | 11626 bp | | |  |  |  |  |
| Genome Start | Genome End | Strand | Div | Repeat Start | Repeat End | Repeat Left | Name | Family | Class |
| 146361368 | 146361534 | - | 23.4 | 6582 | 6761 | -14 | L1MB5 | L1 | LINE |
| 146361544 | 146361922 | - | 13.2 | 1 | 375 | 0 | THE1C | MaLR | LTR |
| 146361923 | 146362671 | - | 12.5 | 1 | 1580 | 0 | THE1C-int | MaLR | LTR |
| 146362672 | 146363039 | - | 13.1 | 1 | 375 | 0 | THE1C | MaLR | LTR |
| 146363040 | 146364178 | - | 19.4 | 5478 | 6584 | -191 | L1MB5 | L1 | LINE |
| 146364179 | 146364388 | - | 18.5 | 218 | 432 | 0 | THE1C | MaLR | LTR |
| 146364430 | 146365484 | + | 13.3 | 2298 | 3355 | -4210 | L1MA2 | L1 | LINE |
| 146365485 | 146365728 | - | 13.8 | 53 | 296 | -16 | AluSx | Alu | SINE |
| 146365729 | 146370038 | + | 10.2 | 3356 | 7713 | -10 | L1MA2 | L1 | LINE |
| 146370058 | 146370703 | - | 23.2 | 4301 | 4936 | -1811 | L1MB5 | L1 | LINE |
| 146370697 | 146373055 | - | 23.1 | 9 | 2537 | -4212 | L1MB5 | L1 | LINE |

  

|  |  |  |  |  |  |  |  |  |  |
| --- | --- | --- | --- | --- | --- | --- | --- | --- | --- |
| chr6:148419392-148428221 | | | 8830 bp | | |  |  |  |  |
| Genome Start | Genome End | Strand | Div | Repeat Start | Repeat End | Repeat Left | Name | Family | Class |
| 148419392 | 148422016 | - | 8.8 | 3686 | 6354 | -5 | L1PA11 | L1 | LINE |
| 148422017 | 148422358 | - | 12.7 | 1 | 375 | 0 | THE1C | MaLR | LTR |
| 148422359 | 148423819 | - | 11.0 | 1 | 1580 | 0 | THE1C-int | MaLR | LTR |
| 148423820 | 148424176 | - | 10.4 | 1 | 375 | 0 | THE1C | MaLR | LTR |
| 148424177 | 148424682 | - | 8.9 | 3184 | 3690 | -2641 | L1PA11 | L1 | LINE |
| 148424683 | 148424995 | - | 7.8 | 1 | 311 | -2 | AluSq | Alu | SINE |
| 148424996 | 148428221 | - | 12.9 | 127 | 3183 | -3148 | L1PA11 | L1 | LINE |

  

|  |  |  |  |  |  |  |  |  |  |
| --- | --- | --- | --- | --- | --- | --- | --- | --- | --- |
| chr6:151490078-151493862 | | | 3749 bp | | |  |  |  |  |
| Genome Start | Genome End | Strand | Div | Repeat Start | Repeat End | Repeat Left | Name | Family | Class |
| 151490078 | 151490235 | + | 33.1 | 1 | 166 | -425 | MLT1K | MaLR | LTR |
| 151490241 | 151490608 | + | 14.2 | 1 | 381 | 0 | THE1D | MaLR | LTR |
| 151490609 | 151490893 | + | 13.3 | 1 | 288 | -1292 | THE1D-int | MaLR | LTR |
| 151490894 | 151491360 | + | 9.4 | 1 | 511 | 0 | LTR10F | ERV1 | LTR |
| 151491361 | 151491773 | + | 13.3 | 289 | 657 | -923 | THE1D-int | MaLR | LTR |
| 151491798 | 151492208 | + | 13.3 | 658 | 1094 | -486 | THE1D-int | MaLR | LTR |
| 151492209 | 151492632 | + | 4.8 | 1 | 456 | -8 | LTR7B | ERV1 | LTR |
| 151492630 | 151493114 | + | 9.6 | 1090 | 1574 | -6 | THE1D-int | MaLR | LTR |
| 151493117 | 151493492 | + | 13.8 | 1 | 381 | 0 | THE1D | MaLR | LTR |
| 151493501 | 151493862 | + | 29.9 | 208 | 566 | -25 | MLT1K | MaLR | LTR |

  

|  |  |  |  |  |  |  |  |  |  |
| --- | --- | --- | --- | --- | --- | --- | --- | --- | --- |
| chr6:155829729-155832482 | | | 2717 bp | | |  |  |  |  |
| Genome Start | Genome End | Strand | Div | Repeat Start | Repeat End | Repeat Left | Name | Family | Class |
| 155829729 | 155829755 | - | 20.6 | 520 | 546 | -13 | MLT2C2 | ERVL | LTR |
| 155829789 | 155830051 | - | 20.6 | 149 | 424 | -40 | MLT2C2 | ERVL | LTR |
| 155830052 | 155830406 | - | 9.3 | 1 | 355 | 0 | THE1A | MaLR | LTR |
| 155830407 | 155831981 | - | 29.8 | 4 | 1735 | 0 | MLT-int | MaLR | LTR |
| 155831986 | 155832339 | - | 9.0 | 1 | 355 | 0 | THE1A | MaLR | LTR |
| 155832340 | 155832482 | - | 20.6 | 5 | 148 | -316 | MLT2C2 | ERVL | LTR |

  

|  |  |  |  |  |  |  |  |  |  |
| --- | --- | --- | --- | --- | --- | --- | --- | --- | --- |
| chr6:156219651-156227750 | | | 8071 bp | | |  |  |  |  |
| Genome Start | Genome End | Strand | Div | Repeat Start | Repeat End | Repeat Left | Name | Family | Class |
| 156219651 | 156220614 | - | 23.0 | 5290 | 6234 | -5 | L1ME1 | L1 | LINE |
| 156220611 | 156220954 | - | 19.7 | 1676 | 2018 | -4382 | L1ME1 | L1 | LINE |
| 156220955 | 156221308 | + | 13.6 | 1 | 364 | 0 | THE1B | MaLR | LTR |
| 156221311 | 156222858 | + | 30.2 | 2 | 1735 | 0 | MLT-int | MaLR | LTR |
| 156222860 | 156223204 | + | 13.0 | 2 | 364 | 0 | THE1B | MaLR | LTR |
| 156223205 | 156223699 | - | 19.7 | 1118 | 1675 | -4725 | L1ME1 | L1 | LINE |
| 156223709 | 156224508 | - | 13.3 | 5353 | 6157 | -9 | L1PA16 | L1 | LINE |
| 156224526 | 156224578 | - | 13.2 | 371 | 423 | -132 | MER66C | ERV1 | LTR |
| 156224585 | 156226051 | - | 12.7 | 3868 | 5359 | -787 | L1PA16 | L1 | LINE |
| 156226056 | 156226740 | + | 16.2 | 3192 | 3863 | -2283 | L1PA16 | L1 | LINE |
| 156226735 | 156227750 | - | 21.2 | 25 | 1130 | -5270 | L1ME1 | L1 | LINE |

  

|  |  |  |  |  |  |  |  |  |  |
| --- | --- | --- | --- | --- | --- | --- | --- | --- | --- |
| chr6:156973752-156979463 | | | 5669 bp | | |  |  |  |  |
| Genome Start | Genome End | Strand | Div | Repeat Start | Repeat End | Repeat Left | Name | Family | Class |
| 156973752 | 156973769 | + | 31.1 | 67 | 85 | -6034 | L1MEd | L1 | LINE |
| 156973770 | 156974108 | + | 22.4 | 1 | 345 | 0 | MER2 | MER2\_type | DNA |
| 156974109 | 156975202 | + | 31.1 | 86 | 1181 | -4938 | L1MEd | L1 | LINE |
| 156975244 | 156975751 | + | 29.1 | 1296 | 1832 | -4317 | L1MEd | L1 | LINE |
| 156975752 | 156976121 | - | 9.7 | 1 | 381 | 0 | THE1D | MaLR | LTR |
| 156976122 | 156977746 | - | 30.0 | 2 | 1735 | 0 | MLT-int | MaLR | LTR |
| 156977749 | 156978091 | - | 11.8 | 1 | 381 | 0 | THE1D | MaLR | LTR |
| 156978092 | 156978530 | + | 29.5 | 1833 | 2279 | -3861 | L1MEd | L1 | LINE |
| 156978531 | 156978842 | + | 13.1 | 1 | 312 | 0 | AluSx | Alu | SINE |
| 156978843 | 156979152 | + | 30.9 | 2269 | 2581 | -3548 | L1MEd | L1 | LINE |
| 156979153 | 156979449 | + | 13.5 | 1 | 303 | -9 | AluSx | Alu | SINE |
| 156979450 | 156979463 | + | 31.9 | 2582 | 2594 | -3535 | L1MEd | L1 | LINE |

  

|  |  |  |  |  |  |  |  |  |  |
| --- | --- | --- | --- | --- | --- | --- | --- | --- | --- |
| chr6:159220147-159233547 | | | 13266 bp | | |  |  |  |  |
| Genome Start | Genome End | Strand | Div | Repeat Start | Repeat End | Repeat Left | Name | Family | Class |
| 159220147 | 159220207 | - | 19.2 | 3200 | 3270 | -3267 | L1MDa | L1 | LINE |
| 159220208 | 159220890 | - | 8.6 | 1 | 691 | 0 | LTR8 | ERV1 | LTR |
| 159220891 | 159220912 | - | 21.4 | 3184 | 3199 | -3338 | L1MDa | L1 | LINE |
| 159220913 | 159221215 | - | 5.0 | 1 | 303 | -8 | AluY | Alu | SINE |
| 159221216 | 159221242 | - | 21.4 | 3157 | 3183 | -3354 | L1MDa | L1 | LINE |
| 159221406 | 159221829 | - | 6.3 | 1 | 456 | -8 | LTR7B | ERV1 | LTR |
| 159221830 | 159223530 | - | 13.4 | 6018 | 7713 | 0 | HERVH | ERV1 | LTR |
| 159223531 | 159223830 | + | 6.0 | 1 | 299 | -12 | AluY | Alu | SINE |
| 159223831 | 159226541 | - | 13.4 | 3357 | 6017 | -1696 | HERVH | ERV1 | LTR |
| 159226535 | 159228941 | - | 20.0 | 825 | 3077 | -3577 | HERVH | ERV1 | LTR |
| 159228942 | 159229251 | + | 7.8 | 1 | 306 | -5 | AluY | Alu | SINE |
| 159229252 | 159229289 | - | 17.0 | 793 | 824 | -6889 | HERVH | ERV1 | LTR |
| 159229257 | 159229771 | - | 24.1 | 1 | 519 | -7194 | HERVH | ERV1 | LTR |
| 159229772 | 159230235 | - | 5.0 | 1 | 464 | 0 | LTR7B | ERV1 | LTR |
| 159230239 | 159230540 | + | 11.9 | 5 | 306 | -6 | AluSx | Alu | SINE |
| 159230543 | 159230691 | + | 11.4 | 1 | 149 | -160 | AluSc | Alu | SINE |
| 159230692 | 159230903 | - | 18.6 | 2882 | 3119 | -3418 | L1MDa | L1 | LINE |
| 159230904 | 159231202 | - | 16.4 | 1 | 304 | -8 | AluJb | Alu | SINE |
| 159231203 | 159231283 | - | 18.6 | 2792 | 2881 | -3656 | L1MDa | L1 | LINE |
| 159231284 | 159231576 | - | 24.6 | 224 | 516 | -3 | LTR40a | ERVL | LTR |
| 159231577 | 159231870 | + | 9.3 | 1 | 289 | -20 | AluSc | Alu | SINE |
| 159231871 | 159232094 | - | 24.6 | 1 | 223 | -296 | LTR40a | ERVL | LTR |
| 159232095 | 159232216 | - | 18.6 | 2681 | 2791 | -3746 | L1MDa | L1 | LINE |
| 159232217 | 159232541 | - | 5.7 | 1 | 321 | 0 | AluY | Alu | SINE |
| 159232542 | 159232687 | - | 18.6 | 2552 | 2680 | -3857 | L1MDa | L1 | LINE |
| 159232688 | 159232775 | + | 20.4 | 4 | 91 | -16 | MER58A | MER1\_type | DNA |
| 159232776 | 159233061 | - | 10.5 | 1 | 294 | -19 | AluSq | Alu | SINE |
| 159233062 | 159233175 | + | 20.4 | 92 | 205 | -19 | MER58A | MER1\_type | DNA |
| 159233183 | 159233547 | - | 26.1 | 2187 | 2556 | -4077 | L1MDa | L1 | LINE |

  

|  |  |  |  |  |  |  |  |  |  |
| --- | --- | --- | --- | --- | --- | --- | --- | --- | --- |
| chr6:159364363-159367740 | | | 3377 bp | | |  |  |  |  |
| Genome Start | Genome End | Strand | Div | Repeat Start | Repeat End | Repeat Left | Name | Family | Class |
| 159364363 | 159364858 | + | 22.3 | 3764 | 4273 | -1873 | L1M5 | L1 | LINE |
| 159364859 | 159365251 | - | 15.7 | 1 | 428 | 0 | MSTA | MaLR | LTR |
| 159365252 | 159366875 | - | 31.0 | 1 | 1735 | 0 | MLT-int | MaLR | LTR |
| 159366877 | 159367272 | - | 13.0 | 1 | 428 | 0 | MSTA | MaLR | LTR |
| 159367273 | 159367328 | + | 22.3 | 4274 | 4327 | -1819 | L1M5 | L1 | LINE |
| 159367329 | 159367625 | - | 15.7 | 1 | 298 | -14 | AluJo | Alu | SINE |
| 159367626 | 159367740 | + | 22.3 | 4328 | 4442 | -1704 | L1M5 | L1 | LINE |

  

|  |  |  |  |  |  |  |  |  |  |
| --- | --- | --- | --- | --- | --- | --- | --- | --- | --- |
| chr6:160566551-160579312 | | | 12710 bp | | |  |  |  |  |
| Genome Start | Genome End | Strand | Div | Repeat Start | Repeat End | Repeat Left | Name | Family | Class |
| 160566551 | 160566678 | + | 29.8 | 21 | 151 | -111 | MIR | MIR | SINE |
| 160566682 | 160566810 | + | 13.9 | 1 | 129 | -274 | MER57A | ERV1 | LTR |
| 160566811 | 160567479 | + | 9.9 | 1 | 691 | 0 | LTR8 | ERV1 | LTR |
| 160567480 | 160567755 | + | 13.9 | 130 | 403 | 0 | MER57A | ERV1 | LTR |
| 160567771 | 160568684 | + | 16.4 | 1 | 900 | 0 | MER4D1 | ERV1 | LTR |
| 160568685 | 160569233 | + | 11.9 | 1 | 554 | 0 | MER41A | ERV1 | LTR |
| 160569234 | 160569664 | + | 13.7 | 1 | 431 | -5515 | MER41A-int | ERV1 | LTR |
| 160569673 | 160570065 | + | 9.6 | 1 | 442 | -8 | LTR7 | ERV1 | LTR |
| 160570074 | 160572763 | + | 12.8 | 1 | 2830 | -4883 | HERVH | ERV1 | LTR |
| 160572764 | 160573731 | + | 13.1 | 3357 | 4290 | -3423 | HERVH | ERV1 | LTR |
| 160573730 | 160574521 | + | 9.3 | 4221 | 5016 | -2697 | HERVH | ERV1 | LTR |
| 160574525 | 160574947 | + | 10.9 | 7283 | 7713 | 0 | HERVH | ERV1 | LTR |
| 160574948 | 160575344 | + | 9.6 | 1 | 448 | -2 | LTR7 | ERV1 | LTR |
| 160575346 | 160575628 | + | 12.3 | 434 | 690 | -5256 | MER41A-int | ERV1 | LTR |
| 160575630 | 160575984 | + | 14.2 | 2005 | 2371 | -5166 | MER41A-int | ERV1 | LTR |
| 160575982 | 160576068 | + | 11.5 | 1898 | 1991 | -3955 | MER41A-int | ERV1 | LTR |
| 160576077 | 160576517 | + | 14.8 | 2167 | 2619 | -3327 | MER41A-int | ERV1 | LTR |
| 160576524 | 160578687 | + | 9.3 | 2955 | 5945 | -1 | MER41A-int | ERV1 | LTR |
| 160578689 | 160579240 | + | 9.3 | 3 | 554 | 0 | MER41A | ERV1 | LTR |
| 160579244 | 160579312 | + | 17.4 | 166 | 235 | -27 | MIR | MIR | SINE |

  

|  |  |  |  |  |  |  |  |  |  |
| --- | --- | --- | --- | --- | --- | --- | --- | --- | --- |
| chr6:161650260-161659077 | | | 8511 bp | | |  |  |  |  |
| Genome Start | Genome End | Strand | Div | Repeat Start | Repeat End | Repeat Left | Name | Family | Class |
| 161650260 | 161650372 | - | 27.7 | 7930 | 8042 | 0 | L1MC4 | L1 | LINE |
| 161650380 | 161650675 | - | 11.7 | 19 | 313 | 0 | AluSp | Alu | SINE |
| 161650676 | 161651180 | - | 9.0 | 1 | 508 | -3 | LTR10F | ERV1 | LTR |
| 161651181 | 161656136 | - | 9.6 | 1 | 5102 | 0 | HERVIP10FH | ERV1 | LTR |
| 161656137 | 161656537 | - | 9.3 | 104 | 511 | 0 | LTR10F | ERV1 | LTR |
| 161656543 | 161656649 | - | 3.0 | 104 | 210 | -301 | LTR10F | ERV1 | LTR |
| 161656655 | 161656684 | - | 0.0 | 127 | 156 | -355 | LTR10F | ERV1 | LTR |
| 161656685 | 161656896 | - | 5.4 | 1 | 210 | -301 | LTR10F | ERV1 | LTR |
| 161656993 | 161657875 | + | 14.7 | 45 | 977 | -254 | Tigger3b | MER2\_type | DNA |
| 161657876 | 161658167 | - | 13.0 | 1 | 295 | -18 | AluSq | Alu | SINE |
| 161658168 | 161658355 | + | 14.7 | 978 | 1170 | -61 | Tigger3b | MER2\_type | DNA |
| 161658356 | 161658644 | - | 8.0 | 10 | 298 | -14 | AluSx | Alu | SINE |
| 161658645 | 161658725 | + | 14.7 | 1171 | 1231 | 0 | Tigger3b | MER2\_type | DNA |
| 161658920 | 161659077 | - | 27.4 | 7742 | 7912 | -130 | L1MC4 | L1 | LINE |

  

|  |  |  |  |  |  |  |  |  |  |
| --- | --- | --- | --- | --- | --- | --- | --- | --- | --- |
| chr6:164498296-164502565 | | | 4168 bp | | |  |  |  |  |
| Genome Start | Genome End | Strand | Div | Repeat Start | Repeat End | Repeat Left | Name | Family | Class |
| 164498296 | 164499466 | - | 30.7 | 2101 | 3378 | -41 | L2 | L2 | LINE |
| 164499507 | 164499882 | - | 8.6 | 2 | 375 | 0 | THE1C | MaLR | LTR |
| 164499884 | 164501426 | - | 10.3 | 1 | 1580 | 0 | THE1C-int | MaLR | LTR |
| 164501427 | 164501802 | - | 9.9 | 1 | 375 | 0 | THE1C | MaLR | LTR |
| 164501804 | 164502131 | - | 28.8 | 1578 | 1973 | -1446 | L2 | L2 | LINE |
| 164502164 | 164502373 | - | 30.1 | 14 | 234 | -28 | MIR | MIR | SINE |
| 164502402 | 164502565 | - | 31.6 | 1407 | 1566 | -1853 | L2 | L2 | LINE |

  

|  |  |  |  |  |  |  |  |  |  |
| --- | --- | --- | --- | --- | --- | --- | --- | --- | --- |
| chr6:164617640-164623195 | | | 5073 bp | | |  |  |  |  |
| Genome Start | Genome End | Strand | Div | Repeat Start | Repeat End | Repeat Left | Name | Family | Class |
| 164617640 | 164617874 | - | 23.3 | 5539 | 5818 | -405 | L1ME3B | L1 | LINE |
| 164617932 | 164618029 | - | 28.6 | 5262 | 5361 | -768 | L1ME3B | L1 | LINE |
| 164618106 | 164618892 | - | 29.9 | 4404 | 5255 | -874 | L1ME3B | L1 | LINE |
| 164618946 | 164619395 | - | 33.1 | 2211 | 2690 | -3439 | L1ME3B | L1 | LINE |
| 164619396 | 164619741 | + | 15.9 | 1 | 375 | 0 | THE1C | MaLR | LTR |
| 164619745 | 164621292 | + | 30.3 | 3 | 1733 | -2 | MLT-int | MaLR | LTR |
| 164621295 | 164621667 | + | 12.7 | 1 | 375 | 0 | THE1C | MaLR | LTR |
| 164621668 | 164621715 | - | 33.1 | 2162 | 2210 | -3919 | L1ME3B | L1 | LINE |
| 164621772 | 164622060 | + | 16.8 | 1 | 289 | -23 | AluSx | Alu | SINE |
| 164622073 | 164622232 | - | 29.9 | 1969 | 2131 | -4018 | L1ME3B | L1 | LINE |
| 164622361 | 164622767 | - | 27.2 | 1343 | 1794 | -4355 | L1ME3B | L1 | LINE |
| 164622783 | 164623000 | + | 20.2 | 1 | 187 | -6 | MER53 | DNA | DNA |
| 164623082 | 164623195 | - | 33.3 | 562 | 675 | -5444 | L1ME3B | L1 | LINE |

  

|  |  |  |  |  |  |  |  |  |  |
| --- | --- | --- | --- | --- | --- | --- | --- | --- | --- |
| chr6:164901838-164904367 | | | 2451 bp | | |  |  |  |  |
| Genome Start | Genome End | Strand | Div | Repeat Start | Repeat End | Repeat Left | Name | Family | Class |
| 164901838 | 164902016 | - | 31.4 | 869 | 1058 | -5365 | L1MCc | L1 | LINE |
| 164902027 | 164902281 | + | 16.9 | 125 | 375 | 0 | THE1C | MaLR | LTR |
| 164902282 | 164903766 | + | 13.6 | 1 | 1580 | 0 | THE1C-int | MaLR | LTR |
| 164903767 | 164904144 | + | 14.1 | 1 | 375 | 0 | THE1C | MaLR | LTR |
| 164904214 | 164904367 | - | 26.6 | 20 | 193 | -6230 | L1MCc | L1 | LINE |

  

|  |  |  |  |  |  |  |  |  |  |
| --- | --- | --- | --- | --- | --- | --- | --- | --- | --- |
| chr6:164992524-164999461 | | | 6930 bp | | |  |  |  |  |
| Genome Start | Genome End | Strand | Div | Repeat Start | Repeat End | Repeat Left | Name | Family | Class |
| 164992524 | 164993139 | - | 21.9 | 3982 | 4604 | -1542 | L1M4 | L1 | LINE |
| 164993165 | 164993574 | - | 9.3 | 1 | 448 | -2 | LTR7 | ERV1 | LTR |
| 164993575 | 164993958 | - | 9.2 | 7315 | 7713 | 0 | HERVH | ERV1 | LTR |
| 164993952 | 164994168 | - | 13.9 | 5600 | 5819 | -1894 | HERVH | ERV1 | LTR |
| 164994170 | 164994532 | - | 16.8 | 4782 | 5149 | -2564 | HERVH | ERV1 | LTR |
| 164994524 | 164994872 | - | 12.9 | 4488 | 4835 | -2878 | HERVH | ERV1 | LTR |
| 164994875 | 164995368 | - | 8.9 | 3500 | 3993 | -3720 | HERVH | ERV1 | LTR |
| 164995368 | 164995515 | - | 15.5 | 3140 | 3290 | -4423 | HERVH | ERV1 | LTR |
| 164995510 | 164998674 | - | 9.8 | 1 | 2987 | -4726 | HERVH | ERV1 | LTR |
| 164998677 | 164999037 | - | 8.6 | 1 | 448 | -2 | LTR7 | ERV1 | LTR |
| 164999039 | 164999461 | - | 21.2 | 3469 | 3944 | -2202 | L1M4 | L1 | LINE |

  

|  |  |  |  |  |  |  |  |  |  |
| --- | --- | --- | --- | --- | --- | --- | --- | --- | --- |
| chr6:165068609-165071652 | | | 3011 bp | | |  |  |  |  |
| Genome Start | Genome End | Strand | Div | Repeat Start | Repeat End | Repeat Left | Name | Family | Class |
| 165068609 | 165068722 | + | 24.8 | 4184 | 4301 | -1845 | L1M4 | L1 | LINE |
| 165068729 | 165069091 | + | 12.1 | 1 | 375 | 0 | THE1C | MaLR | LTR |
| 165069092 | 165070670 | + | 13.1 | 1 | 1574 | -6 | THE1C-int | MaLR | LTR |
| 165070676 | 165071043 | + | 10.9 | 1 | 375 | 0 | THE1C | MaLR | LTR |
| 165071048 | 165071402 | + | 29.6 | 4307 | 4667 | -1479 | L1M4 | L1 | LINE |
| 165071421 | 165071652 | + | 20.3 | 5228 | 5500 | -674 | L1M4 | L1 | LINE |

  

|  |  |  |  |  |  |  |  |  |  |
| --- | --- | --- | --- | --- | --- | --- | --- | --- | --- |
| chr6:165139502-165149314 | | | 9635 bp | | |  |  |  |  |
| Genome Start | Genome End | Strand | Div | Repeat Start | Repeat End | Repeat Left | Name | Family | Class |
| 165139502 | 165139712 | + | 37.7 | 469 | 682 | -5437 | L1MEd | L1 | LINE |
| 165139833 | 165140198 | - | 14.8 | 1 | 381 | 0 | THE1D | MaLR | LTR |
| 165140199 | 165141017 | - | 17.9 | 747 | 1580 | 0 | THE1D-int | MaLR | LTR |
| 165141016 | 165141548 | - | 18.4 | 3 | 550 | -4 | MLT2A2 | ERVL | LTR |
| 165141552 | 165147228 | - | 11.8 | 1 | 5653 | -1 | HERVL-A2 | ERVL | LTR |
| 165147229 | 165147772 | - | 18.2 | 1 | 554 | 0 | MLT2A2 | ERVL | LTR |
| 165147773 | 165148552 | - | 17.0 | 4 | 753 | -827 | THE1D-int | MaLR | LTR |
| 165148556 | 165148914 | - | 16.3 | 1 | 381 | 0 | THE1D | MaLR | LTR |
| 165148969 | 165149314 | + | 32.7 | 743 | 1209 | -4910 | L1MEd | L1 | LINE |

  

|  |  |  |  |  |  |  |  |  |  |
| --- | --- | --- | --- | --- | --- | --- | --- | --- | --- |
| chr6:165270321-165276656 | | | 6304 bp | | |  |  |  |  |
| Genome Start | Genome End | Strand | Div | Repeat Start | Repeat End | Repeat Left | Name | Family | Class |
| 165270321 | 165270884 | + | 23.9 | 251 | 816 | -5935 | L1MCb | L1 | LINE |
| 165270912 | 165271024 | + | 22.1 | 6167 | 6279 | -33 | L1MA9 | L1 | LINE |
| 165271023 | 165272634 | + | 20.7 | 612 | 2355 | -4396 | L1MCb | L1 | LINE |
| 165272635 | 165273058 | + | 14.5 | 1 | 422 | 0 | AluSx | Alu | SINE |
| 165273059 | 165274146 | + | 19.3 | 2356 | 3486 | -3078 | L1MCb | L1 | LINE |
| 165274150 | 165274496 | - | 20.2 | 1 | 391 | -5 | MSTD | MaLR | LTR |
| 165274499 | 165276145 | - | 24.9 | 2 | 1733 | -2 | MSTD-int | MaLR | LTR |
| 165276148 | 165276481 | - | 23.3 | 1 | 396 | 0 | MSTD | MaLR | LTR |
| 165276482 | 165276656 | + | 20.0 | 3480 | 3662 | -2902 | L1MCb | L1 | LINE |

  

|  |  |  |  |  |  |  |  |  |  |
| --- | --- | --- | --- | --- | --- | --- | --- | --- | --- |
| chr6:165785189-165788963 | | | 3683 bp | | |  |  |  |  |
| Genome Start | Genome End | Strand | Div | Repeat Start | Repeat End | Repeat Left | Name | Family | Class |
| 165785189 | 165785338 | - | 29.4 | 3274 | 3411 | -8 | L2 | L2 | LINE |
| 165785389 | 165785826 | + | 27.8 | 1071 | 1548 | -4119 | ERVL-E | ERVL | LTR |
| 165785831 | 165786209 | + | 19.8 | 1 | 396 | 0 | MSTD | MaLR | LTR |
| 165786212 | 165787858 | + | 26.0 | 2 | 1734 | -1 | MSTD-int | MaLR | LTR |
| 165787860 | 165788243 | + | 18.8 | 1 | 385 | -11 | MSTD | MaLR | LTR |
| 165788247 | 165788619 | + | 28.4 | 1544 | 1911 | -3756 | ERVL-E | ERVL | LTR |
| 165788620 | 165788708 | - | 16.9 | 244 | 302 | 0 | Alu | Alu | SINE |
| 165788716 | 165788882 | + | 22.0 | 2000 | 2169 | -3545 | ERVL-E | ERVL | LTR |
| 165788908 | 165788963 | - | 28.6 | 3052 | 3107 | -312 | L2 | L2 | LINE |

  

|  |  |  |  |  |  |  |  |  |  |
| --- | --- | --- | --- | --- | --- | --- | --- | --- | --- |
| chr6:167895694-167898109 | | | 2414 bp | | |  |  |  |  |
| Genome Start | Genome End | Strand | Div | Repeat Start | Repeat End | Repeat Left | Name | Family | Class |
| 167895694 | 167895793 | + | 20.1 | 1 | 102 | -40 | FLAM\_A | Alu | SINE |
| 167895794 | 167896147 | - | 13.3 | 1 | 355 | 0 | THE1A | MaLR | LTR |
| 167896148 | 167897729 | - | 29.4 | 2 | 1735 | 0 | MLT1A0-int | MaLR | LTR |
| 167897732 | 167898076 | - | 7.8 | 1 | 355 | 0 | THE1A | MaLR | LTR |
| 167898077 | 167898109 | + | 20.1 | 103 | 131 | -11 | FLAM\_A | Alu | SINE |

  

|  |  |  |  |  |  |  |  |  |  |
| --- | --- | --- | --- | --- | --- | --- | --- | --- | --- |
| chr6:169414960-169419781 | | | 4824 bp | | |  |  |  |  |
| Genome Start | Genome End | Strand | Div | Repeat Start | Repeat End | Repeat Left | Name | Family | Class |
| 169414960 | 169415514 | + | 15.9 | 3188 | 3742 | -2445 | MER57-int | ERV1 | LTR |
| 169415515 | 169415903 | + | 17.2 | 1 | 428 | 0 | MSTA | MaLR | LTR |
| 169415906 | 169416407 | + | 28.9 | 2 | 546 | -1189 | MLT-int | MaLR | LTR |
| 169416408 | 169416719 | + | 10.3 | 1 | 311 | 0 | AluY | Alu | SINE |
| 169416720 | 169417893 | + | 28.9 | 547 | 1735 | 0 | MLT-int | MaLR | LTR |
| 169417894 | 169418279 | + | 18.1 | 1 | 428 | 0 | MSTA | MaLR | LTR |
| 169418280 | 169419638 | + | 15.9 | 3743 | 5088 | -1099 | MER57-int | ERV1 | LTR |
| 169419635 | 169419781 | + | 8.2 | 6999 | 7146 | -391 | MER57-int | ERV1 | LTR |

  

|  |  |  |  |  |  |  |  |  |  |
| --- | --- | --- | --- | --- | --- | --- | --- | --- | --- |
| chr6:170776257-170779053 | | | 2793 bp | | |  |  |  |  |
| Genome Start | Genome End | Strand | Div | Repeat Start | Repeat End | Repeat Left | Name | Family | Class |
| 170776257 | 170776533 | - | 29.4 | 252 | 526 | -29 | MLT1H1 | MaLR | LTR |
| 170776534 | 170776893 | + | 14.7 | 3 | 364 | 0 | THE1B | MaLR | LTR |
| 170776898 | 170778455 | + | 31.1 | 4 | 1735 | 0 | MLT-int | MaLR | LTR |
| 170778456 | 170778813 | + | 18.1 | 1 | 364 | 0 | THE1B | MaLR | LTR |
| 170778814 | 170779053 | - | 29.4 | 3 | 251 | -304 | MLT1H1 | MaLR | LTR |

  


|  |  |  |  |  |  |  |  |  |  |
| --- | --- | --- | --- | --- | --- | --- | --- | --- | --- |
| chr7:6953844-6968978 | | | 15085 bp | | |  |  |  |  |
| Genome Start | Genome End | Strand | Div | Repeat Start | Repeat End | Repeat Left | Name | Family | Class |
| 6953844 | 6954029 | + | 24.4 | 1 | 185 | -320 | MLT1D | MaLR | LTR |
| 6954040 | 6954342 | + | 8.9 | 3 | 306 | -7 | AluSp | Alu | SINE |
| 6954357 | 6954754 | - | 9.3 | 2 | 448 | -2 | LTR7 | ERV1 | LTR |
| 6954756 | 6955132 | - | 10.5 | 7324 | 7713 | 0 | HERVH | ERV1 | LTR |
| 6955133 | 6955343 | - | 11.8 | 5600 | 5819 | -1894 | HERVH | ERV1 | LTR |
| 6955345 | 6956007 | - | 14.9 | 4488 | 5149 | -2564 | HERVH | ERV1 | LTR |
| 6956010 | 6956564 | - | 8.0 | 3441 | 3993 | -3720 | HERVH | ERV1 | LTR |
| 6956565 | 6957060 | - | 9.7 | 1 | 500 | -1 | LTR2C | ERV1 | LTR |
| 6957061 | 6964901 | - | 6.8 | 3 | 7812 | -1 | HERVE | ERV1 | LTR |
| 6964902 | 6965206 | - | 11.8 | 3140 | 3445 | -4268 | HERVH | ERV1 | LTR |
| 6965193 | 6968066 | - | 9.9 | 1 | 2992 | -4721 | HERVH | ERV1 | LTR |
| 6968069 | 6968419 | - | 10.9 | 1 | 448 | -2 | LTR7 | ERV1 | LTR |
| 6968454 | 6968627 | + | 24.8 | 206 | 431 | -74 | MLT1D | MaLR | LTR |
| 6968628 | 6968934 | - | 13.1 | 1 | 305 | -7 | AluSx | Alu | SINE |
| 6968935 | 6968978 | + | 23.7 | 432 | 484 | -21 | MLT1D | MaLR | LTR |

  

|  |  |  |  |  |  |  |  |  |  |
| --- | --- | --- | --- | --- | --- | --- | --- | --- | --- |
| chr7:7857245-7862888 | | | 5626 bp | | |  |  |  |  |
| Genome Start | Genome End | Strand | Div | Repeat Start | Repeat End | Repeat Left | Name | Family | Class |
| 7857245 | 7858361 | - | 16.9 | 4824 | 5973 | -205 | L1MB8 | L1 | LINE |
| 7858362 | 7859313 | - | 18.2 | 1 | 928 | -3 | MER21A | ERV1 | LTR |
| 7859314 | 7860158 | - | 16.5 | 3958 | 4823 | -1323 | L1MB8 | L1 | LINE |
| 7860159 | 7860229 | - | 13.5 | 314 | 381 | 0 | THE1D | MaLR | LTR |
| 7860230 | 7860535 | + | 11.1 | 1 | 307 | -6 | AluSp | Alu | SINE |
| 7860536 | 7860838 | - | 13.5 | 1 | 313 | -68 | THE1D | MaLR | LTR |
| 7860841 | 7862375 | - | 12.6 | 2 | 1578 | -2 | THE1D-int | MaLR | LTR |
| 7862377 | 7862741 | - | 14.1 | 1 | 381 | 0 | THE1D | MaLR | LTR |
| 7862757 | 7862888 | - | 18.8 | 3839 | 3955 | -2191 | L1MB8 | L1 | LINE |

  

|  |  |  |  |  |  |  |  |  |  |
| --- | --- | --- | --- | --- | --- | --- | --- | --- | --- |
| chr7:8351592-8354222 | | | 2629 bp | | |  |  |  |  |
| Genome Start | Genome End | Strand | Div | Repeat Start | Repeat End | Repeat Left | Name | Family | Class |
| 8351592 | 8351650 | + | 20.9 | 4 | 66 | -308 | MLT1A | MaLR | LTR |
| 8351651 | 8352024 | + | 11.9 | 1 | 364 | 0 | THE1B | MaLR | LTR |
| 8352027 | 8353548 | + | 29.4 | 2 | 1735 | 0 | MLT1A-int | MaLR | LTR |
| 8353549 | 8353926 | + | 13.1 | 1 | 364 | 0 | THE1B | MaLR | LTR |
| 8353927 | 8354222 | + | 20.9 | 67 | 374 | 0 | MLT1A | MaLR | LTR |

  

|  |  |  |  |  |  |  |  |  |  |
| --- | --- | --- | --- | --- | --- | --- | --- | --- | --- |
| chr7:9459088-9471760 | | | 12644 bp | | |  |  |  |  |
| Genome Start | Genome End | Strand | Div | Repeat Start | Repeat End | Repeat Left | Name | Family | Class |
| 9459088 | 9459174 | - | 26.4 | 433 | 530 | -560 | MER97c | MER1\_type? | DNA |
| 9459221 | 9460067 | - | 15.0 | 5310 | 6155 | -11 | L1PA16 | L1 | LINE |
| 9460068 | 9460375 | - | 8.1 | 1 | 308 | -3 | AluY | Alu | SINE |
| 9460376 | 9462497 | - | 17.0 | 3310 | 5434 | -837 | L1P | L1 | LINE |
| 9462498 | 9462804 | - | 7.5 | 1 | 307 | -4 | AluY | Alu | SINE |
| 9462805 | 9463855 | - | 17.1 | 2264 | 3309 | -2962 | L1P | L1 | LINE |
| 9463857 | 9464304 | - | 6.0 | 1 | 448 | -2 | LTR7 | ERV1 | LTR |
| 9464305 | 9464684 | - | 8.5 | 7324 | 7713 | 0 | HERVH | ERV1 | LTR |
| 9464685 | 9464921 | - | 14.5 | 5588 | 5819 | -1894 | HERVH | ERV1 | LTR |
| 9464902 | 9469921 | - | 14.9 | 1 | 5149 | -2564 | HERVH | ERV1 | LTR |
| 9469924 | 9470373 | - | 5.6 | 1 | 448 | -2 | LTR7 | ERV1 | LTR |
| 9470374 | 9471546 | - | 18.8 | 1103 | 2269 | -4022 | L1P4e | L1 | LINE |
| 9471547 | 9471760 | - | 24.8 | 193 | 408 | -682 | MER97c | MER1\_type? | DNA |

  

|  |  |  |  |  |  |  |  |  |  |
| --- | --- | --- | --- | --- | --- | --- | --- | --- | --- |
| chr7:9791654-9795263 | | | 3610 bp | | |  |  |  |  |
| Genome Start | Genome End | Strand | Div | Repeat Start | Repeat End | Repeat Left | Name | Family | Class |
| 9791654 | 9791833 | - | 19.3 | 6101 | 6291 | 0 | L1MA8 | L1 | LINE |
| 9791834 | 9792165 | - | 19.0 | 1 | 346 | 0 | MER7A | MER2\_type | DNA |
| 9792166 | 9792401 | - | 19.3 | 5862 | 6100 | -191 | L1MA8 | L1 | LINE |
| 9792402 | 9793021 | + | 18.2 | 1 | 720 | -73 | MER54B | ERVL | LTR |
| 9793022 | 9793406 | - | 15.4 | 1 | 426 | 0 | MSTB | MaLR | LTR |
| 9793407 | 9794418 | - | 16.7 | 1 | 1651 | 0 | MSTB-int | MaLR | LTR |
| 9794419 | 9794641 | - | 19.9 | 201 | 426 | 0 | MSTB | MaLR | LTR |
| 9794642 | 9794938 | - | 11.8 | 1 | 306 | -4 | AluSg | Alu | SINE |
| 9794939 | 9795127 | - | 19.9 | 1 | 200 | -226 | MSTB | MaLR | LTR |
| 9795128 | 9795213 | + | 18.4 | 717 | 793 | 0 | MER54B | ERVL | LTR |
| 9795214 | 9795263 | - | 16.0 | 5815 | 5865 | -426 | L1MA8 | L1 | LINE |

  

|  |  |  |  |  |  |  |  |  |  |
| --- | --- | --- | --- | --- | --- | --- | --- | --- | --- |
| chr7:10016539-10019894 | | | 3018 bp | | |  |  |  |  |
| Genome Start | Genome End | Strand | Div | Repeat Start | Repeat End | Repeat Left | Name | Family | Class |
| 10016539 | 10016655 | - | 36.5 | 4364 | 4483 | -6 | L3 | CR1 | LINE |
| 10016725 | 10016740 | - | 15.9 | 6136 | 6146 | -20 | L1PA16 | L1 | LINE |
| 10016741 | 10017110 | + | 14.4 | 1 | 381 | 0 | THE1D | MaLR | LTR |
| 10017113 | 10018658 | + | 29.9 | 2 | 1735 | 0 | MLT-int | MaLR | LTR |
| 10018659 | 10019013 | + | 13.3 | 1 | 381 | 0 | THE1D | MaLR | LTR |
| 10019014 | 10019347 | - | 15.9 | 5804 | 6135 | -31 | L1PA16 | L1 | LINE |
| 10019608 | 10019772 | - | 23.9 | 51 | 234 | -34 | MIRb | MIR | SINE |
| 10019780 | 10019894 | - | 33.3 | 3827 | 3944 | -545 | L3 | CR1 | LINE |

  

|  |  |  |  |  |  |  |  |  |  |
| --- | --- | --- | --- | --- | --- | --- | --- | --- | --- |
| chr7:10654370-10657407 | | | 2926 bp | | |  |  |  |  |
| Genome Start | Genome End | Strand | Div | Repeat Start | Repeat End | Repeat Left | Name | Family | Class |
| 10654370 | 10654577 | + | 31.2 | 2 | 208 | -2404 | Charlie7 | MER1\_type | DNA |
| 10654578 | 10654929 | - | 9.1 | 1 | 355 | 0 | THE1A | MaLR | LTR |
| 10654930 | 10656486 | - | 31.1 | 2 | 1735 | 0 | MLT-int | MaLR | LTR |
| 10656489 | 10656842 | - | 10.2 | 1 | 355 | 0 | THE1A | MaLR | LTR |
| 10656843 | 10657235 | + | 31.2 | 209 | 635 | -1977 | Charlie7 | MER1\_type | DNA |
| 10657346 | 10657407 | + | 18.0 | 2518 | 2579 | -33 | Charlie7 | MER1\_type | DNA |

  

|  |  |  |  |  |  |  |  |  |  |
| --- | --- | --- | --- | --- | --- | --- | --- | --- | --- |
| chr7:11874330-11878010 | | | 3679 bp | | |  |  |  |  |
| Genome Start | Genome End | Strand | Div | Repeat Start | Repeat End | Repeat Left | Name | Family | Class |
| 11874330 | 11874447 | - | 18.8 | 7659 | 7775 | -10 | L1MC3 | L1 | LINE |
| 11874448 | 11874834 | + | 18.7 | 1 | 428 | 0 | MSTA | MaLR | LTR |
| 11874837 | 11875714 | + | 16.3 | 3 | 801 | -779 | MSTA-int | MaLR | LTR |
| 11875715 | 11876083 | - | 13.3 | 1 | 364 | 0 | THE1B | MaLR | LTR |
| 11876084 | 11876865 | + | 16.3 | 802 | 1580 | 0 | MSTA-int | MaLR | LTR |
| 11876866 | 11877228 | + | 17.0 | 1 | 428 | 0 | MSTA | MaLR | LTR |
| 11877229 | 11878010 | - | 18.4 | 6884 | 7663 | -122 | L1MC3 | L1 | LINE |

  

|  |  |  |  |  |  |  |  |  |  |
| --- | --- | --- | --- | --- | --- | --- | --- | --- | --- |
| chr7:12195305-12199037 | | | 3725 bp | | |  |  |  |  |
| Genome Start | Genome End | Strand | Div | Repeat Start | Repeat End | Repeat Left | Name | Family | Class |
| 12195305 | 12196333 | + | 25.5 | 3 | 1161 | -490 | MSTB1-int | MaLR | LTR |
| 12196335 | 12196661 | - | 12.7 | 1 | 364 | 0 | THE1B | MaLR | LTR |
| 12196667 | 12198221 | - | 8.6 | 1 | 1574 | -6 | THE1B-int | MaLR | LTR |
| 12198222 | 12198566 | - | 15.4 | 1 | 364 | 0 | THE1B | MaLR | LTR |
| 12198569 | 12199037 | + | 22.1 | 1160 | 1650 | -1 | MSTB1-int | MaLR | LTR |

  

|  |  |  |  |  |  |  |  |  |  |
| --- | --- | --- | --- | --- | --- | --- | --- | --- | --- |
| chr7:12453167-12458401 | | | 5236 bp | | |  |  |  |  |
| Genome Start | Genome End | Strand | Div | Repeat Start | Repeat End | Repeat Left | Name | Family | Class |
| 12453167 | 12453301 | + | 23.7 | 2048 | 2183 | -4666 | L1M1 | L1 | LINE |
| 12453299 | 12455753 | + | 16.9 | 3562 | 6031 | -814 | L1M1 | L1 | LINE |
| 12455754 | 12456174 | + | 17.9 | 1 | 426 | 0 | MSTB | MaLR | LTR |
| 12456177 | 12457661 | + | 27.8 | 2 | 1735 | 0 | MLT-int | MaLR | LTR |
| 12457662 | 12458071 | + | 18.4 | 1 | 426 | 0 | MSTB | MaLR | LTR |
| 12458072 | 12458401 | + | 17.1 | 6032 | 6368 | -637 | L1M1 | L1 | LINE |

  

|  |  |  |  |  |  |  |  |  |  |
| --- | --- | --- | --- | --- | --- | --- | --- | --- | --- |
| chr7:12515648-12526461 | | | 10792 bp | | |  |  |  |  |
| Genome Start | Genome End | Strand | Div | Repeat Start | Repeat End | Repeat Left | Name | Family | Class |
| 12515648 | 12515953 | - | 17.2 | 6897 | 7189 | -176 | L1MA4A | L1 | LINE |
| 12515954 | 12516205 | + | 10.9 | 1 | 299 | -10 | AluSg1 | Alu | SINE |
| 12516206 | 12516318 | - | 17.2 | 6782 | 6896 | -469 | L1MA4A | L1 | LINE |
| 12516339 | 12516563 | - | 17.6 | 73 | 288 | -24 | AluJb | Alu | SINE |
| 12516564 | 12517895 | - | 16.8 | 5345 | 6721 | -644 | L1MA4A | L1 | LINE |
| 12517896 | 12518225 | - | 7.2 | 1 | 306 | -5 | AluY | Alu | SINE |
| 12518226 | 12519869 | - | 18.1 | 3744 | 5344 | -1861 | L1MA4A | L1 | LINE |
| 12519870 | 12520177 | + | 6.5 | 1 | 308 | -3 | AluY | Alu | SINE |
| 12520178 | 12521283 | - | 19.4 | 2589 | 3743 | -3462 | L1MA4A | L1 | LINE |
| 12521284 | 12521634 | + | 9.7 | 3 | 355 | 0 | THE1A | MaLR | LTR |
| 12521637 | 12523211 | + | 29.9 | 2 | 1735 | 0 | MLT-int | MaLR | LTR |
| 12523212 | 12523562 | + | 11.5 | 1 | 353 | -2 | THE1A | MaLR | LTR |
| 12523563 | 12526461 | - | 23.7 | 1 | 2588 | -4621 | L1MA4A | L1 | LINE |

  

|  |  |  |  |  |  |  |  |  |  |
| --- | --- | --- | --- | --- | --- | --- | --- | --- | --- |
| chr7:12540471-12544318 | | | 3766 bp | | |  |  |  |  |
| Genome Start | Genome End | Strand | Div | Repeat Start | Repeat End | Repeat Left | Name | Family | Class |
| 12540471 | 12540815 | + | 22.7 | 4956 | 5321 | -825 | L1ME3A | L1 | LINE |
| 12540824 | 12541104 | + | 21.4 | 1 | 289 | -23 | AluJb | Alu | SINE |
| 12541177 | 12541331 | + | 27.8 | 5345 | 5499 | -671 | L1ME3A | L1 | LINE |
| 12541332 | 12541683 | - | 13.4 | 1 | 362 | -2 | THE1B | MaLR | LTR |
| 12541684 | 12543262 | - | 30.9 | 2 | 1735 | 0 | MLT-int | MaLR | LTR |
| 12543265 | 12543623 | - | 13.6 | 1 | 364 | 0 | THE1B | MaLR | LTR |
| 12543624 | 12544318 | + | 27.8 | 5500 | 6159 | -11 | L1ME3A | L1 | LINE |

  

|  |  |  |  |  |  |  |  |  |  |
| --- | --- | --- | --- | --- | --- | --- | --- | --- | --- |
| chr7:13767097-13780633 | | | 13026 bp | | |  |  |  |  |
| Genome Start | Genome End | Strand | Div | Repeat Start | Repeat End | Repeat Left | Name | Family | Class |
| 13767097 | 13767185 | - | 22.5 | 7437 | 7531 | -6 | MER57A-int | ERV1 | LTR |
| 13767194 | 13767321 | - | 22.1 | 231 | 381 | -117 | LTR54B | ERV1 | LTR |
| 13767330 | 13768415 | - | 16.1 | 4860 | 5973 | -214 | MER57A-int | ERV1 | LTR |
| 13768734 | 13768793 | - | 19.3 | 4055 | 4109 | -762 | MER57A-int | ERV1 | LTR |
| 13768794 | 13769261 | + | 17.9 | 1 | 432 | 0 | MSTB1 | MaLR | LTR |
| 13769264 | 13770826 | + | 27.5 | 2 | 1735 | 0 | MLT-int | MaLR | LTR |
| 13770827 | 13771263 | + | 14.2 | 1 | 432 | 0 | MSTB1 | MaLR | LTR |
| 13771264 | 13771784 | - | 18.2 | 3963 | 4858 | -817 | MER57A-int | ERV1 | LTR |
| 13771795 | 13773231 | + | 3.9 | 3 | 1446 | -4586 | L1HS | L1 | LINE |
| 13773227 | 13776168 | + | 1.7 | 3214 | 6155 | 0 | L1PA2 | L1 | LINE |
| 13776182 | 13778621 | - | 17.6 | 1743 | 4367 | -1820 | MER57A-int | ERV1 | LTR |
| 13778689 | 13778878 | + | 29.6 | 158 | 367 | -420 | LTR48 | ERV1 | LTR |
| 13778913 | 13779159 | - | 25.5 | 155 | 402 | -183 | MER34C\_ | ERV1 | LTR |
| 13779160 | 13779194 | - | 11.4 | 954 | 988 | -4135 | MER57A-int | ERV1 | LTR |
| 13779236 | 13779550 | + | 16.2 | 10 | 346 | 0 | MER7A | MER2\_type | DNA |
| 13779566 | 13780633 | - | 18.3 | 1 | 1064 | -5123 | MER57A-int | ERV1 | LTR |

  

|  |  |  |  |  |  |  |  |  |  |
| --- | --- | --- | --- | --- | --- | --- | --- | --- | --- |
| chr7:14566332-14569500 | | | 3165 bp | | |  |  |  |  |
| Genome Start | Genome End | Strand | Div | Repeat Start | Repeat End | Repeat Left | Name | Family | Class |
| 14566332 | 14567189 | - | 19.9 | 5310 | 6168 | 0 | L1MB1 | L1 | LINE |
| 14567190 | 14567548 | + | 14.5 | 1 | 364 | 0 | THE1B | MaLR | LTR |
| 14567549 | 14569077 | + | 11.8 | 1 | 1580 | 0 | THE1B-int | MaLR | LTR |
| 14569079 | 14569436 | + | 12.9 | 2 | 364 | 0 | THE1B | MaLR | LTR |
| 14569440 | 14569500 | - | 21.3 | 5243 | 5305 | -835 | L1MB1 | L1 | LINE |

  

|  |  |  |  |  |  |  |  |  |  |
| --- | --- | --- | --- | --- | --- | --- | --- | --- | --- |
| chr7:21637074-21639654 | | | 2579 bp | | |  |  |  |  |
| Genome Start | Genome End | Strand | Div | Repeat Start | Repeat End | Repeat Left | Name | Family | Class |
| 21637074 | 21637106 | - | 15.8 | 2739 | 2767 | -3376 | L1M2 | L1 | LINE |
| 21637107 | 21637450 | - | 15.6 | 1 | 364 | 0 | THE1B | MaLR | LTR |
| 21637451 | 21639024 | - | 30.3 | 2 | 1735 | 0 | MLT-int | MaLR | LTR |
| 21639027 | 21639365 | - | 13.9 | 1 | 364 | 0 | THE1B | MaLR | LTR |
| 21639366 | 21639654 | - | 15.8 | 2445 | 2738 | -3405 | L1M2 | L1 | LINE |

  

|  |  |  |  |  |  |  |  |  |  |
| --- | --- | --- | --- | --- | --- | --- | --- | --- | --- |
| chr7:22918192-22937180 | | | 18668 bp | | |  |  |  |  |
| Genome Start | Genome End | Strand | Div | Repeat Start | Repeat End | Repeat Left | Name | Family | Class |
| 22918192 | 22918995 | + | 20.2 | 1 | 921 | -6413 | L1M4b | L1 | LINE |
| 22918996 | 22919285 | + | 11.0 | 2 | 300 | -13 | AluSq | Alu | SINE |
| 22919286 | 22919587 | + | 17.1 | 922 | 1213 | -6121 | L1M4b | L1 | LINE |
| 22919609 | 22919973 | + | 14.4 | 1 | 450 | 0 | LTR7 | ERV1 | LTR |
| 22919974 | 22920401 | + | 19.8 | 1 | 459 | -7254 | HERVH | ERV1 | LTR |
| 22920462 | 22922494 | + | 14.1 | 801 | 2830 | -4883 | HERVH | ERV1 | LTR |
| 22922495 | 22922726 | + | 15.2 | 3357 | 3587 | -4126 | HERVH | ERV1 | LTR |
| 22922721 | 22923889 | + | 12.9 | 3806 | 4910 | -2287 | HERVH | ERV1 | LTR |
| 22923890 | 22924150 | + | 16.9 | 5747 | 6016 | -1697 | HERVH | ERV1 | LTR |
| 22924144 | 22924696 | + | 11.9 | 7157 | 7713 | 0 | HERVH | ERV1 | LTR |
| 22924697 | 22925072 | + | 9.5 | 1 | 450 | 0 | LTR7 | ERV1 | LTR |
| 22925074 | 22925390 | + | 17.9 | 1237 | 1548 | -5786 | L1M4b | L1 | LINE |
| 22925391 | 22925680 | + | 13.1 | 1 | 290 | -22 | AluSx | Alu | SINE |
| 22925681 | 22926191 | + | 17.9 | 1549 | 2086 | -5248 | L1M4b | L1 | LINE |
| 22926192 | 22926504 | + | 19.8 | 3 | 310 | -2 | AluJo | Alu | SINE |
| 22926505 | 22926888 | + | 17.9 | 2087 | 2594 | -4740 | L1M4b | L1 | LINE |
| 22926889 | 22927303 | + | 14.5 | 1 | 426 | 0 | MSTB | MaLR | LTR |
| 22927304 | 22927343 | + | 17.9 | 2595 | 2645 | -4689 | L1M4b | L1 | LINE |
| 22927386 | 22927739 | + | 17.9 | 2646 | 3049 | -4285 | L1M4b | L1 | LINE |
| 22927886 | 22928041 | - | 22.2 | 1 | 159 | -80 | MER8 | MER2\_type | DNA |
| 22928046 | 22928449 | + | 16.8 | 2982 | 3505 | -3927 | L1M4b | L1 | LINE |
| 22928450 | 22928794 | - | 17.7 | 1 | 418 | 0 | Tigger5b | MER2\_type | DNA |
| 22928795 | 22930018 | + | 17.2 | 3080 | 4310 | -2696 | L1M4b | L1 | LINE |
| 22930019 | 22930339 | + | 16.1 | 1 | 300 | -12 | AluSx | Alu | SINE |
| 22930341 | 22936500 | + | 4.2 | 1 | 6155 | 0 | L1PA4 | L1 | LINE |
| 22936560 | 22937180 | + | 17.6 | 4297 | 4895 | -2111 | L1M4b | L1 | LINE |

  

|  |  |  |  |  |  |  |  |  |  |
| --- | --- | --- | --- | --- | --- | --- | --- | --- | --- |
| chr7:24168544-24172537 | | | 3924 bp | | |  |  |  |  |
| Genome Start | Genome End | Strand | Div | Repeat Start | Repeat End | Repeat Left | Name | Family | Class |
| 24168544 | 24168684 | - | 31.6 | 3230 | 3370 | -8 | L2 | L2 | LINE |
| 24168741 | 24169103 | + | 13.4 | 1 | 381 | 0 | THE1D | MaLR | LTR |
| 24169107 | 24170726 | + | 13.6 | 4 | 1580 | 0 | THE1D-int | MaLR | LTR |
| 24170727 | 24170795 | + | 9.0 | 1 | 77 | -304 | THE1D | MaLR | LTR |
| 24170796 | 24171343 | - | 26.1 | 596 | 2123 | 0 | MER52A | ERV1 | LTR |
| 24171346 | 24171989 | - | 23.2 | 1 | 1349 | -147 | LTR27B | ERV1 | LTR |
| 24171990 | 24172283 | + | 14.4 | 74 | 381 | 0 | THE1D | MaLR | LTR |
| 24172293 | 24172537 | - | 39.0 | 2934 | 3173 | -205 | L2 | L2 | LINE |

  

|  |  |  |  |  |  |  |  |  |  |
| --- | --- | --- | --- | --- | --- | --- | --- | --- | --- |
| chr7:25115244-25118702 | | | 3452 bp | | |  |  |  |  |
| Genome Start | Genome End | Strand | Div | Repeat Start | Repeat End | Repeat Left | Name | Family | Class |
| 25115244 | 25115397 | - | 24.2 | 329 | 505 | 0 | MLT1D | MaLR | LTR |
| 25115399 | 25115594 | + | 12.9 | 1 | 187 | -177 | THE1B | MaLR | LTR |
| 25115595 | 25116004 | + | 9.7 | 1 | 472 | 0 | MER4A1 | ERV1 | LTR |
| 25116005 | 25116184 | + | 12.9 | 188 | 364 | 0 | THE1B | MaLR | LTR |
| 25116187 | 25117025 | + | 29.3 | 2 | 975 | -760 | MLT-int | MaLR | LTR |
| 25117026 | 25117319 | + | 10.2 | 1 | 295 | -18 | AluSp | Alu | SINE |
| 25117320 | 25118032 | + | 29.3 | 976 | 1735 | 0 | MLT-int | MaLR | LTR |
| 25118033 | 25118402 | + | 16.4 | 1 | 362 | -2 | THE1B | MaLR | LTR |
| 25118407 | 25118702 | - | 18.8 | 1 | 329 | -176 | MLT1D | MaLR | LTR |

  

|  |  |  |  |  |  |  |  |  |  |
| --- | --- | --- | --- | --- | --- | --- | --- | --- | --- |
| chr7:30954269-30961045 | | | 6736 bp | | |  |  |  |  |
| Genome Start | Genome End | Strand | Div | Repeat Start | Repeat End | Repeat Left | Name | Family | Class |
| 30954269 | 30954898 | - | 23.9 | 5447 | 6005 | -2037 | L1MC3 | L1 | LINE |
| 30954915 | 30955325 | + | 15.5 | 16 | 432 | 0 | MSTB1 | MaLR | LTR |
| 30955328 | 30956981 | + | 27.8 | 2 | 1735 | 0 | MLT-int | MaLR | LTR |
| 30956982 | 30957393 | + | 15.3 | 1 | 427 | -5 | MSTB1 | MaLR | LTR |
| 30957398 | 30957549 | - | 11.2 | 5273 | 5444 | -734 | L1M4 | L1 | LINE |
| 30957553 | 30957927 | - | 15.5 | 1 | 377 | -4 | THE1D | MaLR | LTR |
| 30957938 | 30959418 | - | 12.3 | 6 | 1576 | -4 | THE1D-int | MaLR | LTR |
| 30959423 | 30960039 | - | 23.1 | 1 | 554 | 0 | MLT2B3 | ERVL | LTR |
| 30960042 | 30961045 | - | 21.4 | 4269 | 5278 | -868 | L1MC3 | L1 | LINE |

  

|  |  |  |  |  |  |  |  |  |  |
| --- | --- | --- | --- | --- | --- | --- | --- | --- | --- |
| chr7:31227256-31235822 | | | 8524 bp | | |  |  |  |  |
| Genome Start | Genome End | Strand | Div | Repeat Start | Repeat End | Repeat Left | Name | Family | Class |
| 31227256 | 31230634 | - | 16.2 | 3543 | 7003 | -2 | L1MA4A | L1 | LINE |
| 31230639 | 31230925 | - | 16.7 | 2660 | 2958 | -3890 | L1MA4A | L1 | LINE |
| 31230926 | 31231225 | - | 12.0 | 12 | 310 | -2 | AluSx | Alu | SINE |
| 31231226 | 31232307 | - | 15.6 | 1552 | 2659 | -4190 | L1MA4A | L1 | LINE |
| 31232308 | 31232661 | + | 9.6 | 1 | 355 | 0 | THE1A | MaLR | LTR |
| 31232662 | 31234241 | + | 9.0 | 1 | 1580 | 0 | THE1A-int | MaLR | LTR |
| 31234244 | 31234593 | + | 9.4 | 3 | 355 | 0 | THE1A | MaLR | LTR |
| 31234594 | 31235163 | - | 23.0 | 958 | 1556 | -5293 | L1MA4A | L1 | LINE |
| 31235201 | 31235484 | - | 26.2 | 714 | 997 | -5852 | L1MA4A | L1 | LINE |
| 31235485 | 31235822 | - | 19.2 | 13 | 351 | -6498 | L1MA4A | L1 | LINE |

  

|  |  |  |  |  |  |  |  |  |  |
| --- | --- | --- | --- | --- | --- | --- | --- | --- | --- |
| chr7:34384130-34392821 | | | 8295 bp | | |  |  |  |  |
| Genome Start | Genome End | Strand | Div | Repeat Start | Repeat End | Repeat Left | Name | Family | Class |
| 34384130 | 34384222 | - | 16.9 | 7449 | 7537 | 0 | MER4B-int | ERV1 | LTR |
| 34384266 | 34388147 | - | 17.8 | 1758 | 5945 | -242 | MER4B-int | ERV1 | LTR |
| 34388148 | 34388510 | + | 18.1 | 1 | 364 | 0 | THE1B | MaLR | LTR |
| 34388511 | 34388814 | + | 12.2 | 1 | 310 | -1270 | THE1B-int | MaLR | LTR |
| 34388786 | 34389771 | + | 10.8 | 601 | 1580 | 0 | THE1B-int | MaLR | LTR |
| 34389772 | 34390133 | + | 17.9 | 1 | 364 | 0 | THE1B | MaLR | LTR |
| 34390157 | 34390204 | + | 20.8 | 2 | 49 | -493 | MER34 | ERV1 | LTR |
| 34390465 | 34390753 | - | 24.5 | 4162 | 4465 | -2293 | MER4B-int | ERV1 | LTR |
| 34390823 | 34391073 | - | 28.1 | 174 | 413 | -172 | MER34C\_ | ERV1 | LTR |
| 34391105 | 34391401 | - | 18.6 | 850 | 1132 | -5055 | MER4B-int | ERV1 | LTR |
| 34391402 | 34391821 | - | 12.8 | 1 | 377 | 0 | MER51B | ERV1 | LTR |
| 34391822 | 34391938 | - | 18.6 | 746 | 849 | -5338 | MER4B-int | ERV1 | LTR |
| 34391939 | 34392236 | + | 12.1 | 1 | 298 | -15 | AluSq | Alu | SINE |
| 34392237 | 34392821 | - | 18.6 | 163 | 745 | -5442 | MER4B-int | ERV1 | LTR |

  

|  |  |  |  |  |  |  |  |  |  |
| --- | --- | --- | --- | --- | --- | --- | --- | --- | --- |
| chr7:34900102-34902951 | | | 2837 bp | | |  |  |  |  |
| Genome Start | Genome End | Strand | Div | Repeat Start | Repeat End | Repeat Left | Name | Family | Class |
| 34900102 | 34900430 | + | 18.1 | 2 | 312 | -155 | MLT1C | MaLR | LTR |
| 34900435 | 34900849 | + | 10.9 | 1 | 428 | 0 | MSTA | MaLR | LTR |
| 34900850 | 34902412 | + | 17.3 | 1 | 1649 | -2 | MSTA-int | MaLR | LTR |
| 34902422 | 34902831 | + | 13.5 | 3 | 428 | 0 | MSTA | MaLR | LTR |
| 34902832 | 34902951 | + | 20.6 | 347 | 466 | -1 | MLT1C | MaLR | LTR |

  

|  |  |  |  |  |  |  |  |  |  |
| --- | --- | --- | --- | --- | --- | --- | --- | --- | --- |
| chr7:38083483-38089231 | | | 5536 bp | | |  |  |  |  |
| Genome Start | Genome End | Strand | Div | Repeat Start | Repeat End | Repeat Left | Name | Family | Class |
| 38083483 | 38084427 | + | 22.2 | 3572 | 4529 | -1611 | L1MB3 | L1 | LINE |
| 38084428 | 38084709 | - | 12.4 | 21 | 310 | 0 | AluSg | Alu | SINE |
| 38084710 | 38084866 | + | 22.2 | 4530 | 4682 | -1458 | L1MB3 | L1 | LINE |
| 38084867 | 38085157 | - | 17.2 | 1 | 301 | -11 | AluJo | Alu | SINE |
| 38085158 | 38085946 | + | 20.9 | 4683 | 5470 | -710 | L1MB3 | L1 | LINE |
| 38086158 | 38086612 | + | 17.5 | 5480 | 5937 | -246 | L1MB3 | L1 | LINE |
| 38086613 | 38086973 | - | 12.9 | 1 | 379 | -2 | THE1D | MaLR | LTR |
| 38086974 | 38088603 | - | 30.3 | 2 | 1735 | 0 | MLT-int | MaLR | LTR |
| 38088606 | 38088967 | - | 13.1 | 1 | 381 | 0 | THE1D | MaLR | LTR |
| 38088968 | 38089231 | + | 17.5 | 5938 | 6183 | 0 | L1MB3 | L1 | LINE |

  

|  |  |  |  |  |  |  |  |  |  |
| --- | --- | --- | --- | --- | --- | --- | --- | --- | --- |
| chr7:38103883-38106947 | | | 3002 bp | | |  |  |  |  |
| Genome Start | Genome End | Strand | Div | Repeat Start | Repeat End | Repeat Left | Name | Family | Class |
| 38103883 | 38104043 | - | 29.0 | 425 | 595 | 0 | MLT1H | MaLR | LTR |
| 38104086 | 38104470 | + | 13.9 | 1 | 381 | 0 | THE1D | MaLR | LTR |
| 38104473 | 38106010 | + | 30.3 | 2 | 1735 | 0 | MLT-int | MaLR | LTR |
| 38106011 | 38106368 | + | 14.9 | 1 | 336 | -45 | THE1D | MaLR | LTR |
| 38106369 | 38106666 | + | 14.4 | 1 | 299 | -13 | AluSx | Alu | SINE |
| 38106667 | 38106717 | + | 14.9 | 337 | 381 | 0 | THE1D | MaLR | LTR |
| 38106737 | 38106947 | - | 32.8 | 92 | 305 | -244 | MLT1H | MaLR | LTR |

  

|  |  |  |  |  |  |  |  |  |  |
| --- | --- | --- | --- | --- | --- | --- | --- | --- | --- |
| chr7:38520961-38524986 | | | 4014 bp | | |  |  |  |  |
| Genome Start | Genome End | Strand | Div | Repeat Start | Repeat End | Repeat Left | Name | Family | Class |
| 38520961 | 38521362 | + | 19.3 | 2 | 407 | -1328 | MSTD-int | MaLR | LTR |
| 38521363 | 38521740 | + | 14.9 | 1 | 426 | -2 | MSTA | MaLR | LTR |
| 38521747 | 38523326 | + | 16.6 | 5 | 1645 | -6 | MSTA-int | MaLR | LTR |
| 38523333 | 38523724 | + | 15.9 | 1 | 428 | 0 | MSTA | MaLR | LTR |
| 38523725 | 38524986 | + | 24.8 | 403 | 1728 | -7 | MSTD-int | MaLR | LTR |

  

|  |  |  |  |  |  |  |  |  |  |
| --- | --- | --- | --- | --- | --- | --- | --- | --- | --- |
| chr7:39666761-39671668 | | | 4907 bp | | |  |  |  |  |
| Genome Start | Genome End | Strand | Div | Repeat Start | Repeat End | Repeat Left | Name | Family | Class |
| 39666761 | 39667017 | + | 12.4 | 1 | 281 | -2437 | Tigger2 | MER2\_type | DNA |
| 39667018 | 39667383 | - | 14.3 | 1 | 364 | 0 | THE1B | MaLR | LTR |
| 39667384 | 39668977 | - | 29.7 | 1 | 1735 | 0 | MLT-int | MaLR | LTR |
| 39668979 | 39669307 | - | 16.5 | 1 | 364 | 0 | THE1B | MaLR | LTR |
| 39669308 | 39671668 | + | 12.2 | 282 | 2718 | 0 | Tigger2 | MER2\_type | DNA |

  

|  |  |  |  |  |  |  |  |  |  |
| --- | --- | --- | --- | --- | --- | --- | --- | --- | --- |
| chr7:41292373-41297103 | | | 4172 bp | | |  |  |  |  |
| Genome Start | Genome End | Strand | Div | Repeat Start | Repeat End | Repeat Left | Name | Family | Class |
| 41292373 | 41292916 | + | 33.7 | 3001 | 3638 | -2508 | L1M4 | L1 | LINE |
| 41293167 | 41293587 | + | 17.4 | 1 | 426 | 0 | MSTB | MaLR | LTR |
| 41293590 | 41295387 | + | 27.8 | 2 | 1735 | 0 | MLT-int | MaLR | LTR |
| 41295400 | 41295820 | + | 15.1 | 5 | 426 | 0 | MSTB | MaLR | LTR |
| 41295848 | 41296054 | + | 26.1 | 3967 | 4162 | -1984 | L1M4 | L1 | LINE |
| 41296323 | 41297103 | + | 34.1 | 4505 | 5322 | -824 | L1M4 | L1 | LINE |

  

|  |  |  |  |  |  |  |  |  |  |
| --- | --- | --- | --- | --- | --- | --- | --- | --- | --- |
| chr7:42369342-42378053 | | | 8691 bp | | |  |  |  |  |
| Genome Start | Genome End | Strand | Div | Repeat Start | Repeat End | Repeat Left | Name | Family | Class |
| 42369342 | 42369454 | + | 11.5 | 1 | 113 | -199 | AluSx | Alu | SINE |
| 42369455 | 42369809 | - | 13.5 | 1 | 364 | 0 | THE1B | MaLR | LTR |
| 42369810 | 42371370 | - | 29.0 | 2 | 1735 | 0 | MLT1E-int | MaLR | LTR |
| 42371373 | 42371563 | - | 14.5 | 169 | 364 | 0 | THE1B | MaLR | LTR |
| 42371569 | 42377700 | - | 8.0 | 5 | 6152 | -2 | L1PA6 | L1 | LINE |
| 42377711 | 42377887 | - | 16.0 | 1 | 181 | -183 | THE1B | MaLR | LTR |
| 42377892 | 42378053 | + | 13.8 | 111 | 270 | -39 | AluSx | Alu | SINE |

  

|  |  |  |  |  |  |  |  |  |  |
| --- | --- | --- | --- | --- | --- | --- | --- | --- | --- |
| chr7:43721797-43724701 | | | 2899 bp | | |  |  |  |  |
| Genome Start | Genome End | Strand | Div | Repeat Start | Repeat End | Repeat Left | Name | Family | Class |
| 43721797 | 43721899 | + | 23.8 | 192 | 311 | -517 | MER99 | DNA | DNA |
| 43721900 | 43722257 | + | 11.5 | 1 | 362 | -2 | THE1B | MaLR | LTR |
| 43722262 | 43723827 | + | 29.8 | 2 | 1735 | 0 | MLT-int | MaLR | LTR |
| 43723830 | 43724202 | + | 13.8 | 3 | 364 | 0 | THE1B | MaLR | LTR |
| 43724203 | 43724701 | + | 23.8 | 312 | 825 | -3 | MER99 | DNA | DNA |

  

|  |  |  |  |  |  |  |  |  |  |
| --- | --- | --- | --- | --- | --- | --- | --- | --- | --- |
| chr7:43816009-43831720 | | | 15010 bp | | |  |  |  |  |
| Genome Start | Genome End | Strand | Div | Repeat Start | Repeat End | Repeat Left | Name | Family | Class |
| 43816009 | 43816330 | + | 16.5 | 282 | 607 | -6230 | HERVKC4 | ERVK | LTR |
| 43816336 | 43816783 | + | 7.4 | 1 | 511 | 0 | LTR10F | ERV1 | LTR |
| 43816784 | 43818764 | + | 7.3 | 1 | 1914 | -5823 | HERVIP10F | ERV1 | LTR |
| 43818766 | 43819173 | + | 8.2 | 835 | 1264 | -3838 | HERVIP10FH | ERV1 | LTR |
| 43819168 | 43819385 | + | 8.0 | 1382 | 1595 | -3507 | HERVIP10FH | ERV1 | LTR |
| 43819388 | 43819856 | + | 8.4 | 2 | 511 | 0 | LTR10F | ERV1 | LTR |
| 43819857 | 43820552 | + | 13.0 | 1 | 640 | -4462 | HERVIP10F | ERV1 | LTR |
| 43820534 | 43821574 | + | 5.5 | 360 | 1428 | -6309 | HERVIP10F | ERV1 | LTR |
| 43821552 | 43825658 | + | 6.4 | 687 | 5102 | 0 | HERVIP10F | ERV1 | LTR |
| 43825659 | 43826100 | + | 8.4 | 1 | 511 | 0 | LTR10F | ERV1 | LTR |
| 43826101 | 43829572 | + | 7.8 | 1593 | 5100 | -2 | HERVIP10FH | ERV1 | LTR |
| 43829575 | 43830021 | + | 9.6 | 1 | 511 | 0 | LTR10F | ERV1 | LTR |
| 43830025 | 43830106 | + | 14.6 | 612 | 693 | -6144 | HERVKC4 | ERVK | LTR |
| 43830429 | 43830585 | + | 18.5 | 2327 | 2489 | -4348 | HERVKC4 | ERVK | LTR |
| 43830637 | 43831096 | + | 5.0 | 1 | 463 | 0 | LTR2 | ERV1 | LTR |
| 43831465 | 43831544 | + | 12.5 | 3474 | 3553 | -3284 | HERVKC4 | ERVK | LTR |
| 43831541 | 43831720 | + | 15.3 | 2027 | 3807 | -3030 | HERVKC4 | ERVK | LTR |

  

|  |  |  |  |  |  |  |  |  |  |
| --- | --- | --- | --- | --- | --- | --- | --- | --- | --- |
| chr7:45379134-45383468 | | | 4332 bp | | |  |  |  |  |
| Genome Start | Genome End | Strand | Div | Repeat Start | Repeat End | Repeat Left | Name | Family | Class |
| 45379134 | 45379237 | + | 24.5 | 1006 | 1111 | -5312 | L1MCc | L1 | LINE |
| 45379238 | 45379602 | - | 11.9 | 1 | 362 | -2 | THE1B | MaLR | LTR |
| 45379603 | 45381159 | - | 29.8 | 2 | 1735 | 0 | MLT-int | MaLR | LTR |
| 45381162 | 45381529 | - | 14.0 | 1 | 364 | 0 | THE1B | MaLR | LTR |
| 45381530 | 45381947 | + | 24.5 | 1112 | 1535 | -4888 | L1MCc | L1 | LINE |
| 45381949 | 45382120 | + | 26.8 | 1738 | 1908 | -5214 | L1MCc | L1 | LINE |
| 45382121 | 45382199 | + | 1.3 | 6074 | 6152 | -3 | L1PA4 | L1 | LINE |
| 45382200 | 45382573 | + | 26.8 | 1909 | 2278 | -4844 | L1MCc | L1 | LINE |
| 45382574 | 45382880 | + | 10.5 | 1 | 312 | 0 | AluSx | Alu | SINE |
| 45382881 | 45383468 | + | 26.8 | 2279 | 2944 | -4178 | L1MCc | L1 | LINE |

  

|  |  |  |  |  |  |  |  |  |  |
| --- | --- | --- | --- | --- | --- | --- | --- | --- | --- |
| chr7:46431608-46438045 | | | 6431 bp | | |  |  |  |  |
| Genome Start | Genome End | Strand | Div | Repeat Start | Repeat End | Repeat Left | Name | Family | Class |
| 46431608 | 46432999 | + | 23.0 | 19 | 1494 | -5139 | L1MDa | L1 | LINE |
| 46433000 | 46433293 | + | 15.0 | 1 | 294 | -18 | AluSx | Alu | SINE |
| 46433294 | 46434352 | + | 23.0 | 1495 | 2646 | -3987 | L1MDa | L1 | LINE |
| 46434354 | 46434818 | + | 19.0 | 3130 | 3637 | -2900 | L1MDa | L1 | LINE |
| 46434819 | 46435167 | - | 8.6 | 5 | 355 | 0 | THE1A | MaLR | LTR |
| 46435172 | 46436390 | - | 30.8 | 433 | 1735 | 0 | MLT-int | MaLR | LTR |
| 46436391 | 46436696 | + | 7.9 | 1 | 305 | -6 | AluY | Alu | SINE |
| 46436697 | 46437065 | - | 30.8 | 2 | 432 | -1303 | MLT-int | MaLR | LTR |
| 46437068 | 46437417 | - | 11.1 | 1 | 355 | 0 | THE1A | MaLR | LTR |
| 46437418 | 46438045 | + | 19.0 | 3638 | 4278 | -2259 | L1MDa | L1 | LINE |

  

|  |  |  |  |  |  |  |  |  |  |
| --- | --- | --- | --- | --- | --- | --- | --- | --- | --- |
| chr7:47181817-47184558 | | | 2740 bp | | |  |  |  |  |
| Genome Start | Genome End | Strand | Div | Repeat Start | Repeat End | Repeat Left | Name | Family | Class |
| 47181817 | 47181908 | - | 21.4 | 470 | 567 | 0 | MLT2B1 | ERVL | LTR |
| 47181909 | 47182250 | + | 12.8 | 1 | 364 | 0 | THE1B | MaLR | LTR |
| 47182253 | 47183830 | + | 29.5 | 2 | 1735 | 0 | MLT-int | MaLR | LTR |
| 47183831 | 47184169 | + | 12.0 | 1 | 364 | 0 | THE1B | MaLR | LTR |
| 47184170 | 47184558 | - | 21.4 | 3 | 422 | -98 | MLT2B1 | ERVL | LTR |

  

|  |  |  |  |  |  |  |  |  |  |
| --- | --- | --- | --- | --- | --- | --- | --- | --- | --- |
| chr7:47274439-47279329 | | | 4861 bp | | |  |  |  |  |
| Genome Start | Genome End | Strand | Div | Repeat Start | Repeat End | Repeat Left | Name | Family | Class |
| 47274439 | 47274909 | - | 19.4 | 2834 | 3316 | -3090 | L1MDb | L1 | LINE |
| 47274910 | 47275261 | - | 17.9 | 1 | 365 | 0 | MLT1A0 | MaLR | LTR |
| 47275262 | 47276028 | - | 19.0 | 920 | 1735 | 0 | MLT1A0-int | MaLR | LTR |
| 47276057 | 47276726 | - | 17.0 | 1 | 676 | -1059 | MLT1A0-int | MaLR | LTR |
| 47276727 | 47277076 | - | 18.7 | 1 | 365 | 0 | MLT1A0 | MaLR | LTR |
| 47277079 | 47277176 | - | 28.0 | 2739 | 2836 | -3570 | L1MDb | L1 | LINE |
| 47277177 | 47277380 | - | 22.1 | 1 | 210 | 0 | MER6B | MER2\_type | DNA |
| 47277381 | 47279329 | - | 22.4 | 700 | 2738 | -3668 | L1MDb | L1 | LINE |

  

|  |  |  |  |  |  |  |  |  |  |
| --- | --- | --- | --- | --- | --- | --- | --- | --- | --- |
| chr7:48734419-48737025 | | | 2603 bp | | |  |  |  |  |
| Genome Start | Genome End | Strand | Div | Repeat Start | Repeat End | Repeat Left | Name | Family | Class |
| 48734419 | 48734516 | + | 16.3 | 1 | 98 | -825 | MER49 | ERV1 | LTR |
| 48734517 | 48734912 | - | 16.0 | 1 | 364 | 0 | THE1B | MaLR | LTR |
| 48734913 | 48736481 | - | 11.1 | 1 | 1580 | 0 | THE1B-int | MaLR | LTR |
| 48736484 | 48736839 | - | 16.0 | 3 | 362 | -2 | THE1B | MaLR | LTR |
| 48736842 | 48737025 | + | 17.4 | 94 | 285 | -638 | MER49 | ERV1 | LTR |

  

|  |  |  |  |  |  |  |  |  |  |
| --- | --- | --- | --- | --- | --- | --- | --- | --- | --- |
| chr7:48958157-48961811 | | | 3627 bp | | |  |  |  |  |
| Genome Start | Genome End | Strand | Div | Repeat Start | Repeat End | Repeat Left | Name | Family | Class |
| 48958157 | 48958202 | + | 17.6 | 1 | 40 | -405 | MER65A | ERV1 | LTR |
| 48958223 | 48958460 | + | 17.6 | 41 | 247 | -198 | MER65A | ERV1 | LTR |
| 48958471 | 48958855 | - | 17.7 | 1 | 417 | -11 | MSTA | MaLR | LTR |
| 48958856 | 48959138 | - | 15.3 | 1359 | 1651 | 0 | MSTA-int | MaLR | LTR |
| 48959135 | 48960166 | - | 6.3 | 1 | 1186 | -80 | MER11A | ERVK | LTR |
| 48960169 | 48961219 | - | 20.7 | 1 | 1058 | -593 | MSTA-int | MaLR | LTR |
| 48961220 | 48961617 | - | 20.2 | 1 | 428 | 0 | MSTA | MaLR | LTR |
| 48961618 | 48961811 | + | 18.7 | 244 | 445 | 0 | MER65A | ERV1 | LTR |

  

|  |  |  |  |  |  |  |  |  |  |
| --- | --- | --- | --- | --- | --- | --- | --- | --- | --- |
| chr7:49116632-49119085 | | | 2452 bp | | |  |  |  |  |
| Genome Start | Genome End | Strand | Div | Repeat Start | Repeat End | Repeat Left | Name | Family | Class |
| 49116632 | 49116742 | - | 20.7 | 6219 | 6327 | -7 | L1MA10 | L1 | LINE |
| 49116743 | 49117105 | + | 16.8 | 1 | 364 | 0 | THE1B | MaLR | LTR |
| 49117108 | 49118682 | + | 30.9 | 2 | 1735 | 0 | MLT-int | MaLR | LTR |
| 49118683 | 49119038 | + | 16.3 | 1 | 364 | 0 | THE1B | MaLR | LTR |
| 49119039 | 49119085 | - | 20.7 | 6163 | 6218 | -116 | L1MA10 | L1 | LINE |

  

|  |  |  |  |  |  |  |  |  |  |
| --- | --- | --- | --- | --- | --- | --- | --- | --- | --- |
| chr7:49221510-49225804 | | | 4286 bp | | |  |  |  |  |
| Genome Start | Genome End | Strand | Div | Repeat Start | Repeat End | Repeat Left | Name | Family | Class |
| 49221510 | 49221586 | - | 15.6 | 6231 | 6309 | -3 | L1MA9 | L1 | LINE |
| 49221591 | 49221709 | - | 15.7 | 227 | 343 | -2 | MER2 | MER2\_type | DNA |
| 49221710 | 49222069 | - | 13.4 | 1 | 364 | 0 | THE1B | MaLR | LTR |
| 49222070 | 49223641 | - | 31.0 | 2 | 1735 | 0 | MLT-int | MaLR | LTR |
| 49223644 | 49224007 | - | 14.2 | 1 | 364 | 0 | THE1B | MaLR | LTR |
| 49224008 | 49224226 | - | 15.7 | 2 | 226 | -119 | MER2 | MER2\_type | DNA |
| 49224230 | 49225804 | - | 19.9 | 4600 | 6227 | -85 | L1MA9 | L1 | LINE |

  

|  |  |  |  |  |  |  |  |  |  |
| --- | --- | --- | --- | --- | --- | --- | --- | --- | --- |
| chr7:49373159-49382087 | | | 8593 bp | | |  |  |  |  |
| Genome Start | Genome End | Strand | Div | Repeat Start | Repeat End | Repeat Left | Name | Family | Class |
| 49373159 | 49373244 | - | 20.9 | 6582 | 6670 | -8 | L1MB4 | L1 | LINE |
| 49373246 | 49373342 | + | 22.9 | 32 | 127 | -238 | MLT1A0 | MaLR | LTR |
| 49373343 | 49374505 | - | 21.6 | 5384 | 6585 | -93 | L1MB4 | L1 | LINE |
| 49374516 | 49374853 | - | 22.5 | 92 | 467 | 0 | MLT1C | MaLR | LTR |
| 49374854 | 49375168 | + | 21.2 | 1 | 313 | -52 | MLT1A0 | MaLR | LTR |
| 49375341 | 49375427 | - | 13.8 | 3 | 92 | -375 | MLT1C | MaLR | LTR |
| 49375430 | 49376870 | - | 22.9 | 1 | 1371 | 0 | MLT1C-int | MaLR | LTR |
| 49376871 | 49377299 | - | 19.2 | 1 | 467 | 0 | MLT1C | MaLR | LTR |
| 49377300 | 49378957 | - | 23.9 | 3536 | 5379 | -1265 | L1MB4 | L1 | LINE |
| 49379014 | 49379541 | - | 19.6 | 5797 | 6312 | 0 | L1MA9 | L1 | LINE |
| 49379570 | 49379875 | - | 19.6 | 5476 | 5796 | -516 | L1MA9 | L1 | LINE |
| 49379893 | 49380033 | - | 24.6 | 242 | 388 | -3552 | Arthur1 | Tip100 | DNA |
| 49380047 | 49380151 | + | 18.4 | 6017 | 6151 | -208 | L1MD2 | L1 | LINE |
| 49380204 | 49380300 | + | 18.4 | 6152 | 6248 | -111 | L1MD2 | L1 | LINE |
| 49380297 | 49381260 | - | 16.6 | 4501 | 5529 | -783 | L1M2 | L1 | LINE |
| 49381250 | 49382087 | - | 28.0 | 875 | 1691 | -4955 | L1MB4 | L1 | LINE |

  

|  |  |  |  |  |  |  |  |  |  |
| --- | --- | --- | --- | --- | --- | --- | --- | --- | --- |
| chr7:49397183-49399783 | | | 2598 bp | | |  |  |  |  |
| Genome Start | Genome End | Strand | Div | Repeat Start | Repeat End | Repeat Left | Name | Family | Class |
| 49397183 | 49397292 | + | 17.2 | 3 | 108 | -204 | AluJb | Alu | SINE |
| 49397293 | 49397667 | - | 15.8 | 1 | 364 | 0 | THE1B | MaLR | LTR |
| 49397669 | 49399210 | - | 31.2 | 2 | 1734 | -1 | MLT1D-int | MaLR | LTR |
| 49399213 | 49399587 | - | 17.0 | 1 | 364 | 0 | THE1B | MaLR | LTR |
| 49399588 | 49399783 | + | 17.2 | 109 | 299 | -13 | AluJb | Alu | SINE |

  

|  |  |  |  |  |  |  |  |  |  |
| --- | --- | --- | --- | --- | --- | --- | --- | --- | --- |
| chr7:49940630-49943479 | | | 2686 bp | | |  |  |  |  |
| Genome Start | Genome End | Strand | Div | Repeat Start | Repeat End | Repeat Left | Name | Family | Class |
| 49940630 | 49940980 | + | 33.8 | 5379 | 5756 | -365 | L1ME4a | L1 | LINE |
| 49940999 | 49941358 | + | 12.3 | 1 | 364 | 0 | THE1B | MaLR | LTR |
| 49941359 | 49942863 | + | 10.3 | 1 | 1580 | 0 | THE1B-int | MaLR | LTR |
| 49942866 | 49943207 | + | 11.7 | 3 | 362 | -2 | THE1B | MaLR | LTR |
| 49943352 | 49943479 | + | 27.9 | 5966 | 6093 | -28 | L1ME4a | L1 | LINE |

  

|  |  |  |  |  |  |  |  |  |  |
| --- | --- | --- | --- | --- | --- | --- | --- | --- | --- |
| chr7:49963041-49973812 | | | 9932 bp | | |  |  |  |  |
| Genome Start | Genome End | Strand | Div | Repeat Start | Repeat End | Repeat Left | Name | Family | Class |
| 49963041 | 49963907 | + | 27.7 | 54 | 910 | -5490 | L1ME3 | L1 | LINE |
| 49963917 | 49964635 | + | 21.3 | 4449 | 5192 | -954 | L1M4 | L1 | LINE |
| 49964654 | 49965418 | + | 25.7 | 2348 | 3161 | -3058 | L1ME3 | L1 | LINE |
| 49965419 | 49965720 | + | 15.9 | 1 | 302 | -8 | AluSg | Alu | SINE |
| 49965721 | 49966124 | + | 26.4 | 3162 | 3581 | -2638 | L1ME3 | L1 | LINE |
| 49966385 | 49966519 | + | 25.6 | 3782 | 3915 | -3091 | L1ME1 | L1 | LINE |
| 49966528 | 49967060 | + | 26.0 | 4307 | 4844 | -1375 | L1ME3 | L1 | LINE |
| 49967062 | 49967426 | + | 19.5 | 1 | 396 | 0 | MSTD | MaLR | LTR |
| 49967493 | 49968493 | + | 25.5 | 738 | 1728 | -7 | MSTD-int | MaLR | LTR |
| 49968500 | 49968860 | + | 17.7 | 1 | 396 | 0 | MSTD | MaLR | LTR |
| 49968867 | 49968917 | + | 17.6 | 4847 | 4898 | -1321 | L1ME3 | L1 | LINE |
| 49968919 | 49970279 | + | 28.4 | 4404 | 5910 | -1096 | L1ME1 | L1 | LINE |
| 49970280 | 49970582 | + | 11.6 | 1 | 307 | -5 | AluSx | Alu | SINE |
| 49970583 | 49971738 | + | 23.9 | 5911 | 7003 | -23 | L1ME1 | L1 | LINE |
| 49972191 | 49972942 | + | 26.5 | 3529 | 4313 | -1833 | L1M4 | L1 | LINE |
| 49972938 | 49973663 | + | 25.6 | 5104 | 5856 | -383 | L1ME3 | L1 | LINE |
| 49973682 | 49973812 | + | 23.7 | 6074 | 6193 | -42 | L1ME3 | L1 | LINE |

  

|  |  |  |  |  |  |  |  |  |  |
| --- | --- | --- | --- | --- | --- | --- | --- | --- | --- |
| chr7:53017411-53022116 | | | 4693 bp | | |  |  |  |  |
| Genome Start | Genome End | Strand | Div | Repeat Start | Repeat End | Repeat Left | Name | Family | Class |
| 53017411 | 53018178 | + | 28.0 | 1 | 798 | -5247 | L1MCb | L1 | LINE |
| 53018179 | 53018480 | + | 9.9 | 1 | 305 | -6 | AluY | Alu | SINE |
| 53018481 | 53019098 | + | 25.0 | 799 | 1564 | -5187 | L1MCb | L1 | LINE |
| 53019099 | 53019480 | - | 16.8 | 3 | 381 | 0 | THE1D | MaLR | LTR |
| 53019488 | 53019854 | + | 15.0 | 1 | 364 | 0 | THE1B | MaLR | LTR |
| 53019857 | 53021428 | + | 31.3 | 2 | 1735 | 0 | MLT-int | MaLR | LTR |
| 53021433 | 53021781 | + | 13.2 | 5 | 364 | 0 | THE1B | MaLR | LTR |
| 53021782 | 53022116 | + | 24.3 | 1565 | 1888 | -4863 | L1MCb | L1 | LINE |

  

|  |  |  |  |  |  |  |  |  |  |
| --- | --- | --- | --- | --- | --- | --- | --- | --- | --- |
| chr7:53866976-53875287 | | | 7942 bp | | |  |  |  |  |
| Genome Start | Genome End | Strand | Div | Repeat Start | Repeat End | Repeat Left | Name | Family | Class |
| 53866976 | 53867235 | + | 25.3 | 1076 | 1335 | -4974 | L1M4c | L1 | LINE |
| 53867255 | 53867533 | + | 27.7 | 1611 | 1891 | -4493 | L1M4c | L1 | LINE |
| 53867615 | 53867834 | + | 25.3 | 2130 | 2353 | -4031 | L1M4c | L1 | LINE |
| 53867853 | 53868001 | + | 30.9 | 1792 | 1941 | -1478 | L2 | L2 | LINE |
| 53868097 | 53868483 | - | 25.4 | 5680 | 6117 | -123 | L1ME3B | L1 | LINE |
| 53868484 | 53868572 | - | 25.3 | 225 | 319 | -5 | MER5C | MER1\_type | DNA |
| 53868625 | 53868942 | + | 22.7 | 2346 | 2669 | -3707 | L1M4c | L1 | LINE |
| 53868943 | 53869368 | + | 20.7 | 1 | 426 | 0 | MSTB | MaLR | LTR |
| 53869371 | 53871152 | + | 29.4 | 2 | 1735 | 0 | MLT-int | MaLR | LTR |
| 53871157 | 53871581 | + | 20.6 | 5 | 426 | 0 | MSTB | MaLR | LTR |
| 53871582 | 53872059 | + | 22.7 | 2670 | 3181 | -3195 | L1M4c | L1 | LINE |
| 53872077 | 53872455 | - | 21.4 | 1 | 414 | -12 | MSTB | MaLR | LTR |
| 53872460 | 53873871 | + | 22.8 | 3190 | 4622 | -1754 | L1M4c | L1 | LINE |
| 53873872 | 53873928 | + | 6.1 | 1 | 50 | -314 | THE1B | MaLR | LTR |
| 53873929 | 53874673 | + | 22.8 | 4623 | 5378 | -998 | L1M4c | L1 | LINE |
| 53874687 | 53874895 | + | 19.3 | 4538 | 4753 | -1387 | L1M3 | L1 | LINE |
| 53874961 | 53875287 | + | 19.3 | 5382 | 5722 | -685 | L1M4c | L1 | LINE |

  

|  |  |  |  |  |  |  |  |  |  |
| --- | --- | --- | --- | --- | --- | --- | --- | --- | --- |
| chr7:54228330-54233909 | | | 4893 bp | | |  |  |  |  |
| Genome Start | Genome End | Strand | Div | Repeat Start | Repeat End | Repeat Left | Name | Family | Class |
| 54228330 | 54228393 | - | 21.9 | 3219 | 3283 | -3658 | L1MCc | L1 | LINE |
| 54228415 | 54228986 | - | 27.4 | 2645 | 3203 | -3919 | L1MCc | L1 | LINE |
| 54228994 | 54229525 | + | 34.2 | 2875 | 3431 | -2715 | L1MD | L1 | LINE |
| 54229683 | 54229806 | + | 24.8 | 38 | 156 | 0 | MER58A | MER1\_type | DNA |
| 54229856 | 54230256 | + | 25.5 | 1 | 467 | 0 | MLT1C | MaLR | LTR |
| 54230260 | 54231713 | + | 24.1 | 4 | 1416 | -14 | MLT1C-int | MaLR | LTR |
| 54231728 | 54232167 | + | 26.6 | 1 | 460 | -7 | MLT1C | MaLR | LTR |
| 54232376 | 54233060 | - | 27.2 | 1738 | 2404 | -4633 | L1MCc | L1 | LINE |
| 54233167 | 54233609 | - | 16.5 | 5706 | 6164 | -2 | L1PA16 | L1 | LINE |
| 54233732 | 54233909 | - | 26.4 | 1278 | 1462 | -4961 | L1MCc | L1 | LINE |

  

|  |  |  |  |  |  |  |  |  |  |
| --- | --- | --- | --- | --- | --- | --- | --- | --- | --- |
| chr7:55653027-55655881 | | | 2852 bp | | |  |  |  |  |
| Genome Start | Genome End | Strand | Div | Repeat Start | Repeat End | Repeat Left | Name | Family | Class |
| 55653027 | 55653263 | + | 14.1 | 5594 | 5828 | -338 | L1PA16 | L1 | LINE |
| 55653264 | 55653627 | + | 14.2 | 1 | 375 | 0 | THE1C | MaLR | LTR |
| 55653630 | 55655208 | + | 28.8 | 2 | 1735 | 0 | MLT1G1-int | MaLR | LTR |
| 55655210 | 55655564 | + | 13.1 | 2 | 374 | -1 | THE1C | MaLR | LTR |
| 55655565 | 55655881 | + | 14.1 | 5829 | 6157 | -9 | L1PA16 | L1 | LINE |

  

|  |  |  |  |  |  |  |  |  |  |
| --- | --- | --- | --- | --- | --- | --- | --- | --- | --- |
| chr7:57721768-57725436 | | | 3665 bp | | |  |  |  |  |
| Genome Start | Genome End | Strand | Div | Repeat Start | Repeat End | Repeat Left | Name | Family | Class |
| 57721768 | 57722055 | - | 24.3 | 5497 | 5779 | -461 | L1M5 | L1 | LINE |
| 57722056 | 57722425 | + | 11.9 | 1 | 355 | 0 | THE1A | MaLR | LTR |
| 57722428 | 57724021 | + | 30.5 | 2 | 1735 | 0 | MLT-int | MaLR | LTR |
| 57724024 | 57724384 | + | 14.2 | 3 | 355 | 0 | THE1A | MaLR | LTR |
| 57724385 | 57724670 | - | 25.2 | 5183 | 5496 | -744 | L1M5 | L1 | LINE |
| 57724671 | 57724948 | - | 15.9 | 1 | 306 | -6 | AluSx | Alu | SINE |
| 57724949 | 57725436 | - | 26.6 | 4647 | 5182 | -964 | L1M5 | L1 | LINE |

  

|  |  |  |  |  |  |  |  |  |  |
| --- | --- | --- | --- | --- | --- | --- | --- | --- | --- |
| chr7:66629643-66634765 | | | 5053 bp | | |  |  |  |  |
| Genome Start | Genome End | Strand | Div | Repeat Start | Repeat End | Repeat Left | Name | Family | Class |
| 66629643 | 66629725 | + | 27.2 | 2 | 82 | -2679 | Charlie1 | MER1\_type | DNA |
| 66629726 | 66629805 | - | 12.5 | 18 | 97 | -215 | AluJ/FLAM | Alu | SINE |
| 66629816 | 66629908 | + | 11.8 | 32 | 125 | -185 | AluSg | Alu | SINE |
| 66629932 | 66630731 | + | 19.0 | 1422 | 2274 | -487 | Charlie1 | MER1\_type | DNA |
| 66630732 | 66630863 | + | 21.2 | 1 | 133 | -169 | AluJ | Alu | SINE |
| 66630869 | 66631221 | - | 9.6 | 1 | 355 | 0 | THE1A | MaLR | LTR |
| 66631222 | 66632809 | - | 31.0 | 2 | 1735 | 0 | MLT-int | MaLR | LTR |
| 66632812 | 66633165 | - | 8.8 | 1 | 355 | 0 | THE1A | MaLR | LTR |
| 66633166 | 66633326 | + | 16.1 | 134 | 282 | -30 | AluJ | Alu | SINE |
| 66633327 | 66633731 | + | 19.0 | 2275 | 2672 | -89 | Charlie1 | MER1\_type | DNA |
| 66633758 | 66634237 | - | 21.3 | 1 | 505 | 0 | MLT1D | MaLR | LTR |
| 66634238 | 66634703 | - | 23.8 | 936 | 1371 | 0 | MLT1D-int | MaLR | LTR |
| 66634708 | 66634765 | + | 12.1 | 2697 | 2760 | -1 | Charlie1 | MER1\_type | DNA |

  

|  |  |  |  |  |  |  |  |  |  |
| --- | --- | --- | --- | --- | --- | --- | --- | --- | --- |
| chr7:75244505-75256535 | | | 11652 bp | | |  |  |  |  |
| Genome Start | Genome End | Strand | Div | Repeat Start | Repeat End | Repeat Left | Name | Family | Class |
| 75244505 | 75244719 | + | 22.6 | 5669 | 5894 | -276 | L1ME3A | L1 | LINE |
| 75244720 | 75244996 | + | 16.7 | 1 | 305 | -7 | AluJo | Alu | SINE |
| 75244997 | 75245075 | + | 22.6 | 5895 | 5951 | -219 | L1ME3A | L1 | LINE |
| 75245076 | 75245272 | - | 17.9 | 1 | 162 | -4 | FRAM | Alu | SINE |
| 75245273 | 75245474 | - | 17.9 | 5476 | 5675 | -637 | L1M2 | L1 | LINE |
| 75245481 | 75245712 | - | 14.3 | 87 | 295 | -17 | AluJo | Alu | SINE |
| 75245717 | 75245880 | - | 8.4 | 5326 | 5485 | -806 | L1M2 | L1 | LINE |
| 75245883 | 75246008 | - | 11.9 | 173 | 298 | -14 | AluSg/x | Alu | SINE |
| 75246051 | 75246165 | + | 8.2 | 2 | 111 | -199 | AluSg | Alu | SINE |
| 75246166 | 75246560 | + | 7.7 | 1 | 461 | -3 | LTR7 | ERV1 | LTR |
| 75246564 | 75247031 | + | 12.7 | 1 | 459 | -7254 | HERVH | ERV1 | LTR |
| 75247257 | 75248789 | + | 12.7 | 458 | 1978 | -5735 | HERVH | ERV1 | LTR |
| 75248790 | 75249190 | + | 8.5 | 1 | 450 | 0 | LTR7 | ERV1 | LTR |
| 75249191 | 75249656 | + | 13.6 | 1 | 459 | -7254 | HERVH | ERV1 | LTR |
| 75249717 | 75250771 | + | 21.5 | 663 | 1683 | -6036 | HERVH | ERV1 | LTR |
| 75250772 | 75251065 | + | 6.5 | 1 | 306 | -5 | AluY | Alu | SINE |
| 75251066 | 75254616 | + | 10.8 | 1520 | 4988 | -2725 | HERVH | ERV1 | LTR |
| 75254617 | 75254928 | + | 8.7 | 1 | 311 | 0 | AluY | Alu | SINE |
| 75254929 | 75254967 | + | 10.7 | 4989 | 5014 | -2699 | HERVH | ERV1 | LTR |
| 75254967 | 75255417 | + | 12.0 | 7270 | 7713 | 0 | HERVH | ERV1 | LTR |
| 75255418 | 75255816 | + | 7.1 | 1 | 448 | -2 | LTR7 | ERV1 | LTR |
| 75255847 | 75255998 | + | 17.8 | 1 | 166 | 0 | FRAM | Alu | SINE |
| 75256007 | 75256166 | + | 19.8 | 5962 | 6125 | -45 | L1ME3A | L1 | LINE |
| 75256167 | 75256498 | - | 15.3 | 1 | 308 | -4 | AluJo | Alu | SINE |
| 75256499 | 75256535 | + | 19.8 | 6126 | 6160 | -10 | L1ME3A | L1 | LINE |

  

|  |  |  |  |  |  |  |  |  |  |
| --- | --- | --- | --- | --- | --- | --- | --- | --- | --- |
| chr7:76057498-76066489 | | | 8987 bp | | |  |  |  |  |
| Genome Start | Genome End | Strand | Div | Repeat Start | Repeat End | Repeat Left | Name | Family | Class |
| 76057498 | 76059928 | + | 24.8 | 9 | 2517 | -4811 | L1PA15 | L1 | LINE |
| 76059929 | 76060301 | - | 17.3 | 1 | 375 | 0 | THE1C | MaLR | LTR |
| 76060307 | 76061501 | - | 11.9 | 1 | 1568 | -12 | THE1C-int | MaLR | LTR |
| 76061502 | 76061870 | - | 13.7 | 1 | 375 | 0 | THE1C | MaLR | LTR |
| 76061871 | 76066231 | + | 14.9 | 2514 | 6890 | -443 | L1PA15 | L1 | LINE |
| 76066232 | 76066489 | + | 17.1 | 7069 | 7329 | -4 | L1PA15 | L1 | LINE |

  

|  |  |  |  |  |  |  |  |  |  |
| --- | --- | --- | --- | --- | --- | --- | --- | --- | --- |
| chr7:79510611-79513382 | | | 2765 bp | | |  |  |  |  |
| Genome Start | Genome End | Strand | Div | Repeat Start | Repeat End | Repeat Left | Name | Family | Class |
| 79510611 | 79510940 | - | 23.8 | 74 | 396 | -109 | MLT1D | MaLR | LTR |
| 79510941 | 79511297 | - | 8.7 | 9 | 364 | 0 | THE1B | MaLR | LTR |
| 79511298 | 79512928 | - | 33.0 | 2 | 1733 | -2 | MLT1D-int | MaLR | LTR |
| 79512931 | 79513293 | - | 10.7 | 1 | 364 | 0 | THE1B | MaLR | LTR |
| 79513299 | 79513382 | - | 20.7 | 1 | 87 | -418 | MLT1D | MaLR | LTR |

  

|  |  |  |  |  |  |  |  |  |  |
| --- | --- | --- | --- | --- | --- | --- | --- | --- | --- |
| chr7:79758796-79761499 | | | 2673 bp | | |  |  |  |  |
| Genome Start | Genome End | Strand | Div | Repeat Start | Repeat End | Repeat Left | Name | Family | Class |
| 79758796 | 79758855 | - | 22.2 | 248 | 304 | -8 | AluJb | Alu | SINE |
| 79758856 | 79759225 | - | 13.5 | 1 | 364 | 0 | THE1B | MaLR | LTR |
| 79759226 | 79760840 | - | 30.4 | 2 | 1735 | 0 | MLT1F-int | MaLR | LTR |
| 79760843 | 79761209 | - | 13.6 | 1 | 364 | 0 | THE1B | MaLR | LTR |
| 79761210 | 79761353 | - | 22.2 | 118 | 247 | -65 | AluJb | Alu | SINE |
| 79761383 | 79761499 | - | 22.2 | 1 | 117 | -195 | AluJb | Alu | SINE |

  

|  |  |  |  |  |  |  |  |  |  |
| --- | --- | --- | --- | --- | --- | --- | --- | --- | --- |
| chr7:79998029-80001108 | | | 3070 bp | | |  |  |  |  |
| Genome Start | Genome End | Strand | Div | Repeat Start | Repeat End | Repeat Left | Name | Family | Class |
| 79998029 | 79998108 | + | 20.0 | 1 | 95 | -313 | MLT1A1 | MaLR | LTR |
| 79998109 | 79998475 | + | 13.4 | 1 | 364 | 0 | THE1B | MaLR | LTR |
| 79998476 | 80000064 | + | 9.8 | 1 | 1580 | 0 | THE1B-int | MaLR | LTR |
| 80000065 | 80000409 | + | 14.6 | 1 | 364 | 0 | THE1B | MaLR | LTR |
| 80000411 | 80000822 | - | 9.9 | 3 | 608 | 0 | LTR14B | ERVK | LTR |
| 80000832 | 80001108 | + | 17.6 | 91 | 408 | 0 | MLT1A1 | MaLR | LTR |

  

|  |  |  |  |  |  |  |  |  |  |
| --- | --- | --- | --- | --- | --- | --- | --- | --- | --- |
| chr7:80687250-80691463 | | | 4100 bp | | |  |  |  |  |
| Genome Start | Genome End | Strand | Div | Repeat Start | Repeat End | Repeat Left | Name | Family | Class |
| 80687250 | 80687708 | - | 16.5 | 5809 | 6277 | -23 | L1MA6 | L1 | LINE |
| 80687821 | 80689126 | - | 16.7 | 4534 | 5808 | -492 | L1MA6 | L1 | LINE |
| 80689127 | 80689480 | + | 12.7 | 1 | 364 | 0 | THE1B | MaLR | LTR |
| 80689483 | 80691060 | + | 28.6 | 2 | 1735 | 0 | MLT-int | MaLR | LTR |
| 80691061 | 80691410 | + | 14.3 | 1 | 364 | 0 | THE1B | MaLR | LTR |
| 80691411 | 80691463 | - | 16.8 | 4481 | 4533 | -1610 | L1MA6 | L1 | LINE |

  

|  |  |  |  |  |  |  |  |  |  |
| --- | --- | --- | --- | --- | --- | --- | --- | --- | --- |
| chr7:82040843-82042556 | | | 1656 bp | | |  |  |  |  |
| Genome Start | Genome End | Strand | Div | Repeat Start | Repeat End | Repeat Left | Name | Family | Class |
| 82040843 | 82040913 | + | 13.7 | 181 | 261 | -113 | MLT1A | MaLR | LTR |
| 82040960 | 82041329 | - | 13.3 | 1 | 381 | 0 | THE1D | MaLR | LTR |
| 82041332 | 82041621 | - | 8.3 | 1287 | 1578 | -2 | THE1D-int | MaLR | LTR |
| 82041632 | 82042142 | - | 9.2 | 1 | 467 | -1113 | THE1D-int | MaLR | LTR |
| 82042143 | 82042495 | - | 12.3 | 1 | 381 | 0 | THE1D | MaLR | LTR |
| 82042496 | 82042556 | + | 18.0 | 284 | 344 | -30 | MLT1A | MaLR | LTR |

  

|  |  |  |  |  |  |  |  |  |  |
| --- | --- | --- | --- | --- | --- | --- | --- | --- | --- |
| chr7:84386005-84388580 | | | 2575 bp | | |  |  |  |  |
| Genome Start | Genome End | Strand | Div | Repeat Start | Repeat End | Repeat Left | Name | Family | Class |
| 84386005 | 84386045 | + | 22.2 | 6 | 48 | -419 | MLT1C | MaLR | LTR |
| 84386046 | 84386429 | - | 13.6 | 1 | 428 | 0 | MSTA | MaLR | LTR |
| 84386430 | 84388030 | - | 29.2 | 1 | 1735 | 0 | MLT1H-int | MaLR | LTR |
| 84388032 | 84388411 | - | 15.4 | 3 | 428 | 0 | MSTA | MaLR | LTR |
| 84388412 | 84388580 | + | 22.2 | 49 | 200 | -267 | MLT1C | MaLR | LTR |

  

|  |  |  |  |  |  |  |  |  |  |
| --- | --- | --- | --- | --- | --- | --- | --- | --- | --- |
| chr7:84857653-84865823 | | | 7719 bp | | |  |  |  |  |
| Genome Start | Genome End | Strand | Div | Repeat Start | Repeat End | Repeat Left | Name | Family | Class |
| 84857653 | 84858573 | - | 21.8 | 5718 | 6681 | -1522 | L1MC3 | L1 | LINE |
| 84858573 | 84858820 | - | 17.3 | 3181 | 3443 | -3121 | L1MC3 | L1 | LINE |
| 84858825 | 84859585 | - | 23.7 | 2234 | 3008 | -3556 | L1MC3 | L1 | LINE |
| 84859587 | 84860275 | - | 15.8 | 9 | 711 | -23 | MER50 | ERV1 | LTR |
| 84860595 | 84860982 | - | 19.1 | 1681 | 2089 | -4662 | L1MC3 | L1 | LINE |
| 84860998 | 84861440 | - | 22.1 | 1175 | 1634 | -5117 | L1MC3 | L1 | LINE |
| 84861460 | 84861821 | - | 13.4 | 1 | 364 | 0 | THE1B | MaLR | LTR |
| 84861822 | 84863387 | - | 30.0 | 5 | 1735 | 0 | MLT-int | MaLR | LTR |
| 84863393 | 84863753 | - | 12.0 | 3 | 364 | 0 | THE1B | MaLR | LTR |
| 84863754 | 84864659 | - | 17.7 | 1 | 894 | -9 | MER4D | ERV1 | LTR |
| 84864663 | 84864943 | - | 21.2 | 774 | 1080 | -4965 | L1MC3 | L1 | LINE |
| 84864989 | 84865051 | - | 11.1 | 184 | 246 | -657 | MER4D | ERV1 | LTR |
| 84865094 | 84865823 | - | 27.8 | 40 | 744 | -5301 | L1MC3 | L1 | LINE |

  

|  |  |  |  |  |  |  |  |  |  |
| --- | --- | --- | --- | --- | --- | --- | --- | --- | --- |
| chr7:85686719-85691775 | | | 5007 bp | | |  |  |  |  |
| Genome Start | Genome End | Strand | Div | Repeat Start | Repeat End | Repeat Left | Name | Family | Class |
| 85686719 | 85687113 | - | 27.9 | 2874 | 3273 | -2873 | L1M5 | L1 | LINE |
| 85687155 | 85689224 | + | 19.4 | 2227 | 4350 | -3791 | L1PREC2 | L1 | LINE |
| 85689225 | 85689352 | - | 12.5 | 1 | 131 | -233 | THE1B | MaLR | LTR |
| 85689353 | 85690924 | - | 12.5 | 1 | 1580 | 0 | THE1B-int | MaLR | LTR |
| 85690926 | 85691284 | - | 16.5 | 1 | 363 | -1 | THE1B | MaLR | LTR |
| 85691293 | 85691775 | - | 28.0 | 2320 | 2823 | -3323 | L1M5 | L1 | LINE |

  

|  |  |  |  |  |  |  |  |  |  |
| --- | --- | --- | --- | --- | --- | --- | --- | --- | --- |
| chr7:85854962-85860493 | | | 5220 bp | | |  |  |  |  |
| Genome Start | Genome End | Strand | Div | Repeat Start | Repeat End | Repeat Left | Name | Family | Class |
| 85854962 | 85855081 | - | 20.0 | 6394 | 6506 | -127 | L1MD1 | L1 | LINE |
| 85855110 | 85855689 | - | 20.0 | 5827 | 6393 | -240 | L1MD1 | L1 | LINE |
| 85855684 | 85855840 | - | 20.7 | 3134 | 3292 | -3245 | L1MD1 | L1 | LINE |
| 85855841 | 85856137 | + | 17.8 | 1 | 294 | -18 | AluJb | Alu | SINE |
| 85856138 | 85856279 | - | 20.7 | 2985 | 3133 | -3404 | L1MD1 | L1 | LINE |
| 85856537 | 85856586 | - | 8.2 | 2771 | 2819 | -3718 | L1MD1 | L1 | LINE |
| 85856585 | 85858006 | - | 23.3 | 831 | 2264 | -4369 | L1MD1 | L1 | LINE |
| 85858023 | 85858381 | - | 13.8 | 1 | 364 | 0 | THE1B | MaLR | LTR |
| 85858383 | 85859963 | - | 12.1 | 11 | 1579 | -1 | THE1B-int | MaLR | LTR |
| 85859974 | 85860328 | - | 15.3 | 4 | 364 | 0 | THE1B | MaLR | LTR |
| 85860337 | 85860493 | - | 28.4 | 21 | 185 | -6448 | L1MD1 | L1 | LINE |

  

|  |  |  |  |  |  |  |  |  |  |
| --- | --- | --- | --- | --- | --- | --- | --- | --- | --- |
| chr7:86079926-86088462 | | | 8070 bp | | |  |  |  |  |
| Genome Start | Genome End | Strand | Div | Repeat Start | Repeat End | Repeat Left | Name | Family | Class |
| 86079926 | 86080165 | + | 33.3 | 744 | 1000 | -1507 | HAL1 | L1 | LINE |
| 86080318 | 86080540 | + | 35.1 | 249 | 473 | -5418 | L1M3d | L1 | LINE |
| 86080547 | 86080697 | - | 19.2 | 4159 | 4324 | -1819 | L1M2 | L1 | LINE |
| 86080699 | 86081009 | + | 18.1 | 4329 | 4649 | -1491 | L1MB1 | L1 | LINE |
| 86081020 | 86081393 | + | 10.2 | 1 | 374 | -1 | THE1C | MaLR | LTR |
| 86081395 | 86081513 | + | 19.8 | 6 | 138 | -227 | MLT1A0 | MaLR | LTR |
| 86081514 | 86081878 | + | 12.5 | 1 | 381 | 0 | THE1D | MaLR | LTR |
| 86081879 | 86082102 | + | 19.8 | 139 | 365 | 0 | MLT1A0 | MaLR | LTR |
| 86082103 | 86082956 | + | 18.7 | 4645 | 5534 | -634 | L1MB1 | L1 | LINE |
| 86082958 | 86083331 | + | 11.1 | 1 | 373 | -2 | THE1C | MaLR | LTR |
| 86083334 | 86084872 | + | 13.7 | 1 | 1580 | 0 | THE1C-int | MaLR | LTR |
| 86084873 | 86085241 | + | 11.9 | 1 | 385 | -5 | THE1C | MaLR | LTR |
| 86085242 | 86085662 | + | 17.6 | 5579 | 6017 | -151 | L1MB1 | L1 | LINE |
| 86085680 | 86086094 | - | 14.7 | 5890 | 6290 | -1 | L1MA8 | L1 | LINE |
| 86086096 | 86086286 | - | 19.7 | 1 | 187 | -125 | AluJb | Alu | SINE |
| 86086295 | 86087125 | - | 14.8 | 5053 | 5881 | -410 | L1MA8 | L1 | LINE |
| 86087126 | 86087460 | - | 19.7 | 1 | 306 | -6 | AluJb | Alu | SINE |
| 86087461 | 86087631 | - | 16.0 | 4882 | 5052 | -1091 | L1MA8 | L1 | LINE |
| 86087632 | 86087796 | + | 15.2 | 5998 | 6166 | -2 | L1MB1 | L1 | LINE |
| 86088065 | 86088462 | + | 28.3 | 1386 | 1779 | -728 | HAL1 | L1 | LINE |

  

|  |  |  |  |  |  |  |  |  |  |
| --- | --- | --- | --- | --- | --- | --- | --- | --- | --- |
| chr7:87495336-87502360 | | | 6984 bp | | |  |  |  |  |
| Genome Start | Genome End | Strand | Div | Repeat Start | Repeat End | Repeat Left | Name | Family | Class |
| 87495336 | 87495928 | - | 12.3 | 3867 | 4461 | -3104 | L1M1 | L1 | LINE |
| 87495958 | 87496229 | - | 24.8 | 3593 | 3866 | -3699 | L1M1 | L1 | LINE |
| 87496239 | 87496296 | - | 13.8 | 6247 | 6304 | 0 | L1MA2 | L1 | LINE |
| 87496297 | 87497745 | - | 11.8 | 2115 | 3609 | -3956 | L1M1 | L1 | LINE |
| 87497746 | 87498085 | + | 13.8 | 1 | 366 | -62 | MSTA | MaLR | LTR |
| 87498086 | 87498391 | + | 5.0 | 1 | 306 | 0 | AluY | Alu | SINE |
| 87498392 | 87498462 | + | 13.8 | 367 | 428 | 0 | MSTA | MaLR | LTR |
| 87498465 | 87499913 | + | 16.2 | 3 | 1580 | 0 | MSTA-int | MaLR | LTR |
| 87499915 | 87500300 | + | 17.6 | 2 | 428 | 0 | MSTA | MaLR | LTR |
| 87500301 | 87502360 | - | 15.9 | 12 | 2119 | -5446 | L1M1 | L1 | LINE |

  

|  |  |  |  |  |  |  |  |  |  |
| --- | --- | --- | --- | --- | --- | --- | --- | --- | --- |
| chr7:88603661-88608987 | | | 5201 bp | | |  |  |  |  |
| Genome Start | Genome End | Strand | Div | Repeat Start | Repeat End | Repeat Left | Name | Family | Class |
| 88603661 | 88603973 | - | 20.6 | 5798 | 6113 | -70 | L1MB3 | L1 | LINE |
| 88604030 | 88604369 | + | 16.2 | 5 | 364 | 0 | THE1B | MaLR | LTR |
| 88604372 | 88605874 | + | 15.2 | 2 | 1645 | -6 | THE1-int | MaLR | LTR |
| 88605881 | 88606235 | + | 13.9 | 1 | 363 | -1 | THE1B | MaLR | LTR |
| 88606286 | 88608664 | - | 15.8 | 6 | 2418 | 0 | Tigger1 | MER2\_type | DNA |
| 88608677 | 88608987 | - | 19.7 | 5476 | 5792 | -388 | L1MB3 | L1 | LINE |

  

|  |  |  |  |  |  |  |  |  |  |
| --- | --- | --- | --- | --- | --- | --- | --- | --- | --- |
| chr7:89422104-89424767 | | | 2661 bp | | |  |  |  |  |
| Genome Start | Genome End | Strand | Div | Repeat Start | Repeat End | Repeat Left | Name | Family | Class |
| 89422104 | 89422208 | - | 38.4 | 3246 | 3371 | -7 | L2 | L2 | LINE |
| 89422209 | 89422576 | - | 12.6 | 2 | 364 | 0 | THE1B | MaLR | LTR |
| 89422578 | 89424142 | - | 29.3 | 2 | 1735 | 0 | MLT1J2-int | MaLR | LTR |
| 89424145 | 89424512 | - | 12.6 | 1 | 364 | 0 | THE1B | MaLR | LTR |
| 89424513 | 89424767 | - | 38.4 | 2972 | 3245 | -133 | L2 | L2 | LINE |

  

|  |  |  |  |  |  |  |  |  |  |
| --- | --- | --- | --- | --- | --- | --- | --- | --- | --- |
| chr7:89929909-89935469 | | | 5083 bp | | |  |  |  |  |
| Genome Start | Genome End | Strand | Div | Repeat Start | Repeat End | Repeat Left | Name | Family | Class |
| 89929909 | 89930091 | - | 27.4 | 357 | 565 | -26 | MLT1K | MaLR | LTR |
| 89930092 | 89930131 | - | 15.0 | 413 | 452 | -12 | LTR16B | ERVL | LTR |
| 89930139 | 89930365 | - | 29.1 | 1733 | 1983 | -3013 | HERV16 | ERVL | LTR |
| 89930366 | 89930735 | + | 11.2 | 1 | 375 | 0 | THE1C | MaLR | LTR |
| 89930803 | 89932364 | + | 10.1 | 45 | 1580 | 0 | THE1C-int | MaLR | LTR |
| 89932365 | 89932741 | + | 9.1 | 1 | 375 | 0 | THE1C | MaLR | LTR |
| 89932747 | 89933612 | - | 28.3 | 744 | 1732 | -3264 | HERV16 | ERVL | LTR |
| 89933734 | 89933894 | + | 25.6 | 1248 | 1413 | -3583 | HERV16 | ERVL | LTR |
| 89934085 | 89934170 | - | 26.4 | 378 | 464 | 0 | LTR16B | ERVL | LTR |
| 89934171 | 89934491 | + | 10.3 | 1 | 313 | 0 | AluSp | Alu | SINE |
| 89934492 | 89934815 | - | 26.4 | 51 | 377 | -87 | LTR16B | ERVL | LTR |
| 89934859 | 89935030 | - | 18.3 | 123 | 308 | -4 | AluJb | Alu | SINE |
| 89935057 | 89935173 | - | 18.3 | 6 | 122 | -190 | AluJb | Alu | SINE |
| 89935193 | 89935469 | - | 33.8 | 75 | 353 | -238 | MLT1K | MaLR | LTR |

  

|  |  |  |  |  |  |  |  |  |  |
| --- | --- | --- | --- | --- | --- | --- | --- | --- | --- |
| chr7:91945673-91953831 | | | 8064 bp | | |  |  |  |  |
| Genome Start | Genome End | Strand | Div | Repeat Start | Repeat End | Repeat Left | Name | Family | Class |
| 91945673 | 91945869 | + | 31.1 | 1342 | 1552 | -4115 | ERVL-E | ERVL | LTR |
| 91945878 | 91946442 | + | 16.1 | 10 | 581 | -63 | LTR9B | ERV1 | LTR |
| 91946443 | 91946837 | - | 11.0 | 1 | 450 | 0 | LTR7 | ERV1 | LTR |
| 91946838 | 91947278 | - | 9.9 | 7269 | 7713 | 0 | HERVH | ERV1 | LTR |
| 91947259 | 91952090 | - | 13.1 | 1 | 5275 | -1816 | HERVH | ERV1 | LTR |
| 91952091 | 91952524 | - | 11.4 | 1 | 450 | 0 | LTR7 | ERV1 | LTR |
| 91952525 | 91952591 | + | 7.5 | 577 | 644 | 0 | LTR9B | ERV1 | LTR |
| 91952595 | 91952736 | + | 32.6 | 1651 | 1802 | -3955 | ERVL-E | ERVL | LTR |
| 91952836 | 91952931 | + | 30.2 | 1861 | 1957 | -3710 | ERVL-E | ERVL | LTR |
| 91952929 | 91953719 | + | 29.6 | 3632 | 4477 | -1190 | ERVL-E | ERVL | LTR |
| 91953728 | 91953831 | + | 26.5 | 4678 | 4788 | -879 | ERVL-E | ERVL | LTR |

  

|  |  |  |  |  |  |  |  |  |  |
| --- | --- | --- | --- | --- | --- | --- | --- | --- | --- |
| chr7:92640031-92643858 | | | 3791 bp | | |  |  |  |  |
| Genome Start | Genome End | Strand | Div | Repeat Start | Repeat End | Repeat Left | Name | Family | Class |
| 92640031 | 92640111 | + | 20.4 | 3252 | 3331 | -2815 | L1MC | L1 | LINE |
| 92640145 | 92640586 | + | 20.4 | 3332 | 3793 | -2353 | L1MC | L1 | LINE |
| 92640587 | 92640954 | + | 15.8 | 1 | 379 | -2 | THE1D | MaLR | LTR |
| 92640957 | 92641341 | + | 15.6 | 1 | 375 | -1205 | THE1D-int | MaLR | LTR |
| 92641342 | 92641637 | + | 12.2 | 3 | 299 | -14 | AluSp | Alu | SINE |
| 92641638 | 92641767 | + | 15.6 | 376 | 489 | -1091 | THE1D-int | MaLR | LTR |
| 92641768 | 92642040 | + | 13.9 | 40 | 312 | 0 | AluSx | Alu | SINE |
| 92642041 | 92643086 | + | 14.3 | 490 | 1580 | 0 | THE1D-int | MaLR | LTR |
| 92643089 | 92643457 | + | 11.3 | 3 | 381 | 0 | THE1D | MaLR | LTR |
| 92643458 | 92643858 | + | 20.2 | 3791 | 4197 | -1949 | L1MC | L1 | LINE |

  

|  |  |  |  |  |  |  |  |  |  |
| --- | --- | --- | --- | --- | --- | --- | --- | --- | --- |
| chr7:94189356-94198229 | | | 8873 bp | | |  |  |  |  |
| Genome Start | Genome End | Strand | Div | Repeat Start | Repeat End | Repeat Left | Name | Family | Class |
| 94189356 | 94189540 | + | 8.1 | 2336 | 2536 | -5029 | L1M1 | L1 | LINE |
| 94189541 | 94190049 | - | 12.3 | 1 | 511 | 0 | MER9 | ERVK | LTR |
| 94190050 | 94196048 | - | 9.0 | 1 | 8016 | 0 | HERVK9 | ERVK | LTR |
| 94196049 | 94196557 | - | 11.7 | 1 | 511 | 0 | MER9 | ERVK | LTR |
| 94196559 | 94196950 | + | 12.1 | 2532 | 2914 | -4651 | L1M1 | L1 | LINE |
| 94196951 | 94197198 | + | 16.5 | 1 | 248 | -64 | AluSx | Alu | SINE |
| 94197199 | 94198229 | + | 11.5 | 2915 | 3922 | -3643 | L1M1 | L1 | LINE |

  

|  |  |  |  |  |  |  |  |  |  |
| --- | --- | --- | --- | --- | --- | --- | --- | --- | --- |
| chr7:94238300-94242866 | | | 4157 bp | | |  |  |  |  |
| Genome Start | Genome End | Strand | Div | Repeat Start | Repeat End | Repeat Left | Name | Family | Class |
| 94238300 | 94239369 | + | 19.3 | 6529 | 7590 | -195 | L1MC3 | L1 | LINE |
| 94239375 | 94239602 | + | 9.7 | 5822 | 6058 | -93 | L1PB1 | L1 | LINE |
| 94239770 | 94239849 | + | 10.0 | 6069 | 6151 | 0 | L1PB1 | L1 | LINE |
| 94239959 | 94240314 | + | 13.9 | 1 | 359 | -5 | THE1B | MaLR | LTR |
| 94240323 | 94241896 | + | 30.2 | 2 | 1735 | 0 | MLT1A-int | MaLR | LTR |
| 94241899 | 94242256 | + | 15.0 | 3 | 364 | 0 | THE1B | MaLR | LTR |
| 94242372 | 94242682 | + | 13.8 | 1 | 311 | -2 | AluSq | Alu | SINE |
| 94242687 | 94242866 | + | 13.0 | 7586 | 7773 | -12 | L1MC3 | L1 | LINE |

  

|  |  |  |  |  |  |  |  |  |  |
| --- | --- | --- | --- | --- | --- | --- | --- | --- | --- |
| chr7:94309400-94314747 | | | 4959 bp | | |  |  |  |  |
| Genome Start | Genome End | Strand | Div | Repeat Start | Repeat End | Repeat Left | Name | Family | Class |
| 94309400 | 94309643 | - | 29.9 | 4677 | 4924 | -1156 | L1MEb | L1 | LINE |
| 94309650 | 94309853 | + | 19.8 | 7 | 212 | -7 | MER20 | MER1\_type | DNA |
| 94309864 | 94309981 | - | 22.9 | 4566 | 4681 | -1399 | L1MEb | L1 | LINE |
| 94309982 | 94310279 | + | 11.5 | 6 | 301 | -11 | AluSx | Alu | SINE |
| 94310280 | 94310440 | - | 26.2 | 4427 | 4565 | -1515 | L1MEb | L1 | LINE |
| 94310458 | 94310613 | - | 26.8 | 3375 | 3530 | -2550 | L1MEb | L1 | LINE |
| 94310617 | 94310980 | + | 15.4 | 6 | 364 | 0 | THE1B | MaLR | LTR |
| 94310983 | 94312547 | + | 31.1 | 2 | 1735 | 0 | MLT-int | MaLR | LTR |
| 94312550 | 94312899 | + | 16.4 | 3 | 364 | 0 | THE1B | MaLR | LTR |
| 94312901 | 94313438 | - | 22.5 | 2774 | 3368 | -2712 | L1MEb | L1 | LINE |
| 94313454 | 94313577 | - | 25.8 | 6049 | 6183 | -1 | L1MB7 | L1 | LINE |
| 94313564 | 94314022 | - | 28.3 | 571 | 1012 | -5058 | L1MEb | L1 | LINE |
| 94314370 | 94314747 | - | 33.0 | 12 | 399 | -5671 | L1MEb | L1 | LINE |

  

|  |  |  |  |  |  |  |  |  |  |
| --- | --- | --- | --- | --- | --- | --- | --- | --- | --- |
| chr7:94923375-94929580 | | | 6212 bp | | |  |  |  |  |
| Genome Start | Genome End | Strand | Div | Repeat Start | Repeat End | Repeat Left | Name | Family | Class |
| 94923375 | 94923427 | - | 13.2 | 3355 | 3408 | -3129 | L1MDa | L1 | LINE |
| 94923422 | 94923772 | - | 19.6 | 5980 | 6322 | -37 | L1MD2 | L1 | LINE |
| 94923771 | 94925799 | - | 24.2 | 1298 | 3296 | -3241 | L1MDa | L1 | LINE |
| 94925800 | 94926096 | - | 20.0 | 5 | 299 | -13 | AluJo | Alu | SINE |
| 94926097 | 94927069 | - | 26.2 | 248 | 1297 | -5336 | L1MDa | L1 | LINE |
| 94927070 | 94927432 | + | 12.2 | 1 | 375 | 0 | THE1C | MaLR | LTR |
| 94927435 | 94928945 | + | 28.4 | 2 | 1735 | 0 | MLT-int | MaLR | LTR |
| 94928946 | 94929338 | + | 12.1 | 1 | 375 | 0 | THE1C | MaLR | LTR |
| 94929339 | 94929580 | - | 26.2 | 15 | 247 | -6386 | L1MDa | L1 | LINE |

  

|  |  |  |  |  |  |  |  |  |  |
| --- | --- | --- | --- | --- | --- | --- | --- | --- | --- |
| chr7:96216077-96218669 | | | 2591 bp | | |  |  |  |  |
| Genome Start | Genome End | Strand | Div | Repeat Start | Repeat End | Repeat Left | Name | Family | Class |
| 96216077 | 96216207 | - | 16.5 | 88 | 219 | 0 | MER20 | MER1\_type | DNA |
| 96216208 | 96216581 | - | 10.7 | 1 | 381 | 0 | THE1D | MaLR | LTR |
| 96216582 | 96218204 | - | 30.8 | 2 | 1735 | 0 | MLT-int | MaLR | LTR |
| 96218207 | 96218581 | - | 14.2 | 1 | 381 | 0 | THE1D | MaLR | LTR |
| 96218582 | 96218669 | - | 16.5 | 1 | 87 | -132 | MER20 | MER1\_type | DNA |

  

|  |  |  |  |  |  |  |  |  |  |
| --- | --- | --- | --- | --- | --- | --- | --- | --- | --- |
| chr7:97356067-97364467 | | | 8398 bp | | |  |  |  |  |
| Genome Start | Genome End | Strand | Div | Repeat Start | Repeat End | Repeat Left | Name | Family | Class |
| 97356067 | 97356484 | + | 19.5 | 978 | 1437 | -5412 | L1M2 | L1 | LINE |
| 97356485 | 97356781 | + | 11.1 | 1 | 298 | -12 | AluSg | Alu | SINE |
| 97356782 | 97357848 | + | 19.5 | 1438 | 2520 | -4329 | L1M2 | L1 | LINE |
| 97357849 | 97358005 | + | 20.4 | 1 | 157 | -9 | FRAM | Alu | SINE |
| 97358006 | 97358269 | + | 19.5 | 2521 | 2779 | -4070 | L1M2 | L1 | LINE |
| 97358270 | 97358627 | - | 14.1 | 1 | 364 | 0 | THE1B | MaLR | LTR |
| 97358628 | 97360217 | - | 30.4 | 2 | 1735 | 0 | MLT-int | MaLR | LTR |
| 97360220 | 97360573 | - | 13.8 | 1 | 364 | 0 | THE1B | MaLR | LTR |
| 97360574 | 97362759 | + | 16.1 | 2780 | 4973 | -1872 | L1M2 | L1 | LINE |
| 97362760 | 97363056 | + | 11.8 | 1 | 302 | -10 | AluSx | Alu | SINE |
| 97363057 | 97363554 | + | 16.1 | 4974 | 5449 | -1396 | L1M2 | L1 | LINE |
| 97363556 | 97363863 | + | 7.0 | 1 | 300 | -11 | AluY | Alu | SINE |
| 97363864 | 97364467 | + | 20.6 | 5434 | 5902 | -940 | L1M2 | L1 | LINE |

  

|  |  |  |  |  |  |  |  |  |  |
| --- | --- | --- | --- | --- | --- | --- | --- | --- | --- |
| chr7:100350265-100363656 | | | 12929 bp | | |  |  |  |  |
| Genome Start | Genome End | Strand | Div | Repeat Start | Repeat End | Repeat Left | Name | Family | Class |
| 100350265 | 100350477 | - | 13.6 | 68 | 284 | -28 | AluSx | Alu | SINE |
| 100350553 | 100350644 | - | 16.7 | 2 | 85 | -227 | AluSx | Alu | SINE |
| 100350646 | 100350842 | + | 22.9 | 199 | 381 | -412 | MER54B | ERVL | LTR |
| 100350933 | 100351048 | + | 12.1 | 1 | 116 | -196 | AluJb | Alu | SINE |
| 100351049 | 100351347 | - | 6.0 | 1 | 298 | -13 | AluY | Alu | SINE |
| 100351380 | 100351417 | + | 19.2 | 389 | 426 | -367 | MER54B | ERVL | LTR |
| 100351418 | 100351592 | + | 14.5 | 1 | 181 | -453 | MER41B | ERV1 | LTR |
| 100351593 | 100351878 | + | 9.8 | 1 | 287 | -23 | AluSg | Alu | SINE |
| 100351879 | 100352339 | + | 14.5 | 182 | 567 | -67 | MER41B | ERV1 | LTR |
| 100352340 | 100352648 | - | 5.2 | 1 | 311 | 0 | AluY | Alu | SINE |
| 100352649 | 100352720 | + | 14.5 | 568 | 632 | -2 | MER41B | ERV1 | LTR |
| 100352721 | 100353076 | + | 19.2 | 427 | 792 | -1 | MER54B | ERVL | LTR |
| 100353141 | 100353421 | + | 17.5 | 1 | 280 | -454 | MER50 | ERV1 | LTR |
| 100353422 | 100353724 | - | 10.1 | 1 | 300 | -9 | AluSc | Alu | SINE |
| 100353725 | 100353864 | - | 12.1 | 1 | 141 | -171 | AluSq/x | Alu | SINE |
| 100353873 | 100354139 | + | 13.9 | 281 | 557 | -177 | MER50 | ERV1 | LTR |
| 100354140 | 100354431 | - | 12.1 | 1 | 290 | -22 | AluSx | Alu | SINE |
| 100354432 | 100354586 | + | 13.9 | 558 | 711 | -23 | MER50 | ERV1 | LTR |
| 100354587 | 100354704 | + | 15.2 | 667 | 793 | 0 | MER54B | ERVL | LTR |
| 100354795 | 100355173 | - | 6.6 | 172 | 554 | 0 | MER41A | ERV1 | LTR |
| 100355174 | 100355641 | + | 6.8 | 1 | 463 | 0 | LTR2 | ERV1 | LTR |
| 100355642 | 100355812 | - | 6.6 | 1 | 171 | -383 | MER41A | ERV1 | LTR |
| 100355830 | 100356104 | - | 32.0 | 640 | 902 | -5521 | L1MCc | L1 | LINE |
| 100356124 | 100356276 | - | 24.8 | 574 | 727 | 0 | LTR8A | ERV1 | LTR |
| 100356296 | 100356697 | - | 15.0 | 1 | 420 | -28 | MER83 | ERV1 | LTR |
| 100356698 | 100356899 | - | 16.1 | 350 | 549 | -178 | LTR8A | ERV1 | LTR |
| 100356897 | 100357147 | - | 19.9 | 1 | 249 | -478 | LTR8A | ERV1 | LTR |
| 100357177 | 100357314 | - | 13.0 | 760 | 903 | 0 | MER4D | ERV1 | LTR |
| 100357338 | 100357606 | - | 16.4 | 1 | 278 | -34 | AluSx | Alu | SINE |
| 100357609 | 100357837 | - | 11.8 | 64 | 293 | -19 | AluSx | Alu | SINE |
| 100357838 | 100358225 | + | 8.5 | 8 | 448 | -2 | LTR7 | ERV1 | LTR |
| 100358228 | 100361123 | + | 11.4 | 1 | 2992 | -4721 | HERVH | ERV1 | LTR |
| 100361111 | 100361967 | + | 11.1 | 3141 | 4000 | -3713 | HERVH | ERV1 | LTR |
| 100361963 | 100362569 | + | 13.7 | 4489 | 5149 | -2564 | HERVH | ERV1 | LTR |
| 100362571 | 100362786 | + | 12.5 | 5600 | 5819 | -1894 | HERVH | ERV1 | LTR |
| 100362787 | 100363160 | + | 9.2 | 7324 | 7713 | 0 | HERVH | ERV1 | LTR |
| 100363163 | 100363576 | + | 7.8 | 3 | 440 | -10 | LTR7 | ERV1 | LTR |
| 100363587 | 100363656 | - | 14.3 | 5 | 74 | -238 | AluSx | Alu | SINE |

  

|  |  |  |  |  |  |  |  |  |  |
| --- | --- | --- | --- | --- | --- | --- | --- | --- | --- |
| chr7:103887539-103893836 | | | 6296 bp | | |  |  |  |  |
| Genome Start | Genome End | Strand | Div | Repeat Start | Repeat End | Repeat Left | Name | Family | Class |
| 103887539 | 103888677 | - | 9.0 | 5000 | 6159 | -1 | L1PREC2 | L1 | LINE |
| 103888678 | 103889041 | - | 11.4 | 1 | 375 | 0 | THE1C | MaLR | LTR |
| 103889042 | 103891160 | - | 9.3 | 2910 | 4999 | -1147 | L1PREC2 | L1 | LINE |
| 103891161 | 103891535 | - | 13.2 | 3 | 375 | 0 | THE1C | MaLR | LTR |
| 103891538 | 103893124 | - | 11.8 | 1 | 1579 | -1 | THE1C-int | MaLR | LTR |
| 103893125 | 103893491 | - | 9.3 | 1 | 375 | 0 | THE1C | MaLR | LTR |
| 103893492 | 103893836 | - | 9.6 | 2562 | 2914 | -3232 | L1PREC2 | L1 | LINE |

  

|  |  |  |  |  |  |  |  |  |  |
| --- | --- | --- | --- | --- | --- | --- | --- | --- | --- |
| chr7:105136658-105141011 | | | 4270 bp | | |  |  |  |  |
| Genome Start | Genome End | Strand | Div | Repeat Start | Repeat End | Repeat Left | Name | Family | Class |
| 105136658 | 105136691 | - | 11.8 | 87 | 120 | -40 | MER5A1 | MER1\_type | DNA |
| 105136734 | 105137029 | + | 9.2 | 1 | 296 | -17 | AluSq | Alu | SINE |
| 105137030 | 105137557 | + | 19.7 | 17 | 550 | -8 | MER74A | ERVL | LTR |
| 105137560 | 105137831 | - | 15.4 | 1 | 295 | -17 | AluJo | Alu | SINE |
| 105137863 | 105138083 | - | 13.0 | 200 | 428 | 0 | MSTA | MaLR | LTR |
| 105138084 | 105138386 | - | 7.7 | 7 | 305 | -6 | AluY | Alu | SINE |
| 105138387 | 105138492 | - | 13.0 | 140 | 199 | -229 | MSTA | MaLR | LTR |
| 105138493 | 105138843 | - | 12.7 | 1 | 375 | 0 | THE1C | MaLR | LTR |
| 105138844 | 105138947 | - | 13.0 | 1 | 139 | -289 | MSTA | MaLR | LTR |
| 105138948 | 105140589 | - | 28.8 | 2 | 1734 | -1 | MLT-int | MaLR | LTR |
| 105140592 | 105140957 | - | 13.5 | 1 | 428 | 0 | MSTA | MaLR | LTR |
| 105140965 | 105141011 | - | 19.1 | 2 | 48 | -112 | MER5A1 | MER1\_type | DNA |

  

|  |  |  |  |  |  |  |  |  |  |
| --- | --- | --- | --- | --- | --- | --- | --- | --- | --- |
| chr7:105433524-105436232 | | | 2631 bp | | |  |  |  |  |
| Genome Start | Genome End | Strand | Div | Repeat Start | Repeat End | Repeat Left | Name | Family | Class |
| 105433524 | 105433608 | + | 23.2 | 1 | 93 | -428 | LTR33 | ERVL | LTR |
| 105433609 | 105433964 | - | 12.1 | 1 | 363 | -1 | THE1B | MaLR | LTR |
| 105433965 | 105435537 | - | 31.0 | 2 | 1735 | 0 | MLT-int | MaLR | LTR |
| 105435540 | 105435893 | - | 10.7 | 1 | 364 | 0 | THE1B | MaLR | LTR |
| 105435894 | 105435956 | + | 23.2 | 94 | 159 | -362 | LTR33 | ERVL | LTR |
| 105436033 | 105436232 | + | 31.5 | 301 | 510 | -11 | LTR33 | ERVL | LTR |

  

|  |  |  |  |  |  |  |  |  |  |
| --- | --- | --- | --- | --- | --- | --- | --- | --- | --- |
| chr7:105759899-105771317 | | | 11335 bp | | |  |  |  |  |
| Genome Start | Genome End | Strand | Div | Repeat Start | Repeat End | Repeat Left | Name | Family | Class |
| 105759899 | 105760016 | - | 18.8 | 408 | 542 | 0 | MLT1F | MaLR | LTR |
| 105760087 | 105760476 | + | 10.3 | 1 | 377 | 0 | MER51B | ERV1 | LTR |
| 105760477 | 105760657 | + | 8.9 | 1 | 175 | -4125 | MER51B-int | ERV1 | LTR |
| 105760660 | 105761724 | + | 3.7 | 417 | 1468 | -6348 | MER51B-int | ERV1 | LTR |
| 105761725 | 105762016 | - | 8.2 | 9 | 301 | -10 | AluY | Alu | SINE |
| 105762017 | 105766514 | + | 4.6 | 1469 | 6056 | -1760 | MER51B-int | ERV1 | LTR |
| 105766522 | 105766760 | + | 11.8 | 1 | 237 | -73 | AluSg | Alu | SINE |
| 105766763 | 105767910 | + | 1.8 | 6282 | 7444 | -372 | MER51B-int | ERV1 | LTR |
| 105767911 | 105768266 | - | 13.0 | 1 | 364 | 0 | THE1B | MaLR | LTR |
| 105768267 | 105769848 | - | 30.7 | 2 | 1735 | 0 | MLT1E1-int | MaLR | LTR |
| 105769851 | 105770202 | - | 11.4 | 1 | 364 | 0 | THE1B | MaLR | LTR |
| 105770203 | 105770575 | + | 1.8 | 7445 | 7816 | 0 | MER51B-int | ERV1 | LTR |
| 105770577 | 105770972 | + | 11.9 | 1 | 377 | 0 | MER51B | ERV1 | LTR |
| 105770973 | 105771317 | - | 29.1 | 7 | 364 | -178 | MLT1F | MaLR | LTR |

  

|  |  |  |  |  |  |  |  |  |  |
| --- | --- | --- | --- | --- | --- | --- | --- | --- | --- |
| chr7:105954038-105962499 | | | 8390 bp | | |  |  |  |  |
| Genome Start | Genome End | Strand | Div | Repeat Start | Repeat End | Repeat Left | Name | Family | Class |
| 105954038 | 105954139 | + | 16.0 | 1 | 101 | -533 | MER41B | ERV1 | LTR |
| 105954142 | 105955433 | - | 5.3 | 4844 | 6140 | -14 | L1PA5 | L1 | LINE |
| 105955434 | 105955863 | - | 7.7 | 1 | 464 | 0 | LTR7B | ERV1 | LTR |
| 105955864 | 105956416 | - | 12.2 | 7157 | 7713 | 0 | HERVH | ERV1 | LTR |
| 105956411 | 105956675 | - | 14.7 | 5747 | 6015 | -1698 | HERVH | ERV1 | LTR |
| 105956704 | 105958451 | - | 13.8 | 3140 | 5286 | -1805 | HERVH | ERV1 | LTR |
| 105958431 | 105960646 | - | 13.5 | 801 | 2995 | -4718 | HERVH | ERV1 | LTR |
| 105960707 | 105960872 | - | 16.0 | 304 | 459 | -7254 | HERVH | ERV1 | LTR |
| 105960882 | 105961340 | - | 4.8 | 1 | 464 | 0 | LTR7B | ERV1 | LTR |
| 105961341 | 105961985 | - | 6.1 | 4206 | 4848 | -1298 | L1P1 | L1 | LINE |
| 105961986 | 105962499 | + | 15.8 | 93 | 633 | -1 | MER41B | ERV1 | LTR |

  

|  |  |  |  |  |  |  |  |  |  |
| --- | --- | --- | --- | --- | --- | --- | --- | --- | --- |
| chr7:108663655-108667056 | | | 3330 bp | | |  |  |  |  |
| Genome Start | Genome End | Strand | Div | Repeat Start | Repeat End | Repeat Left | Name | Family | Class |
| 108663655 | 108664112 | + | 18.7 | 5330 | 5787 | -525 | L1MA9 | L1 | LINE |
| 108664182 | 108664288 | + | 18.7 | 5788 | 5893 | -419 | L1MA9 | L1 | LINE |
| 108664289 | 108664658 | + | 13.5 | 1 | 374 | -1 | THE1C | MaLR | LTR |
| 108664662 | 108666271 | + | 30.6 | 2 | 1735 | 0 | MLT-int | MaLR | LTR |
| 108666272 | 108666642 | + | 12.3 | 1 | 375 | 0 | THE1C | MaLR | LTR |
| 108666643 | 108667056 | + | 18.7 | 5894 | 6307 | -5 | L1MA9 | L1 | LINE |

  

|  |  |  |  |  |  |  |  |  |  |
| --- | --- | --- | --- | --- | --- | --- | --- | --- | --- |
| chr7:108956001-108958614 | | | 2510 bp | | |  |  |  |  |
| Genome Start | Genome End | Strand | Div | Repeat Start | Repeat End | Repeat Left | Name | Family | Class |
| 108956001 | 108956295 | + | 30.1 | 98 | 405 | -150 | MLT1H1 | MaLR | LTR |
| 108956304 | 108956655 | - | 20.9 | 1 | 416 | -12 | MSTC | MaLR | LTR |
| 108956657 | 108956867 | - | 24.2 | 1395 | 1650 | -1 | MSTC-int | MaLR | LTR |
| 108956868 | 108956929 | - | 8.1 | 375 | 437 | 0 | LTR23 | ERV1 | LTR |
| 108956991 | 108957109 | - | 20.3 | 1 | 145 | -292 | LTR23 | ERV1 | LTR |
| 108957142 | 108958029 | - | 23.8 | 2 | 911 | -824 | MSTC-int | MaLR | LTR |
| 108958032 | 108958470 | - | 15.4 | 1 | 428 | 0 | MSTC | MaLR | LTR |
| 108958471 | 108958614 | + | 26.4 | 401 | 545 | -10 | MLT1H1 | MaLR | LTR |

  

|  |  |  |  |  |  |  |  |  |  |
| --- | --- | --- | --- | --- | --- | --- | --- | --- | --- |
| chr7:109233920-109236796 | | | 2871 bp | | |  |  |  |  |
| Genome Start | Genome End | Strand | Div | Repeat Start | Repeat End | Repeat Left | Name | Family | Class |
| 109233920 | 109234186 | - | 31.1 | 5785 | 6051 | -189 | L1ME3B | L1 | LINE |
| 109234187 | 109234539 | + | 11.0 | 1 | 364 | 0 | THE1B | MaLR | LTR |
| 109234546 | 109236110 | + | 29.6 | 5 | 1735 | 0 | MLT-int | MaLR | LTR |
| 109236111 | 109236461 | + | 12.8 | 1 | 364 | 0 | THE1B | MaLR | LTR |
| 109236462 | 109236796 | - | 31.1 | 5472 | 5784 | -456 | L1ME3B | L1 | LINE |

  

|  |  |  |  |  |  |  |  |  |  |
| --- | --- | --- | --- | --- | --- | --- | --- | --- | --- |
| chr7:109362198-109368780 | | | 6219 bp | | |  |  |  |  |
| Genome Start | Genome End | Strand | Div | Repeat Start | Repeat End | Repeat Left | Name | Family | Class |
| 109362198 | 109362283 | - | 23.3 | 2763 | 2848 | -571 | L2 | L2 | LINE |
| 109362287 | 109362423 | + | 24.8 | 1 | 149 | -372 | LTR33 | ERVL | LTR |
| 109362595 | 109362909 | + | 36.2 | 118 | 441 | -50 | LTR16C | ERVL | LTR |
| 109362968 | 109363077 | + | 31.3 | 136 | 250 | -271 | LTR33 | ERVL | LTR |
| 109363078 | 109363742 | + | 17.9 | 1 | 711 | 0 | MER67C | ERV1 | LTR |
| 109363743 | 109363991 | + | 31.3 | 251 | 519 | -2 | LTR33 | ERVL | LTR |
| 109364051 | 109364771 | + | 25.2 | 30 | 727 | 0 | LTR8A | ERV1 | LTR |
| 109364776 | 109365275 | - | 33.0 | 2206 | 2720 | -699 | L2 | L2 | LINE |
| 109365318 | 109365726 | - | 20.1 | 1 | 393 | -33 | MSTB | MaLR | LTR |
| 109365728 | 109367383 | - | 29.5 | 2 | 1734 | -1 | MLT1G-int | MaLR | LTR |
| 109367386 | 109367804 | - | 19.6 | 1 | 426 | 0 | MSTB | MaLR | LTR |
| 109367805 | 109368052 | - | 26.4 | 1875 | 2167 | -1252 | L2 | L2 | LINE |
| 109368077 | 109368780 | - | 32.8 | 952 | 1685 | -1734 | L2 | L2 | LINE |

  

|  |  |  |  |  |  |  |  |  |  |
| --- | --- | --- | --- | --- | --- | --- | --- | --- | --- |
| chr7:112311148-112313829 | | | 2680 bp | | |  |  |  |  |
| Genome Start | Genome End | Strand | Div | Repeat Start | Repeat End | Repeat Left | Name | Family | Class |
| 112311148 | 112311170 | + | 27.6 | 5412 | 5433 | -688 | L1M5 | L1 | LINE |
| 112311171 | 112311548 | - | 8.7 | 1 | 375 | 0 | THE1C | MaLR | LTR |
| 112311549 | 112313160 | - | 29.7 | 2 | 1735 | 0 | MLT-int | MaLR | LTR |
| 112313163 | 112313541 | - | 9.3 | 1 | 375 | 0 | THE1C | MaLR | LTR |
| 112313542 | 112313829 | + | 27.6 | 5434 | 5758 | -363 | L1M5 | L1 | LINE |

  

|  |  |  |  |  |  |  |  |  |  |
| --- | --- | --- | --- | --- | --- | --- | --- | --- | --- |
| chr7:112694532-112704480 | | | 9940 bp | | |  |  |  |  |
| Genome Start | Genome End | Strand | Div | Repeat Start | Repeat End | Repeat Left | Name | Family | Class |
| 112694532 | 112694972 | + | 22.2 | 1 | 439 | -6414 | L1PA17 | L1 | LINE |
| 112694973 | 112695326 | + | 15.1 | 1 | 381 | 0 | THE1D | MaLR | LTR |
| 112695327 | 112696760 | + | 24.3 | 440 | 1555 | -5298 | L1PA17 | L1 | LINE |
| 112696755 | 112696944 | + | 23.7 | 1336 | 1527 | -5323 | L1PA17 | L1 | LINE |
| 112696932 | 112696961 | + | 6.7 | 2833 | 2862 | -3284 | L1P1 | L1 | LINE |
| 112696959 | 112697818 | + | 3.4 | 5294 | 6152 | -2 | L1PA5 | L1 | LINE |
| 112697824 | 112699303 | + | 24.0 | 1585 | 3111 | -3738 | L1PA17 | L1 | LINE |
| 112699327 | 112699727 | - | 17.6 | 1 | 428 | 0 | THE1D | MaLR | LTR |
| 112699728 | 112701297 | - | 31.2 | 3 | 1735 | 0 | MLT-int | MaLR | LTR |
| 112701301 | 112701668 | - | 15.1 | 1 | 381 | 0 | THE1D | MaLR | LTR |
| 112701669 | 112704480 | + | 16.6 | 4193 | 6851 | -18 | L1PA17 | L1 | LINE |

  

|  |  |  |  |  |  |  |  |  |  |
| --- | --- | --- | --- | --- | --- | --- | --- | --- | --- |
| chr7:113092330-113099811 | | | 7225 bp | | |  |  |  |  |
| Genome Start | Genome End | Strand | Div | Repeat Start | Repeat End | Repeat Left | Name | Family | Class |
| 113092330 | 113092506 | + | 28.4 | 684 | 871 | -5671 | L1M4 | L1 | LINE |
| 113092760 | 113094517 | + | 22.9 | 1719 | 3582 | -3062 | L1M4 | L1 | LINE |
| 113094501 | 113095186 | + | 14.7 | 4687 | 5367 | -799 | L1PA16 | L1 | LINE |
| 113095187 | 113095469 | + | 7.4 | 2 | 285 | -14 | AluYd8 | Alu | SINE |
| 113095470 | 113096264 | + | 13.6 | 5368 | 6157 | -9 | L1PA16 | L1 | LINE |
| 113096282 | 113097057 | + | 19.2 | 3571 | 4403 | -2241 | L1M4 | L1 | LINE |
| 113097058 | 113097397 | + | 12.0 | 1 | 359 | -5 | THE1B | MaLR | LTR |
| 113097402 | 113098944 | + | 28.6 | 2 | 1735 | 0 | MLT-int | MaLR | LTR |
| 113098945 | 113099090 | + | 14.2 | 1 | 161 | -203 | THE1B | MaLR | LTR |
| 113099091 | 113099393 | - | 10.2 | 1 | 303 | -8 | AluY | Alu | SINE |
| 113099394 | 113099591 | + | 14.2 | 162 | 364 | 0 | THE1B | MaLR | LTR |
| 113099592 | 113099811 | + | 19.6 | 4399 | 4623 | -2021 | L1M4 | L1 | LINE |

  

|  |  |  |  |  |  |  |  |  |  |
| --- | --- | --- | --- | --- | --- | --- | --- | --- | --- |
| chr7:113352990-113365112 | | | 12119 bp | | |  |  |  |  |
| Genome Start | Genome End | Strand | Div | Repeat Start | Repeat End | Repeat Left | Name | Family | Class |
| 113352990 | 113355826 | - | 19.6 | 3527 | 6159 | -4 | L1PA13 | L1 | LINE |
| 113355827 | 113356182 | + | 9.0 | 1 | 355 | 0 | THE1A | MaLR | LTR |
| 113356185 | 113357746 | + | 31.1 | 2 | 1735 | 0 | MLT-int | MaLR | LTR |
| 113357747 | 113358101 | + | 10.1 | 1 | 355 | 0 | THE1A | MaLR | LTR |
| 113358102 | 113358540 | - | 12.3 | 3087 | 3532 | -2614 | L1PREC2 | L1 | LINE |
| 113358541 | 113358910 | - | 18.1 | 1 | 364 | 0 | THE1B | MaLR | LTR |
| 113358911 | 113360054 | - | 29.9 | 521 | 1735 | 0 | MLT-int | MaLR | LTR |
| 113360055 | 113360346 | - | 12.7 | 1 | 295 | -14 | AluSc | Alu | SINE |
| 113360347 | 113360746 | - | 29.9 | 3 | 520 | -1215 | MLT-int | MaLR | LTR |
| 113360749 | 113361086 | - | 14.7 | 1 | 364 | 0 | THE1B | MaLR | LTR |
| 113361087 | 113362921 | - | 13.6 | 1835 | 3706 | -3060 | L1PA13 | L1 | LINE |
| 113362922 | 113363287 | - | 12.5 | 1 | 381 | 0 | THE1D | MaLR | LTR |
| 113363288 | 113365112 | - | 15.1 | 10 | 1834 | -4951 | L1PA13 | L1 | LINE |

  

|  |  |  |  |  |  |  |  |  |  |
| --- | --- | --- | --- | --- | --- | --- | --- | --- | --- |
| chr7:113378581-113387983 | | | 9401 bp | | |  |  |  |  |
| Genome Start | Genome End | Strand | Div | Repeat Start | Repeat End | Repeat Left | Name | Family | Class |
| 113378581 | 113385477 | - | 21.6 | 183 | 6687 | -1454 | L1PREC2 | L1 | LINE |
| 113385478 | 113385860 | - | 15.9 | 1 | 381 | 0 | THE1D | MaLR | LTR |
| 113385861 | 113387428 | - | 31.1 | 2 | 1735 | 0 | MLT-int | MaLR | LTR |
| 113387431 | 113387801 | - | 16.2 | 1 | 381 | 0 | THE1D | MaLR | LTR |
| 113387802 | 113387983 | - | 26.4 | 1 | 182 | -7973 | L1PREC2 | L1 | LINE |

  

|  |  |  |  |  |  |  |  |  |  |
| --- | --- | --- | --- | --- | --- | --- | --- | --- | --- |
| chr7:113592473-113599976 | | | 7501 bp | | |  |  |  |  |
| Genome Start | Genome End | Strand | Div | Repeat Start | Repeat End | Repeat Left | Name | Family | Class |
| 113592473 | 113593712 | + | 17.0 | 4041 | 5307 | -839 | L1MC2 | L1 | LINE |
| 113593713 | 113594025 | + | 13.8 | 1 | 311 | 0 | AluY | Alu | SINE |
| 113594026 | 113594652 | + | 16.3 | 5308 | 5923 | -421 | L1MC2 | L1 | LINE |
| 113594653 | 113594764 | + | 18.0 | 1 | 111 | -2307 | Tigger1 | MER2\_type | DNA |
| 113594766 | 113594945 | + | 12.6 | 1 | 173 | -191 | THE1B | MaLR | LTR |
| 113594946 | 113595245 | + | 9.3 | 1 | 300 | -11 | AluY | Alu | SINE |
| 113595246 | 113595443 | + | 12.6 | 174 | 364 | 0 | THE1B | MaLR | LTR |
| 113595444 | 113596999 | + | 9.8 | 1 | 1580 | 0 | THE1B-int | MaLR | LTR |
| 113597000 | 113597361 | + | 12.5 | 1 | 364 | 0 | THE1B | MaLR | LTR |
| 113597362 | 113598582 | + | 12.9 | 108 | 1340 | -1078 | Tigger1 | MER2\_type | DNA |
| 113598583 | 113598879 | + | 16.2 | 1 | 301 | -11 | AluJo | Alu | SINE |
| 113598880 | 113599366 | + | 12.9 | 1341 | 1820 | -598 | Tigger1 | MER2\_type | DNA |
| 113599369 | 113599582 | + | 14.7 | 2203 | 2418 | 0 | Tigger1 | MER2\_type | DNA |
| 113599583 | 113599976 | + | 17.3 | 5922 | 6335 | -9 | L1MC2 | L1 | LINE |

  

|  |  |  |  |  |  |  |  |  |  |
| --- | --- | --- | --- | --- | --- | --- | --- | --- | --- |
| chr7:113785122-113787940 | | | 2636 bp | | |  |  |  |  |
| Genome Start | Genome End | Strand | Div | Repeat Start | Repeat End | Repeat Left | Name | Family | Class |
| 113785122 | 113785256 | + | 30.4 | 140 | 284 | -331 | MLT1L | MaLR | LTR |
| 113785292 | 113785657 | + | 13.8 | 1 | 364 | 0 | THE1B | MaLR | LTR |
| 113785660 | 113787278 | + | 31.5 | 2 | 1733 | -2 | MLT-int | MaLR | LTR |
| 113787281 | 113787644 | + | 13.2 | 1 | 364 | 0 | THE1B | MaLR | LTR |
| 113787789 | 113787940 | + | 33.3 | 455 | 607 | -8 | MLT1L | MaLR | LTR |

  

|  |  |  |  |  |  |  |  |  |  |
| --- | --- | --- | --- | --- | --- | --- | --- | --- | --- |
| chr7:116417924-116420960 | | | 2827 bp | | |  |  |  |  |
| Genome Start | Genome End | Strand | Div | Repeat Start | Repeat End | Repeat Left | Name | Family | Class |
| 116417924 | 116418106 | + | 27.3 | 3103 | 3270 | -149 | L2 | L2 | LINE |
| 116418164 | 116418330 | - | 22.2 | 5986 | 6152 | -18 | L1ME3A | L1 | LINE |
| 116418345 | 116418464 | - | 16.8 | 5709 | 5827 | -343 | L1ME3A | L1 | LINE |
| 116418596 | 116418950 | - | 10.5 | 1 | 355 | 0 | THE1A | MaLR | LTR |
| 116418951 | 116420503 | - | 10.2 | 1 | 1580 | 0 | THE1A-int | MaLR | LTR |
| 116420504 | 116420858 | - | 9.7 | 1 | 355 | 0 | THE1A | MaLR | LTR |
| 116420867 | 116420960 | + | 37.2 | 3285 | 3386 | -33 | L2 | L2 | LINE |

  

|  |  |  |  |  |  |  |  |  |  |
| --- | --- | --- | --- | --- | --- | --- | --- | --- | --- |
| chr7:117561334-117563995 | | | 2658 bp | | |  |  |  |  |
| Genome Start | Genome End | Strand | Div | Repeat Start | Repeat End | Repeat Left | Name | Family | Class |
| 117561334 | 117561516 | + | 24.2 | 56 | 233 | -193 | LTR37A | ERV1 | LTR |
| 117561517 | 117561883 | + | 15.2 | 1 | 364 | 0 | THE1B | MaLR | LTR |
| 117561886 | 117563460 | + | 29.4 | 2 | 1735 | 0 | MLT-int | MaLR | LTR |
| 117563463 | 117563825 | + | 13.6 | 3 | 362 | -2 | THE1B | MaLR | LTR |
| 117563826 | 117563995 | + | 24.2 | 234 | 425 | -1 | LTR37A | ERV1 | LTR |

  

|  |  |  |  |  |  |  |  |  |  |
| --- | --- | --- | --- | --- | --- | --- | --- | --- | --- |
| chr7:117713964-117719892 | | | 5923 bp | | |  |  |  |  |
| Genome Start | Genome End | Strand | Div | Repeat Start | Repeat End | Repeat Left | Name | Family | Class |
| 117713964 | 117716729 | + | 24.5 | 862 | 3362 | -2985 | L1M3b | L1 | LINE |
| 117716730 | 117717089 | + | 13.7 | 1 | 381 | 0 | THE1D | MaLR | LTR |
| 117717092 | 117717141 | + | 29.0 | 2 | 43 | -1692 | MLT-int | MaLR | LTR |
| 117717142 | 117717431 | + | 14.2 | 3 | 291 | -19 | AluSg | Alu | SINE |
| 117717432 | 117718962 | + | 29.0 | 44 | 1735 | 0 | MLT-int | MaLR | LTR |
| 117718967 | 117719317 | + | 12.3 | 5 | 381 | 0 | THE1D | MaLR | LTR |
| 117719318 | 117719892 | + | 20.4 | 3359 | 3947 | -2400 | L1M3b | L1 | LINE |

  

|  |  |  |  |  |  |  |  |  |  |
| --- | --- | --- | --- | --- | --- | --- | --- | --- | --- |
| chr7:118012658-118016580 | | | 3910 bp | | |  |  |  |  |
| Genome Start | Genome End | Strand | Div | Repeat Start | Repeat End | Repeat Left | Name | Family | Class |
| 118012658 | 118012880 | - | 16.7 | 1422 | 1651 | 0 | MSTB-int | MaLR | LTR |
| 118012882 | 118013252 | + | 18.2 | 1 | 364 | 0 | THE1B | MaLR | LTR |
| 118013253 | 118014792 | + | 12.5 | 1 | 1569 | -11 | THE1B-int | MaLR | LTR |
| 118014803 | 118015163 | + | 17.2 | 1 | 364 | 0 | THE1B | MaLR | LTR |
| 118015166 | 118016580 | - | 21.9 | 1 | 1423 | -228 | MSTB-int | MaLR | LTR |

  

|  |  |  |  |  |  |  |  |  |  |
| --- | --- | --- | --- | --- | --- | --- | --- | --- | --- |
| chr7:118636650-118640281 | | | 3631 bp | | |  |  |  |  |
| Genome Start | Genome End | Strand | Div | Repeat Start | Repeat End | Repeat Left | Name | Family | Class |
| 118636650 | 118637020 | - | 21.3 | 4722 | 5079 | -1061 | L1M3 | L1 | LINE |
| 118637022 | 118637392 | + | 13.2 | 1 | 375 | 0 | THE1C | MaLR | LTR |
| 118637393 | 118638939 | + | 13.6 | 1 | 1580 | 0 | THE1C-int | MaLR | LTR |
| 118638940 | 118639308 | + | 12.8 | 1 | 375 | 0 | THE1C | MaLR | LTR |
| 118639309 | 118640281 | - | 21.6 | 3756 | 4728 | -1415 | L1M3 | L1 | LINE |

  

|  |  |  |  |  |  |  |  |  |  |
| --- | --- | --- | --- | --- | --- | --- | --- | --- | --- |
| chr7:118690275-118699116 | | | 8853 bp | | |  |  |  |  |
| Genome Start | Genome End | Strand | Div | Repeat Start | Repeat End | Repeat Left | Name | Family | Class |
| 118690275 | 118691079 | - | 15.8 | 6054 | 6864 | -5 | L1PA16 | L1 | LINE |
| 118691066 | 118692559 | - | 16.1 | 4112 | 5657 | -1192 | L1PA16 | L1 | LINE |
| 118692560 | 118692921 | - | 14.6 | 1 | 381 | 0 | THE1D | MaLR | LTR |
| 118692922 | 118694546 | - | 31.3 | 3 | 1735 | 0 | MLT-int | MaLR | LTR |
| 118694550 | 118694919 | - | 14.1 | 3 | 381 | 0 | THE1D | MaLR | LTR |
| 118694920 | 118699116 | - | 16.6 | 5 | 4111 | -2738 | L1PA16 | L1 | LINE |

  

|  |  |  |  |  |  |  |  |  |  |
| --- | --- | --- | --- | --- | --- | --- | --- | --- | --- |
| chr7:118852741-118864482 | | | 11678 bp | | |  |  |  |  |
| Genome Start | Genome End | Strand | Div | Repeat Start | Repeat End | Repeat Left | Name | Family | Class |
| 118852741 | 118853608 | - | 20.9 | 3832 | 4701 | -1442 | L1M2 | L1 | LINE |
| 118853615 | 118854751 | - | 18.8 | 2331 | 3532 | -2611 | L1M2 | L1 | LINE |
| 118854752 | 118859757 | + | 17.7 | 2 | 5758 | -1823 | L1PA11 | L1 | LINE |
| 118859758 | 118860066 | + | 11.4 | 1 | 306 | -6 | AluSx | Alu | SINE |
| 118860067 | 118860143 | + | 11.8 | 5759 | 5842 | -1739 | L1PA11 | L1 | LINE |
| 118860187 | 118861746 | + | 12.2 | 5843 | 7395 | -214 | L1PA11 | L1 | LINE |
| 118861755 | 118862123 | - | 21.6 | 1 | 369 | -12 | THE1D | MaLR | LTR |
| 118862124 | 118863738 | - | 18.1 | 4 | 1580 | 0 | THE1D-int | MaLR | LTR |
| 118863742 | 118864099 | - | 15.6 | 8 | 381 | 0 | THE1D | MaLR | LTR |
| 118864103 | 118864321 | + | 10.2 | 5959 | 6174 | 0 | L1PA11 | L1 | LINE |
| 118864323 | 118864482 | - | 18.9 | 2174 | 2336 | -3807 | L1M2 | L1 | LINE |

  

|  |  |  |  |  |  |  |  |  |  |
| --- | --- | --- | --- | --- | --- | --- | --- | --- | --- |
| chr7:118915211-118918141 | | | 2929 bp | | |  |  |  |  |
| Genome Start | Genome End | Strand | Div | Repeat Start | Repeat End | Repeat Left | Name | Family | Class |
| 118915211 | 118915540 | + | 25.0 | 5553 | 5911 | -255 | L1ME1 | L1 | LINE |
| 118915541 | 118915915 | - | 16.7 | 3 | 428 | 0 | MSTA | MaLR | LTR |
| 118915916 | 118917525 | - | 29.7 | 2 | 1735 | 0 | MLT-int | MaLR | LTR |
| 118917528 | 118917911 | - | 19.3 | 1 | 428 | 0 | MSTA | MaLR | LTR |
| 118917912 | 118918141 | + | 25.0 | 5912 | 6139 | -27 | L1ME1 | L1 | LINE |

  

|  |  |  |  |  |  |  |  |  |  |
| --- | --- | --- | --- | --- | --- | --- | --- | --- | --- |
| chr7:118957966-118961954 | | | 3949 bp | | |  |  |  |  |
| Genome Start | Genome End | Strand | Div | Repeat Start | Repeat End | Repeat Left | Name | Family | Class |
| 118957966 | 118959160 | - | 23.6 | 909 | 2165 | -5169 | L1M4b | L1 | LINE |
| 118959166 | 118959568 | + | 19.2 | 1 | 418 | -8 | MSTB | MaLR | LTR |
| 118959584 | 118961166 | + | 20.9 | 19 | 1647 | -4 | MSTB-int | MaLR | LTR |
| 118961179 | 118961592 | + | 20.8 | 3 | 413 | 0 | MSTB | MaLR | LTR |
| 118961601 | 118961954 | - | 25.5 | 500 | 900 | -6434 | L1M4b | L1 | LINE |

  

|  |  |  |  |  |  |  |  |  |  |
| --- | --- | --- | --- | --- | --- | --- | --- | --- | --- |
| chr7:119138769-119145640 | | | 6744 bp | | |  |  |  |  |
| Genome Start | Genome End | Strand | Div | Repeat Start | Repeat End | Repeat Left | Name | Family | Class |
| 119138769 | 119138832 | + | 25.0 | 2 | 67 | -6317 | L1MB4 | L1 | LINE |
| 119138836 | 119139148 | + | 12.6 | 2 | 311 | -1 | AluSx | Alu | SINE |
| 119139152 | 119139296 | - | 17.2 | 1 | 162 | -202 | THE1B | MaLR | LTR |
| 119139297 | 119140869 | - | 32.0 | 2 | 1735 | 0 | MLT-int | MaLR | LTR |
| 119140872 | 119141225 | - | 11.6 | 3 | 364 | 0 | THE1B | MaLR | LTR |
| 119141244 | 119143522 | + | 21.7 | 2264 | 4539 | -1837 | L1MB4 | L1 | LINE |
| 119143550 | 119144675 | + | 22.8 | 4540 | 5704 | -712 | L1MB4 | L1 | LINE |
| 119144733 | 119144954 | - | 21.0 | 5483 | 5707 | -476 | L1M3 | L1 | LINE |
| 119144973 | 119145640 | + | 23.6 | 5782 | 6415 | -1 | L1MB4 | L1 | LINE |

  

|  |  |  |  |  |  |  |  |  |  |
| --- | --- | --- | --- | --- | --- | --- | --- | --- | --- |
| chr7:119244414-119248084 | | | 3627 bp | | |  |  |  |  |
| Genome Start | Genome End | Strand | Div | Repeat Start | Repeat End | Repeat Left | Name | Family | Class |
| 119244414 | 119244934 | - | 23.3 | 5071 | 5613 | -690 | L1M1 | L1 | LINE |
| 119244935 | 119245294 | + | 13.8 | 1 | 362 | -2 | THE1B | MaLR | LTR |
| 119245301 | 119246766 | + | 13.0 | 5 | 1580 | 0 | THE1B-int | MaLR | LTR |
| 119246767 | 119247099 | + | 17.8 | 1 | 336 | -28 | THE1B | MaLR | LTR |
| 119247100 | 119247402 | - | 0.7 | 21 | 318 | 0 | AluYb8 | Alu | SINE |
| 119247403 | 119247444 | + | 17.8 | 337 | 364 | 0 | THE1B | MaLR | LTR |
| 119247483 | 119248084 | - | 19.3 | 4444 | 5056 | -1087 | L1M1 | L1 | LINE |

  

|  |  |  |  |  |  |  |  |  |  |
| --- | --- | --- | --- | --- | --- | --- | --- | --- | --- |
| chr7:119264957-119274695 | | | 9044 bp | | |  |  |  |  |
| Genome Start | Genome End | Strand | Div | Repeat Start | Repeat End | Repeat Left | Name | Family | Class |
| 119264957 | 119265009 | + | 30.8 | 1045 | 1096 | -5831 | L1M4c | L1 | LINE |
| 119265033 | 119265108 | + | 30.8 | 1097 | 1170 | -5757 | L1M4c | L1 | LINE |
| 119265109 | 119265346 | + | 11.8 | 75 | 312 | 0 | AluSg/x | Alu | SINE |
| 119265347 | 119265869 | + | 31.7 | 1171 | 1714 | -5213 | L1M4c | L1 | LINE |
| 119265969 | 119266450 | - | 9.3 | 1 | 464 | 0 | LTR7B | ERV1 | LTR |
| 119266704 | 119267715 | + | 26.2 | 1591 | 2471 | -4618 | L1M4c | L1 | LINE |
| 119267737 | 119269212 | + | 20.2 | 162 | 1651 | 0 | MSTB-int | MaLR | LTR |
| 119269213 | 119269617 | + | 18.8 | 1 | 426 | 0 | MSTB | MaLR | LTR |
| 119269619 | 119271225 | + | 30.9 | 2 | 1734 | -1 | MLT-int | MaLR | LTR |
| 119271226 | 119271643 | + | 21.3 | 1 | 424 | -2 | MSTB | MaLR | LTR |
| 119271647 | 119271799 | - | 19.6 | 1 | 153 | -1498 | MSTB-int | MaLR | LTR |
| 119271800 | 119272241 | - | 17.0 | 1 | 426 | 0 | MSTB | MaLR | LTR |
| 119272397 | 119273703 | + | 21.5 | 2642 | 3995 | -3005 | L1M4c | L1 | LINE |
| 119273812 | 119274341 | + | 15.8 | 4327 | 4880 | -2120 | L1M4c | L1 | LINE |
| 119274374 | 119274695 | + | 21.0 | 5117 | 5457 | -1543 | L1M4c | L1 | LINE |

  

|  |  |  |  |  |  |  |  |  |  |
| --- | --- | --- | --- | --- | --- | --- | --- | --- | --- |
| chr7:119426560-119442899 | | | 15998 bp | | |  |  |  |  |
| Genome Start | Genome End | Strand | Div | Repeat Start | Repeat End | Repeat Left | Name | Family | Class |
| 119426560 | 119426626 | - | 20.1 | 6590 | 6656 | -1547 | L1MC3 | L1 | LINE |
| 119426627 | 119426938 | - | 11.3 | 1 | 311 | -1 | AluSx | Alu | SINE |
| 119426939 | 119429369 | - | 23.0 | 4089 | 6589 | -1614 | L1MC3 | L1 | LINE |
| 119429374 | 119429431 | + | 8.6 | 309 | 375 | 0 | THE1C | MaLR | LTR |
| 119429445 | 119429476 | - | 21.1 | 2698 | 2721 | -3843 | L1MC3 | L1 | LINE |
| 119429477 | 119429776 | - | 14.7 | 1 | 310 | -2 | AluSx | Alu | SINE |
| 119429777 | 119429913 | - | 18.0 | 2566 | 2704 | -3860 | L1MC3 | L1 | LINE |
| 119429896 | 119430038 | - | 19.6 | 1023 | 1194 | -4851 | L1MC3 | L1 | LINE |
| 119430092 | 119432060 | - | 19.9 | 5623 | 7618 | -92 | L1MA8 | L1 | LINE |
| 119432061 | 119432362 | + | 13.3 | 3 | 306 | -6 | AluSx | Alu | SINE |
| 119432363 | 119433110 | - | 22.9 | 4878 | 5622 | -1937 | L1MA8 | L1 | LINE |
| 119433111 | 119433196 | - | 7.0 | 1 | 86 | -224 | AluY | Alu | SINE |
| 119433197 | 119433268 | - | 22.9 | 4807 | 4877 | -2682 | L1MA8 | L1 | LINE |
| 119433392 | 119435909 | - | 22.9 | 2296 | 4806 | -2753 | L1MA8 | L1 | LINE |
| 119435930 | 119436468 | + | 23.1 | 1 | 523 | -44 | MLT2B3 | ERVL | LTR |
| 119436469 | 119436919 | + | 7.2 | 1 | 448 | -2 | LTR7 | ERV1 | LTR |
| 119436922 | 119437296 | + | 10.2 | 1 | 375 | -7338 | HERVH | ERV1 | LTR |
| 119437358 | 119439585 | + | 10.1 | 793 | 2995 | -4718 | HERVH | ERV1 | LTR |
| 119439565 | 119440413 | + | 10.8 | 3140 | 3991 | -3722 | HERVH | ERV1 | LTR |
| 119440413 | 119441079 | + | 14.9 | 4481 | 5149 | -2564 | HERVH | ERV1 | LTR |
| 119441081 | 119441292 | + | 11.3 | 5600 | 5819 | -1894 | HERVH | ERV1 | LTR |
| 119441291 | 119441681 | + | 8.8 | 7322 | 7713 | 0 | HERVH | ERV1 | LTR |
| 119441682 | 119442125 | + | 7.7 | 1 | 448 | -2 | LTR7 | ERV1 | LTR |
| 119442127 | 119442174 | + | 14.6 | 512 | 559 | 0 | MLT2B3 | ERVL | LTR |
| 119442281 | 119442899 | - | 30.2 | 1 | 624 | -5421 | L1MC3 | L1 | LINE |

  

|  |  |  |  |  |  |  |  |  |  |
| --- | --- | --- | --- | --- | --- | --- | --- | --- | --- |
| chr7:119464173-119468062 | | | 3879 bp | | |  |  |  |  |
| Genome Start | Genome End | Strand | Div | Repeat Start | Repeat End | Repeat Left | Name | Family | Class |
| 119464173 | 119464241 | + | 23.2 | 4668 | 4736 | -1410 | L1M5 | L1 | LINE |
| 119464248 | 119465133 | - | 14.4 | 1 | 896 | -684 | THE1B-int | MaLR | LTR |
| 119465134 | 119465440 | - | 12.2 | 66 | 364 | 0 | THE1B | MaLR | LTR |
| 119465441 | 119465803 | - | 16.7 | 1 | 364 | 0 | THE1B | MaLR | LTR |
| 119465804 | 119467368 | - | 31.2 | 2 | 1735 | 0 | MLT-int | MaLR | LTR |
| 119467374 | 119467736 | - | 12.7 | 1 | 361 | -3 | THE1B | MaLR | LTR |
| 119467737 | 119467801 | - | 12.2 | 1 | 65 | -299 | THE1B | MaLR | LTR |
| 119467802 | 119468062 | + | 22.8 | 5489 | 5768 | -398 | L1M5 | L1 | LINE |

  

|  |  |  |  |  |  |  |  |  |  |
| --- | --- | --- | --- | --- | --- | --- | --- | --- | --- |
| chr7:119468188-119471104 | | | 2914 bp | | |  |  |  |  |
| Genome Start | Genome End | Strand | Div | Repeat Start | Repeat End | Repeat Left | Name | Family | Class |
| 119468188 | 119468766 | + | 28.8 | 5237 | 5830 | -336 | L1ME1 | L1 | LINE |
| 119468767 | 119469120 | + | 10.4 | 1 | 355 | 0 | THE1A | MaLR | LTR |
| 119469123 | 119470690 | + | 30.8 | 2 | 1735 | 0 | MLT-int | MaLR | LTR |
| 119470692 | 119471044 | + | 11.4 | 2 | 355 | 0 | THE1A | MaLR | LTR |
| 119471045 | 119471104 | + | 29.3 | 5831 | 5891 | -275 | L1ME1 | L1 | LINE |

  

|  |  |  |  |  |  |  |  |  |  |
| --- | --- | --- | --- | --- | --- | --- | --- | --- | --- |
| chr7:119493884-119503000 | | | 9117 bp | | |  |  |  |  |
| Genome Start | Genome End | Strand | Div | Repeat Start | Repeat End | Repeat Left | Name | Family | Class |
| 119493884 | 119494197 | + | 22.8 | 2 | 315 | -6853 | L1MA5 | L1 | LINE |
| 119494198 | 119494561 | + | 14.3 | 1 | 364 | 0 | THE1B | MaLR | LTR |
| 119494564 | 119496125 | + | 30.8 | 2 | 1735 | 0 | MLT-int | MaLR | LTR |
| 119496126 | 119496494 | + | 12.7 | 1 | 364 | 0 | THE1B | MaLR | LTR |
| 119496495 | 119501979 | + | 22.6 | 316 | 6009 | -1158 | L1MA5 | L1 | LINE |
| 119501978 | 119503000 | + | 17.9 | 6286 | 7315 | -6 | L1MA5 | L1 | LINE |

  

|  |  |  |  |  |  |  |  |  |  |
| --- | --- | --- | --- | --- | --- | --- | --- | --- | --- |
| chr7:122243177-122250453 | | | 7288 bp | | |  |  |  |  |
| Genome Start | Genome End | Strand | Div | Repeat Start | Repeat End | Repeat Left | Name | Family | Class |
| 122243177 | 122244018 | - | 14.9 | 5461 | 6293 | -7 | L1MA4 | L1 | LINE |
| 122244019 | 122244466 | + | 6.5 | 1 | 448 | -2 | LTR7 | ERV1 | LTR |
| 122244469 | 122247758 | + | 9.4 | 1 | 3290 | -4423 | HERVH | ERV1 | LTR |
| 122247749 | 122247839 | + | 11.9 | 3492 | 3578 | -4135 | HERVH | ERV1 | LTR |
| 122247840 | 122248148 | - | 1.0 | 1 | 309 | -2 | AluY | Alu | SINE |
| 122248149 | 122248579 | + | 11.9 | 3579 | 3993 | -3720 | HERVH | ERV1 | LTR |
| 122248576 | 122249245 | + | 14.7 | 4478 | 5149 | -2564 | HERVH | ERV1 | LTR |
| 122249247 | 122249462 | + | 13.4 | 5600 | 5819 | -1894 | HERVH | ERV1 | LTR |
| 122249461 | 122249840 | + | 8.5 | 7322 | 7713 | 0 | HERVH | ERV1 | LTR |
| 122249841 | 122250288 | + | 5.6 | 1 | 448 | -2 | LTR7 | ERV1 | LTR |
| 122250291 | 122250453 | - | 11.1 | 5302 | 5465 | -835 | L1MA4 | L1 | LINE |

  

|  |  |  |  |  |  |  |  |  |  |
| --- | --- | --- | --- | --- | --- | --- | --- | --- | --- |
| chr7:123319828-123334566 | | | 14594 bp | | |  |  |  |  |
| Genome Start | Genome End | Strand | Div | Repeat Start | Repeat End | Repeat Left | Name | Family | Class |
| 123319828 | 123320412 | + | 17.0 | 2300 | 3687 | -2644 | LTR49-int | ERV1 | LTR |
| 123320413 | 123320750 | + | 10.7 | 1 | 339 | -36 | THE1C | MaLR | LTR |
| 123320792 | 123320850 | + | 19.3 | 319 | 375 | 0 | THE1C | MaLR | LTR |
| 123320896 | 123321091 | + | 15.3 | 4024 | 4211 | -2120 | LTR49-int | ERV1 | LTR |
| 123321092 | 123321469 | + | 14.4 | 1 | 381 | 0 | THE1D | MaLR | LTR |
| 123321470 | 123321785 | + | 15.3 | 4212 | 4542 | -1789 | LTR49-int | ERV1 | LTR |
| 123321787 | 123327938 | + | 4.2 | 1 | 6155 | 0 | L1PA3 | L1 | LINE |
| 123327940 | 123328982 | + | 18.2 | 4533 | 5592 | -739 | LTR49-int | ERV1 | LTR |
| 123328983 | 123329247 | + | 12.1 | 44 | 308 | -5 | AluSq | Alu | SINE |
| 123329248 | 123329867 | + | 18.2 | 5593 | 6202 | -129 | LTR49-int | ERV1 | LTR |
| 123329892 | 123330308 | - | 18.0 | 1 | 428 | 0 | MSTA | MaLR | LTR |
| 123330309 | 123331897 | - | 18.3 | 1 | 1651 | 0 | MSTA-int | MaLR | LTR |
| 123331908 | 123332326 | - | 12.7 | 1 | 428 | 0 | MSTA | MaLR | LTR |
| 123332332 | 123333896 | - | 17.3 | 1 | 1645 | -6 | MSTA-int | MaLR | LTR |
| 123333897 | 123334284 | - | 12.4 | 1 | 428 | 0 | MSTA | MaLR | LTR |
| 123334289 | 123334399 | - | 13.5 | 304 | 417 | -9 | MSTA | MaLR | LTR |
| 123334404 | 123334445 | - | 11.9 | 10 | 52 | -374 | MSTB | MaLR | LTR |
| 123334456 | 123334566 | + | 8.7 | 6229 | 6331 | 0 | LTR49-int | ERV1 | LTR |

  

|  |  |  |  |  |  |  |  |  |  |
| --- | --- | --- | --- | --- | --- | --- | --- | --- | --- |
| chr7:123943832-123952502 | | | 8591 bp | | |  |  |  |  |
| Genome Start | Genome End | Strand | Div | Repeat Start | Repeat End | Repeat Left | Name | Family | Class |
| 123943832 | 123944342 | - | 13.2 | 6267 | 6777 | -8 | L1PA15 | L1 | LINE |
| 123944343 | 123944705 | + | 13.9 | 1 | 381 | 0 | THE1D | MaLR | LTR |
| 123944708 | 123944836 | + | 12.4 | 3 | 133 | -1447 | THE1D-int | MaLR | LTR |
| 123944837 | 123944873 | - | 10.3 | 341 | 377 | 0 | MER51B | ERV1 | LTR |
| 123944874 | 123945133 | + | 7.8 | 5890 | 6147 | -7 | L1PA5 | L1 | LINE |
| 123945134 | 123945436 | - | 10.3 | 2 | 340 | -37 | MER51B | ERV1 | LTR |
| 123945437 | 123945715 | + | 11.8 | 129 | 408 | -1172 | THE1D-int | MaLR | LTR |
| 123945716 | 123945936 | + | 13.6 | 72 | 303 | -6 | AluSc | Alu | SINE |
| 123945968 | 123947045 | + | 15.4 | 491 | 1575 | -5 | THE1D-int | MaLR | LTR |
| 123947093 | 123947452 | + | 12.9 | 3 | 381 | 0 | THE1D | MaLR | LTR |
| 123947453 | 123952502 | - | 15.5 | 1175 | 6271 | -514 | L1PA15 | L1 | LINE |

  

|  |  |  |  |  |  |  |  |  |  |
| --- | --- | --- | --- | --- | --- | --- | --- | --- | --- |
| chr7:124383928-124387756 | | | 3691 bp | | |  |  |  |  |
| Genome Start | Genome End | Strand | Div | Repeat Start | Repeat End | Repeat Left | Name | Family | Class |
| 124383928 | 124384148 | - | 36.1 | 3152 | 3362 | -57 | L2 | L2 | LINE |
| 124384153 | 124384373 | - | 13.3 | 248 | 465 | 0 | MER4C | ERV1 | LTR |
| 124384374 | 124384737 | + | 13.8 | 1 | 364 | 0 | THE1B | MaLR | LTR |
| 124384740 | 124386326 | + | 31.1 | 2 | 1735 | 0 | MLT1C-int | MaLR | LTR |
| 124386327 | 124386681 | + | 16.6 | 1 | 364 | 0 | THE1B | MaLR | LTR |
| 124386682 | 124386953 | - | 13.3 | 1 | 247 | -218 | MER4C | ERV1 | LTR |
| 124386955 | 124387123 | - | 42.1 | 2974 | 3139 | -280 | L2 | L2 | LINE |
| 124387214 | 124387576 | + | 35.1 | 52 | 453 | -4 | LTR16A1 | ERVL | LTR |
| 124387618 | 124387756 | - | 31.9 | 2717 | 2858 | -561 | L2 | L2 | LINE |

  

|  |  |  |  |  |  |  |  |  |  |
| --- | --- | --- | --- | --- | --- | --- | --- | --- | --- |
| chr7:124532975-124538207 | | | 5214 bp | | |  |  |  |  |
| Genome Start | Genome End | Strand | Div | Repeat Start | Repeat End | Repeat Left | Name | Family | Class |
| 124532975 | 124533562 | - | 15.3 | 1824 | 2418 | 0 | Tigger1 | MER2\_type | DNA |
| 124533563 | 124533990 | + | 3.0 | 5693 | 6120 | -34 | L1PA5 | L1 | LINE |
| 124534008 | 124535162 | - | 15.2 | 646 | 1825 | -593 | Tigger1 | MER2\_type | DNA |
| 124535163 | 124535524 | - | 13.3 | 1 | 364 | 0 | THE1B | MaLR | LTR |
| 124535525 | 124537188 | - | 32.0 | 2 | 1735 | 0 | MLT-int | MaLR | LTR |
| 124537191 | 124537554 | - | 12.4 | 1 | 364 | 0 | THE1B | MaLR | LTR |
| 124537555 | 124538207 | - | 15.2 | 1 | 645 | -1773 | Tigger1 | MER2\_type | DNA |

  

|  |  |  |  |  |  |  |  |  |  |
| --- | --- | --- | --- | --- | --- | --- | --- | --- | --- |
| chr7:124559246-124563460 | | | 3926 bp | | |  |  |  |  |
| Genome Start | Genome End | Strand | Div | Repeat Start | Repeat End | Repeat Left | Name | Family | Class |
| 124559246 | 124559345 | + | 29.0 | 1894 | 2005 | -1414 | L2 | L2 | LINE |
| 124559362 | 124559665 | + | 25.2 | 66 | 426 | -16 | MLT1J2 | MaLR | LTR |
| 124559683 | 124560605 | + | 33.1 | 1963 | 3022 | -397 | L2 | L2 | LINE |
| 124560841 | 124561208 | + | 17.5 | 3 | 363 | -2 | MLT1A0 | MaLR | LTR |
| 124561209 | 124562993 | + | 20.4 | 3 | 1719 | -16 | MLT1A0-int | MaLR | LTR |
| 124563011 | 124563365 | + | 19.2 | 1 | 365 | 0 | MLT1A0 | MaLR | LTR |
| 124563370 | 124563460 | + | 21.6 | 3328 | 3418 | -1 | L2 | L2 | LINE |

  

|  |  |  |  |  |  |  |  |  |  |
| --- | --- | --- | --- | --- | --- | --- | --- | --- | --- |
| chr7:124981983-124986320 | | | 4324 bp | | |  |  |  |  |
| Genome Start | Genome End | Strand | Div | Repeat Start | Repeat End | Repeat Left | Name | Family | Class |
| 124981983 | 124982576 | + | 24.8 | 4996 | 5672 | -492 | L1ME1 | L1 | LINE |
| 124982586 | 124983011 | - | 15.3 | 3 | 428 | 0 | MSTA | MaLR | LTR |
| 124983015 | 124984506 | - | 19.1 | 89 | 1650 | -1 | MSTA-int | MaLR | LTR |
| 124984507 | 124985039 | + | 12.1 | 1 | 521 | 0 | MER41C | ERV1 | LTR |
| 124985040 | 124985127 | - | 19.1 | 1 | 88 | -1563 | MSTA-int | MaLR | LTR |
| 124985130 | 124985166 | - | 14.6 | 402 | 426 | -2 | MSTA | MaLR | LTR |
| 124985167 | 124985473 | + | 9.1 | 1 | 310 | -2 | AluSx | Alu | SINE |
| 124985474 | 124985858 | - | 14.6 | 1 | 401 | -27 | MSTA | MaLR | LTR |
| 124985859 | 124986320 | + | 30.3 | 5691 | 6166 | 0 | L1ME1 | L1 | LINE |

  

|  |  |  |  |  |  |  |  |  |  |
| --- | --- | --- | --- | --- | --- | --- | --- | --- | --- |
| chr7:128088152-128091037 | | | 2884 bp | | |  |  |  |  |
| Genome Start | Genome End | Strand | Div | Repeat Start | Repeat End | Repeat Left | Name | Family | Class |
| 128088152 | 128088234 | - | 14.5 | 216 | 298 | -14 | AluSx | Alu | SINE |
| 128088235 | 128088386 | + | 13.2 | 1 | 152 | -212 | THE1B | MaLR | LTR |
| 128088387 | 128088698 | - | 12.8 | 1 | 312 | 0 | AluSx | Alu | SINE |
| 128088699 | 128088918 | + | 13.2 | 153 | 364 | 0 | THE1B | MaLR | LTR |
| 128088921 | 128090464 | + | 28.9 | 2 | 1735 | 0 | MLT-int | MaLR | LTR |
| 128090465 | 128090819 | + | 11.0 | 1 | 364 | 0 | THE1B | MaLR | LTR |
| 128090820 | 128091037 | - | 13.4 | 1 | 218 | -94 | AluSx | Alu | SINE |

  

|  |  |  |  |  |  |  |  |  |  |
| --- | --- | --- | --- | --- | --- | --- | --- | --- | --- |
| chr7:129085091-129089803 | | | 4146 bp | | |  |  |  |  |
| Genome Start | Genome End | Strand | Div | Repeat Start | Repeat End | Repeat Left | Name | Family | Class |
| 129085091 | 129085385 | - | 25.7 | 1909 | 2205 | -3944 | L1MEe | L1 | LINE |
| 129085386 | 129085700 | + | 15.6 | 1 | 312 | 0 | AluJo | Alu | SINE |
| 129085701 | 129085798 | - | 25.7 | 1813 | 1908 | -4241 | L1MEe | L1 | LINE |
| 129085820 | 129086129 | + | 22.2 | 1 | 336 | 0 | MER2 | MER2\_type | DNA |
| 129086150 | 129086453 | - | 11.2 | 2 | 305 | -7 | AluSx | Alu | SINE |
| 129086488 | 129086757 | - | 26.2 | 1423 | 1697 | -4452 | L1MEe | L1 | LINE |
| 129086995 | 129087358 | - | 10.0 | 1 | 364 | 0 | THE1B | MaLR | LTR |
| 129087359 | 129088928 | - | 28.9 | 2 | 1735 | 0 | MLT-int | MaLR | LTR |
| 129088931 | 129089295 | - | 13.1 | 1 | 364 | 0 | THE1B | MaLR | LTR |
| 129089549 | 129089803 | - | 30.8 | 561 | 836 | -5283 | L1MEe | L1 | LINE |

  

|  |  |  |  |  |  |  |  |  |  |
| --- | --- | --- | --- | --- | --- | --- | --- | --- | --- |
| chr7:130195211-130197805 | | | 2593 bp | | |  |  |  |  |
| Genome Start | Genome End | Strand | Div | Repeat Start | Repeat End | Repeat Left | Name | Family | Class |
| 130195211 | 130195320 | + | 14.7 | 1 | 107 | -205 | AluSx | Alu | SINE |
| 130195321 | 130195671 | + | 9.2 | 1 | 355 | 0 | THE1A | MaLR | LTR |
| 130195674 | 130197222 | + | 29.3 | 2 | 1735 | 0 | MLT-int | MaLR | LTR |
| 130197223 | 130197575 | + | 8.2 | 1 | 355 | 0 | THE1A | MaLR | LTR |
| 130197576 | 130197805 | + | 14.7 | 108 | 312 | 0 | AluSx | Alu | SINE |

  

|  |  |  |  |  |  |  |  |  |  |
| --- | --- | --- | --- | --- | --- | --- | --- | --- | --- |
| chr7:130308402-130313032 | | | 4514 bp | | |  |  |  |  |
| Genome Start | Genome End | Strand | Div | Repeat Start | Repeat End | Repeat Left | Name | Family | Class |
| 130308402 | 130308980 | + | 17.4 | 4375 | 4952 | -1188 | L1M3 | L1 | LINE |
| 130308984 | 130309381 | + | 14.6 | 1 | 428 | 0 | MSTA | MaLR | LTR |
| 130309384 | 130310949 | + | 28.3 | 2 | 1735 | 0 | MLT-int | MaLR | LTR |
| 130310950 | 130311277 | + | 14.2 | 1 | 354 | -74 | MSTA | MaLR | LTR |
| 130311297 | 130311354 | - | 12.1 | 371 | 428 | 0 | MSTA | MaLR | LTR |
| 130311394 | 130311439 | + | 10.8 | 4954 | 4997 | -1143 | L1M3 | L1 | LINE |
| 130311440 | 130311750 | - | 14.5 | 1 | 292 | -20 | AluSx | Alu | SINE |
| 130311751 | 130312060 | + | 10.8 | 4998 | 5301 | -839 | L1M3 | L1 | LINE |
| 130312095 | 130312138 | + | 10.8 | 5302 | 5328 | -812 | L1M3 | L1 | LINE |
| 130312139 | 130312424 | - | 13.6 | 1 | 295 | -17 | AluSx | Alu | SINE |
| 130312425 | 130312435 | + | 10.8 | 5329 | 5338 | -802 | L1M3 | L1 | LINE |
| 130312456 | 130312734 | - | 9.7 | 2 | 284 | -29 | AluSq | Alu | SINE |
| 130312735 | 130313032 | + | 13.2 | 5339 | 5645 | -523 | L1M3 | L1 | LINE |

  

|  |  |  |  |  |  |  |  |  |  |
| --- | --- | --- | --- | --- | --- | --- | --- | --- | --- |
| chr7:130452967-130457341 | | | 4299 bp | | |  |  |  |  |
| Genome Start | Genome End | Strand | Div | Repeat Start | Repeat End | Repeat Left | Name | Family | Class |
| 130452967 | 130453264 | - | 36.0 | 2475 | 2786 | -633 | L2 | L2 | LINE |
| 130453265 | 130453528 | + | 14.8 | 1 | 281 | -145 | MSTB | MaLR | LTR |
| 130453529 | 130453570 | - | 14.3 | 402 | 428 | 0 | MSTA | MaLR | LTR |
| 130453571 | 130453882 | + | 9.9 | 1 | 313 | 0 | AluSq | Alu | SINE |
| 130453883 | 130454236 | - | 14.3 | 1 | 401 | -27 | MSTA | MaLR | LTR |
| 130454243 | 130455319 | - | 12.8 | 426 | 1573 | -7 | MSTA-int | MaLR | LTR |
| 130455320 | 130455491 | + | 7.1 | 135 | 303 | -8 | AluY | Alu | SINE |
| 130455492 | 130455542 | - | 12.8 | 372 | 425 | -1155 | MSTA-int | MaLR | LTR |
| 130455543 | 130455667 | + | 8.5 | 1 | 124 | -187 | AluY | Alu | SINE |
| 130455697 | 130455876 | + | 8.5 | 125 | 308 | -3 | AluY | Alu | SINE |
| 130455877 | 130456246 | - | 12.8 | 3 | 369 | -1211 | MSTA-int | MaLR | LTR |
| 130456249 | 130456635 | - | 14.4 | 1 | 428 | 0 | MSTA | MaLR | LTR |
| 130456636 | 130456737 | + | 16.3 | 277 | 386 | -40 | MSTB | MaLR | LTR |
| 130456738 | 130456967 | + | 7.0 | 83 | 311 | 0 | AluY | Alu | SINE |
| 130456968 | 130457006 | + | 16.3 | 387 | 414 | -12 | MSTB | MaLR | LTR |
| 130457046 | 130457341 | - | 28.3 | 2049 | 2421 | -998 | L2 | L2 | LINE |

  

|  |  |  |  |  |  |  |  |  |  |
| --- | --- | --- | --- | --- | --- | --- | --- | --- | --- |
| chr7:137463636-137466274 | | | 2638 bp | | |  |  |  |  |
| Genome Start | Genome End | Strand | Div | Repeat Start | Repeat End | Repeat Left | Name | Family | Class |
| 137463636 | 137463697 | - | 20.1 | 6082 | 6137 | -25 | L1ME3 | L1 | LINE |
| 137463698 | 137464128 | - | 11.1 | 1 | 428 | 0 | MSTA | MaLR | LTR |
| 137464129 | 137465730 | - | 27.9 | 2 | 1735 | 0 | MLT-int | MaLR | LTR |
| 137465732 | 137466156 | - | 15.7 | 1 | 428 | 0 | MSTA | MaLR | LTR |
| 137466157 | 137466274 | - | 20.1 | 5963 | 6081 | -81 | L1ME3 | L1 | LINE |

  

|  |  |  |  |  |  |  |  |  |  |
| --- | --- | --- | --- | --- | --- | --- | --- | --- | --- |
| chr7:137846189-137849155 | | | 2962 bp | | |  |  |  |  |
| Genome Start | Genome End | Strand | Div | Repeat Start | Repeat End | Repeat Left | Name | Family | Class |
| 137846189 | 137846334 | - | 31.4 | 5940 | 6088 | -1720 | L1MC4a | L1 | LINE |
| 137846335 | 137846574 | - | 10.3 | 199 | 428 | 0 | MSTA | MaLR | LTR |
| 137846575 | 137846874 | - | 10.0 | 1 | 312 | -1 | AluSq | Alu | SINE |
| 137846875 | 137847049 | - | 10.3 | 1 | 198 | -230 | MSTA | MaLR | LTR |
| 137847050 | 137848652 | - | 28.5 | 2 | 1735 | 0 | MLT-int | MaLR | LTR |
| 137848658 | 137849033 | - | 11.5 | 1 | 426 | -2 | MSTA | MaLR | LTR |
| 137849034 | 137849155 | - | 31.4 | 5809 | 5936 | -1872 | L1MC4a | L1 | LINE |

  

|  |  |  |  |  |  |  |  |  |  |
| --- | --- | --- | --- | --- | --- | --- | --- | --- | --- |
| chr7:138888006-138891601 | | | 3593 bp | | |  |  |  |  |
| Genome Start | Genome End | Strand | Div | Repeat Start | Repeat End | Repeat Left | Name | Family | Class |
| 138888006 | 138888419 | - | 22.0 | 5714 | 6154 | -10 | L1ME2 | L1 | LINE |
| 138888420 | 138888787 | + | 11.5 | 1 | 364 | 0 | THE1B | MaLR | LTR |
| 138888791 | 138890347 | + | 29.2 | 3 | 1735 | 0 | MLT-int | MaLR | LTR |
| 138890348 | 138890728 | + | 12.4 | 1 | 362 | -2 | THE1B | MaLR | LTR |
| 138890729 | 138891601 | - | 23.7 | 4836 | 5713 | -451 | L1ME2 | L1 | LINE |

  

|  |  |  |  |  |  |  |  |  |  |
| --- | --- | --- | --- | --- | --- | --- | --- | --- | --- |
| chr7:141731410-141738353 | | | 6943 bp | | |  |  |  |  |
| Genome Start | Genome End | Strand | Div | Repeat Start | Repeat End | Repeat Left | Name | Family | Class |
| 141731410 | 141734518 | - | 14.3 | 686 | 3830 | -3735 | L1M1 | L1 | LINE |
| 141734519 | 141734738 | - | 15.7 | 207 | 428 | 0 | MSTA | MaLR | LTR |
| 141734739 | 141735103 | - | 14.2 | 2 | 364 | 0 | THE1B | MaLR | LTR |
| 141735104 | 141735294 | - | 15.7 | 1 | 206 | -222 | MSTA | MaLR | LTR |
| 141735295 | 141735615 | - | 17.1 | 1334 | 1651 | 0 | MSTA-int | MaLR | LTR |
| 141735616 | 141735930 | - | 14.1 | 1 | 315 | 0 | AluSx | Alu | SINE |
| 141735931 | 141737262 | - | 17.1 | 1 | 1333 | -318 | MSTA-int | MaLR | LTR |
| 141737263 | 141737673 | - | 18.8 | 2 | 428 | 0 | MSTA | MaLR | LTR |
| 141737675 | 141738353 | - | 15.8 | 7 | 690 | -6875 | L1M1 | L1 | LINE |

  

|  |  |  |  |  |  |  |  |  |  |
| --- | --- | --- | --- | --- | --- | --- | --- | --- | --- |
| chr7:142511612-142517767 | | | 6144 bp | | |  |  |  |  |
| Genome Start | Genome End | Strand | Div | Repeat Start | Repeat End | Repeat Left | Name | Family | Class |
| 142511612 | 142512331 | + | 14.6 | 1 | 709 | -214 | MER49 | ERV1 | LTR |
| 142512332 | 142512720 | + | 4.7 | 1 | 464 | 0 | LTR7B | ERV1 | LTR |
| 142512721 | 142513200 | + | 20.4 | 1 | 459 | -7254 | HERVH | ERV1 | LTR |
| 142513226 | 142515273 | + | 13.4 | 800 | 2830 | -4883 | HERVH | ERV1 | LTR |
| 142515274 | 142515912 | + | 14.1 | 3357 | 3993 | -3720 | HERVH | ERV1 | LTR |
| 142515896 | 142516525 | + | 16.3 | 3993 | 5286 | -1805 | HERVH | ERV1 | LTR |
| 142516527 | 142516784 | + | 14.3 | 5757 | 6014 | -1699 | HERVH | ERV1 | LTR |
| 142516780 | 142517339 | + | 11.7 | 7157 | 7713 | 0 | HERVH | ERV1 | LTR |
| 142517340 | 142517711 | + | 7.2 | 1 | 450 | -14 | LTR7B | ERV1 | LTR |
| 142517720 | 142517767 | + | 14.6 | 705 | 752 | -171 | MER49 | ERV1 | LTR |

  

|  |  |  |  |  |  |  |  |  |  |
| --- | --- | --- | --- | --- | --- | --- | --- | --- | --- |
| chr7:143335585-143340153 | | | 4531 bp | | |  |  |  |  |
| Genome Start | Genome End | Strand | Div | Repeat Start | Repeat End | Repeat Left | Name | Family | Class |
| 143335585 | 143335700 | - | 32.5 | 3283 | 3419 | 0 | L2 | L2 | LINE |
| 143335724 | 143335754 | - | 32.5 | 3254 | 3282 | -137 | L2 | L2 | LINE |
| 143335755 | 143336204 | + | 24.0 | 1 | 505 | 0 | MLT1D | MaLR | LTR |
| 143336205 | 143336493 | - | 32.5 | 2962 | 3253 | -166 | L2 | L2 | LINE |
| 143336502 | 143336870 | + | 12.5 | 1 | 381 | 0 | THE1D | MaLR | LTR |
| 143336873 | 143338000 | + | 15.4 | 3 | 1135 | -445 | THE1D-int | MaLR | LTR |
| 143338002 | 143339003 | - | 7.1 | 1 | 1266 | 0 | MER11A | ERVK | LTR |
| 143339004 | 143339455 | + | 13.6 | 1131 | 1580 | 0 | THE1D-int | MaLR | LTR |
| 143339456 | 143339821 | + | 15.5 | 1 | 381 | 0 | THE1D | MaLR | LTR |
| 143339826 | 143340153 | - | 29.1 | 2621 | 2955 | -464 | L2 | L2 | LINE |

  

|  |  |  |  |  |  |  |  |  |  |
| --- | --- | --- | --- | --- | --- | --- | --- | --- | --- |
| chr7:144043060-144047437 | | | 4159 bp | | |  |  |  |  |
| Genome Start | Genome End | Strand | Div | Repeat Start | Repeat End | Repeat Left | Name | Family | Class |
| 144043060 | 144043487 | + | 22.3 | 5206 | 5683 | -481 | L1ME2 | L1 | LINE |
| 144043490 | 144043547 | - | 17.2 | 6255 | 6312 | 0 | L1MA9 | L1 | LINE |
| 144043548 | 144043599 | - | 11.5 | 510 | 561 | 0 | MLT2A1 | ERVL | LTR |
| 144043697 | 144044086 | - | 7.5 | 2 | 404 | -40 | MLT2A1 | ERVL | LTR |
| 144044088 | 144044183 | - | 7.3 | 6160 | 6259 | -53 | L1MA9 | L1 | LINE |
| 144044188 | 144044311 | + | 18.2 | 5676 | 5806 | -358 | L1ME2 | L1 | LINE |
| 144044327 | 144044679 | + | 16.0 | 8 | 392 | -36 | MSTA | MaLR | LTR |
| 144044680 | 144044857 | - | 11.5 | 132 | 309 | -3 | AluSx | Alu | SINE |
| 144044919 | 144045009 | - | 11.5 | 41 | 131 | -181 | AluSx | Alu | SINE |
| 144045010 | 144045052 | + | 15.7 | 393 | 428 | 0 | MSTA | MaLR | LTR |
| 144045055 | 144046668 | + | 28.9 | 2 | 1735 | 0 | MLT-int | MaLR | LTR |
| 144046669 | 144047066 | + | 13.9 | 1 | 428 | 0 | MSTA | MaLR | LTR |
| 144047104 | 144047437 | + | 23.9 | 5825 | 6163 | -1 | L1ME2 | L1 | LINE |

  

|  |  |  |  |  |  |  |  |  |  |
| --- | --- | --- | --- | --- | --- | --- | --- | --- | --- |
| chr7:144602757-144605810 | | | 3050 bp | | |  |  |  |  |
| Genome Start | Genome End | Strand | Div | Repeat Start | Repeat End | Repeat Left | Name | Family | Class |
| 144602757 | 144603352 | - | 16.3 | 5538 | 6133 | -23 | L1PB4 | L1 | LINE |
| 144603353 | 144603687 | + | 17.0 | 2 | 362 | -2 | THE1B | MaLR | LTR |
| 144603692 | 144605253 | + | 29.5 | 2 | 1735 | 0 | MLT-int | MaLR | LTR |
| 144605254 | 144605591 | + | 13.9 | 1 | 364 | 0 | THE1B | MaLR | LTR |
| 144605592 | 144605810 | - | 16.3 | 5318 | 5537 | -619 | L1PB4 | L1 | LINE |

  

|  |  |  |  |  |  |  |  |  |  |
| --- | --- | --- | --- | --- | --- | --- | --- | --- | --- |
| chr7:145259505-145263445 | | | 3874 bp | | |  |  |  |  |
| Genome Start | Genome End | Strand | Div | Repeat Start | Repeat End | Repeat Left | Name | Family | Class |
| 145259505 | 145259698 | + | 27.1 | 45 | 258 | -515 | LTR41 | ERVL | LTR |
| 145259765 | 145260127 | + | 11.8 | 1 | 364 | 0 | THE1B | MaLR | LTR |
| 145260129 | 145260944 | + | 13.9 | 2 | 865 | -715 | THE1B-int | MaLR | LTR |
| 145260945 | 145261302 | + | 12.8 | 1 | 364 | 0 | THE1B | MaLR | LTR |
| 145261303 | 145262880 | + | 11.8 | 1 | 1580 | 0 | THE1B-int | MaLR | LTR |
| 145262881 | 145263239 | + | 15.0 | 1 | 364 | 0 | THE1B | MaLR | LTR |
| 145263240 | 145263445 | + | 29.6 | 567 | 762 | -11 | LTR41 | ERVL | LTR |

  

|  |  |  |  |  |  |  |  |  |  |
| --- | --- | --- | --- | --- | --- | --- | --- | --- | --- |
| chr7:146712779-146716983 | | | 4130 bp | | |  |  |  |  |
| Genome Start | Genome End | Strand | Div | Repeat Start | Repeat End | Repeat Left | Name | Family | Class |
| 146712779 | 146712889 | - | 18.2 | 7890 | 8042 | 0 | L1MC4 | L1 | LINE |
| 146712913 | 146713283 | - | 12.7 | 1 | 364 | 0 | THE1B | MaLR | LTR |
| 146713284 | 146714853 | - | 10.5 | 1 | 1580 | 0 | THE1B-int | MaLR | LTR |
| 146714854 | 146715224 | - | 13.0 | 1 | 364 | 0 | THE1B | MaLR | LTR |
| 146715225 | 146715606 | - | 29.3 | 7294 | 7700 | -342 | L1MC4 | L1 | LINE |
| 146715624 | 146715823 | - | 11.0 | 6061 | 6289 | -13 | L1MA1 | L1 | LINE |
| 146715859 | 146716602 | - | 25.1 | 6549 | 7296 | -746 | L1MC4 | L1 | LINE |
| 146716603 | 146716890 | - | 12.2 | 15 | 301 | -9 | AluSg | Alu | SINE |
| 146716891 | 146716983 | - | 25.1 | 6460 | 6548 | -1494 | L1MC4 | L1 | LINE |

  

|  |  |  |  |  |  |  |  |  |  |
| --- | --- | --- | --- | --- | --- | --- | --- | --- | --- |
| chr7:146953653-146962625 | | | 8936 bp | | |  |  |  |  |
| Genome Start | Genome End | Strand | Div | Repeat Start | Repeat End | Repeat Left | Name | Family | Class |
| 146953653 | 146955440 | + | 15.3 | 3 | 1864 | -4999 | L1PB2 | L1 | LINE |
| 146955441 | 146955739 | + | 9.4 | 1 | 303 | -7 | AluSg | Alu | SINE |
| 146955740 | 146957144 | + | 14.3 | 1865 | 3327 | -3535 | L1PB2 | L1 | LINE |
| 146957145 | 146957431 | + | 13.3 | 1 | 286 | -27 | AluSq | Alu | SINE |
| 146957475 | 146960308 | + | 12.3 | 3328 | 6165 | -702 | L1PB2 | L1 | LINE |
| 146960309 | 146960674 | + | 12.4 | 1 | 375 | 0 | THE1C | MaLR | LTR |
| 146960675 | 146961456 | + | 13.3 | 1 | 1000 | -580 | THE1C-int | MaLR | LTR |
| 146961449 | 146961579 | + | 16.1 | 1449 | 1580 | 0 | THE1C-int | MaLR | LTR |
| 146961580 | 146961934 | + | 15.7 | 1 | 375 | 0 | THE1C | MaLR | LTR |
| 146961937 | 146962625 | + | 8.6 | 6163 | 6864 | -3 | L1PB2 | L1 | LINE |

  

|  |  |  |  |  |  |  |  |  |  |
| --- | --- | --- | --- | --- | --- | --- | --- | --- | --- |
| chr7:148673966-148679274 | | | 5230 bp | | |  |  |  |  |
| Genome Start | Genome End | Strand | Div | Repeat Start | Repeat End | Repeat Left | Name | Family | Class |
| 148673966 | 148674184 | + | 20.1 | 3088 | 3296 | -2850 | L1M4 | L1 | LINE |
| 148674185 | 148674497 | + | 8.9 | 1 | 313 | 0 | AluY | Alu | SINE |
| 148674498 | 148674924 | + | 20.1 | 3297 | 3716 | -2430 | L1M4 | L1 | LINE |
| 148674931 | 148675170 | - | 11.8 | 125 | 364 | 0 | THE1B | MaLR | LTR |
| 148675172 | 148676015 | + | 18.3 | 3709 | 4559 | -1581 | L1M4 | L1 | LINE |
| 148676016 | 148676394 | - | 15.8 | 1 | 375 | 0 | THE1C | MaLR | LTR |
| 148676395 | 148677833 | - | 13.6 | 1 | 1580 | 0 | THE1C-int | MaLR | LTR |
| 148677834 | 148678212 | - | 13.6 | 1 | 375 | 0 | THE1C | MaLR | LTR |
| 148678285 | 148678748 | + | 19.9 | 4569 | 5025 | -1121 | L1M4 | L1 | LINE |
| 148678749 | 148679053 | - | 14.8 | 1 | 305 | -7 | AluSx | Alu | SINE |
| 148679054 | 148679274 | + | 19.9 | 5026 | 5241 | -905 | L1M4 | L1 | LINE |

  

|  |  |  |  |  |  |  |  |  |  |
| --- | --- | --- | --- | --- | --- | --- | --- | --- | --- |
| chr7:148700218-148703050 | | | 2831 bp | | |  |  |  |  |
| Genome Start | Genome End | Strand | Div | Repeat Start | Repeat End | Repeat Left | Name | Family | Class |
| 148700218 | 148700268 | - | 28.9 | 412 | 456 | -55 | MLT1J | MaLR | LTR |
| 148700269 | 148700653 | - | 15.5 | 1 | 428 | 0 | MSTA | MaLR | LTR |
| 148700654 | 148702269 | - | 28.2 | 2 | 1735 | 0 | MLT1J-int | MaLR | LTR |
| 148702272 | 148702653 | - | 13.7 | 1 | 428 | 0 | MSTA | MaLR | LTR |
| 148702654 | 148703050 | - | 28.9 | 8 | 411 | -100 | MLT1J | MaLR | LTR |

  

|  |  |  |  |  |  |  |  |  |  |
| --- | --- | --- | --- | --- | --- | --- | --- | --- | --- |
| chr7:149500189-149502596 | | | 2405 bp | | |  |  |  |  |
| Genome Start | Genome End | Strand | Div | Repeat Start | Repeat End | Repeat Left | Name | Family | Class |
| 149500189 | 149500228 | + | 15.9 | 461 | 500 | -197 | MER50B | ERV1 | LTR |
| 149500229 | 149500582 | - | 9.6 | 1 | 355 | 0 | THE1A | MaLR | LTR |
| 149500583 | 149502178 | - | 9.7 | 1 | 1580 | 0 | THE1A-int | MaLR | LTR |
| 149502179 | 149502534 | - | 8.2 | 1 | 355 | 0 | THE1A | MaLR | LTR |
| 149502538 | 149502596 | + | 8.5 | 499 | 557 | -140 | MER50B | ERV1 | LTR |

  

|  |  |  |  |  |  |  |  |  |  |
| --- | --- | --- | --- | --- | --- | --- | --- | --- | --- |
| chr7:152898220-152900856 | | | 2635 bp | | |  |  |  |  |
| Genome Start | Genome End | Strand | Div | Repeat Start | Repeat End | Repeat Left | Name | Family | Class |
| 152898220 | 152898402 | - | 11.4 | 191 | 375 | 0 | THE1C | MaLR | LTR |
| 152898403 | 152898738 | + | 11.4 | 1 | 364 | 0 | THE1B | MaLR | LTR |
| 152898741 | 152900295 | + | 32.0 | 2 | 1735 | 0 | MLT-int | MaLR | LTR |
| 152900296 | 152900664 | + | 11.1 | 1 | 362 | -2 | THE1B | MaLR | LTR |
| 152900665 | 152900856 | - | 11.4 | 1 | 190 | -185 | THE1C | MaLR | LTR |

  

|  |  |  |  |  |  |  |  |  |  |
| --- | --- | --- | --- | --- | --- | --- | --- | --- | --- |
| chr7:153120687-153123095 | | | 2406 bp | | |  |  |  |  |
| Genome Start | Genome End | Strand | Div | Repeat Start | Repeat End | Repeat Left | Name | Family | Class |
| 153120687 | 153120748 | - | 12.4 | 501 | 557 | -140 | MER50B | ERV1 | LTR |
| 153120749 | 153121103 | + | 7.7 | 1 | 354 | -1 | THE1A | MaLR | LTR |
| 153121107 | 153122701 | + | 29.3 | 2 | 1735 | 0 | MLT1F2-int | MaLR | LTR |
| 153122702 | 153123055 | + | 9.0 | 1 | 355 | 0 | THE1A | MaLR | LTR |
| 153123056 | 153123095 | - | 12.4 | 461 | 500 | -197 | MER50B | ERV1 | LTR |

  

|  |  |  |  |  |  |  |  |  |  |
| --- | --- | --- | --- | --- | --- | --- | --- | --- | --- |
| chr7:156112415-156118770 | | | 6329 bp | | |  |  |  |  |
| Genome Start | Genome End | Strand | Div | Repeat Start | Repeat End | Repeat Left | Name | Family | Class |
| 156112415 | 156113035 | + | 13.5 | 1 | 618 | -5569 | MER57A-int | ERV1 | LTR |
| 156113036 | 156113310 | + | 12.0 | 1 | 275 | -37 | AluSx | Alu | SINE |
| 156113335 | 156113822 | + | 13.5 | 619 | 1087 | -5100 | MER57A-int | ERV1 | LTR |
| 156113823 | 156114205 | + | 13.1 | 1 | 428 | 0 | MSTA | MaLR | LTR |
| 156114206 | 156114862 | + | 17.7 | 1 | 679 | -972 | MSTA-int | MaLR | LTR |
| 156114863 | 156115219 | - | 17.7 | 1 | 362 | -2 | THE1B | MaLR | LTR |
| 156115220 | 156116184 | + | 17.7 | 680 | 1651 | 0 | MSTA-int | MaLR | LTR |
| 156116187 | 156116384 | + | 12.2 | 3 | 207 | -221 | MSTA | MaLR | LTR |
| 156116391 | 156118383 | + | 13.4 | 1171 | 3464 | -2867 | MER57A-int | ERV1 | LTR |
| 156118379 | 156118770 | + | 13.5 | 3397 | 3782 | -2405 | MER57A-int | ERV1 | LTR |

  

|  |  |  |  |  |  |  |  |  |  |
| --- | --- | --- | --- | --- | --- | --- | --- | --- | --- |
| chr7:156177906-156186972 | | | 8957 bp | | |  |  |  |  |
| Genome Start | Genome End | Strand | Div | Repeat Start | Repeat End | Repeat Left | Name | Family | Class |
| 156177906 | 156178184 | + | 21.3 | 36 | 321 | -6079 | L1ME1 | L1 | LINE |
| 156178258 | 156178726 | + | 23.4 | 894 | 1408 | -4992 | L1ME1 | L1 | LINE |
| 156178697 | 156179620 | + | 23.6 | 3141 | 4066 | -2147 | L1ME1 | L1 | LINE |
| 156179621 | 156179887 | - | 14.7 | 1 | 293 | -19 | AluJo | Alu | SINE |
| 156179888 | 156180485 | + | 23.6 | 4067 | 4676 | -1537 | L1ME1 | L1 | LINE |
| 156180486 | 156180776 | + | 25.1 | 4 | 295 | -17 | AluJo | Alu | SINE |
| 156180779 | 156181197 | - | 14.2 | 1 | 427 | -1 | MSTA | MaLR | LTR |
| 156181198 | 156181890 | + | 23.6 | 4677 | 5431 | -782 | L1ME1 | L1 | LINE |
| 156181894 | 156182231 | - | 13.9 | 1 | 364 | 0 | THE1B | MaLR | LTR |
| 156182232 | 156182439 | - | 5.1 | 1369 | 1580 | 0 | THE1B-int | MaLR | LTR |
| 156182440 | 156183469 | - | 5.8 | 1 | 1266 | 0 | MER11A | ERVK | LTR |
| 156183470 | 156184613 | - | 9.5 | 265 | 1378 | -202 | THE1B-int | MaLR | LTR |
| 156184614 | 156186008 | + | 2.8 | 4745 | 6152 | -3 | L1PA4 | L1 | LINE |
| 156186013 | 156186283 | - | 7.8 | 1 | 271 | -1309 | THE1B-int | MaLR | LTR |
| 156186284 | 156186616 | - | 12.0 | 1 | 364 | 0 | THE1B | MaLR | LTR |
| 156186675 | 156186972 | + | 23.1 | 5738 | 6041 | -198 | L1ME1 | L1 | LINE |

  

|  |  |  |  |  |  |  |  |  |  |
| --- | --- | --- | --- | --- | --- | --- | --- | --- | --- |
| chr7:156339520-156348180 | | | 8621 bp | | |  |  |  |  |
| Genome Start | Genome End | Strand | Div | Repeat Start | Repeat End | Repeat Left | Name | Family | Class |
| 156339520 | 156341400 | + | 19.0 | 1 | 1644 | -4864 | L1MCa | L1 | LINE |
| 156341401 | 156341663 | + | 6.1 | 33 | 295 | -16 | AluY | Alu | SINE |
| 156341702 | 156341906 | + | 19.0 | 1645 | 1785 | -4723 | L1MCa | L1 | LINE |
| 156341907 | 156342184 | + | 15.9 | 14 | 306 | -6 | AluSx | Alu | SINE |
| 156342185 | 156344426 | + | 16.6 | 1786 | 4225 | -2096 | L1MCa | L1 | LINE |
| 156344427 | 156344718 | + | 9.6 | 1 | 298 | -15 | AluSp | Alu | SINE |
| 156344719 | 156345591 | + | 16.1 | 4226 | 5171 | -1150 | L1MCa | L1 | LINE |
| 156345592 | 156345953 | - | 9.6 | 1 | 364 | 0 | THE1B | MaLR | LTR |
| 156345954 | 156347521 | - | 30.9 | 2 | 1735 | 0 | MLT-int | MaLR | LTR |
| 156347524 | 156347891 | - | 10.6 | 3 | 364 | 0 | THE1B | MaLR | LTR |
| 156347892 | 156348180 | + | 16.1 | 5172 | 5456 | -865 | L1MCa | L1 | LINE |

  


|  |  |  |  |  |  |  |  |  |  |
| --- | --- | --- | --- | --- | --- | --- | --- | --- | --- |
| chr8:118589-121388 | | | 2796 bp | | |  |  |  |  |
| Genome Start | Genome End | Strand | Div | Repeat Start | Repeat End | Repeat Left | Name | Family | Class |
| 118589 | 118829 | + | 29.3 | 1 | 249 | -306 | MLT1H1 | MaLR | LTR |
| 118830 | 119187 | - | 18.6 | 1 | 364 | 0 | THE1B | MaLR | LTR |
| 119188 | 120747 | - | 30.7 | 4 | 1735 | 0 | MLT-int | MaLR | LTR |
| 120752 | 121111 | - | 15.5 | 3 | 364 | 0 | THE1B | MaLR | LTR |
| 121112 | 121388 | + | 29.3 | 250 | 522 | -33 | MLT1H1 | MaLR | LTR |

  

|  |  |  |  |  |  |  |  |  |  |
| --- | --- | --- | --- | --- | --- | --- | --- | --- | --- |
| chr8:5689286-5692585 | | | 3293 bp | | |  |  |  |  |
| Genome Start | Genome End | Strand | Div | Repeat Start | Repeat End | Repeat Left | Name | Family | Class |
| 5689286 | 5689634 | + | 27.9 | 5387 | 5723 | -441 | L1ME2 | L1 | LINE |
| 5689635 | 5689939 | - | 12.9 | 1 | 303 | -10 | AluSq | Alu | SINE |
| 5689940 | 5689983 | + | 27.9 | 5724 | 5748 | -416 | L1ME2 | L1 | LINE |
| 5689984 | 5690339 | - | 15.1 | 2 | 375 | 0 | THE1C | MaLR | LTR |
| 5690341 | 5691908 | - | 33.4 | 2 | 1735 | 0 | MLT-int | MaLR | LTR |
| 5691915 | 5692268 | - | 14.6 | 1 | 371 | -4 | THE1C | MaLR | LTR |
| 5692269 | 5692585 | + | 27.9 | 5749 | 6135 | -29 | L1ME2 | L1 | LINE |

  

|  |  |  |  |  |  |  |  |  |  |
| --- | --- | --- | --- | --- | --- | --- | --- | --- | --- |
| chr8:6976865-6985823 | | | 8981 bp | | |  |  |  |  |
| Genome Start | Genome End | Strand | Div | Repeat Start | Repeat End | Repeat Left | Name | Family | Class |
| 6976865 | 6977453 | - | 14.9 | 6037 | 6629 | -376 | L1MA4A | L1 | LINE |
| 6977428 | 6977688 | - | 11.9 | 4958 | 5219 | -927 | L1PREC2 | L1 | LINE |
| 6977691 | 6978152 | - | 16.6 | 5437 | 5903 | -942 | L1MA4A | L1 | LINE |
| 6978153 | 6978424 | - | 8.7 | 1 | 304 | 0 | AluY | Alu | SINE |
| 6978425 | 6978914 | - | 16.6 | 4977 | 5436 | -1409 | L1MA4A | L1 | LINE |
| 6978915 | 6979213 | - | 15.4 | 1 | 302 | -10 | AluSx | Alu | SINE |
| 6979214 | 6981393 | - | 16.7 | 2780 | 4976 | -1869 | L1MA4A | L1 | LINE |
| 6981394 | 6981746 | + | 15.6 | 1 | 364 | 0 | THE1B | MaLR | LTR |
| 6981749 | 6983298 | + | 31.6 | 2 | 1735 | 0 | MLT-int | MaLR | LTR |
| 6983299 | 6983656 | + | 14.4 | 1 | 364 | 0 | THE1B | MaLR | LTR |
| 6983657 | 6983895 | - | 20.8 | 2527 | 2779 | -4070 | L1MA4A | L1 | LINE |
| 6983896 | 6984054 | - | 17.2 | 5 | 161 | -5 | FRAM | Alu | SINE |
| 6984055 | 6985099 | - | 20.8 | 1441 | 2526 | -4323 | L1MA4A | L1 | LINE |
| 6985100 | 6985404 | - | 12.5 | 1 | 306 | -4 | AluSg | Alu | SINE |
| 6985405 | 6985823 | - | 20.8 | 978 | 1440 | -5409 | L1MA4A | L1 | LINE |

  

|  |  |  |  |  |  |  |  |  |  |
| --- | --- | --- | --- | --- | --- | --- | --- | --- | --- |
| chr8:6995315-7004827 | | | 9386 bp | | |  |  |  |  |
| Genome Start | Genome End | Strand | Div | Repeat Start | Repeat End | Repeat Left | Name | Family | Class |
| 6995315 | 6995545 | + | 33.7 | 2233 | 2465 | -954 | L2 | L2 | LINE |
| 6995546 | 6995850 | + | 18.7 | 1 | 294 | -18 | AluJo | Alu | SINE |
| 6995851 | 6996495 | + | 33.0 | 2466 | 3162 | -257 | L2 | L2 | LINE |
| 6996530 | 6997601 | + | 6.3 | 1 | 1266 | 0 | MER11A | ERVK | LTR |
| 6997712 | 6998778 | - | 5.2 | 1 | 1071 | 0 | MER11C | ERVK | LTR |
| 6998779 | 6999783 | - | 7.3 | 6954 | 7953 | 0 | HERVK11 | ERVK | LTR |
| 6999768 | 7000000 | - | 10.7 | 6531 | 6765 | -1188 | HERVK11 | ERVK | LTR |
| 7000001 | 7001005 | - | 7.3 | 5525 | 6530 | -1423 | HERVK11 | ERVK | LTR |
| 7001004 | 7002917 | - | 7.7 | 1165 | 4601 | -3352 | HERVK11 | ERVK | LTR |
| 7002918 | 7003707 | - | 16.5 | 1 | 851 | -7102 | HERVK11 | ERVK | LTR |
| 7003708 | 7004767 | - | 4.3 | 1 | 1071 | 0 | MER11C | ERVK | LTR |
| 7004769 | 7004827 | + | 21.4 | 3364 | 3419 | 0 | L2 | L2 | LINE |

  

|  |  |  |  |  |  |  |  |  |  |
| --- | --- | --- | --- | --- | --- | --- | --- | --- | --- |
| chr8:7018401-7027450 | | | 9055 bp | | |  |  |  |  |
| Genome Start | Genome End | Strand | Div | Repeat Start | Repeat End | Repeat Left | Name | Family | Class |
| 7018401 | 7018818 | + | 19.8 | 978 | 1440 | -5409 | L1MA4A | L1 | LINE |
| 7018819 | 7019129 | + | 11.3 | 1 | 310 | 0 | AluSg | Alu | SINE |
| 7019130 | 7020174 | + | 19.8 | 1441 | 2522 | -4327 | L1MA4A | L1 | LINE |
| 7020175 | 7020346 | + | 20.6 | 1 | 172 | 0 | FRAM | Alu | SINE |
| 7020357 | 7020567 | + | 19.8 | 2523 | 2768 | -4081 | L1MA4A | L1 | LINE |
| 7020568 | 7020925 | - | 13.3 | 1 | 364 | 0 | THE1B | MaLR | LTR |
| 7020926 | 7022496 | - | 31.2 | 2 | 1735 | 0 | MLT-int | MaLR | LTR |
| 7022499 | 7022852 | - | 15.0 | 1 | 364 | 0 | THE1B | MaLR | LTR |
| 7022853 | 7025043 | + | 17.2 | 2769 | 4969 | -1876 | L1MA4A | L1 | LINE |
| 7025044 | 7025351 | + | 13.0 | 1 | 311 | -1 | AluSx | Alu | SINE |
| 7025352 | 7025845 | + | 17.1 | 4970 | 5450 | -1395 | L1MA4A | L1 | LINE |
| 7025846 | 7026165 | + | 8.0 | 1 | 303 | -8 | AluY | Alu | SINE |
| 7026166 | 7026624 | + | 17.1 | 5451 | 5905 | -940 | L1MA4A | L1 | LINE |
| 7026625 | 7026870 | + | 11.4 | 4958 | 5204 | -942 | L1PREC2 | L1 | LINE |
| 7026854 | 7027450 | + | 15.9 | 6029 | 6633 | -372 | L1MA4A | L1 | LINE |

  

|  |  |  |  |  |  |  |  |  |  |
| --- | --- | --- | --- | --- | --- | --- | --- | --- | --- |
| chr8:7246752-7254259 | | | 7510 bp | | |  |  |  |  |
| Genome Start | Genome End | Strand | Div | Repeat Start | Repeat End | Repeat Left | Name | Family | Class |
| 7246752 | 7247221 | + | 20.9 | 1 | 481 | -1170 | MSTB-int | MaLR | LTR |
| 7247222 | 7247672 | + | 8.0 | 1 | 448 | -2 | LTR7 | ERV1 | LTR |
| 7247675 | 7250767 | + | 9.8 | 1 | 2987 | -4726 | HERVH | ERV1 | LTR |
| 7250762 | 7250906 | + | 17.9 | 3140 | 3290 | -4423 | HERVH | ERV1 | LTR |
| 7250906 | 7251396 | + | 11.0 | 3500 | 3993 | -3720 | HERVH | ERV1 | LTR |
| 7251399 | 7252058 | + | 15.0 | 4488 | 5149 | -2564 | HERVH | ERV1 | LTR |
| 7252060 | 7252275 | + | 13.9 | 5600 | 5819 | -1894 | HERVH | ERV1 | LTR |
| 7252274 | 7252653 | + | 7.7 | 7322 | 7713 | 0 | HERVH | ERV1 | LTR |
| 7252654 | 7253106 | + | 6.5 | 1 | 448 | -2 | LTR7 | ERV1 | LTR |
| 7253109 | 7254259 | + | 19.6 | 477 | 1651 | 0 | MSTB-int | MaLR | LTR |

  

|  |  |  |  |  |  |  |  |  |  |
| --- | --- | --- | --- | --- | --- | --- | --- | --- | --- |
| chr8:7342755-7353298 | | | 10230 bp | | |  |  |  |  |
| Genome Start | Genome End | Strand | Div | Repeat Start | Repeat End | Repeat Left | Name | Family | Class |
| 7342755 | 7342796 | - | 17.5 | 4445 | 4484 | -5 | L3 | CR1 | LINE |
| 7342807 | 7343774 | - | 1.0 | 1 | 968 | 0 | LTR5\_Hs | ERVK | LTR |
| 7343775 | 7351309 | - | 0.5 | 1 | 7536 | 0 | HERVK | ERVK | LTR |
| 7351310 | 7352269 | - | 0.9 | 1 | 968 | 0 | LTR5\_Hs | ERVK | LTR |
| 7352273 | 7352371 | + | 39.8 | 16 | 116 | -146 | MIR | MIR | SINE |
| 7352417 | 7352607 | - | 29.3 | 261 | 465 | 0 | MLT1J | MaLR | LTR |
| 7352685 | 7352869 | - | 34.4 | 7 | 195 | -316 | MLT1J | MaLR | LTR |
| 7353049 | 7353298 | - | 29.7 | 4115 | 4397 | -92 | L3 | CR1 | LINE |

  

|  |  |  |  |  |  |  |  |  |  |
| --- | --- | --- | --- | --- | --- | --- | --- | --- | --- |
| chr8:7797198-7804706 | | | 7511 bp | | |  |  |  |  |
| Genome Start | Genome End | Strand | Div | Repeat Start | Repeat End | Repeat Left | Name | Family | Class |
| 7797198 | 7798348 | - | 19.7 | 477 | 1651 | 0 | MSTB-int | MaLR | LTR |
| 7798351 | 7798803 | - | 6.5 | 1 | 448 | -2 | LTR7 | ERV1 | LTR |
| 7798804 | 7799183 | - | 7.7 | 7322 | 7713 | 0 | HERVH | ERV1 | LTR |
| 7799182 | 7799397 | - | 13.9 | 5600 | 5819 | -1894 | HERVH | ERV1 | LTR |
| 7799399 | 7800058 | - | 14.8 | 4488 | 5149 | -2564 | HERVH | ERV1 | LTR |
| 7800061 | 7800551 | - | 11.0 | 3500 | 3993 | -3720 | HERVH | ERV1 | LTR |
| 7800551 | 7800696 | - | 17.9 | 3140 | 3290 | -4423 | HERVH | ERV1 | LTR |
| 7800691 | 7803783 | - | 9.9 | 1 | 2987 | -4726 | HERVH | ERV1 | LTR |
| 7803786 | 7804236 | - | 8.3 | 1 | 448 | -2 | LTR7 | ERV1 | LTR |
| 7804237 | 7804706 | - | 20.9 | 1 | 481 | -1170 | MSTB-int | MaLR | LTR |

  

|  |  |  |  |  |  |  |  |  |  |
| --- | --- | --- | --- | --- | --- | --- | --- | --- | --- |
| chr8:7999914-8008945 | | | 9014 bp | | |  |  |  |  |
| Genome Start | Genome End | Strand | Div | Repeat Start | Repeat End | Repeat Left | Name | Family | Class |
| 7999914 | 8000511 | - | 15.9 | 6029 | 6633 | -372 | L1MA4A | L1 | LINE |
| 8000495 | 8000740 | - | 11.4 | 4958 | 5204 | -942 | L1PREC2 | L1 | LINE |
| 8000741 | 8001199 | - | 17.4 | 5451 | 5905 | -940 | L1MA4A | L1 | LINE |
| 8001200 | 8001514 | - | 8.0 | 1 | 303 | -8 | AluY | Alu | SINE |
| 8001515 | 8002007 | - | 17.4 | 4970 | 5450 | -1395 | L1MA4A | L1 | LINE |
| 8002008 | 8002315 | - | 12.7 | 1 | 311 | -1 | AluSx | Alu | SINE |
| 8002316 | 8004495 | - | 17.4 | 2775 | 4969 | -1876 | L1MA4A | L1 | LINE |
| 8004496 | 8004849 | + | 15.6 | 1 | 364 | 0 | THE1B | MaLR | LTR |
| 8004852 | 8006422 | + | 31.3 | 2 | 1735 | 0 | MLT-int | MaLR | LTR |
| 8006423 | 8006780 | + | 13.6 | 1 | 364 | 0 | THE1B | MaLR | LTR |
| 8006781 | 8006991 | - | 19.8 | 2534 | 2774 | -4075 | L1MA4A | L1 | LINE |
| 8007025 | 8007173 | - | 20.6 | 2 | 150 | -16 | FRAM | Alu | SINE |
| 8007174 | 8008217 | - | 19.8 | 1451 | 2533 | -4316 | L1MA4A | L1 | LINE |
| 8008218 | 8008527 | - | 11.0 | 1 | 309 | -1 | AluSg | Alu | SINE |
| 8008528 | 8008945 | - | 19.8 | 985 | 1450 | -5399 | L1MA4A | L1 | LINE |

  

|  |  |  |  |  |  |  |  |  |  |
| --- | --- | --- | --- | --- | --- | --- | --- | --- | --- |
| chr8:8022513-8031599 | | | 9057 bp | | |  |  |  |  |
| Genome Start | Genome End | Strand | Div | Repeat Start | Repeat End | Repeat Left | Name | Family | Class |
| 8022513 | 8022571 | - | 21.4 | 3364 | 3419 | 0 | L2 | L2 | LINE |
| 8022573 | 8023631 | + | 4.6 | 1 | 1071 | 0 | MER11C | ERVK | LTR |
| 8023632 | 8024421 | + | 15.9 | 1 | 851 | -7102 | HERVK11 | ERVK | LTR |
| 8024422 | 8026332 | + | 7.4 | 1165 | 4601 | -3352 | HERVK11 | ERVK | LTR |
| 8026331 | 8027558 | + | 7.8 | 5525 | 6765 | -1188 | HERVK11 | ERVK | LTR |
| 8027543 | 8028544 | + | 7.2 | 6954 | 7953 | 0 | HERVK11 | ERVK | LTR |
| 8028545 | 8029610 | + | 5.3 | 1 | 1071 | 0 | MER11C | ERVK | LTR |
| 8029611 | 8029695 | - | 35.3 | 3242 | 3327 | -51 | L2 | L2 | LINE |
| 8029723 | 8029918 | - | 11.7 | 1067 | 1266 | 0 | MER11A | ERVK | LTR |
| 8029916 | 8030384 | - | 5.0 | 1 | 578 | -688 | MER11A | ERVK | LTR |
| 8030408 | 8031081 | - | 32.8 | 2466 | 3188 | -190 | L2 | L2 | LINE |
| 8031082 | 8031394 | - | 18.6 | 1 | 296 | -16 | AluJo | Alu | SINE |
| 8031395 | 8031599 | - | 33.2 | 2261 | 2465 | -954 | L2 | L2 | LINE |

  

|  |  |  |  |  |  |  |  |  |  |
| --- | --- | --- | --- | --- | --- | --- | --- | --- | --- |
| chr8:8063068-8079294 | | | 16193 bp | | |  |  |  |  |
| Genome Start | Genome End | Strand | Div | Repeat Start | Repeat End | Repeat Left | Name | Family | Class |
| 8063068 | 8063133 | - | 25.1 | 393 | 484 | 0 | MLT1D | MaLR | LTR |
| 8063134 | 8063442 | + | 15.3 | 2 | 308 | -4 | AluSx | Alu | SINE |
| 8063443 | 8063637 | - | 25.1 | 193 | 432 | -73 | MLT1D | MaLR | LTR |
| 8063655 | 8064006 | + | 8.7 | 1 | 448 | -2 | LTR7 | ERV1 | LTR |
| 8064009 | 8066902 | + | 9.8 | 1 | 2993 | -4720 | HERVH | ERV1 | LTR |
| 8066888 | 8067192 | + | 11.1 | 3140 | 3441 | -4272 | HERVH | ERV1 | LTR |
| 8067193 | 8075019 | + | 6.9 | 3 | 7812 | -1 | HERVE | ERV1 | LTR |
| 8075020 | 8075516 | + | 9.5 | 1 | 501 | 0 | LTR2C | ERV1 | LTR |
| 8075517 | 8076070 | + | 8.9 | 3442 | 3993 | -3720 | HERVH | ERV1 | LTR |
| 8076073 | 8076675 | + | 14.1 | 4488 | 5089 | -2624 | HERVH | ERV1 | LTR |
| 8076676 | 8077742 | + | 4.7 | 1 | 1071 | 0 | MER11C | ERVK | LTR |
| 8077743 | 8077808 | + | 14.1 | 5090 | 5149 | -2564 | HERVH | ERV1 | LTR |
| 8077810 | 8078016 | + | 12.1 | 5600 | 5819 | -1894 | HERVH | ERV1 | LTR |
| 8078017 | 8078393 | + | 9.1 | 7324 | 7713 | 0 | HERVH | ERV1 | LTR |
| 8078395 | 8078788 | + | 9.2 | 2 | 444 | -6 | LTR7 | ERV1 | LTR |
| 8078805 | 8079103 | - | 10.0 | 3 | 302 | -11 | AluSp | Alu | SINE |
| 8079114 | 8079294 | - | 24.0 | 1 | 185 | -320 | MLT1D | MaLR | LTR |

  

|  |  |  |  |  |  |  |  |  |  |
| --- | --- | --- | --- | --- | --- | --- | --- | --- | --- |
| chr8:9695249-9706826 | | | 11592 bp | | |  |  |  |  |
| Genome Start | Genome End | Strand | Div | Repeat Start | Repeat End | Repeat Left | Name | Family | Class |
| 9695249 | 9696027 | + | 10.6 | 1 | 799 | -6038 | HERVK22 | ERVK | LTR |
| 9696006 | 9697902 | + | 9.1 | 2314 | 4222 | -2615 | HERVK22 | ERVK | LTR |
| 9697903 | 9698263 | + | 10.0 | 1 | 450 | 0 | LTR7 | ERV1 | LTR |
| 9698264 | 9698535 | + | 20.7 | 1 | 291 | -7422 | HERVH | ERV1 | LTR |
| 9698536 | 9698805 | - | 6.3 | 37 | 305 | -6 | AluY | Alu | SINE |
| 9698806 | 9699023 | + | 22.3 | 292 | 446 | -7267 | HERVH | ERV1 | LTR |
| 9699051 | 9701872 | + | 17.3 | 801 | 3587 | -4126 | HERVH | ERV1 | LTR |
| 9701861 | 9703016 | + | 13.7 | 3800 | 4910 | -2297 | HERVH | ERV1 | LTR |
| 9703017 | 9703305 | + | 18.2 | 5747 | 6016 | -1697 | HERVH | ERV1 | LTR |
| 9703299 | 9703850 | + | 12.8 | 7157 | 7713 | 0 | HERVH | ERV1 | LTR |
| 9703851 | 9704217 | + | 11.5 | 1 | 450 | 0 | LTR7 | ERV1 | LTR |
| 9704218 | 9706826 | + | 9.4 | 4218 | 6837 | 0 | HERVK22 | ERVK | LTR |

  

|  |  |  |  |  |  |  |  |  |  |
| --- | --- | --- | --- | --- | --- | --- | --- | --- | --- |
| chr8:10465822-10472740 | | | 6918 bp | | |  |  |  |  |
| Genome Start | Genome End | Strand | Div | Repeat Start | Repeat End | Repeat Left | Name | Family | Class |
| 10465822 | 10465960 | + | 16.8 | 2 | 139 | -2279 | Tigger1 | MER2\_type | DNA |
| 10465957 | 10466000 | + | 6.8 | 2200 | 2244 | -174 | Tigger1 | MER2\_type | DNA |
| 10466001 | 10466401 | + | 8.3 | 1 | 448 | -2 | LTR7 | ERV1 | LTR |
| 10466404 | 10470599 | + | 10.4 | 1 | 3993 | -3720 | HERVH | ERV1 | LTR |
| 10470602 | 10471264 | + | 15.4 | 4488 | 5149 | -2564 | HERVH | ERV1 | LTR |
| 10471266 | 10471857 | + | 13.0 | 5600 | 7713 | 0 | HERVH | ERV1 | LTR |
| 10471858 | 10472249 | + | 8.5 | 1 | 448 | -2 | LTR7 | ERV1 | LTR |
| 10472250 | 10472313 | + | 20.3 | 2238 | 2298 | -120 | Tigger1 | MER2\_type | DNA |
| 10472314 | 10472614 | - | 10.7 | 1 | 301 | -12 | AluSq | Alu | SINE |
| 10472615 | 10472740 | + | 20.3 | 2299 | 2417 | -1 | Tigger1 | MER2\_type | DNA |

  

|  |  |  |  |  |  |  |  |  |  |
| --- | --- | --- | --- | --- | --- | --- | --- | --- | --- |
| chr8:12383179-12399410 | | | 16198 bp | | |  |  |  |  |
| Genome Start | Genome End | Strand | Div | Repeat Start | Repeat End | Repeat Left | Name | Family | Class |
| 12383179 | 12383359 | + | 23.4 | 1 | 185 | -320 | MLT1D | MaLR | LTR |
| 12383370 | 12383670 | + | 10.0 | 3 | 304 | -9 | AluSp | Alu | SINE |
| 12383687 | 12384080 | - | 9.2 | 2 | 444 | -6 | LTR7 | ERV1 | LTR |
| 12384082 | 12384458 | - | 9.4 | 7324 | 7713 | 0 | HERVH | ERV1 | LTR |
| 12384459 | 12384665 | - | 11.6 | 5600 | 5819 | -1894 | HERVH | ERV1 | LTR |
| 12384667 | 12384732 | - | 14.8 | 5091 | 5149 | -2564 | HERVH | ERV1 | LTR |
| 12384733 | 12385797 | - | 4.4 | 3 | 1071 | 0 | MER11C | ERVK | LTR |
| 12385798 | 12386402 | - | 14.8 | 4488 | 5090 | -2623 | HERVH | ERV1 | LTR |
| 12386405 | 12386958 | - | 9.4 | 3442 | 3993 | -3720 | HERVH | ERV1 | LTR |
| 12386959 | 12387455 | - | 9.1 | 1 | 501 | 0 | LTR2C | ERV1 | LTR |
| 12387456 | 12395282 | - | 6.8 | 3 | 7812 | -1 | HERVE | ERV1 | LTR |
| 12395283 | 12395587 | - | 11.1 | 3140 | 3441 | -4272 | HERVH | ERV1 | LTR |
| 12395573 | 12398469 | - | 9.9 | 1 | 2993 | -4720 | HERVH | ERV1 | LTR |
| 12398472 | 12398823 | - | 9.0 | 1 | 448 | -2 | LTR7 | ERV1 | LTR |
| 12398841 | 12399035 | + | 25.1 | 193 | 431 | -74 | MLT1D | MaLR | LTR |
| 12399036 | 12399344 | - | 14.7 | 2 | 308 | -4 | AluSx | Alu | SINE |
| 12399345 | 12399410 | + | 25.1 | 393 | 483 | 0 | MLT1D | MaLR | LTR |

  

|  |  |  |  |  |  |  |  |  |  |
| --- | --- | --- | --- | --- | --- | --- | --- | --- | --- |
| chr8:12430885-12439982 | | | 9068 bp | | |  |  |  |  |
| Genome Start | Genome End | Strand | Div | Repeat Start | Repeat End | Repeat Left | Name | Family | Class |
| 12430885 | 12431089 | + | 33.7 | 2261 | 2465 | -954 | L2 | L2 | LINE |
| 12431090 | 12431418 | + | 18.6 | 1 | 301 | -11 | AluJo | Alu | SINE |
| 12431419 | 12432087 | + | 33.1 | 2466 | 3188 | -190 | L2 | L2 | LINE |
| 12432111 | 12432579 | + | 5.2 | 1 | 578 | -688 | MER11A | ERVK | LTR |
| 12432577 | 12432772 | + | 11.7 | 1067 | 1266 | 0 | MER11A | ERVK | LTR |
| 12432800 | 12432884 | + | 35.3 | 3242 | 3327 | -51 | L2 | L2 | LINE |
| 12432885 | 12433950 | - | 5.1 | 1 | 1071 | 0 | MER11C | ERVK | LTR |
| 12433951 | 12434953 | - | 7.2 | 6954 | 7953 | 0 | HERVK11 | ERVK | LTR |
| 12434938 | 12436165 | - | 7.8 | 5525 | 6765 | -1188 | HERVK11 | ERVK | LTR |
| 12436164 | 12438073 | - | 7.4 | 1165 | 4601 | -3352 | HERVK11 | ERVK | LTR |
| 12438074 | 12438863 | - | 16.7 | 1 | 851 | -7102 | HERVK11 | ERVK | LTR |
| 12438864 | 12439922 | - | 4.7 | 1 | 1071 | 0 | MER11C | ERVK | LTR |
| 12439924 | 12439982 | + | 21.4 | 3364 | 3419 | 0 | L2 | L2 | LINE |

  

|  |  |  |  |  |  |  |  |  |  |
| --- | --- | --- | --- | --- | --- | --- | --- | --- | --- |
| chr8:12455911-12461444 | | | 5532 bp | | |  |  |  |  |
| Genome Start | Genome End | Strand | Div | Repeat Start | Repeat End | Repeat Left | Name | Family | Class |
| 12455911 | 12456174 | + | 18.6 | 2883 | 3139 | -4070 | L1MA7 | L1 | LINE |
| 12456175 | 12456532 | - | 13.3 | 1 | 364 | 0 | THE1B | MaLR | LTR |
| 12456533 | 12458122 | - | 29.8 | 2 | 1735 | 0 | MLT-int | MaLR | LTR |
| 12458125 | 12458478 | - | 14.1 | 1 | 364 | 0 | THE1B | MaLR | LTR |
| 12458479 | 12460641 | + | 16.7 | 3140 | 5333 | -1872 | L1MA7 | L1 | LINE |
| 12460642 | 12460945 | + | 12.8 | 1 | 309 | -3 | AluSx | Alu | SINE |
| 12460946 | 12461444 | + | 16.7 | 5334 | 5809 | -1396 | L1MA7 | L1 | LINE |

  

|  |  |  |  |  |  |  |  |  |  |
| --- | --- | --- | --- | --- | --- | --- | --- | --- | --- |
| chr8:12513100-12522113 | | | 9029 bp | | |  |  |  |  |
| Genome Start | Genome End | Strand | Div | Repeat Start | Repeat End | Repeat Left | Name | Family | Class |
| 12513100 | 12513472 | + | 20.1 | 1034 | 1436 | -5413 | L1MA4A | L1 | LINE |
| 12513473 | 12513770 | + | 12.8 | 1 | 299 | -11 | AluSg | Alu | SINE |
| 12513771 | 12514836 | + | 20.1 | 1437 | 2522 | -4327 | L1MA4A | L1 | LINE |
| 12514837 | 12514987 | + | 19.9 | 1 | 153 | -13 | FRAM | Alu | SINE |
| 12514988 | 12515253 | + | 20.1 | 2523 | 2779 | -4070 | L1MA4A | L1 | LINE |
| 12515254 | 12515611 | - | 13.8 | 1 | 364 | 0 | THE1B | MaLR | LTR |
| 12515612 | 12517181 | - | 31.1 | 2 | 1735 | 0 | MLT-int | MaLR | LTR |
| 12517184 | 12517533 | - | 15.0 | 1 | 364 | 0 | THE1B | MaLR | LTR |
| 12517534 | 12519719 | + | 17.2 | 2780 | 4973 | -1872 | L1MA4A | L1 | LINE |
| 12519720 | 12520022 | + | 11.6 | 1 | 306 | -6 | AluSx | Alu | SINE |
| 12520023 | 12520518 | + | 17.1 | 4974 | 5436 | -1409 | L1MA4A | L1 | LINE |
| 12520519 | 12520826 | + | 8.4 | 1 | 297 | -14 | AluY | Alu | SINE |
| 12520827 | 12521289 | + | 17.1 | 5437 | 5905 | -940 | L1MA4A | L1 | LINE |
| 12521290 | 12521535 | + | 11.4 | 4958 | 5204 | -942 | L1PREC2 | L1 | LINE |
| 12521519 | 12522113 | + | 15.0 | 6029 | 6629 | -376 | L1MA4A | L1 | LINE |

  

|  |  |  |  |  |  |  |  |  |  |
| --- | --- | --- | --- | --- | --- | --- | --- | --- | --- |
| chr8:16934579-16941209 | | | 6627 bp | | |  |  |  |  |
| Genome Start | Genome End | Strand | Div | Repeat Start | Repeat End | Repeat Left | Name | Family | Class |
| 16934579 | 16935322 | + | 14.1 | 1 | 755 | -1663 | Tigger1 | MER2\_type | DNA |
| 16935323 | 16935615 | - | 9.3 | 1 | 292 | -19 | AluY | Alu | SINE |
| 16935616 | 16936790 | + | 14.1 | 756 | 1939 | -479 | Tigger1 | MER2\_type | DNA |
| 16936791 | 16937144 | + | 11.9 | 1 | 360 | -4 | THE1B | MaLR | LTR |
| 16937149 | 16937872 | + | 11.4 | 1 | 685 | -895 | THE1B-int | MaLR | LTR |
| 16937873 | 16939262 | - | 7.3 | 1 | 1578 | 0 | LTR12C | ERV1 | LTR |
| 16939263 | 16940070 | + | 11.4 | 686 | 1580 | 0 | THE1B-int | MaLR | LTR |
| 16940071 | 16940430 | + | 10.6 | 1 | 364 | 0 | THE1B | MaLR | LTR |
| 16940431 | 16940574 | + | 11.9 | 1935 | 2075 | -343 | Tigger1 | MER2\_type | DNA |
| 16940575 | 16940855 | + | 7.8 | 24 | 304 | -7 | AluY | Alu | SINE |
| 16940856 | 16941209 | + | 11.9 | 2076 | 2418 | 0 | Tigger1 | MER2\_type | DNA |

  

|  |  |  |  |  |  |  |  |  |  |
| --- | --- | --- | --- | --- | --- | --- | --- | --- | --- |
| chr8:17483113-17486415 | | | 3289 bp | | |  |  |  |  |
| Genome Start | Genome End | Strand | Div | Repeat Start | Repeat End | Repeat Left | Name | Family | Class |
| 17483113 | 17483240 | - | 29.1 | 2801 | 2929 | -490 | L2 | L2 | LINE |
| 17483252 | 17483655 | + | 16.3 | 16 | 432 | 0 | MSTB1 | MaLR | LTR |
| 17483656 | 17484084 | + | 22.1 | 1 | 421 | -1230 | MSTB1-int | MaLR | LTR |
| 17484085 | 17484375 | + | 11.0 | 1 | 297 | -12 | AluSc | Alu | SINE |
| 17484376 | 17485180 | + | 22.1 | 422 | 1260 | -391 | MSTB1-int | MaLR | LTR |
| 17485178 | 17485346 | + | 15.5 | 1470 | 1646 | -5 | MSTB1-int | MaLR | LTR |
| 17485352 | 17485766 | + | 17.3 | 1 | 419 | -13 | MSTB1 | MaLR | LTR |
| 17485768 | 17486415 | - | 29.0 | 2077 | 2773 | -646 | L2 | L2 | LINE |

  

|  |  |  |  |  |  |  |  |  |  |
| --- | --- | --- | --- | --- | --- | --- | --- | --- | --- |
| chr8:18004180-18007157 | | | 2976 bp | | |  |  |  |  |
| Genome Start | Genome End | Strand | Div | Repeat Start | Repeat End | Repeat Left | Name | Family | Class |
| 18004180 | 18004286 | - | 24.6 | 5918 | 6027 | -162 | L1MB1 | L1 | LINE |
| 18004287 | 18004683 | + | 15.3 | 1 | 428 | 0 | MSTA | MaLR | LTR |
| 18004685 | 18006329 | + | 16.9 | 2 | 1651 | 0 | MSTA-int | MaLR | LTR |
| 18006331 | 18006732 | + | 15.6 | 1 | 428 | 0 | MSTA | MaLR | LTR |
| 18006733 | 18007157 | - | 17.7 | 5484 | 5921 | -391 | L1MB1 | L1 | LINE |

  

|  |  |  |  |  |  |  |  |  |  |
| --- | --- | --- | --- | --- | --- | --- | --- | --- | --- |
| chr8:18504961-18508333 | | | 3198 bp | | |  |  |  |  |
| Genome Start | Genome End | Strand | Div | Repeat Start | Repeat End | Repeat Left | Name | Family | Class |
| 18504961 | 18505384 | - | 26.5 | 1449 | 1945 | -4204 | L1MEe | L1 | LINE |
| 18505390 | 18505804 | + | 10.8 | 1 | 428 | 0 | MSTA | MaLR | LTR |
| 18505805 | 18507430 | + | 17.1 | 1 | 1651 | 0 | MSTA-int | MaLR | LTR |
| 18507431 | 18507831 | + | 12.0 | 1 | 428 | 0 | MSTA | MaLR | LTR |
| 18507919 | 18508098 | - | 29.5 | 1020 | 1202 | -4917 | L1MEe | L1 | LINE |
| 18508182 | 18508333 | - | 31.3 | 7 | 159 | -5960 | L1MEe | L1 | LINE |

  

|  |  |  |  |  |  |  |  |  |  |
| --- | --- | --- | --- | --- | --- | --- | --- | --- | --- |
| chr8:20053451-20061614 | | | 8046 bp | | |  |  |  |  |
| Genome Start | Genome End | Strand | Div | Repeat Start | Repeat End | Repeat Left | Name | Family | Class |
| 20053451 | 20053582 | + | 25.5 | 1 | 128 | -140 | MIRb | MIR | SINE |
| 20053627 | 20054609 | - | 29.5 | 2161 | 3385 | -34 | L2 | L2 | LINE |
| 20054610 | 20055186 | - | 31.4 | 1099 | 1690 | -1729 | L2 | L2 | LINE |
| 20055187 | 20055475 | + | 7.6 | 1 | 299 | -12 | AluY | Alu | SINE |
| 20055476 | 20055656 | - | 31.4 | 914 | 1098 | -2321 | L2 | L2 | LINE |
| 20055657 | 20056000 | + | 16.9 | 1 | 426 | 0 | MSTB | MaLR | LTR |
| 20056001 | 20056498 | - | 31.4 | 363 | 913 | -2506 | L2 | L2 | LINE |
| 20056530 | 20057009 | + | 11.7 | 276 | 780 | 0 | LTR17 | ERV1 | LTR |
| 20057032 | 20059041 | + | 20.1 | 22 | 2004 | -6622 | HERV17 | ERV1 | LTR |
| 20059036 | 20060688 | + | 6.2 | 2574 | 4230 | -4396 | HERV17 | ERV1 | LTR |
| 20060670 | 20060818 | + | 11.5 | 7404 | 7552 | -1074 | HERV17 | ERV1 | LTR |
| 20060805 | 20061095 | + | 8.6 | 8335 | 8626 | 0 | HERV17 | ERV1 | LTR |
| 20061100 | 20061424 | + | 10.9 | 5 | 325 | -455 | LTR17 | ERV1 | LTR |
| 20061481 | 20061614 | + | 28.5 | 139 | 268 | 0 | MIRb | MIR | SINE |

  

|  |  |  |  |  |  |  |  |  |  |
| --- | --- | --- | --- | --- | --- | --- | --- | --- | --- |
| chr8:21549025-21554802 | | | 5353 bp | | |  |  |  |  |
| Genome Start | Genome End | Strand | Div | Repeat Start | Repeat End | Repeat Left | Name | Family | Class |
| 21549025 | 21549570 | + | 25.6 | 1931 | 2557 | -862 | L2 | L2 | LINE |
| 21549571 | 21550130 | + | 24.2 | 5022 | 5585 | -577 | L1ME3A | L1 | LINE |
| 21550131 | 21550327 | + | 8.1 | 104 | 302 | -10 | AluSg/x | Alu | SINE |
| 21550328 | 21550650 | + | 26.1 | 5586 | 5938 | -224 | L1ME3A | L1 | LINE |
| 21550651 | 21550818 | + | 16.7 | 1 | 164 | -12 | FRAM | Alu | SINE |
| 21550820 | 21550976 | + | 25.0 | 5930 | 6104 | -66 | L1ME3A | L1 | LINE |
| 21550983 | 21551083 | - | 23.4 | 337 | 446 | -11 | LTR16A1 | ERVL | LTR |
| 21551084 | 21551475 | - | 17.9 | 1 | 426 | 0 | MSTB | MaLR | LTR |
| 21551480 | 21553115 | - | 27.1 | 2 | 1731 | -4 | MLT-int | MaLR | LTR |
| 21553118 | 21553528 | - | 17.9 | 1 | 426 | 0 | MSTB | MaLR | LTR |
| 21553529 | 21553558 | - | 23.4 | 310 | 336 | -121 | LTR16A1 | ERVL | LTR |
| 21553628 | 21554340 | + | 27.8 | 2553 | 3297 | -122 | L2 | L2 | LINE |
| 21554684 | 21554802 | + | 27.3 | 3297 | 3417 | -2 | L2 | L2 | LINE |

  

|  |  |  |  |  |  |  |  |  |  |
| --- | --- | --- | --- | --- | --- | --- | --- | --- | --- |
| chr8:29614364-29620709 | | | 6306 bp | | |  |  |  |  |
| Genome Start | Genome End | Strand | Div | Repeat Start | Repeat End | Repeat Left | Name | Family | Class |
| 29614364 | 29614695 | + | 29.4 | 48 | 369 | -2138 | HAL1 | L1 | LINE |
| 29614696 | 29615081 | + | 13.6 | 1 | 428 | 0 | MSTA | MaLR | LTR |
| 29615084 | 29616707 | + | 29.5 | 2 | 1735 | 0 | MLT-int | MaLR | LTR |
| 29616708 | 29617097 | + | 14.4 | 1 | 426 | -2 | MSTA | MaLR | LTR |
| 29617098 | 29617335 | + | 29.4 | 370 | 647 | -1860 | HAL1 | L1 | LINE |
| 29617336 | 29617710 | - | 14.9 | 1 | 428 | 0 | MSTA | MaLR | LTR |
| 29617711 | 29617789 | + | 29.4 | 648 | 724 | -1783 | HAL1 | L1 | LINE |
| 29617791 | 29618006 | - | 13.4 | 142 | 365 | 0 | MLT1A0 | MaLR | LTR |
| 29618020 | 29618690 | - | 11.0 | 5447 | 6149 | -2 | L1PB2 | L1 | LINE |
| 29618691 | 29618996 | + | 8.2 | 1 | 303 | -8 | AluY | Alu | SINE |
| 29618997 | 29619019 | - | 11.0 | 5441 | 5446 | -705 | L1PB2 | L1 | LINE |
| 29619020 | 29619317 | - | 14.2 | 1 | 301 | -11 | AluSx | Alu | SINE |
| 29619318 | 29619537 | - | 11.2 | 5222 | 5440 | -711 | L1PB2 | L1 | LINE |
| 29619538 | 29619721 | - | 17.5 | 1 | 204 | -186 | MLT1A0 | MaLR | LTR |
| 29619729 | 29619870 | + | 28.6 | 729 | 887 | -1620 | HAL1 | L1 | LINE |
| 29619871 | 29620141 | + | 11.5 | 1382 | 1651 | 0 | MST-int | MaLR | LTR |
| 29620159 | 29620709 | + | 27.1 | 910 | 2024 | -483 | HAL1 | L1 | LINE |

  

|  |  |  |  |  |  |  |  |  |  |
| --- | --- | --- | --- | --- | --- | --- | --- | --- | --- |
| chr8:34571611-34574427 | | | 2622 bp | | |  |  |  |  |
| Genome Start | Genome End | Strand | Div | Repeat Start | Repeat End | Repeat Left | Name | Family | Class |
| 34571611 | 34571716 | + | 35.9 | 3058 | 3165 | -254 | L2 | L2 | LINE |
| 34571717 | 34572129 | - | 19.5 | 1 | 426 | 0 | MSTB | MaLR | LTR |
| 34572130 | 34573739 | - | 19.1 | 37 | 1651 | 0 | MSTB-int | MaLR | LTR |
| 34573798 | 34574221 | - | 18.7 | 1 | 426 | 0 | MSTB | MaLR | LTR |
| 34574359 | 34574427 | + | 25.8 | 3309 | 3377 | -1 | L2 | L2 | LINE |

  

|  |  |  |  |  |  |  |  |  |  |
| --- | --- | --- | --- | --- | --- | --- | --- | --- | --- |
| chr8:34686291-34696160 | | | 8976 bp | | |  |  |  |  |
| Genome Start | Genome End | Strand | Div | Repeat Start | Repeat End | Repeat Left | Name | Family | Class |
| 34686291 | 34686677 | - | 34.2 | 5262 | 5672 | -568 | L1M5 | L1 | LINE |
| 34687106 | 34687632 | - | 33.2 | 4301 | 4846 | -1300 | L1M5 | L1 | LINE |
| 34687649 | 34687950 | - | 9.3 | 1 | 302 | -7 | AluSc | Alu | SINE |
| 34687999 | 34688338 | - | 30.8 | 3896 | 4198 | -1948 | L1M5 | L1 | LINE |
| 34688585 | 34689034 | - | 21.7 | 3288 | 3730 | -2416 | L1MC | L1 | LINE |
| 34689035 | 34689344 | - | 15.2 | 1 | 309 | -3 | AluSx | Alu | SINE |
| 34689345 | 34689858 | - | 21.7 | 2770 | 3287 | -2859 | L1MC | L1 | LINE |
| 34689859 | 34690354 | + | 22.6 | 1 | 518 | -2 | MLT2B1 | ERVL | LTR |
| 34690463 | 34690681 | + | 15.5 | 37 | 312 | 0 | AluSx | Alu | SINE |
| 34690682 | 34691056 | - | 16.8 | 5 | 393 | -35 | MSTA | MaLR | LTR |
| 34691060 | 34692660 | - | 28.7 | 2 | 1735 | 0 | MLT1C-int | MaLR | LTR |
| 34692663 | 34693087 | - | 16.7 | 2 | 428 | 0 | MSTA | MaLR | LTR |
| 34693089 | 34693539 | + | 19.5 | 66 | 511 | -5203 | ERVL-B4 | ERVL | LTR |
| 34693573 | 34693606 | + | 8.8 | 523 | 556 | -4 | MLT2D | ERVL | LTR |
| 34693615 | 34693931 | - | 27.0 | 758 | 1095 | -4950 | L1MCb | L1 | LINE |
| 34693923 | 34695267 | - | 14.4 | 4786 | 6165 | 0 | L1PA15 | L1 | LINE |
| 34695262 | 34696005 | - | 26.6 | 3 | 779 | -5266 | L1MCb | L1 | LINE |
| 34696022 | 34696160 | - | 29.5 | 2464 | 2608 | -3538 | L1M5 | L1 | LINE |

  

|  |  |  |  |  |  |  |  |  |  |
| --- | --- | --- | --- | --- | --- | --- | --- | --- | --- |
| chr8:36154358-36161554 | | | 6923 bp | | |  |  |  |  |
| Genome Start | Genome End | Strand | Div | Repeat Start | Repeat End | Repeat Left | Name | Family | Class |
| 36154358 | 36155493 | - | 25.8 | 3204 | 4385 | -1834 | L1MEc | L1 | LINE |
| 36155500 | 36155793 | - | 18.9 | 1 | 291 | -21 | AluJo | Alu | SINE |
| 36155796 | 36155943 | - | 24.5 | 2057 | 2203 | -4197 | L1MEc | L1 | LINE |
| 36155985 | 36156057 | - | 11.0 | 1907 | 1980 | -4420 | L1MEc | L1 | LINE |
| 36156163 | 36156931 | - | 27.9 | 883 | 1684 | -4722 | L1MEc | L1 | LINE |
| 36157004 | 36157354 | - | 13.2 | 1 | 364 | 0 | THE1B | MaLR | LTR |
| 36157355 | 36157471 | - | 11.0 | 1472 | 1580 | 0 | THE1B-int | MaLR | LTR |
| 36157472 | 36157771 | + | 18.9 | 1 | 299 | -13 | AluSx | Alu | SINE |
| 36157772 | 36159224 | - | 11.0 | 1 | 1471 | -109 | THE1B-int | MaLR | LTR |
| 36159225 | 36159588 | - | 13.5 | 1 | 364 | 0 | THE1B | MaLR | LTR |
| 36159606 | 36159916 | - | 20.1 | 1 | 309 | -3 | AluJb | Alu | SINE |
| 36159948 | 36160016 | - | 19.2 | 5753 | 5821 | -470 | L1M2 | L1 | LINE |
| 36160017 | 36160406 | - | 12.8 | 5856 | 6287 | -4 | L1MA8 | L1 | LINE |
| 36160407 | 36160443 | - | 5.4 | 5716 | 5752 | -539 | L1MA8 | L1 | LINE |
| 36160444 | 36160760 | - | 30.7 | 496 | 838 | -5562 | L1MEc | L1 | LINE |
| 36160761 | 36161076 | - | 13.1 | 1 | 312 | 0 | AluSx | Alu | SINE |
| 36161077 | 36161554 | - | 30.7 | 35 | 495 | -5905 | L1MEc | L1 | LINE |

  

|  |  |  |  |  |  |  |  |  |  |
| --- | --- | --- | --- | --- | --- | --- | --- | --- | --- |
| chr8:39095420-39100042 | | | 4560 bp | | |  |  |  |  |
| Genome Start | Genome End | Strand | Div | Repeat Start | Repeat End | Repeat Left | Name | Family | Class |
| 39095420 | 39096439 | - | 22.6 | 5089 | 6175 | -9 | L1MB7 | L1 | LINE |
| 39096443 | 39096863 | + | 18.3 | 1 | 387 | -3 | MLT1B | MaLR | LTR |
| 39096866 | 39097155 | + | 18.3 | 1 | 291 | -1080 | MLT1B-int | MaLR | LTR |
| 39097156 | 39097456 | + | 10.0 | 1 | 301 | -11 | AluSx | Alu | SINE |
| 39097457 | 39097897 | + | 18.3 | 292 | 731 | -640 | MLT1B-int | MaLR | LTR |
| 39097892 | 39098034 | + | 32.7 | 858 | 1003 | -486 | MLT1F-int | MaLR | LTR |
| 39098035 | 39098288 | + | 5.9 | 5901 | 6154 | 0 | L1PA7 | L1 | LINE |
| 39098289 | 39098309 | + | 32.7 | 1004 | 1029 | -460 | MLT1F-int | MaLR | LTR |
| 39098291 | 39098361 | + | 18.3 | 697 | 767 | -968 | MLT1B-int | MaLR | LTR |
| 39098435 | 39098925 | + | 22.8 | 889 | 1666 | -1 | MLT1B-int | MaLR | LTR |
| 39098927 | 39099248 | + | 21.0 | 1 | 325 | -65 | MLT1B | MaLR | LTR |
| 39099249 | 39099544 | - | 14.9 | 1 | 304 | -8 | AluSx | Alu | SINE |
| 39099545 | 39099611 | + | 21.0 | 326 | 387 | -3 | MLT1B | MaLR | LTR |
| 39099621 | 39100042 | - | 21.8 | 4634 | 5084 | -1062 | L1MB7 | L1 | LINE |

  

|  |  |  |  |  |  |  |  |  |  |
| --- | --- | --- | --- | --- | --- | --- | --- | --- | --- |
| chr8:39213513-39222135 | | | 8611 bp | | |  |  |  |  |
| Genome Start | Genome End | Strand | Div | Repeat Start | Repeat End | Repeat Left | Name | Family | Class |
| 39213513 | 39217179 | + | 11.5 | 1 | 3782 | -3080 | L1PB1 | L1 | LINE |
| 39217187 | 39217512 | - | 11.8 | 1 | 364 | 0 | THE1B | MaLR | LTR |
| 39217518 | 39219054 | - | 10.1 | 1 | 1563 | -17 | THE1B-int | MaLR | LTR |
| 39219055 | 39219390 | - | 14.8 | 1 | 364 | 0 | THE1B | MaLR | LTR |
| 39219391 | 39221341 | + | 12.6 | 3779 | 5713 | -1149 | L1PB1 | L1 | LINE |
| 39221342 | 39221534 | + | 3.6 | 5958 | 6150 | -5 | L1PA4 | L1 | LINE |
| 39221535 | 39221750 | + | 10.4 | 5714 | 5926 | -936 | L1PB1 | L1 | LINE |
| 39221751 | 39222135 | + | 9.6 | 6478 | 6865 | -2 | L1PB1 | L1 | LINE |

  

|  |  |  |  |  |  |  |  |  |  |
| --- | --- | --- | --- | --- | --- | --- | --- | --- | --- |
| chr8:39397697-39401625 | | | 3927 bp | | |  |  |  |  |
| Genome Start | Genome End | Strand | Div | Repeat Start | Repeat End | Repeat Left | Name | Family | Class |
| 39397697 | 39397796 | + | 18.6 | 5366 | 5469 | -831 | L1MA6 | L1 | LINE |
| 39397797 | 39398005 | - | 17.7 | 222 | 459 | -2 | LOR1b | ERV1 | LTR |
| 39398006 | 39398328 | + | 14.9 | 1 | 326 | 0 | AluSx | Alu | SINE |
| 39398329 | 39398562 | - | 17.7 | 1 | 221 | -240 | LOR1b | ERV1 | LTR |
| 39398563 | 39399083 | + | 18.6 | 5470 | 6004 | -296 | L1MA6 | L1 | LINE |
| 39399084 | 39399437 | - | 7.9 | 1 | 355 | 0 | THE1A | MaLR | LTR |
| 39399438 | 39400987 | - | 28.7 | 2 | 1735 | 0 | MLT-int | MaLR | LTR |
| 39400990 | 39401343 | - | 5.7 | 1 | 355 | 0 | THE1A | MaLR | LTR |
| 39401344 | 39401625 | + | 18.6 | 6005 | 6290 | -10 | L1MA6 | L1 | LINE |

  

|  |  |  |  |  |  |  |  |  |  |
| --- | --- | --- | --- | --- | --- | --- | --- | --- | --- |
| chr8:39607217-39612091 | | | 4873 bp | | |  |  |  |  |
| Genome Start | Genome End | Strand | Div | Repeat Start | Repeat End | Repeat Left | Name | Family | Class |
| 39607217 | 39607345 | - | 29.5 | 5769 | 5916 | -246 | L1ME3 | L1 | LINE |
| 39607348 | 39609568 | - | 28.0 | 3414 | 5723 | -439 | L1ME3 | L1 | LINE |
| 39609569 | 39609903 | + | 19.8 | 1 | 408 | 0 | MLT1A1 | MaLR | LTR |
| 39609904 | 39611601 | + | 24.3 | 1 | 1735 | 0 | MLT1A1-int | MaLR | LTR |
| 39611602 | 39611933 | + | 19.7 | 1 | 408 | 0 | MLT1A1 | MaLR | LTR |
| 39611934 | 39612091 | - | 28.0 | 3260 | 3413 | -2733 | L1ME3 | L1 | LINE |

  

|  |  |  |  |  |  |  |  |  |  |
| --- | --- | --- | --- | --- | --- | --- | --- | --- | --- |
| chr8:39846398-39866005 | | | 19494 bp | | |  |  |  |  |
| Genome Start | Genome End | Strand | Div | Repeat Start | Repeat End | Repeat Left | Name | Family | Class |
| 39846398 | 39846835 | + | 33.5 | 102 | 573 | -5827 | L1MEc | L1 | LINE |
| 39846844 | 39847060 | - | 7.8 | 471 | 688 | 0 | LTR12\_ | ERV1 | LTR |
| 39847061 | 39847571 | + | 12.4 | 1 | 511 | 0 | MER9 | ERVK | LTR |
| 39847572 | 39849429 | + | 9.4 | 1 | 1845 | -4071 | HERVK9 | ERVK | LTR |
| 39849430 | 39849738 | + | 12.7 | 1 | 306 | -5 | AluY | Alu | SINE |
| 39849739 | 39850370 | + | 9.4 | 1846 | 2469 | -3447 | HERVK9 | ERVK | LTR |
| 39850370 | 39850499 | + | 4.6 | 2654 | 2784 | -3132 | HERVK9 | ERVK | LTR |
| 39850501 | 39850729 | + | 7.4 | 3711 | 3941 | -1975 | HERVK9 | ERVK | LTR |
| 39850729 | 39851781 | + | 9.8 | 4582 | 5634 | -282 | HERVK9 | ERVK | LTR |
| 39851796 | 39852158 | + | 11.8 | 5635 | 7337 | 0 | HERVK9 | ERVK | LTR |
| 39852159 | 39852682 | + | 10.0 | 1 | 511 | 0 | MER9 | ERVK | LTR |
| 39852683 | 39853304 | - | 9.3 | 1 | 747 | -79 | LTR12 | ERV1 | LTR |
| 39853305 | 39861635 | - | 5.7 | 1 | 8436 | 0 | HERV9 | ERV1 | LTR |
| 39861636 | 39862417 | - | 7.9 | 1 | 826 | 0 | LTR12 | ERV1 | LTR |
| 39862441 | 39862805 | + | 28.3 | 590 | 1006 | -5394 | L1MEc | L1 | LINE |
| 39862806 | 39863101 | + | 10.8 | 1 | 297 | -16 | AluSp | Alu | SINE |
| 39863151 | 39863360 | + | 28.3 | 1007 | 1211 | -5189 | L1MEc | L1 | LINE |
| 39863361 | 39863723 | - | 18.7 | 1 | 396 | 0 | MSTD | MaLR | LTR |
| 39863724 | 39864152 | + | 28.5 | 1212 | 1642 | -4597 | L1MEc | L1 | LINE |
| 39864171 | 39864331 | - | 28.6 | 7 | 180 | -44 | MER58A | MER1\_type | DNA |
| 39864335 | 39864784 | + | 26.8 | 2242 | 2698 | -3521 | L1MEc | L1 | LINE |
| 39864785 | 39865086 | + | 12.9 | 1 | 305 | -7 | AluSx | Alu | SINE |
| 39865087 | 39866005 | + | 26.8 | 2699 | 3678 | -2541 | L1MEc | L1 | LINE |

  

|  |  |  |  |  |  |  |  |  |  |
| --- | --- | --- | --- | --- | --- | --- | --- | --- | --- |
| chr8:43373644-43378262 | | | 4515 bp | | |  |  |  |  |
| Genome Start | Genome End | Strand | Div | Repeat Start | Repeat End | Repeat Left | Name | Family | Class |
| 43373644 | 43374442 | - | 11.3 | 5996 | 6800 | -809 | L1P | L1 | LINE |
| 43374445 | 43374804 | - | 18.6 | 1 | 379 | -2 | THE1D | MaLR | LTR |
| 43374810 | 43376073 | - | 16.5 | 367 | 1575 | -5 | THE1D-int | MaLR | LTR |
| 43376074 | 43376357 | + | 17.6 | 5 | 292 | -20 | AluSx | Alu | SINE |
| 43376358 | 43376723 | - | 19.4 | 2 | 366 | -1214 | THE1D-int | MaLR | LTR |
| 43376725 | 43377082 | - | 19.8 | 1 | 381 | 0 | THE1D | MaLR | LTR |
| 43377083 | 43377589 | - | 13.8 | 5494 | 6000 | -1581 | L1P | L1 | LINE |
| 43377686 | 43377975 | - | 12.8 | 1 | 283 | -30 | AluSp | Alu | SINE |
| 43377976 | 43378262 | - | 13.8 | 5225 | 5493 | -2088 | L1P | L1 | LINE |

  

|  |  |  |  |  |  |  |  |  |  |
| --- | --- | --- | --- | --- | --- | --- | --- | --- | --- |
| chr8:48036128-48045435 | | | 9093 bp | | |  |  |  |  |
| Genome Start | Genome End | Strand | Div | Repeat Start | Repeat End | Repeat Left | Name | Family | Class |
| 48036128 | 48036377 | - | 18.6 | 8659 | 8912 | -7 | HUERS-P3 | ERV1 | LTR |
| 48036534 | 48036821 | + | 11.1 | 1 | 302 | -10 | AluSx | Alu | SINE |
| 48036849 | 48037046 | - | 16.9 | 2421 | 4184 | -2450 | HUERS-P3 | ERV1 | LTR |
| 48037047 | 48037267 | - | 13.0 | 88 | 296 | -13 | AluSg1 | Alu | SINE |
| 48037268 | 48037411 | + | 13.2 | 35 | 179 | -134 | AluSp | Alu | SINE |
| 48037411 | 48037510 | - | 12.0 | 2 | 101 | -208 | AluSg1 | Alu | SINE |
| 48037537 | 48038252 | - | 17.4 | 6270 | 6999 | -1920 | HUERS-P3 | ERV1 | LTR |
| 48038260 | 48038493 | + | 9.8 | 73 | 310 | -3 | AluSp | Alu | SINE |
| 48038498 | 48039712 | - | 12.6 | 4540 | 5817 | -3102 | HUERS-P3 | ERV1 | LTR |
| 48039707 | 48039871 | - | 12.6 | 3127 | 3284 | -5635 | HUERS-P3 | ERV1 | LTR |
| 48039872 | 48040164 | + | 11.4 | 1 | 292 | -19 | AluY | Alu | SINE |
| 48040165 | 48040959 | - | 12.6 | 2330 | 3126 | -5793 | HUERS-P3 | ERV1 | LTR |
| 48040960 | 48041308 | - | 11.3 | 3 | 355 | 0 | THE1A | MaLR | LTR |
| 48041309 | 48042861 | - | 30.2 | 2 | 1735 | 0 | MLT-int | MaLR | LTR |
| 48042864 | 48043213 | - | 12.9 | 1 | 355 | 0 | THE1A | MaLR | LTR |
| 48043214 | 48045053 | - | 17.6 | 97 | 2329 | -5388 | HUERS-P3 | ERV1 | LTR |
| 48045054 | 48045372 | + | 19.1 | 1 | 320 | 0 | AluSx | Alu | SINE |
| 48045373 | 48045435 | - | 19.8 | 47 | 96 | -8823 | HUERS-P3 | ERV1 | LTR |

  

|  |  |  |  |  |  |  |  |  |  |
| --- | --- | --- | --- | --- | --- | --- | --- | --- | --- |
| chr8:49822103-49826892 | | | 4427 bp | | |  |  |  |  |
| Genome Start | Genome End | Strand | Div | Repeat Start | Repeat End | Repeat Left | Name | Family | Class |
| 49822103 | 49822626 | - | 28.6 | 1097 | 1712 | -900 | Charlie7 | MER1\_type | DNA |
| 49822638 | 49823035 | + | 29.9 | 29 | 450 | 0 | MLT1J2 | MaLR | LTR |
| 49823324 | 49823490 | + | 14.5 | 1 | 182 | -372 | MLT2B3 | ERVL | LTR |
| 49823494 | 49823845 | + | 19.1 | 9 | 396 | 0 | MSTD | MaLR | LTR |
| 49823848 | 49824465 | + | 25.8 | 2 | 636 | -1099 | MSTD-int | MaLR | LTR |
| 49824466 | 49824644 | - | 11.2 | 132 | 306 | -3 | AluSc | Alu | SINE |
| 49824702 | 49824841 | - | 11.2 | 3 | 131 | -178 | AluSc | Alu | SINE |
| 49824842 | 49825809 | + | 25.8 | 637 | 1735 | 0 | MSTD-int | MaLR | LTR |
| 49825812 | 49826180 | + | 19.9 | 3 | 396 | 0 | MSTD | MaLR | LTR |
| 49826181 | 49826892 | - | 25.2 | 250 | 983 | -1629 | Charlie7 | MER1\_type | DNA |

  

|  |  |  |  |  |  |  |  |  |  |
| --- | --- | --- | --- | --- | --- | --- | --- | --- | --- |
| chr8:50461727-50464625 | | | 2898 bp | | |  |  |  |  |
| Genome Start | Genome End | Strand | Div | Repeat Start | Repeat End | Repeat Left | Name | Family | Class |
| 50461727 | 50461857 | + | 25.2 | 1 | 133 | -521 | MLT1E | MaLR | LTR |
| 50461858 | 50462231 | + | 13.7 | 1 | 375 | 0 | THE1C | MaLR | LTR |
| 50462232 | 50463833 | + | 11.5 | 1 | 1580 | 0 | THE1C-int | MaLR | LTR |
| 50463834 | 50464192 | + | 12.0 | 1 | 375 | 0 | THE1C | MaLR | LTR |
| 50464194 | 50464625 | + | 22.9 | 177 | 651 | -3 | MLT1E | MaLR | LTR |

  

|  |  |  |  |  |  |  |  |  |  |
| --- | --- | --- | --- | --- | --- | --- | --- | --- | --- |
| chr8:51399724-51406216 | | | 6363 bp | | |  |  |  |  |
| Genome Start | Genome End | Strand | Div | Repeat Start | Repeat End | Repeat Left | Name | Family | Class |
| 51399724 | 51400037 | - | 22.3 | 5152 | 5454 | -867 | L1MCa | L1 | LINE |
| 51400123 | 51400181 | + | 8.5 | 4781 | 4837 | -1309 | L1MD | L1 | LINE |
| 51400225 | 51403479 | - | 19.4 | 1535 | 4939 | -1382 | L1MCa | L1 | LINE |
| 51403480 | 51403860 | - | 15.1 | 1 | 381 | 0 | THE1D | MaLR | LTR |
| 51403861 | 51405172 | - | 14.6 | 192 | 1580 | 0 | THE1D-int | MaLR | LTR |
| 51405173 | 51405530 | + | 12.9 | 1 | 375 | 0 | THE1C | MaLR | LTR |
| 51405531 | 51405721 | - | 14.6 | 1 | 191 | -1389 | THE1D-int | MaLR | LTR |
| 51405722 | 51406084 | - | 13.6 | 1 | 381 | 0 | THE1D | MaLR | LTR |
| 51406087 | 51406216 | - | 20.5 | 1402 | 1537 | -4971 | L1MCa | L1 | LINE |

  

|  |  |  |  |  |  |  |  |  |  |
| --- | --- | --- | --- | --- | --- | --- | --- | --- | --- |
| chr8:51845325-51863129 | | | 17210 bp | | |  |  |  |  |
| Genome Start | Genome End | Strand | Div | Repeat Start | Repeat End | Repeat Left | Name | Family | Class |
| 51845325 | 51845728 | + | 28.2 | 14 | 387 | -6155 | L1MB7 | L1 | LINE |
| 51845729 | 51846165 | + | 20.3 | 1 | 430 | -2 | MSTB1 | MaLR | LTR |
| 51846166 | 51847769 | + | 30.1 | 2 | 1735 | 0 | MLT-int | MaLR | LTR |
| 51847770 | 51848218 | + | 21.2 | 1 | 432 | 0 | MSTB1 | MaLR | LTR |
| 51848219 | 51849978 | + | 27.2 | 388 | 2387 | -4259 | L1MB7 | L1 | LINE |
| 51850219 | 51850340 | + | 25.0 | 2615 | 2737 | -3907 | L1MB7 | L1 | LINE |
| 51850444 | 51851488 | - | 22.3 | 2239 | 3349 | -3188 | L1MDa | L1 | LINE |
| 51851657 | 51852331 | - | 21.3 | 1519 | 2224 | -4409 | L1MDa | L1 | LINE |
| 51852376 | 51852782 | - | 21.3 | 1100 | 1518 | -5115 | L1MDa | L1 | LINE |
| 51852818 | 51858957 | - | 5.0 | 7 | 6152 | -3 | L1PA3 | L1 | LINE |
| 51858963 | 51859286 | - | 29.6 | 802 | 1117 | -5516 | L1MDa | L1 | LINE |
| 51859293 | 51859420 | - | 23.2 | 7 | 132 | -6501 | L1MDa | L1 | LINE |
| 51859415 | 51863129 | + | 25.2 | 2742 | 6672 | -10 | L1MB7 | L1 | LINE |

  

|  |  |  |  |  |  |  |  |  |  |
| --- | --- | --- | --- | --- | --- | --- | --- | --- | --- |
| chr8:52363176-52376863 | | | 13668 bp | | |  |  |  |  |
| Genome Start | Genome End | Strand | Div | Repeat Start | Repeat End | Repeat Left | Name | Family | Class |
| 52363176 | 52363356 | - | 18.7 | 5993 | 6158 | -8 | L1PA16 | L1 | LINE |
| 52363357 | 52363734 | - | 10.8 | 1 | 375 | 0 | THE1C | MaLR | LTR |
| 52363735 | 52363867 | - | 7.5 | 1433 | 1580 | 0 | THE1C-int | MaLR | LTR |
| 52363868 | 52364193 | - | 11.7 | 161 | 436 | -231 | LTR12B | ERV1 | LTR |
| 52364194 | 52364477 | - | 10.9 | 14 | 297 | -14 | AluY | Alu | SINE |
| 52364478 | 52364644 | - | 11.7 | 1 | 160 | -507 | LTR12B | ERV1 | LTR |
| 52364645 | 52364780 | - | 8.1 | 8300 | 8436 | 0 | HERV9 | ERV1 | LTR |
| 52364781 | 52365759 | - | 10.2 | 2 | 1266 | 0 | MER11A | ERVK | LTR |
| 52365762 | 52372941 | - | 8.6 | 1028 | 8304 | -132 | HERV9 | ERV1 | LTR |
| 52372942 | 52373241 | - | 11.4 | 1 | 300 | -11 | AluY | Alu | SINE |
| 52373242 | 52374109 | - | 10.8 | 55 | 1027 | -7409 | HERV9 | ERV1 | LTR |
| 52374128 | 52374691 | - | 16.6 | 1 | 595 | -231 | LTR12 | ERV1 | LTR |
| 52374692 | 52376088 | - | 12.7 | 1 | 1436 | -144 | THE1C-int | MaLR | LTR |
| 52376089 | 52376449 | - | 13.0 | 1 | 375 | 0 | THE1C | MaLR | LTR |
| 52376450 | 52376863 | - | 12.7 | 5584 | 5997 | -169 | L1PA16 | L1 | LINE |

  

|  |  |  |  |  |  |  |  |  |  |
| --- | --- | --- | --- | --- | --- | --- | --- | --- | --- |
| chr8:53954179-53963365 | | | 8770 bp | | |  |  |  |  |
| Genome Start | Genome End | Strand | Div | Repeat Start | Repeat End | Repeat Left | Name | Family | Class |
| 53954179 | 53955277 | - | 18.7 | 5821 | 7033 | -5 | L1MB8 | L1 | LINE |
| 53955309 | 53955865 | - | 12.8 | 147 | 601 | -2 | LTR26 | ERV1 | LTR |
| 53955892 | 53956035 | - | 16.7 | 302 | 448 | -4895 | LTR57-int | ERVL | LTR |
| 53956076 | 53956111 | - | 11.1 | 471 | 506 | -6133 | MER101-int | ERV1 | LTR |
| 53956117 | 53956511 | - | 24.1 | 577 | 1021 | -5044 | MER101-int | ERV1 | LTR |
| 53956708 | 53956859 | - | 35.1 | 25 | 175 | -6088 | LTR26-int | ERV1 | LTR |
| 53956885 | 53957047 | - | 13.5 | 441 | 603 | 0 | LTR26 | ERV1 | LTR |
| 53957048 | 53957335 | - | 13.2 | 1 | 290 | -22 | AluSx | Alu | SINE |
| 53957336 | 53957700 | - | 13.5 | 102 | 440 | -163 | LTR26 | ERV1 | LTR |
| 53957702 | 53957801 | - | 13.7 | 353 | 427 | -1 | MSTA | MaLR | LTR |
| 53957802 | 53958082 | - | 14.9 | 1 | 282 | -30 | AluSx | Alu | SINE |
| 53958083 | 53958403 | - | 13.7 | 1 | 352 | -76 | MSTA | MaLR | LTR |
| 53958404 | 53959771 | - | 15.0 | 3 | 1580 | 0 | MSTA-int | MaLR | LTR |
| 53959774 | 53959958 | - | 14.8 | 224 | 428 | 0 | MSTA | MaLR | LTR |
| 53960040 | 53960229 | - | 14.8 | 1 | 223 | -205 | MSTA | MaLR | LTR |
| 53960230 | 53960335 | - | 11.3 | 1 | 106 | -497 | LTR26 | ERV1 | LTR |
| 53960336 | 53961451 | - | 19.6 | 4635 | 5757 | -1249 | L1MB8 | L1 | LINE |
| 53961462 | 53961613 | - | 19.7 | 2583 | 2732 | -4602 | L1MB8 | L1 | LINE |
| 53961614 | 53962419 | + | 18.5 | 5 | 862 | -1 | MER21B | ERV1 | LTR |
| 53962420 | 53962981 | - | 19.0 | 1939 | 2582 | -4752 | L1MB8 | L1 | LINE |
| 53962982 | 53963304 | + | 18.4 | 1 | 408 | 0 | MLT1A1 | MaLR | LTR |
| 53963305 | 53963365 | - | 19.0 | 1874 | 1938 | -5396 | L1MB8 | L1 | LINE |

  

|  |  |  |  |  |  |  |  |  |  |
| --- | --- | --- | --- | --- | --- | --- | --- | --- | --- |
| chr8:54054456-54060396 | | | 5946 bp | | |  |  |  |  |
| Genome Start | Genome End | Strand | Div | Repeat Start | Repeat End | Repeat Left | Name | Family | Class |
| 54054456 | 54054975 | + | 29.6 | 316 | 867 | -2552 | L2 | L2 | LINE |
| 54054976 | 54055464 | - | 11.8 | 1 | 511 | 0 | MER9 | ERVK | LTR |
| 54055465 | 54055558 | - | 11.7 | 5823 | 5916 | 0 | HERVK9 | ERVK | LTR |
| 54055559 | 54057070 | - | 7.2 | 3601 | 5124 | -792 | HERVK9 | ERVK | LTR |
| 54057068 | 54058117 | - | 8.3 | 1424 | 2483 | -3433 | HERVK9 | ERVK | LTR |
| 54058116 | 54058690 | - | 11.5 | 1 | 568 | -5348 | HERVK9 | ERVK | LTR |
| 54058691 | 54059181 | - | 10.8 | 1 | 511 | 0 | MER9 | ERVK | LTR |
| 54059182 | 54060016 | + | 29.8 | 862 | 1837 | -1582 | L2 | L2 | LINE |
| 54060017 | 54060182 | - | 18.8 | 3876 | 4047 | -2099 | L1MC | L1 | LINE |
| 54060183 | 54060396 | + | 30.2 | 2497 | 2710 | -709 | L2 | L2 | LINE |

  

|  |  |  |  |  |  |  |  |  |  |
| --- | --- | --- | --- | --- | --- | --- | --- | --- | --- |
| chr8:55234206-55239922 | | | 5705 bp | | |  |  |  |  |
| Genome Start | Genome End | Strand | Div | Repeat Start | Repeat End | Repeat Left | Name | Family | Class |
| 55234206 | 55234411 | - | 18.9 | 5972 | 6174 | 0 | L1MB5 | L1 | LINE |
| 55234412 | 55234727 | - | 10.7 | 1 | 309 | -4 | AluSq | Alu | SINE |
| 55234728 | 55235068 | - | 18.9 | 5626 | 5971 | -203 | L1MB5 | L1 | LINE |
| 55235073 | 55235428 | + | 9.0 | 1 | 355 | 0 | THE1A | MaLR | LTR |
| 55235429 | 55236998 | + | 8.3 | 1 | 1580 | 0 | THE1A-int | MaLR | LTR |
| 55236999 | 55237354 | + | 6.5 | 1 | 355 | 0 | THE1A | MaLR | LTR |
| 55237356 | 55238417 | - | 18.2 | 4534 | 5617 | -557 | L1MB5 | L1 | LINE |
| 55238418 | 55238716 | + | 16.5 | 1 | 292 | -20 | AluJb | Alu | SINE |
| 55238717 | 55238747 | - | 18.7 | 4507 | 4533 | -1613 | L1MB5 | L1 | LINE |
| 55238755 | 55239300 | - | 12.8 | 3344 | 3893 | -2253 | L1MB5 | L1 | LINE |
| 55239301 | 55239529 | - | 14.5 | 83 | 310 | -2 | AluSg/x | Alu | SINE |
| 55239530 | 55239546 | - | 12.8 | 3344 | 3343 | -2803 | L1MB5 | L1 | LINE |
| 55239547 | 55239857 | - | 17.8 | 1 | 309 | -3 | AluJb | Alu | SINE |
| 55239858 | 55239922 | - | 12.8 | 3274 | 3343 | -2803 | L1MB5 | L1 | LINE |

  

|  |  |  |  |  |  |  |  |  |  |
| --- | --- | --- | --- | --- | --- | --- | --- | --- | --- |
| chr8:56743873-56746463 | | | 2588 bp | | |  |  |  |  |
| Genome Start | Genome End | Strand | Div | Repeat Start | Repeat End | Repeat Left | Name | Family | Class |
| 56743873 | 56744114 | - | 32.1 | 243 | 514 | -101 | MLT1L | MaLR | LTR |
| 56744115 | 56744442 | + | 15.3 | 1 | 364 | 0 | THE1B | MaLR | LTR |
| 56744446 | 56745994 | + | 28.4 | 3 | 1735 | 0 | MSTD-int | MaLR | LTR |
| 56745995 | 56746335 | + | 13.7 | 1 | 364 | 0 | THE1B | MaLR | LTR |
| 56746336 | 56746463 | - | 32.1 | 114 | 242 | -373 | MLT1L | MaLR | LTR |

  

|  |  |  |  |  |  |  |  |  |  |
| --- | --- | --- | --- | --- | --- | --- | --- | --- | --- |
| chr8:57537809-57542665 | | | 4702 bp | | |  |  |  |  |
| Genome Start | Genome End | Strand | Div | Repeat Start | Repeat End | Repeat Left | Name | Family | Class |
| 57537809 | 57538385 | + | 35.4 | 1715 | 2300 | -1119 | L2 | L2 | LINE |
| 57538397 | 57538811 | + | 21.6 | 1 | 426 | 0 | MSTB | MaLR | LTR |
| 57538812 | 57539700 | + | 20.2 | 1 | 918 | -733 | MSTB-int | MaLR | LTR |
| 57539702 | 57540185 | - | 15.5 | 1 | 480 | -303 | LTR39 | ERV1 | LTR |
| 57540187 | 57540365 | + | 13.0 | 1474 | 1651 | 0 | MSTB-int | MaLR | LTR |
| 57540366 | 57540795 | + | 18.4 | 1 | 426 | 0 | MSTB | MaLR | LTR |
| 57540802 | 57540974 | + | 29.0 | 2319 | 2521 | -898 | L2 | L2 | LINE |
| 57540986 | 57541153 | - | 22.3 | 5318 | 5493 | -662 | L1P1 | L1 | LINE |
| 57541154 | 57541453 | + | 10.7 | 1 | 300 | -12 | AluSx | Alu | SINE |
| 57541454 | 57542474 | - | 26.8 | 4339 | 5319 | -827 | L1M | L1 | LINE |
| 57542600 | 57542665 | + | 32.3 | 3350 | 3414 | -5 | L2 | L2 | LINE |

  

|  |  |  |  |  |  |  |  |  |  |
| --- | --- | --- | --- | --- | --- | --- | --- | --- | --- |
| chr8:58433256-58436063 | | | 2803 bp | | |  |  |  |  |
| Genome Start | Genome End | Strand | Div | Repeat Start | Repeat End | Repeat Left | Name | Family | Class |
| 58433256 | 58433364 | + | 33.3 | 8 | 118 | -150 | MIRb | MIR | SINE |
| 58433365 | 58433711 | - | 15.8 | 71 | 428 | 0 | MSTA | MaLR | LTR |
| 58433713 | 58434094 | + | 15.0 | 1 | 428 | 0 | MSTA | MaLR | LTR |
| 58434097 | 58435553 | + | 16.0 | 3 | 1580 | 0 | MSTA-int | MaLR | LTR |
| 58435554 | 58435925 | + | 13.2 | 1 | 428 | 0 | MSTA | MaLR | LTR |
| 58435926 | 58435999 | - | 10.8 | 1 | 74 | -354 | MSTA | MaLR | LTR |
| 58436002 | 58436063 | + | 25.8 | 116 | 181 | -87 | MIRb | MIR | SINE |

  

|  |  |  |  |  |  |  |  |  |  |
| --- | --- | --- | --- | --- | --- | --- | --- | --- | --- |
| chr8:63475681-63480952 | | | 5270 bp | | |  |  |  |  |
| Genome Start | Genome End | Strand | Div | Repeat Start | Repeat End | Repeat Left | Name | Family | Class |
| 63475681 | 63477519 | - | 16.6 | 1138 | 2987 | -4578 | L1M1 | L1 | LINE |
| 63477520 | 63477899 | + | 11.3 | 1 | 375 | 0 | THE1C | MaLR | LTR |
| 63477902 | 63479467 | + | 30.5 | 2 | 1735 | 0 | MLT-int | MaLR | LTR |
| 63479468 | 63479850 | + | 17.5 | 1 | 375 | 0 | THE1C | MaLR | LTR |
| 63479851 | 63480952 | - | 16.6 | 17 | 1137 | -6428 | L1M1 | L1 | LINE |

  

|  |  |  |  |  |  |  |  |  |  |
| --- | --- | --- | --- | --- | --- | --- | --- | --- | --- |
| chr8:64627828-64630369 | | | 2533 bp | | |  |  |  |  |
| Genome Start | Genome End | Strand | Div | Repeat Start | Repeat End | Repeat Left | Name | Family | Class |
| 64627828 | 64628137 | + | 32.5 | 5488 | 5810 | -1998 | L1MC | L1 | LINE |
| 64628138 | 64628498 | - | 14.7 | 3 | 364 | 0 | THE1B | MaLR | LTR |
| 64628501 | 64630121 | - | 32.3 | 2 | 1735 | 0 | MLT-int | MaLR | LTR |
| 64630125 | 64630265 | - | 11.3 | 222 | 363 | -1 | THE1B | MaLR | LTR |
| 64630270 | 64630369 | + | 30.3 | 5814 | 5912 | -1896 | L1MC | L1 | LINE |

  

|  |  |  |  |  |  |  |  |  |  |
| --- | --- | --- | --- | --- | --- | --- | --- | --- | --- |
| chr8:64736141-64746203 | | | 9900 bp | | |  |  |  |  |
| Genome Start | Genome End | Strand | Div | Repeat Start | Repeat End | Repeat Left | Name | Family | Class |
| 64736141 | 64737859 | - | 25.8 | 4111 | 5932 | -234 | L1ME1 | L1 | LINE |
| 64737860 | 64738163 | + | 12.5 | 5 | 308 | -4 | AluSx | Alu | SINE |
| 64738164 | 64738655 | - | 27.8 | 3662 | 4110 | -2036 | L1ME1 | L1 | LINE |
| 64738656 | 64740459 | + | 9.6 | 3956 | 5786 | -365 | L1PB1 | L1 | LINE |
| 64740502 | 64740861 | + | 9.6 | 5787 | 6150 | -1 | L1PB1 | L1 | LINE |
| 64740978 | 64741075 | - | 24.7 | 3651 | 3745 | -2401 | L1MC | L1 | LINE |
| 64741079 | 64741397 | - | 27.8 | 3299 | 3615 | -2531 | L1ME1 | L1 | LINE |
| 64741398 | 64741747 | + | 12.4 | 3 | 355 | 0 | THE1A | MaLR | LTR |
| 64741750 | 64743325 | + | 31.8 | 2 | 1735 | 0 | MLT-int | MaLR | LTR |
| 64743326 | 64743672 | + | 11.6 | 1 | 355 | 0 | THE1A | MaLR | LTR |
| 64743673 | 64744436 | - | 27.8 | 2454 | 3298 | -2848 | L1ME1 | L1 | LINE |
| 64744437 | 64745904 | - | 17.6 | 1 | 1755 | 0 | MER52A | ERV1 | LTR |
| 64745905 | 64746203 | - | 27.8 | 2159 | 2453 | -3693 | L1ME1 | L1 | LINE |

  

|  |  |  |  |  |  |  |  |  |  |
| --- | --- | --- | --- | --- | --- | --- | --- | --- | --- |
| chr8:65859934-65864390 | | | 4453 bp | | |  |  |  |  |
| Genome Start | Genome End | Strand | Div | Repeat Start | Repeat End | Repeat Left | Name | Family | Class |
| 65859934 | 65860889 | + | 17.4 | 5077 | 6076 | -268 | L1MC2 | L1 | LINE |
| 65860890 | 65861264 | - | 12.4 | 1 | 375 | 0 | THE1C | MaLR | LTR |
| 65861265 | 65862851 | - | 29.6 | 4 | 1735 | 0 | MLT-int | MaLR | LTR |
| 65862856 | 65863220 | - | 14.4 | 5 | 375 | 0 | THE1C | MaLR | LTR |
| 65863221 | 65863410 | + | 17.1 | 6077 | 6253 | -91 | L1MC2 | L1 | LINE |
| 65863411 | 65864006 | + | 16.7 | 1 | 605 | 0 | MER6A | MER2\_type | DNA |
| 65864007 | 65864035 | + | 17.1 | 6254 | 6278 | -66 | L1MC2 | L1 | LINE |
| 65864036 | 65864338 | - | 11.5 | 1 | 296 | -16 | AluSx | Alu | SINE |
| 65864339 | 65864390 | + | 17.1 | 6279 | 6327 | -17 | L1MC2 | L1 | LINE |

  

|  |  |  |  |  |  |  |  |  |  |
| --- | --- | --- | --- | --- | --- | --- | --- | --- | --- |
| chr8:66040148-66044373 | | | 3670 bp | | |  |  |  |  |
| Genome Start | Genome End | Strand | Div | Repeat Start | Repeat End | Repeat Left | Name | Family | Class |
| 66040148 | 66040961 | - | 27.3 | 2528 | 3387 | -3150 | L1MDa | L1 | LINE |
| 66040965 | 66041123 | - | 27.7 | 2395 | 2586 | -4132 | L1MDa | L1 | LINE |
| 66041370 | 66041725 | - | 9.9 | 1 | 355 | 0 | THE1A | MaLR | LTR |
| 66041726 | 66043277 | - | 29.4 | 2 | 1735 | 0 | MLT-int | MaLR | LTR |
| 66043280 | 66043619 | - | 10.0 | 1 | 355 | 0 | THE1A | MaLR | LTR |
| 66043661 | 66043839 | - | 25.0 | 2073 | 2258 | -4460 | L1MDa | L1 | LINE |
| 66044104 | 66044373 | - | 31.1 | 1721 | 1994 | -4639 | L1MDa | L1 | LINE |

  

|  |  |  |  |  |  |  |  |  |  |
| --- | --- | --- | --- | --- | --- | --- | --- | --- | --- |
| chr8:66977988-66981478 | | | 3486 bp | | |  |  |  |  |
| Genome Start | Genome End | Strand | Div | Repeat Start | Repeat End | Repeat Left | Name | Family | Class |
| 66977988 | 66978092 | + | 28.8 | 8 | 113 | -155 | MIRb | MIR | SINE |
| 66978094 | 66978474 | + | 14.8 | 1 | 381 | 0 | THE1D | MaLR | LTR |
| 66978477 | 66980278 | + | 12.0 | 3 | 1580 | 0 | THE1D-int | MaLR | LTR |
| 66980281 | 66980653 | + | 12.9 | 3 | 381 | 0 | THE1D | MaLR | LTR |
| 66980654 | 66980688 | + | 29.4 | 110 | 139 | -129 | MIRb | MIR | SINE |
| 66980689 | 66980887 | - | 19.7 | 2 | 218 | -1 | MER20 | MER1\_type | DNA |
| 66980888 | 66980929 | + | 29.4 | 140 | 173 | -95 | MIRb | MIR | SINE |
| 66980930 | 66981402 | + | 21.1 | 2 | 505 | 0 | MLT1D | MaLR | LTR |
| 66981403 | 66981478 | + | 29.4 | 174 | 254 | -14 | MIRb | MIR | SINE |

  

|  |  |  |  |  |  |  |  |  |  |
| --- | --- | --- | --- | --- | --- | --- | --- | --- | --- |
| chr8:67008890-67011687 | | | 2796 bp | | |  |  |  |  |
| Genome Start | Genome End | Strand | Div | Repeat Start | Repeat End | Repeat Left | Name | Family | Class |
| 67008890 | 67009199 | - | 22.5 | 93 | 365 | -102 | MLT1C | MaLR | LTR |
| 67009200 | 67009576 | + | 13.2 | 1 | 375 | 0 | THE1C | MaLR | LTR |
| 67009579 | 67011234 | + | 30.9 | 2 | 1735 | 0 | MLT-int | MaLR | LTR |
| 67011235 | 67011610 | + | 15.1 | 1 | 375 | 0 | THE1C | MaLR | LTR |
| 67011611 | 67011687 | - | 22.5 | 12 | 92 | -375 | MLT1C | MaLR | LTR |

  

|  |  |  |  |  |  |  |  |  |  |
| --- | --- | --- | --- | --- | --- | --- | --- | --- | --- |
| chr8:67321277-67328439 | | | 6139 bp | | |  |  |  |  |
| Genome Start | Genome End | Strand | Div | Repeat Start | Repeat End | Repeat Left | Name | Family | Class |
| 67321277 | 67321675 | + | 29.4 | 3046 | 3441 | -2705 | L1M5 | L1 | LINE |
| 67321904 | 67321969 | + | 20.9 | 1 | 66 | -386 | LTR47A | ERVL | LTR |
| 67321970 | 67322254 | + | 4.1 | 1 | 385 | -881 | MER11A | ERVK | LTR |
| 67322252 | 67322691 | + | 5.9 | 770 | 1266 | 0 | MER11A | ERVK | LTR |
| 67322692 | 67322956 | + | 6.4 | 7689 | 7953 | 0 | HERVK11 | ERVK | LTR |
| 67322957 | 67323241 | + | 4.1 | 1 | 385 | -881 | MER11A | ERVK | LTR |
| 67323239 | 67323679 | + | 5.1 | 770 | 1266 | 0 | MER11A | ERVK | LTR |
| 67323680 | 67323943 | + | 6.4 | 7689 | 7953 | 0 | HERVK11 | ERVK | LTR |
| 67323944 | 67324902 | + | 5.7 | 1 | 1266 | 0 | MER11A | ERVK | LTR |
| 67324911 | 67325264 | + | 23.3 | 69 | 436 | -7 | LTR47B | ERVL | LTR |
| 67325343 | 67325663 | + | 29.7 | 3697 | 4035 | -2111 | L1M5 | L1 | LINE |
| 67325746 | 67325890 | + | 21.7 | 4166 | 4321 | -1840 | L1M2 | L1 | LINE |
| 67326045 | 67326277 | + | 28.7 | 4299 | 4531 | -1615 | L1M5 | L1 | LINE |
| 67326503 | 67326659 | - | 14.0 | 383 | 536 | -29 | MER34B | ERV1 | LTR |
| 67326660 | 67327495 | - | 13.8 | 1 | 872 | 0 | MER4D0 | ERV1 | LTR |
| 67327496 | 67327861 | - | 14.0 | 1 | 382 | -183 | MER34B | ERV1 | LTR |
| 67328117 | 67328439 | + | 29.7 | 4952 | 5284 | -862 | L1M5 | L1 | LINE |

  

|  |  |  |  |  |  |  |  |  |  |
| --- | --- | --- | --- | --- | --- | --- | --- | --- | --- |
| chr8:69495943-69501458 | | | 5158 bp | | |  |  |  |  |
| Genome Start | Genome End | Strand | Div | Repeat Start | Repeat End | Repeat Left | Name | Family | Class |
| 69495943 | 69496245 | + | 22.3 | 27 | 326 | -6307 | L1MD2 | L1 | LINE |
| 69496537 | 69496899 | - | 15.3 | 1 | 381 | 0 | THE1D | MaLR | LTR |
| 69496900 | 69498486 | - | 29.5 | 2 | 1735 | 0 | MLT-int | MaLR | LTR |
| 69498489 | 69498843 | - | 14.5 | 1 | 381 | 0 | THE1D | MaLR | LTR |
| 69498881 | 69499426 | + | 22.6 | 757 | 1303 | -5237 | L1MD2 | L1 | LINE |
| 69499427 | 69499691 | + | 7.9 | 20 | 284 | -27 | AluY | Alu | SINE |
| 69499720 | 69501025 | + | 22.2 | 4178 | 5586 | -951 | L1MD2 | L1 | LINE |
| 69501026 | 69501335 | + | 16.8 | 1 | 310 | -2 | AluSx | Alu | SINE |
| 69501336 | 69501458 | + | 22.2 | 5587 | 5701 | -836 | L1MD2 | L1 | LINE |

  

|  |  |  |  |  |  |  |  |  |  |
| --- | --- | --- | --- | --- | --- | --- | --- | --- | --- |
| chr8:70047570-70053657 | | | 6090 bp | | |  |  |  |  |
| Genome Start | Genome End | Strand | Div | Repeat Start | Repeat End | Repeat Left | Name | Family | Class |
| 70047570 | 70047732 | + | 19.1 | 5312 | 5484 | -682 | L1M5 | L1 | LINE |
| 70047733 | 70048218 | + | 6.0 | 1 | 448 | -2 | LTR7 | ERV1 | LTR |
| 70048221 | 70051505 | + | 9.1 | 1 | 3290 | -4423 | HERVH | ERV1 | LTR |
| 70051496 | 70051995 | + | 11.6 | 3492 | 3993 | -3720 | HERVH | ERV1 | LTR |
| 70051998 | 70052633 | + | 14.2 | 4488 | 5149 | -2564 | HERVH | ERV1 | LTR |
| 70052635 | 70052784 | + | 9.4 | 5600 | 5748 | -1965 | HERVH | ERV1 | LTR |
| 70052787 | 70053103 | + | 5.5 | 7384 | 7713 | 0 | HERVH | ERV1 | LTR |
| 70053104 | 70053558 | + | 7.1 | 1 | 448 | -2 | LTR7 | ERV1 | LTR |
| 70053560 | 70053657 | + | 17.4 | 5482 | 5585 | -579 | L1M5 | L1 | LINE |

  

|  |  |  |  |  |  |  |  |  |  |
| --- | --- | --- | --- | --- | --- | --- | --- | --- | --- |
| chr8:71586135-71588529 | | | 2391 bp | | |  |  |  |  |
| Genome Start | Genome End | Strand | Div | Repeat Start | Repeat End | Repeat Left | Name | Family | Class |
| 71586135 | 71586205 | + | 20.7 | 120 | 195 | -67 | MIR | MIR | SINE |
| 71586206 | 71586558 | + | 10.5 | 1 | 355 | 0 | THE1A | MaLR | LTR |
| 71586561 | 71588133 | + | 29.1 | 2 | 1733 | -2 | MLT1I-int | MaLR | LTR |
| 71588136 | 71588488 | + | 9.1 | 1 | 355 | 0 | THE1A | MaLR | LTR |
| 71588489 | 71588529 | + | 20.7 | 196 | 235 | -27 | MIR | MIR | SINE |

  

|  |  |  |  |  |  |  |  |  |  |
| --- | --- | --- | --- | --- | --- | --- | --- | --- | --- |
| chr8:71790932-71800388 | | | 9345 bp | | |  |  |  |  |
| Genome Start | Genome End | Strand | Div | Repeat Start | Repeat End | Repeat Left | Name | Family | Class |
| 71790932 | 71791064 | + | 28.5 | 1956 | 2088 | -419 | HAL1 | L1 | LINE |
| 71791123 | 71791493 | - | 11.2 | 1 | 375 | 0 | THE1C | MaLR | LTR |
| 71791494 | 71793054 | - | 11.6 | 35 | 1580 | 0 | THE1C-int | MaLR | LTR |
| 71793094 | 71798676 | - | 11.0 | 894 | 6487 | 0 | L1PA8 | L1 | LINE |
| 71798677 | 71798737 | - | 4.9 | 236 | 296 | -14 | AluY | Alu | SINE |
| 71798738 | 71799617 | - | 11.0 | 13 | 893 | -5594 | L1PA8 | L1 | LINE |
| 71799633 | 71799679 | - | 12.8 | 1 | 49 | -1531 | THE1C-int | MaLR | LTR |
| 71799680 | 71800047 | - | 14.1 | 1 | 375 | 0 | THE1C | MaLR | LTR |
| 71800048 | 71800388 | + | 26.9 | 2140 | 2482 | -25 | HAL1 | L1 | LINE |

  

|  |  |  |  |  |  |  |  |  |  |
| --- | --- | --- | --- | --- | --- | --- | --- | --- | --- |
| chr8:74063067-74067818 | | | 4518 bp | | |  |  |  |  |
| Genome Start | Genome End | Strand | Div | Repeat Start | Repeat End | Repeat Left | Name | Family | Class |
| 74063067 | 74063297 | + | 32.1 | 4 | 234 | -2273 | HAL1 | L1 | LINE |
| 74063392 | 74063688 | + | 16.7 | 1 | 296 | -16 | AluJo | Alu | SINE |
| 74063760 | 74064070 | + | 27.7 | 499 | 802 | -1705 | HAL1 | L1 | LINE |
| 74064071 | 74064375 | - | 11.8 | 1 | 304 | -9 | AluSq | Alu | SINE |
| 74064376 | 74064719 | + | 27.7 | 803 | 1161 | -1346 | HAL1 | L1 | LINE |
| 74064773 | 74065144 | + | 11.2 | 3 | 375 | 0 | THE1C | MaLR | LTR |
| 74065145 | 74066679 | + | 10.9 | 1 | 1580 | 0 | THE1C-int | MaLR | LTR |
| 74066680 | 74067054 | + | 12.6 | 1 | 374 | -1 | THE1C | MaLR | LTR |
| 74067055 | 74067329 | - | 17.1 | 1 | 279 | -33 | AluJo | Alu | SINE |
| 74067346 | 74067818 | + | 28.0 | 1176 | 1675 | -832 | HAL1 | L1 | LINE |

  

|  |  |  |  |  |  |  |  |  |  |
| --- | --- | --- | --- | --- | --- | --- | --- | --- | --- |
| chr8:75299094-75301513 | | | 2418 bp | | |  |  |  |  |
| Genome Start | Genome End | Strand | Div | Repeat Start | Repeat End | Repeat Left | Name | Family | Class |
| 75299094 | 75299189 | + | 15.0 | 6050 | 6143 | -40 | L1MB3 | L1 | LINE |
| 75299190 | 75299543 | - | 8.8 | 1 | 355 | 0 | THE1A | MaLR | LTR |
| 75299544 | 75301112 | - | 30.0 | 2 | 1735 | 0 | MLT-int | MaLR | LTR |
| 75301115 | 75301469 | - | 6.8 | 1 | 355 | 0 | THE1A | MaLR | LTR |
| 75301470 | 75301513 | + | 15.0 | 6144 | 6183 | 0 | L1MB3 | L1 | LINE |

  

|  |  |  |  |  |  |  |  |  |  |
| --- | --- | --- | --- | --- | --- | --- | --- | --- | --- |
| chr8:75693296-75698077 | | | 4741 bp | | |  |  |  |  |
| Genome Start | Genome End | Strand | Div | Repeat Start | Repeat End | Repeat Left | Name | Family | Class |
| 75693296 | 75694220 | + | 14.8 | 3848 | 4801 | -1342 | L1MA8 | L1 | LINE |
| 75694221 | 75694590 | - | 12.8 | 2 | 381 | 0 | THE1D | MaLR | LTR |
| 75694597 | 75696166 | - | 12.0 | 1 | 1574 | -6 | THE1D-int | MaLR | LTR |
| 75696167 | 75696527 | - | 14.2 | 1 | 381 | 0 | THE1D | MaLR | LTR |
| 75696553 | 75697111 | + | 19.1 | 4851 | 5391 | -900 | L1MA8 | L1 | LINE |
| 75697112 | 75697425 | + | 15.6 | 1 | 308 | -4 | AluSx | Alu | SINE |
| 75697426 | 75697612 | + | 17.5 | 5392 | 5577 | -714 | L1MA8 | L1 | LINE |
| 75697623 | 75698077 | + | 15.3 | 5810 | 6289 | -2 | L1MA8 | L1 | LINE |

  

|  |  |  |  |  |  |  |  |  |  |
| --- | --- | --- | --- | --- | --- | --- | --- | --- | --- |
| chr8:76325657-76331292 | | | 5446 bp | | |  |  |  |  |
| Genome Start | Genome End | Strand | Div | Repeat Start | Repeat End | Repeat Left | Name | Family | Class |
| 76325657 | 76325756 | + | 23.5 | 2160 | 2260 | -3883 | L1MA4A | L1 | LINE |
| 76325762 | 76325915 | - | 21.1 | 49 | 200 | -112 | AluJo | Alu | SINE |
| 76325916 | 76326018 | - | 7.8 | 5352 | 5461 | -842 | L1M1 | L1 | LINE |
| 76326012 | 76326287 | + | 18.1 | 3578 | 3848 | -2295 | L1MA4A | L1 | LINE |
| 76326473 | 76326924 | + | 16.2 | 3833 | 4201 | -1942 | L1MA4A | L1 | LINE |
| 76326925 | 76327282 | - | 12.6 | 1 | 362 | -2 | THE1B | MaLR | LTR |
| 76327283 | 76328527 | - | 11.1 | 324 | 1580 | 0 | THE1B-int | MaLR | LTR |
| 76328535 | 76328671 | - | 15.3 | 231 | 364 | 0 | THE1B | MaLR | LTR |
| 76328672 | 76328974 | - | 10.0 | 1 | 300 | -11 | AluY | Alu | SINE |
| 76328975 | 76329209 | - | 15.3 | 1 | 230 | -134 | THE1B | MaLR | LTR |
| 76329210 | 76331292 | + | 15.2 | 4199 | 6295 | -8 | L1MA4A | L1 | LINE |

  

|  |  |  |  |  |  |  |  |  |  |
| --- | --- | --- | --- | --- | --- | --- | --- | --- | --- |
| chr8:76589909-76595081 | | | 5021 bp | | |  |  |  |  |
| Genome Start | Genome End | Strand | Div | Repeat Start | Repeat End | Repeat Left | Name | Family | Class |
| 76589909 | 76590005 | + | 39.6 | 2 | 97 | -165 | MIR | MIR | SINE |
| 76590031 | 76590145 | - | 24.2 | 3093 | 3190 | -3029 | L1MEc | L1 | LINE |
| 76590146 | 76590560 | - | 19.7 | 143 | 515 | 0 | MLT2B2 | ERVL | LTR |
| 76590561 | 76590853 | - | 9.2 | 1 | 295 | -18 | AluSp | Alu | SINE |
| 76590854 | 76590995 | - | 19.7 | 1 | 142 | -373 | MLT2B2 | ERVL | LTR |
| 76590996 | 76591696 | - | 24.2 | 2372 | 3092 | -3127 | L1MEc | L1 | LINE |
| 76591701 | 76592052 | - | 12.6 | 1 | 349 | -15 | THE1B | MaLR | LTR |
| 76592053 | 76592772 | - | 10.7 | 870 | 1580 | 0 | THE1B-int | MaLR | LTR |
| 76592773 | 76593142 | - | 3.0 | 5792 | 6155 | 0 | L1PA4 | L1 | LINE |
| 76593143 | 76594234 | - | 10.7 | 1 | 869 | -711 | THE1B-int | MaLR | LTR |
| 76594235 | 76594530 | - | 10.2 | 3 | 364 | 0 | THE1B | MaLR | LTR |
| 76594533 | 76594616 | - | 24.7 | 2393 | 2476 | -3924 | L1MEc | L1 | LINE |
| 76594652 | 76594867 | - | 24.7 | 2167 | 2392 | -4008 | L1MEc | L1 | LINE |
| 76594954 | 76595081 | + | 26.4 | 134 | 261 | -1 | MIR | MIR | SINE |

  

|  |  |  |  |  |  |  |  |  |  |
| --- | --- | --- | --- | --- | --- | --- | --- | --- | --- |
| chr8:76899688-76903325 | | | 3482 bp | | |  |  |  |  |
| Genome Start | Genome End | Strand | Div | Repeat Start | Repeat End | Repeat Left | Name | Family | Class |
| 76899688 | 76900516 | + | 27.0 | 20 | 890 | -5510 | L1MEc | L1 | LINE |
| 76900657 | 76900757 | + | 17.0 | 1822 | 1929 | -4471 | L1MEc | L1 | LINE |
| 76900762 | 76901185 | + | 13.7 | 2 | 428 | 0 | MSTA | MaLR | LTR |
| 76901186 | 76902788 | + | 18.5 | 1 | 1651 | 0 | MSTA-int | MaLR | LTR |
| 76902789 | 76903209 | + | 12.1 | 1 | 428 | 0 | MSTA | MaLR | LTR |
| 76903222 | 76903325 | + | 30.0 | 1961 | 2060 | -4340 | L1MEc | L1 | LINE |

  

|  |  |  |  |  |  |  |  |  |  |
| --- | --- | --- | --- | --- | --- | --- | --- | --- | --- |
| chr8:76998399-77006974 | | | 8574 bp | | |  |  |  |  |
| Genome Start | Genome End | Strand | Div | Repeat Start | Repeat End | Repeat Left | Name | Family | Class |
| 76998399 | 77002169 | + | 13.3 | 2 | 3690 | -2641 | L1PA10 | L1 | LINE |
| 77002170 | 77002538 | - | 16.7 | 1 | 364 | 0 | THE1B | MaLR | LTR |
| 77002539 | 77004100 | - | 31.6 | 2 | 1735 | 0 | MLT1C-int | MaLR | LTR |
| 77004103 | 77004467 | - | 14.5 | 1 | 364 | 0 | THE1B | MaLR | LTR |
| 77004468 | 77006974 | + | 8.8 | 3506 | 6165 | -3 | L1PA10 | L1 | LINE |

  

|  |  |  |  |  |  |  |  |  |  |
| --- | --- | --- | --- | --- | --- | --- | --- | --- | --- |
| chr8:77130359-77138398 | | | 7998 bp | | |  |  |  |  |
| Genome Start | Genome End | Strand | Div | Repeat Start | Repeat End | Repeat Left | Name | Family | Class |
| 77130359 | 77131118 | + | 23.6 | 2427 | 3208 | -4358 | L1M2 | L1 | LINE |
| 77131119 | 77131427 | + | 6.2 | 1 | 309 | -2 | AluY | Alu | SINE |
| 77131428 | 77132164 | + | 27.1 | 2877 | 3648 | -3914 | L1M2 | L1 | LINE |
| 77132206 | 77132476 | + | 20.0 | 3621 | 3891 | -3668 | L1M2 | L1 | LINE |
| 77132479 | 77132901 | + | 19.1 | 1 | 417 | 0 | MSTB | MaLR | LTR |
| 77132902 | 77134265 | + | 20.4 | 1 | 1283 | -368 | MSTB-int | MaLR | LTR |
| 77134266 | 77135628 | - | 7.3 | 133 | 1578 | 0 | LTR12C | ERV1 | LTR |
| 77135626 | 77135790 | + | 13.5 | 1405 | 1577 | -74 | MSTB-int | MaLR | LTR |
| 77135791 | 77136154 | + | 12.5 | 1 | 379 | -2 | THE1D | MaLR | LTR |
| 77136155 | 77136236 | + | 13.5 | 1578 | 1651 | 0 | MSTB-int | MaLR | LTR |
| 77136237 | 77136639 | + | 17.2 | 1 | 406 | -20 | MSTB | MaLR | LTR |
| 77136642 | 77138398 | + | 21.6 | 3888 | 5701 | -1858 | L1M2 | L1 | LINE |

  

|  |  |  |  |  |  |  |  |  |  |
| --- | --- | --- | --- | --- | --- | --- | --- | --- | --- |
| chr8:78445679-78451997 | | | 5975 bp | | |  |  |  |  |
| Genome Start | Genome End | Strand | Div | Repeat Start | Repeat End | Repeat Left | Name | Family | Class |
| 78445679 | 78445956 | + | 30.8 | 2170 | 2442 | -3773 | L1MC4 | L1 | LINE |
| 78446023 | 78446398 | + | 14.8 | 1 | 428 | 0 | MSTA | MaLR | LTR |
| 78446401 | 78448020 | + | 31.5 | 2 | 1731 | -4 | MLT1J2-int | MaLR | LTR |
| 78448024 | 78448088 | + | 10.9 | 1 | 64 | -364 | MSTA | MaLR | LTR |
| 78448086 | 78448645 | - | 15.7 | 961 | 1509 | -909 | Tigger1 | MER2\_type | DNA |
| 78448646 | 78449136 | + | 11.0 | 1 | 511 | 0 | MER9 | ERVK | LTR |
| 78449137 | 78449157 | - | 15.7 | 940 | 960 | -1458 | Tigger1 | MER2\_type | DNA |
| 78449287 | 78450055 | - | 17.0 | 182 | 951 | -1467 | Tigger1 | MER2\_type | DNA |
| 78450056 | 78450334 | + | 10.4 | 1 | 288 | -21 | AluSc | Alu | SINE |
| 78450335 | 78450516 | - | 17.0 | 1 | 181 | -2237 | Tigger1 | MER2\_type | DNA |
| 78450557 | 78451364 | - | 23.8 | 5319 | 6166 | -12 | L1MB8 | L1 | LINE |
| 78451472 | 78451997 | + | 32.9 | 3027 | 3587 | -2627 | L1MC4 | L1 | LINE |

  

|  |  |  |  |  |  |  |  |  |  |
| --- | --- | --- | --- | --- | --- | --- | --- | --- | --- |
| chr8:78595086-78597130 | | | 1983 bp | | |  |  |  |  |
| Genome Start | Genome End | Strand | Div | Repeat Start | Repeat End | Repeat Left | Name | Family | Class |
| 78595086 | 78595279 | + | 35.2 | 21 | 218 | -232 | LTR16A | ERVL | LTR |
| 78595280 | 78595467 | - | 15.5 | 224 | 428 | 0 | MSTA | MaLR | LTR |
| 78595529 | 78595787 | - | 15.5 | 1 | 223 | -205 | MSTA | MaLR | LTR |
| 78595797 | 78595905 | - | 7.8 | 1465 | 1580 | 0 | MSTA-int | MaLR | LTR |
| 78595898 | 78596511 | - | 18.1 | 1 | 637 | -1014 | MSTA-int | MaLR | LTR |
| 78596512 | 78596896 | - | 17.1 | 1 | 428 | 0 | MSTA | MaLR | LTR |
| 78596897 | 78597130 | + | 31.7 | 215 | 444 | -6 | LTR16A | ERVL | LTR |

  

|  |  |  |  |  |  |  |  |  |  |
| --- | --- | --- | --- | --- | --- | --- | --- | --- | --- |
| chr8:78758494-78766945 | | | 7877 bp | | |  |  |  |  |
| Genome Start | Genome End | Strand | Div | Repeat Start | Repeat End | Repeat Left | Name | Family | Class |
| 78758494 | 78759267 | + | 33.1 | 1 | 754 | -5291 | L1MCb | L1 | LINE |
| 78759495 | 78760346 | + | 26.5 | 772 | 1758 | -4993 | L1MCb | L1 | LINE |
| 78760356 | 78760736 | + | 15.6 | 1 | 421 | -7 | MSTA | MaLR | LTR |
| 78760746 | 78761301 | + | 30.8 | 2 | 585 | -1150 | MLT-int | MaLR | LTR |
| 78761365 | 78762495 | + | 30.8 | 586 | 1735 | 0 | MLT-int | MaLR | LTR |
| 78762496 | 78762889 | + | 13.4 | 1 | 428 | 0 | MSTA | MaLR | LTR |
| 78762890 | 78763338 | + | 24.2 | 1763 | 2343 | -4408 | L1MCb | L1 | LINE |
| 78763339 | 78764191 | + | 15.6 | 5 | 903 | 0 | MER4D | ERV1 | LTR |
| 78764192 | 78764532 | + | 24.7 | 2344 | 2693 | -3871 | L1MCb | L1 | LINE |
| 78764533 | 78764841 | + | 11.4 | 1 | 310 | -3 | AluSq | Alu | SINE |
| 78764842 | 78765240 | + | 25.6 | 2694 | 3105 | -3459 | L1MCb | L1 | LINE |
| 78765226 | 78765978 | + | 7.9 | 5039 | 5791 | -360 | L1PB1 | L1 | LINE |
| 78766258 | 78766621 | + | 14.1 | 5791 | 6151 | 0 | L1PB1 | L1 | LINE |
| 78766625 | 78766945 | + | 17.9 | 3427 | 3764 | -2800 | L1MCb | L1 | LINE |

  

|  |  |  |  |  |  |  |  |  |  |
| --- | --- | --- | --- | --- | --- | --- | --- | --- | --- |
| chr8:79344127-79347438 | | | 3306 bp | | |  |  |  |  |
| Genome Start | Genome End | Strand | Div | Repeat Start | Repeat End | Repeat Left | Name | Family | Class |
| 79344127 | 79344629 | + | 19.4 | 4999 | 5635 | -709 | L1MC2 | L1 | LINE |
| 79344630 | 79345001 | - | 13.0 | 1 | 375 | 0 | THE1C | MaLR | LTR |
| 79345002 | 79346670 | - | 28.9 | 2 | 1735 | 0 | MLT-int | MaLR | LTR |
| 79346677 | 79347048 | - | 10.3 | 1 | 372 | -3 | THE1C | MaLR | LTR |
| 79347049 | 79347438 | + | 20.0 | 5636 | 6034 | -310 | L1MC2 | L1 | LINE |

  

|  |  |  |  |  |  |  |  |  |  |
| --- | --- | --- | --- | --- | --- | --- | --- | --- | --- |
| chr8:79756865-79760189 | | | 3323 bp | | |  |  |  |  |
| Genome Start | Genome End | Strand | Div | Repeat Start | Repeat End | Repeat Left | Name | Family | Class |
| 79756865 | 79757089 | + | 31.6 | 3931 | 4173 | -1973 | L1M5 | L1 | LINE |
| 79757090 | 79757447 | - | 13.0 | 1 | 364 | 0 | THE1B | MaLR | LTR |
| 79757448 | 79758978 | - | 29.2 | 2 | 1735 | 0 | MLT-int | MaLR | LTR |
| 79758981 | 79759341 | - | 14.4 | 1 | 364 | 0 | THE1B | MaLR | LTR |
| 79759342 | 79760189 | + | 31.6 | 4174 | 5078 | -1068 | L1M5 | L1 | LINE |

  

|  |  |  |  |  |  |  |  |  |  |
| --- | --- | --- | --- | --- | --- | --- | --- | --- | --- |
| chr8:83187347-83192087 | | | 4588 bp | | |  |  |  |  |
| Genome Start | Genome End | Strand | Div | Repeat Start | Repeat End | Repeat Left | Name | Family | Class |
| 83187347 | 83187592 | + | 16.6 | 4486 | 4737 | -1403 | L1M2 | L1 | LINE |
| 83187593 | 83187761 | - | 16.9 | 289 | 461 | 0 | LOR1b | ERV1 | LTR |
| 83187800 | 83187981 | - | 9.9 | 105 | 286 | -26 | AluSg/x | Alu | SINE |
| 83187982 | 83188255 | - | 16.9 | 3 | 288 | -173 | LOR1b | ERV1 | LTR |
| 83188256 | 83188591 | + | 16.6 | 4738 | 5070 | -1070 | L1M2 | L1 | LINE |
| 83188595 | 83188975 | - | 21.2 | 1 | 408 | 0 | MLT1A1 | MaLR | LTR |
| 83188976 | 83190637 | - | 22.9 | 2 | 1735 | 0 | MLT1A1-int | MaLR | LTR |
| 83190644 | 83190856 | - | 23.2 | 137 | 361 | -4 | MLT1A1 | MaLR | LTR |
| 83190914 | 83191315 | + | 16.2 | 1 | 428 | 0 | MSTA | MaLR | LTR |
| 83191316 | 83191496 | + | 16.6 | 1 | 185 | -1466 | MSTA-int | MaLR | LTR |
| 83191546 | 83192087 | + | 16.5 | 5072 | 5621 | -691 | L1M2 | L1 | LINE |

  

|  |  |  |  |  |  |  |  |  |  |
| --- | --- | --- | --- | --- | --- | --- | --- | --- | --- |
| chr8:83572377-83575200 | | | 2675 bp | | |  |  |  |  |
| Genome Start | Genome End | Strand | Div | Repeat Start | Repeat End | Repeat Left | Name | Family | Class |
| 83572377 | 83572424 | - | 2.1 | 334 | 381 | 0 | THE1D | MaLR | LTR |
| 83572425 | 83572803 | + | 10.6 | 1 | 375 | 0 | THE1C | MaLR | LTR |
| 83572804 | 83574320 | + | 12.3 | 1 | 1580 | 0 | THE1C-int | MaLR | LTR |
| 83574323 | 83574698 | + | 15.9 | 3 | 375 | 0 | THE1C | MaLR | LTR |
| 83574846 | 83575200 | - | 15.6 | 1 | 348 | -33 | THE1D | MaLR | LTR |

  

|  |  |  |  |  |  |  |  |  |  |
| --- | --- | --- | --- | --- | --- | --- | --- | --- | --- |
| chr8:83649512-83652768 | | | 3242 bp | | |  |  |  |  |
| Genome Start | Genome End | Strand | Div | Repeat Start | Repeat End | Repeat Left | Name | Family | Class |
| 83649512 | 83649827 | + | 26.7 | 6983 | 7406 | -589 | L1MD3 | L1 | LINE |
| 83649833 | 83650199 | - | 26.5 | 1 | 374 | 0 | MLT1A | MaLR | LTR |
| 83650201 | 83651164 | - | 28.3 | 344 | 1370 | -1 | MLT1A-int | MaLR | LTR |
| 83651165 | 83651505 | - | 22.3 | 4 | 408 | 0 | MLT1A1 | MaLR | LTR |
| 83651506 | 83651816 | - | 28.3 | 5 | 343 | -1028 | MLT1A-int | MaLR | LTR |
| 83651820 | 83652211 | - | 26.2 | 1 | 371 | -3 | MLT1A | MaLR | LTR |
| 83652218 | 83652768 | + | 22.4 | 7413 | 7981 | -14 | L1MD3 | L1 | LINE |

  

|  |  |  |  |  |  |  |  |  |  |
| --- | --- | --- | --- | --- | --- | --- | --- | --- | --- |
| chr8:84403854-84407519 | | | 3462 bp | | |  |  |  |  |
| Genome Start | Genome End | Strand | Div | Repeat Start | Repeat End | Repeat Left | Name | Family | Class |
| 84403854 | 84404117 | - | 40.1 | 1299 | 1567 | -3429 | HERV16 | ERVL | LTR |
| 84404118 | 84404473 | - | 13.0 | 1 | 379 | -2 | THE1D | MaLR | LTR |
| 84404474 | 84405984 | - | 30.4 | 2 | 1735 | 0 | MLT-int | MaLR | LTR |
| 84405991 | 84406350 | - | 13.2 | 1 | 377 | -4 | THE1D | MaLR | LTR |
| 84406351 | 84406617 | - | 40.1 | 1010 | 1294 | -3702 | HERV16 | ERVL | LTR |
| 84406724 | 84406950 | - | 27.6 | 629 | 866 | -4130 | HERV16 | ERVL | LTR |
| 84407043 | 84407519 | - | 32.1 | 3 | 497 | -4499 | HERV16 | ERVL | LTR |

  

|  |  |  |  |  |  |  |  |  |  |
| --- | --- | --- | --- | --- | --- | --- | --- | --- | --- |
| chr8:84577401-84586266 | | | 8852 bp | | |  |  |  |  |
| Genome Start | Genome End | Strand | Div | Repeat Start | Repeat End | Repeat Left | Name | Family | Class |
| 84577401 | 84579491 | - | 11.7 | 3199 | 5293 | -1319 | L1PBa1 | L1 | LINE |
| 84579493 | 84579893 | - | 9.0 | 1 | 448 | -2 | LTR7 | ERV1 | LTR |
| 84579894 | 84580271 | - | 9.7 | 7314 | 7713 | 0 | HERVH | ERV1 | LTR |
| 84580264 | 84580483 | - | 15.1 | 5597 | 5819 | -1894 | HERVH | ERV1 | LTR |
| 84580481 | 84582141 | - | 13.6 | 3492 | 5149 | -2564 | HERVH | ERV1 | LTR |
| 84582134 | 84582279 | - | 15.1 | 3140 | 3288 | -4425 | HERVH | ERV1 | LTR |
| 84582277 | 84585309 | - | 9.5 | 1 | 2984 | -4729 | HERVH | ERV1 | LTR |
| 84585312 | 84585710 | - | 8.8 | 1 | 448 | -2 | LTR7 | ERV1 | LTR |
| 84585711 | 84586002 | - | 10.0 | 2913 | 3204 | -3408 | L1PBa1 | L1 | LINE |
| 84586036 | 84586266 | - | 13.9 | 1018 | 1247 | -5365 | L1PBa1 | L1 | LINE |

  

|  |  |  |  |  |  |  |  |  |  |
| --- | --- | --- | --- | --- | --- | --- | --- | --- | --- |
| chr8:84617657-84626507 | | | 8541 bp | | |  |  |  |  |
| Genome Start | Genome End | Strand | Div | Repeat Start | Repeat End | Repeat Left | Name | Family | Class |
| 84617657 | 84618293 | + | 20.7 | 3 | 730 | -6123 | L1PA15-16 | L1 | LINE |
| 84618356 | 84618504 | + | 27.7 | 854 | 1001 | -6148 | L1PA15-16 | L1 | LINE |
| 84618505 | 84618944 | + | 16.2 | 1 | 450 | 0 | LTR7 | ERV1 | LTR |
| 84618968 | 84620207 | + | 19.3 | 22 | 1629 | -6084 | HERVH | ERV1 | LTR |
| 84620233 | 84620763 | - | 11.3 | 1 | 511 | 0 | LTR10F | ERV1 | LTR |
| 84620764 | 84622002 | + | 15.8 | 1627 | 2845 | -4868 | HERVH | ERV1 | LTR |
| 84621993 | 84624470 | + | 21.0 | 3630 | 6016 | -1697 | HERVH | ERV1 | LTR |
| 84624464 | 84625019 | + | 13.8 | 7157 | 7713 | 0 | HERVH | ERV1 | LTR |
| 84625020 | 84625458 | + | 20.7 | 1 | 450 | 0 | LTR7 | ERV1 | LTR |
| 84625676 | 84626507 | + | 22.1 | 1497 | 2290 | -4579 | L1PA15-16 | L1 | LINE |

  

|  |  |  |  |  |  |  |  |  |  |
| --- | --- | --- | --- | --- | --- | --- | --- | --- | --- |
| chr8:84995561-84998084 | | | 2521 bp | | |  |  |  |  |
| Genome Start | Genome End | Strand | Div | Repeat Start | Repeat End | Repeat Left | Name | Family | Class |
| 84995561 | 84995700 | - | 13.0 | 6159 | 6297 | -7 | L1MA2 | L1 | LINE |
| 84995701 | 84996061 | - | 15.4 | 1 | 364 | 0 | THE1B | MaLR | LTR |
| 84996062 | 84997582 | - | 32.7 | 2 | 1735 | 0 | MLT1E-int | MaLR | LTR |
| 84997586 | 84997949 | - | 17.1 | 1 | 363 | -1 | THE1B | MaLR | LTR |
| 84997950 | 84998084 | - | 13.0 | 6025 | 6158 | -146 | L1MA2 | L1 | LINE |

  

|  |  |  |  |  |  |  |  |  |  |
| --- | --- | --- | --- | --- | --- | --- | --- | --- | --- |
| chr8:85383521-85389743 | | | 5221 bp | | |  |  |  |  |
| Genome Start | Genome End | Strand | Div | Repeat Start | Repeat End | Repeat Left | Name | Family | Class |
| 85383521 | 85383881 | + | 32.4 | 2179 | 2555 | -3660 | L1M | L1 | LINE |
| 85383900 | 85384538 | + | 33.5 | 2581 | 3225 | -2989 | L1M | L1 | LINE |
| 85384577 | 85384815 | - | 14.8 | 2 | 301 | -1 | AluSx | Alu | SINE |
| 85384827 | 85385127 | - | 13.0 | 2 | 310 | 0 | AluSg | Alu | SINE |
| 85385185 | 85385235 | + | 11.8 | 6188 | 6242 | -70 | L1MA9 | L1 | LINE |
| 85385556 | 85385873 | + | 1.3 | 1 | 318 | 0 | AluYb8 | Alu | SINE |
| 85385941 | 85386216 | + | 31.5 | 4057 | 4357 | -1857 | L1M | L1 | LINE |
| 85386307 | 85386698 | + | 34.5 | 4553 | 4952 | -1262 | L1M | L1 | LINE |
| 85386743 | 85387111 | - | 14.3 | 1 | 381 | 0 | THE1D | MaLR | LTR |
| 85387112 | 85388722 | - | 13.9 | 6 | 1580 | 0 | THE1D-int | MaLR | LTR |
| 85388728 | 85389099 | - | 14.2 | 1 | 381 | 0 | THE1D | MaLR | LTR |
| 85389362 | 85389519 | + | 18.4 | 1 | 144 | -32 | FRAM | Alu | SINE |
| 85389610 | 85389743 | + | 27.1 | 5337 | 5471 | -2405 | L1M | L1 | LINE |

  

|  |  |  |  |  |  |  |  |  |  |
| --- | --- | --- | --- | --- | --- | --- | --- | --- | --- |
| chr8:87270238-87273075 | | | 2836 bp | | |  |  |  |  |
| Genome Start | Genome End | Strand | Div | Repeat Start | Repeat End | Repeat Left | Name | Family | Class |
| 87270238 | 87270493 | - | 14.2 | 2452 | 2702 | -8 | Charlie3 | MER1\_type | DNA |
| 87270494 | 87270864 | - | 10.5 | 1 | 375 | 0 | THE1C | MaLR | LTR |
| 87270865 | 87272450 | - | 28.0 | 2 | 1735 | 0 | MLT1A0-int | MaLR | LTR |
| 87272453 | 87272824 | - | 13.2 | 1 | 375 | 0 | THE1C | MaLR | LTR |
| 87272825 | 87273075 | - | 14.2 | 28 | 278 | -2432 | Charlie3 | MER1\_type | DNA |

  

|  |  |  |  |  |  |  |  |  |  |
| --- | --- | --- | --- | --- | --- | --- | --- | --- | --- |
| chr8:88293205-88297092 | | | 3851 bp | | |  |  |  |  |
| Genome Start | Genome End | Strand | Div | Repeat Start | Repeat End | Repeat Left | Name | Family | Class |
| 88293205 | 88293396 | + | 26.6 | 3380 | 3579 | -2567 | L1MB7 | L1 | LINE |
| 88293434 | 88294666 | + | 22.2 | 4358 | 5625 | -549 | L1MB7 | L1 | LINE |
| 88294667 | 88295035 | - | 13.7 | 1 | 364 | 0 | THE1B | MaLR | LTR |
| 88295036 | 88295501 | - | 9.0 | 1103 | 1580 | 0 | THE1B-int | MaLR | LTR |
| 88295499 | 88295937 | - | 10.0 | 1 | 491 | -1089 | THE1B-int | MaLR | LTR |
| 88295940 | 88296303 | - | 15.8 | 1 | 362 | -2 | THE1B | MaLR | LTR |
| 88296305 | 88296697 | + | 22.8 | 5622 | 6025 | -159 | L1MB7 | L1 | LINE |
| 88296698 | 88296948 | + | 6.0 | 50 | 298 | -13 | AluY | Alu | SINE |
| 88296949 | 88297092 | + | 22.8 | 6026 | 6183 | -1 | L1MB7 | L1 | LINE |

  

|  |  |  |  |  |  |  |  |  |  |
| --- | --- | --- | --- | --- | --- | --- | --- | --- | --- |
| chr8:89531803-89537131 | | | 5326 bp | | |  |  |  |  |
| Genome Start | Genome End | Strand | Div | Repeat Start | Repeat End | Repeat Left | Name | Family | Class |
| 89531803 | 89532460 | + | 16.6 | 2 | 705 | -2026 | Tigger4(Zombi) | MER2\_type | DNA |
| 89532462 | 89532830 | - | 12.4 | 1 | 364 | 0 | THE1B | MaLR | LTR |
| 89532833 | 89534470 | - | 13.3 | 1 | 1578 | -2 | THE1B-int | MaLR | LTR |
| 89534471 | 89534831 | - | 12.5 | 1 | 364 | 0 | THE1B | MaLR | LTR |
| 89534832 | 89535345 | + | 13.5 | 702 | 1211 | -1520 | Tigger4(Zombi) | MER2\_type | DNA |
| 89535346 | 89535639 | + | 10.9 | 1 | 293 | -18 | AluY | Alu | SINE |
| 89535640 | 89537131 | + | 13.5 | 1212 | 2730 | -1 | Tigger4(Zombi) | MER2\_type | DNA |

  

|  |  |  |  |  |  |  |  |  |  |
| --- | --- | --- | --- | --- | --- | --- | --- | --- | --- |
| chr8:89879216-89884639 | | | 5362 bp | | |  |  |  |  |
| Genome Start | Genome End | Strand | Div | Repeat Start | Repeat End | Repeat Left | Name | Family | Class |
| 89879216 | 89880298 | - | 17.6 | 5276 | 6310 | -2 | L1MA9 | L1 | LINE |
| 89880300 | 89880711 | - | 17.0 | 4774 | 5204 | -936 | L1MA9 | L1 | LINE |
| 89880745 | 89880953 | - | 17.0 | 4555 | 4773 | -1367 | L1MA9 | L1 | LINE |
| 89880954 | 89881326 | + | 14.3 | 1 | 381 | 0 | THE1D | MaLR | LTR |
| 89881329 | 89882926 | + | 14.6 | 3 | 1566 | -14 | THE1D-int | MaLR | LTR |
| 89882935 | 89883295 | + | 12.9 | 3 | 381 | 0 | THE1D | MaLR | LTR |
| 89883297 | 89883860 | - | 20.4 | 3979 | 4558 | -1582 | L1MA9 | L1 | LINE |
| 89883878 | 89884289 | - | 14.5 | 5877 | 6298 | -5 | L1MA4A | L1 | LINE |
| 89884290 | 89884639 | - | 17.9 | 2885 | 3247 | -2893 | L1MA9 | L1 | LINE |

  

|  |  |  |  |  |  |  |  |  |  |
| --- | --- | --- | --- | --- | --- | --- | --- | --- | --- |
| chr8:91696659-91699127 | | | 2458 bp | | |  |  |  |  |
| Genome Start | Genome End | Strand | Div | Repeat Start | Repeat End | Repeat Left | Name | Family | Class |
| 91696659 | 91696766 | + | 26.9 | 6102 | 6212 | -51 | HUERS-P1 | ERV1 | LTR |
| 91696772 | 91697122 | + | 10.3 | 6 | 362 | -2 | THE1B | MaLR | LTR |
| 91697127 | 91698721 | + | 30.0 | 2 | 1735 | 0 | MLT1F2-int | MaLR | LTR |
| 91698724 | 91699072 | + | 10.0 | 3 | 364 | 0 | THE1B | MaLR | LTR |
| 91699073 | 91699127 | + | 20.0 | 6209 | 6263 | 0 | HUERS-P1 | ERV1 | LTR |

  

|  |  |  |  |  |  |  |  |  |  |
| --- | --- | --- | --- | --- | --- | --- | --- | --- | --- |
| chr8:91949238-91952256 | | | 2654 bp | | |  |  |  |  |
| Genome Start | Genome End | Strand | Div | Repeat Start | Repeat End | Repeat Left | Name | Family | Class |
| 91949238 | 91949294 | + | 36.4 | 2710 | 2773 | -646 | L2 | L2 | LINE |
| 91949295 | 91949681 | + | 14.9 | 1 | 427 | -1 | MSTA | MaLR | LTR |
| 91949685 | 91951288 | + | 29.9 | 2 | 1735 | 0 | MLT-int | MaLR | LTR |
| 91951289 | 91951677 | + | 14.1 | 1 | 428 | 0 | MSTA | MaLR | LTR |
| 91951678 | 91951833 | + | 36.4 | 2774 | 2926 | -493 | L2 | L2 | LINE |
| 91952196 | 91952256 | + | 22.9 | 3351 | 3419 | 0 | L2 | L2 | LINE |

  

|  |  |  |  |  |  |  |  |  |  |
| --- | --- | --- | --- | --- | --- | --- | --- | --- | --- |
| chr8:93976043-93979976 | | | 3932 bp | | |  |  |  |  |
| Genome Start | Genome End | Strand | Div | Repeat Start | Repeat End | Repeat Left | Name | Family | Class |
| 93976043 | 93976341 | - | 25.2 | 860 | 1161 | -5381 | L1M4 | L1 | LINE |
| 93976342 | 93976737 | + | 23.3 | 1 | 396 | 0 | MSTD | MaLR | LTR |
| 93976740 | 93978209 | + | 24.4 | 2 | 1484 | -251 | MSTD-int | MaLR | LTR |
| 93978210 | 93978520 | - | 11.9 | 1 | 310 | -2 | AluSx | Alu | SINE |
| 93978521 | 93978760 | + | 24.4 | 1485 | 1735 | 0 | MSTD-int | MaLR | LTR |
| 93978761 | 93979104 | + | 18.7 | 1 | 396 | 0 | MSTD | MaLR | LTR |
| 93979105 | 93979976 | - | 25.2 | 24 | 859 | -5683 | L1M4 | L1 | LINE |

  

|  |  |  |  |  |  |  |  |  |  |
| --- | --- | --- | --- | --- | --- | --- | --- | --- | --- |
| chr8:94080869-94092571 | | | 11544 bp | | |  |  |  |  |
| Genome Start | Genome End | Strand | Div | Repeat Start | Repeat End | Repeat Left | Name | Family | Class |
| 94080869 | 94081624 | - | 25.9 | 823 | 1602 | -2429 | Zaphod | Tip100 | DNA |
| 94081644 | 94082014 | - | 15.4 | 1 | 364 | 0 | THE1B | MaLR | LTR |
| 94082023 | 94082429 | + | 22.9 | 3180 | 3599 | -3963 | L1M1 | L1 | LINE |
| 94082430 | 94082726 | + | 10.1 | 1 | 298 | -13 | AluY | Alu | SINE |
| 94082727 | 94084041 | + | 16.1 | 3600 | 4999 | -2563 | L1M1 | L1 | LINE |
| 94084042 | 94084338 | + | 14.1 | 1 | 297 | -16 | AluSq | Alu | SINE |
| 94084339 | 94084542 | + | 16.1 | 5000 | 5218 | -2344 | L1M1 | L1 | LINE |
| 94084543 | 94084713 | - | 19.3 | 227 | 428 | 0 | MSTA | MaLR | LTR |
| 94084745 | 94084937 | - | 19.3 | 1 | 226 | -202 | MSTA | MaLR | LTR |
| 94084941 | 94086493 | - | 29.9 | 2 | 1733 | -2 | MLT1F2-int | MaLR | LTR |
| 94086496 | 94086880 | - | 14.9 | 1 | 428 | 0 | MSTA | MaLR | LTR |
| 94086881 | 94087552 | + | 16.1 | 5219 | 5888 | -1674 | L1M1 | L1 | LINE |
| 94087561 | 94089218 | + | 10.8 | 1 | 1607 | -4746 | L1PA10 | L1 | LINE |
| 94089251 | 94089289 | - | 5.1 | 1580 | 1618 | -4735 | L1PA10 | L1 | LINE |
| 94089290 | 94089712 | + | 9.8 | 5738 | 6164 | -4 | L1PA10 | L1 | LINE |
| 94089716 | 94090731 | + | 16.9 | 5880 | 6908 | -811 | L1M1 | L1 | LINE |
| 94090732 | 94091037 | + | 12.1 | 1 | 308 | -4 | AluSx | Alu | SINE |
| 94091038 | 94091819 | + | 15.4 | 5490 | 6299 | -1 | L1MA6 | L1 | LINE |
| 94091873 | 94092571 | - | 26.3 | 73 | 872 | -3159 | Zaphod | Tip100 | DNA |

  

|  |  |  |  |  |  |  |  |  |  |
| --- | --- | --- | --- | --- | --- | --- | --- | --- | --- |
| chr8:94461316-94464769 | | | 3452 bp | | |  |  |  |  |
| Genome Start | Genome End | Strand | Div | Repeat Start | Repeat End | Repeat Left | Name | Family | Class |
| 94461316 | 94462059 | + | 14.0 | 2 | 793 | -438 | Tigger3b | MER2\_type | DNA |
| 94462060 | 94462407 | - | 11.0 | 1 | 355 | 0 | THE1A | MaLR | LTR |
| 94462408 | 94463960 | - | 28.7 | 2 | 1735 | 0 | MLT-int | MaLR | LTR |
| 94463963 | 94464317 | - | 13.1 | 1 | 355 | 0 | THE1A | MaLR | LTR |
| 94464318 | 94464769 | + | 14.0 | 794 | 1230 | -1 | Tigger3b | MER2\_type | DNA |

  

|  |  |  |  |  |  |  |  |  |  |
| --- | --- | --- | --- | --- | --- | --- | --- | --- | --- |
| chr8:97757396-97766192 | | | 8770 bp | | |  |  |  |  |
| Genome Start | Genome End | Strand | Div | Repeat Start | Repeat End | Repeat Left | Name | Family | Class |
| 97757396 | 97758067 | - | 13.1 | 6350 | 6996 | -6 | L1MA4 | L1 | LINE |
| 97758086 | 97759806 | - | 10.8 | 4421 | 6149 | -2 | L1PB2 | L1 | LINE |
| 97759799 | 97760553 | - | 13.4 | 5595 | 6378 | -624 | L1MA4 | L1 | LINE |
| 97760554 | 97761672 | - | 16.2 | 3500 | 4622 | -2223 | L1MA4 | L1 | LINE |
| 97761686 | 97762031 | + | 11.4 | 1 | 361 | -3 | THE1B | MaLR | LTR |
| 97762036 | 97763579 | + | 10.5 | 1 | 1580 | 0 | THE1B-int | MaLR | LTR |
| 97763580 | 97763953 | + | 13.8 | 1 | 364 | 0 | THE1B | MaLR | LTR |
| 97763954 | 97766192 | - | 20.4 | 969 | 3242 | -3603 | L1MA4 | L1 | LINE |

  

|  |  |  |  |  |  |  |  |  |  |
| --- | --- | --- | --- | --- | --- | --- | --- | --- | --- |
| chr8:97790517-97797661 | | | 7100 bp | | |  |  |  |  |
| Genome Start | Genome End | Strand | Div | Repeat Start | Repeat End | Repeat Left | Name | Family | Class |
| 97790517 | 97791045 | - | 12.9 | 5659 | 6204 | -7 | L1PB3 | L1 | LINE |
| 97791089 | 97792823 | - | 16.4 | 2053 | 3906 | -2297 | L1PB3 | L1 | LINE |
| 97792824 | 97793197 | - | 10.3 | 1 | 375 | 0 | THE1C | MaLR | LTR |
| 97793198 | 97794772 | - | 30.5 | 2 | 1735 | 0 | MLT-int | MaLR | LTR |
| 97794775 | 97795136 | - | 10.3 | 1 | 375 | 0 | THE1C | MaLR | LTR |
| 97795137 | 97797661 | - | 17.7 | 1 | 2052 | -4152 | L1PB3 | L1 | LINE |

  

|  |  |  |  |  |  |  |  |  |  |
| --- | --- | --- | --- | --- | --- | --- | --- | --- | --- |
| chr8:98463540-98467433 | | | 3872 bp | | |  |  |  |  |
| Genome Start | Genome End | Strand | Div | Repeat Start | Repeat End | Repeat Left | Name | Family | Class |
| 98463540 | 98463740 | + | 25.1 | 15 | 205 | -658 | MER21B | ERV1 | LTR |
| 98463762 | 98464214 | + | 25.1 | 206 | 626 | -237 | MER21B | ERV1 | LTR |
| 98464215 | 98464630 | - | 17.1 | 1 | 428 | 0 | MSTA | MaLR | LTR |
| 98464631 | 98464835 | - | 16.5 | 1444 | 1651 | 0 | MSTA-int | MaLR | LTR |
| 98464836 | 98465468 | - | 17.1 | 1 | 612 | 0 | LTR9 | ERV1 | LTR |
| 98465469 | 98466869 | - | 16.5 | 1 | 1443 | -208 | MSTA-int | MaLR | LTR |
| 98466870 | 98467259 | - | 16.9 | 1 | 428 | 0 | MSTA | MaLR | LTR |
| 98467261 | 98467433 | + | 13.2 | 624 | 803 | -60 | MER21B | ERV1 | LTR |

  

|  |  |  |  |  |  |  |  |  |  |
| --- | --- | --- | --- | --- | --- | --- | --- | --- | --- |
| chr8:98591866-98594824 | | | 2805 bp | | |  |  |  |  |
| Genome Start | Genome End | Strand | Div | Repeat Start | Repeat End | Repeat Left | Name | Family | Class |
| 98591866 | 98591941 | + | 36.8 | 3087 | 3167 | -211 | L2 | L2 | LINE |
| 98591977 | 98592537 | + | 29.9 | 3 | 595 | 0 | MLT1G1 | MaLR | LTR |
| 98592579 | 98593885 | + | 26.0 | 24 | 1489 | 0 | MLT1G1-int | MaLR | LTR |
| 98593886 | 98594454 | + | 24.3 | 1 | 595 | 0 | MLT1G1 | MaLR | LTR |
| 98594491 | 98594561 | + | 39.4 | 27 | 100 | -162 | MIR | MIR | SINE |
| 98594588 | 98594649 | + | 31.1 | 174 | 234 | -34 | MIRb | MIR | SINE |
| 98594666 | 98594824 | + | 27.3 | 3215 | 3378 | -41 | L2 | L2 | LINE |

  

|  |  |  |  |  |  |  |  |  |  |
| --- | --- | --- | --- | --- | --- | --- | --- | --- | --- |
| chr8:99654416-99656640 | | | 2157 bp | | |  |  |  |  |
| Genome Start | Genome End | Strand | Div | Repeat Start | Repeat End | Repeat Left | Name | Family | Class |
| 99654416 | 99654632 | + | 25.8 | 32 | 262 | -1227 | MLT1F1-int | MaLR | LTR |
| 99654636 | 99654983 | - | 12.1 | 1 | 375 | 0 | THE1C | MaLR | LTR |
| 99654984 | 99655131 | - | 4.8 | 1433 | 1580 | 0 | THE1C-int | MaLR | LTR |
| 99655165 | 99655524 | - | 12.6 | 1 | 375 | 0 | THE1C | MaLR | LTR |
| 99655557 | 99656640 | + | 23.5 | 283 | 1487 | -2 | MLT1F1-int | MaLR | LTR |

  

|  |  |  |  |  |  |  |  |  |  |
| --- | --- | --- | --- | --- | --- | --- | --- | --- | --- |
| chr8:101136266-101141176 | | | 4909 bp | | |  |  |  |  |
| Genome Start | Genome End | Strand | Div | Repeat Start | Repeat End | Repeat Left | Name | Family | Class |
| 101136266 | 101136963 | + | 11.9 | 1311 | 2046 | -5519 | L1M1 | L1 | LINE |
| 101136964 | 101137293 | + | 10.4 | 3 | 364 | 0 | THE1B | MaLR | LTR |
| 101137296 | 101138838 | + | 29.8 | 2 | 1735 | 0 | MLT-int | MaLR | LTR |
| 101138839 | 101139192 | + | 12.8 | 1 | 360 | -4 | THE1B | MaLR | LTR |
| 101139193 | 101139952 | + | 11.1 | 2047 | 2825 | -4740 | L1M1 | L1 | LINE |
| 101139953 | 101139998 | - | 4.3 | 1 | 46 | -34 | MADE1 | Mariner | DNA |
| 101139999 | 101141176 | + | 11.1 | 2826 | 4032 | -3533 | L1M1 | L1 | LINE |

  

|  |  |  |  |  |  |  |  |  |  |
| --- | --- | --- | --- | --- | --- | --- | --- | --- | --- |
| chr8:101626447-101629052 | | | 2601 bp | | |  |  |  |  |
| Genome Start | Genome End | Strand | Div | Repeat Start | Repeat End | Repeat Left | Name | Family | Class |
| 101626447 | 101626644 | + | 15.4 | 1 | 198 | -114 | AluJo | Alu | SINE |
| 101626645 | 101627008 | + | 14.9 | 1 | 381 | 0 | THE1D | MaLR | LTR |
| 101627012 | 101628589 | + | 28.4 | 3 | 1735 | 0 | MLT1J-int | MaLR | LTR |
| 101628592 | 101628936 | + | 15.0 | 3 | 381 | 0 | THE1D | MaLR | LTR |
| 101628937 | 101629052 | + | 15.4 | 199 | 309 | -3 | AluJo | Alu | SINE |

  

|  |  |  |  |  |  |  |  |  |  |
| --- | --- | --- | --- | --- | --- | --- | --- | --- | --- |
| chr8:103253859-103256637 | | | 2779 bp | | |  |  |  |  |
| Genome Start | Genome End | Strand | Div | Repeat Start | Repeat End | Repeat Left | Name | Family | Class |
| 103253859 | 103253958 | - | 25.9 | 305 | 409 | 0 | MLT1I | MaLR | LTR |
| 103253959 | 103254362 | - | 16.1 | 1 | 428 | 0 | MSTA | MaLR | LTR |
| 103254363 | 103255973 | - | 28.7 | 1 | 1735 | 0 | MLT1I-int | MaLR | LTR |
| 103255974 | 103256366 | - | 16.7 | 1 | 428 | 0 | MSTA | MaLR | LTR |
| 103256367 | 103256637 | - | 25.9 | 58 | 304 | -105 | MLT1I | MaLR | LTR |

  

|  |  |  |  |  |  |  |  |  |  |
| --- | --- | --- | --- | --- | --- | --- | --- | --- | --- |
| chr8:104351004-104356427 | | | 5383 bp | | |  |  |  |  |
| Genome Start | Genome End | Strand | Div | Repeat Start | Repeat End | Repeat Left | Name | Family | Class |
| 104351004 | 104351603 | - | 18.2 | 6379 | 7010 | -28 | L1MB8 | L1 | LINE |
| 104351604 | 104351975 | + | 15.0 | 1 | 428 | 0 | MSTA | MaLR | LTR |
| 104351978 | 104353594 | + | 29.4 | 2 | 1735 | 0 | MLT-int | MaLR | LTR |
| 104353596 | 104353977 | + | 14.7 | 2 | 428 | 0 | MSTA | MaLR | LTR |
| 104353978 | 104354823 | - | 15.9 | 5487 | 6378 | -660 | L1MB8 | L1 | LINE |
| 104354825 | 104355283 | - | 17.5 | 3388 | 3842 | -3164 | L1MB8 | L1 | LINE |
| 104355320 | 104355507 | - | 17.0 | 92 | 286 | -26 | AluJo | Alu | SINE |
| 104355509 | 104356427 | - | 17.9 | 1205 | 2196 | -5138 | L1MB8 | L1 | LINE |

  

|  |  |  |  |  |  |  |  |  |  |
| --- | --- | --- | --- | --- | --- | --- | --- | --- | --- |
| chr8:104747783-104756411 | | | 8562 bp | | |  |  |  |  |
| Genome Start | Genome End | Strand | Div | Repeat Start | Repeat End | Repeat Left | Name | Family | Class |
| 104747783 | 104747913 | - | 20.9 | 7400 | 7537 | 0 | MER57A-int | ERV1 | LTR |
| 104747924 | 104748174 | - | 19.2 | 2 | 291 | -207 | LTR54 | ERV1 | LTR |
| 104748179 | 104750055 | - | 15.0 | 3864 | 5788 | -399 | MER57A-int | ERV1 | LTR |
| 104750103 | 104750248 | - | 23.3 | 1 | 146 | -30 | FRAM | Alu | SINE |
| 104750250 | 104750411 | - | 19.1 | 3708 | 3866 | -2321 | MER57A-int | ERV1 | LTR |
| 104750416 | 104750770 | - | 14.1 | 1 | 360 | -4 | THE1B | MaLR | LTR |
| 104750772 | 104752350 | - | 9.4 | 1 | 1579 | -1 | THE1B-int | MaLR | LTR |
| 104752351 | 104752709 | - | 13.7 | 1 | 364 | 0 | THE1B | MaLR | LTR |
| 104752710 | 104753739 | - | 14.2 | 2665 | 3712 | -2475 | MER57A-int | ERV1 | LTR |
| 104753740 | 104754158 | - | 18.8 | 1 | 428 | 0 | MSTA | MaLR | LTR |
| 104754159 | 104755753 | - | 18.5 | 7 | 1651 | 0 | MSTA-int | MaLR | LTR |
| 104755754 | 104756411 | - | 17.1 | 2032 | 2669 | -3518 | MER57A-int | ERV1 | LTR |

  

|  |  |  |  |  |  |  |  |  |  |
| --- | --- | --- | --- | --- | --- | --- | --- | --- | --- |
| chr8:105687785-105690814 | | | 3024 bp | | |  |  |  |  |
| Genome Start | Genome End | Strand | Div | Repeat Start | Repeat End | Repeat Left | Name | Family | Class |
| 105687785 | 105687841 | - | 16.4 | 6109 | 6159 | -6 | L1PA15 | L1 | LINE |
| 105687842 | 105688205 | + | 10.5 | 1 | 364 | 0 | THE1B | MaLR | LTR |
| 105688208 | 105689733 | + | 28.3 | 2 | 1735 | 0 | MLT-int | MaLR | LTR |
| 105689734 | 105690093 | + | 11.4 | 1 | 364 | 0 | THE1B | MaLR | LTR |
| 105690094 | 105690202 | - | 16.4 | 6001 | 6108 | -57 | L1PA15 | L1 | LINE |
| 105690207 | 105690814 | - | 10.9 | 5331 | 5934 | -231 | L1PA15 | L1 | LINE |

  

|  |  |  |  |  |  |  |  |  |  |
| --- | --- | --- | --- | --- | --- | --- | --- | --- | --- |
| chr8:106332554-106338898 | | | 6341 bp | | |  |  |  |  |
| Genome Start | Genome End | Strand | Div | Repeat Start | Repeat End | Repeat Left | Name | Family | Class |
| 106332554 | 106333382 | - | 11.1 | 6892 | 7723 | 0 | L1MA2 | L1 | LINE |
| 106333383 | 106333675 | - | 10.6 | 1 | 293 | -19 | AluSx | Alu | SINE |
| 106333676 | 106334659 | - | 10.7 | 5903 | 6891 | -832 | L1MA2 | L1 | LINE |
| 106334660 | 106336293 | - | 14.3 | 3559 | 5220 | -2345 | L1MA2 | L1 | LINE |
| 106336294 | 106336647 | - | 13.9 | 1 | 355 | 0 | THE1A | MaLR | LTR |
| 106336648 | 106338221 | - | 11.3 | 1 | 1580 | 0 | THE1A-int | MaLR | LTR |
| 106338226 | 106338572 | - | 15.7 | 1 | 351 | -4 | THE1A | MaLR | LTR |
| 106338573 | 106338898 | - | 13.2 | 3221 | 3563 | -4002 | L1MA2 | L1 | LINE |

  

|  |  |  |  |  |  |  |  |  |  |
| --- | --- | --- | --- | --- | --- | --- | --- | --- | --- |
| chr8:106982929-106985509 | | | 2579 bp | | |  |  |  |  |
| Genome Start | Genome End | Strand | Div | Repeat Start | Repeat End | Repeat Left | Name | Family | Class |
| 106982929 | 106983105 | + | 18.1 | 1 | 165 | -147 | AluJo | Alu | SINE |
| 106983106 | 106983491 | - | 15.8 | 1 | 426 | -2 | MSTA | MaLR | LTR |
| 106983492 | 106984974 | - | 31.1 | 2 | 1735 | 0 | MLT-int | MaLR | LTR |
| 106984977 | 106985345 | - | 18.6 | 1 | 428 | 0 | MSTA | MaLR | LTR |
| 106985346 | 106985509 | + | 18.1 | 166 | 304 | -8 | AluJo | Alu | SINE |

  

|  |  |  |  |  |  |  |  |  |  |
| --- | --- | --- | --- | --- | --- | --- | --- | --- | --- |
| chr8:106996928-107006100 | | | 9166 bp | | |  |  |  |  |
| Genome Start | Genome End | Strand | Div | Repeat Start | Repeat End | Repeat Left | Name | Family | Class |
| 106996928 | 106998836 | + | 22.1 | 578 | 2293 | -4284 | L1PA17 | L1 | LINE |
| 106998837 | 106999214 | + | 10.7 | 1 | 374 | -1 | THE1C | MaLR | LTR |
| 106999216 | 107000704 | + | 12.4 | 1 | 1535 | -30 | THE1C-int | MaLR | LTR |
| 107000707 | 107001078 | + | 12.5 | 3 | 375 | 0 | THE1C | MaLR | LTR |
| 107001083 | 107002531 | + | 17.5 | 2293 | 3737 | -2836 | L1PA17 | L1 | LINE |
| 107002532 | 107003222 | + | 14.5 | 1 | 691 | 0 | LTR8 | ERV1 | LTR |
| 107003223 | 107006100 | + | 14.9 | 3738 | 6591 | -2 | L1PA17 | L1 | LINE |

  

|  |  |  |  |  |  |  |  |  |  |
| --- | --- | --- | --- | --- | --- | --- | --- | --- | --- |
| chr8:107751714-107756832 | | | 4856 bp | | |  |  |  |  |
| Genome Start | Genome End | Strand | Div | Repeat Start | Repeat End | Repeat Left | Name | Family | Class |
| 107751714 | 107752259 | + | 30.5 | 580 | 1184 | -4935 | L1MEd | L1 | LINE |
| 107752260 | 107752470 | + | 12.3 | 90 | 304 | -8 | AluSg/x | Alu | SINE |
| 107752471 | 107752541 | + | 30.5 | 1185 | 1255 | -4864 | L1MEd | L1 | LINE |
| 107752545 | 107752720 | + | 30.6 | 1353 | 1538 | -4611 | L1MEd | L1 | LINE |
| 107752860 | 107752921 | + | 24.2 | 1862 | 1923 | -4226 | L1MEd | L1 | LINE |
| 107752922 | 107753367 | + | 4.3 | 5692 | 6136 | -18 | L1PA5 | L1 | LINE |
| 107753411 | 107753596 | + | 24.2 | 1924 | 2106 | -4043 | L1MEd | L1 | LINE |
| 107753601 | 107753996 | + | 16.2 | 2 | 390 | 0 | AluSx | Alu | SINE |
| 107754034 | 107754410 | + | 13.2 | 3 | 381 | 0 | THE1D | MaLR | LTR |
| 107754413 | 107756020 | + | 30.8 | 2 | 1735 | 0 | MLT-int | MaLR | LTR |
| 107756021 | 107756389 | + | 13.1 | 1 | 381 | 0 | THE1D | MaLR | LTR |
| 107756425 | 107756832 | + | 26.4 | 2285 | 2701 | -3428 | L1MEd | L1 | LINE |

  

|  |  |  |  |  |  |  |  |  |  |
| --- | --- | --- | --- | --- | --- | --- | --- | --- | --- |
| chr8:108157128-108161909 | | | 4743 bp | | |  |  |  |  |
| Genome Start | Genome End | Strand | Div | Repeat Start | Repeat End | Repeat Left | Name | Family | Class |
| 108157128 | 108157678 | - | 28.5 | 2807 | 3417 | -2 | L2 | L2 | LINE |
| 108157709 | 108159173 | - | 15.4 | 4841 | 6296 | -4 | L1MA4 | L1 | LINE |
| 108159174 | 108159552 | - | 11.3 | 1 | 375 | 0 | THE1C | MaLR | LTR |
| 108159553 | 108161075 | - | 29.3 | 2 | 1735 | 0 | MLT-int | MaLR | LTR |
| 108161078 | 108161442 | - | 11.5 | 7 | 375 | 0 | THE1C | MaLR | LTR |
| 108161447 | 108161709 | - | 14.8 | 4574 | 4842 | -1301 | L1MA4 | L1 | LINE |
| 108161713 | 108161909 | - | 26.2 | 2618 | 2815 | -604 | L2 | L2 | LINE |

  

|  |  |  |  |  |  |  |  |  |  |
| --- | --- | --- | --- | --- | --- | --- | --- | --- | --- |
| chr8:108206535-108210132 | | | 3585 bp | | |  |  |  |  |
| Genome Start | Genome End | Strand | Div | Repeat Start | Repeat End | Repeat Left | Name | Family | Class |
| 108206535 | 108207708 | + | 14.6 | 1 | 1198 | -89 | HSMAR1 | Mariner | DNA |
| 108207709 | 108208083 | - | 12.2 | 2 | 375 | 0 | THE1C | MaLR | LTR |
| 108208096 | 108209658 | - | 12.1 | 1 | 1575 | -5 | THE1C-int | MaLR | LTR |
| 108209659 | 108210039 | - | 16.3 | 1 | 375 | 0 | THE1C | MaLR | LTR |
| 108210041 | 108210132 | + | 15.2 | 1195 | 1286 | -1 | HSMAR1 | Mariner | DNA |

  

|  |  |  |  |  |  |  |  |  |  |
| --- | --- | --- | --- | --- | --- | --- | --- | --- | --- |
| chr8:110092753-110095385 | | | 2551 bp | | |  |  |  |  |
| Genome Start | Genome End | Strand | Div | Repeat Start | Repeat End | Repeat Left | Name | Family | Class |
| 110092753 | 110092878 | - | 22.4 | 7901 | 8038 | -4 | L1MC4 | L1 | LINE |
| 110092933 | 110093247 | + | 11.8 | 1 | 364 | 0 | THE1B | MaLR | LTR |
| 110093250 | 110094823 | + | 29.8 | 2 | 1735 | 0 | MLT-int | MaLR | LTR |
| 110094824 | 110095063 | + | 12.0 | 1 | 250 | -114 | THE1B | MaLR | LTR |
| 110095090 | 110095385 | - | 26.5 | 7599 | 7889 | -153 | L1MC4 | L1 | LINE |

  

|  |  |  |  |  |  |  |  |  |  |
| --- | --- | --- | --- | --- | --- | --- | --- | --- | --- |
| chr8:110379799-110394695 | | | 14636 bp | | |  |  |  |  |
| Genome Start | Genome End | Strand | Div | Repeat Start | Repeat End | Repeat Left | Name | Family | Class |
| 110379799 | 110379953 | + | 31.1 | 4870 | 5025 | -1121 | L1M5 | L1 | LINE |
| 110380011 | 110380109 | + | 29.6 | 5197 | 5298 | -848 | L1MD | L1 | LINE |
| 110380147 | 110382480 | + | 17.0 | 6 | 2618 | -4245 | L1PBa | L1 | LINE |
| 110382482 | 110382902 | + | 6.8 | 1 | 464 | 0 | LTR7B | ERV1 | LTR |
| 110382903 | 110385440 | + | 14.9 | 1 | 2830 | -4883 | HERVH | ERV1 | LTR |
| 110385441 | 110385672 | + | 16.0 | 3357 | 3587 | -4126 | HERVH | ERV1 | LTR |
| 110385667 | 110387861 | + | 13.8 | 3806 | 6016 | -1697 | HERVH | ERV1 | LTR |
| 110387855 | 110388438 | + | 11.9 | 7157 | 7713 | 0 | HERVH | ERV1 | LTR |
| 110388439 | 110388864 | + | 7.7 | 1 | 464 | 0 | LTR7B | ERV1 | LTR |
| 110388865 | 110390094 | + | 8.1 | 2615 | 4181 | -2681 | L1PB1 | L1 | LINE |
| 110390095 | 110390393 | + | 9.4 | 1 | 298 | -13 | AluY | Alu | SINE |
| 110390394 | 110392212 | + | 6.9 | 4182 | 6049 | -813 | L1PB1 | L1 | LINE |
| 110392213 | 110392518 | - | 8.5 | 1 | 306 | -3 | AluSc | Alu | SINE |
| 110392519 | 110393352 | + | 8.5 | 6051 | 6867 | 0 | L1PB1 | L1 | LINE |
| 110393426 | 110393725 | + | 10.0 | 1 | 304 | -8 | AluSx | Alu | SINE |
| 110393832 | 110394087 | + | 28.2 | 5533 | 5759 | -362 | L1M5 | L1 | LINE |
| 110394088 | 110394656 | + | 20.7 | 5 | 614 | 0 | MER90a | ERV1 | LTR |
| 110394657 | 110394695 | + | 28.2 | 5760 | 5801 | -320 | L1M5 | L1 | LINE |

  

|  |  |  |  |  |  |  |  |  |  |
| --- | --- | --- | --- | --- | --- | --- | --- | --- | --- |
| chr8:111026340-111030413 | | | 4072 bp | | |  |  |  |  |
| Genome Start | Genome End | Strand | Div | Repeat Start | Repeat End | Repeat Left | Name | Family | Class |
| 111026340 | 111026996 | - | 20.1 | 6847 | 7474 | -486 | L1MC3 | L1 | LINE |
| 111026997 | 111027307 | + | 10.6 | 1 | 355 | 0 | THE1A | MaLR | LTR |
| 111027310 | 111028865 | + | 30.0 | 2 | 1735 | 0 | MLT-int | MaLR | LTR |
| 111028866 | 111029219 | + | 10.4 | 1 | 355 | 0 | THE1A | MaLR | LTR |
| 111029220 | 111030413 | - | 20.1 | 5557 | 6846 | -1114 | L1MC3 | L1 | LINE |

  

|  |  |  |  |  |  |  |  |  |  |
| --- | --- | --- | --- | --- | --- | --- | --- | --- | --- |
| chr8:111081730-111090452 | | | 8641 bp | | |  |  |  |  |
| Genome Start | Genome End | Strand | Div | Repeat Start | Repeat End | Repeat Left | Name | Family | Class |
| 111081730 | 111081943 | - | 28.0 | 5429 | 5655 | -1910 | L1M1 | L1 | LINE |
| 111081958 | 111082740 | - | 15.3 | 5465 | 6303 | 0 | L1MA4A | L1 | LINE |
| 111082741 | 111083740 | - | 18.1 | 3717 | 4724 | -1419 | L1MA4A | L1 | LINE |
| 111083742 | 111084565 | - | 11.6 | 5489 | 6304 | 0 | L1MA2 | L1 | LINE |
| 111084567 | 111084712 | - | 14.5 | 3811 | 3958 | -2185 | L1M2 | L1 | LINE |
| 111084721 | 111084753 | - | 9.1 | 4841 | 4873 | -1273 | L1MA2 | L1 | LINE |
| 111084754 | 111085155 | - | 15.0 | 3 | 428 | 0 | MSTA | MaLR | LTR |
| 111085217 | 111086065 | - | 16.3 | 1 | 888 | -763 | MSTA-int | MaLR | LTR |
| 111086066 | 111086469 | - | 13.2 | 1 | 428 | 0 | MSTA | MaLR | LTR |
| 111086470 | 111087367 | - | 14.4 | 4005 | 4845 | -1301 | L1MA2 | L1 | LINE |
| 111087365 | 111087404 | - | 13.5 | 5155 | 5192 | -2373 | L1M1 | L1 | LINE |
| 111087405 | 111087700 | - | 11.8 | 1 | 296 | -14 | AluSg | Alu | SINE |
| 111087701 | 111090452 | - | 15.4 | 2223 | 5154 | -2411 | L1M1 | L1 | LINE |

  

|  |  |  |  |  |  |  |  |  |  |
| --- | --- | --- | --- | --- | --- | --- | --- | --- | --- |
| chr8:111090455-111096426 | | | 5941 bp | | |  |  |  |  |
| Genome Start | Genome End | Strand | Div | Repeat Start | Repeat End | Repeat Left | Name | Family | Class |
| 111090455 | 111090844 | - | 12.3 | 5146 | 5547 | -784 | L1PA10 | L1 | LINE |
| 111090845 | 111091202 | + | 20.6 | 1 | 401 | -27 | MSTA | MaLR | LTR |
| 111091231 | 111091251 | + | 20.0 | 295 | 314 | -41 | THE1D | MaLR | LTR |
| 111091252 | 111091561 | - | 15.8 | 1 | 312 | 0 | AluSx | Alu | SINE |
| 111091562 | 111091601 | + | 7.5 | 342 | 381 | 0 | THE1D | MaLR | LTR |
| 111091603 | 111093188 | + | 15.7 | 3 | 1580 | 0 | THE1D-int | MaLR | LTR |
| 111093191 | 111093570 | + | 16.3 | 3 | 381 | 0 | THE1D | MaLR | LTR |
| 111093571 | 111096426 | - | 12.1 | 2257 | 5150 | -1181 | L1PA10 | L1 | LINE |

  

|  |  |  |  |  |  |  |  |  |  |
| --- | --- | --- | --- | --- | --- | --- | --- | --- | --- |
| chr8:112453454-112457581 | | | 3747 bp | | |  |  |  |  |
| Genome Start | Genome End | Strand | Div | Repeat Start | Repeat End | Repeat Left | Name | Family | Class |
| 112453454 | 112453949 | + | 37.4 | 2643 | 3132 | -2156 | ERVL-B4 | ERVL | LTR |
| 112453950 | 112454307 | + | 13.8 | 1 | 364 | 0 | THE1B | MaLR | LTR |
| 112454310 | 112455828 | + | 30.1 | 2 | 1735 | 0 | MLT-int | MaLR | LTR |
| 112455829 | 112456180 | + | 14.6 | 1 | 358 | -6 | THE1B | MaLR | LTR |
| 112456483 | 112456992 | + | 24.6 | 1 | 510 | -4 | MER67D | ERV1 | LTR |
| 112457070 | 112457581 | + | 34.1 | 3720 | 4221 | -1446 | ERVL-B4 | ERVL | LTR |

  

|  |  |  |  |  |  |  |  |  |  |
| --- | --- | --- | --- | --- | --- | --- | --- | --- | --- |
| chr8:112498312-112506998 | | | 8628 bp | | |  |  |  |  |
| Genome Start | Genome End | Strand | Div | Repeat Start | Repeat End | Repeat Left | Name | Family | Class |
| 112498312 | 112498491 | - | 24.7 | 4373 | 4567 | -2641 | L1M2 | L1 | LINE |
| 112498496 | 112498950 | - | 23.6 | 1 | 560 | -7 | MLT2B1 | ERVL | LTR |
| 112498967 | 112499231 | - | 24.8 | 4085 | 4354 | -2854 | L1M2 | L1 | LINE |
| 112499232 | 112499393 | - | 18.0 | 436 | 603 | 0 | LTR26 | ERV1 | LTR |
| 112499394 | 112499569 | + | 11.4 | 5971 | 6146 | -5 | L1PB1 | L1 | LINE |
| 112499570 | 112499601 | - | 27.4 | 347 | 376 | -155 | LTR26B | ERV1 | LTR |
| 112499602 | 112501539 | - | 22.6 | 1680 | 3680 | -3525 | L1M2 | L1 | LINE |
| 112501559 | 112501668 | - | 26.6 | 306 | 414 | -6795 | L1M2 | L1 | LINE |
| 112501670 | 112502016 | + | 13.3 | 1 | 364 | 0 | THE1B | MaLR | LTR |
| 112502017 | 112502359 | + | 11.4 | 1 | 344 | -1236 | THE1B-int | MaLR | LTR |
| 112502374 | 112503964 | - | 9.5 | 4558 | 6143 | -8 | L1PB1 | L1 | LINE |
| 112503966 | 112504734 | + | 8.2 | 2523 | 3285 | -2861 | L1PB | L1 | LINE |
| 112504735 | 112505972 | + | 10.4 | 335 | 1576 | -4 | THE1B-int | MaLR | LTR |
| 112505977 | 112506144 | + | 15.9 | 1 | 182 | -182 | THE1B | MaLR | LTR |
| 112506145 | 112506461 | + | 0.9 | 1 | 317 | -1 | AluYb8 | Alu | SINE |
| 112506462 | 112506650 | + | 15.9 | 183 | 364 | 0 | THE1B | MaLR | LTR |
| 112506651 | 112506998 | - | 28.5 | 14 | 308 | -6901 | L1M2 | L1 | LINE |

  

|  |  |  |  |  |  |  |  |  |  |
| --- | --- | --- | --- | --- | --- | --- | --- | --- | --- |
| chr8:112926163-112942620 | | | 15949 bp | | |  |  |  |  |
| Genome Start | Genome End | Strand | Div | Repeat Start | Repeat End | Repeat Left | Name | Family | Class |
| 112926163 | 112926875 | - | 15.9 | 7002 | 7719 | 0 | L1MA6 | L1 | LINE |
| 112926880 | 112927239 | - | 15.8 | 1 | 428 | 0 | MSTA | MaLR | LTR |
| 112927246 | 112928867 | - | 17.6 | 1 | 1645 | -6 | MSTA-int | MaLR | LTR |
| 112928868 | 112929247 | - | 14.8 | 8 | 428 | 0 | MSTA | MaLR | LTR |
| 112929254 | 112930094 | - | 20.3 | 6165 | 7006 | -704 | L1MA6 | L1 | LINE |
| 112930095 | 112930310 | - | 8.9 | 220 | 444 | 0 | MLT2A1 | ERVL | LTR |
| 112930311 | 112930568 | - | 20.3 | 5907 | 6164 | -1546 | L1MA6 | L1 | LINE |
| 112930569 | 112930878 | - | 8.4 | 1 | 309 | -2 | AluY | Alu | SINE |
| 112930879 | 112932951 | - | 22.4 | 2254 | 4485 | -1655 | L1M3 | L1 | LINE |
| 112932959 | 112933639 | - | 26.2 | 2602 | 3395 | -4289 | L1MA6 | L1 | LINE |
| 112933640 | 112933931 | - | 24.1 | 1 | 302 | 0 | AluJo | Alu | SINE |
| 112933932 | 112934771 | - | 23.7 | 2085 | 2903 | -4663 | L1MA6 | L1 | LINE |
| 112934867 | 112935513 | - | 11.6 | 5648 | 6302 | 0 | L1MA1 | L1 | LINE |
| 112935514 | 112935583 | - | 14.3 | 1 | 70 | -358 | MSTA | MaLR | LTR |
| 112935584 | 112936263 | + | 13.1 | 2167 | 2858 | -4707 | L1M1 | L1 | LINE |
| 112936290 | 112938863 | + | 12.5 | 2859 | 5457 | -2108 | L1M1 | L1 | LINE |
| 112938907 | 112939204 | + | 15.9 | 76 | 428 | 0 | MSTA | MaLR | LTR |
| 112939205 | 112941304 | - | 18.7 | 20 | 2171 | -5394 | L1M1 | L1 | LINE |
| 112941347 | 112941649 | - | 15.5 | 1 | 306 | -6 | AluSx | Alu | SINE |
| 112941930 | 112942620 | - | 31.8 | 517 | 1427 | -6139 | L1MA6 | L1 | LINE |

  

|  |  |  |  |  |  |  |  |  |  |
| --- | --- | --- | --- | --- | --- | --- | --- | --- | --- |
| chr8:112995782-113000582 | | | 4659 bp | | |  |  |  |  |
| Genome Start | Genome End | Strand | Div | Repeat Start | Repeat End | Repeat Left | Name | Family | Class |
| 112995782 | 112996643 | + | 32.7 | 555 | 1441 | -3555 | ERVL-E | ERVL | LTR |
| 112996710 | 112997324 | + | 33.9 | 4350 | 4998 | -669 | ERVL-E | ERVL | LTR |
| 112997344 | 112997393 | - | 26.0 | 120 | 169 | -227 | MSTD | MaLR | LTR |
| 112997400 | 112997777 | - | 25.7 | 3 | 396 | 0 | MSTD | MaLR | LTR |
| 112997791 | 112998219 | - | 20.1 | 1231 | 1722 | -13 | MSTD-int | MaLR | LTR |
| 112998225 | 112998700 | + | 22.6 | 3 | 505 | -4 | LTR22C | ERVK | LTR |
| 112998692 | 112999987 | - | 30.9 | 1 | 1250 | -485 | MSTD-int | MaLR | LTR |
| 112999995 | 113000353 | - | 22.3 | 8 | 390 | -6 | MSTD | MaLR | LTR |
| 113000389 | 113000582 | + | 35.5 | 5144 | 5344 | -310 | ERVL-E | ERVL | LTR |

  

|  |  |  |  |  |  |  |  |  |  |
| --- | --- | --- | --- | --- | --- | --- | --- | --- | --- |
| chr8:113435003-113440325 | | | 5315 bp | | |  |  |  |  |
| Genome Start | Genome End | Strand | Div | Repeat Start | Repeat End | Repeat Left | Name | Family | Class |
| 113435003 | 113436956 | + | 14.0 | 3383 | 5329 | -817 | L1PA16 | L1 | LINE |
| 113436957 | 113437265 | + | 10.0 | 1 | 311 | 0 | AluY | Alu | SINE |
| 113437266 | 113437957 | + | 13.3 | 5330 | 6043 | -123 | L1PA16 | L1 | LINE |
| 113437958 | 113438351 | + | 15.9 | 1 | 362 | -2 | THE1B | MaLR | LTR |
| 113438356 | 113439851 | + | 29.9 | 2 | 1735 | 0 | MLT1D-int | MaLR | LTR |
| 113439852 | 113440206 | + | 12.2 | 1 | 364 | 0 | THE1B | MaLR | LTR |
| 113440211 | 113440325 | + | 17.4 | 6043 | 6157 | -9 | L1PA16 | L1 | LINE |

  

|  |  |  |  |  |  |  |  |  |  |
| --- | --- | --- | --- | --- | --- | --- | --- | --- | --- |
| chr8:114110556-114115633 | | | 5073 bp | | |  |  |  |  |
| Genome Start | Genome End | Strand | Div | Repeat Start | Repeat End | Repeat Left | Name | Family | Class |
| 114110556 | 114113189 | + | 15.1 | 3511 | 6284 | -75 | L1MD2 | L1 | LINE |
| 114113190 | 114113547 | + | 13.0 | 3 | 381 | 0 | THE1D | MaLR | LTR |
| 114113553 | 114115186 | + | 30.9 | 5 | 1735 | 0 | MLT-int | MaLR | LTR |
| 114115187 | 114115563 | + | 13.5 | 1 | 381 | 0 | THE1D | MaLR | LTR |
| 114115564 | 114115633 | + | 20.4 | 6285 | 6355 | -4 | L1MD2 | L1 | LINE |

  

|  |  |  |  |  |  |  |  |  |  |
| --- | --- | --- | --- | --- | --- | --- | --- | --- | --- |
| chr8:114777440-114786212 | | | 7895 bp | | |  |  |  |  |
| Genome Start | Genome End | Strand | Div | Repeat Start | Repeat End | Repeat Left | Name | Family | Class |
| 114777440 | 114777723 | - | 27.6 | 2814 | 3083 | -4039 | L1MCc | L1 | LINE |
| 114777731 | 114778193 | + | 11.6 | 1 | 453 | 0 | MLT2A2 | ERVL | LTR |
| 114778197 | 114779609 | + | 10.6 | 5 | 1571 | -9 | THE1B-int | MaLR | LTR |
| 114779619 | 114779957 | + | 12.7 | 1 | 364 | 0 | THE1B | MaLR | LTR |
| 114779973 | 114780214 | - | 14.9 | 15 | 277 | -6928 | MER50-int | ERV1 | LTR |
| 114780221 | 114780374 | - | 19.5 | 564 | 722 | -12 | MER50 | ERV1 | LTR |
| 114780376 | 114780470 | + | 8.4 | 202 | 296 | -15 | AluY | Alu | SINE |
| 114780471 | 114781043 | - | 17.4 | 1 | 573 | -161 | MER50 | ERV1 | LTR |
| 114781049 | 114781152 | - | 31.7 | 2584 | 2691 | -4437 | L1MCc | L1 | LINE |
| 114781377 | 114781444 | - | 29.4 | 6088 | 6155 | -7 | L1ME3 | L1 | LINE |
| 114781490 | 114781727 | - | 30.2 | 7274 | 7506 | -455 | L1MC5 | L1 | LINE |
| 114781753 | 114782056 | - | 11.2 | 3 | 305 | -6 | AluY | Alu | SINE |
| 114782157 | 114783067 | - | 28.7 | 1134 | 2207 | -4921 | L1MCc | L1 | LINE |
| 114783123 | 114783479 | - | 15.9 | 2 | 364 | 0 | THE1B | MaLR | LTR |
| 114783481 | 114785053 | - | 12.2 | 1 | 1580 | 0 | THE1B-int | MaLR | LTR |
| 114785054 | 114785413 | - | 19.8 | 1 | 364 | 0 | THE1B | MaLR | LTR |
| 114785796 | 114786212 | - | 33.8 | 423 | 846 | -5576 | L1MCc | L1 | LINE |

  

|  |  |  |  |  |  |  |  |  |  |
| --- | --- | --- | --- | --- | --- | --- | --- | --- | --- |
| chr8:115322786-115329890 | | | 7020 bp | | |  |  |  |  |
| Genome Start | Genome End | Strand | Div | Repeat Start | Repeat End | Repeat Left | Name | Family | Class |
| 115322786 | 115322963 | - | 30.6 | 5353 | 5551 | -613 | L1M5 | L1 | LINE |
| 115323009 | 115323459 | + | 4.9 | 1 | 448 | -2 | LTR7 | ERV1 | LTR |
| 115323462 | 115324485 | + | 11.7 | 1 | 1039 | -6674 | HERVH | ERV1 | LTR |
| 115324486 | 115326145 | + | 9.1 | 1330 | 2987 | -4726 | HERVH | ERV1 | LTR |
| 115326140 | 115326286 | + | 12.9 | 3140 | 3290 | -4423 | HERVH | ERV1 | LTR |
| 115326277 | 115326781 | + | 12.2 | 3492 | 3993 | -3720 | HERVH | ERV1 | LTR |
| 115326784 | 115327444 | + | 14.5 | 4488 | 5149 | -2564 | HERVH | ERV1 | LTR |
| 115327442 | 115327662 | + | 11.5 | 5597 | 5819 | -1894 | HERVH | ERV1 | LTR |
| 115327661 | 115328039 | + | 8.8 | 7322 | 7713 | 0 | HERVH | ERV1 | LTR |
| 115328040 | 115328488 | + | 5.4 | 1 | 448 | -2 | LTR7 | ERV1 | LTR |
| 115328524 | 115328819 | - | 18.9 | 1 | 291 | -21 | AluJb | Alu | SINE |
| 115328842 | 115329088 | - | 36.5 | 4981 | 5230 | -916 | L1M5 | L1 | LINE |
| 115329089 | 115329533 | + | 8.8 | 1 | 471 | -1 | MER4A1 | ERV1 | LTR |
| 115329534 | 115329890 | - | 36.5 | 4614 | 4980 | -1166 | L1M5 | L1 | LINE |

  

|  |  |  |  |  |  |  |  |  |  |
| --- | --- | --- | --- | --- | --- | --- | --- | --- | --- |
| chr8:116047832-116050974 | | | 3136 bp | | |  |  |  |  |
| Genome Start | Genome End | Strand | Div | Repeat Start | Repeat End | Repeat Left | Name | Family | Class |
| 116047832 | 116047976 | + | 31.9 | 65 | 259 | -2248 | HAL1 | L1 | LINE |
| 116047977 | 116048345 | + | 10.5 | 1 | 364 | 0 | THE1B | MaLR | LTR |
| 116048348 | 116049922 | + | 30.4 | 2 | 1731 | -4 | MLT-int | MaLR | LTR |
| 116049928 | 116050296 | + | 14.1 | 2 | 364 | 0 | THE1B | MaLR | LTR |
| 116050297 | 116050406 | + | 31.9 | 263 | 362 | -2145 | HAL1 | L1 | LINE |
| 116050407 | 116050682 | + | 9.4 | 1 | 299 | -13 | AluSx | Alu | SINE |
| 116050683 | 116050974 | + | 31.9 | 363 | 681 | -1826 | HAL1 | L1 | LINE |

  

|  |  |  |  |  |  |  |  |  |  |
| --- | --- | --- | --- | --- | --- | --- | --- | --- | --- |
| chr8:117395500-117398111 | | | 2608 bp | | |  |  |  |  |
| Genome Start | Genome End | Strand | Div | Repeat Start | Repeat End | Repeat Left | Name | Family | Class |
| 117395500 | 117395651 | - | 15.9 | 200 | 346 | 0 | MER7A | MER2\_type | DNA |
| 117395652 | 117396055 | - | 9.5 | 3 | 375 | 0 | THE1C | MaLR | LTR |
| 117396058 | 117397544 | - | 29.3 | 2 | 1735 | 0 | MLT-int | MaLR | LTR |
| 117397547 | 117397924 | - | 10.3 | 1 | 375 | 0 | THE1C | MaLR | LTR |
| 117397925 | 117398111 | - | 15.9 | 1 | 199 | -147 | MER7A | MER2\_type | DNA |

  

|  |  |  |  |  |  |  |  |  |  |
| --- | --- | --- | --- | --- | --- | --- | --- | --- | --- |
| chr8:117450282-117457587 | | | 7304 bp | | |  |  |  |  |
| Genome Start | Genome End | Strand | Div | Repeat Start | Repeat End | Repeat Left | Name | Family | Class |
| 117450282 | 117453817 | - | 11.7 | 1800 | 5510 | -1339 | L1PA15-16 | L1 | LINE |
| 117453818 | 117454186 | - | 15.2 | 1 | 364 | 0 | THE1B | MaLR | LTR |
| 117454187 | 117455699 | - | 31.7 | 2 | 1735 | 0 | MLT-int | MaLR | LTR |
| 117455702 | 117456047 | - | 16.3 | 1 | 364 | 0 | THE1B | MaLR | LTR |
| 117456048 | 117457587 | - | 16.0 | 3 | 1799 | -5054 | L1PA15-16 | L1 | LINE |

  

|  |  |  |  |  |  |  |  |  |  |
| --- | --- | --- | --- | --- | --- | --- | --- | --- | --- |
| chr8:118115382-118118339 | | | 2573 bp | | |  |  |  |  |
| Genome Start | Genome End | Strand | Div | Repeat Start | Repeat End | Repeat Left | Name | Family | Class |
| 118115382 | 118115468 | - | 28.7 | 76 | 182 | -26 | MIR3 | MIR | SINE |
| 118115541 | 118115616 | - | 29.3 | 82 | 159 | -109 | MIRb | MIR | SINE |
| 118115889 | 118116074 | - | 26.3 | 1 | 189 | -73 | MIR | MIR | SINE |
| 118116114 | 118116503 | - | 11.3 | 1 | 381 | 0 | THE1D | MaLR | LTR |
| 118116504 | 118117857 | - | 12.8 | 3 | 1580 | 0 | THE1D-int | MaLR | LTR |
| 118117860 | 118118233 | - | 12.7 | 1 | 381 | 0 | THE1D | MaLR | LTR |
| 118118234 | 118118339 | - | 33.3 | 3 | 124 | -84 | MIR3 | MIR | SINE |

  

|  |  |  |  |  |  |  |  |  |  |
| --- | --- | --- | --- | --- | --- | --- | --- | --- | --- |
| chr8:118499778-118507590 | | | 7778 bp | | |  |  |  |  |
| Genome Start | Genome End | Strand | Div | Repeat Start | Repeat End | Repeat Left | Name | Family | Class |
| 118499778 | 118504688 | + | 9.3 | 4 | 4801 | -1530 | L1PA10 | L1 | LINE |
| 118504720 | 118504786 | - | 9.0 | 1 | 69 | -295 | THE1B | MaLR | LTR |
| 118504791 | 118506359 | - | 10.7 | 1 | 1576 | -4 | THE1B-int | MaLR | LTR |
| 118506360 | 118506715 | - | 13.9 | 1 | 364 | 0 | THE1B | MaLR | LTR |
| 118506716 | 118507590 | + | 7.2 | 4797 | 5675 | -641 | L1PA10 | L1 | LINE |

  

|  |  |  |  |  |  |  |  |  |  |
| --- | --- | --- | --- | --- | --- | --- | --- | --- | --- |
| chr8:120480599-120483142 | | | 2542 bp | | |  |  |  |  |
| Genome Start | Genome End | Strand | Div | Repeat Start | Repeat End | Repeat Left | Name | Family | Class |
| 120480599 | 120480632 | - | 20.4 | 171 | 204 | -58 | MIR | MIR | SINE |
| 120480633 | 120481041 | + | 17.4 | 1 | 428 | 0 | MSTA | MaLR | LTR |
| 120481044 | 120482675 | + | 28.6 | 2 | 1735 | 0 | MLT1A0-int | MaLR | LTR |
| 120482676 | 120483073 | + | 15.8 | 1 | 428 | 0 | MSTA | MaLR | LTR |
| 120483074 | 120483142 | - | 20.4 | 112 | 170 | -92 | MIR | MIR | SINE |

  

|  |  |  |  |  |  |  |  |  |  |
| --- | --- | --- | --- | --- | --- | --- | --- | --- | --- |
| chr8:121765311-121768171 | | | 2861 bp | | |  |  |  |  |
| Genome Start | Genome End | Strand | Div | Repeat Start | Repeat End | Repeat Left | Name | Family | Class |
| 121765311 | 121765373 | - | 34.3 | 137 | 202 | -66 | MIRb | MIR | SINE |
| 121765374 | 121765731 | - | 19.0 | 1 | 365 | 0 | MLT1A0 | MaLR | LTR |
| 121765732 | 121766076 | - | 18.5 | 1387 | 1735 | 0 | MLT1A0-int | MaLR | LTR |
| 121766077 | 121766375 | - | 10.1 | 2 | 299 | -13 | AluSx | Alu | SINE |
| 121766376 | 121767688 | - | 18.5 | 1 | 1386 | -349 | MLT1A0-int | MaLR | LTR |
| 121767689 | 121768047 | - | 20.7 | 1 | 363 | -2 | MLT1A0 | MaLR | LTR |
| 121768048 | 121768171 | - | 34.3 | 19 | 136 | -132 | MIRb | MIR | SINE |

  

|  |  |  |  |  |  |  |  |  |  |
| --- | --- | --- | --- | --- | --- | --- | --- | --- | --- |
| chr8:122921628-122924952 | | | 3291 bp | | |  |  |  |  |
| Genome Start | Genome End | Strand | Div | Repeat Start | Repeat End | Repeat Left | Name | Family | Class |
| 122921628 | 122921716 | + | 23.5 | 13 | 110 | -98 | MIRb | MIR | SINE |
| 122921700 | 122921785 | + | 30.6 | 2696 | 2784 | -635 | L2 | L2 | LINE |
| 122921789 | 122922441 | + | 33.6 | 2725 | 3386 | -33 | L2 | L2 | LINE |
| 122922442 | 122922818 | + | 10.4 | 2 | 375 | 0 | THE1C | MaLR | LTR |
| 122922821 | 122924463 | + | 30.4 | 2 | 1735 | 0 | MLT1K-int | MaLR | LTR |
| 122924464 | 122924840 | + | 7.8 | 1 | 375 | 0 | THE1C | MaLR | LTR |
| 122924841 | 122924874 | + | 33.5 | 3387 | 3418 | -1 | L2 | L2 | LINE |
| 122924921 | 122924952 | + | 12.5 | 132 | 166 | -102 | MIRb | MIR | SINE |

  

|  |  |  |  |  |  |  |  |  |  |
| --- | --- | --- | --- | --- | --- | --- | --- | --- | --- |
| chr8:123792061-123795814 | | | 3488 bp | | |  |  |  |  |
| Genome Start | Genome End | Strand | Div | Repeat Start | Repeat End | Repeat Left | Name | Family | Class |
| 123792061 | 123792208 | - | 31.5 | 5784 | 5929 | -1879 | L1MC4a | L1 | LINE |
| 123792210 | 123792267 | + | 22.8 | 1 | 58 | -120 | MER5B | MER1\_type | DNA |
| 123792273 | 123792449 | - | 21.2 | 378 | 589 | -1 | MLT1G | MaLR | LTR |
| 123792560 | 123792916 | + | 11.0 | 1 | 355 | 0 | THE1A | MaLR | LTR |
| 123792917 | 123793279 | + | 9.1 | 1 | 361 | -1219 | THE1A-int | MaLR | LTR |
| 123793280 | 123793591 | - | 9.9 | 1 | 313 | 0 | AluSp | Alu | SINE |
| 123793592 | 123794811 | + | 9.1 | 362 | 1580 | 0 | THE1A-int | MaLR | LTR |
| 123794812 | 123795165 | + | 7.9 | 1 | 355 | 0 | THE1A | MaLR | LTR |
| 123795333 | 123795360 | - | 7.1 | 184 | 211 | -319 | MLT1G3 | MaLR | LTR |
| 123795340 | 123795597 | - | 24.9 | 1 | 266 | -289 | MLT1H1 | MaLR | LTR |
| 123795598 | 123795635 | - | 18.4 | 4 | 41 | -137 | MER5B | MER1\_type | DNA |
| 123795640 | 123795814 | - | 32.0 | 5613 | 5787 | -2021 | L1MC4a | L1 | LINE |

  

|  |  |  |  |  |  |  |  |  |  |
| --- | --- | --- | --- | --- | --- | --- | --- | --- | --- |
| chr8:127120560-127123123 | | | 2561 bp | | |  |  |  |  |
| Genome Start | Genome End | Strand | Div | Repeat Start | Repeat End | Repeat Left | Name | Family | Class |
| 127120560 | 127120819 | - | 12.8 | 49 | 307 | -5 | AluJo | Alu | SINE |
| 127120821 | 127121187 | + | 13.3 | 1 | 362 | -2 | THE1B | MaLR | LTR |
| 127121190 | 127122670 | + | 9.6 | 1 | 1580 | 0 | THE1B-int | MaLR | LTR |
| 127122671 | 127123035 | + | 13.3 | 1 | 364 | 0 | THE1B | MaLR | LTR |
| 127123036 | 127123123 | - | 21.6 | 1 | 52 | -250 | AluJo | Alu | SINE |

  

|  |  |  |  |  |  |  |  |  |  |
| --- | --- | --- | --- | --- | --- | --- | --- | --- | --- |
| chr8:127362458-127365664 | | | 3202 bp | | |  |  |  |  |
| Genome Start | Genome End | Strand | Div | Repeat Start | Repeat End | Repeat Left | Name | Family | Class |
| 127362458 | 127362586 | + | 24.8 | 6707 | 6829 | -979 | L1MC4a | L1 | LINE |
| 127362587 | 127362894 | + | 14.5 | 5 | 312 | 0 | AluJb | Alu | SINE |
| 127362895 | 127363123 | + | 24.8 | 6830 | 7079 | -729 | L1MC4a | L1 | LINE |
| 127363124 | 127363479 | + | 13.3 | 1 | 362 | -2 | THE1B | MaLR | LTR |
| 127363484 | 127365037 | + | 30.6 | 2 | 1735 | 0 | MLT-int | MaLR | LTR |
| 127365039 | 127365399 | + | 15.5 | 1 | 364 | 0 | THE1B | MaLR | LTR |
| 127365400 | 127365664 | + | 24.8 | 7080 | 7340 | -468 | L1MC4a | L1 | LINE |

  

|  |  |  |  |  |  |  |  |  |  |
| --- | --- | --- | --- | --- | --- | --- | --- | --- | --- |
| chr8:128402301-128408202 | | | 5583 bp | | |  |  |  |  |
| Genome Start | Genome End | Strand | Div | Repeat Start | Repeat End | Repeat Left | Name | Family | Class |
| 128402301 | 128402582 | + | 31.2 | 169 | 457 | -5966 | L1MCc | L1 | LINE |
| 128402832 | 128402937 | + | 32.7 | 817 | 921 | -5502 | L1MCc | L1 | LINE |
| 128402948 | 128403132 | + | 35.3 | 955 | 1157 | -5266 | L1MCc | L1 | LINE |
| 128403136 | 128403470 | + | 15.5 | 3 | 359 | -67 | MSTB | MaLR | LTR |
| 128403472 | 128403630 | - | 13.2 | 145 | 304 | -9 | AluSq | Alu | SINE |
| 128403637 | 128403689 | + | 15.1 | 373 | 426 | 0 | MSTB | MaLR | LTR |
| 128403692 | 128405301 | + | 28.2 | 2 | 1735 | 0 | MLT1D-int | MaLR | LTR |
| 128405302 | 128405710 | + | 17.7 | 1 | 425 | -1 | MSTB | MaLR | LTR |
| 128405713 | 128406442 | + | 26.1 | 1155 | 1994 | -5134 | L1MCc | L1 | LINE |
| 128406489 | 128407076 | + | 28.0 | 1995 | 2676 | -4452 | L1MCc | L1 | LINE |
| 128407077 | 128407377 | - | 19.8 | 13 | 306 | -6 | AluJo | Alu | SINE |
| 128407378 | 128408202 | + | 25.1 | 2677 | 3538 | -3403 | L1MCc | L1 | LINE |

  

|  |  |  |  |  |  |  |  |  |  |
| --- | --- | --- | --- | --- | --- | --- | --- | --- | --- |
| chr8:128513079-128519303 | | | 6219 bp | | |  |  |  |  |
| Genome Start | Genome End | Strand | Div | Repeat Start | Repeat End | Repeat Left | Name | Family | Class |
| 128513079 | 128513129 | - | 11.8 | 2933 | 2987 | -3159 | L1P4 | L1 | LINE |
| 128513130 | 128513574 | + | 5.0 | 1 | 448 | -2 | LTR7 | ERV1 | LTR |
| 128513577 | 128516546 | + | 9.8 | 1 | 2987 | -4726 | HERVH | ERV1 | LTR |
| 128516541 | 128516688 | + | 13.5 | 3140 | 3288 | -4425 | HERVH | ERV1 | LTR |
| 128516695 | 128517185 | + | 10.8 | 3505 | 3993 | -3720 | HERVH | ERV1 | LTR |
| 128517188 | 128517844 | + | 14.0 | 4488 | 5149 | -2564 | HERVH | ERV1 | LTR |
| 128517846 | 128518063 | + | 11.1 | 5600 | 5819 | -1894 | HERVH | ERV1 | LTR |
| 128518062 | 128518441 | + | 9.1 | 7322 | 7713 | 0 | HERVH | ERV1 | LTR |
| 128518442 | 128518891 | + | 5.4 | 1 | 448 | -2 | LTR7 | ERV1 | LTR |
| 128518895 | 128519303 | - | 17.7 | 2527 | 2936 | -3210 | L1P4 | L1 | LINE |

  

|  |  |  |  |  |  |  |  |  |  |
| --- | --- | --- | --- | --- | --- | --- | --- | --- | --- |
| chr8:130677960-130691360 | | | 13099 bp | | |  |  |  |  |
| Genome Start | Genome End | Strand | Div | Repeat Start | Repeat End | Repeat Left | Name | Family | Class |
| 130677960 | 130678040 | + | 30.9 | 1464 | 1548 | -1871 | L2 | L2 | LINE |
| 130678041 | 130678105 | + | 24.6 | 1833 | 1901 | -1518 | L2 | L2 | LINE |
| 130678217 | 130678290 | + | 28.4 | 2141 | 2216 | -1203 | L2 | L2 | LINE |
| 130678349 | 130680107 | - | 18.5 | 4395 | 6176 | -8 | L1MB7 | L1 | LINE |
| 130680097 | 130680992 | - | 10.8 | 5322 | 6168 | 0 | L1PA10 | L1 | LINE |
| 130680993 | 130681338 | - | 4.9 | 5808 | 6153 | -1 | L1PA6 | L1 | LINE |
| 130681339 | 130682124 | - | 19.8 | 4462 | 5326 | -820 | L1PA10 | L1 | LINE |
| 130682125 | 130682409 | - | 17.2 | 1 | 292 | -20 | AluJb | Alu | SINE |
| 130682410 | 130682538 | - | 13.9 | 1 | 130 | -13 | FLAM\_C | Alu | SINE |
| 130682539 | 130683430 | - | 19.8 | 3506 | 4461 | -1685 | L1PA10 | L1 | LINE |
| 130683449 | 130684207 | - | 20.5 | 5404 | 6166 | -8 | L1MB5 | L1 | LINE |
| 130684208 | 130684445 | - | 14.3 | 135 | 375 | 0 | THE1C | MaLR | LTR |
| 130684445 | 130684548 | - | 23.1 | 705 | 809 | -842 | MST-int | MaLR | LTR |
| 130684558 | 130684589 | - | 3.1 | 1 | 32 | -343 | THE1C | MaLR | LTR |
| 130684590 | 130686181 | - | 29.3 | 2 | 1735 | 0 | MLT1F2-int | MaLR | LTR |
| 130686184 | 130686557 | - | 11.3 | 1 | 375 | 0 | THE1C | MaLR | LTR |
| 130686558 | 130686678 | - | 15.5 | 6153 | 6268 | -772 | L1M | L1 | LINE |
| 130686682 | 130686843 | - | 19.4 | 4128 | 4309 | -2697 | L1M | L1 | LINE |
| 130686816 | 130687889 | - | 3.7 | 3199 | 6154 | -1 | L1PA4 | L1 | LINE |
| 130687894 | 130688475 | - | 20.1 | 3134 | 3732 | -3274 | L1M | L1 | LINE |
| 130688476 | 130688800 | - | 18.4 | 1 | 309 | -3 | AluJo | Alu | SINE |
| 130688801 | 130689346 | - | 15.3 | 2853 | 3354 | -3980 | L1M4b | L1 | LINE |
| 130689460 | 130690427 | - | 27.5 | 29 | 1022 | -6312 | L1M4b | L1 | LINE |
| 130690452 | 130691360 | + | 29.2 | 2211 | 3183 | -236 | L2 | L2 | LINE |

  

|  |  |  |  |  |  |  |  |  |  |
| --- | --- | --- | --- | --- | --- | --- | --- | --- | --- |
| chr8:130749133-130752975 | | | 3789 bp | | |  |  |  |  |
| Genome Start | Genome End | Strand | Div | Repeat Start | Repeat End | Repeat Left | Name | Family | Class |
| 130749133 | 130749376 | + | 23.2 | 3809 | 4060 | -2086 | L1ME1 | L1 | LINE |
| 130749406 | 130749866 | + | 24.4 | 4962 | 5456 | -714 | L1ME1 | L1 | LINE |
| 130749867 | 130750229 | + | 11.7 | 1 | 364 | 0 | THE1B | MaLR | LTR |
| 130750232 | 130751778 | + | 30.1 | 2 | 1735 | 0 | MLT-int | MaLR | LTR |
| 130751779 | 130752141 | + | 12.3 | 1 | 364 | 0 | THE1B | MaLR | LTR |
| 130752160 | 130752307 | + | 17.4 | 1 | 156 | -20 | FRAM | Alu | SINE |
| 130752313 | 130752975 | + | 23.3 | 5465 | 6161 | -5 | L1ME1 | L1 | LINE |

  

|  |  |  |  |  |  |  |  |  |  |
| --- | --- | --- | --- | --- | --- | --- | --- | --- | --- |
| chr8:130887278-130898549 | | | 10859 bp | | |  |  |  |  |
| Genome Start | Genome End | Strand | Div | Repeat Start | Repeat End | Repeat Left | Name | Family | Class |
| 130887278 | 130887334 | - | 24.3 | 2202 | 2258 | -4126 | L1M4c | L1 | LINE |
| 130887335 | 130887701 | - | 2.8 | 5787 | 6153 | -2 | L1PA4 | L1 | LINE |
| 130887702 | 130887754 | - | 24.3 | 2152 | 2201 | -4183 | L1M4c | L1 | LINE |
| 130887877 | 130888039 | - | 20.6 | 106 | 268 | -44 | AluJo | Alu | SINE |
| 130888040 | 130888446 | + | 19.3 | 1 | 426 | 0 | MSTB | MaLR | LTR |
| 130888452 | 130889960 | + | 28.5 | 1 | 1735 | 0 | MLT-int | MaLR | LTR |
| 130889962 | 130890357 | + | 18.4 | 2 | 420 | -6 | MSTB | MaLR | LTR |
| 130890375 | 130890484 | - | 27.8 | 5 | 112 | -30 | FLAM\_A | Alu | SINE |
| 130890491 | 130890808 | - | 25.2 | 2900 | 3219 | -4346 | L1M1 | L1 | LINE |
| 130890973 | 130891122 | - | 12.0 | 6016 | 6166 | 0 | L1PA16 | L1 | LINE |
| 130891123 | 130891710 | - | 12.1 | 5302 | 5878 | -288 | L1PA16 | L1 | LINE |
| 130891742 | 130892021 | - | 15.0 | 1 | 281 | -32 | AluSq | Alu | SINE |
| 130892022 | 130895614 | - | 13.3 | 2801 | 6469 | -845 | L1P4b | L1 | LINE |
| 130895615 | 130895871 | - | 10.9 | 39 | 294 | -16 | AluSg | Alu | SINE |
| 130895872 | 130896382 | - | 15.9 | 2261 | 2800 | -4518 | L1P4b | L1 | LINE |
| 130896383 | 130896744 | + | 7.3 | 1 | 375 | 0 | THE1C | MaLR | LTR |
| 130896745 | 130897513 | - | 22.2 | 1711 | 2260 | -5058 | L1P4b | L1 | LINE |
| 130897570 | 130898314 | - | 27.0 | 11 | 1331 | -6003 | L1P4b | L1 | LINE |
| 130898326 | 130898549 | - | 22.5 | 1436 | 1657 | -4727 | L1M4c | L1 | LINE |

  

|  |  |  |  |  |  |  |  |  |  |
| --- | --- | --- | --- | --- | --- | --- | --- | --- | --- |
| chr8:130910830-130913889 | | | 3058 bp | | |  |  |  |  |
| Genome Start | Genome End | Strand | Div | Repeat Start | Repeat End | Repeat Left | Name | Family | Class |
| 130910830 | 130910937 | + | 11.7 | 6 | 122 | -345 | MLT1C | MaLR | LTR |
| 130910938 | 130911198 | + | 18.8 | 1 | 242 | -243 | MER31A | ERV1 | LTR |
| 130911199 | 130911553 | - | 11.9 | 1 | 355 | 0 | THE1A | MaLR | LTR |
| 130911554 | 130913075 | - | 30.6 | 2 | 1735 | 0 | MLT-int | MaLR | LTR |
| 130913078 | 130913429 | - | 11.5 | 1 | 355 | 0 | THE1A | MaLR | LTR |
| 130913430 | 130913677 | + | 18.8 | 243 | 485 | 0 | MER31A | ERV1 | LTR |
| 130913678 | 130913889 | + | 20.4 | 119 | 339 | -128 | MLT1C | MaLR | LTR |

  

|  |  |  |  |  |  |  |  |  |  |
| --- | --- | --- | --- | --- | --- | --- | --- | --- | --- |
| chr8:131852997-131857829 | | | 4826 bp | | |  |  |  |  |
| Genome Start | Genome End | Strand | Div | Repeat Start | Repeat End | Repeat Left | Name | Family | Class |
| 131852997 | 131853200 | - | 15.2 | 2226 | 2418 | 0 | Tigger1 | MER2\_type | DNA |
| 131853201 | 131853494 | + | 13.0 | 3 | 294 | -18 | AluSx | Alu | SINE |
| 131853495 | 131853649 | - | 15.2 | 2077 | 2225 | -193 | Tigger1 | MER2\_type | DNA |
| 131853652 | 131855129 | - | 12.9 | 454 | 1905 | -513 | Tigger1 | MER2\_type | DNA |
| 131855130 | 131855438 | + | 15.9 | 1 | 364 | 0 | THE1B | MaLR | LTR |
| 131855440 | 131857019 | + | 11.0 | 2 | 1580 | 0 | THE1B-int | MaLR | LTR |
| 131857020 | 131857386 | + | 13.6 | 1 | 364 | 0 | THE1B | MaLR | LTR |
| 131857391 | 131857829 | - | 15.3 | 1 | 444 | -1974 | Tigger1 | MER2\_type | DNA |

  

|  |  |  |  |  |  |  |  |  |  |
| --- | --- | --- | --- | --- | --- | --- | --- | --- | --- |
| chr8:135849502-135856168 | | | 6667 bp | | |  |  |  |  |
| Genome Start | Genome End | Strand | Div | Repeat Start | Repeat End | Repeat Left | Name | Family | Class |
| 135849502 | 135849843 | + | 21.1 | 33 | 373 | -2045 | Tigger1 | MER2\_type | DNA |
| 135849844 | 135850196 | - | 13.9 | 1 | 364 | 0 | THE1B | MaLR | LTR |
| 135850197 | 135850315 | - | 10.2 | 1473 | 1580 | 0 | THE1B-int | MaLR | LTR |
| 135850316 | 135850599 | + | 13.1 | 1 | 284 | -28 | AluSx | Alu | SINE |
| 135850600 | 135851003 | - | 10.2 | 1092 | 1472 | -108 | THE1B-int | MaLR | LTR |
| 135851004 | 135851687 | - | 8.8 | 426 | 1091 | -489 | THE1B-int | MaLR | LTR |
| 135851688 | 135852758 | + | 7.2 | 1 | 1071 | 0 | MER11C | ERVK | LTR |
| 135852759 | 135853177 | - | 8.8 | 1 | 425 | -1155 | THE1B-int | MaLR | LTR |
| 135853178 | 135853529 | - | 12.2 | 1 | 364 | 0 | THE1B | MaLR | LTR |
| 135853530 | 135854203 | + | 13.1 | 369 | 1072 | -1346 | Tigger1 | MER2\_type | DNA |
| 135854204 | 135854548 | + | 19.7 | 1 | 312 | 0 | AluJo | Alu | SINE |
| 135854549 | 135855409 | + | 13.1 | 1073 | 1947 | -471 | Tigger1 | MER2\_type | DNA |
| 135855410 | 135855719 | - | 9.4 | 3 | 311 | -2 | AluSq | Alu | SINE |
| 135855720 | 135856168 | + | 13.1 | 1948 | 2417 | -1 | Tigger1 | MER2\_type | DNA |

  

|  |  |  |  |  |  |  |  |  |  |
| --- | --- | --- | --- | --- | --- | --- | --- | --- | --- |
| chr8:136218005-136221388 | | | 3286 bp | | |  |  |  |  |
| Genome Start | Genome End | Strand | Div | Repeat Start | Repeat End | Repeat Left | Name | Family | Class |
| 136218005 | 136218212 | + | 23.1 | 3 | 192 | -313 | MLT1D | MaLR | LTR |
| 136218213 | 136218527 | + | 7.1 | 1 | 310 | -1 | AluY | Alu | SINE |
| 136218528 | 136218776 | + | 23.1 | 193 | 436 | -69 | MLT1D | MaLR | LTR |
| 136218780 | 136219113 | - | 16.8 | 1 | 406 | -2 | MLT1A1 | MaLR | LTR |
| 136219114 | 136219311 | - | 20.4 | 1580 | 1735 | 0 | MLT1A1-int | MaLR | LTR |
| 136219312 | 136219615 | + | 16.4 | 1 | 299 | -13 | AluSx | Alu | SINE |
| 136219616 | 136220099 | - | 20.4 | 1092 | 1579 | -156 | MLT1A1-int | MaLR | LTR |
| 136220195 | 136220971 | - | 21.1 | 1 | 794 | -941 | MLT1A1-int | MaLR | LTR |
| 136220972 | 136221315 | - | 14.0 | 1 | 408 | 0 | MLT1A1 | MaLR | LTR |
| 136221316 | 136221388 | + | 20.6 | 433 | 505 | 0 | MLT1D | MaLR | LTR |

  

|  |  |  |  |  |  |  |  |  |  |
| --- | --- | --- | --- | --- | --- | --- | --- | --- | --- |
| chr8:136392287-136395650 | | | 2977 bp | | |  |  |  |  |
| Genome Start | Genome End | Strand | Div | Repeat Start | Repeat End | Repeat Left | Name | Family | Class |
| 136392287 | 136392440 | + | 35.1 | 139 | 291 | -364 | LTR16D | ERVL | LTR |
| 136392704 | 136393162 | - | 14.2 | 15 | 471 | 0 | LTR32 | ERVL | LTR |
| 136393258 | 136393614 | - | 13.9 | 1 | 381 | 0 | THE1D | MaLR | LTR |
| 136393615 | 136395052 | - | 13.5 | 2 | 1542 | -38 | THE1D-int | MaLR | LTR |
| 136395054 | 136395409 | - | 13.3 | 1 | 381 | 0 | THE1D | MaLR | LTR |
| 136395438 | 136395650 | + | 29.1 | 434 | 647 | -8 | LTR16D | ERVL | LTR |

  

|  |  |  |  |  |  |  |  |  |  |
| --- | --- | --- | --- | --- | --- | --- | --- | --- | --- |
| chr8:137774703-137779605 | | | 4900 bp | | |  |  |  |  |
| Genome Start | Genome End | Strand | Div | Repeat Start | Repeat End | Repeat Left | Name | Family | Class |
| 137774703 | 137775401 | - | 13.7 | 5441 | 6158 | -7 | L1PA15 | L1 | LINE |
| 137775402 | 137775779 | + | 10.5 | 1 | 364 | 0 | THE1B | MaLR | LTR |
| 137775780 | 137776058 | + | 12.0 | 1 | 227 | -1353 | THE1B-int | MaLR | LTR |
| 137776059 | 137776905 | - | 17.7 | 1 | 785 | 0 | LTR1 | ERV1 | LTR |
| 137776906 | 137778231 | + | 12.0 | 228 | 1580 | 0 | THE1B-int | MaLR | LTR |
| 137778232 | 137778594 | + | 14.9 | 1 | 364 | 0 | THE1B | MaLR | LTR |
| 137778598 | 137779605 | - | 16.8 | 4408 | 5440 | -725 | L1PA15 | L1 | LINE |

  

|  |  |  |  |  |  |  |  |  |  |
| --- | --- | --- | --- | --- | --- | --- | --- | --- | --- |
| chr8:137987373-138002309 | | | 14649 bp | | |  |  |  |  |
| Genome Start | Genome End | Strand | Div | Repeat Start | Repeat End | Repeat Left | Name | Family | Class |
| 137987373 | 137987596 | + | 22.1 | 1 | 233 | -272 | MLT1D | MaLR | LTR |
| 137987713 | 137988224 | - | 24.3 | 195 | 1452 | -149 | LTR27B | ERV1 | LTR |
| 137988225 | 137988540 | + | 15.5 | 3 | 312 | 0 | AluSx | Alu | SINE |
| 137988541 | 137988740 | - | 20.6 | 1 | 194 | -400 | LTR27B | ERV1 | LTR |
| 137988745 | 137989296 | - | 19.2 | 4918 | 7071 | -4 | LTR25-int | ERV1 | LTR |
| 137989298 | 137989849 | - | 11.9 | 1 | 511 | 0 | MER9 | ERVK | LTR |
| 137989850 | 137990157 | - | 8.5 | 5608 | 5916 | 0 | HERVK9 | ERVK | LTR |
| 137990226 | 137991610 | - | 9.4 | 4246 | 5634 | -282 | HERVK9 | ERVK | LTR |
| 137991604 | 137993176 | - | 7.1 | 1268 | 2860 | -3056 | HERVK9 | ERVK | LTR |
| 137993177 | 137993763 | - | 7.0 | 1 | 592 | -5324 | HERVK9 | ERVK | LTR |
| 137993764 | 137994276 | - | 11.6 | 1 | 511 | 0 | MER9 | ERVK | LTR |
| 137994277 | 137997497 | - | 16.4 | 797 | 6174 | -529 | MER52A-int | ERV1 | LTR |
| 137997500 | 137997635 | - | 14.8 | 990 | 1128 | -4818 | MER52A-int | ERV1 | LTR |
| 137997636 | 137997720 | - | 17.9 | 3912 | 3995 | -885 | MER52A-int | ERV1 | LTR |
| 137997734 | 137997877 | - | 17.5 | 1532 | 4773 | -444 | MER52A-int | ERV1 | LTR |
| 137997910 | 137998838 | - | 17.5 | 642 | 1531 | -5560 | MER52A-int | ERV1 | LTR |
| 137998839 | 137999969 | - | 2.4 | 5024 | 6155 | 0 | L1PA2 | L1 | LINE |
| 137999970 | 138000624 | - | 22.5 | 2 | 655 | -6436 | MER52A-int | ERV1 | LTR |
| 138000631 | 138000784 | - | 27.6 | 1605 | 1750 | -5 | MER52A | ERV1 | LTR |
| 138000808 | 138001089 | - | 30.0 | 1209 | 1459 | -296 | MER52A | ERV1 | LTR |
| 138001071 | 138001640 | - | 22.1 | 1 | 710 | -145 | LTR27B | ERV1 | LTR |
| 138001690 | 138001799 | + | 21.1 | 252 | 379 | -126 | MLT1D | MaLR | LTR |
| 138001800 | 138002174 | - | 20.9 | 1 | 428 | 0 | MSTA | MaLR | LTR |
| 138002175 | 138002309 | + | 21.1 | 380 | 503 | -2 | MLT1D | MaLR | LTR |

  

|  |  |  |  |  |  |  |  |  |  |
| --- | --- | --- | --- | --- | --- | --- | --- | --- | --- |
| chr8:138154949-138160773 | | | 5799 bp | | |  |  |  |  |
| Genome Start | Genome End | Strand | Div | Repeat Start | Repeat End | Repeat Left | Name | Family | Class |
| 138154949 | 138155471 | - | 23.8 | 1254 | 1753 | -1191 | BLACKJACK | AcHobo | DNA |
| 138155476 | 138155809 | + | 24.3 | 1 | 334 | -62 | MSTD | MaLR | LTR |
| 138155817 | 138157382 | - | 8.0 | 4555 | 6143 | -11 | L1PA6 | L1 | LINE |
| 138157385 | 138157676 | + | 17.5 | 1421 | 1735 | 0 | MSTD-int | MaLR | LTR |
| 138157680 | 138158032 | + | 22.4 | 4 | 394 | -2 | MSTD | MaLR | LTR |
| 138158034 | 138158340 | - | 26.5 | 894 | 1255 | -1689 | BLACKJACK | AcHobo | DNA |
| 138158350 | 138158695 | + | 14.2 | 1 | 364 | 0 | THE1B | MaLR | LTR |
| 138158696 | 138160206 | + | 9.6 | 1 | 1580 | 0 | THE1B-int | MaLR | LTR |
| 138160207 | 138160552 | + | 15.8 | 1 | 364 | 0 | THE1B | MaLR | LTR |
| 138160553 | 138160773 | - | 21.2 | 632 | 878 | -2066 | BLACKJACK | AcHobo | DNA |

  

|  |  |  |  |  |  |  |  |  |  |
| --- | --- | --- | --- | --- | --- | --- | --- | --- | --- |
| chr8:138905905-138912117 | | | 6138 bp | | |  |  |  |  |
| Genome Start | Genome End | Strand | Div | Repeat Start | Repeat End | Repeat Left | Name | Family | Class |
| 138905905 | 138906026 | + | 18.0 | 5 | 134 | -240 | MLT1A | MaLR | LTR |
| 138906067 | 138906467 | - | 9.0 | 1 | 448 | -2 | LTR7 | ERV1 | LTR |
| 138906468 | 138906846 | - | 8.9 | 7324 | 7713 | 0 | HERVH | ERV1 | LTR |
| 138906847 | 138907062 | - | 13.0 | 5600 | 5819 | -1894 | HERVH | ERV1 | LTR |
| 138907064 | 138907724 | - | 13.3 | 4489 | 5149 | -2564 | HERVH | ERV1 | LTR |
| 138907728 | 138911589 | - | 9.1 | 1 | 3993 | -3720 | HERVH | ERV1 | LTR |
| 138911598 | 138911942 | - | 7.8 | 1 | 442 | -8 | LTR7 | ERV1 | LTR |
| 138911966 | 138912117 | + | 22.5 | 208 | 358 | -16 | MLT1A | MaLR | LTR |

  

|  |  |  |  |  |  |  |  |  |  |
| --- | --- | --- | --- | --- | --- | --- | --- | --- | --- |
| chr8:140071120-140073756 | | | 2635 bp | | |  |  |  |  |
| Genome Start | Genome End | Strand | Div | Repeat Start | Repeat End | Repeat Left | Name | Family | Class |
| 140071120 | 140071262 | - | 14.2 | 243 | 381 | 0 | THE1D | MaLR | LTR |
| 140071263 | 140071592 | - | 13.2 | 1 | 364 | 0 | THE1B | MaLR | LTR |
| 140071593 | 140073163 | - | 30.0 | 2 | 1735 | 0 | MLT1D-int | MaLR | LTR |
| 140073166 | 140073509 | - | 12.7 | 1 | 364 | 0 | THE1B | MaLR | LTR |
| 140073510 | 140073756 | - | 14.2 | 1 | 242 | -139 | THE1D | MaLR | LTR |

  

|  |  |  |  |  |  |  |  |  |  |
| --- | --- | --- | --- | --- | --- | --- | --- | --- | --- |
| chr8:143965166-143972966 | | | 7725 bp | | |  |  |  |  |
| Genome Start | Genome End | Strand | Div | Repeat Start | Repeat End | Repeat Left | Name | Family | Class |
| 143965166 | 143965208 | - | 8.2 | 7685 | 7722 | -1 | L1MA2 | L1 | LINE |
| 143965209 | 143965515 | - | 14.8 | 1 | 381 | 0 | THE1D | MaLR | LTR |
| 143965516 | 143965623 | - | 8.2 | 7572 | 7684 | -39 | L1MA2 | L1 | LINE |
| 143965624 | 143965817 | - | 11.6 | 208 | 428 | 0 | MSTA | MaLR | LTR |
| 143965870 | 143966007 | - | 11.6 | 58 | 207 | -221 | MSTA | MaLR | LTR |
| 143966017 | 143966078 | - | 16.1 | 5494 | 5555 | -757 | L1M2 | L1 | LINE |
| 143966085 | 143966158 | - | 9.5 | 1 | 65 | -310 | MSTA | MaLR | LTR |
| 143966159 | 143967174 | - | 14.4 | 567 | 1580 | 0 | MSTA-int | MaLR | LTR |
| 143967175 | 143967558 | + | 12.8 | 1 | 375 | 0 | THE1C | MaLR | LTR |
| 143967559 | 143968161 | - | 15.7 | 3 | 566 | -1014 | MSTA-int | MaLR | LTR |
| 143968164 | 143968563 | - | 11.4 | 1 | 428 | 0 | MSTA | MaLR | LTR |
| 143968564 | 143972049 | - | 9.9 | 3927 | 7576 | -147 | L1MA2 | L1 | LINE |
| 143972034 | 143972749 | - | 20.1 | 398 | 1051 | -6514 | L1MA2 | L1 | LINE |
| 143972773 | 143972966 | - | 20.1 | 236 | 397 | -7168 | L1MA2 | L1 | LINE |

  

|  |  |  |  |  |  |  |  |  |  |
| --- | --- | --- | --- | --- | --- | --- | --- | --- | --- |
| chr8:146107237-146114134 | | | 6611 bp | | |  |  |  |  |
| Genome Start | Genome End | Strand | Div | Repeat Start | Repeat End | Repeat Left | Name | Family | Class |
| 146107237 | 146107382 | - | 23.1 | 5922 | 6064 | -694 | MER4B-int | ERV1 | LTR |
| 146107422 | 146107789 | + | 11.7 | 2 | 364 | 0 | THE1B | MaLR | LTR |
| 146107790 | 146108207 | + | 9.8 | 1 | 425 | -1155 | THE1B-int | MaLR | LTR |
| 146108208 | 146108506 | - | 12.9 | 1 | 299 | -13 | AluSx | Alu | SINE |
| 146108507 | 146108860 | + | 9.8 | 426 | 769 | -811 | THE1B-int | MaLR | LTR |
| 146108859 | 146109262 | + | 5.7 | 1174 | 1580 | 0 | THE1B-int | MaLR | LTR |
| 146109263 | 146109628 | + | 10.9 | 1 | 364 | 0 | THE1B | MaLR | LTR |
| 146109736 | 146109849 | + | 21.0 | 1 | 134 | -178 | AluJb | Alu | SINE |
| 146109910 | 146110057 | + | 21.0 | 135 | 282 | -30 | AluJb | Alu | SINE |
| 146110093 | 146110167 | - | 20.0 | 5762 | 5831 | -927 | MER4B-int | ERV1 | LTR |
| 146110174 | 146110335 | - | 10.6 | 1 | 161 | -151 | AluSx | Alu | SINE |
| 146110336 | 146110428 | - | 9.9 | 4202 | 4292 | -2039 | MER4B-int | ERV1 | LTR |
| 146110429 | 146110735 | - | 13.1 | 1 | 307 | -5 | AluSx | Alu | SINE |
| 146110754 | 146110879 | - | 15.1 | 3 | 136 | -176 | AluJo | Alu | SINE |
| 146110900 | 146111156 | - | 14.5 | 1 | 297 | -13 | AluSg | Alu | SINE |
| 146111159 | 146111876 | - | 14.6 | 2469 | 3083 | -3104 | MER4B-int | ERV1 | LTR |
| 146111877 | 146112190 | + | 18.2 | 3 | 311 | -1 | AluJo | Alu | SINE |
| 146112193 | 146112512 | + | 7.4 | 1 | 320 | 0 | AluY | Alu | SINE |
| 146112513 | 146112524 | - | 14.6 | 2457 | 2468 | -3719 | MER4B-int | ERV1 | LTR |
| 146112525 | 146112828 | - | 13.2 | 1 | 305 | -7 | AluSx | Alu | SINE |
| 146112829 | 146112916 | - | 14.6 | 2385 | 2456 | -3731 | MER4B-int | ERV1 | LTR |
| 146112917 | 146113221 | - | 6.2 | 1 | 305 | -6 | AluY | Alu | SINE |
| 146113222 | 146113427 | - | 14.6 | 2164 | 2384 | -3803 | MER4B-int | ERV1 | LTR |
| 146113428 | 146113711 | + | 11.6 | 1 | 284 | -26 | AluSg | Alu | SINE |
| 146113712 | 146114020 | + | 8.8 | 4 | 310 | -1 | AluY | Alu | SINE |
| 146114021 | 146114134 | - | 14.6 | 2066 | 2163 | -4024 | MER4B-int | ERV1 | LTR |

  


|  |  |  |  |  |  |  |  |  |  |
| --- | --- | --- | --- | --- | --- | --- | --- | --- | --- |
| chr9:1760252-1767475 | | | 6993 bp | | |  |  |  |  |
| Genome Start | Genome End | Strand | Div | Repeat Start | Repeat End | Repeat Left | Name | Family | Class |
| 1760252 | 1760393 | - | 27.3 | 5566 | 5713 | -1 | ERVL-E | ERVL | LTR |
| 1760543 | 1760674 | - | 25.2 | 5213 | 5357 | -310 | ERVL-E | ERVL | LTR |
| 1760728 | 1761105 | - | 18.7 | 3 | 408 | 0 | MLT1A1 | MaLR | LTR |
| 1761119 | 1762920 | - | 21.9 | 1 | 1729 | -6 | MLT1A1-int | MaLR | LTR |
| 1762921 | 1763283 | - | 17.6 | 1 | 408 | 0 | MLT1A1 | MaLR | LTR |
| 1763300 | 1765704 | - | 30.8 | 2596 | 5173 | -541 | ERVL-E | ERVL | LTR |
| 1765705 | 1765828 | - | 21.0 | 3 | 158 | -2 | MER5A1 | MER1\_type | DNA |
| 1765829 | 1767475 | - | 30.5 | 981 | 2656 | -3058 | ERVL-E | ERVL | LTR |

  

|  |  |  |  |  |  |  |  |  |  |
| --- | --- | --- | --- | --- | --- | --- | --- | --- | --- |
| chr9:2301382-2308226 | | | 6842 bp | | |  |  |  |  |
| Genome Start | Genome End | Strand | Div | Repeat Start | Repeat End | Repeat Left | Name | Family | Class |
| 2301382 | 2302650 | - | 11.8 | 3089 | 4328 | -3237 | L1M1 | L1 | LINE |
| 2302651 | 2303010 | + | 13.2 | 1 | 375 | 0 | THE1C | MaLR | LTR |
| 2303014 | 2304616 | + | 32.2 | 3 | 1735 | 0 | MLT1I-int | MaLR | LTR |
| 2304617 | 2304986 | + | 10.0 | 1 | 374 | -1 | THE1C | MaLR | LTR |
| 2304987 | 2306014 | - | 15.4 | 2047 | 3088 | -4477 | L1M1 | L1 | LINE |
| 2306015 | 2306315 | + | 11.3 | 1 | 301 | -9 | AluSg | Alu | SINE |
| 2306316 | 2308226 | - | 15.8 | 15 | 2046 | -5519 | L1M1 | L1 | LINE |

  

|  |  |  |  |  |  |  |  |  |  |
| --- | --- | --- | --- | --- | --- | --- | --- | --- | --- |
| chr9:3776940-3780798 | | | 3856 bp | | |  |  |  |  |
| Genome Start | Genome End | Strand | Div | Repeat Start | Repeat End | Repeat Left | Name | Family | Class |
| 3776940 | 3778209 | - | 10.3 | 255 | 1580 | 0 | THE1C-int | MaLR | LTR |
| 3778210 | 3778563 | + | 13.2 | 1 | 364 | 0 | THE1B | MaLR | LTR |
| 3778567 | 3780137 | + | 30.0 | 3 | 1735 | 0 | MLT1I-int | MaLR | LTR |
| 3780138 | 3780489 | + | 15.1 | 1 | 364 | 0 | THE1B | MaLR | LTR |
| 3780490 | 3780798 | - | 10.3 | 1 | 254 | -1326 | THE1C-int | MaLR | LTR |

  

|  |  |  |  |  |  |  |  |  |  |
| --- | --- | --- | --- | --- | --- | --- | --- | --- | --- |
| chr9:3860195-3863675 | | | 3475 bp | | |  |  |  |  |
| Genome Start | Genome End | Strand | Div | Repeat Start | Repeat End | Repeat Left | Name | Family | Class |
| 3860195 | 3860423 | + | 19.1 | 2729 | 2962 | -3181 | L1MA4A | L1 | LINE |
| 3860424 | 3860795 | - | 14.4 | 3 | 364 | 0 | THE1B | MaLR | LTR |
| 3860798 | 3862376 | - | 30.1 | 2 | 1735 | 0 | MLT-int | MaLR | LTR |
| 3862381 | 3862747 | - | 16.0 | 1 | 362 | -2 | THE1B | MaLR | LTR |
| 3862748 | 3863675 | + | 16.6 | 2963 | 6302 | -1 | L1MA4A | L1 | LINE |

  

|  |  |  |  |  |  |  |  |  |  |
| --- | --- | --- | --- | --- | --- | --- | --- | --- | --- |
| chr9:4119191-4121871 | | | 2659 bp | | |  |  |  |  |
| Genome Start | Genome End | Strand | Div | Repeat Start | Repeat End | Repeat Left | Name | Family | Class |
| 4119191 | 4119332 | + | 29.1 | 2 | 149 | -113 | MIR | MIR | SINE |
| 4119348 | 4119754 | + | 11.6 | 1 | 427 | -1 | MSTA | MaLR | LTR |
| 4119757 | 4121357 | + | 16.7 | 15 | 1645 | -6 | MSTA-int | MaLR | LTR |
| 4121363 | 4121770 | + | 14.3 | 1 | 428 | 0 | MSTA | MaLR | LTR |
| 4121771 | 4121871 | + | 32.3 | 161 | 262 | 0 | MIR | MIR | SINE |

  

|  |  |  |  |  |  |  |  |  |  |
| --- | --- | --- | --- | --- | --- | --- | --- | --- | --- |
| chr9:7681855-7684601 | | | 2745 bp | | |  |  |  |  |
| Genome Start | Genome End | Strand | Div | Repeat Start | Repeat End | Repeat Left | Name | Family | Class |
| 7681855 | 7681896 | - | 33.3 | 3378 | 3414 | -5 | L2 | L2 | LINE |
| 7681897 | 7682257 | + | 14.9 | 1 | 381 | 0 | THE1D | MaLR | LTR |
| 7682260 | 7683842 | + | 27.9 | 2 | 1735 | 0 | MLT-int | MaLR | LTR |
| 7683843 | 7684199 | + | 17.1 | 1 | 381 | 0 | THE1D | MaLR | LTR |
| 7684200 | 7684601 | - | 33.3 | 2933 | 3377 | -42 | L2 | L2 | LINE |

  

|  |  |  |  |  |  |  |  |  |  |
| --- | --- | --- | --- | --- | --- | --- | --- | --- | --- |
| chr9:8025306-8028765 | | | 3444 bp | | |  |  |  |  |
| Genome Start | Genome End | Strand | Div | Repeat Start | Repeat End | Repeat Left | Name | Family | Class |
| 8025306 | 8025744 | + | 19.4 | 5128 | 5578 | -596 | L1MB8 | L1 | LINE |
| 8025754 | 8026131 | - | 13.6 | 1 | 428 | 0 | MSTA | MaLR | LTR |
| 8026137 | 8027801 | - | 17.4 | 1 | 1645 | -6 | MSTA-int | MaLR | LTR |
| 8027802 | 8028194 | - | 12.8 | 1 | 428 | 0 | MSTA | MaLR | LTR |
| 8028195 | 8028513 | + | 17.7 | 5583 | 5910 | -268 | L1MB8 | L1 | LINE |
| 8028516 | 8028577 | - | 14.5 | 1 | 62 | -18 | MADE1 | Mariner | DNA |
| 8028578 | 8028765 | + | 22.9 | 5908 | 6172 | -6 | L1MB8 | L1 | LINE |

  

|  |  |  |  |  |  |  |  |  |  |
| --- | --- | --- | --- | --- | --- | --- | --- | --- | --- |
| chr9:9222706-9225594 | | | 2887 bp | | |  |  |  |  |
| Genome Start | Genome End | Strand | Div | Repeat Start | Repeat End | Repeat Left | Name | Family | Class |
| 9222706 | 9223080 | + | 20.1 | 1 | 400 | -307 | MER39 | ERV1 | LTR |
| 9223081 | 9223442 | - | 13.4 | 1 | 375 | 0 | THE1C | MaLR | LTR |
| 9223443 | 9224985 | - | 28.5 | 2 | 1735 | 0 | MLT-int | MaLR | LTR |
| 9224988 | 9225353 | - | 14.3 | 1 | 375 | 0 | THE1C | MaLR | LTR |
| 9225354 | 9225594 | + | 17.5 | 401 | 706 | -1 | MER39 | ERV1 | LTR |

  

|  |  |  |  |  |  |  |  |  |  |
| --- | --- | --- | --- | --- | --- | --- | --- | --- | --- |
| chr9:9626518-9629188 | | | 2670 bp | | |  |  |  |  |
| Genome Start | Genome End | Strand | Div | Repeat Start | Repeat End | Repeat Left | Name | Family | Class |
| 9626518 | 9626767 | + | 32.4 | 220 | 495 | -120 | MLT1L | MaLR | LTR |
| 9626768 | 9627163 | - | 24.6 | 1 | 408 | 0 | MLT1A1 | MaLR | LTR |
| 9627165 | 9628724 | - | 23.8 | 1 | 1734 | -1 | MLT1A1-int | MaLR | LTR |
| 9628725 | 9629090 | - | 20.1 | 1 | 408 | 0 | MLT1A1 | MaLR | LTR |
| 9629091 | 9629188 | + | 32.4 | 496 | 609 | -6 | MLT1L | MaLR | LTR |

  

|  |  |  |  |  |  |  |  |  |  |
| --- | --- | --- | --- | --- | --- | --- | --- | --- | --- |
| chr9:10252732-10255821 | | | 3022 bp | | |  |  |  |  |
| Genome Start | Genome End | Strand | Div | Repeat Start | Repeat End | Repeat Left | Name | Family | Class |
| 10252732 | 10252825 | + | 31.4 | 2 | 94 | -390 | MLT1H2 | MaLR | LTR |
| 10252826 | 10253186 | + | 12.3 | 1 | 364 | 0 | THE1B | MaLR | LTR |
| 10253189 | 10254776 | + | 30.3 | 2 | 1735 | 0 | MLT1H2-int | MaLR | LTR |
| 10254777 | 10255135 | + | 13.8 | 1 | 364 | 0 | THE1B | MaLR | LTR |
| 10255136 | 10255514 | + | 31.4 | 95 | 436 | -48 | MLT1H2 | MaLR | LTR |
| 10255515 | 10255697 | - | 17.5 | 42 | 224 | 0 | MER58A | MER1\_type | DNA |
| 10255764 | 10255821 | + | 20.7 | 491 | 548 | -1 | MLT1H2 | MaLR | LTR |

  

|  |  |  |  |  |  |  |  |  |  |
| --- | --- | --- | --- | --- | --- | --- | --- | --- | --- |
| chr9:10804753-10811105 | | | 5917 bp | | |  |  |  |  |
| Genome Start | Genome End | Strand | Div | Repeat Start | Repeat End | Repeat Left | Name | Family | Class |
| 10804753 | 10804890 | - | 16.7 | 4457 | 4597 | -1549 | L1MC | L1 | LINE |
| 10804890 | 10805162 | + | 12.6 | 5215 | 5482 | -684 | L1PA16 | L1 | LINE |
| 10805213 | 10805505 | + | 11.8 | 5483 | 5783 | -383 | L1PA16 | L1 | LINE |
| 10805506 | 10805823 | + | 20.9 | 1 | 306 | -6 | AluJb | Alu | SINE |
| 10805824 | 10806198 | + | 11.8 | 5784 | 6151 | -15 | L1PA16 | L1 | LINE |
| 10806471 | 10807094 | - | 23.0 | 2 | 669 | -48 | MLT2B5 | ERVL | LTR |
| 10807095 | 10807646 | - | 18.9 | 3887 | 4450 | -1696 | L1MC | L1 | LINE |
| 10807667 | 10807789 | + | 19.6 | 2619 | 2746 | -3394 | L1M3 | L1 | LINE |
| 10807790 | 10808076 | - | 19.6 | 3 | 289 | -23 | AluJb | Alu | SINE |
| 10808077 | 10808179 | + | 19.6 | 2747 | 2837 | -3303 | L1M3 | L1 | LINE |
| 10808270 | 10808614 | - | 13.2 | 1 | 381 | 0 | THE1D | MaLR | LTR |
| 10808618 | 10810039 | - | 13.5 | 1 | 1563 | -17 | THE1D-int | MaLR | LTR |
| 10810040 | 10810408 | - | 11.3 | 3 | 381 | 0 | THE1D | MaLR | LTR |
| 10810411 | 10810518 | - | 13.1 | 3748 | 3852 | -2294 | L1MC | L1 | LINE |
| 10810519 | 10811065 | - | 21.1 | 1 | 550 | 0 | MER44B | MER2\_type | DNA |
| 10811066 | 10811105 | - | 13.1 | 3707 | 3747 | -2399 | L1MC | L1 | LINE |

  

|  |  |  |  |  |  |  |  |  |  |
| --- | --- | --- | --- | --- | --- | --- | --- | --- | --- |
| chr9:11109871-11114045 | | | 4147 bp | | |  |  |  |  |
| Genome Start | Genome End | Strand | Div | Repeat Start | Repeat End | Repeat Left | Name | Family | Class |
| 11109871 | 11110764 | - | 20.4 | 4233 | 5124 | -1022 | L1MC | L1 | LINE |
| 11110765 | 11111190 | - | 8.4 | 5728 | 6154 | 0 | L1PA6 | L1 | LINE |
| 11111217 | 11111284 | - | 12.5 | 304 | 360 | -4 | THE1B | MaLR | LTR |
| 11111285 | 11111651 | + | 7.4 | 5794 | 6168 | -4 | L1PA8 | L1 | LINE |
| 11111652 | 11111968 | - | 12.5 | 1 | 303 | -61 | THE1B | MaLR | LTR |
| 11111969 | 11113529 | - | 30.7 | 2 | 1735 | 0 | MLT1E2-int | MaLR | LTR |
| 11113532 | 11113893 | - | 11.6 | 1 | 364 | 0 | THE1B | MaLR | LTR |
| 11113894 | 11114045 | - | 18.4 | 4051 | 4211 | -1935 | L1MC | L1 | LINE |

  

|  |  |  |  |  |  |  |  |  |  |
| --- | --- | --- | --- | --- | --- | --- | --- | --- | --- |
| chr9:11373282-11378413 | | | 5128 bp | | |  |  |  |  |
| Genome Start | Genome End | Strand | Div | Repeat Start | Repeat End | Repeat Left | Name | Family | Class |
| 11373282 | 11373390 | + | 25.7 | 1360 | 1476 | -5131 | L1M | L1 | LINE |
| 11373377 | 11375485 | + | 21.0 | 3674 | 5841 | -900 | L1M | L1 | LINE |
| 11375486 | 11375847 | + | 13.4 | 1 | 390 | -38 | MSTA | MaLR | LTR |
| 11375848 | 11376150 | - | 14.2 | 1 | 304 | -8 | AluSx | Alu | SINE |
| 11376151 | 11376191 | + | 13.4 | 391 | 428 | 0 | MSTA | MaLR | LTR |
| 11376194 | 11377813 | + | 29.3 | 2 | 1735 | 0 | MLT-int | MaLR | LTR |
| 11377816 | 11378176 | + | 15.6 | 3 | 390 | -38 | MSTA | MaLR | LTR |
| 11378178 | 11378253 | + | 17.1 | 1051 | 1126 | -454 | THE1-int | MaLR | LTR |
| 11378254 | 11378289 | + | 13.9 | 346 | 381 | 0 | MSTA | MaLR | LTR |
| 11378303 | 11378413 | + | 21.6 | 5856 | 5962 | -807 | L1M | L1 | LINE |

  

|  |  |  |  |  |  |  |  |  |  |
| --- | --- | --- | --- | --- | --- | --- | --- | --- | --- |
| chr9:11381308-11384131 | | | 2662 bp | | |  |  |  |  |
| Genome Start | Genome End | Strand | Div | Repeat Start | Repeat End | Repeat Left | Name | Family | Class |
| 11381308 | 11381382 | - | 30.7 | 3335 | 3415 | -4 | L2 | L2 | LINE |
| 11381545 | 11381902 | + | 14.6 | 1 | 364 | 0 | THE1B | MaLR | LTR |
| 11381903 | 11383484 | + | 12.8 | 1 | 1580 | 0 | THE1B-int | MaLR | LTR |
| 11383485 | 11383839 | + | 16.4 | 1 | 364 | 0 | THE1B | MaLR | LTR |
| 11383840 | 11384131 | - | 34.4 | 2962 | 3249 | -170 | L2 | L2 | LINE |

  

|  |  |  |  |  |  |  |  |  |  |
| --- | --- | --- | --- | --- | --- | --- | --- | --- | --- |
| chr9:11438043-11443531 | | | 4925 bp | | |  |  |  |  |
| Genome Start | Genome End | Strand | Div | Repeat Start | Repeat End | Repeat Left | Name | Family | Class |
| 11438043 | 11438422 | + | 30.1 | 1 | 403 | -4593 | HERV16 | ERVL | LTR |
| 11438644 | 11439094 | + | 29.4 | 686 | 1165 | -3831 | HERV16 | ERVL | LTR |
| 11439131 | 11439458 | + | 36.5 | 1243 | 1581 | -3415 | HERV16 | ERVL | LTR |
| 11439534 | 11439782 | + | 31.7 | 1741 | 1994 | -3002 | HERV16 | ERVL | LTR |
| 11439796 | 11439953 | - | 24.7 | 1 | 151 | -214 | MLT1A0 | MaLR | LTR |
| 11440102 | 11440458 | + | 34.4 | 2736 | 3096 | -1900 | HERV16 | ERVL | LTR |
| 11440528 | 11440668 | + | 36.9 | 3129 | 3271 | -2396 | HERV16 | ERVL | LTR |
| 11440669 | 11441045 | + | 12.9 | 1 | 375 | 0 | THE1C | MaLR | LTR |
| 11441048 | 11442645 | + | 31.8 | 2 | 1735 | 0 | MLT-int | MaLR | LTR |
| 11442646 | 11443027 | + | 15.3 | 1 | 375 | 0 | THE1C | MaLR | LTR |
| 11443028 | 11443531 | + | 36.9 | 3272 | 3797 | -1870 | HERV16 | ERVL | LTR |

  

|  |  |  |  |  |  |  |  |  |  |
| --- | --- | --- | --- | --- | --- | --- | --- | --- | --- |
| chr9:11615883-11620656 | | | 4744 bp | | |  |  |  |  |
| Genome Start | Genome End | Strand | Div | Repeat Start | Repeat End | Repeat Left | Name | Family | Class |
| 11615883 | 11616120 | + | 18.5 | 5681 | 5919 | -372 | L1MA7 | L1 | LINE |
| 11616128 | 11616494 | + | 15.7 | 1 | 380 | -1 | THE1D | MaLR | LTR |
| 11616503 | 11617974 | + | 15.9 | 3 | 1478 | -102 | THE1D-int | MaLR | LTR |
| 11617988 | 11619581 | - | 3.0 | 4559 | 6152 | -3 | L1PA3 | L1 | LINE |
| 11619582 | 11619652 | + | 12.1 | 1463 | 1533 | -47 | THE1D-int | MaLR | LTR |
| 11619653 | 11619887 | - | 11.7 | 79 | 308 | -4 | AluSg/x | Alu | SINE |
| 11619888 | 11619947 | + | 12.1 | 1534 | 1580 | 0 | THE1D-int | MaLR | LTR |
| 11619950 | 11620304 | + | 13.6 | 3 | 381 | 0 | THE1D | MaLR | LTR |
| 11620305 | 11620656 | + | 19.3 | 5924 | 6289 | -2 | L1MA7 | L1 | LINE |

  

|  |  |  |  |  |  |  |  |  |  |
| --- | --- | --- | --- | --- | --- | --- | --- | --- | --- |
| chr9:12463344-12466783 | | | 3040 bp | | |  |  |  |  |
| Genome Start | Genome End | Strand | Div | Repeat Start | Repeat End | Repeat Left | Name | Family | Class |
| 12463344 | 12463445 | + | 27.4 | 54 | 175 | -67 | MIRb | MIR | SINE |
| 12463771 | 12464146 | + | 12.9 | 1 | 364 | 0 | THE1B | MaLR | LTR |
| 12464149 | 12465696 | + | 30.6 | 2 | 1735 | 0 | MLT-int | MaLR | LTR |
| 12465698 | 12466049 | + | 11.7 | 2 | 364 | 0 | THE1B | MaLR | LTR |
| 12466079 | 12466111 | + | 28.6 | 33 | 65 | -197 | MIR | MIR | SINE |
| 12466112 | 12466493 | + | 15.9 | 1 | 381 | 0 | THE1D | MaLR | LTR |
| 12466494 | 12466672 | + | 28.6 | 66 | 255 | -7 | MIR | MIR | SINE |
| 12466716 | 12466783 | + | 22.2 | 194 | 257 | -5 | MIRb | MIR | SINE |

  

|  |  |  |  |  |  |  |  |  |  |
| --- | --- | --- | --- | --- | --- | --- | --- | --- | --- |
| chr9:12523694-12526385 | | | 2690 bp | | |  |  |  |  |
| Genome Start | Genome End | Strand | Div | Repeat Start | Repeat End | Repeat Left | Name | Family | Class |
| 12523694 | 12523873 | - | 14.5 | 244 | 426 | 0 | MSTB | MaLR | LTR |
| 12523874 | 12524215 | - | 13.3 | 1 | 364 | 0 | THE1B | MaLR | LTR |
| 12524216 | 12525802 | - | 30.3 | 2 | 1735 | 0 | MLT1A0-int | MaLR | LTR |
| 12525805 | 12526141 | - | 16.6 | 1 | 364 | 0 | THE1B | MaLR | LTR |
| 12526142 | 12526385 | - | 14.5 | 1 | 243 | -183 | MSTB | MaLR | LTR |

  

|  |  |  |  |  |  |  |  |  |  |
| --- | --- | --- | --- | --- | --- | --- | --- | --- | --- |
| chr9:14535965-14539155 | | | 2948 bp | | |  |  |  |  |
| Genome Start | Genome End | Strand | Div | Repeat Start | Repeat End | Repeat Left | Name | Family | Class |
| 14535965 | 14536086 | - | 28.3 | 5793 | 5918 | -203 | L1M5 | L1 | LINE |
| 14536087 | 14536486 | - | 16.6 | 1 | 426 | 0 | MSTB | MaLR | LTR |
| 14536487 | 14538061 | - | 17.3 | 1 | 1651 | 0 | MSTB-int | MaLR | LTR |
| 14538064 | 14538514 | - | 16.5 | 1 | 420 | -6 | MSTB | MaLR | LTR |
| 14538703 | 14538934 | - | 24.5 | 61 | 305 | -209 | MLT1H2 | MaLR | LTR |
| 14538988 | 14539155 | - | 29.4 | 5524 | 5711 | -410 | L1M5 | L1 | LINE |

  

|  |  |  |  |  |  |  |  |  |  |
| --- | --- | --- | --- | --- | --- | --- | --- | --- | --- |
| chr9:15310909-15319241 | | | 8299 bp | | |  |  |  |  |
| Genome Start | Genome End | Strand | Div | Repeat Start | Repeat End | Repeat Left | Name | Family | Class |
| 15310909 | 15311231 | + | 18.7 | 3020 | 3344 | -2802 | L1MB5 | L1 | LINE |
| 15311232 | 15311532 | + | 7.8 | 1 | 294 | -17 | AluY | Alu | SINE |
| 15311533 | 15311826 | + | 15.3 | 3345 | 4687 | -1459 | L1MB5 | L1 | LINE |
| 15311828 | 15311987 | + | 10.8 | 1 | 161 | -214 | THE1C | MaLR | LTR |
| 15311988 | 15312336 | - | 12.9 | 2 | 295 | -5 | AluSg | Alu | SINE |
| 15312337 | 15312557 | + | 10.8 | 162 | 375 | 0 | THE1C | MaLR | LTR |
| 15312558 | 15314088 | + | 11.3 | 1 | 1580 | 0 | THE1C-int | MaLR | LTR |
| 15314089 | 15314464 | + | 10.9 | 1 | 375 | 0 | THE1C | MaLR | LTR |
| 15314465 | 15314932 | + | 17.1 | 4684 | 5157 | -989 | L1MB5 | L1 | LINE |
| 15314933 | 15315074 | + | 18.3 | 3 | 142 | -170 | AluJo | Alu | SINE |
| 15315075 | 15315373 | + | 11.1 | 1 | 297 | -14 | AluY | Alu | SINE |
| 15315374 | 15315536 | + | 18.3 | 143 | 305 | -7 | AluJo | Alu | SINE |
| 15315537 | 15315621 | + | 17.1 | 5158 | 5234 | -912 | L1MB5 | L1 | LINE |
| 15315626 | 15315761 | + | 22.2 | 6 | 140 | -2 | FLAM\_A | Alu | SINE |
| 15315769 | 15315941 | + | 21.8 | 5221 | 5399 | -747 | L1MB5 | L1 | LINE |
| 15315924 | 15316224 | + | 13.5 | 5811 | 6109 | -56 | L1PA15 | L1 | LINE |
| 15316225 | 15316294 | - | 12.9 | 305 | 375 | 0 | THE1B | MaLR | LTR |
| 15316295 | 15317350 | + | 1.2 | 5096 | 6155 | 0 | L1PA2 | L1 | LINE |
| 15317354 | 15317666 | - | 12.5 | 1 | 307 | -57 | THE1B | MaLR | LTR |
| 15317667 | 15317850 | - | 6.8 | 1413 | 1580 | 0 | THE1-int | MaLR | LTR |
| 15317851 | 15318151 | + | 11.6 | 5 | 306 | -7 | AluSp | Alu | SINE |
| 15318152 | 15318426 | - | 6.8 | 1137 | 1412 | -168 | THE1-int | MaLR | LTR |
| 15318464 | 15319241 | + | 15.8 | 5385 | 6174 | 0 | L1MB5 | L1 | LINE |

  

|  |  |  |  |  |  |  |  |  |  |
| --- | --- | --- | --- | --- | --- | --- | --- | --- | --- |
| chr9:15746370-15749052 | | | 2669 bp | | |  |  |  |  |
| Genome Start | Genome End | Strand | Div | Repeat Start | Repeat End | Repeat Left | Name | Family | Class |
| 15746370 | 15746674 | - | 9.3 | 5863 | 6166 | 0 | L1PA16 | L1 | LINE |
| 15746680 | 15747035 | + | 16.1 | 5 | 360 | -4 | THE1B | MaLR | LTR |
| 15747044 | 15748627 | + | 8.2 | 1 | 1580 | 0 | THE1B-int | MaLR | LTR |
| 15748628 | 15748984 | + | 14.7 | 1 | 364 | 0 | THE1B | MaLR | LTR |
| 15748986 | 15749052 | - | 9.0 | 5800 | 5866 | -300 | L1PA16 | L1 | LINE |

  

|  |  |  |  |  |  |  |  |  |  |
| --- | --- | --- | --- | --- | --- | --- | --- | --- | --- |
| chr9:18571427-18575072 | | | 3653 bp | | |  |  |  |  |
| Genome Start | Genome End | Strand | Div | Repeat Start | Repeat End | Repeat Left | Name | Family | Class |
| 18571427 | 18572376 | - | 21.8 | 421 | 1367 | -4 | MLT1C-int | MaLR | LTR |
| 18572381 | 18572564 | + | 16.8 | 1 | 176 | -188 | THE1B | MaLR | LTR |
| 18572565 | 18572910 | + | 10.7 | 1 | 345 | 0 | AluY | Alu | SINE |
| 18572911 | 18573095 | + | 16.8 | 177 | 364 | 0 | THE1B | MaLR | LTR |
| 18573096 | 18573201 | + | 14.3 | 1 | 105 | -1475 | THE1B-int | MaLR | LTR |
| 18573173 | 18574110 | + | 11.0 | 640 | 1580 | 0 | THE1B-int | MaLR | LTR |
| 18574111 | 18574470 | + | 13.8 | 1 | 364 | 0 | THE1B | MaLR | LTR |
| 18574478 | 18574618 | - | 23.6 | 273 | 413 | -958 | MLT1C-int | MaLR | LTR |
| 18574619 | 18574819 | + | 20.9 | 1 | 207 | -347 | MLT2B3 | ERVL | LTR |
| 18574821 | 18574961 | + | 17.0 | 241 | 383 | -13 | MSTD | MaLR | LTR |
| 18574972 | 18575072 | - | 15.0 | 58 | 162 | -1209 | MLT1C-int | MaLR | LTR |

  

|  |  |  |  |  |  |  |  |  |  |
| --- | --- | --- | --- | --- | --- | --- | --- | --- | --- |
| chr9:20528775-20533955 | | | 5057 bp | | |  |  |  |  |
| Genome Start | Genome End | Strand | Div | Repeat Start | Repeat End | Repeat Left | Name | Family | Class |
| 20528775 | 20529349 | - | 13.2 | 1853 | 2418 | 0 | Tigger1 | MER2\_type | DNA |
| 20529350 | 20529718 | - | 10.2 | 1 | 378 | -3 | THE1D | MaLR | LTR |
| 20529719 | 20531356 | - | 29.9 | 2 | 1735 | 0 | MLT-int | MaLR | LTR |
| 20531359 | 20531713 | - | 16.0 | 1 | 381 | 0 | THE1D | MaLR | LTR |
| 20531714 | 20532237 | - | 13.2 | 1325 | 1852 | -566 | Tigger1 | MER2\_type | DNA |
| 20532238 | 20532536 | - | 2.0 | 13 | 311 | -7 | AluYb8 | Alu | SINE |
| 20532537 | 20533380 | - | 13.2 | 436 | 1324 | -1094 | Tigger1 | MER2\_type | DNA |
| 20533390 | 20533486 | - | 17.7 | 187 | 284 | -2134 | Tigger1 | MER2\_type | DNA |
| 20533487 | 20533663 | + | 26.6 | 1 | 169 | -7 | FRAM | Alu | SINE |
| 20533777 | 20533955 | - | 17.9 | 8 | 186 | -2232 | Tigger1 | MER2\_type | DNA |

  

|  |  |  |  |  |  |  |  |  |  |
| --- | --- | --- | --- | --- | --- | --- | --- | --- | --- |
| chr9:20572045-20575718 | | | 3672 bp | | |  |  |  |  |
| Genome Start | Genome End | Strand | Div | Repeat Start | Repeat End | Repeat Left | Name | Family | Class |
| 20572045 | 20572536 | - | 15.4 | 5866 | 6343 | -1 | L1MC2 | L1 | LINE |
| 20572537 | 20572894 | - | 10.0 | 1 | 355 | 0 | THE1A | MaLR | LTR |
| 20572895 | 20574440 | - | 30.3 | 2 | 1735 | 0 | MLT-int | MaLR | LTR |
| 20574443 | 20574804 | - | 9.3 | 1 | 355 | 0 | THE1A | MaLR | LTR |
| 20574805 | 20575054 | - | 15.4 | 5620 | 5865 | -479 | L1MC2 | L1 | LINE |
| 20575055 | 20575456 | - | 24.4 | 2 | 370 | -4 | MLT1A | MaLR | LTR |
| 20575457 | 20575718 | - | 15.4 | 5342 | 5619 | -725 | L1MC2 | L1 | LINE |

  

|  |  |  |  |  |  |  |  |  |  |
| --- | --- | --- | --- | --- | --- | --- | --- | --- | --- |
| chr9:21611711-21614400 | | | 2687 bp | | |  |  |  |  |
| Genome Start | Genome End | Strand | Div | Repeat Start | Repeat End | Repeat Left | Name | Family | Class |
| 21611711 | 21612097 | + | 21.9 | 1 | 419 | -96 | MLT2B2 | ERVL | LTR |
| 21612098 | 21612451 | - | 14.0 | 1 | 355 | 0 | THE1A | MaLR | LTR |
| 21612453 | 21614009 | - | 29.1 | 2 | 1734 | -1 | MLT-int | MaLR | LTR |
| 21614012 | 21614364 | - | 13.1 | 1 | 355 | 0 | THE1A | MaLR | LTR |
| 21614365 | 21614400 | + | 21.9 | 467 | 496 | -66 | MLT2B2 | ERVL | LTR |

  

|  |  |  |  |  |  |  |  |  |  |
| --- | --- | --- | --- | --- | --- | --- | --- | --- | --- |
| chr9:22237573-22242868 | | | 5283 bp | | |  |  |  |  |
| Genome Start | Genome End | Strand | Div | Repeat Start | Repeat End | Repeat Left | Name | Family | Class |
| 22237573 | 22240293 | - | 12.9 | 4072 | 6861 | -8 | L1PA16 | L1 | LINE |
| 22240306 | 22240729 | - | 15.6 | 1 | 428 | 0 | MSTA | MaLR | LTR |
| 22240730 | 22242351 | - | 17.6 | 1 | 1651 | 0 | MSTA-int | MaLR | LTR |
| 22242352 | 22242775 | - | 16.5 | 1 | 428 | 0 | MSTA | MaLR | LTR |
| 22242777 | 22242868 | - | 12.0 | 3959 | 4050 | -2799 | L1PA16 | L1 | LINE |

  

|  |  |  |  |  |  |  |  |  |  |
| --- | --- | --- | --- | --- | --- | --- | --- | --- | --- |
| chr9:22698374-22707653 | | | 9074 bp | | |  |  |  |  |
| Genome Start | Genome End | Strand | Div | Repeat Start | Repeat End | Repeat Left | Name | Family | Class |
| 22698374 | 22699554 | - | 22.2 | 5378 | 6651 | -31 | L1MB7 | L1 | LINE |
| 22699555 | 22699948 | - | 18.8 | 3 | 396 | 0 | MSTD | MaLR | LTR |
| 22699949 | 22699961 | - | 22.4 | 5375 | 5377 | -1267 | L1MB7 | L1 | LINE |
| 22699962 | 22700246 | + | 17.9 | 1 | 296 | -16 | AluJb | Alu | SINE |
| 22700247 | 22701108 | - | 22.4 | 4500 | 5374 | -1270 | L1MB7 | L1 | LINE |
| 22701170 | 22701533 | + | 32.0 | 77 | 450 | -128 | MER70B | ERVL | LTR |
| 22701654 | 22702043 | - | 23.6 | 4111 | 4505 | -2139 | L1MB7 | L1 | LINE |
| 22702044 | 22702401 | + | 17.1 | 3 | 364 | 0 | THE1B | MaLR | LTR |
| 22702404 | 22703976 | + | 32.1 | 2 | 1735 | 0 | MLT-int | MaLR | LTR |
| 22703977 | 22704347 | + | 14.2 | 1 | 364 | 0 | THE1B | MaLR | LTR |
| 22704348 | 22704500 | - | 23.6 | 3950 | 4110 | -2534 | L1MB7 | L1 | LINE |
| 22704524 | 22705745 | - | 25.6 | 1581 | 2895 | -3749 | L1MB7 | L1 | LINE |
| 22705746 | 22706056 | - | 10.6 | 1 | 311 | -1 | AluSx | Alu | SINE |
| 22706057 | 22707653 | - | 26.9 | 1 | 1580 | -4962 | L1MB7 | L1 | LINE |

  

|  |  |  |  |  |  |  |  |  |  |
| --- | --- | --- | --- | --- | --- | --- | --- | --- | --- |
| chr9:23083328-23088391 | | | 4843 bp | | |  |  |  |  |
| Genome Start | Genome End | Strand | Div | Repeat Start | Repeat End | Repeat Left | Name | Family | Class |
| 23083328 | 23085385 | - | 26.5 | 1300 | 3421 | -2798 | L1MEc | L1 | LINE |
| 23085464 | 23085835 | + | 12.1 | 1 | 375 | 0 | THE1C | MaLR | LTR |
| 23085836 | 23087352 | + | 13.3 | 1 | 1580 | 0 | THE1C-int | MaLR | LTR |
| 23087353 | 23087714 | + | 14.1 | 1 | 372 | -3 | THE1C | MaLR | LTR |
| 23087858 | 23088391 | - | 31.9 | 376 | 896 | -5504 | L1MEc | L1 | LINE |

  

|  |  |  |  |  |  |  |  |  |  |
| --- | --- | --- | --- | --- | --- | --- | --- | --- | --- |
| chr9:23317424-23326079 | | | 8395 bp | | |  |  |  |  |
| Genome Start | Genome End | Strand | Div | Repeat Start | Repeat End | Repeat Left | Name | Family | Class |
| 23317424 | 23317567 | + | 30.8 | 1724 | 1872 | -4528 | L1ME3A | L1 | LINE |
| 23317581 | 23318692 | + | 26.9 | 1882 | 3067 | -3152 | L1ME3A | L1 | LINE |
| 23318693 | 23319016 | + | 1.2 | 1 | 318 | 0 | AluYb8 | Alu | SINE |
| 23319017 | 23319107 | + | 25.7 | 3068 | 3136 | -3083 | L1ME3A | L1 | LINE |
| 23319109 | 23319497 | + | 17.2 | 15 | 428 | 0 | MSTA | MaLR | LTR |
| 23319499 | 23321131 | + | 31.1 | 1 | 1734 | -1 | MLT-int | MaLR | LTR |
| 23321133 | 23321539 | + | 18.1 | 1 | 428 | 0 | MSTA | MaLR | LTR |
| 23321543 | 23321994 | + | 23.5 | 3138 | 3621 | -2598 | L1ME3A | L1 | LINE |
| 23322066 | 23322785 | + | 26.9 | 3701 | 4460 | -1759 | L1ME3A | L1 | LINE |
| 23322858 | 23323617 | + | 26.2 | 4698 | 5510 | -725 | L1ME3A | L1 | LINE |
| 23323612 | 23324928 | + | 18.6 | 4822 | 6163 | -21 | L1MB7 | L1 | LINE |
| 23324936 | 23325432 | + | 26.7 | 5490 | 5983 | -260 | L1ME3A | L1 | LINE |
| 23325433 | 23325735 | + | 12.2 | 1 | 294 | -16 | AluSg | Alu | SINE |
| 23325834 | 23326079 | + | 23.8 | 5981 | 6238 | -5 | L1ME3A | L1 | LINE |

  

|  |  |  |  |  |  |  |  |  |  |
| --- | --- | --- | --- | --- | --- | --- | --- | --- | --- |
| chr9:23348264-23352519 | | | 4255 bp | | |  |  |  |  |
| Genome Start | Genome End | Strand | Div | Repeat Start | Repeat End | Repeat Left | Name | Family | Class |
| 23348264 | 23349729 | + | 29.1 | 138 | 1612 | -4703 | L1MEc | L1 | LINE |
| 23349730 | 23350088 | - | 12.7 | 1 | 364 | 0 | THE1B | MaLR | LTR |
| 23350089 | 23350783 | - | 11.1 | 908 | 1580 | 0 | THE1B-int | MaLR | LTR |
| 23350784 | 23351096 | - | 9.4 | 1 | 309 | -4 | AluSp | Alu | SINE |
| 23351097 | 23351976 | - | 11.1 | 1 | 907 | -673 | THE1B-int | MaLR | LTR |
| 23351977 | 23352305 | - | 13.4 | 1 | 364 | 0 | THE1B | MaLR | LTR |
| 23352307 | 23352519 | + | 22.6 | 1695 | 1927 | -4473 | L1MEc | L1 | LINE |

  

|  |  |  |  |  |  |  |  |  |  |
| --- | --- | --- | --- | --- | --- | --- | --- | --- | --- |
| chr9:24185369-24194798 | | | 9148 bp | | |  |  |  |  |
| Genome Start | Genome End | Strand | Div | Repeat Start | Repeat End | Repeat Left | Name | Family | Class |
| 24185369 | 24185887 | - | 19.1 | 5853 | 6351 | -25 | L1MA9 | L1 | LINE |
| 24185928 | 24187296 | - | 17.6 | 4468 | 5852 | -524 | L1MA9 | L1 | LINE |
| 24187337 | 24187916 | - | 13.7 | 7146 | 7724 | 0 | L1MA3 | L1 | LINE |
| 24187917 | 24188266 | + | 10.1 | 1 | 363 | -1 | THE1B | MaLR | LTR |
| 24188267 | 24188756 | - | 13.3 | 6648 | 7145 | -579 | L1MA3 | L1 | LINE |
| 24188757 | 24189069 | - | 9.4 | 1 | 311 | 0 | AluY | Alu | SINE |
| 24189070 | 24189389 | - | 12.6 | 6338 | 6647 | -918 | L1MA3 | L1 | LINE |
| 24189419 | 24189568 | - | 14.4 | 3257 | 3405 | -4160 | L1MA3 | L1 | LINE |
| 24189564 | 24190317 | - | 17.0 | 3707 | 4458 | -1746 | L1MA9 | L1 | LINE |
| 24190318 | 24190745 | - | 8.6 | 1 | 453 | 0 | MLT2A2 | ERVL | LTR |
| 24190746 | 24190847 | - | 17.0 | 3596 | 3706 | -2498 | L1MA9 | L1 | LINE |
| 24190847 | 24191458 | - | 22.6 | 1958 | 2597 | -3610 | L1MA9 | L1 | LINE |
| 24191633 | 24192257 | - | 31.1 | 248 | 931 | -5210 | L1MA9 | L1 | LINE |
| 24192258 | 24192623 | + | 14.7 | 1 | 364 | 0 | THE1B | MaLR | LTR |
| 24192629 | 24194189 | + | 29.9 | 5 | 1735 | 0 | MLT-int | MaLR | LTR |
| 24194190 | 24194551 | + | 16.9 | 1 | 364 | 0 | THE1B | MaLR | LTR |
| 24194552 | 24194798 | - | 32.7 | 1 | 246 | -5895 | L1MA9 | L1 | LINE |

  

|  |  |  |  |  |  |  |  |  |  |
| --- | --- | --- | --- | --- | --- | --- | --- | --- | --- |
| chr9:24279921-24282954 | | | 3030 bp | | |  |  |  |  |
| Genome Start | Genome End | Strand | Div | Repeat Start | Repeat End | Repeat Left | Name | Family | Class |
| 24279921 | 24280052 | - | 20.8 | 650 | 787 | 0 | LTR48 | ERV1 | LTR |
| 24280053 | 24280417 | - | 11.0 | 1 | 364 | 0 | THE1B | MaLR | LTR |
| 24280420 | 24281975 | - | 31.3 | 2 | 1733 | -2 | MLT-int | MaLR | LTR |
| 24281978 | 24282341 | - | 11.3 | 1 | 364 | 0 | THE1B | MaLR | LTR |
| 24282342 | 24282954 | - | 20.8 | 2 | 649 | -138 | LTR48 | ERV1 | LTR |

  

|  |  |  |  |  |  |  |  |  |  |
| --- | --- | --- | --- | --- | --- | --- | --- | --- | --- |
| chr9:24306253-24314211 | | | 6935 bp | | |  |  |  |  |
| Genome Start | Genome End | Strand | Div | Repeat Start | Repeat End | Repeat Left | Name | Family | Class |
| 24306253 | 24306846 | - | 29.1 | 2774 | 3416 | -3 | L2 | L2 | LINE |
| 24306847 | 24307211 | - | 11.3 | 1 | 381 | 0 | THE1D | MaLR | LTR |
| 24307212 | 24307551 | - | 32.3 | 2389 | 2773 | -646 | L2 | L2 | LINE |
| 24307585 | 24307894 | - | 24.8 | 1 | 344 | -1 | MER2 | MER2\_type | DNA |
| 24308080 | 24308234 | - | 30.9 | 21 | 181 | -66 | MIR | MIR | SINE |
| 24308240 | 24308394 | - | 39.6 | 2113 | 2269 | -1150 | L2 | L2 | LINE |
| 24308620 | 24308779 | - | 24.1 | 16 | 190 | -72 | MIR | MIR | SINE |
| 24308796 | 24309000 | + | 26.0 | 5088 | 5303 | -946 | L1M5 | L1 | LINE |
| 24309001 | 24309297 | + | 10.1 | 1 | 298 | -15 | AluSp | Alu | SINE |
| 24309298 | 24309686 | + | 29.5 | 5295 | 5707 | -533 | L1M5 | L1 | LINE |
| 24309687 | 24310088 | - | 15.3 | 1 | 426 | 0 | MSTB | MaLR | LTR |
| 24310100 | 24311720 | - | 19.1 | 1 | 1634 | -17 | MSTB-int | MaLR | LTR |
| 24311721 | 24311912 | - | 21.0 | 217 | 426 | 0 | MSTB | MaLR | LTR |
| 24311939 | 24312122 | - | 21.0 | 1 | 216 | -210 | MSTB | MaLR | LTR |
| 24312161 | 24312237 | + | 22.4 | 5724 | 5799 | -441 | L1M5 | L1 | LINE |
| 24312290 | 24312623 | - | 31.8 | 1418 | 1810 | -1609 | L2 | L2 | LINE |
| 24312900 | 24312972 | - | 23.6 | 3315 | 3418 | -1 | L2 | L2 | LINE |
| 24313056 | 24313149 | - | 23.4 | 3165 | 3272 | -147 | L2 | L2 | LINE |
| 24313154 | 24313319 | - | 26.9 | 1793 | 1956 | -1463 | L2 | L2 | LINE |
| 24313325 | 24313763 | + | 20.6 | 3 | 467 | -53 | MLT2B1 | ERVL | LTR |
| 24313784 | 24313827 | + | 20.6 | 515 | 564 | -3 | MLT2B1 | ERVL | LTR |
| 24313873 | 24314211 | - | 32.6 | 1419 | 1779 | -1640 | L2 | L2 | LINE |

  

|  |  |  |  |  |  |  |  |  |  |
| --- | --- | --- | --- | --- | --- | --- | --- | --- | --- |
| chr9:25277928-25285898 | | | 7611 bp | | |  |  |  |  |
| Genome Start | Genome End | Strand | Div | Repeat Start | Repeat End | Repeat Left | Name | Family | Class |
| 25277928 | 25278140 | - | 29.5 | 5774 | 5990 | -233 | L1ME3B | L1 | LINE |
| 25278174 | 25278589 | + | 8.8 | 3 | 448 | -2 | LTR7 | ERV1 | LTR |
| 25278592 | 25281063 | + | 10.5 | 1 | 2983 | -4730 | HERVH | ERV1 | LTR |
| 25281074 | 25283147 | + | 10.7 | 3140 | 5149 | -2564 | HERVH | ERV1 | LTR |
| 25283149 | 25283364 | + | 17.1 | 5600 | 5819 | -1894 | HERVH | ERV1 | LTR |
| 25283363 | 25283741 | + | 10.2 | 7322 | 7713 | 0 | HERVH | ERV1 | LTR |
| 25283742 | 25284099 | + | 6.8 | 1 | 448 | -2 | LTR7 | ERV1 | LTR |
| 25284200 | 25284365 | - | 31.0 | 5419 | 5599 | -624 | L1ME3B | L1 | LINE |
| 25284356 | 25284603 | - | 32.4 | 1317 | 1572 | -4577 | L1ME3B | L1 | LINE |
| 25284753 | 25284959 | - | 29.0 | 903 | 1108 | -5011 | L1ME3B | L1 | LINE |
| 25285034 | 25285713 | - | 9.5 | 5023 | 5779 | -352 | L1PA8A | L1 | LINE |
| 25285717 | 25285898 | - | 36.1 | 561 | 749 | -5370 | L1ME3B | L1 | LINE |

  

|  |  |  |  |  |  |  |  |  |  |
| --- | --- | --- | --- | --- | --- | --- | --- | --- | --- |
| chr9:25346026-25349258 | | | 3177 bp | | |  |  |  |  |
| Genome Start | Genome End | Strand | Div | Repeat Start | Repeat End | Repeat Left | Name | Family | Class |
| 25346026 | 25346654 | - | 20.8 | 7106 | 7764 | -21 | L1MC3 | L1 | LINE |
| 25346655 | 25347013 | - | 16.9 | 3 | 381 | 0 | THE1D | MaLR | LTR |
| 25347016 | 25348639 | - | 16.3 | 3 | 1580 | 0 | THE1D-int | MaLR | LTR |
| 25348643 | 25348815 | - | 11.8 | 196 | 380 | -1 | THE1D | MaLR | LTR |
| 25348858 | 25349038 | - | 11.8 | 2 | 195 | -186 | THE1D | MaLR | LTR |
| 25349048 | 25349258 | - | 20.4 | 6866 | 7100 | -685 | L1MC3 | L1 | LINE |

  

|  |  |  |  |  |  |  |  |  |  |
| --- | --- | --- | --- | --- | --- | --- | --- | --- | --- |
| chr9:25672434-25675302 | | | 2833 bp | | |  |  |  |  |
| Genome Start | Genome End | Strand | Div | Repeat Start | Repeat End | Repeat Left | Name | Family | Class |
| 25672434 | 25672523 | + | 25.0 | 1 | 94 | -280 | MLT1A | MaLR | LTR |
| 25672524 | 25672938 | + | 15.3 | 1 | 428 | 0 | MSTA | MaLR | LTR |
| 25672941 | 25674558 | + | 28.4 | 2 | 1735 | 0 | MLT1A-int | MaLR | LTR |
| 25674559 | 25674972 | + | 15.2 | 1 | 428 | 0 | MSTA | MaLR | LTR |
| 25674973 | 25675067 | + | 25.0 | 95 | 177 | -197 | MLT1A | MaLR | LTR |
| 25675102 | 25675302 | + | 23.5 | 172 | 370 | -4 | MLT1A | MaLR | LTR |

  

|  |  |  |  |  |  |  |  |  |  |
| --- | --- | --- | --- | --- | --- | --- | --- | --- | --- |
| chr9:25858780-25861926 | | | 3145 bp | | |  |  |  |  |
| Genome Start | Genome End | Strand | Div | Repeat Start | Repeat End | Repeat Left | Name | Family | Class |
| 25858780 | 25859050 | - | 16.1 | 5952 | 6231 | 0 | L1PB3 | L1 | LINE |
| 25859051 | 25859413 | - | 17.3 | 1 | 428 | 0 | MSTA | MaLR | LTR |
| 25859414 | 25861063 | - | 17.2 | 3 | 1580 | 0 | MSTA-int | MaLR | LTR |
| 25861066 | 25861444 | - | 18.6 | 1 | 428 | 0 | MSTA | MaLR | LTR |
| 25861445 | 25861926 | - | 18.2 | 5483 | 5949 | -205 | L1PB3 | L1 | LINE |

  

|  |  |  |  |  |  |  |  |  |  |
| --- | --- | --- | --- | --- | --- | --- | --- | --- | --- |
| chr9:26574377-26586266 | | | 11824 bp | | |  |  |  |  |
| Genome Start | Genome End | Strand | Div | Repeat Start | Repeat End | Repeat Left | Name | Family | Class |
| 26574377 | 26576376 | - | 16.2 | 4350 | 6375 | -1 | L1MA9 | L1 | LINE |
| 26576378 | 26578504 | + | 1.6 | 2134 | 4260 | -1886 | L1P1 | L1 | LINE |
| 26578504 | 26578593 | - | 14.3 | 4255 | 4343 | -1861 | L1MA9 | L1 | LINE |
| 26578594 | 26578974 | - | 8.3 | 5756 | 6130 | -1 | L1PA8A | L1 | LINE |
| 26578975 | 26579397 | - | 14.3 | 3838 | 4254 | -1950 | L1MA9 | L1 | LINE |
| 26579398 | 26579557 | - | 24.4 | 1 | 161 | -5 | FRAM | Alu | SINE |
| 26579584 | 26579939 | - | 14.9 | 1 | 364 | 0 | THE1B | MaLR | LTR |
| 26579940 | 26581362 | - | 11.0 | 104 | 1580 | 0 | THE1B-int | MaLR | LTR |
| 26581363 | 26581726 | - | 15.4 | 1 | 364 | 0 | THE1B | MaLR | LTR |
| 26581727 | 26581829 | - | 11.0 | 1 | 103 | -1477 | THE1B-int | MaLR | LTR |
| 26581830 | 26582188 | - | 13.9 | 1 | 364 | 0 | THE1B | MaLR | LTR |
| 26582189 | 26582298 | - | 17.1 | 1 | 115 | -18 | FLAM\_C | Alu | SINE |
| 26582299 | 26583166 | - | 18.8 | 2910 | 3845 | -2359 | L1MA9 | L1 | LINE |
| 26583167 | 26583459 | - | 15.0 | 1 | 293 | -19 | AluSx | Alu | SINE |
| 26583460 | 26583869 | - | 18.8 | 2498 | 2909 | -3295 | L1MA9 | L1 | LINE |
| 26583878 | 26584195 | - | 26.8 | 1529 | 1854 | -4357 | L1MA9 | L1 | LINE |
| 26584196 | 26584520 | - | 9.4 | 3 | 311 | -2 | AluSq | Alu | SINE |
| 26584521 | 26584531 | - | 26.8 | 1529 | 1528 | -4683 | L1MA9 | L1 | LINE |
| 26584564 | 26584794 | - | 6.9 | 41 | 283 | -28 | AluY | Alu | SINE |
| 26584795 | 26586266 | - | 23.8 | 2 | 1566 | -4575 | L1MA9 | L1 | LINE |

  

|  |  |  |  |  |  |  |  |  |  |
| --- | --- | --- | --- | --- | --- | --- | --- | --- | --- |
| chr9:27565168-27569295 | | | 4127 bp | | |  |  |  |  |
| Genome Start | Genome End | Strand | Div | Repeat Start | Repeat End | Repeat Left | Name | Family | Class |
| 27565168 | 27565332 | + | 23.8 | 5917 | 6098 | -214 | L1MA9 | L1 | LINE |
| 27565333 | 27565729 | - | 14.9 | 1 | 428 | 0 | MSTA | MaLR | LTR |
| 27565730 | 27566551 | - | 17.6 | 827 | 1651 | 0 | MSTA-int | MaLR | LTR |
| 27566552 | 27567260 | - | 18.6 | 1 | 732 | -2 | MER50 | ERV1 | LTR |
| 27567261 | 27567418 | - | 17.6 | 662 | 826 | -825 | MSTA-int | MaLR | LTR |
| 27567419 | 27568050 | + | 2.4 | 5519 | 6152 | -3 | L1PA3 | L1 | LINE |
| 27568051 | 27568716 | - | 17.6 | 1 | 661 | -990 | MSTA-int | MaLR | LTR |
| 27568717 | 27569130 | - | 11.6 | 1 | 428 | 0 | MSTA | MaLR | LTR |
| 27569132 | 27569295 | + | 18.9 | 6103 | 6275 | -37 | L1MA9 | L1 | LINE |

  

|  |  |  |  |  |  |  |  |  |  |
| --- | --- | --- | --- | --- | --- | --- | --- | --- | --- |
| chr9:29498630-29508886 | | | 9770 bp | | |  |  |  |  |
| Genome Start | Genome End | Strand | Div | Repeat Start | Repeat End | Repeat Left | Name | Family | Class |
| 29498630 | 29498813 | + | 26.4 | 45 | 243 | -6157 | L1MEc | L1 | LINE |
| 29499104 | 29499198 | + | 27.4 | 295 | 389 | -5325 | ERVL-B4 | ERVL | LTR |
| 29499199 | 29499562 | - | 10.0 | 1 | 364 | 0 | THE1B | MaLR | LTR |
| 29499563 | 29499991 | - | 14.4 | 1157 | 1580 | 0 | THE1B-int | MaLR | LTR |
| 29500004 | 29501136 | - | 18.9 | 1 | 1149 | -431 | THE1B-int | MaLR | LTR |
| 29501137 | 29501496 | - | 11.8 | 3 | 364 | 0 | THE1B | MaLR | LTR |
| 29501497 | 29502150 | + | 18.9 | 383 | 1021 | -4693 | ERVL-B4 | ERVL | LTR |
| 29502168 | 29502312 | + | 18.3 | 4684 | 4829 | -885 | ERVL-B4 | ERVL | LTR |
| 29502313 | 29502615 | - | 9.2 | 1 | 303 | -8 | AluY | Alu | SINE |
| 29502616 | 29503163 | + | 18.3 | 4830 | 5414 | -300 | ERVL-B4 | ERVL | LTR |
| 29503217 | 29503511 | + | 18.3 | 5415 | 5706 | -8 | ERVL-B4 | ERVL | LTR |
| 29503519 | 29503890 | + | 23.1 | 1 | 377 | -48 | MLT2B4 | ERVL | LTR |
| 29503955 | 29504009 | + | 26.1 | 513 | 558 | -2 | MLT2D | ERVL | LTR |
| 29504010 | 29504359 | + | 24.0 | 5739 | 6068 | -116 | L1MB7 | L1 | LINE |
| 29504362 | 29504764 | - | 18.1 | 576 | 981 | -2 | LTR1D | ERV1 | LTR |
| 29504762 | 29505197 | - | 15.9 | 1 | 435 | -548 | LTR1D | ERV1 | LTR |
| 29505200 | 29505312 | + | 20.4 | 6065 | 6177 | -1 | L1MB8 | L1 | LINE |
| 29505309 | 29505681 | + | 24.9 | 1707 | 2100 | -4300 | L1MEc | L1 | LINE |
| 29505705 | 29505777 | + | 26.1 | 1 | 59 | -2553 | Charlie5 | MER1\_type | DNA |
| 29505778 | 29506130 | + | 11.9 | 5799 | 6159 | -2 | L1PA14 | L1 | LINE |
| 29506131 | 29506663 | + | 25.6 | 56 | 622 | -1990 | Charlie5 | MER1\_type | DNA |
| 29506671 | 29507594 | + | 24.1 | 1101 | 2046 | -566 | Charlie5 | MER1\_type | DNA |
| 29507610 | 29507851 | - | 14.1 | 6062 | 6304 | 0 | L1MA2 | L1 | LINE |
| 29507852 | 29508147 | - | 13.5 | 1 | 298 | -12 | AluSg | Alu | SINE |
| 29508148 | 29508324 | - | 14.1 | 5885 | 6061 | -243 | L1MA2 | L1 | LINE |
| 29508325 | 29508370 | - | 13.0 | 3 | 49 | -253 | Alu | Alu | SINE |
| 29508372 | 29508758 | + | 23.4 | 2239 | 2623 | 0 | Charlie5 | MER1\_type | DNA |
| 29508760 | 29508886 | + | 29.1 | 2123 | 2250 | -4150 | L1MEc | L1 | LINE |

  

|  |  |  |  |  |  |  |  |  |  |
| --- | --- | --- | --- | --- | --- | --- | --- | --- | --- |
| chr9:29798352-29803479 | | | 5112 bp | | |  |  |  |  |
| Genome Start | Genome End | Strand | Div | Repeat Start | Repeat End | Repeat Left | Name | Family | Class |
| 29798352 | 29798920 | + | 18.1 | 3993 | 4548 | -1592 | L1MA9 | L1 | LINE |
| 29798921 | 29799222 | + | 10.1 | 2 | 299 | -12 | AluY | Alu | SINE |
| 29799223 | 29799540 | + | 18.1 | 4549 | 4859 | -1281 | L1MA9 | L1 | LINE |
| 29799552 | 29799928 | + | 15.7 | 1 | 381 | 0 | THE1D | MaLR | LTR |
| 29799929 | 29801387 | + | 14.8 | 1 | 1579 | -1 | THE1D-int | MaLR | LTR |
| 29801389 | 29801767 | + | 14.9 | 1 | 381 | 0 | THE1D | MaLR | LTR |
| 29801772 | 29802673 | + | 21.2 | 4865 | 5780 | -532 | L1MA9 | L1 | LINE |
| 29802674 | 29802964 | + | 10.7 | 1 | 292 | -17 | AluSc | Alu | SINE |
| 29802965 | 29803479 | + | 22.1 | 5781 | 6267 | -45 | L1MA9 | L1 | LINE |

  

|  |  |  |  |  |  |  |  |  |  |
| --- | --- | --- | --- | --- | --- | --- | --- | --- | --- |
| chr9:30588612-30592308 | | | 3696 bp | | |  |  |  |  |
| Genome Start | Genome End | Strand | Div | Repeat Start | Repeat End | Repeat Left | Name | Family | Class |
| 30588612 | 30589378 | - | 20.2 | 5493 | 6264 | -27 | L1MA7 | L1 | LINE |
| 30589379 | 30589742 | - | 15.8 | 1 | 364 | 0 | THE1B | MaLR | LTR |
| 30589743 | 30591309 | - | 30.5 | 2 | 1735 | 0 | MLT-int | MaLR | LTR |
| 30591312 | 30591676 | - | 15.5 | 1 | 364 | 0 | THE1B | MaLR | LTR |
| 30591677 | 30592097 | - | 21.0 | 5066 | 5492 | -799 | L1MA7 | L1 | LINE |
| 30592097 | 30592308 | - | 13.7 | 4533 | 4745 | -1398 | L1MA7 | L1 | LINE |

  

|  |  |  |  |  |  |  |  |  |  |
| --- | --- | --- | --- | --- | --- | --- | --- | --- | --- |
| chr9:30809676-30812433 | | | 2735 bp | | |  |  |  |  |
| Genome Start | Genome End | Strand | Div | Repeat Start | Repeat End | Repeat Left | Name | Family | Class |
| 30809676 | 30810099 | - | 27.5 | 5601 | 6023 | -141 | L1ME2 | L1 | LINE |
| 30810102 | 30810390 | + | 6.9 | 1 | 355 | 0 | THE1A | MaLR | LTR |
| 30810391 | 30811927 | + | 10.9 | 1 | 1574 | -6 | THE1A-int | MaLR | LTR |
| 30811935 | 30812286 | + | 11.7 | 3 | 355 | 0 | THE1A | MaLR | LTR |
| 30812301 | 30812433 | - | 32.3 | 5452 | 5590 | -574 | L1ME2 | L1 | LINE |

  

|  |  |  |  |  |  |  |  |  |  |
| --- | --- | --- | --- | --- | --- | --- | --- | --- | --- |
| chr9:31109683-31115642 | | | 5528 bp | | |  |  |  |  |
| Genome Start | Genome End | Strand | Div | Repeat Start | Repeat End | Repeat Left | Name | Family | Class |
| 31109683 | 31110027 | - | 25.1 | 4527 | 4880 | 0 | MER83C-int | ERV1 | LTR |
| 31110091 | 31110402 | - | 30.2 | 4492 | 4807 | -1732 | MER83C-int | ERV1 | LTR |
| 31110427 | 31110788 | - | 9.6 | 1 | 355 | 0 | THE1A | MaLR | LTR |
| 31110789 | 31112347 | - | 10.9 | 1 | 1580 | 0 | THE1A-int | MaLR | LTR |
| 31112348 | 31112717 | - | 11.3 | 1 | 355 | 0 | THE1A | MaLR | LTR |
| 31112738 | 31113353 | - | 35.6 | 2256 | 2857 | -2097 | MER83C-int | ERV1 | LTR |
| 31113521 | 31114259 | - | 31.3 | 1116 | 1881 | -3073 | MER83C-int | ERV1 | LTR |
| 31114418 | 31115642 | - | 24.9 | 4 | 1742 | -3847 | MER83C-int | ERV1 | LTR |

  

|  |  |  |  |  |  |  |  |  |  |
| --- | --- | --- | --- | --- | --- | --- | --- | --- | --- |
| chr9:31929553-31933550 | | | 3950 bp | | |  |  |  |  |
| Genome Start | Genome End | Strand | Div | Repeat Start | Repeat End | Repeat Left | Name | Family | Class |
| 31929553 | 31929821 | - | 27.4 | 1707 | 1990 | -4271 | L1MEc | L1 | LINE |
| 31929858 | 31930242 | - | 27.4 | 1275 | 1706 | -4555 | L1MEc | L1 | LINE |
| 31930243 | 31930592 | + | 12.1 | 1 | 364 | 0 | THE1B | MaLR | LTR |
| 31930595 | 31932163 | + | 30.7 | 2 | 1735 | 0 | MLT-int | MaLR | LTR |
| 31932164 | 31932519 | + | 13.5 | 1 | 364 | 0 | THE1B | MaLR | LTR |
| 31932520 | 31932886 | - | 27.4 | 948 | 1274 | -4987 | L1MEc | L1 | LINE |
| 31932897 | 31932956 | - | 29.6 | 841 | 893 | -5177 | L1MEc | L1 | LINE |
| 31932957 | 31933266 | - | 14.6 | 1 | 312 | 0 | AluSp | Alu | SINE |
| 31933267 | 31933550 | - | 29.6 | 547 | 840 | -5230 | L1MEc | L1 | LINE |

  

|  |  |  |  |  |  |  |  |  |  |
| --- | --- | --- | --- | --- | --- | --- | --- | --- | --- |
| chr9:32505608-32514268 | | | 8574 bp | | |  |  |  |  |
| Genome Start | Genome End | Strand | Div | Repeat Start | Repeat End | Repeat Left | Name | Family | Class |
| 32505608 | 32505978 | + | 32.1 | 1882 | 2277 | -1142 | L2 | L2 | LINE |
| 32506028 | 32506418 | + | 15.6 | 52 | 461 | 0 | LOR1b | ERV1 | LTR |
| 32506424 | 32506745 | + | 16.8 | 32 | 371 | -7749 | LOR1b-int | ERV1 | LTR |
| 32506756 | 32508821 | + | 10.9 | 201 | 2389 | -5731 | LOR1b-int | ERV1 | LTR |
| 32508827 | 32509007 | - | 13.3 | 1 | 181 | -131 | AluSx | Alu | SINE |
| 32509008 | 32510861 | + | 10.4 | 2397 | 4192 | -3928 | LOR1b-int | ERV1 | LTR |
| 32510862 | 32511160 | + | 8.0 | 1 | 305 | -7 | AluSx | Alu | SINE |
| 32511161 | 32512482 | + | 10.4 | 4193 | 5524 | -2596 | LOR1b-int | ERV1 | LTR |
| 32512473 | 32512736 | + | 12.2 | 5801 | 6064 | -2056 | LOR1b-int | ERV1 | LTR |
| 32512736 | 32512827 | + | 9.9 | 8030 | 8120 | 0 | LOR1b-int | ERV1 | LTR |
| 32512828 | 32513265 | + | 19.4 | 1 | 461 | 0 | LOR1b | ERV1 | LTR |
| 32513295 | 32514268 | + | 35.1 | 2334 | 3359 | -19 | L2 | L2 | LINE |

  

|  |  |  |  |  |  |  |  |  |  |
| --- | --- | --- | --- | --- | --- | --- | --- | --- | --- |
| chr9:32649466-32651885 | | | 2418 bp | | |  |  |  |  |
| Genome Start | Genome End | Strand | Div | Repeat Start | Repeat End | Repeat Left | Name | Family | Class |
| 32649466 | 32649573 | - | 26.6 | 90 | 197 | -71 | MIRb | MIR | SINE |
| 32649574 | 32649923 | - | 12.8 | 1 | 364 | 0 | THE1B | MaLR | LTR |
| 32649924 | 32651454 | - | 29.7 | 2 | 1735 | 0 | MLT1A-int | MaLR | LTR |
| 32651457 | 32651803 | - | 11.2 | 1 | 364 | 0 | THE1B | MaLR | LTR |
| 32651804 | 32651885 | - | 26.6 | 14 | 89 | -179 | MIRb | MIR | SINE |

  

|  |  |  |  |  |  |  |  |  |  |
| --- | --- | --- | --- | --- | --- | --- | --- | --- | --- |
| chr9:34993055-34999486 | | | 6427 bp | | |  |  |  |  |
| Genome Start | Genome End | Strand | Div | Repeat Start | Repeat End | Repeat Left | Name | Family | Class |
| 34993055 | 34993289 | + | 20.6 | 793 | 1035 | -5507 | L1M4 | L1 | LINE |
| 34993290 | 34993701 | + | 8.1 | 1 | 448 | -2 | LTR7 | ERV1 | LTR |
| 34993704 | 34997730 | + | 9.2 | 1 | 3993 | -3720 | HERVH | ERV1 | LTR |
| 34997733 | 34998390 | + | 14.0 | 4488 | 5149 | -2564 | HERVH | ERV1 | LTR |
| 34998392 | 34998603 | + | 11.8 | 5600 | 5819 | -1894 | HERVH | ERV1 | LTR |
| 34998602 | 34998969 | + | 9.9 | 7322 | 7713 | 0 | HERVH | ERV1 | LTR |
| 34998970 | 34999330 | + | 8.4 | 1 | 448 | -2 | LTR7 | ERV1 | LTR |
| 34999333 | 34999486 | + | 21.9 | 1031 | 1185 | -5357 | L1M4 | L1 | LINE |

  

|  |  |  |  |  |  |  |  |  |  |
| --- | --- | --- | --- | --- | --- | --- | --- | --- | --- |
| chr9:35141096-35149712 | | | 8492 bp | | |  |  |  |  |
| Genome Start | Genome End | Strand | Div | Repeat Start | Repeat End | Repeat Left | Name | Family | Class |
| 35141096 | 35141219 | - | 7.3 | 2302 | 2427 | -4171 | MER4-int | ERV1 | LTR |
| 35141220 | 35141593 | - | 11.5 | 1 | 450 | 0 | LTR7 | ERV1 | LTR |
| 35141595 | 35142161 | - | 13.1 | 7157 | 7712 | -1 | HERVH | ERV1 | LTR |
| 35142155 | 35142770 | - | 10.2 | 5400 | 6016 | -1697 | HERVH | ERV1 | LTR |
| 35142765 | 35143042 | - | 25.2 | 4416 | 4693 | -3020 | HERVH | ERV1 | LTR |
| 35143108 | 35143160 | - | 17.4 | 3512 | 3554 | -4159 | HERVH | ERV1 | LTR |
| 35143161 | 35143476 | + | 11.2 | 1 | 307 | -4 | AluY | Alu | SINE |
| 35143477 | 35145738 | - | 14.9 | 830 | 3511 | -4202 | HERVH | ERV1 | LTR |
| 35145807 | 35146231 | - | 20.8 | 1 | 459 | -7254 | HERVH | ERV1 | LTR |
| 35146232 | 35146607 | - | 10.1 | 1 | 450 | 0 | LTR7 | ERV1 | LTR |
| 35146608 | 35146682 | - | 10.0 | 2232 | 2306 | -4292 | MER4-int | ERV1 | LTR |
| 35146683 | 35147443 | + | 8.2 | 1 | 781 | 0 | MER4E1 | ERV1 | LTR |
| 35147444 | 35147645 | - | 10.0 | 2006 | 2231 | -4367 | MER4-int | ERV1 | LTR |
| 35147646 | 35147943 | - | 12.8 | 1 | 300 | -13 | AluSq | Alu | SINE |
| 35147944 | 35148244 | - | 10.0 | 1748 | 2005 | -4593 | MER4-int | ERV1 | LTR |
| 35148249 | 35148847 | - | 12.3 | 522 | 1159 | -5599 | MER4-int | ERV1 | LTR |
| 35148848 | 35149135 | - | 20.2 | 1 | 289 | -23 | AluSx | Alu | SINE |
| 35149136 | 35149424 | - | 12.3 | 232 | 521 | -6237 | MER4-int | ERV1 | LTR |
| 35149425 | 35149670 | - | 12.2 | 1 | 297 | -5 | AluSx | Alu | SINE |
| 35149671 | 35149712 | - | 12.3 | 202 | 231 | -6527 | MER4-int | ERV1 | LTR |

  

|  |  |  |  |  |  |  |  |  |  |
| --- | --- | --- | --- | --- | --- | --- | --- | --- | --- |
| chr9:35442332-35452658 | | | 10302 bp | | |  |  |  |  |
| Genome Start | Genome End | Strand | Div | Repeat Start | Repeat End | Repeat Left | Name | Family | Class |
| 35442332 | 35444792 | + | 13.9 | 18 | 2512 | -5053 | L1M1 | L1 | LINE |
| 35444793 | 35445100 | + | 8.4 | 1 | 308 | -3 | AluY | Alu | SINE |
| 35445101 | 35445413 | + | 13.9 | 2513 | 2821 | -4744 | L1M1 | L1 | LINE |
| 35445414 | 35445906 | - | 10.9 | 1 | 511 | 0 | LTR10F | ERV1 | LTR |
| 35445907 | 35448921 | - | 8.7 | 2055 | 5102 | 0 | HERVIP10FH | ERV1 | LTR |
| 35448922 | 35449228 | - | 4.6 | 1 | 307 | -4 | AluY | Alu | SINE |
| 35449229 | 35451605 | - | 8.7 | 1 | 2054 | -3048 | HERVIP10FH | ERV1 | LTR |
| 35451606 | 35452086 | - | 9.4 | 1 | 511 | 0 | LTR10F | ERV1 | LTR |
| 35452087 | 35452306 | + | 13.7 | 2818 | 3040 | -4525 | L1M1 | L1 | LINE |
| 35452323 | 35452494 | - | 8.7 | 5269 | 5440 | -865 | L1M1 | L1 | LINE |
| 35452504 | 35452658 | + | 7.8 | 5111 | 5264 | -882 | L1M1 | L1 | LINE |

  

|  |  |  |  |  |  |  |  |  |  |
| --- | --- | --- | --- | --- | --- | --- | --- | --- | --- |
| chr9:37661440-37665613 | | | 3894 bp | | |  |  |  |  |
| Genome Start | Genome End | Strand | Div | Repeat Start | Repeat End | Repeat Left | Name | Family | Class |
| 37661440 | 37661670 | - | 35.5 | 3113 | 3377 | -1 | L2 | L2 | LINE |
| 37661686 | 37661982 | + | 11.4 | 6 | 302 | -10 | AluSx | Alu | SINE |
| 37662011 | 37662230 | + | 14.0 | 5964 | 6178 | 0 | L1MB8 | L1 | LINE |
| 37662432 | 37662648 | - | 25.2 | 58 | 262 | 0 | MIR | MIR | SINE |
| 37662649 | 37662980 | - | 7.8 | 1 | 355 | 0 | THE1A | MaLR | LTR |
| 37662981 | 37664556 | - | 30.3 | 2 | 1735 | 0 | MLT-int | MaLR | LTR |
| 37664559 | 37664911 | - | 9.3 | 1 | 355 | 0 | THE1A | MaLR | LTR |
| 37664912 | 37664944 | - | 25.2 | 28 | 57 | -205 | MIR | MIR | SINE |
| 37664979 | 37665141 | - | 31.8 | 2707 | 2863 | -556 | L2 | L2 | LINE |
| 37665142 | 37665431 | + | 13.8 | 5 | 298 | -14 | AluSx | Alu | SINE |
| 37665432 | 37665613 | - | 31.8 | 2524 | 2706 | -713 | L2 | L2 | LINE |

  

|  |  |  |  |  |  |  |  |  |  |
| --- | --- | --- | --- | --- | --- | --- | --- | --- | --- |
| chr9:39332927-39337889 | | | 4928 bp | | |  |  |  |  |
| Genome Start | Genome End | Strand | Div | Repeat Start | Repeat End | Repeat Left | Name | Family | Class |
| 39332927 | 39333442 | - | 15.3 | 5784 | 6312 | 0 | L1MA9 | L1 | LINE |
| 39333476 | 39333920 | - | 15.3 | 5307 | 5783 | -529 | L1MA9 | L1 | LINE |
| 39333921 | 39334280 | + | 16.3 | 1 | 364 | 0 | THE1B | MaLR | LTR |
| 39334283 | 39335846 | + | 31.0 | 2 | 1735 | 0 | MLT-int | MaLR | LTR |
| 39335847 | 39336142 | + | 13.4 | 1 | 299 | -65 | THE1B | MaLR | LTR |
| 39336143 | 39336435 | - | 8.9 | 1 | 293 | -18 | AluY | Alu | SINE |
| 39336436 | 39336506 | + | 13.4 | 300 | 364 | 0 | THE1B | MaLR | LTR |
| 39336507 | 39337371 | - | 20.1 | 4379 | 5306 | -834 | L1MA9 | L1 | LINE |
| 39337372 | 39337634 | - | 8.4 | 1 | 287 | -24 | AluY | Alu | SINE |
| 39337635 | 39337889 | - | 20.1 | 4120 | 4378 | -1762 | L1MA9 | L1 | LINE |

  

|  |  |  |  |  |  |  |  |  |  |
| --- | --- | --- | --- | --- | --- | --- | --- | --- | --- |
| chr9:39627285-39632234 | | | 4625 bp | | |  |  |  |  |
| Genome Start | Genome End | Strand | Div | Repeat Start | Repeat End | Repeat Left | Name | Family | Class |
| 39627285 | 39627509 | - | 33.9 | 2949 | 3176 | -243 | L2 | L2 | LINE |
| 39627724 | 39628044 | + | 30.0 | 4 | 354 | -241 | MLT1H | MaLR | LTR |
| 39628140 | 39628936 | - | 30.4 | 1564 | 2565 | -854 | L2 | L2 | LINE |
| 39628937 | 39629139 | - | 20.7 | 137 | 396 | 0 | MLT1A1 | MaLR | LTR |
| 39629140 | 39629489 | + | 21.0 | 1 | 396 | 0 | MSTD | MaLR | LTR |
| 39629490 | 39629623 | - | 19.1 | 1 | 137 | -271 | MLT1A1 | MaLR | LTR |
| 39629629 | 39631372 | - | 21.6 | 2 | 1729 | -6 | MLT1A1-int | MaLR | LTR |
| 39631375 | 39631440 | - | 19.1 | 343 | 407 | -1 | MLT1A1 | MaLR | LTR |
| 39631441 | 39631749 | + | 13.6 | 1 | 311 | -2 | AluSq | Alu | SINE |
| 39631750 | 39632031 | - | 19.1 | 2 | 342 | -66 | MLT1A1 | MaLR | LTR |
| 39632041 | 39632234 | - | 30.4 | 1351 | 1560 | -1859 | L2 | L2 | LINE |

  

|  |  |  |  |  |  |  |  |  |  |
| --- | --- | --- | --- | --- | --- | --- | --- | --- | --- |
| chr9:41032670-41037620 | | | 4662 bp | | |  |  |  |  |
| Genome Start | Genome End | Strand | Div | Repeat Start | Repeat End | Repeat Left | Name | Family | Class |
| 41032670 | 41032863 | + | 29.9 | 1351 | 1560 | -1859 | L2 | L2 | LINE |
| 41032873 | 41033150 | + | 18.7 | 2 | 341 | -67 | MLT1A1 | MaLR | LTR |
| 41033151 | 41033461 | - | 12.7 | 1 | 313 | 0 | AluSq | Alu | SINE |
| 41033462 | 41033528 | + | 18.7 | 342 | 407 | -1 | MLT1A1 | MaLR | LTR |
| 41033531 | 41035277 | + | 22.2 | 2 | 1735 | 0 | MLT1A1-int | MaLR | LTR |
| 41035278 | 41035411 | + | 19.6 | 1 | 137 | -271 | MLT1A1 | MaLR | LTR |
| 41035412 | 41035761 | - | 20.5 | 1 | 396 | 0 | MSTD | MaLR | LTR |
| 41035762 | 41035964 | + | 20.6 | 138 | 396 | 0 | MLT1A1 | MaLR | LTR |
| 41035965 | 41036796 | + | 30.0 | 1564 | 2598 | -821 | L2 | L2 | LINE |
| 41036861 | 41037181 | - | 29.1 | 4 | 354 | -241 | MLT1H | MaLR | LTR |
| 41037396 | 41037620 | + | 33.5 | 2949 | 3176 | -243 | L2 | L2 | LINE |

  

|  |  |  |  |  |  |  |  |  |  |
| --- | --- | --- | --- | --- | --- | --- | --- | --- | --- |
| chr9:41772314-41777260 | | | 4627 bp | | |  |  |  |  |
| Genome Start | Genome End | Strand | Div | Repeat Start | Repeat End | Repeat Left | Name | Family | Class |
| 41772314 | 41772538 | - | 34.4 | 2949 | 3176 | -243 | L2 | L2 | LINE |
| 41772753 | 41773073 | + | 29.5 | 4 | 354 | -241 | MLT1H | MaLR | LTR |
| 41773169 | 41773964 | - | 30.3 | 1564 | 2565 | -854 | L2 | L2 | LINE |
| 41773965 | 41774167 | - | 20.8 | 137 | 396 | 0 | MLT1A1 | MaLR | LTR |
| 41774168 | 41774517 | + | 20.8 | 1 | 396 | 0 | MSTD | MaLR | LTR |
| 41774518 | 41774651 | - | 19.1 | 1 | 137 | -271 | MLT1A1 | MaLR | LTR |
| 41774652 | 41776400 | - | 21.5 | 2 | 1735 | 0 | MLT1A1-int | MaLR | LTR |
| 41776403 | 41776468 | - | 19.4 | 343 | 407 | -1 | MLT1A1 | MaLR | LTR |
| 41776469 | 41776778 | + | 13.6 | 1 | 312 | -1 | AluSq | Alu | SINE |
| 41776779 | 41777057 | - | 19.4 | 2 | 342 | -66 | MLT1A1 | MaLR | LTR |
| 41777067 | 41777260 | - | 29.9 | 1351 | 1560 | -1859 | L2 | L2 | LINE |

  

|  |  |  |  |  |  |  |  |  |  |
| --- | --- | --- | --- | --- | --- | --- | --- | --- | --- |
| chr9:42000657-42005331 | | | 4630 bp | | |  |  |  |  |
| Genome Start | Genome End | Strand | Div | Repeat Start | Repeat End | Repeat Left | Name | Family | Class |
| 42000657 | 42001165 | - | 14.7 | 5783 | 6312 | 0 | L1MA9 | L1 | LINE |
| 42001201 | 42001640 | - | 14.7 | 5332 | 5782 | -530 | L1MA9 | L1 | LINE |
| 42001641 | 42002000 | + | 16.1 | 1 | 364 | 0 | THE1B | MaLR | LTR |
| 42002001 | 42003568 | + | 12.7 | 1 | 1580 | 0 | THE1B-int | MaLR | LTR |
| 42003569 | 42003932 | + | 14.0 | 1 | 364 | 0 | THE1B | MaLR | LTR |
| 42003943 | 42004789 | - | 19.1 | 4380 | 5286 | -857 | L1MA9 | L1 | LINE |
| 42004790 | 42005066 | - | 8.3 | 1 | 287 | -24 | AluY | Alu | SINE |
| 42005067 | 42005331 | - | 19.1 | 4120 | 4379 | -1764 | L1MA9 | L1 | LINE |

  

|  |  |  |  |  |  |  |  |  |  |
| --- | --- | --- | --- | --- | --- | --- | --- | --- | --- |
| chr9:43353554-43358504 | | | 4619 bp | | |  |  |  |  |
| Genome Start | Genome End | Strand | Div | Repeat Start | Repeat End | Repeat Left | Name | Family | Class |
| 43353554 | 43353747 | + | 29.9 | 1351 | 1560 | -1859 | L2 | L2 | LINE |
| 43353757 | 43354034 | + | 19.0 | 2 | 341 | -67 | MLT1A1 | MaLR | LTR |
| 43354035 | 43354345 | - | 12.7 | 1 | 313 | 0 | AluSq | Alu | SINE |
| 43354346 | 43354412 | + | 19.0 | 342 | 407 | -1 | MLT1A1 | MaLR | LTR |
| 43354415 | 43356156 | + | 22.1 | 2 | 1729 | -6 | MLT1A1-int | MaLR | LTR |
| 43356162 | 43356295 | + | 19.6 | 1 | 137 | -271 | MLT1A1 | MaLR | LTR |
| 43356296 | 43356645 | - | 20.2 | 1 | 396 | 0 | MSTD | MaLR | LTR |
| 43356646 | 43356848 | + | 20.6 | 138 | 396 | 0 | MLT1A1 | MaLR | LTR |
| 43356849 | 43357680 | + | 30.3 | 1564 | 2598 | -821 | L2 | L2 | LINE |
| 43357783 | 43358065 | - | 30.6 | 4 | 290 | -259 | MLT1H | MaLR | LTR |
| 43358280 | 43358504 | + | 33.5 | 2949 | 3176 | -243 | L2 | L2 | LINE |

  

|  |  |  |  |  |  |  |  |  |  |
| --- | --- | --- | --- | --- | --- | --- | --- | --- | --- |
| chr9:43578241-43583203 | | | 4918 bp | | |  |  |  |  |
| Genome Start | Genome End | Strand | Div | Repeat Start | Repeat End | Repeat Left | Name | Family | Class |
| 43578241 | 43578496 | + | 19.9 | 4117 | 4376 | -1764 | L1MA9 | L1 | LINE |
| 43578497 | 43578759 | + | 8.4 | 1 | 287 | -24 | AluY | Alu | SINE |
| 43578760 | 43579618 | + | 19.9 | 4377 | 5283 | -857 | L1MA9 | L1 | LINE |
| 43579629 | 43579699 | - | 14.2 | 300 | 364 | 0 | THE1B | MaLR | LTR |
| 43579700 | 43579989 | + | 9.0 | 1 | 290 | -21 | AluY | Alu | SINE |
| 43579990 | 43580285 | - | 14.2 | 1 | 299 | -65 | THE1B | MaLR | LTR |
| 43580286 | 43581851 | - | 30.8 | 2 | 1735 | 0 | MLT1K-int | MaLR | LTR |
| 43581854 | 43582213 | - | 17.5 | 1 | 364 | 0 | THE1B | MaLR | LTR |
| 43582214 | 43582658 | + | 15.6 | 5332 | 5782 | -530 | L1MA9 | L1 | LINE |
| 43582692 | 43583203 | + | 15.6 | 5783 | 6311 | -1 | L1MA9 | L1 | LINE |

  

|  |  |  |  |  |  |  |  |  |  |
| --- | --- | --- | --- | --- | --- | --- | --- | --- | --- |
| chr9:44144923-44147772 | | | 2846 bp | | |  |  |  |  |
| Genome Start | Genome End | Strand | Div | Repeat Start | Repeat End | Repeat Left | Name | Family | Class |
| 44144923 | 44145023 | - | 30.5 | 106 | 202 | -6 | MIR3 | MIR | SINE |
| 44145024 | 44145074 | - | 15.4 | 391 | 428 | 0 | MSTA | MaLR | LTR |
| 44145075 | 44145367 | - | 13.4 | 3 | 294 | -15 | AluSc | Alu | SINE |
| 44145368 | 44145690 | - | 15.4 | 1 | 390 | -38 | MSTA | MaLR | LTR |
| 44145693 | 44147295 | - | 31.2 | 2 | 1733 | -2 | MLT-int | MaLR | LTR |
| 44147298 | 44147672 | - | 13.8 | 1 | 428 | 0 | MSTA | MaLR | LTR |
| 44147673 | 44147772 | - | 30.5 | 8 | 105 | -103 | MIR3 | MIR | SINE |

  

|  |  |  |  |  |  |  |  |  |  |
| --- | --- | --- | --- | --- | --- | --- | --- | --- | --- |
| chr9:44189673-44194361 | | | 4634 bp | | |  |  |  |  |
| Genome Start | Genome End | Strand | Div | Repeat Start | Repeat End | Repeat Left | Name | Family | Class |
| 44189673 | 44189936 | + | 19.5 | 4120 | 4378 | -1765 | L1MA9 | L1 | LINE |
| 44189937 | 44190212 | + | 9.1 | 1 | 287 | -24 | AluY | Alu | SINE |
| 44190213 | 44191060 | + | 19.5 | 4379 | 5286 | -857 | L1MA9 | L1 | LINE |
| 44191071 | 44191434 | - | 12.5 | 1 | 364 | 0 | THE1B | MaLR | LTR |
| 44191435 | 44192997 | - | 12.2 | 1 | 1580 | 0 | THE1B-int | MaLR | LTR |
| 44192998 | 44193356 | - | 17.2 | 1 | 364 | 0 | THE1B | MaLR | LTR |
| 44193357 | 44193800 | + | 15.4 | 5332 | 5782 | -530 | L1MA9 | L1 | LINE |
| 44193846 | 44194361 | + | 15.4 | 5783 | 6311 | -1 | L1MA9 | L1 | LINE |

  

|  |  |  |  |  |  |  |  |  |  |
| --- | --- | --- | --- | --- | --- | --- | --- | --- | --- |
| chr9:44333510-44338186 | | | 4640 bp | | |  |  |  |  |
| Genome Start | Genome End | Strand | Div | Repeat Start | Repeat End | Repeat Left | Name | Family | Class |
| 44333510 | 44334025 | - | 15.1 | 5784 | 6312 | 0 | L1MA9 | L1 | LINE |
| 44334053 | 44334492 | - | 15.1 | 5333 | 5783 | -529 | L1MA9 | L1 | LINE |
| 44334493 | 44334852 | + | 17.2 | 1 | 364 | 0 | THE1B | MaLR | LTR |
| 44334853 | 44336419 | + | 12.7 | 1 | 1580 | 0 | THE1B-int | MaLR | LTR |
| 44336420 | 44336783 | + | 14.3 | 1 | 364 | 0 | THE1B | MaLR | LTR |
| 44336794 | 44337635 | - | 19.5 | 4380 | 5286 | -857 | L1MA9 | L1 | LINE |
| 44337636 | 44337912 | - | 9.8 | 1 | 287 | -24 | AluY | Alu | SINE |
| 44337913 | 44338186 | - | 19.5 | 4120 | 4379 | -1764 | L1MA9 | L1 | LINE |

  

|  |  |  |  |  |  |  |  |  |  |
| --- | --- | --- | --- | --- | --- | --- | --- | --- | --- |
| chr9:44512832-44517772 | | | 4652 bp | | |  |  |  |  |
| Genome Start | Genome End | Strand | Div | Repeat Start | Repeat End | Repeat Left | Name | Family | Class |
| 44512832 | 44513025 | + | 30.4 | 1351 | 1560 | -1859 | L2 | L2 | LINE |
| 44513035 | 44513312 | + | 18.7 | 2 | 341 | -67 | MLT1A1 | MaLR | LTR |
| 44513313 | 44513613 | - | 12.6 | 1 | 303 | -10 | AluSq | Alu | SINE |
| 44513614 | 44513680 | + | 18.7 | 342 | 407 | -1 | MLT1A1 | MaLR | LTR |
| 44513683 | 44515429 | + | 22.2 | 2 | 1735 | 0 | MLT1A1-int | MaLR | LTR |
| 44515430 | 44515563 | + | 19.6 | 1 | 137 | -271 | MLT1A1 | MaLR | LTR |
| 44515564 | 44515913 | - | 20.5 | 1 | 396 | 0 | MSTD | MaLR | LTR |
| 44515914 | 44516116 | + | 20.6 | 138 | 396 | 0 | MLT1A1 | MaLR | LTR |
| 44516117 | 44516948 | + | 30.7 | 1564 | 2598 | -821 | L2 | L2 | LINE |
| 44517013 | 44517333 | - | 29.1 | 4 | 354 | -241 | MLT1H | MaLR | LTR |
| 44517548 | 44517772 | + | 33.5 | 2949 | 3176 | -243 | L2 | L2 | LINE |

  

|  |  |  |  |  |  |  |  |  |  |
| --- | --- | --- | --- | --- | --- | --- | --- | --- | --- |
| chr9:44757875-44760651 | | | 2775 bp | | |  |  |  |  |
| Genome Start | Genome End | Strand | Div | Repeat Start | Repeat End | Repeat Left | Name | Family | Class |
| 44757875 | 44758044 | + | 25.7 | 5298 | 5496 | -744 | L1M5 | L1 | LINE |
| 44758045 | 44758407 | - | 14.7 | 1 | 355 | 0 | THE1A | MaLR | LTR |
| 44758408 | 44759982 | - | 31.7 | 2 | 1735 | 0 | MLT-int | MaLR | LTR |
| 44759985 | 44760354 | - | 9.0 | 1 | 355 | 0 | THE1A | MaLR | LTR |
| 44760355 | 44760651 | + | 25.7 | 5497 | 5776 | -464 | L1M5 | L1 | LINE |

  

|  |  |  |  |  |  |  |  |  |  |
| --- | --- | --- | --- | --- | --- | --- | --- | --- | --- |
| chr9:44914912-44924527 | | | 9551 bp | | |  |  |  |  |
| Genome Start | Genome End | Strand | Div | Repeat Start | Repeat End | Repeat Left | Name | Family | Class |
| 44914912 | 44915653 | + | 30.9 | 9 | 848 | -5561 | L1MD2 | L1 | LINE |
| 44915654 | 44915898 | + | 23.2 | 1 | 245 | -227 | MER70A | ERVL | LTR |
| 44915899 | 44916207 | + | 8.8 | 1 | 311 | -2 | AluSq | Alu | SINE |
| 44916208 | 44916437 | + | 23.2 | 246 | 472 | 0 | MER70A | ERVL | LTR |
| 44916447 | 44917435 | + | 20.0 | 10 | 1076 | -3947 | MER70A-int | ERVL | LTR |
| 44917455 | 44917538 | + | 19.1 | 2300 | 2384 | -2639 | MER70A-int | ERVL | LTR |
| 44917539 | 44917932 | - | 11.1 | 1 | 450 | 0 | LTR7 | ERV1 | LTR |
| 44917933 | 44918365 | - | 9.8 | 7283 | 7713 | 0 | HERVH | ERV1 | LTR |
| 44918385 | 44919877 | - | 12.1 | 3582 | 5286 | -1805 | HERVH | ERV1 | LTR |
| 44919880 | 44919996 | - | 13.3 | 2613 | 2725 | -4988 | HERVH | ERV1 | LTR |
| 44920004 | 44920394 | - | 12.1 | 1 | 450 | 0 | LTR7 | ERV1 | LTR |
| 44920395 | 44922544 | + | 18.9 | 2380 | 4592 | -431 | MER70A-int | ERVL | LTR |
| 44922545 | 44922841 | - | 13.8 | 1 | 292 | -21 | AluSp | Alu | SINE |
| 44922842 | 44923271 | + | 18.9 | 4593 | 5022 | -1 | MER70A-int | ERVL | LTR |
| 44923280 | 44923783 | + | 24.0 | 1 | 472 | 0 | MER70A | ERVL | LTR |
| 44923785 | 44924527 | + | 24.1 | 845 | 1603 | -4899 | L1MD2 | L1 | LINE |

  

|  |  |  |  |  |  |  |  |  |  |
| --- | --- | --- | --- | --- | --- | --- | --- | --- | --- |
| chr9:45601705-45611318 | | | 9549 bp | | |  |  |  |  |
| Genome Start | Genome End | Strand | Div | Repeat Start | Repeat End | Repeat Left | Name | Family | Class |
| 45601705 | 45602446 | + | 31.2 | 9 | 848 | -5561 | L1MD2 | L1 | LINE |
| 45602447 | 45602691 | + | 23.2 | 1 | 245 | -227 | MER70A | ERVL | LTR |
| 45602692 | 45602994 | + | 8.7 | 1 | 305 | -8 | AluSq | Alu | SINE |
| 45602995 | 45603224 | + | 23.2 | 246 | 472 | 0 | MER70A | ERVL | LTR |
| 45603234 | 45604224 | + | 20.1 | 10 | 1076 | -3947 | MER70A-int | ERVL | LTR |
| 45604244 | 45604327 | + | 17.9 | 2300 | 2384 | -2639 | MER70A-int | ERVL | LTR |
| 45604328 | 45604721 | - | 11.7 | 1 | 450 | 0 | LTR7 | ERV1 | LTR |
| 45604722 | 45605154 | - | 9.6 | 7283 | 7713 | 0 | HERVH | ERV1 | LTR |
| 45605174 | 45606666 | - | 12.6 | 3582 | 5286 | -1805 | HERVH | ERV1 | LTR |
| 45606669 | 45606785 | - | 12.4 | 2613 | 2725 | -4988 | HERVH | ERV1 | LTR |
| 45606793 | 45607183 | - | 11.6 | 1 | 450 | 0 | LTR7 | ERV1 | LTR |
| 45607184 | 45609333 | + | 19.0 | 2380 | 4592 | -431 | MER70A-int | ERVL | LTR |
| 45609334 | 45609632 | - | 13.1 | 1 | 292 | -21 | AluSp | Alu | SINE |
| 45609633 | 45610062 | + | 19.0 | 4593 | 5022 | -1 | MER70A-int | ERVL | LTR |
| 45610071 | 45610574 | + | 24.0 | 1 | 472 | 0 | MER70A | ERVL | LTR |
| 45610576 | 45611318 | + | 24.1 | 845 | 1603 | -4899 | L1MD2 | L1 | LINE |

  

|  |  |  |  |  |  |  |  |  |  |
| --- | --- | --- | --- | --- | --- | --- | --- | --- | --- |
| chr9:45989422-45996249 | | | 6781 bp | | |  |  |  |  |
| Genome Start | Genome End | Strand | Div | Repeat Start | Repeat End | Repeat Left | Name | Family | Class |
| 45989422 | 45990410 | + | 20.0 | 10 | 1076 | -3947 | MER70A-int | ERVL | LTR |
| 45990430 | 45990513 | + | 19.1 | 2300 | 2384 | -2639 | MER70A-int | ERVL | LTR |
| 45990514 | 45990907 | - | 11.4 | 1 | 450 | 0 | LTR7 | ERV1 | LTR |
| 45990908 | 45991340 | - | 9.8 | 7283 | 7713 | 0 | HERVH | ERV1 | LTR |
| 45991360 | 45992852 | - | 12.3 | 3582 | 5010 | -2703 | HERVH | ERV1 | LTR |
| 45992855 | 45992971 | - | 11.5 | 2613 | 2725 | -4988 | HERVH | ERV1 | LTR |
| 45992979 | 45993369 | - | 12.1 | 1 | 450 | 0 | LTR7 | ERV1 | LTR |
| 45993370 | 45995519 | + | 19.0 | 2380 | 4592 | -431 | MER70A-int | ERVL | LTR |
| 45995520 | 45995819 | - | 13.7 | 1 | 292 | -21 | AluSp | Alu | SINE |
| 45995820 | 45996249 | + | 19.0 | 4593 | 5022 | -1 | MER70A-int | ERVL | LTR |

  

|  |  |  |  |  |  |  |  |  |  |
| --- | --- | --- | --- | --- | --- | --- | --- | --- | --- |
| chr9:46061309-46064076 | | | 2766 bp | | |  |  |  |  |
| Genome Start | Genome End | Strand | Div | Repeat Start | Repeat End | Repeat Left | Name | Family | Class |
| 46061309 | 46061596 | - | 25.6 | 5497 | 5776 | -464 | L1M5 | L1 | LINE |
| 46061597 | 46061966 | + | 9.3 | 1 | 355 | 0 | THE1A | MaLR | LTR |
| 46061969 | 46063543 | + | 31.6 | 2 | 1735 | 0 | MLT-int | MaLR | LTR |
| 46063544 | 46063906 | + | 14.4 | 1 | 355 | 0 | THE1A | MaLR | LTR |
| 46063907 | 46064076 | - | 25.6 | 5298 | 5496 | -744 | L1M5 | L1 | LINE |

  

|  |  |  |  |  |  |  |  |  |  |
| --- | --- | --- | --- | --- | --- | --- | --- | --- | --- |
| chr9:46286807-46296419 | | | 9548 bp | | |  |  |  |  |
| Genome Start | Genome End | Strand | Div | Repeat Start | Repeat End | Repeat Left | Name | Family | Class |
| 46286807 | 46287548 | - | 24.4 | 845 | 1603 | -4899 | L1MDb | L1 | LINE |
| 46287550 | 46288051 | - | 24.1 | 1 | 472 | 0 | MER70A | ERVL | LTR |
| 46288060 | 46288489 | - | 18.9 | 4593 | 5022 | -1 | MER70A-int | ERVL | LTR |
| 46288490 | 46288788 | + | 14.1 | 1 | 292 | -21 | AluSp | Alu | SINE |
| 46288789 | 46290938 | - | 18.9 | 2380 | 4592 | -431 | MER70A-int | ERVL | LTR |
| 46290939 | 46291329 | + | 12.1 | 1 | 450 | 0 | LTR7 | ERV1 | LTR |
| 46291337 | 46291453 | + | 11.5 | 2613 | 2725 | -4988 | HERVH | ERV1 | LTR |
| 46291456 | 46292948 | + | 11.8 | 3582 | 5286 | -1805 | HERVH | ERV1 | LTR |
| 46292968 | 46293400 | + | 9.8 | 7283 | 7713 | 0 | HERVH | ERV1 | LTR |
| 46293401 | 46293794 | + | 11.1 | 1 | 450 | 0 | LTR7 | ERV1 | LTR |
| 46293795 | 46293878 | - | 19.1 | 2300 | 2384 | -2639 | MER70A-int | ERVL | LTR |
| 46293898 | 46294887 | - | 20.2 | 10 | 1076 | -3947 | MER70A-int | ERVL | LTR |
| 46294897 | 46295126 | - | 23.4 | 246 | 472 | 0 | MER70A | ERVL | LTR |
| 46295127 | 46295432 | - | 8.9 | 1 | 308 | -5 | AluSq | Alu | SINE |
| 46295433 | 46295677 | - | 23.4 | 1 | 245 | -227 | MER70A | ERVL | LTR |
| 46295678 | 46296419 | - | 31.2 | 9 | 848 | -5561 | L1MDb | L1 | LINE |

  

|  |  |  |  |  |  |  |  |  |  |
| --- | --- | --- | --- | --- | --- | --- | --- | --- | --- |
| chr9:46531707-46536369 | | | 4630 bp | | |  |  |  |  |
| Genome Start | Genome End | Strand | Div | Repeat Start | Repeat End | Repeat Left | Name | Family | Class |
| 46531707 | 46531971 | + | 19.2 | 4120 | 4379 | -1764 | L1MA9 | L1 | LINE |
| 46531972 | 46532248 | + | 8.3 | 1 | 287 | -24 | AluY | Alu | SINE |
| 46532249 | 46533095 | + | 19.2 | 4380 | 5286 | -857 | L1MA9 | L1 | LINE |
| 46533106 | 46533469 | - | 14.0 | 1 | 364 | 0 | THE1B | MaLR | LTR |
| 46533470 | 46535037 | - | 12.7 | 1 | 1580 | 0 | THE1B-int | MaLR | LTR |
| 46535038 | 46535397 | - | 16.1 | 1 | 364 | 0 | THE1B | MaLR | LTR |
| 46535398 | 46535837 | + | 14.7 | 5332 | 5782 | -530 | L1MA9 | L1 | LINE |
| 46535861 | 46536369 | + | 14.7 | 5783 | 6312 | 0 | L1MA9 | L1 | LINE |

  

|  |  |  |  |  |  |  |  |  |  |
| --- | --- | --- | --- | --- | --- | --- | --- | --- | --- |
| chr9:46675411-46680085 | | | 4638 bp | | |  |  |  |  |
| Genome Start | Genome End | Strand | Div | Repeat Start | Repeat End | Repeat Left | Name | Family | Class |
| 46675411 | 46675926 | - | 15.2 | 5784 | 6312 | 0 | L1MA9 | L1 | LINE |
| 46675954 | 46676393 | - | 15.2 | 5333 | 5783 | -529 | L1MA9 | L1 | LINE |
| 46676394 | 46676753 | + | 17.2 | 1 | 364 | 0 | THE1B | MaLR | LTR |
| 46676754 | 46678320 | + | 12.5 | 1 | 1580 | 0 | THE1B-int | MaLR | LTR |
| 46678321 | 46678684 | + | 14.5 | 1 | 364 | 0 | THE1B | MaLR | LTR |
| 46678695 | 46679536 | - | 19.5 | 4380 | 5286 | -857 | L1MA9 | L1 | LINE |
| 46679537 | 46679813 | - | 9.8 | 1 | 287 | -24 | AluY | Alu | SINE |
| 46679814 | 46680085 | - | 19.5 | 4120 | 4379 | -1764 | L1MA9 | L1 | LINE |

  

|  |  |  |  |  |  |  |  |  |  |
| --- | --- | --- | --- | --- | --- | --- | --- | --- | --- |
| chr9:46721749-46724597 | | | 2845 bp | | |  |  |  |  |
| Genome Start | Genome End | Strand | Div | Repeat Start | Repeat End | Repeat Left | Name | Family | Class |
| 46721749 | 46721848 | + | 29.5 | 6 | 105 | -103 | MIR3 | MIR | SINE |
| 46721849 | 46722223 | + | 13.2 | 1 | 428 | 0 | MSTA | MaLR | LTR |
| 46722226 | 46723828 | + | 30.4 | 2 | 1733 | -2 | MLT-int | MaLR | LTR |
| 46723831 | 46724154 | + | 13.7 | 1 | 390 | -38 | MSTA | MaLR | LTR |
| 46724155 | 46724445 | + | 12.4 | 3 | 293 | -16 | AluSc | Alu | SINE |
| 46724446 | 46724496 | + | 13.7 | 391 | 428 | 0 | MSTA | MaLR | LTR |
| 46724497 | 46724597 | + | 29.5 | 106 | 200 | -8 | MIR3 | MIR | SINE |

  

|  |  |  |  |  |  |  |  |  |  |
| --- | --- | --- | --- | --- | --- | --- | --- | --- | --- |
| chr9:46851315-46856261 | | | 4622 bp | | |  |  |  |  |
| Genome Start | Genome End | Strand | Div | Repeat Start | Repeat End | Repeat Left | Name | Family | Class |
| 46851315 | 46851508 | + | 30.4 | 1351 | 1560 | -1859 | L2 | L2 | LINE |
| 46851518 | 46851796 | + | 19.1 | 2 | 342 | -66 | MLT1A1 | MaLR | LTR |
| 46851797 | 46852106 | - | 13.9 | 1 | 312 | -1 | AluSq | Alu | SINE |
| 46852107 | 46852172 | + | 19.1 | 343 | 407 | -1 | MLT1A1 | MaLR | LTR |
| 46852175 | 46853918 | + | 21.6 | 2 | 1729 | -6 | MLT1A1-int | MaLR | LTR |
| 46853924 | 46854057 | + | 19.1 | 1 | 137 | -271 | MLT1A1 | MaLR | LTR |
| 46854058 | 46854407 | - | 20.5 | 1 | 396 | 0 | MSTD | MaLR | LTR |
| 46854408 | 46854610 | + | 20.4 | 138 | 396 | 0 | MLT1A1 | MaLR | LTR |
| 46854611 | 46855406 | + | 30.6 | 1564 | 2565 | -854 | L2 | L2 | LINE |
| 46855502 | 46855822 | - | 30.0 | 4 | 354 | -241 | MLT1H | MaLR | LTR |
| 46856037 | 46856261 | + | 34.8 | 2949 | 3176 | -243 | L2 | L2 | LINE |

  

|  |  |  |  |  |  |  |  |  |  |
| --- | --- | --- | --- | --- | --- | --- | --- | --- | --- |
| chr9:65811953-65816898 | | | 4621 bp | | |  |  |  |  |
| Genome Start | Genome End | Strand | Div | Repeat Start | Repeat End | Repeat Left | Name | Family | Class |
| 65811953 | 65812146 | + | 29.9 | 1351 | 1560 | -1859 | L2 | L2 | LINE |
| 65812156 | 65812434 | + | 19.6 | 2 | 342 | -66 | MLT1A1 | MaLR | LTR |
| 65812435 | 65812743 | - | 13.6 | 1 | 311 | -2 | AluSq | Alu | SINE |
| 65812744 | 65812809 | + | 19.6 | 343 | 407 | -1 | MLT1A1 | MaLR | LTR |
| 65812812 | 65814555 | + | 21.6 | 2 | 1729 | -6 | MLT1A1-int | MaLR | LTR |
| 65814561 | 65814694 | + | 19.1 | 1 | 137 | -271 | MLT1A1 | MaLR | LTR |
| 65814695 | 65815044 | - | 20.8 | 1 | 396 | 0 | MSTD | MaLR | LTR |
| 65815045 | 65815247 | + | 20.4 | 138 | 396 | 0 | MLT1A1 | MaLR | LTR |
| 65815248 | 65816043 | + | 30.3 | 1564 | 2565 | -854 | L2 | L2 | LINE |
| 65816139 | 65816459 | - | 30.0 | 4 | 354 | -241 | MLT1H | MaLR | LTR |
| 65816674 | 65816898 | + | 33.5 | 2949 | 3176 | -243 | L2 | L2 | LINE |

  

|  |  |  |  |  |  |  |  |  |  |
| --- | --- | --- | --- | --- | --- | --- | --- | --- | --- |
| chr9:67390977-67397801 | | | 6778 bp | | |  |  |  |  |
| Genome Start | Genome End | Strand | Div | Repeat Start | Repeat End | Repeat Left | Name | Family | Class |
| 67390977 | 67391406 | - | 19.1 | 4593 | 5022 | -1 | MER70A-int | ERVL | LTR |
| 67391407 | 67391703 | + | 13.8 | 1 | 292 | -21 | AluSp | Alu | SINE |
| 67391704 | 67393853 | - | 19.1 | 2380 | 4592 | -431 | MER70A-int | ERVL | LTR |
| 67393854 | 67394244 | + | 11.8 | 1 | 450 | 0 | LTR7 | ERV1 | LTR |
| 67394252 | 67394368 | + | 13.3 | 2613 | 2725 | -4988 | HERVH | ERV1 | LTR |
| 67394371 | 67395863 | + | 14.0 | 3582 | 5010 | -2287 | HERVH | ERV1 | LTR |
| 67395883 | 67396315 | + | 9.8 | 7283 | 7713 | 0 | HERVH | ERV1 | LTR |
| 67396316 | 67396709 | + | 11.1 | 1 | 450 | 0 | LTR7 | ERV1 | LTR |
| 67396710 | 67396793 | - | 16.7 | 2300 | 2384 | -2639 | MER70A-int | ERVL | LTR |
| 67396813 | 67397801 | - | 20.0 | 10 | 1076 | -3947 | MER70A-int | ERVL | LTR |

  

|  |  |  |  |  |  |  |  |  |  |
| --- | --- | --- | --- | --- | --- | --- | --- | --- | --- |
| chr9:67987289-67992470 | | | 5154 bp | | |  |  |  |  |
| Genome Start | Genome End | Strand | Div | Repeat Start | Repeat End | Repeat Left | Name | Family | Class |
| 67987289 | 67987470 | - | 15.0 | 3123 | 3246 | 0 | Ricksha | MuDR | DNA |
| 67987471 | 67987768 | + | 14.8 | 1 | 298 | -13 | AluY | Alu | SINE |
| 67987769 | 67989659 | - | 15.0 | 1190 | 3122 | -124 | Ricksha | MuDR | DNA |
| 67989662 | 67989988 | - | 14.9 | 79 | 1691 | -1547 | Ricksha | MuDR | DNA |
| 67989992 | 67990098 | + | 14.9 | 5 | 111 | -32 | FLAM\_C | Alu | SINE |
| 67990101 | 67990473 | + | 10.1 | 2 | 375 | 0 | THE1C | MaLR | LTR |
| 67990474 | 67992000 | + | 12.9 | 1 | 1580 | 0 | THE1C-int | MaLR | LTR |
| 67992001 | 67992361 | + | 13.5 | 1 | 375 | 0 | THE1C | MaLR | LTR |
| 67992383 | 67992470 | - | 13.6 | 7 | 94 | -1954 | Ricksha | MuDR | DNA |

  

|  |  |  |  |  |  |  |  |  |  |
| --- | --- | --- | --- | --- | --- | --- | --- | --- | --- |
| chr9:68069285-68074482 | | | 5171 bp | | |  |  |  |  |
| Genome Start | Genome End | Strand | Div | Repeat Start | Repeat End | Repeat Left | Name | Family | Class |
| 68069285 | 68069372 | + | 15.9 | 7 | 94 | -1954 | Ricksha | MuDR | DNA |
| 68069396 | 68069756 | - | 12.4 | 1 | 375 | 0 | THE1C | MaLR | LTR |
| 68069757 | 68071283 | - | 12.6 | 1 | 1580 | 0 | THE1C-int | MaLR | LTR |
| 68071284 | 68071661 | - | 10.5 | 1 | 375 | 0 | THE1C | MaLR | LTR |
| 68071663 | 68071771 | - | 14.7 | 3 | 111 | -32 | FLAM\_C | Alu | SINE |
| 68071773 | 68072100 | + | 14.8 | 79 | 1691 | -1547 | Ricksha | MuDR | DNA |
| 68072103 | 68074002 | + | 14.6 | 1190 | 3122 | -124 | Ricksha | MuDR | DNA |
| 68074003 | 68074300 | - | 12.8 | 1 | 298 | -13 | AluY | Alu | SINE |
| 68074301 | 68074482 | + | 14.6 | 3123 | 3246 | 0 | Ricksha | MuDR | DNA |

  

|  |  |  |  |  |  |  |  |  |  |
| --- | --- | --- | --- | --- | --- | --- | --- | --- | --- |
| chr9:68308309-68313504 | | | 5172 bp | | |  |  |  |  |
| Genome Start | Genome End | Strand | Div | Repeat Start | Repeat End | Repeat Left | Name | Family | Class |
| 68308309 | 68308399 | + | 17.6 | 7 | 97 | -1951 | Ricksha | MuDR | DNA |
| 68308420 | 68308780 | - | 12.4 | 1 | 375 | 0 | THE1C | MaLR | LTR |
| 68308781 | 68310306 | - | 12.6 | 1 | 1580 | 0 | THE1C-int | MaLR | LTR |
| 68310307 | 68310684 | - | 10.5 | 1 | 375 | 0 | THE1C | MaLR | LTR |
| 68310686 | 68310794 | - | 15.6 | 3 | 111 | -32 | FLAM\_C | Alu | SINE |
| 68310796 | 68311122 | + | 14.7 | 79 | 1697 | -1547 | Ricksha | MuDR | DNA |
| 68311125 | 68313024 | + | 15.3 | 1190 | 3122 | -124 | Ricksha | MuDR | DNA |
| 68313025 | 68313312 | - | 11.5 | 11 | 298 | -13 | AluY | Alu | SINE |
| 68313313 | 68313504 | + | 17.5 | 3123 | 3254 | -13 | Ricksha | MuDR | DNA |

  

|  |  |  |  |  |  |  |  |  |  |
| --- | --- | --- | --- | --- | --- | --- | --- | --- | --- |
| chr9:71828229-71831771 | | | 3541 bp | | |  |  |  |  |
| Genome Start | Genome End | Strand | Div | Repeat Start | Repeat End | Repeat Left | Name | Family | Class |
| 71828229 | 71828615 | - | 17.3 | 5780 | 6180 | 0 | L1MB4 | L1 | LINE |
| 71828616 | 71829125 | - | 18.0 | 1 | 520 | 0 | MLT2B1 | ERVL | LTR |
| 71829126 | 71829237 | - | 17.3 | 5673 | 5779 | -401 | L1MB4 | L1 | LINE |
| 71829238 | 71829596 | - | 12.3 | 1 | 375 | 0 | THE1C | MaLR | LTR |
| 71829597 | 71831187 | - | 29.6 | 2 | 1735 | 0 | MLT-int | MaLR | LTR |
| 71831190 | 71831561 | - | 10.0 | 1 | 375 | 0 | THE1C | MaLR | LTR |
| 71831562 | 71831771 | - | 17.3 | 5444 | 5672 | -508 | L1MB4 | L1 | LINE |

  

|  |  |  |  |  |  |  |  |  |  |
| --- | --- | --- | --- | --- | --- | --- | --- | --- | --- |
| chr9:72131863-72136483 | | | 4611 bp | | |  |  |  |  |
| Genome Start | Genome End | Strand | Div | Repeat Start | Repeat End | Repeat Left | Name | Family | Class |
| 72131863 | 72132220 | + | 23.5 | 6689 | 7057 | -904 | L1MC5 | L1 | LINE |
| 72132222 | 72132536 | + | 21.6 | 6931 | 7287 | -674 | L1MC5 | L1 | LINE |
| 72132542 | 72132900 | - | 15.5 | 1 | 420 | -8 | MSTA | MaLR | LTR |
| 72132901 | 72134500 | - | 29.7 | 2 | 1735 | 0 | MLT-int | MaLR | LTR |
| 72134503 | 72134868 | - | 15.0 | 1 | 428 | 0 | MSTA | MaLR | LTR |
| 72134871 | 72134913 | + | 22.6 | 7289 | 7336 | -625 | L1MC5 | L1 | LINE |
| 72134914 | 72135118 | - | 17.4 | 1 | 219 | 0 | MER20 | MER1\_type | DNA |
| 72135119 | 72135237 | + | 22.6 | 7337 | 7452 | -509 | L1MC5 | L1 | LINE |
| 72135238 | 72135280 | - | 19.0 | 2582 | 2624 | 0 | Charlie5 | MER1\_type | DNA |
| 72135281 | 72135578 | - | 11.6 | 1 | 305 | -7 | AluSx | Alu | SINE |
| 72135579 | 72135894 | - | 19.0 | 2234 | 2581 | -31 | Charlie5 | MER1\_type | DNA |
| 72135892 | 72136026 | - | 18.3 | 1 | 127 | -2485 | Charlie5 | MER1\_type | DNA |
| 72136030 | 72136483 | + | 18.0 | 7480 | 7930 | -31 | L1MC5 | L1 | LINE |

  

|  |  |  |  |  |  |  |  |  |  |
| --- | --- | --- | --- | --- | --- | --- | --- | --- | --- |
| chr9:74068682-74072576 | | | 3846 bp | | |  |  |  |  |
| Genome Start | Genome End | Strand | Div | Repeat Start | Repeat End | Repeat Left | Name | Family | Class |
| 74068682 | 74068764 | - | 20.3 | 151 | 237 | -31 | MIRb | MIR | SINE |
| 74068765 | 74068865 | - | 18.9 | 443 | 542 | 0 | MER34 | ERV1 | LTR |
| 74068869 | 74069197 | - | 16.3 | 1 | 301 | -11 | AluJb | Alu | SINE |
| 74069198 | 74069365 | - | 28.9 | 526 | 693 | -94 | LTR48 | ERV1 | LTR |
| 74069397 | 74069885 | - | 21.6 | 48 | 707 | 0 | MER39 | ERV1 | LTR |
| 74069886 | 74070234 | - | 8.0 | 1 | 354 | -1 | THE1A | MaLR | LTR |
| 74070235 | 74071789 | - | 30.4 | 3 | 1735 | 0 | MLT-int | MaLR | LTR |
| 74071793 | 74072145 | - | 8.2 | 1 | 355 | 0 | THE1A | MaLR | LTR |
| 74072146 | 74072185 | - | 20.0 | 8 | 47 | -660 | MER39 | ERV1 | LTR |
| 74072188 | 74072444 | - | 24.8 | 11 | 318 | -326 | LTR48B | ERV1 | LTR |
| 74072455 | 74072576 | - | 26.2 | 3 | 153 | -115 | MIRb | MIR | SINE |

  

|  |  |  |  |  |  |  |  |  |  |
| --- | --- | --- | --- | --- | --- | --- | --- | --- | --- |
| chr9:75022407-75025391 | | | 2909 bp | | |  |  |  |  |
| Genome Start | Genome End | Strand | Div | Repeat Start | Repeat End | Repeat Left | Name | Family | Class |
| 75022407 | 75022521 | + | 17.6 | 14 | 122 | -345 | MLT1C | MaLR | LTR |
| 75022522 | 75022667 | + | 14.8 | 1 | 161 | -203 | THE1B | MaLR | LTR |
| 75022668 | 75022963 | - | 9.5 | 1 | 299 | -12 | AluY | Alu | SINE |
| 75022964 | 75023166 | + | 14.8 | 162 | 362 | -2 | THE1B | MaLR | LTR |
| 75023171 | 75024736 | + | 30.6 | 2 | 1735 | 0 | MLT-int | MaLR | LTR |
| 75024737 | 75025079 | + | 16.3 | 1 | 364 | 0 | THE1B | MaLR | LTR |
| 75025080 | 75025219 | + | 17.6 | 123 | 266 | -201 | MLT1C | MaLR | LTR |
| 75025292 | 75025391 | + | 19.0 | 367 | 467 | 0 | MLT1C | MaLR | LTR |

  

|  |  |  |  |  |  |  |  |  |  |
| --- | --- | --- | --- | --- | --- | --- | --- | --- | --- |
| chr9:75054280-75063842 | | | 9570 bp | | |  |  |  |  |
| Genome Start | Genome End | Strand | Div | Repeat Start | Repeat End | Repeat Left | Name | Family | Class |
| 75054280 | 75056028 | + | 28.9 | 3445 | 5322 | -824 | L1M5 | L1 | LINE |
| 75056041 | 75057202 | + | 17.7 | 1893 | 3067 | -3470 | L1MD1 | L1 | LINE |
| 75057203 | 75057513 | - | 11.9 | 1 | 312 | -1 | AluSq | Alu | SINE |
| 75057514 | 75058195 | + | 18.5 | 3068 | 4037 | -2500 | L1MD1 | L1 | LINE |
| 75058196 | 75058233 | + | 17.9 | 1 | 25 | -339 | THE1B | MaLR | LTR |
| 75058234 | 75058527 | - | 10.9 | 1 | 294 | -15 | AluSc | Alu | SINE |
| 75058528 | 75058859 | + | 17.9 | 26 | 364 | 0 | THE1B | MaLR | LTR |
| 75058862 | 75060501 | + | 31.2 | 2 | 1735 | 0 | MLT-int | MaLR | LTR |
| 75060502 | 75060856 | + | 12.5 | 1 | 364 | 0 | THE1B | MaLR | LTR |
| 75060857 | 75063289 | + | 19.5 | 4038 | 6566 | -67 | L1MD1 | L1 | LINE |
| 75063290 | 75063605 | - | 5.5 | 1 | 309 | -2 | AluY | Alu | SINE |
| 75063606 | 75063669 | + | 19.7 | 6567 | 6631 | -2 | L1MD1 | L1 | LINE |
| 75063649 | 75063842 | + | 22.3 | 5299 | 5494 | -668 | L1M5 | L1 | LINE |

  

|  |  |  |  |  |  |  |  |  |  |
| --- | --- | --- | --- | --- | --- | --- | --- | --- | --- |
| chr9:75182788-75196237 | | | 13393 bp | | |  |  |  |  |
| Genome Start | Genome End | Strand | Div | Repeat Start | Repeat End | Repeat Left | Name | Family | Class |
| 75182788 | 75183511 | - | 19.1 | 5103 | 5841 | -569 | L1M4 | L1 | LINE |
| 75183512 | 75183544 | + | 18.6 | 1 | 33 | -393 | MSTB | MaLR | LTR |
| 75183545 | 75183843 | - | 10.8 | 1 | 297 | -13 | AluSg | Alu | SINE |
| 75183844 | 75184182 | + | 18.6 | 34 | 372 | -54 | MSTB | MaLR | LTR |
| 75184188 | 75184554 | - | 13.8 | 1 | 381 | 0 | THE1D | MaLR | LTR |
| 75184555 | 75186137 | - | 30.2 | 3 | 1735 | 0 | MLT-int | MaLR | LTR |
| 75186141 | 75186456 | - | 14.4 | 53 | 381 | 0 | THE1D | MaLR | LTR |
| 75186451 | 75186502 | + | 15.4 | 376 | 428 | -4 | MSTB | MaLR | LTR |
| 75186511 | 75186793 | - | 27.9 | 4817 | 5103 | -1279 | L1M4 | L1 | LINE |
| 75186824 | 75190986 | - | 12.2 | 3555 | 7723 | 0 | L1MA2 | L1 | LINE |
| 75190987 | 75191296 | - | 0.7 | 1 | 309 | -1 | AluYa5 | Alu | SINE |
| 75191297 | 75194019 | - | 15.4 | 708 | 3568 | -3997 | L1MA2 | L1 | LINE |
| 75194017 | 75194134 | - | 20.9 | 17 | 134 | -7431 | L1MA2 | L1 | LINE |
| 75194135 | 75194453 | - | 23.1 | 4485 | 4805 | -1577 | L1M4 | L1 | LINE |
| 75194487 | 75195928 | - | 23.1 | 2928 | 4484 | -1898 | L1M4 | L1 | LINE |
| 75195916 | 75196237 | - | 23.4 | 1102 | 1428 | -4956 | L1M4 | L1 | LINE |

  

|  |  |  |  |  |  |  |  |  |  |
| --- | --- | --- | --- | --- | --- | --- | --- | --- | --- |
| chr9:75582759-75585673 | | | 2858 bp | | |  |  |  |  |
| Genome Start | Genome End | Strand | Div | Repeat Start | Repeat End | Repeat Left | Name | Family | Class |
| 75582759 | 75583709 | - | 29.5 | 492 | 1489 | 0 | MLT1H-int | MaLR | LTR |
| 75583759 | 75584132 | + | 14.1 | 1 | 381 | 0 | THE1D | MaLR | LTR |
| 75584133 | 75584584 | + | 12.1 | 1 | 490 | -1090 | THE1D-int | MaLR | LTR |
| 75584581 | 75584897 | + | 13.2 | 1269 | 1580 | 0 | THE1D-int | MaLR | LTR |
| 75584898 | 75585267 | + | 14.4 | 1 | 381 | 0 | THE1D | MaLR | LTR |
| 75585280 | 75585673 | - | 30.7 | 2 | 460 | -1012 | MLT1H-int | MaLR | LTR |

  

|  |  |  |  |  |  |  |  |  |  |
| --- | --- | --- | --- | --- | --- | --- | --- | --- | --- |
| chr9:75766760-75776883 | | | 9442 bp | | |  |  |  |  |
| Genome Start | Genome End | Strand | Div | Repeat Start | Repeat End | Repeat Left | Name | Family | Class |
| 75766760 | 75766845 | + | 20.9 | 5480 | 5565 | -768 | L1MC1 | L1 | LINE |
| 75766937 | 75767002 | + | 21.2 | 450 | 519 | -48 | MLT2B1 | ERVL | LTR |
| 75767382 | 75767832 | - | 19.8 | 1 | 534 | -20 | MLT2B1 | ERVL | LTR |
| 75767834 | 75768521 | + | 17.4 | 5576 | 6245 | -88 | L1MC1 | L1 | LINE |
| 75768533 | 75769048 | + | 17.2 | 5 | 550 | -5 | MLT2B1 | ERVL | LTR |
| 75769054 | 75769194 | + | 13.5 | 1 | 145 | -5569 | ERVL-B1 | ERVL | LTR |
| 75769193 | 75771518 | + | 18.1 | 1309 | 3646 | -2068 | ERVL-B1 | ERVL | LTR |
| 75771519 | 75773351 | + | 18.5 | 3885 | 5756 | -1 | ERVL-B1 | ERVL | LTR |
| 75773376 | 75773530 | + | 24.0 | 5 | 165 | -355 | MLT2B1 | ERVL | LTR |
| 75773531 | 75773900 | + | 17.6 | 1 | 428 | 0 | MSTA | MaLR | LTR |
| 75773903 | 75775526 | + | 32.2 | 2 | 1735 | 0 | MLT-int | MaLR | LTR |
| 75775527 | 75775803 | + | 18.4 | 1 | 398 | -30 | MSTA | MaLR | LTR |
| 75775804 | 75776105 | - | 12.9 | 1 | 302 | -10 | AluSx | Alu | SINE |
| 75776106 | 75776146 | + | 18.4 | 399 | 428 | 0 | MSTA | MaLR | LTR |
| 75776147 | 75776458 | + | 24.0 | 166 | 480 | -40 | MLT2B1 | ERVL | LTR |
| 75776559 | 75776651 | - | 19.6 | 2314 | 2406 | 0 | Tigger5 | MER2\_type | DNA |
| 75776647 | 75776711 | - | 20.0 | 1 | 65 | -32 | Tigger5c | MER2\_type | DNA |
| 75776788 | 75776883 | + | 19.6 | 6242 | 6333 | 0 | L1MC1 | L1 | LINE |

  

|  |  |  |  |  |  |  |  |  |  |
| --- | --- | --- | --- | --- | --- | --- | --- | --- | --- |
| chr9:77026442-77032881 | | | 6376 bp | | |  |  |  |  |
| Genome Start | Genome End | Strand | Div | Repeat Start | Repeat End | Repeat Left | Name | Family | Class |
| 77026442 | 77026985 | - | 29.7 | 1782 | 2394 | -113 | HAL1 | L1 | LINE |
| 77026995 | 77027903 | - | 25.0 | 756 | 1749 | -758 | HAL1 | L1 | LINE |
| 77027904 | 77028312 | - | 15.7 | 1 | 428 | 0 | MSTA | MaLR | LTR |
| 77028314 | 77029041 | - | 16.5 | 911 | 1650 | -1 | MSTA-int | MaLR | LTR |
| 77029054 | 77031082 | - | 7.6 | 4140 | 6151 | 0 | L1PB1 | L1 | LINE |
| 77031083 | 77031970 | - | 15.5 | 10 | 932 | -719 | MSTA-int | MaLR | LTR |
| 77031981 | 77032390 | - | 13.7 | 1 | 428 | 0 | MSTA | MaLR | LTR |
| 77032423 | 77032881 | - | 31.8 | 234 | 730 | -1777 | HAL1 | L1 | LINE |

  

|  |  |  |  |  |  |  |  |  |  |
| --- | --- | --- | --- | --- | --- | --- | --- | --- | --- |
| chr9:77155791-77162499 | | | 6764 bp | | |  |  |  |  |
| Genome Start | Genome End | Strand | Div | Repeat Start | Repeat End | Repeat Left | Name | Family | Class |
| 77155791 | 77155878 | - | 15.7 | 6198 | 6293 | -4 | L1MA5 | L1 | LINE |
| 77155881 | 77156292 | - | 10.7 | 1 | 448 | -2 | LTR7 | ERV1 | LTR |
| 77156293 | 77157456 | - | 10.4 | 6529 | 7713 | 0 | HERVH | ERV1 | LTR |
| 77157430 | 77158040 | - | 8.5 | 5769 | 6380 | -1333 | HERVH | ERV1 | LTR |
| 77158037 | 77158216 | - | 11.1 | 4488 | 4667 | -3046 | HERVH | ERV1 | LTR |
| 77158219 | 77158715 | - | 12.6 | 3492 | 3993 | -3720 | HERVH | ERV1 | LTR |
| 77158706 | 77158855 | - | 13.3 | 3140 | 3290 | -4423 | HERVH | ERV1 | LTR |
| 77158835 | 77161897 | - | 11.1 | 1 | 2995 | -4718 | HERVH | ERV1 | LTR |
| 77161900 | 77162311 | - | 9.8 | 1 | 448 | -2 | LTR7 | ERV1 | LTR |
| 77162313 | 77162499 | - | 18.3 | 5988 | 6201 | -99 | L1MA5 | L1 | LINE |

  

|  |  |  |  |  |  |  |  |  |  |
| --- | --- | --- | --- | --- | --- | --- | --- | --- | --- |
| chr9:77326435-77330811 | | | 4367 bp | | |  |  |  |  |
| Genome Start | Genome End | Strand | Div | Repeat Start | Repeat End | Repeat Left | Name | Family | Class |
| 77326435 | 77326539 | + | 23.2 | 1 | 105 | -935 | MER45B | Tip100 | DNA |
| 77326540 | 77326850 | - | 1.3 | 1 | 310 | 0 | AluYa5 | Alu | SINE |
| 77326851 | 77327186 | + | 23.2 | 106 | 455 | -585 | MER45B | Tip100 | DNA |
| 77327188 | 77327339 | + | 23.1 | 1 | 113 | -392 | MLT1D | MaLR | LTR |
| 77327340 | 77327564 | - | 7.1 | 87 | 311 | 0 | AluY | Alu | SINE |
| 77327565 | 77327946 | + | 23.1 | 114 | 498 | -7 | MLT1D | MaLR | LTR |
| 77327954 | 77328363 | + | 20.7 | 452 | 877 | -163 | MER45B | Tip100 | DNA |
| 77328364 | 77328721 | + | 11.1 | 1 | 355 | 0 | THE1A | MaLR | LTR |
| 77328724 | 77330301 | + | 30.4 | 2 | 1735 | 0 | MLT-int | MaLR | LTR |
| 77330302 | 77330655 | + | 9.7 | 1 | 355 | 0 | THE1A | MaLR | LTR |
| 77330656 | 77330811 | + | 20.7 | 878 | 1036 | -4 | MER45B | Tip100 | DNA |

  

|  |  |  |  |  |  |  |  |  |  |
| --- | --- | --- | --- | --- | --- | --- | --- | --- | --- |
| chr9:79486262-79506839 | | | 20352 bp | | |  |  |  |  |
| Genome Start | Genome End | Strand | Div | Repeat Start | Repeat End | Repeat Left | Name | Family | Class |
| 79486262 | 79486323 | - | 16.4 | 6572 | 6633 | 0 | L1MD1 | L1 | LINE |
| 79486324 | 79486623 | + | 12.3 | 2 | 301 | -11 | AluSx | Alu | SINE |
| 79486624 | 79487334 | - | 16.4 | 5875 | 6571 | -62 | L1MD1 | L1 | LINE |
| 79487335 | 79487638 | - | 0.3 | 2 | 305 | -5 | AluYa5 | Alu | SINE |
| 79487639 | 79487794 | - | 16.4 | 5723 | 5874 | -759 | L1MD1 | L1 | LINE |
| 79487795 | 79487867 | - | 23.3 | 357 | 428 | 0 | THE1B | MaLR | LTR |
| 79487868 | 79488176 | + | 10.4 | 1 | 308 | -5 | AluSq | Alu | SINE |
| 79488177 | 79488522 | - | 12.9 | 1 | 356 | -57 | THE1B | MaLR | LTR |
| 79488523 | 79489507 | - | 16.1 | 4708 | 5727 | -810 | L1MD1 | L1 | LINE |
| 79489505 | 79492413 | - | 4.4 | 3238 | 6155 | 0 | L1PA4 | L1 | LINE |
| 79492409 | 79493126 | - | 8.8 | 1 | 722 | -5433 | L1PA4 | L1 | LINE |
| 79493159 | 79493326 | - | 19.2 | 4511 | 4669 | -1868 | L1MD1 | L1 | LINE |
| 79493327 | 79493615 | - | 18.7 | 1 | 290 | -22 | AluJb | Alu | SINE |
| 79493616 | 79493714 | - | 19.2 | 4404 | 4510 | -2027 | L1MD1 | L1 | LINE |
| 79493715 | 79493998 | - | 8.4 | 6 | 289 | -20 | AluSc | Alu | SINE |
| 79493999 | 79494667 | - | 19.2 | 3730 | 4403 | -2134 | L1MD1 | L1 | LINE |
| 79494737 | 79495099 | - | 11.7 | 3 | 362 | -2 | THE1B | MaLR | LTR |
| 79495102 | 79496300 | - | 29.9 | 466 | 1735 | 0 | MLT-int | MaLR | LTR |
| 79496301 | 79496615 | - | 9.7 | 1 | 310 | -1 | AluY | Alu | SINE |
| 79496616 | 79497015 | - | 29.9 | 3 | 465 | -1270 | MLT-int | MaLR | LTR |
| 79497018 | 79497383 | - | 14.4 | 3 | 364 | 0 | THE1B | MaLR | LTR |
| 79497387 | 79500119 | - | 11.4 | 4917 | 7693 | -31 | L1MA3 | L1 | LINE |
| 79500103 | 79500201 | - | 11.2 | 4046 | 4144 | -2002 | L1M1 | L1 | LINE |
| 79500197 | 79504116 | - | 13.0 | 12 | 3928 | -3637 | L1MA3 | L1 | LINE |
| 79504118 | 79504240 | - | 15.1 | 3599 | 3736 | -2801 | L1MD1 | L1 | LINE |
| 79504293 | 79504937 | - | 16.6 | 2984 | 3628 | -2909 | L1MD1 | L1 | LINE |
| 79504938 | 79505226 | - | 12.5 | 1 | 302 | 0 | AluSx | Alu | SINE |
| 79505227 | 79505752 | - | 16.9 | 2459 | 2983 | -3554 | L1MD1 | L1 | LINE |
| 79505753 | 79506041 | - | 12.8 | 3 | 293 | -19 | AluSx | Alu | SINE |
| 79506042 | 79506162 | - | 20.1 | 2324 | 2458 | -4175 | L1MD1 | L1 | LINE |
| 79506258 | 79506839 | - | 20.1 | 1764 | 2323 | -4310 | L1MD1 | L1 | LINE |

  

|  |  |  |  |  |  |  |  |  |  |
| --- | --- | --- | --- | --- | --- | --- | --- | --- | --- |
| chr9:80892400-80895187 | | | 2786 bp | | |  |  |  |  |
| Genome Start | Genome End | Strand | Div | Repeat Start | Repeat End | Repeat Left | Name | Family | Class |
| 80892400 | 80892700 | - | 40.2 | 214 | 545 | -12 | LTR67 | ERVL | LTR |
| 80892701 | 80893062 | - | 12.2 | 1 | 364 | 0 | THE1B | MaLR | LTR |
| 80893063 | 80894634 | - | 31.6 | 2 | 1735 | 0 | MLT-int | MaLR | LTR |
| 80894637 | 80895006 | - | 13.4 | 1 | 364 | 0 | THE1B | MaLR | LTR |
| 80895007 | 80895187 | - | 40.2 | 36 | 213 | -344 | LTR67 | ERVL | LTR |

  

|  |  |  |  |  |  |  |  |  |  |
| --- | --- | --- | --- | --- | --- | --- | --- | --- | --- |
| chr9:82809031-82821557 | | | 12559 bp | | |  |  |  |  |
| Genome Start | Genome End | Strand | Div | Repeat Start | Repeat End | Repeat Left | Name | Family | Class |
| 82809031 | 82809682 | + | 23.3 | 523 | 1282 | -5469 | L1MC2 | L1 | LINE |
| 82809683 | 82810146 | + | 6.2 | 5687 | 6150 | -4 | L1PA7 | L1 | LINE |
| 82810147 | 82811400 | + | 21.7 | 1283 | 2604 | -4147 | L1MC2 | L1 | LINE |
| 82811406 | 82811843 | + | 18.1 | 2662 | 3101 | -3463 | L1MC2 | L1 | LINE |
| 82811844 | 82812148 | - | 9.6 | 1 | 305 | 0 | AluSg1 | Alu | SINE |
| 82812149 | 82813380 | + | 18.1 | 3102 | 4308 | -2256 | L1MC2 | L1 | LINE |
| 82813390 | 82813816 | + | 19.5 | 5055 | 5483 | -1081 | L1MC2 | L1 | LINE |
| 82813819 | 82814216 | + | 8.1 | 1 | 448 | -2 | LTR7 | ERV1 | LTR |
| 82814219 | 82817160 | + | 9.1 | 1 | 2995 | -4718 | HERVH | ERV1 | LTR |
| 82817140 | 82817288 | + | 10.1 | 3140 | 3290 | -4423 | HERVH | ERV1 | LTR |
| 82817279 | 82819011 | + | 11.0 | 3492 | 5149 | -2564 | HERVH | ERV1 | LTR |
| 82818992 | 82819228 | + | 12.3 | 5588 | 5819 | -1894 | HERVH | ERV1 | LTR |
| 82819227 | 82819606 | + | 10.4 | 7322 | 7713 | 0 | HERVH | ERV1 | LTR |
| 82819607 | 82820016 | + | 10.6 | 1 | 448 | -2 | LTR7 | ERV1 | LTR |
| 82820020 | 82820272 | + | 19.6 | 5482 | 5736 | -1028 | L1MC2 | L1 | LINE |
| 82820273 | 82820576 | + | 1.0 | 1 | 304 | -14 | AluYb8 | Alu | SINE |
| 82820577 | 82821557 | + | 19.6 | 5735 | 6754 | -8 | L1MC2 | L1 | LINE |

  

|  |  |  |  |  |  |  |  |  |  |
| --- | --- | --- | --- | --- | --- | --- | --- | --- | --- |
| chr9:84244107-84247059 | | | 2900 bp | | |  |  |  |  |
| Genome Start | Genome End | Strand | Div | Repeat Start | Repeat End | Repeat Left | Name | Family | Class |
| 84244107 | 84244224 | + | 28.1 | 5 | 122 | -399 | LTR33 | ERVL | LTR |
| 84244225 | 84244649 | + | 14.1 | 1 | 426 | 0 | MSTB | MaLR | LTR |
| 84244651 | 84246272 | + | 16.5 | 2 | 1638 | -13 | MSTB-int | MaLR | LTR |
| 84246285 | 84246707 | + | 19.3 | 1 | 426 | 0 | MSTB | MaLR | LTR |
| 84246708 | 84246805 | + | 28.6 | 118 | 217 | -304 | LTR33 | ERVL | LTR |
| 84246846 | 84247059 | + | 33.2 | 307 | 521 | 0 | LTR33 | ERVL | LTR |

  

|  |  |  |  |  |  |  |  |  |  |
| --- | --- | --- | --- | --- | --- | --- | --- | --- | --- |
| chr9:84489281-84491835 | | | 2480 bp | | |  |  |  |  |
| Genome Start | Genome End | Strand | Div | Repeat Start | Repeat End | Repeat Left | Name | Family | Class |
| 84489281 | 84489417 | + | 26.3 | 17 | 780 | -56 | MER63 | AcHobo | DNA |
| 84489427 | 84489797 | - | 12.7 | 1 | 375 | 0 | THE1C | MaLR | LTR |
| 84489798 | 84489838 | - | 12.2 | 1540 | 1580 | 0 | THE1C-int | MaLR | LTR |
| 84489896 | 84491410 | - | 12.3 | 1 | 1552 | -28 | THE1C-int | MaLR | LTR |
| 84491411 | 84491784 | - | 15.3 | 1 | 375 | 0 | THE1C | MaLR | LTR |
| 84491794 | 84491835 | + | 19.1 | 1019 | 1061 | 0 | MER63 | AcHobo | DNA |

  

|  |  |  |  |  |  |  |  |  |  |
| --- | --- | --- | --- | --- | --- | --- | --- | --- | --- |
| chr9:84961426-84967271 | | | 5426 bp | | |  |  |  |  |
| Genome Start | Genome End | Strand | Div | Repeat Start | Repeat End | Repeat Left | Name | Family | Class |
| 84961426 | 84962022 | + | 25.3 | 16 | 664 | -2280 | BLACKJACK | AcHobo | DNA |
| 84962023 | 84962374 | + | 22.8 | 1 | 396 | 0 | MSTD | MaLR | LTR |
| 84962375 | 84963246 | + | 25.3 | 665 | 1586 | -1358 | BLACKJACK | AcHobo | DNA |
| 84963644 | 84963942 | + | 6.7 | 5 | 303 | -8 | AluY | Alu | SINE |
| 84963950 | 84964492 | + | 21.5 | 1690 | 2272 | -672 | BLACKJACK | AcHobo | DNA |
| 84964499 | 84964852 | + | 14.9 | 1 | 364 | 0 | THE1B | MaLR | LTR |
| 84964854 | 84966423 | + | 10.7 | 2 | 1576 | -4 | THE1B-int | MaLR | LTR |
| 84966430 | 84966779 | + | 15.4 | 5 | 364 | 0 | THE1B | MaLR | LTR |
| 84966783 | 84967271 | + | 23.0 | 2301 | 2838 | -106 | BLACKJACK | AcHobo | DNA |

  

|  |  |  |  |  |  |  |  |  |  |
| --- | --- | --- | --- | --- | --- | --- | --- | --- | --- |
| chr9:85568694-85571229 | | | 2534 bp | | |  |  |  |  |
| Genome Start | Genome End | Strand | Div | Repeat Start | Repeat End | Repeat Left | Name | Family | Class |
| 85568694 | 85568904 | - | 32.1 | 1768 | 1950 | -10 | L4 | RTE | LINE |
| 85568905 | 85569241 | + | 13.8 | 1 | 375 | 0 | THE1C | MaLR | LTR |
| 85569244 | 85570736 | + | 29.8 | 2 | 1735 | 0 | MSTD-int | MaLR | LTR |
| 85570737 | 85571091 | + | 14.0 | 1 | 374 | -1 | THE1C | MaLR | LTR |
| 85571092 | 85571229 | - | 32.1 | 1615 | 1767 | -193 | L4 | RTE | LINE |

  

|  |  |  |  |  |  |  |  |  |  |
| --- | --- | --- | --- | --- | --- | --- | --- | --- | --- |
| chr9:87262929-87271976 | | | 9042 bp | | |  |  |  |  |
| Genome Start | Genome End | Strand | Div | Repeat Start | Repeat End | Repeat Left | Name | Family | Class |
| 87262929 | 87263054 | + | 19.0 | 1 | 134 | -333 | MLT1C | MaLR | LTR |
| 87263055 | 87263508 | + | 7.1 | 1 | 448 | -2 | LTR7 | ERV1 | LTR |
| 87263511 | 87271230 | + | 15.7 | 1 | 7713 | 0 | HERVH | ERV1 | LTR |
| 87271231 | 87271679 | + | 5.1 | 1 | 448 | -2 | LTR7 | ERV1 | LTR |
| 87271684 | 87271976 | + | 23.0 | 132 | 465 | -2 | MLT1C | MaLR | LTR |

  

|  |  |  |  |  |  |  |  |  |  |
| --- | --- | --- | --- | --- | --- | --- | --- | --- | --- |
| chr9:88369338-88372241 | | | 2862 bp | | |  |  |  |  |
| Genome Start | Genome End | Strand | Div | Repeat Start | Repeat End | Repeat Left | Name | Family | Class |
| 88369338 | 88369789 | - | 18.3 | 5828 | 6291 | 0 | L1MA7 | L1 | LINE |
| 88369833 | 88370114 | - | 6.2 | 2 | 283 | -28 | AluY | Alu | SINE |
| 88370115 | 88370348 | - | 18.3 | 5586 | 5827 | -464 | L1MA7 | L1 | LINE |
| 88370349 | 88370726 | - | 12.3 | 1 | 381 | 0 | THE1D | MaLR | LTR |
| 88370727 | 88371168 | - | 9.8 | 1140 | 1580 | 0 | THE1D-int | MaLR | LTR |
| 88371159 | 88371431 | - | 11.4 | 1 | 288 | -1292 | THE1D-int | MaLR | LTR |
| 88371441 | 88371819 | - | 13.1 | 1 | 381 | 0 | THE1D | MaLR | LTR |
| 88371820 | 88372241 | - | 12.8 | 5147 | 5587 | -704 | L1MA7 | L1 | LINE |

  

|  |  |  |  |  |  |  |  |  |  |
| --- | --- | --- | --- | --- | --- | --- | --- | --- | --- |
| chr9:89805154-89807858 | | | 2619 bp | | |  |  |  |  |
| Genome Start | Genome End | Strand | Div | Repeat Start | Repeat End | Repeat Left | Name | Family | Class |
| 89805154 | 89805342 | - | 18.4 | 861 | 1060 | 0 | MER63 | AcHobo | DNA |
| 89805427 | 89805533 | - | 15.7 | 87 | 191 | -870 | MER63 | AcHobo | DNA |
| 89805534 | 89805888 | + | 13.0 | 1 | 364 | 0 | THE1B | MaLR | LTR |
| 89805891 | 89807460 | + | 30.0 | 2 | 1735 | 0 | MLT-int | MaLR | LTR |
| 89807461 | 89807806 | + | 14.7 | 1 | 364 | 0 | THE1B | MaLR | LTR |
| 89807807 | 89807858 | - | 15.7 | 36 | 86 | -975 | MER63 | AcHobo | DNA |

  

|  |  |  |  |  |  |  |  |  |  |
| --- | --- | --- | --- | --- | --- | --- | --- | --- | --- |
| chr9:90426693-90429761 | | | 3067 bp | | |  |  |  |  |
| Genome Start | Genome End | Strand | Div | Repeat Start | Repeat End | Repeat Left | Name | Family | Class |
| 90426693 | 90426939 | + | 23.2 | 3 | 239 | -303 | MLT1F | MaLR | LTR |
| 90426940 | 90427344 | - | 13.5 | 1 | 424 | -2 | MSTB | MaLR | LTR |
| 90427345 | 90429049 | - | 28.6 | 2 | 1735 | 0 | MLT-int | MaLR | LTR |
| 90429052 | 90429469 | - | 15.8 | 1 | 426 | 0 | MSTB | MaLR | LTR |
| 90429470 | 90429761 | + | 23.2 | 240 | 536 | -6 | MLT1F | MaLR | LTR |

  

|  |  |  |  |  |  |  |  |  |  |
| --- | --- | --- | --- | --- | --- | --- | --- | --- | --- |
| chr9:90744351-90749953 | | | 5594 bp | | |  |  |  |  |
| Genome Start | Genome End | Strand | Div | Repeat Start | Repeat End | Repeat Left | Name | Family | Class |
| 90744351 | 90744720 | - | 13.8 | 5777 | 6147 | -4 | L1PB1 | L1 | LINE |
| 90744746 | 90745070 | - | 6.8 | 1 | 326 | -454 | LTR17 | ERV1 | LTR |
| 90745071 | 90745361 | - | 7.2 | 8336 | 8626 | 0 | HERV17 | ERV1 | LTR |
| 90745381 | 90745999 | - | 5.3 | 5405 | 6023 | -2603 | HERV17 | ERV1 | LTR |
| 90745979 | 90747251 | - | 5.6 | 2481 | 3754 | -4872 | HERV17 | ERV1 | LTR |
| 90747237 | 90748704 | - | 7.6 | 1 | 1525 | -7101 | HERV17 | ERV1 | LTR |
| 90748705 | 90749159 | - | 9.1 | 256 | 780 | 0 | LTR17 | ERV1 | LTR |
| 90749161 | 90749953 | - | 8.3 | 5017 | 5789 | -362 | L1PB1 | L1 | LINE |

  

|  |  |  |  |  |  |  |  |  |  |
| --- | --- | --- | --- | --- | --- | --- | --- | --- | --- |
| chr9:91079232-91082660 | | | 3427 bp | | |  |  |  |  |
| Genome Start | Genome End | Strand | Div | Repeat Start | Repeat End | Repeat Left | Name | Family | Class |
| 91079232 | 91079711 | - | 23.7 | 737 | 1248 | -4893 | L1M3c | L1 | LINE |
| 91079712 | 91080069 | - | 15.4 | 1 | 379 | -2 | THE1D | MaLR | LTR |
| 91080070 | 91081595 | - | 27.3 | 2 | 1735 | 0 | MLT1J-int | MaLR | LTR |
| 91081598 | 91081955 | - | 15.2 | 1 | 381 | 0 | THE1D | MaLR | LTR |
| 91081956 | 91082660 | - | 23.7 | 2 | 736 | -5405 | L1M3c | L1 | LINE |

  

|  |  |  |  |  |  |  |  |  |  |
| --- | --- | --- | --- | --- | --- | --- | --- | --- | --- |
| chr9:92621348-92624249 | | | 2899 bp | | |  |  |  |  |
| Genome Start | Genome End | Strand | Div | Repeat Start | Repeat End | Repeat Left | Name | Family | Class |
| 92621348 | 92621473 | - | 21.6 | 532 | 651 | -2 | MER82 | MER2\_type | DNA |
| 92621474 | 92621830 | - | 8.2 | 1 | 355 | 0 | THE1A | MaLR | LTR |
| 92621832 | 92623408 | - | 30.0 | 2 | 1733 | -2 | MLT-int | MaLR | LTR |
| 92623411 | 92623749 | - | 8.6 | 1 | 355 | 0 | THE1A | MaLR | LTR |
| 92623750 | 92624249 | - | 21.6 | 3 | 531 | -122 | MER82 | MER2\_type | DNA |

  

|  |  |  |  |  |  |  |  |  |  |
| --- | --- | --- | --- | --- | --- | --- | --- | --- | --- |
| chr9:93920022-93923819 | | | 3525 bp | | |  |  |  |  |
| Genome Start | Genome End | Strand | Div | Repeat Start | Repeat End | Repeat Left | Name | Family | Class |
| 93920022 | 93920219 | + | 31.7 | 73 | 269 | -222 | LTR16C | ERVL | LTR |
| 93920390 | 93920630 | + | 22.9 | 312 | 588 | -2 | MLT1F | MaLR | LTR |
| 93920679 | 93920849 | - | 22.6 | 300 | 467 | 0 | MLT1C | MaLR | LTR |
| 93920852 | 93920974 | - | 29.3 | 1 | 119 | -386 | MLT1C | MaLR | LTR |
| 93920975 | 93922473 | - | 21.7 | 1 | 1371 | 0 | MLT1C-int | MaLR | LTR |
| 93922474 | 93922886 | - | 22.8 | 36 | 467 | 0 | MLT1C | MaLR | LTR |
| 93922882 | 93922960 | - | 18.5 | 483 | 552 | -10 | MLT2B2 | ERVL | LTR |
| 93922961 | 93923260 | + | 9.4 | 1 | 298 | -14 | AluSx | Alu | SINE |
| 93923261 | 93923644 | - | 18.5 | 1 | 435 | -80 | MLT2B2 | ERVL | LTR |
| 93923703 | 93923819 | + | 25.9 | 366 | 484 | -7 | LTR16C | ERVL | LTR |

  

|  |  |  |  |  |  |  |  |  |  |
| --- | --- | --- | --- | --- | --- | --- | --- | --- | --- |
| chr9:95546332-95553166 | | | 6822 bp | | |  |  |  |  |
| Genome Start | Genome End | Strand | Div | Repeat Start | Repeat End | Repeat Left | Name | Family | Class |
| 95546332 | 95550135 | - | 14.3 | 3015 | 6863 | -7 | L1PB3 | L1 | LINE |
| 95550136 | 95550490 | - | 16.4 | 1 | 375 | 0 | THE1C | MaLR | LTR |
| 95550496 | 95552084 | - | 12.1 | 8 | 1575 | -5 | THE1C-int | MaLR | LTR |
| 95552093 | 95552458 | - | 14.8 | 1 | 375 | 0 | THE1C | MaLR | LTR |
| 95552459 | 95553166 | - | 17.2 | 2237 | 3019 | -3843 | L1PB3 | L1 | LINE |

  

|  |  |  |  |  |  |  |  |  |  |
| --- | --- | --- | --- | --- | --- | --- | --- | --- | --- |
| chr9:95771648-95776422 | | | 4772 bp | | |  |  |  |  |
| Genome Start | Genome End | Strand | Div | Repeat Start | Repeat End | Repeat Left | Name | Family | Class |
| 95771648 | 95771764 | + | 6.8 | 1 | 116 | -5538 | HERVL | ERVL | LTR |
| 95771765 | 95772072 | - | 11.0 | 1 | 309 | -2 | AluY | Alu | SINE |
| 95772073 | 95773047 | + | 6.8 | 117 | 1090 | -4564 | HERVL | ERVL | LTR |
| 95773048 | 95773402 | - | 11.6 | 1 | 355 | 0 | THE1A | MaLR | LTR |
| 95773404 | 95774980 | - | 30.0 | 2 | 1734 | -1 | MLT1J2-int | MaLR | LTR |
| 95774983 | 95775337 | - | 8.2 | 1 | 355 | 0 | THE1A | MaLR | LTR |
| 95775338 | 95776422 | + | 6.8 | 1091 | 2180 | -3474 | HERVL | ERVL | LTR |

  

|  |  |  |  |  |  |  |  |  |  |
| --- | --- | --- | --- | --- | --- | --- | --- | --- | --- |
| chr9:95928846-95933083 | | | 4232 bp | | |  |  |  |  |
| Genome Start | Genome End | Strand | Div | Repeat Start | Repeat End | Repeat Left | Name | Family | Class |
| 95928846 | 95929482 | + | 26.2 | 796 | 1578 | -898 | L1MC4 | L1 | LINE |
| 95929486 | 95929898 | + | 15.4 | 1 | 428 | 0 | MSTA | MaLR | LTR |
| 95929899 | 95930267 | + | 16.3 | 1 | 368 | -1283 | MSTA-int | MaLR | LTR |
| 95930268 | 95930550 | - | 10.2 | 1 | 295 | -17 | AluSx | Alu | SINE |
| 95930551 | 95931813 | + | 16.3 | 369 | 1651 | 0 | MSTA-int | MaLR | LTR |
| 95931814 | 95932203 | + | 15.0 | 1 | 428 | 0 | MSTA | MaLR | LTR |
| 95932207 | 95932280 | + | 18.8 | 4535 | 4606 | -1534 | L1M3 | L1 | LINE |
| 95932281 | 95932548 | - | 10.3 | 45 | 309 | -2 | AluY | Alu | SINE |
| 95932549 | 95932580 | + | 18.8 | 4607 | 4630 | -1510 | L1M3 | L1 | LINE |
| 95932581 | 95932644 | + | 27.4 | 1599 | 1664 | -4551 | L1MC4 | L1 | LINE |
| 95932645 | 95933026 | + | 12.7 | 1 | 428 | 0 | MSTA | MaLR | LTR |
| 95933027 | 95933083 | + | 27.4 | 1665 | 1715 | -4500 | L1MC4 | L1 | LINE |

  

|  |  |  |  |  |  |  |  |  |  |
| --- | --- | --- | --- | --- | --- | --- | --- | --- | --- |
| chr9:101482660-101487052 | | | 4085 bp | | |  |  |  |  |
| Genome Start | Genome End | Strand | Div | Repeat Start | Repeat End | Repeat Left | Name | Family | Class |
| 101482660 | 101482919 | - | 33.7 | 4218 | 4481 | -8 | L3 | CR1 | LINE |
| 101482920 | 101483305 | - | 14.8 | 1 | 428 | 0 | MSTA | MaLR | LTR |
| 101483306 | 101484929 | - | 29.7 | 2 | 1735 | 0 | MLT1K-int | MaLR | LTR |
| 101484932 | 101485324 | - | 15.5 | 3 | 428 | 0 | MSTA | MaLR | LTR |
| 101485325 | 101485929 | - | 33.7 | 3532 | 4217 | -272 | L3 | CR1 | LINE |
| 101486014 | 101486326 | + | 30.7 | 1400 | 1759 | -201 | L4 | RTE | LINE |
| 101486549 | 101486690 | - | 33.0 | 3299 | 3418 | -1071 | L3 | CR1 | LINE |
| 101486691 | 101486903 | - | 13.8 | 87 | 297 | -15 | AluJb | Alu | SINE |
| 101486904 | 101487052 | - | 33.0 | 3144 | 3298 | -1191 | L3 | CR1 | LINE |

  

|  |  |  |  |  |  |  |  |  |  |
| --- | --- | --- | --- | --- | --- | --- | --- | --- | --- |
| chr9:101941294-101949144 | | | 7629 bp | | |  |  |  |  |
| Genome Start | Genome End | Strand | Div | Repeat Start | Repeat End | Repeat Left | Name | Family | Class |
| 101941294 | 101941678 | + | 32.8 | 2037 | 2487 | -932 | L2 | L2 | LINE |
| 101941715 | 101941763 | - | 17.3 | 6138 | 6166 | -8 | L1MB5 | L1 | LINE |
| 101941764 | 101942043 | - | 15.1 | 3 | 282 | -30 | AluJb | Alu | SINE |
| 101942044 | 101942281 | - | 17.3 | 5895 | 6137 | -37 | L1MB5 | L1 | LINE |
| 101942285 | 101942693 | - | 8.1 | 1 | 448 | -2 | LTR7 | ERV1 | LTR |
| 101942694 | 101943073 | - | 10.9 | 7321 | 7713 | 0 | HERVH | ERV1 | LTR |
| 101943071 | 101943286 | - | 13.0 | 5600 | 5819 | -1894 | HERVH | ERV1 | LTR |
| 101943285 | 101944978 | - | 11.9 | 3505 | 5147 | -2566 | HERVH | ERV1 | LTR |
| 101944983 | 101945132 | - | 14.0 | 3140 | 3290 | -4423 | HERVH | ERV1 | LTR |
| 101945112 | 101948216 | - | 10.7 | 1 | 2995 | -4718 | HERVH | ERV1 | LTR |
| 101948219 | 101948625 | - | 8.4 | 1 | 448 | -2 | LTR7 | ERV1 | LTR |
| 101948627 | 101948738 | - | 13.5 | 5786 | 5896 | -278 | L1MB5 | L1 | LINE |
| 101948897 | 101949037 | - | 19.4 | 5653 | 5810 | -370 | L1MB5 | L1 | LINE |
| 101949082 | 101949144 | + | 28.6 | 3352 | 3415 | -4 | L2 | L2 | LINE |

  

|  |  |  |  |  |  |  |  |  |  |
| --- | --- | --- | --- | --- | --- | --- | --- | --- | --- |
| chr9:102343369-102348888 | | | 5032 bp | | |  |  |  |  |
| Genome Start | Genome End | Strand | Div | Repeat Start | Repeat End | Repeat Left | Name | Family | Class |
| 102343369 | 102343466 | - | 27.5 | 2488 | 2582 | -3547 | L1MEe | L1 | LINE |
| 102343467 | 102343778 | - | 14.7 | 1 | 308 | -2 | AluSg | Alu | SINE |
| 102343779 | 102345026 | - | 25.8 | 1083 | 2487 | -3642 | L1MEe | L1 | LINE |
| 102345027 | 102345330 | - | 9.9 | 1 | 305 | -8 | AluSp | Alu | SINE |
| 102345331 | 102345519 | - | 30.6 | 893 | 1082 | -5037 | L1MEe | L1 | LINE |
| 102345592 | 102345700 | - | 30.8 | 593 | 693 | -5426 | L1MEe | L1 | LINE |
| 102345701 | 102345807 | - | 7.9 | 104 | 230 | 0 | MER30 | MER1\_type | DNA |
| 102345851 | 102345934 | - | 7.9 | 2 | 103 | -127 | MER30 | MER1\_type | DNA |
| 102345935 | 102346083 | - | 30.8 | 459 | 592 | -5527 | L1MEe | L1 | LINE |
| 102346325 | 102346693 | - | 10.8 | 1 | 364 | 0 | THE1B | MaLR | LTR |
| 102346694 | 102348253 | - | 9.0 | 2 | 1580 | 0 | THE1B-int | MaLR | LTR |
| 102348255 | 102348626 | - | 11.8 | 1 | 364 | 0 | THE1B | MaLR | LTR |
| 102348758 | 102348888 | - | 29.7 | 5 | 135 | -5984 | L1MEe | L1 | LINE |

  

|  |  |  |  |  |  |  |  |  |  |
| --- | --- | --- | --- | --- | --- | --- | --- | --- | --- |
| chr9:102544043-102557018 | | | 12963 bp | | |  |  |  |  |
| Genome Start | Genome End | Strand | Div | Repeat Start | Repeat End | Repeat Left | Name | Family | Class |
| 102544043 | 102544081 | - | 7.7 | 7677 | 7715 | -9 | L1MA3 | L1 | LINE |
| 102544080 | 102544564 | - | 9.7 | 6907 | 7402 | -322 | L1MA3 | L1 | LINE |
| 102544585 | 102545262 | - | 5.3 | 5474 | 6151 | -3 | L1PA6 | L1 | LINE |
| 102545269 | 102547332 | - | 6.2 | 4082 | 6143 | -11 | L1PA5 | L1 | LINE |
| 102547333 | 102547642 | - | 1.6 | 1 | 310 | 0 | AluYa5 | Alu | SINE |
| 102547643 | 102551683 | - | 7.6 | 4 | 4081 | -2065 | L1PA5 | L1 | LINE |
| 102551682 | 102553579 | - | 14.8 | 5202 | 6930 | -794 | L1MA3 | L1 | LINE |
| 102553580 | 102553873 | - | 12.9 | 1 | 294 | -18 | AluSx | Alu | SINE |
| 102553874 | 102554027 | - | 15.5 | 3629 | 3782 | -2361 | L1M2 | L1 | LINE |
| 102554006 | 102554260 | - | 18.9 | 173 | 445 | -7120 | L1MA3 | L1 | LINE |
| 102554266 | 102554626 | - | 14.0 | 1 | 364 | 0 | THE1B | MaLR | LTR |
| 102554633 | 102555839 | - | 10.2 | 359 | 1573 | -7 | THE1B-int | MaLR | LTR |
| 102555840 | 102556120 | + | 15.8 | 1 | 278 | -34 | AluSx | Alu | SINE |
| 102556121 | 102556503 | - | 10.2 | 1 | 358 | -1222 | THE1B-int | MaLR | LTR |
| 102556504 | 102556865 | - | 15.4 | 1 | 364 | 0 | THE1B | MaLR | LTR |
| 102556868 | 102557018 | - | 11.5 | 15 | 170 | -7395 | L1MA3 | L1 | LINE |

  

|  |  |  |  |  |  |  |  |  |  |
| --- | --- | --- | --- | --- | --- | --- | --- | --- | --- |
| chr9:104126703-104132816 | | | 6096 bp | | |  |  |  |  |
| Genome Start | Genome End | Strand | Div | Repeat Start | Repeat End | Repeat Left | Name | Family | Class |
| 104126703 | 104126791 | + | 25.0 | 4114 | 4203 | -2361 | L1MCb | L1 | LINE |
| 104126792 | 104127166 | + | 16.1 | 1 | 375 | 0 | THE1C | MaLR | LTR |
| 104127169 | 104128747 | + | 31.9 | 2 | 1731 | -4 | MLT-int | MaLR | LTR |
| 104128752 | 104129115 | + | 14.2 | 1 | 375 | 0 | THE1C | MaLR | LTR |
| 104129116 | 104129618 | + | 25.0 | 4204 | 4719 | -1845 | L1MCb | L1 | LINE |
| 104129635 | 104129818 | - | 16.9 | 5165 | 5342 | -804 | L1PREC2 | L1 | LINE |
| 104129816 | 104129881 | + | 9.1 | 5099 | 5165 | -981 | L1PREC2 | L1 | LINE |
| 104129880 | 104130149 | - | 14.8 | 3799 | 4069 | -2077 | L1PREC2 | L1 | LINE |
| 104130144 | 104130913 | + | 13.8 | 5371 | 6154 | -11 | L1PA15 | L1 | LINE |
| 104130914 | 104131230 | + | 18.5 | 23 | 324 | 0 | AluJb | Alu | SINE |
| 104131238 | 104132107 | + | 25.2 | 4819 | 5696 | -868 | L1MCb | L1 | LINE |
| 104132108 | 104132400 | + | 7.8 | 1 | 300 | -11 | AluY | Alu | SINE |
| 104132401 | 104132816 | + | 25.4 | 5697 | 6135 | -2068 | L1MCb | L1 | LINE |

  

|  |  |  |  |  |  |  |  |  |  |
| --- | --- | --- | --- | --- | --- | --- | --- | --- | --- |
| chr9:104348898-104351619 | | | 2578 bp | | |  |  |  |  |
| Genome Start | Genome End | Strand | Div | Repeat Start | Repeat End | Repeat Left | Name | Family | Class |
| 104348898 | 104349181 | - | 30.5 | 5620 | 5894 | -346 | L1ME3B | L1 | LINE |
| 104349251 | 104349604 | + | 14.6 | 1 | 373 | -2 | THE1C | MaLR | LTR |
| 104349607 | 104351129 | + | 14.2 | 1 | 1576 | -4 | THE1C-int | MaLR | LTR |
| 104351141 | 104351508 | + | 15.6 | 1 | 375 | 0 | THE1C | MaLR | LTR |
| 104351571 | 104351619 | - | 22.4 | 5398 | 5446 | -675 | L1ME3B | L1 | LINE |

  

|  |  |  |  |  |  |  |  |  |  |
| --- | --- | --- | --- | --- | --- | --- | --- | --- | --- |
| chr9:104426157-104434524 | | | 8366 bp | | |  |  |  |  |
| Genome Start | Genome End | Strand | Div | Repeat Start | Repeat End | Repeat Left | Name | Family | Class |
| 104426157 | 104426529 | - | 21.6 | 6315 | 6648 | -1 | L1PA16 | L1 | LINE |
| 104426530 | 104426878 | - | 17.1 | 1 | 364 | 0 | THE1B | MaLR | LTR |
| 104426879 | 104428476 | - | 30.6 | 2 | 1735 | 0 | MLT-int | MaLR | LTR |
| 104428479 | 104428830 | - | 14.8 | 5 | 364 | 0 | THE1B | MaLR | LTR |
| 104428831 | 104434524 | - | 16.8 | 525 | 6314 | -189 | L1PA16 | L1 | LINE |

  

|  |  |  |  |  |  |  |  |  |  |
| --- | --- | --- | --- | --- | --- | --- | --- | --- | --- |
| chr9:104948905-104954928 | | | 5872 bp | | |  |  |  |  |
| Genome Start | Genome End | Strand | Div | Repeat Start | Repeat End | Repeat Left | Name | Family | Class |
| 104948905 | 104948964 | - | 17.6 | 5404 | 5455 | -2107 | L1M3f | L1 | LINE |
| 104948965 | 104949253 | - | 15.6 | 1 | 291 | -19 | AluSg | Alu | SINE |
| 104949254 | 104951255 | - | 17.5 | 3363 | 5403 | -2159 | L1M3f | L1 | LINE |
| 104951294 | 104951646 | + | 10.8 | 1 | 355 | 0 | THE1A | MaLR | LTR |
| 104951649 | 104953228 | + | 31.3 | 2 | 1735 | 0 | MLT-int | MaLR | LTR |
| 104953229 | 104953578 | + | 11.1 | 1 | 355 | 0 | THE1A | MaLR | LTR |
| 104953691 | 104954928 | - | 28.4 | 1776 | 3023 | -4542 | L1M3f | L1 | LINE |

  

|  |  |  |  |  |  |  |  |  |  |
| --- | --- | --- | --- | --- | --- | --- | --- | --- | --- |
| chr9:105010932-105014569 | | | 3636 bp | | |  |  |  |  |
| Genome Start | Genome End | Strand | Div | Repeat Start | Repeat End | Repeat Left | Name | Family | Class |
| 105010932 | 105011004 | - | 28.9 | 2471 | 2540 | -3127 | ERVL | ERVL | LTR |
| 105011005 | 105011386 | - | 17.3 | 1 | 428 | 0 | MSTA | MaLR | LTR |
| 105011387 | 105012927 | - | 31.9 | 2 | 1735 | 0 | MLT-int | MaLR | LTR |
| 105012930 | 105013294 | - | 15.9 | 1 | 428 | 0 | MSTA | MaLR | LTR |
| 105013295 | 105013329 | - | 28.9 | 2443 | 2470 | -3197 | ERVL | ERVL | LTR |
| 105013330 | 105014389 | - | 11.0 | 1 | 1071 | 0 | MER11C | ERVK | LTR |
| 105014390 | 105014569 | - | 28.9 | 2246 | 2442 | -3225 | ERVL | ERVL | LTR |

  

|  |  |  |  |  |  |  |  |  |  |
| --- | --- | --- | --- | --- | --- | --- | --- | --- | --- |
| chr9:105261445-105274803 | | | 13233 bp | | |  |  |  |  |
| Genome Start | Genome End | Strand | Div | Repeat Start | Repeat End | Repeat Left | Name | Family | Class |
| 105261445 | 105262079 | + | 27.8 | 2 | 645 | -5400 | L1MCb | L1 | LINE |
| 105262139 | 105262529 | - | 23.9 | 1 | 1199 | -123 | LTR12E | ERV1 | LTR |
| 105262532 | 105262804 | + | 23.6 | 671 | 944 | -5101 | L1MCb | L1 | LINE |
| 105262805 | 105263314 | + | 10.9 | 1 | 513 | 0 | LTR10D | ERV1 | LTR |
| 105263315 | 105263533 | + | 23.6 | 945 | 1178 | -4867 | L1MCb | L1 | LINE |
| 105263537 | 105263549 | - | 17.4 | 2482 | 2490 | -1 | Tigger7 | MER2\_type | DNA |
| 105263550 | 105263984 | - | 18.2 | 1 | 510 | 0 | LTR54 | ERV1 | LTR |
| 105263985 | 105264427 | - | 17.4 | 2061 | 2481 | -10 | Tigger7 | MER2\_type | DNA |
| 105264439 | 105264509 | - | 18.3 | 1 | 72 | -267 | Tigger7 | MER2\_type | DNA |
| 105264513 | 105265137 | + | 23.2 | 1268 | 1897 | -4854 | L1MCb | L1 | LINE |
| 105265138 | 105265503 | - | 13.8 | 1 | 364 | 0 | THE1B | MaLR | LTR |
| 105265506 | 105267080 | - | 31.4 | 2 | 1733 | -2 | MLT-int | MaLR | LTR |
| 105267083 | 105267444 | - | 14.7 | 1 | 364 | 0 | THE1B | MaLR | LTR |
| 105267445 | 105267845 | + | 26.5 | 1898 | 2488 | -4257 | L1MCb | L1 | LINE |
| 105267863 | 105274321 | - | 12.2 | 6 | 6866 | -1 | L1PB1 | L1 | LINE |
| 105274349 | 105274803 | + | 21.1 | 2382 | 2850 | -3714 | L1MCb | L1 | LINE |

  

|  |  |  |  |  |  |  |  |  |  |
| --- | --- | --- | --- | --- | --- | --- | --- | --- | --- |
| chr9:108163324-108172857 | | | 9465 bp | | |  |  |  |  |
| Genome Start | Genome End | Strand | Div | Repeat Start | Repeat End | Repeat Left | Name | Family | Class |
| 108163324 | 108165698 | - | 17.0 | 4254 | 6737 | -514 | L1M2 | L1 | LINE |
| 108165699 | 108166101 | - | 21.0 | 1 | 408 | 0 | MLT1A1 | MaLR | LTR |
| 108166102 | 108166486 | - | 18.5 | 1374 | 1735 | 0 | MLT1A1-int | MaLR | LTR |
| 108166506 | 108167470 | - | 6.9 | 5176 | 6149 | -2 | L1PB1 | L1 | LINE |
| 108167476 | 108168797 | - | 24.0 | 24 | 1389 | -346 | MLT1A1-int | MaLR | LTR |
| 108168841 | 108169146 | - | 21.4 | 90 | 408 | 0 | MLT1A1 | MaLR | LTR |
| 108169147 | 108169540 | - | 13.0 | 4 | 428 | 0 | MSTA | MaLR | LTR |
| 108169541 | 108169618 | - | 21.4 | 1 | 89 | -319 | MLT1A1 | MaLR | LTR |
| 108169621 | 108172857 | - | 23.8 | 73 | 4256 | -2844 | L1M2 | L1 | LINE |

  

|  |  |  |  |  |  |  |  |  |  |
| --- | --- | --- | --- | --- | --- | --- | --- | --- | --- |
| chr9:111608450-111613499 | | | 4877 bp | | |  |  |  |  |
| Genome Start | Genome End | Strand | Div | Repeat Start | Repeat End | Repeat Left | Name | Family | Class |
| 111608450 | 111609442 | - | 29.2 | 2396 | 3415 | -4 | L2 | L2 | LINE |
| 111609443 | 111609666 | - | 17.1 | 144 | 365 | 0 | MLT1A0 | MaLR | LTR |
| 111609836 | 111610205 | + | 12.0 | 1 | 375 | 0 | THE1C | MaLR | LTR |
| 111610208 | 111611806 | + | 30.3 | 2 | 1734 | -1 | MLT1D-int | MaLR | LTR |
| 111611808 | 111612147 | + | 9.5 | 1 | 375 | 0 | THE1C | MaLR | LTR |
| 111612148 | 111612250 | - | 13.3 | 1 | 93 | -281 | MLT1A | MaLR | LTR |
| 111612252 | 111613499 | - | 26.9 | 1064 | 2399 | -1020 | L2 | L2 | LINE |

  

|  |  |  |  |  |  |  |  |  |  |
| --- | --- | --- | --- | --- | --- | --- | --- | --- | --- |
| chr9:114188419-114203991 | | | 15027 bp | | |  |  |  |  |
| Genome Start | Genome End | Strand | Div | Repeat Start | Repeat End | Repeat Left | Name | Family | Class |
| 114188419 | 114188597 | + | 26.4 | 4143 | 4326 | -1820 | L1ME3B | L1 | LINE |
| 114188606 | 114188887 | + | 19.6 | 6 | 288 | -24 | AluJb | Alu | SINE |
| 114188964 | 114189314 | + | 22.8 | 7409 | 7767 | -194 | L1MC5 | L1 | LINE |
| 114189316 | 114189615 | - | 14.8 | 6 | 305 | -7 | AluJo | Alu | SINE |
| 114189757 | 114189977 | + | 27.0 | 7298 | 7525 | -517 | L1MC4 | L1 | LINE |
| 114189978 | 114190282 | + | 16.4 | 1 | 305 | -7 | AluJb | Alu | SINE |
| 114190284 | 114190581 | + | 7.7 | 3 | 300 | -10 | AluSg | Alu | SINE |
| 114190585 | 114190866 | + | 13.3 | 2 | 292 | -18 | AluSg | Alu | SINE |
| 114190867 | 114191093 | + | 27.0 | 7526 | 7748 | -294 | L1MC4 | L1 | LINE |
| 114191128 | 114191422 | - | 15.9 | 1 | 301 | -11 | AluJb | Alu | SINE |
| 114191424 | 114191617 | + | 22.3 | 7871 | 8042 | 0 | L1MC4 | L1 | LINE |
| 114191631 | 114191883 | + | 25.4 | 4399 | 4648 | -1498 | L1ME3B | L1 | LINE |
| 114191884 | 114192213 | + | 12.0 | 1 | 330 | 0 | AluSx | Alu | SINE |
| 114192214 | 114192569 | + | 25.4 | 4649 | 5008 | -1138 | L1ME3B | L1 | LINE |
| 114192570 | 114192853 | - | 13.4 | 1 | 286 | -26 | AluJo | Alu | SINE |
| 114192854 | 114192977 | + | 25.4 | 5009 | 5141 | -1005 | L1ME3B | L1 | LINE |
| 114192995 | 114193279 | + | 12.3 | 1 | 285 | -27 | AluSx | Alu | SINE |
| 114193307 | 114193570 | + | 21.8 | 23 | 282 | -30 | AluJo | Alu | SINE |
| 114193576 | 114193724 | + | 22.2 | 5155 | 5331 | -815 | L1ME3B | L1 | LINE |
| 114193725 | 114194015 | + | 11.3 | 1 | 311 | -2 | AluSp | Alu | SINE |
| 114194016 | 114194052 | + | 22.2 | 5332 | 5359 | -787 | L1ME3B | L1 | LINE |
| 114194054 | 114195091 | - | 6.3 | 1 | 1266 | 0 | MER11A | ERVK | LTR |
| 114195092 | 114197620 | - | 6.3 | 5421 | 7953 | 0 | HERVK11 | ERVK | LTR |
| 114197617 | 114201190 | - | 8.3 | 1 | 3539 | -4414 | HERVK11 | ERVK | LTR |
| 114201191 | 114202207 | - | 5.7 | 1 | 1266 | 0 | MER11A | ERVK | LTR |
| 114202208 | 114202407 | + | 25.0 | 5355 | 5544 | -696 | L1ME3B | L1 | LINE |
| 114202428 | 114202723 | + | 8.4 | 1 | 296 | -13 | AluSc | Alu | SINE |
| 114202725 | 114203129 | - | 19.7 | 1 | 437 | -30 | MLT1C | MaLR | LTR |
| 114203306 | 114203569 | + | 15.2 | 26 | 309 | -3 | AluJo | Alu | SINE |
| 114203576 | 114203729 | - | 22.1 | 6 | 161 | -225 | MER58D | MER1\_type | DNA |
| 114203749 | 114203991 | + | 25.3 | 5974 | 6236 | -4 | L1ME3B | L1 | LINE |

  

|  |  |  |  |  |  |  |  |  |  |
| --- | --- | --- | --- | --- | --- | --- | --- | --- | --- |
| chr9:116401007-116404644 | | | 3541 bp | | |  |  |  |  |
| Genome Start | Genome End | Strand | Div | Repeat Start | Repeat End | Repeat Left | Name | Family | Class |
| 116401007 | 116401260 | + | 31.3 | 1828 | 2109 | -1310 | L2 | L2 | LINE |
| 116401261 | 116401550 | - | 17.2 | 1 | 289 | -23 | AluJb | Alu | SINE |
| 116401551 | 116401620 | + | 31.3 | 2110 | 2176 | -1243 | L2 | L2 | LINE |
| 116401624 | 116401925 | - | 10.0 | 1 | 300 | -9 | AluSc | Alu | SINE |
| 116401941 | 116402259 | - | 6.6 | 1 | 353 | -2 | THE1A | MaLR | LTR |
| 116402260 | 116403241 | - | 8.9 | 618 | 1580 | 0 | THE1A-int | MaLR | LTR |
| 116403242 | 116403422 | + | 15.5 | 113 | 294 | -19 | AluSq | Alu | SINE |
| 116403502 | 116404107 | - | 8.2 | 1 | 615 | -965 | THE1A-int | MaLR | LTR |
| 116404108 | 116404457 | - | 6.9 | 1 | 355 | 0 | THE1A | MaLR | LTR |
| 116404458 | 116404644 | + | 22.2 | 2234 | 2452 | -967 | L2 | L2 | LINE |

  

|  |  |  |  |  |  |  |  |  |  |
| --- | --- | --- | --- | --- | --- | --- | --- | --- | --- |
| chr9:116975603-116985037 | | | 8983 bp | | |  |  |  |  |
| Genome Start | Genome End | Strand | Div | Repeat Start | Repeat End | Repeat Left | Name | Family | Class |
| 116975603 | 116976736 | - | 30.8 | 4863 | 5984 | -256 | L1ME3B | L1 | LINE |
| 116976752 | 116977162 | - | 8.8 | 1 | 448 | -2 | LTR7 | ERV1 | LTR |
| 116977163 | 116977753 | - | 12.4 | 5600 | 7713 | 0 | HERVH | ERV1 | LTR |
| 116977755 | 116978416 | - | 16.0 | 4488 | 5149 | -2564 | HERVH | ERV1 | LTR |
| 116978419 | 116978921 | - | 9.8 | 3492 | 3993 | -3720 | HERVH | ERV1 | LTR |
| 116978912 | 116979058 | - | 14.3 | 3140 | 3290 | -4423 | HERVH | ERV1 | LTR |
| 116979053 | 116982023 | - | 9.2 | 1 | 2987 | -4726 | HERVH | ERV1 | LTR |
| 116982026 | 116982435 | - | 10.5 | 1 | 448 | -2 | LTR7 | ERV1 | LTR |
| 116982445 | 116983174 | - | 29.4 | 4038 | 4836 | -1310 | L1ME3B | L1 | LINE |
| 116983185 | 116983371 | + | 31.0 | 6 | 188 | -74 | MIR | MIR | SINE |
| 116983423 | 116983477 | + | 23.3 | 200 | 249 | -17 | MIR | MIR | SINE |
| 116983478 | 116983834 | - | 32.6 | 3673 | 4060 | -2086 | L1ME3B | L1 | LINE |
| 116984213 | 116984285 | - | 28.9 | 3129 | 3199 | -2947 | L1ME3B | L1 | LINE |
| 116984286 | 116984907 | + | 13.8 | 1 | 634 | 0 | MER41B | ERV1 | LTR |
| 116984908 | 116985037 | - | 28.9 | 2997 | 3128 | -3018 | L1ME3B | L1 | LINE |

  

|  |  |  |  |  |  |  |  |  |  |
| --- | --- | --- | --- | --- | --- | --- | --- | --- | --- |
| chr9:117275179-117281425 | | | 6264 bp | | |  |  |  |  |
| Genome Start | Genome End | Strand | Div | Repeat Start | Repeat End | Repeat Left | Name | Family | Class |
| 117275179 | 117275280 | - | 15.7 | 6164 | 6269 | -22 | L1MA8 | L1 | LINE |
| 117275281 | 117275732 | + | 6.2 | 1 | 448 | -2 | LTR7 | ERV1 | LTR |
| 117275735 | 117276355 | + | 9.2 | 1 | 620 | -7093 | HERVH | ERV1 | LTR |
| 117276357 | 117278863 | + | 9.4 | 437 | 2995 | -4718 | HERVH | ERV1 | LTR |
| 117278843 | 117278987 | + | 15.2 | 3140 | 3290 | -4423 | HERVH | ERV1 | LTR |
| 117278978 | 117279480 | + | 12.8 | 3492 | 3992 | -3721 | HERVH | ERV1 | LTR |
| 117279485 | 117280099 | + | 15.5 | 4489 | 5149 | -2564 | HERVH | ERV1 | LTR |
| 117280101 | 117280250 | + | 9.5 | 5600 | 5748 | -1965 | HERVH | ERV1 | LTR |
| 117280252 | 117280569 | + | 6.1 | 7384 | 7713 | 0 | HERVH | ERV1 | LTR |
| 117280570 | 117281018 | + | 5.8 | 1 | 446 | -4 | LTR7 | ERV1 | LTR |
| 117281024 | 117281425 | - | 13.0 | 5771 | 6167 | -124 | L1MA8 | L1 | LINE |

  

|  |  |  |  |  |  |  |  |  |  |
| --- | --- | --- | --- | --- | --- | --- | --- | --- | --- |
| chr9:117366867-117371296 | | | 4434 bp | | |  |  |  |  |
| Genome Start | Genome End | Strand | Div | Repeat Start | Repeat End | Repeat Left | Name | Family | Class |
| 117366867 | 117366920 | + | 13.5 | 5461 | 5515 | -639 | L1PB3 | L1 | LINE |
| 117366921 | 117367231 | - | 14.5 | 1 | 364 | 0 | THE1B | MaLR | LTR |
| 117367232 | 117367391 | - | 8.9 | 1424 | 1580 | 0 | THE1B-int | MaLR | LTR |
| 117367380 | 117367471 | - | 15.2 | 1 | 92 | -1488 | THE1B-int | MaLR | LTR |
| 117367472 | 117367833 | - | 16.2 | 1 | 364 | 0 | THE1B | MaLR | LTR |
| 117367834 | 117367973 | + | 13.6 | 5511 | 5650 | -504 | L1PB3 | L1 | LINE |
| 117367974 | 117368335 | + | 12.5 | 1 | 360 | -4 | THE1B | MaLR | LTR |
| 117368340 | 117368847 | + | 10.7 | 1 | 366 | -1214 | THE1B-int | MaLR | LTR |
| 117368848 | 117369148 | - | 12.4 | 16 | 311 | -1 | AluSx | Alu | SINE |
| 117369149 | 117370360 | + | 10.7 | 367 | 1580 | 0 | THE1B-int | MaLR | LTR |
| 117370363 | 117370726 | + | 14.2 | 3 | 362 | -2 | THE1B | MaLR | LTR |
| 117370729 | 117371296 | + | 16.6 | 5646 | 6148 | -6 | L1PB3 | L1 | LINE |

  

|  |  |  |  |  |  |  |  |  |  |
| --- | --- | --- | --- | --- | --- | --- | --- | --- | --- |
| chr9:119378482-119384601 | | | 6112 bp | | |  |  |  |  |
| Genome Start | Genome End | Strand | Div | Repeat Start | Repeat End | Repeat Left | Name | Family | Class |
| 119378482 | 119378875 | + | 28.5 | 4109 | 4543 | -1603 | L1MC4a | L1 | LINE |
| 119378876 | 119379320 | - | 19.7 | 6185 | 6632 | -1 | L1MD1 | L1 | LINE |
| 119379324 | 119379679 | + | 20.9 | 3 | 365 | 0 | MLT1A0 | MaLR | LTR |
| 119379680 | 119381286 | + | 24.1 | 1 | 1722 | -13 | MLT1A0-int | MaLR | LTR |
| 119381298 | 119381621 | + | 21.7 | 1 | 333 | -32 | MLT1A0 | MaLR | LTR |
| 119381622 | 119381955 | + | 16.5 | 1 | 346 | 0 | MER7A | MER2\_type | DNA |
| 119381956 | 119381988 | + | 21.7 | 334 | 364 | -1 | MLT1A0 | MaLR | LTR |
| 119381989 | 119383722 | - | 20.0 | 4570 | 6189 | -444 | L1MD1 | L1 | LINE |
| 119383706 | 119384079 | - | 25.6 | 1638 | 2008 | -4625 | L1MD1 | L1 | LINE |
| 119384091 | 119384601 | + | 29.7 | 5423 | 5978 | -1830 | L1MC4a | L1 | LINE |

  

|  |  |  |  |  |  |  |  |  |  |
| --- | --- | --- | --- | --- | --- | --- | --- | --- | --- |
| chr9:121918196-121920893 | | | 2692 bp | | |  |  |  |  |
| Genome Start | Genome End | Strand | Div | Repeat Start | Repeat End | Repeat Left | Name | Family | Class |
| 121918196 | 121918320 | + | 15.6 | 14 | 139 | -173 | AluJo | Alu | SINE |
| 121918321 | 121918723 | + | 13.2 | 1 | 428 | 0 | MSTA | MaLR | LTR |
| 121918726 | 121920345 | + | 27.6 | 2 | 1735 | 0 | MLT1I-int | MaLR | LTR |
| 121920350 | 121920744 | + | 14.1 | 5 | 428 | 0 | MSTA | MaLR | LTR |
| 121920745 | 121920893 | + | 15.6 | 140 | 287 | -25 | AluJo | Alu | SINE |

  

|  |  |  |  |  |  |  |  |  |  |
| --- | --- | --- | --- | --- | --- | --- | --- | --- | --- |
| chr9:123315307-123319003 | | | 3693 bp | | |  |  |  |  |
| Genome Start | Genome End | Strand | Div | Repeat Start | Repeat End | Repeat Left | Name | Family | Class |
| 123315307 | 123315335 | + | 12.9 | 1 | 24 | -404 | MSTA | MaLR | LTR |
| 123315336 | 123315698 | - | 11.1 | 1 | 354 | -1 | THE1A | MaLR | LTR |
| 123315699 | 123317274 | - | 29.8 | 2 | 1735 | 0 | MLT-int | MaLR | LTR |
| 123317277 | 123317640 | - | 12.5 | 1 | 355 | 0 | THE1A | MaLR | LTR |
| 123317641 | 123317790 | + | 12.9 | 25 | 169 | -259 | MSTA | MaLR | LTR |
| 123317791 | 123318098 | - | 10.7 | 1 | 310 | 0 | AluSg | Alu | SINE |
| 123318101 | 123318396 | - | 12.2 | 1 | 295 | -15 | AluSg | Alu | SINE |
| 123318397 | 123318423 | + | 12.9 | 170 | 195 | -233 | MSTA | MaLR | LTR |
| 123318424 | 123318733 | + | 17.8 | 1 | 312 | 0 | AluJb | Alu | SINE |
| 123318734 | 123319003 | + | 12.9 | 196 | 428 | 0 | MSTA | MaLR | LTR |

  

|  |  |  |  |  |  |  |  |  |  |
| --- | --- | --- | --- | --- | --- | --- | --- | --- | --- |
| chr9:124235658-124238655 | | | 2942 bp | | |  |  |  |  |
| Genome Start | Genome End | Strand | Div | Repeat Start | Repeat End | Repeat Left | Name | Family | Class |
| 124235658 | 124235919 | - | 28.8 | 5803 | 6064 | -178 | L1ME3A | L1 | LINE |
| 124235922 | 124236286 | + | 7.7 | 1 | 355 | 0 | THE1A | MaLR | LTR |
| 124236289 | 124237860 | + | 29.3 | 2 | 1735 | 0 | MLT-int | MaLR | LTR |
| 124237886 | 124238236 | + | 8.0 | 1 | 355 | 0 | THE1A | MaLR | LTR |
| 124238264 | 124238655 | - | 27.3 | 5423 | 5812 | -428 | L1ME3A | L1 | LINE |

  

|  |  |  |  |  |  |  |  |  |  |
| --- | --- | --- | --- | --- | --- | --- | --- | --- | --- |
| chr9:124269706-124275019 | | | 5310 bp | | |  |  |  |  |
| Genome Start | Genome End | Strand | Div | Repeat Start | Repeat End | Repeat Left | Name | Family | Class |
| 124269706 | 124271290 | + | 34.4 | 3267 | 4860 | -3410 | MER65D-int | ERV1 | LTR |
| 124271291 | 124271645 | + | 12.8 | 1 | 355 | 0 | THE1A | MaLR | LTR |
| 124271650 | 124273234 | + | 30.1 | 4 | 1735 | 0 | MLT-int | MaLR | LTR |
| 124273235 | 124273589 | + | 13.3 | 1 | 355 | 0 | THE1A | MaLR | LTR |
| 124273590 | 124275019 | + | 34.4 | 4861 | 6282 | -1988 | MER65D-int | ERV1 | LTR |

  

|  |  |  |  |  |  |  |  |  |  |
| --- | --- | --- | --- | --- | --- | --- | --- | --- | --- |
| chr9:125523246-125526086 | | | 2746 bp | | |  |  |  |  |
| Genome Start | Genome End | Strand | Div | Repeat Start | Repeat End | Repeat Left | Name | Family | Class |
| 125523246 | 125523605 | + | 34.8 | 2789 | 3148 | -230 | L2 | L2 | LINE |
| 125523621 | 125523979 | + | 13.6 | 1 | 364 | 0 | THE1B | MaLR | LTR |
| 125523982 | 125525557 | + | 30.6 | 2 | 1735 | 0 | MLT1H1-int | MaLR | LTR |
| 125525558 | 125525915 | + | 13.9 | 1 | 364 | 0 | THE1B | MaLR | LTR |
| 125525994 | 125526086 | + | 30.4 | 3300 | 3378 | 0 | L2 | L2 | LINE |

  

|  |  |  |  |  |  |  |  |  |  |
| --- | --- | --- | --- | --- | --- | --- | --- | --- | --- |
| chr9:130650448-130660183 | | | 9586 bp | | |  |  |  |  |
| Genome Start | Genome End | Strand | Div | Repeat Start | Repeat End | Repeat Left | Name | Family | Class |
| 130650448 | 130650635 | + | 18.1 | 4689 | 4884 | -1262 | L1M4 | L1 | LINE |
| 130650636 | 130650942 | + | 10.6 | 1 | 304 | -8 | AluSx | Alu | SINE |
| 130650949 | 130651221 | + | 13.2 | 1 | 291 | -21 | AluSx | Alu | SINE |
| 130651230 | 130651519 | + | 11.0 | 1 | 291 | -21 | AluSx | Alu | SINE |
| 130651520 | 130651613 | + | 18.1 | 4891 | 4945 | -1201 | L1M4 | L1 | LINE |
| 130651614 | 130651925 | + | 12.6 | 1 | 309 | 0 | AluSg1 | Alu | SINE |
| 130651926 | 130651970 | + | 18.1 | 4946 | 4990 | -1156 | L1M4 | L1 | LINE |
| 130651973 | 130652285 | - | 17.9 | 1 | 312 | 0 | AluSx | Alu | SINE |
| 130652287 | 130652335 | - | 18.4 | 46 | 94 | -39 | FLAM\_A | Alu | SINE |
| 130652336 | 130653338 | + | 6.9 | 1 | 1002 | 0 | LTR5B | ERVK | LTR |
| 130653339 | 130654022 | + | 12.2 | 1 | 696 | -6840 | HERVK | ERVK | LTR |
| 130654149 | 130656588 | + | 11.6 | 3007 | 5462 | -2074 | HERVK | ERVK | LTR |
| 130656594 | 130658549 | + | 15.3 | 5587 | 7534 | -2 | HERVK | ERVK | LTR |
| 130658552 | 130659557 | + | 7.2 | 1 | 1002 | 0 | LTR5B | ERVK | LTR |
| 130659558 | 130659607 | - | 16.0 | 1 | 51 | -82 | FLAM\_A | Alu | SINE |
| 130659608 | 130659654 | + | 14.1 | 4972 | 5007 | -1139 | L1M4 | L1 | LINE |
| 130659655 | 130659962 | + | 7.1 | 1 | 308 | 0 | AluY | Alu | SINE |
| 130659963 | 130660183 | + | 14.1 | 5008 | 5246 | -900 | L1M4 | L1 | LINE |

  

|  |  |  |  |  |  |  |  |  |  |
| --- | --- | --- | --- | --- | --- | --- | --- | --- | --- |
| chr9:140174288-140184649 | | | 10353 bp | | |  |  |  |  |
| Genome Start | Genome End | Strand | Div | Repeat Start | Repeat End | Repeat Left | Name | Family | Class |
| 140174288 | 140175187 | + | 16.0 | 1591 | 2560 | -5005 | L1MA2 | L1 | LINE |
| 140175188 | 140175484 | + | 6.5 | 1 | 296 | -15 | AluY | Alu | SINE |
| 140175485 | 140177310 | + | 15.5 | 2561 | 4427 | -3138 | L1MA2 | L1 | LINE |
| 140177313 | 140178304 | - | 1.8 | 5164 | 6155 | 0 | L1PA3 | L1 | LINE |
| 140178302 | 140179063 | + | 16.4 | 4420 | 5299 | -2266 | L1MA2 | L1 | LINE |
| 140179064 | 140179364 | + | 12.6 | 1 | 301 | -11 | AluSx | Alu | SINE |
| 140179365 | 140179724 | + | 16.4 | 5300 | 5669 | -1896 | L1MA2 | L1 | LINE |
| 140179725 | 140180138 | + | 19.6 | 1 | 428 | 0 | MSTA | MaLR | LTR |
| 140180141 | 140181770 | + | 31.6 | 2 | 1735 | 0 | MLT-int | MaLR | LTR |
| 140181771 | 140182177 | + | 21.5 | 1 | 428 | 0 | MSTA | MaLR | LTR |
| 140182178 | 140183164 | + | 16.4 | 5670 | 6679 | -886 | L1MA2 | L1 | LINE |
| 140183169 | 140183496 | + | 15.3 | 1 | 335 | -40 | THE1C | MaLR | LTR |
| 140183497 | 140183776 | + | 12.9 | 2 | 281 | -28 | AluSg1 | Alu | SINE |
| 140183777 | 140183844 | + | 12.1 | 343 | 381 | 0 | THE1C | MaLR | LTR |
| 140183849 | 140184068 | + | 14.6 | 6684 | 6915 | -809 | L1MA2 | L1 | LINE |
| 140184069 | 140184649 | + | 11.9 | 7130 | 7721 | -2 | L1MA2 | L1 | LINE |

  


|  |  |  |  |  |  |  |  |  |  |
| --- | --- | --- | --- | --- | --- | --- | --- | --- | --- |
| chr10:89845-100218 | | | 10358 bp | | |  |  |  |  |
| Genome Start | Genome End | Strand | Div | Repeat Start | Repeat End | Repeat Left | Name | Family | Class |
| 89845 | 90425 | - | 11.2 | 7130 | 7721 | -2 | L1MA2 | L1 | LINE |
| 90426 | 90646 | - | 15.8 | 6684 | 6915 | -809 | L1MA2 | L1 | LINE |
| 90651 | 90714 | - | 12.9 | 343 | 381 | 0 | THE1C | MaLR | LTR |
| 90715 | 90994 | - | 12.1 | 2 | 281 | -28 | AluSg1 | Alu | SINE |
| 90995 | 91322 | - | 14.0 | 1 | 335 | -40 | THE1C | MaLR | LTR |
| 91327 | 92315 | - | 16.5 | 5671 | 6680 | -885 | L1MA2 | L1 | LINE |
| 92316 | 92724 | - | 21.9 | 1 | 428 | 0 | MSTA | MaLR | LTR |
| 92725 | 94351 | - | 31.6 | 2 | 1735 | 0 | MLT-int | MaLR | LTR |
| 94354 | 94764 | - | 19.1 | 1 | 428 | 0 | MSTA | MaLR | LTR |
| 94765 | 95127 | - | 16.5 | 5301 | 5670 | -1895 | L1MA2 | L1 | LINE |
| 95128 | 95428 | - | 13.3 | 1 | 301 | -11 | AluSx | Alu | SINE |
| 95429 | 96191 | - | 16.5 | 4421 | 5300 | -2265 | L1MA2 | L1 | LINE |
| 96189 | 97178 | + | 1.9 | 5164 | 6155 | 0 | L1PA3 | L1 | LINE |
| 97188 | 99022 | - | 15.2 | 2563 | 4425 | -3140 | L1MA2 | L1 | LINE |
| 99023 | 99315 | - | 7.9 | 1 | 292 | -19 | AluY | Alu | SINE |
| 99316 | 100218 | - | 15.7 | 1591 | 2562 | -5003 | L1MA2 | L1 | LINE |

  

|  |  |  |  |  |  |  |  |  |  |
| --- | --- | --- | --- | --- | --- | --- | --- | --- | --- |
| chr10:5240997-5244330 | | | 3191 bp | | |  |  |  |  |
| Genome Start | Genome End | Strand | Div | Repeat Start | Repeat End | Repeat Left | Name | Family | Class |
| 5240997 | 5241200 | - | 24.6 | 7280 | 7484 | -558 | L1MC4 | L1 | LINE |
| 5241057 | 5241260 | - | 28.5 | 7130 | 7340 | -468 | L1MC4a | L1 | LINE |
| 5241488 | 5241826 | - | 13.4 | 1 | 364 | 0 | THE1B | MaLR | LTR |
| 5241829 | 5242569 | - | 9.8 | 858 | 1578 | -2 | THE1B-int | MaLR | LTR |
| 5242570 | 5242872 | - | 8.8 | 1 | 297 | -13 | AluSg | Alu | SINE |
| 5242873 | 5243700 | - | 11.1 | 15 | 857 | -723 | THE1B-int | MaLR | LTR |
| 5243703 | 5244053 | - | 13.4 | 3 | 362 | -2 | THE1B | MaLR | LTR |
| 5244110 | 5244330 | - | 30.3 | 6832 | 7065 | -977 | L1MC4 | L1 | LINE |

  

|  |  |  |  |  |  |  |  |  |  |
| --- | --- | --- | --- | --- | --- | --- | --- | --- | --- |
| chr10:7772077-7775739 | | | 3650 bp | | |  |  |  |  |
| Genome Start | Genome End | Strand | Div | Repeat Start | Repeat End | Repeat Left | Name | Family | Class |
| 7772077 | 7772524 | - | 24.5 | 307 | 783 | 0 | MER20B | MER1\_type | DNA |
| 7772525 | 7772664 | + | 22.8 | 1 | 144 | -230 | MLT1A | MaLR | LTR |
| 7772665 | 7772953 | + | 9.7 | 23 | 310 | -1 | AluY | Alu | SINE |
| 7772954 | 7773148 | + | 22.8 | 145 | 358 | -16 | MLT1A | MaLR | LTR |
| 7773160 | 7773268 | - | 29.6 | 202 | 311 | -472 | MER20B | MER1\_type | DNA |
| 7773269 | 7773629 | - | 11.5 | 1 | 364 | 0 | THE1B | MaLR | LTR |
| 7773630 | 7775193 | - | 30.3 | 2 | 1735 | 0 | MLT-int | MaLR | LTR |
| 7775196 | 7775552 | - | 12.8 | 1 | 364 | 0 | THE1B | MaLR | LTR |
| 7775553 | 7775739 | - | 29.7 | 12 | 201 | -40 | MER20B | MER1\_type | DNA |

  

|  |  |  |  |  |  |  |  |  |  |
| --- | --- | --- | --- | --- | --- | --- | --- | --- | --- |
| chr10:16295394-16304149 | | | 8500 bp | | |  |  |  |  |
| Genome Start | Genome End | Strand | Div | Repeat Start | Repeat End | Repeat Left | Name | Family | Class |
| 16295394 | 16295586 | - | 31.8 | 5449 | 5654 | -586 | L1M5 | L1 | LINE |
| 16295643 | 16296128 | - | 33.5 | 4603 | 5108 | -1038 | L1M5 | L1 | LINE |
| 16296131 | 16296597 | + | 22.1 | 1 | 505 | 0 | MLT1D | MaLR | LTR |
| 16296599 | 16297831 | + | 25.4 | 2 | 1192 | -179 | MLT1D-int | MaLR | LTR |
| 16297830 | 16297877 | - | 22.9 | 656 | 703 | -668 | MLT1-int | MaLR | LTR |
| 16297885 | 16297931 | + | 16.4 | 1187 | 1233 | -138 | MLT1D-int | MaLR | LTR |
| 16297932 | 16298232 | - | 12.6 | 1 | 303 | -9 | AluSx | Alu | SINE |
| 16298233 | 16298263 | + | 16.4 | 1234 | 1262 | -109 | MLT1D-int | MaLR | LTR |
| 16298286 | 16300520 | - | 5.0 | 3899 | 6141 | -13 | L1PA5 | L1 | LINE |
| 16300522 | 16301811 | + | 5.7 | 2594 | 3881 | -2265 | L1PA5 | L1 | LINE |
| 16301842 | 16301959 | + | 22.9 | 1368 | 1488 | -1 | MLT1D-int | MaLR | LTR |
| 16301961 | 16302412 | + | 25.5 | 1 | 486 | -19 | MLT1D | MaLR | LTR |
| 16302433 | 16303074 | - | 27.2 | 3877 | 4597 | -1549 | L1M5 | L1 | LINE |
| 16303097 | 16303352 | - | 27.6 | 993 | 1254 | -4865 | L1MCa | L1 | LINE |
| 16303449 | 16303607 | - | 32.7 | 3091 | 3233 | -2913 | L1M5 | L1 | LINE |
| 16303608 | 16303921 | - | 10.4 | 1 | 311 | 0 | AluY | Alu | SINE |
| 16303922 | 16304149 | - | 32.7 | 2819 | 3090 | -3056 | L1M5 | L1 | LINE |

  

|  |  |  |  |  |  |  |  |  |  |
| --- | --- | --- | --- | --- | --- | --- | --- | --- | --- |
| chr10:22213681-22229413 | | | 15320 bp | | |  |  |  |  |
| Genome Start | Genome End | Strand | Div | Repeat Start | Repeat End | Repeat Left | Name | Family | Class |
| 22213681 | 22213808 | + | 23.0 | 192 | 335 | -553 | Kanga2\_a | Tc2 | DNA |
| 22213809 | 22217831 | + | 16.0 | 3980 | 8108 | -50 | L1PA13 | L1 | LINE |
| 22217945 | 22218350 | - | 14.8 | 3 | 428 | 0 | MSTA | MaLR | LTR |
| 22218476 | 22219064 | + | 25.6 | 94 | 686 | -5947 | L1MDa | L1 | LINE |
| 22219065 | 22219379 | + | 12.1 | 1 | 307 | -5 | AluSx | Alu | SINE |
| 22219380 | 22219843 | + | 25.6 | 687 | 1174 | -5459 | L1MDa | L1 | LINE |
| 22219844 | 22220146 | + | 11.2 | 1 | 304 | -8 | AluSx | Alu | SINE |
| 22220147 | 22220423 | + | 25.6 | 1175 | 1480 | -5153 | L1MDa | L1 | LINE |
| 22220486 | 22221282 | + | 18.8 | 1745 | 2566 | -4067 | L1MDa | L1 | LINE |
| 22221379 | 22222279 | + | 17.9 | 2466 | 3352 | -3185 | L1MDa | L1 | LINE |
| 22222280 | 22222582 | + | 13.9 | 1 | 305 | -7 | AluSx | Alu | SINE |
| 22222583 | 22223223 | + | 17.3 | 3353 | 4005 | -2532 | L1MDa | L1 | LINE |
| 22223224 | 22223523 | + | 14.1 | 1 | 298 | -15 | AluSq | Alu | SINE |
| 22223524 | 22223615 | + | 17.3 | 4006 | 4089 | -2448 | L1MDa | L1 | LINE |
| 22223616 | 22223998 | + | 15.5 | 5 | 408 | 0 | MLT1A1 | MaLR | LTR |
| 22223999 | 22225679 | + | 21.6 | 1 | 1731 | -4 | MLT1A1-int | MaLR | LTR |
| 22225692 | 22226081 | + | 18.3 | 1 | 401 | -7 | MLT1A1 | MaLR | LTR |
| 22226085 | 22226397 | + | 18.5 | 4082 | 4393 | -2144 | L1MDa | L1 | LINE |
| 22226398 | 22226664 | + | 15.5 | 1 | 339 | 0 | MER1B | MER1\_type | DNA |
| 22226665 | 22227237 | + | 18.5 | 4394 | 4968 | -1569 | L1MDa | L1 | LINE |
| 22227239 | 22227486 | + | 16.5 | 2 | 291 | -2738 | Tigger3(Golem) | MER2\_type | DNA |
| 22227487 | 22227717 | - | 13.6 | 88 | 301 | -12 | AluSq | Alu | SINE |
| 22227723 | 22227781 | + | 13.6 | 282 | 340 | -2689 | Tigger3(Golem) | MER2\_type | DNA |
| 22227787 | 22228640 | + | 24.3 | 11 | 892 | -6089 | L1PA12 | L1 | LINE |
| 22228618 | 22228839 | - | 28.9 | 650 | 866 | -5487 | L1PA10 | L1 | LINE |
| 22228849 | 22228995 | - | 11.3 | 2552 | 2704 | -325 | Tigger3(Golem) | MER2\_type | DNA |
| 22228996 | 22229304 | + | 12.3 | 1 | 309 | -3 | AluSx | Alu | SINE |
| 22229310 | 22229413 | + | 23.3 | 751 | 860 | -28 | Kanga2\_a | Tc2 | DNA |

  

|  |  |  |  |  |  |  |  |  |  |
| --- | --- | --- | --- | --- | --- | --- | --- | --- | --- |
| chr10:23871927-23876450 | | | 4127 bp | | |  |  |  |  |
| Genome Start | Genome End | Strand | Div | Repeat Start | Repeat End | Repeat Left | Name | Family | Class |
| 23871927 | 23872389 | - | 27.1 | 2002 | 2494 | -13 | HAL1 | L1 | LINE |
| 23872785 | 23872934 | - | 25.7 | 1707 | 1856 | -651 | HAL1 | L1 | LINE |
| 23872935 | 23873268 | + | 10.5 | 1 | 355 | 0 | THE1A | MaLR | LTR |
| 23873271 | 23874820 | + | 30.2 | 2 | 1735 | 0 | MLT-int | MaLR | LTR |
| 23874821 | 23875165 | + | 8.1 | 1 | 355 | 0 | THE1A | MaLR | LTR |
| 23875166 | 23875710 | - | 25.7 | 1128 | 1706 | -801 | HAL1 | L1 | LINE |
| 23875711 | 23876021 | - | 9.3 | 1 | 311 | 0 | AluY | Alu | SINE |
| 23876022 | 23876450 | - | 25.7 | 684 | 1127 | -1380 | HAL1 | L1 | LINE |

  

|  |  |  |  |  |  |  |  |  |  |
| --- | --- | --- | --- | --- | --- | --- | --- | --- | --- |
| chr10:25336961-25342955 | | | 5713 bp | | |  |  |  |  |
| Genome Start | Genome End | Strand | Div | Repeat Start | Repeat End | Repeat Left | Name | Family | Class |
| 25336961 | 25337544 | + | 32.8 | 1921 | 2513 | -1976 | L3 | CR1 | LINE |
| 25337584 | 25338200 | + | 3.4 | 5531 | 6152 | -3 | L1PA4 | L1 | LINE |
| 25338366 | 25338529 | + | 32.9 | 3312 | 3516 | -973 | L3 | CR1 | LINE |
| 25338551 | 25338906 | + | 11.8 | 1 | 362 | -2 | THE1B | MaLR | LTR |
| 25338909 | 25339338 | + | 9.5 | 1 | 430 | -1150 | THE1B-int | MaLR | LTR |
| 25339379 | 25340388 | - | 2.5 | 5144 | 6155 | 0 | L1PA3 | L1 | LINE |
| 25340389 | 25340669 | + | 4.6 | 4862 | 5142 | -1004 | L1PA3 | L1 | LINE |
| 25340679 | 25341819 | + | 11.4 | 427 | 1580 | 0 | THE1B-int | MaLR | LTR |
| 25341820 | 25342186 | + | 10.5 | 1 | 364 | 0 | THE1B | MaLR | LTR |
| 25342193 | 25342595 | + | 28.2 | 3564 | 4056 | -433 | L3 | CR1 | LINE |
| 25342596 | 25342902 | + | 11.5 | 1 | 306 | -7 | AluSq | Alu | SINE |
| 25342903 | 25342955 | + | 28.2 | 4057 | 4097 | -392 | L3 | CR1 | LINE |

  

|  |  |  |  |  |  |  |  |  |  |
| --- | --- | --- | --- | --- | --- | --- | --- | --- | --- |
| chr10:26043878-26052460 | | | 8582 bp | | |  |  |  |  |
| Genome Start | Genome End | Strand | Div | Repeat Start | Repeat End | Repeat Left | Name | Family | Class |
| 26043878 | 26045355 | + | 22.0 | 2669 | 4239 | -1907 | L1M5 | L1 | LINE |
| 26045356 | 26045767 | + | 9.8 | 1 | 448 | -2 | LTR7 | ERV1 | LTR |
| 26045770 | 26050858 | + | 10.1 | 1 | 5149 | -2564 | HERVH | ERV1 | LTR |
| 26050860 | 26051075 | + | 15.3 | 5600 | 5819 | -1894 | HERVH | ERV1 | LTR |
| 26051074 | 26051451 | + | 10.7 | 7322 | 7713 | 0 | HERVH | ERV1 | LTR |
| 26051452 | 26051861 | + | 8.8 | 1 | 448 | -2 | LTR7 | ERV1 | LTR |
| 26051862 | 26052460 | + | 26.1 | 4233 | 4862 | -1284 | L1M5 | L1 | LINE |

  

|  |  |  |  |  |  |  |  |  |  |
| --- | --- | --- | --- | --- | --- | --- | --- | --- | --- |
| chr10:31873570-31877651 | | | 4079 bp | | |  |  |  |  |
| Genome Start | Genome End | Strand | Div | Repeat Start | Repeat End | Repeat Left | Name | Family | Class |
| 31873570 | 31874171 | + | 15.9 | 1 | 628 | -1023 | MSTA-int | MaLR | LTR |
| 31874172 | 31874525 | - | 13.2 | 1 | 364 | 0 | THE1B | MaLR | LTR |
| 31874526 | 31876091 | - | 8.9 | 1 | 1580 | 0 | THE1B-int | MaLR | LTR |
| 31876092 | 31876213 | - | 15.6 | 236 | 364 | 0 | THE1B | MaLR | LTR |
| 31876214 | 31876389 | - | 11.9 | 1430 | 1612 | -39 | MST-int | MaLR | LTR |
| 31876391 | 31876605 | - | 11.5 | 1 | 214 | -150 | THE1B | MaLR | LTR |
| 31876608 | 31877651 | + | 17.1 | 626 | 1635 | -16 | MSTA-int | MaLR | LTR |

  

|  |  |  |  |  |  |  |  |  |  |
| --- | --- | --- | --- | --- | --- | --- | --- | --- | --- |
| chr10:32030848-32033897 | | | 2812 bp | | |  |  |  |  |
| Genome Start | Genome End | Strand | Div | Repeat Start | Repeat End | Repeat Left | Name | Family | Class |
| 32030848 | 32031037 | + | 20.0 | 32 | 229 | -1477 | MLT-int | MaLR | LTR |
| 32031038 | 32031352 | + | 15.4 | 68 | 428 | 0 | MSTA | MaLR | LTR |
| 32031355 | 32033009 | + | 29.4 | 2 | 1734 | -1 | MLT1L-int | MaLR | LTR |
| 32033011 | 32033162 | + | 15.9 | 1 | 183 | -245 | MSTA | MaLR | LTR |
| 32033398 | 32033644 | + | 11.1 | 161 | 428 | 0 | MSTA | MaLR | LTR |
| 32033645 | 32033897 | + | 23.6 | 387 | 642 | -1093 | MLT-int | MaLR | LTR |

  

|  |  |  |  |  |  |  |  |  |  |
| --- | --- | --- | --- | --- | --- | --- | --- | --- | --- |
| chr10:32735756-32739841 | | | 3669 bp | | |  |  |  |  |
| Genome Start | Genome End | Strand | Div | Repeat Start | Repeat End | Repeat Left | Name | Family | Class |
| 32735756 | 32736002 | - | 35.4 | 3137 | 3379 | -40 | L2 | L2 | LINE |
| 32736010 | 32736401 | + | 18.7 | 1 | 396 | 0 | MSTD | MaLR | LTR |
| 32736405 | 32736869 | + | 24.6 | 2 | 468 | -1267 | MSTD-int | MaLR | LTR |
| 32736870 | 32737006 | - | 16.1 | 228 | 364 | 0 | THE1B | MaLR | LTR |
| 32737007 | 32737314 | - | 13.6 | 1 | 308 | -4 | AluSx | Alu | SINE |
| 32737315 | 32737549 | - | 16.1 | 1 | 227 | -137 | THE1B | MaLR | LTR |
| 32737550 | 32738074 | + | 24.6 | 469 | 1014 | -721 | MSTD-int | MaLR | LTR |
| 32738075 | 32738371 | - | 11.5 | 1 | 296 | -16 | AluSx | Alu | SINE |
| 32738372 | 32739060 | + | 24.6 | 1015 | 1734 | -1 | MSTD-int | MaLR | LTR |
| 32739061 | 32739250 | + | 12.1 | 1 | 210 | -186 | MSTD | MaLR | LTR |
| 32739658 | 32739841 | - | 26.2 | 2910 | 3107 | -312 | L2 | L2 | LINE |

  

|  |  |  |  |  |  |  |  |  |  |
| --- | --- | --- | --- | --- | --- | --- | --- | --- | --- |
| chr10:44028903-44032619 | | | 3517 bp | | |  |  |  |  |
| Genome Start | Genome End | Strand | Div | Repeat Start | Repeat End | Repeat Left | Name | Family | Class |
| 44028903 | 44029039 | + | 22.0 | 1137 | 1283 | -5028 | L1MEc | L1 | LINE |
| 44029240 | 44029554 | + | 26.4 | 1217 | 1676 | -4665 | L1MEc | L1 | LINE |
| 44029555 | 44029923 | + | 14.4 | 1 | 364 | 0 | THE1B | MaLR | LTR |
| 44029924 | 44031508 | + | 29.6 | 1 | 1735 | 0 | MLT-int | MaLR | LTR |
| 44031509 | 44031865 | + | 14.6 | 1 | 364 | 0 | THE1B | MaLR | LTR |
| 44031866 | 44032619 | + | 30.8 | 1564 | 2490 | -3851 | L1MEc | L1 | LINE |

  

|  |  |  |  |  |  |  |  |  |  |
| --- | --- | --- | --- | --- | --- | --- | --- | --- | --- |
| chr10:45290062-45299655 | | | 9592 bp | | |  |  |  |  |
| Genome Start | Genome End | Strand | Div | Repeat Start | Repeat End | Repeat Left | Name | Family | Class |
| 45290062 | 45291737 | + | 16.4 | 1 | 1792 | -5057 | L1MA4A | L1 | LINE |
| 45291738 | 45292015 | + | 15.3 | 3 | 276 | -36 | AluJo | Alu | SINE |
| 45292016 | 45294908 | + | 16.4 | 1793 | 4793 | -2052 | L1MA4A | L1 | LINE |
| 45294909 | 45295271 | + | 13.5 | 1 | 364 | 0 | THE1B | MaLR | LTR |
| 45295274 | 45296866 | + | 29.9 | 2 | 1735 | 0 | MLT-int | MaLR | LTR |
| 45296867 | 45297228 | + | 11.7 | 1 | 364 | 0 | THE1B | MaLR | LTR |
| 45297229 | 45297925 | + | 16.4 | 4794 | 5494 | -1351 | L1MA4A | L1 | LINE |
| 45297926 | 45298236 | + | 12.9 | 1 | 310 | -2 | AluSx | Alu | SINE |
| 45298237 | 45299655 | + | 13.7 | 5495 | 7005 | 0 | L1MA4A | L1 | LINE |

  

|  |  |  |  |  |  |  |  |  |  |
| --- | --- | --- | --- | --- | --- | --- | --- | --- | --- |
| chr10:45379419-45391882 | | | 12440 bp | | |  |  |  |  |
| Genome Start | Genome End | Strand | Div | Repeat Start | Repeat End | Repeat Left | Name | Family | Class |
| 45379419 | 45379447 | + | 29.4 | 1251 | 1279 | -5354 | L1MDa | L1 | LINE |
| 45379448 | 45379733 | + | 9.4 | 1 | 288 | -24 | AluSx | Alu | SINE |
| 45379734 | 45380624 | + | 28.7 | 1280 | 2307 | -4411 | L1MDa | L1 | LINE |
| 45380625 | 45380899 | + | 14.9 | 23 | 299 | -13 | AluJb | Alu | SINE |
| 45380900 | 45381159 | + | 28.7 | 2308 | 2624 | -4094 | L1MDa | L1 | LINE |
| 45381160 | 45381595 | + | 20.1 | 1 | 441 | -212 | MER82 | MER2\_type | DNA |
| 45381597 | 45383813 | - | 9.1 | 4064 | 6297 | -5 | L1MA1 | L1 | LINE |
| 45383814 | 45383892 | - | 16.5 | 156 | 230 | 0 | MER30 | MER1\_type | DNA |
| 45383893 | 45384192 | + | 9.7 | 1 | 302 | -7 | AluSc | Alu | SINE |
| 45384193 | 45384240 | - | 16.5 | 102 | 155 | -75 | MER30 | MER1\_type | DNA |
| 45384241 | 45384552 | + | 7.1 | 1 | 311 | 0 | AluY | Alu | SINE |
| 45384553 | 45384634 | - | 16.5 | 1 | 101 | -129 | MER30 | MER1\_type | DNA |
| 45384635 | 45385029 | - | 8.6 | 3677 | 4063 | -2083 | L1MA1 | L1 | LINE |
| 45385031 | 45385256 | + | 20.3 | 424 | 653 | 0 | MER82 | MER2\_type | DNA |
| 45385270 | 45385407 | + | 19.7 | 2654 | 2796 | -3922 | L1MDa | L1 | LINE |
| 45385424 | 45386626 | + | 28.1 | 3799 | 5111 | -1426 | L1MDa | L1 | LINE |
| 45386628 | 45387022 | + | 8.6 | 1 | 448 | -2 | LTR7 | ERV1 | LTR |
| 45387025 | 45390059 | + | 17.2 | 1 | 2985 | -4728 | HERVH | ERV1 | LTR |
| 45390057 | 45390289 | + | 15.0 | 3504 | 3736 | -3977 | HERVH | ERV1 | LTR |
| 45390311 | 45390610 | + | 14.6 | 3995 | 4288 | -3425 | HERVH | ERV1 | LTR |
| 45390611 | 45390860 | + | 16.9 | 4221 | 4470 | -3243 | HERVH | ERV1 | LTR |
| 45390832 | 45391155 | + | 11.3 | 7365 | 7713 | 0 | HERVH | ERV1 | LTR |
| 45391156 | 45391539 | + | 8.9 | 1 | 448 | -2 | LTR7 | ERV1 | LTR |
| 45391541 | 45391882 | + | 26.5 | 5107 | 5456 | -1081 | L1MDa | L1 | LINE |

  

|  |  |  |  |  |  |  |  |  |  |
| --- | --- | --- | --- | --- | --- | --- | --- | --- | --- |
| chr10:45997244-46000638 | | | 3381 bp | | |  |  |  |  |
| Genome Start | Genome End | Strand | Div | Repeat Start | Repeat End | Repeat Left | Name | Family | Class |
| 45997244 | 45997489 | - | 26.3 | 5885 | 6128 | -38 | L1ME1 | L1 | LINE |
| 45997490 | 45997524 | - | 0.0 | 293 | 330 | -60 | MLT1B | MaLR | LTR |
| 45997530 | 45997945 | + | 16.8 | 1 | 432 | 0 | MSTB1 | MaLR | LTR |
| 45997946 | 45998085 | + | 19.3 | 1 | 140 | -1511 | MSTB1-int | MaLR | LTR |
| 45998082 | 45999054 | + | 22.8 | 637 | 1651 | 0 | MSTB1-int | MaLR | LTR |
| 45999055 | 45999427 | + | 16.9 | 1 | 432 | 0 | MSTB1 | MaLR | LTR |
| 45999429 | 45999736 | - | 19.0 | 1 | 291 | -99 | MLT1B | MaLR | LTR |
| 45999738 | 46000055 | - | 19.8 | 5583 | 5907 | -259 | L1ME1 | L1 | LINE |
| 46000067 | 46000638 | - | 25.3 | 4882 | 5463 | -703 | L1ME1 | L1 | LINE |

  

|  |  |  |  |  |  |  |  |  |  |
| --- | --- | --- | --- | --- | --- | --- | --- | --- | --- |
| chr10:48233793-48236745 | | | 2498 bp | | |  |  |  |  |
| Genome Start | Genome End | Strand | Div | Repeat Start | Repeat End | Repeat Left | Name | Family | Class |
| 48233793 | 48233920 | + | 28.8 | 6 | 131 | -278 | MLT1I | MaLR | LTR |
| 48234078 | 48234209 | + | 12.1 | 2 | 133 | 0 | FLAM\_C | Alu | SINE |
| 48234270 | 48234615 | - | 17.1 | 1 | 365 | 0 | MLT1A0 | MaLR | LTR |
| 48234622 | 48235290 | - | 21.7 | 978 | 1730 | -5 | MLT1A0-int | MaLR | LTR |
| 48235462 | 48236031 | - | 20.7 | 105 | 704 | -1031 | MLT1A0-int | MaLR | LTR |
| 48236032 | 48236133 | + | 23.8 | 6036 | 6145 | -19 | L1ME2 | L1 | LINE |
| 48236193 | 48236315 | - | 7.3 | 2 | 124 | -1611 | MLT1A0-int | MaLR | LTR |
| 48236318 | 48236674 | - | 18.9 | 1 | 365 | 0 | MLT1A0 | MaLR | LTR |
| 48236675 | 48236745 | + | 23.9 | 338 | 409 | 0 | MLT1I | MaLR | LTR |

  

|  |  |  |  |  |  |  |  |  |  |
| --- | --- | --- | --- | --- | --- | --- | --- | --- | --- |
| chr10:48384139-48387532 | | | 3380 bp | | |  |  |  |  |
| Genome Start | Genome End | Strand | Div | Repeat Start | Repeat End | Repeat Left | Name | Family | Class |
| 48384139 | 48384384 | - | 26.3 | 5885 | 6128 | -38 | L1ME1 | L1 | LINE |
| 48384385 | 48384419 | - | 0.0 | 293 | 330 | -60 | MLT1B | MaLR | LTR |
| 48384425 | 48384840 | + | 16.6 | 1 | 432 | 0 | MSTB1 | MaLR | LTR |
| 48384841 | 48384980 | + | 18.6 | 1 | 140 | -1511 | MSTB1-int | MaLR | LTR |
| 48384977 | 48385948 | + | 22.6 | 637 | 1651 | 0 | MSTB1-int | MaLR | LTR |
| 48385949 | 48386321 | + | 16.9 | 1 | 432 | 0 | MSTB1 | MaLR | LTR |
| 48386323 | 48386630 | - | 19.0 | 1 | 291 | -99 | MLT1B | MaLR | LTR |
| 48386632 | 48386949 | - | 19.8 | 5583 | 5907 | -259 | L1ME1 | L1 | LINE |
| 48386961 | 48387532 | - | 25.3 | 4882 | 5463 | -703 | L1ME1 | L1 | LINE |

  

|  |  |  |  |  |  |  |  |  |  |
| --- | --- | --- | --- | --- | --- | --- | --- | --- | --- |
| chr10:48954670-48958063 | | | 3380 bp | | |  |  |  |  |
| Genome Start | Genome End | Strand | Div | Repeat Start | Repeat End | Repeat Left | Name | Family | Class |
| 48954670 | 48954915 | - | 26.3 | 5885 | 6128 | -38 | L1ME1 | L1 | LINE |
| 48954916 | 48954950 | - | 0.0 | 293 | 330 | -60 | MLT1B | MaLR | LTR |
| 48954956 | 48955371 | + | 16.8 | 1 | 432 | 0 | MSTB1 | MaLR | LTR |
| 48955372 | 48955511 | + | 18.6 | 1 | 140 | -1511 | MSTB1-int | MaLR | LTR |
| 48955508 | 48956479 | + | 22.7 | 637 | 1651 | 0 | MSTB1-int | MaLR | LTR |
| 48956480 | 48956852 | + | 16.9 | 1 | 432 | 0 | MSTB1 | MaLR | LTR |
| 48956854 | 48957161 | - | 19.0 | 1 | 291 | -99 | MLT1B | MaLR | LTR |
| 48957163 | 48957480 | - | 19.8 | 5583 | 5907 | -259 | L1ME1 | L1 | LINE |
| 48957492 | 48958063 | - | 25.0 | 4882 | 5463 | -703 | L1ME1 | L1 | LINE |

  

|  |  |  |  |  |  |  |  |  |  |
| --- | --- | --- | --- | --- | --- | --- | --- | --- | --- |
| chr10:49157086-49160220 | | | 3126 bp | | |  |  |  |  |
| Genome Start | Genome End | Strand | Div | Repeat Start | Repeat End | Repeat Left | Name | Family | Class |
| 49157086 | 49157453 | - | 21.0 | 76 | 502 | -13 | MLT2B2 | ERVL | LTR |
| 49157454 | 49157808 | - | 12.5 | 1 | 364 | 0 | THE1B | MaLR | LTR |
| 49157815 | 49158869 | - | 8.9 | 533 | 1573 | -7 | THE1B-int | MaLR | LTR |
| 49158870 | 49159212 | - | 7.2 | 5836 | 6171 | -1 | L1PA8 | L1 | LINE |
| 49159213 | 49159784 | - | 8.9 | 1 | 532 | -1048 | THE1B-int | MaLR | LTR |
| 49159787 | 49160141 | - | 13.1 | 1 | 362 | -2 | THE1B | MaLR | LTR |
| 49160143 | 49160220 | - | 14.1 | 1 | 78 | -478 | MLT2B2 | ERVL | LTR |

  

|  |  |  |  |  |  |  |  |  |  |
| --- | --- | --- | --- | --- | --- | --- | --- | --- | --- |
| chr10:50339710-50344501 | | | 4661 bp | | |  |  |  |  |
| Genome Start | Genome End | Strand | Div | Repeat Start | Repeat End | Repeat Left | Name | Family | Class |
| 50339710 | 50341014 | + | 27.3 | 4220 | 5636 | -604 | L1MC4a | L1 | LINE |
| 50341103 | 50341444 | - | 14.9 | 20 | 236 | 0 | MER46B | MER2\_type | DNA |
| 50341482 | 50341644 | + | 21.7 | 5819 | 5978 | -1830 | L1MC4a | L1 | LINE |
| 50341645 | 50342005 | + | 15.8 | 3 | 362 | -2 | THE1B | MaLR | LTR |
| 50342011 | 50343587 | + | 29.6 | 3 | 1735 | 0 | MLT-int | MaLR | LTR |
| 50343589 | 50343945 | + | 13.4 | 2 | 364 | 0 | THE1B | MaLR | LTR |
| 50343946 | 50344501 | + | 21.7 | 5979 | 6580 | -1228 | L1MC4a | L1 | LINE |

  

|  |  |  |  |  |  |  |  |  |  |
| --- | --- | --- | --- | --- | --- | --- | --- | --- | --- |
| chr10:52881803-52887513 | | | 5691 bp | | |  |  |  |  |
| Genome Start | Genome End | Strand | Div | Repeat Start | Repeat End | Repeat Left | Name | Family | Class |
| 52881803 | 52882065 | - | 22.1 | 5910 | 6177 | -1 | L1MB8 | L1 | LINE |
| 52882065 | 52883520 | - | 13.4 | 2703 | 4145 | -2001 | L1PB2 | L1 | LINE |
| 52883519 | 52884269 | + | 11.5 | 4146 | 4898 | -1248 | L1PB2 | L1 | LINE |
| 52884271 | 52884639 | - | 12.8 | 1 | 364 | 0 | THE1B | MaLR | LTR |
| 52884644 | 52884796 | - | 13.7 | 1424 | 1576 | -4 | THE1B-int | MaLR | LTR |
| 52884800 | 52885739 | - | 9.7 | 1 | 899 | -681 | THE1B-int | MaLR | LTR |
| 52885740 | 52886110 | - | 13.0 | 1 | 364 | 0 | THE1B | MaLR | LTR |
| 52886111 | 52887344 | + | 11.1 | 4895 | 6146 | -5 | L1PB2 | L1 | LINE |
| 52887360 | 52887513 | - | 15.6 | 5751 | 5924 | -250 | L1MB8 | L1 | LINE |

  

|  |  |  |  |  |  |  |  |  |  |
| --- | --- | --- | --- | --- | --- | --- | --- | --- | --- |
| chr10:54009803-54022555 | | | 12671 bp | | |  |  |  |  |
| Genome Start | Genome End | Strand | Div | Repeat Start | Repeat End | Repeat Left | Name | Family | Class |
| 54009803 | 54010330 | + | 15.0 | 1 | 508 | -1910 | Tigger1 | MER2\_type | DNA |
| 54010331 | 54010695 | - | 13.2 | 1 | 364 | 0 | THE1B | MaLR | LTR |
| 54010696 | 54012267 | - | 10.3 | 2 | 1580 | 0 | THE1B-int | MaLR | LTR |
| 54012269 | 54012623 | - | 15.8 | 1 | 364 | 0 | THE1B | MaLR | LTR |
| 54012624 | 54014365 | + | 14.2 | 504 | 2265 | -153 | Tigger1 | MER2\_type | DNA |
| 54014444 | 54020797 | - | 12.9 | 1044 | 7509 | -215 | L1MA3 | L1 | LINE |
| 54020798 | 54021407 | + | 17.2 | 1 | 612 | 0 | LTR9 | ERV1 | LTR |
| 54021408 | 54022437 | - | 14.3 | 13 | 1043 | -6522 | L1MA3 | L1 | LINE |
| 54022441 | 54022555 | + | 18.3 | 2304 | 2418 | 0 | Tigger1 | MER2\_type | DNA |

  

|  |  |  |  |  |  |  |  |  |  |
| --- | --- | --- | --- | --- | --- | --- | --- | --- | --- |
| chr10:54296828-54299787 | | | 2958 bp | | |  |  |  |  |
| Genome Start | Genome End | Strand | Div | Repeat Start | Repeat End | Repeat Left | Name | Family | Class |
| 54296828 | 54296899 | - | 19.2 | 2677 | 2752 | 0 | Charlie9 | MER1\_type | DNA |
| 54296900 | 54297323 | - | 21.3 | 50 | 467 | 0 | MLT1C | MaLR | LTR |
| 54297324 | 54297696 | + | 14.2 | 1 | 381 | 0 | THE1D | MaLR | LTR |
| 54297699 | 54299200 | + | 30.6 | 3 | 1735 | 0 | MLT-int | MaLR | LTR |
| 54299201 | 54299573 | + | 12.9 | 1 | 379 | -2 | THE1D | MaLR | LTR |
| 54299574 | 54299620 | - | 21.3 | 1 | 49 | -418 | MLT1C | MaLR | LTR |
| 54299621 | 54299787 | - | 24.4 | 1 | 2590 | -69 | Charlie9 | MER1\_type | DNA |

  

|  |  |  |  |  |  |  |  |  |  |
| --- | --- | --- | --- | --- | --- | --- | --- | --- | --- |
| chr10:54453860-54462918 | | | 8760 bp | | |  |  |  |  |
| Genome Start | Genome End | Strand | Div | Repeat Start | Repeat End | Repeat Left | Name | Family | Class |
| 54453860 | 54454000 | + | 21.1 | 5149 | 5280 | -866 | L1MC3 | L1 | LINE |
| 54454001 | 54454293 | + | 8.2 | 13 | 304 | -7 | AluY | Alu | SINE |
| 54454294 | 54454447 | + | 18.9 | 5281 | 5453 | -2332 | L1MC3 | L1 | LINE |
| 54454453 | 54454506 | + | 22.1 | 5621 | 5668 | -676 | L1MC3 | L1 | LINE |
| 54454507 | 54454785 | + | 11.5 | 1 | 279 | -32 | AluY | Alu | SINE |
| 54454786 | 54454939 | + | 22.1 | 5660 | 5787 | -1998 | L1MC3 | L1 | LINE |
| 54454940 | 54455654 | - | 9.8 | 1 | 722 | 0 | LTR30 | ERV1 | LTR |
| 54455655 | 54456118 | - | 10.4 | 7796 | 8257 | 0 | HERV30 | ERV1 | LTR |
| 54456121 | 54459709 | - | 9.4 | 1 | 3613 | -4644 | HERV30 | ERV1 | LTR |
| 54459710 | 54460427 | - | 10.2 | 1 | 722 | 0 | LTR30 | ERV1 | LTR |
| 54460428 | 54460790 | + | 21.2 | 5784 | 6158 | -1627 | L1MC3 | L1 | LINE |
| 54460791 | 54461090 | + | 14.7 | 1 | 300 | -12 | AluSx | Alu | SINE |
| 54461091 | 54461330 | + | 21.2 | 6159 | 6405 | -1380 | L1MC3 | L1 | LINE |
| 54461597 | 54462195 | + | 20.7 | 6404 | 7015 | -770 | L1MC3 | L1 | LINE |
| 54462222 | 54462918 | + | 20.7 | 7016 | 7780 | -5 | L1MC3 | L1 | LINE |

  

|  |  |  |  |  |  |  |  |  |  |
| --- | --- | --- | --- | --- | --- | --- | --- | --- | --- |
| chr10:54681844-54684580 | | | 2734 bp | | |  |  |  |  |
| Genome Start | Genome End | Strand | Div | Repeat Start | Repeat End | Repeat Left | Name | Family | Class |
| 54681844 | 54682159 | + | 17.4 | 11 | 325 | -40 | MLT1A0 | MaLR | LTR |
| 54682160 | 54682550 | + | 16.7 | 1 | 428 | 0 | MSTA | MaLR | LTR |
| 54682553 | 54684157 | + | 29.8 | 2 | 1735 | 0 | MLT1A0-int | MaLR | LTR |
| 54684159 | 54684536 | + | 19.3 | 2 | 427 | -1 | MSTA | MaLR | LTR |
| 54684537 | 54684580 | + | 17.4 | 326 | 363 | -2 | MLT1A0 | MaLR | LTR |

  

|  |  |  |  |  |  |  |  |  |  |
| --- | --- | --- | --- | --- | --- | --- | --- | --- | --- |
| chr10:55485877-55489005 | | | 3085 bp | | |  |  |  |  |
| Genome Start | Genome End | Strand | Div | Repeat Start | Repeat End | Repeat Left | Name | Family | Class |
| 55485877 | 55486067 | - | 14.7 | 6097 | 6298 | -5 | L1MA4A | L1 | LINE |
| 55486071 | 55486426 | - | 12.8 | 1 | 364 | 0 | THE1B | MaLR | LTR |
| 55486429 | 55488018 | - | 12.2 | 1 | 1578 | -2 | THE1B-int | MaLR | LTR |
| 55488019 | 55488375 | - | 13.7 | 2 | 364 | 0 | THE1B | MaLR | LTR |
| 55488415 | 55488448 | - | 12.9 | 6061 | 6095 | -208 | L1MA4A | L1 | LINE |
| 55488449 | 55488759 | - | 12.6 | 1 | 311 | -2 | AluSp | Alu | SINE |
| 55488760 | 55489005 | - | 12.9 | 5821 | 6060 | -243 | L1MA4A | L1 | LINE |

  

|  |  |  |  |  |  |  |  |  |  |
| --- | --- | --- | --- | --- | --- | --- | --- | --- | --- |
| chr10:56351090-56359182 | | | 8079 bp | | |  |  |  |  |
| Genome Start | Genome End | Strand | Div | Repeat Start | Repeat End | Repeat Left | Name | Family | Class |
| 56351090 | 56351497 | - | 15.1 | 6376 | 6781 | -2 | L1PA13 | L1 | LINE |
| 56351499 | 56351890 | - | 19.0 | 1 | 428 | 0 | MSTA | MaLR | LTR |
| 56351891 | 56352211 | - | 14.9 | 1263 | 1580 | 0 | MSTA-int | MaLR | LTR |
| 56352218 | 56352389 | + | 12.3 | 1480 | 1651 | 0 | MSTA-int | MaLR | LTR |
| 56352390 | 56352444 | + | 10.9 | 1 | 56 | -372 | MSTA | MaLR | LTR |
| 56352447 | 56353727 | - | 18.8 | 3 | 1254 | -326 | MSTA-int | MaLR | LTR |
| 56353730 | 56354105 | - | 17.7 | 1 | 428 | 0 | MSTA | MaLR | LTR |
| 56354109 | 56359182 | - | 15.3 | 1221 | 6376 | -407 | L1PA13 | L1 | LINE |

  

|  |  |  |  |  |  |  |  |  |  |
| --- | --- | --- | --- | --- | --- | --- | --- | --- | --- |
| chr10:56748588-56756644 | | | 7752 bp | | |  |  |  |  |
| Genome Start | Genome End | Strand | Div | Repeat Start | Repeat End | Repeat Left | Name | Family | Class |
| 56748588 | 56748816 | + | 37.3 | 401 | 616 | -5503 | L1MEd | L1 | LINE |
| 56748849 | 56749041 | + | 37.3 | 617 | 803 | -5316 | L1MEd | L1 | LINE |
| 56749207 | 56751779 | + | 21.3 | 3 | 2765 | -4020 | L1PA13 | L1 | LINE |
| 56751780 | 56752135 | + | 16.3 | 1 | 364 | 0 | THE1B | MaLR | LTR |
| 56752138 | 56753641 | + | 30.0 | 2 | 1735 | 0 | MLT1A0-int | MaLR | LTR |
| 56753642 | 56754009 | + | 18.3 | 1 | 364 | 0 | THE1B | MaLR | LTR |
| 56754010 | 56754527 | + | 14.8 | 2764 | 3280 | -3486 | L1PA15 | L1 | LINE |
| 56754529 | 56756327 | + | 15.0 | 4802 | 6785 | 0 | L1PA15 | L1 | LINE |
| 56756433 | 56756644 | + | 34.6 | 1026 | 1252 | -4867 | L1MEd | L1 | LINE |

  

|  |  |  |  |  |  |  |  |  |  |
| --- | --- | --- | --- | --- | --- | --- | --- | --- | --- |
| chr10:57335716-57338668 | | | 2805 bp | | |  |  |  |  |
| Genome Start | Genome End | Strand | Div | Repeat Start | Repeat End | Repeat Left | Name | Family | Class |
| 57335716 | 57335888 | - | 29.4 | 611 | 771 | -12 | MER20B | MER1\_type | DNA |
| 57336000 | 57336359 | - | 15.1 | 1 | 381 | 0 | THE1D | MaLR | LTR |
| 57336360 | 57337946 | - | 31.8 | 2 | 1735 | 0 | MLT-int | MaLR | LTR |
| 57337950 | 57338115 | - | 15.2 | 212 | 379 | -2 | THE1D | MaLR | LTR |
| 57338150 | 57338309 | - | 15.2 | 1 | 211 | -170 | THE1D | MaLR | LTR |
| 57338310 | 57338668 | - | 31.3 | 64 | 452 | -331 | MER20B | MER1\_type | DNA |

  

|  |  |  |  |  |  |  |  |  |  |
| --- | --- | --- | --- | --- | --- | --- | --- | --- | --- |
| chr10:57593123-57595857 | | | 2732 bp | | |  |  |  |  |
| Genome Start | Genome End | Strand | Div | Repeat Start | Repeat End | Repeat Left | Name | Family | Class |
| 57593123 | 57593308 | - | 15.8 | 157 | 339 | 0 | MER1B | MER1\_type | DNA |
| 57593309 | 57593671 | + | 11.5 | 1 | 381 | 0 | THE1D | MaLR | LTR |
| 57593674 | 57595266 | + | 31.2 | 2 | 1735 | 0 | MLT-int | MaLR | LTR |
| 57595268 | 57595701 | + | 12.1 | 2 | 381 | 0 | THE1D | MaLR | LTR |
| 57595702 | 57595857 | - | 15.8 | 2 | 156 | -141 | MER1B | MER1\_type | DNA |

  

|  |  |  |  |  |  |  |  |  |  |
| --- | --- | --- | --- | --- | --- | --- | --- | --- | --- |
| chr10:58192172-58204870 | | | 12499 bp | | |  |  |  |  |
| Genome Start | Genome End | Strand | Div | Repeat Start | Repeat End | Repeat Left | Name | Family | Class |
| 58192172 | 58192461 | + | 31.3 | 282 | 578 | -5964 | L1M4 | L1 | LINE |
| 58192463 | 58193022 | + | 22.3 | 1 | 1273 | -141 | LTR27 | ERV1 | LTR |
| 58193017 | 58193176 | + | 26.3 | 426 | 594 | 0 | LTR27B | ERV1 | LTR |
| 58193238 | 58193909 | + | 24.6 | 621 | 1319 | -5223 | L1M4 | L1 | LINE |
| 58193916 | 58194021 | + | 27.4 | 1606 | 1713 | -4933 | L1M4 | L1 | LINE |
| 58194023 | 58194485 | - | 14.0 | 132 | 900 | 0 | MER4B | ERV1 | LTR |
| 58194486 | 58194932 | - | 19.7 | 3 | 461 | 0 | LOR1b | ERV1 | LTR |
| 58194935 | 58195025 | - | 16.5 | 8030 | 8120 | 0 | LOR1b-int | ERV1 | LTR |
| 58195025 | 58195281 | - | 14.5 | 5809 | 6064 | -2056 | LOR1b-int | ERV1 | LTR |
| 58195276 | 58198108 | - | 13.1 | 2629 | 5528 | -2592 | LOR1b-int | ERV1 | LTR |
| 58198141 | 58199381 | - | 13.1 | 1340 | 2628 | -5492 | LOR1b-int | ERV1 | LTR |
| 58199382 | 58199672 | - | 14.1 | 1 | 291 | -21 | AluSx | Alu | SINE |
| 58199673 | 58200204 | - | 13.1 | 851 | 1339 | -6781 | LOR1b-int | ERV1 | LTR |
| 58200232 | 58200836 | - | 13.1 | 207 | 850 | -7270 | LOR1b-int | ERV1 | LTR |
| 58200849 | 58201206 | - | 18.8 | 1 | 371 | -7749 | LOR1b-int | ERV1 | LTR |
| 58201207 | 58201662 | - | 20.5 | 1 | 461 | 0 | LOR1b | ERV1 | LTR |
| 58201664 | 58201776 | - | 15.6 | 24 | 134 | -476 | MER4B | ERV1 | LTR |
| 58201789 | 58202846 | + | 22.3 | 1714 | 2900 | -3744 | L1M4 | L1 | LINE |
| 58202907 | 58203460 | - | 33.0 | 3 | 564 | -6299 | L1PBa | L1 | LINE |
| 58203463 | 58203771 | + | 19.5 | 2878 | 3202 | -3442 | L1M4 | L1 | LINE |
| 58203768 | 58204870 | + | 22.3 | 4832 | 5974 | -702 | L1M4 | L1 | LINE |

  

|  |  |  |  |  |  |  |  |  |  |
| --- | --- | --- | --- | --- | --- | --- | --- | --- | --- |
| chr10:58594068-58600856 | | | 6783 bp | | |  |  |  |  |
| Genome Start | Genome End | Strand | Div | Repeat Start | Repeat End | Repeat Left | Name | Family | Class |
| 58594068 | 58594145 | + | 19.0 | 8 | 82 | -7252 | L1P4 | L1 | LINE |
| 58594146 | 58594444 | - | 12.4 | 3 | 301 | -12 | AluSq | Alu | SINE |
| 58594445 | 58594607 | + | 19.0 | 83 | 237 | -7097 | L1P4 | L1 | LINE |
| 58594610 | 58594689 | - | 13.8 | 1 | 80 | -348 | MSTA | MaLR | LTR |
| 58594690 | 58596301 | - | 30.2 | 2 | 1735 | 0 | MLT-int | MaLR | LTR |
| 58596306 | 58596680 | - | 15.2 | 1 | 427 | -1 | MSTA | MaLR | LTR |
| 58596681 | 58600064 | + | 16.9 | 2993 | 6468 | -843 | L1P4 | L1 | LINE |
| 58600065 | 58600380 | + | 10.9 | 1 | 317 | 0 | AluSp | Alu | SINE |
| 58600381 | 58600856 | + | 12.0 | 6469 | 6959 | -374 | L1P4 | L1 | LINE |

  

|  |  |  |  |  |  |  |  |  |  |
| --- | --- | --- | --- | --- | --- | --- | --- | --- | --- |
| chr10:59261948-59272984 | | | 10995 bp | | |  |  |  |  |
| Genome Start | Genome End | Strand | Div | Repeat Start | Repeat End | Repeat Left | Name | Family | Class |
| 59261948 | 59262019 | - | 29.2 | 188 | 259 | -3 | MIR | MIR | SINE |
| 59262033 | 59262129 | - | 24.7 | 164 | 262 | 0 | MIR | MIR | SINE |
| 59262131 | 59262927 | + | 8.0 | 1 | 826 | 0 | LTR12 | ERV1 | LTR |
| 59262941 | 59271326 | + | 5.3 | 40 | 8436 | 0 | HERV9 | ERV1 | LTR |
| 59271327 | 59271970 | + | 7.8 | 1 | 694 | -132 | LTR12 | ERV1 | LTR |
| 59271975 | 59272109 | - | 28.9 | 20 | 156 | -106 | MIR | MIR | SINE |
| 59272121 | 59272173 | - | 27.9 | 103 | 152 | -110 | MIR | MIR | SINE |
| 59272174 | 59272506 | + | 18.3 | 2 | 336 | 0 | MER2B | MER2\_type | DNA |
| 59272507 | 59272539 | - | 27.9 | 74 | 102 | -160 | MIR | MIR | SINE |
| 59272540 | 59272948 | - | 16.8 | 1 | 467 | 0 | MLT1C | MaLR | LTR |
| 59272949 | 59272984 | - | 27.9 | 38 | 73 | -189 | MIR | MIR | SINE |

  

|  |  |  |  |  |  |  |  |  |  |
| --- | --- | --- | --- | --- | --- | --- | --- | --- | --- |
| chr10:59853926-59860937 | | | 6713 bp | | |  |  |  |  |
| Genome Start | Genome End | Strand | Div | Repeat Start | Repeat End | Repeat Left | Name | Family | Class |
| 59853926 | 59854701 | - | 27.3 | 5187 | 5974 | -1834 | L1MC4a | L1 | LINE |
| 59854749 | 59854845 | + | 22.7 | 38 | 134 | -9 | FLAM\_C | Alu | SINE |
| 59855000 | 59855161 | + | 21.6 | 1 | 155 | -21 | FRAM | Alu | SINE |
| 59855191 | 59855543 | - | 8.8 | 1 | 355 | 0 | THE1A | MaLR | LTR |
| 59855544 | 59857082 | - | 29.8 | 2 | 1735 | 0 | MLT1D-int | MaLR | LTR |
| 59857085 | 59857437 | - | 7.7 | 2 | 355 | 0 | THE1A | MaLR | LTR |
| 59857483 | 59857758 | - | 31.9 | 4826 | 5086 | -1060 | L1MC4a | L1 | LINE |
| 59857767 | 59858059 | - | 14.4 | 5386 | 5681 | -610 | L1M2 | L1 | LINE |
| 59858063 | 59859038 | - | 5.7 | 5164 | 6152 | -2 | L1PA5 | L1 | LINE |
| 59859040 | 59859213 | - | 25.1 | 4645 | 4814 | -1332 | L1MC4a | L1 | LINE |
| 59859215 | 59860362 | - | 16.0 | 4971 | 6156 | 0 | L1PB4 | L1 | LINE |
| 59860372 | 59860633 | - | 29.9 | 4381 | 4646 | -1500 | L1MC4a | L1 | LINE |
| 59860634 | 59860922 | - | 19.0 | 1 | 293 | -19 | AluJo | Alu | SINE |
| 59860923 | 59860937 | - | 29.9 | 4366 | 4380 | -1766 | L1MC4a | L1 | LINE |

  

|  |  |  |  |  |  |  |  |  |  |
| --- | --- | --- | --- | --- | --- | --- | --- | --- | --- |
| chr10:60157543-60162868 | | | 5254 bp | | |  |  |  |  |
| Genome Start | Genome End | Strand | Div | Repeat Start | Repeat End | Repeat Left | Name | Family | Class |
| 60157543 | 60159295 | - | 13.6 | 653 | 2418 | 0 | Tigger1 | MER2\_type | DNA |
| 60159299 | 60159709 | - | 16.9 | 2 | 421 | -7 | MSTA | MaLR | LTR |
| 60159711 | 60161264 | - | 30.0 | 2 | 1735 | 0 | MLT-int | MaLR | LTR |
| 60161267 | 60161715 | - | 17.0 | 1 | 428 | 0 | MSTA | MaLR | LTR |
| 60161716 | 60162141 | - | 14.6 | 337 | 657 | -1761 | Tigger1 | MER2\_type | DNA |
| 60162208 | 60162264 | - | 14.6 | 285 | 336 | -2082 | Tigger1 | MER2\_type | DNA |
| 60162265 | 60162548 | + | 14.8 | 1 | 283 | -29 | AluJb | Alu | SINE |
| 60162549 | 60162868 | - | 14.6 | 1 | 284 | -2134 | Tigger1 | MER2\_type | DNA |

  

|  |  |  |  |  |  |  |  |  |  |
| --- | --- | --- | --- | --- | --- | --- | --- | --- | --- |
| chr10:61205964-61209293 | | | 2870 bp | | |  |  |  |  |
| Genome Start | Genome End | Strand | Div | Repeat Start | Repeat End | Repeat Left | Name | Family | Class |
| 61205964 | 61206174 | - | 27.9 | 7707 | 7917 | -44 | L1MC5 | L1 | LINE |
| 61206359 | 61206699 | - | 9.1 | 1 | 355 | 0 | THE1A | MaLR | LTR |
| 61206700 | 61208282 | - | 30.8 | 3 | 1735 | 0 | MLT-int | MaLR | LTR |
| 61208286 | 61208639 | - | 9.0 | 1 | 355 | 0 | THE1A | MaLR | LTR |
| 61208892 | 61209187 | + | 19.6 | 1 | 297 | -15 | AluJb | Alu | SINE |
| 61209209 | 61209293 | - | 19.8 | 7609 | 7692 | -269 | L1MC5 | L1 | LINE |

  

|  |  |  |  |  |  |  |  |  |  |
| --- | --- | --- | --- | --- | --- | --- | --- | --- | --- |
| chr10:65672762-65681540 | | | 8438 bp | | |  |  |  |  |
| Genome Start | Genome End | Strand | Div | Repeat Start | Repeat End | Repeat Left | Name | Family | Class |
| 65672762 | 65673708 | - | 25.1 | 5616 | 6662 | -360 | L1M5 | L1 | LINE |
| 65673709 | 65674009 | - | 12.6 | 1 | 302 | -8 | AluSg | Alu | SINE |
| 65674010 | 65674107 | - | 25.3 | 5524 | 5615 | -1391 | L1M5 | L1 | LINE |
| 65674108 | 65674416 | - | 8.7 | 1 | 309 | 0 | AluSc | Alu | SINE |
| 65674417 | 65674741 | - | 25.3 | 5184 | 5523 | -1483 | L1M5 | L1 | LINE |
| 65674742 | 65674828 | + | 21.5 | 1 | 79 | -1 | MADE2 | Mariner | DNA |
| 65674829 | 65675016 | - | 25.3 | 4987 | 5183 | -1823 | L1M5 | L1 | LINE |
| 65675017 | 65675131 | - | 20.4 | 1 | 133 | 0 | FLAM\_A | Alu | SINE |
| 65675132 | 65676311 | - | 25.3 | 3725 | 4984 | -2022 | L1M5 | L1 | LINE |
| 65676344 | 65676707 | - | 16.8 | 5925 | 6285 | -6 | L1MA7 | L1 | LINE |
| 65676711 | 65677203 | + | 15.9 | 5402 | 5914 | -377 | L1MA7 | L1 | LINE |
| 65677240 | 65677941 | - | 28.4 | 2976 | 3666 | -3340 | L1M5 | L1 | LINE |
| 65677963 | 65678322 | - | 10.8 | 1 | 364 | 0 | THE1B | MaLR | LTR |
| 65678323 | 65679852 | - | 10.8 | 5 | 1580 | 0 | THE1B-int | MaLR | LTR |
| 65679874 | 65680227 | - | 14.2 | 1 | 363 | -1 | THE1B | MaLR | LTR |
| 65680317 | 65680507 | - | 31.9 | 1850 | 2041 | -4365 | L1MEe | L1 | LINE |
| 65680564 | 65681026 | - | 27.6 | 1276 | 1810 | -4590 | L1MEe | L1 | LINE |
| 65681076 | 65681169 | - | 25.4 | 1687 | 1776 | -5558 | L1M5 | L1 | LINE |
| 65681204 | 65681483 | - | 14.5 | 7 | 286 | -26 | AluSx | Alu | SINE |
| 65681484 | 65681540 | - | 25.4 | 1630 | 1686 | -5648 | L1M5 | L1 | LINE |

  

|  |  |  |  |  |  |  |  |  |  |
| --- | --- | --- | --- | --- | --- | --- | --- | --- | --- |
| chr10:65813690-65817092 | | | 3246 bp | | |  |  |  |  |
| Genome Start | Genome End | Strand | Div | Repeat Start | Repeat End | Repeat Left | Name | Family | Class |
| 65813690 | 65813809 | + | 25.6 | 4609 | 4736 | -1410 | L1ME3 | L1 | LINE |
| 65813951 | 65814334 | - | 13.8 | 1 | 416 | -12 | MSTA | MaLR | LTR |
| 65814335 | 65815972 | - | 28.1 | 3 | 1735 | 0 | MLT-int | MaLR | LTR |
| 65815976 | 65816385 | - | 18.1 | 1 | 428 | 0 | MSTA | MaLR | LTR |
| 65816399 | 65816634 | + | 29.4 | 4909 | 5155 | -991 | L1ME3 | L1 | LINE |
| 65816635 | 65816946 | - | 19.9 | 1 | 303 | -9 | AluJb | Alu | SINE |
| 65816947 | 65817092 | + | 29.4 | 5156 | 5300 | -846 | L1ME3 | L1 | LINE |

  

|  |  |  |  |  |  |  |  |  |  |
| --- | --- | --- | --- | --- | --- | --- | --- | --- | --- |
| chr10:66507061-66511495 | | | 4354 bp | | |  |  |  |  |
| Genome Start | Genome End | Strand | Div | Repeat Start | Repeat End | Repeat Left | Name | Family | Class |
| 66507061 | 66507851 | - | 30.6 | 5268 | 6104 | -58 | L1ME3 | L1 | LINE |
| 66507873 | 66508075 | - | 5.9 | 5953 | 6155 | 0 | L1PA4 | L1 | LINE |
| 66508133 | 66508488 | + | 16.9 | 1 | 381 | 0 | THE1D | MaLR | LTR |
| 66508489 | 66510027 | + | 13.0 | 1 | 1580 | 0 | THE1D-int | MaLR | LTR |
| 66510028 | 66510406 | + | 13.0 | 1 | 381 | 0 | THE1D | MaLR | LTR |
| 66510410 | 66511495 | - | 26.9 | 4074 | 5190 | -956 | L1ME3 | L1 | LINE |

  

|  |  |  |  |  |  |  |  |  |  |
| --- | --- | --- | --- | --- | --- | --- | --- | --- | --- |
| chr10:66705077-66710484 | | | 5020 bp | | |  |  |  |  |
| Genome Start | Genome End | Strand | Div | Repeat Start | Repeat End | Repeat Left | Name | Family | Class |
| 66705077 | 66705156 | + | 18.2 | 91 | 178 | -2766 | BLACKJACK | AcHobo | DNA |
| 66705252 | 66705372 | + | 29.8 | 438 | 558 | -2386 | BLACKJACK | AcHobo | DNA |
| 66705460 | 66705546 | + | 20.7 | 734 | 822 | -2122 | BLACKJACK | AcHobo | DNA |
| 66705556 | 66705695 | + | 27.1 | 1060 | 1202 | -1742 | BLACKJACK | AcHobo | DNA |
| 66705773 | 66706062 | - | 20.5 | 1 | 288 | -268 | MLT2B4 | ERVL | LTR |
| 66706063 | 66706489 | + | 25.8 | 1222 | 1683 | -1261 | BLACKJACK | AcHobo | DNA |
| 66706505 | 66707088 | + | 16.8 | 1 | 637 | -34 | LTR51 | ERV1 | LTR |
| 66707097 | 66707168 | + | 18.9 | 1797 | 1865 | -1079 | BLACKJACK | AcHobo | DNA |
| 66707264 | 66707821 | + | 25.6 | 1907 | 2527 | -417 | BLACKJACK | AcHobo | DNA |
| 66707822 | 66708179 | + | 14.7 | 1 | 381 | 0 | THE1D | MaLR | LTR |
| 66708182 | 66709804 | + | 29.7 | 2 | 1735 | 0 | MLT-int | MaLR | LTR |
| 66709805 | 66710173 | + | 12.1 | 1 | 381 | 0 | THE1D | MaLR | LTR |
| 66710174 | 66710484 | + | 25.6 | 2528 | 2857 | -87 | BLACKJACK | AcHobo | DNA |

  

|  |  |  |  |  |  |  |  |  |  |
| --- | --- | --- | --- | --- | --- | --- | --- | --- | --- |
| chr10:67551777-67565203 | | | 12984 bp | | |  |  |  |  |
| Genome Start | Genome End | Strand | Div | Repeat Start | Repeat End | Repeat Left | Name | Family | Class |
| 67551777 | 67552355 | + | 24.6 | 854 | 1477 | -5031 | L1MC4a | L1 | LINE |
| 67552403 | 67552757 | - | 14.1 | 3 | 352 | -12 | THE1B | MaLR | LTR |
| 67552760 | 67554330 | - | 30.9 | 2 | 1735 | 0 | MLT-int | MaLR | LTR |
| 67554333 | 67554703 | - | 13.8 | 1 | 364 | 0 | THE1B | MaLR | LTR |
| 67554707 | 67555399 | + | 21.6 | 1545 | 2465 | -4037 | L1MC4a | L1 | LINE |
| 67555400 | 67556750 | + | 21.5 | 5781 | 7201 | -584 | L1MC3 | L1 | LINE |
| 67556751 | 67557057 | + | 5.3 | 1 | 303 | -8 | AluY | Alu | SINE |
| 67557058 | 67557578 | + | 21.5 | 7202 | 7760 | -25 | L1MC3 | L1 | LINE |
| 67557579 | 67557853 | + | 9.8 | 21 | 298 | -12 | AluSg | Alu | SINE |
| 67558086 | 67558720 | + | 15.8 | 5671 | 6333 | 0 | L1MC1 | L1 | LINE |
| 67558747 | 67559051 | + | 10.2 | 1 | 306 | -7 | AluSp | Alu | SINE |
| 67559065 | 67559935 | + | 21.5 | 237 | 1082 | -5703 | L1PA13 | L1 | LINE |
| 67559935 | 67560326 | + | 12.4 | 3120 | 3514 | -2807 | L1MC4a | L1 | LINE |
| 67560327 | 67560648 | + | 10.4 | 1 | 309 | 0 | AluSc | Alu | SINE |
| 67560649 | 67561195 | + | 12.4 | 3515 | 4031 | -2290 | L1MC4a | L1 | LINE |
| 67561195 | 67562822 | + | 9.3 | 4498 | 6153 | -7 | L1PREC2 | L1 | LINE |
| 67562856 | 67562996 | + | 30.6 | 4672 | 4818 | -1503 | L1MC4a | L1 | LINE |
| 67562997 | 67563306 | + | 13.9 | 1 | 312 | 0 | AluSx | Alu | SINE |
| 67563307 | 67563940 | + | 30.6 | 4819 | 5475 | -846 | L1MC4a | L1 | LINE |
| 67563974 | 67564155 | + | 27.0 | 5597 | 5774 | -522 | L1MC4a | L1 | LINE |
| 67564210 | 67565203 | + | 30.7 | 5967 | 7110 | -873 | L1MC4a | L1 | LINE |

  

|  |  |  |  |  |  |  |  |  |  |
| --- | --- | --- | --- | --- | --- | --- | --- | --- | --- |
| chr10:68826228-68829031 | | | 2792 bp | | |  |  |  |  |
| Genome Start | Genome End | Strand | Div | Repeat Start | Repeat End | Repeat Left | Name | Family | Class |
| 68826228 | 68826455 | - | 15.2 | 2462 | 2718 | 0 | Tigger2 | MER2\_type | DNA |
| 68826456 | 68826818 | + | 7.8 | 1 | 375 | 0 | THE1C | MaLR | LTR |
| 68826823 | 68828403 | + | 29.5 | 4 | 1735 | 0 | MLT1A1-int | MaLR | LTR |
| 68828404 | 68828782 | + | 8.6 | 1 | 375 | 0 | THE1C | MaLR | LTR |
| 68828783 | 68828965 | - | 15.2 | 1543 | 1724 | -257 | Tigger2 | MER2\_type | DNA |
| 68828974 | 68829031 | - | 15.5 | 2 | 59 | -2659 | Tigger2 | MER2\_type | DNA |

  

|  |  |  |  |  |  |  |  |  |  |
| --- | --- | --- | --- | --- | --- | --- | --- | --- | --- |
| chr10:72535399-72545337 | | | 9929 bp | | |  |  |  |  |
| Genome Start | Genome End | Strand | Div | Repeat Start | Repeat End | Repeat Left | Name | Family | Class |
| 72535399 | 72535786 | + | 20.7 | 3030 | 3423 | -2796 | L1MEc | L1 | LINE |
| 72535787 | 72535928 | - | 20.4 | 6153 | 6294 | -6 | L1MA4 | L1 | LINE |
| 72535929 | 72536280 | + | 7.7 | 1 | 355 | 0 | THE1A | MaLR | LTR |
| 72536282 | 72537845 | + | 8.9 | 2 | 1580 | 0 | THE1A-int | MaLR | LTR |
| 72537846 | 72538183 | + | 11.9 | 1 | 338 | -17 | THE1A | MaLR | LTR |
| 72538180 | 72538456 | - | 20.4 | 1 | 277 | -134 | LTR57 | ERVL | LTR |
| 72538468 | 72538711 | - | 13.6 | 1 | 296 | -16 | AluJb | Alu | SINE |
| 72538712 | 72540344 | - | 14.4 | 3564 | 5206 | -2356 | L1M1 | L1 | LINE |
| 72540345 | 72540626 | - | 17.4 | 1 | 297 | -15 | AluJb | Alu | SINE |
| 72540627 | 72541339 | - | 21.1 | 2878 | 3564 | -4001 | L1M1 | L1 | LINE |
| 72541340 | 72541653 | - | 11.4 | 1 | 281 | -29 | AluSg | Alu | SINE |
| 72541654 | 72543252 | - | 17.5 | 10 | 1640 | -4688 | L1M3f | L1 | LINE |
| 72543255 | 72545337 | + | 24.7 | 3413 | 5557 | -682 | L1MEc | L1 | LINE |

  

|  |  |  |  |  |  |  |  |  |  |
| --- | --- | --- | --- | --- | --- | --- | --- | --- | --- |
| chr10:79151999-79156057 | | | 4048 bp | | |  |  |  |  |
| Genome Start | Genome End | Strand | Div | Repeat Start | Repeat End | Repeat Left | Name | Family | Class |
| 79151999 | 79152070 | + | 27.4 | 1902 | 1969 | -5365 | L1MEd | L1 | LINE |
| 79152071 | 79152371 | + | 11.3 | 1 | 302 | -8 | AluSg | Alu | SINE |
| 79152372 | 79152613 | + | 23.4 | 1970 | 2382 | -4644 | L1MEd | L1 | LINE |
| 79152614 | 79153200 | - | 8.2 | 4 | 608 | 0 | LTR14B | ERVK | LTR |
| 79153201 | 79153212 | + | 27.4 | 2228 | 2239 | -5095 | L1MEd | L1 | LINE |
| 79153219 | 79153612 | + | 16.6 | 3 | 425 | -3 | MSTA | MaLR | LTR |
| 79153618 | 79155232 | + | 28.0 | 2 | 1735 | 0 | MLT-int | MaLR | LTR |
| 79155233 | 79155409 | + | 15.4 | 1 | 201 | -227 | MSTA | MaLR | LTR |
| 79155410 | 79155700 | + | 8.6 | 1 | 294 | -19 | AluSq | Alu | SINE |
| 79155701 | 79155939 | + | 15.4 | 202 | 428 | 0 | MSTA | MaLR | LTR |
| 79155940 | 79156057 | + | 22.4 | 2390 | 2492 | -4534 | L1MEd | L1 | LINE |

  

|  |  |  |  |  |  |  |  |  |  |
| --- | --- | --- | --- | --- | --- | --- | --- | --- | --- |
| chr10:79670746-79674743 | | | 3769 bp | | |  |  |  |  |
| Genome Start | Genome End | Strand | Div | Repeat Start | Repeat End | Repeat Left | Name | Family | Class |
| 79670746 | 79671745 | - | 19.2 | 6714 | 7902 | -140 | L1MC4 | L1 | LINE |
| 79671808 | 79672162 | + | 9.6 | 1 | 355 | 0 | THE1A | MaLR | LTR |
| 79672165 | 79673739 | + | 30.6 | 2 | 1735 | 0 | MLT-int | MaLR | LTR |
| 79673740 | 79674094 | + | 11.0 | 1 | 355 | 0 | THE1A | MaLR | LTR |
| 79674260 | 79674340 | - | 23.5 | 6535 | 6602 | -1440 | L1MC4 | L1 | LINE |
| 79674341 | 79674650 | - | 12.9 | 1 | 310 | -2 | AluSx | Alu | SINE |
| 79674651 | 79674743 | - | 23.5 | 6450 | 6534 | -1508 | L1MC4 | L1 | LINE |

  

|  |  |  |  |  |  |  |  |  |  |
| --- | --- | --- | --- | --- | --- | --- | --- | --- | --- |
| chr10:82776885-82782063 | | | 5189 bp | | |  |  |  |  |
| Genome Start | Genome End | Strand | Div | Repeat Start | Repeat End | Repeat Left | Name | Family | Class |
| 82776885 | 82777406 | + | 15.9 | 1 | 528 | -6325 | L1PA15-16 | L1 | LINE |
| 82777407 | 82777471 | - | 7.7 | 96 | 160 | -204 | THE1B | MaLR | LTR |
| 82777472 | 82777917 | + | 22.4 | 543 | 989 | -5864 | L1PA15-16 | L1 | LINE |
| 82777906 | 82778288 | + | 15.2 | 1492 | 1883 | -4970 | L1PA15-16 | L1 | LINE |
| 82778291 | 82778655 | - | 13.5 | 1 | 364 | 0 | THE1B | MaLR | LTR |
| 82778656 | 82780232 | - | 9.5 | 1 | 1579 | -1 | THE1B-int | MaLR | LTR |
| 82780233 | 82780594 | - | 13.3 | 1 | 364 | 0 | THE1B | MaLR | LTR |
| 82780595 | 82782063 | + | 14.2 | 1881 | 4206 | -2643 | L1PA15-16 | L1 | LINE |

  

|  |  |  |  |  |  |  |  |  |  |
| --- | --- | --- | --- | --- | --- | --- | --- | --- | --- |
| chr10:85000835-85005527 | | | 4512 bp | | |  |  |  |  |
| Genome Start | Genome End | Strand | Div | Repeat Start | Repeat End | Repeat Left | Name | Family | Class |
| 85000835 | 85001056 | - | 22.7 | 371 | 595 | 0 | LTR49 | ERV1 | LTR |
| 85001057 | 85001171 | - | 12.2 | 318 | 432 | 0 | MSTB1 | MaLR | LTR |
| 85001182 | 85001682 | - | 20.1 | 1 | 426 | 0 | MSTB | MaLR | LTR |
| 85001683 | 85003221 | - | 29.3 | 2 | 1735 | 0 | MLT-int | MaLR | LTR |
| 85003223 | 85003624 | - | 21.9 | 1 | 426 | 0 | MSTB | MaLR | LTR |
| 85003625 | 85004077 | - | 24.6 | 287 | 722 | -5 | LTR8A | ERV1 | LTR |
| 85004123 | 85004434 | - | 27.1 | 1 | 288 | -439 | LTR8 | ERV1 | LTR |
| 85004435 | 85004700 | - | 23.2 | 41 | 314 | -112 | MSTB | MaLR | LTR |
| 85004826 | 85005122 | - | 12.8 | 1 | 290 | -22 | AluSx | Alu | SINE |
| 85005123 | 85005176 | - | 9.3 | 1 | 54 | -372 | MSTB | MaLR | LTR |
| 85005177 | 85005527 | - | 14.8 | 2 | 375 | -220 | LTR49 | ERV1 | LTR |

  

|  |  |  |  |  |  |  |  |  |  |
| --- | --- | --- | --- | --- | --- | --- | --- | --- | --- |
| chr10:85498041-85512815 | | | 14364 bp | | |  |  |  |  |
| Genome Start | Genome End | Strand | Div | Repeat Start | Repeat End | Repeat Left | Name | Family | Class |
| 85498041 | 85499039 | + | 24.3 | 2110 | 3093 | -3053 | L1MD | L1 | LINE |
| 85499058 | 85500123 | + | 25.0 | 1 | 1266 | -5943 | L1M2b | L1 | LINE |
| 85500156 | 85500521 | + | 16.9 | 1 | 375 | 0 | THE1C | MaLR | LTR |
| 85500522 | 85501027 | + | 10.8 | 1 | 493 | -1087 | THE1C-int | MaLR | LTR |
| 85501031 | 85501096 | - | 13.6 | 14 | 79 | -233 | AluJ/FLAM | Alu | SINE |
| 85501097 | 85501313 | + | 17.7 | 84 | 303 | -9 | AluSg/x | Alu | SINE |
| 85501314 | 85501655 | + | 17.4 | 500 | 834 | -746 | THE1C-int | MaLR | LTR |
| 85501656 | 85501878 | - | 16.7 | 4 | 233 | -148 | THE1D | MaLR | LTR |
| 85501882 | 85503459 | - | 31.9 | 2 | 1735 | 0 | MLT-int | MaLR | LTR |
| 85503462 | 85503803 | - | 18.1 | 1 | 381 | 0 | THE1D | MaLR | LTR |
| 85503807 | 85503987 | + | 18.5 | 933 | 1111 | -540 | THE1C-int | MaLR | LTR |
| 85503988 | 85504345 | - | 16.5 | 1 | 364 | 0 | THE1B | MaLR | LTR |
| 85504346 | 85504887 | + | 18.5 | 1112 | 1651 | 0 | THE1C-int | MaLR | LTR |
| 85504888 | 85505080 | + | 22.4 | 1 | 196 | -230 | MSTB | MaLR | LTR |
| 85505081 | 85505383 | + | 12.2 | 1 | 303 | -9 | AluSx | Alu | SINE |
| 85505384 | 85505613 | + | 22.4 | 197 | 426 | 0 | MSTB | MaLR | LTR |
| 85505614 | 85506256 | + | 17.7 | 2675 | 3337 | -3868 | L1M2b | L1 | LINE |
| 85506252 | 85508714 | + | 19.3 | 3971 | 6462 | -743 | L1M2b | L1 | LINE |
| 85508730 | 85510085 | - | 18.8 | 2305 | 3753 | -3048 | MER57A-int | ERV1 | LTR |
| 85510103 | 85510247 | - | 22.9 | 983 | 1119 | -4004 | MER57A-int | ERV1 | LTR |
| 85510241 | 85510332 | - | 20.1 | 866 | 1064 | -4169 | MER57A-int | ERV1 | LTR |
| 85510349 | 85510942 | - | 26.4 | 2009 | 2999 | -3787 | MER57A-int | ERV1 | LTR |
| 85511257 | 85511289 | - | 18.3 | 419 | 451 | -1 | LTR47A | ERVL | LTR |
| 85511290 | 85511658 | + | 13.2 | 1 | 381 | 0 | THE1D | MaLR | LTR |
| 85511659 | 85511803 | - | 18.3 | 277 | 418 | -34 | LTR47A | ERVL | LTR |
| 85511804 | 85512815 | + | 24.8 | 3625 | 4686 | -1460 | L1MD | L1 | LINE |

  

|  |  |  |  |  |  |  |  |  |  |
| --- | --- | --- | --- | --- | --- | --- | --- | --- | --- |
| chr10:85857931-85867698 | | | 9677 bp | | |  |  |  |  |
| Genome Start | Genome End | Strand | Div | Repeat Start | Repeat End | Repeat Left | Name | Family | Class |
| 85857931 | 85858180 | - | 22.2 | 419 | 666 | -1 | PABL\_B | ERV1 | LTR |
| 85858181 | 85858646 | + | 6.5 | 1 | 463 | 0 | LTR2 | ERV1 | LTR |
| 85858647 | 85866463 | + | 19.0 | 1 | 7811 | -2 | HERVE | ERV1 | LTR |
| 85866468 | 85866929 | + | 7.4 | 1 | 463 | 0 | LTR2 | ERV1 | LTR |
| 85866957 | 85867431 | + | 10.9 | 1 | 511 | 0 | LTR10F | ERV1 | LTR |
| 85867492 | 85867698 | - | 37.0 | 1 | 205 | -462 | PABL\_B | ERV1 | LTR |

  

|  |  |  |  |  |  |  |  |  |  |
| --- | --- | --- | --- | --- | --- | --- | --- | --- | --- |
| chr10:86274429-86285455 | | | 11064 bp | | |  |  |  |  |
| Genome Start | Genome End | Strand | Div | Repeat Start | Repeat End | Repeat Left | Name | Family | Class |
| 86274429 | 86274826 | - | 22.6 | 5667 | 6105 | -73 | L1MB7 | L1 | LINE |
| 86274827 | 86275106 | + | 11.8 | 1 | 280 | -289 | MER61E | ERV1 | LTR |
| 86275107 | 86275865 | - | 6.8 | 1 | 780 | 0 | LTR17 | ERV1 | LTR |
| 86275866 | 86276157 | - | 6.2 | 8335 | 8626 | 0 | HERV17 | ERV1 | LTR |
| 86276148 | 86276186 | - | 5.1 | 7510 | 7548 | -1078 | HERV17 | ERV1 | LTR |
| 86276180 | 86276791 | - | 5.2 | 5405 | 6019 | -2607 | HERV17 | ERV1 | LTR |
| 86276771 | 86278096 | - | 5.5 | 2430 | 3754 | -4872 | HERV17 | ERV1 | LTR |
| 86278089 | 86280044 | - | 6.4 | 1 | 2007 | -6619 | HERV17 | ERV1 | LTR |
| 86280045 | 86280805 | - | 7.5 | 1 | 780 | 0 | LTR17 | ERV1 | LTR |
| 86280806 | 86281062 | + | 16.7 | 277 | 529 | -40 | MER61E | ERV1 | LTR |
| 86281070 | 86282429 | - | 20.9 | 4285 | 5670 | -514 | L1MB7 | L1 | LINE |
| 86282430 | 86282771 | + | 12.1 | 2 | 364 | 0 | THE1B | MaLR | LTR |
| 86282774 | 86283187 | + | 28.8 | 2 | 432 | -1303 | MLT-int | MaLR | LTR |
| 86283188 | 86283483 | - | 9.1 | 5 | 301 | -12 | AluSp | Alu | SINE |
| 86283484 | 86284635 | + | 28.8 | 433 | 1734 | -1 | MLT1H2-int | MaLR | LTR |
| 86284636 | 86284865 | + | 14.8 | 1 | 227 | -137 | THE1B | MaLR | LTR |
| 86284866 | 86285177 | + | 5.8 | 1 | 311 | 0 | AluY | Alu | SINE |
| 86285178 | 86285311 | + | 14.8 | 228 | 362 | -2 | THE1B | MaLR | LTR |
| 86285312 | 86285455 | - | 21.2 | 4145 | 4284 | -1862 | L1MB7 | L1 | LINE |

  

|  |  |  |  |  |  |  |  |  |  |
| --- | --- | --- | --- | --- | --- | --- | --- | --- | --- |
| chr10:86367909-86372989 | | | 4237 bp | | |  |  |  |  |
| Genome Start | Genome End | Strand | Div | Repeat Start | Repeat End | Repeat Left | Name | Family | Class |
| 86367909 | 86368013 | - | 22.5 | 3046 | 3168 | -218 | L2 | L2 | LINE |
| 86368138 | 86368324 | - | 18.3 | 234 | 423 | -45 | LTR37B | ERV1 | LTR |
| 86368325 | 86368522 | + | 14.5 | 1 | 189 | -239 | MSTA | MaLR | LTR |
| 86368523 | 86368821 | + | 9.4 | 2 | 299 | -14 | AluSq | Alu | SINE |
| 86368822 | 86369061 | + | 14.5 | 190 | 428 | 0 | MSTA | MaLR | LTR |
| 86369062 | 86369640 | + | 16.0 | 1 | 560 | -1091 | MSTA-int | MaLR | LTR |
| 86369704 | 86369895 | + | 19.5 | 231 | 423 | -45 | LTR37B | ERV1 | LTR |
| 86370009 | 86370124 | + | 23.8 | 3057 | 3168 | -251 | L2 | L2 | LINE |
| 86370199 | 86371051 | + | 16.4 | 635 | 1537 | -114 | MSTA-int | MaLR | LTR |
| 86371052 | 86371357 | - | 5.0 | 1 | 300 | -11 | AluY | Alu | SINE |
| 86371358 | 86371483 | + | 16.4 | 1538 | 1651 | 0 | MSTA-int | MaLR | LTR |
| 86371484 | 86371898 | + | 13.5 | 1 | 428 | 0 | MSTA | MaLR | LTR |
| 86371964 | 86372145 | - | 36.0 | 10 | 193 | -275 | LTR37B | ERV1 | LTR |
| 86372171 | 86372299 | - | 29.6 | 2819 | 2960 | -459 | L2 | L2 | LINE |
| 86372402 | 86372532 | - | 27.2 | 3278 | 3413 | -6 | L2 | L2 | LINE |
| 86372811 | 86372989 | - | 30.9 | 2675 | 2849 | -570 | L2 | L2 | LINE |

  

|  |  |  |  |  |  |  |  |  |  |
| --- | --- | --- | --- | --- | --- | --- | --- | --- | --- |
| chr10:86961703-86972323 | | | 10388 bp | | |  |  |  |  |
| Genome Start | Genome End | Strand | Div | Repeat Start | Repeat End | Repeat Left | Name | Family | Class |
| 86961703 | 86962011 | - | 30.4 | 7676 | 8016 | -26 | L1MC4 | L1 | LINE |
| 86962018 | 86962361 | + | 23.4 | 7343 | 7689 | -353 | L1MC4 | L1 | LINE |
| 86962362 | 86962569 | - | 19.2 | 1 | 224 | 0 | MER58A | MER1\_type | DNA |
| 86962570 | 86962666 | + | 23.4 | 7690 | 7782 | -260 | L1MC4 | L1 | LINE |
| 86962674 | 86967186 | + | 17.2 | 5 | 4510 | -1973 | L1PA16 | L1 | LINE |
| 86967187 | 86967539 | + | 9.1 | 1 | 355 | 0 | THE1A | MaLR | LTR |
| 86967543 | 86969144 | + | 30.8 | 3 | 1735 | 0 | MLT-int | MaLR | LTR |
| 86969145 | 86969499 | + | 10.4 | 1 | 355 | 0 | THE1A | MaLR | LTR |
| 86969500 | 86971347 | + | 13.9 | 4511 | 6398 | -105 | L1PA16 | L1 | LINE |
| 86971349 | 86971406 | + | 17.2 | 6435 | 6492 | -11 | L1PA16 | L1 | LINE |
| 86971557 | 86971927 | + | 11.2 | 1 | 375 | 0 | THE1C | MaLR | LTR |
| 86971994 | 86972323 | - | 26.0 | 7326 | 7677 | -365 | L1MC4 | L1 | LINE |

  

|  |  |  |  |  |  |  |  |  |  |
| --- | --- | --- | --- | --- | --- | --- | --- | --- | --- |
| chr10:87079884-87087584 | | | 7353 bp | | |  |  |  |  |
| Genome Start | Genome End | Strand | Div | Repeat Start | Repeat End | Repeat Left | Name | Family | Class |
| 87079884 | 87080947 | - | 26.6 | 3195 | 4320 | -2217 | L1MDa | L1 | LINE |
| 87081094 | 87081214 | - | 27.7 | 3043 | 3164 | -3373 | L1MDa | L1 | LINE |
| 87081229 | 87081359 | - | 25.6 | 2861 | 2992 | -3545 | L1MDa | L1 | LINE |
| 87081360 | 87081762 | + | 25.9 | 1 | 429 | -38 | MLT1C | MaLR | LTR |
| 87081777 | 87082576 | - | 29.9 | 2002 | 2865 | -3672 | L1MDa | L1 | LINE |
| 87082705 | 87083005 | - | 27.0 | 1 | 291 | -117 | MLT1A1 | MaLR | LTR |
| 87083006 | 87084536 | - | 22.2 | 255 | 1735 | 0 | MLT1A1-int | MaLR | LTR |
| 87084537 | 87086264 | + | 10.5 | 4566 | 6302 | 0 | L1MA1 | L1 | LINE |
| 87086297 | 87086559 | - | 19.8 | 1 | 269 | -1466 | MLT1A1-int | MaLR | LTR |
| 87086560 | 87086962 | - | 20.1 | 1 | 408 | 0 | MLT1A1 | MaLR | LTR |
| 87086977 | 87087584 | - | 28.2 | 1316 | 1964 | -4754 | L1MDa | L1 | LINE |

  

|  |  |  |  |  |  |  |  |  |  |
| --- | --- | --- | --- | --- | --- | --- | --- | --- | --- |
| chr10:87466447-87469782 | | | 3338 bp | | |  |  |  |  |
| Genome Start | Genome End | Strand | Div | Repeat Start | Repeat End | Repeat Left | Name | Family | Class |
| 87466447 | 87466941 | + | 17.4 | 5774 | 6235 | -77 | L1MA9 | L1 | LINE |
| 87466938 | 87467005 | + | 18.6 | 4869 | 4929 | -1217 | L1MD | L1 | LINE |
| 87467006 | 87467354 | - | 8.7 | 1 | 355 | 0 | THE1A | MaLR | LTR |
| 87467355 | 87469111 | - | 29.5 | 2 | 1735 | 0 | MLT-int | MaLR | LTR |
| 87469114 | 87469471 | - | 8.2 | 1 | 355 | 0 | THE1A | MaLR | LTR |
| 87469472 | 87469722 | + | 18.6 | 4930 | 5185 | -961 | L1MD | L1 | LINE |
| 87469723 | 87469782 | + | 11.7 | 6234 | 6303 | -9 | L1MA9 | L1 | LINE |

  

|  |  |  |  |  |  |  |  |  |  |
| --- | --- | --- | --- | --- | --- | --- | --- | --- | --- |
| chr10:89313313-89322884 | | | 9565 bp | | |  |  |  |  |
| Genome Start | Genome End | Strand | Div | Repeat Start | Repeat End | Repeat Left | Name | Family | Class |
| 89313313 | 89316158 | + | 16.7 | 15 | 3220 | -3625 | L1MA4A | L1 | LINE |
| 89316161 | 89316284 | + | 20.6 | 1 | 124 | -52 | FRAM | Alu | SINE |
| 89316285 | 89316684 | + | 18.1 | 1 | 428 | 0 | MSTA | MaLR | LTR |
| 89316687 | 89318323 | + | 29.6 | 2 | 1735 | 0 | MLT-int | MaLR | LTR |
| 89318326 | 89318729 | + | 17.2 | 3 | 426 | -2 | MSTA | MaLR | LTR |
| 89318730 | 89318772 | + | 20.6 | 261 | 296 | -16 | AluJ/monomer | Alu | SINE |
| 89318774 | 89321803 | + | 18.3 | 3210 | 6205 | -800 | L1MA4A | L1 | LINE |
| 89321804 | 89322094 | + | 11.3 | 1 | 291 | -19 | AluSg | Alu | SINE |
| 89322095 | 89322884 | + | 12.8 | 6206 | 7005 | 0 | L1MA4A | L1 | LINE |

  

|  |  |  |  |  |  |  |  |  |  |
| --- | --- | --- | --- | --- | --- | --- | --- | --- | --- |
| chr10:91002055-91006325 | | | 4220 bp | | |  |  |  |  |
| Genome Start | Genome End | Strand | Div | Repeat Start | Repeat End | Repeat Left | Name | Family | Class |
| 91002055 | 91002216 | - | 29.9 | 3231 | 3407 | -12 | L2 | L2 | LINE |
| 91002263 | 91002559 | + | 11.8 | 1 | 299 | -13 | AluSx | Alu | SINE |
| 91002560 | 91002954 | - | 31.1 | 2729 | 3230 | -189 | L2 | L2 | LINE |
| 91002955 | 91003313 | - | 13.3 | 1 | 364 | 0 | THE1B | MaLR | LTR |
| 91003314 | 91004893 | - | 29.7 | 2 | 1735 | 0 | MLT1B-int | MaLR | LTR |
| 91004899 | 91005256 | - | 13.6 | 1 | 357 | -7 | THE1B | MaLR | LTR |
| 91005257 | 91005541 | - | 26.8 | 2425 | 2733 | -686 | L2 | L2 | LINE |
| 91005542 | 91005967 | - | 23.7 | 3 | 523 | 0 | LTR33A | ERVL | LTR |
| 91005968 | 91006325 | - | 26.8 | 2014 | 2424 | -995 | L2 | L2 | LINE |

  

|  |  |  |  |  |  |  |  |  |  |
| --- | --- | --- | --- | --- | --- | --- | --- | --- | --- |
| chr10:91403926-91407374 | | | 3447 bp | | |  |  |  |  |
| Genome Start | Genome End | Strand | Div | Repeat Start | Repeat End | Repeat Left | Name | Family | Class |
| 91403926 | 91404318 | + | 19.8 | 1 | 530 | -405 | MER21C | ERV1 | LTR |
| 91404319 | 91404730 | - | 11.9 | 1 | 428 | 0 | MSTA | MaLR | LTR |
| 91404731 | 91404888 | + | 19.7 | 531 | 684 | -251 | MER21C | ERV1 | LTR |
| 91404889 | 91405236 | + | 13.7 | 1 | 428 | 0 | MSTA | MaLR | LTR |
| 91405239 | 91406805 | + | 27.8 | 2 | 1735 | 0 | MLT-int | MaLR | LTR |
| 91406806 | 91407139 | + | 12.7 | 1 | 428 | 0 | MSTA | MaLR | LTR |
| 91407140 | 91407374 | + | 19.7 | 685 | 933 | -2 | MER21C | ERV1 | LTR |

  

|  |  |  |  |  |  |  |  |  |  |
| --- | --- | --- | --- | --- | --- | --- | --- | --- | --- |
| chr10:91829986-91841970 | | | 11851 bp | | |  |  |  |  |
| Genome Start | Genome End | Strand | Div | Repeat Start | Repeat End | Repeat Left | Name | Family | Class |
| 91829986 | 91830474 | + | 11.6 | 1 | 504 | -6162 | MER4A1-int | ERV1 | LTR |
| 91830501 | 91830586 | + | 23.2 | 951 | 1033 | -7237 | MER4A1-int | ERV1 | LTR |
| 91830663 | 91830754 | + | 13.3 | 1262 | 1357 | -5241 | MER4A1-int | ERV1 | LTR |
| 91830755 | 91831206 | - | 7.1 | 1 | 448 | -2 | LTR7 | ERV1 | LTR |
| 91831207 | 91831584 | - | 7.8 | 7324 | 7713 | 0 | HERVH | ERV1 | LTR |
| 91831587 | 91831800 | - | 11.7 | 5600 | 5817 | -1896 | HERVH | ERV1 | LTR |
| 91831802 | 91832464 | - | 14.9 | 4488 | 5149 | -2564 | HERVH | ERV1 | LTR |
| 91832459 | 91832971 | - | 12.0 | 3492 | 4000 | -3713 | HERVH | ERV1 | LTR |
| 91832962 | 91833111 | - | 14.0 | 3140 | 3290 | -4423 | HERVH | ERV1 | LTR |
| 91833091 | 91836075 | - | 10.2 | 1 | 2995 | -4718 | HERVH | ERV1 | LTR |
| 91836078 | 91836529 | - | 6.7 | 1 | 448 | -2 | LTR7 | ERV1 | LTR |
| 91836530 | 91836745 | + | 27.4 | 1940 | 2160 | -4171 | LTR49-int | ERV1 | LTR |
| 91836790 | 91837701 | + | 20.3 | 196 | 1189 | -5569 | MER4A1-int | ERV1 | LTR |
| 91837714 | 91838388 | + | 10.2 | 1756 | 2502 | -4096 | MER4A1-int | ERV1 | LTR |
| 91838389 | 91838681 | - | 13.0 | 1 | 297 | -15 | AluSx | Alu | SINE |
| 91838686 | 91838909 | - | 15.6 | 1 | 257 | -434 | LTR8 | ERV1 | LTR |
| 91838910 | 91839433 | + | 12.7 | 2737 | 3320 | -3278 | MER4A1-int | ERV1 | LTR |
| 91839438 | 91841970 | + | 11.2 | 3919 | 6593 | -5 | MER4A1-int | ERV1 | LTR |

  

|  |  |  |  |  |  |  |  |  |  |
| --- | --- | --- | --- | --- | --- | --- | --- | --- | --- |
| chr10:91850987-91858784 | | | 7699 bp | | |  |  |  |  |
| Genome Start | Genome End | Strand | Div | Repeat Start | Repeat End | Repeat Left | Name | Family | Class |
| 91850987 | 91851093 | + | 17.9 | 19 | 134 | -7431 | L1MA3 | L1 | LINE |
| 91851089 | 91851746 | + | 15.0 | 1103 | 1767 | -5798 | L1MA3 | L1 | LINE |
| 91851784 | 91855124 | + | 14.6 | 1768 | 5206 | -2359 | L1MA3 | L1 | LINE |
| 91855153 | 91856200 | + | 9.6 | 6129 | 7168 | -555 | L1MA3 | L1 | LINE |
| 91856201 | 91856562 | + | 13.1 | 1 | 381 | 0 | THE1D | MaLR | LTR |
| 91856563 | 91857087 | + | 12.0 | 1 | 577 | -1003 | THE1D-int | MaLR | LTR |
| 91857095 | 91857388 | + | 11.9 | 1287 | 1580 | 0 | THE1D-int | MaLR | LTR |
| 91857389 | 91857726 | + | 10.5 | 1 | 353 | -28 | THE1D | MaLR | LTR |
| 91857727 | 91858181 | - | 7.9 | 7 | 463 | 0 | LTR2 | ERV1 | LTR |
| 91858214 | 91858784 | + | 11.4 | 7163 | 7724 | 0 | L1MA3 | L1 | LINE |

  

|  |  |  |  |  |  |  |  |  |  |
| --- | --- | --- | --- | --- | --- | --- | --- | --- | --- |
| chr10:92570757-92579829 | | | 8988 bp | | |  |  |  |  |
| Genome Start | Genome End | Strand | Div | Repeat Start | Repeat End | Repeat Left | Name | Family | Class |
| 92570757 | 92570842 | + | 13.4 | 8 | 97 | -7468 | L1M1 | L1 | LINE |
| 92570892 | 92571407 | + | 13.4 | 98 | 626 | -6939 | L1M1 | L1 | LINE |
| 92571408 | 92571789 | + | 14.8 | 1 | 428 | 0 | MSTA | MaLR | LTR |
| 92571792 | 92573410 | + | 28.9 | 2 | 1735 | 0 | MSTD-int | MaLR | LTR |
| 92573411 | 92573788 | + | 16.9 | 1 | 428 | 0 | MSTA | MaLR | LTR |
| 92573789 | 92576416 | + | 13.4 | 627 | 3341 | -4224 | L1M1 | L1 | LINE |
| 92576417 | 92576702 | + | 8.4 | 25 | 310 | -1 | AluY | Alu | SINE |
| 92576703 | 92576761 | + | 13.4 | 3342 | 3376 | -4189 | L1M1 | L1 | LINE |
| 92576784 | 92576999 | + | 13.4 | 3377 | 3606 | -3959 | L1M1 | L1 | LINE |
| 92577001 | 92577134 | + | 16.4 | 1 | 136 | -176 | AluJo | Alu | SINE |
| 92577146 | 92579118 | + | 11.1 | 3591 | 5520 | -2045 | L1M1 | L1 | LINE |
| 92579119 | 92579304 | - | 4.8 | 5956 | 6152 | -2 | L1PA7 | L1 | LINE |
| 92579305 | 92579829 | + | 11.1 | 5521 | 6069 | -1496 | L1M1 | L1 | LINE |

  

|  |  |  |  |  |  |  |  |  |  |
| --- | --- | --- | --- | --- | --- | --- | --- | --- | --- |
| chr10:93050511-93057118 | | | 6537 bp | | |  |  |  |  |
| Genome Start | Genome End | Strand | Div | Repeat Start | Repeat End | Repeat Left | Name | Family | Class |
| 93050511 | 93050540 | - | 18.9 | 517 | 546 | -8 | MLT2B3 | ERVL | LTR |
| 93050581 | 93050601 | - | 18.9 | 505 | 516 | -38 | MLT2B3 | ERVL | LTR |
| 93050654 | 93051069 | - | 18.9 | 59 | 504 | -50 | MLT2B3 | ERVL | LTR |
| 93051087 | 93051439 | - | 6.5 | 1 | 448 | -2 | LTR7 | ERV1 | LTR |
| 93051440 | 93051807 | - | 13.2 | 7322 | 7713 | 0 | HERVH | ERV1 | LTR |
| 93051792 | 93052021 | - | 11.7 | 5600 | 5826 | -1887 | HERVH | ERV1 | LTR |
| 93052023 | 93052177 | - | 20.0 | 4994 | 5149 | -2564 | HERVH | ERV1 | LTR |
| 93052178 | 93053673 | - | 11.9 | 3492 | 5286 | -1805 | HERVH | ERV1 | LTR |
| 93053664 | 93053822 | - | 13.5 | 3134 | 3290 | -4423 | HERVH | ERV1 | LTR |
| 93053799 | 93054098 | - | 11.9 | 2701 | 2995 | -4718 | HERVH | ERV1 | LTR |
| 93054107 | 93056656 | - | 10.1 | 1 | 2445 | -5268 | HERVH | ERV1 | LTR |
| 93056659 | 93057066 | - | 8.6 | 1 | 448 | -2 | LTR7 | ERV1 | LTR |
| 93057068 | 93057118 | - | 19.6 | 1 | 51 | -503 | MLT2B3 | ERVL | LTR |

  

|  |  |  |  |  |  |  |  |  |  |
| --- | --- | --- | --- | --- | --- | --- | --- | --- | --- |
| chr10:95582060-95585061 | | | 3000 bp | | |  |  |  |  |
| Genome Start | Genome End | Strand | Div | Repeat Start | Repeat End | Repeat Left | Name | Family | Class |
| 95582060 | 95582254 | - | 27.5 | 3208 | 3419 | 0 | L2 | L2 | LINE |
| 95582255 | 95582668 | - | 13.1 | 1 | 428 | 0 | MSTA | MaLR | LTR |
| 95582669 | 95584258 | - | 28.4 | 2 | 1735 | 0 | MLT1H-int | MaLR | LTR |
| 95584261 | 95584395 | - | 14.0 | 293 | 428 | 0 | MSTA | MaLR | LTR |
| 95584396 | 95584681 | - | 8.8 | 22 | 306 | -5 | AluY | Alu | SINE |
| 95584682 | 95584967 | - | 14.0 | 1 | 292 | -136 | MSTA | MaLR | LTR |
| 95584968 | 95585061 | - | 27.5 | 3122 | 3207 | -212 | L2 | L2 | LINE |

  

|  |  |  |  |  |  |  |  |  |  |
| --- | --- | --- | --- | --- | --- | --- | --- | --- | --- |
| chr10:96491739-96496058 | | | 4314 bp | | |  |  |  |  |
| Genome Start | Genome End | Strand | Div | Repeat Start | Repeat End | Repeat Left | Name | Family | Class |
| 96491739 | 96493271 | - | 17.5 | 2019 | 3564 | -3109 | L1PA14 | L1 | LINE |
| 96493272 | 96493631 | + | 12.8 | 1 | 360 | -4 | THE1B | MaLR | LTR |
| 96493638 | 96494443 | + | 29.5 | 2 | 980 | -755 | MLT-int | MaLR | LTR |
| 96494444 | 96494738 | + | 14.0 | 1 | 293 | -19 | AluSx | Alu | SINE |
| 96494739 | 96495473 | + | 29.5 | 981 | 1735 | 0 | MLT-int | MaLR | LTR |
| 96495474 | 96495828 | + | 14.2 | 1 | 362 | -2 | THE1B | MaLR | LTR |
| 96495829 | 96496058 | - | 13.2 | 1784 | 2012 | -4677 | L1PA14 | L1 | LINE |

  

|  |  |  |  |  |  |  |  |  |  |
| --- | --- | --- | --- | --- | --- | --- | --- | --- | --- |
| chr10:96580509-96592361 | | | 11861 bp | | |  |  |  |  |
| Genome Start | Genome End | Strand | Div | Repeat Start | Repeat End | Repeat Left | Name | Family | Class |
| 96580509 | 96581936 | - | 11.3 | 6291 | 7721 | 0 | L1MA1 | L1 | LINE |
| 96581937 | 96582290 | - | 13.7 | 1 | 364 | 0 | THE1B | MaLR | LTR |
| 96582291 | 96583196 | - | 11.6 | 5385 | 6290 | -1275 | L1MA1 | L1 | LINE |
| 96583197 | 96583296 | - | 2.0 | 6056 | 6155 | 0 | L1PA2 | L1 | LINE |
| 96583297 | 96584315 | - | 11.6 | 4492 | 5384 | -2181 | L1MA1 | L1 | LINE |
| 96584316 | 96584637 | - | 6.2 | 3 | 327 | -453 | LTR17 | ERV1 | LTR |
| 96584640 | 96584931 | - | 6.2 | 8335 | 8626 | 0 | HERV17 | ERV1 | LTR |
| 96584922 | 96584955 | - | 5.9 | 7515 | 7548 | -1078 | HERV17 | ERV1 | LTR |
| 96584956 | 96590752 | - | 11.1 | 1 | 6005 | -2621 | HERV17 | ERV1 | LTR |
| 96590753 | 96591281 | - | 9.3 | 253 | 780 | 0 | LTR17 | ERV1 | LTR |
| 96591282 | 96592361 | - | 13.3 | 3440 | 4525 | -3040 | L1MA1 | L1 | LINE |

  

|  |  |  |  |  |  |  |  |  |  |
| --- | --- | --- | --- | --- | --- | --- | --- | --- | --- |
| chr10:97861805-97867160 | | | 4894 bp | | |  |  |  |  |
| Genome Start | Genome End | Strand | Div | Repeat Start | Repeat End | Repeat Left | Name | Family | Class |
| 97861805 | 97862079 | + | 25.5 | 1072 | 1498 | -5017 | L1M4c | L1 | LINE |
| 97862083 | 97862494 | + | 24.6 | 2 | 418 | -124 | MLT1F | MaLR | LTR |
| 97862863 | 97863004 | + | 21.2 | 403 | 542 | 0 | MLT1F | MaLR | LTR |
| 97863008 | 97864367 | + | 23.5 | 3 | 1474 | -15 | MLT1F-int | MaLR | LTR |
| 97864383 | 97864818 | + | 24.9 | 1 | 480 | -62 | MLT1F | MaLR | LTR |
| 97864820 | 97864944 | - | 16.8 | 1 | 130 | -12 | FLAM\_A | Alu | SINE |
| 97864947 | 97865020 | + | 15.5 | 489 | 560 | -2 | MLT1F | MaLR | LTR |
| 97865091 | 97867160 | + | 25.8 | 1585 | 3684 | -2698 | L1M4c | L1 | LINE |

  

|  |  |  |  |  |  |  |  |  |  |
| --- | --- | --- | --- | --- | --- | --- | --- | --- | --- |
| chr10:99866678-99876763 | | | 10009 bp | | |  |  |  |  |
| Genome Start | Genome End | Strand | Div | Repeat Start | Repeat End | Repeat Left | Name | Family | Class |
| 99866678 | 99866772 | - | 16.3 | 252 | 345 | 0 | MER2 | MER2\_type | DNA |
| 99866773 | 99867069 | + | 21.0 | 2 | 296 | -16 | AluJo | Alu | SINE |
| 99867070 | 99867119 | - | 16.3 | 210 | 251 | -94 | MER2 | MER2\_type | DNA |
| 99867150 | 99867461 | - | 15.1 | 1 | 312 | 0 | AluSx | Alu | SINE |
| 99867473 | 99867849 | - | 9.4 | 6485 | 6865 | -2 | L1PB1 | L1 | LINE |
| 99867857 | 99868100 | - | 3.7 | 5909 | 6152 | -3 | L1PA4 | L1 | LINE |
| 99868102 | 99869626 | - | 9.3 | 4932 | 6501 | -366 | L1PB1 | L1 | LINE |
| 99869627 | 99869983 | + | 13.2 | 1 | 364 | 0 | THE1B | MaLR | LTR |
| 99869984 | 99873538 | - | 9.2 | 1311 | 4931 | -1931 | L1PB1 | L1 | LINE |
| 99873562 | 99873919 | - | 11.1 | 1 | 362 | -2 | THE1B | MaLR | LTR |
| 99873920 | 99875445 | - | 11.0 | 1 | 1580 | 0 | THE1B-int | MaLR | LTR |
| 99875446 | 99875807 | - | 11.3 | 1 | 364 | 0 | THE1B | MaLR | LTR |
| 99875808 | 99876333 | - | 13.6 | 634 | 1145 | -5718 | L1PB1 | L1 | LINE |
| 99876334 | 99876530 | - | 16.8 | 3 | 200 | -6663 | L1PB1 | L1 | LINE |
| 99876536 | 99876763 | - | 16.1 | 1 | 225 | -120 | MER2 | MER2\_type | DNA |

  

|  |  |  |  |  |  |  |  |  |  |
| --- | --- | --- | --- | --- | --- | --- | --- | --- | --- |
| chr10:100816009-100818878 | | | 2861 bp | | |  |  |  |  |
| Genome Start | Genome End | Strand | Div | Repeat Start | Repeat End | Repeat Left | Name | Family | Class |
| 100816009 | 100816172 | + | 17.8 | 2984 | 3143 | -3003 | L1M5 | L1 | LINE |
| 100816173 | 100816508 | - | 14.0 | 1 | 364 | 0 | THE1B | MaLR | LTR |
| 100816516 | 100818039 | - | 29.7 | 2 | 1734 | -1 | MLT-int | MaLR | LTR |
| 100818042 | 100818375 | - | 14.1 | 2 | 364 | 0 | THE1B | MaLR | LTR |
| 100818376 | 100818878 | + | 17.8 | 3144 | 3657 | -2489 | L1M5 | L1 | LINE |

  

|  |  |  |  |  |  |  |  |  |  |
| --- | --- | --- | --- | --- | --- | --- | --- | --- | --- |
| chr10:100997134-101001489 | | | 4354 bp | | |  |  |  |  |
| Genome Start | Genome End | Strand | Div | Repeat Start | Repeat End | Repeat Left | Name | Family | Class |
| 100997134 | 100997444 | + | 11.7 | 2683 | 2989 | -3860 | L1PA15-16 | L1 | LINE |
| 100997445 | 100997818 | + | 13.4 | 1 | 428 | 0 | MSTA | MaLR | LTR |
| 100997819 | 100999435 | + | 17.4 | 1 | 1651 | 0 | MSTA-int | MaLR | LTR |
| 100999436 | 100999608 | + | 13.6 | 1 | 196 | -232 | MSTA | MaLR | LTR |
| 100999609 | 100999906 | + | 9.8 | 1 | 300 | -13 | AluSq | Alu | SINE |
| 100999907 | 101000119 | + | 13.6 | 197 | 426 | -2 | MSTA | MaLR | LTR |
| 101000122 | 101001489 | + | 10.1 | 3201 | 4587 | -2262 | L1PA15-16 | L1 | LINE |

  

|  |  |  |  |  |  |  |  |  |  |
| --- | --- | --- | --- | --- | --- | --- | --- | --- | --- |
| chr10:101508171-101511582 | | | 3390 bp | | |  |  |  |  |
| Genome Start | Genome End | Strand | Div | Repeat Start | Repeat End | Repeat Left | Name | Family | Class |
| 101508171 | 101508263 | - | 27.2 | 2627 | 2718 | 0 | Tigger2 | MER2\_type | DNA |
| 101508266 | 101508664 | - | 13.1 | 722 | 1129 | -852 | Tigger2 | MER2\_type | DNA |
| 101508665 | 101508758 | - | 8.6 | 339 | 432 | 0 | MSTB1 | MaLR | LTR |
| 101508762 | 101509170 | - | 23.9 | 1 | 443 | 0 | LTR47B | ERVL | LTR |
| 101509171 | 101509507 | - | 20.3 | 1 | 340 | -92 | MSTB1 | MaLR | LTR |
| 101509508 | 101510672 | - | 19.5 | 402 | 1651 | 0 | MSTB1-int | MaLR | LTR |
| 101510673 | 101510967 | - | 15.3 | 1 | 295 | -17 | AluJo | Alu | SINE |
| 101510968 | 101511000 | - | 19.5 | 370 | 401 | -1250 | MSTB1-int | MaLR | LTR |
| 101511002 | 101511203 | - | 17.4 | 5 | 208 | -1443 | MSTB1-int | MaLR | LTR |
| 101511211 | 101511316 | - | 12.3 | 315 | 429 | -3 | MSTB1 | MaLR | LTR |
| 101511326 | 101511582 | - | 11.3 | 469 | 731 | -1250 | Tigger2 | MER2\_type | DNA |

  

|  |  |  |  |  |  |  |  |  |  |
| --- | --- | --- | --- | --- | --- | --- | --- | --- | --- |
| chr10:104882639-104885943 | | | 3302 bp | | |  |  |  |  |
| Genome Start | Genome End | Strand | Div | Repeat Start | Repeat End | Repeat Left | Name | Family | Class |
| 104882639 | 104882737 | + | 19.0 | 5814 | 5911 | -253 | L1ME2 | L1 | LINE |
| 104882738 | 104883020 | + | 13.3 | 1 | 296 | -68 | THE1B | MaLR | LTR |
| 104883021 | 104883307 | - | 12.5 | 1 | 294 | -19 | AluSq | Alu | SINE |
| 104883308 | 104883380 | + | 13.3 | 297 | 364 | 0 | THE1B | MaLR | LTR |
| 104883383 | 104884955 | + | 29.8 | 2 | 1735 | 0 | MLT1D-int | MaLR | LTR |
| 104884956 | 104885239 | + | 12.8 | 1 | 296 | -68 | THE1B | MaLR | LTR |
| 104885240 | 104885548 | + | 10.5 | 1 | 296 | -16 | AluSx | Alu | SINE |
| 104885550 | 104885844 | + | 10.9 | 1 | 293 | -19 | AluSx | Alu | SINE |
| 104885845 | 104885912 | + | 12.8 | 297 | 364 | 0 | THE1B | MaLR | LTR |
| 104885913 | 104885943 | + | 19.0 | 5912 | 5936 | -228 | L1ME2 | L1 | LINE |

  

|  |  |  |  |  |  |  |  |  |  |
| --- | --- | --- | --- | --- | --- | --- | --- | --- | --- |
| chr10:107766390-107773181 | | | 6818 bp | | |  |  |  |  |
| Genome Start | Genome End | Strand | Div | Repeat Start | Repeat End | Repeat Left | Name | Family | Class |
| 107766390 | 107766487 | - | 32.0 | 341 | 438 | -6351 | L1M2c | L1 | LINE |
| 107766488 | 107766937 | - | 5.8 | 1 | 448 | -2 | LTR7 | ERV1 | LTR |
| 107766944 | 107768891 | - | 8.2 | 5769 | 7707 | -6 | HERVH | ERV1 | LTR |
| 107768878 | 107769067 | - | 10.5 | 4488 | 4680 | -3033 | HERVH | ERV1 | LTR |
| 107769070 | 107769572 | - | 11.2 | 3492 | 3993 | -3720 | HERVH | ERV1 | LTR |
| 107769563 | 107769709 | - | 10.2 | 3140 | 3290 | -4423 | HERVH | ERV1 | LTR |
| 107769696 | 107772391 | - | 12.2 | 3 | 2992 | -4721 | HERVH | ERV1 | LTR |
| 107772396 | 107772846 | - | 6.0 | 1 | 448 | -2 | LTR7 | ERV1 | LTR |
| 107772847 | 107773181 | - | 36.9 | 12 | 346 | -6443 | L1M2c | L1 | LINE |

  

|  |  |  |  |  |  |  |  |  |  |
| --- | --- | --- | --- | --- | --- | --- | --- | --- | --- |
| chr10:108857315-108866468 | | | 9161 bp | | |  |  |  |  |
| Genome Start | Genome End | Strand | Div | Repeat Start | Repeat End | Repeat Left | Name | Family | Class |
| 108857315 | 108857754 | + | 29.9 | 11 | 450 | -5505 | L1MB1 | L1 | LINE |
| 108857746 | 108857977 | + | 28.9 | 253 | 702 | -5564 | L1M3a | L1 | LINE |
| 108857978 | 108858388 | - | 15.9 | 1 | 428 | 0 | MSTA | MaLR | LTR |
| 108858389 | 108860025 | - | 28.2 | 2 | 1735 | 0 | MLT-int | MaLR | LTR |
| 108860028 | 108860451 | - | 17.9 | 1 | 428 | 0 | MSTA | MaLR | LTR |
| 108860452 | 108863154 | + | 23.6 | 392 | 2927 | -3101 | L1MB1 | L1 | LINE |
| 108863155 | 108863466 | + | 10.1 | 1 | 308 | -1 | AluSc | Alu | SINE |
| 108863467 | 108866468 | + | 18.0 | 2928 | 5954 | -123 | L1MB1 | L1 | LINE |

  

|  |  |  |  |  |  |  |  |  |  |
| --- | --- | --- | --- | --- | --- | --- | --- | --- | --- |
| chr10:110301309-110309146 | | | 7789 bp | | |  |  |  |  |
| Genome Start | Genome End | Strand | Div | Repeat Start | Repeat End | Repeat Left | Name | Family | Class |
| 110301309 | 110303146 | + | 20.1 | 6 | 1870 | -4638 | L1MC1 | L1 | LINE |
| 110303147 | 110303451 | + | 10.4 | 5983 | 6295 | -7 | L1MA1 | L1 | LINE |
| 110303452 | 110304043 | + | 20.1 | 1871 | 2398 | -4110 | L1MC1 | L1 | LINE |
| 110304051 | 110304415 | + | 17.2 | 1 | 381 | 0 | THE1D | MaLR | LTR |
| 110304416 | 110306008 | + | 13.1 | 1 | 1580 | 0 | THE1D-int | MaLR | LTR |
| 110306009 | 110306365 | + | 19.0 | 1 | 381 | 0 | THE1D | MaLR | LTR |
| 110306367 | 110307599 | + | 16.5 | 2401 | 3721 | -2600 | L1MC1 | L1 | LINE |
| 110307626 | 110307892 | + | 17.3 | 5510 | 5787 | -721 | L1MC1 | L1 | LINE |
| 110307889 | 110307974 | - | 14.1 | 6044 | 6128 | -163 | L1MA8 | L1 | LINE |
| 110307971 | 110308105 | + | 17.8 | 6145 | 6289 | -2 | L1MA8 | L1 | LINE |
| 110308129 | 110308801 | + | 16.9 | 5770 | 6452 | -56 | L1MC1 | L1 | LINE |
| 110308802 | 110309092 | - | 19.5 | 1 | 287 | -25 | AluJb | Alu | SINE |
| 110309093 | 110309146 | + | 16.9 | 6453 | 6503 | -5 | L1MC1 | L1 | LINE |

  

|  |  |  |  |  |  |  |  |  |  |
| --- | --- | --- | --- | --- | --- | --- | --- | --- | --- |
| chr10:110574991-110585315 | | | 10251 bp | | |  |  |  |  |
| Genome Start | Genome End | Strand | Div | Repeat Start | Repeat End | Repeat Left | Name | Family | Class |
| 110574991 | 110577820 | + | 28.0 | 14 | 3123 | -3521 | L1MB8 | L1 | LINE |
| 110577825 | 110577999 | + | 31.1 | 3 | 163 | -342 | MLT1D | MaLR | LTR |
| 110578000 | 110578379 | - | 14.3 | 1 | 428 | 0 | MSTA | MaLR | LTR |
| 110578397 | 110579997 | - | 19.5 | 1 | 1634 | -17 | MSTA-int | MaLR | LTR |
| 110579998 | 110580381 | - | 14.4 | 1 | 428 | 0 | MSTA | MaLR | LTR |
| 110580430 | 110580700 | + | 22.9 | 228 | 502 | -3 | MLT1D | MaLR | LTR |
| 110580706 | 110581321 | + | 23.2 | 3128 | 3687 | -2957 | L1MB8 | L1 | LINE |
| 110581322 | 110581931 | + | 10.3 | 5556 | 6168 | 0 | L1PA12 | L1 | LINE |
| 110581932 | 110582138 | + | 23.2 | 3688 | 3876 | -2768 | L1MB8 | L1 | LINE |
| 110582139 | 110582420 | + | 12.1 | 1 | 284 | -26 | AluSg | Alu | SINE |
| 110582421 | 110584326 | + | 23.2 | 3877 | 5816 | -828 | L1MB8 | L1 | LINE |
| 110584327 | 110584636 | + | 8.4 | 1 | 311 | 0 | AluY | Alu | SINE |
| 110584637 | 110585315 | + | 22.6 | 5817 | 6507 | -169 | L1MB8 | L1 | LINE |

  

|  |  |  |  |  |  |  |  |  |  |
| --- | --- | --- | --- | --- | --- | --- | --- | --- | --- |
| chr10:111043043-111046801 | | | 3307 bp | | |  |  |  |  |
| Genome Start | Genome End | Strand | Div | Repeat Start | Repeat End | Repeat Left | Name | Family | Class |
| 111043043 | 111043161 | + | 36.0 | 2475 | 2577 | -842 | L2 | L2 | LINE |
| 111043162 | 111043297 | - | 14.8 | 6031 | 6168 | 0 | L1MB1 | L1 | LINE |
| 111043298 | 111043584 | + | 36.0 | 2578 | 2873 | -546 | L2 | L2 | LINE |
| 111043626 | 111043976 | + | 10.0 | 6 | 364 | 0 | THE1B | MaLR | LTR |
| 111043979 | 111045524 | + | 30.2 | 2 | 1735 | 0 | MLT1L-int | MaLR | LTR |
| 111045525 | 111045871 | + | 11.8 | 1 | 364 | 0 | THE1B | MaLR | LTR |
| 111046048 | 111046182 | + | 27.9 | 120 | 245 | -23 | MIRb | MIR | SINE |
| 111046240 | 111046516 | + | 17.7 | 2 | 298 | -14 | AluJb | Alu | SINE |
| 111046693 | 111046801 | + | 34.3 | 3244 | 3361 | -17 | L2 | L2 | LINE |

  

|  |  |  |  |  |  |  |  |  |  |
| --- | --- | --- | --- | --- | --- | --- | --- | --- | --- |
| chr10:111558207-111561983 | | | 3779 bp | | |  |  |  |  |
| Genome Start | Genome End | Strand | Div | Repeat Start | Repeat End | Repeat Left | Name | Family | Class |
| 111558207 | 111558622 | + | 18.5 | 5209 | 5643 | -523 | L1ME2 | L1 | LINE |
| 111558623 | 111558979 | - | 14.7 | 7 | 364 | 0 | THE1B | MaLR | LTR |
| 111558978 | 111560135 | - | 8.8 | 426 | 1574 | -6 | THE1B-int | MaLR | LTR |
| 111560136 | 111560428 | + | 11.3 | 1 | 296 | -17 | AluSp | Alu | SINE |
| 111560429 | 111560849 | - | 8.8 | 1 | 425 | -1155 | THE1B-int | MaLR | LTR |
| 111560850 | 111561211 | - | 11.6 | 1 | 364 | 0 | THE1B | MaLR | LTR |
| 111561212 | 111561662 | + | 22.2 | 5642 | 6099 | -65 | L1ME2 | L1 | LINE |
| 111561663 | 111561930 | + | 13.6 | 33 | 297 | -15 | AluSx | Alu | SINE |
| 111561931 | 111561983 | + | 22.2 | 6100 | 6152 | -12 | L1ME2 | L1 | LINE |

  

|  |  |  |  |  |  |  |  |  |  |
| --- | --- | --- | --- | --- | --- | --- | --- | --- | --- |
| chr10:112781311-112784295 | | | 2978 bp | | |  |  |  |  |
| Genome Start | Genome End | Strand | Div | Repeat Start | Repeat End | Repeat Left | Name | Family | Class |
| 112781311 | 112781571 | - | 22.8 | 5587 | 5860 | -380 | L1ME3B | L1 | LINE |
| 112781577 | 112781929 | - | 8.2 | 3 | 355 | 0 | THE1A | MaLR | LTR |
| 112781932 | 112783499 | - | 8.8 | 1 | 1580 | 0 | THE1A-int | MaLR | LTR |
| 112783500 | 112783615 | - | 9.7 | 239 | 355 | 0 | THE1A | MaLR | LTR |
| 112783616 | 112783918 | + | 6.6 | 1 | 301 | -10 | AluY | Alu | SINE |
| 112783919 | 112784163 | - | 9.7 | 1 | 238 | -117 | THE1A | MaLR | LTR |
| 112784164 | 112784295 | - | 26.0 | 5454 | 5586 | -578 | L1ME3B | L1 | LINE |

  

|  |  |  |  |  |  |  |  |  |  |
| --- | --- | --- | --- | --- | --- | --- | --- | --- | --- |
| chr10:116887044-116889615 | | | 2512 bp | | |  |  |  |  |
| Genome Start | Genome End | Strand | Div | Repeat Start | Repeat End | Repeat Left | Name | Family | Class |
| 116887044 | 116887177 | + | 27.1 | 4 | 145 | -366 | MLT1J | MaLR | LTR |
| 116887178 | 116887521 | - | 12.1 | 1 | 364 | 0 | THE1B | MaLR | LTR |
| 116887522 | 116889090 | - | 30.0 | 2 | 1735 | 0 | MLT1A-int | MaLR | LTR |
| 116889093 | 116889434 | - | 13.3 | 1 | 364 | 0 | THE1B | MaLR | LTR |
| 116889435 | 116889482 | + | 27.1 | 146 | 191 | -320 | MLT1J | MaLR | LTR |
| 116889541 | 116889615 | + | 26.7 | 391 | 468 | -43 | MLT1J | MaLR | LTR |

  

|  |  |  |  |  |  |  |  |  |  |
| --- | --- | --- | --- | --- | --- | --- | --- | --- | --- |
| chr10:118229938-118236435 | | | 6425 bp | | |  |  |  |  |
| Genome Start | Genome End | Strand | Div | Repeat Start | Repeat End | Repeat Left | Name | Family | Class |
| 118229938 | 118230165 | - | 30.9 | 7706 | 7911 | -50 | L1MC5 | L1 | LINE |
| 118230177 | 118230585 | + | 8.6 | 1 | 448 | -2 | LTR7 | ERV1 | LTR |
| 118230588 | 118233860 | + | 8.9 | 1 | 3290 | -4423 | HERVH | ERV1 | LTR |
| 118233851 | 118234353 | + | 9.4 | 3492 | 3993 | -3720 | HERVH | ERV1 | LTR |
| 118234356 | 118235016 | + | 12.7 | 4488 | 5149 | -2564 | HERVH | ERV1 | LTR |
| 118235018 | 118235234 | + | 13.4 | 5600 | 5819 | -1894 | HERVH | ERV1 | LTR |
| 118235233 | 118235611 | + | 10.2 | 7322 | 7713 | 0 | HERVH | ERV1 | LTR |
| 118235612 | 118236020 | + | 7.8 | 1 | 448 | -2 | LTR7 | ERV1 | LTR |
| 118236090 | 118236435 | - | 27.6 | 7309 | 7685 | -123 | L1MC5 | L1 | LINE |

  

|  |  |  |  |  |  |  |  |  |  |
| --- | --- | --- | --- | --- | --- | --- | --- | --- | --- |
| chr10:120238232-120240782 | | | 2549 bp | | |  |  |  |  |
| Genome Start | Genome End | Strand | Div | Repeat Start | Repeat End | Repeat Left | Name | Family | Class |
| 120238232 | 120238452 | - | 22.8 | 99 | 331 | -211 | MLT1F | MaLR | LTR |
| 120238453 | 120238795 | + | 15.7 | 1 | 364 | 0 | THE1B | MaLR | LTR |
| 120238798 | 120240359 | + | 29.5 | 2 | 1735 | 0 | MLT-int | MaLR | LTR |
| 120240360 | 120240694 | + | 11.2 | 1 | 364 | 0 | THE1B | MaLR | LTR |
| 120240695 | 120240782 | - | 22.8 | 1 | 98 | -444 | MLT1F | MaLR | LTR |

  

|  |  |  |  |  |  |  |  |  |  |
| --- | --- | --- | --- | --- | --- | --- | --- | --- | --- |
| chr10:124973342-124977232 | | | 3860 bp | | |  |  |  |  |
| Genome Start | Genome End | Strand | Div | Repeat Start | Repeat End | Repeat Left | Name | Family | Class |
| 124973342 | 124973826 | + | 12.5 | 59 | 545 | -686 | Tigger3b | MER2\_type | DNA |
| 124973852 | 124974213 | - | 15.0 | 1 | 364 | 0 | THE1B | MaLR | LTR |
| 124974214 | 124975787 | - | 30.5 | 2 | 1735 | 0 | MLT-int | MaLR | LTR |
| 124975790 | 124975950 | - | 11.2 | 195 | 364 | 0 | THE1B | MaLR | LTR |
| 124975954 | 124976222 | - | 17.4 | 1 | 283 | -29 | AluJo | Alu | SINE |
| 124976224 | 124976267 | + | 11.9 | 534 | 575 | -657 | Tigger3b | MER2\_type | DNA |
| 124976268 | 124976581 | + | 17.6 | 1 | 312 | 0 | AluJb | Alu | SINE |
| 124976582 | 124977232 | + | 11.9 | 576 | 1231 | -1 | Tigger3b | MER2\_type | DNA |

  

|  |  |  |  |  |  |  |  |  |  |
| --- | --- | --- | --- | --- | --- | --- | --- | --- | --- |
| chr10:125085168-125088274 | | | 3096 bp | | |  |  |  |  |
| Genome Start | Genome End | Strand | Div | Repeat Start | Repeat End | Repeat Left | Name | Family | Class |
| 125085168 | 125085770 | + | 11.6 | 2849 | 3463 | -2683 | L1PREC2 | L1 | LINE |
| 125085771 | 125086124 | - | 8.8 | 1 | 355 | 0 | THE1A | MaLR | LTR |
| 125086133 | 125087679 | - | 8.9 | 1 | 1556 | -24 | THE1A-int | MaLR | LTR |
| 125087680 | 125088029 | - | 7.4 | 5 | 355 | 0 | THE1A | MaLR | LTR |
| 125088033 | 125088274 | + | 10.4 | 3458 | 3698 | -2448 | L1PREC2 | L1 | LINE |

  

|  |  |  |  |  |  |  |  |  |  |
| --- | --- | --- | --- | --- | --- | --- | --- | --- | --- |
| chr10:125136469-125139275 | | | 2803 bp | | |  |  |  |  |
| Genome Start | Genome End | Strand | Div | Repeat Start | Repeat End | Repeat Left | Name | Family | Class |
| 125136469 | 125136576 | - | 23.2 | 402 | 503 | -2 | MLT1D | MaLR | LTR |
| 125136577 | 125136938 | + | 13.2 | 1 | 364 | 0 | THE1B | MaLR | LTR |
| 125136942 | 125138514 | + | 29.1 | 3 | 1735 | 0 | MLT-int | MaLR | LTR |
| 125138516 | 125138881 | + | 13.5 | 2 | 364 | 0 | THE1B | MaLR | LTR |
| 125138882 | 125139275 | - | 23.2 | 4 | 401 | -104 | MLT1D | MaLR | LTR |

  

|  |  |  |  |  |  |  |  |  |  |
| --- | --- | --- | --- | --- | --- | --- | --- | --- | --- |
| chr10:129315645-129326364 | | | 10715 bp | | |  |  |  |  |
| Genome Start | Genome End | Strand | Div | Repeat Start | Repeat End | Repeat Left | Name | Family | Class |
| 129315645 | 129317217 | - | 14.5 | 5407 | 7005 | 0 | L1MA4A | L1 | LINE |
| 129317218 | 129318314 | - | 10.6 | 1 | 1266 | 0 | MER11A | ERVK | LTR |
| 129318316 | 129320812 | - | 16.7 | 2898 | 5411 | -1434 | L1MA4A | L1 | LINE |
| 129320813 | 129321099 | - | 11.2 | 1 | 290 | -23 | AluSq | Alu | SINE |
| 129321100 | 129321679 | - | 16.9 | 2293 | 2897 | -3952 | L1MA4A | L1 | LINE |
| 129321680 | 129322052 | - | 11.1 | 1 | 428 | 0 | MSTA | MaLR | LTR |
| 129322053 | 129323628 | - | 29.7 | 4 | 1735 | 0 | MLT-int | MaLR | LTR |
| 129323633 | 129324017 | - | 15.3 | 1 | 428 | 0 | MSTA | MaLR | LTR |
| 129324018 | 129326364 | - | 16.9 | 2 | 2292 | -4557 | L1MA4A | L1 | LINE |

  

|  |  |  |  |  |  |  |  |  |  |
| --- | --- | --- | --- | --- | --- | --- | --- | --- | --- |
| chr10:133875606-133882736 | | | 6964 bp | | |  |  |  |  |
| Genome Start | Genome End | Strand | Div | Repeat Start | Repeat End | Repeat Left | Name | Family | Class |
| 133875606 | 133876189 | + | 26.9 | 4647 | 5276 | -870 | L1MB7 | L1 | LINE |
| 133876247 | 133878518 | + | 23.6 | 7 | 2664 | -4081 | L1MCb | L1 | LINE |
| 133878519 | 133878777 | - | 17.0 | 18 | 294 | -18 | AluJo | Alu | SINE |
| 133878821 | 133879180 | + | 9.5 | 1 | 364 | 0 | THE1B | MaLR | LTR |
| 133879181 | 133879676 | + | 8.3 | 1 | 492 | -1088 | THE1B-int | MaLR | LTR |
| 133879677 | 133879991 | + | 10.0 | 1 | 312 | 0 | AluSx | Alu | SINE |
| 133879992 | 133881054 | + | 8.3 | 493 | 1580 | 0 | THE1B-int | MaLR | LTR |
| 133881055 | 133881420 | + | 11.4 | 1 | 364 | 0 | THE1B | MaLR | LTR |
| 133881424 | 133881501 | - | 15.4 | 32 | 109 | -33 | FLAM\_A | Alu | SINE |
| 133881534 | 133881836 | + | 19.8 | 1 | 306 | -6 | AluJb | Alu | SINE |
| 133881869 | 133882736 | + | 22.1 | 5295 | 6184 | 0 | L1MB7 | L1 | LINE |

  

|  |  |  |  |  |  |  |  |  |  |
| --- | --- | --- | --- | --- | --- | --- | --- | --- | --- |
| chr10:134261724-134266338 | | | 4612 bp | | |  |  |  |  |
| Genome Start | Genome End | Strand | Div | Repeat Start | Repeat End | Repeat Left | Name | Family | Class |
| 134261724 | 134262205 | - | 17.8 | 5651 | 6181 | -2 | L1MB3 | L1 | LINE |
| 134262206 | 134262558 | - | 9.1 | 1 | 355 | 0 | THE1A | MaLR | LTR |
| 134262559 | 134264128 | - | 30.1 | 2 | 1735 | 0 | MLT-int | MaLR | LTR |
| 134264132 | 134264484 | - | 7.1 | 1 | 354 | -1 | THE1A | MaLR | LTR |
| 134264485 | 134265802 | - | 19.7 | 4268 | 5650 | -533 | L1MB3 | L1 | LINE |
| 134265803 | 134266088 | - | 12.3 | 1 | 287 | -25 | AluSx | Alu | SINE |
| 134266089 | 134266338 | - | 20.6 | 4012 | 4267 | -1873 | L1MB3 | L1 | LINE |

  


|  |  |  |  |  |  |  |  |  |  |
| --- | --- | --- | --- | --- | --- | --- | --- | --- | --- |
| chr11:89455-92271 | | | 2813 bp | | |  |  |  |  |
| Genome Start | Genome End | Strand | Div | Repeat Start | Repeat End | Repeat Left | Name | Family | Class |
| 89455 | 89695 | + | 29.3 | 1 | 249 | -306 | MLT1H1 | MaLR | LTR |
| 89696 | 90053 | - | 18.1 | 1 | 364 | 0 | THE1B | MaLR | LTR |
| 90054 | 91613 | - | 30.4 | 4 | 1735 | 0 | MLT-int | MaLR | LTR |
| 91618 | 91977 | - | 15.0 | 3 | 364 | 0 | THE1B | MaLR | LTR |
| 91978 | 92271 | + | 29.3 | 250 | 540 | -15 | MLT1H1 | MaLR | LTR |

  

|  |  |  |  |  |  |  |  |  |  |
| --- | --- | --- | --- | --- | --- | --- | --- | --- | --- |
| chr11:2321753-2339401 | | | 16830 bp | | |  |  |  |  |
| Genome Start | Genome End | Strand | Div | Repeat Start | Repeat End | Repeat Left | Name | Family | Class |
| 2321753 | 2321873 | + | 29.4 | 1585 | 1706 | -3290 | ERVL-B3 | ERVL | LTR |
| 2321874 | 2322251 | + | 15.7 | 1 | 390 | 0 | MLT1B | MaLR | LTR |
| 2322252 | 2322851 | + | 29.4 | 1707 | 2357 | -2639 | ERVL-B3 | ERVL | LTR |
| 2322891 | 2323118 | + | 18.4 | 1 | 243 | -313 | MLT2B4 | ERVL | LTR |
| 2323119 | 2323502 | - | 14.1 | 34 | 432 | 0 | MSTB1 | MaLR | LTR |
| 2323529 | 2323689 | + | 19.3 | 244 | 517 | -50 | MLT2B1 | ERVL | LTR |
| 2323895 | 2324231 | + | 33.5 | 3860 | 4203 | -1464 | ERVL-B3 | ERVL | LTR |
| 2324638 | 2325185 | + | 38.3 | 4732 | 5279 | -435 | ERVL-B3 | ERVL | LTR |
| 2325265 | 2325779 | - | 16.9 | 55 | 553 | -1 | MLT2B3 | ERVL | LTR |
| 2325781 | 2325864 | + | 9.2 | 1 | 84 | -470 | MLT2B3 | ERVL | LTR |
| 2325865 | 2326240 | + | 12.9 | 1 | 401 | -27 | MSTA | MaLR | LTR |
| 2326241 | 2326593 | - | 16.8 | 1 | 367 | 0 | AluSx | Alu | SINE |
| 2326635 | 2328283 | + | 17.1 | 1 | 1651 | 0 | MSTB-int | MaLR | LTR |
| 2328284 | 2328668 | + | 15.4 | 1 | 426 | 0 | MSTB | MaLR | LTR |
| 2328669 | 2328719 | + | 13.7 | 80 | 130 | -424 | MLT2B5 | ERVL | LTR |
| 2328720 | 2329117 | - | 21.0 | 1 | 408 | 0 | MLT1A1 | MaLR | LTR |
| 2329118 | 2330603 | - | 24.6 | 213 | 1735 | 0 | MLT1A1-int | MaLR | LTR |
| 2330604 | 2330899 | - | 5.7 | 1 | 296 | -15 | AluY | Alu | SINE |
| 2330900 | 2331096 | - | 24.6 | 20 | 212 | -1523 | MLT1A1-int | MaLR | LTR |
| 2331116 | 2331490 | - | 25.3 | 35 | 408 | 0 | MLT1A1 | MaLR | LTR |
| 2331491 | 2331780 | + | 11.5 | 1 | 300 | -12 | AluSx | Alu | SINE |
| 2331781 | 2331809 | - | 25.3 | 2 | 34 | -374 | MLT1A1 | MaLR | LTR |
| 2331810 | 2332239 | + | 19.6 | 130 | 552 | -49 | MLT2B5 | ERVL | LTR |
| 2332240 | 2332541 | - | 8.6 | 1 | 301 | -8 | AluSc | Alu | SINE |
| 2332542 | 2332590 | + | 19.6 | 507 | 548 | -7 | MLT2B5 | ERVL | LTR |
| 2332594 | 2333888 | + | 19.7 | 1 | 1321 | -4393 | ERVL-B3 | ERVL | LTR |
| 2333889 | 2334413 | + | 12.6 | 1 | 529 | 0 | MER1A | MER1\_type | DNA |
| 2334414 | 2336368 | + | 19.7 | 1322 | 3337 | -2377 | ERVL-B3 | ERVL | LTR |
| 2336369 | 2336657 | + | 11.1 | 23 | 312 | 0 | AluSx | Alu | SINE |
| 2336658 | 2338513 | + | 19.7 | 3338 | 5131 | -583 | ERVL-B3 | ERVL | LTR |
| 2338514 | 2338824 | + | 5.8 | 1 | 310 | -1 | AluY | Alu | SINE |
| 2338825 | 2339401 | + | 19.7 | 5132 | 5714 | 0 | ERVL-B3 | ERVL | LTR |

  

|  |  |  |  |  |  |  |  |  |  |
| --- | --- | --- | --- | --- | --- | --- | --- | --- | --- |
| chr11:2455579-2458830 | | | 3187 bp | | |  |  |  |  |
| Genome Start | Genome End | Strand | Div | Repeat Start | Repeat End | Repeat Left | Name | Family | Class |
| 2455579 | 2455697 | + | 27.8 | 7006 | 7130 | -912 | L1MC4 | L1 | LINE |
| 2455761 | 2456131 | + | 30.8 | 7269 | 7658 | -384 | L1MC4 | L1 | LINE |
| 2456132 | 2456503 | - | 13.8 | 1 | 364 | 0 | THE1B | MaLR | LTR |
| 2456504 | 2458074 | - | 30.4 | 2 | 1735 | 0 | MLT-int | MaLR | LTR |
| 2458077 | 2458449 | - | 10.5 | 1 | 364 | 0 | THE1B | MaLR | LTR |
| 2458450 | 2458830 | + | 30.8 | 7659 | 8026 | -16 | L1MC4 | L1 | LINE |

  

|  |  |  |  |  |  |  |  |  |  |
| --- | --- | --- | --- | --- | --- | --- | --- | --- | --- |
| chr11:3447568-3463566 | | | 15976 bp | | |  |  |  |  |
| Genome Start | Genome End | Strand | Div | Repeat Start | Repeat End | Repeat Left | Name | Family | Class |
| 3447568 | 3447743 | + | 22.3 | 1 | 185 | -320 | MLT1D | MaLR | LTR |
| 3447754 | 3448050 | + | 9.1 | 3 | 300 | -13 | AluSp | Alu | SINE |
| 3448063 | 3448459 | - | 8.6 | 1 | 448 | -2 | LTR7 | ERV1 | LTR |
| 3448460 | 3448837 | - | 10.7 | 7324 | 7713 | 0 | HERVH | ERV1 | LTR |
| 3448838 | 3449048 | - | 12.3 | 5600 | 5819 | -1894 | HERVH | ERV1 | LTR |
| 3449050 | 3449115 | - | 14.5 | 5090 | 5149 | -2564 | HERVH | ERV1 | LTR |
| 3449116 | 3450181 | - | 4.2 | 1 | 1071 | 0 | MER11C | ERVK | LTR |
| 3450182 | 3450782 | - | 14.5 | 4489 | 5089 | -2624 | HERVH | ERV1 | LTR |
| 3450778 | 3451334 | - | 8.6 | 3442 | 4000 | -3713 | HERVH | ERV1 | LTR |
| 3451335 | 3451831 | - | 8.7 | 1 | 501 | 0 | LTR2C | ERV1 | LTR |
| 3451832 | 3457043 | - | 6.6 | 2611 | 7812 | -1 | HERVE | ERV1 | LTR |
| 3457044 | 3459517 | - | 7.3 | 3 | 2493 | -5320 | HERVE | ERV1 | LTR |
| 3459518 | 3459822 | - | 11.1 | 3140 | 3441 | -4272 | HERVH | ERV1 | LTR |
| 3459809 | 3462641 | - | 10.5 | 1 | 2992 | -4721 | HERVH | ERV1 | LTR |
| 3462644 | 3462993 | - | 9.8 | 1 | 448 | -2 | LTR7 | ERV1 | LTR |
| 3463011 | 3463201 | + | 24.8 | 193 | 428 | -76 | MLT1D | MaLR | LTR |
| 3463202 | 3463501 | - | 14.8 | 1 | 298 | -14 | AluSx | Alu | SINE |
| 3463502 | 3463566 | + | 24.9 | 392 | 483 | -1 | MLT1D | MaLR | LTR |

  

|  |  |  |  |  |  |  |  |  |  |
| --- | --- | --- | --- | --- | --- | --- | --- | --- | --- |
| chr11:3514098-3521435 | | | 7297 bp | | |  |  |  |  |
| Genome Start | Genome End | Strand | Div | Repeat Start | Repeat End | Repeat Left | Name | Family | Class |
| 3514098 | 3514336 | + | 19.4 | 2887 | 3139 | -4070 | L1MA4 | L1 | LINE |
| 3514337 | 3514694 | - | 13.0 | 1 | 364 | 0 | THE1B | MaLR | LTR |
| 3514695 | 3516247 | - | 11.2 | 1 | 1580 | 0 | THE1B-int | MaLR | LTR |
| 3516248 | 3516600 | - | 14.7 | 1 | 364 | 0 | THE1B | MaLR | LTR |
| 3516601 | 3518808 | + | 16.2 | 3135 | 5336 | -1869 | L1MA4 | L1 | LINE |
| 3518809 | 3519119 | + | 12.4 | 1 | 310 | -2 | AluSx | Alu | SINE |
| 3519120 | 3519604 | + | 16.3 | 5337 | 5796 | -1409 | L1MA4 | L1 | LINE |
| 3519605 | 3519882 | + | 13.0 | 1 | 304 | 0 | AluY | Alu | SINE |
| 3519883 | 3520348 | + | 16.3 | 5797 | 6265 | -940 | L1MA4 | L1 | LINE |
| 3520349 | 3520594 | + | 10.2 | 4958 | 5204 | -942 | L1PREC2 | L1 | LINE |
| 3520578 | 3521170 | + | 13.5 | 6389 | 6989 | -373 | L1MA4 | L1 | LINE |
| 3521229 | 3521435 | + | 15.5 | 6961 | 7184 | -178 | L1MA4 | L1 | LINE |

  

|  |  |  |  |  |  |  |  |  |  |
| --- | --- | --- | --- | --- | --- | --- | --- | --- | --- |
| chr11:4759466-4762928 | | | 3374 bp | | |  |  |  |  |
| Genome Start | Genome End | Strand | Div | Repeat Start | Repeat End | Repeat Left | Name | Family | Class |
| 4759466 | 4759499 | - | 29.3 | 561 | 593 | -2 | MLT1G1 | MaLR | LTR |
| 4759500 | 4759872 | + | 13.2 | 1 | 364 | 0 | THE1B | MaLR | LTR |
| 4759875 | 4761443 | + | 30.0 | 2 | 1735 | 0 | MLT-int | MaLR | LTR |
| 4761444 | 4761811 | + | 12.8 | 1 | 363 | -1 | THE1B | MaLR | LTR |
| 4761812 | 4762116 | - | 29.3 | 223 | 560 | -35 | MLT1G1 | MaLR | LTR |
| 4762154 | 4762588 | + | 26.6 | 3 | 505 | 0 | MLT1D | MaLR | LTR |
| 4762615 | 4762712 | - | 18.4 | 6203 | 6300 | 0 | L1MA6 | L1 | LINE |
| 4762718 | 4762770 | + | 18.9 | 6108 | 6160 | -140 | L1MA6 | L1 | LINE |
| 4762790 | 4762928 | - | 25.9 | 2 | 144 | -451 | MLT1G1 | MaLR | LTR |

  

|  |  |  |  |  |  |  |  |  |  |
| --- | --- | --- | --- | --- | --- | --- | --- | --- | --- |
| chr11:5802986-5805572 | | | 2584 bp | | |  |  |  |  |
| Genome Start | Genome End | Strand | Div | Repeat Start | Repeat End | Repeat Left | Name | Family | Class |
| 5802986 | 5803150 | + | 14.9 | 1 | 169 | -143 | AluSx | Alu | SINE |
| 5803151 | 5803509 | + | 14.4 | 1 | 364 | 0 | THE1B | MaLR | LTR |
| 5803513 | 5805077 | + | 30.2 | 3 | 1735 | 0 | MLT1F1-int | MaLR | LTR |
| 5805078 | 5805431 | + | 12.6 | 1 | 364 | 0 | THE1B | MaLR | LTR |
| 5805432 | 5805572 | + | 14.9 | 170 | 307 | -5 | AluSx | Alu | SINE |

  

|  |  |  |  |  |  |  |  |  |  |
| --- | --- | --- | --- | --- | --- | --- | --- | --- | --- |
| chr11:6342220-6355906 | | | 12913 bp | | |  |  |  |  |
| Genome Start | Genome End | Strand | Div | Repeat Start | Repeat End | Repeat Left | Name | Family | Class |
| 6342220 | 6342656 | - | 30.8 | 2733 | 3158 | -220 | L2 | L2 | LINE |
| 6342663 | 6342691 | - | 33.9 | 3388 | 3416 | -3 | L2 | L2 | LINE |
| 6342692 | 6343115 | + | 22.2 | 1 | 505 | 0 | MLT1D | MaLR | LTR |
| 6343116 | 6343194 | - | 33.9 | 3306 | 3387 | -32 | L2 | L2 | LINE |
| 6343214 | 6343407 | - | 27.4 | 3205 | 3419 | 0 | L2 | L2 | LINE |
| 6343542 | 6343810 | + | 27.9 | 13 | 305 | -218 | LTR33A | ERVL | LTR |
| 6343834 | 6344237 | + | 7.2 | 1 | 441 | -9 | LTR7 | ERV1 | LTR |
| 6344248 | 6347030 | + | 11.2 | 1 | 2995 | -4718 | HERVH | ERV1 | LTR |
| 6347010 | 6347857 | + | 10.7 | 3140 | 4000 | -3713 | HERVH | ERV1 | LTR |
| 6347853 | 6348463 | + | 13.3 | 4489 | 5149 | -2564 | HERVH | ERV1 | LTR |
| 6348465 | 6348684 | + | 12.3 | 5600 | 5819 | -1894 | HERVH | ERV1 | LTR |
| 6348685 | 6349055 | + | 7.9 | 7324 | 7713 | 0 | HERVH | ERV1 | LTR |
| 6349056 | 6349466 | + | 8.8 | 1 | 448 | -2 | LTR7 | ERV1 | LTR |
| 6349469 | 6349637 | + | 23.2 | 339 | 513 | -10 | LTR33A | ERVL | LTR |
| 6349645 | 6349835 | - | 26.9 | 2994 | 3195 | -224 | L2 | L2 | LINE |
| 6349948 | 6350175 | + | 23.8 | 2910 | 3135 | -284 | L2 | L2 | LINE |
| 6350176 | 6350466 | - | 11.0 | 1 | 290 | -19 | AluSg1 | Alu | SINE |
| 6350467 | 6350728 | + | 23.8 | 3136 | 3419 | 0 | L2 | L2 | LINE |
| 6350728 | 6351006 | - | 31.8 | 2556 | 2844 | -575 | L2 | L2 | LINE |
| 6351007 | 6351138 | + | 16.7 | 1 | 132 | -1 | FLAM\_A | Alu | SINE |
| 6351139 | 6351304 | - | 31.8 | 2430 | 2555 | -864 | L2 | L2 | LINE |
| 6351505 | 6351826 | + | 15.7 | 1 | 312 | 0 | AluJb | Alu | SINE |
| 6351839 | 6352137 | + | 9.4 | 1 | 299 | -14 | AluSp | Alu | SINE |
| 6352154 | 6352353 | + | 32.0 | 1 | 209 | -299 | FordPrefect\_a | Tip100 | DNA |
| 6352371 | 6352684 | + | 17.3 | 1 | 312 | 0 | AluSx | Alu | SINE |
| 6352693 | 6352866 | + | 31.8 | 5040 | 5225 | -921 | L1M4 | L1 | LINE |
| 6352896 | 6353087 | - | 10.9 | 258 | 434 | 0 | MER57B | ERV1 | LTR |
| 6353088 | 6353376 | + | 6.6 | 15 | 302 | -9 | AluY | Alu | SINE |
| 6353377 | 6353633 | - | 10.9 | 1 | 257 | -177 | MER57B | ERV1 | LTR |
| 6353639 | 6353946 | - | 28.9 | 1805 | 2151 | -1268 | L2 | L2 | LINE |
| 6354040 | 6354496 | + | 26.9 | 1 | 505 | -6 | MLT1J | MaLR | LTR |
| 6354504 | 6354808 | + | 9.8 | 1 | 305 | -7 | AluSx | Alu | SINE |
| 6354915 | 6355008 | + | 20.6 | 63 | 155 | -107 | MIR | MIR | SINE |
| 6355003 | 6355303 | - | 37.5 | 1311 | 1611 | -1808 | L2 | L2 | LINE |
| 6355304 | 6355614 | - | 6.1 | 1 | 311 | 0 | AluY | Alu | SINE |
| 6355615 | 6355906 | - | 37.5 | 1014 | 1310 | -2109 | L2 | L2 | LINE |

  

|  |  |  |  |  |  |  |  |  |  |
| --- | --- | --- | --- | --- | --- | --- | --- | --- | --- |
| chr11:7311590-7324384 | | | 12350 bp | | |  |  |  |  |
| Genome Start | Genome End | Strand | Div | Repeat Start | Repeat End | Repeat Left | Name | Family | Class |
| 7311590 | 7312208 | - | 33.2 | 2724 | 3372 | -6 | L2 | L2 | LINE |
| 7312266 | 7313151 | - | 22.3 | 5355 | 6235 | -4 | L1ME1 | L1 | LINE |
| 7313121 | 7319040 | - | 12.6 | 479 | 6343 | -2 | L1PREC2 | L1 | LINE |
| 7319106 | 7319467 | + | 12.5 | 1 | 364 | 0 | THE1B | MaLR | LTR |
| 7319612 | 7319965 | - | 16.6 | 1 | 364 | 0 | THE1B | MaLR | LTR |
| 7319966 | 7321535 | - | 30.4 | 2 | 1735 | 0 | MLT1J-int | MaLR | LTR |
| 7321538 | 7321898 | - | 11.2 | 1 | 364 | 0 | THE1B | MaLR | LTR |
| 7322063 | 7322736 | - | 24.1 | 4698 | 5373 | -846 | L1ME1 | L1 | LINE |
| 7322771 | 7322920 | - | 24.1 | 4538 | 4697 | -1522 | L1ME1 | L1 | LINE |
| 7322931 | 7323271 | - | 26.3 | 44 | 395 | -6005 | L1ME1 | L1 | LINE |
| 7323272 | 7324384 | - | 33.5 | 1470 | 2610 | -809 | L2 | L2 | LINE |

  

|  |  |  |  |  |  |  |  |  |  |
| --- | --- | --- | --- | --- | --- | --- | --- | --- | --- |
| chr11:7890144-7896226 | | | 5964 bp | | |  |  |  |  |
| Genome Start | Genome End | Strand | Div | Repeat Start | Repeat End | Repeat Left | Name | Family | Class |
| 7890144 | 7890269 | - | 25.8 | 278 | 402 | 0 | MER93a | ERV1 | LTR |
| 7890274 | 7890724 | - | 5.8 | 1 | 448 | -2 | LTR7 | ERV1 | LTR |
| 7890725 | 7891099 | - | 7.5 | 7322 | 7713 | 0 | HERVH | ERV1 | LTR |
| 7891098 | 7891315 | - | 12.5 | 5600 | 5819 | -1894 | HERVH | ERV1 | LTR |
| 7891317 | 7891978 | - | 13.9 | 4488 | 5149 | -2564 | HERVH | ERV1 | LTR |
| 7891981 | 7892481 | - | 12.4 | 3492 | 3993 | -3720 | HERVH | ERV1 | LTR |
| 7892472 | 7895588 | - | 10.0 | 1 | 3290 | -4423 | HERVH | ERV1 | LTR |
| 7895591 | 7896030 | - | 6.0 | 1 | 448 | -2 | LTR7 | ERV1 | LTR |
| 7896153 | 7896226 | - | 21.9 | 1 | 73 | -329 | MER93a | ERV1 | LTR |

  

|  |  |  |  |  |  |  |  |  |  |
| --- | --- | --- | --- | --- | --- | --- | --- | --- | --- |
| chr11:10108537-10117043 | | | 8335 bp | | |  |  |  |  |
| Genome Start | Genome End | Strand | Div | Repeat Start | Repeat End | Repeat Left | Name | Family | Class |
| 10108537 | 10108982 | - | 22.3 | 5915 | 7992 | -3 | L1MD2 | L1 | LINE |
| 10108983 | 10109345 | - | 32.0 | 5465 | 5836 | -319 | L1HS | L1 | LINE |
| 10109350 | 10109464 | - | 10.5 | 186 | 299 | -13 | AluJb | Alu | SINE |
| 10109469 | 10109581 | - | 19.1 | 5283 | 5361 | -785 | L1MD2 | L1 | LINE |
| 10109582 | 10109863 | - | 9.4 | 1 | 275 | -38 | AluSp | Alu | SINE |
| 10109864 | 10110340 | - | 19.1 | 4796 | 5282 | -864 | L1MD2 | L1 | LINE |
| 10110364 | 10110443 | + | 12.9 | 17 | 94 | -841 | MER21C | ERV1 | LTR |
| 10110444 | 10111993 | - | 7.9 | 1 | 1578 | 0 | LTR12C | ERV1 | LTR |
| 10111994 | 10112089 | + | 12.9 | 95 | 193 | -742 | MER21C | ERV1 | LTR |
| 10112118 | 10112479 | - | 11.9 | 1 | 364 | 0 | THE1B | MaLR | LTR |
| 10112480 | 10114053 | - | 9.4 | 2 | 1580 | 0 | THE1B-int | MaLR | LTR |
| 10114055 | 10114423 | - | 13.6 | 1 | 364 | 0 | THE1B | MaLR | LTR |
| 10114433 | 10114635 | + | 19.3 | 177 | 415 | -520 | MER21C | ERV1 | LTR |
| 10114674 | 10114935 | + | 20.4 | 631 | 931 | -4 | MER21C | ERV1 | LTR |
| 10114970 | 10115847 | - | 18.2 | 3626 | 4725 | -1421 | L1MD2 | L1 | LINE |
| 10115848 | 10116122 | + | 9.1 | 1 | 282 | -30 | AluSx | Alu | SINE |
| 10116154 | 10117043 | - | 20.9 | 2659 | 3625 | -2521 | L1MD2 | L1 | LINE |

  

|  |  |  |  |  |  |  |  |  |  |
| --- | --- | --- | --- | --- | --- | --- | --- | --- | --- |
| chr11:11658464-11666968 | | | 8002 bp | | |  |  |  |  |
| Genome Start | Genome End | Strand | Div | Repeat Start | Repeat End | Repeat Left | Name | Family | Class |
| 11658464 | 11658845 | + | 24.2 | 3437 | 3827 | -2319 | L1ME2 | L1 | LINE |
| 11658846 | 11659129 | - | 15.5 | 2 | 298 | -14 | AluJb | Alu | SINE |
| 11659130 | 11659248 | + | 24.2 | 3828 | 3955 | -2191 | L1ME2 | L1 | LINE |
| 11659266 | 11659555 | + | 12.1 | 1 | 293 | -20 | AluSp | Alu | SINE |
| 11659592 | 11660001 | - | 29.4 | 4 | 462 | -2 | LTR16B | ERVL | LTR |
| 11660023 | 11660253 | + | 23.5 | 4361 | 4589 | -1557 | L1ME2 | L1 | LINE |
| 11660254 | 11660644 | + | 19.2 | 1 | 390 | 0 | MLT1B | MaLR | LTR |
| 11660645 | 11660732 | + | 23.5 | 4590 | 4675 | -1471 | L1ME2 | L1 | LINE |
| 11660717 | 11661166 | + | 12.5 | 4856 | 5325 | -815 | L1MA8 | L1 | LINE |
| 11661296 | 11661650 | + | 13.0 | 1 | 364 | 0 | THE1B | MaLR | LTR |
| 11661736 | 11662707 | + | 14.6 | 5300 | 6291 | 0 | L1MA8 | L1 | LINE |
| 11662710 | 11662895 | + | 21.7 | 4653 | 4838 | -1308 | L1ME2 | L1 | LINE |
| 11662909 | 11663206 | + | 15.8 | 12 | 307 | -5 | AluJb | Alu | SINE |
| 11663421 | 11664467 | + | 22.6 | 4831 | 5919 | -245 | L1ME2 | L1 | LINE |
| 11664468 | 11664819 | + | 11.5 | 1 | 355 | 0 | THE1A | MaLR | LTR |
| 11664822 | 11666383 | + | 30.2 | 2 | 1735 | 0 | MLT-int | MaLR | LTR |
| 11666384 | 11666735 | + | 9.6 | 1 | 355 | 0 | THE1A | MaLR | LTR |
| 11666736 | 11666968 | + | 22.0 | 5920 | 6154 | -10 | L1ME2 | L1 | LINE |

  

|  |  |  |  |  |  |  |  |  |  |
| --- | --- | --- | --- | --- | --- | --- | --- | --- | --- |
| chr11:11801377-11806014 | | | 4125 bp | | |  |  |  |  |
| Genome Start | Genome End | Strand | Div | Repeat Start | Repeat End | Repeat Left | Name | Family | Class |
| 11801377 | 11801452 | + | 27.6 | 5 | 80 | -2532 | Charlie7 | MER1\_type | DNA |
| 11801737 | 11801943 | + | 16.4 | 1 | 207 | -139 | MER7A | MER2\_type | DNA |
| 11801944 | 11802300 | - | 9.9 | 1 | 355 | 0 | THE1A | MaLR | LTR |
| 11802303 | 11803882 | - | 31.4 | 2 | 1733 | -2 | MLT-int | MaLR | LTR |
| 11803885 | 11804235 | - | 8.9 | 1 | 355 | 0 | THE1A | MaLR | LTR |
| 11804236 | 11804383 | + | 16.4 | 208 | 346 | 0 | MER7A | MER2\_type | DNA |
| 11804450 | 11804541 | + | 26.7 | 90 | 184 | -2428 | Charlie7 | MER1\_type | DNA |
| 11804636 | 11804727 | + | 25.0 | 327 | 419 | -2193 | Charlie7 | MER1\_type | DNA |
| 11804791 | 11805235 | + | 19.4 | 2 | 433 | -1 | Tigger2a | MER2\_type | DNA |
| 11805236 | 11805469 | - | 27.8 | 2 | 251 | -11 | MIR | MIR | SINE |
| 11805472 | 11805657 | + | 25.2 | 446 | 627 | -1985 | Charlie7 | MER1\_type | DNA |
| 11805658 | 11805944 | - | 19.0 | 1 | 288 | -24 | AluJo | Alu | SINE |
| 11805945 | 11806014 | + | 25.2 | 628 | 692 | -1920 | Charlie7 | MER1\_type | DNA |

  

|  |  |  |  |  |  |  |  |  |  |
| --- | --- | --- | --- | --- | --- | --- | --- | --- | --- |
| chr11:12000002-12003213 | | | 3208 bp | | |  |  |  |  |
| Genome Start | Genome End | Strand | Div | Repeat Start | Repeat End | Repeat Left | Name | Family | Class |
| 12000002 | 12000292 | + | 12.1 | 5254 | 5545 | -629 | L1MB5 | L1 | LINE |
| 12000293 | 12000664 | + | 11.3 | 1 | 375 | 0 | THE1C | MaLR | LTR |
| 12000665 | 12002244 | + | 12.9 | 1 | 1575 | -5 | THE1C-int | MaLR | LTR |
| 12002248 | 12002620 | + | 15.2 | 1 | 375 | 0 | THE1C | MaLR | LTR |
| 12002622 | 12003213 | + | 18.3 | 5542 | 6166 | -8 | L1MB5 | L1 | LINE |

  

|  |  |  |  |  |  |  |  |  |  |
| --- | --- | --- | --- | --- | --- | --- | --- | --- | --- |
| chr11:12017619-12020411 | | | 2774 bp | | |  |  |  |  |
| Genome Start | Genome End | Strand | Div | Repeat Start | Repeat End | Repeat Left | Name | Family | Class |
| 12017619 | 12017724 | + | 22.1 | 5406 | 5511 | -672 | L1M3 | L1 | LINE |
| 12017728 | 12018092 | - | 14.2 | 1 | 362 | -2 | THE1B | MaLR | LTR |
| 12018096 | 12019122 | - | 9.9 | 532 | 1552 | -28 | THE1B-int | MaLR | LTR |
| 12019123 | 12019419 | + | 13.5 | 1 | 308 | -2 | AluSg | Alu | SINE |
| 12019420 | 12019935 | - | 9.9 | 7 | 531 | -1049 | THE1B-int | MaLR | LTR |
| 12019947 | 12020310 | - | 15.5 | 3 | 364 | 0 | THE1B | MaLR | LTR |
| 12020313 | 12020411 | + | 19.2 | 5507 | 5605 | -566 | L1M3 | L1 | LINE |

  

|  |  |  |  |  |  |  |  |  |  |
| --- | --- | --- | --- | --- | --- | --- | --- | --- | --- |
| chr11:13523019-13528731 | | | 5738 bp | | |  |  |  |  |
| Genome Start | Genome End | Strand | Div | Repeat Start | Repeat End | Repeat Left | Name | Family | Class |
| 13523019 | 13523398 | - | 12.3 | 2119 | 2514 | -4349 | L1PBa | L1 | LINE |
| 13523399 | 13523761 | + | 15.3 | 1 | 364 | 0 | THE1B | MaLR | LTR |
| 13523764 | 13525339 | + | 31.2 | 2 | 1735 | 0 | MLT1J-int | MaLR | LTR |
| 13525342 | 13525703 | + | 14.3 | 3 | 364 | 0 | THE1B | MaLR | LTR |
| 13525704 | 13527131 | - | 14.8 | 8 | 1459 | -4745 | L1PBb | L1 | LINE |
| 13527103 | 13528731 | - | 21.3 | 4 | 1640 | -5223 | L1PBa | L1 | LINE |

  

|  |  |  |  |  |  |  |  |  |  |
| --- | --- | --- | --- | --- | --- | --- | --- | --- | --- |
| chr11:13787970-13796599 | | | 8149 bp | | |  |  |  |  |
| Genome Start | Genome End | Strand | Div | Repeat Start | Repeat End | Repeat Left | Name | Family | Class |
| 13787970 | 13788394 | - | 27.1 | 2772 | 3247 | -172 | L2 | L2 | LINE |
| 13788408 | 13788585 | + | 21.4 | 525 | 703 | -3237 | Arthur1 | Tip100 | DNA |
| 13788601 | 13789455 | + | 20.6 | 2853 | 3773 | -167 | Arthur1 | Tip100 | DNA |
| 13789456 | 13789763 | - | 17.2 | 1 | 300 | -12 | AluJo | Alu | SINE |
| 13789764 | 13789916 | + | 20.6 | 3774 | 3935 | -5 | Arthur1 | Tip100 | DNA |
| 13789946 | 13789999 | - | 21.6 | 2366 | 2415 | -1004 | L2 | L2 | LINE |
| 13790000 | 13790349 | - | 9.1 | 1 | 355 | 0 | THE1A | MaLR | LTR |
| 13790350 | 13791915 | - | 31.1 | 2 | 1735 | 0 | MLT1F1-int | MaLR | LTR |
| 13791920 | 13792268 | - | 7.5 | 1 | 353 | -2 | THE1A | MaLR | LTR |
| 13792269 | 13792342 | - | 21.6 | 2299 | 2365 | -1054 | L2 | L2 | LINE |
| 13792343 | 13792416 | - | 16.4 | 203 | 275 | 0 | ORSL | Tip100 | DNA |
| 13792419 | 13792649 | + | 21.7 | 1 | 238 | -2085 | Tigger5 | MER2\_type | DNA |
| 13792640 | 13792810 | + | 11.1 | 2168 | 2338 | 0 | Tigger5 | MER2\_type | DNA |
| 13792816 | 13792935 | - | 28.2 | 3297 | 3419 | 0 | L2 | L2 | LINE |
| 13792963 | 13793013 | + | 17.6 | 14 | 65 | -32 | Tigger5c | MER2\_type | DNA |
| 13793014 | 13793091 | + | 14.1 | 2320 | 2406 | 0 | Tigger5 | MER2\_type | DNA |
| 13793186 | 13793289 | - | 17.3 | 4 | 107 | -168 | ORSL | Tip100 | DNA |
| 13793290 | 13793395 | - | 27.4 | 2198 | 2306 | -1113 | L2 | L2 | LINE |
| 13793682 | 13794377 | - | 26.4 | 1369 | 2196 | -1223 | L2 | L2 | LINE |
| 13794378 | 13794564 | + | 23.9 | 1 | 1591 | 0 | MER69A | Tip100 | DNA |
| 13794565 | 13794983 | - | 26.4 | 871 | 1368 | -2051 | L2 | L2 | LINE |
| 13794985 | 13795226 | + | 18.4 | 1 | 337 | -222 | MER89 | ERV1 | LTR |
| 13795227 | 13795532 | + | 11.1 | 1 | 306 | -4 | AluSg | Alu | SINE |
| 13795533 | 13795712 | + | 18.8 | 338 | 506 | -53 | MER89 | ERV1 | LTR |
| 13795728 | 13796599 | - | 30.7 | 2 | 871 | -2548 | L2 | L2 | LINE |

  

|  |  |  |  |  |  |  |  |  |  |
| --- | --- | --- | --- | --- | --- | --- | --- | --- | --- |
| chr11:14990983-15004980 | | | 13892 bp | | |  |  |  |  |
| Genome Start | Genome End | Strand | Div | Repeat Start | Repeat End | Repeat Left | Name | Family | Class |
| 14990983 | 14991470 | - | 17.1 | 5921 | 6402 | -516 | HUERS-P1 | ERV1 | LTR |
| 14991472 | 14991640 | - | 7.7 | 131 | 299 | -11 | AluYa5 | Alu | SINE |
| 14991641 | 14991835 | - | 16.8 | 5425 | 5633 | -630 | HUERS-P1 | ERV1 | LTR |
| 14991836 | 14992132 | - | 12.8 | 2 | 301 | -10 | AluY | Alu | SINE |
| 14992133 | 14992744 | - | 16.8 | 4865 | 5424 | -839 | HUERS-P1 | ERV1 | LTR |
| 14992771 | 14994674 | + | 11.6 | 669 | 2526 | -3805 | L1PA10 | L1 | LINE |
| 14994675 | 14995030 | + | 13.6 | 1 | 375 | 0 | THE1C | MaLR | LTR |
| 14995031 | 14996385 | + | 13.3 | 1 | 1580 | 0 | THE1C-int | MaLR | LTR |
| 14996386 | 14996743 | + | 17.3 | 1 | 375 | 0 | THE1C | MaLR | LTR |
| 14996745 | 15000575 | + | 9.5 | 2523 | 6346 | -7 | L1PA10 | L1 | LINE |
| 15000580 | 15002614 | - | 22.8 | 2226 | 4261 | -1685 | HUERS-P1 | ERV1 | LTR |
| 15002654 | 15003720 | - | 21.2 | 1227 | 2373 | -3890 | HUERS-P1 | ERV1 | LTR |
| 15003756 | 15004980 | - | 21.2 | 3 | 1226 | -4862 | HUERS-P1 | ERV1 | LTR |

  

|  |  |  |  |  |  |  |  |  |  |
| --- | --- | --- | --- | --- | --- | --- | --- | --- | --- |
| chr11:16472657-16487096 | | | 14431 bp | | |  |  |  |  |
| Genome Start | Genome End | Strand | Div | Repeat Start | Repeat End | Repeat Left | Name | Family | Class |
| 16472657 | 16474457 | + | 20.8 | 4 | 1844 | -5005 | L1MA4A | L1 | LINE |
| 16474456 | 16480594 | + | 6.1 | 1 | 6153 | -2 | L1PA4 | L1 | LINE |
| 16480581 | 16481885 | + | 14.9 | 1825 | 3154 | -3691 | L1MA4A | L1 | LINE |
| 16481886 | 16482221 | + | 9.6 | 1 | 337 | 0 | AluY | Alu | SINE |
| 16482222 | 16482284 | + | 17.1 | 3155 | 3211 | -3634 | L1MA4A | L1 | LINE |
| 16482287 | 16482768 | + | 14.6 | 4759 | 5252 | -1593 | L1MA4A | L1 | LINE |
| 16482773 | 16483138 | + | 15.8 | 2 | 428 | 0 | MSTA | MaLR | LTR |
| 16483152 | 16484733 | + | 14.9 | 14 | 1578 | -2 | MSTA-int | MaLR | LTR |
| 16484736 | 16484904 | + | 15.1 | 1 | 195 | -233 | MSTA | MaLR | LTR |
| 16484905 | 16485191 | + | 14.6 | 1 | 288 | -25 | AluSq | Alu | SINE |
| 16485192 | 16485447 | + | 15.1 | 196 | 428 | 0 | MSTA | MaLR | LTR |
| 16485452 | 16487096 | + | 14.0 | 5255 | 6997 | -8 | L1MA4A | L1 | LINE |

  

|  |  |  |  |  |  |  |  |  |  |
| --- | --- | --- | --- | --- | --- | --- | --- | --- | --- |
| chr11:16602014-16622961 | | | 20525 bp | | |  |  |  |  |
| Genome Start | Genome End | Strand | Div | Repeat Start | Repeat End | Repeat Left | Name | Family | Class |
| 16602014 | 16602294 | - | 22.1 | 5804 | 6078 | -69 | L1ME2 | L1 | LINE |
| 16602295 | 16602598 | - | 10.0 | 1 | 314 | 0 | AluSc | Alu | SINE |
| 16602599 | 16603468 | - | 23.4 | 4942 | 5803 | -344 | L1ME2 | L1 | LINE |
| 16603469 | 16604335 | + | 20.2 | 1 | 927 | -8 | MER21C | ERV1 | LTR |
| 16604383 | 16604962 | - | 24.1 | 4328 | 4934 | -1195 | L1ME2 | L1 | LINE |
| 16604941 | 16606464 | - | 11.6 | 5324 | 6868 | -1 | L1PA16 | L1 | LINE |
| 16606465 | 16606772 | + | 12.1 | 1 | 311 | -1 | AluSx | Alu | SINE |
| 16606773 | 16611300 | - | 11.9 | 734 | 5323 | -1526 | L1PA16 | L1 | LINE |
| 16611331 | 16611504 | - | 27.0 | 3572 | 3738 | -2391 | L1ME2 | L1 | LINE |
| 16611517 | 16612617 | + | 27.4 | 4 | 1177 | -4999 | L1MEb | L1 | LINE |
| 16612692 | 16612864 | - | 26.6 | 3303 | 3486 | -2643 | L1ME2 | L1 | LINE |
| 16612865 | 16613240 | + | 10.7 | 1 | 375 | 0 | THE1C | MaLR | LTR |
| 16613243 | 16614745 | + | 30.2 | 2 | 1735 | 0 | MLT-int | MaLR | LTR |
| 16614746 | 16615116 | + | 12.0 | 1 | 373 | -2 | THE1C | MaLR | LTR |
| 16615117 | 16615351 | - | 26.6 | 3040 | 3302 | -2827 | L1ME2 | L1 | LINE |
| 16615352 | 16615641 | - | 11.4 | 1 | 290 | -20 | AluSg | Alu | SINE |
| 16615642 | 16615713 | - | 26.6 | 2951 | 3039 | -3090 | L1ME2 | L1 | LINE |
| 16615847 | 16616023 | - | 32.5 | 118 | 300 | -146 | MLT1J1 | MaLR | LTR |
| 16616037 | 16622171 | - | 5.6 | 11 | 6153 | -1 | L1PA5 | L1 | LINE |
| 16622181 | 16622460 | - | 17.5 | 1 | 283 | -29 | AluJb | Alu | SINE |
| 16622493 | 16622541 | - | 20.4 | 100 | 149 | -362 | MLT1J | MaLR | LTR |
| 16622635 | 16622961 | - | 30.2 | 2550 | 2903 | -3226 | L1ME2 | L1 | LINE |

  

|  |  |  |  |  |  |  |  |  |  |
| --- | --- | --- | --- | --- | --- | --- | --- | --- | --- |
| chr11:17844646-17847211 | | | 2564 bp | | |  |  |  |  |
| Genome Start | Genome End | Strand | Div | Repeat Start | Repeat End | Repeat Left | Name | Family | Class |
| 17844646 | 17844833 | - | 13.8 | 103 | 282 | -30 | AluSx | Alu | SINE |
| 17844834 | 17845179 | - | 10.6 | 1 | 362 | -2 | THE1B | MaLR | LTR |
| 17845180 | 17846751 | - | 30.5 | 2 | 1735 | 0 | MLT1A0-int | MaLR | LTR |
| 17846754 | 17847108 | - | 9.5 | 1 | 364 | 0 | THE1B | MaLR | LTR |
| 17847109 | 17847211 | - | 13.8 | 1 | 102 | -210 | AluSx | Alu | SINE |

  

|  |  |  |  |  |  |  |  |  |  |
| --- | --- | --- | --- | --- | --- | --- | --- | --- | --- |
| chr11:18177612-18181164 | | | 3551 bp | | |  |  |  |  |
| Genome Start | Genome End | Strand | Div | Repeat Start | Repeat End | Repeat Left | Name | Family | Class |
| 18177612 | 18178154 | - | 24.0 | 5818 | 6346 | -13 | L1MD2 | L1 | LINE |
| 18178155 | 18178462 | - | 6.2 | 1 | 308 | -3 | AluY | Alu | SINE |
| 18178463 | 18178585 | - | 24.0 | 5708 | 5817 | -542 | L1MD2 | L1 | LINE |
| 18178586 | 18178957 | - | 12.2 | 1 | 364 | 0 | THE1B | MaLR | LTR |
| 18178958 | 18180531 | - | 30.2 | 2 | 1735 | 0 | MLT-int | MaLR | LTR |
| 18180534 | 18180903 | - | 13.0 | 1 | 364 | 0 | THE1B | MaLR | LTR |
| 18180904 | 18181164 | - | 24.0 | 5437 | 5707 | -652 | L1MD2 | L1 | LINE |

  

|  |  |  |  |  |  |  |  |  |  |
| --- | --- | --- | --- | --- | --- | --- | --- | --- | --- |
| chr11:18978883-18985699 | | | 6731 bp | | |  |  |  |  |
| Genome Start | Genome End | Strand | Div | Repeat Start | Repeat End | Repeat Left | Name | Family | Class |
| 18978883 | 18980757 | + | 15.6 | 4 | 1963 | -455 | Tigger1 | MER2\_type | DNA |
| 18980771 | 18980985 | - | 9.8 | 5936 | 6157 | -4 | L1PA14 | L1 | LINE |
| 18980986 | 18982052 | - | 6.4 | 1 | 1071 | 0 | MER11C | ERVK | LTR |
| 18982053 | 18982097 | - | 9.8 | 5891 | 5935 | -226 | L1PA14 | L1 | LINE |
| 18982098 | 18982415 | + | 13.4 | 1 | 340 | -35 | THE1C | MaLR | LTR |
| 18982416 | 18982668 | - | 14.2 | 109 | 364 | 0 | THE1B | MaLR | LTR |
| 18982659 | 18982979 | + | 15.4 | 75 | 381 | 0 | THE1D | MaLR | LTR |
| 18982983 | 18984580 | + | 29.9 | 3 | 1735 | 0 | MLT1K-int | MaLR | LTR |
| 18984583 | 18984966 | + | 14.4 | 3 | 381 | 0 | THE1D | MaLR | LTR |
| 18984982 | 18985107 | + | 19.8 | 1950 | 2075 | -343 | Tigger1 | MER2\_type | DNA |
| 18985170 | 18985452 | - | 15.9 | 1 | 286 | -26 | AluJb | Alu | SINE |
| 18985454 | 18985699 | + | 14.7 | 2064 | 2312 | -106 | Tigger1 | MER2\_type | DNA |

  

|  |  |  |  |  |  |  |  |  |  |
| --- | --- | --- | --- | --- | --- | --- | --- | --- | --- |
| chr11:20802035-20804668 | | | 2484 bp | | |  |  |  |  |
| Genome Start | Genome End | Strand | Div | Repeat Start | Repeat End | Repeat Left | Name | Family | Class |
| 20802035 | 20802102 | - | 27.9 | 399 | 466 | -45 | MLT1J | MaLR | LTR |
| 20802103 | 20802481 | + | 15.0 | 1 | 375 | 0 | THE1C | MaLR | LTR |
| 20802482 | 20803978 | + | 12.9 | 1 | 1580 | 0 | THE1C-int | MaLR | LTR |
| 20803979 | 20804340 | + | 11.7 | 1 | 375 | 0 | THE1C | MaLR | LTR |
| 20804491 | 20804668 | - | 27.0 | 4 | 191 | -320 | MLT1J | MaLR | LTR |

  

|  |  |  |  |  |  |  |  |  |  |
| --- | --- | --- | --- | --- | --- | --- | --- | --- | --- |
| chr11:21303753-21310696 | | | 6524 bp | | |  |  |  |  |
| Genome Start | Genome End | Strand | Div | Repeat Start | Repeat End | Repeat Left | Name | Family | Class |
| 21303753 | 21304257 | - | 22.9 | 5848 | 6329 | -10 | L1ME2 | L1 | LINE |
| 21304254 | 21304928 | - | 27.2 | 1541 | 2405 | -4097 | L1ME2 | L1 | LINE |
| 21304951 | 21305064 | - | 13.2 | 2970 | 3326 | -3958 | L1M4b | L1 | LINE |
| 21305127 | 21305305 | + | 9.2 | 1 | 163 | -212 | THE1C | MaLR | LTR |
| 21305306 | 21305475 | + | 4.7 | 5981 | 6154 | -1 | L1PA4 | L1 | LINE |
| 21305476 | 21305695 | + | 9.2 | 164 | 375 | 0 | THE1C | MaLR | LTR |
| 21305698 | 21307237 | + | 30.3 | 2 | 1735 | 0 | MLT1C-int | MaLR | LTR |
| 21307238 | 21307602 | + | 11.8 | 1 | 375 | 0 | THE1C | MaLR | LTR |
| 21307610 | 21307728 | - | 24.8 | 6094 | 6224 | -88 | L1MA9 | L1 | LINE |
| 21307782 | 21310088 | - | 21.1 | 702 | 3207 | -4127 | L1M4b | L1 | LINE |
| 21310367 | 21310696 | - | 33.6 | 183 | 518 | -5984 | L1ME2 | L1 | LINE |

  

|  |  |  |  |  |  |  |  |  |  |
| --- | --- | --- | --- | --- | --- | --- | --- | --- | --- |
| chr11:21570406-21577585 | | | 7088 bp | | |  |  |  |  |
| Genome Start | Genome End | Strand | Div | Repeat Start | Repeat End | Repeat Left | Name | Family | Class |
| 21570406 | 21570600 | + | 30.8 | 2663 | 2865 | -3281 | L1ME3B | L1 | LINE |
| 21570601 | 21570909 | + | 10.4 | 1 | 309 | 0 | AluSc | Alu | SINE |
| 21570910 | 21570923 | + | 30.8 | 2866 | 2872 | -3274 | L1ME3B | L1 | LINE |
| 21570945 | 21571092 | + | 30.8 | 2873 | 3047 | -3099 | L1ME3B | L1 | LINE |
| 21571102 | 21571446 | + | 14.0 | 1 | 359 | -5 | THE1B | MaLR | LTR |
| 21571458 | 21573056 | + | 30.2 | 2 | 1735 | 0 | MLT-int | MaLR | LTR |
| 21573058 | 21573413 | + | 14.1 | 2 | 364 | 0 | THE1B | MaLR | LTR |
| 21573420 | 21576411 | + | 16.4 | 3059 | 6149 | -2 | L1PB2 | L1 | LINE |
| 21576427 | 21576669 | + | 25.4 | 3865 | 4114 | -2032 | L1ME3B | L1 | LINE |
| 21576671 | 21577443 | - | 3.5 | 5375 | 6154 | -1 | L1PA3 | L1 | LINE |
| 21577472 | 21577585 | + | 22.1 | 4152 | 4269 | -1877 | L1ME3B | L1 | LINE |

  

|  |  |  |  |  |  |  |  |  |  |
| --- | --- | --- | --- | --- | --- | --- | --- | --- | --- |
| chr11:21716942-21720215 | | | 3264 bp | | |  |  |  |  |
| Genome Start | Genome End | Strand | Div | Repeat Start | Repeat End | Repeat Left | Name | Family | Class |
| 21716942 | 21717186 | - | 23.7 | 126 | 369 | -5 | MLT1A | MaLR | LTR |
| 21717187 | 21717596 | - | 15.4 | 1 | 428 | 0 | MSTA | MaLR | LTR |
| 21717597 | 21717776 | - | 14.3 | 1404 | 1580 | 0 | MSTA-int | MaLR | LTR |
| 21717782 | 21717919 | - | 18.8 | 1049 | 1185 | -466 | MSTA-int | MaLR | LTR |
| 21717920 | 21718314 | - | 16.6 | 2 | 428 | 0 | MSTA | MaLR | LTR |
| 21718315 | 21718995 | - | 18.8 | 372 | 1048 | -603 | MSTA-int | MaLR | LTR |
| 21718996 | 21719297 | + | 11.9 | 1 | 302 | -7 | AluSc | Alu | SINE |
| 21719298 | 21719678 | - | 18.8 | 1 | 371 | -1280 | MSTA-int | MaLR | LTR |
| 21719684 | 21720102 | - | 16.7 | 1 | 428 | 0 | MSTA | MaLR | LTR |
| 21720103 | 21720215 | - | 20.4 | 3 | 130 | -244 | MLT1A | MaLR | LTR |

  

|  |  |  |  |  |  |  |  |  |  |
| --- | --- | --- | --- | --- | --- | --- | --- | --- | --- |
| chr11:22820889-22824885 | | | 3966 bp | | |  |  |  |  |
| Genome Start | Genome End | Strand | Div | Repeat Start | Repeat End | Repeat Left | Name | Family | Class |
| 22820889 | 22821067 | - | 23.6 | 1320 | 1513 | -4887 | L1MEc | L1 | LINE |
| 22821073 | 22821344 | + | 10.7 | 1 | 228 | -136 | THE1B | MaLR | LTR |
| 22821345 | 22821631 | + | 15.7 | 11 | 299 | -13 | AluSx | Alu | SINE |
| 22821632 | 22821775 | + | 10.7 | 229 | 364 | 0 | THE1B | MaLR | LTR |
| 22821776 | 22823297 | + | 13.0 | 1 | 1580 | 0 | THE1B-int | MaLR | LTR |
| 22823298 | 22823655 | + | 14.9 | 1 | 364 | 0 | THE1B | MaLR | LTR |
| 22823682 | 22824885 | - | 30.1 | 34 | 1261 | -5139 | L1MEc | L1 | LINE |

  

|  |  |  |  |  |  |  |  |  |  |
| --- | --- | --- | --- | --- | --- | --- | --- | --- | --- |
| chr11:23610256-23621004 | | | 10750 bp | | |  |  |  |  |
| Genome Start | Genome End | Strand | Div | Repeat Start | Repeat End | Repeat Left | Name | Family | Class |
| 23610256 | 23611036 | + | 24.0 | 17 | 798 | -6767 | L1MA1 | L1 | LINE |
| 23611028 | 23611316 | - | 12.5 | 2492 | 2774 | -4791 | L1M1 | L1 | LINE |
| 23611313 | 23611821 | - | 7.1 | 3164 | 3672 | -2474 | L1P1 | L1 | LINE |
| 23611825 | 23613496 | + | 8.3 | 4473 | 6150 | -4 | L1PA6 | L1 | LINE |
| 23613502 | 23614743 | + | 13.4 | 3347 | 4590 | -2975 | L1MA1 | L1 | LINE |
| 23614744 | 23615130 | - | 17.2 | 1 | 428 | 0 | MSTA | MaLR | LTR |
| 23615131 | 23616610 | - | 16.4 | 3 | 1580 | 0 | MSTA-int | MaLR | LTR |
| 23616613 | 23616837 | - | 20.1 | 199 | 428 | 0 | MSTA | MaLR | LTR |
| 23616838 | 23617139 | - | 6.6 | 1 | 301 | -10 | AluY | Alu | SINE |
| 23617140 | 23617316 | - | 20.1 | 1 | 198 | -230 | MSTA | MaLR | LTR |
| 23617317 | 23617912 | + | 11.7 | 4593 | 5214 | -2351 | L1MA1 | L1 | LINE |
| 23617913 | 23618264 | + | 13.8 | 1 | 375 | 0 | THE1C | MaLR | LTR |
| 23618265 | 23619621 | + | 11.7 | 5215 | 6589 | -976 | L1MA1 | L1 | LINE |
| 23619622 | 23619697 | - | 13.2 | 17 | 92 | -220 | AluS | Alu | SINE |
| 23619698 | 23619905 | + | 12.5 | 93 | 304 | -8 | AluSg/x | Alu | SINE |
| 23619908 | 23621004 | + | 11.8 | 6584 | 7721 | 0 | L1MA1 | L1 | LINE |

  

|  |  |  |  |  |  |  |  |  |  |
| --- | --- | --- | --- | --- | --- | --- | --- | --- | --- |
| chr11:23971092-23981007 | | | 9858 bp | | |  |  |  |  |
| Genome Start | Genome End | Strand | Div | Repeat Start | Repeat End | Repeat Left | Name | Family | Class |
| 23971092 | 23971541 | + | 28.4 | 4092 | 4542 | -1604 | L1MD | L1 | LINE |
| 23971542 | 23975650 | + | 22.5 | 2 | 4644 | -2670 | L1P4b | L1 | LINE |
| 23975651 | 23975970 | + | 14.1 | 1 | 302 | -10 | AluSx | Alu | SINE |
| 23975971 | 23976921 | + | 19.0 | 4645 | 5592 | -1722 | L1P4b | L1 | LINE |
| 23976924 | 23977284 | - | 15.3 | 3 | 364 | 0 | THE1B | MaLR | LTR |
| 23977287 | 23978862 | - | 12.0 | 1 | 1580 | 0 | THE1B-int | MaLR | LTR |
| 23978863 | 23979224 | - | 14.1 | 1 | 364 | 0 | THE1B | MaLR | LTR |
| 23979225 | 23980134 | + | 17.3 | 5590 | 6515 | -799 | L1P4b | L1 | LINE |
| 23980189 | 23980600 | + | 29.9 | 4546 | 4972 | -1174 | L1MD | L1 | LINE |
| 23980601 | 23980951 | + | 13.4 | 1 | 364 | 0 | THE1B | MaLR | LTR |
| 23980952 | 23981007 | + | 29.9 | 4973 | 5031 | -1115 | L1MD | L1 | LINE |

  

|  |  |  |  |  |  |  |  |  |  |
| --- | --- | --- | --- | --- | --- | --- | --- | --- | --- |
| chr11:24419181-24425827 | | | 5928 bp | | |  |  |  |  |
| Genome Start | Genome End | Strand | Div | Repeat Start | Repeat End | Repeat Left | Name | Family | Class |
| 24419181 | 24419377 | - | 24.3 | 5888 | 6066 | -87 | L1ME3A | L1 | LINE |
| 24419452 | 24419720 | - | 28.2 | 5628 | 5910 | -243 | L1ME3A | L1 | LINE |
| 24419721 | 24419893 | - | 19.2 | 140 | 311 | -1 | AluJb | Alu | SINE |
| 24419903 | 24420078 | - | 28.4 | 5445 | 5632 | -472 | L1ME3A | L1 | LINE |
| 24420433 | 24420728 | - | 11.5 | 1 | 288 | -21 | AluSc | Alu | SINE |
| 24420731 | 24421278 | - | 33.4 | 2568 | 3138 | -2991 | L1ME3A | L1 | LINE |
| 24421327 | 24421695 | + | 15.3 | 1 | 381 | 0 | THE1D | MaLR | LTR |
| 24421700 | 24423282 | + | 29.9 | 4 | 1733 | -2 | MLT-int | MaLR | LTR |
| 24423285 | 24423651 | + | 16.3 | 1 | 381 | 0 | THE1D | MaLR | LTR |
| 24423653 | 24423773 | - | 33.9 | 571 | 685 | -5434 | L1ME3A | L1 | LINE |
| 24423926 | 24424215 | - | 26.4 | 188 | 483 | -84 | MLT1F1 | MaLR | LTR |
| 24424216 | 24425174 | + | 1.5 | 1 | 968 | 0 | LTR5\_Hs | ERVK | LTR |
| 24425175 | 24425361 | - | 26.4 | 1 | 187 | -380 | MLT1F1 | MaLR | LTR |
| 24425435 | 24425827 | - | 34.0 | 56 | 469 | -5650 | L1ME3A | L1 | LINE |

  

|  |  |  |  |  |  |  |  |  |  |
| --- | --- | --- | --- | --- | --- | --- | --- | --- | --- |
| chr11:24451873-24455107 | | | 3233 bp | | |  |  |  |  |
| Genome Start | Genome End | Strand | Div | Repeat Start | Repeat End | Repeat Left | Name | Family | Class |
| 24451873 | 24452125 | + | 30.8 | 21 | 257 | -193 | LTR16A | ERVL | LTR |
| 24452126 | 24452542 | - | 20.0 | 1 | 426 | 0 | MSTB | MaLR | LTR |
| 24452543 | 24454218 | - | 30.0 | 2 | 1735 | 0 | MLT-int | MaLR | LTR |
| 24454221 | 24454471 | - | 14.4 | 178 | 426 | 0 | MSTB | MaLR | LTR |
| 24454472 | 24454769 | + | 15.3 | 1 | 312 | 0 | AluSx | Alu | SINE |
| 24454770 | 24454780 | + | 15.3 | 290 | 300 | -12 | AluSx | Alu | SINE |
| 24454781 | 24454951 | - | 14.4 | 1 | 177 | -249 | MSTB | MaLR | LTR |
| 24454952 | 24455107 | + | 30.8 | 258 | 423 | -27 | LTR16A | ERVL | LTR |

  

|  |  |  |  |  |  |  |  |  |  |
| --- | --- | --- | --- | --- | --- | --- | --- | --- | --- |
| chr11:25342111-25346665 | | | 4505 bp | | |  |  |  |  |
| Genome Start | Genome End | Strand | Div | Repeat Start | Repeat End | Repeat Left | Name | Family | Class |
| 25342111 | 25342377 | - | 15.7 | 5200 | 5462 | -829 | L1M2 | L1 | LINE |
| 25342378 | 25342741 | - | 17.2 | 1 | 364 | 0 | THE1B | MaLR | LTR |
| 25342742 | 25343389 | - | 12.3 | 940 | 1580 | 0 | THE1B-int | MaLR | LTR |
| 25343392 | 25344883 | + | 5.3 | 4657 | 6151 | -3 | L1PA5 | L1 | LINE |
| 25344931 | 25345861 | - | 10.8 | 1 | 935 | -645 | THE1B-int | MaLR | LTR |
| 25345862 | 25346226 | - | 17.1 | 1 | 364 | 0 | THE1B | MaLR | LTR |
| 25346228 | 25346665 | - | 17.2 | 4756 | 5203 | -937 | L1M2 | L1 | LINE |

  

|  |  |  |  |  |  |  |  |  |  |
| --- | --- | --- | --- | --- | --- | --- | --- | --- | --- |
| chr11:25583051-25591340 | | | 8248 bp | | |  |  |  |  |
| Genome Start | Genome End | Strand | Div | Repeat Start | Repeat End | Repeat Left | Name | Family | Class |
| 25583051 | 25583398 | - | 11.7 | 6500 | 6866 | -1 | L1PB1 | L1 | LINE |
| 25583441 | 25587619 | - | 12.1 | 2057 | 6499 | -368 | L1PB1 | L1 | LINE |
| 25587620 | 25587975 | - | 18.7 | 1 | 364 | 0 | THE1B | MaLR | LTR |
| 25587976 | 25589514 | - | 11.1 | 15 | 1580 | 0 | THE1B-int | MaLR | LTR |
| 25589515 | 25589870 | - | 17.4 | 1 | 364 | 0 | THE1B | MaLR | LTR |
| 25589871 | 25591340 | - | 15.3 | 3 | 2061 | -4802 | L1PB1 | L1 | LINE |

  

|  |  |  |  |  |  |  |  |  |  |
| --- | --- | --- | --- | --- | --- | --- | --- | --- | --- |
| chr11:25898470-25902545 | | | 4060 bp | | |  |  |  |  |
| Genome Start | Genome End | Strand | Div | Repeat Start | Repeat End | Repeat Left | Name | Family | Class |
| 25898470 | 25898979 | + | 31.3 | 260 | 793 | -696 | MLT1G-int | MaLR | LTR |
| 25898981 | 25899336 | - | 12.5 | 3 | 364 | 0 | THE1B | MaLR | LTR |
| 25899345 | 25900936 | - | 10.4 | 1 | 1573 | -7 | THE1B-int | MaLR | LTR |
| 25900937 | 25901293 | - | 13.6 | 1 | 364 | 0 | THE1B | MaLR | LTR |
| 25901301 | 25901730 | + | 30.4 | 804 | 1260 | -229 | MLT1G-int | MaLR | LTR |
| 25901731 | 25901983 | - | 14.5 | 5929 | 6165 | -1 | L1PA16 | L1 | LINE |
| 25901984 | 25902296 | - | 11.2 | 1 | 311 | -1 | AluSx | Alu | SINE |
| 25902297 | 25902352 | - | 14.5 | 5881 | 5928 | -238 | L1PA16 | L1 | LINE |
| 25902353 | 25902545 | + | 30.4 | 1261 | 1470 | -19 | MLT1G-int | MaLR | LTR |

  

|  |  |  |  |  |  |  |  |  |  |
| --- | --- | --- | --- | --- | --- | --- | --- | --- | --- |
| chr11:27976701-27986041 | | | 9212 bp | | |  |  |  |  |
| Genome Start | Genome End | Strand | Div | Repeat Start | Repeat End | Repeat Left | Name | Family | Class |
| 27976701 | 27977169 | + | 22.5 | 1 | 519 | -402 | MER21C | ERV1 | LTR |
| 27977183 | 27977636 | + | 15.9 | 1 | 444 | -1 | MER65A | ERV1 | LTR |
| 27977721 | 27978040 | + | 26.8 | 82 | 378 | -4493 | MER65A-int | ERV1 | LTR |
| 27978051 | 27979186 | + | 21.1 | 483 | 1634 | -3237 | MER65A-int | ERV1 | LTR |
| 27979187 | 27979549 | + | 11.1 | 1 | 364 | 0 | THE1B | MaLR | LTR |
| 27979553 | 27981101 | + | 29.8 | 3 | 1735 | 0 | MLT-int | MaLR | LTR |
| 27981102 | 27981432 | + | 11.1 | 1 | 352 | -12 | THE1B | MaLR | LTR |
| 27981444 | 27984799 | + | 20.6 | 1546 | 4603 | -353 | MER65A-int | ERV1 | LTR |
| 27984804 | 27985271 | + | 23.6 | 4402 | 4871 | 0 | MER65A-int | ERV1 | LTR |
| 27985272 | 27985705 | + | 19.1 | 1 | 443 | -2 | MER65A | ERV1 | LTR |
| 27985710 | 27986041 | + | 19.8 | 587 | 930 | -5 | MER21C | ERV1 | LTR |

  

|  |  |  |  |  |  |  |  |  |  |
| --- | --- | --- | --- | --- | --- | --- | --- | --- | --- |
| chr11:28742709-28751068 | | | 8326 bp | | |  |  |  |  |
| Genome Start | Genome End | Strand | Div | Repeat Start | Repeat End | Repeat Left | Name | Family | Class |
| 28742709 | 28743264 | - | 18.3 | 3748 | 4329 | -3230 | L1M2 | L1 | LINE |
| 28743266 | 28743622 | + | 21.4 | 1 | 396 | 0 | MSTD | MaLR | LTR |
| 28743625 | 28743789 | + | 19.9 | 2 | 175 | -1560 | MSTD-int | MaLR | LTR |
| 28743790 | 28747129 | + | 13.3 | 31 | 3461 | -4104 | L1M1 | L1 | LINE |
| 28747153 | 28747713 | + | 11.9 | 3462 | 4041 | -3524 | L1M1 | L1 | LINE |
| 28747715 | 28749336 | + | 25.6 | 144 | 1735 | 0 | MSTD-int | MaLR | LTR |
| 28749344 | 28749682 | + | 20.3 | 9 | 392 | -4 | MSTD | MaLR | LTR |
| 28749683 | 28751068 | - | 27.3 | 2298 | 3756 | -3803 | L1M2 | L1 | LINE |

  

|  |  |  |  |  |  |  |  |  |  |
| --- | --- | --- | --- | --- | --- | --- | --- | --- | --- |
| chr11:29692808-29702206 | | | 9097 bp | | |  |  |  |  |
| Genome Start | Genome End | Strand | Div | Repeat Start | Repeat End | Repeat Left | Name | Family | Class |
| 29692808 | 29694303 | - | 18.0 | 4956 | 6471 | -24 | L1MA7 | L1 | LINE |
| 29694304 | 29694635 | - | 14.2 | 1 | 364 | 0 | THE1B | MaLR | LTR |
| 29694637 | 29696198 | - | 31.0 | 2 | 1734 | -1 | MLT-int | MaLR | LTR |
| 29696201 | 29696522 | - | 12.7 | 4 | 364 | 0 | THE1B | MaLR | LTR |
| 29696523 | 29698228 | - | 18.9 | 3133 | 4955 | -1392 | L1MA7 | L1 | LINE |
| 29698229 | 29698525 | - | 16.8 | 1 | 286 | -26 | AluJo | Alu | SINE |
| 29698526 | 29699518 | - | 20.8 | 2089 | 3132 | -3215 | L1MA7 | L1 | LINE |
| 29699818 | 29702206 | - | 26.2 | 1 | 2177 | -4345 | L1MA7 | L1 | LINE |

  

|  |  |  |  |  |  |  |  |  |  |
| --- | --- | --- | --- | --- | --- | --- | --- | --- | --- |
| chr11:31248573-31253255 | | | 4174 bp | | |  |  |  |  |
| Genome Start | Genome End | Strand | Div | Repeat Start | Repeat End | Repeat Left | Name | Family | Class |
| 31248573 | 31248787 | + | 32.7 | 294 | 532 | -2887 | L2 | L2 | LINE |
| 31248975 | 31249775 | + | 35.1 | 1209 | 2058 | -1361 | L2 | L2 | LINE |
| 31249786 | 31250003 | - | 13.0 | 152 | 364 | 0 | THE1B | MaLR | LTR |
| 31250004 | 31250300 | + | 10.8 | 1 | 296 | -14 | AluSg | Alu | SINE |
| 31250301 | 31250449 | - | 13.0 | 1 | 151 | -213 | THE1B | MaLR | LTR |
| 31250465 | 31251853 | - | 10.1 | 1 | 1575 | -5 | THE1B-int | MaLR | LTR |
| 31251854 | 31252203 | - | 11.4 | 1 | 364 | 0 | THE1B | MaLR | LTR |
| 31252414 | 31252758 | - | 29.2 | 2944 | 3419 | 0 | L2 | L2 | LINE |
| 31252823 | 31253118 | - | 13.5 | 4 | 299 | -11 | AluSg | Alu | SINE |
| 31253142 | 31253255 | + | 23.7 | 3290 | 3419 | 0 | L2 | L2 | LINE |

  

|  |  |  |  |  |  |  |  |  |  |
| --- | --- | --- | --- | --- | --- | --- | --- | --- | --- |
| chr11:34688411-34692966 | | | 4298 bp | | |  |  |  |  |
| Genome Start | Genome End | Strand | Div | Repeat Start | Repeat End | Repeat Left | Name | Family | Class |
| 34688411 | 34689960 | - | 28.5 | 645 | 2295 | -4020 | L1MEc | L1 | LINE |
| 34690198 | 34690577 | + | 17.3 | 1 | 428 | 0 | MSTA | MaLR | LTR |
| 34690580 | 34692207 | + | 14.1 | 3 | 1573 | -7 | MSTA-int | MaLR | LTR |
| 34692214 | 34692589 | + | 18.8 | 1 | 428 | 0 | MSTA | MaLR | LTR |
| 34692603 | 34692966 | - | 34.6 | 8 | 396 | -6004 | L1MEc | L1 | LINE |

  

|  |  |  |  |  |  |  |  |  |  |
| --- | --- | --- | --- | --- | --- | --- | --- | --- | --- |
| chr11:36498992-36501767 | | | 2774 bp | | |  |  |  |  |
| Genome Start | Genome End | Strand | Div | Repeat Start | Repeat End | Repeat Left | Name | Family | Class |
| 36498992 | 36499050 | - | 25.7 | 7827 | 7896 | -146 | L1MC4 | L1 | LINE |
| 36499051 | 36499086 | + | 16.1 | 3 | 37 | -327 | THE1B | MaLR | LTR |
| 36499087 | 36499384 | - | 11.7 | 1 | 299 | -14 | AluSp | Alu | SINE |
| 36499385 | 36499709 | + | 16.1 | 38 | 363 | -1 | THE1B | MaLR | LTR |
| 36499712 | 36501287 | + | 30.9 | 2 | 1735 | 0 | MLT1I-int | MaLR | LTR |
| 36501288 | 36501652 | + | 12.6 | 1 | 364 | 0 | THE1B | MaLR | LTR |
| 36501653 | 36501767 | - | 25.7 | 7712 | 7826 | -216 | L1MC4 | L1 | LINE |

  

|  |  |  |  |  |  |  |  |  |  |
| --- | --- | --- | --- | --- | --- | --- | --- | --- | --- |
| chr11:37104045-37109862 | | | 5816 bp | | |  |  |  |  |
| Genome Start | Genome End | Strand | Div | Repeat Start | Repeat End | Repeat Left | Name | Family | Class |
| 37104045 | 37104358 | - | 25.5 | 2573 | 2941 | -3 | BLACKJACK | AcHobo | DNA |
| 37104359 | 37104654 | - | 21.1 | 1 | 295 | -17 | AluJb | Alu | SINE |
| 37104655 | 37105685 | - | 25.5 | 1488 | 2572 | -372 | BLACKJACK | AcHobo | DNA |
| 37105686 | 37106260 | - | 30.5 | 1 | 595 | 0 | MLT1G1 | MaLR | LTR |
| 37106261 | 37107347 | - | 25.5 | 329 | 1487 | -1457 | BLACKJACK | AcHobo | DNA |
| 37107348 | 37107690 | + | 13.2 | 1 | 364 | 0 | THE1B | MaLR | LTR |
| 37107693 | 37109234 | + | 30.4 | 2 | 1735 | 0 | MLT-int | MaLR | LTR |
| 37109235 | 37109587 | + | 14.8 | 1 | 364 | 0 | THE1B | MaLR | LTR |
| 37109588 | 37109862 | - | 25.5 | 44 | 328 | -2616 | BLACKJACK | AcHobo | DNA |

  

|  |  |  |  |  |  |  |  |  |  |
| --- | --- | --- | --- | --- | --- | --- | --- | --- | --- |
| chr11:37122738-37128825 | | | 5829 bp | | |  |  |  |  |
| Genome Start | Genome End | Strand | Div | Repeat Start | Repeat End | Repeat Left | Name | Family | Class |
| 37122738 | 37122820 | - | 17.9 | 3265 | 3350 | -69 | L2 | L2 | LINE |
| 37122828 | 37122874 | + | 19.1 | 1 | 47 | -255 | AluJo | Alu | SINE |
| 37122875 | 37123222 | - | 13.6 | 1 | 364 | 0 | THE1B | MaLR | LTR |
| 37123223 | 37124775 | - | 30.5 | 2 | 1735 | 0 | MLT1A0-int | MaLR | LTR |
| 37124781 | 37125128 | - | 15.3 | 3 | 361 | -3 | THE1B | MaLR | LTR |
| 37125130 | 37125394 | + | 17.8 | 42 | 300 | -12 | AluJo | Alu | SINE |
| 37125610 | 37125798 | - | 36.8 | 2819 | 3008 | -411 | L2 | L2 | LINE |
| 37125813 | 37126232 | - | 15.7 | 5748 | 6183 | 0 | L1MB3 | L1 | LINE |
| 37126233 | 37126344 | - | 10.8 | 164 | 274 | -38 | AluSg/x | Alu | SINE |
| 37126345 | 37127960 | + | 4.3 | 4537 | 6153 | -2 | L1PA4 | L1 | LINE |
| 37127962 | 37128084 | + | 18.9 | 5652 | 5773 | -410 | L1M3 | L1 | LINE |
| 37128086 | 37128236 | + | 12.6 | 1 | 151 | -161 | AluSq/x | Alu | SINE |
| 37128252 | 37128825 | - | 31.9 | 2150 | 2810 | -609 | L2 | L2 | LINE |

  

|  |  |  |  |  |  |  |  |  |  |
| --- | --- | --- | --- | --- | --- | --- | --- | --- | --- |
| chr11:37182278-37198930 | | | 16613 bp | | |  |  |  |  |
| Genome Start | Genome End | Strand | Div | Repeat Start | Repeat End | Repeat Left | Name | Family | Class |
| 37182278 | 37183198 | - | 13.1 | 6708 | 7622 | -102 | L1MA3 | L1 | LINE |
| 37183186 | 37189697 | - | 10.4 | 8 | 6468 | -1 | L1PA7 | L1 | LINE |
| 37189706 | 37189857 | - | 14.5 | 6575 | 6713 | -852 | L1MA3 | L1 | LINE |
| 37189882 | 37194444 | - | 14.4 | 1980 | 6574 | -991 | L1MA3 | L1 | LINE |
| 37194447 | 37194808 | - | 12.2 | 1 | 364 | 0 | THE1B | MaLR | LTR |
| 37194809 | 37194986 | - | 11.8 | 1407 | 1580 | 0 | THE1B-int | MaLR | LTR |
| 37194987 | 37195292 | + | 10.5 | 1 | 305 | -6 | AluY | Alu | SINE |
| 37195293 | 37196654 | - | 11.8 | 1 | 1406 | -174 | THE1B-int | MaLR | LTR |
| 37196655 | 37197005 | - | 12.0 | 9 | 364 | 0 | THE1B | MaLR | LTR |
| 37197025 | 37198930 | - | 18.2 | 72 | 1954 | -5611 | L1MA3 | L1 | LINE |

  

|  |  |  |  |  |  |  |  |  |  |
| --- | --- | --- | --- | --- | --- | --- | --- | --- | --- |
| chr11:37354201-37356767 | | | 2565 bp | | |  |  |  |  |
| Genome Start | Genome End | Strand | Div | Repeat Start | Repeat End | Repeat Left | Name | Family | Class |
| 37354201 | 37354348 | - | 34.1 | 305 | 450 | 0 | LTR16A | ERVL | LTR |
| 37354349 | 37354727 | - | 14.6 | 1 | 364 | 0 | THE1B | MaLR | LTR |
| 37354728 | 37356343 | - | 30.6 | 2 | 1735 | 0 | MLT-int | MaLR | LTR |
| 37356346 | 37356702 | - | 13.3 | 1 | 364 | 0 | THE1B | MaLR | LTR |
| 37356703 | 37356767 | - | 34.1 | 242 | 304 | -146 | LTR16A | ERVL | LTR |

  

|  |  |  |  |  |  |  |  |  |  |
| --- | --- | --- | --- | --- | --- | --- | --- | --- | --- |
| chr11:37400629-37405647 | | | 5019 bp | | |  |  |  |  |
| Genome Start | Genome End | Strand | Div | Repeat Start | Repeat End | Repeat Left | Name | Family | Class |
| 37400629 | 37401048 | + | 14.3 | 3576 | 4008 | -2138 | L1MA2 | L1 | LINE |
| 37401049 | 37401408 | - | 17.3 | 1 | 364 | 0 | THE1B | MaLR | LTR |
| 37401409 | 37402981 | - | 10.5 | 1 | 1580 | 0 | THE1B-int | MaLR | LTR |
| 37402982 | 37403339 | - | 15.6 | 1 | 364 | 0 | THE1B | MaLR | LTR |
| 37403340 | 37405647 | + | 12.2 | 4004 | 6304 | 0 | L1MA2 | L1 | LINE |

  

|  |  |  |  |  |  |  |  |  |  |
| --- | --- | --- | --- | --- | --- | --- | --- | --- | --- |
| chr11:37501065-37509562 | | | 8378 bp | | |  |  |  |  |
| Genome Start | Genome End | Strand | Div | Repeat Start | Repeat End | Repeat Left | Name | Family | Class |
| 37501065 | 37501640 | - | 24.5 | 6172 | 6783 | -1500 | L1MC3 | L1 | LINE |
| 37501676 | 37504792 | - | 24.6 | 2960 | 6171 | -2112 | L1MC3 | L1 | LINE |
| 37504806 | 37505291 | - | 23.7 | 4761 | 5250 | -896 | L1M4 | L1 | LINE |
| 37505321 | 37506426 | - | 22.9 | 2828 | 3955 | -2191 | L1M4 | L1 | LINE |
| 37506427 | 37506803 | - | 17.2 | 1 | 428 | 0 | MSTA | MaLR | LTR |
| 37506804 | 37508426 | - | 17.1 | 3 | 1580 | 0 | MSTA-int | MaLR | LTR |
| 37508429 | 37508810 | - | 16.1 | 1 | 428 | 0 | MSTA | MaLR | LTR |
| 37508827 | 37508960 | - | 29.1 | 826 | 970 | -5572 | L1MC3 | L1 | LINE |
| 37508984 | 37509443 | - | 29.1 | 362 | 825 | -5717 | L1MC3 | L1 | LINE |
| 37509446 | 37509562 | - | 29.8 | 153 | 273 | -6269 | L1MC3 | L1 | LINE |

  

|  |  |  |  |  |  |  |  |  |  |
| --- | --- | --- | --- | --- | --- | --- | --- | --- | --- |
| chr11:37522753-37525091 | | | 2263 bp | | |  |  |  |  |
| Genome Start | Genome End | Strand | Div | Repeat Start | Repeat End | Repeat Left | Name | Family | Class |
| 37522753 | 37522961 | - | 25.4 | 184 | 386 | 0 | MLT1A | MaLR | LTR |
| 37523054 | 37523382 | - | 14.1 | 3 | 339 | -25 | THE1B | MaLR | LTR |
| 37523384 | 37523422 | - | 7.7 | 1542 | 1580 | 0 | THE1B-int | MaLR | LTR |
| 37523406 | 37524641 | - | 11.5 | 1 | 1244 | -336 | THE1B-int | MaLR | LTR |
| 37524642 | 37524996 | - | 13.0 | 1 | 364 | 0 | THE1B | MaLR | LTR |
| 37524997 | 37525091 | - | 13.2 | 4 | 94 | -280 | MLT1A | MaLR | LTR |

  

|  |  |  |  |  |  |  |  |  |  |
| --- | --- | --- | --- | --- | --- | --- | --- | --- | --- |
| chr11:37748709-37755292 | | | 5451 bp | | |  |  |  |  |
| Genome Start | Genome End | Strand | Div | Repeat Start | Repeat End | Repeat Left | Name | Family | Class |
| 37748709 | 37748790 | - | 34.1 | 3335 | 3417 | -2 | L2 | L2 | LINE |
| 37749213 | 37749312 | - | 38.0 | 2676 | 2779 | -640 | L2 | L2 | LINE |
| 37749441 | 37749872 | + | 17.5 | 4585 | 5019 | -1127 | L1MC | L1 | LINE |
| 37749874 | 37750182 | + | 22.0 | 5457 | 5770 | -2015 | L1MC | L1 | LINE |
| 37750189 | 37750556 | + | 23.7 | 1 | 514 | -42 | MLT2D | ERVL | LTR |
| 37750855 | 37750889 | + | 5.7 | 1 | 35 | -45 | MADE1 | Mariner | DNA |
| 37750903 | 37751077 | - | 39.3 | 2308 | 2482 | -937 | L2 | L2 | LINE |
| 37751096 | 37751442 | - | 11.2 | 1 | 355 | 0 | THE1A | MaLR | LTR |
| 37751443 | 37752328 | - | 10.7 | 696 | 1580 | 0 | THE1A-int | MaLR | LTR |
| 37752329 | 37752747 | + | 7.7 | 1 | 464 | 0 | LTR7B | ERV1 | LTR |
| 37752748 | 37753435 | - | 10.7 | 8 | 695 | -885 | THE1A-int | MaLR | LTR |
| 37753436 | 37753786 | - | 10.5 | 2 | 352 | -3 | THE1A | MaLR | LTR |
| 37754006 | 37754465 | + | 34.2 | 24 | 511 | 0 | MLT1J | MaLR | LTR |
| 37754477 | 37754920 | + | 31.2 | 2910 | 3417 | -2 | L2 | L2 | LINE |
| 37754938 | 37755292 | - | 39.3 | 1936 | 2313 | -1106 | L2 | L2 | LINE |

  

|  |  |  |  |  |  |  |  |  |  |
| --- | --- | --- | --- | --- | --- | --- | --- | --- | --- |
| chr11:37934191-37939497 | | | 4854 bp | | |  |  |  |  |
| Genome Start | Genome End | Strand | Div | Repeat Start | Repeat End | Repeat Left | Name | Family | Class |
| 37934191 | 37934323 | - | 21.8 | 3229 | 3340 | -79 | L2 | L2 | LINE |
| 37934436 | 37934520 | - | 14.9 | 196 | 280 | -32 | AluSg/x | Alu | SINE |
| 37934521 | 37934757 | - | 30.0 | 2933 | 3187 | -232 | L2 | L2 | LINE |
| 37934801 | 37935097 | + | 16.4 | 9 | 301 | -11 | AluJb | Alu | SINE |
| 37935108 | 37935312 | + | 29.3 | 8 | 239 | -714 | MER45C | Tip100 | DNA |
| 37935351 | 37935518 | - | 9.1 | 127 | 291 | -20 | AluY | Alu | SINE |
| 37935524 | 37935598 | + | 6.7 | 51 | 126 | -185 | AluY | Alu | SINE |
| 37935764 | 37935903 | + | 32.0 | 412 | 549 | -404 | MER45C | Tip100 | DNA |
| 37935904 | 37936271 | - | 15.4 | 1 | 360 | -4 | THE1B | MaLR | LTR |
| 37936272 | 37937837 | - | 29.6 | 2 | 1735 | 0 | MLT-int | MaLR | LTR |
| 37937840 | 37938205 | - | 12.3 | 1 | 364 | 0 | THE1B | MaLR | LTR |
| 37938206 | 37938256 | + | 32.0 | 550 | 600 | -353 | MER45C | Tip100 | DNA |
| 37938335 | 37938860 | - | 31.7 | 2304 | 2850 | -569 | L2 | L2 | LINE |
| 37938861 | 37939212 | - | 16.5 | 1 | 364 | 0 | THE1B | MaLR | LTR |
| 37939213 | 37939497 | - | 31.7 | 2032 | 2303 | -1116 | L2 | L2 | LINE |

  

|  |  |  |  |  |  |  |  |  |  |
| --- | --- | --- | --- | --- | --- | --- | --- | --- | --- |
| chr11:39076930-39079820 | | | 2889 bp | | |  |  |  |  |
| Genome Start | Genome End | Strand | Div | Repeat Start | Repeat End | Repeat Left | Name | Family | Class |
| 39076930 | 39077282 | + | 21.3 | 6637 | 7009 | -776 | L1MC3 | L1 | LINE |
| 39077283 | 39077643 | - | 14.3 | 1 | 364 | 0 | THE1B | MaLR | LTR |
| 39077644 | 39079202 | - | 30.9 | 2 | 1735 | 0 | MLT-int | MaLR | LTR |
| 39079205 | 39079564 | - | 12.9 | 1 | 364 | 0 | THE1B | MaLR | LTR |
| 39079565 | 39079820 | + | 21.3 | 7010 | 7254 | -531 | L1MC3 | L1 | LINE |

  

|  |  |  |  |  |  |  |  |  |  |
| --- | --- | --- | --- | --- | --- | --- | --- | --- | --- |
| chr11:39426677-39430189 | | | 3488 bp | | |  |  |  |  |
| Genome Start | Genome End | Strand | Div | Repeat Start | Repeat End | Repeat Left | Name | Family | Class |
| 39426677 | 39427057 | - | 27.9 | 212 | 600 | -7 | MER77 | ERVL | LTR |
| 39427058 | 39427278 | - | 12.1 | 158 | 375 | 0 | THE1C | MaLR | LTR |
| 39427279 | 39427556 | + | 10.8 | 1 | 278 | -31 | AluSc | Alu | SINE |
| 39427580 | 39427733 | - | 12.1 | 1 | 157 | -218 | THE1C | MaLR | LTR |
| 39427734 | 39429320 | - | 30.3 | 2 | 1735 | 0 | MLT1C-int | MaLR | LTR |
| 39429323 | 39429702 | - | 11.5 | 37 | 375 | 0 | THE1C | MaLR | LTR |
| 39429703 | 39429982 | + | 11.8 | 1 | 284 | -26 | AluSg | Alu | SINE |
| 39429983 | 39430029 | - | 11.5 | 1 | 36 | -339 | THE1C | MaLR | LTR |
| 39430030 | 39430189 | - | 27.9 | 48 | 211 | -396 | MER77 | ERVL | LTR |

  

|  |  |  |  |  |  |  |  |  |  |
| --- | --- | --- | --- | --- | --- | --- | --- | --- | --- |
| chr11:39487910-39493396 | | | 5230 bp | | |  |  |  |  |
| Genome Start | Genome End | Strand | Div | Repeat Start | Repeat End | Repeat Left | Name | Family | Class |
| 39487910 | 39488549 | - | 31.3 | 1228 | 1906 | -601 | HAL1 | L1 | LINE |
| 39488550 | 39488867 | - | 8.4 | 1 | 318 | 0 | AluY | Alu | SINE |
| 39488868 | 39489747 | - | 31.3 | 359 | 1227 | -1280 | HAL1 | L1 | LINE |
| 39489752 | 39490128 | + | 27.7 | 1 | 374 | 0 | MLT1A | MaLR | LTR |
| 39490129 | 39490469 | + | 22.0 | 1 | 347 | -1024 | MLT1A-int | MaLR | LTR |
| 39490483 | 39491215 | - | 20.4 | 2 | 761 | -974 | MLT1A0-int | MaLR | LTR |
| 39491216 | 39491578 | - | 19.1 | 1 | 365 | 0 | MLT1A0 | MaLR | LTR |
| 39491583 | 39491921 | + | 22.0 | 347 | 680 | -691 | MLT1A-int | MaLR | LTR |
| 39491917 | 39491980 | + | 20.3 | 858 | 921 | -568 | MLT1F-int | MaLR | LTR |
| 39492066 | 39492150 | + | 24.1 | 686 | 769 | -966 | MLT1A-int | MaLR | LTR |
| 39492226 | 39492692 | + | 22.2 | 895 | 1735 | 0 | MLT1A-int | MaLR | LTR |
| 39492693 | 39493048 | + | 23.3 | 1 | 368 | -6 | MLT1A | MaLR | LTR |
| 39493130 | 39493396 | - | 34.0 | 4 | 283 | -2224 | HAL1 | L1 | LINE |

  

|  |  |  |  |  |  |  |  |  |  |
| --- | --- | --- | --- | --- | --- | --- | --- | --- | --- |
| chr11:39693108-39695460 | | | 2350 bp | | |  |  |  |  |
| Genome Start | Genome End | Strand | Div | Repeat Start | Repeat End | Repeat Left | Name | Family | Class |
| 39693108 | 39693144 | - | 26.6 | 3152 | 3183 | -236 | L2 | L2 | LINE |
| 39693145 | 39693492 | + | 10.1 | 1 | 355 | 0 | THE1A | MaLR | LTR |
| 39693495 | 39695055 | + | 30.8 | 2 | 1734 | -1 | MLT1H-int | MaLR | LTR |
| 39695057 | 39695412 | + | 11.8 | 1 | 355 | 0 | THE1A | MaLR | LTR |
| 39695413 | 39695460 | - | 26.6 | 3105 | 3151 | -268 | L2 | L2 | LINE |

  

|  |  |  |  |  |  |  |  |  |  |
| --- | --- | --- | --- | --- | --- | --- | --- | --- | --- |
| chr11:39712062-39719554 | | | 7500 bp | | |  |  |  |  |
| Genome Start | Genome End | Strand | Div | Repeat Start | Repeat End | Repeat Left | Name | Family | Class |
| 39712062 | 39712248 | - | 16.6 | 519 | 711 | -23 | MER50 | ERV1 | LTR |
| 39712252 | 39712683 | + | 5.3 | 1 | 464 | 0 | LTR7B | ERV1 | LTR |
| 39712684 | 39717929 | + | 18.0 | 1 | 4915 | -2287 | HERVH | ERV1 | LTR |
| 39717927 | 39718193 | + | 18.0 | 5749 | 6016 | -1697 | HERVH | ERV1 | LTR |
| 39718187 | 39718757 | + | 10.5 | 7157 | 7713 | 0 | HERVH | ERV1 | LTR |
| 39718758 | 39719189 | + | 6.0 | 1 | 464 | 0 | LTR7B | ERV1 | LTR |
| 39719190 | 39719554 | - | 17.9 | 158 | 520 | -214 | MER50 | ERV1 | LTR |

  

|  |  |  |  |  |  |  |  |  |  |
| --- | --- | --- | --- | --- | --- | --- | --- | --- | --- |
| chr11:41053605-41057288 | | | 3682 bp | | |  |  |  |  |
| Genome Start | Genome End | Strand | Div | Repeat Start | Repeat End | Repeat Left | Name | Family | Class |
| 41053605 | 41054440 | + | 31.8 | 1669 | 2529 | -890 | L2 | L2 | LINE |
| 41054441 | 41054814 | - | 17.9 | 1 | 428 | 0 | MSTA | MaLR | LTR |
| 41054815 | 41056492 | - | 31.6 | 2 | 1735 | 0 | MLT-int | MaLR | LTR |
| 41056495 | 41056878 | - | 17.6 | 1 | 428 | 0 | MSTA | MaLR | LTR |
| 41056879 | 41057288 | + | 31.9 | 2530 | 2955 | -464 | L2 | L2 | LINE |

  

|  |  |  |  |  |  |  |  |  |  |
| --- | --- | --- | --- | --- | --- | --- | --- | --- | --- |
| chr11:41610989-41622199 | | | 10915 bp | | |  |  |  |  |
| Genome Start | Genome End | Strand | Div | Repeat Start | Repeat End | Repeat Left | Name | Family | Class |
| 41610989 | 41611537 | - | 22.9 | 5759 | 6291 | 0 | L1MA7 | L1 | LINE |
| 41611623 | 41614651 | - | 17.2 | 3696 | 6779 | -6 | L1PA15 | L1 | LINE |
| 41614652 | 41615005 | - | 13.1 | 1 | 364 | 0 | THE1B | MaLR | LTR |
| 41615006 | 41616598 | - | 30.2 | 4 | 1735 | 0 | MLT-int | MaLR | LTR |
| 41616604 | 41616694 | - | 13.9 | 278 | 364 | 0 | THE1B | MaLR | LTR |
| 41616695 | 41617333 | + | 14.4 | 1 | 689 | -2 | LTR8 | ERV1 | LTR |
| 41617334 | 41617604 | - | 13.9 | 1 | 277 | -87 | THE1B | MaLR | LTR |
| 41617605 | 41620061 | - | 19.5 | 1237 | 3695 | -3071 | L1PA15 | L1 | LINE |
| 41620268 | 41622199 | - | 19.8 | 3860 | 5777 | -514 | L1MA7 | L1 | LINE |

  

|  |  |  |  |  |  |  |  |  |  |
| --- | --- | --- | --- | --- | --- | --- | --- | --- | --- |
| chr11:42343444-42351745 | | | 8148 bp | | |  |  |  |  |
| Genome Start | Genome End | Strand | Div | Repeat Start | Repeat End | Repeat Left | Name | Family | Class |
| 42343444 | 42345051 | - | 16.5 | 5687 | 7307 | -5 | L1MA8 | L1 | LINE |
| 42345052 | 42345427 | + | 14.3 | 1 | 364 | 0 | THE1B | MaLR | LTR |
| 42345428 | 42345613 | + | 9.9 | 1 | 186 | -1394 | THE1B-int | MaLR | LTR |
| 42345614 | 42345916 | + | 13.0 | 1 | 299 | -14 | AluSq | Alu | SINE |
| 42345917 | 42346537 | + | 9.9 | 187 | 798 | -782 | THE1B-int | MaLR | LTR |
| 42346537 | 42346881 | + | 8.4 | 1233 | 1577 | -3 | THE1B-int | MaLR | LTR |
| 42346885 | 42347260 | + | 14.9 | 1 | 364 | 0 | THE1B | MaLR | LTR |
| 42347413 | 42350448 | - | 21.0 | 2217 | 5455 | -1709 | L1MA8 | L1 | LINE |
| 42350449 | 42350815 | + | 13.2 | 5 | 379 | -2 | THE1D | MaLR | LTR |
| 42350816 | 42351745 | - | 28.9 | 1591 | 2216 | -4951 | L1MA8 | L1 | LINE |

  

|  |  |  |  |  |  |  |  |  |  |
| --- | --- | --- | --- | --- | --- | --- | --- | --- | --- |
| chr11:43061628-43065711 | | | 4076 bp | | |  |  |  |  |
| Genome Start | Genome End | Strand | Div | Repeat Start | Repeat End | Repeat Left | Name | Family | Class |
| 43061628 | 43061727 | - | 14.6 | 5306 | 5400 | -743 | L1M2 | L1 | LINE |
| 43061728 | 43062205 | - | 15.8 | 1 | 431 | -3 | Tigger2a | MER2\_type | DNA |
| 43062206 | 43062909 | - | 14.6 | 4600 | 5305 | -838 | L1M2 | L1 | LINE |
| 43062911 | 43063293 | - | 14.0 | 2 | 428 | 0 | MSTA | MaLR | LTR |
| 43063299 | 43064899 | - | 14.7 | 3 | 1576 | -4 | MSTA-int | MaLR | LTR |
| 43064902 | 43065284 | - | 13.4 | 1 | 428 | 0 | MSTA | MaLR | LTR |
| 43065285 | 43065711 | - | 18.5 | 4186 | 4603 | -1540 | L1M2 | L1 | LINE |

  

|  |  |  |  |  |  |  |  |  |  |
| --- | --- | --- | --- | --- | --- | --- | --- | --- | --- |
| chr11:43435541-43442663 | | | 6698 bp | | |  |  |  |  |
| Genome Start | Genome End | Strand | Div | Repeat Start | Repeat End | Repeat Left | Name | Family | Class |
| 43435541 | 43436127 | + | 31.3 | 1048 | 1804 | -4936 | L1M4 | L1 | LINE |
| 43436170 | 43436711 | - | 26.2 | 4 | 549 | 0 | MLT1H | MaLR | LTR |
| 43436714 | 43436828 | - | 25.1 | 1347 | 1472 | 0 | MLT1H-int | MaLR | LTR |
| 43436829 | 43437186 | + | 13.8 | 1 | 339 | 0 | MER44A | MER2\_type | DNA |
| 43437187 | 43437799 | - | 25.1 | 605 | 1346 | -126 | MLT1H-int | MaLR | LTR |
| 43437800 | 43438152 | + | 15.6 | 1 | 366 | 0 | Tigger5a | MER2\_type | DNA |
| 43438153 | 43438684 | - | 26.4 | 5 | 604 | -868 | MLT1H-int | MaLR | LTR |
| 43438693 | 43439127 | - | 27.1 | 83 | 545 | -4 | MLT1H | MaLR | LTR |
| 43439180 | 43439228 | + | 29.2 | 1897 | 1978 | -4686 | L1M4 | L1 | LINE |
| 43439229 | 43439542 | + | 1.9 | 1 | 314 | -4 | AluYb8 | Alu | SINE |
| 43439543 | 43440171 | + | 28.5 | 1948 | 2657 | -4007 | L1M4 | L1 | LINE |
| 43440219 | 43440327 | + | 26.4 | 2809 | 2915 | -3729 | L1M4 | L1 | LINE |
| 43440328 | 43440805 | + | 21.4 | 1 | 505 | 0 | MLT1D | MaLR | LTR |
| 43440806 | 43441044 | + | 26.4 | 2916 | 3172 | -3472 | L1M4 | L1 | LINE |
| 43441045 | 43441339 | - | 10.2 | 1 | 295 | -15 | AluSg | Alu | SINE |
| 43441340 | 43441524 | + | 26.4 | 3173 | 3343 | -3301 | L1M4 | L1 | LINE |
| 43441685 | 43442009 | + | 29.4 | 4491 | 4817 | -1329 | L1M4 | L1 | LINE |
| 43442124 | 43442421 | + | 10.5 | 5 | 298 | -14 | AluSx | Alu | SINE |
| 43442422 | 43442663 | + | 31.6 | 4954 | 5201 | -945 | L1M4 | L1 | LINE |

  

|  |  |  |  |  |  |  |  |  |  |
| --- | --- | --- | --- | --- | --- | --- | --- | --- | --- |
| chr11:45612811-45615368 | | | 2556 bp | | |  |  |  |  |
| Genome Start | Genome End | Strand | Div | Repeat Start | Repeat End | Repeat Left | Name | Family | Class |
| 45612811 | 45612861 | + | 14.8 | 1 | 51 | -261 | AluJb | Alu | SINE |
| 45612862 | 45613237 | + | 13.3 | 1 | 381 | 0 | THE1D | MaLR | LTR |
| 45613240 | 45614766 | + | 30.8 | 2 | 1735 | 0 | MLT1J-int | MaLR | LTR |
| 45614767 | 45615130 | + | 13.2 | 1 | 381 | 0 | THE1D | MaLR | LTR |
| 45615131 | 45615368 | + | 14.8 | 52 | 298 | -14 | AluJb | Alu | SINE |

  

|  |  |  |  |  |  |  |  |  |  |
| --- | --- | --- | --- | --- | --- | --- | --- | --- | --- |
| chr11:54870182-54875751 | | | 5537 bp | | |  |  |  |  |
| Genome Start | Genome End | Strand | Div | Repeat Start | Repeat End | Repeat Left | Name | Family | Class |
| 54870182 | 54870963 | - | 29.9 | 5163 | 5992 | -248 | L1ME3B | L1 | LINE |
| 54870996 | 54871635 | - | 17.7 | 5661 | 6284 | -28 | L1MA9 | L1 | LINE |
| 54871636 | 54871938 | - | 18.9 | 5 | 301 | -12 | AluSq | Alu | SINE |
| 54871939 | 54872275 | - | 17.7 | 5332 | 5660 | -652 | L1MA9 | L1 | LINE |
| 54872276 | 54872633 | + | 15.0 | 1 | 364 | 0 | THE1B | MaLR | LTR |
| 54872634 | 54874135 | + | 11.7 | 1 | 1580 | 0 | THE1B-int | MaLR | LTR |
| 54874136 | 54874480 | + | 12.8 | 1 | 353 | -11 | THE1B | MaLR | LTR |
| 54874488 | 54875067 | - | 16.8 | 4730 | 5333 | -810 | L1MA9 | L1 | LINE |
| 54875062 | 54875751 | - | 32.5 | 4422 | 5177 | -969 | L1ME3B | L1 | LINE |

  

|  |  |  |  |  |  |  |  |  |  |
| --- | --- | --- | --- | --- | --- | --- | --- | --- | --- |
| chr11:55329032-55332207 | | | 3025 bp | | |  |  |  |  |
| Genome Start | Genome End | Strand | Div | Repeat Start | Repeat End | Repeat Left | Name | Family | Class |
| 55329032 | 55329142 | - | 31.5 | 545 | 655 | -2127 | Charlie2b | MER1\_type | DNA |
| 55329236 | 55329587 | - | 13.9 | 2 | 364 | 0 | THE1B | MaLR | LTR |
| 55329589 | 55331163 | - | 31.2 | 2 | 1735 | 0 | MLT-int | MaLR | LTR |
| 55331166 | 55331522 | - | 12.7 | 1 | 364 | 0 | THE1B | MaLR | LTR |
| 55331567 | 55331863 | - | 22.3 | 1 | 297 | -15 | AluJo | Alu | SINE |
| 55331875 | 55332207 | - | 32.3 | 110 | 449 | -2412 | Charlie2b | MER1\_type | DNA |

  

|  |  |  |  |  |  |  |  |  |  |
| --- | --- | --- | --- | --- | --- | --- | --- | --- | --- |
| chr11:55846767-55849800 | | | 3027 bp | | |  |  |  |  |
| Genome Start | Genome End | Strand | Div | Repeat Start | Repeat End | Repeat Left | Name | Family | Class |
| 55846767 | 55847191 | + | 21.5 | 3040 | 3480 | -2660 | L1M3 | L1 | LINE |
| 55847196 | 55847447 | + | 15.6 | 1 | 260 | -121 | THE1D | MaLR | LTR |
| 55847448 | 55847887 | + | 16.0 | 1 | 470 | -2 | LTR7Y | ERV1 | LTR |
| 55847888 | 55848013 | + | 15.6 | 261 | 381 | 0 | THE1D | MaLR | LTR |
| 55848018 | 55848894 | + | 15.2 | 5 | 831 | -749 | THE1D-int | MaLR | LTR |
| 55848890 | 55849361 | + | 15.0 | 1092 | 1580 | 0 | THE1D-int | MaLR | LTR |
| 55849362 | 55849684 | + | 13.9 | 1 | 381 | 0 | THE1D | MaLR | LTR |
| 55849689 | 55849800 | + | 16.2 | 3484 | 3599 | -2541 | L1M3 | L1 | LINE |

  

|  |  |  |  |  |  |  |  |  |  |
| --- | --- | --- | --- | --- | --- | --- | --- | --- | --- |
| chr11:56083605-56093257 | | | 9620 bp | | |  |  |  |  |
| Genome Start | Genome End | Strand | Div | Repeat Start | Repeat End | Repeat Left | Name | Family | Class |
| 56083605 | 56083989 | + | 26.5 | 2371 | 2769 | -4796 | L1M1 | L1 | LINE |
| 56083990 | 56084378 | + | 16.0 | 1 | 428 | 0 | MSTA | MaLR | LTR |
| 56084381 | 56085997 | + | 30.2 | 2 | 1735 | 0 | MLT-int | MaLR | LTR |
| 56085998 | 56086391 | + | 14.9 | 1 | 428 | 0 | MSTA | MaLR | LTR |
| 56086392 | 56087350 | + | 25.4 | 2770 | 3714 | -3848 | L1M1 | L1 | LINE |
| 56087326 | 56088455 | + | 14.4 | 3604 | 4773 | -2792 | L1M1 | L1 | LINE |
| 56088453 | 56088830 | + | 18.4 | 6391 | 6766 | -796 | L1M1 | L1 | LINE |
| 56088871 | 56088995 | - | 16.4 | 6023 | 6149 | -14 | L1PA13 | L1 | LINE |
| 56089008 | 56089845 | - | 10.8 | 5333 | 6152 | -9 | L1PA14 | L1 | LINE |
| 56089846 | 56090159 | - | 12.8 | 2 | 315 | 0 | AluSx | Alu | SINE |
| 56090160 | 56090697 | - | 11.7 | 4797 | 5332 | -814 | L1P | L1 | LINE |
| 56090703 | 56091395 | - | 14.6 | 5323 | 6028 | -138 | L1PA16 | L1 | LINE |
| 56091396 | 56091606 | - | 9.0 | 5937 | 6147 | -7 | L1PA7 | L1 | LINE |
| 56091607 | 56092997 | - | 14.1 | 3918 | 5322 | -824 | L1P | L1 | LINE |
| 56093000 | 56093257 | + | 23.3 | 6771 | 7024 | -695 | L1M1 | L1 | LINE |

  

|  |  |  |  |  |  |  |  |  |  |
| --- | --- | --- | --- | --- | --- | --- | --- | --- | --- |
| chr11:56361245-56364378 | | | 3132 bp | | |  |  |  |  |
| Genome Start | Genome End | Strand | Div | Repeat Start | Repeat End | Repeat Left | Name | Family | Class |
| 56361245 | 56361872 | + | 35.6 | 3725 | 4339 | -1328 | ERVL-E | ERVL | LTR |
| 56361873 | 56362242 | - | 13.8 | 1 | 375 | 0 | THE1C | MaLR | LTR |
| 56362243 | 56363797 | - | 32.1 | 2 | 1735 | 0 | MLT-int | MaLR | LTR |
| 56363800 | 56364096 | - | 12.8 | 1 | 375 | 0 | THE1C | MaLR | LTR |
| 56364097 | 56364378 | + | 35.6 | 4340 | 4624 | -1043 | ERVL-E | ERVL | LTR |

  

|  |  |  |  |  |  |  |  |  |  |
| --- | --- | --- | --- | --- | --- | --- | --- | --- | --- |
| chr11:56513983-56527681 | | | 13641 bp | | |  |  |  |  |
| Genome Start | Genome End | Strand | Div | Repeat Start | Repeat End | Repeat Left | Name | Family | Class |
| 56513983 | 56515774 | + | 15.6 | 3035 | 4834 | -1312 | L1PA16 | L1 | LINE |
| 56515825 | 56517016 | + | 15.3 | 4835 | 6046 | -120 | L1PA16 | L1 | LINE |
| 56517019 | 56517377 | - | 18.6 | 1 | 364 | 0 | THE1B | MaLR | LTR |
| 56517378 | 56518792 | - | 11.8 | 1 | 1580 | 0 | THE1B-int | MaLR | LTR |
| 56518793 | 56519138 | - | 15.9 | 1 | 364 | 0 | THE1B | MaLR | LTR |
| 56519142 | 56519943 | - | 15.8 | 1 | 826 | 0 | LTR1B-int | ERV1 | LTR |
| 56519945 | 56526764 | - | 18.7 | 3 | 7090 | -1 | LTR1B-int | ERV1 | LTR |
| 56526767 | 56527567 | - | 16.8 | 1 | 826 | 0 | LTR1B | ERV1 | LTR |
| 56527568 | 56527681 | + | 16.5 | 6043 | 6157 | -9 | L1PA16 | L1 | LINE |

  

|  |  |  |  |  |  |  |  |  |  |
| --- | --- | --- | --- | --- | --- | --- | --- | --- | --- |
| chr11:56819946-56823507 | | | 3501 bp | | |  |  |  |  |
| Genome Start | Genome End | Strand | Div | Repeat Start | Repeat End | Repeat Left | Name | Family | Class |
| 56819946 | 56820070 | + | 25.0 | 3 | 134 | -316 | MLT1J2 | MaLR | LTR |
| 56820076 | 56820282 | + | 13.4 | 1 | 221 | -144 | MLT1A0 | MaLR | LTR |
| 56820283 | 56820644 | + | 14.3 | 1 | 364 | 0 | THE1B | MaLR | LTR |
| 56820645 | 56820797 | + | 13.4 | 222 | 361 | -4 | MLT1A0 | MaLR | LTR |
| 56820803 | 56822302 | + | 21.9 | 1 | 1589 | -146 | MLT1A0-int | MaLR | LTR |
| 56822303 | 56822595 | - | 15.2 | 2 | 294 | -18 | AluSx | Alu | SINE |
| 56822596 | 56822624 | + | 21.9 | 1590 | 1620 | -115 | MLT1A0-int | MaLR | LTR |
| 56822635 | 56822729 | + | 19.2 | 2 | 87 | -278 | MLT1A0 | MaLR | LTR |
| 56822730 | 56823017 | + | 11.5 | 11 | 299 | -13 | AluSx | Alu | SINE |
| 56823018 | 56823298 | + | 19.2 | 88 | 363 | -2 | MLT1A0 | MaLR | LTR |
| 56823340 | 56823507 | + | 24.4 | 241 | 418 | -32 | MLT1J2 | MaLR | LTR |

  

|  |  |  |  |  |  |  |  |  |  |
| --- | --- | --- | --- | --- | --- | --- | --- | --- | --- |
| chr11:58122838-58130097 | | | 7241 bp | | |  |  |  |  |
| Genome Start | Genome End | Strand | Div | Repeat Start | Repeat End | Repeat Left | Name | Family | Class |
| 58122838 | 58125973 | - | 19.6 | 3290 | 6766 | -9 | L1MB5 | L1 | LINE |
| 58125974 | 58126271 | - | 11.8 | 1 | 297 | -15 | AluSx | Alu | SINE |
| 58126272 | 58126653 | - | 20.7 | 2930 | 3289 | -3458 | L1MB5 | L1 | LINE |
| 58126656 | 58126743 | + | 23.9 | 3 | 99 | -368 | MLT1C | MaLR | LTR |
| 58126754 | 58126794 | + | 7.3 | 427 | 467 | 0 | MLT1C | MaLR | LTR |
| 58126794 | 58126890 | + | 20.8 | 2 | 104 | -1267 | MLT1C-int | MaLR | LTR |
| 58126891 | 58127049 | + | 10.8 | 148 | 304 | -9 | AluSp | Alu | SINE |
| 58127053 | 58128155 | + | 21.9 | 236 | 1371 | 0 | MLT1C-int | MaLR | LTR |
| 58128159 | 58128594 | + | 23.1 | 4 | 467 | 0 | MLT1C | MaLR | LTR |
| 58128597 | 58128683 | - | 20.1 | 2846 | 2931 | -3816 | L1MB5 | L1 | LINE |
| 58128684 | 58128893 | + | 13.8 | 1 | 227 | -3 | MER30 | MER1\_type | DNA |
| 58128894 | 58130097 | - | 22.6 | 1415 | 2845 | -3904 | L1MB5 | L1 | LINE |

  

|  |  |  |  |  |  |  |  |  |  |
| --- | --- | --- | --- | --- | --- | --- | --- | --- | --- |
| chr11:58292746-58307236 | | | 14459 bp | | |  |  |  |  |
| Genome Start | Genome End | Strand | Div | Repeat Start | Repeat End | Repeat Left | Name | Family | Class |
| 58292746 | 58293226 | + | 18.2 | 2658 | 3128 | -5033 | L1PREC2 | L1 | LINE |
| 58293227 | 58293491 | + | 18.9 | 3 | 278 | -34 | AluSx | Alu | SINE |
| 58293494 | 58293877 | + | 13.8 | 5 | 381 | 0 | THE1D | MaLR | LTR |
| 58293878 | 58294165 | + | 17.2 | 3129 | 3391 | -4770 | L1PREC2 | L1 | LINE |
| 58294166 | 58294652 | + | 8.6 | 1 | 491 | 0 | HERV1\_LTRc | ERV1 | LTR |
| 58294653 | 58295032 | + | 20.2 | 1 | 379 | -8422 | HERV1\_I | ERV1 | LTR |
| 58295062 | 58295536 | + | 19.5 | 368 | 1021 | -7780 | HERV1\_I | ERV1 | LTR |
| 58295533 | 58296746 | + | 25.9 | 1154 | 2359 | -6073 | HERV1\_I | ERV1 | LTR |
| 58296754 | 58298517 | + | 20.9 | 3508 | 5480 | -3321 | HERV1\_I | ERV1 | LTR |
| 58298518 | 58299874 | + | 18.7 | 5586 | 6990 | -1811 | HERV1\_I | ERV1 | LTR |
| 58299875 | 58299963 | + | 16.9 | 7513 | 7602 | -1199 | HERV1\_I | ERV1 | LTR |
| 58299960 | 58300898 | + | 22.0 | 7861 | 8801 | 0 | HERV1\_I | ERV1 | LTR |
| 58300899 | 58301383 | + | 7.8 | 1 | 491 | 0 | HERV1\_LTRc | ERV1 | LTR |
| 58301384 | 58303549 | + | 17.8 | 3390 | 5500 | -2641 | L1PREC2 | L1 | LINE |
| 58303550 | 58303908 | - | 14.2 | 1 | 364 | 0 | THE1B | MaLR | LTR |
| 58303909 | 58305422 | - | 30.2 | 2 | 1735 | 0 | MLT-int | MaLR | LTR |
| 58305425 | 58305795 | - | 14.1 | 1 | 364 | 0 | THE1B | MaLR | LTR |
| 58305796 | 58306473 | + | 17.9 | 5501 | 6151 | -1990 | L1PREC2 | L1 | LINE |
| 58306474 | 58306758 | + | 17.7 | 5 | 290 | -22 | AluSx | Alu | SINE |
| 58306759 | 58307236 | + | 17.9 | 6152 | 6617 | -1524 | L1PREC2 | L1 | LINE |

  

|  |  |  |  |  |  |  |  |  |  |
| --- | --- | --- | --- | --- | --- | --- | --- | --- | --- |
| chr11:59438242-59442466 | | | 4222 bp | | |  |  |  |  |
| Genome Start | Genome End | Strand | Div | Repeat Start | Repeat End | Repeat Left | Name | Family | Class |
| 59438242 | 59438301 | + | 24.5 | 2394 | 2450 | -969 | L2 | L2 | LINE |
| 59438302 | 59438591 | + | 9.4 | 1 | 289 | -20 | AluSc | Alu | SINE |
| 59438592 | 59438880 | + | 30.8 | 2451 | 2814 | -605 | L2 | L2 | LINE |
| 59438881 | 59439366 | - | 21.7 | 1 | 467 | 0 | MLT1C | MaLR | LTR |
| 59439367 | 59439649 | + | 31.0 | 2815 | 3082 | -337 | L2 | L2 | LINE |
| 59439650 | 59439788 | - | 13.1 | 227 | 364 | 0 | THE1B | MaLR | LTR |
| 59439789 | 59440090 | - | 3.7 | 1 | 299 | -12 | AluY | Alu | SINE |
| 59440091 | 59440313 | - | 13.1 | 1 | 226 | -138 | THE1B | MaLR | LTR |
| 59440314 | 59441893 | - | 29.4 | 3 | 1735 | 0 | MLT-int | MaLR | LTR |
| 59441897 | 59442246 | - | 12.8 | 1 | 364 | 0 | THE1B | MaLR | LTR |
| 59442247 | 59442466 | + | 29.7 | 3083 | 3315 | -104 | L2 | L2 | LINE |

  

|  |  |  |  |  |  |  |  |  |  |
| --- | --- | --- | --- | --- | --- | --- | --- | --- | --- |
| chr11:60200966-60203776 | | | 2810 bp | | |  |  |  |  |
| Genome Start | Genome End | Strand | Div | Repeat Start | Repeat End | Repeat Left | Name | Family | Class |
| 60200966 | 60201180 | + | 24.0 | 1 | 213 | -254 | MLT1C | MaLR | LTR |
| 60201181 | 60201543 | - | 11.1 | 1 | 381 | 0 | THE1D | MaLR | LTR |
| 60201544 | 60203169 | - | 29.6 | 1 | 1735 | 0 | MLT-int | MaLR | LTR |
| 60203171 | 60203536 | - | 12.4 | 1 | 381 | 0 | THE1D | MaLR | LTR |
| 60203537 | 60203776 | + | 24.0 | 214 | 467 | 0 | MLT1C | MaLR | LTR |

  

|  |  |  |  |  |  |  |  |  |  |
| --- | --- | --- | --- | --- | --- | --- | --- | --- | --- |
| chr11:62682739-62686542 | | | 3797 bp | | |  |  |  |  |
| Genome Start | Genome End | Strand | Div | Repeat Start | Repeat End | Repeat Left | Name | Family | Class |
| 62682739 | 62683320 | - | 9.5 | 5641 | 6224 | -80 | L1MA2 | L1 | LINE |
| 62683323 | 62683679 | + | 12.1 | 1 | 364 | 0 | THE1B | MaLR | LTR |
| 62683680 | 62685470 | + | 13.5 | 1 | 1651 | 0 | THE1B-int | MaLR | LTR |
| 62685471 | 62685828 | + | 13.0 | 1 | 364 | 0 | THE1B | MaLR | LTR |
| 62685834 | 62686542 | - | 9.1 | 4922 | 5638 | -666 | L1MA2 | L1 | LINE |

  

|  |  |  |  |  |  |  |  |  |  |
| --- | --- | --- | --- | --- | --- | --- | --- | --- | --- |
| chr11:62973831-62984342 | | | 10526 bp | | |  |  |  |  |
| Genome Start | Genome End | Strand | Div | Repeat Start | Repeat End | Repeat Left | Name | Family | Class |
| 62973831 | 62976682 | - | 8.0 | 3618 | 6473 | -14 | L1PA8 | L1 | LINE |
| 62976687 | 62977137 | + | 9.5 | 1 | 448 | -2 | LTR7 | ERV1 | LTR |
| 62977141 | 62977835 | + | 16.1 | 1 | 917 | -6796 | HERVH | ERV1 | LTR |
| 62977836 | 62978249 | + | 10.0 | 1 | 448 | -2 | LTR7 | ERV1 | LTR |
| 62978253 | 62980373 | + | 9.7 | 913 | 2995 | -4718 | HERVH | ERV1 | LTR |
| 62980353 | 62980500 | + | 10.8 | 3140 | 3288 | -4425 | HERVH | ERV1 | LTR |
| 62980493 | 62980997 | + | 11.4 | 3492 | 3993 | -3720 | HERVH | ERV1 | LTR |
| 62981000 | 62981189 | + | 10.5 | 4488 | 4680 | -3033 | HERVH | ERV1 | LTR |
| 62981176 | 62983104 | + | 7.8 | 5769 | 7713 | 0 | HERVH | ERV1 | LTR |
| 62983105 | 62983555 | + | 7.9 | 1 | 448 | -2 | LTR7 | ERV1 | LTR |
| 62983558 | 62983748 | - | 11.2 | 3423 | 3618 | -2843 | L1PA8 | L1 | LINE |
| 62983764 | 62984342 | - | 12.8 | 6 | 605 | -5856 | L1PA8 | L1 | LINE |

  

|  |  |  |  |  |  |  |  |  |  |
| --- | --- | --- | --- | --- | --- | --- | --- | --- | --- |
| chr11:67365457-67381588 | | | 16101 bp | | |  |  |  |  |
| Genome Start | Genome End | Strand | Div | Repeat Start | Repeat End | Repeat Left | Name | Family | Class |
| 67365457 | 67365632 | + | 21.7 | 1 | 185 | -320 | MLT1D | MaLR | LTR |
| 67365643 | 67365940 | + | 9.4 | 3 | 301 | -12 | AluSp | Alu | SINE |
| 67365953 | 67366348 | - | 8.4 | 2 | 448 | -2 | LTR7 | ERV1 | LTR |
| 67366350 | 67366705 | - | 10.8 | 7324 | 7713 | 0 | HERVH | ERV1 | LTR |
| 67366706 | 67366916 | - | 10.9 | 5600 | 5819 | -1894 | HERVH | ERV1 | LTR |
| 67366918 | 67366983 | - | 14.2 | 5090 | 5149 | -2564 | HERVH | ERV1 | LTR |
| 67366984 | 67368051 | - | 4.5 | 1 | 1071 | 0 | MER11C | ERVK | LTR |
| 67368052 | 67368653 | - | 14.2 | 4488 | 5089 | -2624 | HERVH | ERV1 | LTR |
| 67368656 | 67369204 | - | 8.7 | 3442 | 3993 | -3720 | HERVH | ERV1 | LTR |
| 67369205 | 67369701 | - | 9.1 | 1 | 501 | 0 | LTR2C | ERV1 | LTR |
| 67369702 | 67377532 | - | 6.6 | 3 | 7812 | -1 | HERVE | ERV1 | LTR |
| 67377533 | 67377837 | - | 10.4 | 3140 | 3441 | -4272 | HERVH | ERV1 | LTR |
| 67377824 | 67380656 | - | 9.7 | 1 | 2992 | -4721 | HERVH | ERV1 | LTR |
| 67380659 | 67381008 | - | 9.2 | 1 | 448 | -2 | LTR7 | ERV1 | LTR |
| 67381026 | 67381217 | + | 26.5 | 193 | 428 | -76 | MLT1D | MaLR | LTR |
| 67381218 | 67381523 | - | 13.5 | 1 | 305 | -8 | AluSq | Alu | SINE |
| 67381524 | 67381588 | + | 26.6 | 392 | 483 | -1 | MLT1D | MaLR | LTR |

  

|  |  |  |  |  |  |  |  |  |  |
| --- | --- | --- | --- | --- | --- | --- | --- | --- | --- |
| chr11:67432396-67444011 | | | 11479 bp | | |  |  |  |  |
| Genome Start | Genome End | Strand | Div | Repeat Start | Repeat End | Repeat Left | Name | Family | Class |
| 67432396 | 67432806 | + | 19.8 | 1056 | 1440 | -5409 | L1MA4 | L1 | LINE |
| 67432807 | 67433110 | + | 11.2 | 1 | 305 | -5 | AluSg | Alu | SINE |
| 67433111 | 67434157 | + | 19.8 | 1441 | 2526 | -4323 | L1MA4 | L1 | LINE |
| 67434158 | 67434316 | + | 17.8 | 5 | 161 | -5 | FRAM | Alu | SINE |
| 67434317 | 67434551 | + | 19.8 | 2527 | 2779 | -4070 | L1MA4 | L1 | LINE |
| 67434552 | 67434909 | - | 13.0 | 1 | 364 | 0 | THE1B | MaLR | LTR |
| 67434910 | 67436459 | - | 30.4 | 2 | 1735 | 0 | MLT-int | MaLR | LTR |
| 67436462 | 67436813 | - | 16.1 | 1 | 364 | 0 | THE1B | MaLR | LTR |
| 67436814 | 67437964 | + | 16.2 | 2780 | 3954 | -2891 | L1MA4 | L1 | LINE |
| 67437989 | 67439003 | + | 16.1 | 3955 | 4974 | -1871 | L1MA4 | L1 | LINE |
| 67439004 | 67439313 | + | 11.1 | 1 | 310 | -2 | AluSx | Alu | SINE |
| 67439314 | 67439795 | + | 16.1 | 4975 | 5443 | -1402 | L1MA4 | L1 | LINE |
| 67439796 | 67440068 | + | 8.3 | 1 | 302 | -9 | AluY | Alu | SINE |
| 67440126 | 67440557 | + | 16.1 | 5444 | 5904 | -941 | L1MA4 | L1 | LINE |
| 67440558 | 67440803 | + | 11.0 | 4958 | 5204 | -942 | L1PREC2 | L1 | LINE |
| 67440787 | 67441381 | + | 13.8 | 6029 | 6629 | -373 | L1MA4 | L1 | LINE |
| 67441441 | 67441647 | + | 16.6 | 6601 | 6824 | -178 | L1MA4 | L1 | LINE |
| 67441653 | 67442368 | - | 9.7 | 5486 | 6150 | -1 | L1PB1 | L1 | LINE |
| 67442369 | 67442678 | - | 10.7 | 1 | 310 | 0 | AluSg | Alu | SINE |
| 67442679 | 67443830 | - | 9.8 | 4294 | 5485 | -666 | L1PB1 | L1 | LINE |
| 67443838 | 67444011 | + | 13.2 | 6812 | 6990 | -12 | L1MA4 | L1 | LINE |

  

|  |  |  |  |  |  |  |  |  |  |
| --- | --- | --- | --- | --- | --- | --- | --- | --- | --- |
| chr11:68156897-68165320 | | | 8339 bp | | |  |  |  |  |
| Genome Start | Genome End | Strand | Div | Repeat Start | Repeat End | Repeat Left | Name | Family | Class |
| 68156897 | 68157104 | + | 29.1 | 2290 | 2509 | -2487 | HERV16 | ERVL | LTR |
| 68157158 | 68157223 | - | 20.9 | 99 | 160 | -433 | MLT1E | MaLR | LTR |
| 68157224 | 68157580 | - | 22.4 | 1 | 365 | 0 | MLT1A0 | MaLR | LTR |
| 68157581 | 68157676 | - | 20.9 | 1 | 98 | -495 | MLT1E | MaLR | LTR |
| 68157697 | 68157821 | + | 14.5 | 2 | 148 | -164 | AluJo | Alu | SINE |
| 68157822 | 68158214 | + | 5.8 | 1 | 448 | -2 | LTR7 | ERV1 | LTR |
| 68158217 | 68161166 | + | 9.7 | 1 | 2995 | -4718 | HERVH | ERV1 | LTR |
| 68161146 | 68161290 | + | 14.5 | 3140 | 3290 | -4423 | HERVH | ERV1 | LTR |
| 68161281 | 68161783 | + | 11.0 | 3492 | 3993 | -3720 | HERVH | ERV1 | LTR |
| 68161786 | 68162442 | + | 13.4 | 4488 | 5149 | -2564 | HERVH | ERV1 | LTR |
| 68162444 | 68162660 | + | 11.6 | 5600 | 5819 | -1894 | HERVH | ERV1 | LTR |
| 68162659 | 68163038 | + | 8.3 | 7322 | 7713 | 0 | HERVH | ERV1 | LTR |
| 68163039 | 68163483 | + | 6.5 | 1 | 448 | -2 | LTR7 | ERV1 | LTR |
| 68163486 | 68163639 | + | 17.5 | 144 | 300 | -12 | AluJo | Alu | SINE |
| 68163651 | 68163943 | + | 12.7 | 1 | 284 | -28 | AluSx | Alu | SINE |
| 68163971 | 68164174 | - | 10.8 | 389 | 594 | -8 | LTR43 | ERV1 | LTR |
| 68164175 | 68164468 | - | 9.0 | 320 | 610 | 0 | LTR71B | ERV1 | LTR |
| 68164469 | 68165320 | + | 35.4 | 3938 | 4834 | -833 | HERV16 | ERVL | LTR |

  

|  |  |  |  |  |  |  |  |  |  |
| --- | --- | --- | --- | --- | --- | --- | --- | --- | --- |
| chr11:71064683-71076401 | | | 11561 bp | | |  |  |  |  |
| Genome Start | Genome End | Strand | Div | Repeat Start | Repeat End | Repeat Left | Name | Family | Class |
| 71064683 | 71064856 | - | 12.6 | 6812 | 6990 | -12 | L1MA4 | L1 | LINE |
| 71064864 | 71066010 | + | 10.3 | 4294 | 5484 | -667 | L1PB1 | L1 | LINE |
| 71066011 | 71066320 | + | 10.0 | 1 | 310 | 0 | AluSg | Alu | SINE |
| 71066321 | 71067098 | + | 10.4 | 5485 | 6150 | -1 | L1PB1 | L1 | LINE |
| 71067105 | 71067311 | - | 15.5 | 5899 | 6122 | -178 | L1MA4 | L1 | LINE |
| 71067371 | 71067964 | - | 15.0 | 6032 | 6629 | -373 | L1MA4 | L1 | LINE |
| 71067949 | 71068194 | - | 10.6 | 4958 | 5204 | -942 | L1PREC2 | L1 | LINE |
| 71068195 | 71068633 | - | 16.4 | 5451 | 5905 | -940 | L1MA4 | L1 | LINE |
| 71068690 | 71068959 | - | 7.9 | 1 | 307 | -4 | AluY | Alu | SINE |
| 71068960 | 71069259 | - | 16.4 | 5146 | 5450 | -1395 | L1MA4 | L1 | LINE |
| 71069304 | 71069486 | - | 16.4 | 4979 | 5145 | -1700 | L1MA4 | L1 | LINE |
| 71069487 | 71069783 | - | 13.1 | 1 | 300 | -12 | AluSx | Alu | SINE |
| 71069784 | 71071979 | - | 16.5 | 2780 | 4978 | -1867 | L1MA4 | L1 | LINE |
| 71071980 | 71072333 | + | 15.3 | 1 | 364 | 0 | THE1B | MaLR | LTR |
| 71072336 | 71073885 | + | 31.4 | 2 | 1735 | 0 | MLT-int | MaLR | LTR |
| 71073886 | 71074243 | + | 13.8 | 1 | 364 | 0 | THE1B | MaLR | LTR |
| 71074244 | 71074478 | - | 19.9 | 2527 | 2779 | -4070 | L1MA4 | L1 | LINE |
| 71074479 | 71074637 | - | 17.8 | 5 | 161 | -5 | FRAM | Alu | SINE |
| 71074638 | 71075684 | - | 19.9 | 1441 | 2526 | -4323 | L1MA4 | L1 | LINE |
| 71075685 | 71075989 | - | 11.5 | 1 | 306 | -4 | AluSg | Alu | SINE |
| 71075990 | 71076401 | - | 19.9 | 1056 | 1440 | -5409 | L1MA4 | L1 | LINE |

  

|  |  |  |  |  |  |  |  |  |  |
| --- | --- | --- | --- | --- | --- | --- | --- | --- | --- |
| chr11:71125675-71141878 | | | 16122 bp | | |  |  |  |  |
| Genome Start | Genome End | Strand | Div | Repeat Start | Repeat End | Repeat Left | Name | Family | Class |
| 71125675 | 71125739 | - | 25.2 | 393 | 484 | 0 | MLT1D | MaLR | LTR |
| 71125740 | 71126049 | + | 15.3 | 1 | 308 | -4 | AluSx | Alu | SINE |
| 71126050 | 71126204 | - | 24.4 | 229 | 437 | -68 | MLT1D | MaLR | LTR |
| 71126269 | 71126618 | + | 9.8 | 1 | 448 | -2 | LTR7 | ERV1 | LTR |
| 71126621 | 71129517 | + | 10.1 | 1 | 2992 | -4721 | HERVH | ERV1 | LTR |
| 71129504 | 71129808 | + | 10.8 | 3140 | 3441 | -4272 | HERVH | ERV1 | LTR |
| 71129809 | 71137624 | + | 6.5 | 3 | 7812 | -1 | HERVE | ERV1 | LTR |
| 71137625 | 71138120 | + | 8.1 | 1 | 501 | 0 | LTR2C | ERV1 | LTR |
| 71138121 | 71138670 | + | 9.3 | 3442 | 3993 | -3720 | HERVH | ERV1 | LTR |
| 71138673 | 71139275 | + | 14.8 | 4488 | 5089 | -2624 | HERVH | ERV1 | LTR |
| 71139276 | 71140343 | + | 4.5 | 1 | 1071 | 0 | MER11C | ERVK | LTR |
| 71140344 | 71140409 | + | 14.8 | 5090 | 5149 | -2564 | HERVH | ERV1 | LTR |
| 71140411 | 71140615 | + | 11.2 | 5600 | 5817 | -1896 | HERVH | ERV1 | LTR |
| 71140618 | 71140994 | + | 8.9 | 7324 | 7713 | 0 | HERVH | ERV1 | LTR |
| 71140996 | 71141389 | + | 9.9 | 2 | 448 | -2 | LTR7 | ERV1 | LTR |
| 71141402 | 71141693 | - | 10.6 | 3 | 295 | -18 | AluSp | Alu | SINE |
| 71141706 | 71141878 | - | 25.8 | 1 | 183 | -322 | MLT1D | MaLR | LTR |

  

|  |  |  |  |  |  |  |  |  |  |
| --- | --- | --- | --- | --- | --- | --- | --- | --- | --- |
| chr11:74042026-74053136 | | | 10828 bp | | |  |  |  |  |
| Genome Start | Genome End | Strand | Div | Repeat Start | Repeat End | Repeat Left | Name | Family | Class |
| 74042026 | 74042479 | + | 23.9 | 1957 | 2398 | -4002 | L1MEc | L1 | LINE |
| 74042480 | 74042881 | - | 17.3 | 1 | 426 | 0 | MSTB | MaLR | LTR |
| 74042882 | 74043156 | + | 24.3 | 2297 | 2617 | -3602 | L1MEc | L1 | LINE |
| 74043172 | 74043367 | + | 25.4 | 11 | 246 | -7088 | L1M4b | L1 | LINE |
| 74043607 | 74044179 | + | 25.4 | 926 | 1465 | -5439 | L1M4b | L1 | LINE |
| 74044189 | 74044305 | + | 27.0 | 2666 | 2780 | -3439 | L1MEc | L1 | LINE |
| 74044322 | 74044865 | + | 24.5 | 3995 | 4559 | -1660 | L1MEc | L1 | LINE |
| 74044868 | 74044999 | - | 10.4 | 4333 | 4471 | -3094 | L1M1 | L1 | LINE |
| 74045000 | 74045290 | + | 9.6 | 1 | 291 | -19 | AluSg | Alu | SINE |
| 74045291 | 74046743 | - | 10.8 | 2621 | 4332 | -3233 | L1M1 | L1 | LINE |
| 74046744 | 74047089 | - | 7.8 | 1 | 353 | -2 | THE1A | MaLR | LTR |
| 74047090 | 74048662 | - | 29.4 | 2 | 1735 | 0 | MLT1D-int | MaLR | LTR |
| 74048665 | 74049019 | - | 7.3 | 1 | 355 | 0 | THE1A | MaLR | LTR |
| 74049020 | 74049210 | - | 11.3 | 2440 | 2620 | -4945 | L1M1 | L1 | LINE |
| 74049211 | 74049591 | + | 11.3 | 1 | 428 | 0 | MSTA | MaLR | LTR |
| 74049592 | 74051903 | - | 11.3 | 17 | 2439 | -5126 | L1M1 | L1 | LINE |
| 74051904 | 74053136 | + | 21.2 | 4545 | 5892 | -345 | L1MEc | L1 | LINE |

  

|  |  |  |  |  |  |  |  |  |  |
| --- | --- | --- | --- | --- | --- | --- | --- | --- | --- |
| chr11:77119069-77122076 | | | 3001 bp | | |  |  |  |  |
| Genome Start | Genome End | Strand | Div | Repeat Start | Repeat End | Repeat Left | Name | Family | Class |
| 77119069 | 77119283 | - | 23.9 | 5952 | 6163 | -1 | L1ME2 | L1 | LINE |
| 77119284 | 77119642 | + | 11.3 | 1 | 364 | 0 | THE1B | MaLR | LTR |
| 77119645 | 77121192 | + | 30.4 | 2 | 1735 | 0 | MLT-int | MaLR | LTR |
| 77121195 | 77121550 | + | 13.6 | 3 | 364 | 0 | THE1B | MaLR | LTR |
| 77121551 | 77121701 | - | 23.9 | 5784 | 5951 | -213 | L1ME2 | L1 | LINE |
| 77121705 | 77122076 | - | 24.5 | 5335 | 5719 | -445 | L1ME2 | L1 | LINE |

  

|  |  |  |  |  |  |  |  |  |  |
| --- | --- | --- | --- | --- | --- | --- | --- | --- | --- |
| chr11:77177483-77186996 | | | 9205 bp | | |  |  |  |  |
| Genome Start | Genome End | Strand | Div | Repeat Start | Repeat End | Repeat Left | Name | Family | Class |
| 77177483 | 77177590 | + | 13.6 | 2 | 377 | -7003 | L1M2 | L1 | LINE |
| 77177598 | 77177705 | + | 24.1 | 13 | 121 | -21 | FLAM\_A | Alu | SINE |
| 77177728 | 77178793 | + | 29.4 | 372 | 1451 | -5665 | L1M2 | L1 | LINE |
| 77178794 | 77179143 | - | 13.9 | 1 | 364 | 0 | THE1B | MaLR | LTR |
| 77179146 | 77180683 | - | 31.1 | 5 | 1733 | -2 | MLT-int | MaLR | LTR |
| 77180689 | 77181040 | - | 11.3 | 2 | 364 | 0 | THE1B | MaLR | LTR |
| 77181041 | 77181377 | + | 29.4 | 1457 | 1795 | -5321 | L1M2 | L1 | LINE |
| 77181376 | 77181891 | + | 14.9 | 2668 | 3179 | -3933 | L1M2 | L1 | LINE |
| 77181892 | 77182200 | + | 17.2 | 1 | 305 | -7 | AluJo | Alu | SINE |
| 77182201 | 77182333 | + | 17.0 | 3180 | 3319 | -3793 | L1M2 | L1 | LINE |
| 77182577 | 77183093 | + | 17.1 | 3298 | 3828 | -3284 | L1M2 | L1 | LINE |
| 77183094 | 77183234 | + | 16.4 | 1 | 140 | -3 | FLAM\_C | Alu | SINE |
| 77183245 | 77183339 | + | 15.8 | 3810 | 3912 | -3203 | L1M2 | L1 | LINE |
| 77183342 | 77184484 | + | 17.1 | 4904 | 6050 | -1062 | L1M2 | L1 | LINE |
| 77184506 | 77184808 | - | 8.9 | 3246 | 3557 | -2589 | L1P3 | L1 | LINE |
| 77184808 | 77186996 | + | 8.8 | 4562 | 6754 | -369 | L1M2 | L1 | LINE |

  

|  |  |  |  |  |  |  |  |  |  |
| --- | --- | --- | --- | --- | --- | --- | --- | --- | --- |
| chr11:77246939-77252382 | | | 5411 bp | | |  |  |  |  |
| Genome Start | Genome End | Strand | Div | Repeat Start | Repeat End | Repeat Left | Name | Family | Class |
| 77246939 | 77247000 | - | 17.7 | 189 | 250 | -52 | AluSx | Alu | SINE |
| 77247021 | 77247317 | - | 4.7 | 1 | 326 | -454 | LTR17 | ERV1 | LTR |
| 77247318 | 77247609 | - | 5.1 | 8335 | 8626 | 0 | HERV17 | ERV1 | LTR |
| 77247600 | 77247638 | - | 7.7 | 7510 | 7548 | -1078 | HERV17 | ERV1 | LTR |
| 77247632 | 77251647 | - | 4.4 | 1953 | 6019 | -2607 | HERV17 | ERV1 | LTR |
| 77251678 | 77252172 | - | 4.9 | 258 | 780 | 0 | LTR17 | ERV1 | LTR |
| 77252173 | 77252382 | - | 14.8 | 1 | 210 | -102 | AluSx | Alu | SINE |

  

|  |  |  |  |  |  |  |  |  |  |
| --- | --- | --- | --- | --- | --- | --- | --- | --- | --- |
| chr11:77660405-77662945 | | | 2539 bp | | |  |  |  |  |
| Genome Start | Genome End | Strand | Div | Repeat Start | Repeat End | Repeat Left | Name | Family | Class |
| 77660405 | 77660498 | + | 15.6 | 1 | 94 | -218 | AluJb | Alu | SINE |
| 77660499 | 77660863 | + | 10.7 | 1 | 364 | 0 | THE1B | MaLR | LTR |
| 77660866 | 77662427 | + | 29.4 | 2 | 1735 | 0 | MLT1L-int | MaLR | LTR |
| 77662428 | 77662775 | + | 14.7 | 1 | 364 | 0 | THE1B | MaLR | LTR |
| 77662776 | 77662945 | + | 15.6 | 95 | 273 | -39 | AluJb | Alu | SINE |

  

|  |  |  |  |  |  |  |  |  |  |
| --- | --- | --- | --- | --- | --- | --- | --- | --- | --- |
| chr11:80161294-80164554 | | | 3258 bp | | |  |  |  |  |
| Genome Start | Genome End | Strand | Div | Repeat Start | Repeat End | Repeat Left | Name | Family | Class |
| 80161294 | 80161868 | + | 18.5 | 5049 | 5655 | -636 | L1MA8 | L1 | LINE |
| 80161869 | 80162223 | + | 15.7 | 5 | 364 | 0 | THE1B | MaLR | LTR |
| 80162227 | 80163795 | + | 31.4 | 3 | 1735 | 0 | MLT-int | MaLR | LTR |
| 80163796 | 80164168 | + | 16.3 | 1 | 364 | 0 | THE1B | MaLR | LTR |
| 80164169 | 80164554 | + | 17.9 | 5656 | 6053 | -238 | L1MA8 | L1 | LINE |

  

|  |  |  |  |  |  |  |  |  |  |
| --- | --- | --- | --- | --- | --- | --- | --- | --- | --- |
| chr11:81042204-81051132 | | | 8773 bp | | |  |  |  |  |
| Genome Start | Genome End | Strand | Div | Repeat Start | Repeat End | Repeat Left | Name | Family | Class |
| 81042204 | 81047624 | - | 18.4 | 1502 | 6863 | -6 | L1PA17 | L1 | LINE |
| 81047765 | 81048148 | - | 16.2 | 1 | 428 | 0 | MSTA | MaLR | LTR |
| 81048150 | 81049780 | - | 30.4 | 2 | 1734 | -1 | MLT-int | MaLR | LTR |
| 81049783 | 81050154 | - | 16.2 | 1 | 428 | 0 | MSTA | MaLR | LTR |
| 81050168 | 81051132 | - | 23.4 | 1 | 1394 | -5475 | L1PA17 | L1 | LINE |

  

|  |  |  |  |  |  |  |  |  |  |
| --- | --- | --- | --- | --- | --- | --- | --- | --- | --- |
| chr11:81179925-81182968 | | | 3039 bp | | |  |  |  |  |
| Genome Start | Genome End | Strand | Div | Repeat Start | Repeat End | Repeat Left | Name | Family | Class |
| 81179925 | 81180048 | + | 17.7 | 1 | 125 | -301 | MSTB | MaLR | LTR |
| 81180050 | 81180423 | - | 13.7 | 3 | 381 | 0 | THE1D | MaLR | LTR |
| 81180424 | 81181986 | - | 14.7 | 1 | 1579 | -1 | THE1D-int | MaLR | LTR |
| 81181987 | 81182365 | - | 15.7 | 1 | 381 | 0 | THE1D | MaLR | LTR |
| 81182370 | 81182440 | + | 18.4 | 126 | 196 | -230 | MSTB | MaLR | LTR |
| 81182441 | 81182731 | + | 13.8 | 1 | 292 | -20 | AluSx | Alu | SINE |
| 81182732 | 81182968 | + | 18.4 | 197 | 426 | 0 | MSTB | MaLR | LTR |

  

|  |  |  |  |  |  |  |  |  |  |
| --- | --- | --- | --- | --- | --- | --- | --- | --- | --- |
| chr11:81433090-81445088 | | | 11921 bp | | |  |  |  |  |
| Genome Start | Genome End | Strand | Div | Repeat Start | Repeat End | Repeat Left | Name | Family | Class |
| 81433090 | 81433138 | + | 12.2 | 9 | 59 | -306 | MLT1A0 | MaLR | LTR |
| 81433142 | 81434054 | + | 16.9 | 1 | 939 | -1479 | Tigger1 | MER2\_type | DNA |
| 81434055 | 81434260 | + | 15.1 | 1 | 224 | -379 | LTR26 | ERV1 | LTR |
| 81434272 | 81434784 | + | 24.9 | 19 | 539 | -7026 | L1MA1 | L1 | LINE |
| 81434793 | 81435184 | + | 13.9 | 1 | 375 | 0 | THE1C | MaLR | LTR |
| 81435185 | 81436702 | + | 14.2 | 1 | 1580 | 0 | THE1C-int | MaLR | LTR |
| 81436703 | 81437088 | + | 13.8 | 1 | 375 | 0 | THE1C | MaLR | LTR |
| 81437089 | 81439450 | + | 17.4 | 543 | 3040 | -4525 | L1MA1 | L1 | LINE |
| 81439449 | 81442989 | + | 11.1 | 4118 | 7721 | 0 | L1MA1 | L1 | LINE |
| 81443048 | 81443398 | + | 20.8 | 226 | 603 | 0 | LTR26 | ERV1 | LTR |
| 81443399 | 81444891 | + | 14.3 | 936 | 2416 | -2 | Tigger1 | MER2\_type | DNA |
| 81444892 | 81445088 | + | 23.7 | 167 | 361 | -4 | MLT1A0 | MaLR | LTR |

  

|  |  |  |  |  |  |  |  |  |  |
| --- | --- | --- | --- | --- | --- | --- | --- | --- | --- |
| chr11:82039731-82044158 | | | 4426 bp | | |  |  |  |  |
| Genome Start | Genome End | Strand | Div | Repeat Start | Repeat End | Repeat Left | Name | Family | Class |
| 82039731 | 82040656 | + | 29.5 | 637 | 1548 | -959 | HAL1 | L1 | LINE |
| 82040657 | 82040966 | - | 13.9 | 2 | 312 | 0 | AluSx | Alu | SINE |
| 82040967 | 82041569 | + | 29.5 | 1549 | 2203 | -304 | HAL1 | L1 | LINE |
| 82041570 | 82041917 | + | 11.8 | 2 | 355 | 0 | THE1A | MaLR | LTR |
| 82041920 | 82043482 | + | 30.0 | 2 | 1735 | 0 | MLT-int | MaLR | LTR |
| 82043483 | 82043832 | + | 13.1 | 1 | 355 | 0 | THE1A | MaLR | LTR |
| 82043833 | 82044158 | + | 29.5 | 2204 | 2506 | -1 | HAL1 | L1 | LINE |

  

|  |  |  |  |  |  |  |  |  |  |
| --- | --- | --- | --- | --- | --- | --- | --- | --- | --- |
| chr11:82331046-82334997 | | | 3950 bp | | |  |  |  |  |
| Genome Start | Genome End | Strand | Div | Repeat Start | Repeat End | Repeat Left | Name | Family | Class |
| 82331046 | 82331849 | - | 17.8 | 1296 | 2121 | -2 | MER52D | ERV1 | LTR |
| 82331850 | 82332183 | - | 12.4 | 1 | 364 | 0 | THE1B | MaLR | LTR |
| 82332184 | 82333752 | - | 30.9 | 2 | 1735 | 0 | MLT-int | MaLR | LTR |
| 82333755 | 82334115 | - | 15.1 | 1 | 364 | 0 | THE1B | MaLR | LTR |
| 82334116 | 82334997 | - | 18.2 | 134 | 1295 | -745 | MER52D | ERV1 | LTR |

  

|  |  |  |  |  |  |  |  |  |  |
| --- | --- | --- | --- | --- | --- | --- | --- | --- | --- |
| chr11:82817451-82825739 | | | 8022 bp | | |  |  |  |  |
| Genome Start | Genome End | Strand | Div | Repeat Start | Repeat End | Repeat Left | Name | Family | Class |
| 82817451 | 82817518 | - | 21.9 | 3354 | 3417 | -2 | L2 | L2 | LINE |
| 82817511 | 82818629 | - | 31.3 | 1817 | 3109 | -310 | L2 | L2 | LINE |
| 82818630 | 82818871 | + | 28.3 | 1 | 278 | -233 | MLT1J | MaLR | LTR |
| 82818876 | 82819293 | + | 7.7 | 1 | 429 | -238 | PABL\_B | ERV1 | LTR |
| 82819294 | 82819741 | - | 8.9 | 1 | 448 | -2 | LTR7 | ERV1 | LTR |
| 82819742 | 82820112 | - | 8.1 | 7324 | 7713 | 0 | HERVH | ERV1 | LTR |
| 82820113 | 82820329 | - | 13.8 | 5600 | 5819 | -1894 | HERVH | ERV1 | LTR |
| 82820331 | 82821034 | - | 11.7 | 4429 | 5149 | -2564 | HERVH | ERV1 | LTR |
| 82821226 | 82821278 | + | 20.8 | 3302 | 3358 | -20 | L2 | L2 | LINE |
| 82821306 | 82822633 | - | 11.4 | 3140 | 4437 | -3276 | HERVH | ERV1 | LTR |
| 82822613 | 82824720 | - | 10.7 | 894 | 2995 | -4718 | HERVH | ERV1 | LTR |
| 82824696 | 82824807 | - | 17.8 | 1 | 110 | -7603 | HERVH | ERV1 | LTR |
| 82824811 | 82825266 | - | 9.0 | 3 | 448 | -2 | LTR7 | ERV1 | LTR |
| 82825268 | 82825512 | + | 10.2 | 424 | 667 | 0 | PABL\_B | ERV1 | LTR |
| 82825607 | 82825739 | - | 21.8 | 1634 | 1807 | -1612 | L2 | L2 | LINE |

  

|  |  |  |  |  |  |  |  |  |  |
| --- | --- | --- | --- | --- | --- | --- | --- | --- | --- |
| chr11:84904779-84909803 | | | 4985 bp | | |  |  |  |  |
| Genome Start | Genome End | Strand | Div | Repeat Start | Repeat End | Repeat Left | Name | Family | Class |
| 84904779 | 84906293 | + | 19.2 | 2773 | 4326 | -1817 | L1M2 | L1 | LINE |
| 84906294 | 84906454 | - | 14.8 | 138 | 306 | -6 | AluJo | Alu | SINE |
| 84906455 | 84906811 | + | 11.0 | 1 | 362 | -2 | THE1B | MaLR | LTR |
| 84906816 | 84908401 | + | 30.6 | 2 | 1735 | 0 | MLT-int | MaLR | LTR |
| 84908402 | 84908724 | + | 13.6 | 1 | 364 | 0 | THE1B | MaLR | LTR |
| 84908735 | 84908871 | - | 18.2 | 1 | 133 | -169 | AluJo | Alu | SINE |
| 84908872 | 84909207 | + | 19.2 | 4327 | 4656 | -1487 | L1M2 | L1 | LINE |
| 84909234 | 84909803 | + | 19.2 | 4657 | 5234 | -909 | L1M2 | L1 | LINE |

  

|  |  |  |  |  |  |  |  |  |  |
| --- | --- | --- | --- | --- | --- | --- | --- | --- | --- |
| chr11:86930032-86934067 | | | 4013 bp | | |  |  |  |  |
| Genome Start | Genome End | Strand | Div | Repeat Start | Repeat End | Repeat Left | Name | Family | Class |
| 86930032 | 86930180 | - | 12.8 | 6154 | 6303 | -2 | L1MA3 | L1 | LINE |
| 86930198 | 86930699 | - | 7.9 | 1 | 585 | -9 | PRIMA4\_LTR | ERV1 | LTR |
| 86930700 | 86930718 | - | 12.0 | 6141 | 6157 | -148 | L1MA3 | L1 | LINE |
| 86930719 | 86931093 | - | 14.2 | 1 | 381 | 0 | THE1D | MaLR | LTR |
| 86931096 | 86932627 | - | 30.9 | 4 | 1733 | -2 | MLT-int | MaLR | LTR |
| 86932632 | 86933002 | - | 11.9 | 1 | 381 | 0 | THE1D | MaLR | LTR |
| 86933003 | 86933177 | - | 12.0 | 5966 | 6140 | -165 | L1MA3 | L1 | LINE |
| 86933178 | 86933582 | - | 16.5 | 1 | 428 | 0 | MSTA | MaLR | LTR |
| 86933583 | 86934067 | - | 12.0 | 5476 | 5965 | -340 | L1MA3 | L1 | LINE |

  

|  |  |  |  |  |  |  |  |  |  |
| --- | --- | --- | --- | --- | --- | --- | --- | --- | --- |
| chr11:87713750-87716287 | | | 2536 bp | | |  |  |  |  |
| Genome Start | Genome End | Strand | Div | Repeat Start | Repeat End | Repeat Left | Name | Family | Class |
| 87713750 | 87713793 | + | 24.0 | 1 | 45 | -144 | MER5A | MER1\_type | DNA |
| 87713794 | 87714180 | - | 18.1 | 1 | 428 | 0 | MSTC | MaLR | LTR |
| 87714181 | 87715702 | - | 23.8 | 2 | 1735 | 0 | MSTC-int | MaLR | LTR |
| 87715705 | 87716141 | - | 19.2 | 1 | 428 | 0 | MSTC | MaLR | LTR |
| 87716142 | 87716287 | + | 24.0 | 46 | 189 | 0 | MER5A | MER1\_type | DNA |

  

|  |  |  |  |  |  |  |  |  |  |
| --- | --- | --- | --- | --- | --- | --- | --- | --- | --- |
| chr11:90065352-90068109 | | | 2742 bp | | |  |  |  |  |
| Genome Start | Genome End | Strand | Div | Repeat Start | Repeat End | Repeat Left | Name | Family | Class |
| 90065352 | 90065597 | + | 20.7 | 1 | 292 | -264 | MLT2B4 | ERVL | LTR |
| 90065613 | 90065985 | + | 12.2 | 1 | 375 | 0 | THE1C | MaLR | LTR |
| 90065986 | 90067514 | + | 14.5 | 1 | 1580 | 0 | THE1C-int | MaLR | LTR |
| 90067515 | 90067885 | + | 11.9 | 1 | 375 | 0 | THE1C | MaLR | LTR |
| 90067887 | 90068109 | + | 19.7 | 355 | 546 | -10 | MLT2B4 | ERVL | LTR |

  

|  |  |  |  |  |  |  |  |  |  |
| --- | --- | --- | --- | --- | --- | --- | --- | --- | --- |
| chr11:91047894-91050738 | | | 2825 bp | | |  |  |  |  |
| Genome Start | Genome End | Strand | Div | Repeat Start | Repeat End | Repeat Left | Name | Family | Class |
| 91047894 | 91048225 | + | 30.9 | 5255 | 5590 | -531 | L1M5 | L1 | LINE |
| 91048226 | 91048591 | + | 10.5 | 3 | 355 | 0 | THE1A | MaLR | LTR |
| 91048594 | 91050167 | + | 30.8 | 2 | 1735 | 0 | MLT-int | MaLR | LTR |
| 91050168 | 91050522 | + | 8.2 | 1 | 355 | 0 | THE1A | MaLR | LTR |
| 91050523 | 91050653 | + | 33.2 | 5591 | 5731 | -390 | L1M5 | L1 | LINE |
| 91050672 | 91050738 | + | 22.4 | 5790 | 5859 | -262 | L1M5 | L1 | LINE |

  

|  |  |  |  |  |  |  |  |  |  |
| --- | --- | --- | --- | --- | --- | --- | --- | --- | --- |
| chr11:91154424-91157360 | | | 2935 bp | | |  |  |  |  |
| Genome Start | Genome End | Strand | Div | Repeat Start | Repeat End | Repeat Left | Name | Family | Class |
| 91154424 | 91154852 | + | 30.6 | 4489 | 4916 | -1230 | L1M5 | L1 | LINE |
| 91154853 | 91155193 | - | 13.7 | 1 | 364 | 0 | THE1B | MaLR | LTR |
| 91155194 | 91156834 | - | 30.1 | 2 | 1735 | 0 | MLT-int | MaLR | LTR |
| 91156837 | 91157180 | - | 13.3 | 1 | 364 | 0 | THE1B | MaLR | LTR |
| 91157181 | 91157360 | + | 30.6 | 4917 | 5096 | -1050 | L1M5 | L1 | LINE |

  

|  |  |  |  |  |  |  |  |  |  |
| --- | --- | --- | --- | --- | --- | --- | --- | --- | --- |
| chr11:91286833-91293289 | | | 6439 bp | | |  |  |  |  |
| Genome Start | Genome End | Strand | Div | Repeat Start | Repeat End | Repeat Left | Name | Family | Class |
| 91286833 | 91287317 | + | 18.1 | 5038 | 5515 | -659 | L1MB7 | L1 | LINE |
| 91287318 | 91287679 | - | 20.4 | 1 | 365 | 0 | MLT1A0 | MaLR | LTR |
| 91287680 | 91288382 | - | 24.6 | 1003 | 1735 | 0 | MLT1A0-int | MaLR | LTR |
| 91288383 | 91288685 | + | 15.6 | 1 | 304 | -8 | AluSx | Alu | SINE |
| 91288686 | 91289146 | - | 24.6 | 523 | 1002 | -733 | MLT1A0-int | MaLR | LTR |
| 91289148 | 91291817 | + | 4.4 | 3481 | 6151 | -4 | L1PA4 | L1 | LINE |
| 91291834 | 91292310 | - | 16.4 | 2 | 497 | -1238 | MLT1A0-int | MaLR | LTR |
| 91292312 | 91292669 | - | 16.7 | 1 | 365 | 0 | MLT1A0 | MaLR | LTR |
| 91292670 | 91293289 | + | 19.9 | 5511 | 6165 | -19 | L1MB7 | L1 | LINE |

  

|  |  |  |  |  |  |  |  |  |  |
| --- | --- | --- | --- | --- | --- | --- | --- | --- | --- |
| chr11:93717804-93730900 | | | 13100 bp | | |  |  |  |  |
| Genome Start | Genome End | Strand | Div | Repeat Start | Repeat End | Repeat Left | Name | Family | Class |
| 93717804 | 93718537 | + | 17.1 | 5069 | 5801 | -1984 | L1MC3 | L1 | LINE |
| 93718539 | 93718932 | + | 7.1 | 1 | 443 | -7 | LTR7 | ERV1 | LTR |
| 93718940 | 93721934 | + | 10.6 | 1 | 2987 | -4726 | HERVH | ERV1 | LTR |
| 93721929 | 93722076 | + | 14.2 | 3140 | 3290 | -4423 | HERVH | ERV1 | LTR |
| 93722067 | 93722567 | + | 11.8 | 3492 | 3993 | -3720 | HERVH | ERV1 | LTR |
| 93722570 | 93723232 | + | 14.3 | 4488 | 5149 | -2564 | HERVH | ERV1 | LTR |
| 93723234 | 93723451 | + | 12.5 | 5600 | 5819 | -1894 | HERVH | ERV1 | LTR |
| 93723450 | 93723825 | + | 10.8 | 7322 | 7713 | 0 | HERVH | ERV1 | LTR |
| 93723826 | 93724229 | + | 8.9 | 1 | 448 | -2 | LTR7 | ERV1 | LTR |
| 93724232 | 93725847 | + | 8.4 | 1377 | 2987 | -4726 | HERVH | ERV1 | LTR |
| 93725842 | 93726693 | + | 8.2 | 3140 | 3993 | -3720 | HERVH | ERV1 | LTR |
| 93726696 | 93727358 | + | 13.5 | 4488 | 5149 | -2564 | HERVH | ERV1 | LTR |
| 93727360 | 93727575 | + | 13.4 | 5600 | 5819 | -1894 | HERVH | ERV1 | LTR |
| 93727574 | 93727952 | + | 9.1 | 7322 | 7713 | 0 | HERVH | ERV1 | LTR |
| 93727953 | 93728348 | + | 8.1 | 1 | 448 | -2 | LTR7 | ERV1 | LTR |
| 93728351 | 93729747 | + | 16.2 | 5798 | 7219 | -566 | L1MC3 | L1 | LINE |
| 93729748 | 93730312 | + | 23.0 | 1 | 547 | -7 | MLT2B3 | ERVL | LTR |
| 93730318 | 93730900 | + | 17.6 | 7217 | 7784 | -1 | L1MC3 | L1 | LINE |

  

|  |  |  |  |  |  |  |  |  |  |
| --- | --- | --- | --- | --- | --- | --- | --- | --- | --- |
| chr11:94763766-94771150 | | | 6959 bp | | |  |  |  |  |
| Genome Start | Genome End | Strand | Div | Repeat Start | Repeat End | Repeat Left | Name | Family | Class |
| 94763766 | 94763937 | + | 25.6 | 34 | 305 | -6427 | L1MDb | L1 | LINE |
| 94763938 | 94764327 | - | 22.4 | 1 | 394 | -2 | MSTD | MaLR | LTR |
| 94764342 | 94764835 | - | 24.2 | 1212 | 1720 | -15 | MSTD-int | MaLR | LTR |
| 94764832 | 94765025 | - | 18.9 | 2 | 189 | -1546 | MSTD-int | MaLR | LTR |
| 94765028 | 94765421 | - | 20.3 | 1 | 396 | 0 | MSTD | MaLR | LTR |
| 94765423 | 94765504 | + | 28.1 | 197 | 279 | -6354 | L1MDb | L1 | LINE |
| 94765890 | 94766349 | + | 20.2 | 844 | 1309 | -5324 | L1MDb | L1 | LINE |
| 94766350 | 94766594 | + | 15.6 | 6 | 249 | -63 | AluSx | Alu | SINE |
| 94766595 | 94767097 | + | 21.6 | 1309 | 1850 | -4783 | L1MDb | L1 | LINE |
| 94767098 | 94767500 | + | 13.6 | 1 | 403 | 0 | AluSx | Alu | SINE |
| 94767501 | 94769913 | + | 20.3 | 1851 | 4186 | -2351 | L1MDb | L1 | LINE |
| 94769942 | 94771150 | + | 19.3 | 4187 | 5411 | -1126 | L1MDb | L1 | LINE |

  

|  |  |  |  |  |  |  |  |  |  |
| --- | --- | --- | --- | --- | --- | --- | --- | --- | --- |
| chr11:95315858-95320090 | | | 4231 bp | | |  |  |  |  |
| Genome Start | Genome End | Strand | Div | Repeat Start | Repeat End | Repeat Left | Name | Family | Class |
| 95315858 | 95316427 | - | 33.1 | 5045 | 5616 | -51 | HERVL | ERVL | LTR |
| 95316428 | 95316805 | + | 14.1 | 1 | 381 | 0 | THE1D | MaLR | LTR |
| 95316808 | 95318443 | + | 31.5 | 2 | 1735 | 0 | MLT-int | MaLR | LTR |
| 95318444 | 95318824 | + | 15.6 | 1 | 381 | 0 | THE1D | MaLR | LTR |
| 95318825 | 95320090 | - | 32.8 | 3786 | 5107 | -547 | HERVL | ERVL | LTR |

  

|  |  |  |  |  |  |  |  |  |  |
| --- | --- | --- | --- | --- | --- | --- | --- | --- | --- |
| chr11:95320305-95322886 | | | 2579 bp | | |  |  |  |  |
| Genome Start | Genome End | Strand | Div | Repeat Start | Repeat End | Repeat Left | Name | Family | Class |
| 95320305 | 95320439 | + | 18.0 | 5909 | 6043 | -113 | L1PB4 | L1 | LINE |
| 95320440 | 95320806 | - | 11.6 | 2 | 364 | 0 | THE1B | MaLR | LTR |
| 95320808 | 95322391 | - | 30.4 | 2 | 1735 | 0 | MLT1H-int | MaLR | LTR |
| 95322394 | 95322768 | - | 14.9 | 1 | 364 | 0 | THE1B | MaLR | LTR |
| 95322769 | 95322886 | + | 18.0 | 6044 | 6153 | -3 | L1PB4 | L1 | LINE |

  

|  |  |  |  |  |  |  |  |  |  |
| --- | --- | --- | --- | --- | --- | --- | --- | --- | --- |
| chr11:95963552-95970496 | | | 6692 bp | | |  |  |  |  |
| Genome Start | Genome End | Strand | Div | Repeat Start | Repeat End | Repeat Left | Name | Family | Class |
| 95963552 | 95963675 | - | 20.2 | 2028 | 2154 | -3995 | L1MEe | L1 | LINE |
| 95963845 | 95964295 | + | 7.2 | 1 | 448 | -2 | LTR7 | ERV1 | LTR |
| 95964298 | 95967681 | + | 9.1 | 1 | 3290 | -4423 | HERVH | ERV1 | LTR |
| 95967672 | 95968176 | + | 12.2 | 3492 | 3993 | -3720 | HERVH | ERV1 | LTR |
| 95968179 | 95968838 | + | 14.1 | 4488 | 5149 | -2564 | HERVH | ERV1 | LTR |
| 95968840 | 95969055 | + | 11.6 | 5600 | 5819 | -1894 | HERVH | ERV1 | LTR |
| 95969054 | 95969433 | + | 8.0 | 7322 | 7713 | 0 | HERVH | ERV1 | LTR |
| 95969434 | 95969884 | + | 6.2 | 1 | 448 | -2 | LTR7 | ERV1 | LTR |
| 95969937 | 95970304 | - | 19.1 | 5913 | 6289 | -11 | L1MA4 | L1 | LINE |
| 95970344 | 95970496 | - | 34.7 | 4 | 153 | -5966 | L1MEe | L1 | LINE |

  

|  |  |  |  |  |  |  |  |  |  |
| --- | --- | --- | --- | --- | --- | --- | --- | --- | --- |
| chr11:96838961-96841732 | | | 2768 bp | | |  |  |  |  |
| Genome Start | Genome End | Strand | Div | Repeat Start | Repeat End | Repeat Left | Name | Family | Class |
| 96838961 | 96839009 | - | 30.2 | 415 | 462 | -2 | LTR16B | ERVL | LTR |
| 96839010 | 96839382 | + | 10.2 | 1 | 373 | -2 | THE1C | MaLR | LTR |
| 96839387 | 96840952 | + | 29.9 | 2 | 1735 | 0 | MLT-int | MaLR | LTR |
| 96840953 | 96841327 | + | 14.3 | 1 | 375 | 0 | THE1C | MaLR | LTR |
| 96841328 | 96841732 | - | 30.2 | 8 | 414 | -50 | LTR16B | ERVL | LTR |

  

|  |  |  |  |  |  |  |  |  |  |
| --- | --- | --- | --- | --- | --- | --- | --- | --- | --- |
| chr11:97525809-97528512 | | | 2702 bp | | |  |  |  |  |
| Genome Start | Genome End | Strand | Div | Repeat Start | Repeat End | Repeat Left | Name | Family | Class |
| 97525809 | 97525868 | - | 21.9 | 348 | 403 | 0 | MER57A | ERV1 | LTR |
| 97525869 | 97526226 | + | 14.8 | 3 | 364 | 0 | THE1B | MaLR | LTR |
| 97526229 | 97527810 | + | 31.3 | 2 | 1735 | 0 | MLT-int | MaLR | LTR |
| 97527811 | 97528166 | + | 14.2 | 1 | 364 | 0 | THE1B | MaLR | LTR |
| 97528167 | 97528512 | - | 21.9 | 1 | 347 | -56 | MER57A | ERV1 | LTR |

  

|  |  |  |  |  |  |  |  |  |  |
| --- | --- | --- | --- | --- | --- | --- | --- | --- | --- |
| chr11:97641800-97647794 | | | 5901 bp | | |  |  |  |  |
| Genome Start | Genome End | Strand | Div | Repeat Start | Repeat End | Repeat Left | Name | Family | Class |
| 97641800 | 97642299 | - | 23.9 | 6312 | 6799 | -239 | L1MB8 | L1 | LINE |
| 97642327 | 97642759 | - | 23.1 | 4169 | 4550 | -2456 | L1MB8 | L1 | LINE |
| 97642760 | 97643324 | + | 16.2 | 1 | 595 | 0 | MER41E | ERV1 | LTR |
| 97643325 | 97643698 | - | 23.1 | 3756 | 4168 | -2838 | L1MB8 | L1 | LINE |
| 97643699 | 97644075 | - | 13.2 | 1 | 375 | 0 | THE1C | MaLR | LTR |
| 97644076 | 97645584 | - | 29.3 | 2 | 1735 | 0 | MLT-int | MaLR | LTR |
| 97645587 | 97645961 | - | 14.6 | 1 | 375 | 0 | THE1C | MaLR | LTR |
| 97645962 | 97646498 | - | 23.1 | 3134 | 3755 | -3251 | L1MB8 | L1 | LINE |
| 97646516 | 97646658 | - | 25.2 | 1040 | 1187 | -6147 | L1MB8 | L1 | LINE |
| 97646662 | 97646879 | + | 21.9 | 1 | 264 | -407 | LTR51 | ERV1 | LTR |
| 97646904 | 97646977 | + | 21.9 | 265 | 344 | -327 | LTR51 | ERV1 | LTR |
| 97646976 | 97647148 | + | 18.6 | 487 | 671 | 0 | LTR51 | ERV1 | LTR |
| 97647172 | 97647794 | - | 24.9 | 384 | 1040 | -6294 | L1MB8 | L1 | LINE |

  

|  |  |  |  |  |  |  |  |  |  |
| --- | --- | --- | --- | --- | --- | --- | --- | --- | --- |
| chr11:97836833-97841150 | | | 4293 bp | | |  |  |  |  |
| Genome Start | Genome End | Strand | Div | Repeat Start | Repeat End | Repeat Left | Name | Family | Class |
| 97836833 | 97836972 | - | 23.7 | 7625 | 7760 | -25 | L1MC3 | L1 | LINE |
| 97836976 | 97837106 | - | 8.4 | 168 | 298 | -14 | AluSg/x | Alu | SINE |
| 97837107 | 97837299 | - | 17.6 | 7372 | 7554 | -231 | L1MC3 | L1 | LINE |
| 97837303 | 97837690 | + | 23.4 | 2 | 408 | 0 | MLT1A1 | MaLR | LTR |
[truncated: 1,079,556 more chars]
