# Supplementary material for: Evolutionary History of Mammalian Transposons Determined by Genome-Wide Defragmentation
Source: PLoS Comput Biol. 2007 Jul 13;3(7):e137. doi: 10.1371/journal.pcbi.0030137 (PMC1914374; doi:10.1371/journal.pcbi.0030137)
Supplement: Supplementary file 5 [file pcbi.0030137.sd002.htm]

Clusters with Inverted L1s

**Supplementary Table S1b: Clusters containing L1 5' inversions**  
  
To load the custom track into the UCSC Genome Browser, click here. The custom track must be loaded in order to view the clusters in Supplementary Table S1. It may take a minute to load, so please be patient.  
  
Jump to:  
chr1  
chr2  
chr3  
chr4  
chr5  
chr6  
chr7  
chr8  
chr9  
chr10  
chr11  
chr12  
chr13  
chr14  
chr15  
chr16  
chr17  
chr18  
chr19  
chr20  
chr21  
chr22  
chrX  
chrY  
  


|  |  |  |  |  |  |  |  |  |  |
| --- | --- | --- | --- | --- | --- | --- | --- | --- | --- |
| chr1:63551-67251 | | | 3703 bp | | |  |  |  |  |
| Genome Start | Genome End | Strand | Div | Repeat Start | Repeat End | Repeat Left | Name | Family | Class |
| 63551 | 63706 | + | 24.2 | 5372 | 5525 | -2283 | L1MC5 | L1 | LINE |
| 63708 | 64758 | - | 1.6 | 5105 | 6155 | 0 | L1PA2 | L1 | LINE |
| 64761 | 66560 | + | 2.1 | 3311 | 5110 | -1036 | L1PA2 | L1 | LINE |
| 66556 | 66755 | + | 23.9 | 5512 | 5734 | -2074 | L1MC5 | L1 | LINE |
| 66756 | 67064 | + | 13.3 | 1 | 309 | -3 | AluSx | Alu | SINE |
| 67065 | 67251 | + | 23.9 | 5735 | 5942 | -1866 | L1MC5 | L1 | LINE |

  

|  |  |  |  |  |  |  |  |  |  |
| --- | --- | --- | --- | --- | --- | --- | --- | --- | --- |
| chr1:369434-371928 | | | 2460 bp | | |  |  |  |  |
| Genome Start | Genome End | Strand | Div | Repeat Start | Repeat End | Repeat Left | Name | Family | Class |
| 369434 | 370518 | - | 16.4 | 3657 | 4741 | -1399 | L1M3 | L1 | LINE |
| 370538 | 370715 | - | 4.5 | 5977 | 6155 | 0 | L1PA4 | L1 | LINE |
| 370716 | 371437 | + | 2.2 | 5254 | 5975 | -180 | L1PA4 | L1 | LINE |
| 371454 | 371928 | - | 18.7 | 3167 | 3646 | -2494 | L1M3 | L1 | LINE |

  

|  |  |  |  |  |  |  |  |  |  |
| --- | --- | --- | --- | --- | --- | --- | --- | --- | --- |
| chr1:597494-599988 | | | 2460 bp | | |  |  |  |  |
| Genome Start | Genome End | Strand | Div | Repeat Start | Repeat End | Repeat Left | Name | Family | Class |
| 597494 | 597968 | + | 18.7 | 3167 | 3646 | -2494 | L1M3 | L1 | LINE |
| 597985 | 598706 | - | 2.4 | 5254 | 5975 | -180 | L1PA4 | L1 | LINE |
| 598707 | 598884 | + | 3.9 | 5977 | 6155 | 0 | L1PA4 | L1 | LINE |
| 598904 | 599988 | + | 16.5 | 3657 | 4741 | -1399 | L1M3 | L1 | LINE |

  

|  |  |  |  |  |  |  |  |  |  |
| --- | --- | --- | --- | --- | --- | --- | --- | --- | --- |
| chr1:5682257-5684373 | | | 2102 bp | | |  |  |  |  |
| Genome Start | Genome End | Strand | Div | Repeat Start | Repeat End | Repeat Left | Name | Family | Class |
| 5682257 | 5682344 | + | 20.8 | 3850 | 3935 | -2211 | L1MD | L1 | LINE |
| 5682345 | 5682589 | - | 20.7 | 2 | 239 | 0 | MER8 | MER2\_type | DNA |
| 5682590 | 5683029 | + | 20.8 | 3936 | 4385 | -1761 | L1MD | L1 | LINE |
| 5683030 | 5683330 | + | 12.7 | 1 | 301 | -12 | AluSq | Alu | SINE |
| 5683335 | 5683448 | - | 13.2 | 6096 | 6215 | -97 | L1MA9 | L1 | LINE |
| 5683451 | 5683555 | + | 17.1 | 6203 | 6307 | -5 | L1MA9 | L1 | LINE |
| 5683564 | 5683824 | + | 14.4 | 4377 | 4642 | -1504 | L1MD | L1 | LINE |
| 5683826 | 5684001 | + | 16.5 | 130 | 305 | -7 | AluSg/x | Alu | SINE |
| 5684002 | 5684060 | + | 20.0 | 4648 | 4706 | -1440 | L1MD | L1 | LINE |
| 5684061 | 5684361 | + | 12.3 | 1 | 306 | -7 | AluSq | Alu | SINE |
| 5684362 | 5684373 | + | 20.0 | 4707 | 4717 | -1429 | L1MD | L1 | LINE |

  

|  |  |  |  |  |  |  |  |  |  |
| --- | --- | --- | --- | --- | --- | --- | --- | --- | --- |
| chr1:7225024-7227853 | | | 2777 bp | | |  |  |  |  |
| Genome Start | Genome End | Strand | Div | Repeat Start | Repeat End | Repeat Left | Name | Family | Class |
| 7225024 | 7225293 | - | 34.9 | 4901 | 5164 | -982 | L1M5 | L1 | LINE |
| 7225294 | 7225418 | + | 8.1 | 1 | 125 | -186 | AluY | Alu | SINE |
| 7225458 | 7225638 | + | 8.1 | 126 | 309 | -2 | AluY | Alu | SINE |
| 7225639 | 7225979 | - | 34.9 | 4566 | 4900 | -1246 | L1M5 | L1 | LINE |
| 7225974 | 7226457 | - | 4.1 | 4572 | 5054 | -1092 | L1PA4 | L1 | LINE |
| 7226459 | 7227552 | + | 3.1 | 5063 | 6155 | 0 | L1PA4 | L1 | LINE |
| 7227572 | 7227853 | - | 32.0 | 4310 | 4583 | -1563 | L1M5 | L1 | LINE |

  

|  |  |  |  |  |  |  |  |  |  |
| --- | --- | --- | --- | --- | --- | --- | --- | --- | --- |
| chr1:12917372-12917949 | | | 578 bp | | |  |  |  |  |
| Genome Start | Genome End | Strand | Div | Repeat Start | Repeat End | Repeat Left | Name | Family | Class |
| 12917372 | 12917542 | - | 14.0 | 126 | 300 | -12 | AluSx | Alu | SINE |
| 12917543 | 12917767 | - | 5.3 | 5923 | 6151 | -4 | L1PA4 | L1 | LINE |
| 12917768 | 12917814 | + | 6.4 | 5885 | 5931 | -223 | L1PA4 | L1 | LINE |
| 12917815 | 12917949 | - | 14.1 | 1 | 135 | -177 | AluSx | Alu | SINE |

  

|  |  |  |  |  |  |  |  |  |  |
| --- | --- | --- | --- | --- | --- | --- | --- | --- | --- |
| chr1:13027700-13028282 | | | 583 bp | | |  |  |  |  |
| Genome Start | Genome End | Strand | Div | Repeat Start | Repeat End | Repeat Left | Name | Family | Class |
| 13027700 | 13027875 | - | 13.6 | 126 | 305 | -7 | AluSx | Alu | SINE |
| 13027876 | 13028100 | - | 5.3 | 5923 | 6151 | -4 | L1PA4 | L1 | LINE |
| 13028101 | 13028147 | + | 4.3 | 5885 | 5931 | -223 | L1PA4 | L1 | LINE |
| 13028148 | 13028282 | - | 14.1 | 1 | 135 | -177 | AluSx | Alu | SINE |

  

|  |  |  |  |  |  |  |  |  |  |
| --- | --- | --- | --- | --- | --- | --- | --- | --- | --- |
| chr1:13244463-13245044 | | | 582 bp | | |  |  |  |  |
| Genome Start | Genome End | Strand | Div | Repeat Start | Repeat End | Repeat Left | Name | Family | Class |
| 13244463 | 13244597 | + | 14.1 | 1 | 135 | -177 | AluSx | Alu | SINE |
| 13244598 | 13244644 | - | 4.3 | 5885 | 5931 | -223 | L1PA4 | L1 | LINE |
| 13244645 | 13244869 | + | 5.3 | 5923 | 6151 | -4 | L1PA4 | L1 | LINE |
| 13244870 | 13245044 | + | 13.7 | 126 | 304 | -8 | AluSx | Alu | SINE |

  

|  |  |  |  |  |  |  |  |  |  |
| --- | --- | --- | --- | --- | --- | --- | --- | --- | --- |
| chr1:15496661-15497841 | | | 1080 bp | | |  |  |  |  |
| Genome Start | Genome End | Strand | Div | Repeat Start | Repeat End | Repeat Left | Name | Family | Class |
| 15496661 | 15496874 | - | 21.1 | 286 | 550 | -4 | MLT1H | MaLR | LTR |
| 15496964 | 15497021 | - | 14.5 | 6097 | 6151 | 0 | L1PB1 | L1 | LINE |
| 15497018 | 15497571 | + | 13.7 | 5549 | 6096 | -55 | L1PB1 | L1 | LINE |
| 15497588 | 15497841 | - | 24.9 | 1 | 305 | -244 | MLT1H | MaLR | LTR |

  

|  |  |  |  |  |  |  |  |  |  |
| --- | --- | --- | --- | --- | --- | --- | --- | --- | --- |
| chr1:17369125-17382426 | | | 12333 bp | | |  |  |  |  |
| Genome Start | Genome End | Strand | Div | Repeat Start | Repeat End | Repeat Left | Name | Family | Class |
| 17369125 | 17369385 | + | 30.8 | 244 | 520 | -5599 | L1MEd | L1 | LINE |
| 17369405 | 17369629 | - | 7.6 | 5948 | 6171 | -1 | L1PA8 | L1 | LINE |
| 17369658 | 17369762 | - | 13.3 | 25 | 134 | -178 | AluJo | Alu | SINE |
| 17369768 | 17369963 | + | 30.9 | 528 | 724 | -5395 | L1MEd | L1 | LINE |
| 17370064 | 17370369 | + | 13.8 | 2 | 312 | 0 | AluJo | Alu | SINE |
| 17370424 | 17370657 | + | 22.4 | 5583 | 5833 | -333 | L1ME1 | L1 | LINE |
| 17370664 | 17370972 | - | 6.2 | 1 | 309 | -2 | AluY | Alu | SINE |
| 17370986 | 17371033 | - | 10.4 | 499 | 546 | -188 | MER50 | ERV1 | LTR |
| 17371036 | 17371365 | + | 21.2 | 5829 | 6151 | -15 | L1ME1 | L1 | LINE |
| 17371398 | 17371700 | - | 11.0 | 1 | 301 | -11 | AluSx | Alu | SINE |
| 17371807 | 17372078 | - | 21.9 | 51 | 308 | -4 | AluJo | Alu | SINE |
| 17372090 | 17372861 | + | 23.9 | 2084 | 2959 | -460 | L2 | L2 | LINE |
| 17372862 | 17373225 | + | 11.1 | 1 | 364 | 0 | THE1B | MaLR | LTR |
| 17373226 | 17373441 | + | 28.1 | 2960 | 3192 | -227 | L2 | L2 | LINE |
| 17373471 | 17373509 | - | 12.8 | 7222 | 7260 | -525 | L1MC3 | L1 | LINE |
| 17373513 | 17373782 | + | 16.3 | 1 | 258 | -132 | MLT1B | MaLR | LTR |
| 17373783 | 17374163 | + | 11.4 | 1 | 381 | 0 | THE1D | MaLR | LTR |
| 17374164 | 17374322 | + | 16.3 | 259 | 390 | 0 | MLT1B | MaLR | LTR |
| 17374384 | 17374576 | + | 25.3 | 3220 | 3418 | -1 | L2 | L2 | LINE |
| 17374587 | 17374698 | + | 31.7 | 2118 | 2224 | -3905 | L1MEd | L1 | LINE |
| 17374699 | 17374987 | + | 9.4 | 1 | 289 | -23 | AluSx | Alu | SINE |
| 17375037 | 17375291 | + | 31.7 | 2225 | 2471 | -3658 | L1MEd | L1 | LINE |
| 17375279 | 17378827 | + | 18.5 | 175 | 3889 | -2648 | L1MDa | L1 | LINE |
| 17378858 | 17379345 | - | 9.0 | 5674 | 6151 | 0 | L1PB1 | L1 | LINE |
| 17379346 | 17379992 | + | 8.9 | 5000 | 5658 | -493 | L1PB1 | L1 | LINE |
| 17379989 | 17380167 | + | 14.5 | 3874 | 4057 | -2480 | L1MDa | L1 | LINE |
| 17380162 | 17380886 | + | 26.7 | 2456 | 3249 | -2880 | L1MEd | L1 | LINE |
| 17381246 | 17381652 | - | 16.2 | 5167 | 5576 | -608 | L1M4 | L1 | LINE |
| 17381653 | 17381958 | - | 8.5 | 1 | 306 | 0 | AluY | Alu | SINE |
| 17381959 | 17382011 | - | 17.1 | 5115 | 5166 | -980 | L1M4 | L1 | LINE |
| 17382087 | 17382160 | + | 25.6 | 3347 | 3413 | -2716 | L1MEd | L1 | LINE |
| 17382161 | 17382363 | - | 20.2 | 1 | 208 | -2 | MER63A | AcHobo | DNA |
| 17382364 | 17382426 | + | 25.6 | 3414 | 3474 | -2655 | L1MEd | L1 | LINE |

  

|  |  |  |  |  |  |  |  |  |  |
| --- | --- | --- | --- | --- | --- | --- | --- | --- | --- |
| chr1:34142105-34144030 | | | 1758 bp | | |  |  |  |  |
| Genome Start | Genome End | Strand | Div | Repeat Start | Repeat End | Repeat Left | Name | Family | Class |
| 34142105 | 34142407 | - | 29.0 | 2737 | 3082 | -337 | L2 | L2 | LINE |
| 34142537 | 34142655 | + | 10.8 | 5723 | 5837 | -294 | L1MB5 | L1 | LINE |
| 34142645 | 34142702 | - | 24.1 | 5930 | 5989 | -191 | L1MB3 | L1 | LINE |
| 34142698 | 34142840 | + | 21.1 | 6005 | 6150 | -33 | L1MB3 | L1 | LINE |
| 34142841 | 34143133 | - | 9.4 | 1 | 311 | 0 | AluY | Alu | SINE |
| 34143134 | 34143155 | + | 21.1 | 6151 | 6163 | -20 | L1MB3 | L1 | LINE |
| 34143156 | 34143291 | - | 16.6 | 1 | 133 | 0 | FLAM\_A | Alu | SINE |
| 34143347 | 34144030 | - | 30.7 | 1592 | 2359 | -1060 | L2 | L2 | LINE |

  

|  |  |  |  |  |  |  |  |  |  |
| --- | --- | --- | --- | --- | --- | --- | --- | --- | --- |
| chr1:34744740-34747351 | | | 2593 bp | | |  |  |  |  |
| Genome Start | Genome End | Strand | Div | Repeat Start | Repeat End | Repeat Left | Name | Family | Class |
| 34744740 | 34745322 | - | 33.3 | 2803 | 3418 | -1 | L2 | L2 | LINE |
| 34745333 | 34745400 | - | 10.3 | 5790 | 5857 | -434 | L1MA7 | L1 | LINE |
| 34745396 | 34745701 | + | 16.8 | 5837 | 6114 | -177 | L1MA7 | L1 | LINE |
| 34745702 | 34746003 | + | 1.0 | 1 | 299 | -11 | AluYa5 | Alu | SINE |
| 34746004 | 34746146 | + | 16.8 | 6115 | 6258 | -33 | L1MA7 | L1 | LINE |
| 34746147 | 34746458 | - | 21.3 | 1 | 345 | 0 | MER2 | MER2\_type | DNA |
| 34746459 | 34746491 | + | 16.8 | 6259 | 6287 | -4 | L1MA7 | L1 | LINE |
| 34746506 | 34747351 | - | 28.8 | 1811 | 2748 | -671 | L2 | L2 | LINE |

  

|  |  |  |  |  |  |  |  |  |  |
| --- | --- | --- | --- | --- | --- | --- | --- | --- | --- |
| chr1:35514527-35524686 | | | 9568 bp | | |  |  |  |  |
| Genome Start | Genome End | Strand | Div | Repeat Start | Repeat End | Repeat Left | Name | Family | Class |
| 35514527 | 35514797 | - | 23.7 | 7502 | 7779 | -182 | L1MC5 | L1 | LINE |
| 35514798 | 35515094 | - | 13.8 | 1 | 298 | -15 | AluSq | Alu | SINE |
| 35515095 | 35515282 | - | 23.7 | 7330 | 7501 | -460 | L1MC5 | L1 | LINE |
| 35515283 | 35515455 | + | 27.9 | 1 | 174 | -4 | MER5B | MER1\_type | DNA |
| 35515456 | 35515509 | - | 23.7 | 7283 | 7329 | -632 | L1MC5 | L1 | LINE |
| 35515514 | 35515641 | - | 26.0 | 1 | 133 | -1 | MER94 | AcHobo | DNA |
| 35515786 | 35516120 | - | 23.1 | 6882 | 7217 | -744 | L1MC5 | L1 | LINE |
| 35516140 | 35516499 | - | 21.8 | 11 | 464 | -3 | MLT1C | MaLR | LTR |
| 35516518 | 35516614 | - | 26.9 | 1387 | 1481 | -8 | MLT1F-int | MaLR | LTR |
| 35516615 | 35516908 | - | 11.6 | 1 | 298 | -13 | AluY | Alu | SINE |
| 35516909 | 35518272 | - | 23.1 | 44 | 1386 | -103 | MLT1C-int | MaLR | LTR |
| 35518501 | 35518624 | - | 18.6 | 2 | 125 | -187 | AluJb | Alu | SINE |
| 35518625 | 35518679 | - | 14.6 | 1 | 59 | -1312 | MLT1C-int | MaLR | LTR |
| 35518680 | 35519125 | - | 24.1 | 1 | 467 | 0 | MLT1C | MaLR | LTR |
| 35519191 | 35519486 | + | 9.5 | 1 | 296 | -15 | AluY | Alu | SINE |
| 35519528 | 35519698 | + | 16.8 | 5874 | 6041 | -250 | L1MA7 | L1 | LINE |
| 35519708 | 35519830 | + | 16.4 | 6164 | 6279 | -12 | L1MA7 | L1 | LINE |
| 35519876 | 35520337 | - | 15.9 | 5646 | 6147 | -33 | L1MB4 | L1 | LINE |
| 35520342 | 35520684 | + | 21.1 | 5 | 457 | 0 | MSTB2 | MaLR | LTR |
| 35520685 | 35520739 | - | 15.9 | 5608 | 5650 | -530 | L1MB4 | L1 | LINE |
| 35520740 | 35521043 | - | 18.8 | 1 | 304 | -8 | AluJb | Alu | SINE |
| 35521044 | 35521174 | - | 15.9 | 5481 | 5607 | -573 | L1MB4 | L1 | LINE |
| 35521175 | 35521467 | - | 14.4 | 36 | 312 | 0 | AluSx | Alu | SINE |
| 35521468 | 35521514 | - | 15.9 | 5453 | 5480 | -700 | L1MB4 | L1 | LINE |
| 35521515 | 35521824 | - | 6.8 | 1 | 310 | -1 | AluY | Alu | SINE |
| 35521825 | 35522148 | - | 16.7 | 5168 | 5452 | -728 | L1MB4 | L1 | LINE |
| 35522149 | 35522463 | - | 2.2 | 1 | 315 | -3 | AluYb8 | Alu | SINE |
| 35522464 | 35522783 | - | 18.6 | 4844 | 5167 | -979 | L1MB4 | L1 | LINE |
| 35522788 | 35523173 | - | 22.9 | 1 | 366 | -8 | MLT1A | MaLR | LTR |
| 35523174 | 35523608 | - | 18.3 | 4410 | 4848 | -1298 | L1MB4 | L1 | LINE |
| 35523607 | 35523661 | + | 21.0 | 3837 | 3888 | -2252 | L1M3 | L1 | LINE |
| 35523662 | 35523983 | + | 16.4 | 1 | 344 | -1 | MER2 | MER2\_type | DNA |
| 35523984 | 35524501 | + | 21.0 | 3889 | 4404 | -1736 | L1M3 | L1 | LINE |
| 35524515 | 35524686 | - | 29.0 | 6695 | 6866 | -1095 | L1MC5 | L1 | LINE |

  

|  |  |  |  |  |  |  |  |  |  |
| --- | --- | --- | --- | --- | --- | --- | --- | --- | --- |
| chr1:37000891-37002618 | | | 1566 bp | | |  |  |  |  |
| Genome Start | Genome End | Strand | Div | Repeat Start | Repeat End | Repeat Left | Name | Family | Class |
| 37000891 | 37001030 | - | 31.1 | 3052 | 3198 | -221 | L2 | L2 | LINE |
| 37001193 | 37001627 | - | 9.9 | 5712 | 6174 | 0 | L1PA11 | L1 | LINE |
| 37001628 | 37002069 | + | 7.3 | 5268 | 5711 | -463 | L1PA11 | L1 | LINE |
| 37002070 | 37002618 | - | 31.2 | 2234 | 2850 | -569 | L2 | L2 | LINE |

  

|  |  |  |  |  |  |  |  |  |  |
| --- | --- | --- | --- | --- | --- | --- | --- | --- | --- |
| chr1:42489079-42494342 | | | 5264 bp | | |  |  |  |  |
| Genome Start | Genome End | Strand | Div | Repeat Start | Repeat End | Repeat Left | Name | Family | Class |
| 42489079 | 42489751 | + | 26.7 | 1333 | 2244 | -4581 | L1M4 | L1 | LINE |
| 42489747 | 42489964 | - | 2.6 | 3758 | 3963 | -2183 | L1P1 | L1 | LINE |
| 42489965 | 42490274 | - | 1.6 | 1 | 311 | 0 | AluYg6 | Alu | SINE |
| 42490275 | 42491048 | - | 2.6 | 2988 | 3757 | -2389 | L1P1 | L1 | LINE |
| 42491048 | 42493238 | + | 2.7 | 3967 | 6155 | 0 | L1PA3 | L1 | LINE |
| 42493245 | 42493999 | + | 28.4 | 2221 | 2901 | -3757 | L1M4 | L1 | LINE |
| 42494000 | 42494291 | + | 10.0 | 2 | 292 | -18 | AluSg | Alu | SINE |
| 42494292 | 42494342 | + | 30.0 | 2888 | 2935 | -3709 | L1M4 | L1 | LINE |

  

|  |  |  |  |  |  |  |  |  |  |
| --- | --- | --- | --- | --- | --- | --- | --- | --- | --- |
| chr1:45192065-45195751 | | | 3574 bp | | |  |  |  |  |
| Genome Start | Genome End | Strand | Div | Repeat Start | Repeat End | Repeat Left | Name | Family | Class |
| 45192065 | 45192435 | + | 21.6 | 2169 | 2555 | -3660 | L1M | L1 | LINE |
| 45192463 | 45193346 | - | 7.6 | 3786 | 4692 | -1454 | L1PA7 | L1 | LINE |
| 45193346 | 45194756 | + | 5.8 | 4708 | 6151 | -3 | L1PA7 | L1 | LINE |
| 45194742 | 45194888 | + | 27.6 | 2569 | 2721 | -3493 | L1M | L1 | LINE |
| 45194928 | 45195237 | + | 31.6 | 4913 | 5231 | -983 | L1M | L1 | LINE |
| 45195240 | 45195517 | + | 17.9 | 1 | 275 | -37 | AluJb | Alu | SINE |
| 45195579 | 45195751 | + | 20.9 | 5342 | 5488 | -2388 | L1M | L1 | LINE |

  

|  |  |  |  |  |  |  |  |  |  |
| --- | --- | --- | --- | --- | --- | --- | --- | --- | --- |
| chr1:46324969-46328402 | | | 3367 bp | | |  |  |  |  |
| Genome Start | Genome End | Strand | Div | Repeat Start | Repeat End | Repeat Left | Name | Family | Class |
| 46324969 | 46325216 | + | 32.8 | 244 | 488 | -2019 | HAL1 | L1 | LINE |
| 46325217 | 46325243 | - | 0.0 | 5830 | 5856 | -298 | L1PA5 | L1 | LINE |
| 46325244 | 46325535 | + | 6.5 | 5861 | 6152 | -2 | L1PA5 | L1 | LINE |
| 46325590 | 46325858 | + | 28.4 | 468 | 740 | -1767 | HAL1 | L1 | LINE |
| 46325859 | 46325991 | - | 13.0 | 2 | 133 | 0 | FLAM\_C | Alu | SINE |
| 46325992 | 46326068 | + | 28.4 | 741 | 801 | -1706 | HAL1 | L1 | LINE |
| 46326053 | 46326359 | + | 15.2 | 5834 | 6140 | -43 | L1MB3 | L1 | LINE |
| 46326362 | 46326494 | + | 19.6 | 1 | 133 | -10 | FLAM\_C | Alu | SINE |
| 46326514 | 46326566 | + | 9.6 | 6128 | 6180 | -3 | L1MB3 | L1 | LINE |
| 46326575 | 46327692 | + | 23.8 | 850 | 2086 | -421 | HAL1 | L1 | LINE |
| 46327693 | 46327993 | + | 10.3 | 1 | 301 | -10 | AluY | Alu | SINE |
| 46327994 | 46328402 | + | 23.8 | 2087 | 2507 | 0 | HAL1 | L1 | LINE |

  

|  |  |  |  |  |  |  |  |  |  |
| --- | --- | --- | --- | --- | --- | --- | --- | --- | --- |
| chr1:46888636-46890454 | | | 1809 bp | | |  |  |  |  |
| Genome Start | Genome End | Strand | Div | Repeat Start | Repeat End | Repeat Left | Name | Family | Class |
| 46888636 | 46889247 | + | 10.4 | 5497 | 6125 | -175 | L1MA5 | L1 | LINE |
| 46889258 | 46889950 | - | 1.9 | 5463 | 6155 | 0 | L1PA3 | L1 | LINE |
| 46889951 | 46890269 | + | 2.8 | 5152 | 5470 | -685 | L1PA3 | L1 | LINE |
| 46890270 | 46890454 | + | 9.2 | 6111 | 6298 | -2 | L1MA5 | L1 | LINE |

  

|  |  |  |  |  |  |  |  |  |  |
| --- | --- | --- | --- | --- | --- | --- | --- | --- | --- |
| chr1:47046188-47047634 | | | 1371 bp | | |  |  |  |  |
| Genome Start | Genome End | Strand | Div | Repeat Start | Repeat End | Repeat Left | Name | Family | Class |
| 47046188 | 47046438 | - | 13.3 | 174 | 432 | 0 | MSTB1 | MaLR | LTR |
| 47046439 | 47046859 | - | 0.8 | 5199 | 5619 | -536 | L1PA2 | L1 | LINE |
| 47046860 | 47047394 | + | 1.1 | 5624 | 6155 | 0 | L1PA2 | L1 | LINE |
| 47047471 | 47047634 | - | 13.8 | 5 | 173 | -253 | MSTB1 | MaLR | LTR |

  

|  |  |  |  |  |  |  |  |  |  |
| --- | --- | --- | --- | --- | --- | --- | --- | --- | --- |
| chr1:48699252-48702662 | | | 2794 bp | | |  |  |  |  |
| Genome Start | Genome End | Strand | Div | Repeat Start | Repeat End | Repeat Left | Name | Family | Class |
| 48699252 | 48699360 | + | 23.0 | 1081 | 1186 | -4933 | L1MEd | L1 | LINE |
| 48699352 | 48699863 | - | 3.1 | 4888 | 5401 | -745 | L1PA3 | L1 | LINE |
| 48699865 | 48700615 | + | 3.3 | 5404 | 6154 | -1 | L1PA3 | L1 | LINE |
| 48700756 | 48700991 | + | 30.3 | 2490 | 2740 | -3389 | L1MEd | L1 | LINE |
| 48701022 | 48701531 | + | 16.4 | 1 | 515 | 0 | MLT2B2 | ERVL | LTR |
| 48701666 | 48701852 | + | 27.2 | 2839 | 3033 | -3096 | L1MEd | L1 | LINE |
| 48702062 | 48702363 | + | 29.6 | 3683 | 4020 | -2109 | L1MEd | L1 | LINE |
| 48702449 | 48702568 | + | 33.0 | 4021 | 4153 | -1976 | L1MEd | L1 | LINE |
| 48702596 | 48702662 | + | 33.0 | 4154 | 4225 | -1904 | L1MEd | L1 | LINE |

  

|  |  |  |  |  |  |  |  |  |  |
| --- | --- | --- | --- | --- | --- | --- | --- | --- | --- |
| chr1:49332319-49335729 | | | 3341 bp | | |  |  |  |  |
| Genome Start | Genome End | Strand | Div | Repeat Start | Repeat End | Repeat Left | Name | Family | Class |
| 49332319 | 49332423 | + | 26.5 | 5458 | 5559 | -605 | L1ME3A | L1 | LINE |
| 49332492 | 49332656 | + | 15.2 | 21 | 187 | -318 | MLT1D | MaLR | LTR |
| 49332663 | 49332906 | - | 6.2 | 3692 | 3935 | -2211 | L1PA4 | L1 | LINE |
| 49332902 | 49335111 | + | 3.3 | 3946 | 6155 | 0 | L1PA4 | L1 | LINE |
| 49335112 | 49335395 | + | 21.6 | 205 | 505 | 0 | MLT1D | MaLR | LTR |
| 49335396 | 49335562 | + | 22.2 | 5605 | 5784 | -386 | L1ME3A | L1 | LINE |
| 49335564 | 49335729 | + | 18.9 | 5965 | 6153 | -17 | L1ME3A | L1 | LINE |

  

|  |  |  |  |  |  |  |  |  |  |
| --- | --- | --- | --- | --- | --- | --- | --- | --- | --- |
| chr1:49550791-49554697 | | | 3907 bp | | |  |  |  |  |
| Genome Start | Genome End | Strand | Div | Repeat Start | Repeat End | Repeat Left | Name | Family | Class |
| 49550791 | 49551130 | + | 15.9 | 5196 | 5539 | -805 | L1MC2 | L1 | LINE |
| 49551131 | 49552494 | - | 6.9 | 4786 | 6154 | 0 | L1PA7 | L1 | LINE |
| 49552497 | 49553244 | - | 6.4 | 5400 | 6150 | -4 | L1PA7 | L1 | LINE |
| 49553243 | 49553901 | + | 7.1 | 4717 | 5393 | -753 | L1PA7 | L1 | LINE |
| 49553902 | 49554697 | + | 15.9 | 5525 | 6332 | -12 | L1MC2 | L1 | LINE |

  

|  |  |  |  |  |  |  |  |  |  |
| --- | --- | --- | --- | --- | --- | --- | --- | --- | --- |
| chr1:49752083-49754955 | | | 2805 bp | | |  |  |  |  |
| Genome Start | Genome End | Strand | Div | Repeat Start | Repeat End | Repeat Left | Name | Family | Class |
| 49752083 | 49752136 | - | 27.8 | 3319 | 3372 | -6 | L2 | L2 | LINE |
| 49752142 | 49752190 | + | 24.5 | 1 | 49 | -147 | MER91A | Tip100 | DNA |
| 49752213 | 49752583 | - | 3.2 | 4048 | 4418 | -1728 | L1PA4 | L1 | LINE |
| 49752576 | 49754309 | + | 3.8 | 4421 | 6155 | 0 | L1PA4 | L1 | LINE |
| 49754357 | 49754415 | + | 25.4 | 132 | 190 | 0 | MER91A | Tip100 | DNA |
| 49754418 | 49754955 | - | 34.6 | 2790 | 3318 | -60 | L2 | L2 | LINE |

  

|  |  |  |  |  |  |  |  |  |  |
| --- | --- | --- | --- | --- | --- | --- | --- | --- | --- |
| chr1:50026554-50029457 | | | 2837 bp | | |  |  |  |  |
| Genome Start | Genome End | Strand | Div | Repeat Start | Repeat End | Repeat Left | Name | Family | Class |
| 50026554 | 50027108 | + | 26.9 | 1335 | 1953 | -4951 | L1MEb | L1 | LINE |
| 50027102 | 50027608 | - | 7.9 | 3644 | 4151 | -1995 | L1P3 | L1 | LINE |
| 50027608 | 50028355 | + | 8.2 | 4155 | 4907 | -1239 | L1P3 | L1 | LINE |
| 50028356 | 50028644 | + | 11.1 | 1 | 302 | -8 | AluSg | Alu | SINE |
| 50028645 | 50029311 | + | 26.6 | 2158 | 2855 | -4332 | L1MEb | L1 | LINE |
| 50029387 | 50029457 | + | 25.4 | 2847 | 2921 | -4266 | L1MEb | L1 | LINE |

  

|  |  |  |  |  |  |  |  |  |  |
| --- | --- | --- | --- | --- | --- | --- | --- | --- | --- |
| chr1:50038351-50039774 | | | 1355 bp | | |  |  |  |  |
| Genome Start | Genome End | Strand | Div | Repeat Start | Repeat End | Repeat Left | Name | Family | Class |
| 50038351 | 50038580 | + | 25.0 | 1 | 243 | -350 | MLT1E | MaLR | LTR |
| 50038624 | 50039332 | - | 1.1 | 5447 | 6155 | 0 | L1PA2 | L1 | LINE |
| 50039333 | 50039440 | + | 1.9 | 5364 | 5470 | -685 | L1PA2 | L1 | LINE |
| 50039467 | 50039774 | + | 22.5 | 276 | 593 | 0 | MLT1E | MaLR | LTR |

  

|  |  |  |  |  |  |  |  |  |  |
| --- | --- | --- | --- | --- | --- | --- | --- | --- | --- |
| chr1:50204095-50206129 | | | 1846 bp | | |  |  |  |  |
| Genome Start | Genome End | Strand | Div | Repeat Start | Repeat End | Repeat Left | Name | Family | Class |
| 50204095 | 50204198 | - | 32.7 | 6012 | 6119 | -2 | L1ME4a | L1 | LINE |
| 50204199 | 50204500 | - | 7.7 | 1 | 301 | -10 | AluY | Alu | SINE |
| 50204501 | 50204726 | - | 32.7 | 5751 | 6011 | -110 | L1ME4a | L1 | LINE |
| 50204775 | 50205030 | + | 33.2 | 1 | 261 | -1 | MIR | MIR | SINE |
| 50205031 | 50205163 | - | 29.3 | 5554 | 5687 | -434 | L1ME4a | L1 | LINE |
| 50205164 | 50205517 | - | 3.7 | 5402 | 5754 | -400 | L1PA7 | L1 | LINE |
| 50205518 | 50205908 | + | 5.4 | 5738 | 6148 | -6 | L1PA7 | L1 | LINE |
| 50206050 | 50206129 | - | 28.8 | 5348 | 5430 | -810 | L1ME4a | L1 | LINE |

  

|  |  |  |  |  |  |  |  |  |  |
| --- | --- | --- | --- | --- | --- | --- | --- | --- | --- |
| chr1:55350319-55357850 | | | 7533 bp | | |  |  |  |  |
| Genome Start | Genome End | Strand | Div | Repeat Start | Repeat End | Repeat Left | Name | Family | Class |
| 55350319 | 55350457 | + | 18.2 | 2888 | 3027 | -3113 | L1MB3 | L1 | LINE |
| 55350455 | 55351389 | - | 8.3 | 2287 | 3231 | -2915 | L1PA7 | L1 | LINE |
| 55351389 | 55353497 | + | 12.1 | 3231 | 5488 | -666 | L1PA7 | L1 | LINE |
| 55353498 | 55353782 | + | 2.5 | 1 | 307 | -3 | AluYa5 | Alu | SINE |
| 55353783 | 55354438 | + | 6.8 | 5489 | 6152 | -2 | L1PA7 | L1 | LINE |
| 55354442 | 55355707 | + | 17.5 | 3013 | 4254 | -1886 | L1MB3 | L1 | LINE |
| 55355708 | 55356031 | + | 0.6 | 1 | 316 | -2 | AluYb8 | Alu | SINE |
| 55356032 | 55357850 | + | 16.3 | 4255 | 6183 | 0 | L1MB3 | L1 | LINE |

  

|  |  |  |  |  |  |  |  |  |  |
| --- | --- | --- | --- | --- | --- | --- | --- | --- | --- |
| chr1:56285473-56289952 | | | 4442 bp | | |  |  |  |  |
| Genome Start | Genome End | Strand | Div | Repeat Start | Repeat End | Repeat Left | Name | Family | Class |
| 56285473 | 56286487 | - | 12.4 | 1364 | 2418 | 0 | Tigger1 | MER2\_type | DNA |
| 56286524 | 56287845 | - | 4.3 | 4808 | 6134 | -20 | L1PA5 | L1 | LINE |
| 56287848 | 56288609 | + | 4.9 | 4061 | 4820 | -1326 | L1PA5 | L1 | LINE |
| 56288610 | 56289952 | - | 12.5 | 1 | 1375 | -1043 | Tigger1 | MER2\_type | DNA |

  

|  |  |  |  |  |  |  |  |  |  |
| --- | --- | --- | --- | --- | --- | --- | --- | --- | --- |
| chr1:59404017-59407876 | | | 3793 bp | | |  |  |  |  |
| Genome Start | Genome End | Strand | Div | Repeat Start | Repeat End | Repeat Left | Name | Family | Class |
| 59404017 | 59404084 | + | 19.1 | 7712 | 7779 | -182 | L1MC5 | L1 | LINE |
| 59404126 | 59405155 | - | 9.0 | 5120 | 6162 | -1 | L1PA13 | L1 | LINE |
| 59405155 | 59405921 | + | 9.6 | 4371 | 5135 | -1011 | L1PREC2 | L1 | LINE |
| 59405913 | 59406921 | + | 16.8 | 4847 | 5891 | -287 | L1MB8 | L1 | LINE |
| 59406922 | 59407413 | + | 7.7 | 5681 | 6171 | -1 | L1PA8 | L1 | LINE |
| 59407414 | 59407699 | + | 18.1 | 5878 | 6172 | -6 | L1MB8 | L1 | LINE |
| 59407736 | 59407876 | + | 21.9 | 7794 | 7936 | -25 | L1MC5 | L1 | LINE |

  

|  |  |  |  |  |  |  |  |  |  |
| --- | --- | --- | --- | --- | --- | --- | --- | --- | --- |
| chr1:59698358-59705767 | | | 7362 bp | | |  |  |  |  |
| Genome Start | Genome End | Strand | Div | Repeat Start | Repeat End | Repeat Left | Name | Family | Class |
| 59698358 | 59700030 | + | 7.7 | 175 | 1856 | -419 | L1PA6 | L1 | LINE |
| 59700091 | 59700370 | - | 3.9 | 5871 | 6150 | -5 | L1PA3 | L1 | LINE |
| 59700371 | 59701477 | + | 3.3 | 4794 | 5875 | -280 | L1PA3 | L1 | LINE |
| 59701466 | 59705767 | + | 7.0 | 1848 | 6151 | -3 | L1PA6 | L1 | LINE |

  

|  |  |  |  |  |  |  |  |  |  |
| --- | --- | --- | --- | --- | --- | --- | --- | --- | --- |
| chr1:60120088-60122868 | | | 2546 bp | | |  |  |  |  |
| Genome Start | Genome End | Strand | Div | Repeat Start | Repeat End | Repeat Left | Name | Family | Class |
| 60120088 | 60120444 | + | 34.4 | 34 | 409 | -5991 | L1MEc | L1 | LINE |
| 60120572 | 60121440 | - | 5.3 | 5296 | 6168 | -4 | L1PA8 | L1 | LINE |
| 60121440 | 60121769 | + | 7.0 | 4967 | 5296 | -850 | L1PA8 | L1 | LINE |
| 60121879 | 60122868 | + | 28.8 | 642 | 1651 | -4664 | L1MEc | L1 | LINE |

  

|  |  |  |  |  |  |  |  |  |  |
| --- | --- | --- | --- | --- | --- | --- | --- | --- | --- |
| chr1:60515394-60518002 | | | 2600 bp | | |  |  |  |  |
| Genome Start | Genome End | Strand | Div | Repeat Start | Repeat End | Repeat Left | Name | Family | Class |
| 60515394 | 60515473 | - | 24.5 | 4341 | 4425 | -1721 | L1M5 | L1 | LINE |
| 60515474 | 60515876 | - | 22.0 | 1 | 390 | 0 | MLT1B | MaLR | LTR |
| 60515877 | 60515910 | - | 24.5 | 4311 | 4340 | -1806 | L1M5 | L1 | LINE |
| 60515911 | 60516166 | + | 19.6 | 1 | 251 | -88 | MER44A | MER2\_type | DNA |
| 60516167 | 60516472 | + | 11.4 | 1 | 306 | -6 | AluSx | Alu | SINE |
| 60516473 | 60516566 | + | 19.6 | 252 | 339 | 0 | MER44A | MER2\_type | DNA |
| 60516567 | 60516652 | - | 24.5 | 4225 | 4310 | -1836 | L1M5 | L1 | LINE |
| 60516646 | 60516902 | - | 1.2 | 5075 | 5331 | -815 | L1HS | L1 | LINE |
| 60516902 | 60517716 | + | 1.0 | 5340 | 6154 | -1 | L1HS | L1 | LINE |
| 60517734 | 60518002 | - | 25.9 | 3963 | 4242 | -1904 | L1M5 | L1 | LINE |

  

|  |  |  |  |  |  |  |  |  |  |
| --- | --- | --- | --- | --- | --- | --- | --- | --- | --- |
| chr1:60616482-60621174 | | | 4282 bp | | |  |  |  |  |
| Genome Start | Genome End | Strand | Div | Repeat Start | Repeat End | Repeat Left | Name | Family | Class |
| 60616482 | 60616652 | - | 15.2 | 6124 | 6297 | -3 | L1MA6 | L1 | LINE |
| 60617068 | 60618870 | - | 3.0 | 4349 | 6152 | -3 | L1PA3 | L1 | LINE |
| 60618870 | 60621060 | + | 3.5 | 2182 | 4374 | -1772 | L1PA3 | L1 | LINE |
| 60621058 | 60621174 | - | 18.8 | 6016 | 6133 | -167 | L1MA6 | L1 | LINE |

  

|  |  |  |  |  |  |  |  |  |  |
| --- | --- | --- | --- | --- | --- | --- | --- | --- | --- |
| chr1:60674977-60679661 | | | 4687 bp | | |  |  |  |  |
| Genome Start | Genome End | Strand | Div | Repeat Start | Repeat End | Repeat Left | Name | Family | Class |
| 60674977 | 60675309 | + | 12.5 | 2 | 340 | -351 | LTR8 | ERV1 | LTR |
| 60675310 | 60675436 | - | 6.3 | 2521 | 2647 | -3499 | L1PA5 | L1 | LINE |
| 60675435 | 60678952 | + | 5.0 | 2635 | 6151 | -3 | L1PA5 | L1 | LINE |
| 60678953 | 60679228 | + | 8.8 | 313 | 582 | -109 | LTR8 | ERV1 | LTR |
| 60679229 | 60679528 | + | 13.0 | 2 | 301 | -11 | AluSx | Alu | SINE |
| 60679529 | 60679661 | + | 8.8 | 583 | 689 | -2 | LTR8 | ERV1 | LTR |

  

|  |  |  |  |  |  |  |  |  |  |
| --- | --- | --- | --- | --- | --- | --- | --- | --- | --- |
| chr1:67031289-67033707 | | | 2417 bp | | |  |  |  |  |
| Genome Start | Genome End | Strand | Div | Repeat Start | Repeat End | Repeat Left | Name | Family | Class |
| 67031289 | 67031487 | - | 18.2 | 2551 | 2760 | -1 | Charlie1 | MER1\_type | DNA |
| 67031488 | 67031768 | + | 12.8 | 1 | 291 | -21 | AluSx | Alu | SINE |
| 67031769 | 67032729 | - | 17.8 | 1562 | 2550 | -211 | Charlie1 | MER1\_type | DNA |
| 67032732 | 67033341 | - | 3.0 | 5546 | 6155 | 0 | L1PA4 | L1 | LINE |
| 67033342 | 67033607 | + | 1.5 | 5275 | 5540 | -615 | L1PA4 | L1 | LINE |
| 67033608 | 67033707 | - | 24.5 | 1475 | 1574 | -1187 | Charlie1 | MER1\_type | DNA |

  

|  |  |  |  |  |  |  |  |  |  |
| --- | --- | --- | --- | --- | --- | --- | --- | --- | --- |
| chr1:67785922-67790288 | | | 4040 bp | | |  |  |  |  |
| Genome Start | Genome End | Strand | Div | Repeat Start | Repeat End | Repeat Left | Name | Family | Class |
| 67785922 | 67785986 | + | 29.7 | 2746 | 2811 | -608 | L2 | L2 | LINE |
| 67785989 | 67788061 | - | 4.1 | 580 | 2650 | -3496 | L1P1 | L1 | LINE |
| 67788062 | 67788771 | + | 2.8 | 2654 | 3363 | -2783 | L1P1 | L1 | LINE |
| 67788772 | 67789346 | + | 1.8 | 5598 | 6173 | 0 | L1PA3 | L1 | LINE |
| 67789404 | 67789711 | - | 13.0 | 1 | 308 | -2 | AluSg | Alu | SINE |
| 67789980 | 67790288 | + | 33.9 | 3092 | 3418 | -1 | L2 | L2 | LINE |

  

|  |  |  |  |  |  |  |  |  |  |
| --- | --- | --- | --- | --- | --- | --- | --- | --- | --- |
| chr1:68265298-68275665 | | | 10350 bp | | |  |  |  |  |
| Genome Start | Genome End | Strand | Div | Repeat Start | Repeat End | Repeat Left | Name | Family | Class |
| 68265298 | 68266673 | + | 21.4 | 2841 | 4278 | -1868 | L1MD2 | L1 | LINE |
| 68266674 | 68267044 | - | 10.2 | 1 | 375 | 0 | THE1C | MaLR | LTR |
| 68267045 | 68267466 | + | 21.4 | 4279 | 4703 | -1443 | L1MD2 | L1 | LINE |
| 68267471 | 68267876 | - | 22.6 | 74 | 505 | 0 | MLT1D | MaLR | LTR |
| 68267877 | 68267949 | - | 27.4 | 2645 | 2717 | -1 | Tigger2 | MER2\_type | DNA |
| 68267956 | 68269650 | - | 8.7 | 4439 | 6146 | -5 | L1PB1 | L1 | LINE |
| 68269649 | 68270269 | + | 8.7 | 3812 | 4425 | -1721 | L1PB1 | L1 | LINE |
| 68270270 | 68272642 | - | 15.2 | 343 | 2659 | -59 | Tigger2 | MER2\_type | DNA |
| 68272643 | 68272998 | - | 12.5 | 1 | 355 | 0 | THE1A | MaLR | LTR |
| 68272999 | 68273320 | - | 15.2 | 1 | 342 | -2376 | Tigger2 | MER2\_type | DNA |
| 68273321 | 68273389 | - | 15.9 | 3 | 76 | -429 | MLT1D | MaLR | LTR |
| 68273392 | 68273594 | + | 18.6 | 4705 | 4898 | -1248 | L1MD2 | L1 | LINE |
| 68273603 | 68273955 | - | 13.9 | 30 | 381 | 0 | THE1D | MaLR | LTR |
| 68273956 | 68274313 | + | 14.2 | 29 | 428 | 0 | MSTA | MaLR | LTR |
| 68274314 | 68275665 | + | 18.0 | 4895 | 6255 | -104 | L1MD2 | L1 | LINE |

  

|  |  |  |  |  |  |  |  |  |  |
| --- | --- | --- | --- | --- | --- | --- | --- | --- | --- |
| chr1:69273471-69281584 | | | 8118 bp | | |  |  |  |  |
| Genome Start | Genome End | Strand | Div | Repeat Start | Repeat End | Repeat Left | Name | Family | Class |
| 69273471 | 69273554 | + | 28.6 | 18 | 108 | -7457 | L1MA1 | L1 | LINE |
| 69273560 | 69274464 | - | 2.6 | 4055 | 4959 | -1187 | L1PA3 | L1 | LINE |
| 69274462 | 69275635 | + | 2.2 | 4973 | 6154 | -1 | L1PA3 | L1 | LINE |
| 69275630 | 69279999 | + | 15.1 | 102 | 4505 | -3060 | L1MA1 | L1 | LINE |
| 69280000 | 69280306 | + | 9.8 | 1 | 306 | -4 | AluSg | Alu | SINE |
| 69280307 | 69281044 | + | 9.2 | 4506 | 5249 | -2316 | L1MA1 | L1 | LINE |
| 69281045 | 69281584 | + | 10.6 | 7156 | 7720 | -1 | L1MA1 | L1 | LINE |

  

|  |  |  |  |  |  |  |  |  |  |
| --- | --- | --- | --- | --- | --- | --- | --- | --- | --- |
| chr1:71031734-71038847 | | | 7051 bp | | |  |  |  |  |
| Genome Start | Genome End | Strand | Div | Repeat Start | Repeat End | Repeat Left | Name | Family | Class |
| 71031734 | 71031969 | - | 19.5 | 183 | 419 | -48 | MLT1C | MaLR | LTR |
| 71031983 | 71032922 | + | 10.5 | 4130 | 5045 | -1101 | L1PA16 | L1 | LINE |
| 71032923 | 71033233 | + | 13.8 | 1 | 312 | 0 | AluSx | Alu | SINE |
| 71033234 | 71033645 | + | 9.2 | 5046 | 5476 | -690 | L1PA16 | L1 | LINE |
| 71033651 | 71033914 | - | 7.7 | 5901 | 6165 | -7 | L1PA8 | L1 | LINE |
| 71033915 | 71034229 | - | 10.0 | 1 | 309 | -2 | AluY | Alu | SINE |
| 71034230 | 71035467 | - | 8.3 | 4651 | 5900 | -272 | L1PA4 | L1 | LINE |
| 71035468 | 71035727 | + | 3.1 | 5895 | 6155 | 0 | L1PA4 | L1 | LINE |
| 71035728 | 71037634 | - | 8.8 | 2735 | 4650 | -1496 | L1PA8 | L1 | LINE |
| 71037638 | 71038127 | + | 13.0 | 5464 | 5948 | -218 | L1PA16 | L1 | LINE |
| 71038128 | 71038433 | - | 11.1 | 1 | 309 | -1 | AluSg | Alu | SINE |
| 71038434 | 71038636 | + | 13.0 | 5949 | 6155 | -11 | L1PA16 | L1 | LINE |
| 71038679 | 71038847 | - | 13.2 | 1 | 180 | -287 | MLT1C | MaLR | LTR |

  

|  |  |  |  |  |  |  |  |  |  |
| --- | --- | --- | --- | --- | --- | --- | --- | --- | --- |
| chr1:71346424-71351143 | | | 4722 bp | | |  |  |  |  |
| Genome Start | Genome End | Strand | Div | Repeat Start | Repeat End | Repeat Left | Name | Family | Class |
| 71346424 | 71346988 | - | 25.0 | 2139 | 2786 | -76 | Charlie2 | MER1\_type | DNA |
| 71346989 | 71347314 | - | 3.1 | 2514 | 2839 | -3307 | L1PA3 | L1 | LINE |
| 71347311 | 71350636 | + | 3.2 | 2826 | 6153 | -2 | L1PA3 | L1 | LINE |
| 71350637 | 71350920 | - | 14.4 | 1856 | 2150 | -712 | Charlie2 | MER1\_type | DNA |
| 71350923 | 71351143 | - | 24.4 | 67 | 325 | -2457 | Charlie2 | MER1\_type | DNA |

  

|  |  |  |  |  |  |  |  |  |  |
| --- | --- | --- | --- | --- | --- | --- | --- | --- | --- |
| chr1:71357776-71365369 | | | 7532 bp | | |  |  |  |  |
| Genome Start | Genome End | Strand | Div | Repeat Start | Repeat End | Repeat Left | Name | Family | Class |
| 71357776 | 71358036 | - | 19.1 | 2078 | 2346 | -3982 | L1M3f | L1 | LINE |
| 71358068 | 71360611 | - | 6.6 | 918 | 3488 | -2658 | L1PA5 | L1 | LINE |
| 71360610 | 71363285 | + | 6.2 | 3474 | 6149 | -5 | L1PA5 | L1 | LINE |
| 71363289 | 71363589 | - | 24.6 | 1780 | 2060 | -4268 | L1M3f | L1 | LINE |
| 71363620 | 71365369 | - | 18.6 | 2 | 1779 | -4549 | L1M3f | L1 | LINE |

  

|  |  |  |  |  |  |  |  |  |  |
| --- | --- | --- | --- | --- | --- | --- | --- | --- | --- |
| chr1:72621502-72631330 | | | 9778 bp | | |  |  |  |  |
| Genome Start | Genome End | Strand | Div | Repeat Start | Repeat End | Repeat Left | Name | Family | Class |
| 72621502 | 72621625 | + | 17.0 | 4676 | 4798 | -1348 | L1PA16 | L1 | LINE |
| 72621626 | 72621927 | + | 10.6 | 1 | 302 | -7 | AluSc | Alu | SINE |
| 72621928 | 72622610 | + | 15.5 | 4799 | 5476 | -690 | L1PA16 | L1 | LINE |
| 72622617 | 72626140 | - | 9.7 | 2632 | 6168 | 0 | L1PA10 | L1 | LINE |
| 72626188 | 72628309 | - | 4.3 | 4009 | 6135 | -19 | L1PA5 | L1 | LINE |
| 72628307 | 72630169 | + | 5.6 | 2137 | 3999 | -2147 | L1PA5 | L1 | LINE |
| 72630171 | 72630619 | - | 8.7 | 2189 | 2637 | -3509 | L1PA10 | L1 | LINE |
| 72630620 | 72631330 | + | 13.0 | 5460 | 6157 | -9 | L1PA16 | L1 | LINE |

  

|  |  |  |  |  |  |  |  |  |  |
| --- | --- | --- | --- | --- | --- | --- | --- | --- | --- |
| chr1:72659055-72667979 | | | 8853 bp | | |  |  |  |  |
| Genome Start | Genome End | Strand | Div | Repeat Start | Repeat End | Repeat Left | Name | Family | Class |
| 72659055 | 72659688 | + | 23.2 | 5218 | 5860 | -1146 | L1MB8 | L1 | LINE |
| 72659689 | 72659989 | + | 10.6 | 1 | 301 | -9 | AluSg | Alu | SINE |
| 72659990 | 72660003 | + | 23.2 | 5861 | 5864 | -1142 | L1MB8 | L1 | LINE |
| 72660042 | 72660913 | + | 22.7 | 5865 | 6736 | -302 | L1MB8 | L1 | LINE |
| 72660909 | 72661742 | - | 11.8 | 4005 | 4825 | -1321 | L1PREC2 | L1 | LINE |
| 72661744 | 72663049 | + | 12.0 | 4840 | 6150 | -15 | L1PA15 | L1 | LINE |
| 72663056 | 72663232 | + | 24.1 | 6724 | 6898 | -140 | L1MB8 | L1 | LINE |
| 72663251 | 72666714 | - | 8.4 | 2722 | 6170 | -2 | L1PA8 | L1 | LINE |
| 72666723 | 72666736 | + | 7.6 | 1840 | 1853 | -422 | L1P2 | L1 | LINE |
| 72666737 | 72667040 | - | 9.2 | 1 | 303 | -10 | AluSp | Alu | SINE |
| 72667041 | 72667904 | + | 7.4 | 1854 | 2685 | -3461 | L1P2 | L1 | LINE |
| 72667911 | 72667979 | + | 21.7 | 6959 | 7033 | -5 | L1MB8 | L1 | LINE |

  

|  |  |  |  |  |  |  |  |  |  |
| --- | --- | --- | --- | --- | --- | --- | --- | --- | --- |
| chr1:72767852-72771213 | | | 3356 bp | | |  |  |  |  |
| Genome Start | Genome End | Strand | Div | Repeat Start | Repeat End | Repeat Left | Name | Family | Class |
| 72767852 | 72769388 | + | 15.4 | 31 | 1619 | -799 | Tigger1 | MER2\_type | DNA |
| 72769389 | 72769902 | - | 1.8 | 5527 | 6040 | -115 | L1PA3 | L1 | LINE |
| 72769903 | 72770021 | + | 3.4 | 6038 | 6155 | 0 | L1PA3 | L1 | LINE |
| 72770028 | 72770123 | + | 14.1 | 1607 | 1702 | -716 | Tigger1 | MER2\_type | DNA |
| 72770124 | 72770532 | - | 10.5 | 1 | 375 | 0 | THE1C | MaLR | LTR |
| 72770533 | 72771213 | + | 14.1 | 1703 | 2417 | -1 | Tigger1 | MER2\_type | DNA |

  

|  |  |  |  |  |  |  |  |  |  |
| --- | --- | --- | --- | --- | --- | --- | --- | --- | --- |
| chr1:72949326-72957121 | | | 7512 bp | | |  |  |  |  |
| Genome Start | Genome End | Strand | Div | Repeat Start | Repeat End | Repeat Left | Name | Family | Class |
| 72949326 | 72949473 | + | 34.5 | 132 | 279 | -4575 | L1MEe | L1 | LINE |
| 72949618 | 72950411 | - | 16.4 | 5494 | 6300 | 0 | L1MA6 | L1 | LINE |
| 72950413 | 72950899 | + | 15.3 | 4991 | 5489 | -811 | L1M1 | L1 | LINE |
| 72950928 | 72951027 | + | 19.4 | 1465 | 1567 | -3267 | L1MEe | L1 | LINE |
| 72951032 | 72955933 | - | 11.7 | 3229 | 8176 | 0 | L1PA14 | L1 | LINE |
| 72956041 | 72957121 | + | 27.1 | 1552 | 2732 | -2102 | L1MEe | L1 | LINE |

  

|  |  |  |  |  |  |  |  |  |  |
| --- | --- | --- | --- | --- | --- | --- | --- | --- | --- |
| chr1:73047323-73057337 | | | 9872 bp | | |  |  |  |  |
| Genome Start | Genome End | Strand | Div | Repeat Start | Repeat End | Repeat Left | Name | Family | Class |
| 73047323 | 73047431 | - | 31.5 | 3307 | 3414 | -5 | L2 | L2 | LINE |
| 73047533 | 73047957 | - | 13.9 | 1 | 427 | -7 | MER57B | ERV1 | LTR |
| 73047958 | 73050101 | - | 12.0 | 5307 | 7537 | 0 | MER57B-int | ERV1 | LTR |
| 73050093 | 73051119 | - | 16.8 | 3010 | 4041 | -1905 | MER57B-int | ERV1 | LTR |
| 73051164 | 73053556 | - | 3.2 | 3744 | 6137 | -18 | L1PA3 | L1 | LINE |
| 73053551 | 73053792 | + | 4.5 | 3483 | 3725 | -2421 | L1PA3 | L1 | LINE |
| 73053793 | 73055027 | - | 16.2 | 1371 | 4170 | -2230 | MER57B-int | ERV1 | LTR |
| 73055018 | 73055716 | - | 14.1 | 288 | 1136 | -4810 | MER57B-int | ERV1 | LTR |
| 73055714 | 73056374 | - | 14.3 | 1 | 672 | -6865 | MER57B-int | ERV1 | LTR |
| 73056375 | 73056806 | - | 14.6 | 1 | 434 | 0 | MER57B | ERV1 | LTR |
| 73056833 | 73057337 | - | 36.4 | 2670 | 3160 | -218 | L2 | L2 | LINE |

  

|  |  |  |  |  |  |  |  |  |  |
| --- | --- | --- | --- | --- | --- | --- | --- | --- | --- |
| chr1:73281636-73291978 | | | 10131 bp | | |  |  |  |  |
| Genome Start | Genome End | Strand | Div | Repeat Start | Repeat End | Repeat Left | Name | Family | Class |
| 73281636 | 73281668 | - | 23.3 | 524 | 560 | 0 | MLT2D | ERVL | LTR |
| 73281710 | 73281963 | - | 23.3 | 97 | 377 | -37 | MLT2D | ERVL | LTR |
| 73282070 | 73289662 | + | 17.5 | 43 | 6865 | -2 | L1PB2 | L1 | LINE |
| 73289708 | 73290002 | - | 29.1 | 7561 | 7849 | -193 | L1MC4 | L1 | LINE |
| 73290020 | 73290371 | - | 27.1 | 5925 | 6274 | -38 | L1MA9 | L1 | LINE |
| 73290374 | 73290781 | + | 21.9 | 4787 | 5189 | -951 | L1MA9 | L1 | LINE |
| 73290782 | 73291090 | + | 12.0 | 1 | 309 | -1 | AluSg | Alu | SINE |
| 73291091 | 73291860 | + | 19.6 | 5190 | 5938 | -374 | L1MA9 | L1 | LINE |
| 73291862 | 73291978 | - | 19.8 | 1 | 118 | -438 | MLT2D | ERVL | LTR |

  

|  |  |  |  |  |  |  |  |  |  |
| --- | --- | --- | --- | --- | --- | --- | --- | --- | --- |
| chr1:73665633-73670817 | | | 4897 bp | | |  |  |  |  |
| Genome Start | Genome End | Strand | Div | Repeat Start | Repeat End | Repeat Left | Name | Family | Class |
| 73665633 | 73666023 | - | 24.0 | 2824 | 3209 | -3335 | L1MD2 | L1 | LINE |
| 73666043 | 73667192 | - | 30.0 | 1383 | 2650 | -3990 | L1MD2 | L1 | LINE |
| 73667185 | 73669352 | - | 5.2 | 3842 | 6154 | 0 | L1PA5 | L1 | LINE |
| 73669353 | 73669746 | + | 6.3 | 3438 | 3831 | -2315 | L1PA5 | L1 | LINE |
| 73669745 | 73670384 | - | 30.2 | 763 | 1391 | -5143 | L1MD2 | L1 | LINE |
| 73670664 | 73670817 | - | 34.9 | 25 | 179 | -6368 | L1MD2 | L1 | LINE |

  

|  |  |  |  |  |  |  |  |  |  |
| --- | --- | --- | --- | --- | --- | --- | --- | --- | --- |
| chr1:74202171-74207097 | | | 4906 bp | | |  |  |  |  |
| Genome Start | Genome End | Strand | Div | Repeat Start | Repeat End | Repeat Left | Name | Family | Class |
| 74202171 | 74203782 | - | 36.4 | 1569 | 3359 | -60 | L2 | L2 | LINE |
| 74203796 | 74205397 | - | 9.6 | 3202 | 4842 | -1304 | L1PA8 | L1 | LINE |
| 74205394 | 74206703 | + | 9.7 | 4844 | 6159 | -13 | L1PA8 | L1 | LINE |
| 74206716 | 74207097 | - | 34.7 | 1139 | 1562 | -1857 | L2 | L2 | LINE |

  

|  |  |  |  |  |  |  |  |  |  |
| --- | --- | --- | --- | --- | --- | --- | --- | --- | --- |
| chr1:75603249-75606226 | | | 2950 bp | | |  |  |  |  |
| Genome Start | Genome End | Strand | Div | Repeat Start | Repeat End | Repeat Left | Name | Family | Class |
| 75603249 | 75603398 | - | 21.0 | 1928 | 2073 | -3961 | L1M3a | L1 | LINE |
| 75603432 | 75603764 | - | 10.8 | 5835 | 6167 | -1 | L1PA12 | L1 | LINE |
| 75603765 | 75604003 | + | 8.0 | 5597 | 5838 | -325 | L1PA12 | L1 | LINE |
| 75603999 | 75606226 | - | 27.0 | 1 | 1937 | -4099 | L1M3a | L1 | LINE |

  

|  |  |  |  |  |  |  |  |  |  |
| --- | --- | --- | --- | --- | --- | --- | --- | --- | --- |
| chr1:75910246-75922214 | | | 11954 bp | | |  |  |  |  |
| Genome Start | Genome End | Strand | Div | Repeat Start | Repeat End | Repeat Left | Name | Family | Class |
| 75910246 | 75912657 | + | 15.6 | 16 | 2452 | -5113 | L1M1 | L1 | LINE |
| 75912658 | 75912954 | - | 12.2 | 1 | 295 | -18 | AluSq | Alu | SINE |
| 75912955 | 75913083 | + | 15.6 | 2453 | 2572 | -4993 | L1M1 | L1 | LINE |
| 75913079 | 75913795 | - | 9.0 | 4243 | 4958 | -1188 | L1PA10 | L1 | LINE |
| 75913785 | 75914973 | + | 9.4 | 4947 | 6153 | -15 | L1PA10 | L1 | LINE |
| 75914970 | 75916020 | + | 9.9 | 2560 | 3666 | -3899 | L1M1 | L1 | LINE |
| 75916024 | 75922058 | + | 3.1 | 3 | 6048 | 0 | L1PA3 | L1 | LINE |
| 75922091 | 75922214 | + | 15.3 | 3653 | 3776 | -3789 | L1M1 | L1 | LINE |

  

|  |  |  |  |  |  |  |  |  |  |
| --- | --- | --- | --- | --- | --- | --- | --- | --- | --- |
| chr1:76058544-76060232 | | | 1671 bp | | |  |  |  |  |
| Genome Start | Genome End | Strand | Div | Repeat Start | Repeat End | Repeat Left | Name | Family | Class |
| 76058544 | 76058599 | + | 28.6 | 7 | 69 | -120 | MER5A | MER1\_type | DNA |
| 76058601 | 76059055 | - | 4.8 | 3189 | 3643 | -2503 | L1P1 | L1 | LINE |
| 76059056 | 76059878 | + | 3.6 | 2366 | 3188 | -2958 | L1P1 | L1 | LINE |
| 76059894 | 76060183 | - | 20.2 | 1 | 289 | -23 | AluJb | Alu | SINE |
| 76060186 | 76060232 | + | 31.9 | 63 | 109 | -80 | MER5A | MER1\_type | DNA |

  

|  |  |  |  |  |  |  |  |  |  |
| --- | --- | --- | --- | --- | --- | --- | --- | --- | --- |
| chr1:76549374-76551180 | | | 1569 bp | | |  |  |  |  |
| Genome Start | Genome End | Strand | Div | Repeat Start | Repeat End | Repeat Left | Name | Family | Class |
| 76549374 | 76549505 | + | 38.8 | 2655 | 2785 | -634 | L2 | L2 | LINE |
| 76549548 | 76550341 | - | 7.1 | 5367 | 6154 | 0 | L1PA7 | L1 | LINE |
| 76550343 | 76550945 | + | 9.7 | 4782 | 5375 | -771 | L1PA7 | L1 | LINE |
| 76551141 | 76551180 | + | 22.5 | 3050 | 3089 | -330 | L2 | L2 | LINE |

  

|  |  |  |  |  |  |  |  |  |  |
| --- | --- | --- | --- | --- | --- | --- | --- | --- | --- |
| chr1:77202753-77209112 | | | 6365 bp | | |  |  |  |  |
| Genome Start | Genome End | Strand | Div | Repeat Start | Repeat End | Repeat Left | Name | Family | Class |
| 77202753 | 77202888 | - | 25.4 | 5895 | 6051 | -189 | L1ME3B | L1 | LINE |
| 77202883 | 77207318 | - | 7.1 | 1701 | 6153 | -1 | L1PA6 | L1 | LINE |
| 77207324 | 77209023 | + | 7.7 | 5 | 1696 | -579 | L1PA6 | L1 | LINE |
| 77209020 | 77209112 | - | 24.7 | 5811 | 5913 | -257 | L1ME3B | L1 | LINE |

  

|  |  |  |  |  |  |  |  |  |  |
| --- | --- | --- | --- | --- | --- | --- | --- | --- | --- |
| chr1:78716408-78720161 | | | 3703 bp | | |  |  |  |  |
| Genome Start | Genome End | Strand | Div | Repeat Start | Repeat End | Repeat Left | Name | Family | Class |
| 78716408 | 78717086 | + | 25.7 | 4891 | 5661 | -579 | L1ME3B | L1 | LINE |
| 78717090 | 78717836 | - | 5.0 | 4003 | 4749 | -1397 | L1PA4 | L1 | LINE |
| 78717836 | 78719224 | + | 3.5 | 4746 | 6147 | -8 | L1PA4 | L1 | LINE |
| 78719239 | 78719466 | + | 33.6 | 5653 | 5899 | -341 | L1ME3B | L1 | LINE |
| 78719502 | 78719652 | + | 29.9 | 5969 | 6119 | -121 | L1ME3B | L1 | LINE |
| 78719653 | 78720117 | + | 19.9 | 1 | 467 | 0 | MLT1C | MaLR | LTR |
| 78720118 | 78720161 | + | 29.9 | 6120 | 6164 | -76 | L1ME3B | L1 | LINE |

  

|  |  |  |  |  |  |  |  |  |  |
| --- | --- | --- | --- | --- | --- | --- | --- | --- | --- |
| chr1:82271957-82273590 | | | 1558 bp | | |  |  |  |  |
| Genome Start | Genome End | Strand | Div | Repeat Start | Repeat End | Repeat Left | Name | Family | Class |
| 82271957 | 82272216 | - | 19.8 | 2034 | 2304 | -3845 | L1MEe | L1 | LINE |
| 82272279 | 82272460 | - | 9.3 | 5304 | 5485 | -675 | L1PREC2 | L1 | LINE |
| 82272459 | 82273168 | + | 9.5 | 4582 | 5301 | -845 | L1PREC2 | L1 | LINE |
| 82273176 | 82273439 | + | 22.3 | 163 | 396 | 0 | MSTD | MaLR | LTR |
| 82273449 | 82273590 | - | 21.3 | 1852 | 2007 | -4142 | L1MEe | L1 | LINE |

  

|  |  |  |  |  |  |  |  |  |  |
| --- | --- | --- | --- | --- | --- | --- | --- | --- | --- |
| chr1:86069932-86072828 | | | 2690 bp | | |  |  |  |  |
| Genome Start | Genome End | Strand | Div | Repeat Start | Repeat End | Repeat Left | Name | Family | Class |
| 86069932 | 86070038 | - | 30.8 | 3042 | 3164 | -214 | L2 | L2 | LINE |
| 86070039 | 86070158 | - | 31.9 | 2694 | 2807 | -612 | L2 | L2 | LINE |
| 86070159 | 86071438 | - | 3.0 | 3912 | 5192 | -954 | L1PA4 | L1 | LINE |
| 86071438 | 86072397 | + | 3.5 | 5189 | 6152 | -3 | L1PA4 | L1 | LINE |
| 86072606 | 86072828 | - | 34.7 | 2353 | 2587 | -832 | L2 | L2 | LINE |

  

|  |  |  |  |  |  |  |  |  |  |
| --- | --- | --- | --- | --- | --- | --- | --- | --- | --- |
| chr1:91463258-91468827 | | | 5526 bp | | |  |  |  |  |
| Genome Start | Genome End | Strand | Div | Repeat Start | Repeat End | Repeat Left | Name | Family | Class |
| 91463258 | 91463764 | + | 18.5 | 985 | 1656 | -4675 | MER51B-int | ERV1 | LTR |
| 91463766 | 91463810 | + | 23.4 | 1882 | 1926 | -4405 | MER51B-int | ERV1 | LTR |
| 91463811 | 91464119 | + | 11.3 | 1 | 309 | -4 | AluSq | Alu | SINE |
| 91464120 | 91464403 | + | 23.4 | 1927 | 2210 | -4121 | MER51B-int | ERV1 | LTR |
| 91464404 | 91465134 | + | 19.6 | 1 | 691 | 0 | LTR8 | ERV1 | LTR |
| 91465135 | 91465187 | + | 23.4 | 2211 | 2262 | -4069 | MER51B-int | ERV1 | LTR |
| 91465198 | 91465283 | - | 8.6 | 6086 | 6167 | -1 | L1PA10 | L1 | LINE |
| 91465284 | 91465423 | - | 17.5 | 575 | 691 | 0 | LTR8 | ERV1 | LTR |
| 91465424 | 91465736 | - | 9.1 | 1 | 309 | -4 | AluSq | Alu | SINE |
| 91465737 | 91466342 | - | 17.5 | 1 | 574 | -117 | LTR8 | ERV1 | LTR |
| 91466343 | 91467356 | - | 7.6 | 5048 | 6085 | -83 | L1PA10 | L1 | LINE |
| 91467355 | 91467392 | + | 8.5 | 4552 | 4574 | -1572 | L1P3 | L1 | LINE |
| 91467393 | 91467697 | - | 10.5 | 1 | 306 | -7 | AluSp | Alu | SINE |
| 91467698 | 91468169 | + | 8.5 | 4575 | 5044 | -1102 | L1P3 | L1 | LINE |
| 91468171 | 91468474 | + | 12.2 | 3 | 305 | -7 | AluSx | Alu | SINE |
| 91468509 | 91468827 | + | 19.4 | 3011 | 3401 | -3647 | MER51B-int | ERV1 | LTR |

  

|  |  |  |  |  |  |  |  |  |  |
| --- | --- | --- | --- | --- | --- | --- | --- | --- | --- |
| chr1:92209718-92213875 | | | 3835 bp | | |  |  |  |  |
| Genome Start | Genome End | Strand | Div | Repeat Start | Repeat End | Repeat Left | Name | Family | Class |
| 92209718 | 92209858 | - | 28.3 | 3268 | 3414 | -5 | L2 | L2 | LINE |
| 92209881 | 92209932 | - | 23.1 | 3038 | 3092 | -327 | L2 | L2 | LINE |
| 92209966 | 92210447 | + | 33.0 | 1644 | 2207 | -1212 | L2 | L2 | LINE |
| 92210425 | 92210723 | + | 30.4 | 3091 | 3408 | -11 | L2 | L2 | LINE |
| 92210704 | 92210832 | - | 31.8 | 3242 | 3371 | -7 | L2 | L2 | LINE |
| 92211069 | 92211335 | - | 29.9 | 2949 | 3262 | -157 | L2 | L2 | LINE |
| 92211336 | 92212016 | - | 4.0 | 4060 | 4744 | -1402 | L1PA5 | L1 | LINE |
| 92212022 | 92213434 | + | 4.0 | 4732 | 6149 | -5 | L1PA5 | L1 | LINE |
| 92213505 | 92213875 | - | 25.9 | 2473 | 2895 | -524 | L2 | L2 | LINE |

  

|  |  |  |  |  |  |  |  |  |  |
| --- | --- | --- | --- | --- | --- | --- | --- | --- | --- |
| chr1:95369200-95372754 | | | 3371 bp | | |  |  |  |  |
| Genome Start | Genome End | Strand | Div | Repeat Start | Repeat End | Repeat Left | Name | Family | Class |
| 95369200 | 95369573 | - | 35.8 | 2962 | 3373 | -5 | L2 | L2 | LINE |
| 95369728 | 95369906 | - | 27.9 | 2547 | 2747 | -672 | L2 | L2 | LINE |
| 95369927 | 95370320 | + | 17.2 | 1 | 415 | -42 | MSTB2 | MaLR | LTR |
| 95370321 | 95370622 | + | 11.0 | 5 | 304 | -8 | AluSx | Alu | SINE |
| 95370623 | 95370668 | + | 17.2 | 416 | 457 | 0 | MSTB2 | MaLR | LTR |
| 95370679 | 95371330 | - | 31.6 | 1749 | 2521 | -898 | L2 | L2 | LINE |
| 95371331 | 95371372 | - | 9.5 | 5950 | 5991 | -164 | L1PA4 | L1 | LINE |
| 95371373 | 95371539 | + | 2.4 | 5987 | 6155 | 0 | L1PA4 | L1 | LINE |
| 95371540 | 95372754 | - | 31.6 | 336 | 1748 | -1671 | L2 | L2 | LINE |

  

|  |  |  |  |  |  |  |  |  |  |
| --- | --- | --- | --- | --- | --- | --- | --- | --- | --- |
| chr1:98522018-98526322 | | | 4079 bp | | |  |  |  |  |
| Genome Start | Genome End | Strand | Div | Repeat Start | Repeat End | Repeat Left | Name | Family | Class |
| 98522018 | 98522700 | + | 32.2 | 417 | 1064 | -5055 | L1MEd | L1 | LINE |
| 98522926 | 98523649 | - | 6.1 | 5428 | 6151 | -3 | L1PA6 | L1 | LINE |
| 98523650 | 98524939 | + | 8.2 | 4133 | 5432 | -722 | L1PA6 | L1 | LINE |
| 98524941 | 98524984 | + | 30.6 | 1437 | 1480 | -4669 | L1MEd | L1 | LINE |
| 98524985 | 98525061 | - | 9.1 | 1 | 78 | -2 | MADE1 | Mariner | DNA |
| 98525062 | 98526322 | + | 31.2 | 1481 | 2806 | -3323 | L1MEd | L1 | LINE |

  

|  |  |  |  |  |  |  |  |  |  |
| --- | --- | --- | --- | --- | --- | --- | --- | --- | --- |
| chr1:98603041-98604213 | | | 1173 bp | | |  |  |  |  |
| Genome Start | Genome End | Strand | Div | Repeat Start | Repeat End | Repeat Left | Name | Family | Class |
| 98603041 | 98603213 | + | 10.4 | 1 | 189 | -175 | THE1B | MaLR | LTR |
| 98603214 | 98603760 | - | 2.2 | 5338 | 5884 | -271 | L1PA4 | L1 | LINE |
| 98603761 | 98604018 | + | 3.9 | 5894 | 6151 | -4 | L1PA4 | L1 | LINE |
| 98604019 | 98604213 | + | 17.3 | 174 | 361 | -3 | THE1B | MaLR | LTR |

  

|  |  |  |  |  |  |  |  |  |  |
| --- | --- | --- | --- | --- | --- | --- | --- | --- | --- |
| chr1:99444174-99445645 | | | 1458 bp | | |  |  |  |  |
| Genome Start | Genome End | Strand | Div | Repeat Start | Repeat End | Repeat Left | Name | Family | Class |
| 99444174 | 99444395 | - | 17.9 | 91 | 390 | -6 | MSTD | MaLR | LTR |
| 99444413 | 99444999 | - | 13.9 | 5493 | 6157 | -9 | L1PA16 | L1 | LINE |
| 99444997 | 99445559 | + | 12.2 | 4917 | 5482 | -684 | L1PA16 | L1 | LINE |
| 99445560 | 99445645 | - | 15.1 | 1 | 101 | -307 | MSTD | MaLR | LTR |

  

|  |  |  |  |  |  |  |  |  |  |
| --- | --- | --- | --- | --- | --- | --- | --- | --- | --- |
| chr1:99556671-99561269 | | | 4293 bp | | |  |  |  |  |
| Genome Start | Genome End | Strand | Div | Repeat Start | Repeat End | Repeat Left | Name | Family | Class |
| 99556671 | 99557300 | + | 34.7 | 16 | 670 | -1837 | HAL1 | L1 | LINE |
| 99557421 | 99557815 | + | 29.4 | 776 | 1171 | -1336 | HAL1 | L1 | LINE |
| 99557862 | 99558305 | + | 29.4 | 1172 | 1684 | -823 | HAL1 | L1 | LINE |
| 99558353 | 99558912 | - | 14.6 | 4222 | 4794 | -1349 | L1M3 | L1 | LINE |
| 99558909 | 99559450 | + | 18.4 | 4781 | 5331 | -960 | L1M3 | L1 | LINE |
| 99559451 | 99559762 | + | 11.8 | 1 | 312 | 0 | AluSx | Alu | SINE |
| 99559763 | 99560717 | + | 14.4 | 5332 | 6290 | -1 | L1MA7 | L1 | LINE |
| 99560815 | 99561269 | + | 28.9 | 1685 | 2174 | -333 | HAL1 | L1 | LINE |

  

|  |  |  |  |  |  |  |  |  |  |
| --- | --- | --- | --- | --- | --- | --- | --- | --- | --- |
| chr1:99625434-99630316 | | | 4879 bp | | |  |  |  |  |
| Genome Start | Genome End | Strand | Div | Repeat Start | Repeat End | Repeat Left | Name | Family | Class |
| 99625434 | 99626677 | + | 13.9 | 1 | 1165 | -1253 | Tigger1 | MER2\_type | DNA |
| 99626683 | 99627665 | - | 0.9 | 5173 | 6155 | 0 | L1HS | L1 | LINE |
| 99627665 | 99629036 | + | 0.2 | 3790 | 5174 | -972 | L1HS | L1 | LINE |
| 99629037 | 99630316 | + | 13.3 | 1150 | 2418 | 0 | Tigger1 | MER2\_type | DNA |

  

|  |  |  |  |  |  |  |  |  |  |
| --- | --- | --- | --- | --- | --- | --- | --- | --- | --- |
| chr1:100787509-100796350 | | | 8424 bp | | |  |  |  |  |
| Genome Start | Genome End | Strand | Div | Repeat Start | Repeat End | Repeat Left | Name | Family | Class |
| 100787509 | 100787776 | - | 24.3 | 311 | 505 | 0 | MLT1D | MaLR | LTR |
| 100787783 | 100788001 | - | 13.7 | 6320 | 6534 | -1031 | L1M1 | L1 | LINE |
| 100788002 | 100788295 | + | 11.6 | 1 | 293 | -16 | AluSc | Alu | SINE |
| 100788296 | 100788709 | - | 13.7 | 5910 | 6319 | -1246 | L1M1 | L1 | LINE |
| 100788905 | 100789204 | - | 20.4 | 2 | 302 | -10 | AluJb | Alu | SINE |
| 100789205 | 100789267 | - | 13.7 | 5854 | 5909 | -1656 | L1M1 | L1 | LINE |
| 100789268 | 100789755 | - | 9.9 | 2 | 510 | -4 | MER75 | PiggyBac | DNA |
| 100789756 | 100792013 | - | 13.7 | 3625 | 5853 | -1712 | L1M1 | L1 | LINE |
| 100792019 | 100792369 | - | 9.1 | 5799 | 6150 | -1 | L1PB1 | L1 | LINE |
| 100792493 | 100793050 | - | 9.1 | 5213 | 5798 | -353 | L1PB1 | L1 | LINE |
| 100793048 | 100793296 | + | 6.8 | 4953 | 5201 | -945 | L1PB1 | L1 | LINE |
| 100793296 | 100796162 | - | 22.2 | 16 | 3634 | -3931 | L1M1 | L1 | LINE |
| 100796256 | 100796350 | - | 18.5 | 1 | 95 | -410 | MLT1D | MaLR | LTR |

  

|  |  |  |  |  |  |  |  |  |  |
| --- | --- | --- | --- | --- | --- | --- | --- | --- | --- |
| chr1:101039141-101041982 | | | 2818 bp | | |  |  |  |  |
| Genome Start | Genome End | Strand | Div | Repeat Start | Repeat End | Repeat Left | Name | Family | Class |
| 101039141 | 101039769 | + | 9.6 | 2 | 722 | -514 | MER11A | ERVK | LTR |
| 101039795 | 101040572 | - | 2.8 | 5373 | 6153 | -2 | L1PA4 | L1 | LINE |
| 101040572 | 101041580 | + | 4.5 | 4361 | 5372 | -774 | L1PA4 | L1 | LINE |
| 101041581 | 101041982 | + | 7.2 | 878 | 1266 | 0 | MER11A | ERVK | LTR |

  

|  |  |  |  |  |  |  |  |  |  |
| --- | --- | --- | --- | --- | --- | --- | --- | --- | --- |
| chr1:101848804-101849838 | | | 1035 bp | | |  |  |  |  |
| Genome Start | Genome End | Strand | Div | Repeat Start | Repeat End | Repeat Left | Name | Family | Class |
| 101848804 | 101849067 | + | 14.5 | 5953 | 6224 | -88 | L1MA9 | L1 | LINE |
| 101849068 | 101849611 | - | 13.4 | 5614 | 6157 | -8 | L1PA15 | L1 | LINE |
| 101849612 | 101849748 | + | 10.9 | 5468 | 5605 | -560 | L1PA15 | L1 | LINE |
| 101849749 | 101849838 | + | 21.1 | 6221 | 6310 | -2 | L1MA9 | L1 | LINE |

  

|  |  |  |  |  |  |  |  |  |  |
| --- | --- | --- | --- | --- | --- | --- | --- | --- | --- |
| chr1:101929872-101933780 | | | 3837 bp | | |  |  |  |  |
| Genome Start | Genome End | Strand | Div | Repeat Start | Repeat End | Repeat Left | Name | Family | Class |
| 101929872 | 101929976 | - | 22.3 | 3274 | 3380 | -2833 | L1MEc | L1 | LINE |
| 101929979 | 101930293 | - | 7.0 | 5834 | 6154 | 0 | L1PA7 | L1 | LINE |
| 101930294 | 101931405 | + | 7.0 | 4701 | 5819 | -335 | L1PA7 | L1 | LINE |
| 101931402 | 101931863 | - | 26.0 | 2842 | 3282 | -2937 | L1MEc | L1 | LINE |
| 101931867 | 101932005 | - | 21.9 | 160 | 297 | -15 | AluJ | Alu | SINE |
| 101932018 | 101932089 | - | 20.8 | 3047 | 3118 | -3028 | L1M4 | L1 | LINE |
| 101932108 | 101932264 | - | 16.6 | 1 | 157 | -155 | AluJ | Alu | SINE |
| 101932289 | 101932409 | - | 29.4 | 2709 | 2832 | -3387 | L1MEc | L1 | LINE |
| 101932410 | 101932715 | + | 17.0 | 1 | 309 | -3 | AluSx | Alu | SINE |
| 101932716 | 101933536 | - | 27.5 | 2040 | 2708 | -3511 | L1MEc | L1 | LINE |
| 101933554 | 101933780 | - | 35.1 | 268 | 503 | -5706 | L1MEc | L1 | LINE |

  

|  |  |  |  |  |  |  |  |  |  |
| --- | --- | --- | --- | --- | --- | --- | --- | --- | --- |
| chr1:102579422-102584266 | | | 4844 bp | | |  |  |  |  |
| Genome Start | Genome End | Strand | Div | Repeat Start | Repeat End | Repeat Left | Name | Family | Class |
| 102579422 | 102579825 | + | 16.8 | 3709 | 4155 | -1988 | L1M2 | L1 | LINE |
| 102579827 | 102580702 | - | 3.3 | 3581 | 4454 | -1692 | L1PA3 | L1 | LINE |
| 102580697 | 102582413 | + | 2.2 | 4440 | 6155 | 0 | L1PA3 | L1 | LINE |
| 102582420 | 102582589 | + | 18.6 | 4148 | 4275 | -1868 | L1M2 | L1 | LINE |
| 102582590 | 102583464 | + | 20.8 | 1 | 901 | 0 | MER54A | ERVL | LTR |
| 102583465 | 102584266 | + | 18.6 | 4276 | 5122 | -1021 | L1M2 | L1 | LINE |

  

|  |  |  |  |  |  |  |  |  |  |
| --- | --- | --- | --- | --- | --- | --- | --- | --- | --- |
| chr1:102718767-102721007 | | | 2188 bp | | |  |  |  |  |
| Genome Start | Genome End | Strand | Div | Repeat Start | Repeat End | Repeat Left | Name | Family | Class |
| 102718767 | 102718997 | - | 19.7 | 139 | 381 | 0 | THE1D | MaLR | LTR |
| 102718998 | 102719865 | - | 5.0 | 4324 | 5191 | -955 | L1PA5 | L1 | LINE |
| 102719867 | 102720795 | + | 4.4 | 5214 | 6147 | -7 | L1PA5 | L1 | LINE |
| 102720848 | 102721007 | - | 16.5 | 1 | 162 | -219 | THE1D | MaLR | LTR |

  

|  |  |  |  |  |  |  |  |  |  |
| --- | --- | --- | --- | --- | --- | --- | --- | --- | --- |
| chr1:102750757-102754083 | | | 3295 bp | | |  |  |  |  |
| Genome Start | Genome End | Strand | Div | Repeat Start | Repeat End | Repeat Left | Name | Family | Class |
| 102750757 | 102750882 | + | 16.9 | 3545 | 3673 | -2470 | L1MA6 | L1 | LINE |
| 102750925 | 102751586 | + | 16.9 | 3674 | 4345 | -1798 | L1MA6 | L1 | LINE |
| 102751587 | 102751880 | + | 12.9 | 6 | 299 | -13 | AluSx | Alu | SINE |
| 102751881 | 102753162 | + | 17.1 | 4346 | 5674 | -626 | L1MA6 | L1 | LINE |
| 102753161 | 102753294 | - | 3.7 | 5835 | 5968 | -204 | L1PA8 | L1 | LINE |
| 102753295 | 102753486 | + | 8.5 | 5981 | 6169 | -3 | L1PA8 | L1 | LINE |
| 102753479 | 102754083 | + | 18.7 | 5657 | 6299 | -1 | L1MA6 | L1 | LINE |

  

|  |  |  |  |  |  |  |  |  |  |
| --- | --- | --- | --- | --- | --- | --- | --- | --- | --- |
| chr1:102787772-102799708 | | | 11401 bp | | |  |  |  |  |
| Genome Start | Genome End | Strand | Div | Repeat Start | Repeat End | Repeat Left | Name | Family | Class |
| 102787772 | 102788104 | - | 33.7 | 3074 | 3419 | 0 | L2 | L2 | LINE |
| 102788197 | 102788563 | - | 16.7 | 2 | 375 | 0 | THE1C | MaLR | LTR |
| 102788565 | 102789169 | - | 13.5 | 983 | 1580 | 0 | THE1B-int | MaLR | LTR |
| 102789158 | 102789449 | - | 18.1 | 432 | 718 | -862 | THE1B-int | MaLR | LTR |
| 102789451 | 102790528 | - | 8.0 | 5064 | 6149 | -5 | L1PA7 | L1 | LINE |
| 102790521 | 102790853 | + | 7.8 | 4723 | 5060 | -1086 | L1PA7 | L1 | LINE |
| 102790854 | 102791339 | - | 13.7 | 6 | 487 | -1150 | THE1B-int | MaLR | LTR |
| 102791345 | 102791711 | - | 15.3 | 1 | 364 | 0 | THE1B | MaLR | LTR |
| 102791712 | 102791774 | - | 30.2 | 2700 | 2762 | -657 | L2 | L2 | LINE |
| 102791775 | 102791934 | + | 18.8 | 1 | 169 | -814 | LTR1D | ERV1 | LTR |
| 102791935 | 102792149 | + | 8.4 | 1 | 221 | -1045 | MER11A | ERVK | LTR |
| 102792150 | 102792762 | + | 9.7 | 630 | 1266 | 0 | MER11A | ERVK | LTR |
| 102792763 | 102793457 | + | 21.7 | 169 | 887 | -96 | LTR1D | ERV1 | LTR |
| 102793484 | 102794366 | - | 34.7 | 1785 | 2704 | -715 | L2 | L2 | LINE |
| 102794367 | 102794669 | - | 13.9 | 1 | 307 | -4 | AluY | Alu | SINE |
| 102794670 | 102794924 | - | 34.7 | 1520 | 1784 | -1635 | L2 | L2 | LINE |
| 102794933 | 102795022 | - | 18.9 | 6069 | 6165 | -9 | L1MB5 | L1 | LINE |
| 102795090 | 102795428 | - | 11.3 | 1 | 347 | -106 | MLT2A2 | ERVL | LTR |
| 102795429 | 102796134 | - | 7.4 | 4926 | 5654 | 0 | HERVL-A2 | ERVL | LTR |
| 102796148 | 102797129 | - | 24.9 | 3974 | 5008 | -1138 | L1M4 | L1 | LINE |
| 102797402 | 102799015 | - | 27.6 | 174 | 1899 | -4747 | L1M4c | L1 | LINE |
| 102799058 | 102799112 | - | 18.2 | 20 | 75 | -6532 | L1MB4 | L1 | LINE |
| 102799142 | 102799708 | - | 31.9 | 922 | 1477 | -1942 | L2 | L2 | LINE |

  

|  |  |  |  |  |  |  |  |  |  |
| --- | --- | --- | --- | --- | --- | --- | --- | --- | --- |
| chr1:103007697-103010175 | | | 2292 bp | | |  |  |  |  |
| Genome Start | Genome End | Strand | Div | Repeat Start | Repeat End | Repeat Left | Name | Family | Class |
| 103007697 | 103008032 | + | 13.8 | 14 | 348 | -238 | LTR60 | ERV1 | LTR |
| 103008064 | 103008554 | - | 7.3 | 4463 | 4959 | -1187 | L1PB1 | L1 | LINE |
| 103008554 | 103009416 | + | 8.0 | 4949 | 5791 | -360 | L1PB1 | L1 | LINE |
| 103009574 | 103009919 | + | 12.1 | 5791 | 6148 | -3 | L1PB1 | L1 | LINE |
| 103009920 | 103010175 | + | 16.0 | 332 | 586 | 0 | LTR60 | ERV1 | LTR |

  

|  |  |  |  |  |  |  |  |  |  |
| --- | --- | --- | --- | --- | --- | --- | --- | --- | --- |
| chr1:103369873-103373397 | | | 3497 bp | | |  |  |  |  |
| Genome Start | Genome End | Strand | Div | Repeat Start | Repeat End | Repeat Left | Name | Family | Class |
| 103369873 | 103370534 | - | 11.2 | 5492 | 6141 | -10 | L1PB1 | L1 | LINE |
| 103370581 | 103371387 | - | 4.0 | 5344 | 6148 | -7 | L1PA4 | L1 | LINE |
| 103371375 | 103372820 | + | 5.4 | 3920 | 5368 | -778 | L1PA4 | L1 | LINE |
| 103372816 | 103373397 | - | 7.8 | 4909 | 5492 | -659 | L1PB1 | L1 | LINE |

  

|  |  |  |  |  |  |  |  |  |  |
| --- | --- | --- | --- | --- | --- | --- | --- | --- | --- |
| chr1:104684213-104686912 | | | 2610 bp | | |  |  |  |  |
| Genome Start | Genome End | Strand | Div | Repeat Start | Repeat End | Repeat Left | Name | Family | Class |
| 104684213 | 104684409 | - | 29.6 | 2469 | 2670 | -749 | L2 | L2 | LINE |
| 104684500 | 104685002 | - | 11.2 | 4366 | 4868 | -1278 | L1PA7 | L1 | LINE |
| 104684999 | 104686307 | + | 8.9 | 4869 | 6184 | 0 | L1PA7 | L1 | LINE |
| 104686312 | 104686912 | - | 28.7 | 1692 | 2302 | -1117 | L2 | L2 | LINE |

  

|  |  |  |  |  |  |  |  |  |  |
| --- | --- | --- | --- | --- | --- | --- | --- | --- | --- |
| chr1:104769774-104774850 | | | 4968 bp | | |  |  |  |  |
| Genome Start | Genome End | Strand | Div | Repeat Start | Repeat End | Repeat Left | Name | Family | Class |
| 104769774 | 104769904 | + | 19.7 | 2193 | 2322 | -3824 | L1MC1 | L1 | LINE |
| 104769905 | 104770421 | + | 11.5 | 1 | 453 | 0 | MLT2A2 | ERVL | LTR |
| 104770422 | 104771330 | + | 21.1 | 2323 | 3286 | -2860 | L1M5 | L1 | LINE |
| 104771346 | 104772193 | + | 18.1 | 4089 | 4952 | -1194 | L1MC1 | L1 | LINE |
| 104772289 | 104772538 | - | 8.8 | 1 | 250 | -60 | AluSg | Alu | SINE |
| 104772539 | 104772721 | + | 18.6 | 5703 | 5892 | -441 | L1MC1 | L1 | LINE |
| 104772719 | 104773373 | - | 8.4 | 4704 | 5357 | -789 | L1PA8A | L1 | LINE |
| 104773370 | 104774127 | + | 8.8 | 5363 | 6131 | 0 | L1PA8A | L1 | LINE |
| 104774134 | 104774533 | + | 20.0 | 5878 | 6316 | -17 | L1MC1 | L1 | LINE |
| 104774534 | 104774833 | + | 0.7 | 1 | 300 | -10 | AluYa5 | Alu | SINE |
| 104774834 | 104774850 | + | 20.0 | 6317 | 6333 | 0 | L1MC1 | L1 | LINE |

  

|  |  |  |  |  |  |  |  |  |  |
| --- | --- | --- | --- | --- | --- | --- | --- | --- | --- |
| chr1:105510866-105513472 | | | 2541 bp | | |  |  |  |  |
| Genome Start | Genome End | Strand | Div | Repeat Start | Repeat End | Repeat Left | Name | Family | Class |
| 105510866 | 105511683 | + | 12.7 | 4656 | 5475 | -676 | L1PB1 | L1 | LINE |
| 105511679 | 105511746 | - | 7.3 | 5117 | 5185 | -961 | L1PA7 | L1 | LINE |
| 105511745 | 105512680 | + | 7.6 | 5195 | 6154 | 0 | L1PA7 | L1 | LINE |
| 105512724 | 105513040 | + | 11.4 | 5460 | 5771 | -380 | L1PB1 | L1 | LINE |
| 105513071 | 105513472 | + | 11.4 | 5772 | 6150 | -1 | L1PB1 | L1 | LINE |

  

|  |  |  |  |  |  |  |  |  |  |
| --- | --- | --- | --- | --- | --- | --- | --- | --- | --- |
| chr1:105557916-105560760 | | | 2816 bp | | |  |  |  |  |
| Genome Start | Genome End | Strand | Div | Repeat Start | Repeat End | Repeat Left | Name | Family | Class |
| 105557916 | 105558047 | + | 5.3 | 1 | 132 | -179 | AluY | Alu | SINE |
| 105558051 | 105558299 | - | 2.4 | 3636 | 3884 | -2262 | L1PA2 | L1 | LINE |
| 105558292 | 105560556 | + | 1.8 | 3888 | 6150 | -5 | L1PA2 | L1 | LINE |
| 105560591 | 105560760 | + | 5.3 | 130 | 299 | -12 | AluY | Alu | SINE |

  

|  |  |  |  |  |  |  |  |  |  |
| --- | --- | --- | --- | --- | --- | --- | --- | --- | --- |
| chr1:106088713-106094784 | | | 5524 bp | | |  |  |  |  |
| Genome Start | Genome End | Strand | Div | Repeat Start | Repeat End | Repeat Left | Name | Family | Class |
| 106088713 | 106089032 | - | 36.1 | 2567 | 2894 | -3320 | L1MC4 | L1 | LINE |
| 106089053 | 106089292 | - | 26.8 | 2249 | 2487 | -3728 | L1MC4 | L1 | LINE |
| 106089294 | 106089878 | + | 17.8 | 5219 | 5780 | -376 | L1PB4 | L1 | LINE |
| 106089914 | 106090285 | + | 17.6 | 5781 | 6155 | -1 | L1PB4 | L1 | LINE |
| 106090307 | 106090373 | - | 14.9 | 2175 | 2243 | -3972 | L1MC4 | L1 | LINE |
| 106090381 | 106090419 | - | 29.2 | 509 | 553 | -1 | MLT2B2 | ERVL | LTR |
| 106090456 | 106090910 | - | 23.6 | 6 | 508 | -46 | MLT2B2 | ERVL | LTR |
| 106091027 | 106092398 | - | 10.1 | 4779 | 6158 | -14 | L1PA8 | L1 | LINE |
| 106092399 | 106093204 | + | 9.1 | 3949 | 4761 | -1385 | L1PA8 | L1 | LINE |
| 106093236 | 106093577 | + | 3.8 | 5810 | 6152 | -3 | L1PA4 | L1 | LINE |
| 106093757 | 106093961 | - | 28.4 | 1616 | 1830 | -4385 | L1MC4 | L1 | LINE |
| 106094064 | 106094784 | - | 28.7 | 888 | 1608 | -4607 | L1MC4 | L1 | LINE |

  

|  |  |  |  |  |  |  |  |  |  |
| --- | --- | --- | --- | --- | --- | --- | --- | --- | --- |
| chr1:106291512-106294062 | | | 2530 bp | | |  |  |  |  |
| Genome Start | Genome End | Strand | Div | Repeat Start | Repeat End | Repeat Left | Name | Family | Class |
| 106291512 | 106292009 | + | 7.0 | 1361 | 1858 | -417 | L1P2 | L1 | LINE |
| 106292035 | 106292798 | - | 2.6 | 5391 | 6155 | 0 | L1PA3 | L1 | LINE |
| 106292798 | 106293864 | + | 2.8 | 4334 | 5400 | -746 | L1PA3 | L1 | LINE |
| 106293862 | 106294062 | + | 8.5 | 1848 | 2047 | -228 | L1P2 | L1 | LINE |

  

|  |  |  |  |  |  |  |  |  |  |
| --- | --- | --- | --- | --- | --- | --- | --- | --- | --- |
| chr1:106532304-106535936 | | | 3643 bp | | |  |  |  |  |
| Genome Start | Genome End | Strand | Div | Repeat Start | Repeat End | Repeat Left | Name | Family | Class |
| 106532304 | 106532442 | + | 21.9 | 13 | 156 | -7409 | L1M1 | L1 | LINE |
| 106532443 | 106532843 | - | 8.5 | 5732 | 6131 | 0 | L1PA8A | L1 | LINE |
| 106532844 | 106532908 | + | 10.9 | 5022 | 5085 | -1061 | L1PA8A | L1 | LINE |
| 106532911 | 106533320 | + | 8.8 | 5266 | 5683 | -448 | L1PA8A | L1 | LINE |
| 106533321 | 106533625 | + | 7.2 | 1 | 305 | -6 | AluY | Alu | SINE |
| 106533626 | 106533676 | + | 8.8 | 5684 | 5733 | -398 | L1PA8A | L1 | LINE |
| 106533665 | 106535936 | + | 21.9 | 143 | 2730 | -4835 | L1M1 | L1 | LINE |

  

|  |  |  |  |  |  |  |  |  |  |
| --- | --- | --- | --- | --- | --- | --- | --- | --- | --- |
| chr1:107165249-107175568 | | | 9918 bp | | |  |  |  |  |
| Genome Start | Genome End | Strand | Div | Repeat Start | Repeat End | Repeat Left | Name | Family | Class |
| 107165249 | 107165361 | - | 29.2 | 4482 | 4597 | -1622 | L1MEc | L1 | LINE |
| 107165451 | 107167257 | - | 14.4 | 4481 | 6293 | -4 | L1MA5A | L1 | LINE |
| 107167258 | 107167434 | - | 11.0 | 135 | 303 | -9 | AluSx | Alu | SINE |
| 107167490 | 107167630 | - | 11.0 | 1 | 134 | -178 | AluSx | Alu | SINE |
| 107167631 | 107168469 | - | 16.5 | 3632 | 4480 | -1663 | L1MA5A | L1 | LINE |
| 107168474 | 107169903 | - | 27.1 | 2959 | 4441 | -1778 | L1MEc | L1 | LINE |
| 107169921 | 107170052 | - | 25.3 | 2551 | 2709 | -3510 | L1MEc | L1 | LINE |
| 107170046 | 107170164 | - | 27.5 | 1131 | 1254 | -5146 | L1MEc | L1 | LINE |
| 107170165 | 107170556 | - | 15.4 | 1 | 428 | 0 | MSTA | MaLR | LTR |
| 107170557 | 107170901 | - | 27.5 | 767 | 1130 | -5270 | L1MEc | L1 | LINE |
| 107170923 | 107170965 | - | 16.3 | 2427 | 2469 | -3674 | L1MA9 | L1 | LINE |
| 107170971 | 107171497 | + | 19.1 | 2461 | 2905 | -3235 | L1MA9 | L1 | LINE |
| 107171554 | 107171822 | - | 12.2 | 1 | 279 | -31 | AluSg | Alu | SINE |
| 107171823 | 107174029 | + | 19.1 | 2906 | 5292 | -848 | L1MA9 | L1 | LINE |
| 107174053 | 107174859 | + | 17.1 | 5293 | 6085 | -227 | L1MA9 | L1 | LINE |
| 107174879 | 107175051 | - | 26.9 | 585 | 766 | -5634 | L1MEc | L1 | LINE |
| 107175172 | 107175568 | - | 28.1 | 17 | 449 | -5951 | L1MEc | L1 | LINE |

  

|  |  |  |  |  |  |  |  |  |  |
| --- | --- | --- | --- | --- | --- | --- | --- | --- | --- |
| chr1:107994584-107995415 | | | 821 bp | | |  |  |  |  |
| Genome Start | Genome End | Strand | Div | Repeat Start | Repeat End | Repeat Left | Name | Family | Class |
| 107994584 | 107994669 | + | 13.9 | 1 | 87 | -278 | MLT1A0 | MaLR | LTR |
| 107994681 | 107995004 | - | 3.8 | 5856 | 6178 | 0 | L1PA3 | L1 | LINE |
| 107995005 | 107995143 | + | 0.7 | 5715 | 5854 | -301 | L1PA3 | L1 | LINE |
| 107995144 | 107995415 | + | 19.6 | 72 | 365 | 0 | MLT1A0 | MaLR | LTR |

  

|  |  |  |  |  |  |  |  |  |  |
| --- | --- | --- | --- | --- | --- | --- | --- | --- | --- |
| chr1:108060491-108064833 | | | 4306 bp | | |  |  |  |  |
| Genome Start | Genome End | Strand | Div | Repeat Start | Repeat End | Repeat Left | Name | Family | Class |
| 108060491 | 108060574 | + | 8.3 | 1 | 84 | -228 | AluSp | Alu | SINE |
| 108060575 | 108062186 | - | 7.6 | 2348 | 3956 | -2190 | L1PA6 | L1 | LINE |
| 108062185 | 108064367 | + | 6.7 | 3973 | 6154 | 0 | L1PA6 | L1 | LINE |
| 108064369 | 108064576 | + | 15.5 | 123 | 331 | 0 | AluJb | Alu | SINE |
| 108064615 | 108064833 | + | 11.0 | 74 | 293 | -20 | AluSp | Alu | SINE |

  

|  |  |  |  |  |  |  |  |  |  |
| --- | --- | --- | --- | --- | --- | --- | --- | --- | --- |
| chr1:108629696-108638004 | | | 7868 bp | | |  |  |  |  |
| Genome Start | Genome End | Strand | Div | Repeat Start | Repeat End | Repeat Left | Name | Family | Class |
| 108629696 | 108630590 | - | 32.7 | 4696 | 5603 | -637 | L1M5 | L1 | LINE |
| 108630621 | 108631868 | - | 7.3 | 4892 | 6170 | 0 | L1PA7 | L1 | LINE |
| 108631867 | 108633682 | + | 8.5 | 3079 | 4896 | -1250 | L1PA7 | L1 | LINE |
| 108633825 | 108634179 | - | 21.1 | 1 | 358 | -7 | MLT1A0 | MaLR | LTR |
| 108634181 | 108635641 | - | 24.4 | 5 | 1370 | -1 | MLT1A0-int | MaLR | LTR |
| 108635646 | 108635974 | - | 23.7 | 1 | 374 | 0 | MLT1A0 | MaLR | LTR |
| 108635975 | 108636569 | - | 33.0 | 3979 | 4589 | -1557 | L1M5 | L1 | LINE |
| 108636588 | 108636795 | - | 23.9 | 5956 | 6165 | -13 | L1MB8 | L1 | LINE |
| 108636883 | 108637038 | + | 22.6 | 2 | 161 | -101 | MIR | MIR | SINE |
| 108637042 | 108637100 | + | 15.2 | 5 | 63 | -17 | MADE2 | Mariner | DNA |
| 108637092 | 108637181 | + | 17.8 | 264 | 354 | -74 | MSTC | MaLR | LTR |
| 108637154 | 108637209 | + | 18.9 | 455 | 514 | -40 | MLT2B3 | ERVL | LTR |
| 108637283 | 108637321 | + | 18.9 | 515 | 553 | -1 | MLT2B3 | ERVL | LTR |
| 108637444 | 108638004 | - | 31.6 | 2405 | 2966 | -3180 | L1M5 | L1 | LINE |

  

|  |  |  |  |  |  |  |  |  |  |
| --- | --- | --- | --- | --- | --- | --- | --- | --- | --- |
| chr1:108742704-108752973 | | | 9327 bp | | |  |  |  |  |
| Genome Start | Genome End | Strand | Div | Repeat Start | Repeat End | Repeat Left | Name | Family | Class |
| 108742704 | 108742786 | + | 21.7 | 1296 | 1387 | -5246 | L1MDa | L1 | LINE |
| 108742810 | 108743041 | + | 28.9 | 1429 | 1704 | -4834 | L1MDa | L1 | LINE |
| 108743191 | 108743484 | - | 21.3 | 1 | 305 | -7 | AluJb | Alu | SINE |
| 108743570 | 108743868 | + | 20.4 | 1 | 239 | 0 | MER8 | MER2\_type | DNA |
| 108743904 | 108743979 | + | 36.6 | 1872 | 1952 | -4605 | L1MDa | L1 | LINE |
| 108743980 | 108744287 | + | 8.1 | 1 | 308 | -3 | AluY | Alu | SINE |
| 108744288 | 108744454 | + | 36.6 | 1953 | 2119 | -4438 | L1MDa | L1 | LINE |
| 108744665 | 108745225 | + | 31.6 | 2796 | 3357 | -3180 | L1MDa | L1 | LINE |
| 108745348 | 108745386 | - | 18.9 | 516 | 554 | 0 | MLT2B3 | ERVL | LTR |
| 108745460 | 108745515 | - | 18.9 | 456 | 515 | -39 | MLT2B3 | ERVL | LTR |
| 108745488 | 108745577 | - | 17.8 | 264 | 354 | -74 | MSTC | MaLR | LTR |
| 108745569 | 108745627 | - | 15.2 | 5 | 63 | -17 | MADE2 | Mariner | DNA |
| 108745631 | 108745786 | - | 22.6 | 2 | 161 | -101 | MIR | MIR | SINE |
| 108745874 | 108746081 | + | 23.9 | 5956 | 6165 | -13 | L1MB8 | L1 | LINE |
| 108746100 | 108746694 | + | 33.0 | 4370 | 4980 | -1557 | L1MDa | L1 | LINE |
| 108746695 | 108747023 | + | 23.7 | 1 | 374 | 0 | MLT1A | MaLR | LTR |
| 108747028 | 108748488 | + | 24.4 | 5 | 1370 | -1 | MLT1A-int | MaLR | LTR |
| 108748490 | 108748844 | + | 21.1 | 1 | 358 | -7 | MLT1A0 | MaLR | LTR |
| 108748987 | 108750802 | - | 8.5 | 3079 | 4896 | -1250 | L1PA7 | L1 | LINE |
| 108750801 | 108752048 | + | 7.3 | 4892 | 6170 | 0 | L1PA7 | L1 | LINE |
| 108752079 | 108752973 | + | 32.7 | 5087 | 5994 | -637 | L1MDa | L1 | LINE |

  

|  |  |  |  |  |  |  |  |  |  |
| --- | --- | --- | --- | --- | --- | --- | --- | --- | --- |
| chr1:111149620-111156167 | | | 6432 bp | | |  |  |  |  |
| Genome Start | Genome End | Strand | Div | Repeat Start | Repeat End | Repeat Left | Name | Family | Class |
| 111149620 | 111151312 | + | 18.3 | 2913 | 4604 | -2958 | L1MA9 | L1 | LINE |
| 111151309 | 111152038 | - | 2.2 | 4219 | 4949 | -1197 | L1PA3 | L1 | LINE |
| 111152039 | 111153238 | + | 2.3 | 4954 | 6154 | -1 | L1PA3 | L1 | LINE |
| 111153226 | 111153846 | + | 14.3 | 4584 | 5132 | -2430 | L1MA9 | L1 | LINE |
| 111153942 | 111154089 | + | 7.4 | 401 | 561 | 0 | MLT2A2 | ERVL | LTR |
| 111154095 | 111154891 | + | 18.8 | 5764 | 6563 | -996 | L1MA9 | L1 | LINE |
| 111154892 | 111155020 | + | 18.3 | 2 | 127 | -6 | FLAM\_A | Alu | SINE |
| 111155021 | 111155633 | + | 19.5 | 6564 | 7207 | -524 | L1MA9 | L1 | LINE |
| 111155667 | 111156167 | + | 20.1 | 7208 | 7693 | -38 | L1MA9 | L1 | LINE |

  

|  |  |  |  |  |  |  |  |  |  |
| --- | --- | --- | --- | --- | --- | --- | --- | --- | --- |
| chr1:111272618-111287482 | | | 14622 bp | | |  |  |  |  |
| Genome Start | Genome End | Strand | Div | Repeat Start | Repeat End | Repeat Left | Name | Family | Class |
| 111272618 | 111273114 | - | 29.1 | 2804 | 3357 | -62 | L2 | L2 | LINE |
| 111273125 | 111273233 | - | 24.1 | 7878 | 7987 | -8 | L1MD3 | L1 | LINE |
| 111273246 | 111273827 | - | 8.2 | 5230 | 5819 | -341 | L1PREC2 | L1 | LINE |
| 111273828 | 111274162 | + | 11.0 | 5814 | 6153 | -7 | L1PREC2 | L1 | LINE |
| 111274173 | 111274448 | - | 22.6 | 7580 | 7853 | -142 | L1MD3 | L1 | LINE |
| 111274457 | 111274863 | - | 23.4 | 1 | 369 | -5 | MLT1A | MaLR | LTR |
| 111274866 | 111274956 | - | 26.1 | 7314 | 7404 | -404 | L1MC4a | L1 | LINE |
| 111275134 | 111276773 | - | 6.6 | 4508 | 6152 | -2 | L1PA6 | L1 | LINE |
| 111276768 | 111277405 | - | 22.7 | 6690 | 7318 | -490 | L1MC4a | L1 | LINE |
| 111277407 | 111283253 | + | 13.3 | 17 | 5976 | -1589 | L1MA2 | L1 | LINE |
| 111283254 | 111283543 | + | 4.5 | 13 | 302 | -9 | AluY | Alu | SINE |
| 111283544 | 111285275 | + | 9.7 | 5977 | 7723 | 0 | L1MA2 | L1 | LINE |
| 111285304 | 111286609 | - | 22.7 | 5281 | 6704 | -1104 | L1MC4a | L1 | LINE |
| 111286611 | 111287482 | - | 26.5 | 1868 | 2803 | -616 | L2 | L2 | LINE |

  

|  |  |  |  |  |  |  |  |  |  |
| --- | --- | --- | --- | --- | --- | --- | --- | --- | --- |
| chr1:115207031-115208556 | | | 1514 bp | | |  |  |  |  |
| Genome Start | Genome End | Strand | Div | Repeat Start | Repeat End | Repeat Left | Name | Family | Class |
| 115207031 | 115207123 | - | 19.8 | 3314 | 3419 | 0 | L2 | L2 | LINE |
| 115207134 | 115207416 | - | 5.7 | 5526 | 5801 | -371 | L1P3 | L1 | LINE |
| 115207417 | 115207706 | + | 9.0 | 1 | 290 | -19 | AluSc | Alu | SINE |
| 115207707 | 115207937 | - | 5.7 | 5294 | 5525 | -647 | L1P3 | L1 | LINE |
| 115207940 | 115208286 | + | 4.0 | 5816 | 6169 | -3 | L1PA8 | L1 | LINE |
| 115208287 | 115208556 | - | 27.2 | 2986 | 3311 | -108 | L2 | L2 | LINE |

  

|  |  |  |  |  |  |  |  |  |  |
| --- | --- | --- | --- | --- | --- | --- | --- | --- | --- |
| chr1:115943183-115947913 | | | 4625 bp | | |  |  |  |  |
| Genome Start | Genome End | Strand | Div | Repeat Start | Repeat End | Repeat Left | Name | Family | Class |
| 115943183 | 115943297 | + | 25.5 | 1329 | 1461 | -5172 | L1MDa | L1 | LINE |
| 115943298 | 115943603 | + | 19.3 | 1 | 306 | -6 | AluJo | Alu | SINE |
| 115943604 | 115944390 | + | 25.5 | 1462 | 2314 | -4319 | L1MDa | L1 | LINE |
| 115944454 | 115944487 | + | 21.4 | 2531 | 2559 | -3978 | L1MDa | L1 | LINE |
| 115944528 | 115944645 | - | 16.9 | 1 | 118 | -15 | FLAM\_C | Alu | SINE |
| 115944646 | 115944703 | + | 21.4 | 2560 | 2602 | -3935 | L1MDa | L1 | LINE |
| 115944704 | 115944994 | + | 19.2 | 1 | 291 | -21 | AluJb | Alu | SINE |
| 115944995 | 115945389 | + | 21.4 | 2603 | 2998 | -3539 | L1MDa | L1 | LINE |
| 115945390 | 115945702 | + | 11.8 | 1 | 313 | 0 | AluSq | Alu | SINE |
| 115945703 | 115946030 | + | 21.4 | 2999 | 3313 | -3224 | L1MDa | L1 | LINE |
| 115946027 | 115946954 | - | 1.5 | 4734 | 5662 | -493 | L1PA3 | L1 | LINE |
| 115946955 | 115947481 | + | 2.5 | 5662 | 6152 | -3 | L1PA3 | L1 | LINE |
| 115947489 | 115947913 | + | 22.3 | 3301 | 3872 | -2665 | L1MDa | L1 | LINE |

  

|  |  |  |  |  |  |  |  |  |  |
| --- | --- | --- | --- | --- | --- | --- | --- | --- | --- |
| chr1:117818620-117824949 | | | 6189 bp | | |  |  |  |  |
| Genome Start | Genome End | Strand | Div | Repeat Start | Repeat End | Repeat Left | Name | Family | Class |
| 117818620 | 117818821 | - | 29.5 | 786 | 996 | -3858 | L1MEe | L1 | LINE |
| 117818833 | 117819494 | - | 21.8 | 5530 | 6234 | -5 | L1ME1 | L1 | LINE |
| 117819516 | 117821197 | - | 7.0 | 4458 | 6152 | -2 | L1PA7 | L1 | LINE |
| 117821191 | 117822255 | + | 8.4 | 3399 | 4466 | -1680 | L1PA7 | L1 | LINE |
| 117822254 | 117823049 | - | 24.6 | 4728 | 5545 | -694 | L1ME1 | L1 | LINE |
| 117823061 | 117823854 | - | 8.6 | 5390 | 6174 | 0 | L1PA11 | L1 | LINE |
| 117823875 | 117824682 | - | 23.3 | 42 | 910 | -5490 | L1ME1 | L1 | LINE |
| 117824770 | 117824949 | - | 29.1 | 548 | 720 | -4134 | L1MEe | L1 | LINE |

  

|  |  |  |  |  |  |  |  |  |  |
| --- | --- | --- | --- | --- | --- | --- | --- | --- | --- |
| chr1:118779574-118783701 | | | 3953 bp | | |  |  |  |  |
| Genome Start | Genome End | Strand | Div | Repeat Start | Repeat End | Repeat Left | Name | Family | Class |
| 118779574 | 118780079 | - | 20.8 | 2998 | 3508 | -3036 | L1MEa | L1 | LINE |
| 118780091 | 118780277 | - | 26.2 | 5 | 204 | -11 | MER58C | MER1\_type | DNA |
| 118780441 | 118782144 | - | 24.3 | 668 | 2558 | -4082 | L1MEa | L1 | LINE |
| 118782150 | 118782390 | - | 5.0 | 5914 | 6154 | 0 | L1PA5 | L1 | LINE |
| 118782391 | 118783066 | + | 4.2 | 5225 | 5900 | -255 | L1PA5 | L1 | LINE |
| 118783063 | 118783701 | - | 28.6 | 41 | 681 | -5866 | L1MEa | L1 | LINE |

  

|  |  |  |  |  |  |  |  |  |  |
| --- | --- | --- | --- | --- | --- | --- | --- | --- | --- |
| chr1:119022301-119025637 | | | 3336 bp | | |  |  |  |  |
| Genome Start | Genome End | Strand | Div | Repeat Start | Repeat End | Repeat Left | Name | Family | Class |
| 119022301 | 119022598 | + | 28.0 | 2280 | 2601 | -818 | L2 | L2 | LINE |
| 119022600 | 119024478 | - | 5.2 | 4281 | 6154 | 0 | L1PA5 | L1 | LINE |
| 119024478 | 119024936 | + | 5.5 | 3815 | 4275 | -1871 | L1PA5 | L1 | LINE |
| 119024938 | 119025637 | + | 28.3 | 2593 | 3403 | -16 | L2 | L2 | LINE |

  

|  |  |  |  |  |  |  |  |  |  |
| --- | --- | --- | --- | --- | --- | --- | --- | --- | --- |
| chr1:119064388-119073930 | | | 9196 bp | | |  |  |  |  |
| Genome Start | Genome End | Strand | Div | Repeat Start | Repeat End | Repeat Left | Name | Family | Class |
| 119064388 | 119064676 | + | 31.4 | 1 | 286 | -5855 | L1MB1 | L1 | LINE |
| 119065004 | 119065197 | + | 25.5 | 943 | 1134 | -5001 | L1MB1 | L1 | LINE |
| 119065197 | 119067071 | + | 3.6 | 4273 | 6147 | -8 | L1PA4 | L1 | LINE |
| 119067088 | 119067861 | + | 17.5 | 2863 | 3662 | -2542 | L1MB1 | L1 | LINE |
| 119067862 | 119068163 | + | 8.3 | 1 | 302 | -9 | AluY | Alu | SINE |
| 119068164 | 119068653 | + | 17.5 | 3663 | 4120 | -2084 | L1MB1 | L1 | LINE |
| 119068654 | 119069007 | - | 10.8 | 1 | 355 | 0 | THE1A | MaLR | LTR |
| 119069008 | 119069232 | + | 17.5 | 4121 | 4344 | -1860 | L1MB1 | L1 | LINE |
| 119069229 | 119069841 | - | 9.0 | 3661 | 4273 | -1873 | L1PA8 | L1 | LINE |
| 119069846 | 119071730 | + | 8.0 | 4287 | 6170 | -2 | L1PA8 | L1 | LINE |
| 119071736 | 119073484 | + | 17.5 | 4330 | 6084 | -169 | L1MB1 | L1 | LINE |
| 119073485 | 119073839 | + | 20.7 | 1 | 365 | 0 | MLT1A0 | MaLR | LTR |
| 119073840 | 119073930 | + | 19.0 | 6085 | 6174 | -79 | L1MB1 | L1 | LINE |

  

|  |  |  |  |  |  |  |  |  |  |
| --- | --- | --- | --- | --- | --- | --- | --- | --- | --- |
| chr1:144484896-144488570 | | | 3606 bp | | |  |  |  |  |
| Genome Start | Genome End | Strand | Div | Repeat Start | Repeat End | Repeat Left | Name | Family | Class |
| 144484896 | 144484939 | + | 11.6 | 5439 | 5481 | -703 | L1MB8 | L1 | LINE |
| 144484951 | 144486305 | - | 3.0 | 4801 | 6152 | -3 | L1PA3 | L1 | LINE |
| 144486301 | 144486802 | + | 3.0 | 4308 | 4811 | -1335 | L1PA3 | L1 | LINE |
| 144486806 | 144486932 | + | 18.0 | 5479 | 5607 | -571 | L1MB8 | L1 | LINE |
| 144486933 | 144487240 | + | 10.7 | 1 | 306 | 0 | AluSg | Alu | SINE |
| 144487241 | 144487340 | + | 18.0 | 5608 | 5694 | -484 | L1MB8 | L1 | LINE |
| 144487341 | 144487639 | + | 11.7 | 1 | 300 | -12 | AluSx | Alu | SINE |
| 144487640 | 144487782 | + | 18.0 | 5695 | 5818 | -360 | L1MB8 | L1 | LINE |
| 144487783 | 144488094 | - | 17.7 | 1 | 292 | -20 | AluJb | Alu | SINE |
| 144488095 | 144488153 | + | 18.0 | 5819 | 5876 | -302 | L1MB8 | L1 | LINE |
| 144488170 | 144488211 | + | 11.9 | 86 | 128 | -5 | FLAM | Alu | SINE |
| 144488256 | 144488570 | + | 17.4 | 5865 | 6173 | -5 | L1MB8 | L1 | LINE |

  

|  |  |  |  |  |  |  |  |  |  |
| --- | --- | --- | --- | --- | --- | --- | --- | --- | --- |
| chr1:144622730-144626275 | | | 3444 bp | | |  |  |  |  |
| Genome Start | Genome End | Strand | Div | Repeat Start | Repeat End | Repeat Left | Name | Family | Class |
| 144622730 | 144622908 | - | 17.6 | 5865 | 6034 | -150 | L1MB7 | L1 | LINE |
| 144622955 | 144622993 | - | 12.8 | 86 | 125 | -177 | Alu | Alu | SINE |
| 144623010 | 144623068 | - | 17.8 | 5819 | 5876 | -302 | L1MB7 | L1 | LINE |
| 144623069 | 144623201 | + | 17.1 | 1 | 130 | -182 | AluJb | Alu | SINE |
| 144623233 | 144623392 | + | 17.1 | 131 | 292 | -20 | AluJb | Alu | SINE |
| 144623393 | 144623535 | - | 17.8 | 5694 | 5818 | -360 | L1MB7 | L1 | LINE |
| 144623536 | 144623834 | - | 10.4 | 1 | 300 | -12 | AluSx | Alu | SINE |
| 144623835 | 144623934 | - | 17.8 | 5609 | 5693 | -485 | L1MB7 | L1 | LINE |
| 144623935 | 144624242 | - | 11.4 | 1 | 306 | 0 | AluSg | Alu | SINE |
| 144624243 | 144624369 | - | 17.8 | 5482 | 5608 | -570 | L1MB7 | L1 | LINE |
| 144624373 | 144624874 | - | 3.4 | 4308 | 4811 | -1335 | L1PA3 | L1 | LINE |
| 144624870 | 144626220 | + | 3.1 | 4801 | 6152 | -3 | L1PA3 | L1 | LINE |
| 144626232 | 144626275 | - | 11.6 | 5439 | 5481 | -703 | L1MB7 | L1 | LINE |

  

|  |  |  |  |  |  |  |  |  |  |
| --- | --- | --- | --- | --- | --- | --- | --- | --- | --- |
| chr1:145919398-145923248 | | | 3758 bp | | |  |  |  |  |
| Genome Start | Genome End | Strand | Div | Repeat Start | Repeat End | Repeat Left | Name | Family | Class |
| 145919398 | 145919546 | - | 16.7 | 6029 | 6173 | -5 | L1MB8 | L1 | LINE |
| 145919547 | 145919702 | + | 0.0 | 154 | 309 | -1 | AluYa5 | Alu | SINE |
| 145919703 | 145919881 | - | 16.7 | 5865 | 6028 | -150 | L1MB8 | L1 | LINE |
| 145919928 | 145919966 | - | 12.8 | 86 | 125 | -8 | FLAM | Alu | SINE |
| 145919983 | 145920041 | - | 17.8 | 5819 | 5876 | -302 | L1MB8 | L1 | LINE |
| 145920042 | 145920174 | + | 17.1 | 1 | 130 | -182 | AluJb | Alu | SINE |
| 145920206 | 145920365 | + | 17.1 | 131 | 292 | -20 | AluJb | Alu | SINE |
| 145920366 | 145920508 | - | 17.8 | 5694 | 5818 | -360 | L1MB8 | L1 | LINE |
| 145920509 | 145920807 | - | 10.4 | 1 | 300 | -12 | AluSx | Alu | SINE |
| 145920808 | 145920907 | - | 17.8 | 5609 | 5693 | -485 | L1MB8 | L1 | LINE |
| 145920908 | 145921215 | - | 11.4 | 1 | 306 | 0 | AluSg | Alu | SINE |
| 145921216 | 145921342 | - | 17.8 | 5482 | 5608 | -570 | L1MB8 | L1 | LINE |
| 145921340 | 145921847 | - | 3.9 | 4308 | 4817 | -1329 | L1PA3 | L1 | LINE |
| 145921843 | 145923196 | + | 3.1 | 4801 | 6155 | 0 | L1PA3 | L1 | LINE |
| 145923205 | 145923248 | - | 11.6 | 5439 | 5481 | -703 | L1MB8 | L1 | LINE |

  

|  |  |  |  |  |  |  |  |  |  |
| --- | --- | --- | --- | --- | --- | --- | --- | --- | --- |
| chr1:151037989-151040352 | | | 2310 bp | | |  |  |  |  |
| Genome Start | Genome End | Strand | Div | Repeat Start | Repeat End | Repeat Left | Name | Family | Class |
| 151037989 | 151038090 | - | 22.0 | 6061 | 6162 | -4 | L1ME1 | L1 | LINE |
| 151038154 | 151038376 | - | 17.6 | 4614 | 4846 | -1297 | L1M2 | L1 | LINE |
| 151038377 | 151038460 | - | 9.5 | 478 | 561 | 0 | MLT2A2 | ERVL | LTR |
| 151038461 | 151039142 | - | 17.6 | 3900 | 4613 | -1530 | L1M2 | L1 | LINE |
| 151039138 | 151039779 | + | 15.3 | 3226 | 3878 | -2265 | L1M2 | L1 | LINE |
| 151039776 | 151040352 | - | 17.9 | 5237 | 5831 | -481 | L1ME1 | L1 | LINE |

  

|  |  |  |  |  |  |  |  |  |  |
| --- | --- | --- | --- | --- | --- | --- | --- | --- | --- |
| chr1:151175540-151180298 | | | 4331 bp | | |  |  |  |  |
| Genome Start | Genome End | Strand | Div | Repeat Start | Repeat End | Repeat Left | Name | Family | Class |
| 151175540 | 151175730 | - | 33.5 | 2803 | 3012 | -407 | L2 | L2 | LINE |
| 151175731 | 151175809 | - | 19.0 | 7960 | 8042 | 0 | L1MC4 | L1 | LINE |
| 151175967 | 151178081 | - | 2.8 | 4061 | 6155 | 0 | L1PA3 | L1 | LINE |
| 151178069 | 151179615 | + | 3.5 | 2501 | 4080 | -2066 | L1PA3 | L1 | LINE |
| 151179624 | 151179722 | - | 14.1 | 7803 | 7902 | -140 | L1MC4 | L1 | LINE |
| 151179731 | 151179876 | - | 36.6 | 2665 | 2810 | -609 | L2 | L2 | LINE |
| 151180145 | 151180298 | - | 31.7 | 2279 | 2420 | -999 | L2 | L2 | LINE |

  

|  |  |  |  |  |  |  |  |  |  |
| --- | --- | --- | --- | --- | --- | --- | --- | --- | --- |
| chr1:156672271-156677640 | | | 4851 bp | | |  |  |  |  |
| Genome Start | Genome End | Strand | Div | Repeat Start | Repeat End | Repeat Left | Name | Family | Class |
| 156672271 | 156672748 | - | 27.0 | 5611 | 6161 | -9 | L1ME3A | L1 | LINE |
| 156672767 | 156672957 | + | 29.4 | 23 | 223 | -326 | MLT1H | MaLR | LTR |
| 156673169 | 156673329 | - | 16.2 | 5998 | 6161 | -10 | L1MB2 | L1 | LINE |
| 156673447 | 156673828 | - | 26.5 | 5167 | 5586 | -578 | L1ME3A | L1 | LINE |
| 156673827 | 156673883 | - | 12.3 | 5897 | 5953 | -203 | L1PB4 | L1 | LINE |
| 156673942 | 156674099 | - | 21.4 | 4235 | 4378 | -1768 | L1ME3A | L1 | LINE |
| 156674128 | 156675250 | - | 17.7 | 5163 | 6302 | -10 | L1MA9 | L1 | LINE |
| 156675253 | 156675606 | + | 19.2 | 3952 | 4317 | -1823 | L1M3 | L1 | LINE |
| 156675600 | 156676149 | + | 15.5 | 5661 | 6164 | -2 | L1PA16 | L1 | LINE |
| 156676154 | 156677011 | + | 18.0 | 4310 | 5184 | -956 | L1M3 | L1 | LINE |
| 156677012 | 156677325 | + | 12.5 | 1 | 313 | 0 | AluSq | Alu | SINE |
| 156677416 | 156677640 | - | 30.9 | 3890 | 4127 | -2019 | L1ME3A | L1 | LINE |

  

|  |  |  |  |  |  |  |  |  |  |
| --- | --- | --- | --- | --- | --- | --- | --- | --- | --- |
| chr1:157527990-157531114 | | | 2875 bp | | |  |  |  |  |
| Genome Start | Genome End | Strand | Div | Repeat Start | Repeat End | Repeat Left | Name | Family | Class |
| 157527990 | 157528974 | + | 35.0 | 1675 | 2713 | -706 | L2 | L2 | LINE |
| 157529104 | 157529528 | + | 15.4 | 3 | 428 | 0 | MSTC | MaLR | LTR |
| 157529621 | 157529789 | + | 33.3 | 2828 | 2998 | -421 | L2 | L2 | LINE |
| 157529810 | 157530220 | - | 3.2 | 5742 | 6152 | -3 | L1PA3 | L1 | LINE |
| 157530221 | 157530728 | + | 1.3 | 5231 | 5737 | -418 | L1PA3 | L1 | LINE |
| 157530738 | 157531114 | + | 34.3 | 3003 | 3419 | 0 | L2 | L2 | LINE |

  

|  |  |  |  |  |  |  |  |  |  |
| --- | --- | --- | --- | --- | --- | --- | --- | --- | --- |
| chr1:157625748-157628691 | | | 2963 bp | | |  |  |  |  |
| Genome Start | Genome End | Strand | Div | Repeat Start | Repeat End | Repeat Left | Name | Family | Class |
| 157625748 | 157626065 | + | 26.7 | 3080 | 3393 | -2753 | L1M5 | L1 | LINE |
| 157626066 | 157627176 | - | 4.5 | 4203 | 5313 | -833 | L1PA4 | L1 | LINE |
| 157627163 | 157628004 | + | 3.2 | 5313 | 6155 | 0 | L1PA4 | L1 | LINE |
| 157628000 | 157628691 | + | 30.0 | 3379 | 4092 | -2054 | L1M5 | L1 | LINE |

  

|  |  |  |  |  |  |  |  |  |  |
| --- | --- | --- | --- | --- | --- | --- | --- | --- | --- |
| chr1:157890485-157894033 | | | 3102 bp | | |  |  |  |  |
| Genome Start | Genome End | Strand | Div | Repeat Start | Repeat End | Repeat Left | Name | Family | Class |
| 157890485 | 157890963 | + | 25.5 | 606 | 1098 | -3736 | L1MEe | L1 | LINE |
| 157891310 | 157891406 | + | 26.6 | 1410 | 1507 | -3324 | L1MEe | L1 | LINE |
| 157891449 | 157891563 | - | 9.6 | 6017 | 6132 | -159 | L1MA8 | L1 | LINE |
| 157891565 | 157891731 | + | 14.4 | 6113 | 6290 | -1 | L1MA8 | L1 | LINE |
| 157891751 | 157892324 | + | 35.1 | 1609 | 2239 | -2595 | L1MEe | L1 | LINE |
| 157892327 | 157892624 | - | 12.1 | 1 | 299 | -13 | AluSx | Alu | SINE |
| 157892625 | 157893420 | + | 32.2 | 2242 | 3098 | -1736 | L1MEe | L1 | LINE |
| 157893458 | 157894033 | + | 29.6 | 3413 | 4019 | -815 | L1MEe | L1 | LINE |

  

|  |  |  |  |  |  |  |  |  |  |
| --- | --- | --- | --- | --- | --- | --- | --- | --- | --- |
| chr1:157917205-157917574 | | | 365 bp | | |  |  |  |  |
| Genome Start | Genome End | Strand | Div | Repeat Start | Repeat End | Repeat Left | Name | Family | Class |
| 157917205 | 157917374 | - | 18.2 | 30 | 208 | -1 | MER3 | MER1\_type | DNA |
| 157917375 | 157917426 | - | 1.9 | 5991 | 6042 | -113 | L1PA5 | L1 | LINE |
| 157917427 | 157917526 | + | 5.0 | 6054 | 6153 | -1 | L1PA5 | L1 | LINE |
| 157917532 | 157917574 | - | 18.6 | 1 | 43 | -166 | MER3 | MER1\_type | DNA |

  

|  |  |  |  |  |  |  |  |  |  |
| --- | --- | --- | --- | --- | --- | --- | --- | --- | --- |
| chr1:160973246-160976417 | | | 3064 bp | | |  |  |  |  |
| Genome Start | Genome End | Strand | Div | Repeat Start | Repeat End | Repeat Left | Name | Family | Class |
| 160973246 | 160973353 | - | 22.6 | 2935 | 3056 | -363 | L2 | L2 | LINE |
| 160973369 | 160973412 | - | 9.1 | 5689 | 5738 | -428 | L1P4 | L1 | LINE |
| 160973418 | 160973483 | - | 9.2 | 5769 | 5834 | -297 | L1PA8A | L1 | LINE |
| 160973574 | 160974393 | - | 1.5 | 5334 | 6153 | -2 | L1PA2 | L1 | LINE |
| 160974391 | 160975732 | + | 0.6 | 4010 | 5351 | -795 | L1PA2 | L1 | LINE |
| 160975734 | 160976020 | - | 11.5 | 1 | 295 | -17 | AluSx | Alu | SINE |
| 160976021 | 160976093 | - | 9.6 | 5708 | 5780 | -386 | L1P4 | L1 | LINE |
| 160976094 | 160976417 | - | 30.2 | 2475 | 2841 | -578 | L2 | L2 | LINE |

  

|  |  |  |  |  |  |  |  |  |  |
| --- | --- | --- | --- | --- | --- | --- | --- | --- | --- |
| chr1:162352194-162362933 | | | 10738 bp | | |  |  |  |  |
| Genome Start | Genome End | Strand | Div | Repeat Start | Repeat End | Repeat Left | Name | Family | Class |
| 162352194 | 162352866 | - | 6.5 | 5476 | 6152 | -2 | L1PA5 | L1 | LINE |
| 162352875 | 162354011 | - | 4.5 | 5016 | 6152 | -3 | L1PA4 | L1 | LINE |
| 162354007 | 162357492 | + | 5.8 | 1505 | 5005 | -1141 | L1PA4 | L1 | LINE |
| 162357492 | 162362933 | - | 7.2 | 4 | 5490 | -664 | L1PA5 | L1 | LINE |

  

|  |  |  |  |  |  |  |  |  |  |
| --- | --- | --- | --- | --- | --- | --- | --- | --- | --- |
| chr1:164732417-164740877 | | | 8476 bp | | |  |  |  |  |
| Genome Start | Genome End | Strand | Div | Repeat Start | Repeat End | Repeat Left | Name | Family | Class |
| 164732417 | 164733677 | + | 14.9 | 3395 | 4668 | -1475 | L1MA6 | L1 | LINE |
| 164733678 | 164733866 | - | 14.8 | 230 | 428 | 0 | MSTA | MaLR | LTR |
| 164733868 | 164734306 | + | 13.1 | 5211 | 5650 | -650 | L1MA6 | L1 | LINE |
| 164734307 | 164734658 | + | 13.7 | 1 | 352 | -12 | THE1B | MaLR | LTR |
| 164734659 | 164734764 | + | 9.4 | 5654 | 5761 | -530 | L1MA6 | L1 | LINE |
| 164734764 | 164735832 | + | 7.7 | 4455 | 5503 | -669 | L1PA8 | L1 | LINE |
| 164735812 | 164737199 | - | 5.0 | 2281 | 3663 | -2483 | L1PA4 | L1 | LINE |
| 164737203 | 164739647 | + | 4.8 | 3674 | 6149 | -6 | L1PA4 | L1 | LINE |
| 164739651 | 164740348 | + | 6.3 | 5475 | 6172 | 0 | L1PA8 | L1 | LINE |
| 164740349 | 164740877 | + | 16.0 | 5769 | 6297 | -3 | L1MA6 | L1 | LINE |

  

|  |  |  |  |  |  |  |  |  |  |
| --- | --- | --- | --- | --- | --- | --- | --- | --- | --- |
| chr1:166798099-166800892 | | | 2791 bp | | |  |  |  |  |
| Genome Start | Genome End | Strand | Div | Repeat Start | Repeat End | Repeat Left | Name | Family | Class |
| 166798099 | 166798894 | + | 17.7 | 5284 | 6042 | -126 | L1MB1 | L1 | LINE |
| 166798891 | 166799554 | - | 5.2 | 4211 | 4881 | -1265 | L1PB1 | L1 | LINE |
| 166799553 | 166800742 | + | 9.4 | 4892 | 6150 | -1 | L1PB1 | L1 | LINE |
| 166800752 | 166800892 | + | 10.2 | 6028 | 6167 | -1 | L1MB1 | L1 | LINE |

  

|  |  |  |  |  |  |  |  |  |  |
| --- | --- | --- | --- | --- | --- | --- | --- | --- | --- |
| chr1:166980197-166983850 | | | 3529 bp | | |  |  |  |  |
| Genome Start | Genome End | Strand | Div | Repeat Start | Repeat End | Repeat Left | Name | Family | Class |
| 166980197 | 166980427 | + | 30.7 | 2975 | 3192 | -186 | L2 | L2 | LINE |
| 166980539 | 166982803 | - | 3.7 | 3860 | 6155 | 0 | L1PA4 | L1 | LINE |
| 166982803 | 166983647 | + | 4.6 | 3004 | 3845 | -2301 | L1PA4 | L1 | LINE |
| 166983663 | 166983742 | - | 8.8 | 1 | 80 | 0 | MADE1 | Mariner | DNA |
| 166983743 | 166983850 | + | 32.8 | 3297 | 3407 | -12 | L2 | L2 | LINE |

  

|  |  |  |  |  |  |  |  |  |  |
| --- | --- | --- | --- | --- | --- | --- | --- | --- | --- |
| chr1:167118812-167122725 | | | 3862 bp | | |  |  |  |  |
| Genome Start | Genome End | Strand | Div | Repeat Start | Repeat End | Repeat Left | Name | Family | Class |
| 167118812 | 167119080 | + | 18.9 | 3093 | 3398 | -2748 | L1M5 | L1 | LINE |
| 167119106 | 167119487 | - | 5.2 | 5771 | 6153 | -2 | L1PA4 | L1 | LINE |
| 167119510 | 167120237 | - | 2.5 | 5410 | 6137 | -18 | L1PA3 | L1 | LINE |
| 167120238 | 167121947 | - | 4.6 | 4077 | 5788 | -366 | L1PA4 | L1 | LINE |
| 167121948 | 167122547 | + | 5.3 | 3477 | 4076 | -2070 | L1PA4 | L1 | LINE |
| 167122553 | 167122725 | + | 24.2 | 3403 | 3572 | -2574 | L1M5 | L1 | LINE |

  

|  |  |  |  |  |  |  |  |  |  |
| --- | --- | --- | --- | --- | --- | --- | --- | --- | --- |
| chr1:167160937-167168333 | | | 6544 bp | | |  |  |  |  |
| Genome Start | Genome End | Strand | Div | Repeat Start | Repeat End | Repeat Left | Name | Family | Class |
| 167160937 | 167161294 | + | 34.0 | 742 | 1124 | -4633 | ERVL | ERVL | LTR |
| 167161361 | 167161807 | - | 7.2 | 5720 | 6170 | -2 | L1PA8 | L1 | LINE |
| 167161896 | 167162058 | + | 31.0 | 1284 | 1442 | -4315 | ERVL | ERVL | LTR |
| 167162238 | 167162617 | + | 26.5 | 31 | 422 | -161 | MER119 | MER1\_type | DNA |
| 167162619 | 167162858 | + | 20.4 | 334 | 582 | -1 | MER119 | MER1\_type | DNA |
| 167162922 | 167163735 | - | 11.5 | 5366 | 6172 | 0 | L1PA10 | L1 | LINE |
| 167163738 | 167165800 | + | 7.3 | 3278 | 5358 | -788 | L1PA10 | L1 | LINE |
| 167165823 | 167165935 | + | 30.3 | 1614 | 1726 | -3941 | ERVL | ERVL | LTR |
| 167166001 | 167166384 | + | 38.5 | 2575 | 2964 | -2703 | ERVL | ERVL | LTR |
| 167166663 | 167166958 | + | 30.2 | 530 | 874 | -5245 | L1MEd | L1 | LINE |
| 167167026 | 167168043 | + | 30.2 | 875 | 2002 | -4147 | L1MEd | L1 | LINE |
| 167168066 | 167168333 | + | 30.8 | 3268 | 3546 | -2121 | ERVL | ERVL | LTR |

  

|  |  |  |  |  |  |  |  |  |  |
| --- | --- | --- | --- | --- | --- | --- | --- | --- | --- |
| chr1:168622299-168631310 | | | 8960 bp | | |  |  |  |  |
| Genome Start | Genome End | Strand | Div | Repeat Start | Repeat End | Repeat Left | Name | Family | Class |
| 168622299 | 168622370 | - | 22.2 | 5354 | 5425 | -957 | L1M4c | L1 | LINE |
| 168622399 | 168623617 | - | 13.9 | 3117 | 8151 | -9 | L1PA15 | L1 | LINE |
| 168623618 | 168623931 | + | 14.5 | 1 | 312 | 0 | AluJb | Alu | SINE |
| 168623932 | 168625078 | - | 19.8 | 1936 | 3117 | -5038 | L1PA15 | L1 | LINE |
| 168625076 | 168625125 | + | 28.0 | 1882 | 1931 | -6224 | L1PREC2 | L1 | LINE |
| 168625131 | 168627465 | - | 20.3 | 2960 | 5345 | -1037 | L1M4c | L1 | LINE |
| 168627466 | 168627885 | + | 18.4 | 1 | 426 | 0 | MSTB | MaLR | LTR |
| 168627886 | 168628469 | - | 20.3 | 2374 | 2959 | -3423 | L1M4c | L1 | LINE |
| 168628470 | 168628795 | - | 23.5 | 1 | 345 | 0 | MER2 | MER2\_type | DNA |
| 168628796 | 168629480 | - | 16.4 | 1598 | 2375 | -4009 | L1M4c | L1 | LINE |
| 168629503 | 168630547 | - | 16.4 | 487 | 1597 | -4787 | L1M4c | L1 | LINE |
| 168630548 | 168630840 | - | 7.9 | 1 | 292 | -19 | AluY | Alu | SINE |
| 168630841 | 168631310 | - | 16.4 | 17 | 486 | -5898 | L1M4c | L1 | LINE |

  

|  |  |  |  |  |  |  |  |  |  |
| --- | --- | --- | --- | --- | --- | --- | --- | --- | --- |
| chr1:170162500-170167042 | | | 4518 bp | | |  |  |  |  |
| Genome Start | Genome End | Strand | Div | Repeat Start | Repeat End | Repeat Left | Name | Family | Class |
| 170162500 | 170162999 | + | 19.6 | 3805 | 4329 | -1817 | L1M4 | L1 | LINE |
| 170163000 | 170163310 | - | 9.9 | 5858 | 6159 | -2 | L1PA14 | L1 | LINE |
| 170163311 | 170163494 | + | 13.7 | 5676 | 5860 | -300 | L1PA14 | L1 | LINE |
| 170163507 | 170166626 | - | 21.1 | 1 | 3236 | -4919 | L1PREC2 | L1 | LINE |
| 170166627 | 170166725 | + | 16.2 | 4320 | 4418 | -1728 | L1M4 | L1 | LINE |
| 170166730 | 170166832 | + | 24.3 | 6 | 127 | -6 | FLAM\_C | Alu | SINE |
| 170166842 | 170167042 | + | 17.4 | 4412 | 4612 | -1534 | L1M4 | L1 | LINE |

  

|  |  |  |  |  |  |  |  |  |  |
| --- | --- | --- | --- | --- | --- | --- | --- | --- | --- |
| chr1:170534675-170536882 | | | 1998 bp | | |  |  |  |  |
| Genome Start | Genome End | Strand | Div | Repeat Start | Repeat End | Repeat Left | Name | Family | Class |
| 170534675 | 170535103 | - | 32.2 | 2841 | 3285 | -134 | L2 | L2 | LINE |
| 170535177 | 170536271 | - | 12.2 | 5033 | 6129 | -37 | L1PA16 | L1 | LINE |
| 170536274 | 170536472 | + | 8.5 | 4839 | 5037 | -1109 | L1PA16 | L1 | LINE |
| 170536505 | 170536556 | - | 19.1 | 2700 | 2746 | -673 | L2 | L2 | LINE |
| 170536660 | 170536882 | - | 31.4 | 2258 | 2515 | -904 | L2 | L2 | LINE |

  

|  |  |  |  |  |  |  |  |  |  |
| --- | --- | --- | --- | --- | --- | --- | --- | --- | --- |
| chr1:172411722-172415583 | | | 3752 bp | | |  |  |  |  |
| Genome Start | Genome End | Strand | Div | Repeat Start | Repeat End | Repeat Left | Name | Family | Class |
| 172411722 | 172412188 | - | 18.7 | 742 | 1221 | -234 | Charlie1a | MER1\_type | DNA |
| 172412189 | 172412502 | + | 14.4 | 1 | 312 | 0 | AluJb | Alu | SINE |
| 172412503 | 172412558 | - | 18.7 | 697 | 741 | -714 | Charlie1a | MER1\_type | DNA |
| 172412559 | 172412856 | + | 15.3 | 1 | 302 | 0 | AluSx | Alu | SINE |
| 172412857 | 172414137 | - | 6.6 | 4245 | 5540 | -632 | L1PA8 | L1 | LINE |
| 172414140 | 172414773 | + | 7.6 | 5539 | 6172 | 0 | L1PA8 | L1 | LINE |
| 172414830 | 172414927 | - | 13.3 | 43 | 140 | -2 | FLAM\_A | Alu | SINE |
| 172414941 | 172415305 | - | 20.4 | 260 | 642 | -813 | Charlie1a | MER1\_type | DNA |
| 172415345 | 172415583 | - | 20.4 | 3 | 259 | -1196 | Charlie1a | MER1\_type | DNA |

  

|  |  |  |  |  |  |  |  |  |  |
| --- | --- | --- | --- | --- | --- | --- | --- | --- | --- |
| chr1:172465164-172466445 | | | 1274 bp | | |  |  |  |  |
| Genome Start | Genome End | Strand | Div | Repeat Start | Repeat End | Repeat Left | Name | Family | Class |
| 172465164 | 172465433 | - | 23.1 | 406 | 695 | -4 | LTR34 | ERV1 | LTR |
| 172465435 | 172465747 | - | 1.9 | 5435 | 5747 | -408 | L1PA4 | L1 | LINE |
| 172465748 | 172466169 | + | 2.8 | 5731 | 6153 | -2 | L1PA4 | L1 | LINE |
| 172466177 | 172466445 | - | 26.5 | 104 | 401 | -298 | LTR34 | ERV1 | LTR |

  

|  |  |  |  |  |  |  |  |  |  |
| --- | --- | --- | --- | --- | --- | --- | --- | --- | --- |
| chr1:172718751-172727926 | | | 9169 bp | | |  |  |  |  |
| Genome Start | Genome End | Strand | Div | Repeat Start | Repeat End | Repeat Left | Name | Family | Class |
| 172718751 | 172719336 | - | 10.1 | 7141 | 7724 | 0 | L1MA3 | L1 | LINE |
| 172719337 | 172719594 | + | 13.9 | 17 | 294 | -16 | AluSg | Alu | SINE |
| 172719595 | 172722112 | - | 11.1 | 4566 | 7140 | -584 | L1MA3 | L1 | LINE |
| 172722113 | 172722406 | - | 8.2 | 1 | 294 | -17 | AluY | Alu | SINE |
| 172722407 | 172724522 | - | 12.1 | 2404 | 4565 | -3000 | L1MA3 | L1 | LINE |
| 172724532 | 172724863 | - | 6.7 | 5820 | 6149 | -5 | L1PA5 | L1 | LINE |
| 172724864 | 172725607 | + | 4.7 | 5066 | 5808 | -346 | L1PA5 | L1 | LINE |
| 172725606 | 172727926 | - | 17.4 | 11 | 2418 | -5147 | L1MA3 | L1 | LINE |

  

|  |  |  |  |  |  |  |  |  |  |
| --- | --- | --- | --- | --- | --- | --- | --- | --- | --- |
| chr1:173670643-173676952 | | | 6255 bp | | |  |  |  |  |
| Genome Start | Genome End | Strand | Div | Repeat Start | Repeat End | Repeat Left | Name | Family | Class |
| 173670643 | 173670819 | + | 34.8 | 3125 | 3286 | -92 | L2 | L2 | LINE |
| 173670820 | 173671432 | - | 25.5 | 2216 | 2871 | -548 | L2 | L2 | LINE |
| 173671437 | 173672266 | - | 3.7 | 1281 | 2113 | -4042 | L1PA4 | L1 | LINE |
| 173672266 | 173676299 | + | 4.2 | 2114 | 6155 | 0 | L1PA4 | L1 | LINE |
| 173676347 | 173676573 | - | 28.0 | 2005 | 2206 | -1213 | L2 | L2 | LINE |
| 173676575 | 173676853 | - | 27.3 | 1464 | 1762 | -1657 | L2 | L2 | LINE |
| 173676858 | 173676952 | + | 29.8 | 3292 | 3377 | -1 | L2 | L2 | LINE |

  

|  |  |  |  |  |  |  |  |  |  |
| --- | --- | --- | --- | --- | --- | --- | --- | --- | --- |
| chr1:174741077-174747259 | | | 5741 bp | | |  |  |  |  |
| Genome Start | Genome End | Strand | Div | Repeat Start | Repeat End | Repeat Left | Name | Family | Class |
| 174741077 | 174741351 | + | 27.7 | 40 | 317 | -6083 | L1ME1 | L1 | LINE |
| 174741379 | 174741451 | + | 9.7 | 4619 | 4680 | -1466 | L1P4 | L1 | LINE |
| 174741512 | 174741640 | + | 32.8 | 323 | 458 | -5942 | L1ME1 | L1 | LINE |
| 174741761 | 174742573 | - | 13.8 | 4190 | 4997 | -1149 | L1PA16 | L1 | LINE |
| 174742574 | 174743682 | + | 11.9 | 5013 | 6165 | -1 | L1PA16 | L1 | LINE |
| 174743738 | 174744380 | + | 27.3 | 563 | 1219 | -5181 | L1ME1 | L1 | LINE |
| 174744381 | 174744748 | + | 13.8 | 1 | 381 | 0 | THE1D | MaLR | LTR |
| 174744749 | 174744789 | + | 27.3 | 1220 | 1256 | -5144 | L1ME1 | L1 | LINE |
| 174744869 | 174745026 | - | 18.3 | 1 | 162 | -213 | THE1C | MaLR | LTR |
| 174745075 | 174745394 | - | 16.1 | 58 | 381 | 0 | THE1D | MaLR | LTR |
| 174745411 | 174745480 | + | 29.1 | 1244 | 1306 | -5094 | L1ME1 | L1 | LINE |
| 174745481 | 174745826 | + | 16.1 | 2 | 312 | 0 | AluJb | Alu | SINE |
| 174745827 | 174746563 | + | 23.9 | 1307 | 2132 | -4268 | L1ME1 | L1 | LINE |
| 174746612 | 174746960 | + | 23.9 | 2133 | 2480 | -3920 | L1ME1 | L1 | LINE |
| 174746950 | 174747259 | + | 21.8 | 3904 | 4190 | -2029 | L1ME1 | L1 | LINE |

  

|  |  |  |  |  |  |  |  |  |  |
| --- | --- | --- | --- | --- | --- | --- | --- | --- | --- |
| chr1:175527715-175536403 | | | 8659 bp | | |  |  |  |  |
| Genome Start | Genome End | Strand | Div | Repeat Start | Repeat End | Repeat Left | Name | Family | Class |
| 175527715 | 175528943 | - | 33.9 | 2803 | 4127 | -1540 | HERV16 | ERVL | LTR |
| 175528944 | 175529329 | - | 18.4 | 3 | 396 | 0 | MSTD | MaLR | LTR |
| 175529330 | 175530007 | - | 32.8 | 2162 | 2875 | -2121 | HERV16 | ERVL | LTR |
| 175530008 | 175531271 | - | 13.9 | 2712 | 3991 | -2155 | L1P4 | L1 | LINE |
| 175531272 | 175531518 | + | 6.9 | 34 | 280 | -31 | AluY | Alu | SINE |
| 175531546 | 175531557 | + | 6.9 | 281 | 292 | -19 | AluY | Alu | SINE |
| 175531558 | 175532105 | - | 13.9 | 2128 | 2711 | -3435 | L1P4 | L1 | LINE |
| 175532106 | 175534229 | + | 11.8 | 4000 | 6165 | -1 | L1PA16 | L1 | LINE |
| 175534233 | 175536403 | - | 33.3 | 2 | 2173 | -2823 | HERV16 | ERVL | LTR |

  

|  |  |  |  |  |  |  |  |  |  |
| --- | --- | --- | --- | --- | --- | --- | --- | --- | --- |
| chr1:177131134-177133392 | | | 2017 bp | | |  |  |  |  |
| Genome Start | Genome End | Strand | Div | Repeat Start | Repeat End | Repeat Left | Name | Family | Class |
| 177131134 | 177131221 | - | 23.0 | 3278 | 3369 | -50 | L2 | L2 | LINE |
| 177131288 | 177131343 | - | 26.8 | 3079 | 3134 | -285 | L2 | L2 | LINE |
| 177131430 | 177132189 | - | 27.9 | 1722 | 2615 | -804 | L2 | L2 | LINE |
| 177132226 | 177132838 | - | 1.3 | 5536 | 6155 | 0 | L1PA2 | L1 | LINE |
| 177132839 | 177133106 | + | 0.8 | 5286 | 5553 | -602 | L1PA2 | L1 | LINE |
| 177133113 | 177133272 | - | 32.7 | 1540 | 1696 | -1723 | L2 | L2 | LINE |
| 177133321 | 177133392 | - | 28.2 | 815 | 888 | -2531 | L2 | L2 | LINE |

  

|  |  |  |  |  |  |  |  |  |  |
| --- | --- | --- | --- | --- | --- | --- | --- | --- | --- |
| chr1:178537007-178542111 | | | 5077 bp | | |  |  |  |  |
| Genome Start | Genome End | Strand | Div | Repeat Start | Repeat End | Repeat Left | Name | Family | Class |
| 178537007 | 178537799 | - | 15.2 | 245 | 1068 | 0 | Tigger2b\_Pri | MER2\_type | DNA |
| 178537800 | 178537951 | + | 6.6 | 5998 | 6149 | -5 | L1PA5 | L1 | LINE |
| 178537952 | 178538147 | - | 15.2 | 43 | 244 | -824 | Tigger2b\_Pri | MER2\_type | DNA |
| 178538148 | 178538362 | - | 3.7 | 2263 | 2477 | -3669 | L1PA4 | L1 | LINE |
| 178538362 | 178542019 | + | 4.5 | 2487 | 6155 | 0 | L1PA4 | L1 | LINE |
| 178542049 | 178542111 | - | 11.3 | 1 | 62 | -1006 | Tigger2b\_Pri | MER2\_type | DNA |

  

|  |  |  |  |  |  |  |  |  |  |
| --- | --- | --- | --- | --- | --- | --- | --- | --- | --- |
| chr1:180117374-180129765 | | | 12359 bp | | |  |  |  |  |
| Genome Start | Genome End | Strand | Div | Repeat Start | Repeat End | Repeat Left | Name | Family | Class |
| 180117374 | 180117981 | + | 24.4 | 2582 | 3166 | -2980 | L1M5 | L1 | LINE |
| 180117986 | 180121826 | + | 7.9 | 1 | 3846 | -2300 | L1P2 | L1 | LINE |
| 180121824 | 180123500 | - | 3.0 | 3517 | 5197 | -949 | L1PA4 | L1 | LINE |
| 180123503 | 180124465 | + | 4.5 | 5191 | 6155 | 0 | L1PA4 | L1 | LINE |
| 180124448 | 180124744 | + | 20.3 | 3127 | 3439 | -2707 | L1M5 | L1 | LINE |
| 180124745 | 180124840 | + | 11.5 | 189 | 293 | -19 | AluSg/x | Alu | SINE |
| 180124849 | 180128028 | + | 14.8 | 7 | 2886 | -3260 | L1P2 | L1 | LINE |
| 180128028 | 180129386 | + | 6.6 | 4761 | 6134 | -20 | L1PA7 | L1 | LINE |
| 180129428 | 180129765 | + | 21.6 | 5464 | 5805 | -361 | L1M5 | L1 | LINE |

  

|  |  |  |  |  |  |  |  |  |  |
| --- | --- | --- | --- | --- | --- | --- | --- | --- | --- |
| chr1:183200663-183201768 | | | 1044 bp | | |  |  |  |  |
| Genome Start | Genome End | Strand | Div | Repeat Start | Repeat End | Repeat Left | Name | Family | Class |
| 183200663 | 183200729 | + | 16.1 | 7178 | 7239 | -722 | L1MC5 | L1 | LINE |
| 183200773 | 183201073 | + | 6.3 | 1 | 301 | -10 | AluY | Alu | SINE |
| 183201083 | 183201235 | - | 11.2 | 5686 | 5854 | -309 | L1PA12 | L1 | LINE |
| 183201236 | 183201578 | + | 11.0 | 5832 | 6168 | 0 | L1PA12 | L1 | LINE |
| 183201589 | 183201768 | + | 24.7 | 7314 | 7515 | -446 | L1MC5 | L1 | LINE |

  

|  |  |  |  |  |  |  |  |  |  |
| --- | --- | --- | --- | --- | --- | --- | --- | --- | --- |
| chr1:183457977-183461319 | | | 3328 bp | | |  |  |  |  |
| Genome Start | Genome End | Strand | Div | Repeat Start | Repeat End | Repeat Left | Name | Family | Class |
| 183457977 | 183458149 | - | 12.7 | 5714 | 5886 | -414 | L1MA6 | L1 | LINE |
| 183458178 | 183458229 | - | 15.5 | 6151 | 6195 | -105 | L1MA6 | L1 | LINE |
| 183458230 | 183458542 | + | 13.4 | 1 | 307 | -5 | AluSx | Alu | SINE |
| 183458543 | 183458748 | - | 15.5 | 5932 | 6150 | -150 | L1MA6 | L1 | LINE |
| 183458738 | 183458970 | - | 13.8 | 5492 | 5722 | -578 | L1MA6 | L1 | LINE |
| 183458971 | 183459235 | - | 18.2 | 1 | 302 | 0 | AluJo | Alu | SINE |
| 183459236 | 183459405 | - | 13.8 | 5323 | 5491 | -809 | L1MA6 | L1 | LINE |
| 183459406 | 183459698 | - | 8.9 | 5861 | 6158 | -5 | L1PA13 | L1 | LINE |
| 183459700 | 183459789 | + | 5.6 | 5786 | 5874 | -289 | L1PA13 | L1 | LINE |
| 183459787 | 183459948 | - | 12.4 | 5160 | 5329 | -814 | L1MA6 | L1 | LINE |
| 183459949 | 183461319 | - | 13.7 | 3495 | 4891 | -1252 | L1MA6 | L1 | LINE |

  

|  |  |  |  |  |  |  |  |  |  |
| --- | --- | --- | --- | --- | --- | --- | --- | --- | --- |
| chr1:184903317-184907155 | | | 3721 bp | | |  |  |  |  |
| Genome Start | Genome End | Strand | Div | Repeat Start | Repeat End | Repeat Left | Name | Family | Class |
| 184903317 | 184903863 | - | 31.5 | 2795 | 3417 | -2 | L2 | L2 | LINE |
| 184903867 | 184904916 | - | 7.8 | 3908 | 4980 | -1166 | L1PA7 | L1 | LINE |
| 184904918 | 184906059 | + | 6.6 | 4994 | 6140 | -14 | L1PA7 | L1 | LINE |
| 184906172 | 184906714 | - | 31.4 | 2050 | 2678 | -741 | L2 | L2 | LINE |
| 184906717 | 184907155 | - | 33.6 | 767 | 1255 | -2164 | L2 | L2 | LINE |

  

|  |  |  |  |  |  |  |  |  |  |
| --- | --- | --- | --- | --- | --- | --- | --- | --- | --- |
| chr1:185550063-185554660 | | | 4574 bp | | |  |  |  |  |
| Genome Start | Genome End | Strand | Div | Repeat Start | Repeat End | Repeat Left | Name | Family | Class |
| 185550063 | 185550493 | - | 28.9 | 3117 | 3539 | -2607 | L1M | L1 | LINE |
| 185550521 | 185551901 | - | 19.9 | 4948 | 6327 | -7 | L1MA10 | L1 | LINE |
| 185551905 | 185553175 | + | 19.6 | 3385 | 4682 | -1458 | L1M3 | L1 | LINE |
| 185553176 | 185553497 | - | 9.5 | 9 | 321 | 0 | AluSx | Alu | SINE |
| 185553498 | 185553766 | + | 19.6 | 4683 | 4961 | -1179 | L1M3 | L1 | LINE |
| 185553761 | 185554660 | - | 26.4 | 2245 | 3130 | -3016 | L1M | L1 | LINE |

  

|  |  |  |  |  |  |  |  |  |  |
| --- | --- | --- | --- | --- | --- | --- | --- | --- | --- |
| chr1:186055098-186062518 | | | 7469 bp | | |  |  |  |  |
| Genome Start | Genome End | Strand | Div | Repeat Start | Repeat End | Repeat Left | Name | Family | Class |
| 186055098 | 186059119 | - | 13.6 | 1773 | 5959 | -903 | L1PBa | L1 | LINE |
| 186059096 | 186060026 | - | 2.7 | 5225 | 6155 | 0 | L1PA2 | L1 | LINE |
| 186060019 | 186061352 | + | 2.2 | 3909 | 5223 | -923 | L1PA2 | L1 | LINE |
| 186061351 | 186061948 | - | 15.1 | 1205 | 1809 | -5054 | L1PBa | L1 | LINE |
| 186061952 | 186062017 | - | 22.7 | 989 | 1054 | -5809 | L1PBa | L1 | LINE |
| 186062001 | 186062518 | - | 21.9 | 1 | 527 | -6336 | L1PBa | L1 | LINE |

  

|  |  |  |  |  |  |  |  |  |  |
| --- | --- | --- | --- | --- | --- | --- | --- | --- | --- |
| chr1:187152765-187154272 | | | 1382 bp | | |  |  |  |  |
| Genome Start | Genome End | Strand | Div | Repeat Start | Repeat End | Repeat Left | Name | Family | Class |
| 187152765 | 187153041 | - | 31.5 | 1487 | 1782 | -725 | HAL1 | L1 | LINE |
| 187153117 | 187153200 | - | 11.9 | 6202 | 6288 | -3 | L1MA7 | L1 | LINE |
| 187153201 | 187153488 | + | 19.7 | 5940 | 6227 | -64 | L1MA7 | L1 | LINE |
| 187153540 | 187154272 | - | 32.2 | 290 | 1042 | -1465 | HAL1 | L1 | LINE |

  

|  |  |  |  |  |  |  |  |  |  |
| --- | --- | --- | --- | --- | --- | --- | --- | --- | --- |
| chr1:188533203-188537294 | | | 3882 bp | | |  |  |  |  |
| Genome Start | Genome End | Strand | Div | Repeat Start | Repeat End | Repeat Left | Name | Family | Class |
| 188533203 | 188533532 | - | 27.7 | 4114 | 4473 | -2064 | L1MDa | L1 | LINE |
| 188533582 | 188535161 | - | 20.5 | 2416 | 4113 | -2424 | L1MDa | L1 | LINE |
| 188535186 | 188535432 | - | 1.2 | 5909 | 6155 | 0 | L1PA3 | L1 | LINE |
| 188535444 | 188536207 | - | 3.4 | 1123 | 1887 | -4145 | L1HS | L1 | LINE |
| 188536207 | 188536797 | + | 3.7 | 541 | 1132 | -4900 | L1HS | L1 | LINE |
| 188536794 | 188536880 | - | 21.9 | 2345 | 2435 | -4198 | L1MDa | L1 | LINE |
| 188537012 | 188537294 | - | 21.9 | 2026 | 2344 | -4289 | L1MDa | L1 | LINE |

  

|  |  |  |  |  |  |  |  |  |  |
| --- | --- | --- | --- | --- | --- | --- | --- | --- | --- |
| chr1:189912974-189921865 | | | 8905 bp | | |  |  |  |  |
| Genome Start | Genome End | Strand | Div | Repeat Start | Repeat End | Repeat Left | Name | Family | Class |
| 189912974 | 189915750 | - | 13.0 | 4905 | 7723 | -1 | L1MA3 | L1 | LINE |
| 189915755 | 189916369 | - | 14.8 | 3138 | 3745 | -3820 | L1MA3 | L1 | LINE |
| 189916368 | 189916514 | + | 12.5 | 2810 | 2953 | -4612 | L1M1 | L1 | LINE |
| 189916505 | 189916650 | - | 17.8 | 2977 | 3129 | -4436 | L1MA3 | L1 | LINE |
| 189916651 | 189916748 | - | 13.0 | 6040 | 6131 | 0 | L1PA8A | L1 | LINE |
| 189916750 | 189918116 | - | 8.9 | 4588 | 5995 | -136 | L1PA8A | L1 | LINE |
| 189918114 | 189919159 | + | 9.8 | 3517 | 4566 | -1580 | L1PA8A | L1 | LINE |
| 189919157 | 189921865 | - | 16.4 | 147 | 2994 | -4571 | L1MA3 | L1 | LINE |

  

|  |  |  |  |  |  |  |  |  |  |
| --- | --- | --- | --- | --- | --- | --- | --- | --- | --- |
| chr1:190067127-190070853 | | | 3649 bp | | |  |  |  |  |
| Genome Start | Genome End | Strand | Div | Repeat Start | Repeat End | Repeat Left | Name | Family | Class |
| 190067127 | 190067315 | + | 19.7 | 6868 | 7097 | -743 | L1MC3 | L1 | LINE |
| 190067316 | 190067544 | - | 23.4 | 182 | 408 | 0 | MLT1A1 | MaLR | LTR |
| 190067571 | 190067639 | - | 20.3 | 36 | 117 | -291 | MSTD | MaLR | LTR |
| 190067647 | 190068565 | - | 4.6 | 4485 | 5403 | -743 | L1PA4 | L1 | LINE |
| 190068566 | 190069292 | + | 3.0 | 5425 | 6151 | -4 | L1PA4 | L1 | LINE |
| 190069310 | 190069353 | - | 7.2 | 1 | 42 | -354 | MSTD | MaLR | LTR |
| 190069358 | 190069562 | + | 19.1 | 7052 | 7254 | -531 | L1MC3 | L1 | LINE |
| 190069587 | 190069610 | + | 19.1 | 7255 | 7277 | -508 | L1MC3 | L1 | LINE |
| 190069611 | 190069944 | + | 21.0 | 4 | 396 | 0 | MSTD | MaLR | LTR |
| 190069945 | 190070321 | + | 19.1 | 7278 | 7636 | -149 | L1MC3 | L1 | LINE |
| 190070322 | 190070693 | + | 11.6 | 1 | 375 | 0 | THE1C | MaLR | LTR |
| 190070694 | 190070853 | + | 19.1 | 7637 | 7772 | -13 | L1MC3 | L1 | LINE |

  

|  |  |  |  |  |  |  |  |  |  |
| --- | --- | --- | --- | --- | --- | --- | --- | --- | --- |
| chr1:190163720-190173547 | | | 9821 bp | | |  |  |  |  |
| Genome Start | Genome End | Strand | Div | Repeat Start | Repeat End | Repeat Left | Name | Family | Class |
| 190163720 | 190164208 | + | 19.4 | 22 | 505 | -7060 | L1MA1 | L1 | LINE |
| 190164209 | 190164506 | + | 15.2 | 1 | 296 | -16 | AluSx | Alu | SINE |
| 190164507 | 190166917 | + | 19.4 | 506 | 2956 | -4609 | L1MA1 | L1 | LINE |
| 190166912 | 190167436 | - | 5.1 | 5194 | 5724 | -430 | L1PA5 | L1 | LINE |
| 190167437 | 190167855 | + | 4.3 | 5731 | 6149 | -5 | L1PA5 | L1 | LINE |
| 190167861 | 190170754 | + | 12.6 | 2935 | 5879 | -1686 | L1MA1 | L1 | LINE |
| 190170755 | 190171060 | + | 14.1 | 1 | 306 | -4 | AluSg | Alu | SINE |
| 190171061 | 190171314 | + | 12.2 | 5880 | 6141 | -1424 | L1MA1 | L1 | LINE |
| 190171316 | 190171985 | - | 7.8 | 18 | 688 | 0 | LTR12\_ | ERV1 | LTR |
| 190171993 | 190173547 | + | 11.0 | 6166 | 7717 | -4 | L1MA1 | L1 | LINE |

  

|  |  |  |  |  |  |  |  |  |  |
| --- | --- | --- | --- | --- | --- | --- | --- | --- | --- |
| chr1:190444751-190455256 | | | 10407 bp | | |  |  |  |  |
| Genome Start | Genome End | Strand | Div | Repeat Start | Repeat End | Repeat Left | Name | Family | Class |
| 190444751 | 190444884 | + | 26.7 | 2 | 137 | -6371 | L1MC2 | L1 | LINE |
| 190444876 | 190445398 | - | 16.2 | 3692 | 4214 | -1932 | L1PB | L1 | LINE |
| 190445399 | 190445697 | - | 10.7 | 21 | 319 | 0 | AluSp | Alu | SINE |
| 190445727 | 190445872 | - | 16.2 | 3539 | 3691 | -2455 | L1PB | L1 | LINE |
| 190445871 | 190447813 | + | 13.0 | 4220 | 6148 | -3 | L1PB2 | L1 | LINE |
| 190447824 | 190447907 | + | 26.5 | 125 | 208 | -6300 | L1MC2 | L1 | LINE |
| 190447934 | 190448319 | + | 12.9 | 5289 | 5712 | -453 | L1P4 | L1 | LINE |
| 190448315 | 190449447 | + | 20.7 | 3325 | 4487 | -1834 | L1MC2 | L1 | LINE |
| 190449448 | 190449759 | + | 6.1 | 1 | 311 | 0 | AluY | Alu | SINE |
| 190449760 | 190450694 | + | 20.7 | 4488 | 5466 | -855 | L1MC2 | L1 | LINE |
| 190450695 | 190451004 | + | 5.5 | 1 | 311 | 0 | AluY | Alu | SINE |
| 190451005 | 190451334 | + | 17.0 | 5458 | 5784 | -2176 | L1MC2 | L1 | LINE |
| 190451347 | 190452323 | - | 18.1 | 5331 | 6305 | -7 | L1MA9 | L1 | LINE |
| 190452324 | 190452726 | - | 24.6 | 3 | 405 | -3 | MLT1A1 | MaLR | LTR |
| 190452727 | 190453764 | - | 19.5 | 4275 | 5330 | -982 | L1M | L1 | LINE |
| 190453760 | 190454772 | + | 19.6 | 2612 | 3697 | -2443 | L1M3 | L1 | LINE |
| 190454810 | 190455174 | + | 19.6 | 3698 | 4074 | -2066 | L1M3 | L1 | LINE |
| 190455181 | 190455256 | + | 18.4 | 6432 | 6510 | -9 | L1MC2 | L1 | LINE |

  

|  |  |  |  |  |  |  |  |  |  |
| --- | --- | --- | --- | --- | --- | --- | --- | --- | --- |
| chr1:192106312-192114386 | | | 8079 bp | | |  |  |  |  |
| Genome Start | Genome End | Strand | Div | Repeat Start | Repeat End | Repeat Left | Name | Family | Class |
| 192106312 | 192107616 | - | 9.3 | 4892 | 6168 | 0 | L1PA10 | L1 | LINE |
| 192107615 | 192107734 | - | 7.5 | 955 | 1074 | -5081 | L1PA5 | L1 | LINE |
| 192107723 | 192112764 | + | 5.9 | 1081 | 6150 | -4 | L1PA5 | L1 | LINE |
| 192112775 | 192114386 | - | 8.0 | 3296 | 4906 | -1240 | L1PA10 | L1 | LINE |

  

|  |  |  |  |  |  |  |  |  |  |
| --- | --- | --- | --- | --- | --- | --- | --- | --- | --- |
| chr1:192441837-192448564 | | | 6599 bp | | |  |  |  |  |
| Genome Start | Genome End | Strand | Div | Repeat Start | Repeat End | Repeat Left | Name | Family | Class |
| 192441837 | 192442700 | + | 24.8 | 3 | 960 | -1801 | Charlie1 | MER1\_type | DNA |
| 192442701 | 192443398 | - | 6.4 | 3912 | 4601 | -1545 | L1PB1 | L1 | LINE |
| 192443399 | 192444166 | + | 9.6 | 4592 | 5363 | -783 | L1PB1 | L1 | LINE |
| 192444167 | 192444447 | + | 11.5 | 1 | 280 | -29 | AluSc | Alu | SINE |
| 192444481 | 192445046 | + | 12.6 | 5364 | 5793 | -510 | L1M1 | L1 | LINE |
| 192445080 | 192445437 | + | 12.3 | 5794 | 6150 | -1 | L1PB1 | L1 | LINE |
| 192445448 | 192446155 | + | 21.7 | 944 | 1653 | -1108 | Charlie1 | MER1\_type | DNA |
| 192446159 | 192447176 | - | 26.6 | 5126 | 6159 | -7 | L1ME1 | L1 | LINE |
| 192447195 | 192448104 | + | 16.6 | 5325 | 6167 | -1 | L1PA15 | L1 | LINE |
| 192448105 | 192448452 | + | 21.8 | 2264 | 2659 | -102 | Charlie1 | MER1\_type | DNA |
| 192448485 | 192448564 | + | 21.8 | 2660 | 2759 | -2 | Charlie1 | MER1\_type | DNA |

  

|  |  |  |  |  |  |  |  |  |  |
| --- | --- | --- | --- | --- | --- | --- | --- | --- | --- |
| chr1:193489916-193492207 | | | 2066 bp | | |  |  |  |  |
| Genome Start | Genome End | Strand | Div | Repeat Start | Repeat End | Repeat Left | Name | Family | Class |
| 193489916 | 193490042 | + | 34.6 | 28 | 160 | -108 | MIRb | MIR | SINE |
| 193490055 | 193490286 | + | 30.9 | 13 | 291 | -258 | MLT1H | MaLR | LTR |
| 193490349 | 193491025 | - | 14.6 | 5221 | 5911 | -380 | L1MA8 | L1 | LINE |
| 193491022 | 193491212 | + | 23.5 | 5925 | 6114 | -186 | L1MA5 | L1 | LINE |
| 193491260 | 193491449 | + | 23.9 | 5991 | 6178 | 0 | L1MB8 | L1 | LINE |
| 193491490 | 193491628 | + | 31.6 | 405 | 548 | -1 | MLT1H | MaLR | LTR |
| 193491629 | 193492077 | + | 12.7 | 5851 | 6304 | 0 | L1MA2 | L1 | LINE |
| 193492147 | 193492207 | + | 21.3 | 172 | 241 | -27 | MIRb | MIR | SINE |

  

|  |  |  |  |  |  |  |  |  |  |
| --- | --- | --- | --- | --- | --- | --- | --- | --- | --- |
| chr1:193786279-193789944 | | | 3520 bp | | |  |  |  |  |
| Genome Start | Genome End | Strand | Div | Repeat Start | Repeat End | Repeat Left | Name | Family | Class |
| 193786279 | 193786788 | + | 22.0 | 2 | 540 | -691 | Tigger3b | MER2\_type | DNA |
| 193786856 | 193787651 | - | 1.9 | 5359 | 6155 | 0 | L1PA2 | L1 | LINE |
| 193787646 | 193788999 | + | 1.9 | 4027 | 5382 | -764 | L1PA2 | L1 | LINE |
| 193789000 | 193789090 | + | 12.9 | 533 | 628 | -604 | Tigger3b | MER2\_type | DNA |
| 193789176 | 193789447 | - | 9.2 | 1 | 273 | -38 | AluY | Alu | SINE |
| 193789448 | 193789944 | + | 12.9 | 629 | 1120 | -112 | Tigger3b | MER2\_type | DNA |

  

|  |  |  |  |  |  |  |  |  |  |
| --- | --- | --- | --- | --- | --- | --- | --- | --- | --- |
| chr1:194054150-194057205 | | | 2961 bp | | |  |  |  |  |
| Genome Start | Genome End | Strand | Div | Repeat Start | Repeat End | Repeat Left | Name | Family | Class |
| 194054150 | 194054284 | + | 8.9 | 5350 | 5484 | -682 | L1PA16 | L1 | LINE |
| 194054286 | 194055338 | - | 9.0 | 3979 | 5041 | -1105 | L1PA8 | L1 | LINE |
| 194055338 | 194056434 | + | 9.3 | 5060 | 6172 | 0 | L1PA8 | L1 | LINE |
| 194056530 | 194057205 | + | 16.1 | 5476 | 6149 | -17 | L1PA16 | L1 | LINE |

  

|  |  |  |  |  |  |  |  |  |  |
| --- | --- | --- | --- | --- | --- | --- | --- | --- | --- |
| chr1:194220386-194223107 | | | 2686 bp | | |  |  |  |  |
| Genome Start | Genome End | Strand | Div | Repeat Start | Repeat End | Repeat Left | Name | Family | Class |
| 194220386 | 194220710 | + | 29.9 | 775 | 1137 | -5263 | L1MEc | L1 | LINE |
| 194220712 | 194221401 | - | 18.5 | 4253 | 4956 | -1184 | L1M3 | L1 | LINE |
| 194221430 | 194221588 | - | 18.5 | 4083 | 4252 | -1888 | L1M3 | L1 | LINE |
| 194221588 | 194221650 | + | 15.9 | 4964 | 5027 | -1113 | L1MA9 | L1 | LINE |
| 194221649 | 194222288 | + | 22.3 | 5676 | 6298 | -14 | L1MA9 | L1 | LINE |
| 194222299 | 194222493 | + | 23.1 | 1119 | 1320 | -5080 | L1MEc | L1 | LINE |
| 194222494 | 194223051 | + | 21.5 | 1 | 552 | -2 | MLT2B3 | ERVL | LTR |
| 194223052 | 194223107 | + | 23.1 | 1321 | 1370 | -5030 | L1MEc | L1 | LINE |

  

|  |  |  |  |  |  |  |  |  |  |
| --- | --- | --- | --- | --- | --- | --- | --- | --- | --- |
| chr1:194931791-194934858 | | | 3081 bp | | |  |  |  |  |
| Genome Start | Genome End | Strand | Div | Repeat Start | Repeat End | Repeat Left | Name | Family | Class |
| 194931791 | 194932309 | + | 13.9 | 2558 | 3084 | -3062 | L1P3 | L1 | LINE |
| 194932308 | 194932359 | - | 5.8 | 4552 | 4604 | -1542 | L1PA4 | L1 | LINE |
| 194932353 | 194933920 | + | 3.9 | 4586 | 6155 | 0 | L1PA4 | L1 | LINE |
| 194933917 | 194934858 | + | 11.2 | 3072 | 4041 | -2105 | L1P3 | L1 | LINE |

  

|  |  |  |  |  |  |  |  |  |  |
| --- | --- | --- | --- | --- | --- | --- | --- | --- | --- |
| chr1:197337747-197344940 | | | 7176 bp | | |  |  |  |  |
| Genome Start | Genome End | Strand | Div | Repeat Start | Repeat End | Repeat Left | Name | Family | Class |
| 197337747 | 197337792 | - | 16.9 | 7094 | 7128 | 0 | L1MB1 | L1 | LINE |
| 197337793 | 197338096 | - | 15.8 | 3 | 307 | -5 | AluSx | Alu | SINE |
| 197338097 | 197338508 | - | 16.9 | 6687 | 7093 | -35 | L1MB1 | L1 | LINE |
| 197338537 | 197340973 | - | 16.4 | 3231 | 5798 | -1302 | L1MB1 | L1 | LINE |
| 197340970 | 197341980 | - | 11.4 | 5156 | 6165 | 0 | L1PA15 | L1 | LINE |
| 197341975 | 197343197 | + | 13.7 | 3899 | 5144 | -1002 | L1PREC2 | L1 | LINE |
| 197343198 | 197344940 | - | 26.7 | 1525 | 3245 | -3855 | L1MB1 | L1 | LINE |

  

|  |  |  |  |  |  |  |  |  |  |
| --- | --- | --- | --- | --- | --- | --- | --- | --- | --- |
| chr1:197430269-197431897 | | | 1627 bp | | |  |  |  |  |
| Genome Start | Genome End | Strand | Div | Repeat Start | Repeat End | Repeat Left | Name | Family | Class |
| 197430269 | 197430433 | + | 16.3 | 1 | 202 | -224 | MSTA | MaLR | LTR |
| 197430434 | 197430498 | - | 10.8 | 389 | 453 | 0 | MLT2B3 | ERVL | LTR |
| 197430500 | 197430857 | - | 10.6 | 5795 | 6170 | -4 | L1PA11 | L1 | LINE |
| 197430858 | 197431170 | + | 11.0 | 5467 | 5778 | -390 | L1PA11 | L1 | LINE |
| 197431171 | 197431674 | - | 25.7 | -107 | 400 | -46 | MLT2B3 | ERVL | LTR |
| 197431676 | 197431897 | + | 13.5 | 202 | 428 | 0 | MSTA | MaLR | LTR |

  

|  |  |  |  |  |  |  |  |  |  |
| --- | --- | --- | --- | --- | --- | --- | --- | --- | --- |
| chr1:197705043-197706920 | | | 1878 bp | | |  |  |  |  |
| Genome Start | Genome End | Strand | Div | Repeat Start | Repeat End | Repeat Left | Name | Family | Class |
| 197705043 | 197705361 | - | 19.0 | 52 | 408 | 0 | MLT1A1 | MaLR | LTR |
| 197705362 | 197706199 | - | 0.8 | 4709 | 5546 | -609 | L1HS | L1 | LINE |
| 197706200 | 197706860 | + | 0.3 | 5533 | 6193 | 0 | L1HS | L1 | LINE |
| 197706861 | 197706920 | - | 23.7 | 1 | 59 | -369 | MLT1A1 | MaLR | LTR |

  

|  |  |  |  |  |  |  |  |  |  |
| --- | --- | --- | --- | --- | --- | --- | --- | --- | --- |
| chr1:197832032-197832839 | | | 808 bp | | |  |  |  |  |
| Genome Start | Genome End | Strand | Div | Repeat Start | Repeat End | Repeat Left | Name | Family | Class |
| 197832032 | 197832191 | + | 13.3 | 2201 | 2352 | 0 | Tigger5 | MER2\_type | DNA |
| 197832192 | 197832630 | - | 8.7 | 5568 | 6015 | -159 | L1PA11 | L1 | LINE |
| 197832631 | 197832770 | + | 8.6 | 6029 | 6173 | -1 | L1PA11 | L1 | LINE |
| 197832771 | 197832839 | + | 24.6 | 2337 | 2405 | 0 | Tigger5 | MER2\_type | DNA |

  

|  |  |  |  |  |  |  |  |  |  |
| --- | --- | --- | --- | --- | --- | --- | --- | --- | --- |
| chr1:200843638-200849415 | | | 5773 bp | | |  |  |  |  |
| Genome Start | Genome End | Strand | Div | Repeat Start | Repeat End | Repeat Left | Name | Family | Class |
| 200843638 | 200844034 | - | 13.7 | 5786 | 6184 | 0 | L1MB7 | L1 | LINE |
| 200844035 | 200844326 | - | 14.4 | 1 | 297 | -15 | AluJo | Alu | SINE |
| 200844327 | 200844646 | - | 13.7 | 5469 | 5785 | -399 | L1MB7 | L1 | LINE |
| 200844631 | 200846089 | - | 3.3 | 4702 | 6152 | -3 | L1PA4 | L1 | LINE |
| 200846087 | 200846790 | + | 2.7 | 4003 | 4706 | -1440 | L1PA4 | L1 | LINE |
| 200846788 | 200847103 | - | 15.4 | 5193 | 5501 | -673 | L1MB7 | L1 | LINE |
| 200847104 | 200847406 | - | 10.4 | 1 | 299 | -14 | AluSq | Alu | SINE |
| 200847407 | 200847609 | - | 16.9 | 4996 | 5192 | -954 | L1MB7 | L1 | LINE |
| 200847611 | 200847806 | - | 19.4 | 96 | 291 | -21 | AluJb | Alu | SINE |
| 200847807 | 200848126 | - | 16.1 | 4708 | 5006 | -1140 | L1MB7 | L1 | LINE |
| 200848127 | 200848417 | - | 14.1 | 1 | 292 | -20 | AluJb | Alu | SINE |
| 200848418 | 200848819 | - | 16.1 | 4278 | 4707 | -1439 | L1MB7 | L1 | LINE |
| 200848820 | 200849131 | - | 9.3 | 1 | 312 | 0 | AluSx | Alu | SINE |
| 200849132 | 200849154 | - | 16.1 | 4260 | 4277 | -1869 | L1MB7 | L1 | LINE |
| 200849181 | 200849415 | - | 16.1 | 3991 | 4259 | -1887 | L1MB7 | L1 | LINE |

  

|  |  |  |  |  |  |  |  |  |  |
| --- | --- | --- | --- | --- | --- | --- | --- | --- | --- |
| chr1:207152657-207155591 | | | 2814 bp | | |  |  |  |  |
| Genome Start | Genome End | Strand | Div | Repeat Start | Repeat End | Repeat Left | Name | Family | Class |
| 207152657 | 207152774 | + | 13.4 | 5714 | 5831 | -353 | L1MB7 | L1 | LINE |
| 207152773 | 207153027 | - | 11.4 | 5387 | 5655 | -511 | L1P4 | L1 | LINE |
| 207153026 | 207153066 | + | 4.9 | 5635 | 5675 | -485 | L1PREC2 | L1 | LINE |
| 207153082 | 207153594 | + | 15.9 | 5658 | 6157 | -9 | L1PA16 | L1 | LINE |
| 207153608 | 207153671 | + | 12.5 | 6057 | 6124 | -42 | L1PA16 | L1 | LINE |
| 207153672 | 207153972 | - | 9.3 | 1 | 301 | -10 | AluY | Alu | SINE |
| 207154039 | 207154352 | + | 15.7 | 1 | 312 | 0 | AluSx | Alu | SINE |
| 207154367 | 207154627 | + | 20.7 | 5817 | 6086 | -98 | L1MB7 | L1 | LINE |
| 207154645 | 207154790 | - | 24.5 | 538 | 682 | -25 | MER39 | ERV1 | LTR |
| 207154791 | 207155085 | - | 10.2 | 2 | 296 | -16 | AluSx | Alu | SINE |
| 207155086 | 207155399 | - | 24.5 | 216 | 537 | -170 | MER39 | ERV1 | LTR |
| 207155400 | 207155506 | - | 22.6 | 4 | 114 | -428 | MER39 | ERV1 | LTR |
| 207155507 | 207155591 | + | 16.5 | 6079 | 6175 | -9 | L1MB7 | L1 | LINE |

  

|  |  |  |  |  |  |  |  |  |  |
| --- | --- | --- | --- | --- | --- | --- | --- | --- | --- |
| chr1:207955095-207961442 | | | 6310 bp | | |  |  |  |  |
| Genome Start | Genome End | Strand | Div | Repeat Start | Repeat End | Repeat Left | Name | Family | Class |
| 207955095 | 207955863 | + | 19.9 | 6458 | 7288 | -754 | L1MC4 | L1 | LINE |
| 207955871 | 207955919 | + | 6.7 | 5289 | 5333 | -850 | L1M | L1 | LINE |
| 207955914 | 207957540 | + | 14.3 | 3996 | 5665 | -635 | L1MA4A | L1 | LINE |
| 207957542 | 207957866 | + | 12.3 | 1 | 309 | -3 | AluSx | Alu | SINE |
| 207957874 | 207958360 | + | 14.6 | 5812 | 6302 | -1 | L1MA4A | L1 | LINE |
| 207958364 | 207958721 | + | 14.7 | 5321 | 5669 | -514 | L1MB3 | L1 | LINE |
| 207958722 | 207959017 | + | 10.1 | 1 | 312 | 0 | AluSx | Alu | SINE |
| 207959018 | 207959544 | + | 14.7 | 5670 | 6143 | -40 | L1MB3 | L1 | LINE |
| 207959540 | 207959924 | - | 6.8 | 5597 | 5990 | -164 | L1PA6 | L1 | LINE |
| 207959926 | 207960073 | + | 9.5 | 6002 | 6149 | -5 | L1PA6 | L1 | LINE |
| 207960094 | 207960352 | + | 20.2 | 7277 | 7533 | -509 | L1MC4 | L1 | LINE |
| 207960353 | 207960545 | - | 28.2 | 4 | 210 | 0 | MER63A | AcHobo | DNA |
| 207960546 | 207960770 | + | 20.2 | 7534 | 7768 | -274 | L1MC4 | L1 | LINE |
| 207960777 | 207961225 | + | 19.5 | 6 | 465 | -2 | MLT1C | MaLR | LTR |
| 207961226 | 207961385 | + | 20.1 | 7768 | 7907 | -135 | L1MC4 | L1 | LINE |
| 207961390 | 207961442 | + | 5.8 | 7991 | 8042 | 0 | L1MC4 | L1 | LINE |

  

|  |  |  |  |  |  |  |  |  |  |
| --- | --- | --- | --- | --- | --- | --- | --- | --- | --- |
| chr1:208327912-208330367 | | | 2448 bp | | |  |  |  |  |
| Genome Start | Genome End | Strand | Div | Repeat Start | Repeat End | Repeat Left | Name | Family | Class |
| 208327912 | 208328154 | - | 19.1 | 3073 | 3337 | -2809 | L1M5 | L1 | LINE |
| 208328168 | 208328462 | - | 6.1 | 5860 | 6154 | 0 | L1PA7 | L1 | LINE |
| 208328463 | 208328686 | + | 3.6 | 5652 | 5875 | -297 | L1PA7 | L1 | LINE |
| 208328682 | 208328850 | - | 21.9 | 2935 | 3092 | -3054 | L1M5 | L1 | LINE |
| 208328851 | 208329534 | - | 5.0 | 5468 | 6151 | -3 | L1PA7 | L1 | LINE |
| 208329535 | 208329692 | - | 21.9 | 2794 | 2934 | -3212 | L1M5 | L1 | LINE |
| 208329693 | 208330235 | - | 11.6 | 1 | 634 | 0 | MER51A | ERV1 | LTR |
| 208330236 | 208330367 | - | 21.9 | 2663 | 2793 | -3353 | L1M5 | L1 | LINE |

  

|  |  |  |  |  |  |  |  |  |  |
| --- | --- | --- | --- | --- | --- | --- | --- | --- | --- |
| chr1:208898234-208902127 | | | 3807 bp | | |  |  |  |  |
| Genome Start | Genome End | Strand | Div | Repeat Start | Repeat End | Repeat Left | Name | Family | Class |
| 208898234 | 208898417 | - | 29.4 | 4075 | 4267 | -222 | L3 | CR1 | LINE |
| 208898466 | 208898538 | - | 8.2 | 2548 | 2620 | -3526 | L1PA6 | L1 | LINE |
| 208898536 | 208901963 | + | 6.6 | 2620 | 6127 | -4 | L1PA6 | L1 | LINE |
| 208902006 | 208902127 | - | 26.1 | 3599 | 3730 | -759 | L3 | CR1 | LINE |

  

|  |  |  |  |  |  |  |  |  |  |
| --- | --- | --- | --- | --- | --- | --- | --- | --- | --- |
| chr1:212162243-212164077 | | | 1823 bp | | |  |  |  |  |
| Genome Start | Genome End | Strand | Div | Repeat Start | Repeat End | Repeat Left | Name | Family | Class |
| 212162243 | 212162792 | - | 22.7 | 5595 | 6163 | -1 | L1ME2 | L1 | LINE |
| 212162804 | 212162987 | - | 2.2 | 5964 | 6150 | -5 | L1PA2 | L1 | LINE |
| 212162988 | 212163375 | + | 1.8 | 5593 | 5980 | -175 | L1PA2 | L1 | LINE |
| 212163377 | 212163496 | - | 18.7 | 5485 | 5589 | -575 | L1ME2 | L1 | LINE |
| 212163497 | 212163797 | - | 9.6 | 1 | 303 | -10 | AluSq | Alu | SINE |
| 212163798 | 212164077 | - | 19.4 | 5182 | 5484 | -680 | L1ME2 | L1 | LINE |

  

|  |  |  |  |  |  |  |  |  |  |
| --- | --- | --- | --- | --- | --- | --- | --- | --- | --- |
| chr1:212319082-212322106 | | | 3013 bp | | |  |  |  |  |
| Genome Start | Genome End | Strand | Div | Repeat Start | Repeat End | Repeat Left | Name | Family | Class |
| 212319082 | 212319203 | + | 22.1 | 1 | 123 | -439 | MLT1F2 | MaLR | LTR |
| 212319204 | 212319613 | + | 17.9 | 1 | 486 | -379 | MER6 | MER2\_type | DNA |
| 212319625 | 212319661 | - | 12.7 | 6128 | 6158 | -2 | L1PREC2 | L1 | LINE |
| 212319662 | 212320026 | + | 15.0 | 1 | 364 | 0 | THE1B | MaLR | LTR |
| 212320027 | 212320825 | - | 12.7 | 5315 | 6127 | -33 | L1PREC2 | L1 | LINE |
| 212320824 | 212321000 | + | 7.9 | 5108 | 5297 | -849 | L1PREC2 | L1 | LINE |
| 212321003 | 212321144 | + | 12.2 | 478 | 617 | -248 | MER6 | MER2\_type | DNA |
| 212321145 | 212321444 | - | 11.0 | 1 | 300 | -10 | AluSg | Alu | SINE |
| 212321445 | 212321694 | + | 12.2 | 618 | 865 | 0 | MER6 | MER2\_type | DNA |
| 212321696 | 212322106 | + | 19.2 | 123 | 530 | -32 | MLT1F2 | MaLR | LTR |

  

|  |  |  |  |  |  |  |  |  |  |
| --- | --- | --- | --- | --- | --- | --- | --- | --- | --- |
| chr1:212476720-212478949 | | | 1976 bp | | |  |  |  |  |
| Genome Start | Genome End | Strand | Div | Repeat Start | Repeat End | Repeat Left | Name | Family | Class |
| 212476720 | 212476894 | + | 21.3 | 56 | 236 | -2525 | Charlie1 | MER1\_type | DNA |
| 212476880 | 212476944 | + | 18.5 | 2619 | 2683 | -78 | Charlie1 | MER1\_type | DNA |
| 212476945 | 212477331 | - | 10.0 | 4945 | 5333 | -813 | L1PREC2 | L1 | LINE |
| 212477335 | 212478139 | + | 9.9 | 5356 | 6156 | -4 | L1PREC2 | L1 | LINE |
| 212478190 | 212478265 | + | 8.8 | 6096 | 6163 | -9 | L1PA8 | L1 | LINE |
| 212478482 | 212478531 | + | 13.9 | 2672 | 2711 | -50 | Charlie1 | MER1\_type | DNA |
| 212478532 | 212478894 | - | 17.5 | 1 | 299 | -3 | AluSx | Alu | SINE |
| 212478895 | 212478949 | + | 13.9 | 2712 | 2759 | -2 | Charlie1 | MER1\_type | DNA |

  

|  |  |  |  |  |  |  |  |  |  |
| --- | --- | --- | --- | --- | --- | --- | --- | --- | --- |
| chr1:213118730-213123504 | | | 4674 bp | | |  |  |  |  |
| Genome Start | Genome End | Strand | Div | Repeat Start | Repeat End | Repeat Left | Name | Family | Class |
| 213118730 | 213118848 | - | 31.1 | 2954 | 3082 | -2572 | HERVL | ERVL | LTR |
| 213118849 | 213120049 | - | 4.2 | 4261 | 5466 | -689 | L1PA4 | L1 | LINE |
| 213120050 | 213120742 | + | 3.8 | 5462 | 6152 | -3 | L1PA4 | L1 | LINE |
| 213120808 | 213122016 | - | 34.3 | 1655 | 2898 | -2756 | HERVL | ERVL | LTR |
| 213122033 | 213123305 | + | 18.4 | 4856 | 6181 | -3 | L1MB7 | L1 | LINE |
| 213123326 | 213123504 | - | 25.1 | 1377 | 1560 | -4107 | HERVL | ERVL | LTR |

  

|  |  |  |  |  |  |  |  |  |  |
| --- | --- | --- | --- | --- | --- | --- | --- | --- | --- |
| chr1:218014631-218017439 | | | 2811 bp | | |  |  |  |  |
| Genome Start | Genome End | Strand | Div | Repeat Start | Repeat End | Repeat Left | Name | Family | Class |
| 218014631 | 218014845 | + | 7.1 | 2 | 217 | -158 | THE1C | MaLR | LTR |
| 218014846 | 218016682 | - | 3.0 | 4318 | 6154 | -1 | L1PA3 | L1 | LINE |
| 218016681 | 218017270 | + | 2.9 | 3722 | 4312 | -1834 | L1PA3 | L1 | LINE |
| 218017271 | 218017439 | + | 11.8 | 206 | 375 | 0 | THE1C | MaLR | LTR |

  

|  |  |  |  |  |  |  |  |  |  |
| --- | --- | --- | --- | --- | --- | --- | --- | --- | --- |
| chr1:219209978-219214258 | | | 3984 bp | | |  |  |  |  |
| Genome Start | Genome End | Strand | Div | Repeat Start | Repeat End | Repeat Left | Name | Family | Class |
| 219209978 | 219210103 | + | 21.0 | 52 | 195 | -2312 | HAL1 | L1 | LINE |
| 219210212 | 219210596 | + | 27.1 | 378 | 777 | -1730 | HAL1 | L1 | LINE |
| 219210597 | 219210907 | - | 8.4 | 1 | 312 | -1 | AluSq | Alu | SINE |
| 219210908 | 219211108 | + | 27.1 | 778 | 955 | -1552 | HAL1 | L1 | LINE |
| 219211129 | 219211558 | + | 27.1 | 956 | 1464 | -1043 | HAL1 | L1 | LINE |
| 219211592 | 219211645 | - | 5.6 | 499 | 552 | 0 | MLT2A1 | ERVL | LTR |
| 219211771 | 219212210 | - | 9.3 | 1 | 515 | -37 | MLT2A1 | ERVL | LTR |
| 219212212 | 219212377 | + | 27.1 | 1466 | 1672 | -835 | HAL1 | L1 | LINE |
| 219212382 | 219212685 | - | 14.1 | 5461 | 5771 | -397 | L1M3 | L1 | LINE |
| 219212686 | 219213060 | + | 18.4 | 3 | 365 | 0 | MLT1A0 | MaLR | LTR |
| 219213061 | 219213113 | - | 14.1 | 5412 | 5460 | -708 | L1M3 | L1 | LINE |
| 219213112 | 219213469 | + | 12.9 | 5777 | 6131 | -37 | L1MB1 | L1 | LINE |
| 219213470 | 219213695 | + | 8.8 | 5939 | 6164 | -8 | L1PA8 | L1 | LINE |
| 219213690 | 219213742 | + | 13.2 | 6115 | 6167 | -1 | L1MB1 | L1 | LINE |
| 219213757 | 219214258 | + | 27.8 | 1657 | 2186 | -321 | HAL1 | L1 | LINE |

  

|  |  |  |  |  |  |  |  |  |  |
| --- | --- | --- | --- | --- | --- | --- | --- | --- | --- |
| chr1:219374408-219375205 | | | 792 bp | | |  |  |  |  |
| Genome Start | Genome End | Strand | Div | Repeat Start | Repeat End | Repeat Left | Name | Family | Class |
| 219374408 | 219374763 | - | 20.1 | 1144 | 1410 | -5341 | L1MC3 | L1 | LINE |
| 219374769 | 219374945 | - | 14.1 | 5795 | 5971 | -185 | L1PB4 | L1 | LINE |
| 219374947 | 219375110 | + | 13.4 | 5981 | 6156 | 0 | L1PB4 | L1 | LINE |
| 219375111 | 219375205 | - | 27.0 | 984 | 1146 | -5605 | L1MC3 | L1 | LINE |

  

|  |  |  |  |  |  |  |  |  |  |
| --- | --- | --- | --- | --- | --- | --- | --- | --- | --- |
| chr1:219601878-219605984 | | | 3926 bp | | |  |  |  |  |
| Genome Start | Genome End | Strand | Div | Repeat Start | Repeat End | Repeat Left | Name | Family | Class |
| 219601878 | 219603535 | + | 32.5 | 1478 | 3158 | -944 | MER101-int | ERV1 | LTR |
| 219603698 | 219603824 | - | 12.6 | 5268 | 5398 | -748 | L1PREC2 | L1 | LINE |
| 219603816 | 219604739 | + | 11.4 | 4172 | 5098 | -1048 | L1PREC2 | L1 | LINE |
| 219604740 | 219605526 | - | 6.9 | 1 | 781 | 0 | MER4E1 | ERV1 | LTR |
| 219605527 | 219605685 | + | 11.4 | 5099 | 5270 | -876 | L1PREC2 | L1 | LINE |
| 219605714 | 219605984 | + | 34.6 | 5970 | 6246 | -2024 | MER101-int | ERV1 | LTR |

  

|  |  |  |  |  |  |  |  |  |  |
| --- | --- | --- | --- | --- | --- | --- | --- | --- | --- |
| chr1:220409606-220420827 | | | 11185 bp | | |  |  |  |  |
| Genome Start | Genome End | Strand | Div | Repeat Start | Repeat End | Repeat Left | Name | Family | Class |
| 220409606 | 220413358 | + | 15.8 | 1 | 3659 | -3190 | L1PA16 | L1 | LINE |
| 220413363 | 220413780 | - | 12.1 | 1 | 423 | -5 | MSTA | MaLR | LTR |
| 220413781 | 220414713 | - | 17.1 | 706 | 1651 | 0 | MSTA-int | MaLR | LTR |
| 220414720 | 220414973 | - | 14.2 | 1 | 257 | -1394 | MSTA-int | MaLR | LTR |
| 220414974 | 220415204 | - | 13.4 | 203 | 428 | 0 | MSTA | MaLR | LTR |
| 220415205 | 220415517 | - | 8.5 | 1 | 313 | 0 | AluY | Alu | SINE |
| 220415518 | 220415710 | - | 13.4 | 1 | 202 | -226 | MSTA | MaLR | LTR |
| 220415711 | 220416250 | + | 12.5 | 3655 | 4194 | -2655 | L1PA16 | L1 | LINE |
| 220416276 | 220416723 | + | 12.5 | 4195 | 4693 | -2156 | L1PA16 | L1 | LINE |
| 220416722 | 220417332 | - | 8.5 | 4570 | 5180 | -966 | L1PA8 | L1 | LINE |
| 220417331 | 220418327 | + | 9.3 | 5170 | 6172 | 0 | L1PA8 | L1 | LINE |
| 220418334 | 220418756 | + | 12.2 | 4680 | 5100 | -1749 | L1PA16 | L1 | LINE |
| 220418757 | 220419057 | + | 9.5 | 1 | 311 | 0 | AluSq | Alu | SINE |
| 220419058 | 220420827 | + | 12.3 | 5101 | 6860 | -9 | L1PA16 | L1 | LINE |

  

|  |  |  |  |  |  |  |  |  |  |
| --- | --- | --- | --- | --- | --- | --- | --- | --- | --- |
| chr1:220589503-220598971 | | | 9469 bp | | |  |  |  |  |
| Genome Start | Genome End | Strand | Div | Repeat Start | Repeat End | Repeat Left | Name | Family | Class |
| 220589503 | 220591534 | + | 12.7 | 3014 | 5003 | -2562 | L1MA3 | L1 | LINE |
| 220591527 | 220594369 | - | 3.0 | 1784 | 4630 | -1516 | L1PA3 | L1 | LINE |
| 220594369 | 220595873 | + | 2.8 | 4648 | 6152 | -3 | L1PA3 | L1 | LINE |
| 220595883 | 220597300 | + | 12.2 | 4981 | 6391 | -1174 | L1MA3 | L1 | LINE |
| 220597301 | 220597641 | + | 15.9 | 1 | 343 | 0 | MER61A | ERV1 | LTR |
| 220597642 | 220598971 | + | 13.6 | 6392 | 7724 | 0 | L1MA3 | L1 | LINE |

  

|  |  |  |  |  |  |  |  |  |  |
| --- | --- | --- | --- | --- | --- | --- | --- | --- | --- |
| chr1:222356059-222360444 | | | 4189 bp | | |  |  |  |  |
| Genome Start | Genome End | Strand | Div | Repeat Start | Repeat End | Repeat Left | Name | Family | Class |
| 222356059 | 222356087 | - | 28.3 | 2573 | 2588 | -831 | L2 | L2 | LINE |
| 222356088 | 222356208 | + | 14.4 | 6048 | 6169 | -9 | L1MB8 | L1 | LINE |
| 222356209 | 222356462 | - | 28.3 | 2329 | 2572 | -847 | L2 | L2 | LINE |
| 222356463 | 222356770 | + | 17.7 | 1 | 299 | -13 | AluJo | Alu | SINE |
| 222356771 | 222356864 | - | 28.3 | 2261 | 2328 | -1091 | L2 | L2 | LINE |
| 222356865 | 222357163 | + | 11.0 | 11 | 310 | 0 | AluSg | Alu | SINE |
| 222357164 | 222357221 | - | 28.3 | 2205 | 2260 | -1159 | L2 | L2 | LINE |
| 222357222 | 222357380 | - | 11.3 | 4609 | 4767 | -1379 | L1PB3 | L1 | LINE |
| 222357371 | 222357943 | + | 16.2 | 4762 | 5375 | -779 | L1PB3 | L1 | LINE |
| 222357944 | 222358245 | + | 14.6 | 1 | 303 | -9 | AluSx | Alu | SINE |
| 222358246 | 222358363 | + | 11.3 | 5376 | 5480 | -674 | L1PB3 | L1 | LINE |
| 222358364 | 222358670 | + | 12.1 | 1 | 307 | 0 | AluSx | Alu | SINE |
| 222358671 | 222359316 | + | 11.3 | 5481 | 6151 | -3 | L1PB3 | L1 | LINE |
| 222359524 | 222360444 | - | 32.3 | 1229 | 2175 | -1244 | L2 | L2 | LINE |

  

|  |  |  |  |  |  |  |  |  |  |
| --- | --- | --- | --- | --- | --- | --- | --- | --- | --- |
| chr1:223233397-223241413 | | | 7937 bp | | |  |  |  |  |
| Genome Start | Genome End | Strand | Div | Repeat Start | Repeat End | Repeat Left | Name | Family | Class |
| 223233397 | 223235167 | + | 15.5 | 17 | 1742 | -5823 | L1M1 | L1 | LINE |
| 223235169 | 223235325 | + | 2.5 | 5996 | 6154 | 0 | L1PA7 | L1 | LINE |
| 223235340 | 223236155 | - | 6.7 | 3110 | 3935 | -2211 | L1PB | L1 | LINE |
| 223236156 | 223236712 | + | 5.6 | 1 | 556 | -15 | LTR22 | ERVK | LTR |
| 223236804 | 223237682 | - | 8.4 | 2240 | 3115 | -3031 | L1PB | L1 | LINE |
| 223237684 | 223239941 | + | 8.8 | 3944 | 6145 | -6 | L1PB1 | L1 | LINE |
| 223239937 | 223240409 | + | 8.1 | 2177 | 2666 | -4899 | L1M1 | L1 | LINE |
| 223240388 | 223241413 | + | 12.7 | 4853 | 5817 | -1748 | L1M1 | L1 | LINE |

  

|  |  |  |  |  |  |  |  |  |  |
| --- | --- | --- | --- | --- | --- | --- | --- | --- | --- |
| chr1:223241726-223243445 | | | 1693 bp | | |  |  |  |  |
| Genome Start | Genome End | Strand | Div | Repeat Start | Repeat End | Repeat Left | Name | Family | Class |
| 223241726 | 223241909 | - | 23.0 | 222 | 396 | 0 | MSTD | MaLR | LTR |
| 223241946 | 223242238 | - | 13.4 | 13 | 304 | -6 | AluSg | Alu | SINE |
| 223242239 | 223242691 | - | 7.7 | 5690 | 6147 | -21 | L1PA10 | L1 | LINE |
| 223242682 | 223242984 | + | 9.3 | 5378 | 5683 | -485 | L1PA10 | L1 | LINE |
| 223242985 | 223243259 | - | 19.6 | 1 | 288 | -24 | AluJo | Alu | SINE |
| 223243261 | 223243445 | - | 20.4 | 4 | 191 | -205 | MSTD | MaLR | LTR |

  

|  |  |  |  |  |  |  |  |  |  |
| --- | --- | --- | --- | --- | --- | --- | --- | --- | --- |
| chr1:223537429-223542866 | | | 5369 bp | | |  |  |  |  |
| Genome Start | Genome End | Strand | Div | Repeat Start | Repeat End | Repeat Left | Name | Family | Class |
| 223537429 | 223539566 | + | 18.6 | 2259 | 4483 | -1899 | L1MD3 | L1 | LINE |
| 223539592 | 223539891 | + | 12.7 | 1 | 304 | -8 | AluSx | Alu | SINE |
| 223539900 | 223540198 | + | 15.8 | 1 | 299 | -13 | AluJo | Alu | SINE |
| 223540213 | 223540319 | - | 15.9 | 5699 | 5820 | -480 | L1MA5 | L1 | LINE |
| 223540320 | 223540773 | + | 18.3 | 5839 | 6300 | 0 | L1MA5 | L1 | LINE |
| 223540796 | 223542866 | + | 20.8 | 5613 | 8043 | -235 | L1MD3 | L1 | LINE |

  

|  |  |  |  |  |  |  |  |  |  |
| --- | --- | --- | --- | --- | --- | --- | --- | --- | --- |
| chr1:235747864-235752598 | | | 4675 bp | | |  |  |  |  |
| Genome Start | Genome End | Strand | Div | Repeat Start | Repeat End | Repeat Left | Name | Family | Class |
| 235747864 | 235748138 | + | 34.0 | 2476 | 2742 | -677 | L2 | L2 | LINE |
| 235748198 | 235750583 | - | 18.0 | 3792 | 6147 | -9 | L1PB4 | L1 | LINE |
| 235750582 | 235750676 | + | 16.8 | 3401 | 3480 | -2666 | L1P4 | L1 | LINE |
| 235750677 | 235750968 | + | 13.1 | 1 | 291 | -21 | AluSx | Alu | SINE |
| 235750969 | 235751260 | + | 16.8 | 3481 | 3774 | -2372 | L1P4 | L1 | LINE |
| 235751264 | 235751346 | + | 30.3 | 2773 | 2847 | -572 | L2 | L2 | LINE |
| 235751347 | 235751677 | + | 10.6 | 1 | 312 | 0 | AluSx | Alu | SINE |
| 235751678 | 235751897 | + | 30.3 | 2848 | 3075 | -304 | L2 | L2 | LINE |
| 235751898 | 235752079 | - | 5.0 | 5969 | 6154 | -1 | L1PA4 | L1 | LINE |
| 235752080 | 235752118 | + | 36.0 | 3075 | 3114 | -264 | L2 | L2 | LINE |
| 235752119 | 235752347 | - | 8.7 | 1 | 230 | 0 | MER30 | MER1\_type | DNA |
| 235752348 | 235752598 | + | 36.0 | 3115 | 3378 | 0 | L2 | L2 | LINE |

  

|  |  |  |  |  |  |  |  |  |  |
| --- | --- | --- | --- | --- | --- | --- | --- | --- | --- |
| chr1:236732188-236732825 | | | 589 bp | | |  |  |  |  |
| Genome Start | Genome End | Strand | Div | Repeat Start | Repeat End | Repeat Left | Name | Family | Class |
| 236732188 | 236732369 | + | 18.5 | 6032 | 6250 | -84 | L1MA10 | L1 | LINE |
| 236732382 | 236732445 | - | 3.1 | 5797 | 5860 | -295 | L1PA3 | L1 | LINE |
| 236732446 | 236732731 | + | 1.8 | 5865 | 6151 | -4 | L1PA3 | L1 | LINE |
| 236732769 | 236732825 | + | 14.3 | 6278 | 6333 | -1 | L1MA10 | L1 | LINE |

  

|  |  |  |  |  |  |  |  |  |  |
| --- | --- | --- | --- | --- | --- | --- | --- | --- | --- |
| chr1:236774871-236779167 | | | 4270 bp | | |  |  |  |  |
| Genome Start | Genome End | Strand | Div | Repeat Start | Repeat End | Repeat Left | Name | Family | Class |
| 236774871 | 236774952 | + | 23.2 | 1 | 83 | -263 | MER7A | MER2\_type | DNA |
| 236774980 | 236775328 | - | 9.5 | 1 | 355 | 0 | THE1A | MaLR | LTR |
| 236775329 | 236776411 | - | 31.4 | 598 | 1735 | 0 | MLT-int | MaLR | LTR |
| 236776412 | 236776699 | - | 9.0 | 15 | 305 | -4 | AluSc | Alu | SINE |
| 236776700 | 236777183 | - | 31.4 | 3 | 597 | -1138 | MLT-int | MaLR | LTR |
| 236777186 | 236777531 | - | 10.4 | 1 | 355 | 0 | THE1A | MaLR | LTR |
| 236777532 | 236778300 | - | 11.4 | 5357 | 6138 | -13 | L1PB1 | L1 | LINE |
| 236778299 | 236778884 | + | 11.1 | 4757 | 5356 | -790 | L1PB1 | L1 | LINE |
| 236778885 | 236779167 | + | 18.8 | 65 | 345 | -1 | MER7A | MER2\_type | DNA |

  

|  |  |  |  |  |  |  |  |  |  |
| --- | --- | --- | --- | --- | --- | --- | --- | --- | --- |
| chr1:241876871-241885226 | | | 7779 bp | | |  |  |  |  |
| Genome Start | Genome End | Strand | Div | Repeat Start | Repeat End | Repeat Left | Name | Family | Class |
| 241876871 | 241877110 | - | 23.8 | 4909 | 5157 | -1487 | L1M4 | L1 | LINE |
| 241877111 | 241877442 | + | 16.0 | 1 | 346 | 0 | MER7A | MER2\_type | DNA |
| 241877443 | 241877900 | - | 23.8 | 4419 | 4908 | -1736 | L1M4 | L1 | LINE |
| 241877901 | 241878211 | + | 13.5 | 1 | 311 | -1 | AluSx | Alu | SINE |
| 241878212 | 241879227 | - | 23.8 | 3225 | 4418 | -2226 | L1M4 | L1 | LINE |
| 241879356 | 241881807 | - | 7.7 | 3664 | 6136 | -18 | L1PA7 | L1 | LINE |
| 241881798 | 241881969 | + | 8.0 | 1699 | 1859 | -416 | L1P2 | L1 | LINE |
| 241881970 | 241882254 | - | 13.1 | 21 | 304 | -8 | AluSx | Alu | SINE |
| 241882255 | 241882939 | + | 8.5 | 1860 | 2543 | -3603 | L1P2 | L1 | LINE |
| 241882935 | 241883442 | + | 7.2 | 3153 | 3660 | -2486 | L1P2 | L1 | LINE |
| 241883417 | 241883856 | - | 27.8 | 2752 | 3255 | -3389 | L1M4 | L1 | LINE |
| 241884011 | 241884180 | - | 28.1 | 2365 | 2540 | -4124 | L1M4 | L1 | LINE |
| 241884425 | 241884543 | - | 15.1 | 1 | 119 | -24 | FLAM\_C | Alu | SINE |
| 241884544 | 241884672 | - | 23.2 | 1966 | 2112 | -4552 | L1M4 | L1 | LINE |
| 241884765 | 241885226 | - | 31.7 | 1289 | 1912 | -4913 | L1M4 | L1 | LINE |

  

|  |  |  |  |  |  |  |  |  |  |
| --- | --- | --- | --- | --- | --- | --- | --- | --- | --- |
| chr1:243510128-243512743 | | | 2616 bp | | |  |  |  |  |
| Genome Start | Genome End | Strand | Div | Repeat Start | Repeat End | Repeat Left | Name | Family | Class |
| 243510128 | 243510156 | + | 2.6 | 1 | 29 | -615 | LTR9B | ERV1 | LTR |
| 243510157 | 243510475 | + | 7.0 | 1 | 301 | -10 | AluY | Alu | SINE |
| 243510476 | 243510530 | + | 2.6 | 30 | 77 | -567 | LTR9B | ERV1 | LTR |
| 243510531 | 243511943 | - | 1.3 | 4740 | 6153 | -2 | L1PA2 | L1 | LINE |
| 243511944 | 243512264 | + | 1.2 | 4415 | 4735 | -1411 | L1PA2 | L1 | LINE |
| 243512265 | 243512743 | + | 12.6 | 61 | 557 | -87 | LTR9B | ERV1 | LTR |

  

|  |  |  |  |  |  |  |  |  |  |
| --- | --- | --- | --- | --- | --- | --- | --- | --- | --- |
| chr1:245979175-245985763 | | | 6336 bp | | |  |  |  |  |
| Genome Start | Genome End | Strand | Div | Repeat Start | Repeat End | Repeat Left | Name | Family | Class |
| 245979175 | 245981224 | + | 25.2 | 41 | 2521 | -4516 | L1M2 | L1 | LINE |
| 245981254 | 245981498 | + | 16.3 | 5402 | 5655 | -1448 | L1M2 | L1 | LINE |
| 245981553 | 245981596 | + | 18.2 | 5661 | 5704 | -1399 | L1M2 | L1 | LINE |
| 245981603 | 245982189 | - | 17.6 | 4733 | 5327 | -816 | L1M2 | L1 | LINE |
| 245982182 | 245982408 | - | 14.4 | 5958 | 6160 | -5 | L1PA15 | L1 | LINE |
| 245982400 | 245982714 | - | 16.2 | 4412 | 4739 | -1404 | L1M2 | L1 | LINE |
| 245982746 | 245982964 | - | 18.9 | 2387 | 2622 | -139 | Charlie1 | MER1\_type | DNA |
| 245983098 | 245983985 | - | 2.8 | 5262 | 6152 | -3 | L1PA4 | L1 | LINE |
| 245983983 | 245984387 | + | 3.7 | 4868 | 5271 | -875 | L1PA4 | L1 | LINE |
| 245984393 | 245984653 | - | 23.9 | 1913 | 2180 | -581 | Charlie1 | MER1\_type | DNA |
| 245984668 | 245984854 | + | 18.2 | 6096 | 6290 | -7 | L1MA5A | L1 | LINE |
| 245984856 | 245985763 | + | 18.0 | 5689 | 6636 | -615 | L1M2 | L1 | LINE |

  

|  |  |  |  |  |  |  |  |  |  |
| --- | --- | --- | --- | --- | --- | --- | --- | --- | --- |
| chr1:246756312-246767103 | | | 10581 bp | | |  |  |  |  |
| Genome Start | Genome End | Strand | Div | Repeat Start | Repeat End | Repeat Left | Name | Family | Class |
| 246756312 | 246756603 | + | 30.7 | 209 | 510 | -5998 | L1MCa | L1 | LINE |
| 246756593 | 246757591 | - | 6.0 | 4472 | 5484 | -670 | L1PA5 | L1 | LINE |
| 246757592 | 246757883 | + | 4.1 | 5491 | 5782 | -372 | L1PA5 | L1 | LINE |
| 246757882 | 246763865 | + | 3.1 | 10 | 6032 | 0 | L1PA3 | L1 | LINE |
| 246763900 | 246764283 | + | 5.2 | 5770 | 6154 | 0 | L1PA5 | L1 | LINE |
| 246764284 | 246764754 | + | 26.5 | 502 | 979 | -5113 | L1MCa | L1 | LINE |
| 246764758 | 246764919 | + | 17.8 | 3 | 160 | -307 | MLT1C | MaLR | LTR |
| 246764925 | 246765034 | - | 24.6 | 402 | 512 | -31 | MER67A | ERV1 | LTR |
| 246765035 | 246765207 | + | 19.1 | 17 | 188 | -355 | MER67A | ERV1 | LTR |
| 246765217 | 246765545 | - | 20.1 | 1 | 349 | -16 | MLT1A0 | MaLR | LTR |
| 246765546 | 246765882 | + | 24.8 | 201 | 543 | 0 | MER67A | ERV1 | LTR |
| 246765966 | 246766724 | + | 28.3 | 49 | 778 | -4158 | MER67A-int | ERV1 | LTR |
| 246766772 | 246766841 | + | 21.4 | 468 | 541 | -2 | MER67A | ERV1 | LTR |
| 246766843 | 246766979 | + | 20.1 | 157 | 301 | -166 | MLT1C | MaLR | LTR |
| 246767022 | 246767103 | + | 26.8 | 1038 | 1121 | -5630 | L1MCa | L1 | LINE |

  


|  |  |  |  |  |  |  |  |  |  |
| --- | --- | --- | --- | --- | --- | --- | --- | --- | --- |
| chr2:689641-696133 | | | 6233 bp | | |  |  |  |  |
| Genome Start | Genome End | Strand | Div | Repeat Start | Repeat End | Repeat Left | Name | Family | Class |
| 689641 | 689926 | + | 26.2 | 3276 | 3580 | -2566 | L1M5 | L1 | LINE |
| 689926 | 690081 | - | 9.6 | 5609 | 5764 | -390 | L1PA7 | L1 | LINE |
| 690082 | 690461 | + | 6.2 | 5763 | 6136 | -18 | L1PA7 | L1 | LINE |
| 690543 | 691579 | + | 28.4 | 3634 | 4807 | -1339 | L1M5 | L1 | LINE |
| 691629 | 691750 | - | 5.7 | 4437 | 4558 | -1588 | L1PB | L1 | LINE |
| 691746 | 692409 | + | 14.0 | 4550 | 5329 | -817 | L1PB1 | L1 | LINE |
| 692412 | 692544 | + | 9.0 | 1 | 133 | -179 | AluSx | Alu | SINE |
| 692549 | 693588 | - | 6.6 | 1 | 1033 | 0 | LTR5A | ERVK | LTR |
| 693589 | 693766 | + | 9.5 | 134 | 304 | -8 | AluSx | Alu | SINE |
| 693775 | 694546 | + | 6.6 | 5406 | 6168 | -4 | L1PA8 | L1 | LINE |
| 694580 | 695409 | + | 10.9 | 5314 | 6143 | -8 | L1PB1 | L1 | LINE |
| 695499 | 696133 | + | 28.9 | 4975 | 5653 | -509 | L1M5 | L1 | LINE |

  

|  |  |  |  |  |  |  |  |  |  |
| --- | --- | --- | --- | --- | --- | --- | --- | --- | --- |
| chr2:776252-777535 | | | 1247 bp | | |  |  |  |  |
| Genome Start | Genome End | Strand | Div | Repeat Start | Repeat End | Repeat Left | Name | Family | Class |
| 776252 | 776561 | + | 24.3 | 4549 | 4870 | -1276 | L1M5 | L1 | LINE |
| 776582 | 776808 | - | 0.9 | 5570 | 5796 | -359 | L1PA4 | L1 | LINE |
| 776809 | 777158 | + | 5.1 | 5801 | 6150 | -5 | L1PA4 | L1 | LINE |
| 777163 | 777309 | + | 21.8 | 5327 | 5473 | -697 | L1M5 | L1 | LINE |
| 777323 | 777535 | + | 35.0 | 4937 | 5152 | -994 | L1M5 | L1 | LINE |

  

|  |  |  |  |  |  |  |  |  |  |
| --- | --- | --- | --- | --- | --- | --- | --- | --- | --- |
| chr2:5335901-5338371 | | | 2229 bp | | |  |  |  |  |
| Genome Start | Genome End | Strand | Div | Repeat Start | Repeat End | Repeat Left | Name | Family | Class |
| 5335901 | 5336190 | + | 27.1 | 277 | 540 | -4314 | L1MEe | L1 | LINE |
| 5336231 | 5336479 | + | 26.7 | 1493 | 1768 | -3066 | L1MEe | L1 | LINE |
| 5336684 | 5336809 | + | 19.7 | 2886 | 3012 | -1816 | L1MEe | L1 | LINE |
| 5336810 | 5337104 | - | 12.2 | 11 | 305 | -7 | AluSx | Alu | SINE |
| 5337105 | 5337299 | + | 19.7 | 3013 | 3204 | -1624 | L1MEe | L1 | LINE |
| 5337296 | 5337682 | - | 1.5 | 5227 | 5613 | -542 | L1PA2 | L1 | LINE |
| 5337683 | 5338241 | + | 1.7 | 5621 | 6179 | 0 | L1PA2 | L1 | LINE |
| 5338244 | 5338371 | + | 19.4 | 3193 | 3314 | -1514 | L1MEe | L1 | LINE |

  

|  |  |  |  |  |  |  |  |  |  |
| --- | --- | --- | --- | --- | --- | --- | --- | --- | --- |
| chr2:8987351-8988634 | | | 1284 bp | | |  |  |  |  |
| Genome Start | Genome End | Strand | Div | Repeat Start | Repeat End | Repeat Left | Name | Family | Class |
| 8987351 | 8987867 | + | 4.5 | 1 | 552 | -170 | LTR30 | ERV1 | LTR |
| 8987868 | 8988253 | - | 1.6 | 5561 | 5947 | -208 | L1PA4 | L1 | LINE |
| 8988254 | 8988455 | + | 4.5 | 5952 | 6153 | -2 | L1PA4 | L1 | LINE |
| 8988456 | 8988634 | + | 7.8 | 543 | 722 | 0 | LTR30 | ERV1 | LTR |

  

|  |  |  |  |  |  |  |  |  |  |
| --- | --- | --- | --- | --- | --- | --- | --- | --- | --- |
| chr2:11380785-11382342 | | | 1163 bp | | |  |  |  |  |
| Genome Start | Genome End | Strand | Div | Repeat Start | Repeat End | Repeat Left | Name | Family | Class |
| 11380785 | 11380905 | + | 29.8 | 8 | 143 | -125 | MIRb | MIR | SINE |
| 11381249 | 11381665 | - | 17.2 | 5909 | 6333 | 0 | L1MC1 | L1 | LINE |
| 11381666 | 11381731 | + | 11.3 | 5609 | 5674 | -659 | L1MC1 | L1 | LINE |
| 11381732 | 11382030 | + | 7.8 | 1 | 309 | 0 | AluSc | Alu | SINE |
| 11382031 | 11382261 | + | 11.3 | 5675 | 5898 | -435 | L1MC1 | L1 | LINE |
| 11382314 | 11382342 | + | 1.9 | 202 | 241 | -27 | MIRb | MIR | SINE |

  

|  |  |  |  |  |  |  |  |  |  |
| --- | --- | --- | --- | --- | --- | --- | --- | --- | --- |
| chr2:11901515-11904050 | | | 2355 bp | | |  |  |  |  |
| Genome Start | Genome End | Strand | Div | Repeat Start | Repeat End | Repeat Left | Name | Family | Class |
| 11901515 | 11901689 | - | 10.3 | 119 | 294 | -17 | AluY | Alu | SINE |
| 11901882 | 11903849 | - | 4.1 | 4169 | 6143 | -11 | L1PA5 | L1 | LINE |
| 11903836 | 11903915 | + | 5.1 | 4106 | 4184 | -1962 | L1PA5 | L1 | LINE |
| 11903919 | 11904050 | - | 8.3 | 1 | 132 | -179 | AluY | Alu | SINE |

  

|  |  |  |  |  |  |  |  |  |  |
| --- | --- | --- | --- | --- | --- | --- | --- | --- | --- |
| chr2:17035057-17044291 | | | 9252 bp | | |  |  |  |  |
| Genome Start | Genome End | Strand | Div | Repeat Start | Repeat End | Repeat Left | Name | Family | Class |
| 17035057 | 17035222 | - | 9.0 | 7533 | 7713 | -11 | L1MA3 | L1 | LINE |
| 17035223 | 17035514 | + | 12.3 | 3 | 294 | -16 | AluSg | Alu | SINE |
| 17035515 | 17039145 | - | 10.2 | 3866 | 7532 | -192 | L1MA3 | L1 | LINE |
| 17039129 | 17040839 | - | 12.2 | 1782 | 3638 | -3927 | L1MA3 | L1 | LINE |
| 17040832 | 17042812 | - | 8.6 | 4198 | 6172 | 0 | L1PA8 | L1 | LINE |
| 17042806 | 17043186 | + | 10.5 | 3814 | 4194 | -1952 | L1PA8 | L1 | LINE |
| 17043202 | 17044291 | - | 18.1 | 605 | 1777 | -5788 | L1MA3 | L1 | LINE |

  

|  |  |  |  |  |  |  |  |  |  |
| --- | --- | --- | --- | --- | --- | --- | --- | --- | --- |
| chr2:17409780-17417431 | | | 7623 bp | | |  |  |  |  |
| Genome Start | Genome End | Strand | Div | Repeat Start | Repeat End | Repeat Left | Name | Family | Class |
| 17409780 | 17414599 | - | 9.8 | 1838 | 6863 | -4 | L1PB1 | L1 | LINE |
| 17414636 | 17415399 | - | 2.1 | 5377 | 6143 | -12 | L1PA3 | L1 | LINE |
| 17415402 | 17416430 | + | 2.5 | 4328 | 5356 | -790 | L1PA3 | L1 | LINE |
| 17416418 | 17417242 | - | 15.0 | 1098 | 1870 | -4993 | L1PB1 | L1 | LINE |
| 17417247 | 17417431 | - | 6.0 | 7 | 191 | -6672 | L1PB1 | L1 | LINE |

  

|  |  |  |  |  |  |  |  |  |  |
| --- | --- | --- | --- | --- | --- | --- | --- | --- | --- |
| chr2:19273097-19275994 | | | 2898 bp | | |  |  |  |  |
| Genome Start | Genome End | Strand | Div | Repeat Start | Repeat End | Repeat Left | Name | Family | Class |
| 19273097 | 19273163 | + | 34.9 | 10 | 75 | -187 | MIR | MIR | SINE |
| 19273164 | 19273518 | + | 15.4 | 3 | 372 | -56 | MSTA | MaLR | LTR |
| 19273519 | 19274476 | - | 3.1 | 5198 | 6151 | -3 | L1PA5 | L1 | LINE |
| 19274477 | 19275818 | + | 5.1 | 3839 | 5184 | -962 | L1PA5 | L1 | LINE |
| 19275819 | 19275900 | + | 5.0 | 357 | 457 | 0 | MSTA | MaLR | LTR |
| 19275901 | 19275994 | + | 31.9 | 69 | 170 | -92 | MIR | MIR | SINE |

  

|  |  |  |  |  |  |  |  |  |  |
| --- | --- | --- | --- | --- | --- | --- | --- | --- | --- |
| chr2:19316365-19318333 | | | 1679 bp | | |  |  |  |  |
| Genome Start | Genome End | Strand | Div | Repeat Start | Repeat End | Repeat Left | Name | Family | Class |
| 19316365 | 19316706 | + | 32.7 | 2210 | 2594 | -825 | L2 | L2 | LINE |
| 19316822 | 19316983 | - | 8.0 | 5208 | 5374 | -772 | L1PA7 | L1 | LINE |
| 19316982 | 19317103 | + | 4.9 | 5365 | 5487 | -667 | L1PA7 | L1 | LINE |
| 19317104 | 19317414 | + | 9.7 | 1 | 300 | -11 | AluY | Alu | SINE |
| 19317415 | 19318080 | + | 4.9 | 5488 | 6152 | -2 | L1PA7 | L1 | LINE |
| 19318258 | 19318333 | + | 24.3 | 3299 | 3374 | -4 | L2 | L2 | LINE |

  

|  |  |  |  |  |  |  |  |  |  |
| --- | --- | --- | --- | --- | --- | --- | --- | --- | --- |
| chr2:21155145-21157452 | | | 2267 bp | | |  |  |  |  |
| Genome Start | Genome End | Strand | Div | Repeat Start | Repeat End | Repeat Left | Name | Family | Class |
| 21155145 | 21155247 | + | 15.6 | 2 | 98 | -1273 | MLT1A-int | MaLR | LTR |
| 21155272 | 21155387 | - | 19.0 | 16 | 134 | -178 | AluJo | Alu | SINE |
| 21155414 | 21155983 | - | 5.3 | 5569 | 6147 | -7 | L1PA7 | L1 | LINE |
| 21155984 | 21156981 | + | 7.3 | 4553 | 5554 | -600 | L1PA7 | L1 | LINE |
| 21156982 | 21157015 | + | 8.8 | 82 | 115 | -1620 | MLT1A-int | MaLR | LTR |
| 21157007 | 21157452 | + | 20.7 | 260 | 732 | -639 | MLT1A-int | MaLR | LTR |

  

|  |  |  |  |  |  |  |  |  |  |
| --- | --- | --- | --- | --- | --- | --- | --- | --- | --- |
| chr2:23513654-23523059 | | | 9022 bp | | |  |  |  |  |
| Genome Start | Genome End | Strand | Div | Repeat Start | Repeat End | Repeat Left | Name | Family | Class |
| 23513654 | 23513981 | + | 35.6 | 435 | 774 | -1186 | L4 | RTE | LINE |
| 23513983 | 23514033 | - | 18.0 | 435 | 484 | 0 | MLT1H2 | MaLR | LTR |
| 23514034 | 23516524 | - | 3.3 | 2314 | 4805 | -1341 | L1PA3 | L1 | LINE |
| 23516524 | 23517850 | + | 3.3 | 4811 | 6137 | -18 | L1PA3 | L1 | LINE |
| 23517901 | 23518299 | - | 24.7 | 135 | 558 | -37 | MLT1G1 | MaLR | LTR |
| 23518300 | 23518471 | + | 19.4 | 1 | 175 | -330 | MLT1D | MaLR | LTR |
| 23518472 | 23518768 | - | 17.4 | 1 | 298 | -14 | AluJb | Alu | SINE |
| 23518769 | 23519109 | + | 19.4 | 176 | 505 | 0 | MLT1D | MaLR | LTR |
| 23519110 | 23519200 | - | 22.3 | 13 | 134 | -389 | MLT1G1 | MaLR | LTR |
| 23519207 | 23519380 | + | 37.2 | 793 | 974 | -986 | L4 | RTE | LINE |
| 23519445 | 23520408 | - | 29.3 | 7104 | 8042 | 0 | L1MC4 | L1 | LINE |
| 23520417 | 23520725 | - | 28.8 | 6492 | 6833 | -975 | L1MC4a | L1 | LINE |
| 23520726 | 23521000 | - | 9.5 | 2 | 274 | -39 | AluSq | Alu | SINE |
| 23521001 | 23521340 | - | 28.8 | 6144 | 6491 | -1317 | L1MC4a | L1 | LINE |
| 23521341 | 23521539 | - | 15.7 | 5 | 209 | -1 | MER6B | MER2\_type | DNA |
| 23521540 | 23522355 | - | 28.8 | 5255 | 6143 | -1665 | L1MC4a | L1 | LINE |
| 23522476 | 23522742 | - | 27.1 | 4795 | 5092 | -1054 | L1MC4a | L1 | LINE |
| 23522879 | 23523059 | + | 41.3 | 1353 | 1532 | -428 | L4 | RTE | LINE |

  

|  |  |  |  |  |  |  |  |  |  |
| --- | --- | --- | --- | --- | --- | --- | --- | --- | --- |
| chr2:27887964-27890200 | | | 1931 bp | | |  |  |  |  |
| Genome Start | Genome End | Strand | Div | Repeat Start | Repeat End | Repeat Left | Name | Family | Class |
| 27887964 | 27888269 | + | 28.4 | 3 | 333 | -5786 | L1MEd | L1 | LINE |
| 27888390 | 27888459 | - | 2.9 | 5283 | 5356 | -775 | L1P | L1 | LINE |
| 27888460 | 27889238 | + | 3.3 | 5357 | 6152 | -2 | L1PA5 | L1 | LINE |
| 27889425 | 27890200 | + | 24.9 | 510 | 1257 | -4862 | L1MEd | L1 | LINE |

  

|  |  |  |  |  |  |  |  |  |  |
| --- | --- | --- | --- | --- | --- | --- | --- | --- | --- |
| chr2:30917877-30921907 | | | 3659 bp | | |  |  |  |  |
| Genome Start | Genome End | Strand | Div | Repeat Start | Repeat End | Repeat Left | Name | Family | Class |
| 30917877 | 30918327 | - | 38.7 | 2914 | 3378 | 0 | L2 | L2 | LINE |
| 30918426 | 30918653 | - | 27.2 | 2905 | 3155 | -2991 | L1MC4a | L1 | LINE |
| 30918649 | 30918948 | + | 30.0 | 3145 | 3486 | -2660 | L1MC4a | L1 | LINE |
| 30919076 | 30919216 | + | 24.5 | 3647 | 3789 | -2357 | L1MC4a | L1 | LINE |
| 30919210 | 30920956 | + | 18.8 | 4334 | 6168 | -16 | L1MB7 | L1 | LINE |
| 30921069 | 30921737 | + | 28.6 | 5284 | 5976 | -326 | L1MC4a | L1 | LINE |
| 30921785 | 30921907 | - | 30.4 | 2680 | 2806 | -613 | L2 | L2 | LINE |

  

|  |  |  |  |  |  |  |  |  |  |
| --- | --- | --- | --- | --- | --- | --- | --- | --- | --- |
| chr2:31257652-31261839 | | | 3495 bp | | |  |  |  |  |
| Genome Start | Genome End | Strand | Div | Repeat Start | Repeat End | Repeat Left | Name | Family | Class |
| 31257652 | 31257731 | - | 28.2 | 4408 | 4488 | -1 | L3 | CR1 | LINE |
| 31257890 | 31258484 | - | 3.0 | 5563 | 6155 | 0 | L1PA3 | L1 | LINE |
| 31258485 | 31260125 | + | 2.7 | 3920 | 5560 | -595 | L1PA3 | L1 | LINE |
| 31260244 | 31260552 | - | 10.8 | 1 | 310 | -2 | AluSx | Alu | SINE |
| 31260708 | 31260923 | - | 30.2 | 3934 | 4164 | -325 | L3 | CR1 | LINE |
| 31261186 | 31261839 | - | 31.9 | 3269 | 3918 | -571 | L3 | CR1 | LINE |

  

|  |  |  |  |  |  |  |  |  |  |
| --- | --- | --- | --- | --- | --- | --- | --- | --- | --- |
| chr2:31512117-31516381 | | | 4231 bp | | |  |  |  |  |
| Genome Start | Genome End | Strand | Div | Repeat Start | Repeat End | Repeat Left | Name | Family | Class |
| 31512117 | 31512481 | - | 18.0 | 4442 | 4833 | -19 | L1ME2 | L1 | LINE |
| 31512483 | 31512980 | - | 25.4 | 3773 | 4316 | -536 | L1ME2 | L1 | LINE |
| 31512983 | 31514372 | - | 2.5 | 3809 | 5198 | -948 | L1PA3 | L1 | LINE |
| 31514372 | 31515390 | + | 3.4 | 5185 | 6205 | 0 | L1PA3 | L1 | LINE |
| 31515423 | 31515625 | - | 23.2 | 3219 | 3439 | -1395 | L1ME2 | L1 | LINE |
| 31515626 | 31515935 | + | 5.5 | 1 | 311 | 0 | AluY | Alu | SINE |
| 31515936 | 31516381 | - | 23.2 | 2749 | 3218 | -1616 | L1ME2 | L1 | LINE |

  

|  |  |  |  |  |  |  |  |  |  |
| --- | --- | --- | --- | --- | --- | --- | --- | --- | --- |
| chr2:31902405-31908569 | | | 6181 bp | | |  |  |  |  |
| Genome Start | Genome End | Strand | Div | Repeat Start | Repeat End | Repeat Left | Name | Family | Class |
| 31902405 | 31902879 | - | 10.6 | 3786 | 4270 | -3295 | L1MA2 | L1 | LINE |
| 31902875 | 31903918 | - | 2.9 | 5103 | 6150 | -5 | L1PA4 | L1 | LINE |
| 31903917 | 31904820 | + | 4.5 | 4192 | 5096 | -1050 | L1PA4 | L1 | LINE |
| 31904812 | 31905476 | - | 10.0 | 3129 | 3808 | -3757 | L1MA2 | L1 | LINE |
| 31905477 | 31905782 | + | 8.6 | 1 | 304 | -7 | AluY | Alu | SINE |
| 31905783 | 31908569 | - | 17.9 | 25 | 3128 | -4437 | L1MA2 | L1 | LINE |

  

|  |  |  |  |  |  |  |  |  |  |
| --- | --- | --- | --- | --- | --- | --- | --- | --- | --- |
| chr2:31973470-31981047 | | | 7517 bp | | |  |  |  |  |
| Genome Start | Genome End | Strand | Div | Repeat Start | Repeat End | Repeat Left | Name | Family | Class |
| 31973470 | 31973555 | + | 35.3 | 792 | 876 | -5666 | L1MB7 | L1 | LINE |
| 31973619 | 31974410 | + | 23.6 | 844 | 1723 | -5249 | L1MB7 | L1 | LINE |
| 31974411 | 31974714 | - | 4.0 | 2 | 306 | -5 | AluY | Alu | SINE |
| 31974715 | 31974768 | + | 23.6 | 1724 | 1772 | -5200 | L1MB7 | L1 | LINE |
| 31974770 | 31974924 | + | 21.2 | 5399 | 5541 | -1103 | L1MB7 | L1 | LINE |
| 31974925 | 31975138 | - | 26.6 | 1 | 224 | 0 | MER58A | MER1\_type | DNA |
| 31975139 | 31975482 | + | 21.2 | 5542 | 5898 | -746 | L1MB7 | L1 | LINE |
| 31975478 | 31977598 | - | 9.6 | 2337 | 4473 | -1673 | L1PA7 | L1 | LINE |
| 31977594 | 31979267 | + | 7.3 | 4476 | 6154 | 0 | L1PA7 | L1 | LINE |
| 31979267 | 31979662 | + | 20.6 | 5884 | 6322 | -360 | L1MB7 | L1 | LINE |
| 31979663 | 31979974 | + | 12.2 | 1 | 311 | -1 | AluSx | Alu | SINE |
| 31979975 | 31980197 | + | 20.6 | 6323 | 6551 | -131 | L1MB7 | L1 | LINE |
| 31980198 | 31980494 | + | 11.2 | 1 | 295 | -17 | AluSx | Alu | SINE |
| 31980495 | 31980565 | + | 20.6 | 6552 | 6616 | -66 | L1MB7 | L1 | LINE |
| 31980566 | 31980699 | + | 18.4 | 2 | 126 | -7 | FLAM\_A | Alu | SINE |
| 31980708 | 31980982 | + | 13.4 | 1 | 294 | -18 | AluSx | Alu | SINE |
| 31980983 | 31981047 | + | 20.6 | 6617 | 6672 | -10 | L1MB7 | L1 | LINE |

  

|  |  |  |  |  |  |  |  |  |  |
| --- | --- | --- | --- | --- | --- | --- | --- | --- | --- |
| chr2:33228964-33231376 | | | 2322 bp | | |  |  |  |  |
| Genome Start | Genome End | Strand | Div | Repeat Start | Repeat End | Repeat Left | Name | Family | Class |
| 33228964 | 33229277 | - | 17.6 | 5872 | 6180 | 0 | L1MB4 | L1 | LINE |
| 33229278 | 33229610 | - | 10.6 | 1 | 312 | 0 | AluSx | Alu | SINE |
| 33229611 | 33229999 | - | 17.6 | 5476 | 5871 | -309 | L1MB4 | L1 | LINE |
| 33230041 | 33230394 | - | 12.6 | 5783 | 6149 | -2 | L1PB1 | L1 | LINE |
| 33230450 | 33230549 | - | 12.6 | 5683 | 5782 | -369 | L1PB1 | L1 | LINE |
| 33230550 | 33231182 | + | 7.2 | 5052 | 5686 | -465 | L1PB1 | L1 | LINE |
| 33231178 | 33231376 | - | 12.2 | 5297 | 5491 | -689 | L1MB4 | L1 | LINE |

  

|  |  |  |  |  |  |  |  |  |  |
| --- | --- | --- | --- | --- | --- | --- | --- | --- | --- |
| chr2:34598371-34600802 | | | 2294 bp | | |  |  |  |  |
| Genome Start | Genome End | Strand | Div | Repeat Start | Repeat End | Repeat Left | Name | Family | Class |
| 34598371 | 34599231 | + | 30.0 | 78 | 1056 | -3959 | L1ME3A | L1 | LINE |
| 34599245 | 34599629 | + | 25.1 | 3815 | 4295 | -633 | L1ME3A | L1 | LINE |
| 34599755 | 34600063 | + | 31.4 | 4366 | 4689 | -169 | L1ME3A | L1 | LINE |
| 34600064 | 34600191 | - | 5.5 | 5594 | 5721 | -433 | L1PA6 | L1 | LINE |
| 34600192 | 34600619 | + | 5.8 | 5725 | 6152 | -2 | L1PA6 | L1 | LINE |
| 34600620 | 34600802 | + | 32.2 | 4678 | 4854 | -4 | L1ME3A | L1 | LINE |

  

|  |  |  |  |  |  |  |  |  |  |
| --- | --- | --- | --- | --- | --- | --- | --- | --- | --- |
| chr2:35942782-35948441 | | | 5461 bp | | |  |  |  |  |
| Genome Start | Genome End | Strand | Div | Repeat Start | Repeat End | Repeat Left | Name | Family | Class |
| 35942782 | 35943003 | + | 27.2 | 92 | 341 | -6059 | L1MEc | L1 | LINE |
| 35943037 | 35944026 | + | 26.8 | 467 | 1450 | -4950 | L1MEc | L1 | LINE |
| 35944027 | 35944336 | + | 0.3 | 1 | 310 | 0 | AluYa5 | Alu | SINE |
| 35944337 | 35944740 | + | 26.8 | 1451 | 1946 | -4454 | L1MEc | L1 | LINE |
| 35944875 | 35946198 | + | 25.2 | 2168 | 3420 | -2799 | L1MEc | L1 | LINE |
| 35946199 | 35946493 | - | 12.9 | 1 | 295 | -18 | AluSq | Alu | SINE |
| 35946494 | 35946633 | + | 25.2 | 3421 | 3558 | -2661 | L1MEc | L1 | LINE |
| 35946655 | 35947192 | - | 16.4 | 5143 | 5689 | -623 | L1MA9 | L1 | LINE |
| 35947196 | 35947830 | + | 18.2 | 5664 | 6277 | -35 | L1MA9 | L1 | LINE |
| 35947839 | 35948441 | + | 22.0 | 3571 | 4189 | -2030 | L1MEc | L1 | LINE |

  

|  |  |  |  |  |  |  |  |  |  |
| --- | --- | --- | --- | --- | --- | --- | --- | --- | --- |
| chr2:40052633-40055889 | | | 3257 bp | | |  |  |  |  |
| Genome Start | Genome End | Strand | Div | Repeat Start | Repeat End | Repeat Left | Name | Family | Class |
| 40052633 | 40052815 | + | 15.0 | 5949 | 6139 | -152 | L1MA8 | L1 | LINE |
| 40052824 | 40054843 | - | 4.5 | 4128 | 6152 | -3 | L1PA4 | L1 | LINE |
| 40054836 | 40055736 | + | 4.4 | 3221 | 4124 | -2022 | L1PA4 | L1 | LINE |
| 40055737 | 40055889 | + | 13.2 | 6127 | 6285 | -6 | L1MA8 | L1 | LINE |

  

|  |  |  |  |  |  |  |  |  |  |
| --- | --- | --- | --- | --- | --- | --- | --- | --- | --- |
| chr2:41234918-41236044 | | | 1126 bp | | |  |  |  |  |
| Genome Start | Genome End | Strand | Div | Repeat Start | Repeat End | Repeat Left | Name | Family | Class |
| 41234918 | 41235608 | - | 26.8 | 2556 | 3419 | 0 | L2 | L2 | LINE |
| 41235609 | 41235812 | - | 2.9 | 5948 | 6153 | -2 | L1PA4 | L1 | LINE |
| 41235813 | 41235913 | + | 5.0 | 5854 | 5954 | -201 | L1PA4 | L1 | LINE |
| 41235915 | 41236044 | - | 23.9 | 2420 | 2565 | -854 | L2 | L2 | LINE |

  

|  |  |  |  |  |  |  |  |  |  |
| --- | --- | --- | --- | --- | --- | --- | --- | --- | --- |
| chr2:41308076-41314061 | | | 5980 bp | | |  |  |  |  |
| Genome Start | Genome End | Strand | Div | Repeat Start | Repeat End | Repeat Left | Name | Family | Class |
| 41308076 | 41308641 | + | 23.7 | 24 | 644 | -5756 | L1MEc | L1 | LINE |
| 41308642 | 41308857 | + | 26.4 | 1 | 219 | 0 | MER20 | MER1\_type | DNA |
| 41308858 | 41309257 | + | 23.7 | 645 | 1080 | -5320 | L1MEc | L1 | LINE |
| 41309258 | 41309563 | + | 12.8 | 1 | 307 | -3 | AluSg | Alu | SINE |
| 41309564 | 41310284 | + | 23.7 | 1081 | 1728 | -4672 | L1MEc | L1 | LINE |
| 41310279 | 41312708 | - | 17.0 | 2718 | 5229 | -914 | L1MA4 | L1 | LINE |
| 41312703 | 41313703 | + | 16.6 | 5254 | 6278 | -22 | L1MA4 | L1 | LINE |
| 41313722 | 41314061 | + | 21.8 | 1688 | 5431 | -788 | L1MEc | L1 | LINE |

  

|  |  |  |  |  |  |  |  |  |  |
| --- | --- | --- | --- | --- | --- | --- | --- | --- | --- |
| chr2:41352138-41357225 | | | 5076 bp | | |  |  |  |  |
| Genome Start | Genome End | Strand | Div | Repeat Start | Repeat End | Repeat Left | Name | Family | Class |
| 41352138 | 41353232 | - | 15.2 | 1295 | 2417 | -1 | Tigger1 | MER2\_type | DNA |
| 41353233 | 41354888 | - | 3.1 | 3427 | 5082 | -1064 | L1PA4 | L1 | LINE |
| 41354889 | 41355951 | + | 3.9 | 5086 | 6152 | -3 | L1PA4 | L1 | LINE |
| 41355964 | 41357225 | - | 13.5 | 26 | 1310 | -1108 | Tigger1 | MER2\_type | DNA |

  

|  |  |  |  |  |  |  |  |  |  |
| --- | --- | --- | --- | --- | --- | --- | --- | --- | --- |
| chr2:41595303-41599522 | | | 4225 bp | | |  |  |  |  |
| Genome Start | Genome End | Strand | Div | Repeat Start | Repeat End | Repeat Left | Name | Family | Class |
| 41595303 | 41595491 | - | 30.1 | 243 | 445 | -5 | LTR16A | ERVL | LTR |
| 41595492 | 41597589 | - | 3.1 | 2294 | 4391 | -1755 | L1PA3 | L1 | LINE |
| 41597585 | 41599333 | + | 2.5 | 4405 | 6154 | -1 | L1PA3 | L1 | LINE |
| 41599334 | 41599522 | - | 28.2 | 66 | 255 | -195 | LTR16A | ERVL | LTR |

  

|  |  |  |  |  |  |  |  |  |  |
| --- | --- | --- | --- | --- | --- | --- | --- | --- | --- |
| chr2:41754904-41758659 | | | 3327 bp | | |  |  |  |  |
| Genome Start | Genome End | Strand | Div | Repeat Start | Repeat End | Repeat Left | Name | Family | Class |
| 41754904 | 41755030 | + | 32.8 | 27 | 150 | -5969 | L1MEd | L1 | LINE |
| 41755186 | 41756201 | - | 16.4 | 4738 | 5694 | -606 | L1M2 | L1 | LINE |
| 41756204 | 41756817 | + | 15.2 | 5710 | 6334 | 0 | L1MA10 | L1 | LINE |
| 41756901 | 41757195 | + | 14.6 | 5 | 299 | -10 | AluSc | Alu | SINE |
| 41757261 | 41757586 | - | 25.3 | 114 | 459 | -9 | LTR37B | ERV1 | LTR |
| 41757584 | 41757707 | - | 16.5 | 10 | 133 | -76 | MER3 | MER1\_type | DNA |
| 41757737 | 41758043 | + | 4.9 | 1 | 308 | -3 | AluY | Alu | SINE |
| 41758068 | 41758376 | - | 9.1 | 1 | 309 | -1 | AluSg | Alu | SINE |
| 41758377 | 41758481 | - | 14.9 | 1 | 97 | -371 | LTR37B | ERV1 | LTR |
| 41758556 | 41758659 | + | 26.7 | 498 | 607 | -5512 | L1MEd | L1 | LINE |

  

|  |  |  |  |  |  |  |  |  |  |
| --- | --- | --- | --- | --- | --- | --- | --- | --- | --- |
| chr2:44670566-44674377 | | | 3794 bp | | |  |  |  |  |
| Genome Start | Genome End | Strand | Div | Repeat Start | Repeat End | Repeat Left | Name | Family | Class |
| 44670566 | 44670843 | - | 24.1 | 4154 | 4442 | -1777 | L1MEc | L1 | LINE |
| 44670834 | 44671057 | - | 20.6 | 3561 | 3793 | -2420 | L1MEc | L1 | LINE |
| 44671066 | 44672208 | - | 3.0 | 4342 | 5488 | -667 | L1PA3 | L1 | LINE |
| 44672209 | 44672868 | + | 2.3 | 5495 | 6154 | -1 | L1PA3 | L1 | LINE |
| 44672879 | 44673110 | - | 24.7 | 3337 | 3568 | -2651 | L1MEc | L1 | LINE |
| 44673112 | 44673411 | + | 11.1 | 243 | 550 | 0 | MER44B | MER2\_type | DNA |
| 44673416 | 44673664 | - | 22.6 | 2603 | 2874 | -3345 | L1MEc | L1 | LINE |
| 44673670 | 44674377 | - | 22.5 | 33 | 769 | -5631 | L1MEc | L1 | LINE |

  

|  |  |  |  |  |  |  |  |  |  |
| --- | --- | --- | --- | --- | --- | --- | --- | --- | --- |
| chr2:51620049-51623453 | | | 3271 bp | | |  |  |  |  |
| Genome Start | Genome End | Strand | Div | Repeat Start | Repeat End | Repeat Left | Name | Family | Class |
| 51620049 | 51620206 | + | 31.2 | 63 | 233 | -4621 | L1MEe | L1 | LINE |
| 51620215 | 51620457 | + | 30.7 | 8 | 252 | -212 | LTR16B | ERVL | LTR |
| 51620458 | 51621211 | - | 5.3 | 5392 | 6146 | -8 | L1PA5 | L1 | LINE |
| 51621210 | 51622068 | + | 7.5 | 4539 | 5391 | -755 | L1PA5 | L1 | LINE |
| 51622070 | 51622294 | + | 30.7 | 233 | 454 | -10 | LTR16B | ERVL | LTR |
| 51622301 | 51622598 | + | 30.7 | 241 | 536 | -4318 | L1MEe | L1 | LINE |
| 51622654 | 51623203 | + | 27.2 | 75 | 638 | -16 | MLT1E1 | MaLR | LTR |
| 51623270 | 51623453 | + | 23.5 | 1049 | 1246 | -3588 | L1MEe | L1 | LINE |

  

|  |  |  |  |  |  |  |  |  |  |
| --- | --- | --- | --- | --- | --- | --- | --- | --- | --- |
| chr2:52227103-52228869 | | | 1694 bp | | |  |  |  |  |
| Genome Start | Genome End | Strand | Div | Repeat Start | Repeat End | Repeat Left | Name | Family | Class |
| 52227103 | 52227902 | - | 20.0 | 1951 | 2761 | 0 | Charlie1 | MER1\_type | DNA |
| 52227965 | 52228511 | - | 6.0 | 5587 | 6154 | 0 | L1PA7 | L1 | LINE |
| 52228512 | 52228618 | + | 4.7 | 5481 | 5587 | -567 | L1PA7 | L1 | LINE |
| 52228630 | 52228869 | - | 27.7 | 1673 | 1891 | -870 | Charlie1 | MER1\_type | DNA |

  

|  |  |  |  |  |  |  |  |  |  |
| --- | --- | --- | --- | --- | --- | --- | --- | --- | --- |
| chr2:55473385-55475991 | | | 2565 bp | | |  |  |  |  |
| Genome Start | Genome End | Strand | Div | Repeat Start | Repeat End | Repeat Left | Name | Family | Class |
| 55473385 | 55473929 | - | 29.1 | 4333 | 4945 | -1201 | L1M5 | L1 | LINE |
| 55473930 | 55474233 | - | 11.7 | 1 | 302 | -7 | AluSc | Alu | SINE |
| 55474234 | 55474582 | - | 29.1 | 3926 | 4332 | -1814 | L1M5 | L1 | LINE |
| 55474591 | 55474764 | - | 20.8 | 1 | 173 | -3 | FRAM | Alu | SINE |
| 55474774 | 55475247 | - | 2.5 | 5479 | 5949 | -206 | L1PA4 | L1 | LINE |
| 55475248 | 55475461 | + | 3.7 | 5942 | 6155 | 0 | L1PA4 | L1 | LINE |
| 55475477 | 55475785 | - | 11.3 | 1 | 309 | -3 | AluSx | Alu | SINE |
| 55475795 | 55475918 | - | 22.5 | 1 | 120 | -13 | FLAM\_A | Alu | SINE |
| 55475920 | 55475991 | - | 20.8 | 3849 | 3928 | -2218 | L1M5 | L1 | LINE |

  

|  |  |  |  |  |  |  |  |  |  |
| --- | --- | --- | --- | --- | --- | --- | --- | --- | --- |
| chr2:57317657-57323560 | | | 5896 bp | | |  |  |  |  |
| Genome Start | Genome End | Strand | Div | Repeat Start | Repeat End | Repeat Left | Name | Family | Class |
| 57317657 | 57318187 | + | 27.4 | 1 | 585 | -6445 | L1MA8 | L1 | LINE |
| 57318199 | 57319403 | + | 18.9 | 3886 | 5084 | -2019 | L1MA8 | L1 | LINE |
| 57319404 | 57319709 | - | 10.8 | 1 | 297 | -13 | AluSg | Alu | SINE |
| 57319710 | 57321381 | + | 17.3 | 5085 | 6794 | -457 | L1MA8 | L1 | LINE |
| 57321375 | 57321412 | - | 5.3 | 4455 | 4493 | -1653 | L1P1 | L1 | LINE |
| 57321410 | 57323090 | + | 8.5 | 4493 | 6176 | 0 | L1PA8 | L1 | LINE |
| 57323098 | 57323560 | + | 17.4 | 6774 | 7245 | -6 | L1MA8 | L1 | LINE |

  

|  |  |  |  |  |  |  |  |  |  |
| --- | --- | --- | --- | --- | --- | --- | --- | --- | --- |
| chr2:57343031-57345694 | | | 2519 bp | | |  |  |  |  |
| Genome Start | Genome End | Strand | Div | Repeat Start | Repeat End | Repeat Left | Name | Family | Class |
| 57343031 | 57343228 | - | 33.3 | 464 | 651 | -4 | LTR16D | ERVL | LTR |
| 57343376 | 57344109 | - | 10.1 | 4090 | 4826 | -1320 | L1PA8 | L1 | LINE |
| 57344108 | 57345437 | + | 9.3 | 4845 | 6171 | -1 | L1PA8 | L1 | LINE |
| 57345438 | 57345694 | - | 36.3 | 30 | 289 | -366 | LTR16D | ERVL | LTR |

  

|  |  |  |  |  |  |  |  |  |  |
| --- | --- | --- | --- | --- | --- | --- | --- | --- | --- |
| chr2:57725376-57731026 | | | 5632 bp | | |  |  |  |  |
| Genome Start | Genome End | Strand | Div | Repeat Start | Repeat End | Repeat Left | Name | Family | Class |
| 57725376 | 57726587 | - | 20.7 | 4892 | 6176 | -2 | L1MB8 | L1 | LINE |
| 57726588 | 57727005 | - | 1.7 | 5739 | 6155 | 0 | L1PA2 | L1 | LINE |
| 57727006 | 57727307 | + | 5.2 | 5428 | 5729 | -426 | L1PA2 | L1 | LINE |
| 57727303 | 57727554 | - | 22.9 | 4644 | 4904 | -1242 | L1MB8 | L1 | LINE |
| 57727563 | 57727850 | - | 15.3 | 1 | 289 | -23 | AluSx | Alu | SINE |
| 57727885 | 57728901 | - | 11.1 | 5118 | 6147 | -4 | L1PB1 | L1 | LINE |
| 57728884 | 57730006 | - | 10.6 | 4981 | 6168 | -4 | L1PA8 | L1 | LINE |
| 57730004 | 57730432 | - | 22.1 | 4187 | 4646 | -1500 | L1MB8 | L1 | LINE |
| 57730436 | 57730824 | + | 17.6 | 1 | 428 | 0 | MSTA | MaLR | LTR |
| 57730825 | 57731026 | - | 21.4 | 3981 | 4185 | -1961 | L1MB8 | L1 | LINE |

  

|  |  |  |  |  |  |  |  |  |  |
| --- | --- | --- | --- | --- | --- | --- | --- | --- | --- |
| chr2:59510595-59513856 | | | 3240 bp | | |  |  |  |  |
| Genome Start | Genome End | Strand | Div | Repeat Start | Repeat End | Repeat Left | Name | Family | Class |
| 59510595 | 59510651 | - | 21.1 | 319 | 375 | -15 | MLT1A | MaLR | LTR |
| 59510683 | 59511968 | - | 6.5 | 4882 | 6152 | -2 | L1PA7 | L1 | LINE |
| 59511960 | 59513523 | + | 8.8 | 3308 | 4869 | -1277 | L1PA7 | L1 | LINE |
| 59513524 | 59513856 | - | 22.3 | 1 | 318 | -56 | MLT1A | MaLR | LTR |

  

|  |  |  |  |  |  |  |  |  |  |
| --- | --- | --- | --- | --- | --- | --- | --- | --- | --- |
| chr2:59717754-59724447 | | | 6682 bp | | |  |  |  |  |
| Genome Start | Genome End | Strand | Div | Repeat Start | Repeat End | Repeat Left | Name | Family | Class |
| 59717754 | 59718558 | - | 29.0 | 5688 | 6561 | 0 | L1ME3A | L1 | LINE |
| 59718563 | 59718724 | - | 25.6 | 5241 | 5406 | -1131 | L1ME3A | L1 | LINE |
| 59718725 | 59719400 | - | 4.0 | 4486 | 5162 | -984 | L1PA4 | L1 | LINE |
| 59719401 | 59720390 | + | 3.6 | 5162 | 6152 | -3 | L1PA4 | L1 | LINE |
| 59720399 | 59721830 | - | 26.2 | 3776 | 5251 | -1286 | L1ME3A | L1 | LINE |
| 59721831 | 59722081 | - | 11.9 | 40 | 292 | -20 | AluSx | Alu | SINE |
| 59722082 | 59724447 | - | 28.3 | 1320 | 3775 | -2762 | L1ME3A | L1 | LINE |

  

|  |  |  |  |  |  |  |  |  |  |
| --- | --- | --- | --- | --- | --- | --- | --- | --- | --- |
| chr2:61386234-61388799 | | | 2352 bp | | |  |  |  |  |
| Genome Start | Genome End | Strand | Div | Repeat Start | Repeat End | Repeat Left | Name | Family | Class |
| 61386234 | 61386408 | + | 17.8 | 1276 | 1466 | -4934 | L1MEc | L1 | LINE |
| 61386532 | 61387303 | - | 5.0 | 4553 | 5350 | -796 | L1PA5 | L1 | LINE |
| 61387296 | 61388108 | + | 3.6 | 5334 | 6150 | -4 | L1PA5 | L1 | LINE |
| 61388208 | 61388404 | + | 25.9 | 1467 | 1790 | -4610 | L1MEc | L1 | LINE |
| 61388405 | 61388712 | + | 16.9 | 1 | 307 | -5 | AluJb | Alu | SINE |
| 61388713 | 61388799 | + | 27.3 | 1791 | 1870 | -4530 | L1MEc | L1 | LINE |

  

|  |  |  |  |  |  |  |  |  |  |
| --- | --- | --- | --- | --- | --- | --- | --- | --- | --- |
| chr2:67585582-67589227 | | | 3650 bp | | |  |  |  |  |
| Genome Start | Genome End | Strand | Div | Repeat Start | Repeat End | Repeat Left | Name | Family | Class |
| 67585582 | 67585817 | + | 19.7 | 1 | 247 | -134 | THE1D | MaLR | LTR |
| 67585823 | 67587748 | - | 3.3 | 4226 | 6154 | -1 | L1PA4 | L1 | LINE |
| 67587740 | 67589073 | + | 4.7 | 2901 | 4232 | -1914 | L1PA4 | L1 | LINE |
| 67589074 | 67589227 | + | 12.3 | 226 | 379 | -2 | THE1D | MaLR | LTR |

  

|  |  |  |  |  |  |  |  |  |  |
| --- | --- | --- | --- | --- | --- | --- | --- | --- | --- |
| chr2:68015062-68017978 | | | 2839 bp | | |  |  |  |  |
| Genome Start | Genome End | Strand | Div | Repeat Start | Repeat End | Repeat Left | Name | Family | Class |
| 68015062 | 68015142 | + | 27.5 | 1 | 80 | -316 | MSTD | MaLR | LTR |
| 68015144 | 68015854 | - | 7.2 | 3946 | 4665 | -1481 | L1PA7 | L1 | LINE |
| 68015851 | 68017327 | + | 7.5 | 4673 | 6154 | 0 | L1PA7 | L1 | LINE |
| 68017409 | 68017669 | + | 12.6 | 32 | 292 | -20 | AluSx | Alu | SINE |
| 68017670 | 68017978 | + | 22.5 | 69 | 396 | 0 | MSTD | MaLR | LTR |

  

|  |  |  |  |  |  |  |  |  |  |
| --- | --- | --- | --- | --- | --- | --- | --- | --- | --- |
| chr2:70509681-70513321 | | | 3459 bp | | |  |  |  |  |
| Genome Start | Genome End | Strand | Div | Repeat Start | Repeat End | Repeat Left | Name | Family | Class |
| 70509681 | 70510109 | - | 32.0 | 1965 | 2411 | -6 | Charlie8 | MER1\_type | DNA |
| 70510125 | 70511802 | - | 1.5 | 4214 | 5895 | -260 | L1PA2 | L1 | LINE |
| 70511803 | 70512051 | + | 3.2 | 5907 | 6155 | 0 | L1PA2 | L1 | LINE |
| 70512219 | 70513321 | - | 28.0 | 812 | 1963 | -454 | Charlie8 | MER1\_type | DNA |

  

|  |  |  |  |  |  |  |  |  |  |
| --- | --- | --- | --- | --- | --- | --- | --- | --- | --- |
| chr2:73826229-73827067 | | | 834 bp | | |  |  |  |  |
| Genome Start | Genome End | Strand | Div | Repeat Start | Repeat End | Repeat Left | Name | Family | Class |
| 73826229 | 73826294 | - | 21.2 | 590 | 655 | 0 | LTR16D | ERVL | LTR |
| 73826299 | 73826364 | - | 6.1 | 6085 | 6150 | -4 | L1PA5 | L1 | LINE |
| 73826366 | 73826712 | + | 4.6 | 5737 | 6083 | -71 | L1PA5 | L1 | LINE |
| 73826713 | 73827067 | - | 30.4 | 265 | 608 | -47 | LTR16D | ERVL | LTR |

  

|  |  |  |  |  |  |  |  |  |  |
| --- | --- | --- | --- | --- | --- | --- | --- | --- | --- |
| chr2:75607627-75619083 | | | 11092 bp | | |  |  |  |  |
| Genome Start | Genome End | Strand | Div | Repeat Start | Repeat End | Repeat Left | Name | Family | Class |
| 75607627 | 75608186 | + | 27.8 | 234 | 809 | -5824 | L1MD1 | L1 | LINE |
| 75608187 | 75608407 | + | 17.1 | 1125 | 1346 | -5287 | L1MD1 | L1 | LINE |
| 75608413 | 75609811 | + | 12.2 | 59 | 1485 | -5378 | L1PB1 | L1 | LINE |
| 75609842 | 75611835 | + | 11.8 | 3163 | 5153 | -1709 | L1PB1 | L1 | LINE |
| 75611840 | 75612511 | + | 10.8 | 6162 | 6819 | -48 | L1PB1 | L1 | LINE |
| 75612512 | 75612822 | + | 6.5 | 1 | 310 | -1 | AluY | Alu | SINE |
| 75612823 | 75612877 | + | 10.8 | 6820 | 6866 | -1 | L1PB1 | L1 | LINE |
| 75613090 | 75613494 | + | 17.1 | 1334 | 1731 | -4902 | L1MD1 | L1 | LINE |
| 75613495 | 75613810 | + | 16.4 | 1 | 310 | -2 | AluJb | Alu | SINE |
| 75613811 | 75614288 | + | 17.1 | 1732 | 2204 | -4429 | L1MD1 | L1 | LINE |
| 75614289 | 75614429 | + | 17.6 | 1 | 127 | -185 | AluJo | Alu | SINE |
| 75614430 | 75614707 | - | 3.7 | 39 | 283 | -28 | AluY | Alu | SINE |
| 75614708 | 75614887 | + | 17.6 | 128 | 297 | -15 | AluJo | Alu | SINE |
| 75614888 | 75615294 | + | 17.1 | 2205 | 2606 | -4027 | L1MD1 | L1 | LINE |
| 75615276 | 75615683 | - | 16.0 | 4852 | 5263 | -883 | L1PB3 | L1 | LINE |
| 75615684 | 75616585 | + | 11.0 | 5260 | 6300 | -3 | L1PB3 | L1 | LINE |
| 75616670 | 75616732 | - | 16.1 | 2202 | 2267 | -3879 | L1MD | L1 | LINE |
| 75616735 | 75616988 | + | 14.6 | 46 | 303 | -9 | AluJb | Alu | SINE |
| 75617020 | 75617152 | + | 13.0 | 2645 | 2774 | -3763 | L1MD1 | L1 | LINE |
| 75617152 | 75618222 | + | 15.5 | 3594 | 4655 | -1882 | L1MD1 | L1 | LINE |
| 75618239 | 75618620 | + | 17.8 | 18 | 426 | 0 | MSTB | MaLR | LTR |
| 75618622 | 75618641 | + | 18.0 | 4652 | 4669 | -1868 | L1MD1 | L1 | LINE |
| 75618642 | 75618956 | + | 13.6 | 1 | 309 | -3 | AluSx | Alu | SINE |
| 75618957 | 75619083 | + | 18.0 | 4670 | 4804 | -1733 | L1MD1 | L1 | LINE |

  

|  |  |  |  |  |  |  |  |  |  |
| --- | --- | --- | --- | --- | --- | --- | --- | --- | --- |
| chr2:76103255-76108383 | | | 5104 bp | | |  |  |  |  |
| Genome Start | Genome End | Strand | Div | Repeat Start | Repeat End | Repeat Left | Name | Family | Class |
| 76103255 | 76103610 | - | 21.3 | 2252 | 2624 | 0 | Charlie5 | MER1\_type | DNA |
| 76103609 | 76104132 | - | 19.0 | 154 | 680 | -1932 | Charlie5 | MER1\_type | DNA |
| 76104134 | 76104562 | - | 15.5 | 2880 | 3294 | -2852 | L1P4 | L1 | LINE |
| 76104563 | 76104870 | - | 14.0 | 1 | 309 | -1 | AluSg | Alu | SINE |
| 76104871 | 76105470 | - | 15.5 | 2255 | 2879 | -3267 | L1P4 | L1 | LINE |
| 76105472 | 76105770 | + | 14.6 | 3298 | 3600 | -2546 | L1PA16 | L1 | LINE |
| 76105794 | 76108322 | + | 14.3 | 3601 | 6164 | -2 | L1PA16 | L1 | LINE |
| 76108325 | 76108383 | - | 20.3 | 108 | 167 | -2445 | Charlie5 | MER1\_type | DNA |

  

|  |  |  |  |  |  |  |  |  |  |
| --- | --- | --- | --- | --- | --- | --- | --- | --- | --- |
| chr2:76670673-76673499 | | | 2824 bp | | |  |  |  |  |
| Genome Start | Genome End | Strand | Div | Repeat Start | Repeat End | Repeat Left | Name | Family | Class |
| 76670673 | 76670859 | - | 8.0 | 119 | 305 | -6 | AluY | Alu | SINE |
| 76670862 | 76672799 | - | 3.3 | 4207 | 6144 | -11 | L1PA3 | L1 | LINE |
| 76672801 | 76673370 | + | 3.3 | 3626 | 4195 | -1951 | L1PA3 | L1 | LINE |
| 76673371 | 76673499 | - | 5.5 | 1 | 134 | -177 | AluY | Alu | SINE |

  

|  |  |  |  |  |  |  |  |  |  |
| --- | --- | --- | --- | --- | --- | --- | --- | --- | --- |
| chr2:77711356-77730142 | | | 18570 bp | | |  |  |  |  |
| Genome Start | Genome End | Strand | Div | Repeat Start | Repeat End | Repeat Left | Name | Family | Class |
| 77711356 | 77712143 | - | 21.8 | 5815 | 6628 | -54 | L1MB7 | L1 | LINE |
| 77712157 | 77712320 | - | 14.4 | 6149 | 6304 | 0 | L1MA2 | L1 | LINE |
| 77712321 | 77712619 | - | 10.7 | 1 | 300 | -12 | AluSx | Alu | SINE |
| 77712620 | 77713243 | - | 14.4 | 5517 | 6148 | -156 | L1MA2 | L1 | LINE |
| 77713244 | 77713490 | - | 14.7 | 2698 | 2945 | -4620 | L1M1 | L1 | LINE |
| 77713491 | 77713837 | - | 16.1 | 3 | 364 | 0 | THE1B | MaLR | LTR |
| 77713838 | 77714924 | - | 14.7 | 1595 | 2697 | -4868 | L1M1 | L1 | LINE |
| 77714917 | 77715352 | - | 22.4 | 5400 | 5836 | -808 | L1MB7 | L1 | LINE |
| 77715353 | 77715649 | - | 14.3 | 1 | 294 | -18 | AluSx | Alu | SINE |
| 77715650 | 77716693 | - | 22.4 | 4331 | 5399 | -1245 | L1MB7 | L1 | LINE |
| 77716768 | 77718682 | - | 18.1 | 5097 | 6955 | -47 | L1MA4 | L1 | LINE |
| 77718689 | 77719142 | + | 19.8 | 1 | 423 | -3 | MSTB | MaLR | LTR |
| 77719145 | 77724341 | - | 20.8 | 4 | 5139 | -1706 | L1MA4 | L1 | LINE |
| 77724336 | 77724749 | - | 21.4 | 3766 | 4118 | -2526 | L1MB7 | L1 | LINE |
| 77724750 | 77725057 | - | 20.7 | 1 | 283 | -29 | AluJb | Alu | SINE |
| 77725058 | 77725752 | - | 21.4 | 3111 | 3765 | -2879 | L1MB7 | L1 | LINE |
| 77725753 | 77726075 | - | 11.5 | 1 | 313 | 0 | AluSq | Alu | SINE |
| 77726076 | 77726303 | - | 21.4 | 2866 | 3110 | -3534 | L1MB7 | L1 | LINE |
| 77726325 | 77726560 | - | 27.4 | 1309 | 1543 | -4999 | L1MB7 | L1 | LINE |
| 77726648 | 77726711 | - | 21.9 | 1218 | 1283 | -5259 | L1MB7 | L1 | LINE |
| 77726737 | 77726805 | - | 24.6 | 499 | 563 | 0 | MLT2C1 | ERVL | LTR |
| 77726806 | 77727492 | - | 2.0 | 3977 | 4663 | -1483 | L1PA2 | L1 | LINE |
| 77727490 | 77728966 | + | 1.6 | 4674 | 6150 | -5 | L1PA2 | L1 | LINE |
| 77728967 | 77729255 | - | 19.5 | 1 | 332 | -65 | MLT2C1 | ERVL | LTR |
| 77729261 | 77729560 | - | 24.7 | 871 | 1198 | -5344 | L1MB7 | L1 | LINE |
| 77729550 | 77729910 | - | 31.1 | 153 | 503 | -6039 | L1MB7 | L1 | LINE |
| 77729916 | 77730001 | - | 12.8 | 395 | 481 | -114 | MER41E | ERV1 | LTR |
| 77730009 | 77730142 | - | 20.9 | 16 | 160 | -6382 | L1MB7 | L1 | LINE |

  

|  |  |  |  |  |  |  |  |  |  |
| --- | --- | --- | --- | --- | --- | --- | --- | --- | --- |
| chr2:78010395-78016624 | | | 6173 bp | | |  |  |  |  |
| Genome Start | Genome End | Strand | Div | Repeat Start | Repeat End | Repeat Left | Name | Family | Class |
| 78010395 | 78010482 | + | 25.0 | 2626 | 2713 | -3427 | L1MB5 | L1 | LINE |
| 78010484 | 78010797 | - | 18.7 | 1 | 314 | -25 | MER1B | MER1\_type | DNA |
| 78010833 | 78011699 | + | 23.4 | 2757 | 3566 | -2580 | L1MB5 | L1 | LINE |
| 78011700 | 78012037 | + | 5.3 | 1 | 338 | 0 | AluY | Alu | SINE |
| 78012038 | 78012808 | + | 23.4 | 3567 | 4339 | -1807 | L1MB5 | L1 | LINE |
| 78012835 | 78013262 | - | 7.7 | 4116 | 4544 | -1602 | L1PA8 | L1 | LINE |
| 78013260 | 78014841 | + | 9.9 | 4552 | 6171 | -1 | L1PA8 | L1 | LINE |
| 78014840 | 78016624 | + | 22.0 | 4369 | 6166 | -8 | L1MB5 | L1 | LINE |

  

|  |  |  |  |  |  |  |  |  |  |
| --- | --- | --- | --- | --- | --- | --- | --- | --- | --- |
| chr2:78389823-78395247 | | | 4951 bp | | |  |  |  |  |
| Genome Start | Genome End | Strand | Div | Repeat Start | Repeat End | Repeat Left | Name | Family | Class |
| 78389823 | 78390363 | + | 35.2 | 1283 | 1900 | -1519 | L2 | L2 | LINE |
| 78390592 | 78391025 | + | 37.5 | 2325 | 2760 | -659 | L2 | L2 | LINE |
| 78391094 | 78391140 | - | 20.5 | 6130 | 6168 | 0 | L1MB1 | L1 | LINE |
| 78391141 | 78391403 | - | 6.5 | 1 | 298 | -15 | AluSp | Alu | SINE |
| 78391404 | 78391753 | - | 20.5 | 5787 | 6129 | -39 | L1MB1 | L1 | LINE |
| 78391754 | 78392237 | + | 20.2 | 1 | 519 | -1 | MLT2B1 | ERVL | LTR |
| 78392238 | 78392915 | - | 19.2 | 5139 | 5786 | -382 | L1MB1 | L1 | LINE |
| 78392907 | 78393052 | + | 19.6 | 4465 | 4609 | -1531 | L1M3 | L1 | LINE |
| 78393184 | 78393463 | + | 16.0 | 5993 | 6295 | -17 | L1MA9 | L1 | LINE |
| 78393461 | 78393519 | + | 16.9 | 4599 | 4657 | -1483 | L1M3 | L1 | LINE |
| 78393514 | 78393866 | + | 19.3 | 5486 | 5852 | -481 | L1MC1 | L1 | LINE |
| 78393867 | 78394156 | + | 22.9 | 1 | 287 | -25 | AluJo | Alu | SINE |
| 78394185 | 78394402 | + | 12.0 | 1 | 216 | -96 | AluSx | Alu | SINE |
| 78394429 | 78394884 | + | 19.5 | 4670 | 5118 | -1022 | L1M3 | L1 | LINE |
| 78394896 | 78395247 | + | 35.7 | 2792 | 3160 | -259 | L2 | L2 | LINE |

  

|  |  |  |  |  |  |  |  |  |  |
| --- | --- | --- | --- | --- | --- | --- | --- | --- | --- |
| chr2:79022464-79028036 | | | 4997 bp | | |  |  |  |  |
| Genome Start | Genome End | Strand | Div | Repeat Start | Repeat End | Repeat Left | Name | Family | Class |
| 79022464 | 79023043 | + | 31.0 | 483 | 1119 | -5000 | L1ME3B | L1 | LINE |
| 79023060 | 79023723 | + | 30.1 | 2946 | 3572 | -2557 | L1ME3B | L1 | LINE |
| 79023724 | 79024029 | - | 13.4 | 1 | 306 | -6 | AluSx | Alu | SINE |
| 79024030 | 79024739 | + | 30.1 | 3573 | 4331 | -1798 | L1ME3B | L1 | LINE |
| 79025156 | 79025247 | + | 30.4 | 4501 | 4595 | -1534 | L1ME3B | L1 | LINE |
| 79025270 | 79025597 | + | 31.6 | 4750 | 5114 | -1015 | L1ME3B | L1 | LINE |
| 79025653 | 79025815 | - | 19.4 | 130 | 289 | -23 | AluJb | Alu | SINE |
| 79025866 | 79026024 | - | 19.4 | 1 | 129 | -183 | AluJb | Alu | SINE |
| 79026035 | 79026750 | + | 29.3 | 5114 | 5853 | -370 | L1ME3B | L1 | LINE |
| 79026736 | 79027375 | - | 8.0 | 5178 | 5843 | -311 | L1PA7 | L1 | LINE |
| 79027376 | 79027706 | + | 6.8 | 5823 | 6148 | -6 | L1PA7 | L1 | LINE |
| 79027729 | 79028036 | + | 29.9 | 5849 | 6185 | -38 | L1ME3B | L1 | LINE |

  

|  |  |  |  |  |  |  |  |  |  |
| --- | --- | --- | --- | --- | --- | --- | --- | --- | --- |
| chr2:79109551-79115103 | | | 5481 bp | | |  |  |  |  |
| Genome Start | Genome End | Strand | Div | Repeat Start | Repeat End | Repeat Left | Name | Family | Class |
| 79109551 | 79110749 | + | 26.0 | 1 | 1301 | -5450 | L1MCb | L1 | LINE |
| 79110750 | 79111014 | - | 9.2 | 38 | 297 | -15 | AluSx | Alu | SINE |
| 79111015 | 79111800 | + | 24.3 | 1302 | 2130 | -4621 | L1MCb | L1 | LINE |
| 79111823 | 79113316 | + | 20.8 | 2196 | 3721 | -2843 | L1MCb | L1 | LINE |
| 79113322 | 79113760 | + | 19.9 | 4 | 505 | 0 | MLT1D | MaLR | LTR |
| 79113800 | 79113881 | - | 3.7 | 5129 | 5210 | -936 | L1PA7 | L1 | LINE |
| 79113883 | 79114828 | + | 5.3 | 5203 | 6146 | -8 | L1PA7 | L1 | LINE |
| 79114834 | 79115103 | + | 20.4 | 3750 | 4030 | -2534 | L1MCb | L1 | LINE |

  

|  |  |  |  |  |  |  |  |  |  |
| --- | --- | --- | --- | --- | --- | --- | --- | --- | --- |
| chr2:80146684-80148735 | | | 1699 bp | | |  |  |  |  |
| Genome Start | Genome End | Strand | Div | Repeat Start | Repeat End | Repeat Left | Name | Family | Class |
| 80146684 | 80146815 | + | 23.4 | 1 | 131 | -411 | MLT1F | MaLR | LTR |
| 80146817 | 80146880 | - | 12.5 | 5102 | 5165 | -978 | L1MA8 | L1 | LINE |
| 80146875 | 80147967 | + | 12.1 | 5160 | 6249 | -42 | L1MA8 | L1 | LINE |
| 80148326 | 80148735 | + | 21.0 | 130 | 540 | -2 | MLT1F | MaLR | LTR |

  

|  |  |  |  |  |  |  |  |  |  |
| --- | --- | --- | --- | --- | --- | --- | --- | --- | --- |
| chr2:80674075-80679940 | | | 5764 bp | | |  |  |  |  |
| Genome Start | Genome End | Strand | Div | Repeat Start | Repeat End | Repeat Left | Name | Family | Class |
| 80674075 | 80674122 | - | 12.5 | 161 | 208 | -1 | MER3 | MER1\_type | DNA |
| 80674124 | 80676248 | - | 18.2 | 4008 | 6182 | -1 | L1MB3 | L1 | LINE |
| 80676315 | 80676933 | - | 9.2 | 4243 | 4838 | -1308 | L1P3 | L1 | LINE |
| 80676934 | 80677247 | - | 5.8 | 1 | 311 | 0 | AluY | Alu | SINE |
| 80677248 | 80677795 | - | 9.2 | 3700 | 4242 | -1904 | L1P3 | L1 | LINE |
| 80677795 | 80677982 | + | 8.5 | 4841 | 5027 | -1119 | L1PA8 | L1 | LINE |
| 80678003 | 80679109 | + | 8.3 | 5028 | 6170 | -2 | L1PA8 | L1 | LINE |
| 80679113 | 80679792 | + | 25.3 | 5589 | 6231 | -11 | L1MD1 | L1 | LINE |
| 80679806 | 80679940 | - | 16.5 | 12 | 150 | -59 | MER3 | MER1\_type | DNA |

  

|  |  |  |  |  |  |  |  |  |  |
| --- | --- | --- | --- | --- | --- | --- | --- | --- | --- |
| chr2:80829866-80840442 | | | 10476 bp | | |  |  |  |  |
| Genome Start | Genome End | Strand | Div | Repeat Start | Repeat End | Repeat Left | Name | Family | Class |
| 80829866 | 80834050 | - | 20.4 | 2384 | 6633 | 0 | L1MD1 | L1 | LINE |
| 80834081 | 80834211 | - | 20.1 | 6163 | 6274 | -38 | L1MA9 | L1 | LINE |
| 80834212 | 80834509 | + | 10.1 | 1 | 298 | -11 | AluSc | Alu | SINE |
| 80834510 | 80834791 | - | 20.6 | 5877 | 6162 | -150 | L1MA9 | L1 | LINE |
| 80834791 | 80834914 | - | 18.6 | 5619 | 5742 | -562 | L1M1 | L1 | LINE |
| 80834920 | 80835664 | + | 16.2 | 4852 | 5614 | -698 | L1M1 | L1 | LINE |
| 80835667 | 80836383 | - | 21.2 | 1590 | 2393 | -4240 | L1MD1 | L1 | LINE |
| 80836412 | 80838020 | - | 16.6 | 4504 | 6290 | 0 | L1PA15 | L1 | LINE |
| 80838026 | 80838770 | - | 23.3 | 1 | 750 | -5541 | L1PA15 | L1 | LINE |
| 80838803 | 80838911 | - | 25.0 | 1485 | 1604 | -5029 | L1MD1 | L1 | LINE |
| 80838912 | 80839072 | - | 9.3 | 6119 | 6285 | -12 | L1MA5A | L1 | LINE |
| 80839073 | 80840442 | - | 25.0 | 17 | 1484 | -5149 | L1MD1 | L1 | LINE |

  

|  |  |  |  |  |  |  |  |  |  |
| --- | --- | --- | --- | --- | --- | --- | --- | --- | --- |
| chr2:81634474-81640746 | | | 6245 bp | | |  |  |  |  |
| Genome Start | Genome End | Strand | Div | Repeat Start | Repeat End | Repeat Left | Name | Family | Class |
| 81634474 | 81635684 | + | 22.1 | 10 | 1182 | -5326 | L1MC1 | L1 | LINE |
| 81635691 | 81635895 | - | 15.2 | 1 | 205 | -223 | MSTA | MaLR | LTR |
| 81635896 | 81637856 | + | 19.3 | 3653 | 5674 | -834 | L1MC1 | L1 | LINE |
| 81637854 | 81639004 | - | 4.4 | 4123 | 5272 | -874 | L1PA4 | L1 | LINE |
| 81638995 | 81639900 | + | 2.9 | 5275 | 6180 | 0 | L1PA4 | L1 | LINE |
| 81639936 | 81640746 | + | 18.0 | 5660 | 6499 | -9 | L1MC1 | L1 | LINE |

  

|  |  |  |  |  |  |  |  |  |  |
| --- | --- | --- | --- | --- | --- | --- | --- | --- | --- |
| chr2:82595246-82598339 | | | 3095 bp | | |  |  |  |  |
| Genome Start | Genome End | Strand | Div | Repeat Start | Repeat End | Repeat Left | Name | Family | Class |
| 82595246 | 82595429 | - | 6.5 | 119 | 302 | -9 | AluY | Alu | SINE |
| 82595431 | 82597301 | - | 3.4 | 4272 | 6144 | -11 | L1PA4 | L1 | LINE |
| 82597300 | 82598205 | + | 5.0 | 3362 | 4267 | -1879 | L1PA4 | L1 | LINE |
| 82598206 | 82598339 | - | 7.5 | 1 | 134 | -177 | AluY | Alu | SINE |

  

|  |  |  |  |  |  |  |  |  |  |
| --- | --- | --- | --- | --- | --- | --- | --- | --- | --- |
| chr2:83572945-83580053 | | | 6586 bp | | |  |  |  |  |
| Genome Start | Genome End | Strand | Div | Repeat Start | Repeat End | Repeat Left | Name | Family | Class |
| 83572945 | 83575697 | + | 21.3 | 2206 | 4992 | -1154 | L1MC2 | L1 | LINE |
| 83575696 | 83575841 | - | 4.1 | 5652 | 5796 | -359 | L1PA5 | L1 | LINE |
| 83575842 | 83576204 | + | 5.0 | 5779 | 6141 | -13 | L1PA5 | L1 | LINE |
| 83576666 | 83576883 | + | 29.0 | 5962 | 6191 | -49 | L1ME3B | L1 | LINE |
| 83576880 | 83576950 | + | 21.1 | 4980 | 5053 | -1093 | L1MC2 | L1 | LINE |
| 83576987 | 83578566 | - | 4.5 | 4571 | 6141 | -13 | L1PA5 | L1 | LINE |
| 83578567 | 83578813 | - | 15.5 | 2150 | 2406 | 0 | Tigger5 | MER2\_type | DNA |
| 83578846 | 83580053 | + | 20.3 | 5050 | 6332 | -12 | L1MC2 | L1 | LINE |

  

|  |  |  |  |  |  |  |  |  |  |
| --- | --- | --- | --- | --- | --- | --- | --- | --- | --- |
| chr2:83919645-83921207 | | | 1395 bp | | |  |  |  |  |
| Genome Start | Genome End | Strand | Div | Repeat Start | Repeat End | Repeat Left | Name | Family | Class |
| 83919645 | 83919744 | - | 41.0 | 1336 | 1436 | -524 | L4 | RTE | LINE |
| 83919767 | 83920277 | - | 1.8 | 5698 | 6208 | 0 | L1PA2 | L1 | LINE |
| 83920278 | 83920831 | + | 1.4 | 5157 | 5710 | -445 | L1PA2 | L1 | LINE |
| 83920978 | 83921207 | - | 38.0 | 916 | 1157 | -803 | L4 | RTE | LINE |

  

|  |  |  |  |  |  |  |  |  |  |
| --- | --- | --- | --- | --- | --- | --- | --- | --- | --- |
| chr2:84220331-84222869 | | | 2241 bp | | |  |  |  |  |
| Genome Start | Genome End | Strand | Div | Repeat Start | Repeat End | Repeat Left | Name | Family | Class |
| 84220331 | 84220478 | + | 18.2 | 1 | 140 | -496 | MER92B | ERV1 | LTR |
| 84220479 | 84220590 | - | 16.1 | 5036 | 5148 | -992 | L1M3 | L1 | LINE |
| 84220591 | 84221382 | + | 13.8 | 5156 | 5989 | -311 | L1MA6 | L1 | LINE |
| 84221465 | 84221581 | + | 21.6 | 7533 | 7663 | -298 | L1MC5 | L1 | LINE |
| 84221712 | 84221798 | - | 16.3 | 2405 | 2491 | 0 | Tigger7 | MER2\_type | DNA |
| 84221799 | 84222015 | - | 20.0 | 1 | 239 | 0 | MER8 | MER2\_type | DNA |
| 84222016 | 84222026 | - | 16.3 | 2395 | 2404 | -87 | Tigger7 | MER2\_type | DNA |
| 84222028 | 84222269 | - | 24.0 | 8 | 267 | -2224 | Tigger7 | MER2\_type | DNA |
| 84222286 | 84222342 | + | 19.3 | 7804 | 7864 | -97 | L1MC5 | L1 | LINE |
| 84222412 | 84222869 | + | 20.6 | 131 | 614 | -22 | MER92B | ERV1 | LTR |

  

|  |  |  |  |  |  |  |  |  |  |
| --- | --- | --- | --- | --- | --- | --- | --- | --- | --- |
| chr2:84420393-84422127 | | | 1693 bp | | |  |  |  |  |
| Genome Start | Genome End | Strand | Div | Repeat Start | Repeat End | Repeat Left | Name | Family | Class |
| 84420393 | 84420513 | + | 18.2 | 1 | 139 | -50 | MER5A | MER1\_type | DNA |
| 84420539 | 84421291 | - | 13.5 | 5477 | 6279 | -12 | L1MA8 | L1 | LINE |
| 84421292 | 84421906 | + | 15.6 | 4841 | 5473 | -839 | L1MA8 | L1 | LINE |
| 84421907 | 84422072 | + | 10.2 | 6114 | 6288 | -3 | L1MA7 | L1 | LINE |
| 84422090 | 84422127 | + | 13.2 | 134 | 171 | -18 | MER5A | MER1\_type | DNA |

  

|  |  |  |  |  |  |  |  |  |  |
| --- | --- | --- | --- | --- | --- | --- | --- | --- | --- |
| chr2:84639785-84643147 | | | 3241 bp | | |  |  |  |  |
| Genome Start | Genome End | Strand | Div | Repeat Start | Repeat End | Repeat Left | Name | Family | Class |
| 84639785 | 84640068 | + | 27.2 | 314 | 587 | -5628 | L1MC4 | L1 | LINE |
| 84640069 | 84640190 | + | 19.7 | 170 | 291 | -21 | AluJb | Alu | SINE |
| 84640255 | 84640314 | + | 20.0 | 660 | 720 | -5495 | L1MC4 | L1 | LINE |
| 84640361 | 84641077 | + | 18.2 | 841 | 1655 | -4560 | L1MC4 | L1 | LINE |
| 84641094 | 84641792 | - | 2.4 | 5458 | 6155 | 0 | L1PA3 | L1 | LINE |
| 84641793 | 84642480 | + | 2.7 | 4761 | 5445 | -710 | L1PA3 | L1 | LINE |
| 84642477 | 84643147 | + | 21.7 | 1640 | 2390 | -3825 | L1MC4 | L1 | LINE |

  

|  |  |  |  |  |  |  |  |  |  |
| --- | --- | --- | --- | --- | --- | --- | --- | --- | --- |
| chr2:87349359-87355366 | | | 5993 bp | | |  |  |  |  |
| Genome Start | Genome End | Strand | Div | Repeat Start | Repeat End | Repeat Left | Name | Family | Class |
| 87349359 | 87350594 | - | 12.1 | 1183 | 2418 | 0 | Tigger1 | MER2\_type | DNA |
| 87350603 | 87353389 | - | 3.0 | 3357 | 6172 | 0 | L1PA4 | L1 | LINE |
| 87353390 | 87353954 | + | 4.3 | 2798 | 3359 | -2787 | L1PA4 | L1 | LINE |
| 87353962 | 87354241 | - | 11.7 | 882 | 1173 | -1245 | Tigger1 | MER2\_type | DNA |
| 87354242 | 87354544 | - | 11.5 | 1 | 301 | -12 | AluSq | Alu | SINE |
| 87354545 | 87355366 | - | 11.7 | 24 | 881 | -1537 | Tigger1 | MER2\_type | DNA |

  

|  |  |  |  |  |  |  |  |  |  |
| --- | --- | --- | --- | --- | --- | --- | --- | --- | --- |
| chr2:88227743-88236491 | | | 8774 bp | | |  |  |  |  |
| Genome Start | Genome End | Strand | Div | Repeat Start | Repeat End | Repeat Left | Name | Family | Class |
| 88227743 | 88230472 | + | 22.3 | 231 | 3447 | -3758 | L1MA5 | L1 | LINE |
| 88230467 | 88231382 | - | 4.8 | 4252 | 5167 | -979 | L1PA4 | L1 | LINE |
| 88231378 | 88232369 | + | 3.1 | 5159 | 6155 | 0 | L1PA4 | L1 | LINE |
| 88232356 | 88234691 | + | 15.8 | 3429 | 5848 | -1357 | L1MA5 | L1 | LINE |
| 88234692 | 88234998 | + | 4.6 | 1 | 308 | -3 | AluY | Alu | SINE |
| 88234999 | 88236491 | + | 15.3 | 5849 | 7362 | 0 | L1MA5 | L1 | LINE |

  

|  |  |  |  |  |  |  |  |  |  |
| --- | --- | --- | --- | --- | --- | --- | --- | --- | --- |
| chr2:89101977-89103291 | | | 1310 bp | | |  |  |  |  |
| Genome Start | Genome End | Strand | Div | Repeat Start | Repeat End | Repeat Left | Name | Family | Class |
| 89101977 | 89102501 | - | 29.5 | 1930 | 2518 | -4675 | L1M4b | L1 | LINE |
| 89102503 | 89102708 | - | 19.5 | 5962 | 6166 | 0 | L1PA16 | L1 | LINE |
| 89102709 | 89102928 | + | 12.7 | 5749 | 5966 | -200 | L1PA16 | L1 | LINE |
| 89102933 | 89103291 | - | 30.1 | 1512 | 1928 | -4976 | L1M4b | L1 | LINE |

  

|  |  |  |  |  |  |  |  |  |  |
| --- | --- | --- | --- | --- | --- | --- | --- | --- | --- |
| chr2:89160053-89161354 | | | 1316 bp | | |  |  |  |  |
| Genome Start | Genome End | Strand | Div | Repeat Start | Repeat End | Repeat Left | Name | Family | Class |
| 89160053 | 89160580 | - | 29.0 | 1556 | 2152 | -4679 | L1M4b | L1 | LINE |
| 89160573 | 89160772 | - | 22.1 | 5962 | 6166 | 0 | L1PA16 | L1 | LINE |
| 89160764 | 89160989 | + | 13.7 | 5745 | 5971 | -195 | L1PA16 | L1 | LINE |
| 89160993 | 89161354 | - | 28.3 | 1194 | 1566 | -4976 | L1M4b | L1 | LINE |

  

|  |  |  |  |  |  |  |  |  |  |
| --- | --- | --- | --- | --- | --- | --- | --- | --- | --- |
| chr2:89795993-89797287 | | | 1309 bp | | |  |  |  |  |
| Genome Start | Genome End | Strand | Div | Repeat Start | Repeat End | Repeat Left | Name | Family | Class |
| 89795993 | 89796354 | + | 28.1 | 1194 | 1566 | -4976 | L1M4 | L1 | LINE |
| 89796358 | 89796583 | - | 13.7 | 5745 | 5971 | -195 | L1PA16 | L1 | LINE |
| 89796575 | 89796774 | + | 22.1 | 5962 | 6166 | 0 | L1PA16 | L1 | LINE |
| 89796767 | 89797287 | + | 29.0 | 1556 | 2152 | -4679 | L1M4 | L1 | LINE |

  

|  |  |  |  |  |  |  |  |  |  |
| --- | --- | --- | --- | --- | --- | --- | --- | --- | --- |
| chr2:89854049-89855314 | | | 1261 bp | | |  |  |  |  |
| Genome Start | Genome End | Strand | Div | Repeat Start | Repeat End | Repeat Left | Name | Family | Class |
| 89854049 | 89854410 | + | 29.6 | 1512 | 1928 | -4976 | L1M4b | L1 | LINE |
| 89854415 | 89854634 | - | 12.7 | 5754 | 5971 | -195 | L1PA16 | L1 | LINE |
| 89854635 | 89854840 | + | 19.5 | 5962 | 6166 | 0 | L1PA16 | L1 | LINE |
| 89854842 | 89855314 | + | 28.7 | 1930 | 2473 | -4861 | L1M4b | L1 | LINE |

  

|  |  |  |  |  |  |  |  |  |  |
| --- | --- | --- | --- | --- | --- | --- | --- | --- | --- |
| chr2:97300735-97314384 | | | 13393 bp | | |  |  |  |  |
| Genome Start | Genome End | Strand | Div | Repeat Start | Repeat End | Repeat Left | Name | Family | Class |
| 97300735 | 97301245 | + | 26.9 | 799 | 1173 | -5810 | L1PA12 | L1 | LINE |
| 97301261 | 97301846 | + | 25.3 | 802 | 1360 | -5891 | L1PA12 | L1 | LINE |
| 97301851 | 97302551 | - | 19.4 | 5 | 1166 | -5815 | L1PA12 | L1 | LINE |
| 97302730 | 97302801 | + | 28.2 | 100 | 175 | -87 | MIR | MIR | SINE |
| 97302840 | 97306620 | + | 14.5 | 3 | 4391 | -3750 | L1PREC2 | L1 | LINE |
| 97306614 | 97308239 | + | 18.7 | 1252 | 2650 | -4953 | L1PA12 | L1 | LINE |
| 97308246 | 97308407 | + | 11.1 | 2808 | 2977 | -4004 | L1PA12 | L1 | LINE |
| 97308408 | 97308482 | - | 8.0 | 1330 | 1404 | -4949 | L1PA10 | L1 | LINE |
| 97308483 | 97310427 | + | 9.7 | 1402 | 3369 | -2962 | L1PA10 | L1 | LINE |
| 97310429 | 97311210 | + | 8.9 | 4336 | 5080 | -1251 | L1PA10 | L1 | LINE |
| 97311211 | 97311515 | - | 11.8 | 1 | 305 | 0 | AluSc | Alu | SINE |
| 97311516 | 97312776 | + | 8.7 | 5081 | 6353 | 0 | L1PA10 | L1 | LINE |
| 97312764 | 97314041 | + | 11.7 | 3806 | 5111 | -2470 | L1PA12 | L1 | LINE |
| 97314077 | 97314384 | + | 11.7 | 5112 | 5436 | -2145 | L1PA12 | L1 | LINE |

  

|  |  |  |  |  |  |  |  |  |  |
| --- | --- | --- | --- | --- | --- | --- | --- | --- | --- |
| chr2:97456932-97468475 | | | 11524 bp | | |  |  |  |  |
| Genome Start | Genome End | Strand | Div | Repeat Start | Repeat End | Repeat Left | Name | Family | Class |
| 97456932 | 97457239 | - | 11.7 | 5672 | 5996 | -2145 | L1PA12 | L1 | LINE |
| 97457275 | 97458552 | - | 11.7 | 4366 | 5671 | -2470 | L1PA12 | L1 | LINE |
| 97458540 | 97459800 | - | 8.7 | 5081 | 6353 | 0 | L1PA10 | L1 | LINE |
| 97459801 | 97460105 | + | 11.8 | 1 | 305 | 0 | AluSc | Alu | SINE |
| 97460106 | 97460888 | - | 9.0 | 4336 | 5080 | -1251 | L1PA10 | L1 | LINE |
| 97460888 | 97462832 | - | 9.8 | 1402 | 3369 | -2962 | L1PA10 | L1 | LINE |
| 97462833 | 97462907 | + | 8.0 | 1330 | 1404 | -4949 | L1PA10 | L1 | LINE |
| 97462908 | 97463069 | - | 11.1 | 2808 | 2977 | -4004 | L1PA10 | L1 | LINE |
| 97463076 | 97464701 | - | 19.1 | 1252 | 2650 | -4953 | L1PA10 | L1 | LINE |
| 97464695 | 97468475 | - | 14.7 | 3 | 4391 | -3750 | L1PA12 | L1 | LINE |

  

|  |  |  |  |  |  |  |  |  |  |
| --- | --- | --- | --- | --- | --- | --- | --- | --- | --- |
| chr2:99208146-99219587 | | | 11264 bp | | |  |  |  |  |
| Genome Start | Genome End | Strand | Div | Repeat Start | Repeat End | Repeat Left | Name | Family | Class |
| 99208146 | 99208725 | + | 16.6 | 1615 | 2207 | -4296 | L1PA16 | L1 | LINE |
| 99208723 | 99210548 | - | 3.8 | 2405 | 4235 | -1911 | L1PA4 | L1 | LINE |
| 99210548 | 99212464 | + | 3.5 | 4236 | 6152 | -3 | L1PA4 | L1 | LINE |
| 99212653 | 99213570 | + | 11.6 | 2209 | 3104 | -3379 | L1PA16 | L1 | LINE |
| 99213571 | 99213856 | + | 14.3 | 3 | 297 | -15 | AluSx | Alu | SINE |
| 99213857 | 99214719 | + | 10.7 | 3105 | 3901 | -2582 | L1PA16 | L1 | LINE |
| 99214713 | 99216497 | + | 10.9 | 4286 | 6123 | -380 | L1PA16 | L1 | LINE |
| 99216498 | 99218514 | + | 7.5 | 3771 | 5786 | -368 | L1PA7 | L1 | LINE |
| 99218515 | 99218818 | + | 8.6 | 1 | 303 | -8 | AluY | Alu | SINE |
| 99218819 | 99219193 | + | 6.1 | 5787 | 6152 | -2 | L1PA7 | L1 | LINE |
| 99219195 | 99219587 | + | 11.0 | 6106 | 6503 | 0 | L1PA16 | L1 | LINE |

  

|  |  |  |  |  |  |  |  |  |  |
| --- | --- | --- | --- | --- | --- | --- | --- | --- | --- |
| chr2:101335288-101337123 | | | 1827 bp | | |  |  |  |  |
| Genome Start | Genome End | Strand | Div | Repeat Start | Repeat End | Repeat Left | Name | Family | Class |
| 101335288 | 101335413 | + | 27.8 | 5 | 133 | -1339 | MLT1H-int | MaLR | LTR |
| 101335420 | 101335706 | + | 12.9 | 1 | 289 | -23 | AluSx | Alu | SINE |
| 101335707 | 101335869 | + | 21.1 | 146 | 306 | -6 | AluJo/FRAM | Alu | SINE |
| 101335872 | 101335965 | + | 24.7 | 206 | 298 | -1191 | MLT1H-int | MaLR | LTR |
| 101335966 | 101336217 | - | 8.8 | 5089 | 5338 | -808 | L1PA8 | L1 | LINE |
| 101336219 | 101337027 | + | 5.3 | 5351 | 6166 | -6 | L1PA8 | L1 | LINE |
| 101337028 | 101337123 | + | 18.9 | 284 | 407 | -1082 | MLT1H-int | MaLR | LTR |

  

|  |  |  |  |  |  |  |  |  |  |
| --- | --- | --- | --- | --- | --- | --- | --- | --- | --- |
| chr2:103515077-103521461 | | | 6307 bp | | |  |  |  |  |
| Genome Start | Genome End | Strand | Div | Repeat Start | Repeat End | Repeat Left | Name | Family | Class |
| 103515077 | 103517507 | - | 17.3 | 3572 | 6204 | -7 | L1PB3 | L1 | LINE |
| 103517508 | 103517947 | + | 18.2 | 1 | 450 | -104 | MLT2A2 | ERVL | LTR |
| 103517955 | 103518987 | - | 3.9 | 5117 | 6154 | -1 | L1PA4 | L1 | LINE |
| 103518967 | 103519018 | - | 9.6 | 5140 | 5191 | -955 | L1P4 | L1 | LINE |
| 103519015 | 103519090 | + | 5.3 | 5049 | 5124 | -1022 | L1P1 | L1 | LINE |
| 103519091 | 103519213 | + | 10.6 | 439 | 561 | 0 | MLT2A2 | ERVL | LTR |
| 103519232 | 103519516 | - | 14.0 | 3259 | 3557 | -2646 | L1PB3 | L1 | LINE |
| 103519518 | 103519663 | - | 22.6 | 6013 | 6160 | -6 | L1PA16 | L1 | LINE |
| 103519683 | 103520652 | - | 16.0 | 4661 | 5644 | -522 | L1PA16 | L1 | LINE |
| 103520671 | 103520912 | - | 16.2 | 1 | 290 | -12 | AluJb | Alu | SINE |
| 103520953 | 103521461 | - | 20.3 | 1 | 985 | -5219 | L1PB3 | L1 | LINE |

  

|  |  |  |  |  |  |  |  |  |  |
| --- | --- | --- | --- | --- | --- | --- | --- | --- | --- |
| chr2:103614326-103618130 | | | 3795 bp | | |  |  |  |  |
| Genome Start | Genome End | Strand | Div | Repeat Start | Repeat End | Repeat Left | Name | Family | Class |
| 103614326 | 103614735 | - | 25.3 | 6320 | 6731 | -1054 | L1MC3 | L1 | LINE |
| 103614736 | 103615191 | + | 20.1 | 1 | 441 | -2 | LTR47B | ERVL | LTR |
| 103615192 | 103615363 | - | 25.3 | 6151 | 6319 | -1466 | L1MC3 | L1 | LINE |
| 103615359 | 103615929 | - | 7.5 | 4924 | 5492 | -680 | L1PA7 | L1 | LINE |
| 103615930 | 103616598 | + | 5.5 | 5481 | 6151 | -3 | L1PA7 | L1 | LINE |
| 103616614 | 103618130 | - | 23.0 | 4515 | 6146 | -1639 | L1MC3 | L1 | LINE |

  

|  |  |  |  |  |  |  |  |  |  |
| --- | --- | --- | --- | --- | --- | --- | --- | --- | --- |
| chr2:106880995-106881952 | | | 947 bp | | |  |  |  |  |
| Genome Start | Genome End | Strand | Div | Repeat Start | Repeat End | Repeat Left | Name | Family | Class |
| 106880995 | 106881439 | - | 17.6 | 1536 | 2718 | 0 | Tigger2 | MER2\_type | DNA |
| 106881442 | 106881744 | - | 9.0 | 5835 | 6153 | -15 | L1PA10 | L1 | LINE |
| 106881745 | 106881876 | + | 6.1 | 5681 | 5812 | -356 | L1PA10 | L1 | LINE |
| 106881886 | 106881952 | - | 17.9 | 3 | 69 | -2649 | Tigger2 | MER2\_type | DNA |

  

|  |  |  |  |  |  |  |  |  |  |
| --- | --- | --- | --- | --- | --- | --- | --- | --- | --- |
| chr2:112060919-112066924 | | | 5991 bp | | |  |  |  |  |
| Genome Start | Genome End | Strand | Div | Repeat Start | Repeat End | Repeat Left | Name | Family | Class |
| 112060919 | 112061740 | + | 11.7 | 24 | 881 | -1537 | Tigger1 | MER2\_type | DNA |
| 112061741 | 112062043 | + | 10.7 | 1 | 303 | -10 | AluSq | Alu | SINE |
| 112062044 | 112062323 | + | 11.7 | 882 | 1173 | -1245 | Tigger1 | MER2\_type | DNA |
| 112062331 | 112062895 | - | 3.7 | 2798 | 3359 | -2787 | L1PA4 | L1 | LINE |
| 112062896 | 112065680 | + | 2.9 | 3357 | 6172 | 0 | L1PA4 | L1 | LINE |
| 112065689 | 112066924 | + | 12.3 | 1183 | 2418 | 0 | Tigger1 | MER2\_type | DNA |

  

|  |  |  |  |  |  |  |  |  |  |
| --- | --- | --- | --- | --- | --- | --- | --- | --- | --- |
| chr2:113425088-113428062 | | | 2872 bp | | |  |  |  |  |
| Genome Start | Genome End | Strand | Div | Repeat Start | Repeat End | Repeat Left | Name | Family | Class |
| 113425088 | 113425557 | - | 18.0 | 1334 | 1889 | -4511 | L1MEc | L1 | LINE |
| 113425559 | 113425726 | - | 11.9 | 712 | 885 | 0 | MER41G | ERV1 | LTR |
| 113425735 | 113426179 | - | 8.6 | 5712 | 6158 | -10 | L1PA10 | L1 | LINE |
| 113426180 | 113426632 | + | 10.0 | 5268 | 5722 | -446 | L1PA10 | L1 | LINE |
| 113426644 | 113426979 | - | 16.3 | 1 | 725 | -160 | MER41G | ERV1 | LTR |
| 113427063 | 113427405 | - | 18.2 | 858 | 1198 | -5202 | L1MEc | L1 | LINE |
| 113427406 | 113427696 | - | 12.1 | 1 | 290 | -23 | AluSq | Alu | SINE |
| 113427697 | 113428062 | - | 18.2 | 502 | 857 | -5543 | L1MEc | L1 | LINE |

  

|  |  |  |  |  |  |  |  |  |  |
| --- | --- | --- | --- | --- | --- | --- | --- | --- | --- |
| chr2:115485695-115498205 | | | 12091 bp | | |  |  |  |  |
| Genome Start | Genome End | Strand | Div | Repeat Start | Repeat End | Repeat Left | Name | Family | Class |
| 115485695 | 115485876 | + | 17.7 | 3 | 183 | -6680 | L1PB1 | L1 | LINE |
| 115485877 | 115486984 | + | 13.3 | 625 | 1890 | -4973 | L1PB1 | L1 | LINE |
| 115486989 | 115487598 | - | 8.7 | 1609 | 2220 | -55 | L1P2 | L1 | LINE |
| 115487596 | 115490771 | + | 9.7 | 2203 | 5363 | -783 | L1P3 | L1 | LINE |
| 115490772 | 115491264 | + | 5.1 | 5665 | 6152 | -3 | L1PA3 | L1 | LINE |
| 115491265 | 115492062 | + | 6.7 | 5364 | 6154 | 0 | L1PA5 | L1 | LINE |
| 115492058 | 115492910 | + | 7.0 | 5293 | 6143 | -11 | L1PA7 | L1 | LINE |
| 115493333 | 115494153 | + | 9.5 | 1882 | 2744 | -4119 | L1PB1 | L1 | LINE |
| 115494156 | 115498205 | + | 10.6 | 2799 | 6859 | -8 | L1PB1 | L1 | LINE |

  

|  |  |  |  |  |  |  |  |  |  |
| --- | --- | --- | --- | --- | --- | --- | --- | --- | --- |
| chr2:116001182-116003171 | | | 1950 bp | | |  |  |  |  |
| Genome Start | Genome End | Strand | Div | Repeat Start | Repeat End | Repeat Left | Name | Family | Class |
| 116001182 | 116002019 | + | 17.1 | 4749 | 5606 | -636 | L1MD1 | L1 | LINE |
| 116002020 | 116002231 | - | 6.6 | 5533 | 5748 | -406 | L1P2 | L1 | LINE |
| 116002221 | 116002250 | + | 0.0 | 5747 | 5776 | -379 | L1P2 | L1 | LINE |
| 116002251 | 116002445 | + | 3.6 | 5987 | 6170 | 0 | L1PA5 | L1 | LINE |
| 116002497 | 116003171 | + | 20.7 | 5593 | 6242 | 0 | L1MD1 | L1 | LINE |

  

|  |  |  |  |  |  |  |  |  |  |
| --- | --- | --- | --- | --- | --- | --- | --- | --- | --- |
| chr2:116848497-116849967 | | | 1387 bp | | |  |  |  |  |
| Genome Start | Genome End | Strand | Div | Repeat Start | Repeat End | Repeat Left | Name | Family | Class |
| 116848497 | 116848843 | + | 35.3 | 2472 | 2851 | -568 | L2 | L2 | LINE |
| 116848864 | 116849040 | - | 20.4 | 5687 | 5869 | -305 | L1MB5 | L1 | LINE |
| 116849024 | 116849346 | + | 24.3 | 5849 | 6179 | -36 | L1MB5 | L1 | LINE |
| 116849345 | 116849459 | + | 20.0 | 6061 | 6182 | -2 | L1MB7 | L1 | LINE |
| 116849543 | 116849967 | + | 36.5 | 2943 | 3406 | -13 | L2 | L2 | LINE |

  

|  |  |  |  |  |  |  |  |  |  |
| --- | --- | --- | --- | --- | --- | --- | --- | --- | --- |
| chr2:117040711-117051847 | | | 11137 bp | | |  |  |  |  |
| Genome Start | Genome End | Strand | Div | Repeat Start | Repeat End | Repeat Left | Name | Family | Class |
| 117040711 | 117044666 | - | 12.3 | 3706 | 7715 | -6 | L1MA1 | L1 | LINE |
| 117044677 | 117045541 | - | 11.4 | 5302 | 6171 | -1 | L1PA8 | L1 | LINE |
| 117045542 | 117045851 | - | 13.3 | 1 | 309 | -1 | AluSg | Alu | SINE |
| 117045852 | 117046796 | - | 10.7 | 4375 | 5301 | -845 | L1P | L1 | LINE |
| 117046791 | 117047406 | + | 11.3 | 3758 | 4371 | -1775 | L1P | L1 | LINE |
| 117047403 | 117049663 | - | 16.9 | 1547 | 3721 | -3844 | L1MA1 | L1 | LINE |
| 117049664 | 117050039 | - | 16.8 | 1 | 381 | 0 | THE1D | MaLR | LTR |
| 117050040 | 117051406 | - | 18.0 | 155 | 1546 | -6019 | L1MA1 | L1 | LINE |
| 117051407 | 117051714 | + | 1.3 | 1 | 307 | -3 | AluYa5 | Alu | SINE |
| 117051715 | 117051847 | - | 18.0 | 17 | 154 | -7411 | L1MA1 | L1 | LINE |

  

|  |  |  |  |  |  |  |  |  |  |
| --- | --- | --- | --- | --- | --- | --- | --- | --- | --- |
| chr2:117358855-117361546 | | | 2593 bp | | |  |  |  |  |
| Genome Start | Genome End | Strand | Div | Repeat Start | Repeat End | Repeat Left | Name | Family | Class |
| 117358855 | 117359018 | + | 33.3 | 5419 | 5588 | -578 | L1ME1 | L1 | LINE |
| 117359014 | 117359311 | - | 11.4 | 4324 | 4633 | -1513 | L1P3 | L1 | LINE |
| 117359309 | 117359437 | + | 10.5 | 4653 | 4790 | -1356 | L1P3 | L1 | LINE |
| 117359478 | 117360435 | + | 9.2 | 4791 | 5731 | -441 | L1P3 | L1 | LINE |
| 117360481 | 117361047 | + | 9.5 | 5589 | 6155 | -17 | L1PA8 | L1 | LINE |
| 117361070 | 117361546 | + | 24.6 | 5583 | 6039 | -127 | L1ME1 | L1 | LINE |

  

|  |  |  |  |  |  |  |  |  |  |
| --- | --- | --- | --- | --- | --- | --- | --- | --- | --- |
| chr2:117697661-117707719 | | | 10055 bp | | |  |  |  |  |
| Genome Start | Genome End | Strand | Div | Repeat Start | Repeat End | Repeat Left | Name | Family | Class |
| 117697661 | 117698042 | - | 16.4 | 6486 | 6860 | -7 | L1PB2 | L1 | LINE |
| 117698043 | 117698348 | - | 8.2 | 6 | 311 | 0 | AluY | Alu | SINE |
| 117698349 | 117699787 | - | 16.1 | 5132 | 6485 | -382 | L1PB2 | L1 | LINE |
| 117699786 | 117700858 | - | 3.7 | 5078 | 6153 | -2 | L1PA4 | L1 | LINE |
| 117700859 | 117701879 | + | 4.3 | 4054 | 5074 | -1072 | L1PA4 | L1 | LINE |
| 117701882 | 117704155 | - | 16.2 | 2878 | 5143 | -1719 | L1PB2 | L1 | LINE |
| 117704160 | 117707719 | - | 21.8 | 41 | 2681 | -4182 | L1PB2 | L1 | LINE |

  

|  |  |  |  |  |  |  |  |  |  |
| --- | --- | --- | --- | --- | --- | --- | --- | --- | --- |
| chr2:117920624-117922175 | | | 1551 bp | | |  |  |  |  |
| Genome Start | Genome End | Strand | Div | Repeat Start | Repeat End | Repeat Left | Name | Family | Class |
| 117920624 | 117920939 | + | 11.8 | 1 | 304 | -60 | THE1B | MaLR | LTR |
| 117920941 | 117921253 | - | 11.2 | 5802 | 6128 | -3 | L1PA8A | L1 | LINE |
| 117921254 | 117922102 | + | 7.4 | 4951 | 5799 | -332 | L1PA8A | L1 | LINE |
| 117922103 | 117922175 | + | 12.5 | 292 | 364 | 0 | THE1B | MaLR | LTR |

  

|  |  |  |  |  |  |  |  |  |  |
| --- | --- | --- | --- | --- | --- | --- | --- | --- | --- |
| chr2:118277218-118284734 | | | 7519 bp | | |  |  |  |  |
| Genome Start | Genome End | Strand | Div | Repeat Start | Repeat End | Repeat Left | Name | Family | Class |
| 118277218 | 118277277 | + | 13.9 | 7730 | 7788 | -207 | L1MD3 | L1 | LINE |
| 118277267 | 118277308 | - | 9.5 | 4936 | 4977 | -1169 | L1PA8A | L1 | LINE |
| 118277306 | 118277825 | + | 7.5 | 4975 | 5491 | -640 | L1PA8A | L1 | LINE |
| 118277827 | 118283855 | + | 2.6 | 22 | 6032 | 0 | L1PA3 | L1 | LINE |
| 118283857 | 118284514 | + | 8.5 | 5476 | 6131 | 0 | L1PA8A | L1 | LINE |
| 118284525 | 118284734 | + | 16.2 | 7765 | 7994 | -1 | L1MD3 | L1 | LINE |

  

|  |  |  |  |  |  |  |  |  |  |
| --- | --- | --- | --- | --- | --- | --- | --- | --- | --- |
| chr2:119174577-119178271 | | | 3526 bp | | |  |  |  |  |
| Genome Start | Genome End | Strand | Div | Repeat Start | Repeat End | Repeat Left | Name | Family | Class |
| 119174577 | 119174669 | - | 22.6 | 1384 | 1495 | -1012 | HAL1 | L1 | LINE |
| 119174790 | 119175701 | - | 26.8 | 351 | 1363 | -1144 | HAL1 | L1 | LINE |
| 119175736 | 119176784 | - | 4.3 | 5083 | 6142 | -12 | L1PA5 | L1 | LINE |
| 119176785 | 119177925 | + | 4.9 | 3956 | 5096 | -1050 | L1PA5 | L1 | LINE |
| 119177941 | 119178271 | - | 28.8 | 11 | 327 | -2180 | HAL1 | L1 | LINE |

  

|  |  |  |  |  |  |  |  |  |  |
| --- | --- | --- | --- | --- | --- | --- | --- | --- | --- |
| chr2:120278170-120282763 | | | 4598 bp | | |  |  |  |  |
| Genome Start | Genome End | Strand | Div | Repeat Start | Repeat End | Repeat Left | Name | Family | Class |
| 120278170 | 120278337 | - | 17.3 | 122 | 289 | -23 | AluJo | Alu | SINE |
| 120278339 | 120279996 | - | 5.9 | 4484 | 6141 | -13 | L1PA6 | L1 | LINE |
| 120279992 | 120282620 | + | 6.7 | 1871 | 4491 | -1655 | L1PA6 | L1 | LINE |
| 120282621 | 120282763 | - | 19.0 | 1 | 143 | -169 | AluJo | Alu | SINE |

  

|  |  |  |  |  |  |  |  |  |  |
| --- | --- | --- | --- | --- | --- | --- | --- | --- | --- |
| chr2:123712721-123717587 | | | 4764 bp | | |  |  |  |  |
| Genome Start | Genome End | Strand | Div | Repeat Start | Repeat End | Repeat Left | Name | Family | Class |
| 123712721 | 123712852 | - | 21.4 | 6995 | 7146 | -639 | L1MC3 | L1 | LINE |
| 123712861 | 123713159 | - | 19.2 | 6637 | 6952 | -833 | L1MC3 | L1 | LINE |
| 123713224 | 123713599 | + | 23.5 | 86 | 505 | 0 | MLT1D | MaLR | LTR |
| 123713612 | 123714283 | - | 24.3 | 5811 | 6558 | -1227 | L1MC3 | L1 | LINE |
| 123714284 | 123714584 | - | 12.0 | 1 | 306 | -7 | AluSp | Alu | SINE |
| 123714585 | 123714681 | - | 24.3 | 5722 | 5810 | -1975 | L1MC3 | L1 | LINE |
| 123714682 | 123714994 | + | 4.5 | 1 | 311 | 0 | AluY | Alu | SINE |
| 123714995 | 123715674 | - | 24.2 | 4993 | 5721 | -2064 | L1MC3 | L1 | LINE |
| 123715685 | 123715898 | + | 20.2 | 5930 | 6143 | -40 | L1MB3 | L1 | LINE |
| 123715896 | 123716212 | - | 7.8 | 5451 | 5743 | -429 | L1PA8 | L1 | LINE |
| 123716213 | 123716606 | + | 9.1 | 5760 | 6154 | -18 | L1PA8 | L1 | LINE |
| 123716614 | 123716668 | + | 18.2 | 6126 | 6180 | 0 | L1MB4 | L1 | LINE |
| 123716674 | 123716851 | - | 21.2 | 4856 | 5031 | -1115 | L1MC3 | L1 | LINE |
| 123716852 | 123717145 | + | 22.9 | 1 | 290 | -12 | AluJo | Alu | SINE |
| 123717146 | 123717587 | - | 21.2 | 4369 | 4855 | -1291 | L1MC3 | L1 | LINE |

  

|  |  |  |  |  |  |  |  |  |  |
| --- | --- | --- | --- | --- | --- | --- | --- | --- | --- |
| chr2:123881988-123890666 | | | 7919 bp | | |  |  |  |  |
| Genome Start | Genome End | Strand | Div | Repeat Start | Repeat End | Repeat Left | Name | Family | Class |
| 123881988 | 123882794 | + | 26.0 | 1044 | 1963 | -4670 | L1MD1 | L1 | LINE |
| 123882815 | 123883128 | + | 16.2 | 1 | 310 | -2 | AluSx | Alu | SINE |
| 123883145 | 123883174 | - | 21.4 | 5866 | 5896 | -258 | L1PA6 | L1 | LINE |
| 123883175 | 123883476 | + | 18.1 | 5 | 289 | -13 | AluJb | Alu | SINE |
| 123883477 | 123883769 | - | 12.2 | 5567 | 5865 | -289 | L1PA11 | L1 | LINE |
| 123883771 | 123884077 | + | 11.7 | 5858 | 6171 | -3 | L1PA11 | L1 | LINE |
| 123884176 | 123884332 | + | 26.0 | 2026 | 2180 | -4453 | L1MD1 | L1 | LINE |
| 123884529 | 123885029 | + | 17.7 | 1 | 482 | -245 | LTR8A | ERV1 | LTR |
| 123885030 | 123885292 | - | 16.0 | 3 | 298 | -14 | AluJb | Alu | SINE |
| 123885293 | 123885477 | + | 17.7 | 483 | 678 | -49 | LTR8A | ERV1 | LTR |
| 123885507 | 123885583 | - | 22.1 | 2 | 78 | -198 | MER6B | MER2\_type | DNA |
| 123885593 | 123885735 | + | 22.9 | 2311 | 2478 | -4155 | L1MD1 | L1 | LINE |
| 123885736 | 123885854 | + | 4.2 | 6034 | 6154 | -1 | L1PA4 | L1 | LINE |
| 123885855 | 123887251 | + | 26.7 | 2479 | 4046 | -2491 | L1MD1 | L1 | LINE |
| 123887257 | 123887912 | - | 23.0 | 4 | 642 | -11 | MER39 | ERV1 | LTR |
| 123888283 | 123888545 | + | 27.2 | 4080 | 4341 | -2196 | L1MD1 | L1 | LINE |
| 123888562 | 123890666 | + | 26.0 | 4452 | 6610 | -23 | L1MD1 | L1 | LINE |

  

|  |  |  |  |  |  |  |  |  |  |
| --- | --- | --- | --- | --- | --- | --- | --- | --- | --- |
| chr2:125574585-125583935 | | | 9211 bp | | |  |  |  |  |
| Genome Start | Genome End | Strand | Div | Repeat Start | Repeat End | Repeat Left | Name | Family | Class |
| 125574585 | 125575414 | - | 21.9 | 7029 | 7919 | -41 | L1MC3 | L1 | LINE |
| 125575435 | 125576285 | - | 21.9 | 6160 | 7028 | -932 | L1MC3 | L1 | LINE |
| 125576286 | 125576564 | + | 5.4 | 5868 | 6149 | -5 | L1PA6 | L1 | LINE |
| 125576565 | 125577664 | - | 22.4 | 5029 | 6159 | -1801 | L1MC3 | L1 | LINE |
| 125577665 | 125577977 | - | 10.8 | 1 | 314 | 0 | AluSx | Alu | SINE |
| 125577978 | 125580253 | - | 23.2 | 2670 | 5028 | -1293 | L1MC3 | L1 | LINE |
| 125580251 | 125580320 | - | 25.7 | 1995 | 2067 | -4441 | L1MC3 | L1 | LINE |
| 125580361 | 125580453 | + | 33.7 | 162 | 258 | -107 | MLT1A0 | MaLR | LTR |
| 125580527 | 125580690 | + | 25.1 | 197 | 365 | 0 | MLT1A0 | MaLR | LTR |
| 125580693 | 125580927 | - | 24.9 | 1672 | 1914 | -4594 | L1MC3 | L1 | LINE |
| 125580928 | 125581777 | - | 17.2 | 5463 | 6286 | -5 | L1MA7 | L1 | LINE |
| 125581778 | 125582180 | + | 18.7 | 5071 | 5475 | -837 | L1MA7 | L1 | LINE |
| 125582189 | 125583935 | - | 27.3 | 1 | 1669 | -4839 | L1MC3 | L1 | LINE |

  

|  |  |  |  |  |  |  |  |  |  |
| --- | --- | --- | --- | --- | --- | --- | --- | --- | --- |
| chr2:125878476-125880248 | | | 1754 bp | | |  |  |  |  |
| Genome Start | Genome End | Strand | Div | Repeat Start | Repeat End | Repeat Left | Name | Family | Class |
| 125878476 | 125878576 | - | 24.2 | 2395 | 2491 | 0 | Tigger7 | MER2\_type | DNA |
| 125878588 | 125879674 | - | 14.7 | 5171 | 6295 | -5 | L1MA4 | L1 | LINE |
| 125879673 | 125879969 | + | 16.2 | 4882 | 5172 | -971 | L1MA4 | L1 | LINE |
| 125879980 | 125880248 | - | 22.1 | 1 | 267 | -2224 | Tigger7 | MER2\_type | DNA |

  

|  |  |  |  |  |  |  |  |  |  |
| --- | --- | --- | --- | --- | --- | --- | --- | --- | --- |
| chr2:126334044-126335023 | | | 969 bp | | |  |  |  |  |
| Genome Start | Genome End | Strand | Div | Repeat Start | Repeat End | Repeat Left | Name | Family | Class |
| 126334044 | 126334235 | + | 24.7 | 1 | 191 | -351 | MER34 | ERV1 | LTR |
| 126334246 | 126334591 | - | 14.4 | 5804 | 6145 | -16 | L1PA14 | L1 | LINE |
| 126334592 | 126334689 | + | 11.2 | 5711 | 5808 | -352 | L1PA14 | L1 | LINE |
| 126334691 | 126335023 | + | 22.7 | 169 | 541 | -1 | MER34 | ERV1 | LTR |

  

|  |  |  |  |  |  |  |  |  |  |
| --- | --- | --- | --- | --- | --- | --- | --- | --- | --- |
| chr2:126460630-126468762 | | | 8085 bp | | |  |  |  |  |
| Genome Start | Genome End | Strand | Div | Repeat Start | Repeat End | Repeat Left | Name | Family | Class |
| 126460630 | 126462020 | - | 11.8 | 6325 | 7723 | -1 | L1MA3 | L1 | LINE |
| 126462019 | 126462887 | - | 11.9 | 5285 | 6151 | 0 | L1PB1 | L1 | LINE |
| 126462916 | 126463729 | - | 11.6 | 4466 | 5284 | -862 | L1PB | L1 | LINE |
| 126463731 | 126463823 | + | 10.0 | 4305 | 4397 | -1749 | L1PB | L1 | LINE |
| 126463851 | 126463896 | + | 10.0 | 4398 | 4453 | -1693 | L1PB | L1 | LINE |
| 126463891 | 126468762 | - | 14.8 | 1323 | 6341 | -1224 | L1MA3 | L1 | LINE |

  

|  |  |  |  |  |  |  |  |  |  |
| --- | --- | --- | --- | --- | --- | --- | --- | --- | --- |
| chr2:126634534-126643558 | | | 9025 bp | | |  |  |  |  |
| Genome Start | Genome End | Strand | Div | Repeat Start | Repeat End | Repeat Left | Name | Family | Class |
| 126634534 | 126639822 | + | 9.4 | 1 | 5308 | -1153 | L1PA7 | L1 | LINE |
| 126639823 | 126640124 | + | 12.1 | 2 | 303 | 0 | AluSg | Alu | SINE |
| 126640125 | 126640498 | + | 8.8 | 5309 | 5679 | -782 | L1PA7 | L1 | LINE |
| 126640500 | 126641318 | - | 3.5 | 3861 | 4680 | -1466 | L1PA3 | L1 | LINE |
| 126641318 | 126642810 | + | 3.6 | 4680 | 6173 | 0 | L1PA3 | L1 | LINE |
| 126642811 | 126643558 | + | 7.5 | 5671 | 6469 | 0 | L1PA7 | L1 | LINE |

  

|  |  |  |  |  |  |  |  |  |  |
| --- | --- | --- | --- | --- | --- | --- | --- | --- | --- |
| chr2:126923923-126931893 | | | 7802 bp | | |  |  |  |  |
| Genome Start | Genome End | Strand | Div | Repeat Start | Repeat End | Repeat Left | Name | Family | Class |
| 126923923 | 126924293 | + | 31.1 | 9 | 405 | -1067 | MLT1H-int | MaLR | LTR |
| 126924406 | 126924521 | + | 30.7 | 784 | 898 | -574 | MLT1H-int | MaLR | LTR |
| 126924522 | 126925413 | - | 16.0 | 4594 | 5547 | -744 | L1M2 | L1 | LINE |
| 126925420 | 126930726 | - | 3.5 | 841 | 6150 | -5 | L1PA3 | L1 | LINE |
| 126930724 | 126931085 | - | 14.3 | 4232 | 4600 | -1540 | L1M2 | L1 | LINE |
| 126931148 | 126931169 | - | 14.3 | 4198 | 4231 | -1909 | L1M2 | L1 | LINE |
| 126931167 | 126931575 | + | 16.7 | 2255 | 2719 | -3421 | L1M3 | L1 | LINE |
| 126931567 | 126931830 | + | 15.7 | 3803 | 4191 | -1949 | L1M3 | L1 | LINE |
| 126931835 | 126931893 | + | 22.0 | 885 | 944 | -528 | MLT1H-int | MaLR | LTR |

  

|  |  |  |  |  |  |  |  |  |  |
| --- | --- | --- | --- | --- | --- | --- | --- | --- | --- |
| chr2:131491788-131497189 | | | 5336 bp | | |  |  |  |  |
| Genome Start | Genome End | Strand | Div | Repeat Start | Repeat End | Repeat Left | Name | Family | Class |
| 131491788 | 131492241 | + | 18.3 | 4436 | 4860 | -1286 | L1M | L1 | LINE |
| 131492287 | 131493173 | - | 19.3 | 4607 | 5495 | -688 | L1M3 | L1 | LINE |
| 131493174 | 131493466 | + | 9.9 | 1 | 294 | -19 | AluSp | Alu | SINE |
| 131493467 | 131493669 | - | 19.5 | 4415 | 4606 | -1540 | L1M3 | L1 | LINE |
| 131493670 | 131493976 | - | 4.9 | 1 | 306 | -5 | AluY | Alu | SINE |
| 131493977 | 131494229 | - | 19.5 | 4151 | 4414 | -1732 | L1M3 | L1 | LINE |
| 131494232 | 131495219 | - | 2.5 | 5164 | 6154 | -1 | L1PA4 | L1 | LINE |
| 131495218 | 131495699 | + | 3.3 | 4686 | 5167 | -979 | L1PA4 | L1 | LINE |
| 131495699 | 131496544 | - | 17.8 | 3304 | 4166 | -1980 | L1M3 | L1 | LINE |
| 131496567 | 131497003 | + | 18.6 | 4853 | 5271 | -875 | L1M | L1 | LINE |
| 131497004 | 131497135 | + | 15.2 | 1 | 133 | 0 | FLAM\_A | Alu | SINE |
| 131497136 | 131497189 | + | 17.8 | 5272 | 5322 | -920 | L1M | L1 | LINE |

  

|  |  |  |  |  |  |  |  |  |  |
| --- | --- | --- | --- | --- | --- | --- | --- | --- | --- |
| chr2:137079547-137080610 | | | 901 bp | | |  |  |  |  |
| Genome Start | Genome End | Strand | Div | Repeat Start | Repeat End | Repeat Left | Name | Family | Class |
| 137079547 | 137079667 | + | 34.7 | 2988 | 3116 | -262 | L2 | L2 | LINE |
| 137079678 | 137080017 | - | 1.8 | 5816 | 6155 | 0 | L1PA2 | L1 | LINE |
| 137080018 | 137080380 | + | 1.9 | 5436 | 5799 | -356 | L1PA2 | L1 | LINE |
| 137080534 | 137080610 | + | 29.2 | 3240 | 3312 | -66 | L2 | L2 | LINE |

  

|  |  |  |  |  |  |  |  |  |  |
| --- | --- | --- | --- | --- | --- | --- | --- | --- | --- |
| chr2:138169318-138170205 | | | 844 bp | | |  |  |  |  |
| Genome Start | Genome End | Strand | Div | Repeat Start | Repeat End | Repeat Left | Name | Family | Class |
| 138169318 | 138169366 | - | 18.4 | 125 | 173 | -16 | MER5A | MER1\_type | DNA |
| 138169411 | 138169903 | - | 2.4 | 5655 | 6154 | -1 | L1PA4 | L1 | LINE |
| 138169904 | 138170099 | + | 1.5 | 5454 | 5649 | -506 | L1PA4 | L1 | LINE |
| 138170100 | 138170205 | - | 26.4 | 31 | 141 | -48 | MER5A | MER1\_type | DNA |

  

|  |  |  |  |  |  |  |  |  |  |
| --- | --- | --- | --- | --- | --- | --- | --- | --- | --- |
| chr2:138281323-138285958 | | | 4105 bp | | |  |  |  |  |
| Genome Start | Genome End | Strand | Div | Repeat Start | Repeat End | Repeat Left | Name | Family | Class |
| 138281323 | 138281877 | - | 36.7 | 2821 | 3413 | -6 | L2 | L2 | LINE |
| 138281884 | 138281974 | - | 23.0 | 190 | 275 | -115 | MLT1B | MaLR | LTR |
| 138282019 | 138282049 | - | 23.0 | 159 | 189 | -201 | MLT1A | MaLR | LTR |
| 138282151 | 138282280 | - | 24.4 | 2 | 133 | -241 | MLT1A | MaLR | LTR |
| 138282332 | 138282637 | - | 34.3 | 2326 | 2703 | -716 | L2 | L2 | LINE |
| 138282754 | 138283046 | - | 12.7 | 5493 | 5794 | -372 | L1P4 | L1 | LINE |
| 138283095 | 138283458 | - | 11.1 | 5099 | 5492 | -674 | L1P4 | L1 | LINE |
| 138283460 | 138283831 | + | 18.0 | 5794 | 6165 | -1 | L1PA16 | L1 | LINE |
| 138283958 | 138284391 | - | 21.3 | 131 | 563 | 0 | MER68 | ERVL | LTR |
| 138284422 | 138285085 | - | 17.2 | 5658 | 6326 | -8 | L1MA10 | L1 | LINE |
| 138285086 | 138285211 | - | 21.3 | 1 | 130 | -433 | MER68 | ERVL | LTR |
| 138285220 | 138285958 | - | 37.4 | 1273 | 2080 | -1339 | L2 | L2 | LINE |

  

|  |  |  |  |  |  |  |  |  |  |
| --- | --- | --- | --- | --- | --- | --- | --- | --- | --- |
| chr2:139035704-139037235 | | | 1532 bp | | |  |  |  |  |
| Genome Start | Genome End | Strand | Div | Repeat Start | Repeat End | Repeat Left | Name | Family | Class |
| 139035704 | 139035810 | + | 21.9 | 4358 | 4467 | -1679 | L1M | L1 | LINE |
| 139035804 | 139035968 | - | 13.0 | 5950 | 6113 | -53 | L1PA16 | L1 | LINE |
| 139035969 | 139036046 | + | 14.3 | 6090 | 6166 | 0 | L1PA16 | L1 | LINE |
| 139036047 | 139036542 | + | 24.3 | 4456 | 4985 | -1161 | L1M | L1 | LINE |
| 139036543 | 139036843 | + | 12.3 | 1 | 302 | -10 | AluSx | Alu | SINE |
| 139036844 | 139037102 | + | 24.3 | 4986 | 5271 | -875 | L1M | L1 | LINE |
| 139037110 | 139037235 | + | 23.8 | 5255 | 5382 | -784 | L1M | L1 | LINE |

  

|  |  |  |  |  |  |  |  |  |  |
| --- | --- | --- | --- | --- | --- | --- | --- | --- | --- |
| chr2:139484661-139486742 | | | 2021 bp | | |  |  |  |  |
| Genome Start | Genome End | Strand | Div | Repeat Start | Repeat End | Repeat Left | Name | Family | Class |
| 139484661 | 139484720 | - | 20.0 | 6156 | 6219 | -93 | L1MA9 | L1 | LINE |
| 139484782 | 139484882 | - | 3.0 | 6051 | 6154 | 0 | L1PA7 | L1 | LINE |
| 139484883 | 139485617 | + | 6.1 | 5319 | 6063 | -91 | L1PA7 | L1 | LINE |
| 139485618 | 139486742 | - | 19.3 | 4962 | 6106 | -206 | L1MA9 | L1 | LINE |

  

|  |  |  |  |  |  |  |  |  |  |
| --- | --- | --- | --- | --- | --- | --- | --- | --- | --- |
| chr2:139576367-139587705 | | | 11266 bp | | |  |  |  |  |
| Genome Start | Genome End | Strand | Div | Repeat Start | Repeat End | Repeat Left | Name | Family | Class |
| 139576367 | 139576858 | - | 18.7 | 5476 | 5989 | -3 | L1MB1 | L1 | LINE |
| 139576887 | 139577131 | - | 4.5 | 5908 | 6152 | -3 | L1PA3 | L1 | LINE |
| 139577134 | 139577411 | + | 2.2 | 5214 | 5491 | -664 | L1P2 | L1 | LINE |
| 139577411 | 139577463 | - | 9.4 | 5437 | 5489 | -503 | L1MB1 | L1 | LINE |
| 139577459 | 139577561 | - | 17.6 | 5061 | 5165 | -799 | L1MB1 | L1 | LINE |
| 139577562 | 139578267 | + | 1.7 | 5448 | 6153 | -2 | L1PA2 | L1 | LINE |
| 139578268 | 139579167 | - | 17.6 | 4217 | 5060 | -904 | L1MB1 | L1 | LINE |
| 139579213 | 139581607 | - | 8.9 | 3883 | 6289 | -27 | L1PA8A | L1 | LINE |
| 139581608 | 139583315 | + | 10.6 | 2169 | 3896 | -2435 | L1PA8A | L1 | LINE |
| 139583320 | 139587705 | - | 24.3 | 13 | 4225 | -1739 | L1MB1 | L1 | LINE |

  

|  |  |  |  |  |  |  |  |  |  |
| --- | --- | --- | --- | --- | --- | --- | --- | --- | --- |
| chr2:139604637-139611186 | | | 5444 bp | | |  |  |  |  |
| Genome Start | Genome End | Strand | Div | Repeat Start | Repeat End | Repeat Left | Name | Family | Class |
| 139604637 | 139604714 | - | 28.6 | 6040 | 6118 | -105 | L1ME3B | L1 | LINE |
| 139604804 | 139604870 | - | 15.2 | 1 | 66 | -401 | MLT1C | MaLR | LTR |
| 139604877 | 139605169 | - | 23.9 | 5630 | 5960 | -263 | L1ME3B | L1 | LINE |
| 139605336 | 139605887 | - | 31.0 | 5000 | 5612 | -611 | L1ME3B | L1 | LINE |
| 139605953 | 139606752 | - | 12.6 | 5326 | 6157 | -9 | L1PA16 | L1 | LINE |
| 139606753 | 139607020 | - | 15.3 | 1 | 276 | -37 | AluSp | Alu | SINE |
| 139607021 | 139607754 | - | 14.3 | 4596 | 5325 | -821 | L1P | L1 | LINE |
| 139607750 | 139608335 | + | 14.9 | 3995 | 4586 | -1560 | L1P | L1 | LINE |
| 139608395 | 139608851 | + | 21.7 | 2 | 456 | -64 | MLT2B1 | ERVL | LTR |
| 139608880 | 139608934 | + | 21.7 | 504 | 567 | 0 | MLT2B1 | ERVL | LTR |
| 139608936 | 139609571 | - | 30.0 | 4244 | 4923 | -1206 | L1ME3B | L1 | LINE |
| 139609860 | 139610349 | - | 28.2 | 2214 | 2747 | -3382 | L1ME3B | L1 | LINE |
| 139610639 | 139610740 | - | 20.8 | 1639 | 1744 | -4405 | L1ME3B | L1 | LINE |
| 139610752 | 139610900 | - | 29.7 | 1426 | 1583 | -4566 | L1ME3B | L1 | LINE |
| 139611010 | 139611186 | - | 33.1 | 1020 | 1204 | -4915 | L1ME3B | L1 | LINE |

  

|  |  |  |  |  |  |  |  |  |  |
| --- | --- | --- | --- | --- | --- | --- | --- | --- | --- |
| chr2:139884122-139893617 | | | 8900 bp | | |  |  |  |  |
| Genome Start | Genome End | Strand | Div | Repeat Start | Repeat End | Repeat Left | Name | Family | Class |
| 139884122 | 139884198 | - | 26.1 | 6146 | 6218 | -19 | L1ME2 | L1 | LINE |
| 139884199 | 139885407 | + | 5.7 | 2 | 1578 | 0 | LTR12C | ERV1 | LTR |
| 139885408 | 139885711 | - | 26.1 | 5876 | 6145 | -92 | L1ME2 | L1 | LINE |
| 139885712 | 139886024 | - | 11.9 | 1 | 236 | 0 | MER46B | MER2\_type | DNA |
| 139886025 | 139886256 | - | 26.1 | 5637 | 5875 | -362 | L1ME2 | L1 | LINE |
| 139886257 | 139886729 | + | 20.1 | 1 | 502 | -3 | MLT1D | MaLR | LTR |
| 139886730 | 139887075 | - | 26.6 | 5271 | 5636 | -601 | L1ME2 | L1 | LINE |
| 139887076 | 139887380 | - | 10.8 | 1 | 306 | -7 | AluSp | Alu | SINE |
| 139887381 | 139887481 | - | 28.5 | 5167 | 5270 | -949 | L1ME2 | L1 | LINE |
| 139887489 | 139887797 | - | 10.7 | 1 | 310 | 0 | AluSg | Alu | SINE |
| 139887978 | 139889406 | - | 9.2 | 4694 | 6115 | -36 | L1PB1 | L1 | LINE |
| 139889406 | 139889737 | + | 7.9 | 4351 | 4684 | -1462 | L1PB1 | L1 | LINE |
| 139889738 | 139889910 | - | 24.7 | 4783 | 4955 | -1191 | L1MC | L1 | LINE |
| 139890106 | 139891132 | - | 28.3 | 3311 | 4334 | -1885 | L1ME2 | L1 | LINE |
| 139891126 | 139891256 | - | 23.1 | 2959 | 3089 | -3475 | L1MCb | L1 | LINE |
| 139891314 | 139891719 | - | 27.1 | 1 | 415 | -5630 | L1MCb | L1 | LINE |
| 139891739 | 139891987 | - | 24.2 | 1676 | 1928 | -4472 | L1ME2 | L1 | LINE |
| 139892016 | 139892146 | - | 28.6 | 1513 | 1640 | -4862 | L1MDb | L1 | LINE |
| 139892156 | 139892291 | - | 12.6 | 297 | 432 | 0 | MSTB1 | MaLR | LTR |
| 139892306 | 139892373 | - | 10.3 | 1 | 68 | -358 | MSTB | MaLR | LTR |
| 139892374 | 139892652 | - | 28.1 | 1194 | 1508 | -4994 | L1MDb | L1 | LINE |
| 139892748 | 139893617 | - | 34.9 | 37 | 919 | -5481 | L1ME2 | L1 | LINE |

  

|  |  |  |  |  |  |  |  |  |  |
| --- | --- | --- | --- | --- | --- | --- | --- | --- | --- |
| chr2:140444089-140451680 | | | 7378 bp | | |  |  |  |  |
| Genome Start | Genome End | Strand | Div | Repeat Start | Repeat End | Repeat Left | Name | Family | Class |
| 140444089 | 140449104 | + | 16.2 | 16 | 5099 | -2466 | L1M1 | L1 | LINE |
| 140449250 | 140449608 | + | 12.3 | 5100 | 5481 | -2084 | L1M1 | L1 | LINE |
| 140449602 | 140450128 | + | 18.3 | 5747 | 6281 | -22 | L1MA4A | L1 | LINE |
| 140450179 | 140450350 | + | 22.1 | 5728 | 5914 | -1651 | L1M1 | L1 | LINE |
| 140450349 | 140450410 | - | 13.6 | 5304 | 5365 | -775 | L1M3 | L1 | LINE |
| 140450431 | 140450499 | - | 13.6 | 5223 | 5303 | -837 | L1M3 | L1 | LINE |
| 140450498 | 140450953 | + | 28.0 | 5367 | 5839 | -345 | L1MB7 | L1 | LINE |
| 140450964 | 140451132 | + | 25.3 | 6427 | 6611 | -954 | L1M1 | L1 | LINE |
| 140451133 | 140451492 | - | 13.4 | 1 | 428 | 0 | MSTA | MaLR | LTR |
| 140451493 | 140451680 | + | 25.3 | 6612 | 6798 | -767 | L1M1 | L1 | LINE |

  

|  |  |  |  |  |  |  |  |  |  |
| --- | --- | --- | --- | --- | --- | --- | --- | --- | --- |
| chr2:141599752-141602586 | | | 2675 bp | | |  |  |  |  |
| Genome Start | Genome End | Strand | Div | Repeat Start | Repeat End | Repeat Left | Name | Family | Class |
| 141599752 | 141600063 | + | 32.7 | 209 | 521 | -2898 | L2 | L2 | LINE |
| 141600064 | 141600372 | - | 12.1 | 1 | 308 | -4 | AluSx | Alu | SINE |
| 141600373 | 141600877 | + | 32.7 | 522 | 1041 | -2378 | L2 | L2 | LINE |
| 141600906 | 141601189 | - | 7.8 | 5864 | 6153 | -1 | L1PA7 | L1 | LINE |
| 141601193 | 141601640 | + | 5.8 | 5407 | 5855 | -299 | L1PA7 | L1 | LINE |
| 141601645 | 141601920 | + | 31.6 | 1074 | 1416 | -2003 | L2 | L2 | LINE |
| 141601921 | 141602027 | + | 31.8 | 2 | 111 | -2650 | Charlie10 | MER1\_type | DNA |
| 141602033 | 141602185 | - | 24.5 | 1 | 160 | -16 | FRAM | Alu | SINE |
| 141602230 | 141602387 | + | 20.4 | 2668 | 2822 | 0 | Charlie10 | MER1\_type | DNA |
| 141602464 | 141602586 | + | 25.8 | 2905 | 3032 | -387 | L2 | L2 | LINE |

  

|  |  |  |  |  |  |  |  |  |  |
| --- | --- | --- | --- | --- | --- | --- | --- | --- | --- |
| chr2:142407344-142414044 | | | 5582 bp | | |  |  |  |  |
| Genome Start | Genome End | Strand | Div | Repeat Start | Repeat End | Repeat Left | Name | Family | Class |
| 142407344 | 142407636 | + | 36.0 | 115 | 419 | -5700 | L1MEd | L1 | LINE |
| 142407871 | 142408507 | + | 28.5 | 639 | 1254 | -4839 | L1MEd | L1 | LINE |
| 142408589 | 142408956 | + | 10.1 | 1 | 375 | 0 | THE1C | MaLR | LTR |
| 142408965 | 142409078 | - | 29.8 | 18 | 134 | 0 | MER94 | AcHobo | DNA |
| 142409400 | 142409566 | + | 30.0 | 1852 | 2019 | -4130 | L1MEd | L1 | LINE |
| 142409586 | 142409873 | + | 28.6 | 2074 | 2381 | -3768 | L1MEd | L1 | LINE |
| 142409876 | 142409964 | + | 15.7 | 31 | 122 | -20 | FLAM\_A | Alu | SINE |
| 142409967 | 142410469 | + | 29.6 | 3829 | 4332 | -1797 | L1MEd | L1 | LINE |
| 142410495 | 142410670 | + | 22.3 | 204 | 389 | -1 | MLT1B | MaLR | LTR |
| 142410672 | 142410746 | - | 16.4 | 5542 | 5618 | -556 | L1M4 | L1 | LINE |
| 142411095 | 142411147 | + | 27.4 | 4395 | 4444 | -1685 | L1MEd | L1 | LINE |
| 142411148 | 142411453 | + | 8.7 | 2 | 311 | 0 | AluY | Alu | SINE |
| 142411454 | 142411690 | + | 27.4 | 4445 | 4664 | -1465 | L1MEd | L1 | LINE |
| 142411723 | 142412161 | - | 18.0 | 4718 | 5154 | -986 | L1M4 | L1 | LINE |
| 142412159 | 142413295 | + | 17.1 | 5151 | 6272 | -40 | L1MA9 | L1 | LINE |
| 142413345 | 142413499 | + | 33.8 | 4728 | 4881 | -1248 | L1MEd | L1 | LINE |
| 142413500 | 142413912 | + | 24.0 | 4 | 436 | 0 | MER63B | AcHobo | DNA |
| 142413913 | 142414044 | + | 33.8 | 4882 | 5008 | -1121 | L1MEd | L1 | LINE |

  

|  |  |  |  |  |  |  |  |  |  |
| --- | --- | --- | --- | --- | --- | --- | --- | --- | --- |
| chr2:145190923-145200720 | | | 9430 bp | | |  |  |  |  |
| Genome Start | Genome End | Strand | Div | Repeat Start | Repeat End | Repeat Left | Name | Family | Class |
| 145190923 | 145191070 | + | 27.6 | 341 | 489 | -5934 | L1MCc | L1 | LINE |
| 145191300 | 145193478 | + | 27.8 | 656 | 3150 | -3791 | L1MCc | L1 | LINE |
| 145193534 | 145195708 | - | 3.1 | 3971 | 6152 | -3 | L1PA3 | L1 | LINE |
| 145195709 | 145197395 | + | 3.7 | 2283 | 3969 | -2177 | L1PA3 | L1 | LINE |
| 145197387 | 145198542 | + | 26.0 | 3142 | 4347 | -2594 | L1MCc | L1 | LINE |
| 145198543 | 145198904 | - | 11.5 | 5 | 364 | 0 | THE1B | MaLR | LTR |
| 145198905 | 145200005 | + | 26.0 | 4348 | 5493 | -1448 | L1MCc | L1 | LINE |
| 145200009 | 145200250 | + | 17.8 | 2931 | 3203 | -2943 | L1MD | L1 | LINE |
| 145200244 | 145200384 | + | 27.7 | 5500 | 5641 | -1300 | L1MCc | L1 | LINE |
| 145200482 | 145200720 | + | 27.7 | 5627 | 5879 | -1062 | L1MCc | L1 | LINE |

  

|  |  |  |  |  |  |  |  |  |  |
| --- | --- | --- | --- | --- | --- | --- | --- | --- | --- |
| chr2:145910984-145916847 | | | 5863 bp | | |  |  |  |  |
| Genome Start | Genome End | Strand | Div | Repeat Start | Repeat End | Repeat Left | Name | Family | Class |
| 145910984 | 145911186 | - | 14.8 | 2225 | 2418 | 0 | Tigger1 | MER2\_type | DNA |
| 145911187 | 145911505 | + | 12.2 | 1 | 311 | -1 | AluSx | Alu | SINE |
| 145911506 | 145913640 | - | 14.8 | 45 | 2224 | -194 | Tigger1 | MER2\_type | DNA |
| 145913641 | 145913867 | - | 4.9 | 2989 | 3214 | -2932 | L1PA4 | L1 | LINE |
| 145913869 | 145916790 | + | 4.9 | 3216 | 6141 | -14 | L1PA4 | L1 | LINE |
| 145916791 | 145916847 | - | 15.8 | 1 | 61 | -2357 | Tigger1 | MER2\_type | DNA |

  

|  |  |  |  |  |  |  |  |  |  |
| --- | --- | --- | --- | --- | --- | --- | --- | --- | --- |
| chr2:148216940-148221213 | | | 4250 bp | | |  |  |  |  |
| Genome Start | Genome End | Strand | Div | Repeat Start | Repeat End | Repeat Left | Name | Family | Class |
| 148216940 | 148217463 | + | 24.2 | 2 | 547 | -1188 | MSTD-int | MaLR | LTR |
| 148217464 | 148219046 | - | 2.5 | 3531 | 5105 | -1041 | L1PA3 | L1 | LINE |
| 148219044 | 148220066 | + | 1.9 | 5104 | 6129 | -26 | L1PA3 | L1 | LINE |
| 148220067 | 148220109 | + | 30.8 | 493 | 548 | -823 | MSTD-int | MaLR | LTR |
| 148220137 | 148221213 | + | 25.0 | 474 | 1735 | 0 | MSTD-int | MaLR | LTR |

  

|  |  |  |  |  |  |  |  |  |  |
| --- | --- | --- | --- | --- | --- | --- | --- | --- | --- |
| chr2:154512108-154514038 | | | 1914 bp | | |  |  |  |  |
| Genome Start | Genome End | Strand | Div | Repeat Start | Repeat End | Repeat Left | Name | Family | Class |
| 154512108 | 154512180 | + | 21.9 | 2 | 74 | -274 | MER7A | MER2\_type | DNA |
| 154512193 | 154513001 | - | 9.3 | 4590 | 5403 | -743 | L1PA13 | L1 | LINE |
| 154513006 | 154513764 | + | 10.5 | 5386 | 6155 | -8 | L1PA13 | L1 | LINE |
| 154513766 | 154514038 | + | 13.9 | 66 | 346 | 0 | MER7A | MER2\_type | DNA |

  

|  |  |  |  |  |  |  |  |  |  |
| --- | --- | --- | --- | --- | --- | --- | --- | --- | --- |
| chr2:155301456-155303713 | | | 2253 bp | | |  |  |  |  |
| Genome Start | Genome End | Strand | Div | Repeat Start | Repeat End | Repeat Left | Name | Family | Class |
| 155301456 | 155302751 | + | 21.2 | 155 | 1554 | -4948 | L1MEc | L1 | LINE |
| 155302749 | 155302854 | - | 5.7 | 5700 | 5805 | -497 | L1MA1 | L1 | LINE |
| 155302856 | 155303327 | + | 10.0 | 5819 | 6302 | 0 | L1MA1 | L1 | LINE |
| 155303335 | 155303713 | + | 21.4 | 1425 | 1981 | -4521 | L1MEc | L1 | LINE |

  

|  |  |  |  |  |  |  |  |  |  |
| --- | --- | --- | --- | --- | --- | --- | --- | --- | --- |
| chr2:155852370-155859240 | | | 6808 bp | | |  |  |  |  |
| Genome Start | Genome End | Strand | Div | Repeat Start | Repeat End | Repeat Left | Name | Family | Class |
| 155852370 | 155855122 | + | 15.9 | 901 | 3737 | -3828 | L1M1 | L1 | LINE |
| 155855124 | 155855297 | + | 12.6 | 6112 | 6303 | -1 | L1MA2 | L1 | LINE |
| 155855336 | 155855502 | + | 25.0 | 4609 | 4776 | -2789 | L1M1 | L1 | LINE |
| 155855510 | 155857822 | - | 8.0 | 3560 | 5895 | -259 | L1PA7 | L1 | LINE |
| 155857823 | 155858071 | + | 6.9 | 5919 | 6166 | 0 | L1PA7 | L1 | LINE |
| 155858074 | 155858154 | + | 17.9 | 5652 | 5738 | -1827 | L1M1 | L1 | LINE |
| 155858155 | 155858231 | + | 22.1 | 6084 | 6165 | -5 | L1ME3A | L1 | LINE |
| 155858247 | 155859240 | + | 30.9 | 5745 | 6766 | -799 | L1M1 | L1 | LINE |

  

|  |  |  |  |  |  |  |  |  |  |
| --- | --- | --- | --- | --- | --- | --- | --- | --- | --- |
| chr2:156008444-156011931 | | | 3475 bp | | |  |  |  |  |
| Genome Start | Genome End | Strand | Div | Repeat Start | Repeat End | Repeat Left | Name | Family | Class |
| 156008444 | 156008581 | + | 35.0 | 34 | 176 | -86 | MIR | MIR | SINE |
| 156008595 | 156011308 | - | 0.7 | 3438 | 6151 | -4 | L1PA2 | L1 | LINE |
| 156011307 | 156011884 | + | 1.2 | 2852 | 3429 | -2717 | L1PA2 | L1 | LINE |
| 156011887 | 156011931 | + | 24.4 | 166 | 211 | -51 | MIR | MIR | SINE |

  

|  |  |  |  |  |  |  |  |  |  |
| --- | --- | --- | --- | --- | --- | --- | --- | --- | --- |
| chr2:156134117-156139640 | | | 5497 bp | | |  |  |  |  |
| Genome Start | Genome End | Strand | Div | Repeat Start | Repeat End | Repeat Left | Name | Family | Class |
| 156134117 | 156134994 | - | 37.7 | 1499 | 2479 | -940 | L2 | L2 | LINE |
| 156134995 | 156138294 | - | 5.9 | 2834 | 6154 | 0 | L1PA5 | L1 | LINE |
| 156138295 | 156138600 | - | 10.5 | 1 | 306 | -5 | AluY | Alu | SINE |
| 156138601 | 156139360 | - | 6.1 | 2090 | 2833 | -3313 | L1PA5 | L1 | LINE |
| 156139354 | 156139406 | - | 7.5 | 2072 | 2124 | -4031 | L1HS | L1 | LINE |
| 156139403 | 156139439 | + | 5.4 | 2033 | 2069 | -4086 | L1HS | L1 | LINE |
| 156139478 | 156139640 | - | 32.9 | 1295 | 1453 | -1966 | L2 | L2 | LINE |

  

|  |  |  |  |  |  |  |  |  |  |
| --- | --- | --- | --- | --- | --- | --- | --- | --- | --- |
| chr2:163726144-163728738 | | | 2090 bp | | |  |  |  |  |
| Genome Start | Genome End | Strand | Div | Repeat Start | Repeat End | Repeat Left | Name | Family | Class |
| 163726144 | 163726252 | - | 22.4 | 5369 | 5501 | -739 | L1M5 | L1 | LINE |
| 163726288 | 163726642 | - | 29.4 | 4928 | 5329 | -817 | L1M5 | L1 | LINE |
| 163726721 | 163727019 | - | 12.7 | 348 | 634 | 0 | MER51A | ERV1 | LTR |
| 163727047 | 163727443 | - | 19.5 | 6588 | 7006 | -779 | L1MC3 | L1 | LINE |
| 163727446 | 163727759 | + | 22.1 | 6290 | 6604 | -1181 | L1MC3 | L1 | LINE |
| 163727761 | 163727929 | - | 31.3 | 2958 | 3122 | -3024 | L1M5 | L1 | LINE |
| 163728059 | 163728372 | + | 25.8 | 3 | 331 | -231 | LTR75 | ERVL | LTR |
| 163728606 | 163728738 | - | 22.6 | 2475 | 2611 | -3535 | L1M5 | L1 | LINE |

  

|  |  |  |  |  |  |  |  |  |  |
| --- | --- | --- | --- | --- | --- | --- | --- | --- | --- |
| chr2:165826873-165830227 | | | 3347 bp | | |  |  |  |  |
| Genome Start | Genome End | Strand | Div | Repeat Start | Repeat End | Repeat Left | Name | Family | Class |
| 165826873 | 165826975 | - | 11.7 | 6066 | 6168 | 0 | L1PA12 | L1 | LINE |
| 165826988 | 165828961 | - | 13.7 | 4090 | 6144 | -7 | L1PB1 | L1 | LINE |
| 165828960 | 165829293 | + | 9.9 | 3741 | 4075 | -2071 | L1PB1 | L1 | LINE |
| 165829292 | 165830227 | - | 9.1 | 5177 | 6083 | -85 | L1PA12 | L1 | LINE |

  

|  |  |  |  |  |  |  |  |  |  |
| --- | --- | --- | --- | --- | --- | --- | --- | --- | --- |
| chr2:165992975-165996107 | | | 2962 bp | | |  |  |  |  |
| Genome Start | Genome End | Strand | Div | Repeat Start | Repeat End | Repeat Left | Name | Family | Class |
| 165992975 | 165994083 | - | 18.3 | 313 | 1455 | 0 | Charlie1a | MER1\_type | DNA |
| 165994098 | 165994715 | - | 2.3 | 4873 | 5490 | -665 | L1PA3 | L1 | LINE |
| 165994717 | 165995369 | + | 1.7 | 5503 | 6155 | 0 | L1PA3 | L1 | LINE |
| 165995390 | 165995655 | - | 21.8 | 22 | 282 | -30 | AluJo | Alu | SINE |
| 165995687 | 165995751 | - | 12.3 | 6099 | 6163 | -9 | L1PA8 | L1 | LINE |
| 165995857 | 165996107 | - | 21.2 | 1 | 247 | -1208 | Charlie1a | MER1\_type | DNA |

  

|  |  |  |  |  |  |  |  |  |  |
| --- | --- | --- | --- | --- | --- | --- | --- | --- | --- |
| chr2:167065417-167077767 | | | 12037 bp | | |  |  |  |  |
| Genome Start | Genome End | Strand | Div | Repeat Start | Repeat End | Repeat Left | Name | Family | Class |
| 167065417 | 167066127 | + | 32.7 | 319 | 1042 | -1465 | HAL1 | L1 | LINE |
| 167066121 | 167066551 | + | 20.6 | 5888 | 6309 | -3 | L1MA9 | L1 | LINE |
| 167066656 | 167067259 | + | 20.3 | 7183 | 7775 | -10 | L1MC3 | L1 | LINE |
| 167067258 | 167068890 | - | 2.0 | 3605 | 5237 | -909 | L1PA2 | L1 | LINE |
| 167068890 | 167069813 | + | 1.7 | 5233 | 6155 | 0 | L1PA2 | L1 | LINE |
| 167069857 | 167069924 | + | 19.1 | 7171 | 7255 | -740 | L1MD3 | L1 | LINE |
| 167069892 | 167070305 | + | 24.3 | 7172 | 7587 | -221 | L1MC4a | L1 | LINE |
| 167070337 | 167076762 | - | 11.5 | 6 | 6487 | 0 | L1PA8 | L1 | LINE |
| 167076767 | 167076839 | + | 23.3 | 7748 | 7822 | -173 | L1MD3 | L1 | LINE |
| 167076849 | 167077264 | + | 31.0 | 1473 | 1884 | -623 | HAL1 | L1 | LINE |
| 167077431 | 167077767 | + | 31.9 | 2128 | 2507 | 0 | HAL1 | L1 | LINE |

  

|  |  |  |  |  |  |  |  |  |  |
| --- | --- | --- | --- | --- | --- | --- | --- | --- | --- |
| chr2:167273935-167283214 | | | 9267 bp | | |  |  |  |  |
| Genome Start | Genome End | Strand | Div | Repeat Start | Repeat End | Repeat Left | Name | Family | Class |
| 167273935 | 167275291 | - | 17.8 | 4999 | 6403 | -1 | L1MB1 | L1 | LINE |
| 167275330 | 167276041 | - | 13.5 | 5558 | 6291 | -12 | L1MA4A | L1 | LINE |
| 167276042 | 167276348 | - | 9.4 | 1 | 308 | -3 | AluY | Alu | SINE |
| 167276349 | 167276676 | - | 14.0 | 5237 | 5557 | -746 | L1MA4A | L1 | LINE |
| 167276685 | 167279047 | - | 18.6 | 2705 | 5001 | -1375 | L1MB1 | L1 | LINE |
| 167279023 | 167280098 | - | 4.1 | 5065 | 6149 | -5 | L1PA5 | L1 | LINE |
| 167280095 | 167280907 | + | 6.3 | 4203 | 5079 | -1067 | L1PA5 | L1 | LINE |
| 167280903 | 167282853 | - | 25.9 | 810 | 2758 | -3618 | L1MB1 | L1 | LINE |
| 167282855 | 167283214 | - | 33.1 | 652 | 924 | -5379 | L1MB1 | L1 | LINE |

  

|  |  |  |  |  |  |  |  |  |  |
| --- | --- | --- | --- | --- | --- | --- | --- | --- | --- |
| chr2:167593077-167599573 | | | 6500 bp | | |  |  |  |  |
| Genome Start | Genome End | Strand | Div | Repeat Start | Repeat End | Repeat Left | Name | Family | Class |
| 167593077 | 167593912 | + | 15.2 | 1721 | 2572 | -4993 | L1M1 | L1 | LINE |
| 167593910 | 167596277 | - | 10.0 | 1838 | 4206 | -1940 | L1PA7 | L1 | LINE |
| 167596271 | 167598197 | + | 8.9 | 4198 | 6144 | -10 | L1PA7 | L1 | LINE |
| 167598205 | 167599573 | + | 12.4 | 2560 | 3935 | -3630 | L1M1 | L1 | LINE |

  

|  |  |  |  |  |  |  |  |  |  |
| --- | --- | --- | --- | --- | --- | --- | --- | --- | --- |
| chr2:167625202-167632761 | | | 7389 bp | | |  |  |  |  |
| Genome Start | Genome End | Strand | Div | Repeat Start | Repeat End | Repeat Left | Name | Family | Class |
| 167625202 | 167626263 | - | 28.8 | 4377 | 5450 | -1087 | L1MEc | L1 | LINE |
| 167626264 | 167626721 | + | 23.9 | 1 | 467 | 0 | MLT1C | MaLR | LTR |
| 167626722 | 167626914 | - | 26.0 | 4194 | 4376 | -2161 | L1MEc | L1 | LINE |
| 167626915 | 167627092 | + | 22.3 | 40 | 209 | -1 | MER63A | AcHobo | DNA |
| 167627093 | 167628843 | - | 26.0 | 2526 | 4193 | -2344 | L1MEc | L1 | LINE |
| 167628844 | 167628970 | - | 15.8 | 6175 | 6312 | 0 | L1MA9 | L1 | LINE |
| 167628971 | 167629245 | - | 26.7 | 2232 | 2523 | -4195 | L1MEc | L1 | LINE |
| 167629251 | 167630365 | - | 18.7 | 5059 | 6169 | -2 | L1MB2 | L1 | LINE |
| 167630365 | 167630685 | - | 28.2 | 1810 | 2247 | -4471 | L1MEc | L1 | LINE |
| 167630682 | 167630813 | - | 16.9 | 6155 | 6327 | -7 | L1MA10 | L1 | LINE |
| 167630838 | 167631080 | - | 16.9 | 5913 | 6154 | -35 | L1MB1 | L1 | LINE |
| 167631081 | 167631639 | + | 19.6 | 5339 | 5894 | -274 | L1MB1 | L1 | LINE |
| 167631640 | 167632134 | - | 21.8 | 1349 | 1869 | -4849 | L1MEc | L1 | LINE |
| 167632215 | 167632410 | - | 16.8 | 2201 | 2418 | 0 | Tigger1 | MER2\_type | DNA |
| 167632399 | 167632554 | - | 16.1 | 1 | 157 | -2261 | Tigger1 | MER2\_type | DNA |
| 167632634 | 167632761 | - | 29.1 | 677 | 795 | -5923 | L1MEc | L1 | LINE |

  

|  |  |  |  |  |  |  |  |  |  |
| --- | --- | --- | --- | --- | --- | --- | --- | --- | --- |
| chr2:170532838-170543717 | | | 10845 bp | | |  |  |  |  |
| Genome Start | Genome End | Strand | Div | Repeat Start | Repeat End | Repeat Left | Name | Family | Class |
| 170532838 | 170534304 | - | 14.8 | 5814 | 7310 | -11 | L1MA4 | L1 | LINE |
| 170534305 | 170534604 | - | 9.0 | 1 | 299 | -12 | AluY | Alu | SINE |
| 170534605 | 170535087 | - | 14.8 | 5334 | 5813 | -1351 | L1MA4 | L1 | LINE |
| 170535083 | 170536486 | - | 2.2 | 4749 | 6155 | 0 | L1PA3 | L1 | LINE |
| 170536488 | 170538279 | + | 2.7 | 2956 | 4748 | -1398 | L1PA3 | L1 | LINE |
| 170538275 | 170539655 | - | 14.3 | 3933 | 5353 | -1811 | L1MA4 | L1 | LINE |
| 170539656 | 170539944 | + | 15.9 | 1 | 301 | -11 | AluJb | Alu | SINE |
| 170539945 | 170540423 | - | 14.3 | 3446 | 3932 | -3232 | L1MA4 | L1 | LINE |
| 170540424 | 170540569 | + | 21.4 | 2 | 127 | -6 | FLAM\_A | Alu | SINE |
| 170540570 | 170540641 | - | 14.3 | 3446 | 3445 | -3719 | L1MA4 | L1 | LINE |
| 170540664 | 170542009 | - | 18.4 | 2038 | 3445 | -3719 | L1MA4 | L1 | LINE |
| 170542032 | 170542596 | - | 24.3 | 3 | 546 | -8 | MLT2B3 | ERVL | LTR |
| 170542597 | 170543717 | - | 18.2 | 853 | 2029 | -5139 | L1MA4 | L1 | LINE |

  

|  |  |  |  |  |  |  |  |  |  |
| --- | --- | --- | --- | --- | --- | --- | --- | --- | --- |
| chr2:179949120-179952544 | | | 3320 bp | | |  |  |  |  |
| Genome Start | Genome End | Strand | Div | Repeat Start | Repeat End | Repeat Left | Name | Family | Class |
| 179949120 | 179949390 | + | 27.6 | 1890 | 2211 | -1208 | L2 | L2 | LINE |
| 179949430 | 179950908 | - | 12.3 | 4674 | 6162 | -4 | L1PA16 | L1 | LINE |
| 179950909 | 179951214 | - | 15.5 | 1 | 299 | -13 | AluJb | Alu | SINE |
| 179951215 | 179951237 | - | 11.0 | 4651 | 4673 | -1473 | L1PA16 | L1 | LINE |
| 179951240 | 179951521 | + | 10.8 | 4359 | 4641 | -1505 | L1PA16 | L1 | LINE |
| 179951586 | 179952544 | + | 32.9 | 2324 | 3415 | -4 | L2 | L2 | LINE |

  

|  |  |  |  |  |  |  |  |  |  |
| --- | --- | --- | --- | --- | --- | --- | --- | --- | --- |
| chr2:180666420-180677840 | | | 11370 bp | | |  |  |  |  |
| Genome Start | Genome End | Strand | Div | Repeat Start | Repeat End | Repeat Left | Name | Family | Class |
| 180666420 | 180666910 | - | 24.7 | 4072 | 4761 | -97 | L1ME3A | L1 | LINE |
| 180666912 | 180667878 | - | 11.5 | 5146 | 6159 | -6 | L1PA15 | L1 | LINE |
| 180667880 | 180670565 | + | 14.2 | 2329 | 5137 | -1009 | L1PREC2 | L1 | LINE |
| 180670560 | 180671453 | - | 24.8 | 3243 | 4086 | -748 | L1ME3A | L1 | LINE |
| 180671454 | 180671754 | - | 9.4 | 1 | 298 | -13 | AluY | Alu | SINE |
| 180671755 | 180672372 | - | 24.8 | 2524 | 3242 | -1592 | L1ME3A | L1 | LINE |
| 180672364 | 180673664 | - | 9.8 | 4845 | 6160 | 0 | L1PREC2 | L1 | LINE |
| 180673670 | 180673755 | - | 24.2 | 2472 | 2536 | -2298 | L1ME3A | L1 | LINE |
| 180673756 | 180674037 | - | 7.8 | 1 | 282 | -29 | AluY | Alu | SINE |
| 180674038 | 180674939 | - | 24.2 | 1532 | 2471 | -2363 | L1ME3A | L1 | LINE |
| 180674959 | 180676432 | + | 11.7 | 1200 | 2731 | 0 | Tigger4(Zombi) | MER2\_type | DNA |
| 180676468 | 180676759 | - | 11.0 | 1 | 291 | -22 | AluSq | Alu | SINE |
| 180676765 | 180677219 | - | 24.3 | 709 | 1179 | -3655 | L1ME3A | L1 | LINE |
| 180677220 | 180677529 | - | 13.3 | 1 | 309 | -4 | AluSq | Alu | SINE |
| 180677530 | 180677840 | - | 27.0 | 397 | 708 | -4146 | L1ME3A | L1 | LINE |

  

|  |  |  |  |  |  |  |  |  |  |
| --- | --- | --- | --- | --- | --- | --- | --- | --- | --- |
| chr2:182904180-182910071 | | | 5816 bp | | |  |  |  |  |
| Genome Start | Genome End | Strand | Div | Repeat Start | Repeat End | Repeat Left | Name | Family | Class |
| 182904180 | 182904240 | + | 22.9 | 86 | 147 | -115 | MIR | MIR | SINE |
| 182904255 | 182905820 | - | 9.7 | 665 | 2234 | -41 | L1PA7 | L1 | LINE |
| 182905801 | 182907239 | + | 9.3 | 2241 | 3676 | -2470 | L1PA7 | L1 | LINE |
| 182907280 | 182909737 | + | 8.0 | 3677 | 6139 | -15 | L1PA7 | L1 | LINE |
| 182909758 | 182909932 | + | 5.7 | 5928 | 6103 | -28 | L1PA8A | L1 | LINE |
| 182909955 | 182910071 | + | 36.1 | 144 | 257 | -11 | MIR | MIR | SINE |

  

|  |  |  |  |  |  |  |  |  |  |
| --- | --- | --- | --- | --- | --- | --- | --- | --- | --- |
| chr2:183066195-183066909 | | | 708 bp | | |  |  |  |  |
| Genome Start | Genome End | Strand | Div | Repeat Start | Repeat End | Repeat Left | Name | Family | Class |
| 183066195 | 183066255 | - | 23.9 | 83 | 142 | -160 | AluJo | Alu | SINE |
| 183066256 | 183066505 | - | 5.7 | 5903 | 6151 | -3 | L1PA5 | L1 | LINE |
| 183066512 | 183066813 | + | 6.3 | 5607 | 5908 | -246 | L1PA5 | L1 | LINE |
| 183066815 | 183066909 | - | 11.6 | 3 | 101 | -211 | AluJo | Alu | SINE |

  

|  |  |  |  |  |  |  |  |  |  |
| --- | --- | --- | --- | --- | --- | --- | --- | --- | --- |
| chr2:185689843-185693950 | | | 3571 bp | | |  |  |  |  |
| Genome Start | Genome End | Strand | Div | Repeat Start | Repeat End | Repeat Left | Name | Family | Class |
| 185689843 | 185690145 | - | 34.9 | 3125 | 3419 | 0 | L2 | L2 | LINE |
| 185690274 | 185691051 | - | 33.3 | 1958 | 2813 | -606 | L2 | L2 | LINE |
| 185691137 | 185691576 | + | 14.6 | 5823 | 6285 | -6 | L1MA8 | L1 | LINE |
| 185691785 | 185692298 | - | 5.2 | 4244 | 4766 | -1380 | L1PA4 | L1 | LINE |
| 185692298 | 185693692 | + | 4.0 | 4753 | 6154 | -1 | L1PA4 | L1 | LINE |
| 185693807 | 185693882 | + | 23.7 | 183 | 260 | -2 | MIR | MIR | SINE |
| 185693886 | 185693950 | - | 23.4 | 1451 | 1514 | -1905 | L2 | L2 | LINE |

  

|  |  |  |  |  |  |  |  |  |  |
| --- | --- | --- | --- | --- | --- | --- | --- | --- | --- |
| chr2:186299107-186300458 | | | 1345 bp | | |  |  |  |  |
| Genome Start | Genome End | Strand | Div | Repeat Start | Repeat End | Repeat Left | Name | Family | Class |
| 186299107 | 186299384 | - | 24.7 | 270 | 563 | 0 | MER68 | ERVL | LTR |
| 186299385 | 186299701 | - | 17.7 | 1 | 388 | -2 | MLT1B | MaLR | LTR |
| 186299702 | 186299918 | - | 24.7 | 46 | 269 | -294 | MER68 | ERVL | LTR |
| 186299924 | 186300222 | - | 5.7 | 5871 | 6170 | -2 | L1PA8 | L1 | LINE |
| 186300223 | 186300394 | + | 4.7 | 5682 | 5853 | -319 | L1PA8 | L1 | LINE |
| 186300397 | 186300458 | - | 26.2 | 1 | 61 | -502 | MER68 | ERVL | LTR |

  

|  |  |  |  |  |  |  |  |  |  |
| --- | --- | --- | --- | --- | --- | --- | --- | --- | --- |
| chr2:186338208-186341512 | | | 3265 bp | | |  |  |  |  |
| Genome Start | Genome End | Strand | Div | Repeat Start | Repeat End | Repeat Left | Name | Family | Class |
| 186338208 | 186338324 | - | 14.3 | 6125 | 6241 | -71 | L1MA9 | L1 | LINE |
| 186338322 | 186339125 | - | 3.1 | 4144 | 4947 | -1199 | L1PA2 | L1 | LINE |
| 186339099 | 186340302 | + | 1.8 | 4951 | 6154 | -1 | L1PA2 | L1 | LINE |
| 186340325 | 186340625 | - | 17.4 | 5793 | 6139 | -173 | L1MA9 | L1 | LINE |
| 186340659 | 186341078 | - | 17.4 | 5417 | 5792 | -520 | L1MA9 | L1 | LINE |
| 186341094 | 186341512 | - | 21.2 | 4867 | 5296 | -844 | L1MA9 | L1 | LINE |

  

|  |  |  |  |  |  |  |  |  |  |
| --- | --- | --- | --- | --- | --- | --- | --- | --- | --- |
| chr2:186836001-186841804 | | | 5343 bp | | |  |  |  |  |
| Genome Start | Genome End | Strand | Div | Repeat Start | Repeat End | Repeat Left | Name | Family | Class |
| 186836001 | 186836062 | - | 30.6 | 4424 | 4486 | -3 | L3 | CR1 | LINE |
| 186836253 | 186836419 | + | 24.6 | 1 | 168 | -8 | FRAM | Alu | SINE |
| 186836644 | 186836727 | + | 21.4 | 268 | 351 | -205 | MLT2B4 | ERVL | LTR |
| 186836731 | 186838145 | + | 15.6 | 718 | 2164 | -3550 | ERVL-B4 | ERVL | LTR |
| 186838158 | 186838273 | - | 28.9 | 4058 | 4175 | -314 | L3 | CR1 | LINE |
| 186838286 | 186839923 | - | 8.2 | 4504 | 6149 | -5 | L1PA7 | L1 | LINE |
| 186839923 | 186841592 | + | 9.3 | 2816 | 4496 | -1650 | L1PA7 | L1 | LINE |
| 186841614 | 186841804 | - | 34.7 | 3661 | 3858 | -631 | L3 | CR1 | LINE |

  

|  |  |  |  |  |  |  |  |  |  |
| --- | --- | --- | --- | --- | --- | --- | --- | --- | --- |
| chr2:188304821-188308871 | | | 3560 bp | | |  |  |  |  |
| Genome Start | Genome End | Strand | Div | Repeat Start | Repeat End | Repeat Left | Name | Family | Class |
| 188304821 | 188305044 | + | 33.6 | 105 | 323 | -6310 | L1MDa | L1 | LINE |
| 188305349 | 188305560 | + | 29.7 | 850 | 1090 | -5450 | L1MDa | L1 | LINE |
| 188305689 | 188305792 | + | 18.4 | 1099 | 1208 | -5332 | L1MDa | L1 | LINE |
| 188305851 | 188307179 | - | 4.5 | 3792 | 5126 | -1020 | L1PA5 | L1 | LINE |
| 188307178 | 188308222 | + | 4.5 | 5117 | 6150 | -4 | L1PA5 | L1 | LINE |
| 188308226 | 188308871 | + | 23.2 | 1233 | 1909 | -4724 | L1MDa | L1 | LINE |

  

|  |  |  |  |  |  |  |  |  |  |
| --- | --- | --- | --- | --- | --- | --- | --- | --- | --- |
| chr2:188392830-188396175 | | | 3004 bp | | |  |  |  |  |
| Genome Start | Genome End | Strand | Div | Repeat Start | Repeat End | Repeat Left | Name | Family | Class |
| 188392830 | 188392996 | + | 28.0 | 2224 | 2388 | -3758 | L1M5 | L1 | LINE |
| 188392997 | 188393296 | - | 12.0 | 2 | 301 | -11 | AluSx | Alu | SINE |
| 188393297 | 188393805 | + | 28.0 | 2389 | 2923 | -3223 | L1M5 | L1 | LINE |
| 188393806 | 188394077 | + | 15.1 | 6015 | 6296 | -4 | L1MA4 | L1 | LINE |
| 188394078 | 188394269 | + | 28.0 | 2924 | 3150 | -2996 | L1M5 | L1 | LINE |
| 188394373 | 188394724 | + | 23.1 | 3656 | 3938 | -2208 | L1M5 | L1 | LINE |
| 188394863 | 188395026 | + | 25.0 | 4719 | 4898 | -1248 | L1M5 | L1 | LINE |
| 188395108 | 188395498 | - | 16.9 | 5897 | 6318 | -15 | L1MC1 | L1 | LINE |
| 188395494 | 188395886 | + | 17.3 | 5496 | 5897 | -436 | L1MC1 | L1 | LINE |
| 188395912 | 188396175 | + | 21.5 | 5075 | 5334 | -812 | L1M5 | L1 | LINE |

  

|  |  |  |  |  |  |  |  |  |  |
| --- | --- | --- | --- | --- | --- | --- | --- | --- | --- |
| chr2:188541673-188545474 | | | 3777 bp | | |  |  |  |  |
| Genome Start | Genome End | Strand | Div | Repeat Start | Repeat End | Repeat Left | Name | Family | Class |
| 188541673 | 188541890 | + | 28.0 | 2334 | 2578 | -3568 | L1M4 | L1 | LINE |
| 188541891 | 188542245 | - | 17.9 | 1 | 428 | 0 | MSTA | MaLR | LTR |
| 188542246 | 188543350 | + | 28.0 | 2579 | 3794 | -2352 | L1M4 | L1 | LINE |
| 188543352 | 188543589 | - | 18.0 | 184 | 426 | 0 | MSTB | MaLR | LTR |
| 188543614 | 188543645 | - | 12.8 | 6120 | 6158 | -3 | L1PA14 | L1 | LINE |
| 188543646 | 188543940 | + | 13.6 | 4 | 300 | -12 | AluSx | Alu | SINE |
| 188543941 | 188544620 | - | 12.8 | 5455 | 6119 | -42 | L1PA14 | L1 | LINE |
| 188544621 | 188544991 | + | 9.0 | 5083 | 5454 | -706 | L1PA14 | L1 | LINE |
| 188544992 | 188545188 | - | 13.8 | 1 | 192 | -234 | MSTB | MaLR | LTR |
| 188545189 | 188545474 | + | 28.9 | 3791 | 4082 | -2064 | L1M4 | L1 | LINE |

  

|  |  |  |  |  |  |  |  |  |  |
| --- | --- | --- | --- | --- | --- | --- | --- | --- | --- |
| chr2:191692441-191695961 | | | 3312 bp | | |  |  |  |  |
| Genome Start | Genome End | Strand | Div | Repeat Start | Repeat End | Repeat Left | Name | Family | Class |
| 191692441 | 191692925 | + | 24.0 | 4429 | 4932 | -1214 | L1ME3 | L1 | LINE |
| 191692966 | 191693331 | + | 24.0 | 4933 | 5312 | -834 | L1ME3 | L1 | LINE |
| 191693347 | 191693490 | + | 15.0 | 87 | 284 | -28 | AluJo | Alu | SINE |
| 191693538 | 191693591 | - | 5.6 | 4777 | 4830 | -1316 | L1PA5 | L1 | LINE |
| 191693587 | 191694920 | + | 4.1 | 4832 | 6157 | -145 | L1PA5 | L1 | LINE |
| 191695028 | 191695175 | + | 21.8 | 6158 | 6302 | 0 | L1MA1 | L1 | LINE |
| 191695181 | 191695961 | + | 24.8 | 5353 | 6159 | -3 | L1ME3 | L1 | LINE |

  

|  |  |  |  |  |  |  |  |  |  |
| --- | --- | --- | --- | --- | --- | --- | --- | --- | --- |
| chr2:192889260-192894575 | | | 5193 bp | | |  |  |  |  |
| Genome Start | Genome End | Strand | Div | Repeat Start | Repeat End | Repeat Left | Name | Family | Class |
| 192889260 | 192889272 | + | 32.5 | 2344 | 2355 | -4029 | L1ME1 | L1 | LINE |
| 192889354 | 192892367 | + | 29.2 | 2356 | 5679 | -723 | L1ME1 | L1 | LINE |
| 192892381 | 192892689 | - | 7.1 | 4981 | 5289 | -857 | L1PA4 | L1 | LINE |
| 192892695 | 192893565 | + | 2.4 | 5285 | 6149 | -6 | L1PA4 | L1 | LINE |
| 192893590 | 192894038 | + | 30.2 | 5684 | 6160 | -242 | L1ME1 | L1 | LINE |
| 192894039 | 192894344 | - | 12.3 | 1 | 304 | -8 | AluSx | Alu | SINE |
| 192894345 | 192894575 | + | 30.2 | 6161 | 6395 | -7 | L1ME1 | L1 | LINE |

  

|  |  |  |  |  |  |  |  |  |  |
| --- | --- | --- | --- | --- | --- | --- | --- | --- | --- |
| chr2:193636791-193646827 | | | 10034 bp | | |  |  |  |  |
| Genome Start | Genome End | Strand | Div | Repeat Start | Repeat End | Repeat Left | Name | Family | Class |
| 193636791 | 193636855 | - | 20.6 | 6891 | 6953 | -52 | L1MA4A | L1 | LINE |
| 193636863 | 193638261 | - | 21.7 | 5395 | 6820 | -185 | L1MA4A | L1 | LINE |
| 193638269 | 193639064 | - | 3.4 | 5360 | 6155 | 0 | L1PA3 | L1 | LINE |
| 193639057 | 193640546 | + | 3.1 | 3847 | 5342 | -804 | L1PA3 | L1 | LINE |
| 193640544 | 193643259 | - | 20.5 | 2743 | 5410 | -1435 | L1MA4A | L1 | LINE |
| 193643238 | 193643388 | - | 17.2 | 1730 | 1884 | -4965 | L1MA4A | L1 | LINE |
| 193643410 | 193644657 | + | 20.1 | 370 | 1651 | 0 | MSTA-int | MaLR | LTR |
| 193644658 | 193645067 | + | 19.6 | 1 | 428 | 0 | MSTA | MaLR | LTR |
| 193645069 | 193646827 | - | 28.7 | 4 | 1733 | -5116 | L1MA4A | L1 | LINE |

  

|  |  |  |  |  |  |  |  |  |  |
| --- | --- | --- | --- | --- | --- | --- | --- | --- | --- |
| chr2:194324018-194331634 | | | 6362 bp | | |  |  |  |  |
| Genome Start | Genome End | Strand | Div | Repeat Start | Repeat End | Repeat Left | Name | Family | Class |
| 194324018 | 194324242 | - | 29.1 | 3580 | 3789 | -3152 | L1MCc | L1 | LINE |
| 194324358 | 194324703 | - | 32.9 | 21 | 386 | -23 | MLT1I | MaLR | LTR |
| 194324819 | 194325185 | - | 17.7 | 5789 | 6154 | -2 | L1PB4 | L1 | LINE |
| 194325241 | 194325315 | - | 9.0 | 5718 | 5793 | -510 | L1M1 | L1 | LINE |
| 194325344 | 194326047 | - | 16.8 | 5057 | 5769 | -387 | L1PB4 | L1 | LINE |
| 194326048 | 194326258 | - | 34.5 | 3130 | 3353 | -3588 | L1MCc | L1 | LINE |
| 194326315 | 194326659 | - | 24.2 | 1 | 501 | -4 | MLT1A0 | MaLR | LTR |
| 194326728 | 194326815 | + | 23.0 | 2 | 88 | -214 | AluS | Alu | SINE |
| 194326819 | 194328129 | - | 14.3 | 4883 | 6236 | -69 | L1MA3 | L1 | LINE |
| 194328137 | 194328387 | - | 28.4 | 2737 | 2988 | -3973 | L1MCc | L1 | LINE |
| 194328470 | 194329328 | - | 27.1 | 2300 | 3240 | -2906 | L1M4 | L1 | LINE |
| 194329574 | 194329894 | - | 30.2 | 1763 | 2206 | -4916 | L1MCc | L1 | LINE |
| 194329937 | 194329982 | - | 15.2 | 6252 | 6297 | -6 | L1MA4A | L1 | LINE |
| 194329983 | 194330360 | + | 19.2 | 5853 | 6238 | -62 | L1MA4A | L1 | LINE |
| 194330360 | 194330725 | - | 30.2 | 1348 | 1796 | -5332 | L1MCc | L1 | LINE |
| 194330773 | 194330856 | - | 26.2 | 1175 | 1254 | -5169 | L1MCc | L1 | LINE |
| 194331250 | 194331634 | - | 30.4 | 35 | 442 | -5981 | L1MCc | L1 | LINE |

  

|  |  |  |  |  |  |  |  |  |  |
| --- | --- | --- | --- | --- | --- | --- | --- | --- | --- |
| chr2:194571645-194574501 | | | 2538 bp | | |  |  |  |  |
| Genome Start | Genome End | Strand | Div | Repeat Start | Repeat End | Repeat Left | Name | Family | Class |
| 194571645 | 194572208 | + | 35.2 | -225 | 226 | -4816 | L1MCa | L1 | LINE |
| 194572355 | 194572460 | - | 15.7 | 6142 | 6338 | -21 | L1MD1 | L1 | LINE |
| 194572474 | 194572662 | + | 17.5 | 140 | 337 | -8 | MER2 | MER2\_type | DNA |
| 194572677 | 194573385 | - | 21.4 | 5217 | 5907 | -335 | L1MD1 | L1 | LINE |
| 194573389 | 194573924 | + | 18.6 | 4626 | 5215 | -931 | L1MD1 | L1 | LINE |
| 194574095 | 194574158 | + | 11.1 | 6115 | 6177 | -135 | L1MA9 | L1 | LINE |
| 194574132 | 194574501 | + | 28.2 | 799 | 1189 | -3645 | L1MCa | L1 | LINE |

  

|  |  |  |  |  |  |  |  |  |  |
| --- | --- | --- | --- | --- | --- | --- | --- | --- | --- |
| chr2:199505525-199509960 | | | 3937 bp | | |  |  |  |  |
| Genome Start | Genome End | Strand | Div | Repeat Start | Repeat End | Repeat Left | Name | Family | Class |
| 199505525 | 199505662 | + | 32.4 | 344 | 484 | -5916 | L1MEc | L1 | LINE |
| 199505716 | 199506526 | + | 25.9 | 593 | 1417 | -4983 | L1MEc | L1 | LINE |
| 199506741 | 199507327 | + | 24.8 | 1460 | 2098 | -4302 | L1MEc | L1 | LINE |
| 199507334 | 199507439 | + | 20.3 | 6179 | 6283 | -8 | L1MA8 | L1 | LINE |
| 199507452 | 199507934 | - | 0.8 | 5413 | 5895 | -260 | L1PA2 | L1 | LINE |
| 199507936 | 199508205 | + | 1.5 | 5886 | 6155 | 0 | L1PA2 | L1 | LINE |
| 199508378 | 199508615 | - | 8.4 | 1 | 239 | -74 | AluSp | Alu | SINE |
| 199508629 | 199509352 | + | 27.1 | 2201 | 2793 | -3426 | L1MEc | L1 | LINE |
| 199509353 | 199509392 | - | 19.9 | 511 | 554 | 0 | MLT2B3 | ERVL | LTR |
| 199509421 | 199509928 | - | 19.9 | 4 | 510 | -44 | MLT2B3 | ERVL | LTR |
| 199509929 | 199509960 | + | 26.7 | 2794 | 2822 | -3397 | L1MEc | L1 | LINE |

  

|  |  |  |  |  |  |  |  |  |  |
| --- | --- | --- | --- | --- | --- | --- | --- | --- | --- |
| chr2:205370951-205379868 | | | 8898 bp | | |  |  |  |  |
| Genome Start | Genome End | Strand | Div | Repeat Start | Repeat End | Repeat Left | Name | Family | Class |
| 205370951 | 205373579 | + | 16.3 | 22 | 2718 | -4847 | L1M1 | L1 | LINE |
| 205373578 | 205375085 | - | 8.8 | 3740 | 5255 | -891 | L1PA7 | L1 | LINE |
| 205375086 | 205375951 | + | 5.9 | 5265 | 6136 | -18 | L1PA7 | L1 | LINE |
| 205375974 | 205376907 | + | 13.2 | 2710 | 3639 | -3926 | L1M1 | L1 | LINE |
| 205376908 | 205377203 | + | 17.1 | 1 | 289 | -23 | AluJb | Alu | SINE |
| 205377204 | 205379868 | + | 12.3 | 3635 | 6822 | -743 | L1M1 | L1 | LINE |

  

|  |  |  |  |  |  |  |  |  |  |
| --- | --- | --- | --- | --- | --- | --- | --- | --- | --- |
| chr2:209854692-209861773 | | | 7085 bp | | |  |  |  |  |
| Genome Start | Genome End | Strand | Div | Repeat Start | Repeat End | Repeat Left | Name | Family | Class |
| 209854692 | 209856197 | + | 16.6 | 3832 | 5336 | -810 | L1MC1 | L1 | LINE |
| 209856192 | 209857698 | - | 6.8 | 1518 | 3014 | -3132 | L1PA5 | L1 | LINE |
| 209857697 | 209860818 | + | 5.1 | 3022 | 6153 | -1 | L1PA5 | L1 | LINE |
| 209860824 | 209861773 | + | 17.1 | 5319 | 6292 | -41 | L1MC1 | L1 | LINE |

  

|  |  |  |  |  |  |  |  |  |  |
| --- | --- | --- | --- | --- | --- | --- | --- | --- | --- |
| chr2:211331617-211336702 | | | 5066 bp | | |  |  |  |  |
| Genome Start | Genome End | Strand | Div | Repeat Start | Repeat End | Repeat Left | Name | Family | Class |
| 211331617 | 211331914 | - | 14.2 | 1334 | 1645 | -6 | MSTA-int | MaLR | LTR |
| 211331936 | 211333520 | - | 2.8 | 4567 | 6152 | -3 | L1PA3 | L1 | LINE |
| 211333520 | 211335368 | + | 3.0 | 2680 | 4545 | -1601 | L1PA3 | L1 | LINE |
| 211335369 | 211336702 | - | 17.5 | 1 | 1346 | -305 | MSTA-int | MaLR | LTR |

  

|  |  |  |  |  |  |  |  |  |  |
| --- | --- | --- | --- | --- | --- | --- | --- | --- | --- |
| chr2:211574852-211577000 | | | 2018 bp | | |  |  |  |  |
| Genome Start | Genome End | Strand | Div | Repeat Start | Repeat End | Repeat Left | Name | Family | Class |
| 211574852 | 211575530 | + | 28.0 | 2173 | 2852 | -3294 | L1M5 | L1 | LINE |
| 211575645 | 211575833 | - | 3.2 | 5967 | 6155 | 0 | L1PA2 | L1 | LINE |
| 211575834 | 211576395 | + | 1.4 | 5400 | 5964 | -191 | L1PA2 | L1 | LINE |
| 211576413 | 211576713 | + | 23.0 | 2850 | 3135 | -3011 | L1M5 | L1 | LINE |
| 211576714 | 211576879 | + | 14.6 | 6119 | 6287 | -4 | L1MA7 | L1 | LINE |
| 211576880 | 211577000 | + | 23.6 | 3136 | 3249 | -2897 | L1M5 | L1 | LINE |

  

|  |  |  |  |  |  |  |  |  |  |
| --- | --- | --- | --- | --- | --- | --- | --- | --- | --- |
| chr2:212977389-212978617 | | | 1107 bp | | |  |  |  |  |
| Genome Start | Genome End | Strand | Div | Repeat Start | Repeat End | Repeat Left | Name | Family | Class |
| 212977389 | 212977447 | - | 20.2 | 3320 | 3418 | -1 | L2 | L2 | LINE |
| 212977559 | 212977615 | + | 11.8 | 5172 | 5231 | -915 | L1PREC2 | L1 | LINE |
| 212977616 | 212978054 | - | 9.1 | 5722 | 6159 | -2 | L1PA14 | L1 | LINE |
| 212978055 | 212978519 | + | 9.0 | 5232 | 5708 | -453 | L1PA14 | L1 | LINE |
| 212978531 | 212978617 | - | 34.9 | 3104 | 3190 | -229 | L2 | L2 | LINE |

  

|  |  |  |  |  |  |  |  |  |  |
| --- | --- | --- | --- | --- | --- | --- | --- | --- | --- |
| chr2:213939136-213942310 | | | 3146 bp | | |  |  |  |  |
| Genome Start | Genome End | Strand | Div | Repeat Start | Repeat End | Repeat Left | Name | Family | Class |
| 213939136 | 213939350 | + | 31.6 | 1455 | 1710 | -4832 | L1MEc | L1 | LINE |
| 213939376 | 213939697 | - | 8.3 | 1 | 315 | -49 | THE1B | MaLR | LTR |
| 213939698 | 213939760 | + | 14.3 | 5741 | 5803 | -380 | L1M3 | L1 | LINE |
| 213939759 | 213941334 | - | 4.6 | 4576 | 6152 | -2 | L1PA5 | L1 | LINE |
| 213941334 | 213941968 | + | 5.8 | 3939 | 4576 | -1570 | L1PA5 | L1 | LINE |
| 213941976 | 213942310 | + | 31.5 | 1897 | 2329 | -4496 | L1MEc | L1 | LINE |

  

|  |  |  |  |  |  |  |  |  |  |
| --- | --- | --- | --- | --- | --- | --- | --- | --- | --- |
| chr2:213974522-213978180 | | | 2852 bp | | |  |  |  |  |
| Genome Start | Genome End | Strand | Div | Repeat Start | Repeat End | Repeat Left | Name | Family | Class |
| 213974522 | 213974816 | - | 22.3 | 2029 | 2317 | -3806 | L1MEe | L1 | LINE |
| 213975152 | 213975361 | - | 30.6 | 1042 | 1248 | -4871 | L1MEe | L1 | LINE |
| 213975398 | 213976386 | - | 2.4 | 5164 | 6152 | -3 | L1PA4 | L1 | LINE |
| 213976386 | 213977692 | + | 3.6 | 3849 | 5156 | -990 | L1PA4 | L1 | LINE |
| 213978130 | 213978180 | - | 20.0 | 12 | 61 | -6058 | L1MEe | L1 | LINE |

  

|  |  |  |  |  |  |  |  |  |  |
| --- | --- | --- | --- | --- | --- | --- | --- | --- | --- |
| chr2:214606562-214614765 | | | 7872 bp | | |  |  |  |  |
| Genome Start | Genome End | Strand | Div | Repeat Start | Repeat End | Repeat Left | Name | Family | Class |
| 214606562 | 214607347 | - | 23.6 | 7235 | 8039 | -3 | L1MC4 | L1 | LINE |
| 214607348 | 214608946 | - | 19.6 | 3 | 1755 | 0 | MER52A | ERV1 | LTR |
| 214608947 | 214609978 | - | 24.1 | 6113 | 7234 | -808 | L1MC4 | L1 | LINE |
| 214609979 | 214610158 | - | 35.8 | 3026 | 3215 | -2999 | L1MC4 | L1 | LINE |
| 214610387 | 214610833 | - | 29.7 | 2366 | 2820 | -3394 | L1MC4 | L1 | LINE |
| 214610884 | 214611507 | - | 24.3 | 1737 | 2365 | -3850 | L1MC4 | L1 | LINE |
| 214611518 | 214612363 | - | 2.8 | 5303 | 6153 | -2 | L1PA4 | L1 | LINE |
| 214612363 | 214612576 | + | 3.7 | 5085 | 5298 | -848 | L1PA4 | L1 | LINE |
| 214612605 | 214612736 | - | 23.5 | 1523 | 1657 | -4558 | L1MC4 | L1 | LINE |
| 214612743 | 214613253 | - | 15.3 | 163 | 672 | -55 | LTR8A | ERV1 | LTR |
| 214613265 | 214613647 | - | 8.0 | 5773 | 6160 | -3 | L1PA13 | L1 | LINE |
| 214613648 | 214614765 | - | 26.6 | 368 | 1536 | -4679 | L1MC4 | L1 | LINE |

  

|  |  |  |  |  |  |  |  |  |  |
| --- | --- | --- | --- | --- | --- | --- | --- | --- | --- |
| chr2:221132720-221137666 | | | 4741 bp | | |  |  |  |  |
| Genome Start | Genome End | Strand | Div | Repeat Start | Repeat End | Repeat Left | Name | Family | Class |
| 221132720 | 221132860 | + | 23.4 | 2084 | 2235 | -1184 | L2 | L2 | LINE |
| 221132900 | 221132985 | + | 24.7 | 23 | 104 | -417 | LTR33 | ERVL | LTR |
| 221132997 | 221133228 | + | 18.4 | 10 | 250 | -217 | MLT1C | MaLR | LTR |
| 221133229 | 221133699 | - | 14.3 | 14 | 471 | 0 | LTR32 | ERVL | LTR |
| 221133704 | 221133773 | + | 15.7 | 251 | 322 | -145 | MLT1C | MaLR | LTR |
| 221133840 | 221134021 | - | 7.2 | 5006 | 5187 | -959 | L1PA10 | L1 | LINE |
| 221134022 | 221134201 | + | 8.5 | 5198 | 5365 | -803 | L1PA10 | L1 | LINE |
| 221134202 | 221134548 | + | 6.6 | 5800 | 6148 | -6 | L1PA6 | L1 | LINE |
| 221134549 | 221135348 | + | 10.3 | 5366 | 6167 | -1 | L1PA10 | L1 | LINE |
| 221135418 | 221135791 | + | 26.8 | 2251 | 2618 | -801 | L2 | L2 | LINE |
| 221135792 | 221136338 | + | 21.3 | 5511 | 6060 | -120 | L1MB4 | L1 | LINE |
| 221136339 | 221136634 | + | 8.8 | 1 | 296 | -16 | AluSx | Alu | SINE |
| 221136635 | 221136758 | + | 21.3 | 6061 | 6177 | -3 | L1MB4 | L1 | LINE |
| 221136759 | 221137195 | + | 26.8 | 2619 | 2999 | -420 | L2 | L2 | LINE |
| 221137213 | 221137666 | + | 29.6 | 2915 | 3419 | 0 | L2 | L2 | LINE |

  

|  |  |  |  |  |  |  |  |  |  |
| --- | --- | --- | --- | --- | --- | --- | --- | --- | --- |
| chr2:224119076-224121706 | | | 2482 bp | | |  |  |  |  |
| Genome Start | Genome End | Strand | Div | Repeat Start | Repeat End | Repeat Left | Name | Family | Class |
| 224119076 | 224119274 | + | 35.2 | 4167 | 4364 | -1782 | L1MC4a | L1 | LINE |
| 224119381 | 224119688 | + | 31.4 | 4499 | 4832 | -1314 | L1MC4a | L1 | LINE |
| 224119660 | 224119909 | + | 30.9 | 5092 | 5332 | -814 | L1MC4a | L1 | LINE |
| 224119909 | 224119978 | - | 15.7 | 5519 | 5592 | -579 | L1MA10 | L1 | LINE |
| 224119979 | 224120138 | + | 15.8 | 5587 | 5768 | -566 | L1MA10 | L1 | LINE |
| 224120173 | 224120675 | + | 15.8 | 5769 | 6266 | -68 | L1MA10 | L1 | LINE |
| 224120676 | 224120976 | - | 14.3 | 1 | 294 | -18 | AluSx | Alu | SINE |
| 224120977 | 224121057 | + | 15.8 | 6267 | 6333 | -1 | L1MA10 | L1 | LINE |
| 224121097 | 224121706 | + | 25.9 | 5317 | 5925 | -1883 | L1MC4a | L1 | LINE |

  

|  |  |  |  |  |  |  |  |  |  |
| --- | --- | --- | --- | --- | --- | --- | --- | --- | --- |
| chr2:224162543-224167488 | | | 4857 bp | | |  |  |  |  |
| Genome Start | Genome End | Strand | Div | Repeat Start | Repeat End | Repeat Left | Name | Family | Class |
| 224162543 | 224165220 | - | 30.5 | 623 | 3419 | 0 | L2 | L2 | LINE |
| 224165232 | 224165494 | - | 2.3 | 5891 | 6153 | -2 | L1PA4 | L1 | LINE |
| 224165495 | 224165935 | + | 3.6 | 5460 | 5900 | -254 | L1PA4 | L1 | LINE |
| 224165998 | 224166170 | - | 25.0 | 333 | 507 | -2912 | L2 | L2 | LINE |
| 224166181 | 224166606 | - | 8.8 | 1 | 435 | -9 | MLT2A1 | ERVL | LTR |
| 224166613 | 224166682 | - | 31.4 | 247 | 322 | -3097 | L2 | L2 | LINE |
| 224166683 | 224166951 | + | 6.5 | 1 | 309 | 0 | AluY | Alu | SINE |
| 224166952 | 224166964 | - | 31.4 | 233 | 246 | -3173 | L2 | L2 | LINE |
| 224166965 | 224167283 | + | 8.2 | 1 | 309 | 0 | AluSc | Alu | SINE |
| 224167284 | 224167488 | - | 31.4 | 32 | 232 | -3187 | L2 | L2 | LINE |

  

|  |  |  |  |  |  |  |  |  |  |
| --- | --- | --- | --- | --- | --- | --- | --- | --- | --- |
| chr2:224890538-224891155 | | | 614 bp | | |  |  |  |  |
| Genome Start | Genome End | Strand | Div | Repeat Start | Repeat End | Repeat Left | Name | Family | Class |
| 224890538 | 224890726 | - | 27.5 | 3094 | 3268 | -151 | L2 | L2 | LINE |
| 224890727 | 224890781 | - | 3.6 | 5850 | 5904 | -250 | L1PA5 | L1 | LINE |
| 224890782 | 224891025 | + | 5.7 | 5910 | 6154 | 0 | L1PA5 | L1 | LINE |
| 224891030 | 224891155 | - | 34.4 | 2977 | 3106 | -313 | L2 | L2 | LINE |

  

|  |  |  |  |  |  |  |  |  |  |
| --- | --- | --- | --- | --- | --- | --- | --- | --- | --- |
| chr2:227072945-227077282 | | | 4338 bp | | |  |  |  |  |
| Genome Start | Genome End | Strand | Div | Repeat Start | Repeat End | Repeat Left | Name | Family | Class |
| 227072945 | 227074298 | + | 15.7 | 2855 | 4242 | -1901 | L1MA5 | L1 | LINE |
| 227074299 | 227074928 | - | 10.5 | 5527 | 6157 | -4 | L1PA14 | L1 | LINE |
| 227074929 | 227076179 | + | 15.3 | 4243 | 5527 | -773 | L1PA14 | L1 | LINE |
| 227076180 | 227076483 | - | 5.3 | 1 | 306 | -5 | AluY | Alu | SINE |
| 227076484 | 227077282 | + | 14.2 | 5528 | 6299 | -1 | L1MA5 | L1 | LINE |

  

|  |  |  |  |  |  |  |  |  |  |
| --- | --- | --- | --- | --- | --- | --- | --- | --- | --- |
| chr2:227260888-227263183 | | | 2244 bp | | |  |  |  |  |
| Genome Start | Genome End | Strand | Div | Repeat Start | Repeat End | Repeat Left | Name | Family | Class |
| 227260888 | 227261122 | - | 19.8 | 141 | 370 | -4 | MLT1A | MaLR | LTR |
| 227261123 | 227261534 | - | 5.6 | 4609 | 5020 | -1126 | L1PB1 | L1 | LINE |
| 227261535 | 227262667 | + | 9.3 | 5031 | 6150 | -1 | L1PB1 | L1 | LINE |
| 227262689 | 227262996 | + | 10.7 | 1 | 308 | -5 | AluSp | Alu | SINE |
| 227263028 | 227263183 | - | 23.4 | 1 | 157 | -217 | MLT1A | MaLR | LTR |

  

|  |  |  |  |  |  |  |  |  |  |
| --- | --- | --- | --- | --- | --- | --- | --- | --- | --- |
| chr2:228847866-228852045 | | | 4112 bp | | |  |  |  |  |
| Genome Start | Genome End | Strand | Div | Repeat Start | Repeat End | Repeat Left | Name | Family | Class |
| 228847866 | 228848110 | + | 23.1 | 2 | 260 | -85 | MER2 | MER2\_type | DNA |
| 228848139 | 228848432 | - | 15.7 | 1 | 293 | -19 | AluSx | Alu | SINE |
| 228848437 | 228849527 | - | 13.3 | 5057 | 6149 | -17 | L1PA16 | L1 | LINE |
| 228849526 | 228850242 | + | 13.9 | 2958 | 3673 | -2473 | L1P4 | L1 | LINE |
| 228850280 | 228850571 | + | 16.8 | 1 | 291 | -21 | AluJb | Alu | SINE |
| 228850572 | 228851950 | + | 13.9 | 3674 | 5069 | -1077 | L1P4 | L1 | LINE |
| 228851952 | 228852045 | + | 17.0 | 251 | 345 | 0 | MER2 | MER2\_type | DNA |

  

|  |  |  |  |  |  |  |  |  |  |
| --- | --- | --- | --- | --- | --- | --- | --- | --- | --- |
| chr2:229265818-229270949 | | | 5129 bp | | |  |  |  |  |
| Genome Start | Genome End | Strand | Div | Repeat Start | Repeat End | Repeat Left | Name | Family | Class |
| 229265818 | 229265961 | - | 22.8 | 640 | 762 | -11 | LTR41 | ERVL | LTR |
| 229265962 | 229268594 | - | 8.2 | 1798 | 4425 | -1721 | L1PA6 | L1 | LINE |
| 229268587 | 229270317 | + | 7.9 | 4416 | 6152 | -2 | L1PA6 | L1 | LINE |
| 229270329 | 229270949 | - | 28.7 | 1 | 653 | -120 | LTR41 | ERVL | LTR |

  

|  |  |  |  |  |  |  |  |  |  |
| --- | --- | --- | --- | --- | --- | --- | --- | --- | --- |
| chr2:229479964-229484562 | | | 4592 bp | | |  |  |  |  |
| Genome Start | Genome End | Strand | Div | Repeat Start | Repeat End | Repeat Left | Name | Family | Class |
| 229479964 | 229480650 | + | 16.2 | 5088 | 5783 | -508 | L1MA8 | L1 | LINE |
| 229480651 | 229480952 | + | 12.2 | 1 | 302 | -11 | AluSq | Alu | SINE |
| 229480953 | 229481076 | + | 16.3 | 5784 | 5903 | -388 | L1MA8 | L1 | LINE |
| 229481077 | 229481434 | + | 10.1 | 5814 | 6172 | 0 | L1PA8 | L1 | LINE |
| 229481435 | 229481616 | + | 16.3 | 5904 | 6089 | -202 | L1MA8 | L1 | LINE |
| 229481627 | 229483380 | - | 8.1 | 4417 | 6164 | -4 | L1PA10 | L1 | LINE |
| 229483378 | 229483981 | + | 7.8 | 3785 | 4395 | -1751 | L1PA10 | L1 | LINE |
| 229483982 | 229484076 | + | 15.7 | 6078 | 6167 | -124 | L1MA8 | L1 | LINE |
| 229484077 | 229484442 | + | 14.1 | 1 | 362 | -2 | THE1B | MaLR | LTR |
| 229484443 | 229484562 | + | 15.7 | 6168 | 6288 | -3 | L1MA8 | L1 | LINE |

  

|  |  |  |  |  |  |  |  |  |  |
| --- | --- | --- | --- | --- | --- | --- | --- | --- | --- |
| chr2:230759473-230766682 | | | 7202 bp | | |  |  |  |  |
| Genome Start | Genome End | Strand | Div | Repeat Start | Repeat End | Repeat Left | Name | Family | Class |
| 230759473 | 230760654 | - | 12.5 | 5529 | 6868 | -2 | L1PB3 | L1 | LINE |
| 230760655 | 230760724 | - | 22.1 | 128 | 195 | -126 | MER7D | MER2\_type | DNA |
| 230760725 | 230762532 | - | 12.5 | 3479 | 5528 | -1342 | L1PB3 | L1 | LINE |
| 230762555 | 230764060 | - | 8.5 | 4612 | 6103 | -28 | L1PA8A | L1 | LINE |
| 230764054 | 230765710 | + | 8.1 | 2942 | 4610 | -1536 | L1PA8A | L1 | LINE |
| 230765704 | 230766682 | - | 15.0 | 2438 | 3494 | -3368 | L1PB3 | L1 | LINE |

  

|  |  |  |  |  |  |  |  |  |  |
| --- | --- | --- | --- | --- | --- | --- | --- | --- | --- |
| chr2:231007966-231009154 | | | 868 bp | | |  |  |  |  |
| Genome Start | Genome End | Strand | Div | Repeat Start | Repeat End | Repeat Left | Name | Family | Class |
| 231007966 | 231008308 | - | 29.9 | 5281 | 5659 | -581 | L1M5 | L1 | LINE |
| 231008514 | 231008774 | - | 14.2 | 5906 | 6170 | -4 | L1PA11 | L1 | LINE |
| 231008775 | 231008965 | + | 5.8 | 5705 | 5894 | -269 | L1PA13 | L1 | LINE |
| 231009082 | 231009154 | - | 23.6 | 4438 | 4509 | -1637 | L1M5 | L1 | LINE |

  

|  |  |  |  |  |  |  |  |  |  |
| --- | --- | --- | --- | --- | --- | --- | --- | --- | --- |
| chr2:233394526-233402648 | | | 7919 bp | | |  |  |  |  |
| Genome Start | Genome End | Strand | Div | Repeat Start | Repeat End | Repeat Left | Name | Family | Class |
| 233394526 | 233395354 | - | 25.7 | 4303 | 5204 | -876 | L1MEb | L1 | LINE |
| 233395355 | 233395631 | + | 9.4 | 1 | 280 | -30 | AluSg | Alu | SINE |
| 233395632 | 233396309 | - | 25.7 | 3625 | 4302 | -1778 | L1MEb | L1 | LINE |
| 233396310 | 233396609 | - | 19.2 | 1 | 312 | 0 | AluJo | Alu | SINE |
| 233396610 | 233396738 | - | 25.7 | 3493 | 3624 | -2456 | L1MEb | L1 | LINE |
| 233396742 | 233398672 | + | 11.1 | 2 | 1963 | -455 | Tigger1 | MER2\_type | DNA |
| 233398681 | 233399469 | - | 3.6 | 5343 | 6133 | -21 | L1PA6 | L1 | LINE |
| 233399470 | 233399679 | + | 6.7 | 5119 | 5336 | -810 | L1PA6 | L1 | LINE |
| 233399680 | 233399942 | + | 11.2 | 1948 | 2219 | -199 | Tigger1 | MER2\_type | DNA |
| 233399936 | 233400137 | + | 8.9 | 961 | 1164 | -1254 | Tigger1 | MER2\_type | DNA |
| 233400148 | 233400325 | + | 18.1 | 2237 | 2418 | 0 | Tigger1 | MER2\_type | DNA |
| 233400326 | 233400935 | - | 26.5 | 2777 | 3492 | -2588 | L1MEb | L1 | LINE |
| 233400936 | 233401240 | - | 18.1 | 1 | 307 | -5 | AluJb | Alu | SINE |
| 233401241 | 233401384 | - | 26.5 | 2628 | 2776 | -3304 | L1MEb | L1 | LINE |
| 233401432 | 233401605 | - | 21.1 | 105 | 275 | -37 | AluJo | Alu | SINE |
| 233401740 | 233401859 | - | 18.3 | 1 | 120 | -192 | AluJo | Alu | SINE |
| 233401863 | 233402307 | - | 28.8 | 2221 | 2634 | -3446 | L1MEb | L1 | LINE |
| 233402314 | 233402648 | - | 27.8 | 342 | 669 | -5401 | L1MEb | L1 | LINE |

  

|  |  |  |  |  |  |  |  |  |  |
| --- | --- | --- | --- | --- | --- | --- | --- | --- | --- |
| chr2:234238332-234242985 | | | 4415 bp | | |  |  |  |  |
| Genome Start | Genome End | Strand | Div | Repeat Start | Repeat End | Repeat Left | Name | Family | Class |
| 234238332 | 234238752 | - | 34.9 | 5652 | 6077 | -44 | L1MC4a | L1 | LINE |
| 234238887 | 234239141 | - | 24.9 | 15 | 402 | -156 | MER74A | ERVL | LTR |
| 234239167 | 234239330 | - | 26.7 | 5493 | 5655 | -511 | L1MC4a | L1 | LINE |
| 234239331 | 234239741 | - | 18.6 | 1 | 432 | 0 | MSTB1 | MaLR | LTR |
| 234239742 | 234239974 | - | 26.1 | 5243 | 5492 | -674 | L1MC4a | L1 | LINE |
| 234240034 | 234240651 | - | 3.9 | 5538 | 6155 | 0 | L1PA4 | L1 | LINE |
| 234240652 | 234241330 | + | 3.7 | 4876 | 5555 | -600 | L1PA4 | L1 | LINE |
| 234241325 | 234241444 | - | 20.7 | 7659 | 7775 | -10 | L1MC3 | L1 | LINE |
| 234241445 | 234242103 | - | 5.6 | 5492 | 6150 | -4 | L1PA6 | L1 | LINE |
| 234242104 | 234242437 | - | 20.7 | 7324 | 7658 | -127 | L1MC3 | L1 | LINE |
| 234242465 | 234242985 | - | 23.0 | 4338 | 4914 | -1232 | L1MC4a | L1 | LINE |

  

|  |  |  |  |  |  |  |  |  |  |
| --- | --- | --- | --- | --- | --- | --- | --- | --- | --- |
| chr2:234621597-234623432 | | | 1742 bp | | |  |  |  |  |
| Genome Start | Genome End | Strand | Div | Repeat Start | Repeat End | Repeat Left | Name | Family | Class |
| 234621597 | 234621644 | - | 25.0 | 209 | 259 | -9 | MIRb | MIR | SINE |
| 234621660 | 234622014 | + | 18.4 | 1 | 366 | -48 | MLT2D | ERVL | LTR |
| 234622015 | 234622381 | - | 8.7 | 4927 | 5293 | -853 | L1PA7 | L1 | LINE |
| 234622381 | 234623240 | + | 5.8 | 5291 | 6151 | -3 | L1PA7 | L1 | LINE |
| 234623321 | 234623432 | - | 25.0 | 45 | 156 | -112 | MIRb | MIR | SINE |

  

|  |  |  |  |  |  |  |  |  |  |
| --- | --- | --- | --- | --- | --- | --- | --- | --- | --- |
| chr2:234763594-234767752 | | | 4141 bp | | |  |  |  |  |
| Genome Start | Genome End | Strand | Div | Repeat Start | Repeat End | Repeat Left | Name | Family | Class |
| 234763594 | 234765323 | + | 29.7 | 2214 | 3991 | -2155 | L1M5 | L1 | LINE |
| 234765324 | 234765692 | + | 17.6 | 29 | 378 | -184 | LTR75 | ERVL | LTR |
| 234765698 | 234766467 | - | 2.6 | 5386 | 6155 | 0 | L1PA3 | L1 | LINE |
| 234766472 | 234767227 | + | 2.4 | 4613 | 5368 | -778 | L1PA3 | L1 | LINE |
| 234767229 | 234767382 | + | 20.8 | 365 | 518 | -44 | LTR75 | ERVL | LTR |
| 234767391 | 234767752 | + | 30.6 | 5106 | 5469 | -652 | L1M5 | L1 | LINE |

  

|  |  |  |  |  |  |  |  |  |  |
| --- | --- | --- | --- | --- | --- | --- | --- | --- | --- |
| chr2:236982491-236985575 | | | 3060 bp | | |  |  |  |  |
| Genome Start | Genome End | Strand | Div | Repeat Start | Repeat End | Repeat Left | Name | Family | Class |
| 236982491 | 236982657 | - | 15.6 | 130 | 298 | -14 | AluJ | Alu | SINE |
| 236982674 | 236983752 | - | 2.2 | 3391 | 4469 | -1677 | L1PA3 | L1 | LINE |
| 236983753 | 236985441 | + | 1.7 | 4466 | 6155 | 0 | L1PA3 | L1 | LINE |
| 236985451 | 236985575 | - | 14.5 | 2 | 126 | -186 | AluJ | Alu | SINE |

  

|  |  |  |  |  |  |  |  |  |  |
| --- | --- | --- | --- | --- | --- | --- | --- | --- | --- |
| chr2:238376195-238379825 | | | 3498 bp | | |  |  |  |  |
| Genome Start | Genome End | Strand | Div | Repeat Start | Repeat End | Repeat Left | Name | Family | Class |
| 238376195 | 238376366 | - | 23.3 | 5461 | 5641 | -521 | L1M5 | L1 | LINE |
| 238376367 | 238376640 | + | 11.7 | 1 | 274 | -38 | AluSx | Alu | SINE |
| 238376760 | 238376865 | - | 13.3 | 5367 | 5475 | -687 | L1M5 | L1 | LINE |
| 238376876 | 238377253 | - | 25.8 | 4903 | 5288 | -858 | L1M5 | L1 | LINE |
| 238377267 | 238377551 | - | 10.5 | 1 | 303 | -9 | AluSx | Alu | SINE |
| 238377554 | 238378252 | - | 11.0 | 5075 | 5783 | -383 | L1P4 | L1 | LINE |
| 238378250 | 238379064 | + | 13.5 | 4012 | 5023 | -1123 | L1P4 | L1 | LINE |
| 238379065 | 238379391 | + | 15.2 | 1 | 310 | -2 | AluJo | Alu | SINE |
| 238379392 | 238379459 | + | 12.4 | 5024 | 5079 | -1067 | L1P4 | L1 | LINE |
| 238379452 | 238379825 | - | 22.0 | 3061 | 3457 | -2689 | L1M5 | L1 | LINE |

  

|  |  |  |  |  |  |  |  |  |  |
| --- | --- | --- | --- | --- | --- | --- | --- | --- | --- |
| chr2:240422661-240426277 | | | 3611 bp | | |  |  |  |  |
| Genome Start | Genome End | Strand | Div | Repeat Start | Repeat End | Repeat Left | Name | Family | Class |
| 240422661 | 240423851 | + | 18.6 | 2 | 1531 | -522 | MER52D | ERV1 | LTR |
| 240423852 | 240424305 | - | 2.9 | 4254 | 4709 | -1437 | L1PA5 | L1 | LINE |
| 240424302 | 240425757 | + | 4.0 | 4684 | 6148 | -6 | L1PA5 | L1 | LINE |
| 240425768 | 240426277 | + | 18.4 | 1558 | 2123 | 0 | MER52D | ERV1 | LTR |

  

|  |  |  |  |  |  |  |  |  |  |
| --- | --- | --- | --- | --- | --- | --- | --- | --- | --- |
| chr2:241245690-241250145 | | | 4389 bp | | |  |  |  |  |
| Genome Start | Genome End | Strand | Div | Repeat Start | Repeat End | Repeat Left | Name | Family | Class |
| 241245690 | 241246253 | - | 14.7 | 5798 | 6332 | -1 | L1MC1 | L1 | LINE |
| 241246263 | 241246776 | - | 10.1 | 5641 | 6150 | -1 | L1PB1 | L1 | LINE |
| 241246777 | 241246982 | + | 6.8 | 5447 | 5653 | -498 | L1PB1 | L1 | LINE |
| 241247037 | 241248152 | - | 13.8 | 4651 | 5772 | -561 | L1MC1 | L1 | LINE |
| 241248153 | 241248455 | - | 11.4 | 1 | 323 | 0 | AluSx | Alu | SINE |
| 241248456 | 241248793 | - | 15.3 | 4322 | 4650 | -1496 | L1MC1 | L1 | LINE |
| 241248794 | 241249088 | - | 8.5 | 1 | 304 | -7 | AluY | Alu | SINE |
| 241249089 | 241249134 | - | 15.3 | 4277 | 4321 | -1825 | L1MC1 | L1 | LINE |
| 241249136 | 241249576 | - | 11.7 | 18 | 428 | 0 | MSTA | MaLR | LTR |
| 241249580 | 241250145 | - | 15.4 | 3720 | 4281 | -1865 | L1MC1 | L1 | LINE |

  


|  |  |  |  |  |  |  |  |  |  |
| --- | --- | --- | --- | --- | --- | --- | --- | --- | --- |
| chr3:776212-780953 | | | 3956 bp | | |  |  |  |  |
| Genome Start | Genome End | Strand | Div | Repeat Start | Repeat End | Repeat Left | Name | Family | Class |
| 776212 | 776558 | - | 38.5 | 4828 | 5180 | -966 | L1M5 | L1 | LINE |
| 776637 | 778424 | - | 2.6 | 4365 | 6153 | -2 | L1PA3 | L1 | LINE |
| 778425 | 779495 | + | 3.7 | 3292 | 4364 | -1782 | L1PA3 | L1 | LINE |
| 779543 | 779708 | - | 30.9 | 4545 | 4716 | -1430 | L1M5 | L1 | LINE |
| 779819 | 779897 | - | 25.6 | 2 | 79 | -1 | MADE2 | Mariner | DNA |
| 780056 | 780147 | - | 28.3 | 4131 | 4223 | -1923 | L1M5 | L1 | LINE |
| 780416 | 780568 | + | 33.1 | 52 | 199 | -312 | MLT1J | MaLR | LTR |
| 780674 | 780800 | + | 27.8 | 374 | 508 | -3 | MLT1J | MaLR | LTR |
| 780821 | 780953 | - | 28.9 | 3681 | 3809 | -2337 | L1M5 | L1 | LINE |

  

|  |  |  |  |  |  |  |  |  |  |
| --- | --- | --- | --- | --- | --- | --- | --- | --- | --- |
| chr3:1670701-1677866 | | | 7193 bp | | |  |  |  |  |
| Genome Start | Genome End | Strand | Div | Repeat Start | Repeat End | Repeat Left | Name | Family | Class |
| 1670701 | 1671340 | + | 13.4 | 1075 | 1925 | -350 | L1P2 | L1 | LINE |
| 1671332 | 1671464 | + | 6.1 | 2380 | 2511 | -3635 | L1P2 | L1 | LINE |
| 1671459 | 1673505 | - | 3.2 | 1967 | 4012 | -2134 | L1PA2 | L1 | LINE |
| 1673495 | 1675670 | + | 2.6 | 4001 | 6167 | 0 | L1PA2 | L1 | LINE |
| 1675670 | 1677866 | + | 9.8 | 2497 | 4709 | -1437 | L1P2 | L1 | LINE |

  

|  |  |  |  |  |  |  |  |  |  |
| --- | --- | --- | --- | --- | --- | --- | --- | --- | --- |
| chr3:2076702-2078093 | | | 1393 bp | | |  |  |  |  |
| Genome Start | Genome End | Strand | Div | Repeat Start | Repeat End | Repeat Left | Name | Family | Class |
| 2076702 | 2077124 | + | 32.9 | 63 | 529 | -62 | MLT1K | MaLR | LTR |
| 2077130 | 2077930 | - | 1.8 | 5355 | 6155 | 0 | L1PA3 | L1 | LINE |
| 2077925 | 2078016 | + | 1.1 | 5239 | 5334 | -812 | L1PA3 | L1 | LINE |
| 2078017 | 2078093 | + | 25.0 | 515 | 590 | -1 | MLT1K | MaLR | LTR |

  

|  |  |  |  |  |  |  |  |  |  |
| --- | --- | --- | --- | --- | --- | --- | --- | --- | --- |
| chr3:3574958-3576204 | | | 1239 bp | | |  |  |  |  |
| Genome Start | Genome End | Strand | Div | Repeat Start | Repeat End | Repeat Left | Name | Family | Class |
| 3574958 | 3575237 | + | 34.4 | 2883 | 3168 | -251 | L2 | L2 | LINE |
| 3575241 | 3575367 | - | 12.6 | 5405 | 5531 | -637 | L1PA10 | L1 | LINE |
| 3575368 | 3575995 | + | 8.3 | 5541 | 6168 | 0 | L1PA10 | L1 | LINE |
| 3575996 | 3576008 | - | 20.4 | 5386 | 5405 | -897 | L1M | L1 | LINE |
| 3576014 | 3576204 | + | 26.8 | 3211 | 3413 | -6 | L2 | L2 | LINE |

  

|  |  |  |  |  |  |  |  |  |  |
| --- | --- | --- | --- | --- | --- | --- | --- | --- | --- |
| chr3:3925273-3931889 | | | 6587 bp | | |  |  |  |  |
| Genome Start | Genome End | Strand | Div | Repeat Start | Repeat End | Repeat Left | Name | Family | Class |
| 3925273 | 3925465 | + | 8.3 | 3 | 204 | -240 | MLT2A1 | ERVL | LTR |
| 3925466 | 3926160 | - | 8.3 | 2 | 696 | -5459 | L1PA4 | L1 | LINE |
| 3926154 | 3931626 | + | 4.2 | 685 | 6152 | -3 | L1PA4 | L1 | LINE |
| 3931664 | 3931889 | + | 9.8 | 214 | 444 | 0 | MLT2A1 | ERVL | LTR |

  

|  |  |  |  |  |  |  |  |  |  |
| --- | --- | --- | --- | --- | --- | --- | --- | --- | --- |
| chr3:4041112-4044757 | | | 3492 bp | | |  |  |  |  |
| Genome Start | Genome End | Strand | Div | Repeat Start | Repeat End | Repeat Left | Name | Family | Class |
| 4041112 | 4041207 | - | 28.4 | 635 | 738 | -1222 | L4 | RTE | LINE |
| 4041266 | 4041346 | + | 25.4 | 1 | 83 | -241 | MER33 | MER1\_type | DNA |
| 4041347 | 4041796 | + | 20.2 | 1 | 443 | 0 | LTR47B | ERVL | LTR |
| 4041797 | 4041925 | + | 25.4 | 84 | 220 | -104 | MER33 | MER1\_type | DNA |
| 4041982 | 4043071 | - | 0.6 | 5066 | 6155 | 0 | L1HS | L1 | LINE |
| 4043068 | 4044558 | + | 0.3 | 3581 | 5071 | -1075 | L1HS | L1 | LINE |
| 4044563 | 4044651 | + | 18.0 | 213 | 301 | -23 | MER33 | MER1\_type | DNA |
| 4044692 | 4044757 | - | 21.9 | 501 | 569 | -1391 | L4 | RTE | LINE |

  

|  |  |  |  |  |  |  |  |  |  |
| --- | --- | --- | --- | --- | --- | --- | --- | --- | --- |
| chr3:6200732-6211298 | | | 9651 bp | | |  |  |  |  |
| Genome Start | Genome End | Strand | Div | Repeat Start | Repeat End | Repeat Left | Name | Family | Class |
| 6200732 | 6200861 | + | 38.0 | 2343 | 2473 | -946 | L2 | L2 | LINE |
| 6200897 | 6201340 | + | 28.7 | 10 | 467 | 0 | MLT1C | MaLR | LTR |
| 6201395 | 6201746 | + | 36.6 | 2585 | 2952 | -467 | L2 | L2 | LINE |
| 6201750 | 6203751 | - | 18.2 | 4711 | 6752 | -10 | L1MC2 | L1 | LINE |
| 6203854 | 6203970 | - | 16.2 | 6047 | 6164 | -19 | L1MB3 | L1 | LINE |
| 6203972 | 6204280 | - | 5.2 | 1 | 309 | -2 | AluY | Alu | SINE |
| 6204290 | 6204767 | - | 18.7 | 5677 | 6182 | -1 | L1MB3 | L1 | LINE |
| 6205144 | 6205194 | + | 31.4 | 2646 | 2697 | -722 | L2 | L2 | LINE |
| 6205365 | 6205470 | - | 16.1 | 2344 | 2461 | -4290 | L1MC2 | L1 | LINE |
| 6205471 | 6205853 | + | 24.1 | 1 | 373 | -1 | MLT1A | MaLR | LTR |
| 6205854 | 6205902 | - | 16.1 | 2297 | 2343 | -4408 | L1MC2 | L1 | LINE |
| 6205913 | 6207235 | - | 15.1 | 4810 | 6166 | 0 | L1PA16 | L1 | LINE |
| 6207234 | 6207829 | + | 10.9 | 4261 | 4816 | -1330 | L1PA16 | L1 | LINE |
| 6207820 | 6208009 | - | 21.2 | 2073 | 2304 | -4447 | L1MC2 | L1 | LINE |
| 6208010 | 6208317 | + | 13.6 | 1 | 315 | 0 | AluSq | Alu | SINE |
| 6208318 | 6208551 | - | 21.2 | 1834 | 2072 | -4679 | L1MC2 | L1 | LINE |
| 6208552 | 6208644 | - | 5.5 | 6082 | 6172 | 0 | L1PA8 | L1 | LINE |
| 6208645 | 6210345 | - | 23.8 | 3 | 1833 | -4918 | L1MC2 | L1 | LINE |
| 6210399 | 6210486 | + | 36.8 | 2974 | 3061 | -358 | L2 | L2 | LINE |
| 6210487 | 6211037 | + | 13.8 | 1 | 580 | 0 | LTR19B | ERV1 | LTR |
| 6211038 | 6211061 | + | 36.8 | 3062 | 3079 | -340 | L2 | L2 | LINE |
| 6211177 | 6211298 | + | 29.8 | 3250 | 3389 | -30 | L2 | L2 | LINE |

  

|  |  |  |  |  |  |  |  |  |  |
| --- | --- | --- | --- | --- | --- | --- | --- | --- | --- |
| chr3:6377695-6382037 | | | 4344 bp | | |  |  |  |  |
| Genome Start | Genome End | Strand | Div | Repeat Start | Repeat End | Repeat Left | Name | Family | Class |
| 6377695 | 6377991 | + | 12.2 | 4125 | 4412 | -1734 | L1PA16 | L1 | LINE |
| 6377992 | 6378302 | - | 11.6 | 1 | 312 | 0 | AluSx | Alu | SINE |
| 6378303 | 6379484 | + | 11.2 | 4413 | 5607 | -559 | L1PA16 | L1 | LINE |
| 6379484 | 6379716 | - | 6.4 | 4271 | 4504 | -1642 | L1PA8 | L1 | LINE |
| 6379717 | 6381417 | + | 10.2 | 4522 | 6166 | -6 | L1PA8 | L1 | LINE |
| 6381418 | 6382037 | + | 14.0 | 5593 | 6157 | -9 | L1PA16 | L1 | LINE |

  

|  |  |  |  |  |  |  |  |  |  |
| --- | --- | --- | --- | --- | --- | --- | --- | --- | --- |
| chr3:7080559-7084518 | | | 3809 bp | | |  |  |  |  |
| Genome Start | Genome End | Strand | Div | Repeat Start | Repeat End | Repeat Left | Name | Family | Class |
| 7080559 | 7080731 | - | 29.6 | 264 | 444 | -2 | MLT1J1 | MaLR | LTR |
| 7080787 | 7081150 | - | 15.1 | 5781 | 6143 | -8 | L1PB1 | L1 | LINE |
| 7081247 | 7082507 | - | 8.2 | 4523 | 5791 | -360 | L1PB1 | L1 | LINE |
| 7082508 | 7084236 | + | 6.7 | 2777 | 4514 | -1632 | L1PB1 | L1 | LINE |
| 7084237 | 7084518 | - | 34.9 | 1 | 293 | -153 | MLT1J1 | MaLR | LTR |

  

|  |  |  |  |  |  |  |  |  |  |
| --- | --- | --- | --- | --- | --- | --- | --- | --- | --- |
| chr3:8076185-8085425 | | | 9233 bp | | |  |  |  |  |
| Genome Start | Genome End | Strand | Div | Repeat Start | Repeat End | Repeat Left | Name | Family | Class |
| 8076185 | 8077532 | - | 7.1 | 3996 | 5356 | -790 | L1P2 | L1 | LINE |
| 8077527 | 8078722 | - | 4.1 | 2251 | 3445 | -2701 | L1PA4 | L1 | LINE |
| 8078725 | 8081431 | + | 2.9 | 3448 | 6155 | 0 | L1PA4 | L1 | LINE |
| 8081444 | 8085425 | - | 7.9 | 4 | 4011 | -2135 | L1P2 | L1 | LINE |

  

|  |  |  |  |  |  |  |  |  |  |
| --- | --- | --- | --- | --- | --- | --- | --- | --- | --- |
| chr3:17359295-17363337 | | | 3749 bp | | |  |  |  |  |
| Genome Start | Genome End | Strand | Div | Repeat Start | Repeat End | Repeat Left | Name | Family | Class |
| 17359295 | 17359426 | - | 31.0 | 5738 | 5867 | -254 | L1M5 | L1 | LINE |
| 17359444 | 17360615 | - | 2.2 | 2998 | 4171 | -1975 | L1PA3 | L1 | LINE |
| 17360615 | 17362578 | + | 2.8 | 4194 | 6155 | 0 | L1PA3 | L1 | LINE |
| 17362857 | 17363337 | - | 31.7 | 4888 | 5391 | -755 | L1M5 | L1 | LINE |

  

|  |  |  |  |  |  |  |  |  |  |
| --- | --- | --- | --- | --- | --- | --- | --- | --- | --- |
| chr3:18336574-18348084 | | | 11400 bp | | |  |  |  |  |
| Genome Start | Genome End | Strand | Div | Repeat Start | Repeat End | Repeat Left | Name | Family | Class |
| 18336574 | 18336637 | - | 21.7 | 5377 | 5449 | -1088 | L1MDa | L1 | LINE |
| 18336638 | 18336934 | - | 16.2 | 1 | 292 | -20 | AluJo | Alu | SINE |
| 18336935 | 18336988 | - | 21.7 | 5333 | 5376 | -1161 | L1MDa | L1 | LINE |
| 18336982 | 18339206 | - | 12.1 | 3915 | 6160 | -5 | L1PA15 | L1 | LINE |
| 18339211 | 18340342 | + | 12.6 | 2761 | 3921 | -2225 | L1PREC2 | L1 | LINE |
| 18340337 | 18341222 | - | 21.3 | 4408 | 5347 | -1190 | L1MDa | L1 | LINE |
| 18341323 | 18342214 | - | 21.3 | 3471 | 4407 | -2130 | L1MDa | L1 | LINE |
| 18342250 | 18344564 | - | 2.8 | 3713 | 6019 | -13 | L1PA3 | L1 | LINE |
| 18344553 | 18347783 | - | 4.0 | 3 | 3252 | -2771 | L1PA3 | L1 | LINE |
| 18347781 | 18348084 | - | 22.7 | 3146 | 3484 | -3053 | L1MDa | L1 | LINE |

  

|  |  |  |  |  |  |  |  |  |  |
| --- | --- | --- | --- | --- | --- | --- | --- | --- | --- |
| chr3:19651405-19655696 | | | 4263 bp | | |  |  |  |  |
| Genome Start | Genome End | Strand | Div | Repeat Start | Repeat End | Repeat Left | Name | Family | Class |
| 19651405 | 19651538 | - | 19.6 | 373 | 505 | 0 | MLT1D | MaLR | LTR |
| 19651540 | 19653166 | - | 8.2 | 2381 | 4010 | -2136 | L1PA5 | L1 | LINE |
| 19653164 | 19655305 | + | 6.1 | 4018 | 6154 | 0 | L1PA5 | L1 | LINE |
| 19655337 | 19655696 | - | 23.2 | 3 | 386 | -119 | MLT1D | MaLR | LTR |

  

|  |  |  |  |  |  |  |  |  |  |
| --- | --- | --- | --- | --- | --- | --- | --- | --- | --- |
| chr3:20846045-20849094 | | | 2722 bp | | |  |  |  |  |
| Genome Start | Genome End | Strand | Div | Repeat Start | Repeat End | Repeat Left | Name | Family | Class |
| 20846045 | 20846519 | - | 27.4 | 3404 | 5923 | -300 | L1ME3B | L1 | LINE |
| 20846522 | 20846768 | - | 28.4 | 2708 | 2958 | -3171 | L1ME3B | L1 | LINE |
| 20846876 | 20847290 | - | 29.1 | 2065 | 2497 | -3632 | L1ME3B | L1 | LINE |
| 20847404 | 20847919 | - | 11.8 | 5509 | 6028 | -137 | L1PA15 | L1 | LINE |
| 20847920 | 20848051 | + | 10.6 | 6031 | 6163 | -2 | L1PA15 | L1 | LINE |
| 20848098 | 20848419 | - | 28.8 | 1310 | 1660 | -4489 | L1ME3B | L1 | LINE |
| 20848425 | 20848901 | - | 29.2 | 757 | 1257 | -4862 | L1ME3B | L1 | LINE |
| 20848957 | 20849094 | - | 31.1 | 516 | 653 | -5466 | L1ME3B | L1 | LINE |

  

|  |  |  |  |  |  |  |  |  |  |
| --- | --- | --- | --- | --- | --- | --- | --- | --- | --- |
| chr3:21784853-21788149 | | | 3290 bp | | |  |  |  |  |
| Genome Start | Genome End | Strand | Div | Repeat Start | Repeat End | Repeat Left | Name | Family | Class |
| 21784853 | 21785472 | + | 18.5 | 20 | 626 | -5758 | L1M4c | L1 | LINE |
| 21785494 | 21785619 | - | 7.1 | 3895 | 4020 | -2126 | L1PA5 | L1 | LINE |
| 21785613 | 21787748 | + | 4.3 | 4023 | 6154 | 0 | L1PA5 | L1 | LINE |
| 21787742 | 21788149 | + | 19.9 | 621 | 1021 | -5363 | L1M4c | L1 | LINE |

  

|  |  |  |  |  |  |  |  |  |  |
| --- | --- | --- | --- | --- | --- | --- | --- | --- | --- |
| chr3:24646402-24656111 | | | 9586 bp | | |  |  |  |  |
| Genome Start | Genome End | Strand | Div | Repeat Start | Repeat End | Repeat Left | Name | Family | Class |
| 24646402 | 24647860 | - | 22.2 | 5955 | 7273 | -944 | L1MC3 | L1 | LINE |
| 24647885 | 24648032 | - | 21.1 | 136 | 282 | -30 | AluJb | Alu | SINE |
| 24648033 | 24649235 | - | 18.0 | 4691 | 5962 | -1998 | L1MC3 | L1 | LINE |
| 24649294 | 24649550 | - | 15.6 | 9 | 276 | -36 | AluJb | Alu | SINE |
| 24649551 | 24649697 | - | 19.1 | 4493 | 4690 | -1631 | L1MC3 | L1 | LINE |
| 24649747 | 24652573 | - | 6.4 | 3325 | 6172 | 0 | L1PA8 | L1 | LINE |
| 24652567 | 24653092 | + | 8.2 | 2786 | 3315 | -2831 | L1PA8 | L1 | LINE |
| 24653103 | 24653553 | - | 20.6 | 4034 | 4485 | -1836 | L1MC3 | L1 | LINE |
| 24653544 | 24656111 | - | 21.6 | 1 | 2617 | -3704 | L1MC3 | L1 | LINE |

  

|  |  |  |  |  |  |  |  |  |  |
| --- | --- | --- | --- | --- | --- | --- | --- | --- | --- |
| chr3:26061112-26062423 | | | 1306 bp | | |  |  |  |  |
| Genome Start | Genome End | Strand | Div | Repeat Start | Repeat End | Repeat Left | Name | Family | Class |
| 26061112 | 26061230 | - | 21.9 | 62 | 189 | 0 | MER5A | MER1\_type | DNA |
| 26061231 | 26062154 | - | 11.2 | 5215 | 6161 | 0 | L1PB2 | L1 | LINE |
| 26062154 | 26062336 | + | 16.4 | 5048 | 5230 | -916 | L1PB2 | L1 | LINE |
| 26062344 | 26062378 | - | 11.4 | 6263 | 6297 | -8 | L1MA3 | L1 | LINE |
| 26062379 | 26062423 | - | 13.3 | 1 | 45 | -144 | MER5A | MER1\_type | DNA |

  

|  |  |  |  |  |  |  |  |  |  |
| --- | --- | --- | --- | --- | --- | --- | --- | --- | --- |
| chr3:26332990-26341319 | | | 8326 bp | | |  |  |  |  |
| Genome Start | Genome End | Strand | Div | Repeat Start | Repeat End | Repeat Left | Name | Family | Class |
| 26332990 | 26333414 | - | 14.7 | 294 | 722 | 0 | MER72 | ERV1 | LTR |
| 26333418 | 26337573 | - | 9.6 | 2330 | 6469 | 0 | L1PA7 | L1 | LINE |
| 26337574 | 26338101 | - | 2.5 | 5628 | 6155 | 0 | L1PA3 | L1 | LINE |
| 26338102 | 26338705 | + | 2.6 | 5015 | 5622 | -533 | L1PA3 | L1 | LINE |
| 26338707 | 26341023 | - | 11.3 | 14 | 2338 | -252 | L1PA7 | L1 | LINE |
| 26341024 | 26341319 | - | 13.9 | 1 | 327 | -395 | MER72 | ERV1 | LTR |

  

|  |  |  |  |  |  |  |  |  |  |
| --- | --- | --- | --- | --- | --- | --- | --- | --- | --- |
| chr3:26588391-26591034 | | | 2607 bp | | |  |  |  |  |
| Genome Start | Genome End | Strand | Div | Repeat Start | Repeat End | Repeat Left | Name | Family | Class |
| 26588391 | 26588559 | - | 21.1 | 399 | 567 | 0 | MLT1F1 | MaLR | LTR |
| 26588560 | 26588646 | + | 9.2 | 214 | 300 | -11 | AluY | Alu | SINE |
| 26588647 | 26588848 | - | 27.4 | 153 | 386 | -156 | MLT1F1 | MaLR | LTR |
| 26588895 | 26590477 | - | 3.1 | 4571 | 6152 | -3 | L1PA3 | L1 | LINE |
| 26590468 | 26590877 | + | 4.2 | 4170 | 4587 | -1559 | L1PA3 | L1 | LINE |
| 26590879 | 26591034 | - | 27.7 | 10 | 167 | -375 | MLT1F1 | MaLR | LTR |

  

|  |  |  |  |  |  |  |  |  |  |
| --- | --- | --- | --- | --- | --- | --- | --- | --- | --- |
| chr3:28976180-28981298 | | | 5070 bp | | |  |  |  |  |
| Genome Start | Genome End | Strand | Div | Repeat Start | Repeat End | Repeat Left | Name | Family | Class |
| 28976180 | 28976349 | - | 22.9 | 5758 | 5931 | -2300 | L1M4 | L1 | LINE |
| 28976347 | 28976541 | - | 3.1 | 3127 | 3327 | -2819 | L1PA4 | L1 | LINE |
| 28976540 | 28979372 | + | 3.7 | 3317 | 6154 | -1 | L1PA4 | L1 | LINE |
| 28979384 | 28979504 | - | 22.5 | 5655 | 5775 | -644 | L1M4 | L1 | LINE |
| 28979501 | 28979923 | - | 27.8 | 2034 | 2462 | -3922 | L1M4 | L1 | LINE |
| 28979925 | 28980311 | - | 19.4 | 4 | 355 | -10 | MLT1A0 | MaLR | LTR |
| 28980358 | 28980446 | - | 22.4 | 1799 | 1887 | -4497 | L1M4 | L1 | LINE |
| 28980447 | 28980766 | - | 4.5 | 1 | 311 | 0 | AluY | Alu | SINE |
| 28980767 | 28981298 | - | 22.4 | 1262 | 1798 | -4586 | L1M4 | L1 | LINE |

  

|  |  |  |  |  |  |  |  |  |  |
| --- | --- | --- | --- | --- | --- | --- | --- | --- | --- |
| chr3:29009406-29015786 | | | 5792 bp | | |  |  |  |  |
| Genome Start | Genome End | Strand | Div | Repeat Start | Repeat End | Repeat Left | Name | Family | Class |
| 29009406 | 29009588 | - | 28.3 | 2175 | 2797 | -4164 | L1MCa | L1 | LINE |
| 29009589 | 29009895 | + | 10.4 | 1 | 308 | -5 | AluSq | Alu | SINE |
| 29009896 | 29010517 | - | 28.0 | 1526 | 2174 | -4954 | L1MCa | L1 | LINE |
| 29010556 | 29010785 | - | 30.8 | 1225 | 1455 | -4968 | L1MCa | L1 | LINE |
| 29011256 | 29011358 | - | 28.7 | 1022 | 1129 | -5294 | L1MCa | L1 | LINE |
| 29011362 | 29011550 | - | 25.4 | 249 | 442 | -5981 | L1MCa | L1 | LINE |
| 29011562 | 29012403 | - | 13.1 | 4371 | 5227 | -919 | L1PA16 | L1 | LINE |
| 29012403 | 29013225 | + | 13.9 | 5327 | 6158 | -8 | L1PA16 | L1 | LINE |
| 29013286 | 29014177 | - | 5.2 | 3878 | 4770 | -1376 | L1PA5 | L1 | LINE |
| 29014178 | 29015563 | + | 5.4 | 4761 | 6154 | 0 | L1PA5 | L1 | LINE |
| 29015572 | 29015786 | - | 26.3 | 19 | 223 | -6200 | L1MCa | L1 | LINE |

  

|  |  |  |  |  |  |  |  |  |  |
| --- | --- | --- | --- | --- | --- | --- | --- | --- | --- |
| chr3:30435300-30437123 | | | 1787 bp | | |  |  |  |  |
| Genome Start | Genome End | Strand | Div | Repeat Start | Repeat End | Repeat Left | Name | Family | Class |
| 30435300 | 30435381 | + | 24.4 | 33 | 121 | -2296 | Charlie8 | MER1\_type | DNA |
| 30435383 | 30435971 | - | 19.0 | 4770 | 5358 | -782 | L1MB1 | L1 | LINE |
| 30435969 | 30436753 | + | 18.9 | 5376 | 6161 | -28 | L1MB1 | L1 | LINE |
| 30436790 | 30436954 | + | 18.9 | 6162 | 6333 | -1 | L1MA10 | L1 | LINE |
| 30436958 | 30437123 | + | 30.8 | 108 | 273 | -2144 | Charlie8 | MER1\_type | DNA |

  

|  |  |  |  |  |  |  |  |  |  |
| --- | --- | --- | --- | --- | --- | --- | --- | --- | --- |
| chr3:30604128-30611898 | | | 7605 bp | | |  |  |  |  |
| Genome Start | Genome End | Strand | Div | Repeat Start | Repeat End | Repeat Left | Name | Family | Class |
| 30604128 | 30604892 | - | 19.6 | 5866 | 6633 | 0 | L1MD1 | L1 | LINE |
| 30604923 | 30605217 | - | 6.6 | 1 | 291 | -20 | AluY | Alu | SINE |
| 30605219 | 30605600 | - | 13.8 | 5914 | 6312 | 0 | L1MA9 | L1 | LINE |
| 30605602 | 30605771 | + | 16.8 | 5612 | 5783 | -529 | L1MA9 | L1 | LINE |
| 30605815 | 30605936 | + | 16.8 | 5784 | 5903 | -409 | L1MA9 | L1 | LINE |
| 30605946 | 30606464 | - | 18.2 | 5341 | 5869 | -764 | L1MD1 | L1 | LINE |
| 30606507 | 30607231 | - | 18.2 | 4559 | 5340 | -1197 | L1MD1 | L1 | LINE |
| 30607272 | 30607860 | - | 18.2 | 3898 | 4558 | -1979 | L1MD1 | L1 | LINE |
| 30607861 | 30608157 | - | 4.4 | 1 | 301 | -10 | AluY | Alu | SINE |
| 30608158 | 30611898 | - | 21.3 | 17 | 3897 | -2640 | L1MD1 | L1 | LINE |

  

|  |  |  |  |  |  |  |  |  |  |
| --- | --- | --- | --- | --- | --- | --- | --- | --- | --- |
| chr3:34715605-34720142 | | | 4436 bp | | |  |  |  |  |
| Genome Start | Genome End | Strand | Div | Repeat Start | Repeat End | Repeat Left | Name | Family | Class |
| 34715605 | 34716330 | + | 22.2 | 2097 | 2836 | -3546 | L1M4c | L1 | LINE |
| 34716330 | 34716377 | - | 10.4 | 5320 | 5367 | -779 | L1PREC2 | L1 | LINE |
| 34716380 | 34717131 | + | 12.0 | 5374 | 6159 | -1 | L1PREC2 | L1 | LINE |
| 34717164 | 34717221 | + | 8.6 | 2827 | 2884 | -3498 | L1M4c | L1 | LINE |
| 34717222 | 34717588 | - | 16.5 | 1 | 364 | 0 | THE1B | MaLR | LTR |
| 34717589 | 34717641 | - | 7.5 | 1528 | 1580 | 0 | THE1B-int | MaLR | LTR |
| 34717682 | 34718778 | - | 10.8 | 435 | 1541 | -39 | THE1B-int | MaLR | LTR |
| 34718779 | 34719091 | - | 14.4 | 1 | 312 | -1 | AluSp | Alu | SINE |
| 34719092 | 34719532 | - | 10.8 | 1 | 434 | -1146 | THE1B-int | MaLR | LTR |
| 34719535 | 34719901 | - | 17.8 | 1 | 362 | -2 | THE1B | MaLR | LTR |
| 34719902 | 34719950 | + | 12.2 | 2880 | 2928 | -3454 | L1M4c | L1 | LINE |
| 34719978 | 34720142 | + | 15.4 | 2920 | 3078 | -3304 | L1M4c | L1 | LINE |

  

|  |  |  |  |  |  |  |  |  |  |
| --- | --- | --- | --- | --- | --- | --- | --- | --- | --- |
| chr3:35338254-35343805 | | | 5358 bp | | |  |  |  |  |
| Genome Start | Genome End | Strand | Div | Repeat Start | Repeat End | Repeat Left | Name | Family | Class |
| 35338254 | 35338373 | - | 36.8 | 134 | 251 | -17 | MIRb | MIR | SINE |
| 35338393 | 35339129 | - | 21.7 | 1 | 727 | 0 | LTR8A | ERV1 | LTR |
| 35339132 | 35339436 | - | 28.9 | 5952 | 6262 | -1 | HUERS-P1 | ERV1 | LTR |
| 35339542 | 35340158 | - | 9.2 | 4841 | 5451 | -700 | L1P5 | L1 | LINE |
| 35340159 | 35340878 | + | 9.3 | 5438 | 6159 | -2 | L1PA14 | L1 | LINE |
| 35340932 | 35342105 | - | 19.3 | 4447 | 6123 | -411 | HUERS-P1 | ERV1 | LTR |
| 35342106 | 35342274 | + | 19.5 | 1 | 173 | -3 | FRAM | Alu | SINE |
| 35342275 | 35343681 | - | 19.3 | 2437 | 4446 | -2088 | HUERS-P1 | ERV1 | LTR |
| 35343697 | 35343805 | - | 41.3 | 2 | 112 | -156 | MIRb | MIR | SINE |

  

|  |  |  |  |  |  |  |  |  |  |
| --- | --- | --- | --- | --- | --- | --- | --- | --- | --- |
| chr3:35626031-35631381 | | | 5304 bp | | |  |  |  |  |
| Genome Start | Genome End | Strand | Div | Repeat Start | Repeat End | Repeat Left | Name | Family | Class |
| 35626031 | 35626118 | + | 30.7 | 844 | 933 | -539 | MLT1H-int | MaLR | LTR |
| 35626158 | 35626942 | - | 5.1 | 1221 | 2005 | -4150 | L1PA4 | L1 | LINE |
| 35626943 | 35631078 | + | 4.6 | 2021 | 6155 | 0 | L1PA4 | L1 | LINE |
| 35631087 | 35631381 | + | 26.0 | 1006 | 1281 | -191 | MLT1H-int | MaLR | LTR |

  

|  |  |  |  |  |  |  |  |  |  |
| --- | --- | --- | --- | --- | --- | --- | --- | --- | --- |
| chr3:38355767-38361576 | | | 5383 bp | | |  |  |  |  |
| Genome Start | Genome End | Strand | Div | Repeat Start | Repeat End | Repeat Left | Name | Family | Class |
| 38355767 | 38356547 | + | 33.0 | 4986 | 5784 | -456 | L1ME3 | L1 | LINE |
| 38356588 | 38357161 | - | 20.0 | 1 | 583 | 0 | MER119 | MER1\_type | DNA |
| 38357228 | 38357616 | + | 20.4 | 1 | 372 | -2 | MLT1A | MaLR | LTR |
| 38357751 | 38357837 | - | 8.1 | 81 | 190 | 0 | MER30B | MER1\_type | DNA |
| 38357880 | 38359774 | - | 1.8 | 4243 | 6137 | -18 | L1PA3 | L1 | LINE |
| 38359772 | 38360261 | + | 1.8 | 3744 | 4242 | -1904 | L1PA3 | L1 | LINE |
| 38360262 | 38360365 | - | 13.5 | 1 | 104 | -126 | MER30B | MER1\_type | DNA |
| 38360511 | 38360632 | - | 9.8 | 384 | 510 | 0 | LTR54 | ERV1 | LTR |
| 38360635 | 38361352 | - | 10.5 | 1 | 691 | 0 | LTR8 | ERV1 | LTR |
| 38361353 | 38361415 | + | 19.7 | 91 | 152 | -110 | MIR | MIR | SINE |
| 38361417 | 38361576 | + | 17.9 | 5921 | 6101 | -61 | L1ME3 | L1 | LINE |

  

|  |  |  |  |  |  |  |  |  |  |
| --- | --- | --- | --- | --- | --- | --- | --- | --- | --- |
| chr3:38705194-38710558 | | | 5354 bp | | |  |  |  |  |
| Genome Start | Genome End | Strand | Div | Repeat Start | Repeat End | Repeat Left | Name | Family | Class |
| 38705194 | 38705282 | - | 25.3 | 4815 | 4897 | -31 | L1ME3B | L1 | LINE |
| 38705283 | 38705581 | - | 10.4 | 1 | 298 | -11 | AluSc | Alu | SINE |
| 38705582 | 38706068 | - | 25.3 | 4253 | 4814 | -114 | L1ME3B | L1 | LINE |
| 38706069 | 38707000 | + | 12.8 | 1 | 975 | -256 | Tigger3b | MER2\_type | DNA |
| 38707001 | 38707304 | - | 12.2 | 1 | 310 | -1 | AluY | Alu | SINE |
| 38707305 | 38707549 | + | 12.8 | 976 | 1231 | 0 | Tigger3b | MER2\_type | DNA |
| 38707550 | 38708227 | - | 25.4 | 3513 | 4252 | -676 | L1ME3B | L1 | LINE |
| 38708226 | 38708364 | - | 28.3 | 1381 | 1521 | -3313 | L1ME3B | L1 | LINE |
| 38708365 | 38708551 | - | 26.4 | 770 | 979 | -3875 | L1ME3B | L1 | LINE |
| 38708572 | 38710020 | - | 2.8 | 4702 | 6154 | -1 | L1PA4 | L1 | LINE |
| 38710012 | 38710382 | + | 4.6 | 4326 | 4696 | -1450 | L1PA4 | L1 | LINE |
| 38710385 | 38710558 | - | 25.9 | 592 | 772 | -4082 | L1ME3B | L1 | LINE |

  

|  |  |  |  |  |  |  |  |  |  |
| --- | --- | --- | --- | --- | --- | --- | --- | --- | --- |
| chr3:39326716-39329974 | | | 3229 bp | | |  |  |  |  |
| Genome Start | Genome End | Strand | Div | Repeat Start | Repeat End | Repeat Left | Name | Family | Class |
| 39326716 | 39326849 | + | 9.0 | 1 | 135 | -177 | AluSx | Alu | SINE |
| 39326850 | 39328138 | - | 5.5 | 3193 | 4481 | -1665 | L1PA5 | L1 | LINE |
| 39328140 | 39329783 | + | 5.2 | 4497 | 6138 | -16 | L1PA5 | L1 | LINE |
| 39329813 | 39329974 | + | 14.8 | 124 | 295 | -17 | AluSx | Alu | SINE |

  

|  |  |  |  |  |  |  |  |  |  |
| --- | --- | --- | --- | --- | --- | --- | --- | --- | --- |
| chr3:39901103-39906390 | | | 5204 bp | | |  |  |  |  |
| Genome Start | Genome End | Strand | Div | Repeat Start | Repeat End | Repeat Left | Name | Family | Class |
| 39901103 | 39901732 | - | 24.3 | 5297 | 5961 | -1847 | L1MC4a | L1 | LINE |
| 39901807 | 39902547 | - | 4.5 | 972 | 1712 | -4320 | L1HS | L1 | LINE |
| 39902548 | 39903951 | + | 2.6 | 1729 | 3132 | -2891 | L1HS | L1 | LINE |
| 39903948 | 39906187 | + | 2.8 | 3915 | 6155 | 0 | L1PA3 | L1 | LINE |
| 39906202 | 39906390 | - | 35.1 | 5000 | 5202 | -944 | L1MC4a | L1 | LINE |

  

|  |  |  |  |  |  |  |  |  |  |
| --- | --- | --- | --- | --- | --- | --- | --- | --- | --- |
| chr3:40360297-40368398 | | | 8069 bp | | |  |  |  |  |
| Genome Start | Genome End | Strand | Div | Repeat Start | Repeat End | Repeat Left | Name | Family | Class |
| 40360297 | 40360489 | + | 11.4 | 1 | 193 | -5753 | MER41B-int | ERV1 | LTR |
| 40360490 | 40360775 | - | 15.4 | 3 | 292 | -20 | AluSx | Alu | SINE |
| 40360776 | 40361229 | + | 11.4 | 194 | 638 | -5308 | MER41B-int | ERV1 | LTR |
| 40361219 | 40361480 | + | 13.5 | 1080 | 1352 | -4594 | MER41B-int | ERV1 | LTR |
| 40361504 | 40361910 | + | 13.5 | 1353 | 1774 | -4172 | MER41B-int | ERV1 | LTR |
| 40361898 | 40361983 | + | 12.8 | 2476 | 2561 | -4976 | MER41B-int | ERV1 | LTR |
| 40361992 | 40362754 | - | 4.2 | 4074 | 4836 | -1310 | L1PA4 | L1 | LINE |
| 40362750 | 40364054 | + | 3.2 | 4832 | 6146 | -9 | L1PA4 | L1 | LINE |
| 40364086 | 40366675 | + | 10.5 | 500 | 4534 | -1412 | MER41B-int | ERV1 | LTR |
| 40366676 | 40366962 | + | 16.0 | 1 | 288 | -24 | AluSx | Alu | SINE |
| 40366963 | 40368398 | + | 10.4 | 4535 | 5946 | 0 | MER41B-int | ERV1 | LTR |

  

|  |  |  |  |  |  |  |  |  |  |
| --- | --- | --- | --- | --- | --- | --- | --- | --- | --- |
| chr3:40774050-40780158 | | | 6126 bp | | |  |  |  |  |
| Genome Start | Genome End | Strand | Div | Repeat Start | Repeat End | Repeat Left | Name | Family | Class |
| 40774050 | 40774811 | + | 9.8 | 2167 | 2962 | -3184 | L1MA2 | L1 | LINE |
| 40774808 | 40775526 | - | 2.6 | 4457 | 5175 | -971 | L1PA3 | L1 | LINE |
| 40775523 | 40776511 | + | 1.9 | 5164 | 6153 | -2 | L1PA3 | L1 | LINE |
| 40776503 | 40780158 | + | 9.0 | 2931 | 6304 | 0 | L1MA2 | L1 | LINE |

  

|  |  |  |  |  |  |  |  |  |  |
| --- | --- | --- | --- | --- | --- | --- | --- | --- | --- |
| chr3:40943695-40951700 | | | 7644 bp | | |  |  |  |  |
| Genome Start | Genome End | Strand | Div | Repeat Start | Repeat End | Repeat Left | Name | Family | Class |
| 40943695 | 40944901 | - | 28.9 | 1160 | 2491 | -16 | HAL1 | L1 | LINE |
| 40945170 | 40946381 | + | 14.5 | 4134 | 5330 | -813 | L1M2 | L1 | LINE |
| 40946379 | 40949684 | + | 14.8 | 12 | 3615 | -3950 | L1M1 | L1 | LINE |
| 40949755 | 40951214 | + | 10.4 | 3616 | 5128 | -2437 | L1M1 | L1 | LINE |
| 40951214 | 40951319 | - | 9.9 | 5889 | 5989 | -302 | L1MA7 | L1 | LINE |
| 40951320 | 40951592 | + | 17.6 | 6011 | 6291 | 0 | L1MA8 | L1 | LINE |
| 40951621 | 40951700 | - | 22.8 | 735 | 826 | -1681 | HAL1 | L1 | LINE |

  

|  |  |  |  |  |  |  |  |  |  |
| --- | --- | --- | --- | --- | --- | --- | --- | --- | --- |
| chr3:43502444-43506062 | | | 3603 bp | | |  |  |  |  |
| Genome Start | Genome End | Strand | Div | Repeat Start | Repeat End | Repeat Left | Name | Family | Class |
| 43502444 | 43502794 | + | 15.6 | 1 | 361 | -234 | LTR49 | ERV1 | LTR |
| 43502795 | 43504316 | - | 2.0 | 3145 | 4661 | -1485 | L1PA3 | L1 | LINE |
| 43504318 | 43505830 | + | 2.1 | 4644 | 6154 | -1 | L1PA3 | L1 | LINE |
| 43505846 | 43506062 | + | 15.9 | 370 | 593 | -2 | LTR49 | ERV1 | LTR |

  

|  |  |  |  |  |  |  |  |  |  |
| --- | --- | --- | --- | --- | --- | --- | --- | --- | --- |
| chr3:50778060-50781743 | | | 3693 bp | | |  |  |  |  |
| Genome Start | Genome End | Strand | Div | Repeat Start | Repeat End | Repeat Left | Name | Family | Class |
| 50778060 | 50778562 | - | 10.6 | 5797 | 6300 | 0 | L1MA6 | L1 | LINE |
| 50778563 | 50780787 | - | 5.4 | 3918 | 6149 | -5 | L1PA5 | L1 | LINE |
| 50780779 | 50781035 | + | 3.1 | 3661 | 3917 | -2229 | L1PA5 | L1 | LINE |
| 50781036 | 50781743 | - | 12.8 | 5032 | 5811 | -489 | L1MA6 | L1 | LINE |

  

|  |  |  |  |  |  |  |  |  |  |
| --- | --- | --- | --- | --- | --- | --- | --- | --- | --- |
| chr3:50843243-50845129 | | | 1835 bp | | |  |  |  |  |
| Genome Start | Genome End | Strand | Div | Repeat Start | Repeat End | Repeat Left | Name | Family | Class |
| 50843243 | 50843620 | - | 8.7 | 164 | 548 | -86 | MER41B | ERV1 | LTR |
| 50843673 | 50844515 | - | 6.9 | 5304 | 6154 | 0 | L1PA7 | L1 | LINE |
| 50844514 | 50844955 | + | 7.7 | 4858 | 5299 | -847 | L1PA7 | L1 | LINE |
| 50844958 | 50845129 | - | 7.0 | 1 | 172 | -462 | MER41B | ERV1 | LTR |

  

|  |  |  |  |  |  |  |  |  |  |
| --- | --- | --- | --- | --- | --- | --- | --- | --- | --- |
| chr3:51072011-51075176 | | | 3121 bp | | |  |  |  |  |
| Genome Start | Genome End | Strand | Div | Repeat Start | Repeat End | Repeat Left | Name | Family | Class |
| 51072011 | 51072579 | + | 29.4 | 1360 | 1966 | -1453 | L2 | L2 | LINE |
| 51072580 | 51072633 | + | 0.0 | 6099 | 6152 | -3 | L1PA3 | L1 | LINE |
| 51072634 | 51072768 | + | 29.4 | 1967 | 2083 | -1336 | L2 | L2 | LINE |
| 51072783 | 51073292 | - | 5.1 | 4726 | 5236 | -910 | L1PA4 | L1 | LINE |
| 51073295 | 51074193 | + | 3.7 | 5254 | 6152 | -3 | L1PA4 | L1 | LINE |
| 51074215 | 51074329 | + | 23.5 | 2112 | 2228 | -1191 | L2 | L2 | LINE |
| 51074338 | 51075176 | + | 26.6 | 2437 | 3419 | 0 | L2 | L2 | LINE |

  

|  |  |  |  |  |  |  |  |  |  |
| --- | --- | --- | --- | --- | --- | --- | --- | --- | --- |
| chr3:51923841-51935992 | | | 11905 bp | | |  |  |  |  |
| Genome Start | Genome End | Strand | Div | Repeat Start | Repeat End | Repeat Left | Name | Family | Class |
| 51923841 | 51923945 | + | 19.4 | 38 | 149 | -6398 | L1MD2 | L1 | LINE |
| 51923946 | 51924254 | + | 8.7 | 1 | 311 | -2 | AluSp | Alu | SINE |
| 51924359 | 51924838 | + | 30.0 | 294 | 789 | -5758 | L1MD2 | L1 | LINE |
| 51924839 | 51925433 | + | 11.7 | 3 | 610 | 0 | MER4B | ERV1 | LTR |
| 51925434 | 51925835 | + | 27.3 | 790 | 1236 | -5404 | L1MD2 | L1 | LINE |
| 51925836 | 51926138 | - | 12.0 | 1 | 301 | -12 | AluSq | Alu | SINE |
| 51926139 | 51926302 | + | 25.1 | 1237 | 1340 | -5300 | L1MD2 | L1 | LINE |
| 51926303 | 51926604 | + | 5.8 | 2 | 297 | -14 | AluY | Alu | SINE |
| 51926605 | 51926989 | + | 25.1 | 1341 | 1738 | -4902 | L1MD2 | L1 | LINE |
| 51927013 | 51927970 | + | 25.1 | 1739 | 2708 | -3836 | L1MD2 | L1 | LINE |
| 51927990 | 51928234 | + | 21.2 | 348 | 618 | -6127 | L1ME1 | L1 | LINE |
| 51928225 | 51928734 | + | 22.6 | 5766 | 6278 | -306 | L1ME1 | L1 | LINE |
| 51928763 | 51929063 | + | 6.8 | 1 | 293 | -16 | AluSc | Alu | SINE |
| 51929103 | 51929359 | - | 6.2 | 1 | 295 | -16 | AluY | Alu | SINE |
| 51929360 | 51929426 | - | 10.9 | 6112 | 6182 | -118 | L1MA4 | L1 | LINE |
| 51929427 | 51929730 | - | 11.0 | 1 | 301 | -11 | AluSx | Alu | SINE |
| 51929731 | 51930002 | - | 10.9 | 5841 | 6111 | -189 | L1MA4 | L1 | LINE |
| 51930006 | 51930125 | + | 11.7 | 6176 | 6295 | -5 | L1MA4 | L1 | LINE |
| 51930130 | 51930293 | + | 23.3 | 6316 | 6461 | -123 | L1ME1 | L1 | LINE |
| 51930310 | 51930590 | + | 18.0 | 1 | 301 | -11 | AluJb | Alu | SINE |
| 51930592 | 51930685 | + | 16.0 | 848 | 1019 | -5732 | L1MEc | L1 | LINE |
| 51930686 | 51930983 | - | 16.5 | 1 | 302 | -10 | AluJo | Alu | SINE |
| 51930984 | 51931753 | + | 17.7 | 928 | 1832 | -4919 | L1MEc | L1 | LINE |
| 51931759 | 51931795 | - | 10.8 | 6 | 42 | -260 | Alu | Alu | SINE |
| 51931798 | 51932092 | + | 18.0 | 1 | 310 | -2 | AluJo | Alu | SINE |
| 51932093 | 51932500 | + | 22.2 | 3478 | 3898 | -2248 | L1MD2 | L1 | LINE |
| 51932501 | 51932808 | + | 11.8 | 1 | 309 | 0 | AluSx | Alu | SINE |
| 51932822 | 51933123 | + | 11.3 | 1 | 304 | -8 | AluSx | Alu | SINE |
| 51933124 | 51933186 | + | 22.2 | 3899 | 3958 | -2188 | L1MD2 | L1 | LINE |
| 51933187 | 51933483 | + | 9.1 | 1 | 298 | -11 | AluSg1 | Alu | SINE |
| 51933484 | 51934643 | + | 22.2 | 3959 | 5175 | -971 | L1MD2 | L1 | LINE |
| 51934644 | 51934938 | + | 8.8 | 1 | 301 | -12 | AluSp | Alu | SINE |
| 51934939 | 51935667 | + | 20.7 | 5176 | 5930 | -429 | L1MD2 | L1 | LINE |
| 51935668 | 51935969 | - | 6.6 | 1 | 301 | -10 | AluY | Alu | SINE |
| 51935970 | 51935992 | + | 20.5 | 5931 | 5946 | -413 | L1MD2 | L1 | LINE |

  

|  |  |  |  |  |  |  |  |  |  |
| --- | --- | --- | --- | --- | --- | --- | --- | --- | --- |
| chr3:53399134-53408030 | | | 8682 bp | | |  |  |  |  |
| Genome Start | Genome End | Strand | Div | Repeat Start | Repeat End | Repeat Left | Name | Family | Class |
| 53399134 | 53399322 | + | 23.5 | 6668 | 6851 | -1110 | L1MC5 | L1 | LINE |
| 53399344 | 53399464 | - | 15.7 | 2 | 123 | -20 | FLAM\_C | Alu | SINE |
| 53399593 | 53399901 | - | 12.6 | 1 | 311 | -2 | AluSq | Alu | SINE |
| 53399934 | 53400188 | - | 13.8 | 1 | 224 | -88 | AluSx | Alu | SINE |
| 53400223 | 53406351 | + | 7.3 | 4 | 6151 | -3 | L1PA6 | L1 | LINE |
| 53406369 | 53406479 | + | 31.8 | 6955 | 7065 | -896 | L1MC5 | L1 | LINE |
| 53406479 | 53407491 | - | 5.5 | 5140 | 6154 | 0 | L1PA6 | L1 | LINE |
| 53407477 | 53407599 | + | 6.5 | 5007 | 5132 | -1014 | L1PA6 | L1 | LINE |
| 53407599 | 53408030 | + | 24.9 | 7051 | 7485 | -476 | L1MC5 | L1 | LINE |

  

|  |  |  |  |  |  |  |  |  |  |
| --- | --- | --- | --- | --- | --- | --- | --- | --- | --- |
| chr3:56488253-56491929 | | | 3183 bp | | |  |  |  |  |
| Genome Start | Genome End | Strand | Div | Repeat Start | Repeat End | Repeat Left | Name | Family | Class |
| 56488253 | 56488420 | - | 31.3 | 4220 | 4387 | -102 | L3 | CR1 | LINE |
| 56488479 | 56488617 | + | 25.6 | 81 | 236 | -32 | MIRb | MIR | SINE |
| 56488643 | 56488685 | - | 23.3 | 4159 | 4201 | -288 | L3 | CR1 | LINE |
| 56488702 | 56489443 | - | 2.8 | 5431 | 6155 | 0 | L1PA4 | L1 | LINE |
| 56489444 | 56490097 | + | 4.3 | 4775 | 5428 | -727 | L1PA4 | L1 | LINE |
| 56490247 | 56490351 | + | 14.3 | 28 | 132 | -10 | FLAM\_A | Alu | SINE |
| 56490549 | 56490687 | - | 28.1 | 5970 | 6115 | -6 | L1ME4a | L1 | LINE |
| 56490715 | 56490880 | - | 24.5 | 5 | 180 | -331 | MLT1J | MaLR | LTR |
| 56490891 | 56491059 | - | 28.0 | 5582 | 5755 | -366 | L1MC4a | L1 | LINE |
| 56491061 | 56491212 | - | 31.4 | 3567 | 3768 | -721 | L3 | CR1 | LINE |
| 56491213 | 56491526 | + | 16.3 | 1 | 312 | 0 | AluJb | Alu | SINE |
| 56491527 | 56491654 | - | 31.4 | 3432 | 3566 | -923 | L3 | CR1 | LINE |
| 56491660 | 56491753 | - | 34.0 | 3274 | 3372 | -6 | L2 | L2 | LINE |
| 56491760 | 56491929 | - | 31.7 | 3259 | 3433 | -1056 | L3 | CR1 | LINE |

  

|  |  |  |  |  |  |  |  |  |  |
| --- | --- | --- | --- | --- | --- | --- | --- | --- | --- |
| chr3:67883931-67885180 | | | 1244 bp | | |  |  |  |  |
| Genome Start | Genome End | Strand | Div | Repeat Start | Repeat End | Repeat Left | Name | Family | Class |
| 67883931 | 67883982 | - | 5.9 | 30 | 80 | 0 | MADE1 | Mariner | DNA |
| 67883983 | 67884062 | - | 7.6 | 4972 | 5050 | -1096 | L1PB1 | L1 | LINE |
| 67884063 | 67885134 | + | 10.6 | 5065 | 6138 | -13 | L1PB1 | L1 | LINE |
| 67885141 | 67885180 | - | 12.5 | 4 | 43 | -37 | MADE1 | Mariner | DNA |

  

|  |  |  |  |  |  |  |  |  |  |
| --- | --- | --- | --- | --- | --- | --- | --- | --- | --- |
| chr3:68941344-68951356 | | | 10025 bp | | |  |  |  |  |
| Genome Start | Genome End | Strand | Div | Repeat Start | Repeat End | Repeat Left | Name | Family | Class |
| 68941344 | 68942033 | - | 14.7 | 4531 | 5201 | -942 | L1M2 | L1 | LINE |
| 68942032 | 68942064 | - | 3.0 | 7 | 39 | -6116 | L1PA4 | L1 | LINE |
| 68942060 | 68948166 | + | 5.5 | 33 | 6154 | -1 | L1PA4 | L1 | LINE |
| 68948162 | 68948530 | - | 17.2 | 4160 | 4545 | -1598 | L1M2 | L1 | LINE |
| 68948531 | 68950829 | - | 13.0 | 49 | 2418 | 0 | Tigger1 | MER2\_type | DNA |
| 68950830 | 68951140 | + | 17.6 | 1 | 310 | -2 | AluSx | Alu | SINE |
| 68951141 | 68951189 | - | 13.0 | 1 | 48 | -2370 | Tigger1 | MER2\_type | DNA |
| 68951190 | 68951356 | - | 17.2 | 3984 | 4159 | -1984 | L1M2 | L1 | LINE |

  

|  |  |  |  |  |  |  |  |  |  |
| --- | --- | --- | --- | --- | --- | --- | --- | --- | --- |
| chr3:74889603-74892560 | | | 2929 bp | | |  |  |  |  |
| Genome Start | Genome End | Strand | Div | Repeat Start | Repeat End | Repeat Left | Name | Family | Class |
| 74889603 | 74890086 | - | 26.8 | 1112 | 1599 | -4577 | L1MEb | L1 | LINE |
| 74890075 | 74890389 | - | 8.0 | 4321 | 4633 | -1513 | L1PB1 | L1 | LINE |
| 74890379 | 74891869 | + | 9.2 | 4635 | 6151 | 0 | L1PB1 | L1 | LINE |
| 74891922 | 74892560 | - | 29.8 | 447 | 1089 | -4981 | L1MEb | L1 | LINE |

  

|  |  |  |  |  |  |  |  |  |  |
| --- | --- | --- | --- | --- | --- | --- | --- | --- | --- |
| chr3:75084583-75088115 | | | 3405 bp | | |  |  |  |  |
| Genome Start | Genome End | Strand | Div | Repeat Start | Repeat End | Repeat Left | Name | Family | Class |
| 75084583 | 75084672 | + | 24.5 | 2673 | 2764 | -3382 | L1MD | L1 | LINE |
| 75084673 | 75084982 | + | 7.1 | 1 | 311 | 0 | AluY | Alu | SINE |
| 75084983 | 75087073 | + | 24.7 | 2756 | 4655 | -1491 | L1MD | L1 | LINE |
| 75087074 | 75087318 | - | 1.2 | 5430 | 5674 | -481 | L1HS | L1 | LINE |
| 75087319 | 75087810 | + | 0.6 | 5665 | 6155 | 0 | L1HS | L1 | LINE |
| 75087939 | 75088115 | + | 24.0 | 4730 | 4911 | -1235 | L1MD | L1 | LINE |

  

|  |  |  |  |  |  |  |  |  |  |
| --- | --- | --- | --- | --- | --- | --- | --- | --- | --- |
| chr3:75649985-75650923 | | | 931 bp | | |  |  |  |  |
| Genome Start | Genome End | Strand | Div | Repeat Start | Repeat End | Repeat Left | Name | Family | Class |
| 75649985 | 75650110 | + | 19.5 | 5 | 128 | -50 | MER5B | MER1\_type | DNA |
| 75650129 | 75650431 | - | 14.5 | 2 | 304 | -8 | AluSx | Alu | SINE |
| 75650437 | 75650718 | - | 18.0 | 5902 | 6174 | 0 | L1MB5 | L1 | LINE |
| 75650689 | 75650864 | + | 19.4 | 5684 | 5887 | -297 | L1MB5 | L1 | LINE |
| 75650880 | 75650923 | + | 11.4 | 135 | 178 | 0 | MER5B | MER1\_type | DNA |

  

|  |  |  |  |  |  |  |  |  |  |
| --- | --- | --- | --- | --- | --- | --- | --- | --- | --- |
| chr3:76303703-76304690 | | | 855 bp | | |  |  |  |  |
| Genome Start | Genome End | Strand | Div | Repeat Start | Repeat End | Repeat Left | Name | Family | Class |
| 76303703 | 76303821 | + | 33.0 | 323 | 445 | -5625 | L1MEb | L1 | LINE |
| 76303940 | 76304028 | - | 4.5 | 5807 | 5894 | -261 | L1PA5 | L1 | LINE |
| 76304032 | 76304286 | + | 4.3 | 5895 | 6154 | 0 | L1PA5 | L1 | LINE |
| 76304299 | 76304690 | + | 31.3 | 484 | 881 | -5189 | L1MEb | L1 | LINE |

  

|  |  |  |  |  |  |  |  |  |  |
| --- | --- | --- | --- | --- | --- | --- | --- | --- | --- |
| chr3:77533479-77534869 | | | 1386 bp | | |  |  |  |  |
| Genome Start | Genome End | Strand | Div | Repeat Start | Repeat End | Repeat Left | Name | Family | Class |
| 77533479 | 77533617 | + | 18.7 | 2 | 136 | -598 | MER50 | ERV1 | LTR |
| 77533618 | 77533769 | - | 5.3 | 5494 | 5644 | -510 | L1PA7 | L1 | LINE |
| 77533770 | 77534289 | + | 7.2 | 5642 | 6154 | 0 | L1PA7 | L1 | LINE |
| 77534295 | 77534869 | + | 17.3 | 117 | 697 | -37 | MER50 | ERV1 | LTR |

  

|  |  |  |  |  |  |  |  |  |  |
| --- | --- | --- | --- | --- | --- | --- | --- | --- | --- |
| chr3:79131880-79133308 | | | 1429 bp | | |  |  |  |  |
| Genome Start | Genome End | Strand | Div | Repeat Start | Repeat End | Repeat Left | Name | Family | Class |
| 79131880 | 79132098 | - | 11.4 | 153 | 375 | 0 | THE1C | MaLR | LTR |
| 79132099 | 79132277 | - | 6.2 | 5132 | 5319 | -827 | L1PA8 | L1 | LINE |
| 79132275 | 79133136 | + | 4.9 | 5303 | 6167 | -5 | L1PA8 | L1 | LINE |
| 79133140 | 79133308 | - | 8.3 | 2 | 170 | -205 | THE1C | MaLR | LTR |

  

|  |  |  |  |  |  |  |  |  |  |
| --- | --- | --- | --- | --- | --- | --- | --- | --- | --- |
| chr3:80160019-80161788 | | | 1748 bp | | |  |  |  |  |
| Genome Start | Genome End | Strand | Div | Repeat Start | Repeat End | Repeat Left | Name | Family | Class |
| 80160019 | 80160307 | - | 30.4 | 3699 | 4012 | -2134 | L1ME3B | L1 | LINE |
| 80160329 | 80161365 | - | 6.5 | 5098 | 6136 | -18 | L1PA7 | L1 | LINE |
| 80161369 | 80161719 | + | 10.8 | 4746 | 5096 | -1050 | L1PA7 | L1 | LINE |
| 80161718 | 80161788 | - | 22.5 | 3633 | 3707 | -2439 | L1ME3B | L1 | LINE |

  

|  |  |  |  |  |  |  |  |  |  |
| --- | --- | --- | --- | --- | --- | --- | --- | --- | --- |
| chr3:81156893-81158609 | | | 1637 bp | | |  |  |  |  |
| Genome Start | Genome End | Strand | Div | Repeat Start | Repeat End | Repeat Left | Name | Family | Class |
| 81156893 | 81157923 | + | 15.7 | 5113 | 6140 | -151 | L1MA8 | L1 | LINE |
| 81157921 | 81158015 | - | 5.3 | 6006 | 6100 | -55 | L1PA4 | L1 | LINE |
| 81158019 | 81158058 | + | 0.0 | 6109 | 6148 | -7 | L1PA4 | L1 | LINE |
| 81158138 | 81158448 | + | 8.6 | 1 | 303 | -8 | AluY | Alu | SINE |
| 81158450 | 81158609 | + | 18.6 | 6131 | 6291 | 0 | L1MA8 | L1 | LINE |

  

|  |  |  |  |  |  |  |  |  |  |
| --- | --- | --- | --- | --- | --- | --- | --- | --- | --- |
| chr3:81655195-81664143 | | | 8884 bp | | |  |  |  |  |
| Genome Start | Genome End | Strand | Div | Repeat Start | Repeat End | Repeat Left | Name | Family | Class |
| 81655195 | 81656811 | - | 12.1 | 6009 | 7719 | -5 | L1MA3 | L1 | LINE |
| 81656850 | 81657047 | - | 14.1 | 4121 | 4319 | -3246 | L1MA3 | L1 | LINE |
| 81657028 | 81658084 | - | 5.9 | 2940 | 3996 | -2150 | L1PA4 | L1 | LINE |
| 81658079 | 81660225 | + | 4.0 | 4007 | 6152 | -3 | L1PA4 | L1 | LINE |
| 81660259 | 81661759 | - | 16.8 | 2539 | 4131 | -3434 | L1MA3 | L1 | LINE |
| 81661780 | 81664143 | - | 17.5 | 25 | 2538 | -5027 | L1MA3 | L1 | LINE |

  

|  |  |  |  |  |  |  |  |  |  |
| --- | --- | --- | --- | --- | --- | --- | --- | --- | --- |
| chr3:82430725-82433527 | | | 2756 bp | | |  |  |  |  |
| Genome Start | Genome End | Strand | Div | Repeat Start | Repeat End | Repeat Left | Name | Family | Class |
| 82430725 | 82431274 | + | 15.1 | 20 | 586 | -716 | HSMAR2 | Mariner | DNA |
| 82431323 | 82433136 | - | 2.3 | 4338 | 6155 | 0 | L1PA3 | L1 | LINE |
| 82433136 | 82433458 | + | 2.5 | 4003 | 4325 | -1821 | L1PA3 | L1 | LINE |
| 82433459 | 82433527 | + | 11.6 | 589 | 657 | -645 | HSMAR2 | Mariner | DNA |

  

|  |  |  |  |  |  |  |  |  |  |
| --- | --- | --- | --- | --- | --- | --- | --- | --- | --- |
| chr3:82564452-82568854 | | | 4382 bp | | |  |  |  |  |
| Genome Start | Genome End | Strand | Div | Repeat Start | Repeat End | Repeat Left | Name | Family | Class |
| 82564452 | 82565090 | + | 25.8 | 1026 | 1794 | -4721 | L1M3d | L1 | LINE |
| 82565093 | 82565219 | + | 14.7 | 2357 | 2484 | -3892 | L1M3d | L1 | LINE |
| 82565220 | 82565531 | + | 14.2 | 1 | 310 | -3 | AluSq | Alu | SINE |
| 82565532 | 82565609 | + | 14.7 | 2485 | 2551 | -3825 | L1M3d | L1 | LINE |
| 82565608 | 82566681 | - | 11.5 | 4270 | 5337 | -809 | L1P | L1 | LINE |
| 82566681 | 82566699 | + | 12.1 | 5332 | 5350 | -813 | L1P | L1 | LINE |
| 82566700 | 82567043 | + | 14.4 | 1 | 364 | 0 | THE1B | MaLR | LTR |
| 82567044 | 82567866 | + | 12.1 | 5351 | 6163 | 0 | L1PA13 | L1 | LINE |
| 82567865 | 82568061 | + | 19.0 | 2540 | 2691 | -3685 | L1M3d | L1 | LINE |
| 82568095 | 82568655 | + | 19.0 | 2692 | 3261 | -3115 | L1M3d | L1 | LINE |
| 82568647 | 82568854 | + | 22.0 | 3595 | 3810 | -2566 | L1M3d | L1 | LINE |

  

|  |  |  |  |  |  |  |  |  |  |
| --- | --- | --- | --- | --- | --- | --- | --- | --- | --- |
| chr3:83200544-83210234 | | | 9221 bp | | |  |  |  |  |
| Genome Start | Genome End | Strand | Div | Repeat Start | Repeat End | Repeat Left | Name | Family | Class |
| 83200544 | 83200915 | + | 29.8 | 2637 | 3032 | -3114 | L1M5 | L1 | LINE |
| 83201052 | 83201456 | - | 11.6 | 5452 | 5858 | -310 | L1PA13 | L1 | LINE |
| 83201462 | 83201755 | + | 12.9 | 5862 | 6156 | -7 | L1PA13 | L1 | LINE |
| 83201879 | 83206334 | + | 21.5 | 3 | 4408 | -1917 | L1M3f | L1 | LINE |
| 83206339 | 83207543 | + | 14.0 | 4922 | 6163 | -3 | L1PA16 | L1 | LINE |
| 83207543 | 83208065 | + | 18.9 | 4490 | 4994 | -1328 | L1M3f | L1 | LINE |
| 83208066 | 83208224 | - | 25.2 | 5 | 163 | -13 | FRAM | Alu | SINE |
| 83208225 | 83208514 | + | 18.9 | 4995 | 5274 | -1048 | L1M3f | L1 | LINE |
| 83208548 | 83209560 | + | 20.2 | 5276 | 6202 | -280 | L1MA4 | L1 | LINE |
| 83209575 | 83209624 | + | 18.0 | 6099 | 6148 | -32 | L1MB4 | L1 | LINE |
| 83209781 | 83210234 | + | 29.7 | 5333 | 5817 | -347 | L1M5 | L1 | LINE |

  

|  |  |  |  |  |  |  |  |  |  |
| --- | --- | --- | --- | --- | --- | --- | --- | --- | --- |
| chr3:83416932-83424941 | | | 7583 bp | | |  |  |  |  |
| Genome Start | Genome End | Strand | Div | Repeat Start | Repeat End | Repeat Left | Name | Family | Class |
| 83416932 | 83417047 | - | 23.0 | 4695 | 4812 | -1375 | MER34B-int | ERV1 | LTR |
| 83417048 | 83417407 | - | 20.6 | 1 | 408 | 0 | MLT1A1 | MaLR | LTR |
| 83417408 | 83417492 | - | 23.0 | 4612 | 4694 | -1493 | MER34B-int | ERV1 | LTR |
| 83417520 | 83417754 | - | 27.7 | 4390 | 4622 | -1565 | MER34B-int | ERV1 | LTR |
| 83417845 | 83419777 | - | 23.1 | 2455 | 4273 | -2 | MER34B-int | ERV1 | LTR |
| 83419778 | 83419896 | - | 11.8 | 1 | 124 | -348 | MER4A1 | ERV1 | LTR |
| 83419959 | 83420307 | + | 12.0 | 120 | 472 | 0 | MER4A1 | ERV1 | LTR |
| 83420311 | 83420514 | + | 11.3 | 1 | 232 | -6366 | MER4A1-int | ERV1 | LTR |
| 83420629 | 83421920 | - | 8.5 | 2514 | 3800 | -2346 | L1PA6 | L1 | LINE |
| 83421920 | 83424236 | + | 7.3 | 3814 | 6147 | -7 | L1PA6 | L1 | LINE |
| 83424369 | 83424410 | - | 28.2 | 2175 | 2214 | -4425 | MER34B-int | ERV1 | LTR |
| 83424411 | 83424701 | - | 15.5 | 1 | 291 | -21 | AluSx | Alu | SINE |
| 83424702 | 83424941 | - | 28.2 | 1942 | 2174 | -4465 | MER34B-int | ERV1 | LTR |

  

|  |  |  |  |  |  |  |  |  |  |
| --- | --- | --- | --- | --- | --- | --- | --- | --- | --- |
| chr3:84838987-84848446 | | | 9450 bp | | |  |  |  |  |
| Genome Start | Genome End | Strand | Div | Repeat Start | Repeat End | Repeat Left | Name | Family | Class |
| 84838987 | 84840049 | + | 24.8 | 287 | 1340 | -5809 | L1PA16 | L1 | LINE |
| 84840050 | 84840214 | + | 21.5 | 258 | 426 | 0 | MSTB | MaLR | LTR |
| 84840216 | 84841966 | + | 20.8 | 2393 | 4187 | -2662 | L1PA16 | L1 | LINE |
| 84841964 | 84842724 | - | 8.6 | 3401 | 4175 | -2674 | L1PB1 | L1 | LINE |
| 84842724 | 84845399 | + | 10.1 | 4167 | 6853 | -1 | L1PB1 | L1 | LINE |
| 84845413 | 84847044 | + | 15.5 | 4177 | 5848 | -1001 | L1PA16 | L1 | LINE |
| 84847045 | 84847448 | + | 13.8 | 1 | 428 | 0 | MSTA | MaLR | LTR |
| 84847449 | 84848446 | + | 15.4 | 5849 | 6859 | -10 | L1PA16 | L1 | LINE |

  

|  |  |  |  |  |  |  |  |  |  |
| --- | --- | --- | --- | --- | --- | --- | --- | --- | --- |
| chr3:86074042-86079075 | | | 5036 bp | | |  |  |  |  |
| Genome Start | Genome End | Strand | Div | Repeat Start | Repeat End | Repeat Left | Name | Family | Class |
| 86074042 | 86074163 | + | 9.0 | 2104 | 2245 | -215 | L1PA10 | L1 | LINE |
| 86074174 | 86075439 | - | 2.8 | 4892 | 6155 | 0 | L1PA2 | L1 | LINE |
| 86075430 | 86075655 | + | 2.6 | 4682 | 4907 | -1239 | L1PA2 | L1 | LINE |
| 86075654 | 86079075 | + | 5.5 | 2230 | 5616 | -724 | L1PA10 | L1 | LINE |

  

|  |  |  |  |  |  |  |  |  |  |
| --- | --- | --- | --- | --- | --- | --- | --- | --- | --- |
| chr3:86259668-86265788 | | | 6020 bp | | |  |  |  |  |
| Genome Start | Genome End | Strand | Div | Repeat Start | Repeat End | Repeat Left | Name | Family | Class |
| 86259668 | 86261458 | + | 19.8 | 1768 | 3604 | -2110 | ERVL-B4 | ERVL | LTR |
| 86261559 | 86261971 | + | 19.4 | 3581 | 4007 | -1707 | ERVL-B4 | ERVL | LTR |
| 86261972 | 86262854 | - | 5.7 | 4114 | 4998 | -1148 | L1P1 | L1 | LINE |
| 86262855 | 86263923 | - | 9.4 | 1 | 1071 | 0 | MER11C | ERVK | LTR |
| 86263924 | 86264279 | - | 5.7 | 3758 | 4113 | -2033 | L1P1 | L1 | LINE |
| 86264276 | 86265437 | + | 4.8 | 4988 | 6153 | -1 | L1PA5 | L1 | LINE |
| 86265443 | 86265788 | + | 24.0 | 4002 | 4357 | -1357 | ERVL-B4 | ERVL | LTR |

  

|  |  |  |  |  |  |  |  |  |  |
| --- | --- | --- | --- | --- | --- | --- | --- | --- | --- |
| chr3:86314991-86322584 | | | 7601 bp | | |  |  |  |  |
| Genome Start | Genome End | Strand | Div | Repeat Start | Repeat End | Repeat Left | Name | Family | Class |
| 86314991 | 86318866 | + | 22.2 | 9 | 3952 | -2531 | L1PA15 | L1 | LINE |
| 86318861 | 86319005 | - | 10.3 | 5207 | 5351 | -795 | L1PA7 | L1 | LINE |
| 86318998 | 86319776 | + | 6.0 | 5364 | 6152 | -2 | L1PA7 | L1 | LINE |
| 86319784 | 86320077 | + | 16.4 | 3942 | 4234 | -2249 | L1PA15 | L1 | LINE |
| 86320078 | 86320370 | - | 7.5 | 1 | 293 | -18 | AluY | Alu | SINE |
| 86320371 | 86322584 | + | 16.5 | 4235 | 6499 | -3 | L1PA15 | L1 | LINE |

  

|  |  |  |  |  |  |  |  |  |  |
| --- | --- | --- | --- | --- | --- | --- | --- | --- | --- |
| chr3:86418978-86422504 | | | 3009 bp | | |  |  |  |  |
| Genome Start | Genome End | Strand | Div | Repeat Start | Repeat End | Repeat Left | Name | Family | Class |
| 86418978 | 86419179 | + | 35.3 | 1985 | 2202 | -1217 | L2 | L2 | LINE |
| 86419180 | 86419477 | - | 12.1 | 1 | 298 | -14 | AluSx | Alu | SINE |
| 86419478 | 86419980 | + | 35.3 | 2203 | 2736 | -683 | L2 | L2 | LINE |
| 86420128 | 86420404 | + | 29.9 | 115 | 427 | -23 | MLT1J2 | MaLR | LTR |
| 86420437 | 86420759 | + | 24.2 | 2119 | 2757 | -3790 | L1MEc | L1 | LINE |
| 86420760 | 86420882 | + | 18.6 | 6034 | 6151 | -15 | L1PA17 | L1 | LINE |
| 86421111 | 86421171 | + | 23.3 | 10 | 69 | -120 | MER5A | MER1\_type | DNA |
| 86421223 | 86421636 | + | 36.3 | 2910 | 3326 | -93 | L2 | L2 | LINE |
| 86421637 | 86421967 | - | 2.1 | 5560 | 5898 | -257 | L1PA2 | L1 | LINE |
| 86421968 | 86422211 | + | 5.3 | 5911 | 6154 | -1 | L1PA2 | L1 | LINE |
| 86422251 | 86422441 | + | 12.6 | 184 | 378 | -48 | MSTB | MaLR | LTR |
| 86422463 | 86422504 | + | 26.2 | 3343 | 3384 | -35 | L2 | L2 | LINE |

  

|  |  |  |  |  |  |  |  |  |  |
| --- | --- | --- | --- | --- | --- | --- | --- | --- | --- |
| chr3:86806859-86831031 | | | 23844 bp | | |  |  |  |  |
| Genome Start | Genome End | Strand | Div | Repeat Start | Repeat End | Repeat Left | Name | Family | Class |
| 86806859 | 86807032 | - | 24.0 | 3576 | 3754 | -2375 | L1MEe | L1 | LINE |
| 86807056 | 86807312 | - | 14.8 | 6034 | 6299 | -6 | L1MA3 | L1 | LINE |
| 86807313 | 86807696 | + | 16.4 | 1 | 428 | 0 | MSTA | MaLR | LTR |
| 86807697 | 86807944 | + | 11.3 | 1 | 249 | -1402 | MSTA-int | MaLR | LTR |
| 86807972 | 86808244 | - | 16.4 | 5771 | 6039 | -266 | L1MA3 | L1 | LINE |
| 86808246 | 86809082 | - | 14.9 | 5327 | 6163 | -3 | L1PA16 | L1 | LINE |
| 86809083 | 86809381 | - | 11.7 | 9 | 308 | -2 | AluSg | Alu | SINE |
| 86809382 | 86813359 | - | 16.2 | 1631 | 5663 | -820 | L1P4a | L1 | LINE |
| 86813360 | 86813658 | - | 20.6 | 13 | 308 | -4 | AluJo | Alu | SINE |
| 86813659 | 86815257 | - | 20.0 | 3 | 1630 | -4873 | L1P4a | L1 | LINE |
| 86815279 | 86815721 | - | 29.0 | 3069 | 3538 | -2591 | L1MEe | L1 | LINE |
| 86815786 | 86816467 | - | 25.6 | 5646 | 6342 | -17 | L1MD2 | L1 | LINE |
| 86816468 | 86816810 | + | 25.7 | 4 | 374 | 0 | MLT1A | MaLR | LTR |
| 86816811 | 86816853 | - | 25.6 | 5611 | 5645 | -714 | L1MD2 | L1 | LINE |
| 86816859 | 86817041 | + | 24.9 | 4946 | 5125 | -1021 | L1MD | L1 | LINE |
| 86817042 | 86817352 | + | 9.0 | 1 | 310 | -1 | AluY | Alu | SINE |
| 86817353 | 86817804 | + | 23.0 | 5126 | 5594 | -765 | L1MD | L1 | LINE |
| 86817860 | 86818076 | - | 32.8 | 1535 | 1751 | -4398 | L1MEe | L1 | LINE |
| 86818077 | 86818196 | + | 13.2 | 1 | 120 | -190 | AluSg | Alu | SINE |
| 86818232 | 86818398 | + | 13.2 | 121 | 298 | -12 | AluSg | Alu | SINE |
| 86818399 | 86818446 | - | 32.8 | 1497 | 1534 | -4615 | L1MEe | L1 | LINE |
| 86818515 | 86820680 | + | 15.1 | 808 | 3017 | -4548 | L1MA1 | L1 | LINE |
| 86820668 | 86823391 | + | 17.6 | 3475 | 6134 | -1428 | L1MA1 | L1 | LINE |
| 86823392 | 86824089 | - | 10.2 | 1 | 722 | 0 | LTR30 | ERV1 | LTR |
| 86824090 | 86824554 | - | 10.0 | 7797 | 8257 | 0 | HERV30 | ERV1 | LTR |
| 86824551 | 86828159 | - | 11.3 | 1 | 3621 | -4636 | HERV30 | ERV1 | LTR |
| 86828161 | 86828863 | - | 9.6 | 1 | 721 | -1 | LTR30 | ERV1 | LTR |
| 86828875 | 86830349 | + | 10.3 | 6223 | 7711 | -10 | L1MA1 | L1 | LINE |
| 86830385 | 86830492 | - | 31.3 | 1085 | 1193 | -4926 | L1MEe | L1 | LINE |
| 86830493 | 86830801 | - | 6.5 | 1 | 308 | -3 | AluY | Alu | SINE |
| 86830802 | 86831031 | - | 31.3 | 829 | 1084 | -5035 | L1MEe | L1 | LINE |

  

|  |  |  |  |  |  |  |  |  |  |
| --- | --- | --- | --- | --- | --- | --- | --- | --- | --- |
| chr3:87144008-87145519 | | | 1429 bp | | |  |  |  |  |
| Genome Start | Genome End | Strand | Div | Repeat Start | Repeat End | Repeat Left | Name | Family | Class |
| 87144008 | 87144111 | + | 28.4 | 82 | 184 | -225 | MLT1I | MaLR | LTR |
| 87144112 | 87144184 | - | 11.0 | 5162 | 5237 | -909 | L1MC1 | L1 | LINE |
| 87144177 | 87145281 | + | 15.6 | 5230 | 6331 | -2 | L1MC1 | L1 | LINE |
| 87145373 | 87145519 | + | 30.1 | 240 | 393 | -16 | MLT1I | MaLR | LTR |

  

|  |  |  |  |  |  |  |  |  |  |
| --- | --- | --- | --- | --- | --- | --- | --- | --- | --- |
| chr3:88682621-88686803 | | | 4142 bp | | |  |  |  |  |
| Genome Start | Genome End | Strand | Div | Repeat Start | Repeat End | Repeat Left | Name | Family | Class |
| 88682621 | 88682845 | + | 6.7 | 4391 | 4615 | -1531 | L1PB1 | L1 | LINE |
| 88682843 | 88683700 | - | 2.5 | 3798 | 4655 | -1491 | L1PA3 | L1 | LINE |
| 88683698 | 88685239 | + | 2.8 | 4636 | 6178 | 0 | L1PA3 | L1 | LINE |
| 88685240 | 88686409 | + | 10.7 | 4602 | 5766 | -385 | L1PB1 | L1 | LINE |
| 88686457 | 88686803 | + | 11.8 | 5767 | 6147 | -4 | L1PB1 | L1 | LINE |

  

|  |  |  |  |  |  |  |  |  |  |
| --- | --- | --- | --- | --- | --- | --- | --- | --- | --- |
| chr3:90028208-90034629 | | | 6363 bp | | |  |  |  |  |
| Genome Start | Genome End | Strand | Div | Repeat Start | Repeat End | Repeat Left | Name | Family | Class |
| 90028208 | 90028325 | - | 14.5 | 1043 | 1159 | -72 | Tigger3b | MER2\_type | DNA |
| 90028390 | 90031946 | - | 6.0 | 2578 | 6146 | -8 | L1PA5 | L1 | LINE |
| 90031942 | 90033825 | + | 7.7 | 689 | 2589 | -3557 | L1PA5 | L1 | LINE |
| 90033826 | 90034629 | - | 13.9 | 132 | 959 | -272 | Tigger3b | MER2\_type | DNA |

  

|  |  |  |  |  |  |  |  |  |  |
| --- | --- | --- | --- | --- | --- | --- | --- | --- | --- |
| chr3:95749916-95757501 | | | 7436 bp | | |  |  |  |  |
| Genome Start | Genome End | Strand | Div | Repeat Start | Repeat End | Repeat Left | Name | Family | Class |
| 95749916 | 95752313 | + | 24.3 | 1 | 2463 | -4040 | L1PA15 | L1 | LINE |
| 95752312 | 95752349 | + | 7.9 | 5450 | 5487 | -685 | L1PA7 | L1 | LINE |
| 95752359 | 95753206 | - | 5.5 | 2360 | 3210 | -2936 | L1PA4 | L1 | LINE |
| 95753200 | 95756120 | + | 5.6 | 3215 | 6144 | -11 | L1PA4 | L1 | LINE |
| 95756261 | 95756913 | + | 5.8 | 5478 | 6146 | -8 | L1PA7 | L1 | LINE |
| 95756918 | 95757030 | + | 15.0 | 2460 | 2572 | -3931 | L1PA15 | L1 | LINE |
| 95757037 | 95757501 | + | 15.8 | 5814 | 6298 | -204 | L1PA15 | L1 | LINE |

  

|  |  |  |  |  |  |  |  |  |  |
| --- | --- | --- | --- | --- | --- | --- | --- | --- | --- |
| chr3:95943313-95944821 | | | 1404 bp | | |  |  |  |  |
| Genome Start | Genome End | Strand | Div | Repeat Start | Repeat End | Repeat Left | Name | Family | Class |
| 95943313 | 95943465 | - | 36.2 | 3208 | 3378 | 0 | L2 | L2 | LINE |
| 95943567 | 95943885 | - | 12.8 | 5105 | 5425 | -741 | L1P | L1 | LINE |
| 95943886 | 95944629 | + | 16.1 | 5414 | 6163 | -3 | L1PA16 | L1 | LINE |
| 95944634 | 95944821 | - | 29.3 | 2941 | 3163 | -256 | L2 | L2 | LINE |

  

|  |  |  |  |  |  |  |  |  |  |
| --- | --- | --- | --- | --- | --- | --- | --- | --- | --- |
| chr3:96214767-96220286 | | | 5281 bp | | |  |  |  |  |
| Genome Start | Genome End | Strand | Div | Repeat Start | Repeat End | Repeat Left | Name | Family | Class |
| 96214767 | 96214954 | + | 36.0 | 167 | 360 | -3671 | Zaphod | Tip100 | DNA |
| 96214968 | 96217958 | - | 4.9 | 1934 | 4919 | -1227 | L1PA4 | L1 | LINE |
| 96217951 | 96219204 | + | 4.3 | 4937 | 6144 | -11 | L1PA4 | L1 | LINE |
| 96219214 | 96219308 | + | 24.2 | 367 | 478 | -3553 | Zaphod | Tip100 | DNA |
| 96219350 | 96219373 | + | 24.2 | 479 | 503 | -3528 | Zaphod | Tip100 | DNA |
| 96219439 | 96219796 | + | 17.4 | 66 | 2406 | 0 | Tigger5 | MER2\_type | DNA |
| 96219916 | 96220286 | + | 24.5 | 3620 | 4023 | -8 | Zaphod | Tip100 | DNA |

  

|  |  |  |  |  |  |  |  |  |  |
| --- | --- | --- | --- | --- | --- | --- | --- | --- | --- |
| chr3:97824169-97830457 | | | 6267 bp | | |  |  |  |  |
| Genome Start | Genome End | Strand | Div | Repeat Start | Repeat End | Repeat Left | Name | Family | Class |
| 97824169 | 97824848 | + | 31.8 | 12 | 724 | -6610 | L1M4b | L1 | LINE |
| 97824849 | 97826078 | - | 6.1 | 2834 | 4016 | -2130 | L1P1 | L1 | LINE |
| 97826079 | 97826359 | - | 4.6 | 1 | 293 | -18 | AluY | Alu | SINE |
| 97826360 | 97826875 | - | 6.1 | 2325 | 2833 | -3313 | L1P1 | L1 | LINE |
| 97826876 | 97829012 | + | 5.8 | 4015 | 6150 | -4 | L1PA5 | L1 | LINE |
| 97829035 | 97829148 | + | 21.9 | 710 | 809 | -6525 | L1M4b | L1 | LINE |
| 97829149 | 97829299 | + | 21.0 | 3 | 126 | -7 | FLAM\_C | Alu | SINE |
| 97829300 | 97830457 | + | 21.9 | 810 | 2098 | -5236 | L1M4b | L1 | LINE |

  

|  |  |  |  |  |  |  |  |  |  |
| --- | --- | --- | --- | --- | --- | --- | --- | --- | --- |
| chr3:98147339-98149596 | | | 2279 bp | | |  |  |  |  |
| Genome Start | Genome End | Strand | Div | Repeat Start | Repeat End | Repeat Left | Name | Family | Class |
| 98147339 | 98147713 | - | 17.9 | 5814 | 6190 | -143 | L1MC1 | L1 | LINE |
| 98147709 | 98149100 | - | 5.0 | 4768 | 6153 | -1 | L1PA5 | L1 | LINE |
| 98149100 | 98149552 | + | 6.2 | 4308 | 4760 | -1386 | L1PA5 | L1 | LINE |
| 98149538 | 98149596 | - | 8.6 | 5780 | 5839 | -494 | L1MC1 | L1 | LINE |

  

|  |  |  |  |  |  |  |  |  |  |
| --- | --- | --- | --- | --- | --- | --- | --- | --- | --- |
| chr3:102642723-102648688 | | | 5972 bp | | |  |  |  |  |
| Genome Start | Genome End | Strand | Div | Repeat Start | Repeat End | Repeat Left | Name | Family | Class |
| 102642723 | 102643779 | + | 25.3 | 1154 | 2148 | -5061 | L1M2b | L1 | LINE |
| 102643780 | 102644126 | - | 15.8 | 3 | 339 | 0 | MER1B | MER1\_type | DNA |
| 102644127 | 102644513 | + | 24.9 | 2149 | 2552 | -4657 | L1M2b | L1 | LINE |
| 102644509 | 102645440 | - | 3.3 | 4191 | 5128 | -1018 | L1PA3 | L1 | LINE |
| 102645421 | 102646438 | + | 2.5 | 5136 | 6154 | -1 | L1PA3 | L1 | LINE |
| 102646458 | 102647493 | + | 18.7 | 2543 | 3616 | -3589 | L1M2b | L1 | LINE |
| 102647494 | 102647878 | + | 14.6 | 3 | 428 | 0 | MSTA | MaLR | LTR |
| 102647879 | 102648688 | + | 19.6 | 3617 | 4461 | -2744 | L1M2b | L1 | LINE |

  

|  |  |  |  |  |  |  |  |  |  |
| --- | --- | --- | --- | --- | --- | --- | --- | --- | --- |
| chr3:102648996-102656550 | | | 7568 bp | | |  |  |  |  |
| Genome Start | Genome End | Strand | Div | Repeat Start | Repeat End | Repeat Left | Name | Family | Class |
| 102648996 | 102650004 | + | 17.5 | 4352 | 5380 | -766 | L1MB8 | L1 | LINE |
| 102650004 | 102650179 | - | 9.1 | 709 | 885 | -5270 | L1PA4 | L1 | LINE |
| 102650177 | 102655419 | + | 4.4 | 904 | 6155 | 0 | L1PA4 | L1 | LINE |
| 102655411 | 102655767 | + | 18.1 | 5353 | 5672 | -506 | L1MB8 | L1 | LINE |
| 102655768 | 102656074 | + | 16.3 | 5 | 312 | 0 | AluJb | Alu | SINE |
| 102656075 | 102656550 | + | 18.1 | 5673 | 6169 | -9 | L1MB8 | L1 | LINE |

  

|  |  |  |  |  |  |  |  |  |  |
| --- | --- | --- | --- | --- | --- | --- | --- | --- | --- |
| chr3:105320617-105323666 | | | 3039 bp | | |  |  |  |  |
| Genome Start | Genome End | Strand | Div | Repeat Start | Repeat End | Repeat Left | Name | Family | Class |
| 105320617 | 105320989 | - | 21.3 | 31 | 365 | 0 | MLT1A0 | MaLR | LTR |
| 105321012 | 105322853 | - | 4.6 | 4305 | 6147 | -7 | L1PA5 | L1 | LINE |
| 105322843 | 105323618 | + | 5.7 | 3526 | 4301 | -1845 | L1PA5 | L1 | LINE |
| 105323619 | 105323666 | - | 13.6 | 1 | 44 | -330 | MLT1A0 | MaLR | LTR |

  

|  |  |  |  |  |  |  |  |  |  |
| --- | --- | --- | --- | --- | --- | --- | --- | --- | --- |
| chr3:105805724-105812464 | | | 6441 bp | | |  |  |  |  |
| Genome Start | Genome End | Strand | Div | Repeat Start | Repeat End | Repeat Left | Name | Family | Class |
| 105805724 | 105805916 | - | 27.5 | 3224 | 3419 | 0 | L2 | L2 | LINE |
| 105805931 | 105806226 | - | 32.1 | 134 | 413 | -78 | LTR16C | ERVL | LTR |
| 105806285 | 105806456 | - | 37.8 | 2984 | 3161 | -258 | L2 | L2 | LINE |
| 105806485 | 105806664 | - | 41.8 | 2723 | 2901 | -518 | L2 | L2 | LINE |
| 105806678 | 105806786 | - | 15.6 | 2635 | 2760 | -1 | Charlie1 | MER1\_type | DNA |
| 105806788 | 105807092 | + | 11.7 | 1 | 301 | -11 | AluSx | Alu | SINE |
| 105807136 | 105807198 | - | 14.3 | 6117 | 6179 | -5 | L1MB7 | L1 | LINE |
| 105807199 | 105807248 | - | 12.0 | 6025 | 6074 | -89 | L1PA13 | L1 | LINE |
| 105807249 | 105807319 | + | 8.4 | 6074 | 6144 | -19 | L1PA13 | L1 | LINE |
| 105807329 | 105807845 | - | 19.3 | 5568 | 6127 | -51 | L1MB8 | L1 | LINE |
| 105807860 | 105808012 | - | 24.7 | 77 | 232 | -2529 | Charlie1 | MER1\_type | DNA |
| 105808060 | 105808884 | - | 31.9 | 1624 | 2478 | -941 | L2 | L2 | LINE |
| 105808940 | 105809038 | - | 17.2 | 5704 | 5802 | -376 | L1M4 | L1 | LINE |
| 105809054 | 105809469 | + | 18.0 | 5 | 421 | -7 | MSTA | MaLR | LTR |
| 105809470 | 105812234 | + | 12.1 | 3313 | 6152 | -11 | L1PA13 | L1 | LINE |
| 105812238 | 105812464 | - | 33.6 | 1352 | 1600 | -1819 | L2 | L2 | LINE |

  

|  |  |  |  |  |  |  |  |  |  |
| --- | --- | --- | --- | --- | --- | --- | --- | --- | --- |
| chr3:106387239-106389808 | | | 2554 bp | | |  |  |  |  |
| Genome Start | Genome End | Strand | Div | Repeat Start | Repeat End | Repeat Left | Name | Family | Class |
| 106387239 | 106387683 | - | 13.6 | 5718 | 6166 | 0 | L1PA16 | L1 | LINE |
| 106387702 | 106388113 | - | 4.1 | 5737 | 6153 | -1 | L1PA5 | L1 | LINE |
| 106388114 | 106388737 | + | 4.3 | 5107 | 5737 | -417 | L1PA5 | L1 | LINE |
| 106388736 | 106389808 | - | 13.3 | 4672 | 5728 | -438 | L1PA16 | L1 | LINE |

  

|  |  |  |  |  |  |  |  |  |  |
| --- | --- | --- | --- | --- | --- | --- | --- | --- | --- |
| chr3:107191619-107200232 | | | 8629 bp | | |  |  |  |  |
| Genome Start | Genome End | Strand | Div | Repeat Start | Repeat End | Repeat Left | Name | Family | Class |
| 107191619 | 107192994 | - | 11.1 | 6314 | 7682 | -41 | L1MA2 | L1 | LINE |
| 107192992 | 107193057 | + | 10.3 | 4553 | 4610 | -1536 | L1M1 | L1 | LINE |
| 107193053 | 107195140 | - | 12.9 | 2699 | 4798 | -2767 | L1MA2 | L1 | LINE |
| 107195141 | 107196041 | - | 2.2 | 5274 | 6174 | 0 | L1PA3 | L1 | LINE |
| 107196034 | 107197757 | + | 2.5 | 3531 | 5253 | -893 | L1PA3 | L1 | LINE |
| 107197759 | 107200232 | - | 15.9 | 11 | 2706 | -4859 | L1MA2 | L1 | LINE |

  

|  |  |  |  |  |  |  |  |  |  |
| --- | --- | --- | --- | --- | --- | --- | --- | --- | --- |
| chr3:107797746-107806700 | | | 8966 bp | | |  |  |  |  |
| Genome Start | Genome End | Strand | Div | Repeat Start | Repeat End | Repeat Left | Name | Family | Class |
| 107797746 | 107798552 | - | 18.1 | 6120 | 6937 | -5 | L1MA6 | L1 | LINE |
| 107798547 | 107798802 | - | 14.1 | 5278 | 5535 | -1250 | L1MA6 | L1 | LINE |
| 107798797 | 107799711 | - | 19.8 | 3950 | 4866 | -1919 | L1MA6 | L1 | LINE |
| 107799712 | 107799985 | - | 9.2 | 1 | 274 | -39 | AluSq | Alu | SINE |
| 107799986 | 107800366 | - | 19.8 | 3537 | 3949 | -2836 | L1MA6 | L1 | LINE |
| 107800366 | 107801310 | - | 13.7 | 3776 | 4721 | -1425 | L1PA16 | L1 | LINE |
| 107801309 | 107802729 | + | 16.1 | 4723 | 6150 | -16 | L1PA16 | L1 | LINE |
| 107802734 | 107802874 | - | 16.9 | 3411 | 3547 | -3238 | L1MA6 | L1 | LINE |
| 107802875 | 107803174 | - | 7.7 | 1 | 299 | -12 | AluY | Alu | SINE |
| 107803175 | 107806700 | - | 26.4 | 79 | 3410 | -3375 | L1MA6 | L1 | LINE |

  

|  |  |  |  |  |  |  |  |  |  |
| --- | --- | --- | --- | --- | --- | --- | --- | --- | --- |
| chr3:109103384-109105724 | | | 2367 bp | | |  |  |  |  |
| Genome Start | Genome End | Strand | Div | Repeat Start | Repeat End | Repeat Left | Name | Family | Class |
| 109103384 | 109103447 | - | 15.9 | 7483 | 7546 | -415 | L1MC5 | L1 | LINE |
| 109103432 | 109104773 | - | 11.9 | 3883 | 5225 | -921 | L1P4 | L1 | LINE |
| 109104766 | 109105418 | + | 10.2 | 3202 | 3889 | -2257 | L1P4 | L1 | LINE |
| 109105417 | 109105724 | - | 26.6 | 6555 | 6866 | -1095 | L1MC5 | L1 | LINE |

  

|  |  |  |  |  |  |  |  |  |  |
| --- | --- | --- | --- | --- | --- | --- | --- | --- | --- |
| chr3:109474249-109482057 | | | 7090 bp | | |  |  |  |  |
| Genome Start | Genome End | Strand | Div | Repeat Start | Repeat End | Repeat Left | Name | Family | Class |
| 109474249 | 109474475 | - | 35.3 | 2420 | 2657 | -3057 | HERVL | ERVL | LTR |
| 109474476 | 109474679 | - | 10.9 | 516 | 722 | 0 | LTR30 | ERV1 | LTR |
| 109475025 | 109477027 | - | 3.5 | 4120 | 6152 | -3 | L1PA4 | L1 | LINE |
| 109477014 | 109477296 | + | 4.2 | 3823 | 4106 | -2040 | L1PA4 | L1 | LINE |
| 109477297 | 109477825 | - | 7.2 | 1 | 530 | -192 | LTR30 | ERV1 | LTR |
| 109477826 | 109477979 | - | 37.7 | 2269 | 2423 | -3291 | HERVL | ERVL | LTR |
| 109477980 | 109478283 | - | 12.9 | 1 | 304 | -8 | AluSx | Alu | SINE |
| 109478284 | 109478442 | - | 37.7 | 2104 | 2268 | -3446 | HERVL | ERVL | LTR |
| 109478759 | 109478841 | - | 31.3 | 1908 | 1990 | -3664 | HERVL | ERVL | LTR |
| 109478896 | 109479254 | - | 14.0 | 1 | 362 | -2 | THE1B | MaLR | LTR |
| 109479261 | 109480827 | - | 11.3 | 1 | 1574 | -6 | THE1B-int | MaLR | LTR |
| 109480831 | 109481182 | - | 11.4 | 1 | 357 | -7 | THE1B | MaLR | LTR |
| 109481192 | 109482057 | - | 36.8 | 6 | 865 | -4131 | HERVL | ERVL | LTR |

  

|  |  |  |  |  |  |  |  |  |  |
| --- | --- | --- | --- | --- | --- | --- | --- | --- | --- |
| chr3:111400240-111407256 | | | 7021 bp | | |  |  |  |  |
| Genome Start | Genome End | Strand | Div | Repeat Start | Repeat End | Repeat Left | Name | Family | Class |
| 111400240 | 111401657 | + | 22.1 | 171 | 1597 | -5188 | L1PA13 | L1 | LINE |
| 111401663 | 111401734 | + | 18.1 | 1703 | 1776 | -5009 | L1PA13 | L1 | LINE |
| 111401739 | 111403170 | + | 12.0 | 2364 | 3831 | -2935 | L1PA13 | L1 | LINE |
| 111403177 | 111405508 | - | 5.7 | 3821 | 6154 | 0 | L1PA5 | L1 | LINE |
| 111405507 | 111406562 | + | 5.4 | 2774 | 3830 | -2316 | L1PA5 | L1 | LINE |
| 111406546 | 111407256 | + | 13.6 | 3810 | 4567 | -2199 | L1PA13 | L1 | LINE |

  

|  |  |  |  |  |  |  |  |  |  |
| --- | --- | --- | --- | --- | --- | --- | --- | --- | --- |
| chr3:112099573-112100997 | | | 1415 bp | | |  |  |  |  |
| Genome Start | Genome End | Strand | Div | Repeat Start | Repeat End | Repeat Left | Name | Family | Class |
| 112099573 | 112099692 | - | 20.8 | 61 | 188 | -1 | MER5A | MER1\_type | DNA |
| 112099706 | 112100482 | - | 6.4 | 5393 | 6171 | -1 | L1PA8 | L1 | LINE |
| 112100480 | 112100925 | + | 7.6 | 4932 | 5382 | -764 | L1PA8 | L1 | LINE |
| 112100926 | 112100997 | - | 20.8 | 1 | 78 | -111 | MER5A | MER1\_type | DNA |

  

|  |  |  |  |  |  |  |  |  |  |
| --- | --- | --- | --- | --- | --- | --- | --- | --- | --- |
| chr3:112859461-112866807 | | | 7215 bp | | |  |  |  |  |
| Genome Start | Genome End | Strand | Div | Repeat Start | Repeat End | Repeat Left | Name | Family | Class |
| 112859461 | 112860346 | + | 28.5 | 6658 | 7535 | -507 | L1MC4 | L1 | LINE |
| 112860347 | 112861165 | - | 14.6 | 2857 | 3730 | -2416 | L1P4 | L1 | LINE |
| 112861166 | 112862492 | + | 13.7 | 3742 | 5079 | -1067 | L1P4 | L1 | LINE |
| 112862493 | 112862874 | - | 14.4 | 1 | 426 | -2 | MSTA | MaLR | LTR |
| 112862875 | 112864481 | - | 30.4 | 2 | 1735 | 0 | MLT-int | MaLR | LTR |
| 112864484 | 112864895 | - | 15.6 | 1 | 428 | 0 | MSTA | MaLR | LTR |
| 112864896 | 112865289 | + | 13.9 | 5080 | 5525 | -641 | L1PA16 | L1 | LINE |
| 112865290 | 112865586 | - | 8.8 | 1 | 295 | -16 | AluY | Alu | SINE |
| 112865587 | 112866215 | + | 14.3 | 5526 | 6166 | 0 | L1PA16 | L1 | LINE |
| 112866240 | 112866549 | + | 24.6 | 7546 | 7889 | -153 | L1MC4 | L1 | LINE |
| 112866618 | 112866709 | + | 22.4 | 7902 | 7984 | -58 | L1MC4 | L1 | LINE |
| 112866748 | 112866807 | + | 22.4 | 7985 | 8041 | -1 | L1MC4 | L1 | LINE |

  

|  |  |  |  |  |  |  |  |  |  |
| --- | --- | --- | --- | --- | --- | --- | --- | --- | --- |
| chr3:114611147-114613921 | | | 2777 bp | | |  |  |  |  |
| Genome Start | Genome End | Strand | Div | Repeat Start | Repeat End | Repeat Left | Name | Family | Class |
| 114611147 | 114611701 | + | 12.2 | 1 | 554 | -80 | MER41B | ERV1 | LTR |
| 114611702 | 114613564 | - | 4.7 | 4264 | 6139 | -15 | L1PA5 | L1 | LINE |
| 114613563 | 114613811 | + | 5.2 | 4002 | 4252 | -1894 | L1PA5 | L1 | LINE |
| 114613812 | 114613921 | + | 13.6 | 525 | 634 | 0 | MER41B | ERV1 | LTR |

  

|  |  |  |  |  |  |  |  |  |  |
| --- | --- | --- | --- | --- | --- | --- | --- | --- | --- |
| chr3:115186396-115199257 | | | 12474 bp | | |  |  |  |  |
| Genome Start | Genome End | Strand | Div | Repeat Start | Repeat End | Repeat Left | Name | Family | Class |
| 115186396 | 115186808 | + | 29.7 | 33 | 441 | -5604 | L1MC3 | L1 | LINE |
| 115187092 | 115188139 | + | 24.3 | 614 | 1734 | -5017 | L1MC3 | L1 | LINE |
| 115188140 | 115188570 | + | 24.3 | 1 | 374 | 0 | MLT1A | MaLR | LTR |
| 115188571 | 115189119 | + | 23.9 | 1735 | 2329 | -4422 | L1MC3 | L1 | LINE |
| 115189120 | 115189233 | - | 23.7 | 2 | 115 | -18 | FLAM\_A | Alu | SINE |
| 115189234 | 115189298 | + | 23.9 | 2330 | 2380 | -4371 | L1MC3 | L1 | LINE |
| 115189299 | 115189580 | + | 9.6 | 1 | 282 | -28 | AluSg | Alu | SINE |
| 115189581 | 115189745 | + | 23.9 | 2381 | 2545 | -4206 | L1MC3 | L1 | LINE |
| 115189751 | 115189883 | + | 6.8 | 1 | 133 | -176 | AluSg1 | Alu | SINE |
| 115189884 | 115190186 | + | 11.3 | 1 | 306 | -7 | AluSp | Alu | SINE |
| 115190187 | 115190331 | + | 23.4 | 2534 | 2683 | -3881 | L1MC3 | L1 | LINE |
| 115190339 | 115190632 | - | 8.2 | 1 | 291 | -20 | AluY | Alu | SINE |
| 115190648 | 115191240 | + | 22.3 | 2864 | 3456 | -3108 | L1MC3 | L1 | LINE |
| 115191241 | 115191547 | + | 17.6 | 1 | 306 | -6 | AluJo | Alu | SINE |
| 115191548 | 115192008 | + | 22.3 | 3457 | 3924 | -2640 | L1MC3 | L1 | LINE |
| 115192009 | 115192151 | + | 12.6 | 6008 | 6150 | -1 | L1PB1 | L1 | LINE |
| 115192152 | 115192360 | + | 22.3 | 3925 | 4121 | -2443 | L1MC3 | L1 | LINE |
| 115192361 | 115192486 | + | 23.0 | 5 | 133 | 0 | FLAM\_A | Alu | SINE |
| 115192493 | 115192795 | + | 11.9 | 1 | 308 | -2 | AluSg | Alu | SINE |
| 115192796 | 115193559 | + | 22.3 | 4126 | 4932 | -1632 | L1MC3 | L1 | LINE |
| 115193554 | 115194009 | + | 23.8 | 5 | 484 | -5657 | L1M3c | L1 | LINE |
| 115194008 | 115194101 | + | 12.1 | 3456 | 3558 | -2582 | L1M3 | L1 | LINE |
| 115194102 | 115194276 | + | 5.1 | 5980 | 6154 | -1 | L1PA4 | L1 | LINE |
| 115194277 | 115194657 | + | 12.1 | 3559 | 3947 | -2193 | L1M3 | L1 | LINE |
| 115194690 | 115194804 | + | 12.2 | 3 | 118 | -194 | AluJb | Alu | SINE |
| 115194808 | 115195134 | + | 19.8 | 5066 | 5415 | -1149 | L1MC3 | L1 | LINE |
| 115195135 | 115195447 | + | 11.7 | 1 | 310 | -2 | AluSx | Alu | SINE |
| 115195448 | 115196114 | + | 21.2 | 5416 | 6071 | -2132 | L1MC3 | L1 | LINE |
| 115196115 | 115196404 | - | 14.1 | 15 | 306 | -6 | AluJo | Alu | SINE |
| 115196405 | 115197106 | + | 23.3 | 6072 | 6699 | -1504 | L1MC3 | L1 | LINE |
| 115197143 | 115198092 | - | 8.1 | 4707 | 5675 | -456 | L1PA8 | L1 | LINE |
| 115198092 | 115198567 | + | 6.5 | 5676 | 6151 | -21 | L1PA8 | L1 | LINE |
| 115198578 | 115199257 | + | 19.8 | 6722 | 7415 | -788 | L1MC3 | L1 | LINE |

  

|  |  |  |  |  |  |  |  |  |  |
| --- | --- | --- | --- | --- | --- | --- | --- | --- | --- |
| chr3:115239816-115240760 | | | 909 bp | | |  |  |  |  |
| Genome Start | Genome End | Strand | Div | Repeat Start | Repeat End | Repeat Left | Name | Family | Class |
| 115239816 | 115240022 | + | 16.4 | 1 | 210 | -2819 | Tigger3(Golem) | MER2\_type | DNA |
| 115240024 | 115240222 | - | 6.2 | 5591 | 5785 | -383 | L1PA11 | L1 | LINE |
| 115240223 | 115240588 | + | 7.7 | 5796 | 6174 | 0 | L1PA11 | L1 | LINE |
| 115240624 | 115240760 | + | 12.3 | 198 | 340 | -2689 | Tigger3(Golem) | MER2\_type | DNA |

  

|  |  |  |  |  |  |  |  |  |  |
| --- | --- | --- | --- | --- | --- | --- | --- | --- | --- |
| chr3:116670050-116672454 | | | 2097 bp | | |  |  |  |  |
| Genome Start | Genome End | Strand | Div | Repeat Start | Repeat End | Repeat Left | Name | Family | Class |
| 116670050 | 116670122 | + | 37.0 | 150 | 222 | -369 | MLT1K | MaLR | LTR |
| 116670296 | 116670588 | - | 1.0 | 5262 | 5554 | -601 | L1PA2 | L1 | LINE |
| 116670589 | 116671204 | + | 1.9 | 5540 | 6155 | 0 | L1PA2 | L1 | LINE |
| 116671228 | 116671776 | - | 17.2 | 1 | 563 | 0 | MER68 | ERVL | LTR |
| 116671839 | 116672095 | - | 31.2 | 1 | 247 | -244 | LTR16C | ERVL | LTR |
| 116672146 | 116672454 | + | 31.2 | 298 | 591 | 0 | MLT1K | MaLR | LTR |

  

|  |  |  |  |  |  |  |  |  |  |
| --- | --- | --- | --- | --- | --- | --- | --- | --- | --- |
| chr3:116948264-116952229 | | | 3958 bp | | |  |  |  |  |
| Genome Start | Genome End | Strand | Div | Repeat Start | Repeat End | Repeat Left | Name | Family | Class |
| 116948264 | 116948455 | + | 10.5 | 1 | 192 | -588 | LTR17 | ERV1 | LTR |
| 116948456 | 116949198 | - | 4.3 | 2519 | 3262 | -2884 | L1PA4 | L1 | LINE |
| 116949200 | 116952065 | + | 4.3 | 3270 | 6139 | -16 | L1PA4 | L1 | LINE |
| 116952073 | 116952229 | + | 7.2 | 171 | 326 | -454 | LTR17 | ERV1 | LTR |

  

|  |  |  |  |  |  |  |  |  |  |
| --- | --- | --- | --- | --- | --- | --- | --- | --- | --- |
| chr3:119249475-119252547 | | | 2697 bp | | |  |  |  |  |
| Genome Start | Genome End | Strand | Div | Repeat Start | Repeat End | Repeat Left | Name | Family | Class |
| 119249475 | 119249566 | - | 20.9 | 5998 | 6100 | -66 | L1ME1 | L1 | LINE |
| 119249641 | 119249774 | + | 27.1 | 5689 | 5821 | -303 | L1M5 | L1 | LINE |
| 119250049 | 119250367 | - | 4.1 | 5831 | 6154 | 0 | L1PA5 | L1 | LINE |
| 119250369 | 119251820 | + | 2.4 | 4365 | 5814 | -341 | L1PA5 | L1 | LINE |
| 119251848 | 119252547 | - | 23.7 | 5210 | 5950 | -214 | L1ME1 | L1 | LINE |

  

|  |  |  |  |  |  |  |  |  |  |
| --- | --- | --- | --- | --- | --- | --- | --- | --- | --- |
| chr3:119335672-119349022 | | | 13304 bp | | |  |  |  |  |
| Genome Start | Genome End | Strand | Div | Repeat Start | Repeat End | Repeat Left | Name | Family | Class |
| 119335672 | 119335823 | - | 18.9 | 2833 | 3010 | -4324 | L1M4b | L1 | LINE |
| 119335824 | 119338198 | + | 15.0 | 2 | 2417 | -1 | Tigger1 | MER2\_type | DNA |
| 119338199 | 119338711 | - | 18.9 | 2211 | 2832 | -4502 | L1M4b | L1 | LINE |
| 119338719 | 119339749 | - | 22.2 | 973 | 2041 | -5293 | L1M4b | L1 | LINE |
| 119339778 | 119342317 | - | 9.5 | 3468 | 6012 | -304 | L1PA8A | L1 | LINE |
| 119342308 | 119343364 | - | 3.8 | 5091 | 6150 | -5 | L1PA4 | L1 | LINE |
| 119343363 | 119343664 | + | 5.6 | 4777 | 5078 | -1068 | L1PA4 | L1 | LINE |
| 119343663 | 119347057 | - | 15.1 | 63 | 3491 | -2840 | L1PA8A | L1 | LINE |
| 119347093 | 119347134 | - | 18.2 | 16 | 62 | -6291 | L1PA8A | L1 | LINE |
| 119347126 | 119347302 | - | 28.4 | 811 | 983 | -6351 | L1M4b | L1 | LINE |
| 119347303 | 119347738 | - | 11.6 | 1 | 444 | 0 | MLT2A1 | ERVL | LTR |
| 119347739 | 119347990 | - | 28.4 | 557 | 810 | -6524 | L1M4b | L1 | LINE |
| 119347991 | 119348456 | - | 19.5 | 1 | 470 | -2 | LTR7Y | ERV1 | LTR |
| 119348457 | 119349022 | - | 28.4 | 11 | 556 | -6778 | L1M4b | L1 | LINE |

  

|  |  |  |  |  |  |  |  |  |  |
| --- | --- | --- | --- | --- | --- | --- | --- | --- | --- |
| chr3:119425018-119426994 | | | 1976 bp | | |  |  |  |  |
| Genome Start | Genome End | Strand | Div | Repeat Start | Repeat End | Repeat Left | Name | Family | Class |
| 119425018 | 119425111 | - | 28.3 | 61 | 155 | -34 | MER5A | MER1\_type | DNA |
| 119425117 | 119425840 | - | 13.7 | 5400 | 6149 | -7 | L1PB4 | L1 | LINE |
| 119425841 | 119426223 | + | 15.8 | 3869 | 4258 | -1888 | L1P4 | L1 | LINE |
| 119426218 | 119426922 | + | 19.7 | 4679 | 5403 | -743 | L1P4 | L1 | LINE |
| 119426925 | 119426994 | - | 26.5 | 4 | 73 | -116 | MER5A | MER1\_type | DNA |

  

|  |  |  |  |  |  |  |  |  |  |
| --- | --- | --- | --- | --- | --- | --- | --- | --- | --- |
| chr3:119936618-119941385 | | | 4049 bp | | |  |  |  |  |
| Genome Start | Genome End | Strand | Div | Repeat Start | Repeat End | Repeat Left | Name | Family | Class |
| 119936618 | 119937420 | - | 28.4 | 4786 | 5666 | -1 | ERVL-E | ERVL | LTR |
| 119937529 | 119938299 | - | 22.8 | 3742 | 4512 | -1155 | ERVL-E | ERVL | LTR |
| 119938333 | 119938766 | - | 28.8 | 3231 | 3681 | -1986 | ERVL-E | ERVL | LTR |
| 119938895 | 119939232 | - | 29.6 | 2701 | 3069 | -2598 | ERVL-E | ERVL | LTR |
| 119939352 | 119940235 | - | 33.3 | 924 | 1833 | -3834 | ERVL-E | ERVL | LTR |
| 119940464 | 119940778 | - | 5.1 | 5879 | 6193 | 0 | L1PA3 | L1 | LINE |
| 119940779 | 119941000 | + | 3.6 | 5652 | 5873 | -282 | L1PA3 | L1 | LINE |
| 119941104 | 119941385 | - | 34.5 | 19 | 302 | -5365 | ERVL-E | ERVL | LTR |

  

|  |  |  |  |  |  |  |  |  |  |
| --- | --- | --- | --- | --- | --- | --- | --- | --- | --- |
| chr3:119974747-119979797 | | | 4997 bp | | |  |  |  |  |
| Genome Start | Genome End | Strand | Div | Repeat Start | Repeat End | Repeat Left | Name | Family | Class |
| 119974747 | 119974770 | + | 23.1 | 3 | 26 | -1014 | MER45B | Tip100 | DNA |
| 119974771 | 119975083 | - | 8.4 | 1 | 309 | -4 | AluSq | Alu | SINE |
| 119975084 | 119975669 | + | 23.1 | 27 | 646 | -394 | MER45B | Tip100 | DNA |
| 119975670 | 119976224 | + | 16.9 | 1 | 555 | -6318 | L1PA17 | L1 | LINE |
| 119976223 | 119977546 | + | 13.6 | 4596 | 6007 | -586 | L1PA17 | L1 | LINE |
| 119977547 | 119977906 | + | 12.0 | 1 | 364 | 0 | THE1B | MaLR | LTR |
| 119977907 | 119978125 | + | 14.3 | 6008 | 6229 | -364 | L1PA17 | L1 | LINE |
| 119978162 | 119978491 | + | 14.3 | 6230 | 6576 | -17 | L1PA17 | L1 | LINE |
| 119978490 | 119978825 | - | 9.5 | 5276 | 5601 | -564 | L1PA15 | L1 | LINE |
| 119978826 | 119979397 | + | 11.9 | 5599 | 6165 | 0 | L1PA15 | L1 | LINE |
| 119979420 | 119979797 | + | 17.0 | 633 | 1021 | -19 | MER45B | Tip100 | DNA |

  

|  |  |  |  |  |  |  |  |  |  |
| --- | --- | --- | --- | --- | --- | --- | --- | --- | --- |
| chr3:122148996-122154043 | | | 5066 bp | | |  |  |  |  |
| Genome Start | Genome End | Strand | Div | Repeat Start | Repeat End | Repeat Left | Name | Family | Class |
| 122148996 | 122149954 | - | 17.7 | 5188 | 6163 | -15 | L1MB8 | L1 | LINE |
| 122149950 | 122151413 | - | 10.0 | 4696 | 6149 | -2 | L1PB2 | L1 | LINE |
| 122151403 | 122151536 | - | 19.7 | 5078 | 5205 | -941 | L1MB8 | L1 | LINE |
| 122151537 | 122151608 | + | 15.3 | 6210 | 6286 | -5 | L1MA7 | L1 | LINE |
| 122151609 | 122152076 | - | 19.7 | 4628 | 5077 | -1069 | L1MB8 | L1 | LINE |
| 122152077 | 122152173 | - | 4.1 | 6054 | 6150 | -5 | L1PA2 | L1 | LINE |
| 122152174 | 122153743 | + | 2.1 | 4473 | 6043 | -112 | L1PA2 | L1 | LINE |
| 122153742 | 122154043 | - | 18.9 | 4316 | 4637 | -1509 | L1MB8 | L1 | LINE |

  

|  |  |  |  |  |  |  |  |  |  |
| --- | --- | --- | --- | --- | --- | --- | --- | --- | --- |
| chr3:122270528-122291949 | | | 21159 bp | | |  |  |  |  |
| Genome Start | Genome End | Strand | Div | Repeat Start | Repeat End | Repeat Left | Name | Family | Class |
| 122270528 | 122271191 | - | 21.9 | 5881 | 6554 | -196 | L1MD2 | L1 | LINE |
| 122271213 | 122271436 | - | 18.0 | 5239 | 5468 | -1069 | L1MD2 | L1 | LINE |
| 122271438 | 122271591 | - | 15.6 | 2949 | 3102 | -3101 | L1PBa | L1 | LINE |
| 122271592 | 122272076 | - | 6.2 | 1 | 491 | 0 | HERV1\_LTRc | ERV1 | LTR |
| 122272077 | 122272195 | - | 23.5 | 8683 | 8801 | 0 | HERV1\_I | ERV1 | LTR |
| 122272225 | 122278652 | - | 21.5 | 580 | 7068 | -1733 | HERV1\_I | ERV1 | LTR |
| 122278790 | 122278816 | - | 6.2 | 469 | 491 | 0 | HERV1\_LTRc | ERV1 | LTR |
| 122278817 | 122280655 | - | 7.3 | 2 | 1578 | 0 | LTR12C | ERV1 | LTR |
| 122280656 | 122281117 | - | 6.2 | 1 | 468 | -23 | HERV1\_LTRc | ERV1 | LTR |
| 122281119 | 122283468 | - | 13.3 | 373 | 2951 | -3252 | L1PBa | L1 | LINE |
| 122283456 | 122284017 | - | 18.4 | 4451 | 5249 | -1288 | L1MD2 | L1 | LINE |
| 122284018 | 122284217 | + | 31.5 | 1 | 202 | -838 | MER45B | Tip100 | DNA |
| 122284218 | 122284453 | - | 18.4 | 4116 | 4450 | -2087 | L1MD2 | L1 | LINE |
| 122284454 | 122284762 | + | 9.2 | 1 | 307 | -2 | AluSc | Alu | SINE |
| 122284763 | 122284920 | - | 18.4 | 3971 | 4112 | -2425 | L1MD2 | L1 | LINE |
| 122284921 | 122285214 | + | 11.3 | 5 | 298 | -15 | AluSq | Alu | SINE |
| 122285215 | 122285732 | - | 18.4 | 3423 | 3970 | -2567 | L1MD2 | L1 | LINE |
| 122285743 | 122286893 | - | 10.0 | 5013 | 6164 | -4 | L1PA10 | L1 | LINE |
| 122286892 | 122287717 | + | 7.7 | 4174 | 5009 | -1137 | L1PA10 | L1 | LINE |
| 122287703 | 122287860 | - | 22.3 | 3274 | 3445 | -3092 | L1MD2 | L1 | LINE |
| 122287881 | 122287990 | + | 21.1 | 21 | 144 | -2885 | Tigger3(Golem) | MER2\_type | DNA |
| 122287989 | 122288106 | + | 14.2 | 2773 | 2892 | -137 | Tigger3(Golem) | MER2\_type | DNA |
| 122288107 | 122288335 | - | 19.2 | 1 | 236 | 0 | MER46A | MER2\_type | DNA |
| 122288336 | 122288485 | + | 14.2 | 2893 | 3028 | -1 | Tigger3(Golem) | MER2\_type | DNA |
| 122288486 | 122289596 | - | 20.5 | 2132 | 3275 | -3262 | L1MD2 | L1 | LINE |
| 122289602 | 122290642 | + | 0.8 | 5115 | 6155 | 0 | L1HS | L1 | LINE |
| 122290714 | 122291949 | - | 24.8 | 630 | 2102 | -4531 | L1MD2 | L1 | LINE |

  

|  |  |  |  |  |  |  |  |  |  |
| --- | --- | --- | --- | --- | --- | --- | --- | --- | --- |
| chr3:124035806-124043201 | | | 7270 bp | | |  |  |  |  |
| Genome Start | Genome End | Strand | Div | Repeat Start | Repeat End | Repeat Left | Name | Family | Class |
| 124035806 | 124035862 | - | 17.5 | 2524 | 2580 | -32 | Charlie5 | MER1\_type | DNA |
| 124035864 | 124037640 | + | 14.0 | 2750 | 4492 | -1654 | L1PB3 | L1 | LINE |
| 124037639 | 124038164 | - | 3.6 | 3520 | 4046 | -2100 | L1PA4 | L1 | LINE |
| 124038161 | 124040253 | + | 3.4 | 4051 | 6152 | -3 | L1PA4 | L1 | LINE |
| 124040262 | 124041922 | + | 12.3 | 4477 | 6147 | -7 | L1PB3 | L1 | LINE |
| 124042056 | 124042306 | - | 17.7 | 2298 | 2545 | -67 | Charlie5 | MER1\_type | DNA |
| 124042301 | 124042876 | - | 16.9 | 587 | 1186 | -1426 | Charlie5 | MER1\_type | DNA |
| 124042873 | 124043201 | - | 19.2 | 45 | 384 | -2228 | Charlie5 | MER1\_type | DNA |

  

|  |  |  |  |  |  |  |  |  |  |
| --- | --- | --- | --- | --- | --- | --- | --- | --- | --- |
| chr3:125442312-125443190 | | | 518 bp | | |  |  |  |  |
| Genome Start | Genome End | Strand | Div | Repeat Start | Repeat End | Repeat Left | Name | Family | Class |
| 125442312 | 125442403 | + | 32.6 | 94 | 187 | -81 | MIRb | MIR | SINE |
| 125442563 | 125442860 | - | 30.2 | 5881 | 6185 | -55 | L1ME3B | L1 | LINE |
| 125442860 | 125442924 | + | 19.1 | 5823 | 5886 | -354 | L1ME3B | L1 | LINE |
| 125443128 | 125443190 | + | 23.8 | 187 | 252 | -16 | MIRb | MIR | SINE |

  

|  |  |  |  |  |  |  |  |  |  |
| --- | --- | --- | --- | --- | --- | --- | --- | --- | --- |
| chr3:125515417-125516671 | | | 1252 bp | | |  |  |  |  |
| Genome Start | Genome End | Strand | Div | Repeat Start | Repeat End | Repeat Left | Name | Family | Class |
| 125515417 | 125515455 | - | 10.3 | 417 | 456 | -1 | MSTB | MaLR | LTR |
| 125515458 | 125515539 | - | 3.7 | 5554 | 5635 | -519 | L1PA5 | L1 | LINE |
| 125515541 | 125516049 | + | 5.1 | 5646 | 6154 | 0 | L1PA5 | L1 | LINE |
| 125516050 | 125516211 | - | 19.1 | 226 | 392 | -34 | MSTB | MaLR | LTR |
| 125516212 | 125516487 | - | 15.6 | 1 | 282 | -30 | AluJb | Alu | SINE |
| 125516488 | 125516671 | - | 19.1 | 5 | 225 | -201 | MSTB | MaLR | LTR |

  

|  |  |  |  |  |  |  |  |  |  |
| --- | --- | --- | --- | --- | --- | --- | --- | --- | --- |
| chr3:127021894-127022823 | | | 907 bp | | |  |  |  |  |
| Genome Start | Genome End | Strand | Div | Repeat Start | Repeat End | Repeat Left | Name | Family | Class |
| 127021894 | 127021937 | - | 6.8 | 135 | 178 | 0 | MER5B | MER1\_type | DNA |
| 127021953 | 127022128 | - | 24.4 | 5684 | 5887 | -287 | L1MB5 | L1 | LINE |
| 127022099 | 127022381 | + | 19.1 | 5902 | 6174 | 0 | L1MB5 | L1 | LINE |
| 127022387 | 127022665 | + | 12.9 | 2 | 286 | -26 | AluSx | Alu | SINE |
| 127022699 | 127022823 | - | 20.5 | 5 | 127 | -51 | MER5B | MER1\_type | DNA |

  

|  |  |  |  |  |  |  |  |  |  |
| --- | --- | --- | --- | --- | --- | --- | --- | --- | --- |
| chr3:132110932-132114956 | | | 3842 bp | | |  |  |  |  |
| Genome Start | Genome End | Strand | Div | Repeat Start | Repeat End | Repeat Left | Name | Family | Class |
| 132110932 | 132111368 | - | 33.8 | 3753 | 4225 | -283 | L3 | CR1 | LINE |
| 132111409 | 132111539 | - | 16.5 | 1 | 127 | -16 | FLAM\_C | Alu | SINE |
| 132111664 | 132113133 | - | 1.6 | 3002 | 4470 | -1676 | L1PA2 | L1 | LINE |
| 132113134 | 132114790 | + | 1.5 | 4489 | 6152 | -3 | L1PA2 | L1 | LINE |
| 132114810 | 132114956 | - | 32.4 | 3517 | 3665 | -824 | L3 | CR1 | LINE |

  

|  |  |  |  |  |  |  |  |  |  |
| --- | --- | --- | --- | --- | --- | --- | --- | --- | --- |
| chr3:132420079-132421325 | | | 1244 bp | | |  |  |  |  |
| Genome Start | Genome End | Strand | Div | Repeat Start | Repeat End | Repeat Left | Name | Family | Class |
| 132420079 | 132420258 | + | 15.7 | 1 | 180 | -210 | MLT1B | MaLR | LTR |
| 132420259 | 132420431 | - | 5.2 | 5309 | 5481 | -691 | L1PA7 | L1 | LINE |
| 132420432 | 132421102 | + | 5.3 | 5497 | 6152 | -2 | L1PA7 | L1 | LINE |
| 132421106 | 132421325 | + | 17.6 | 166 | 390 | 0 | MLT1B | MaLR | LTR |

  

|  |  |  |  |  |  |  |  |  |  |
| --- | --- | --- | --- | --- | --- | --- | --- | --- | --- |
| chr3:132492330-132507573 | | | 14913 bp | | |  |  |  |  |
| Genome Start | Genome End | Strand | Div | Repeat Start | Repeat End | Repeat Left | Name | Family | Class |
| 132492330 | 132494049 | - | 12.7 | 4742 | 6480 | -28 | L1MC1 | L1 | LINE |
| 132494050 | 132494217 | - | 14.3 | 1 | 164 | -12 | FRAM | Alu | SINE |
| 132494218 | 132496727 | - | 12.7 | 2203 | 4741 | -1767 | L1MC1 | L1 | LINE |
| 132496773 | 132497000 | - | 14.5 | 1 | 235 | -1 | MER46A | MER2\_type | DNA |
| 132497244 | 132497386 | - | 14.4 | 2079 | 2216 | -4292 | L1MC1 | L1 | LINE |
| 132497387 | 132497674 | - | 17.4 | 1 | 289 | -23 | AluJb | Alu | SINE |
| 132497675 | 132498185 | - | 14.4 | 1532 | 2078 | -4430 | L1MC1 | L1 | LINE |
| 132498197 | 132499998 | - | 2.6 | 4354 | 6152 | -3 | L1PA3 | L1 | LINE |
| 132499998 | 132500087 | + | 0.0 | 4249 | 4338 | -1808 | L1PA3 | L1 | LINE |
| 132500085 | 132500222 | - | 14.2 | 1407 | 1545 | -4963 | L1MC1 | L1 | LINE |
| 132500263 | 132500614 | - | 14.2 | 1035 | 1406 | -5102 | L1MC1 | L1 | LINE |
| 132500610 | 132500740 | - | 11.2 | 6661 | 6779 | -6 | L1PA15 | L1 | LINE |
| 132500741 | 132501026 | - | 15.8 | 1 | 302 | -10 | AluSx | Alu | SINE |
| 132501027 | 132507527 | - | 16.5 | 4 | 6660 | -125 | L1PA15 | L1 | LINE |
| 132507529 | 132507573 | - | 6.7 | 995 | 1039 | -5469 | L1MC1 | L1 | LINE |

  

|  |  |  |  |  |  |  |  |  |  |
| --- | --- | --- | --- | --- | --- | --- | --- | --- | --- |
| chr3:132794552-132798469 | | | 3880 bp | | |  |  |  |  |
| Genome Start | Genome End | Strand | Div | Repeat Start | Repeat End | Repeat Left | Name | Family | Class |
| 132794552 | 132794685 | + | 15.8 | 1 | 142 | -255 | MLT2C1 | ERVL | LTR |
| 132794686 | 132795235 | - | 15.3 | 4124 | 4658 | -1482 | L1M3 | L1 | LINE |
| 132795262 | 132795551 | - | 9.2 | 1 | 284 | -25 | AluSc | Alu | SINE |
| 132795552 | 132795762 | - | 15.2 | 3917 | 4123 | -2017 | L1M3 | L1 | LINE |
| 132795762 | 132796961 | + | 16.5 | 4662 | 5851 | -338 | L1MB1 | L1 | LINE |
| 132796962 | 132797373 | - | 15.5 | 1 | 426 | 0 | MSTB | MaLR | LTR |
| 132797374 | 132797863 | + | 16.4 | 5852 | 6331 | -3 | L1MA10 | L1 | LINE |
| 132797875 | 132798184 | + | 0.0 | 1 | 310 | 0 | AluYa5 | Alu | SINE |
| 132798187 | 132798469 | + | 19.5 | 127 | 397 | 0 | MLT2C1 | ERVL | LTR |

  

|  |  |  |  |  |  |  |  |  |  |
| --- | --- | --- | --- | --- | --- | --- | --- | --- | --- |
| chr3:133357641-133358526 | | | 704 bp | | |  |  |  |  |
| Genome Start | Genome End | Strand | Div | Repeat Start | Repeat End | Repeat Left | Name | Family | Class |
| 133357641 | 133358118 | + | 25.4 | 5 | 495 | -35 | MLT1G3 | MaLR | LTR |
| 133358119 | 133358221 | - | 5.8 | 5990 | 6092 | -63 | L1PA4 | L1 | LINE |
| 133358223 | 133358271 | + | 4.1 | 6103 | 6151 | -3 | L1PA6 | L1 | LINE |
| 133358453 | 133358526 | + | 15.5 | 518 | 589 | -1 | MLT1G3 | MaLR | LTR |

  

|  |  |  |  |  |  |  |  |  |  |
| --- | --- | --- | --- | --- | --- | --- | --- | --- | --- |
| chr3:135755512-135758131 | | | 2607 bp | | |  |  |  |  |
| Genome Start | Genome End | Strand | Div | Repeat Start | Repeat End | Repeat Left | Name | Family | Class |
| 135755512 | 135755592 | + | 25.0 | 10 | 90 | -2417 | HAL1 | L1 | LINE |
| 135755615 | 135756544 | - | 2.0 | 5226 | 6155 | 0 | L1PA3 | L1 | LINE |
| 135756539 | 135757898 | + | 2.1 | 3874 | 5233 | -913 | L1PA3 | L1 | LINE |
| 135757896 | 135758131 | + | 30.6 | 78 | 299 | -2208 | HAL1 | L1 | LINE |

  

|  |  |  |  |  |  |  |  |  |  |
| --- | --- | --- | --- | --- | --- | --- | --- | --- | --- |
| chr3:135939605-135941225 | | | 1576 bp | | |  |  |  |  |
| Genome Start | Genome End | Strand | Div | Repeat Start | Repeat End | Repeat Left | Name | Family | Class |
| 135939605 | 135939677 | + | 20.6 | 5417 | 5496 | -744 | L1ME3B | L1 | LINE |
| 135939679 | 135939778 | - | 11.5 | 5794 | 5893 | -258 | L1PB1 | L1 | LINE |
| 135939822 | 135940042 | - | 11.5 | 5546 | 5793 | -358 | L1PB1 | L1 | LINE |
| 135940043 | 135940307 | + | 15.1 | 5885 | 6150 | -1 | L1PB1 | L1 | LINE |
| 135940308 | 135940828 | + | 17.5 | 5271 | 5806 | -378 | L1M4 | L1 | LINE |
| 135940830 | 135941225 | + | 28.2 | 5505 | 5946 | -294 | L1ME3B | L1 | LINE |

  

|  |  |  |  |  |  |  |  |  |  |
| --- | --- | --- | --- | --- | --- | --- | --- | --- | --- |
| chr3:136483183-136486407 | | | 3189 bp | | |  |  |  |  |
| Genome Start | Genome End | Strand | Div | Repeat Start | Repeat End | Repeat Left | Name | Family | Class |
| 136483183 | 136483325 | - | 29.8 | 118 | 273 | -276 | MLT1H | MaLR | LTR |
| 136483334 | 136484426 | - | 2.5 | 3168 | 4260 | -1886 | L1PA3 | L1 | LINE |
| 136484428 | 136486310 | + | 2.5 | 4276 | 6155 | 0 | L1PA3 | L1 | LINE |
| 136486338 | 136486407 | - | 21.2 | 55 | 122 | -427 | MLT1H | MaLR | LTR |

  

|  |  |  |  |  |  |  |  |  |  |
| --- | --- | --- | --- | --- | --- | --- | --- | --- | --- |
| chr3:136597403-136604464 | | | 7063 bp | | |  |  |  |  |
| Genome Start | Genome End | Strand | Div | Repeat Start | Repeat End | Repeat Left | Name | Family | Class |
| 136597403 | 136601459 | - | 5.6 | 2135 | 6150 | -4 | L1PA5 | L1 | LINE |
| 136601474 | 136602277 | - | 1.8 | 5359 | 6155 | 0 | L1PA2 | L1 | LINE |
| 136602266 | 136603311 | + | 2.0 | 4311 | 5356 | -790 | L1PA2 | L1 | LINE |
| 136603309 | 136604464 | - | 6.1 | 1 | 2163 | -112 | L1PA5 | L1 | LINE |

  

|  |  |  |  |  |  |  |  |  |  |
| --- | --- | --- | --- | --- | --- | --- | --- | --- | --- |
| chr3:138507327-138513969 | | | 6384 bp | | |  |  |  |  |
| Genome Start | Genome End | Strand | Div | Repeat Start | Repeat End | Repeat Left | Name | Family | Class |
| 138507327 | 138508465 | - | 29.2 | 4963 | 6140 | -1736 | L1MC4a | L1 | LINE |
| 138508469 | 138508608 | + | 18.6 | 1 | 146 | -30 | FRAM | Alu | SINE |
| 138508667 | 138509490 | - | 31.3 | 4125 | 4948 | -1266 | L1MC4a | L1 | LINE |
| 138509491 | 138509662 | + | 20.9 | 1 | 175 | -3 | MER5B | MER1\_type | DNA |
| 138509663 | 138510416 | - | 31.3 | 3392 | 4124 | -2090 | L1MC4a | L1 | LINE |
| 138510519 | 138511897 | - | 2.7 | 4796 | 6172 | 0 | L1PA3 | L1 | LINE |
| 138511899 | 138512757 | + | 2.7 | 3938 | 4796 | -1350 | L1PA3 | L1 | LINE |
| 138512757 | 138513792 | - | 31.1 | 2164 | 3282 | -2932 | L1MC4a | L1 | LINE |
| 138513889 | 138513969 | - | 24.7 | 2022 | 2104 | -4111 | L1MC4a | L1 | LINE |

  

|  |  |  |  |  |  |  |  |  |  |
| --- | --- | --- | --- | --- | --- | --- | --- | --- | --- |
| chr3:138569354-138572006 | | | 2650 bp | | |  |  |  |  |
| Genome Start | Genome End | Strand | Div | Repeat Start | Repeat End | Repeat Left | Name | Family | Class |
| 138569354 | 138570685 | - | 19.4 | 2504 | 3851 | -2686 | L1MDa | L1 | LINE |
| 138570689 | 138571404 | - | 1.8 | 5457 | 6173 | 0 | L1PA4 | L1 | LINE |
| 138571405 | 138571782 | + | 4.9 | 5098 | 5476 | -678 | L1PA4 | L1 | LINE |
| 138571783 | 138572006 | - | 18.8 | 2276 | 2495 | -4138 | L1MDa | L1 | LINE |

  

|  |  |  |  |  |  |  |  |  |  |
| --- | --- | --- | --- | --- | --- | --- | --- | --- | --- |
| chr3:138917489-138921766 | | | 3785 bp | | |  |  |  |  |
| Genome Start | Genome End | Strand | Div | Repeat Start | Repeat End | Repeat Left | Name | Family | Class |
| 138917489 | 138917601 | + | 22.2 | 3550 | 3662 | -827 | L3 | CR1 | LINE |
| 138917749 | 138917815 | + | 27.3 | 3351 | 3417 | -2 | L2 | L2 | LINE |
| 138918112 | 138918572 | - | 9.1 | 5680 | 6143 | -25 | L1PA12 | L1 | LINE |
| 138918573 | 138918871 | - | 14.1 | 1 | 300 | -12 | AluSx | Alu | SINE |
| 138918872 | 138920479 | - | 9.3 | 4027 | 5679 | -489 | L1PA12 | L1 | LINE |
| 138920477 | 138921636 | + | 11.3 | 2836 | 4014 | -2132 | L1PREC2 | L1 | LINE |
| 138921690 | 138921766 | + | 31.6 | 3683 | 3758 | -731 | L3 | CR1 | LINE |

  

|  |  |  |  |  |  |  |  |  |  |
| --- | --- | --- | --- | --- | --- | --- | --- | --- | --- |
| chr3:139084799-139090486 | | | 5161 bp | | |  |  |  |  |
| Genome Start | Genome End | Strand | Div | Repeat Start | Repeat End | Repeat Left | Name | Family | Class |
| 139084799 | 139085533 | - | 25.8 | 2147 | 2908 | -3311 | L1MEe | L1 | LINE |
| 139085525 | 139086950 | - | 8.1 | 4708 | 6154 | 0 | L1PA7 | L1 | LINE |
| 139086982 | 139087162 | - | 9.1 | 4512 | 4707 | -1439 | L1PA7 | L1 | LINE |
| 139087163 | 139088846 | + | 8.6 | 2829 | 4519 | -1627 | L1PA7 | L1 | LINE |
| 139088884 | 139089183 | - | 10.7 | 1 | 301 | -12 | AluSp | Alu | SINE |
| 139089184 | 139089471 | - | 24.7 | 1913 | 2218 | -4182 | L1MEe | L1 | LINE |
| 139089524 | 139089687 | - | 17.1 | 6143 | 6315 | -18 | L1MC1 | L1 | LINE |
| 139089688 | 139089821 | - | 30.3 | 933 | 1071 | -5138 | L1MEe | L1 | LINE |
| 139090199 | 139090311 | - | 37.5 | 600 | 712 | -5497 | L1MEe | L1 | LINE |
| 139090351 | 139090486 | - | 25.2 | 92 | 224 | -5985 | L1MEe | L1 | LINE |

  

|  |  |  |  |  |  |  |  |  |  |
| --- | --- | --- | --- | --- | --- | --- | --- | --- | --- |
| chr3:139152743-139161471 | | | 7909 bp | | |  |  |  |  |
| Genome Start | Genome End | Strand | Div | Repeat Start | Repeat End | Repeat Left | Name | Family | Class |
| 139152743 | 139152870 | + | 30.7 | 2479 | 2609 | -4024 | L1MDa | L1 | LINE |
| 139152931 | 139153221 | + | 10.7 | 13 | 310 | 0 | AluSg | Alu | SINE |
| 139153259 | 139156974 | - | 8.2 | 2435 | 6163 | -5 | L1PA10 | L1 | LINE |
| 139156971 | 139157240 | + | 6.3 | 2160 | 2429 | -3717 | L1PA10 | L1 | LINE |
| 139157412 | 139157850 | + | 28.1 | 108 | 545 | -45 | MLT1G | MaLR | LTR |
| 139157959 | 139158213 | + | 29.3 | 2913 | 3155 | -3382 | L1MDa | L1 | LINE |
| 139158598 | 139158737 | + | 28.3 | 3275 | 3413 | -3124 | L1MDa | L1 | LINE |
| 139158739 | 139159874 | - | 23.3 | 6719 | 7925 | -36 | L1MC5 | L1 | LINE |
| 139159938 | 139161471 | + | 27.2 | 3523 | 5079 | -1458 | L1MDa | L1 | LINE |

  

|  |  |  |  |  |  |  |  |  |  |
| --- | --- | --- | --- | --- | --- | --- | --- | --- | --- |
| chr3:145220490-145229125 | | | 8607 bp | | |  |  |  |  |
| Genome Start | Genome End | Strand | Div | Repeat Start | Repeat End | Repeat Left | Name | Family | Class |
| 145220490 | 145220626 | - | 18.8 | 137 | 302 | 0 | AluJo | Alu | SINE |
| 145220627 | 145220980 | - | 11.9 | 1 | 355 | 0 | THE1A | MaLR | LTR |
| 145220981 | 145222537 | - | 11.8 | 14 | 1580 | 0 | THE1A-int | MaLR | LTR |
| 145222538 | 145222885 | - | 8.1 | 7 | 355 | 0 | THE1A | MaLR | LTR |
| 145222909 | 145225651 | - | 7.4 | 11 | 2769 | -3377 | L1PA5 | L1 | LINE |
| 145225653 | 145228993 | + | 5.5 | 2786 | 6147 | -7 | L1PA5 | L1 | LINE |
| 145228999 | 145229125 | - | 18.9 | 5 | 133 | -169 | AluJo | Alu | SINE |

  

|  |  |  |  |  |  |  |  |  |  |
| --- | --- | --- | --- | --- | --- | --- | --- | --- | --- |
| chr3:145845850-145849711 | | | 3325 bp | | |  |  |  |  |
| Genome Start | Genome End | Strand | Div | Repeat Start | Repeat End | Repeat Left | Name | Family | Class |
| 145845850 | 145846016 | + | 28.2 | 6461 | 6628 | -1414 | L1MC4 | L1 | LINE |
| 145846382 | 145846543 | + | 17.1 | 5745 | 5902 | -281 | L1MB8 | L1 | LINE |
| 145846576 | 145846687 | + | 9.8 | 6059 | 6173 | -5 | L1MB8 | L1 | LINE |
| 145846717 | 145846766 | + | 18.0 | 7000 | 7049 | -993 | L1MC4 | L1 | LINE |
| 145846806 | 145846819 | + | 23.0 | 7239 | 7249 | -793 | L1MC4 | L1 | LINE |
| 145846820 | 145847309 | - | 26.7 | 1 | 593 | 0 | MLT1E | MaLR | LTR |
| 145847310 | 145847708 | + | 23.0 | 7250 | 7664 | -378 | L1MC4 | L1 | LINE |
| 145847707 | 145848298 | - | 17.6 | 5232 | 5814 | -498 | L1M2 | L1 | LINE |
| 145848299 | 145848661 | + | 26.3 | 3 | 365 | 0 | MLT1A0 | MaLR | LTR |
| 145848662 | 145849000 | - | 14.2 | 4886 | 5231 | -909 | L1M2 | L1 | LINE |
| 145848988 | 145849236 | + | 19.0 | 5817 | 6067 | -101 | L1MB1 | L1 | LINE |
| 145849270 | 145849533 | + | 22.5 | 6060 | 6318 | -16 | L1MA10 | L1 | LINE |
| 145849588 | 145849711 | + | 23.4 | 7689 | 7817 | -225 | L1MC4 | L1 | LINE |

  

|  |  |  |  |  |  |  |  |  |  |
| --- | --- | --- | --- | --- | --- | --- | --- | --- | --- |
| chr3:146232570-146233542 | | | 835 bp | | |  |  |  |  |
| Genome Start | Genome End | Strand | Div | Repeat Start | Repeat End | Repeat Left | Name | Family | Class |
| 146232570 | 146232715 | + | 31.9 | 14 | 162 | -359 | LTR33 | ERVL | LTR |
| 146232734 | 146232872 | + | 13.9 | 2209 | 2356 | -62 | Tigger1 | MER2\_type | DNA |
| 146232882 | 146233009 | - | 14.1 | 6023 | 6157 | -9 | L1PA16 | L1 | LINE |
| 146233010 | 146233108 | + | 17.4 | 5916 | 6014 | -152 | L1PA16 | L1 | LINE |
| 146233220 | 146233542 | + | 38.4 | 194 | 504 | -17 | LTR33 | ERVL | LTR |

  

|  |  |  |  |  |  |  |  |  |  |
| --- | --- | --- | --- | --- | --- | --- | --- | --- | --- |
| chr3:146384561-146386091 | | | 1511 bp | | |  |  |  |  |
| Genome Start | Genome End | Strand | Div | Repeat Start | Repeat End | Repeat Left | Name | Family | Class |
| 146384561 | 146385233 | - | 27.5 | 5458 | 6162 | -4 | L1ME1 | L1 | LINE |
| 146385229 | 146385470 | - | 11.6 | 5731 | 5972 | -330 | L1MA1 | L1 | LINE |
| 146385474 | 146385793 | + | 11.4 | 5970 | 6295 | -7 | L1MA1 | L1 | LINE |
| 146385816 | 146386091 | - | 23.3 | 5176 | 5465 | -701 | L1ME1 | L1 | LINE |

  

|  |  |  |  |  |  |  |  |  |  |
| --- | --- | --- | --- | --- | --- | --- | --- | --- | --- |
| chr3:146479397-146481278 | | | 1814 bp | | |  |  |  |  |
| Genome Start | Genome End | Strand | Div | Repeat Start | Repeat End | Repeat Left | Name | Family | Class |
| 146479397 | 146479791 | + | 24.1 | 4735 | 5139 | -1007 | L1MB8 | L1 | LINE |
| 146479796 | 146480009 | - | 18.6 | 5509 | 5739 | -573 | L1MA9 | L1 | LINE |
| 146479996 | 146480038 | + | 17.4 | 5740 | 5788 | -524 | L1MA9 | L1 | LINE |
| 146480087 | 146480474 | + | 19.5 | 5789 | 6292 | -20 | L1MA9 | L1 | LINE |
| 146480497 | 146480663 | + | 18.6 | 5135 | 5305 | -841 | L1MB8 | L1 | LINE |
| 146480672 | 146481278 | + | 23.8 | 5560 | 6177 | -1 | L1MB8 | L1 | LINE |

  

|  |  |  |  |  |  |  |  |  |  |
| --- | --- | --- | --- | --- | --- | --- | --- | --- | --- |
| chr3:146992570-146998137 | | | 5254 bp | | |  |  |  |  |
| Genome Start | Genome End | Strand | Div | Repeat Start | Repeat End | Repeat Left | Name | Family | Class |
| 146992570 | 146993109 | + | 18.3 | 1 | 555 | -156 | MER67C | ERV1 | LTR |
| 146993110 | 146993923 | - | 14.2 | 4566 | 5352 | -794 | L1P4 | L1 | LINE |
| 146993924 | 146994139 | - | 15.0 | 156 | 364 | 0 | THE1B | MaLR | LTR |
| 146994140 | 146994427 | + | 13.4 | 1 | 297 | -13 | AluSg | Alu | SINE |
| 146994428 | 146994598 | - | 15.0 | 1 | 155 | -209 | THE1B | MaLR | LTR |
| 146994599 | 146996176 | - | 32.3 | 2 | 1735 | 0 | MLT1A-int | MaLR | LTR |
| 146996179 | 146996547 | - | 13.3 | 1 | 364 | 0 | THE1B | MaLR | LTR |
| 146996548 | 146996792 | - | 14.2 | 4317 | 4565 | -1581 | L1P4 | L1 | LINE |
| 146996793 | 146997529 | + | 14.9 | 5373 | 6157 | -9 | L1PA16 | L1 | LINE |
| 146997680 | 146997800 | - | 18.2 | 4675 | 4798 | -1348 | L1P4 | L1 | LINE |
| 146997963 | 146998137 | + | 17.4 | 537 | 711 | 0 | MER67C | ERV1 | LTR |

  

|  |  |  |  |  |  |  |  |  |  |
| --- | --- | --- | --- | --- | --- | --- | --- | --- | --- |
| chr3:147034010-147036155 | | | 2146 bp | | |  |  |  |  |
| Genome Start | Genome End | Strand | Div | Repeat Start | Repeat End | Repeat Left | Name | Family | Class |
| 147034010 | 147034165 | + | 19.0 | 1 | 144 | -320 | MLT2C2 | ERVL | LTR |
| 147034166 | 147034550 | - | 15.2 | 1 | 381 | 0 | THE1D | MaLR | LTR |
| 147034551 | 147034669 | + | 19.0 | 145 | 266 | -198 | MLT2C2 | ERVL | LTR |
| 147034670 | 147034868 | - | 3.0 | 5344 | 5542 | -613 | L1PA4 | L1 | LINE |
| 147034869 | 147035493 | + | 3.8 | 5529 | 6155 | 0 | L1PA4 | L1 | LINE |
| 147035494 | 147035548 | + | 22.2 | 247 | 321 | -143 | MLT2C2 | ERVL | LTR |
| 147035549 | 147036042 | - | 17.9 | 5676 | 6168 | 0 | L1MB1 | L1 | LINE |
| 147036043 | 147036155 | + | 22.2 | 417 | 517 | -42 | MLT2C2 | ERVL | LTR |

  

|  |  |  |  |  |  |  |  |  |  |
| --- | --- | --- | --- | --- | --- | --- | --- | --- | --- |
| chr3:147380008-147381203 | | | 1030 bp | | |  |  |  |  |
| Genome Start | Genome End | Strand | Div | Repeat Start | Repeat End | Repeat Left | Name | Family | Class |
| 147380008 | 147380131 | - | 29.0 | 3268 | 3416 | -3 | L2 | L2 | LINE |
| 147380136 | 147380669 | - | 12.6 | 5617 | 6151 | 0 | L1PB1 | L1 | LINE |
| 147380670 | 147380990 | + | 9.3 | 5284 | 5609 | -542 | L1PB1 | L1 | LINE |
| 147381153 | 147381203 | - | 21.6 | 2818 | 2871 | -548 | L2 | L2 | LINE |

  

|  |  |  |  |  |  |  |  |  |  |
| --- | --- | --- | --- | --- | --- | --- | --- | --- | --- |
| chr3:147866479-147878374 | | | 11487 bp | | |  |  |  |  |
| Genome Start | Genome End | Strand | Div | Repeat Start | Repeat End | Repeat Left | Name | Family | Class |
| 147866479 | 147867348 | + | 33.5 | 1014 | 1918 | -1501 | L2 | L2 | LINE |
| 147867378 | 147867715 | - | 27.9 | 5813 | 6135 | -35 | L1ME3A | L1 | LINE |
| 147867716 | 147868540 | + | 31.8 | 1941 | 2815 | -604 | L2 | L2 | LINE |
| 147868541 | 147870537 | - | 14.8 | 4839 | 6853 | -16 | L1PA16 | L1 | LINE |
| 147870533 | 147873484 | - | 17.7 | 1 | 3150 | -3699 | L1PA16 | L1 | LINE |
| 147873610 | 147874604 | - | 27.4 | 3496 | 4550 | -308 | L1ME3A | L1 | LINE |
| 147874642 | 147874925 | - | 21.0 | 1 | 281 | -31 | AluJb | Alu | SINE |
| 147874926 | 147875074 | - | 27.7 | 3344 | 3495 | -1339 | L1ME3A | L1 | LINE |
| 147875128 | 147875626 | - | 25.9 | 899 | 1407 | -3427 | L1ME3A | L1 | LINE |
| 147875674 | 147875940 | - | 29.2 | 661 | 909 | -3945 | L1ME3A | L1 | LINE |
| 147875941 | 147876135 | - | 20.1 | 3 | 207 | -2 | MER3 | MER1\_type | DNA |
| 147876136 | 147876599 | - | 31.4 | 173 | 660 | -4194 | L1ME3A | L1 | LINE |
| 147876619 | 147876758 | - | 27.9 | 1 | 146 | -449 | MLT1G1 | MaLR | LTR |
| 147876760 | 147876836 | - | 18.1 | 3278 | 3350 | -2796 | L1M5 | L1 | LINE |
| 147876827 | 147877071 | - | 12.4 | 5871 | 6111 | -50 | L1PA15 | L1 | LINE |
| 147877072 | 147877121 | + | 10.0 | 6110 | 6159 | -6 | L1PA15 | L1 | LINE |
| 147877132 | 147877231 | - | 24.8 | 36 | 147 | -121 | MIR | MIR | SINE |
| 147877309 | 147877484 | - | 24.3 | 3106 | 3294 | -2852 | L1M5 | L1 | LINE |
| 147877511 | 147877642 | + | 32.4 | 2947 | 3096 | -323 | L2 | L2 | LINE |
| 147877643 | 147878009 | - | 13.0 | 1 | 381 | 0 | THE1D | MaLR | LTR |
| 147878010 | 147878374 | + | 32.4 | 3097 | 3418 | -1 | L2 | L2 | LINE |

  

|  |  |  |  |  |  |  |  |  |  |
| --- | --- | --- | --- | --- | --- | --- | --- | --- | --- |
| chr3:148150196-148151684 | | | 1373 bp | | |  |  |  |  |
| Genome Start | Genome End | Strand | Div | Repeat Start | Repeat End | Repeat Left | Name | Family | Class |
| 148150196 | 148150241 | + | 15.2 | 5809 | 5854 | -479 | L1MC1 | L1 | LINE |
| 148150239 | 148150539 | - | 14.0 | 4328 | 4638 | -1505 | L1M2 | L1 | LINE |
| 148150535 | 148150959 | + | 17.2 | 4651 | 5085 | -1058 | L1M2 | L1 | LINE |
| 148150967 | 148151123 | + | 16.9 | 6145 | 6298 | -2 | L1MA6 | L1 | LINE |
| 148151241 | 148151684 | + | 15.2 | 5840 | 6328 | -5 | L1MC1 | L1 | LINE |

  

|  |  |  |  |  |  |  |  |  |  |
| --- | --- | --- | --- | --- | --- | --- | --- | --- | --- |
| chr3:152535288-152535929 | | | 644 bp | | |  |  |  |  |
| Genome Start | Genome End | Strand | Div | Repeat Start | Repeat End | Repeat Left | Name | Family | Class |
| 152535288 | 152535513 | - | 19.7 | 87 | 327 | -14 | MER58B | MER1\_type | DNA |
| 152535514 | 152535793 | - | 16.1 | 5960 | 6260 | -40 | L1MA3 | L1 | LINE |
| 152535791 | 152535834 | + | 18.2 | 6262 | 6305 | 0 | L1MA3 | L1 | LINE |
| 152535836 | 152535929 | - | 20.9 | 1 | 91 | -250 | MER58B | MER1\_type | DNA |

  

|  |  |  |  |  |  |  |  |  |  |
| --- | --- | --- | --- | --- | --- | --- | --- | --- | --- |
| chr3:153448838-153452593 | | | 3710 bp | | |  |  |  |  |
| Genome Start | Genome End | Strand | Div | Repeat Start | Repeat End | Repeat Left | Name | Family | Class |
| 153448838 | 153449073 | - | 23.7 | 7803 | 8039 | -3 | L1MC4 | L1 | LINE |
| 153449103 | 153451395 | - | 2.4 | 3856 | 6148 | -7 | L1PA3 | L1 | LINE |
| 153451385 | 153451553 | + | 2.4 | 3706 | 3874 | -2272 | L1PA3 | L1 | LINE |
| 153451558 | 153451742 | - | 27.8 | 2369 | 2556 | -233 | L1MC4\_3endX | L1 | LINE |
| 153451753 | 153451977 | - | 17.4 | 6056 | 6286 | -5 | L1MA8 | L1 | LINE |
| 153451978 | 153452291 | - | 26.2 | 7331 | 7636 | -406 | L1MC4 | L1 | LINE |
| 153452306 | 153452593 | - | 22.3 | 6956 | 7252 | -790 | L1MC4 | L1 | LINE |

  

|  |  |  |  |  |  |  |  |  |  |
| --- | --- | --- | --- | --- | --- | --- | --- | --- | --- |
| chr3:154544721-154546866 | | | 1892 bp | | |  |  |  |  |
| Genome Start | Genome End | Strand | Div | Repeat Start | Repeat End | Repeat Left | Name | Family | Class |
| 154544721 | 154545154 | + | 29.4 | 3 | 439 | -84 | LTR33A | ERVL | LTR |
| 154545279 | 154545445 | + | 30.1 | 162 | 336 | -121 | LTR16A1 | ERVL | LTR |
| 154545448 | 154546349 | - | 2.8 | 5268 | 6173 | 0 | L1PA3 | L1 | LINE |
| 154546349 | 154546676 | + | 1.8 | 4928 | 5255 | -891 | L1PA3 | L1 | LINE |
| 154546806 | 154546866 | + | 21.3 | 463 | 523 | 0 | LTR33A | ERVL | LTR |

  

|  |  |  |  |  |  |  |  |  |  |
| --- | --- | --- | --- | --- | --- | --- | --- | --- | --- |
| chr3:154715995-154726931 | | | 10882 bp | | |  |  |  |  |
| Genome Start | Genome End | Strand | Div | Repeat Start | Repeat End | Repeat Left | Name | Family | Class |
| 154715995 | 154718115 | - | 16.3 | 4381 | 6476 | -6 | L1MA4 | L1 | LINE |
| 154718116 | 154718470 | - | 15.5 | 1 | 355 | 0 | THE1A | MaLR | LTR |
| 154718471 | 154719658 | - | 10.2 | 362 | 1580 | 0 | THE1A-int | MaLR | LTR |
| 154719659 | 154721149 | - | 3.1 | 2400 | 3890 | -2256 | L1PA3 | L1 | LINE |
| 154721141 | 154723406 | + | 3.5 | 3887 | 6152 | -3 | L1PA3 | L1 | LINE |
| 154723418 | 154723782 | - | 6.6 | 1 | 375 | -1205 | THE1A-int | MaLR | LTR |
| 154723846 | 154724142 | - | 15.3 | 1 | 298 | -57 | THE1A | MaLR | LTR |
| 154724143 | 154724334 | - | 20.3 | 4194 | 4385 | -1940 | L1MA4 | L1 | LINE |
| 154724325 | 154726931 | - | 20.9 | 182 | 2779 | -3546 | L1MA4 | L1 | LINE |

  

|  |  |  |  |  |  |  |  |  |  |
| --- | --- | --- | --- | --- | --- | --- | --- | --- | --- |
| chr3:154909098-154913778 | | | 4683 bp | | |  |  |  |  |
| Genome Start | Genome End | Strand | Div | Repeat Start | Repeat End | Repeat Left | Name | Family | Class |
| 154909098 | 154909215 | - | 21.4 | 6104 | 6222 | -20 | L1MD1 | L1 | LINE |
| 154909220 | 154909645 | - | 7.4 | 4917 | 5337 | -809 | L1PB | L1 | LINE |
| 154909642 | 154910440 | + | 8.0 | 4136 | 4927 | -1219 | L1PB | L1 | LINE |
| 154910439 | 154911823 | - | 18.6 | 4589 | 5963 | -279 | L1MD1 | L1 | LINE |
| 154911824 | 154912135 | - | 11.9 | 1 | 311 | 0 | AluY | Alu | SINE |
| 154912136 | 154913367 | - | 19.0 | 3352 | 4588 | -1558 | L1MD1 | L1 | LINE |
| 154913368 | 154913700 | + | 18.1 | 1 | 342 | -3 | MER2 | MER2\_type | DNA |
| 154913701 | 154913778 | - | 19.0 | 3249 | 3351 | -2795 | L1MD1 | L1 | LINE |

  

|  |  |  |  |  |  |  |  |  |  |
| --- | --- | --- | --- | --- | --- | --- | --- | --- | --- |
| chr3:160360790-160364147 | | | 3314 bp | | |  |  |  |  |
| Genome Start | Genome End | Strand | Div | Repeat Start | Repeat End | Repeat Left | Name | Family | Class |
| 160360790 | 160360965 | - | 13.1 | 121 | 296 | -16 | AluSx | Alu | SINE |
| 160361018 | 160363073 | - | 3.4 | 4094 | 6152 | -3 | L1PA3 | L1 | LINE |
| 160363066 | 160364010 | + | 3.6 | 3149 | 4093 | -2053 | L1PA3 | L1 | LINE |
| 160364011 | 160364147 | - | 10.2 | 1 | 137 | -175 | AluSx | Alu | SINE |

  

|  |  |  |  |  |  |  |  |  |  |
| --- | --- | --- | --- | --- | --- | --- | --- | --- | --- |
| chr3:164345660-164353349 | | | 7645 bp | | |  |  |  |  |
| Genome Start | Genome End | Strand | Div | Repeat Start | Repeat End | Repeat Left | Name | Family | Class |
| 164345660 | 164346227 | - | 12.8 | 4399 | 4929 | -1217 | L1PB | L1 | LINE |
| 164346251 | 164346791 | - | 13.9 | 3749 | 4398 | -1748 | L1P4 | L1 | LINE |
| 164346792 | 164346865 | + | 13.5 | 1 | 80 | 0 | MADE1 | Mariner | DNA |
| 164346866 | 164346933 | - | 18.4 | 3682 | 3748 | -2398 | L1P4 | L1 | LINE |
| 164346965 | 164351261 | - | 3.4 | 1845 | 6152 | -3 | L1PA3 | L1 | LINE |
| 164351255 | 164352262 | + | 5.1 | 838 | 1846 | -4309 | L1PA3 | L1 | LINE |
| 164352261 | 164353349 | - | 15.8 | 2581 | 3697 | -2449 | L1PB | L1 | LINE |

  

|  |  |  |  |  |  |  |  |  |  |
| --- | --- | --- | --- | --- | --- | --- | --- | --- | --- |
| chr3:164516935-164531101 | | | 14014 bp | | |  |  |  |  |
| Genome Start | Genome End | Strand | Div | Repeat Start | Repeat End | Repeat Left | Name | Family | Class |
| 164516935 | 164517629 | - | 22.0 | 5680 | 6374 | -631 | L1M1 | L1 | LINE |
| 164517645 | 164520175 | - | 4.6 | 3574 | 6154 | -1 | L1PA4 | L1 | LINE |
| 164520174 | 164521201 | + | 5.3 | 2543 | 3573 | -2573 | L1PA4 | L1 | LINE |
| 164521199 | 164524552 | - | 23.8 | 2290 | 5692 | -1153 | L1M1 | L1 | LINE |
| 164524553 | 164524746 | + | 24.2 | 1 | 193 | -150 | MER61A | ERV1 | LTR |
| 164524728 | 164524771 | + | 9.1 | 300 | 343 | 0 | MER61A | ERV1 | LTR |
| 164524773 | 164525244 | + | 23.7 | 1 | 483 | -4734 | MER61A-int | ERV1 | LTR |
| 164525245 | 164525350 | - | 2.8 | 5716 | 5821 | -334 | L1P2 | L1 | LINE |
| 164525351 | 164525452 | + | 1.0 | 6051 | 6152 | -3 | L1PA2 | L1 | LINE |
| 164525458 | 164525521 | + | 20.3 | 486 | 549 | -4668 | MER61A-int | ERV1 | LTR |
| 164525667 | 164526119 | + | 12.7 | 1406 | 1946 | -3271 | MER61A-int | ERV1 | LTR |
| 164526120 | 164526413 | - | 9.6 | 1 | 295 | -16 | AluY | Alu | SINE |
| 164526414 | 164529283 | + | 11.5 | 1947 | 4796 | -421 | MER61A-int | ERV1 | LTR |
| 164529274 | 164529463 | + | 15.3 | 5021 | 5216 | -1 | MER61A-int | ERV1 | LTR |
| 164529464 | 164529800 | + | 16.0 | 1 | 343 | 0 | MER61A | ERV1 | LTR |
| 164529822 | 164531101 | - | 31.3 | 824 | 2274 | -4575 | L1M1 | L1 | LINE |

  

|  |  |  |  |  |  |  |  |  |  |
| --- | --- | --- | --- | --- | --- | --- | --- | --- | --- |
| chr3:164670815-164673422 | | | 2568 bp | | |  |  |  |  |
| Genome Start | Genome End | Strand | Div | Repeat Start | Repeat End | Repeat Left | Name | Family | Class |
| 164670815 | 164671277 | - | 9.1 | 3877 | 4342 | -1804 | L1P3 | L1 | LINE |
| 164671326 | 164672550 | - | 2.3 | 4912 | 6153 | -2 | L1PA2 | L1 | LINE |
| 164672547 | 164672850 | + | 1.6 | 4605 | 4908 | -1238 | L1PA2 | L1 | LINE |
| 164672847 | 164673422 | - | 11.7 | 3311 | 3898 | -2248 | L1P3 | L1 | LINE |

  

|  |  |  |  |  |  |  |  |  |  |
| --- | --- | --- | --- | --- | --- | --- | --- | --- | --- |
| chr3:164833918-164837713 | | | 3749 bp | | |  |  |  |  |
| Genome Start | Genome End | Strand | Div | Repeat Start | Repeat End | Repeat Left | Name | Family | Class |
| 164833918 | 164834874 | - | 19.3 | 4392 | 5389 | -794 | L1M | L1 | LINE |
| 164834864 | 164836598 | - | 1.3 | 4421 | 6155 | 0 | L1PA2 | L1 | LINE |
| 164836596 | 164837132 | + | 1.9 | 3871 | 4411 | -1735 | L1PA2 | L1 | LINE |
| 164837146 | 164837207 | + | 24.2 | 1 | 63 | -273 | MER2B | MER2\_type | DNA |
| 164837256 | 164837713 | - | 19.3 | 3956 | 4421 | -1719 | L1M | L1 | LINE |

  

|  |  |  |  |  |  |  |  |  |  |
| --- | --- | --- | --- | --- | --- | --- | --- | --- | --- |
| chr3:164959885-164962523 | | | 2616 bp | | |  |  |  |  |
| Genome Start | Genome End | Strand | Div | Repeat Start | Repeat End | Repeat Left | Name | Family | Class |
| 164959885 | 164960702 | - | 20.8 | 5476 | 6290 | -1 | L1MA7 | L1 | LINE |
| 164960732 | 164961206 | - | 2.7 | 5676 | 6150 | -5 | L1PA3 | L1 | LINE |
| 164961207 | 164962352 | + | 2.1 | 4531 | 5676 | -479 | L1PA3 | L1 | LINE |
| 164962347 | 164962523 | - | 17.5 | 5317 | 5494 | -797 | L1MA7 | L1 | LINE |

  

|  |  |  |  |  |  |  |  |  |  |
| --- | --- | --- | --- | --- | --- | --- | --- | --- | --- |
| chr3:165048558-165052498 | | | 3500 bp | | |  |  |  |  |
| Genome Start | Genome End | Strand | Div | Repeat Start | Repeat End | Repeat Left | Name | Family | Class |
| 165048558 | 165048658 | - | 33.0 | 2574 | 2673 | -271 | BLACKJACK | AcHobo | DNA |
| 165048870 | 165049275 | - | 21.8 | 37 | 467 | 0 | MLT1C | MaLR | LTR |
| 165049314 | 165049386 | + | 19.7 | 76 | 141 | -197 | MER46C | MER2\_type | DNA |
| 165049534 | 165049656 | - | 16.3 | 6021 | 6143 | -8 | L1PB1 | L1 | LINE |
| 165049657 | 165050320 | + | 8.3 | 4916 | 5607 | -544 | L1PB1 | L1 | LINE |
| 165050321 | 165050612 | + | 11.4 | 1 | 294 | -15 | AluSc | Alu | SINE |
| 165050613 | 165050789 | + | 9.5 | 5608 | 5792 | -359 | L1PB1 | L1 | LINE |
| 165050824 | 165050909 | - | 18.8 | 483 | 567 | -7703 | PRIMA4-int | ERV1 | LTR |
| 165050921 | 165051151 | + | 15.7 | 5795 | 6023 | -128 | L1PB1 | L1 | LINE |
| 165051152 | 165052498 | - | 25.7 | 1149 | 2589 | -355 | BLACKJACK | AcHobo | DNA |

  

|  |  |  |  |  |  |  |  |  |  |
| --- | --- | --- | --- | --- | --- | --- | --- | --- | --- |
| chr3:165197104-165198599 | | | 1451 bp | | |  |  |  |  |
| Genome Start | Genome End | Strand | Div | Repeat Start | Repeat End | Repeat Left | Name | Family | Class |
| 165197104 | 165197392 | - | 29.6 | 2922 | 3215 | -3004 | L1MEc | L1 | LINE |
| 165197396 | 165197661 | - | 33.6 | 1742 | 2006 | -4233 | L1MEc | L1 | LINE |
| 165197711 | 165197911 | - | 9.0 | 5780 | 5980 | -194 | L1PA13 | L1 | LINE |
| 165197911 | 165198048 | + | 16.8 | 5985 | 6161 | -2 | L1PA13 | L1 | LINE |
| 165198043 | 165198599 | - | 28.4 | 1135 | 1716 | -4684 | L1MEc | L1 | LINE |

  

|  |  |  |  |  |  |  |  |  |  |
| --- | --- | --- | --- | --- | --- | --- | --- | --- | --- |
| chr3:165333821-165338038 | | | 4075 bp | | |  |  |  |  |
| Genome Start | Genome End | Strand | Div | Repeat Start | Repeat End | Repeat Left | Name | Family | Class |
| 165333821 | 165334097 | + | 22.9 | 4389 | 4663 | -1483 | L1ME2 | L1 | LINE |
| 165334242 | 165334535 | + | 31.9 | 5351 | 5641 | -523 | L1ME2 | L1 | LINE |
| 165334537 | 165334829 | - | 5.8 | 3169 | 3462 | -2684 | L1PA4 | L1 | LINE |
| 165334830 | 165337540 | + | 4.6 | 3438 | 6151 | -4 | L1PA4 | L1 | LINE |
| 165337539 | 165338038 | + | 26.2 | 5628 | 6156 | -8 | L1ME2 | L1 | LINE |

  

|  |  |  |  |  |  |  |  |  |  |
| --- | --- | --- | --- | --- | --- | --- | --- | --- | --- |
| chr3:166170535-166172158 | | | 1546 bp | | |  |  |  |  |
| Genome Start | Genome End | Strand | Div | Repeat Start | Repeat End | Repeat Left | Name | Family | Class |
| 166170535 | 166170878 | - | 29.9 | 5790 | 6140 | -26 | L1ME1 | L1 | LINE |
| 166170952 | 166171276 | - | 7.7 | 5812 | 6136 | -18 | L1PA7 | L1 | LINE |
| 166171277 | 166171921 | + | 7.5 | 5133 | 5787 | -385 | L1PA7 | L1 | LINE |
| 166171927 | 166172158 | - | 26.4 | 5540 | 5771 | -395 | L1ME1 | L1 | LINE |

  

|  |  |  |  |  |  |  |  |  |  |
| --- | --- | --- | --- | --- | --- | --- | --- | --- | --- |
| chr3:166560984-166563471 | | | 2440 bp | | |  |  |  |  |
| Genome Start | Genome End | Strand | Div | Repeat Start | Repeat End | Repeat Left | Name | Family | Class |
| 166560984 | 166561377 | - | 15.1 | 5892 | 6291 | 0 | L1MA7 | L1 | LINE |
| 166561430 | 166561798 | - | 3.5 | 5786 | 6155 | 0 | L1PA4 | L1 | LINE |
| 166561799 | 166562035 | + | 3.4 | 5552 | 5788 | -367 | L1PA4 | L1 | LINE |
| 166562032 | 166563471 | - | 18.9 | 4447 | 5907 | -384 | L1MA7 | L1 | LINE |

  

|  |  |  |  |  |  |  |  |  |  |
| --- | --- | --- | --- | --- | --- | --- | --- | --- | --- |
| chr3:166859232-166862823 | | | 3193 bp | | |  |  |  |  |
| Genome Start | Genome End | Strand | Div | Repeat Start | Repeat End | Repeat Left | Name | Family | Class |
| 166859232 | 166859382 | + | 21.2 | 4689 | 4845 | -1301 | L1ME1 | L1 | LINE |
| 166859395 | 166859511 | - | 20.6 | 249 | 353 | -12 | MLT1A0 | MaLR | LTR |
| 166859512 | 166859812 | + | 10.3 | 1 | 301 | -8 | AluSc | Alu | SINE |
| 166859813 | 166860063 | - | 20.6 | 2 | 248 | -117 | MLT1A0 | MaLR | LTR |
| 166860065 | 166860556 | + | 28.5 | 4842 | 5331 | -815 | L1MD | L1 | LINE |
| 166860559 | 166861612 | - | 15.7 | 5037 | 6117 | -49 | L1PA16 | L1 | LINE |
| 166861613 | 166861661 | + | 6.1 | 6108 | 6157 | -9 | L1PA16 | L1 | LINE |
| 166862046 | 166862823 | + | 28.6 | 5335 | 6112 | -54 | L1ME1 | L1 | LINE |

  

|  |  |  |  |  |  |  |  |  |  |
| --- | --- | --- | --- | --- | --- | --- | --- | --- | --- |
| chr3:167354982-167359823 | | | 4696 bp | | |  |  |  |  |
| Genome Start | Genome End | Strand | Div | Repeat Start | Repeat End | Repeat Left | Name | Family | Class |
| 167354982 | 167355786 | - | 23.9 | 5077 | 5994 | -170 | L1ME2 | L1 | LINE |
| 167355818 | 167356101 | + | 14.6 | 1 | 296 | -16 | AluSx | Alu | SINE |
| 167356209 | 167358257 | - | 5.1 | 4099 | 6153 | -1 | L1PA5 | L1 | LINE |
| 167358259 | 167358353 | + | 1.1 | 4001 | 4095 | -2051 | L1PA5 | L1 | LINE |
| 167358354 | 167358695 | + | 22.7 | 25 | 365 | 0 | MLT1A0 | MaLR | LTR |
| 167358703 | 167359823 | - | 29.7 | 3884 | 5071 | -1075 | L1ME2 | L1 | LINE |

  

|  |  |  |  |  |  |  |  |  |  |
| --- | --- | --- | --- | --- | --- | --- | --- | --- | --- |
| chr3:167478386-167479931 | | | 1466 bp | | |  |  |  |  |
| Genome Start | Genome End | Strand | Div | Repeat Start | Repeat End | Repeat Left | Name | Family | Class |
| 167478386 | 167478434 | + | 24.5 | 1 | 49 | -466 | MLT2B2 | ERVL | LTR |
| 167478435 | 167478744 | - | 11.7 | 5119 | 5437 | -728 | L1PA15 | L1 | LINE |
| 167478745 | 167479476 | + | 16.9 | 5420 | 6148 | -17 | L1PA15 | L1 | LINE |
| 167479484 | 167479822 | + | 27.2 | 50 | 499 | -63 | MLT2B2 | ERVL | LTR |
| 167479896 | 167479931 | + | 25.4 | 500 | 554 | -8 | MLT2B2 | ERVL | LTR |

  

|  |  |  |  |  |  |  |  |  |  |
| --- | --- | --- | --- | --- | --- | --- | --- | --- | --- |
| chr3:167751122-167755172 | | | 2978 bp | | |  |  |  |  |
| Genome Start | Genome End | Strand | Div | Repeat Start | Repeat End | Repeat Left | Name | Family | Class |
| 167751122 | 167751434 | - | 37.2 | 4028 | 4394 | -878 | HERVL | ERVL | LTR |
| 167751586 | 167751705 | - | 35.0 | 3523 | 3644 | -1352 | HERVL | ERVL | LTR |
| 167751987 | 167752741 | - | 34.3 | 2336 | 3072 | -1924 | HERVL | ERVL | LTR |
| 167752742 | 167753047 | + | 9.8 | 1 | 306 | -5 | AluY | Alu | SINE |
| 167753048 | 167753245 | - | 34.3 | 2110 | 2335 | -2661 | HERVL | ERVL | LTR |
| 167753275 | 167753636 | - | 32.3 | 1600 | 2039 | -2957 | HERVL | ERVL | LTR |
| 167753754 | 167754102 | - | 34.3 | 1066 | 1421 | -3575 | HERVL | ERVL | LTR |
| 167754105 | 167754508 | - | 18.9 | 5899 | 6308 | -4 | L1MA9 | L1 | LINE |
| 167754511 | 167754614 | + | 8.7 | 5810 | 5918 | -394 | L1MA9 | L1 | LINE |
| 167755106 | 167755172 | - | 28.4 | 600 | 667 | -4329 | HERVL | ERVL | LTR |

  

|  |  |  |  |  |  |  |  |  |  |
| --- | --- | --- | --- | --- | --- | --- | --- | --- | --- |
| chr3:167915928-167926542 | | | 10202 bp | | |  |  |  |  |
| Genome Start | Genome End | Strand | Div | Repeat Start | Repeat End | Repeat Left | Name | Family | Class |
| 167915928 | 167916138 | + | 11.4 | 1 | 216 | -684 | MER4D1 | ERV1 | LTR |
| 167916153 | 167916549 | - | 22.2 | 14 | 399 | -4 | MER57A | ERV1 | LTR |
| 167916566 | 167916635 | - | 8.6 | 7463 | 7532 | -5 | MER57A-int | ERV1 | LTR |
| 167916643 | 167917515 | - | 2.6 | 5281 | 6155 | 0 | L1PA4 | L1 | LINE |
| 167917514 | 167919680 | + | 4.5 | 3102 | 5271 | -875 | L1PA4 | L1 | LINE |
| 167919684 | 167920537 | - | 11.8 | 6541 | 7446 | -91 | MER57A-int | ERV1 | LTR |
| 167920565 | 167920577 | - | 11.8 | 6541 | 6540 | -997 | MER57A-int | ERV1 | LTR |
| 167920578 | 167921083 | - | 7.9 | 5633 | 6141 | -13 | L1PA6 | L1 | LINE |
| 167921084 | 167921909 | - | 11.8 | 5737 | 6540 | -997 | MER57A-int | ERV1 | LTR |
| 167922266 | 167922420 | - | 16.1 | 3927 | 4083 | -2104 | MER57A-int | ERV1 | LTR |
| 167922411 | 167922974 | - | 12.1 | 3153 | 3770 | -3767 | MER57A-int | ERV1 | LTR |
| 167922958 | 167923046 | - | 14.2 | 2955 | 3040 | -4497 | MER57A-int | ERV1 | LTR |
| 167923047 | 167923460 | + | 11.4 | 1 | 472 | 0 | MER4A1 | ERV1 | LTR |
| 167923461 | 167924177 | - | 14.2 | 2218 | 2954 | -4583 | MER57A-int | ERV1 | LTR |
| 167924169 | 167925531 | - | 16.0 | 3 | 1451 | -4495 | MER57A-int | ERV1 | LTR |
| 167925534 | 167925919 | - | 19.5 | 1 | 403 | 0 | MER57A | ERV1 | LTR |
| 167925946 | 167926542 | + | 11.9 | 279 | 883 | -17 | MER4D1 | ERV1 | LTR |

  

|  |  |  |  |  |  |  |  |  |  |
| --- | --- | --- | --- | --- | --- | --- | --- | --- | --- |
| chr3:168053306-168055883 | | | 2341 bp | | |  |  |  |  |
| Genome Start | Genome End | Strand | Div | Repeat Start | Repeat End | Repeat Left | Name | Family | Class |
| 168053306 | 168053518 | - | 25.6 | 7315 | 7525 | -436 | L1MC5 | L1 | LINE |
| 168053786 | 168054635 | - | 14.8 | 4478 | 5316 | -830 | L1PA16 | L1 | LINE |
| 168054636 | 168055517 | + | 16.1 | 5306 | 6161 | -5 | L1PA16 | L1 | LINE |
| 168055488 | 168055883 | - | 23.4 | 6633 | 7033 | -928 | L1MC5 | L1 | LINE |

  

|  |  |  |  |  |  |  |  |  |  |
| --- | --- | --- | --- | --- | --- | --- | --- | --- | --- |
| chr3:173700711-173704863 | | | 3955 bp | | |  |  |  |  |
| Genome Start | Genome End | Strand | Div | Repeat Start | Repeat End | Repeat Left | Name | Family | Class |
| 173700711 | 173700963 | + | 33.3 | 3611 | 3852 | -2294 | L1M5 | L1 | LINE |
| 173700959 | 173701113 | - | 3.9 | 5617 | 5771 | -384 | L1PA3 | L1 | LINE |
| 173701114 | 173701537 | + | 1.8 | 5764 | 6188 | 0 | L1PA3 | L1 | LINE |
| 173701538 | 173701710 | + | 17.9 | 3841 | 4022 | -2124 | L1M5 | L1 | LINE |
| 173701695 | 173702026 | + | 18.5 | 7413 | 7751 | -34 | L1MC3 | L1 | LINE |
| 173702038 | 173702791 | - | 20.6 | 1777 | 2625 | -3751 | L1M4c | L1 | LINE |
| 173702853 | 173703145 | - | 15.7 | 1 | 294 | -18 | AluJb | Alu | SINE |
| 173703166 | 173703386 | - | 28.3 | 1 | 240 | -22 | MIR | MIR | SINE |
| 173703485 | 173703637 | + | 31.4 | 4467 | 4614 | -1532 | L1M5 | L1 | LINE |
| 173703667 | 173703827 | + | 31.4 | 4615 | 4776 | -1370 | L1M5 | L1 | LINE |
| 173703828 | 173704233 | + | 17.3 | 5751 | 6176 | -7 | L1MB3 | L1 | LINE |
| 173704234 | 173704421 | + | 31.4 | 4777 | 4955 | -1191 | L1M5 | L1 | LINE |
| 173704422 | 173704712 | + | 12.2 | 1 | 289 | -20 | AluSg1 | Alu | SINE |
| 173704713 | 173704863 | + | 31.4 | 4956 | 5112 | -1034 | L1M5 | L1 | LINE |

  

|  |  |  |  |  |  |  |  |  |  |
| --- | --- | --- | --- | --- | --- | --- | --- | --- | --- |
| chr3:174472446-174483228 | | | 10702 bp | | |  |  |  |  |
| Genome Start | Genome End | Strand | Div | Repeat Start | Repeat End | Repeat Left | Name | Family | Class |
| 174472446 | 174472737 | + | 12.2 | 4 | 305 | -132 | LTR23 | ERV1 | LTR |
| 174472740 | 174474075 | - | 15.7 | 3725 | 5078 | -1068 | L1P4 | L1 | LINE |
| 174474075 | 174474535 | + | 13.3 | 5101 | 5575 | -581 | L1PB4 | L1 | LINE |
| 174474539 | 174474882 | + | 10.6 | 1 | 335 | -72 | MER48 | ERV1 | LTR |
| 174474883 | 174475191 | + | 9.9 | 1 | 310 | 0 | AluY | Alu | SINE |
| 174475192 | 174475266 | + | 10.6 | 336 | 407 | 0 | MER48 | ERV1 | LTR |
| 174475267 | 174478975 | + | 11.1 | 1 | 3820 | -2834 | HERVH48 | ERV1 | LTR |
| 174478980 | 174479240 | + | 7.3 | 2957 | 3216 | -3438 | HERVH48 | ERV1 | LTR |
| 174479236 | 174481993 | + | 9.4 | 3822 | 6649 | -5 | HERVH48 | ERV1 | LTR |
| 174481999 | 174482457 | + | 8.9 | 1 | 407 | 0 | MER48 | ERV1 | LTR |
| 174482458 | 174483035 | + | 14.5 | 5570 | 6151 | -5 | L1PB4 | L1 | LINE |
| 174483109 | 174483228 | + | 17.5 | 300 | 437 | 0 | LTR23 | ERV1 | LTR |

  

|  |  |  |  |  |  |  |  |  |  |
| --- | --- | --- | --- | --- | --- | --- | --- | --- | --- |
| chr3:174677073-174679762 | | | 2514 bp | | |  |  |  |  |
| Genome Start | Genome End | Strand | Div | Repeat Start | Repeat End | Repeat Left | Name | Family | Class |
| 174677073 | 174677192 | - | 29.8 | 2837 | 2957 | -462 | L2 | L2 | LINE |
| 174677353 | 174677742 | - | 33.1 | 2237 | 2608 | -811 | L2 | L2 | LINE |
| 174677743 | 174678043 | + | 19.5 | 1 | 298 | -14 | AluJo | Alu | SINE |
| 174678044 | 174678185 | - | 33.1 | 2097 | 2236 | -1183 | L2 | L2 | LINE |
| 174678193 | 174678332 | - | 2.9 | 5278 | 5423 | -745 | L1P | L1 | LINE |
| 174678333 | 174679053 | + | 9.0 | 5438 | 6167 | -1 | L1PA10 | L1 | LINE |
| 174679063 | 174679122 | - | 33.9 | 2035 | 2087 | -1332 | L2 | L2 | LINE |
| 174679123 | 174679406 | + | 12.0 | 1 | 311 | -1 | AluSx | Alu | SINE |
| 174679407 | 174679762 | - | 33.9 | 1626 | 2034 | -1385 | L2 | L2 | LINE |

  

|  |  |  |  |  |  |  |  |  |  |
| --- | --- | --- | --- | --- | --- | --- | --- | --- | --- |
| chr3:175906646-175908384 | | | 1701 bp | | |  |  |  |  |
| Genome Start | Genome End | Strand | Div | Repeat Start | Repeat End | Repeat Left | Name | Family | Class |
| 175906646 | 175906995 | + | 21.6 | 4849 | 5200 | -946 | L1M4 | L1 | LINE |
| 175906996 | 175907415 | - | 6.0 | 4859 | 5279 | -867 | L1PA5 | L1 | LINE |
| 175907414 | 175908287 | + | 4.0 | 5273 | 6150 | -4 | L1PA5 | L1 | LINE |
| 175908328 | 175908384 | + | 22.8 | 5197 | 5253 | -893 | L1M4 | L1 | LINE |

  

|  |  |  |  |  |  |  |  |  |  |
| --- | --- | --- | --- | --- | --- | --- | --- | --- | --- |
| chr3:177316180-177320544 | | | 4312 bp | | |  |  |  |  |
| Genome Start | Genome End | Strand | Div | Repeat Start | Repeat End | Repeat Left | Name | Family | Class |
| 177316180 | 177316376 | - | 33.3 | 1607 | 1807 | -1612 | L2 | L2 | LINE |
| 177316383 | 177316556 | - | 14.6 | 4312 | 4495 | -1651 | L1P4 | L1 | LINE |
| 177316557 | 177316867 | - | 14.3 | 1 | 308 | -2 | AluSg | Alu | SINE |
| 177316868 | 177317273 | - | 14.6 | 3906 | 4311 | -1835 | L1P4 | L1 | LINE |
| 177317274 | 177318265 | + | 13.7 | 4496 | 5363 | -783 | L1PA16 | L1 | LINE |
| 177318266 | 177318557 | + | 13.4 | 5 | 297 | -16 | AluSp | Alu | SINE |
| 177318558 | 177318817 | + | 11.8 | 5364 | 5602 | -564 | L1PA16 | L1 | LINE |
| 177318818 | 177318961 | + | 19.6 | 1 | 146 | -30 | FRAM | Alu | SINE |
| 177318976 | 177319540 | + | 12.9 | 5587 | 6157 | -9 | L1PA16 | L1 | LINE |
| 177319549 | 177320295 | - | 32.6 | 836 | 1603 | -1816 | L2 | L2 | LINE |
| 177320298 | 177320394 | + | 9.8 | 219 | 312 | 0 | AluSg/x | Alu | SINE |
| 177320418 | 177320544 | - | 30.7 | 674 | 802 | -2617 | L2 | L2 | LINE |

  

|  |  |  |  |  |  |  |  |  |  |
| --- | --- | --- | --- | --- | --- | --- | --- | --- | --- |
| chr3:177837845-177849416 | | | 11500 bp | | |  |  |  |  |
| Genome Start | Genome End | Strand | Div | Repeat Start | Repeat End | Repeat Left | Name | Family | Class |
| 177837845 | 177837983 | + | 25.6 | 65 | 225 | -5820 | L1MC3 | L1 | LINE |
| 177837984 | 177838193 | + | 11.5 | 1 | 230 | 0 | MER30 | MER1\_type | DNA |
| 177838194 | 177838219 | + | 25.6 | 226 | 243 | -5802 | L1MC3 | L1 | LINE |
| 177838225 | 177839066 | + | 21.6 | 1263 | 2105 | -4646 | L1MC3 | L1 | LINE |
| 177839107 | 177840133 | + | 21.0 | 2193 | 3297 | -3267 | L1MC3 | L1 | LINE |
| 177840131 | 177841429 | - | 8.4 | 4853 | 6156 | -12 | L1PA10 | L1 | LINE |
| 177841424 | 177842571 | + | 8.1 | 3700 | 4846 | -1300 | L1PA10 | L1 | LINE |
| 177842604 | 177843436 | - | 13.6 | 5304 | 6157 | -9 | L1PA16 | L1 | LINE |
| 177843437 | 177843771 | + | 20.5 | 3285 | 3626 | -2938 | L1MC3 | L1 | LINE |
| 177843772 | 177844069 | + | 16.0 | 1 | 288 | -24 | AluJb | Alu | SINE |
| 177844070 | 177844352 | + | 20.5 | 3627 | 3912 | -2652 | L1MC3 | L1 | LINE |
| 177844353 | 177844759 | + | 22.1 | 1 | 374 | 0 | MLT1A | MaLR | LTR |
| 177844760 | 177845463 | + | 20.5 | 3913 | 4592 | -1972 | L1MC3 | L1 | LINE |
| 177845464 | 177845824 | + | 19.6 | 1 | 365 | 0 | MLT1A0 | MaLR | LTR |
| 177845825 | 177845978 | + | 20.5 | 4593 | 4742 | -1822 | L1MC3 | L1 | LINE |
| 177845983 | 177846491 | - | 20.1 | 1 | 497 | -8 | MLT1D | MaLR | LTR |
| 177846492 | 177847444 | + | 21.6 | 4757 | 5738 | -826 | L1MC3 | L1 | LINE |
| 177847445 | 177847776 | + | 13.2 | 5813 | 6157 | -9 | L1PA16 | L1 | LINE |
| 177847777 | 177849416 | + | 19.8 | 5738 | 7474 | -729 | L1MC3 | L1 | LINE |

  

|  |  |  |  |  |  |  |  |  |  |
| --- | --- | --- | --- | --- | --- | --- | --- | --- | --- |
| chr3:178036417-178039294 | | | 2389 bp | | |  |  |  |  |
| Genome Start | Genome End | Strand | Div | Repeat Start | Repeat End | Repeat Left | Name | Family | Class |
| 178036417 | 178036855 | - | 22.5 | 5010 | 5462 | -778 | L1M5 | L1 | LINE |
| 178036939 | 178037295 | + | 16.0 | 1 | 364 | 0 | THE1B | MaLR | LTR |
| 178037450 | 178037540 | - | 15.6 | 6219 | 6312 | 0 | L1MA9 | L1 | LINE |
| 178037574 | 178037626 | - | 15.1 | 5720 | 5776 | -515 | L1M2 | L1 | LINE |
| 178037627 | 178038577 | + | 15.6 | 4744 | 5727 | -564 | L1M2 | L1 | LINE |
| 178038773 | 178039152 | + | 22.8 | 181 | 554 | 0 | MLT2B3 | ERVL | LTR |
| 178039177 | 178039294 | - | 22.9 | 4727 | 4844 | -1302 | L1M5 | L1 | LINE |

  

|  |  |  |  |  |  |  |  |  |  |
| --- | --- | --- | --- | --- | --- | --- | --- | --- | --- |
| chr3:178107940-178111127 | | | 2707 bp | | |  |  |  |  |
| Genome Start | Genome End | Strand | Div | Repeat Start | Repeat End | Repeat Left | Name | Family | Class |
| 178107940 | 178108021 | - | 32.1 | 3292 | 3374 | -4 | L2 | L2 | LINE |
| 178108031 | 178108678 | - | 9.3 | 4958 | 5605 | -555 | L1PREC2 | L1 | LINE |
| 178108680 | 178109225 | + | 11.5 | 5615 | 6159 | -1 | L1PREC2 | L1 | LINE |
| 178109237 | 178109781 | - | 36.7 | 2684 | 3280 | -98 | L2 | L2 | LINE |
| 178109797 | 178110177 | + | 32.7 | 2011 | 2412 | -1007 | L2 | L2 | LINE |
| 178110619 | 178111059 | + | 38.8 | 2910 | 3373 | -5 | L2 | L2 | LINE |
| 178111064 | 178111127 | - | 27.0 | 1509 | 1574 | -1845 | L2 | L2 | LINE |

  

|  |  |  |  |  |  |  |  |  |  |
| --- | --- | --- | --- | --- | --- | --- | --- | --- | --- |
| chr3:179211790-179216454 | | | 4606 bp | | |  |  |  |  |
| Genome Start | Genome End | Strand | Div | Repeat Start | Repeat End | Repeat Left | Name | Family | Class |
| 179211790 | 179212229 | - | 29.1 | 1684 | 2121 | -1910 | Zaphod | Tip100 | DNA |
| 179212241 | 179212551 | - | 7.7 | 5845 | 6162 | -1 | L1PA13 | L1 | LINE |
| 179212552 | 179212800 | + | 7.4 | 5605 | 5837 | -326 | L1PA13 | L1 | LINE |
| 179212835 | 179214148 | - | 24.9 | 244 | 1670 | -2361 | Zaphod | Tip100 | DNA |
| 179214163 | 179216283 | - | 1.9 | 4029 | 6155 | 0 | L1PA3 | L1 | LINE |
| 179216284 | 179216454 | - | 24.2 | 11 | 250 | -3781 | Zaphod | Tip100 | DNA |

  

|  |  |  |  |  |  |  |  |  |  |
| --- | --- | --- | --- | --- | --- | --- | --- | --- | --- |
| chr3:180505422-180508735 | | | 3247 bp | | |  |  |  |  |
| Genome Start | Genome End | Strand | Div | Repeat Start | Repeat End | Repeat Left | Name | Family | Class |
| 180505422 | 180505480 | - | 27.2 | 2469 | 2506 | -1 | HAL1 | L1 | LINE |
| 180505481 | 180505786 | - | 9.0 | 2 | 309 | 0 | AluSx | Alu | SINE |
| 180505787 | 180506998 | - | 27.2 | 1152 | 2468 | -39 | HAL1 | L1 | LINE |
| 180507002 | 180507249 | - | 17.7 | 1 | 265 | -47 | AluJb | Alu | SINE |
| 180507271 | 180507713 | - | 26.4 | 579 | 1087 | -1420 | HAL1 | L1 | LINE |
| 180507734 | 180508013 | - | 5.7 | 5484 | 5772 | -396 | L1PA10 | L1 | LINE |
| 180508014 | 180508417 | + | 7.2 | 5761 | 6168 | 0 | L1PA10 | L1 | LINE |
| 180508441 | 180508735 | - | 33.0 | 236 | 561 | -1946 | HAL1 | L1 | LINE |

  

|  |  |  |  |  |  |  |  |  |  |
| --- | --- | --- | --- | --- | --- | --- | --- | --- | --- |
| chr3:181660394-181663970 | | | 3439 bp | | |  |  |  |  |
| Genome Start | Genome End | Strand | Div | Repeat Start | Repeat End | Repeat Left | Name | Family | Class |
| 181660394 | 181660500 | - | 26.2 | 3961 | 4114 | -375 | L3 | CR1 | LINE |
| 181660532 | 181661076 | - | 14.8 | 5601 | 6156 | 0 | L1PB4 | L1 | LINE |
| 181661076 | 181661735 | - | 15.5 | 5660 | 6333 | 0 | L1MC1 | L1 | LINE |
| 181661736 | 181662529 | - | 2.4 | 5354 | 6151 | -4 | L1PA3 | L1 | LINE |
| 181662527 | 181663487 | + | 2.9 | 4394 | 5356 | -790 | L1PA3 | L1 | LINE |
| 181663599 | 181663970 | - | 30.1 | 3466 | 3879 | -610 | L3 | CR1 | LINE |

  

|  |  |  |  |  |  |  |  |  |  |
| --- | --- | --- | --- | --- | --- | --- | --- | --- | --- |
| chr3:181684061-181691315 | | | 7254 bp | | |  |  |  |  |
| Genome Start | Genome End | Strand | Div | Repeat Start | Repeat End | Repeat Left | Name | Family | Class |
| 181684061 | 181684147 | + | 12.6 | 12 | 98 | -6057 | L1PA5 | L1 | LINE |
| 181684146 | 181685025 | + | 8.1 | 605 | 1504 | -771 | L1PA5 | L1 | LINE |
| 181685024 | 181685639 | - | 1.9 | 4833 | 5447 | -708 | L1PA3 | L1 | LINE |
| 181685640 | 181686336 | + | 1.0 | 5458 | 6155 | 0 | L1PA3 | L1 | LINE |
| 181686342 | 181687232 | + | 4.4 | 1485 | 2362 | -3784 | L1PA5 | L1 | LINE |
| 181687233 | 181687531 | + | 8.7 | 1 | 299 | -12 | AluY | Alu | SINE |
| 181687532 | 181691315 | + | 6.1 | 2363 | 6149 | -5 | L1PA5 | L1 | LINE |

  

|  |  |  |  |  |  |  |  |  |  |
| --- | --- | --- | --- | --- | --- | --- | --- | --- | --- |
| chr3:182356678-182367924 | | | 11230 bp | | |  |  |  |  |
| Genome Start | Genome End | Strand | Div | Repeat Start | Repeat End | Repeat Left | Name | Family | Class |
| 182356678 | 182356762 | - | 25.9 | 3335 | 3419 | 0 | L2 | L2 | LINE |
| 182356740 | 182356879 | - | 39.1 | 2807 | 2946 | -473 | L2 | L2 | LINE |
| 182356904 | 182357245 | - | 17.9 | 5816 | 6166 | -2 | L1MB1 | L1 | LINE |
| 182357246 | 182357555 | - | 10.8 | 1 | 308 | -1 | AluSc | Alu | SINE |
| 182357556 | 182357760 | - | 17.9 | 5616 | 5815 | -353 | L1MB1 | L1 | LINE |
| 182357761 | 182358066 | - | 11.7 | 1 | 306 | 0 | AluSx | Alu | SINE |
| 182358067 | 182358267 | - | 17.9 | 5413 | 5615 | -553 | L1MB1 | L1 | LINE |
| 182358267 | 182359581 | - | 2.6 | 4839 | 6153 | -2 | L1PA4 | L1 | LINE |
| 182359579 | 182360419 | + | 4.0 | 3997 | 4837 | -1309 | L1PA4 | L1 | LINE |
| 182360420 | 182360836 | - | 17.0 | 6432 | 6843 | -744 | L1M | L1 | LINE |
| 182360837 | 182361125 | - | 4.5 | 5863 | 6151 | -3 | L1PA5 | L1 | LINE |
| 182361126 | 182361389 | - | 16.8 | 6034 | 6431 | -1128 | L1M | L1 | LINE |
| 182361390 | 182361903 | - | 12.7 | 5782 | 6305 | -7 | L1MA9 | L1 | LINE |
| 182361904 | 182361950 | - | 16.8 | 5919 | 6033 | -1526 | L1M | L1 | LINE |
| 182361951 | 182362028 | + | 23.4 | 2 | 78 | -2245 | Tigger5 | MER2\_type | DNA |
| 182362032 | 182362104 | - | 24.7 | 2328 | 2400 | 0 | Tigger5 | MER2\_type | DNA |
| 182362098 | 182362172 | + | 24.0 | 2325 | 2399 | 0 | Tigger5 | MER2\_type | DNA |
| 182362174 | 182362561 | - | 16.3 | 1 | 426 | 0 | MSTB | MaLR | LTR |
| 182362562 | 182362830 | - | 16.8 | 5462 | 5930 | -1629 | L1M | L1 | LINE |
| 182362854 | 182363675 | - | 16.8 | 4619 | 5461 | -2098 | L1M | L1 | LINE |
| 182363676 | 182363990 | + | 12.2 | 1 | 310 | 0 | AluSg | Alu | SINE |
| 182363991 | 182366088 | - | 22.1 | 2464 | 4618 | -2941 | L1M1 | L1 | LINE |
| 182366089 | 182366445 | - | 23.3 | 1 | 365 | 0 | MLT1A0 | MaLR | LTR |
| 182366446 | 182367664 | - | 27.5 | 1 | 1201 | -4690 | L1M3d | L1 | LINE |
| 182367665 | 182367924 | - | 30.6 | 2551 | 2808 | -611 | L2 | L2 | LINE |

  

|  |  |  |  |  |  |  |  |  |  |
| --- | --- | --- | --- | --- | --- | --- | --- | --- | --- |
| chr3:182670173-182683665 | | | 13058 bp | | |  |  |  |  |
| Genome Start | Genome End | Strand | Div | Repeat Start | Repeat End | Repeat Left | Name | Family | Class |
| 182670173 | 182670240 | - | 29.4 | 4306 | 4373 | -116 | L3 | CR1 | LINE |
| 182670275 | 182670449 | - | 36.3 | 2608 | 2784 | -635 | L2 | L2 | LINE |
| 182670456 | 182670540 | - | 27.1 | 1 | 89 | -466 | MLT1H1 | MaLR | LTR |
| 182670542 | 182670591 | - | 14.9 | 2556 | 2604 | -1885 | L3 | CR1 | LINE |
| 182670636 | 182670867 | - | 19.8 | 5923 | 6154 | -2 | L1PB4 | L1 | LINE |
| 182670950 | 182671278 | - | 12.3 | 5592 | 5907 | -249 | L1PB4 | L1 | LINE |
| 182671353 | 182672187 | - | 9.2 | 5647 | 6489 | 0 | L1PA11 | L1 | LINE |
| 182672184 | 182673434 | - | 9.4 | 4161 | 5371 | -1090 | L1PA11 | L1 | LINE |
| 182673435 | 182674169 | - | 6.0 | 5424 | 6159 | -13 | L1PA8 | L1 | LINE |
| 182674170 | 182678391 | - | 15.0 | 10 | 4160 | -2301 | L1PA11 | L1 | LINE |
| 182678513 | 182678665 | - | 20.0 | 9 | 160 | 0 | MER5A1 | MER1\_type | DNA |
| 182678677 | 182679535 | - | 7.3 | 5285 | 6149 | -5 | L1PA7 | L1 | LINE |
| 182679531 | 182683391 | + | 8.2 | 1416 | 5293 | -853 | L1PA7 | L1 | LINE |
| 182683463 | 182683665 | - | 31.7 | 2040 | 2266 | -2223 | L3 | CR1 | LINE |

  

|  |  |  |  |  |  |  |  |  |  |
| --- | --- | --- | --- | --- | --- | --- | --- | --- | --- |
| chr3:182726594-182729846 | | | 2718 bp | | |  |  |  |  |
| Genome Start | Genome End | Strand | Div | Repeat Start | Repeat End | Repeat Left | Name | Family | Class |
| 182726594 | 182726918 | + | 32.2 | 2306 | 2651 | -3495 | L1M4 | L1 | LINE |
| 182727103 | 182727234 | + | 26.7 | 2691 | 2831 | -3315 | L1M4 | L1 | LINE |
| 182727342 | 182727425 | + | 20.7 | 3043 | 3116 | -3030 | L1M4 | L1 | LINE |
| 182727426 | 182727539 | + | 14.0 | 6190 | 6304 | -8 | L1MA9 | L1 | LINE |
| 182727540 | 182727628 | + | 20.7 | 3117 | 3209 | -2937 | L1M4 | L1 | LINE |
| 182727763 | 182728070 | - | 12.4 | 1 | 308 | -1 | AluSc | Alu | SINE |
| 182728102 | 182729087 | - | 7.4 | 4589 | 5579 | -572 | L1PB1 | L1 | LINE |
| 182729088 | 182729667 | + | 12.4 | 5570 | 6148 | -3 | L1PB1 | L1 | LINE |
| 182729747 | 182729846 | + | 26.0 | 3379 | 3479 | -2667 | L1M4 | L1 | LINE |

  

|  |  |  |  |  |  |  |  |  |  |
| --- | --- | --- | --- | --- | --- | --- | --- | --- | --- |
| chr3:186437636-186445612 | | | 7852 bp | | |  |  |  |  |
| Genome Start | Genome End | Strand | Div | Repeat Start | Repeat End | Repeat Left | Name | Family | Class |
| 186437636 | 186438213 | - | 25.3 | 3758 | 4367 | -1779 | L1M5 | L1 | LINE |
| 186438244 | 186441222 | - | 13.7 | 3641 | 6617 | -5 | L1PB4 | L1 | LINE |
| 186441292 | 186442516 | - | 7.8 | 4916 | 6151 | 0 | L1PB1 | L1 | LINE |
| 186442512 | 186443066 | + | 7.1 | 4340 | 4908 | -1238 | L1PB1 | L1 | LINE |
| 186443069 | 186443874 | - | 12.3 | 5 | 818 | -5794 | L1PB4 | L1 | LINE |
| 186443877 | 186444893 | - | 20.8 | 2779 | 3894 | -2952 | L1PA15-16 | L1 | LINE |
| 186444899 | 186445017 | - | 17.6 | 3619 | 3738 | -2408 | L1M5 | L1 | LINE |
| 186445038 | 186445400 | + | 23.8 | 53 | 465 | -2 | MLT1C | MaLR | LTR |
| 186445403 | 186445612 | - | 26.2 | 3410 | 3626 | -2520 | L1M5 | L1 | LINE |

  

|  |  |  |  |  |  |  |  |  |  |
| --- | --- | --- | --- | --- | --- | --- | --- | --- | --- |
| chr3:187579377-187581038 | | | 1656 bp | | |  |  |  |  |
| Genome Start | Genome End | Strand | Div | Repeat Start | Repeat End | Repeat Left | Name | Family | Class |
| 187579377 | 187579609 | - | 24.4 | 237 | 567 | 0 | MLT2B1 | ERVL | LTR |
| 187579610 | 187579654 | - | 8.9 | 5003 | 5049 | -1097 | L1PA3 | L1 | LINE |
| 187579643 | 187580767 | + | 2.5 | 5028 | 6155 | 0 | L1PA3 | L1 | LINE |
| 187580786 | 187581038 | - | 17.1 | 7 | 257 | -263 | MLT2B1 | ERVL | LTR |

  

|  |  |  |  |  |  |  |  |  |  |
| --- | --- | --- | --- | --- | --- | --- | --- | --- | --- |
| chr3:190717206-190725772 | | | 8530 bp | | |  |  |  |  |
| Genome Start | Genome End | Strand | Div | Repeat Start | Repeat End | Repeat Left | Name | Family | Class |
| 190717206 | 190720003 | + | 21.5 | 3 | 2930 | -4186 | L1M2a1 | L1 | LINE |
| 190720005 | 190720346 | - | 16.1 | 15 | 375 | 0 | THE1C | MaLR | LTR |
| 190720370 | 190720419 | - | 10.0 | 5032 | 5081 | -1065 | L1P1 | L1 | LINE |
| 190720418 | 190721505 | + | 1.4 | 5063 | 6152 | -3 | L1PA3 | L1 | LINE |
| 190721521 | 190725772 | + | 19.4 | 2911 | 6731 | -404 | L1M2a1 | L1 | LINE |

  

|  |  |  |  |  |  |  |  |  |  |
| --- | --- | --- | --- | --- | --- | --- | --- | --- | --- |
| chr3:192665303-192668216 | | | 2903 bp | | |  |  |  |  |
| Genome Start | Genome End | Strand | Div | Repeat Start | Repeat End | Repeat Left | Name | Family | Class |
| 192665303 | 192665576 | - | 12.6 | 100 | 375 | 0 | THE1C | MaLR | LTR |
| 192665593 | 192667533 | - | 3.7 | 4213 | 6155 | 0 | L1PA3 | L1 | LINE |
| 192667529 | 192668112 | + | 3.2 | 3650 | 4233 | -1913 | L1PA3 | L1 | LINE |
| 192668113 | 192668216 | - | 18.8 | 1 | 101 | -274 | THE1C | MaLR | LTR |

  

|  |  |  |  |  |  |  |  |  |  |
| --- | --- | --- | --- | --- | --- | --- | --- | --- | --- |
| chr3:192822443-192826444 | | | 3798 bp | | |  |  |  |  |
| Genome Start | Genome End | Strand | Div | Repeat Start | Repeat End | Repeat Left | Name | Family | Class |
| 192822443 | 192822687 | - | 30.2 | 3652 | 3882 | -2247 | L1MEd | L1 | LINE |
| 192822666 | 192823220 | - | 32.9 | 2253 | 2827 | -3302 | L1MEd | L1 | LINE |
| 192823260 | 192823697 | - | 9.6 | 4929 | 5366 | -780 | L1PA7 | L1 | LINE |
| 192823687 | 192824472 | + | 6.0 | 5366 | 6153 | -1 | L1PA7 | L1 | LINE |
| 192824505 | 192824555 | + | 25.5 | 201 | 251 | -11 | MIR | MIR | SINE |
| 192824564 | 192824774 | + | 29.1 | 3 | 226 | -36 | MIR | MIR | SINE |
| 192824775 | 192824868 | - | 28.7 | 2155 | 2247 | -3902 | L1MEd | L1 | LINE |
| 192824869 | 192825351 | - | 31.2 | 1 | 555 | 0 | MLT1H1 | MaLR | LTR |
| 192825352 | 192825529 | - | 28.7 | 1984 | 2154 | -3995 | L1MEd | L1 | LINE |
| 192825600 | 192825718 | - | 32.2 | 1744 | 1865 | -4284 | L1MEd | L1 | LINE |
| 192825784 | 192826071 | - | 27.5 | 989 | 1211 | -4908 | L1MEd | L1 | LINE |
| 192826095 | 192826444 | - | 34.2 | 444 | 796 | -5323 | L1MEd | L1 | LINE |

  

|  |  |  |  |  |  |  |  |  |  |
| --- | --- | --- | --- | --- | --- | --- | --- | --- | --- |
| chr3:193800118-193802936 | | | 2754 bp | | |  |  |  |  |
| Genome Start | Genome End | Strand | Div | Repeat Start | Repeat End | Repeat Left | Name | Family | Class |
| 193800118 | 193800310 | - | 26.7 | 3218 | 3419 | 0 | L2 | L2 | LINE |
| 193800313 | 193800368 | + | 17.9 | 5916 | 5976 | -368 | L1MC1 | L1 | LINE |
| 193800368 | 193800966 | - | 15.7 | 4555 | 5154 | -989 | L1MA5A | L1 | LINE |
| 193800968 | 193802069 | + | 12.2 | 5177 | 6297 | 0 | L1MA5A | L1 | LINE |
| 193802070 | 193802153 | + | 17.4 | 5961 | 6044 | -289 | L1MC1 | L1 | LINE |
| 193802154 | 193802475 | - | 17.7 | 1 | 345 | 0 | MER2 | MER2\_type | DNA |
| 193802476 | 193802760 | + | 17.4 | 6045 | 6327 | -6 | L1MC1 | L1 | LINE |
| 193802824 | 193802936 | - | 31.2 | 3087 | 3203 | -216 | L2 | L2 | LINE |

  

|  |  |  |  |  |  |  |  |  |  |
| --- | --- | --- | --- | --- | --- | --- | --- | --- | --- |
| chr3:194726465-194727385 | | | 764 bp | | |  |  |  |  |
| Genome Start | Genome End | Strand | Div | Repeat Start | Repeat End | Repeat Left | Name | Family | Class |
| 194726465 | 194726544 | + | 26.2 | 5532 | 5614 | -626 | L1ME3B | L1 | LINE |
| 194726648 | 194726683 | - | 5.6 | 5697 | 5732 | -440 | L1PA8 | L1 | LINE |
| 194726684 | 194727112 | + | 7.8 | 5745 | 6168 | -4 | L1PA8 | L1 | LINE |
| 194727167 | 194727385 | + | 30.3 | 5955 | 6165 | -75 | L1ME3B | L1 | LINE |

  


|  |  |  |  |  |  |  |  |  |  |
| --- | --- | --- | --- | --- | --- | --- | --- | --- | --- |
| chr4:1120734-1126793 | | | 5788 bp | | |  |  |  |  |
| Genome Start | Genome End | Strand | Div | Repeat Start | Repeat End | Repeat Left | Name | Family | Class |
| 1120734 | 1121536 | - | 23.6 | 1300 | 2348 | -3913 | L1MEb | L1 | LINE |
| 1121533 | 1122114 | - | 16.4 | 5597 | 6167 | -1 | L1MB1 | L1 | LINE |
| 1122115 | 1122399 | - | 10.6 | 1 | 308 | -1 | AluSg1 | Alu | SINE |
| 1122400 | 1123226 | - | 15.3 | 4762 | 5596 | -572 | L1MB1 | L1 | LINE |
| 1123227 | 1123540 | - | 13.8 | 1 | 312 | 0 | AluSx | Alu | SINE |
| 1123541 | 1123584 | - | 15.0 | 4723 | 4761 | -1379 | L1MB1 | L1 | LINE |
| 1123589 | 1124597 | + | 14.5 | 3242 | 4265 | -1875 | L1M3 | L1 | LINE |
| 1124598 | 1124900 | + | 9.7 | 1 | 306 | 0 | AluY | Alu | SINE |
| 1124901 | 1125362 | + | 14.5 | 4266 | 4744 | -1396 | L1M3 | L1 | LINE |
| 1125399 | 1126203 | - | 26.6 | 454 | 1255 | -5153 | L1MEb | L1 | LINE |
| 1126440 | 1126793 | - | 33.0 | 90 | 463 | -5607 | L1MEb | L1 | LINE |

  

|  |  |  |  |  |  |  |  |  |  |
| --- | --- | --- | --- | --- | --- | --- | --- | --- | --- |
| chr4:4106858-4107799 | | | 904 bp | | |  |  |  |  |
| Genome Start | Genome End | Strand | Div | Repeat Start | Repeat End | Repeat Left | Name | Family | Class |
| 4106858 | 4106984 | + | 19.4 | 5 | 129 | -49 | MER5B | MER1\_type | DNA |
| 4107003 | 4107310 | - | 14.3 | 2 | 308 | -4 | AluSx | Alu | SINE |
| 4107316 | 4107594 | - | 18.4 | 5902 | 6174 | 0 | L1MB5 | L1 | LINE |
| 4107595 | 4107740 | + | 20.8 | 5716 | 5887 | -287 | L1MB5 | L1 | LINE |
| 4107756 | 4107799 | + | 6.8 | 135 | 178 | 0 | MER5B | MER1\_type | DNA |

  

|  |  |  |  |  |  |  |  |  |  |
| --- | --- | --- | --- | --- | --- | --- | --- | --- | --- |
| chr4:5184058-5185879 | | | 1814 bp | | |  |  |  |  |
| Genome Start | Genome End | Strand | Div | Repeat Start | Repeat End | Repeat Left | Name | Family | Class |
| 5184058 | 5184449 | + | 32.6 | 2808 | 3197 | -222 | L2 | L2 | LINE |
| 5184472 | 5184653 | + | 21.7 | 1 | 191 | -314 | MLT1D | MaLR | LTR |
| 5184654 | 5184927 | - | 11.7 | 5624 | 5904 | -261 | L1PA15 | L1 | LINE |
| 5184908 | 5185120 | + | 15.0 | 5917 | 6154 | -11 | L1PA15 | L1 | LINE |
| 5185121 | 5185392 | + | 21.0 | 178 | 505 | 0 | MLT1D | MaLR | LTR |
| 5185399 | 5185425 | + | 34.8 | 3219 | 3245 | -174 | L2 | L2 | LINE |
| 5185426 | 5185725 | - | 12.9 | 1 | 296 | -16 | AluSx | Alu | SINE |
| 5185726 | 5185879 | + | 34.8 | 3246 | 3404 | -15 | L2 | L2 | LINE |

  

|  |  |  |  |  |  |  |  |  |  |
| --- | --- | --- | --- | --- | --- | --- | --- | --- | --- |
| chr4:5322379-5326978 | | | 4579 bp | | |  |  |  |  |
| Genome Start | Genome End | Strand | Div | Repeat Start | Repeat End | Repeat Left | Name | Family | Class |
| 5322379 | 5322752 | + | 24.7 | 3604 | 4007 | -2139 | L1M5 | L1 | LINE |
| 5322753 | 5323199 | - | 8.3 | 1 | 453 | 0 | MLT2A2 | ERVL | LTR |
| 5323200 | 5323586 | + | 24.7 | 4008 | 4376 | -1770 | L1M5 | L1 | LINE |
| 5323587 | 5323864 | - | 11.6 | 1 | 280 | -30 | AluSg | Alu | SINE |
| 5323865 | 5324035 | + | 24.7 | 4377 | 4546 | -1600 | L1M5 | L1 | LINE |
| 5324036 | 5325223 | - | 1.5 | 3562 | 4749 | -1397 | L1PA2 | L1 | LINE |
| 5325221 | 5326632 | + | 1.5 | 4743 | 6155 | 0 | L1PA2 | L1 | LINE |
| 5326657 | 5326978 | + | 22.1 | 4533 | 4865 | -1281 | L1M5 | L1 | LINE |

  

|  |  |  |  |  |  |  |  |  |  |
| --- | --- | --- | --- | --- | --- | --- | --- | --- | --- |
| chr4:5394225-5395336 | | | 1110 bp | | |  |  |  |  |
| Genome Start | Genome End | Strand | Div | Repeat Start | Repeat End | Repeat Left | Name | Family | Class |
| 5394225 | 5394477 | - | 12.7 | 189 | 428 | 0 | MSTA | MaLR | LTR |
| 5394478 | 5394658 | - | 9.4 | 5970 | 6150 | -4 | L1PA5 | L1 | LINE |
| 5394661 | 5395139 | + | 8.2 | 5477 | 5953 | -201 | L1PA5 | L1 | LINE |
| 5395140 | 5395336 | - | 14.2 | 1 | 197 | -231 | MSTA | MaLR | LTR |

  

|  |  |  |  |  |  |  |  |  |  |
| --- | --- | --- | --- | --- | --- | --- | --- | --- | --- |
| chr4:9198938-9199870 | | | 903 bp | | |  |  |  |  |
| Genome Start | Genome End | Strand | Div | Repeat Start | Repeat End | Repeat Left | Name | Family | Class |
| 9198938 | 9198981 | - | 9.1 | 135 | 178 | 0 | MER5B | MER1\_type | DNA |
| 9198998 | 9199147 | - | 21.5 | 5711 | 5887 | -287 | L1MB5 | L1 | LINE |
| 9199143 | 9199425 | + | 20.7 | 5902 | 6174 | 0 | L1MB5 | L1 | LINE |
| 9199430 | 9199729 | + | 15.0 | 2 | 301 | -11 | AluSx | Alu | SINE |
| 9199745 | 9199870 | - | 20.3 | 5 | 128 | -50 | MER5B | MER1\_type | DNA |

  

|  |  |  |  |  |  |  |  |  |  |
| --- | --- | --- | --- | --- | --- | --- | --- | --- | --- |
| chr4:11286969-11289734 | | | 2553 bp | | |  |  |  |  |
| Genome Start | Genome End | Strand | Div | Repeat Start | Repeat End | Repeat Left | Name | Family | Class |
| 11286969 | 11287432 | - | 31.0 | 5426 | 5934 | -289 | L1ME3B | L1 | LINE |
| 11287433 | 11287598 | - | 22.9 | 135 | 303 | -9 | AluJ | Alu | SINE |
| 11287599 | 11288155 | - | 9.8 | 5610 | 6165 | -7 | L1PA8 | L1 | LINE |
| 11288157 | 11288770 | + | 9.7 | 4957 | 5598 | -533 | L1PA8 | L1 | LINE |
| 11288772 | 11288904 | - | 19.6 | 1 | 133 | -179 | AluJ | Alu | SINE |
| 11288998 | 11289266 | - | 29.2 | 5073 | 5378 | -751 | L1ME3B | L1 | LINE |
| 11289385 | 11289734 | - | 34.0 | 54 | 394 | -5725 | L1ME3B | L1 | LINE |

  

|  |  |  |  |  |  |  |  |  |  |
| --- | --- | --- | --- | --- | --- | --- | --- | --- | --- |
| chr4:12594272-12597139 | | | 2804 bp | | |  |  |  |  |
| Genome Start | Genome End | Strand | Div | Repeat Start | Repeat End | Repeat Left | Name | Family | Class |
| 12594272 | 12594915 | - | 23.5 | 180 | 875 | -60 | MER21C | ERV1 | LTR |
| 12594920 | 12595299 | - | 7.4 | 5786 | 6169 | -3 | L1PA8 | L1 | LINE |
| 12595304 | 12595884 | - | 8.6 | 4800 | 5388 | -758 | L1PA8 | L1 | LINE |
| 12595932 | 12596117 | - | 8.6 | 4604 | 4799 | -1347 | L1PA8 | L1 | LINE |
| 12596121 | 12597071 | + | 11.6 | 3661 | 4619 | -1527 | L1PA8 | L1 | LINE |
| 12597078 | 12597139 | - | 21.0 | 2 | 65 | -870 | MER21C | ERV1 | LTR |

  

|  |  |  |  |  |  |  |  |  |  |
| --- | --- | --- | --- | --- | --- | --- | --- | --- | --- |
| chr4:14234258-14235441 | | | 1158 bp | | |  |  |  |  |
| Genome Start | Genome End | Strand | Div | Repeat Start | Repeat End | Repeat Left | Name | Family | Class |
| 14234258 | 14234331 | - | 17.6 | 317 | 390 | 0 | MLT1B | MaLR | LTR |
| 14234334 | 14234504 | - | 8.2 | 5982 | 6152 | -2 | L1PA6 | L1 | LINE |
| 14234505 | 14234724 | + | 2.3 | 5751 | 5970 | -184 | L1PA6 | L1 | LINE |
| 14234725 | 14234997 | - | 27.4 | 66 | 323 | -67 | MLT1B | MaLR | LTR |
| 14235022 | 14235386 | + | 13.6 | 15 | 381 | 0 | THE1D | MaLR | LTR |
| 14235387 | 14235441 | - | 20.3 | 1 | 55 | -335 | MLT1B | MaLR | LTR |

  

|  |  |  |  |  |  |  |  |  |  |
| --- | --- | --- | --- | --- | --- | --- | --- | --- | --- |
| chr4:14266433-14268994 | | | 2530 bp | | |  |  |  |  |
| Genome Start | Genome End | Strand | Div | Repeat Start | Repeat End | Repeat Left | Name | Family | Class |
| 14266433 | 14266821 | - | 29.8 | 2142 | 2558 | -861 | L2 | L2 | LINE |
| 14266851 | 14267713 | - | 20.4 | 5288 | 6173 | -7 | L1MB4 | L1 | LINE |
| 14267713 | 14267875 | + | 18.5 | 4864 | 5035 | -1111 | L1M5 | L1 | LINE |
| 14267876 | 14267933 | - | 9.3 | 242 | 295 | -4 | AluSc | Alu | SINE |
| 14267934 | 14267970 | + | 21.0 | 5027 | 5063 | -1083 | L1M5 | L1 | LINE |
| 14267971 | 14268380 | + | 18.4 | 1 | 426 | 0 | MSTB | MaLR | LTR |
| 14268381 | 14268630 | + | 22.5 | 5062 | 5302 | -844 | L1M5 | L1 | LINE |
| 14268635 | 14268994 | - | 24.5 | 1694 | 2076 | -1343 | L2 | L2 | LINE |

  

|  |  |  |  |  |  |  |  |  |  |
| --- | --- | --- | --- | --- | --- | --- | --- | --- | --- |
| chr4:14383680-14386111 | | | 2332 bp | | |  |  |  |  |
| Genome Start | Genome End | Strand | Div | Repeat Start | Repeat End | Repeat Left | Name | Family | Class |
| 14383680 | 14383926 | - | 32.2 | 249 | 511 | 0 | MLT1J | MaLR | LTR |
| 14384025 | 14384155 | - | 20.6 | 4162 | 4294 | -1852 | L1PB4 | L1 | LINE |
| 14384153 | 14385934 | + | 17.6 | 4319 | 6156 | 0 | L1PB4 | L1 | LINE |
| 14385940 | 14386111 | - | 27.8 | 3 | 211 | -300 | MLT1J | MaLR | LTR |

  

|  |  |  |  |  |  |  |  |  |  |
| --- | --- | --- | --- | --- | --- | --- | --- | --- | --- |
| chr4:16913840-16920071 | | | 6148 bp | | |  |  |  |  |
| Genome Start | Genome End | Strand | Div | Repeat Start | Repeat End | Repeat Left | Name | Family | Class |
| 16913840 | 16914054 | + | 33.0 | 2471 | 2695 | -724 | L2 | L2 | LINE |
| 16914056 | 16914129 | - | 23.0 | 141 | 214 | -5 | MER20 | MER1\_type | DNA |
| 16914130 | 16915670 | + | 21.8 | 411 | 2074 | -4677 | L1MCb | L1 | LINE |
| 16915672 | 16916334 | - | 15.4 | 5480 | 6156 | 0 | L1PB4 | L1 | LINE |
| 16916338 | 16917025 | + | 9.7 | 5465 | 6157 | -11 | L1PA10 | L1 | LINE |
| 16917033 | 16917481 | - | 14.3 | 5017 | 5476 | -690 | L1P4 | L1 | LINE |
| 16917480 | 16918356 | + | 14.6 | 4132 | 5030 | -1116 | L1P4 | L1 | LINE |
| 16918357 | 16918988 | + | 20.9 | 2073 | 2766 | -3798 | L1MCb | L1 | LINE |
| 16919063 | 16919197 | + | 33.0 | 2685 | 2809 | -610 | L2 | L2 | LINE |
| 16919198 | 16919495 | + | 15.8 | 3 | 309 | -3 | AluSx | Alu | SINE |
| 16919496 | 16920071 | + | 35.2 | 2810 | 3378 | 0 | L2 | L2 | LINE |

  

|  |  |  |  |  |  |  |  |  |  |
| --- | --- | --- | --- | --- | --- | --- | --- | --- | --- |
| chr4:17061306-17064241 | | | 2905 bp | | |  |  |  |  |
| Genome Start | Genome End | Strand | Div | Repeat Start | Repeat End | Repeat Left | Name | Family | Class |
| 17061306 | 17061601 | + | 15.9 | 1 | 310 | -246 | MLT2B4 | ERVL | LTR |
| 17061624 | 17061761 | - | 14.0 | 6156 | 6294 | -3 | L1MA5A | L1 | LINE |
| 17061763 | 17062347 | - | 15.3 | 3 | 656 | 0 | LTR1C | ERV1 | LTR |
| 17062355 | 17063090 | - | 10.7 | 5411 | 6167 | -130 | L1MA5A | L1 | LINE |
| 17063091 | 17063417 | + | 9.3 | 1 | 324 | -40 | THE1B | MaLR | LTR |
| 17063418 | 17063744 | + | 13.4 | 1 | 309 | -4 | AluSp | Alu | SINE |
| 17063745 | 17063914 | + | 10.0 | 132 | 305 | -4 | AluSc | Alu | SINE |
| 17063915 | 17063987 | - | 5.5 | 5142 | 5218 | -925 | L1MA5A | L1 | LINE |
| 17063986 | 17064020 | + | 8.6 | 5115 | 5149 | -994 | L1MA5A | L1 | LINE |
| 17064024 | 17064241 | + | 22.7 | 365 | 567 | 0 | MLT2B4 | ERVL | LTR |

  

|  |  |  |  |  |  |  |  |  |  |
| --- | --- | --- | --- | --- | --- | --- | --- | --- | --- |
| chr4:18156880-18162999 | | | 6097 bp | | |  |  |  |  |
| Genome Start | Genome End | Strand | Div | Repeat Start | Repeat End | Repeat Left | Name | Family | Class |
| 18156880 | 18157355 | - | 16.8 | 5790 | 6312 | 0 | L1MA9 | L1 | LINE |
| 18157378 | 18158910 | - | 16.9 | 4210 | 5789 | -523 | L1MA9 | L1 | LINE |
| 18158912 | 18160488 | - | 3.1 | 3299 | 4877 | -1269 | L1PA3 | L1 | LINE |
| 18160491 | 18161763 | + | 3.5 | 4879 | 6155 | 0 | L1PA3 | L1 | LINE |
| 18161762 | 18162999 | - | 15.5 | 2963 | 4220 | -1920 | L1MA9 | L1 | LINE |

  

|  |  |  |  |  |  |  |  |  |  |
| --- | --- | --- | --- | --- | --- | --- | --- | --- | --- |
| chr4:19342650-19345053 | | | 2302 bp | | |  |  |  |  |
| Genome Start | Genome End | Strand | Div | Repeat Start | Repeat End | Repeat Left | Name | Family | Class |
| 19342650 | 19342713 | - | 28.1 | 164 | 227 | -35 | MIR | MIR | SINE |
| 19342756 | 19343548 | - | 3.8 | 3992 | 4785 | -1361 | L1PA4 | L1 | LINE |
| 19343549 | 19344911 | + | 4.4 | 4786 | 6155 | 0 | L1PA4 | L1 | LINE |
| 19344972 | 19345053 | - | 32.5 | 1 | 87 | -175 | MIR | MIR | SINE |

  

|  |  |  |  |  |  |  |  |  |  |
| --- | --- | --- | --- | --- | --- | --- | --- | --- | --- |
| chr4:19681370-19682153 | | | 761 bp | | |  |  |  |  |
| Genome Start | Genome End | Strand | Div | Repeat Start | Repeat End | Repeat Left | Name | Family | Class |
| 19681370 | 19681491 | + | 19.7 | 1 | 125 | -265 | MLT1B | MaLR | LTR |
| 19681492 | 19681628 | - | 1.5 | 5774 | 5910 | -245 | L1PA5 | L1 | LINE |
| 19681629 | 19681844 | + | 3.7 | 5928 | 6143 | -11 | L1PA5 | L1 | LINE |
| 19681868 | 19682153 | + | 22.1 | 108 | 389 | -1 | MLT1B | MaLR | LTR |

  

|  |  |  |  |  |  |  |  |  |  |
| --- | --- | --- | --- | --- | --- | --- | --- | --- | --- |
| chr4:21945212-21953263 | | | 7679 bp | | |  |  |  |  |
| Genome Start | Genome End | Strand | Div | Repeat Start | Repeat End | Repeat Left | Name | Family | Class |
| 21945212 | 21945418 | - | 34.2 | 4255 | 4459 | -30 | L3 | CR1 | LINE |
| 21945565 | 21946154 | - | 6.6 | 1 | 610 | 0 | LTR66 | ERVL | LTR |
| 21946155 | 21946391 | - | 8.6 | 3988 | 4329 | 0 | HERVL66 | ERVL | LTR |
| 21946411 | 21947282 | - | 4.5 | 5269 | 6153 | -1 | L1PA5 | L1 | LINE |
| 21947281 | 21947571 | + | 5.2 | 4968 | 5258 | -888 | L1PA5 | L1 | LINE |
| 21947572 | 21947999 | - | 14.1 | 3569 | 4000 | -329 | HERVL66 | ERVL | LTR |
| 21947998 | 21948679 | - | 11.2 | 2655 | 3352 | -977 | HERVL66 | ERVL | LTR |
| 21948661 | 21949008 | - | 8.9 | 1436 | 1797 | -2532 | HERVL66 | ERVL | LTR |
| 21949009 | 21950103 | - | 8.9 | 1 | 1130 | -3199 | HERVL66 | ERVL | LTR |
| 21950104 | 21950692 | - | 6.5 | 1 | 610 | 0 | LTR66 | ERVL | LTR |
| 21950693 | 21950740 | - | 10.4 | 326 | 373 | -17 | MLT1H2 | MaLR | LTR |
| 21950742 | 21951141 | - | 12.5 | 1597 | 2718 | 0 | Tigger2 | MER2\_type | DNA |
| 21951247 | 21951303 | + | 18.5 | 500 | 553 | -3 | MLT2B4 | ERVL | LTR |
| 21951311 | 21952862 | + | 10.7 | 1 | 1574 | -4080 | HERVL-B4 | ERVL | LTR |
| 21952897 | 21953040 | + | 4.2 | 6008 | 6151 | -3 | L1PA7 | L1 | LINE |
| 21953125 | 21953263 | - | 38.1 | 2969 | 3110 | -1379 | L3 | CR1 | LINE |

  

|  |  |  |  |  |  |  |  |  |  |
| --- | --- | --- | --- | --- | --- | --- | --- | --- | --- |
| chr4:21984900-21992907 | | | 7953 bp | | |  |  |  |  |
| Genome Start | Genome End | Strand | Div | Repeat Start | Repeat End | Repeat Left | Name | Family | Class |
| 21984900 | 21986122 | + | 12.9 | 3 | 1363 | -4583 | MER41A-int | ERV1 | LTR |
| 21986123 | 21986433 | + | 10.6 | 1 | 310 | 0 | AluSg | Alu | SINE |
| 21986434 | 21986651 | + | 7.9 | 1364 | 1621 | -4473 | HUERS-P1 | ERV1 | LTR |
| 21986711 | 21988035 | + | 7.9 | 1622 | 3193 | -2901 | HUERS-P1 | ERV1 | LTR |
| 21988043 | 21990208 | - | 8.6 | 2786 | 4998 | -1148 | L1P3 | L1 | LINE |
| 21990197 | 21990484 | + | 9.7 | 2491 | 2782 | -3364 | L1P3 | L1 | LINE |
| 21990486 | 21992907 | + | 9.9 | 2660 | 5944 | -2 | MER41A-int | ERV1 | LTR |

  

|  |  |  |  |  |  |  |  |  |  |
| --- | --- | --- | --- | --- | --- | --- | --- | --- | --- |
| chr4:22311082-22312393 | | | 1306 bp | | |  |  |  |  |
| Genome Start | Genome End | Strand | Div | Repeat Start | Repeat End | Repeat Left | Name | Family | Class |
| 22311082 | 22311187 | + | 16.2 | 1 | 100 | -265 | MLT1A0 | MaLR | LTR |
| 22311188 | 22311547 | + | 14.7 | 1 | 364 | 0 | THE1B | MaLR | LTR |
| 22311548 | 22311735 | + | 16.2 | 101 | 304 | -61 | MLT1A0 | MaLR | LTR |
| 22311741 | 22311959 | - | 7.6 | 5958 | 6172 | 0 | L1PA8 | L1 | LINE |
| 22311960 | 22312327 | + | 5.7 | 5572 | 5939 | -233 | L1PA8 | L1 | LINE |
| 22312329 | 22312393 | + | 16.9 | 299 | 363 | -2 | MLT1A0 | MaLR | LTR |

  

|  |  |  |  |  |  |  |  |  |  |
| --- | --- | --- | --- | --- | --- | --- | --- | --- | --- |
| chr4:24657217-24661039 | | | 3823 bp | | |  |  |  |  |
| Genome Start | Genome End | Strand | Div | Repeat Start | Repeat End | Repeat Left | Name | Family | Class |
| 24657217 | 24657341 | - | 21.4 | 2498 | 2623 | 0 | Charlie5 | MER1\_type | DNA |
| 24657342 | 24657803 | - | 17.4 | 1 | 467 | 0 | MLT1C | MaLR | LTR |
| 24657804 | 24658018 | - | 21.4 | 2308 | 2497 | -115 | Charlie5 | MER1\_type | DNA |
| 24658019 | 24658054 | - | 13.1 | 5481 | 5516 | -650 | L1P4 | L1 | LINE |
| 24658055 | 24658372 | - | 17.7 | 1 | 300 | -12 | AluJo | Alu | SINE |
| 24658373 | 24660310 | - | 20.4 | 3726 | 5480 | -686 | L1P4 | L1 | LINE |
| 24660311 | 24660945 | + | 12.8 | 5532 | 6159 | -7 | L1PA16 | L1 | LINE |
| 24660946 | 24661039 | - | 22.6 | 2208 | 2300 | -312 | Charlie5 | MER1\_type | DNA |

  

|  |  |  |  |  |  |  |  |  |  |
| --- | --- | --- | --- | --- | --- | --- | --- | --- | --- |
| chr4:24810329-24812989 | | | 2353 bp | | |  |  |  |  |
| Genome Start | Genome End | Strand | Div | Repeat Start | Repeat End | Repeat Left | Name | Family | Class |
| 24810329 | 24810521 | + | 26.6 | 5684 | 5907 | -214 | L1ME4a | L1 | LINE |
| 24810606 | 24811007 | - | 7.8 | 4105 | 4506 | -1640 | L1PB1 | L1 | LINE |
| 24811007 | 24812635 | + | 7.0 | 4520 | 6151 | 0 | L1PB1 | L1 | LINE |
| 24812861 | 24812989 | + | 28.4 | 5967 | 6105 | -16 | L1ME4a | L1 | LINE |

  

|  |  |  |  |  |  |  |  |  |  |
| --- | --- | --- | --- | --- | --- | --- | --- | --- | --- |
| chr4:27002283-27005902 | | | 3521 bp | | |  |  |  |  |
| Genome Start | Genome End | Strand | Div | Repeat Start | Repeat End | Repeat Left | Name | Family | Class |
| 27002283 | 27002846 | - | 26.5 | 5250 | 5830 | -332 | L1ME3 | L1 | LINE |
| 27002847 | 27003112 | - | 19.0 | 1 | 264 | -48 | AluJo | Alu | SINE |
| 27003113 | 27004649 | - | 27.4 | 3601 | 5249 | -897 | L1ME3 | L1 | LINE |
| 27004716 | 27005205 | - | 14.8 | 5670 | 6155 | -11 | L1PA16 | L1 | LINE |
| 27005208 | 27005775 | + | 10.8 | 5083 | 5660 | -506 | L1PA16 | L1 | LINE |
| 27005807 | 27005902 | - | 29.5 | 3437 | 3531 | -2615 | L1ME3 | L1 | LINE |

  

|  |  |  |  |  |  |  |  |  |  |
| --- | --- | --- | --- | --- | --- | --- | --- | --- | --- |
| chr4:27641879-27652149 | | | 10202 bp | | |  |  |  |  |
| Genome Start | Genome End | Strand | Div | Repeat Start | Repeat End | Repeat Left | Name | Family | Class |
| 27641879 | 27641995 | - | 36.8 | 3303 | 3419 | 0 | L2 | L2 | LINE |
| 27641996 | 27643164 | - | 17.7 | 2484 | 3666 | -2480 | L1PA15 | L1 | LINE |
| 27643171 | 27643295 | + | 14.5 | 3658 | 3785 | -2361 | L1PA15 | L1 | LINE |
| 27643301 | 27644180 | + | 15.5 | 2958 | 3856 | -2290 | L1MA2 | L1 | LINE |
| 27644179 | 27645693 | + | 14.2 | 4712 | 6297 | -7 | L1MA2 | L1 | LINE |
| 27645703 | 27646042 | + | 17.4 | 3775 | 4131 | -2015 | L1PA15 | L1 | LINE |
| 27646043 | 27646454 | - | 18.4 | 5 | 428 | 0 | MSTA | MaLR | LTR |
| 27646457 | 27646540 | - | 18.7 | 1570 | 1651 | 0 | MSTA-int | MaLR | LTR |
| 27646541 | 27646711 | - | 11.9 | 130 | 296 | -17 | AluSp | Alu | SINE |
| 27646732 | 27646861 | - | 11.9 | 3 | 129 | -184 | AluSp | Alu | SINE |
| 27646862 | 27648385 | - | 18.7 | 1 | 1569 | -82 | MSTA-int | MaLR | LTR |
| 27648386 | 27648785 | - | 16.9 | 3 | 428 | 0 | MSTA | MaLR | LTR |
| 27648788 | 27648883 | + | 15.5 | 4127 | 4211 | -1935 | L1PA15 | L1 | LINE |
| 27648884 | 27649165 | - | 13.5 | 12 | 295 | -17 | AluSx | Alu | SINE |
| 27649166 | 27649485 | + | 15.5 | 4212 | 4511 | -1635 | L1PA15 | L1 | LINE |
| 27649486 | 27649775 | + | 15.9 | 3 | 299 | -13 | AluSx | Alu | SINE |
| 27649776 | 27650749 | + | 15.6 | 4512 | 5492 | -673 | L1PA15 | L1 | LINE |
| 27650750 | 27651060 | + | 8.8 | 1 | 307 | -4 | AluY | Alu | SINE |
| 27651061 | 27651731 | + | 16.9 | 5493 | 6159 | -6 | L1PA15 | L1 | LINE |
| 27651759 | 27652149 | - | 32.2 | 2838 | 3284 | -135 | L2 | L2 | LINE |

  

|  |  |  |  |  |  |  |  |  |  |
| --- | --- | --- | --- | --- | --- | --- | --- | --- | --- |
| chr4:28892385-28895859 | | | 3413 bp | | |  |  |  |  |
| Genome Start | Genome End | Strand | Div | Repeat Start | Repeat End | Repeat Left | Name | Family | Class |
| 28892385 | 28892530 | - | 24.1 | 2030 | 2176 | -4224 | L1MEc | L1 | LINE |
| 28892530 | 28892655 | - | 11.1 | 4703 | 4828 | -1318 | L1PA7 | L1 | LINE |
| 28892658 | 28893976 | + | 7.0 | 4834 | 6154 | 0 | L1PA7 | L1 | LINE |
| 28894002 | 28895200 | - | 24.8 | 747 | 2041 | -4359 | L1MEc | L1 | LINE |
| 28895237 | 28895859 | - | 24.3 | 153 | 746 | -5654 | L1MEc | L1 | LINE |

  

|  |  |  |  |  |  |  |  |  |  |
| --- | --- | --- | --- | --- | --- | --- | --- | --- | --- |
| chr4:29050611-29051670 | | | 1053 bp | | |  |  |  |  |
| Genome Start | Genome End | Strand | Div | Repeat Start | Repeat End | Repeat Left | Name | Family | Class |
| 29050611 | 29050853 | + | 10.5 | 1 | 235 | -120 | THE1A | MaLR | LTR |
| 29050854 | 29051248 | - | 1.0 | 5457 | 5851 | -304 | L1PA3 | L1 | LINE |
| 29051249 | 29051528 | + | 2.5 | 5876 | 6155 | 0 | L1PA3 | L1 | LINE |
| 29051536 | 29051670 | + | 13.5 | 223 | 355 | 0 | THE1A | MaLR | LTR |

  

|  |  |  |  |  |  |  |  |  |  |
| --- | --- | --- | --- | --- | --- | --- | --- | --- | --- |
| chr4:31404888-31413980 | | | 8956 bp | | |  |  |  |  |
| Genome Start | Genome End | Strand | Div | Repeat Start | Repeat End | Repeat Left | Name | Family | Class |
| 31404888 | 31405452 | - | 13.1 | 7180 | 7724 | 0 | L1MA3 | L1 | LINE |
| 31405457 | 31407982 | - | 13.8 | 4561 | 7079 | -642 | L1MA3 | L1 | LINE |
| 31408034 | 31408318 | - | 13.3 | 1 | 287 | -26 | AluSp | Alu | SINE |
| 31408319 | 31409017 | - | 14.3 | 3876 | 4560 | -3005 | L1MA3 | L1 | LINE |
| 31409057 | 31409501 | - | 14.1 | 3411 | 3875 | -3690 | L1MA3 | L1 | LINE |
| 31409534 | 31409869 | - | 14.0 | 3074 | 3410 | -4155 | L1MA3 | L1 | LINE |
| 31409870 | 31410163 | - | 10.2 | 1 | 295 | -18 | AluSq | Alu | SINE |
| 31410164 | 31411670 | - | 14.0 | 1557 | 3073 | -4492 | L1MA3 | L1 | LINE |
| 31411668 | 31412680 | - | 9.1 | 4394 | 5423 | -745 | L1P | L1 | LINE |
| 31412681 | 31413413 | + | 11.4 | 5435 | 6163 | -5 | L1PA10 | L1 | LINE |
| 31413430 | 31413885 | - | 21.5 | 1100 | 1568 | -5997 | L1MA3 | L1 | LINE |
| 31413884 | 31413980 | - | 19.6 | 12 | 133 | -7432 | L1MA3 | L1 | LINE |

  

|  |  |  |  |  |  |  |  |  |  |
| --- | --- | --- | --- | --- | --- | --- | --- | --- | --- |
| chr4:31690163-31691915 | | | 1769 bp | | |  |  |  |  |
| Genome Start | Genome End | Strand | Div | Repeat Start | Repeat End | Repeat Left | Name | Family | Class |
| 31690163 | 31690512 | - | 10.2 | 5813 | 6160 | -3 | L1PA13 | L1 | LINE |
| 31690514 | 31691275 | - | 10.1 | 5383 | 6164 | -4 | L1PA10 | L1 | LINE |
| 31691270 | 31691477 | + | 12.6 | 5184 | 5386 | -760 | L1PA10 | L1 | LINE |
| 31691467 | 31691915 | - | 11.8 | 5386 | 5838 | -325 | L1PA13 | L1 | LINE |

  

|  |  |  |  |  |  |  |  |  |  |
| --- | --- | --- | --- | --- | --- | --- | --- | --- | --- |
| chr4:33131544-33136120 | | | 4485 bp | | |  |  |  |  |
| Genome Start | Genome End | Strand | Div | Repeat Start | Repeat End | Repeat Left | Name | Family | Class |
| 33131544 | 33133266 | + | 17.9 | 709 | 2421 | -4432 | L1PA15-16 | L1 | LINE |
| 33133267 | 33133574 | + | 15.2 | 1 | 300 | -10 | AluSg | Alu | SINE |
| 33133575 | 33133829 | + | 17.9 | 2422 | 2664 | -4189 | L1PA15-16 | L1 | LINE |
| 33133830 | 33134134 | - | 5.9 | 1 | 305 | -6 | AluY | Alu | SINE |
| 33134135 | 33134699 | + | 15.5 | 2665 | 3229 | -3620 | L1PA15-16 | L1 | LINE |
| 33134694 | 33134768 | - | 6.7 | 4857 | 4931 | -1215 | L1P3 | L1 | LINE |
| 33134765 | 33135955 | + | 3.8 | 4944 | 6151 | -3 | L1PA5 | L1 | LINE |
| 33136058 | 33136120 | + | 15.3 | 3211 | 3270 | -3579 | L1PA15-16 | L1 | LINE |

  

|  |  |  |  |  |  |  |  |  |  |
| --- | --- | --- | --- | --- | --- | --- | --- | --- | --- |
| chr4:33889279-33891830 | | | 2542 bp | | |  |  |  |  |
| Genome Start | Genome End | Strand | Div | Repeat Start | Repeat End | Repeat Left | Name | Family | Class |
| 33889279 | 33890433 | - | 21.3 | 5010 | 6173 | -11 | L1MB7 | L1 | LINE |
| 33890441 | 33891049 | - | 10.0 | 5553 | 6164 | -4 | L1PA10 | L1 | LINE |
| 33891050 | 33891743 | + | 9.8 | 4852 | 5551 | -617 | L1PA10 | L1 | LINE |
| 33891747 | 33891830 | - | 23.2 | 4936 | 5018 | -1128 | L1MB7 | L1 | LINE |

  

|  |  |  |  |  |  |  |  |  |  |
| --- | --- | --- | --- | --- | --- | --- | --- | --- | --- |
| chr4:35294119-35296667 | | | 2462 bp | | |  |  |  |  |
| Genome Start | Genome End | Strand | Div | Repeat Start | Repeat End | Repeat Left | Name | Family | Class |
| 35294119 | 35294278 | - | 22.6 | 5996 | 6160 | -2 | L1ME2 | L1 | LINE |
| 35294309 | 35294587 | - | 25.9 | 5520 | 5777 | -389 | L1ME2 | L1 | LINE |
| 35294586 | 35294788 | - | 30.1 | 4549 | 4749 | -1397 | L1ME2 | L1 | LINE |
| 35294855 | 35295493 | - | 15.8 | 5469 | 6129 | -37 | L1PA16 | L1 | LINE |
| 35295484 | 35296192 | + | 14.4 | 4780 | 5493 | -673 | L1PA16 | L1 | LINE |
| 35296196 | 35296667 | - | 27.6 | 3784 | 4318 | -1828 | L1ME2 | L1 | LINE |

  

|  |  |  |  |  |  |  |  |  |  |
| --- | --- | --- | --- | --- | --- | --- | --- | --- | --- |
| chr4:37548384-37550479 | | | 2096 bp | | |  |  |  |  |
| Genome Start | Genome End | Strand | Div | Repeat Start | Repeat End | Repeat Left | Name | Family | Class |
| 37548384 | 37548508 | + | 13.6 | 5217 | 5336 | -976 | L1MA9 | L1 | LINE |
| 37548509 | 37548855 | - | 9.1 | 5951 | 6298 | -5 | L1MA4A | L1 | LINE |
| 37548856 | 37549087 | - | 4.3 | 64 | 295 | -16 | AluY | Alu | SINE |
| 37549088 | 37549235 | - | 9.1 | 5801 | 5950 | -353 | L1MA4A | L1 | LINE |
| 37549236 | 37549702 | + | 13.1 | 5331 | 5822 | -490 | L1MA4A | L1 | LINE |
| 37549703 | 37550009 | + | 4.6 | 1 | 307 | -4 | AluY | Alu | SINE |
| 37550010 | 37550479 | + | 13.1 | 5823 | 6308 | -4 | L1MA9 | L1 | LINE |

  

|  |  |  |  |  |  |  |  |  |  |
| --- | --- | --- | --- | --- | --- | --- | --- | --- | --- |
| chr4:42836112-42839650 | | | 3404 bp | | |  |  |  |  |
| Genome Start | Genome End | Strand | Div | Repeat Start | Repeat End | Repeat Left | Name | Family | Class |
| 42836112 | 42836239 | - | 27.0 | 328 | 458 | -63 | LTR33 | ERVL | LTR |
| 42836371 | 42837128 | - | 9.0 | 3016 | 3780 | -2366 | L1PA10 | L1 | LINE |
| 42837129 | 42839465 | + | 9.2 | 3799 | 6171 | 0 | L1PA10 | L1 | LINE |
| 42839470 | 42839650 | - | 32.4 | 3 | 188 | -333 | LTR33 | ERVL | LTR |

  

|  |  |  |  |  |  |  |  |  |  |
| --- | --- | --- | --- | --- | --- | --- | --- | --- | --- |
| chr4:42846505-42856084 | | | 9550 bp | | |  |  |  |  |
| Genome Start | Genome End | Strand | Div | Repeat Start | Repeat End | Repeat Left | Name | Family | Class |
| 42846505 | 42846668 | - | 17.1 | 2574 | 2730 | -1 | Tigger4(Zombi) | MER2\_type | DNA |
| 42846669 | 42847055 | - | 13.5 | 1 | 428 | 0 | MSTA | MaLR | LTR |
| 42847056 | 42847180 | - | 17.1 | 2449 | 2573 | -158 | Tigger4(Zombi) | MER2\_type | DNA |
| 42847189 | 42847310 | - | 12.3 | 1175 | 1296 | -1435 | Tigger4(Zombi) | MER2\_type | DNA |
| 42847311 | 42848368 | - | 8.3 | 2502 | 3558 | -2588 | L1PA6 | L1 | LINE |
| 42848366 | 42850947 | + | 7.4 | 3566 | 6148 | -6 | L1PA6 | L1 | LINE |
| 42850948 | 42851407 | - | 13.8 | 727 | 1189 | -1542 | Tigger4(Zombi) | MER2\_type | DNA |
| 42851433 | 42855356 | - | 16.1 | 2192 | 6165 | 0 | L1PA15 | L1 | LINE |
| 42855357 | 42856084 | - | 16.5 | 1 | 738 | -1993 | Tigger4(Zombi) | MER2\_type | DNA |

  

|  |  |  |  |  |  |  |  |  |  |
| --- | --- | --- | --- | --- | --- | --- | --- | --- | --- |
| chr4:43271521-43275712 | | | 4190 bp | | |  |  |  |  |
| Genome Start | Genome End | Strand | Div | Repeat Start | Repeat End | Repeat Left | Name | Family | Class |
| 43271521 | 43271707 | + | 17.1 | 3 | 197 | -5131 | L1MA9 | L1 | LINE |
| 43271704 | 43271794 | - | 5.5 | 3500 | 3590 | -2556 | L1PA4 | L1 | LINE |
| 43271796 | 43274360 | + | 4.4 | 3589 | 6152 | -3 | L1PA4 | L1 | LINE |
| 43274371 | 43274758 | + | 24.8 | 181 | 582 | -4746 | L1MA9 | L1 | LINE |
| 43274754 | 43275712 | + | 18.6 | 3318 | 4295 | -2030 | L1MA9 | L1 | LINE |

  

|  |  |  |  |  |  |  |  |  |  |
| --- | --- | --- | --- | --- | --- | --- | --- | --- | --- |
| chr4:44086549-44088553 | | | 1993 bp | | |  |  |  |  |
| Genome Start | Genome End | Strand | Div | Repeat Start | Repeat End | Repeat Left | Name | Family | Class |
| 44086549 | 44086618 | + | 13.1 | 1 | 69 | -2649 | Tigger2 | MER2\_type | DNA |
| 44086631 | 44087454 | - | 7.6 | 4610 | 5434 | -717 | L1PB1 | L1 | LINE |
| 44087445 | 44088100 | + | 7.9 | 5430 | 6144 | -7 | L1PB1 | L1 | LINE |
| 44088111 | 44088553 | + | 15.9 | 1545 | 2718 | 0 | Tigger2 | MER2\_type | DNA |

  

|  |  |  |  |  |  |  |  |  |  |
| --- | --- | --- | --- | --- | --- | --- | --- | --- | --- |
| chr4:45026483-45027930 | | | 1320 bp | | |  |  |  |  |
| Genome Start | Genome End | Strand | Div | Repeat Start | Repeat End | Repeat Left | Name | Family | Class |
| 45026483 | 45026518 | - | 25.0 | 3274 | 3309 | -69 | L2 | L2 | LINE |
| 45026635 | 45027073 | - | 31.6 | 2091 | 2578 | -841 | L2 | L2 | LINE |
| 45027074 | 45027282 | - | 14.0 | 5952 | 6154 | -137 | L1MA8 | L1 | LINE |
| 45027282 | 45027395 | + | 17.7 | 6160 | 6273 | -18 | L1MA8 | L1 | LINE |
| 45027409 | 45027930 | - | 31.7 | 1592 | 2123 | -1296 | L2 | L2 | LINE |

  

|  |  |  |  |  |  |  |  |  |  |
| --- | --- | --- | --- | --- | --- | --- | --- | --- | --- |
| chr4:45207293-45211627 | | | 4252 bp | | |  |  |  |  |
| Genome Start | Genome End | Strand | Div | Repeat Start | Repeat End | Repeat Left | Name | Family | Class |
| 45207293 | 45207971 | - | 13.6 | 5428 | 6151 | 0 | L1PB1 | L1 | LINE |
| 45208060 | 45208858 | - | 1.8 | 5356 | 6155 | 0 | L1PA2 | L1 | LINE |
| 45208855 | 45209125 | + | 4.8 | 5088 | 5358 | -788 | L1PA2 | L1 | LINE |
| 45209125 | 45211627 | - | 10.9 | 2943 | 5438 | -713 | L1PB1 | L1 | LINE |

  

|  |  |  |  |  |  |  |  |  |  |
| --- | --- | --- | --- | --- | --- | --- | --- | --- | --- |
| chr4:45805658-45810589 | | | 4599 bp | | |  |  |  |  |
| Genome Start | Genome End | Strand | Div | Repeat Start | Repeat End | Repeat Left | Name | Family | Class |
| 45805658 | 45805824 | + | 30.1 | 2724 | 2878 | -541 | L2 | L2 | LINE |
| 45805831 | 45807260 | - | 14.6 | 2172 | 3612 | -2534 | L1P4 | L1 | LINE |
| 45807260 | 45809720 | + | 16.4 | 3620 | 6160 | 0 | L1PREC2 | L1 | LINE |
| 45809934 | 45810309 | - | 14.3 | 1 | 381 | 0 | THE1D | MaLR | LTR |
| 45810425 | 45810589 | + | 30.9 | 3247 | 3419 | 0 | L2 | L2 | LINE |

  

|  |  |  |  |  |  |  |  |  |  |
| --- | --- | --- | --- | --- | --- | --- | --- | --- | --- |
| chr4:46373285-46376055 | | | 2763 bp | | |  |  |  |  |
| Genome Start | Genome End | Strand | Div | Repeat Start | Repeat End | Repeat Left | Name | Family | Class |
| 46373285 | 46374447 | + | 27.8 | 1911 | 3115 | -304 | L2 | L2 | LINE |
| 46374456 | 46375550 | - | 2.6 | 5058 | 6155 | 0 | L1PA3 | L1 | LINE |
| 46375551 | 46375747 | + | 3.0 | 4861 | 5057 | -1089 | L1PA3 | L1 | LINE |
| 46375748 | 46376055 | + | 34.5 | 3104 | 3407 | -12 | L2 | L2 | LINE |

  

|  |  |  |  |  |  |  |  |  |  |
| --- | --- | --- | --- | --- | --- | --- | --- | --- | --- |
| chr4:46448307-46454778 | | | 6237 bp | | |  |  |  |  |
| Genome Start | Genome End | Strand | Div | Repeat Start | Repeat End | Repeat Left | Name | Family | Class |
| 46448307 | 46448513 | - | 31.0 | 3118 | 3343 | -76 | L2 | L2 | LINE |
| 46448563 | 46450146 | - | 6.2 | 3197 | 4779 | -1367 | L1PA5 | L1 | LINE |
| 46450147 | 46451468 | + | 4.9 | 4801 | 6145 | -9 | L1PA5 | L1 | LINE |
| 46451528 | 46454239 | + | 16.6 | 3448 | 6151 | -5 | L1PB4 | L1 | LINE |
| 46454336 | 46454578 | + | 34.4 | 3139 | 3419 | 0 | L2 | L2 | LINE |
| 46454610 | 46454778 | - | 31.3 | 2873 | 3081 | -338 | L2 | L2 | LINE |

  

|  |  |  |  |  |  |  |  |  |  |
| --- | --- | --- | --- | --- | --- | --- | --- | --- | --- |
| chr4:48424006-48438957 | | | 14900 bp | | |  |  |  |  |
| Genome Start | Genome End | Strand | Div | Repeat Start | Repeat End | Repeat Left | Name | Family | Class |
| 48424006 | 48425520 | + | 14.4 | 1 | 1554 | -4954 | L1MC1 | L1 | LINE |
| 48425527 | 48428051 | + | 8.3 | 5 | 2543 | -3603 | L1PA7 | L1 | LINE |
| 48428047 | 48429713 | + | 7.7 | 4479 | 6154 | 0 | L1PA7 | L1 | LINE |
| 48429722 | 48429767 | + | 13.9 | 1539 | 1587 | -4921 | L1MC1 | L1 | LINE |
| 48429768 | 48430080 | + | 10.6 | 1 | 312 | 0 | AluSx | Alu | SINE |
| 48430081 | 48431453 | + | 12.5 | 1588 | 2949 | -3372 | L1MC1 | L1 | LINE |
| 48431454 | 48431620 | - | 0.0 | 4849 | 5015 | -1131 | L1HS | L1 | LINE |
| 48431619 | 48432758 | + | 0.7 | 5016 | 6155 | 0 | L1HS | L1 | LINE |
| 48432766 | 48433341 | + | 14.0 | 2936 | 3381 | -2940 | L1MC1 | L1 | LINE |
| 48433342 | 48433651 | + | 10.0 | 1 | 310 | -2 | AluSx | Alu | SINE |
| 48433652 | 48433771 | + | 14.0 | 3382 | 3477 | -2844 | L1MC1 | L1 | LINE |
| 48433772 | 48434061 | + | 11.1 | 1 | 312 | 0 | AluSx | Alu | SINE |
| 48434062 | 48435290 | + | 14.0 | 3478 | 4716 | -1605 | L1MC1 | L1 | LINE |
| 48435291 | 48435577 | - | 19.4 | 3 | 282 | -30 | AluJo | Alu | SINE |
| 48435578 | 48435892 | + | 14.0 | 4717 | 5023 | -1298 | L1MC1 | L1 | LINE |
| 48435893 | 48436195 | + | 13.2 | 1 | 303 | -9 | AluSx | Alu | SINE |
| 48436196 | 48436483 | + | 14.0 | 5024 | 5318 | -1003 | L1MC1 | L1 | LINE |
| 48436490 | 48436894 | + | 16.3 | 7 | 428 | 0 | MSTC | MaLR | LTR |
| 48436895 | 48437063 | + | 11.9 | 5314 | 5480 | -1028 | L1MC1 | L1 | LINE |
| 48437064 | 48437368 | - | 10.2 | 1 | 305 | -7 | AluSx | Alu | SINE |
| 48437369 | 48437899 | + | 14.9 | 5481 | 6019 | -489 | L1MC1 | L1 | LINE |
| 48437900 | 48438035 | + | 17.3 | 1 | 134 | -178 | AluJb | Alu | SINE |
| 48438036 | 48438327 | + | 14.4 | 1 | 295 | -16 | AluY | Alu | SINE |
| 48438328 | 48438471 | + | 17.3 | 135 | 284 | -28 | AluJb | Alu | SINE |
| 48438504 | 48438957 | + | 14.9 | 5845 | 6315 | -18 | L1MC1 | L1 | LINE |

  

|  |  |  |  |  |  |  |  |  |  |
| --- | --- | --- | --- | --- | --- | --- | --- | --- | --- |
| chr4:53040719-53043514 | | | 2796 bp | | |  |  |  |  |
| Genome Start | Genome End | Strand | Div | Repeat Start | Repeat End | Repeat Left | Name | Family | Class |
| 53040719 | 53040942 | + | 27.8 | 1882 | 2115 | -1304 | L2 | L2 | LINE |
| 53040943 | 53041399 | - | 4.6 | 5699 | 6155 | 0 | L1PA3 | L1 | LINE |
| 53041400 | 53043172 | + | 3.3 | 3907 | 5685 | -470 | L1PA3 | L1 | LINE |
| 53043173 | 53043514 | + | 30.4 | 2109 | 2462 | -957 | L2 | L2 | LINE |

  

|  |  |  |  |  |  |  |  |  |  |
| --- | --- | --- | --- | --- | --- | --- | --- | --- | --- |
| chr4:53343913-53347236 | | | 3105 bp | | |  |  |  |  |
| Genome Start | Genome End | Strand | Div | Repeat Start | Repeat End | Repeat Left | Name | Family | Class |
| 53343913 | 53344461 | + | 26.6 | 1529 | 2093 | -1326 | L2 | L2 | LINE |
| 53344463 | 53344727 | - | 5.3 | 5890 | 6155 | 0 | L1PA4 | L1 | LINE |
| 53344734 | 53345379 | + | 3.1 | 5254 | 5901 | -254 | L1PA4 | L1 | LINE |
| 53345391 | 53345500 | + | 19.6 | 2109 | 2216 | -1203 | L2 | L2 | LINE |
| 53345542 | 53345808 | - | 17.3 | 1 | 268 | -44 | AluJo | Alu | SINE |
| 53345903 | 53345960 | + | 19.2 | 1 | 49 | -316 | MLT1A0 | MaLR | LTR |
| 53345961 | 53346250 | + | 9.7 | 1 | 290 | -20 | AluSg | Alu | SINE |
| 53346251 | 53346530 | + | 19.2 | 50 | 353 | -12 | MLT1A0 | MaLR | LTR |
| 53346544 | 53346880 | + | 21.2 | 2305 | 2701 | -718 | L2 | L2 | LINE |
| 53346919 | 53347074 | - | 20.1 | 122 | 285 | -27 | AluJo | Alu | SINE |
| 53347090 | 53347236 | + | 20.8 | 3274 | 3418 | -1 | L2 | L2 | LINE |

  

|  |  |  |  |  |  |  |  |  |  |
| --- | --- | --- | --- | --- | --- | --- | --- | --- | --- |
| chr4:55156592-55158795 | | | 1831 bp | | |  |  |  |  |
| Genome Start | Genome End | Strand | Div | Repeat Start | Repeat End | Repeat Left | Name | Family | Class |
| 55156592 | 55156777 | + | 36.0 | 5320 | 5506 | -734 | L1M5 | L1 | LINE |
| 55156793 | 55156892 | - | 31.3 | 336 | 435 | -15 | LTR16A | ERVL | LTR |
| 55156927 | 55157170 | - | 14.8 | 5217 | 5469 | -843 | L1MA9 | L1 | LINE |
| 55157172 | 55158009 | + | 11.4 | 5445 | 6312 | 0 | L1MA9 | L1 | LINE |
| 55158328 | 55158483 | - | 17.4 | 6163 | 6318 | -16 | L1MA10 | L1 | LINE |
| 55158489 | 55158648 | - | 14.8 | 1 | 172 | -245 | MER96B | MER1\_type? | DNA |
| 55158649 | 55158795 | + | 40.1 | 5535 | 5687 | -553 | L1M5 | L1 | LINE |

  

|  |  |  |  |  |  |  |  |  |  |
| --- | --- | --- | --- | --- | --- | --- | --- | --- | --- |
| chr4:57817819-57823678 | | | 5857 bp | | |  |  |  |  |
| Genome Start | Genome End | Strand | Div | Repeat Start | Repeat End | Repeat Left | Name | Family | Class |
| 57817819 | 57818509 | - | 9.5 | 6089 | 6772 | -11 | L1PA13 | L1 | LINE |
| 57818510 | 57818795 | + | 15.8 | 1 | 286 | -26 | AluSx | Alu | SINE |
| 57818796 | 57822192 | - | 11.0 | 2685 | 6088 | -695 | L1PA13 | L1 | LINE |
| 57822201 | 57822446 | - | 5.3 | 5909 | 6152 | -3 | L1PA4 | L1 | LINE |
| 57822447 | 57822690 | + | 2.9 | 5671 | 5914 | -241 | L1PA4 | L1 | LINE |
| 57822686 | 57823678 | - | 13.0 | 1689 | 2698 | -4087 | L1PA13 | L1 | LINE |

  

|  |  |  |  |  |  |  |  |  |  |
| --- | --- | --- | --- | --- | --- | --- | --- | --- | --- |
| chr4:58567553-58574348 | | | 6774 bp | | |  |  |  |  |
| Genome Start | Genome End | Strand | Div | Repeat Start | Repeat End | Repeat Left | Name | Family | Class |
| 58567553 | 58568164 | + | 19.8 | 3617 | 4222 | -1918 | L1MB3 | L1 | LINE |
| 58568194 | 58571799 | - | 7.1 | 2529 | 6146 | -8 | L1PA6 | L1 | LINE |
| 58571795 | 58572454 | + | 5.6 | 1885 | 2520 | -3626 | L1PA6 | L1 | LINE |
| 58572453 | 58574348 | + | 20.9 | 4211 | 6182 | -1 | L1MB3 | L1 | LINE |

  

|  |  |  |  |  |  |  |  |  |  |
| --- | --- | --- | --- | --- | --- | --- | --- | --- | --- |
| chr4:59846924-59854340 | | | 7336 bp | | |  |  |  |  |
| Genome Start | Genome End | Strand | Div | Repeat Start | Repeat End | Repeat Left | Name | Family | Class |
| 59846924 | 59847358 | + | 27.6 | 5 | 423 | -6911 | L1MB7 | L1 | LINE |
| 59847360 | 59848318 | + | 21.1 | 2056 | 3155 | -4179 | L1MB7 | L1 | LINE |
| 59848406 | 59848669 | + | 11.4 | 1 | 266 | -47 | AluSq | Alu | SINE |
| 59848679 | 59849147 | + | 17.7 | 5819 | 6295 | -5 | L1MA4 | L1 | LINE |
| 59849162 | 59849308 | + | 20.4 | 5845 | 6009 | -997 | L1MB7 | L1 | LINE |
| 59849315 | 59851563 | - | 2.4 | 2144 | 4392 | -1754 | L1PA2 | L1 | LINE |
| 59851548 | 59853314 | + | 2.5 | 4381 | 6152 | -3 | L1PA2 | L1 | LINE |
| 59853295 | 59854340 | + | 22.9 | 5967 | 7037 | -7 | L1MB7 | L1 | LINE |

  

|  |  |  |  |  |  |  |  |  |  |
| --- | --- | --- | --- | --- | --- | --- | --- | --- | --- |
| chr4:60265874-60273190 | | | 7302 bp | | |  |  |  |  |
| Genome Start | Genome End | Strand | Div | Repeat Start | Repeat End | Repeat Left | Name | Family | Class |
| 60265874 | 60268706 | - | 6.5 | 3122 | 6150 | -4 | L1PA5 | L1 | LINE |
| 60268724 | 60269366 | - | 1.1 | 5512 | 6154 | -1 | L1HS | L1 | LINE |
| 60269367 | 60269749 | + | 0.0 | 5128 | 5510 | -645 | L1HS | L1 | LINE |
| 60269748 | 60271039 | - | 8.3 | 1875 | 3139 | -3007 | L1PA5 | L1 | LINE |
| 60271040 | 60271298 | + | 4.2 | 5893 | 6152 | -3 | L1PA4 | L1 | LINE |
| 60271299 | 60273190 | - | 9.7 | 4 | 1874 | -401 | L1PA5 | L1 | LINE |

  

|  |  |  |  |  |  |  |  |  |  |
| --- | --- | --- | --- | --- | --- | --- | --- | --- | --- |
| chr4:60822624-60828449 | | | 5782 bp | | |  |  |  |  |
| Genome Start | Genome End | Strand | Div | Repeat Start | Repeat End | Repeat Left | Name | Family | Class |
| 60822624 | 60824007 | - | 23.3 | 6076 | 7693 | -38 | L1MA9 | L1 | LINE |
| 60824054 | 60825810 | - | 23.0 | 4314 | 6075 | -1484 | L1MA9 | L1 | LINE |
| 60825808 | 60826726 | - | 4.8 | 4814 | 5739 | -416 | L1PA4 | L1 | LINE |
| 60826727 | 60827173 | + | 3.3 | 5722 | 6168 | 0 | L1PA4 | L1 | LINE |
| 60827175 | 60828449 | - | 24.8 | 3045 | 4325 | -3237 | L1MA9 | L1 | LINE |

  

|  |  |  |  |  |  |  |  |  |  |
| --- | --- | --- | --- | --- | --- | --- | --- | --- | --- |
| chr4:60862480-60872579 | | | 9974 bp | | |  |  |  |  |
| Genome Start | Genome End | Strand | Div | Repeat Start | Repeat End | Repeat Left | Name | Family | Class |
| 60862480 | 60862842 | + | 20.5 | 15 | 392 | -6241 | L1MD1 | L1 | LINE |
| 60862905 | 60863286 | + | 18.2 | 2 | 465 | -102 | MLT2B4 | ERVL | LTR |
| 60863287 | 60863567 | - | 21.9 | 1 | 239 | 0 | MER8 | MER2\_type | DNA |
| 60863568 | 60863652 | + | 24.0 | 466 | 565 | -2 | MLT2B1 | ERVL | LTR |
| 60863673 | 60864705 | + | 25.6 | 487 | 1572 | -5061 | L1MD1 | L1 | LINE |
| 60864680 | 60865288 | + | 22.8 | 1781 | 2379 | -4254 | L1MD1 | L1 | LINE |
| 60865289 | 60865653 | + | 26.1 | 1 | 365 | 0 | MLT1A0 | MaLR | LTR |
| 60865654 | 60865941 | + | 22.8 | 2380 | 2645 | -3988 | L1MD1 | L1 | LINE |
| 60865937 | 60866099 | - | 7.8 | 5600 | 5768 | -388 | L1P5 | L1 | LINE |
| 60866100 | 60866172 | + | 8.4 | 5757 | 5828 | -344 | L1PA8 | L1 | LINE |
| 60866173 | 60866484 | + | 8.4 | 1 | 311 | 0 | AluY | Alu | SINE |
| 60866485 | 60866841 | + | 8.4 | 5829 | 6172 | 0 | L1PA8 | L1 | LINE |
| 60866842 | 60867984 | - | 17.8 | 3 | 1215 | -16 | Tigger3b | MER2\_type | DNA |
| 60867989 | 60869029 | + | 19.5 | 2644 | 3619 | -2918 | L1MD1 | L1 | LINE |
| 60869066 | 60869921 | + | 19.5 | 3620 | 4502 | -2035 | L1MD1 | L1 | LINE |
| 60869922 | 60870306 | - | 15.6 | 1 | 381 | 0 | THE1D | MaLR | LTR |
| 60870307 | 60870348 | + | 19.5 | 4503 | 4539 | -1998 | L1MD1 | L1 | LINE |
| 60870359 | 60870582 | - | 19.1 | 2483 | 2718 | 0 | Tigger2 | MER2\_type | DNA |
| 60870585 | 60871202 | + | 11.9 | 4915 | 5514 | -649 | L1P4 | L1 | LINE |
| 60871226 | 60871919 | + | 20.3 | 5468 | 6215 | -418 | L1MD1 | L1 | LINE |
| 60871920 | 60872282 | - | 22.7 | 1 | 365 | 0 | MLT1A0 | MaLR | LTR |
| 60872283 | 60872579 | + | 20.0 | 6216 | 6533 | -100 | L1MD1 | L1 | LINE |

  

|  |  |  |  |  |  |  |  |  |  |
| --- | --- | --- | --- | --- | --- | --- | --- | --- | --- |
| chr4:61177772-61186924 | | | 9147 bp | | |  |  |  |  |
| Genome Start | Genome End | Strand | Div | Repeat Start | Repeat End | Repeat Left | Name | Family | Class |
| 61177772 | 61180413 | - | 17.3 | 3630 | 6281 | -10 | L1PA16 | L1 | LINE |
| 61180441 | 61181645 | - | 5.0 | 3802 | 5006 | -1140 | L1PA4 | L1 | LINE |
| 61181625 | 61182767 | + | 4.9 | 5008 | 6152 | -3 | L1PA4 | L1 | LINE |
| 61182777 | 61184265 | - | 19.1 | 2095 | 3619 | -2652 | L1PA16 | L1 | LINE |
| 61184274 | 61185252 | - | 4.5 | 5174 | 6152 | -3 | L1PA4 | L1 | LINE |
| 61185247 | 61186413 | - | 19.4 | 945 | 2109 | -4178 | L1PA16 | L1 | LINE |
| 61186403 | 61186924 | - | 24.6 | 1 | 524 | -5767 | L1PA16 | L1 | LINE |

  

|  |  |  |  |  |  |  |  |  |  |
| --- | --- | --- | --- | --- | --- | --- | --- | --- | --- |
| chr4:61423774-61428267 | | | 3964 bp | | |  |  |  |  |
| Genome Start | Genome End | Strand | Div | Repeat Start | Repeat End | Repeat Left | Name | Family | Class |
| 61423774 | 61423906 | + | 22.5 | 4422 | 4555 | -1591 | L1MB7 | L1 | LINE |
| 61423907 | 61424068 | + | 18.5 | 1 | 168 | -299 | MLT1C | MaLR | LTR |
| 61424099 | 61424374 | - | 12.8 | 6 | 279 | -33 | AluSx | Alu | SINE |
| 61424405 | 61424967 | - | 10.6 | 4569 | 5155 | -991 | L1PREC2 | L1 | LINE |
| 61424967 | 61425964 | + | 11.4 | 5151 | 6163 | 0 | L1PA13 | L1 | LINE |
| 61425992 | 61426256 | + | 21.1 | 192 | 467 | 0 | MLT1C | MaLR | LTR |
| 61426261 | 61426900 | + | 20.9 | 4555 | 5174 | -972 | L1MB7 | L1 | LINE |
| 61427137 | 61427376 | + | 17.8 | 5188 | 5439 | -745 | L1MB7 | L1 | LINE |
| 61427377 | 61427455 | + | 11.4 | 1 | 80 | 0 | MADE1 | Mariner | DNA |
| 61427456 | 61427847 | + | 20.5 | 5440 | 5828 | -356 | L1MB7 | L1 | LINE |
| 61428052 | 61428267 | + | 21.8 | 5797 | 6014 | -170 | L1MB7 | L1 | LINE |

  

|  |  |  |  |  |  |  |  |  |  |
| --- | --- | --- | --- | --- | --- | --- | --- | --- | --- |
| chr4:64618653-64626208 | | | 7347 bp | | |  |  |  |  |
| Genome Start | Genome End | Strand | Div | Repeat Start | Repeat End | Repeat Left | Name | Family | Class |
| 64618653 | 64618777 | + | 26.4 | 1515 | 1652 | -4015 | ERVL-E | ERVL | LTR |
| 64618818 | 64618956 | + | 22.6 | 1 | 149 | -232 | THE1D | MaLR | LTR |
| 64618946 | 64619536 | + | 16.2 | 18 | 591 | -989 | THE1-int | MaLR | LTR |
| 64619559 | 64619945 | + | 15.2 | 1 | 428 | 0 | MSTA | MaLR | LTR |
| 64619946 | 64621549 | + | 32.6 | 1744 | 3385 | -2329 | ERVL-E | ERVL | LTR |
| 64621623 | 64621850 | - | 4.4 | 3166 | 3399 | -2747 | L1PA5 | L1 | LINE |
| 64621849 | 64622951 | + | 5.8 | 3395 | 4483 | -1663 | L1PA5 | L1 | LINE |
| 64622952 | 64623260 | + | 10.0 | 1 | 309 | -2 | AluY | Alu | SINE |
| 64623261 | 64624920 | + | 5.3 | 4484 | 6149 | -5 | L1PA5 | L1 | LINE |
| 64625008 | 64626208 | + | 35.9 | 3790 | 4962 | -705 | ERVL-E | ERVL | LTR |

  

|  |  |  |  |  |  |  |  |  |  |
| --- | --- | --- | --- | --- | --- | --- | --- | --- | --- |
| chr4:65278498-65283186 | | | 4646 bp | | |  |  |  |  |
| Genome Start | Genome End | Strand | Div | Repeat Start | Repeat End | Repeat Left | Name | Family | Class |
| 65278498 | 65278946 | + | 18.0 | 1474 | 3172 | -2774 | HERV4\_I | ERV1 | LTR |
| 65278976 | 65279066 | + | 8.8 | 2210 | 2301 | -2916 | HERV4\_I | ERV1 | LTR |
| 65279064 | 65279280 | + | 10.2 | 5081 | 5297 | -2121 | HERV4\_I | ERV1 | LTR |
| 65279312 | 65280106 | - | 4.3 | 5361 | 6143 | -11 | L1PA5 | L1 | LINE |
| 65280107 | 65280426 | + | 8.2 | 1 | 317 | -1 | AluYb8 | Alu | SINE |
| 65280427 | 65281089 | - | 7.1 | 4700 | 5360 | -786 | L1P | L1 | LINE |
| 65281087 | 65281537 | + | 5.5 | 4247 | 4702 | -1444 | L1P | L1 | LINE |
| 65281538 | 65281635 | + | 13.4 | 4906 | 5004 | -2533 | HERV4\_I | ERV1 | LTR |
| 65281635 | 65281726 | + | 14.3 | 5249 | 5349 | -2188 | HERV4\_I | ERV1 | LTR |
| 65281717 | 65283186 | + | 13.9 | 5620 | 7147 | -390 | HERV4\_I | ERV1 | LTR |

  

|  |  |  |  |  |  |  |  |  |  |
| --- | --- | --- | --- | --- | --- | --- | --- | --- | --- |
| chr4:66944222-66952273 | | | 8078 bp | | |  |  |  |  |
| Genome Start | Genome End | Strand | Div | Repeat Start | Repeat End | Repeat Left | Name | Family | Class |
| 66944222 | 66944407 | - | 12.9 | 7534 | 7715 | -9 | L1MA3 | L1 | LINE |
| 66944408 | 66944718 | + | 0.6 | 1 | 311 | -7 | AluYb8 | Alu | SINE |
| 66944719 | 66946203 | - | 12.9 | 6076 | 7533 | -191 | L1MA3 | L1 | LINE |
| 66946181 | 66948160 | - | 14.9 | 2840 | 4892 | -2673 | L1MA3 | L1 | LINE |
| 66948157 | 66948921 | - | 16.2 | 1444 | 2220 | -5345 | L1MA3 | L1 | LINE |
| 66948919 | 66950353 | - | 5.7 | 3262 | 4695 | -1451 | L1PA4 | L1 | LINE |
| 66950354 | 66951815 | + | 3.7 | 4694 | 6155 | 0 | L1PA4 | L1 | LINE |
| 66951820 | 66952273 | - | 16.8 | 997 | 1444 | -6121 | L1MA3 | L1 | LINE |

  

|  |  |  |  |  |  |  |  |  |  |
| --- | --- | --- | --- | --- | --- | --- | --- | --- | --- |
| chr4:66959108-66963645 | | | 4507 bp | | |  |  |  |  |
| Genome Start | Genome End | Strand | Div | Repeat Start | Repeat End | Repeat Left | Name | Family | Class |
| 66959108 | 66960590 | + | 30.6 | 3750 | 5278 | -868 | L1ME1 | L1 | LINE |
| 66960626 | 66960909 | + | 24.5 | 5282 | 5584 | -578 | L1ME1 | L1 | LINE |
| 66960904 | 66961569 | - | 9.9 | 3992 | 4663 | -1483 | L1PA7 | L1 | LINE |
| 66961569 | 66963036 | + | 9.3 | 4676 | 6154 | 0 | L1PA7 | L1 | LINE |
| 66963040 | 66963645 | + | 27.4 | 5569 | 6162 | -4 | L1ME1 | L1 | LINE |

  

|  |  |  |  |  |  |  |  |  |  |
| --- | --- | --- | --- | --- | --- | --- | --- | --- | --- |
| chr4:67683374-67685054 | | | 1690 bp | | |  |  |  |  |
| Genome Start | Genome End | Strand | Div | Repeat Start | Repeat End | Repeat Left | Name | Family | Class |
| 67683374 | 67683615 | - | 30.8 | 5145 | 5392 | -754 | L1M5 | L1 | LINE |
| 67683601 | 67684622 | - | 16.6 | 5141 | 6154 | -2 | L1PB4 | L1 | LINE |
| 67684628 | 67684820 | + | 18.1 | 4927 | 5132 | -1014 | L1P4 | L1 | LINE |
| 67684822 | 67685054 | - | 38.8 | 4915 | 5159 | -987 | L1M5 | L1 | LINE |

  

|  |  |  |  |  |  |  |  |  |  |
| --- | --- | --- | --- | --- | --- | --- | --- | --- | --- |
| chr4:67793090-67794338 | | | 936 bp | | |  |  |  |  |
| Genome Start | Genome End | Strand | Div | Repeat Start | Repeat End | Repeat Left | Name | Family | Class |
| 67793090 | 67793375 | + | 29.2 | 2109 | 2420 | -3795 | L1MEe | L1 | LINE |
| 67793382 | 67793481 | + | 22.0 | 70 | 170 | -298 | MER110A | ERV1 | LTR |
| 67793487 | 67793584 | - | 15.3 | 5933 | 6033 | -128 | L1PA14 | L1 | LINE |
| 67793585 | 67793722 | + | 11.6 | 6021 | 6158 | -2 | L1PREC2 | L1 | LINE |
| 67793852 | 67794017 | + | 25.8 | 177 | 372 | -96 | MER110A | ERV1 | LTR |
| 67794191 | 67794338 | + | 35.2 | 2473 | 2618 | -3596 | L1MEe | L1 | LINE |

  

|  |  |  |  |  |  |  |  |  |  |
| --- | --- | --- | --- | --- | --- | --- | --- | --- | --- |
| chr4:67800057-67810190 | | | 9639 bp | | |  |  |  |  |
| Genome Start | Genome End | Strand | Div | Repeat Start | Repeat End | Repeat Left | Name | Family | Class |
| 67800057 | 67800626 | - | 29.2 | 5121 | 5667 | 0 | ERVL-E | ERVL | LTR |
| 67800646 | 67801123 | - | 10.1 | 5690 | 6157 | -11 | L1PA10 | L1 | LINE |
| 67801124 | 67801411 | - | 12.9 | 1 | 291 | -21 | AluSx | Alu | SINE |
| 67801412 | 67804093 | - | 8.9 | 3014 | 5689 | -479 | L1PA10 | L1 | LINE |
| 67804089 | 67804627 | + | 15.1 | 2513 | 2998 | -3148 | L1PREC2 | L1 | LINE |
| 67804638 | 67804730 | - | 29.0 | 4978 | 5074 | -593 | ERVL-E | ERVL | LTR |
| 67804733 | 67805293 | - | 15.0 | 1 | 541 | -14 | MER66C | ERV1 | LTR |
| 67805302 | 67805501 | - | 25.9 | 4757 | 4971 | -696 | ERVL-E | ERVL | LTR |
| 67805540 | 67805690 | - | 33.0 | 3199 | 3352 | -2315 | ERVL-E | ERVL | LTR |
| 67805691 | 67806097 | + | 20.4 | 1 | 397 | 0 | MLT2C1 | ERVL | LTR |
| 67806098 | 67806564 | - | 33.0 | 2721 | 3198 | -2469 | ERVL-E | ERVL | LTR |
| 67806573 | 67806702 | + | 21.8 | 1 | 131 | -193 | MER5C | MER1\_type | DNA |
| 67806727 | 67806835 | + | 20.9 | 2704 | 2822 | 0 | Charlie10 | MER1\_type | DNA |
| 67806838 | 67807343 | - | 29.7 | 2116 | 2715 | -2952 | ERVL-E | ERVL | LTR |
| 67807333 | 67807987 | - | 32.9 | 1325 | 1982 | -3685 | ERVL-E | ERVL | LTR |
| 67807988 | 67808388 | + | 15.8 | 1 | 390 | 0 | MLT1B | MaLR | LTR |
| 67808389 | 67808429 | - | 32.9 | 1276 | 1324 | -4343 | ERVL-E | ERVL | LTR |
| 67808498 | 67808661 | - | 32.7 | 1019 | 1200 | -4467 | ERVL-E | ERVL | LTR |
| 67808804 | 67809196 | - | 22.9 | 1 | 389 | -1 | MLT1B | MaLR | LTR |
| 67809280 | 67809883 | - | 32.4 | 445 | 1063 | -4604 | ERVL-E | ERVL | LTR |
| 67809991 | 67810190 | - | 28.6 | 22 | 215 | -5452 | ERVL-E | ERVL | LTR |

  

|  |  |  |  |  |  |  |  |  |  |
| --- | --- | --- | --- | --- | --- | --- | --- | --- | --- |
| chr4:69657318-69666880 | | | 8527 bp | | |  |  |  |  |
| Genome Start | Genome End | Strand | Div | Repeat Start | Repeat End | Repeat Left | Name | Family | Class |
| 69657318 | 69657491 | + | 34.7 | 5 | 181 | -4815 | HERV16 | ERVL | LTR |
| 69657691 | 69658156 | - | 20.1 | 2 | 557 | 0 | MER41D | ERV1 | LTR |
| 69658369 | 69658492 | + | 27.4 | 589 | 720 | -4276 | HERV16 | ERVL | LTR |
| 69658514 | 69659569 | - | 20.4 | 2073 | 3124 | -3359 | L1P4a | L1 | LINE |
| 69659568 | 69659751 | + | 12.0 | 1727 | 1912 | -4591 | L1P4a | L1 | LINE |
| 69659761 | 69660695 | - | 31.0 | 9 | 967 | -5536 | L1P4a | L1 | LINE |
| 69660708 | 69660976 | - | 19.7 | 5018 | 5366 | -780 | L1P4 | L1 | LINE |
| 69660977 | 69661340 | - | 15.4 | 1 | 364 | 0 | THE1B | MaLR | LTR |
| 69661341 | 69662991 | - | 31.4 | 2 | 1735 | 0 | MLT-int | MaLR | LTR |
| 69662994 | 69663353 | - | 14.4 | 1 | 364 | 0 | THE1B | MaLR | LTR |
| 69663354 | 69664410 | - | 19.7 | 3943 | 5017 | -1129 | L1P4 | L1 | LINE |
| 69664405 | 69665192 | + | 22.3 | 3151 | 3944 | -2202 | L1P4 | L1 | LINE |
| 69665199 | 69665413 | + | 37.7 | 976 | 1190 | -3806 | HERV16 | ERVL | LTR |
| 69665662 | 69665857 | + | 34.0 | 1608 | 1808 | -3188 | HERV16 | ERVL | LTR |
| 69666102 | 69666290 | - | 33.3 | 226 | 418 | -49 | MLT1C | MaLR | LTR |
| 69666342 | 69666417 | - | 20.0 | 50 | 117 | -16 | FLAM | Alu | SINE |
| 69666405 | 69666597 | - | 27.7 | 6 | 199 | -285 | MLT1C | MaLR | LTR |
| 69666651 | 69666880 | + | 27.8 | 2255 | 2495 | -2501 | HERV16 | ERVL | LTR |

  

|  |  |  |  |  |  |  |  |  |  |
| --- | --- | --- | --- | --- | --- | --- | --- | --- | --- |
| chr4:70272941-70286282 | | | 12613 bp | | |  |  |  |  |
| Genome Start | Genome End | Strand | Div | Repeat Start | Repeat End | Repeat Left | Name | Family | Class |
| 70272941 | 70273170 | - | 31.4 | 2254 | 2494 | -2502 | HERV16 | ERVL | LTR |
| 70273264 | 70273402 | + | 27.3 | 48 | 181 | -286 | MLT1C | MaLR | LTR |
| 70273403 | 70273485 | + | 19.3 | 50 | 133 | -10 | FLAM | Alu | SINE |
| 70273548 | 70273747 | + | 29.3 | 219 | 425 | -42 | MLT1C | MaLR | LTR |
| 70273768 | 70273888 | - | 30.8 | 2104 | 2226 | -3428 | HERV16 | ERVL | LTR |
| 70273961 | 70274172 | - | 38.6 | 1608 | 1824 | -3172 | HERV16 | ERVL | LTR |
| 70274425 | 70274831 | + | 5.9 | 5745 | 6152 | -3 | L1PA4 | L1 | LINE |
| 70274843 | 70275060 | - | 39.4 | 972 | 1188 | -3808 | HERV16 | ERVL | LTR |
| 70275064 | 70275850 | - | 22.5 | 3150 | 3944 | -2202 | L1P4 | L1 | LINE |
| 70275853 | 70276208 | + | 19.4 | 3951 | 4318 | -1828 | L1P4 | L1 | LINE |
| 70276209 | 70276914 | + | 8.3 | 1 | 722 | 0 | LTR30 | ERV1 | LTR |
| 70276916 | 70280356 | + | 9.3 | 1 | 3462 | -4795 | HERV30 | ERV1 | LTR |
| 70280350 | 70281250 | + | 10.8 | 7344 | 8257 | 0 | HERV30 | ERV1 | LTR |
| 70281257 | 70281948 | + | 8.6 | 9 | 720 | -2 | LTR30 | ERV1 | LTR |
| 70281950 | 70282647 | + | 19.2 | 4314 | 5015 | -1131 | L1P4 | L1 | LINE |
| 70282648 | 70283008 | + | 17.8 | 1 | 364 | 0 | THE1B | MaLR | LTR |
| 70283011 | 70284658 | + | 32.8 | 2 | 1735 | 0 | MLT1A0-int | MaLR | LTR |
| 70284659 | 70285021 | + | 16.0 | 1 | 364 | 0 | THE1B | MaLR | LTR |
| 70285022 | 70285288 | + | 23.6 | 5013 | 5363 | -780 | L1M2 | L1 | LINE |
| 70285301 | 70285699 | + | 30.1 | 9 | 406 | -6097 | L1P4a | L1 | LINE |
| 70285701 | 70285910 | + | 21.5 | 342 | 557 | 0 | MER41D | ERV1 | LTR |
| 70286109 | 70286282 | - | 28.4 | 5 | 181 | -4815 | HERV16 | ERVL | LTR |

  

|  |  |  |  |  |  |  |  |  |  |
| --- | --- | --- | --- | --- | --- | --- | --- | --- | --- |
| chr4:70440323-70445405 | | | 3923 bp | | |  |  |  |  |
| Genome Start | Genome End | Strand | Div | Repeat Start | Repeat End | Repeat Left | Name | Family | Class |
| 70440323 | 70440709 | + | 36.7 | 1233 | 1584 | -4083 | ERVL-E | ERVL | LTR |
| 70441047 | 70441344 | + | 35.0 | 3168 | 3474 | -2193 | ERVL-E | ERVL | LTR |
| 70441475 | 70441784 | + | 8.7 | 1 | 311 | 0 | AluY | Alu | SINE |
| 70441809 | 70442031 | + | 30.6 | 3681 | 3893 | -1774 | ERVL-E | ERVL | LTR |
| 70442376 | 70442542 | - | 3.6 | 5989 | 6154 | -1 | L1PA4 | L1 | LINE |
| 70442543 | 70444507 | + | 3.1 | 4017 | 5986 | -169 | L1PA4 | L1 | LINE |
| 70444526 | 70444970 | + | 31.3 | 4054 | 4509 | -1158 | ERVL-E | ERVL | LTR |
| 70445278 | 70445405 | + | 34.4 | 4905 | 5036 | -678 | ERVL-E | ERVL | LTR |

  

|  |  |  |  |  |  |  |  |  |  |
| --- | --- | --- | --- | --- | --- | --- | --- | --- | --- |
| chr4:70778467-70780965 | | | 2136 bp | | |  |  |  |  |
| Genome Start | Genome End | Strand | Div | Repeat Start | Repeat End | Repeat Left | Name | Family | Class |
| 70778467 | 70778660 | - | 30.1 | 5828 | 6071 | -1737 | L1MC4a | L1 | LINE |
| 70778661 | 70778971 | - | 23.0 | 1 | 312 | 0 | AluJb | Alu | SINE |
| 70778972 | 70779214 | - | 30.1 | 5555 | 5827 | -1981 | L1MC4a | L1 | LINE |
| 70779239 | 70779450 | - | 27.3 | 5255 | 5470 | -2338 | L1MC4a | L1 | LINE |
| 70779589 | 70780174 | - | 7.9 | 5573 | 6168 | 0 | L1PA12 | L1 | LINE |
| 70780175 | 70780381 | + | 10.9 | 5388 | 5597 | -643 | L1PA12 | L1 | LINE |
| 70780514 | 70780665 | - | 30.7 | 332 | 485 | -36 | LTR33 | ERVL | LTR |
| 70780735 | 70780965 | - | 29.0 | 4439 | 4681 | -1465 | L1MC4a | L1 | LINE |

  

|  |  |  |  |  |  |  |  |  |  |
| --- | --- | --- | --- | --- | --- | --- | --- | --- | --- |
| chr4:71006187-71007292 | | | 1110 bp | | |  |  |  |  |
| Genome Start | Genome End | Strand | Div | Repeat Start | Repeat End | Repeat Left | Name | Family | Class |
| 71006187 | 71006417 | - | 17.3 | 6053 | 6284 | -16 | L1MA6 | L1 | LINE |
| 71006422 | 71006591 | - | 3.6 | 5988 | 6155 | 0 | L1PA2 | L1 | LINE |
| 71006592 | 71006948 | + | 0.8 | 5639 | 5995 | -160 | L1PA2 | L1 | LINE |
| 71006949 | 71007105 | + | 0.6 | 5191 | 5348 | -798 | L1P1 | L1 | LINE |
| 71007098 | 71007292 | - | 16.8 | 5874 | 6069 | -231 | L1MA6 | L1 | LINE |

  

|  |  |  |  |  |  |  |  |  |  |
| --- | --- | --- | --- | --- | --- | --- | --- | --- | --- |
| chr4:71044228-71045775 | | | 1528 bp | | |  |  |  |  |
| Genome Start | Genome End | Strand | Div | Repeat Start | Repeat End | Repeat Left | Name | Family | Class |
| 71044228 | 71044342 | - | 28.7 | 147 | 262 | 0 | MIR | MIR | SINE |
| 71044348 | 71045019 | - | 12.1 | 5471 | 6148 | -3 | L1PB1 | L1 | LINE |
| 71045020 | 71045623 | + | 7.8 | 4867 | 5478 | -673 | L1PB1 | L1 | LINE |
| 71045639 | 71045775 | - | 34.4 | 2 | 134 | -128 | MIR | MIR | SINE |

  

|  |  |  |  |  |  |  |  |  |  |
| --- | --- | --- | --- | --- | --- | --- | --- | --- | --- |
| chr4:71206548-71211813 | | | 5250 bp | | |  |  |  |  |
| Genome Start | Genome End | Strand | Div | Repeat Start | Repeat End | Repeat Left | Name | Family | Class |
| 71206548 | 71207010 | - | 28.8 | 2526 | 3004 | -415 | L2 | L2 | LINE |
| 71207011 | 71207297 | - | 23.2 | 2 | 370 | -4 | MLT1A | MaLR | LTR |
| 71207298 | 71207924 | - | 28.8 | 1855 | 2525 | -894 | L2 | L2 | LINE |
| 71207925 | 71208013 | - | 6.7 | 4126 | 4214 | -1932 | L1PA4 | L1 | LINE |
| 71208017 | 71209946 | + | 3.3 | 4221 | 6152 | -3 | L1PA4 | L1 | LINE |
| 71209960 | 71210435 | - | 31.2 | 1394 | 1872 | -1547 | L2 | L2 | LINE |
| 71210436 | 71210729 | + | 10.7 | 4 | 296 | -16 | AluSx | Alu | SINE |
| 71210730 | 71211813 | - | 31.2 | 131 | 1393 | -2026 | L2 | L2 | LINE |

  

|  |  |  |  |  |  |  |  |  |  |
| --- | --- | --- | --- | --- | --- | --- | --- | --- | --- |
| chr4:72002542-72004028 | | | 1486 bp | | |  |  |  |  |
| Genome Start | Genome End | Strand | Div | Repeat Start | Repeat End | Repeat Left | Name | Family | Class |
| 72002542 | 72002790 | - | 10.5 | 2365 | 2645 | -2572 | MER61-int | ERV1 | LTR |
| 72002791 | 72003394 | - | 1.2 | 5032 | 5634 | -521 | L1PA3 | L1 | LINE |
| 72003395 | 72003902 | + | 1.8 | 5645 | 6155 | 0 | L1PA3 | L1 | LINE |
| 72003904 | 72004028 | - | 7.7 | 2210 | 2378 | -2839 | MER61-int | ERV1 | LTR |

  

|  |  |  |  |  |  |  |  |  |  |
| --- | --- | --- | --- | --- | --- | --- | --- | --- | --- |
| chr4:73777229-73780583 | | | 3345 bp | | |  |  |  |  |
| Genome Start | Genome End | Strand | Div | Repeat Start | Repeat End | Repeat Left | Name | Family | Class |
| 73777229 | 73777341 | + | 22.1 | 39 | 155 | -329 | MLT1H | MaLR | LTR |
| 73777347 | 73779377 | - | 3.5 | 4130 | 6153 | -2 | L1PA4 | L1 | LINE |
| 73779372 | 73780290 | + | 5.8 | 3194 | 4115 | -2031 | L1PA4 | L1 | LINE |
| 73780302 | 73780583 | + | 25.0 | 200 | 516 | -33 | MLT1H | MaLR | LTR |

  

|  |  |  |  |  |  |  |  |  |  |
| --- | --- | --- | --- | --- | --- | --- | --- | --- | --- |
| chr4:73881838-73887326 | | | 5486 bp | | |  |  |  |  |
| Genome Start | Genome End | Strand | Div | Repeat Start | Repeat End | Repeat Left | Name | Family | Class |
| 73881838 | 73882962 | - | 14.5 | 427 | 1580 | 0 | THE1D-int | MaLR | LTR |
| 73882991 | 73884301 | - | 9.1 | 2233 | 3540 | -2606 | L1PA6 | L1 | LINE |
| 73884277 | 73886899 | + | 6.7 | 3517 | 6153 | -1 | L1PA6 | L1 | LINE |
| 73886900 | 73887326 | - | 12.0 | 1 | 429 | -1151 | THE1D-int | MaLR | LTR |

  

|  |  |  |  |  |  |  |  |  |  |
| --- | --- | --- | --- | --- | --- | --- | --- | --- | --- |
| chr4:73914039-73927034 | | | 12993 bp | | |  |  |  |  |
| Genome Start | Genome End | Strand | Div | Repeat Start | Repeat End | Repeat Left | Name | Family | Class |
| 73914039 | 73914853 | + | 18.8 | 3211 | 4030 | -2116 | L1M4 | L1 | LINE |
| 73914849 | 73917903 | + | 9.1 | 2973 | 6001 | -153 | L1PA7 | L1 | LINE |
| 73917899 | 73918728 | - | 11.8 | 4281 | 5106 | -1040 | L1PREC2 | L1 | LINE |
| 73918726 | 73919757 | + | 11.0 | 5123 | 6159 | -2 | L1PA14 | L1 | LINE |
| 73919768 | 73920086 | + | 18.4 | 4014 | 4334 | -1812 | L1M4 | L1 | LINE |
| 73920086 | 73926243 | - | 8.2 | 11 | 6152 | -2 | L1PA5 | L1 | LINE |
| 73926251 | 73927034 | + | 19.5 | 4319 | 5069 | -1077 | L1M4 | L1 | LINE |

  

|  |  |  |  |  |  |  |  |  |  |
| --- | --- | --- | --- | --- | --- | --- | --- | --- | --- |
| chr4:74876848-74883529 | | | 6606 bp | | |  |  |  |  |
| Genome Start | Genome End | Strand | Div | Repeat Start | Repeat End | Repeat Left | Name | Family | Class |
| 74876848 | 74877014 | - | 21.3 | 7600 | 7765 | -20 | L1MC3 | L1 | LINE |
| 74877022 | 74877070 | - | 16.3 | 7387 | 7435 | -350 | L1MC3 | L1 | LINE |
| 74877066 | 74877747 | - | 7.9 | 1848 | 2499 | -3647 | L1PA7 | L1 | LINE |
| 74877746 | 74881381 | + | 9.1 | 2498 | 6151 | -3 | L1PA7 | L1 | LINE |
| 74881392 | 74881696 | - | 5.2 | 2 | 306 | -5 | AluY | Alu | SINE |
| 74881762 | 74881924 | - | 24.8 | 7249 | 7397 | -388 | L1MC3 | L1 | LINE |
| 74881947 | 74882381 | - | 25.5 | 6330 | 6861 | -944 | L1MC3 | L1 | LINE |
| 74882361 | 74882907 | - | 19.1 | 5529 | 6124 | -1661 | L1MC3 | L1 | LINE |
| 74882908 | 74883223 | - | 6.8 | 1 | 311 | 0 | AluY | Alu | SINE |
| 74883224 | 74883529 | - | 18.8 | 5235 | 5528 | -2257 | L1MC3 | L1 | LINE |

  

|  |  |  |  |  |  |  |  |  |  |
| --- | --- | --- | --- | --- | --- | --- | --- | --- | --- |
| chr4:75368753-75370370 | | | 1612 bp | | |  |  |  |  |
| Genome Start | Genome End | Strand | Div | Repeat Start | Repeat End | Repeat Left | Name | Family | Class |
| 75368753 | 75368950 | + | 14.1 | 1059 | 1265 | -332 | Tigger6 | MER2\_type | DNA |
| 75368951 | 75369259 | - | 4.1 | 5159 | 5464 | -690 | L1PA5 | L1 | LINE |
| 75369260 | 75369930 | + | 4.5 | 5477 | 6150 | -4 | L1PA5 | L1 | LINE |
| 75369937 | 75370370 | + | 15.5 | 1248 | 1597 | 0 | Tigger6 | MER2\_type | DNA |

  

|  |  |  |  |  |  |  |  |  |  |
| --- | --- | --- | --- | --- | --- | --- | --- | --- | --- |
| chr4:76435901-76445359 | | | 9346 bp | | |  |  |  |  |
| Genome Start | Genome End | Strand | Div | Repeat Start | Repeat End | Repeat Left | Name | Family | Class |
| 76435901 | 76439776 | - | 7.6 | 2272 | 6149 | -5 | L1PA6 | L1 | LINE |
| 76439886 | 76442182 | - | 4.7 | 3854 | 6153 | -2 | L1PA4 | L1 | LINE |
| 76442186 | 76443079 | + | 5.2 | 2943 | 3843 | -2303 | L1PA4 | L1 | LINE |
| 76443081 | 76445359 | - | 7.7 | 1 | 2286 | -3860 | L1PA6 | L1 | LINE |

  

|  |  |  |  |  |  |  |  |  |  |
| --- | --- | --- | --- | --- | --- | --- | --- | --- | --- |
| chr4:76677780-76679517 | | | 1710 bp | | |  |  |  |  |
| Genome Start | Genome End | Strand | Div | Repeat Start | Repeat End | Repeat Left | Name | Family | Class |
| 76677780 | 76677971 | - | 11.2 | 176 | 364 | 0 | THE1B | MaLR | LTR |
| 76678000 | 76678456 | - | 3.5 | 5693 | 6150 | -4 | L1PA5 | L1 | LINE |
| 76678457 | 76679340 | + | 4.4 | 4802 | 5696 | -458 | L1PA5 | L1 | LINE |
| 76679341 | 76679517 | - | 13.9 | 1 | 185 | -179 | THE1B | MaLR | LTR |

  

|  |  |  |  |  |  |  |  |  |  |
| --- | --- | --- | --- | --- | --- | --- | --- | --- | --- |
| chr4:78397022-78400311 | | | 3252 bp | | |  |  |  |  |
| Genome Start | Genome End | Strand | Div | Repeat Start | Repeat End | Repeat Left | Name | Family | Class |
| 78397022 | 78397179 | + | 27.9 | 182 | 356 | -206 | MLT1E | MaLR | LTR |
| 78397183 | 78397535 | - | 25.9 | 168 | 548 | -2 | MER44B | MER2\_type | DNA |
| 78397536 | 78397580 | - | 2.2 | 3744 | 3788 | -2358 | L1P3 | L1 | LINE |
| 78397577 | 78399909 | + | 4.6 | 3783 | 6152 | -3 | L1PA4 | L1 | LINE |
| 78399917 | 78400076 | - | 23.6 | 1 | 163 | -387 | MER44B | MER2\_type | DNA |
| 78400109 | 78400311 | + | 26.3 | 375 | 585 | -8 | MLT1E | MaLR | LTR |

  

|  |  |  |  |  |  |  |  |  |  |
| --- | --- | --- | --- | --- | --- | --- | --- | --- | --- |
| chr4:78824985-78827778 | | | 2783 bp | | |  |  |  |  |
| Genome Start | Genome End | Strand | Div | Repeat Start | Repeat End | Repeat Left | Name | Family | Class |
| 78824985 | 78825703 | + | 16.5 | 5271 | 5974 | -210 | L1MB7 | L1 | LINE |
| 78825692 | 78826601 | - | 2.5 | 4330 | 5239 | -907 | L1PA3 | L1 | LINE |
| 78826598 | 78827530 | + | 2.5 | 5219 | 6152 | -3 | L1PA3 | L1 | LINE |
| 78827558 | 78827778 | + | 24.4 | 5946 | 6173 | -11 | L1MB7 | L1 | LINE |

  

|  |  |  |  |  |  |  |  |  |  |
| --- | --- | --- | --- | --- | --- | --- | --- | --- | --- |
| chr4:79554914-79556120 | | | 1141 bp | | |  |  |  |  |
| Genome Start | Genome End | Strand | Div | Repeat Start | Repeat End | Repeat Left | Name | Family | Class |
| 79554914 | 79555173 | - | 34.9 | 3120 | 3372 | -6 | L2 | L2 | LINE |
| 79555199 | 79555257 | - | 23.7 | 126 | 185 | -4 | MER5A | MER1\_type | DNA |
| 79555259 | 79555407 | - | 4.0 | 5866 | 6013 | -138 | L1PB1 | L1 | LINE |
| 79555409 | 79555553 | + | 11.9 | 6008 | 6150 | -1 | L1PB1 | L1 | LINE |
| 79555579 | 79555718 | - | 17.6 | 1 | 140 | -49 | MER5A | MER1\_type | DNA |
| 79555733 | 79556120 | - | 34.9 | 2682 | 3082 | -337 | L2 | L2 | LINE |

  

|  |  |  |  |  |  |  |  |  |  |
| --- | --- | --- | --- | --- | --- | --- | --- | --- | --- |
| chr4:80159203-80161832 | | | 2585 bp | | |  |  |  |  |
| Genome Start | Genome End | Strand | Div | Repeat Start | Repeat End | Repeat Left | Name | Family | Class |
| 80159203 | 80159527 | - | 36.0 | 3022 | 3371 | -7 | L2 | L2 | LINE |
| 80159532 | 80160363 | - | 2.5 | 4568 | 5399 | -747 | L1PA3 | L1 | LINE |
| 80160364 | 80161114 | + | 3.2 | 5405 | 6155 | 0 | L1PA3 | L1 | LINE |
| 80161156 | 80161832 | - | 31.9 | 2256 | 2997 | -422 | L2 | L2 | LINE |

  

|  |  |  |  |  |  |  |  |  |  |
| --- | --- | --- | --- | --- | --- | --- | --- | --- | --- |
| chr4:80307791-80310072 | | | 2266 bp | | |  |  |  |  |
| Genome Start | Genome End | Strand | Div | Repeat Start | Repeat End | Repeat Left | Name | Family | Class |
| 80307791 | 80308030 | - | 16.7 | 185 | 427 | -1 | MSTA | MaLR | LTR |
| 80308051 | 80309053 | - | 7.3 | 5149 | 6153 | -1 | L1PA7 | L1 | LINE |
| 80309050 | 80309906 | + | 8.6 | 4257 | 5124 | -1022 | L1PA7 | L1 | LINE |
| 80309907 | 80310072 | - | 15.1 | 1 | 198 | -230 | MSTA | MaLR | LTR |

  

|  |  |  |  |  |  |  |  |  |  |
| --- | --- | --- | --- | --- | --- | --- | --- | --- | --- |
| chr4:81873498-81882480 | | | 8974 bp | | |  |  |  |  |
| Genome Start | Genome End | Strand | Div | Repeat Start | Repeat End | Repeat Left | Name | Family | Class |
| 81873498 | 81876209 | - | 11.5 | 4974 | 7723 | 0 | L1MA2 | L1 | LINE |
| 81876190 | 81876575 | - | 3.1 | 4838 | 5223 | -923 | L1PA3 | L1 | LINE |
| 81876574 | 81877500 | + | 2.3 | 5224 | 6151 | -4 | L1PA3 | L1 | LINE |
| 81877532 | 81881412 | - | 16.6 | 969 | 5003 | -2562 | L1MA2 | L1 | LINE |
| 81881413 | 81881551 | - | 10.2 | 176 | 312 | 0 | AluSg/x | Alu | SINE |
| 81881552 | 81882480 | - | 16.6 | 3 | 968 | -6597 | L1MA2 | L1 | LINE |

  

|  |  |  |  |  |  |  |  |  |  |
| --- | --- | --- | --- | --- | --- | --- | --- | --- | --- |
| chr4:82022840-82035518 | | | 12443 bp | | |  |  |  |  |
| Genome Start | Genome End | Strand | Div | Repeat Start | Repeat End | Repeat Left | Name | Family | Class |
| 82022840 | 82024020 | + | 26.2 | 2736 | 3970 | -3589 | L1MA9 | L1 | LINE |
| 82024050 | 82024293 | - | 11.1 | 5907 | 6160 | -8 | L1PA12 | L1 | LINE |
| 82024294 | 82024942 | + | 20.4 | 3971 | 4684 | -2875 | L1MA9 | L1 | LINE |
| 82024937 | 82030657 | + | 12.4 | 13 | 5910 | -1655 | L1M1 | L1 | LINE |
| 82030659 | 82031535 | - | 0.8 | 4857 | 5734 | -421 | L1HS | L1 | LINE |
| 82031536 | 82032024 | + | 1.2 | 5735 | 6223 | 0 | L1HS | L1 | LINE |
| 82032025 | 82032742 | + | 11.3 | 5896 | 6618 | -947 | L1M1 | L1 | LINE |
| 82032740 | 82034438 | + | 18.6 | 5234 | 6957 | -774 | L1MA9 | L1 | LINE |
| 82034453 | 82034575 | + | 34.1 | 431 | 557 | 0 | LTR67 | ERVL | LTR |
| 82034748 | 82035348 | + | 17.8 | 6944 | 7570 | -161 | L1MA9 | L1 | LINE |
| 82035378 | 82035518 | + | 17.8 | 7571 | 7727 | -4 | L1MA9 | L1 | LINE |

  

|  |  |  |  |  |  |  |  |  |  |
| --- | --- | --- | --- | --- | --- | --- | --- | --- | --- |
| chr4:88702153-88705572 | | | 3321 bp | | |  |  |  |  |
| Genome Start | Genome End | Strand | Div | Repeat Start | Repeat End | Repeat Left | Name | Family | Class |
| 88702153 | 88702832 | + | 26.3 | 5135 | 5823 | -417 | L1ME3B | L1 | LINE |
| 88702922 | 88703131 | - | 0.9 | 3741 | 3950 | -2196 | L1HS | L1 | LINE |
| 88703122 | 88705318 | + | 0.2 | 3960 | 6155 | 0 | L1HS | L1 | LINE |
| 88705339 | 88705572 | + | 26.9 | 5927 | 6162 | -78 | L1ME3B | L1 | LINE |

  

|  |  |  |  |  |  |  |  |  |  |
| --- | --- | --- | --- | --- | --- | --- | --- | --- | --- |
| chr4:88775880-88784605 | | | 8537 bp | | |  |  |  |  |
| Genome Start | Genome End | Strand | Div | Repeat Start | Repeat End | Repeat Left | Name | Family | Class |
| 88775880 | 88776052 | - | 26.2 | 7750 | 7928 | -33 | L1MC5 | L1 | LINE |
| 88776076 | 88776172 | - | 13.4 | 6 | 102 | -6359 | L1PA8 | L1 | LINE |
| 88776177 | 88778207 | + | 10.3 | 100 | 2164 | -426 | L1PA8 | L1 | LINE |
| 88778208 | 88778291 | - | 6.0 | 6068 | 6151 | -4 | L1PA4 | L1 | LINE |
| 88778292 | 88782599 | + | 8.6 | 2165 | 6487 | 0 | L1PA8 | L1 | LINE |
| 88782604 | 88782743 | + | 14.3 | 6023 | 6163 | -3 | L1PA16 | L1 | LINE |
| 88782758 | 88783053 | - | 22.4 | 7400 | 7691 | -270 | L1MC5 | L1 | LINE |
| 88783063 | 88783235 | - | 18.7 | 6990 | 7184 | -777 | L1MC5 | L1 | LINE |
| 88783325 | 88783594 | + | 15.2 | 1 | 289 | -23 | AluJb | Alu | SINE |
| 88783634 | 88783910 | - | 30.5 | 5937 | 6225 | -15 | L1ME3B | L1 | LINE |
| 88783918 | 88784305 | - | 25.2 | 7142 | 7533 | -428 | L1MC5 | L1 | LINE |
| 88784306 | 88784588 | - | 12.1 | 1 | 282 | -28 | AluSg | Alu | SINE |
| 88784589 | 88784605 | - | 25.2 | 7126 | 7141 | -820 | L1MC5 | L1 | LINE |

  

|  |  |  |  |  |  |  |  |  |  |
| --- | --- | --- | --- | --- | --- | --- | --- | --- | --- |
| chr4:91524763-91527235 | | | 2452 bp | | |  |  |  |  |
| Genome Start | Genome End | Strand | Div | Repeat Start | Repeat End | Repeat Left | Name | Family | Class |
| 91524763 | 91524892 | + | 32.8 | 2881 | 3022 | -397 | L2 | L2 | LINE |
| 91524915 | 91525634 | - | 12.1 | 5441 | 6152 | -2 | L1PB3 | L1 | LINE |
| 91525635 | 91525876 | - | 6.2 | 59 | 298 | -13 | AluY | Alu | SINE |
| 91525877 | 91526099 | - | 11.9 | 5215 | 5440 | -714 | L1PB3 | L1 | LINE |
| 91526099 | 91526909 | + | 16.1 | 4408 | 5232 | -914 | L1PB3 | L1 | LINE |
| 91526910 | 91527235 | + | 31.7 | 3028 | 3419 | 0 | L2 | L2 | LINE |

  

|  |  |  |  |  |  |  |  |  |  |
| --- | --- | --- | --- | --- | --- | --- | --- | --- | --- |
| chr4:92552015-92562401 | | | 10259 bp | | |  |  |  |  |
| Genome Start | Genome End | Strand | Div | Repeat Start | Repeat End | Repeat Left | Name | Family | Class |
| 92552015 | 92552135 | + | 20.7 | 60 | 184 | -442 | MLT1E2 | MaLR | LTR |
| 92552143 | 92553334 | + | 29.3 | 1532 | 2706 | -5152 | L1MA9 | L1 | LINE |
| 92553335 | 92553647 | + | 19.7 | 1 | 296 | -16 | AluJo | Alu | SINE |
| 92553648 | 92555342 | + | 27.7 | 2414 | 4141 | -3418 | L1M1 | L1 | LINE |
| 92555328 | 92556303 | - | 10.2 | 3419 | 4407 | -1739 | L1PA11 | L1 | LINE |
| 92556300 | 92558037 | + | 9.4 | 4415 | 6169 | -5 | L1PA11 | L1 | LINE |
| 92558043 | 92561320 | + | 17.1 | 4123 | 7500 | -231 | L1MA9 | L1 | LINE |
| 92561325 | 92561841 | - | 25.1 | 1 | 554 | 0 | MLT2B3 | ERVL | LTR |
| 92561846 | 92561951 | + | 22.6 | 7600 | 7709 | -22 | L1MA9 | L1 | LINE |
| 92562079 | 92562401 | + | 23.4 | 270 | 618 | -8 | MLT1E2 | MaLR | LTR |

  

|  |  |  |  |  |  |  |  |  |  |
| --- | --- | --- | --- | --- | --- | --- | --- | --- | --- |
| chr4:93295000-93297958 | | | 2729 bp | | |  |  |  |  |
| Genome Start | Genome End | Strand | Div | Repeat Start | Repeat End | Repeat Left | Name | Family | Class |
| 93295000 | 93295056 | - | 30.4 | 151 | 206 | -2 | MIR3 | MIR | SINE |
| 93295133 | 93296333 | - | 15.3 | 3968 | 5176 | -967 | L1M | L1 | LINE |
| 93296325 | 93296756 | + | 16.2 | 5160 | 5593 | -698 | L1MA8 | L1 | LINE |
| 93296757 | 93297009 | - | 12.7 | 47 | 300 | -12 | AluSx | Alu | SINE |
| 93297010 | 93297653 | + | 16.2 | 5594 | 6230 | -61 | L1MA8 | L1 | LINE |
| 93297719 | 93297791 | + | 18.3 | 6217 | 6288 | -3 | L1MA8 | L1 | LINE |
| 93297890 | 93297958 | - | 33.8 | 6 | 77 | -131 | MIR3 | MIR | SINE |

  

|  |  |  |  |  |  |  |  |  |  |
| --- | --- | --- | --- | --- | --- | --- | --- | --- | --- |
| chr4:93781479-93783933 | | | 2378 bp | | |  |  |  |  |
| Genome Start | Genome End | Strand | Div | Repeat Start | Repeat End | Repeat Left | Name | Family | Class |
| 93781479 | 93781645 | - | 19.9 | 110 | 289 | -23 | AluJo | Alu | SINE |
| 93781695 | 93781833 | + | 20.9 | 5980 | 6128 | -184 | L1MA9 | L1 | LINE |
| 93781849 | 93783488 | - | 14.5 | 4439 | 6154 | -12 | L1PA16 | L1 | LINE |
| 93783485 | 93783799 | + | 12.7 | 4111 | 4432 | -1714 | L1PA16 | L1 | LINE |
| 93783817 | 93783933 | - | 22.4 | 1 | 122 | -180 | AluJo | Alu | SINE |

  

|  |  |  |  |  |  |  |  |  |  |
| --- | --- | --- | --- | --- | --- | --- | --- | --- | --- |
| chr4:95289570-95297185 | | | 7612 bp | | |  |  |  |  |
| Genome Start | Genome End | Strand | Div | Repeat Start | Repeat End | Repeat Left | Name | Family | Class |
| 95289570 | 95289695 | + | 14.3 | 1 | 126 | -5528 | HERVL-B3 | ERVL | LTR |
| 95289698 | 95291416 | - | 9.4 | 4508 | 6171 | -1 | L1PA8 | L1 | LINE |
| 95291413 | 95292290 | + | 7.1 | 3634 | 4511 | -1635 | L1PA8 | L1 | LINE |
| 95292291 | 95293320 | + | 13.7 | 114 | 1118 | -4536 | HERVL-B3 | ERVL | LTR |
| 95293321 | 95293622 | - | 15.1 | 1 | 299 | -13 | AluSx | Alu | SINE |
| 95293623 | 95295048 | + | 13.7 | 1119 | 2558 | -3096 | HERVL-B3 | ERVL | LTR |
| 95295049 | 95295351 | + | 7.0 | 1 | 298 | -13 | AluY | Alu | SINE |
| 95295358 | 95297185 | + | 12.1 | 2825 | 4709 | -1048 | HERVL-B3 | ERVL | LTR |

  

|  |  |  |  |  |  |  |  |  |  |
| --- | --- | --- | --- | --- | --- | --- | --- | --- | --- |
| chr4:97541323-97543963 | | | 2621 bp | | |  |  |  |  |
| Genome Start | Genome End | Strand | Div | Repeat Start | Repeat End | Repeat Left | Name | Family | Class |
| 97541323 | 97541447 | + | 34.4 | 52 | 184 | -358 | MLT1F1 | MaLR | LTR |
| 97541456 | 97542571 | - | 4.1 | 5058 | 6176 | 0 | L1PA3 | L1 | LINE |
| 97542567 | 97543641 | + | 2.5 | 3969 | 5043 | -1103 | L1PA3 | L1 | LINE |
| 97543659 | 97543963 | + | 23.8 | 218 | 561 | -6 | MLT1F1 | MaLR | LTR |

  

|  |  |  |  |  |  |  |  |  |  |
| --- | --- | --- | --- | --- | --- | --- | --- | --- | --- |
| chr4:97634356-97642333 | | | 7945 bp | | |  |  |  |  |
| Genome Start | Genome End | Strand | Div | Repeat Start | Repeat End | Repeat Left | Name | Family | Class |
| 97634356 | 97637285 | + | 21.8 | 1 | 3981 | -3224 | L1M2b | L1 | LINE |
| 97637286 | 97637622 | - | 8.7 | 1 | 310 | -1 | AluY | Alu | SINE |
| 97637623 | 97637759 | + | 19.9 | 3982 | 4104 | -3101 | L1M2b | L1 | LINE |
| 97637760 | 97638088 | + | 23.6 | 3 | 299 | -3 | AluJo | Alu | SINE |
| 97638089 | 97638503 | + | 19.9 | 4105 | 4517 | -2688 | L1M2b | L1 | LINE |
| 97638492 | 97638610 | + | 17.9 | 4808 | 4922 | -2283 | L1M2b | L1 | LINE |
| 97638611 | 97639107 | - | 12.3 | 1 | 499 | 0 | LTR3B\_ | ERVK | LTR |
| 97639108 | 97639469 | + | 17.9 | 4923 | 5285 | -1920 | L1M2b | L1 | LINE |
| 97639466 | 97640750 | - | 6.8 | 4883 | 6164 | -8 | L1PA8 | L1 | LINE |
| 97640753 | 97641928 | + | 8.7 | 3695 | 4873 | -1273 | L1PA8 | L1 | LINE |
| 97641976 | 97642333 | + | 19.7 | 5780 | 6150 | -1055 | L1M2b | L1 | LINE |

  

|  |  |  |  |  |  |  |  |  |  |
| --- | --- | --- | --- | --- | --- | --- | --- | --- | --- |
| chr4:98040708-98044891 | | | 3731 bp | | |  |  |  |  |
| Genome Start | Genome End | Strand | Div | Repeat Start | Repeat End | Repeat Left | Name | Family | Class |
| 98040708 | 98041014 | + | 12.1 | 4735 | 5043 | -1103 | L1PA16 | L1 | LINE |
| 98041012 | 98041626 | - | 4.5 | 3836 | 4452 | -1694 | L1PA5 | L1 | LINE |
| 98041626 | 98042942 | + | 5.0 | 4467 | 5783 | -371 | L1PA5 | L1 | LINE |
| 98043375 | 98043757 | + | 5.2 | 5770 | 6153 | -1 | L1PA5 | L1 | LINE |
| 98043783 | 98044891 | + | 14.5 | 5031 | 6158 | -8 | L1PA16 | L1 | LINE |

  

|  |  |  |  |  |  |  |  |  |  |
| --- | --- | --- | --- | --- | --- | --- | --- | --- | --- |
| chr4:98100590-98109183 | | | 8445 bp | | |  |  |  |  |
| Genome Start | Genome End | Strand | Div | Repeat Start | Repeat End | Repeat Left | Name | Family | Class |
| 98100590 | 98100796 | - | 16.9 | 3416 | 3651 | -3683 | L1M4b | L1 | LINE |
| 98100917 | 98100956 | - | 28.2 | 6287 | 6326 | -8 | L1MA10 | L1 | LINE |
| 98100957 | 98102419 | - | 28.0 | 4684 | 6193 | -46 | L1ME1 | L1 | LINE |
| 98102421 | 98103104 | - | 22.9 | 1486 | 2237 | -4163 | L1ME1 | L1 | LINE |
| 98103108 | 98103462 | - | 19.3 | 2942 | 3299 | -4035 | L1M4b | L1 | LINE |
| 98103501 | 98103845 | - | 19.3 | 2562 | 2941 | -4393 | L1M4b | L1 | LINE |
| 98103846 | 98104158 | - | 8.2 | 1 | 305 | -6 | AluY | Alu | SINE |
| 98104159 | 98105032 | - | 19.3 | 1548 | 2561 | -4773 | L1M4b | L1 | LINE |
| 98105033 | 98105159 | + | 5.5 | 1 | 137 | -2 | MER85 | PiggyBac | DNA |
| 98105160 | 98105959 | - | 19.3 | 740 | 1547 | -5787 | L1M4b | L1 | LINE |
| 98105959 | 98107161 | - | 6.3 | 4949 | 6154 | 0 | L1PA6 | L1 | LINE |
| 98107157 | 98108494 | + | 6.5 | 3634 | 4952 | -1194 | L1PA6 | L1 | LINE |
| 98108488 | 98109183 | - | 29.3 | 3 | 756 | -6578 | L1M4b | L1 | LINE |

  

|  |  |  |  |  |  |  |  |  |  |
| --- | --- | --- | --- | --- | --- | --- | --- | --- | --- |
| chr4:98186465-98192123 | | | 4309 bp | | |  |  |  |  |
| Genome Start | Genome End | Strand | Div | Repeat Start | Repeat End | Repeat Left | Name | Family | Class |
| 98186465 | 98186939 | - | 27.5 | 2943 | 3421 | -2708 | L1MEe | L1 | LINE |
| 98186992 | 98187484 | - | 31.0 | 5664 | 6240 | 0 | L1ME3A | L1 | LINE |
| 98187510 | 98187659 | - | 22.0 | 305 | 460 | -4 | MER88 | ERVL | LTR |
| 98187965 | 98188088 | - | 26.0 | 5523 | 5647 | -523 | L1ME3A | L1 | LINE |
| 98188091 | 98188781 | + | 26.6 | 4813 | 5524 | -638 | L1ME3A | L1 | LINE |
| 98188777 | 98188854 | - | 20.8 | 2782 | 2863 | -3266 | L1MEe | L1 | LINE |
| 98188935 | 98189131 | - | 25.1 | 2446 | 2643 | -3486 | L1MEe | L1 | LINE |
| 98189134 | 98189279 | - | 20.9 | 2258 | 2422 | -3727 | L1MEe | L1 | LINE |
| 98189735 | 98189795 | - | 28.9 | 2089 | 2148 | -4001 | L1MEe | L1 | LINE |
| 98189796 | 98190138 | - | 18.9 | 1 | 405 | -3 | MLT1A1 | MaLR | LTR |
| 98190139 | 98190722 | - | 28.9 | 1433 | 2088 | -4061 | L1MEe | L1 | LINE |
| 98190723 | 98191047 | - | 16.3 | 1 | 306 | -6 | AluSx | Alu | SINE |
| 98191048 | 98191148 | - | 28.9 | 1337 | 1432 | -4717 | L1MEe | L1 | LINE |
| 98191553 | 98191729 | + | 33.7 | 301 | 491 | 0 | LTR16C | ERVL | LTR |
| 98191739 | 98191922 | - | 1.6 | 128 | 311 | 0 | AluYg6 | Alu | SINE |
| 98191944 | 98192123 | - | 32.4 | 916 | 1096 | -5023 | L1MEe | L1 | LINE |

  

|  |  |  |  |  |  |  |  |  |  |
| --- | --- | --- | --- | --- | --- | --- | --- | --- | --- |
| chr4:98870004-98874822 | | | 4831 bp | | |  |  |  |  |
| Genome Start | Genome End | Strand | Div | Repeat Start | Repeat End | Repeat Left | Name | Family | Class |
| 98870004 | 98870170 | - | 13.8 | 4236 | 4401 | -1742 | L1M2 | L1 | LINE |
| 98870172 | 98870770 | - | 14.4 | 5562 | 6164 | -2 | L1PA16 | L1 | LINE |
| 98870773 | 98872451 | - | 23.2 | 744 | 2341 | -4594 | L1P4a | L1 | LINE |
| 98872430 | 98872683 | - | 15.9 | 4831 | 5083 | -1063 | L1PB3 | L1 | LINE |
| 98872677 | 98873766 | + | 14.9 | 5072 | 6152 | -2 | L1PB3 | L1 | LINE |
| 98873778 | 98874091 | - | 15.0 | 4 | 317 | -6618 | L1P4a | L1 | LINE |
| 98874095 | 98874440 | - | 18.2 | 3913 | 4233 | -1907 | L1M2 | L1 | LINE |
| 98874441 | 98874690 | - | 10.4 | 5909 | 6158 | -5 | L1PA13 | L1 | LINE |
| 98874691 | 98874822 | - | 18.2 | 3797 | 3912 | -2228 | L1M2 | L1 | LINE |

  

|  |  |  |  |  |  |  |  |  |  |
| --- | --- | --- | --- | --- | --- | --- | --- | --- | --- |
| chr4:98988643-98991078 | | | 2002 bp | | |  |  |  |  |
| Genome Start | Genome End | Strand | Div | Repeat Start | Repeat End | Repeat Left | Name | Family | Class |
| 98988643 | 98988794 | - | 33.6 | 258 | 407 | -104 | MLT1J | MaLR | LTR |
| 98988805 | 98989462 | - | 1.8 | 4365 | 5022 | -1124 | L1PA2 | L1 | LINE |
| 98989461 | 98990471 | + | 2.7 | 5008 | 6018 | -137 | L1PA2 | L1 | LINE |
| 98990898 | 98991078 | - | 28.6 | 1 | 180 | -331 | MLT1J | MaLR | LTR |

  

|  |  |  |  |  |  |  |  |  |  |
| --- | --- | --- | --- | --- | --- | --- | --- | --- | --- |
| chr4:100615332-100616822 | | | 1385 bp | | |  |  |  |  |
| Genome Start | Genome End | Strand | Div | Repeat Start | Repeat End | Repeat Left | Name | Family | Class |
| 100615332 | 100615775 | + | 27.9 | 2424 | 2913 | -506 | L2 | L2 | LINE |
| 100615868 | 100616030 | - | 11.0 | 5984 | 6163 | 0 | L1PA13 | L1 | LINE |
| 100616037 | 100616677 | + | 10.2 | 5341 | 5988 | -180 | L1PA13 | L1 | LINE |
| 100616686 | 100616822 | + | 33.8 | 3273 | 3417 | -2 | L2 | L2 | LINE |

  

|  |  |  |  |  |  |  |  |  |  |
| --- | --- | --- | --- | --- | --- | --- | --- | --- | --- |
| chr4:104327153-104332618 | | | 5394 bp | | |  |  |  |  |
| Genome Start | Genome End | Strand | Div | Repeat Start | Repeat End | Repeat Left | Name | Family | Class |
| 104327153 | 104327715 | - | 32.5 | 2825 | 3417 | -2 | L2 | L2 | LINE |
| 104327720 | 104327881 | - | 4.9 | 1444 | 1606 | -4549 | L1PA5 | L1 | LINE |
| 104327881 | 104332411 | + | 4.6 | 1614 | 6152 | -2 | L1PA5 | L1 | LINE |
| 104332481 | 104332618 | - | 30.6 | 2669 | 2805 | -614 | L2 | L2 | LINE |

  

|  |  |  |  |  |  |  |  |  |  |
| --- | --- | --- | --- | --- | --- | --- | --- | --- | --- |
| chr4:104944496-104955088 | | | 10313 bp | | |  |  |  |  |
| Genome Start | Genome End | Strand | Div | Repeat Start | Repeat End | Repeat Left | Name | Family | Class |
| 104944496 | 104944784 | - | 21.0 | 4207 | 4504 | -2502 | L1M4b | L1 | LINE |
| 104944829 | 104946985 | - | 22.1 | 3943 | 6117 | -61 | L1MB8 | L1 | LINE |
| 104946986 | 104947288 | - | 21.3 | 1 | 297 | -15 | AluJo | Alu | SINE |
| 104947289 | 104948692 | - | 22.4 | 2505 | 3942 | -2204 | L1MB8 | L1 | LINE |
| 104948689 | 104951377 | - | 9.0 | 9 | 2882 | -3264 | L1P2 | L1 | LINE |
| 104951358 | 104951515 | - | 25.9 | 3522 | 3682 | -3652 | L1M4b | L1 | LINE |
| 104951513 | 104951994 | - | 16.0 | 5377 | 5862 | -429 | L1MA7 | L1 | LINE |
| 104951995 | 104952410 | + | 19.0 | 5885 | 6290 | -1 | L1MA7 | L1 | LINE |
| 104952438 | 104952599 | + | 20.5 | 2 | 196 | -145 | MER58 | MER1\_type | DNA |
| 104952769 | 104953930 | - | 22.5 | 2005 | 3323 | -4011 | L1M4b | L1 | LINE |
| 104953962 | 104954525 | - | 19.8 | 355 | 905 | -1143 | Ricksha | MuDR | DNA |
| 104954535 | 104954913 | - | 29.9 | 365 | 781 | -6553 | L1M4b | L1 | LINE |
| 104954941 | 104955088 | - | 29.9 | 229 | 364 | -6970 | L1M4b | L1 | LINE |

  

|  |  |  |  |  |  |  |  |  |  |
| --- | --- | --- | --- | --- | --- | --- | --- | --- | --- |
| chr4:105216317-105219570 | | | 2858 bp | | |  |  |  |  |
| Genome Start | Genome End | Strand | Div | Repeat Start | Repeat End | Repeat Left | Name | Family | Class |
| 105216317 | 105217149 | + | 24.9 | 2291 | 3164 | -2982 | L1M4 | L1 | LINE |
| 105217220 | 105217782 | - | 8.2 | 4627 | 5188 | -958 | L1PB1 | L1 | LINE |
| 105217781 | 105218375 | + | 8.5 | 5178 | 5792 | -359 | L1PB1 | L1 | LINE |
| 105218661 | 105219020 | + | 12.3 | 5783 | 6150 | -1 | L1PB1 | L1 | LINE |
| 105219064 | 105219570 | + | 26.2 | 4408 | 4929 | -1217 | L1M4 | L1 | LINE |

  

|  |  |  |  |  |  |  |  |  |  |
| --- | --- | --- | --- | --- | --- | --- | --- | --- | --- |
| chr4:105284442-105290199 | | | 5803 bp | | |  |  |  |  |
| Genome Start | Genome End | Strand | Div | Repeat Start | Repeat End | Repeat Left | Name | Family | Class |
| 105284442 | 105285265 | - | 25.0 | 4118 | 4963 | -1443 | L1MDa | L1 | LINE |
| 105285304 | 105286740 | - | 17.2 | 4678 | 6148 | -8 | L1PB4 | L1 | LINE |
| 105286740 | 105287360 | + | 12.5 | 4092 | 4699 | -1447 | L1P4 | L1 | LINE |
| 105287349 | 105287454 | - | 15.2 | 6241 | 7994 | -1 | L1MC1 | L1 | LINE |
| 105287364 | 105287577 | - | 16.3 | 6117 | 6320 | -13 | L1MC1 | L1 | LINE |
| 105287580 | 105288109 | - | 17.0 | 5457 | 5995 | -338 | L1MC1 | L1 | LINE |
| 105288127 | 105289191 | - | 24.9 | 2959 | 4067 | -2339 | L1MDa | L1 | LINE |
| 105289185 | 105289388 | - | 27.0 | 1495 | 1708 | -4794 | L1MDa | L1 | LINE |
| 105289405 | 105290021 | - | 24.2 | 788 | 1420 | -4989 | L1MDa | L1 | LINE |
| 105290015 | 105290199 | - | 25.1 | 20 | 203 | -6206 | L1MDa | L1 | LINE |

  

|  |  |  |  |  |  |  |  |  |  |
| --- | --- | --- | --- | --- | --- | --- | --- | --- | --- |
| chr4:107519492-107522530 | | | 2790 bp | | |  |  |  |  |
| Genome Start | Genome End | Strand | Div | Repeat Start | Repeat End | Repeat Left | Name | Family | Class |
| 107519492 | 107519666 | + | 16.9 | 4777 | 4953 | -1193 | L1MB7 | L1 | LINE |
| 107519895 | 107520205 | + | 18.8 | 4961 | 5268 | -878 | L1MB7 | L1 | LINE |
| 107520206 | 107520503 | + | 18.8 | 1 | 345 | -1 | MER7A | MER2\_type | DNA |
| 107520504 | 107520830 | + | 19.0 | 5266 | 5603 | -581 | L1MB7 | L1 | LINE |
| 107520838 | 107521125 | - | 11.0 | 5192 | 5485 | -815 | L1MA4 | L1 | LINE |
| 107521126 | 107521936 | + | 14.0 | 5498 | 6298 | -2 | L1MA4 | L1 | LINE |
| 107521951 | 107522530 | + | 19.3 | 5600 | 6174 | -10 | L1MB7 | L1 | LINE |

  

|  |  |  |  |  |  |  |  |  |  |
| --- | --- | --- | --- | --- | --- | --- | --- | --- | --- |
| chr4:109438789-109442272 | | | 3079 bp | | |  |  |  |  |
| Genome Start | Genome End | Strand | Div | Repeat Start | Repeat End | Repeat Left | Name | Family | Class |
| 109438789 | 109439076 | - | 28.3 | 5263 | 5548 | -573 | L1M5 | L1 | LINE |
| 109439265 | 109439530 | - | 17.1 | 44 | 308 | -4 | AluJb | Alu | SINE |
| 109439532 | 109439623 | - | 15.2 | 1 | 92 | -220 | AluJ/FLAM | Alu | SINE |
| 109439658 | 109441014 | - | 7.3 | 4792 | 6145 | -6 | L1PB1 | L1 | LINE |
| 109441015 | 109441186 | + | 5.2 | 4621 | 4792 | -1354 | L1PB1 | L1 | LINE |
| 109441354 | 109441657 | + | 13.5 | 1 | 306 | -6 | AluSx | Alu | SINE |
| 109441673 | 109442272 | - | 36.8 | 4184 | 4821 | -1325 | L1M5 | L1 | LINE |

  

|  |  |  |  |  |  |  |  |  |  |
| --- | --- | --- | --- | --- | --- | --- | --- | --- | --- |
| chr4:112333653-112338733 | | | 5034 bp | | |  |  |  |  |
| Genome Start | Genome End | Strand | Div | Repeat Start | Repeat End | Repeat Left | Name | Family | Class |
| 112333653 | 112333949 | + | 32.2 | 44 | 332 | -5738 | L1MEa | L1 | LINE |
| 112333930 | 112334086 | - | 7.3 | 4057 | 4214 | -1932 | L1P3 | L1 | LINE |
| 112334087 | 112334569 | + | 6.8 | 5658 | 6141 | -13 | L1PA7 | L1 | LINE |
| 112334582 | 112335028 | + | 15.1 | 5878 | 6325 | -19 | L1MC2 | L1 | LINE |
| 112335027 | 112335151 | + | 20.8 | 323 | 459 | -5611 | L1MEa | L1 | LINE |
| 112335156 | 112335562 | + | 18.4 | 4114 | 4514 | -1632 | L1P4 | L1 | LINE |
| 112335564 | 112335878 | + | 13.6 | 1 | 325 | -56 | THE1D | MaLR | LTR |
| 112335888 | 112336492 | - | 3.6 | 5548 | 6152 | -3 | L1PA4 | L1 | LINE |
| 112336493 | 112336735 | + | 2.9 | 5286 | 5529 | -626 | L1PA4 | L1 | LINE |
| 112336736 | 112336807 | + | 5.6 | 309 | 381 | 0 | THE1D | MaLR | LTR |
| 112336808 | 112338476 | + | 16.9 | 4511 | 6116 | -40 | L1PB4 | L1 | LINE |
| 112338520 | 112338733 | + | 27.1 | 466 | 679 | -5391 | L1MEa | L1 | LINE |

  

|  |  |  |  |  |  |  |  |  |  |
| --- | --- | --- | --- | --- | --- | --- | --- | --- | --- |
| chr4:112698324-112702533 | | | 4230 bp | | |  |  |  |  |
| Genome Start | Genome End | Strand | Div | Repeat Start | Repeat End | Repeat Left | Name | Family | Class |
| 112698324 | 112698557 | + | 15.0 | 5380 | 5608 | -692 | L1MA6 | L1 | LINE |
| 112698557 | 112699920 | - | 8.4 | 3424 | 4801 | -1345 | L1P3 | L1 | LINE |
| 112699919 | 112700431 | - | 10.9 | 2521 | 3057 | -3089 | L1P3 | L1 | LINE |
| 112700427 | 112701781 | + | 7.5 | 4804 | 6154 | 0 | L1PA7 | L1 | LINE |
| 112701770 | 112702533 | + | 19.6 | 5594 | 6296 | -4 | L1MA6 | L1 | LINE |

  

|  |  |  |  |  |  |  |  |  |  |
| --- | --- | --- | --- | --- | --- | --- | --- | --- | --- |
| chr4:113318761-113321535 | | | 2580 bp | | |  |  |  |  |
| Genome Start | Genome End | Strand | Div | Repeat Start | Repeat End | Repeat Left | Name | Family | Class |
| 113318761 | 113318940 | + | 17.5 | 1 | 178 | -327 | MLT1D | MaLR | LTR |
| 113318950 | 113319112 | - | 11.8 | 4851 | 5008 | -1138 | L1P4 | L1 | LINE |
| 113319106 | 113320316 | + | 10.6 | 4998 | 6156 | -9 | L1PA5 | L1 | LINE |
| 113320336 | 113320969 | + | 17.2 | 5593 | 6157 | -9 | L1PA16 | L1 | LINE |
| 113321130 | 113321262 | - | 20.2 | 2691 | 2822 | 0 | Charlie10 | MER1\_type | DNA |
| 113321277 | 113321535 | + | 20.0 | 202 | 503 | -2 | MLT1D | MaLR | LTR |

  

|  |  |  |  |  |  |  |  |  |  |
| --- | --- | --- | --- | --- | --- | --- | --- | --- | --- |
| chr4:115454111-115457185 | | | 3047 bp | | |  |  |  |  |
| Genome Start | Genome End | Strand | Div | Repeat Start | Repeat End | Repeat Left | Name | Family | Class |
| 115454111 | 115454785 | + | 28.4 | 4267 | 4996 | -1150 | L1M4 | L1 | LINE |
| 115454798 | 115455618 | - | 10.3 | 4400 | 5215 | -931 | L1PA16 | L1 | LINE |
| 115455621 | 115456211 | + | 15.1 | 5198 | 5785 | -381 | L1PA16 | L1 | LINE |
| 115456212 | 115456494 | + | 5.7 | 1 | 308 | -3 | AluY | Alu | SINE |
| 115456495 | 115456861 | + | 15.1 | 5786 | 6149 | -17 | L1PA16 | L1 | LINE |
| 115456876 | 115457185 | + | 33.1 | 4992 | 5309 | -837 | L1M4 | L1 | LINE |

  

|  |  |  |  |  |  |  |  |  |  |
| --- | --- | --- | --- | --- | --- | --- | --- | --- | --- |
| chr4:116476199-116478674 | | | 2489 bp | | |  |  |  |  |
| Genome Start | Genome End | Strand | Div | Repeat Start | Repeat End | Repeat Left | Name | Family | Class |
| 116476199 | 116476700 | - | 24.2 | 5621 | 6146 | -20 | L1ME1 | L1 | LINE |
| 116476695 | 116477815 | - | 4.4 | 5033 | 6154 | -1 | L1PA4 | L1 | LINE |
| 116477812 | 116478631 | + | 3.8 | 4206 | 5029 | -1117 | L1PA4 | L1 | LINE |
| 116478629 | 116478674 | - | 23.9 | 5593 | 5638 | -528 | L1ME1 | L1 | LINE |

  

|  |  |  |  |  |  |  |  |  |  |
| --- | --- | --- | --- | --- | --- | --- | --- | --- | --- |
| chr4:118747727-118750500 | | | 2746 bp | | |  |  |  |  |
| Genome Start | Genome End | Strand | Div | Repeat Start | Repeat End | Repeat Left | Name | Family | Class |
| 118747727 | 118748145 | - | 26.6 | 104 | 514 | 0 | MER67D | ERV1 | LTR |
| 118748153 | 118748534 | - | 5.5 | 5770 | 6151 | -3 | L1PA5 | L1 | LINE |
| 118748549 | 118750293 | - | 3.8 | 4400 | 6152 | -3 | L1PA4 | L1 | LINE |
| 118750295 | 118750384 | + | 4.4 | 4289 | 4378 | -1768 | L1P3 | L1 | LINE |
| 118750391 | 118750500 | - | 16.8 | 3 | 111 | -403 | MER67D | ERV1 | LTR |

  

|  |  |  |  |  |  |  |  |  |  |
| --- | --- | --- | --- | --- | --- | --- | --- | --- | --- |
| chr4:118996741-119001713 | | | 4960 bp | | |  |  |  |  |
| Genome Start | Genome End | Strand | Div | Repeat Start | Repeat End | Repeat Left | Name | Family | Class |
| 118996741 | 118997202 | + | 24.5 | 4928 | 5495 | -667 | L1ME3A | L1 | LINE |
| 118997204 | 118997397 | + | 22.9 | 1 | 222 | -432 | MLT1E | MaLR | LTR |
| 118997398 | 118998857 | - | 10.6 | 2990 | 4484 | -1662 | L1PREC2 | L1 | LINE |
| 118998856 | 119000485 | + | 9.9 | 4485 | 6142 | -19 | L1PA14 | L1 | LINE |
| 119000486 | 119000696 | - | 11.7 | 93 | 298 | -15 | AluSp | Alu | SINE |
| 119000698 | 119001059 | + | 26.6 | 217 | 593 | 0 | MLT1E | MaLR | LTR |
| 119001073 | 119001713 | + | 30.3 | 5505 | 6166 | -4 | L1ME3A | L1 | LINE |

  

|  |  |  |  |  |  |  |  |  |  |
| --- | --- | --- | --- | --- | --- | --- | --- | --- | --- |
| chr4:119003202-119006092 | | | 2890 bp | | |  |  |  |  |
| Genome Start | Genome End | Strand | Div | Repeat Start | Repeat End | Repeat Left | Name | Family | Class |
| 119003202 | 119003246 | + | 12.7 | 4 | 47 | -1255 | HSMAR2 | Mariner | DNA |
| 119003247 | 119003557 | + | 11.9 | 1 | 311 | 0 | AluY | Alu | SINE |
| 119003558 | 119003569 | + | 12.7 | 48 | 49 | -1253 | HSMAR2 | Mariner | DNA |
| 119003570 | 119003879 | + | 10.7 | 1 | 311 | -2 | AluSp | Alu | SINE |
| 119003880 | 119004448 | + | 12.7 | 50 | 617 | -685 | HSMAR2 | Mariner | DNA |
| 119004451 | 119004951 | - | 12.9 | 5125 | 5643 | -513 | L1PB4 | L1 | LINE |
| 119004951 | 119005404 | + | 19.6 | 5643 | 6156 | 0 | L1PB4 | L1 | LINE |
| 119005405 | 119006092 | + | 12.3 | 607 | 1302 | 0 | HSMAR2 | Mariner | DNA |

  

|  |  |  |  |  |  |  |  |  |  |
| --- | --- | --- | --- | --- | --- | --- | --- | --- | --- |
| chr4:119100832-119102693 | | | 1748 bp | | |  |  |  |  |
| Genome Start | Genome End | Strand | Div | Repeat Start | Repeat End | Repeat Left | Name | Family | Class |
| 119100832 | 119101320 | - | 21.6 | 57 | 558 | -78 | MER92B | ERV1 | LTR |
| 119101325 | 119101369 | - | 4.4 | 4932 | 4976 | -1170 | L1PA5 | L1 | LINE |
| 119101369 | 119102541 | + | 5.0 | 4964 | 6142 | -12 | L1PA5 | L1 | LINE |
| 119102653 | 119102693 | - | 14.6 | 3 | 44 | -592 | MER92B | ERV1 | LTR |

  

|  |  |  |  |  |  |  |  |  |  |
| --- | --- | --- | --- | --- | --- | --- | --- | --- | --- |
| chr4:122225812-122229720 | | | 3852 bp | | |  |  |  |  |
| Genome Start | Genome End | Strand | Div | Repeat Start | Repeat End | Repeat Left | Name | Family | Class |
| 122225812 | 122227573 | + | 17.1 | 3540 | 5329 | -811 | L1MA9 | L1 | LINE |
| 122227568 | 122227888 | - | 6.8 | 5029 | 5350 | -796 | L1PB1 | L1 | LINE |
| 122227886 | 122228312 | + | 11.7 | 5361 | 5785 | -366 | L1PB1 | L1 | LINE |
| 122228371 | 122228723 | + | 11.7 | 5786 | 6146 | -5 | L1PB1 | L1 | LINE |
| 122228732 | 122229720 | + | 15.4 | 5316 | 6307 | -5 | L1MA9 | L1 | LINE |

  

|  |  |  |  |  |  |  |  |  |  |
| --- | --- | --- | --- | --- | --- | --- | --- | --- | --- |
| chr4:122635528-122640134 | | | 4542 bp | | |  |  |  |  |
| Genome Start | Genome End | Strand | Div | Repeat Start | Repeat End | Repeat Left | Name | Family | Class |
| 122635528 | 122635895 | - | 20.9 | 5798 | 6281 | -31 | L1MA9 | L1 | LINE |
| 122635961 | 122638163 | - | 7.2 | 3942 | 6154 | 0 | L1PA7 | L1 | LINE |
| 122638157 | 122638956 | + | 8.8 | 3138 | 3938 | -2208 | L1PA7 | L1 | LINE |
| 122638964 | 122640134 | - | 14.1 | 4571 | 5774 | -538 | L1MA9 | L1 | LINE |

  

|  |  |  |  |  |  |  |  |  |  |
| --- | --- | --- | --- | --- | --- | --- | --- | --- | --- |
| chr4:122799016-122802940 | | | 3659 bp | | |  |  |  |  |
| Genome Start | Genome End | Strand | Div | Repeat Start | Repeat End | Repeat Left | Name | Family | Class |
| 122799016 | 122799939 | - | 29.6 | 5260 | 6226 | -1582 | L1MC4a | L1 | LINE |
| 122799949 | 122800457 | - | 30.7 | 4712 | 5239 | -907 | L1MC4a | L1 | LINE |
| 122800639 | 122802032 | - | 6.1 | 4740 | 6152 | -2 | L1PA6 | L1 | LINE |
| 122802029 | 122802792 | + | 6.5 | 3971 | 4730 | -1416 | L1PA6 | L1 | LINE |
| 122802873 | 122802940 | - | 20.9 | 4542 | 4609 | -1537 | L1MC4a | L1 | LINE |

  

|  |  |  |  |  |  |  |  |  |  |
| --- | --- | --- | --- | --- | --- | --- | --- | --- | --- |
| chr4:126701849-126715457 | | | 13468 bp | | |  |  |  |  |
| Genome Start | Genome End | Strand | Div | Repeat Start | Repeat End | Repeat Left | Name | Family | Class |
| 126701849 | 126702222 | - | 14.5 | 6253 | 6638 | -927 | L1M1 | L1 | LINE |
| 126702225 | 126702675 | - | 5.4 | 1 | 448 | -2 | LTR7 | ERV1 | LTR |
| 126702676 | 126703055 | - | 8.3 | 7322 | 7713 | 0 | HERVH | ERV1 | LTR |
| 126703054 | 126703265 | - | 10.8 | 5600 | 5819 | -1894 | HERVH | ERV1 | LTR |
| 126703267 | 126703928 | - | 14.2 | 4488 | 5149 | -2564 | HERVH | ERV1 | LTR |
| 126703931 | 126704435 | - | 10.8 | 3492 | 3993 | -3720 | HERVH | ERV1 | LTR |
| 126704414 | 126707610 | - | 9.4 | 1 | 3308 | -4405 | HERVH | ERV1 | LTR |
| 126707613 | 126708061 | - | 3.4 | 1 | 448 | -2 | LTR7 | ERV1 | LTR |
| 126708062 | 126708112 | - | 8.2 | 6216 | 6257 | -1308 | L1M1 | L1 | LINE |
| 126708113 | 126708400 | - | 9.7 | 1 | 288 | -22 | AluSg | Alu | SINE |
| 126708401 | 126708457 | - | 8.2 | 6158 | 6215 | -1350 | L1M1 | L1 | LINE |
| 126708530 | 126710236 | - | 11.9 | 4523 | 6171 | -1394 | L1M1 | L1 | LINE |
| 126710237 | 126710542 | + | 19.2 | 1 | 299 | -13 | AluJo | Alu | SINE |
| 126710543 | 126710595 | - | 11.9 | 4473 | 4522 | -3043 | L1M1 | L1 | LINE |
| 126710581 | 126711661 | - | 7.5 | 3721 | 4815 | -1331 | L1PA5 | L1 | LINE |
| 126711659 | 126712985 | + | 6.3 | 4830 | 6153 | -1 | L1PA5 | L1 | LINE |
| 126712984 | 126713385 | - | 14.7 | 4079 | 4491 | -3074 | L1M1 | L1 | LINE |
| 126713382 | 126713757 | - | 18.6 | 1363 | 1742 | -5823 | L1M1 | L1 | LINE |
| 126713758 | 126714049 | - | 19.7 | 118 | 437 | 0 | LTR21B | ERV1 | LTR |
| 126714160 | 126715457 | - | 20.9 | 5 | 1367 | -6198 | L1M1 | L1 | LINE |

  

|  |  |  |  |  |  |  |  |  |  |
| --- | --- | --- | --- | --- | --- | --- | --- | --- | --- |
| chr4:127186681-127192184 | | | 5511 bp | | |  |  |  |  |
| Genome Start | Genome End | Strand | Div | Repeat Start | Repeat End | Repeat Left | Name | Family | Class |
| 127186681 | 127187848 | + | 23.4 | 502 | 1631 | -5703 | L1MB8 | L1 | LINE |
| 127187846 | 127188474 | - | 9.5 | 4769 | 5403 | -743 | L1PB | L1 | LINE |
| 127188482 | 127189026 | + | 8.0 | 5662 | 6146 | -5 | L1PB1 | L1 | LINE |
| 127189027 | 127189557 | + | 22.3 | 1593 | 2186 | -5148 | L1MB8 | L1 | LINE |
| 127189555 | 127189621 | - | 14.9 | 4844 | 4911 | -1232 | L1M1 | L1 | LINE |
| 127189623 | 127190250 | + | 13.7 | 4919 | 5561 | -744 | L1M1 | L1 | LINE |
| 127190248 | 127190747 | + | 19.2 | 3560 | 4062 | -2944 | L1MB8 | L1 | LINE |
| 127190746 | 127190946 | - | 19.4 | 5608 | 5804 | -1202 | L1MB8 | L1 | LINE |
| 127190943 | 127192184 | + | 21.3 | 5776 | 7033 | -5 | L1MB8 | L1 | LINE |

  

|  |  |  |  |  |  |  |  |  |  |
| --- | --- | --- | --- | --- | --- | --- | --- | --- | --- |
| chr4:130725192-130736227 | | | 11040 bp | | |  |  |  |  |
| Genome Start | Genome End | Strand | Div | Repeat Start | Repeat End | Repeat Left | Name | Family | Class |
| 130725192 | 130728474 | + | 24.0 | 887 | 4236 | -2140 | L1M3c | L1 | LINE |
| 130728475 | 130728892 | - | 19.9 | 1 | 431 | -1 | MSTB1 | MaLR | LTR |
| 130728893 | 130728946 | + | 19.3 | 4237 | 4285 | -2091 | L1M3c | L1 | LINE |
| 130728949 | 130731197 | - | 7.0 | 572 | 2842 | -3304 | L1PA5 | L1 | LINE |
| 130731196 | 130734521 | + | 7.2 | 2857 | 6172 | 0 | L1PA5 | L1 | LINE |
| 130734518 | 130736227 | + | 18.5 | 4275 | 6027 | -521 | L1M3c | L1 | LINE |

  

|  |  |  |  |  |  |  |  |  |  |
| --- | --- | --- | --- | --- | --- | --- | --- | --- | --- |
| chr4:132276583-132285194 | | | 8219 bp | | |  |  |  |  |
| Genome Start | Genome End | Strand | Div | Repeat Start | Repeat End | Repeat Left | Name | Family | Class |
| 132276583 | 132277320 | + | 29.8 | 1216 | 1950 | -5546 | L1M2 | L1 | LINE |
| 132277414 | 132278272 | + | 34.8 | 1781 | 2412 | -5084 | L1M2 | L1 | LINE |
| 132278329 | 132278655 | + | 22.6 | 3296 | 3626 | -3939 | L1M2 | L1 | LINE |
| 132278656 | 132278743 | + | 2.3 | 6058 | 6145 | -9 | L1PA5 | L1 | LINE |
| 132278880 | 132281245 | + | 20.7 | 3627 | 6075 | -1487 | L1M2 | L1 | LINE |
| 132281246 | 132281731 | - | 14.5 | 4 | 497 | 0 | LOR1a | ERV1 | LTR |
| 132281800 | 132282272 | + | 20.7 | 6086 | 6531 | -1031 | L1M2 | L1 | LINE |
| 132282273 | 132282582 | + | 5.8 | 1 | 309 | -2 | AluY | Alu | SINE |
| 132282583 | 132282825 | + | 20.7 | 6532 | 6785 | -777 | L1M2 | L1 | LINE |
| 132282854 | 132284144 | - | 3.4 | 4858 | 6152 | -3 | L1PA4 | L1 | LINE |
| 132284149 | 132284915 | + | 4.6 | 4103 | 4869 | -1277 | L1PA4 | L1 | LINE |
| 132284924 | 132285194 | + | 17.1 | 6801 | 7072 | -638 | L1M2 | L1 | LINE |

  

|  |  |  |  |  |  |  |  |  |  |
| --- | --- | --- | --- | --- | --- | --- | --- | --- | --- |
| chr4:133624132-133626952 | | | 2815 bp | | |  |  |  |  |
| Genome Start | Genome End | Strand | Div | Repeat Start | Repeat End | Repeat Left | Name | Family | Class |
| 133624132 | 133624533 | + | 10.2 | 1 | 395 | -245 | MER51C | ERV1 | LTR |
| 133624545 | 133624832 | - | 2.1 | 3964 | 4253 | -1893 | L1PA2 | L1 | LINE |
| 133624828 | 133626720 | + | 2.1 | 4258 | 6150 | -5 | L1PA2 | L1 | LINE |
| 133626721 | 133626952 | + | 14.2 | 407 | 640 | 0 | MER51C | ERV1 | LTR |

  

|  |  |  |  |  |  |  |  |  |  |
| --- | --- | --- | --- | --- | --- | --- | --- | --- | --- |
| chr4:134847897-134852763 | | | 4344 bp | | |  |  |  |  |
| Genome Start | Genome End | Strand | Div | Repeat Start | Repeat End | Repeat Left | Name | Family | Class |
| 134847897 | 134848830 | - | 23.3 | 4718 | 5624 | -792 | L1M | L1 | LINE |
| 134848831 | 134848877 | - | 2.1 | 33 | 79 | -1 | MADE1 | Mariner | DNA |
| 134848878 | 134849648 | - | 23.3 | 3970 | 4717 | -1699 | L1M | L1 | LINE |
| 134849774 | 134849853 | - | 25.0 | 3850 | 3931 | -2451 | L1M | L1 | LINE |
| 134850167 | 134850296 | - | 23.1 | 3708 | 3845 | -2531 | L1M | L1 | LINE |
| 134850297 | 134850359 | + | 14.3 | 5730 | 5792 | -359 | L1PB1 | L1 | LINE |
| 134850417 | 134850753 | + | 12.2 | 5782 | 6151 | 0 | L1PB1 | L1 | LINE |
| 134850786 | 134851758 | - | 19.6 | 5167 | 6127 | -62 | L1MB1 | L1 | LINE |
| 134851758 | 134852236 | + | 18.2 | 4698 | 5181 | -959 | L1MB1 | L1 | LINE |
| 134852234 | 134852763 | - | 27.0 | 26 | 636 | -5748 | L1M | L1 | LINE |

  

|  |  |  |  |  |  |  |  |  |  |
| --- | --- | --- | --- | --- | --- | --- | --- | --- | --- |
| chr4:136797914-136803828 | | | 5884 bp | | |  |  |  |  |
| Genome Start | Genome End | Strand | Div | Repeat Start | Repeat End | Repeat Left | Name | Family | Class |
| 136797914 | 136799088 | - | 16.8 | 1026 | 2198 | -220 | Tigger1 | MER2\_type | DNA |
| 136799110 | 136802322 | - | 11.9 | 2230 | 5497 | -663 | L1PA14 | L1 | LINE |
| 136802327 | 136802996 | + | 14.4 | 5499 | 6159 | -2 | L1PA14 | L1 | LINE |
| 136802997 | 136803122 | - | 17.1 | 857 | 981 | -1437 | Tigger1 | MER2\_type | DNA |
| 136803123 | 136803489 | + | 16.3 | 1 | 381 | 0 | THE1D | MaLR | LTR |
| 136803490 | 136803626 | - | 17.1 | 721 | 856 | -1562 | Tigger1 | MER2\_type | DNA |
| 136803633 | 136803828 | - | 17.0 | 15 | 209 | -2209 | Tigger1 | MER2\_type | DNA |

  

|  |  |  |  |  |  |  |  |  |  |
| --- | --- | --- | --- | --- | --- | --- | --- | --- | --- |
| chr4:141457821-141459496 | | | 1603 bp | | |  |  |  |  |
| Genome Start | Genome End | Strand | Div | Repeat Start | Repeat End | Repeat Left | Name | Family | Class |
| 141457821 | 141458236 | + | 8.2 | 3 | 419 | -44 | LTR2 | ERV1 | LTR |
| 141458309 | 141458695 | - | 1.8 | 5762 | 6148 | -7 | L1PA2 | L1 | LINE |
| 141458697 | 141459435 | + | 0.9 | 5005 | 5743 | -412 | L1PA2 | L1 | LINE |
| 141459436 | 141459496 | + | 4.9 | 403 | 463 | 0 | LTR2 | ERV1 | LTR |

  

|  |  |  |  |  |  |  |  |  |  |
| --- | --- | --- | --- | --- | --- | --- | --- | --- | --- |
| chr4:142409670-142413728 | | | 4043 bp | | |  |  |  |  |
| Genome Start | Genome End | Strand | Div | Repeat Start | Repeat End | Repeat Left | Name | Family | Class |
| 142409670 | 142410159 | - | 27.3 | 7016 | 7531 | -430 | L1MC5 | L1 | LINE |
| 142410179 | 142412068 | - | 3.2 | 4286 | 6152 | -3 | L1PA3 | L1 | LINE |
| 142412067 | 142413326 | + | 3.1 | 3010 | 4274 | -1872 | L1PA3 | L1 | LINE |
| 142413326 | 142413728 | - | 22.7 | 6650 | 7031 | -930 | L1MC5 | L1 | LINE |

  

|  |  |  |  |  |  |  |  |  |  |
| --- | --- | --- | --- | --- | --- | --- | --- | --- | --- |
| chr4:142602840-142605140 | | | 2271 bp | | |  |  |  |  |
| Genome Start | Genome End | Strand | Div | Repeat Start | Repeat End | Repeat Left | Name | Family | Class |
| 142602840 | 142603235 | - | 27.7 | 78 | 457 | 0 | LTR16A1 | ERVL | LTR |
| 142603239 | 142604473 | - | 10.1 | 4357 | 5586 | -588 | L1PA11 | L1 | LINE |
| 142604476 | 142605039 | + | 11.9 | 5598 | 6166 | -8 | L1PA11 | L1 | LINE |
| 142605065 | 142605140 | - | 32.9 | 7 | 82 | -375 | LTR16A1 | ERVL | LTR |

  

|  |  |  |  |  |  |  |  |  |  |
| --- | --- | --- | --- | --- | --- | --- | --- | --- | --- |
| chr4:143082577-143096323 | | | 13184 bp | | |  |  |  |  |
| Genome Start | Genome End | Strand | Div | Repeat Start | Repeat End | Repeat Left | Name | Family | Class |
| 143082577 | 143082634 | - | 32.8 | 2661 | 2718 | 0 | Tigger2 | MER2\_type | DNA |
| 143082818 | 143084727 | + | 19.9 | 70 | 1875 | -4978 | L1PA15-16 | L1 | LINE |
| 143084732 | 143087009 | - | 12.9 | 2990 | 5392 | -754 | L1PA13 | L1 | LINE |
| 143087008 | 143087745 | + | 9.5 | 5396 | 6150 | -13 | L1PA13 | L1 | LINE |
| 143087819 | 143087890 | + | 13.7 | 1865 | 1934 | -4919 | L1PA15-16 | L1 | LINE |
| 143087891 | 143088198 | - | 14.0 | 1 | 310 | -2 | AluSx | Alu | SINE |
| 143088199 | 143089167 | + | 13.7 | 1935 | 2912 | -3937 | L1PA15-16 | L1 | LINE |
| 143089226 | 143090354 | + | 13.2 | 2913 | 4073 | -2776 | L1PA15-16 | L1 | LINE |
| 143090355 | 143090765 | + | 19.2 | 1 | 426 | 0 | MSTB | MaLR | LTR |
| 143090766 | 143091818 | + | 13.2 | 4074 | 5097 | -1752 | L1PA16 | L1 | LINE |
| 143091847 | 143093626 | + | 14.1 | 5098 | 6867 | -2 | L1PA16 | L1 | LINE |
| 143093694 | 143094909 | - | 15.4 | 716 | 2665 | -53 | Tigger2 | MER2\_type | DNA |
| 143095055 | 143096144 | - | 16.6 | 350 | 704 | -1277 | Tigger2 | MER2\_type | DNA |
| 143096152 | 143096323 | - | 18.6 | 1 | 175 | -2543 | Tigger2 | MER2\_type | DNA |

  

|  |  |  |  |  |  |  |  |  |  |
| --- | --- | --- | --- | --- | --- | --- | --- | --- | --- |
| chr4:143583173-143586748 | | | 3285 bp | | |  |  |  |  |
| Genome Start | Genome End | Strand | Div | Repeat Start | Repeat End | Repeat Left | Name | Family | Class |
| 143583173 | 143583369 | + | 29.9 | 25 | 252 | -6171 | L1MCc | L1 | LINE |
| 143583370 | 143583727 | - | 12.6 | 1 | 364 | 0 | THE1B | MaLR | LTR |
| 143583728 | 143583986 | + | 29.9 | 253 | 517 | -5906 | L1MCc | L1 | LINE |
| 143584122 | 143584397 | + | 35.2 | 819 | 1102 | -5321 | L1MCc | L1 | LINE |
| 143584521 | 143584825 | + | 25.7 | 1435 | 1761 | -5367 | L1MCc | L1 | LINE |
| 143584836 | 143584918 | - | 7.2 | 5201 | 5283 | -863 | L1PA8 | L1 | LINE |
| 143584919 | 143585797 | + | 8.1 | 5301 | 6172 | 0 | L1PA8 | L1 | LINE |
| 143585799 | 143585986 | + | 27.3 | 1726 | 1914 | -4925 | L1MCc | L1 | LINE |
| 143586009 | 143586748 | + | 24.5 | 3112 | 3923 | -3018 | L1MCc | L1 | LINE |

  

|  |  |  |  |  |  |  |  |  |  |
| --- | --- | --- | --- | --- | --- | --- | --- | --- | --- |
| chr4:143764095-143767338 | | | 3239 bp | | |  |  |  |  |
| Genome Start | Genome End | Strand | Div | Repeat Start | Repeat End | Repeat Left | Name | Family | Class |
| 143764095 | 143764168 | + | 24.3 | 2 | 78 | -576 | MLT1E3 | MaLR | LTR |
| 143764174 | 143765107 | - | 2.4 | 5218 | 6152 | -3 | L1PA3 | L1 | LINE |
| 143765108 | 143766788 | + | 3.1 | 3514 | 5193 | -953 | L1PA3 | L1 | LINE |
| 143766789 | 143767338 | + | 25.7 | 48 | 615 | -9 | MLT1E3 | MaLR | LTR |

  

|  |  |  |  |  |  |  |  |  |  |
| --- | --- | --- | --- | --- | --- | --- | --- | --- | --- |
| chr4:144904430-144907681 | | | 3213 bp | | |  |  |  |  |
| Genome Start | Genome End | Strand | Div | Repeat Start | Repeat End | Repeat Left | Name | Family | Class |
| 144904430 | 144905237 | - | 18.0 | 6970 | 7785 | 0 | L1MC3 | L1 | LINE |
| 144905238 | 144905615 | + | 22.1 | 1 | 408 | 0 | MLT1A1 | MaLR | LTR |
| 144905625 | 144906042 | - | 18.8 | 1 | 432 | 0 | MSTB1 | MaLR | LTR |
| 144906043 | 144906402 | - | 18.0 | 6595 | 6969 | -816 | L1MC3 | L1 | LINE |
| 144906437 | 144906798 | - | 15.4 | 5792 | 6157 | -9 | L1PA16 | L1 | LINE |
| 144906799 | 144907074 | + | 11.6 | 5514 | 5780 | -386 | L1PA16 | L1 | LINE |
| 144907071 | 144907681 | - | 19.6 | 5500 | 6023 | -1762 | L1MC3 | L1 | LINE |

  

|  |  |  |  |  |  |  |  |  |  |
| --- | --- | --- | --- | --- | --- | --- | --- | --- | --- |
| chr4:145962156-145967545 | | | 5296 bp | | |  |  |  |  |
| Genome Start | Genome End | Strand | Div | Repeat Start | Repeat End | Repeat Left | Name | Family | Class |
| 145962156 | 145962388 | + | 21.9 | 4097 | 4326 | -1820 | L1ME2 | L1 | LINE |
| 145962389 | 145962644 | + | 13.4 | 38 | 290 | -22 | AluSx | Alu | SINE |
| 145962645 | 145962829 | + | 21.9 | 4327 | 4521 | -1625 | L1ME2 | L1 | LINE |
| 145962862 | 145963027 | + | 18.7 | 11 | 195 | -117 | AluJo | Alu | SINE |
| 145963041 | 145963166 | + | 21.1 | 4555 | 4691 | -1455 | L1ME2 | L1 | LINE |
| 145963167 | 145963470 | + | 12.2 | 1 | 305 | -7 | AluSx | Alu | SINE |
| 145963471 | 145963580 | + | 21.1 | 4692 | 4798 | -1348 | L1ME2 | L1 | LINE |
| 145963588 | 145964267 | - | 12.2 | 5476 | 6164 | -1 | L1PA15 | L1 | LINE |
| 145964272 | 145965638 | - | 2.8 | 4777 | 6141 | -14 | L1PA3 | L1 | LINE |
| 145965632 | 145965897 | + | 2.6 | 4527 | 4792 | -1354 | L1PA3 | L1 | LINE |
| 145965898 | 145966157 | - | 9.8 | 5236 | 5488 | -677 | L1PA15 | L1 | LINE |
| 145966199 | 145967109 | + | 23.6 | 4864 | 5832 | -330 | L1ME2 | L1 | LINE |
| 145967113 | 145967301 | - | 23.3 | 1 | 199 | -11 | MER63A | AcHobo | DNA |
| 145967303 | 145967545 | + | 30.3 | 5826 | 6075 | -89 | L1ME2 | L1 | LINE |

  

|  |  |  |  |  |  |  |  |  |  |
| --- | --- | --- | --- | --- | --- | --- | --- | --- | --- |
| chr4:147224623-147228953 | | | 4328 bp | | |  |  |  |  |
| Genome Start | Genome End | Strand | Div | Repeat Start | Repeat End | Repeat Left | Name | Family | Class |
| 147224623 | 147224743 | - | 16.5 | 2290 | 2404 | -14 | Tigger1 | MER2\_type | DNA |
| 147224744 | 147225204 | - | 12.9 | 4477 | 4937 | -1206 | L1M2 | L1 | LINE |
| 147225206 | 147225396 | - | 20.9 | 4204 | 4394 | -1749 | L1M2 | L1 | LINE |
| 147225395 | 147226748 | + | 12.9 | 4941 | 6301 | -2 | L1MA4A | L1 | LINE |
| 147226753 | 147228953 | - | 11.9 | 24 | 2314 | -104 | Tigger1 | MER2\_type | DNA |

  

|  |  |  |  |  |  |  |  |  |  |
| --- | --- | --- | --- | --- | --- | --- | --- | --- | --- |
| chr4:149899768-149905982 | | | 6216 bp | | |  |  |  |  |
| Genome Start | Genome End | Strand | Div | Repeat Start | Repeat End | Repeat Left | Name | Family | Class |
| 149899768 | 149900311 | - | 12.8 | 1433 | 2718 | 0 | Tigger2 | MER2\_type | DNA |
| 149900312 | 149900675 | - | 13.4 | 1 | 364 | 0 | THE1B | MaLR | LTR |
| 149900676 | 149901707 | - | 12.8 | 325 | 1432 | -549 | Tigger2 | MER2\_type | DNA |
| 149901711 | 149903497 | - | 8.3 | 4333 | 6143 | -11 | L1PA7 | L1 | LINE |
| 149903494 | 149904662 | + | 8.5 | 3139 | 4309 | -1837 | L1PA7 | L1 | LINE |
| 149904663 | 149905478 | - | 11.7 | 263 | 340 | -1641 | Tigger2 | MER2\_type | DNA |
| 149905479 | 149905790 | - | 17.5 | 1 | 296 | -16 | AluJb | Alu | SINE |
| 149905791 | 149905982 | - | 11.7 | 64 | 262 | -2456 | Tigger2 | MER2\_type | DNA |

  

|  |  |  |  |  |  |  |  |  |  |
| --- | --- | --- | --- | --- | --- | --- | --- | --- | --- |
| chr4:149956082-149959901 | | | 3793 bp | | |  |  |  |  |
| Genome Start | Genome End | Strand | Div | Repeat Start | Repeat End | Repeat Left | Name | Family | Class |
| 149956082 | 149956243 | + | 15.6 | 1 | 160 | -205 | MLT1A0 | MaLR | LTR |
| 149956244 | 149956452 | - | 5.3 | 2758 | 2966 | -3180 | L1PA5 | L1 | LINE |
| 149956452 | 149959649 | + | 6.6 | 2945 | 6146 | -8 | L1PA5 | L1 | LINE |
| 149959678 | 149959901 | + | 27.8 | 142 | 365 | 0 | MLT1A0 | MaLR | LTR |

  

|  |  |  |  |  |  |  |  |  |  |
| --- | --- | --- | --- | --- | --- | --- | --- | --- | --- |
| chr4:150115999-150119134 | | | 3136 bp | | |  |  |  |  |
| Genome Start | Genome End | Strand | Div | Repeat Start | Repeat End | Repeat Left | Name | Family | Class |
| 150115999 | 150116831 | - | 20.1 | 138 | 997 | -43 | MER45B | Tip100 | DNA |
| 150116835 | 150118144 | - | 3.7 | 4841 | 6155 | 0 | L1PA4 | L1 | LINE |
| 150118142 | 150118985 | + | 3.2 | 4000 | 4844 | -1302 | L1PA4 | L1 | LINE |
| 150118986 | 150119134 | - | 24.2 | 1 | 149 | -891 | MER45B | Tip100 | DNA |

  

|  |  |  |  |  |  |  |  |  |  |
| --- | --- | --- | --- | --- | --- | --- | --- | --- | --- |
| chr4:150226537-150236223 | | | 9439 bp | | |  |  |  |  |
| Genome Start | Genome End | Strand | Div | Repeat Start | Repeat End | Repeat Left | Name | Family | Class |
| 150226537 | 150226579 | - | 25.8 | 5406 | 5451 | -1086 | L1MDa | L1 | LINE |
| 150226689 | 150226823 | - | 10.4 | 6157 | 6291 | -9 | L1MA4 | L1 | LINE |
| 150226826 | 150226956 | + | 14.5 | 6024 | 6158 | -142 | L1MA4 | L1 | LINE |
| 150226962 | 150227353 | - | 25.4 | 4987 | 5390 | -1147 | L1MDa | L1 | LINE |
| 150227367 | 150227769 | - | 24.2 | 3900 | 4334 | -2203 | L1MDa | L1 | LINE |
| 150227795 | 150227859 | - | 24.2 | 3833 | 3899 | -2638 | L1MDa | L1 | LINE |
| 150227832 | 150228441 | - | 27.5 | 2678 | 3327 | -3210 | L1MDa | L1 | LINE |
| 150228452 | 150228814 | - | 21.7 | 5 | 382 | -8 | MLT1B | MaLR | LTR |
| 150228819 | 150229160 | - | 28.7 | 2427 | 2779 | -3939 | L1MDa | L1 | LINE |
| 150229197 | 150235336 | - | 5.4 | 1 | 6152 | -3 | L1PA4 | L1 | LINE |
| 150235340 | 150235516 | - | 29.8 | 2261 | 2435 | -4283 | L1MDa | L1 | LINE |
| 150235543 | 150235753 | - | 22.9 | 11 | 211 | -4 | MER58C | MER1\_type | DNA |
| 150235768 | 150235995 | - | 26.8 | 2015 | 2252 | -4466 | L1MDa | L1 | LINE |
| 150236025 | 150236223 | - | 29.4 | 1752 | 1954 | -4679 | L1MDa | L1 | LINE |

  

|  |  |  |  |  |  |  |  |  |  |
| --- | --- | --- | --- | --- | --- | --- | --- | --- | --- |
| chr4:150365513-150368176 | | | 2635 bp | | |  |  |  |  |
| Genome Start | Genome End | Strand | Div | Repeat Start | Repeat End | Repeat Left | Name | Family | Class |
| 150365513 | 150366466 | - | 27.8 | 5161 | 6133 | -107 | L1ME3B | L1 | LINE |
| 150366501 | 150367022 | - | 3.1 | 5630 | 6155 | 0 | L1PA4 | L1 | LINE |
| 150367023 | 150367442 | + | 1.7 | 5220 | 5640 | -515 | L1PA4 | L1 | LINE |
| 150367438 | 150368176 | - | 33.2 | 4362 | 5108 | -1038 | L1ME3B | L1 | LINE |

  

|  |  |  |  |  |  |  |  |  |  |
| --- | --- | --- | --- | --- | --- | --- | --- | --- | --- |
| chr4:151974032-151981456 | | | 7433 bp | | |  |  |  |  |
| Genome Start | Genome End | Strand | Div | Repeat Start | Repeat End | Repeat Left | Name | Family | Class |
| 151974032 | 151975436 | - | 16.3 | 982 | 2418 | 0 | Tigger1 | MER2\_type | DNA |
| 151975437 | 151979489 | - | 5.8 | 1063 | 5123 | -1023 | L1PA5 | L1 | LINE |
| 151979482 | 151980494 | + | 4.5 | 5130 | 6149 | -5 | L1PA5 | L1 | LINE |
| 151980495 | 151981456 | - | 15.1 | 1 | 998 | -1420 | Tigger1 | MER2\_type | DNA |

  

|  |  |  |  |  |  |  |  |  |  |
| --- | --- | --- | --- | --- | --- | --- | --- | --- | --- |
| chr4:157259066-157268389 | | | 9290 bp | | |  |  |  |  |
| Genome Start | Genome End | Strand | Div | Repeat Start | Repeat End | Repeat Left | Name | Family | Class |
| 157259066 | 157260987 | + | 18.6 | 1 | 2200 | -4308 | L1MC1 | L1 | LINE |
| 157261001 | 157262057 | - | 16.5 | 4075 | 5152 | -994 | L1M1 | L1 | LINE |
| 157262058 | 157262367 | + | 12.9 | 1 | 310 | -2 | AluSx | Alu | SINE |
| 157262368 | 157262561 | - | 16.5 | 3876 | 4074 | -2072 | L1M1 | L1 | LINE |
| 157262563 | 157263026 | + | 15.6 | 5145 | 5625 | -680 | L1MA4 | L1 | LINE |
| 157263030 | 157263602 | + | 19.2 | 5714 | 6299 | -1 | L1MA4 | L1 | LINE |
| 157263620 | 157266077 | + | 17.6 | 2202 | 4646 | -1675 | L1MC1 | L1 | LINE |
| 157266078 | 157266374 | + | 8.1 | 13 | 310 | -1 | AluY | Alu | SINE |
| 157266375 | 157268389 | + | 17.6 | 4647 | 6507 | -1 | L1MC1 | L1 | LINE |

  

|  |  |  |  |  |  |  |  |  |  |
| --- | --- | --- | --- | --- | --- | --- | --- | --- | --- |
| chr4:157373430-157378376 | | | 4695 bp | | |  |  |  |  |
| Genome Start | Genome End | Strand | Div | Repeat Start | Repeat End | Repeat Left | Name | Family | Class |
| 157373430 | 157373604 | - | 30.1 | 5557 | 5747 | -374 | L1M5 | L1 | LINE |
| 157373787 | 157374284 | - | 7.3 | 5654 | 6151 | -3 | L1PA7 | L1 | LINE |
| 157374285 | 157374744 | + | 6.9 | 5188 | 5661 | -493 | L1PA7 | L1 | LINE |
| 157374771 | 157375601 | - | 15.7 | 6825 | 7706 | -4 | L1MA8 | L1 | LINE |
| 157375594 | 157375741 | - | 22.4 | 5949 | 6097 | -1465 | L1MA8 | L1 | LINE |
| 157375736 | 157377845 | - | 20.3 | 2415 | 4604 | -2958 | L1MA8 | L1 | LINE |
| 157377846 | 157378149 | - | 15.1 | 65 | 376 | -236 | LTR9 | ERV1 | LTR |
| 157378200 | 157378263 | - | 15.1 | 5 | 64 | -548 | LTR9 | ERV1 | LTR |
| 157378272 | 157378376 | - | 23.5 | 4401 | 4511 | -1635 | L1M5 | L1 | LINE |

  

|  |  |  |  |  |  |  |  |  |  |
| --- | --- | --- | --- | --- | --- | --- | --- | --- | --- |
| chr4:158761294-158768185 | | | 6735 bp | | |  |  |  |  |
| Genome Start | Genome End | Strand | Div | Repeat Start | Repeat End | Repeat Left | Name | Family | Class |
| 158761294 | 158761587 | - | 39.4 | 2595 | 2898 | -3316 | L1MC4 | L1 | LINE |
| 158761630 | 158762145 | - | 23.1 | 1941 | 2496 | -3719 | L1MC4 | L1 | LINE |
| 158762146 | 158762562 | - | 14.2 | 452 | 900 | 0 | MER4D1 | ERV1 | LTR |
| 158762557 | 158762593 | - | 8.1 | 183 | 219 | -653 | MER4D0 | ERV1 | LTR |
| 158762609 | 158762758 | - | 26.4 | 6020 | 6175 | -65 | L1ME3B | L1 | LINE |
| 158762820 | 158763580 | - | 3.8 | 5392 | 6149 | -6 | L1PA4 | L1 | LINE |
| 158763569 | 158765826 | + | 4.9 | 3133 | 5390 | -756 | L1PA4 | L1 | LINE |
| 158765827 | 158766018 | - | 15.6 | 1 | 197 | -703 | MER4D1 | ERV1 | LTR |
| 158766019 | 158766402 | - | 21.4 | 1591 | 1943 | -4272 | L1MC4 | L1 | LINE |
| 158766403 | 158766710 | + | 12.0 | 1 | 309 | -1 | AluSg | Alu | SINE |
| 158766724 | 158766865 | + | 14.8 | 157 | 298 | -15 | AluSp/q | Alu | SINE |
| 158766868 | 158767545 | - | 25.3 | 826 | 1554 | -4661 | L1MC4 | L1 | LINE |
| 158767588 | 158768185 | - | 31.4 | 1 | 606 | -5609 | L1MC4 | L1 | LINE |

  

|  |  |  |  |  |  |  |  |  |  |
| --- | --- | --- | --- | --- | --- | --- | --- | --- | --- |
| chr4:159445884-159455105 | | | 9204 bp | | |  |  |  |  |
| Genome Start | Genome End | Strand | Div | Repeat Start | Repeat End | Repeat Left | Name | Family | Class |
| 159445884 | 159446237 | - | 13.3 | 6166 | 6494 | -9 | L1PA16 | L1 | LINE |
| 159446238 | 159446548 | - | 7.8 | 1 | 308 | -3 | AluY | Alu | SINE |
| 159446549 | 159447060 | - | 13.3 | 5654 | 6165 | -338 | L1PA16 | L1 | LINE |
| 159447055 | 159448344 | - | 7.6 | 4853 | 6150 | -4 | L1PA7 | L1 | LINE |
| 159448335 | 159449331 | + | 9.1 | 3844 | 4843 | -1303 | L1PA7 | L1 | LINE |
| 159449331 | 159451088 | - | 15.2 | 4023 | 5667 | -816 | L1PA16 | L1 | LINE |
| 159451124 | 159455105 | - | 17.1 | 9 | 4022 | -2461 | L1PA16 | L1 | LINE |

  

|  |  |  |  |  |  |  |  |  |  |
| --- | --- | --- | --- | --- | --- | --- | --- | --- | --- |
| chr4:161132310-161137837 | | | 4853 bp | | |  |  |  |  |
| Genome Start | Genome End | Strand | Div | Repeat Start | Repeat End | Repeat Left | Name | Family | Class |
| 161132310 | 161132665 | - | 24.2 | 1820 | 2198 | -2636 | L1MEe | L1 | LINE |
| 161132782 | 161133078 | - | 20.7 | 762 | 1060 | -3774 | L1MEe | L1 | LINE |
| 161133307 | 161133374 | - | 17.9 | 411 | 477 | -4377 | L1MEe | L1 | LINE |
| 161133667 | 161136223 | - | 10.0 | 3643 | 6169 | -3 | L1PA8 | L1 | LINE |
| 161136224 | 161136870 | + | 6.8 | 2351 | 3007 | -3139 | L1P3 | L1 | LINE |
| 161136873 | 161136938 | + | 9.3 | 3237 | 3294 | -2852 | L1P3 | L1 | LINE |
| 161136939 | 161137243 | + | 6.2 | 1 | 307 | -4 | AluY | Alu | SINE |
| 161137244 | 161137582 | + | 9.3 | 3295 | 3633 | -2513 | L1P3 | L1 | LINE |
| 161137620 | 161137837 | - | 27.6 | 14 | 231 | -4623 | L1MEe | L1 | LINE |

  

|  |  |  |  |  |  |  |  |  |  |
| --- | --- | --- | --- | --- | --- | --- | --- | --- | --- |
| chr4:162265299-162267126 | | | 1797 bp | | |  |  |  |  |
| Genome Start | Genome End | Strand | Div | Repeat Start | Repeat End | Repeat Left | Name | Family | Class |
| 162265299 | 162265509 | - | 35.6 | 1230 | 1441 | -4226 | HERVL | ERVL | LTR |
| 162265533 | 162266004 | - | 19.8 | 5836 | 6312 | 0 | L1MA9 | L1 | LINE |
| 162266005 | 162266806 | + | 20.3 | 5028 | 5829 | -354 | L1MA9 | L1 | LINE |
| 162266815 | 162267126 | - | 36.4 | 887 | 1207 | -4460 | HERVL | ERVL | LTR |

  

|  |  |  |  |  |  |  |  |  |  |
| --- | --- | --- | --- | --- | --- | --- | --- | --- | --- |
| chr4:162353793-162357653 | | | 3549 bp | | |  |  |  |  |
| Genome Start | Genome End | Strand | Div | Repeat Start | Repeat End | Repeat Left | Name | Family | Class |
| 162353793 | 162355030 | - | 30.3 | 814 | 2130 | -4998 | L1MCc | L1 | LINE |
| 162355249 | 162355399 | - | 27.8 | 337 | 512 | -5911 | L1MCc | L1 | LINE |
| 162355433 | 162356456 | - | 17.6 | 3684 | 4731 | -1412 | L1M2 | L1 | LINE |
| 162356486 | 162356601 | - | 17.6 | 3555 | 3683 | -2460 | L1M2 | L1 | LINE |
| 162356600 | 162357452 | + | 20.1 | 4728 | 5644 | -647 | L1M2 | L1 | LINE |
| 162357487 | 162357653 | - | 25.2 | 18 | 174 | -6249 | L1MCc | L1 | LINE |

  

|  |  |  |  |  |  |  |  |  |  |
| --- | --- | --- | --- | --- | --- | --- | --- | --- | --- |
| chr4:162757491-162763959 | | | 6083 bp | | |  |  |  |  |
| Genome Start | Genome End | Strand | Div | Repeat Start | Repeat End | Repeat Left | Name | Family | Class |
| 162757491 | 162758485 | + | 30.8 | 1333 | 2479 | -4623 | L1M4b | L1 | LINE |
| 162758475 | 162759385 | - | 3.1 | 3693 | 4603 | -1543 | L1PA3 | L1 | LINE |
| 162759380 | 162760956 | + | 2.9 | 4579 | 6155 | 0 | L1PA3 | L1 | LINE |
| 162760959 | 162760974 | + | 22.7 | 2392 | 2407 | -4927 | L1M4b | L1 | LINE |
| 162760975 | 162761288 | + | 13.7 | 1 | 313 | 0 | AluSp | Alu | SINE |
| 162761289 | 162761394 | + | 23.8 | 2408 | 2667 | -4520 | L1M4b | L1 | LINE |
| 162761402 | 162761869 | + | 28.4 | 3263 | 3739 | -3267 | L1M4b | L1 | LINE |
| 162761915 | 162762297 | + | 28.4 | 3740 | 4149 | -2857 | L1M4b | L1 | LINE |
| 162762298 | 162762625 | + | 13.7 | 1 | 310 | 0 | AluSq | Alu | SINE |
| 162762626 | 162763120 | + | 28.4 | 4150 | 4633 | -2373 | L1M4b | L1 | LINE |
| 162763166 | 162763254 | + | 29.9 | 4643 | 4732 | -2274 | L1M4b | L1 | LINE |
| 162763559 | 162763959 | + | 28.2 | 4665 | 5089 | -1917 | L1M4b | L1 | LINE |

  

|  |  |  |  |  |  |  |  |  |  |
| --- | --- | --- | --- | --- | --- | --- | --- | --- | --- |
| chr4:163334667-163338050 | | | 3398 bp | | |  |  |  |  |
| Genome Start | Genome End | Strand | Div | Repeat Start | Repeat End | Repeat Left | Name | Family | Class |
| 163334667 | 163335083 | + | 22.1 | 1 | 423 | -660 | Tigger6a | MER2\_type | DNA |
| 163335084 | 163335533 | - | 9.1 | 4646 | 5103 | -1043 | L1PREC2 | L1 | LINE |
| 163335534 | 163335601 | + | 4.6 | 5110 | 5175 | -971 | L1PA13 | L1 | LINE |
| 163335582 | 163335681 | + | 9.1 | 5248 | 5349 | -797 | L1PREC2 | L1 | LINE |
| 163335688 | 163336620 | - | 11.2 | 5202 | 6151 | 0 | L1PB1 | L1 | LINE |
| 163336621 | 163337420 | + | 11.4 | 5336 | 6147 | -16 | L1PA13 | L1 | LINE |
| 163337421 | 163338050 | + | 21.0 | 394 | 1083 | 0 | Tigger6a | MER2\_type | DNA |

  

|  |  |  |  |  |  |  |  |  |  |
| --- | --- | --- | --- | --- | --- | --- | --- | --- | --- |
| chr4:164453269-164460942 | | | 7677 bp | | |  |  |  |  |
| Genome Start | Genome End | Strand | Div | Repeat Start | Repeat End | Repeat Left | Name | Family | Class |
| 164453269 | 164454424 | - | 18.0 | 5402 | 6504 | -15 | L1MC2 | L1 | LINE |
| 164454428 | 164454973 | - | 8.6 | 5608 | 6156 | -12 | L1PA10 | L1 | LINE |
| 164454974 | 164455276 | + | 9.6 | 5319 | 5621 | -510 | L1PA10 | L1 | LINE |
| 164455265 | 164455325 | - | 18.5 | 4140 | 4196 | -2125 | L1MC2 | L1 | LINE |
| 164455326 | 164455605 | - | 16.8 | 5874 | 6158 | -8 | L1PA16 | L1 | LINE |
| 164455606 | 164456521 | - | 18.1 | 2053 | 4139 | -2182 | L1MC2 | L1 | LINE |
| 164456522 | 164456932 | - | 15.3 | 5 | 428 | 0 | MSTA | MaLR | LTR |
| 164456937 | 164458554 | - | 27.9 | 2 | 1735 | 0 | MLT-int | MaLR | LTR |
| 164458557 | 164458975 | - | 16.6 | 1 | 428 | 0 | MSTA | MaLR | LTR |
| 164458976 | 164460942 | - | 17.6 | 4 | 2052 | -4456 | L1MC2 | L1 | LINE |

  

|  |  |  |  |  |  |  |  |  |  |
| --- | --- | --- | --- | --- | --- | --- | --- | --- | --- |
| chr4:167375458-167379294 | | | 3811 bp | | |  |  |  |  |
| Genome Start | Genome End | Strand | Div | Repeat Start | Repeat End | Repeat Left | Name | Family | Class |
| 167375458 | 167375713 | - | 16.1 | 2145 | 2405 | 0 | Tigger5 | MER2\_type | DNA |
| 167375743 | 167377621 | - | 1.8 | 4272 | 6151 | -4 | L1PA3 | L1 | LINE |
| 167377619 | 167379215 | + | 3.4 | 2663 | 4259 | -1887 | L1PA3 | L1 | LINE |
| 167379216 | 167379294 | - | 23.1 | 1 | 81 | -2242 | Tigger5 | MER2\_type | DNA |

  

|  |  |  |  |  |  |  |  |  |  |
| --- | --- | --- | --- | --- | --- | --- | --- | --- | --- |
| chr4:168105450-168112394 | | | 6962 bp | | |  |  |  |  |
| Genome Start | Genome End | Strand | Div | Repeat Start | Repeat End | Repeat Left | Name | Family | Class |
| 168105450 | 168106410 | - | 13.8 | 5336 | 6300 | 0 | L1MA6 | L1 | LINE |
| 168106403 | 168107478 | - | 5.8 | 739 | 1817 | -4338 | L1PA3 | L1 | LINE |
| 168107475 | 168111823 | + | 3.1 | 1801 | 6155 | 0 | L1PA3 | L1 | LINE |
| 168111819 | 168112394 | - | 13.6 | 4786 | 5347 | -796 | L1MA6 | L1 | LINE |

  

|  |  |  |  |  |  |  |  |  |  |
| --- | --- | --- | --- | --- | --- | --- | --- | --- | --- |
| chr4:170088249-170100890 | | | 12522 bp | | |  |  |  |  |
| Genome Start | Genome End | Strand | Div | Repeat Start | Repeat End | Repeat Left | Name | Family | Class |
| 170088249 | 170091835 | + | 18.1 | 3 | 3653 | -3132 | L1M2 | L1 | LINE |
| 170091836 | 170091953 | + | 25.2 | 2 | 121 | -64 | FAM | Alu | SINE |
| 170091954 | 170092727 | + | 18.1 | 3654 | 4440 | -2345 | L1M2 | L1 | LINE |
| 170092711 | 170098760 | + | 8.2 | 11 | 6062 | -407 | L1PA7 | L1 | LINE |
| 170098763 | 170099055 | + | 8.2 | 6161 | 6463 | -6 | L1PA7 | L1 | LINE |
| 170099061 | 170099231 | + | 12.9 | 4412 | 4582 | -2203 | L1M2 | L1 | LINE |
| 170099297 | 170099616 | - | 8.1 | 5329 | 5650 | -504 | L1PA8 | L1 | LINE |
| 170099617 | 170100142 | + | 5.4 | 5651 | 6172 | 0 | L1PA8 | L1 | LINE |
| 170100208 | 170100890 | + | 15.8 | 5633 | 6334 | -605 | L1M2 | L1 | LINE |

  

|  |  |  |  |  |  |  |  |  |  |
| --- | --- | --- | --- | --- | --- | --- | --- | --- | --- |
| chr4:171411944-171414646 | | | 2569 bp | | |  |  |  |  |
| Genome Start | Genome End | Strand | Div | Repeat Start | Repeat End | Repeat Left | Name | Family | Class |
| 171411944 | 171412277 | + | 16.0 | 1 | 350 | -76 | MSTB | MaLR | LTR |
| 171412410 | 171412459 | - | 16.0 | 241 | 290 | -9 | Alu | Alu | SINE |
| 171412467 | 171414360 | - | 1.9 | 4338 | 6231 | 0 | L1PA3 | L1 | LINE |
| 171414356 | 171414561 | + | 3.9 | 4133 | 4338 | -1808 | L1PA3 | L1 | LINE |
| 171414562 | 171414646 | + | 16.0 | 342 | 426 | 0 | MSTB | MaLR | LTR |

  

|  |  |  |  |  |  |  |  |  |  |
| --- | --- | --- | --- | --- | --- | --- | --- | --- | --- |
| chr4:171628598-171632280 | | | 3424 bp | | |  |  |  |  |
| Genome Start | Genome End | Strand | Div | Repeat Start | Repeat End | Repeat Left | Name | Family | Class |
| 171628598 | 171628985 | + | 24.5 | 4374 | 4773 | -1373 | L1M4 | L1 | LINE |
| 171629001 | 171629179 | - | 22.6 | 529 | 707 | 0 | MER39 | ERV1 | LTR |
| 171629360 | 171630457 | - | 3.5 | 3870 | 4969 | -1177 | L1PA3 | L1 | LINE |
| 171630455 | 171631645 | + | 2.3 | 4954 | 6154 | -1 | L1PA3 | L1 | LINE |
| 171631646 | 171631897 | - | 23.2 | 250 | 512 | -195 | MER39 | ERV1 | LTR |
| 171631965 | 171632280 | + | 29.9 | 4787 | 5112 | -1034 | L1M4 | L1 | LINE |

  

|  |  |  |  |  |  |  |  |  |  |
| --- | --- | --- | --- | --- | --- | --- | --- | --- | --- |
| chr4:171861115-171863645 | | | 2363 bp | | |  |  |  |  |
| Genome Start | Genome End | Strand | Div | Repeat Start | Repeat End | Repeat Left | Name | Family | Class |
| 171861115 | 171861506 | + | 27.6 | 1412 | 1824 | -1595 | L2 | L2 | LINE |
| 171861622 | 171862789 | - | 9.5 | 4947 | 6158 | -2 | L1PREC2 | L1 | LINE |
| 171862814 | 171863163 | - | 21.1 | 5809 | 6170 | -130 | L1MA4 | L1 | LINE |
| 171863163 | 171863294 | + | 23.5 | 6162 | 6295 | -5 | L1MA4 | L1 | LINE |
| 171863325 | 171863645 | + | 30.9 | 1843 | 2205 | -1214 | L2 | L2 | LINE |

  

|  |  |  |  |  |  |  |  |  |  |
| --- | --- | --- | --- | --- | --- | --- | --- | --- | --- |
| chr4:172385682-172388102 | | | 2332 bp | | |  |  |  |  |
| Genome Start | Genome End | Strand | Div | Repeat Start | Repeat End | Repeat Left | Name | Family | Class |
| 172385682 | 172385847 | + | 27.7 | 2337 | 2508 | -911 | L2 | L2 | LINE |
| 172385880 | 172386353 | - | 7.0 | 4165 | 4656 | -1490 | L1PA8A | L1 | LINE |
| 172386350 | 172387794 | + | 11.5 | 4640 | 6121 | -10 | L1PA8A | L1 | LINE |
| 172387856 | 172388102 | + | 33.8 | 2591 | 2864 | -555 | L2 | L2 | LINE |

  

|  |  |  |  |  |  |  |  |  |  |
| --- | --- | --- | --- | --- | --- | --- | --- | --- | --- |
| chr4:176046384-176052635 | | | 6252 bp | | |  |  |  |  |
| Genome Start | Genome End | Strand | Div | Repeat Start | Repeat End | Repeat Left | Name | Family | Class |
| 176046384 | 176046811 | + | 23.1 | 2448 | 2847 | -3690 | L1MDa | L1 | LINE |
| 176046812 | 176047157 | + | 25.3 | 1 | 372 | -2 | MLT1A | MaLR | LTR |
| 176047158 | 176047254 | + | 24.5 | 2848 | 3003 | -3534 | L1MDa | L1 | LINE |
| 176047255 | 176047558 | + | 12.2 | 4 | 312 | 0 | AluSx | Alu | SINE |
| 176047559 | 176047938 | + | 24.5 | 3004 | 3409 | -3128 | L1MDa | L1 | LINE |
| 176047945 | 176048196 | + | 19.5 | 1 | 254 | -266 | MLT2B1 | ERVL | LTR |
| 176048197 | 176049115 | - | 15.8 | 239 | 1175 | 0 | Merlin1\_HS | Merlin | DNA |
| 176049110 | 176049225 | - | 12.9 | 4 | 124 | -1051 | Merlin1\_HS | Merlin | DNA |
| 176049226 | 176049534 | + | 25.2 | 249 | 554 | 0 | MLT2B1 | ERVL | LTR |
| 176049537 | 176049847 | + | 22.7 | 3413 | 3731 | -2806 | L1MDa | L1 | LINE |
| 176049848 | 176050149 | + | 5.0 | 1 | 301 | -10 | AluY | Alu | SINE |
| 176050150 | 176050969 | + | 22.7 | 3732 | 4571 | -1966 | L1MDa | L1 | LINE |
| 176050966 | 176051471 | - | 3.4 | 5551 | 6056 | -99 | L1PA4 | L1 | LINE |
| 176051472 | 176051559 | + | 5.7 | 6068 | 6155 | 0 | L1PA4 | L1 | LINE |
| 176051562 | 176052635 | + | 21.8 | 4561 | 5583 | -954 | L1MDa | L1 | LINE |

  

|  |  |  |  |  |  |  |  |  |  |
| --- | --- | --- | --- | --- | --- | --- | --- | --- | --- |
| chr4:176363687-176367313 | | | 3636 bp | | |  |  |  |  |
| Genome Start | Genome End | Strand | Div | Repeat Start | Repeat End | Repeat Left | Name | Family | Class |
| 176363687 | 176364045 | - | 23.4 | 218 | 538 | -4 | MER34D | ERV1 | LTR |
| 176364046 | 176366277 | - | 1.5 | 3918 | 6149 | -6 | L1PA2 | L1 | LINE |
| 176366269 | 176367092 | + | 1.0 | 3103 | 3926 | -2220 | L1PA2 | L1 | LINE |
| 176367093 | 176367313 | - | 27.6 | 1 | 227 | -352 | MER34D | ERV1 | LTR |

  

|  |  |  |  |  |  |  |  |  |  |
| --- | --- | --- | --- | --- | --- | --- | --- | --- | --- |
| chr4:176440429-176444589 | | | 3992 bp | | |  |  |  |  |
| Genome Start | Genome End | Strand | Div | Repeat Start | Repeat End | Repeat Left | Name | Family | Class |
| 176440429 | 176440879 | - | 35.1 | 2924 | 3419 | 0 | L2 | L2 | LINE |
| 176440880 | 176441097 | + | 4.6 | 1 | 228 | -83 | AluY | Alu | SINE |
| 176441098 | 176441248 | - | 33.5 | 2792 | 2939 | -480 | L2 | L2 | LINE |
| 176441409 | 176441776 | - | 31.2 | 2109 | 2479 | -940 | L2 | L2 | LINE |
| 176441777 | 176442262 | + | 13.0 | 5791 | 6297 | 0 | L1MA5A | L1 | LINE |
| 176442263 | 176442367 | - | 31.2 | 2003 | 2108 | -1311 | L2 | L2 | LINE |
| 176442376 | 176442879 | - | 14.2 | 5769 | 6281 | -10 | L1MA7 | L1 | LINE |
| 176442882 | 176443134 | - | 18.8 | 49 | 312 | 0 | AluSx | Alu | SINE |
| 176443135 | 176443426 | - | 18.5 | 5486 | 5780 | -511 | L1MA7 | L1 | LINE |
| 176443425 | 176443552 | + | 12.5 | 5346 | 5480 | -688 | L1M3 | L1 | LINE |
| 176443554 | 176444589 | - | 32.3 | 771 | 2014 | -1405 | L2 | L2 | LINE |

  

|  |  |  |  |  |  |  |  |  |  |
| --- | --- | --- | --- | --- | --- | --- | --- | --- | --- |
| chr4:176690375-176694270 | | | 3864 bp | | |  |  |  |  |
| Genome Start | Genome End | Strand | Div | Repeat Start | Repeat End | Repeat Left | Name | Family | Class |
| 176690375 | 176690738 | - | 17.9 | 82 | 467 | 0 | MLT1C | MaLR | LTR |
| 176690779 | 176692166 | - | 2.8 | 4756 | 6152 | -3 | L1PA3 | L1 | LINE |
| 176692159 | 176694171 | + | 3.5 | 2720 | 4737 | -1409 | L1PA3 | L1 | LINE |
| 176694172 | 176694270 | - | 15.2 | 1 | 99 | -368 | MLT1C | MaLR | LTR |

  

|  |  |  |  |  |  |  |  |  |  |
| --- | --- | --- | --- | --- | --- | --- | --- | --- | --- |
| chr4:179073790-179078641 | | | 4852 bp | | |  |  |  |  |
| Genome Start | Genome End | Strand | Div | Repeat Start | Repeat End | Repeat Left | Name | Family | Class |
| 179073790 | 179074208 | - | 19.4 | 6797 | 7237 | -2177 | L1M3 | L1 | LINE |
| 179074193 | 179074521 | - | 21.8 | 4447 | 4795 | -2770 | L1M3 | L1 | LINE |
| 179074536 | 179076045 | - | 6.1 | 4615 | 6151 | -3 | L1PA7 | L1 | LINE |
| 179076040 | 179077354 | + | 8.4 | 3267 | 4592 | -1554 | L1PA7 | L1 | LINE |
| 179077363 | 179078641 | - | 22.2 | 3156 | 4455 | -3104 | L1M3 | L1 | LINE |

  

|  |  |  |  |  |  |  |  |  |  |
| --- | --- | --- | --- | --- | --- | --- | --- | --- | --- |
| chr4:181286053-181287988 | | | 1938 bp | | |  |  |  |  |
| Genome Start | Genome End | Strand | Div | Repeat Start | Repeat End | Repeat Left | Name | Family | Class |
| 181286053 | 181286466 | + | 23.8 | 5642 | 6070 | -172 | L1MD1 | L1 | LINE |
| 181286466 | 181287239 | - | 0.7 | 5380 | 6154 | -1 | L1PA2 | L1 | LINE |
| 181287239 | 181287814 | + | 2.4 | 4798 | 5373 | -773 | L1PA2 | L1 | LINE |
| 181287815 | 181287988 | + | 20.9 | 6060 | 6235 | -7 | L1MD1 | L1 | LINE |

  

|  |  |  |  |  |  |  |  |  |  |
| --- | --- | --- | --- | --- | --- | --- | --- | --- | --- |
| chr4:182705153-182706813 | | | 1457 bp | | |  |  |  |  |
| Genome Start | Genome End | Strand | Div | Repeat Start | Repeat End | Repeat Left | Name | Family | Class |
| 182705153 | 182705851 | + | 30.5 | 772 | 1551 | -956 | HAL1 | L1 | LINE |
| 182705954 | 182706267 | - | 5.4 | 5844 | 6155 | 0 | L1PA4 | L1 | LINE |
| 182706268 | 182706640 | + | 3.5 | 5452 | 5823 | -332 | L1PA4 | L1 | LINE |
| 182706743 | 182706813 | + | 23.9 | 1682 | 1752 | -755 | HAL1 | L1 | LINE |

  

|  |  |  |  |  |  |  |  |  |  |
| --- | --- | --- | --- | --- | --- | --- | --- | --- | --- |
| chr4:188731366-188733651 | | | 2270 bp | | |  |  |  |  |
| Genome Start | Genome End | Strand | Div | Repeat Start | Repeat End | Repeat Left | Name | Family | Class |
| 188731366 | 188731503 | + | 21.2 | 1 | 142 | -223 | MLT1A0 | MaLR | LTR |
| 188731508 | 188731820 | - | 14.3 | 5830 | 6153 | -3 | L1PB4 | L1 | LINE |
| 188731821 | 188732125 | - | 8.8 | 1 | 305 | -6 | AluY | Alu | SINE |
| 188732126 | 188732784 | - | 14.4 | 5162 | 5829 | -327 | L1PB4 | L1 | LINE |
| 188732779 | 188733475 | + | 16.1 | 4454 | 5158 | -988 | L1P4 | L1 | LINE |
| 188733494 | 188733651 | + | 23.7 | 197 | 355 | -10 | MLT1A0 | MaLR | LTR |

  

|  |  |  |  |  |  |  |  |  |  |
| --- | --- | --- | --- | --- | --- | --- | --- | --- | --- |
| chr4:189033133-189036981 | | | 3865 bp | | |  |  |  |  |
| Genome Start | Genome End | Strand | Div | Repeat Start | Repeat End | Repeat Left | Name | Family | Class |
| 189033133 | 189034648 | - | 9.3 | 4629 | 6168 | 0 | L1PA12 | L1 | LINE |
| 189034643 | 189035637 | - | 2.9 | 5157 | 6155 | 0 | L1PA4 | L1 | LINE |
| 189035633 | 189035936 | + | 4.6 | 4856 | 5159 | -987 | L1PA4 | L1 | LINE |
| 189035932 | 189036981 | - | 9.2 | 3604 | 4645 | -1501 | L1PA12 | L1 | LINE |

  

|  |  |  |  |  |  |  |  |  |  |
| --- | --- | --- | --- | --- | --- | --- | --- | --- | --- |
| chr4:190505063-190510571 | | | 5397 bp | | |  |  |  |  |
| Genome Start | Genome End | Strand | Div | Repeat Start | Repeat End | Repeat Left | Name | Family | Class |
| 190505063 | 190505632 | + | 31.7 | 1 | 563 | -5482 | L1MCb | L1 | LINE |
| 190505742 | 190506870 | + | 26.2 | 576 | 1858 | -4893 | L1MCb | L1 | LINE |
| 190506879 | 190509206 | - | 3.8 | 3825 | 6152 | -3 | L1PA4 | L1 | LINE |
| 190509205 | 190510021 | + | 4.7 | 2995 | 3805 | -2341 | L1PA4 | L1 | LINE |
| 190510019 | 190510571 | + | 22.7 | 1860 | 2477 | -4274 | L1MCb | L1 | LINE |

  

|  |  |  |  |  |  |  |  |  |  |
| --- | --- | --- | --- | --- | --- | --- | --- | --- | --- |
| chr4:191143153-191151280 | | | 8126 bp | | |  |  |  |  |
| Genome Start | Genome End | Strand | Div | Repeat Start | Repeat End | Repeat Left | Name | Family | Class |
| 191143153 | 191144734 | - | 31.5 | 4518 | 6163 | -1593 | MER41D-int | ERV1 | LTR |
| 191144735 | 191144897 | + | 11.3 | 1 | 162 | -202 | THE1B | MaLR | LTR |
| 191144898 | 191145176 | - | 14.7 | 23 | 303 | -9 | AluSx | Alu | SINE |
| 191145177 | 191145385 | + | 11.3 | 163 | 364 | 0 | THE1B | MaLR | LTR |
| 191145386 | 191147963 | - | 31.5 | 1904 | 4517 | -3239 | MER41D-int | ERV1 | LTR |
| 191147969 | 191149718 | - | 4.9 | 4405 | 6155 | 0 | L1PA4 | L1 | LINE |
| 191149711 | 191150625 | + | 5.2 | 3498 | 4413 | -1733 | L1PA4 | L1 | LINE |
| 191150631 | 191151280 | - | 34.2 | 1269 | 1904 | -5852 | MER41D-int | ERV1 | LTR |

  


|  |  |  |  |  |  |  |  |  |  |
| --- | --- | --- | --- | --- | --- | --- | --- | --- | --- |
| chr5:185037-190539 | | | 5337 bp | | |  |  |  |  |
| Genome Start | Genome End | Strand | Div | Repeat Start | Repeat End | Repeat Left | Name | Family | Class |
| 185037 | 185411 | + | 16.4 | 3887 | 4261 | -1879 | L1MB3 | L1 | LINE |
| 185396 | 186513 | - | 9.9 | 4143 | 5269 | -877 | L1PB1 | L1 | LINE |
| 186512 | 187028 | + | 8.2 | 5254 | 5780 | -371 | L1PB1 | L1 | LINE |
| 187058 | 187142 | + | 8.2 | 5781 | 5795 | -356 | L1PB1 | L1 | LINE |
| 187238 | 187592 | + | 8.2 | 5796 | 6151 | 0 | L1PB1 | L1 | LINE |
| 187601 | 187906 | + | 13.7 | 3 | 308 | -4 | AluSx | Alu | SINE |
| 187909 | 188022 | + | 19.1 | 4250 | 4376 | -1764 | L1MB3 | L1 | LINE |
| 188023 | 188324 | + | 7.0 | 1 | 303 | -8 | AluY | Alu | SINE |
| 188325 | 188499 | + | 19.1 | 4377 | 4548 | -1592 | L1MB3 | L1 | LINE |
| 188500 | 188797 | + | 10.1 | 1 | 298 | -13 | AluY | Alu | SINE |
| 188848 | 190539 | + | 19.2 | 4549 | 6166 | -17 | L1MB3 | L1 | LINE |

  

|  |  |  |  |  |  |  |  |  |  |
| --- | --- | --- | --- | --- | --- | --- | --- | --- | --- |
| chr5:2285969-2291596 | | | 5638 bp | | |  |  |  |  |
| Genome Start | Genome End | Strand | Div | Repeat Start | Repeat End | Repeat Left | Name | Family | Class |
| 2285969 | 2287037 | + | 15.4 | 4688 | 5780 | -523 | L1MA4A | L1 | LINE |
| 2287036 | 2288532 | - | 9.9 | 2330 | 3832 | -2314 | L1PREC2 | L1 | LINE |
| 2288528 | 2290855 | + | 11.6 | 3818 | 6159 | -2 | L1PA14 | L1 | LINE |
| 2290853 | 2291122 | + | 16.7 | 5769 | 6061 | -242 | L1MA4A | L1 | LINE |
| 2291123 | 2291424 | - | 10.9 | 1 | 304 | -5 | AluSc | Alu | SINE |
| 2291425 | 2291596 | + | 16.7 | 6062 | 6232 | -71 | L1MA4A | L1 | LINE |

  

|  |  |  |  |  |  |  |  |  |  |
| --- | --- | --- | --- | --- | --- | --- | --- | --- | --- |
| chr5:2458513-2460870 | | | 2309 bp | | |  |  |  |  |
| Genome Start | Genome End | Strand | Div | Repeat Start | Repeat End | Repeat Left | Name | Family | Class |
| 2458513 | 2458840 | + | 26.6 | 2 | 383 | -6125 | L1MCa | L1 | LINE |
| 2458841 | 2459187 | - | 13.3 | 1 | 426 | -2 | MSTA | MaLR | LTR |
| 2459188 | 2459225 | + | 26.6 | 384 | 415 | -6093 | L1MCa | L1 | LINE |
| 2459226 | 2459557 | + | 16.3 | 265 | 605 | 0 | MER6A | MER2\_type | DNA |
| 2459558 | 2459679 | - | 15.2 | 5255 | 5375 | -765 | L1M3 | L1 | LINE |
| 2459680 | 2459976 | - | 15.2 | 1 | 300 | -12 | AluSx | Alu | SINE |
| 2459977 | 2460076 | - | 15.2 | 5165 | 5254 | -886 | L1M3 | L1 | LINE |
| 2460074 | 2460161 | + | 17.2 | 5072 | 5158 | -982 | L1M3 | L1 | LINE |
| 2460187 | 2460258 | + | 25.0 | 2051 | 2131 | -4377 | L1MCa | L1 | LINE |
| 2460259 | 2460419 | - | 14.0 | 134 | 295 | -17 | AluSx | Alu | SINE |
| 2460447 | 2460562 | - | 14.0 | 4 | 133 | -179 | AluSx | Alu | SINE |
| 2460563 | 2460870 | + | 24.8 | 2132 | 2459 | -3862 | L1MCa | L1 | LINE |

  

|  |  |  |  |  |  |  |  |  |  |
| --- | --- | --- | --- | --- | --- | --- | --- | --- | --- |
| chr5:3458396-3463023 | | | 4629 bp | | |  |  |  |  |
| Genome Start | Genome End | Strand | Div | Repeat Start | Repeat End | Repeat Left | Name | Family | Class |
| 3458396 | 3460387 | - | 10.2 | 3915 | 5947 | -213 | L1PREC2 | L1 | LINE |
| 3460396 | 3461226 | - | 2.8 | 5326 | 6155 | 0 | L1PA3 | L1 | LINE |
| 3461224 | 3461997 | + | 3.2 | 4559 | 5332 | -814 | L1PA3 | L1 | LINE |
| 3461992 | 3462184 | - | 9.6 | 3742 | 3925 | -2221 | L1PREC2 | L1 | LINE |
| 3462185 | 3462483 | - | 10.0 | 1 | 301 | -9 | AluSg | Alu | SINE |
| 3462484 | 3463023 | - | 9.6 | 3192 | 3741 | -2405 | L1PREC2 | L1 | LINE |

  

|  |  |  |  |  |  |  |  |  |  |
| --- | --- | --- | --- | --- | --- | --- | --- | --- | --- |
| chr5:7108856-7116445 | | | 7580 bp | | |  |  |  |  |
| Genome Start | Genome End | Strand | Div | Repeat Start | Repeat End | Repeat Left | Name | Family | Class |
| 7108856 | 7109152 | - | 10.7 | 7282 | 7579 | -134 | HERVH | ERV1 | LTR |
| 7109178 | 7109302 | - | 10.7 | 7158 | 7281 | -432 | HERVH | ERV1 | LTR |
| 7109273 | 7109353 | - | 23.5 | 5959 | 6043 | -1670 | HERVH | ERV1 | LTR |
| 7109359 | 7109921 | - | 2.1 | 4834 | 5396 | -750 | L1PA2 | L1 | LINE |
| 7109912 | 7110690 | + | 1.4 | 5377 | 6155 | 0 | L1PA2 | L1 | LINE |
| 7110711 | 7116445 | - | 12.0 | 1 | 5968 | -1745 | HERVH | ERV1 | LTR |

  

|  |  |  |  |  |  |  |  |  |  |
| --- | --- | --- | --- | --- | --- | --- | --- | --- | --- |
| chr5:7159641-7161211 | | | 1569 bp | | |  |  |  |  |
| Genome Start | Genome End | Strand | Div | Repeat Start | Repeat End | Repeat Left | Name | Family | Class |
| 7159641 | 7159978 | + | 9.2 | 4773 | 5110 | -1036 | L1P3 | L1 | LINE |
| 7159981 | 7160397 | - | 2.6 | 5285 | 5702 | -453 | L1PA4 | L1 | LINE |
| 7160398 | 7160835 | + | 3.2 | 5719 | 6154 | -1 | L1PA4 | L1 | LINE |
| 7160836 | 7161211 | + | 6.3 | 5105 | 5483 | -689 | L1P3 | L1 | LINE |

  

|  |  |  |  |  |  |  |  |  |  |
| --- | --- | --- | --- | --- | --- | --- | --- | --- | --- |
| chr5:7255329-7259516 | | | 4099 bp | | |  |  |  |  |
| Genome Start | Genome End | Strand | Div | Repeat Start | Repeat End | Repeat Left | Name | Family | Class |
| 7255329 | 7256035 | - | 31.4 | 1924 | 2755 | -664 | L2 | L2 | LINE |
| 7256050 | 7256131 | + | 23.3 | 1 | 73 | -87 | MER5A1 | MER1\_type | DNA |
| 7256182 | 7256319 | - | 16.7 | 21 | 158 | -27 | FAM | Alu | SINE |
| 7256328 | 7256391 | + | 29.7 | 86 | 149 | -11 | MER5A1 | MER1\_type | DNA |
| 7256405 | 7256563 | - | 22.9 | 1741 | 1896 | -1523 | L2 | L2 | LINE |
| 7256566 | 7256705 | - | 16.4 | 5650 | 5819 | -347 | L1PA16 | L1 | LINE |
| 7256705 | 7257015 | + | 15.4 | 5820 | 6166 | 0 | L1PA16 | L1 | LINE |
| 7257016 | 7257671 | - | 34.1 | 1013 | 1748 | -1671 | L2 | L2 | LINE |
| 7257672 | 7257860 | + | 17.5 | 1 | 191 | -2 | MER53 | DNA | DNA |
| 7257861 | 7257952 | - | 34.1 | 925 | 1012 | -2407 | L2 | L2 | LINE |
| 7257953 | 7258498 | - | 18.4 | 2 | 554 | 0 | MLT2A2 | ERVL | LTR |
| 7258502 | 7259025 | - | 31.2 | 421 | 927 | -2492 | L2 | L2 | LINE |
| 7259026 | 7259335 | + | 14.3 | 1 | 308 | -4 | AluSx | Alu | SINE |
| 7259336 | 7259516 | - | 31.2 | 223 | 420 | -2999 | L2 | L2 | LINE |

  

|  |  |  |  |  |  |  |  |  |  |
| --- | --- | --- | --- | --- | --- | --- | --- | --- | --- |
| chr5:8802961-8805537 | | | 2542 bp | | |  |  |  |  |
| Genome Start | Genome End | Strand | Div | Repeat Start | Repeat End | Repeat Left | Name | Family | Class |
| 8802961 | 8803601 | + | 21.6 | 6813 | 7474 | -311 | L1MC3 | L1 | LINE |
| 8803599 | 8804103 | - | 14.6 | 4593 | 5096 | -1050 | L1PA15 | L1 | LINE |
| 8804109 | 8805160 | + | 14.8 | 5105 | 6161 | -4 | L1PA15 | L1 | LINE |
| 8805194 | 8805537 | + | 21.8 | 7461 | 7785 | 0 | L1MC3 | L1 | LINE |

  

|  |  |  |  |  |  |  |  |  |  |
| --- | --- | --- | --- | --- | --- | --- | --- | --- | --- |
| chr5:8982574-8986101 | | | 3540 bp | | |  |  |  |  |
| Genome Start | Genome End | Strand | Div | Repeat Start | Repeat End | Repeat Left | Name | Family | Class |
| 8982574 | 8984017 | - | 9.6 | 4696 | 6168 | 0 | L1PA10 | L1 | LINE |
| 8984008 | 8984374 | - | 17.9 | 7379 | 7770 | -15 | L1MC3 | L1 | LINE |
| 8984375 | 8984539 | - | 6.1 | 5990 | 6154 | 0 | L1PA5 | L1 | LINE |
| 8984540 | 8985581 | + | 4.1 | 4919 | 5967 | -187 | L1PA5 | L1 | LINE |
| 8985580 | 8986101 | - | 8.5 | 4187 | 4719 | -1427 | L1PA10 | L1 | LINE |

  

|  |  |  |  |  |  |  |  |  |  |
| --- | --- | --- | --- | --- | --- | --- | --- | --- | --- |
| chr5:12334806-12336818 | | | 2010 bp | | |  |  |  |  |
| Genome Start | Genome End | Strand | Div | Repeat Start | Repeat End | Repeat Left | Name | Family | Class |
| 12334806 | 12335696 | - | 21.6 | 1780 | 2809 | -3512 | L1MCa | L1 | LINE |
| 12335700 | 12336299 | - | 19.4 | 5742 | 6332 | -2 | L1MA10 | L1 | LINE |
| 12336300 | 12336682 | + | 20.9 | 5350 | 5729 | -439 | L1MA10 | L1 | LINE |
| 12336683 | 12336818 | - | 21.3 | 1656 | 1791 | -4717 | L1MCa | L1 | LINE |

  

|  |  |  |  |  |  |  |  |  |  |
| --- | --- | --- | --- | --- | --- | --- | --- | --- | --- |
| chr5:12904010-12905129 | | | 1037 bp | | |  |  |  |  |
| Genome Start | Genome End | Strand | Div | Repeat Start | Repeat End | Repeat Left | Name | Family | Class |
| 12904010 | 12904109 | + | 32.5 | 1 | 109 | -348 | LTR16A1 | ERVL | LTR |
| 12904110 | 12904456 | + | 12.3 | 3 | 364 | 0 | THE1B | MaLR | LTR |
| 12904457 | 12904477 | + | 32.5 | 110 | 123 | -334 | LTR16A1 | ERVL | LTR |
| 12904499 | 12904770 | - | 17.6 | 5960 | 6217 | -74 | L1MA8 | L1 | LINE |
| 12904771 | 12904831 | + | 14.8 | 6226 | 6288 | -3 | L1MA8 | L1 | LINE |
| 12904894 | 12905129 | + | 27.7 | 170 | 431 | -26 | LTR16A1 | ERVL | LTR |

  

|  |  |  |  |  |  |  |  |  |  |
| --- | --- | --- | --- | --- | --- | --- | --- | --- | --- |
| chr5:15150939-15154664 | | | 3731 bp | | |  |  |  |  |
| Genome Start | Genome End | Strand | Div | Repeat Start | Repeat End | Repeat Left | Name | Family | Class |
| 15150939 | 15151037 | - | 25.2 | 148 | 246 | -16 | MIR | MIR | SINE |
| 15151048 | 15152391 | - | 5.3 | 4804 | 6143 | -11 | L1PA6 | L1 | LINE |
| 15152377 | 15154559 | + | 6.1 | 2616 | 4802 | -1344 | L1PA6 | L1 | LINE |
| 15154560 | 15154664 | - | 29.1 | 50 | 159 | -103 | MIR | MIR | SINE |

  

|  |  |  |  |  |  |  |  |  |  |
| --- | --- | --- | --- | --- | --- | --- | --- | --- | --- |
| chr5:15365777-15373399 | | | 7463 bp | | |  |  |  |  |
| Genome Start | Genome End | Strand | Div | Repeat Start | Repeat End | Repeat Left | Name | Family | Class |
| 15365777 | 15367020 | + | 33.2 | 345 | 1627 | -4040 | ERVL-E | ERVL | LTR |
| 15367114 | 15368072 | - | 9.0 | 5174 | 6150 | -1 | L1PB1 | L1 | LINE |
| 15368072 | 15368113 | + | 9.0 | 3233 | 3274 | -2872 | L1PB | L1 | LINE |
| 15368114 | 15368420 | + | 3.9 | 1 | 308 | -3 | AluY | Alu | SINE |
| 15368421 | 15370381 | + | 9.0 | 3275 | 5161 | -985 | L1PB | L1 | LINE |
| 15370385 | 15372406 | + | 31.3 | 1769 | 3860 | -1807 | ERVL-E | ERVL | LTR |
| 15372472 | 15373399 | + | 28.9 | 3984 | 5029 | -638 | ERVL-E | ERVL | LTR |

  

|  |  |  |  |  |  |  |  |  |  |
| --- | --- | --- | --- | --- | --- | --- | --- | --- | --- |
| chr5:15431941-15433231 | | | 1115 bp | | |  |  |  |  |
| Genome Start | Genome End | Strand | Div | Repeat Start | Repeat End | Repeat Left | Name | Family | Class |
| 15431941 | 15432384 | + | 17.3 | 4866 | 5316 | -840 | L1M2 | L1 | LINE |
| 15432378 | 15432407 | - | 0.0 | 5868 | 5897 | -254 | L1PB1 | L1 | LINE |
| 15432408 | 15432641 | + | 7.3 | 5911 | 6151 | 0 | L1PB1 | L1 | LINE |
| 15432645 | 15432772 | + | 24.3 | 5172 | 5301 | -845 | L1M4 | L1 | LINE |
| 15432953 | 15433231 | + | 16.0 | 5302 | 5573 | -718 | L1M2 | L1 | LINE |

  

|  |  |  |  |  |  |  |  |  |  |
| --- | --- | --- | --- | --- | --- | --- | --- | --- | --- |
| chr5:15613719-15616263 | | | 2409 bp | | |  |  |  |  |
| Genome Start | Genome End | Strand | Div | Repeat Start | Repeat End | Repeat Left | Name | Family | Class |
| 15613719 | 15614010 | - | 37.2 | 5894 | 6186 | -54 | L1ME3B | L1 | LINE |
| 15614030 | 15614909 | - | 17.2 | 5410 | 6270 | -42 | L1MA9 | L1 | LINE |
| 15614908 | 15615627 | + | 17.7 | 4652 | 5397 | -743 | L1MA9 | L1 | LINE |
| 15615630 | 15615728 | - | 26.5 | 5808 | 5905 | -335 | L1ME3B | L1 | LINE |
| 15615846 | 15616117 | - | 30.4 | 3153 | 3417 | -2 | L2 | L2 | LINE |
| 15616118 | 15616263 | - | 18.3 | 5628 | 5787 | -453 | L1ME3B | L1 | LINE |

  

|  |  |  |  |  |  |  |  |  |  |
| --- | --- | --- | --- | --- | --- | --- | --- | --- | --- |
| chr5:18136752-18139719 | | | 2900 bp | | |  |  |  |  |
| Genome Start | Genome End | Strand | Div | Repeat Start | Repeat End | Repeat Left | Name | Family | Class |
| 18136752 | 18136805 | - | 31.5 | 3097 | 3150 | -269 | L2 | L2 | LINE |
| 18136863 | 18137493 | - | 34.8 | 2325 | 2995 | -424 | L2 | L2 | LINE |
| 18137494 | 18137818 | - | 18.9 | 5760 | 6079 | -221 | L1MA6 | L1 | LINE |
| 18137819 | 18138042 | + | 14.9 | 6074 | 6300 | 0 | L1MA6 | L1 | LINE |
| 18138054 | 18139719 | - | 35.7 | 438 | 2330 | -1089 | L2 | L2 | LINE |

  

|  |  |  |  |  |  |  |  |  |  |
| --- | --- | --- | --- | --- | --- | --- | --- | --- | --- |
| chr5:18543288-18547742 | | | 4436 bp | | |  |  |  |  |
| Genome Start | Genome End | Strand | Div | Repeat Start | Repeat End | Repeat Left | Name | Family | Class |
| 18543288 | 18544823 | + | 14.1 | 2252 | 3825 | -2848 | L1PA15 | L1 | LINE |
| 18544824 | 18545424 | + | 12.7 | 5699 | 6298 | -4 | L1MA1 | L1 | LINE |
| 18545425 | 18546130 | + | 14.6 | 3826 | 4486 | -2187 | L1PA15 | L1 | LINE |
| 18546126 | 18546160 | - | 11.4 | 5648 | 5682 | -492 | L1PA10 | L1 | LINE |
| 18546161 | 18546643 | + | 9.3 | 5681 | 6164 | -4 | L1PA10 | L1 | LINE |
| 18546668 | 18547742 | + | 13.6 | 5603 | 6688 | -4 | L1PA15 | L1 | LINE |

  

|  |  |  |  |  |  |  |  |  |  |
| --- | --- | --- | --- | --- | --- | --- | --- | --- | --- |
| chr5:18922968-18928863 | | | 5841 bp | | |  |  |  |  |
| Genome Start | Genome End | Strand | Div | Repeat Start | Repeat End | Repeat Left | Name | Family | Class |
| 18922968 | 18923483 | - | 27.3 | 4822 | 5326 | -893 | L1ME1 | L1 | LINE |
| 18923516 | 18923883 | - | 17.8 | 1 | 375 | 0 | THE1C | MaLR | LTR |
| 18923884 | 18925436 | - | 30.4 | 2 | 1735 | 0 | MLT-int | MaLR | LTR |
| 18925439 | 18925656 | - | 14.8 | 156 | 381 | 0 | THE1D | MaLR | LTR |
| 18925659 | 18926299 | - | 10.9 | 3550 | 4197 | -1949 | L1PA8A | L1 | LINE |
| 18926299 | 18928188 | + | 11.6 | 4197 | 6119 | -12 | L1PA8A | L1 | LINE |
| 18928189 | 18928371 | - | 12.5 | 1 | 208 | -147 | THE1D | MaLR | LTR |
| 18928372 | 18928586 | - | 27.1 | 4546 | 4789 | -1430 | L1ME1 | L1 | LINE |
| 18928607 | 18928863 | - | 28.6 | 35 | 301 | -6099 | L1ME1 | L1 | LINE |

  

|  |  |  |  |  |  |  |  |  |  |
| --- | --- | --- | --- | --- | --- | --- | --- | --- | --- |
| chr5:19219956-19226049 | | | 6065 bp | | |  |  |  |  |
| Genome Start | Genome End | Strand | Div | Repeat Start | Repeat End | Repeat Left | Name | Family | Class |
| 19219956 | 19222931 | + | 24.1 | 6 | 2928 | -3645 | L1PA17 | L1 | LINE |
| 19222925 | 19223111 | - | 10.2 | 5043 | 5232 | -914 | L1PA7 | L1 | LINE |
| 19223115 | 19224040 | + | 7.7 | 5223 | 6146 | -8 | L1PA7 | L1 | LINE |
| 19224074 | 19226049 | + | 18.7 | 2944 | 4937 | -1636 | L1PA17 | L1 | LINE |

  

|  |  |  |  |  |  |  |  |  |  |
| --- | --- | --- | --- | --- | --- | --- | --- | --- | --- |
| chr5:19415347-19417122 | | | 1739 bp | | |  |  |  |  |
| Genome Start | Genome End | Strand | Div | Repeat Start | Repeat End | Repeat Left | Name | Family | Class |
| 19415347 | 19415547 | + | 12.4 | 5580 | 5780 | -376 | L1PB4 | L1 | LINE |
| 19415543 | 19415748 | - | 7.8 | 5483 | 5686 | -477 | L1P4 | L1 | LINE |
| 19415749 | 19416064 | - | 11.0 | 1 | 301 | -9 | AluSg | Alu | SINE |
| 19416065 | 19416189 | - | 7.8 | 5365 | 5482 | -681 | L1P4 | L1 | LINE |
| 19416190 | 19416679 | + | 9.8 | 5676 | 6157 | -6 | L1PA13 | L1 | LINE |
| 19416689 | 19416725 | + | 20.9 | 5769 | 5793 | -363 | L1PB4 | L1 | LINE |
| 19416759 | 19417122 | + | 20.9 | 5794 | 6155 | -1 | L1PB4 | L1 | LINE |

  

|  |  |  |  |  |  |  |  |  |  |
| --- | --- | --- | --- | --- | --- | --- | --- | --- | --- |
| chr5:20071911-20076751 | | | 4511 bp | | |  |  |  |  |
| Genome Start | Genome End | Strand | Div | Repeat Start | Repeat End | Repeat Left | Name | Family | Class |
| 20071911 | 20072052 | + | 36.4 | 3335 | 3477 | -1012 | L3 | CR1 | LINE |
| 20072118 | 20072249 | + | 28.5 | 112 | 253 | -15 | MIRb | MIR | SINE |
| 20072304 | 20072417 | + | 19.1 | 3498 | 3625 | -864 | L3 | CR1 | LINE |
| 20072518 | 20073202 | - | 6.9 | 1945 | 2613 | -3533 | L1P3 | L1 | LINE |
| 20073203 | 20076078 | + | 9.8 | 2618 | 5504 | -650 | L1P3 | L1 | LINE |
| 20076079 | 20076163 | + | 11.8 | 6070 | 6154 | 0 | L1PA7 | L1 | LINE |
| 20076275 | 20076751 | + | 32.2 | 3973 | 4484 | -5 | L3 | CR1 | LINE |

  

|  |  |  |  |  |  |  |  |  |  |
| --- | --- | --- | --- | --- | --- | --- | --- | --- | --- |
| chr5:20170888-20176659 | | | 5613 bp | | |  |  |  |  |
| Genome Start | Genome End | Strand | Div | Repeat Start | Repeat End | Repeat Left | Name | Family | Class |
| 20170888 | 20171116 | - | 30.2 | 330 | 757 | -5994 | L1MC3 | L1 | LINE |
| 20171119 | 20173691 | - | 7.6 | 899 | 3489 | -2657 | L1PA5 | L1 | LINE |
| 20173689 | 20176342 | + | 6.7 | 3499 | 6151 | -3 | L1PA5 | L1 | LINE |
| 20176503 | 20176659 | - | 30.1 | 245 | 409 | -6342 | L1MC3 | L1 | LINE |

  

|  |  |  |  |  |  |  |  |  |  |
| --- | --- | --- | --- | --- | --- | --- | --- | --- | --- |
| chr5:22061162-22067948 | | | 6510 bp | | |  |  |  |  |
| Genome Start | Genome End | Strand | Div | Repeat Start | Repeat End | Repeat Left | Name | Family | Class |
| 22061162 | 22062038 | - | 16.2 | 1506 | 2392 | -26 | Tigger1 | MER2\_type | DNA |
| 22062200 | 22062455 | + | 27.9 | 314 | 619 | -198 | MER41G | ERV1 | LTR |
| 22062558 | 22062740 | + | 27.4 | 366 | 569 | -3 | LTR43B | ERV1 | LTR |
| 22062741 | 22062787 | - | 17.0 | 1465 | 1512 | -906 | Tigger1 | MER2\_type | DNA |
| 22062788 | 22063000 | - | 3.3 | 3832 | 4045 | -2101 | L1P1 | L1 | LINE |
| 22063001 | 22064030 | - | 3.1 | 2802 | 3831 | -2315 | L1P1 | L1 | LINE |
| 22064031 | 22066140 | + | 3.5 | 4044 | 6152 | -3 | L1PA3 | L1 | LINE |
| 22066155 | 22066918 | - | 13.6 | 723 | 1478 | -940 | Tigger1 | MER2\_type | DNA |
| 22066919 | 22067225 | + | 11.3 | 1 | 310 | 0 | AluSg | Alu | SINE |
| 22067226 | 22067948 | - | 13.6 | 5 | 722 | -1696 | Tigger1 | MER2\_type | DNA |

  

|  |  |  |  |  |  |  |  |  |  |
| --- | --- | --- | --- | --- | --- | --- | --- | --- | --- |
| chr5:23768993-23772656 | | | 3087 bp | | |  |  |  |  |
| Genome Start | Genome End | Strand | Div | Repeat Start | Repeat End | Repeat Left | Name | Family | Class |
| 23768993 | 23769457 | - | 32.7 | 4188 | 4660 | -268 | L1ME3B | L1 | LINE |
| 23769845 | 23770148 | - | 30.9 | 1497 | 1832 | -3002 | L1ME3B | L1 | LINE |
| 23770160 | 23770657 | - | 6.2 | 5651 | 6152 | -2 | L1PA5 | L1 | LINE |
| 23770658 | 23771837 | + | 6.2 | 4464 | 5649 | -505 | L1PA5 | L1 | LINE |
| 23771978 | 23772220 | - | 16.1 | 1 | 243 | -70 | AluSq | Alu | SINE |
| 23772260 | 23772656 | - | 30.4 | 804 | 1221 | -3613 | L1ME3B | L1 | LINE |

  

|  |  |  |  |  |  |  |  |  |  |
| --- | --- | --- | --- | --- | --- | --- | --- | --- | --- |
| chr5:23887473-23895211 | | | 7764 bp | | |  |  |  |  |
| Genome Start | Genome End | Strand | Div | Repeat Start | Repeat End | Repeat Left | Name | Family | Class |
| 23887473 | 23888878 | - | 16.3 | 5466 | 6866 | -3 | L1PA16 | L1 | LINE |
| 23888865 | 23889222 | - | 17.2 | 4478 | 4826 | -2023 | L1PA16 | L1 | LINE |
| 23889218 | 23892762 | - | 9.9 | 2603 | 6152 | -2 | L1PA7 | L1 | LINE |
| 23892761 | 23893342 | + | 9.0 | 2062 | 2611 | -3535 | L1PA7 | L1 | LINE |
| 23893339 | 23894489 | - | 16.3 | 3058 | 4213 | -2636 | L1PA16 | L1 | LINE |
| 23894490 | 23894786 | + | 15.2 | 1 | 297 | -15 | AluSx | Alu | SINE |
| 23894787 | 23895211 | - | 16.8 | 2681 | 3057 | -3792 | L1PA16 | L1 | LINE |

  

|  |  |  |  |  |  |  |  |  |  |
| --- | --- | --- | --- | --- | --- | --- | --- | --- | --- |
| chr5:26024408-26026451 | | | 2013 bp | | |  |  |  |  |
| Genome Start | Genome End | Strand | Div | Repeat Start | Repeat End | Repeat Left | Name | Family | Class |
| 26024408 | 26024708 | + | 18.2 | 1 | 301 | -2117 | Tigger1 | MER2\_type | DNA |
| 26024710 | 26024743 | + | 17.9 | 1349 | 1374 | -1044 | Tigger1 | MER2\_type | DNA |
| 26024744 | 26025035 | - | 11.6 | 14 | 308 | -4 | AluSx | Alu | SINE |
| 26025036 | 26025534 | + | 17.9 | 1375 | 1870 | -548 | Tigger1 | MER2\_type | DNA |
| 26025534 | 26025753 | + | 17.4 | 2092 | 2313 | -105 | Tigger1 | MER2\_type | DNA |
| 26025785 | 26026222 | - | 7.5 | 5717 | 6154 | 0 | L1PA5 | L1 | LINE |
| 26026223 | 26026328 | + | 3.8 | 5590 | 5695 | -459 | L1PA5 | L1 | LINE |
| 26026329 | 26026451 | + | 20.0 | 2298 | 2418 | 0 | Tigger1 | MER2\_type | DNA |

  

|  |  |  |  |  |  |  |  |  |  |
| --- | --- | --- | --- | --- | --- | --- | --- | --- | --- |
| chr5:27199506-27202689 | | | 2526 bp | | |  |  |  |  |
| Genome Start | Genome End | Strand | Div | Repeat Start | Repeat End | Repeat Left | Name | Family | Class |
| 27199506 | 27200129 | + | 15.0 | 4 | 671 | -560 | Tigger3b | MER2\_type | DNA |
| 27200358 | 27200631 | + | 12.8 | 683 | 949 | -283 | Tigger3b | MER2\_type | DNA |
| 27200722 | 27200954 | - | 27.7 | 10 | 262 | 0 | MIR | MIR | SINE |
| 27201294 | 27202325 | - | 6.2 | 5124 | 6154 | 0 | L1PA7 | L1 | LINE |
| 27202327 | 27202406 | + | 7.5 | 5042 | 5121 | -1025 | L1PA7 | L1 | LINE |
| 27202407 | 27202689 | + | 16.2 | 945 | 1231 | 0 | Tigger3b | MER2\_type | DNA |

  

|  |  |  |  |  |  |  |  |  |  |
| --- | --- | --- | --- | --- | --- | --- | --- | --- | --- |
| chr5:27572955-27577707 | | | 4609 bp | | |  |  |  |  |
| Genome Start | Genome End | Strand | Div | Repeat Start | Repeat End | Repeat Left | Name | Family | Class |
| 27572955 | 27573986 | + | 25.2 | 1766 | 2896 | -3425 | L1MC3 | L1 | LINE |
| 27573986 | 27574085 | + | 24.5 | 4444 | 4548 | -1773 | L1MC3 | L1 | LINE |
| 27574089 | 27574469 | - | 14.9 | 5204 | 5574 | -592 | L1PA16 | L1 | LINE |
| 27574473 | 27575066 | + | 16.6 | 5581 | 6160 | -6 | L1PA16 | L1 | LINE |
| 27575081 | 27575508 | + | 24.1 | 4576 | 5014 | -1307 | L1MC3 | L1 | LINE |
| 27575533 | 27575877 | + | 21.6 | 5861 | 6196 | -1764 | L1MC3 | L1 | LINE |
| 27575902 | 27576028 | - | 27.1 | 2858 | 2989 | -3157 | L1M5 | L1 | LINE |
| 27576105 | 27577484 | + | 22.3 | 6222 | 7563 | -397 | L1MC3 | L1 | LINE |
| 27577486 | 27577707 | + | 23.2 | 7714 | 7929 | -31 | L1MC3 | L1 | LINE |

  

|  |  |  |  |  |  |  |  |  |  |
| --- | --- | --- | --- | --- | --- | --- | --- | --- | --- |
| chr5:28035549-28041755 | | | 6206 bp | | |  |  |  |  |
| Genome Start | Genome End | Strand | Div | Repeat Start | Repeat End | Repeat Left | Name | Family | Class |
| 28035549 | 28037120 | - | 15.1 | 2752 | 4327 | -1819 | L1P4 | L1 | LINE |
| 28037128 | 28038850 | - | 8.1 | 4428 | 6146 | -8 | L1PA7 | L1 | LINE |
| 28038848 | 28039763 | - | 9.8 | 3758 | 4670 | -1476 | L1P1 | L1 | LINE |
| 28039760 | 28041575 | + | 9.0 | 1930 | 3758 | -2388 | L1P2 | L1 | LINE |
| 28041577 | 28041755 | - | 16.2 | 2585 | 2775 | -3371 | L1P4 | L1 | LINE |

  

|  |  |  |  |  |  |  |  |  |  |
| --- | --- | --- | --- | --- | --- | --- | --- | --- | --- |
| chr5:28079880-28081028 | | | 1184 bp | | |  |  |  |  |
| Genome Start | Genome End | Strand | Div | Repeat Start | Repeat End | Repeat Left | Name | Family | Class |
| 28079880 | 28080247 | + | 16.2 | 5418 | 5784 | -519 | L1MA5 | L1 | LINE |
| 28080230 | 28080300 | - | 8.4 | 5851 | 5921 | -233 | L1PA5 | L1 | LINE |
| 28080297 | 28080530 | + | 5.6 | 5921 | 6154 | 0 | L1PA5 | L1 | LINE |
| 28080518 | 28081028 | + | 21.6 | 5762 | 6300 | 0 | L1MA5 | L1 | LINE |

  

|  |  |  |  |  |  |  |  |  |  |
| --- | --- | --- | --- | --- | --- | --- | --- | --- | --- |
| chr5:28351970-28355626 | | | 3335 bp | | |  |  |  |  |
| Genome Start | Genome End | Strand | Div | Repeat Start | Repeat End | Repeat Left | Name | Family | Class |
| 28351970 | 28352939 | - | 29.6 | 2368 | 3340 | -2327 | ERVL-B4 | ERVL | LTR |
| 28352976 | 28353441 | - | 6.1 | 5683 | 6143 | -11 | L1PA7 | L1 | LINE |
| 28353442 | 28353508 | + | 11.9 | 5631 | 5697 | -475 | L1PA7 | L1 | LINE |
| 28353539 | 28354925 | - | 30.0 | 856 | 2348 | -3319 | ERVL-B4 | ERVL | LTR |
| 28355198 | 28355298 | - | 32.7 | 714 | 814 | -4853 | ERVL-B4 | ERVL | LTR |
| 28355283 | 28355626 | - | 30.2 | 281 | 607 | -5060 | ERVL-B4 | ERVL | LTR |

  

|  |  |  |  |  |  |  |  |  |  |
| --- | --- | --- | --- | --- | --- | --- | --- | --- | --- |
| chr5:28537175-28546668 | | | 9396 bp | | |  |  |  |  |
| Genome Start | Genome End | Strand | Div | Repeat Start | Repeat End | Repeat Left | Name | Family | Class |
| 28537175 | 28537209 | - | 8.8 | 523 | 561 | 0 | MLT2A2 | ERVL | LTR |
| 28537283 | 28537673 | - | 8.8 | 1 | 414 | -39 | MLT2A2 | ERVL | LTR |
| 28537674 | 28537916 | - | 10.7 | 5412 | 5654 | 0 | HERVL-A2 | ERVL | LTR |
| 28537917 | 28539743 | - | 2.1 | 4329 | 6155 | 0 | L1PA2 | L1 | LINE |
| 28539741 | 28540818 | + | 2.3 | 3249 | 4326 | -1820 | L1PA2 | L1 | LINE |
| 28540819 | 28541021 | - | 10.5 | 5222 | 5422 | -232 | HERVL-A2 | ERVL | LTR |
| 28541050 | 28546243 | - | 10.5 | 1 | 5221 | -433 | HERVL-A2 | ERVL | LTR |
| 28546244 | 28546668 | - | 10.3 | 1 | 453 | 0 | MLT2A2 | ERVL | LTR |

  

|  |  |  |  |  |  |  |  |  |  |
| --- | --- | --- | --- | --- | --- | --- | --- | --- | --- |
| chr5:29018841-29021647 | | | 2796 bp | | |  |  |  |  |
| Genome Start | Genome End | Strand | Div | Repeat Start | Repeat End | Repeat Left | Name | Family | Class |
| 29018841 | 29018945 | - | 27.8 | 950 | 1056 | -5590 | L1M4c | L1 | LINE |
| 29018959 | 29020310 | - | 9.8 | 4768 | 6129 | -2 | L1PA8A | L1 | LINE |
| 29020312 | 29021455 | + | 10.3 | 3624 | 4790 | -1356 | L1PA8A | L1 | LINE |
| 29021453 | 29021647 | - | 28.3 | 764 | 949 | -5593 | L1M4c | L1 | LINE |

  

|  |  |  |  |  |  |  |  |  |  |
| --- | --- | --- | --- | --- | --- | --- | --- | --- | --- |
| chr5:30089264-30093979 | | | 4393 bp | | |  |  |  |  |
| Genome Start | Genome End | Strand | Div | Repeat Start | Repeat End | Repeat Left | Name | Family | Class |
| 30089264 | 30089882 | - | 29.4 | 4330 | 4983 | -1163 | L1M5 | L1 | LINE |
| 30089883 | 30090175 | + | 10.9 | 1 | 294 | -18 | AluSx | Alu | SINE |
| 30090176 | 30090636 | - | 29.6 | 3805 | 4329 | -1817 | L1M5 | L1 | LINE |
| 30090843 | 30091052 | - | 27.7 | 2826 | 3050 | -3096 | L1MC | L1 | LINE |
| 30091093 | 30091543 | - | 14.5 | 5427 | 5889 | -416 | L1MA3 | L1 | LINE |
| 30091543 | 30091922 | + | 12.2 | 5890 | 6260 | -45 | L1MA3 | L1 | LINE |
| 30091923 | 30092295 | + | 11.6 | 4986 | 5365 | -766 | L1PA8A | L1 | LINE |
| 30092296 | 30092583 | + | 8.2 | 1 | 310 | -1 | AluY | Alu | SINE |
| 30092584 | 30093351 | + | 9.4 | 5366 | 6128 | -3 | L1PA8A | L1 | LINE |
| 30093375 | 30093658 | - | 20.1 | 3 | 300 | -2 | AluJo | Alu | SINE |
| 30093666 | 30093711 | + | 8.7 | 6259 | 6304 | -1 | L1MA3 | L1 | LINE |
| 30093760 | 30093979 | - | 31.3 | 2585 | 2809 | -3337 | L1M5 | L1 | LINE |

  

|  |  |  |  |  |  |  |  |  |  |
| --- | --- | --- | --- | --- | --- | --- | --- | --- | --- |
| chr5:30095303-30097212 | | | 1862 bp | | |  |  |  |  |
| Genome Start | Genome End | Strand | Div | Repeat Start | Repeat End | Repeat Left | Name | Family | Class |
| 30095303 | 30095622 | - | 22.4 | 5659 | 5994 | -350 | L1MC2 | L1 | LINE |
| 30095625 | 30095801 | - | 11.4 | 5985 | 6160 | -3 | L1PA13 | L1 | LINE |
| 30095806 | 30096447 | - | 10.8 | 5158 | 5812 | -356 | L1PA13 | L1 | LINE |
| 30096453 | 30096511 | + | 13.4 | 4638 | 4696 | -1450 | L1PREC2 | L1 | LINE |
| 30096556 | 30097002 | + | 13.4 | 4697 | 5155 | -991 | L1PREC2 | L1 | LINE |
| 30096996 | 30097212 | - | 25.4 | 5450 | 5669 | -2116 | L1MC2 | L1 | LINE |

  

|  |  |  |  |  |  |  |  |  |  |
| --- | --- | --- | --- | --- | --- | --- | --- | --- | --- |
| chr5:30105020-30114119 | | | 9016 bp | | |  |  |  |  |
| Genome Start | Genome End | Strand | Div | Repeat Start | Repeat End | Repeat Left | Name | Family | Class |
| 30105020 | 30106290 | + | 20.0 | 125 | 1408 | -5377 | L1PA13 | L1 | LINE |
| 30106389 | 30106584 | - | 11.2 | 5925 | 6121 | -33 | L1PB3 | L1 | LINE |
| 30106583 | 30107481 | - | 3.2 | 5254 | 6151 | -3 | L1PA5 | L1 | LINE |
| 30107489 | 30108094 | - | 5.3 | 2863 | 3465 | -2681 | L1P1 | L1 | LINE |
| 30108080 | 30108352 | + | 8.1 | 2593 | 2865 | -3281 | L1P1 | L1 | LINE |
| 30108349 | 30112060 | + | 13.2 | 1393 | 5151 | -1615 | L1PA13 | L1 | LINE |
| 30112061 | 30112432 | + | 16.1 | 1 | 381 | 0 | THE1D | MaLR | LTR |
| 30112433 | 30114119 | + | 13.2 | 5152 | 6783 | 0 | L1PA13 | L1 | LINE |

  

|  |  |  |  |  |  |  |  |  |  |
| --- | --- | --- | --- | --- | --- | --- | --- | --- | --- |
| chr5:30499993-30504499 | | | 4392 bp | | |  |  |  |  |
| Genome Start | Genome End | Strand | Div | Repeat Start | Repeat End | Repeat Left | Name | Family | Class |
| 30499993 | 30500429 | + | 37.5 | 1356 | 1798 | -5698 | L1M3c | L1 | LINE |
| 30500423 | 30502908 | + | 24.4 | 2018 | 4463 | -3096 | L1M3c | L1 | LINE |
| 30502906 | 30503093 | - | 17.6 | 5701 | 5915 | -253 | L1MA10 | L1 | LINE |
| 30503094 | 30503532 | + | 16.4 | 5898 | 6321 | -13 | L1MA10 | L1 | LINE |
| 30503654 | 30504210 | + | 27.3 | 27 | 626 | 0 | MLT1E2 | MaLR | LTR |
| 30504215 | 30504499 | + | 26.1 | 5236 | 5528 | -2037 | L1M3c | L1 | LINE |

  

|  |  |  |  |  |  |  |  |  |  |
| --- | --- | --- | --- | --- | --- | --- | --- | --- | --- |
| chr5:30759265-30767631 | | | 8195 bp | | |  |  |  |  |
| Genome Start | Genome End | Strand | Div | Repeat Start | Repeat End | Repeat Left | Name | Family | Class |
| 30759265 | 30761842 | - | 21.3 | 5737 | 8202 | -1 | L1MC3 | L1 | LINE |
| 30761839 | 30762596 | - | 18.6 | 5379 | 6150 | -16 | L1PA17 | L1 | LINE |
| 30762589 | 30763072 | + | 15.5 | 4688 | 5102 | -1044 | L1P4 | L1 | LINE |
| 30763073 | 30763462 | + | 16.4 | 1 | 425 | -1 | MSTB | MaLR | LTR |
| 30763463 | 30763730 | + | 15.5 | 5103 | 5373 | -773 | L1P4 | L1 | LINE |
| 30763733 | 30763893 | - | 19.1 | 5152 | 5305 | -841 | L1MD | L1 | LINE |
| 30763926 | 30764238 | + | 15.7 | 1 | 220 | -16 | MER46B | MER2\_type | DNA |
| 30764320 | 30765079 | - | 24.2 | 4714 | 5519 | -1045 | L1MC3 | L1 | LINE |
| 30765080 | 30765641 | - | 19.7 | 3236 | 3796 | -2768 | L1MC3 | L1 | LINE |
| 30765681 | 30765882 | - | 23.8 | 1862 | 2096 | -4655 | L1MC3 | L1 | LINE |
| 30765913 | 30767631 | - | 24.8 | 1 | 1861 | -4890 | L1MC3 | L1 | LINE |

  

|  |  |  |  |  |  |  |  |  |  |
| --- | --- | --- | --- | --- | --- | --- | --- | --- | --- |
| chr5:36054315-36061404 | | | 7102 bp | | |  |  |  |  |
| Genome Start | Genome End | Strand | Div | Repeat Start | Repeat End | Repeat Left | Name | Family | Class |
| 36054315 | 36054646 | - | 20.0 | 6999 | 7327 | -715 | L1MC4 | L1 | LINE |
| 36054636 | 36055289 | - | 9.5 | 5988 | 6617 | 0 | L1PB1 | L1 | LINE |
| 36055290 | 36055575 | + | 9.1 | 1 | 294 | -15 | AluSc | Alu | SINE |
| 36055576 | 36055618 | - | 9.5 | 5961 | 5987 | -630 | L1PB1 | L1 | LINE |
| 36055619 | 36055950 | - | 13.5 | 1 | 333 | 0 | AluSx | Alu | SINE |
| 36055951 | 36058126 | - | 11.3 | 3760 | 5960 | -657 | L1PB1 | L1 | LINE |
| 36058127 | 36058540 | - | 10.6 | 1 | 608 | 0 | LTR14B | ERVK | LTR |
| 36058544 | 36059841 | - | 8.4 | 2384 | 3765 | -2847 | L1PB1 | L1 | LINE |
| 36059841 | 36061247 | + | 10.7 | 942 | 2382 | -4231 | L1PB1 | L1 | LINE |
| 36061245 | 36061404 | - | 24.1 | 6871 | 7016 | -1026 | L1MC4 | L1 | LINE |

  

|  |  |  |  |  |  |  |  |  |  |
| --- | --- | --- | --- | --- | --- | --- | --- | --- | --- |
| chr5:36349259-36361318 | | | 11589 bp | | |  |  |  |  |
| Genome Start | Genome End | Strand | Div | Repeat Start | Repeat End | Repeat Left | Name | Family | Class |
| 36349259 | 36351210 | - | 27.8 | 1301 | 3303 | -3234 | L1MEb | L1 | LINE |
| 36351335 | 36354064 | - | 9.6 | 4979 | 7721 | -2 | L1MA2 | L1 | LINE |
| 36354065 | 36354469 | + | 12.9 | 1 | 417 | -97 | MER75 | PiggyBac | DNA |
| 36354470 | 36354970 | + | 9.8 | 2460 | 2943 | -4622 | L1M1 | L1 | LINE |
| 36354971 | 36355084 | + | 12.5 | 393 | 504 | 0 | MER75 | PiggyBac | DNA |
| 36355085 | 36356860 | - | 9.7 | 3174 | 4982 | -2583 | L1MA2 | L1 | LINE |
| 36356859 | 36357270 | - | 0.2 | 4979 | 5390 | -756 | L1PA2 | L1 | LINE |
| 36357269 | 36358040 | + | 1.7 | 5384 | 6155 | 0 | L1PA2 | L1 | LINE |
| 36358058 | 36360830 | - | 16.9 | 15 | 3189 | -4376 | L1MA2 | L1 | LINE |
| 36361165 | 36361318 | - | 35.1 | 469 | 626 | -5901 | L1MEb | L1 | LINE |

  

|  |  |  |  |  |  |  |  |  |  |
| --- | --- | --- | --- | --- | --- | --- | --- | --- | --- |
| chr5:36590284-36596396 | | | 6051 bp | | |  |  |  |  |
| Genome Start | Genome End | Strand | Div | Repeat Start | Repeat End | Repeat Left | Name | Family | Class |
| 36590284 | 36590758 | - | 16.8 | 5693 | 6172 | -6 | L1MB8 | L1 | LINE |
| 36590759 | 36592088 | - | 11.7 | 4817 | 6158 | -7 | L1PA15 | L1 | LINE |
| 36592087 | 36593213 | + | 10.9 | 3748 | 4851 | -1295 | L1PREC2 | L1 | LINE |
| 36593248 | 36593552 | - | 11.2 | 1 | 311 | -2 | AluSq | Alu | SINE |
| 36593553 | 36594366 | - | 16.9 | 4848 | 5668 | -510 | L1MB3 | L1 | LINE |
| 36594373 | 36594891 | + | 20.0 | 5689 | 6180 | -3 | L1MB3 | L1 | LINE |
| 36594890 | 36595188 | - | 19.7 | 4553 | 4859 | -1287 | L1MB8 | L1 | LINE |
| 36595189 | 36595580 | + | 26.5 | 1 | 390 | -7 | MLT2C1 | ERVL | LTR |
| 36595607 | 36595849 | - | 17.1 | 4304 | 4551 | -1595 | L1MB8 | L1 | LINE |
| 36595850 | 36596318 | - | 3.6 | 5709 | 6155 | 0 | L1PA4 | L1 | LINE |
| 36596319 | 36596396 | - | 17.1 | 4233 | 4303 | -1843 | L1MB8 | L1 | LINE |

  

|  |  |  |  |  |  |  |  |  |  |
| --- | --- | --- | --- | --- | --- | --- | --- | --- | --- |
| chr5:37481001-37484354 | | | 3365 bp | | |  |  |  |  |
| Genome Start | Genome End | Strand | Div | Repeat Start | Repeat End | Repeat Left | Name | Family | Class |
| 37481001 | 37481331 | - | 31.6 | 7099 | 7430 | -612 | L1MC4 | L1 | LINE |
| 37481326 | 37481388 | - | 9.5 | 3670 | 3732 | -2414 | L1PA5 | L1 | LINE |
| 37481385 | 37483808 | + | 5.3 | 3726 | 6154 | 0 | L1PA5 | L1 | LINE |
| 37483808 | 37484354 | - | 23.3 | 6625 | 7118 | -843 | L1MC4 | L1 | LINE |

  

|  |  |  |  |  |  |  |  |  |  |
| --- | --- | --- | --- | --- | --- | --- | --- | --- | --- |
| chr5:39789125-39790897 | | | 1326 bp | | |  |  |  |  |
| Genome Start | Genome End | Strand | Div | Repeat Start | Repeat End | Repeat Left | Name | Family | Class |
| 39789125 | 39789418 | - | 25.9 | 3030 | 3358 | -61 | L2 | L2 | LINE |
| 39789424 | 39789745 | - | 5.3 | 5384 | 5705 | -449 | L1PA7 | L1 | LINE |
| 39789746 | 39790183 | + | 7.1 | 5698 | 6135 | -19 | L1PA7 | L1 | LINE |
| 39790412 | 39790565 | - | 22.1 | 1501 | 1682 | -1 | FordPrefect | Tip100 | DNA |
| 39790780 | 39790830 | - | 17.6 | 2699 | 2750 | -669 | L2 | L2 | LINE |
| 39790831 | 39790897 | - | 20.9 | 2024 | 2090 | -1329 | L2 | L2 | LINE |

  

|  |  |  |  |  |  |  |  |  |  |
| --- | --- | --- | --- | --- | --- | --- | --- | --- | --- |
| chr5:40529165-40530720 | | | 1467 bp | | |  |  |  |  |
| Genome Start | Genome End | Strand | Div | Repeat Start | Repeat End | Repeat Left | Name | Family | Class |
| 40529165 | 40529322 | + | 13.4 | 1 | 159 | -222 | THE1D | MaLR | LTR |
| 40529330 | 40529478 | - | 24.2 | 187 | 337 | -53 | MLT1B | MaLR | LTR |
| 40529520 | 40529781 | - | 9.5 | 5889 | 6150 | -1 | L1PB1 | L1 | LINE |
| 40529782 | 40529988 | + | 7.3 | 5577 | 5785 | -366 | L1PB1 | L1 | LINE |
| 40529989 | 40530223 | + | 8.1 | 5909 | 6152 | -16 | L1PA10 | L1 | LINE |
| 40530261 | 40530295 | + | 20.2 | 5754 | 5787 | -513 | L1M2 | L1 | LINE |
| 40530296 | 40530382 | - | 12.5 | 1 | 80 | 0 | MADE1 | Mariner | DNA |
| 40530383 | 40530506 | + | 7.0 | 5788 | 5896 | -255 | L1PB1 | L1 | LINE |
| 40530511 | 40530720 | + | 14.9 | 158 | 373 | -8 | THE1D | MaLR | LTR |

  

|  |  |  |  |  |  |  |  |  |  |
| --- | --- | --- | --- | --- | --- | --- | --- | --- | --- |
| chr5:44187916-44195148 | | | 7118 bp | | |  |  |  |  |
| Genome Start | Genome End | Strand | Div | Repeat Start | Repeat End | Repeat Left | Name | Family | Class |
| 44187916 | 44188060 | + | 15.0 | 2 | 153 | -5064 | MER61B-int | ERV1 | LTR |
| 44188145 | 44188777 | - | 0.8 | 4000 | 4632 | -1514 | L1HS | L1 | LINE |
| 44188770 | 44190314 | + | 0.4 | 4609 | 6155 | 0 | L1HS | L1 | LINE |
| 44190321 | 44191180 | + | 13.7 | 379 | 1213 | -4004 | MER61B-int | ERV1 | LTR |
| 44191176 | 44193586 | + | 10.8 | 1361 | 3853 | -1364 | MER61B-int | ERV1 | LTR |
| 44193589 | 44193824 | + | 9.5 | 4497 | 4735 | -482 | MER61B-int | ERV1 | LTR |
| 44193812 | 44194756 | + | 12.3 | 3857 | 4812 | -405 | MER61B-int | ERV1 | LTR |
| 44194806 | 44195148 | + | 13.1 | 4868 | 5216 | -1 | MER61B-int | ERV1 | LTR |

  

|  |  |  |  |  |  |  |  |  |  |
| --- | --- | --- | --- | --- | --- | --- | --- | --- | --- |
| chr5:44558616-44562265 | | | 3646 bp | | |  |  |  |  |
| Genome Start | Genome End | Strand | Div | Repeat Start | Repeat End | Repeat Left | Name | Family | Class |
| 44558616 | 44559062 | - | 29.3 | 1434 | 1925 | -5203 | L1MCa | L1 | LINE |
| 44559066 | 44560440 | - | 3.2 | 4777 | 6151 | -4 | L1PA3 | L1 | LINE |
| 44560440 | 44560928 | + | 3.1 | 4267 | 4754 | -1392 | L1PA3 | L1 | LINE |
| 44560931 | 44562265 | - | 28.0 | 17 | 1354 | -5069 | L1MCa | L1 | LINE |

  

|  |  |  |  |  |  |  |  |  |  |
| --- | --- | --- | --- | --- | --- | --- | --- | --- | --- |
| chr5:49598649-49601244 | | | 2535 bp | | |  |  |  |  |
| Genome Start | Genome End | Strand | Div | Repeat Start | Repeat End | Repeat Left | Name | Family | Class |
| 49598649 | 49599279 | + | 25.2 | 551 | 1194 | -4851 | L1MCb | L1 | LINE |
| 49599304 | 49599956 | - | 2.0 | 5118 | 5777 | -378 | L1PA2 | L1 | LINE |
| 49599957 | 49600335 | + | 2.9 | 5772 | 6152 | -3 | L1PA2 | L1 | LINE |
| 49600338 | 49600570 | + | 20.7 | 1297 | 1531 | -5220 | L1MCb | L1 | LINE |
| 49600580 | 49600720 | + | 23.0 | 20 | 154 | -22 | FRAM | Alu | SINE |
| 49600723 | 49600785 | + | 22.4 | 1566 | 1644 | -5107 | L1MCb | L1 | LINE |
| 49600810 | 49601244 | + | 22.4 | 1645 | 2138 | -4613 | L1MCb | L1 | LINE |

  

|  |  |  |  |  |  |  |  |  |  |
| --- | --- | --- | --- | --- | --- | --- | --- | --- | --- |
| chr5:53513239-53516055 | | | 2794 bp | | |  |  |  |  |
| Genome Start | Genome End | Strand | Div | Repeat Start | Repeat End | Repeat Left | Name | Family | Class |
| 53513239 | 53513367 | + | 20.2 | 1 | 129 | -173 | AluJo | Alu | SINE |
| 53513370 | 53515203 | - | 1.4 | 3686 | 5520 | -635 | L1PA2 | L1 | LINE |
| 53515204 | 53515846 | + | 1.4 | 5509 | 6152 | -3 | L1PA2 | L1 | LINE |
| 53515868 | 53516055 | + | 12.2 | 121 | 312 | 0 | AluJo | Alu | SINE |

  

|  |  |  |  |  |  |  |  |  |  |
| --- | --- | --- | --- | --- | --- | --- | --- | --- | --- |
| chr5:55806728-55807629 | | | 898 bp | | |  |  |  |  |
| Genome Start | Genome End | Strand | Div | Repeat Start | Repeat End | Repeat Left | Name | Family | Class |
| 55806728 | 55806870 | - | 18.9 | 384 | 539 | -3 | MER34 | ERV1 | LTR |
| 55806871 | 55807002 | - | 3.0 | 5725 | 5857 | -298 | L1PA4 | L1 | LINE |
| 55807003 | 55807277 | + | 5.8 | 5852 | 6155 | 0 | L1PA4 | L1 | LINE |
| 55807282 | 55807629 | - | 22.7 | 11 | 400 | -142 | MER34 | ERV1 | LTR |

  

|  |  |  |  |  |  |  |  |  |  |
| --- | --- | --- | --- | --- | --- | --- | --- | --- | --- |
| chr5:59267270-59268052 | | | 783 bp | | |  |  |  |  |
| Genome Start | Genome End | Strand | Div | Repeat Start | Repeat End | Repeat Left | Name | Family | Class |
| 59267270 | 59267342 | + | 23.3 | 1 | 73 | -87 | MER5A1 | MER1\_type | DNA |
| 59267343 | 59267660 | - | 3.5 | 5535 | 5852 | -303 | L1PA4 | L1 | LINE |
| 59267661 | 59267956 | + | 4.4 | 5858 | 6153 | -2 | L1PA4 | L1 | LINE |
| 59267957 | 59268052 | + | 21.9 | 59 | 160 | 0 | MER5A1 | MER1\_type | DNA |

  

|  |  |  |  |  |  |  |  |  |  |
| --- | --- | --- | --- | --- | --- | --- | --- | --- | --- |
| chr5:60437295-60448765 | | | 11204 bp | | |  |  |  |  |
| Genome Start | Genome End | Strand | Div | Repeat Start | Repeat End | Repeat Left | Name | Family | Class |
| 60437295 | 60437654 | + | 26.8 | 1 | 375 | -2132 | HAL1 | L1 | LINE |
| 60437654 | 60437977 | + | 27.7 | 7123 | 7466 | -576 | L1MC4 | L1 | LINE |
| 60437985 | 60438630 | - | 9.5 | 5649 | 6305 | 0 | L1MA3 | L1 | LINE |
| 60438676 | 60439230 | - | 9.7 | 5094 | 5648 | -657 | L1MA3 | L1 | LINE |
| 60439268 | 60440057 | - | 1.1 | 5382 | 6137 | -18 | L1PA3 | L1 | LINE |
| 60440054 | 60440092 | + | 0.0 | 5334 | 5372 | -783 | L1P | L1 | LINE |
| 60440091 | 60441150 | - | 12.1 | 4036 | 5111 | -1035 | L1MA3 | L1 | LINE |
| 60441151 | 60441398 | + | 16.0 | 4 | 260 | -115 | THE1C | MaLR | LTR |
| 60441399 | 60441513 | + | 10.5 | 268 | 381 | 0 | THE1D | MaLR | LTR |
| 60441514 | 60443009 | - | 12.0 | 3961 | 5456 | -2109 | L1M1 | L1 | LINE |
| 60443045 | 60443190 | - | 16.1 | 134 | 276 | -36 | AluJb | Alu | SINE |
| 60443191 | 60443501 | - | 9.3 | 1 | 311 | 0 | AluY | Alu | SINE |
| 60443502 | 60443640 | - | 16.1 | 1 | 133 | -179 | AluJb | Alu | SINE |
| 60443641 | 60443803 | - | 12.0 | 3810 | 3960 | -3605 | L1M1 | L1 | LINE |
| 60443804 | 60444095 | - | 11.0 | 1 | 292 | -17 | AluSc | Alu | SINE |
| 60444096 | 60445018 | - | 11.4 | 2867 | 3809 | -3756 | L1M1 | L1 | LINE |
| 60445019 | 60445389 | + | 10.0 | 1 | 375 | 0 | THE1C | MaLR | LTR |
| 60445390 | 60447017 | - | 12.6 | 1099 | 2866 | -4699 | L1M1 | L1 | LINE |
| 60447016 | 60447133 | - | 12.8 | 12 | 132 | -7433 | L1M1 | L1 | LINE |
| 60447130 | 60447420 | + | 25.1 | 7451 | 7775 | -267 | L1MC4 | L1 | LINE |
| 60447467 | 60447751 | + | 31.7 | 404 | 699 | -1808 | HAL1 | L1 | LINE |
| 60447827 | 60448143 | + | 25.5 | 842 | 1208 | -1299 | HAL1 | L1 | LINE |
| 60448141 | 60448361 | + | 23.4 | 1513 | 1765 | -742 | HAL1 | L1 | LINE |
| 60448400 | 60448765 | + | 24.0 | 1857 | 2345 | -162 | HAL1 | L1 | LINE |

  

|  |  |  |  |  |  |  |  |  |  |
| --- | --- | --- | --- | --- | --- | --- | --- | --- | --- |
| chr5:61022239-61028912 | | | 6603 bp | | |  |  |  |  |
| Genome Start | Genome End | Strand | Div | Repeat Start | Repeat End | Repeat Left | Name | Family | Class |
| 61022239 | 61022624 | + | 24.5 | 38 | 465 | -6082 | L1MD2 | L1 | LINE |
| 61022597 | 61023261 | + | 22.4 | 3506 | 4063 | -2481 | L1MD2 | L1 | LINE |
| 61023268 | 61023465 | - | 16.7 | 2974 | 3166 | -2980 | L1MC | L1 | LINE |
| 61023510 | 61024473 | + | 22.9 | 4055 | 5057 | -1487 | L1MD2 | L1 | LINE |
| 61024470 | 61025574 | - | 15.1 | 3767 | 4896 | -1244 | L1MB1 | L1 | LINE |
| 61025578 | 61026011 | + | 14.1 | 4909 | 5359 | -781 | L1MB1 | L1 | LINE |
| 61026012 | 61026498 | + | 16.8 | 5677 | 6168 | 0 | L1MB1 | L1 | LINE |
| 61026549 | 61027660 | + | 22.5 | 5042 | 6106 | -651 | L1MD2 | L1 | LINE |
| 61027661 | 61027886 | - | 15.2 | 4 | 292 | -10 | AluJb | Alu | SINE |
| 61027887 | 61028067 | + | 21.4 | 6107 | 6283 | -474 | L1MD2 | L1 | LINE |
| 61028068 | 61028370 | + | 7.3 | 1 | 303 | -8 | AluY | Alu | SINE |
| 61028371 | 61028464 | + | 21.4 | 6284 | 6428 | -329 | L1MD2 | L1 | LINE |
| 61028465 | 61028786 | + | 14.1 | 1 | 321 | 0 | AluSx | Alu | SINE |
| 61028787 | 61028912 | + | 21.4 | 6429 | 6547 | -210 | L1MD2 | L1 | LINE |

  

|  |  |  |  |  |  |  |  |  |  |
| --- | --- | --- | --- | --- | --- | --- | --- | --- | --- |
| chr5:61354540-61359518 | | | 4973 bp | | |  |  |  |  |
| Genome Start | Genome End | Strand | Div | Repeat Start | Repeat End | Repeat Left | Name | Family | Class |
| 61354540 | 61358422 | - | 9.1 | 903 | 4769 | -1692 | L1P2 | L1 | LINE |
| 61358431 | 61358973 | - | 3.7 | 5608 | 6152 | -3 | L1PA4 | L1 | LINE |
| 61358974 | 61359458 | + | 2.4 | 5121 | 5608 | -547 | L1PA4 | L1 | LINE |
| 61359457 | 61359518 | - | 8.1 | 844 | 905 | -5556 | L1P2 | L1 | LINE |

  

|  |  |  |  |  |  |  |  |  |  |
| --- | --- | --- | --- | --- | --- | --- | --- | --- | --- |
| chr5:62818896-62827222 | | | 8315 bp | | |  |  |  |  |
| Genome Start | Genome End | Strand | Div | Repeat Start | Repeat End | Repeat Left | Name | Family | Class |
| 62818896 | 62819461 | - | 20.2 | 3039 | 3643 | -2678 | L1MCa | L1 | LINE |
| 62819461 | 62820125 | - | 4.8 | 1784 | 2450 | -3696 | L1PA4 | L1 | LINE |
| 62820125 | 62823821 | + | 3.8 | 2444 | 6147 | -8 | L1PA4 | L1 | LINE |
| 62823836 | 62824317 | - | 20.3 | 2570 | 3053 | -3268 | L1MCa | L1 | LINE |
| 62824318 | 62824660 | + | 17.2 | 1 | 339 | 0 | MER1B | MER1\_type | DNA |
| 62824661 | 62827222 | - | 22.4 | 2 | 2569 | -3752 | L1MCa | L1 | LINE |

  

|  |  |  |  |  |  |  |  |  |  |
| --- | --- | --- | --- | --- | --- | --- | --- | --- | --- |
| chr5:62936180-62938692 | | | 2393 bp | | |  |  |  |  |
| Genome Start | Genome End | Strand | Div | Repeat Start | Repeat End | Repeat Left | Name | Family | Class |
| 62936180 | 62936561 | - | 20.8 | 1315 | 1830 | -4570 | L1MEc | L1 | LINE |
| 62936626 | 62937060 | - | 15.7 | 5846 | 6284 | -16 | L1MA4 | L1 | LINE |
| 62937061 | 62937685 | + | 13.6 | 5214 | 5841 | -459 | L1MA4 | L1 | LINE |
| 62937696 | 62937967 | - | 23.4 | 996 | 1292 | -5108 | L1MEc | L1 | LINE |
| 62938014 | 62938692 | - | 25.8 | 207 | 916 | -5484 | L1MEc | L1 | LINE |

  

|  |  |  |  |  |  |  |  |  |  |
| --- | --- | --- | --- | --- | --- | --- | --- | --- | --- |
| chr5:63437246-63440312 | | | 3072 bp | | |  |  |  |  |
| Genome Start | Genome End | Strand | Div | Repeat Start | Repeat End | Repeat Left | Name | Family | Class |
| 63437246 | 63437413 | + | 34.4 | 4096 | 4261 | -228 | L3 | CR1 | LINE |
| 63437414 | 63438834 | - | 6.3 | 3493 | 4918 | -1228 | L1PA4 | L1 | LINE |
| 63438830 | 63440081 | + | 4.4 | 4904 | 6155 | 0 | L1PA4 | L1 | LINE |
| 63440082 | 63440312 | + | 33.2 | 4249 | 4488 | -1 | L3 | CR1 | LINE |

  

|  |  |  |  |  |  |  |  |  |  |
| --- | --- | --- | --- | --- | --- | --- | --- | --- | --- |
| chr5:66025890-66031011 | | | 5048 bp | | |  |  |  |  |
| Genome Start | Genome End | Strand | Div | Repeat Start | Repeat End | Repeat Left | Name | Family | Class |
| 66025890 | 66026012 | + | 32.5 | 493 | 615 | -2804 | L2 | L2 | LINE |
| 66026018 | 66026060 | - | 9.0 | 5387 | 5426 | -742 | L1P | L1 | LINE |
| 66026061 | 66026370 | - | 12.3 | 1 | 310 | -2 | AluSx | Alu | SINE |
| 66026371 | 66027223 | - | 6.9 | 4531 | 5401 | -745 | L1P | L1 | LINE |
| 66027226 | 66027954 | + | 8.0 | 5431 | 6168 | 0 | L1PA10 | L1 | LINE |
| 66027965 | 66028238 | + | 30.9 | 637 | 911 | -2508 | L2 | L2 | LINE |
| 66028239 | 66029501 | + | 17.7 | 5 | 1455 | 0 | Charlie1a | MER1\_type | DNA |
| 66029502 | 66029855 | + | 30.9 | 912 | 1314 | -2105 | L2 | L2 | LINE |
| 66029913 | 66031011 | + | 33.9 | 2038 | 3304 | -74 | L2 | L2 | LINE |

  

|  |  |  |  |  |  |  |  |  |  |
| --- | --- | --- | --- | --- | --- | --- | --- | --- | --- |
| chr5:66687804-66690908 | | | 2680 bp | | |  |  |  |  |
| Genome Start | Genome End | Strand | Div | Repeat Start | Repeat End | Repeat Left | Name | Family | Class |
| 66687804 | 66688208 | + | 35.8 | 1568 | 1976 | -1443 | L2 | L2 | LINE |
| 66688209 | 66688523 | - | 17.1 | 1 | 310 | -2 | AluJb | Alu | SINE |
| 66688524 | 66688644 | + | 35.8 | 1977 | 2104 | -1315 | L2 | L2 | LINE |
| 66689055 | 66689089 | - | 8.6 | 5864 | 5898 | -233 | L1PA10 | L1 | LINE |
| 66689090 | 66689336 | + | 8.9 | 5916 | 6168 | 0 | L1PA10 | L1 | LINE |
| 66689352 | 66689739 | + | 34.9 | 2242 | 2608 | -811 | L2 | L2 | LINE |
| 66689740 | 66690179 | - | 12.8 | 5710 | 6154 | 0 | L1PB3 | L1 | LINE |
| 66690180 | 66690908 | + | 35.3 | 2609 | 3372 | -6 | L2 | L2 | LINE |

  

|  |  |  |  |  |  |  |  |  |  |
| --- | --- | --- | --- | --- | --- | --- | --- | --- | --- |
| chr5:69412034-69416359 | | | 4290 bp | | |  |  |  |  |
| Genome Start | Genome End | Strand | Div | Repeat Start | Repeat End | Repeat Left | Name | Family | Class |
| 69412034 | 69412083 | - | 16.0 | 5816 | 5869 | -1939 | L1MC | L1 | LINE |
| 69412082 | 69414939 | - | 2.5 | 2655 | 5524 | -631 | L1PA3 | L1 | LINE |
| 69414940 | 69415547 | + | 3.3 | 5547 | 6154 | -1 | L1PA3 | L1 | LINE |
| 69415586 | 69415771 | - | 28.8 | 5599 | 5824 | -1984 | L1MC | L1 | LINE |
| 69415772 | 69416069 | - | 8.4 | 1 | 296 | -14 | AluSg | Alu | SINE |
| 69416070 | 69416359 | - | 28.8 | 5273 | 5598 | -2210 | L1MC | L1 | LINE |

  

|  |  |  |  |  |  |  |  |  |  |
| --- | --- | --- | --- | --- | --- | --- | --- | --- | --- |
| chr5:70287488-70291813 | | | 4290 bp | | |  |  |  |  |
| Genome Start | Genome End | Strand | Div | Repeat Start | Repeat End | Repeat Left | Name | Family | Class |
| 70287488 | 70287537 | - | 16.0 | 5816 | 5869 | -1939 | L1MC | L1 | LINE |
| 70287536 | 70290393 | - | 2.2 | 2655 | 5524 | -631 | L1PA3 | L1 | LINE |
| 70290394 | 70291001 | + | 3.1 | 5547 | 6154 | -1 | L1PA3 | L1 | LINE |
| 70291040 | 70291225 | - | 28.6 | 5599 | 5824 | -1984 | L1MC | L1 | LINE |
| 70291226 | 70291523 | - | 8.4 | 1 | 296 | -14 | AluSg | Alu | SINE |
| 70291524 | 70291813 | - | 28.6 | 5273 | 5598 | -2210 | L1MC | L1 | LINE |

  

|  |  |  |  |  |  |  |  |  |  |
| --- | --- | --- | --- | --- | --- | --- | --- | --- | --- |
| chr5:75327365-75330179 | | | 2610 bp | | |  |  |  |  |
| Genome Start | Genome End | Strand | Div | Repeat Start | Repeat End | Repeat Left | Name | Family | Class |
| 75327365 | 75327507 | + | 37.0 | 7 | 147 | -6068 | L1MC4 | L1 | LINE |
| 75327665 | 75328847 | - | 3.7 | 3870 | 5054 | -1092 | L1PA4 | L1 | LINE |
| 75328847 | 75329935 | + | 4.2 | 5068 | 6155 | 0 | L1PA4 | L1 | LINE |
| 75329985 | 75330179 | + | 33.5 | 7380 | 7563 | -479 | L1MC4 | L1 | LINE |

  

|  |  |  |  |  |  |  |  |  |  |
| --- | --- | --- | --- | --- | --- | --- | --- | --- | --- |
| chr5:75451387-75455366 | | | 3924 bp | | |  |  |  |  |
| Genome Start | Genome End | Strand | Div | Repeat Start | Repeat End | Repeat Left | Name | Family | Class |
| 75451387 | 75451517 | - | 15.1 | 2632 | 2760 | -1 | Charlie1 | MER1\_type | DNA |
| 75451546 | 75451729 | + | 15.2 | 6103 | 6288 | -12 | L1MA6 | L1 | LINE |
| 75451730 | 75451864 | - | 25.2 | 96 | 237 | -2524 | Charlie1 | MER1\_type | DNA |
| 75451893 | 75454142 | - | 3.0 | 3962 | 6210 | 0 | L1PA3 | L1 | LINE |
| 75454142 | 75455261 | + | 3.6 | 2826 | 3948 | -2198 | L1PA3 | L1 | LINE |
| 75455263 | 75455366 | - | 26.2 | 2 | 107 | -2654 | Charlie1 | MER1\_type | DNA |

  

|  |  |  |  |  |  |  |  |  |  |
| --- | --- | --- | --- | --- | --- | --- | --- | --- | --- |
| chr5:80198017-80200482 | | | 2177 bp | | |  |  |  |  |
| Genome Start | Genome End | Strand | Div | Repeat Start | Repeat End | Repeat Left | Name | Family | Class |
| 80198017 | 80198695 | - | 28.9 | 1779 | 2507 | 0 | HAL1 | L1 | LINE |
| 80198875 | 80199186 | - | 28.5 | 54 | 376 | -239 | MLT1L | MaLR | LTR |
| 80199270 | 80199592 | - | 9.6 | 5058 | 5388 | -758 | L1PREC2 | L1 | LINE |
| 80199592 | 80200357 | + | 10.2 | 5389 | 6163 | 0 | L1PA13 | L1 | LINE |
| 80200386 | 80200482 | - | 21.6 | 1513 | 1611 | -896 | HAL1 | L1 | LINE |

  

|  |  |  |  |  |  |  |  |  |  |
| --- | --- | --- | --- | --- | --- | --- | --- | --- | --- |
| chr5:81044211-81047937 | | | 3642 bp | | |  |  |  |  |
| Genome Start | Genome End | Strand | Div | Repeat Start | Repeat End | Repeat Left | Name | Family | Class |
| 81044211 | 81044295 | + | 28.6 | 1 | 90 | -2417 | HAL1 | L1 | LINE |
| 81044296 | 81044602 | - | 14.2 | 1 | 312 | 0 | AluJb | Alu | SINE |
| 81044603 | 81045336 | + | 28.6 | 91 | 894 | -1613 | HAL1 | L1 | LINE |
| 81045364 | 81045476 | + | 28.6 | 895 | 1025 | -1482 | HAL1 | L1 | LINE |
| 81045485 | 81046425 | - | 5.2 | 5198 | 6152 | -2 | L1PA7 | L1 | LINE |
| 81046426 | 81046500 | + | 5.3 | 5107 | 5181 | -965 | L1PA7 | L1 | LINE |
| 81046556 | 81046836 | + | 26.7 | 1106 | 1359 | -1148 | HAL1 | L1 | LINE |
| 81046832 | 81047347 | + | 26.2 | 1685 | 2235 | -272 | HAL1 | L1 | LINE |
| 81047348 | 81047661 | + | 15.8 | 1 | 312 | 0 | AluJo | Alu | SINE |
| 81047662 | 81047937 | + | 26.2 | 2236 | 2501 | -6 | HAL1 | L1 | LINE |

  

|  |  |  |  |  |  |  |  |  |  |
| --- | --- | --- | --- | --- | --- | --- | --- | --- | --- |
| chr5:81768685-81770712 | | | 2031 bp | | |  |  |  |  |
| Genome Start | Genome End | Strand | Div | Repeat Start | Repeat End | Repeat Left | Name | Family | Class |
| 81768685 | 81769006 | - | 27.6 | 1896 | 2218 | -5116 | L1MEd | L1 | LINE |
| 81769014 | 81769514 | - | 8.9 | 5647 | 6153 | -15 | L1PA10 | L1 | LINE |
| 81769515 | 81769702 | + | 8.0 | 5445 | 5632 | -536 | L1PA10 | L1 | LINE |
| 81769693 | 81770712 | - | 29.5 | 976 | 2033 | -4963 | L1MEd | L1 | LINE |

  

|  |  |  |  |  |  |  |  |  |  |
| --- | --- | --- | --- | --- | --- | --- | --- | --- | --- |
| chr5:84709985-84714601 | | | 4395 bp | | |  |  |  |  |
| Genome Start | Genome End | Strand | Div | Repeat Start | Repeat End | Repeat Left | Name | Family | Class |
| 84709985 | 84711415 | - | 16.6 | 5265 | 6737 | -828 | L1M | L1 | LINE |
| 84711423 | 84712927 | - | 3.2 | 4653 | 6155 | 0 | L1PA3 | L1 | LINE |
| 84712925 | 84713355 | + | 3.5 | 4218 | 4648 | -1498 | L1PA3 | L1 | LINE |
| 84713354 | 84714054 | - | 19.2 | 4548 | 5275 | -2287 | L1M | L1 | LINE |
| 84714272 | 84714329 | - | 8.6 | 6100 | 6157 | -9 | L1PA16 | L1 | LINE |
| 84714333 | 84714601 | - | 25.8 | 4295 | 4560 | -3005 | L1M | L1 | LINE |

  

|  |  |  |  |  |  |  |  |  |  |
| --- | --- | --- | --- | --- | --- | --- | --- | --- | --- |
| chr5:85706277-85707358 | | | 1077 bp | | |  |  |  |  |
| Genome Start | Genome End | Strand | Div | Repeat Start | Repeat End | Repeat Left | Name | Family | Class |
| 85706277 | 85706488 | - | 20.9 | 83 | 297 | -117 | MLT2D | ERVL | LTR |
| 85706489 | 85706897 | - | 3.9 | 5380 | 5771 | -384 | L1PA4 | L1 | LINE |
| 85706898 | 85707275 | + | 4.2 | 5776 | 6155 | 0 | L1PA4 | L1 | LINE |
| 85707281 | 85707358 | - | 12.8 | 7 | 85 | -379 | MLT2D | ERVL | LTR |

  

|  |  |  |  |  |  |  |  |  |  |
| --- | --- | --- | --- | --- | --- | --- | --- | --- | --- |
| chr5:86791272-86799271 | | | 7990 bp | | |  |  |  |  |
| Genome Start | Genome End | Strand | Div | Repeat Start | Repeat End | Repeat Left | Name | Family | Class |
| 86791272 | 86791405 | - | 16.5 | 6872 | 7005 | 0 | L1MA4A | L1 | LINE |
| 86791411 | 86791601 | - | 13.2 | 5962 | 6151 | 0 | L1PB1 | L1 | LINE |
| 86791602 | 86792493 | - | 8.3 | 4874 | 5773 | -378 | L1PB1 | L1 | LINE |
| 86792492 | 86793758 | + | 6.9 | 3586 | 4867 | -1279 | L1PB1 | L1 | LINE |
| 86793758 | 86796847 | - | 16.4 | 3729 | 6888 | -117 | L1MA4A | L1 | LINE |
| 86796857 | 86798948 | - | 8.2 | 4054 | 6154 | 0 | L1PA7 | L1 | LINE |
| 86798948 | 86799271 | - | 26.6 | 10 | 337 | -6512 | L1MA4A | L1 | LINE |

  

|  |  |  |  |  |  |  |  |  |  |
| --- | --- | --- | --- | --- | --- | --- | --- | --- | --- |
| chr5:87023353-87033141 | | | 9759 bp | | |  |  |  |  |
| Genome Start | Genome End | Strand | Div | Repeat Start | Repeat End | Repeat Left | Name | Family | Class |
| 87023353 | 87025817 | - | 9.3 | 3792 | 6275 | -78 | L1PA10 | L1 | LINE |
| 87025813 | 87027208 | - | 4.4 | 4755 | 6153 | -1 | L1PA5 | L1 | LINE |
| 87027206 | 87028853 | + | 5.8 | 3097 | 4749 | -1397 | L1PA5 | L1 | LINE |
| 87028849 | 87030366 | - | 9.1 | 2258 | 3808 | -2523 | L1PA10 | L1 | LINE |
| 87030367 | 87030419 | - | 9.4 | 2258 | 2310 | -4043 | L1PA10 | L1 | LINE |
| 87030420 | 87030463 | - | 9.1 | 2267 | 2310 | -4043 | L1PA10 | L1 | LINE |
| 87030464 | 87031358 | - | 13.8 | 1421 | 2322 | -4031 | L1PA10 | L1 | LINE |
| 87031402 | 87031714 | - | 10.5 | 1 | 313 | 0 | AluSp | Alu | SINE |
| 87031715 | 87033141 | - | 13.8 | 8 | 1420 | -4933 | L1PA10 | L1 | LINE |

  

|  |  |  |  |  |  |  |  |  |  |
| --- | --- | --- | --- | --- | --- | --- | --- | --- | --- |
| chr5:87191208-87193772 | | | 2559 bp | | |  |  |  |  |
| Genome Start | Genome End | Strand | Div | Repeat Start | Repeat End | Repeat Left | Name | Family | Class |
| 87191208 | 87191424 | - | 20.1 | 264 | 497 | -8 | MLT1E2 | MaLR | LTR |
| 87191432 | 87192510 | - | 13.6 | 5065 | 6151 | -3 | L1PB3 | L1 | LINE |
| 87192510 | 87193491 | + | 15.2 | 4091 | 5072 | -1074 | L1PB3 | L1 | LINE |
| 87193492 | 87193772 | - | 23.8 | 1 | 295 | -331 | MLT1E2 | MaLR | LTR |

  

|  |  |  |  |  |  |  |  |  |  |
| --- | --- | --- | --- | --- | --- | --- | --- | --- | --- |
| chr5:87663804-87677666 | | | 13813 bp | | |  |  |  |  |
| Genome Start | Genome End | Strand | Div | Repeat Start | Repeat End | Repeat Left | Name | Family | Class |
| 87663804 | 87664122 | - | 16.3 | 6321 | 6628 | -5 | L1MD1 | L1 | LINE |
| 87664123 | 87664425 | + | 14.4 | 1 | 292 | -20 | AluSx | Alu | SINE |
| 87664426 | 87664760 | - | 16.3 | 5978 | 6320 | -313 | L1MD1 | L1 | LINE |
| 87664761 | 87665051 | + | 1.4 | 15 | 305 | -6 | AluY | Alu | SINE |
| 87665052 | 87665335 | - | 16.3 | 5690 | 5977 | -656 | L1MD1 | L1 | LINE |
| 87665352 | 87665436 | - | 17.3 | 49 | 129 | -14 | FLAM | Alu | SINE |
| 87665437 | 87666779 | - | 3.0 | 3836 | 5178 | -968 | L1PA3 | L1 | LINE |
| 87666778 | 87667749 | + | 2.3 | 5184 | 6155 | 0 | L1PA3 | L1 | LINE |
| 87667768 | 87667849 | - | 27.0 | 1 | 66 | -236 | AluJ/FLAM | Alu | SINE |
| 87667850 | 87668561 | - | 17.3 | 4988 | 5687 | -850 | L1MD1 | L1 | LINE |
| 87668562 | 87671460 | + | 11.1 | 2141 | 5037 | -2687 | L1MA3 | L1 | LINE |
| 87671461 | 87671491 | - | 3.2 | 38 | 68 | -12 | MADE1 | Mariner | DNA |
| 87671492 | 87674177 | + | 11.1 | 5038 | 7721 | -3 | L1MA3 | L1 | LINE |
| 87674221 | 87674745 | - | 14.9 | 4218 | 4723 | -1814 | L1MD1 | L1 | LINE |
| 87674746 | 87675055 | + | 14.9 | 1 | 311 | -1 | AluSx | Alu | SINE |
| 87675056 | 87675788 | - | 14.9 | 3427 | 4217 | -2320 | L1MD1 | L1 | LINE |
| 87675789 | 87676093 | - | 12.5 | 1 | 304 | -9 | AluSq | Alu | SINE |
| 87676094 | 87676205 | - | 14.9 | 3309 | 3426 | -3111 | L1MD1 | L1 | LINE |
| 87676181 | 87676475 | - | 28.1 | 913 | 1208 | -5425 | L1MD1 | L1 | LINE |
| 87676476 | 87676787 | + | 20.4 | 1 | 311 | -1 | AluJo | Alu | SINE |
| 87676788 | 87677666 | - | 28.1 | 3 | 912 | -5721 | L1MD1 | L1 | LINE |

  

|  |  |  |  |  |  |  |  |  |  |
| --- | --- | --- | --- | --- | --- | --- | --- | --- | --- |
| chr5:89923618-89925318 | | | 1693 bp | | |  |  |  |  |
| Genome Start | Genome End | Strand | Div | Repeat Start | Repeat End | Repeat Left | Name | Family | Class |
| 89923618 | 89924067 | + | 5.1 | 5565 | 6036 | -115 | L1PB1 | L1 | LINE |
| 89924066 | 89924188 | - | 5.9 | 5037 | 5155 | -991 | L1PA6 | L1 | LINE |
| 89924189 | 89925174 | + | 6.1 | 5157 | 6143 | -11 | L1PA6 | L1 | LINE |
| 89925185 | 89925318 | + | 10.5 | 6018 | 6151 | 0 | L1PB1 | L1 | LINE |

  

|  |  |  |  |  |  |  |  |  |  |
| --- | --- | --- | --- | --- | --- | --- | --- | --- | --- |
| chr5:91387568-91399298 | | | 11468 bp | | |  |  |  |  |
| Genome Start | Genome End | Strand | Div | Repeat Start | Repeat End | Repeat Left | Name | Family | Class |
| 91387568 | 91388437 | - | 15.8 | 5604 | 6502 | -6 | L1MC1 | L1 | LINE |
| 91388429 | 91388658 | - | 5.7 | 5922 | 6150 | -4 | L1PA5 | L1 | LINE |
| 91388659 | 91388776 | + | 4.2 | 5807 | 5924 | -230 | L1PA5 | L1 | LINE |
| 91388772 | 91390280 | - | 16.5 | 4015 | 5616 | -892 | L1MC1 | L1 | LINE |
| 91390440 | 91391636 | - | 10.5 | 5106 | 6303 | -1 | L1MA2 | L1 | LINE |
| 91391694 | 91391917 | + | 9.7 | 1577 | 1793 | -3861 | HERVL-A2 | ERVL | LTR |
| 91391918 | 91392475 | + | 10.3 | 1 | 554 | 0 | MER41A | ERV1 | LTR |
| 91392476 | 91396316 | + | 9.7 | 1794 | 5654 | 0 | HERVL-A2 | ERVL | LTR |
| 91396317 | 91396858 | + | 22.2 | 1 | 547 | -7 | MLT2A2 | ERVL | LTR |
| 91396859 | 91397261 | + | 19.9 | 1 | 426 | 0 | MSTB | MaLR | LTR |
| 91397263 | 91398810 | + | 28.6 | 1 | 1735 | 0 | MLT1I-int | MaLR | LTR |
| 91398871 | 91399298 | - | 17.9 | 1 | 458 | -6050 | L1MC1 | L1 | LINE |

  

|  |  |  |  |  |  |  |  |  |  |
| --- | --- | --- | --- | --- | --- | --- | --- | --- | --- |
| chr5:91599066-91618424 | | | 19302 bp | | |  |  |  |  |
| Genome Start | Genome End | Strand | Div | Repeat Start | Repeat End | Repeat Left | Name | Family | Class |
| 91599066 | 91602739 | + | 16.4 | 100 | 3808 | -2958 | L1PA13 | L1 | LINE |
| 91602738 | 91603121 | + | 12.0 | 4352 | 4739 | -2027 | L1PA13 | L1 | LINE |
| 91603119 | 91603239 | - | 18.2 | 4105 | 4228 | -1918 | L1PREC2 | L1 | LINE |
| 91603239 | 91603467 | + | 22.3 | 459 | 691 | -5513 | L1PBa | L1 | LINE |
| 91603534 | 91605432 | - | 3.6 | 4259 | 6152 | -3 | L1PA3 | L1 | LINE |
| 91605433 | 91607011 | + | 3.4 | 2678 | 4257 | -1889 | L1PA3 | L1 | LINE |
| 91607007 | 91613075 | + | 14.5 | 1 | 6115 | -93 | L1PB2 | L1 | LINE |
| 91613078 | 91614731 | + | 4.9 | 4494 | 6152 | -2 | L1PA5 | L1 | LINE |
| 91614727 | 91614800 | + | 7.5 | 6134 | 6201 | -7 | L1PB2 | L1 | LINE |
| 91614806 | 91615668 | + | 11.8 | 3179 | 4096 | -2050 | L1PREC2 | L1 | LINE |
| 91615669 | 91616039 | - | 12.4 | 1 | 381 | 0 | THE1D | MaLR | LTR |
| 91616040 | 91617422 | + | 11.7 | 4097 | 5465 | -698 | L1PA13 | L1 | LINE |
| 91617423 | 91617731 | + | 13.3 | 1 | 310 | 0 | AluSg | Alu | SINE |
| 91617732 | 91618424 | + | 11.4 | 5466 | 6159 | -4 | L1PA13 | L1 | LINE |

  

|  |  |  |  |  |  |  |  |  |  |
| --- | --- | --- | --- | --- | --- | --- | --- | --- | --- |
| chr5:91716963-91722727 | | | 5629 bp | | |  |  |  |  |
| Genome Start | Genome End | Strand | Div | Repeat Start | Repeat End | Repeat Left | Name | Family | Class |
| 91716963 | 91717423 | - | 26.1 | 3714 | 4191 | -2028 | L1MEc | L1 | LINE |
| 91717489 | 91717984 | - | 27.5 | 2923 | 3434 | -2785 | L1MEc | L1 | LINE |
| 91718056 | 91718315 | - | 25.9 | 2275 | 2577 | -3642 | L1MEc | L1 | LINE |
| 91718316 | 91718632 | + | 1.3 | 1 | 317 | -1 | AluYb8 | Alu | SINE |
| 91718633 | 91719054 | - | 26.1 | 1923 | 2375 | -4025 | L1MEc | L1 | LINE |
| 91719058 | 91719229 | + | 13.7 | 35 | 203 | -106 | AluSc | Alu | SINE |
| 91719232 | 91719448 | - | 17.0 | 5608 | 5829 | -337 | L1PA16 | L1 | LINE |
| 91719449 | 91719946 | + | 15.5 | 1 | 592 | -234 | LTR12 | ERV1 | LTR |
| 91719947 | 91720258 | - | 9.7 | 5301 | 5612 | -554 | L1PA16 | L1 | LINE |
| 91720259 | 91721203 | + | 12.8 | 4353 | 5297 | -859 | L1PA16 | L1 | LINE |
| 91721203 | 91722073 | - | 23.0 | 928 | 1930 | -4470 | L1MEc | L1 | LINE |
| 91722089 | 91722276 | - | 28.3 | 616 | 808 | -5592 | L1MEc | L1 | LINE |
| 91722275 | 91722664 | + | 27.6 | 310 | 667 | -5733 | L1MEc | L1 | LINE |
| 91722648 | 91722727 | - | 22.7 | 74 | 151 | -6249 | L1MEc | L1 | LINE |

  

|  |  |  |  |  |  |  |  |  |  |
| --- | --- | --- | --- | --- | --- | --- | --- | --- | --- |
| chr5:93691629-93695838 | | | 4195 bp | | |  |  |  |  |
| Genome Start | Genome End | Strand | Div | Repeat Start | Repeat End | Repeat Left | Name | Family | Class |
| 93691629 | 93692313 | + | 23.5 | 2570 | 3299 | -2847 | L1MD | L1 | LINE |
| 93692314 | 93692614 | + | 13.8 | 1 | 302 | -11 | AluSq | Alu | SINE |
| 93692615 | 93693324 | + | 23.5 | 3300 | 3975 | -2171 | L1MD | L1 | LINE |
| 93693326 | 93693649 | + | 10.7 | 1 | 310 | -2 | AluSx | Alu | SINE |
| 93693690 | 93695185 | - | 8.3 | 4656 | 6154 | 0 | L1PA7 | L1 | LINE |
| 93695183 | 93695567 | + | 7.8 | 4262 | 4645 | -1501 | L1PA7 | L1 | LINE |
| 93695545 | 93695838 | + | 26.1 | 3969 | 4264 | -1882 | L1MD | L1 | LINE |

  

|  |  |  |  |  |  |  |  |  |  |
| --- | --- | --- | --- | --- | --- | --- | --- | --- | --- |
| chr5:96651096-96660062 | | | 8936 bp | | |  |  |  |  |
| Genome Start | Genome End | Strand | Div | Repeat Start | Repeat End | Repeat Left | Name | Family | Class |
| 96651096 | 96653452 | - | 15.2 | 5360 | 7710 | -14 | L1MA3 | L1 | LINE |
| 96653453 | 96653586 | - | 16.8 | 1 | 131 | -2 | FLAM\_C | Alu | SINE |
| 96653587 | 96654634 | - | 14.6 | 4313 | 5359 | -2206 | L1MA3 | L1 | LINE |
| 96654668 | 96655568 | - | 15.9 | 2571 | 3464 | -4101 | L1MA3 | L1 | LINE |
| 96655571 | 96657010 | - | 1.8 | 4716 | 6155 | 0 | L1PA3 | L1 | LINE |
| 96657009 | 96658049 | + | 2.6 | 3656 | 4696 | -1450 | L1PA3 | L1 | LINE |
| 96658048 | 96660062 | - | 21.0 | 17 | 2588 | -4977 | L1MA3 | L1 | LINE |

  

|  |  |  |  |  |  |  |  |  |  |
| --- | --- | --- | --- | --- | --- | --- | --- | --- | --- |
| chr5:97002651-97006995 | | | 4297 bp | | |  |  |  |  |
| Genome Start | Genome End | Strand | Div | Repeat Start | Repeat End | Repeat Left | Name | Family | Class |
| 97002651 | 97002928 | - | 37.2 | 2911 | 3216 | -162 | L2 | L2 | LINE |
| 97002934 | 97003085 | - | 16.4 | 6024 | 6166 | 0 | L1PA16 | L1 | LINE |
| 97003119 | 97004601 | - | 2.0 | 4673 | 6155 | 0 | L1PA3 | L1 | LINE |
| 97004600 | 97005312 | - | 15.6 | 5307 | 6029 | -137 | L1PA16 | L1 | LINE |
| 97005312 | 97005850 | + | 13.7 | 4748 | 5297 | -849 | L1PA16 | L1 | LINE |
| 97005864 | 97006995 | - | 33.8 | 1687 | 2907 | -512 | L2 | L2 | LINE |

  

|  |  |  |  |  |  |  |  |  |  |
| --- | --- | --- | --- | --- | --- | --- | --- | --- | --- |
| chr5:97551050-97553830 | | | 2753 bp | | |  |  |  |  |
| Genome Start | Genome End | Strand | Div | Repeat Start | Repeat End | Repeat Left | Name | Family | Class |
| 97551050 | 97551220 | - | 24.9 | 298 | 467 | 0 | MLT1C | MaLR | LTR |
| 97551243 | 97552951 | - | 2.9 | 4443 | 6155 | 0 | L1PA4 | L1 | LINE |
| 97552949 | 97553593 | + | 3.6 | 3809 | 4452 | -1694 | L1PA4 | L1 | LINE |
| 97553603 | 97553830 | - | 18.8 | 1 | 225 | -242 | MLT1C | MaLR | LTR |

  

|  |  |  |  |  |  |  |  |  |  |
| --- | --- | --- | --- | --- | --- | --- | --- | --- | --- |
| chr5:98864567-98867928 | | | 3376 bp | | |  |  |  |  |
| Genome Start | Genome End | Strand | Div | Repeat Start | Repeat End | Repeat Left | Name | Family | Class |
| 98864567 | 98864916 | - | 29.5 | 7096 | 7439 | -603 | L1MC4 | L1 | LINE |
| 98864908 | 98864970 | - | 7.9 | 3670 | 3732 | -2414 | L1PA5 | L1 | LINE |
| 98864967 | 98867382 | + | 5.6 | 3726 | 6154 | 0 | L1PA5 | L1 | LINE |
| 98867382 | 98867928 | - | 23.9 | 6625 | 7118 | -843 | L1MC4 | L1 | LINE |

  

|  |  |  |  |  |  |  |  |  |  |
| --- | --- | --- | --- | --- | --- | --- | --- | --- | --- |
| chr5:99103715-99112640 | | | 8893 bp | | |  |  |  |  |
| Genome Start | Genome End | Strand | Div | Repeat Start | Repeat End | Repeat Left | Name | Family | Class |
| 99103715 | 99106268 | - | 18.2 | 5192 | 7709 | -1 | L1MA8 | L1 | LINE |
| 99106294 | 99106973 | - | 18.1 | 5610 | 6291 | -9 | L1MA4 | L1 | LINE |
| 99106974 | 99107288 | - | 1.3 | 1 | 315 | -3 | AluYb8 | Alu | SINE |
| 99107289 | 99107804 | - | 16.7 | 5048 | 5609 | -691 | L1MA4 | L1 | LINE |
| 99107806 | 99108319 | + | 17.8 | 4533 | 5043 | -1103 | L1MA4 | L1 | LINE |
| 99108325 | 99109108 | - | 18.4 | 4439 | 5198 | -2364 | L1MA8 | L1 | LINE |
| 99109109 | 99109535 | - | 20.3 | 3 | 428 | 0 | MSTC | MaLR | LTR |
| 99109536 | 99112026 | - | 23.3 | 1970 | 4438 | -3124 | L1MA8 | L1 | LINE |
| 99112029 | 99112640 | - | 27.9 | 466 | 1091 | -6398 | L1MA8 | L1 | LINE |

  

|  |  |  |  |  |  |  |  |  |  |
| --- | --- | --- | --- | --- | --- | --- | --- | --- | --- |
| chr5:99434441-99437794 | | | 3368 bp | | |  |  |  |  |
| Genome Start | Genome End | Strand | Div | Repeat Start | Repeat End | Repeat Left | Name | Family | Class |
| 99434441 | 99434987 | + | 23.2 | 6625 | 7118 | -843 | L1MC4 | L1 | LINE |
| 99434987 | 99437410 | - | 5.4 | 3726 | 6154 | 0 | L1PA5 | L1 | LINE |
| 99437407 | 99437468 | + | 8.1 | 3670 | 3732 | -2414 | L1PA5 | L1 | LINE |
| 99437460 | 99437794 | + | 30.6 | 7096 | 7430 | -612 | L1MC4 | L1 | LINE |

  

|  |  |  |  |  |  |  |  |  |  |
| --- | --- | --- | --- | --- | --- | --- | --- | --- | --- |
| chr5:99539397-99547174 | | | 7528 bp | | |  |  |  |  |
| Genome Start | Genome End | Strand | Div | Repeat Start | Repeat End | Repeat Left | Name | Family | Class |
| 99539397 | 99539817 | + | 33.8 | 1 | 445 | -1027 | MLT1H-int | MaLR | LTR |
| 99539818 | 99540110 | + | 23.2 | 3 | 296 | -16 | AluJo | Alu | SINE |
| 99540111 | 99540230 | + | 33.8 | 446 | 555 | -917 | MLT1H-int | MaLR | LTR |
| 99540235 | 99540753 | + | 14.5 | 3161 | 3695 | -2451 | L1PREC2 | L1 | LINE |
| 99540817 | 99541068 | + | 14.5 | 3696 | 3957 | -2189 | L1PREC2 | L1 | LINE |
| 99541069 | 99541367 | + | 9.4 | 1 | 303 | -6 | AluSc | Alu | SINE |
| 99541368 | 99543612 | + | 15.4 | 3958 | 6156 | -4 | L1PREC2 | L1 | LINE |
| 99543653 | 99543730 | + | 24.6 | 525 | 601 | -871 | MLT1H-int | MaLR | LTR |
| 99543731 | 99544084 | + | 22.1 | 2 | 236 | 0 | MER46B | MER2\_type | DNA |
| 99544085 | 99544150 | + | 24.6 | 602 | 668 | -804 | MLT1H-int | MaLR | LTR |
| 99544154 | 99544661 | - | 20.3 | 5628 | 6164 | -20 | L1MB7 | L1 | LINE |
| 99544664 | 99544826 | + | 22.7 | 3585 | 3754 | -2392 | L1M4 | L1 | LINE |
| 99544836 | 99544961 | + | 23.8 | 6 | 132 | -10 | FLAM\_A | Alu | SINE |
| 99544962 | 99545108 | - | 1.4 | 5785 | 5933 | -222 | L1PA4 | L1 | LINE |
| 99545109 | 99545325 | + | 4.2 | 5937 | 6153 | -2 | L1PA4 | L1 | LINE |
| 99545391 | 99545924 | - | 25.1 | 440 | 1016 | -5526 | L1M4 | L1 | LINE |
| 99545945 | 99546374 | - | 25.1 | 8 | 439 | -6103 | L1M4 | L1 | LINE |
| 99546419 | 99547174 | + | 31.6 | 692 | 1483 | -6 | MLT1H-int | MaLR | LTR |

  

|  |  |  |  |  |  |  |  |  |  |
| --- | --- | --- | --- | --- | --- | --- | --- | --- | --- |
| chr5:99991412-99994767 | | | 3204 bp | | |  |  |  |  |
| Genome Start | Genome End | Strand | Div | Repeat Start | Repeat End | Repeat Left | Name | Family | Class |
| 99991412 | 99991818 | + | 33.8 | 1956 | 2374 | -1045 | L2 | L2 | LINE |
| 99991977 | 99992648 | - | 6.4 | 5481 | 6152 | -2 | L1PA7 | L1 | LINE |
| 99992649 | 99992953 | - | 13.1 | 1 | 306 | -7 | AluSp | Alu | SINE |
| 99992954 | 99993705 | - | 8.4 | 4727 | 5480 | -674 | L1PA7 | L1 | LINE |
| 99993700 | 99994389 | + | 10.0 | 4023 | 4718 | -1428 | L1PA7 | L1 | LINE |
| 99994390 | 99994767 | + | 34.5 | 2563 | 2960 | -459 | L2 | L2 | LINE |

  

|  |  |  |  |  |  |  |  |  |  |
| --- | --- | --- | --- | --- | --- | --- | --- | --- | --- |
| chr5:100653008-100655905 | | | 2877 bp | | |  |  |  |  |
| Genome Start | Genome End | Strand | Div | Repeat Start | Repeat End | Repeat Left | Name | Family | Class |
| 100653008 | 100653057 | - | 10.0 | 4784 | 4833 | -1313 | L1PB | L1 | LINE |
| 100653098 | 100653734 | - | 1.9 | 5519 | 6155 | 0 | L1PA3 | L1 | LINE |
| 100653735 | 100654579 | + | 2.2 | 4693 | 5535 | -620 | L1PA3 | L1 | LINE |
| 100654561 | 100655905 | - | 9.8 | 3460 | 4797 | -1349 | L1PB | L1 | LINE |

  

|  |  |  |  |  |  |  |  |  |  |
| --- | --- | --- | --- | --- | --- | --- | --- | --- | --- |
| chr5:101474136-101478615 | | | 4358 bp | | |  |  |  |  |
| Genome Start | Genome End | Strand | Div | Repeat Start | Repeat End | Repeat Left | Name | Family | Class |
| 101474136 | 101474217 | + | 19.8 | 9 | 89 | -6892 | L1PA12 | L1 | LINE |
| 101474233 | 101474329 | + | 24.7 | 2347 | 2448 | -4886 | L1M4b | L1 | LINE |
| 101474447 | 101477115 | - | 5.5 | 3473 | 6147 | -7 | L1PA5 | L1 | LINE |
| 101477106 | 101478233 | + | 5.0 | 2357 | 3484 | -2662 | L1PA5 | L1 | LINE |
| 101478234 | 101478615 | + | 19.5 | 76 | 494 | -6487 | L1PA12 | L1 | LINE |

  

|  |  |  |  |  |  |  |  |  |  |
| --- | --- | --- | --- | --- | --- | --- | --- | --- | --- |
| chr5:101569018-101575103 | | | 5980 bp | | |  |  |  |  |
| Genome Start | Genome End | Strand | Div | Repeat Start | Repeat End | Repeat Left | Name | Family | Class |
| 101569018 | 101569794 | + | 17.3 | 3621 | 4319 | -1821 | L1MA9 | L1 | LINE |
| 101569795 | 101570102 | - | 15.4 | 1 | 301 | -11 | AluSx | Alu | SINE |
| 101570103 | 101570403 | + | 17.3 | 4320 | 4630 | -1510 | L1MA9 | L1 | LINE |
| 101570404 | 101570832 | - | 0.7 | 3402 | 3830 | -2316 | L1PA2 | L1 | LINE |
| 101570833 | 101573172 | + | 1.8 | 3813 | 6153 | -2 | L1PA2 | L1 | LINE |
| 101573172 | 101573822 | + | 19.7 | 4617 | 5270 | -870 | L1MA9 | L1 | LINE |
| 101573824 | 101574110 | + | 16.4 | 1 | 287 | -25 | AluSx | Alu | SINE |
| 101574217 | 101575103 | + | 17.4 | 5266 | 6150 | -162 | L1MA9 | L1 | LINE |

  

|  |  |  |  |  |  |  |  |  |  |
| --- | --- | --- | --- | --- | --- | --- | --- | --- | --- |
| chr5:101995425-101999458 | | | 3812 bp | | |  |  |  |  |
| Genome Start | Genome End | Strand | Div | Repeat Start | Repeat End | Repeat Left | Name | Family | Class |
| 101995425 | 101996055 | + | 5.6 | 2862 | 3513 | -2633 | L1PB | L1 | LINE |
| 101996056 | 101996332 | - | 6.2 | 5058 | 5332 | -814 | L1PA5 | L1 | LINE |
| 101996332 | 101997138 | + | 3.6 | 5333 | 6139 | -15 | L1PA5 | L1 | LINE |
| 101997311 | 101998198 | + | 7.9 | 3497 | 4398 | -1748 | L1PB | L1 | LINE |
| 101998250 | 101998729 | + | 7.9 | 4399 | 4885 | -1261 | L1PB | L1 | LINE |
| 101998730 | 101999042 | + | 15.2 | 1 | 303 | -9 | AluSx | Alu | SINE |
| 101999043 | 101999458 | + | 7.9 | 4886 | 5298 | -848 | L1PB | L1 | LINE |

  

|  |  |  |  |  |  |  |  |  |  |
| --- | --- | --- | --- | --- | --- | --- | --- | --- | --- |
| chr5:104240721-104248586 | | | 7842 bp | | |  |  |  |  |
| Genome Start | Genome End | Strand | Div | Repeat Start | Repeat End | Repeat Left | Name | Family | Class |
| 104240721 | 104244631 | + | 25.2 | 320 | 4561 | -2083 | L1MB7 | L1 | LINE |
| 104244632 | 104244922 | + | 10.7 | 1 | 291 | -21 | AluSx | Alu | SINE |
| 104244923 | 104245473 | + | 23.7 | 4562 | 5114 | -1530 | L1MB7 | L1 | LINE |
| 104245470 | 104246085 | - | 4.5 | 4468 | 5086 | -1060 | L1PA4 | L1 | LINE |
| 104246078 | 104247106 | + | 2.6 | 5109 | 6137 | -18 | L1PA4 | L1 | LINE |
| 104247143 | 104248586 | + | 22.5 | 5106 | 6672 | -10 | L1MB7 | L1 | LINE |

  

|  |  |  |  |  |  |  |  |  |  |
| --- | --- | --- | --- | --- | --- | --- | --- | --- | --- |
| chr5:104675459-104681849 | | | 6309 bp | | |  |  |  |  |
| Genome Start | Genome End | Strand | Div | Repeat Start | Repeat End | Repeat Left | Name | Family | Class |
| 104675459 | 104676694 | - | 23.1 | 5132 | 6406 | -8 | L1MB8 | L1 | LINE |
| 104676728 | 104677296 | - | 21.2 | 4590 | 5131 | -1251 | L1MB8 | L1 | LINE |
| 104677297 | 104677606 | + | 11.1 | 5 | 282 | -30 | AluSx | Alu | SINE |
| 104677607 | 104678062 | - | 21.2 | 4196 | 4589 | -1793 | L1MB8 | L1 | LINE |
| 104678102 | 104678875 | - | 21.2 | 3273 | 4195 | -2187 | L1MB8 | L1 | LINE |
| 104678889 | 104679145 | - | 18.6 | 5910 | 6165 | -1 | L1PA16 | L1 | LINE |
| 104679146 | 104679965 | - | 2.0 | 4916 | 5735 | -420 | L1PA2 | L1 | LINE |
| 104679966 | 104680060 | + | 1.9 | 5735 | 5829 | -326 | L1PA2 | L1 | LINE |
| 104680061 | 104680323 | + | 0.4 | 35 | 297 | -13 | AluYa5 | Alu | SINE |
| 104680324 | 104680659 | + | 1.9 | 5830 | 6155 | 0 | L1PA2 | L1 | LINE |
| 104680662 | 104681285 | - | 14.4 | 5281 | 5918 | -248 | L1PA16 | L1 | LINE |
| 104681281 | 104681849 | - | 21.6 | 2269 | 2883 | -3499 | L1MB8 | L1 | LINE |

  

|  |  |  |  |  |  |  |  |  |  |
| --- | --- | --- | --- | --- | --- | --- | --- | --- | --- |
| chr5:104732355-104735304 | | | 2811 bp | | |  |  |  |  |
| Genome Start | Genome End | Strand | Div | Repeat Start | Repeat End | Repeat Left | Name | Family | Class |
| 104732355 | 104733734 | + | 22.2 | 3766 | 5170 | -1072 | L1MD1 | L1 | LINE |
| 104733781 | 104734270 | + | 22.2 | 5171 | 5668 | -574 | L1MD1 | L1 | LINE |
| 104734365 | 104734465 | + | 23.8 | 5694 | 5795 | -564 | L1MD1 | L1 | LINE |
| 104734470 | 104734737 | - | 3.7 | 5888 | 6155 | 0 | L1PA4 | L1 | LINE |
| 104734738 | 104734887 | + | 2.0 | 5721 | 5870 | -285 | L1PA4 | L1 | LINE |
| 104734883 | 104735304 | + | 19.7 | 5791 | 6359 | 0 | L1MD1 | L1 | LINE |

  

|  |  |  |  |  |  |  |  |  |  |
| --- | --- | --- | --- | --- | --- | --- | --- | --- | --- |
| chr5:105077087-105084307 | | | 7020 bp | | |  |  |  |  |
| Genome Start | Genome End | Strand | Div | Repeat Start | Repeat End | Repeat Left | Name | Family | Class |
| 105077087 | 105078595 | + | 24.0 | 54 | 1599 | -4835 | L1MEc | L1 | LINE |
| 105078564 | 105079254 | + | 25.4 | 3663 | 4255 | -1891 | L1MD2 | L1 | LINE |
| 105079308 | 105080997 | + | 22.1 | 4256 | 6062 | -104 | L1MD2 | L1 | LINE |
| 105080953 | 105081241 | + | 22.5 | 6036 | 6335 | -24 | L1MD2 | L1 | LINE |
| 105081264 | 105081583 | - | 10.0 | 5830 | 6150 | -1 | L1PB1 | L1 | LINE |
| 105081731 | 105081962 | - | 6.5 | 5560 | 5791 | -360 | L1PB1 | L1 | LINE |
| 105081959 | 105083456 | + | 9.7 | 4050 | 5558 | -593 | L1PB1 | L1 | LINE |
| 105083516 | 105083678 | - | 30.7 | 1 | 175 | 0 | MER96 | MER1\_type? | DNA |
| 105083680 | 105084307 | + | 22.3 | 1731 | 2361 | -4039 | L1MEc | L1 | LINE |

  

|  |  |  |  |  |  |  |  |  |  |
| --- | --- | --- | --- | --- | --- | --- | --- | --- | --- |
| chr5:107592608-107594653 | | | 2019 bp | | |  |  |  |  |
| Genome Start | Genome End | Strand | Div | Repeat Start | Repeat End | Repeat Left | Name | Family | Class |
| 107592608 | 107592677 | + | 24.3 | 3 | 72 | -88 | MER5A1 | MER1\_type | DNA |
| 107592678 | 107592755 | - | 7.7 | 4501 | 4578 | -1568 | L1PB1 | L1 | LINE |
| 107592748 | 107594325 | + | 7.8 | 4582 | 6147 | -4 | L1PB1 | L1 | LINE |
| 107594352 | 107594544 | + | 13.5 | 5951 | 6144 | -7 | L1PB2 | L1 | LINE |
| 107594554 | 107594653 | + | 26.0 | 58 | 157 | -3 | MER5A1 | MER1\_type | DNA |

  

|  |  |  |  |  |  |  |  |  |  |
| --- | --- | --- | --- | --- | --- | --- | --- | --- | --- |
| chr5:108630086-108639828 | | | 9470 bp | | |  |  |  |  |
| Genome Start | Genome End | Strand | Div | Repeat Start | Repeat End | Repeat Left | Name | Family | Class |
| 108630086 | 108630157 | - | 27.8 | 156 | 236 | -26 | MIR | MIR | SINE |
| 108630160 | 108630231 | + | 25.4 | 1 | 73 | -324 | MLT2C1 | ERVL | LTR |
| 108630238 | 108631899 | + | 23.8 | 460 | 2241 | -5248 | L1M3e | L1 | LINE |
| 108631900 | 108632514 | + | 4.9 | 5539 | 6154 | 0 | L1PA5 | L1 | LINE |
| 108632515 | 108634826 | + | 20.9 | 2009 | 4654 | -2908 | L1MA8 | L1 | LINE |
| 108634905 | 108636488 | - | 7.1 | 3943 | 5557 | -597 | L1PA7 | L1 | LINE |
| 108636489 | 108637065 | + | 5.0 | 5570 | 6148 | -6 | L1PA7 | L1 | LINE |
| 108637066 | 108639371 | + | 17.5 | 4792 | 7710 | 0 | L1MA8 | L1 | LINE |
| 108639382 | 108639487 | + | 24.0 | 60 | 168 | -229 | MLT2C1 | ERVL | LTR |
| 108639665 | 108639828 | - | 28.5 | 9 | 172 | -90 | MIR | MIR | SINE |

  

|  |  |  |  |  |  |  |  |  |  |
| --- | --- | --- | --- | --- | --- | --- | --- | --- | --- |
| chr5:109490917-109502740 | | | 11828 bp | | |  |  |  |  |
| Genome Start | Genome End | Strand | Div | Repeat Start | Repeat End | Repeat Left | Name | Family | Class |
| 109490917 | 109493181 | - | 9.1 | 4195 | 6469 | 0 | L1PA7 | L1 | LINE |
| 109493173 | 109493820 | - | 10.0 | 4450 | 5102 | -1044 | L1P3 | L1 | LINE |
| 109493807 | 109494530 | + | 9.8 | 3731 | 4460 | -1686 | L1P3 | L1 | LINE |
| 109494539 | 109496446 | - | 9.2 | 4535 | 6469 | 0 | L1PA7 | L1 | LINE |
| 109496447 | 109497540 | - | 10.5 | 2932 | 4042 | -2419 | L1PA7 | L1 | LINE |
| 109497543 | 109500008 | - | 13.4 | 8 | 2676 | -3785 | L1PA7 | L1 | LINE |
| 109500018 | 109502740 | - | 12.6 | 383 | 3089 | -3372 | L1PA7 | L1 | LINE |

  

|  |  |  |  |  |  |  |  |  |  |
| --- | --- | --- | --- | --- | --- | --- | --- | --- | --- |
| chr5:110150964-110154338 | | | 3380 bp | | |  |  |  |  |
| Genome Start | Genome End | Strand | Div | Repeat Start | Repeat End | Repeat Left | Name | Family | Class |
| 110150964 | 110151494 | - | 16.1 | 2502 | 3029 | 0 | Tigger3(Golem) | MER2\_type | DNA |
| 110151496 | 110152238 | - | 6.5 | 5401 | 6141 | -13 | L1PA7 | L1 | LINE |
| 110152239 | 110152529 | - | 8.3 | 3 | 293 | -18 | AluY | Alu | SINE |
| 110152530 | 110153415 | - | 9.2 | 4504 | 5400 | -746 | L1P | L1 | LINE |
| 110153410 | 110154140 | + | 8.5 | 3759 | 4493 | -1653 | L1P | L1 | LINE |
| 110154141 | 110154338 | - | 9.1 | 2310 | 2520 | -509 | Tigger3(Golem) | MER2\_type | DNA |

  

|  |  |  |  |  |  |  |  |  |  |
| --- | --- | --- | --- | --- | --- | --- | --- | --- | --- |
| chr5:113201104-113203350 | | | 2233 bp | | |  |  |  |  |
| Genome Start | Genome End | Strand | Div | Repeat Start | Repeat End | Repeat Left | Name | Family | Class |
| 113201104 | 113201179 | + | 21.3 | 1 | 76 | -113 | MER5A | MER1\_type | DNA |
| 113201180 | 113201485 | + | 6.9 | 1 | 306 | -5 | AluY | Alu | SINE |
| 113201491 | 113202649 | - | 14.8 | 4563 | 5723 | -568 | L1MA7 | L1 | LINE |
| 113202650 | 113203212 | + | 14.8 | 5706 | 6286 | -5 | L1MA7 | L1 | LINE |
| 113203222 | 113203350 | + | 22.5 | 60 | 189 | 0 | MER5A | MER1\_type | DNA |

  

|  |  |  |  |  |  |  |  |  |  |
| --- | --- | --- | --- | --- | --- | --- | --- | --- | --- |
| chr5:114207533-114219334 | | | 11819 bp | | |  |  |  |  |
| Genome Start | Genome End | Strand | Div | Repeat Start | Repeat End | Repeat Left | Name | Family | Class |
| 114207533 | 114208017 | + | 14.8 | 3878 | 4383 | -1760 | L1MA4A | L1 | LINE |
| 114208021 | 114214154 | + | 6.3 | 13 | 6155 | 0 | L1PA4 | L1 | LINE |
| 114214139 | 114215224 | + | 17.0 | 4358 | 5450 | -862 | L1MA4A | L1 | LINE |
| 114215224 | 114217277 | - | 9.8 | 4087 | 6148 | -3 | L1PB1 | L1 | LINE |
| 114217277 | 114218557 | + | 9.2 | 2796 | 4077 | -2069 | L1PB1 | L1 | LINE |
| 114218556 | 114219334 | + | 15.6 | 5439 | 6250 | -53 | L1MA4A | L1 | LINE |

  

|  |  |  |  |  |  |  |  |  |  |
| --- | --- | --- | --- | --- | --- | --- | --- | --- | --- |
| chr5:114275762-114286623 | | | 10815 bp | | |  |  |  |  |
| Genome Start | Genome End | Strand | Div | Repeat Start | Repeat End | Repeat Left | Name | Family | Class |
| 114275762 | 114275952 | + | 20.4 | 4 | 194 | -6669 | L1PBa | L1 | LINE |
| 114275953 | 114276527 | + | 11.5 | 903 | 1666 | -5197 | L1PBa | L1 | LINE |
| 114276548 | 114278266 | + | 11.6 | 1667 | 3459 | -3403 | L1PBa | L1 | LINE |
| 114278284 | 114283159 | - | 4.1 | 1250 | 6152 | -3 | L1PA3 | L1 | LINE |
| 114283159 | 114283561 | + | 5.2 | 841 | 1243 | -4912 | L1PA3 | L1 | LINE |
| 114283573 | 114286623 | + | 9.3 | 3470 | 6118 | -744 | L1PBa | L1 | LINE |

  

|  |  |  |  |  |  |  |  |  |  |
| --- | --- | --- | --- | --- | --- | --- | --- | --- | --- |
| chr5:116955629-116956723 | | | 1093 bp | | |  |  |  |  |
| Genome Start | Genome End | Strand | Div | Repeat Start | Repeat End | Repeat Left | Name | Family | Class |
| 116955629 | 116955758 | - | 23.1 | 6128 | 6265 | -26 | L1MA8 | L1 | LINE |
| 116955764 | 116956230 | - | 6.2 | 5685 | 6154 | 0 | L1PA7 | L1 | LINE |
| 116956232 | 116956337 | + | 6.6 | 5566 | 5671 | -501 | L1PA7 | L1 | LINE |
| 116956334 | 116956363 | - | 13.0 | 6100 | 6129 | -162 | L1MA8 | L1 | LINE |
| 116956364 | 116956666 | + | 16.7 | 1 | 301 | -11 | AluSx | Alu | SINE |
| 116956667 | 116956723 | - | 13.0 | 6052 | 6099 | -192 | L1MA8 | L1 | LINE |

  

|  |  |  |  |  |  |  |  |  |  |
| --- | --- | --- | --- | --- | --- | --- | --- | --- | --- |
| chr5:117294140-117295760 | | | 1618 bp | | |  |  |  |  |
| Genome Start | Genome End | Strand | Div | Repeat Start | Repeat End | Repeat Left | Name | Family | Class |
| 117294140 | 117294341 | - | 14.8 | 127 | 304 | -8 | AluSx | Alu | SINE |
| 117294344 | 117295005 | - | 1.7 | 5491 | 6155 | 0 | L1PA2 | L1 | LINE |
| 117295007 | 117295625 | + | 1.0 | 4868 | 5486 | -669 | L1PA2 | L1 | LINE |
| 117295626 | 117295760 | - | 14.1 | 1 | 135 | -177 | AluSx | Alu | SINE |

  

|  |  |  |  |  |  |  |  |  |  |
| --- | --- | --- | --- | --- | --- | --- | --- | --- | --- |
| chr5:117635269-117640111 | | | 4839 bp | | |  |  |  |  |
| Genome Start | Genome End | Strand | Div | Repeat Start | Repeat End | Repeat Left | Name | Family | Class |
| 117635269 | 117637330 | + | 21.4 | 31 | 2393 | -4240 | L1MDa | L1 | LINE |
| 117637330 | 117637523 | - | 6.7 | 5281 | 5477 | -695 | L1PA7 | L1 | LINE |
| 117637524 | 117638194 | + | 6.1 | 5479 | 6149 | -5 | L1PA7 | L1 | LINE |
| 117638200 | 117640111 | + | 17.8 | 2389 | 4321 | -2216 | L1MDa | L1 | LINE |

  

|  |  |  |  |  |  |  |  |  |  |
| --- | --- | --- | --- | --- | --- | --- | --- | --- | --- |
| chr5:117765243-117769940 | | | 4729 bp | | |  |  |  |  |
| Genome Start | Genome End | Strand | Div | Repeat Start | Repeat End | Repeat Left | Name | Family | Class |
| 117765243 | 117766447 | - | 9.5 | 5093 | 6353 | 0 | L1PA10 | L1 | LINE |
| 117766448 | 117766994 | - | 3.5 | 5611 | 6154 | -1 | L1PA3 | L1 | LINE |
| 117766995 | 117767407 | + | 3.9 | 5198 | 5608 | -547 | L1PA3 | L1 | LINE |
| 117767406 | 117767538 | - | 14.6 | 4973 | 5103 | -1228 | L1PA10 | L1 | LINE |
| 117767510 | 117768846 | - | 13.0 | 572 | 1958 | -4395 | L1PA10 | L1 | LINE |
| 117768847 | 117769129 | - | 17.3 | 9 | 292 | -20 | AluSx | Alu | SINE |
| 117769130 | 117769940 | - | 13.0 | 4 | 571 | -5782 | L1PA10 | L1 | LINE |

  

|  |  |  |  |  |  |  |  |  |  |
| --- | --- | --- | --- | --- | --- | --- | --- | --- | --- |
| chr5:117820927-117823029 | | | 2076 bp | | |  |  |  |  |
| Genome Start | Genome End | Strand | Div | Repeat Start | Repeat End | Repeat Left | Name | Family | Class |
| 117820927 | 117821836 | + | 30.3 | 43 | 1039 | -1468 | HAL1 | L1 | LINE |
| 117821839 | 117821992 | - | 6.5 | 5801 | 5955 | -199 | L1PA5 | L1 | LINE |
| 117821993 | 117822190 | + | 10.1 | 5946 | 6143 | -11 | L1PA5 | L1 | LINE |
| 117822216 | 117823029 | + | 28.1 | 1042 | 1879 | -628 | HAL1 | L1 | LINE |

  

|  |  |  |  |  |  |  |  |  |  |
| --- | --- | --- | --- | --- | --- | --- | --- | --- | --- |
| chr5:119322259-119325439 | | | 3165 bp | | |  |  |  |  |
| Genome Start | Genome End | Strand | Div | Repeat Start | Repeat End | Repeat Left | Name | Family | Class |
| 119322259 | 119322535 | - | 21.0 | 139 | 407 | -19 | LTR37A | ERV1 | LTR |
| 119322536 | 119322629 | - | 21.3 | 3428 | 3521 | -2622 | L1MA4A | L1 | LINE |
| 119322621 | 119322847 | + | 14.3 | 3523 | 3753 | -2390 | L1MA4A | L1 | LINE |
| 119322864 | 119323155 | - | 11.6 | 1 | 308 | -5 | AluSq | Alu | SINE |
| 119323157 | 119325302 | + | 14.9 | 4090 | 6299 | -4 | L1MA4A | L1 | LINE |
| 119325311 | 119325439 | - | 21.6 | 20 | 149 | -277 | LTR37A | ERV1 | LTR |

  

|  |  |  |  |  |  |  |  |  |  |
| --- | --- | --- | --- | --- | --- | --- | --- | --- | --- |
| chr5:120399311-120401583 | | | 1797 bp | | |  |  |  |  |
| Genome Start | Genome End | Strand | Div | Repeat Start | Repeat End | Repeat Left | Name | Family | Class |
| 120399311 | 120399718 | + | 29.5 | 3777 | 4180 | -1966 | L1MC4a | L1 | LINE |
| 120399805 | 120400246 | + | 34.3 | 4382 | 4833 | -1313 | L1MC4a | L1 | LINE |
| 120400317 | 120400504 | - | 4.3 | 5842 | 6030 | -124 | L1PA5 | L1 | LINE |
| 120400505 | 120400631 | + | 3.9 | 6025 | 6151 | -3 | L1PA5 | L1 | LINE |
| 120400952 | 120401583 | + | 28.8 | 5267 | 5946 | -1862 | L1MC4a | L1 | LINE |

  

|  |  |  |  |  |  |  |  |  |  |
| --- | --- | --- | --- | --- | --- | --- | --- | --- | --- |
| chr5:121042059-121045335 | | | 3224 bp | | |  |  |  |  |
| Genome Start | Genome End | Strand | Div | Repeat Start | Repeat End | Repeat Left | Name | Family | Class |
| 121042059 | 121042169 | + | 15.3 | 3 | 104 | -465 | MER61E | ERV1 | LTR |
| 121042157 | 121042200 | + | 22.7 | 246 | 289 | -555 | LTR25 | ERV1 | LTR |
| 121042201 | 121042749 | - | 10.1 | 3474 | 4038 | -2108 | L1PA7 | L1 | LINE |
| 121042747 | 121044823 | + | 8.2 | 4054 | 6146 | -8 | L1PA7 | L1 | LINE |
| 121044893 | 121045335 | + | 20.8 | 85 | 526 | -43 | MER61E | ERV1 | LTR |

  

|  |  |  |  |  |  |  |  |  |  |
| --- | --- | --- | --- | --- | --- | --- | --- | --- | --- |
| chr5:121407299-121418223 | | | 10911 bp | | |  |  |  |  |
| Genome Start | Genome End | Strand | Div | Repeat Start | Repeat End | Repeat Left | Name | Family | Class |
| 121407299 | 121407433 | - | 20.0 | 89 | 223 | -1 | MER58A | MER1\_type | DNA |
| 121407445 | 121409650 | - | 12.4 | 3951 | 6164 | -1 | L1PA15 | L1 | LINE |
| 121409646 | 121412743 | - | 4.3 | 3049 | 6152 | -3 | L1PA4 | L1 | LINE |
| 121412742 | 121413527 | + | 5.5 | 2260 | 3046 | -3100 | L1PA4 | L1 | LINE |
| 121413526 | 121418129 | - | 14.1 | 11 | 4491 | -2182 | L1PA14 | L1 | LINE |
| 121418142 | 121418223 | - | 26.8 | 1 | 84 | -23 | MER58A | MER1\_type | DNA |

  

|  |  |  |  |  |  |  |  |  |  |
| --- | --- | --- | --- | --- | --- | --- | --- | --- | --- |
| chr5:122714644-122720990 | | | 6372 bp | | |  |  |  |  |
| Genome Start | Genome End | Strand | Div | Repeat Start | Repeat End | Repeat Left | Name | Family | Class |
| 122714644 | 122715174 | - | 25.4 | 5662 | 6243 | 0 | L1ME3A | L1 | LINE |
| 122715175 | 122715474 | - | 13.0 | 1 | 232 | -4 | MER46B | MER2\_type | DNA |
| 122715475 | 122718381 | - | 27.2 | 2318 | 5661 | -582 | L1ME3A | L1 | LINE |
| 122718369 | 122719945 | - | 3.5 | 4582 | 6155 | 0 | L1PA4 | L1 | LINE |
| 122719934 | 122720001 | + | 1.5 | 4502 | 4567 | -1579 | L1PA4 | L1 | LINE |
| 122720002 | 122720990 | - | 30.0 | 1249 | 2352 | -3887 | L1ME3A | L1 | LINE |

  

|  |  |  |  |  |  |  |  |  |  |
| --- | --- | --- | --- | --- | --- | --- | --- | --- | --- |
| chr5:124987183-124991924 | | | 4711 bp | | |  |  |  |  |
| Genome Start | Genome End | Strand | Div | Repeat Start | Repeat End | Repeat Left | Name | Family | Class |
| 124987183 | 124987801 | + | 20.1 | 1522 | 2216 | -5118 | L1M4b | L1 | LINE |
| 124987819 | 124988383 | - | 6.6 | 3415 | 3981 | -2165 | L1PA5 | L1 | LINE |
| 124988383 | 124990529 | + | 5.0 | 4000 | 6147 | -7 | L1PA5 | L1 | LINE |
| 124990545 | 124991924 | + | 22.9 | 2258 | 3999 | -3007 | L1M4b | L1 | LINE |

  

|  |  |  |  |  |  |  |  |  |  |
| --- | --- | --- | --- | --- | --- | --- | --- | --- | --- |
| chr5:125090933-125108683 | | | 17524 bp | | |  |  |  |  |
| Genome Start | Genome End | Strand | Div | Repeat Start | Repeat End | Repeat Left | Name | Family | Class |
| 125090933 | 125091153 | - | 28.9 | 5610 | 5818 | -1990 | L1MC | L1 | LINE |
| 125091213 | 125091478 | - | 18.9 | 3 | 268 | -44 | AluJb | Alu | SINE |
| 125091647 | 125092189 | - | 33.6 | 2858 | 3419 | 0 | L2 | L2 | LINE |
| 125092201 | 125092585 | + | 19.8 | 1 | 396 | 0 | MSTD | MaLR | LTR |
| 125092588 | 125092988 | + | 22.2 | 2 | 410 | -1325 | MSTD-int | MaLR | LTR |
| 125092993 | 125093380 | - | 7.6 | 4539 | 4916 | -1230 | L1PB | L1 | LINE |
| 125093379 | 125093426 | + | 8.3 | 4939 | 4991 | -1155 | L1PB | L1 | LINE |
| 125093431 | 125093981 | + | 21.2 | 5 | 578 | -5297 | L1P3b | L1 | LINE |
| 125093969 | 125100044 | + | 11.1 | 383 | 6483 | 0 | L1PA10 | L1 | LINE |
| 125100035 | 125100651 | + | 9.9 | 4974 | 5607 | -544 | L1PB1 | L1 | LINE |
| 125100648 | 125104145 | - | 7.1 | 445 | 3950 | -2196 | L1PA5 | L1 | LINE |
| 125104149 | 125106361 | + | 6.5 | 3935 | 6153 | -1 | L1PA5 | L1 | LINE |
| 125106362 | 125106914 | + | 9.4 | 5593 | 6151 | 0 | L1PB1 | L1 | LINE |
| 125106920 | 125108176 | + | 25.9 | 402 | 1735 | 0 | MSTD-int | MaLR | LTR |
| 125108177 | 125108578 | + | 17.5 | 1 | 408 | 0 | MSTD | MaLR | LTR |
| 125108579 | 125108683 | - | 25.0 | 5370 | 5483 | -2325 | L1MC | L1 | LINE |

  

|  |  |  |  |  |  |  |  |  |  |
| --- | --- | --- | --- | --- | --- | --- | --- | --- | --- |
| chr5:125212798-125215706 | | | 2894 bp | | |  |  |  |  |
| Genome Start | Genome End | Strand | Div | Repeat Start | Repeat End | Repeat Left | Name | Family | Class |
| 125212798 | 125212901 | - | 18.8 | 3288 | 3394 | -25 | L2 | L2 | LINE |
| 125212916 | 125213892 | - | 9.4 | 3481 | 4480 | -1666 | L1PA8 | L1 | LINE |
| 125213893 | 125215515 | + | 8.8 | 4490 | 6166 | -6 | L1PA8 | L1 | LINE |
| 125215517 | 125215706 | - | 29.8 | 3083 | 3284 | -135 | L2 | L2 | LINE |

  

|  |  |  |  |  |  |  |  |  |  |
| --- | --- | --- | --- | --- | --- | --- | --- | --- | --- |
| chr5:126416717-126419083 | | | 1966 bp | | |  |  |  |  |
| Genome Start | Genome End | Strand | Div | Repeat Start | Repeat End | Repeat Left | Name | Family | Class |
| 126416717 | 126416959 | + | 33.2 | 18 | 306 | -1654 | L4 | RTE | LINE |
| 126417033 | 126417528 | - | 6.2 | 5651 | 6146 | -8 | L1PA6 | L1 | LINE |
| 126417833 | 126418735 | - | 5.6 | 4746 | 5648 | -506 | L1PA6 | L1 | LINE |
| 126418737 | 126418962 | + | 4.4 | 4530 | 4755 | -1391 | L1PA6 | L1 | LINE |
| 126418986 | 126419083 | + | 32.6 | 326 | 423 | -1537 | L4 | RTE | LINE |

  

|  |  |  |  |  |  |  |  |  |  |
| --- | --- | --- | --- | --- | --- | --- | --- | --- | --- |
| chr5:126628398-126636593 | | | 8206 bp | | |  |  |  |  |
| Genome Start | Genome End | Strand | Div | Repeat Start | Repeat End | Repeat Left | Name | Family | Class |
| 126628398 | 126628512 | - | 7.8 | 6052 | 6166 | -8 | L1PA11 | L1 | LINE |
| 126628513 | 126632103 | - | 5.3 | 2557 | 6150 | -4 | L1PA5 | L1 | LINE |
| 126632104 | 126634259 | + | 7.0 | 411 | 2573 | -3573 | L1PA5 | L1 | LINE |
| 126634250 | 126635113 | - | 8.8 | 5188 | 6066 | -108 | L1PA11 | L1 | LINE |
| 126635114 | 126635479 | + | 10.1 | 1 | 375 | 0 | THE1C | MaLR | LTR |
| 126635480 | 126636593 | - | 8.8 | 4090 | 5187 | -959 | L1PA11 | L1 | LINE |

  

|  |  |  |  |  |  |  |  |  |  |
| --- | --- | --- | --- | --- | --- | --- | --- | --- | --- |
| chr5:126903928-126909966 | | | 6066 bp | | |  |  |  |  |
| Genome Start | Genome End | Strand | Div | Repeat Start | Repeat End | Repeat Left | Name | Family | Class |
| 126903928 | 126904350 | - | 27.9 | 1489 | 1948 | -4291 | L1MEc | L1 | LINE |
| 126904318 | 126904506 | - | 3.7 | 5182 | 5371 | -775 | L1P1 | L1 | LINE |
| 126904492 | 126908622 | - | 3.9 | 3 | 4122 | -2024 | L1P1 | L1 | LINE |
| 126908629 | 126909382 | + | 2.8 | 5393 | 6155 | 0 | L1PA3 | L1 | LINE |
| 126909398 | 126909966 | - | 27.1 | 926 | 1511 | -4889 | L1MEc | L1 | LINE |

  

|  |  |  |  |  |  |  |  |  |  |
| --- | --- | --- | --- | --- | --- | --- | --- | --- | --- |
| chr5:130256094-130270492 | | | 14108 bp | | |  |  |  |  |
| Genome Start | Genome End | Strand | Div | Repeat Start | Repeat End | Repeat Left | Name | Family | Class |
| 130256094 | 130256476 | - | 31.4 | 2876 | 3318 | -101 | L2 | L2 | LINE |
| 130256477 | 130256768 | + | 12.3 | 1 | 303 | -9 | AluSx | Alu | SINE |
| 130256769 | 130257469 | - | 32.0 | 2140 | 2875 | -544 | L2 | L2 | LINE |
| 130257471 | 130259286 | + | 6.8 | 632 | 2410 | -3736 | L1PA6 | L1 | LINE |
| 130259287 | 130259997 | + | 5.8 | 5457 | 6152 | -2 | L1PA6 | L1 | LINE |
| 130260017 | 130260617 | + | 17.9 | 2317 | 2957 | -4236 | L1MCa | L1 | LINE |
| 130260672 | 130260722 | + | 7.8 | 3558 | 3609 | -3725 | L1MCa | L1 | LINE |
| 130260731 | 130261157 | + | 9.8 | 4858 | 5285 | -861 | L1PA6 | L1 | LINE |
| 130261159 | 130267261 | + | 4.5 | 13 | 6152 | -3 | L1PA3 | L1 | LINE |
| 130267271 | 130268148 | + | 5.7 | 5270 | 6152 | -2 | L1PA6 | L1 | LINE |
| 130268203 | 130269008 | + | 23.5 | 4629 | 5483 | -683 | L1M5 | L1 | LINE |
| 130269014 | 130269679 | - | 1.6 | 5364 | 6028 | -127 | L1PA3 | L1 | LINE |
| 130269680 | 130269805 | + | 3.2 | 6030 | 6155 | 0 | L1PA3 | L1 | LINE |
| 130269899 | 130270003 | + | 24.8 | 5479 | 5583 | -583 | L1M5 | L1 | LINE |
| 130270051 | 130270492 | - | 26.4 | 1373 | 1859 | -1560 | L2 | L2 | LINE |

  

|  |  |  |  |  |  |  |  |  |  |
| --- | --- | --- | --- | --- | --- | --- | --- | --- | --- |
| chr5:131946965-131949182 | | | 2238 bp | | |  |  |  |  |
| Genome Start | Genome End | Strand | Div | Repeat Start | Repeat End | Repeat Left | Name | Family | Class |
| 131946965 | 131947281 | - | 20.5 | 5826 | 6161 | -5 | L1ME1 | L1 | LINE |
| 131947282 | 131947586 | - | 7.6 | 2 | 303 | -10 | AluSp | Alu | SINE |
| 131947587 | 131947694 | - | 20.5 | 5725 | 5825 | -341 | L1ME1 | L1 | LINE |
| 131947690 | 131948117 | - | 4.0 | 5137 | 5565 | -589 | L1PA5 | L1 | LINE |
| 131948118 | 131948698 | + | 3.8 | 5574 | 6150 | -4 | L1PA5 | L1 | LINE |
| 131948699 | 131948716 | - | 7.1 | 5122 | 5136 | -1010 | L1P2 | L1 | LINE |
| 131948702 | 131949182 | - | 24.8 | 5275 | 5737 | -429 | L1ME1 | L1 | LINE |

  

|  |  |  |  |  |  |  |  |  |  |
| --- | --- | --- | --- | --- | --- | --- | --- | --- | --- |
| chr5:132708342-132710737 | | | 2310 bp | | |  |  |  |  |
| Genome Start | Genome End | Strand | Div | Repeat Start | Repeat End | Repeat Left | Name | Family | Class |
| 132708342 | 132708542 | - | 31.0 | 1700 | 1917 | -590 | HAL1 | L1 | LINE |
| 132708560 | 132708711 | - | 4.6 | 146 | 299 | -12 | AluY | Alu | SINE |
| 132708774 | 132708823 | - | 20.0 | 1546 | 1595 | -912 | HAL1 | L1 | LINE |
| 132708823 | 132709213 | - | 8.2 | 4954 | 5348 | -798 | L1PA8 | L1 | LINE |
| 132709211 | 132710013 | + | 6.5 | 5369 | 6172 | 0 | L1PA8 | L1 | LINE |
| 132710025 | 132710737 | - | 27.2 | 833 | 1560 | -947 | HAL1 | L1 | LINE |

  

|  |  |  |  |  |  |  |  |  |  |
| --- | --- | --- | --- | --- | --- | --- | --- | --- | --- |
| chr5:133000800-133002939 | | | 2000 bp | | |  |  |  |  |
| Genome Start | Genome End | Strand | Div | Repeat Start | Repeat End | Repeat Left | Name | Family | Class |
| 133000800 | 133000965 | + | 21.9 | 1897 | 2060 | -1359 | L2 | L2 | LINE |
| 133000970 | 133001112 | - | 25.6 | 3279 | 3417 | -2 | L2 | L2 | LINE |
| 133001096 | 133001471 | + | 26.1 | 2063 | 2440 | -979 | L2 | L2 | LINE |
| 133001472 | 133001533 | + | 9.7 | 222 | 284 | -27 | Alu | Alu | SINE |
| 133001570 | 133002365 | + | 26.1 | 2441 | 3323 | -96 | L2 | L2 | LINE |
| 133002381 | 133002462 | - | 25.0 | 5833 | 5913 | -208 | L1ME4a | L1 | LINE |
| 133002557 | 133002777 | - | 9.9 | 5943 | 6169 | -3 | L1PA8 | L1 | LINE |
| 133002779 | 133002856 | + | 7.7 | 5876 | 5954 | -218 | L1PA8 | L1 | LINE |
| 133002864 | 133002939 | + | 34.2 | 3327 | 3403 | -16 | L2 | L2 | LINE |

  

|  |  |  |  |  |  |  |  |  |  |
| --- | --- | --- | --- | --- | --- | --- | --- | --- | --- |
| chr5:135900898-135903492 | | | 2617 bp | | |  |  |  |  |
| Genome Start | Genome End | Strand | Div | Repeat Start | Repeat End | Repeat Left | Name | Family | Class |
| 135900898 | 135901690 | + | 19.0 | 2179 | 3043 | -3103 | L1M4 | L1 | LINE |
| 135901693 | 135902384 | - | 12.3 | 4493 | 5192 | -954 | L1PREC2 | L1 | LINE |
| 135902383 | 135903353 | + | 11.7 | 5194 | 6159 | -6 | L1PA15 | L1 | LINE |
| 135903332 | 135903492 | + | 15.8 | 3020 | 3187 | -2959 | L1M4 | L1 | LINE |

  

|  |  |  |  |  |  |  |  |  |  |
| --- | --- | --- | --- | --- | --- | --- | --- | --- | --- |
| chr5:136369197-136372778 | | | 3277 bp | | |  |  |  |  |
| Genome Start | Genome End | Strand | Div | Repeat Start | Repeat End | Repeat Left | Name | Family | Class |
| 136369197 | 136369572 | + | 30.0 | 1052 | 1447 | -4768 | L1MC4 | L1 | LINE |
| 136369573 | 136370608 | - | 8.2 | 3826 | 4873 | -1273 | L1PA7 | L1 | LINE |
| 136370610 | 136371882 | + | 7.3 | 4872 | 6147 | -7 | L1PA7 | L1 | LINE |
| 136371935 | 136372179 | + | 27.2 | 1470 | 1719 | -4496 | L1MC4 | L1 | LINE |
| 136372191 | 136372360 | + | 30.3 | 1819 | 1986 | -4229 | L1MC4 | L1 | LINE |
| 136372602 | 136372778 | + | 26.1 | 2175 | 2337 | -3878 | L1MC4 | L1 | LINE |

  

|  |  |  |  |  |  |  |  |  |  |
| --- | --- | --- | --- | --- | --- | --- | --- | --- | --- |
| chr5:141400418-141404756 | | | 4345 bp | | |  |  |  |  |
| Genome Start | Genome End | Strand | Div | Repeat Start | Repeat End | Repeat Left | Name | Family | Class |
| 141400418 | 141400821 | + | 12.2 | 1 | 418 | -235 | MER39B | ERV1 | LTR |
| 141400822 | 141401126 | - | 13.8 | 1 | 306 | 0 | AluSx | Alu | SINE |
| 141401127 | 141401158 | + | 12.2 | 419 | 450 | -203 | MER39B | ERV1 | LTR |
| 141401177 | 141402417 | - | 2.3 | 4912 | 6155 | 0 | L1PA3 | L1 | LINE |
| 141402394 | 141404547 | + | 2.6 | 2775 | 4928 | -1218 | L1PA3 | L1 | LINE |
| 141404548 | 141404756 | + | 10.1 | 435 | 653 | 0 | MER39B | ERV1 | LTR |

  

|  |  |  |  |  |  |  |  |  |  |
| --- | --- | --- | --- | --- | --- | --- | --- | --- | --- |
| chr5:144288422-144297717 | | | 9310 bp | | |  |  |  |  |
| Genome Start | Genome End | Strand | Div | Repeat Start | Repeat End | Repeat Left | Name | Family | Class |
| 144288422 | 144290137 | + | 23.8 | 1171 | 3001 | -3375 | L1MA9 | L1 | LINE |
| 144290138 | 144290434 | + | 6.8 | 1 | 295 | -16 | AluY | Alu | SINE |
| 144290435 | 144290550 | + | 19.5 | 3002 | 3116 | -3260 | L1MA9 | L1 | LINE |
| 144290548 | 144292055 | - | 3.4 | 2524 | 4024 | -2122 | L1PA3 | L1 | LINE |
| 144292054 | 144294164 | + | 3.4 | 4037 | 6154 | -1 | L1PA3 | L1 | LINE |
| 144294156 | 144295633 | + | 18.8 | 3097 | 4658 | -1718 | L1MA9 | L1 | LINE |
| 144295634 | 144295947 | + | 13.6 | 1 | 308 | -2 | AluSg | Alu | SINE |
| 144295948 | 144297717 | + | 18.4 | 4659 | 6526 | -22 | L1MA9 | L1 | LINE |

  

|  |  |  |  |  |  |  |  |  |  |
| --- | --- | --- | --- | --- | --- | --- | --- | --- | --- |
| chr5:144352060-144357417 | | | 5290 bp | | |  |  |  |  |
| Genome Start | Genome End | Strand | Div | Repeat Start | Repeat End | Repeat Left | Name | Family | Class |
| 144352060 | 144353298 | - | 27.9 | 4117 | 5498 | -741 | L1M | L1 | LINE |
| 144353333 | 144353491 | - | 30.0 | 3793 | 3953 | -2266 | L1M | L1 | LINE |
| 144353521 | 144354311 | - | 30.0 | 2969 | 3792 | -2427 | L1M | L1 | LINE |
| 144354321 | 144356060 | - | 3.3 | 4415 | 6155 | 0 | L1PA3 | L1 | LINE |
| 144356059 | 144357184 | + | 2.8 | 3287 | 4412 | -1734 | L1PA3 | L1 | LINE |
| 144357183 | 144357417 | - | 28.4 | 2806 | 3000 | -3219 | L1M | L1 | LINE |

  

|  |  |  |  |  |  |  |  |  |  |
| --- | --- | --- | --- | --- | --- | --- | --- | --- | --- |
| chr5:144446580-144451559 | | | 4909 bp | | |  |  |  |  |
| Genome Start | Genome End | Strand | Div | Repeat Start | Repeat End | Repeat Left | Name | Family | Class |
| 144446580 | 144446697 | - | 24.8 | 6030 | 6161 | -3 | L1ME2 | L1 | LINE |
| 144446746 | 144448491 | - | 15.0 | 4395 | 6161 | -5 | L1PA16 | L1 | LINE |
| 144448526 | 144448597 | - | 15.6 | 4323 | 4394 | -1752 | L1PA16 | L1 | LINE |
| 144448595 | 144448705 | - | 8.1 | 5228 | 5342 | -804 | L1PA7 | L1 | LINE |
| 144448704 | 144449515 | + | 7.3 | 5338 | 6149 | -5 | L1PA7 | L1 | LINE |
| 144449510 | 144450264 | - | 14.1 | 3574 | 4335 | -1811 | L1PA16 | L1 | LINE |
| 144450265 | 144450550 | - | 22.7 | 1 | 291 | -21 | AluJb | Alu | SINE |
| 144450551 | 144450812 | - | 14.1 | 3314 | 3573 | -2573 | L1PA16 | L1 | LINE |
| 144450813 | 144451559 | - | 25.8 | 5164 | 5988 | -176 | L1ME2 | L1 | LINE |

  

|  |  |  |  |  |  |  |  |  |  |
| --- | --- | --- | --- | --- | --- | --- | --- | --- | --- |
| chr5:144695308-144697096 | | | 1761 bp | | |  |  |  |  |
| Genome Start | Genome End | Strand | Div | Repeat Start | Repeat End | Repeat Left | Name | Family | Class |
| 144695308 | 144695515 | + | 20.5 | 2841 | 3052 | -3088 | L1M3 | L1 | LINE |
| 144695522 | 144696393 | - | 2.7 | 4790 | 5661 | -494 | L1PA3 | L1 | LINE |
| 144696394 | 144696863 | + | 3.4 | 5668 | 6137 | -18 | L1PA3 | L1 | LINE |
| 144696886 | 144697096 | + | 16.8 | 3051 | 3266 | -2874 | L1M3 | L1 | LINE |

  

|  |  |  |  |  |  |  |  |  |  |
| --- | --- | --- | --- | --- | --- | --- | --- | --- | --- |
| chr5:144970528-144979916 | | | 9234 bp | | |  |  |  |  |
| Genome Start | Genome End | Strand | Div | Repeat Start | Repeat End | Repeat Left | Name | Family | Class |
| 144970528 | 144970720 | + | 31.9 | 3963 | 4164 | -1982 | L1M5 | L1 | LINE |
| 144970787 | 144970928 | + | 25.7 | 4159 | 4305 | -1841 | L1M5 | L1 | LINE |
| 144970929 | 144971219 | - | 15.1 | 1 | 291 | -21 | AluSx | Alu | SINE |
| 144971220 | 144972206 | + | 25.7 | 4306 | 5313 | -833 | L1M5 | L1 | LINE |
| 144972221 | 144973038 | - | 12.6 | 5818 | 6616 | -1 | L1PB1 | L1 | LINE |
| 144973053 | 144973269 | - | 2.8 | 5936 | 6152 | -2 | L1PA5 | L1 | LINE |
| 144973273 | 144973376 | + | 5.8 | 5850 | 5953 | -201 | L1PA5 | L1 | LINE |
| 144973371 | 144974684 | - | 7.8 | 4519 | 5836 | -776 | L1PB1 | L1 | LINE |
| 144974684 | 144975074 | - | 7.0 | 2702 | 3092 | -3520 | L1PB1 | L1 | LINE |
| 144975075 | 144975381 | - | 2.9 | 1 | 308 | -3 | AluY | Alu | SINE |
| 144975382 | 144976144 | - | 10.7 | 1891 | 2710 | -3903 | L1PB1 | L1 | LINE |
| 144976186 | 144977257 | - | 9.6 | 1 | 1057 | -14 | MER11C | ERVK | LTR |
| 144977258 | 144977473 | - | 17.9 | 1678 | 1894 | -4719 | L1PB1 | L1 | LINE |
| 144977474 | 144977788 | - | 10.9 | 1 | 313 | 0 | AluSq | Alu | SINE |
| 144977789 | 144979490 | - | 13.6 | 2 | 1677 | -4936 | L1PB1 | L1 | LINE |
| 144979515 | 144979916 | + | 28.8 | 5315 | 5724 | -516 | L1M5 | L1 | LINE |

  

|  |  |  |  |  |  |  |  |  |  |
| --- | --- | --- | --- | --- | --- | --- | --- | --- | --- |
| chr5:146257191-146259822 | | | 2631 bp | | |  |  |  |  |
| Genome Start | Genome End | Strand | Div | Repeat Start | Repeat End | Repeat Left | Name | Family | Class |
| 146257191 | 146257661 | + | 35.1 | 684 | 1150 | -1357 | HAL1 | L1 | LINE |
| 146257664 | 146257964 | + | 10.6 | 1 | 306 | -7 | AluSq | Alu | SINE |
| 146257967 | 146258097 | - | 1.5 | 4730 | 4860 | -1286 | L1PA3 | L1 | LINE |
| 146258097 | 146259393 | + | 2.4 | 4858 | 6155 | 0 | L1PA3 | L1 | LINE |
| 146259392 | 146259822 | + | 27.0 | 1140 | 1600 | -907 | HAL1 | L1 | LINE |

  

|  |  |  |  |  |  |  |  |  |  |
| --- | --- | --- | --- | --- | --- | --- | --- | --- | --- |
| chr5:146369954-146374954 | | | 4932 bp | | |  |  |  |  |
| Genome Start | Genome End | Strand | Div | Repeat Start | Repeat End | Repeat Left | Name | Family | Class |
| 146369954 | 146369991 | + | 27.3 | 2538 | 2567 | -852 | L2 | L2 | LINE |
| 146369992 | 146370530 | - | 12.4 | 4 | 528 | -1 | MER1A | MER1\_type | DNA |
| 146370531 | 146371143 | + | 28.4 | 2568 | 3262 | -157 | L2 | L2 | LINE |
| 146371198 | 146371285 | + | 11.6 | 2511 | 2598 | -133 | Tigger4(Zombi) | MER2\_type | DNA |
| 146371286 | 146372710 | - | 2.3 | 2780 | 4200 | -1946 | L1PA2 | L1 | LINE |
| 146372703 | 146374653 | + | 1.8 | 4203 | 6154 | -1 | L1PA2 | L1 | LINE |
| 146374663 | 146374803 | + | 10.7 | 2587 | 2731 | 0 | Tigger4(Zombi) | MER2\_type | DNA |
| 146374818 | 146374954 | + | 22.1 | 3277 | 3417 | -2 | L2 | L2 | LINE |

  

|  |  |  |  |  |  |  |  |  |  |
| --- | --- | --- | --- | --- | --- | --- | --- | --- | --- |
| chr5:147611747-147619664 | | | 7951 bp | | |  |  |  |  |
| Genome Start | Genome End | Strand | Div | Repeat Start | Repeat End | Repeat Left | Name | Family | Class |
| 147611747 | 147611883 | - | 21.1 | 6982 | 7114 | -1027 | L1PREC2 | L1 | LINE |
| 147611858 | 147613973 | - | 1.7 | 4037 | 6153 | -2 | L1PA2 | L1 | LINE |
| 147613971 | 147614741 | + | 1.8 | 3273 | 4044 | -2102 | L1PA2 | L1 | LINE |
| 147614738 | 147619664 | - | 15.9 | 1 | 7018 | -1123 | L1PREC2 | L1 | LINE |

  

|  |  |  |  |  |  |  |  |  |  |
| --- | --- | --- | --- | --- | --- | --- | --- | --- | --- |
| chr5:148061435-148070221 | | | 8759 bp | | |  |  |  |  |
| Genome Start | Genome End | Strand | Div | Repeat Start | Repeat End | Repeat Left | Name | Family | Class |
| 148061435 | 148062538 | + | 20.3 | 1213 | 2618 | -4716 | L1MB8 | L1 | LINE |
| 148062533 | 148064032 | - | 10.5 | 2994 | 4505 | -1641 | L1PB1 | L1 | LINE |
| 148064028 | 148064970 | + | 9.5 | 4521 | 5468 | -683 | L1PB1 | L1 | LINE |
| 148064997 | 148065755 | + | 9.1 | 5469 | 6150 | -1 | L1PB1 | L1 | LINE |
| 148065769 | 148065990 | + | 15.2 | 2607 | 2819 | -4515 | L1MB8 | L1 | LINE |
| 148065991 | 148066213 | - | 12.3 | 1 | 230 | 0 | MER30 | MER1\_type | DNA |
| 148066214 | 148068946 | + | 18.0 | 2820 | 6506 | -532 | L1MB8 | L1 | LINE |
| 148068947 | 148069299 | + | 16.5 | 1 | 346 | 0 | MER7A | MER2\_type | DNA |
| 148069300 | 148069745 | + | 19.9 | 6507 | 6968 | -70 | L1MB8 | L1 | LINE |
| 148069746 | 148070148 | - | 18.1 | 1 | 390 | 0 | MLT1B | MaLR | LTR |
| 148070149 | 148070221 | + | 19.9 | 6969 | 7037 | -1 | L1MB8 | L1 | LINE |

  

|  |  |  |  |  |  |  |  |  |  |
| --- | --- | --- | --- | --- | --- | --- | --- | --- | --- |
| chr5:151667254-151669720 | | | 2467 bp | | |  |  |  |  |
| Genome Start | Genome End | Strand | Div | Repeat Start | Repeat End | Repeat Left | Name | Family | Class |
| 151667254 | 151667885 | - | 18.6 | 299 | 923 | 0 | MER49 | ERV1 | LTR |
| 151667886 | 151668425 | - | 3.0 | 5616 | 6155 | 0 | L1PA3 | L1 | LINE |
| 151668426 | 151669418 | + | 2.6 | 4628 | 5620 | -535 | L1PA3 | L1 | LINE |
| 151669419 | 151669720 | - | 17.9 | 1 | 313 | -610 | MER49 | ERV1 | LTR |

  

|  |  |  |  |  |  |  |  |  |  |
| --- | --- | --- | --- | --- | --- | --- | --- | --- | --- |
| chr5:152073986-152076339 | | | 1897 bp | | |  |  |  |  |
| Genome Start | Genome End | Strand | Div | Repeat Start | Repeat End | Repeat Left | Name | Family | Class |
| 152073986 | 152074299 | - | 31.6 | 6475 | 6825 | -983 | L1MC4a | L1 | LINE |
| 152074673 | 152074790 | - | 30.5 | 5817 | 5937 | -1871 | L1MC4a | L1 | LINE |
| 152074794 | 152075172 | - | 1.6 | 5778 | 6151 | -4 | L1PA2 | L1 | LINE |
| 152075174 | 152075825 | + | 1.7 | 5141 | 5792 | -363 | L1PA2 | L1 | LINE |
| 152075855 | 152075999 | - | 24.0 | 5618 | 5784 | -2024 | L1MC4a | L1 | LINE |
| 152076027 | 152076142 | + | 17.2 | 1 | 123 | -20 | FLAM\_C | Alu | SINE |
| 152076167 | 152076339 | - | 31.2 | 5437 | 5593 | -2215 | L1MC4a | L1 | LINE |

  

|  |  |  |  |  |  |  |  |  |  |
| --- | --- | --- | --- | --- | --- | --- | --- | --- | --- |
| chr5:152098126-152109804 | | | 11596 bp | | |  |  |  |  |
| Genome Start | Genome End | Strand | Div | Repeat Start | Repeat End | Repeat Left | Name | Family | Class |
| 152098126 | 152100696 | + | 28.7 | 1 | 2067 | -4166 | L1M4c | L1 | LINE |
| 152100690 | 152106721 | + | 2.8 | 1 | 6027 | -5 | L1PA2 | L1 | LINE |
| 152106813 | 152107297 | - | 13.0 | 3968 | 4478 | -1668 | L1PA12 | L1 | LINE |
| 152107300 | 152108966 | + | 9.8 | 4493 | 6152 | -16 | L1PA12 | L1 | LINE |
| 152108964 | 152109804 | + | 20.9 | 2062 | 2972 | -3232 | L1M4c | L1 | LINE |

  

|  |  |  |  |  |  |  |  |  |  |
| --- | --- | --- | --- | --- | --- | --- | --- | --- | --- |
| chr5:152380372-152386669 | | | 6303 bp | | |  |  |  |  |
| Genome Start | Genome End | Strand | Div | Repeat Start | Repeat End | Repeat Left | Name | Family | Class |
| 152380372 | 152382215 | + | 28.1 | 1621 | 3212 | -3952 | L1M2a | L1 | LINE |
| 152382216 | 152382488 | + | 15.4 | 1 | 291 | -21 | AluJb | Alu | SINE |
| 152382489 | 152384263 | + | 20.3 | 3213 | 5187 | -1977 | L1M2a | L1 | LINE |
| 152384264 | 152384520 | + | 14.4 | 6032 | 6304 | -1 | L1MA3 | L1 | LINE |
| 152384521 | 152384684 | + | 20.3 | 5188 | 5371 | -1793 | L1M2a | L1 | LINE |
| 152384685 | 152385328 | - | 2.2 | 4715 | 5360 | -786 | L1PA3 | L1 | LINE |
| 152385328 | 152385860 | + | 2.1 | 5361 | 5893 | -262 | L1PA3 | L1 | LINE |
| 152385857 | 152386669 | + | 19.1 | 5366 | 6193 | -971 | L1M2a | L1 | LINE |

  

|  |  |  |  |  |  |  |  |  |  |
| --- | --- | --- | --- | --- | --- | --- | --- | --- | --- |
| chr5:152439222-152440311 | | | 1087 bp | | |  |  |  |  |
| Genome Start | Genome End | Strand | Div | Repeat Start | Repeat End | Repeat Left | Name | Family | Class |
| 152439222 | 152439783 | + | 9.8 | 1 | 572 | -62 | MER41B | ERV1 | LTR |
| 152439785 | 152440119 | - | 8.8 | 5826 | 6154 | 0 | L1PA6 | L1 | LINE |
| 152440122 | 152440237 | + | 0.9 | 5693 | 5808 | -346 | L1P2 | L1 | LINE |
| 152440238 | 152440311 | + | 8.1 | 561 | 634 | 0 | MER41B | ERV1 | LTR |

  

|  |  |  |  |  |  |  |  |  |  |
| --- | --- | --- | --- | --- | --- | --- | --- | --- | --- |
| chr5:156231495-156236799 | | | 4968 bp | | |  |  |  |  |
| Genome Start | Genome End | Strand | Div | Repeat Start | Repeat End | Repeat Left | Name | Family | Class |
| 156231495 | 156231758 | + | 29.7 | 3865 | 4170 | -1976 | L1M5 | L1 | LINE |
| 156231762 | 156232527 | + | 27.4 | 6711 | 7532 | -510 | L1MC4 | L1 | LINE |
| 156232547 | 156233843 | - | 5.3 | 4845 | 6152 | -2 | L1PA7 | L1 | LINE |
| 156233843 | 156234230 | + | 9.3 | 4458 | 4862 | -1284 | L1PA7 | L1 | LINE |
| 156234275 | 156234506 | + | 20.2 | 7730 | 7965 | -30 | L1MD3 | L1 | LINE |
| 156234507 | 156234801 | - | 16.9 | 1 | 296 | -16 | AluSx | Alu | SINE |
| 156234802 | 156234840 | + | 20.2 | 7966 | 7986 | -9 | L1MD3 | L1 | LINE |
| 156234876 | 156235145 | + | 26.8 | 7563 | 7850 | -192 | L1MC4 | L1 | LINE |
| 156235233 | 156235457 | - | 27.1 | 578 | 794 | -137 | MER21A | ERV1 | LTR |
| 156235479 | 156235672 | + | 17.1 | 140 | 344 | -1 | MER2 | MER2\_type | DNA |
| 156235696 | 156236176 | - | 28.5 | 1 | 448 | -415 | MER21B | ERV1 | LTR |
| 156236219 | 156236552 | + | 28.8 | 4198 | 4571 | -1575 | L1M5 | L1 | LINE |
| 156236617 | 156236799 | + | 30.2 | 4613 | 4793 | -1353 | L1M5 | L1 | LINE |

  

|  |  |  |  |  |  |  |  |  |  |
| --- | --- | --- | --- | --- | --- | --- | --- | --- | --- |
| chr5:159803429-159809762 | | | 6216 bp | | |  |  |  |  |
| Genome Start | Genome End | Strand | Div | Repeat Start | Repeat End | Repeat Left | Name | Family | Class |
| 159803429 | 159803835 | - | 29.6 | 2936 | 3419 | 0 | L2 | L2 | LINE |
| 159803941 | 159804037 | + | 15.3 | 7505 | 7576 | -466 | L1MC4 | L1 | LINE |
| 159804015 | 159804325 | - | 15.2 | 5043 | 5353 | -790 | L1MA5 | L1 | LINE |
| 159804323 | 159805280 | + | 15.1 | 5334 | 6300 | 0 | L1MA5 | L1 | LINE |
| 159805285 | 159805710 | + | 22.7 | 7560 | 8012 | -30 | L1MC4 | L1 | LINE |
| 159805719 | 159805784 | - | 20.3 | 2261 | 2318 | -4082 | L1MEc | L1 | LINE |
| 159805785 | 159806087 | - | 14.5 | 1 | 303 | -9 | AluSx | Alu | SINE |
| 159806088 | 159806538 | - | 20.3 | 1780 | 2260 | -4140 | L1MEc | L1 | LINE |
| 159806539 | 159806846 | - | 9.8 | 1 | 305 | -5 | AluSg | Alu | SINE |
| 159806847 | 159807032 | - | 20.3 | 1593 | 1779 | -4621 | L1MEc | L1 | LINE |
| 159807033 | 159807295 | - | 12.9 | 1 | 263 | -49 | AluSx | Alu | SINE |
| 159807296 | 159807934 | - | 20.3 | 949 | 1592 | -4808 | L1MEc | L1 | LINE |
| 159807935 | 159808243 | - | 11.8 | 1 | 297 | -14 | AluY | Alu | SINE |
| 159808244 | 159808742 | - | 20.3 | 470 | 948 | -5452 | L1MEc | L1 | LINE |
| 159808743 | 159809158 | + | 16.5 | 1 | 425 | -1 | MSTB | MaLR | LTR |
| 159809159 | 159809590 | - | 20.3 | 30 | 469 | -5931 | L1MEc | L1 | LINE |
| 159809618 | 159809762 | - | 25.5 | 2629 | 2809 | -610 | L2 | L2 | LINE |

  

|  |  |  |  |  |  |  |  |  |  |
| --- | --- | --- | --- | --- | --- | --- | --- | --- | --- |
| chr5:160323608-160324518 | | | 885 bp | | |  |  |  |  |
| Genome Start | Genome End | Strand | Div | Repeat Start | Repeat End | Repeat Left | Name | Family | Class |
| 160323608 | 160323790 | + | 30.8 | 2613 | 2815 | -604 | L2 | L2 | LINE |
| 160323791 | 160323839 | - | 4.1 | 5999 | 6047 | -108 | L1PA3 | L1 | LINE |
| 160323840 | 160323976 | + | 2.9 | 6048 | 6184 | 0 | L1PA3 | L1 | LINE |
| 160323977 | 160324368 | + | 33.2 | 2814 | 3265 | -154 | L2 | L2 | LINE |
| 160324395 | 160324518 | + | 30.8 | 3266 | 3409 | -10 | L2 | L2 | LINE |

  

|  |  |  |  |  |  |  |  |  |  |
| --- | --- | --- | --- | --- | --- | --- | --- | --- | --- |
| chr5:160348859-160365855 | | | 16678 bp | | |  |  |  |  |
| Genome Start | Genome End | Strand | Div | Repeat Start | Repeat End | Repeat Left | Name | Family | Class |
| 160348859 | 160349805 | - | 19.4 | 6462 | 7352 | -1 | L1MA7 | L1 | LINE |
| 160349808 | 160350244 | + | 7.9 | 3 | 444 | 0 | MLT2A1 | ERVL | LTR |
| 160350245 | 160350886 | + | 7.8 | 1 | 649 | -5005 | HERVL-A1 | ERVL | LTR |
| 160350910 | 160355083 | + | 7.4 | 650 | 4859 | -795 | HERVL-A1 | ERVL | LTR |
| 160355084 | 160355346 | - | 0.8 | 44 | 307 | -3 | AluYa5 | Alu | SINE |
| 160355347 | 160355983 | + | 6.9 | 4860 | 5492 | -162 | HERVL-A1 | ERVL | LTR |
| 160355985 | 160357227 | - | 8.7 | 4906 | 6151 | -3 | L1PA7 | L1 | LINE |
| 160357232 | 160357512 | + | 10.0 | 4639 | 4919 | -1227 | L1PA7 | L1 | LINE |
| 160357506 | 160358289 | - | 12.0 | 8 | 803 | -5658 | L1P3 | L1 | LINE |
| 160358296 | 160358469 | + | 5.8 | 5480 | 5654 | 0 | HERVL-A1 | ERVL | LTR |
| 160358470 | 160358860 | + | 7.4 | 1 | 431 | -13 | MLT2A1 | ERVL | LTR |
| 160358861 | 160359218 | + | 16.2 | 4071 | 4438 | -1705 | L1M2 | L1 | LINE |
| 160359378 | 160359574 | - | 15.6 | 3566 | 3757 | -2386 | L1M2 | L1 | LINE |
| 160359644 | 160359698 | + | 9.1 | 498 | 552 | 0 | MLT2A1 | ERVL | LTR |
| 160359700 | 160363903 | - | 21.9 | 2113 | 6462 | -743 | L1MA7 | L1 | LINE |
| 160363904 | 160364214 | - | 0.6 | 1 | 310 | 0 | AluYa5 | Alu | SINE |
| 160364215 | 160364609 | - | 22.4 | 1718 | 2112 | -5097 | L1MA7 | L1 | LINE |
| 160364671 | 160365283 | - | 25.5 | 161 | 817 | -6392 | L1MA7 | L1 | LINE |
| 160365284 | 160365693 | + | 15.5 | 1 | 428 | 0 | MSTA | MaLR | LTR |
| 160365694 | 160365855 | - | 25.5 | 15 | 160 | -7049 | L1MA7 | L1 | LINE |

  

|  |  |  |  |  |  |  |  |  |  |
| --- | --- | --- | --- | --- | --- | --- | --- | --- | --- |
| chr5:160721330-160723303 | | | 1966 bp | | |  |  |  |  |
| Genome Start | Genome End | Strand | Div | Repeat Start | Repeat End | Repeat Left | Name | Family | Class |
| 160721330 | 160721428 | + | 22.2 | 38 | 136 | -176 | AluJo | Alu | SINE |
| 160721429 | 160722047 | - | 9.2 | 4468 | 5085 | -1061 | L1PA7 | L1 | LINE |
| 160722043 | 160723119 | + | 5.7 | 5070 | 6148 | -6 | L1PA7 | L1 | LINE |
| 160723133 | 160723303 | + | 12.3 | 129 | 299 | -13 | AluJo | Alu | SINE |

  

|  |  |  |  |  |  |  |  |  |  |
| --- | --- | --- | --- | --- | --- | --- | --- | --- | --- |
| chr5:161261545-161265827 | | | 4282 bp | | |  |  |  |  |
| Genome Start | Genome End | Strand | Div | Repeat Start | Repeat End | Repeat Left | Name | Family | Class |
| 161261545 | 161262437 | - | 14.3 | 307 | 1248 | -54 | HSMAR2 | Mariner | DNA |
| 161262438 | 161264869 | - | 8.2 | 2908 | 5343 | -803 | L1PA6 | L1 | LINE |
| 161264866 | 161265638 | + | 7.8 | 5361 | 6143 | -11 | L1PA6 | L1 | LINE |
| 161265644 | 161265827 | - | 7.7 | 118 | 332 | -970 | HSMAR2 | Mariner | DNA |

  

|  |  |  |  |  |  |  |  |  |  |
| --- | --- | --- | --- | --- | --- | --- | --- | --- | --- |
| chr5:161776937-161778970 | | | 2016 bp | | |  |  |  |  |
| Genome Start | Genome End | Strand | Div | Repeat Start | Repeat End | Repeat Left | Name | Family | Class |
| 161776937 | 161777042 | + | 26.4 | 1 | 106 | -599 | Tigger7 | MER2\_type | DNA |
| 161777072 | 161778252 | - | 13.1 | 4953 | 6157 | -9 | L1PA16 | L1 | LINE |
| 161778247 | 161778418 | + | 15.7 | 4799 | 4970 | -1176 | L1PA16 | L1 | LINE |
| 161778421 | 161778856 | + | 15.8 | 110 | 546 | -1945 | Tigger7 | MER2\_type | DNA |
| 161778850 | 161778970 | + | 22.3 | 2363 | 2491 | 0 | Tigger7 | MER2\_type | DNA |

  

|  |  |  |  |  |  |  |  |  |  |
| --- | --- | --- | --- | --- | --- | --- | --- | --- | --- |
| chr5:162083760-162089959 | | | 5691 bp | | |  |  |  |  |
| Genome Start | Genome End | Strand | Div | Repeat Start | Repeat End | Repeat Left | Name | Family | Class |
| 162083760 | 162083861 | + | 34.3 | 403 | 514 | -2905 | L2 | L2 | LINE |
| 162083863 | 162085488 | - | 7.8 | 4510 | 6167 | -5 | L1PA8 | L1 | LINE |
| 162085489 | 162085754 | + | 8.3 | 2957 | 3224 | -2922 | L1P3 | L1 | LINE |
| 162085778 | 162086037 | + | 33.3 | 547 | 826 | -2593 | L2 | L2 | LINE |
| 162086197 | 162086495 | + | 17.1 | 1 | 286 | -26 | AluJb | Alu | SINE |
| 162086567 | 162086631 | + | 32.8 | 69 | 133 | -45 | MER5B | MER1\_type | DNA |
| 162086790 | 162087006 | + | 31.2 | 960 | 1192 | -2227 | L2 | L2 | LINE |
| 162087037 | 162087849 | + | 30.6 | 1652 | 2554 | -865 | L2 | L2 | LINE |
| 162087901 | 162088406 | - | 16.1 | 5806 | 6331 | -3 | L1MA10 | L1 | LINE |
| 162088408 | 162088483 | + | 20.3 | 5713 | 5782 | -552 | L1MA10 | L1 | LINE |
| 162088492 | 162088570 | + | 29.1 | 2600 | 2678 | -741 | L2 | L2 | LINE |
| 162088580 | 162088925 | - | 11.3 | 50 | 377 | 0 | MER51B | ERV1 | LTR |
| 162088920 | 162088999 | - | 14.1 | 1 | 103 | -531 | MER51A | ERV1 | LTR |
| 162089004 | 162089442 | + | 33.9 | 2696 | 3180 | -239 | L2 | L2 | LINE |
| 162089443 | 162089742 | - | 19.4 | 1 | 289 | -23 | AluJo | Alu | SINE |
| 162089743 | 162089959 | + | 36.3 | 3181 | 3394 | -25 | L2 | L2 | LINE |

  

|  |  |  |  |  |  |  |  |  |  |
| --- | --- | --- | --- | --- | --- | --- | --- | --- | --- |
| chr5:162215348-162221560 | | | 5964 bp | | |  |  |  |  |
| Genome Start | Genome End | Strand | Div | Repeat Start | Repeat End | Repeat Left | Name | Family | Class |
| 162215348 | 162215511 | + | 28.4 | 335 | 510 | -6824 | L1MB8 | L1 | LINE |
| 162215715 | 162216579 | - | 4.5 | 4075 | 4953 | -1193 | L1PA3 | L1 | LINE |
| 162216555 | 162217757 | + | 4.0 | 4949 | 6151 | -4 | L1PA3 | L1 | LINE |
| 162217811 | 162218663 | + | 19.7 | 1346 | 2326 | -5008 | L1MB8 | L1 | LINE |
| 162218659 | 162219431 | + | 20.4 | 4636 | 5416 | -1590 | L1MB8 | L1 | LINE |
| 162219432 | 162219819 | + | 17.6 | 1 | 390 | 0 | MLT1B | MaLR | LTR |
| 162219820 | 162221070 | + | 20.5 | 5417 | 6678 | -360 | L1MB8 | L1 | LINE |
| 162221082 | 162221220 | + | 12.2 | 172 | 312 | 0 | AluSg/x | Alu | SINE |
| 162221233 | 162221560 | + | 21.3 | 6690 | 7038 | 0 | L1MB8 | L1 | LINE |

  

|  |  |  |  |  |  |  |  |  |  |
| --- | --- | --- | --- | --- | --- | --- | --- | --- | --- |
| chr5:162400168-162414068 | | | 13814 bp | | |  |  |  |  |
| Genome Start | Genome End | Strand | Div | Repeat Start | Repeat End | Repeat Left | Name | Family | Class |
| 162400168 | 162405820 | + | 19.8 | 5 | 6263 | -1299 | L1MA4 | L1 | LINE |
| 162405852 | 162406149 | + | 14.8 | 3 | 335 | -40 | THE1C | MaLR | LTR |
| 162406150 | 162408684 | - | 11.8 | 2 | 2566 | -24 | L1PA7 | L1 | LINE |
| 162408689 | 162412554 | + | 8.3 | 2568 | 6462 | -7 | L1PA7 | L1 | LINE |
| 162412607 | 162412662 | + | 5.5 | 326 | 381 | 0 | THE1C | MaLR | LTR |
| 162412663 | 162414068 | + | 15.0 | 6263 | 7710 | -9 | L1MA4 | L1 | LINE |

  

|  |  |  |  |  |  |  |  |  |  |
| --- | --- | --- | --- | --- | --- | --- | --- | --- | --- |
| chr5:162748476-162752200 | | | 3529 bp | | |  |  |  |  |
| Genome Start | Genome End | Strand | Div | Repeat Start | Repeat End | Repeat Left | Name | Family | Class |
| 162748476 | 162749855 | - | 15.2 | 4941 | 6254 | -46 | L1MA4 | L1 | LINE |
| 162749856 | 162750029 | - | 11.6 | 5985 | 6156 | -7 | L1PA13 | L1 | LINE |
| 162750030 | 162750324 | - | 23.5 | 4661 | 4960 | -1186 | L1PREC2 | L1 | LINE |
| 162750523 | 162751236 | - | 7.0 | 5436 | 6152 | -2 | L1PA7 | L1 | LINE |
| 162751238 | 162751799 | + | 8.4 | 4857 | 5428 | -744 | L1PA7 | L1 | LINE |
| 162751797 | 162752200 | - | 16.9 | 4317 | 4694 | -1449 | L1MA4 | L1 | LINE |

  

|  |  |  |  |  |  |  |  |  |  |
| --- | --- | --- | --- | --- | --- | --- | --- | --- | --- |
| chr5:163329957-163332242 | | | 2239 bp | | |  |  |  |  |
| Genome Start | Genome End | Strand | Div | Repeat Start | Repeat End | Repeat Left | Name | Family | Class |
| 163329957 | 163330205 | + | 31.6 | 2831 | 3077 | -342 | L2 | L2 | LINE |
| 163330223 | 163330973 | - | 3.9 | 5401 | 6151 | -4 | L1PA4 | L1 | LINE |
| 163330973 | 163331890 | + | 5.0 | 4467 | 5399 | -747 | L1PA4 | L1 | LINE |
| 163331922 | 163332242 | + | 30.3 | 3103 | 3409 | -10 | L2 | L2 | LINE |

  

|  |  |  |  |  |  |  |  |  |  |
| --- | --- | --- | --- | --- | --- | --- | --- | --- | --- |
| chr5:164844984-164847220 | | | 1320 bp | | |  |  |  |  |
| Genome Start | Genome End | Strand | Div | Repeat Start | Repeat End | Repeat Left | Name | Family | Class |
| 164844984 | 164845227 | - | 32.7 | 3356 | 3585 | -904 | L3 | CR1 | LINE |
| 164845337 | 164845412 | - | 6.6 | 6077 | 6152 | -2 | L1PA5 | L1 | LINE |
| 164845413 | 164845656 | + | 5.8 | 5850 | 6091 | -63 | L1PA5 | L1 | LINE |
| 164845789 | 164846222 | - | 36.4 | 2574 | 3040 | -1449 | L3 | CR1 | LINE |
| 164846483 | 164846598 | + | 32.0 | 52 | 162 | -114 | MIRm | MIR | SINE |
| 164846734 | 164846824 | - | 34.1 | 2131 | 2227 | -2262 | L3 | CR1 | LINE |
| 164847106 | 164847220 | - | 35.1 | 2040 | 2152 | -2337 | L3 | CR1 | LINE |

  

|  |  |  |  |  |  |  |  |  |  |
| --- | --- | --- | --- | --- | --- | --- | --- | --- | --- |
| chr5:165236149-165238828 | | | 2791 bp | | |  |  |  |  |
| Genome Start | Genome End | Strand | Div | Repeat Start | Repeat End | Repeat Left | Name | Family | Class |
| 165236149 | 165237569 | - | 30.7 | 1932 | 3404 | -15 | L2 | L2 | LINE |
| 165237646 | 165238352 | - | 7.7 | 5447 | 6153 | -1 | L1PA7 | L1 | LINE |
| 165238173 | 165238358 | - | 10.2 | 5441 | 5626 | -528 | L1PA7 | L1 | LINE |
| 165238343 | 165238735 | + | 9.6 | 5018 | 5419 | -759 | L1PA7 | L1 | LINE |
| 165238745 | 165238828 | - | 27.7 | 1632 | 1719 | -1700 | L2 | L2 | LINE |

  

|  |  |  |  |  |  |  |  |  |  |
| --- | --- | --- | --- | --- | --- | --- | --- | --- | --- |
| chr5:166047971-166049356 | | | 1067 bp | | |  |  |  |  |
| Genome Start | Genome End | Strand | Div | Repeat Start | Repeat End | Repeat Left | Name | Family | Class |
| 166047971 | 166048020 | + | 26.0 | 1732 | 1781 | -179 | L4 | RTE | LINE |
| 166048071 | 166048109 | - | 20.1 | 6226 | 6272 | -40 | L1MA9 | L1 | LINE |
| 166048110 | 166048446 | + | 16.5 | 1 | 346 | 0 | MER7A | MER2\_type | DNA |
| 166048491 | 166048685 | - | 20.1 | 6027 | 6225 | -87 | L1MA9 | L1 | LINE |
| 166048687 | 166048928 | - | 17.4 | 1 | 260 | -52 | AluJb | Alu | SINE |
| 166048945 | 166049019 | - | 18.7 | 5861 | 5935 | -377 | L1MA9 | L1 | LINE |
| 166049022 | 166049082 | + | 11.5 | 5818 | 5878 | -311 | L1MB1 | L1 | LINE |
| 166049289 | 166049356 | + | 30.9 | 1889 | 1958 | -2 | L4 | RTE | LINE |

  

|  |  |  |  |  |  |  |  |  |  |
| --- | --- | --- | --- | --- | --- | --- | --- | --- | --- |
| chr5:166276061-166281491 | | | 4817 bp | | |  |  |  |  |
| Genome Start | Genome End | Strand | Div | Repeat Start | Repeat End | Repeat Left | Name | Family | Class |
| 166276061 | 166276266 | + | 25.4 | 2825 | 3067 | -352 | L2 | L2 | LINE |
| 166276267 | 166276575 | - | 8.4 | 1 | 310 | 0 | AluSg | Alu | SINE |
| 166276576 | 166276755 | + | 28.8 | 3055 | 3261 | -158 | L2 | L2 | LINE |
| 166276821 | 166276905 | + | 21.2 | 944 | 1036 | -26 | MER63 | AcHobo | DNA |
| 166276916 | 166277045 | + | 31.5 | 3250 | 3381 | -38 | L2 | L2 | LINE |
| 166277151 | 166277214 | - | 18.6 | 3352 | 3413 | -6 | L2 | L2 | LINE |
| 166277216 | 166277386 | - | 14.0 | 137 | 307 | -5 | AluSg/x | Alu | SINE |
| 166277399 | 166277816 | - | 26.9 | 2982 | 3345 | -74 | L2 | L2 | LINE |
| 166277817 | 166278135 | + | 10.3 | 1 | 310 | 0 | AluSg | Alu | SINE |
| 166278136 | 166278201 | - | 30.3 | 2915 | 2981 | -438 | L2 | L2 | LINE |
| 166278374 | 166278882 | + | 28.2 | 3523 | 4048 | -2098 | L1M5 | L1 | LINE |
| 166278946 | 166279263 | - | 13.1 | 1 | 312 | 0 | AluSx | Alu | SINE |
| 166279379 | 166279927 | - | 23.2 | 5773 | 6314 | -20 | L1MA10 | L1 | LINE |
| 166279931 | 166280285 | + | 14.2 | 5395 | 5753 | -559 | L1M2 | L1 | LINE |
| 166280312 | 166280604 | + | 29.9 | 2783 | 3173 | -246 | L2 | L2 | LINE |
| 166280605 | 166280902 | - | 9.2 | 3 | 300 | -10 | AluSg | Alu | SINE |
| 166280903 | 166280955 | + | 32.2 | 3174 | 3218 | -201 | L2 | L2 | LINE |
| 166280998 | 166281299 | - | 8.2 | 2 | 297 | -14 | AluY | Alu | SINE |
| 166281300 | 166281491 | + | 32.2 | 3219 | 3417 | -2 | L2 | L2 | LINE |

  

|  |  |  |  |  |  |  |  |  |  |
| --- | --- | --- | --- | --- | --- | --- | --- | --- | --- |
| chr5:166752826-166756491 | | | 3628 bp | | |  |  |  |  |
| Genome Start | Genome End | Strand | Div | Repeat Start | Repeat End | Repeat Left | Name | Family | Class |
| 166752826 | 166753499 | - | 31.2 | 2589 | 3271 | -148 | L2 | L2 | LINE |
| 166753527 | 166754182 | - | 16.0 | 4741 | 5332 | -808 | L1M3 | L1 | LINE |
| 166754183 | 166754479 | - | 18.0 | 1 | 294 | -18 | AluJb | Alu | SINE |
| 166754480 | 166755188 | - | 16.0 | 4040 | 4740 | -1400 | L1M3 | L1 | LINE |
| 166755184 | 166755219 | + | 16.9 | 5346 | 5378 | -790 | L1M | L1 | LINE |
| 166755220 | 166755424 | + | 23.4 | 2 | 209 | -1 | MER6B | MER2\_type | DNA |
| 166755425 | 166756073 | + | 16.9 | 5379 | 6024 | -144 | L1MB1 | L1 | LINE |
| 166756077 | 166756393 | - | 32.1 | 364 | 699 | -2720 | L2 | L2 | LINE |
| 166756407 | 166756491 | - | 20.2 | 83 | 191 | -3228 | L2 | L2 | LINE |

  

|  |  |  |  |  |  |  |  |  |  |
| --- | --- | --- | --- | --- | --- | --- | --- | --- | --- |
| chr5:168570864-168573416 | | | 2533 bp | | |  |  |  |  |
| Genome Start | Genome End | Strand | Div | Repeat Start | Repeat End | Repeat Left | Name | Family | Class |
| 168570864 | 168571198 | + | 24.5 | 2728 | 3058 | -361 | L2 | L2 | LINE |
| 168571227 | 168572615 | - | 7.2 | 4763 | 6149 | -5 | L1PA7 | L1 | LINE |
| 168572606 | 168573111 | + | 9.3 | 4200 | 4740 | -1406 | L1PA7 | L1 | LINE |
| 168573114 | 168573416 | + | 24.9 | 3096 | 3419 | 0 | L2 | L2 | LINE |

  

|  |  |  |  |  |  |  |  |  |  |
| --- | --- | --- | --- | --- | --- | --- | --- | --- | --- |
| chr5:168970218-168979457 | | | 8841 bp | | |  |  |  |  |
| Genome Start | Genome End | Strand | Div | Repeat Start | Repeat End | Repeat Left | Name | Family | Class |
| 168970218 | 168971061 | + | 19.7 | 825 | 1785 | -4966 | L1MCb | L1 | LINE |
| 168971062 | 168972993 | - | 8.3 | 2763 | 4691 | -1455 | L1PB1 | L1 | LINE |
| 168972995 | 168973265 | + | 8.1 | 4709 | 4980 | -1166 | L1PB1 | L1 | LINE |
| 168973370 | 168973742 | + | 9.8 | 5782 | 6151 | 0 | L1PB1 | L1 | LINE |
| 168973754 | 168974214 | + | 19.0 | 1786 | 2439 | -4432 | L1MCb | L1 | LINE |
| 168974215 | 168974527 | + | 7.4 | 1 | 311 | 0 | AluY | Alu | SINE |
| 168974528 | 168974876 | + | 19.8 | 2320 | 2676 | -4075 | L1MCb | L1 | LINE |
| 168974884 | 168975097 | + | 16.5 | 2885 | 3105 | -3459 | L1MCb | L1 | LINE |
| 168975098 | 168975463 | + | 13.6 | 1 | 381 | 0 | THE1D | MaLR | LTR |
| 168975464 | 168976825 | + | 16.5 | 3106 | 4484 | -2080 | L1MCb | L1 | LINE |
| 168976823 | 168977056 | + | 7.3 | 5558 | 5791 | -360 | L1P5 | L1 | LINE |
| 168977298 | 168977834 | + | 20.3 | 4446 | 4952 | -1612 | L1MCb | L1 | LINE |
| 168977835 | 168978898 | + | 6.5 | 1 | 1222 | -14 | MER11B | ERVK | LTR |
| 168978937 | 168979457 | + | 17.7 | 4946 | 5460 | -1104 | L1MCb | L1 | LINE |

  

|  |  |  |  |  |  |  |  |  |  |
| --- | --- | --- | --- | --- | --- | --- | --- | --- | --- |
| chr5:168986769-168988651 | | | 1725 bp | | |  |  |  |  |
| Genome Start | Genome End | Strand | Div | Repeat Start | Repeat End | Repeat Left | Name | Family | Class |
| 168986769 | 168986977 | + | 30.6 | 2611 | 2856 | -563 | L2 | L2 | LINE |
| 168986980 | 168987492 | - | 18.8 | 72 | 614 | 0 | MER90a | ERV1 | LTR |
| 168987507 | 168987722 | + | 35.9 | 2856 | 3080 | -339 | L2 | L2 | LINE |
| 168987747 | 168988312 | - | 6.5 | 5592 | 6158 | -14 | L1PA8 | L1 | LINE |
| 168988313 | 168988409 | + | 6.2 | 5503 | 5599 | -573 | L1PA8 | L1 | LINE |
| 168988528 | 168988651 | + | 37.7 | 3243 | 3376 | -2 | L2 | L2 | LINE |

  

|  |  |  |  |  |  |  |  |  |  |
| --- | --- | --- | --- | --- | --- | --- | --- | --- | --- |
| chr5:170322749-170326812 | | | 3791 bp | | |  |  |  |  |
| Genome Start | Genome End | Strand | Div | Repeat Start | Repeat End | Repeat Left | Name | Family | Class |
| 170322749 | 170322981 | + | 31.7 | 4804 | 5034 | -1112 | L1MC4a | L1 | LINE |
| 170323107 | 170323422 | - | 13.0 | 1 | 319 | -20 | MER1B | MER1\_type | DNA |
| 170323552 | 170323986 | + | 29.8 | 5347 | 5798 | -2010 | L1MC4a | L1 | LINE |
| 170324009 | 170325612 | - | 3.3 | 4549 | 6152 | -3 | L1PA3 | L1 | LINE |
| 170325613 | 170326688 | + | 2.9 | 3450 | 4525 | -1621 | L1PA3 | L1 | LINE |
| 170326686 | 170326812 | + | 29.1 | 5786 | 5913 | -1895 | L1MC4a | L1 | LINE |

  

|  |  |  |  |  |  |  |  |  |  |
| --- | --- | --- | --- | --- | --- | --- | --- | --- | --- |
| chr5:174782697-174790289 | | | 7544 bp | | |  |  |  |  |
| Genome Start | Genome End | Strand | Div | Repeat Start | Repeat End | Repeat Left | Name | Family | Class |
| 174782697 | 174783865 | + | 17.7 | 2633 | 3853 | -2293 | L1MC3 | L1 | LINE |
| 174783863 | 174784006 | - | 13.9 | 4873 | 5030 | -1116 | L1PA17 | L1 | LINE |
| 174784008 | 174785092 | + | 13.4 | 5083 | 6152 | -14 | L1PA17 | L1 | LINE |
| 174785095 | 174785219 | - | 8.8 | 162 | 290 | -22 | AluSg/x | Alu | SINE |
| 174785224 | 174786392 | + | 20.0 | 3840 | 5045 | -1101 | L1MC3 | L1 | LINE |
| 174786393 | 174786699 | + | 14.3 | 1 | 304 | -8 | AluJo | Alu | SINE |
| 174786700 | 174788097 | + | 17.8 | 5046 | 6428 | -1357 | L1MC3 | L1 | LINE |
| 174788109 | 174788541 | - | 11.9 | 5529 | 5957 | -209 | L1PA16 | L1 | LINE |
| 174788546 | 174788632 | + | 14.9 | 5975 | 6061 | -105 | L1PA16 | L1 | LINE |
| 174788634 | 174788700 | + | 16.4 | 6098 | 6165 | -1 | L1PA16 | L1 | LINE |
| 174788711 | 174788999 | + | 11.0 | 1 | 285 | -26 | AluY | Alu | SINE |
| 174789019 | 174790289 | + | 15.8 | 6423 | 7779 | -6 | L1MC3 | L1 | LINE |

  

|  |  |  |  |  |  |  |  |  |  |
| --- | --- | --- | --- | --- | --- | --- | --- | --- | --- |
| chr5:180366039-180377894 | | | 11689 bp | | |  |  |  |  |
| Genome Start | Genome End | Strand | Div | Repeat Start | Repeat End | Repeat Left | Name | Family | Class |
| 180366039 | 180366403 | + | 23.6 | 3598 | 3965 | -2181 | L1ME1 | L1 | LINE |
| 180366397 | 180366878 | + | 21.2 | 5345 | 5867 | -299 | L1ME1 | L1 | LINE |
| 180367024 | 180367334 | + | 7.1 | 1 | 311 | 0 | AluY | Alu | SINE |
| 180367358 | 180371972 | + | 11.7 | 8 | 4346 | -1800 | L1PA8 | L1 | LINE |
| 180371962 | 180373250 | + | 9.7 | 3840 | 5133 | -1013 | L1PA7 | L1 | LINE |
| 180373248 | 180374056 | - | 6.8 | 4607 | 5426 | -728 | L1P | L1 | LINE |
| 180374057 | 180374771 | + | 6.5 | 5441 | 6154 | 0 | L1PA6 | L1 | LINE |
| 180374774 | 180375800 | + | 7.3 | 5128 | 6153 | -1 | L1PA7 | L1 | LINE |
| 180375796 | 180377591 | + | 7.1 | 4316 | 6159 | -13 | L1PA8 | L1 | LINE |
| 180377615 | 180377894 | + | 28.0 | 5863 | 6163 | -1 | L1ME1 | L1 | LINE |

  

|  |  |  |  |  |  |  |  |  |  |
| --- | --- | --- | --- | --- | --- | --- | --- | --- | --- |
| chr5:180738806-180741300 | | | 2460 bp | | |  |  |  |  |
| Genome Start | Genome End | Strand | Div | Repeat Start | Repeat End | Repeat Left | Name | Family | Class |
| 180738806 | 180739890 | - | 16.4 | 3657 | 4741 | -1399 | L1M3 | L1 | LINE |
| 180739910 | 180740087 | - | 4.5 | 5977 | 6155 | 0 | L1PA4 | L1 | LINE |
| 180740088 | 180740809 | + | 2.2 | 5254 | 5975 | -180 | L1PA4 | L1 | LINE |
| 180740826 | 180741300 | - | 18.7 | 3167 | 3646 | -2494 | L1M3 | L1 | LINE |

  


|  |  |  |  |  |  |  |  |  |  |
| --- | --- | --- | --- | --- | --- | --- | --- | --- | --- |
| chr6:8688-12390 | | | 3703 bp | | |  |  |  |  |
| Genome Start | Genome End | Strand | Div | Repeat Start | Repeat End | Repeat Left | Name | Family | Class |
| 8688 | 8843 | + | 23.5 | 5372 | 5525 | -2283 | L1MC5 | L1 | LINE |
| 8847 | 9896 | - | 1.9 | 5105 | 6155 | 0 | L1PA2 | L1 | LINE |
| 9899 | 11698 | + | 2.1 | 3311 | 5110 | -1036 | L1PA2 | L1 | LINE |
| 11694 | 11893 | + | 24.1 | 5512 | 5734 | -2074 | L1MC5 | L1 | LINE |
| 11894 | 12203 | + | 12.9 | 1 | 310 | -2 | AluSx | Alu | SINE |
| 12204 | 12390 | + | 24.1 | 5735 | 5942 | -1866 | L1MC5 | L1 | LINE |

  

|  |  |  |  |  |  |  |  |  |  |
| --- | --- | --- | --- | --- | --- | --- | --- | --- | --- |
| chr6:37439-39945 | | | 2472 bp | | |  |  |  |  |
| Genome Start | Genome End | Strand | Div | Repeat Start | Repeat End | Repeat Left | Name | Family | Class |
| 37439 | 37927 | + | 19.5 | 3150 | 3646 | -2494 | L1M3 | L1 | LINE |
| 37944 | 38664 | - | 2.6 | 5254 | 5975 | -180 | L1PA4 | L1 | LINE |
| 38665 | 38842 | + | 5.1 | 5977 | 6155 | 0 | L1PA4 | L1 | LINE |
| 38862 | 39945 | + | 16.4 | 3657 | 4741 | -1399 | L1M3 | L1 | LINE |

  

|  |  |  |  |  |  |  |  |  |  |
| --- | --- | --- | --- | --- | --- | --- | --- | --- | --- |
| chr6:959667-961102 | | | 1401 bp | | |  |  |  |  |
| Genome Start | Genome End | Strand | Div | Repeat Start | Repeat End | Repeat Left | Name | Family | Class |
| 959667 | 959923 | + | 22.8 | 70 | 341 | -24 | MLT1A0 | MaLR | LTR |
| 959924 | 960119 | - | 8.7 | 5084 | 5279 | -867 | L1PA8 | L1 | LINE |
| 960120 | 961018 | + | 7.3 | 5276 | 6172 | 0 | L1PA8 | L1 | LINE |
| 961054 | 961102 | + | 18.4 | 358 | 406 | -2 | MLT1A0 | MaLR | LTR |

  

|  |  |  |  |  |  |  |  |  |  |
| --- | --- | --- | --- | --- | --- | --- | --- | --- | --- |
| chr6:1944987-1947394 | | | 2176 bp | | |  |  |  |  |
| Genome Start | Genome End | Strand | Div | Repeat Start | Repeat End | Repeat Left | Name | Family | Class |
| 1944987 | 1945097 | - | 26.9 | 1377 | 1487 | -522 | HAL1b | L1 | LINE |
| 1945269 | 1945565 | - | 6.8 | 3 | 298 | -13 | AluY | Alu | SINE |
| 1945569 | 1945849 | - | 20.1 | 5817 | 6106 | -72 | L1MB8 | L1 | LINE |
| 1945913 | 1946058 | - | 17.8 | 1 | 146 | -30 | FRAM | Alu | SINE |
| 1946059 | 1946768 | - | 16.9 | 5000 | 5827 | -351 | L1MB8 | L1 | LINE |
| 1946762 | 1947144 | + | 17.3 | 4616 | 5016 | -1130 | L1MB8 | L1 | LINE |
| 1947147 | 1947394 | - | 29.3 | 1138 | 1388 | -621 | HAL1b | L1 | LINE |

  

|  |  |  |  |  |  |  |  |  |  |
| --- | --- | --- | --- | --- | --- | --- | --- | --- | --- |
| chr6:2477227-2481193 | | | 3528 bp | | |  |  |  |  |
| Genome Start | Genome End | Strand | Div | Repeat Start | Repeat End | Repeat Left | Name | Family | Class |
| 2477227 | 2477358 | + | 28.2 | 1 | 144 | -118 | MIR | MIR | SINE |
| 2477400 | 2477445 | - | 10.9 | 5388 | 5433 | -735 | L1M3 | L1 | LINE |
| 2477461 | 2478376 | - | 15.1 | 4477 | 5418 | -766 | L1M | L1 | LINE |
| 2478374 | 2478441 | + | 13.2 | 5424 | 5491 | -821 | L1M2 | L1 | LINE |
| 2478442 | 2478566 | + | 16.8 | 4 | 128 | -174 | AluJ | Alu | SINE |
| 2478577 | 2478668 | + | 21.2 | 183 | 267 | -35 | AluJ | Alu | SINE |
| 2478669 | 2478836 | + | 14.3 | 4576 | 4743 | -1397 | L1MA9 | L1 | LINE |
| 2478837 | 2478894 | + | 12.5 | 246 | 301 | -1 | AluJ | Alu | SINE |
| 2478904 | 2479267 | + | 15.8 | 5784 | 6166 | -146 | L1MA9 | L1 | LINE |
| 2479302 | 2479444 | + | 15.8 | 6167 | 6311 | -1 | L1MA9 | L1 | LINE |
| 2479482 | 2479853 | - | 17.9 | 3 | 393 | -74 | MLT1C | MaLR | LTR |
| 2479874 | 2480297 | - | 25.8 | 31 | 519 | -276 | LTR50 | ERVL | LTR |
| 2480315 | 2480492 | - | 25.9 | 5479 | 5667 | 0 | ERVL-E | ERVL | LTR |
| 2480721 | 2481046 | - | 19.1 | 5833 | 6174 | -10 | L1MB7 | L1 | LINE |
| 2481078 | 2481193 | + | 21.6 | 144 | 262 | 0 | MIR | MIR | SINE |

  

|  |  |  |  |  |  |  |  |  |  |
| --- | --- | --- | --- | --- | --- | --- | --- | --- | --- |
| chr6:6384898-6390896 | | | 6253 bp | | |  |  |  |  |
| Genome Start | Genome End | Strand | Div | Repeat Start | Repeat End | Repeat Left | Name | Family | Class |
| 6384898 | 6386105 | + | 25.7 | 28 | 1237 | -5305 | L1MB7 | L1 | LINE |
| 6386120 | 6387389 | + | 19.0 | 4370 | 5645 | -999 | L1MB7 | L1 | LINE |
| 6387390 | 6387518 | + | 22.5 | 1 | 129 | -425 | MLT2B5 | ERVL | LTR |
| 6387519 | 6387914 | - | 2.7 | 5031 | 5425 | -730 | L1P | L1 | LINE |
| 6387915 | 6388647 | + | 2.2 | 5419 | 6154 | -1 | L1PA3 | L1 | LINE |
| 6388648 | 6389123 | + | 20.7 | 120 | 597 | -4 | MLT2B5 | ERVL | LTR |
| 6388843 | 6389124 | + | 19.5 | 647 | 902 | -1146 | Ricksha | MuDR | DNA |
| 6389128 | 6389340 | + | 13.6 | 5641 | 5828 | -816 | L1MB7 | L1 | LINE |
| 6389349 | 6389555 | + | 17.3 | 95 | 291 | -21 | AluJo | Alu | SINE |
| 6389558 | 6389885 | + | 16.0 | 5821 | 6143 | -539 | L1MB7 | L1 | LINE |
| 6389886 | 6390435 | + | 22.2 | 1 | 505 | 0 | MLT1D | MaLR | LTR |
| 6390436 | 6390896 | + | 17.6 | 6144 | 6663 | -19 | L1MB7 | L1 | LINE |

  

|  |  |  |  |  |  |  |  |  |  |
| --- | --- | --- | --- | --- | --- | --- | --- | --- | --- |
| chr6:6434932-6435986 | | | 1036 bp | | |  |  |  |  |
| Genome Start | Genome End | Strand | Div | Repeat Start | Repeat End | Repeat Left | Name | Family | Class |
| 6434932 | 6435132 | + | 18.5 | 53 | 252 | -215 | MLT1C | MaLR | LTR |
| 6435133 | 6435299 | - | 9.6 | 5512 | 5678 | -485 | L1PA13 | L1 | LINE |
| 6435304 | 6435770 | + | 12.0 | 5694 | 6161 | -2 | L1PA13 | L1 | LINE |
| 6435786 | 6435986 | + | 25.0 | 278 | 502 | -3 | MLT1C | MaLR | LTR |

  

|  |  |  |  |  |  |  |  |  |  |
| --- | --- | --- | --- | --- | --- | --- | --- | --- | --- |
| chr6:12729623-12730308 | | | 685 bp | | |  |  |  |  |
| Genome Start | Genome End | Strand | Div | Repeat Start | Repeat End | Repeat Left | Name | Family | Class |
| 12729623 | 12729763 | - | 7.1 | 241 | 381 | 0 | THE1D | MaLR | LTR |
| 12729765 | 12729843 | - | 2.5 | 5876 | 5955 | -199 | L1PA4 | L1 | LINE |
| 12729844 | 12730051 | + | 3.4 | 5942 | 6154 | -1 | L1PA4 | L1 | LINE |
| 12730052 | 12730308 | - | 12.4 | 1 | 253 | -128 | THE1D | MaLR | LTR |

  

|  |  |  |  |  |  |  |  |  |  |
| --- | --- | --- | --- | --- | --- | --- | --- | --- | --- |
| chr6:13190534-13196497 | | | 5428 bp | | |  |  |  |  |
| Genome Start | Genome End | Strand | Div | Repeat Start | Repeat End | Repeat Left | Name | Family | Class |
| 13190534 | 13190705 | + | 28.0 | 185 | 368 | -4486 | L1MEe | L1 | LINE |
| 13190701 | 13192180 | - | 3.2 | 4136 | 5619 | -536 | L1PA4 | L1 | LINE |
| 13192181 | 13192750 | + | 3.1 | 5599 | 6153 | -2 | L1PA4 | L1 | LINE |
| 13192764 | 13193275 | + | 28.0 | 350 | 883 | -3971 | L1MEe | L1 | LINE |
| 13193560 | 13193937 | + | 26.2 | 895 | 1321 | -3513 | L1MEe | L1 | LINE |
| 13193964 | 13194535 | + | 30.2 | 1543 | 2193 | -2641 | L1MEe | L1 | LINE |
| 13194552 | 13195346 | - | 24.0 | 5266 | 6161 | -1 | L1ME3 | L1 | LINE |
| 13195540 | 13195866 | + | 27.4 | 2275 | 2670 | -2164 | L1MEe | L1 | LINE |
| 13195867 | 13196164 | - | 15.2 | 1 | 298 | -14 | AluJb | Alu | SINE |
| 13196165 | 13196330 | + | 27.4 | 2671 | 2839 | -1995 | L1MEe | L1 | LINE |
| 13196340 | 13196497 | + | 27.8 | 2918 | 3073 | -1761 | L1MEe | L1 | LINE |

  

|  |  |  |  |  |  |  |  |  |  |
| --- | --- | --- | --- | --- | --- | --- | --- | --- | --- |
| chr6:18651225-18657964 | | | 6731 bp | | |  |  |  |  |
| Genome Start | Genome End | Strand | Div | Repeat Start | Repeat End | Repeat Left | Name | Family | Class |
| 18651225 | 18652628 | + | 13.3 | 18 | 1428 | -4956 | L1M4c | L1 | LINE |
| 18652651 | 18652949 | + | 12.4 | 1 | 291 | -20 | AluY | Alu | SINE |
| 18652951 | 18653865 | - | 8.1 | 5235 | 6150 | -11 | L1PA14 | L1 | LINE |
| 18653861 | 18654062 | + | 5.7 | 5070 | 5246 | -900 | L1PREC2 | L1 | LINE |
| 18654059 | 18654905 | + | 12.2 | 1418 | 2271 | -4113 | L1M4c | L1 | LINE |
| 18654904 | 18656684 | - | 4.7 | 3205 | 4978 | -1168 | L1PA5 | L1 | LINE |
| 18656686 | 18657855 | + | 3.8 | 4978 | 6149 | -5 | L1PA5 | L1 | LINE |
| 18657852 | 18657964 | + | 12.6 | 2258 | 2371 | -4013 | L1M4c | L1 | LINE |

  

|  |  |  |  |  |  |  |  |  |  |
| --- | --- | --- | --- | --- | --- | --- | --- | --- | --- |
| chr6:19553959-19556342 | | | 2369 bp | | |  |  |  |  |
| Genome Start | Genome End | Strand | Div | Repeat Start | Repeat End | Repeat Left | Name | Family | Class |
| 19553959 | 19554074 | - | 22.4 | 50 | 166 | -12 | MER5B | MER1\_type | DNA |
| 19554092 | 19554222 | - | 12.6 | 6034 | 6160 | -5 | L1PA15 | L1 | LINE |
| 19554221 | 19554289 | + | 15.9 | 4427 | 4495 | -1651 | L1PB | L1 | LINE |
| 19554289 | 19554903 | - | 9.9 | 5390 | 6034 | -131 | L1PA15 | L1 | LINE |
| 19554901 | 19556281 | + | 11.7 | 3955 | 5394 | -752 | L1PREC2 | L1 | LINE |
| 19556286 | 19556342 | - | 22.8 | 1 | 57 | -121 | MER5B | MER1\_type | DNA |

  

|  |  |  |  |  |  |  |  |  |  |
| --- | --- | --- | --- | --- | --- | --- | --- | --- | --- |
| chr6:21345625-21349029 | | | 3405 bp | | |  |  |  |  |
| Genome Start | Genome End | Strand | Div | Repeat Start | Repeat End | Repeat Left | Name | Family | Class |
| 21345625 | 21346995 | + | 19.3 | 4089 | 5490 | -690 | L1MB4 | L1 | LINE |
| 21346993 | 21347950 | - | 11.0 | 4642 | 5618 | -548 | L1PA16 | L1 | LINE |
| 21347953 | 21348472 | + | 10.5 | 5643 | 6166 | 0 | L1PA16 | L1 | LINE |
| 21348474 | 21349029 | + | 16.3 | 5486 | 6058 | -122 | L1MB4 | L1 | LINE |

  

|  |  |  |  |  |  |  |  |  |  |
| --- | --- | --- | --- | --- | --- | --- | --- | --- | --- |
| chr6:23340543-23344751 | | | 4205 bp | | |  |  |  |  |
| Genome Start | Genome End | Strand | Div | Repeat Start | Repeat End | Repeat Left | Name | Family | Class |
| 23340543 | 23340919 | - | 20.9 | 4762 | 5162 | -984 | L1MC | L1 | LINE |
| 23340937 | 23343196 | - | 2.0 | 3893 | 6155 | 0 | L1PA3 | L1 | LINE |
| 23343189 | 23344194 | + | 2.7 | 2901 | 3906 | -2240 | L1PA3 | L1 | LINE |
| 23344190 | 23344751 | - | 20.0 | 4200 | 4778 | -1368 | L1MC | L1 | LINE |

  

|  |  |  |  |  |  |  |  |  |  |
| --- | --- | --- | --- | --- | --- | --- | --- | --- | --- |
| chr6:23851828-23853739 | | | 1888 bp | | |  |  |  |  |
| Genome Start | Genome End | Strand | Div | Repeat Start | Repeat End | Repeat Left | Name | Family | Class |
| 23851828 | 23852209 | - | 23.7 | 156 | 561 | -6 | MLT1F1 | MaLR | LTR |
| 23852221 | 23852370 | + | 24.7 | 13 | 158 | -207 | MLT1A0 | MaLR | LTR |
| 23852371 | 23852705 | - | 7.4 | 5142 | 5481 | -691 | L1PA8 | L1 | LINE |
| 23852706 | 23853386 | + | 7.7 | 5493 | 6171 | -1 | L1PA8 | L1 | LINE |
| 23853395 | 23853614 | + | 22.8 | 144 | 365 | 0 | MLT1A0 | MaLR | LTR |
| 23853620 | 23853739 | - | 21.0 | 10 | 139 | -428 | MLT1F1 | MaLR | LTR |

  

|  |  |  |  |  |  |  |  |  |  |
| --- | --- | --- | --- | --- | --- | --- | --- | --- | --- |
| chr6:25050262-25054248 | | | 3988 bp | | |  |  |  |  |
| Genome Start | Genome End | Strand | Div | Repeat Start | Repeat End | Repeat Left | Name | Family | Class |
| 25050262 | 25050412 | + | 36.1 | 198 | 348 | -2513 | Charlie2 | MER1\_type | DNA |
| 25050381 | 25050580 | + | 31.3 | 1855 | 2058 | -804 | Charlie2 | MER1\_type | DNA |
| 25050617 | 25051214 | - | 4.3 | 5060 | 5657 | -498 | L1PA4 | L1 | LINE |
| 25051215 | 25051703 | + | 5.9 | 5659 | 6147 | -8 | L1PA4 | L1 | LINE |
| 25051704 | 25051725 | - | 6.6 | 5039 | 5059 | -1087 | L1P2 | L1 | LINE |
| 25051719 | 25052854 | + | 19.3 | 409 | 1519 | -5385 | L1M4 | L1 | LINE |
| 25052852 | 25053082 | + | 20.4 | 1941 | 2184 | -5150 | L1M4 | L1 | LINE |
| 25053083 | 25053366 | + | 13.0 | 6009 | 6300 | 0 | L1MA4 | L1 | LINE |
| 25053367 | 25054012 | + | 22.4 | 2185 | 3000 | -4187 | L1M4 | L1 | LINE |
| 25054018 | 25054248 | + | 19.8 | 2040 | 2276 | -586 | Charlie2 | MER1\_type | DNA |

  

|  |  |  |  |  |  |  |  |  |  |
| --- | --- | --- | --- | --- | --- | --- | --- | --- | --- |
| chr6:28354036-28356479 | | | 2401 bp | | |  |  |  |  |
| Genome Start | Genome End | Strand | Div | Repeat Start | Repeat End | Repeat Left | Name | Family | Class |
| 28354036 | 28354334 | - | 30.2 | 5458 | 5760 | -361 | L1M5 | L1 | LINE |
| 28354341 | 28354353 | + | 9.5 | 5221 | 5231 | -915 | L1MC | L1 | LINE |
| 28354354 | 28354745 | - | 12.0 | 5924 | 6333 | 0 | L1MC1 | L1 | LINE |
| 28354746 | 28354891 | + | 13.8 | 1 | 26 | -210 | MER46B | MER2\_type | DNA |
| 28354892 | 28355182 | + | 11.7 | 1 | 291 | -21 | AluSx | Alu | SINE |
| 28355183 | 28355381 | + | 13.8 | 27 | 236 | 0 | MER46B | MER2\_type | DNA |
| 28355382 | 28355481 | - | 12.0 | 5826 | 5923 | -410 | L1MC1 | L1 | LINE |
| 28355482 | 28355792 | - | 14.9 | 1 | 309 | -3 | AluSx | Alu | SINE |
| 28355793 | 28355845 | - | 12.0 | 5778 | 5825 | -508 | L1MC1 | L1 | LINE |
| 28355846 | 28356363 | + | 10.6 | 5232 | 5774 | -559 | L1MC1 | L1 | LINE |
| 28356401 | 28356479 | - | 24.1 | 5313 | 5399 | -747 | L1M5 | L1 | LINE |

  

|  |  |  |  |  |  |  |  |  |  |
| --- | --- | --- | --- | --- | --- | --- | --- | --- | --- |
| chr6:32332212-32341955 | | | 9651 bp | | |  |  |  |  |
| Genome Start | Genome End | Strand | Div | Repeat Start | Repeat End | Repeat Left | Name | Family | Class |
| 32332212 | 32332534 | - | 22.8 | 1584 | 1920 | -4720 | L1MD2 | L1 | LINE |
| 32332537 | 32332742 | + | 15.8 | 82 | 284 | -28 | AluJb | Alu | SINE |
| 32332751 | 32332872 | - | 22.9 | 1456 | 1588 | -5052 | L1MD2 | L1 | LINE |
| 32332866 | 32337509 | - | 8.8 | 1815 | 6468 | -1 | L1PA7 | L1 | LINE |
| 32337510 | 32337803 | - | 7.5 | 5 | 298 | -13 | AluY | Alu | SINE |
| 32337804 | 32339504 | - | 12.6 | 10 | 1814 | -776 | L1PA7 | L1 | LINE |
| 32339523 | 32339703 | - | 17.8 | 1310 | 1492 | -5148 | L1MD2 | L1 | LINE |
| 32339706 | 32339799 | - | 5.8 | 6089 | 6174 | 0 | L1PA11 | L1 | LINE |
| 32339874 | 32341020 | - | 3.5 | 5008 | 6155 | 0 | L1PA4 | L1 | LINE |
| 32341019 | 32341527 | + | 3.0 | 4487 | 5014 | -1132 | L1PA4 | L1 | LINE |
| 32341526 | 32341955 | - | 26.2 | 853 | 1312 | -5235 | L1MD2 | L1 | LINE |

  

|  |  |  |  |  |  |  |  |  |  |
| --- | --- | --- | --- | --- | --- | --- | --- | --- | --- |
| chr6:34028746-34030493 | | | 1733 bp | | |  |  |  |  |
| Genome Start | Genome End | Strand | Div | Repeat Start | Repeat End | Repeat Left | Name | Family | Class |
| 34028746 | 34028809 | + | 21.9 | 2575 | 2639 | -780 | L2 | L2 | LINE |
| 34028810 | 34028933 | - | 10.5 | 5322 | 5445 | -855 | L1MA6 | L1 | LINE |
| 34028932 | 34029843 | + | 16.8 | 5425 | 6332 | -2 | L1MA6 | L1 | LINE |
| 34029857 | 34030148 | + | 10.0 | 1 | 289 | -20 | AluSc | Alu | SINE |
| 34030153 | 34030493 | + | 26.5 | 2644 | 3067 | -352 | L2 | L2 | LINE |

  

|  |  |  |  |  |  |  |  |  |  |
| --- | --- | --- | --- | --- | --- | --- | --- | --- | --- |
| chr6:38366666-38373590 | | | 6686 bp | | |  |  |  |  |
| Genome Start | Genome End | Strand | Div | Repeat Start | Repeat End | Repeat Left | Name | Family | Class |
| 38366666 | 38366946 | - | 24.2 | 4228 | 4513 | -508 | L1M5 | L1 | LINE |
| 38366942 | 38367703 | - | 6.5 | 3700 | 4459 | -1687 | L1P3 | L1 | LINE |
| 38367704 | 38368017 | - | 10.3 | 1 | 312 | -1 | AluSq | Alu | SINE |
| 38368018 | 38368895 | - | 6.5 | 2828 | 3699 | -2447 | L1P3 | L1 | LINE |
| 38368896 | 38370568 | + | 6.2 | 4468 | 6154 | 0 | L1PA7 | L1 | LINE |
| 38370618 | 38370808 | - | 23.3 | 4000 | 4206 | -722 | L1M5 | L1 | LINE |
| 38370935 | 38371218 | - | 15.9 | 2 | 284 | -28 | AluJo | Alu | SINE |
| 38371222 | 38371517 | - | 29.0 | 3511 | 3798 | -1036 | L1M5 | L1 | LINE |
| 38371518 | 38371828 | + | 13.3 | 1 | 309 | -3 | AluSx | Alu | SINE |
| 38371829 | 38371993 | - | 29.0 | 3351 | 3510 | -1324 | L1M5 | L1 | LINE |
| 38372060 | 38372839 | - | 26.7 | 1226 | 1989 | -2845 | L1M5 | L1 | LINE |
| 38372840 | 38373131 | + | 10.6 | 1 | 294 | -16 | AluSg | Alu | SINE |
| 38373132 | 38373590 | - | 24.7 | 760 | 1225 | -3609 | L1M5 | L1 | LINE |

  

|  |  |  |  |  |  |  |  |  |  |
| --- | --- | --- | --- | --- | --- | --- | --- | --- | --- |
| chr6:39750326-39754167 | | | 3478 bp | | |  |  |  |  |
| Genome Start | Genome End | Strand | Div | Repeat Start | Repeat End | Repeat Left | Name | Family | Class |
| 39750326 | 39750715 | + | 32.0 | 3385 | 3785 | -704 | L3 | CR1 | LINE |
| 39750730 | 39750930 | + | 30.5 | 1 | 207 | -5912 | L1MEd | L1 | LINE |
| 39751058 | 39751908 | + | 27.8 | 413 | 1257 | -4862 | L1MEd | L1 | LINE |
| 39751914 | 39752654 | + | 27.7 | 1308 | 2127 | -4022 | L1MEd | L1 | LINE |
| 39752775 | 39752893 | + | 23.5 | 126 | 246 | -92 | MER46C | MER2\_type | DNA |
| 39752935 | 39753225 | + | 30.3 | 2333 | 2646 | -3483 | L1MEd | L1 | LINE |
| 39753262 | 39753783 | - | 0.4 | 5658 | 6179 | 0 | L1HS | L1 | LINE |
| 39753784 | 39754081 | + | 0.3 | 5370 | 5667 | -488 | L1HS | L1 | LINE |
| 39754103 | 39754167 | + | 26.6 | 4412 | 4476 | -13 | L3 | CR1 | LINE |

  

|  |  |  |  |  |  |  |  |  |  |
| --- | --- | --- | --- | --- | --- | --- | --- | --- | --- |
| chr6:40138993-40141690 | | | 2672 bp | | |  |  |  |  |
| Genome Start | Genome End | Strand | Div | Repeat Start | Repeat End | Repeat Left | Name | Family | Class |
| 40138993 | 40139118 | + | 27.4 | 29 | 162 | -726 | Kanga2\_a | Tc2 | DNA |
| 40139122 | 40140371 | - | 2.4 | 4902 | 6155 | 0 | L1PA3 | L1 | LINE |
| 40140366 | 40141065 | + | 3.6 | 4193 | 4892 | -1254 | L1PA3 | L1 | LINE |
| 40141066 | 40141512 | + | 28.9 | 154 | 645 | -243 | Kanga2\_a | Tc2 | DNA |
| 40141542 | 40141690 | + | 26.9 | 704 | 881 | -7 | Kanga2\_a | Tc2 | DNA |

  

|  |  |  |  |  |  |  |  |  |  |
| --- | --- | --- | --- | --- | --- | --- | --- | --- | --- |
| chr6:45023153-45025788 | | | 2602 bp | | |  |  |  |  |
| Genome Start | Genome End | Strand | Div | Repeat Start | Repeat End | Repeat Left | Name | Family | Class |
| 45023153 | 45023692 | + | 23.9 | 4501 | 5131 | -1015 | L1M5 | L1 | LINE |
| 45023696 | 45023914 | - | 5.9 | 4754 | 4972 | -1174 | L1PA5 | L1 | LINE |
| 45023912 | 45025114 | + | 5.1 | 4948 | 6150 | -4 | L1PA5 | L1 | LINE |
| 45025149 | 45025788 | + | 22.9 | 5105 | 5789 | -375 | L1M5 | L1 | LINE |

  

|  |  |  |  |  |  |  |  |  |  |
| --- | --- | --- | --- | --- | --- | --- | --- | --- | --- |
| chr6:45767401-45772995 | | | 5230 bp | | |  |  |  |  |
| Genome Start | Genome End | Strand | Div | Repeat Start | Repeat End | Repeat Left | Name | Family | Class |
| 45767401 | 45767560 | - | 27.1 | 3259 | 3419 | 0 | L2 | L2 | LINE |
| 45767565 | 45768496 | - | 18.8 | 1761 | 2761 | 0 | Charlie1 | MER1\_type | DNA |
| 45768497 | 45768581 | + | 9.9 | 5950 | 6033 | -118 | L1PB1 | L1 | LINE |
| 45768582 | 45768884 | + | 7.7 | 1 | 299 | -12 | AluY | Alu | SINE |
| 45768885 | 45769013 | + | 9.9 | 6034 | 6150 | -1 | L1PB1 | L1 | LINE |
| 45769027 | 45769354 | + | 23.7 | 833 | 1191 | -4928 | L1MEd | L1 | LINE |
| 45769506 | 45769748 | - | 11.5 | 76 | 307 | 0 | AluSx | Alu | SINE |
| 45769759 | 45770712 | - | 3.7 | 5198 | 6155 | 0 | L1PA4 | L1 | LINE |
| 45770712 | 45772005 | + | 3.2 | 3893 | 5189 | -957 | L1PA4 | L1 | LINE |
| 45772006 | 45772096 | - | 12.1 | 2 | 92 | -220 | AluSx | Alu | SINE |
| 45772136 | 45772287 | + | 28.4 | 47 | 210 | -58 | MIR | MIR | SINE |
| 45772299 | 45772434 | - | 20.0 | 1627 | 1776 | -985 | Charlie1 | MER1\_type | DNA |
| 45772573 | 45772995 | - | 28.5 | 2818 | 3249 | -170 | L2 | L2 | LINE |

  

|  |  |  |  |  |  |  |  |  |  |
| --- | --- | --- | --- | --- | --- | --- | --- | --- | --- |
| chr6:46238857-46240100 | | | 1239 bp | | |  |  |  |  |
| Genome Start | Genome End | Strand | Div | Repeat Start | Repeat End | Repeat Left | Name | Family | Class |
| 46238857 | 46238912 | - | 16.1 | 62 | 124 | -65 | MER5A | MER1\_type | DNA |
| 46238913 | 46239226 | - | 1.6 | 5841 | 6154 | -1 | L1PA2 | L1 | LINE |
| 46239236 | 46239510 | - | 2.2 | 5374 | 5648 | -507 | L1PA2 | L1 | LINE |
| 46239507 | 46240022 | + | 2.1 | 4856 | 5371 | -775 | L1PA2 | L1 | LINE |
| 46240023 | 46240100 | - | 16.7 | 1 | 78 | -111 | MER5A | MER1\_type | DNA |

  

|  |  |  |  |  |  |  |  |  |  |
| --- | --- | --- | --- | --- | --- | --- | --- | --- | --- |
| chr6:48912528-48926091 | | | 13271 bp | | |  |  |  |  |
| Genome Start | Genome End | Strand | Div | Repeat Start | Repeat End | Repeat Left | Name | Family | Class |
| 48912528 | 48912597 | + | 18.6 | 1133 | 1213 | -5118 | MER57A-int | ERV1 | LTR |
| 48912627 | 48912878 | + | 23.0 | 1017 | 1399 | -4932 | MER57A-int | ERV1 | LTR |
| 48912879 | 48913342 | + | 5.2 | 1 | 464 | 0 | LTR7B | ERV1 | LTR |
| 48913343 | 48913378 | + | 23.9 | 1218 | 1248 | -7796 | MER57A-int | ERV1 | LTR |
| 48913401 | 48913617 | - | 20.6 | 348 | 572 | -23 | LTR49 | ERV1 | LTR |
| 48913618 | 48914152 | - | 10.8 | 3 | 521 | 0 | MER41C | ERV1 | LTR |
| 48914153 | 48914480 | - | 20.6 | 1 | 347 | -248 | LTR49 | ERV1 | LTR |
| 48914481 | 48914577 | + | 15.1 | 1445 | 1537 | -4794 | MER57A-int | ERV1 | LTR |
| 48914579 | 48914705 | + | 18.8 | 878 | 1007 | -5180 | MER57A-int | ERV1 | LTR |
| 48914733 | 48914817 | + | 18.8 | 1008 | 1103 | -5084 | MER57A-int | ERV1 | LTR |
| 48914818 | 48915022 | + | 27.1 | 160 | 408 | -177 | MER34C\_ | ERV1 | LTR |
| 48915024 | 48915196 | + | 23.1 | 1209 | 1395 | -3728 | MER57A-int | ERV1 | LTR |
| 48915251 | 48915398 | - | 21.6 | 2357 | 2506 | -2617 | LTR23-int | ERV1 | LTR |
| 48915400 | 48915651 | - | 11.2 | 5585 | 5836 | -2305 | L1P4b | L1 | LINE |
| 48915622 | 48917578 | - | 17.8 | 3285 | 5261 | -2880 | L1P4b | L1 | LINE |
| 48917607 | 48918910 | - | 21.1 | 1954 | 3284 | -4871 | L1P4b | L1 | LINE |
| 48918911 | 48919005 | + | 17.0 | 1 | 99 | -513 | LTR9 | ERV1 | LTR |
| 48919009 | 48919770 | - | 5.5 | 5376 | 6154 | 0 | L1PA5 | L1 | LINE |
| 48919771 | 48920294 | + | 22.1 | 98 | 596 | -16 | LTR9 | ERV1 | LTR |
| 48920337 | 48921502 | - | 29.1 | 836 | 2007 | -6154 | L1P4b | L1 | LINE |
| 48921565 | 48921685 | + | 18.6 | 4108 | 4221 | -2110 | MER57A-int | ERV1 | LTR |
| 48921686 | 48922258 | - | 14.4 | 5 | 634 | 0 | MER51A | ERV1 | LTR |
| 48922259 | 48922882 | + | 18.6 | 4222 | 4852 | -1479 | MER57A-int | ERV1 | LTR |
| 48922884 | 48924182 | - | 7.8 | 3083 | 4419 | -1727 | L1PB1 | L1 | LINE |
| 48924181 | 48925545 | + | 10.1 | 4413 | 5795 | -356 | L1PB1 | L1 | LINE |
| 48925600 | 48925958 | + | 13.2 | 5796 | 6151 | 0 | L1PB1 | L1 | LINE |
| 48925959 | 48926091 | + | 19.7 | 4845 | 4976 | -1355 | MER57A-int | ERV1 | LTR |

  

|  |  |  |  |  |  |  |  |  |  |
| --- | --- | --- | --- | --- | --- | --- | --- | --- | --- |
| chr6:50968814-50972043 | | | 3182 bp | | |  |  |  |  |
| Genome Start | Genome End | Strand | Div | Repeat Start | Repeat End | Repeat Left | Name | Family | Class |
| 50968814 | 50969370 | - | 24.9 | 2967 | 3540 | -2679 | L1MEc | L1 | LINE |
| 50969424 | 50970043 | - | 0.8 | 5552 | 6171 | 0 | L1PA2 | L1 | LINE |
| 50970044 | 50970646 | + | 0.9 | 4947 | 5549 | -606 | L1PA2 | L1 | LINE |
| 50970642 | 50971456 | - | 25.3 | 691 | 1476 | -4924 | L1MEc | L1 | LINE |
| 50971457 | 50971769 | - | 13.1 | 1 | 312 | -1 | AluSq | Alu | SINE |
| 50971770 | 50972043 | - | 25.3 | 388 | 690 | -5710 | L1MEc | L1 | LINE |

  

|  |  |  |  |  |  |  |  |  |  |
| --- | --- | --- | --- | --- | --- | --- | --- | --- | --- |
| chr6:54476996-54481333 | | | 4320 bp | | |  |  |  |  |
| Genome Start | Genome End | Strand | Div | Repeat Start | Repeat End | Repeat Left | Name | Family | Class |
| 54476996 | 54478605 | - | 14.2 | 914 | 2418 | 0 | Tigger1 | MER2\_type | DNA |
| 54478623 | 54479328 | - | 1.7 | 5449 | 6155 | 0 | L1PA3 | L1 | LINE |
| 54479329 | 54480472 | + | 2.7 | 4298 | 5444 | -711 | L1PA3 | L1 | LINE |
| 54480474 | 54481333 | - | 14.1 | 2 | 873 | -1545 | Tigger1 | MER2\_type | DNA |

  

|  |  |  |  |  |  |  |  |  |  |
| --- | --- | --- | --- | --- | --- | --- | --- | --- | --- |
| chr6:54576591-54580558 | | | 3642 bp | | |  |  |  |  |
| Genome Start | Genome End | Strand | Div | Repeat Start | Repeat End | Repeat Left | Name | Family | Class |
| 54576591 | 54577676 | - | 14.7 | 5294 | 6333 | 0 | L1MC1 | L1 | LINE |
| 54578006 | 54579070 | - | 8.2 | 5038 | 6111 | -20 | L1PA8A | L1 | LINE |
| 54579068 | 54579304 | + | 12.2 | 4796 | 5034 | -1112 | L1PA8A | L1 | LINE |
| 54579305 | 54580558 | - | 13.6 | 3932 | 5332 | -814 | L1MC1 | L1 | LINE |

  

|  |  |  |  |  |  |  |  |  |  |
| --- | --- | --- | --- | --- | --- | --- | --- | --- | --- |
| chr6:55279950-55291519 | | | 11501 bp | | |  |  |  |  |
| Genome Start | Genome End | Strand | Div | Repeat Start | Repeat End | Repeat Left | Name | Family | Class |
| 55279950 | 55281160 | - | 19.2 | 6495 | 7710 | 0 | L1MA7 | L1 | LINE |
| 55281156 | 55284670 | - | 4.1 | 355 | 3863 | -2283 | L1PA3 | L1 | LINE |
| 55284668 | 55287010 | + | 3.9 | 3857 | 6203 | 0 | L1PA3 | L1 | LINE |
| 55287011 | 55287681 | - | 18.9 | 5831 | 6509 | -1053 | L1MA7 | L1 | LINE |
| 55287731 | 55287806 | - | 18.9 | 5798 | 5830 | -1732 | L1MA7 | L1 | LINE |
| 55287835 | 55288447 | - | 18.9 | 5258 | 5797 | -1765 | L1MA7 | L1 | LINE |
| 55288448 | 55288732 | - | 15.4 | 6019 | 6305 | 0 | L1MA3 | L1 | LINE |
| 55288733 | 55288898 | - | 22.2 | 3677 | 3845 | -2301 | L1PB | L1 | LINE |
| 55288899 | 55289153 | - | 5.5 | 5902 | 6155 | 0 | L1PA4 | L1 | LINE |
| 55289154 | 55291519 | - | 23.7 | 2663 | 5094 | -2468 | L1MA7 | L1 | LINE |

  

|  |  |  |  |  |  |  |  |  |  |
| --- | --- | --- | --- | --- | --- | --- | --- | --- | --- |
| chr6:56393598-56395778 | | | 2111 bp | | |  |  |  |  |
| Genome Start | Genome End | Strand | Div | Repeat Start | Repeat End | Repeat Left | Name | Family | Class |
| 56393598 | 56393659 | - | 30.0 | 194 | 253 | -9 | MIR | MIR | SINE |
| 56393688 | 56393878 | + | 27.9 | 4 | 225 | -37 | MIR | MIR | SINE |
| 56393879 | 56394458 | - | 12.7 | 4427 | 4954 | -1192 | L1PB2 | L1 | LINE |
| 56394456 | 56395649 | + | 11.0 | 4962 | 6151 | 0 | L1PB2 | L1 | LINE |
| 56395695 | 56395778 | - | 27.4 | 69 | 157 | -105 | MIR | MIR | SINE |

  

|  |  |  |  |  |  |  |  |  |  |
| --- | --- | --- | --- | --- | --- | --- | --- | --- | --- |
| chr6:56554536-56561378 | | | 6596 bp | | |  |  |  |  |
| Genome Start | Genome End | Strand | Div | Repeat Start | Repeat End | Repeat Left | Name | Family | Class |
| 56554536 | 56554956 | + | 33.8 | 2391 | 2833 | -586 | L2 | L2 | LINE |
| 56554957 | 56555151 | + | 16.2 | 1 | 215 | -213 | MSTA | MaLR | LTR |
| 56555175 | 56555356 | + | 16.2 | 216 | 401 | -27 | MSTA | MaLR | LTR |
| 56555359 | 56559343 | - | 3.6 | 2161 | 6154 | -1 | L1PA4 | L1 | LINE |
| 56559343 | 56560854 | + | 4.8 | 669 | 2173 | -3982 | L1PA4 | L1 | LINE |
| 56560855 | 56560886 | + | 9.4 | 386 | 417 | -9 | MSTA | MaLR | LTR |
| 56561110 | 56561378 | + | 34.5 | 3082 | 3415 | -4 | L2 | L2 | LINE |

  

|  |  |  |  |  |  |  |  |  |  |
| --- | --- | --- | --- | --- | --- | --- | --- | --- | --- |
| chr6:57959164-57963347 | | | 4192 bp | | |  |  |  |  |
| Genome Start | Genome End | Strand | Div | Repeat Start | Repeat End | Repeat Left | Name | Family | Class |
| 57959164 | 57959244 | + | 18.5 | 5 | 88 | -426 | MER67D | ERV1 | LTR |
| 57959245 | 57961617 | - | 7.3 | 3824 | 6201 | 0 | L1PA8 | L1 | LINE |
| 57961609 | 57962978 | + | 8.9 | 2430 | 3812 | -2334 | L1PA8 | L1 | LINE |
| 57962980 | 57963347 | + | 21.2 | 63 | 436 | -78 | MER67D | ERV1 | LTR |

  

|  |  |  |  |  |  |  |  |  |  |
| --- | --- | --- | --- | --- | --- | --- | --- | --- | --- |
| chr6:57996518-57997379 | | | 834 bp | | |  |  |  |  |
| Genome Start | Genome End | Strand | Div | Repeat Start | Repeat End | Repeat Left | Name | Family | Class |
| 57996518 | 57996674 | + | 24.0 | 2 | 155 | -107 | MIR | MIR | SINE |
| 57996690 | 57997024 | - | 2.4 | 5539 | 5873 | -282 | L1PA5 | L1 | LINE |
| 57997025 | 57997309 | + | 4.6 | 5866 | 6150 | -4 | L1PA5 | L1 | LINE |
| 57997323 | 57997379 | + | 23.2 | 196 | 257 | -10 | MIR | MIR | SINE |

  

|  |  |  |  |  |  |  |  |  |  |
| --- | --- | --- | --- | --- | --- | --- | --- | --- | --- |
| chr6:58130260-58132732 | | | 2447 bp | | |  |  |  |  |
| Genome Start | Genome End | Strand | Div | Repeat Start | Repeat End | Repeat Left | Name | Family | Class |
| 58130260 | 58130584 | - | 17.3 | 127 | 449 | -3 | LTR47A | ERVL | LTR |
| 58130609 | 58132131 | - | 13.1 | 4625 | 6160 | -6 | L1PA16 | L1 | LINE |
| 58132133 | 58132634 | + | 13.7 | 4126 | 4627 | -1519 | L1PA16 | L1 | LINE |
| 58132636 | 58132732 | - | 19.6 | 1 | 97 | -355 | LTR47A | ERVL | LTR |

  

|  |  |  |  |  |  |  |  |  |  |
| --- | --- | --- | --- | --- | --- | --- | --- | --- | --- |
| chr6:58531448-58537623 | | | 6168 bp | | |  |  |  |  |
| Genome Start | Genome End | Strand | Div | Repeat Start | Repeat End | Repeat Left | Name | Family | Class |
| 58531448 | 58531650 | + | 21.7 | 23 | 235 | -6268 | L1P4a | L1 | LINE |
| 58531649 | 58532261 | + | 15.1 | 1085 | 1691 | -4812 | L1P4a | L1 | LINE |
| 58532262 | 58532533 | + | 16.7 | 12 | 281 | -31 | AluJb | Alu | SINE |
| 58532534 | 58532660 | + | 15.1 | 1692 | 1785 | -4718 | L1P4a | L1 | LINE |
| 58532659 | 58533212 | - | 1.4 | 5000 | 5553 | -602 | L1PA3 | L1 | LINE |
| 58533213 | 58533839 | + | 1.9 | 5529 | 6155 | 0 | L1PA3 | L1 | LINE |
| 58533852 | 58536791 | + | 13.9 | 1771 | 4728 | -1755 | L1P4a | L1 | LINE |
| 58536792 | 58537103 | + | 5.3 | 1 | 307 | -4 | AluY | Alu | SINE |
| 58537104 | 58537623 | + | 15.1 | 4729 | 5266 | -1217 | L1P4a | L1 | LINE |

  

|  |  |  |  |  |  |  |  |  |  |
| --- | --- | --- | --- | --- | --- | --- | --- | --- | --- |
| chr6:58564090-58568256 | | | 4150 bp | | |  |  |  |  |
| Genome Start | Genome End | Strand | Div | Repeat Start | Repeat End | Repeat Left | Name | Family | Class |
| 58564090 | 58564170 | + | 16.1 | 5 | 88 | -426 | MER67D | ERV1 | LTR |
| 58564197 | 58566533 | - | 7.5 | 3824 | 6171 | -1 | L1PA8 | L1 | LINE |
| 58566525 | 58567890 | + | 9.6 | 2430 | 3809 | -2337 | L1PA8 | L1 | LINE |
| 58567891 | 58568256 | + | 20.7 | 71 | 436 | -78 | MER67D | ERV1 | LTR |

  

|  |  |  |  |  |  |  |  |  |  |
| --- | --- | --- | --- | --- | --- | --- | --- | --- | --- |
| chr6:58600192-58601053 | | | 834 bp | | |  |  |  |  |
| Genome Start | Genome End | Strand | Div | Repeat Start | Repeat End | Repeat Left | Name | Family | Class |
| 58600192 | 58600348 | + | 24.0 | 2 | 155 | -107 | MIR | MIR | SINE |
| 58600364 | 58600698 | - | 2.1 | 5539 | 5873 | -282 | L1PA5 | L1 | LINE |
| 58600699 | 58600983 | + | 3.2 | 5866 | 6150 | -4 | L1PA5 | L1 | LINE |
| 58600997 | 58601053 | + | 23.2 | 196 | 257 | -10 | MIR | MIR | SINE |

  

|  |  |  |  |  |  |  |  |  |  |
| --- | --- | --- | --- | --- | --- | --- | --- | --- | --- |
| chr6:58824639-58827039 | | | 2370 bp | | |  |  |  |  |
| Genome Start | Genome End | Strand | Div | Repeat Start | Repeat End | Repeat Left | Name | Family | Class |
| 58824639 | 58824735 | + | 20.6 | 1 | 97 | -355 | LTR47A | ERVL | LTR |
| 58824737 | 58825167 | - | 13.5 | 4126 | 4627 | -1519 | L1PA16 | L1 | LINE |
| 58825169 | 58826690 | + | 13.2 | 4625 | 6160 | -6 | L1PA16 | L1 | LINE |
| 58826720 | 58827039 | + | 18.6 | 132 | 449 | -3 | LTR47A | ERVL | LTR |

  

|  |  |  |  |  |  |  |  |  |  |
| --- | --- | --- | --- | --- | --- | --- | --- | --- | --- |
| chr6:62043015-62048900 | | | 5881 bp | | |  |  |  |  |
| Genome Start | Genome End | Strand | Div | Repeat Start | Repeat End | Repeat Left | Name | Family | Class |
| 62043015 | 62044120 | - | 12.4 | 5094 | 6173 | -1 | L1PA11 | L1 | LINE |
| 62044128 | 62044857 | - | 5.6 | 5419 | 6150 | -5 | L1PA4 | L1 | LINE |
| 62044860 | 62047851 | + | 6.8 | 2392 | 5395 | -751 | L1PA4 | L1 | LINE |
| 62047848 | 62048900 | - | 9.8 | 4067 | 5112 | -1034 | L1PA11 | L1 | LINE |

  

|  |  |  |  |  |  |  |  |  |  |
| --- | --- | --- | --- | --- | --- | --- | --- | --- | --- |
| chr6:62871692-62878610 | | | 6746 bp | | |  |  |  |  |
| Genome Start | Genome End | Strand | Div | Repeat Start | Repeat End | Repeat Left | Name | Family | Class |
| 62871692 | 62871996 | + | 21.1 | 1639 | 1957 | -4443 | L1ME1 | L1 | LINE |
| 62872001 | 62872157 | + | 27.1 | 2663 | 2822 | -3397 | L1ME1 | L1 | LINE |
| 62872158 | 62872285 | - | 2.3 | 6026 | 6153 | -2 | L1PA2 | L1 | LINE |
| 62872286 | 62873250 | + | 27.1 | 2823 | 3839 | -2380 | L1ME1 | L1 | LINE |
| 62873249 | 62874389 | - | 3.9 | 2405 | 3544 | -2602 | L1PA3 | L1 | LINE |
| 62874383 | 62876974 | + | 3.4 | 3548 | 6155 | 0 | L1PA3 | L1 | LINE |
| 62876977 | 62877544 | + | 25.0 | 3831 | 4475 | -1744 | L1ME1 | L1 | LINE |
| 62877718 | 62877990 | + | 20.0 | 5040 | 5322 | -897 | L1ME1 | L1 | LINE |
| 62877994 | 62878610 | + | 24.5 | 5511 | 6086 | -153 | L1ME1 | L1 | LINE |

  

|  |  |  |  |  |  |  |  |  |  |
| --- | --- | --- | --- | --- | --- | --- | --- | --- | --- |
| chr6:63458743-63465657 | | | 6880 bp | | |  |  |  |  |
| Genome Start | Genome End | Strand | Div | Repeat Start | Repeat End | Repeat Left | Name | Family | Class |
| 63458743 | 63459388 | + | 27.1 | 44 | 764 | -5636 | L1MEc | L1 | LINE |
| 63459389 | 63459709 | + | 12.2 | 1 | 317 | 0 | AluSx | Alu | SINE |
| 63459710 | 63460389 | + | 27.1 | 765 | 1479 | -4921 | L1MEc | L1 | LINE |
| 63460386 | 63462265 | + | 18.0 | 4208 | 6113 | -184 | L1MA5A | L1 | LINE |
| 63462271 | 63462553 | + | 16.0 | 27 | 308 | -4 | AluJb | Alu | SINE |
| 63462554 | 63463016 | + | 25.7 | 1741 | 2214 | -4186 | L1MEc | L1 | LINE |
| 63463041 | 63463613 | - | 3.3 | 5572 | 6144 | -11 | L1PA4 | L1 | LINE |
| 63463615 | 63465118 | + | 4.4 | 4063 | 5562 | -592 | L1PA4 | L1 | LINE |
| 63465128 | 63465657 | + | 25.6 | 2148 | 2681 | -3538 | L1MEc | L1 | LINE |

  

|  |  |  |  |  |  |  |  |  |  |
| --- | --- | --- | --- | --- | --- | --- | --- | --- | --- |
| chr6:64503163-64507060 | | | 3856 bp | | |  |  |  |  |
| Genome Start | Genome End | Strand | Div | Repeat Start | Repeat End | Repeat Left | Name | Family | Class |
| 64503163 | 64503345 | - | 26.0 | 7881 | 8035 | -5 | L1MC4 | L1 | LINE |
| 64503369 | 64503961 | - | 26.6 | 7303 | 7909 | -133 | L1MC4 | L1 | LINE |
| 64503947 | 64505594 | - | 2.9 | 3498 | 5144 | -1002 | L1PA3 | L1 | LINE |
| 64505592 | 64506600 | + | 2.8 | 5146 | 6153 | -2 | L1PA3 | L1 | LINE |
| 64506638 | 64507060 | - | 26.9 | 6938 | 7322 | -720 | L1MC4 | L1 | LINE |

  

|  |  |  |  |  |  |  |  |  |  |
| --- | --- | --- | --- | --- | --- | --- | --- | --- | --- |
| chr6:64896588-64899094 | | | 2303 bp | | |  |  |  |  |
| Genome Start | Genome End | Strand | Div | Repeat Start | Repeat End | Repeat Left | Name | Family | Class |
| 64896588 | 64896644 | - | 31.6 | 3363 | 3419 | 0 | L2 | L2 | LINE |
| 64896770 | 64896806 | - | 21.6 | 3127 | 3164 | -255 | L2 | L2 | LINE |
| 64896840 | 64897044 | + | 20.2 | 9 | 213 | -11 | MER58A | MER1\_type | DNA |
| 64897048 | 64897927 | - | 4.0 | 4708 | 5582 | -573 | L1PA4 | L1 | LINE |
| 64897928 | 64898489 | + | 4.3 | 5588 | 6155 | 0 | L1PA4 | L1 | LINE |
| 64898533 | 64898715 | - | 29.5 | 2882 | 3082 | -337 | L2 | L2 | LINE |
| 64898716 | 64899016 | - | 8.9 | 3 | 302 | 0 | AluSg | Alu | SINE |
| 64899017 | 64899094 | - | 29.5 | 2809 | 2881 | -538 | L2 | L2 | LINE |

  

|  |  |  |  |  |  |  |  |  |  |
| --- | --- | --- | --- | --- | --- | --- | --- | --- | --- |
| chr6:64958771-64962703 | | | 3820 bp | | |  |  |  |  |
| Genome Start | Genome End | Strand | Div | Repeat Start | Repeat End | Repeat Left | Name | Family | Class |
| 64958771 | 64959182 | - | 25.8 | 5009 | 5456 | -784 | L1M5 | L1 | LINE |
| 64959190 | 64960261 | - | 2.5 | 5074 | 6149 | -6 | L1PA3 | L1 | LINE |
| 64960257 | 64960607 | + | 2.6 | 4715 | 5065 | -1081 | L1PA3 | L1 | LINE |
| 64960592 | 64961129 | - | 27.7 | 4496 | 5041 | -1105 | L1M5 | L1 | LINE |
| 64961163 | 64961352 | - | 10.4 | 127 | 299 | -12 | AluY | Alu | SINE |
| 64961353 | 64961896 | - | 5.9 | 5611 | 6154 | 0 | L1PA5 | L1 | LINE |
| 64961897 | 64962022 | - | 10.4 | 1 | 126 | -185 | AluY | Alu | SINE |
| 64962117 | 64962703 | - | 30.6 | 2527 | 3146 | -3000 | L1M5 | L1 | LINE |

  

|  |  |  |  |  |  |  |  |  |  |
| --- | --- | --- | --- | --- | --- | --- | --- | --- | --- |
| chr6:65012164-65013631 | | | 1143 bp | | |  |  |  |  |
| Genome Start | Genome End | Strand | Div | Repeat Start | Repeat End | Repeat Left | Name | Family | Class |
| 65012164 | 65012335 | - | 28.1 | 1324 | 1495 | -4654 | L1MEe | L1 | LINE |
| 65012488 | 65012671 | - | 26.2 | 895 | 1078 | -5041 | L1MEe | L1 | LINE |
| 65012693 | 65012982 | - | 6.2 | 5852 | 6141 | -13 | L1PA5 | L1 | LINE |
| 65012983 | 65013106 | + | 2.4 | 5717 | 5839 | -315 | L1PA5 | L1 | LINE |
| 65013171 | 65013331 | - | 21.8 | 136 | 308 | -4 | AluJb | Alu | SINE |
| 65013420 | 65013631 | - | 27.2 | 604 | 815 | -5304 | L1MEe | L1 | LINE |

  

|  |  |  |  |  |  |  |  |  |  |
| --- | --- | --- | --- | --- | --- | --- | --- | --- | --- |
| chr6:65330637-65331992 | | | 1357 bp | | |  |  |  |  |
| Genome Start | Genome End | Strand | Div | Repeat Start | Repeat End | Repeat Left | Name | Family | Class |
| 65330637 | 65330881 | - | 12.7 | 5481 | 5732 | -559 | L1M2 | L1 | LINE |
| 65330883 | 65331194 | - | 8.2 | 1 | 306 | -3 | AluSc | Alu | SINE |
| 65331195 | 65331266 | - | 11.1 | 6076 | 6150 | -4 | L1PA7 | L1 | LINE |
| 65331267 | 65331470 | + | 4.1 | 5858 | 6053 | -101 | L1PA7 | L1 | LINE |
| 65331469 | 65331992 | - | 15.8 | 4959 | 5484 | -828 | L1M2 | L1 | LINE |

  

|  |  |  |  |  |  |  |  |  |  |
| --- | --- | --- | --- | --- | --- | --- | --- | --- | --- |
| chr6:65360824-65362464 | | | 1627 bp | | |  |  |  |  |
| Genome Start | Genome End | Strand | Div | Repeat Start | Repeat End | Repeat Left | Name | Family | Class |
| 65360824 | 65360925 | + | 20.8 | 3486 | 3584 | -2561 | L1M2 | L1 | LINE |
| 65360917 | 65361048 | - | 1.5 | 5602 | 5733 | -421 | L1PA4 | L1 | LINE |
| 65361050 | 65361464 | + | 2.9 | 5737 | 6152 | -3 | L1PA4 | L1 | LINE |
| 65361487 | 65362464 | + | 17.4 | 3559 | 4547 | -1596 | L1M2 | L1 | LINE |

  

|  |  |  |  |  |  |  |  |  |  |
| --- | --- | --- | --- | --- | --- | --- | --- | --- | --- |
| chr6:65622112-65629050 | | | 6741 bp | | |  |  |  |  |
| Genome Start | Genome End | Strand | Div | Repeat Start | Repeat End | Repeat Left | Name | Family | Class |
| 65622112 | 65622891 | - | 20.3 | 5907 | 6736 | -26 | L1MC2 | L1 | LINE |
| 65622921 | 65623212 | - | 19.2 | 1 | 293 | -19 | AluJb | Alu | SINE |
| 65623214 | 65623770 | - | 21.2 | 5187 | 5745 | -819 | L1MC2 | L1 | LINE |
| 65623787 | 65623885 | - | 19.2 | 3381 | 3483 | -3081 | L1MC2 | L1 | LINE |
| 65623906 | 65625268 | - | 18.5 | 4598 | 5990 | -301 | L1MA7 | L1 | LINE |
| 65625248 | 65625801 | + | 22.2 | 3784 | 4344 | -1799 | L1M3 | L1 | LINE |
| 65625939 | 65626078 | + | 19.2 | 4371 | 4515 | -1625 | L1M3 | L1 | LINE |
| 65626079 | 65626422 | + | 15.1 | 1 | 364 | 0 | THE1B | MaLR | LTR |
| 65626423 | 65626513 | + | 19.2 | 4516 | 4602 | -1538 | L1M3 | L1 | LINE |
| 65626508 | 65626577 | - | 14.5 | 2687 | 2748 | -3816 | L1MC2 | L1 | LINE |
| 65626596 | 65626816 | - | 23.4 | 2318 | 2546 | -4205 | L1MC2 | L1 | LINE |
| 65626817 | 65628259 | - | 18.4 | 97 | 1755 | 0 | MER52A | ERV1 | LTR |
| 65628265 | 65628872 | - | 22.3 | 1662 | 2321 | -4430 | L1MC2 | L1 | LINE |
| 65628872 | 65629050 | - | 28.1 | 9 | 445 | -6306 | L1MC2 | L1 | LINE |

  

|  |  |  |  |  |  |  |  |  |  |
| --- | --- | --- | --- | --- | --- | --- | --- | --- | --- |
| chr6:66161175-66165791 | | | 4395 bp | | |  |  |  |  |
| Genome Start | Genome End | Strand | Div | Repeat Start | Repeat End | Repeat Left | Name | Family | Class |
| 66161175 | 66161351 | + | 34.3 | 112 | 299 | -3120 | L2 | L2 | LINE |
| 66161359 | 66161653 | + | 36.1 | 428 | 763 | -2656 | L2 | L2 | LINE |
| 66161691 | 66161975 | - | 9.1 | 2 | 287 | -26 | AluSp | Alu | SINE |
| 66161999 | 66162262 | - | 22.2 | 6026 | 6312 | 0 | L1MA9 | L1 | LINE |
| 66162263 | 66162447 | - | 14.1 | 6116 | 6303 | 0 | L1MA4A | L1 | LINE |
| 66162448 | 66162538 | - | 22.2 | 5935 | 6025 | -287 | L1MA9 | L1 | LINE |
| 66162539 | 66162738 | - | 28.6 | 5739 | 5928 | -238 | L1PA16 | L1 | LINE |
| 66162741 | 66163555 | + | 33.5 | 783 | 1690 | -1729 | L2 | L2 | LINE |
| 66163628 | 66163771 | + | 30.5 | 2148 | 2307 | -1112 | L2 | L2 | LINE |
| 66163807 | 66164197 | - | 14.3 | 5793 | 6156 | 0 | L1PB4 | L1 | LINE |
| 66164246 | 66164838 | - | 14.5 | 5186 | 5792 | -364 | L1PB4 | L1 | LINE |
| 66164837 | 66165269 | + | 17.8 | 4729 | 5176 | -970 | L1P4 | L1 | LINE |
| 66165270 | 66165791 | + | 28.4 | 2300 | 2812 | -607 | L2 | L2 | LINE |

  

|  |  |  |  |  |  |  |  |  |  |
| --- | --- | --- | --- | --- | --- | --- | --- | --- | --- |
| chr6:66250894-66256974 | | | 6071 bp | | |  |  |  |  |
| Genome Start | Genome End | Strand | Div | Repeat Start | Repeat End | Repeat Left | Name | Family | Class |
| 66250894 | 66251425 | + | 25.9 | 78 | 687 | -2257 | BLACKJACK | AcHobo | DNA |
| 66251426 | 66251644 | + | 23.3 | 1 | 224 | 0 | MER58A | MER1\_type | DNA |
| 66251645 | 66252136 | + | 25.9 | 688 | 1216 | -1728 | BLACKJACK | AcHobo | DNA |
| 66252137 | 66252430 | + | 17.7 | 1 | 297 | -15 | AluSx | Alu | SINE |
| 66252431 | 66253482 | + | 25.9 | 1217 | 2321 | -623 | BLACKJACK | AcHobo | DNA |
| 66253485 | 66255039 | - | 8.8 | 2900 | 4457 | -1689 | L1PA7 | L1 | LINE |
| 66255038 | 66256747 | + | 9.7 | 4438 | 6149 | -5 | L1PA7 | L1 | LINE |
| 66256758 | 66256974 | + | 27.1 | 2332 | 2556 | -388 | BLACKJACK | AcHobo | DNA |

  

|  |  |  |  |  |  |  |  |  |  |
| --- | --- | --- | --- | --- | --- | --- | --- | --- | --- |
| chr6:67384782-67390199 | | | 5305 bp | | |  |  |  |  |
| Genome Start | Genome End | Strand | Div | Repeat Start | Repeat End | Repeat Left | Name | Family | Class |
| 67384782 | 67385473 | + | 25.4 | 5269 | 5963 | -211 | L1MB5 | L1 | LINE |
| 67385466 | 67385968 | + | 20.6 | 5768 | 6290 | -10 | L1MA4 | L1 | LINE |
| 67385995 | 67386125 | + | 18.1 | 2390 | 2528 | -84 | Charlie5 | MER1\_type | DNA |
| 67386144 | 67386500 | - | 14.6 | 1 | 381 | 0 | THE1D | MaLR | LTR |
| 67386512 | 67386607 | + | 31.2 | 5938 | 6041 | -133 | L1MB5 | L1 | LINE |
| 67386674 | 67386957 | - | 20.7 | 5769 | 6042 | -258 | L1MA4 | L1 | LINE |
| 67386955 | 67387979 | - | 9.4 | 5139 | 6171 | -1 | L1PA8 | L1 | LINE |
| 67387980 | 67389163 | + | 10.2 | 3920 | 5114 | -1032 | L1PA8 | L1 | LINE |
| 67389162 | 67390074 | - | 16.5 | 4889 | 5788 | -512 | L1MA4 | L1 | LINE |
| 67390080 | 67390199 | + | 20.2 | 6031 | 6151 | -23 | L1MB5 | L1 | LINE |

  

|  |  |  |  |  |  |  |  |  |  |
| --- | --- | --- | --- | --- | --- | --- | --- | --- | --- |
| chr6:67606930-67611269 | | | 4287 bp | | |  |  |  |  |
| Genome Start | Genome End | Strand | Div | Repeat Start | Repeat End | Repeat Left | Name | Family | Class |
| 67606930 | 67607097 | + | 30.9 | 1 | 177 | -328 | MLT1D | MaLR | LTR |
| 67607118 | 67608645 | - | 2.9 | 4647 | 6180 | 0 | L1PA3 | L1 | LINE |
| 67608643 | 67610980 | + | 4.0 | 2312 | 4663 | -1483 | L1PA3 | L1 | LINE |
| 67611017 | 67611269 | + | 27.6 | 250 | 505 | 0 | MLT1D | MaLR | LTR |

  

|  |  |  |  |  |  |  |  |  |  |
| --- | --- | --- | --- | --- | --- | --- | --- | --- | --- |
| chr6:67794546-67800366 | | | 5773 bp | | |  |  |  |  |
| Genome Start | Genome End | Strand | Div | Repeat Start | Repeat End | Repeat Left | Name | Family | Class |
| 67794546 | 67795800 | + | 34.4 | 1482 | 2799 | -620 | L2 | L2 | LINE |
| 67795809 | 67796655 | - | 13.1 | 5333 | 6162 | -1 | L1PA13 | L1 | LINE |
| 67796656 | 67796955 | - | 12.3 | 1 | 317 | 0 | AluSq | Alu | SINE |
| 67796956 | 67797523 | - | 14.1 | 4775 | 5332 | -814 | L1P | L1 | LINE |
| 67797524 | 67798672 | + | 10.1 | 1 | 1254 | 0 | LTR12D | ERV1 | LTR |
| 67798673 | 67799740 | - | 14.3 | 3685 | 4774 | -1372 | L1P | L1 | LINE |
| 67799766 | 67799777 | - | 14.3 | 3669 | 3684 | -2462 | L1P | L1 | LINE |
| 67799774 | 67800258 | + | 13.8 | 3121 | 3666 | -2480 | L1P | L1 | LINE |
| 67800278 | 67800366 | + | 34.1 | 2818 | 2906 | -513 | L2 | L2 | LINE |

  

|  |  |  |  |  |  |  |  |  |  |
| --- | --- | --- | --- | --- | --- | --- | --- | --- | --- |
| chr6:67959843-67961291 | | | 1398 bp | | |  |  |  |  |
| Genome Start | Genome End | Strand | Div | Repeat Start | Repeat End | Repeat Left | Name | Family | Class |
| 67959843 | 67959997 | - | 32.2 | 3242 | 3419 | 0 | L2 | L2 | LINE |
| 67960000 | 67960285 | - | 7.0 | 5477 | 5764 | -390 | L1PA7 | L1 | LINE |
| 67960287 | 67960653 | + | 7.9 | 5787 | 6154 | 0 | L1PA7 | L1 | LINE |
| 67960702 | 67961291 | - | 37.3 | 2534 | 3181 | -238 | L2 | L2 | LINE |

  

|  |  |  |  |  |  |  |  |  |  |
| --- | --- | --- | --- | --- | --- | --- | --- | --- | --- |
| chr6:68250951-68259888 | | | 8288 bp | | |  |  |  |  |
| Genome Start | Genome End | Strand | Div | Repeat Start | Repeat End | Repeat Left | Name | Family | Class |
| 68250951 | 68251296 | + | 30.5 | 51 | 443 | -5980 | L1MCc | L1 | LINE |
| 68251607 | 68252026 | + | 31.9 | 1035 | 1475 | -4948 | L1MCc | L1 | LINE |
| 68252023 | 68253299 | - | 6.6 | 2447 | 3723 | -2423 | L1PA5 | L1 | LINE |
| 68253292 | 68255734 | + | 5.2 | 3715 | 6152 | -2 | L1PA5 | L1 | LINE |
| 68255736 | 68256277 | + | 26.7 | 1554 | 2101 | -5027 | L1MCc | L1 | LINE |
| 68256453 | 68256905 | + | 27.5 | 1 | 503 | -2 | MLT1D | MaLR | LTR |
| 68256910 | 68257068 | + | 29.9 | 2485 | 2653 | -4469 | L1MCc | L1 | LINE |
| 68257186 | 68257716 | + | 30.2 | 2860 | 3402 | -3539 | L1MCc | L1 | LINE |
| 68257720 | 68258499 | + | 13.6 | 5368 | 6160 | 0 | L1PREC2 | L1 | LINE |
| 68258552 | 68259888 | + | 28.7 | 3453 | 4862 | -2079 | L1MCc | L1 | LINE |

  

|  |  |  |  |  |  |  |  |  |  |
| --- | --- | --- | --- | --- | --- | --- | --- | --- | --- |
| chr6:68960937-68968499 | | | 7509 bp | | |  |  |  |  |
| Genome Start | Genome End | Strand | Div | Repeat Start | Repeat End | Repeat Left | Name | Family | Class |
| 68960937 | 68961780 | + | 24.0 | 5 | 851 | -5783 | L1PA16 | L1 | LINE |
| 68961793 | 68962119 | + | 18.8 | 918 | 1251 | -5383 | L1PA16 | L1 | LINE |
| 68962121 | 68962313 | - | 5.2 | 4918 | 5111 | -1035 | L1PA3 | L1 | LINE |
| 68962314 | 68963353 | + | 4.0 | 5097 | 6173 | -1 | L1PA3 | L1 | LINE |
| 68963395 | 68968499 | + | 16.1 | 1245 | 6624 | -10 | L1PA16 | L1 | LINE |

  

|  |  |  |  |  |  |  |  |  |  |
| --- | --- | --- | --- | --- | --- | --- | --- | --- | --- |
| chr6:69059033-69073524 | | | 14443 bp | | |  |  |  |  |
| Genome Start | Genome End | Strand | Div | Repeat Start | Repeat End | Repeat Left | Name | Family | Class |
| 69059033 | 69060244 | + | 20.6 | 24 | 1286 | -6279 | L1MA1 | L1 | LINE |
| 69060240 | 69061074 | - | 13.9 | 3170 | 3962 | -2184 | L1PREC2 | L1 | LINE |
| 69061108 | 69061482 | - | 13.9 | 2790 | 3169 | -2977 | L1PREC2 | L1 | LINE |
| 69061488 | 69063663 | + | 11.6 | 3969 | 6166 | -8 | L1PA11 | L1 | LINE |
| 69063683 | 69066248 | + | 16.0 | 1273 | 3871 | -3694 | L1MA1 | L1 | LINE |
| 69066255 | 69070229 | - | 10.3 | 2143 | 6148 | -6 | L1PA7 | L1 | LINE |
| 69070221 | 69073524 | + | 12.4 | 4028 | 7377 | -344 | L1MA1 | L1 | LINE |

  

|  |  |  |  |  |  |  |  |  |  |
| --- | --- | --- | --- | --- | --- | --- | --- | --- | --- |
| chr6:69227596-69232191 | | | 4489 bp | | |  |  |  |  |
| Genome Start | Genome End | Strand | Div | Repeat Start | Repeat End | Repeat Left | Name | Family | Class |
| 69227596 | 69228057 | - | 28.2 | 2208 | 2905 | -4282 | L1M4b | L1 | LINE |
| 69228171 | 69230518 | - | 4.6 | 3799 | 6147 | -8 | L1PA4 | L1 | LINE |
| 69230509 | 69231379 | + | 4.4 | 2931 | 3801 | -2345 | L1PA4 | L1 | LINE |
| 69231384 | 69232191 | - | 31.2 | 1325 | 2175 | -5159 | L1M4b | L1 | LINE |

  

|  |  |  |  |  |  |  |  |  |  |
| --- | --- | --- | --- | --- | --- | --- | --- | --- | --- |
| chr6:69301506-69307436 | | | 5810 bp | | |  |  |  |  |
| Genome Start | Genome End | Strand | Div | Repeat Start | Repeat End | Repeat Left | Name | Family | Class |
| 69301506 | 69301686 | + | 32.9 | 48 | 220 | -6180 | L1ME1 | L1 | LINE |
| 69301683 | 69301986 | + | 28.1 | 3811 | 4123 | -2023 | L1MD | L1 | LINE |
| 69301987 | 69302303 | + | 9.1 | 1 | 310 | -1 | AluY | Alu | SINE |
| 69302304 | 69302353 | + | 28.1 | 4124 | 4173 | -1973 | L1MD | L1 | LINE |
| 69302497 | 69302989 | + | 29.9 | 4759 | 5298 | -921 | L1ME1 | L1 | LINE |
| 69302957 | 69303008 | + | 9.6 | 3663 | 3715 | -2431 | L1PREC2 | L1 | LINE |
| 69303024 | 69304233 | - | 8.0 | 4938 | 6151 | -3 | L1PA7 | L1 | LINE |
| 69304235 | 69305703 | + | 8.0 | 3448 | 4925 | -1221 | L1PA7 | L1 | LINE |
| 69305697 | 69306028 | + | 21.0 | 5291 | 5674 | -563 | L1ME1 | L1 | LINE |
| 69306037 | 69306621 | + | 21.7 | 7197 | 7777 | -8 | L1MC3 | L1 | LINE |
| 69306620 | 69306921 | + | 27.6 | 5669 | 5961 | -278 | L1ME1 | L1 | LINE |
| 69306922 | 69307248 | - | 17.4 | 2 | 404 | -4 | MLT1A1 | MaLR | LTR |
| 69307249 | 69307436 | + | 27.6 | 5962 | 6147 | -92 | L1ME1 | L1 | LINE |

  

|  |  |  |  |  |  |  |  |  |  |
| --- | --- | --- | --- | --- | --- | --- | --- | --- | --- |
| chr6:69353096-69365927 | | | 12442 bp | | |  |  |  |  |
| Genome Start | Genome End | Strand | Div | Repeat Start | Repeat End | Repeat Left | Name | Family | Class |
| 69353096 | 69354223 | + | 17.4 | 3210 | 4423 | -1720 | L1MA7 | L1 | LINE |
| 69354225 | 69354720 | + | 18.2 | 5148 | 5646 | -645 | L1MA7 | L1 | LINE |
| 69354721 | 69355002 | - | 18.9 | 58 | 345 | 0 | MER2 | MER2\_type | DNA |
| 69355003 | 69356267 | - | 15.8 | 4894 | 6161 | -4 | L1PA15 | L1 | LINE |
| 69356268 | 69356619 | - | 13.7 | 6 | 375 | 0 | THE1C | MaLR | LTR |
| 69356625 | 69358199 | - | 30.4 | 2 | 1735 | 0 | MLT-int | MaLR | LTR |
| 69358203 | 69358573 | - | 15.1 | 1 | 374 | -1 | THE1C | MaLR | LTR |
| 69358574 | 69361253 | - | 16.4 | 2423 | 4898 | -1248 | L1PA15 | L1 | LINE |
| 69361249 | 69361920 | - | 6.5 | 5490 | 6154 | 0 | L1PA7 | L1 | LINE |
| 69361921 | 69362214 | - | 8.2 | 1 | 305 | -6 | AluY | Alu | SINE |
| 69362215 | 69363040 | - | 9.0 | 4665 | 5489 | -665 | L1PA7 | L1 | LINE |
| 69363041 | 69363106 | + | 3.0 | 4590 | 4655 | -1491 | L1PA7 | L1 | LINE |
| 69363106 | 69364804 | - | 18.2 | 1340 | 3058 | -3708 | L1PA13 | L1 | LINE |
| 69365190 | 69365239 | - | 20.0 | 20 | 69 | -276 | MER2 | MER2\_type | DNA |
| 69365239 | 69365295 | - | 28.1 | 2 | 58 | -287 | MER2 | MER2\_type | DNA |
| 69365299 | 69365927 | + | 21.2 | 5650 | 6285 | -6 | L1MA7 | L1 | LINE |

  

|  |  |  |  |  |  |  |  |  |  |
| --- | --- | --- | --- | --- | --- | --- | --- | --- | --- |
| chr6:69366868-69369996 | | | 3115 bp | | |  |  |  |  |
| Genome Start | Genome End | Strand | Div | Repeat Start | Repeat End | Repeat Left | Name | Family | Class |
| 69366868 | 69366942 | + | 9.3 | 1 | 81 | -372 | MLT2A2 | ERVL | LTR |
| 69366962 | 69369047 | - | 3.1 | 4108 | 6155 | 0 | L1PA4 | L1 | LINE |
| 69369043 | 69369656 | + | 2.6 | 3496 | 4120 | -2026 | L1PA4 | L1 | LINE |
| 69369657 | 69369996 | + | 12.4 | 70 | 398 | -55 | MLT2A2 | ERVL | LTR |

  

|  |  |  |  |  |  |  |  |  |  |
| --- | --- | --- | --- | --- | --- | --- | --- | --- | --- |
| chr6:70064685-70067603 | | | 2888 bp | | |  |  |  |  |
| Genome Start | Genome End | Strand | Div | Repeat Start | Repeat End | Repeat Left | Name | Family | Class |
| 70064685 | 70064943 | - | 20.2 | 218 | 514 | 0 | MER67D | ERV1 | LTR |
| 70064965 | 70066607 | - | 15.3 | 4541 | 6294 | -6 | L1MA4 | L1 | LINE |
| 70066606 | 70067470 | + | 17.8 | 3655 | 4528 | -1615 | L1MA4 | L1 | LINE |
| 70067483 | 70067603 | - | 12.6 | 98 | 214 | -300 | MER67D | ERV1 | LTR |

  

|  |  |  |  |  |  |  |  |  |  |
| --- | --- | --- | --- | --- | --- | --- | --- | --- | --- |
| chr6:70133443-70135529 | | | 1953 bp | | |  |  |  |  |
| Genome Start | Genome End | Strand | Div | Repeat Start | Repeat End | Repeat Left | Name | Family | Class |
| 70133443 | 70133910 | - | 32.5 | 2889 | 3418 | -1 | L2 | L2 | LINE |
| 70133944 | 70134295 | - | 2.0 | 5335 | 5686 | -469 | L1PA2 | L1 | LINE |
| 70134296 | 70134770 | + | 2.1 | 5680 | 6154 | -1 | L1PA2 | L1 | LINE |
| 70134872 | 70135529 | - | 30.4 | 1917 | 2769 | -650 | L2 | L2 | LINE |

  

|  |  |  |  |  |  |  |  |  |  |
| --- | --- | --- | --- | --- | --- | --- | --- | --- | --- |
| chr6:72575405-72581377 | | | 5952 bp | | |  |  |  |  |
| Genome Start | Genome End | Strand | Div | Repeat Start | Repeat End | Repeat Left | Name | Family | Class |
| 72575405 | 72576097 | + | 24.5 | 2048 | 2760 | -4806 | L1M1 | L1 | LINE |
| 72576098 | 72576794 | - | 3.3 | 5459 | 6155 | 0 | L1PA4 | L1 | LINE |
| 72576795 | 72577125 | + | 3.2 | 5109 | 5466 | -689 | L1PA4 | L1 | LINE |
| 72577124 | 72577926 | + | 24.9 | 2402 | 3395 | -4367 | L1M2c | L1 | LINE |
| 72577927 | 72578237 | + | 13.2 | 1 | 310 | 0 | AluY | Alu | SINE |
| 72578238 | 72580595 | + | 22.3 | 3199 | 5673 | -1886 | L1M1 | L1 | LINE |
| 72580619 | 72581377 | + | 21.0 | 5674 | 6465 | -1094 | L1M1 | L1 | LINE |

  

|  |  |  |  |  |  |  |  |  |  |
| --- | --- | --- | --- | --- | --- | --- | --- | --- | --- |
| chr6:75380103-75384180 | | | 3412 bp | | |  |  |  |  |
| Genome Start | Genome End | Strand | Div | Repeat Start | Repeat End | Repeat Left | Name | Family | Class |
| 75380103 | 75380229 | + | 37.3 | 2658 | 2784 | -635 | L2 | L2 | LINE |
| 75380246 | 75381107 | - | 13.2 | 5307 | 6163 | 0 | L1PA13 | L1 | LINE |
| 75381449 | 75381964 | - | 29.0 | 2819 | 3364 | -55 | L2 | L2 | LINE |
| 75382027 | 75382081 | + | 25.4 | 3022 | 3078 | -300 | L2 | L2 | LINE |
| 75382217 | 75382608 | - | 33.2 | 219 | 611 | -4 | MLT1L | MaLR | LTR |
| 75382711 | 75383644 | - | 12.6 | 5254 | 6157 | -3 | L1PREC2 | L1 | LINE |
| 75383643 | 75384063 | + | 10.6 | 4819 | 5246 | -900 | L1PREC2 | L1 | LINE |
| 75384076 | 75384180 | + | 28.4 | 3313 | 3419 | 0 | L2 | L2 | LINE |

  

|  |  |  |  |  |  |  |  |  |  |
| --- | --- | --- | --- | --- | --- | --- | --- | --- | --- |
| chr6:75769951-75777792 | | | 7825 bp | | |  |  |  |  |
| Genome Start | Genome End | Strand | Div | Repeat Start | Repeat End | Repeat Left | Name | Family | Class |
| 75769951 | 75770134 | + | 24.2 | 5992 | 6181 | -131 | L1MA9 | L1 | LINE |
| 75770131 | 75773436 | + | 5.9 | 1 | 3303 | -2843 | L1PA4 | L1 | LINE |
| 75773436 | 75773497 | - | 4.8 | 4791 | 4852 | -1294 | L1PA3 | L1 | LINE |
| 75773488 | 75774784 | + | 4.2 | 4840 | 6137 | -18 | L1PA3 | L1 | LINE |
| 75774807 | 75777662 | + | 4.5 | 3293 | 6151 | -4 | L1PA4 | L1 | LINE |
| 75777673 | 75777792 | + | 25.0 | 6161 | 6274 | -38 | L1MA9 | L1 | LINE |

  

|  |  |  |  |  |  |  |  |  |  |
| --- | --- | --- | --- | --- | --- | --- | --- | --- | --- |
| chr6:77500982-77504887 | | | 3929 bp | | |  |  |  |  |
| Genome Start | Genome End | Strand | Div | Repeat Start | Repeat End | Repeat Left | Name | Family | Class |
| 77500982 | 77501267 | - | 19.0 | 7636 | 7917 | -44 | L1MC5 | L1 | LINE |
| 77501248 | 77501770 | - | 7.1 | 2884 | 3407 | -2739 | L1PA5 | L1 | LINE |
| 77501771 | 77504512 | + | 5.4 | 3411 | 6150 | -4 | L1PA5 | L1 | LINE |
| 77504510 | 77504887 | - | 22.8 | 7277 | 7663 | -298 | L1MC5 | L1 | LINE |

  

|  |  |  |  |  |  |  |  |  |  |
| --- | --- | --- | --- | --- | --- | --- | --- | --- | --- |
| chr6:78553398-78564730 | | | 11167 bp | | |  |  |  |  |
| Genome Start | Genome End | Strand | Div | Repeat Start | Repeat End | Repeat Left | Name | Family | Class |
| 78553398 | 78554296 | + | 28.5 | 1289 | 2063 | -4864 | L1M4b | L1 | LINE |
| 78554283 | 78554518 | - | 6.8 | 3571 | 3813 | -2333 | L1PA4 | L1 | LINE |
| 78554518 | 78556844 | + | 4.6 | 3819 | 6154 | -1 | L1PA4 | L1 | LINE |
| 78556865 | 78557005 | + | 20.6 | 6016 | 6156 | 0 | L1PB4 | L1 | LINE |
| 78557013 | 78557160 | + | 27.9 | 2337 | 2488 | -4519 | L1M4b | L1 | LINE |
| 78557249 | 78558128 | + | 2.0 | 5273 | 6153 | -2 | L1PA2 | L1 | LINE |
| 78558129 | 78558962 | - | 18.3 | 5435 | 6231 | -60 | L1MA8 | L1 | LINE |
| 78558956 | 78559153 | - | 20.5 | 4921 | 5107 | -1036 | L1MA8 | L1 | LINE |
| 78559154 | 78559447 | - | 8.5 | 1 | 294 | -17 | AluY | Alu | SINE |
| 78559448 | 78559561 | - | 20.5 | 4809 | 4920 | -1223 | L1MA8 | L1 | LINE |
| 78559561 | 78560717 | + | 25.3 | 2714 | 3876 | -3683 | L1M1 | L1 | LINE |
| 78560718 | 78561013 | + | 7.1 | 1 | 297 | -14 | AluY | Alu | SINE |
| 78561014 | 78563860 | + | 18.1 | 3877 | 6646 | -913 | L1M1 | L1 | LINE |
| 78563861 | 78564381 | - | 23.1 | 1 | 501 | -53 | MLT2B3 | ERVL | LTR |
| 78564456 | 78564730 | + | 25.2 | 2969 | 3287 | -4047 | L1M4b | L1 | LINE |

  

|  |  |  |  |  |  |  |  |  |  |
| --- | --- | --- | --- | --- | --- | --- | --- | --- | --- |
| chr6:78673420-78683004 | | | 9517 bp | | |  |  |  |  |
| Genome Start | Genome End | Strand | Div | Repeat Start | Repeat End | Repeat Left | Name | Family | Class |
| 78673420 | 78673473 | - | 20.4 | 6420 | 6473 | -529 | L1M2 | L1 | LINE |
| 78673577 | 78675100 | - | 5.0 | 4637 | 6153 | -2 | L1PA4 | L1 | LINE |
| 78675100 | 78677129 | + | 9.7 | 2302 | 4630 | -1516 | L1PA4 | L1 | LINE |
| 78677133 | 78677752 | - | 18.1 | 5805 | 6428 | -577 | L1M2 | L1 | LINE |
| 78677733 | 78678295 | - | 17.4 | 5672 | 6166 | 0 | L1PA16 | L1 | LINE |
| 78678280 | 78680190 | - | 19.9 | 3896 | 5840 | -1005 | L1M2 | L1 | LINE |
| 78680191 | 78680484 | + | 9.6 | 3 | 298 | -13 | AluY | Alu | SINE |
| 78680485 | 78682019 | - | 18.9 | 2301 | 3895 | -2950 | L1M2 | L1 | LINE |
| 78682035 | 78682149 | - | 14.8 | 2375 | 2490 | -3656 | L1M1 | L1 | LINE |
| 78682134 | 78683004 | - | 19.7 | 1453 | 2326 | -4523 | L1M2 | L1 | LINE |

  

|  |  |  |  |  |  |  |  |  |  |
| --- | --- | --- | --- | --- | --- | --- | --- | --- | --- |
| chr6:78683009-78688545 | | | 5538 bp | | |  |  |  |  |
| Genome Start | Genome End | Strand | Div | Repeat Start | Repeat End | Repeat Left | Name | Family | Class |
| 78683009 | 78683212 | - | 17.2 | 1720 | 1929 | -489 | Tigger1 | MER2\_type | DNA |
| 78683217 | 78685427 | - | 2.1 | 3930 | 6143 | -12 | L1PA2 | L1 | LINE |
| 78685423 | 78686854 | + | 2.5 | 2477 | 3908 | -2238 | L1PA2 | L1 | LINE |
| 78686855 | 78688545 | - | 15.4 | 1 | 1743 | -675 | Tigger1 | MER2\_type | DNA |

  

|  |  |  |  |  |  |  |  |  |  |
| --- | --- | --- | --- | --- | --- | --- | --- | --- | --- |
| chr6:78693811-78695720 | | | 1912 bp | | |  |  |  |  |
| Genome Start | Genome End | Strand | Div | Repeat Start | Repeat End | Repeat Left | Name | Family | Class |
| 78693811 | 78693889 | + | 27.9 | 1 | 80 | -109 | MER5A | MER1\_type | DNA |
| 78693890 | 78694003 | - | 3.5 | 4449 | 4562 | -1584 | L1PA4 | L1 | LINE |
| 78693994 | 78695581 | + | 3.9 | 4561 | 6155 | 0 | L1PA4 | L1 | LINE |
| 78695590 | 78695720 | + | 21.1 | 61 | 188 | -1 | MER5A | MER1\_type | DNA |

  

|  |  |  |  |  |  |  |  |  |  |
| --- | --- | --- | --- | --- | --- | --- | --- | --- | --- |
| chr6:80125360-80136125 | | | 10769 bp | | |  |  |  |  |
| Genome Start | Genome End | Strand | Div | Repeat Start | Repeat End | Repeat Left | Name | Family | Class |
| 80125360 | 80127377 | - | 14.0 | 676 | 2716 | -2 | Tigger2 | MER2\_type | DNA |
| 80127378 | 80129089 | - | 5.3 | 4441 | 6153 | -1 | L1PA5 | L1 | LINE |
| 80129089 | 80129795 | + | 5.8 | 3749 | 4461 | -1685 | L1PA5 | L1 | LINE |
| 80129794 | 80135456 | - | 7.7 | 1 | 5688 | -466 | L1P2 | L1 | LINE |
| 80135457 | 80136125 | - | 16.5 | 3 | 688 | -2030 | Tigger2 | MER2\_type | DNA |

  

|  |  |  |  |  |  |  |  |  |  |
| --- | --- | --- | --- | --- | --- | --- | --- | --- | --- |
| chr6:80156129-80158134 | | | 1962 bp | | |  |  |  |  |
| Genome Start | Genome End | Strand | Div | Repeat Start | Repeat End | Repeat Left | Name | Family | Class |
| 80156129 | 80156216 | - | 12.6 | 294 | 396 | 0 | MLT1A0 | MaLR | LTR |
| 80156217 | 80156331 | - | 4.3 | 4571 | 4685 | -1461 | L1PA2 | L1 | LINE |
| 80156331 | 80157781 | + | 2.8 | 4705 | 6155 | 0 | L1PA2 | L1 | LINE |
| 80157827 | 80158134 | - | 15.6 | 1 | 311 | -54 | MLT1A0 | MaLR | LTR |

  

|  |  |  |  |  |  |  |  |  |  |
| --- | --- | --- | --- | --- | --- | --- | --- | --- | --- |
| chr6:80721886-80727481 | | | 5569 bp | | |  |  |  |  |
| Genome Start | Genome End | Strand | Div | Repeat Start | Repeat End | Repeat Left | Name | Family | Class |
| 80721886 | 80722216 | + | 27.2 | 2451 | 2836 | -3310 | L1M5 | L1 | LINE |
| 80722235 | 80722354 | + | 21.7 | 1 | 121 | -22 | FLAM\_C | Alu | SINE |
| 80722376 | 80724512 | - | 5.3 | 1832 | 3970 | -2176 | L1PA5 | L1 | LINE |
| 80724509 | 80726675 | + | 4.1 | 3974 | 6154 | 0 | L1PA5 | L1 | LINE |
| 80726668 | 80727481 | + | 24.2 | 2919 | 3790 | -2356 | L1M5 | L1 | LINE |

  

|  |  |  |  |  |  |  |  |  |  |
| --- | --- | --- | --- | --- | --- | --- | --- | --- | --- |
| chr6:81561263-81564122 | | | 2876 bp | | |  |  |  |  |
| Genome Start | Genome End | Strand | Div | Repeat Start | Repeat End | Repeat Left | Name | Family | Class |
| 81561263 | 81561649 | + | 10.3 | 5535 | 5922 | -252 | L1PA11 | L1 | LINE |
| 81561657 | 81563032 | - | 2.7 | 4779 | 6155 | 0 | L1PA3 | L1 | LINE |
| 81563033 | 81563904 | + | 3.7 | 3909 | 4780 | -1366 | L1PA3 | L1 | LINE |
| 81563882 | 81564122 | + | 13.3 | 5898 | 6158 | -16 | L1PA11 | L1 | LINE |

  

|  |  |  |  |  |  |  |  |  |  |
| --- | --- | --- | --- | --- | --- | --- | --- | --- | --- |
| chr6:81974198-81977857 | | | 3673 bp | | |  |  |  |  |
| Genome Start | Genome End | Strand | Div | Repeat Start | Repeat End | Repeat Left | Name | Family | Class |
| 81974198 | 81974482 | + | 16.1 | 5052 | 5338 | -808 | L1MA2 | L1 | LINE |
| 81974480 | 81975079 | - | 3.8 | 3723 | 4323 | -1823 | L1PA3 | L1 | LINE |
| 81975076 | 81976906 | + | 3.3 | 4320 | 6154 | -1 | L1PA3 | L1 | LINE |
| 81976901 | 81977857 | + | 10.4 | 5328 | 6304 | 0 | L1MA2 | L1 | LINE |

  

|  |  |  |  |  |  |  |  |  |  |
| --- | --- | --- | --- | --- | --- | --- | --- | --- | --- |
| chr6:82110294-82118319 | | | 7973 bp | | |  |  |  |  |
| Genome Start | Genome End | Strand | Div | Repeat Start | Repeat End | Repeat Left | Name | Family | Class |
| 82110294 | 82110363 | + | 5.4 | 1 | 70 | -710 | LTR17 | ERV1 | LTR |
| 82110364 | 82110795 | - | 8.3 | 1 | 472 | 0 | LTR7Y | ERV1 | LTR |
| 82110800 | 82111168 | - | 8.5 | 7324 | 7709 | -4 | HERVH | ERV1 | LTR |
| 82111169 | 82111385 | - | 13.0 | 5600 | 5819 | -1894 | HERVH | ERV1 | LTR |
| 82111429 | 82111548 | - | 0.8 | 4926 | 5045 | -1101 | L1PA3 | L1 | LINE |
| 82111545 | 82112639 | + | 2.0 | 5050 | 6135 | -20 | L1PA3 | L1 | LINE |
| 82112648 | 82113277 | - | 14.3 | 4488 | 5116 | -2597 | HERVH | ERV1 | LTR |
| 82113280 | 82117162 | - | 9.3 | 1 | 3993 | -3720 | HERVH | ERV1 | LTR |
| 82117163 | 82117596 | - | 9.2 | 1 | 472 | 0 | LTR7Y | ERV1 | LTR |
| 82117597 | 82118319 | + | 8.6 | 66 | 780 | 0 | LTR17 | ERV1 | LTR |

  

|  |  |  |  |  |  |  |  |  |  |
| --- | --- | --- | --- | --- | --- | --- | --- | --- | --- |
| chr6:82193356-82209828 | | | 16421 bp | | |  |  |  |  |
| Genome Start | Genome End | Strand | Div | Repeat Start | Repeat End | Repeat Left | Name | Family | Class |
| 82193356 | 82193553 | - | 16.0 | 6821 | 7000 | -5 | L1MA4A | L1 | LINE |
| 82193605 | 82193856 | - | 5.6 | 31 | 283 | -28 | AluY | Alu | SINE |
| 82193857 | 82194053 | - | 16.0 | 6647 | 6820 | -185 | L1MA4A | L1 | LINE |
| 82194054 | 82194172 | - | 22.7 | 1 | 119 | -14 | FLAM\_A | Alu | SINE |
| 82194173 | 82197281 | - | 19.0 | 3462 | 6646 | -359 | L1MA4A | L1 | LINE |
| 82197282 | 82199790 | - | 4.4 | 3648 | 6152 | -3 | L1PA3 | L1 | LINE |
| 82199795 | 82203434 | + | 4.5 | 11 | 3642 | -2504 | L1PA3 | L1 | LINE |
| 82203432 | 82203603 | - | 17.4 | 3318 | 3494 | -3351 | L1MA4A | L1 | LINE |
| 82203605 | 82205227 | - | 18.6 | 4578 | 6191 | 0 | L1PB4 | L1 | LINE |
| 82205227 | 82207177 | - | 19.8 | 1328 | 3315 | -3530 | L1MA4A | L1 | LINE |
| 82207178 | 82208051 | + | 5.7 | 1 | 897 | 0 | MER11D | ERVK | LTR |
| 82208052 | 82208215 | - | 19.9 | 1167 | 1327 | -5522 | L1MA4A | L1 | LINE |
| 82208216 | 82208875 | + | 9.1 | 1 | 660 | 0 | PABL\_A | ERV1 | LTR |
| 82208876 | 82209828 | - | 19.9 | 154 | 1166 | -5683 | L1MA4A | L1 | LINE |

  

|  |  |  |  |  |  |  |  |  |  |
| --- | --- | --- | --- | --- | --- | --- | --- | --- | --- |
| chr6:82366341-82371973 | | | 5555 bp | | |  |  |  |  |
| Genome Start | Genome End | Strand | Div | Repeat Start | Repeat End | Repeat Left | Name | Family | Class |
| 82366341 | 82366477 | + | 24.1 | 2 | 139 | -50 | MER5A | MER1\_type | DNA |
| 82366492 | 82366654 | - | 15.4 | 6132 | 6296 | -4 | L1MA6 | L1 | LINE |
| 82366655 | 82366880 | + | 9.8 | 4944 | 5174 | -972 | L1PREC2 | L1 | LINE |
| 82366874 | 82367739 | + | 10.5 | 5304 | 6160 | -1 | L1PA14 | L1 | LINE |
| 82367779 | 82368769 | - | 20.5 | 5131 | 6140 | -151 | L1MA7 | L1 | LINE |
| 82368791 | 82369353 | - | 10.6 | 5568 | 6131 | 0 | L1PA8A | L1 | LINE |
| 82369354 | 82370008 | + | 7.9 | 3315 | 3975 | -2171 | L1PA8A | L1 | LINE |
| 82370009 | 82370324 | + | 10.8 | 5 | 311 | 0 | AluY | Alu | SINE |
| 82370325 | 82371911 | + | 8.0 | 3976 | 5554 | -577 | L1PA8A | L1 | LINE |
| 82371923 | 82371973 | + | 15.7 | 135 | 189 | 0 | MER5A | MER1\_type | DNA |

  

|  |  |  |  |  |  |  |  |  |  |
| --- | --- | --- | --- | --- | --- | --- | --- | --- | --- |
| chr6:82858274-82860615 | | | 2335 bp | | |  |  |  |  |
| Genome Start | Genome End | Strand | Div | Repeat Start | Repeat End | Repeat Left | Name | Family | Class |
| 82858274 | 82858439 | + | 26.7 | 9 | 187 | -263 | LTR16A | ERVL | LTR |
| 82858442 | 82858804 | - | 3.6 | 4207 | 4569 | -1577 | L1PA3 | L1 | LINE |
| 82858793 | 82860390 | + | 3.1 | 4555 | 6153 | -2 | L1PA3 | L1 | LINE |
| 82860408 | 82860615 | + | 23.1 | 205 | 441 | -9 | LTR16A | ERVL | LTR |

  

|  |  |  |  |  |  |  |  |  |  |
| --- | --- | --- | --- | --- | --- | --- | --- | --- | --- |
| chr6:83305308-83318801 | | | 13382 bp | | |  |  |  |  |
| Genome Start | Genome End | Strand | Div | Repeat Start | Repeat End | Repeat Left | Name | Family | Class |
| 83305308 | 83305683 | + | 32.4 | 5270 | 5686 | -1255 | L1MC4a | L1 | LINE |
| 83305684 | 83305994 | - | 15.6 | 1 | 309 | -3 | AluSx | Alu | SINE |
| 83305995 | 83306096 | + | 32.4 | 5687 | 5797 | -1144 | L1MC4a | L1 | LINE |
| 83306131 | 83306437 | - | 13.7 | 1 | 312 | -1 | AluSp | Alu | SINE |
| 83306446 | 83306569 | - | 8.1 | 3 | 126 | -187 | AluSp | Alu | SINE |
| 83306583 | 83306679 | + | 29.2 | 5856 | 5953 | -988 | L1MC4a | L1 | LINE |
| 83306680 | 83306979 | - | 12.7 | 1 | 302 | -10 | AluSx | Alu | SINE |
| 83306990 | 83307313 | - | 13.6 | 5833 | 6158 | -2 | L1PREC2 | L1 | LINE |
| 83307314 | 83307453 | + | 28.0 | 5963 | 6091 | -2512 | L1MC4a | L1 | LINE |
| 83307454 | 83307755 | + | 9.9 | 1 | 303 | -7 | AluSg | Alu | SINE |
| 83307756 | 83308066 | + | 25.4 | 6085 | 6408 | -2195 | L1MC4a | L1 | LINE |
| 83308074 | 83309421 | + | 22.1 | 6552 | 8017 | -586 | L1MC4a | L1 | LINE |
| 83309454 | 83313755 | - | 7.7 | 1849 | 6150 | -4 | L1PA6 | L1 | LINE |
| 83313757 | 83314081 | - | 6.5 | 3487 | 3811 | -2335 | L1PA4 | L1 | LINE |
| 83314079 | 83316415 | + | 4.6 | 3818 | 6155 | 0 | L1PA4 | L1 | LINE |
| 83316418 | 83316805 | - | 7.4 | 1470 | 1858 | -417 | L1PA6 | L1 | LINE |
| 83316806 | 83317116 | - | 10.9 | 1 | 311 | 0 | AluY | Alu | SINE |
| 83317117 | 83318572 | - | 7.4 | 7 | 1469 | -806 | L1PA6 | L1 | LINE |
| 83318581 | 83318801 | + | 19.2 | 8004 | 8222 | -381 | L1MC4a | L1 | LINE |

  

|  |  |  |  |  |  |  |  |  |  |
| --- | --- | --- | --- | --- | --- | --- | --- | --- | --- |
| chr6:83520466-83528032 | | | 7465 bp | | |  |  |  |  |
| Genome Start | Genome End | Strand | Div | Repeat Start | Repeat End | Repeat Left | Name | Family | Class |
| 83520466 | 83520570 | + | 22.1 | 94 | 204 | -2303 | HAL1 | L1 | LINE |
| 83520573 | 83521169 | + | 29.3 | 469 | 1082 | -1425 | HAL1 | L1 | LINE |
| 83521168 | 83524265 | - | 6.7 | 910 | 4015 | -2131 | L1PA5 | L1 | LINE |
| 83524259 | 83524288 | + | 3.3 | 4021 | 4050 | -2096 | L1PA5 | L1 | LINE |
| 83524344 | 83526443 | + | 5.9 | 4054 | 6154 | 0 | L1PA5 | L1 | LINE |
| 83526485 | 83527204 | + | 11.4 | 5430 | 6149 | -2 | L1PB2 | L1 | LINE |
| 83527218 | 83528032 | + | 26.2 | 1419 | 2311 | -196 | HAL1 | L1 | LINE |

  

|  |  |  |  |  |  |  |  |  |  |
| --- | --- | --- | --- | --- | --- | --- | --- | --- | --- |
| chr6:84083408-84086536 | | | 3124 bp | | |  |  |  |  |
| Genome Start | Genome End | Strand | Div | Repeat Start | Repeat End | Repeat Left | Name | Family | Class |
| 84083408 | 84083737 | + | 21.3 | 261 | 653 | -819 | MLT1H-int | MaLR | LTR |
| 84083742 | 84084120 | - | 2.4 | 4135 | 4513 | -1633 | L1PA3 | L1 | LINE |
| 84084115 | 84085802 | + | 1.8 | 4516 | 6203 | 0 | L1PA3 | L1 | LINE |
| 84085810 | 84086536 | + | 26.2 | 679 | 1457 | -15 | MLT1H-int | MaLR | LTR |

  

|  |  |  |  |  |  |  |  |  |  |
| --- | --- | --- | --- | --- | --- | --- | --- | --- | --- |
| chr6:84307376-84312727 | | | 5347 bp | | |  |  |  |  |
| Genome Start | Genome End | Strand | Div | Repeat Start | Repeat End | Repeat Left | Name | Family | Class |
| 84307376 | 84307852 | + | 15.3 | 5591 | 6059 | -232 | L1MA7 | L1 | LINE |
| 84307867 | 84311029 | - | 5.4 | 2960 | 6145 | -9 | L1PA5 | L1 | LINE |
| 84311026 | 84312653 | + | 5.7 | 1348 | 2945 | -3201 | L1PA5 | L1 | LINE |
| 84312649 | 84312727 | + | 19.0 | 6046 | 6124 | -167 | L1MA7 | L1 | LINE |

  

|  |  |  |  |  |  |  |  |  |  |
| --- | --- | --- | --- | --- | --- | --- | --- | --- | --- |
| chr6:84433489-84435276 | | | 1774 bp | | |  |  |  |  |
| Genome Start | Genome End | Strand | Div | Repeat Start | Repeat End | Repeat Left | Name | Family | Class |
| 84433489 | 84434066 | + | 19.4 | 2064 | 2650 | -111 | Charlie1 | MER1\_type | DNA |
| 84434081 | 84434201 | - | 4.1 | 6028 | 6148 | -6 | L1PA5 | L1 | LINE |
| 84434202 | 84435175 | + | 4.1 | 5075 | 6050 | -104 | L1PA5 | L1 | LINE |
| 84435176 | 84435276 | + | 18.8 | 2642 | 2760 | -1 | Charlie1 | MER1\_type | DNA |

  

|  |  |  |  |  |  |  |  |  |  |
| --- | --- | --- | --- | --- | --- | --- | --- | --- | --- |
| chr6:85237558-85257343 | | | 19736 bp | | |  |  |  |  |
| Genome Start | Genome End | Strand | Div | Repeat Start | Repeat End | Repeat Left | Name | Family | Class |
| 85237558 | 85237900 | + | 24.3 | 8 | 325 | -6217 | L1MB7 | L1 | LINE |
| 85237901 | 85238268 | + | 14.1 | 1 | 364 | 0 | THE1B | MaLR | LTR |
| 85238269 | 85241127 | + | 22.9 | 326 | 3374 | -3270 | L1MB7 | L1 | LINE |
| 85241133 | 85241310 | - | 21.9 | 272 | 452 | -53 | MLT1D | MaLR | LTR |
| 85241370 | 85241553 | + | 16.7 | 3363 | 3543 | -3101 | L1MB7 | L1 | LINE |
| 85241549 | 85242237 | - | 3.3 | 3777 | 4469 | -1677 | L1PA4 | L1 | LINE |
| 85242234 | 85243937 | + | 5.2 | 4446 | 6150 | -5 | L1PA4 | L1 | LINE |
| 85243932 | 85245077 | + | 20.3 | 3526 | 4686 | -1958 | L1MB7 | L1 | LINE |
| 85245080 | 85251647 | + | 11.1 | 5 | 6620 | 0 | L1PB3 | L1 | LINE |
| 85251641 | 85253087 | + | 20.0 | 4649 | 6103 | -573 | L1MB7 | L1 | LINE |
| 85253090 | 85254571 | + | 15.1 | 4819 | 6333 | 0 | L1MC1 | L1 | LINE |
| 85254572 | 85254993 | + | 19.5 | 6091 | 6532 | -150 | L1MB7 | L1 | LINE |
| 85254994 | 85255377 | - | 13.7 | 1 | 375 | 0 | THE1C | MaLR | LTR |
| 85255380 | 85256859 | - | 12.2 | 4 | 1578 | -2 | THE1C-int | MaLR | LTR |
| 85256862 | 85257240 | - | 14.7 | 1 | 375 | 0 | THE1C | MaLR | LTR |
| 85257241 | 85257343 | + | 16.5 | 6528 | 6631 | -51 | L1MB7 | L1 | LINE |

  

|  |  |  |  |  |  |  |  |  |  |
| --- | --- | --- | --- | --- | --- | --- | --- | --- | --- |
| chr6:85350954-85353426 | | | 2446 bp | | |  |  |  |  |
| Genome Start | Genome End | Strand | Div | Repeat Start | Repeat End | Repeat Left | Name | Family | Class |
| 85350954 | 85351346 | - | 17.1 | 28 | 432 | 0 | MSTB1 | MaLR | LTR |
| 85351347 | 85351947 | - | 3.8 | 4117 | 4715 | -1431 | L1PA4 | L1 | LINE |
| 85351937 | 85353349 | + | 3.6 | 4731 | 6152 | -3 | L1PA4 | L1 | LINE |
| 85353388 | 85353426 | - | 5.1 | 2 | 40 | -392 | MSTB1 | MaLR | LTR |

  

|  |  |  |  |  |  |  |  |  |  |
| --- | --- | --- | --- | --- | --- | --- | --- | --- | --- |
| chr6:86003526-86003980 | | | 447 bp | | |  |  |  |  |
| Genome Start | Genome End | Strand | Div | Repeat Start | Repeat End | Repeat Left | Name | Family | Class |
| 86003526 | 86003710 | + | 22.2 | 3 | 198 | -143 | MER58B | MER1\_type | DNA |
| 86003719 | 86003784 | - | 4.5 | 6090 | 6155 | 0 | L1PA4 | L1 | LINE |
| 86003785 | 86003848 | + | 4.7 | 6024 | 6087 | -68 | L1PA4 | L1 | LINE |
| 86003849 | 86003980 | + | 20.4 | 205 | 337 | -4 | MER58B | MER1\_type | DNA |

  

|  |  |  |  |  |  |  |  |  |  |
| --- | --- | --- | --- | --- | --- | --- | --- | --- | --- |
| chr6:87308335-87313755 | | | 5319 bp | | |  |  |  |  |
| Genome Start | Genome End | Strand | Div | Repeat Start | Repeat End | Repeat Left | Name | Family | Class |
| 87308335 | 87308672 | - | 19.6 | 7426 | 7732 | -263 | L1MD3 | L1 | LINE |
| 87308675 | 87308782 | - | 25.9 | 38 | 147 | -420 | MLT1F1 | MaLR | LTR |
| 87308785 | 87308929 | - | 23.9 | 6984 | 7118 | -877 | L1MD3 | L1 | LINE |
| 87308929 | 87309198 | - | 11.5 | 5093 | 5371 | -775 | L1PA15 | L1 | LINE |
| 87309195 | 87309991 | + | 17.0 | 5371 | 6165 | 0 | L1PA15 | L1 | LINE |
| 87310004 | 87310670 | + | 13.7 | 5485 | 6159 | -7 | L1PA16 | L1 | LINE |
| 87310672 | 87312002 | - | 26.9 | 5680 | 6971 | -814 | L1MC3 | L1 | LINE |
| 87312008 | 87312167 | - | 20.0 | 1 | 168 | -17 | FAM | Alu | SINE |
| 87312220 | 87312372 | - | 18.1 | 5523 | 5666 | -2329 | L1MD3 | L1 | LINE |
| 87312374 | 87312459 | - | 23.6 | 4941 | 5043 | -1103 | L1MD3 | L1 | LINE |
| 87312492 | 87312695 | - | 23.6 | 4743 | 4940 | -1206 | L1MD3 | L1 | LINE |
| 87312696 | 87312996 | - | 9.3 | 6 | 306 | -5 | AluY | Alu | SINE |
| 87312997 | 87313755 | - | 23.6 | 4013 | 4742 | -1404 | L1MD3 | L1 | LINE |

  

|  |  |  |  |  |  |  |  |  |  |
| --- | --- | --- | --- | --- | --- | --- | --- | --- | --- |
| chr6:91971491-91981462 | | | 9598 bp | | |  |  |  |  |
| Genome Start | Genome End | Strand | Div | Repeat Start | Repeat End | Repeat Left | Name | Family | Class |
| 91971491 | 91971641 | - | 32.7 | 3266 | 3415 | -4 | L2 | L2 | LINE |
| 91971686 | 91971989 | + | 12.5 | 1 | 296 | -14 | AluSg | Alu | SINE |
| 91971990 | 91972385 | - | 10.5 | 6228 | 6608 | -9 | L1PB1 | L1 | LINE |
| 91972405 | 91973241 | - | 8.8 | 5398 | 6261 | -356 | L1PB1 | L1 | LINE |
| 91973246 | 91975232 | - | 8.5 | 2814 | 4802 | -1810 | L1PB1 | L1 | LINE |
| 91975220 | 91977427 | - | 1.9 | 3948 | 6153 | -2 | L1PA2 | L1 | LINE |
| 91977428 | 91978076 | + | 1.9 | 3302 | 3950 | -2196 | L1PA2 | L1 | LINE |
| 91978073 | 91980808 | - | 14.2 | 8 | 2839 | -3773 | L1PB1 | L1 | LINE |
| 91980819 | 91980914 | - | 24.0 | 1 | 97 | 0 | Tigger5c | MER2\_type | DNA |
| 91981229 | 91981462 | - | 39.3 | 2726 | 2965 | -454 | L2 | L2 | LINE |

  

|  |  |  |  |  |  |  |  |  |  |
| --- | --- | --- | --- | --- | --- | --- | --- | --- | --- |
| chr6:92737501-92742071 | | | 4544 bp | | |  |  |  |  |
| Genome Start | Genome End | Strand | Div | Repeat Start | Repeat End | Repeat Left | Name | Family | Class |
| 92737501 | 92737962 | - | 7.3 | 1127 | 1580 | 0 | THE1B-int | MaLR | LTR |
| 92737958 | 92738116 | - | 13.2 | 846 | 1006 | -574 | THE1B-int | MaLR | LTR |
| 92738152 | 92739610 | - | 4.0 | 4690 | 6139 | -16 | L1PA4 | L1 | LINE |
| 92739608 | 92741229 | + | 4.3 | 3080 | 4703 | -1443 | L1PA4 | L1 | LINE |
| 92741230 | 92742071 | - | 13.2 | 1 | 860 | -720 | THE1B-int | MaLR | LTR |

  

|  |  |  |  |  |  |  |  |  |  |
| --- | --- | --- | --- | --- | --- | --- | --- | --- | --- |
| chr6:93167524-93171452 | | | 3933 bp | | |  |  |  |  |
| Genome Start | Genome End | Strand | Div | Repeat Start | Repeat End | Repeat Left | Name | Family | Class |
| 93167524 | 93168467 | - | 18.0 | 1465 | 2417 | -1 | Tigger1 | MER2\_type | DNA |
| 93168468 | 93169933 | - | 7.1 | 4683 | 6150 | -4 | L1PA6 | L1 | LINE |
| 93169930 | 93170060 | + | 3.9 | 4553 | 4683 | -1463 | L1PA6 | L1 | LINE |
| 93170061 | 93171452 | - | 15.5 | 1 | 1480 | -938 | Tigger1 | MER2\_type | DNA |

  

|  |  |  |  |  |  |  |  |  |  |
| --- | --- | --- | --- | --- | --- | --- | --- | --- | --- |
| chr6:94673447-94676503 | | | 3052 bp | | |  |  |  |  |
| Genome Start | Genome End | Strand | Div | Repeat Start | Repeat End | Repeat Left | Name | Family | Class |
| 94673447 | 94673687 | - | 15.0 | 1068 | 1302 | 0 | HSMAR2 | Mariner | DNA |
| 94673688 | 94674057 | + | 13.6 | 1 | 362 | -2 | THE1B | MaLR | LTR |
| 94674058 | 94674501 | - | 15.0 | 625 | 1067 | -235 | HSMAR2 | Mariner | DNA |
| 94674503 | 94674655 | - | 8.7 | 5524 | 5670 | -461 | L1PA8A | L1 | LINE |
| 94674656 | 94674966 | + | 9.0 | 1 | 311 | 0 | AluY | Alu | SINE |
| 94674967 | 94675412 | - | 9.1 | 5080 | 5523 | -608 | L1PA8A | L1 | LINE |
| 94675414 | 94675893 | + | 13.3 | 5667 | 6156 | -12 | L1PA10 | L1 | LINE |
| 94675897 | 94676503 | - | 14.0 | 1 | 641 | -661 | HSMAR2 | Mariner | DNA |

  

|  |  |  |  |  |  |  |  |  |  |
| --- | --- | --- | --- | --- | --- | --- | --- | --- | --- |
| chr6:95254416-95257579 | | | 3030 bp | | |  |  |  |  |
| Genome Start | Genome End | Strand | Div | Repeat Start | Repeat End | Repeat Left | Name | Family | Class |
| 95254416 | 95254674 | - | 30.5 | 4774 | 5030 | -1911 | L1MCc | L1 | LINE |
| 95254687 | 95256349 | - | 28.8 | 2049 | 3785 | -3156 | L1MCc | L1 | LINE |
| 95256368 | 95256556 | - | 24.3 | 1106 | 1284 | -5139 | L1MCc | L1 | LINE |
| 95256566 | 95256900 | - | 5.4 | 5403 | 5737 | -417 | L1PA4 | L1 | LINE |
| 95256901 | 95257293 | + | 3.0 | 5743 | 6144 | -11 | L1PA4 | L1 | LINE |
| 95257389 | 95257579 | - | 33.9 | 819 | 1017 | -5406 | L1MCc | L1 | LINE |

  

|  |  |  |  |  |  |  |  |  |  |
| --- | --- | --- | --- | --- | --- | --- | --- | --- | --- |
| chr6:95676615-95680223 | | | 3393 bp | | |  |  |  |  |
| Genome Start | Genome End | Strand | Div | Repeat Start | Repeat End | Repeat Left | Name | Family | Class |
| 95676615 | 95677244 | - | 28.8 | 4194 | 4842 | -1304 | L1M5 | L1 | LINE |
| 95677228 | 95677806 | - | 1.6 | 4825 | 5403 | -743 | L1PA3 | L1 | LINE |
| 95677806 | 95678579 | + | 1.6 | 5382 | 6155 | 0 | L1PA3 | L1 | LINE |
| 95678598 | 95678813 | - | 33.2 | 4018 | 4228 | -1918 | L1MD | L1 | LINE |
| 95679030 | 95680223 | - | 26.7 | 2607 | 3873 | -2273 | L1M5 | L1 | LINE |

  

|  |  |  |  |  |  |  |  |  |  |
| --- | --- | --- | --- | --- | --- | --- | --- | --- | --- |
| chr6:96361419-96368420 | | | 7009 bp | | |  |  |  |  |
| Genome Start | Genome End | Strand | Div | Repeat Start | Repeat End | Repeat Left | Name | Family | Class |
| 96361419 | 96362928 | - | 21.1 | 5121 | 6598 | -35 | L1MD1 | L1 | LINE |
| 96362928 | 96364828 | - | 12.9 | 4241 | 6162 | -3 | L1PA15 | L1 | LINE |
| 96364827 | 96365440 | + | 13.9 | 3617 | 4226 | -1920 | L1PREC2 | L1 | LINE |
| 96365440 | 96366648 | - | 19.9 | 3868 | 5133 | -1404 | L1MD1 | L1 | LINE |
| 96366646 | 96367245 | - | 27.9 | 891 | 1559 | -5074 | L1MD1 | L1 | LINE |
| 96367246 | 96367544 | - | 7.0 | 1 | 310 | -1 | AluY | Alu | SINE |
| 96367545 | 96368420 | - | 27.9 | 16 | 890 | -5743 | L1MD1 | L1 | LINE |

  

|  |  |  |  |  |  |  |  |  |  |
| --- | --- | --- | --- | --- | --- | --- | --- | --- | --- |
| chr6:98058338-98060601 | | | 2057 bp | | |  |  |  |  |
| Genome Start | Genome End | Strand | Div | Repeat Start | Repeat End | Repeat Left | Name | Family | Class |
| 98058338 | 98058743 | - | 38.5 | 1537 | 1957 | -3 | L4 | RTE | LINE |
| 98058813 | 98058959 | - | 33.6 | 3260 | 3418 | -1 | L2 | L2 | LINE |
| 98059106 | 98060013 | - | 10.7 | 5258 | 6159 | -1 | L1PREC2 | L1 | LINE |
| 98060006 | 98060392 | + | 7.8 | 4867 | 5250 | -896 | L1PREC2 | L1 | LINE |
| 98060393 | 98060601 | - | 36.4 | 1112 | 1320 | -640 | L4 | RTE | LINE |

  

|  |  |  |  |  |  |  |  |  |  |
| --- | --- | --- | --- | --- | --- | --- | --- | --- | --- |
| chr6:100623320-100628229 | | | 4908 bp | | |  |  |  |  |
| Genome Start | Genome End | Strand | Div | Repeat Start | Repeat End | Repeat Left | Name | Family | Class |
| 100623320 | 100624188 | - | 28.3 | 2145 | 3002 | -4563 | L1M3e | L1 | LINE |
| 100624192 | 100626329 | - | 2.5 | 4022 | 6155 | 0 | L1PA3 | L1 | LINE |
| 100626329 | 100627072 | + | 2.3 | 3270 | 4013 | -2133 | L1PA3 | L1 | LINE |
| 100627073 | 100628229 | - | 30.6 | 1217 | 2456 | -5040 | L1M3e | L1 | LINE |

  

|  |  |  |  |  |  |  |  |  |  |
| --- | --- | --- | --- | --- | --- | --- | --- | --- | --- |
| chr6:101366396-101376792 | | | 10239 bp | | |  |  |  |  |
| Genome Start | Genome End | Strand | Div | Repeat Start | Repeat End | Repeat Left | Name | Family | Class |
| 101366396 | 101367244 | + | 23.9 | 890 | 1768 | -5566 | L1MB8 | L1 | LINE |
| 101367245 | 101367530 | + | 17.1 | 1 | 286 | -26 | AluJo | Alu | SINE |
| 101367531 | 101367768 | + | 23.9 | 1769 | 2018 | -5316 | L1MB8 | L1 | LINE |
| 101367771 | 101368080 | + | 14.3 | 1 | 309 | -4 | AluSp | Alu | SINE |
| 101368082 | 101368190 | + | 14.9 | 2351 | 2460 | -4874 | L1MB8 | L1 | LINE |
| 101368296 | 101368416 | + | 20.9 | 3357 | 3472 | -3534 | L1MB8 | L1 | LINE |
| 101368417 | 101369658 | - | 11.3 | 3025 | 4277 | -1869 | L1PREC2 | L1 | LINE |
| 101369658 | 101371515 | + | 11.7 | 4283 | 6158 | -5 | L1PA13 | L1 | LINE |
| 101371542 | 101372883 | + | 17.4 | 3453 | 4886 | -2120 | L1MB8 | L1 | LINE |
| 101372884 | 101373193 | + | 14.1 | 1 | 308 | -2 | AluSg | Alu | SINE |
| 101373194 | 101373513 | + | 17.4 | 4887 | 5218 | -1788 | L1MB8 | L1 | LINE |
| 101373514 | 101373918 | + | 4.7 | 5743 | 6149 | -5 | L1PA6 | L1 | LINE |
| 101373919 | 101373942 | + | 17.4 | 5219 | 5261 | -1745 | L1MB8 | L1 | LINE |
| 101373948 | 101374497 | - | 18.1 | 5 | 554 | 0 | MLT2A2 | ERVL | LTR |
| 101374518 | 101375628 | + | 20.9 | 5282 | 6449 | -589 | L1MB8 | L1 | LINE |
| 101375629 | 101375912 | - | 19.7 | 1 | 298 | -14 | AluJo | Alu | SINE |
| 101375913 | 101376140 | + | 20.5 | 6450 | 6690 | -348 | L1MB8 | L1 | LINE |
| 101376141 | 101376447 | + | 12.7 | 1 | 307 | -3 | AluSg | Alu | SINE |
| 101376448 | 101376792 | + | 20.5 | 6691 | 7030 | -8 | L1MB8 | L1 | LINE |

  

|  |  |  |  |  |  |  |  |  |  |
| --- | --- | --- | --- | --- | --- | --- | --- | --- | --- |
| chr6:101447105-101453922 | | | 6452 bp | | |  |  |  |  |
| Genome Start | Genome End | Strand | Div | Repeat Start | Repeat End | Repeat Left | Name | Family | Class |
| 101447105 | 101447194 | + | 25.0 | 1 | 92 | -5125 | MER61B-int | ERV1 | LTR |
| 101447538 | 101447953 | + | 16.6 | 356 | 755 | -4462 | MER61B-int | ERV1 | LTR |
| 101447954 | 101448171 | - | 2.8 | 5689 | 5906 | -249 | L1PA2 | L1 | LINE |
| 101448172 | 101448418 | + | 2.0 | 5909 | 6155 | 0 | L1PA2 | L1 | LINE |
| 101448457 | 101448569 | + | 17.7 | 743 | 855 | -4362 | MER61B-int | ERV1 | LTR |
| 101448555 | 101448743 | + | 10.1 | 958 | 1143 | -4074 | MER61B-int | ERV1 | LTR |
| 101448744 | 101449013 | - | 11.7 | 40 | 309 | 0 | AluSg | Alu | SINE |
| 101449014 | 101449481 | + | 10.1 | 1144 | 1608 | -3609 | MER61B-int | ERV1 | LTR |
| 101449482 | 101449781 | + | 12.8 | 1 | 298 | -15 | AluSp | Alu | SINE |
| 101449782 | 101451248 | + | 10.1 | 1609 | 3085 | -2132 | MER61B-int | ERV1 | LTR |
| 101451249 | 101451539 | - | 11.7 | 1 | 297 | -15 | AluSx | Alu | SINE |
| 101451540 | 101452649 | + | 10.1 | 3086 | 4193 | -1024 | MER61B-int | ERV1 | LTR |
| 101452650 | 101452952 | + | 5.9 | 1 | 303 | -8 | AluY | Alu | SINE |
| 101452953 | 101453922 | + | 10.1 | 4194 | 5217 | 0 | MER61B-int | ERV1 | LTR |

  

|  |  |  |  |  |  |  |  |  |  |
| --- | --- | --- | --- | --- | --- | --- | --- | --- | --- |
| chr6:101545619-101547839 | | | 2223 bp | | |  |  |  |  |
| Genome Start | Genome End | Strand | Div | Repeat Start | Repeat End | Repeat Left | Name | Family | Class |
| 101545619 | 101545979 | - | 26.5 | 1821 | 2162 | -4395 | L1MDa | L1 | LINE |
| 101545972 | 101546607 | - | 8.5 | 4617 | 5252 | -894 | L1PA7 | L1 | LINE |
| 101546608 | 101547496 | + | 6.2 | 5260 | 6154 | 0 | L1PA7 | L1 | LINE |
| 101547503 | 101547839 | - | 29.6 | 1428 | 1815 | -4903 | L1MDa | L1 | LINE |

  

|  |  |  |  |  |  |  |  |  |  |
| --- | --- | --- | --- | --- | --- | --- | --- | --- | --- |
| chr6:102810077-102812224 | | | 2113 bp | | |  |  |  |  |
| Genome Start | Genome End | Strand | Div | Repeat Start | Repeat End | Repeat Left | Name | Family | Class |
| 102810077 | 102810265 | + | 27.7 | 35 | 206 | -1020 | Arthur1 | Tip100 | DNA |
| 102810276 | 102810932 | - | 7.9 | 5499 | 6162 | -10 | L1PA8 | L1 | LINE |
| 102810933 | 102811582 | + | 7.9 | 4826 | 5481 | -691 | L1PA8 | L1 | LINE |
| 102811583 | 102812131 | + | 32.0 | 195 | 775 | -3165 | Arthur1 | Tip100 | DNA |
| 102812157 | 102812224 | + | 22.1 | 3431 | 3500 | -440 | Arthur1 | Tip100 | DNA |

  

|  |  |  |  |  |  |  |  |  |  |
| --- | --- | --- | --- | --- | --- | --- | --- | --- | --- |
| chr6:103379144-103380797 | | | 1655 bp | | |  |  |  |  |
| Genome Start | Genome End | Strand | Div | Repeat Start | Repeat End | Repeat Left | Name | Family | Class |
| 103379144 | 103379312 | + | 8.8 | 1 | 164 | -217 | THE1D | MaLR | LTR |
| 103379313 | 103380172 | - | 4.0 | 5312 | 6170 | 0 | L1PA5 | L1 | LINE |
| 103380172 | 103380566 | + | 6.8 | 4894 | 5288 | -858 | L1PA5 | L1 | LINE |
| 103380567 | 103380797 | + | 16.6 | 149 | 381 | 0 | THE1D | MaLR | LTR |

  

|  |  |  |  |  |  |  |  |  |  |
| --- | --- | --- | --- | --- | --- | --- | --- | --- | --- |
| chr6:103868486-103873116 | | | 4626 bp | | |  |  |  |  |
| Genome Start | Genome End | Strand | Div | Repeat Start | Repeat End | Repeat Left | Name | Family | Class |
| 103868486 | 103868895 | + | 16.7 | 70 | 475 | -1150 | THE1D-int | MaLR | LTR |
| 103868896 | 103871112 | - | 8.4 | 3857 | 6139 | -15 | L1PA7 | L1 | LINE |
| 103871112 | 103871660 | + | 10.9 | 3298 | 3847 | -2299 | L1PA7 | L1 | LINE |
| 103871667 | 103872505 | + | 16.2 | 426 | 1263 | -317 | THE1D-int | MaLR | LTR |
| 103872506 | 103872781 | + | 18.5 | 1 | 276 | -36 | AluSx | Alu | SINE |
| 103872782 | 103873116 | + | 16.2 | 1264 | 1580 | 0 | THE1D-int | MaLR | LTR |

  

|  |  |  |  |  |  |  |  |  |  |
| --- | --- | --- | --- | --- | --- | --- | --- | --- | --- |
| chr6:104739598-104754363 | | | 14333 bp | | |  |  |  |  |
| Genome Start | Genome End | Strand | Div | Repeat Start | Repeat End | Repeat Left | Name | Family | Class |
| 104739598 | 104739812 | + | 36.5 | 413 | 625 | -2794 | L2 | L2 | LINE |
| 104739973 | 104740316 | + | 31.5 | 3 | 356 | -6507 | L1PB3 | L1 | LINE |
| 104740317 | 104741291 | + | 17.1 | 1 | 983 | 0 | LTR1D | ERV1 | LTR |
| 104741292 | 104743787 | + | 24.7 | 357 | 1905 | -4958 | L1PB3 | L1 | LINE |
| 104743847 | 104743987 | + | 34.8 | 1 | 147 | -129 | MIRm | MIR | SINE |
| 104744177 | 104744324 | - | 2.0 | 6001 | 6148 | -6 | L1PA5 | L1 | LINE |
| 104744326 | 104745249 | + | 3.6 | 5058 | 5987 | -167 | L1PA5 | L1 | LINE |
| 104745250 | 104745532 | + | 18.3 | 1902 | 2190 | -4673 | L1PB3 | L1 | LINE |
| 104745530 | 104745681 | - | 21.1 | 2437 | 2588 | -3558 | L1PB | L1 | LINE |
| 104745700 | 104746034 | + | 19.3 | 2184 | 2542 | -4321 | L1PB3 | L1 | LINE |
| 104746035 | 104746337 | + | 10.6 | 1 | 303 | -8 | AluY | Alu | SINE |
| 104746338 | 104749776 | + | 19.1 | 2543 | 6080 | -782 | L1PB3 | L1 | LINE |
| 104749784 | 104750352 | + | 23.1 | 1144 | 1721 | -5064 | L1PA15 | L1 | LINE |
| 104750358 | 104751041 | + | 16.5 | 2730 | 3437 | -3329 | L1PA15 | L1 | LINE |
| 104751028 | 104753454 | + | 13.9 | 4300 | 6769 | -16 | L1PA15 | L1 | LINE |
| 104753458 | 104754248 | + | 13.4 | 6064 | 6861 | -9 | L1PB3 | L1 | LINE |
| 104754257 | 104754363 | + | 34.9 | 779 | 891 | -2528 | L2 | L2 | LINE |

  

|  |  |  |  |  |  |  |  |  |  |
| --- | --- | --- | --- | --- | --- | --- | --- | --- | --- |
| chr6:104990180-104991801 | | | 1604 bp | | |  |  |  |  |
| Genome Start | Genome End | Strand | Div | Repeat Start | Repeat End | Repeat Left | Name | Family | Class |
| 104990180 | 104990504 | + | 20.2 | 20 | 335 | -110 | LTR56 | ERV1 | LTR |
| 104990505 | 104991175 | - | 7.2 | 4992 | 5658 | -510 | L1PA13 | L1 | LINE |
| 104991176 | 104991645 | + | 8.3 | 5679 | 6161 | -2 | L1PA13 | L1 | LINE |
| 104991664 | 104991801 | + | 25.9 | 292 | 445 | 0 | LTR56 | ERV1 | LTR |

  

|  |  |  |  |  |  |  |  |  |  |
| --- | --- | --- | --- | --- | --- | --- | --- | --- | --- |
| chr6:105072264-105073232 | | | 966 bp | | |  |  |  |  |
| Genome Start | Genome End | Strand | Div | Repeat Start | Repeat End | Repeat Left | Name | Family | Class |
| 105072264 | 105072318 | + | 21.8 | 1 | 55 | -2557 | Charlie5 | MER1\_type | DNA |
| 105072321 | 105072521 | - | 3.2 | 5704 | 5892 | -276 | L1PA10 | L1 | LINE |
| 105072522 | 105072781 | + | 13.1 | 5867 | 6136 | -32 | L1PA10 | L1 | LINE |
| 105072783 | 105072901 | + | 14.0 | 2409 | 2517 | -95 | Charlie5 | MER1\_type | DNA |
| 105072902 | 105073157 | - | 10.2 | 21 | 295 | -17 | AluJb | Alu | SINE |
| 105073158 | 105073232 | + | 14.0 | 2518 | 2584 | -28 | Charlie5 | MER1\_type | DNA |

  

|  |  |  |  |  |  |  |  |  |  |
| --- | --- | --- | --- | --- | --- | --- | --- | --- | --- |
| chr6:110270252-110273701 | | | 2976 bp | | |  |  |  |  |
| Genome Start | Genome End | Strand | Div | Repeat Start | Repeat End | Repeat Left | Name | Family | Class |
| 110270252 | 110270462 | + | 18.0 | 4 | 226 | -119 | MER2B | MER2\_type | DNA |
| 110270463 | 110271251 | - | 7.9 | 4530 | 5349 | -797 | L1PB1 | L1 | LINE |
| 110271253 | 110271330 | + | 3.9 | 5363 | 5440 | -711 | L1PB1 | L1 | LINE |
| 110271328 | 110271389 | - | 3.2 | 5362 | 5423 | -728 | L1P | L1 | LINE |
| 110271385 | 110271440 | + | 5.4 | 4525 | 4580 | -1566 | L1PB | L1 | LINE |
| 110271438 | 110271471 | + | 2.9 | 4539 | 4572 | -1574 | L1PB | L1 | LINE |
| 110271518 | 110271712 | + | 4.8 | 100 | 287 | -24 | AluY | Alu | SINE |
| 110271739 | 110272055 | + | 9.6 | 1 | 313 | 0 | AluSq | Alu | SINE |
| 110272064 | 110272413 | + | 8.7 | 5432 | 5780 | -371 | L1PB1 | L1 | LINE |
| 110272778 | 110273145 | + | 8.2 | 5782 | 6151 | 0 | L1PB1 | L1 | LINE |
| 110273185 | 110273564 | + | 3.7 | 5775 | 6154 | 0 | L1PA5 | L1 | LINE |
| 110273566 | 110273701 | + | 16.2 | 201 | 336 | 0 | MER2B | MER2\_type | DNA |

  

|  |  |  |  |  |  |  |  |  |  |
| --- | --- | --- | --- | --- | --- | --- | --- | --- | --- |
| chr6:112062597-112067127 | | | 4522 bp | | |  |  |  |  |
| Genome Start | Genome End | Strand | Div | Repeat Start | Repeat End | Repeat Left | Name | Family | Class |
| 112062597 | 112062755 | - | 19.0 | 5167 | 5324 | -822 | L1M | L1 | LINE |
| 112062763 | 112065238 | - | 4.9 | 3690 | 6154 | 0 | L1PA5 | L1 | LINE |
| 112065240 | 112066198 | + | 5.8 | 2733 | 3690 | -2456 | L1PA5 | L1 | LINE |
| 112066200 | 112066588 | - | 24.1 | 4770 | 5175 | -971 | L1M | L1 | LINE |
| 112066589 | 112066885 | - | 10.2 | 1 | 294 | -18 | AluSx | Alu | SINE |
| 112066886 | 112067127 | - | 24.1 | 4505 | 4769 | -1377 | L1M | L1 | LINE |

  

|  |  |  |  |  |  |  |  |  |  |
| --- | --- | --- | --- | --- | --- | --- | --- | --- | --- |
| chr6:113469031-113474037 | | | 4682 bp | | |  |  |  |  |
| Genome Start | Genome End | Strand | Div | Repeat Start | Repeat End | Repeat Left | Name | Family | Class |
| 113469031 | 113469161 | - | 32.2 | 3242 | 3378 | 0 | L2 | L2 | LINE |
| 113469374 | 113470150 | - | 8.8 | 4251 | 5023 | -1123 | L1PA8 | L1 | LINE |
| 113470151 | 113471276 | + | 6.8 | 5020 | 6159 | -13 | L1PA8 | L1 | LINE |
| 113471279 | 113471626 | + | 22.6 | 2129 | 2485 | -377 | Charlie2 | MER1\_type | DNA |
| 113471627 | 113471915 | + | 13.8 | 1 | 299 | -13 | AluSx | Alu | SINE |
| 113471916 | 113471954 | + | 22.6 | 2486 | 2525 | -337 | Charlie2 | MER1\_type | DNA |
| 113471955 | 113472415 | + | 24.5 | 1 | 414 | 0 | MLT2D | ERVL | LTR |
| 113472416 | 113472596 | + | 22.6 | 2526 | 2705 | -157 | Charlie2 | MER1\_type | DNA |
| 113472598 | 113472791 | - | 24.2 | 28 | 223 | -1 | MER58A | MER1\_type | DNA |
| 113472836 | 113472995 | + | 30.2 | 2704 | 2862 | 0 | Charlie2 | MER1\_type | DNA |
| 113473062 | 113474037 | - | 38.0 | 2022 | 3069 | -350 | L2 | L2 | LINE |

  

|  |  |  |  |  |  |  |  |  |  |
| --- | --- | --- | --- | --- | --- | --- | --- | --- | --- |
| chr6:115342227-115348544 | | | 6317 bp | | |  |  |  |  |
| Genome Start | Genome End | Strand | Div | Repeat Start | Repeat End | Repeat Left | Name | Family | Class |
| 115342227 | 115342485 | - | 20.2 | 7477 | 7776 | -9 | L1MC3 | L1 | LINE |
| 115342493 | 115345656 | - | 4.1 | 2989 | 6152 | -3 | L1PA4 | L1 | LINE |
| 115345658 | 115347029 | + | 4.0 | 1624 | 2993 | -3153 | L1PA4 | L1 | LINE |
| 115347023 | 115347636 | - | 22.6 | 6876 | 7492 | -293 | L1MC3 | L1 | LINE |
| 115347637 | 115347991 | - | 21.0 | 5 | 365 | 0 | MLT1A0 | MaLR | LTR |
| 115347992 | 115348544 | - | 22.6 | 6248 | 6875 | -910 | L1MC3 | L1 | LINE |

  

|  |  |  |  |  |  |  |  |  |  |
| --- | --- | --- | --- | --- | --- | --- | --- | --- | --- |
| chr6:115723460-115728865 | | | 5029 bp | | |  |  |  |  |
| Genome Start | Genome End | Strand | Div | Repeat Start | Repeat End | Repeat Left | Name | Family | Class |
| 115723460 | 115723594 | + | 34.0 | 549 | 679 | -5440 | L1MEd | L1 | LINE |
| 115723644 | 115724215 | + | 34.0 | 680 | 1248 | -4871 | L1MEd | L1 | LINE |
| 115724261 | 115724669 | + | 29.7 | 1373 | 1787 | -4362 | L1MEd | L1 | LINE |
| 115724670 | 115724936 | + | 15.8 | 1 | 291 | -19 | AluSg | Alu | SINE |
| 115724937 | 115725132 | + | 29.7 | 1788 | 1952 | -4197 | L1MEd | L1 | LINE |
| 115725439 | 115726659 | - | 8.6 | 3234 | 4452 | -1694 | L1PA7 | L1 | LINE |
| 115726660 | 115727687 | + | 8.6 | 4465 | 5489 | -665 | L1PA7 | L1 | LINE |
| 115727688 | 115728002 | + | 9.7 | 1 | 309 | -2 | AluY | Alu | SINE |
| 115728003 | 115728683 | + | 6.4 | 5490 | 6153 | -1 | L1PA7 | L1 | LINE |
| 115728661 | 115728865 | + | 25.1 | 2243 | 2446 | -3703 | L1MEd | L1 | LINE |

  

|  |  |  |  |  |  |  |  |  |  |
| --- | --- | --- | --- | --- | --- | --- | --- | --- | --- |
| chr6:116376757-116379104 | | | 2329 bp | | |  |  |  |  |
| Genome Start | Genome End | Strand | Div | Repeat Start | Repeat End | Repeat Left | Name | Family | Class |
| 116376757 | 116376850 | + | 27.7 | 89 | 187 | -75 | MIR | MIR | SINE |
| 116376856 | 116377338 | - | 9.3 | 3954 | 4437 | -1709 | L1PA7 | L1 | LINE |
| 116377336 | 116379036 | + | 8.0 | 4438 | 6154 | 0 | L1PA7 | L1 | LINE |
| 116379054 | 116379104 | + | 22.0 | 185 | 235 | -27 | MIR | MIR | SINE |

  

|  |  |  |  |  |  |  |  |  |  |
| --- | --- | --- | --- | --- | --- | --- | --- | --- | --- |
| chr6:117477985-117482931 | | | 4707 bp | | |  |  |  |  |
| Genome Start | Genome End | Strand | Div | Repeat Start | Repeat End | Repeat Left | Name | Family | Class |
| 117477985 | 117478602 | - | 33.5 | 4640 | 5261 | -406 | HERV16 | ERVL | LTR |
| 117478748 | 117478889 | - | 29.8 | 4449 | 4609 | -1148 | HERV16 | ERVL | LTR |
| 117478893 | 117479244 | - | 20.3 | 7 | 354 | -11 | MLT1A0 | MaLR | LTR |
| 117479250 | 117479791 | - | 30.2 | 3779 | 4347 | -1320 | HERV16 | ERVL | LTR |
| 117479800 | 117480115 | - | 6.2 | 1 | 307 | -4 | AluY | Alu | SINE |
| 117480157 | 117481473 | - | 32.1 | 2323 | 3698 | -1911 | HERV16 | ERVL | LTR |
| 117481527 | 117482609 | - | 11.1 | 5072 | 6154 | -12 | L1PA16 | L1 | LINE |
| 117482594 | 117482817 | + | 16.1 | 4856 | 5079 | -1067 | L1PA16 | L1 | LINE |
| 117482819 | 117482931 | - | 26.6 | 1665 | 1790 | -3206 | HERV16 | ERVL | LTR |

  

|  |  |  |  |  |  |  |  |  |  |
| --- | --- | --- | --- | --- | --- | --- | --- | --- | --- |
| chr6:117519864-117521715 | | | 1819 bp | | |  |  |  |  |
| Genome Start | Genome End | Strand | Div | Repeat Start | Repeat End | Repeat Left | Name | Family | Class |
| 117519864 | 117520045 | - | 8.8 | 122 | 302 | -9 | AluY | Alu | SINE |
| 117520083 | 117520866 | - | 1.7 | 5356 | 6139 | -16 | L1PA2 | L1 | LINE |
| 117520863 | 117521583 | + | 2.2 | 4647 | 5367 | -779 | L1PA2 | L1 | LINE |
| 117521584 | 117521715 | - | 6.8 | 1 | 132 | -179 | AluY | Alu | SINE |

  

|  |  |  |  |  |  |  |  |  |  |
| --- | --- | --- | --- | --- | --- | --- | --- | --- | --- |
| chr6:118277305-118287476 | | | 10146 bp | | |  |  |  |  |
| Genome Start | Genome End | Strand | Div | Repeat Start | Repeat End | Repeat Left | Name | Family | Class |
| 118277305 | 118278353 | - | 11.2 | 5540 | 6596 | -2 | MER4A1-int | ERV1 | LTR |
| 118278349 | 118279540 | - | 11.6 | 4144 | 5390 | -1208 | MER4A1-int | ERV1 | LTR |
| 118279541 | 118282655 | - | 5.2 | 3038 | 6155 | 0 | L1PA4 | L1 | LINE |
| 118282650 | 118284711 | + | 5.9 | 981 | 3039 | -3107 | L1PA4 | L1 | LINE |
| 118284712 | 118285301 | - | 12.1 | 2971 | 4152 | -2446 | MER4A1-int | ERV1 | LTR |
| 118285341 | 118285616 | - | 11.3 | 9 | 283 | -28 | AluY | Alu | SINE |
| 118285617 | 118285873 | - | 12.8 | 2737 | 2970 | -3628 | MER4A1-int | ERV1 | LTR |
| 118285874 | 118286116 | + | 16.1 | 1 | 257 | -434 | LTR8 | ERV1 | LTR |
| 118286117 | 118286300 | - | 8.2 | 2309 | 2495 | -4103 | MER4A1-int | ERV1 | LTR |
| 118286301 | 118286598 | + | 9.7 | 1 | 299 | -11 | AluSg | Alu | SINE |
| 118286599 | 118287131 | - | 8.2 | 1771 | 2308 | -4290 | MER4A1-int | ERV1 | LTR |
| 118287130 | 118287476 | - | 8.2 | 503 | 1268 | -4266 | MER4A1-int | ERV1 | LTR |

  

|  |  |  |  |  |  |  |  |  |  |
| --- | --- | --- | --- | --- | --- | --- | --- | --- | --- |
| chr6:120109340-120121247 | | | 11761 bp | | |  |  |  |  |
| Genome Start | Genome End | Strand | Div | Repeat Start | Repeat End | Repeat Left | Name | Family | Class |
| 120109340 | 120109590 | - | 14.3 | 6660 | 6915 | -20 | L1PA16 | L1 | LINE |
| 120109588 | 120111555 | - | 4.4 | 4183 | 6154 | 0 | L1PA5 | L1 | LINE |
| 120111557 | 120111896 | + | 4.4 | 3850 | 4189 | -1957 | L1PA5 | L1 | LINE |
| 120111882 | 120115323 | - | 16.1 | 3230 | 6690 | -245 | L1PA16 | L1 | LINE |
| 120115332 | 120115645 | - | 20.7 | 440 | 763 | -6353 | L1M2a1 | L1 | LINE |
| 120115661 | 120115702 | - | 14.3 | 1 | 42 | -38 | MADE1 | Mariner | DNA |
| 120115703 | 120116842 | - | 16.5 | 2071 | 3218 | -3697 | L1PA16 | L1 | LINE |
| 120116864 | 120117529 | - | 6.0 | 5491 | 6151 | -3 | L1PA7 | L1 | LINE |
| 120117571 | 120117851 | - | 7.5 | 3 | 281 | -30 | AluY | Alu | SINE |
| 120117852 | 120118286 | - | 6.8 | 5059 | 5490 | -664 | L1PA7 | L1 | LINE |
| 120118306 | 120119962 | + | 8.3 | 2983 | 4631 | -1515 | L1P3 | L1 | LINE |
| 120119966 | 120120887 | - | 24.5 | 1270 | 2080 | -4855 | L1PA16 | L1 | LINE |
| 120120945 | 120121247 | - | 26.2 | 510 | 799 | -6120 | L1PA16 | L1 | LINE |

  

|  |  |  |  |  |  |  |  |  |  |
| --- | --- | --- | --- | --- | --- | --- | --- | --- | --- |
| chr6:120729726-120734906 | | | 5190 bp | | |  |  |  |  |
| Genome Start | Genome End | Strand | Div | Repeat Start | Repeat End | Repeat Left | Name | Family | Class |
| 120729726 | 120729886 | + | 18.6 | 2449 | 2618 | -3758 | L1MB1 | L1 | LINE |
| 120729884 | 120730882 | - | 10.5 | 4158 | 5167 | -979 | L1PA8 | L1 | LINE |
| 120730877 | 120731830 | + | 9.6 | 5162 | 6132 | -40 | L1PA8 | L1 | LINE |
| 120731838 | 120733470 | + | 18.7 | 2603 | 4280 | -2096 | L1MB1 | L1 | LINE |
| 120733464 | 120734906 | + | 17.8 | 4921 | 6397 | -7 | L1MB1 | L1 | LINE |

  

|  |  |  |  |  |  |  |  |  |  |
| --- | --- | --- | --- | --- | --- | --- | --- | --- | --- |
| chr6:120779543-120784410 | | | 4793 bp | | |  |  |  |  |
| Genome Start | Genome End | Strand | Div | Repeat Start | Repeat End | Repeat Left | Name | Family | Class |
| 120779543 | 120779966 | - | 27.4 | 4429 | 4888 | -2053 | L1MCc | L1 | LINE |
| 120780013 | 120781223 | - | 5.1 | 5118 | 6296 | -7 | L1PA4 | L1 | LINE |
| 120781222 | 120782518 | + | 4.1 | 3805 | 5094 | -1052 | L1PA4 | L1 | LINE |
| 120782521 | 120782597 | - | 15.6 | 6090 | 6166 | -137 | L1MA4A | L1 | LINE |
| 120782596 | 120783178 | - | 27.5 | 3774 | 4357 | -2584 | L1MCc | L1 | LINE |
| 120783210 | 120783493 | - | 28.0 | 1940 | 2325 | -4636 | L1MCc | L1 | LINE |
| 120783494 | 120783879 | - | 10.4 | 2 | 381 | 0 | THE1D | MaLR | LTR |
| 120783880 | 120784410 | - | 27.1 | 1253 | 1939 | -5189 | L1MCc | L1 | LINE |

  

|  |  |  |  |  |  |  |  |  |  |
| --- | --- | --- | --- | --- | --- | --- | --- | --- | --- |
| chr6:122684041-122690652 | | | 6049 bp | | |  |  |  |  |
| Genome Start | Genome End | Strand | Div | Repeat Start | Repeat End | Repeat Left | Name | Family | Class |
| 122684041 | 122684533 | + | 28.9 | 4018 | 4517 | -1629 | L1M5 | L1 | LINE |
| 122684657 | 122684791 | + | 28.8 | 4651 | 4783 | -1363 | L1M5 | L1 | LINE |
| 122685181 | 122687551 | - | 15.0 | 249 | 2689 | -29 | Tigger2 | MER2\_type | DNA |
| 122687578 | 122688467 | - | 3.6 | 5263 | 6152 | -3 | L1PA4 | L1 | LINE |
| 122688468 | 122689710 | + | 3.3 | 4017 | 5262 | -884 | L1PA4 | L1 | LINE |
| 122689711 | 122689979 | - | 16.8 | 1 | 262 | -2456 | Tigger2 | MER2\_type | DNA |
| 122690003 | 122690287 | + | 15.1 | 1 | 302 | -10 | AluJo | Alu | SINE |
| 122690290 | 122690652 | + | 24.5 | 5306 | 5718 | -522 | L1M5 | L1 | LINE |

  

|  |  |  |  |  |  |  |  |  |  |
| --- | --- | --- | --- | --- | --- | --- | --- | --- | --- |
| chr6:123806101-123809663 | | | 3539 bp | | |  |  |  |  |
| Genome Start | Genome End | Strand | Div | Repeat Start | Repeat End | Repeat Left | Name | Family | Class |
| 123806101 | 123806351 | - | 36.8 | 2996 | 3251 | -168 | L2 | L2 | LINE |
| 123806355 | 123806495 | + | 8.5 | 1 | 141 | -177 | AluYb | Alu | SINE |
| 123806496 | 123808104 | - | 2.3 | 3412 | 5021 | -1125 | L1PA3 | L1 | LINE |
| 123808101 | 123809242 | + | 2.1 | 5012 | 6154 | -1 | L1PA3 | L1 | LINE |
| 123809263 | 123809456 | + | 11.0 | 117 | 309 | -2 | AluY | Alu | SINE |
| 123809462 | 123809663 | - | 29.6 | 2785 | 2995 | -424 | L2 | L2 | LINE |

  

|  |  |  |  |  |  |  |  |  |  |
| --- | --- | --- | --- | --- | --- | --- | --- | --- | --- |
| chr6:126826250-126831560 | | | 5152 bp | | |  |  |  |  |
| Genome Start | Genome End | Strand | Div | Repeat Start | Repeat End | Repeat Left | Name | Family | Class |
| 126826250 | 126827178 | + | 25.7 | 3081 | 4007 | -2139 | L1MC3 | L1 | LINE |
| 126827179 | 126827717 | + | 13.0 | 1 | 444 | 0 | MLT2A1 | ERVL | LTR |
| 126827718 | 126828640 | + | 25.7 | 4008 | 4928 | -1218 | L1MC3 | L1 | LINE |
| 126828736 | 126828812 | + | 23.4 | 4959 | 5036 | -1110 | L1MC3 | L1 | LINE |
| 126828800 | 126829502 | + | 20.9 | 5937 | 6653 | -1132 | L1MC3 | L1 | LINE |
| 126829499 | 126829572 | - | 6.8 | 4952 | 5025 | -1121 | L1PREC2 | L1 | LINE |
| 126829573 | 126830267 | + | 9.3 | 4236 | 4927 | -1219 | L1PREC2 | L1 | LINE |
| 126830315 | 126830460 | + | 18.7 | 6859 | 7015 | -770 | L1MC3 | L1 | LINE |
| 126830495 | 126830976 | + | 18.7 | 7016 | 7526 | -259 | L1MC3 | L1 | LINE |
| 126830977 | 126831283 | + | 10.8 | 1 | 307 | -6 | AluSp | Alu | SINE |
| 126831284 | 126831560 | + | 18.7 | 7527 | 7784 | -1 | L1MC3 | L1 | LINE |

  

|  |  |  |  |  |  |  |  |  |  |
| --- | --- | --- | --- | --- | --- | --- | --- | --- | --- |
| chr6:127199829-127204089 | | | 4213 bp | | |  |  |  |  |
| Genome Start | Genome End | Strand | Div | Repeat Start | Repeat End | Repeat Left | Name | Family | Class |
| 127199829 | 127199995 | - | 32.5 | 5954 | 6120 | -46 | L1ME1 | L1 | LINE |
| 127200013 | 127200790 | - | 4.9 | 5373 | 6151 | -3 | L1PA7 | L1 | LINE |
| 127200791 | 127202565 | - | 8.9 | 3223 | 5004 | -1142 | L1PA7 | L1 | LINE |
| 127202565 | 127203606 | + | 9.0 | 2166 | 3218 | -2928 | L1PA7 | L1 | LINE |
| 127203639 | 127204089 | - | 22.5 | 5453 | 5876 | -290 | L1ME1 | L1 | LINE |

  

|  |  |  |  |  |  |  |  |  |  |
| --- | --- | --- | --- | --- | --- | --- | --- | --- | --- |
| chr6:128208174-128215430 | | | 7153 bp | | |  |  |  |  |
| Genome Start | Genome End | Strand | Div | Repeat Start | Repeat End | Repeat Left | Name | Family | Class |
| 128208174 | 128209223 | + | 25.1 | 90 | 1139 | -5403 | L1M4 | L1 | LINE |
| 128209227 | 128210727 | + | 13.7 | 3240 | 4736 | -1410 | L1PB2 | L1 | LINE |
| 128210774 | 128212280 | - | 4.0 | 4637 | 6137 | -18 | L1PA4 | L1 | LINE |
| 128212281 | 128212323 | + | 0.0 | 4586 | 4628 | -1518 | L1PA4 | L1 | LINE |
| 128212321 | 128213713 | + | 13.8 | 4730 | 6132 | -19 | L1PB2 | L1 | LINE |
| 128213745 | 128213902 | + | 23.2 | 1128 | 1282 | -5260 | L1M4 | L1 | LINE |
| 128213917 | 128214200 | + | 23.5 | 1423 | 1710 | -4832 | L1M4 | L1 | LINE |
| 128214223 | 128214700 | + | 15.5 | 5186 | 5666 | -667 | L1MC | L1 | LINE |
| 128214703 | 128214761 | - | 7.4 | 2235 | 2288 | -35 | Tigger5 | MER2\_type | DNA |
| 128214751 | 128214861 | - | 19.8 | 1 | 114 | -2209 | Tigger5 | MER2\_type | DNA |
| 128214862 | 128215430 | + | 15.8 | 2763 | 3351 | -3293 | L1M4 | L1 | LINE |

  

|  |  |  |  |  |  |  |  |  |  |
| --- | --- | --- | --- | --- | --- | --- | --- | --- | --- |
| chr6:133878747-133880932 | | | 1917 bp | | |  |  |  |  |
| Genome Start | Genome End | Strand | Div | Repeat Start | Repeat End | Repeat Left | Name | Family | Class |
| 133878747 | 133878996 | + | 35.2 | 3356 | 3599 | -890 | L3 | CR1 | LINE |
| 133878997 | 133879224 | + | 10.1 | 5923 | 6160 | 0 | L1PREC2 | L1 | LINE |
| 133879262 | 133879577 | + | 10.9 | 1 | 303 | -6 | AluSc | Alu | SINE |
| 133879582 | 133879960 | - | 8.2 | 5543 | 5921 | -242 | L1PA13 | L1 | LINE |
| 133879961 | 133880224 | + | 9.8 | 5899 | 6163 | 0 | L1PA13 | L1 | LINE |
| 133880453 | 133880932 | + | 30.8 | 3900 | 4420 | -69 | L3 | CR1 | LINE |

  

|  |  |  |  |  |  |  |  |  |  |
| --- | --- | --- | --- | --- | --- | --- | --- | --- | --- |
| chr6:134498674-134500355 | | | 1458 bp | | |  |  |  |  |
| Genome Start | Genome End | Strand | Div | Repeat Start | Repeat End | Repeat Left | Name | Family | Class |
| 134498674 | 134498987 | + | 13.9 | 1 | 314 | -50 | THE1B | MaLR | LTR |
| 134499213 | 134499987 | - | 3.5 | 5373 | 6146 | -8 | L1PA5 | L1 | LINE |
| 134499987 | 134500282 | + | 6.8 | 5075 | 5369 | -777 | L1PA5 | L1 | LINE |
| 134500283 | 134500355 | + | 6.2 | 292 | 355 | 0 | THE1B | MaLR | LTR |

  

|  |  |  |  |  |  |  |  |  |  |
| --- | --- | --- | --- | --- | --- | --- | --- | --- | --- |
| chr6:135871491-135876248 | | | 4707 bp | | |  |  |  |  |
| Genome Start | Genome End | Strand | Div | Repeat Start | Repeat End | Repeat Left | Name | Family | Class |
| 135871491 | 135872238 | - | 16.5 | 3031 | 3796 | -2344 | L1M3 | L1 | LINE |
| 135872282 | 135874475 | - | 2.7 | 3947 | 6137 | -18 | L1PA3 | L1 | LINE |
| 135874474 | 135876077 | + | 3.6 | 2333 | 3934 | -2212 | L1PA3 | L1 | LINE |
| 135876088 | 135876248 | - | 14.9 | 2866 | 3029 | -3111 | L1M3 | L1 | LINE |

  

|  |  |  |  |  |  |  |  |  |  |
| --- | --- | --- | --- | --- | --- | --- | --- | --- | --- |
| chr6:136226452-136228603 | | | 2001 bp | | |  |  |  |  |
| Genome Start | Genome End | Strand | Div | Repeat Start | Repeat End | Repeat Left | Name | Family | Class |
| 136226452 | 136227093 | - | 29.4 | 1817 | 2454 | -3695 | L1ME3 | L1 | LINE |
| 136227197 | 136227622 | - | 31.9 | 1142 | 1638 | -4511 | L1ME3 | L1 | LINE |
| 136227623 | 136227895 | - | 6.2 | 5881 | 6154 | 0 | L1PA5 | L1 | LINE |
| 136227899 | 136228022 | + | 0.8 | 5751 | 5874 | -281 | L1PA5 | L1 | LINE |
| 136228026 | 136228446 | - | 35.5 | 685 | 1146 | -4973 | L1ME3 | L1 | LINE |
| 136228489 | 136228603 | - | 35.5 | 573 | 684 | -5435 | L1ME3 | L1 | LINE |

  

|  |  |  |  |  |  |  |  |  |  |
| --- | --- | --- | --- | --- | --- | --- | --- | --- | --- |
| chr6:137213423-137216226 | | | 2629 bp | | |  |  |  |  |
| Genome Start | Genome End | Strand | Div | Repeat Start | Repeat End | Repeat Left | Name | Family | Class |
| 137213423 | 137213677 | - | 31.9 | 2280 | 2570 | -849 | L2 | L2 | LINE |
| 137213831 | 137215044 | - | 0.7 | 4942 | 6155 | 0 | L1HS | L1 | LINE |
| 137215051 | 137215952 | + | 0.4 | 4050 | 4951 | -1195 | L1HS | L1 | LINE |
| 137215955 | 137216130 | + | 19.5 | 49 | 224 | 0 | MER58A | MER1\_type | DNA |
| 137216145 | 137216226 | - | 24.7 | 1828 | 1914 | -1505 | L2 | L2 | LINE |

  

|  |  |  |  |  |  |  |  |  |  |
| --- | --- | --- | --- | --- | --- | --- | --- | --- | --- |
| chr6:138030688-138036040 | | | 5347 bp | | |  |  |  |  |
| Genome Start | Genome End | Strand | Div | Repeat Start | Repeat End | Repeat Left | Name | Family | Class |
| 138030688 | 138030791 | - | 22.1 | 3806 | 3894 | -137 | Zaphod | Tip100 | DNA |
| 138030792 | 138031046 | - | 9.8 | 41 | 296 | -14 | AluSg | Alu | SINE |
| 138031047 | 138032664 | - | 22.1 | 2096 | 3805 | -226 | Zaphod | Tip100 | DNA |
| 138032672 | 138033963 | - | 8.3 | 4889 | 6162 | -6 | L1PA10 | L1 | LINE |
| 138033963 | 138034026 | + | 12.5 | 4801 | 4864 | -1282 | L1PA10 | L1 | LINE |
| 138034027 | 138036040 | - | 26.3 | 37 | 2110 | -1921 | Zaphod | Tip100 | DNA |

  

|  |  |  |  |  |  |  |  |  |  |
| --- | --- | --- | --- | --- | --- | --- | --- | --- | --- |
| chr6:141071131-141073782 | | | 2611 bp | | |  |  |  |  |
| Genome Start | Genome End | Strand | Div | Repeat Start | Repeat End | Repeat Left | Name | Family | Class |
| 141071131 | 141071831 | + | 34.2 | 21 | 735 | -5665 | L1MEc | L1 | LINE |
| 141071832 | 141072133 | + | 13.7 | 1 | 300 | -13 | AluSq | Alu | SINE |
| 141072134 | 141072502 | + | 29.3 | 736 | 4932 | -1287 | L1MEc | L1 | LINE |
| 141072514 | 141072986 | - | 16.9 | 5683 | 6167 | -1 | L1MB1 | L1 | LINE |
| 141072987 | 141073134 | - | 17.7 | 5440 | 5589 | -745 | L1MB1 | L1 | LINE |
| 141073131 | 141073401 | + | 14.4 | 5179 | 5439 | -729 | L1MB1 | L1 | LINE |
| 141073377 | 141073560 | + | 30.5 | 4889 | 5094 | -1125 | L1MEc | L1 | LINE |
| 141073620 | 141073782 | + | 30.5 | 5095 | 5277 | -942 | L1MEc | L1 | LINE |

  

|  |  |  |  |  |  |  |  |  |  |
| --- | --- | --- | --- | --- | --- | --- | --- | --- | --- |
| chr6:141980861-141984989 | | | 4131 bp | | |  |  |  |  |
| Genome Start | Genome End | Strand | Div | Repeat Start | Repeat End | Repeat Left | Name | Family | Class |
| 141980861 | 141982003 | + | 6.1 | 2 | 1448 | -130 | LTR12C | ERV1 | LTR |
| 141982004 | 141983150 | - | 1.1 | 3316 | 4462 | -1684 | L1PA2 | L1 | LINE |
| 141983149 | 141984843 | + | 1.8 | 4475 | 6170 | 0 | L1PA2 | L1 | LINE |
| 141984844 | 141984989 | + | 4.8 | 1433 | 1578 | 0 | LTR12C | ERV1 | LTR |

  

|  |  |  |  |  |  |  |  |  |  |
| --- | --- | --- | --- | --- | --- | --- | --- | --- | --- |
| chr6:143050405-143054303 | | | 3335 bp | | |  |  |  |  |
| Genome Start | Genome End | Strand | Div | Repeat Start | Repeat End | Repeat Left | Name | Family | Class |
| 143050405 | 143050600 | + | 29.6 | 3480 | 3707 | -782 | L3 | CR1 | LINE |
| 143050606 | 143050851 | + | 34.2 | 3098 | 3414 | -5 | L2 | L2 | LINE |
| 143051104 | 143051169 | + | 30.3 | 4017 | 4084 | -405 | L3 | CR1 | LINE |
| 143051215 | 143051577 | - | 17.1 | 4656 | 5020 | -1120 | L1MB1 | L1 | LINE |
| 143051578 | 143052679 | + | 14.7 | 5026 | 6133 | -56 | L1MB1 | L1 | LINE |
| 143052680 | 143052972 | + | 9.6 | 3 | 295 | -17 | AluSx | Alu | SINE |
| 143052973 | 143053017 | + | 15.3 | 6128 | 6166 | -2 | L1MB1 | L1 | LINE |
| 143053052 | 143053428 | - | 10.4 | 5781 | 6157 | -11 | L1PA10 | L1 | LINE |
| 143053471 | 143053611 | + | 16.6 | 6007 | 6293 | -41 | L1MA10 | L1 | LINE |
| 143053616 | 143053817 | + | 31.3 | 4130 | 4329 | -160 | L3 | CR1 | LINE |
| 143053990 | 143054125 | + | 27.3 | 5984 | 6118 | -3 | L1ME4a | L1 | LINE |
| 143054136 | 143054303 | + | 30.1 | 4313 | 4480 | -9 | L3 | CR1 | LINE |

  

|  |  |  |  |  |  |  |  |  |  |
| --- | --- | --- | --- | --- | --- | --- | --- | --- | --- |
| chr6:144087899-144092479 | | | 4597 bp | | |  |  |  |  |
| Genome Start | Genome End | Strand | Div | Repeat Start | Repeat End | Repeat Left | Name | Family | Class |
| 144087899 | 144088028 | - | 23.1 | 6044 | 6167 | -192 | L1MD2 | L1 | LINE |
| 144088029 | 144088329 | - | 4.7 | 1 | 308 | -3 | AluY | Alu | SINE |
| 144088330 | 144088428 | - | 23.1 | 5931 | 6043 | -316 | L1MD2 | L1 | LINE |
| 144088429 | 144088669 | + | 8.8 | 44 | 298 | -11 | AluSc | Alu | SINE |
| 144088670 | 144089106 | - | 23.1 | 5472 | 5930 | -429 | L1MD2 | L1 | LINE |
| 144089098 | 144089549 | - | 15.2 | 5687 | 6169 | -2 | L1MB2 | L1 | LINE |
| 144089546 | 144089645 | - | 17.2 | 5387 | 5492 | -867 | L1MD2 | L1 | LINE |
| 144089640 | 144090667 | - | 12.4 | 5266 | 6298 | -5 | L1MA4A | L1 | LINE |
| 144090664 | 144090967 | + | 10.2 | 3979 | 4324 | -1819 | L1M2 | L1 | LINE |
| 144090975 | 144091295 | - | 12.4 | 49 | 373 | 0 | AluJb | Alu | SINE |
| 144091296 | 144091929 | + | 14.2 | 4606 | 5246 | -897 | L1M2 | L1 | LINE |
| 144091930 | 144092479 | - | 20.8 | 4838 | 5397 | -749 | L1MD2 | L1 | LINE |

  

|  |  |  |  |  |  |  |  |  |  |
| --- | --- | --- | --- | --- | --- | --- | --- | --- | --- |
| chr6:144371728-144374428 | | | 2660 bp | | |  |  |  |  |
| Genome Start | Genome End | Strand | Div | Repeat Start | Repeat End | Repeat Left | Name | Family | Class |
| 144371728 | 144372314 | - | 28.3 | 1137 | 1818 | -191 | HAL1b | L1 | LINE |
| 144372348 | 144373066 | - | 5.2 | 5437 | 6152 | -2 | L1PA7 | L1 | LINE |
| 144373067 | 144374210 | + | 7.0 | 4275 | 5422 | -750 | L1PA7 | L1 | LINE |
| 144374219 | 144374428 | - | 26.4 | 885 | 1089 | -920 | HAL1b | L1 | LINE |

  

|  |  |  |  |  |  |  |  |  |  |
| --- | --- | --- | --- | --- | --- | --- | --- | --- | --- |
| chr6:146214515-146221561 | | | 7041 bp | | |  |  |  |  |
| Genome Start | Genome End | Strand | Div | Repeat Start | Repeat End | Repeat Left | Name | Family | Class |
| 146214515 | 146215304 | + | 25.7 | 36 | 840 | -5560 | L1ME1 | L1 | LINE |
| 146215303 | 146217199 | - | 1.8 | 2473 | 4375 | -1771 | L1PA2 | L1 | LINE |
| 146217199 | 146218959 | + | 2.0 | 4393 | 6153 | -2 | L1PA2 | L1 | LINE |
| 146218968 | 146219236 | + | 19.2 | 833 | 1130 | -5270 | L1ME1 | L1 | LINE |
| 146219237 | 146219538 | - | 5.7 | 1 | 297 | -14 | AluY | Alu | SINE |
| 146219539 | 146220690 | + | 22.2 | 1131 | 2422 | -3978 | L1ME1 | L1 | LINE |
| 146220692 | 146221561 | + | 23.8 | 5359 | 6235 | -4 | L1ME1 | L1 | LINE |

  

|  |  |  |  |  |  |  |  |  |  |
| --- | --- | --- | --- | --- | --- | --- | --- | --- | --- |
| chr6:146929184-146930776 | | | 1552 bp | | |  |  |  |  |
| Genome Start | Genome End | Strand | Div | Repeat Start | Repeat End | Repeat Left | Name | Family | Class |
| 146929184 | 146929227 | + | 10.8 | 1 | 46 | -421 | MLT1C | MaLR | LTR |
| 146929228 | 146929579 | + | 17.8 | 1 | 364 | 0 | THE1B | MaLR | LTR |
| 146929580 | 146929633 | + | 10.8 | 47 | 95 | -372 | MLT1C | MaLR | LTR |
| 146929634 | 146929713 | - | 3.8 | 5400 | 5479 | -675 | L1PA5 | L1 | LINE |
| 146929714 | 146930386 | + | 4.6 | 5481 | 6154 | 0 | L1PA5 | L1 | LINE |
| 146930428 | 146930776 | + | 22.4 | 81 | 467 | 0 | MLT1C | MaLR | LTR |

  

|  |  |  |  |  |  |  |  |  |  |
| --- | --- | --- | --- | --- | --- | --- | --- | --- | --- |
| chr6:152283169-152286873 | | | 3706 bp | | |  |  |  |  |
| Genome Start | Genome End | Strand | Div | Repeat Start | Repeat End | Repeat Left | Name | Family | Class |
| 152283169 | 152283749 | - | 15.7 | 7147 | 7753 | -32 | L1MC3 | L1 | LINE |
| 152283752 | 152284878 | - | 8.9 | 5036 | 6168 | 0 | L1PA10 | L1 | LINE |
| 152284879 | 152285164 | + | 6.6 | 4729 | 5014 | -1132 | L1PA10 | L1 | LINE |
| 152285162 | 152286873 | - | 19.4 | 5424 | 7156 | -629 | L1MC3 | L1 | LINE |

  

|  |  |  |  |  |  |  |  |  |  |
| --- | --- | --- | --- | --- | --- | --- | --- | --- | --- |
| chr6:153661980-153663592 | | | 1247 bp | | |  |  |  |  |
| Genome Start | Genome End | Strand | Div | Repeat Start | Repeat End | Repeat Left | Name | Family | Class |
| 153661980 | 153662365 | - | 27.1 | 665 | 1059 | -5150 | L1MEb | L1 | LINE |
| 153662399 | 153662854 | - | 5.3 | 5703 | 6154 | 0 | L1PA7 | L1 | LINE |
| 153662855 | 153663084 | + | 3.9 | 5450 | 5680 | -474 | L1PA7 | L1 | LINE |
| 153663418 | 153663592 | - | 26.9 | 108 | 277 | -6123 | L1MEb | L1 | LINE |

  

|  |  |  |  |  |  |  |  |  |  |
| --- | --- | --- | --- | --- | --- | --- | --- | --- | --- |
| chr6:154316606-154319672 | | | 3044 bp | | |  |  |  |  |
| Genome Start | Genome End | Strand | Div | Repeat Start | Repeat End | Repeat Left | Name | Family | Class |
| 154316606 | 154316845 | - | 36.6 | 2708 | 2951 | -468 | L2 | L2 | LINE |
| 154316847 | 154317738 | - | 6.4 | 5254 | 6149 | -5 | L1PA7 | L1 | LINE |
| 154317744 | 154319539 | + | 9.3 | 3456 | 5249 | -897 | L1PA7 | L1 | LINE |
| 154319557 | 154319672 | - | 34.8 | 2508 | 2628 | -791 | L2 | L2 | LINE |

  

|  |  |  |  |  |  |  |  |  |  |
| --- | --- | --- | --- | --- | --- | --- | --- | --- | --- |
| chr6:156064657-156068519 | | | 3748 bp | | |  |  |  |  |
| Genome Start | Genome End | Strand | Div | Repeat Start | Repeat End | Repeat Left | Name | Family | Class |
| 156064657 | 156064831 | - | 34.1 | 4219 | 4411 | -78 | L3 | CR1 | LINE |
| 156064855 | 156066188 | - | 12.6 | 3065 | 4427 | -1719 | L1P4 | L1 | LINE |
| 156066189 | 156066542 | + | 9.6 | 4427 | 4798 | -1348 | L1P4 | L1 | LINE |
| 156066561 | 156066600 | - | 5.0 | 5302 | 5343 | -823 | L1P | L1 | LINE |
| 156066599 | 156066805 | + | 10.6 | 3277 | 3487 | -2659 | L1PA16 | L1 | LINE |
| 156066809 | 156068164 | + | 11.7 | 4800 | 6157 | -9 | L1PA16 | L1 | LINE |
| 156068238 | 156068519 | - | 34.9 | 3606 | 3888 | -601 | L3 | CR1 | LINE |

  

|  |  |  |  |  |  |  |  |  |  |
| --- | --- | --- | --- | --- | --- | --- | --- | --- | --- |
| chr6:156219651-156227750 | | | 8071 bp | | |  |  |  |  |
| Genome Start | Genome End | Strand | Div | Repeat Start | Repeat End | Repeat Left | Name | Family | Class |
| 156219651 | 156220614 | - | 23.0 | 5290 | 6234 | -5 | L1ME1 | L1 | LINE |
| 156220611 | 156220954 | - | 19.7 | 1676 | 2018 | -4382 | L1ME1 | L1 | LINE |
| 156220955 | 156221308 | + | 13.6 | 1 | 364 | 0 | THE1B | MaLR | LTR |
| 156221311 | 156222858 | + | 30.2 | 2 | 1735 | 0 | MLT-int | MaLR | LTR |
| 156222860 | 156223204 | + | 13.0 | 2 | 364 | 0 | THE1B | MaLR | LTR |
| 156223205 | 156223699 | - | 19.7 | 1118 | 1675 | -4725 | L1ME1 | L1 | LINE |
| 156223709 | 156224508 | - | 13.3 | 5353 | 6157 | -9 | L1PA16 | L1 | LINE |
| 156224526 | 156224578 | - | 13.2 | 371 | 423 | -132 | MER66C | ERV1 | LTR |
| 156224585 | 156226051 | - | 12.7 | 3868 | 5359 | -787 | L1PA16 | L1 | LINE |
| 156226056 | 156226740 | + | 16.2 | 3192 | 3863 | -2283 | L1PA16 | L1 | LINE |
| 156226735 | 156227750 | - | 21.2 | 25 | 1130 | -5270 | L1ME1 | L1 | LINE |

  

|  |  |  |  |  |  |  |  |  |  |
| --- | --- | --- | --- | --- | --- | --- | --- | --- | --- |
| chr6:160505175-160507573 | | | 2385 bp | | |  |  |  |  |
| Genome Start | Genome End | Strand | Div | Repeat Start | Repeat End | Repeat Left | Name | Family | Class |
| 160505175 | 160505509 | - | 17.8 | 178 | 528 | 0 | LTR64 | ERV1 | LTR |
| 160505523 | 160506323 | - | 2.4 | 5353 | 6152 | -3 | L1PA4 | L1 | LINE |
| 160506323 | 160507053 | + | 3.7 | 4604 | 5335 | -811 | L1PA4 | L1 | LINE |
| 160507054 | 160507383 | - | 17.1 | 1 | 318 | 0 | AluJb | Alu | SINE |
| 160507386 | 160507573 | - | 15.2 | 12 | 191 | -337 | LTR64 | ERV1 | LTR |

  

|  |  |  |  |  |  |  |  |  |  |
| --- | --- | --- | --- | --- | --- | --- | --- | --- | --- |
| chr6:163322869-163329239 | | | 6358 bp | | |  |  |  |  |
| Genome Start | Genome End | Strand | Div | Repeat Start | Repeat End | Repeat Left | Name | Family | Class |
| 163322869 | 163325042 | - | 11.1 | 2074 | 4304 | -2558 | L1PBa | L1 | LINE |
| 163325049 | 163326023 | - | 8.5 | 5161 | 6151 | 0 | L1PB1 | L1 | LINE |
| 163326020 | 163326986 | + | 9.6 | 3427 | 4398 | -1748 | L1PB | L1 | LINE |
| 163327008 | 163327742 | + | 9.6 | 4399 | 5150 | -996 | L1PB | L1 | LINE |
| 163327741 | 163328403 | - | 13.5 | 1395 | 2085 | -4778 | L1PBa | L1 | LINE |
| 163328404 | 163328758 | + | 10.7 | 1 | 364 | 0 | THE1B | MaLR | LTR |
| 163328759 | 163329052 | - | 13.5 | 1108 | 1394 | -5469 | L1PBa | L1 | LINE |
| 163329045 | 163329239 | - | 12.8 | 1 | 195 | -6668 | L1PBa | L1 | LINE |

  

|  |  |  |  |  |  |  |  |  |  |
| --- | --- | --- | --- | --- | --- | --- | --- | --- | --- |
| chr6:165579268-165583221 | | | 3949 bp | | |  |  |  |  |
| Genome Start | Genome End | Strand | Div | Repeat Start | Repeat End | Repeat Left | Name | Family | Class |
| 165579268 | 165580207 | - | 25.0 | 5264 | 6204 | -35 | L1ME1 | L1 | LINE |
| 165580213 | 165580343 | - | 28.9 | 2391 | 2520 | -3699 | L1ME1 | L1 | LINE |
| 165580344 | 165581052 | - | 6.1 | 5429 | 6154 | 0 | L1PA6 | L1 | LINE |
| 165581053 | 165581434 | + | 4.8 | 5052 | 5433 | -721 | L1PA6 | L1 | LINE |
| 165581435 | 165582405 | - | 25.9 | 1306 | 2435 | -3965 | L1ME1 | L1 | LINE |
| 165582406 | 165582707 | - | 10.6 | 1 | 302 | -10 | AluSx | Alu | SINE |
| 165582708 | 165583221 | - | 25.0 | 682 | 1305 | -5095 | L1ME1 | L1 | LINE |

  

|  |  |  |  |  |  |  |  |  |  |
| --- | --- | --- | --- | --- | --- | --- | --- | --- | --- |
| chr6:170802529-170805035 | | | 2473 bp | | |  |  |  |  |
| Genome Start | Genome End | Strand | Div | Repeat Start | Repeat End | Repeat Left | Name | Family | Class |
| 170802529 | 170803612 | - | 16.7 | 3657 | 4741 | -1399 | L1M3 | L1 | LINE |
| 170803632 | 170803809 | - | 4.5 | 5977 | 6155 | 0 | L1PA4 | L1 | LINE |
| 170803810 | 170804531 | + | 2.8 | 5254 | 5975 | -180 | L1PA4 | L1 | LINE |
| 170804547 | 170805035 | - | 19.7 | 3150 | 3646 | -2494 | L1M3 | L1 | LINE |

  


|  |  |  |  |  |  |  |  |  |  |
| --- | --- | --- | --- | --- | --- | --- | --- | --- | --- |
| chr7:4062497-4071369 | | | 8842 bp | | |  |  |  |  |
| Genome Start | Genome End | Strand | Div | Repeat Start | Repeat End | Repeat Left | Name | Family | Class |
| 4062497 | 4066035 | + | 16.8 | 3 | 3626 | -3219 | L1MA4A | L1 | LINE |
| 4066036 | 4066338 | - | 5.7 | 1 | 301 | -10 | AluY | Alu | SINE |
| 4066339 | 4066569 | + | 14.7 | 3627 | 3881 | -2964 | L1MA4A | L1 | LINE |
| 4066568 | 4067526 | - | 2.8 | 4427 | 5385 | -761 | L1PA3 | L1 | LINE |
| 4067524 | 4068285 | + | 2.0 | 5376 | 6137 | -18 | L1PA3 | L1 | LINE |
| 4068322 | 4071369 | + | 15.7 | 3893 | 7004 | -1 | L1MA4A | L1 | LINE |

  

|  |  |  |  |  |  |  |  |  |  |
| --- | --- | --- | --- | --- | --- | --- | --- | --- | --- |
| chr7:6867914-6870325 | | | 2391 bp | | |  |  |  |  |
| Genome Start | Genome End | Strand | Div | Repeat Start | Repeat End | Repeat Left | Name | Family | Class |
| 6867914 | 6868025 | - | 21.6 | 6042 | 6153 | -30 | L1MB3 | L1 | LINE |
| 6868047 | 6868254 | - | 5.8 | 5946 | 6153 | -2 | L1PA2 | L1 | LINE |
| 6868255 | 6868486 | + | 0.9 | 5714 | 5945 | -210 | L1PA2 | L1 | LINE |
| 6868487 | 6868637 | - | 24.5 | 5909 | 6052 | -131 | L1MB3 | L1 | LINE |
| 6868638 | 6868920 | - | 12.4 | 3 | 295 | -17 | AluSx | Alu | SINE |
| 6868921 | 6869013 | - | 24.5 | 5821 | 5908 | -275 | L1MB3 | L1 | LINE |
| 6869014 | 6869341 | - | 13.5 | 1 | 312 | 0 | AluSx | Alu | SINE |
| 6869342 | 6870325 | - | 25.1 | 4818 | 5820 | -363 | L1MB3 | L1 | LINE |

  

|  |  |  |  |  |  |  |  |  |  |
| --- | --- | --- | --- | --- | --- | --- | --- | --- | --- |
| chr7:7823980-7825002 | | | 1009 bp | | |  |  |  |  |
| Genome Start | Genome End | Strand | Div | Repeat Start | Repeat End | Repeat Left | Name | Family | Class |
| 7823980 | 7824140 | - | 19.1 | 130 | 290 | -22 | AluJ | Alu | SINE |
| 7824151 | 7824516 | - | 9.6 | 5778 | 6153 | -15 | L1PA12 | L1 | LINE |
| 7824518 | 7824951 | + | 7.4 | 5336 | 5776 | -392 | L1PA12 | L1 | LINE |
| 7824955 | 7825002 | - | 6.2 | 91 | 138 | -164 | AluJ | Alu | SINE |

  

|  |  |  |  |  |  |  |  |  |  |
| --- | --- | --- | --- | --- | --- | --- | --- | --- | --- |
| chr7:8417790-8419447 | | | 1613 bp | | |  |  |  |  |
| Genome Start | Genome End | Strand | Div | Repeat Start | Repeat End | Repeat Left | Name | Family | Class |
| 8417790 | 8417891 | - | 30.7 | 3268 | 3378 | 0 | L2 | L2 | LINE |
| 8417906 | 8418127 | - | 6.8 | 5076 | 5297 | -849 | L1P3 | L1 | LINE |
| 8418128 | 8418275 | + | 2.0 | 5295 | 5444 | -728 | L1P3 | L1 | LINE |
| 8418259 | 8418334 | - | 20.3 | 4998 | 5073 | -1073 | L1M5 | L1 | LINE |
| 8418343 | 8418512 | + | 17.2 | 172 | 341 | 0 | MER58B | MER1\_type | DNA |
| 8418553 | 8418969 | - | 30.1 | 2847 | 3293 | -126 | L2 | L2 | LINE |
| 8418970 | 8419290 | + | 18.3 | 1 | 300 | -12 | AluJb | Alu | SINE |
| 8419291 | 8419447 | - | 29.6 | 2686 | 2846 | -573 | L2 | L2 | LINE |

  

|  |  |  |  |  |  |  |  |  |  |
| --- | --- | --- | --- | --- | --- | --- | --- | --- | --- |
| chr7:9432771-9436870 | | | 4103 bp | | |  |  |  |  |
| Genome Start | Genome End | Strand | Div | Repeat Start | Repeat End | Repeat Left | Name | Family | Class |
| 9432771 | 9432884 | + | 15.0 | 5914 | 6022 | -269 | L1MA8 | L1 | LINE |
| 9432886 | 9433041 | - | 17.1 | 565 | 711 | -23 | MER50 | ERV1 | LTR |
| 9433044 | 9434133 | - | 2.1 | 3155 | 4244 | -1902 | L1PA2 | L1 | LINE |
| 9434127 | 9436054 | + | 2.1 | 4226 | 6153 | -2 | L1PA2 | L1 | LINE |
| 9436056 | 9436622 | - | 15.9 | 1 | 575 | -159 | MER50 | ERV1 | LTR |
| 9436623 | 9436870 | + | 13.0 | 6019 | 6285 | -6 | L1MA8 | L1 | LINE |

  

|  |  |  |  |  |  |  |  |  |  |
| --- | --- | --- | --- | --- | --- | --- | --- | --- | --- |
| chr7:10532044-10542660 | | | 10599 bp | | |  |  |  |  |
| Genome Start | Genome End | Strand | Div | Repeat Start | Repeat End | Repeat Left | Name | Family | Class |
| 10532044 | 10532179 | + | 13.3 | 3 | 137 | -5738 | L1PA8A | L1 | LINE |
| 10532179 | 10532581 | - | 5.5 | 4750 | 5152 | -994 | L1PA4 | L1 | LINE |
| 10532581 | 10533587 | + | 3.2 | 5146 | 6152 | -3 | L1PA4 | L1 | LINE |
| 10533610 | 10539380 | + | 12.5 | 253 | 5921 | -525 | L1PA8A | L1 | LINE |
| 10539376 | 10542139 | + | 8.7 | 8 | 2788 | -3358 | L1P1 | L1 | LINE |
| 10542143 | 10542660 | + | 8.5 | 5921 | 6445 | -1 | L1PA8A | L1 | LINE |

  

|  |  |  |  |  |  |  |  |  |  |
| --- | --- | --- | --- | --- | --- | --- | --- | --- | --- |
| chr7:12186396-12191324 | | | 4941 bp | | |  |  |  |  |
| Genome Start | Genome End | Strand | Div | Repeat Start | Repeat End | Repeat Left | Name | Family | Class |
| 12186396 | 12187491 | - | 13.4 | 846 | 1952 | -466 | Tigger1 | MER2\_type | DNA |
| 12187492 | 12188587 | - | 3.1 | 5082 | 6145 | -10 | L1PA3 | L1 | LINE |
| 12188576 | 12190467 | + | 3.0 | 3188 | 5078 | -1068 | L1PA3 | L1 | LINE |
| 12190468 | 12191324 | - | 15.2 | 1 | 859 | -1559 | Tigger1 | MER2\_type | DNA |

  

|  |  |  |  |  |  |  |  |  |  |
| --- | --- | --- | --- | --- | --- | --- | --- | --- | --- |
| chr7:13311232-13313462 | | | 2223 bp | | |  |  |  |  |
| Genome Start | Genome End | Strand | Div | Repeat Start | Repeat End | Repeat Left | Name | Family | Class |
| 13311232 | 13311412 | + | 12.2 | 1 | 181 | -183 | THE1B | MaLR | LTR |
| 13311417 | 13312082 | - | 8.3 | 4624 | 5308 | -838 | L1PA8 | L1 | LINE |
| 13312081 | 13312454 | + | 8.5 | 5315 | 5687 | -485 | L1PA8 | L1 | LINE |
| 13312455 | 13312752 | + | 8.7 | 1 | 298 | -13 | AluY | Alu | SINE |
| 13312753 | 13313253 | + | 8.5 | 5688 | 6172 | 0 | L1PA8 | L1 | LINE |
| 13313260 | 13313462 | + | 17.2 | 171 | 364 | 0 | THE1B | MaLR | LTR |

  

|  |  |  |  |  |  |  |  |  |  |
| --- | --- | --- | --- | --- | --- | --- | --- | --- | --- |
| chr7:15125584-15129612 | | | 3958 bp | | |  |  |  |  |
| Genome Start | Genome End | Strand | Div | Repeat Start | Repeat End | Repeat Left | Name | Family | Class |
| 15125584 | 15126520 | + | 28.0 | 1065 | 2104 | -1315 | L2 | L2 | LINE |
| 15126533 | 15126896 | + | 24.0 | 1 | 374 | 0 | MLT1A | MaLR | LTR |
| 15126957 | 15127766 | - | 7.3 | 5360 | 6168 | -4 | L1PA8 | L1 | LINE |
| 15127766 | 15128413 | + | 9.3 | 4676 | 5351 | -795 | L1PA8 | L1 | LINE |
| 15128414 | 15129612 | + | 30.5 | 2172 | 3414 | -5 | L2 | L2 | LINE |

  

|  |  |  |  |  |  |  |  |  |  |
| --- | --- | --- | --- | --- | --- | --- | --- | --- | --- |
| chr7:18433634-18435430 | | | 2050 bp | | |  |  |  |  |
| Genome Start | Genome End | Strand | Div | Repeat Start | Repeat End | Repeat Left | Name | Family | Class |
| 18433634 | 18433801 | + | 28.6 | 2561 | 2769 | -650 | L2 | L2 | LINE |
| 18433872 | 18434304 | - | 12.9 | 5731 | 6167 | -1 | L1MB1 | L1 | LINE |
| 18433980 | 18434427 | - | 24.9 | 5532 | 6062 | -116 | L1MB8 | L1 | LINE |
| 18434429 | 18434812 | + | 16.8 | 5142 | 5531 | -637 | L1M3 | L1 | LINE |
| 18434814 | 18435430 | + | 25.5 | 2774 | 3419 | 0 | L2 | L2 | LINE |

  

|  |  |  |  |  |  |  |  |  |  |
| --- | --- | --- | --- | --- | --- | --- | --- | --- | --- |
| chr7:18869421-18871508 | | | 1860 bp | | |  |  |  |  |
| Genome Start | Genome End | Strand | Div | Repeat Start | Repeat End | Repeat Left | Name | Family | Class |
| 18869421 | 18869464 | - | 28.9 | 3375 | 3412 | -7 | L2 | L2 | LINE |
| 18869465 | 18869709 | + | 8.2 | 3 | 230 | 0 | MER30 | MER1\_type | DNA |
| 18869710 | 18870048 | - | 28.9 | 3037 | 3374 | -45 | L2 | L2 | LINE |
| 18870051 | 18870267 | - | 14.8 | 5508 | 5725 | -443 | L1MB1 | L1 | LINE |
| 18870269 | 18870764 | + | 12.1 | 5734 | 6168 | 0 | L1MB1 | L1 | LINE |
| 18870847 | 18871092 | + | 14.9 | 5949 | 6179 | -4 | L1MB3 | L1 | LINE |
| 18871236 | 18871508 | - | 26.8 | 2563 | 2839 | -580 | L2 | L2 | LINE |

  

|  |  |  |  |  |  |  |  |  |  |
| --- | --- | --- | --- | --- | --- | --- | --- | --- | --- |
| chr7:19972752-19976833 | | | 3969 bp | | |  |  |  |  |
| Genome Start | Genome End | Strand | Div | Repeat Start | Repeat End | Repeat Left | Name | Family | Class |
| 19972752 | 19974544 | + | 33.3 | 444 | 2436 | -983 | L2 | L2 | LINE |
| 19974545 | 19974820 | - | 21.9 | 232 | 514 | 0 | MER67D | ERV1 | LTR |
| 19974934 | 19975329 | - | 11.1 | 5867 | 6273 | -27 | L1MA6 | L1 | LINE |
| 19975329 | 19975708 | + | 15.5 | 5484 | 5858 | -442 | L1MA6 | L1 | LINE |
| 19975709 | 19975940 | - | 24.3 | 1 | 370 | -291 | MER67D | ERV1 | LTR |
| 19975942 | 19976833 | + | 31.2 | 2434 | 3419 | 0 | L2 | L2 | LINE |

  

|  |  |  |  |  |  |  |  |  |  |
| --- | --- | --- | --- | --- | --- | --- | --- | --- | --- |
| chr7:21698401-21702225 | | | 3800 bp | | |  |  |  |  |
| Genome Start | Genome End | Strand | Div | Repeat Start | Repeat End | Repeat Left | Name | Family | Class |
| 21698401 | 21699121 | - | 23.7 | 5344 | 6088 | -78 | L1ME1 | L1 | LINE |
| 21699128 | 21699358 | - | 24.9 | 5022 | 5268 | -878 | L1ME1 | L1 | LINE |
| 21699365 | 21699898 | - | 26.2 | 3358 | 3895 | -2251 | L1ME1 | L1 | LINE |
| 21699899 | 21700295 | - | 15.6 | 1 | 428 | 0 | MSTA | MaLR | LTR |
| 21700296 | 21700322 | - | 26.2 | 3331 | 3357 | -2789 | L1ME1 | L1 | LINE |
| 21700328 | 21701121 | - | 6.4 | 5376 | 6169 | -3 | L1PA8 | L1 | LINE |
| 21701120 | 21702111 | + | 8.7 | 4374 | 5377 | -769 | L1PA8 | L1 | LINE |
| 21702122 | 21702225 | - | 17.5 | 3220 | 3318 | -2828 | L1ME1 | L1 | LINE |

  

|  |  |  |  |  |  |  |  |  |  |
| --- | --- | --- | --- | --- | --- | --- | --- | --- | --- |
| chr7:23403918-23405803 | | | 1842 bp | | |  |  |  |  |
| Genome Start | Genome End | Strand | Div | Repeat Start | Repeat End | Repeat Left | Name | Family | Class |
| 23403918 | 23404152 | + | 21.3 | 2 | 244 | -261 | MLT1D | MaLR | LTR |
| 23404174 | 23405425 | - | 3.6 | 4782 | 6039 | -116 | L1PA3 | L1 | LINE |
| 23405426 | 23405543 | + | 4.2 | 6031 | 6152 | -3 | L1PA3 | L1 | LINE |
| 23405567 | 23405803 | + | 19.3 | 263 | 505 | 0 | MLT1D | MaLR | LTR |

  

|  |  |  |  |  |  |  |  |  |  |
| --- | --- | --- | --- | --- | --- | --- | --- | --- | --- |
| chr7:24327893-24329874 | | | 1892 bp | | |  |  |  |  |
| Genome Start | Genome End | Strand | Div | Repeat Start | Repeat End | Repeat Left | Name | Family | Class |
| 24327893 | 24328004 | + | 23.4 | 24 | 146 | -2361 | HAL1 | L1 | LINE |
| 24328010 | 24329122 | + | 27.0 | 176 | 1371 | -1136 | HAL1 | L1 | LINE |
| 24329196 | 24329344 | - | 15.0 | 294 | 445 | 0 | MER65A | ERV1 | LTR |
| 24329345 | 24329409 | - | 9.4 | 1 | 64 | -467 | MER65B | ERV1 | LTR |
| 24329410 | 24329500 | + | 24.7 | 1471 | 1566 | -941 | HAL1 | L1 | LINE |
| 24329499 | 24329528 | - | 3.3 | 5944 | 5973 | -181 | L1PA5 | L1 | LINE |
| 24329529 | 24329699 | + | 5.3 | 5977 | 6147 | -7 | L1PA5 | L1 | LINE |
| 24329714 | 24329874 | + | 26.1 | 1553 | 1727 | -780 | HAL1 | L1 | LINE |

  

|  |  |  |  |  |  |  |  |  |  |
| --- | --- | --- | --- | --- | --- | --- | --- | --- | --- |
| chr7:27858949-27869126 | | | 9807 bp | | |  |  |  |  |
| Genome Start | Genome End | Strand | Div | Repeat Start | Repeat End | Repeat Left | Name | Family | Class |
| 27858949 | 27859085 | - | 24.1 | 6914 | 7060 | -982 | L1MC4 | L1 | LINE |
| 27859107 | 27859374 | - | 26.1 | 2198 | 2501 | -6 | HAL1 | L1 | LINE |
| 27859402 | 27859725 | + | 18.8 | 24 | 363 | -502 | MER6 | MER2\_type | DNA |
| 27859727 | 27861314 | - | 8.8 | 1357 | 2943 | -3203 | L1PA7 | L1 | LINE |
| 27861316 | 27862191 | + | 7.5 | 2937 | 3823 | -2323 | L1PA7 | L1 | LINE |
| 27862192 | 27863717 | - | 6.8 | 1 | 1578 | 0 | LTR12C | ERV1 | LTR |
| 27863718 | 27866021 | + | 6.3 | 3824 | 6151 | -3 | L1PA7 | L1 | LINE |
| 27866022 | 27866501 | + | 14.2 | 365 | 865 | 0 | MER6 | MER2\_type | DNA |
| 27866684 | 27866903 | - | 24.6 | 2 | 238 | -24 | MIR | MIR | SINE |
| 27867026 | 27868356 | - | 25.4 | 280 | 1917 | -590 | HAL1 | L1 | LINE |
| 27868357 | 27868566 | - | 14.3 | 5939 | 6158 | -8 | L1PA16 | L1 | LINE |
| 27868574 | 27868735 | - | 18.6 | 6006 | 6169 | -9 | L1MB8 | L1 | LINE |
| 27868736 | 27868985 | - | 25.4 | 2 | 279 | -2228 | HAL1 | L1 | LINE |
| 27868996 | 27869126 | - | 27.1 | 6768 | 6917 | -1125 | L1MC4 | L1 | LINE |

  

|  |  |  |  |  |  |  |  |  |  |
| --- | --- | --- | --- | --- | --- | --- | --- | --- | --- |
| chr7:28862491-28864588 | | | 1639 bp | | |  |  |  |  |
| Genome Start | Genome End | Strand | Div | Repeat Start | Repeat End | Repeat Left | Name | Family | Class |
| 28862491 | 28863226 | + | 35.2 | 1978 | 2754 | -665 | L2 | L2 | LINE |
| 28863303 | 28863358 | - | 16.1 | 5775 | 5837 | -454 | L1MA7 | L1 | LINE |
| 28863359 | 28863824 | + | 15.1 | 5828 | 6290 | -1 | L1MA7 | L1 | LINE |
| 28864093 | 28864359 | - | 14.9 | 33 | 293 | -19 | AluJb | Alu | SINE |
| 28864475 | 28864588 | + | 31.6 | 3248 | 3369 | -9 | L2 | L2 | LINE |

  

|  |  |  |  |  |  |  |  |  |  |
| --- | --- | --- | --- | --- | --- | --- | --- | --- | --- |
| chr7:29524584-29531240 | | | 6629 bp | | |  |  |  |  |
| Genome Start | Genome End | Strand | Div | Repeat Start | Repeat End | Repeat Left | Name | Family | Class |
| 29524584 | 29524877 | - | 32.9 | 5681 | 6006 | -168 | L1ME3B | L1 | LINE |
| 29524888 | 29525287 | - | 26.8 | 5215 | 5637 | -603 | L1ME3B | L1 | LINE |
| 29525297 | 29525518 | - | 27.8 | 4902 | 5136 | -1010 | L1ME3B | L1 | LINE |
| 29525519 | 29525656 | + | 16.8 | 1 | 121 | -203 | MER33 | MER1\_type | DNA |
| 29525657 | 29525913 | + | 7.8 | 12 | 269 | -44 | AluSp | Alu | SINE |
| 29525914 | 29526121 | + | 16.8 | 122 | 322 | -2 | MER33 | MER1\_type | DNA |
| 29526122 | 29526813 | - | 27.8 | 4163 | 4901 | -1245 | L1ME3B | L1 | LINE |
| 29526825 | 29526903 | - | 12.7 | 6214 | 6292 | -5 | L1MA5A | L1 | LINE |
| 29526904 | 29527181 | + | 6.8 | 4013 | 4294 | -1852 | L1MA1 | L1 | LINE |
| 29527171 | 29527707 | + | 8.6 | 5705 | 6253 | -49 | L1MA1 | L1 | LINE |
| 29527702 | 29528320 | - | 13.7 | 4670 | 5301 | -842 | L1M2 | L1 | LINE |
| 29528317 | 29530718 | + | 14.5 | 2165 | 4658 | -1485 | L1M2 | L1 | LINE |
| 29530716 | 29530856 | - | 29.1 | 4020 | 4181 | -1965 | L1ME3B | L1 | LINE |
| 29530868 | 29530993 | - | 32.0 | 29 | 177 | -1 | MER5B | MER1\_type | DNA |
| 29531005 | 29531240 | - | 25.8 | 3764 | 3990 | -2156 | L1ME3B | L1 | LINE |

  

|  |  |  |  |  |  |  |  |  |  |
| --- | --- | --- | --- | --- | --- | --- | --- | --- | --- |
| chr7:33165328-33180481 | | | 15147 bp | | |  |  |  |  |
| Genome Start | Genome End | Strand | Div | Repeat Start | Repeat End | Repeat Left | Name | Family | Class |
| 33165328 | 33165492 | - | 14.6 | 7371 | 7537 | 0 | MER57A-int | ERV1 | LTR |
| 33165494 | 33165708 | - | 14.3 | 5 | 239 | -271 | LTR54 | ERV1 | LTR |
| 33165710 | 33167256 | - | 12.8 | 5600 | 7147 | -390 | MER57A-int | ERV1 | LTR |
| 33167263 | 33173149 | - | 6.6 | 9 | 6151 | -3 | L1PA6 | L1 | LINE |
| 33173157 | 33173537 | - | 10.4 | 3543 | 3919 | -2268 | MER57A-int | ERV1 | LTR |
| 33173538 | 33173835 | - | 12.8 | 1 | 300 | -10 | AluSg | Alu | SINE |
| 33173836 | 33174949 | - | 10.4 | 2413 | 3542 | -2645 | MER57A-int | ERV1 | LTR |
| 33174950 | 33176135 | - | 2.1 | 4967 | 6152 | -3 | L1PA2 | L1 | LINE |
| 33176128 | 33177554 | + | 2.1 | 3528 | 4954 | -1192 | L1PA2 | L1 | LINE |
| 33177555 | 33178235 | - | 9.7 | 1741 | 2430 | -3757 | MER57A-int | ERV1 | LTR |
| 33178236 | 33178543 | + | 8.2 | 1 | 307 | -4 | AluY | Alu | SINE |
| 33178544 | 33179435 | - | 9.7 | 736 | 1740 | -4447 | MER57A-int | ERV1 | LTR |
| 33179436 | 33179726 | + | 8.6 | 1 | 299 | -12 | AluY | Alu | SINE |
| 33179727 | 33180481 | - | 9.5 | 1 | 735 | -5452 | MER57A-int | ERV1 | LTR |

  

|  |  |  |  |  |  |  |  |  |  |
| --- | --- | --- | --- | --- | --- | --- | --- | --- | --- |
| chr7:34634686-34645141 | | | 10469 bp | | |  |  |  |  |
| Genome Start | Genome End | Strand | Div | Repeat Start | Repeat End | Repeat Left | Name | Family | Class |
| 34634686 | 34635720 | + | 8.5 | 748 | 1777 | -4703 | L1PA7 | L1 | LINE |
| 34635720 | 34638029 | - | 4.4 | 1386 | 3696 | -2450 | L1PA4 | L1 | LINE |
| 34638024 | 34640477 | + | 4.4 | 3702 | 6155 | 0 | L1PA4 | L1 | LINE |
| 34640472 | 34645141 | + | 8.3 | 1763 | 6465 | -4 | L1PA7 | L1 | LINE |

  

|  |  |  |  |  |  |  |  |  |  |
| --- | --- | --- | --- | --- | --- | --- | --- | --- | --- |
| chr7:37362118-37363484 | | | 1283 bp | | |  |  |  |  |
| Genome Start | Genome End | Strand | Div | Repeat Start | Repeat End | Repeat Left | Name | Family | Class |
| 37362118 | 37362290 | + | 16.6 | 1 | 187 | -520 | MER39 | ERV1 | LTR |
| 37362291 | 37362574 | - | 6.4 | 5512 | 5795 | -359 | L1PA7 | L1 | LINE |
| 37362575 | 37362901 | + | 5.2 | 5817 | 6154 | 0 | L1PA7 | L1 | LINE |
| 37362986 | 37363484 | + | 16.1 | 180 | 697 | -10 | MER39 | ERV1 | LTR |

  

|  |  |  |  |  |  |  |  |  |  |
| --- | --- | --- | --- | --- | --- | --- | --- | --- | --- |
| chr7:37807258-37822881 | | | 15231 bp | | |  |  |  |  |
| Genome Start | Genome End | Strand | Div | Repeat Start | Repeat End | Repeat Left | Name | Family | Class |
| 37807258 | 37808988 | - | 15.3 | 2461 | 4182 | -2139 | L1MCa | L1 | LINE |
| 37808989 | 37809283 | - | 16.7 | 1 | 299 | -13 | AluJo | Alu | SINE |
| 37809284 | 37809875 | - | 12.3 | 1861 | 2460 | -3861 | L1MCa | L1 | LINE |
| 37809877 | 37810905 | + | 22.9 | 2049 | 3106 | -5049 | L1PREC2 | L1 | LINE |
| 37810906 | 37811544 | + | 19.7 | 1 | 612 | 0 | LTR9 | ERV1 | LTR |
| 37811545 | 37812665 | + | 22.9 | 3107 | 4212 | -3943 | L1PREC2 | L1 | LINE |
| 37812663 | 37814087 | - | 2.6 | 2586 | 4013 | -2133 | L1PA3 | L1 | LINE |
| 37814087 | 37816210 | + | 2.2 | 4025 | 6153 | -2 | L1PA3 | L1 | LINE |
| 37816537 | 37817135 | + | 15.6 | 4197 | 4802 | -3339 | L1PREC2 | L1 | LINE |
| 37817136 | 37817357 | + | 9.1 | 83 | 303 | -8 | AluY | Alu | SINE |
| 37817358 | 37819548 | + | 15.6 | 4803 | 6992 | -1149 | L1PREC2 | L1 | LINE |
| 37819549 | 37819852 | + | 9.9 | 1 | 304 | -7 | AluY | Alu | SINE |
| 37819853 | 37821012 | + | 12.9 | 6993 | 8158 | -2 | L1PA15 | L1 | LINE |
| 37821051 | 37821111 | - | 15.8 | 1793 | 1843 | -4665 | L1MCa | L1 | LINE |
| 37821112 | 37821472 | - | 16.8 | 1 | 310 | -1 | AluY | Alu | SINE |
| 37821473 | 37821681 | - | 15.8 | 1586 | 1792 | -4716 | L1MCa | L1 | LINE |
| 37821677 | 37822629 | - | 16.1 | 378 | 1331 | -5177 | L1MCa | L1 | LINE |
| 37822667 | 37822881 | - | 22.6 | 13 | 228 | -6280 | L1MCa | L1 | LINE |

  

|  |  |  |  |  |  |  |  |  |  |
| --- | --- | --- | --- | --- | --- | --- | --- | --- | --- |
| chr7:38032194-38036357 | | | 3949 bp | | |  |  |  |  |
| Genome Start | Genome End | Strand | Div | Repeat Start | Repeat End | Repeat Left | Name | Family | Class |
| 38032194 | 38033096 | + | 26.0 | 3495 | 4383 | -1763 | L1M5 | L1 | LINE |
| 38033097 | 38033402 | + | 14.1 | 5 | 310 | -2 | AluSx | Alu | SINE |
| 38033403 | 38033606 | + | 26.0 | 4384 | 4595 | -1551 | L1M5 | L1 | LINE |
| 38033616 | 38033698 | - | 7.8 | 3 | 80 | 0 | MADE1 | Mariner | DNA |
| 38033729 | 38034063 | + | 33.0 | 854 | 1202 | -3794 | HERV16 | ERVL | LTR |
| 38034232 | 38034403 | + | 18.3 | 214 | 432 | 0 | MLT1A0 | MaLR | LTR |
| 38034404 | 38034497 | + | 36.5 | 54 | 148 | -309 | LTR16A1 | ERVL | LTR |
| 38034507 | 38035695 | - | 1.1 | 4963 | 6150 | -5 | L1PA2 | L1 | LINE |
| 38035675 | 38035749 | + | 0.0 | 4903 | 4977 | -1169 | L1PA2 | L1 | LINE |
| 38035750 | 38036036 | + | 33.1 | 138 | 441 | -9 | LTR16A | ERVL | LTR |
| 38036057 | 38036357 | + | 30.0 | 4622 | 4913 | -1233 | L1M5 | L1 | LINE |

  

|  |  |  |  |  |  |  |  |  |  |
| --- | --- | --- | --- | --- | --- | --- | --- | --- | --- |
| chr7:39683007-39684442 | | | 1434 bp | | |  |  |  |  |
| Genome Start | Genome End | Strand | Div | Repeat Start | Repeat End | Repeat Left | Name | Family | Class |
| 39683007 | 39683385 | + | 24.0 | 5 | 407 | -98 | MLT1D | MaLR | LTR |
| 39683386 | 39684113 | - | 0.4 | 5217 | 5947 | -208 | L1PA2 | L1 | LINE |
| 39684114 | 39684330 | + | 1.4 | 5939 | 6155 | 0 | L1PA2 | L1 | LINE |
| 39684333 | 39684442 | + | 14.6 | 396 | 505 | 0 | MLT1D | MaLR | LTR |

  

|  |  |  |  |  |  |  |  |  |  |
| --- | --- | --- | --- | --- | --- | --- | --- | --- | --- |
| chr7:40944381-40954027 | | | 9589 bp | | |  |  |  |  |
| Genome Start | Genome End | Strand | Div | Repeat Start | Repeat End | Repeat Left | Name | Family | Class |
| 40944381 | 40944600 | - | 21.5 | 3040 | 3279 | -4055 | L1M4b | L1 | LINE |
| 40944615 | 40944629 | - | 24.1 | 6284 | 6298 | -5 | L1MA4A | L1 | LINE |
| 40944630 | 40945157 | - | 13.9 | 5704 | 6210 | -1 | L1PB3 | L1 | LINE |
| 40945158 | 40945526 | - | 14.5 | 1 | 364 | 0 | THE1B | MaLR | LTR |
| 40945527 | 40947294 | - | 15.0 | 3907 | 5703 | -508 | L1PB3 | L1 | LINE |
| 40947305 | 40949387 | - | 16.9 | 913 | 3106 | -3097 | L1PB3 | L1 | LINE |
| 40949422 | 40950879 | - | 2.7 | 4666 | 6137 | -18 | L1PA3 | L1 | LINE |
| 40950878 | 40950940 | + | 1.6 | 4606 | 4668 | -1478 | L1PA3 | L1 | LINE |
| 40950939 | 40951028 | - | 14.4 | 835 | 924 | -5280 | L1PB3 | L1 | LINE |
| 40951049 | 40951828 | - | 17.2 | 1 | 781 | -5423 | L1PB3 | L1 | LINE |
| 40951796 | 40952651 | - | 27.6 | 762 | 1637 | -5226 | L1PBa | L1 | LINE |
| 40952669 | 40954027 | - | 20.4 | 1058 | 2474 | -4860 | L1M4b | L1 | LINE |

  

|  |  |  |  |  |  |  |  |  |  |
| --- | --- | --- | --- | --- | --- | --- | --- | --- | --- |
| chr7:41185764-41186401 | | | 548 bp | | |  |  |  |  |
| Genome Start | Genome End | Strand | Div | Repeat Start | Repeat End | Repeat Left | Name | Family | Class |
| 41185764 | 41185979 | - | 13.9 | 136 | 345 | 0 | MER2B | MER2\_type | DNA |
| 41186059 | 41186226 | - | 6.0 | 5986 | 6153 | -2 | L1PA4 | L1 | LINE |
| 41186227 | 41186322 | + | 3.1 | 5894 | 5989 | -166 | L1PA4 | L1 | LINE |
| 41186334 | 41186401 | - | 20.6 | 1 | 75 | -261 | MER2B | MER2\_type | DNA |

  

|  |  |  |  |  |  |  |  |  |  |
| --- | --- | --- | --- | --- | --- | --- | --- | --- | --- |
| chr7:46146046-46150211 | | | 4164 bp | | |  |  |  |  |
| Genome Start | Genome End | Strand | Div | Repeat Start | Repeat End | Repeat Left | Name | Family | Class |
| 46146046 | 46146119 | - | 20.3 | 5156 | 5237 | -909 | L1M1 | L1 | LINE |
| 46146125 | 46146260 | - | 9.2 | 181 | 311 | 0 | AluY | Alu | SINE |
| 46146261 | 46146445 | - | 12.4 | 4977 | 5162 | -981 | L1M1 | L1 | LINE |
| 46146447 | 46147312 | - | 2.9 | 5290 | 6155 | 0 | L1PA2 | L1 | LINE |
| 46147311 | 46149241 | + | 2.2 | 3364 | 5296 | -850 | L1PA2 | L1 | LINE |
| 46149240 | 46149476 | - | 14.8 | 4738 | 4988 | -1155 | L1M1 | L1 | LINE |
| 46149477 | 46150145 | - | 12.8 | 1 | 653 | 0 | MER39B | ERV1 | LTR |
| 46150146 | 46150211 | - | 14.8 | 4681 | 4737 | -1406 | L1M1 | L1 | LINE |

  

|  |  |  |  |  |  |  |  |  |  |
| --- | --- | --- | --- | --- | --- | --- | --- | --- | --- |
| chr7:46300718-46307804 | | | 6865 bp | | |  |  |  |  |
| Genome Start | Genome End | Strand | Div | Repeat Start | Repeat End | Repeat Left | Name | Family | Class |
| 46300718 | 46301566 | + | 26.8 | 88 | 1087 | -5322 | L1MD2 | L1 | LINE |
| 46301581 | 46301850 | + | 13.7 | 1 | 263 | -47 | AluSg | Alu | SINE |
| 46301911 | 46302625 | - | 19.4 | 5567 | 6270 | -42 | L1MA9 | L1 | LINE |
| 46302699 | 46302756 | + | 17.2 | 5508 | 5565 | -747 | L1M2 | L1 | LINE |
| 46302758 | 46303277 | - | 21.0 | 5024 | 5555 | -757 | L1MA9 | L1 | LINE |
| 46303283 | 46303482 | + | 22.7 | 4840 | 5037 | -1103 | L1MA9 | L1 | LINE |
| 46303489 | 46304264 | + | 27.4 | 1103 | 1940 | -4562 | L1MD2 | L1 | LINE |
| 46304269 | 46304980 | + | 22.4 | 3163 | 3929 | -2477 | L1MD2 | L1 | LINE |
| 46305013 | 46307579 | + | 22.2 | 3930 | 6394 | -225 | L1MD2 | L1 | LINE |
| 46307607 | 46307804 | + | 21.7 | 6395 | 6610 | -9 | L1MD2 | L1 | LINE |

  

|  |  |  |  |  |  |  |  |  |  |
| --- | --- | --- | --- | --- | --- | --- | --- | --- | --- |
| chr7:47142189-47147964 | | | 5778 bp | | |  |  |  |  |
| Genome Start | Genome End | Strand | Div | Repeat Start | Repeat End | Repeat Left | Name | Family | Class |
| 47142189 | 47144555 | - | 27.8 | 1914 | 4348 | -1871 | L1MEc | L1 | LINE |
| 47144559 | 47145201 | - | 17.8 | 5644 | 6290 | -1 | L1MA7 | L1 | LINE |
| 47145203 | 47146464 | + | 17.6 | 4326 | 5641 | -650 | L1MA7 | L1 | LINE |
| 47146466 | 47147173 | - | 21.0 | 1200 | 1934 | -4466 | L1MEc | L1 | LINE |
| 47147167 | 47147964 | - | 25.6 | 36 | 881 | -5519 | L1MEc | L1 | LINE |

  

|  |  |  |  |  |  |  |  |  |  |
| --- | --- | --- | --- | --- | --- | --- | --- | --- | --- |
| chr7:49468103-49469505 | | | 1337 bp | | |  |  |  |  |
| Genome Start | Genome End | Strand | Div | Repeat Start | Repeat End | Repeat Left | Name | Family | Class |
| 49468103 | 49468625 | - | 23.3 | 4985 | 5554 | -2254 | L1M5 | L1 | LINE |
| 49468627 | 49468954 | - | 4.6 | 5824 | 6150 | -4 | L1PA5 | L1 | LINE |
| 49468957 | 49469227 | + | 7.1 | 5565 | 5833 | -469 | L1PA5 | L1 | LINE |
| 49469291 | 49469505 | - | 26.6 | 4733 | 4947 | -1199 | L1M5 | L1 | LINE |

  

|  |  |  |  |  |  |  |  |  |  |
| --- | --- | --- | --- | --- | --- | --- | --- | --- | --- |
| chr7:50907402-50914793 | | | 7345 bp | | |  |  |  |  |
| Genome Start | Genome End | Strand | Div | Repeat Start | Repeat End | Repeat Left | Name | Family | Class |
| 50907402 | 50911063 | + | 9.1 | 8 | 3688 | -2643 | L1PA10 | L1 | LINE |
| 50911087 | 50912074 | + | 8.0 | 3689 | 4680 | -1651 | L1PA10 | L1 | LINE |
| 50912072 | 50912132 | - | 1.6 | 5136 | 5196 | -950 | L1PA3 | L1 | LINE |
| 50912133 | 50913085 | + | 3.3 | 5187 | 6139 | -16 | L1PA3 | L1 | LINE |
| 50913113 | 50914793 | + | 7.2 | 4665 | 6353 | 0 | L1PA10 | L1 | LINE |

  

|  |  |  |  |  |  |  |  |  |  |
| --- | --- | --- | --- | --- | --- | --- | --- | --- | --- |
| chr7:51125375-51134934 | | | 9331 bp | | |  |  |  |  |
| Genome Start | Genome End | Strand | Div | Repeat Start | Repeat End | Repeat Left | Name | Family | Class |
| 51125375 | 51125975 | + | 33.0 | 1597 | 2104 | -5547 | L1MB2 | L1 | LINE |
| 51126189 | 51127117 | + | 22.3 | 1961 | 2965 | -4764 | L1MB2 | L1 | LINE |
| 51127119 | 51127147 | + | 16.4 | 1598 | 1622 | -4762 | L1M4c | L1 | LINE |
| 51127148 | 51127485 | + | 16.3 | 1 | 339 | 0 | MER1B | MER1\_type | DNA |
| 51127486 | 51127519 | + | 16.4 | 1623 | 1652 | -4732 | L1M4c | L1 | LINE |
| 51127515 | 51128194 | - | 16.3 | 4420 | 5103 | -1040 | L1MA6 | L1 | LINE |
| 51128195 | 51128800 | + | 19.9 | 5112 | 5718 | -582 | L1MA6 | L1 | LINE |
| 51128801 | 51129103 | + | 20.3 | 1 | 301 | -11 | AluJo | Alu | SINE |
| 51129104 | 51129710 | + | 20.3 | 5719 | 6299 | -1 | L1MA6 | L1 | LINE |
| 51129705 | 51130227 | + | 25.3 | 2885 | 3432 | -4137 | L1MB2 | L1 | LINE |
| 51130228 | 51130597 | - | 11.0 | 1 | 364 | 0 | THE1B | MaLR | LTR |
| 51130598 | 51131237 | + | 19.1 | 3433 | 4101 | -3458 | L1MB2 | L1 | LINE |
| 51131264 | 51132932 | + | 17.7 | 4102 | 5845 | -1714 | L1MB2 | L1 | LINE |
| 51132933 | 51133233 | + | 7.0 | 1 | 300 | -11 | AluY | Alu | SINE |
| 51133234 | 51134934 | + | 17.8 | 5846 | 7583 | -7 | L1MB2 | L1 | LINE |

  

|  |  |  |  |  |  |  |  |  |  |
| --- | --- | --- | --- | --- | --- | --- | --- | --- | --- |
| chr7:51704211-51713708 | | | 9524 bp | | |  |  |  |  |
| Genome Start | Genome End | Strand | Div | Repeat Start | Repeat End | Repeat Left | Name | Family | Class |
| 51704211 | 51704944 | + | 17.5 | 3266 | 3976 | -2164 | L1MA9 | L1 | LINE |
| 51704951 | 51706327 | - | 4.6 | 1032 | 2411 | -3735 | L1PA4 | L1 | LINE |
| 51706328 | 51710190 | + | 4.6 | 2417 | 6155 | 0 | L1PA4 | L1 | LINE |
| 51710161 | 51710540 | + | 19.5 | 3946 | 4328 | -1812 | L1MA9 | L1 | LINE |
| 51710537 | 51711617 | - | 9.2 | 5067 | 6163 | 0 | L1PA13 | L1 | LINE |
| 51711620 | 51713362 | + | 17.2 | 4318 | 6155 | -157 | L1MA9 | L1 | LINE |
| 51713363 | 51713582 | + | 6.4 | 5932 | 6152 | -2 | L1PA6 | L1 | LINE |
| 51713583 | 51713708 | + | 16.4 | 6156 | 6282 | -30 | L1MA9 | L1 | LINE |

  

|  |  |  |  |  |  |  |  |  |  |
| --- | --- | --- | --- | --- | --- | --- | --- | --- | --- |
| chr7:52254601-52255283 | | | 658 bp | | |  |  |  |  |
| Genome Start | Genome End | Strand | Div | Repeat Start | Repeat End | Repeat Left | Name | Family | Class |
| 52254601 | 52254735 | + | 23.3 | 56 | 213 | -196 | MLT1I | MaLR | LTR |
| 52254751 | 52254981 | - | 8.2 | 5934 | 6168 | -4 | L1PA8 | L1 | LINE |
| 52254982 | 52255132 | + | 9.9 | 5777 | 5927 | -245 | L1PA8 | L1 | LINE |
| 52255143 | 52255283 | + | 31.4 | 228 | 370 | -39 | MLT1I | MaLR | LTR |

  

|  |  |  |  |  |  |  |  |  |  |
| --- | --- | --- | --- | --- | --- | --- | --- | --- | --- |
| chr7:52927564-52931838 | | | 4266 bp | | |  |  |  |  |
| Genome Start | Genome End | Strand | Div | Repeat Start | Repeat End | Repeat Left | Name | Family | Class |
| 52927564 | 52927769 | + | 18.5 | 1 | 211 | -1020 | Tigger3b | MER2\_type | DNA |
| 52927770 | 52929248 | - | 2.2 | 3379 | 4859 | -1287 | L1PA3 | L1 | LINE |
| 52929227 | 52930521 | + | 2.5 | 4839 | 6137 | -18 | L1PA3 | L1 | LINE |
| 52930553 | 52930889 | + | 13.1 | 202 | 578 | -653 | Tigger3b | MER2\_type | DNA |
| 52930890 | 52931188 | + | 11.2 | 1 | 295 | -16 | AluY | Alu | SINE |
| 52931189 | 52931838 | + | 13.1 | 579 | 1231 | 0 | Tigger3b | MER2\_type | DNA |

  

|  |  |  |  |  |  |  |  |  |  |
| --- | --- | --- | --- | --- | --- | --- | --- | --- | --- |
| chr7:54260843-54272792 | | | 11898 bp | | |  |  |  |  |
| Genome Start | Genome End | Strand | Div | Repeat Start | Repeat End | Repeat Left | Name | Family | Class |
| 54260843 | 54265290 | + | 21.4 | 3 | 3705 | -3154 | L1PB3 | L1 | LINE |
| 54265322 | 54267682 | - | 3.9 | 3789 | 6150 | -5 | L1PA4 | L1 | LINE |
| 54267681 | 54269209 | + | 3.7 | 2267 | 3794 | -2352 | L1PA4 | L1 | LINE |
| 54269202 | 54270603 | + | 15.8 | 3706 | 5115 | -1747 | L1PB3 | L1 | LINE |
| 54270635 | 54272215 | + | 15.4 | 5116 | 6701 | -169 | L1PB3 | L1 | LINE |
| 54272216 | 54272634 | + | 14.6 | 1 | 428 | 0 | MSTA | MaLR | LTR |
| 54272635 | 54272792 | + | 15.0 | 6702 | 6857 | -13 | L1PB3 | L1 | LINE |

  

|  |  |  |  |  |  |  |  |  |  |
| --- | --- | --- | --- | --- | --- | --- | --- | --- | --- |
| chr7:64442124-64444224 | | | 2028 bp | | |  |  |  |  |
| Genome Start | Genome End | Strand | Div | Repeat Start | Repeat End | Repeat Left | Name | Family | Class |
| 64442124 | 64442320 | - | 25.4 | 2454 | 2651 | -3725 | L1M4c | L1 | LINE |
| 64442327 | 64442386 | - | 5.0 | 242 | 301 | -9 | Alu | Alu | SINE |
| 64442391 | 64442937 | - | 1.6 | 5646 | 6191 | 0 | L1PA2 | L1 | LINE |
| 64442938 | 64443564 | + | 1.3 | 5020 | 5646 | -509 | L1PA2 | L1 | LINE |
| 64443567 | 64443952 | - | 26.5 | 2039 | 2450 | -3934 | L1M4c | L1 | LINE |
| 64444014 | 64444224 | - | 33.5 | 1648 | 1856 | -4528 | L1M4c | L1 | LINE |

  

|  |  |  |  |  |  |  |  |  |  |
| --- | --- | --- | --- | --- | --- | --- | --- | --- | --- |
| chr7:65094800-65098481 | | | 3592 bp | | |  |  |  |  |
| Genome Start | Genome End | Strand | Div | Repeat Start | Repeat End | Repeat Left | Name | Family | Class |
| 65094800 | 65094981 | + | 10.6 | 1 | 186 | -307 | LTR15 | ERV1 | LTR |
| 65095000 | 65095245 | - | 20.5 | 18 | 265 | -78 | MER61A | ERV1 | LTR |
| 65095253 | 65095335 | - | 19.3 | 2 | 90 | -222 | AluJ/FLAM | Alu | SINE |
| 65095401 | 65095833 | - | 3.0 | 5706 | 6139 | -15 | L1PA5 | L1 | LINE |
| 65095834 | 65097853 | + | 4.4 | 3705 | 5724 | -430 | L1PA5 | L1 | LINE |
| 65097854 | 65098043 | + | 10.9 | 172 | 347 | -146 | LTR15 | ERV1 | LTR |
| 65098044 | 65098334 | - | 10.3 | 6 | 297 | -15 | AluSx | Alu | SINE |
| 65098335 | 65098481 | + | 12.1 | 348 | 675 | 0 | LTR15 | ERV1 | LTR |

  

|  |  |  |  |  |  |  |  |  |  |
| --- | --- | --- | --- | --- | --- | --- | --- | --- | --- |
| chr7:69431703-69437275 | | | 5576 bp | | |  |  |  |  |
| Genome Start | Genome End | Strand | Div | Repeat Start | Repeat End | Repeat Left | Name | Family | Class |
| 69431703 | 69432693 | - | 19.7 | 5148 | 6165 | -9 | L1MB5 | L1 | LINE |
| 69432694 | 69433053 | - | 20.1 | 1 | 407 | -1 | MLT1A1 | MaLR | LTR |
| 69433054 | 69433883 | - | 18.2 | 4319 | 5147 | -999 | L1MB5 | L1 | LINE |
| 69433882 | 69434497 | - | 6.9 | 3134 | 3740 | -2406 | L1PA7 | L1 | LINE |
| 69434498 | 69436891 | + | 7.3 | 3745 | 6154 | 0 | L1PA7 | L1 | LINE |
| 69436891 | 69437275 | - | 18.8 | 3887 | 4334 | -1812 | L1MB5 | L1 | LINE |

  

|  |  |  |  |  |  |  |  |  |  |
| --- | --- | --- | --- | --- | --- | --- | --- | --- | --- |
| chr7:75172254-75177845 | | | 5590 bp | | |  |  |  |  |
| Genome Start | Genome End | Strand | Div | Repeat Start | Repeat End | Repeat Left | Name | Family | Class |
| 75172254 | 75172615 | - | 13.5 | 5824 | 6180 | -3 | L1MB3 | L1 | LINE |
| 75172616 | 75172908 | - | 9.6 | 1 | 306 | -7 | AluSq | Alu | SINE |
| 75172909 | 75173133 | - | 13.5 | 5608 | 5823 | -360 | L1MB3 | L1 | LINE |
| 75173134 | 75173446 | - | 11.3 | 3 | 312 | 0 | AluSx | Alu | SINE |
| 75173447 | 75173730 | - | 13.5 | 5353 | 5607 | -576 | L1MB3 | L1 | LINE |
| 75173731 | 75173880 | - | 7.1 | 6041 | 6190 | 0 | L1PA6 | L1 | LINE |
| 75173886 | 75174857 | - | 0.4 | 5184 | 6155 | 0 | L1HS | L1 | LINE |
| 75174856 | 75175402 | + | 0.4 | 4643 | 5189 | -957 | L1HS | L1 | LINE |
| 75175402 | 75176359 | - | 17.6 | 4410 | 5358 | -782 | L1MB3 | L1 | LINE |
| 75176360 | 75176666 | - | 11.4 | 1 | 313 | 0 | AluSq | Alu | SINE |
| 75176667 | 75177131 | - | 17.6 | 3953 | 4409 | -1731 | L1MB3 | L1 | LINE |
| 75177132 | 75177450 | - | 13.3 | 2 | 303 | -9 | AluJb | Alu | SINE |
| 75177451 | 75177845 | - | 17.6 | 3561 | 3952 | -2188 | L1MB3 | L1 | LINE |

  

|  |  |  |  |  |  |  |  |  |  |
| --- | --- | --- | --- | --- | --- | --- | --- | --- | --- |
| chr7:76606969-76618870 | | | 11699 bp | | |  |  |  |  |
| Genome Start | Genome End | Strand | Div | Repeat Start | Repeat End | Repeat Left | Name | Family | Class |
| 76606969 | 76607140 | + | 24.6 | 4178 | 4326 | -1820 | L1ME1 | L1 | LINE |
| 76607141 | 76607447 | - | 9.1 | 1 | 312 | 0 | AluSx | Alu | SINE |
| 76607448 | 76607568 | + | 24.6 | 4327 | 4450 | -1696 | L1ME1 | L1 | LINE |
| 76607569 | 76607934 | - | 20.0 | 1 | 374 | 0 | MLT1A | MaLR | LTR |
| 76607935 | 76608048 | + | 24.6 | 4451 | 4563 | -1583 | L1ME1 | L1 | LINE |
| 76608176 | 76609302 | - | 6.1 | 5019 | 6151 | -3 | L1PA7 | L1 | LINE |
| 76609298 | 76609553 | + | 5.9 | 3968 | 4223 | -1923 | L1P3 | L1 | LINE |
| 76609559 | 76609667 | - | 0.0 | 6071 | 6179 | 0 | L1PA5 | L1 | LINE |
| 76609700 | 76615811 | - | 6.4 | 1 | 6120 | -34 | L1PA5 | L1 | LINE |
| 76615812 | 76616597 | + | 8.9 | 4220 | 5007 | -1139 | L1P3 | L1 | LINE |
| 76616616 | 76616736 | + | 23.1 | 4623 | 4747 | -1399 | L1ME1 | L1 | LINE |
| 76616737 | 76617037 | + | 14.7 | 1 | 302 | -10 | AluSx | Alu | SINE |
| 76617064 | 76618215 | + | 23.2 | 4748 | 5909 | -257 | L1ME1 | L1 | LINE |
| 76618216 | 76618568 | - | 22.0 | 1 | 365 | 0 | MLT1A0 | MaLR | LTR |
| 76618569 | 76618870 | + | 23.3 | 5910 | 6161 | -5 | L1ME1 | L1 | LINE |

  

|  |  |  |  |  |  |  |  |  |  |
| --- | --- | --- | --- | --- | --- | --- | --- | --- | --- |
| chr7:79349275-79356365 | | | 6556 bp | | |  |  |  |  |
| Genome Start | Genome End | Strand | Div | Repeat Start | Repeat End | Repeat Left | Name | Family | Class |
| 79349275 | 79349607 | - | 25.1 | 5908 | 6235 | -4 | L1ME1 | L1 | LINE |
| 79349612 | 79350077 | + | 23.6 | 6 | 462 | 0 | LTR40b | ERVL | LTR |
| 79350083 | 79351465 | - | 26.4 | 4428 | 5896 | -343 | L1ME1 | L1 | LINE |
| 79351481 | 79351676 | + | 17.9 | 6093 | 6288 | -12 | L1MA6 | L1 | LINE |
| 79352014 | 79352151 | - | 18.0 | 2200 | 2341 | -4059 | L1ME1 | L1 | LINE |
| 79352152 | 79352695 | - | 2.4 | 5612 | 6155 | 0 | L1PA2 | L1 | LINE |
| 79352698 | 79354306 | + | 1.8 | 4002 | 5610 | -545 | L1PA2 | L1 | LINE |
| 79354417 | 79354751 | - | 23.9 | 1672 | 2036 | -4364 | L1ME1 | L1 | LINE |
| 79354781 | 79355443 | - | 23.8 | 787 | 1511 | -4889 | L1ME1 | L1 | LINE |
| 79355471 | 79355694 | + | 15.9 | 15 | 188 | -158 | MER7A | MER2\_type | DNA |
| 79355701 | 79356365 | - | 26.1 | 76 | 773 | -5627 | L1ME1 | L1 | LINE |

  

|  |  |  |  |  |  |  |  |  |  |
| --- | --- | --- | --- | --- | --- | --- | --- | --- | --- |
| chr7:79804705-79806180 | | | 1453 bp | | |  |  |  |  |
| Genome Start | Genome End | Strand | Div | Repeat Start | Repeat End | Repeat Left | Name | Family | Class |
| 79804705 | 79804908 | + | 30.2 | 1 | 215 | -306 | LTR33 | ERVL | LTR |
| 79804930 | 79805799 | - | 6.2 | 5285 | 6152 | -2 | L1PA6 | L1 | LINE |
| 79805793 | 79805921 | + | 8.5 | 5151 | 5279 | -867 | L1PA6 | L1 | LINE |
| 79805931 | 79806180 | + | 24.4 | 227 | 498 | -23 | LTR33 | ERVL | LTR |

  

|  |  |  |  |  |  |  |  |  |  |
| --- | --- | --- | --- | --- | --- | --- | --- | --- | --- |
| chr7:81867172-81869231 | | | 2000 bp | | |  |  |  |  |
| Genome Start | Genome End | Strand | Div | Repeat Start | Repeat End | Repeat Left | Name | Family | Class |
| 81867172 | 81867240 | - | 31.9 | 3350 | 3419 | 0 | L2 | L2 | LINE |
| 81867248 | 81867688 | - | 1.1 | 4419 | 4859 | -1287 | L1PA3 | L1 | LINE |
| 81867689 | 81868971 | + | 2.3 | 4873 | 6155 | 0 | L1PA3 | L1 | LINE |
| 81869025 | 81869231 | - | 32.0 | 3155 | 3367 | -52 | L2 | L2 | LINE |

  

|  |  |  |  |  |  |  |  |  |  |
| --- | --- | --- | --- | --- | --- | --- | --- | --- | --- |
| chr7:86013092-86026422 | | | 13179 bp | | |  |  |  |  |
| Genome Start | Genome End | Strand | Div | Repeat Start | Repeat End | Repeat Left | Name | Family | Class |
| 86013092 | 86013718 | + | 25.4 | 1689 | 2395 | -4005 | L1ME1 | L1 | LINE |
| 86013717 | 86015663 | - | 8.0 | 3 | 1956 | -319 | L1P2 | L1 | LINE |
| 86015669 | 86017699 | + | 7.6 | 1972 | 4249 | -1897 | L1P2 | L1 | LINE |
| 86017698 | 86018103 | - | 3.2 | 1497 | 1903 | -4252 | L1PA6 | L1 | LINE |
| 86018103 | 86022347 | + | 4.6 | 1910 | 6166 | 0 | L1PA6 | L1 | LINE |
| 86022420 | 86024080 | + | 27.8 | 2300 | 4157 | -2062 | L1ME1 | L1 | LINE |
| 86024081 | 86024361 | + | 11.8 | 1 | 280 | -33 | AluSq | Alu | SINE |
| 86024405 | 86025562 | + | 27.8 | 4158 | 5341 | -878 | L1ME1 | L1 | LINE |
| 86025600 | 86026422 | + | 26.9 | 5342 | 6234 | -5 | L1ME1 | L1 | LINE |

  

|  |  |  |  |  |  |  |  |  |  |
| --- | --- | --- | --- | --- | --- | --- | --- | --- | --- |
| chr7:86079926-86088462 | | | 8070 bp | | |  |  |  |  |
| Genome Start | Genome End | Strand | Div | Repeat Start | Repeat End | Repeat Left | Name | Family | Class |
| 86079926 | 86080165 | + | 33.3 | 744 | 1000 | -1507 | HAL1 | L1 | LINE |
| 86080318 | 86080540 | + | 35.1 | 249 | 473 | -5418 | L1M3d | L1 | LINE |
| 86080547 | 86080697 | - | 19.2 | 4159 | 4324 | -1819 | L1M2 | L1 | LINE |
| 86080699 | 86081009 | + | 18.1 | 4329 | 4649 | -1491 | L1MB1 | L1 | LINE |
| 86081020 | 86081393 | + | 10.2 | 1 | 374 | -1 | THE1C | MaLR | LTR |
| 86081395 | 86081513 | + | 19.8 | 6 | 138 | -227 | MLT1A0 | MaLR | LTR |
| 86081514 | 86081878 | + | 12.5 | 1 | 381 | 0 | THE1D | MaLR | LTR |
| 86081879 | 86082102 | + | 19.8 | 139 | 365 | 0 | MLT1A0 | MaLR | LTR |
| 86082103 | 86082956 | + | 18.7 | 4645 | 5534 | -634 | L1MB1 | L1 | LINE |
| 86082958 | 86083331 | + | 11.1 | 1 | 373 | -2 | THE1C | MaLR | LTR |
| 86083334 | 86084872 | + | 13.7 | 1 | 1580 | 0 | THE1C-int | MaLR | LTR |
| 86084873 | 86085241 | + | 11.9 | 1 | 385 | -5 | THE1C | MaLR | LTR |
| 86085242 | 86085662 | + | 17.6 | 5579 | 6017 | -151 | L1MB1 | L1 | LINE |
| 86085680 | 86086094 | - | 14.7 | 5890 | 6290 | -1 | L1MA8 | L1 | LINE |
| 86086096 | 86086286 | - | 19.7 | 1 | 187 | -125 | AluJb | Alu | SINE |
| 86086295 | 86087125 | - | 14.8 | 5053 | 5881 | -410 | L1MA8 | L1 | LINE |
| 86087126 | 86087460 | - | 19.7 | 1 | 306 | -6 | AluJb | Alu | SINE |
| 86087461 | 86087631 | - | 16.0 | 4882 | 5052 | -1091 | L1MA8 | L1 | LINE |
| 86087632 | 86087796 | + | 15.2 | 5998 | 6166 | -2 | L1MB1 | L1 | LINE |
| 86088065 | 86088462 | + | 28.3 | 1386 | 1779 | -728 | HAL1 | L1 | LINE |

  

|  |  |  |  |  |  |  |  |  |  |
| --- | --- | --- | --- | --- | --- | --- | --- | --- | --- |
| chr7:88411763-88415175 | | | 3413 bp | | |  |  |  |  |
| Genome Start | Genome End | Strand | Div | Repeat Start | Repeat End | Repeat Left | Name | Family | Class |
| 88411763 | 88411976 | + | 21.6 | 14 | 249 | -96 | MER2 | MER2\_type | DNA |
| 88411978 | 88412182 | + | 15.7 | 2 | 103 | -133 | MER46B | MER2\_type | DNA |
| 88412183 | 88414087 | - | 8.4 | 3416 | 5314 | -832 | L1PA7 | L1 | LINE |
| 88414087 | 88414933 | + | 7.3 | 5315 | 6154 | 0 | L1PA7 | L1 | LINE |
| 88414934 | 88415078 | + | 12.5 | 2587 | 2730 | -1 | Tigger4(Zombi) | MER2\_type | DNA |
| 88415079 | 88415175 | + | 21.6 | 247 | 345 | 0 | MER2 | MER2\_type | DNA |

  

|  |  |  |  |  |  |  |  |  |  |
| --- | --- | --- | --- | --- | --- | --- | --- | --- | --- |
| chr7:88760262-88761482 | | | 963 bp | | |  |  |  |  |
| Genome Start | Genome End | Strand | Div | Repeat Start | Repeat End | Repeat Left | Name | Family | Class |
| 88760262 | 88760453 | - | 26.3 | 2737 | 2954 | -4007 | L1MCc | L1 | LINE |
| 88760459 | 88760510 | - | 21.1 | 6092 | 6143 | -157 | L1MA6 | L1 | LINE |
| 88760511 | 88760660 | + | 11.5 | 6140 | 6291 | 0 | L1MA7 | L1 | LINE |
| 88760753 | 88761062 | + | 7.5 | 1 | 305 | -6 | AluY | Alu | SINE |
| 88761224 | 88761482 | - | 33.2 | 25 | 284 | -6139 | L1MCc | L1 | LINE |

  

|  |  |  |  |  |  |  |  |  |  |
| --- | --- | --- | --- | --- | --- | --- | --- | --- | --- |
| chr7:88826783-88829237 | | | 2388 bp | | |  |  |  |  |
| Genome Start | Genome End | Strand | Div | Repeat Start | Repeat End | Repeat Left | Name | Family | Class |
| 88826783 | 88826981 | + | 34.0 | 3027 | 3242 | -177 | L2 | L2 | LINE |
| 88826988 | 88828609 | - | 4.8 | 4541 | 6155 | 0 | L1PA4 | L1 | LINE |
| 88828604 | 88829118 | + | 4.7 | 4026 | 4546 | -1600 | L1PA4 | L1 | LINE |
| 88829186 | 88829237 | + | 26.9 | 3335 | 3386 | -33 | L2 | L2 | LINE |

  

|  |  |  |  |  |  |  |  |  |  |
| --- | --- | --- | --- | --- | --- | --- | --- | --- | --- |
| chr7:88908140-88910644 | | | 2509 bp | | |  |  |  |  |
| Genome Start | Genome End | Strand | Div | Repeat Start | Repeat End | Repeat Left | Name | Family | Class |
| 88908140 | 88908249 | + | 18.2 | 8 | 113 | -96 | MER3 | MER1\_type | DNA |
| 88908250 | 88908558 | + | 0.3 | 1 | 309 | -1 | AluYa5 | Alu | SINE |
| 88908559 | 88908617 | + | 18.2 | 114 | 155 | -54 | MER3 | MER1\_type | DNA |
| 88908618 | 88909640 | - | 3.6 | 5118 | 6138 | -17 | L1PA4 | L1 | LINE |
| 88909637 | 88910569 | + | 4.4 | 4170 | 5106 | -1040 | L1PA4 | L1 | LINE |
| 88910570 | 88910644 | + | 18.9 | 135 | 209 | 0 | MER3 | MER1\_type | DNA |

  

|  |  |  |  |  |  |  |  |  |  |
| --- | --- | --- | --- | --- | --- | --- | --- | --- | --- |
| chr7:88988758-88993537 | | | 4779 bp | | |  |  |  |  |
| Genome Start | Genome End | Strand | Div | Repeat Start | Repeat End | Repeat Left | Name | Family | Class |
| 88988758 | 88989211 | - | 18.6 | 44 | 482 | -32 | MER67D | ERV1 | LTR |
| 88989212 | 88991009 | - | 6.1 | 1890 | 3673 | -2473 | L1PA4 | L1 | LINE |
| 88991011 | 88993485 | + | 5.0 | 3678 | 6155 | 0 | L1PA4 | L1 | LINE |
| 88993486 | 88993537 | - | 15.4 | 1 | 57 | -457 | MER67D | ERV1 | LTR |

  

|  |  |  |  |  |  |  |  |  |  |
| --- | --- | --- | --- | --- | --- | --- | --- | --- | --- |
| chr7:89134871-89146258 | | | 11358 bp | | |  |  |  |  |
| Genome Start | Genome End | Strand | Div | Repeat Start | Repeat End | Repeat Left | Name | Family | Class |
| 89134871 | 89136188 | + | 13.6 | 1326 | 2656 | -4129 | L1PA13 | L1 | LINE |
| 89136223 | 89140509 | - | 8.7 | 2473 | 6763 | -11 | L1PA7 | L1 | LINE |
| 89140510 | 89140808 | + | 15.9 | 1 | 296 | -13 | AluSc | Alu | SINE |
| 89140809 | 89141289 | - | 9.1 | 1991 | 2472 | -423 | L1PA7 | L1 | LINE |
| 89141289 | 89142148 | + | 7.6 | 1112 | 1981 | -4785 | L1PA7 | L1 | LINE |
| 89142146 | 89146258 | + | 13.2 | 2648 | 6780 | -3 | L1PA13 | L1 | LINE |

  

|  |  |  |  |  |  |  |  |  |  |
| --- | --- | --- | --- | --- | --- | --- | --- | --- | --- |
| chr7:89244794-89255024 | | | 9836 bp | | |  |  |  |  |
| Genome Start | Genome End | Strand | Div | Repeat Start | Repeat End | Repeat Left | Name | Family | Class |
| 89244794 | 89246168 | - | 19.6 | 6171 | 7677 | -176 | L1MC3 | L1 | LINE |
| 89246169 | 89246468 | - | 10.8 | 1 | 298 | -15 | AluSq | Alu | SINE |
| 89246469 | 89247063 | - | 19.6 | 5677 | 6170 | -1683 | L1MC3 | L1 | LINE |
| 89247064 | 89247992 | + | 19.3 | 1 | 983 | 0 | LTR1D | ERV1 | LTR |
| 89247993 | 89249499 | - | 22.8 | 4106 | 5676 | -2177 | L1MC3 | L1 | LINE |
| 89249489 | 89249576 | - | 23.3 | 3710 | 3798 | -2342 | L1M3 | L1 | LINE |
| 89249686 | 89251755 | - | 13.5 | 2213 | 4244 | -1902 | L1P4 | L1 | LINE |
| 89251756 | 89253168 | + | 11.4 | 4228 | 5682 | -479 | L1P4 | L1 | LINE |
| 89253367 | 89253930 | - | 31.4 | 2736 | 3350 | -2864 | L1MC3 | L1 | LINE |
| 89254030 | 89255024 | - | 28.0 | 1563 | 2527 | -3688 | L1MC3 | L1 | LINE |

  

|  |  |  |  |  |  |  |  |  |  |
| --- | --- | --- | --- | --- | --- | --- | --- | --- | --- |
| chr7:89508589-89518146 | | | 9241 bp | | |  |  |  |  |
| Genome Start | Genome End | Strand | Div | Repeat Start | Repeat End | Repeat Left | Name | Family | Class |
| 89508589 | 89509020 | + | 27.2 | 1 | 453 | -52 | MLT1D | MaLR | LTR |
| 89509032 | 89509083 | - | 13.5 | 6351 | 6403 | -1 | L1MB1 | L1 | LINE |
| 89509090 | 89509289 | - | 4.0 | 5950 | 6153 | -2 | L1PA4 | L1 | LINE |
| 89509290 | 89509427 | + | 2.9 | 5822 | 5959 | -196 | L1PA4 | L1 | LINE |
| 89509431 | 89510041 | - | 19.8 | 5730 | 6356 | -48 | L1MB1 | L1 | LINE |
| 89510088 | 89510586 | - | 18.4 | 5073 | 5593 | -783 | L1MB1 | L1 | LINE |
| 89510587 | 89511104 | + | 16.8 | 1 | 518 | -1900 | Tigger1 | MER2\_type | DNA |
| 89511106 | 89512676 | - | 18.7 | 2877 | 4445 | -1931 | L1MB1 | L1 | LINE |
| 89512829 | 89513178 | - | 21.4 | 2512 | 2861 | -3515 | L1MB1 | L1 | LINE |
| 89513193 | 89515331 | + | 16.7 | 3464 | 5606 | -706 | L1M2 | L1 | LINE |
| 89515338 | 89515537 | + | 25.5 | 1 | 210 | -6143 | L1PA10 | L1 | LINE |
| 89515545 | 89515898 | + | 14.2 | 3 | 364 | 0 | THE1B | MaLR | LTR |
| 89515899 | 89515995 | + | 13.4 | 6187 | 6290 | -10 | L1MA4 | L1 | LINE |
| 89516088 | 89516147 | - | 9.9 | 2157 | 2214 | -3926 | L1M3 | L1 | LINE |
| 89516148 | 89516498 | - | 11.7 | 1 | 355 | 0 | THE1A | MaLR | LTR |
| 89516499 | 89516673 | - | 21.5 | 2216 | 2392 | -3992 | L1MB1 | L1 | LINE |
| 89516649 | 89518077 | - | 23.5 | 414 | 1787 | -4921 | L1MB1 | L1 | LINE |
| 89518082 | 89518146 | + | 15.4 | 440 | 504 | -1 | MLT1D | MaLR | LTR |

  

|  |  |  |  |  |  |  |  |  |  |
| --- | --- | --- | --- | --- | --- | --- | --- | --- | --- |
| chr7:89846838-89849874 | | | 2955 bp | | |  |  |  |  |
| Genome Start | Genome End | Strand | Div | Repeat Start | Repeat End | Repeat Left | Name | Family | Class |
| 89846838 | 89847319 | - | 19.0 | 5254 | 5736 | -448 | L1M4 | L1 | LINE |
| 89847320 | 89847627 | - | 11.0 | 1 | 309 | -3 | AluSx | Alu | SINE |
| 89847628 | 89847737 | - | 20.4 | 5164 | 5257 | -889 | L1M4 | L1 | LINE |
| 89847757 | 89848727 | - | 13.9 | 5308 | 6311 | -1 | L1MA9 | L1 | LINE |
| 89848712 | 89849469 | + | 14.8 | 4540 | 5302 | -841 | L1MA9 | L1 | LINE |
| 89849542 | 89849667 | - | 16.7 | 1 | 126 | -17 | FLAM\_C | Alu | SINE |
| 89849675 | 89849789 | - | 21.4 | 49 | 163 | -13 | FRAM | Alu | SINE |
| 89849790 | 89849874 | - | 17.6 | 5048 | 5139 | -1007 | L1M4 | L1 | LINE |

  

|  |  |  |  |  |  |  |  |  |  |
| --- | --- | --- | --- | --- | --- | --- | --- | --- | --- |
| chr7:91082525-91085874 | | | 3301 bp | | |  |  |  |  |
| Genome Start | Genome End | Strand | Div | Repeat Start | Repeat End | Repeat Left | Name | Family | Class |
| 91082525 | 91082683 | - | 24.4 | 5880 | 6062 | -178 | L1ME3B | L1 | LINE |
| 91082689 | 91083296 | - | 14.2 | 5671 | 6287 | -4 | L1MA7 | L1 | LINE |
| 91083297 | 91083642 | + | 11.6 | 5322 | 5673 | -618 | L1MA7 | L1 | LINE |
| 91083640 | 91084062 | - | 29.7 | 5485 | 5894 | -346 | L1ME3B | L1 | LINE |
| 91084095 | 91084189 | - | 21.1 | 6194 | 6290 | -22 | L1MA9 | L1 | LINE |
| 91084189 | 91084723 | - | 27.1 | 4921 | 5482 | -758 | L1ME3B | L1 | LINE |
| 91084724 | 91084830 | + | 11.9 | 1 | 103 | -90 | MER53 | DNA | DNA |
| 91084831 | 91085145 | - | 6.5 | 1 | 309 | -2 | AluY | Alu | SINE |
| 91085146 | 91085242 | + | 11.9 | 104 | 190 | -3 | MER53 | DNA | DNA |
| 91085243 | 91085437 | - | 29.0 | 4698 | 4920 | -1226 | L1ME3B | L1 | LINE |
| 91085454 | 91085874 | - | 33.0 | 3685 | 4130 | -2016 | L1ME3B | L1 | LINE |

  

|  |  |  |  |  |  |  |  |  |  |
| --- | --- | --- | --- | --- | --- | --- | --- | --- | --- |
| chr7:91306391-91311010 | | | 4123 bp | | |  |  |  |  |
| Genome Start | Genome End | Strand | Div | Repeat Start | Repeat End | Repeat Left | Name | Family | Class |
| 91306391 | 91306679 | + | 31.9 | 2197 | 2492 | -927 | L2 | L2 | LINE |
| 91306692 | 91307640 | - | 3.0 | 5201 | 6154 | -1 | L1PA4 | L1 | LINE |
| 91307638 | 91308483 | + | 3.2 | 4361 | 5213 | -933 | L1PA4 | L1 | LINE |
| 91308497 | 91308582 | + | 33.7 | 2626 | 2713 | -706 | L2 | L2 | LINE |
| 91308840 | 91308891 | + | 25.0 | 3026 | 3079 | -340 | L2 | L2 | LINE |
| 91309053 | 91309284 | - | 24.1 | 5942 | 6184 | 0 | L1ME1 | L1 | LINE |
| 91309285 | 91310024 | + | 18.1 | 1 | 783 | -22 | LTR1D | ERV1 | LTR |
| 91310037 | 91310825 | - | 20.3 | 5126 | 5946 | -220 | L1ME1 | L1 | LINE |
| 91310871 | 91311010 | + | 29.4 | 3286 | 3416 | -3 | L2 | L2 | LINE |

  

|  |  |  |  |  |  |  |  |  |  |
| --- | --- | --- | --- | --- | --- | --- | --- | --- | --- |
| chr7:92880779-92884514 | | | 3711 bp | | |  |  |  |  |
| Genome Start | Genome End | Strand | Div | Repeat Start | Repeat End | Repeat Left | Name | Family | Class |
| 92880779 | 92881297 | - | 16.5 | 5662 | 6167 | -1 | L1MB1 | L1 | LINE |
| 92881327 | 92882587 | - | 13.6 | 4958 | 6285 | -6 | L1MA8 | L1 | LINE |
| 92882593 | 92883210 | + | 17.4 | 4380 | 4965 | -1178 | L1MA8 | L1 | LINE |
| 92883202 | 92884514 | - | 15.2 | 4342 | 5669 | -499 | L1MB1 | L1 | LINE |

  

|  |  |  |  |  |  |  |  |  |  |
| --- | --- | --- | --- | --- | --- | --- | --- | --- | --- |
| chr7:94544689-94548322 | | | 3633 bp | | |  |  |  |  |
| Genome Start | Genome End | Strand | Div | Repeat Start | Repeat End | Repeat Left | Name | Family | Class |
| 94544689 | 94544792 | - | 19.6 | 323 | 425 | -1 | LTR37A | ERV1 | LTR |
| 94544793 | 94545228 | - | 15.3 | 138 | 585 | 0 | MER34C\_ | ERV1 | LTR |
| 94545229 | 94546640 | - | 2.0 | 4743 | 6155 | 0 | L1PA3 | L1 | LINE |
| 94546633 | 94547938 | + | 2.8 | 3435 | 4741 | -1405 | L1PA3 | L1 | LINE |
| 94547939 | 94548075 | - | 8.4 | 1 | 153 | -432 | MER34C\_ | ERV1 | LTR |
| 94548085 | 94548322 | - | 23.8 | 48 | 317 | -109 | LTR37A | ERV1 | LTR |

  

|  |  |  |  |  |  |  |  |  |  |
| --- | --- | --- | --- | --- | --- | --- | --- | --- | --- |
| chr7:95480025-95481019 | | | 985 bp | | |  |  |  |  |
| Genome Start | Genome End | Strand | Div | Repeat Start | Repeat End | Repeat Left | Name | Family | Class |
| 95480025 | 95480129 | + | 17.1 | 12 | 118 | -387 | MLT1D | MaLR | LTR |
| 95480139 | 95480216 | - | 11.0 | 6079 | 6151 | 0 | L1PB1 | L1 | LINE |
| 95480217 | 95480303 | + | 9.2 | 5990 | 6077 | -74 | L1PB1 | L1 | LINE |
| 95480305 | 95480696 | + | 16.4 | 105 | 479 | -26 | MLT1D | MaLR | LTR |
| 95480697 | 95480986 | - | 12.3 | 22 | 310 | 0 | AluJb | Alu | SINE |
| 95480987 | 95481019 | + | 16.4 | 480 | 504 | -1 | MLT1D | MaLR | LTR |

  

|  |  |  |  |  |  |  |  |  |  |
| --- | --- | --- | --- | --- | --- | --- | --- | --- | --- |
| chr7:96148443-96152176 | | | 3402 bp | | |  |  |  |  |
| Genome Start | Genome End | Strand | Div | Repeat Start | Repeat End | Repeat Left | Name | Family | Class |
| 96148443 | 96148674 | - | 30.8 | 2670 | 2926 | -493 | L2 | L2 | LINE |
| 96148688 | 96148752 | - | 24.6 | 546 | 613 | -2 | MLT1L | MaLR | LTR |
| 96148757 | 96149798 | - | 7.5 | 3988 | 5029 | -1117 | L1P2 | L1 | LINE |
| 96149799 | 96150424 | + | 7.2 | 5019 | 5614 | -540 | L1P2 | L1 | LINE |
| 96150457 | 96151282 | + | 5.5 | 5318 | 6152 | -2 | L1PA7 | L1 | LINE |
| 96151504 | 96151665 | - | 32.3 | 101 | 268 | -347 | MLT1L | MaLR | LTR |
| 96151728 | 96152176 | - | 30.4 | 2244 | 2713 | -706 | L2 | L2 | LINE |

  

|  |  |  |  |  |  |  |  |  |  |
| --- | --- | --- | --- | --- | --- | --- | --- | --- | --- |
| chr7:97176088-97184450 | | | 8426 bp | | |  |  |  |  |
| Genome Start | Genome End | Strand | Div | Repeat Start | Repeat End | Repeat Left | Name | Family | Class |
| 97176088 | 97176302 | + | 19.7 | 2 | 216 | -6647 | L1PB1 | L1 | LINE |
| 97176280 | 97177294 | + | 11.9 | 1392 | 2422 | -4441 | L1PB1 | L1 | LINE |
| 97177292 | 97177898 | - | 8.7 | 2514 | 3120 | -3026 | L1PA6 | L1 | LINE |
| 97177894 | 97178816 | + | 7.8 | 3131 | 4050 | -2096 | L1PA6 | L1 | LINE |
| 97178813 | 97179974 | + | 6.1 | 4980 | 6146 | -8 | L1PA6 | L1 | LINE |
| 97179988 | 97180317 | + | 10.9 | 2411 | 2742 | -4121 | L1PB1 | L1 | LINE |
| 97180321 | 97180660 | + | 8.5 | 2799 | 3094 | -3754 | L1M2 | L1 | LINE |
| 97180617 | 97180698 | + | 6.8 | 228 | 311 | 0 | AluYc3 | Alu | SINE |
| 97180699 | 97184450 | + | 8.1 | 3109 | 6859 | -8 | L1PB1 | L1 | LINE |

  

|  |  |  |  |  |  |  |  |  |  |
| --- | --- | --- | --- | --- | --- | --- | --- | --- | --- |
| chr7:97245021-97251750 | | | 6624 bp | | |  |  |  |  |
| Genome Start | Genome End | Strand | Div | Repeat Start | Repeat End | Repeat Left | Name | Family | Class |
| 97245021 | 97245147 | - | 16.7 | 299 | 426 | 0 | LTR37A | ERV1 | LTR |
| 97245148 | 97246727 | - | 5.8 | 4 | 1579 | -4576 | L1PA3 | L1 | LINE |
| 97246721 | 97251269 | + | 3.7 | 1584 | 6152 | -3 | L1PA3 | L1 | LINE |
| 97251275 | 97251390 | + | 17.5 | 3282 | 3387 | -2759 | L1M4 | L1 | LINE |
| 97251499 | 97251750 | - | 24.5 | 54 | 306 | -120 | LTR37A | ERV1 | LTR |

  

|  |  |  |  |  |  |  |  |  |  |
| --- | --- | --- | --- | --- | --- | --- | --- | --- | --- |
| chr7:97348031-97348981 | | | 936 bp | | |  |  |  |  |
| Genome Start | Genome End | Strand | Div | Repeat Start | Repeat End | Repeat Left | Name | Family | Class |
| 97348031 | 97348157 | + | 20.2 | 5 | 129 | -49 | MER5B | MER1\_type | DNA |
| 97348183 | 97348488 | - | 15.0 | 2 | 306 | 0 | AluSx | Alu | SINE |
| 97348494 | 97348776 | - | 18.7 | 5902 | 6174 | 0 | L1MB5 | L1 | LINE |
| 97348747 | 97348922 | + | 22.7 | 5684 | 5887 | -287 | L1MB5 | L1 | LINE |
| 97348938 | 97348981 | + | 6.8 | 135 | 178 | 0 | MER5B | MER1\_type | DNA |

  

|  |  |  |  |  |  |  |  |  |  |
| --- | --- | --- | --- | --- | --- | --- | --- | --- | --- |
| chr7:98663969-98667153 | | | 3176 bp | | |  |  |  |  |
| Genome Start | Genome End | Strand | Div | Repeat Start | Repeat End | Repeat Left | Name | Family | Class |
| 98663969 | 98664065 | + | 26.8 | 4997 | 5093 | -1053 | L1MB5 | L1 | LINE |
| 98664074 | 98664169 | - | 5.2 | 6060 | 6155 | 0 | L1PA2 | L1 | LINE |
| 98664170 | 98664406 | + | 1.7 | 5842 | 6078 | -77 | L1PA2 | L1 | LINE |
| 98664404 | 98664511 | + | 12.3 | 5080 | 5189 | -957 | L1MB5 | L1 | LINE |
| 98664512 | 98664660 | + | 11.4 | 1 | 149 | -163 | AluSq/x | Alu | SINE |
| 98664665 | 98664956 | + | 17.4 | 15 | 304 | 0 | AluJb | Alu | SINE |
| 98664957 | 98665179 | + | 20.1 | 5325 | 5539 | -635 | L1MB5 | L1 | LINE |
| 98665180 | 98665316 | - | 11.3 | 6030 | 6165 | 0 | L1PA15 | L1 | LINE |
| 98665317 | 98665617 | - | 10.7 | 2 | 300 | -12 | AluSx | Alu | SINE |
| 98665618 | 98665841 | - | 11.3 | 5813 | 6029 | -136 | L1PA15 | L1 | LINE |
| 98665842 | 98666139 | - | 6.1 | 1 | 296 | -15 | AluY | Alu | SINE |
| 98666140 | 98666216 | - | 11.3 | 5729 | 5812 | -353 | L1PA15 | L1 | LINE |
| 98666217 | 98666507 | + | 20.1 | 5540 | 5798 | -376 | L1MB5 | L1 | LINE |
| 98666508 | 98666796 | - | 11.5 | 1 | 299 | -13 | AluSx | Alu | SINE |
| 98666797 | 98667153 | + | 20.1 | 5799 | 6164 | -10 | L1MB5 | L1 | LINE |

  

|  |  |  |  |  |  |  |  |  |  |
| --- | --- | --- | --- | --- | --- | --- | --- | --- | --- |
| chr7:102328952-102338858 | | | 9609 bp | | |  |  |  |  |
| Genome Start | Genome End | Strand | Div | Repeat Start | Repeat End | Repeat Left | Name | Family | Class |
| 102328952 | 102333597 | - | 7.5 | 3268 | 7721 | 0 | L1MA1 | L1 | LINE |
| 102333598 | 102333923 | - | 7.1 | 1 | 355 | 0 | THE1A | MaLR | LTR |
| 102333924 | 102334643 | - | 10.1 | 2570 | 3267 | -4298 | L1MA1 | L1 | LINE |
| 102334644 | 102334870 | - | 11.4 | 86 | 312 | 0 | AluSg/x | Alu | SINE |
| 102334871 | 102335003 | - | 10.1 | 2452 | 2585 | -4980 | L1MA1 | L1 | LINE |
| 102335004 | 102335462 | - | 4.6 | 5696 | 6153 | -1 | L1PA5 | L1 | LINE |
| 102335463 | 102335791 | + | 5.2 | 5358 | 5686 | -468 | L1PA5 | L1 | LINE |
| 102335790 | 102338058 | - | 16.4 | 103 | 2455 | -5110 | L1MA1 | L1 | LINE |
| 102338351 | 102338456 | - | 12.5 | 595 | 703 | -4 | MER39 | ERV1 | LTR |
| 102338457 | 102338743 | - | 17.2 | 1 | 285 | -27 | AluJb | Alu | SINE |
| 102338752 | 102338858 | - | 18.9 | 7 | 118 | -7447 | L1MA1 | L1 | LINE |

  

|  |  |  |  |  |  |  |  |  |  |
| --- | --- | --- | --- | --- | --- | --- | --- | --- | --- |
| chr7:103428980-103432218 | | | 3081 bp | | |  |  |  |  |
| Genome Start | Genome End | Strand | Div | Repeat Start | Repeat End | Repeat Left | Name | Family | Class |
| 103428980 | 103429498 | + | 27.9 | 3670 | 4199 | -1947 | L1ME3B | L1 | LINE |
| 103429495 | 103429672 | - | 23.7 | 50 | 228 | -4626 | L1MEe | L1 | LINE |
| 103429886 | 103429991 | - | 27.7 | 2 | 114 | -148 | MIR | MIR | SINE |
| 103430028 | 103430384 | + | 25.5 | 5407 | 5798 | -442 | L1ME3B | L1 | LINE |
| 103430399 | 103430544 | - | 25.0 | 6034 | 6178 | 0 | L1MB8 | L1 | LINE |
| 103430568 | 103430868 | - | 22.0 | 5779 | 6105 | -79 | L1MB7 | L1 | LINE |
| 103430617 | 103430983 | - | 20.7 | 5623 | 6028 | -138 | L1ME1 | L1 | LINE |
| 103430996 | 103431089 | - | 23.3 | 416 | 503 | -5 | Charlie4a | MER1\_type | DNA |
| 103431090 | 103431380 | + | 8.6 | 1 | 291 | -20 | AluY | Alu | SINE |
| 103431381 | 103431698 | - | 23.3 | 79 | 415 | -93 | Charlie4a | MER1\_type | DNA |
| 103431804 | 103431874 | - | 2.8 | 6082 | 6152 | -3 | L1PA4 | L1 | LINE |
| 103431875 | 103431976 | + | 2.9 | 5960 | 6061 | -94 | L1PA4 | L1 | LINE |
| 103431988 | 103432218 | + | 28.2 | 5844 | 6082 | -158 | L1ME3B | L1 | LINE |

  

|  |  |  |  |  |  |  |  |  |  |
| --- | --- | --- | --- | --- | --- | --- | --- | --- | --- |
| chr7:104197079-104197616 | | | 538 bp | | |  |  |  |  |
| Genome Start | Genome End | Strand | Div | Repeat Start | Repeat End | Repeat Left | Name | Family | Class |
| 104197079 | 104197141 | + | 19.1 | 1 | 64 | -16 | MADE2 | Mariner | DNA |
| 104197142 | 104197439 | - | 4.0 | 5858 | 6154 | 0 | L1PA7 | L1 | LINE |
| 104197440 | 104197585 | + | 7.6 | 5717 | 5861 | -293 | L1PA7 | L1 | LINE |
| 104197586 | 104197616 | + | 6.5 | 50 | 80 | 0 | MADE2 | Mariner | DNA |

  

|  |  |  |  |  |  |  |  |  |  |
| --- | --- | --- | --- | --- | --- | --- | --- | --- | --- |
| chr7:105636413-105643757 | | | 7336 bp | | |  |  |  |  |
| Genome Start | Genome End | Strand | Div | Repeat Start | Repeat End | Repeat Left | Name | Family | Class |
| 105636413 | 105638242 | + | 8.6 | 11 | 1842 | -4313 | L1PA5 | L1 | LINE |
| 105638277 | 105639125 | - | 2.2 | 5345 | 6155 | 0 | L1PA3 | L1 | LINE |
| 105639126 | 105639499 | + | 5.9 | 4983 | 5365 | -781 | L1PA3 | L1 | LINE |
| 105639475 | 105643757 | + | 5.3 | 1848 | 6154 | 0 | L1PA5 | L1 | LINE |

  

|  |  |  |  |  |  |  |  |  |  |
| --- | --- | --- | --- | --- | --- | --- | --- | --- | --- |
| chr7:106582952-106584117 | | | 1152 bp | | |  |  |  |  |
| Genome Start | Genome End | Strand | Div | Repeat Start | Repeat End | Repeat Left | Name | Family | Class |
| 106582952 | 106583085 | + | 8.3 | 1 | 133 | -179 | AluSx | Alu | SINE |
| 106583100 | 106583400 | - | 9.2 | 5312 | 5606 | -525 | L1PA8 | L1 | LINE |
| 106583401 | 106583964 | + | 7.3 | 5606 | 6172 | 0 | L1PA8 | L1 | LINE |
| 106583965 | 106584117 | + | 13.7 | 129 | 281 | -31 | AluSx | Alu | SINE |

  

|  |  |  |  |  |  |  |  |  |  |
| --- | --- | --- | --- | --- | --- | --- | --- | --- | --- |
| chr7:108385929-108391141 | | | 4886 bp | | |  |  |  |  |
| Genome Start | Genome End | Strand | Div | Repeat Start | Repeat End | Repeat Left | Name | Family | Class |
| 108385929 | 108386338 | + | 30.2 | 138 | 573 | -4281 | L1ME3A | L1 | LINE |
| 108386467 | 108386754 | + | 26.0 | 760 | 1066 | -3788 | L1ME3A | L1 | LINE |
| 108386769 | 108386937 | + | 26.0 | 1414 | 1570 | -3264 | L1ME3A | L1 | LINE |
| 108386938 | 108387243 | + | 13.4 | 1 | 309 | -3 | AluSx | Alu | SINE |
| 108387244 | 108387333 | + | 26.0 | 1571 | 1667 | -3167 | L1ME3A | L1 | LINE |
| 108387369 | 108387730 | + | 29.7 | 3512 | 3924 | -910 | L1ME3A | L1 | LINE |
| 108387760 | 108388050 | - | 19.3 | 2 | 282 | -30 | AluJo | Alu | SINE |
| 108388121 | 108388315 | - | 19.1 | 5676 | 5898 | -414 | L1MA9 | L1 | LINE |
| 108388341 | 108388828 | - | 6.2 | 5665 | 6154 | 0 | L1PA7 | L1 | LINE |
| 108388829 | 108389783 | + | 7.7 | 4699 | 5662 | -492 | L1PA7 | L1 | LINE |
| 108389799 | 108390065 | + | 28.4 | 3951 | 4214 | -636 | L1ME3A | L1 | LINE |
| 108390070 | 108390254 | - | 17.3 | 128 | 312 | 0 | AluSg/x | Alu | SINE |
| 108390257 | 108390549 | - | 9.6 | 1 | 293 | -18 | AluY | Alu | SINE |
| 108390555 | 108391141 | + | 25.9 | 4212 | 4819 | -39 | L1ME3A | L1 | LINE |

  

|  |  |  |  |  |  |  |  |  |  |
| --- | --- | --- | --- | --- | --- | --- | --- | --- | --- |
| chr7:108508969-108517517 | | | 8261 bp | | |  |  |  |  |
| Genome Start | Genome End | Strand | Div | Repeat Start | Repeat End | Repeat Left | Name | Family | Class |
| 108508969 | 108509571 | + | 21.0 | 1 | 665 | -265 | MER63C | AcHobo | DNA |
| 108509619 | 108509778 | - | 10.3 | 3514 | 3680 | -2466 | L1P3 | L1 | LINE |
| 108509802 | 108510500 | - | 10.3 | 2804 | 3513 | -2633 | L1P3 | L1 | LINE |
| 108510500 | 108510778 | + | 10.4 | 3696 | 3965 | -2181 | L1PREC2 | L1 | LINE |
| 108510797 | 108512458 | + | 12.7 | 8 | 2264 | -326 | L1P3 | L1 | LINE |
| 108512446 | 108514089 | + | 7.6 | 4495 | 6143 | -11 | L1PA7 | L1 | LINE |
| 108514146 | 108514317 | + | 23.4 | 5625 | 5836 | -1972 | L1MC4a | L1 | LINE |
| 108514434 | 108515514 | + | 10.2 | 3961 | 5053 | -1093 | L1PA6 | L1 | LINE |
| 108515515 | 108515977 | + | 8.6 | 5704 | 6154 | 0 | L1PA6 | L1 | LINE |
| 108515978 | 108516269 | + | 8.6 | 5432 | 5728 | -446 | L1P4 | L1 | LINE |
| 108516275 | 108516814 | - | 19.4 | 4 | 554 | 0 | MLT2A2 | ERVL | LTR |
| 108516815 | 108517251 | + | 12.1 | 5728 | 6171 | -3 | L1PA11 | L1 | LINE |
| 108517289 | 108517517 | + | 24.0 | 686 | 931 | 0 | MER63C | AcHobo | DNA |

  

|  |  |  |  |  |  |  |  |  |  |
| --- | --- | --- | --- | --- | --- | --- | --- | --- | --- |
| chr7:109332720-109341518 | | | 8801 bp | | |  |  |  |  |
| Genome Start | Genome End | Strand | Div | Repeat Start | Repeat End | Repeat Left | Name | Family | Class |
| 109332720 | 109333312 | + | 7.4 | 1 | 593 | -5562 | L1PA3 | L1 | LINE |
| 109333309 | 109334809 | - | 0.5 | 3517 | 5017 | -1129 | L1HS | L1 | LINE |
| 109334809 | 109335934 | + | 0.3 | 5030 | 6155 | 0 | L1HS | L1 | LINE |
| 109335938 | 109341518 | + | 4.2 | 573 | 6153 | -2 | L1PA3 | L1 | LINE |

  

|  |  |  |  |  |  |  |  |  |  |
| --- | --- | --- | --- | --- | --- | --- | --- | --- | --- |
| chr7:109567041-109569571 | | | 2517 bp | | |  |  |  |  |
| Genome Start | Genome End | Strand | Div | Repeat Start | Repeat End | Repeat Left | Name | Family | Class |
| 109567041 | 109567773 | + | 19.3 | 4894 | 5621 | -553 | L1MB5 | L1 | LINE |
| 109567794 | 109568266 | + | 19.4 | 5622 | 6047 | -127 | L1MB5 | L1 | LINE |
| 109568272 | 109569077 | - | 2.9 | 5353 | 6153 | -2 | L1PA4 | L1 | LINE |
| 109569067 | 109569462 | + | 6.6 | 4958 | 5353 | -793 | L1PA4 | L1 | LINE |
| 109569463 | 109569571 | + | 18.4 | 6050 | 6159 | -15 | L1MB5 | L1 | LINE |

  

|  |  |  |  |  |  |  |  |  |  |
| --- | --- | --- | --- | --- | --- | --- | --- | --- | --- |
| chr7:109760809-109763030 | | | 2206 bp | | |  |  |  |  |
| Genome Start | Genome End | Strand | Div | Repeat Start | Repeat End | Repeat Left | Name | Family | Class |
| 109760809 | 109761193 | - | 23.8 | 501 | 864 | -507 | MLT1C-int | MaLR | LTR |
| 109761209 | 109761503 | - | 8.1 | 1 | 298 | -13 | AluY | Alu | SINE |
| 109761504 | 109762055 | - | 8.4 | 5609 | 6160 | -8 | L1PA10 | L1 | LINE |
| 109762057 | 109762525 | + | 9.3 | 5135 | 5604 | -564 | L1PA10 | L1 | LINE |
| 109762526 | 109763030 | - | 20.0 | 2 | 514 | -857 | MLT1C-int | MaLR | LTR |

  

|  |  |  |  |  |  |  |  |  |  |
| --- | --- | --- | --- | --- | --- | --- | --- | --- | --- |
| chr7:109775595-109784530 | | | 8935 bp | | |  |  |  |  |
| Genome Start | Genome End | Strand | Div | Repeat Start | Repeat End | Repeat Left | Name | Family | Class |
| 109775595 | 109776401 | - | 7.4 | 5648 | 6454 | -15 | L1PA7 | L1 | LINE |
| 109776394 | 109776706 | - | 3.8 | 3692 | 4004 | -2142 | L1PA3 | L1 | LINE |
| 109776708 | 109778864 | + | 3.7 | 3999 | 6155 | 0 | L1PA3 | L1 | LINE |
| 109778873 | 109784530 | - | 10.6 | 8 | 5666 | -795 | L1PA7 | L1 | LINE |

  

|  |  |  |  |  |  |  |  |  |  |
| --- | --- | --- | --- | --- | --- | --- | --- | --- | --- |
| chr7:109935689-109947956 | | | 11993 bp | | |  |  |  |  |
| Genome Start | Genome End | Strand | Div | Repeat Start | Repeat End | Repeat Left | Name | Family | Class |
| 109935689 | 109935836 | - | 26.7 | 6094 | 6224 | -15 | L1ME1 | L1 | LINE |
| 109935837 | 109936188 | - | 14.3 | 5 | 364 | 0 | THE1B | MaLR | LTR |
| 109936189 | 109937216 | - | 26.5 | 5058 | 6093 | -146 | L1ME1 | L1 | LINE |
| 109937217 | 109937470 | - | 9.1 | 44 | 297 | -12 | AluSc | Alu | SINE |
| 109937471 | 109937656 | - | 26.2 | 4868 | 5057 | -1162 | L1ME1 | L1 | LINE |
| 109937657 | 109938172 | + | 20.6 | 1 | 523 | 0 | Charlie1b | MER1\_type | DNA |
| 109938173 | 109938276 | - | 26.2 | 4770 | 4867 | -1352 | L1ME1 | L1 | LINE |
| 109938361 | 109938820 | - | 26.6 | 24 | 489 | -137 | MLT1E2 | MaLR | LTR |
| 109938875 | 109939347 | - | 23.7 | 4225 | 4750 | -1469 | L1ME1 | L1 | LINE |
| 109939415 | 109939831 | - | 14.4 | 1 | 423 | -5 | MSTA | MaLR | LTR |
| 109939854 | 109940779 | - | 26.0 | 1690 | 2505 | -3895 | L1ME1 | L1 | LINE |
| 109940811 | 109940953 | - | 24.6 | 1339 | 1482 | -4918 | L1ME1 | L1 | LINE |
| 109940972 | 109941755 | - | 13.6 | 3287 | 4076 | -2070 | L1PA15 | L1 | LINE |
| 109941755 | 109942383 | + | 12.6 | 4081 | 4722 | -1424 | L1PA15 | L1 | LINE |
| 109942384 | 109943181 | - | 20.0 | 1 | 819 | -7 | LTR1B | ERV1 | LTR |
| 109943182 | 109944598 | + | 12.4 | 4727 | 6164 | -1 | L1PA15 | L1 | LINE |
| 109944599 | 109945134 | - | 28.4 | 811 | 1325 | -5075 | L1ME1 | L1 | LINE |
| 109945135 | 109945409 | - | 12.9 | 2 | 298 | -14 | AluSx | Alu | SINE |
| 109945410 | 109945728 | - | 28.4 | 507 | 810 | -5590 | L1ME1 | L1 | LINE |
| 109945729 | 109947505 | - | 22.2 | 1 | 1755 | 0 | MER52A | ERV1 | LTR |
| 109947506 | 109947956 | - | 28.4 | 40 | 506 | -5894 | L1ME1 | L1 | LINE |

  

|  |  |  |  |  |  |  |  |  |  |
| --- | --- | --- | --- | --- | --- | --- | --- | --- | --- |
| chr7:111116330-111119346 | | | 2776 bp | | |  |  |  |  |
| Genome Start | Genome End | Strand | Div | Repeat Start | Repeat End | Repeat Left | Name | Family | Class |
| 111116330 | 111116480 | + | 36.9 | 3619 | 3768 | -721 | L3 | CR1 | LINE |
| 111116504 | 111116643 | + | 28.7 | 4690 | 4842 | -1304 | L1ME3B | L1 | LINE |
| 111116673 | 111116868 | - | 14.8 | 11 | 210 | -156 | Tigger5a | MER2\_type | DNA |
| 111116871 | 111117598 | + | 28.6 | 4924 | 5712 | -528 | L1ME3B | L1 | LINE |
| 111117599 | 111117883 | - | 15.6 | 5 | 288 | -24 | AluJo | Alu | SINE |
| 111117884 | 111118373 | + | 28.9 | 5713 | 6235 | -5 | L1ME3B | L1 | LINE |
| 111118435 | 111118576 | - | 11.5 | 5783 | 5923 | -245 | L1MB1 | L1 | LINE |
| 111118577 | 111118833 | + | 16.5 | 5904 | 6167 | -1 | L1MB1 | L1 | LINE |
| 111118960 | 111119346 | + | 33.0 | 3953 | 4424 | -65 | L3 | CR1 | LINE |

  

|  |  |  |  |  |  |  |  |  |  |
| --- | --- | --- | --- | --- | --- | --- | --- | --- | --- |
| chr7:112181737-112184041 | | | 1762 bp | | |  |  |  |  |
| Genome Start | Genome End | Strand | Div | Repeat Start | Repeat End | Repeat Left | Name | Family | Class |
| 112181737 | 112181905 | + | 28.5 | 801 | 965 | -1542 | HAL1 | L1 | LINE |
| 112181909 | 112182018 | - | 6.4 | 5919 | 6028 | -126 | L1PA7 | L1 | LINE |
| 112182019 | 112182147 | + | 6.2 | 6024 | 6154 | 0 | L1PA7 | L1 | LINE |
| 112182285 | 112182455 | - | 27.8 | 5803 | 5978 | -262 | L1ME3B | L1 | LINE |
| 112182594 | 112183555 | + | 27.8 | 1032 | 2147 | -360 | HAL1 | L1 | LINE |
| 112183821 | 112184041 | + | 29.9 | 2245 | 2467 | -40 | HAL1 | L1 | LINE |

  

|  |  |  |  |  |  |  |  |  |  |
| --- | --- | --- | --- | --- | --- | --- | --- | --- | --- |
| chr7:113165656-113169419 | | | 3740 bp | | |  |  |  |  |
| Genome Start | Genome End | Strand | Div | Repeat Start | Repeat End | Repeat Left | Name | Family | Class |
| 113165656 | 113165871 | + | 16.0 | 1 | 238 | -143 | THE1D | MaLR | LTR |
| 113165872 | 113168109 | - | 3.2 | 2738 | 4977 | -1169 | L1PA3 | L1 | LINE |
| 113168108 | 113169241 | + | 2.5 | 4995 | 6129 | -26 | L1PA3 | L1 | LINE |
| 113169268 | 113169419 | + | 7.9 | 225 | 381 | 0 | THE1D | MaLR | LTR |

  

|  |  |  |  |  |  |  |  |  |  |
| --- | --- | --- | --- | --- | --- | --- | --- | --- | --- |
| chr7:113373380-113377102 | | | 3431 bp | | |  |  |  |  |
| Genome Start | Genome End | Strand | Div | Repeat Start | Repeat End | Repeat Left | Name | Family | Class |
| 113373380 | 113374333 | + | 16.8 | 1 | 972 | -5232 | L1PBb | L1 | LINE |
| 113374335 | 113374529 | - | 10.3 | 5791 | 5984 | -167 | L1PB1 | L1 | LINE |
| 113374714 | 113374952 | - | 11.3 | 5552 | 5791 | -360 | L1PB1 | L1 | LINE |
| 113374953 | 113375110 | + | 13.3 | 5993 | 6150 | -1 | L1PB1 | L1 | LINE |
| 113375133 | 113376212 | + | 19.6 | 961 | 2089 | -4115 | L1PBb | L1 | LINE |
| 113376300 | 113376486 | - | 18.3 | 1982 | 2239 | -4023 | L1PBa | L1 | LINE |
| 113376485 | 113377102 | + | 20.3 | 2185 | 2824 | -3379 | L1PBb | L1 | LINE |

  

|  |  |  |  |  |  |  |  |  |  |
| --- | --- | --- | --- | --- | --- | --- | --- | --- | --- |
| chr7:113676253-113685582 | | | 9286 bp | | |  |  |  |  |
| Genome Start | Genome End | Strand | Div | Repeat Start | Repeat End | Repeat Left | Name | Family | Class |
| 113676253 | 113676490 | - | 32.9 | 3148 | 3413 | -6 | L2 | L2 | LINE |
| 113676491 | 113677065 | - | 7.8 | 5595 | 6172 | 0 | L1PA10 | L1 | LINE |
| 113677090 | 113683230 | - | 6.8 | 2 | 6154 | 0 | L1PA5 | L1 | LINE |
| 113683229 | 113683397 | - | 9.4 | 5451 | 5608 | -560 | L1PA10 | L1 | LINE |
| 113683398 | 113683697 | - | 9.8 | 1 | 296 | -17 | AluSp | Alu | SINE |
| 113683698 | 113684050 | - | 8.9 | 5096 | 5450 | -718 | L1PA10 | L1 | LINE |
| 113684041 | 113684704 | + | 6.5 | 4444 | 5110 | -1036 | L1PA10 | L1 | LINE |
| 113684734 | 113685043 | - | 10.7 | 1 | 310 | -1 | AluY | Alu | SINE |
| 113685047 | 113685582 | - | 29.9 | 2595 | 3139 | -280 | L2 | L2 | LINE |

  

|  |  |  |  |  |  |  |  |  |  |
| --- | --- | --- | --- | --- | --- | --- | --- | --- | --- |
| chr7:113941453-113945379 | | | 3908 bp | | |  |  |  |  |
| Genome Start | Genome End | Strand | Div | Repeat Start | Repeat End | Repeat Left | Name | Family | Class |
| 113941453 | 113941971 | - | 16.8 | 5666 | 6254 | -37 | L1MA8 | L1 | LINE |
| 113942001 | 113942013 | - | 18.8 | 5653 | 5665 | -635 | L1M1 | L1 | LINE |
| 113942015 | 113943101 | - | 2.4 | 5062 | 6152 | -3 | L1PA3 | L1 | LINE |
| 113943099 | 113943764 | + | 3.5 | 4389 | 5054 | -1092 | L1PA3 | L1 | LINE |
| 113943757 | 113943974 | - | 15.2 | 5493 | 5699 | -592 | L1MA8 | L1 | LINE |
| 113943975 | 113944284 | - | 12.0 | 1 | 309 | 0 | AluSc | Alu | SINE |
| 113944285 | 113945379 | - | 16.8 | 4381 | 5492 | -799 | L1MA8 | L1 | LINE |

  

|  |  |  |  |  |  |  |  |  |  |
| --- | --- | --- | --- | --- | --- | --- | --- | --- | --- |
| chr7:116013818-116015210 | | | 1357 bp | | |  |  |  |  |
| Genome Start | Genome End | Strand | Div | Repeat Start | Repeat End | Repeat Left | Name | Family | Class |
| 116013818 | 116014143 | - | 13.0 | 5827 | 6171 | 0 | L1MB2 | L1 | LINE |
| 116014150 | 116014447 | + | 14.2 | 1 | 307 | -57 | THE1B | MaLR | LTR |
| 116014474 | 116014568 | - | 9.5 | 6077 | 6172 | 0 | L1PA8 | L1 | LINE |
| 116014569 | 116014928 | + | 8.3 | 5707 | 6072 | -100 | L1PA8 | L1 | LINE |
| 116014929 | 116015015 | + | 13.4 | 283 | 364 | 0 | THE1B | MaLR | LTR |
| 116015020 | 116015210 | - | 17.3 | 5627 | 5821 | -350 | L1MB2 | L1 | LINE |

  

|  |  |  |  |  |  |  |  |  |  |
| --- | --- | --- | --- | --- | --- | --- | --- | --- | --- |
| chr7:117392828-117397564 | | | 4379 bp | | |  |  |  |  |
| Genome Start | Genome End | Strand | Div | Repeat Start | Repeat End | Repeat Left | Name | Family | Class |
| 117392828 | 117392947 | + | 24.4 | 2495 | 2615 | -3531 | L1M5 | L1 | LINE |
| 117392999 | 117393096 | + | 28.6 | 78 | 188 | -1 | MER5A | MER1\_type | DNA |
| 117393163 | 117393284 | + | 27.7 | 2704 | 2823 | -3323 | L1M5 | L1 | LINE |
| 117393434 | 117394103 | - | 5.7 | 2602 | 3270 | -2876 | L1PA4 | L1 | LINE |
| 117394108 | 117397002 | + | 4.2 | 3256 | 6154 | -1 | L1PA4 | L1 | LINE |
| 117397091 | 117397564 | + | 30.1 | 3198 | 3729 | -2417 | L1M5 | L1 | LINE |

  

|  |  |  |  |  |  |  |  |  |  |
| --- | --- | --- | --- | --- | --- | --- | --- | --- | --- |
| chr7:117411407-117414846 | | | 3277 bp | | |  |  |  |  |
| Genome Start | Genome End | Strand | Div | Repeat Start | Repeat End | Repeat Left | Name | Family | Class |
| 117411407 | 117411556 | - | 26.4 | 3052 | 3214 | -164 | L2 | L2 | LINE |
| 117411593 | 117411966 | - | 3.1 | 4896 | 5269 | -877 | L1PA3 | L1 | LINE |
| 117411967 | 117412848 | + | 3.0 | 5272 | 6152 | -3 | L1PA3 | L1 | LINE |
| 117412931 | 117413246 | - | 11.0 | 1 | 311 | -2 | AluSp | Alu | SINE |
| 117413278 | 117413481 | + | 28.7 | 1 | 219 | -372 | MLT1K | MaLR | LTR |
| 117413482 | 117413875 | - | 17.4 | 1 | 505 | 0 | MLT1D | MaLR | LTR |
| 117413876 | 117414227 | + | 28.7 | 220 | 591 | 0 | MLT1K | MaLR | LTR |
| 117414242 | 117414401 | - | 33.1 | 2686 | 2844 | -575 | L2 | L2 | LINE |
| 117414402 | 117414700 | + | 13.3 | 1 | 297 | -13 | AluSg | Alu | SINE |
| 117414701 | 117414846 | - | 33.1 | 2529 | 2685 | -734 | L2 | L2 | LINE |

  

|  |  |  |  |  |  |  |  |  |  |
| --- | --- | --- | --- | --- | --- | --- | --- | --- | --- |
| chr7:118651894-118653751 | | | 1466 bp | | |  |  |  |  |
| Genome Start | Genome End | Strand | Div | Repeat Start | Repeat End | Repeat Left | Name | Family | Class |
| 118651894 | 118652056 | + | 39.0 | 96 | 261 | -6000 | L1MEc | L1 | LINE |
| 118652284 | 118652497 | - | 31.9 | 1 | 208 | -16 | MER58A | MER1\_type | DNA |
| 118652605 | 118652942 | + | 28.4 | 529 | 888 | -5182 | L1MEc | L1 | LINE |
| 118652940 | 118653022 | + | 21.2 | 2689 | 2768 | -3312 | L1MEc | L1 | LINE |
| 118653079 | 118653172 | - | 9.6 | 6062 | 6161 | -2 | L1PA13 | L1 | LINE |
| 118653179 | 118653433 | + | 8.6 | 5786 | 6041 | -127 | L1PA13 | L1 | LINE |
| 118653433 | 118653751 | + | 27.2 | 2784 | 3117 | -2963 | L1MEc | L1 | LINE |

  

|  |  |  |  |  |  |  |  |  |  |
| --- | --- | --- | --- | --- | --- | --- | --- | --- | --- |
| chr7:119083333-119085600 | | | 2230 bp | | |  |  |  |  |
| Genome Start | Genome End | Strand | Div | Repeat Start | Repeat End | Repeat Left | Name | Family | Class |
| 119083333 | 119083959 | - | 22.3 | 5593 | 6224 | -88 | L1MA9 | L1 | LINE |
| 119084003 | 119085059 | - | 1.3 | 5099 | 6155 | 0 | L1HS | L1 | LINE |
| 119085058 | 119085505 | + | 0.7 | 4648 | 5095 | -1051 | L1HS | L1 | LINE |
| 119085503 | 119085600 | - | 19.4 | 5509 | 5607 | -705 | L1MA9 | L1 | LINE |

  

|  |  |  |  |  |  |  |  |  |  |
| --- | --- | --- | --- | --- | --- | --- | --- | --- | --- |
| chr7:119580569-119583133 | | | 2318 bp | | |  |  |  |  |
| Genome Start | Genome End | Strand | Div | Repeat Start | Repeat End | Repeat Left | Name | Family | Class |
| 119580569 | 119581272 | - | 22.8 | 7019 | 7758 | -27 | L1MC3 | L1 | LINE |
| 119581387 | 119581428 | + | 11.9 | 7011 | 7052 | -943 | L1MD3 | L1 | LINE |
| 119581559 | 119581732 | - | 16.7 | 6882 | 7037 | -748 | L1MC3 | L1 | LINE |
| 119581726 | 119582102 | - | 8.5 | 5033 | 5416 | -738 | L1P | L1 | LINE |
| 119582099 | 119582842 | + | 8.7 | 5393 | 6131 | 0 | L1PA8A | L1 | LINE |
| 119582857 | 119583133 | - | 23.1 | 6571 | 6853 | -932 | L1MC3 | L1 | LINE |

  

|  |  |  |  |  |  |  |  |  |  |
| --- | --- | --- | --- | --- | --- | --- | --- | --- | --- |
| chr7:120581028-120584457 | | | 3431 bp | | |  |  |  |  |
| Genome Start | Genome End | Strand | Div | Repeat Start | Repeat End | Repeat Left | Name | Family | Class |
| 120581028 | 120581552 | + | 15.3 | 1 | 543 | -2175 | Tigger2 | MER2\_type | DNA |
| 120581553 | 120581830 | - | 15.3 | 3941 | 4181 | -1962 | L1M2 | L1 | LINE |
| 120581831 | 120582133 | - | 8.6 | 1 | 303 | -6 | AluSc | Alu | SINE |
| 120582134 | 120582229 | - | 15.3 | 3834 | 3940 | -2203 | L1M2 | L1 | LINE |
| 120582227 | 120584002 | + | 15.0 | 4202 | 6021 | -279 | L1MA4 | L1 | LINE |
| 120584000 | 120584092 | + | 23.7 | 6087 | 6183 | 0 | L1MB3 | L1 | LINE |
| 120584099 | 120584287 | + | 12.9 | 526 | 714 | -2004 | Tigger2 | MER2\_type | DNA |
| 120584287 | 120584457 | + | 20.1 | 2562 | 2718 | 0 | Tigger2 | MER2\_type | DNA |

  

|  |  |  |  |  |  |  |  |  |  |
| --- | --- | --- | --- | --- | --- | --- | --- | --- | --- |
| chr7:122083187-122088538 | | | 5178 bp | | |  |  |  |  |
| Genome Start | Genome End | Strand | Div | Repeat Start | Repeat End | Repeat Left | Name | Family | Class |
| 122083187 | 122083695 | + | 29.3 | 2 | 530 | -5515 | L1MCb | L1 | LINE |
| 122083837 | 122084070 | + | 23.6 | 570 | 812 | -5233 | L1MCb | L1 | LINE |
| 122084072 | 122085213 | - | 5.0 | 4039 | 5180 | -966 | L1PA5 | L1 | LINE |
| 122085216 | 122086187 | + | 4.2 | 5178 | 6153 | -1 | L1PA5 | L1 | LINE |
| 122086218 | 122087918 | + | 21.4 | 813 | 2626 | -4125 | L1MCb | L1 | LINE |
| 122087919 | 122088215 | + | 11.5 | 1 | 296 | -14 | AluSg | Alu | SINE |
| 122088216 | 122088538 | + | 20.7 | 2627 | 2950 | -3614 | L1MCb | L1 | LINE |

  

|  |  |  |  |  |  |  |  |  |  |
| --- | --- | --- | --- | --- | --- | --- | --- | --- | --- |
| chr7:124421410-124424594 | | | 3186 bp | | |  |  |  |  |
| Genome Start | Genome End | Strand | Div | Repeat Start | Repeat End | Repeat Left | Name | Family | Class |
| 124421410 | 124421683 | + | 24.6 | 1 | 280 | -187 | MLT1C | MaLR | LTR |
| 124421685 | 124421776 | - | 20.9 | 336 | 428 | 0 | MSTC | MaLR | LTR |
| 124421777 | 124422168 | - | 7.9 | 4162 | 4553 | -1593 | L1PA7 | L1 | LINE |
| 124422167 | 124423752 | + | 8.4 | 4558 | 6152 | -2 | L1PA7 | L1 | LINE |
| 124423753 | 124423767 | - | 16.5 | 329 | 340 | -88 | MSTC | MaLR | LTR |
| 124423768 | 124424069 | + | 13.6 | 1 | 302 | -10 | AluSx | Alu | SINE |
| 124424070 | 124424413 | - | 16.5 | 1 | 328 | -100 | MSTC | MaLR | LTR |
| 124424414 | 124424594 | + | 24.9 | 277 | 467 | 0 | MLT1C | MaLR | LTR |

  

|  |  |  |  |  |  |  |  |  |  |
| --- | --- | --- | --- | --- | --- | --- | --- | --- | --- |
| chr7:125771344-125774149 | | | 2785 bp | | |  |  |  |  |
| Genome Start | Genome End | Strand | Div | Repeat Start | Repeat End | Repeat Left | Name | Family | Class |
| 125771344 | 125771573 | - | 22.1 | 354 | 563 | -2 | MER34 | ERV1 | LTR |
| 125771601 | 125772184 | - | 3.6 | 3808 | 4394 | -1752 | L1PA3 | L1 | LINE |
| 125772174 | 125773914 | + | 2.9 | 4411 | 6152 | -3 | L1PA3 | L1 | LINE |
| 125773920 | 125774149 | - | 17.0 | 11 | 245 | -297 | MER34 | ERV1 | LTR |

  

|  |  |  |  |  |  |  |  |  |  |
| --- | --- | --- | --- | --- | --- | --- | --- | --- | --- |
| chr7:126095615-126100296 | | | 4659 bp | | |  |  |  |  |
| Genome Start | Genome End | Strand | Div | Repeat Start | Repeat End | Repeat Left | Name | Family | Class |
| 126095615 | 126096095 | + | 15.9 | 1 | 500 | -4717 | MER61A-int | ERV1 | LTR |
| 126096105 | 126096167 | + | 11.1 | 1408 | 1484 | -3733 | MER61A-int | ERV1 | LTR |
| 126096168 | 126096230 | - | 4.8 | 5698 | 5760 | -412 | L1PA8 | L1 | LINE |
| 126096231 | 126096641 | + | 8.6 | 5766 | 6172 | 0 | L1PA8 | L1 | LINE |
| 126096656 | 126100296 | + | 10.1 | 1655 | 5215 | -2 | MER61A-int | ERV1 | LTR |

  

|  |  |  |  |  |  |  |  |  |  |
| --- | --- | --- | --- | --- | --- | --- | --- | --- | --- |
| chr7:126946466-126950516 | | | 4051 bp | | |  |  |  |  |
| Genome Start | Genome End | Strand | Div | Repeat Start | Repeat End | Repeat Left | Name | Family | Class |
| 126946466 | 126946948 | + | 7.9 | 4163 | 4660 | -1486 | L1PREC2 | L1 | LINE |
| 126946949 | 126947259 | + | 5.8 | 1 | 311 | 0 | AluY | Alu | SINE |
| 126947260 | 126947790 | + | 7.9 | 4661 | 5187 | -959 | L1PREC2 | L1 | LINE |
| 126947788 | 126949013 | - | 5.8 | 4424 | 5658 | -496 | L1PA7 | L1 | LINE |
| 126949016 | 126949538 | + | 5.2 | 5634 | 6154 | 0 | L1PA7 | L1 | LINE |
| 126949540 | 126950516 | + | 9.1 | 5175 | 6159 | -1 | L1PREC2 | L1 | LINE |

  

|  |  |  |  |  |  |  |  |  |  |
| --- | --- | --- | --- | --- | --- | --- | --- | --- | --- |
| chr7:131248167-131254352 | | | 6212 bp | | |  |  |  |  |
| Genome Start | Genome End | Strand | Div | Repeat Start | Repeat End | Repeat Left | Name | Family | Class |
| 131248167 | 131248837 | + | 28.4 | 50 | 743 | -5657 | L1ME1 | L1 | LINE |
| 131248848 | 131249169 | + | 23.8 | 819 | 1156 | -5244 | L1ME1 | L1 | LINE |
| 131249170 | 131249484 | + | 10.7 | 1 | 317 | 0 | AluSx | Alu | SINE |
| 131249485 | 131249755 | + | 23.8 | 1157 | 1438 | -4962 | L1ME1 | L1 | LINE |
| 131249777 | 131250589 | - | 3.2 | 5344 | 6155 | 0 | L1PA3 | L1 | LINE |
| 131250569 | 131251313 | + | 4.0 | 4608 | 5356 | -790 | L1PA3 | L1 | LINE |
| 131251295 | 131252167 | + | 23.4 | 1425 | 2280 | -4120 | L1ME1 | L1 | LINE |
| 131252168 | 131252477 | + | 6.5 | 1 | 310 | -1 | AluY | Alu | SINE |
| 131252478 | 131253074 | + | 24.6 | 2183 | 2773 | -3446 | L1ME1 | L1 | LINE |
| 131253074 | 131253192 | + | 25.2 | 4501 | 4619 | -1600 | L1ME1 | L1 | LINE |
| 131253177 | 131253501 | + | 24.6 | 5347 | 5657 | -582 | L1ME1 | L1 | LINE |
| 131253502 | 131253794 | - | 10.3 | 2 | 302 | -10 | AluSx | Alu | SINE |
| 131253795 | 131254352 | + | 24.6 | 5658 | 6234 | -5 | L1ME1 | L1 | LINE |

  

|  |  |  |  |  |  |  |  |  |  |
| --- | --- | --- | --- | --- | --- | --- | --- | --- | --- |
| chr7:131996974-132001014 | | | 3830 bp | | |  |  |  |  |
| Genome Start | Genome End | Strand | Div | Repeat Start | Repeat End | Repeat Left | Name | Family | Class |
| 131996974 | 131997026 | + | 28.3 | 4077 | 4129 | -379 | L3b | CR1 | LINE |
| 131997057 | 131997406 | + | 26.0 | 1 | 357 | -105 | LTR40b | ERVL | LTR |
| 131997442 | 131999790 | - | 3.2 | 3799 | 6155 | 0 | L1PA3 | L1 | LINE |
| 131999781 | 132000647 | + | 2.4 | 2931 | 3796 | -2350 | L1PA3 | L1 | LINE |
| 132000648 | 132000769 | + | 19.7 | 335 | 462 | 0 | LTR40b | ERVL | LTR |
| 132000926 | 132001014 | + | 31.4 | 4327 | 4415 | -93 | L3b | CR1 | LINE |

  

|  |  |  |  |  |  |  |  |  |  |
| --- | --- | --- | --- | --- | --- | --- | --- | --- | --- |
| chr7:135775485-135779558 | | | 4058 bp | | |  |  |  |  |
| Genome Start | Genome End | Strand | Div | Repeat Start | Repeat End | Repeat Left | Name | Family | Class |
| 135775485 | 135775984 | - | 16.7 | 5817 | 6315 | -18 | L1MC1 | L1 | LINE |
| 135776004 | 135777703 | - | 7.9 | 4494 | 6150 | -1 | L1PB1 | L1 | LINE |
| 135777704 | 135777880 | + | 10.0 | 3343 | 3517 | -2629 | L1PB | L1 | LINE |
| 135777881 | 135778181 | + | 11.0 | 1 | 300 | -12 | AluSx | Alu | SINE |
| 135778182 | 135779160 | + | 10.0 | 3518 | 4477 | -1669 | L1PB | L1 | LINE |
| 135779158 | 135779558 | - | 13.7 | 5421 | 5832 | -501 | L1MC1 | L1 | LINE |

  

|  |  |  |  |  |  |  |  |  |  |
| --- | --- | --- | --- | --- | --- | --- | --- | --- | --- |
| chr7:136515206-136523824 | | | 7619 bp | | |  |  |  |  |
| Genome Start | Genome End | Strand | Div | Repeat Start | Repeat End | Repeat Left | Name | Family | Class |
| 136515206 | 136515700 | + | 25.0 | 2039 | 2555 | -3660 | L1MC4 | L1 | LINE |
| 136516008 | 136516203 | + | 31.8 | 2937 | 3126 | -3088 | L1MC4 | L1 | LINE |
| 136516204 | 136516291 | + | 14.3 | 6068 | 6151 | -17 | L1PA12 | L1 | LINE |
| 136516331 | 136516582 | + | 31.8 | 3127 | 3377 | -2837 | L1MC4 | L1 | LINE |
| 136516663 | 136516770 | - | 22.2 | 6090 | 6200 | -91 | L1MA7 | L1 | LINE |
| 136516772 | 136516875 | + | 15.4 | 6186 | 6289 | -2 | L1MA7 | L1 | LINE |
| 136516896 | 136522888 | + | 2.9 | 1 | 6029 | -3 | L1PA3 | L1 | LINE |
| 136523117 | 136523402 | + | 30.8 | 3592 | 3928 | -2286 | L1MC4 | L1 | LINE |
| 136523728 | 136523824 | + | 26.6 | 7923 | 8020 | -22 | L1MC4 | L1 | LINE |

  

|  |  |  |  |  |  |  |  |  |  |
| --- | --- | --- | --- | --- | --- | --- | --- | --- | --- |
| chr7:136545044-136547746 | | | 2713 bp | | |  |  |  |  |
| Genome Start | Genome End | Strand | Div | Repeat Start | Repeat End | Repeat Left | Name | Family | Class |
| 136545044 | 136545110 | - | 20.9 | 124 | 190 | -3 | MER53 | DNA | DNA |
| 136545114 | 136546329 | - | 3.2 | 4926 | 6152 | -3 | L1PA4 | L1 | LINE |
| 136546317 | 136547608 | + | 3.3 | 3637 | 4927 | -1219 | L1PA4 | L1 | LINE |
| 136547609 | 136547746 | - | 14.6 | 1 | 139 | -54 | MER53 | DNA | DNA |

  

|  |  |  |  |  |  |  |  |  |  |
| --- | --- | --- | --- | --- | --- | --- | --- | --- | --- |
| chr7:141223935-141230192 | | | 6167 bp | | |  |  |  |  |
| Genome Start | Genome End | Strand | Div | Repeat Start | Repeat End | Repeat Left | Name | Family | Class |
| 141223935 | 141224703 | - | 17.4 | 3200 | 3991 | -2756 | L1MB4 | L1 | LINE |
| 141224704 | 141225007 | + | 8.9 | 2 | 305 | -6 | AluY | Alu | SINE |
| 141225008 | 141228314 | - | 21.2 | 261 | 3199 | -3548 | L1MB4 | L1 | LINE |
| 141228428 | 141229185 | - | 1.3 | 5398 | 6155 | 0 | L1HS | L1 | LINE |
| 141229172 | 141230017 | + | 1.2 | 4552 | 5396 | -750 | L1HS | L1 | LINE |
| 141230010 | 141230192 | - | 15.3 | 8 | 194 | -6413 | L1MB4 | L1 | LINE |

  

|  |  |  |  |  |  |  |  |  |  |
| --- | --- | --- | --- | --- | --- | --- | --- | --- | --- |
| chr7:141547298-141550559 | | | 3228 bp | | |  |  |  |  |
| Genome Start | Genome End | Strand | Div | Repeat Start | Repeat End | Repeat Left | Name | Family | Class |
| 141547298 | 141547337 | - | 28.8 | 5705 | 5742 | -379 | L1M5 | L1 | LINE |
| 141547338 | 141547638 | - | 12.3 | 1 | 312 | 0 | AluSx | Alu | SINE |
| 141547639 | 141547721 | - | 28.8 | 5624 | 5704 | -417 | L1M5 | L1 | LINE |
| 141547743 | 141547881 | - | 23.7 | 6003 | 6154 | -12 | L1ME1 | L1 | LINE |
| 141547884 | 141548105 | + | 20.6 | 33 | 257 | -88 | MER2 | MER2\_type | DNA |
| 141548106 | 141548483 | + | 22.4 | 5676 | 6018 | -148 | L1ME1 | L1 | LINE |
| 141548489 | 141548586 | - | 15.3 | 240 | 340 | -5 | MER2 | MER2\_type | DNA |
| 141548588 | 141549635 | - | 14.0 | 5063 | 6157 | -9 | L1PA16 | L1 | LINE |
| 141549632 | 141550171 | + | 9.9 | 4479 | 5048 | -1098 | L1PA16 | L1 | LINE |
| 141550181 | 141550559 | - | 26.4 | 4335 | 4705 | -1441 | L1M5 | L1 | LINE |

  

|  |  |  |  |  |  |  |  |  |  |
| --- | --- | --- | --- | --- | --- | --- | --- | --- | --- |
| chr7:143392771-143396895 | | | 3983 bp | | |  |  |  |  |
| Genome Start | Genome End | Strand | Div | Repeat Start | Repeat End | Repeat Left | Name | Family | Class |
| 143392771 | 143393449 | + | 34.2 | 2208 | 2919 | -3227 | L1MEe | L1 | LINE |
| 143393540 | 143393835 | + | 29.9 | 3052 | 3386 | -2760 | L1MEe | L1 | LINE |
| 143393841 | 143394460 | - | 7.6 | 3458 | 4080 | -2066 | L1PB1 | L1 | LINE |
| 143394461 | 143396136 | + | 8.9 | 4094 | 5784 | -367 | L1PB1 | L1 | LINE |
| 143396181 | 143396544 | + | 12.2 | 5785 | 6149 | -2 | L1PB1 | L1 | LINE |
| 143396548 | 143396895 | + | 31.8 | 3407 | 3767 | -2379 | L1MEe | L1 | LINE |

  

|  |  |  |  |  |  |  |  |  |  |
| --- | --- | --- | --- | --- | --- | --- | --- | --- | --- |
| chr7:144017889-144022385 | | | 4435 bp | | |  |  |  |  |
| Genome Start | Genome End | Strand | Div | Repeat Start | Repeat End | Repeat Left | Name | Family | Class |
| 144017889 | 144018724 | - | 15.9 | 5319 | 6168 | 0 | L1MB1 | L1 | LINE |
| 144018726 | 144018765 | + | 19.1 | 6125 | 6164 | -127 | L1MA7 | L1 | LINE |
| 144018787 | 144018851 | + | 19.1 | 6165 | 6248 | -43 | L1MA7 | L1 | LINE |
| 144018849 | 144019654 | + | 12.3 | 5345 | 6161 | -4 | L1PA15 | L1 | LINE |
| 144019695 | 144021483 | - | 2.0 | 4359 | 6151 | -4 | L1PA2 | L1 | LINE |
| 144021483 | 144022043 | + | 2.0 | 3790 | 4350 | -1796 | L1PA2 | L1 | LINE |
| 144022048 | 144022385 | - | 13.3 | 4979 | 5320 | -820 | L1MB1 | L1 | LINE |

  

|  |  |  |  |  |  |  |  |  |  |
| --- | --- | --- | --- | --- | --- | --- | --- | --- | --- |
| chr7:144395694-144403119 | | | 7250 bp | | |  |  |  |  |
| Genome Start | Genome End | Strand | Div | Repeat Start | Repeat End | Repeat Left | Name | Family | Class |
| 144395694 | 144396181 | - | 28.8 | 3344 | 3844 | -2302 | L1M5 | L1 | LINE |
| 144396211 | 144396350 | - | 25.7 | 6175 | 6318 | -16 | L1MA10 | L1 | LINE |
| 144396377 | 144396569 | - | 22.4 | 4806 | 5011 | -1526 | L1MEb | L1 | LINE |
| 144396570 | 144397252 | + | 16.4 | 4 | 703 | -4 | MER39 | ERV1 | LTR |
| 144397253 | 144397271 | - | 31.5 | 4789 | 4805 | -1732 | L1MEb | L1 | LINE |
| 144397291 | 144397746 | - | 2.0 | 5693 | 6149 | -5 | L1PA5 | L1 | LINE |
| 144397747 | 144398578 | + | 4.1 | 4857 | 5694 | -460 | L1PA5 | L1 | LINE |
| 144398577 | 144398838 | - | 25.5 | 4511 | 4812 | -1725 | L1MEb | L1 | LINE |
| 144398839 | 144399240 | + | 11.8 | 1 | 381 | 0 | THE1D | MaLR | LTR |
| 144399241 | 144399785 | - | 25.5 | 3888 | 4510 | -2027 | L1MEb | L1 | LINE |
| 144399791 | 144400359 | - | 27.4 | 2568 | 3117 | -3420 | L1MEb | L1 | LINE |
| 144400363 | 144400606 | - | 26.7 | 2409 | 2706 | -4012 | L1MEb | L1 | LINE |
| 144400624 | 144400765 | - | 12.9 | 4611 | 4747 | -1393 | L1M3 | L1 | LINE |
| 144400766 | 144401128 | - | 15.2 | 1 | 364 | 0 | THE1B | MaLR | LTR |
| 144401129 | 144401192 | - | 12.9 | 4546 | 4610 | -1530 | L1M3 | L1 | LINE |
| 144401194 | 144401295 | - | 19.8 | 1265 | 1370 | -5263 | L1MEb | L1 | LINE |
| 144401344 | 144401819 | - | 34.2 | 811 | 1288 | -5239 | L1MEb | L1 | LINE |
| 144401820 | 144402185 | - | 15.3 | 1 | 381 | 0 | THE1D | MaLR | LTR |
| 144402186 | 144402534 | - | 34.2 | 463 | 810 | -5717 | L1MEb | L1 | LINE |
| 144402565 | 144403119 | - | 31.4 | 2709 | 3318 | -2828 | L1M5 | L1 | LINE |

  

|  |  |  |  |  |  |  |  |  |  |
| --- | --- | --- | --- | --- | --- | --- | --- | --- | --- |
| chr7:145338791-145340267 | | | 1309 bp | | |  |  |  |  |
| Genome Start | Genome End | Strand | Div | Repeat Start | Repeat End | Repeat Left | Name | Family | Class |
| 145338791 | 145339157 | + | 29.4 | 2474 | 2885 | -534 | L2 | L2 | LINE |
| 145339314 | 145339386 | - | 9.9 | 6088 | 6151 | 0 | L1PB1 | L1 | LINE |
| 145339387 | 145339684 | - | 12.8 | 1 | 298 | -14 | AluSx | Alu | SINE |
| 145339685 | 145339888 | - | 9.9 | 5890 | 6087 | -64 | L1PB1 | L1 | LINE |
| 145339889 | 145339930 | + | 2.4 | 5844 | 5885 | -266 | L1PB1 | L1 | LINE |
| 145339943 | 145340267 | + | 32.4 | 3094 | 3419 | 0 | L2 | L2 | LINE |

  

|  |  |  |  |  |  |  |  |  |  |
| --- | --- | --- | --- | --- | --- | --- | --- | --- | --- |
| chr7:145372061-145375475 | | | 3119 bp | | |  |  |  |  |
| Genome Start | Genome End | Strand | Div | Repeat Start | Repeat End | Repeat Left | Name | Family | Class |
| 145372061 | 145372610 | - | 17.6 | 98 | 703 | -2 | MER44D | MER2\_type | DNA |
| 145372611 | 145373414 | - | 5.0 | 3893 | 4701 | -1445 | L1PA4 | L1 | LINE |
| 145373414 | 145374856 | + | 4.1 | 4706 | 6152 | -3 | L1PA4 | L1 | LINE |
| 145374949 | 145375158 | - | 33.3 | 39 | 258 | -10 | MIRb | MIR | SINE |
| 145375364 | 145375475 | - | 18.8 | 1 | 114 | -591 | MER44D | MER2\_type | DNA |

  

|  |  |  |  |  |  |  |  |  |  |
| --- | --- | --- | --- | --- | --- | --- | --- | --- | --- |
| chr7:146704468-146708076 | | | 3147 bp | | |  |  |  |  |
| Genome Start | Genome End | Strand | Div | Repeat Start | Repeat End | Repeat Left | Name | Family | Class |
| 146704468 | 146704590 | - | 26.8 | 2359 | 2468 | -3661 | L1MEe | L1 | LINE |
| 146704644 | 146704852 | - | 27.7 | 21 | 233 | -29 | MIR | MIR | SINE |
| 146704917 | 146705867 | - | 29.0 | 1377 | 2341 | -3808 | L1MEe | L1 | LINE |
| 146705937 | 146706055 | - | 18.8 | 1124 | 1249 | -4870 | L1MEe | L1 | LINE |
| 146706059 | 146706133 | + | 9.3 | 6 | 80 | 0 | MADE1 | Mariner | DNA |
| 146706314 | 146706422 | - | 15.6 | 6175 | 6303 | -2 | L1MA3 | L1 | LINE |
| 146706420 | 146706798 | + | 16.1 | 5784 | 6166 | -139 | L1MA3 | L1 | LINE |
| 146706892 | 146707258 | + | 10.1 | 1 | 375 | 0 | THE1C | MaLR | LTR |
| 146707262 | 146708076 | - | 32.7 | 16 | 889 | -5230 | L1MEe | L1 | LINE |

  

|  |  |  |  |  |  |  |  |  |  |
| --- | --- | --- | --- | --- | --- | --- | --- | --- | --- |
| chr7:147391108-147397245 | | | 6078 bp | | |  |  |  |  |
| Genome Start | Genome End | Strand | Div | Repeat Start | Repeat End | Repeat Left | Name | Family | Class |
| 147391108 | 147391298 | - | 17.1 | 1463 | 1696 | -4704 | L1MEc | L1 | LINE |
| 147391345 | 147391600 | - | 19.1 | 1069 | 1328 | -5072 | L1MEc | L1 | LINE |
| 147391616 | 147391682 | - | 10.4 | 6085 | 6151 | 0 | L1PB1 | L1 | LINE |
| 147391690 | 147392432 | - | 9.2 | 5190 | 5934 | -928 | L1PB | L1 | LINE |
| 147392431 | 147392785 | + | 11.2 | 4844 | 5191 | -1671 | L1PB | L1 | LINE |
| 147392785 | 147394508 | - | 8.7 | 2090 | 3902 | -2960 | L1PBa | L1 | LINE |
| 147394509 | 147394822 | - | 7.1 | 1 | 311 | 0 | AluY | Alu | SINE |
| 147394823 | 147396540 | - | 11.0 | 192 | 2089 | -4774 | L1PBa | L1 | LINE |
| 147396536 | 147397245 | - | 23.3 | 34 | 808 | -5592 | L1MEc | L1 | LINE |

  

|  |  |  |  |  |  |  |  |  |  |
| --- | --- | --- | --- | --- | --- | --- | --- | --- | --- |
| chr7:150086814-150090749 | | | 3956 bp | | |  |  |  |  |
| Genome Start | Genome End | Strand | Div | Repeat Start | Repeat End | Repeat Left | Name | Family | Class |
| 150086814 | 150087982 | + | 25.5 | 2021 | 2992 | -3227 | L1MEc | L1 | LINE |
| 150087981 | 150089641 | - | 4.8 | 4472 | 6150 | -4 | L1PA5 | L1 | LINE |
| 150089640 | 150090451 | + | 4.6 | 3659 | 4466 | -1680 | L1PA5 | L1 | LINE |
| 150090436 | 150090749 | + | 23.3 | 2967 | 3285 | -2934 | L1MEc | L1 | LINE |

  

|  |  |  |  |  |  |  |  |  |  |
| --- | --- | --- | --- | --- | --- | --- | --- | --- | --- |
| chr7:150978576-150981001 | | | 2347 bp | | |  |  |  |  |
| Genome Start | Genome End | Strand | Div | Repeat Start | Repeat End | Repeat Left | Name | Family | Class |
| 150978576 | 150979343 | + | 20.2 | 18 | 872 | -583 | Charlie1a | MER1\_type | DNA |
| 150979344 | 150979647 | + | 10.5 | 1 | 304 | -8 | AluSx | Alu | SINE |
| 150979648 | 150979766 | + | 16.1 | 873 | 975 | -480 | Charlie1a | MER1\_type | DNA |
| 150979767 | 150979798 | - | 9.4 | 5831 | 5862 | -429 | L1MA7 | L1 | LINE |
| 150979792 | 150980193 | + | 16.2 | 5860 | 6284 | -7 | L1MA7 | L1 | LINE |
| 150980196 | 150980530 | + | 16.9 | 955 | 1355 | -100 | Charlie1a | MER1\_type | DNA |
| 150980615 | 150980670 | + | 22.0 | 1344 | 1394 | -61 | Charlie1a | MER1\_type | DNA |
| 150980671 | 150980937 | - | 18.1 | 1 | 294 | -18 | AluJb | Alu | SINE |
| 150980938 | 150981001 | + | 22.0 | 1395 | 1455 | 0 | Charlie1a | MER1\_type | DNA |

  

|  |  |  |  |  |  |  |  |  |  |
| --- | --- | --- | --- | --- | --- | --- | --- | --- | --- |
| chr7:152597145-152600504 | | | 3304 bp | | |  |  |  |  |
| Genome Start | Genome End | Strand | Div | Repeat Start | Repeat End | Repeat Left | Name | Family | Class |
| 152597145 | 152597355 | + | 28.0 | 5158 | 5386 | -760 | L1M5 | L1 | LINE |
| 152597388 | 152597495 | + | 31.1 | 5470 | 5575 | -587 | L1M5 | L1 | LINE |
| 152597505 | 152598170 | - | 6.3 | 3321 | 3986 | -2160 | L1PA4 | L1 | LINE |
| 152598161 | 152600306 | + | 3.8 | 4003 | 6153 | -2 | L1PA4 | L1 | LINE |
| 152600332 | 152600504 | + | 24.1 | 5596 | 5767 | -473 | L1M5 | L1 | LINE |

  

|  |  |  |  |  |  |  |  |  |  |
| --- | --- | --- | --- | --- | --- | --- | --- | --- | --- |
| chr7:152814458-152819497 | | | 4641 bp | | |  |  |  |  |
| Genome Start | Genome End | Strand | Div | Repeat Start | Repeat End | Repeat Left | Name | Family | Class |
| 152814458 | 152814836 | + | 23.8 | 2429 | 2864 | -3282 | L1M4 | L1 | LINE |
| 152814852 | 152816890 | - | 8.4 | 4109 | 6167 | -1 | L1PA10 | L1 | LINE |
| 152816888 | 152816996 | + | 10.1 | 4006 | 4114 | -2032 | L1PA10 | L1 | LINE |
| 152817087 | 152817231 | + | 26.2 | 1 | 150 | -623 | LTR41 | ERVL | LTR |
| 152817525 | 152817868 | + | 32.6 | 337 | 669 | -104 | LTR41 | ERVL | LTR |
| 152817873 | 152819497 | + | 25.9 | 3235 | 4948 | -1198 | L1M4 | L1 | LINE |

  

|  |  |  |  |  |  |  |  |  |  |
| --- | --- | --- | --- | --- | --- | --- | --- | --- | --- |
| chr7:152825902-152830854 | | | 4960 bp | | |  |  |  |  |
| Genome Start | Genome End | Strand | Div | Repeat Start | Repeat End | Repeat Left | Name | Family | Class |
| 152825902 | 152827387 | + | 14.2 | 3 | 1450 | -968 | Tigger1 | MER2\_type | DNA |
| 152827390 | 152828772 | - | 9.1 | 4768 | 6168 | 0 | L1PA10 | L1 | LINE |
| 152828764 | 152829554 | + | 9.1 | 3979 | 4776 | -1370 | L1PA10 | L1 | LINE |
| 152829555 | 152830206 | + | 11.8 | 1437 | 2065 | -353 | Tigger1 | MER2\_type | DNA |
| 152830207 | 152830513 | - | 10.8 | 3 | 308 | -4 | AluSx | Alu | SINE |
| 152830514 | 152830854 | + | 11.8 | 2066 | 2418 | 0 | Tigger1 | MER2\_type | DNA |

  

|  |  |  |  |  |  |  |  |  |  |
| --- | --- | --- | --- | --- | --- | --- | --- | --- | --- |
| chr7:152838969-152840681 | | | 1425 bp | | |  |  |  |  |
| Genome Start | Genome End | Strand | Div | Repeat Start | Repeat End | Repeat Left | Name | Family | Class |
| 152838969 | 152839162 | - | 27.9 | 2649 | 2852 | -92 | BLACKJACK | AcHobo | DNA |
| 152839151 | 152839625 | - | 22.3 | 1846 | 2439 | -505 | BLACKJACK | AcHobo | DNA |
| 152839626 | 152839804 | - | 15.1 | 5971 | 6142 | -149 | L1MA7 | L1 | LINE |
| 152839805 | 152839938 | + | 12.7 | 6158 | 6291 | 0 | L1MA7 | L1 | LINE |
| 152839947 | 152840199 | - | 24.0 | 1629 | 1877 | -1067 | BLACKJACK | AcHobo | DNA |
| 152840275 | 152840362 | - | 14.1 | 1403 | 1497 | -1447 | BLACKJACK | AcHobo | DNA |
| 152840580 | 152840681 | - | 24.8 | 984 | 1096 | -1848 | BLACKJACK | AcHobo | DNA |

  

|  |  |  |  |  |  |  |  |  |  |
| --- | --- | --- | --- | --- | --- | --- | --- | --- | --- |
| chr7:153657250-153658311 | | | 1068 bp | | |  |  |  |  |
| Genome Start | Genome End | Strand | Div | Repeat Start | Repeat End | Repeat Left | Name | Family | Class |
| 153657250 | 153657362 | + | 25.0 | 1 | 113 | -96 | MER3 | MER1\_type | DNA |
| 153657365 | 153657472 | - | 12.0 | 5298 | 5406 | -748 | L1P | L1 | LINE |
| 153657461 | 153658215 | + | 4.8 | 5401 | 6158 | -14 | L1PA8 | L1 | LINE |
| 153658220 | 153658311 | + | 24.2 | 97 | 190 | -19 | MER3 | MER1\_type | DNA |

  


|  |  |  |  |  |  |  |  |  |  |
| --- | --- | --- | --- | --- | --- | --- | --- | --- | --- |
| chr8:92607-95114 | | | 2473 bp | | |  |  |  |  |
| Genome Start | Genome End | Strand | Div | Repeat Start | Repeat End | Repeat Left | Name | Family | Class |
| 92607 | 93095 | + | 19.5 | 3150 | 3646 | -2494 | L1M3 | L1 | LINE |
| 93112 | 93833 | - | 2.6 | 5254 | 5975 | -180 | L1PA4 | L1 | LINE |
| 93834 | 94011 | + | 4.5 | 5977 | 6155 | 0 | L1PA4 | L1 | LINE |
| 94031 | 95114 | + | 16.5 | 3657 | 4741 | -1399 | L1M3 | L1 | LINE |

  

|  |  |  |  |  |  |  |  |  |  |
| --- | --- | --- | --- | --- | --- | --- | --- | --- | --- |
| chr8:496323-502680 | | | 6018 bp | | |  |  |  |  |
| Genome Start | Genome End | Strand | Div | Repeat Start | Repeat End | Repeat Left | Name | Family | Class |
| 496323 | 496581 | - | 23.5 | 4572 | 5021 | -224 | MER31A-int | ERV1 | LTR |
| 496623 | 497263 | - | 19.5 | 3337 | 4023 | -913 | MER31A-int | ERV1 | LTR |
| 497264 | 497782 | + | 15.5 | 1 | 554 | 0 | MLT2B3 | ERVL | LTR |
| 497783 | 497800 | - | 26.6 | 3493 | 3510 | -2677 | MER57A-int | ERV1 | LTR |
| 497814 | 498205 | - | 25.6 | 2870 | 3274 | -1662 | MER57A-int | ERV1 | LTR |
| 498206 | 498534 | - | 16.5 | 1 | 396 | 0 | MSTD | MaLR | LTR |
| 498535 | 498645 | - | 26.7 | 2821 | 3196 | -2067 | MER57A-int | ERV1 | LTR |
| 498736 | 500000 | + | 9.6 | 2980 | 4249 | -1897 | L1P3 | L1 | LINE |
| 500001 | 500457 | + | 8.6 | 4250 | 4713 | -1433 | L1PA8A | L1 | LINE |
| 500457 | 500580 | - | 4.8 | 5747 | 5870 | -302 | L1PA8 | L1 | LINE |
| 500581 | 500864 | + | 7.1 | 5883 | 6166 | -6 | L1PA8 | L1 | LINE |
| 500881 | 501835 | + | 7.5 | 4696 | 5666 | -465 | L1PA8A | L1 | LINE |
| 501846 | 502224 | - | 18.8 | 1198 | 1786 | -3150 | MER31A-int | ERV1 | LTR |
| 502396 | 502680 | - | 25.0 | 168 | 482 | -4454 | MER31A-int | ERV1 | LTR |

  

|  |  |  |  |  |  |  |  |  |  |
| --- | --- | --- | --- | --- | --- | --- | --- | --- | --- |
| chr8:1510820-1512848 | | | 2010 bp | | |  |  |  |  |
| Genome Start | Genome End | Strand | Div | Repeat Start | Repeat End | Repeat Left | Name | Family | Class |
| 1510820 | 1511028 | + | 25.2 | 5845 | 6052 | -239 | L1MA7 | L1 | LINE |
| 1511064 | 1512418 | - | 4.7 | 4773 | 6132 | -22 | L1PA5 | L1 | LINE |
| 1512418 | 1512628 | + | 6.2 | 4566 | 4777 | -1369 | L1PA5 | L1 | LINE |
| 1512614 | 1512848 | + | 25.0 | 6040 | 6291 | 0 | L1MA7 | L1 | LINE |

  

|  |  |  |  |  |  |  |  |  |  |
| --- | --- | --- | --- | --- | --- | --- | --- | --- | --- |
| chr8:2247543-2248369 | | | 767 bp | | |  |  |  |  |
| Genome Start | Genome End | Strand | Div | Repeat Start | Repeat End | Repeat Left | Name | Family | Class |
| 2247543 | 2247712 | + | 22.9 | 7 | 179 | -474 | MER82 | MER2\_type | DNA |
| 2247768 | 2247908 | - | 22.0 | 6028 | 6168 | 0 | L1MB1 | L1 | LINE |
| 2247914 | 2248067 | + | 21.4 | 5853 | 6013 | -155 | L1MB1 | L1 | LINE |
| 2248068 | 2248369 | + | 19.4 | 158 | 497 | -156 | MER82 | MER2\_type | DNA |

  

|  |  |  |  |  |  |  |  |  |  |
| --- | --- | --- | --- | --- | --- | --- | --- | --- | --- |
| chr8:5173813-5177457 | | | 3594 bp | | |  |  |  |  |
| Genome Start | Genome End | Strand | Div | Repeat Start | Repeat End | Repeat Left | Name | Family | Class |
| 5173813 | 5173982 | - | 19.0 | 5076 | 5236 | -1864 | L1M3e | L1 | LINE |
| 5173983 | 5174355 | - | 13.7 | 3 | 375 | 0 | THE1C | MaLR | LTR |
| 5174356 | 5174496 | - | 19.0 | 4939 | 5075 | -2025 | L1M3e | L1 | LINE |
| 5174476 | 5174646 | - | 18.9 | 3146 | 3322 | -3781 | L1M3e | L1 | LINE |
| 5174722 | 5175121 | - | 18.6 | 5909 | 6289 | -2 | L1MA7 | L1 | LINE |
| 5175121 | 5175431 | + | 20.1 | 5590 | 5907 | -384 | L1MA7 | L1 | LINE |
| 5175430 | 5177457 | - | 29.1 | 282 | 3149 | -3957 | L1M3e | L1 | LINE |

  

|  |  |  |  |  |  |  |  |  |  |
| --- | --- | --- | --- | --- | --- | --- | --- | --- | --- |
| chr8:5305404-5310968 | | | 5532 bp | | |  |  |  |  |
| Genome Start | Genome End | Strand | Div | Repeat Start | Repeat End | Repeat Left | Name | Family | Class |
| 5305404 | 5305620 | + | 28.7 | 6776 | 6994 | -814 | L1MC4a | L1 | LINE |
| 5305648 | 5305764 | + | 23.9 | 7119 | 7236 | -759 | L1MD3 | L1 | LINE |
| 5305777 | 5309263 | - | 3.3 | 2659 | 6151 | -4 | L1PA3 | L1 | LINE |
| 5309262 | 5310296 | + | 5.3 | 1622 | 2656 | -3490 | L1PA3 | L1 | LINE |
| 5310293 | 5310968 | + | 21.5 | 7171 | 7808 | 0 | L1MC4a | L1 | LINE |

  

|  |  |  |  |  |  |  |  |  |  |
| --- | --- | --- | --- | --- | --- | --- | --- | --- | --- |
| chr8:5772494-5777591 | | | 5058 bp | | |  |  |  |  |
| Genome Start | Genome End | Strand | Div | Repeat Start | Repeat End | Repeat Left | Name | Family | Class |
| 5772494 | 5773227 | + | 26.5 | 4759 | 5553 | -1453 | L1MDb | L1 | LINE |
| 5773228 | 5773364 | + | 12.5 | 6160 | 6295 | -2 | L1MA5A | L1 | LINE |
| 5773370 | 5773615 | - | 5.3 | 2263 | 2508 | -3638 | L1PA3 | L1 | LINE |
| 5773615 | 5777215 | + | 4.2 | 2518 | 6137 | -18 | L1PA3 | L1 | LINE |
| 5777252 | 5777267 | + | 3.7 | 6138 | 6151 | -4 | L1PA3 | L1 | LINE |
| 5777268 | 5777591 | + | 27.9 | 5552 | 5890 | -1116 | L1MDb | L1 | LINE |

  

|  |  |  |  |  |  |  |  |  |  |
| --- | --- | --- | --- | --- | --- | --- | --- | --- | --- |
| chr8:9836117-9836932 | | | 814 bp | | |  |  |  |  |
| Genome Start | Genome End | Strand | Div | Repeat Start | Repeat End | Repeat Left | Name | Family | Class |
| 9836117 | 9836435 | + | 12.5 | 1 | 323 | -41 | THE1B | MaLR | LTR |
| 9836436 | 9836503 | - | 2.9 | 5703 | 5770 | -384 | L1PA5 | L1 | LINE |
| 9836504 | 9836875 | + | 5.1 | 5783 | 6154 | 0 | L1PA5 | L1 | LINE |
| 9836878 | 9836932 | + | 10.9 | 310 | 364 | 0 | THE1B | MaLR | LTR |

  

|  |  |  |  |  |  |  |  |  |  |
| --- | --- | --- | --- | --- | --- | --- | --- | --- | --- |
| chr8:10674353-10678293 | | | 3817 bp | | |  |  |  |  |
| Genome Start | Genome End | Strand | Div | Repeat Start | Repeat End | Repeat Left | Name | Family | Class |
| 10674353 | 10674943 | + | 28.4 | 6279 | 6866 | -1095 | L1MC5 | L1 | LINE |
| 10674941 | 10675476 | - | 19.7 | 3797 | 4349 | -1794 | L1MA9 | L1 | LINE |
| 10675478 | 10677291 | + | 18.5 | 4333 | 6177 | -135 | L1MA9 | L1 | LINE |
| 10677374 | 10677665 | + | 11.6 | 1 | 294 | -18 | AluSx | Alu | SINE |
| 10677668 | 10677913 | + | 19.6 | 6860 | 7109 | -852 | L1MC5 | L1 | LINE |
| 10677956 | 10678293 | + | 24.8 | 7477 | 7804 | -157 | L1MC5 | L1 | LINE |

  

|  |  |  |  |  |  |  |  |  |  |
| --- | --- | --- | --- | --- | --- | --- | --- | --- | --- |
| chr8:12446061-12447004 | | | 933 bp | | |  |  |  |  |
| Genome Start | Genome End | Strand | Div | Repeat Start | Repeat End | Repeat Left | Name | Family | Class |
| 12446061 | 12446188 | + | 16.3 | 5 | 129 | -49 | MER5B | MER1\_type | DNA |
| 12446210 | 12446512 | - | 13.9 | 2 | 304 | -8 | AluSx | Alu | SINE |
| 12446518 | 12446799 | - | 18.7 | 5902 | 6174 | 0 | L1MB5 | L1 | LINE |
| 12446770 | 12446945 | + | 23.3 | 5684 | 5887 | -287 | L1MB5 | L1 | LINE |
| 12446961 | 12447004 | + | 9.1 | 135 | 178 | 0 | MER5B | MER1\_type | DNA |

  

|  |  |  |  |  |  |  |  |  |  |
| --- | --- | --- | --- | --- | --- | --- | --- | --- | --- |
| chr8:14190138-14193470 | | | 3320 bp | | |  |  |  |  |
| Genome Start | Genome End | Strand | Div | Repeat Start | Repeat End | Repeat Left | Name | Family | Class |
| 14190138 | 14191476 | - | 24.4 | 4824 | 6174 | 0 | L1MB5 | L1 | LINE |
| 14191453 | 14191515 | - | 11.1 | 4744 | 4806 | -1340 | L1M1 | L1 | LINE |
| 14191515 | 14191650 | + | 17.0 | 4810 | 4946 | -1197 | L1MA6 | L1 | LINE |
| 14191651 | 14192025 | - | 15.4 | 1 | 381 | 0 | THE1D | MaLR | LTR |
| 14192026 | 14193310 | + | 17.4 | 4947 | 6296 | -4 | L1MA6 | L1 | LINE |
| 14193349 | 14193470 | - | 28.7 | 4668 | 4800 | -1346 | L1MB5 | L1 | LINE |

  

|  |  |  |  |  |  |  |  |  |  |
| --- | --- | --- | --- | --- | --- | --- | --- | --- | --- |
| chr8:14738943-14741379 | | | 2010 bp | | |  |  |  |  |
| Genome Start | Genome End | Strand | Div | Repeat Start | Repeat End | Repeat Left | Name | Family | Class |
| 14738943 | 14739066 | - | 29.8 | 3279 | 3404 | -15 | L2 | L2 | LINE |
| 14739094 | 14739269 | - | 33.1 | 2999 | 3181 | -238 | L2 | L2 | LINE |
| 14739350 | 14739462 | + | 23.4 | 65 | 188 | -287 | MLT1J | MaLR | LTR |
| 14739674 | 14739851 | - | 16.4 | 6107 | 6291 | -9 | L1MA4 | L1 | LINE |
| 14739857 | 14740162 | + | 14.6 | 5782 | 6110 | -190 | L1MA4 | L1 | LINE |
| 14740163 | 14740638 | + | 25.8 | 81 | 548 | -74 | MER74B | ERVL | LTR |
| 14740743 | 14741379 | - | 31.6 | 2330 | 2999 | -420 | L2 | L2 | LINE |

  

|  |  |  |  |  |  |  |  |  |  |
| --- | --- | --- | --- | --- | --- | --- | --- | --- | --- |
| chr8:15327954-15334209 | | | 6201 bp | | |  |  |  |  |
| Genome Start | Genome End | Strand | Div | Repeat Start | Repeat End | Repeat Left | Name | Family | Class |
| 15327954 | 15329107 | - | 18.0 | 5151 | 6331 | -3 | L1MA10 | L1 | LINE |
| 15329144 | 15329362 | + | 15.3 | 1 | 222 | -512 | MER50 | ERV1 | LTR |
| 15329384 | 15329639 | + | 14.6 | 452 | 711 | -23 | MER50 | ERV1 | LTR |
| 15329640 | 15329841 | - | 15.3 | 4936 | 5140 | -1000 | L1MA10 | L1 | LINE |
| 15329848 | 15331630 | - | 3.6 | 4378 | 6155 | 0 | L1PA4 | L1 | LINE |
| 15331627 | 15332734 | + | 5.4 | 3255 | 4365 | -1781 | L1PA4 | L1 | LINE |
| 15332731 | 15334209 | - | 17.7 | 3430 | 4951 | -1189 | L1MA10 | L1 | LINE |

  

|  |  |  |  |  |  |  |  |  |  |
| --- | --- | --- | --- | --- | --- | --- | --- | --- | --- |
| chr8:15731026-15736173 | | | 4808 bp | | |  |  |  |  |
| Genome Start | Genome End | Strand | Div | Repeat Start | Repeat End | Repeat Left | Name | Family | Class |
| 15731026 | 15731394 | - | 30.7 | 5388 | 5793 | -2015 | L1MC | L1 | LINE |
| 15731397 | 15731597 | - | 28.9 | 5039 | 5258 | -888 | L1M5 | L1 | LINE |
| 15731655 | 15732320 | - | 14.3 | 4998 | 5679 | -477 | L1PB4 | L1 | LINE |
| 15732321 | 15732794 | + | 18.8 | 5674 | 6154 | -2 | L1PB4 | L1 | LINE |
| 15732818 | 15734940 | + | 12.5 | 1242 | 3391 | -3375 | L1PA13 | L1 | LINE |
| 15734914 | 15735733 | + | 11.5 | 5937 | 6783 | 0 | L1PA13 | L1 | LINE |
| 15736019 | 15736173 | - | 30.5 | 4406 | 4566 | -1580 | L1MC | L1 | LINE |

  

|  |  |  |  |  |  |  |  |  |  |
| --- | --- | --- | --- | --- | --- | --- | --- | --- | --- |
| chr8:15806626-15808023 | | | 1383 bp | | |  |  |  |  |
| Genome Start | Genome End | Strand | Div | Repeat Start | Repeat End | Repeat Left | Name | Family | Class |
| 15806626 | 15806872 | - | 16.5 | 185 | 428 | 0 | MSTA | MaLR | LTR |
| 15806889 | 15807740 | - | 5.6 | 5277 | 6145 | -9 | L1PA5 | L1 | LINE |
| 15807740 | 15807850 | + | 6.3 | 5167 | 5277 | -869 | L1P3 | L1 | LINE |
| 15807851 | 15808023 | - | 16.9 | 1 | 195 | -233 | MSTA | MaLR | LTR |

  

|  |  |  |  |  |  |  |  |  |  |
| --- | --- | --- | --- | --- | --- | --- | --- | --- | --- |
| chr8:16742850-16745219 | | | 2331 bp | | |  |  |  |  |
| Genome Start | Genome End | Strand | Div | Repeat Start | Repeat End | Repeat Left | Name | Family | Class |
| 16742850 | 16743047 | - | 33.9 | 300 | 491 | 0 | LTR16C | ERVL | LTR |
| 16743052 | 16743091 | + | 12.8 | 2 | 33 | -160 | MER53 | DNA | DNA |
| 16743092 | 16743374 | + | 16.0 | 6 | 306 | -6 | AluJb | Alu | SINE |
| 16743375 | 16743426 | + | 12.8 | 34 | 79 | -114 | MER53 | DNA | DNA |
| 16743462 | 16743673 | - | 4.7 | 5943 | 6154 | -1 | L1PA4 | L1 | LINE |
| 16743674 | 16744970 | + | 3.7 | 4638 | 5934 | -221 | L1PA4 | L1 | LINE |
| 16744971 | 16745114 | - | 18.4 | 2 | 143 | -50 | MER53 | DNA | DNA |
| 16745115 | 16745219 | - | 30.1 | 178 | 291 | -200 | LTR16C | ERVL | LTR |

  

|  |  |  |  |  |  |  |  |  |  |
| --- | --- | --- | --- | --- | --- | --- | --- | --- | --- |
| chr8:18108217-18110333 | | | 2101 bp | | |  |  |  |  |
| Genome Start | Genome End | Strand | Div | Repeat Start | Repeat End | Repeat Left | Name | Family | Class |
| 18108217 | 18108366 | + | 20.4 | 4021 | 4170 | -1970 | L1M3 | L1 | LINE |
| 18108371 | 18109307 | - | 11.7 | 4520 | 5391 | -755 | L1PA10 | L1 | LINE |
| 18109305 | 18110075 | + | 9.9 | 5392 | 6156 | -12 | L1PA10 | L1 | LINE |
| 18110091 | 18110333 | + | 19.0 | 4145 | 4406 | -1734 | L1M3 | L1 | LINE |

  

|  |  |  |  |  |  |  |  |  |  |
| --- | --- | --- | --- | --- | --- | --- | --- | --- | --- |
| chr8:18469405-18473465 | | | 3580 bp | | |  |  |  |  |
| Genome Start | Genome End | Strand | Div | Repeat Start | Repeat End | Repeat Left | Name | Family | Class |
| 18469405 | 18469604 | + | 32.5 | 1804 | 2001 | -1418 | L2 | L2 | LINE |
| 18469605 | 18470010 | + | 18.5 | 1 | 422 | -4 | MSTB | MaLR | LTR |
| 18470011 | 18470066 | + | 32.5 | 2002 | 2050 | -1369 | L2 | L2 | LINE |
| 18470497 | 18471219 | - | 2.5 | 5432 | 6154 | -1 | L1PA2 | L1 | LINE |
| 18471220 | 18471423 | + | 2.0 | 4316 | 4519 | -1627 | L1P1 | L1 | LINE |
| 18471423 | 18471652 | - | 4.3 | 5906 | 6135 | -20 | L1PA2 | L1 | LINE |
| 18471650 | 18472545 | + | 1.9 | 4527 | 5424 | -731 | L1P1 | L1 | LINE |
| 18472567 | 18472633 | + | 34.0 | 2776 | 2845 | -574 | L2 | L2 | LINE |
| 18472668 | 18472950 | - | 13.5 | 1 | 282 | -30 | AluSx | Alu | SINE |
| 18472951 | 18473465 | + | 34.2 | 2846 | 3415 | -4 | L2 | L2 | LINE |

  

|  |  |  |  |  |  |  |  |  |  |
| --- | --- | --- | --- | --- | --- | --- | --- | --- | --- |
| chr8:20298125-20302087 | | | 3860 bp | | |  |  |  |  |
| Genome Start | Genome End | Strand | Div | Repeat Start | Repeat End | Repeat Left | Name | Family | Class |
| 20298125 | 20298260 | - | 27.3 | 5794 | 5934 | -187 | L1M5 | L1 | LINE |
| 20298367 | 20300462 | - | 15.2 | 4130 | 6312 | 0 | L1MA9 | L1 | LINE |
| 20300463 | 20300747 | + | 15.8 | 1 | 300 | -12 | AluJo | Alu | SINE |
| 20300748 | 20300970 | - | 14.9 | 3916 | 4129 | -2011 | L1MA9 | L1 | LINE |
| 20300969 | 20301773 | + | 15.1 | 3100 | 3918 | -2222 | L1MA9 | L1 | LINE |
| 20301773 | 20301812 | - | 32.5 | 5623 | 5662 | -459 | L1M5 | L1 | LINE |
| 20301813 | 20302005 | + | 29.9 | 1 | 210 | 0 | MER63A | AcHobo | DNA |
| 20302006 | 20302087 | - | 32.5 | 5546 | 5622 | -499 | L1M5 | L1 | LINE |

  

|  |  |  |  |  |  |  |  |  |  |
| --- | --- | --- | --- | --- | --- | --- | --- | --- | --- |
| chr8:21439767-21444104 | | | 4329 bp | | |  |  |  |  |
| Genome Start | Genome End | Strand | Div | Repeat Start | Repeat End | Repeat Left | Name | Family | Class |
| 21439767 | 21440399 | - | 16.0 | 5972 | 6633 | 0 | L1MD1 | L1 | LINE |
| 21440400 | 21441056 | - | 2.8 | 5060 | 5719 | -436 | L1PA4 | L1 | LINE |
| 21441057 | 21441486 | + | 4.0 | 5720 | 6152 | -3 | L1PA4 | L1 | LINE |
| 21441496 | 21442038 | - | 16.0 | 5440 | 5983 | -650 | L1MD1 | L1 | LINE |
| 21442039 | 21442341 | - | 11.7 | 1 | 300 | -10 | AluSg | Alu | SINE |
| 21442342 | 21442984 | - | 17.3 | 4772 | 5439 | -1098 | L1MD1 | L1 | LINE |
| 21442985 | 21443328 | - | 17.8 | 1 | 418 | -1317 | MSTB1-int | MaLR | LTR |
| 21443329 | 21443762 | - | 13.1 | 1 | 432 | 0 | MSTB1 | MaLR | LTR |
| 21443763 | 21444104 | - | 22.1 | 26 | 369 | -6264 | L1MD1 | L1 | LINE |

  

|  |  |  |  |  |  |  |  |  |  |
| --- | --- | --- | --- | --- | --- | --- | --- | --- | --- |
| chr8:24134258-24135774 | | | 1251 bp | | |  |  |  |  |
| Genome Start | Genome End | Strand | Div | Repeat Start | Repeat End | Repeat Left | Name | Family | Class |
| 24134258 | 24134390 | + | 25.0 | 17 | 180 | -435 | MLT1L | MaLR | LTR |
| 24134401 | 24134649 | - | 12.9 | 5871 | 6118 | -13 | L1PA8A | L1 | LINE |
| 24134650 | 24134719 | - | 7.8 | 5557 | 5620 | -511 | L1PA8A | L1 | LINE |
| 24134718 | 24135266 | + | 7.6 | 4971 | 5537 | -594 | L1PA8A | L1 | LINE |
| 24135267 | 24135404 | + | 30.6 | 127 | 269 | -346 | MLT1L | MaLR | LTR |
| 24135663 | 24135774 | + | 29.7 | 487 | 605 | -10 | MLT1L | MaLR | LTR |

  

|  |  |  |  |  |  |  |  |  |  |
| --- | --- | --- | --- | --- | --- | --- | --- | --- | --- |
| chr8:26978786-26983243 | | | 4229 bp | | |  |  |  |  |
| Genome Start | Genome End | Strand | Div | Repeat Start | Repeat End | Repeat Left | Name | Family | Class |
| 26978786 | 26979007 | + | 19.6 | 4897 | 5104 | -1042 | L1MC2 | L1 | LINE |
| 26979024 | 26979572 | - | 23.7 | 3 | 529 | -19 | LTR55 | ERV | LTR |
| 26979666 | 26980278 | + | 16.1 | 5150 | 5756 | -588 | L1MC2 | L1 | LINE |
| 26980294 | 26981418 | - | 15.4 | 5122 | 6294 | -6 | L1MA4 | L1 | LINE |
| 26981390 | 26981921 | + | 15.3 | 4180 | 4713 | -1430 | L1M2 | L1 | LINE |
| 26981927 | 26981968 | - | 14.3 | 4312 | 4353 | -1793 | L1MA4 | L1 | LINE |
| 26982027 | 26982158 | + | 18.2 | 1 | 132 | -44 | FRAM | Alu | SINE |
| 26982230 | 26982619 | + | 14.4 | 4741 | 5119 | -1024 | L1M2 | L1 | LINE |
| 26982620 | 26983243 | + | 15.1 | 5738 | 6335 | -9 | L1MC2 | L1 | LINE |

  

|  |  |  |  |  |  |  |  |  |  |
| --- | --- | --- | --- | --- | --- | --- | --- | --- | --- |
| chr8:27725074-27728307 | | | 3173 bp | | |  |  |  |  |
| Genome Start | Genome End | Strand | Div | Repeat Start | Repeat End | Repeat Left | Name | Family | Class |
| 27725074 | 27725750 | - | 23.2 | 3898 | 4661 | -1485 | L1M5 | L1 | LINE |
| 27725759 | 27726890 | + | 5.3 | 4989 | 6134 | -20 | L1PA6 | L1 | LINE |
| 27726936 | 27727119 | - | 11.4 | 6103 | 6298 | -14 | L1MA9 | L1 | LINE |
| 27727124 | 27727190 | + | 14.9 | 6018 | 6084 | -228 | L1MA9 | L1 | LINE |
| 27727195 | 27727361 | - | 21.1 | 3682 | 3848 | -2298 | L1M5 | L1 | LINE |
| 27727362 | 27727464 | - | 19.4 | 6196 | 6299 | -6 | L1MA3 | L1 | LINE |
| 27727465 | 27728307 | - | 21.1 | 2770 | 3681 | -2465 | L1M5 | L1 | LINE |

  

|  |  |  |  |  |  |  |  |  |  |
| --- | --- | --- | --- | --- | --- | --- | --- | --- | --- |
| chr8:31482765-31490255 | | | 7479 bp | | |  |  |  |  |
| Genome Start | Genome End | Strand | Div | Repeat Start | Repeat End | Repeat Left | Name | Family | Class |
| 31482765 | 31484645 | - | 11.7 | 4976 | 6866 | -3 | L1PA16 | L1 | LINE |
| 31484660 | 31486662 | - | 5.7 | 4137 | 6145 | -9 | L1PA5 | L1 | LINE |
| 31486657 | 31487359 | + | 6.0 | 3451 | 4151 | -1995 | L1PA5 | L1 | LINE |
| 31487364 | 31490255 | - | 12.6 | 2062 | 4987 | -1862 | L1PA16 | L1 | LINE |

  

|  |  |  |  |  |  |  |  |  |  |
| --- | --- | --- | --- | --- | --- | --- | --- | --- | --- |
| chr8:31734812-31737065 | | | 2236 bp | | |  |  |  |  |
| Genome Start | Genome End | Strand | Div | Repeat Start | Repeat End | Repeat Left | Name | Family | Class |
| 31734812 | 31735109 | - | 21.9 | 5886 | 6155 | -9 | L1ME2 | L1 | LINE |
| 31735137 | 31735407 | - | 6.3 | 5865 | 6135 | -19 | L1PA7 | L1 | LINE |
| 31735408 | 31735645 | + | 6.0 | 5627 | 5861 | -293 | L1PA7 | L1 | LINE |
| 31735637 | 31737065 | - | 25.7 | 4348 | 5893 | -271 | L1ME2 | L1 | LINE |

  

|  |  |  |  |  |  |  |  |  |  |
| --- | --- | --- | --- | --- | --- | --- | --- | --- | --- |
| chr8:31737079-31738986 | | | 1878 bp | | |  |  |  |  |
| Genome Start | Genome End | Strand | Div | Repeat Start | Repeat End | Repeat Left | Name | Family | Class |
| 31737079 | 31737175 | - | 13.4 | 1605 | 1701 | -717 | Tigger1 | MER2\_type | DNA |
| 31737190 | 31737425 | - | 2.5 | 4836 | 5071 | -1075 | L1PA3 | L1 | LINE |
| 31737426 | 31738523 | + | 3.3 | 5058 | 6155 | 0 | L1PA3 | L1 | LINE |
| 31738540 | 31738986 | - | 18.6 | 24 | 475 | -1943 | Tigger1 | MER2\_type | DNA |

  

|  |  |  |  |  |  |  |  |  |  |
| --- | --- | --- | --- | --- | --- | --- | --- | --- | --- |
| chr8:32099330-32100243 | | | 859 bp | | |  |  |  |  |
| Genome Start | Genome End | Strand | Div | Repeat Start | Repeat End | Repeat Left | Name | Family | Class |
| 32099330 | 32099442 | + | 25.2 | 4 | 116 | -667 | MER20B | MER1\_type | DNA |
| 32099472 | 32099674 | + | 26.0 | 178 | 382 | -401 | MER20B | MER1\_type | DNA |
| 32099700 | 32100009 | - | 4.2 | 5828 | 6137 | -18 | L1PA3 | L1 | LINE |
| 32100010 | 32100060 | + | 3.9 | 5772 | 5822 | -333 | L1PA3 | L1 | LINE |
| 32100062 | 32100243 | + | 29.7 | 376 | 558 | -225 | MER20B | MER1\_type | DNA |

  

|  |  |  |  |  |  |  |  |  |  |
| --- | --- | --- | --- | --- | --- | --- | --- | --- | --- |
| chr8:32793180-32797420 | | | 4183 bp | | |  |  |  |  |
| Genome Start | Genome End | Strand | Div | Repeat Start | Repeat End | Repeat Left | Name | Family | Class |
| 32793180 | 32793250 | - | 8.4 | 359 | 430 | -2 | MSTB1 | MaLR | LTR |
| 32793309 | 32795124 | - | 0.7 | 4338 | 6153 | -2 | L1HS | L1 | LINE |
| 32795125 | 32796724 | + | 0.2 | 2725 | 4324 | -1822 | L1HS | L1 | LINE |
| 32796725 | 32796904 | - | 14.0 | 202 | 376 | -56 | MSTB1 | MaLR | LTR |
| 32796905 | 32797210 | - | 6.2 | 1 | 305 | -6 | AluY | Alu | SINE |
| 32797211 | 32797420 | - | 14.0 | 1 | 201 | -231 | MSTB1 | MaLR | LTR |

  

|  |  |  |  |  |  |  |  |  |  |
| --- | --- | --- | --- | --- | --- | --- | --- | --- | --- |
| chr8:35846009-35847822 | | | 1797 bp | | |  |  |  |  |
| Genome Start | Genome End | Strand | Div | Repeat Start | Repeat End | Repeat Left | Name | Family | Class |
| 35846009 | 35846159 | + | 27.9 | 2772 | 2906 | -513 | L2 | L2 | LINE |
| 35846160 | 35846799 | + | 12.3 | 1 | 640 | 0 | MER51C | ERV1 | LTR |
| 35846800 | 35847056 | + | 29.5 | 2907 | 3176 | -243 | L2 | L2 | LINE |
| 35847072 | 35847371 | - | 9.2 | 5851 | 6151 | 0 | L1PB1 | L1 | LINE |
| 35847373 | 35847575 | + | 11.4 | 5647 | 5847 | -304 | L1PB1 | L1 | LINE |
| 35847577 | 35847822 | + | 34.6 | 3168 | 3415 | -4 | L2 | L2 | LINE |

  

|  |  |  |  |  |  |  |  |  |  |
| --- | --- | --- | --- | --- | --- | --- | --- | --- | --- |
| chr8:36233985-36238569 | | | 4562 bp | | |  |  |  |  |
| Genome Start | Genome End | Strand | Div | Repeat Start | Repeat End | Repeat Left | Name | Family | Class |
| 36233985 | 36234664 | + | 19.4 | 6709 | 7339 | -446 | L1MC3 | L1 | LINE |
| 36234665 | 36235220 | - | 15.9 | 3 | 585 | 0 | MER34C\_ | ERV1 | LTR |
| 36235221 | 36235297 | + | 19.4 | 7340 | 7408 | -377 | L1MC3 | L1 | LINE |
| 36235298 | 36236014 | - | 17.3 | 1 | 722 | 0 | MER72 | ERV1 | LTR |
| 36236015 | 36236122 | + | 19.4 | 7409 | 7512 | -273 | L1MC3 | L1 | LINE |
| 36236126 | 36236755 | - | 8.2 | 3989 | 4614 | -1532 | L1PA7 | L1 | LINE |
| 36236745 | 36238289 | + | 7.7 | 4602 | 6152 | -2 | L1PA7 | L1 | LINE |
| 36238321 | 36238569 | + | 21.8 | 7527 | 7777 | -8 | L1MC3 | L1 | LINE |

  

|  |  |  |  |  |  |  |  |  |  |
| --- | --- | --- | --- | --- | --- | --- | --- | --- | --- |
| chr8:37168415-37169850 | | | 1433 bp | | |  |  |  |  |
| Genome Start | Genome End | Strand | Div | Repeat Start | Repeat End | Repeat Left | Name | Family | Class |
| 37168415 | 37168466 | + | 17.6 | 49 | 99 | -291 | MLT1B | MaLR | LTR |
| 37168467 | 37168586 | - | 1.7 | 5427 | 5546 | -608 | L1PA4 | L1 | LINE |
| 37168587 | 37169186 | + | 2.3 | 5556 | 6155 | 0 | L1PA4 | L1 | LINE |
| 37169190 | 37169333 | + | 19.7 | 81 | 216 | -174 | MLT1B | MaLR | LTR |
| 37169334 | 37169677 | + | 14.3 | 1 | 364 | 0 | THE1B | MaLR | LTR |
| 37169678 | 37169850 | + | 19.7 | 217 | 385 | -5 | MLT1B | MaLR | LTR |

  

|  |  |  |  |  |  |  |  |  |  |
| --- | --- | --- | --- | --- | --- | --- | --- | --- | --- |
| chr8:39435560-39441616 | | | 5964 bp | | |  |  |  |  |
| Genome Start | Genome End | Strand | Div | Repeat Start | Repeat End | Repeat Left | Name | Family | Class |
| 39435560 | 39435854 | + | 24.9 | 3400 | 3702 | -2444 | L1MD2 | L1 | LINE |
| 39435899 | 39437100 | + | 24.9 | 3703 | 4921 | -1225 | L1MD2 | L1 | LINE |
| 39437096 | 39437811 | - | 6.1 | 4308 | 5041 | -1105 | L1PB | L1 | LINE |
| 39437812 | 39438344 | + | 10.9 | 5657 | 6150 | -1 | L1PB1 | L1 | LINE |
| 39438345 | 39438676 | + | 28.9 | 4902 | 5273 | -873 | L1MD2 | L1 | LINE |
| 39438682 | 39438899 | - | 13.2 | 4262 | 4528 | -1618 | L1MA3 | L1 | LINE |
| 39438896 | 39440615 | + | 13.2 | 4538 | 6305 | 0 | L1MA3 | L1 | LINE |
| 39440638 | 39441460 | + | 24.8 | 5278 | 6129 | -230 | L1MD2 | L1 | LINE |
| 39441492 | 39441616 | + | 24.8 | 6130 | 6275 | -84 | L1MD2 | L1 | LINE |

  

|  |  |  |  |  |  |  |  |  |  |
| --- | --- | --- | --- | --- | --- | --- | --- | --- | --- |
| chr8:39465530-39466173 | | | 597 bp | | |  |  |  |  |
| Genome Start | Genome End | Strand | Div | Repeat Start | Repeat End | Repeat Left | Name | Family | Class |
| 39465530 | 39465710 | + | 28.0 | 4563 | 4742 | -1404 | L1ME3B | L1 | LINE |
| 39465717 | 39465825 | - | 4.5 | 5866 | 5980 | -194 | L1PA5 | L1 | LINE |
| 39465826 | 39466015 | + | 4.2 | 5964 | 6153 | -1 | L1PA5 | L1 | LINE |
| 39466057 | 39466173 | + | 35.9 | 4823 | 4940 | -1206 | L1ME3B | L1 | LINE |

  

|  |  |  |  |  |  |  |  |  |  |
| --- | --- | --- | --- | --- | --- | --- | --- | --- | --- |
| chr8:39802231-39805223 | | | 2902 bp | | |  |  |  |  |
| Genome Start | Genome End | Strand | Div | Repeat Start | Repeat End | Repeat Left | Name | Family | Class |
| 39802231 | 39802481 | + | 27.8 | 1994 | 2217 | -5117 | L1M | L1 | LINE |
| 39802548 | 39802802 | - | 15.6 | 4405 | 4656 | -1484 | L1MA8 | L1 | LINE |
| 39802796 | 39803117 | + | 16.7 | 4668 | 4985 | -1158 | L1MA8 | L1 | LINE |
| 39803118 | 39803418 | + | 8.6 | 1 | 310 | -1 | AluY | Alu | SINE |
| 39803419 | 39804671 | + | 16.7 | 4986 | 6281 | -10 | L1MA8 | L1 | LINE |
| 39804704 | 39805223 | + | 31.0 | 2434 | 2985 | -4041 | L1M | L1 | LINE |

  

|  |  |  |  |  |  |  |  |  |  |
| --- | --- | --- | --- | --- | --- | --- | --- | --- | --- |
| chr8:40214893-40223447 | | | 7828 bp | | |  |  |  |  |
| Genome Start | Genome End | Strand | Div | Repeat Start | Repeat End | Repeat Left | Name | Family | Class |
| 40214893 | 40215095 | - | 25.4 | 1658 | 1899 | -608 | HAL1 | L1 | LINE |
| 40215219 | 40218134 | - | 17.9 | 1902 | 4950 | -2615 | L1M1 | L1 | LINE |
| 40218132 | 40219031 | + | 16.3 | 4047 | 5013 | -1133 | L1MA4 | L1 | LINE |
| 40219018 | 40219140 | - | 7.1 | 4072 | 4184 | -1962 | L1PB1 | L1 | LINE |
| 40219136 | 40220746 | + | 8.3 | 4178 | 5782 | -369 | L1PB1 | L1 | LINE |
| 40220996 | 40221364 | + | 12.6 | 5783 | 6149 | -2 | L1PB1 | L1 | LINE |
| 40221396 | 40222690 | + | 17.0 | 4986 | 6299 | -1 | L1MA4 | L1 | LINE |
| 40222720 | 40222887 | - | 31.7 | 1472 | 1646 | -861 | HAL1 | L1 | LINE |
| 40222988 | 40223107 | + | 12.5 | 190 | 309 | 0 | AluSc | Alu | SINE |
| 40223325 | 40223447 | - | 22.9 | 5 | 141 | -2366 | HAL1 | L1 | LINE |

  

|  |  |  |  |  |  |  |  |  |  |
| --- | --- | --- | --- | --- | --- | --- | --- | --- | --- |
| chr8:40225197-40225933 | | | 685 bp | | |  |  |  |  |
| Genome Start | Genome End | Strand | Div | Repeat Start | Repeat End | Repeat Left | Name | Family | Class |
| 40225197 | 40225392 | - | 24.0 | 200 | 396 | 0 | MSTD | MaLR | LTR |
| 40225432 | 40225689 | - | 3.5 | 5895 | 6152 | -3 | L1PA4 | L1 | LINE |
| 40225690 | 40225774 | + | 1.2 | 5817 | 5901 | -254 | L1PA4 | L1 | LINE |
| 40225788 | 40225933 | - | 16.7 | 1 | 138 | -258 | MSTD | MaLR | LTR |

  

|  |  |  |  |  |  |  |  |  |  |
| --- | --- | --- | --- | --- | --- | --- | --- | --- | --- |
| chr8:47631807-47636053 | | | 4202 bp | | |  |  |  |  |
| Genome Start | Genome End | Strand | Div | Repeat Start | Repeat End | Repeat Left | Name | Family | Class |
| 47631807 | 47632512 | - | 11.5 | 2002 | 2731 | 0 | Tigger4(Zombi) | MER2\_type | DNA |
| 47632513 | 47634510 | - | 2.4 | 3207 | 5203 | -943 | L1PA2 | L1 | LINE |
| 47634508 | 47635431 | + | 2.8 | 5217 | 6144 | -11 | L1PA2 | L1 | LINE |
| 47635481 | 47635582 | - | 12.8 | 1911 | 2012 | -719 | Tigger4(Zombi) | MER2\_type | DNA |
| 47635582 | 47636053 | - | 16.7 | 3 | 530 | -2201 | Tigger4(Zombi) | MER2\_type | DNA |

  

|  |  |  |  |  |  |  |  |  |  |
| --- | --- | --- | --- | --- | --- | --- | --- | --- | --- |
| chr8:47756304-47766633 | | | 9663 bp | | |  |  |  |  |
| Genome Start | Genome End | Strand | Div | Repeat Start | Repeat End | Repeat Left | Name | Family | Class |
| 47756304 | 47756761 | - | 30.7 | 5653 | 6129 | -1747 | L1MC4a | L1 | LINE |
| 47757103 | 47757394 | - | 23.3 | 7719 | 8041 | -1 | L1MC4 | L1 | LINE |
| 47757493 | 47757753 | - | 28.5 | 3798 | 4065 | -2149 | L1MC4a | L1 | LINE |
| 47757760 | 47758181 | + | 9.5 | 3 | 423 | -5732 | L1PA4 | L1 | LINE |
| 47758181 | 47762337 | + | 5.3 | 1974 | 6152 | -3 | L1PA4 | L1 | LINE |
| 47762351 | 47762852 | - | 34.5 | 3321 | 3805 | -2409 | L1MC4a | L1 | LINE |
| 47762869 | 47763658 | - | 4.2 | 5360 | 6152 | -3 | L1PA3 | L1 | LINE |
| 47763655 | 47765157 | + | 3.7 | 3863 | 5365 | -781 | L1PA3 | L1 | LINE |
| 47765220 | 47765530 | - | 8.7 | 1 | 311 | -2 | AluSp | Alu | SINE |
| 47765605 | 47765979 | - | 26.5 | 2813 | 3207 | -3007 | L1MC4a | L1 | LINE |
| 47765986 | 47766244 | - | 15.8 | 1 | 293 | -17 | AluSg | Alu | SINE |
| 47766245 | 47766308 | - | 27.5 | 2757 | 2811 | -3403 | L1MC4a | L1 | LINE |
| 47766365 | 47766633 | - | 24.1 | 2250 | 2545 | -3670 | L1MC4a | L1 | LINE |

  

|  |  |  |  |  |  |  |  |  |  |
| --- | --- | --- | --- | --- | --- | --- | --- | --- | --- |
| chr8:48369773-48384149 | | | 14582 bp | | |  |  |  |  |
| Genome Start | Genome End | Strand | Div | Repeat Start | Repeat End | Repeat Left | Name | Family | Class |
| 48369773 | 48369956 | - | 15.8 | 117 | 300 | -12 | AluJo | Alu | SINE |
| 48369979 | 48370476 | - | 19.9 | 823 | 1360 | -5040 | L1MEc | L1 | LINE |
| 48370483 | 48372601 | - | 17.5 | 3900 | 6117 | -174 | L1MA7 | L1 | LINE |
| 48372600 | 48378724 | - | 5.6 | 4 | 6154 | -1 | L1PA4 | L1 | LINE |
| 48378861 | 48379377 | - | 23.5 | 5589 | 6143 | -21 | L1ME2 | L1 | LINE |
| 48378977 | 48380242 | - | 27.7 | 4490 | 5952 | -1977 | L1MD3 | L1 | LINE |
| 48380246 | 48380947 | - | 26.9 | 2044 | 2803 | -3277 | L1MD3 | L1 | LINE |
| 48380948 | 48381319 | + | 8.7 | 1 | 375 | 0 | THE1C | MaLR | LTR |
| 48381320 | 48381912 | - | 27.9 | 1380 | 2120 | -4141 | L1MEe | L1 | LINE |
| 48381901 | 48382710 | - | 3.8 | 4060 | 4871 | -1275 | L1PA4 | L1 | LINE |
| 48382706 | 48383978 | + | 2.9 | 4887 | 6155 | 0 | L1PA4 | L1 | LINE |
| 48384027 | 48384149 | - | 18.7 | 9 | 132 | -180 | AluJo | Alu | SINE |

  

|  |  |  |  |  |  |  |  |  |  |
| --- | --- | --- | --- | --- | --- | --- | --- | --- | --- |
| chr8:49256593-49261700 | | | 4915 bp | | |  |  |  |  |
| Genome Start | Genome End | Strand | Div | Repeat Start | Repeat End | Repeat Left | Name | Family | Class |
| 49256593 | 49256657 | - | 21.5 | 3402 | 3466 | -2663 | L1MEd | L1 | LINE |
| 49256665 | 49256904 | - | 32.5 | 333 | 580 | -15 | MLT1G | MaLR | LTR |
| 49256952 | 49258326 | - | 4.5 | 2868 | 4276 | -1870 | L1PA5 | L1 | LINE |
| 49258327 | 49260162 | + | 5.0 | 4296 | 6141 | -13 | L1PA5 | L1 | LINE |
| 49260177 | 49260278 | - | 28.4 | 125 | 227 | -363 | MLT1G | MaLR | LTR |
| 49260342 | 49260663 | - | 27.1 | 3064 | 3406 | -2723 | L1MEd | L1 | LINE |
| 49260682 | 49261017 | - | 19.1 | 1 | 289 | -13 | AluJo | Alu | SINE |
| 49261041 | 49261238 | - | 30.6 | 1335 | 1539 | -4610 | L1MEd | L1 | LINE |
| 49261260 | 49261700 | - | 24.3 | 805 | 1297 | -5013 | L1MEd | L1 | LINE |

  

|  |  |  |  |  |  |  |  |  |  |
| --- | --- | --- | --- | --- | --- | --- | --- | --- | --- |
| chr8:49612417-49615400 | | | 2535 bp | | |  |  |  |  |
| Genome Start | Genome End | Strand | Div | Repeat Start | Repeat End | Repeat Left | Name | Family | Class |
| 49612417 | 49612924 | - | 31.6 | 1274 | 2009 | -4393 | L1MEd | L1 | LINE |
| 49612945 | 49613069 | - | 26.5 | 392 | 509 | -2 | MLT1J | MaLR | LTR |
| 49613425 | 49613593 | - | 23.2 | 1157 | 1328 | -5044 | L1MEd | L1 | LINE |
| 49613650 | 49614582 | - | 4.5 | 5223 | 6154 | 0 | L1PA5 | L1 | LINE |
| 49614583 | 49614807 | + | 4.9 | 4995 | 5219 | -927 | L1PA5 | L1 | LINE |
| 49614826 | 49614844 | - | 28.7 | 1070 | 1088 | -5284 | L1MEd | L1 | LINE |
| 49614845 | 49615137 | + | 9.2 | 1 | 293 | -18 | AluY | Alu | SINE |
| 49615138 | 49615400 | - | 28.7 | 819 | 1069 | -5303 | L1MEd | L1 | LINE |

  

|  |  |  |  |  |  |  |  |  |  |
| --- | --- | --- | --- | --- | --- | --- | --- | --- | --- |
| chr8:50014195-50021908 | | | 7714 bp | | |  |  |  |  |
| Genome Start | Genome End | Strand | Div | Repeat Start | Repeat End | Repeat Left | Name | Family | Class |
| 50014195 | 50018677 | - | 7.9 | 1673 | 6152 | -2 | L1PA6 | L1 | LINE |
| 50018684 | 50019798 | - | 1.3 | 5041 | 6155 | 0 | L1PA2 | L1 | LINE |
| 50019798 | 50020210 | + | 2.7 | 4607 | 5019 | -1127 | L1PA2 | L1 | LINE |
| 50020206 | 50021908 | - | 8.1 | 1 | 1688 | -587 | L1PA6 | L1 | LINE |

  

|  |  |  |  |  |  |  |  |  |  |
| --- | --- | --- | --- | --- | --- | --- | --- | --- | --- |
| chr8:50068016-50081266 | | | 13209 bp | | |  |  |  |  |
| Genome Start | Genome End | Strand | Div | Repeat Start | Repeat End | Repeat Left | Name | Family | Class |
| 50068016 | 50068990 | - | 5.3 | 5178 | 6152 | -2 | L1PA7 | L1 | LINE |
| 50069025 | 50072743 | - | 4.8 | 2392 | 6137 | -18 | L1PA4 | L1 | LINE |
| 50072735 | 50075119 | + | 7.4 | 13 | 2409 | -3737 | L1PA4 | L1 | LINE |
| 50075112 | 50075804 | - | 9.9 | 4499 | 5191 | -955 | L1PA7 | L1 | LINE |
| 50075840 | 50077009 | - | 3.1 | 4967 | 6137 | -18 | L1PA2 | L1 | LINE |
| 50077003 | 50078286 | + | 2.4 | 3724 | 4997 | -1149 | L1PA2 | L1 | LINE |
| 50078284 | 50081266 | - | 7.9 | 1554 | 4514 | -1632 | L1PA7 | L1 | LINE |

  

|  |  |  |  |  |  |  |  |  |  |
| --- | --- | --- | --- | --- | --- | --- | --- | --- | --- |
| chr8:50338765-50350103 | | | 11064 bp | | |  |  |  |  |
| Genome Start | Genome End | Strand | Div | Repeat Start | Repeat End | Repeat Left | Name | Family | Class |
| 50338765 | 50339049 | + | 23.4 | 8 | 267 | -142 | MLT1D | MaLR | LTR |
| 50339111 | 50339236 | - | 7.9 | 4157 | 4282 | -1864 | L1P3 | L1 | LINE |
| 50339300 | 50339850 | - | 8.2 | 3730 | 4282 | -1864 | L1P3 | L1 | LINE |
| 50339879 | 50340265 | - | 11.2 | 6499 | 6859 | -8 | L1PB1 | L1 | LINE |
| 50340300 | 50341674 | - | 9.4 | 5114 | 6498 | -369 | L1PB1 | L1 | LINE |
| 50341695 | 50346427 | - | 13.2 | 7 | 5113 | -1749 | L1PB1 | L1 | LINE |
| 50346440 | 50347462 | - | 9.8 | 2706 | 3748 | -2398 | L1P3 | L1 | LINE |
| 50347462 | 50349310 | + | 9.5 | 4298 | 6164 | -4 | L1PA10 | L1 | LINE |
| 50349369 | 50349421 | + | 23.3 | 249 | 297 | -208 | MLT1D | MaLR | LTR |
| 50349422 | 50349892 | - | 25.4 | 5 | 504 | -1 | MLT1D | MaLR | LTR |
| 50349893 | 50350103 | + | 23.3 | 298 | 505 | 0 | MLT1D | MaLR | LTR |

  

|  |  |  |  |  |  |  |  |  |  |
| --- | --- | --- | --- | --- | --- | --- | --- | --- | --- |
| chr8:51831130-51836719 | | | 5582 bp | | |  |  |  |  |
| Genome Start | Genome End | Strand | Div | Repeat Start | Repeat End | Repeat Left | Name | Family | Class |
| 51831130 | 51832107 | - | 17.8 | 4594 | 5567 | -754 | L1MCa | L1 | LINE |
| 51832124 | 51832401 | - | 20.6 | 3989 | 4277 | -2044 | L1MCa | L1 | LINE |
| 51832401 | 51833196 | - | 19.3 | 1845 | 2714 | -3607 | L1MCa | L1 | LINE |
| 51833196 | 51833432 | - | 21.6 | 791 | 1026 | -5482 | L1MCa | L1 | LINE |
| 51833427 | 51835714 | - | 8.8 | 3891 | 6154 | 0 | L1PA7 | L1 | LINE |
| 51835715 | 51836374 | + | 8.5 | 3217 | 3878 | -2268 | L1PA7 | L1 | LINE |
| 51836375 | 51836719 | - | 17.5 | 451 | 804 | -5704 | L1MCa | L1 | LINE |

  

|  |  |  |  |  |  |  |  |  |  |
| --- | --- | --- | --- | --- | --- | --- | --- | --- | --- |
| chr8:52076748-52085072 | | | 8020 bp | | |  |  |  |  |
| Genome Start | Genome End | Strand | Div | Repeat Start | Repeat End | Repeat Left | Name | Family | Class |
| 52076748 | 52076917 | + | 31.8 | 3658 | 3829 | -2317 | L1M5 | L1 | LINE |
| 52076933 | 52077150 | - | 2.8 | 5351 | 5568 | -587 | L1PA3 | L1 | LINE |
| 52077151 | 52077724 | + | 1.1 | 5570 | 6144 | -11 | L1PA3 | L1 | LINE |
| 52078001 | 52078927 | + | 29.0 | 20 | 1061 | -5546 | L1MB4 | L1 | LINE |
| 52078914 | 52080797 | + | 25.1 | 801 | 2868 | -3514 | L1M4c | L1 | LINE |
| 52080798 | 52080903 | - | 28.2 | 146 | 239 | 0 | MER8 | MER2\_type | DNA |
| 52080904 | 52081207 | + | 12.9 | 3 | 305 | -5 | AluSg | Alu | SINE |
| 52081208 | 52081293 | - | 28.2 | 61 | 145 | -94 | MER8 | MER2\_type | DNA |
| 52081294 | 52084925 | + | 21.7 | 2633 | 6336 | -8 | L1MC2 | L1 | LINE |
| 52084954 | 52085072 | + | 32.2 | 4075 | 4197 | -1949 | L1M5 | L1 | LINE |

  

|  |  |  |  |  |  |  |  |  |  |
| --- | --- | --- | --- | --- | --- | --- | --- | --- | --- |
| chr8:54155060-54158064 | | | 3063 bp | | |  |  |  |  |
| Genome Start | Genome End | Strand | Div | Repeat Start | Repeat End | Repeat Left | Name | Family | Class |
| 54155060 | 54155570 | + | 9.7 | 1 | 504 | -1076 | THE1B-int | MaLR | LTR |
| 54155584 | 54155657 | - | 1.4 | 6082 | 6155 | 0 | L1PA3 | L1 | LINE |
| 54155587 | 54155986 | - | 2.2 | 5753 | 6152 | -3 | L1PA3 | L1 | LINE |
| 54155987 | 54156984 | + | 2.6 | 4773 | 5769 | -386 | L1PA3 | L1 | LINE |
| 54156985 | 54158064 | + | 11.6 | 487 | 1575 | -5 | THE1B-int | MaLR | LTR |

  

|  |  |  |  |  |  |  |  |  |  |
| --- | --- | --- | --- | --- | --- | --- | --- | --- | --- |
| chr8:57418525-57424095 | | | 5505 bp | | |  |  |  |  |
| Genome Start | Genome End | Strand | Div | Repeat Start | Repeat End | Repeat Left | Name | Family | Class |
| 57418525 | 57418550 | - | 14.7 | 5781 | 5806 | -350 | L1P5 | L1 | LINE |
| 57418584 | 57418887 | - | 14.7 | 5476 | 5780 | -376 | L1P5 | L1 | LINE |
| 57418921 | 57420110 | - | 3.7 | 4957 | 6151 | -4 | L1PA4 | L1 | LINE |
| 57420110 | 57421123 | + | 5.7 | 3920 | 4937 | -1209 | L1PA4 | L1 | LINE |
| 57421125 | 57424095 | - | 18.0 | 2484 | 5489 | -667 | L1P5 | L1 | LINE |

  

|  |  |  |  |  |  |  |  |  |  |
| --- | --- | --- | --- | --- | --- | --- | --- | --- | --- |
| chr8:57878960-57894618 | | | 15428 bp | | |  |  |  |  |
| Genome Start | Genome End | Strand | Div | Repeat Start | Repeat End | Repeat Left | Name | Family | Class |
| 57878960 | 57879594 | + | 29.2 | 35 | 684 | -5716 | L1ME1 | L1 | LINE |
| 57879588 | 57879771 | + | 25.6 | 348 | 516 | -6117 | L1MDa | L1 | LINE |
| 57879760 | 57881434 | + | 20.0 | 1264 | 3001 | -3536 | L1MDa | L1 | LINE |
| 57881437 | 57881668 | - | 24.3 | 278 | 695 | 0 | MER4C | ERV1 | LTR |
| 57881687 | 57887838 | - | 5.0 | 1 | 6155 | 0 | L1PA4 | L1 | LINE |
| 57887848 | 57888109 | - | 22.2 | 1 | 486 | -205 | MER4C | ERV1 | LTR |
| 57888114 | 57888214 | + | 21.0 | 3007 | 3112 | -3425 | L1MDa | L1 | LINE |
| 57888230 | 57888702 | + | 25.0 | 1646 | 2107 | -4293 | L1ME1 | L1 | LINE |
| 57888703 | 57888889 | + | 23.0 | 1 | 212 | -3 | MER58C | MER1\_type | DNA |
| 57888890 | 57889440 | + | 26.2 | 2108 | 2562 | -3657 | L1ME1 | L1 | LINE |
| 57889441 | 57889732 | + | 11.0 | 1 | 293 | -17 | AluSg | Alu | SINE |
| 57889733 | 57890252 | + | 28.8 | 2563 | 3082 | -3137 | L1ME1 | L1 | LINE |
| 57890308 | 57890913 | - | 1.5 | 5375 | 5980 | -175 | L1PA2 | L1 | LINE |
| 57890914 | 57891083 | + | 3.5 | 5986 | 6155 | 0 | L1PA2 | L1 | LINE |
| 57891147 | 57892211 | + | 25.4 | 3103 | 4224 | -1995 | L1ME1 | L1 | LINE |
| 57892296 | 57893003 | + | 25.4 | 4225 | 4948 | -1271 | L1ME1 | L1 | LINE |
| 57893004 | 57893370 | + | 15.7 | 1 | 381 | 0 | THE1D | MaLR | LTR |
| 57893371 | 57894618 | + | 24.0 | 4949 | 6407 | 0 | L1ME1 | L1 | LINE |

  

|  |  |  |  |  |  |  |  |  |  |
| --- | --- | --- | --- | --- | --- | --- | --- | --- | --- |
| chr8:58942902-58946016 | | | 3029 bp | | |  |  |  |  |
| Genome Start | Genome End | Strand | Div | Repeat Start | Repeat End | Repeat Left | Name | Family | Class |
| 58942902 | 58943513 | - | 26.0 | 5533 | 6170 | 0 | L1ME3A | L1 | LINE |
| 58943519 | 58943687 | - | 28.7 | 5290 | 5464 | -776 | L1ME3A | L1 | LINE |
| 58943706 | 58943983 | - | 7.2 | 5908 | 6168 | -4 | L1PA8 | L1 | LINE |
| 58943984 | 58944552 | - | 10.9 | 1 | 571 | 0 | LTR22 | ERVK | LTR |
| 58944553 | 58945567 | - | 7.7 | 4895 | 5907 | -265 | L1PA8 | L1 | LINE |
| 58945564 | 58945798 | + | 11.1 | 4667 | 4901 | -1245 | L1PA8 | L1 | LINE |
| 58945866 | 58946016 | - | 22.5 | 5071 | 5233 | -913 | L1ME3A | L1 | LINE |

  

|  |  |  |  |  |  |  |  |  |  |
| --- | --- | --- | --- | --- | --- | --- | --- | --- | --- |
| chr8:59699057-59705420 | | | 6118 bp | | |  |  |  |  |
| Genome Start | Genome End | Strand | Div | Repeat Start | Repeat End | Repeat Left | Name | Family | Class |
| 59699057 | 59699148 | - | 14.2 | 2882 | 2973 | -2991 | L1M4c | L1 | LINE |
| 59699149 | 59699459 | - | 8.2 | 1 | 306 | -6 | AluSx | Alu | SINE |
| 59699460 | 59700058 | - | 14.2 | 2271 | 2881 | -3083 | L1M4c | L1 | LINE |
| 59700060 | 59700456 | + | 19.6 | 2 | 503 | -40 | MLT2B4 | ERVL | LTR |
| 59700553 | 59700600 | + | 4.2 | 513 | 560 | 0 | MLT2B4 | ERVL | LTR |
| 59700601 | 59701055 | - | 16.4 | 1778 | 2271 | -3693 | L1M4c | L1 | LINE |
| 59701033 | 59701201 | - | 11.6 | 5966 | 6147 | -4 | L1PB2 | L1 | LINE |
| 59701202 | 59701506 | - | 8.3 | 1 | 305 | -6 | AluY | Alu | SINE |
| 59701507 | 59702402 | - | 11.7 | 5074 | 5965 | -186 | L1PB2 | L1 | LINE |
| 59702401 | 59703424 | + | 11.9 | 4063 | 5066 | -1080 | L1PB2 | L1 | LINE |
| 59703421 | 59704735 | - | 24.2 | 391 | 1815 | -4157 | L1M3d | L1 | LINE |
| 59704762 | 59704854 | - | 21.9 | 676 | 767 | -5124 | L1M3d | L1 | LINE |
| 59705007 | 59705420 | - | 28.4 | 1 | 393 | -5498 | L1M4c | L1 | LINE |

  

|  |  |  |  |  |  |  |  |  |  |
| --- | --- | --- | --- | --- | --- | --- | --- | --- | --- |
| chr8:60487450-60490364 | | | 2897 bp | | |  |  |  |  |
| Genome Start | Genome End | Strand | Div | Repeat Start | Repeat End | Repeat Left | Name | Family | Class |
| 60487450 | 60487660 | + | 12.9 | 4293 | 4509 | -1637 | L1MC2 | L1 | LINE |
| 60487662 | 60488100 | - | 18.9 | 5001 | 5441 | -725 | L1PA17 | L1 | LINE |
| 60488101 | 60488798 | + | 16.0 | 5447 | 6166 | 0 | L1PA17 | L1 | LINE |
| 60488808 | 60488913 | + | 23.6 | 4506 | 4611 | -1535 | L1MC2 | L1 | LINE |
| 60488924 | 60489346 | + | 23.5 | 5617 | 6044 | -300 | L1MC2 | L1 | LINE |
| 60489345 | 60490167 | + | 20.8 | 6754 | 7586 | -199 | L1MC3 | L1 | LINE |
| 60490168 | 60490364 | + | 15.0 | 6127 | 6334 | -10 | L1MC2 | L1 | LINE |

  

|  |  |  |  |  |  |  |  |  |  |
| --- | --- | --- | --- | --- | --- | --- | --- | --- | --- |
| chr8:60538091-60543372 | | | 5196 bp | | |  |  |  |  |
| Genome Start | Genome End | Strand | Div | Repeat Start | Repeat End | Repeat Left | Name | Family | Class |
| 60538091 | 60539241 | - | 28.3 | 2345 | 3499 | -2720 | L1MEc | L1 | LINE |
| 60539252 | 60539378 | - | 4.8 | 6030 | 6155 | 0 | L1PA4 | L1 | LINE |
| 60539379 | 60541333 | + | 3.4 | 4073 | 6047 | -108 | L1PA4 | L1 | LINE |
| 60541334 | 60541650 | - | 22.0 | 2079 | 2431 | -3969 | L1MEc | L1 | LINE |
| 60541727 | 60543372 | - | 23.4 | 21 | 1943 | -4457 | L1MEc | L1 | LINE |

  

|  |  |  |  |  |  |  |  |  |  |
| --- | --- | --- | --- | --- | --- | --- | --- | --- | --- |
| chr8:62117900-62123320 | | | 5389 bp | | |  |  |  |  |
| Genome Start | Genome End | Strand | Div | Repeat Start | Repeat End | Repeat Left | Name | Family | Class |
| 62117900 | 62120973 | + | 34.7 | 1706 | 4914 | -753 | HERV16 | ERVL | LTR |
| 62120981 | 62122210 | - | 2.8 | 4045 | 5274 | -872 | L1PA3 | L1 | LINE |
| 62122211 | 62123068 | + | 3.1 | 5283 | 6155 | 0 | L1PA3 | L1 | LINE |
| 62123094 | 62123320 | + | 34.2 | 4904 | 5187 | -527 | HERV16 | ERVL | LTR |

  

|  |  |  |  |  |  |  |  |  |  |
| --- | --- | --- | --- | --- | --- | --- | --- | --- | --- |
| chr8:63065989-63069282 | | | 3303 bp | | |  |  |  |  |
| Genome Start | Genome End | Strand | Div | Repeat Start | Repeat End | Repeat Left | Name | Family | Class |
| 63065989 | 63066637 | - | 20.0 | 5476 | 6169 | -9 | L1MB8 | L1 | LINE |
| 63066639 | 63067128 | - | 6.3 | 5617 | 6116 | -38 | L1PA7 | L1 | LINE |
| 63067118 | 63069214 | + | 8.5 | 3520 | 5616 | -556 | L1PA7 | L1 | LINE |
| 63069216 | 63069282 | - | 17.9 | 5417 | 5483 | -691 | L1MB8 | L1 | LINE |

  

|  |  |  |  |  |  |  |  |  |  |
| --- | --- | --- | --- | --- | --- | --- | --- | --- | --- |
| chr8:63916124-63918883 | | | 2697 bp | | |  |  |  |  |
| Genome Start | Genome End | Strand | Div | Repeat Start | Repeat End | Repeat Left | Name | Family | Class |
| 63916124 | 63916287 | + | 22.1 | 1 | 164 | -341 | MLT1D | MaLR | LTR |
| 63916312 | 63917331 | - | 17.3 | 5234 | 6273 | -18 | L1MA7 | L1 | LINE |
| 63917332 | 63918204 | + | 18.3 | 4345 | 5232 | -911 | L1MA7 | L1 | LINE |
| 63918205 | 63918407 | + | 20.9 | 167 | 366 | -139 | MLT1D | MaLR | LTR |
| 63918447 | 63918747 | - | 15.8 | 1 | 303 | -9 | AluSx | Alu | SINE |
| 63918748 | 63918883 | + | 20.9 | 367 | 501 | -4 | MLT1D | MaLR | LTR |

  

|  |  |  |  |  |  |  |  |  |  |
| --- | --- | --- | --- | --- | --- | --- | --- | --- | --- |
| chr8:65384265-65389772 | | | 5263 bp | | |  |  |  |  |
| Genome Start | Genome End | Strand | Div | Repeat Start | Repeat End | Repeat Left | Name | Family | Class |
| 65384265 | 65384662 | - | 35.3 | 2978 | 3414 | -5 | L2 | L2 | LINE |
| 65384717 | 65384851 | + | 24.6 | 194 | 338 | -6164 | L1MEc | L1 | LINE |
| 65384871 | 65385233 | + | 34.6 | 398 | 774 | -5728 | L1MEc | L1 | LINE |
| 65385325 | 65386530 | + | 16.0 | 4872 | 6163 | -3 | L1PB4 | L1 | LINE |
| 65386527 | 65387145 | + | 25.7 | 974 | 1623 | -4879 | L1MEc | L1 | LINE |
| 65387170 | 65387471 | - | 9.9 | 5841 | 6143 | -20 | L1PA13 | L1 | LINE |
| 65387470 | 65387585 | + | 9.5 | 5710 | 5825 | -338 | L1PA13 | L1 | LINE |
| 65387616 | 65388146 | + | 24.6 | 1543 | 2262 | -4240 | L1MEc | L1 | LINE |
| 65388147 | 65388438 | + | 16.1 | 1 | 298 | -14 | AluSx | Alu | SINE |
| 65388439 | 65389006 | + | 26.1 | 2263 | 2753 | -3568 | L1MEc | L1 | LINE |
| 65389039 | 65389435 | + | 25.1 | 3230 | 3636 | -2685 | L1MEc | L1 | LINE |
| 65389437 | 65389772 | - | 36.2 | 2636 | 2982 | -437 | L2 | L2 | LINE |

  

|  |  |  |  |  |  |  |  |  |  |
| --- | --- | --- | --- | --- | --- | --- | --- | --- | --- |
| chr8:65718142-65723028 | | | 4748 bp | | |  |  |  |  |
| Genome Start | Genome End | Strand | Div | Repeat Start | Repeat End | Repeat Left | Name | Family | Class |
| 65718142 | 65718219 | - | 17.2 | 478 | 560 | 0 | MLT2B1 | ERVL | LTR |
| 65718329 | 65719103 | - | 12.4 | 1649 | 2418 | 0 | Tigger1 | MER2\_type | DNA |
| 65719104 | 65719401 | + | 9.1 | 1 | 299 | -11 | AluSg | Alu | SINE |
| 65719402 | 65719862 | - | 12.4 | 1178 | 1648 | -770 | Tigger1 | MER2\_type | DNA |
| 65719871 | 65720621 | - | 2.8 | 5405 | 6155 | 0 | L1PA3 | L1 | LINE |
| 65720622 | 65721551 | + | 2.7 | 4454 | 5382 | -764 | L1PA3 | L1 | LINE |
| 65721555 | 65722582 | - | 12.4 | 34 | 1171 | -1247 | Tigger1 | MER2\_type | DNA |
| 65722602 | 65723028 | - | 18.4 | 1 | 456 | -64 | MLT2B1 | ERVL | LTR |

  

|  |  |  |  |  |  |  |  |  |  |
| --- | --- | --- | --- | --- | --- | --- | --- | --- | --- |
| chr8:70976038-70980664 | | | 4478 bp | | |  |  |  |  |
| Genome Start | Genome End | Strand | Div | Repeat Start | Repeat End | Repeat Left | Name | Family | Class |
| 70976038 | 70976317 | - | 30.1 | 3083 | 3419 | 0 | L2 | L2 | LINE |
| 70976332 | 70976410 | - | 19.7 | 5487 | 5563 | -749 | L1M2 | L1 | LINE |
| 70976411 | 70976685 | - | 11.3 | 6 | 300 | -13 | AluSq | Alu | SINE |
| 70976686 | 70976861 | - | 19.7 | 5327 | 5486 | -826 | L1M2 | L1 | LINE |
| 70976909 | 70977186 | - | 10.0 | 3 | 282 | -30 | AluSx | Alu | SINE |
| 70977190 | 70977484 | - | 13.6 | 1 | 301 | -11 | AluSx | Alu | SINE |
| 70977485 | 70977614 | - | 14.6 | 4987 | 5139 | -1004 | L1M2 | L1 | LINE |
| 70977604 | 70977821 | + | 20.4 | 5561 | 5783 | -517 | L1MA6 | L1 | LINE |
| 70977822 | 70978136 | + | 17.1 | 1 | 306 | -6 | AluJb | Alu | SINE |
| 70978137 | 70978461 | + | 20.4 | 5784 | 6102 | -198 | L1MA6 | L1 | LINE |
| 70978462 | 70978765 | - | 19.3 | 6 | 312 | 0 | AluJo | Alu | SINE |
| 70978779 | 70979086 | - | 12.2 | 1 | 302 | -10 | AluSx | Alu | SINE |
| 70979092 | 70979381 | - | 10.0 | 11 | 300 | -9 | AluSc | Alu | SINE |
| 70979382 | 70979438 | + | 20.4 | 6107 | 6144 | -156 | L1MA6 | L1 | LINE |
| 70979439 | 70979724 | + | 10.6 | 9 | 291 | -21 | AluSx | Alu | SINE |
| 70979725 | 70979900 | + | 20.4 | 6145 | 6299 | -1 | L1MA6 | L1 | LINE |
| 70979910 | 70980203 | + | 11.0 | 5 | 307 | 0 | AluSp | Alu | SINE |
| 70980213 | 70980501 | + | 12.6 | 11 | 300 | -13 | AluSq | Alu | SINE |
| 70980562 | 70980664 | - | 28.4 | 2958 | 3062 | -316 | L2 | L2 | LINE |

  

|  |  |  |  |  |  |  |  |  |  |
| --- | --- | --- | --- | --- | --- | --- | --- | --- | --- |
| chr8:72044544-72057621 | | | 13055 bp | | |  |  |  |  |
| Genome Start | Genome End | Strand | Div | Repeat Start | Repeat End | Repeat Left | Name | Family | Class |
| 72044544 | 72045091 | - | 9.9 | 6311 | 6867 | 0 | L1PB1 | L1 | LINE |
| 72045109 | 72045550 | - | 8.1 | 5709 | 6153 | -1 | L1PA7 | L1 | LINE |
| 72045553 | 72045814 | + | 5.7 | 5452 | 5714 | -440 | L1PA7 | L1 | LINE |
| 72045808 | 72046986 | - | 10.1 | 5129 | 6323 | -544 | L1PB1 | L1 | LINE |
| 72047008 | 72049036 | - | 10.8 | 3080 | 5128 | -1734 | L1PB1 | L1 | LINE |
| 72049034 | 72055185 | - | 8.0 | 4 | 6151 | -3 | L1PA6 | L1 | LINE |
| 72055195 | 72057442 | - | 10.5 | 625 | 3090 | -3772 | L1PB1 | L1 | LINE |
| 72057427 | 72057621 | - | 17.9 | 3 | 200 | -6663 | L1PB1 | L1 | LINE |

  

|  |  |  |  |  |  |  |  |  |  |
| --- | --- | --- | --- | --- | --- | --- | --- | --- | --- |
| chr8:72949372-72966167 | | | 16697 bp | | |  |  |  |  |
| Genome Start | Genome End | Strand | Div | Repeat Start | Repeat End | Repeat Left | Name | Family | Class |
| 72949372 | 72949427 | - | 13.9 | 6110 | 6160 | -3 | L1PA13 | L1 | LINE |
| 72949428 | 72949805 | + | 19.2 | 1 | 428 | 0 | MSTA | MaLR | LTR |
| 72949806 | 72950568 | - | 13.9 | 5342 | 6109 | -54 | L1PA13 | L1 | LINE |
| 72950570 | 72953427 | - | 8.1 | 1610 | 4458 | -1688 | L1PA7 | L1 | LINE |
| 72953428 | 72955067 | + | 8.5 | 4467 | 6114 | -40 | L1PA7 | L1 | LINE |
| 72955057 | 72955856 | - | 12.0 | 4395 | 5218 | -928 | L1PREC2 | L1 | LINE |
| 72955858 | 72956149 | - | 10.6 | 7426 | 7721 | 0 | L1MA1 | L1 | LINE |
| 72956150 | 72957019 | - | 8.9 | 6400 | 7294 | -427 | L1MA1 | L1 | LINE |
| 72957020 | 72957260 | - | 12.0 | 5903 | 6146 | -5 | L1PB2 | L1 | LINE |
| 72957337 | 72957564 | - | 9.4 | 6392 | 6399 | -1166 | L1MA1 | L1 | LINE |
| 72957599 | 72960808 | - | 10.1 | 3199 | 6391 | -1174 | L1MA1 | L1 | LINE |
| 72960809 | 72961169 | - | 14.9 | 1 | 362 | -2 | THE1B | MaLR | LTR |
| 72961170 | 72964315 | - | 17.3 | 13 | 3198 | -4367 | L1MA1 | L1 | LINE |
| 72964317 | 72964510 | - | 12.9 | 4225 | 4418 | -1728 | L1PREC2 | L1 | LINE |
| 72964508 | 72964791 | - | 16.8 | 118 | 420 | -6365 | L1PA13 | L1 | LINE |
| 72964792 | 72964891 | + | 13.8 | 1 | 100 | -534 | MER41B | ERV1 | LTR |
| 72964892 | 72965198 | - | 9.8 | 5 | 311 | -2 | AluSq | Alu | SINE |
| 72965199 | 72965320 | + | 13.8 | 101 | 205 | -429 | MER41B | ERV1 | LTR |
| 72965321 | 72965620 | + | 9.3 | 1 | 300 | -11 | AluY | Alu | SINE |
| 72965621 | 72966060 | + | 13.8 | 206 | 633 | -1 | MER41B | ERV1 | LTR |
| 72966061 | 72966167 | - | 16.8 | 15 | 117 | -6668 | L1PA13 | L1 | LINE |

  

|  |  |  |  |  |  |  |  |  |  |
| --- | --- | --- | --- | --- | --- | --- | --- | --- | --- |
| chr8:72972401-72976574 | | | 4123 bp | | |  |  |  |  |
| Genome Start | Genome End | Strand | Div | Repeat Start | Repeat End | Repeat Left | Name | Family | Class |
| 72972401 | 72974287 | + | 24.1 | 3559 | 5556 | -2439 | L1MD3 | L1 | LINE |
| 72974286 | 72974876 | - | 2.7 | 5126 | 5718 | -437 | L1PA3 | L1 | LINE |
| 72974877 | 72975304 | + | 4.0 | 5710 | 6137 | -18 | L1PA3 | L1 | LINE |
| 72975354 | 72975431 | + | 16.7 | 5548 | 5627 | -539 | L1M5 | L1 | LINE |
| 72975436 | 72976574 | + | 22.2 | 6808 | 7983 | -12 | L1MD3 | L1 | LINE |

  

|  |  |  |  |  |  |  |  |  |  |
| --- | --- | --- | --- | --- | --- | --- | --- | --- | --- |
| chr8:73772797-73776024 | | | 3205 bp | | |  |  |  |  |
| Genome Start | Genome End | Strand | Div | Repeat Start | Repeat End | Repeat Left | Name | Family | Class |
| 73772797 | 73773978 | + | 10.5 | 4141 | 5330 | -816 | L1PA16 | L1 | LINE |
| 73773977 | 73774542 | - | 6.8 | 4995 | 5559 | -613 | L1PA8 | L1 | LINE |
| 73774544 | 73775148 | + | 6.0 | 5554 | 6156 | -16 | L1PA8 | L1 | LINE |
| 73775173 | 73776024 | + | 13.7 | 5315 | 6157 | -9 | L1PA16 | L1 | LINE |

  

|  |  |  |  |  |  |  |  |  |  |
| --- | --- | --- | --- | --- | --- | --- | --- | --- | --- |
| chr8:74936906-74938684 | | | 1770 bp | | |  |  |  |  |
| Genome Start | Genome End | Strand | Div | Repeat Start | Repeat End | Repeat Left | Name | Family | Class |
| 74936906 | 74936934 | + | 10.3 | 11 | 39 | -139 | MER5B | MER1\_type | DNA |
| 74936937 | 74937072 | + | 10.3 | 1 | 137 | -175 | AluSx | Alu | SINE |
| 74937073 | 74937234 | - | 0.6 | 4865 | 5026 | -1120 | L1PA2 | L1 | LINE |
| 74937233 | 74938379 | + | 1.0 | 5008 | 6154 | -1 | L1PA2 | L1 | LINE |
| 74938385 | 74938552 | + | 14.9 | 121 | 292 | -20 | AluSx | Alu | SINE |
| 74938557 | 74938684 | + | 25.8 | 37 | 178 | 0 | MER5B | MER1\_type | DNA |

  

|  |  |  |  |  |  |  |  |  |  |
| --- | --- | --- | --- | --- | --- | --- | --- | --- | --- |
| chr8:78046209-78051941 | | | 5743 bp | | |  |  |  |  |
| Genome Start | Genome End | Strand | Div | Repeat Start | Repeat End | Repeat Left | Name | Family | Class |
| 78046209 | 78047939 | + | 17.4 | 9 | 1652 | -5201 | L1PA16 | L1 | LINE |
| 78047935 | 78048311 | - | 5.0 | 3748 | 4124 | -2022 | L1P2 | L1 | LINE |
| 78048309 | 78049887 | + | 5.6 | 4146 | 5749 | -406 | L1P2 | L1 | LINE |
| 78049886 | 78051941 | + | 12.0 | 4802 | 6859 | -10 | L1PA16 | L1 | LINE |

  

|  |  |  |  |  |  |  |  |  |  |
| --- | --- | --- | --- | --- | --- | --- | --- | --- | --- |
| chr8:78094752-78103570 | | | 8340 bp | | |  |  |  |  |
| Genome Start | Genome End | Strand | Div | Repeat Start | Repeat End | Repeat Left | Name | Family | Class |
| 78094752 | 78095059 | + | 33.4 | 102 | 407 | -4589 | HERV16 | ERVL | LTR |
| 78095060 | 78095350 | + | 12.4 | 1 | 292 | -21 | AluSp | Alu | SINE |
| 78095351 | 78095723 | + | 33.4 | 408 | 783 | -4213 | HERV16 | ERVL | LTR |
| 78095721 | 78095889 | + | 32.1 | 910 | 1081 | -3915 | HERV16 | ERVL | LTR |
| 78095891 | 78096410 | - | 10.2 | 4401 | 4919 | -1227 | L1PA8A | L1 | LINE |
| 78096405 | 78096930 | + | 7.4 | 4895 | 5427 | -704 | L1PA8A | L1 | LINE |
| 78096932 | 78097253 | + | 9.0 | 1 | 310 | -1 | AluY | Alu | SINE |
| 78097254 | 78100203 | + | 2.7 | 3255 | 6208 | 0 | L1PA3 | L1 | LINE |
| 78100206 | 78100924 | + | 9.9 | 5413 | 6131 | 0 | L1PA8A | L1 | LINE |
| 78100928 | 78101145 | + | 25.2 | 1067 | 1286 | -3710 | HERV16 | ERVL | LTR |
| 78101463 | 78101548 | + | 31.5 | 1671 | 1747 | -3249 | HERV16 | ERVL | LTR |
| 78101549 | 78101852 | - | 10.2 | 1 | 305 | -7 | AluSx | Alu | SINE |
| 78101853 | 78102698 | + | 31.5 | 1748 | 2617 | -2379 | HERV16 | ERVL | LTR |
| 78102863 | 78103145 | + | 30.4 | 3770 | 4053 | -1661 | HERV16 | ERVL | LTR |
| 78103146 | 78103447 | + | 9.3 | 1 | 302 | -10 | AluSx | Alu | SINE |
| 78103448 | 78103570 | + | 30.4 | 4054 | 4171 | -1543 | HERV16 | ERVL | LTR |

  

|  |  |  |  |  |  |  |  |  |  |
| --- | --- | --- | --- | --- | --- | --- | --- | --- | --- |
| chr8:78150248-78151937 | | | 1398 bp | | |  |  |  |  |
| Genome Start | Genome End | Strand | Div | Repeat Start | Repeat End | Repeat Left | Name | Family | Class |
| 78150248 | 78150390 | - | 28.5 | 484 | 629 | -24 | MER82 | MER2\_type | DNA |
| 78150419 | 78150733 | - | 17.5 | 178 | 492 | -161 | MER82 | MER2\_type | DNA |
| 78150734 | 78150891 | - | 5.7 | 5543 | 5701 | -471 | L1PA8 | L1 | LINE |
| 78150892 | 78151363 | + | 7.2 | 5701 | 6172 | 0 | L1PA8 | L1 | LINE |
| 78151598 | 78151760 | - | 33.8 | 3219 | 3382 | -37 | L2 | L2 | LINE |
| 78151791 | 78151937 | - | 23.8 | 38 | 184 | -469 | MER82 | MER2\_type | DNA |

  

|  |  |  |  |  |  |  |  |  |  |
| --- | --- | --- | --- | --- | --- | --- | --- | --- | --- |
| chr8:79509107-79512442 | | | 2906 bp | | |  |  |  |  |
| Genome Start | Genome End | Strand | Div | Repeat Start | Repeat End | Repeat Left | Name | Family | Class |
| 79509107 | 79510005 | - | 15.0 | 278 | 1226 | -5 | Tigger3b | MER2\_type | DNA |
| 79510006 | 79510936 | - | 6.8 | 4403 | 5346 | -800 | L1PB1 | L1 | LINE |
| 79510936 | 79511358 | + | 9.1 | 5363 | 5786 | -365 | L1PB1 | L1 | LINE |
| 79511418 | 79511774 | + | 9.1 | 5787 | 6148 | -3 | L1PB1 | L1 | LINE |
| 79512147 | 79512442 | - | 17.1 | 4 | 296 | -935 | Tigger3b | MER2\_type | DNA |

  

|  |  |  |  |  |  |  |  |  |  |
| --- | --- | --- | --- | --- | --- | --- | --- | --- | --- |
| chr8:79575424-79580074 | | | 4596 bp | | |  |  |  |  |
| Genome Start | Genome End | Strand | Div | Repeat Start | Repeat End | Repeat Left | Name | Family | Class |
| 79575424 | 79575963 | + | 22.7 | 5834 | 6374 | -1434 | L1MC4a | L1 | LINE |
| 79575964 | 79576339 | - | 17.5 | 1 | 428 | 0 | MSTA | MaLR | LTR |
| 79576340 | 79576472 | + | 22.7 | 6375 | 6527 | -1281 | L1MC4a | L1 | LINE |
| 79576452 | 79577481 | - | 8.2 | 3949 | 4979 | -1167 | L1PA7 | L1 | LINE |
| 79577480 | 79578651 | + | 6.6 | 4980 | 6154 | 0 | L1PA7 | L1 | LINE |
| 79578660 | 79578834 | + | 24.2 | 6521 | 6681 | -1361 | L1MC4 | L1 | LINE |
| 79578903 | 79579094 | - | 17.5 | 1 | 166 | -146 | AluJo | Alu | SINE |
| 79579097 | 79580074 | + | 26.2 | 6703 | 7754 | -54 | L1MC4a | L1 | LINE |

  

|  |  |  |  |  |  |  |  |  |  |
| --- | --- | --- | --- | --- | --- | --- | --- | --- | --- |
| chr8:79826413-79834012 | | | 7606 bp | | |  |  |  |  |
| Genome Start | Genome End | Strand | Div | Repeat Start | Repeat End | Repeat Left | Name | Family | Class |
| 79826413 | 79827395 | - | 17.0 | 6380 | 7362 | 0 | L1MA5 | L1 | LINE |
| 79827392 | 79828756 | - | 7.5 | 4756 | 6153 | -1 | L1PA7 | L1 | LINE |
| 79828756 | 79829441 | + | 7.3 | 4058 | 4752 | -1394 | L1PA7 | L1 | LINE |
| 79829441 | 79832567 | - | 17.0 | 3324 | 6389 | -816 | L1MA5 | L1 | LINE |
| 79832568 | 79832931 | + | 13.5 | 1 | 364 | 0 | THE1B | MaLR | LTR |
| 79832932 | 79834012 | - | 19.2 | 2255 | 3323 | -3882 | L1MA5 | L1 | LINE |

  

|  |  |  |  |  |  |  |  |  |  |
| --- | --- | --- | --- | --- | --- | --- | --- | --- | --- |
| chr8:80023864-80028867 | | | 5036 bp | | |  |  |  |  |
| Genome Start | Genome End | Strand | Div | Repeat Start | Repeat End | Repeat Left | Name | Family | Class |
| 80023864 | 80025044 | + | 17.6 | 3263 | 4486 | -1660 | L1MA4 | L1 | LINE |
| 80025041 | 80025968 | - | 8.3 | 4127 | 5058 | -1088 | L1PA7 | L1 | LINE |
| 80025964 | 80027069 | + | 7.6 | 5047 | 6154 | 0 | L1PA7 | L1 | LINE |
| 80027047 | 80028867 | + | 14.3 | 4458 | 6291 | -9 | L1MA4 | L1 | LINE |

  

|  |  |  |  |  |  |  |  |  |  |
| --- | --- | --- | --- | --- | --- | --- | --- | --- | --- |
| chr8:80294194-80305369 | | | 11147 bp | | |  |  |  |  |
| Genome Start | Genome End | Strand | Div | Repeat Start | Repeat End | Repeat Left | Name | Family | Class |
| 80294194 | 80297039 | - | 11.0 | 4614 | 7421 | -300 | L1MA1 | L1 | LINE |
| 80297037 | 80298640 | - | 8.6 | 2624 | 4238 | -1908 | L1PA6 | L1 | LINE |
| 80298632 | 80300530 | + | 8.4 | 4248 | 6153 | -1 | L1PA6 | L1 | LINE |
| 80300571 | 80301044 | - | 12.8 | 4148 | 4621 | -2944 | L1MA1 | L1 | LINE |
| 80301046 | 80301391 | + | 14.9 | 15 | 381 | 0 | THE1D | MaLR | LTR |
| 80301392 | 80305369 | - | 16.4 | 19 | 4153 | -3412 | L1MA1 | L1 | LINE |

  

|  |  |  |  |  |  |  |  |  |  |
| --- | --- | --- | --- | --- | --- | --- | --- | --- | --- |
| chr8:80753132-80756080 | | | 2606 bp | | |  |  |  |  |
| Genome Start | Genome End | Strand | Div | Repeat Start | Repeat End | Repeat Left | Name | Family | Class |
| 80753132 | 80753349 | + | 31.2 | 825 | 1040 | -1467 | HAL1 | L1 | LINE |
| 80753673 | 80753685 | - | 4.2 | 6143 | 6154 | 0 | L1PA5 | L1 | LINE |
| 80753709 | 80754192 | - | 4.2 | 5659 | 6142 | -12 | L1PA5 | L1 | LINE |
| 80754194 | 80755945 | + | 4.9 | 3893 | 5644 | -510 | L1PA5 | L1 | LINE |
| 80755942 | 80756080 | + | 27.3 | 1533 | 1674 | -833 | HAL1 | L1 | LINE |

  

|  |  |  |  |  |  |  |  |  |  |
| --- | --- | --- | --- | --- | --- | --- | --- | --- | --- |
| chr8:83652882-83662624 | | | 9724 bp | | |  |  |  |  |
| Genome Start | Genome End | Strand | Div | Repeat Start | Repeat End | Repeat Left | Name | Family | Class |
| 83652882 | 83656127 | + | 20.2 | 4 | 3189 | -3659 | L1MA4A | L1 | LINE |
| 83656143 | 83656892 | + | 21.2 | 5124 | 5895 | -950 | L1MA4A | L1 | LINE |
| 83656891 | 83657582 | - | 4.2 | 1903 | 2596 | -3550 | L1PA4 | L1 | LINE |
| 83657581 | 83661107 | + | 3.5 | 2603 | 6133 | -22 | L1PA4 | L1 | LINE |
| 83661115 | 83661181 | + | 0.0 | 6115 | 6181 | 0 | L1PA3 | L1 | LINE |
| 83661183 | 83661440 | + | 16.8 | 5885 | 6141 | -864 | L1MA4A | L1 | LINE |
| 83661441 | 83661734 | + | 12.2 | 1 | 295 | -15 | AluSg | Alu | SINE |
| 83661735 | 83662624 | + | 16.3 | 6142 | 7023 | 0 | L1MA4A | L1 | LINE |

  

|  |  |  |  |  |  |  |  |  |  |
| --- | --- | --- | --- | --- | --- | --- | --- | --- | --- |
| chr8:83913567-83915835 | | | 2237 bp | | |  |  |  |  |
| Genome Start | Genome End | Strand | Div | Repeat Start | Repeat End | Repeat Left | Name | Family | Class |
| 83913567 | 83913843 | + | 28.1 | 1420 | 1719 | -1700 | L2 | L2 | LINE |
| 83913848 | 83914337 | + | 33.1 | 2555 | 3062 | -357 | L2 | L2 | LINE |
| 83914357 | 83915287 | - | 16.0 | 5357 | 6300 | 0 | L1MA6 | L1 | LINE |
| 83915287 | 83915495 | + | 19.6 | 5142 | 5351 | -792 | L1MA6 | L1 | LINE |
| 83915506 | 83915835 | + | 32.4 | 3074 | 3415 | -4 | L2 | L2 | LINE |

  

|  |  |  |  |  |  |  |  |  |  |
| --- | --- | --- | --- | --- | --- | --- | --- | --- | --- |
| chr8:84674981-84683254 | | | 7835 bp | | |  |  |  |  |
| Genome Start | Genome End | Strand | Div | Repeat Start | Repeat End | Repeat Left | Name | Family | Class |
| 84674981 | 84675818 | + | 33.4 | 77 | 944 | -5456 | L1MEc | L1 | LINE |
| 84675868 | 84675962 | - | 15.6 | 5803 | 5891 | -414 | L1MA3 | L1 | LINE |
| 84675963 | 84676420 | + | 11.5 | 5873 | 6305 | 0 | L1MA3 | L1 | LINE |
| 84676519 | 84682363 | + | 8.5 | 9 | 6154 | 0 | L1PA6 | L1 | LINE |
| 84682656 | 84683254 | + | 27.4 | 1039 | 1705 | -4695 | L1MEc | L1 | LINE |

  

|  |  |  |  |  |  |  |  |  |  |
| --- | --- | --- | --- | --- | --- | --- | --- | --- | --- |
| chr8:85202360-85206734 | | | 3918 bp | | |  |  |  |  |
| Genome Start | Genome End | Strand | Div | Repeat Start | Repeat End | Repeat Left | Name | Family | Class |
| 85202360 | 85202602 | + | 31.0 | 1822 | 2087 | -1332 | L2 | L2 | LINE |
| 85202686 | 85202776 | - | 18.3 | 6066 | 6159 | -9 | L1MB1 | L1 | LINE |
| 85202777 | 85203129 | - | 13.6 | 1 | 364 | 0 | THE1B | MaLR | LTR |
| 85203130 | 85203309 | - | 18.3 | 5888 | 6065 | -103 | L1MB1 | L1 | LINE |
| 85203310 | 85203641 | + | 19.7 | 5529 | 5876 | -313 | L1MB1 | L1 | LINE |
| 85203754 | 85204028 | - | 32.8 | 7505 | 7783 | -178 | L1MC5 | L1 | LINE |
| 85204146 | 85204456 | - | 32.0 | 5327 | 5641 | -2167 | L1MC | L1 | LINE |
| 85204463 | 85205157 | + | 30.3 | 2083 | 2824 | -595 | L2 | L2 | LINE |
| 85205159 | 85205708 | + | 14.5 | 5621 | 6170 | -1 | L1MB2 | L1 | LINE |
| 85205716 | 85206158 | + | 31.9 | 2805 | 3309 | -110 | L2 | L2 | LINE |
| 85206216 | 85206506 | + | 8.2 | 1 | 297 | -14 | AluY | Alu | SINE |
| 85206532 | 85206609 | - | 27.3 | 2084 | 2162 | -1257 | L2 | L2 | LINE |
| 85206659 | 85206734 | + | 31.6 | 3343 | 3419 | 0 | L2 | L2 | LINE |

  

|  |  |  |  |  |  |  |  |  |  |
| --- | --- | --- | --- | --- | --- | --- | --- | --- | --- |
| chr8:86479668-86482655 | | | 2944 bp | | |  |  |  |  |
| Genome Start | Genome End | Strand | Div | Repeat Start | Repeat End | Repeat Left | Name | Family | Class |
| 86479668 | 86479975 | - | 30.6 | 4372 | 4689 | -996 | HERVL74 | ERVL | LTR |
| 86479976 | 86481522 | - | 8.6 | 4297 | 5848 | -320 | L1PA10 | L1 | LINE |
| 86481523 | 86481844 | + | 9.2 | 5852 | 6168 | 0 | L1PA10 | L1 | LINE |
| 86481845 | 86482062 | - | 17.4 | 4165 | 4384 | -1301 | HERVL74 | ERVL | LTR |
| 86482107 | 86482655 | - | 26.6 | 1 | 528 | -5157 | HERVL74 | ERVL | LTR |

  

|  |  |  |  |  |  |  |  |  |  |
| --- | --- | --- | --- | --- | --- | --- | --- | --- | --- |
| chr8:86943332-86943940 | | | 575 bp | | |  |  |  |  |
| Genome Start | Genome End | Strand | Div | Repeat Start | Repeat End | Repeat Left | Name | Family | Class |
| 86943332 | 86943394 | + | 27.4 | 12 | 73 | -87 | MER5A1 | MER1\_type | DNA |
| 86943395 | 86943586 | - | 17.7 | 5848 | 6054 | -246 | L1MA6 | L1 | LINE |
| 86943589 | 86943808 | + | 12.7 | 6065 | 6288 | -12 | L1MA6 | L1 | LINE |
| 86943841 | 86943940 | + | 22.4 | 58 | 157 | -3 | MER5A1 | MER1\_type | DNA |

  

|  |  |  |  |  |  |  |  |  |  |
| --- | --- | --- | --- | --- | --- | --- | --- | --- | --- |
| chr8:88450716-88453764 | | | 3061 bp | | |  |  |  |  |
| Genome Start | Genome End | Strand | Div | Repeat Start | Repeat End | Repeat Left | Name | Family | Class |
| 88450716 | 88451081 | - | 28.8 | 1455 | 1833 | -4551 | L1M4c | L1 | LINE |
| 88451070 | 88452817 | - | 3.2 | 4408 | 6155 | 0 | L1PA3 | L1 | LINE |
| 88452818 | 88453609 | + | 3.5 | 3626 | 4417 | -1729 | L1PA3 | L1 | LINE |
| 88453610 | 88453764 | - | 22.9 | 1315 | 1480 | -4904 | L1M4c | L1 | LINE |

  

|  |  |  |  |  |  |  |  |  |  |
| --- | --- | --- | --- | --- | --- | --- | --- | --- | --- |
| chr8:89239018-89243372 | | | 4317 bp | | |  |  |  |  |
| Genome Start | Genome End | Strand | Div | Repeat Start | Repeat End | Repeat Left | Name | Family | Class |
| 89239018 | 89241289 | - | 5.2 | 3840 | 6153 | -1 | L1PA5 | L1 | LINE |
| 89241334 | 89241778 | - | 4.7 | 5693 | 6137 | -18 | L1PA3 | L1 | LINE |
| 89241779 | 89242192 | + | 2.4 | 5293 | 5706 | -449 | L1PA3 | L1 | LINE |
| 89242187 | 89243372 | - | 6.0 | 2679 | 3857 | -2289 | L1PA5 | L1 | LINE |

  

|  |  |  |  |  |  |  |  |  |  |
| --- | --- | --- | --- | --- | --- | --- | --- | --- | --- |
| chr8:90210618-90212514 | | | 1887 bp | | |  |  |  |  |
| Genome Start | Genome End | Strand | Div | Repeat Start | Repeat End | Repeat Left | Name | Family | Class |
| 90210618 | 90210960 | - | 20.2 | 254 | 630 | -14 | MER73 | ERVL | LTR |
| 90210961 | 90211405 | - | 16.0 | 4973 | 5429 | -868 | L1M | L1 | LINE |
| 90211404 | 90212253 | + | 14.5 | 5410 | 6227 | -70 | L1MA5A | L1 | LINE |
| 90212266 | 90212514 | - | 17.7 | 1 | 265 | -379 | MER73 | ERVL | LTR |

  

|  |  |  |  |  |  |  |  |  |  |
| --- | --- | --- | --- | --- | --- | --- | --- | --- | --- |
| chr8:93842973-93851292 | | | 8151 bp | | |  |  |  |  |
| Genome Start | Genome End | Strand | Div | Repeat Start | Repeat End | Repeat Left | Name | Family | Class |
| 93842973 | 93843219 | - | 7.3 | 5378 | 5625 | -29 | HERVL | ERVL | LTR |
| 93843274 | 93843558 | - | 9.8 | 1 | 284 | -29 | AluSq | Alu | SINE |
| 93843675 | 93844777 | - | 7.5 | 5043 | 6148 | -6 | L1PA7 | L1 | LINE |
| 93844777 | 93846520 | + | 9.3 | 3308 | 5048 | -1098 | L1PA7 | L1 | LINE |
| 93846521 | 93851003 | - | 7.7 | 852 | 5381 | -273 | HERVL | ERVL | LTR |
| 93851004 | 93851292 | - | 9.3 | 563 | 851 | -4803 | HERVL | ERVL | LTR |

  

|  |  |  |  |  |  |  |  |  |  |
| --- | --- | --- | --- | --- | --- | --- | --- | --- | --- |
| chr8:96449745-96457139 | | | 7337 bp | | |  |  |  |  |
| Genome Start | Genome End | Strand | Div | Repeat Start | Repeat End | Repeat Left | Name | Family | Class |
| 96449745 | 96451423 | - | 1.6 | 4354 | 6032 | 0 | L1PA2 | L1 | LINE |
| 96451485 | 96452285 | - | 1.1 | 5355 | 6155 | 0 | L1HS | L1 | LINE |
| 96452285 | 96452764 | + | 0.0 | 4885 | 5364 | -782 | L1HS | L1 | LINE |
| 96452763 | 96457139 | - | 2.9 | 1 | 4375 | -1648 | L1PA2 | L1 | LINE |

  

|  |  |  |  |  |  |  |  |  |  |
| --- | --- | --- | --- | --- | --- | --- | --- | --- | --- |
| chr8:96647035-96654745 | | | 7702 bp | | |  |  |  |  |
| Genome Start | Genome End | Strand | Div | Repeat Start | Repeat End | Repeat Left | Name | Family | Class |
| 96647035 | 96647224 | - | 15.8 | 7516 | 7698 | -33 | L1MA9 | L1 | LINE |
| 96647227 | 96647525 | - | 13.7 | 6900 | 7181 | -550 | L1MA9 | L1 | LINE |
| 96647526 | 96647803 | - | 10.1 | 1 | 280 | -30 | AluSg | Alu | SINE |
| 96647804 | 96647954 | - | 13.7 | 6759 | 6899 | -832 | L1MA9 | L1 | LINE |
| 96647961 | 96648197 | - | 14.3 | 5892 | 6136 | -176 | L1MA9 | L1 | LINE |
| 96648182 | 96648557 | - | 17.6 | 6404 | 6780 | -779 | L1MA9 | L1 | LINE |
| 96648566 | 96649444 | - | 17.7 | 5393 | 6284 | -7 | L1MA8 | L1 | LINE |
| 96649446 | 96649684 | + | 16.1 | 5137 | 5390 | -753 | L1MA8 | L1 | LINE |
| 96649689 | 96652735 | - | 17.8 | 3287 | 6414 | -1145 | L1MA9 | L1 | LINE |
| 96652736 | 96653105 | + | 15.0 | 1 | 428 | 0 | MSTA | MaLR | LTR |
| 96653106 | 96653642 | - | 26.2 | 2781 | 3395 | -4279 | L1MA9 | L1 | LINE |
| 96653643 | 96654414 | + | 14.7 | 5 | 785 | 0 | LTR1 | ERV1 | LTR |
| 96654415 | 96654654 | - | 25.7 | 2797 | 3077 | -4489 | L1MA9 | L1 | LINE |
| 96654659 | 96654745 | - | 30.6 | 464 | 548 | -6941 | L1MA9 | L1 | LINE |

  

|  |  |  |  |  |  |  |  |  |  |
| --- | --- | --- | --- | --- | --- | --- | --- | --- | --- |
| chr8:96884754-96886482 | | | 1710 bp | | |  |  |  |  |
| Genome Start | Genome End | Strand | Div | Repeat Start | Repeat End | Repeat Left | Name | Family | Class |
| 96884754 | 96885402 | + | 19.4 | 1 | 750 | -290 | MER45B | Tip100 | DNA |
| 96885419 | 96885557 | - | 14.5 | 6026 | 6165 | -1 | L1PA16 | L1 | LINE |
| 96885558 | 96885977 | - | 10.5 | 5512 | 5935 | -231 | L1PA16 | L1 | LINE |
| 96885981 | 96886189 | + | 9.6 | 5309 | 5516 | -649 | L1PA16 | L1 | LINE |
| 96886190 | 96886482 | + | 17.6 | 738 | 1040 | 0 | MER45B | Tip100 | DNA |

  

|  |  |  |  |  |  |  |  |  |  |
| --- | --- | --- | --- | --- | --- | --- | --- | --- | --- |
| chr8:97038147-97041307 | | | 3127 bp | | |  |  |  |  |
| Genome Start | Genome End | Strand | Div | Repeat Start | Repeat End | Repeat Left | Name | Family | Class |
| 97038147 | 97038359 | + | 23.8 | 1 | 205 | -2577 | Charlie2 | MER1\_type | DNA |
| 97038360 | 97038822 | + | 18.6 | 1 | 432 | 0 | MSTB1 | MaLR | LTR |
| 97038823 | 97038872 | + | 23.8 | 206 | 259 | -2523 | Charlie2 | MER1\_type | DNA |
| 97038873 | 97039220 | + | 25.9 | 1 | 369 | -221 | MLT1G | MaLR | LTR |
| 97039212 | 97039546 | + | 19.2 | 9 | 365 | 0 | MLT1A0 | MaLR | LTR |
| 97039547 | 97039744 | + | 24.6 | 370 | 578 | -12 | MLT1G | MaLR | LTR |
| 97039744 | 97039808 | + | 15.4 | 255 | 319 | -2463 | Charlie2 | MER1\_type | DNA |
| 97039811 | 97040256 | + | 23.5 | 1850 | 2306 | -556 | Charlie2 | MER1\_type | DNA |
| 97040257 | 97040544 | + | 16.3 | 1 | 299 | -13 | AluJb | Alu | SINE |
| 97040545 | 97040649 | + | 23.5 | 2307 | 2412 | -450 | Charlie2 | MER1\_type | DNA |
| 97040650 | 97040785 | - | 16.2 | 6170 | 6311 | -1 | L1MA9 | L1 | LINE |
| 97040788 | 97040954 | + | 18.0 | 5982 | 6151 | -161 | L1MA9 | L1 | LINE |
| 97040995 | 97041307 | + | 22.4 | 2418 | 2763 | -99 | Charlie2 | MER1\_type | DNA |

  

|  |  |  |  |  |  |  |  |  |  |
| --- | --- | --- | --- | --- | --- | --- | --- | --- | --- |
| chr8:97773714-97781042 | | | 7337 bp | | |  |  |  |  |
| Genome Start | Genome End | Strand | Div | Repeat Start | Repeat End | Repeat Left | Name | Family | Class |
| 97773714 | 97775785 | + | 11.1 | 1578 | 3662 | -3903 | L1MA2 | L1 | LINE |
| 97775778 | 97776272 | + | 13.3 | 4005 | 4508 | -3057 | L1MA2 | L1 | LINE |
| 97776267 | 97776340 | - | 2.7 | 4570 | 4643 | -1503 | L1PA3 | L1 | LINE |
| 97776339 | 97777854 | + | 2.2 | 4636 | 6151 | -4 | L1PA3 | L1 | LINE |
| 97777863 | 97781042 | + | 11.2 | 4492 | 7723 | 0 | L1MA2 | L1 | LINE |

  

|  |  |  |  |  |  |  |  |  |  |
| --- | --- | --- | --- | --- | --- | --- | --- | --- | --- |
| chr8:98337763-98338618 | | | 834 bp | | |  |  |  |  |
| Genome Start | Genome End | Strand | Div | Repeat Start | Repeat End | Repeat Left | Name | Family | Class |
| 98337763 | 98337823 | - | 15.2 | 530 | 588 | -7 | MLT1H2 | MaLR | LTR |
| 98337830 | 98337960 | - | 15.3 | 5678 | 5809 | -365 | L1MB5 | L1 | LINE |
| 98337966 | 98338050 | + | 10.6 | 5814 | 5898 | -280 | L1MB5 | L1 | LINE |
| 98338055 | 98338281 | + | 15.1 | 5948 | 6174 | 0 | L1MB5 | L1 | LINE |
| 98338289 | 98338618 | - | 22.9 | 1 | 430 | -54 | MLT1H2 | MaLR | LTR |

  

|  |  |  |  |  |  |  |  |  |  |
| --- | --- | --- | --- | --- | --- | --- | --- | --- | --- |
| chr8:100415224-100419766 | | | 4509 bp | | |  |  |  |  |
| Genome Start | Genome End | Strand | Div | Repeat Start | Repeat End | Repeat Left | Name | Family | Class |
| 100415224 | 100415779 | - | 23.1 | 5341 | 5919 | -245 | L1ME2 | L1 | LINE |
| 100415794 | 100416911 | - | 3.5 | 3001 | 4116 | -2030 | L1PA3 | L1 | LINE |
| 100416897 | 100418919 | + | 3.5 | 4118 | 6144 | -11 | L1PA3 | L1 | LINE |
| 100418955 | 100419766 | - | 27.1 | 4487 | 5336 | -810 | L1ME2 | L1 | LINE |

  

|  |  |  |  |  |  |  |  |  |  |
| --- | --- | --- | --- | --- | --- | --- | --- | --- | --- |
| chr8:104906506-104912598 | | | 5963 bp | | |  |  |  |  |
| Genome Start | Genome End | Strand | Div | Repeat Start | Repeat End | Repeat Left | Name | Family | Class |
| 104906506 | 104906677 | - | 23.6 | 5800 | 5956 | -142 | L1ME2 | L1 | LINE |
| 104906678 | 104906987 | - | 18.0 | 1 | 312 | 0 | AluSx | Alu | SINE |
| 104906988 | 104908050 | - | 24.9 | 4624 | 5799 | -299 | L1ME2 | L1 | LINE |
| 104908140 | 104908379 | - | 17.4 | 1 | 246 | -66 | AluJo | Alu | SINE |
| 104908381 | 104908958 | - | 27.3 | 4029 | 4655 | -1491 | L1MD | L1 | LINE |
| 104908959 | 104909260 | + | 12.1 | 1 | 298 | -12 | AluSg | Alu | SINE |
| 104909261 | 104909768 | - | 27.3 | 3477 | 4028 | -2118 | L1MD | L1 | LINE |
| 104909767 | 104910541 | - | 12.6 | 4598 | 5375 | -771 | L1PREC2 | L1 | LINE |
| 104910540 | 104911274 | + | 14.0 | 5386 | 6165 | 0 | L1PA15 | L1 | LINE |
| 104911319 | 104912218 | - | 26.9 | 2373 | 3421 | -2659 | L1ME2 | L1 | LINE |
| 104912219 | 104912510 | - | 17.5 | 3 | 294 | -18 | AluJb | Alu | SINE |
| 104912511 | 104912598 | - | 26.9 | 2289 | 2372 | -3708 | L1ME2 | L1 | LINE |

  

|  |  |  |  |  |  |  |  |  |  |
| --- | --- | --- | --- | --- | --- | --- | --- | --- | --- |
| chr8:106278954-106293681 | | | 14486 bp | | |  |  |  |  |
| Genome Start | Genome End | Strand | Div | Repeat Start | Repeat End | Repeat Left | Name | Family | Class |
| 106278954 | 106280948 | - | 16.6 | 3656 | 5629 | -935 | L1MCa | L1 | LINE |
| 106280949 | 106281277 | - | 13.4 | 1 | 305 | -5 | AluSg | Alu | SINE |
| 106281278 | 106281692 | - | 16.6 | 3242 | 3655 | -2909 | L1MCa | L1 | LINE |
| 106281705 | 106282648 | - | 9.1 | 5224 | 6159 | -9 | L1PA10 | L1 | LINE |
| 106282645 | 106283874 | + | 7.8 | 3355 | 4594 | -1552 | L1P3 | L1 | LINE |
| 106283862 | 106284768 | - | 16.2 | 2320 | 3260 | -3304 | L1MCa | L1 | LINE |
| 106284769 | 106285071 | - | 9.3 | 1 | 305 | -4 | AluSc | Alu | SINE |
| 106285072 | 106285218 | - | 16.8 | 2175 | 2319 | -4432 | L1MCa | L1 | LINE |
| 106285219 | 106285414 | - | 15.2 | 2225 | 2418 | 0 | Tigger1 | MER2\_type | DNA |
| 106285415 | 106285725 | + | 6.9 | 1 | 303 | -8 | AluY | Alu | SINE |
| 106285726 | 106287924 | - | 15.2 | 1 | 2224 | -194 | Tigger1 | MER2\_type | DNA |
| 106287925 | 106288365 | - | 16.8 | 1719 | 2174 | -4577 | L1MCa | L1 | LINE |
| 106288380 | 106288592 | - | 16.5 | 4628 | 4826 | -1320 | L1PREC2 | L1 | LINE |
| 106288591 | 106289525 | + | 16.7 | 4829 | 5775 | -381 | L1P5 | L1 | LINE |
| 106289532 | 106289674 | - | 12.6 | 5481 | 5623 | -533 | L1P5 | L1 | LINE |
| 106289729 | 106289766 | - | 5.4 | 5751 | 5787 | -369 | L1P5 | L1 | LINE |
| 106289762 | 106290214 | - | 15.6 | 1102 | 1563 | -5188 | L1MCa | L1 | LINE |
| 106290233 | 106290417 | - | 19.9 | 481 | 936 | -5815 | L1MCa | L1 | LINE |
| 106290503 | 106290657 | - | 24.7 | 1520 | 1675 | -4564 | L1MEc | L1 | LINE |
| 106290667 | 106291281 | - | 25.8 | 2400 | 3022 | -3299 | L1MCa | L1 | LINE |
| 106291274 | 106292230 | + | 10.6 | 5199 | 6174 | 0 | L1PA11 | L1 | LINE |
| 106292229 | 106292463 | - | 29.9 | 2295 | 2524 | -3978 | L1MCa | L1 | LINE |
| 106292464 | 106292734 | - | 11.8 | 37 | 309 | -1 | AluSg | Alu | SINE |
| 106292735 | 106293457 | - | 29.9 | 1522 | 2294 | -4208 | L1MCa | L1 | LINE |
| 106293536 | 106293681 | - | 27.3 | 1154 | 1314 | -5194 | L1MCa | L1 | LINE |

  

|  |  |  |  |  |  |  |  |  |  |
| --- | --- | --- | --- | --- | --- | --- | --- | --- | --- |
| chr8:107050820-107053857 | | | 2995 bp | | |  |  |  |  |
| Genome Start | Genome End | Strand | Div | Repeat Start | Repeat End | Repeat Left | Name | Family | Class |
| 107050820 | 107050925 | + | 11.1 | 2838 | 2938 | -3202 | L1M2 | L1 | LINE |
| 107050923 | 107051589 | - | 8.4 | 3341 | 3976 | -2170 | L1PREC2 | L1 | LINE |
| 107051589 | 107053755 | + | 10.4 | 3966 | 6157 | -3 | L1PREC2 | L1 | LINE |
| 107053803 | 107053857 | + | 10.9 | 2914 | 2974 | -3169 | L1M2 | L1 | LINE |

  

|  |  |  |  |  |  |  |  |  |  |
| --- | --- | --- | --- | --- | --- | --- | --- | --- | --- |
| chr8:108121121-108128107 | | | 6354 bp | | |  |  |  |  |
| Genome Start | Genome End | Strand | Div | Repeat Start | Repeat End | Repeat Left | Name | Family | Class |
| 108121121 | 108121368 | + | 38.5 | 341 | 587 | -5628 | L1MC4 | L1 | LINE |
| 108121430 | 108121643 | - | 12.2 | 76 | 304 | -9 | AluSq | Alu | SINE |
| 108121691 | 108122520 | + | 25.4 | 736 | 1627 | -4588 | L1MC4 | L1 | LINE |
| 108122550 | 108122867 | + | 25.4 | 1628 | 1968 | -4247 | L1MC4 | L1 | LINE |
| 108122868 | 108123167 | - | 10.4 | 1 | 304 | -8 | AluSx | Alu | SINE |
| 108123168 | 108123387 | + | 25.4 | 1971 | 2201 | -4014 | L1MC4 | L1 | LINE |
| 108123396 | 108123689 | + | 18.1 | 1 | 289 | -23 | AluJo | Alu | SINE |
| 108123698 | 108124724 | - | 7.9 | 2707 | 3750 | -2396 | L1PB1 | L1 | LINE |
| 108124725 | 108127166 | + | 9.6 | 3725 | 6150 | -1 | L1PB1 | L1 | LINE |
| 108127647 | 108128107 | + | 28.7 | 7163 | 7669 | -373 | L1MC4 | L1 | LINE |

  

|  |  |  |  |  |  |  |  |  |  |
| --- | --- | --- | --- | --- | --- | --- | --- | --- | --- |
| chr8:110709466-110712679 | | | 3098 bp | | |  |  |  |  |
| Genome Start | Genome End | Strand | Div | Repeat Start | Repeat End | Repeat Left | Name | Family | Class |
| 110709466 | 110709628 | - | 36.1 | 3250 | 3409 | -10 | L2 | L2 | LINE |
| 110709629 | 110709747 | + | 18.8 | 1 | 116 | -186 | AluJb | Alu | SINE |
| 110709748 | 110710047 | - | 18.1 | 1 | 296 | -16 | AluJb | Alu | SINE |
| 110710048 | 110710235 | + | 18.8 | 117 | 292 | -10 | AluJb | Alu | SINE |
| 110710236 | 110710603 | - | 36.0 | 2857 | 3249 | -170 | L2 | L2 | LINE |
| 110710687 | 110710956 | - | 30.3 | 8 | 257 | -5 | MIR | MIR | SINE |
| 110710983 | 110711414 | - | 1.4 | 5290 | 5721 | -434 | L1PA2 | L1 | LINE |
| 110711415 | 110711841 | + | 1.2 | 5728 | 6155 | 0 | L1PA2 | L1 | LINE |
| 110711849 | 110712679 | - | 30.8 | 1748 | 2759 | -660 | L2 | L2 | LINE |

  

|  |  |  |  |  |  |  |  |  |  |
| --- | --- | --- | --- | --- | --- | --- | --- | --- | --- |
| chr8:110899769-110906803 | | | 7041 bp | | |  |  |  |  |
| Genome Start | Genome End | Strand | Div | Repeat Start | Repeat End | Repeat Left | Name | Family | Class |
| 110899769 | 110901246 | - | 10.9 | 4807 | 6304 | 0 | L1MA2 | L1 | LINE |
| 110901247 | 110903250 | - | 3.2 | 4136 | 6155 | 0 | L1PA3 | L1 | LINE |
| 110903250 | 110904181 | + | 3.9 | 3203 | 4134 | -2012 | L1PA3 | L1 | LINE |
| 110904177 | 110906803 | - | 13.1 | 2197 | 4825 | -1321 | L1MA2 | L1 | LINE |

  

|  |  |  |  |  |  |  |  |  |  |
| --- | --- | --- | --- | --- | --- | --- | --- | --- | --- |
| chr8:111108320-111112006 | | | 3002 bp | | |  |  |  |  |
| Genome Start | Genome End | Strand | Div | Repeat Start | Repeat End | Repeat Left | Name | Family | Class |
| 111108320 | 111108534 | - | 28.5 | 2288 | 2507 | 0 | HAL1 | L1 | LINE |
| 111108654 | 111109011 | - | 28.2 | 1646 | 2043 | -464 | HAL1 | L1 | LINE |
| 111109442 | 111109611 | - | 24.4 | 1409 | 1605 | -902 | HAL1 | L1 | LINE |
| 111109620 | 111110148 | - | 3.0 | 4738 | 5281 | -865 | L1PA4 | L1 | LINE |
| 111110149 | 111111014 | + | 3.6 | 5285 | 6155 | 0 | L1PA4 | L1 | LINE |
| 111111034 | 111111655 | - | 30.1 | 730 | 1386 | -1121 | HAL1 | L1 | LINE |
| 111111765 | 111112006 | - | 31.2 | 23 | 308 | -2199 | HAL1 | L1 | LINE |

  

|  |  |  |  |  |  |  |  |  |  |
| --- | --- | --- | --- | --- | --- | --- | --- | --- | --- |
| chr8:111536157-111539429 | | | 3015 bp | | |  |  |  |  |
| Genome Start | Genome End | Strand | Div | Repeat Start | Repeat End | Repeat Left | Name | Family | Class |
| 111536157 | 111536390 | + | 34.1 | 8 | 279 | -4210 | L3 | CR1 | LINE |
| 111536416 | 111537768 | - | 18.9 | 3952 | 5325 | -815 | L1MA10 | L1 | LINE |
| 111537770 | 111538833 | + | 19.6 | 5307 | 6333 | -1 | L1MA10 | L1 | LINE |
| 111539066 | 111539429 | + | 35.0 | 726 | 1129 | -3360 | L3 | CR1 | LINE |

  

|  |  |  |  |  |  |  |  |  |  |
| --- | --- | --- | --- | --- | --- | --- | --- | --- | --- |
| chr8:112073775-112074761 | | | 934 bp | | |  |  |  |  |
| Genome Start | Genome End | Strand | Div | Repeat Start | Repeat End | Repeat Left | Name | Family | Class |
| 112073775 | 112073974 | + | 14.8 | 1 | 184 | -330 | MER67D | ERV1 | LTR |
| 112073981 | 112074119 | - | 16.6 | 5771 | 5911 | -257 | L1MB1 | L1 | LINE |
| 112074120 | 112074351 | + | 16.2 | 5921 | 6158 | -31 | L1MB1 | L1 | LINE |
| 112074397 | 112074587 | + | 16.2 | 6159 | 6323 | -11 | L1MA10 | L1 | LINE |
| 112074590 | 112074761 | + | 22.6 | 214 | 395 | -119 | MER67D | ERV1 | LTR |

  

|  |  |  |  |  |  |  |  |  |  |
| --- | --- | --- | --- | --- | --- | --- | --- | --- | --- |
| chr8:112656180-112657737 | | | 1509 bp | | |  |  |  |  |
| Genome Start | Genome End | Strand | Div | Repeat Start | Repeat End | Repeat Left | Name | Family | Class |
| 112656180 | 112656512 | - | 29.1 | 5813 | 6151 | -13 | L1ME1 | L1 | LINE |
| 112656559 | 112657347 | - | 4.9 | 5355 | 6146 | -8 | L1PA5 | L1 | LINE |
| 112657352 | 112657381 | + | 0.0 | 5323 | 5352 | -820 | L1P | L1 | LINE |
| 112657381 | 112657737 | - | 24.1 | 5454 | 5831 | -335 | L1ME1 | L1 | LINE |

  

|  |  |  |  |  |  |  |  |  |  |
| --- | --- | --- | --- | --- | --- | --- | --- | --- | --- |
| chr8:114598941-114600033 | | | 1084 bp | | |  |  |  |  |
| Genome Start | Genome End | Strand | Div | Repeat Start | Repeat End | Repeat Left | Name | Family | Class |
| 114598941 | 114599504 | + | 18.0 | 22 | 588 | -146 | MER50 | ERV1 | LTR |
| 114599514 | 114599860 | - | 2.6 | 5802 | 6147 | -8 | L1PA4 | L1 | LINE |
| 114599861 | 114599896 | + | 2.8 | 5782 | 5817 | -355 | L1P3 | L1 | LINE |
| 114599897 | 114600033 | + | 19.7 | 582 | 722 | -12 | MER50 | ERV1 | LTR |

  

|  |  |  |  |  |  |  |  |  |  |
| --- | --- | --- | --- | --- | --- | --- | --- | --- | --- |
| chr8:114933574-114937270 | | | 3220 bp | | |  |  |  |  |
| Genome Start | Genome End | Strand | Div | Repeat Start | Repeat End | Repeat Left | Name | Family | Class |
| 114933574 | 114933666 | + | 31.5 | 362 | 451 | -2968 | L2 | L2 | LINE |
| 114933688 | 114934077 | - | 19.8 | 4625 | 5016 | -1127 | L1M2 | L1 | LINE |
| 114934073 | 114934328 | - | 20.8 | 4004 | 4266 | -1877 | L1M2 | L1 | LINE |
| 114934332 | 114935573 | + | 18.2 | 5013 | 6308 | -4 | L1MA9 | L1 | LINE |
| 114935849 | 114936239 | + | 37.9 | 521 | 929 | -2490 | L2 | L2 | LINE |
| 114936423 | 114937270 | + | 37.4 | 965 | 1807 | -1612 | L2 | L2 | LINE |

  

|  |  |  |  |  |  |  |  |  |  |
| --- | --- | --- | --- | --- | --- | --- | --- | --- | --- |
| chr8:115209325-115216482 | | | 7170 bp | | |  |  |  |  |
| Genome Start | Genome End | Strand | Div | Repeat Start | Repeat End | Repeat Left | Name | Family | Class |
| 115209325 | 115212844 | - | 18.6 | 2741 | 6372 | -993 | L1M2 | L1 | LINE |
| 115212845 | 115213207 | - | 11.7 | 3 | 364 | 0 | THE1B | MaLR | LTR |
| 115213208 | 115213981 | - | 17.6 | 1970 | 2740 | -4469 | L1M2 | L1 | LINE |
| 115213977 | 115215161 | - | 3.0 | 4970 | 6155 | 0 | L1PA3 | L1 | LINE |
| 115215158 | 115215765 | + | 4.0 | 4372 | 4979 | -1167 | L1PA3 | L1 | LINE |
| 115215763 | 115216482 | - | 25.3 | 1169 | 1977 | -5232 | L1M2 | L1 | LINE |

  

|  |  |  |  |  |  |  |  |  |  |
| --- | --- | --- | --- | --- | --- | --- | --- | --- | --- |
| chr8:115518208-115519857 | | | 1633 bp | | |  |  |  |  |
| Genome Start | Genome End | Strand | Div | Repeat Start | Repeat End | Repeat Left | Name | Family | Class |
| 115518208 | 115518419 | - | 27.3 | 1602 | 1812 | -4690 | L1MDb | L1 | LINE |
| 115518420 | 115518724 | - | 12.8 | 1 | 305 | -7 | AluSx | Alu | SINE |
| 115518725 | 115519499 | - | 26.5 | 776 | 1601 | -4901 | L1MDb | L1 | LINE |
| 115519518 | 115519681 | - | 1.8 | 5989 | 6152 | -3 | L1PA4 | L1 | LINE |
| 115519682 | 115519736 | + | 1.8 | 5942 | 5996 | -159 | L1PA4 | L1 | LINE |
| 115519736 | 115519857 | - | 34.5 | 652 | 775 | -5634 | L1MDb | L1 | LINE |

  

|  |  |  |  |  |  |  |  |  |  |
| --- | --- | --- | --- | --- | --- | --- | --- | --- | --- |
| chr8:115848249-115850974 | | | 2592 bp | | |  |  |  |  |
| Genome Start | Genome End | Strand | Div | Repeat Start | Repeat End | Repeat Left | Name | Family | Class |
| 115848249 | 115849751 | + | 26.7 | 15 | 1677 | -4865 | L1M4 | L1 | LINE |
| 115849868 | 115850310 | - | 15.8 | 5447 | 5877 | -414 | L1MA8 | L1 | LINE |
| 115850306 | 115850678 | + | 18.4 | 5901 | 6280 | -11 | L1MA8 | L1 | LINE |
| 115850702 | 115850974 | + | 30.6 | 1766 | 2040 | -4606 | L1M4 | L1 | LINE |

  

|  |  |  |  |  |  |  |  |  |  |
| --- | --- | --- | --- | --- | --- | --- | --- | --- | --- |
| chr8:115989705-116001965 | | | 11929 bp | | |  |  |  |  |
| Genome Start | Genome End | Strand | Div | Repeat Start | Repeat End | Repeat Left | Name | Family | Class |
| 115989705 | 115991983 | + | 27.4 | 837 | 2862 | -5293 | L1PA15 | L1 | LINE |
| 115991987 | 115992258 | + | 13.6 | 36 | 307 | -3 | AluSg | Alu | SINE |
| 115992259 | 115996133 | + | 19.8 | 3323 | 7330 | -811 | L1PA15 | L1 | LINE |
| 115996129 | 115996217 | - | 12.4 | 4478 | 4566 | -1580 | L1P3 | L1 | LINE |
| 115996209 | 115996856 | - | 14.1 | 3527 | 4195 | -1951 | L1P3 | L1 | LINE |
| 115996855 | 115997117 | + | 10.5 | 4587 | 4847 | -1299 | L1PA11 | L1 | LINE |
| 115997119 | 115997677 | - | 19.5 | 84 | 612 | 0 | LTR9 | ERV1 | LTR |
| 115997678 | 115997813 | - | 11.0 | 164 | 299 | -12 | AluY | Alu | SINE |
| 115997814 | 115997902 | - | 19.1 | 3 | 92 | -520 | LTR9 | ERV1 | LTR |
| 115997907 | 115998116 | - | 16.7 | 8706 | 8916 | -3 | HUERS-P3 | ERV1 | LTR |
| 115998454 | 115998633 | - | 18.4 | 2384 | 2565 | -2450 | PABL\_A-int | ERV1 | LTR |
| 115998628 | 116000053 | - | 17.9 | 5572 | 6983 | -1936 | PABL\_A-int | ERV1 | LTR |
| 116000054 | 116000264 | + | 10.5 | 96 | 305 | -8 | AluSq | Alu | SINE |
| 116000265 | 116001120 | + | 11.2 | 5313 | 6171 | -3 | L1PA11 | L1 | LINE |
| 116001130 | 116001965 | + | 15.6 | 7313 | 8143 | -17 | L1PA15 | L1 | LINE |

  

|  |  |  |  |  |  |  |  |  |  |
| --- | --- | --- | --- | --- | --- | --- | --- | --- | --- |
| chr8:116398845-116402728 | | | 3858 bp | | |  |  |  |  |
| Genome Start | Genome End | Strand | Div | Repeat Start | Repeat End | Repeat Left | Name | Family | Class |
| 116398845 | 116399184 | + | 30.0 | 2457 | 2796 | -623 | L2 | L2 | LINE |
| 116399185 | 116399363 | - | 12.6 | 132 | 304 | -8 | AluSx | Alu | SINE |
| 116399364 | 116399664 | - | 12.5 | 1 | 295 | -15 | AluSg | Alu | SINE |
| 116399665 | 116399782 | - | 12.6 | 17 | 131 | -181 | AluSx | Alu | SINE |
| 116399783 | 116400053 | + | 30.0 | 2797 | 3065 | -354 | L2 | L2 | LINE |
| 116400080 | 116401902 | - | 4.7 | 4322 | 6151 | -3 | L1PA5 | L1 | LINE |
| 116401902 | 116402377 | + | 4.6 | 3828 | 4303 | -1843 | L1PA5 | L1 | LINE |
| 116402379 | 116402728 | + | 25.2 | 3105 | 3418 | -1 | L2 | L2 | LINE |

  

|  |  |  |  |  |  |  |  |  |  |
| --- | --- | --- | --- | --- | --- | --- | --- | --- | --- |
| chr8:117271228-117274051 | | | 2723 bp | | |  |  |  |  |
| Genome Start | Genome End | Strand | Div | Repeat Start | Repeat End | Repeat Left | Name | Family | Class |
| 117271228 | 117271350 | - | 28.1 | 140 | 260 | -8 | MIRb | MIR | SINE |
| 117271357 | 117271598 | - | 3.7 | 4173 | 4414 | -1732 | L1PA3 | L1 | LINE |
| 117271600 | 117273365 | + | 2.6 | 4405 | 6171 | -3 | L1PA3 | L1 | LINE |
| 117273429 | 117273618 | - | 26.1 | 416 | 624 | 0 | MLT1E1 | MaLR | LTR |
| 117273645 | 117273964 | - | 22.4 | 1 | 333 | -321 | MLT1E1 | MaLR | LTR |
| 117273970 | 117274051 | - | 29.3 | 56 | 138 | -130 | MIRb | MIR | SINE |

  

|  |  |  |  |  |  |  |  |  |  |
| --- | --- | --- | --- | --- | --- | --- | --- | --- | --- |
| chr8:118334296-118338995 | | | 4695 bp | | |  |  |  |  |
| Genome Start | Genome End | Strand | Div | Repeat Start | Repeat End | Repeat Left | Name | Family | Class |
| 118334296 | 118337317 | + | 11.2 | 2612 | 5669 | -497 | L1PA16 | L1 | LINE |
| 118337315 | 118337772 | - | 8.3 | 4693 | 5150 | -996 | L1PA7 | L1 | LINE |
| 118337771 | 118338766 | + | 5.3 | 5146 | 6148 | -6 | L1PA7 | L1 | LINE |
| 118338777 | 118338995 | + | 12.8 | 5658 | 5876 | -290 | L1PA16 | L1 | LINE |

  

|  |  |  |  |  |  |  |  |  |  |
| --- | --- | --- | --- | --- | --- | --- | --- | --- | --- |
| chr8:118427136-118434221 | | | 7036 bp | | |  |  |  |  |
| Genome Start | Genome End | Strand | Div | Repeat Start | Repeat End | Repeat Left | Name | Family | Class |
| 118427136 | 118428644 | + | 24.7 | 2547 | 4027 | -3535 | L1MA9 | L1 | LINE |
| 118428648 | 118429024 | + | 13.5 | 1 | 375 | 0 | THE1C | MaLR | LTR |
| 118429025 | 118430367 | + | 18.9 | 4055 | 5610 | -1952 | L1MA9 | L1 | LINE |
| 118430368 | 118430657 | + | 13.4 | 1 | 293 | -19 | AluSx | Alu | SINE |
| 118430658 | 118430963 | + | 18.9 | 5611 | 5921 | -1641 | L1MA9 | L1 | LINE |
| 118430956 | 118431784 | - | 3.8 | 4793 | 5621 | -534 | L1PA4 | L1 | LINE |
| 118431786 | 118432321 | + | 3.8 | 5623 | 6155 | 0 | L1PA4 | L1 | LINE |
| 118432347 | 118433417 | + | 16.6 | 5890 | 7140 | -591 | L1MA9 | L1 | LINE |
| 118433447 | 118433495 | + | 17.0 | 7204 | 7272 | -459 | L1MA9 | L1 | LINE |
| 118433496 | 118433782 | + | 18.9 | 1 | 296 | -16 | AluJo | Alu | SINE |
| 118433783 | 118434221 | + | 17.0 | 7273 | 7724 | -7 | L1MA9 | L1 | LINE |

  

|  |  |  |  |  |  |  |  |  |  |
| --- | --- | --- | --- | --- | --- | --- | --- | --- | --- |
| chr8:119780196-119782747 | | | 2369 bp | | |  |  |  |  |
| Genome Start | Genome End | Strand | Div | Repeat Start | Repeat End | Repeat Left | Name | Family | Class |
| 119780196 | 119780517 | - | 28.9 | 289 | 608 | -55 | MLT2F | ERVL | LTR |
| 119780555 | 119780710 | + | 22.2 | 14 | 166 | -255 | LTR52 | ERVL | LTR |
| 119780760 | 119781741 | - | 2.9 | 5173 | 6153 | -2 | L1PA4 | L1 | LINE |
| 119781739 | 119782323 | + | 3.2 | 4587 | 5171 | -975 | L1PA4 | L1 | LINE |
| 119782324 | 119782522 | + | 19.2 | 217 | 414 | -7 | LTR52 | ERVL | LTR |
| 119782623 | 119782747 | - | 20.2 | 1 | 133 | -530 | MLT2F | ERVL | LTR |

  

|  |  |  |  |  |  |  |  |  |  |
| --- | --- | --- | --- | --- | --- | --- | --- | --- | --- |
| chr8:120330317-120339642 | | | 8239 bp | | |  |  |  |  |
| Genome Start | Genome End | Strand | Div | Repeat Start | Repeat End | Repeat Left | Name | Family | Class |
| 120330317 | 120331094 | - | 35.9 | 4475 | 5261 | -406 | ERVL-E | ERVL | LTR |
| 120331240 | 120331466 | + | 17.7 | 5032 | 5273 | -873 | L1MB5 | L1 | LINE |
| 120331467 | 120331568 | - | 2.0 | 5673 | 5774 | -381 | L1PA2 | L1 | LINE |
| 120331569 | 120331964 | + | 2.8 | 5753 | 6155 | 0 | L1PA2 | L1 | LINE |
| 120331965 | 120332830 | + | 17.6 | 5275 | 6173 | -1 | L1MB5 | L1 | LINE |
| 120332962 | 120333292 | - | 26.3 | 3178 | 3543 | -2124 | ERVL-E | ERVL | LTR |
| 120333337 | 120333654 | + | 21.5 | 22 | 371 | -19 | MLT1B | MaLR | LTR |
| 120333687 | 120334003 | - | 36.4 | 2720 | 3042 | -2625 | ERVL-E | ERVL | LTR |
| 120334430 | 120335172 | - | 34.5 | 1720 | 2475 | -2521 | ERVL-E | ERVL | LTR |
| 120335374 | 120335604 | - | 33.9 | 1182 | 1423 | -3573 | ERVL-E | ERVL | LTR |
| 120335611 | 120338782 | + | 3.6 | 2974 | 6155 | 0 | L1PA4 | L1 | LINE |
| 120338885 | 120339642 | - | 34.2 | 198 | 980 | -4016 | ERVL-E | ERVL | LTR |

  

|  |  |  |  |  |  |  |  |  |  |
| --- | --- | --- | --- | --- | --- | --- | --- | --- | --- |
| chr8:120372948-120396493 | | | 23378 bp | | |  |  |  |  |
| Genome Start | Genome End | Strand | Div | Repeat Start | Repeat End | Repeat Left | Name | Family | Class |
| 120372948 | 120373175 | + | 33.9 | 2228 | 2464 | -955 | L2 | L2 | LINE |
| 120373200 | 120373379 | + | 19.0 | 1 | 159 | -6871 | L1M3e | L1 | LINE |
| 120373380 | 120373454 | - | 13.3 | 352 | 428 | 0 | THE1C | MaLR | LTR |
| 120373455 | 120379608 | + | 4.5 | 2 | 6150 | -5 | L1PA3 | L1 | LINE |
| 120379614 | 120379917 | - | 11.0 | 8 | 318 | -57 | THE1C | MaLR | LTR |
| 120379923 | 120381434 | - | 14.0 | 1 | 1580 | 0 | THE1C-int | MaLR | LTR |
| 120381435 | 120381805 | - | 11.9 | 3 | 375 | 0 | THE1C | MaLR | LTR |
| 120381806 | 120382025 | + | 21.9 | 153 | 382 | -6648 | L1M3e | L1 | LINE |
| 120382088 | 120384260 | + | 23.6 | 1070 | 3113 | -3987 | L1M3e | L1 | LINE |
| 120384261 | 120384706 | - | 15.9 | 1 | 424 | -4 | MSTC | MaLR | LTR |
| 120384707 | 120385365 | + | 17.3 | 3114 | 3793 | -3307 | L1M3e | L1 | LINE |
| 120385366 | 120385686 | + | 18.1 | 1 | 322 | -24 | MER7A | MER2\_type | DNA |
| 120385687 | 120387433 | + | 17.3 | 3794 | 5597 | -1503 | L1M3e | L1 | LINE |
| 120387433 | 120387556 | - | 4.0 | 5456 | 5579 | -576 | L1PA2 | L1 | LINE |
| 120387557 | 120388130 | + | 2.8 | 5580 | 6153 | -2 | L1PA2 | L1 | LINE |
| 120388139 | 120388225 | + | 18.4 | 5580 | 5670 | -1433 | L1MA8 | L1 | LINE |
| 120388226 | 120394342 | + | 8.1 | 3 | 6151 | -3 | L1PA6 | L1 | LINE |
| 120394338 | 120395856 | + | 16.2 | 5653 | 7220 | -31 | L1MA8 | L1 | LINE |
| 120395892 | 120396194 | + | 34.5 | 2535 | 2844 | -534 | L2 | L2 | LINE |
| 120396230 | 120396493 | + | 34.5 | 2845 | 3114 | -264 | L2 | L2 | LINE |

  

|  |  |  |  |  |  |  |  |  |  |
| --- | --- | --- | --- | --- | --- | --- | --- | --- | --- |
| chr8:120629094-120634334 | | | 5189 bp | | |  |  |  |  |
| Genome Start | Genome End | Strand | Div | Repeat Start | Repeat End | Repeat Left | Name | Family | Class |
| 120629094 | 120630482 | + | 21.5 | 37 | 1498 | -4902 | L1ME1 | L1 | LINE |
| 120630483 | 120630611 | + | 16.4 | 2 | 132 | -1 | FLAM\_C | Alu | SINE |
| 120630612 | 120630680 | + | 21.5 | 1499 | 1571 | -4829 | L1ME1 | L1 | LINE |
| 120630736 | 120631559 | - | 4.0 | 5318 | 6141 | -14 | L1PA3 | L1 | LINE |
| 120631559 | 120632625 | + | 3.0 | 4274 | 5339 | -961 | L1PA3 | L1 | LINE |
| 120632632 | 120632725 | + | 23.4 | 1639 | 1734 | -4666 | L1ME1 | L1 | LINE |
| 120632718 | 120634334 | + | 25.0 | 4559 | 6190 | -49 | L1ME1 | L1 | LINE |

  

|  |  |  |  |  |  |  |  |  |  |
| --- | --- | --- | --- | --- | --- | --- | --- | --- | --- |
| chr8:122229825-122232761 | | | 2947 bp | | |  |  |  |  |
| Genome Start | Genome End | Strand | Div | Repeat Start | Repeat End | Repeat Left | Name | Family | Class |
| 122229825 | 122230141 | + | 30.6 | 2525 | 2820 | -599 | L2 | L2 | LINE |
| 122230142 | 122231220 | - | 8.6 | 4004 | 5096 | -1050 | L1PA8 | L1 | LINE |
| 122231219 | 122232287 | + | 6.4 | 5085 | 6158 | -14 | L1PA8 | L1 | LINE |
| 122232290 | 122232433 | + | 30.6 | 2808 | 2956 | -463 | L2 | L2 | LINE |
| 122232424 | 122232761 | + | 33.8 | 3074 | 3418 | -1 | L2 | L2 | LINE |

  

|  |  |  |  |  |  |  |  |  |  |
| --- | --- | --- | --- | --- | --- | --- | --- | --- | --- |
| chr8:123744212-123744966 | | | 755 bp | | |  |  |  |  |
| Genome Start | Genome End | Strand | Div | Repeat Start | Repeat End | Repeat Left | Name | Family | Class |
| 123744212 | 123744346 | + | 15.6 | 1 | 137 | -175 | AluSx | Alu | SINE |
| 123744347 | 123744511 | - | 0.6 | 5707 | 5871 | -284 | L1PA2 | L1 | LINE |
| 123744512 | 123744779 | + | 1.9 | 5885 | 6152 | -3 | L1PA2 | L1 | LINE |
| 123744780 | 123744966 | + | 14.4 | 126 | 312 | 0 | AluSx | Alu | SINE |

  

|  |  |  |  |  |  |  |  |  |  |
| --- | --- | --- | --- | --- | --- | --- | --- | --- | --- |
| chr8:130796562-130801079 | | | 4555 bp | | |  |  |  |  |
| Genome Start | Genome End | Strand | Div | Repeat Start | Repeat End | Repeat Left | Name | Family | Class |
| 130796562 | 130796826 | + | 20.9 | 528 | 796 | -5604 | L1MEc | L1 | LINE |
| 130796823 | 130796972 | + | 19.5 | 1254 | 1410 | -4990 | L1MEc | L1 | LINE |
| 130796961 | 130797775 | + | 20.1 | 1640 | 2472 | -3823 | L1MEc | L1 | LINE |
| 130797784 | 130798881 | - | 8.5 | 3304 | 4414 | -1732 | L1PA7 | L1 | LINE |
| 130798876 | 130800623 | + | 7.6 | 4419 | 6154 | 0 | L1PA7 | L1 | LINE |
| 130800601 | 130801079 | + | 25.9 | 2359 | 2837 | -3382 | L1MEc | L1 | LINE |

  

|  |  |  |  |  |  |  |  |  |  |
| --- | --- | --- | --- | --- | --- | --- | --- | --- | --- |
| chr8:132144165-132148984 | | | 4816 bp | | |  |  |  |  |
| Genome Start | Genome End | Strand | Div | Repeat Start | Repeat End | Repeat Left | Name | Family | Class |
| 132144165 | 132144261 | - | 33.0 | 993 | 1089 | -1 | MER97c | MER1\_type? | DNA |
| 132144268 | 132144357 | - | 26.7 | 598 | 689 | -401 | MER97c | MER1\_type? | DNA |
| 132144358 | 132147419 | - | 2.5 | 3138 | 6206 | 0 | L1PA3 | L1 | LINE |
| 132147418 | 132148400 | + | 2.3 | 2157 | 3140 | -3006 | L1PA3 | L1 | LINE |
| 132148401 | 132148984 | - | 23.6 | 2 | 613 | -477 | MER97c | MER1\_type? | DNA |

  

|  |  |  |  |  |  |  |  |  |  |
| --- | --- | --- | --- | --- | --- | --- | --- | --- | --- |
| chr8:132177481-132179278 | | | 1297 bp | | |  |  |  |  |
| Genome Start | Genome End | Strand | Div | Repeat Start | Repeat End | Repeat Left | Name | Family | Class |
| 132177481 | 132177604 | - | 23.1 | 118 | 238 | -30 | MIRb | MIR | SINE |
| 132178031 | 132178175 | - | 31.2 | 102 | 256 | -12 | MIRb | MIR | SINE |
| 132178221 | 132178345 | - | 29.8 | 130 | 257 | -5 | MIR | MIR | SINE |
| 132178348 | 132178361 | - | 3.4 | 5800 | 5811 | -320 | L1PA8A | L1 | LINE |
| 132178362 | 132178722 | + | 14.7 | 1 | 364 | 0 | THE1B | MaLR | LTR |
| 132178723 | 132178773 | - | 3.4 | 5753 | 5799 | -332 | L1PA13 | L1 | LINE |
| 132178774 | 132179143 | + | 7.6 | 5787 | 6157 | -6 | L1PA13 | L1 | LINE |
| 132179172 | 132179278 | - | 21.9 | 25 | 143 | -125 | MIRb | MIR | SINE |

  

|  |  |  |  |  |  |  |  |  |  |
| --- | --- | --- | --- | --- | --- | --- | --- | --- | --- |
| chr8:132526214-132534170 | | | 7505 bp | | |  |  |  |  |
| Genome Start | Genome End | Strand | Div | Repeat Start | Repeat End | Repeat Left | Name | Family | Class |
| 132526214 | 132526775 | + | 33.9 | 90 | 699 | -2720 | L2 | L2 | LINE |
| 132526791 | 132527116 | - | 24.6 | 5176 | 5513 | -649 | L1M5 | L1 | LINE |
| 132527112 | 132527543 | - | 25.1 | 5822 | 6236 | -3 | L1ME1 | L1 | LINE |
| 132527542 | 132528252 | - | 25.4 | 3573 | 4277 | -1942 | L1ME1 | L1 | LINE |
| 132528339 | 132528499 | - | 14.4 | 6074 | 6240 | -2 | L1MD1 | L1 | LINE |
| 132528510 | 132529031 | - | 25.9 | 18 | 619 | -6014 | L1MDa | L1 | LINE |
| 132529033 | 132529694 | - | 26.5 | 2897 | 3581 | -2638 | L1ME1 | L1 | LINE |
| 132529695 | 132529948 | - | 14.2 | 44 | 299 | -13 | AluSx | Alu | SINE |
| 132529949 | 132530270 | - | 26.5 | 2568 | 2896 | -3323 | L1ME1 | L1 | LINE |
| 132530242 | 132530683 | - | 12.8 | 4879 | 5330 | -816 | L1M1 | L1 | LINE |
| 132530697 | 132530800 | + | 11.5 | 6050 | 6153 | -1 | L1PB3 | L1 | LINE |
| 132530826 | 132531345 | - | 11.9 | 3016 | 3546 | -2600 | L1M1 | L1 | LINE |
| 132531371 | 132531440 | + | 20.9 | 855 | 921 | -14 | MER21C | ERV1 | LTR |
| 132531499 | 132531676 | - | 25.4 | 1941 | 2132 | -4268 | L1ME1 | L1 | LINE |
| 132531715 | 132531821 | - | 28.0 | 1768 | 1878 | -4522 | L1ME1 | L1 | LINE |
| 132531958 | 132532758 | - | 10.1 | 5362 | 6163 | 0 | L1PA13 | L1 | LINE |
| 132532765 | 132532849 | + | 30.6 | 2704 | 2788 | -631 | L2 | L2 | LINE |
| 132532858 | 132532903 | - | 17.4 | 221 | 266 | -34 | Alu | Alu | SINE |
| 132532906 | 132532950 | - | 24.4 | 2865 | 2909 | -510 | L2 | L2 | LINE |
| 132532974 | 132533392 | - | 6.7 | 5527 | 5947 | -207 | L1PA7 | L1 | LINE |
| 132533393 | 132533589 | + | 7.1 | 5956 | 6153 | -1 | L1PA7 | L1 | LINE |
| 132533632 | 132534170 | + | 32.0 | 2806 | 3411 | -8 | L2 | L2 | LINE |

  

|  |  |  |  |  |  |  |  |  |  |
| --- | --- | --- | --- | --- | --- | --- | --- | --- | --- |
| chr8:132932397-132937515 | | | 5115 bp | | |  |  |  |  |
| Genome Start | Genome End | Strand | Div | Repeat Start | Repeat End | Repeat Left | Name | Family | Class |
| 132932397 | 132933608 | + | 27.4 | 776 | 2068 | -1351 | L2 | L2 | LINE |
| 132933609 | 132933913 | - | 10.8 | 1 | 305 | -7 | AluSx | Alu | SINE |
| 132933914 | 132934334 | + | 27.4 | 2069 | 2491 | -928 | L2 | L2 | LINE |
| 132934335 | 132934933 | + | 6.3 | 5546 | 6154 | 0 | L1PA7 | L1 | LINE |
| 132934934 | 132935187 | + | 27.4 | 2492 | 2733 | -686 | L2 | L2 | LINE |
| 132935189 | 132935362 | + | 15.6 | 2 | 186 | -242 | MSTA | MaLR | LTR |
| 132935363 | 132935526 | - | 3.7 | 5966 | 6129 | -25 | L1PA5 | L1 | LINE |
| 132935527 | 132935797 | + | 16.6 | 114 | 428 | 0 | MSTA | MaLR | LTR |
| 132935799 | 132936213 | + | 29.9 | 2730 | 3126 | -293 | L2 | L2 | LINE |
| 132936214 | 132936884 | - | 15.3 | 5440 | 6164 | -2 | L1PA16 | L1 | LINE |
| 132936885 | 132936963 | + | 31.6 | 3127 | 3191 | -226 | L2 | L2 | LINE |
| 132936964 | 132936997 | - | 0.0 | 5871 | 5904 | -250 | L1PA5 | L1 | LINE |
| 132937000 | 132937259 | + | 2.7 | 5894 | 6153 | -1 | L1PA5 | L1 | LINE |
| 132937260 | 132937515 | + | 31.6 | 3194 | 3412 | -7 | L2 | L2 | LINE |

  

|  |  |  |  |  |  |  |  |  |  |
| --- | --- | --- | --- | --- | --- | --- | --- | --- | --- |
| chr8:133697765-133703027 | | | 5183 bp | | |  |  |  |  |
| Genome Start | Genome End | Strand | Div | Repeat Start | Repeat End | Repeat Left | Name | Family | Class |
| 133697765 | 133698137 | - | 30.1 | 2400 | 2788 | -631 | L2 | L2 | LINE |
| 133698143 | 133698494 | - | 24.1 | 188 | 547 | 0 | LTR10G | ERV1 | LTR |
| 133698574 | 133698826 | - | 29.2 | 2092 | 2394 | -1025 | L2 | L2 | LINE |
| 133698830 | 133699140 | - | 3.5 | 4059 | 4398 | -1748 | L1PA3 | L1 | LINE |
| 133699125 | 133700885 | + | 1.6 | 4391 | 6151 | -4 | L1PA3 | L1 | LINE |
| 133700895 | 133701759 | - | 30.5 | 1158 | 2102 | -1317 | L2 | L2 | LINE |
| 133701760 | 133702062 | - | 11.6 | 1 | 303 | -9 | AluSx | Alu | SINE |
| 133702063 | 133703027 | - | 30.5 | 70 | 1157 | -2262 | L2 | L2 | LINE |

  

|  |  |  |  |  |  |  |  |  |  |
| --- | --- | --- | --- | --- | --- | --- | --- | --- | --- |
| chr8:135496066-135502604 | | | 6093 bp | | |  |  |  |  |
| Genome Start | Genome End | Strand | Div | Repeat Start | Repeat End | Repeat Left | Name | Family | Class |
| 135496066 | 135496275 | - | 27.4 | 4615 | 4837 | -830 | ERVL-E | ERVL | LTR |
| 135496350 | 135496855 | - | 29.9 | 4089 | 4599 | -1068 | ERVL-E | ERVL | LTR |
| 135496856 | 135497521 | - | 30.1 | 2818 | 3513 | -2154 | ERVL-E | ERVL | LTR |
| 135497608 | 135498139 | - | 0.2 | 4751 | 5282 | -864 | L1HS | L1 | LINE |
| 135498138 | 135499009 | + | 1.1 | 5281 | 6153 | -2 | L1HS | L1 | LINE |
| 135499149 | 135499763 | + | 11.1 | 4882 | 5489 | -679 | L1PA12 | L1 | LINE |
| 135499764 | 135500061 | + | 13.5 | 1 | 298 | -14 | AluSx | Alu | SINE |
| 135500062 | 135500611 | + | 9.3 | 5490 | 6014 | -154 | L1PA12 | L1 | LINE |
| 135500619 | 135502267 | - | 31.0 | 902 | 2554 | -3113 | ERVL-E | ERVL | LTR |
| 135502410 | 135502604 | - | 31.4 | 509 | 712 | -4955 | ERVL-E | ERVL | LTR |

  

|  |  |  |  |  |  |  |  |  |  |
| --- | --- | --- | --- | --- | --- | --- | --- | --- | --- |
| chr8:136177324-136193588 | | | 15789 bp | | |  |  |  |  |
| Genome Start | Genome End | Strand | Div | Repeat Start | Repeat End | Repeat Left | Name | Family | Class |
| 136177324 | 136177462 | + | 28.7 | 307 | 456 | -2051 | HAL1 | L1 | LINE |
| 136177569 | 136178154 | + | 29.3 | 672 | 1262 | -1245 | HAL1 | L1 | LINE |
| 136178171 | 136179838 | + | 21.7 | 15 | 1732 | -4901 | L1MDa | L1 | LINE |
| 136179837 | 136180660 | - | 13.5 | 4371 | 5193 | -950 | L1MA8 | L1 | LINE |
| 136180660 | 136181736 | + | 17.2 | 5205 | 6291 | 0 | L1MA8 | L1 | LINE |
| 136181751 | 136181931 | + | 21.1 | 1718 | 1909 | -4724 | L1MDa | L1 | LINE |
| 136181923 | 136182374 | + | 17.6 | 5767 | 6242 | 0 | L1MD1 | L1 | LINE |
| 136182531 | 136182615 | + | 27.2 | 1607 | 1675 | -832 | HAL1 | L1 | LINE |
| 136182616 | 136183475 | - | 15.7 | 5420 | 6289 | -2 | L1MA7 | L1 | LINE |
| 136183476 | 136183780 | - | 10.7 | 1 | 300 | -9 | AluSc | Alu | SINE |
| 136183781 | 136184226 | + | 27.2 | 1676 | 2129 | -378 | HAL1 | L1 | LINE |
| 136184231 | 136184633 | + | 25.3 | 2021 | 2426 | -81 | HAL1 | L1 | LINE |
| 136184685 | 136186398 | - | 15.3 | 4407 | 6147 | -19 | L1PA17 | L1 | LINE |
| 136186430 | 136187873 | - | 12.7 | 4854 | 6302 | 0 | L1MA1 | L1 | LINE |
| 136187874 | 136188172 | + | 8.7 | 11 | 310 | -3 | AluSp | Alu | SINE |
| 136188173 | 136190113 | - | 17.1 | 3148 | 5280 | -1293 | L1PA17 | L1 | LINE |
| 136190114 | 136190807 | - | 12.8 | 5615 | 6304 | 0 | L1MA2 | L1 | LINE |
| 136190808 | 136191069 | - | 16.6 | 2882 | 3148 | -3425 | L1PA17 | L1 | LINE |
| 136191128 | 136192735 | - | 19.8 | 1298 | 2881 | -3692 | L1PA17 | L1 | LINE |
| 136192758 | 136193170 | - | 25.9 | 697 | 1114 | -5759 | L1PA17 | L1 | LINE |
| 136193192 | 136193530 | - | 18.0 | 2 | 355 | -6518 | L1PA17 | L1 | LINE |
| 136193540 | 136193588 | + | 18.4 | 2459 | 2507 | 0 | HAL1 | L1 | LINE |

  

|  |  |  |  |  |  |  |  |  |  |
| --- | --- | --- | --- | --- | --- | --- | --- | --- | --- |
| chr8:137826780-137834395 | | | 7556 bp | | |  |  |  |  |
| Genome Start | Genome End | Strand | Div | Repeat Start | Repeat End | Repeat Left | Name | Family | Class |
| 137826780 | 137828508 | - | 19.8 | 4668 | 6432 | -1130 | L1M3b | L1 | LINE |
| 137828516 | 137828614 | + | 13.6 | 1 | 94 | -287 | THE1D | MaLR | LTR |
| 137828615 | 137829183 | - | 14.5 | 1 | 611 | -1 | LTR9 | ERV1 | LTR |
| 137829184 | 137829452 | + | 13.6 | 95 | 381 | 0 | THE1D | MaLR | LTR |
| 137829455 | 137829531 | - | 13.2 | 4587 | 4663 | -2899 | L1M3b | L1 | LINE |
| 137829529 | 137830494 | - | 1.1 | 5190 | 6155 | 0 | L1PA2 | L1 | LINE |
| 137830494 | 137830987 | + | 1.2 | 4715 | 5208 | -938 | L1PA2 | L1 | LINE |
| 137830986 | 137831096 | - | 15.3 | 4479 | 4600 | -2962 | L1M3b | L1 | LINE |
| 137831092 | 137832331 | - | 25.2 | 2857 | 4129 | -3433 | L1M3b | L1 | LINE |
| 137832363 | 137834284 | - | 29.0 | 1296 | 2980 | -4709 | L1M3b | L1 | LINE |
| 137834316 | 137834395 | - | 28.8 | 1216 | 1295 | -6201 | L1M3b | L1 | LINE |

  

|  |  |  |  |  |  |  |  |  |  |
| --- | --- | --- | --- | --- | --- | --- | --- | --- | --- |
| chr8:139079093-139086388 | | | 7257 bp | | |  |  |  |  |
| Genome Start | Genome End | Strand | Div | Repeat Start | Repeat End | Repeat Left | Name | Family | Class |
| 139079093 | 139080337 | + | 26.0 | 1651 | 2824 | -3395 | L1ME1 | L1 | LINE |
| 139080338 | 139080619 | + | 11.0 | 1 | 283 | -27 | AluSg | Alu | SINE |
| 139080620 | 139081456 | + | 29.4 | 2825 | 3657 | -2562 | L1ME1 | L1 | LINE |
| 139081452 | 139082520 | - | 10.0 | 4324 | 5395 | -751 | L1PREC2 | L1 | LINE |
| 139082521 | 139083273 | + | 10.6 | 5404 | 6168 | 0 | L1PA12 | L1 | LINE |
| 139083274 | 139084950 | + | 27.9 | 3635 | 5428 | -791 | L1ME1 | L1 | LINE |
| 139084992 | 139085299 | + | 16.8 | 67 | 428 | 0 | MSTA | MaLR | LTR |
| 139085303 | 139085735 | + | 26.7 | 5524 | 5955 | -284 | L1ME1 | L1 | LINE |
| 139085736 | 139086086 | - | 12.7 | 1 | 364 | 0 | THE1B | MaLR | LTR |
| 139086087 | 139086388 | + | 26.7 | 5956 | 6234 | -5 | L1ME1 | L1 | LINE |

  

|  |  |  |  |  |  |  |  |  |  |
| --- | --- | --- | --- | --- | --- | --- | --- | --- | --- |
| chr8:139485501-139491018 | | | 5515 bp | | |  |  |  |  |
| Genome Start | Genome End | Strand | Div | Repeat Start | Repeat End | Repeat Left | Name | Family | Class |
| 139485501 | 139486639 | - | 13.2 | 1291 | 2417 | -1 | Tigger1 | MER2\_type | DNA |
| 139486645 | 139487668 | - | 4.1 | 5126 | 6155 | 0 | L1PA3 | L1 | LINE |
| 139487667 | 139489334 | + | 3.2 | 3472 | 5142 | -1004 | L1PA3 | L1 | LINE |
| 139489335 | 139489958 | - | 13.7 | 721 | 1324 | -1094 | Tigger1 | MER2\_type | DNA |
| 139489959 | 139490318 | - | 13.3 | 1 | 377 | -4 | THE1D | MaLR | LTR |
| 139490319 | 139491018 | - | 13.7 | 20 | 720 | -1698 | Tigger1 | MER2\_type | DNA |

  

|  |  |  |  |  |  |  |  |  |  |
| --- | --- | --- | --- | --- | --- | --- | --- | --- | --- |
| chr8:141978620-141986213 | | | 7568 bp | | |  |  |  |  |
| Genome Start | Genome End | Strand | Div | Repeat Start | Repeat End | Repeat Left | Name | Family | Class |
| 141978620 | 141979720 | + | 14.9 | 3310 | 4489 | -1654 | L1MA4 | L1 | LINE |
| 141979721 | 141980026 | + | 15.4 | 1 | 308 | -4 | AluSx | Alu | SINE |
| 141980027 | 141980509 | + | 14.9 | 4490 | 4968 | -1175 | L1MA4 | L1 | LINE |
| 141980530 | 141980856 | + | 14.9 | 4969 | 5291 | -852 | L1MA4 | L1 | LINE |
| 141980858 | 141980931 | + | 20.6 | 72 | 145 | -21 | FRAM | Alu | SINE |
| 141980932 | 141981316 | - | 4.7 | 5778 | 6154 | 0 | L1PA7 | L1 | LINE |
| 141981317 | 141981615 | - | 10.7 | 1 | 298 | -14 | AluSx | Alu | SINE |
| 141981616 | 141984457 | - | 7.4 | 2969 | 5777 | -377 | L1PA7 | L1 | LINE |
| 141984457 | 141985275 | + | 8.5 | 2139 | 2965 | -3181 | L1PA7 | L1 | LINE |
| 141985282 | 141986213 | + | 13.8 | 5287 | 6294 | -6 | L1MA4 | L1 | LINE |

  


|  |  |  |  |  |  |  |  |  |  |
| --- | --- | --- | --- | --- | --- | --- | --- | --- | --- |
| chr9:1199195-1201079 | | | 1891 bp | | |  |  |  |  |
| Genome Start | Genome End | Strand | Div | Repeat Start | Repeat End | Repeat Left | Name | Family | Class |
| 1199195 | 1199460 | - | 27.6 | 180 | 455 | -66 | LTR33 | ERVL | LTR |
| 1199461 | 1199557 | - | 4.1 | 4728 | 4824 | -1322 | L1PA5 | L1 | LINE |
| 1199552 | 1200891 | + | 5.4 | 4831 | 6149 | -5 | L1PA5 | L1 | LINE |
| 1200892 | 1201079 | - | 23.5 | 1 | 200 | -321 | LTR33 | ERVL | LTR |

  

|  |  |  |  |  |  |  |  |  |  |
| --- | --- | --- | --- | --- | --- | --- | --- | --- | --- |
| chr9:1487281-1494818 | | | 7002 bp | | |  |  |  |  |
| Genome Start | Genome End | Strand | Div | Repeat Start | Repeat End | Repeat Left | Name | Family | Class |
| 1487281 | 1487391 | - | 24.8 | 2838 | 2944 | 0 | BLACKJACK | AcHobo | DNA |
| 1487674 | 1487867 | - | 11.9 | 5909 | 6105 | -55 | L1PREC2 | L1 | LINE |
| 1487869 | 1488808 | - | 23.3 | 1582 | 2545 | -399 | BLACKJACK | AcHobo | DNA |
| 1488809 | 1489051 | - | 26.0 | 6096 | 7765 | -20 | L1MD2 | L1 | LINE |
| 1489053 | 1489383 | - | 22.3 | 2 | 2491 | 0 | Tigger7 | MER2\_type | DNA |
| 1489385 | 1489881 | - | 25.4 | 5577 | 6096 | -263 | L1MD2 | L1 | LINE |
| 1490085 | 1490372 | - | 23.8 | 5302 | 5591 | -651 | L1MD2 | L1 | LINE |
| 1490375 | 1490656 | - | 5.0 | 5868 | 6155 | 0 | L1PA4 | L1 | LINE |
| 1490657 | 1490722 | + | 0.0 | 5797 | 5863 | -291 | L1PA4 | L1 | LINE |
| 1490722 | 1491401 | - | 22.8 | 4604 | 5310 | -836 | L1MD2 | L1 | LINE |
| 1491419 | 1492476 | - | 19.9 | 5136 | 6178 | 0 | L1MB8 | L1 | LINE |
| 1492501 | 1492555 | - | 17.1 | 5063 | 5135 | -1011 | L1MB8 | L1 | LINE |
| 1492552 | 1493740 | + | 18.7 | 4699 | 5917 | -257 | L1MB5 | L1 | LINE |
| 1493741 | 1494534 | + | 15.7 | 1 | 826 | 0 | LTR1B | ERV1 | LTR |
| 1494535 | 1494746 | + | 21.6 | 5918 | 6159 | -19 | L1MB5 | L1 | LINE |
| 1494757 | 1494818 | - | 27.4 | 41 | 102 | -2842 | BLACKJACK | AcHobo | DNA |

  

|  |  |  |  |  |  |  |  |  |  |
| --- | --- | --- | --- | --- | --- | --- | --- | --- | --- |
| chr9:1750277-1751447 | | | 1036 bp | | |  |  |  |  |
| Genome Start | Genome End | Strand | Div | Repeat Start | Repeat End | Repeat Left | Name | Family | Class |
| 1750277 | 1750426 | + | 32.2 | 13 | 164 | -104 | MIRb | MIR | SINE |
| 1750542 | 1750676 | + | 26.7 | 14 | 160 | -102 | MIR | MIR | SINE |
| 1750692 | 1750935 | - | 4.5 | 5503 | 5746 | -409 | L1PA2 | L1 | LINE |
| 1750936 | 1751354 | + | 2.1 | 5737 | 6155 | 0 | L1PA2 | L1 | LINE |
| 1751360 | 1751447 | + | 24.4 | 178 | 267 | -1 | MIRb | MIR | SINE |

  

|  |  |  |  |  |  |  |  |  |  |
| --- | --- | --- | --- | --- | --- | --- | --- | --- | --- |
| chr9:3628866-3633036 | | | 4078 bp | | |  |  |  |  |
| Genome Start | Genome End | Strand | Div | Repeat Start | Repeat End | Repeat Left | Name | Family | Class |
| 3628866 | 3629270 | + | 23.6 | 1 | 424 | -616 | MER45B | Tip100 | DNA |
| 3629271 | 3630219 | + | 12.2 | 5192 | 6154 | -137 | L1MA8 | L1 | LINE |
| 3630232 | 3631179 | - | 9.0 | 4437 | 5388 | -758 | L1PA8 | L1 | LINE |
| 3631163 | 3631951 | + | 6.9 | 5388 | 6171 | -1 | L1PA8 | L1 | LINE |
| 3631964 | 3632272 | + | 10.7 | 1 | 308 | -1 | AluSc | Alu | SINE |
| 3632312 | 3632442 | + | 13.7 | 6153 | 6291 | 0 | L1MA8 | L1 | LINE |
| 3632490 | 3633036 | + | 16.7 | 411 | 1006 | -34 | MER45B | Tip100 | DNA |

  

|  |  |  |  |  |  |  |  |  |  |
| --- | --- | --- | --- | --- | --- | --- | --- | --- | --- |
| chr9:4455976-4466108 | | | 10022 bp | | |  |  |  |  |
| Genome Start | Genome End | Strand | Div | Repeat Start | Repeat End | Repeat Left | Name | Family | Class |
| 4455976 | 4456647 | + | 20.3 | 3161 | 3852 | -2294 | L1MC4a | L1 | LINE |
| 4456666 | 4462767 | + | 6.9 | 4 | 6152 | -2 | L1PA6 | L1 | LINE |
| 4462759 | 4462955 | + | 23.2 | 4183 | 4389 | -1757 | L1MC4a | L1 | LINE |
| 4462929 | 4463114 | - | 10.6 | 5499 | 5686 | -485 | L1PA13 | L1 | LINE |
| 4463115 | 4463243 | + | 8.3 | 5699 | 5823 | -340 | L1PA13 | L1 | LINE |
| 4463244 | 4463469 | + | 12.4 | 81 | 306 | -6 | AluSg/x | Alu | SINE |
| 4463470 | 4463794 | + | 8.3 | 5824 | 6160 | -3 | L1PA13 | L1 | LINE |
| 4463800 | 4464269 | + | 23.4 | 4359 | 4821 | -1325 | L1MC4a | L1 | LINE |
| 4464271 | 4464355 | + | 15.5 | 48 | 131 | -181 | AluJb | Alu | SINE |
| 4464356 | 4464651 | + | 22.2 | 4761 | 5093 | -1053 | L1MC4a | L1 | LINE |
| 4464698 | 4464915 | - | 16.4 | 87 | 299 | -13 | AluJb | Alu | SINE |
| 4464917 | 4464985 | + | 21.7 | 5081 | 5153 | -993 | L1MC4a | L1 | LINE |
| 4464986 | 4465160 | - | 12.0 | 134 | 308 | -4 | AluSg/x | Alu | SINE |
| 4465172 | 4465377 | + | 22.8 | 5173 | 5412 | -2373 | L1MC4a | L1 | LINE |
| 4465443 | 4465914 | + | 25.3 | 5692 | 6101 | -1707 | L1MC4a | L1 | LINE |
| 4465915 | 4466048 | - | 16.1 | 1 | 130 | -3 | FLAM\_C | Alu | SINE |
| 4466049 | 4466108 | + | 25.3 | 6102 | 6166 | -1642 | L1MC4a | L1 | LINE |

  

|  |  |  |  |  |  |  |  |  |  |
| --- | --- | --- | --- | --- | --- | --- | --- | --- | --- |
| chr9:5228903-5233474 | | | 4519 bp | | |  |  |  |  |
| Genome Start | Genome End | Strand | Div | Repeat Start | Repeat End | Repeat Left | Name | Family | Class |
| 5228903 | 5229325 | + | 26.6 | 7 | 497 | -6110 | L1MB4 | L1 | LINE |
| 5229373 | 5229634 | + | 26.6 | 498 | 790 | -5817 | L1MB4 | L1 | LINE |
| 5229641 | 5229781 | + | 26.9 | 1147 | 1301 | -5306 | L1MB4 | L1 | LINE |
| 5229799 | 5231054 | - | 0.7 | 4900 | 6155 | 0 | L1HS | L1 | LINE |
| 5231050 | 5233281 | + | 0.6 | 2673 | 4904 | -1242 | L1HS | L1 | LINE |
| 5233270 | 5233474 | + | 23.8 | 1280 | 1473 | -5134 | L1MB4 | L1 | LINE |

  

|  |  |  |  |  |  |  |  |  |  |
| --- | --- | --- | --- | --- | --- | --- | --- | --- | --- |
| chr9:5700362-5702674 | | | 2093 bp | | |  |  |  |  |
| Genome Start | Genome End | Strand | Div | Repeat Start | Repeat End | Repeat Left | Name | Family | Class |
| 5700362 | 5701239 | + | 28.8 | 586 | 1533 | -974 | HAL1 | L1 | LINE |
| 5701322 | 5701486 | - | 27.8 | 7602 | 7773 | -188 | L1MC5 | L1 | LINE |
| 5701584 | 5702356 | - | 4.0 | 5374 | 6146 | -8 | L1PA5 | L1 | LINE |
| 5702356 | 5702559 | + | 5.9 | 5156 | 5363 | -783 | L1PA5 | L1 | LINE |
| 5702602 | 5702674 | + | 24.7 | 2432 | 2507 | 0 | HAL1 | L1 | LINE |

  

|  |  |  |  |  |  |  |  |  |  |
| --- | --- | --- | --- | --- | --- | --- | --- | --- | --- |
| chr9:7895436-7896797 | | | 1276 bp | | |  |  |  |  |
| Genome Start | Genome End | Strand | Div | Repeat Start | Repeat End | Repeat Left | Name | Family | Class |
| 7895436 | 7895560 | - | 20.3 | 350 | 467 | 0 | MLT1C | MaLR | LTR |
| 7895611 | 7896108 | - | 14.2 | 5787 | 6291 | 0 | L1MA7 | L1 | LINE |
| 7896109 | 7896184 | + | 14.5 | 5732 | 5807 | -505 | L1MA7 | L1 | LINE |
| 7896221 | 7896393 | - | 28.1 | 98 | 262 | -205 | MLT1C | MaLR | LTR |
| 7896394 | 7896703 | - | 8.7 | 1 | 313 | 0 | AluSq | Alu | SINE |
| 7896704 | 7896797 | - | 28.1 | 1 | 97 | -370 | MLT1C | MaLR | LTR |

  

|  |  |  |  |  |  |  |  |  |  |
| --- | --- | --- | --- | --- | --- | --- | --- | --- | --- |
| chr9:8030079-8031623 | | | 1541 bp | | |  |  |  |  |
| Genome Start | Genome End | Strand | Div | Repeat Start | Repeat End | Repeat Left | Name | Family | Class |
| 8030079 | 8030124 | - | 17.4 | 6122 | 6167 | -1 | L1MB1 | L1 | LINE |
| 8030127 | 8030554 | - | 11.4 | 5737 | 6166 | 0 | L1PA16 | L1 | LINE |
| 8030555 | 8031349 | + | 12.3 | 4949 | 5737 | -429 | L1PA16 | L1 | LINE |
| 8031348 | 8031458 | - | 16.2 | 6007 | 6129 | -39 | L1MB1 | L1 | LINE |
| 8031463 | 8031623 | - | 18.0 | 5769 | 5932 | -236 | L1MB1 | L1 | LINE |

  

|  |  |  |  |  |  |  |  |  |  |
| --- | --- | --- | --- | --- | --- | --- | --- | --- | --- |
| chr9:10447814-10452198 | | | 4242 bp | | |  |  |  |  |
| Genome Start | Genome End | Strand | Div | Repeat Start | Repeat End | Repeat Left | Name | Family | Class |
| 10447814 | 10448868 | + | 20.0 | 3205 | 4317 | -1823 | L1MA9 | L1 | LINE |
| 10448903 | 10449828 | - | 8.4 | 5226 | 6150 | -4 | L1PA7 | L1 | LINE |
| 10449931 | 10450032 | - | 14.7 | 6077 | 6180 | 0 | L1MB4 | L1 | LINE |
| 10450034 | 10450071 | + | 15.8 | 6052 | 6089 | -94 | L1MB4 | L1 | LINE |
| 10450078 | 10451618 | + | 20.2 | 4321 | 5919 | -393 | L1MA9 | L1 | LINE |
| 10451619 | 10451921 | - | 10.6 | 1 | 304 | -8 | AluSx | Alu | SINE |
| 10451922 | 10452198 | + | 22.5 | 5920 | 6184 | -128 | L1MA9 | L1 | LINE |

  

|  |  |  |  |  |  |  |  |  |  |
| --- | --- | --- | --- | --- | --- | --- | --- | --- | --- |
| chr9:10496211-10501768 | | | 5482 bp | | |  |  |  |  |
| Genome Start | Genome End | Strand | Div | Repeat Start | Repeat End | Repeat Left | Name | Family | Class |
| 10496211 | 10496439 | - | 22.6 | 7769 | 7994 | -1 | L1MD3 | L1 | LINE |
| 10496440 | 10496624 | + | 8.7 | 6112 | 6305 | 0 | L1MA3 | L1 | LINE |
| 10496625 | 10496914 | - | 22.6 | 7504 | 7768 | -227 | L1MD3 | L1 | LINE |
| 10496915 | 10497059 | - | 4.1 | 4033 | 4177 | -1969 | L1PA5 | L1 | LINE |
| 10497011 | 10498955 | + | 5.8 | 4198 | 6153 | -1 | L1PA5 | L1 | LINE |
| 10498976 | 10499466 | - | 26.0 | 6847 | 7338 | -470 | L1MC4a | L1 | LINE |
| 10499504 | 10499530 | - | 26.0 | 6832 | 6846 | -962 | L1MC4a | L1 | LINE |
| 10499581 | 10499997 | - | 26.0 | 6488 | 6831 | -977 | L1MC4a | L1 | LINE |
| 10500016 | 10500575 | - | 25.8 | 5878 | 6475 | -1520 | L1MD3 | L1 | LINE |
| 10500576 | 10501024 | - | 17.9 | 5834 | 6294 | -6 | L1MA4 | L1 | LINE |
| 10501025 | 10501420 | - | 25.8 | 5488 | 5877 | -2118 | L1MD3 | L1 | LINE |
| 10501421 | 10501713 | - | 14.9 | 1 | 281 | -29 | AluSg | Alu | SINE |
| 10501714 | 10501768 | - | 25.8 | 5436 | 5487 | -2508 | L1MD3 | L1 | LINE |

  

|  |  |  |  |  |  |  |  |  |  |
| --- | --- | --- | --- | --- | --- | --- | --- | --- | --- |
| chr9:10777762-10781990 | | | 3727 bp | | |  |  |  |  |
| Genome Start | Genome End | Strand | Div | Repeat Start | Repeat End | Repeat Left | Name | Family | Class |
| 10777762 | 10778710 | + | 31.4 | 3553 | 4541 | -1605 | L1M5 | L1 | LINE |
| 10778711 | 10778969 | - | 3.5 | 5895 | 6153 | -1 | L1PA5 | L1 | LINE |
| 10778970 | 10779503 | + | 4.8 | 5362 | 5895 | -260 | L1PA5 | L1 | LINE |
| 10779501 | 10779626 | + | 28.6 | 4532 | 4660 | -1486 | L1M5 | L1 | LINE |
| 10779976 | 10780178 | + | 31.9 | 5120 | 5340 | -806 | L1M5 | L1 | LINE |
| 10780179 | 10781707 | + | 20.3 | 1 | 1755 | 0 | MER52A | ERV1 | LTR |
| 10781708 | 10781784 | + | 27.8 | 5340 | 5417 | -745 | L1M5 | L1 | LINE |
| 10781941 | 10781990 | + | 16.7 | 5648 | 5697 | -543 | L1M5 | L1 | LINE |

  

|  |  |  |  |  |  |  |  |  |  |
| --- | --- | --- | --- | --- | --- | --- | --- | --- | --- |
| chr9:10890390-10893005 | | | 2618 bp | | |  |  |  |  |
| Genome Start | Genome End | Strand | Div | Repeat Start | Repeat End | Repeat Left | Name | Family | Class |
| 10890390 | 10890599 | - | 14.0 | 155 | 361 | -3 | THE1B | MaLR | LTR |
| 10890600 | 10890834 | - | 7.7 | 3870 | 4109 | -2037 | L1PA6 | L1 | LINE |
| 10890833 | 10892843 | + | 6.4 | 4124 | 6153 | -1 | L1PA6 | L1 | LINE |
| 10892844 | 10893005 | - | 17.9 | 1 | 170 | -211 | THE1B | MaLR | LTR |

  

|  |  |  |  |  |  |  |  |  |  |
| --- | --- | --- | --- | --- | --- | --- | --- | --- | --- |
| chr9:11203979-11207411 | | | 3261 bp | | |  |  |  |  |
| Genome Start | Genome End | Strand | Div | Repeat Start | Repeat End | Repeat Left | Name | Family | Class |
| 11203979 | 11204118 | - | 14.2 | 6166 | 6299 | -6 | L1MA3 | L1 | LINE |
| 11204119 | 11205798 | - | 4.6 | 4211 | 5884 | -270 | L1PA5 | L1 | LINE |
| 11205799 | 11206047 | + | 6.8 | 5901 | 6149 | -5 | L1PA5 | L1 | LINE |
| 11206220 | 11207411 | - | 14.6 | 5024 | 6187 | -118 | L1MA3 | L1 | LINE |

  

|  |  |  |  |  |  |  |  |  |  |
| --- | --- | --- | --- | --- | --- | --- | --- | --- | --- |
| chr9:11464907-11467330 | | | 2389 bp | | |  |  |  |  |
| Genome Start | Genome End | Strand | Div | Repeat Start | Repeat End | Repeat Left | Name | Family | Class |
| 11464907 | 11464998 | - | 28.3 | 145 | 236 | -26 | MIR | MIR | SINE |
| 11465011 | 11465384 | - | 8.0 | 4332 | 4685 | -1461 | L1P3 | L1 | LINE |
| 11465385 | 11465679 | - | 13.6 | 1 | 297 | -16 | AluSq | Alu | SINE |
| 11465680 | 11465717 | - | 8.0 | 4294 | 4331 | -1815 | L1P3 | L1 | LINE |
| 11465716 | 11466329 | + | 9.1 | 4683 | 5297 | -875 | L1PA8 | L1 | LINE |
| 11466352 | 11467193 | + | 6.9 | 5298 | 6171 | -1 | L1PA8 | L1 | LINE |
| 11467197 | 11467330 | - | 29.7 | 2 | 156 | -112 | MIR | MIR | SINE |

  

|  |  |  |  |  |  |  |  |  |  |
| --- | --- | --- | --- | --- | --- | --- | --- | --- | --- |
| chr9:11913532-11917275 | | | 3435 bp | | |  |  |  |  |
| Genome Start | Genome End | Strand | Div | Repeat Start | Repeat End | Repeat Left | Name | Family | Class |
| 11913532 | 11913966 | + | 20.1 | 2 | 431 | -222 | MER82 | MER2\_type | DNA |
| 11913968 | 11914565 | - | 11.8 | 4330 | 4930 | -1216 | L1PREC2 | L1 | LINE |
| 11914566 | 11914874 | - | 9.7 | 1 | 310 | -3 | AluSp | Alu | SINE |
| 11914875 | 11915311 | - | 11.8 | 3889 | 4329 | -1817 | L1PREC2 | L1 | LINE |
| 11915297 | 11916638 | + | 10.6 | 4918 | 6161 | 0 | L1PA14 | L1 | LINE |
| 11916726 | 11916821 | + | 29.1 | 132 | 217 | -51 | MIRb | MIR | SINE |
| 11917058 | 11917275 | + | 25.8 | 424 | 653 | 0 | MER82 | MER2\_type | DNA |

  

|  |  |  |  |  |  |  |  |  |  |
| --- | --- | --- | --- | --- | --- | --- | --- | --- | --- |
| chr9:13680623-13693656 | | | 13002 bp | | |  |  |  |  |
| Genome Start | Genome End | Strand | Div | Repeat Start | Repeat End | Repeat Left | Name | Family | Class |
| 13680623 | 13681811 | - | 15.9 | 5195 | 6400 | -6 | L1MC1 | L1 | LINE |
| 13681805 | 13684717 | - | 12.4 | 3247 | 6163 | 0 | L1PA13 | L1 | LINE |
| 13684768 | 13688543 | - | 15.7 | 1 | 3866 | -2900 | L1PA13 | L1 | LINE |
| 13688534 | 13689419 | - | 16.4 | 4307 | 5217 | -1002 | L1MC1 | L1 | LINE |
| 13689420 | 13689941 | - | 4.0 | 5631 | 6154 | -1 | L1PA4 | L1 | LINE |
| 13689942 | 13691246 | + | 3.4 | 4298 | 5607 | -548 | L1PA4 | L1 | LINE |
| 13691246 | 13693656 | - | 16.3 | 1980 | 4322 | -1897 | L1MC1 | L1 | LINE |

  

|  |  |  |  |  |  |  |  |  |  |
| --- | --- | --- | --- | --- | --- | --- | --- | --- | --- |
| chr9:18014258-18023059 | | | 8817 bp | | |  |  |  |  |
| Genome Start | Genome End | Strand | Div | Repeat Start | Repeat End | Repeat Left | Name | Family | Class |
| 18014258 | 18018548 | - | 12.4 | 2477 | 6932 | -3 | L1PA16 | L1 | LINE |
| 18018539 | 18019281 | - | 3.6 | 4129 | 4877 | -1269 | L1PA4 | L1 | LINE |
| 18019270 | 18020565 | + | 3.6 | 4867 | 6155 | 0 | L1PA4 | L1 | LINE |
| 18020573 | 18023059 | - | 20.0 | 1 | 2485 | -4450 | L1PA16 | L1 | LINE |

  

|  |  |  |  |  |  |  |  |  |  |
| --- | --- | --- | --- | --- | --- | --- | --- | --- | --- |
| chr9:18328040-18331498 | | | 3429 bp | | |  |  |  |  |
| Genome Start | Genome End | Strand | Div | Repeat Start | Repeat End | Repeat Left | Name | Family | Class |
| 18328040 | 18328416 | + | 35.8 | 1284 | 1705 | -1714 | L2 | L2 | LINE |
| 18328437 | 18328609 | - | 17.3 | 136 | 309 | -3 | AluJo | Alu | SINE |
| 18328612 | 18329108 | - | 12.7 | 5668 | 6165 | 0 | L1PA15 | L1 | LINE |
| 18329115 | 18329620 | + | 8.4 | 5150 | 5660 | -505 | L1PA15 | L1 | LINE |
| 18329621 | 18329737 | - | 21.1 | 1 | 130 | -172 | AluJo | Alu | SINE |
| 18329740 | 18330542 | + | 34.9 | 1710 | 2561 | -858 | L2 | L2 | LINE |
| 18330543 | 18330906 | + | 9.5 | 1 | 364 | 0 | THE1B | MaLR | LTR |
| 18330907 | 18331498 | + | 36.1 | 2562 | 3186 | -192 | L2 | L2 | LINE |

  

|  |  |  |  |  |  |  |  |  |  |
| --- | --- | --- | --- | --- | --- | --- | --- | --- | --- |
| chr9:19248957-19258101 | | | 9077 bp | | |  |  |  |  |
| Genome Start | Genome End | Strand | Div | Repeat Start | Repeat End | Repeat Left | Name | Family | Class |
| 19248957 | 19249491 | - | 12.2 | 6484 | 7006 | -1 | L1MA3 | L1 | LINE |
| 19249492 | 19249802 | - | 7.7 | 1 | 311 | -2 | AluSp | Alu | SINE |
| 19249803 | 19250369 | - | 12.2 | 5964 | 6483 | -524 | L1MA3 | L1 | LINE |
| 19250410 | 19250694 | - | 14.0 | 1 | 286 | -26 | AluSx | Alu | SINE |
| 19250695 | 19250876 | - | 13.1 | 5791 | 5972 | -876 | L1MA3 | L1 | LINE |
| 19250877 | 19250929 | + | 17.0 | 250 | 302 | 0 | Alu | Alu | SINE |
| 19250930 | 19251502 | - | 13.1 | 5216 | 5790 | -1058 | L1MA3 | L1 | LINE |
| 19251503 | 19251801 | - | 21.0 | 1 | 298 | -14 | AluJo | Alu | SINE |
| 19251802 | 19252074 | - | 13.1 | 4978 | 5213 | -1635 | L1MA3 | L1 | LINE |
| 19252075 | 19252385 | - | 11.3 | 1 | 311 | -1 | AluSx | Alu | SINE |
| 19252386 | 19252446 | - | 13.1 | 4925 | 4977 | -1871 | L1MA3 | L1 | LINE |
| 19252447 | 19252729 | - | 14.8 | 1 | 295 | -17 | AluSx | Alu | SINE |
| 19252732 | 19252866 | - | 8.2 | 1 | 134 | -178 | AluSq/x | Alu | SINE |
| 19252867 | 19253338 | - | 13.1 | 4452 | 4922 | -1924 | L1MA3 | L1 | LINE |
| 19253339 | 19253581 | - | 13.1 | 76 | 316 | 0 | AluSq | Alu | SINE |
| 19253582 | 19253655 | - | 13.1 | 4400 | 4451 | -2397 | L1MA3 | L1 | LINE |
| 19253656 | 19253957 | - | 9.1 | 1 | 297 | -16 | AluSq | Alu | SINE |
| 19253958 | 19254303 | - | 13.1 | 4048 | 4399 | -2449 | L1MA3 | L1 | LINE |
| 19254304 | 19254604 | - | 11.9 | 1 | 294 | -18 | AluSx | Alu | SINE |
| 19254605 | 19255015 | - | 13.1 | 3630 | 4047 | -2801 | L1MA3 | L1 | LINE |
| 19255016 | 19255314 | - | 19.9 | 1 | 298 | -14 | AluJb | Alu | SINE |
| 19255315 | 19255438 | - | 13.1 | 3507 | 3629 | -3219 | L1MA3 | L1 | LINE |
| 19255468 | 19256494 | - | 5.2 | 5116 | 6138 | -16 | L1PA5 | L1 | LINE |
| 19256494 | 19256936 | + | 6.5 | 4660 | 5102 | -1044 | L1PA5 | L1 | LINE |
| 19256935 | 19257536 | - | 14.9 | 2944 | 3525 | -3323 | L1MA3 | L1 | LINE |
| 19257537 | 19257836 | - | 20.8 | 1 | 298 | -14 | AluJo | Alu | SINE |
| 19257837 | 19258101 | - | 21.1 | 2727 | 2943 | -3905 | L1MA3 | L1 | LINE |

  

|  |  |  |  |  |  |  |  |  |  |
| --- | --- | --- | --- | --- | --- | --- | --- | --- | --- |
| chr9:21259551-21266501 | | | 6953 bp | | |  |  |  |  |
| Genome Start | Genome End | Strand | Div | Repeat Start | Repeat End | Repeat Left | Name | Family | Class |
| 21259551 | 21262335 | - | 8.4 | 3440 | 6167 | 0 | L1PA7 | L1 | LINE |
| 21262347 | 21263509 | - | 6.2 | 4978 | 6151 | -3 | L1PA5 | L1 | LINE |
| 21263512 | 21264385 | + | 6.2 | 4090 | 4967 | -1179 | L1PA5 | L1 | LINE |
| 21264371 | 21266501 | - | 8.8 | 1357 | 3454 | -2692 | L1PA7 | L1 | LINE |

  

|  |  |  |  |  |  |  |  |  |  |
| --- | --- | --- | --- | --- | --- | --- | --- | --- | --- |
| chr9:22003360-22007132 | | | 3737 bp | | |  |  |  |  |
| Genome Start | Genome End | Strand | Div | Repeat Start | Repeat End | Repeat Left | Name | Family | Class |
| 22003360 | 22003450 | - | 24.2 | 7512 | 7586 | -375 | L1MC5 | L1 | LINE |
| 22003451 | 22003749 | - | 20.1 | 2 | 295 | -17 | AluJo | Alu | SINE |
| 22003750 | 22004154 | - | 24.2 | 7133 | 7511 | -450 | L1MC5 | L1 | LINE |
| 22004155 | 22004463 | - | 15.7 | 1 | 307 | 0 | AluJo | Alu | SINE |
| 22004464 | 22004600 | - | 24.2 | 6989 | 7132 | -829 | L1MC5 | L1 | LINE |
| 22004613 | 22005287 | - | 2.7 | 5476 | 6150 | -5 | L1PA3 | L1 | LINE |
| 22005291 | 22005619 | - | 24.8 | 4497 | 4832 | -1314 | L1MC | L1 | LINE |
| 22005637 | 22005758 | - | 4.1 | 6034 | 6155 | 0 | L1PA3 | L1 | LINE |
| 22005759 | 22006233 | - | 1.8 | 5015 | 5489 | -666 | L1P2 | L1 | LINE |
| 22006234 | 22006812 | + | 2.9 | 4428 | 5006 | -1140 | L1P2 | L1 | LINE |
| 22006817 | 22007132 | - | 23.0 | 6707 | 6995 | -966 | L1MC5 | L1 | LINE |

  

|  |  |  |  |  |  |  |  |  |  |
| --- | --- | --- | --- | --- | --- | --- | --- | --- | --- |
| chr9:22616651-22623894 | | | 6867 bp | | |  |  |  |  |
| Genome Start | Genome End | Strand | Div | Repeat Start | Repeat End | Repeat Left | Name | Family | Class |
| 22616651 | 22616938 | + | 36.8 | 92 | 393 | -6007 | L1ME2 | L1 | LINE |
| 22617227 | 22617706 | + | 27.8 | 721 | 1231 | -5299 | L1ME2 | L1 | LINE |
| 22617730 | 22618421 | + | 28.2 | 1232 | 2011 | -4389 | L1ME2 | L1 | LINE |
| 22618449 | 22619730 | - | 30.4 | 73 | 1404 | -4996 | L1MEc | L1 | LINE |
| 22619762 | 22621151 | + | 26.6 | 4112 | 5591 | -648 | L1ME2 | L1 | LINE |
| 22621167 | 22621382 | - | 6.0 | 3984 | 4199 | -1947 | L1PA8 | L1 | LINE |
| 22621376 | 22623352 | + | 9.5 | 4186 | 6179 | 0 | L1PA8 | L1 | LINE |
| 22623353 | 22623894 | + | 27.8 | 5622 | 6125 | -112 | L1ME2 | L1 | LINE |

  

|  |  |  |  |  |  |  |  |  |  |
| --- | --- | --- | --- | --- | --- | --- | --- | --- | --- |
| chr9:22748596-22754063 | | | 5464 bp | | |  |  |  |  |
| Genome Start | Genome End | Strand | Div | Repeat Start | Repeat End | Repeat Left | Name | Family | Class |
| 22748596 | 22748950 | + | 21.1 | 4562 | 4916 | -1224 | L1MA9 | L1 | LINE |
| 22748947 | 22750809 | - | 5.7 | 2867 | 4727 | -1419 | L1PA5 | L1 | LINE |
| 22750809 | 22752238 | + | 4.3 | 4724 | 6153 | -1 | L1PA5 | L1 | LINE |
| 22752251 | 22752342 | + | 21.7 | 4903 | 4997 | -1143 | L1MA9 | L1 | LINE |
| 22752339 | 22752478 | - | 2.9 | 5323 | 5462 | -692 | L1P2 | L1 | LINE |
| 22752480 | 22752624 | + | 19.2 | 5011 | 5156 | -984 | L1MA9 | L1 | LINE |
| 22752625 | 22752927 | - | 14.2 | 1 | 303 | -9 | AluSx | Alu | SINE |
| 22752928 | 22754063 | + | 15.9 | 5157 | 6292 | -20 | L1MA9 | L1 | LINE |

  

|  |  |  |  |  |  |  |  |  |  |
| --- | --- | --- | --- | --- | --- | --- | --- | --- | --- |
| chr9:23381903-23391455 | | | 9560 bp | | |  |  |  |  |
| Genome Start | Genome End | Strand | Div | Repeat Start | Repeat End | Repeat Left | Name | Family | Class |
| 23381903 | 23383340 | + | 21.7 | 2977 | 4457 | -3102 | L1M1 | L1 | LINE |
| 23383341 | 23383640 | + | 9.0 | 1 | 300 | -11 | AluY | Alu | SINE |
| 23383641 | 23385206 | + | 19.1 | 4458 | 6050 | -1509 | L1M1 | L1 | LINE |
| 23385204 | 23385783 | - | 6.9 | 1025 | 1604 | -4551 | L1PA4 | L1 | LINE |
| 23385783 | 23390345 | + | 4.1 | 1579 | 6152 | -3 | L1PA4 | L1 | LINE |
| 23390343 | 23391455 | + | 19.2 | 6038 | 7214 | -517 | L1M1 | L1 | LINE |

  

|  |  |  |  |  |  |  |  |  |  |
| --- | --- | --- | --- | --- | --- | --- | --- | --- | --- |
| chr9:23536481-23539090 | | | 2324 bp | | |  |  |  |  |
| Genome Start | Genome End | Strand | Div | Repeat Start | Repeat End | Repeat Left | Name | Family | Class |
| 23536481 | 23536929 | - | 26.1 | 2944 | 3413 | -6 | L2 | L2 | LINE |
| 23536941 | 23537212 | + | 19.4 | 7 | 260 | -52 | AluJo | Alu | SINE |
| 23537451 | 23538028 | - | 5.5 | 4774 | 5351 | -795 | L1PA4 | L1 | LINE |
| 23538028 | 23538830 | + | 3.6 | 5353 | 6155 | 0 | L1PA4 | L1 | LINE |
| 23538869 | 23539090 | - | 32.4 | 2831 | 3051 | -368 | L2 | L2 | LINE |

  

|  |  |  |  |  |  |  |  |  |  |
| --- | --- | --- | --- | --- | --- | --- | --- | --- | --- |
| chr9:24082812-24085469 | | | 2442 bp | | |  |  |  |  |
| Genome Start | Genome End | Strand | Div | Repeat Start | Repeat End | Repeat Left | Name | Family | Class |
| 24082812 | 24082971 | - | 33.1 | 2279 | 2442 | -977 | L2 | L2 | LINE |
| 24082974 | 24083455 | - | 27.5 | 65 | 555 | 0 | MLT1H1 | MaLR | LTR |
| 24083582 | 24084922 | - | 2.3 | 4643 | 5983 | -172 | L1PA2 | L1 | LINE |
| 24084923 | 24085091 | + | 0.6 | 5984 | 6152 | -3 | L1PA2 | L1 | LINE |
| 24085180 | 24085469 | - | 31.4 | 1839 | 2183 | -1236 | L2 | L2 | LINE |

  

|  |  |  |  |  |  |  |  |  |  |
| --- | --- | --- | --- | --- | --- | --- | --- | --- | --- |
| chr9:24326883-24329947 | | | 2884 bp | | |  |  |  |  |
| Genome Start | Genome End | Strand | Div | Repeat Start | Repeat End | Repeat Left | Name | Family | Class |
| 24326883 | 24327252 | - | 14.1 | 5783 | 6150 | -1 | L1PB1 | L1 | LINE |
| 24327444 | 24328175 | - | 1.9 | 5398 | 6155 | 0 | L1PA3 | L1 | LINE |
| 24328170 | 24329389 | + | 3.1 | 4163 | 5383 | -763 | L1PA3 | L1 | LINE |
| 24329386 | 24329947 | - | 7.8 | 5187 | 5780 | -371 | L1PB1 | L1 | LINE |

  

|  |  |  |  |  |  |  |  |  |  |
| --- | --- | --- | --- | --- | --- | --- | --- | --- | --- |
| chr9:25549615-25553339 | | | 3650 bp | | |  |  |  |  |
| Genome Start | Genome End | Strand | Div | Repeat Start | Repeat End | Repeat Left | Name | Family | Class |
| 25549615 | 25549802 | + | 21.7 | 959 | 1151 | -5249 | L1MEc | L1 | LINE |
| 25549777 | 25550046 | - | 19.8 | 3852 | 4114 | -2026 | L1MA10 | L1 | LINE |
| 25550042 | 25551378 | + | 17.9 | 4120 | 5474 | -860 | L1MA10 | L1 | LINE |
| 25551379 | 25551691 | + | 13.8 | 1 | 312 | -1 | AluSq | Alu | SINE |
| 25551692 | 25551872 | + | 19.8 | 5475 | 5651 | -683 | L1MA10 | L1 | LINE |
| 25551873 | 25552482 | - | 12.7 | 1 | 600 | 0 | MER4A1\_ | ERV1 | LTR |
| 25552483 | 25553144 | + | 19.8 | 5652 | 6323 | -11 | L1MA10 | L1 | LINE |
| 25553251 | 25553339 | + | 13.5 | 1853 | 1945 | -4455 | L1MEc | L1 | LINE |

  

|  |  |  |  |  |  |  |  |  |  |
| --- | --- | --- | --- | --- | --- | --- | --- | --- | --- |
| chr9:26333373-26345638 | | | 12257 bp | | |  |  |  |  |
| Genome Start | Genome End | Strand | Div | Repeat Start | Repeat End | Repeat Left | Name | Family | Class |
| 26333373 | 26336324 | + | 21.2 | 2533 | 5595 | -1964 | L1MA9 | L1 | LINE |
| 26336322 | 26336966 | - | 12.9 | 6 | 636 | -5976 | L1PB1 | L1 | LINE |
| 26336967 | 26338862 | + | 12.1 | 622 | 2722 | -3890 | L1PB1 | L1 | LINE |
| 26338863 | 26339566 | + | 5.9 | 1 | 722 | 0 | LTR30 | ERV1 | LTR |
| 26339567 | 26343473 | + | 8.7 | 2723 | 6609 | -8 | L1PB1 | L1 | LINE |
| 26343486 | 26345638 | + | 16.9 | 5588 | 7731 | 0 | L1MA9 | L1 | LINE |

  

|  |  |  |  |  |  |  |  |  |  |
| --- | --- | --- | --- | --- | --- | --- | --- | --- | --- |
| chr9:27186504-27187273 | | | 674 bp | | |  |  |  |  |
| Genome Start | Genome End | Strand | Div | Repeat Start | Repeat End | Repeat Left | Name | Family | Class |
| 27186504 | 27186764 | - | 16.0 | 185 | 426 | 0 | MSTA | MaLR | LTR |
| 27186861 | 27187017 | - | 3.2 | 5999 | 6155 | 0 | L1PA4 | L1 | LINE |
| 27187018 | 27187075 | + | 6.9 | 5929 | 5986 | -169 | L1PA4 | L1 | LINE |
| 27187076 | 27187273 | - | 13.2 | 1 | 198 | -230 | MSTA | MaLR | LTR |

  

|  |  |  |  |  |  |  |  |  |  |
| --- | --- | --- | --- | --- | --- | --- | --- | --- | --- |
| chr9:27709992-27710850 | | | 815 bp | | |  |  |  |  |
| Genome Start | Genome End | Strand | Div | Repeat Start | Repeat End | Repeat Left | Name | Family | Class |
| 27709992 | 27710143 | - | 13.8 | 541 | 694 | -4 | MLT1E1 | MaLR | LTR |
| 27710146 | 27710272 | - | 16.5 | 6096 | 6223 | -77 | L1MA6 | L1 | LINE |
| 27710273 | 27710331 | + | 6.8 | 6230 | 6291 | 0 | L1MA7 | L1 | LINE |
| 27710374 | 27710850 | - | 23.8 | 1 | 474 | -180 | MLT1E1 | MaLR | LTR |

  

|  |  |  |  |  |  |  |  |  |  |
| --- | --- | --- | --- | --- | --- | --- | --- | --- | --- |
| chr9:28997747-29000472 | | | 2717 bp | | |  |  |  |  |
| Genome Start | Genome End | Strand | Div | Repeat Start | Repeat End | Repeat Left | Name | Family | Class |
| 28997747 | 28997916 | - | 31.8 | 2299 | 2475 | -944 | L2 | L2 | LINE |
| 28997926 | 28998792 | - | 5.4 | 5288 | 6153 | -1 | L1PA5 | L1 | LINE |
| 28998793 | 29000000 | + | 5.9 | 3753 | 4963 | -1183 | L1P1 | L1 | LINE |
| 29000001 | 29000315 | + | 7.6 | 4964 | 5277 | -869 | L1P1 | L1 | LINE |
| 29000316 | 29000472 | - | 33.1 | 2150 | 2310 | -1109 | L2 | L2 | LINE |

  

|  |  |  |  |  |  |  |  |  |  |
| --- | --- | --- | --- | --- | --- | --- | --- | --- | --- |
| chr9:29034475-29040551 | | | 6080 bp | | |  |  |  |  |
| Genome Start | Genome End | Strand | Div | Repeat Start | Repeat End | Repeat Left | Name | Family | Class |
| 29034475 | 29034725 | + | 28.0 | 5521 | 5781 | -2027 | L1MC4a | L1 | LINE |
| 29034726 | 29035337 | - | 23.1 | 1 | 549 | -1 | MER44B | MER2\_type | DNA |
| 29035338 | 29035513 | + | 28.0 | 5782 | 5950 | -1858 | L1MC4a | L1 | LINE |
| 29035507 | 29035614 | + | 9.1 | 3877 | 3976 | -2170 | L1MA1 | L1 | LINE |
| 29035614 | 29036279 | - | 12.5 | 4003 | 4680 | -1466 | L1PREC2 | L1 | LINE |
| 29036277 | 29036794 | + | 9.7 | 4668 | 5186 | -960 | L1PREC2 | L1 | LINE |
| 29036795 | 29037068 | + | 12.4 | 1 | 312 | 0 | AluSx | Alu | SINE |
| 29037069 | 29037470 | + | 8.6 | 5187 | 5576 | -585 | L1P4 | L1 | LINE |
| 29037472 | 29037816 | + | 9.4 | 3988 | 4326 | -1820 | L1MA1 | L1 | LINE |
| 29037817 | 29038138 | + | 16.7 | 1 | 312 | 0 | AluSx | Alu | SINE |
| 29038139 | 29040031 | + | 10.0 | 4327 | 6217 | -85 | L1MA1 | L1 | LINE |
| 29040032 | 29040321 | - | 11.0 | 1 | 294 | -18 | AluSx | Alu | SINE |
| 29040322 | 29040413 | + | 10.6 | 6218 | 6302 | 0 | L1MA1 | L1 | LINE |
| 29040421 | 29040551 | + | 24.8 | 5925 | 6049 | -1759 | L1MC4a | L1 | LINE |

  

|  |  |  |  |  |  |  |  |  |  |
| --- | --- | --- | --- | --- | --- | --- | --- | --- | --- |
| chr9:29416056-29420385 | | | 4178 bp | | |  |  |  |  |
| Genome Start | Genome End | Strand | Div | Repeat Start | Repeat End | Repeat Left | Name | Family | Class |
| 29416056 | 29416923 | + | 33.2 | 816 | 1606 | -4615 | L1MEb | L1 | LINE |
| 29417017 | 29417731 | - | 7.6 | 3988 | 4701 | -1445 | L1PA7 | L1 | LINE |
| 29417730 | 29419187 | + | 7.6 | 4700 | 6146 | -8 | L1PA7 | L1 | LINE |
| 29419203 | 29419357 | + | 16.2 | 153 | 308 | -4 | AluSg/x | Alu | SINE |
| 29419404 | 29420385 | + | 29.3 | 1978 | 2881 | -3338 | L1MEb | L1 | LINE |

  

|  |  |  |  |  |  |  |  |  |  |
| --- | --- | --- | --- | --- | --- | --- | --- | --- | --- |
| chr9:29594567-29598550 | | | 3962 bp | | |  |  |  |  |
| Genome Start | Genome End | Strand | Div | Repeat Start | Repeat End | Repeat Left | Name | Family | Class |
| 29594567 | 29594707 | + | 25.2 | 469 | 618 | -6871 | L1M3e | L1 | LINE |
| 29594703 | 29596106 | - | 17.6 | 2851 | 4314 | -1832 | L1MA4 | L1 | LINE |
| 29596108 | 29598081 | + | 17.5 | 4298 | 6299 | -1 | L1MA4 | L1 | LINE |
| 29598108 | 29598550 | + | 31.8 | 617 | 1042 | -6447 | L1M3e | L1 | LINE |

  

|  |  |  |  |  |  |  |  |  |  |
| --- | --- | --- | --- | --- | --- | --- | --- | --- | --- |
| chr9:29659106-29663043 | | | 3808 bp | | |  |  |  |  |
| Genome Start | Genome End | Strand | Div | Repeat Start | Repeat End | Repeat Left | Name | Family | Class |
| 29659106 | 29659761 | - | 23.7 | 4989 | 5672 | -512 | L1M4 | L1 | LINE |
| 29659791 | 29660813 | - | 26.0 | 3897 | 4988 | -1158 | L1M4 | L1 | LINE |
| 29660814 | 29660859 | + | 28.3 | 129 | 174 | -125 | Alu | Alu | SINE |
| 29660861 | 29660949 | + | 17.2 | 40 | 128 | -14 | FLAM\_A | Alu | SINE |
| 29660950 | 29661871 | - | 26.0 | 2913 | 3896 | -2250 | L1M4 | L1 | LINE |
| 29661872 | 29662437 | - | 12.1 | 5600 | 6168 | 0 | L1PA12 | L1 | LINE |
| 29662438 | 29662772 | + | 10.2 | 5260 | 5603 | -565 | L1PA12 | L1 | LINE |
| 29662873 | 29663043 | - | 25.7 | 2699 | 2870 | -3276 | L1M4 | L1 | LINE |

  

|  |  |  |  |  |  |  |  |  |  |
| --- | --- | --- | --- | --- | --- | --- | --- | --- | --- |
| chr9:30470788-30475167 | | | 4332 bp | | |  |  |  |  |
| Genome Start | Genome End | Strand | Div | Repeat Start | Repeat End | Repeat Left | Name | Family | Class |
| 30470788 | 30471118 | - | 19.3 | 5357 | 5674 | -494 | L1M3 | L1 | LINE |
| 30471136 | 30471470 | - | 13.1 | 5823 | 6157 | -6 | L1PA13 | L1 | LINE |
| 30471473 | 30473695 | + | 14.8 | 3050 | 5343 | -803 | L1PREC2 | L1 | LINE |
| 30473694 | 30473756 | - | 12.9 | 5348 | 5409 | -765 | L1P | L1 | LINE |
| 30473764 | 30473854 | + | 17.8 | 5216 | 5307 | -833 | L1M3 | L1 | LINE |
| 30473870 | 30474088 | - | 14.9 | 4916 | 5132 | -1008 | L1M3 | L1 | LINE |
| 30474105 | 30474566 | + | 12.6 | 5347 | 5813 | -347 | L1PREC2 | L1 | LINE |
| 30474560 | 30474612 | - | 20.2 | 5317 | 5368 | -772 | L1M3 | L1 | LINE |
| 30474613 | 30474706 | - | 13.8 | 6066 | 6163 | 0 | L1PA13 | L1 | LINE |
| 30474707 | 30475167 | - | 20.2 | 4830 | 5316 | -824 | L1M3 | L1 | LINE |

  

|  |  |  |  |  |  |  |  |  |  |
| --- | --- | --- | --- | --- | --- | --- | --- | --- | --- |
| chr9:30697435-30698552 | | | 1112 bp | | |  |  |  |  |
| Genome Start | Genome End | Strand | Div | Repeat Start | Repeat End | Repeat Left | Name | Family | Class |
| 30697435 | 30697751 | - | 11.4 | 178 | 511 | 0 | MER9 | ERVK | LTR |
| 30697758 | 30698215 | - | 2.4 | 5697 | 6155 | 0 | L1PA2 | L1 | LINE |
| 30698216 | 30698366 | + | 1.3 | 5528 | 5678 | -477 | L1PA2 | L1 | LINE |
| 30698367 | 30698552 | - | 10.2 | 1 | 189 | -322 | MER9 | ERVK | LTR |

  

|  |  |  |  |  |  |  |  |  |  |
| --- | --- | --- | --- | --- | --- | --- | --- | --- | --- |
| chr9:32111528-32116103 | | | 4501 bp | | |  |  |  |  |
| Genome Start | Genome End | Strand | Div | Repeat Start | Repeat End | Repeat Left | Name | Family | Class |
| 32111528 | 32112867 | - | 24.1 | 3350 | 4784 | -74 | L1ME3A | L1 | LINE |
| 32112902 | 32113303 | - | 5.5 | 4523 | 4924 | -1222 | L1PA5 | L1 | LINE |
| 32113310 | 32114499 | + | 4.3 | 4939 | 6149 | -5 | L1PA5 | L1 | LINE |
| 32114514 | 32114668 | + | 27.6 | 1 | 155 | -21 | FRAM | Alu | SINE |
| 32114670 | 32114899 | + | 24.4 | 4020 | 4263 | -1906 | L1MD | L1 | LINE |
| 32114900 | 32115018 | + | 16.9 | 1 | 134 | -178 | AluJo | Alu | SINE |
| 32115039 | 32116103 | - | 26.4 | 563 | 1623 | -3211 | L1ME3A | L1 | LINE |

  

|  |  |  |  |  |  |  |  |  |  |
| --- | --- | --- | --- | --- | --- | --- | --- | --- | --- |
| chr9:35211367-35216044 | | | 4651 bp | | |  |  |  |  |
| Genome Start | Genome End | Strand | Div | Repeat Start | Repeat End | Repeat Left | Name | Family | Class |
| 35211367 | 35212431 | - | 21.5 | 5435 | 6423 | -785 | L1M | L1 | LINE |
| 35212433 | 35212564 | - | 21.0 | 586 | 713 | -74 | LTR48 | ERV1 | LTR |
| 35212570 | 35214698 | - | 13.8 | 4078 | 6296 | -7 | L1MA4A | L1 | LINE |
| 35214702 | 35215138 | + | 14.4 | 3661 | 4092 | -2051 | L1MA4A | L1 | LINE |
| 35215150 | 35215451 | - | 15.6 | 1 | 308 | -4 | AluSx | Alu | SINE |
| 35215459 | 35216044 | - | 25.5 | 1369 | 2020 | -5189 | L1M | L1 | LINE |

  

|  |  |  |  |  |  |  |  |  |  |
| --- | --- | --- | --- | --- | --- | --- | --- | --- | --- |
| chr9:38784495-38795018 | | | 10478 bp | | |  |  |  |  |
| Genome Start | Genome End | Strand | Div | Repeat Start | Repeat End | Repeat Left | Name | Family | Class |
| 38784495 | 38786650 | - | 18.1 | 4922 | 7128 | -6 | L1MB5 | L1 | LINE |
| 38786651 | 38786959 | - | 12.5 | 1 | 295 | -15 | AluSg | Alu | SINE |
| 38786960 | 38787366 | - | 18.8 | 4520 | 4921 | -2185 | L1MB5 | L1 | LINE |
| 38787373 | 38787945 | - | 9.0 | 5591 | 6149 | -2 | L1PB2 | L1 | LINE |
| 38787946 | 38788258 | + | 8.0 | 1 | 311 | 0 | AluY | Alu | SINE |
| 38788259 | 38789524 | - | 11.2 | 4380 | 5590 | -561 | L1PB2 | L1 | LINE |
| 38789525 | 38789832 | - | 13.7 | 1 | 307 | -5 | AluSx | Alu | SINE |
| 38789833 | 38790285 | - | 12.1 | 3929 | 4379 | -1767 | L1PB2 | L1 | LINE |
| 38790287 | 38791795 | + | 12.8 | 2398 | 3910 | -2236 | L1PB2 | L1 | LINE |
| 38791792 | 38792017 | - | 16.7 | 4312 | 4538 | -2568 | L1MB5 | L1 | LINE |
| 38792019 | 38792315 | - | 9.1 | 1 | 297 | -15 | AluSx | Alu | SINE |
| 38792318 | 38792625 | - | 18.5 | 3625 | 3953 | -3153 | L1MB5 | L1 | LINE |
| 38792626 | 38792933 | - | 6.0 | 1 | 302 | -9 | AluY | Alu | SINE |
| 38792934 | 38793045 | - | 18.5 | 3515 | 3624 | -3482 | L1MB5 | L1 | LINE |
| 38793048 | 38793381 | - | 19.2 | 10 | 432 | 0 | MSTB1 | MaLR | LTR |
| 38793412 | 38793622 | - | 21.7 | 3306 | 3518 | -3582 | L1MB5 | L1 | LINE |
| 38793597 | 38794415 | - | 27.2 | 2565 | 3427 | -4138 | L1M1 | L1 | LINE |
| 38794422 | 38794837 | - | 28.1 | 649 | 1100 | -5930 | L1MB5 | L1 | LINE |
| 38794866 | 38795018 | - | 15.2 | 7 | 184 | -6846 | L1MB5 | L1 | LINE |

  

|  |  |  |  |  |  |  |  |  |  |
| --- | --- | --- | --- | --- | --- | --- | --- | --- | --- |
| chr9:39362196-39363005 | | | 810 bp | | |  |  |  |  |
| Genome Start | Genome End | Strand | Div | Repeat Start | Repeat End | Repeat Left | Name | Family | Class |
| 39362196 | 39362266 | - | 9.9 | 320 | 390 | 0 | MLT1B | MaLR | LTR |
| 39362267 | 39362628 | - | 1.2 | 5815 | 6176 | 0 | L1PA3 | L1 | LINE |
| 39362629 | 39362673 | + | 0.0 | 5770 | 5814 | -358 | L1P3 | L1 | LINE |
| 39362674 | 39363005 | - | 18.0 | 1 | 331 | -59 | MLT1B | MaLR | LTR |

  

|  |  |  |  |  |  |  |  |  |  |
| --- | --- | --- | --- | --- | --- | --- | --- | --- | --- |
| chr9:39891480-39892289 | | | 810 bp | | |  |  |  |  |
| Genome Start | Genome End | Strand | Div | Repeat Start | Repeat End | Repeat Left | Name | Family | Class |
| 39891480 | 39891543 | - | 9.4 | 327 | 390 | 0 | MLT1B | MaLR | LTR |
| 39891544 | 39891912 | - | 0.9 | 5815 | 6183 | 0 | L1PA3 | L1 | LINE |
| 39891913 | 39891957 | + | 0.0 | 5770 | 5814 | -358 | L1P3 | L1 | LINE |
| 39891958 | 39892289 | - | 18.4 | 1 | 331 | -59 | MLT1B | MaLR | LTR |

  

|  |  |  |  |  |  |  |  |  |  |
| --- | --- | --- | --- | --- | --- | --- | --- | --- | --- |
| chr9:41300041-41300851 | | | 793 bp | | |  |  |  |  |
| Genome Start | Genome End | Strand | Div | Repeat Start | Repeat End | Repeat Left | Name | Family | Class |
| 41300041 | 41300373 | + | 18.0 | 1 | 331 | -59 | MLT1B | MaLR | LTR |
| 41300374 | 41300418 | - | 0.0 | 5770 | 5814 | -358 | L1P3 | L1 | LINE |
| 41300419 | 41300759 | + | 1.5 | 5815 | 6155 | 0 | L1PA3 | L1 | LINE |
| 41300778 | 41300851 | + | 10.8 | 317 | 390 | 0 | MLT1B | MaLR | LTR |

  

|  |  |  |  |  |  |  |  |  |  |
| --- | --- | --- | --- | --- | --- | --- | --- | --- | --- |
| chr9:41507256-41508066 | | | 793 bp | | |  |  |  |  |
| Genome Start | Genome End | Strand | Div | Repeat Start | Repeat End | Repeat Left | Name | Family | Class |
| 41507256 | 41507329 | - | 10.8 | 317 | 390 | 0 | MLT1B | MaLR | LTR |
| 41507348 | 41507688 | - | 1.5 | 5815 | 6155 | 0 | L1PA3 | L1 | LINE |
| 41507689 | 41507733 | + | 0.0 | 5770 | 5814 | -358 | L1P3 | L1 | LINE |
| 41507734 | 41508066 | - | 18.0 | 1 | 331 | -59 | MLT1B | MaLR | LTR |

  

|  |  |  |  |  |  |  |  |  |  |
| --- | --- | --- | --- | --- | --- | --- | --- | --- | --- |
| chr9:42599328-42603951 | | | 4632 bp | | |  |  |  |  |
| Genome Start | Genome End | Strand | Div | Repeat Start | Repeat End | Repeat Left | Name | Family | Class |
| 42599328 | 42601327 | + | 12.7 | 4 | 2027 | -4326 | L1PA10 | L1 | LINE |
| 42601326 | 42602368 | - | 1.6 | 5107 | 6149 | -6 | L1PA2 | L1 | LINE |
| 42602365 | 42603837 | + | 2.2 | 3624 | 5096 | -1050 | L1PA2 | L1 | LINE |
| 42603836 | 42603951 | + | 7.8 | 2017 | 2132 | -4221 | L1PA10 | L1 | LINE |

  

|  |  |  |  |  |  |  |  |  |  |
| --- | --- | --- | --- | --- | --- | --- | --- | --- | --- |
| chr9:42873772-42887518 | | | 13683 bp | | |  |  |  |  |
| Genome Start | Genome End | Strand | Div | Repeat Start | Repeat End | Repeat Left | Name | Family | Class |
| 42873772 | 42873859 | + | 20.5 | 3 | 81 | -5964 | L1MC3 | L1 | LINE |
| 42873892 | 42874110 | - | 32.9 | 4087 | 4331 | -158 | L3 | CR1 | LINE |
| 42874116 | 42878069 | - | 9.2 | 2017 | 6086 | -267 | L1PA10 | L1 | LINE |
| 42878068 | 42879540 | - | 2.2 | 3624 | 5096 | -1050 | L1PA2 | L1 | LINE |
| 42879537 | 42880581 | + | 1.8 | 5107 | 6151 | -4 | L1PA2 | L1 | LINE |
| 42880578 | 42882577 | - | 12.8 | 4 | 2027 | -4326 | L1PA10 | L1 | LINE |
| 42882574 | 42882614 | + | 25.2 | 525 | 561 | -5484 | L1MC3 | L1 | LINE |
| 42882615 | 42883201 | + | 25.4 | 1 | 603 | 0 | LTR26 | ERV1 | LTR |
| 42883202 | 42884247 | + | 25.4 | 562 | 1671 | -5080 | L1MC3 | L1 | LINE |
| 42884269 | 42884789 | + | 26.5 | 1672 | 2387 | -4358 | L1MC3 | L1 | LINE |
| 42884790 | 42886028 | - | 12.9 | 2 | 1577 | -1 | LTR12C | ERV1 | LTR |
| 42886029 | 42886144 | + | 30.9 | 2388 | 2500 | -4245 | L1MC3 | L1 | LINE |
| 42886165 | 42887518 | + | 23.7 | 5485 | 6868 | -1335 | L1MC3 | L1 | LINE |

  

|  |  |  |  |  |  |  |  |  |  |
| --- | --- | --- | --- | --- | --- | --- | --- | --- | --- |
| chr9:43553322-43554127 | | | 792 bp | | |  |  |  |  |
| Genome Start | Genome End | Strand | Div | Repeat Start | Repeat End | Repeat Left | Name | Family | Class |
| 43553322 | 43553653 | + | 17.7 | 1 | 331 | -59 | MLT1B | MaLR | LTR |
| 43553654 | 43553698 | - | 0.0 | 5770 | 5814 | -358 | L1P3 | L1 | LINE |
| 43553699 | 43554039 | + | 1.2 | 5815 | 6155 | 0 | L1PA3 | L1 | LINE |
| 43554054 | 43554127 | + | 10.8 | 317 | 390 | 0 | MLT1B | MaLR | LTR |

  

|  |  |  |  |  |  |  |  |  |  |
| --- | --- | --- | --- | --- | --- | --- | --- | --- | --- |
| chr9:45095068-45105588 | | | 10471 bp | | |  |  |  |  |
| Genome Start | Genome End | Strand | Div | Repeat Start | Repeat End | Repeat Left | Name | Family | Class |
| 45095068 | 45097224 | - | 18.2 | 4922 | 7128 | -6 | L1MB5 | L1 | LINE |
| 45097225 | 45097530 | - | 12.5 | 1 | 295 | -15 | AluSg | Alu | SINE |
| 45097531 | 45097937 | - | 18.8 | 4520 | 4921 | -2185 | L1MB5 | L1 | LINE |
| 45097944 | 45098516 | - | 9.0 | 5591 | 6149 | -2 | L1PB2 | L1 | LINE |
| 45098517 | 45098829 | + | 8.0 | 1 | 311 | 0 | AluY | Alu | SINE |
| 45098830 | 45100092 | - | 11.2 | 4380 | 5590 | -561 | L1PB2 | L1 | LINE |
| 45100093 | 45100404 | - | 13.5 | 1 | 311 | -1 | AluSx | Alu | SINE |
| 45100405 | 45100857 | - | 12.1 | 3929 | 4379 | -1767 | L1PB2 | L1 | LINE |
| 45100859 | 45102362 | + | 13.1 | 2398 | 3910 | -2236 | L1PB2 | L1 | LINE |
| 45102359 | 45102584 | - | 16.3 | 4312 | 4538 | -2568 | L1MB5 | L1 | LINE |
| 45102586 | 45102884 | - | 8.0 | 1 | 299 | -13 | AluSx | Alu | SINE |
| 45102887 | 45103194 | - | 18.8 | 3626 | 3953 | -3153 | L1MB5 | L1 | LINE |
| 45103195 | 45103502 | - | 5.3 | 1 | 302 | -9 | AluY | Alu | SINE |
| 45103503 | 45103615 | - | 18.8 | 3515 | 3625 | -3481 | L1MB5 | L1 | LINE |
| 45103618 | 45103951 | - | 19.5 | 10 | 432 | 0 | MSTB1 | MaLR | LTR |
| 45103982 | 45104192 | - | 22.2 | 3306 | 3518 | -3582 | L1MB5 | L1 | LINE |
| 45104167 | 45104985 | - | 26.2 | 2565 | 3427 | -4138 | L1M1 | L1 | LINE |
| 45104992 | 45105403 | - | 29.7 | 714 | 1100 | -5930 | L1MB5 | L1 | LINE |
| 45105436 | 45105588 | - | 15.2 | 7 | 184 | -6846 | L1MB5 | L1 | LINE |

  

|  |  |  |  |  |  |  |  |  |  |
| --- | --- | --- | --- | --- | --- | --- | --- | --- | --- |
| chr9:45632528-45633524 | | | 997 bp | | |  |  |  |  |
| Genome Start | Genome End | Strand | Div | Repeat Start | Repeat End | Repeat Left | Name | Family | Class |
| 45632528 | 45632569 | - | 19.5 | 137 | 177 | -1 | MER5B | MER1\_type | DNA |
| 45632570 | 45632879 | - | 12.9 | 1 | 310 | -3 | AluSq | Alu | SINE |
| 45632880 | 45632959 | - | 19.5 | 60 | 136 | -42 | MER5B | MER1\_type | DNA |
| 45632960 | 45633266 | - | 1.6 | 5784 | 6090 | -65 | L1PA2 | L1 | LINE |
| 45633267 | 45633469 | + | 1.5 | 5593 | 5795 | -360 | L1PA2 | L1 | LINE |
| 45633470 | 45633524 | - | 5.5 | 8 | 64 | -114 | MER5B | MER1\_type | DNA |

  

|  |  |  |  |  |  |  |  |  |  |
| --- | --- | --- | --- | --- | --- | --- | --- | --- | --- |
| chr9:65232048-65232858 | | | 793 bp | | |  |  |  |  |
| Genome Start | Genome End | Strand | Div | Repeat Start | Repeat End | Repeat Left | Name | Family | Class |
| 65232048 | 65232380 | + | 18.0 | 1 | 331 | -59 | MLT1B | MaLR | LTR |
| 65232381 | 65232425 | - | 0.0 | 5770 | 5814 | -358 | L1P3 | L1 | LINE |
| 65232426 | 65232766 | + | 1.5 | 5815 | 6155 | 0 | L1PA3 | L1 | LINE |
| 65232785 | 65232858 | + | 10.8 | 317 | 390 | 0 | MLT1B | MaLR | LTR |

  

|  |  |  |  |  |  |  |  |  |  |
| --- | --- | --- | --- | --- | --- | --- | --- | --- | --- |
| chr9:66796638-66807655 | | | 10995 bp | | |  |  |  |  |
| Genome Start | Genome End | Strand | Div | Repeat Start | Repeat End | Repeat Left | Name | Family | Class |
| 66796638 | 66796725 | + | 20.5 | 3 | 81 | -5964 | L1MC3 | L1 | LINE |
| 66796758 | 66796976 | - | 33.3 | 4087 | 4331 | -158 | L3 | CR1 | LINE |
| 66796982 | 66800936 | - | 9.5 | 2017 | 6086 | -267 | L1PA10 | L1 | LINE |
| 66800935 | 66802407 | - | 2.1 | 3624 | 5096 | -1050 | L1PA2 | L1 | LINE |
| 66802404 | 66803447 | + | 1.7 | 5107 | 6151 | -4 | L1PA2 | L1 | LINE |
| 66803444 | 66805442 | - | 12.9 | 4 | 2027 | -4326 | L1PA10 | L1 | LINE |
| 66805439 | 66805479 | + | 25.4 | 525 | 561 | -5484 | L1MC3 | L1 | LINE |
| 66805480 | 66806066 | + | 26.3 | 1 | 603 | 0 | LTR26 | ERV1 | LTR |
| 66806067 | 66807655 | + | 26.5 | 562 | 2387 | -4358 | L1MC3 | L1 | LINE |

  

|  |  |  |  |  |  |  |  |  |  |
| --- | --- | --- | --- | --- | --- | --- | --- | --- | --- |
| chr9:66941090-66953449 | | | 12311 bp | | |  |  |  |  |
| Genome Start | Genome End | Strand | Div | Repeat Start | Repeat End | Repeat Left | Name | Family | Class |
| 66941090 | 66941191 | - | 31.5 | 2388 | 2489 | -4256 | L1MCb | L1 | LINE |
| 66941192 | 66942430 | + | 13.0 | 2 | 1577 | -1 | LTR12C | ERV1 | LTR |
| 66942431 | 66942949 | - | 27.0 | 1672 | 2387 | -4358 | L1MCb | L1 | LINE |
| 66942974 | 66944019 | - | 27.5 | 562 | 1782 | -4963 | L1MCb | L1 | LINE |
| 66944020 | 66944606 | - | 25.0 | 1 | 603 | 0 | LTR26 | ERV1 | LTR |
| 66944607 | 66944647 | - | 25.2 | 525 | 561 | -5484 | L1MCb | L1 | LINE |
| 66944644 | 66946643 | + | 12.8 | 4 | 2027 | -4326 | L1PA10 | L1 | LINE |
| 66946642 | 66947684 | - | 1.7 | 5107 | 6149 | -6 | L1PA2 | L1 | LINE |
| 66947681 | 66949153 | + | 2.1 | 3624 | 5096 | -1050 | L1PA2 | L1 | LINE |
| 66949152 | 66953105 | + | 9.2 | 2017 | 6086 | -267 | L1PA10 | L1 | LINE |
| 66953111 | 66953329 | + | 32.4 | 4087 | 4331 | -158 | L3 | CR1 | LINE |
| 66953362 | 66953449 | - | 20.5 | 3 | 81 | -5964 | L1MCb | L1 | LINE |

  

|  |  |  |  |  |  |  |  |  |  |
| --- | --- | --- | --- | --- | --- | --- | --- | --- | --- |
| chr9:68903076-68915369 | | | 12269 bp | | |  |  |  |  |
| Genome Start | Genome End | Strand | Div | Repeat Start | Repeat End | Repeat Left | Name | Family | Class |
| 68903076 | 68903177 | - | 31.0 | 2388 | 2489 | -4256 | L1MCb | L1 | LINE |
| 68903178 | 68904419 | + | 13.3 | 2 | 1577 | -1 | LTR12C | ERV1 | LTR |
| 68904420 | 68906008 | - | 26.5 | 562 | 2387 | -4358 | L1MCb | L1 | LINE |
| 68906009 | 68906595 | - | 25.6 | 1 | 603 | 0 | LTR26 | ERV1 | LTR |
| 68906596 | 68906636 | - | 25.2 | 525 | 561 | -5484 | L1MCb | L1 | LINE |
| 68906633 | 68908631 | + | 12.8 | 4 | 2027 | -4326 | L1PA10 | L1 | LINE |
| 68908630 | 68909672 | - | 1.7 | 5107 | 6150 | -5 | L1PA2 | L1 | LINE |
| 68909669 | 68911071 | + | 2.1 | 3624 | 5096 | -1050 | L1PA2 | L1 | LINE |
| 68911070 | 68915025 | + | 9.5 | 2017 | 6086 | -267 | L1PA10 | L1 | LINE |
| 68915031 | 68915249 | + | 32.4 | 4087 | 4331 | -158 | L3 | CR1 | LINE |
| 68915282 | 68915369 | - | 20.5 | 3 | 81 | -5964 | L1MCb | L1 | LINE |

  

|  |  |  |  |  |  |  |  |  |  |
| --- | --- | --- | --- | --- | --- | --- | --- | --- | --- |
| chr9:70391917-70399352 | | | 7455 bp | | |  |  |  |  |
| Genome Start | Genome End | Strand | Div | Repeat Start | Repeat End | Repeat Left | Name | Family | Class |
| 70391917 | 70397072 | - | 8.0 | 1320 | 6468 | -1 | L1PA7 | L1 | LINE |
| 70397061 | 70397826 | - | 1.2 | 5389 | 6155 | 0 | L1PA2 | L1 | LINE |
| 70397826 | 70398017 | + | 2.6 | 5195 | 5387 | -759 | L1PA2 | L1 | LINE |
| 70398012 | 70399352 | - | 7.5 | 2 | 1341 | -5120 | L1PA7 | L1 | LINE |

  

|  |  |  |  |  |  |  |  |  |  |
| --- | --- | --- | --- | --- | --- | --- | --- | --- | --- |
| chr9:74995221-74995881 | | | 614 bp | | |  |  |  |  |
| Genome Start | Genome End | Strand | Div | Repeat Start | Repeat End | Repeat Left | Name | Family | Class |
| 74995221 | 74995345 | + | 28.2 | 4615 | 4742 | -1404 | L1M5 | L1 | LINE |
| 74995352 | 74995460 | - | 5.5 | 5866 | 5980 | -194 | L1PA5 | L1 | LINE |
| 74995461 | 74995650 | + | 6.8 | 5964 | 6153 | -1 | L1PA5 | L1 | LINE |
| 74995692 | 74995881 | + | 34.7 | 4823 | 5026 | -1120 | L1M5 | L1 | LINE |

  

|  |  |  |  |  |  |  |  |  |  |
| --- | --- | --- | --- | --- | --- | --- | --- | --- | --- |
| chr9:76025289-76034856 | | | 9556 bp | | |  |  |  |  |
| Genome Start | Genome End | Strand | Div | Repeat Start | Repeat End | Repeat Left | Name | Family | Class |
| 76025289 | 76026722 | + | 12.5 | 670 | 2119 | -4744 | L1PB1 | L1 | LINE |
| 76026707 | 76026747 | - | 4.9 | 2825 | 2865 | -3281 | L1PA6 | L1 | LINE |
| 76026748 | 76029398 | + | 8.8 | 2866 | 5513 | -641 | L1PA6 | L1 | LINE |
| 76029396 | 76029561 | - | 10.8 | 4516 | 4681 | -1465 | L1P1 | L1 | LINE |
| 76029562 | 76030192 | + | 6.2 | 5518 | 6150 | -4 | L1PA6 | L1 | LINE |
| 76030199 | 76031269 | + | 9.2 | 2091 | 3233 | -3629 | L1PB1 | L1 | LINE |
| 76031270 | 76031555 | + | 12.9 | 1 | 299 | -11 | AluSg | Alu | SINE |
| 76031556 | 76033298 | + | 10.8 | 3234 | 4963 | -1899 | L1PB1 | L1 | LINE |
| 76033299 | 76033601 | + | 12.9 | 1 | 303 | -10 | AluSq | Alu | SINE |
| 76033602 | 76034472 | + | 10.8 | 4964 | 5831 | -1031 | L1PB1 | L1 | LINE |
| 76034498 | 76034856 | + | 9.2 | 6501 | 6864 | -3 | L1PB1 | L1 | LINE |

  

|  |  |  |  |  |  |  |  |  |  |
| --- | --- | --- | --- | --- | --- | --- | --- | --- | --- |
| chr9:76748625-76750873 | | | 2171 bp | | |  |  |  |  |
| Genome Start | Genome End | Strand | Div | Repeat Start | Repeat End | Repeat Left | Name | Family | Class |
| 76748625 | 76748790 | + | 18.1 | 6853 | 7039 | -769 | L1MC4a | L1 | LINE |
| 76748791 | 76749312 | + | 10.9 | 1 | 529 | 0 | MER1A | MER1\_type | DNA |
| 76749313 | 76749359 | + | 18.1 | 7040 | 7084 | -724 | L1MC4a | L1 | LINE |
| 76749359 | 76749533 | - | 10.3 | 5853 | 6031 | -132 | L1PA12 | L1 | LINE |
| 76749534 | 76749662 | + | 6.3 | 6038 | 6168 | 0 | L1PA12 | L1 | LINE |
| 76749668 | 76749699 | + | 18.7 | 3567 | 3595 | -345 | Arthur1 | Tip100 | DNA |
| 76749700 | 76750002 | - | 13.9 | 1 | 303 | -9 | AluSx | Alu | SINE |
| 76750003 | 76750167 | + | 18.7 | 3596 | 3755 | -185 | Arthur1 | Tip100 | DNA |
| 76750172 | 76750705 | + | 23.8 | 7072 | 7649 | -159 | L1MC4a | L1 | LINE |
| 76750776 | 76750873 | + | 16.6 | 7654 | 7807 | -66 | L1MC4a | L1 | LINE |

  

|  |  |  |  |  |  |  |  |  |  |
| --- | --- | --- | --- | --- | --- | --- | --- | --- | --- |
| chr9:80538766-80544726 | | | 5775 bp | | |  |  |  |  |
| Genome Start | Genome End | Strand | Div | Repeat Start | Repeat End | Repeat Left | Name | Family | Class |
| 80538766 | 80538885 | - | 22.4 | 6026 | 6144 | -3 | L1ME2 | L1 | LINE |
| 80538886 | 80539199 | + | 6.8 | 1 | 327 | 0 | AluY | Alu | SINE |
| 80539200 | 80539923 | - | 22.7 | 5241 | 6025 | -122 | L1ME2 | L1 | LINE |
| 80540080 | 80540543 | - | 9.5 | 5689 | 6143 | -20 | L1PA13 | L1 | LINE |
| 80540544 | 80540846 | - | 1.0 | 1 | 304 | -7 | AluYg6 | Alu | SINE |
| 80540847 | 80541154 | - | 9.5 | 5387 | 5688 | -475 | L1PA13 | L1 | LINE |
| 80541180 | 80541975 | + | 11.1 | 4558 | 5375 | -771 | L1PREC2 | L1 | LINE |
| 80541983 | 80542192 | - | 26.7 | 4954 | 5164 | -965 | L1ME2 | L1 | LINE |
| 80542181 | 80542565 | - | 2.1 | 5058 | 5442 | -713 | L1PA2 | L1 | LINE |
| 80542566 | 80543277 | + | 1.5 | 5442 | 6153 | -2 | L1PA2 | L1 | LINE |
| 80543278 | 80543482 | - | 24.9 | 4764 | 5007 | -1122 | L1ME2 | L1 | LINE |
| 80543493 | 80544726 | - | 27.2 | 894 | 2306 | -4004 | L1ME2 | L1 | LINE |

  

|  |  |  |  |  |  |  |  |  |  |
| --- | --- | --- | --- | --- | --- | --- | --- | --- | --- |
| chr9:80878143-80882497 | | | 4003 bp | | |  |  |  |  |
| Genome Start | Genome End | Strand | Div | Repeat Start | Repeat End | Repeat Left | Name | Family | Class |
| 80878143 | 80878547 | + | 33.8 | 1960 | 2368 | -1051 | L2 | L2 | LINE |
| 80878588 | 80878753 | + | 15.7 | 6166 | 6333 | 0 | L1MC1 | L1 | LINE |
| 80878770 | 80880144 | - | 2.8 | 3379 | 4761 | -1385 | L1PA3 | L1 | LINE |
| 80880145 | 80881503 | + | 1.6 | 4778 | 6137 | -18 | L1PA3 | L1 | LINE |
| 80881535 | 80881885 | + | 13.2 | 5816 | 6179 | -4 | L1MB3 | L1 | LINE |
| 80882151 | 80882497 | + | 34.9 | 3027 | 3419 | 0 | L2 | L2 | LINE |

  

|  |  |  |  |  |  |  |  |  |  |
| --- | --- | --- | --- | --- | --- | --- | --- | --- | --- |
| chr9:81222757-81232781 | | | 9824 bp | | |  |  |  |  |
| Genome Start | Genome End | Strand | Div | Repeat Start | Repeat End | Repeat Left | Name | Family | Class |
| 81222757 | 81222801 | - | 26.7 | 3593 | 3637 | -852 | L3 | CR1 | LINE |
| 81222808 | 81223259 | + | 17.0 | 4 | 556 | 0 | MLT2B4 | ERVL | LTR |
| 81223273 | 81226519 | - | 15.8 | 3542 | 6940 | -2 | L1MA5 | L1 | LINE |
| 81226518 | 81227838 | - | 7.0 | 4827 | 6152 | -2 | L1PA7 | L1 | LINE |
| 81227839 | 81228627 | + | 7.8 | 4021 | 4817 | -1329 | L1PA7 | L1 | LINE |
| 81228628 | 81228820 | + | 4.7 | 5953 | 6146 | -8 | L1PA5 | L1 | LINE |
| 81228823 | 81229355 | - | 16.5 | 3032 | 3559 | -3226 | L1MA5 | L1 | LINE |
| 81229356 | 81229663 | - | 8.1 | 1 | 308 | -3 | AluY | Alu | SINE |
| 81229664 | 81232337 | - | 23.0 | 8 | 3031 | -3754 | L1MA5 | L1 | LINE |
| 81232447 | 81232524 | - | 26.0 | 2323 | 2410 | -1009 | L2 | L2 | LINE |
| 81232598 | 81232781 | - | 33.9 | 2840 | 3009 | -1480 | L3 | CR1 | LINE |

  

|  |  |  |  |  |  |  |  |  |  |
| --- | --- | --- | --- | --- | --- | --- | --- | --- | --- |
| chr9:82349653-82352606 | | | 2661 bp | | |  |  |  |  |
| Genome Start | Genome End | Strand | Div | Repeat Start | Repeat End | Repeat Left | Name | Family | Class |
| 82349653 | 82349720 | - | 32.4 | 2794 | 2864 | -555 | L2 | L2 | LINE |
| 82349721 | 82350673 | - | 2.5 | 4341 | 5293 | -853 | L1PA3 | L1 | LINE |
| 82350674 | 82351528 | + | 2.9 | 5299 | 6152 | -3 | L1PA3 | L1 | LINE |
| 82351679 | 82351775 | - | 13.4 | 6202 | 6299 | -1 | L1MA5 | L1 | LINE |
| 82351847 | 82352156 | + | 18.7 | 1 | 310 | -2 | AluJb | Alu | SINE |
| 82352229 | 82352606 | - | 28.5 | 2056 | 2478 | -941 | L2 | L2 | LINE |

  

|  |  |  |  |  |  |  |  |  |  |
| --- | --- | --- | --- | --- | --- | --- | --- | --- | --- |
| chr9:82470081-82475507 | | | 5410 bp | | |  |  |  |  |
| Genome Start | Genome End | Strand | Div | Repeat Start | Repeat End | Repeat Left | Name | Family | Class |
| 82470081 | 82471396 | - | 16.9 | 5600 | 6981 | -24 | L1MA4A | L1 | LINE |
| 82471397 | 82471781 | - | 13.7 | 1 | 375 | 0 | THE1C | MaLR | LTR |
| 82471782 | 82472520 | - | 19.1 | 4856 | 5599 | -1246 | L1MA4A | L1 | LINE |
| 82472522 | 82472725 | - | 7.8 | 5948 | 6151 | -3 | L1PA7 | L1 | LINE |
| 82472726 | 82472924 | + | 3.0 | 5758 | 5956 | -198 | L1PA7 | L1 | LINE |
| 82472924 | 82473046 | - | 13.0 | 4745 | 4867 | -1978 | L1MA4A | L1 | LINE |
| 82473043 | 82473433 | - | 16.3 | 3471 | 3876 | -2969 | L1MA4A | L1 | LINE |
| 82473434 | 82473742 | - | 8.8 | 6 | 311 | 0 | AluY | Alu | SINE |
| 82473743 | 82474260 | - | 16.3 | 2956 | 3470 | -3375 | L1MA4A | L1 | LINE |
| 82474282 | 82475507 | - | 26.8 | 3 | 1046 | -5803 | L1MA4A | L1 | LINE |

  

|  |  |  |  |  |  |  |  |  |  |
| --- | --- | --- | --- | --- | --- | --- | --- | --- | --- |
| chr9:82583248-82587019 | | | 3739 bp | | |  |  |  |  |
| Genome Start | Genome End | Strand | Div | Repeat Start | Repeat End | Repeat Left | Name | Family | Class |
| 82583248 | 82583932 | - | 19.4 | 5539 | 6213 | -420 | L1MD | L1 | LINE |
| 82583933 | 82584287 | + | 13.7 | 1 | 364 | 0 | THE1B | MaLR | LTR |
| 82584288 | 82586269 | - | 19.8 | 3450 | 5538 | -999 | L1MD | L1 | LINE |
| 82586311 | 82586900 | - | 3.0 | 5548 | 6137 | -18 | L1PA4 | L1 | LINE |
| 82586901 | 82586937 | + | 2.7 | 5492 | 5529 | -625 | L1P3 | L1 | LINE |
| 82586930 | 82587019 | - | 25.9 | 3366 | 3458 | -3079 | L1MD | L1 | LINE |

  

|  |  |  |  |  |  |  |  |  |  |
| --- | --- | --- | --- | --- | --- | --- | --- | --- | --- |
| chr9:83155110-83161640 | | | 6503 bp | | |  |  |  |  |
| Genome Start | Genome End | Strand | Div | Repeat Start | Repeat End | Repeat Left | Name | Family | Class |
| 83155110 | 83155488 | + | 20.4 | 15 | 389 | -7176 | L1M1 | L1 | LINE |
| 83155485 | 83156183 | + | 10.5 | 2351 | 3043 | -4522 | L1M1 | L1 | LINE |
| 83156219 | 83157781 | + | 12.1 | 3029 | 4467 | -3098 | L1M1 | L1 | LINE |
| 83157775 | 83159261 | - | 9.0 | 2973 | 4444 | -1702 | L1PB1 | L1 | LINE |
| 83159261 | 83160980 | + | 9.0 | 4452 | 6165 | 0 | L1PB1 | L1 | LINE |
| 83160974 | 83161564 | + | 11.4 | 4456 | 5052 | -2513 | L1M1 | L1 | LINE |
| 83161577 | 83161640 | + | 9.4 | 6854 | 6917 | -806 | L1M1 | L1 | LINE |

  

|  |  |  |  |  |  |  |  |  |  |
| --- | --- | --- | --- | --- | --- | --- | --- | --- | --- |
| chr9:84332128-84336890 | | | 4352 bp | | |  |  |  |  |
| Genome Start | Genome End | Strand | Div | Repeat Start | Repeat End | Repeat Left | Name | Family | Class |
| 84332128 | 84332753 | - | 23.4 | 2552 | 3239 | -2980 | L1MEc | L1 | LINE |
| 84332754 | 84333163 | - | 6.8 | 5757 | 6168 | -4 | L1PA8 | L1 | LINE |
| 84333165 | 84333756 | + | 6.5 | 5153 | 5747 | -425 | L1PA8 | L1 | LINE |
| 84333760 | 84334493 | - | 22.7 | 1890 | 2528 | -3691 | L1MEc | L1 | LINE |
| 84334494 | 84334782 | + | 12.2 | 1 | 288 | -24 | AluSx | Alu | SINE |
| 84334786 | 84335089 | + | 12.2 | 1 | 306 | -7 | AluSq | Alu | SINE |
| 84335090 | 84335242 | - | 21.6 | 1733 | 1889 | -4511 | L1MEc | L1 | LINE |
| 84335360 | 84335592 | - | 21.6 | 6868 | 7103 | -682 | L1MC3 | L1 | LINE |
| 84335683 | 84336100 | - | 25.1 | 34 | 467 | 0 | MLT1C | MaLR | LTR |
| 84336163 | 84336405 | - | 22.1 | 1079 | 1354 | -5046 | L1MEc | L1 | LINE |
| 84336541 | 84336890 | - | 27.9 | 526 | 889 | -5511 | L1MEc | L1 | LINE |

  

|  |  |  |  |  |  |  |  |  |  |
| --- | --- | --- | --- | --- | --- | --- | --- | --- | --- |
| chr9:88856786-88863734 | | | 6030 bp | | |  |  |  |  |
| Genome Start | Genome End | Strand | Div | Repeat Start | Repeat End | Repeat Left | Name | Family | Class |
| 88856786 | 88856893 | - | 21.9 | 3233 | 3333 | -86 | L2 | L2 | LINE |
| 88856923 | 88857071 | - | 20.9 | 2491 | 2658 | -286 | BLACKJACK | AcHobo | DNA |
| 88857201 | 88857472 | + | 18.4 | 1169 | 1451 | -1493 | BLACKJACK | AcHobo | DNA |
| 88857474 | 88857513 | - | 12.5 | 463 | 502 | -3 | MLT1D | MaLR | LTR |
| 88857514 | 88857830 | + | 16.3 | 1 | 307 | -5 | AluJb | Alu | SINE |
| 88857859 | 88858283 | - | 21.9 | 1 | 477 | -28 | MLT1D | MaLR | LTR |
| 88858284 | 88858381 | + | 24.0 | 1450 | 1549 | -1395 | BLACKJACK | AcHobo | DNA |
| 88858380 | 88859035 | - | 25.7 | 286 | 992 | -1952 | BLACKJACK | AcHobo | DNA |
| 88859057 | 88859389 | - | 17.8 | 1 | 338 | -27 | MLT1A0 | MaLR | LTR |
| 88859407 | 88859666 | - | 28.6 | 2 | 267 | -2677 | BLACKJACK | AcHobo | DNA |
| 88859672 | 88859804 | - | 34.4 | 3026 | 3165 | -254 | L2 | L2 | LINE |
| 88859900 | 88860974 | - | 17.0 | 4866 | 5934 | -378 | L1MA9 | L1 | LINE |
| 88860975 | 88861350 | - | 15.5 | 1 | 381 | 0 | THE1D | MaLR | LTR |
| 88861351 | 88861743 | - | 17.0 | 4467 | 4865 | -1275 | L1MA9 | L1 | LINE |
| 88861739 | 88862102 | + | 17.9 | 5912 | 6272 | -40 | L1MA9 | L1 | LINE |
| 88862334 | 88862543 | + | 33.3 | 4430 | 4652 | -1494 | L1M5 | L1 | LINE |
| 88862689 | 88863074 | - | 30.4 | 2480 | 2888 | -531 | L2 | L2 | LINE |
| 88863129 | 88863413 | - | 8.6 | 39 | 323 | 0 | AluY | Alu | SINE |
| 88863585 | 88863734 | - | 27.3 | 2318 | 2468 | -951 | L2 | L2 | LINE |

  

|  |  |  |  |  |  |  |  |  |  |
| --- | --- | --- | --- | --- | --- | --- | --- | --- | --- |
| chr9:90099486-90106869 | | | 7350 bp | | |  |  |  |  |
| Genome Start | Genome End | Strand | Div | Repeat Start | Repeat End | Repeat Left | Name | Family | Class |
| 90099486 | 90100022 | - | 16.8 | 3561 | 4127 | -64 | MER21C-int | ERV1 | LTR |
| 90100024 | 90100167 | + | 5.6 | 1 | 143 | -168 | AluY | Alu | SINE |
| 90100168 | 90100200 | + | 3.0 | 250 | 282 | -17 | Alu | Alu | SINE |
| 90100202 | 90100419 | - | 16.5 | 3336 | 3574 | -617 | MER21C-int | ERV1 | LTR |
| 90100420 | 90100738 | + | 15.5 | 1 | 309 | -3 | AluSx | Alu | SINE |
| 90100754 | 90100926 | - | 19.6 | 129 | 301 | -11 | AluJ | Alu | SINE |
| 90100939 | 90102169 | - | 4.5 | 1658 | 2889 | -3257 | L1PA4 | L1 | LINE |
| 90102171 | 90105404 | + | 3.9 | 2905 | 6155 | 0 | L1PA4 | L1 | LINE |
| 90105407 | 90105526 | - | 14.3 | 2 | 121 | -191 | AluJ | Alu | SINE |
| 90105529 | 90105655 | - | 21.7 | 3219 | 3330 | -861 | MER21C-int | ERV1 | LTR |
| 90105656 | 90105951 | - | 11.0 | 1 | 294 | -15 | AluSc | Alu | SINE |
| 90105952 | 90106301 | - | 21.7 | 2874 | 3218 | -973 | MER21C-int | ERV1 | LTR |
| 90106302 | 90106613 | - | 11.8 | 2 | 312 | 0 | AluSc | Alu | SINE |
| 90106614 | 90106869 | - | 21.7 | 2637 | 2873 | -1318 | MER21C-int | ERV1 | LTR |

  

|  |  |  |  |  |  |  |  |  |  |
| --- | --- | --- | --- | --- | --- | --- | --- | --- | --- |
| chr9:103891171-103897548 | | | 6302 bp | | |  |  |  |  |
| Genome Start | Genome End | Strand | Div | Repeat Start | Repeat End | Repeat Left | Name | Family | Class |
| 103891171 | 103891466 | + | 24.6 | 1432 | 1733 | -4775 | L1MC3 | L1 | LINE |
| 103891462 | 103892266 | - | 14.9 | 5207 | 6042 | -255 | L1MA5A | L1 | LINE |
| 103892267 | 103892501 | + | 10.2 | 6050 | 6285 | -12 | L1MA5A | L1 | LINE |
| 103892512 | 103892899 | + | 25.7 | 1704 | 2255 | -4247 | L1MC3 | L1 | LINE |
| 103892905 | 103893059 | + | 21.3 | 2382 | 2539 | -3782 | L1MC3 | L1 | LINE |
| 103893061 | 103893610 | + | 24.9 | 1 | 583 | -61 | LTR48B | ERV1 | LTR |
| 103893611 | 103894551 | - | 1.5 | 5214 | 6154 | -1 | L1PA3 | L1 | LINE |
| 103894552 | 103894648 | + | 20.4 | 500 | 595 | 0 | LTR49 | ERV1 | LTR |
| 103894650 | 103895412 | + | 27.2 | 2538 | 3323 | -2998 | L1MC3 | L1 | LINE |
| 103895407 | 103896970 | + | 26.6 | 3681 | 5390 | -931 | L1MC3 | L1 | LINE |
| 103896983 | 103897190 | + | 18.1 | 5577 | 5795 | -2165 | L1MC3 | L1 | LINE |
| 103897249 | 103897548 | + | 23.9 | 5966 | 7171 | -789 | L1MC3 | L1 | LINE |

  

|  |  |  |  |  |  |  |  |  |  |
| --- | --- | --- | --- | --- | --- | --- | --- | --- | --- |
| chr9:104474966-104478061 | | | 2688 bp | | |  |  |  |  |
| Genome Start | Genome End | Strand | Div | Repeat Start | Repeat End | Repeat Left | Name | Family | Class |
| 104474966 | 104475349 | + | 32.7 | 798 | 1173 | -4946 | L1MEd | L1 | LINE |
| 104475387 | 104475641 | + | 34.0 | 1611 | 1871 | -4278 | L1MEd | L1 | LINE |
| 104475642 | 104475937 | - | 13.8 | 1 | 296 | -16 | AluSx | Alu | SINE |
| 104475938 | 104475991 | + | 34.0 | 1872 | 1919 | -4230 | L1MEd | L1 | LINE |
| 104476152 | 104476600 | - | 14.2 | 4972 | 5441 | -725 | L1PA16 | L1 | LINE |
| 104476599 | 104476759 | + | 16.3 | 5461 | 5612 | -554 | L1PA16 | L1 | LINE |
| 104476760 | 104477056 | + | 11.3 | 5 | 310 | 0 | AluSq | Alu | SINE |
| 104477057 | 104477069 | + | 11.3 | 285 | 296 | -17 | AluSq | Alu | SINE |
| 104477070 | 104477575 | + | 16.3 | 5613 | 6146 | -20 | L1PA16 | L1 | LINE |
| 104477571 | 104477675 | + | 22.8 | 2925 | 3035 | -3094 | L1MEd | L1 | LINE |
| 104477894 | 104478061 | + | 27.7 | 3323 | 3500 | -2629 | L1MEd | L1 | LINE |

  

|  |  |  |  |  |  |  |  |  |  |
| --- | --- | --- | --- | --- | --- | --- | --- | --- | --- |
| chr9:104495855-104500477 | | | 4615 bp | | |  |  |  |  |
| Genome Start | Genome End | Strand | Div | Repeat Start | Repeat End | Repeat Left | Name | Family | Class |
| 104495855 | 104496245 | + | 16.1 | 2 | 397 | -1338 | MLT1A0-int | MaLR | LTR |
| 104496254 | 104496714 | - | 3.2 | 3231 | 3692 | -2454 | L1PA4 | L1 | LINE |
| 104496714 | 104499182 | + | 4.6 | 3690 | 6152 | -3 | L1PA4 | L1 | LINE |
| 104499184 | 104500477 | + | 24.7 | 393 | 1735 | 0 | MLT1A0-int | MaLR | LTR |

  

|  |  |  |  |  |  |  |  |  |  |
| --- | --- | --- | --- | --- | --- | --- | --- | --- | --- |
| chr9:104592182-104596926 | | | 4672 bp | | |  |  |  |  |
| Genome Start | Genome End | Strand | Div | Repeat Start | Repeat End | Repeat Left | Name | Family | Class |
| 104592182 | 104592466 | - | 24.7 | 2469 | 2772 | -3447 | L1MEc | L1 | LINE |
| 104592466 | 104593934 | - | 2.3 | 4688 | 6155 | 0 | L1PA3 | L1 | LINE |
| 104593934 | 104594435 | + | 2.0 | 4212 | 4713 | -1433 | L1PA3 | L1 | LINE |
| 104594439 | 104595051 | - | 1.3 | 5604 | 6216 | 0 | L1PA2 | L1 | LINE |
| 104595052 | 104595983 | + | 1.0 | 4440 | 5371 | -775 | L1P1 | L1 | LINE |
| 104595983 | 104596102 | - | 24.2 | 2376 | 2498 | -3721 | L1MEc | L1 | LINE |
| 104596132 | 104596528 | - | 28.3 | 1914 | 2337 | -4063 | L1MEc | L1 | LINE |
| 104596558 | 104596764 | - | 18.2 | 47 | 253 | -59 | AluJo | Alu | SINE |
| 104596780 | 104596926 | - | 26.7 | 1704 | 1863 | -4537 | L1MEc | L1 | LINE |

  

|  |  |  |  |  |  |  |  |  |  |
| --- | --- | --- | --- | --- | --- | --- | --- | --- | --- |
| chr9:104651823-104656435 | | | 4380 bp | | |  |  |  |  |
| Genome Start | Genome End | Strand | Div | Repeat Start | Repeat End | Repeat Left | Name | Family | Class |
| 104651823 | 104652072 | - | 20.8 | 2560 | 2817 | 0 | Charlie10 | MER1\_type | DNA |
| 104652091 | 104652626 | + | 10.1 | 5618 | 6159 | -1 | L1PREC2 | L1 | LINE |
| 104652809 | 104654298 | - | 24.8 | 404 | 2046 | -715 | Charlie10 | MER1\_type | DNA |
| 104654314 | 104654479 | - | 12.1 | 125 | 290 | -22 | AluSx | Alu | SINE |
| 104654490 | 104655666 | - | 7.5 | 4962 | 6141 | -13 | L1PA7 | L1 | LINE |
| 104655664 | 104655932 | + | 6.7 | 4699 | 4967 | -1179 | L1PA7 | L1 | LINE |
| 104655933 | 104656068 | - | 11.0 | 1 | 136 | -176 | AluSx | Alu | SINE |
| 104656080 | 104656435 | - | 30.0 | 1 | 381 | -2380 | Charlie10 | MER1\_type | DNA |

  

|  |  |  |  |  |  |  |  |  |  |
| --- | --- | --- | --- | --- | --- | --- | --- | --- | --- |
| chr9:105733624-105738317 | | | 3775 bp | | |  |  |  |  |
| Genome Start | Genome End | Strand | Div | Repeat Start | Repeat End | Repeat Left | Name | Family | Class |
| 105733624 | 105733829 | + | 27.7 | 166 | 374 | -4480 | L1ME4a | L1 | LINE |
| 105733844 | 105733990 | + | 23.7 | 6113 | 6270 | -42 | L1MA9 | L1 | LINE |
| 105734085 | 105734147 | - | 30.0 | 3310 | 3369 | -9 | L2 | L2 | LINE |
| 105734201 | 105734304 | + | 25.0 | 391 | 506 | -4348 | L1ME4a | L1 | LINE |
| 105734372 | 105734564 | + | 28.0 | 520 | 732 | -4122 | L1ME4a | L1 | LINE |
| 105734766 | 105737004 | + | 25.8 | 756 | 3378 | -1456 | L1ME4a | L1 | LINE |
| 105737212 | 105737407 | + | 15.7 | 1 | 199 | -76 | ORSL | Tip100 | DNA |
| 105737409 | 105737522 | - | 12.3 | 5686 | 5800 | -366 | L1PA17 | L1 | LINE |
| 105737523 | 105737884 | + | 14.8 | 5780 | 6166 | 0 | L1PA17 | L1 | LINE |
| 105738167 | 105738317 | + | 28.4 | 3471 | 3625 | -1209 | L1ME4a | L1 | LINE |

  

|  |  |  |  |  |  |  |  |  |  |
| --- | --- | --- | --- | --- | --- | --- | --- | --- | --- |
| chr9:105863822-105866037 | | | 2212 bp | | |  |  |  |  |
| Genome Start | Genome End | Strand | Div | Repeat Start | Repeat End | Repeat Left | Name | Family | Class |
| 105863822 | 105863955 | + | 9.8 | 1 | 133 | -177 | AluSg | Alu | SINE |
| 105863956 | 105864578 | - | 1.8 | 4223 | 4845 | -1301 | L1PA2 | L1 | LINE |
| 105864578 | 105865858 | + | 2.1 | 4862 | 6142 | -13 | L1PA2 | L1 | LINE |
| 105865864 | 105866037 | + | 9.8 | 121 | 294 | -18 | AluSg | Alu | SINE |

  

|  |  |  |  |  |  |  |  |  |  |
| --- | --- | --- | --- | --- | --- | --- | --- | --- | --- |
| chr9:106229192-106230646 | | | 1439 bp | | |  |  |  |  |
| Genome Start | Genome End | Strand | Div | Repeat Start | Repeat End | Repeat Left | Name | Family | Class |
| 106229192 | 106229363 | + | 16.4 | 1 | 201 | -227 | MSTA | MaLR | LTR |
| 106229364 | 106229526 | - | 4.3 | 5086 | 5248 | -898 | L1PA5 | L1 | LINE |
| 106229526 | 106230406 | + | 4.1 | 5271 | 6151 | -3 | L1PA5 | L1 | LINE |
| 106230424 | 106230646 | + | 18.7 | 187 | 428 | 0 | MSTA | MaLR | LTR |

  

|  |  |  |  |  |  |  |  |  |  |
| --- | --- | --- | --- | --- | --- | --- | --- | --- | --- |
| chr9:107965213-107973228 | | | 7741 bp | | |  |  |  |  |
| Genome Start | Genome End | Strand | Div | Repeat Start | Repeat End | Repeat Left | Name | Family | Class |
| 107965213 | 107965362 | + | 33.8 | 24 | 175 | -6040 | L1MC4 | L1 | LINE |
| 107965367 | 107965616 | + | 20.6 | 2 | 221 | -284 | MLT1D | MaLR | LTR |
| 107965617 | 107965890 | + | 12.0 | 1 | 286 | -26 | AluSx | Alu | SINE |
| 107965891 | 107966203 | + | 20.6 | 222 | 501 | -4 | MLT1D | MaLR | LTR |
| 107966444 | 107966520 | + | 19.5 | 72 | 162 | -4 | FRAM | Alu | SINE |
| 107966546 | 107967976 | + | 27.2 | 448 | 1911 | -4304 | L1MC4 | L1 | LINE |
| 107967945 | 107968239 | + | 13.3 | 5399 | 5700 | -451 | L1PB1 | L1 | LINE |
| 107968247 | 107970840 | - | 3.1 | 3563 | 6155 | 0 | L1PA3 | L1 | LINE |
| 107970841 | 107971551 | + | 2.8 | 2850 | 3560 | -2586 | L1PA3 | L1 | LINE |
| 107971558 | 107972010 | + | 9.1 | 5696 | 6150 | -1 | L1PB1 | L1 | LINE |
| 107972035 | 107972306 | - | 5.4 | 5167 | 5441 | -733 | L1PA11 | L1 | LINE |
| 107972307 | 107973020 | + | 10.1 | 5447 | 6174 | 0 | L1PA11 | L1 | LINE |
| 107973022 | 107973228 | + | 23.9 | 1927 | 2141 | -4074 | L1MC4 | L1 | LINE |

  

|  |  |  |  |  |  |  |  |  |  |
| --- | --- | --- | --- | --- | --- | --- | --- | --- | --- |
| chr9:108349470-108355788 | | | 6112 bp | | |  |  |  |  |
| Genome Start | Genome End | Strand | Div | Repeat Start | Repeat End | Repeat Left | Name | Family | Class |
| 108349470 | 108349597 | + | 33.3 | -573 | -455 | -5291 | L1M4c | L1 | LINE |
| 108349598 | 108349884 | + | 8.2 | 1 | 303 | 0 | AluSp | Alu | SINE |
| 108349885 | 108350177 | + | 33.3 | -454 | -51 | -4981 | L1M4c | L1 | LINE |
| 108350193 | 108350423 | + | 29.2 | 18 | 253 | -4601 | L1M4c | L1 | LINE |
| 108350482 | 108351569 | - | 2.4 | 5055 | 6150 | -5 | L1PA4 | L1 | LINE |
| 108351568 | 108351728 | + | 3.1 | 4912 | 5072 | -1074 | L1PA4 | L1 | LINE |
| 108351804 | 108352501 | + | 27.6 | 5369 | 6095 | -67 | L1ME3 | L1 | LINE |
| 108352547 | 108353559 | + | 26.1 | 265 | 1377 | -3457 | L1M4c | L1 | LINE |
| 108353560 | 108353874 | - | 7.7 | 1 | 311 | 0 | AluY | Alu | SINE |
| 108353875 | 108355122 | + | 25.7 | 1378 | 2643 | -2191 | L1M4c | L1 | LINE |
| 108355139 | 108355788 | + | 29.6 | 3279 | 4008 | -826 | L1M4c | L1 | LINE |

  

|  |  |  |  |  |  |  |  |  |  |
| --- | --- | --- | --- | --- | --- | --- | --- | --- | --- |
| chr9:110052768-110055219 | | | 2173 bp | | |  |  |  |  |
| Genome Start | Genome End | Strand | Div | Repeat Start | Repeat End | Repeat Left | Name | Family | Class |
| 110052768 | 110052985 | - | 30.5 | 3611 | 3826 | -2303 | L1MEd | L1 | LINE |
| 110053037 | 110054198 | - | 14.8 | 4503 | 5664 | -669 | L1MC1 | L1 | LINE |
| 110054200 | 110054863 | + | 14.8 | 5643 | 6333 | 0 | L1MC1 | L1 | LINE |
| 110055091 | 110055219 | - | 29.7 | 2171 | 2301 | -3848 | L1MEd | L1 | LINE |

  

|  |  |  |  |  |  |  |  |  |  |
| --- | --- | --- | --- | --- | --- | --- | --- | --- | --- |
| chr9:110529528-110539573 | | | 10035 bp | | |  |  |  |  |
| Genome Start | Genome End | Strand | Div | Repeat Start | Repeat End | Repeat Left | Name | Family | Class |
| 110529528 | 110531487 | + | 15.8 | 16 | 2330 | -5235 | L1M1 | L1 | LINE |
| 110531488 | 110531751 | + | 18.7 | 42 | 303 | -9 | AluJb | Alu | SINE |
| 110531752 | 110531859 | + | 18.3 | 2331 | 2430 | -5135 | L1M1 | L1 | LINE |
| 110531860 | 110532164 | + | 13.4 | 1 | 310 | -2 | AluSx | Alu | SINE |
| 110532165 | 110532299 | + | 18.3 | 2431 | 2549 | -5016 | L1M1 | L1 | LINE |
| 110532300 | 110532578 | + | 15.5 | 7 | 290 | -22 | AluSx | Alu | SINE |
| 110532579 | 110534253 | + | 15.0 | 2550 | 4284 | -3281 | L1M1 | L1 | LINE |
| 110534255 | 110534563 | + | 0.3 | 1 | 309 | -1 | AluYa5 | Alu | SINE |
| 110534578 | 110535688 | - | 2.7 | 3797 | 4883 | -1263 | L1PA3 | L1 | LINE |
| 110535687 | 110536963 | + | 3.2 | 4877 | 6153 | -2 | L1PA3 | L1 | LINE |
| 110536962 | 110539573 | + | 10.9 | 4270 | 6943 | -781 | L1M1 | L1 | LINE |

  

|  |  |  |  |  |  |  |  |  |  |
| --- | --- | --- | --- | --- | --- | --- | --- | --- | --- |
| chr9:111496522-111499396 | | | 2722 bp | | |  |  |  |  |
| Genome Start | Genome End | Strand | Div | Repeat Start | Repeat End | Repeat Left | Name | Family | Class |
| 111496522 | 111497242 | + | 29.8 | 4335 | 5108 | -1038 | L1MC4a | L1 | LINE |
| 111497333 | 111497727 | - | 10.9 | 5322 | 5724 | -576 | L1MA4 | L1 | LINE |
| 111497729 | 111498264 | + | 12.7 | 5746 | 6295 | -5 | L1MA4 | L1 | LINE |
| 111498324 | 111498781 | + | 29.5 | 5270 | 5751 | -2059 | L1MC4a | L1 | LINE |
| 111498785 | 111499220 | + | 24.1 | 16 | 457 | 0 | LTR16A1 | ERVL | LTR |
| 111499221 | 111499396 | + | 29.5 | 5746 | 5929 | -1879 | L1MC4a | L1 | LINE |

  

|  |  |  |  |  |  |  |  |  |  |
| --- | --- | --- | --- | --- | --- | --- | --- | --- | --- |
| chr9:116776185-116779440 | | | 3274 bp | | |  |  |  |  |
| Genome Start | Genome End | Strand | Div | Repeat Start | Repeat End | Repeat Left | Name | Family | Class |
| 116776185 | 116776360 | - | 15.9 | 373 | 550 | -6 | LTR38 | ERV1 | LTR |
| 116776361 | 116777364 | - | 5.3 | 3404 | 4419 | -1727 | L1PA4 | L1 | LINE |
| 116777347 | 116779055 | + | 4.8 | 4439 | 6153 | -2 | L1PA4 | L1 | LINE |
| 116779056 | 116779440 | - | 12.3 | 1 | 388 | -168 | LTR38 | ERV1 | LTR |

  

|  |  |  |  |  |  |  |  |  |  |
| --- | --- | --- | --- | --- | --- | --- | --- | --- | --- |
| chr9:117507737-117512198 | | | 4394 bp | | |  |  |  |  |
| Genome Start | Genome End | Strand | Div | Repeat Start | Repeat End | Repeat Left | Name | Family | Class |
| 117507737 | 117508080 | - | 10.1 | 5793 | 6150 | -1 | L1PB1 | L1 | LINE |
| 117508137 | 117508452 | - | 10.1 | 5474 | 5792 | -359 | L1PB1 | L1 | LINE |
| 117508465 | 117509704 | - | 7.5 | 3122 | 4362 | -1784 | L1PA6 | L1 | LINE |
| 117509704 | 117511493 | + | 5.8 | 4367 | 6154 | 0 | L1PA6 | L1 | LINE |
| 117511495 | 117512198 | - | 11.6 | 4751 | 5465 | -686 | L1PB1 | L1 | LINE |

  

|  |  |  |  |  |  |  |  |  |  |
| --- | --- | --- | --- | --- | --- | --- | --- | --- | --- |
| chr9:117602529-117609609 | | | 6784 bp | | |  |  |  |  |
| Genome Start | Genome End | Strand | Div | Repeat Start | Repeat End | Repeat Left | Name | Family | Class |
| 117602529 | 117602628 | - | 18.0 | 2301 | 2417 | -1 | Tigger1 | MER2\_type | DNA |
| 117602629 | 117604566 | - | 3.3 | 2720 | 4664 | -1482 | L1PA4 | L1 | LINE |
| 117604562 | 117606033 | + | 4.0 | 4678 | 6152 | -3 | L1PA4 | L1 | LINE |
| 117606039 | 117606136 | - | 15.4 | 2215 | 2313 | -105 | Tigger1 | MER2\_type | DNA |
| 117606137 | 117606431 | + | 19.3 | 1 | 290 | -22 | AluSx | Alu | SINE |
| 117606432 | 117606868 | - | 15.4 | 1761 | 2214 | -204 | Tigger1 | MER2\_type | DNA |
| 117606870 | 117606993 | + | 11.5 | 1 | 122 | -190 | AluSq/x | Alu | SINE |
| 117606996 | 117607058 | + | 17.7 | 504 | 565 | -7705 | PRIMA4-int | ERV1 | LTR |
| 117607328 | 117607477 | + | 18.1 | 134 | 285 | -27 | AluSg/x | Alu | SINE |
| 117607498 | 117607811 | - | 11.5 | 1454 | 1769 | -649 | Tigger1 | MER2\_type | DNA |
| 117607815 | 117608204 | + | 17.7 | 5759 | 6166 | 0 | L1PA17 | L1 | LINE |
| 117608207 | 117609609 | - | 15.8 | 2 | 1450 | -968 | Tigger1 | MER2\_type | DNA |

  

|  |  |  |  |  |  |  |  |  |  |
| --- | --- | --- | --- | --- | --- | --- | --- | --- | --- |
| chr9:119710072-119718616 | | | 8349 bp | | |  |  |  |  |
| Genome Start | Genome End | Strand | Div | Repeat Start | Repeat End | Repeat Left | Name | Family | Class |
| 119710072 | 119711001 | + | 18.0 | 17 | 922 | -5462 | L1M4c | L1 | LINE |
| 119711032 | 119711382 | - | 10.0 | 4060 | 4410 | -1736 | L1PB1 | L1 | LINE |
| 119711385 | 119713111 | + | 9.6 | 4409 | 6151 | 0 | L1PB1 | L1 | LINE |
| 119713121 | 119713195 | + | 16.1 | 935 | 1009 | -5375 | L1M4c | L1 | LINE |
| 119713256 | 119713311 | + | 16.1 | 1010 | 1099 | -5285 | L1M4c | L1 | LINE |
| 119713312 | 119713654 | - | 16.0 | 1 | 428 | 0 | MSTA | MaLR | LTR |
| 119713655 | 119716223 | + | 18.4 | 1100 | 3723 | -2659 | L1M4c | L1 | LINE |
| 119716322 | 119716437 | + | 21.3 | 3724 | 3855 | -2527 | L1M4c | L1 | LINE |
| 119716435 | 119717016 | + | 21.5 | 4396 | 4923 | -1453 | L1M4c | L1 | LINE |
| 119717017 | 119717813 | + | 15.1 | 5528 | 6301 | -2 | L1MA4A | L1 | LINE |
| 119717814 | 119718616 | + | 20.4 | 4924 | 5668 | -748 | L1M4c | L1 | LINE |

  

|  |  |  |  |  |  |  |  |  |  |
| --- | --- | --- | --- | --- | --- | --- | --- | --- | --- |
| chr9:119863152-119872185 | | | 8633 bp | | |  |  |  |  |
| Genome Start | Genome End | Strand | Div | Repeat Start | Repeat End | Repeat Left | Name | Family | Class |
| 119863152 | 119864020 | - | 28.2 | 5291 | 6221 | -18 | L1ME1 | L1 | LINE |
| 119864242 | 119865007 | - | 26.4 | 5396 | 6173 | -66 | L1ME1 | L1 | LINE |
| 119865003 | 119865494 | - | 29.6 | 37 | 599 | -5801 | L1ME1 | L1 | LINE |
| 119865505 | 119866927 | - | 28.3 | 2772 | 4238 | -1981 | L1ME1 | L1 | LINE |
| 119866945 | 119867994 | - | 10.1 | 4099 | 5155 | -991 | L1PB1 | L1 | LINE |
| 119867990 | 119868624 | + | 12.2 | 5130 | 5791 | -360 | L1PB1 | L1 | LINE |
| 119868727 | 119869081 | + | 11.9 | 5792 | 6146 | -5 | L1PB1 | L1 | LINE |
| 119869091 | 119869176 | - | 28.6 | 2676 | 2764 | -3455 | L1ME1 | L1 | LINE |
| 119869200 | 119869490 | - | 12.0 | 1 | 291 | -18 | AluSc | Alu | SINE |
| 119869491 | 119870797 | - | 28.8 | 1404 | 2675 | -3544 | L1ME1 | L1 | LINE |
| 119870827 | 119872185 | - | 28.9 | 42 | 1403 | -4997 | L1ME1 | L1 | LINE |

  

|  |  |  |  |  |  |  |  |  |  |
| --- | --- | --- | --- | --- | --- | --- | --- | --- | --- |
| chr9:120373218-120381032 | | | 7296 bp | | |  |  |  |  |
| Genome Start | Genome End | Strand | Div | Repeat Start | Repeat End | Repeat Left | Name | Family | Class |
| 120373218 | 120374029 | - | 26.3 | 5226 | 6090 | -8 | L1ME2 | L1 | LINE |
| 120374093 | 120374379 | - | 27.1 | 4887 | 5173 | -907 | L1ME2 | L1 | LINE |
| 120374400 | 120376933 | - | 27.1 | 2144 | 4886 | -1194 | L1ME2 | L1 | LINE |
| 120376949 | 120378311 | - | 12.7 | 4784 | 6157 | -9 | L1PA16 | L1 | LINE |
| 120378311 | 120378807 | + | 11.5 | 4311 | 4799 | -1347 | L1PA16 | L1 | LINE |
| 120378810 | 120379985 | - | 27.7 | 1061 | 2259 | -4002 | L1ME2 | L1 | LINE |
| 120380124 | 120380322 | - | 31.2 | 580 | 778 | -5483 | L1ME2 | L1 | LINE |
| 120380605 | 120381032 | - | 31.8 | 11 | 453 | -5617 | L1ME2 | L1 | LINE |

  

|  |  |  |  |  |  |  |  |  |  |
| --- | --- | --- | --- | --- | --- | --- | --- | --- | --- |
| chr9:120716237-120726975 | | | 10557 bp | | |  |  |  |  |
| Genome Start | Genome End | Strand | Div | Repeat Start | Repeat End | Repeat Left | Name | Family | Class |
| 120716237 | 120716300 | - | 6.0 | 4729 | 4791 | -1352 | L1M2 | L1 | LINE |
| 120716313 | 120717763 | - | 11.8 | 4846 | 6304 | 0 | L1MA2 | L1 | LINE |
| 120717765 | 120718074 | + | 14.2 | 1 | 311 | -1 | AluSx | Alu | SINE |
| 120718209 | 120718363 | - | 12.8 | 4707 | 4859 | -1287 | L1MA2 | L1 | LINE |
| 120718393 | 120718616 | - | 12.8 | 4481 | 4706 | -1440 | L1MA2 | L1 | LINE |
| 120718617 | 120724362 | - | 15.2 | 146 | 5894 | -1671 | L1M1 | L1 | LINE |
| 120724361 | 120724560 | - | 1.0 | 4502 | 4701 | -1445 | L1PA3 | L1 | LINE |
| 120724562 | 120726014 | + | 2.8 | 4698 | 6155 | 0 | L1PA3 | L1 | LINE |
| 120726017 | 120726155 | - | 17.2 | 19 | 158 | -7407 | L1M1 | L1 | LINE |
| 120726161 | 120726975 | - | 16.6 | 3914 | 4743 | -1400 | L1M2 | L1 | LINE |

  

|  |  |  |  |  |  |  |  |  |  |
| --- | --- | --- | --- | --- | --- | --- | --- | --- | --- |
| chr9:121382247-121387283 | | | 4539 bp | | |  |  |  |  |
| Genome Start | Genome End | Strand | Div | Repeat Start | Repeat End | Repeat Left | Name | Family | Class |
| 121382247 | 121382823 | - | 27.3 | 2930 | 3532 | -2682 | L1MC4 | L1 | LINE |
| 121383159 | 121383221 | - | 14.5 | 1 | 63 | -51 | MER81 | AcHobo | DNA |
| 121383290 | 121383364 | - | 20.0 | 6075 | 6151 | -27 | L1MB8 | L1 | LINE |
| 121383379 | 121383625 | + | 20.8 | 5787 | 6037 | -141 | L1MB8 | L1 | LINE |
| 121383681 | 121384965 | - | 7.7 | 4863 | 6148 | -6 | L1PA7 | L1 | LINE |
| 121384965 | 121385816 | + | 7.6 | 4003 | 4862 | -1284 | L1PA7 | L1 | LINE |
| 121385821 | 121387015 | - | 27.6 | 1235 | 2384 | -3831 | L1MC4 | L1 | LINE |
| 121387039 | 121387283 | - | 27.6 | 957 | 1234 | -4981 | L1MC4 | L1 | LINE |

  

|  |  |  |  |  |  |  |  |  |  |
| --- | --- | --- | --- | --- | --- | --- | --- | --- | --- |
| chr9:121923573-121927285 | | | 3681 bp | | |  |  |  |  |
| Genome Start | Genome End | Strand | Div | Repeat Start | Repeat End | Repeat Left | Name | Family | Class |
| 121923573 | 121924692 | - | 19.0 | 4210 | 5354 | -786 | L1M3 | L1 | LINE |
| 121924687 | 121925956 | - | 3.9 | 3804 | 5073 | -1073 | L1PA4 | L1 | LINE |
| 121925955 | 121927050 | + | 3.7 | 5073 | 6155 | 0 | L1PA4 | L1 | LINE |
| 121927091 | 121927285 | - | 18.3 | 4012 | 4220 | -1920 | L1M3 | L1 | LINE |

  

|  |  |  |  |  |  |  |  |  |  |
| --- | --- | --- | --- | --- | --- | --- | --- | --- | --- |
| chr9:122001298-122003583 | | | 1930 bp | | |  |  |  |  |
| Genome Start | Genome End | Strand | Div | Repeat Start | Repeat End | Repeat Left | Name | Family | Class |
| 122001298 | 122001493 | + | 29.8 | 2752 | 2952 | -467 | L2 | L2 | LINE |
| 122001516 | 122001973 | - | 6.3 | 5695 | 6154 | 0 | L1PA7 | L1 | LINE |
| 122001974 | 122002257 | + | 4.6 | 5402 | 5685 | -469 | L1PA7 | L1 | LINE |
| 122002534 | 122003068 | - | 21.8 | 5583 | 6161 | -5 | L1ME1 | L1 | LINE |
| 122003069 | 122003369 | + | 9.4 | 1 | 297 | -14 | AluY | Alu | SINE |
| 122003370 | 122003418 | - | 21.8 | 5536 | 5582 | -584 | L1ME1 | L1 | LINE |
| 122003477 | 122003583 | + | 16.5 | 3299 | 3418 | -1 | L2 | L2 | LINE |

  

|  |  |  |  |  |  |  |  |  |  |
| --- | --- | --- | --- | --- | --- | --- | --- | --- | --- |
| chr9:128804090-128806012 | | | 1911 bp | | |  |  |  |  |
| Genome Start | Genome End | Strand | Div | Repeat Start | Repeat End | Repeat Left | Name | Family | Class |
| 128804090 | 128804535 | - | 22.5 | 5691 | 6148 | -22 | L1ME3A | L1 | LINE |
| 128804546 | 128805205 | - | 1.8 | 5496 | 6155 | 0 | L1PA2 | L1 | LINE |
| 128805206 | 128805646 | + | 2.2 | 5044 | 5484 | -671 | L1PA2 | L1 | LINE |
| 128805649 | 128805810 | - | 26.0 | 5570 | 5699 | -463 | L1ME3A | L1 | LINE |
| 128805811 | 128805924 | - | 6.1 | 6053 | 6167 | -1 | L1PA10 | L1 | LINE |
| 128805925 | 128806012 | - | 26.0 | 5483 | 5569 | -593 | L1ME3A | L1 | LINE |

  

|  |  |  |  |  |  |  |  |  |  |
| --- | --- | --- | --- | --- | --- | --- | --- | --- | --- |
| chr9:138056989-138075203 | | | 16772 bp | | |  |  |  |  |
| Genome Start | Genome End | Strand | Div | Repeat Start | Repeat End | Repeat Left | Name | Family | Class |
| 138056989 | 138057102 | + | 32.6 | 711 | 810 | -5500 | L1MEc | L1 | LINE |
| 138057136 | 138057298 | + | 32.6 | 780 | 969 | -5189 | L1MEc | L1 | LINE |
| 138057299 | 138057597 | + | 14.1 | 1 | 300 | -12 | AluSx | Alu | SINE |
| 138057598 | 138057724 | + | 36.3 | 931 | 1055 | -5064 | L1MEc | L1 | LINE |
| 138057725 | 138058040 | + | 10.5 | 1 | 313 | 0 | AluSq | Alu | SINE |
| 138058041 | 138058236 | + | 34.3 | 1056 | 1255 | -4864 | L1MEc | L1 | LINE |
| 138058212 | 138058370 | + | 28.8 | 1652 | 1817 | -4332 | L1ME1 | L1 | LINE |
| 138058374 | 138058613 | + | 32.8 | 2009 | 2277 | -4033 | L1ME1 | L1 | LINE |
| 138058614 | 138058908 | + | 11.5 | 1 | 299 | -13 | AluSx | Alu | SINE |
| 138058909 | 138058944 | + | 32.8 | 2278 | 2303 | -4007 | L1ME1 | L1 | LINE |
| 138058968 | 138059048 | + | 28.8 | 2261 | 2340 | -3789 | L1ME1 | L1 | LINE |
| 138059103 | 138059642 | + | 22.0 | 2669 | 3266 | -2863 | L1ME1 | L1 | LINE |
| 138059645 | 138059896 | + | 10.8 | 1 | 301 | -1 | AluSx | Alu | SINE |
| 138059945 | 138060028 | - | 23.8 | 127 | 212 | -3 | MER58C | MER1\_type | DNA |
| 138060035 | 138060186 | - | 15.8 | 157 | 309 | -3 | AluSx | Alu | SINE |
| 138060187 | 138061690 | + | 16.9 | 1 | 1755 | 0 | MER52A | ERV1 | LTR |
| 138061691 | 138061841 | - | 15.8 | 1 | 156 | -156 | AluSx | Alu | SINE |
| 138061862 | 138061931 | - | 24.3 | 30 | 99 | -116 | MER58C | MER1\_type | DNA |
| 138061973 | 138061994 | + | 26.3 | 3307 | 3327 | -2802 | L1ME1 | L1 | LINE |
| 138061995 | 138062294 | + | 6.7 | 1 | 299 | -12 | AluY | Alu | SINE |
| 138062295 | 138062338 | + | 26.3 | 3328 | 3363 | -2766 | L1ME1 | L1 | LINE |
| 138062339 | 138062627 | - | 15.0 | 1 | 289 | -23 | AluSx | Alu | SINE |
| 138062628 | 138062930 | + | 26.3 | 3364 | 3658 | -2471 | L1ME1 | L1 | LINE |
| 138063022 | 138063304 | - | 11.3 | 1 | 283 | -29 | AluSx | Alu | SINE |
| 138063361 | 138063441 | + | 18.6 | 3670 | 3739 | -2390 | L1ME1 | L1 | LINE |
| 138063843 | 138064281 | + | 30.4 | 3727 | 4221 | -1908 | L1ME1 | L1 | LINE |
| 138064282 | 138064419 | + | 13.8 | 1 | 137 | -175 | AluSx | Alu | SINE |
| 138064420 | 138064696 | + | 10.9 | 3 | 292 | -18 | AluSg | Alu | SINE |
| 138064697 | 138064873 | + | 13.8 | 138 | 313 | 0 | AluSg/x | Alu | SINE |
| 138064874 | 138065028 | + | 30.4 | 4222 | 4395 | -1734 | L1ME1 | L1 | LINE |
| 138065029 | 138065338 | + | 8.6 | 1 | 305 | -7 | AluSx | Alu | SINE |
| 138065339 | 138065561 | + | 30.4 | 4396 | 4604 | -1525 | L1ME1 | L1 | LINE |
| 138065609 | 138065981 | + | 30.4 | 4605 | 4995 | -1134 | L1ME1 | L1 | LINE |
| 138066038 | 138066328 | + | 24.0 | 6499 | 6834 | -974 | L1MC4a | L1 | LINE |
| 138066130 | 138066478 | + | 25.3 | 6695 | 7176 | -866 | L1MC4 | L1 | LINE |
| 138066479 | 138066776 | + | 11.7 | 1 | 298 | -14 | AluSx | Alu | SINE |
| 138066777 | 138067593 | + | 25.4 | 7177 | 8039 | -6 | L1MC4 | L1 | LINE |
| 138067604 | 138067956 | + | 30.5 | 5029 | 5425 | -720 | L1ME1 | L1 | LINE |
| 138067983 | 138068822 | + | 27.3 | 784 | 1635 | -4722 | L1MEc | L1 | LINE |
| 138068897 | 138069064 | - | 10.1 | 124 | 296 | -16 | AluSg/x | Alu | SINE |
| 138069065 | 138069173 | - | 22.2 | 5666 | 5794 | -372 | L1M5 | L1 | LINE |
| 138069174 | 138069475 | - | 11.3 | 1 | 301 | -11 | AluSx | Alu | SINE |
| 138069476 | 138069524 | - | 22.2 | 5593 | 5648 | -501 | L1ME1 | L1 | LINE |
| 138069528 | 138069920 | + | 23.8 | 5632 | 6083 | -66 | L1ME1 | L1 | LINE |
| 138069921 | 138070227 | + | 17.2 | 1 | 303 | -9 | AluJb | Alu | SINE |
| 138070228 | 138070299 | + | 23.8 | 6084 | 6146 | -3 | L1ME1 | L1 | LINE |
| 138070358 | 138070863 | + | 28.9 | 1985 | 2467 | -3784 | L1MEc | L1 | LINE |
| 138070864 | 138071730 | + | 24.1 | 3464 | 4397 | -1822 | L1MEc | L1 | LINE |
| 138071731 | 138072023 | + | 7.8 | 1 | 293 | -18 | AluY | Alu | SINE |
| 138072024 | 138072147 | + | 27.4 | 4398 | 4513 | -1706 | L1MEc | L1 | LINE |
| 138072143 | 138072344 | + | 19.4 | 5616 | 5818 | -360 | L1MB8 | L1 | LINE |
| 138072345 | 138072659 | + | 16.9 | 1 | 311 | -1 | AluJb | Alu | SINE |
| 138072807 | 138073114 | + | 13.3 | 1 | 313 | 0 | AluSq | Alu | SINE |
| 138073115 | 138073446 | + | 23.2 | 5822 | 6173 | -5 | L1MB8 | L1 | LINE |
| 138073497 | 138073666 | + | 27.5 | 4571 | 4751 | -1468 | L1MEc | L1 | LINE |
| 138073667 | 138073954 | - | 11.1 | 1 | 295 | -17 | AluSx | Alu | SINE |
| 138073955 | 138074299 | + | 27.5 | 4752 | 5124 | -1095 | L1MEc | L1 | LINE |
| 138074723 | 138075203 | + | 29.2 | 5141 | 5641 | -594 | L1MEc | L1 | LINE |

  


|  |  |  |  |  |  |  |  |  |  |
| --- | --- | --- | --- | --- | --- | --- | --- | --- | --- |
| chr10:331558-333871 | | | 2247 bp | | |  |  |  |  |
| Genome Start | Genome End | Strand | Div | Repeat Start | Repeat End | Repeat Left | Name | Family | Class |
| 331558 | 331696 | + | 10.1 | 5461 | 5599 | -698 | L1MA5A | L1 | LINE |
| 331705 | 331815 | + | 9.2 | 39 | 147 | -163 | AluSg | Alu | SINE |
| 331816 | 332245 | - | 6.5 | 4968 | 5398 | -748 | L1PA5 | L1 | LINE |
| 332246 | 332990 | + | 4.2 | 5410 | 6154 | 0 | L1PA5 | L1 | LINE |
| 332991 | 333157 | + | 9.2 | 134 | 308 | 0 | AluSg/x | Alu | SINE |
| 333217 | 333871 | + | 17.0 | 5596 | 6296 | -1 | L1MA5A | L1 | LINE |

  

|  |  |  |  |  |  |  |  |  |  |
| --- | --- | --- | --- | --- | --- | --- | --- | --- | --- |
| chr10:3358203-3360594 | | | 2283 bp | | |  |  |  |  |
| Genome Start | Genome End | Strand | Div | Repeat Start | Repeat End | Repeat Left | Name | Family | Class |
| 3358203 | 3358423 | - | 24.6 | 6147 | 6345 | -14 | L1MD2 | L1 | LINE |
| 3358450 | 3359588 | - | 23.5 | 4943 | 6146 | -213 | L1MD2 | L1 | LINE |
| 3359653 | 3360060 | - | 16.2 | 5767 | 6174 | -9 | L1MB3 | L1 | LINE |
| 3360066 | 3360163 | + | 19.4 | 5652 | 5749 | -422 | L1MB3 | L1 | LINE |
| 3360178 | 3360594 | - | 25.8 | 4481 | 4912 | -1234 | L1MD2 | L1 | LINE |

  

|  |  |  |  |  |  |  |  |  |  |
| --- | --- | --- | --- | --- | --- | --- | --- | --- | --- |
| chr10:4927141-4933492 | | | 6292 bp | | |  |  |  |  |
| Genome Start | Genome End | Strand | Div | Repeat Start | Repeat End | Repeat Left | Name | Family | Class |
| 4927141 | 4928110 | - | 8.9 | 6757 | 7719 | -4 | L1MA2 | L1 | LINE |
| 4928105 | 4929091 | - | 4.9 | 3703 | 4702 | -1444 | L1PA4 | L1 | LINE |
| 4929090 | 4930529 | + | 3.6 | 4714 | 6151 | -4 | L1PA4 | L1 | LINE |
| 4930580 | 4931209 | - | 9.6 | 6143 | 6772 | -793 | L1MA2 | L1 | LINE |
| 4931208 | 4932563 | + | 6.6 | 4744 | 6103 | -51 | L1PA6 | L1 | LINE |
| 4932583 | 4932655 | - | 8.2 | 6085 | 6157 | -1408 | L1MA2 | L1 | LINE |
| 4932657 | 4933492 | - | 10.1 | 2504 | 3333 | -4232 | L1MA2 | L1 | LINE |

  

|  |  |  |  |  |  |  |  |  |  |
| --- | --- | --- | --- | --- | --- | --- | --- | --- | --- |
| chr10:5175989-5178710 | | | 2712 bp | | |  |  |  |  |
| Genome Start | Genome End | Strand | Div | Repeat Start | Repeat End | Repeat Left | Name | Family | Class |
| 5175989 | 5176259 | + | 28.5 | 1 | 278 | -5436 | ERVL-B3 | ERVL | LTR |
| 5176260 | 5177382 | - | 2.6 | 3833 | 4959 | -1187 | L1PA3 | L1 | LINE |
| 5177381 | 5178552 | + | 2.5 | 4984 | 6155 | 0 | L1PA3 | L1 | LINE |
| 5178565 | 5178710 | + | 30.1 | 303 | 458 | -5299 | ERVL-B3 | ERVL | LTR |

  

|  |  |  |  |  |  |  |  |  |  |
| --- | --- | --- | --- | --- | --- | --- | --- | --- | --- |
| chr10:9450864-9455332 | | | 4424 bp | | |  |  |  |  |
| Genome Start | Genome End | Strand | Div | Repeat Start | Repeat End | Repeat Left | Name | Family | Class |
| 9450864 | 9451969 | - | 26.2 | 4921 | 5958 | -2428 | L1MD | L1 | LINE |
| 9451991 | 9452873 | - | 27.0 | 3684 | 4529 | -1617 | L1M5 | L1 | LINE |
| 9452888 | 9453730 | - | 2.4 | 5310 | 6152 | -3 | L1PA3 | L1 | LINE |
| 9453729 | 9454906 | + | 2.6 | 4125 | 5302 | -844 | L1PA3 | L1 | LINE |
| 9454907 | 9455078 | - | 24.9 | 3520 | 3697 | -2449 | L1MC | L1 | LINE |
| 9455091 | 9455332 | - | 24.2 | 1511 | 1746 | -4887 | L1MD | L1 | LINE |

  

|  |  |  |  |  |  |  |  |  |  |
| --- | --- | --- | --- | --- | --- | --- | --- | --- | --- |
| chr10:9918156-9925770 | | | 7359 bp | | |  |  |  |  |
| Genome Start | Genome End | Strand | Div | Repeat Start | Repeat End | Repeat Left | Name | Family | Class |
| 9918156 | 9918548 | + | 33.9 | 1857 | 2271 | -1148 | L2 | L2 | LINE |
| 9918632 | 9918973 | + | 20.3 | 90 | 437 | -30 | MLT1C | MaLR | LTR |
| 9918974 | 9920567 | + | 15.5 | 3 | 1619 | -799 | Tigger1 | MER2\_type | DNA |
| 9920568 | 9921007 | + | 8.2 | 5362 | 5790 | -361 | L1PB1 | L1 | LINE |
| 9921037 | 9921404 | + | 8.2 | 5791 | 6151 | 0 | L1PB1 | L1 | LINE |
| 9921451 | 9921617 | + | 16.8 | 1621 | 1788 | -630 | Tigger1 | MER2\_type | DNA |
| 9921618 | 9922531 | - | 2.6 | 5062 | 5974 | -181 | L1PA4 | L1 | LINE |
| 9922532 | 9922699 | + | 3.0 | 5982 | 6152 | -3 | L1PA4 | L1 | LINE |
| 9922751 | 9923155 | + | 7.7 | 5769 | 6181 | 0 | L1PB1 | L1 | LINE |
| 9923156 | 9923776 | + | 15.4 | 1778 | 2418 | 0 | Tigger1 | MER2\_type | DNA |
| 9923824 | 9924358 | + | 33.7 | 2332 | 2906 | -513 | L2 | L2 | LINE |
| 9924359 | 9924568 | + | 21.8 | 1 | 219 | 0 | MER20 | MER1\_type | DNA |
| 9924569 | 9924886 | + | 32.4 | 2907 | 3262 | -157 | L2 | L2 | LINE |
| 9924887 | 9925121 | + | 13.7 | 1 | 1731 | 0 | Tigger2a | MER2\_type | DNA |
| 9925122 | 9925433 | - | 12.5 | 1 | 312 | 0 | AluSx | Alu | SINE |
| 9925434 | 9925657 | + | 13.7 | 195 | 434 | 0 | Tigger2a | MER2\_type | DNA |
| 9925658 | 9925770 | + | 32.4 | 3263 | 3363 | -56 | L2 | L2 | LINE |

  

|  |  |  |  |  |  |  |  |  |  |
| --- | --- | --- | --- | --- | --- | --- | --- | --- | --- |
| chr10:15853965-15859156 | | | 5127 bp | | |  |  |  |  |
| Genome Start | Genome End | Strand | Div | Repeat Start | Repeat End | Repeat Left | Name | Family | Class |
| 15853965 | 15854386 | - | 20.4 | 3953 | 4422 | -1724 | L1M5 | L1 | LINE |
| 15854415 | 15854923 | - | 3.0 | 5646 | 6153 | -2 | L1PA3 | L1 | LINE |
| 15854924 | 15856863 | + | 2.8 | 3697 | 5644 | -511 | L1PA3 | L1 | LINE |
| 15856887 | 15857616 | - | 21.0 | 3132 | 3928 | -2218 | L1M5 | L1 | LINE |
| 15857624 | 15857955 | - | 18.6 | 5782 | 6119 | -65 | L1MB7 | L1 | LINE |
| 15857958 | 15858042 | - | 16.5 | 2320 | 2404 | 0 | Tigger5 | MER2\_type | DNA |
| 15858043 | 15858109 | - | 15.6 | 2 | 65 | -32 | Tigger5c | MER2\_type | DNA |
| 15858118 | 15858897 | - | 18.0 | 4928 | 5775 | -403 | L1MB7 | L1 | LINE |
| 15858895 | 15859156 | - | 25.0 | 2826 | 3112 | -3034 | L1M5 | L1 | LINE |

  

|  |  |  |  |  |  |  |  |  |  |
| --- | --- | --- | --- | --- | --- | --- | --- | --- | --- |
| chr10:16208610-16213022 | | | 4257 bp | | |  |  |  |  |
| Genome Start | Genome End | Strand | Div | Repeat Start | Repeat End | Repeat Left | Name | Family | Class |
| 16208610 | 16208733 | + | 39.8 | 2886 | 3014 | -1475 | L3 | CR1 | LINE |
| 16208816 | 16209122 | + | 13.2 | 1 | 306 | -4 | AluSg | Alu | SINE |
| 16209197 | 16212052 | - | 3.1 | 3296 | 6152 | -3 | L1PA3 | L1 | LINE |
| 16212052 | 16212936 | + | 3.2 | 2398 | 3282 | -2864 | L1PA3 | L1 | LINE |
| 16212938 | 16213022 | + | 22.2 | 3171 | 3260 | -1229 | L3 | CR1 | LINE |

  

|  |  |  |  |  |  |  |  |  |  |
| --- | --- | --- | --- | --- | --- | --- | --- | --- | --- |
| chr10:16295394-16304149 | | | 8500 bp | | |  |  |  |  |
| Genome Start | Genome End | Strand | Div | Repeat Start | Repeat End | Repeat Left | Name | Family | Class |
| 16295394 | 16295586 | - | 31.8 | 5449 | 5654 | -586 | L1M5 | L1 | LINE |
| 16295643 | 16296128 | - | 33.5 | 4603 | 5108 | -1038 | L1M5 | L1 | LINE |
| 16296131 | 16296597 | + | 22.1 | 1 | 505 | 0 | MLT1D | MaLR | LTR |
| 16296599 | 16297831 | + | 25.4 | 2 | 1192 | -179 | MLT1D-int | MaLR | LTR |
| 16297830 | 16297877 | - | 22.9 | 656 | 703 | -668 | MLT1-int | MaLR | LTR |
| 16297885 | 16297931 | + | 16.4 | 1187 | 1233 | -138 | MLT1D-int | MaLR | LTR |
| 16297932 | 16298232 | - | 12.6 | 1 | 303 | -9 | AluSx | Alu | SINE |
| 16298233 | 16298263 | + | 16.4 | 1234 | 1262 | -109 | MLT1D-int | MaLR | LTR |
| 16298286 | 16300520 | - | 5.0 | 3899 | 6141 | -13 | L1PA5 | L1 | LINE |
| 16300522 | 16301811 | + | 5.7 | 2594 | 3881 | -2265 | L1PA5 | L1 | LINE |
| 16301842 | 16301959 | + | 22.9 | 1368 | 1488 | -1 | MLT1D-int | MaLR | LTR |
| 16301961 | 16302412 | + | 25.5 | 1 | 486 | -19 | MLT1D | MaLR | LTR |
| 16302433 | 16303074 | - | 27.2 | 3877 | 4597 | -1549 | L1M5 | L1 | LINE |
| 16303097 | 16303352 | - | 27.6 | 993 | 1254 | -4865 | L1MCa | L1 | LINE |
| 16303449 | 16303607 | - | 32.7 | 3091 | 3233 | -2913 | L1M5 | L1 | LINE |
| 16303608 | 16303921 | - | 10.4 | 1 | 311 | 0 | AluY | Alu | SINE |
| 16303922 | 16304149 | - | 32.7 | 2819 | 3090 | -3056 | L1M5 | L1 | LINE |

  

|  |  |  |  |  |  |  |  |  |  |
| --- | --- | --- | --- | --- | --- | --- | --- | --- | --- |
| chr10:18461230-18462891 | | | 1635 bp | | |  |  |  |  |
| Genome Start | Genome End | Strand | Div | Repeat Start | Repeat End | Repeat Left | Name | Family | Class |
| 18461230 | 18461738 | + | 22.6 | 1410 | 1999 | -4401 | L1MEc | L1 | LINE |
| 18461768 | 18462041 | - | 7.3 | 5897 | 6171 | -1 | L1PA8 | L1 | LINE |
| 18462045 | 18462241 | + | 6.1 | 5682 | 5878 | -294 | L1PA8 | L1 | LINE |
| 18462237 | 18462891 | + | 25.7 | 2027 | 2604 | -3615 | L1MEc | L1 | LINE |

  

|  |  |  |  |  |  |  |  |  |  |
| --- | --- | --- | --- | --- | --- | --- | --- | --- | --- |
| chr10:19316607-19320249 | | | 3642 bp | | |  |  |  |  |
| Genome Start | Genome End | Strand | Div | Repeat Start | Repeat End | Repeat Left | Name | Family | Class |
| 19316607 | 19318076 | - | 11.7 | 113 | 1545 | -35 | THE1B-int | MaLR | LTR |
| 19318080 | 19318441 | - | 4.5 | 5787 | 6139 | -15 | L1PA5 | L1 | LINE |
| 19318442 | 19318752 | - | 8.0 | 1 | 311 | 0 | AluY | Alu | SINE |
| 19318753 | 19319411 | - | 5.0 | 5127 | 5786 | -368 | L1PA5 | L1 | LINE |
| 19319410 | 19320118 | + | 7.4 | 4437 | 5124 | -1022 | L1PA5 | L1 | LINE |
| 19320119 | 19320249 | - | 11.4 | 1 | 131 | -1449 | THE1B-int | MaLR | LTR |

  

|  |  |  |  |  |  |  |  |  |  |
| --- | --- | --- | --- | --- | --- | --- | --- | --- | --- |
| chr10:20747723-20757369 | | | 9604 bp | | |  |  |  |  |
| Genome Start | Genome End | Strand | Div | Repeat Start | Repeat End | Repeat Left | Name | Family | Class |
| 20747723 | 20748423 | + | 20.3 | 4594 | 5296 | -850 | L1MC | L1 | LINE |
| 20748422 | 20749291 | - | 5.3 | 4235 | 5098 | -1048 | L1PA5 | L1 | LINE |
| 20749292 | 20750337 | + | 4.4 | 5106 | 6152 | -2 | L1PA5 | L1 | LINE |
| 20750372 | 20752288 | - | 15.7 | 4248 | 6163 | -2 | L1PA15 | L1 | LINE |
| 20752289 | 20752584 | - | 7.4 | 1 | 300 | -11 | AluY | Alu | SINE |
| 20752585 | 20756969 | - | 18.9 | 1 | 4372 | -1899 | L1P4e | L1 | LINE |
| 20756981 | 20757251 | + | 18.9 | 5311 | 5581 | -763 | L1MC | L1 | LINE |
| 20757252 | 20757351 | + | 20.0 | 6242 | 6344 | 0 | L1MC2 | L1 | LINE |
| 20757352 | 20757369 | + | 18.9 | 5582 | 5601 | -743 | L1MC | L1 | LINE |

  

|  |  |  |  |  |  |  |  |  |  |
| --- | --- | --- | --- | --- | --- | --- | --- | --- | --- |
| chr10:22440815-22446289 | | | 4860 bp | | |  |  |  |  |
| Genome Start | Genome End | Strand | Div | Repeat Start | Repeat End | Repeat Left | Name | Family | Class |
| 22440815 | 22441042 | + | 37.9 | 816 | 1049 | -911 | L4 | RTE | LINE |
| 22441158 | 22441269 | + | 20.9 | 109 | 215 | -47 | MIR | MIR | SINE |
| 22441270 | 22441554 | + | 17.6 | 1 | 279 | -33 | AluJo | Alu | SINE |
| 22441682 | 22441828 | + | 36.7 | 1278 | 1442 | -518 | L4 | RTE | LINE |
| 22441903 | 22442204 | - | 7.3 | 1 | 302 | -9 | AluY | Alu | SINE |
| 22442274 | 22442666 | - | 8.3 | 5792 | 6146 | -5 | L1PB1 | L1 | LINE |
| 22442696 | 22444358 | - | 7.2 | 4116 | 5791 | -360 | L1PB1 | L1 | LINE |
| 22444353 | 22445123 | + | 7.9 | 3350 | 4120 | -2026 | L1PB1 | L1 | LINE |
| 22445143 | 22445424 | - | 17.5 | 1 | 291 | -21 | AluJb | Alu | SINE |
| 22445560 | 22446014 | - | 36.2 | 1303 | 1759 | -201 | L4 | RTE | LINE |
| 22446068 | 22446289 | + | 33.9 | 1436 | 1696 | -264 | L4 | RTE | LINE |

  

|  |  |  |  |  |  |  |  |  |  |
| --- | --- | --- | --- | --- | --- | --- | --- | --- | --- |
| chr10:24312652-24314490 | | | 1830 bp | | |  |  |  |  |
| Genome Start | Genome End | Strand | Div | Repeat Start | Repeat End | Repeat Left | Name | Family | Class |
| 24312652 | 24313327 | - | 26.8 | 794 | 1471 | -1 | MLT1H-int | MaLR | LTR |
| 24313328 | 24313379 | - | 3.9 | 5614 | 5665 | -507 | L1PA8 | L1 | LINE |
| 24313380 | 24313873 | + | 5.9 | 5674 | 6171 | -1 | L1PA8 | L1 | LINE |
| 24313883 | 24314490 | - | 30.6 | 198 | 790 | -682 | MLT1H-int | MaLR | LTR |

  

|  |  |  |  |  |  |  |  |  |  |
| --- | --- | --- | --- | --- | --- | --- | --- | --- | --- |
| chr10:25336961-25342955 | | | 5713 bp | | |  |  |  |  |
| Genome Start | Genome End | Strand | Div | Repeat Start | Repeat End | Repeat Left | Name | Family | Class |
| 25336961 | 25337544 | + | 32.8 | 1921 | 2513 | -1976 | L3 | CR1 | LINE |
| 25337584 | 25338200 | + | 3.4 | 5531 | 6152 | -3 | L1PA4 | L1 | LINE |
| 25338366 | 25338529 | + | 32.9 | 3312 | 3516 | -973 | L3 | CR1 | LINE |
| 25338551 | 25338906 | + | 11.8 | 1 | 362 | -2 | THE1B | MaLR | LTR |
| 25338909 | 25339338 | + | 9.5 | 1 | 430 | -1150 | THE1B-int | MaLR | LTR |
| 25339379 | 25340388 | - | 2.5 | 5144 | 6155 | 0 | L1PA3 | L1 | LINE |
| 25340389 | 25340669 | + | 4.6 | 4862 | 5142 | -1004 | L1PA3 | L1 | LINE |
| 25340679 | 25341819 | + | 11.4 | 427 | 1580 | 0 | THE1B-int | MaLR | LTR |
| 25341820 | 25342186 | + | 10.5 | 1 | 364 | 0 | THE1B | MaLR | LTR |
| 25342193 | 25342595 | + | 28.2 | 3564 | 4056 | -433 | L3 | CR1 | LINE |
| 25342596 | 25342902 | + | 11.5 | 1 | 306 | -7 | AluSq | Alu | SINE |
| 25342903 | 25342955 | + | 28.2 | 4057 | 4097 | -392 | L3 | CR1 | LINE |

  

|  |  |  |  |  |  |  |  |  |  |
| --- | --- | --- | --- | --- | --- | --- | --- | --- | --- |
| chr10:26516230-26518315 | | | 2085 bp | | |  |  |  |  |
| Genome Start | Genome End | Strand | Div | Repeat Start | Repeat End | Repeat Left | Name | Family | Class |
| 26516230 | 26516423 | - | 13.4 | 183 | 390 | 0 | MLT1B | MaLR | LTR |
| 26516426 | 26518002 | - | 3.5 | 4574 | 6155 | 0 | L1PA4 | L1 | LINE |
| 26518002 | 26518125 | + | 4.8 | 4463 | 4586 | -1560 | L1PA4 | L1 | LINE |
| 26518126 | 26518315 | - | 11.6 | 1 | 195 | -195 | MLT1B | MaLR | LTR |

  

|  |  |  |  |  |  |  |  |  |  |
| --- | --- | --- | --- | --- | --- | --- | --- | --- | --- |
| chr10:26933565-26934483 | | | 905 bp | | |  |  |  |  |
| Genome Start | Genome End | Strand | Div | Repeat Start | Repeat End | Repeat Left | Name | Family | Class |
| 26933565 | 26933846 | + | 12.4 | 5541 | 5833 | -333 | L1PA16 | L1 | LINE |
| 26933842 | 26934064 | - | 4.0 | 5854 | 6076 | -79 | L1PA4 | L1 | LINE |
| 26934065 | 26934145 | + | 3.7 | 6067 | 6147 | -8 | L1PA4 | L1 | LINE |
| 26934165 | 26934483 | + | 11.3 | 5814 | 6154 | -12 | L1PA16 | L1 | LINE |

  

|  |  |  |  |  |  |  |  |  |  |
| --- | --- | --- | --- | --- | --- | --- | --- | --- | --- |
| chr10:26962443-26964241 | | | 1654 bp | | |  |  |  |  |
| Genome Start | Genome End | Strand | Div | Repeat Start | Repeat End | Repeat Left | Name | Family | Class |
| 26962443 | 26962674 | + | 25.0 | 7382 | 7582 | -379 | L1MC5 | L1 | LINE |
| 26962675 | 26962957 | + | 13.8 | 1 | 283 | -26 | AluSg1 | Alu | SINE |
| 26962958 | 26962981 | + | 25.0 | 7583 | 7606 | -355 | L1MC5 | L1 | LINE |
| 26963065 | 26963231 | - | 5.4 | 5305 | 5471 | -684 | L1PA4 | L1 | LINE |
| 26963232 | 26963912 | + | 4.1 | 5474 | 6154 | -1 | L1PA4 | L1 | LINE |
| 26963975 | 26964241 | + | 23.8 | 7701 | 7925 | -36 | L1MC5 | L1 | LINE |

  

|  |  |  |  |  |  |  |  |  |  |
| --- | --- | --- | --- | --- | --- | --- | --- | --- | --- |
| chr10:27820815-27826439 | | | 5468 bp | | |  |  |  |  |
| Genome Start | Genome End | Strand | Div | Repeat Start | Repeat End | Repeat Left | Name | Family | Class |
| 27820815 | 27820957 | + | 25.9 | 760 | 930 | -5617 | L1MD2 | L1 | LINE |
| 27820984 | 27821159 | + | 22.3 | 996 | 1176 | -5464 | L1MD2 | L1 | LINE |
| 27821160 | 27821449 | - | 18.7 | 239 | 510 | 0 | LTR54 | ERV1 | LTR |
| 27821456 | 27822155 | - | 14.0 | 5594 | 6299 | -4 | L1MA4A | L1 | LINE |
| 27822156 | 27822307 | + | 15.4 | 4247 | 4396 | -1747 | L1M1 | L1 | LINE |
| 27822332 | 27823476 | + | 14.3 | 4397 | 5477 | -826 | L1M1 | L1 | LINE |
| 27823498 | 27823630 | + | 9.8 | 5478 | 5578 | -725 | L1M1 | L1 | LINE |
| 27823681 | 27823909 | - | 20.3 | 1 | 239 | -271 | LTR54 | ERV1 | LTR |
| 27823928 | 27824049 | + | 18.0 | 10 | 131 | -54 | FAM | Alu | SINE |
| 27824052 | 27824836 | + | 24.4 | 1314 | 2182 | -4458 | L1MD2 | L1 | LINE |
| 27824837 | 27825533 | + | 10.5 | 1 | 691 | 0 | LTR8 | ERV1 | LTR |
| 27825534 | 27825610 | + | 24.4 | 2183 | 2255 | -4385 | L1MD2 | L1 | LINE |
| 27825621 | 27826439 | + | 19.3 | 3341 | 4080 | -2464 | L1MD2 | L1 | LINE |

  

|  |  |  |  |  |  |  |  |  |  |
| --- | --- | --- | --- | --- | --- | --- | --- | --- | --- |
| chr10:30521900-30526073 | | | 4208 bp | | |  |  |  |  |
| Genome Start | Genome End | Strand | Div | Repeat Start | Repeat End | Repeat Left | Name | Family | Class |
| 30521900 | 30524090 | + | 12.9 | 1 | 2226 | -192 | Tigger1 | MER2\_type | DNA |
| 30524091 | 30524379 | - | 14.5 | 3 | 294 | -18 | AluSx | Alu | SINE |
| 30524380 | 30524396 | + | 12.9 | 2227 | 2233 | -185 | Tigger1 | MER2\_type | DNA |
| 30524397 | 30524622 | - | 8.9 | 5927 | 6151 | 0 | L1PB1 | L1 | LINE |
| 30524623 | 30524715 | + | 12.9 | 2234 | 2315 | -103 | Tigger1 | MER2\_type | DNA |
| 30524717 | 30524902 | - | 25.0 | 5299 | 5492 | -692 | L1MB8 | L1 | LINE |
| 30524879 | 30525775 | - | 11.4 | 5264 | 6164 | -1 | L1PA15 | L1 | LINE |
| 30525765 | 30525966 | + | 5.9 | 5080 | 5282 | -864 | L1PREC2 | L1 | LINE |
| 30525967 | 30526073 | + | 13.2 | 2307 | 2418 | 0 | Tigger1 | MER2\_type | DNA |

  

|  |  |  |  |  |  |  |  |  |  |
| --- | --- | --- | --- | --- | --- | --- | --- | --- | --- |
| chr10:31067875-31070055 | | | 2061 bp | | |  |  |  |  |
| Genome Start | Genome End | Strand | Div | Repeat Start | Repeat End | Repeat Left | Name | Family | Class |
| 31067875 | 31067895 | + | 17.7 | 1 | 17 | -918 | MER21C | ERV1 | LTR |
| 31067896 | 31068031 | + | 16.9 | 1 | 127 | -185 | AluJb | Alu | SINE |
| 31068032 | 31068311 | + | 8.9 | 1 | 281 | -32 | AluSp | Alu | SINE |
| 31068418 | 31068598 | + | 16.9 | 128 | 295 | -17 | AluJb | Alu | SINE |
| 31068599 | 31068848 | + | 19.7 | 18 | 375 | -560 | MER21C | ERV1 | LTR |
| 31068849 | 31069143 | + | 8.5 | 1 | 296 | -13 | AluSc | Alu | SINE |
| 31069144 | 31069318 | + | 20.9 | 376 | 541 | -394 | MER21C | ERV1 | LTR |
| 31069324 | 31069481 | + | 17.8 | 658 | 828 | -107 | MER21C | ERV1 | LTR |
| 31069491 | 31069769 | - | 10.0 | 5874 | 6153 | -15 | L1PA12 | L1 | LINE |
| 31069770 | 31069975 | + | 5.3 | 5684 | 5889 | -274 | L1PA12 | L1 | LINE |
| 31069976 | 31070055 | + | 19.2 | 814 | 895 | -40 | MER21C | ERV1 | LTR |

  

|  |  |  |  |  |  |  |  |  |  |
| --- | --- | --- | --- | --- | --- | --- | --- | --- | --- |
| chr10:31273546-31275977 | | | 2323 bp | | |  |  |  |  |
| Genome Start | Genome End | Strand | Div | Repeat Start | Repeat End | Repeat Left | Name | Family | Class |
| 31273546 | 31273646 | - | 23.8 | 3478 | 3582 | -2637 | L1MEc | L1 | LINE |
| 31273734 | 31273966 | - | 20.6 | 3226 | 3485 | -2734 | L1MEc | L1 | LINE |
| 31273994 | 31274342 | - | 25.9 | 2345 | 2917 | -3630 | L1MEc | L1 | LINE |
| 31274342 | 31275106 | - | 1.7 | 5386 | 6155 | 0 | L1PA2 | L1 | LINE |
| 31275101 | 31275524 | + | 3.1 | 4963 | 5386 | -760 | L1PA2 | L1 | LINE |
| 31275527 | 31275977 | - | 23.9 | 1991 | 2456 | -3944 | L1MEc | L1 | LINE |

  

|  |  |  |  |  |  |  |  |  |  |
| --- | --- | --- | --- | --- | --- | --- | --- | --- | --- |
| chr10:31734645-31740019 | | | 5358 bp | | |  |  |  |  |
| Genome Start | Genome End | Strand | Div | Repeat Start | Repeat End | Repeat Left | Name | Family | Class |
| 31734645 | 31736000 | - | 6.3 | 4783 | 6153 | -1 | L1PA7 | L1 | LINE |
| 31736019 | 31736568 | - | 1.9 | 5617 | 6166 | 0 | L1PA3 | L1 | LINE |
| 31736569 | 31738301 | + | 2.1 | 3880 | 5612 | -543 | L1PA3 | L1 | LINE |
| 31738301 | 31740019 | - | 8.1 | 3062 | 4802 | -1344 | L1PA7 | L1 | LINE |

  

|  |  |  |  |  |  |  |  |  |  |
| --- | --- | --- | --- | --- | --- | --- | --- | --- | --- |
| chr10:32129871-32131345 | | | 1467 bp | | |  |  |  |  |
| Genome Start | Genome End | Strand | Div | Repeat Start | Repeat End | Repeat Left | Name | Family | Class |
| 32129871 | 32129952 | + | 32.1 | 2 | 82 | -2530 | Charlie5 | MER1\_type | DNA |
| 32129953 | 32130213 | - | 6.3 | 37 | 290 | -21 | AluY | Alu | SINE |
| 32130253 | 32130867 | - | 15.6 | 5047 | 5664 | -519 | L1MB3 | L1 | LINE |
| 32130836 | 32131298 | + | 14.1 | 5666 | 6176 | -7 | L1MB3 | L1 | LINE |
| 32131300 | 32131345 | + | 15.6 | 163 | 207 | -2405 | Charlie5 | MER1\_type | DNA |

  

|  |  |  |  |  |  |  |  |  |  |
| --- | --- | --- | --- | --- | --- | --- | --- | --- | --- |
| chr10:32974203-32991375 | | | 17216 bp | | |  |  |  |  |
| Genome Start | Genome End | Strand | Div | Repeat Start | Repeat End | Repeat Left | Name | Family | Class |
| 32974203 | 32975516 | - | 16.1 | 4004 | 5323 | -2239 | L1M1 | L1 | LINE |
| 32975511 | 32976158 | - | 14.8 | 5843 | 6502 | -1 | L1PA16 | L1 | LINE |
| 32976159 | 32976420 | + | 16.7 | 1 | 257 | -55 | AluSx | Alu | SINE |
| 32976421 | 32977378 | - | 14.4 | 4874 | 5842 | -661 | L1PA16 | L1 | LINE |
| 32977380 | 32977792 | - | 9.3 | 5308 | 5717 | -457 | L1PA11 | L1 | LINE |
| 32977779 | 32977793 | - | 21.9 | 2171 | 2185 | -4318 | L1PA16 | L1 | LINE |
| 32977794 | 32978268 | + | 11.6 | 5694 | 6174 | 0 | L1PA11 | L1 | LINE |
| 32978269 | 32980405 | - | 21.9 | 9 | 2170 | -4333 | L1PA16 | L1 | LINE |
| 32980411 | 32980892 | - | 9.8 | 1 | 486 | 0 | LTR19A | ERV1 | LTR |
| 32980905 | 32982081 | - | 20.4 | 2804 | 4022 | -3540 | L1M1 | L1 | LINE |
| 32982096 | 32982557 | - | 26.2 | 21 | 496 | -7069 | L1M1 | L1 | LINE |
| 32982713 | 32984206 | - | 24.7 | 193 | 1703 | -4839 | L1M4 | L1 | LINE |
| 32984243 | 32984456 | - | 18.3 | 5364 | 5573 | -739 | L1M2 | L1 | LINE |
| 32984457 | 32984780 | - | 15.1 | 1 | 304 | -8 | AluSx | Alu | SINE |
| 32984781 | 32985099 | - | 17.8 | 5021 | 5366 | -946 | L1M | L1 | LINE |
| 32985109 | 32985279 | + | 15.6 | 5579 | 5749 | -563 | L1MA9 | L1 | LINE |
| 32985280 | 32985645 | - | 11.2 | 1 | 375 | 0 | THE1C | MaLR | LTR |
| 32985646 | 32987170 | - | 30.1 | 2 | 1735 | 0 | MLT1H-int | MaLR | LTR |
| 32986907 | 32987172 | - | 9.8 | 1 | 269 | -1311 | THE1C-int | MaLR | LTR |
| 32987173 | 32987544 | - | 14.3 | 1 | 375 | 0 | THE1C | MaLR | LTR |
| 32987545 | 32987707 | + | 15.6 | 5750 | 5911 | -401 | L1MA9 | L1 | LINE |
| 32987707 | 32988391 | - | 16.4 | 2722 | 3425 | -2715 | L1M3 | L1 | LINE |
| 32988394 | 32988508 | - | 14.8 | 2300 | 2417 | -1 | Tigger1 | MER2\_type | DNA |
| 32988513 | 32988964 | + | 11.2 | 5254 | 5698 | -465 | L1P4 | L1 | LINE |
| 32988965 | 32989049 | - | 20.0 | 1061 | 1146 | -6419 | L1M1 | L1 | LINE |
| 32989060 | 32989623 | - | 1.4 | 5591 | 6154 | -1 | L1PA3 | L1 | LINE |
| 32989624 | 32990356 | + | 1.5 | 4856 | 5592 | -563 | L1PA3 | L1 | LINE |
| 32990351 | 32991375 | - | 18.4 | 19 | 1078 | -6487 | L1M1 | L1 | LINE |

  

|  |  |  |  |  |  |  |  |  |  |
| --- | --- | --- | --- | --- | --- | --- | --- | --- | --- |
| chr10:33326894-33330782 | | | 3864 bp | | |  |  |  |  |
| Genome Start | Genome End | Strand | Div | Repeat Start | Repeat End | Repeat Left | Name | Family | Class |
| 33326894 | 33327219 | - | 17.9 | 78 | 408 | 0 | MLT1A1 | MaLR | LTR |
| 33327245 | 33327548 | - | 12.5 | 1 | 304 | -9 | AluSp | Alu | SINE |
| 33327555 | 33327838 | - | 15.2 | 5874 | 6155 | -11 | L1PA16 | L1 | LINE |
| 33327839 | 33330183 | - | 10.4 | 3268 | 5658 | -508 | L1PA16 | L1 | LINE |
| 33330178 | 33330703 | + | 11.8 | 2718 | 3260 | -2886 | L1PA16 | L1 | LINE |
| 33330704 | 33330782 | - | 17.7 | 6 | 88 | -320 | MLT1A1 | MaLR | LTR |

  

|  |  |  |  |  |  |  |  |  |  |
| --- | --- | --- | --- | --- | --- | --- | --- | --- | --- |
| chr10:33449096-33450679 | | | 1508 bp | | |  |  |  |  |
| Genome Start | Genome End | Strand | Div | Repeat Start | Repeat End | Repeat Left | Name | Family | Class |
| 33449096 | 33449460 | - | 24.4 | 195 | 589 | -1 | MLT1G | MaLR | LTR |
| 33449537 | 33450387 | - | 18.4 | 5498 | 6334 | 0 | L1MA10 | L1 | LINE |
| 33450388 | 33450499 | + | 14.3 | 5370 | 5481 | -831 | L1M2 | L1 | LINE |
| 33450500 | 33450679 | - | 32.4 | 9 | 191 | -364 | MLT1G | MaLR | LTR |

  

|  |  |  |  |  |  |  |  |  |  |
| --- | --- | --- | --- | --- | --- | --- | --- | --- | --- |
| chr10:36144307-36146607 | | | 2307 bp | | |  |  |  |  |
| Genome Start | Genome End | Strand | Div | Repeat Start | Repeat End | Repeat Left | Name | Family | Class |
| 36144307 | 36144357 | - | 5.9 | 25 | 75 | -5 | MADE1 | Mariner | DNA |
| 36144358 | 36144858 | - | 4.8 | 3941 | 4442 | -1704 | L1PA5 | L1 | LINE |
| 36144853 | 36146564 | + | 4.3 | 4439 | 6152 | -2 | L1PA5 | L1 | LINE |
| 36146565 | 36146607 | - | 4.7 | 1 | 43 | -37 | MADE1 | Mariner | DNA |

  

|  |  |  |  |  |  |  |  |  |  |
| --- | --- | --- | --- | --- | --- | --- | --- | --- | --- |
| chr10:45972987-45976427 | | | 3402 bp | | |  |  |  |  |
| Genome Start | Genome End | Strand | Div | Repeat Start | Repeat End | Repeat Left | Name | Family | Class |
| 45972987 | 45973330 | + | 21.1 | 15 | 396 | -2365 | Charlie10 | MER1\_type | DNA |
| 45973341 | 45973620 | - | 16.9 | 11 | 295 | -17 | AluJo | Alu | SINE |
| 45973631 | 45974896 | - | 17.8 | 5153 | 6353 | -6 | L1MD2 | L1 | LINE |
| 45974896 | 45975993 | + | 20.1 | 4056 | 5164 | -982 | L1MD2 | L1 | LINE |
| 45976014 | 45976427 | + | 23.2 | 399 | 838 | -1923 | Charlie10 | MER1\_type | DNA |

  

|  |  |  |  |  |  |  |  |  |  |
| --- | --- | --- | --- | --- | --- | --- | --- | --- | --- |
| chr10:48084029-48087095 | | | 3016 bp | | |  |  |  |  |
| Genome Start | Genome End | Strand | Div | Repeat Start | Repeat End | Repeat Left | Name | Family | Class |
| 48084029 | 48084321 | - | 25.6 | 1245 | 1530 | -977 | HAL1 | L1 | LINE |
| 48084336 | 48085027 | - | 15.2 | 5414 | 6159 | -7 | L1PA16 | L1 | LINE |
| 48085028 | 48085429 | + | 12.2 | 3991 | 4393 | -1753 | L1P4 | L1 | LINE |
| 48085465 | 48086147 | + | 12.2 | 4394 | 5118 | -1028 | L1P4 | L1 | LINE |
| 48086148 | 48086461 | + | 13.4 | 1 | 313 | 0 | AluSq | Alu | SINE |
| 48086462 | 48086757 | + | 12.2 | 5119 | 5393 | -753 | L1P4 | L1 | LINE |
| 48086760 | 48087095 | - | 29.0 | 807 | 1167 | -1340 | HAL1 | L1 | LINE |

  

|  |  |  |  |  |  |  |  |  |  |
| --- | --- | --- | --- | --- | --- | --- | --- | --- | --- |
| chr10:48187823-48190592 | | | 2472 bp | | |  |  |  |  |
| Genome Start | Genome End | Strand | Div | Repeat Start | Repeat End | Repeat Left | Name | Family | Class |
| 48187823 | 48188308 | + | 42.1 | 501 | 1045 | -915 | L4 | RTE | LINE |
| 48188309 | 48189376 | + | 8.4 | 1 | 1232 | -4 | MER11B | ERVK | LTR |
| 48189377 | 48189595 | + | 42.1 | 1046 | 1279 | -681 | L4 | RTE | LINE |
| 48189844 | 48189917 | - | 21.6 | 6077 | 6152 | -139 | L1MA7 | L1 | LINE |
| 48189918 | 48189986 | + | 15.1 | 6147 | 6221 | -70 | L1MA7 | L1 | LINE |
| 48189987 | 48190417 | - | 16.3 | 3 | 434 | 0 | Tigger2a | MER2\_type | DNA |
| 48190418 | 48190491 | + | 15.1 | 6222 | 6291 | 0 | L1MA7 | L1 | LINE |
| 48190542 | 48190592 | + | 29.4 | 1632 | 1682 | -278 | L4 | RTE | LINE |

  

|  |  |  |  |  |  |  |  |  |  |
| --- | --- | --- | --- | --- | --- | --- | --- | --- | --- |
| chr10:48359906-48363324 | | | 3366 bp | | |  |  |  |  |
| Genome Start | Genome End | Strand | Div | Repeat Start | Repeat End | Repeat Left | Name | Family | Class |
| 48359906 | 48360249 | + | 21.1 | 15 | 396 | -2365 | Charlie10 | MER1\_type | DNA |
| 48360266 | 48360518 | - | 18.4 | 11 | 289 | -23 | AluJo | Alu | SINE |
| 48360537 | 48361793 | - | 18.3 | 5153 | 6342 | -17 | L1MD2 | L1 | LINE |
| 48361793 | 48362890 | + | 20.2 | 4056 | 5164 | -982 | L1MD2 | L1 | LINE |
| 48362911 | 48363324 | + | 23.2 | 399 | 838 | -1923 | Charlie10 | MER1\_type | DNA |

  

|  |  |  |  |  |  |  |  |  |  |
| --- | --- | --- | --- | --- | --- | --- | --- | --- | --- |
| chr10:51723223-51733393 | | | 9913 bp | | |  |  |  |  |
| Genome Start | Genome End | Strand | Div | Repeat Start | Repeat End | Repeat Left | Name | Family | Class |
| 51723223 | 51723467 | + | 25.6 | 445 | 695 | -5792 | L1MCb | L1 | LINE |
| 51723676 | 51726062 | + | 17.7 | 7 | 2476 | -4373 | L1M2 | L1 | LINE |
| 51726057 | 51726508 | - | 2.3 | 4690 | 5119 | -1027 | L1PA3 | L1 | LINE |
| 51726507 | 51727549 | + | 2.8 | 5109 | 6155 | 0 | L1PA3 | L1 | LINE |
| 51727545 | 51728001 | + | 14.8 | 2220 | 3050 | -3953 | L1MCb | L1 | LINE |
| 51728002 | 51728302 | + | 6.0 | 1 | 301 | -10 | AluY | Alu | SINE |
| 51728303 | 51731419 | + | 16.6 | 3051 | 6258 | -745 | L1MCb | L1 | LINE |
| 51731459 | 51732111 | + | 13.4 | 5593 | 6299 | -4 | L1MA4A | L1 | LINE |
| 51732118 | 51732296 | + | 23.1 | 614 | 792 | -5253 | L1MCb | L1 | LINE |
| 51732315 | 51733393 | + | 23.4 | 1249 | 2681 | -4064 | L1MCb | L1 | LINE |

  

|  |  |  |  |  |  |  |  |  |  |
| --- | --- | --- | --- | --- | --- | --- | --- | --- | --- |
| chr10:52259534-52262228 | | | 2640 bp | | |  |  |  |  |
| Genome Start | Genome End | Strand | Div | Repeat Start | Repeat End | Repeat Left | Name | Family | Class |
| 52259534 | 52259599 | - | 13.4 | 104 | 178 | 0 | MER5B | MER1\_type | DNA |
| 52259600 | 52259655 | + | 30.4 | 1 | 56 | -874 | MER63 | AcHobo | DNA |
| 52259688 | 52260156 | - | 12.2 | 5791 | 6281 | -10 | L1MA8 | L1 | LINE |
| 52260157 | 52261212 | + | 15.8 | 4265 | 5328 | -815 | L1M2 | L1 | LINE |
| 52261213 | 52261512 | + | 8.7 | 1 | 301 | -12 | AluSp | Alu | SINE |
| 52261513 | 52261966 | + | 13.3 | 5329 | 5779 | -512 | L1M2 | L1 | LINE |
| 52261990 | 52262119 | + | 19.2 | 933 | 1062 | 0 | MER63 | AcHobo | DNA |
| 52262120 | 52262228 | - | 24.8 | 1 | 119 | -59 | MER5B | MER1\_type | DNA |

  

|  |  |  |  |  |  |  |  |  |  |
| --- | --- | --- | --- | --- | --- | --- | --- | --- | --- |
| chr10:52705574-52712314 | | | 6448 bp | | |  |  |  |  |
| Genome Start | Genome End | Strand | Div | Repeat Start | Repeat End | Repeat Left | Name | Family | Class |
| 52705574 | 52705707 | - | 29.9 | 3018 | 3167 | -211 | L2 | L2 | LINE |
| 52705731 | 52711159 | - | 6.0 | 618 | 6154 | 0 | L1PA5 | L1 | LINE |
| 52711142 | 52711452 | + | 11.6 | 298 | 620 | -5535 | L1PA5 | L1 | LINE |
| 52711625 | 52711793 | + | 28.5 | 1 | 177 | -1 | MER5B | MER1\_type | DNA |
| 52711910 | 52712314 | - | 33.6 | 2173 | 2586 | -833 | L2 | L2 | LINE |

  

|  |  |  |  |  |  |  |  |  |  |
| --- | --- | --- | --- | --- | --- | --- | --- | --- | --- |
| chr10:52881803-52887513 | | | 5691 bp | | |  |  |  |  |
| Genome Start | Genome End | Strand | Div | Repeat Start | Repeat End | Repeat Left | Name | Family | Class |
| 52881803 | 52882065 | - | 22.1 | 5910 | 6177 | -1 | L1MB8 | L1 | LINE |
| 52882065 | 52883520 | - | 13.4 | 2703 | 4145 | -2001 | L1PB2 | L1 | LINE |
| 52883519 | 52884269 | + | 11.5 | 4146 | 4898 | -1248 | L1PB2 | L1 | LINE |
| 52884271 | 52884639 | - | 12.8 | 1 | 364 | 0 | THE1B | MaLR | LTR |
| 52884644 | 52884796 | - | 13.7 | 1424 | 1576 | -4 | THE1B-int | MaLR | LTR |
| 52884800 | 52885739 | - | 9.7 | 1 | 899 | -681 | THE1B-int | MaLR | LTR |
| 52885740 | 52886110 | - | 13.0 | 1 | 364 | 0 | THE1B | MaLR | LTR |
| 52886111 | 52887344 | + | 11.1 | 4895 | 6146 | -5 | L1PB2 | L1 | LINE |
| 52887360 | 52887513 | - | 15.6 | 5751 | 5924 | -250 | L1MB8 | L1 | LINE |

  

|  |  |  |  |  |  |  |  |  |  |
| --- | --- | --- | --- | --- | --- | --- | --- | --- | --- |
| chr10:54034594-54038558 | | | 3959 bp | | |  |  |  |  |
| Genome Start | Genome End | Strand | Div | Repeat Start | Repeat End | Repeat Left | Name | Family | Class |
| 54034594 | 54034813 | + | 19.6 | 4679 | 4905 | -1241 | L1MB7 | L1 | LINE |
| 54034809 | 54035945 | - | 4.2 | 3919 | 5048 | -1098 | L1PA5 | L1 | LINE |
| 54035942 | 54037035 | + | 4.6 | 5052 | 6147 | -7 | L1PA5 | L1 | LINE |
| 54037051 | 54037094 | + | 15.9 | 4889 | 4932 | -1214 | L1MB7 | L1 | LINE |
| 54037095 | 54037147 | - | 1.9 | 246 | 298 | -1 | AluYc5 | Alu | SINE |
| 54037148 | 54037623 | + | 18.9 | 4921 | 5430 | -754 | L1MB7 | L1 | LINE |
| 54037624 | 54037874 | + | 8.0 | 5896 | 6150 | -4 | L1PA7 | L1 | LINE |
| 54037875 | 54038558 | + | 20.4 | 5431 | 6155 | -29 | L1MB7 | L1 | LINE |

  

|  |  |  |  |  |  |  |  |  |  |
| --- | --- | --- | --- | --- | --- | --- | --- | --- | --- |
| chr10:54510157-54519139 | | | 8985 bp | | |  |  |  |  |
| Genome Start | Genome End | Strand | Div | Repeat Start | Repeat End | Repeat Left | Name | Family | Class |
| 54510157 | 54512311 | - | 13.4 | 4662 | 6864 | -5 | L1PA16 | L1 | LINE |
| 54512312 | 54512614 | - | 11.4 | 3 | 299 | -11 | AluSg | Alu | SINE |
| 54512615 | 54513607 | - | 12.8 | 3763 | 4661 | -2188 | L1PA16 | L1 | LINE |
| 54513608 | 54513906 | - | 11.8 | 1 | 297 | -13 | AluSg | Alu | SINE |
| 54513907 | 54516095 | - | 12.8 | 1546 | 3762 | -3087 | L1PA16 | L1 | LINE |
| 54516108 | 54516880 | - | 9.1 | 5361 | 6131 | 0 | L1PA8A | L1 | LINE |
| 54516878 | 54517848 | + | 9.2 | 4369 | 5356 | -790 | L1PA8A | L1 | LINE |
| 54517838 | 54519139 | - | 21.5 | 2 | 1570 | -5283 | L1PA16 | L1 | LINE |

  

|  |  |  |  |  |  |  |  |  |  |
| --- | --- | --- | --- | --- | --- | --- | --- | --- | --- |
| chr10:56148073-56154299 | | | 6112 bp | | |  |  |  |  |
| Genome Start | Genome End | Strand | Div | Repeat Start | Repeat End | Repeat Left | Name | Family | Class |
| 56148073 | 56148447 | + | 25.6 | 690 | 1159 | -5048 | L1PB4 | L1 | LINE |
| 56148448 | 56148760 | - | 11.0 | 1 | 308 | -2 | AluSg | Alu | SINE |
| 56148761 | 56149230 | + | 25.6 | 1160 | 1643 | -4564 | L1PB4 | L1 | LINE |
| 56149278 | 56149739 | - | 10.6 | 5708 | 6171 | -1 | L1PA8 | L1 | LINE |
| 56149741 | 56150029 | + | 7.6 | 5423 | 5711 | -420 | L1PA8 | L1 | LINE |
| 56150037 | 56150405 | + | 26.0 | 1649 | 2032 | -4175 | L1PB4 | L1 | LINE |
| 56150466 | 56154299 | + | 23.4 | 2501 | 6210 | -3 | L1PB4 | L1 | LINE |

  

|  |  |  |  |  |  |  |  |  |  |
| --- | --- | --- | --- | --- | --- | --- | --- | --- | --- |
| chr10:56496909-56511100 | | | 13883 bp | | |  |  |  |  |
| Genome Start | Genome End | Strand | Div | Repeat Start | Repeat End | Repeat Left | Name | Family | Class |
| 56496909 | 56498535 | - | 17.5 | 5518 | 7168 | -197 | L1MA4A | L1 | LINE |
| 56498569 | 56498664 | - | 21.0 | 5418 | 5517 | -1688 | L1MA4A | L1 | LINE |
| 56498677 | 56498964 | - | 16.9 | 3 | 287 | -25 | AluSx | Alu | SINE |
| 56499057 | 56500749 | - | 2.2 | 4447 | 6141 | -14 | L1PA3 | L1 | LINE |
| 56500750 | 56503316 | + | 3.4 | 1873 | 4440 | -1706 | L1PA3 | L1 | LINE |
| 56503315 | 56503591 | - | 17.0 | 4531 | 4809 | -2396 | L1MA4A | L1 | LINE |
| 56503603 | 56504881 | - | 8.5 | 4753 | 6032 | -136 | L1PA10 | L1 | LINE |
| 56504869 | 56505132 | - | 19.0 | 4280 | 4558 | -2647 | L1MA4A | L1 | LINE |
| 56505133 | 56505428 | - | 12.5 | 1 | 297 | -12 | AluSc | Alu | SINE |
| 56505429 | 56506197 | - | 19.0 | 3481 | 4279 | -2926 | L1MA4A | L1 | LINE |
| 56506198 | 56506523 | - | 19.5 | 1 | 312 | 0 | AluSx | Alu | SINE |
| 56506531 | 56506827 | - | 10.4 | 6 | 303 | -7 | AluSg | Alu | SINE |
| 56506828 | 56507164 | - | 20.0 | 3083 | 3453 | -3752 | L1MA4A | L1 | LINE |
| 56507334 | 56507612 | - | 12.2 | 1 | 282 | -30 | AluSx | Alu | SINE |
| 56507613 | 56508395 | - | 22.1 | 2311 | 3082 | -4127 | L1MA4A | L1 | LINE |
| 56508396 | 56508694 | - | 12.8 | 5851 | 6151 | 0 | L1PB2 | L1 | LINE |
| 56508695 | 56509944 | - | 22.2 | 933 | 2310 | -4899 | L1MA4A | L1 | LINE |
| 56509945 | 56510244 | + | 17.6 | 1 | 339 | 0 | MER1B | MER1\_type | DNA |
| 56510245 | 56511100 | - | 31.9 | 1 | 932 | -6277 | L1MA4A | L1 | LINE |

  

|  |  |  |  |  |  |  |  |  |  |
| --- | --- | --- | --- | --- | --- | --- | --- | --- | --- |
| chr10:57230990-57239583 | | | 8603 bp | | |  |  |  |  |
| Genome Start | Genome End | Strand | Div | Repeat Start | Repeat End | Repeat Left | Name | Family | Class |
| 57230990 | 57231685 | + | 24.9 | 7 | 712 | -5895 | L1MB4 | L1 | LINE |
| 57231686 | 57232109 | - | 11.1 | 5759 | 6173 | -1 | L1PA11 | L1 | LINE |
| 57232110 | 57236990 | + | 23.0 | 713 | 5907 | -840 | L1MB4 | L1 | LINE |
| 57236998 | 57238019 | - | 10.0 | 5159 | 6151 | 0 | L1PB1 | L1 | LINE |
| 57238018 | 57238403 | + | 8.6 | 4776 | 5165 | -981 | L1PB1 | L1 | LINE |
| 57238390 | 57238552 | + | 21.2 | 5901 | 6056 | -725 | L1MB4 | L1 | LINE |
| 57238553 | 57238869 | - | 16.2 | 1 | 303 | -9 | AluSx | Alu | SINE |
| 57238870 | 57239583 | + | 21.2 | 6057 | 6760 | -21 | L1MB4 | L1 | LINE |

  

|  |  |  |  |  |  |  |  |  |  |
| --- | --- | --- | --- | --- | --- | --- | --- | --- | --- |
| chr10:57810423-57818829 | | | 8092 bp | | |  |  |  |  |
| Genome Start | Genome End | Strand | Div | Repeat Start | Repeat End | Repeat Left | Name | Family | Class |
| 57810423 | 57815026 | - | 18.2 | 1261 | 5987 | -5 | L1MB1 | L1 | LINE |
| 57815020 | 57815480 | - | 22.4 | 2573 | 3036 | -3104 | L1M3 | L1 | LINE |
| 57815516 | 57816518 | - | 4.9 | 5139 | 6153 | -1 | L1PA5 | L1 | LINE |
| 57816517 | 57817155 | + | 5.2 | 4503 | 5141 | -1005 | L1PA5 | L1 | LINE |
| 57817153 | 57817452 | - | 21.8 | 2283 | 2578 | -3562 | L1M3 | L1 | LINE |
| 57817462 | 57817525 | - | 26.9 | 1195 | 1271 | -4620 | L1MB1 | L1 | LINE |
| 57817567 | 57818031 | - | 26.9 | 694 | 1194 | -4697 | L1MB1 | L1 | LINE |
| 57818179 | 57818333 | - | 37.1 | 522 | 678 | -5213 | L1MB1 | L1 | LINE |
| 57818429 | 57818829 | - | 35.6 | 2 | 430 | -5461 | L1MB1 | L1 | LINE |

  

|  |  |  |  |  |  |  |  |  |  |
| --- | --- | --- | --- | --- | --- | --- | --- | --- | --- |
| chr10:57955566-57959390 | | | 3816 bp | | |  |  |  |  |
| Genome Start | Genome End | Strand | Div | Repeat Start | Repeat End | Repeat Left | Name | Family | Class |
| 57955566 | 57955798 | + | 37.2 | 16 | 256 | -335 | MLT1K | MaLR | LTR |
| 57955805 | 57957840 | - | 2.6 | 4183 | 6182 | 0 | L1PA3 | L1 | LINE |
| 57957836 | 57959086 | + | 3.0 | 2927 | 4179 | -1967 | L1PA3 | L1 | LINE |
| 57959087 | 57959217 | - | 16.0 | 1 | 131 | -2 | FLAM\_C | Alu | SINE |
| 57959226 | 57959390 | + | 28.8 | 263 | 432 | -159 | MLT1K | MaLR | LTR |

  

|  |  |  |  |  |  |  |  |  |  |
| --- | --- | --- | --- | --- | --- | --- | --- | --- | --- |
| chr10:58066194-58077146 | | | 10940 bp | | |  |  |  |  |
| Genome Start | Genome End | Strand | Div | Repeat Start | Repeat End | Repeat Left | Name | Family | Class |
| 58066194 | 58068736 | - | 9.5 | 3920 | 6467 | -16 | L1PA10 | L1 | LINE |
| 58068737 | 58068874 | - | 5.1 | 6015 | 6152 | -3 | L1PA4 | L1 | LINE |
| 58068875 | 58069631 | - | 9.4 | 3149 | 3919 | -2542 | L1PA10 | L1 | LINE |
| 58069656 | 58071938 | - | 7.7 | 3845 | 6136 | -18 | L1PA7 | L1 | LINE |
| 58071929 | 58073632 | + | 14.9 | 2219 | 3826 | -2320 | L1PREC2 | L1 | LINE |
| 58073632 | 58075384 | - | 13.4 | 1492 | 3156 | -3305 | L1PA10 | L1 | LINE |
| 58075385 | 58075663 | - | 9.7 | 1 | 295 | -17 | AluSx | Alu | SINE |
| 58075664 | 58077146 | - | 16.3 | 10 | 1500 | -4961 | L1PA10 | L1 | LINE |

  

|  |  |  |  |  |  |  |  |  |  |
| --- | --- | --- | --- | --- | --- | --- | --- | --- | --- |
| chr10:59291665-59293399 | | | 1691 bp | | |  |  |  |  |
| Genome Start | Genome End | Strand | Div | Repeat Start | Repeat End | Repeat Left | Name | Family | Class |
| 59291665 | 59291795 | + | 19.2 | 297 | 424 | -267 | MER76 | ERVL | LTR |
| 59291800 | 59291951 | - | 13.4 | 5784 | 5945 | -367 | L1MA9 | L1 | LINE |
| 59291987 | 59292397 | - | 13.4 | 5373 | 5783 | -529 | L1MA9 | L1 | LINE |
| 59292398 | 59292612 | + | 14.4 | 5945 | 6159 | -153 | L1MA9 | L1 | LINE |
| 59292613 | 59293009 | + | 16.6 | 1 | 403 | 0 | MER57A | ERV1 | LTR |
| 59293010 | 59293161 | + | 14.4 | 6160 | 6309 | -3 | L1MA9 | L1 | LINE |
| 59293167 | 59293399 | + | 27.5 | 415 | 656 | -35 | MER76 | ERVL | LTR |

  

|  |  |  |  |  |  |  |  |  |  |
| --- | --- | --- | --- | --- | --- | --- | --- | --- | --- |
| chr10:59726300-59728929 | | | 2600 bp | | |  |  |  |  |
| Genome Start | Genome End | Strand | Div | Repeat Start | Repeat End | Repeat Left | Name | Family | Class |
| 59726300 | 59726415 | + | 4.4 | 21 | 134 | -177 | AluY | Alu | SINE |
| 59726416 | 59727613 | - | 3.8 | 3844 | 5041 | -1105 | L1PA3 | L1 | LINE |
| 59727615 | 59728732 | + | 3.1 | 5031 | 6152 | -3 | L1PA3 | L1 | LINE |
| 59728762 | 59728929 | + | 3.6 | 128 | 295 | -16 | AluY | Alu | SINE |

  

|  |  |  |  |  |  |  |  |  |  |
| --- | --- | --- | --- | --- | --- | --- | --- | --- | --- |
| chr10:65672762-65681540 | | | 8438 bp | | |  |  |  |  |
| Genome Start | Genome End | Strand | Div | Repeat Start | Repeat End | Repeat Left | Name | Family | Class |
| 65672762 | 65673708 | - | 25.1 | 5616 | 6662 | -360 | L1M5 | L1 | LINE |
| 65673709 | 65674009 | - | 12.6 | 1 | 302 | -8 | AluSg | Alu | SINE |
| 65674010 | 65674107 | - | 25.3 | 5524 | 5615 | -1391 | L1M5 | L1 | LINE |
| 65674108 | 65674416 | - | 8.7 | 1 | 309 | 0 | AluSc | Alu | SINE |
| 65674417 | 65674741 | - | 25.3 | 5184 | 5523 | -1483 | L1M5 | L1 | LINE |
| 65674742 | 65674828 | + | 21.5 | 1 | 79 | -1 | MADE2 | Mariner | DNA |
| 65674829 | 65675016 | - | 25.3 | 4987 | 5183 | -1823 | L1M5 | L1 | LINE |
| 65675017 | 65675131 | - | 20.4 | 1 | 133 | 0 | FLAM\_A | Alu | SINE |
| 65675132 | 65676311 | - | 25.3 | 3725 | 4984 | -2022 | L1M5 | L1 | LINE |
| 65676344 | 65676707 | - | 16.8 | 5925 | 6285 | -6 | L1MA7 | L1 | LINE |
| 65676711 | 65677203 | + | 15.9 | 5402 | 5914 | -377 | L1MA7 | L1 | LINE |
| 65677240 | 65677941 | - | 28.4 | 2976 | 3666 | -3340 | L1M5 | L1 | LINE |
| 65677963 | 65678322 | - | 10.8 | 1 | 364 | 0 | THE1B | MaLR | LTR |
| 65678323 | 65679852 | - | 10.8 | 5 | 1580 | 0 | THE1B-int | MaLR | LTR |
| 65679874 | 65680227 | - | 14.2 | 1 | 363 | -1 | THE1B | MaLR | LTR |
| 65680317 | 65680507 | - | 31.9 | 1850 | 2041 | -4365 | L1MEe | L1 | LINE |
| 65680564 | 65681026 | - | 27.6 | 1276 | 1810 | -4590 | L1MEe | L1 | LINE |
| 65681076 | 65681169 | - | 25.4 | 1687 | 1776 | -5558 | L1M5 | L1 | LINE |
| 65681204 | 65681483 | - | 14.5 | 7 | 286 | -26 | AluSx | Alu | SINE |
| 65681484 | 65681540 | - | 25.4 | 1630 | 1686 | -5648 | L1M5 | L1 | LINE |

  

|  |  |  |  |  |  |  |  |  |  |
| --- | --- | --- | --- | --- | --- | --- | --- | --- | --- |
| chr10:65940330-65940813 | | | 435 bp | | |  |  |  |  |
| Genome Start | Genome End | Strand | Div | Repeat Start | Repeat End | Repeat Left | Name | Family | Class |
| 65940330 | 65940464 | - | 22.2 | 3972 | 4113 | -823 | MER31-int | ERV1 | LTR |
| 65940465 | 65940589 | - | 6.4 | 5864 | 5986 | -169 | L1PA7 | L1 | LINE |
| 65940588 | 65940674 | + | 6.0 | 5982 | 6064 | -90 | L1PA7 | L1 | LINE |
| 65940726 | 65940813 | - | 22.4 | 3822 | 3896 | -1040 | MER31-int | ERV1 | LTR |

  

|  |  |  |  |  |  |  |  |  |  |
| --- | --- | --- | --- | --- | --- | --- | --- | --- | --- |
| chr10:66342761-66360402 | | | 17316 bp | | |  |  |  |  |
| Genome Start | Genome End | Strand | Div | Repeat Start | Repeat End | Repeat Left | Name | Family | Class |
| 66342761 | 66343545 | - | 29.9 | 4649 | 5462 | -778 | L1M5 | L1 | LINE |
| 66343564 | 66344872 | - | 19.1 | 4967 | 6291 | 0 | L1MA7 | L1 | LINE |
| 66344873 | 66345236 | - | 9.6 | 1 | 364 | 0 | THE1B | MaLR | LTR |
| 66345237 | 66345925 | - | 21.0 | 4259 | 4966 | -1177 | L1MA7 | L1 | LINE |
| 66345923 | 66346367 | + | 23.3 | 2738 | 3181 | -2962 | L1M2 | L1 | LINE |
| 66346378 | 66346508 | + | 15.3 | 11 | 142 | -1 | FLAM\_C | Alu | SINE |
| 66346524 | 66349986 | + | 17.7 | 15 | 3739 | -3826 | L1M1 | L1 | LINE |
| 66349960 | 66351703 | + | 11.7 | 4244 | 6067 | -1498 | L1M1 | L1 | LINE |
| 66351698 | 66351807 | + | 13.8 | 5715 | 5823 | -479 | L1MA1 | L1 | LINE |
| 66351811 | 66351866 | - | 12.2 | 5774 | 5827 | -339 | L1P4 | L1 | LINE |
| 66351940 | 66351991 | - | 4.3 | 5403 | 5454 | -714 | L1M3 | L1 | LINE |
| 66352049 | 66352514 | + | 10.2 | 5828 | 6295 | -7 | L1MA1 | L1 | LINE |
| 66352532 | 66352693 | + | 21.6 | 1 | 154 | -22 | FRAM | Alu | SINE |
| 66352712 | 66353761 | + | 17.6 | 3179 | 4248 | -1895 | L1M2 | L1 | LINE |
| 66353877 | 66354053 | - | 28.5 | 4026 | 4204 | -1942 | L1M5 | L1 | LINE |
| 66354050 | 66360025 | + | 2.8 | 46 | 6032 | 0 | L1PA3 | L1 | LINE |
| 66360066 | 66360402 | - | 27.3 | 3699 | 4038 | -2108 | L1M5 | L1 | LINE |

  

|  |  |  |  |  |  |  |  |  |  |
| --- | --- | --- | --- | --- | --- | --- | --- | --- | --- |
| chr10:68906505-68915284 | | | 8769 bp | | |  |  |  |  |
| Genome Start | Genome End | Strand | Div | Repeat Start | Repeat End | Repeat Left | Name | Family | Class |
| 68906505 | 68910114 | + | 14.6 | 734 | 4449 | -2400 | L1PA16 | L1 | LINE |
| 68910115 | 68910624 | + | 12.2 | 1 | 499 | 0 | MER9B | ERVK | LTR |
| 68910625 | 68911391 | + | 13.0 | 4450 | 5178 | -1671 | L1PA16 | L1 | LINE |
| 68911389 | 68912693 | - | 2.8 | 3972 | 5278 | -868 | L1PA3 | L1 | LINE |
| 68912690 | 68913532 | + | 2.5 | 5301 | 6152 | -3 | L1PA3 | L1 | LINE |
| 68913551 | 68915284 | + | 11.7 | 5154 | 6869 | 0 | L1PA16 | L1 | LINE |

  

|  |  |  |  |  |  |  |  |  |  |
| --- | --- | --- | --- | --- | --- | --- | --- | --- | --- |
| chr10:75928165-75929559 | | | 1290 bp | | |  |  |  |  |
| Genome Start | Genome End | Strand | Div | Repeat Start | Repeat End | Repeat Left | Name | Family | Class |
| 75928165 | 75928290 | + | 34.9 | 1254 | 1382 | -578 | L4 | RTE | LINE |
| 75928291 | 75928534 | - | 4.9 | 5910 | 6154 | -1 | L1PA4 | L1 | LINE |
| 75928537 | 75928729 | + | 1.6 | 5708 | 5900 | -255 | L1PA4 | L1 | LINE |
| 75928828 | 75929002 | - | 13.9 | 117 | 288 | -24 | AluJo | Alu | SINE |
| 75929008 | 75929170 | + | 32.0 | 1482 | 1676 | -284 | L4 | RTE | LINE |
| 75929171 | 75929463 | + | 4.4 | 1 | 293 | -18 | AluY | Alu | SINE |
| 75929464 | 75929559 | + | 32.0 | 1677 | 1764 | -196 | L4 | RTE | LINE |

  

|  |  |  |  |  |  |  |  |  |  |
| --- | --- | --- | --- | --- | --- | --- | --- | --- | --- |
| chr10:78030696-78034495 | | | 3813 bp | | |  |  |  |  |
| Genome Start | Genome End | Strand | Div | Repeat Start | Repeat End | Repeat Left | Name | Family | Class |
| 78030696 | 78031467 | + | 29.6 | 100 | 892 | -3962 | L1MEe | L1 | LINE |
| 78031465 | 78032748 | - | 4.8 | 4034 | 5318 | -828 | L1PA5 | L1 | LINE |
| 78032738 | 78033588 | + | 4.9 | 5311 | 6154 | 0 | L1PA5 | L1 | LINE |
| 78033582 | 78034033 | + | 26.7 | 864 | 1448 | -3386 | L1MEe | L1 | LINE |
| 78034034 | 78034229 | - | 19.0 | 22 | 219 | 0 | MER20 | MER1\_type | DNA |
| 78034238 | 78034495 | + | 24.8 | 1468 | 1741 | -3093 | L1MEe | L1 | LINE |

  

|  |  |  |  |  |  |  |  |  |  |
| --- | --- | --- | --- | --- | --- | --- | --- | --- | --- |
| chr10:81073332-81079509 | | | 6156 bp | | |  |  |  |  |
| Genome Start | Genome End | Strand | Div | Repeat Start | Repeat End | Repeat Left | Name | Family | Class |
| 81073332 | 81073962 | - | 21.1 | 5728 | 6365 | -51 | L1MB4 | L1 | LINE |
| 81073983 | 81074052 | - | 21.1 | 5684 | 5727 | -689 | L1MB4 | L1 | LINE |
| 81074053 | 81074365 | + | 14.8 | 1 | 290 | -22 | AluSx | Alu | SINE |
| 81074366 | 81074533 | - | 21.1 | 5535 | 5683 | -733 | L1MB4 | L1 | LINE |
| 81074522 | 81074905 | - | 7.5 | 4776 | 5163 | -983 | L1PB | L1 | LINE |
| 81074904 | 81075147 | + | 5.7 | 5159 | 5403 | -743 | L1PB | L1 | LINE |
| 81075160 | 81075254 | + | 6.5 | 6057 | 6150 | -1 | L1PB1 | L1 | LINE |
| 81075259 | 81077015 | - | 23.2 | 3761 | 5545 | -837 | L1MB4 | L1 | LINE |
| 81077016 | 81077312 | + | 5.4 | 8 | 304 | -7 | AluY | Alu | SINE |
| 81077313 | 81079509 | - | 23.4 | 1434 | 3760 | -2622 | L1MB4 | L1 | LINE |

  

|  |  |  |  |  |  |  |  |  |  |
| --- | --- | --- | --- | --- | --- | --- | --- | --- | --- |
| chr10:81393326-81399539 | | | 6198 bp | | |  |  |  |  |
| Genome Start | Genome End | Strand | Div | Repeat Start | Repeat End | Repeat Left | Name | Family | Class |
| 81393326 | 81393996 | - | 21.1 | 5728 | 6415 | -1 | L1MB4 | L1 | LINE |
| 81394017 | 81394084 | - | 21.1 | 5684 | 5727 | -689 | L1MB4 | L1 | LINE |
| 81394085 | 81394394 | + | 14.8 | 1 | 290 | -22 | AluSx | Alu | SINE |
| 81394395 | 81394562 | - | 21.1 | 5535 | 5683 | -733 | L1MB4 | L1 | LINE |
| 81394549 | 81394934 | - | 7.8 | 4776 | 5165 | -981 | L1PB | L1 | LINE |
| 81394933 | 81395176 | + | 5.7 | 5159 | 5403 | -743 | L1PB | L1 | LINE |
| 81395189 | 81395283 | + | 6.5 | 6057 | 6150 | -1 | L1PB1 | L1 | LINE |
| 81395284 | 81397044 | - | 22.0 | 3761 | 5549 | -833 | L1MB4 | L1 | LINE |
| 81397045 | 81397341 | + | 6.1 | 8 | 304 | -7 | AluY | Alu | SINE |
| 81397342 | 81399539 | - | 23.5 | 1434 | 3760 | -2622 | L1MB4 | L1 | LINE |

  

|  |  |  |  |  |  |  |  |  |  |
| --- | --- | --- | --- | --- | --- | --- | --- | --- | --- |
| chr10:82227112-82231416 | | | 3919 bp | | |  |  |  |  |
| Genome Start | Genome End | Strand | Div | Repeat Start | Repeat End | Repeat Left | Name | Family | Class |
| 82227112 | 82227278 | + | 27.9 | 3 | 174 | -2333 | HAL1 | L1 | LINE |
| 82227545 | 82228495 | + | 27.9 | 579 | 1625 | -882 | HAL1 | L1 | LINE |
| 82228497 | 82228610 | + | 10.5 | 22 | 135 | -177 | AluSx | Alu | SINE |
| 82228611 | 82230096 | - | 2.6 | 4086 | 5571 | -584 | L1PA3 | L1 | LINE |
| 82230097 | 82230679 | + | 3.8 | 5573 | 6155 | 0 | L1PA3 | L1 | LINE |
| 82230680 | 82230869 | + | 18.4 | 121 | 311 | -1 | AluSx | Alu | SINE |
| 82230989 | 82231416 | + | 30.5 | 1860 | 2322 | -185 | HAL1 | L1 | LINE |

  

|  |  |  |  |  |  |  |  |  |  |
| --- | --- | --- | --- | --- | --- | --- | --- | --- | --- |
| chr10:83054530-83056667 | | | 1928 bp | | |  |  |  |  |
| Genome Start | Genome End | Strand | Div | Repeat Start | Repeat End | Repeat Left | Name | Family | Class |
| 83054530 | 83055516 | + | 26.7 | 6625 | 7595 | -366 | L1MC5 | L1 | LINE |
| 83055675 | 83055901 | - | 7.5 | 5928 | 6154 | 0 | L1PA5 | L1 | LINE |
| 83055902 | 83055931 | + | 6.7 | 5904 | 5933 | -221 | L1PA5 | L1 | LINE |
| 83055944 | 83056073 | + | 32.3 | 5474 | 5619 | -621 | L1ME3 | L1 | LINE |
| 83056089 | 83056432 | + | 28.2 | 5733 | 6087 | -75 | L1ME3 | L1 | LINE |
| 83056458 | 83056667 | + | 22.9 | 7570 | 7780 | -181 | L1MC5 | L1 | LINE |

  

|  |  |  |  |  |  |  |  |  |  |
| --- | --- | --- | --- | --- | --- | --- | --- | --- | --- |
| chr10:83098874-83105997 | | | 7080 bp | | |  |  |  |  |
| Genome Start | Genome End | Strand | Div | Repeat Start | Repeat End | Repeat Left | Name | Family | Class |
| 83098874 | 83100130 | - | 19.3 | 5063 | 6333 | -1 | L1MA10 | L1 | LINE |
| 83100115 | 83101953 | + | 2.8 | 4299 | 6137 | -18 | L1PA3 | L1 | LINE |
| 83101975 | 83103240 | - | 19.3 | 3927 | 5078 | -1062 | L1MA10 | L1 | LINE |
| 83103241 | 83103555 | + | 5.4 | 5835 | 6150 | -4 | L1PA5 | L1 | LINE |
| 83103556 | 83104293 | - | 19.3 | 3199 | 3926 | -2214 | L1MA10 | L1 | LINE |
| 83104321 | 83104421 | - | 16.8 | 191 | 291 | -21 | AluJ | Alu | SINE |
| 83104423 | 83104537 | - | 5.2 | 4900 | 5014 | -1132 | L1PA5 | L1 | LINE |
| 83104536 | 83105687 | + | 5.8 | 5004 | 6149 | -5 | L1PA5 | L1 | LINE |
| 83105701 | 83105902 | - | 21.5 | 1 | 201 | -111 | AluJ | Alu | SINE |
| 83105903 | 83105997 | - | 15.8 | 3070 | 3179 | -2961 | L1MA10 | L1 | LINE |

  

|  |  |  |  |  |  |  |  |  |  |
| --- | --- | --- | --- | --- | --- | --- | --- | --- | --- |
| chr10:83288397-83292952 | | | 4505 bp | | |  |  |  |  |
| Genome Start | Genome End | Strand | Div | Repeat Start | Repeat End | Repeat Left | Name | Family | Class |
| 83288397 | 83288904 | + | 31.6 | 4523 | 5027 | -1119 | L1ME3A | L1 | LINE |
| 83288904 | 83289147 | - | 9.8 | 2157 | 2406 | -3740 | L1PB1 | L1 | LINE |
| 83289144 | 83289359 | + | 10.7 | 2423 | 2639 | -3507 | L1PB1 | L1 | LINE |
| 83289347 | 83291454 | + | 10.6 | 4157 | 6192 | 0 | L1PB1 | L1 | LINE |
| 83291459 | 83292163 | + | 28.7 | 5036 | 5814 | -426 | L1ME3A | L1 | LINE |
| 83292164 | 83292472 | - | 12.0 | 1 | 309 | 0 | AluSq | Alu | SINE |
| 83292536 | 83292631 | - | 34.4 | 10 | 106 | -83 | MER5A | MER1\_type | DNA |
| 83292634 | 83292952 | + | 28.0 | 5791 | 6121 | -49 | L1ME3A | L1 | LINE |

  

|  |  |  |  |  |  |  |  |  |  |
| --- | --- | --- | --- | --- | --- | --- | --- | --- | --- |
| chr10:84991967-84995995 | | | 3661 bp | | |  |  |  |  |
| Genome Start | Genome End | Strand | Div | Repeat Start | Repeat End | Repeat Left | Name | Family | Class |
| 84991967 | 84992349 | + | 31.8 | 2502 | 2897 | -522 | L2 | L2 | LINE |
| 84992377 | 84992482 | - | 25.0 | 442 | 546 | -16 | MLT1F2 | MaLR | LTR |
| 84992621 | 84992999 | - | 34.2 | 44 | 443 | -21 | LTR16B | ERVL | LTR |
| 84993145 | 84993900 | - | 8.3 | 3644 | 4406 | -1740 | L1PA8 | L1 | LINE |
| 84993898 | 84994210 | + | 10.6 | 4416 | 4703 | -1443 | L1PA8 | L1 | LINE |
| 84994234 | 84995709 | + | 8.6 | 4704 | 6161 | -11 | L1PA8 | L1 | LINE |
| 84995748 | 84995995 | + | 31.7 | 2910 | 3176 | -243 | L2 | L2 | LINE |

  

|  |  |  |  |  |  |  |  |  |  |
| --- | --- | --- | --- | --- | --- | --- | --- | --- | --- |
| chr10:85130890-85144741 | | | 13852 bp | | |  |  |  |  |
| Genome Start | Genome End | Strand | Div | Repeat Start | Repeat End | Repeat Left | Name | Family | Class |
| 85130890 | 85132714 | + | 19.9 | 3830 | 5754 | -590 | L1MC2 | L1 | LINE |
| 85132715 | 85133004 | + | 17.3 | 1 | 290 | -22 | AluJb | Alu | SINE |
| 85133005 | 85133304 | + | 21.0 | 5755 | 6032 | -312 | L1MC2 | L1 | LINE |
| 85133315 | 85134166 | - | 11.7 | 6911 | 7722 | -1 | L1MA2 | L1 | LINE |
| 85134167 | 85134477 | - | 8.0 | 1 | 311 | 0 | AluY | Alu | SINE |
| 85134478 | 85136917 | - | 12.4 | 4420 | 6910 | -813 | L1MA2 | L1 | LINE |
| 85136914 | 85137536 | - | 14.9 | 3219 | 3861 | -3698 | L1MA2 | L1 | LINE |
| 85137537 | 85137817 | - | 17.6 | 1 | 296 | -16 | AluJo | Alu | SINE |
| 85137818 | 85137896 | - | 13.4 | 3143 | 3218 | -4347 | L1MA2 | L1 | LINE |
| 85137873 | 85139221 | - | 11.3 | 4801 | 6158 | -3 | L1PA14 | L1 | LINE |
| 85139221 | 85140988 | + | 11.2 | 3000 | 4787 | -1359 | L1PREC2 | L1 | LINE |
| 85140995 | 85141142 | - | 17.8 | 3037 | 3171 | -4394 | L1MA2 | L1 | LINE |
| 85141143 | 85141442 | - | 13.1 | 1 | 310 | 0 | AluSg | Alu | SINE |
| 85141443 | 85144403 | - | 17.8 | 22 | 3036 | -4529 | L1MA2 | L1 | LINE |
| 85144417 | 85144741 | + | 13.4 | 6019 | 6330 | -14 | L1MC2 | L1 | LINE |

  

|  |  |  |  |  |  |  |  |  |  |
| --- | --- | --- | --- | --- | --- | --- | --- | --- | --- |
| chr10:87142776-87143417 | | | 625 bp | | |  |  |  |  |
| Genome Start | Genome End | Strand | Div | Repeat Start | Repeat End | Repeat Left | Name | Family | Class |
| 87142776 | 87142841 | + | 21.2 | 5051 | 5118 | -1028 | L1M5 | L1 | LINE |
| 87142836 | 87143080 | - | 3.7 | 5756 | 5999 | -156 | L1PA4 | L1 | LINE |
| 87143081 | 87143244 | + | 3.0 | 5989 | 6152 | -3 | L1PA4 | L1 | LINE |
| 87143268 | 87143417 | + | 25.7 | 5177 | 5335 | -811 | L1M5 | L1 | LINE |

  

|  |  |  |  |  |  |  |  |  |  |
| --- | --- | --- | --- | --- | --- | --- | --- | --- | --- |
| chr10:87266581-87272296 | | | 4867 bp | | |  |  |  |  |
| Genome Start | Genome End | Strand | Div | Repeat Start | Repeat End | Repeat Left | Name | Family | Class |
| 87266581 | 87266654 | - | 21.9 | 3049 | 3121 | -4066 | L1MEa | L1 | LINE |
| 87266657 | 87268364 | - | 3.0 | 4443 | 6150 | -5 | L1PA3 | L1 | LINE |
| 87268364 | 87269751 | + | 3.1 | 3067 | 4451 | -1695 | L1PA3 | L1 | LINE |
| 87270099 | 87271120 | - | 30.5 | 1488 | 2798 | -4389 | L1MEa | L1 | LINE |
| 87271170 | 87271227 | - | 30.4 | 1427 | 1487 | -5847 | L1MEa | L1 | LINE |
| 87271291 | 87271356 | - | 19.7 | 1457 | 1524 | -5472 | L1MEa | L1 | LINE |
| 87271572 | 87271936 | - | 10.5 | 1 | 363 | -1 | THE1B | MaLR | LTR |
| 87272111 | 87272296 | - | 30.4 | 981 | 1167 | -6020 | L1MEa | L1 | LINE |

  

|  |  |  |  |  |  |  |  |  |  |
| --- | --- | --- | --- | --- | --- | --- | --- | --- | --- |
| chr10:96368808-96372934 | | | 4098 bp | | |  |  |  |  |
| Genome Start | Genome End | Strand | Div | Repeat Start | Repeat End | Repeat Left | Name | Family | Class |
| 96368808 | 96369206 | - | 13.3 | 99 | 507 | 0 | MER84 | ERV1 | LTR |
| 96369229 | 96371186 | - | 6.6 | 4177 | 6145 | -9 | L1PA7 | L1 | LINE |
| 96371183 | 96371929 | + | 9.4 | 3430 | 4180 | -1966 | L1PA7 | L1 | LINE |
| 96371933 | 96372144 | - | 15.2 | 1 | 224 | -6 | MER30 | MER1\_type | DNA |
| 96372153 | 96372835 | - | 9.7 | 1 | 664 | 0 | MER4A | ERV1 | LTR |
| 96372836 | 96372934 | - | 10.2 | 1 | 100 | -407 | MER84 | ERV1 | LTR |

  

|  |  |  |  |  |  |  |  |  |  |
| --- | --- | --- | --- | --- | --- | --- | --- | --- | --- |
| chr10:97467434-97474109 | | | 6664 bp | | |  |  |  |  |
| Genome Start | Genome End | Strand | Div | Repeat Start | Repeat End | Repeat Left | Name | Family | Class |
| 97467434 | 97467726 | - | 6.2 | 8335 | 8626 | 0 | HERV17 | ERV1 | LTR |
| 97467720 | 97467755 | - | 0.0 | 7510 | 7545 | -1081 | HERV17 | ERV1 | LTR |
| 97467752 | 97467915 | - | 5.6 | 6986 | 7147 | -1479 | HERV17 | ERV1 | LTR |
| 97467908 | 97471384 | - | 4.3 | 2430 | 5902 | -2724 | HERV17 | ERV1 | LTR |
| 97471368 | 97471992 | - | 8.2 | 1396 | 2016 | -6610 | HERV17 | ERV1 | LTR |
| 97472041 | 97472141 | - | 2.0 | 6055 | 6155 | 0 | L1PA3 | L1 | LINE |
| 97472142 | 97472435 | + | 2.4 | 5741 | 6034 | -121 | L1PA3 | L1 | LINE |
| 97472436 | 97473629 | - | 5.4 | 181 | 1412 | -7214 | HERV17 | ERV1 | LTR |
| 97473630 | 97473933 | + | 9.2 | 4 | 306 | -5 | AluY | Alu | SINE |
| 97473934 | 97474109 | - | 5.6 | 1 | 180 | -8446 | HERV17 | ERV1 | LTR |

  

|  |  |  |  |  |  |  |  |  |  |
| --- | --- | --- | --- | --- | --- | --- | --- | --- | --- |
| chr10:99966885-99973995 | | | 7058 bp | | |  |  |  |  |
| Genome Start | Genome End | Strand | Div | Repeat Start | Repeat End | Repeat Left | Name | Family | Class |
| 99966885 | 99968058 | - | 15.2 | 4548 | 5735 | -1110 | L1M2 | L1 | LINE |
| 99968061 | 99968172 | + | 14.3 | 4425 | 4545 | -2300 | L1M2 | L1 | LINE |
| 99968192 | 99968374 | - | 18.6 | 3755 | 3952 | -2893 | L1M2 | L1 | LINE |
| 99968372 | 99968668 | - | 16.0 | 3101 | 3404 | -3441 | L1M2 | L1 | LINE |
| 99968669 | 99968958 | - | 14.8 | 1 | 300 | -12 | AluSx | Alu | SINE |
| 99968959 | 99969487 | - | 14.5 | 2525 | 3100 | -3745 | L1M2 | L1 | LINE |
| 99969522 | 99970117 | - | 14.7 | 1740 | 2375 | -4474 | L1M2 | L1 | LINE |
| 99970134 | 99972464 | - | 2.3 | 3815 | 6153 | -2 | L1PA2 | L1 | LINE |
| 99972465 | 99973123 | + | 2.6 | 3168 | 3826 | -2320 | L1PA2 | L1 | LINE |
| 99973109 | 99973995 | - | 25.0 | 840 | 1764 | -5085 | L1M2 | L1 | LINE |

  

|  |  |  |  |  |  |  |  |  |  |
| --- | --- | --- | --- | --- | --- | --- | --- | --- | --- |
| chr10:100622845-100626054 | | | 3128 bp | | |  |  |  |  |
| Genome Start | Genome End | Strand | Div | Repeat Start | Repeat End | Repeat Left | Name | Family | Class |
| 100622845 | 100622960 | + | 26.3 | 4 | 119 | -402 | MER113 | MER1\_type | DNA |
| 100622997 | 100624125 | - | 7.9 | 4983 | 6112 | -19 | L1PA8A | L1 | LINE |
| 100624098 | 100624808 | - | 9.1 | 3701 | 4416 | -1730 | L1PA8A | L1 | LINE |
| 100624806 | 100625488 | + | 9.1 | 3019 | 3702 | -2444 | L1PA8A | L1 | LINE |
| 100625566 | 100625653 | + | 24.1 | 243 | 321 | -200 | MER113 | MER1\_type | DNA |
| 100625654 | 100625897 | + | 29.4 | 3 | 259 | -3 | MIR | MIR | SINE |
| 100625898 | 100626054 | + | 24.1 | 322 | 491 | -30 | MER113 | MER1\_type | DNA |

  

|  |  |  |  |  |  |  |  |  |  |
| --- | --- | --- | --- | --- | --- | --- | --- | --- | --- |
| chr10:108150987-108153164 | | | 2187 bp | | |  |  |  |  |
| Genome Start | Genome End | Strand | Div | Repeat Start | Repeat End | Repeat Left | Name | Family | Class |
| 108150987 | 108151476 | - | 34.6 | 2456 | 2956 | -463 | L2 | L2 | LINE |
| 108151478 | 108151569 | - | 6.9 | 5698 | 5786 | -518 | L1MA2 | L1 | LINE |
| 108151547 | 108152090 | + | 10.7 | 5794 | 6304 | 0 | L1MA2 | L1 | LINE |
| 108152104 | 108153164 | - | 32.8 | 1399 | 2456 | -963 | L2 | L2 | LINE |

  

|  |  |  |  |  |  |  |  |  |  |
| --- | --- | --- | --- | --- | --- | --- | --- | --- | --- |
| chr10:108191178-108195386 | | | 4003 bp | | |  |  |  |  |
| Genome Start | Genome End | Strand | Div | Repeat Start | Repeat End | Repeat Left | Name | Family | Class |
| 108191178 | 108191274 | - | 15.5 | 7580 | 7677 | -108 | L1MC3 | L1 | LINE |
| 108191275 | 108192805 | - | 19.9 | 5846 | 7360 | -425 | L1MC3 | L1 | LINE |
| 108192806 | 108193773 | + | 11.2 | 5220 | 6161 | -141 | L1MA1 | L1 | LINE |
| 108193741 | 108193796 | + | 17.9 | 6190 | 6247 | -1538 | L1MC3 | L1 | LINE |
| 108193798 | 108193987 | + | 11.2 | 6101 | 6296 | -8 | L1MA2 | L1 | LINE |
| 108194084 | 108194366 | - | 18.1 | 5564 | 5858 | -1927 | L1MA8 | L1 | LINE |
| 108194371 | 108194656 | + | 17.0 | 5835 | 6151 | -140 | L1MA8 | L1 | LINE |
| 108194657 | 108195072 | + | 18.8 | 1 | 426 | 0 | MSTB | MaLR | LTR |
| 108195073 | 108195210 | + | 15.6 | 6152 | 6289 | -2 | L1MA8 | L1 | LINE |
| 108195349 | 108195386 | - | 10.5 | 5492 | 5529 | -2256 | L1MC3 | L1 | LINE |

  

|  |  |  |  |  |  |  |  |  |  |
| --- | --- | --- | --- | --- | --- | --- | --- | --- | --- |
| chr10:108547737-108549646 | | | 1967 bp | | |  |  |  |  |
| Genome Start | Genome End | Strand | Div | Repeat Start | Repeat End | Repeat Left | Name | Family | Class |
| 108547737 | 108548042 | + | 14.3 | 1 | 325 | -39 | THE1B | MaLR | LTR |
| 108548045 | 108548582 | - | 5.0 | 5608 | 6146 | -8 | L1PA5 | L1 | LINE |
| 108548537 | 108548584 | - | 8.3 | 5606 | 5653 | -501 | L1PA5 | L1 | LINE |
| 108548572 | 108549604 | + | 5.3 | 4585 | 5617 | -537 | L1PA5 | L1 | LINE |
| 108549605 | 108549646 | + | 14.3 | 314 | 355 | 0 | THE1B | MaLR | LTR |

  

|  |  |  |  |  |  |  |  |  |  |
| --- | --- | --- | --- | --- | --- | --- | --- | --- | --- |
| chr10:108927015-108930498 | | | 3466 bp | | |  |  |  |  |
| Genome Start | Genome End | Strand | Div | Repeat Start | Repeat End | Repeat Left | Name | Family | Class |
| 108927015 | 108927222 | - | 16.9 | 6029 | 6238 | -4 | L1MD1 | L1 | LINE |
| 108927231 | 108927361 | - | 19.7 | 6135 | 6288 | -3 | L1MA7 | L1 | LINE |
| 108927362 | 108927525 | - | 14.6 | 6121 | 6292 | -8 | L1MA4 | L1 | LINE |
| 108927536 | 108927923 | + | 9.0 | 5779 | 6174 | 0 | L1PA11 | L1 | LINE |
| 108927924 | 108928250 | - | 13.2 | 5820 | 6128 | -175 | L1MA4A | L1 | LINE |
| 108928251 | 108928530 | - | 13.8 | 6 | 291 | -21 | AluSx | Alu | SINE |
| 108928531 | 108929007 | - | 13.2 | 5359 | 5819 | -484 | L1MA4A | L1 | LINE |
| 108928995 | 108929112 | + | 17.8 | 6110 | 6232 | -59 | L1MA7 | L1 | LINE |
| 108929110 | 108929296 | - | 20.4 | 5833 | 6042 | -200 | L1MD1 | L1 | LINE |
| 108929305 | 108929652 | - | 17.9 | 5150 | 5527 | -715 | L1MD1 | L1 | LINE |
| 108929653 | 108929963 | - | 15.4 | 1 | 312 | 0 | AluJo | Alu | SINE |
| 108929964 | 108930218 | - | 18.1 | 4897 | 5149 | -997 | L1MD1 | L1 | LINE |
| 108930227 | 108930498 | - | 15.9 | 3026 | 3300 | -2846 | L1MD1 | L1 | LINE |

  

|  |  |  |  |  |  |  |  |  |  |
| --- | --- | --- | --- | --- | --- | --- | --- | --- | --- |
| chr10:109118611-109121254 | | | 2527 bp | | |  |  |  |  |
| Genome Start | Genome End | Strand | Div | Repeat Start | Repeat End | Repeat Left | Name | Family | Class |
| 109118611 | 109119255 | + | 26.5 | 3881 | 4576 | -1570 | L1ME3A | L1 | LINE |
| 109119282 | 109119601 | - | 13.8 | 5850 | 6173 | -127 | L1MA4 | L1 | LINE |
| 109119604 | 109119731 | + | 18.0 | 6172 | 6299 | -1 | L1MA4 | L1 | LINE |
| 109119772 | 109120120 | + | 29.9 | 4632 | 4987 | -1159 | L1ME3A | L1 | LINE |
| 109120123 | 109120340 | + | 28.0 | 5035 | 5272 | -874 | L1ME3A | L1 | LINE |
| 109120388 | 109121254 | + | 28.8 | 5222 | 6135 | -35 | L1ME3A | L1 | LINE |

  

|  |  |  |  |  |  |  |  |  |  |
| --- | --- | --- | --- | --- | --- | --- | --- | --- | --- |
| chr10:110301309-110309146 | | | 7789 bp | | |  |  |  |  |
| Genome Start | Genome End | Strand | Div | Repeat Start | Repeat End | Repeat Left | Name | Family | Class |
| 110301309 | 110303146 | + | 20.1 | 6 | 1870 | -4638 | L1MC1 | L1 | LINE |
| 110303147 | 110303451 | + | 10.4 | 5983 | 6295 | -7 | L1MA1 | L1 | LINE |
| 110303452 | 110304043 | + | 20.1 | 1871 | 2398 | -4110 | L1MC1 | L1 | LINE |
| 110304051 | 110304415 | + | 17.2 | 1 | 381 | 0 | THE1D | MaLR | LTR |
| 110304416 | 110306008 | + | 13.1 | 1 | 1580 | 0 | THE1D-int | MaLR | LTR |
| 110306009 | 110306365 | + | 19.0 | 1 | 381 | 0 | THE1D | MaLR | LTR |
| 110306367 | 110307599 | + | 16.5 | 2401 | 3721 | -2600 | L1MC1 | L1 | LINE |
| 110307626 | 110307892 | + | 17.3 | 5510 | 5787 | -721 | L1MC1 | L1 | LINE |
| 110307889 | 110307974 | - | 14.1 | 6044 | 6128 | -163 | L1MA8 | L1 | LINE |
| 110307971 | 110308105 | + | 17.8 | 6145 | 6289 | -2 | L1MA8 | L1 | LINE |
| 110308129 | 110308801 | + | 16.9 | 5770 | 6452 | -56 | L1MC1 | L1 | LINE |
| 110308802 | 110309092 | - | 19.5 | 1 | 287 | -25 | AluJb | Alu | SINE |
| 110309093 | 110309146 | + | 16.9 | 6453 | 6503 | -5 | L1MC1 | L1 | LINE |

  

|  |  |  |  |  |  |  |  |  |  |
| --- | --- | --- | --- | --- | --- | --- | --- | --- | --- |
| chr10:112916858-112921588 | | | 4707 bp | | |  |  |  |  |
| Genome Start | Genome End | Strand | Div | Repeat Start | Repeat End | Repeat Left | Name | Family | Class |
| 112916858 | 112917160 | - | 18.7 | 3760 | 4083 | -2923 | L1M4b | L1 | LINE |
| 112917155 | 112918365 | - | 7.2 | 4909 | 6150 | -4 | L1PA7 | L1 | LINE |
| 112918363 | 112918628 | - | 7.8 | 4237 | 4499 | -1647 | L1PA7 | L1 | LINE |
| 112918631 | 112919097 | + | 9.0 | 3759 | 4234 | -1912 | L1PA7 | L1 | LINE |
| 112919119 | 112919992 | - | 24.3 | 2906 | 3730 | -3276 | L1M4b | L1 | LINE |
| 112920003 | 112920908 | - | 24.1 | 1876 | 2847 | -4340 | L1M4b | L1 | LINE |
| 112920909 | 112921350 | + | 17.9 | 4 | 414 | 0 | MLT2D | ERVL | LTR |
| 112921351 | 112921588 | - | 24.2 | 1388 | 1875 | -5312 | L1M4b | L1 | LINE |

  

|  |  |  |  |  |  |  |  |  |  |
| --- | --- | --- | --- | --- | --- | --- | --- | --- | --- |
| chr10:117135860-117136898 | | | 1024 bp | | |  |  |  |  |
| Genome Start | Genome End | Strand | Div | Repeat Start | Repeat End | Repeat Left | Name | Family | Class |
| 117135860 | 117136151 | - | 13.4 | 214 | 489 | -3 | LTR22B | ERVK | LTR |
| 117136167 | 117136437 | - | 3.3 | 5882 | 6152 | -3 | L1PA3 | L1 | LINE |
| 117136438 | 117136681 | + | 1.3 | 5663 | 5894 | -261 | L1PA3 | L1 | LINE |
| 117136682 | 117136898 | - | 11.5 | 1 | 226 | -266 | LTR22B | ERVK | LTR |

  

|  |  |  |  |  |  |  |  |  |  |
| --- | --- | --- | --- | --- | --- | --- | --- | --- | --- |
| chr10:117448403-117453608 | | | 4773 bp | | |  |  |  |  |
| Genome Start | Genome End | Strand | Div | Repeat Start | Repeat End | Repeat Left | Name | Family | Class |
| 117448403 | 117449037 | + | 27.8 | 4280 | 4888 | -1258 | L1M5 | L1 | LINE |
| 117449194 | 117450173 | - | 12.9 | 5179 | 6157 | -9 | L1PA16 | L1 | LINE |
| 117450173 | 117452951 | + | 11.3 | 2354 | 5182 | -964 | L1PA16 | L1 | LINE |
| 117452991 | 117453112 | + | 31.7 | 6029 | 6156 | -84 | L1ME3B | L1 | LINE |
| 117453352 | 117453608 | + | 28.3 | 4803 | 5063 | -1083 | L1M5 | L1 | LINE |

  

|  |  |  |  |  |  |  |  |  |  |
| --- | --- | --- | --- | --- | --- | --- | --- | --- | --- |
| chr10:118051589-118055304 | | | 3706 bp | | |  |  |  |  |
| Genome Start | Genome End | Strand | Div | Repeat Start | Repeat End | Repeat Left | Name | Family | Class |
| 118051589 | 118051693 | - | 12.0 | 2310 | 2418 | 0 | Tigger1 | MER2\_type | DNA |
| 118051694 | 118051941 | + | 7.3 | 52 | 299 | -13 | AluSx | Alu | SINE |
| 118051942 | 118052709 | - | 12.0 | 1537 | 2309 | -109 | Tigger1 | MER2\_type | DNA |
| 118052718 | 118053116 | - | 4.5 | 5755 | 6154 | 0 | L1PA5 | L1 | LINE |
| 118053119 | 118053751 | + | 3.7 | 5101 | 5730 | -424 | L1PA5 | L1 | LINE |
| 118053752 | 118055304 | - | 14.2 | 1 | 1559 | -859 | Tigger1 | MER2\_type | DNA |

  

|  |  |  |  |  |  |  |  |  |  |
| --- | --- | --- | --- | --- | --- | --- | --- | --- | --- |
| chr10:120609815-120615049 | | | 5235 bp | | |  |  |  |  |
| Genome Start | Genome End | Strand | Div | Repeat Start | Repeat End | Repeat Left | Name | Family | Class |
| 120609815 | 120611864 | + | 12.0 | 2 | 2077 | -341 | Tigger1 | MER2\_type | DNA |
| 120611865 | 120613573 | - | 7.0 | 4443 | 6145 | -9 | L1PA6 | L1 | LINE |
| 120613574 | 120614610 | + | 7.9 | 3390 | 4434 | -1712 | L1PA6 | L1 | LINE |
| 120614611 | 120615049 | + | 13.0 | 2065 | 2417 | -1 | Tigger1 | MER2\_type | DNA |

  

|  |  |  |  |  |  |  |  |  |  |
| --- | --- | --- | --- | --- | --- | --- | --- | --- | --- |
| chr10:125467616-125469557 | | | 1836 bp | | |  |  |  |  |
| Genome Start | Genome End | Strand | Div | Repeat Start | Repeat End | Repeat Left | Name | Family | Class |
| 125467616 | 125467707 | - | 27.8 | 179 | 268 | 0 | MIRb | MIR | SINE |
| 125467822 | 125468123 | + | 36.0 | 26 | 337 | -254 | MLT1K | MaLR | LTR |
| 125468139 | 125468492 | - | 21.3 | 140 | 476 | -8 | MLT1H2 | MaLR | LTR |
| 125468497 | 125469206 | - | 22.6 | 5214 | 5935 | -231 | L1ME1 | L1 | LINE |
| 125469180 | 125469413 | + | 28.1 | 4969 | 5202 | -944 | L1MD | L1 | LINE |
| 125469414 | 125469557 | - | 35.4 | 13 | 162 | -100 | MIRb | MIR | SINE |

  

|  |  |  |  |  |  |  |  |  |  |
| --- | --- | --- | --- | --- | --- | --- | --- | --- | --- |
| chr10:129354527-129360934 | | | 6031 bp | | |  |  |  |  |
| Genome Start | Genome End | Strand | Div | Repeat Start | Repeat End | Repeat Left | Name | Family | Class |
| 129354527 | 129356563 | + | 25.8 | 341 | 2518 | -3697 | L1MC4 | L1 | LINE |
| 129356630 | 129357512 | - | 4.5 | 5263 | 6151 | -3 | L1PA6 | L1 | LINE |
| 129357511 | 129357695 | + | 7.0 | 5088 | 5272 | -874 | L1PA6 | L1 | LINE |
| 129357912 | 129358883 | + | 16.5 | 4 | 1022 | -1469 | Tigger7 | MER2\_type | DNA |
| 129358888 | 129358965 | + | 17.9 | 2407 | 2484 | -7 | Tigger7 | MER2\_type | DNA |
| 129358978 | 129359003 | + | 31.4 | 2756 | 2780 | -3434 | L1MC4 | L1 | LINE |
| 129359004 | 129359378 | + | 18.7 | 1 | 390 | 0 | MLT1B | MaLR | LTR |
| 129359379 | 129360185 | + | 31.4 | 2781 | 3634 | -2580 | L1MC4 | L1 | LINE |
| 129360191 | 129360491 | + | 24.3 | 3260 | 3568 | -2578 | L1M5 | L1 | LINE |
| 129360502 | 129360650 | + | 25.5 | 137 | 285 | -27 | AluJb | Alu | SINE |
| 129360717 | 129360934 | + | 28.0 | 3639 | 3866 | -2348 | L1MC4 | L1 | LINE |

  

|  |  |  |  |  |  |  |  |  |  |
| --- | --- | --- | --- | --- | --- | --- | --- | --- | --- |
| chr10:133281412-133288352 | | | 6763 bp | | |  |  |  |  |
| Genome Start | Genome End | Strand | Div | Repeat Start | Repeat End | Repeat Left | Name | Family | Class |
| 133281412 | 133282339 | - | 20.2 | 3556 | 4476 | -2265 | L1M4c | L1 | LINE |
| 133282327 | 133283125 | - | 10.8 | 3979 | 4766 | -1380 | L1PREC2 | L1 | LINE |
| 133283124 | 133284465 | + | 8.9 | 4760 | 6112 | -49 | L1PA14 | L1 | LINE |
| 133284539 | 133284605 | + | 14.9 | 6099 | 6165 | 0 | L1PA15 | L1 | LINE |
| 133284606 | 133286296 | - | 20.5 | 1825 | 3568 | -3173 | L1M4c | L1 | LINE |
| 133286296 | 133287244 | + | 17.5 | 4289 | 5282 | -858 | L1MA9 | L1 | LINE |
| 133287272 | 133287808 | + | 20.0 | 5289 | 5844 | -468 | L1MA9 | L1 | LINE |
| 133287903 | 133288352 | - | 21.7 | 1367 | 1821 | -4928 | L1M4c | L1 | LINE |

  

|  |  |  |  |  |  |  |  |  |  |
| --- | --- | --- | --- | --- | --- | --- | --- | --- | --- |
| chr10:135166718-135169794 | | | 2925 bp | | |  |  |  |  |
| Genome Start | Genome End | Strand | Div | Repeat Start | Repeat End | Repeat Left | Name | Family | Class |
| 135166718 | 135166799 | - | 23.2 | 1521 | 1612 | -397 | HAL1b | L1 | LINE |
| 135166968 | 135168857 | - | 1.0 | 4265 | 6154 | -1 | L1PA2 | L1 | LINE |
| 135168856 | 135169734 | + | 2.0 | 3374 | 4252 | -1894 | L1PA2 | L1 | LINE |
| 135169721 | 135169794 | - | 19.4 | 1254 | 1328 | -681 | HAL1b | L1 | LINE |

  


|  |  |  |  |  |  |  |  |  |  |
| --- | --- | --- | --- | --- | --- | --- | --- | --- | --- |
| chr11:63171-65677 | | | 2473 bp | | |  |  |  |  |
| Genome Start | Genome End | Strand | Div | Repeat Start | Repeat End | Repeat Left | Name | Family | Class |
| 63171 | 63659 | + | 19.5 | 3150 | 3646 | -2494 | L1M3 | L1 | LINE |
| 63675 | 64396 | - | 2.6 | 5254 | 5975 | -180 | L1PA4 | L1 | LINE |
| 64397 | 64574 | + | 5.1 | 5977 | 6155 | 0 | L1PA4 | L1 | LINE |
| 64594 | 65677 | + | 16.5 | 3657 | 4741 | -1399 | L1M3 | L1 | LINE |

  

|  |  |  |  |  |  |  |  |  |  |
| --- | --- | --- | --- | --- | --- | --- | --- | --- | --- |
| chr11:4010691-4016516 | | | 5558 bp | | |  |  |  |  |
| Genome Start | Genome End | Strand | Div | Repeat Start | Repeat End | Repeat Left | Name | Family | Class |
| 4010691 | 4011406 | - | 10.1 | 4143 | 5202 | -1017 | L1MEc | L1 | LINE |
| 4011407 | 4011704 | - | 7.5 | 1 | 294 | -17 | AluY | Alu | SINE |
| 4011705 | 4011754 | - | 23.1 | 4100 | 4142 | -2077 | L1MEc | L1 | LINE |
| 4011755 | 4012059 | + | 10.5 | 1 | 305 | -5 | AluSg | Alu | SINE |
| 4012060 | 4013076 | - | 23.1 | 3021 | 4099 | -2120 | L1MEc | L1 | LINE |
| 4013323 | 4013644 | - | 22.3 | 2703 | 3036 | -3183 | L1MEc | L1 | LINE |
| 4013662 | 4014607 | - | 8.9 | 5208 | 6174 | 0 | L1PA11 | L1 | LINE |
| 4014606 | 4014768 | + | 8.6 | 5058 | 5225 | -921 | L1PA11 | L1 | LINE |
| 4014767 | 4014850 | - | 20.9 | 2655 | 2715 | -3504 | L1MEc | L1 | LINE |
| 4014851 | 4015156 | - | 12.8 | 1 | 311 | -1 | AluSx | Alu | SINE |
| 4015157 | 4015844 | - | 20.0 | 2091 | 2654 | -3565 | L1MEc | L1 | LINE |
| 4015845 | 4016140 | - | 14.5 | 1 | 296 | -16 | AluSx | Alu | SINE |
| 4016141 | 4016159 | - | 19.8 | 2053 | 2090 | -4310 | L1MEc | L1 | LINE |
| 4016160 | 4016236 | + | 10.5 | 6071 | 6155 | 0 | L1PA4 | L1 | LINE |
| 4016237 | 4016279 | - | 19.8 | 2009 | 2052 | -4348 | L1MEc | L1 | LINE |
| 4016289 | 4016516 | - | 19.3 | 34 | 256 | -6144 | L1MEc | L1 | LINE |

  

|  |  |  |  |  |  |  |  |  |  |
| --- | --- | --- | --- | --- | --- | --- | --- | --- | --- |
| chr11:4415145-4419548 | | | 4292 bp | | |  |  |  |  |
| Genome Start | Genome End | Strand | Div | Repeat Start | Repeat End | Repeat Left | Name | Family | Class |
| 4415145 | 4415321 | - | 28.5 | 3229 | 3411 | -8 | L2 | L2 | LINE |
| 4415323 | 4415564 | + | 16.5 | 5459 | 5712 | -579 | L1M2 | L1 | LINE |
| 4415577 | 4415909 | + | 15.1 | 5458 | 5793 | -498 | L1MA8 | L1 | LINE |
| 4415934 | 4416412 | + | 15.1 | 5794 | 6290 | -1 | L1MA8 | L1 | LINE |
| 4416419 | 4417217 | - | 31.2 | 2397 | 3240 | -179 | L2 | L2 | LINE |
| 4417223 | 4418130 | - | 5.7 | 4199 | 5119 | -1027 | L1PA5 | L1 | LINE |
| 4418127 | 4419134 | + | 4.8 | 5141 | 6150 | -4 | L1PA5 | L1 | LINE |
| 4419135 | 4419206 | - | 17.1 | 9 | 94 | -95 | MER5A | MER1\_type | DNA |
| 4419275 | 4419548 | - | 26.2 | 2039 | 2391 | -1028 | L2 | L2 | LINE |

  

|  |  |  |  |  |  |  |  |  |  |
| --- | --- | --- | --- | --- | --- | --- | --- | --- | --- |
| chr11:5032159-5033412 | | | 1237 bp | | |  |  |  |  |
| Genome Start | Genome End | Strand | Div | Repeat Start | Repeat End | Repeat Left | Name | Family | Class |
| 5032159 | 5032349 | - | 23.7 | 4182 | 4383 | -1763 | L1MD | L1 | LINE |
| 5032379 | 5032934 | - | 5.4 | 5598 | 6152 | -20 | L1PA8 | L1 | LINE |
| 5032923 | 5033101 | + | 7.3 | 5404 | 5582 | -590 | L1PA8 | L1 | LINE |
| 5033102 | 5033412 | - | 22.7 | 3846 | 4166 | -1980 | L1MD | L1 | LINE |

  

|  |  |  |  |  |  |  |  |  |  |
| --- | --- | --- | --- | --- | --- | --- | --- | --- | --- |
| chr11:5649069-5655641 | | | 6469 bp | | |  |  |  |  |
| Genome Start | Genome End | Strand | Div | Repeat Start | Repeat End | Repeat Left | Name | Family | Class |
| 5649069 | 5650008 | + | 24.3 | 1421 | 2471 | -4631 | L1M4 | L1 | LINE |
| 5650009 | 5650308 | - | 14.0 | 9 | 309 | -3 | AluSx | Alu | SINE |
| 5650309 | 5652033 | + | 26.2 | 2472 | 4192 | -2808 | L1M4 | L1 | LINE |
| 5652042 | 5652500 | + | 25.7 | 1 | 466 | -6564 | L1M3e | L1 | LINE |
| 5652519 | 5652566 | + | 12.5 | 4914 | 4975 | -1171 | L1M4 | L1 | LINE |
| 5652566 | 5652837 | + | 19.9 | 5858 | 6134 | -157 | L1MA8 | L1 | LINE |
| 5652838 | 5653012 | - | 8.0 | 122 | 296 | -15 | AluY | Alu | SINE |
| 5653023 | 5653122 | - | 18.0 | 63 | 162 | -16 | MER45A | Tip100 | DNA |
| 5653192 | 5653468 | - | 30.6 | 159 | 466 | -25 | LTR16C | ERVL | LTR |
| 5653469 | 5654051 | - | 1.6 | 5611 | 6193 | 0 | L1PA2 | L1 | LINE |
| 5654052 | 5655370 | + | 1.9 | 4280 | 5602 | -553 | L1PA2 | L1 | LINE |
| 5655371 | 5655534 | - | 4.5 | 1 | 134 | -177 | AluY | Alu | SINE |
| 5655535 | 5655641 | + | 29.9 | 4395 | 4503 | -2503 | L1M4 | L1 | LINE |

  

|  |  |  |  |  |  |  |  |  |  |
| --- | --- | --- | --- | --- | --- | --- | --- | --- | --- |
| chr11:8987291-8991402 | | | 3946 bp | | |  |  |  |  |
| Genome Start | Genome End | Strand | Div | Repeat Start | Repeat End | Repeat Left | Name | Family | Class |
| 8987291 | 8987411 | - | 17.4 | 7928 | 8041 | -1 | L1MC4 | L1 | LINE |
| 8987409 | 8987525 | - | 10.3 | 5272 | 5389 | -757 | L1M4 | L1 | LINE |
| 8987523 | 8989139 | - | 7.2 | 4525 | 6153 | -1 | L1PA7 | L1 | LINE |
| 8989139 | 8989205 | + | 3.0 | 4441 | 4507 | -1639 | L1PA7 | L1 | LINE |
| 8989205 | 8989331 | - | 17.3 | 5153 | 5281 | -865 | L1M4 | L1 | LINE |
| 8989382 | 8989710 | - | 23.0 | 7558 | 7899 | -143 | L1MC4 | L1 | LINE |
| 8989810 | 8990637 | - | 17.5 | 5322 | 6161 | -17 | L1MB8 | L1 | LINE |
| 8990638 | 8990942 | - | 8.8 | 1 | 305 | -6 | AluY | Alu | SINE |
| 8990943 | 8991142 | - | 17.0 | 5133 | 5321 | -825 | L1M | L1 | LINE |
| 8991168 | 8991402 | - | 26.3 | 7298 | 7535 | -507 | L1MC4 | L1 | LINE |

  

|  |  |  |  |  |  |  |  |  |  |
| --- | --- | --- | --- | --- | --- | --- | --- | --- | --- |
| chr11:10312932-10316571 | | | 3395 bp | | |  |  |  |  |
| Genome Start | Genome End | Strand | Div | Repeat Start | Repeat End | Repeat Left | Name | Family | Class |
| 10312932 | 10313088 | - | 31.9 | 2946 | 3100 | -278 | L2 | L2 | LINE |
| 10313165 | 10313327 | - | 14.1 | 7623 | 7785 | 0 | L1MC3 | L1 | LINE |
| 10313328 | 10313627 | - | 18.0 | 1 | 300 | -12 | AluJb | Alu | SINE |
| 10313628 | 10313901 | - | 14.1 | 7354 | 7622 | -163 | L1MC3 | L1 | LINE |
| 10313986 | 10314157 | + | 30.8 | 2813 | 2978 | -441 | L2 | L2 | LINE |
| 10314176 | 10314454 | - | 19.1 | 54 | 308 | -4 | AluJo | Alu | SINE |
| 10314455 | 10314577 | + | 13.8 | 1 | 124 | -188 | AluJb | Alu | SINE |
| 10314581 | 10315590 | - | 4.6 | 4736 | 5745 | -410 | L1PA4 | L1 | LINE |
| 10315591 | 10315985 | + | 3.5 | 5756 | 6150 | -5 | L1PA4 | L1 | LINE |
| 10315991 | 10316166 | + | 21.0 | 131 | 312 | 0 | AluJb | Alu | SINE |
| 10316168 | 10316248 | - | 17.7 | 1 | 62 | -240 | AluJo | Alu | SINE |
| 10316307 | 10316571 | - | 31.2 | 2362 | 2643 | -776 | L2 | L2 | LINE |

  

|  |  |  |  |  |  |  |  |  |  |
| --- | --- | --- | --- | --- | --- | --- | --- | --- | --- |
| chr11:11300639-11303851 | | | 3172 bp | | |  |  |  |  |
| Genome Start | Genome End | Strand | Div | Repeat Start | Repeat End | Repeat Left | Name | Family | Class |
| 11300639 | 11300863 | + | 21.2 | 5356 | 5589 | -573 | L1ME3A | L1 | LINE |
| 11300865 | 11301228 | - | 22.2 | 1 | 370 | -4 | MLT1A | MaLR | LTR |
| 11301229 | 11301637 | - | 19.9 | 1315 | 1735 | 0 | MLT1A-int | MaLR | LTR |
| 11301654 | 11301919 | - | 28.6 | 679 | 1030 | -459 | MLT1A-int | MaLR | LTR |
| 11301915 | 11302086 | - | 26.0 | 553 | 731 | -640 | MLT1A-int | MaLR | LTR |
| 11302079 | 11302359 | + | 20.4 | 98 | 370 | -4 | MLT1A | MaLR | LTR |
| 11302364 | 11302655 | + | 25.0 | 5582 | 5875 | -295 | L1ME3A | L1 | LINE |
| 11302653 | 11303005 | - | 7.5 | 5169 | 5532 | -619 | L1PB2 | L1 | LINE |
| 11303007 | 11303648 | + | 7.5 | 5521 | 6149 | -2 | L1PB2 | L1 | LINE |
| 11303672 | 11303748 | + | 29.9 | 5877 | 5954 | -210 | L1ME3A | L1 | LINE |
| 11303761 | 11303851 | + | 24.2 | 6077 | 6170 | 0 | L1ME3A | L1 | LINE |

  

|  |  |  |  |  |  |  |  |  |  |
| --- | --- | --- | --- | --- | --- | --- | --- | --- | --- |
| chr11:21462941-21472018 | | | 9087 bp | | |  |  |  |  |
| Genome Start | Genome End | Strand | Div | Repeat Start | Repeat End | Repeat Left | Name | Family | Class |
| 21462941 | 21469247 | + | 20.0 | 13 | 6020 | -825 | L1MA4A | L1 | LINE |
| 21469245 | 21470100 | - | 3.9 | 4373 | 5228 | -918 | L1PA3 | L1 | LINE |
| 21470089 | 21471025 | + | 2.7 | 5214 | 6150 | -5 | L1PA3 | L1 | LINE |
| 21471032 | 21472018 | + | 15.5 | 6019 | 6998 | -7 | L1MA4A | L1 | LINE |

  

|  |  |  |  |  |  |  |  |  |  |
| --- | --- | --- | --- | --- | --- | --- | --- | --- | --- |
| chr11:21499186-21502139 | | | 2861 bp | | |  |  |  |  |
| Genome Start | Genome End | Strand | Div | Repeat Start | Repeat End | Repeat Left | Name | Family | Class |
| 21499186 | 21499755 | - | 27.6 | 2696 | 3324 | -3058 | L1MEc | L1 | LINE |
| 21499770 | 21500189 | - | 15.5 | 5184 | 5630 | -526 | L1P5 | L1 | LINE |
| 21500192 | 21500228 | + | 5.4 | 5649 | 5686 | -468 | L1P5 | L1 | LINE |
| 21500229 | 21500352 | + | 6.5 | 155 | 278 | -33 | AluY | Alu | SINE |
| 21500353 | 21500841 | + | 18.7 | 5649 | 6153 | -3 | L1PB4 | L1 | LINE |
| 21500852 | 21500951 | - | 28.1 | 2587 | 2689 | -3693 | L1MEc | L1 | LINE |
| 21500952 | 21501268 | - | 6.5 | 1 | 310 | -1 | AluY | Alu | SINE |
| 21501269 | 21501537 | - | 28.2 | 2316 | 2586 | -3796 | L1MEc | L1 | LINE |
| 21501547 | 21502018 | - | 26.8 | 268 | 763 | -5800 | L1MEc | L1 | LINE |
| 21502077 | 21502139 | - | 31.8 | 198 | 260 | -6303 | L1MEc | L1 | LINE |

  

|  |  |  |  |  |  |  |  |  |  |
| --- | --- | --- | --- | --- | --- | --- | --- | --- | --- |
| chr11:21669339-21677981 | | | 8212 bp | | |  |  |  |  |
| Genome Start | Genome End | Strand | Div | Repeat Start | Repeat End | Repeat Left | Name | Family | Class |
| 21669339 | 21669908 | + | 23.4 | 909 | 1485 | -5148 | L1MD | L1 | LINE |
| 21669947 | 21670556 | + | 24.2 | 1646 | 3837 | -2700 | L1MD | L1 | LINE |
| 21670679 | 21674194 | - | 9.8 | 2615 | 6152 | -2 | L1PA7 | L1 | LINE |
| 21674195 | 21675506 | + | 7.7 | 1308 | 2591 | -3555 | L1PA7 | L1 | LINE |
| 21675530 | 21675562 | + | 9.1 | 3899 | 3931 | -2606 | L1MD | L1 | LINE |
| 21675585 | 21675651 | + | 14.9 | 235 | 301 | -11 | Alu | Alu | SINE |
| 21675669 | 21675800 | + | 17.9 | 4014 | 4144 | -2393 | L1MD | L1 | LINE |
| 21675801 | 21676095 | + | 8.9 | 1 | 291 | -20 | AluY | Alu | SINE |
| 21676234 | 21677183 | + | 19.2 | 4136 | 5163 | -1374 | L1MD | L1 | LINE |
| 21677209 | 21677551 | + | 22.2 | 5417 | 5769 | -864 | L1MD | L1 | LINE |
| 21677598 | 21677981 | + | 19.9 | 5770 | 6165 | -468 | L1MD | L1 | LINE |

  

|  |  |  |  |  |  |  |  |  |  |
| --- | --- | --- | --- | --- | --- | --- | --- | --- | --- |
| chr11:21825872-21830748 | | | 4877 bp | | |  |  |  |  |
| Genome Start | Genome End | Strand | Div | Repeat Start | Repeat End | Repeat Left | Name | Family | Class |
| 21825872 | 21826056 | + | 13.6 | 1 | 208 | -468 | MER61F | ERV1 | LTR |
| 21826059 | 21827161 | - | 7.2 | 5070 | 6146 | -8 | L1PA7 | L1 | LINE |
| 21827160 | 21829185 | + | 9.0 | 2346 | 4381 | -1765 | L1P3 | L1 | LINE |
| 21829186 | 21829624 | - | 13.3 | 2 | 440 | -44 | LTR3A | ERVK | LTR |
| 21829625 | 21830308 | + | 9.5 | 4375 | 5060 | -1086 | L1P3 | L1 | LINE |
| 21830309 | 21830748 | + | 13.9 | 194 | 639 | -37 | MER61F | ERV1 | LTR |

  

|  |  |  |  |  |  |  |  |  |  |
| --- | --- | --- | --- | --- | --- | --- | --- | --- | --- |
| chr11:22269040-22271060 | | | 2015 bp | | |  |  |  |  |
| Genome Start | Genome End | Strand | Div | Repeat Start | Repeat End | Repeat Left | Name | Family | Class |
| 22269040 | 22269166 | - | 32.4 | 493 | 619 | -9 | MLT2E | ERVL | LTR |
| 22269167 | 22269532 | + | 13.5 | 1 | 364 | 0 | THE1B | MaLR | LTR |
| 22269533 | 22269688 | - | 32.4 | 328 | 492 | -136 | MLT2E | ERVL | LTR |
| 22269695 | 22270145 | - | 3.1 | 5707 | 6155 | 0 | L1PA2 | L1 | LINE |
| 22270146 | 22270786 | + | 2.2 | 5051 | 5695 | -460 | L1PA2 | L1 | LINE |
| 22270787 | 22271060 | - | 34.1 | 24 | 337 | -291 | MLT2E | ERVL | LTR |

  

|  |  |  |  |  |  |  |  |  |  |
| --- | --- | --- | --- | --- | --- | --- | --- | --- | --- |
| chr11:22379151-22381269 | | | 1816 bp | | |  |  |  |  |
| Genome Start | Genome End | Strand | Div | Repeat Start | Repeat End | Repeat Left | Name | Family | Class |
| 22379151 | 22379302 | - | 34.2 | 2758 | 2921 | -498 | L2 | L2 | LINE |
| 22379404 | 22379798 | - | 29.9 | 5760 | 6160 | -2 | L1ME3 | L1 | LINE |
| 22379833 | 22379954 | - | 22.1 | 5168 | 5296 | -850 | L1ME3 | L1 | LINE |
| 22379948 | 22380116 | + | 27.4 | 4478 | 4663 | -1483 | L1M5 | L1 | LINE |
| 22380117 | 22380420 | + | 6.2 | 1 | 305 | -6 | AluY | Alu | SINE |
| 22380421 | 22380659 | + | 27.4 | 4664 | 4910 | -1236 | L1M5 | L1 | LINE |
| 22380736 | 22380810 | + | 20.0 | 5079 | 5157 | -989 | L1M5 | L1 | LINE |
| 22380868 | 22380987 | + | 21.1 | 6029 | 6158 | -154 | L1MA9 | L1 | LINE |
| 22381016 | 22381152 | + | 21.1 | 6159 | 6305 | -7 | L1MA9 | L1 | LINE |
| 22381167 | 22381269 | - | 34.3 | 2469 | 2569 | -850 | L2 | L2 | LINE |

  

|  |  |  |  |  |  |  |  |  |  |
| --- | --- | --- | --- | --- | --- | --- | --- | --- | --- |
| chr11:22396681-22398753 | | | 2002 bp | | |  |  |  |  |
| Genome Start | Genome End | Strand | Div | Repeat Start | Repeat End | Repeat Left | Name | Family | Class |
| 22396681 | 22396782 | - | 18.6 | 400 | 501 | -62 | MLT2C2 | ERVL | LTR |
| 22396790 | 22397266 | - | 20.8 | 5782 | 6274 | -38 | L1MA9 | L1 | LINE |
| 22397336 | 22397763 | - | 20.8 | 5384 | 5781 | -531 | L1MA9 | L1 | LINE |
| 22397759 | 22398442 | + | 17.6 | 4642 | 5380 | -760 | L1MA9 | L1 | LINE |
| 22398443 | 22398753 | - | 21.0 | 1 | 314 | -150 | MLT2C2 | ERVL | LTR |

  

|  |  |  |  |  |  |  |  |  |  |
| --- | --- | --- | --- | --- | --- | --- | --- | --- | --- |
| chr11:24462220-24468252 | | | 5703 bp | | |  |  |  |  |
| Genome Start | Genome End | Strand | Div | Repeat Start | Repeat End | Repeat Left | Name | Family | Class |
| 24462220 | 24463147 | - | 28.5 | 2715 | 3731 | -2651 | L1MEc | L1 | LINE |
| 24463148 | 24463788 | - | 9.1 | 5657 | 6302 | 0 | L1MA1 | L1 | LINE |
| 24463789 | 24464207 | - | 29.1 | 2270 | 2714 | -3668 | L1MEc | L1 | LINE |
| 24464228 | 24464807 | - | 31.9 | 1496 | 2269 | -4133 | L1MEc | L1 | LINE |
| 24464819 | 24465024 | - | 5.3 | 4056 | 4262 | -1884 | L1PA4 | L1 | LINE |
| 24465022 | 24466875 | + | 4.3 | 4269 | 6154 | -1 | L1PA4 | L1 | LINE |
| 24466878 | 24467308 | - | 31.5 | 1133 | 1586 | -4892 | L1MEc | L1 | LINE |
| 24467310 | 24467526 | - | 32.6 | 875 | 1115 | -5448 | L1MEc | L1 | LINE |
| 24467826 | 24468252 | - | 35.4 | 177 | 620 | -5943 | L1MEc | L1 | LINE |

  

|  |  |  |  |  |  |  |  |  |  |
| --- | --- | --- | --- | --- | --- | --- | --- | --- | --- |
| chr11:25634464-25635816 | | | 1269 bp | | |  |  |  |  |
| Genome Start | Genome End | Strand | Div | Repeat Start | Repeat End | Repeat Left | Name | Family | Class |
| 25634464 | 25634568 | + | 17.3 | 1 | 113 | -47 | MER5A1 | MER1\_type | DNA |
| 25634601 | 25634663 | - | 12.9 | 1 | 62 | -250 | AluJ/FLAM | Alu | SINE |
| 25634715 | 25635248 | - | 4.5 | 5614 | 6145 | -9 | L1PA5 | L1 | LINE |
| 25635249 | 25635746 | + | 3.2 | 5117 | 5615 | -539 | L1PA5 | L1 | LINE |
| 25635748 | 25635816 | + | 18.3 | 101 | 160 | 0 | MER5A1 | MER1\_type | DNA |

  

|  |  |  |  |  |  |  |  |  |  |
| --- | --- | --- | --- | --- | --- | --- | --- | --- | --- |
| chr11:27145778-27148486 | | | 2663 bp | | |  |  |  |  |
| Genome Start | Genome End | Strand | Div | Repeat Start | Repeat End | Repeat Left | Name | Family | Class |
| 27145778 | 27146113 | + | 35.6 | 44 | 384 | -80 | LTR16B | ERVL | LTR |
| 27146143 | 27146991 | - | 19.7 | 5497 | 6290 | -13 | L1MA4A | L1 | LINE |
| 27146995 | 27148160 | - | 12.8 | 4967 | 6153 | -13 | L1PA16 | L1 | LINE |
| 27148158 | 27148258 | + | 8.0 | 4875 | 4974 | -1172 | L1PA16 | L1 | LINE |
| 27148247 | 27148360 | - | 12.6 | 5380 | 5491 | -800 | L1MA4A | L1 | LINE |
| 27148390 | 27148486 | + | 21.9 | 369 | 464 | 0 | LTR16B | ERVL | LTR |

  

|  |  |  |  |  |  |  |  |  |  |
| --- | --- | --- | --- | --- | --- | --- | --- | --- | --- |
| chr11:28243760-28245756 | | | 1997 bp | | |  |  |  |  |
| Genome Start | Genome End | Strand | Div | Repeat Start | Repeat End | Repeat Left | Name | Family | Class |
| 28243760 | 28243937 | + | 28.9 | 2245 | 2440 | -979 | L2 | L2 | LINE |
| 28243938 | 28244171 | - | 6.0 | 5916 | 6152 | -3 | L1PA4 | L1 | LINE |
| 28244172 | 28244591 | + | 3.3 | 5482 | 5901 | -254 | L1PA4 | L1 | LINE |
| 28244592 | 28245000 | + | 27.4 | 2437 | 2882 | -537 | L2 | L2 | LINE |
| 28245001 | 28245306 | + | 10.1 | 1 | 306 | -7 | AluSq | Alu | SINE |
| 28245307 | 28245756 | + | 28.9 | 2883 | 3376 | -43 | L2 | L2 | LINE |

  

|  |  |  |  |  |  |  |  |  |  |
| --- | --- | --- | --- | --- | --- | --- | --- | --- | --- |
| chr11:29115519-29122439 | | | 6502 bp | | |  |  |  |  |
| Genome Start | Genome End | Strand | Div | Repeat Start | Repeat End | Repeat Left | Name | Family | Class |
| 29115519 | 29115704 | + | 26.2 | 1 | 193 | -374 | MLT1F | MaLR | LTR |
| 29115741 | 29116815 | - | 30.4 | 165 | 1289 | -5134 | L1MCc | L1 | LINE |
| 29116940 | 29117030 | - | 35.2 | 4374 | 4465 | -24 | L3 | CR1 | LINE |
| 29117034 | 29118215 | - | 20.3 | 6570 | 7783 | -2 | L1MC3 | L1 | LINE |
| 29118251 | 29118812 | - | 20.3 | 6006 | 6569 | -1216 | L1MC3 | L1 | LINE |
| 29118821 | 29119048 | - | 17.4 | 3991 | 4235 | -2086 | L1MCa | L1 | LINE |
| 29119051 | 29120381 | - | 22.5 | 1804 | 3211 | -3110 | L1MCa | L1 | LINE |
| 29120400 | 29120680 | - | 21.6 | 1 | 326 | -48 | MLT1A0 | MaLR | LTR |
| 29120683 | 29120860 | - | 33.3 | 9 | 189 | -79 | MIRb | MIR | SINE |
| 29120878 | 29121182 | - | 14.8 | 1672 | 2718 | 0 | Tigger2 | MER2\_type | DNA |
| 29121197 | 29121355 | - | 0.6 | 5997 | 6155 | 0 | L1HS | L1 | LINE |
| 29121356 | 29121892 | + | 1.7 | 5481 | 6017 | -138 | L1HS | L1 | LINE |
| 29122033 | 29122232 | + | 29.5 | 2 | 209 | -15 | MER58A | MER1\_type | DNA |
| 29122253 | 29122439 | + | 20.6 | 338 | 531 | -11 | MLT1F | MaLR | LTR |

  

|  |  |  |  |  |  |  |  |  |  |
| --- | --- | --- | --- | --- | --- | --- | --- | --- | --- |
| chr11:29762936-29770455 | | | 7300 bp | | |  |  |  |  |
| Genome Start | Genome End | Strand | Div | Repeat Start | Repeat End | Repeat Left | Name | Family | Class |
| 29762936 | 29763679 | + | 22.4 | 1 | 748 | -707 | Charlie1a | MER1\_type | DNA |
| 29763680 | 29765204 | - | 19.0 | 4693 | 6338 | -6 | L1MC2 | L1 | LINE |
| 29765204 | 29766105 | - | 3.2 | 5254 | 6155 | 0 | L1PA3 | L1 | LINE |
| 29766097 | 29767659 | + | 3.2 | 3693 | 5263 | -883 | L1PA3 | L1 | LINE |
| 29767660 | 29768156 | - | 21.7 | 4212 | 4699 | -1447 | L1MC2 | L1 | LINE |
| 29768364 | 29769395 | - | 18.4 | 3089 | 4201 | -1945 | L1MC2 | L1 | LINE |
| 29769417 | 29769760 | + | 23.6 | 1 | 365 | 0 | MLT1A0 | MaLR | LTR |
| 29769763 | 29770455 | + | 22.7 | 767 | 1452 | -3 | Charlie1a | MER1\_type | DNA |

  

|  |  |  |  |  |  |  |  |  |  |
| --- | --- | --- | --- | --- | --- | --- | --- | --- | --- |
| chr11:30177110-30178337 | | | 1179 bp | | |  |  |  |  |
| Genome Start | Genome End | Strand | Div | Repeat Start | Repeat End | Repeat Left | Name | Family | Class |
| 30177110 | 30177301 | + | 20.8 | 1 | 168 | -337 | MLT1D | MaLR | LTR |
| 30177311 | 30177813 | - | 4.0 | 5653 | 6155 | 0 | L1PA2 | L1 | LINE |
| 30177814 | 30178008 | + | 1.0 | 5476 | 5670 | -485 | L1PA2 | L1 | LINE |
| 30178049 | 30178337 | + | 16.1 | 202 | 502 | -3 | MLT1D | MaLR | LTR |

  

|  |  |  |  |  |  |  |  |  |  |
| --- | --- | --- | --- | --- | --- | --- | --- | --- | --- |
| chr11:30942220-30945749 | | | 3443 bp | | |  |  |  |  |
| Genome Start | Genome End | Strand | Div | Repeat Start | Repeat End | Repeat Left | Name | Family | Class |
| 30942220 | 30943603 | - | 25.0 | 177 | 1683 | 0 | FordPrefect\_a | Tip100 | DNA |
| 30943671 | 30943700 | + | 6.7 | 4519 | 4548 | -1598 | L1M4 | L1 | LINE |
| 30943728 | 30945514 | - | 0.8 | 4365 | 6151 | -4 | L1HS | L1 | LINE |
| 30945508 | 30945636 | + | 0.0 | 4218 | 4346 | -1800 | L1HS | L1 | LINE |
| 30945637 | 30945749 | - | 25.2 | 1 | 116 | -392 | FordPrefect\_a | Tip100 | DNA |

  

|  |  |  |  |  |  |  |  |  |  |
| --- | --- | --- | --- | --- | --- | --- | --- | --- | --- |
| chr11:32684804-32690275 | | | 4703 bp | | |  |  |  |  |
| Genome Start | Genome End | Strand | Div | Repeat Start | Repeat End | Repeat Left | Name | Family | Class |
| 32684804 | 32684997 | + | 32.1 | 2117 | 2311 | -4490 | MER110-int | ERV1 | LTR |
| 32685347 | 32685646 | + | 30.4 | 180 | 470 | -4177 | MER110-int | ERV1 | LTR |
| 32686066 | 32687416 | - | 9.5 | 3771 | 5108 | -1038 | L1PB | L1 | LINE |
| 32687417 | 32687717 | - | 15.6 | 1 | 304 | -8 | AluSx | Alu | SINE |
| 32687718 | 32688946 | - | 9.5 | 2510 | 3770 | -2376 | L1PB | L1 | LINE |
| 32688940 | 32689892 | + | 9.5 | 5087 | 6151 | 0 | L1PB1 | L1 | LINE |
| 32689901 | 32690275 | + | 33.0 | 3078 | 3460 | -3341 | MER110-int | ERV1 | LTR |

  

|  |  |  |  |  |  |  |  |  |  |
| --- | --- | --- | --- | --- | --- | --- | --- | --- | --- |
| chr11:35373759-35376054 | | | 2294 bp | | |  |  |  |  |
| Genome Start | Genome End | Strand | Div | Repeat Start | Repeat End | Repeat Left | Name | Family | Class |
| 35373759 | 35373794 | + | 8.3 | 1 | 36 | -338 | MLT1A0 | MaLR | LTR |
| 35373799 | 35374897 | - | 1.2 | 5057 | 6155 | 0 | L1PA2 | L1 | LINE |
| 35374896 | 35375720 | + | 1.6 | 4235 | 5059 | -1087 | L1PA2 | L1 | LINE |
| 35375721 | 35376054 | + | 16.2 | 24 | 365 | 0 | MLT1A0 | MaLR | LTR |

  

|  |  |  |  |  |  |  |  |  |  |
| --- | --- | --- | --- | --- | --- | --- | --- | --- | --- |
| chr11:36871297-36873524 | | | 2218 bp | | |  |  |  |  |
| Genome Start | Genome End | Strand | Div | Repeat Start | Repeat End | Repeat Left | Name | Family | Class |
| 36871297 | 36871701 | + | 24.5 | 6740 | 7141 | -644 | L1MC3 | L1 | LINE |
| 36871715 | 36872065 | - | 1.7 | 5804 | 6154 | -1 | L1HS | L1 | LINE |
| 36872066 | 36872906 | + | 1.0 | 4960 | 5800 | -355 | L1HS | L1 | LINE |
| 36872904 | 36873524 | + | 18.4 | 7127 | 7770 | -15 | L1MC3 | L1 | LINE |

  

|  |  |  |  |  |  |  |  |  |  |
| --- | --- | --- | --- | --- | --- | --- | --- | --- | --- |
| chr11:36994036-36994975 | | | 916 bp | | |  |  |  |  |
| Genome Start | Genome End | Strand | Div | Repeat Start | Repeat End | Repeat Left | Name | Family | Class |
| 36994036 | 36994223 | - | 30.9 | 3223 | 3419 | 0 | L2 | L2 | LINE |
| 36994230 | 36994371 | - | 15.5 | 5560 | 5701 | -467 | L1MB1 | L1 | LINE |
| 36994370 | 36994824 | + | 17.4 | 5696 | 6146 | -22 | L1MB1 | L1 | LINE |
| 36994845 | 36994975 | - | 31.8 | 3086 | 3224 | -195 | L2 | L2 | LINE |

  

|  |  |  |  |  |  |  |  |  |  |
| --- | --- | --- | --- | --- | --- | --- | --- | --- | --- |
| chr11:37007919-37011736 | | | 3785 bp | | |  |  |  |  |
| Genome Start | Genome End | Strand | Div | Repeat Start | Repeat End | Repeat Left | Name | Family | Class |
| 37007919 | 37008075 | - | 25.5 | 412 | 582 | -8 | MLT1G1 | MaLR | LTR |
| 37008106 | 37010659 | - | 7.5 | 3535 | 6154 | 0 | L1PA7 | L1 | LINE |
| 37010658 | 37011341 | + | 10.1 | 2831 | 3517 | -2629 | L1PA7 | L1 | LINE |
| 37011347 | 37011736 | - | 29.4 | 10 | 388 | -207 | MLT1G1 | MaLR | LTR |

  

|  |  |  |  |  |  |  |  |  |  |
| --- | --- | --- | --- | --- | --- | --- | --- | --- | --- |
| chr11:39285340-39289301 | | | 3912 bp | | |  |  |  |  |
| Genome Start | Genome End | Strand | Div | Repeat Start | Repeat End | Repeat Left | Name | Family | Class |
| 39285340 | 39286824 | - | 7.7 | 4661 | 6152 | -2 | L1PA7 | L1 | LINE |
| 39286877 | 39287108 | - | 5.6 | 5919 | 6150 | -4 | L1PA5 | L1 | LINE |
| 39287110 | 39287287 | + | 3.4 | 5717 | 5894 | -260 | L1PA5 | L1 | LINE |
| 39287285 | 39288275 | - | 10.6 | 3691 | 4676 | -1470 | L1PA7 | L1 | LINE |
| 39288276 | 39288591 | - | 10.4 | 3 | 311 | 0 | AluY | Alu | SINE |
| 39288592 | 39289301 | - | 10.6 | 2997 | 3690 | -2456 | L1PA7 | L1 | LINE |

  

|  |  |  |  |  |  |  |  |  |  |
| --- | --- | --- | --- | --- | --- | --- | --- | --- | --- |
| chr11:39774968-39780064 | | | 4854 bp | | |  |  |  |  |
| Genome Start | Genome End | Strand | Div | Repeat Start | Repeat End | Repeat Left | Name | Family | Class |
| 39774968 | 39775097 | + | 25.8 | 5350 | 5478 | -2330 | L1MC4a | L1 | LINE |
| 39775280 | 39775596 | + | 30.4 | 5630 | 5957 | -1851 | L1MC4a | L1 | LINE |
| 39775599 | 39777337 | - | 4.5 | 4410 | 6152 | -2 | L1PA5 | L1 | LINE |
| 39777338 | 39778990 | + | 6.5 | 2749 | 4405 | -1741 | L1PA5 | L1 | LINE |
| 39779018 | 39779951 | + | 30.8 | 5954 | 6982 | -826 | L1MC4a | L1 | LINE |
| 39779984 | 39780064 | + | 30.8 | 6983 | 7076 | -732 | L1MC4a | L1 | LINE |

  

|  |  |  |  |  |  |  |  |  |  |
| --- | --- | --- | --- | --- | --- | --- | --- | --- | --- |
| chr11:40308310-40310108 | | | 1769 bp | | |  |  |  |  |
| Genome Start | Genome End | Strand | Div | Repeat Start | Repeat End | Repeat Left | Name | Family | Class |
| 40308310 | 40308421 | + | 12.5 | 1 | 113 | -402 | MLT2B2 | ERVL | LTR |
| 40308452 | 40308611 | - | 12.5 | 6144 | 6298 | -2 | L1MA5 | L1 | LINE |
| 40308612 | 40308973 | - | 10.5 | 1 | 375 | 0 | THE1C | MaLR | LTR |
| 40308974 | 40309211 | - | 12.5 | 5888 | 6143 | -157 | L1MA5 | L1 | LINE |
| 40309212 | 40309682 | + | 12.4 | 5451 | 5894 | -406 | L1MA5 | L1 | LINE |
| 40309683 | 40310108 | + | 18.9 | 98 | 515 | 0 | MLT2B2 | ERVL | LTR |

  

|  |  |  |  |  |  |  |  |  |  |
| --- | --- | --- | --- | --- | --- | --- | --- | --- | --- |
| chr11:41622208-41634900 | | | 12216 bp | | |  |  |  |  |
| Genome Start | Genome End | Strand | Div | Repeat Start | Repeat End | Repeat Left | Name | Family | Class |
| 41622208 | 41622313 | - | 26.7 | 3608 | 3715 | -2822 | L1MDa | L1 | LINE |
| 41622317 | 41622891 | - | 9.9 | 5590 | 6147 | -4 | L1PB1 | L1 | LINE |
| 41622892 | 41623116 | - | 16.7 | 5456 | 5681 | -470 | L1P5 | L1 | LINE |
| 41623221 | 41629651 | - | 10.9 | 8 | 6454 | -15 | L1PA7 | L1 | LINE |
| 41629657 | 41629875 | - | 10.5 | 5266 | 5487 | -664 | L1P5 | L1 | LINE |
| 41629871 | 41630957 | + | 10.0 | 4163 | 5247 | -899 | L1PB | L1 | LINE |
| 41630959 | 41631054 | - | 21.1 | 3551 | 3631 | -2906 | L1MDa | L1 | LINE |
| 41631055 | 41631408 | - | 19.8 | 5823 | 6180 | -3 | L1MB3 | L1 | LINE |
| 41631409 | 41633109 | - | 22.1 | 1795 | 3550 | -2987 | L1MDa | L1 | LINE |
| 41633280 | 41633838 | - | 23.8 | 1269 | 1794 | -4839 | L1MDa | L1 | LINE |
| 41633881 | 41634109 | + | 23.1 | 1 | 231 | -317 | LTR55 | ERV | LTR |
| 41634110 | 41634233 | - | 33.1 | 842 | 981 | -5652 | L1MDa | L1 | LINE |
| 41634290 | 41634507 | - | 31.8 | 604 | 842 | -5791 | L1MDa | L1 | LINE |
| 41634609 | 41634900 | - | 21.4 | 16 | 302 | -6331 | L1MDa | L1 | LINE |

  

|  |  |  |  |  |  |  |  |  |  |
| --- | --- | --- | --- | --- | --- | --- | --- | --- | --- |
| chr11:42280982-42283426 | | | 2366 bp | | |  |  |  |  |
| Genome Start | Genome End | Strand | Div | Repeat Start | Repeat End | Repeat Left | Name | Family | Class |
| 42280982 | 42281049 | - | 26.9 | 3350 | 3418 | -1 | L2 | L2 | LINE |
| 42281115 | 42281510 | - | 14.7 | 4215 | 4611 | -1535 | L1PA16 | L1 | LINE |
| 42281503 | 42283043 | + | 15.1 | 4612 | 6157 | -9 | L1PA16 | L1 | LINE |
| 42283065 | 42283365 | + | 16.0 | 1 | 301 | 0 | AluJb | Alu | SINE |
| 42283367 | 42283426 | - | 33.3 | 3300 | 3360 | -18 | L2 | L2 | LINE |

  

|  |  |  |  |  |  |  |  |  |  |
| --- | --- | --- | --- | --- | --- | --- | --- | --- | --- |
| chr11:42543166-42546493 | | | 3096 bp | | |  |  |  |  |
| Genome Start | Genome End | Strand | Div | Repeat Start | Repeat End | Repeat Left | Name | Family | Class |
| 42543166 | 42543267 | - | 27.4 | 3299 | 3402 | -17 | L2 | L2 | LINE |
| 42543274 | 42543432 | - | 21.1 | 16 | 215 | -4 | MER20 | MER1\_type | DNA |
| 42543551 | 42544124 | - | 35.6 | 2577 | 3183 | -236 | L2 | L2 | LINE |
| 42544225 | 42544794 | - | 9.3 | 4230 | 4799 | -1347 | L1PA10 | L1 | LINE |
| 42544791 | 42546147 | + | 10.0 | 4814 | 6167 | -1 | L1PA10 | L1 | LINE |
| 42546160 | 42546493 | - | 31.6 | 2134 | 2480 | -939 | L2 | L2 | LINE |

  

|  |  |  |  |  |  |  |  |  |  |
| --- | --- | --- | --- | --- | --- | --- | --- | --- | --- |
| chr11:42631825-42636131 | | | 4104 bp | | |  |  |  |  |
| Genome Start | Genome End | Strand | Div | Repeat Start | Repeat End | Repeat Left | Name | Family | Class |
| 42631825 | 42632358 | - | 36.0 | 4417 | 4951 | -716 | HERV16 | ERVL | LTR |
| 42632455 | 42633404 | - | 8.8 | 4311 | 5258 | -888 | L1PA10 | L1 | LINE |
| 42633405 | 42634288 | + | 7.2 | 5262 | 6159 | -9 | L1PA10 | L1 | LINE |
| 42634384 | 42635732 | - | 33.3 | 2914 | 4275 | -1392 | HERV16 | ERVL | LTR |
| 42635745 | 42636131 | - | 31.9 | 39 | 436 | -4560 | HERV16 | ERVL | LTR |

  

|  |  |  |  |  |  |  |  |  |  |
| --- | --- | --- | --- | --- | --- | --- | --- | --- | --- |
| chr11:42662493-42675469 | | | 12821 bp | | |  |  |  |  |
| Genome Start | Genome End | Strand | Div | Repeat Start | Repeat End | Repeat Left | Name | Family | Class |
| 42662493 | 42662718 | + | 21.7 | 6459 | 6703 | -1339 | L1MC4 | L1 | LINE |
| 42662897 | 42663939 | + | 27.1 | 6640 | 7715 | -327 | L1MC4 | L1 | LINE |
| 42663927 | 42664715 | - | 16.5 | 2746 | 3575 | -2565 | L1M3 | L1 | LINE |
| 42664684 | 42666142 | + | 17.9 | 3568 | 5055 | -1085 | L1M3 | L1 | LINE |
| 42666154 | 42669335 | + | 6.9 | 9 | 3190 | -2956 | L1P1 | L1 | LINE |
| 42669336 | 42672291 | + | 4.9 | 3191 | 6151 | -4 | L1PA4 | L1 | LINE |
| 42672281 | 42672761 | + | 14.8 | 5044 | 5566 | -746 | L1MA9 | L1 | LINE |
| 42672762 | 42673117 | + | 13.2 | 1 | 358 | -6 | THE1B | MaLR | LTR |
| 42673120 | 42673333 | + | 14.0 | 5558 | 5776 | -536 | L1MA9 | L1 | LINE |
| 42673318 | 42674561 | + | 14.9 | 4863 | 6151 | -3 | L1PB3 | L1 | LINE |
| 42674563 | 42674644 | + | 26.0 | 5314 | 5387 | -853 | L1M | L1 | LINE |
| 42674674 | 42674724 | + | 26.0 | 5388 | 5437 | -803 | L1M5 | L1 | LINE |
| 42674725 | 42675256 | + | 18.1 | 5769 | 6308 | -4 | L1MA9 | L1 | LINE |
| 42675264 | 42675469 | + | 23.3 | 7695 | 7901 | -141 | L1MC4 | L1 | LINE |

  

|  |  |  |  |  |  |  |  |  |  |
| --- | --- | --- | --- | --- | --- | --- | --- | --- | --- |
| chr11:42710535-42716971 | | | 6126 bp | | |  |  |  |  |
| Genome Start | Genome End | Strand | Div | Repeat Start | Repeat End | Repeat Left | Name | Family | Class |
| 42710535 | 42710719 | - | 34.2 | 3221 | 3410 | -9 | L2 | L2 | LINE |
| 42710724 | 42711085 | + | 24.7 | 125 | 500 | -5900 | L1MEc | L1 | LINE |
| 42711074 | 42713313 | + | 9.9 | 3869 | 6157 | -6 | L1PA13 | L1 | LINE |
| 42713405 | 42713769 | + | 24.4 | 527 | 881 | -5519 | L1MEc | L1 | LINE |
| 42713786 | 42715154 | + | 23.7 | 1631 | 2988 | -3231 | L1MEc | L1 | LINE |
| 42715157 | 42715277 | - | 22.3 | 5347 | 5467 | -703 | L1M5 | L1 | LINE |
| 42715360 | 42715414 | + | 16.4 | 5248 | 5304 | -842 | L1M4 | L1 | LINE |
| 42715511 | 42715883 | - | 6.4 | 5386 | 5789 | -365 | L1PA7 | L1 | LINE |
| 42715888 | 42715927 | + | 5.0 | 5789 | 5829 | -325 | L1PA7 | L1 | LINE |
| 42715936 | 42716191 | - | 8.3 | 4446 | 4703 | -1443 | L1PREC2 | L1 | LINE |
| 42716205 | 42716528 | + | 6.8 | 5833 | 6154 | 0 | L1PA7 | L1 | LINE |
| 42716536 | 42716834 | + | 6.0 | 1 | 299 | -12 | AluY | Alu | SINE |
| 42716835 | 42716971 | - | 31.9 | 2882 | 3022 | -397 | L2 | L2 | LINE |

  

|  |  |  |  |  |  |  |  |  |  |
| --- | --- | --- | --- | --- | --- | --- | --- | --- | --- |
| chr11:42874337-42877090 | | | 2746 bp | | |  |  |  |  |
| Genome Start | Genome End | Strand | Div | Repeat Start | Repeat End | Repeat Left | Name | Family | Class |
| 42874337 | 42875388 | - | 4.0 | 5098 | 6153 | -2 | L1PA4 | L1 | LINE |
| 42875403 | 42875909 | - | 1.4 | 5647 | 6153 | -2 | L1PA2 | L1 | LINE |
| 42875910 | 42876209 | + | 2.3 | 5338 | 5640 | -515 | L1PA2 | L1 | LINE |
| 42876204 | 42877090 | - | 4.4 | 4229 | 5116 | -1030 | L1PA4 | L1 | LINE |

  

|  |  |  |  |  |  |  |  |  |  |
| --- | --- | --- | --- | --- | --- | --- | --- | --- | --- |
| chr11:44989730-44996720 | | | 6972 bp | | |  |  |  |  |
| Genome Start | Genome End | Strand | Div | Repeat Start | Repeat End | Repeat Left | Name | Family | Class |
| 44989730 | 44989799 | - | 14.3 | 356 | 426 | 0 | MSTB | MaLR | LTR |
| 44989800 | 44989934 | - | 6.9 | 5757 | 5896 | -276 | L1PA8 | L1 | LINE |
| 44989943 | 44996072 | - | 7.2 | 5 | 6149 | -5 | L1PA6 | L1 | LINE |
| 44996084 | 44996167 | - | 3.6 | 5689 | 5772 | -400 | L1PA8 | L1 | LINE |
| 44996168 | 44996432 | + | 5.3 | 5909 | 6172 | 0 | L1PA8 | L1 | LINE |
| 44996433 | 44996720 | - | 17.2 | 18 | 369 | -63 | MSTB | MaLR | LTR |

  

|  |  |  |  |  |  |  |  |  |  |
| --- | --- | --- | --- | --- | --- | --- | --- | --- | --- |
| chr11:48558621-48560772 | | | 2094 bp | | |  |  |  |  |
| Genome Start | Genome End | Strand | Div | Repeat Start | Repeat End | Repeat Left | Name | Family | Class |
| 48558621 | 48558845 | - | 27.3 | 1172 | 1390 | -82 | MLT1H-int | MaLR | LTR |
| 48558882 | 48559253 | - | 3.5 | 4985 | 5356 | -790 | L1PA3 | L1 | LINE |
| 48559252 | 48560084 | + | 2.1 | 5332 | 6166 | 0 | L1PA3 | L1 | LINE |
| 48560109 | 48560772 | - | 32.8 | 6 | 753 | -719 | MLT1H-int | MaLR | LTR |

  

|  |  |  |  |  |  |  |  |  |  |
| --- | --- | --- | --- | --- | --- | --- | --- | --- | --- |
| chr11:48570398-48572864 | | | 2388 bp | | |  |  |  |  |
| Genome Start | Genome End | Strand | Div | Repeat Start | Repeat End | Repeat Left | Name | Family | Class |
| 48570398 | 48570774 | - | 29.3 | 4277 | 4679 | -1540 | L1MEc | L1 | LINE |
| 48570808 | 48570931 | - | 25.8 | 3397 | 3524 | -2695 | L1MEc | L1 | LINE |
| 48570932 | 48571276 | - | 3.2 | 5275 | 5619 | -536 | L1PA5 | L1 | LINE |
| 48571277 | 48571809 | + | 5.1 | 5611 | 6145 | -9 | L1PA5 | L1 | LINE |
| 48571846 | 48572317 | - | 28.5 | 2911 | 3404 | -2815 | L1MEc | L1 | LINE |
| 48572318 | 48572554 | - | 24.2 | 313 | 562 | 0 | MLT1F2 | MaLR | LTR |
| 48572565 | 48572864 | - | 29.8 | 1718 | 2052 | -4348 | L1MEc | L1 | LINE |

  

|  |  |  |  |  |  |  |  |  |  |
| --- | --- | --- | --- | --- | --- | --- | --- | --- | --- |
| chr11:48574489-48582214 | | | 7738 bp | | |  |  |  |  |
| Genome Start | Genome End | Strand | Div | Repeat Start | Repeat End | Repeat Left | Name | Family | Class |
| 48574489 | 48576403 | - | 12.4 | 4926 | 6859 | -8 | L1PB1 | L1 | LINE |
| 48576408 | 48576743 | - | 12.8 | 3908 | 4242 | -2620 | L1PB1 | L1 | LINE |
| 48576736 | 48578069 | - | 4.6 | 4821 | 6153 | -2 | L1PA4 | L1 | LINE |
| 48578068 | 48578761 | + | 5.6 | 4128 | 4822 | -1324 | L1PA4 | L1 | LINE |
| 48578756 | 48582214 | - | 15.8 | 6 | 3918 | -2944 | L1PB1 | L1 | LINE |

  

|  |  |  |  |  |  |  |  |  |  |
| --- | --- | --- | --- | --- | --- | --- | --- | --- | --- |
| chr11:49447410-49451167 | | | 3612 bp | | |  |  |  |  |
| Genome Start | Genome End | Strand | Div | Repeat Start | Repeat End | Repeat Left | Name | Family | Class |
| 49447410 | 49447459 | - | 31.4 | 5795 | 5844 | -326 | L1M5 | L1 | LINE |
| 49447503 | 49447688 | - | 31.4 | 5599 | 5794 | -376 | L1M5 | L1 | LINE |
| 49447705 | 49448810 | - | 5.0 | 5051 | 6155 | 0 | L1PA4 | L1 | LINE |
| 49448797 | 49449036 | + | 5.0 | 4827 | 5066 | -1080 | L1PA4 | L1 | LINE |
| 49449038 | 49450166 | - | 30.5 | 4374 | 5552 | -612 | L1M5 | L1 | LINE |
| 49450181 | 49450316 | + | 20.0 | 3 | 138 | -227 | MLT1A1 | MaLR | LTR |
| 49450317 | 49450455 | - | 8.6 | 171 | 309 | -2 | AluY | Alu | SINE |
| 49450506 | 49450681 | + | 16.8 | 228 | 408 | 0 | MLT1A1 | MaLR | LTR |
| 49450718 | 49451167 | - | 26.1 | 3836 | 4313 | -1833 | L1M5 | L1 | LINE |

  

|  |  |  |  |  |  |  |  |  |  |
| --- | --- | --- | --- | --- | --- | --- | --- | --- | --- |
| chr11:49510582-49513279 | | | 2665 bp | | |  |  |  |  |
| Genome Start | Genome End | Strand | Div | Repeat Start | Repeat End | Repeat Left | Name | Family | Class |
| 49510582 | 49511609 | - | 24.1 | 1457 | 2622 | -3757 | L1M2 | L1 | LINE |
| 49511618 | 49511971 | - | 18.4 | 5939 | 6298 | -2 | L1MA5 | L1 | LINE |
| 49511972 | 49512094 | + | 15.9 | 5701 | 5793 | -507 | L1MA5 | L1 | LINE |
| 49512120 | 49512258 | + | 15.9 | 5794 | 5939 | -361 | L1MA5 | L1 | LINE |
| 49512259 | 49513279 | - | 26.0 | 828 | 1862 | -4521 | L1M2 | L1 | LINE |

  

|  |  |  |  |  |  |  |  |  |  |
| --- | --- | --- | --- | --- | --- | --- | --- | --- | --- |
| chr11:49550185-49552317 | | | 2015 bp | | |  |  |  |  |
| Genome Start | Genome End | Strand | Div | Repeat Start | Repeat End | Repeat Left | Name | Family | Class |
| 49550185 | 49550909 | + | 33.4 | 2100 | 2876 | -543 | L2 | L2 | LINE |
| 49550910 | 49551202 | - | 10.6 | 1 | 293 | -19 | AluSx | Alu | SINE |
| 49551203 | 49551295 | + | 33.4 | 2877 | 2967 | -452 | L2 | L2 | LINE |
| 49551357 | 49551498 | - | 6.3 | 160 | 301 | -10 | AluY | Alu | SINE |
| 49551502 | 49552060 | - | 10.7 | 5607 | 6163 | 0 | L1PA13 | L1 | LINE |
| 49552064 | 49552146 | + | 8.4 | 5541 | 5623 | -540 | L1PA13 | L1 | LINE |
| 49552198 | 49552317 | + | 31.4 | 3296 | 3419 | 0 | L2 | L2 | LINE |

  

|  |  |  |  |  |  |  |  |  |  |
| --- | --- | --- | --- | --- | --- | --- | --- | --- | --- |
| chr11:55229244-55230260 | | | 1015 bp | | |  |  |  |  |
| Genome Start | Genome End | Strand | Div | Repeat Start | Repeat End | Repeat Left | Name | Family | Class |
| 55229244 | 55229366 | + | 25.4 | 1 | 131 | -214 | MER2 | MER2\_type | DNA |
| 55229367 | 55229951 | - | 3.0 | 5578 | 6155 | 0 | L1PA4 | L1 | LINE |
| 55229954 | 55230044 | + | 1.1 | 5496 | 5586 | -569 | L1PA4 | L1 | LINE |
| 55230045 | 55230260 | + | 18.1 | 117 | 344 | -1 | MER2 | MER2\_type | DNA |

  

|  |  |  |  |  |  |  |  |  |  |
| --- | --- | --- | --- | --- | --- | --- | --- | --- | --- |
| chr11:56208899-56214216 | | | 4898 bp | | |  |  |  |  |
| Genome Start | Genome End | Strand | Div | Repeat Start | Repeat End | Repeat Left | Name | Family | Class |
| 56208899 | 56209297 | - | 30.5 | 2988 | 3417 | -2 | L2 | L2 | LINE |
| 56209343 | 56209715 | + | 19.9 | 4107 | 4475 | -1671 | L1MC1 | L1 | LINE |
| 56209723 | 56209836 | - | 13.2 | 5233 | 5350 | -796 | L1PB1 | L1 | LINE |
| 56209825 | 56210666 | + | 9.1 | 5358 | 6179 | 0 | L1PB1 | L1 | LINE |
| 56210680 | 56211039 | + | 14.8 | 4477 | 4854 | -1292 | L1MC1 | L1 | LINE |
| 56211051 | 56211198 | + | 26.2 | 10 | 132 | -1 | FLAM\_A | Alu | SINE |
| 56211257 | 56211349 | + | 18.8 | 22 | 107 | -59 | FRAM | Alu | SINE |
| 56211457 | 56212658 | + | 17.9 | 4848 | 6069 | -264 | L1MC1 | L1 | LINE |
| 56212653 | 56212869 | + | 20.0 | 6009 | 6227 | -106 | L1MC1 | L1 | LINE |
| 56212928 | 56212991 | - | 7.9 | 5193 | 5257 | -889 | L1MC | L1 | LINE |
| 56213008 | 56213107 | + | 20.2 | 6234 | 6333 | 0 | L1MC1 | L1 | LINE |
| 56213108 | 56213782 | + | 29.0 | 5438 | 6117 | -53 | L1ME3A | L1 | LINE |
| 56213899 | 56214052 | - | 24.7 | 1 | 155 | -5 | MER5A1 | MER1\_type | DNA |
| 56214060 | 56214216 | - | 33.1 | 2729 | 2901 | -518 | L2 | L2 | LINE |

  

|  |  |  |  |  |  |  |  |  |  |
| --- | --- | --- | --- | --- | --- | --- | --- | --- | --- |
| chr11:56777149-56783652 | | | 6357 bp | | |  |  |  |  |
| Genome Start | Genome End | Strand | Div | Repeat Start | Repeat End | Repeat Left | Name | Family | Class |
| 56777149 | 56777384 | - | 32.0 | 2256 | 2478 | -941 | L2 | L2 | LINE |
| 56777385 | 56777973 | + | 23.3 | 1 | 562 | 0 | MLT1F2 | MaLR | LTR |
| 56777974 | 56778074 | - | 32.0 | 2148 | 2255 | -1164 | L2 | L2 | LINE |
| 56778075 | 56778496 | - | 17.1 | 1 | 432 | 0 | MSTB1 | MaLR | LTR |
| 56778497 | 56778647 | - | 32.0 | 1986 | 2147 | -1272 | L2 | L2 | LINE |
| 56778674 | 56780539 | + | 22.8 | 90 | 1954 | -5380 | L1M4b | L1 | LINE |
| 56780573 | 56780780 | - | 23.6 | 2 | 221 | -91 | AluJo | Alu | SINE |
| 56780781 | 56781073 | + | 19.7 | 1946 | 2257 | -4751 | L1M4b | L1 | LINE |
| 56781073 | 56781430 | + | 16.4 | 5290 | 5653 | -525 | L1MB8 | L1 | LINE |
| 56781431 | 56781789 | + | 19.1 | 3 | 365 | 0 | MLT1A0 | MaLR | LTR |
| 56781790 | 56782258 | + | 16.4 | 5654 | 6125 | -53 | L1MB8 | L1 | LINE |
| 56782255 | 56782411 | - | 10.8 | 5388 | 5553 | -613 | L1PA15 | L1 | LINE |
| 56782412 | 56782959 | + | 12.8 | 5558 | 6158 | -7 | L1PA15 | L1 | LINE |
| 56782962 | 56783269 | + | 9.2 | 1 | 309 | -3 | AluSx | Alu | SINE |
| 56783293 | 56783361 | + | 11.6 | 6100 | 6173 | -5 | L1MB8 | L1 | LINE |
| 56783430 | 56783652 | - | 25.7 | 1396 | 1680 | -1739 | L2 | L2 | LINE |

  

|  |  |  |  |  |  |  |  |  |  |
| --- | --- | --- | --- | --- | --- | --- | --- | --- | --- |
| chr11:57690578-57693093 | | | 1814 bp | | |  |  |  |  |
| Genome Start | Genome End | Strand | Div | Repeat Start | Repeat End | Repeat Left | Name | Family | Class |
| 57690578 | 57690693 | - | 30.1 | 5625 | 5739 | -382 | L1M5 | L1 | LINE |
| 57690916 | 57691172 | - | 20.5 | 5901 | 6152 | -4 | L1PB4 | L1 | LINE |
| 57691173 | 57691226 | + | 16.7 | 5840 | 5893 | -263 | L1PB4 | L1 | LINE |
| 57691235 | 57691530 | - | 31.2 | 4630 | 4946 | -1200 | L1M5 | L1 | LINE |
| 57691543 | 57692043 | - | 21.9 | 41 | 542 | 0 | MLT1H | MaLR | LTR |
| 57692258 | 57692543 | - | 34.1 | 5807 | 6098 | -142 | L1ME3B | L1 | LINE |
| 57692790 | 57693093 | - | 30.8 | 2772 | 3085 | -3061 | L1M5 | L1 | LINE |

  

|  |  |  |  |  |  |  |  |  |  |
| --- | --- | --- | --- | --- | --- | --- | --- | --- | --- |
| chr11:58475008-58477685 | | | 2540 bp | | |  |  |  |  |
| Genome Start | Genome End | Strand | Div | Repeat Start | Repeat End | Repeat Left | Name | Family | Class |
| 58475008 | 58476470 | - | 32.8 | 3687 | 5244 | -902 | L1M5 | L1 | LINE |
| 58476602 | 58476899 | - | 18.2 | 5863 | 6165 | -1 | L1PA16 | L1 | LINE |
| 58476900 | 58477203 | + | 15.8 | 5565 | 5867 | -299 | L1PA16 | L1 | LINE |
| 58477211 | 58477685 | - | 31.5 | 3071 | 3518 | -2628 | L1M5 | L1 | LINE |

  

|  |  |  |  |  |  |  |  |  |  |
| --- | --- | --- | --- | --- | --- | --- | --- | --- | --- |
| chr11:59996309-60000071 | | | 3745 bp | | |  |  |  |  |
| Genome Start | Genome End | Strand | Div | Repeat Start | Repeat End | Repeat Left | Name | Family | Class |
| 59996309 | 59996392 | + | 27.7 | 17 | 103 | -75 | MER5B | MER1\_type | DNA |
| 59996396 | 59996510 | - | 6.1 | 2538 | 2652 | -3494 | L1PA3 | L1 | LINE |
| 59996505 | 59999991 | + | 3.0 | 2661 | 6152 | -3 | L1PA3 | L1 | LINE |
| 60000013 | 60000071 | + | 20.9 | 92 | 152 | -26 | MER5B | MER1\_type | DNA |

  

|  |  |  |  |  |  |  |  |  |  |
| --- | --- | --- | --- | --- | --- | --- | --- | --- | --- |
| chr11:60124347-60128457 | | | 4117 bp | | |  |  |  |  |
| Genome Start | Genome End | Strand | Div | Repeat Start | Repeat End | Repeat Left | Name | Family | Class |
| 60124347 | 60125014 | - | 9.9 | 5483 | 6155 | -8 | L1PA13 | L1 | LINE |
| 60125021 | 60126579 | - | 3.1 | 4580 | 6155 | 0 | L1PA3 | L1 | LINE |
| 60126576 | 60128004 | + | 2.5 | 3135 | 4565 | -1581 | L1PA3 | L1 | LINE |
| 60127997 | 60128457 | - | 8.7 | 5045 | 5498 | -663 | L1PA13 | L1 | LINE |

  

|  |  |  |  |  |  |  |  |  |  |
| --- | --- | --- | --- | --- | --- | --- | --- | --- | --- |
| chr11:62592173-62594742 | | | 2299 bp | | |  |  |  |  |
| Genome Start | Genome End | Strand | Div | Repeat Start | Repeat End | Repeat Left | Name | Family | Class |
| 62592173 | 62592405 | + | 29.6 | 3512 | 3756 | -2390 | L1M4 | L1 | LINE |
| 62592406 | 62592779 | + | 20.1 | 1 | 365 | 0 | MLT1A0 | MaLR | LTR |
| 62592780 | 62592903 | + | 29.6 | 3757 | 3878 | -2268 | L1M4 | L1 | LINE |
| 62592931 | 62593237 | - | 11.6 | 1 | 303 | -9 | AluSx | Alu | SINE |
| 62593262 | 62593570 | + | 10.8 | 5473 | 5782 | -384 | L1PA16 | L1 | LINE |
| 62593566 | 62593698 | - | 10.7 | 5958 | 6088 | -63 | L1PB1 | L1 | LINE |
| 62593695 | 62593750 | + | 14.3 | 6096 | 6151 | 0 | L1PB1 | L1 | LINE |
| 62593969 | 62594109 | + | 19.1 | 482 | 617 | -90 | MER39 | ERV1 | LTR |
| 62594110 | 62594473 | + | 15.4 | 5771 | 6163 | -3 | L1PA16 | L1 | LINE |
| 62594485 | 62594742 | + | 22.9 | 3968 | 4231 | -1915 | L1M4 | L1 | LINE |

  

|  |  |  |  |  |  |  |  |  |  |
| --- | --- | --- | --- | --- | --- | --- | --- | --- | --- |
| chr11:70509973-70511643 | | | 1676 bp | | |  |  |  |  |
| Genome Start | Genome End | Strand | Div | Repeat Start | Repeat End | Repeat Left | Name | Family | Class |
| 70509973 | 70510155 | + | 16.0 | 1 | 190 | -277 | MLT1C | MaLR | LTR |
| 70510156 | 70510470 | - | 8.2 | 4939 | 5257 | -889 | L1PA6 | L1 | LINE |
| 70510465 | 70511340 | + | 4.5 | 5267 | 6141 | -13 | L1PA6 | L1 | LINE |
| 70511342 | 70511643 | + | 20.7 | 167 | 465 | -2 | MLT1C | MaLR | LTR |

  

|  |  |  |  |  |  |  |  |  |  |
| --- | --- | --- | --- | --- | --- | --- | --- | --- | --- |
| chr11:76301507-76304320 | | | 2772 bp | | |  |  |  |  |
| Genome Start | Genome End | Strand | Div | Repeat Start | Repeat End | Repeat Left | Name | Family | Class |
| 76301507 | 76301731 | - | 10.7 | 448 | 697 | 0 | MER50B | ERV1 | LTR |
| 76301739 | 76302251 | - | 4.3 | 3963 | 4475 | -1671 | L1PA5 | L1 | LINE |
| 76302251 | 76303894 | + | 4.5 | 4499 | 6154 | 0 | L1PA5 | L1 | LINE |
| 76303931 | 76304320 | - | 10.5 | 1 | 460 | -237 | MER50B | ERV1 | LTR |

  

|  |  |  |  |  |  |  |  |  |  |
| --- | --- | --- | --- | --- | --- | --- | --- | --- | --- |
| chr11:79137298-79142657 | | | 5330 bp | | |  |  |  |  |
| Genome Start | Genome End | Strand | Div | Repeat Start | Repeat End | Repeat Left | Name | Family | Class |
| 79137298 | 79137931 | + | 24.4 | 45 | 751 | -5649 | L1MEc | L1 | LINE |
| 79137969 | 79140043 | + | 22.5 | 752 | 3050 | -3169 | L1MEc | L1 | LINE |
| 79140045 | 79140279 | - | 18.0 | 4115 | 4350 | -1793 | L1MA4A | L1 | LINE |
| 79140278 | 79140729 | + | 17.9 | 4349 | 4772 | -1371 | L1MA4A | L1 | LINE |
| 79140730 | 79141084 | + | 13.6 | 3 | 381 | 0 | THE1D | MaLR | LTR |
| 79141085 | 79142562 | + | 15.3 | 4773 | 6291 | -12 | L1MA4A | L1 | LINE |
| 79142557 | 79142657 | + | 18.2 | 3032 | 3147 | -3072 | L1MEc | L1 | LINE |

  

|  |  |  |  |  |  |  |  |  |  |
| --- | --- | --- | --- | --- | --- | --- | --- | --- | --- |
| chr11:79323595-79325488 | | | 1821 bp | | |  |  |  |  |
| Genome Start | Genome End | Strand | Div | Repeat Start | Repeat End | Repeat Left | Name | Family | Class |
| 79323595 | 79324170 | + | 30.2 | 1637 | 2281 | -1138 | L2 | L2 | LINE |
| 79324183 | 79324446 | - | 15.2 | 5907 | 6163 | -5 | L1PA10 | L1 | LINE |
| 79324450 | 79324913 | + | 8.3 | 5266 | 5902 | -266 | L1PA10 | L1 | LINE |
| 79324972 | 79325488 | + | 30.2 | 2849 | 3416 | -3 | L2 | L2 | LINE |

  

|  |  |  |  |  |  |  |  |  |  |
| --- | --- | --- | --- | --- | --- | --- | --- | --- | --- |
| chr11:79659535-79667039 | | | 7469 bp | | |  |  |  |  |
| Genome Start | Genome End | Strand | Div | Repeat Start | Repeat End | Repeat Left | Name | Family | Class |
| 79659535 | 79661619 | + | 28.5 | 183 | 2380 | -4566 | L1MA9 | L1 | LINE |
| 79661620 | 79661972 | - | 6.8 | 5801 | 6153 | -1 | L1PA6 | L1 | LINE |
| 79661973 | 79662601 | + | 6.0 | 5166 | 5794 | -360 | L1PA6 | L1 | LINE |
| 79662598 | 79662944 | + | 19.4 | 2369 | 2687 | -3689 | L1MA9 | L1 | LINE |
| 79662945 | 79663117 | - | 11.4 | 5993 | 6159 | -1 | L1PREC2 | L1 | LINE |
| 79663118 | 79664400 | + | 19.4 | 2688 | 3909 | -2467 | L1MA9 | L1 | LINE |
| 79664441 | 79667039 | + | 19.4 | 3910 | 6389 | -159 | L1MA9 | L1 | LINE |

  

|  |  |  |  |  |  |  |  |  |  |
| --- | --- | --- | --- | --- | --- | --- | --- | --- | --- |
| chr11:81313434-81315699 | | | 2177 bp | | |  |  |  |  |
| Genome Start | Genome End | Strand | Div | Repeat Start | Repeat End | Repeat Left | Name | Family | Class |
| 81313434 | 81313978 | - | 20.9 | 7227 | 7785 | 0 | L1MC3 | L1 | LINE |
| 81313979 | 81314101 | + | 12.2 | 6178 | 6300 | -3 | L1MA4A | L1 | LINE |
| 81314102 | 81314716 | - | 20.9 | 6646 | 7226 | -559 | L1MC3 | L1 | LINE |
| 81314767 | 81314892 | - | 20.9 | 6524 | 6645 | -1140 | L1MC3 | L1 | LINE |
| 81314925 | 81315007 | - | 6.0 | 6086 | 6170 | -2 | L1PA8 | L1 | LINE |
| 81315008 | 81315141 | + | 9.0 | 5955 | 6088 | -84 | L1PA8 | L1 | LINE |
| 81315149 | 81315699 | - | 24.3 | 5996 | 6543 | -1242 | L1MC3 | L1 | LINE |

  

|  |  |  |  |  |  |  |  |  |  |
| --- | --- | --- | --- | --- | --- | --- | --- | --- | --- |
| chr11:81525818-81528865 | | | 2744 bp | | |  |  |  |  |
| Genome Start | Genome End | Strand | Div | Repeat Start | Repeat End | Repeat Left | Name | Family | Class |
| 81525818 | 81525956 | + | 32.3 | 84 | 217 | -5828 | L1MCb | L1 | LINE |
| 81526246 | 81526311 | + | 26.1 | 570 | 637 | -5408 | L1MCb | L1 | LINE |
| 81526312 | 81526614 | + | 24.2 | 1 | 297 | -5 | AluJb | Alu | SINE |
| 81526615 | 81526701 | + | 26.1 | 638 | 735 | -5310 | L1MCb | L1 | LINE |
| 81526734 | 81527087 | - | 21.4 | 9 | 365 | 0 | MLT1A0 | MaLR | LTR |
| 81527088 | 81527374 | + | 23.4 | 2494 | 2790 | -3774 | L1MCb | L1 | LINE |
| 81527375 | 81528447 | - | 15.6 | 5049 | 6165 | -1 | L1PA17 | L1 | LINE |
| 81528448 | 81528710 | + | 14.9 | 4786 | 5049 | -1097 | L1PA17 | L1 | LINE |
| 81528694 | 81528865 | + | 30.1 | 2773 | 2939 | -3625 | L1MCb | L1 | LINE |

  

|  |  |  |  |  |  |  |  |  |  |
| --- | --- | --- | --- | --- | --- | --- | --- | --- | --- |
| chr11:81665789-81674009 | | | 8010 bp | | |  |  |  |  |
| Genome Start | Genome End | Strand | Div | Repeat Start | Repeat End | Repeat Left | Name | Family | Class |
| 81665789 | 81665953 | + | 30.6 | 5296 | 5468 | -772 | L1MC4a | L1 | LINE |
| 81666048 | 81666991 | - | 4.0 | 4331 | 5274 | -872 | L1P1 | L1 | LINE |
| 81666991 | 81672179 | + | 3.5 | 964 | 6152 | -3 | L1PA3 | L1 | LINE |
| 81672188 | 81672263 | - | 2.6 | 4259 | 4334 | -1812 | L1P1 | L1 | LINE |
| 81672264 | 81673136 | + | 3.8 | 5282 | 6153 | -2 | L1PA4 | L1 | LINE |
| 81673146 | 81673227 | + | 29.3 | 5567 | 5649 | -521 | L1MC4a | L1 | LINE |
| 81673326 | 81673804 | + | 21.7 | 1 | 472 | 0 | MER70A | ERVL | LTR |
| 81673808 | 81674009 | + | 29.6 | 5706 | 5920 | -1888 | L1MC4a | L1 | LINE |

  

|  |  |  |  |  |  |  |  |  |  |
| --- | --- | --- | --- | --- | --- | --- | --- | --- | --- |
| chr11:81797940-81805001 | | | 6731 bp | | |  |  |  |  |
| Genome Start | Genome End | Strand | Div | Repeat Start | Repeat End | Repeat Left | Name | Family | Class |
| 81797940 | 81798202 | + | 33.1 | 806 | 1050 | -6516 | L1M1 | L1 | LINE |
| 81798203 | 81798509 | + | 15.3 | 1 | 308 | -5 | AluSq | Alu | SINE |
| 81798510 | 81798667 | + | 33.1 | 1051 | 1201 | -6365 | L1M1 | L1 | LINE |
| 81798680 | 81798837 | + | 32.0 | 1280 | 1434 | -6132 | L1M1 | L1 | LINE |
| 81799132 | 81800651 | + | 24.0 | 2121 | 3395 | -4228 | L1M1 | L1 | LINE |
| 81800678 | 81801576 | - | 2.5 | 5260 | 6155 | 0 | L1PA3 | L1 | LINE |
| 81801574 | 81801613 | + | 7.5 | 5217 | 5256 | -890 | L1PA3 | L1 | LINE |
| 81801612 | 81802077 | + | 20.8 | 3344 | 3832 | -3730 | L1M1 | L1 | LINE |
| 81802078 | 81802228 | + | 0.7 | 158 | 308 | -10 | AluYb8 | Alu | SINE |
| 81802232 | 81802899 | + | 16.9 | 3827 | 4459 | -3103 | L1M1 | L1 | LINE |
| 81802903 | 81803977 | + | 15.5 | 4 | 1104 | -4224 | L1M3f | L1 | LINE |
| 81803976 | 81804064 | + | 13.3 | 6449 | 6537 | -1025 | L1M1 | L1 | LINE |
| 81804065 | 81804322 | + | 17.4 | 35 | 301 | -11 | AluSx | Alu | SINE |
| 81804323 | 81805001 | + | 15.3 | 6538 | 7205 | -514 | L1M1 | L1 | LINE |

  

|  |  |  |  |  |  |  |  |  |  |
| --- | --- | --- | --- | --- | --- | --- | --- | --- | --- |
| chr11:81852936-81854398 | | | 1365 bp | | |  |  |  |  |
| Genome Start | Genome End | Strand | Div | Repeat Start | Repeat End | Repeat Left | Name | Family | Class |
| 81852936 | 81853063 | - | 28.6 | 129 | 268 | 0 | MIR | MIR | SINE |
| 81853091 | 81854093 | - | 11.4 | 5138 | 6165 | -1 | L1PA16 | L1 | LINE |
| 81854100 | 81854206 | + | 16.8 | 5022 | 5128 | -1018 | L1PA16 | L1 | LINE |
| 81854272 | 81854398 | - | 31.9 | 2 | 121 | -141 | MIR | MIR | SINE |

  

|  |  |  |  |  |  |  |  |  |  |
| --- | --- | --- | --- | --- | --- | --- | --- | --- | --- |
| chr11:82056425-82059952 | | | 3488 bp | | |  |  |  |  |
| Genome Start | Genome End | Strand | Div | Repeat Start | Repeat End | Repeat Left | Name | Family | Class |
| 82056425 | 82056643 | - | 22.3 | 1028 | 1257 | -4862 | L1MEd | L1 | LINE |
| 82056661 | 82058218 | - | 13.8 | 4614 | 6155 | -1 | L1PB4 | L1 | LINE |
| 82058214 | 82058985 | + | 15.4 | 3832 | 4624 | -1522 | L1P4 | L1 | LINE |
| 82059014 | 82059952 | - | 37.1 | 19 | 968 | -5151 | L1MEd | L1 | LINE |

  

|  |  |  |  |  |  |  |  |  |  |
| --- | --- | --- | --- | --- | --- | --- | --- | --- | --- |
| chr11:83374030-83377337 | | | 3285 bp | | |  |  |  |  |
| Genome Start | Genome End | Strand | Div | Repeat Start | Repeat End | Repeat Left | Name | Family | Class |
| 83374030 | 83374724 | - | 11.8 | 5476 | 6174 | 0 | L1PA11 | L1 | LINE |
| 83374753 | 83375818 | - | 2.6 | 5087 | 6152 | -3 | L1PA3 | L1 | LINE |
| 83375814 | 83376487 | + | 3.7 | 4417 | 5090 | -1056 | L1PA3 | L1 | LINE |
| 83376486 | 83377049 | - | 10.1 | 4927 | 5489 | -685 | L1PA11 | L1 | LINE |
| 83377052 | 83377337 | - | 6.3 | 4415 | 4719 | -1427 | L1PA11 | L1 | LINE |

  

|  |  |  |  |  |  |  |  |  |  |
| --- | --- | --- | --- | --- | --- | --- | --- | --- | --- |
| chr11:83588860-83596635 | | | 7654 bp | | |  |  |  |  |
| Genome Start | Genome End | Strand | Div | Repeat Start | Repeat End | Repeat Left | Name | Family | Class |
| 83588860 | 83589056 | + | 34.0 | 2278 | 2482 | -4852 | L1MDa | L1 | LINE |
| 83589066 | 83589125 | - | 11.1 | 97 | 144 | -158 | AluSq/x | Alu | SINE |
| 83589204 | 83590451 | - | 10.1 | 3344 | 4586 | -1560 | L1PA11 | L1 | LINE |
| 83590436 | 83590499 | + | 9.5 | 4581 | 4643 | -1503 | L1PA11 | L1 | LINE |
| 83590500 | 83590807 | - | 11.7 | 1 | 309 | -1 | AluSg | Alu | SINE |
| 83590808 | 83591992 | + | 9.7 | 4644 | 5824 | -350 | L1PA11 | L1 | LINE |
| 83591993 | 83592290 | + | 6.4 | 2 | 299 | -12 | AluY | Alu | SINE |
| 83592291 | 83592644 | + | 10.0 | 5825 | 6170 | -4 | L1PA11 | L1 | LINE |
| 83592647 | 83592944 | - | 8.7 | 4 | 302 | -11 | AluSp | Alu | SINE |
| 83592947 | 83593399 | + | 26.9 | 2705 | 3194 | -3914 | L1MDa | L1 | LINE |
| 83593400 | 83593693 | - | 15.8 | 2 | 309 | -3 | AluSx | Alu | SINE |
| 83593694 | 83595750 | + | 25.6 | 3093 | 5278 | -1722 | L1MDa | L1 | LINE |
| 83595798 | 83596635 | + | 30.8 | 5279 | 6166 | -834 | L1MDa | L1 | LINE |

  

|  |  |  |  |  |  |  |  |  |  |
| --- | --- | --- | --- | --- | --- | --- | --- | --- | --- |
| chr11:83665515-83671197 | | | 5363 bp | | |  |  |  |  |
| Genome Start | Genome End | Strand | Div | Repeat Start | Repeat End | Repeat Left | Name | Family | Class |
| 83665515 | 83665798 | + | 27.3 | 44 | 337 | -6063 | L1ME1 | L1 | LINE |
| 83665796 | 83666558 | + | 28.2 | 2580 | 3397 | -2822 | L1ME1 | L1 | LINE |
| 83666648 | 83666879 | - | 18.4 | 1 | 237 | -75 | AluJo | Alu | SINE |
| 83666881 | 83667392 | + | 21.6 | 3416 | 3933 | -2286 | L1ME1 | L1 | LINE |
| 83667495 | 83667616 | - | 8.1 | 6044 | 6165 | 0 | L1PA7 | L1 | LINE |
| 83667619 | 83668535 | + | 6.8 | 5098 | 6027 | -127 | L1PA7 | L1 | LINE |
| 83668582 | 83668641 | + | 26.7 | 3965 | 4024 | -2195 | L1ME1 | L1 | LINE |
| 83668659 | 83668997 | + | 24.0 | 4149 | 4502 | -1717 | L1ME1 | L1 | LINE |
| 83668998 | 83669236 | - | 13.0 | 24 | 294 | -18 | AluSx | Alu | SINE |
| 83669237 | 83669306 | + | 24.0 | 4503 | 4571 | -1648 | L1ME1 | L1 | LINE |
| 83669373 | 83669383 | + | 23.1 | 4911 | 4917 | -1302 | L1ME1 | L1 | LINE |
| 83669384 | 83669711 | - | 5.3 | 1 | 304 | -7 | AluY | Alu | SINE |
| 83669712 | 83670128 | + | 23.1 | 4918 | 5343 | -876 | L1ME1 | L1 | LINE |
| 83670129 | 83670339 | - | 3.4 | 5948 | 6155 | 0 | L1PA3 | L1 | LINE |
| 83670340 | 83671197 | + | 21.3 | 5343 | 6187 | -52 | L1ME1 | L1 | LINE |

  

|  |  |  |  |  |  |  |  |  |  |
| --- | --- | --- | --- | --- | --- | --- | --- | --- | --- |
| chr11:84455721-84459266 | | | 3529 bp | | |  |  |  |  |
| Genome Start | Genome End | Strand | Div | Repeat Start | Repeat End | Repeat Left | Name | Family | Class |
| 84455721 | 84455784 | + | 12.5 | 5627 | 5692 | -464 | L1PB4 | L1 | LINE |
| 84455783 | 84456257 | - | 5.7 | 2931 | 3410 | -2736 | L1PA4 | L1 | LINE |
| 84456256 | 84459032 | + | 4.4 | 3417 | 6180 | 0 | L1PA4 | L1 | LINE |
| 84459036 | 84459133 | + | 17.4 | 5683 | 5780 | -376 | L1PB4 | L1 | LINE |
| 84459152 | 84459266 | + | 13.0 | 6021 | 6149 | -7 | L1PB4 | L1 | LINE |

  

|  |  |  |  |  |  |  |  |  |  |
| --- | --- | --- | --- | --- | --- | --- | --- | --- | --- |
| chr11:84606389-84608162 | | | 1729 bp | | |  |  |  |  |
| Genome Start | Genome End | Strand | Div | Repeat Start | Repeat End | Repeat Left | Name | Family | Class |
| 84606389 | 84606468 | + | 28.8 | 3003 | 3084 | -294 | L2 | L2 | LINE |
| 84606504 | 84606856 | - | 16.7 | 4771 | 5113 | -1033 | L1PA16 | L1 | LINE |
| 84606856 | 84607926 | + | 13.3 | 5108 | 6157 | -9 | L1PA16 | L1 | LINE |
| 84607938 | 84608162 | + | 32.9 | 3129 | 3376 | -43 | L2 | L2 | LINE |

  

|  |  |  |  |  |  |  |  |  |  |
| --- | --- | --- | --- | --- | --- | --- | --- | --- | --- |
| chr11:86000560-86002978 | | | 2361 bp | | |  |  |  |  |
| Genome Start | Genome End | Strand | Div | Repeat Start | Repeat End | Repeat Left | Name | Family | Class |
| 86000560 | 86000608 | - | 32.6 | 208 | 256 | -12 | MIRb | MIR | SINE |
| 86000626 | 86001969 | - | 6.7 | 4781 | 6131 | 0 | L1PA8A | L1 | LINE |
| 86001959 | 86002813 | + | 7.3 | 3939 | 4793 | -1353 | L1PA8A | L1 | LINE |
| 86002866 | 86002978 | - | 30.5 | 32 | 137 | -131 | MIRb | MIR | SINE |

  

|  |  |  |  |  |  |  |  |  |  |
| --- | --- | --- | --- | --- | --- | --- | --- | --- | --- |
| chr11:86269550-86272571 | | | 3029 bp | | |  |  |  |  |
| Genome Start | Genome End | Strand | Div | Repeat Start | Repeat End | Repeat Left | Name | Family | Class |
| 86269550 | 86270910 | - | 15.3 | 3026 | 4334 | -1812 | L1M4 | L1 | LINE |
| 86270896 | 86271861 | - | 9.9 | 5186 | 6167 | -1 | L1PA12 | L1 | LINE |
| 86271861 | 86272373 | + | 8.4 | 4661 | 5180 | -966 | L1PREC2 | L1 | LINE |
| 86272383 | 86272571 | - | 11.2 | 2843 | 3033 | -3113 | L1M4 | L1 | LINE |

  

|  |  |  |  |  |  |  |  |  |  |
| --- | --- | --- | --- | --- | --- | --- | --- | --- | --- |
| chr11:86550916-86559684 | | | 8754 bp | | |  |  |  |  |
| Genome Start | Genome End | Strand | Div | Repeat Start | Repeat End | Repeat Left | Name | Family | Class |
| 86550916 | 86551711 | - | 4.9 | 5667 | 6465 | -4 | L1PA7 | L1 | LINE |
| 86551730 | 86554369 | - | 1.5 | 3513 | 6152 | -3 | L1PA2 | L1 | LINE |
| 86554370 | 86554804 | + | 3.2 | 3078 | 3513 | -2633 | L1PA2 | L1 | LINE |
| 86554802 | 86559684 | - | 9.2 | 763 | 5680 | -781 | L1PA7 | L1 | LINE |

  

|  |  |  |  |  |  |  |  |  |  |
| --- | --- | --- | --- | --- | --- | --- | --- | --- | --- |
| chr11:87180424-87190185 | | | 9736 bp | | |  |  |  |  |
| Genome Start | Genome End | Strand | Div | Repeat Start | Repeat End | Repeat Left | Name | Family | Class |
| 87180424 | 87182994 | - | 8.6 | 3876 | 6456 | -13 | L1PA7 | L1 | LINE |
| 87183024 | 87185261 | - | 5.8 | 3909 | 6153 | -1 | L1PA5 | L1 | LINE |
| 87185260 | 87186304 | + | 8.0 | 2868 | 3913 | -2233 | L1PA5 | L1 | LINE |
| 87186304 | 87190185 | - | 11.1 | 2 | 3893 | -2568 | L1PA7 | L1 | LINE |

  

|  |  |  |  |  |  |  |  |  |  |
| --- | --- | --- | --- | --- | --- | --- | --- | --- | --- |
| chr11:88009430-88011941 | | | 2475 bp | | |  |  |  |  |
| Genome Start | Genome End | Strand | Div | Repeat Start | Repeat End | Repeat Left | Name | Family | Class |
| 88009430 | 88009523 | - | 20.2 | 3773 | 3871 | -2275 | L1M5 | L1 | LINE |
| 88009532 | 88009980 | - | 2.7 | 4541 | 4989 | -1157 | L1PA3 | L1 | LINE |
| 88009971 | 88011131 | + | 2.8 | 4989 | 6152 | -3 | L1PA3 | L1 | LINE |
| 88011144 | 88011232 | - | 25.0 | 3694 | 3770 | -2376 | L1M5 | L1 | LINE |
| 88011260 | 88011941 | - | 25.0 | 2981 | 3693 | -2453 | L1M5 | L1 | LINE |

  

|  |  |  |  |  |  |  |  |  |  |
| --- | --- | --- | --- | --- | --- | --- | --- | --- | --- |
| chr11:88423141-88425256 | | | 1993 bp | | |  |  |  |  |
| Genome Start | Genome End | Strand | Div | Repeat Start | Repeat End | Repeat Left | Name | Family | Class |
| 88423141 | 88423254 | - | 34.5 | 3302 | 3419 | 0 | L2 | L2 | LINE |
| 88423312 | 88423394 | - | 8.4 | 5541 | 5623 | -540 | L1PA13 | L1 | LINE |
| 88423397 | 88423946 | + | 11.1 | 5606 | 6163 | 0 | L1PA13 | L1 | LINE |
| 88423950 | 88424089 | + | 7.9 | 160 | 301 | -10 | AluY | Alu | SINE |
| 88424151 | 88424243 | - | 34.6 | 2877 | 2967 | -452 | L2 | L2 | LINE |
| 88424244 | 88424532 | + | 10.0 | 1 | 289 | -23 | AluSx | Alu | SINE |
| 88424533 | 88425256 | - | 34.6 | 2100 | 2876 | -543 | L2 | L2 | LINE |

  

|  |  |  |  |  |  |  |  |  |  |
| --- | --- | --- | --- | --- | --- | --- | --- | --- | --- |
| chr11:88462174-88468839 | | | 6419 bp | | |  |  |  |  |
| Genome Start | Genome End | Strand | Div | Repeat Start | Repeat End | Repeat Left | Name | Family | Class |
| 88462174 | 88463149 | + | 24.5 | 2047 | 3045 | -4521 | L1M2c | L1 | LINE |
| 88463150 | 88463299 | - | 15.3 | 5783 | 5939 | -361 | L1MA5 | L1 | LINE |
| 88463401 | 88463493 | - | 18.3 | 5701 | 5793 | -507 | L1MA5 | L1 | LINE |
| 88463495 | 88463856 | + | 19.0 | 5940 | 6298 | -2 | L1MA5 | L1 | LINE |
| 88463865 | 88464923 | + | 26.4 | 2714 | 3805 | -3757 | L1M2c | L1 | LINE |
| 88464928 | 88467133 | + | 19.1 | 3867 | 6168 | -3 | L1MB2 | L1 | LINE |
| 88467209 | 88468045 | - | 26.1 | 2380 | 3298 | -2848 | L1MD | L1 | LINE |
| 88468097 | 88468683 | + | 29.0 | 5166 | 5767 | -1805 | L1M2c | L1 | LINE |
| 88468689 | 88468754 | + | 31.8 | 11 | 76 | -99 | MER96 | MER1\_type? | DNA |
| 88468757 | 88468839 | + | 22.9 | 5757 | 5846 | -1719 | L1M2c | L1 | LINE |

  

|  |  |  |  |  |  |  |  |  |  |
| --- | --- | --- | --- | --- | --- | --- | --- | --- | --- |
| chr11:88525031-88528746 | | | 3606 bp | | |  |  |  |  |
| Genome Start | Genome End | Strand | Div | Repeat Start | Repeat End | Repeat Left | Name | Family | Class |
| 88525031 | 88525480 | + | 26.5 | 3836 | 4313 | -1833 | L1M5 | L1 | LINE |
| 88525494 | 88525678 | - | 17.1 | 157 | 408 | 0 | MLT1A1 | MaLR | LTR |
| 88525717 | 88525850 | + | 8.2 | 171 | 304 | -7 | AluY | Alu | SINE |
| 88525851 | 88525992 | - | 19.9 | 3 | 144 | -221 | MLT1A1 | MaLR | LTR |
| 88526007 | 88527137 | + | 30.4 | 4374 | 5557 | -605 | L1M5 | L1 | LINE |
| 88527134 | 88527374 | - | 5.4 | 4827 | 5067 | -1079 | L1PA4 | L1 | LINE |
| 88527366 | 88528452 | + | 3.8 | 5053 | 6155 | 0 | L1PA4 | L1 | LINE |
| 88528470 | 88528655 | + | 32.3 | 5599 | 5794 | -376 | L1M5 | L1 | LINE |
| 88528697 | 88528746 | + | 32.3 | 5795 | 5844 | -326 | L1M5 | L1 | LINE |

  

|  |  |  |  |  |  |  |  |  |  |
| --- | --- | --- | --- | --- | --- | --- | --- | --- | --- |
| chr11:88837649-88839023 | | | 1363 bp | | |  |  |  |  |
| Genome Start | Genome End | Strand | Div | Repeat Start | Repeat End | Repeat Left | Name | Family | Class |
| 88837649 | 88837801 | + | 22.2 | 1 | 154 | -220 | MLT1A0 | MaLR | LTR |
| 88837817 | 88838759 | - | 6.9 | 5187 | 6143 | -11 | L1PA7 | L1 | LINE |
| 88838757 | 88838796 | + | 5.0 | 5139 | 5178 | -968 | L1PA7 | L1 | LINE |
| 88838797 | 88839023 | + | 23.4 | 128 | 358 | -7 | MLT1A0 | MaLR | LTR |

  

|  |  |  |  |  |  |  |  |  |  |
| --- | --- | --- | --- | --- | --- | --- | --- | --- | --- |
| chr11:90229498-90234428 | | | 4930 bp | | |  |  |  |  |
| Genome Start | Genome End | Strand | Div | Repeat Start | Repeat End | Repeat Left | Name | Family | Class |
| 90229498 | 90229706 | + | 20.2 | 5211 | 5424 | -750 | L1ME1 | L1 | LINE |
| 90229702 | 90230311 | - | 16.5 | 4752 | 5319 | -827 | L1PREC2 | L1 | LINE |
| 90230309 | 90230744 | + | 12.9 | 5326 | 5785 | -375 | L1PREC2 | L1 | LINE |
| 90230746 | 90233317 | + | 5.1 | 3561 | 6141 | -13 | L1PA5 | L1 | LINE |
| 90233325 | 90233727 | + | 14.6 | 5769 | 6160 | 0 | L1PREC2 | L1 | LINE |
| 90233729 | 90234428 | + | 24.5 | 5412 | 6082 | -84 | L1ME1 | L1 | LINE |

  

|  |  |  |  |  |  |  |  |  |  |
| --- | --- | --- | --- | --- | --- | --- | --- | --- | --- |
| chr11:90948911-90955887 | | | 6982 bp | | |  |  |  |  |
| Genome Start | Genome End | Strand | Div | Repeat Start | Repeat End | Repeat Left | Name | Family | Class |
| 90948911 | 90951173 | + | 6.9 | 829 | 3081 | -3065 | L1PA6 | L1 | LINE |
| 90951171 | 90951661 | - | 1.8 | 4452 | 4943 | -1203 | L1PA3 | L1 | LINE |
| 90951656 | 90952832 | + | 2.0 | 4964 | 6141 | -14 | L1PA3 | L1 | LINE |
| 90952837 | 90955887 | + | 7.5 | 3072 | 6125 | -29 | L1PA6 | L1 | LINE |

  

|  |  |  |  |  |  |  |  |  |  |
| --- | --- | --- | --- | --- | --- | --- | --- | --- | --- |
| chr11:92364045-92374303 | | | 9831 bp | | |  |  |  |  |
| Genome Start | Genome End | Strand | Div | Repeat Start | Repeat End | Repeat Left | Name | Family | Class |
| 92364045 | 92364280 | + | 21.6 | 2846 | 3102 | -3044 | L1M5 | L1 | LINE |
| 92364292 | 92364484 | - | 18.6 | 1 | 204 | -359 | MER68 | ERVL | LTR |
| 92364486 | 92364712 | + | 21.6 | 2846 | 3090 | -3056 | L1M5 | L1 | LINE |
| 92364733 | 92364911 | - | 16.8 | 16 | 204 | -359 | MER68 | ERVL | LTR |
| 92364912 | 92365120 | + | 24.4 | 3274 | 3478 | -2668 | L1M5 | L1 | LINE |
| 92365121 | 92365460 | - | 8.8 | 5821 | 6161 | -2 | L1PA13 | L1 | LINE |
| 92365461 | 92366184 | + | 24.4 | 3479 | 4251 | -1895 | L1M5 | L1 | LINE |
| 92366185 | 92366637 | - | 8.2 | 5710 | 6174 | 0 | L1PA11 | L1 | LINE |
| 92366638 | 92367558 | + | 24.4 | 4252 | 5174 | -972 | L1M5 | L1 | LINE |
| 92367559 | 92367883 | + | 8.9 | 5969 | 6295 | -7 | L1MA1 | L1 | LINE |
| 92367884 | 92368066 | + | 24.4 | 5175 | 5333 | -813 | L1M5 | L1 | LINE |
| 92368058 | 92368404 | + | 19.5 | 4939 | 5283 | -863 | L1MD | L1 | LINE |
| 92368405 | 92368738 | + | 12.3 | 5841 | 6168 | -3 | L1MB2 | L1 | LINE |
| 92368739 | 92369031 | - | 14.4 | 1 | 318 | 0 | AluSx | Alu | SINE |
| 92369124 | 92369781 | - | 8.6 | 5496 | 6156 | -12 | L1PA10 | L1 | LINE |
| 92369782 | 92370443 | + | 7.7 | 4815 | 5484 | -690 | L1PA10 | L1 | LINE |
| 92370444 | 92370598 | - | 23.4 | 8 | 161 | -15 | FRAM | Alu | SINE |
| 92370629 | 92371284 | + | 17.3 | 5244 | 5933 | -426 | L1MD2 | L1 | LINE |
| 92371285 | 92371368 | - | 17.4 | 6090 | 6164 | -7 | L1MB2 | L1 | LINE |
| 92371369 | 92371390 | - | 14.8 | 284 | 312 | 0 | AluSx | Alu | SINE |
| 92371425 | 92371582 | - | 14.8 | 138 | 283 | -29 | AluSx | Alu | SINE |
| 92371583 | 92371881 | - | 8.0 | 1 | 303 | -10 | AluSp | Alu | SINE |
| 92371882 | 92372017 | - | 14.8 | 2 | 137 | -175 | AluSx | Alu | SINE |
| 92372018 | 92372336 | - | 17.4 | 5760 | 6089 | -82 | L1MB2 | L1 | LINE |
| 92372337 | 92372727 | + | 16.7 | 5934 | 6336 | -23 | L1MD2 | L1 | LINE |
| 92372728 | 92373030 | - | 5.7 | 1 | 301 | -10 | AluY | Alu | SINE |
| 92373055 | 92373217 | + | 21.4 | 5322 | 5478 | -692 | L1M5 | L1 | LINE |
| 92373352 | 92373819 | + | 16.1 | 32 | 525 | -1966 | Tigger7 | MER2\_type | DNA |
| 92373814 | 92373894 | + | 21.8 | 2400 | 2477 | -14 | Tigger7 | MER2\_type | DNA |
| 92373960 | 92374115 | - | 24.7 | 1 | 154 | -22 | FRAM | Alu | SINE |
| 92374148 | 92374303 | + | 21.9 | 5618 | 5774 | -390 | L1M5 | L1 | LINE |

  

|  |  |  |  |  |  |  |  |  |  |
| --- | --- | --- | --- | --- | --- | --- | --- | --- | --- |
| chr11:92473909-92485426 | | | 11329 bp | | |  |  |  |  |
| Genome Start | Genome End | Strand | Div | Repeat Start | Repeat End | Repeat Left | Name | Family | Class |
| 92473909 | 92474407 | - | 29.7 | 2877 | 3419 | 0 | L2 | L2 | LINE |
| 92474409 | 92474522 | - | 23.2 | 123 | 239 | 0 | MER8 | MER2\_type | DNA |
| 92474523 | 92474830 | + | 13.2 | 1 | 309 | 0 | AluSx | Alu | SINE |
| 92474831 | 92474952 | - | 23.2 | 1 | 122 | -117 | MER8 | MER2\_type | DNA |
| 92474961 | 92476337 | - | 9.3 | 4773 | 6168 | -4 | L1PA8 | L1 | LINE |
| 92476338 | 92476800 | - | 7.6 | 1 | 463 | 0 | LTR2 | ERV1 | LTR |
| 92476801 | 92481707 | - | 5.8 | 1966 | 6896 | 0 | Harlequin | ERV1 | LTR |
| 92481707 | 92483233 | - | 6.6 | 1 | 1568 | -3534 | Harlequin | ERV1 | LTR |
| 92483234 | 92483671 | - | 7.5 | 1 | 463 | 0 | LTR2 | ERV1 | LTR |
| 92483672 | 92483740 | - | 5.8 | 4708 | 4776 | -1370 | L1PA8 | L1 | LINE |
| 92483740 | 92484650 | + | 8.1 | 3787 | 4710 | -1436 | L1PA8 | L1 | LINE |
| 92484663 | 92484729 | - | 26.9 | 2803 | 2871 | -548 | L2 | L2 | LINE |
| 92484735 | 92485019 | + | 22.4 | 5843 | 6174 | -66 | L1ME3B | L1 | LINE |
| 92485185 | 92485426 | - | 27.8 | 2573 | 2828 | -591 | L2 | L2 | LINE |

  

|  |  |  |  |  |  |  |  |  |  |
| --- | --- | --- | --- | --- | --- | --- | --- | --- | --- |
| chr11:94272275-94279813 | | | 7487 bp | | |  |  |  |  |
| Genome Start | Genome End | Strand | Div | Repeat Start | Repeat End | Repeat Left | Name | Family | Class |
| 94272275 | 94273307 | + | 15.8 | 3529 | 4561 | -1585 | L1MB7 | L1 | LINE |
| 94273310 | 94274023 | - | 12.3 | 1676 | 2406 | -12 | Tigger1 | MER2\_type | DNA |
| 94274073 | 94275280 | - | 13.4 | 4850 | 6053 | -113 | L1PA16 | L1 | LINE |
| 94275279 | 94276519 | + | 11.1 | 3580 | 4833 | -1313 | L1PA16 | L1 | LINE |
| 94276520 | 94278257 | - | 12.4 | 1 | 1703 | -715 | Tigger1 | MER2\_type | DNA |
| 94278261 | 94279813 | + | 15.5 | 4563 | 6184 | 0 | L1MB7 | L1 | LINE |

  

|  |  |  |  |  |  |  |  |  |  |
| --- | --- | --- | --- | --- | --- | --- | --- | --- | --- |
| chr11:96752197-96756802 | | | 4370 bp | | |  |  |  |  |
| Genome Start | Genome End | Strand | Div | Repeat Start | Repeat End | Repeat Left | Name | Family | Class |
| 96752197 | 96752620 | + | 22.8 | 1 | 512 | -549 | MER63D | AcHobo | DNA |
| 96752676 | 96753433 | - | 9.9 | 5400 | 6160 | -3 | L1PA13 | L1 | LINE |
| 96753434 | 96753730 | - | 12.5 | 1 | 298 | -14 | AluSx | Alu | SINE |
| 96753731 | 96754434 | - | 10.7 | 4691 | 5399 | -764 | L1P | L1 | LINE |
| 96754433 | 96755849 | + | 14.2 | 3306 | 4687 | -1459 | L1P | L1 | LINE |
| 96755861 | 96755972 | - | 37.8 | 35 | 145 | -131 | MIRm | MIR | SINE |
| 96756003 | 96756275 | - | 10.7 | 5 | 300 | -13 | AluSq | Alu | SINE |
| 96756418 | 96756802 | + | 25.7 | 671 | 1060 | 0 | MER63D | AcHobo | DNA |

  

|  |  |  |  |  |  |  |  |  |  |
| --- | --- | --- | --- | --- | --- | --- | --- | --- | --- |
| chr11:96853383-96857189 | | | 3040 bp | | |  |  |  |  |
| Genome Start | Genome End | Strand | Div | Repeat Start | Repeat End | Repeat Left | Name | Family | Class |
| 96853383 | 96853571 | + | 28.6 | 347 | 544 | -6174 | L1MEc | L1 | LINE |
| 96853994 | 96854294 | + | 29.8 | 847 | 1165 | -5553 | L1MEc | L1 | LINE |
| 96854351 | 96854785 | + | 27.5 | 1705 | 2245 | -4473 | L1MEc | L1 | LINE |
| 96855066 | 96855901 | + | 24.9 | 2482 | 3138 | -3399 | L1MEc | L1 | LINE |
| 96855910 | 96856170 | - | 18.2 | 5151 | 5434 | -878 | L1MA8 | L1 | LINE |
| 96856171 | 96857009 | + | 16.5 | 5440 | 6286 | -5 | L1MA8 | L1 | LINE |
| 96857011 | 96857189 | + | 25.3 | 3130 | 3318 | -3219 | L1MEc | L1 | LINE |

  

|  |  |  |  |  |  |  |  |  |  |
| --- | --- | --- | --- | --- | --- | --- | --- | --- | --- |
| chr11:97199268-97205222 | | | 5956 bp | | |  |  |  |  |
| Genome Start | Genome End | Strand | Div | Repeat Start | Repeat End | Repeat Left | Name | Family | Class |
| 97199268 | 97200030 | - | 20.8 | 5440 | 6175 | -3 | L1MB8 | L1 | LINE |
| 97200031 | 97200329 | - | 16.4 | 1 | 300 | -12 | AluSx | Alu | SINE |
| 97200330 | 97200889 | - | 21.1 | 4844 | 5439 | -739 | L1MB8 | L1 | LINE |
| 97200890 | 97201200 | + | 5.1 | 1 | 311 | 0 | AluY | Alu | SINE |
| 97201201 | 97201440 | - | 21.2 | 4605 | 4843 | -1303 | L1MB8 | L1 | LINE |
| 97201445 | 97201578 | - | 13.4 | 4938 | 5072 | -1074 | L1PREC2 | L1 | LINE |
| 97201574 | 97202437 | + | 13.5 | 5089 | 5937 | -228 | L1PA15 | L1 | LINE |
| 97202438 | 97202758 | + | 14.0 | 1 | 323 | 0 | AluSx | Alu | SINE |
| 97202759 | 97202986 | + | 14.3 | 5938 | 6165 | 0 | L1PA15 | L1 | LINE |
| 97202987 | 97204556 | - | 22.6 | 3063 | 4620 | -1526 | L1MB8 | L1 | LINE |
| 97204557 | 97204856 | - | 13.1 | 1 | 310 | 0 | AluSg | Alu | SINE |
| 97204857 | 97205222 | - | 22.6 | 2701 | 3062 | -3084 | L1MB8 | L1 | LINE |

  

|  |  |  |  |  |  |  |  |  |  |
| --- | --- | --- | --- | --- | --- | --- | --- | --- | --- |
| chr11:97409528-97412490 | | | 2873 bp | | |  |  |  |  |
| Genome Start | Genome End | Strand | Div | Repeat Start | Repeat End | Repeat Left | Name | Family | Class |
| 97409528 | 97409695 | + | 22.5 | 1 | 169 | -216 | MLT1A0 | MaLR | LTR |
| 97409696 | 97410649 | - | 10.9 | 3713 | 4506 | -1640 | L1PB1 | L1 | LINE |
| 97410648 | 97411903 | + | 10.4 | 4512 | 5778 | -373 | L1PB1 | L1 | LINE |
| 97411938 | 97412280 | + | 9.1 | 5779 | 6148 | -3 | L1PB1 | L1 | LINE |
| 97412339 | 97412490 | + | 22.0 | 163 | 374 | 0 | MLT1A0 | MaLR | LTR |

  

|  |  |  |  |  |  |  |  |  |  |
| --- | --- | --- | --- | --- | --- | --- | --- | --- | --- |
| chr11:97649728-97653024 | | | 3301 bp | | |  |  |  |  |
| Genome Start | Genome End | Strand | Div | Repeat Start | Repeat End | Repeat Left | Name | Family | Class |
| 97649728 | 97649857 | + | 6.2 | 1 | 130 | -225 | THE1A | MaLR | LTR |
| 97649861 | 97651373 | - | 2.2 | 4637 | 6155 | 0 | L1PA2 | L1 | LINE |
| 97651367 | 97652783 | + | 2.5 | 3223 | 4639 | -1507 | L1PA2 | L1 | LINE |
| 97652784 | 97653024 | + | 8.5 | 114 | 355 | 0 | THE1A | MaLR | LTR |

  

|  |  |  |  |  |  |  |  |  |  |
| --- | --- | --- | --- | --- | --- | --- | --- | --- | --- |
| chr11:98417849-98418725 | | | 665 bp | | |  |  |  |  |
| Genome Start | Genome End | Strand | Div | Repeat Start | Repeat End | Repeat Left | Name | Family | Class |
| 98417849 | 98417979 | + | 32.3 | 3011 | 3148 | -230 | L2 | L2 | LINE |
| 98418154 | 98418196 | - | 11.6 | 6108 | 6150 | -4 | L1PA6 | L1 | LINE |
| 98418193 | 98418291 | + | 6.1 | 6026 | 6124 | -30 | L1PA6 | L1 | LINE |
| 98418303 | 98418614 | - | 6.1 | 1 | 311 | 0 | AluY | Alu | SINE |
| 98418646 | 98418725 | + | 24.7 | 3294 | 3419 | 0 | L2 | L2 | LINE |

  

|  |  |  |  |  |  |  |  |  |  |
| --- | --- | --- | --- | --- | --- | --- | --- | --- | --- |
| chr11:103489380-103494570 | | | 5009 bp | | |  |  |  |  |
| Genome Start | Genome End | Strand | Div | Repeat Start | Repeat End | Repeat Left | Name | Family | Class |
| 103489380 | 103490814 | - | 12.6 | 4371 | 5828 | -505 | L1MC1 | L1 | LINE |
| 103490840 | 103491207 | - | 13.0 | 5782 | 6150 | -1 | L1PB1 | L1 | LINE |
| 103491357 | 103492417 | - | 7.5 | 4704 | 5771 | -380 | L1PB1 | L1 | LINE |
| 103492412 | 103492618 | + | 8.2 | 3579 | 3782 | -2364 | L1PB | L1 | LINE |
| 103492619 | 103492960 | + | 7.9 | 1 | 355 | 0 | THE1A | MaLR | LTR |
| 103492961 | 103493886 | + | 8.2 | 3783 | 4705 | -1441 | L1PB | L1 | LINE |
| 103493885 | 103494075 | - | 15.2 | 4185 | 4375 | -1771 | L1MC1 | L1 | LINE |
| 103494076 | 103494419 | + | 22.4 | 1 | 339 | -26 | MLT1A0 | MaLR | LTR |
| 103494436 | 103494570 | - | 18.5 | 4050 | 4185 | -1961 | L1MC1 | L1 | LINE |

  

|  |  |  |  |  |  |  |  |  |  |
| --- | --- | --- | --- | --- | --- | --- | --- | --- | --- |
| chr11:103642319-103647803 | | | 5092 bp | | |  |  |  |  |
| Genome Start | Genome End | Strand | Div | Repeat Start | Repeat End | Repeat Left | Name | Family | Class |
| 103642319 | 103642488 | - | 31.1 | 5857 | 6026 | -140 | L1ME1 | L1 | LINE |
| 103642484 | 103644106 | - | 8.0 | 4523 | 6153 | -1 | L1PA7 | L1 | LINE |
| 103644107 | 103645168 | + | 9.5 | 3463 | 4515 | -1631 | L1PA7 | L1 | LINE |
| 103645165 | 103646521 | - | 28.1 | 4419 | 5875 | -291 | L1ME1 | L1 | LINE |
| 103646521 | 103646597 | - | 26.8 | 3849 | 3931 | -2215 | L1ME1 | L1 | LINE |
| 103646598 | 103646901 | - | 11.6 | 1 | 303 | -10 | AluSq | Alu | SINE |
| 103646902 | 103647136 | - | 26.8 | 3615 | 3848 | -2298 | L1ME1 | L1 | LINE |
| 103647523 | 103647632 | + | 29.9 | 603 | 713 | -27 | LTR68 | ERV1 | LTR |
| 103647650 | 103647803 | - | 22.4 | 3460 | 3614 | -2532 | L1ME1 | L1 | LINE |

  

|  |  |  |  |  |  |  |  |  |  |
| --- | --- | --- | --- | --- | --- | --- | --- | --- | --- |
| chr11:104720724-104728581 | | | 7730 bp | | |  |  |  |  |
| Genome Start | Genome End | Strand | Div | Repeat Start | Repeat End | Repeat Left | Name | Family | Class |
| 104720724 | 104722826 | + | 25.5 | 402 | 2737 | -4012 | L1MB4 | L1 | LINE |
| 104722827 | 104723732 | + | 20.0 | 3980 | 4898 | -816 | ERVL-B4 | ERVL | LTR |
| 104723733 | 104723793 | + | 19.7 | 493 | 556 | 0 | MLT2B4 | ERVL | LTR |
| 104723805 | 104724735 | + | 23.1 | 4037 | 4991 | -1756 | L1MB4 | L1 | LINE |
| 104724737 | 104724952 | - | 12.6 | 154 | 364 | 0 | THE1B | MaLR | LTR |
| 104724954 | 104725189 | - | 1.7 | 5152 | 5387 | -759 | L1PA3 | L1 | LINE |
| 104725188 | 104725946 | + | 2.9 | 5394 | 6152 | -3 | L1PA3 | L1 | LINE |
| 104726041 | 104726206 | - | 16.9 | 1 | 168 | -196 | THE1B | MaLR | LTR |
| 104726207 | 104727539 | - | 10.3 | 244 | 1580 | 0 | THE1-int | MaLR | LTR |
| 104727541 | 104727993 | + | 25.9 | 5274 | 5743 | -1004 | L1MB4 | L1 | LINE |
| 104728016 | 104728581 | + | 21.6 | 5744 | 6367 | -414 | L1MB4 | L1 | LINE |

  

|  |  |  |  |  |  |  |  |  |  |
| --- | --- | --- | --- | --- | --- | --- | --- | --- | --- |
| chr11:108704966-108707234 | | | 2221 bp | | |  |  |  |  |
| Genome Start | Genome End | Strand | Div | Repeat Start | Repeat End | Repeat Left | Name | Family | Class |
| 108704966 | 108705243 | + | 25.4 | 4915 | 5196 | -950 | L1M5 | L1 | LINE |
| 108705248 | 108705452 | - | 10.8 | 5677 | 5884 | -282 | L1PA16 | L1 | LINE |
| 108705453 | 108705711 | + | 15.4 | 5881 | 6157 | -9 | L1PA16 | L1 | LINE |
| 108705756 | 108706176 | + | 31.4 | 18 | 450 | 0 | LTR16A | ERVL | LTR |
| 108706177 | 108706465 | + | 28.7 | 5190 | 5481 | -759 | L1M5 | L1 | LINE |
| 108706466 | 108707045 | + | 6.0 | 5567 | 6146 | -8 | L1PA7 | L1 | LINE |
| 108707046 | 108707234 | + | 32.3 | 5481 | 5656 | -510 | L1M5 | L1 | LINE |

  

|  |  |  |  |  |  |  |  |  |  |
| --- | --- | --- | --- | --- | --- | --- | --- | --- | --- |
| chr11:108889827-108893266 | | | 3422 bp | | |  |  |  |  |
| Genome Start | Genome End | Strand | Div | Repeat Start | Repeat End | Repeat Left | Name | Family | Class |
| 108889827 | 108891119 | + | 31.4 | 22 | 1426 | -1993 | L2 | L2 | LINE |
| 108891121 | 108892168 | - | 13.7 | 5056 | 6143 | -22 | L1PA15 | L1 | LINE |
| 108892166 | 108892479 | + | 12.2 | 4737 | 5052 | -1094 | L1PREC2 | L1 | LINE |
| 108892500 | 108893266 | + | 27.1 | 1441 | 2230 | -1189 | L2 | L2 | LINE |

  

|  |  |  |  |  |  |  |  |  |  |
| --- | --- | --- | --- | --- | --- | --- | --- | --- | --- |
| chr11:108996469-108999485 | | | 2534 bp | | |  |  |  |  |
| Genome Start | Genome End | Strand | Div | Repeat Start | Repeat End | Repeat Left | Name | Family | Class |
| 108996469 | 108996656 | + | 36.3 | 2970 | 3170 | -208 | L2 | L2 | LINE |
| 108996706 | 108997005 | - | 14.3 | 1 | 300 | -12 | AluSx | Alu | SINE |
| 108997116 | 108997249 | - | 32.1 | 3275 | 3406 | -13 | L2 | L2 | LINE |
| 108997327 | 108998016 | - | 2.3 | 5458 | 6154 | -1 | L1PA4 | L1 | LINE |
| 108998018 | 108998711 | + | 4.8 | 4747 | 5448 | -706 | L1PA4 | L1 | LINE |
| 108998744 | 108998956 | + | 30.7 | 23 | 252 | -16 | MIR | MIR | SINE |
| 108999171 | 108999485 | + | 33.7 | 3090 | 3419 | 0 | L2 | L2 | LINE |

  

|  |  |  |  |  |  |  |  |  |  |
| --- | --- | --- | --- | --- | --- | --- | --- | --- | --- |
| chr11:109379286-109383681 | | | 4070 bp | | |  |  |  |  |
| Genome Start | Genome End | Strand | Div | Repeat Start | Repeat End | Repeat Left | Name | Family | Class |
| 109379286 | 109379483 | + | 29.1 | 2091 | 2289 | -3926 | L1MC4 | L1 | LINE |
| 109379484 | 109379901 | - | 12.3 | 770 | 1231 | 0 | Tigger3b | MER2\_type | DNA |
| 109379906 | 109380689 | - | 13.6 | 5409 | 6163 | -2 | L1PA15 | L1 | LINE |
| 109380690 | 109381743 | + | 10.5 | 4344 | 5392 | -754 | L1PREC2 | L1 | LINE |
| 109381747 | 109382492 | - | 13.7 | 1 | 764 | -467 | Tigger3b | MER2\_type | DNA |
| 109382506 | 109382527 | - | 11.1 | 285 | 306 | -5 | AluY | Alu | SINE |
| 109382606 | 109382889 | - | 11.1 | 2 | 284 | -27 | AluY | Alu | SINE |
| 109382892 | 109383144 | + | 27.9 | 2288 | 2539 | -3676 | L1M5 | L1 | LINE |
| 109383297 | 109383467 | + | 27.1 | 2759 | 2938 | -3276 | L1MC4 | L1 | LINE |
| 109383542 | 109383681 | + | 32.6 | 2945 | 3083 | -3131 | L1MC4 | L1 | LINE |

  

|  |  |  |  |  |  |  |  |  |  |
| --- | --- | --- | --- | --- | --- | --- | --- | --- | --- |
| chr11:109924722-109933894 | | | 9177 bp | | |  |  |  |  |
| Genome Start | Genome End | Strand | Div | Repeat Start | Repeat End | Repeat Left | Name | Family | Class |
| 109924722 | 109925768 | - | 5.5 | 5106 | 6152 | -2 | L1PA6 | L1 | LINE |
| 109925777 | 109929908 | - | 3.8 | 2025 | 6152 | -3 | L1PA4 | L1 | LINE |
| 109929901 | 109931807 | + | 5.7 | 123 | 2012 | -4143 | L1PA4 | L1 | LINE |
| 109931804 | 109933894 | - | 6.5 | 3023 | 5117 | -1029 | L1PA6 | L1 | LINE |

  

|  |  |  |  |  |  |  |  |  |  |
| --- | --- | --- | --- | --- | --- | --- | --- | --- | --- |
| chr11:114030174-114032544 | | | 2373 bp | | |  |  |  |  |
| Genome Start | Genome End | Strand | Div | Repeat Start | Repeat End | Repeat Left | Name | Family | Class |
| 114030174 | 114030270 | + | 34.0 | 31 | 129 | -139 | MIRb | MIR | SINE |
| 114030275 | 114031666 | - | 8.3 | 4744 | 6129 | -2 | L1PA8A | L1 | LINE |
| 114031659 | 114032436 | + | 8.5 | 3919 | 4727 | -1419 | L1PA8A | L1 | LINE |
| 114032439 | 114032544 | + | 27.0 | 122 | 224 | -44 | MIRb | MIR | SINE |

  

|  |  |  |  |  |  |  |  |  |  |
| --- | --- | --- | --- | --- | --- | --- | --- | --- | --- |
| chr11:114342594-114345890 | | | 3146 bp | | |  |  |  |  |
| Genome Start | Genome End | Strand | Div | Repeat Start | Repeat End | Repeat Left | Name | Family | Class |
| 114342594 | 114343121 | - | 34.9 | 2347 | 2898 | -521 | L2 | L2 | LINE |
| 114343155 | 114344328 | - | 15.8 | 4384 | 5591 | -563 | L1P5 | L1 | LINE |
| 114344321 | 114344510 | + | 19.5 | 5598 | 5791 | -363 | L1P5 | L1 | LINE |
| 114344598 | 114344641 | + | 9.1 | 5630 | 5673 | -481 | L1PB3 | L1 | LINE |
| 114344652 | 114345008 | + | 14.6 | 5782 | 6154 | 0 | L1PB3 | L1 | LINE |
| 114345038 | 114345890 | - | 35.3 | 1390 | 2313 | -1106 | L2 | L2 | LINE |

  

|  |  |  |  |  |  |  |  |  |  |
| --- | --- | --- | --- | --- | --- | --- | --- | --- | --- |
| chr11:116942264-116945487 | | | 3151 bp | | |  |  |  |  |
| Genome Start | Genome End | Strand | Div | Repeat Start | Repeat End | Repeat Left | Name | Family | Class |
| 116942264 | 116942469 | - | 20.1 | 105 | 312 | 0 | AluJb | Alu | SINE |
| 116942503 | 116942794 | - | 2.4 | 5864 | 6155 | 0 | L1PA3 | L1 | LINE |
| 116942795 | 116943503 | - | 2.4 | 5155 | 5863 | -292 | L1PA3 | L1 | LINE |
| 116943500 | 116945328 | + | 2.7 | 3341 | 5174 | -972 | L1PA3 | L1 | LINE |
| 116945373 | 116945487 | - | 13.9 | 6 | 134 | -178 | AluJb | Alu | SINE |

  

|  |  |  |  |  |  |  |  |  |  |
| --- | --- | --- | --- | --- | --- | --- | --- | --- | --- |
| chr11:119534910-119537111 | | | 2201 bp | | |  |  |  |  |
| Genome Start | Genome End | Strand | Div | Repeat Start | Repeat End | Repeat Left | Name | Family | Class |
| 119534910 | 119535413 | - | 23.2 | 1535 | 2056 | -4344 | L1MEc | L1 | LINE |
| 119535414 | 119535475 | - | 19.4 | 228 | 293 | -7 | Alu | Alu | SINE |
| 119535476 | 119536476 | - | 23.2 | 499 | 1534 | -4866 | L1MEc | L1 | LINE |
| 119536484 | 119536532 | - | 14.3 | 6104 | 6152 | -2 | L1PB3 | L1 | LINE |
| 119536521 | 119536659 | + | 14.5 | 5965 | 6102 | -52 | L1PB3 | L1 | LINE |
| 119536666 | 119537111 | - | 26.5 | 32 | 493 | -5907 | L1MEc | L1 | LINE |

  

|  |  |  |  |  |  |  |  |  |  |
| --- | --- | --- | --- | --- | --- | --- | --- | --- | --- |
| chr11:121127099-121130123 | | | 2947 bp | | |  |  |  |  |
| Genome Start | Genome End | Strand | Div | Repeat Start | Repeat End | Repeat Left | Name | Family | Class |
| 121127099 | 121127339 | - | 23.1 | 3149 | 3417 | -2 | L2 | L2 | LINE |
| 121127341 | 121127439 | - | 25.8 | 353 | 450 | 0 | LTR16A | ERVL | LTR |
| 121127472 | 121127992 | - | 2.9 | 4244 | 4764 | -1382 | L1PA3 | L1 | LINE |
| 121127995 | 121129385 | + | 1.7 | 4765 | 6155 | 0 | L1PA3 | L1 | LINE |
| 121129411 | 121129756 | + | 13.1 | 20 | 366 | 0 | Tigger5a | MER2\_type | DNA |
| 121129760 | 121130064 | - | 27.3 | 16 | 344 | -106 | LTR16A | ERVL | LTR |
| 121130080 | 121130123 | - | 20.4 | 3108 | 3151 | -268 | L2 | L2 | LINE |

  

|  |  |  |  |  |  |  |  |  |  |
| --- | --- | --- | --- | --- | --- | --- | --- | --- | --- |
| chr11:123651961-123660350 | | | 8302 bp | | |  |  |  |  |
| Genome Start | Genome End | Strand | Div | Repeat Start | Repeat End | Repeat Left | Name | Family | Class |
| 123651961 | 123654007 | + | 19.1 | 2 | 2054 | -4449 | L1P4a | L1 | LINE |
| 123654008 | 123654307 | - | 12.0 | 1 | 312 | -1 | AluSq | Alu | SINE |
| 123654308 | 123655472 | + | 17.3 | 2055 | 3188 | -3295 | L1P4a | L1 | LINE |
| 123655470 | 123655767 | - | 9.7 | 4095 | 4396 | -1750 | L1PB1 | L1 | LINE |
| 123655769 | 123657526 | + | 9.2 | 4394 | 6151 | 0 | L1PB1 | L1 | LINE |
| 123657530 | 123657880 | + | 18.7 | 3177 | 3537 | -2946 | L1P4a | L1 | LINE |
| 123657868 | 123658079 | + | 7.7 | 5098 | 5309 | -845 | L1PA7 | L1 | LINE |
| 123658080 | 123658228 | + | 11.6 | 1 | 149 | -163 | AluSx | Alu | SINE |
| 123658268 | 123658409 | + | 11.6 | 150 | 296 | -16 | AluSx | Alu | SINE |
| 123658410 | 123659217 | + | 6.5 | 5310 | 6132 | -22 | L1PA7 | L1 | LINE |
| 123659255 | 123659570 | + | 14.4 | 3523 | 3850 | -2633 | L1P4a | L1 | LINE |
| 123659595 | 123660350 | + | 14.4 | 3851 | 4647 | -1836 | L1P4a | L1 | LINE |

  

|  |  |  |  |  |  |  |  |  |  |
| --- | --- | --- | --- | --- | --- | --- | --- | --- | --- |
| chr11:123862917-123866331 | | | 3398 bp | | |  |  |  |  |
| Genome Start | Genome End | Strand | Div | Repeat Start | Repeat End | Repeat Left | Name | Family | Class |
| 123862917 | 123863007 | - | 22.0 | 3406 | 3499 | -2581 | L1MEc | L1 | LINE |
| 123863021 | 123863656 | - | 2.8 | 4546 | 5181 | -965 | L1PA3 | L1 | LINE |
| 123863656 | 123864624 | + | 2.3 | 5187 | 6155 | 0 | L1PA3 | L1 | LINE |
| 123864630 | 123866331 | - | 25.8 | 1751 | 3394 | -2686 | L1MEc | L1 | LINE |

  

|  |  |  |  |  |  |  |  |  |  |
| --- | --- | --- | --- | --- | --- | --- | --- | --- | --- |
| chr11:127124220-127138539 | | | 13890 bp | | |  |  |  |  |
| Genome Start | Genome End | Strand | Div | Repeat Start | Repeat End | Repeat Left | Name | Family | Class |
| 127124220 | 127124967 | - | 26.8 | 1916 | 2573 | -3646 | L1MEc | L1 | LINE |
| 127124963 | 127126216 | - | 22.8 | 3864 | 5180 | -1826 | L1M4b | L1 | LINE |
| 127126228 | 127127607 | - | 9.2 | 4821 | 6164 | -10 | L1PA11 | L1 | LINE |
| 127127609 | 127128453 | - | 23.1 | 2954 | 3831 | -3175 | L1M4b | L1 | LINE |
| 127128462 | 127129029 | - | 20.6 | 1626 | 2227 | -5107 | L1M4b | L1 | LINE |
| 127129030 | 127129318 | - | 10.0 | 1 | 289 | -22 | AluY | Alu | SINE |
| 127129319 | 127129476 | - | 20.6 | 1487 | 1625 | -5709 | L1M4b | L1 | LINE |
| 127129494 | 127130280 | - | 11.9 | 6068 | 6860 | -7 | L1PB2 | L1 | LINE |
| 127130608 | 127130673 | - | 18.2 | 233 | 298 | -5 | AluSp/q | Alu | SINE |
| 127130676 | 127133705 | - | 13.6 | 2213 | 5363 | -783 | L1P1 | L1 | LINE |
| 127133703 | 127133721 | - | 13.8 | 5129 | 5147 | -1715 | L1PB2 | L1 | LINE |
| 127133742 | 127135538 | - | 13.8 | 3301 | 5128 | -1734 | L1PB2 | L1 | LINE |
| 127135540 | 127136750 | + | 10.0 | 2005 | 3280 | -3582 | L1PB2 | L1 | LINE |
| 127136757 | 127137286 | - | 25.5 | 917 | 1472 | -5862 | L1M4b | L1 | LINE |
| 127137289 | 127137341 | + | 5.7 | 842 | 895 | -6 | MER54A | ERVL | LTR |
| 127137349 | 127137638 | - | 23.6 | 3837 | 4131 | -2015 | L1M5 | L1 | LINE |
| 127137661 | 127138005 | - | 40.8 | 37 | 385 | -6015 | L1MEc | L1 | LINE |
| 127138020 | 127138539 | - | 30.7 | 1314 | 1920 | -4713 | L1MEc | L1 | LINE |

  

|  |  |  |  |  |  |  |  |  |  |
| --- | --- | --- | --- | --- | --- | --- | --- | --- | --- |
| chr11:130547197-130550730 | | | 2841 bp | | |  |  |  |  |
| Genome Start | Genome End | Strand | Div | Repeat Start | Repeat End | Repeat Left | Name | Family | Class |
| 130547197 | 130547608 | + | 34.6 | 2331 | 2731 | -688 | L2 | L2 | LINE |
| 130547924 | 130548001 | + | 24.4 | 1 | 79 | -1 | MADE2 | Mariner | DNA |
| 130548007 | 130548315 | - | 25.7 | 5671 | 5990 | -1818 | L1MC4a | L1 | LINE |
| 130548395 | 130548409 | + | 2.0 | 4603 | 4617 | -1529 | L1P1 | L1 | LINE |
| 130548410 | 130549089 | - | 1.5 | 5476 | 6155 | 0 | L1PA2 | L1 | LINE |
| 130549090 | 130549942 | + | 1.7 | 4618 | 5471 | -684 | L1PA2 | L1 | LINE |
| 130549943 | 130550140 | + | 30.1 | 2826 | 3039 | -380 | L2 | L2 | LINE |
| 130550221 | 130550405 | - | 26.8 | 11 | 210 | 0 | MER63A | AcHobo | DNA |
| 130550620 | 130550730 | + | 30.9 | 3305 | 3415 | -4 | L2 | L2 | LINE |

  

|  |  |  |  |  |  |  |  |  |  |
| --- | --- | --- | --- | --- | --- | --- | --- | --- | --- |
| chr11:131895654-131897382 | | | 1596 bp | | |  |  |  |  |
| Genome Start | Genome End | Strand | Div | Repeat Start | Repeat End | Repeat Left | Name | Family | Class |
| 131895654 | 131895770 | - | 23.1 | 6234 | 6358 | -1 | L1MD2 | L1 | LINE |
| 131895849 | 131896537 | - | 8.0 | 5098 | 5787 | -385 | L1PA8 | L1 | LINE |
| 131896540 | 131896878 | + | 7.4 | 5788 | 6163 | -9 | L1PA8 | L1 | LINE |
| 131896932 | 131897382 | - | 23.4 | 5693 | 6184 | -175 | L1MD2 | L1 | LINE |

  


|  |  |  |  |  |  |  |  |  |  |
| --- | --- | --- | --- | --- | --- | --- | --- | --- | --- |
| chr12:5086477-5090929 | | | 4436 bp | | |  |  |  |  |
| Genome Start | Genome End | Strand | Div | Repeat Start | Repeat End | Repeat Left | Name | Family | Class |
| 5086477 | 5086571 | + | 37.9 | 22 | 116 | -152 | MIRb | MIR | SINE |
| 5086578 | 5087315 | + | 23.9 | 3728 | 4491 | -1655 | L1ME2 | L1 | LINE |
| 5087318 | 5087899 | - | 10.9 | 5015 | 5609 | -547 | L1PB4 | L1 | LINE |
| 5087904 | 5088416 | + | 14.7 | 5622 | 6144 | -12 | L1PB4 | L1 | LINE |
| 5088422 | 5089209 | + | 26.0 | 4477 | 5252 | -894 | L1ME2 | L1 | LINE |
| 5089210 | 5089505 | - | 18.6 | 1 | 298 | -14 | AluJb | Alu | SINE |
| 5089506 | 5090166 | + | 23.8 | 5253 | 5942 | -222 | L1ME2 | L1 | LINE |
| 5090167 | 5090558 | + | 11.0 | 1 | 390 | 0 | MLT1B | MaLR | LTR |
| 5090559 | 5090784 | + | 23.7 | 5943 | 6161 | -3 | L1ME2 | L1 | LINE |
| 5090785 | 5090929 | + | 30.1 | 92 | 249 | -17 | MIRb | MIR | SINE |

  

|  |  |  |  |  |  |  |  |  |  |
| --- | --- | --- | --- | --- | --- | --- | --- | --- | --- |
| chr12:5519186-5524846 | | | 5606 bp | | |  |  |  |  |
| Genome Start | Genome End | Strand | Div | Repeat Start | Repeat End | Repeat Left | Name | Family | Class |
| 5519186 | 5519461 | + | 12.5 | 1 | 289 | -145 | Tigger2a | MER2\_type | DNA |
| 5519496 | 5521225 | - | 10.8 | 5114 | 6867 | 0 | L1PB2 | L1 | LINE |
| 5521246 | 5522869 | - | 11.1 | 3487 | 5113 | -1749 | L1PB2 | L1 | LINE |
| 5522871 | 5524699 | + | 10.7 | 1664 | 3504 | -3358 | L1PB2 | L1 | LINE |
| 5524700 | 5524846 | + | 18.4 | 294 | 443 | -1 | Tigger2a | MER2\_type | DNA |

  

|  |  |  |  |  |  |  |  |  |  |
| --- | --- | --- | --- | --- | --- | --- | --- | --- | --- |
| chr12:11330849-11348309 | | | 17405 bp | | |  |  |  |  |
| Genome Start | Genome End | Strand | Div | Repeat Start | Repeat End | Repeat Left | Name | Family | Class |
| 11330849 | 11332780 | + | 9.4 | 3047 | 4990 | -1156 | L1PA8 | L1 | LINE |
| 11332783 | 11333107 | - | 4.9 | 4125 | 4454 | -1692 | L1P2 | L1 | LINE |
| 11333107 | 11334430 | + | 6.0 | 4474 | 5781 | -373 | L1P2 | L1 | LINE |
| 11334438 | 11340562 | + | 4.8 | 7 | 6145 | -10 | L1PA4 | L1 | LINE |
| 11340568 | 11341072 | + | 8.8 | 4982 | 5491 | -681 | L1PA8 | L1 | LINE |
| 11341073 | 11341356 | + | 14.5 | 1 | 293 | -20 | AluSq | Alu | SINE |
| 11341370 | 11341548 | + | 13.4 | 127 | 305 | -7 | AluSg/x | Alu | SINE |
| 11341549 | 11341848 | + | 7.7 | 5481 | 5781 | -391 | L1PA8 | L1 | LINE |
| 11341858 | 11347886 | + | 1.8 | 2 | 6032 | 0 | L1PA2 | L1 | LINE |
| 11347908 | 11348309 | + | 7.2 | 5769 | 6170 | -2 | L1PA8 | L1 | LINE |

  

|  |  |  |  |  |  |  |  |  |  |
| --- | --- | --- | --- | --- | --- | --- | --- | --- | --- |
| chr12:11978733-11985091 | | | 6362 bp | | |  |  |  |  |
| Genome Start | Genome End | Strand | Div | Repeat Start | Repeat End | Repeat Left | Name | Family | Class |
| 11978733 | 11979951 | + | 15.8 | 4072 | 5299 | -847 | L1MC3 | L1 | LINE |
| 11979968 | 11980053 | + | 24.4 | 5997 | 6083 | -217 | L1MA6 | L1 | LINE |
| 11980035 | 11981677 | - | 1.9 | 4507 | 6154 | -1 | L1PA2 | L1 | LINE |
| 11981676 | 11982971 | + | 1.4 | 3205 | 4500 | -1646 | L1PA2 | L1 | LINE |
| 11982972 | 11983213 | + | 15.4 | 6059 | 6303 | 0 | L1MA4A | L1 | LINE |
| 11983215 | 11983434 | + | 21.8 | 2313 | 2546 | -66 | Charlie7 | MER1\_type | DNA |
| 11983436 | 11985091 | + | 19.8 | 5316 | 6962 | -6 | L1MC3 | L1 | LINE |

  

|  |  |  |  |  |  |  |  |  |  |
| --- | --- | --- | --- | --- | --- | --- | --- | --- | --- |
| chr12:15326364-15328750 | | | 2352 bp | | |  |  |  |  |
| Genome Start | Genome End | Strand | Div | Repeat Start | Repeat End | Repeat Left | Name | Family | Class |
| 15326364 | 15326582 | + | 27.4 | 51 | 274 | -2338 | Charlie7 | MER1\_type | DNA |
| 15326602 | 15326924 | + | 25.2 | 407 | 778 | -1834 | Charlie7 | MER1\_type | DNA |
| 15326935 | 15327081 | + | 20.1 | 1644 | 1789 | -823 | Charlie7 | MER1\_type | DNA |
| 15327088 | 15327799 | - | 0.6 | 5442 | 6152 | -3 | L1PA2 | L1 | LINE |
| 15327800 | 15328287 | + | 1.3 | 4940 | 5427 | -728 | L1PA2 | L1 | LINE |
| 15328288 | 15328750 | + | 26.6 | 1773 | 2260 | -352 | Charlie7 | MER1\_type | DNA |

  

|  |  |  |  |  |  |  |  |  |  |
| --- | --- | --- | --- | --- | --- | --- | --- | --- | --- |
| chr12:16438057-16442290 | | | 3718 bp | | |  |  |  |  |
| Genome Start | Genome End | Strand | Div | Repeat Start | Repeat End | Repeat Left | Name | Family | Class |
| 16438057 | 16438542 | - | 27.5 | 5300 | 5814 | -409 | L1ME3B | L1 | LINE |
| 16438791 | 16439097 | - | 13.9 | 1 | 303 | -9 | AluSx | Alu | SINE |
| 16439108 | 16439616 | - | 29.2 | 4718 | 5282 | -847 | L1ME3B | L1 | LINE |
| 16439629 | 16439981 | - | 9.2 | 5781 | 6147 | -4 | L1PB1 | L1 | LINE |
| 16440028 | 16440392 | - | 9.2 | 5410 | 5780 | -371 | L1PB1 | L1 | LINE |
| 16440393 | 16441923 | + | 8.9 | 3897 | 5421 | -912 | L1PB1 | L1 | LINE |
| 16442124 | 16442290 | - | 23.9 | 4405 | 4588 | -1541 | L1ME3B | L1 | LINE |

  

|  |  |  |  |  |  |  |  |  |  |
| --- | --- | --- | --- | --- | --- | --- | --- | --- | --- |
| chr12:16869031-16872641 | | | 3575 bp | | |  |  |  |  |
| Genome Start | Genome End | Strand | Div | Repeat Start | Repeat End | Repeat Left | Name | Family | Class |
| 16869031 | 16870491 | + | 15.3 | 1 | 1479 | -939 | Tigger1 | MER2\_type | DNA |
| 16870492 | 16870778 | + | 9.0 | 6077 | 6367 | 0 | L1MA2 | L1 | LINE |
| 16870779 | 16870900 | + | 15.3 | 1480 | 1592 | -826 | Tigger1 | MER2\_type | DNA |
| 16870902 | 16871022 | + | 19.8 | 2 | 122 | -21 | FLAM\_C | Alu | SINE |
| 16871024 | 16871125 | + | 10.8 | 1580 | 1681 | -737 | Tigger1 | MER2\_type | DNA |
| 16871160 | 16871656 | - | 9.7 | 5665 | 6166 | -8 | L1PA11 | L1 | LINE |
| 16871657 | 16871890 | + | 9.8 | 5418 | 5653 | -521 | L1PA11 | L1 | LINE |
| 16871891 | 16872641 | + | 14.0 | 1668 | 2418 | 0 | Tigger1 | MER2\_type | DNA |

  

|  |  |  |  |  |  |  |  |  |  |
| --- | --- | --- | --- | --- | --- | --- | --- | --- | --- |
| chr12:17446944-17453873 | | | 6851 bp | | |  |  |  |  |
| Genome Start | Genome End | Strand | Div | Repeat Start | Repeat End | Repeat Left | Name | Family | Class |
| 17446944 | 17447867 | + | 29.1 | 37 | 1133 | -5500 | L1MD1 | L1 | LINE |
| 17447871 | 17447964 | + | 17.9 | 219 | 312 | 0 | AluSg/x | Alu | SINE |
| 17448019 | 17448375 | + | 13.3 | 1 | 364 | 0 | THE1B | MaLR | LTR |
| 17448376 | 17450079 | + | 10.4 | 1 | 1573 | -7 | THE1B-int | MaLR | LTR |
| 17450086 | 17450441 | + | 15.0 | 1 | 364 | 0 | THE1B | MaLR | LTR |
| 17450442 | 17451803 | + | 20.2 | 4182 | 5559 | -978 | L1MD1 | L1 | LINE |
| 17451798 | 17452303 | - | 17.7 | 4142 | 4664 | -1479 | L1MA8 | L1 | LINE |
| 17452302 | 17452666 | + | 18.4 | 4662 | 5045 | -1098 | L1MA8 | L1 | LINE |
| 17452667 | 17453540 | + | 17.6 | 5409 | 6286 | -5 | L1MA8 | L1 | LINE |
| 17453565 | 17453873 | + | 23.8 | 5567 | 5884 | -749 | L1MD1 | L1 | LINE |

  

|  |  |  |  |  |  |  |  |  |  |
| --- | --- | --- | --- | --- | --- | --- | --- | --- | --- |
| chr12:17818657-17820735 | | | 1992 bp | | |  |  |  |  |
| Genome Start | Genome End | Strand | Div | Repeat Start | Repeat End | Repeat Left | Name | Family | Class |
| 17818657 | 17819243 | + | 33.2 | 2625 | 3231 | -188 | L2 | L2 | LINE |
| 17819245 | 17820200 | - | 16.0 | 5213 | 6160 | -6 | L1PA16 | L1 | LINE |
| 17820203 | 17820504 | + | 12.9 | 4899 | 5201 | -945 | L1PA16 | L1 | LINE |
| 17820589 | 17820735 | + | 30.0 | 3229 | 3419 | 0 | L2 | L2 | LINE |

  

|  |  |  |  |  |  |  |  |  |  |
| --- | --- | --- | --- | --- | --- | --- | --- | --- | --- |
| chr12:18316910-18319276 | | | 2172 bp | | |  |  |  |  |
| Genome Start | Genome End | Strand | Div | Repeat Start | Repeat End | Repeat Left | Name | Family | Class |
| 18316910 | 18317225 | - | 34.6 | 6661 | 6977 | -1065 | L1MC4 | L1 | LINE |
| 18317403 | 18317767 | - | 3.0 | 4560 | 4924 | -1222 | L1PA4 | L1 | LINE |
| 18317767 | 18319017 | + | 3.7 | 4903 | 6155 | 0 | L1PA4 | L1 | LINE |
| 18319037 | 18319276 | - | 26.0 | 1657 | 1900 | -4315 | L1MC4 | L1 | LINE |

  

|  |  |  |  |  |  |  |  |  |  |
| --- | --- | --- | --- | --- | --- | --- | --- | --- | --- |
| chr12:18451666-18454918 | | | 3235 bp | | |  |  |  |  |
| Genome Start | Genome End | Strand | Div | Repeat Start | Repeat End | Repeat Left | Name | Family | Class |
| 18451666 | 18452315 | - | 37.3 | 2751 | 3419 | 0 | L2 | L2 | LINE |
| 18452324 | 18452736 | - | 9.7 | 4681 | 5094 | -1052 | L1PA7 | L1 | LINE |
| 18452738 | 18453766 | + | 6.5 | 5118 | 6154 | 0 | L1PA7 | L1 | LINE |
| 18453776 | 18453934 | - | 34.1 | 2566 | 2740 | -679 | L2 | L2 | LINE |
| 18453935 | 18454310 | - | 10.8 | 1 | 428 | 0 | MSTA | MaLR | LTR |
| 18454311 | 18454418 | - | 34.1 | 2459 | 2565 | -854 | L2 | L2 | LINE |
| 18454419 | 18454729 | + | 11.1 | 1 | 306 | -6 | AluSx | Alu | SINE |
| 18454730 | 18454918 | - | 34.1 | 2255 | 2458 | -961 | L2 | L2 | LINE |

  

|  |  |  |  |  |  |  |  |  |  |
| --- | --- | --- | --- | --- | --- | --- | --- | --- | --- |
| chr12:21139974-21145783 | | | 5627 bp | | |  |  |  |  |
| Genome Start | Genome End | Strand | Div | Repeat Start | Repeat End | Repeat Left | Name | Family | Class |
| 21139974 | 21140235 | - | 16.5 | 5797 | 6059 | -238 | L1MA5A | L1 | LINE |
| 21140310 | 21141928 | - | 4.2 | 4521 | 6145 | -9 | L1PA5 | L1 | LINE |
| 21141919 | 21142625 | + | 5.0 | 3807 | 4516 | -1630 | L1PA5 | L1 | LINE |
| 21142745 | 21145783 | - | 20.9 | 2612 | 5779 | -518 | L1MA5A | L1 | LINE |

  

|  |  |  |  |  |  |  |  |  |  |
| --- | --- | --- | --- | --- | --- | --- | --- | --- | --- |
| chr12:23055935-23058442 | | | 2449 bp | | |  |  |  |  |
| Genome Start | Genome End | Strand | Div | Repeat Start | Repeat End | Repeat Left | Name | Family | Class |
| 23055935 | 23056081 | - | 19.6 | 324 | 505 | 0 | MLT1D | MaLR | LTR |
| 23056084 | 23057367 | - | 8.9 | 4865 | 6157 | -6 | L1PA13 | L1 | LINE |
| 23057367 | 23057675 | + | 10.1 | 4562 | 4859 | -1287 | L1PREC2 | L1 | LINE |
| 23057677 | 23057767 | - | 20.6 | 251 | 342 | -163 | MLT1D | MaLR | LTR |
| 23057825 | 23058039 | - | 20.6 | 32 | 250 | -255 | MLT1D | MaLR | LTR |
| 23058040 | 23058405 | - | 21.4 | 5 | 363 | -2 | MLT1A0 | MaLR | LTR |
| 23058406 | 23058442 | - | 20.6 | 3 | 31 | -474 | MLT1D | MaLR | LTR |

  

|  |  |  |  |  |  |  |  |  |  |
| --- | --- | --- | --- | --- | --- | --- | --- | --- | --- |
| chr12:23132091-23139294 | | | 7159 bp | | |  |  |  |  |
| Genome Start | Genome End | Strand | Div | Repeat Start | Repeat End | Repeat Left | Name | Family | Class |
| 23132091 | 23132228 | - | 29.3 | 6018 | 6160 | -2 | L1ME3 | L1 | LINE |
| 23132251 | 23132292 | + | 23.8 | 19 | 60 | -2969 | Tigger3(Golem) | MER2\_type | DNA |
| 23132298 | 23132337 | + | 7.5 | 66 | 105 | -2924 | Tigger3(Golem) | MER2\_type | DNA |
| 23132343 | 23132545 | + | 15.3 | 2822 | 3028 | -1 | Tigger3(Golem) | MER2\_type | DNA |
| 23132600 | 23132624 | - | 26.6 | 6271 | 6295 | -17 | L1MA9 | L1 | LINE |
| 23132625 | 23133148 | - | 23.2 | 5568 | 6123 | -43 | L1ME1 | L1 | LINE |
| 23133152 | 23135717 | - | 4.9 | 3583 | 6154 | 0 | L1PA5 | L1 | LINE |
| 23135715 | 23136142 | + | 6.2 | 2466 | 2881 | -3265 | L1P1 | L1 | LINE |
| 23136131 | 23136261 | - | 8.3 | 2466 | 2586 | -3560 | L1PA5 | L1 | LINE |
| 23136259 | 23136308 | + | 6.0 | 3583 | 3632 | -2514 | L1P1 | L1 | LINE |
| 23136303 | 23137013 | + | 5.9 | 2883 | 3593 | -2553 | L1P1 | L1 | LINE |
| 23137008 | 23137535 | - | 20.6 | 5040 | 5587 | -579 | L1ME1 | L1 | LINE |
| 23137536 | 23137591 | - | 19.6 | 4888 | 4943 | -1203 | L1MC | L1 | LINE |
| 23137578 | 23139294 | - | 23.7 | 4077 | 5954 | -208 | L1ME3 | L1 | LINE |

  

|  |  |  |  |  |  |  |  |  |  |
| --- | --- | --- | --- | --- | --- | --- | --- | --- | --- |
| chr12:28561870-28572856 | | | 10954 bp | | |  |  |  |  |
| Genome Start | Genome End | Strand | Div | Repeat Start | Repeat End | Repeat Left | Name | Family | Class |
| 28561870 | 28563494 | - | 13.9 | 5115 | 6836 | -31 | L1PB1 | L1 | LINE |
| 28563522 | 28563761 | - | 17.7 | 4148 | 4398 | -1748 | L1P4 | L1 | LINE |
| 28563772 | 28568148 | - | 5.4 | 1793 | 6152 | -2 | L1PA5 | L1 | LINE |
| 28568149 | 28568317 | + | 7.1 | 1634 | 1802 | -4353 | L1PA5 | L1 | LINE |
| 28568314 | 28572856 | - | 12.2 | 6 | 4877 | -1985 | L1PB1 | L1 | LINE |

  

|  |  |  |  |  |  |  |  |  |  |
| --- | --- | --- | --- | --- | --- | --- | --- | --- | --- |
| chr12:28877300-28886085 | | | 8795 bp | | |  |  |  |  |
| Genome Start | Genome End | Strand | Div | Repeat Start | Repeat End | Repeat Left | Name | Family | Class |
| 28877300 | 28878965 | + | 20.4 | 1105 | 2769 | -4016 | L1PA15 | L1 | LINE |
| 28878968 | 28880018 | - | 18.3 | 2277 | 3360 | -2786 | L1MD | L1 | LINE |
| 28880024 | 28880477 | - | 4.9 | 5689 | 6141 | -13 | L1PA5 | L1 | LINE |
| 28880478 | 28881376 | + | 5.5 | 4797 | 5697 | -457 | L1PA5 | L1 | LINE |
| 28881361 | 28884089 | + | 14.2 | 2747 | 5487 | -1279 | L1PA15 | L1 | LINE |
| 28884090 | 28884444 | + | 14.9 | 1 | 364 | 0 | THE1B | MaLR | LTR |
| 28884445 | 28885624 | + | 12.6 | 5488 | 6659 | -126 | L1PA15 | L1 | LINE |
| 28885625 | 28885934 | + | 11.9 | 1 | 311 | -2 | AluSp | Alu | SINE |
| 28885935 | 28886085 | + | 11.9 | 6660 | 6780 | -5 | L1PA15 | L1 | LINE |

  

|  |  |  |  |  |  |  |  |  |  |
| --- | --- | --- | --- | --- | --- | --- | --- | --- | --- |
| chr12:29045004-29045941 | | | 905 bp | | |  |  |  |  |
| Genome Start | Genome End | Strand | Div | Repeat Start | Repeat End | Repeat Left | Name | Family | Class |
| 29045004 | 29045371 | + | 24.3 | 52 | 462 | -100 | MLT1G | MaLR | LTR |
| 29045373 | 29045578 | - | 6.0 | 5722 | 5927 | -245 | L1PA8 | L1 | LINE |
| 29045579 | 29045818 | + | 11.9 | 5932 | 6166 | -6 | L1PA8 | L1 | LINE |
| 29045851 | 29045941 | + | 16.5 | 447 | 543 | -12 | MLT1G | MaLR | LTR |

  

|  |  |  |  |  |  |  |  |  |  |
| --- | --- | --- | --- | --- | --- | --- | --- | --- | --- |
| chr12:31253894-31254480 | | | 582 bp | | |  |  |  |  |
| Genome Start | Genome End | Strand | Div | Repeat Start | Repeat End | Repeat Left | Name | Family | Class |
| 31253894 | 31253958 | + | 30.8 | 10 | 74 | -115 | MER5A | MER1\_type | DNA |
| 31253959 | 31254009 | - | 3.9 | 5749 | 5799 | -356 | L1PA5 | L1 | LINE |
| 31254010 | 31254363 | + | 3.4 | 5789 | 6142 | -12 | L1PA5 | L1 | LINE |
| 31254369 | 31254480 | + | 24.1 | 65 | 189 | 0 | MER5A | MER1\_type | DNA |

  

|  |  |  |  |  |  |  |  |  |  |
| --- | --- | --- | --- | --- | --- | --- | --- | --- | --- |
| chr12:33285882-33291289 | | | 5287 bp | | |  |  |  |  |
| Genome Start | Genome End | Strand | Div | Repeat Start | Repeat End | Repeat Left | Name | Family | Class |
| 33285882 | 33286749 | - | 22.7 | 1390 | 2393 | -114 | HAL1 | L1 | LINE |
| 33286755 | 33286795 | + | 9.8 | 285 | 325 | -547 | MER4D0 | ERV1 | LTR |
| 33286796 | 33288262 | - | 2.5 | 4684 | 6152 | -3 | L1PA3 | L1 | LINE |
| 33288261 | 33289631 | + | 3.0 | 3280 | 4677 | -1469 | L1PA3 | L1 | LINE |
| 33289633 | 33290209 | + | 18.3 | 25 | 595 | 0 | LTR49 | ERV1 | LTR |
| 33290327 | 33290498 | - | 29.4 | 1084 | 1253 | -1254 | HAL1 | L1 | LINE |
| 33290499 | 33290791 | - | 10.2 | 2 | 295 | -18 | AluSp | Alu | SINE |
| 33290792 | 33291289 | - | 29.4 | 576 | 1083 | -1424 | HAL1 | L1 | LINE |

  

|  |  |  |  |  |  |  |  |  |  |
| --- | --- | --- | --- | --- | --- | --- | --- | --- | --- |
| chr12:33923205-33929981 | | | 6757 bp | | |  |  |  |  |
| Genome Start | Genome End | Strand | Div | Repeat Start | Repeat End | Repeat Left | Name | Family | Class |
| 33923205 | 33923565 | + | 28.6 | 542 | 895 | -5175 | L1ME2 | L1 | LINE |
| 33923572 | 33924988 | - | 8.8 | 1783 | 3204 | -3127 | L1PA10 | L1 | LINE |
| 33924985 | 33928086 | + | 10.6 | 3219 | 6341 | -12 | L1PA10 | L1 | LINE |
| 33928102 | 33929397 | + | 31.7 | 878 | 2250 | -3904 | L1ME2 | L1 | LINE |
| 33929401 | 33929981 | + | 26.6 | 3564 | 4136 | -1944 | L1ME2 | L1 | LINE |

  

|  |  |  |  |  |  |  |  |  |  |
| --- | --- | --- | --- | --- | --- | --- | --- | --- | --- |
| chr12:34015430-34017707 | | | 2276 bp | | |  |  |  |  |
| Genome Start | Genome End | Strand | Div | Repeat Start | Repeat End | Repeat Left | Name | Family | Class |
| 34015430 | 34015612 | + | 18.7 | 5189 | 5371 | -775 | L1MC | L1 | LINE |
| 34015609 | 34016529 | - | 8.5 | 4101 | 5030 | -1116 | L1PA7 | L1 | LINE |
| 34016532 | 34017625 | + | 7.5 | 5041 | 6141 | -13 | L1PA7 | L1 | LINE |
| 34017630 | 34017707 | + | 5.1 | 5354 | 5431 | -902 | L1MC | L1 | LINE |

  

|  |  |  |  |  |  |  |  |  |  |
| --- | --- | --- | --- | --- | --- | --- | --- | --- | --- |
| chr12:36782247-36787721 | | | 5375 bp | | |  |  |  |  |
| Genome Start | Genome End | Strand | Div | Repeat Start | Repeat End | Repeat Left | Name | Family | Class |
| 36782247 | 36782371 | - | 25.9 | 5826 | 5946 | -205 | L1MB5 | L1 | LINE |
| 36782372 | 36782671 | - | 22.7 | 2 | 292 | -20 | AluJb | Alu | SINE |
| 36782672 | 36782894 | - | 23.7 | 5670 | 5825 | -349 | L1MB5 | L1 | LINE |
| 36782895 | 36783119 | + | 17.3 | 1 | 225 | -87 | AluJb | Alu | SINE |
| 36783120 | 36783648 | - | 22.3 | 5148 | 5669 | -505 | L1MB5 | L1 | LINE |
| 36783715 | 36783870 | - | 26.9 | 5017 | 5173 | -973 | L1MB5 | L1 | LINE |
| 36783875 | 36784292 | - | 14.9 | 5730 | 6152 | -9 | L1PA14 | L1 | LINE |
| 36784333 | 36784647 | - | 9.6 | 5435 | 5744 | -417 | L1PA14 | L1 | LINE |
| 36784648 | 36784950 | - | 12.2 | 1 | 303 | -6 | AluSc | Alu | SINE |
| 36784951 | 36786969 | - | 16.5 | 3347 | 5434 | -727 | L1PA14 | L1 | LINE |
| 36786962 | 36787010 | + | 16.3 | 3305 | 3353 | -2793 | L1PREC2 | L1 | LINE |
| 36787009 | 36787721 | - | 25.6 | 4308 | 5031 | -1115 | L1MB5 | L1 | LINE |

  

|  |  |  |  |  |  |  |  |  |  |
| --- | --- | --- | --- | --- | --- | --- | --- | --- | --- |
| chr12:36945168-36947443 | | | 2274 bp | | |  |  |  |  |
| Genome Start | Genome End | Strand | Div | Repeat Start | Repeat End | Repeat Left | Name | Family | Class |
| 36945168 | 36945351 | + | 19.7 | 5188 | 5371 | -775 | L1MC | L1 | LINE |
| 36945348 | 36946270 | - | 8.8 | 4101 | 5030 | -1116 | L1PA7 | L1 | LINE |
| 36946273 | 36947361 | + | 8.0 | 5041 | 6141 | -13 | L1PA7 | L1 | LINE |
| 36947366 | 36947443 | + | 7.7 | 5354 | 5431 | -902 | L1MC | L1 | LINE |

  

|  |  |  |  |  |  |  |  |  |  |
| --- | --- | --- | --- | --- | --- | --- | --- | --- | --- |
| chr12:37055811-37066813 | | | 10877 bp | | |  |  |  |  |
| Genome Start | Genome End | Strand | Div | Repeat Start | Repeat End | Repeat Left | Name | Family | Class |
| 37055811 | 37056112 | + | 21.7 | 4585 | 4873 | -1273 | L1M4 | L1 | LINE |
| 37056184 | 37057930 | - | 10.2 | 4879 | 6617 | 0 | L1PB1 | L1 | LINE |
| 37057974 | 37061684 | - | 11.9 | 1055 | 4878 | -1734 | L1PB1 | L1 | LINE |
| 37061693 | 37063754 | - | 9.7 | 4090 | 6156 | -16 | L1PA8 | L1 | LINE |
| 37063749 | 37064903 | + | 9.5 | 2908 | 4070 | -2076 | L1PA8 | L1 | LINE |
| 37064899 | 37065975 | - | 11.0 | 2 | 1080 | -5532 | L1PB1 | L1 | LINE |
| 37065991 | 37066813 | + | 20.1 | 4955 | 5821 | -357 | L1M4 | L1 | LINE |

  

|  |  |  |  |  |  |  |  |  |  |
| --- | --- | --- | --- | --- | --- | --- | --- | --- | --- |
| chr12:38462663-38466198 | | | 3516 bp | | |  |  |  |  |
| Genome Start | Genome End | Strand | Div | Repeat Start | Repeat End | Repeat Left | Name | Family | Class |
| 38462663 | 38462939 | + | 9.8 | 1 | 277 | -1303 | THE1A-int | MaLR | LTR |
| 38462954 | 38463834 | - | 4.2 | 5254 | 6140 | -14 | L1PA5 | L1 | LINE |
| 38463841 | 38464885 | + | 5.3 | 4187 | 5232 | -914 | L1PA5 | L1 | LINE |
| 38464886 | 38466198 | + | 7.6 | 262 | 1580 | 0 | THE1A-int | MaLR | LTR |

  

|  |  |  |  |  |  |  |  |  |  |
| --- | --- | --- | --- | --- | --- | --- | --- | --- | --- |
| chr12:38469072-38473144 | | | 4009 bp | | |  |  |  |  |
| Genome Start | Genome End | Strand | Div | Repeat Start | Repeat End | Repeat Left | Name | Family | Class |
| 38469072 | 38469993 | - | 28.7 | 5259 | 6231 | -9 | L1ME3B | L1 | LINE |
| 38470015 | 38470190 | - | 11.9 | 2388 | 2567 | -45 | Charlie5 | MER1\_type | DNA |
| 38470215 | 38470277 | + | 4.8 | 2537 | 2609 | -420 | Tigger3(Golem) | MER2\_type | DNA |
| 38470289 | 38471229 | - | 1.5 | 5214 | 6155 | 0 | L1PA2 | L1 | LINE |
| 38471230 | 38472507 | + | 0.9 | 3927 | 5204 | -942 | L1PA2 | L1 | LINE |
| 38472508 | 38472625 | - | 19.7 | 1 | 136 | -104 | MER33 | MER1\_type | DNA |
| 38472634 | 38472651 | - | 30.2 | 5236 | 5251 | -895 | L1ME3B | L1 | LINE |
| 38472652 | 38472938 | - | 20.2 | 1 | 239 | 0 | MER8 | MER2\_type | DNA |
| 38472939 | 38473144 | - | 30.2 | 5024 | 5235 | -911 | L1ME3B | L1 | LINE |

  

|  |  |  |  |  |  |  |  |  |  |
| --- | --- | --- | --- | --- | --- | --- | --- | --- | --- |
| chr12:39836620-39842256 | | | 5639 bp | | |  |  |  |  |
| Genome Start | Genome End | Strand | Div | Repeat Start | Repeat End | Repeat Left | Name | Family | Class |
| 39836620 | 39838275 | - | 10.0 | 4497 | 6164 | -4 | L1PA10 | L1 | LINE |
| 39838275 | 39841137 | - | 3.9 | 3286 | 6155 | 0 | L1PA4 | L1 | LINE |
| 39841138 | 39841903 | + | 5.0 | 2511 | 3280 | -2866 | L1PA4 | L1 | LINE |
| 39841903 | 39842256 | - | 7.1 | 4142 | 4512 | -1634 | L1PA10 | L1 | LINE |

  

|  |  |  |  |  |  |  |  |  |  |
| --- | --- | --- | --- | --- | --- | --- | --- | --- | --- |
| chr12:41551132-41558561 | | | 7363 bp | | |  |  |  |  |
| Genome Start | Genome End | Strand | Div | Repeat Start | Repeat End | Repeat Left | Name | Family | Class |
| 41551132 | 41551229 | - | 22.2 | 6188 | 6285 | -737 | L1M | L1 | LINE |
| 41551257 | 41551741 | - | 23.3 | 5456 | 6009 | -997 | L1M | L1 | LINE |
| 41551734 | 41552372 | - | 24.9 | 7118 | 7770 | -15 | L1MC3 | L1 | LINE |
| 41552373 | 41552673 | - | 11.7 | 1 | 301 | -11 | AluSx | Alu | SINE |
| 41552674 | 41553313 | - | 24.9 | 6462 | 7117 | -668 | L1MC3 | L1 | LINE |
| 41553314 | 41553654 | + | 15.8 | 3 | 346 | 0 | MER7A | MER2\_type | DNA |
| 41553655 | 41554881 | - | 24.7 | 5266 | 6461 | -1324 | L1MC3 | L1 | LINE |
| 41554882 | 41554938 | - | 1.8 | 6089 | 6144 | -11 | L1PA4 | L1 | LINE |
| 41554939 | 41555357 | - | 23.9 | 4824 | 5265 | -881 | L1MC3 | L1 | LINE |
| 41555367 | 41555917 | - | 27.6 | 4872 | 5468 | -1538 | L1M | L1 | LINE |
| 41555922 | 41557460 | - | 3.0 | 4614 | 6152 | -3 | L1PA3 | L1 | LINE |
| 41557457 | 41557993 | + | 4.7 | 4068 | 4604 | -1542 | L1PA3 | L1 | LINE |
| 41557990 | 41558503 | - | 25.2 | 4315 | 4886 | -2120 | L1M | L1 | LINE |
| 41558547 | 41558561 | - | 25.2 | 4300 | 4314 | -2692 | L1M | L1 | LINE |

  

|  |  |  |  |  |  |  |  |  |  |
| --- | --- | --- | --- | --- | --- | --- | --- | --- | --- |
| chr12:43540889-43550480 | | | 9533 bp | | |  |  |  |  |
| Genome Start | Genome End | Strand | Div | Repeat Start | Repeat End | Repeat Left | Name | Family | Class |
| 43540889 | 43541008 | - | 22.8 | 1229 | 1489 | 0 | MLT1D-int | MaLR | LTR |
| 43541025 | 43541836 | + | 25.0 | 461 | 1251 | -6238 | L1MA8 | L1 | LINE |
| 43541837 | 43542212 | + | 22.2 | 2008 | 2572 | -5279 | L1MA8 | L1 | LINE |
| 43542213 | 43542721 | + | 11.4 | 1 | 513 | -1 | MER75 | PiggyBac | DNA |
| 43542722 | 43543008 | + | 25.4 | 2287 | 2568 | -4997 | L1M1 | L1 | LINE |
| 43543009 | 43543296 | - | 4.9 | 5853 | 6139 | -15 | L1PA7 | L1 | LINE |
| 43543297 | 43544888 | + | 21.1 | 2569 | 4175 | -3387 | L1M1 | L1 | LINE |
| 43544888 | 43545384 | - | 0.6 | 5190 | 5686 | -469 | L1HS | L1 | LINE |
| 43545385 | 43545874 | + | 2.0 | 5664 | 6153 | -2 | L1HS | L1 | LINE |
| 43545881 | 43545948 | + | 11.8 | 4159 | 4226 | -3336 | L1M1 | L1 | LINE |
| 43545949 | 43546320 | + | 21.4 | 1 | 422 | -4 | MLT1A1 | MaLR | LTR |
| 43546325 | 43547892 | + | 14.9 | 4222 | 5765 | -1797 | L1MA8 | L1 | LINE |
| 43547893 | 43548193 | + | 13.0 | 6 | 306 | -6 | AluSx | Alu | SINE |
| 43548194 | 43548465 | + | 14.9 | 5766 | 6036 | -1526 | L1MA8 | L1 | LINE |
| 43548461 | 43548552 | - | 19.1 | 5688 | 5776 | -515 | L1M2 | L1 | LINE |
| 43548553 | 43549063 | + | 12.1 | 7175 | 7706 | -4 | L1MA8 | L1 | LINE |
| 43549103 | 43550480 | - | 25.1 | 2 | 1264 | -107 | MLT1D-int | MaLR | LTR |

  

|  |  |  |  |  |  |  |  |  |  |
| --- | --- | --- | --- | --- | --- | --- | --- | --- | --- |
| chr12:45618893-45621687 | | | 2703 bp | | |  |  |  |  |
| Genome Start | Genome End | Strand | Div | Repeat Start | Repeat End | Repeat Left | Name | Family | Class |
| 45618893 | 45619169 | + | 29.2 | 5588 | 5881 | -240 | L1ME4a | L1 | LINE |
| 45619184 | 45619484 | + | 4.4 | 1 | 300 | -11 | AluY | Alu | SINE |
| 45619500 | 45620370 | - | 1.1 | 4203 | 5073 | -1073 | L1PA2 | L1 | LINE |
| 45620368 | 45621446 | + | 1.3 | 5073 | 6151 | -4 | L1PA2 | L1 | LINE |
| 45621513 | 45621687 | + | 22.4 | 5900 | 6101 | -20 | L1ME4a | L1 | LINE |

  

|  |  |  |  |  |  |  |  |  |  |
| --- | --- | --- | --- | --- | --- | --- | --- | --- | --- |
| chr12:46022211-46024501 | | | 2151 bp | | |  |  |  |  |
| Genome Start | Genome End | Strand | Div | Repeat Start | Repeat End | Repeat Left | Name | Family | Class |
| 46022211 | 46022476 | - | 19.4 | 1357 | 1631 | -52 | FordPrefect | Tip100 | DNA |
| 46022481 | 46022681 | - | 9.0 | 5193 | 5393 | -753 | L1P3 | L1 | LINE |
| 46022682 | 46023429 | + | 4.1 | 5404 | 6151 | -3 | L1PA5 | L1 | LINE |
| 46023453 | 46023807 | + | 22.3 | 1526 | 2057 | -4445 | L1MCa | L1 | LINE |
| 46023925 | 46023991 | + | 23.9 | 2164 | 2230 | -4272 | L1MCa | L1 | LINE |
| 46023985 | 46024082 | + | 24.5 | 6054 | 6152 | -14 | L1ME1 | L1 | LINE |
| 46024086 | 46024501 | - | 20.9 | 927 | 1380 | -303 | FordPrefect | Tip100 | DNA |

  

|  |  |  |  |  |  |  |  |  |  |
| --- | --- | --- | --- | --- | --- | --- | --- | --- | --- |
| chr12:51160958-51165941 | | | 4963 bp | | |  |  |  |  |
| Genome Start | Genome End | Strand | Div | Repeat Start | Repeat End | Repeat Left | Name | Family | Class |
| 51160958 | 51161305 | - | 22.3 | 4327 | 4670 | -1476 | L1MC | L1 | LINE |
| 51161306 | 51161618 | + | 20.2 | 1 | 312 | 0 | AluJb | Alu | SINE |
| 51161619 | 51162578 | - | 22.3 | 3371 | 4326 | -1820 | L1MC | L1 | LINE |
| 51162603 | 51165301 | - | 13.5 | 3571 | 6297 | 0 | L1MA5A | L1 | LINE |
| 51165302 | 51165879 | + | 13.5 | 2995 | 3591 | -2552 | L1MA5A | L1 | LINE |
| 51165877 | 51165941 | - | 20.0 | 3321 | 3388 | -2758 | L1MC | L1 | LINE |

  

|  |  |  |  |  |  |  |  |  |  |
| --- | --- | --- | --- | --- | --- | --- | --- | --- | --- |
| chr12:57467079-57469732 | | | 2521 bp | | |  |  |  |  |
| Genome Start | Genome End | Strand | Div | Repeat Start | Repeat End | Repeat Left | Name | Family | Class |
| 57467079 | 57467561 | + | 19.9 | 1 | 480 | -2251 | Tigger4(Zombi) | MER2\_type | DNA |
| 57467562 | 57467799 | - | 14.1 | 4881 | 5122 | -1024 | L1P5 | L1 | LINE |
| 57467772 | 57468312 | + | 10.6 | 5147 | 5698 | -458 | L1P5 | L1 | LINE |
| 57468313 | 57468478 | - | 6.0 | 105 | 270 | -41 | AluY | Alu | SINE |
| 57468479 | 57468579 | + | 17.3 | 5687 | 5784 | -2024 | L1MC | L1 | LINE |
| 57468624 | 57468687 | - | 17.5 | 5729 | 5800 | -365 | L1P4 | L1 | LINE |
| 57468680 | 57468732 | - | 13.7 | 5747 | 5799 | -381 | L1M3 | L1 | LINE |
| 57468756 | 57469131 | + | 17.7 | 5791 | 6165 | -1 | L1PA17 | L1 | LINE |
| 57469234 | 57469732 | + | 14.0 | 451 | 965 | -1766 | Tigger4(Zombi) | MER2\_type | DNA |

  

|  |  |  |  |  |  |  |  |  |  |
| --- | --- | --- | --- | --- | --- | --- | --- | --- | --- |
| chr12:58268945-58270743 | | | 1793 bp | | |  |  |  |  |
| Genome Start | Genome End | Strand | Div | Repeat Start | Repeat End | Repeat Left | Name | Family | Class |
| 58268945 | 58269896 | + | 11.4 | 1267 | 2221 | -197 | Tigger1 | MER2\_type | DNA |
| 58269903 | 58270097 | - | 0.0 | 5958 | 6152 | -3 | L1PA2 | L1 | LINE |
| 58270098 | 58270532 | + | 0.9 | 5499 | 5933 | -222 | L1PA2 | L1 | LINE |
| 58270533 | 58270743 | + | 17.6 | 2212 | 2418 | 0 | Tigger1 | MER2\_type | DNA |

  

|  |  |  |  |  |  |  |  |  |  |
| --- | --- | --- | --- | --- | --- | --- | --- | --- | --- |
| chr12:59453641-59461367 | | | 7390 bp | | |  |  |  |  |
| Genome Start | Genome End | Strand | Div | Repeat Start | Repeat End | Repeat Left | Name | Family | Class |
| 59453641 | 59454662 | - | 10.8 | 5335 | 6337 | -16 | L1PA10 | L1 | LINE |
| 59454666 | 59456473 | - | 10.5 | 2472 | 4285 | -2046 | L1PA10 | L1 | LINE |
| 59456811 | 59458355 | - | 3.3 | 4605 | 6151 | -4 | L1PA3 | L1 | LINE |
| 59458356 | 59459041 | + | 4.5 | 3922 | 4608 | -1538 | L1PA3 | L1 | LINE |
| 59459039 | 59461367 | - | 17.0 | 194 | 2494 | -3837 | L1PA10 | L1 | LINE |

  

|  |  |  |  |  |  |  |  |  |  |
| --- | --- | --- | --- | --- | --- | --- | --- | --- | --- |
| chr12:59576210-59578264 | | | 2088 bp | | |  |  |  |  |
| Genome Start | Genome End | Strand | Div | Repeat Start | Repeat End | Repeat Left | Name | Family | Class |
| 59576210 | 59576503 | - | 18.4 | 6007 | 6303 | -9 | L1MA9 | L1 | LINE |
| 59576504 | 59577400 | - | 3.2 | 5255 | 6154 | -1 | L1PA3 | L1 | LINE |
| 59577382 | 59578052 | + | 4.2 | 4575 | 5244 | -902 | L1PA3 | L1 | LINE |
| 59578039 | 59578264 | - | 19.0 | 5793 | 6016 | -296 | L1MA9 | L1 | LINE |

  

|  |  |  |  |  |  |  |  |  |  |
| --- | --- | --- | --- | --- | --- | --- | --- | --- | --- |
| chr12:59734536-59741018 | | | 6434 bp | | |  |  |  |  |
| Genome Start | Genome End | Strand | Div | Repeat Start | Repeat End | Repeat Left | Name | Family | Class |
| 59734536 | 59734769 | + | 19.0 | 2 | 238 | -2180 | Tigger1 | MER2\_type | DNA |
| 59734800 | 59735310 | - | 3.1 | 5627 | 6137 | -18 | L1PA3 | L1 | LINE |
| 59735316 | 59736014 | + | 2.3 | 3615 | 4316 | -1830 | L1P1 | L1 | LINE |
| 59736015 | 59737758 | + | 14.8 | 232 | 1994 | -424 | Tigger1 | MER2\_type | DNA |
| 59737773 | 59739233 | - | 14.2 | 3506 | 4853 | -1293 | L1PREC2 | L1 | LINE |
| 59739235 | 59739310 | - | 10.5 | 3262 | 3337 | -2809 | L1PREC2 | L1 | LINE |
| 59739306 | 59740594 | + | 15.6 | 4856 | 6152 | -13 | L1PA15 | L1 | LINE |
| 59740599 | 59741018 | + | 16.7 | 1986 | 2418 | 0 | Tigger1 | MER2\_type | DNA |

  

|  |  |  |  |  |  |  |  |  |  |
| --- | --- | --- | --- | --- | --- | --- | --- | --- | --- |
| chr12:59833558-59835471 | | | 1601 bp | | |  |  |  |  |
| Genome Start | Genome End | Strand | Div | Repeat Start | Repeat End | Repeat Left | Name | Family | Class |
| 59833558 | 59833681 | + | 31.7 | 545 | 669 | -1291 | L4 | RTE | LINE |
| 59833985 | 59835131 | - | 3.2 | 5000 | 6152 | -3 | L1PA4 | L1 | LINE |
| 59835133 | 59835252 | + | 3.3 | 4873 | 4992 | -1154 | L1PA4 | L1 | LINE |
| 59835262 | 59835471 | + | 31.4 | 832 | 1088 | -872 | L4 | RTE | LINE |

  

|  |  |  |  |  |  |  |  |  |  |
| --- | --- | --- | --- | --- | --- | --- | --- | --- | --- |
| chr12:64075605-64089153 | | | 13490 bp | | |  |  |  |  |
| Genome Start | Genome End | Strand | Div | Repeat Start | Repeat End | Repeat Left | Name | Family | Class |
| 64075605 | 64076455 | - | 14.1 | 3155 | 4020 | -2544 | L1MCb | L1 | LINE |
| 64076456 | 64076802 | + | 12.3 | 1 | 347 | 0 | AluSq | Alu | SINE |
| 64076803 | 64077667 | - | 14.4 | 2361 | 3154 | -3410 | L1MCb | L1 | LINE |
| 64077658 | 64084106 | - | 9.4 | 8 | 6152 | -2 | L1PA7 | L1 | LINE |
| 64084118 | 64084284 | - | 25.1 | 1 | 170 | -15 | FAM | Alu | SINE |
| 64084290 | 64084697 | - | 16.9 | 1915 | 2375 | -4376 | L1MCb | L1 | LINE |
| 64084715 | 64085006 | - | 13.7 | 5963 | 6254 | -37 | L1MA8 | L1 | LINE |
| 64085007 | 64085298 | - | 12.4 | 1 | 296 | -16 | AluSx | Alu | SINE |
| 64085299 | 64085469 | - | 13.7 | 5784 | 5962 | -329 | L1MA8 | L1 | LINE |
| 64085505 | 64086332 | - | 14.3 | 4934 | 5783 | -508 | L1MA8 | L1 | LINE |
| 64086334 | 64086748 | + | 15.0 | 4330 | 4750 | -1393 | L1M2 | L1 | LINE |
| 64086749 | 64087114 | + | 19.9 | 1 | 408 | 0 | MLT1A1 | MaLR | LTR |
| 64087115 | 64087287 | + | 15.0 | 4751 | 4921 | -1222 | L1M2 | L1 | LINE |
| 64087288 | 64088146 | - | 18.1 | 1049 | 1922 | -4829 | L1MCb | L1 | LINE |
| 64088147 | 64088433 | - | 10.8 | 6 | 293 | -17 | AluSg | Alu | SINE |
| 64088434 | 64089153 | - | 20.2 | 227 | 1048 | -5703 | L1MCb | L1 | LINE |

  

|  |  |  |  |  |  |  |  |  |  |
| --- | --- | --- | --- | --- | --- | --- | --- | --- | --- |
| chr12:65833368-65835206 | | | 1550 bp | | |  |  |  |  |
| Genome Start | Genome End | Strand | Div | Repeat Start | Repeat End | Repeat Left | Name | Family | Class |
| 65833368 | 65833681 | + | 24.4 | 5001 | 5332 | -814 | L1M5 | L1 | LINE |
| 65833684 | 65833833 | - | 3.4 | 5799 | 5947 | -207 | L1PA7 | L1 | LINE |
| 65833835 | 65834016 | + | 9.3 | 5960 | 6150 | -4 | L1PA7 | L1 | LINE |
| 65834090 | 65834220 | + | 32.3 | 5360 | 5501 | -739 | L1M5 | L1 | LINE |
| 65834276 | 65834526 | - | 28.9 | 1 | 256 | -6 | MIR | MIR | SINE |
| 65834680 | 65834941 | - | 28.0 | 2 | 303 | -254 | LTR67 | ERVL | LTR |
| 65834947 | 65835206 | + | 32.7 | 5484 | 5784 | -456 | L1M5 | L1 | LINE |

  

|  |  |  |  |  |  |  |  |  |  |
| --- | --- | --- | --- | --- | --- | --- | --- | --- | --- |
| chr12:68839000-68842808 | | | 3788 bp | | |  |  |  |  |
| Genome Start | Genome End | Strand | Div | Repeat Start | Repeat End | Repeat Left | Name | Family | Class |
| 68839000 | 68841179 | + | 14.1 | 3010 | 5299 | -844 | L1MA4 | L1 | LINE |
| 68841178 | 68841513 | - | 11.6 | 5487 | 5838 | -322 | L1PA13 | L1 | LINE |
| 68841514 | 68841805 | + | 11.3 | 5845 | 6144 | -19 | L1PA13 | L1 | LINE |
| 68841825 | 68842464 | + | 13.7 | 5298 | 5939 | -361 | L1MA4 | L1 | LINE |
| 68842469 | 68842607 | + | 12.9 | 174 | 312 | 0 | AluSg/x | Alu | SINE |
| 68842608 | 68842808 | + | 15.4 | 6066 | 6271 | -29 | L1MA4 | L1 | LINE |

  

|  |  |  |  |  |  |  |  |  |  |
| --- | --- | --- | --- | --- | --- | --- | --- | --- | --- |
| chr12:69147946-69157393 | | | 9425 bp | | |  |  |  |  |
| Genome Start | Genome End | Strand | Div | Repeat Start | Repeat End | Repeat Left | Name | Family | Class |
| 69147946 | 69148008 | + | 9.8 | 204 | 264 | -117 | THE1D | MaLR | LTR |
| 69148009 | 69148341 | + | 17.2 | 1 | 343 | 0 | MER61A | ERV1 | LTR |
| 69148342 | 69149034 | + | 18.1 | 1 | 707 | -4510 | MER61A-int | ERV1 | LTR |
| 69149073 | 69149371 | - | 8.0 | 1 | 313 | 0 | AluSp | Alu | SINE |
| 69149375 | 69149417 | + | 13.9 | 1320 | 1362 | -3855 | MER61A-int | ERV1 | LTR |
| 69149401 | 69152066 | + | 9.8 | 1655 | 4816 | -1062 | MER61A-int | ERV1 | LTR |
| 69152067 | 69154410 | - | 4.9 | 2282 | 4631 | -1515 | L1PA5 | L1 | LINE |
| 69154410 | 69155923 | + | 6.0 | 4637 | 6154 | 0 | L1PA5 | L1 | LINE |
| 69155924 | 69156922 | + | 11.7 | 4150 | 5217 | 0 | MER61A-int | ERV1 | LTR |
| 69156923 | 69157257 | + | 13.8 | 1 | 343 | 0 | MER61A | ERV1 | LTR |
| 69157258 | 69157393 | + | 8.3 | 261 | 381 | 0 | THE1D | MaLR | LTR |

  

|  |  |  |  |  |  |  |  |  |  |
| --- | --- | --- | --- | --- | --- | --- | --- | --- | --- |
| chr12:69949568-69951524 | | | 1685 bp | | |  |  |  |  |
| Genome Start | Genome End | Strand | Div | Repeat Start | Repeat End | Repeat Left | Name | Family | Class |
| 69949568 | 69950395 | - | 14.1 | 330 | 1225 | -6 | Tigger3b | MER2\_type | DNA |
| 69950645 | 69950659 | - | 13.3 | 6148 | 6168 | 0 | L1PA10 | L1 | LINE |
| 69950681 | 69950840 | - | 13.3 | 5983 | 6147 | -21 | L1PA10 | L1 | LINE |
| 69950842 | 69951193 | + | 7.7 | 5618 | 5973 | -195 | L1PA10 | L1 | LINE |
| 69951195 | 69951524 | - | 17.9 | 1 | 335 | -896 | Tigger3b | MER2\_type | DNA |

  

|  |  |  |  |  |  |  |  |  |  |
| --- | --- | --- | --- | --- | --- | --- | --- | --- | --- |
| chr12:71740511-71743723 | | | 2997 bp | | |  |  |  |  |
| Genome Start | Genome End | Strand | Div | Repeat Start | Repeat End | Repeat Left | Name | Family | Class |
| 71740511 | 71740720 | + | 18.8 | 1 | 224 | -290 | MER67D | ERV1 | LTR |
| 71740726 | 71741496 | - | 19.6 | 3782 | 4561 | -1579 | L1M3 | L1 | LINE |
| 71741645 | 71741788 | - | 19.4 | 3621 | 3782 | -2358 | L1M3 | L1 | LINE |
| 71741790 | 71743442 | + | 17.4 | 4584 | 6168 | 0 | L1MB1 | L1 | LINE |
| 71743479 | 71743603 | + | 28.0 | 237 | 364 | -150 | MER67D | ERV1 | LTR |
| 71743630 | 71743723 | + | 15.8 | 431 | 514 | 0 | MER67D | ERV1 | LTR |

  

|  |  |  |  |  |  |  |  |  |  |
| --- | --- | --- | --- | --- | --- | --- | --- | --- | --- |
| chr12:72526112-72528996 | | | 2762 bp | | |  |  |  |  |
| Genome Start | Genome End | Strand | Div | Repeat Start | Repeat End | Repeat Left | Name | Family | Class |
| 72526112 | 72526369 | - | 25.7 | 3729 | 4001 | -30 | Zaphod | Tip100 | DNA |
| 72526373 | 72526821 | - | 9.4 | 4585 | 5044 | -1102 | L1PA7 | L1 | LINE |
| 72526815 | 72527898 | + | 6.8 | 5058 | 6139 | -15 | L1PA7 | L1 | LINE |
| 72527902 | 72528672 | - | 24.6 | 2884 | 3738 | -293 | Zaphod | Tip100 | DNA |
| 72528797 | 72528996 | - | 35.6 | 33 | 232 | -3799 | Zaphod | Tip100 | DNA |

  

|  |  |  |  |  |  |  |  |  |  |
| --- | --- | --- | --- | --- | --- | --- | --- | --- | --- |
| chr12:72649771-72653085 | | | 3255 bp | | |  |  |  |  |
| Genome Start | Genome End | Strand | Div | Repeat Start | Repeat End | Repeat Left | Name | Family | Class |
| 72649771 | 72649890 | + | 33.0 | 2 | 124 | -91 | MER58 | MER1\_type | DNA |
| 72649930 | 72650662 | - | 7.2 | 3078 | 3812 | -2334 | L1PA7 | L1 | LINE |
| 72650657 | 72653008 | + | 8.3 | 3795 | 6154 | 0 | L1PA7 | L1 | LINE |
| 72653036 | 72653085 | + | 16.0 | 2365 | 2417 | -3 | MER58 | MER1\_type | DNA |

  

|  |  |  |  |  |  |  |  |  |  |
| --- | --- | --- | --- | --- | --- | --- | --- | --- | --- |
| chr12:72766794-72774406 | | | 7494 bp | | |  |  |  |  |
| Genome Start | Genome End | Strand | Div | Repeat Start | Repeat End | Repeat Left | Name | Family | Class |
| 72766794 | 72768207 | - | 20.4 | 4973 | 6402 | -2 | L1MB1 | L1 | LINE |
| 72768209 | 72768641 | + | 23.8 | 1 | 434 | -467 | MER54A | ERVL | LTR |
| 72768773 | 72770570 | - | 19.0 | 3156 | 4976 | -1400 | L1MB1 | L1 | LINE |
| 72770572 | 72770678 | + | 12.3 | 3469 | 3579 | -2567 | L1M2 | L1 | LINE |
| 72770674 | 72771629 | - | 3.0 | 4060 | 5017 | -1129 | L1PA3 | L1 | LINE |
| 72771622 | 72772733 | + | 3.7 | 5040 | 6152 | -3 | L1PA3 | L1 | LINE |
| 72772748 | 72773386 | + | 13.6 | 3563 | 4205 | -1938 | L1M2 | L1 | LINE |
| 72773372 | 72774406 | - | 21.0 | 2101 | 3158 | -3218 | L1MB1 | L1 | LINE |

  

|  |  |  |  |  |  |  |  |  |  |
| --- | --- | --- | --- | --- | --- | --- | --- | --- | --- |
| chr12:73107209-73108719 | | | 1503 bp | | |  |  |  |  |
| Genome Start | Genome End | Strand | Div | Repeat Start | Repeat End | Repeat Left | Name | Family | Class |
| 73107209 | 73107565 | + | 15.2 | 14 | 376 | -56 | MSTB1 | MaLR | LTR |
| 73107574 | 73108283 | - | 9.0 | 5455 | 6164 | -8 | L1PA8 | L1 | LINE |
| 73108284 | 73108643 | + | 9.9 | 5069 | 5438 | -734 | L1PA8 | L1 | LINE |
| 73108644 | 73108719 | + | 9.3 | 352 | 429 | -3 | MSTB1 | MaLR | LTR |

  

|  |  |  |  |  |  |  |  |  |  |
| --- | --- | --- | --- | --- | --- | --- | --- | --- | --- |
| chr12:77676191-77681676 | | | 5407 bp | | |  |  |  |  |
| Genome Start | Genome End | Strand | Div | Repeat Start | Repeat End | Repeat Left | Name | Family | Class |
| 77676191 | 77676413 | - | 19.5 | 7533 | 7770 | -15 | L1MC3 | L1 | LINE |
| 77676485 | 77676860 | - | 25.5 | 1 | 396 | 0 | MSTD | MaLR | LTR |
| 77676861 | 77677108 | - | 19.5 | 7267 | 7532 | -253 | L1MC3 | L1 | LINE |
| 77677113 | 77677276 | - | 24.2 | 342 | 505 | 0 | MLT1D | MaLR | LTR |
| 77677277 | 77677608 | - | 14.5 | 1 | 355 | -2 | LTR18A | ERVL | LTR |
| 77677609 | 77677953 | - | 24.2 | 5 | 341 | -164 | MLT1D | MaLR | LTR |
| 77677959 | 77678737 | - | 26.1 | 6456 | 7262 | -523 | L1MC3 | L1 | LINE |
| 77678738 | 77679089 | - | 14.4 | 1 | 381 | 0 | THE1D | MaLR | LTR |
| 77679090 | 77679208 | - | 26.1 | 6340 | 6455 | -1330 | L1MC3 | L1 | LINE |
| 77679209 | 77679493 | - | 13.7 | 1 | 306 | -6 | AluSx | Alu | SINE |
| 77679494 | 77680727 | - | 6.2 | 5134 | 6339 | -1446 | L1PA3 | L1 | LINE |
| 77680728 | 77680934 | + | 2.4 | 4912 | 5118 | -1028 | L1PA3 | L1 | LINE |
| 77680934 | 77681676 | - | 23.1 | 5393 | 6147 | -1638 | L1MC3 | L1 | LINE |

  

|  |  |  |  |  |  |  |  |  |  |
| --- | --- | --- | --- | --- | --- | --- | --- | --- | --- |
| chr12:78644888-78652782 | | | 7887 bp | | |  |  |  |  |
| Genome Start | Genome End | Strand | Div | Repeat Start | Repeat End | Repeat Left | Name | Family | Class |
| 78644888 | 78648062 | + | 17.7 | 24 | 3305 | -3232 | L1MDa | L1 | LINE |
| 78648063 | 78648383 | - | 10.3 | 1 | 317 | 0 | AluSg | Alu | SINE |
| 78648384 | 78648645 | + | 15.8 | 3306 | 3573 | -2964 | L1MDa | L1 | LINE |
| 78648641 | 78649081 | - | 13.0 | 5508 | 5950 | -350 | L1MA8 | L1 | LINE |
| 78649080 | 78649397 | + | 18.2 | 5969 | 6287 | -4 | L1MA8 | L1 | LINE |
| 78649413 | 78650266 | + | 16.9 | 3524 | 4334 | -2203 | L1MDa | L1 | LINE |
| 78650267 | 78650537 | + | 15.2 | 1 | 266 | -46 | AluJo | Alu | SINE |
| 78650538 | 78651475 | + | 16.9 | 4335 | 5242 | -1295 | L1MDa | L1 | LINE |
| 78651476 | 78651773 | - | 14.9 | 1 | 297 | -13 | AluSg | Alu | SINE |
| 78651774 | 78652184 | + | 16.9 | 5243 | 5694 | -843 | L1MDa | L1 | LINE |
| 78652185 | 78652453 | + | 16.5 | 5896 | 6155 | -1 | L1PB4 | L1 | LINE |
| 78652454 | 78652782 | + | 17.0 | 5695 | 5998 | -635 | L1MDa | L1 | LINE |

  

|  |  |  |  |  |  |  |  |  |  |
| --- | --- | --- | --- | --- | --- | --- | --- | --- | --- |
| chr12:79502953-79505447 | | | 2028 bp | | |  |  |  |  |
| Genome Start | Genome End | Strand | Div | Repeat Start | Repeat End | Repeat Left | Name | Family | Class |
| 79502953 | 79503448 | + | 28.4 | 2331 | 2843 | -576 | L2 | L2 | LINE |
| 79503496 | 79503613 | + | 29.9 | 2980 | 3111 | -308 | L2 | L2 | LINE |
| 79503643 | 79504106 | - | 1.7 | 5693 | 6155 | 0 | L1PA2 | L1 | LINE |
| 79504107 | 79504494 | + | 0.8 | 5293 | 5680 | -475 | L1PA2 | L1 | LINE |
| 79504615 | 79504944 | + | 22.2 | 1 | 344 | -1 | MER2 | MER2\_type | DNA |
| 79504967 | 79505095 | + | 23.2 | 3281 | 3412 | -7 | L2 | L2 | LINE |
| 79505345 | 79505447 | + | 33.0 | 3248 | 3358 | -20 | L2 | L2 | LINE |

  

|  |  |  |  |  |  |  |  |  |  |
| --- | --- | --- | --- | --- | --- | --- | --- | --- | --- |
| chr12:79920084-79922735 | | | 2670 bp | | |  |  |  |  |
| Genome Start | Genome End | Strand | Div | Repeat Start | Repeat End | Repeat Left | Name | Family | Class |
| 79920084 | 79920132 | - | 14.3 | 6120 | 6168 | 0 | L1MB1 | L1 | LINE |
| 79920133 | 79920801 | - | 2.5 | 5487 | 6155 | 0 | L1PA3 | L1 | LINE |
| 79920802 | 79922681 | + | 3.1 | 3606 | 5482 | -673 | L1PA3 | L1 | LINE |
| 79922664 | 79922735 | - | 14.1 | 6059 | 6135 | -54 | L1MB1 | L1 | LINE |

  

|  |  |  |  |  |  |  |  |  |  |
| --- | --- | --- | --- | --- | --- | --- | --- | --- | --- |
| chr12:80825503-80828530 | | | 2973 bp | | |  |  |  |  |
| Genome Start | Genome End | Strand | Div | Repeat Start | Repeat End | Repeat Left | Name | Family | Class |
| 80825503 | 80826231 | - | 18.2 | 4453 | 5190 | -953 | L1M2 | L1 | LINE |
| 80826288 | 80826778 | - | 8.9 | 5666 | 6151 | -3 | L1PA7 | L1 | LINE |
| 80826779 | 80827644 | + | 8.4 | 4806 | 5680 | -474 | L1PA7 | L1 | LINE |
| 80827644 | 80828530 | - | 17.4 | 3565 | 4466 | -1677 | L1M2 | L1 | LINE |

  

|  |  |  |  |  |  |  |  |  |  |
| --- | --- | --- | --- | --- | --- | --- | --- | --- | --- |
| chr12:81097247-81100723 | | | 2887 bp | | |  |  |  |  |
| Genome Start | Genome End | Strand | Div | Repeat Start | Repeat End | Repeat Left | Name | Family | Class |
| 81097247 | 81097382 | - | 35.5 | 3225 | 3378 | 0 | L2 | L2 | LINE |
| 81097419 | 81097482 | + | 18.8 | 6 | 69 | -233 | AluJo | Alu | SINE |
| 81097489 | 81098482 | - | 4.9 | 4350 | 5343 | -803 | L1PA4 | L1 | LINE |
| 81098482 | 81099302 | + | 4.2 | 5336 | 6155 | 0 | L1PA4 | L1 | LINE |
| 81099313 | 81099372 | + | 19.6 | 78 | 137 | -175 | AluJo | Alu | SINE |
| 81099373 | 81099677 | + | 12.1 | 1 | 305 | -5 | AluSg | Alu | SINE |
| 81099678 | 81099817 | + | 19.6 | 138 | 287 | -25 | AluJo | Alu | SINE |
| 81099884 | 81099977 | - | 29.1 | 3098 | 3186 | -192 | L2 | L2 | LINE |
| 81100348 | 81100465 | + | 30.4 | 393 | 510 | -47 | LTR67 | ERVL | LTR |
| 81100569 | 81100723 | - | 29.3 | 2801 | 2975 | -444 | L2 | L2 | LINE |

  

|  |  |  |  |  |  |  |  |  |  |
| --- | --- | --- | --- | --- | --- | --- | --- | --- | --- |
| chr12:81221050-81233442 | | | 12303 bp | | |  |  |  |  |
| Genome Start | Genome End | Strand | Div | Repeat Start | Repeat End | Repeat Left | Name | Family | Class |
| 81221050 | 81221650 | + | 17.9 | 27 | 628 | -5880 | L1MCa | L1 | LINE |
| 81221648 | 81222860 | - | 3.3 | 2777 | 3989 | -2157 | L1PA3 | L1 | LINE |
| 81222858 | 81225030 | + | 3.6 | 3967 | 6155 | 0 | L1PA3 | L1 | LINE |
| 81225101 | 81226598 | + | 17.8 | 620 | 2133 | -4375 | L1MCa | L1 | LINE |
| 81226599 | 81226900 | + | 10.9 | 1 | 302 | -8 | AluSg | Alu | SINE |
| 81226901 | 81228405 | + | 15.4 | 2134 | 3583 | -2738 | L1MCa | L1 | LINE |
| 81228401 | 81231769 | - | 7.2 | 2774 | 6154 | 0 | L1PA6 | L1 | LINE |
| 81231799 | 81232298 | - | 7.5 | 2255 | 2773 | -3373 | L1PA6 | L1 | LINE |
| 81232295 | 81232909 | - | 10.9 | 1 | 618 | -1657 | L1P2 | L1 | LINE |
| 81232916 | 81233442 | + | 15.4 | 3573 | 4092 | -2229 | L1MCa | L1 | LINE |

  

|  |  |  |  |  |  |  |  |  |  |
| --- | --- | --- | --- | --- | --- | --- | --- | --- | --- |
| chr12:83529157-83532909 | | | 3752 bp | | |  |  |  |  |
| Genome Start | Genome End | Strand | Div | Repeat Start | Repeat End | Repeat Left | Name | Family | Class |
| 83529157 | 83530140 | + | 17.2 | 1 | 998 | -1420 | Tigger1 | MER2\_type | DNA |
| 83530141 | 83530441 | + | 11.8 | 1 | 298 | -15 | AluSq | Alu | SINE |
| 83530442 | 83530549 | + | 17.2 | 999 | 1088 | -1330 | Tigger1 | MER2\_type | DNA |
| 83530551 | 83531253 | - | 3.3 | 5450 | 6154 | -1 | L1PA4 | L1 | LINE |
| 83531254 | 83531590 | + | 4.1 | 5119 | 5460 | -695 | L1PA4 | L1 | LINE |
| 83531591 | 83532909 | + | 16.4 | 1071 | 2417 | -1 | Tigger1 | MER2\_type | DNA |

  

|  |  |  |  |  |  |  |  |  |  |
| --- | --- | --- | --- | --- | --- | --- | --- | --- | --- |
| chr12:86625361-86635984 | | | 9919 bp | | |  |  |  |  |
| Genome Start | Genome End | Strand | Div | Repeat Start | Repeat End | Repeat Left | Name | Family | Class |
| 86625361 | 86625474 | + | 22.9 | 2336 | 2451 | -3695 | L1MC3 | L1 | LINE |
| 86625475 | 86625820 | - | 22.4 | 1 | 396 | 0 | MSTD | MaLR | LTR |
| 86625821 | 86626792 | + | 22.9 | 2452 | 3435 | -2711 | L1MC3 | L1 | LINE |
| 86626793 | 86627167 | - | 15.8 | 1 | 375 | 0 | THE1C | MaLR | LTR |
| 86627168 | 86627362 | + | 22.9 | 3436 | 3627 | -2519 | L1MC3 | L1 | LINE |
| 86627859 | 86628235 | - | 3.7 | 5776 | 6152 | -3 | L1PA3 | L1 | LINE |
| 86628236 | 86628724 | + | 2.5 | 5287 | 5776 | -379 | L1PA3 | L1 | LINE |
| 86628719 | 86629279 | + | 21.8 | 3614 | 4166 | -1980 | L1MC3 | L1 | LINE |
| 86629320 | 86630022 | + | 21.8 | 4167 | 4897 | -1249 | L1MC3 | L1 | LINE |
| 86630023 | 86630358 | - | 20.9 | 29 | 365 | 0 | MLT1A0 | MaLR | LTR |
| 86630360 | 86631696 | + | 21.0 | 5237 | 6618 | -1167 | L1MC3 | L1 | LINE |
| 86631699 | 86631743 | - | 11.1 | 368 | 412 | -2 | MLT2B4 | ERVL | LTR |
| 86631750 | 86631809 | + | 21.7 | 498 | 557 | -7713 | PRIMA4-int | ERV1 | LTR |
| 86631842 | 86632334 | - | 22.6 | -145 | 356 | -54 | MLT2B4 | ERVL | LTR |
| 86632335 | 86632712 | - | 15.3 | 5233 | 5714 | 0 | ERVL-B4 | ERVL | LTR |
| 86632703 | 86633136 | - | 19.5 | 3153 | 3613 | -2101 | ERVL-B4 | ERVL | LTR |
| 86633135 | 86633381 | - | 18.0 | 2671 | 2919 | -2795 | ERVL-B4 | ERVL | LTR |
| 86633386 | 86634341 | + | 13.9 | 4339 | 5305 | -841 | L1P4 | L1 | LINE |
| 86634363 | 86634981 | - | 18.4 | 992 | 1625 | -4089 | ERVL-B4 | ERVL | LTR |
| 86634994 | 86635369 | + | 25.4 | 1 | 363 | -11 | MLT1A | MaLR | LTR |
| 86635466 | 86635784 | + | 21.6 | 6572 | 6912 | -924 | L1MC3 | L1 | LINE |
| 86635798 | 86635984 | + | 19.2 | 7542 | 7741 | -44 | L1MC3 | L1 | LINE |

  

|  |  |  |  |  |  |  |  |  |  |
| --- | --- | --- | --- | --- | --- | --- | --- | --- | --- |
| chr12:87261959-87267443 | | | 5362 bp | | |  |  |  |  |
| Genome Start | Genome End | Strand | Div | Repeat Start | Repeat End | Repeat Left | Name | Family | Class |
| 87261959 | 87262097 | - | 26.3 | 7772 | 7913 | -48 | L1MC5 | L1 | LINE |
| 87262156 | 87262263 | - | 18.5 | 6221 | 6331 | -2 | L1MC1 | L1 | LINE |
| 87262274 | 87263266 | - | 14.5 | 4839 | 5825 | -508 | L1MC1 | L1 | LINE |
| 87263252 | 87263334 | - | 24.1 | 494 | 576 | -5932 | L1MCa | L1 | LINE |
| 87263336 | 87263727 | - | 20.5 | 2 | 398 | -6110 | L1MCa | L1 | LINE |
| 87263728 | 87264324 | - | 24.5 | 6927 | 7485 | -476 | L1MC5 | L1 | LINE |
| 87264354 | 87264516 | - | 17.7 | 127 | 282 | -30 | AluJb | Alu | SINE |
| 87264558 | 87264683 | - | 17.7 | 1 | 126 | -186 | AluJb | Alu | SINE |
| 87264684 | 87264916 | - | 24.5 | 6712 | 6926 | -1035 | L1MC5 | L1 | LINE |
| 87264917 | 87266431 | - | 6.7 | 4657 | 6154 | 0 | L1PA6 | L1 | LINE |
| 87266433 | 87267341 | + | 5.4 | 3750 | 4657 | -1489 | L1PA6 | L1 | LINE |
| 87267340 | 87267443 | - | 25.5 | 6629 | 6723 | -1238 | L1MC5 | L1 | LINE |

  

|  |  |  |  |  |  |  |  |  |  |
| --- | --- | --- | --- | --- | --- | --- | --- | --- | --- |
| chr12:87539384-87541041 | | | 1660 bp | | |  |  |  |  |
| Genome Start | Genome End | Strand | Div | Repeat Start | Repeat End | Repeat Left | Name | Family | Class |
| 87539384 | 87539578 | + | 10.5 | 1 | 193 | -235 | MSTA | MaLR | LTR |
| 87539579 | 87539639 | - | 0.0 | 4967 | 5027 | -1119 | L1PA2 | L1 | LINE |
| 87539637 | 87540803 | + | 1.0 | 5022 | 6188 | 0 | L1PA2 | L1 | LINE |
| 87540805 | 87541041 | + | 19.1 | 185 | 428 | 0 | MSTA | MaLR | LTR |

  

|  |  |  |  |  |  |  |  |  |  |
| --- | --- | --- | --- | --- | --- | --- | --- | --- | --- |
| chr12:88744487-88746436 | | | 1708 bp | | |  |  |  |  |
| Genome Start | Genome End | Strand | Div | Repeat Start | Repeat End | Repeat Left | Name | Family | Class |
| 88744487 | 88744890 | + | 27.9 | 437 | 870 | -1090 | L4 | RTE | LINE |
| 88744892 | 88745009 | - | 11.9 | 5789 | 5906 | -245 | L1PB1 | L1 | LINE |
| 88745010 | 88745265 | + | 6.3 | 5898 | 6151 | 0 | L1PB1 | L1 | LINE |
| 88745431 | 88745817 | - | 31.4 | 185 | 549 | 0 | MLT1H | MaLR | LTR |
| 88745822 | 88745886 | + | 29.7 | 1287 | 1350 | -610 | L4 | RTE | LINE |
| 88745893 | 88746006 | + | 16.7 | 78 | 204 | -58 | MIR | MIR | SINE |
| 88746029 | 88746335 | + | 36.2 | 1364 | 1696 | -264 | L4 | RTE | LINE |
| 88746380 | 88746436 | + | 19.2 | 1706 | 1757 | -203 | L4 | RTE | LINE |

  

|  |  |  |  |  |  |  |  |  |  |
| --- | --- | --- | --- | --- | --- | --- | --- | --- | --- |
| chr12:90435647-90439906 | | | 4250 bp | | |  |  |  |  |
| Genome Start | Genome End | Strand | Div | Repeat Start | Repeat End | Repeat Left | Name | Family | Class |
| 90435647 | 90436804 | + | 11.6 | 4822 | 5986 | -180 | L1PA16 | L1 | LINE |
| 90436804 | 90438606 | - | 5.3 | 3592 | 5393 | -753 | L1PA5 | L1 | LINE |
| 90438606 | 90438923 | + | 5.1 | 5393 | 5691 | -463 | L1PA5 | L1 | LINE |
| 90438924 | 90439239 | - | 6.5 | 1 | 310 | -1 | AluY | Alu | SINE |
| 90439240 | 90439709 | + | 5.1 | 5692 | 6154 | 0 | L1PA5 | L1 | LINE |
| 90439722 | 90439906 | + | 14.6 | 5968 | 6156 | -10 | L1PA16 | L1 | LINE |

  

|  |  |  |  |  |  |  |  |  |  |
| --- | --- | --- | --- | --- | --- | --- | --- | --- | --- |
| chr12:91484291-91487662 | | | 2242 bp | | |  |  |  |  |
| Genome Start | Genome End | Strand | Div | Repeat Start | Repeat End | Repeat Left | Name | Family | Class |
| 91484291 | 91484398 | + | 29.2 | 1 | 112 | -2395 | HAL1 | L1 | LINE |
| 91484738 | 91485081 | + | 31.7 | 814 | 1167 | -1340 | HAL1 | L1 | LINE |
| 91485090 | 91485124 | - | 5.7 | 5061 | 5095 | -1051 | L1PA7 | L1 | LINE |
| 91485125 | 91486137 | + | 6.6 | 5106 | 6138 | -16 | L1PA7 | L1 | LINE |
| 91486377 | 91486689 | + | 31.4 | 1450 | 1749 | -758 | HAL1 | L1 | LINE |
| 91486872 | 91487008 | + | 27.9 | 2027 | 2175 | -332 | HAL1 | L1 | LINE |
| 91487222 | 91487322 | + | 26.0 | 1450 | 1551 | -956 | HAL1 | L1 | LINE |
| 91487472 | 91487662 | + | 25.8 | 2246 | 2439 | -68 | HAL1 | L1 | LINE |

  

|  |  |  |  |  |  |  |  |  |  |
| --- | --- | --- | --- | --- | --- | --- | --- | --- | --- |
| chr12:92126700-92133625 | | | 6896 bp | | |  |  |  |  |
| Genome Start | Genome End | Strand | Div | Repeat Start | Repeat End | Repeat Left | Name | Family | Class |
| 92126700 | 92127073 | - | 10.5 | 6255 | 6616 | -1 | L1PB1 | L1 | LINE |
| 92127104 | 92127991 | - | 8.9 | 5364 | 6254 | -363 | L1PA5 | L1 | LINE |
| 92127992 | 92128347 | + | 5.6 | 6258 | 6616 | -4 | L1PA5 | L1 | LINE |
| 92128348 | 92133625 | - | 9.5 | 50 | 5363 | -1249 | L1PB1 | L1 | LINE |

  

|  |  |  |  |  |  |  |  |  |  |
| --- | --- | --- | --- | --- | --- | --- | --- | --- | --- |
| chr12:93400613-93404822 | | | 4122 bp | | |  |  |  |  |
| Genome Start | Genome End | Strand | Div | Repeat Start | Repeat End | Repeat Left | Name | Family | Class |
| 93400613 | 93401119 | + | 16.5 | 1679 | 2264 | -497 | Charlie1 | MER1\_type | DNA |
| 93401141 | 93401419 | - | 14.9 | 9 | 295 | -17 | AluJo | Alu | SINE |
| 93401465 | 93402503 | - | 3.0 | 3266 | 4305 | -1841 | L1PA3 | L1 | LINE |
| 93402504 | 93404350 | + | 2.8 | 4309 | 6155 | 0 | L1PA3 | L1 | LINE |
| 93404373 | 93404822 | + | 16.1 | 2264 | 2730 | -31 | Charlie1 | MER1\_type | DNA |

  

|  |  |  |  |  |  |  |  |  |  |
| --- | --- | --- | --- | --- | --- | --- | --- | --- | --- |
| chr12:95357384-95359750 | | | 2354 bp | | |  |  |  |  |
| Genome Start | Genome End | Strand | Div | Repeat Start | Repeat End | Repeat Left | Name | Family | Class |
| 95357384 | 95357558 | + | 25.4 | 5337 | 5509 | -2299 | L1MC4a | L1 | LINE |
| 95357586 | 95357627 | + | 30.1 | 5606 | 5637 | -2171 | L1MC4a | L1 | LINE |
| 95357628 | 95357768 | - | 20.1 | 135 | 290 | -22 | AluJo | Alu | SINE |
| 95357769 | 95358076 | - | 12.0 | 1 | 308 | -5 | AluSq | Alu | SINE |
| 95358077 | 95358218 | - | 20.1 | 3 | 134 | -178 | AluJo | Alu | SINE |
| 95358219 | 95358347 | + | 30.1 | 5638 | 5769 | -2039 | L1MC4a | L1 | LINE |
| 95358333 | 95359031 | - | 4.9 | 4874 | 5572 | -582 | L1PA5 | L1 | LINE |
| 95359032 | 95359603 | + | 3.9 | 5579 | 6150 | -4 | L1PA5 | L1 | LINE |
| 95359605 | 95359750 | + | 32.9 | 5745 | 5895 | -1913 | L1MC4a | L1 | LINE |

  

|  |  |  |  |  |  |  |  |  |  |
| --- | --- | --- | --- | --- | --- | --- | --- | --- | --- |
| chr12:95772593-95777292 | | | 4692 bp | | |  |  |  |  |
| Genome Start | Genome End | Strand | Div | Repeat Start | Repeat End | Repeat Left | Name | Family | Class |
| 95772593 | 95772704 | - | 26.8 | 146 | 262 | 0 | MIR | MIR | SINE |
| 95772708 | 95772820 | - | 7.9 | 6049 | 6160 | -1 | L1PA14 | L1 | LINE |
| 95772821 | 95773189 | - | 12.5 | 1 | 375 | 0 | THE1C | MaLR | LTR |
| 95773190 | 95774779 | - | 28.8 | 2 | 1735 | 0 | MLT1K-int | MaLR | LTR |
| 95774782 | 95775154 | - | 9.2 | 1 | 375 | 0 | THE1C | MaLR | LTR |
| 95775155 | 95775475 | - | 7.9 | 5730 | 6048 | -113 | L1PA14 | L1 | LINE |
| 95775476 | 95775832 | + | 14.1 | 1 | 381 | 0 | THE1D | MaLR | LTR |
| 95775833 | 95776531 | - | 9.2 | 5022 | 5729 | -432 | L1PA14 | L1 | LINE |
| 95776534 | 95777174 | + | 11.9 | 4372 | 5000 | -1146 | L1PREC2 | L1 | LINE |
| 95777176 | 95777292 | - | 35.9 | 30 | 147 | -115 | MIR | MIR | SINE |

  

|  |  |  |  |  |  |  |  |  |  |
| --- | --- | --- | --- | --- | --- | --- | --- | --- | --- |
| chr12:96922500-96925244 | | | 2590 bp | | |  |  |  |  |
| Genome Start | Genome End | Strand | Div | Repeat Start | Repeat End | Repeat Left | Name | Family | Class |
| 96922500 | 96922615 | - | 28.8 | 3244 | 3378 | 0 | L2 | L2 | LINE |
| 96922623 | 96922718 | - | 18.8 | 1 | 97 | -96 | MER53 | DNA | DNA |
| 96922745 | 96922910 | - | 18.1 | 1 | 166 | 0 | FRAM | Alu | SINE |
| 96922956 | 96923556 | - | 13.3 | 5081 | 5701 | -611 | L1MA9 | L1 | LINE |
| 96923557 | 96924182 | + | 16.0 | 5688 | 6307 | -5 | L1MA9 | L1 | LINE |
| 96924221 | 96924675 | - | 34.9 | 2664 | 3135 | -243 | L2 | L2 | LINE |
| 96924715 | 96924844 | - | 23.4 | 39 | 177 | -85 | MIR | MIR | SINE |
| 96924845 | 96925244 | - | 34.9 | 2121 | 2656 | -763 | L2 | L2 | LINE |

  

|  |  |  |  |  |  |  |  |  |  |
| --- | --- | --- | --- | --- | --- | --- | --- | --- | --- |
| chr12:97492764-97496362 | | | 3433 bp | | |  |  |  |  |
| Genome Start | Genome End | Strand | Div | Repeat Start | Repeat End | Repeat Left | Name | Family | Class |
| 97492764 | 97492869 | + | 20.7 | 6895 | 7006 | -1036 | L1MC4 | L1 | LINE |
| 97492870 | 97493171 | + | 16.1 | 1 | 302 | -10 | AluJo | Alu | SINE |
| 97493172 | 97493274 | + | 20.7 | 7007 | 7100 | -942 | L1MC4 | L1 | LINE |
| 97493275 | 97493574 | - | 15.8 | 3 | 301 | 0 | AluSx | Alu | SINE |
| 97493575 | 97493637 | + | 20.7 | 7101 | 7159 | -883 | L1MC4 | L1 | LINE |
| 97493638 | 97493949 | - | 13.2 | 1 | 309 | -3 | AluSx | Alu | SINE |
| 97493950 | 97494365 | + | 20.7 | 7160 | 7576 | -466 | L1MC4 | L1 | LINE |
| 97494361 | 97494565 | - | 3.4 | 5264 | 5468 | -704 | L1PA7 | L1 | LINE |
| 97494566 | 97495249 | + | 4.8 | 5465 | 6152 | -2 | L1PA7 | L1 | LINE |
| 97495292 | 97495549 | + | 17.3 | 1 | 254 | -58 | AluSx | Alu | SINE |
| 97495557 | 97495779 | + | 20.9 | 7613 | 7839 | -203 | L1MC4 | L1 | LINE |
| 97495780 | 97496056 | + | 14.1 | 1 | 276 | -36 | AluSx | Alu | SINE |
| 97496083 | 97496150 | + | 15.2 | 7824 | 7894 | -148 | L1MC4 | L1 | LINE |
| 97496247 | 97496362 | + | 17.5 | 7927 | 8042 | 0 | L1MC4 | L1 | LINE |

  

|  |  |  |  |  |  |  |  |  |  |
| --- | --- | --- | --- | --- | --- | --- | --- | --- | --- |
| chr12:99218266-99227142 | | | 8852 bp | | |  |  |  |  |
| Genome Start | Genome End | Strand | Div | Repeat Start | Repeat End | Repeat Left | Name | Family | Class |
| 99218266 | 99219490 | - | 9.8 | 6488 | 7723 | 0 | L1MA2 | L1 | LINE |
| 99219486 | 99219606 | - | 8.3 | 3986 | 4106 | -3459 | L1MA2 | L1 | LINE |
| 99219601 | 99220269 | - | 17.6 | 521 | 1197 | -6368 | L1MA2 | L1 | LINE |
| 99220267 | 99222508 | - | 9.8 | 125 | 2379 | -211 | L1PA7 | L1 | LINE |
| 99222493 | 99226559 | + | 7.5 | 2401 | 6469 | 0 | L1PA7 | L1 | LINE |
| 99226573 | 99227015 | - | 20.5 | 114 | 536 | -7029 | L1MA2 | L1 | LINE |
| 99227058 | 99227142 | - | 20.5 | 13 | 113 | -7452 | L1MA2 | L1 | LINE |

  

|  |  |  |  |  |  |  |  |  |  |
| --- | --- | --- | --- | --- | --- | --- | --- | --- | --- |
| chr12:100502833-100505336 | | | 2431 bp | | |  |  |  |  |
| Genome Start | Genome End | Strand | Div | Repeat Start | Repeat End | Repeat Left | Name | Family | Class |
| 100502833 | 100502866 | - | 25.2 | 2828 | 2862 | 0 | Charlie2 | MER1\_type | DNA |
| 100502867 | 100503235 | + | 18.2 | 1 | 370 | -4 | MLT1A | MaLR | LTR |
| 100503236 | 100503825 | - | 25.2 | 2243 | 2827 | -35 | Charlie2 | MER1\_type | DNA |
| 100503826 | 100504022 | - | 1.5 | 4786 | 4982 | -1164 | L1PA2 | L1 | LINE |
| 100504023 | 100505194 | + | 1.4 | 4983 | 6154 | -1 | L1PA2 | L1 | LINE |
| 100505268 | 100505336 | - | 24.6 | 2151 | 2225 | -636 | Charlie2 | MER1\_type | DNA |

  

|  |  |  |  |  |  |  |  |  |  |
| --- | --- | --- | --- | --- | --- | --- | --- | --- | --- |
| chr12:101169363-101179328 | | | 9581 bp | | |  |  |  |  |
| Genome Start | Genome End | Strand | Div | Repeat Start | Repeat End | Repeat Left | Name | Family | Class |
| 101169363 | 101170370 | - | 33.3 | 4144 | 5208 | -1011 | L1MEc | L1 | LINE |
| 101170488 | 101172685 | - | 8.8 | 2514 | 4703 | -1443 | L1PA7 | L1 | LINE |
| 101172683 | 101174108 | + | 6.5 | 4706 | 6141 | -13 | L1PA7 | L1 | LINE |
| 101174177 | 101174449 | - | 31.6 | 2314 | 2600 | -4033 | L1MDa | L1 | LINE |
| 101174467 | 101174599 | - | 32.6 | 2736 | 2870 | -549 | L2 | L2 | LINE |
| 101174611 | 101175125 | - | 24.8 | 3543 | 4060 | -2159 | L1MEc | L1 | LINE |
| 101175126 | 101175683 | + | 20.8 | 1 | 548 | -2 | MER44B | MER2\_type | DNA |
| 101175684 | 101176511 | - | 24.8 | 2692 | 3542 | -2677 | L1MEc | L1 | LINE |
| 101176514 | 101177046 | - | 27.7 | 1 | 595 | 0 | MLT1G1 | MaLR | LTR |
| 101177141 | 101177269 | - | 26.8 | 2498 | 2632 | -3587 | L1MEc | L1 | LINE |
| 101177277 | 101177428 | - | 25.0 | 1800 | 1980 | -4420 | L1MEc | L1 | LINE |
| 101177429 | 101177737 | - | 12.0 | 1 | 309 | 0 | AluSc | Alu | SINE |
| 101177810 | 101179328 | - | 30.5 | 50 | 1664 | -4575 | L1MEc | L1 | LINE |

  

|  |  |  |  |  |  |  |  |  |  |
| --- | --- | --- | --- | --- | --- | --- | --- | --- | --- |
| chr12:101971081-101979235 | | | 8163 bp | | |  |  |  |  |
| Genome Start | Genome End | Strand | Div | Repeat Start | Repeat End | Repeat Left | Name | Family | Class |
| 101971081 | 101971226 | - | 19.2 | 6473 | 6619 | -14 | L1MD1 | L1 | LINE |
| 101971227 | 101974166 | - | 16.8 | 2817 | 5831 | -919 | L1MD1 | L1 | LINE |
| 101974163 | 101975077 | - | 20.3 | 966 | 1878 | -4755 | L1MD1 | L1 | LINE |
| 101975092 | 101975869 | - | 8.4 | 5400 | 6172 | 0 | L1PA8 | L1 | LINE |
| 101975870 | 101976162 | - | 11.6 | 1 | 294 | -15 | AluSc | Alu | SINE |
| 101976163 | 101976349 | - | 3.3 | 5187 | 5399 | -773 | L1P | L1 | LINE |
| 101976350 | 101978919 | + | 8.5 | 2595 | 5181 | -965 | L1P | L1 | LINE |
| 101978902 | 101979235 | - | 28.2 | 702 | 984 | -5649 | L1MD1 | L1 | LINE |

  

|  |  |  |  |  |  |  |  |  |  |
| --- | --- | --- | --- | --- | --- | --- | --- | --- | --- |
| chr12:107332806-107338440 | | | 5135 bp | | |  |  |  |  |
| Genome Start | Genome End | Strand | Div | Repeat Start | Repeat End | Repeat Left | Name | Family | Class |
| 107332806 | 107333678 | - | 34.9 | 3983 | 4902 | -752 | ERVL-E | ERVL | LTR |
| 107333679 | 107333977 | + | 12.1 | 1 | 298 | -14 | AluSx | Alu | SINE |
| 107333978 | 107334108 | - | 34.9 | 3849 | 3982 | -1672 | ERVL-E | ERVL | LTR |
| 107334127 | 107334165 | - | 12.8 | 390 | 428 | 0 | MSTB | MaLR | LTR |
| 107334166 | 107334505 | + | 13.7 | 1 | 345 | 0 | AluSq | Alu | SINE |
| 107334807 | 107335159 | - | 17.7 | 55 | 397 | -29 | MSTB | MaLR | LTR |
| 107335160 | 107335451 | - | 15.8 | 1 | 312 | 0 | AluJb | Alu | SINE |
| 107335452 | 107335507 | - | 17.7 | 1 | 54 | -372 | MSTB | MaLR | LTR |
| 107335514 | 107335652 | - | 28.8 | 3614 | 3763 | -1904 | ERVL-E | ERVL | LTR |
| 107335709 | 107335766 | + | 26.3 | 72 | 130 | -3289 | L2 | L2 | LINE |
| 107335767 | 107335976 | - | 29.9 | 3288 | 3491 | -2163 | ERVL-E | ERVL | LTR |
| 107336003 | 107336458 | - | 18.2 | 8 | 467 | 0 | MLT1C | MaLR | LTR |
| 107336492 | 107336915 | - | 30.3 | 2695 | 3126 | -2541 | ERVL-E | ERVL | LTR |
| 107336945 | 107337021 | - | 13.1 | 6225 | 6294 | -3 | L1MA5A | L1 | LINE |
| 107337022 | 107337349 | + | 20.0 | 1 | 295 | -17 | AluJo | Alu | SINE |
| 107337350 | 107337456 | - | 13.1 | 6117 | 6224 | -73 | L1MA5A | L1 | LINE |
| 107337496 | 107337773 | - | 18.2 | 1 | 280 | -32 | AluJo | Alu | SINE |
| 107337775 | 107337811 | - | 10.8 | 6092 | 6128 | -169 | L1MA5A | L1 | LINE |
| 107337800 | 107338048 | + | 12.8 | 5822 | 6097 | -200 | L1MA5A | L1 | LINE |
| 107338052 | 107338440 | - | 30.2 | 2379 | 2770 | -2226 | ERVL-E | ERVL | LTR |

  

|  |  |  |  |  |  |  |  |  |  |
| --- | --- | --- | --- | --- | --- | --- | --- | --- | --- |
| chr12:113004160-113009514 | | | 5013 bp | | |  |  |  |  |
| Genome Start | Genome End | Strand | Div | Repeat Start | Repeat End | Repeat Left | Name | Family | Class |
| 113004160 | 113004531 | - | 22.6 | 1949 | 2447 | -3953 | L1MEc | L1 | LINE |
| 113004801 | 113004861 | + | 11.5 | 1517 | 1582 | -4818 | L1MEc | L1 | LINE |
| 113004888 | 113005009 | - | 19.7 | 1253 | 1405 | -4995 | L1MEc | L1 | LINE |
| 113005010 | 113005947 | + | 17.5 | 10 | 1150 | -81 | Tigger3b | MER2\_type | DNA |
| 113006001 | 113007330 | - | 2.0 | 4811 | 6154 | -1 | L1PA3 | L1 | LINE |
| 113007326 | 113008350 | + | 3.4 | 3792 | 4816 | -1330 | L1PA3 | L1 | LINE |
| 113008350 | 113008499 | - | 25.3 | 868 | 1023 | -5377 | L1MEc | L1 | LINE |
| 113008500 | 113008781 | - | 21.7 | 1 | 286 | -26 | AluJo | Alu | SINE |
| 113008782 | 113009514 | - | 25.3 | 41 | 867 | -5533 | L1MEc | L1 | LINE |

  

|  |  |  |  |  |  |  |  |  |  |
| --- | --- | --- | --- | --- | --- | --- | --- | --- | --- |
| chr12:117814429-117817592 | | | 3173 bp | | |  |  |  |  |
| Genome Start | Genome End | Strand | Div | Repeat Start | Repeat End | Repeat Left | Name | Family | Class |
| 117814429 | 117815979 | + | 15.3 | 2110 | 3675 | -2471 | L1PREC2 | L1 | LINE |
| 117815975 | 117816004 | - | 3.3 | 5242 | 5271 | -875 | L1PA10 | L1 | LINE |
| 117816004 | 117816862 | + | 9.6 | 5273 | 6158 | -10 | L1PA10 | L1 | LINE |
| 117816860 | 117817592 | + | 11.3 | 3663 | 4411 | -1735 | L1PREC2 | L1 | LINE |

  

|  |  |  |  |  |  |  |  |  |  |
| --- | --- | --- | --- | --- | --- | --- | --- | --- | --- |
| chr12:118820630-118821765 | | | 1137 bp | | |  |  |  |  |
| Genome Start | Genome End | Strand | Div | Repeat Start | Repeat End | Repeat Left | Name | Family | Class |
| 118820630 | 118820800 | - | 23.4 | 5643 | 5820 | -1988 | L1MC | L1 | LINE |
| 118820801 | 118821343 | - | 2.6 | 5609 | 6150 | -5 | L1PA4 | L1 | LINE |
| 118821344 | 118821492 | + | 0.7 | 5476 | 5624 | -531 | L1PA4 | L1 | LINE |
| 118821492 | 118821765 | - | 26.0 | 5350 | 5653 | -2155 | L1MC | L1 | LINE |

  

|  |  |  |  |  |  |  |  |  |  |
| --- | --- | --- | --- | --- | --- | --- | --- | --- | --- |
| chr12:126098595-126100545 | | | 1951 bp | | |  |  |  |  |
| Genome Start | Genome End | Strand | Div | Repeat Start | Repeat End | Repeat Left | Name | Family | Class |
| 126098595 | 126098869 | + | 19.1 | 5845 | 6123 | -61 | L1MB7 | L1 | LINE |
| 126098871 | 126099665 | - | 4.5 | 5354 | 6149 | -5 | L1PA5 | L1 | LINE |
| 126099665 | 126100482 | + | 6.1 | 4519 | 5336 | -810 | L1PA5 | L1 | LINE |
| 126100483 | 126100545 | + | 12.7 | 6114 | 6176 | -8 | L1MB7 | L1 | LINE |

  

|  |  |  |  |  |  |  |  |  |  |
| --- | --- | --- | --- | --- | --- | --- | --- | --- | --- |
| chr12:127045778-127048361 | | | 2306 bp | | |  |  |  |  |
| Genome Start | Genome End | Strand | Div | Repeat Start | Repeat End | Repeat Left | Name | Family | Class |
| 127045778 | 127045937 | + | 30.8 | 3995 | 4158 | -1988 | L1ME3B | L1 | LINE |
| 127045942 | 127046202 | - | 8.1 | 5563 | 5823 | -331 | L1PA7 | L1 | LINE |
| 127046205 | 127046524 | + | 4.1 | 5832 | 6152 | -2 | L1PA7 | L1 | LINE |
| 127046797 | 127048361 | + | 27.9 | 4228 | 5870 | -370 | L1ME3B | L1 | LINE |

  

|  |  |  |  |  |  |  |  |  |  |
| --- | --- | --- | --- | --- | --- | --- | --- | --- | --- |
| chr12:128337038-128340921 | | | 3675 bp | | |  |  |  |  |
| Genome Start | Genome End | Strand | Div | Repeat Start | Repeat End | Repeat Left | Name | Family | Class |
| 128337038 | 128337157 | + | 22.6 | 15 | 137 | -313 | MLT1J2 | MaLR | LTR |
| 128337184 | 128337263 | + | 24.5 | 6665 | 6734 | -1080 | L1MC4a | L1 | LINE |
| 128337264 | 128337564 | + | 13.9 | 1 | 303 | -9 | AluSx | Alu | SINE |
| 128337565 | 128337980 | + | 23.6 | 6729 | 7201 | -607 | L1MC4a | L1 | LINE |
| 128337981 | 128338055 | + | 14.7 | 1 | 79 | -1 | MADE1 | Mariner | DNA |
| 128338056 | 128338243 | + | 23.6 | 7202 | 7394 | -414 | L1MC4a | L1 | LINE |
| 128338251 | 128338553 | - | 18.1 | 4638 | 4941 | -1199 | L1MB1 | L1 | LINE |
| 128338556 | 128338877 | + | 17.5 | 4922 | 5288 | -858 | L1MB1 | L1 | LINE |
| 128338908 | 128339759 | + | 16.8 | 5283 | 6154 | -14 | L1MB1 | L1 | LINE |
| 128339760 | 128340071 | + | 9.6 | 1 | 312 | -1 | AluSq | Alu | SINE |
| 128340133 | 128340233 | + | 27.7 | 7468 | 7575 | -467 | L1MC4 | L1 | LINE |
| 128340249 | 128340344 | + | 17.7 | 21 | 116 | -26 | FLAM\_A | Alu | SINE |
| 128340368 | 128340671 | + | 27.3 | 7487 | 7808 | 0 | L1MC4a | L1 | LINE |
| 128340717 | 128340921 | + | 27.9 | 238 | 409 | 0 | MLT1J2 | MaLR | LTR |

  

|  |  |  |  |  |  |  |  |  |  |
| --- | --- | --- | --- | --- | --- | --- | --- | --- | --- |
| chr12:128466478-128469651 | | | 3146 bp | | |  |  |  |  |
| Genome Start | Genome End | Strand | Div | Repeat Start | Repeat End | Repeat Left | Name | Family | Class |
| 128466478 | 128466552 | + | 22.7 | 6 | 92 | -375 | MLT1C | MaLR | LTR |
| 128466554 | 128467723 | - | 8.2 | 3474 | 4653 | -1493 | L1PA8 | L1 | LINE |
| 128467720 | 128469220 | + | 6.6 | 4663 | 6154 | -18 | L1PA8 | L1 | LINE |
| 128469252 | 128469651 | + | 25.1 | 83 | 467 | 0 | MLT1C | MaLR | LTR |

  

|  |  |  |  |  |  |  |  |  |  |
| --- | --- | --- | --- | --- | --- | --- | --- | --- | --- |
| chr12:128839163-128840200 | | | 1012 bp | | |  |  |  |  |
| Genome Start | Genome End | Strand | Div | Repeat Start | Repeat End | Repeat Left | Name | Family | Class |
| 128839163 | 128839267 | - | 32.4 | 3309 | 3419 | 0 | L2 | L2 | LINE |
| 128839270 | 128839552 | - | 27.1 | 2835 | 3154 | -265 | L2 | L2 | LINE |
| 128839568 | 128839902 | - | 10.6 | 5827 | 6149 | -2 | L1PB2 | L1 | LINE |
| 128839905 | 128840064 | + | 10.5 | 5674 | 5826 | -325 | L1PB2 | L1 | LINE |
| 128840072 | 128840200 | - | 27.3 | 2682 | 2823 | -596 | L2 | L2 | LINE |

  

|  |  |  |  |  |  |  |  |  |  |
| --- | --- | --- | --- | --- | --- | --- | --- | --- | --- |
| chr12:129658902-129660042 | | | 1116 bp | | |  |  |  |  |
| Genome Start | Genome End | Strand | Div | Repeat Start | Repeat End | Repeat Left | Name | Family | Class |
| 129658902 | 129659154 | - | 30.0 | 384 | 646 | -5763 | L1MDb | L1 | LINE |
| 129659182 | 129659592 | - | 20.7 | 5836 | 6265 | -26 | L1MA8 | L1 | LINE |
| 129659589 | 129659888 | + | 17.9 | 5571 | 5837 | -463 | L1MA8 | L1 | LINE |
| 129659891 | 129660042 | - | 25.0 | 196 | 371 | -6038 | L1MDb | L1 | LINE |

  

|  |  |  |  |  |  |  |  |  |  |
| --- | --- | --- | --- | --- | --- | --- | --- | --- | --- |
| chr12:130572341-130575537 | | | 3125 bp | | |  |  |  |  |
| Genome Start | Genome End | Strand | Div | Repeat Start | Repeat End | Repeat Left | Name | Family | Class |
| 130572341 | 130572465 | + | 21.6 | 1121 | 1241 | -5182 | L1MCc | L1 | LINE |
| 130572516 | 130572799 | + | 22.0 | 1858 | 2164 | -4964 | L1MCc | L1 | LINE |
| 130572795 | 130572846 | - | 13.7 | 5347 | 5397 | -749 | L1PREC2 | L1 | LINE |
| 130572877 | 130573262 | - | 8.1 | 5000 | 5406 | -766 | L1P | L1 | LINE |
| 130573261 | 130574036 | + | 8.5 | 5390 | 6173 | -1 | L1PA11 | L1 | LINE |
| 130574036 | 130574359 | + | 26.4 | 2150 | 2500 | -4628 | L1MCc | L1 | LINE |
| 130574360 | 130574726 | + | 18.8 | 1 | 365 | 0 | MLT1A0 | MaLR | LTR |
| 130574727 | 130575537 | + | 26.8 | 2501 | 3279 | -3662 | L1MCc | L1 | LINE |

  


|  |  |  |  |  |  |  |  |  |  |
| --- | --- | --- | --- | --- | --- | --- | --- | --- | --- |
| chr13:21023129-21028662 | | | 5381 bp | | |  |  |  |  |
| Genome Start | Genome End | Strand | Div | Repeat Start | Repeat End | Repeat Left | Name | Family | Class |
| 21023129 | 21023411 | + | 25.6 | 1020 | 1309 | -2110 | L2 | L2 | LINE |
| 21023584 | 21025637 | - | 3.2 | 4086 | 6147 | -8 | L1PA4 | L1 | LINE |
| 21025619 | 21027742 | + | 4.5 | 1934 | 4072 | -2074 | L1PA4 | L1 | LINE |
| 21027743 | 21028662 | + | 25.8 | 1307 | 2355 | -1064 | L2 | L2 | LINE |

  

|  |  |  |  |  |  |  |  |  |  |
| --- | --- | --- | --- | --- | --- | --- | --- | --- | --- |
| chr13:25065608-25067493 | | | 1632 bp | | |  |  |  |  |
| Genome Start | Genome End | Strand | Div | Repeat Start | Repeat End | Repeat Left | Name | Family | Class |
| 25065608 | 25066373 | + | 28.9 | 1 | 827 | -1785 | Charlie7 | MER1\_type | DNA |
| 25066568 | 25066872 | - | 9.2 | 5838 | 6150 | -1 | L1PB1 | L1 | LINE |
| 25066873 | 25067122 | + | 8.9 | 5544 | 5793 | -358 | L1PB1 | L1 | LINE |
| 25067183 | 25067225 | + | 8.9 | 5794 | 5836 | -315 | L1PB1 | L1 | LINE |
| 25067226 | 25067493 | + | 26.8 | 1020 | 1322 | -1290 | Charlie7 | MER1\_type | DNA |

  

|  |  |  |  |  |  |  |  |  |  |
| --- | --- | --- | --- | --- | --- | --- | --- | --- | --- |
| chr13:25942273-25943498 | | | 1150 bp | | |  |  |  |  |
| Genome Start | Genome End | Strand | Div | Repeat Start | Repeat End | Repeat Left | Name | Family | Class |
| 25942273 | 25942423 | - | 25.2 | 5942 | 6102 | -19 | L1ME4a | L1 | LINE |
| 25942485 | 25942654 | - | 16.5 | 5996 | 6166 | 0 | L1PA16 | L1 | LINE |
| 25942661 | 25942987 | + | 13.8 | 5639 | 5972 | -194 | L1PA16 | L1 | LINE |
| 25942997 | 25943498 | - | 30.0 | 5352 | 5892 | -229 | L1ME4a | L1 | LINE |

  

|  |  |  |  |  |  |  |  |  |  |
| --- | --- | --- | --- | --- | --- | --- | --- | --- | --- |
| chr13:28371227-28380277 | | | 9043 bp | | |  |  |  |  |
| Genome Start | Genome End | Strand | Div | Repeat Start | Repeat End | Repeat Left | Name | Family | Class |
| 28371227 | 28371346 | - | 19.0 | 6610 | 6741 | -9 | L1MD2 | L1 | LINE |
| 28371347 | 28371545 | + | 15.8 | 1 | 202 | -201 | MER57A | ERV1 | LTR |
| 28371546 | 28371845 | - | 7.3 | 2 | 300 | -11 | AluY | Alu | SINE |
| 28371846 | 28372005 | + | 15.8 | 203 | 364 | -39 | MER57A | ERV1 | LTR |
| 28372006 | 28372324 | - | 15.1 | 1 | 311 | -1 | AluSx | Alu | SINE |
| 28372325 | 28372362 | + | 15.8 | 365 | 403 | 0 | MER57A | ERV1 | LTR |
| 28372363 | 28372433 | - | 19.0 | 6519 | 6609 | -141 | L1MD2 | L1 | LINE |
| 28372461 | 28372871 | - | 19.5 | 6102 | 6518 | -232 | L1MD2 | L1 | LINE |
| 28372872 | 28372934 | - | 14.3 | 400 | 467 | 0 | MLT1C | MaLR | LTR |
| 28372935 | 28375222 | - | 19.5 | 3778 | 6101 | -649 | L1MD2 | L1 | LINE |
| 28375225 | 28375389 | - | 21.3 | 1868 | 2031 | -4602 | L1MD2 | L1 | LINE |
| 28375398 | 28378927 | - | 3.4 | 2616 | 6155 | 0 | L1PA4 | L1 | LINE |
| 28378906 | 28379998 | + | 5.0 | 1505 | 2596 | -3550 | L1PA4 | L1 | LINE |
| 28379992 | 28380277 | - | 17.9 | 1589 | 1887 | -4746 | L1MD2 | L1 | LINE |

  

|  |  |  |  |  |  |  |  |  |  |
| --- | --- | --- | --- | --- | --- | --- | --- | --- | --- |
| chr13:30418896-30423168 | | | 4291 bp | | |  |  |  |  |
| Genome Start | Genome End | Strand | Div | Repeat Start | Repeat End | Repeat Left | Name | Family | Class |
| 30418896 | 30419887 | - | 21.5 | 1681 | 2764 | -97 | Charlie2a | MER1\_type | DNA |
| 30419888 | 30421335 | - | 4.4 | 4699 | 6143 | -12 | L1PA4 | L1 | LINE |
| 30421318 | 30421414 | + | 4.1 | 4598 | 4698 | -1448 | L1PA4 | L1 | LINE |
| 30421415 | 30422262 | - | 24.4 | 786 | 1698 | -1163 | Charlie2a | MER1\_type | DNA |
| 30422263 | 30422483 | + | 12.7 | 3 | 223 | -89 | AluSx | Alu | SINE |
| 30422484 | 30423168 | - | 24.4 | 79 | 785 | -2076 | Charlie2a | MER1\_type | DNA |

  

|  |  |  |  |  |  |  |  |  |  |
| --- | --- | --- | --- | --- | --- | --- | --- | --- | --- |
| chr13:30461201-30463904 | | | 2324 bp | | |  |  |  |  |
| Genome Start | Genome End | Strand | Div | Repeat Start | Repeat End | Repeat Left | Name | Family | Class |
| 30461201 | 30461733 | + | 30.9 | 4532 | 5045 | -1101 | L1ME4a | L1 | LINE |
| 30461736 | 30462224 | - | 8.4 | 5686 | 6172 | 0 | L1PA8 | L1 | LINE |
| 30462225 | 30462344 | + | 4.2 | 5562 | 5681 | -491 | L1PA8 | L1 | LINE |
| 30462598 | 30462723 | + | 26.5 | 5279 | 5540 | -581 | L1ME4a | L1 | LINE |
| 30462724 | 30463178 | - | 22.5 | 1 | 467 | 0 | MLT1C | MaLR | LTR |
| 30463179 | 30463229 | + | 29.8 | 5541 | 5590 | -531 | L1ME4a | L1 | LINE |
| 30463331 | 30463686 | - | 28.1 | 2 | 363 | -10 | MER83C | ERV1 | LTR |
| 30463711 | 30463904 | + | 21.8 | 5725 | 5950 | -171 | L1ME4a | L1 | LINE |

  

|  |  |  |  |  |  |  |  |  |  |
| --- | --- | --- | --- | --- | --- | --- | --- | --- | --- |
| chr13:32973216-32976979 | | | 3753 bp | | |  |  |  |  |
| Genome Start | Genome End | Strand | Div | Repeat Start | Repeat End | Repeat Left | Name | Family | Class |
| 32973216 | 32973474 | - | 15.9 | 41 | 306 | -7 | AluSq | Alu | SINE |
| 32973475 | 32975937 | - | 6.3 | 2733 | 5200 | -946 | L1PA6 | L1 | LINE |
| 32975931 | 32976902 | + | 5.8 | 5182 | 6151 | -3 | L1PA6 | L1 | LINE |
| 32976921 | 32976979 | - | 22.0 | 1 | 59 | -241 | AluSq | Alu | SINE |

  

|  |  |  |  |  |  |  |  |  |  |
| --- | --- | --- | --- | --- | --- | --- | --- | --- | --- |
| chr13:37283418-37305008 | | | 21350 bp | | |  |  |  |  |
| Genome Start | Genome End | Strand | Div | Repeat Start | Repeat End | Repeat Left | Name | Family | Class |
| 37283418 | 37284017 | + | 26.4 | 78 | 697 | -5703 | L1MEc | L1 | LINE |
| 37284018 | 37284332 | + | 14.7 | 1 | 301 | -11 | AluSx | Alu | SINE |
| 37284333 | 37284599 | + | 26.4 | 698 | 975 | -5425 | L1MEc | L1 | LINE |
| 37284601 | 37285198 | + | 21.1 | 1045 | 1651 | -4588 | L1MEc | L1 | LINE |
| 37285202 | 37287355 | + | 22.2 | 2795 | 5031 | -1115 | L1M4 | L1 | LINE |
| 37287354 | 37289717 | + | 15.0 | 2613 | 5233 | -1440 | L1PA15 | L1 | LINE |
| 37289741 | 37290356 | + | 15.0 | 5234 | 5873 | -800 | L1PA15 | L1 | LINE |
| 37290357 | 37291096 | + | 6.6 | 5411 | 6152 | -2 | L1PA7 | L1 | LINE |
| 37291097 | 37291126 | + | 15.0 | 5874 | 5917 | -756 | L1PA15 | L1 | LINE |
| 37291138 | 37291977 | + | 12.5 | 5312 | 6163 | 0 | L1PA13 | L1 | LINE |
| 37291981 | 37293327 | + | 14.4 | 3036 | 4394 | -1752 | L1PREC2 | L1 | LINE |
| 37293370 | 37294413 | + | 13.8 | 4395 | 5469 | -691 | L1PREC2 | L1 | LINE |
| 37294412 | 37300436 | - | 2.9 | 1 | 6032 | 0 | L1PA3 | L1 | LINE |
| 37300439 | 37301121 | + | 13.6 | 5987 | 6689 | -3 | L1PA15 | L1 | LINE |
| 37301128 | 37302298 | + | 19.0 | 5016 | 6165 | -19 | L1MB7 | L1 | LINE |
| 37302319 | 37302561 | + | 25.0 | 1682 | 1927 | -4473 | L1MEc | L1 | LINE |
| 37302565 | 37302861 | + | 12.5 | 2 | 295 | -18 | AluSq | Alu | SINE |
| 37302862 | 37302913 | + | 23.5 | 1929 | 1998 | -4402 | L1MEc | L1 | LINE |
| 37302964 | 37303083 | + | 23.5 | 1999 | 2117 | -4283 | L1MEc | L1 | LINE |
| 37303115 | 37303316 | - | 14.9 | 5069 | 5274 | -866 | L1M3 | L1 | LINE |
| 37303310 | 37304325 | + | 16.5 | 5278 | 6289 | -2 | L1MA7 | L1 | LINE |
| 37304355 | 37304402 | + | 23.1 | 2168 | 2214 | -4186 | L1MEc | L1 | LINE |
| 37304403 | 37304672 | + | 11.4 | 1 | 279 | -33 | AluSx | Alu | SINE |
| 37304701 | 37305008 | + | 23.5 | 2206 | 2481 | -3810 | L1MEc | L1 | LINE |

  

|  |  |  |  |  |  |  |  |  |  |
| --- | --- | --- | --- | --- | --- | --- | --- | --- | --- |
| chr13:37736488-37742857 | | | 6362 bp | | |  |  |  |  |
| Genome Start | Genome End | Strand | Div | Repeat Start | Repeat End | Repeat Left | Name | Family | Class |
| 37736488 | 37737954 | + | 16.5 | 5 | 1349 | -5004 | L1PA10 | L1 | LINE |
| 37737955 | 37738262 | + | 12.7 | 1 | 308 | -4 | AluSx | Alu | SINE |
| 37738263 | 37739143 | + | 16.5 | 1350 | 2230 | -4123 | L1PA10 | L1 | LINE |
| 37739136 | 37739413 | - | 5.8 | 4100 | 4381 | -1765 | L1PA7 | L1 | LINE |
| 37739414 | 37741150 | + | 7.9 | 4384 | 6149 | -5 | L1PA7 | L1 | LINE |
| 37741167 | 37742857 | + | 9.2 | 2212 | 3907 | -2424 | L1PA10 | L1 | LINE |

  

|  |  |  |  |  |  |  |  |  |  |
| --- | --- | --- | --- | --- | --- | --- | --- | --- | --- |
| chr13:38608462-38613071 | | | 4602 bp | | |  |  |  |  |
| Genome Start | Genome End | Strand | Div | Repeat Start | Repeat End | Repeat Left | Name | Family | Class |
| 38608462 | 38609995 | - | 20.4 | 2933 | 6269 | 0 | PRIMA4-int | ERV1 | LTR |
| 38609994 | 38610474 | - | 25.1 | 2304 | 2793 | -1309 | PRIMA4-int | ERV1 | LTR |
| 38610475 | 38610778 | + | 1.0 | 1 | 304 | -14 | AluYb8 | Alu | SINE |
| 38610784 | 38611107 | - | 2.5 | 5339 | 5662 | -492 | L1PA5 | L1 | LINE |
| 38611108 | 38611601 | + | 3.4 | 5658 | 6151 | -3 | L1PA5 | L1 | LINE |
| 38611607 | 38613071 | - | 23.0 | 855 | 2316 | -1786 | PRIMA4-int | ERV1 | LTR |

  

|  |  |  |  |  |  |  |  |  |  |
| --- | --- | --- | --- | --- | --- | --- | --- | --- | --- |
| chr13:41263697-41271785 | | | 7614 bp | | |  |  |  |  |
| Genome Start | Genome End | Strand | Div | Repeat Start | Repeat End | Repeat Left | Name | Family | Class |
| 41263697 | 41264421 | - | 28.5 | 5185 | 5980 | -243 | L1ME3B | L1 | LINE |
| 41264501 | 41265129 | - | 29.9 | 4195 | 4898 | -1231 | L1ME3B | L1 | LINE |
| 41265147 | 41265295 | - | 26.9 | 3938 | 4093 | -2036 | L1ME3B | L1 | LINE |
| 41265348 | 41265478 | - | 24.6 | 3772 | 3925 | -2204 | L1ME3B | L1 | LINE |
| 41265668 | 41265970 | + | 10.4 | 1 | 299 | -10 | AluSc | Alu | SINE |
| 41265982 | 41266903 | - | 7.7 | 4019 | 4948 | -1198 | L1PA11 | L1 | LINE |
| 41266901 | 41268098 | + | 9.0 | 4955 | 6174 | 0 | L1PA11 | L1 | LINE |
| 41268229 | 41269162 | - | 29.2 | 2730 | 3740 | -2389 | L1ME3B | L1 | LINE |
| 41269163 | 41269473 | - | 14.0 | 3 | 302 | -8 | AluSg | Alu | SINE |
| 41269474 | 41271785 | - | 29.1 | 249 | 2729 | -3400 | L1ME3B | L1 | LINE |

  

|  |  |  |  |  |  |  |  |  |  |
| --- | --- | --- | --- | --- | --- | --- | --- | --- | --- |
| chr13:43666123-43667977 | | | 1517 bp | | |  |  |  |  |
| Genome Start | Genome End | Strand | Div | Repeat Start | Repeat End | Repeat Left | Name | Family | Class |
| 43666123 | 43666273 | + | 30.9 | 2057 | 2210 | -1209 | L2 | L2 | LINE |
| 43666351 | 43667173 | - | 4.0 | 5333 | 6155 | 0 | L1PA4 | L1 | LINE |
| 43667170 | 43667446 | + | 2.9 | 5069 | 5342 | -804 | L1PA4 | L1 | LINE |
| 43667712 | 43667977 | + | 38.6 | 3039 | 3362 | -16 | L2 | L2 | LINE |

  

|  |  |  |  |  |  |  |  |  |  |
| --- | --- | --- | --- | --- | --- | --- | --- | --- | --- |
| chr13:45453290-45455536 | | | 1952 bp | | |  |  |  |  |
| Genome Start | Genome End | Strand | Div | Repeat Start | Repeat End | Repeat Left | Name | Family | Class |
| 45453290 | 45453411 | + | 26.4 | 590 | 721 | -5398 | L1MEd | L1 | LINE |
| 45453405 | 45453876 | - | 1.3 | 5399 | 5870 | -285 | L1PA3 | L1 | LINE |
| 45453877 | 45454195 | + | 3.1 | 5865 | 6183 | 0 | L1PA3 | L1 | LINE |
| 45454196 | 45454206 | + | 3.1 | 6146 | 6155 | 0 | L1PA3 | L1 | LINE |
| 45454459 | 45454615 | + | 26.1 | 958 | 1126 | -4993 | L1MEd | L1 | LINE |
| 45454616 | 45454709 | + | 22.3 | 2200 | 2309 | -3820 | L1MEd | L1 | LINE |
| 45454737 | 45454905 | - | 13.7 | 47 | 236 | 0 | MER46A | MER2\_type | DNA |
| 45454946 | 45455007 | + | 23.3 | 82 | 142 | -94 | MER46A | MER2\_type | DNA |
| 45454960 | 45455009 | - | 8.0 | 1 | 65 | -171 | MER46A | MER2\_type | DNA |
| 45455044 | 45455404 | + | 25.1 | 2409 | 2782 | -3347 | L1MEd | L1 | LINE |
| 45455402 | 45455536 | + | 27.9 | 3948 | 4085 | -2044 | L1MEd | L1 | LINE |

  

|  |  |  |  |  |  |  |  |  |  |
| --- | --- | --- | --- | --- | --- | --- | --- | --- | --- |
| chr13:46787309-46789756 | | | 2416 bp | | |  |  |  |  |
| Genome Start | Genome End | Strand | Div | Repeat Start | Repeat End | Repeat Left | Name | Family | Class |
| 46787309 | 46787542 | + | 27.4 | 5051 | 5299 | -847 | L1ME3A | L1 | LINE |
| 46787543 | 46787605 | + | 11.1 | 6275 | 6343 | -1 | L1MC2 | L1 | LINE |
| 46787634 | 46787725 | + | 22.0 | 5308 | 5401 | -745 | L1ME3A | L1 | LINE |
| 46787719 | 46787899 | - | 7.2 | 4808 | 4988 | -1158 | L1PA8 | L1 | LINE |
| 46787900 | 46789070 | + | 6.7 | 4988 | 6155 | -17 | L1PA8 | L1 | LINE |
| 46789082 | 46789756 | + | 24.4 | 5384 | 6143 | -27 | L1ME3A | L1 | LINE |

  

|  |  |  |  |  |  |  |  |  |  |
| --- | --- | --- | --- | --- | --- | --- | --- | --- | --- |
| chr13:47154129-47158728 | | | 4341 bp | | |  |  |  |  |
| Genome Start | Genome End | Strand | Div | Repeat Start | Repeat End | Repeat Left | Name | Family | Class |
| 47154129 | 47154226 | + | 16.5 | 5044 | 5150 | -996 | L1P4 | L1 | LINE |
| 47154275 | 47155094 | - | 23.1 | 84 | 952 | -946 | Kanga1 | Tc2 | DNA |
| 47155095 | 47155707 | - | 6.1 | 5536 | 6165 | -7 | L1PA8 | L1 | LINE |
| 47155708 | 47155769 | - | 23.1 | 16 | 83 | -1815 | Kanga1 | Tc2 | DNA |
| 47155967 | 47156205 | - | 26.1 | 8 | 262 | 0 | MIR | MIR | SINE |
| 47156211 | 47156678 | - | 10.7 | 5687 | 6161 | -4 | L1PA15 | L1 | LINE |
| 47156679 | 47157588 | + | 9.9 | 4716 | 5615 | -550 | L1PA15 | L1 | LINE |
| 47157585 | 47157935 | - | 7.1 | 5080 | 5444 | -728 | L1PA7 | L1 | LINE |
| 47157936 | 47158629 | + | 6.5 | 5445 | 6138 | -16 | L1PA7 | L1 | LINE |
| 47158643 | 47158728 | + | 10.6 | 5603 | 5687 | -478 | L1P4 | L1 | LINE |

  

|  |  |  |  |  |  |  |  |  |  |
| --- | --- | --- | --- | --- | --- | --- | --- | --- | --- |
| chr13:53841395-53844017 | | | 2604 bp | | |  |  |  |  |
| Genome Start | Genome End | Strand | Div | Repeat Start | Repeat End | Repeat Left | Name | Family | Class |
| 53841395 | 53841583 | - | 20.6 | 120 | 310 | -2 | AluJo | Alu | SINE |
| 53841591 | 53843300 | - | 13.5 | 4479 | 6150 | -4 | L1PB3 | L1 | LINE |
| 53843301 | 53843878 | + | 19.6 | 3883 | 4460 | -1686 | L1PB3 | L1 | LINE |
| 53843891 | 53844017 | - | 17.8 | 4 | 123 | -189 | AluJo | Alu | SINE |

  

|  |  |  |  |  |  |  |  |  |  |
| --- | --- | --- | --- | --- | --- | --- | --- | --- | --- |
| chr13:54744427-54752227 | | | 7559 bp | | |  |  |  |  |
| Genome Start | Genome End | Strand | Div | Repeat Start | Repeat End | Repeat Left | Name | Family | Class |
| 54744427 | 54744556 | + | 23.9 | 1 | 139 | -235 | MLT1A | MaLR | LTR |
| 54744680 | 54750846 | - | 7.4 | 4 | 6141 | -13 | L1PA5 | L1 | LINE |
| 54750866 | 54750964 | - | 7.2 | 34 | 130 | -180 | AluSg | Alu | SINE |
| 54750967 | 54751281 | - | 15.9 | 5299 | 5620 | -677 | L1MA5A | L1 | LINE |
| 54751282 | 54751631 | + | 12.3 | 5608 | 5969 | -328 | L1MA5A | L1 | LINE |
| 54751632 | 54751926 | + | 11.9 | 1 | 296 | -16 | AluSx | Alu | SINE |
| 54751927 | 54752013 | + | 12.3 | 5970 | 6045 | -252 | L1MA5A | L1 | LINE |
| 54752112 | 54752227 | + | 22.6 | 197 | 332 | -96 | MLT1A | MaLR | LTR |

  

|  |  |  |  |  |  |  |  |  |  |
| --- | --- | --- | --- | --- | --- | --- | --- | --- | --- |
| chr13:55106344-55108853 | | | 2484 bp | | |  |  |  |  |
| Genome Start | Genome End | Strand | Div | Repeat Start | Repeat End | Repeat Left | Name | Family | Class |
| 55106344 | 55106535 | - | 23.2 | 1723 | 1919 | -1025 | BLACKJACK | AcHobo | DNA |
| 55106538 | 55108288 | - | 8.0 | 3952 | 5715 | -457 | L1PA8 | L1 | LINE |
| 55108289 | 55108743 | + | 7.8 | 5720 | 6172 | 0 | L1PA8 | L1 | LINE |
| 55108768 | 55108853 | - | 26.2 | 1630 | 1713 | -1231 | BLACKJACK | AcHobo | DNA |

  

|  |  |  |  |  |  |  |  |  |  |
| --- | --- | --- | --- | --- | --- | --- | --- | --- | --- |
| chr13:55277192-55287336 | | | 9796 bp | | |  |  |  |  |
| Genome Start | Genome End | Strand | Div | Repeat Start | Repeat End | Repeat Left | Name | Family | Class |
| 55277192 | 55277892 | + | 29.7 | 10 | 772 | -6562 | L1MB8 | L1 | LINE |
| 55277893 | 55278170 | - | 11.2 | 3 | 280 | -30 | AluSg | Alu | SINE |
| 55278171 | 55278422 | + | 29.7 | 773 | 1020 | -6314 | L1MB8 | L1 | LINE |
| 55278425 | 55278797 | - | 15.2 | 1 | 381 | 0 | THE1D | MaLR | LTR |
| 55278802 | 55280305 | - | 15.5 | 4 | 1575 | -5 | THE1D-int | MaLR | LTR |
| 55280309 | 55280677 | - | 17.3 | 1 | 381 | 0 | THE1D | MaLR | LTR |
| 55280678 | 55281184 | + | 28.8 | 1018 | 1533 | -5801 | L1MB8 | L1 | LINE |
| 55281181 | 55282675 | + | 22.9 | 2622 | 3884 | -3122 | L1MB8 | L1 | LINE |
| 55282676 | 55282973 | - | 14.0 | 1 | 308 | 0 | AluSg | Alu | SINE |
| 55282974 | 55283520 | + | 22.4 | 3885 | 4474 | -2532 | L1MB8 | L1 | LINE |
| 55283532 | 55283673 | + | 21.5 | 4689 | 4819 | -2187 | L1MB8 | L1 | LINE |
| 55283677 | 55283744 | + | 19.1 | 5378 | 5445 | -1561 | L1MB8 | L1 | LINE |
| 55283896 | 55284236 | + | 21.1 | 5818 | 6164 | -842 | L1MB8 | L1 | LINE |
| 55284238 | 55285405 | - | 1.4 | 4676 | 5843 | -312 | L1PA2 | L1 | LINE |
| 55285406 | 55285723 | + | 0.9 | 5838 | 6155 | 0 | L1PA2 | L1 | LINE |
| 55285847 | 55286410 | + | 20.2 | 7260 | 7889 | -72 | L1MC5 | L1 | LINE |
| 55286466 | 55287336 | + | 24.3 | 6150 | 7038 | 0 | L1MB8 | L1 | LINE |

  

|  |  |  |  |  |  |  |  |  |  |
| --- | --- | --- | --- | --- | --- | --- | --- | --- | --- |
| chr13:55430406-55439795 | | | 9313 bp | | |  |  |  |  |
| Genome Start | Genome End | Strand | Div | Repeat Start | Repeat End | Repeat Left | Name | Family | Class |
| 55430406 | 55432828 | + | 25.1 | 803 | 3273 | -3103 | L1MB2 | L1 | LINE |
| 55432828 | 55433619 | - | 16.2 | 4635 | 5460 | -705 | L1PA16 | L1 | LINE |
| 55433620 | 55434343 | + | 15.8 | 5438 | 6166 | 0 | L1PA16 | L1 | LINE |
| 55434348 | 55434878 | + | 20.0 | 3268 | 3811 | -2565 | L1MB2 | L1 | LINE |
| 55434895 | 55435446 | - | 25.7 | 2 | 547 | -7 | MLT2B3 | ERVL | LTR |
| 55435493 | 55435549 | + | 21.1 | 3850 | 3907 | -2472 | L1MB2 | L1 | LINE |
| 55435554 | 55436610 | + | 21.3 | 94 | 1245 | -5537 | L1PA14 | L1 | LINE |
| 55436630 | 55436739 | + | 20.3 | 1909 | 2018 | -4767 | L1PA14 | L1 | LINE |
| 55436740 | 55437064 | + | 15.0 | 5832 | 6157 | -3 | L1PREC2 | L1 | LINE |
| 55437065 | 55437098 | + | 20.3 | 2019 | 2056 | -4729 | L1PA14 | L1 | LINE |
| 55437088 | 55438830 | + | 21.9 | 3912 | 5588 | -788 | L1MB2 | L1 | LINE |
| 55438831 | 55439128 | + | 9.4 | 1 | 298 | -15 | AluSq | Alu | SINE |
| 55439129 | 55439795 | + | 22.1 | 5595 | 6291 | -116 | L1MB2 | L1 | LINE |

  

|  |  |  |  |  |  |  |  |  |  |
| --- | --- | --- | --- | --- | --- | --- | --- | --- | --- |
| chr13:55908051-55910366 | | | 2250 bp | | |  |  |  |  |
| Genome Start | Genome End | Strand | Div | Repeat Start | Repeat End | Repeat Left | Name | Family | Class |
| 55908051 | 55908548 | + | 27.7 | 2021 | 2457 | -3762 | L1MEc | L1 | LINE |
| 55908576 | 55908884 | - | 23.5 | 5467 | 5777 | -535 | L1M2 | L1 | LINE |
| 55908885 | 55908964 | + | 12.5 | 6050 | 6136 | -155 | L1MA8 | L1 | LINE |
| 55908964 | 55909128 | - | 17.9 | 5329 | 5491 | -677 | L1M2 | L1 | LINE |
| 55909130 | 55909619 | + | 24.0 | 5785 | 6267 | -45 | L1MA9 | L1 | LINE |
| 55909659 | 55910366 | + | 26.2 | 2739 | 3536 | -2683 | L1MEc | L1 | LINE |

  

|  |  |  |  |  |  |  |  |  |  |
| --- | --- | --- | --- | --- | --- | --- | --- | --- | --- |
| chr13:56131896-56135738 | | | 3834 bp | | |  |  |  |  |
| Genome Start | Genome End | Strand | Div | Repeat Start | Repeat End | Repeat Left | Name | Family | Class |
| 56131896 | 56133488 | + | 27.5 | 37 | 1732 | -4507 | L1MEc | L1 | LINE |
| 56133483 | 56134115 | - | 10.5 | 4182 | 4843 | -1303 | L1PA7 | L1 | LINE |
| 56134113 | 56135388 | + | 8.4 | 4858 | 6143 | -11 | L1PA7 | L1 | LINE |
| 56135407 | 56135738 | + | 21.9 | 1768 | 2154 | -4246 | L1MEc | L1 | LINE |

  

|  |  |  |  |  |  |  |  |  |  |
| --- | --- | --- | --- | --- | --- | --- | --- | --- | --- |
| chr13:56827256-56832016 | | | 4526 bp | | |  |  |  |  |
| Genome Start | Genome End | Strand | Div | Repeat Start | Repeat End | Repeat Left | Name | Family | Class |
| 56827256 | 56827353 | + | 18.3 | 5489 | 5581 | -763 | L1MC2 | L1 | LINE |
| 56827363 | 56827563 | - | 20.1 | 6163 | 6359 | 0 | L1MD2 | L1 | LINE |
| 56827790 | 56828719 | - | 1.7 | 5227 | 6155 | 0 | L1PA3 | L1 | LINE |
| 56828718 | 56831325 | + | 2.1 | 2596 | 5210 | -936 | L1PA3 | L1 | LINE |
| 56831328 | 56832016 | + | 20.9 | 5585 | 6323 | -21 | L1MC2 | L1 | LINE |

  

|  |  |  |  |  |  |  |  |  |  |
| --- | --- | --- | --- | --- | --- | --- | --- | --- | --- |
| chr13:56952060-56958028 | | | 5558 bp | | |  |  |  |  |
| Genome Start | Genome End | Strand | Div | Repeat Start | Repeat End | Repeat Left | Name | Family | Class |
| 56952060 | 56952451 | + | 25.5 | 1493 | 1875 | -4509 | L1M4c | L1 | LINE |
| 56952818 | 56953366 | + | 25.4 | 1979 | 2562 | -3814 | L1M4c | L1 | LINE |
| 56953367 | 56955240 | - | 4.7 | 3830 | 5668 | -486 | L1PA5 | L1 | LINE |
| 56955241 | 56955735 | + | 4.4 | 5648 | 6143 | -11 | L1PA5 | L1 | LINE |
| 56955766 | 56956611 | + | 20.0 | 2522 | 3409 | -2967 | L1M4c | L1 | LINE |
| 56956627 | 56958028 | + | 17.2 | 3814 | 5227 | -1149 | L1M4c | L1 | LINE |

  

|  |  |  |  |  |  |  |  |  |  |
| --- | --- | --- | --- | --- | --- | --- | --- | --- | --- |
| chr13:57287541-57289644 | | | 2092 bp | | |  |  |  |  |
| Genome Start | Genome End | Strand | Div | Repeat Start | Repeat End | Repeat Left | Name | Family | Class |
| 57287541 | 57288122 | - | 30.9 | 2613 | 3248 | -171 | L2 | L2 | LINE |
| 57288123 | 57288561 | - | 5.2 | 4932 | 5381 | -765 | L1PA5 | L1 | LINE |
| 57288562 | 57289345 | + | 3.9 | 5378 | 6154 | 0 | L1PA5 | L1 | LINE |
| 57289358 | 57289644 | - | 26.7 | 2260 | 2585 | -834 | L2 | L2 | LINE |

  

|  |  |  |  |  |  |  |  |  |  |
| --- | --- | --- | --- | --- | --- | --- | --- | --- | --- |
| chr13:57982378-57988631 | | | 6258 bp | | |  |  |  |  |
| Genome Start | Genome End | Strand | Div | Repeat Start | Repeat End | Repeat Left | Name | Family | Class |
| 57982378 | 57984472 | + | 8.0 | 2827 | 4957 | -1905 | L1PB1 | L1 | LINE |
| 57984468 | 57986004 | - | 0.6 | 4233 | 5769 | -386 | L1HS | L1 | LINE |
| 57986005 | 57986389 | + | 0.3 | 5771 | 6155 | 0 | L1HS | L1 | LINE |
| 57986391 | 57987069 | + | 8.0 | 4949 | 5609 | -1253 | L1PB1 | L1 | LINE |
| 57987070 | 57987364 | - | 9.3 | 1 | 289 | -22 | AluY | Alu | SINE |
| 57987365 | 57988631 | + | 9.7 | 5610 | 6866 | -1 | L1PB1 | L1 | LINE |

  

|  |  |  |  |  |  |  |  |  |  |
| --- | --- | --- | --- | --- | --- | --- | --- | --- | --- |
| chr13:58245253-58251752 | | | 5835 bp | | |  |  |  |  |
| Genome Start | Genome End | Strand | Div | Repeat Start | Repeat End | Repeat Left | Name | Family | Class |
| 58245253 | 58245358 | - | 20.8 | 2414 | 2519 | -425 | BLACKJACK | AcHobo | DNA |
| 58245356 | 58246562 | + | 7.7 | 88 | 1578 | 0 | LTR12C | ERV1 | LTR |
| 58246563 | 58246838 | - | 23.1 | 2119 | 2417 | -527 | BLACKJACK | AcHobo | DNA |
| 58246917 | 58246988 | + | 14.5 | 333 | 405 | -116 | MER113 | MER1\_type | DNA |
| 58246990 | 58247197 | - | 15.4 | 4071 | 4280 | -1866 | L1PA16 | L1 | LINE |
| 58247198 | 58249025 | + | 14.1 | 4271 | 6165 | -1 | L1PA16 | L1 | LINE |
| 58249218 | 58249349 | - | 23.7 | 2001 | 2112 | -832 | BLACKJACK | AcHobo | DNA |
| 58249350 | 58249661 | - | 12.7 | 1 | 306 | -7 | AluSq | Alu | SINE |
| 58249662 | 58249786 | - | 23.7 | 1872 | 2000 | -944 | BLACKJACK | AcHobo | DNA |
| 58250184 | 58251752 | - | 25.3 | 1 | 1619 | -1325 | BLACKJACK | AcHobo | DNA |

  

|  |  |  |  |  |  |  |  |  |  |
| --- | --- | --- | --- | --- | --- | --- | --- | --- | --- |
| chr13:59319967-59324177 | | | 4130 bp | | |  |  |  |  |
| Genome Start | Genome End | Strand | Div | Repeat Start | Repeat End | Repeat Left | Name | Family | Class |
| 59319967 | 59320445 | + | 26.9 | 2339 | 2891 | -528 | L2 | L2 | LINE |
| 59320449 | 59322819 | - | 1.0 | 3788 | 6155 | 0 | L1PA2 | L1 | LINE |
| 59322815 | 59323322 | + | 1.6 | 3293 | 3800 | -2346 | L1PA2 | L1 | LINE |
| 59323324 | 59323698 | + | 24.6 | 2884 | 3338 | -81 | L2 | L2 | LINE |
| 59323771 | 59324052 | - | 10.6 | 1 | 301 | -8 | AluSc | Alu | SINE |
| 59324063 | 59324131 | + | 24.6 | 443 | 511 | 0 | MLT1J | MaLR | LTR |
| 59324132 | 59324177 | + | 20.0 | 3316 | 3363 | -15 | L2 | L2 | LINE |

  

|  |  |  |  |  |  |  |  |  |  |
| --- | --- | --- | --- | --- | --- | --- | --- | --- | --- |
| chr13:62373281-62376442 | | | 3149 bp | | |  |  |  |  |
| Genome Start | Genome End | Strand | Div | Repeat Start | Repeat End | Repeat Left | Name | Family | Class |
| 62373281 | 62373384 | - | 21.2 | 7680 | 7784 | -1 | L1MC3 | L1 | LINE |
| 62373385 | 62374680 | + | 11.9 | 2 | 1286 | -1 | HSMAR1 | Mariner | DNA |
| 62374681 | 62375146 | - | 21.2 | 7200 | 7679 | -106 | L1MC3 | L1 | LINE |
| 62375154 | 62375282 | - | 24.8 | 6150 | 6280 | -11 | L1MA7 | L1 | LINE |
| 62375282 | 62375369 | - | 24.1 | 7123 | 7209 | -576 | L1MC3 | L1 | LINE |
| 62375360 | 62375409 | - | 20.0 | 5517 | 5566 | -617 | L1M3 | L1 | LINE |
| 62375406 | 62376144 | + | 15.6 | 5552 | 6279 | -21 | L1MA4 | L1 | LINE |
| 62376166 | 62376442 | - | 20.6 | 6845 | 7137 | -648 | L1MC3 | L1 | LINE |

  

|  |  |  |  |  |  |  |  |  |  |
| --- | --- | --- | --- | --- | --- | --- | --- | --- | --- |
| chr13:62739007-62743206 | | | 4162 bp | | |  |  |  |  |
| Genome Start | Genome End | Strand | Div | Repeat Start | Repeat End | Repeat Left | Name | Family | Class |
| 62739007 | 62739392 | - | 16.1 | 1256 | 1644 | -7 | MSTA-int | MaLR | LTR |
| 62739404 | 62739601 | + | 7.1 | 3978 | 4177 | -1969 | L1PB | L1 | LINE |
| 62739602 | 62739929 | - | 22.9 | 852 | 1188 | -392 | MSTA-int | MaLR | LTR |
| 62739930 | 62740289 | - | 13.9 | 5794 | 6150 | -1 | L1PB1 | L1 | LINE |
| 62740317 | 62740596 | - | 13.9 | 5474 | 5793 | -358 | L1PB1 | L1 | LINE |
| 62740597 | 62742279 | + | 8.6 | 3756 | 5478 | -673 | L1PB1 | L1 | LINE |
| 62742280 | 62743206 | - | 19.7 | 1 | 927 | -724 | MSTA-int | MaLR | LTR |

  

|  |  |  |  |  |  |  |  |  |  |
| --- | --- | --- | --- | --- | --- | --- | --- | --- | --- |
| chr13:62969187-62973921 | | | 4688 bp | | |  |  |  |  |
| Genome Start | Genome End | Strand | Div | Repeat Start | Repeat End | Repeat Left | Name | Family | Class |
| 62969187 | 62970800 | + | 24.7 | 656 | 2401 | -4933 | L1M4b | L1 | LINE |
| 62970801 | 62971086 | + | 13.0 | 1 | 285 | -25 | AluSg | Alu | SINE |
| 62971087 | 62971497 | + | 24.7 | 2402 | 2852 | -4482 | L1M4b | L1 | LINE |
| 62971540 | 62971672 | + | 29.8 | 2907 | 3039 | -4154 | L1M4b | L1 | LINE |
| 62971678 | 62973216 | - | 9.1 | 4628 | 6168 | 0 | L1PA10 | L1 | LINE |
| 62973207 | 62973256 | + | 12.0 | 4588 | 4637 | -1509 | L1PA10 | L1 | LINE |
| 62973267 | 62973921 | + | 26.1 | 3045 | 3722 | -3284 | L1M4b | L1 | LINE |

  

|  |  |  |  |  |  |  |  |  |  |
| --- | --- | --- | --- | --- | --- | --- | --- | --- | --- |
| chr13:62980536-62981706 | | | 1165 bp | | |  |  |  |  |
| Genome Start | Genome End | Strand | Div | Repeat Start | Repeat End | Repeat Left | Name | Family | Class |
| 62980536 | 62980627 | - | 23.9 | 299 | 390 | 0 | MLT1B | MaLR | LTR |
| 62980633 | 62981118 | - | 9.5 | 5667 | 6158 | -5 | L1PA13 | L1 | LINE |
| 62981120 | 62981422 | + | 7.0 | 5369 | 5677 | -486 | L1PA13 | L1 | LINE |
| 62981423 | 62981706 | - | 25.1 | 1 | 306 | -84 | MLT1B | MaLR | LTR |

  

|  |  |  |  |  |  |  |  |  |  |
| --- | --- | --- | --- | --- | --- | --- | --- | --- | --- |
| chr13:63406043-63411379 | | | 5265 bp | | |  |  |  |  |
| Genome Start | Genome End | Strand | Div | Repeat Start | Repeat End | Repeat Left | Name | Family | Class |
| 63406043 | 63406600 | - | 29.7 | 1252 | 1885 | -4515 | L1MEc | L1 | LINE |
| 63406650 | 63407349 | - | 29.7 | 512 | 1251 | -5149 | L1MEc | L1 | LINE |
| 63407376 | 63407390 | - | 15.1 | 3158 | 3172 | -2974 | L1P3 | L1 | LINE |
| 63407391 | 63407777 | - | 12.2 | 5723 | 6118 | -13 | L1PA8A | L1 | LINE |
| 63407778 | 63407856 | - | 15.1 | 3079 | 3157 | -2989 | L1P3 | L1 | LINE |
| 63407855 | 63408045 | - | 15.2 | 590 | 780 | -5095 | L1P3b | L1 | LINE |
| 63408043 | 63408973 | - | 9.0 | 4848 | 5788 | -343 | L1PA8A | L1 | LINE |
| 63408975 | 63410983 | + | 9.6 | 2858 | 4857 | -1289 | L1PA8A | L1 | LINE |
| 63410985 | 63411379 | - | 35.7 | 44 | 499 | -5901 | L1MEc | L1 | LINE |

  

|  |  |  |  |  |  |  |  |  |  |
| --- | --- | --- | --- | --- | --- | --- | --- | --- | --- |
| chr13:63552682-63557926 | | | 5235 bp | | |  |  |  |  |
| Genome Start | Genome End | Strand | Div | Repeat Start | Repeat End | Repeat Left | Name | Family | Class |
| 63552682 | 63552878 | - | 15.9 | 6138 | 6333 | 0 | L1MC1 | L1 | LINE |
| 63552881 | 63553595 | - | 17.1 | 5374 | 6097 | -236 | L1MC1 | L1 | LINE |
| 63553607 | 63555367 | - | 15.6 | 4385 | 6156 | -10 | L1PA16 | L1 | LINE |
| 63555368 | 63556419 | + | 14.9 | 3316 | 4369 | -1777 | L1PA16 | L1 | LINE |
| 63556417 | 63557926 | - | 18.6 | 3865 | 5386 | -760 | L1MC1 | L1 | LINE |

  

|  |  |  |  |  |  |  |  |  |  |
| --- | --- | --- | --- | --- | --- | --- | --- | --- | --- |
| chr13:63602296-63611326 | | | 8894 bp | | |  |  |  |  |
| Genome Start | Genome End | Strand | Div | Repeat Start | Repeat End | Repeat Left | Name | Family | Class |
| 63602296 | 63605438 | + | 20.0 | 16 | 2848 | -4717 | L1M1 | L1 | LINE |
| 63605439 | 63605739 | + | 17.9 | 1 | 301 | -11 | AluSx | Alu | SINE |
| 63605740 | 63605926 | + | 17.9 | 2849 | 3050 | -4515 | L1M1 | L1 | LINE |
| 63605924 | 63607209 | - | 10.0 | 2140 | 3374 | -2772 | L1PB1 | L1 | LINE |
| 63607207 | 63609596 | + | 11.3 | 3386 | 5771 | -380 | L1PB1 | L1 | LINE |
| 63609731 | 63610099 | + | 13.9 | 5782 | 6151 | 0 | L1PB1 | L1 | LINE |
| 63610109 | 63611326 | + | 13.5 | 3032 | 4279 | -3286 | L1M1 | L1 | LINE |

  

|  |  |  |  |  |  |  |  |  |  |
| --- | --- | --- | --- | --- | --- | --- | --- | --- | --- |
| chr13:63703151-63707949 | | | 4593 bp | | |  |  |  |  |
| Genome Start | Genome End | Strand | Div | Repeat Start | Repeat End | Repeat Left | Name | Family | Class |
| 63703151 | 63703271 | - | 24.2 | 5894 | 6009 | -180 | L1MB1 | L1 | LINE |
| 63703301 | 63704886 | - | 1.4 | 4569 | 6154 | -1 | L1PA2 | L1 | LINE |
| 63704884 | 63705091 | + | 1.4 | 4339 | 4546 | -1600 | L1PA2 | L1 | LINE |
| 63705066 | 63705807 | - | 21.6 | 5194 | 5929 | -239 | L1MB1 | L1 | LINE |
| 63705846 | 63707542 | + | 20.3 | 4610 | 6312 | 0 | L1MA9 | L1 | LINE |
| 63707711 | 63707949 | - | 19.7 | 4826 | 5070 | -1070 | L1MB1 | L1 | LINE |

  

|  |  |  |  |  |  |  |  |  |  |
| --- | --- | --- | --- | --- | --- | --- | --- | --- | --- |
| chr13:64489788-64496501 | | | 6705 bp | | |  |  |  |  |
| Genome Start | Genome End | Strand | Div | Repeat Start | Repeat End | Repeat Left | Name | Family | Class |
| 64489788 | 64490162 | - | 17.1 | 6297 | 6692 | 0 | L1PA15 | L1 | LINE |
| 64490163 | 64490861 | - | 8.6 | 3988 | 4691 | -1455 | L1PA8 | L1 | LINE |
| 64490864 | 64492348 | + | 10.4 | 4679 | 6168 | -4 | L1PA8 | L1 | LINE |
| 64492356 | 64496501 | - | 16.7 | 2140 | 6297 | -395 | L1PA15 | L1 | LINE |

  

|  |  |  |  |  |  |  |  |  |  |
| --- | --- | --- | --- | --- | --- | --- | --- | --- | --- |
| chr13:65494488-65500564 | | | 6023 bp | | |  |  |  |  |
| Genome Start | Genome End | Strand | Div | Repeat Start | Repeat End | Repeat Left | Name | Family | Class |
| 65494488 | 65495015 | + | 15.6 | 185 | 725 | -1693 | Tigger1 | MER2\_type | DNA |
| 65495028 | 65495599 | - | 3.0 | 2647 | 3218 | -2928 | L1PA3 | L1 | LINE |
| 65495596 | 65498505 | + | 3.3 | 3224 | 6137 | -18 | L1PA3 | L1 | LINE |
| 65498552 | 65499022 | + | 15.3 | 727 | 1172 | -1246 | Tigger1 | MER2\_type | DNA |
| 65499023 | 65499324 | - | 16.2 | 1 | 302 | -11 | AluSq | Alu | SINE |
| 65499325 | 65500564 | + | 15.3 | 1173 | 2418 | 0 | Tigger1 | MER2\_type | DNA |

  

|  |  |  |  |  |  |  |  |  |  |
| --- | --- | --- | --- | --- | --- | --- | --- | --- | --- |
| chr13:69554654-69555643 | | | 961 bp | | |  |  |  |  |
| Genome Start | Genome End | Strand | Div | Repeat Start | Repeat End | Repeat Left | Name | Family | Class |
| 69554654 | 69554748 | + | 26.9 | 163 | 260 | -4594 | L1MEe | L1 | LINE |
| 69554749 | 69554852 | - | 12.9 | 6053 | 6157 | -9 | L1PA16 | L1 | LINE |
| 69554853 | 69555139 | + | 16.9 | 5740 | 6031 | -135 | L1PA16 | L1 | LINE |
| 69555169 | 69555470 | + | 31.9 | 279 | 575 | -4279 | L1MEe | L1 | LINE |
| 69555471 | 69555597 | - | 25.2 | 1 | 133 | 0 | FLAM\_A | Alu | SINE |
| 69555598 | 69555643 | + | 31.9 | 576 | 622 | -4232 | L1MEe | L1 | LINE |

  

|  |  |  |  |  |  |  |  |  |  |
| --- | --- | --- | --- | --- | --- | --- | --- | --- | --- |
| chr13:69913754-69914860 | | | 1095 bp | | |  |  |  |  |
| Genome Start | Genome End | Strand | Div | Repeat Start | Repeat End | Repeat Left | Name | Family | Class |
| 69913754 | 69913970 | - | 15.1 | 155 | 381 | 0 | THE1D | MaLR | LTR |
| 69913971 | 69914263 | - | 6.5 | 5439 | 5732 | -440 | L1PA8 | L1 | LINE |
| 69914264 | 69914688 | + | 8.5 | 5744 | 6171 | -1 | L1PA8 | L1 | LINE |
| 69914701 | 69914860 | - | 13.9 | 1 | 168 | -207 | THE1D | MaLR | LTR |

  

|  |  |  |  |  |  |  |  |  |  |
| --- | --- | --- | --- | --- | --- | --- | --- | --- | --- |
| chr13:70597676-70607598 | | | 9421 bp | | |  |  |  |  |
| Genome Start | Genome End | Strand | Div | Repeat Start | Repeat End | Repeat Left | Name | Family | Class |
| 70597676 | 70598938 | + | 24.1 | 1206 | 2517 | -4817 | L1M4b | L1 | LINE |
| 70599115 | 70599243 | + | 25.6 | 6 | 138 | -252 | MLT1C | MaLR | LTR |
| 70599244 | 70601363 | - | 7.9 | 4046 | 6152 | -2 | L1PA7 | L1 | LINE |
| 70601355 | 70604515 | + | 9.7 | 909 | 4030 | -2116 | L1PA7 | L1 | LINE |
| 70604516 | 70604874 | + | 24.1 | 121 | 464 | -3 | MLT1C | MaLR | LTR |
| 70605039 | 70605408 | - | 20.7 | 20 | 390 | 0 | MLT1B | MaLR | LTR |
| 70605425 | 70605533 | + | 20.6 | 2664 | 2777 | -4557 | L1M4b | L1 | LINE |
| 70605567 | 70605894 | + | 27.6 | 150 | 482 | -23 | MLT1D | MaLR | LTR |
| 70605927 | 70605991 | - | 14.1 | 404 | 467 | 0 | MLT1C | MaLR | LTR |
| 70606019 | 70606561 | + | 18.0 | 5614 | 6170 | -1 | L1MB2 | L1 | LINE |
| 70606621 | 70607017 | - | 24.6 | 11 | 417 | -50 | MLT1C | MaLR | LTR |
| 70607022 | 70607598 | + | 23.9 | 2960 | 3326 | -3753 | L1M4b | L1 | LINE |

  

|  |  |  |  |  |  |  |  |  |  |
| --- | --- | --- | --- | --- | --- | --- | --- | --- | --- |
| chr13:73561625-73565012 | | | 3371 bp | | |  |  |  |  |
| Genome Start | Genome End | Strand | Div | Repeat Start | Repeat End | Repeat Left | Name | Family | Class |
| 73561625 | 73562491 | + | 19.0 | 3670 | 4557 | -1589 | L1M4 | L1 | LINE |
| 73562484 | 73562582 | - | 20.2 | 6169 | 6267 | -66 | L1MC1 | L1 | LINE |
| 73562580 | 73562677 | + | 20.4 | 4559 | 4657 | -1489 | L1M4 | L1 | LINE |
| 73562679 | 73563000 | - | 21.0 | 5132 | 5463 | -779 | L1MD | L1 | LINE |
| 73563019 | 73563492 | + | 21.3 | 4643 | 5124 | -1022 | L1M4 | L1 | LINE |
| 73563499 | 73563766 | - | 14.1 | 5709 | 5972 | -361 | L1MC1 | L1 | LINE |
| 73563768 | 73564120 | + | 20.3 | 5959 | 6314 | -19 | L1MC1 | L1 | LINE |
| 73564123 | 73564147 | + | 15.3 | 5114 | 5133 | -1013 | L1M4 | L1 | LINE |
| 73564148 | 73564449 | + | 8.5 | 3 | 296 | -15 | AluY | Alu | SINE |
| 73564450 | 73564513 | + | 15.3 | 5134 | 5199 | -947 | L1M4 | L1 | LINE |
| 73564514 | 73564787 | + | 12.4 | 35 | 312 | 0 | AluSx | Alu | SINE |
| 73564788 | 73565012 | + | 15.5 | 5200 | 5424 | -742 | L1M4 | L1 | LINE |

  

|  |  |  |  |  |  |  |  |  |  |
| --- | --- | --- | --- | --- | --- | --- | --- | --- | --- |
| chr13:74070985-74074029 | | | 2923 bp | | |  |  |  |  |
| Genome Start | Genome End | Strand | Div | Repeat Start | Repeat End | Repeat Left | Name | Family | Class |
| 74070985 | 74071116 | + | 27.4 | 4788 | 4918 | -1228 | L1ME3B | L1 | LINE |
| 74071221 | 74071601 | + | 28.7 | 5133 | 5556 | -684 | L1ME3B | L1 | LINE |
| 74071619 | 74072831 | - | 8.8 | 5058 | 6284 | -7 | L1PA7 | L1 | LINE |
| 74072831 | 74073147 | + | 9.5 | 4747 | 5063 | -1083 | L1PA7 | L1 | LINE |
| 74073152 | 74073433 | - | 13.0 | 5921 | 6164 | -127 | L1MA7 | L1 | LINE |
| 74073432 | 74074029 | + | 28.2 | 5561 | 6231 | -9 | L1ME3B | L1 | LINE |

  

|  |  |  |  |  |  |  |  |  |  |
| --- | --- | --- | --- | --- | --- | --- | --- | --- | --- |
| chr13:78576079-78580184 | | | 3991 bp | | |  |  |  |  |
| Genome Start | Genome End | Strand | Div | Repeat Start | Repeat End | Repeat Left | Name | Family | Class |
| 78576079 | 78576807 | + | 27.9 | 1455 | 2157 | -3970 | L1MEd | L1 | LINE |
| 78576820 | 78576891 | + | 22.2 | 8 | 79 | -2244 | Tigger5 | MER2\_type | DNA |
| 78576898 | 78576954 | + | 17.9 | 2351 | 2406 | 0 | Tigger5 | MER2\_type | DNA |
| 78577001 | 78577528 | + | 37.3 | 2212 | 2751 | -3378 | L1MEd | L1 | LINE |
| 78577531 | 78578290 | - | 15.4 | 4636 | 5420 | -913 | L1M | L1 | LINE |
| 78578295 | 78579201 | + | 16.0 | 5403 | 6327 | -6 | L1MC1 | L1 | LINE |
| 78579202 | 78579998 | + | 7.2 | 5360 | 6158 | -14 | L1PA8 | L1 | LINE |
| 78580044 | 78580184 | + | 28.1 | 2768 | 2923 | -3206 | L1MEd | L1 | LINE |

  

|  |  |  |  |  |  |  |  |  |  |
| --- | --- | --- | --- | --- | --- | --- | --- | --- | --- |
| chr13:79328670-79331977 | | | 3123 bp | | |  |  |  |  |
| Genome Start | Genome End | Strand | Div | Repeat Start | Repeat End | Repeat Left | Name | Family | Class |
| 79328670 | 79329643 | + | 13.5 | 3948 | 4935 | -719 | HERVL-B3 | ERVL | LTR |
| 79329647 | 79329893 | - | 3.6 | 5551 | 5797 | -357 | L1PA6 | L1 | LINE |
| 79329894 | 79330263 | + | 6.8 | 5783 | 6152 | -2 | L1PA6 | L1 | LINE |
| 79330270 | 79330645 | + | 9.4 | 1 | 448 | -2 | LTR7 | ERV1 | LTR |
| 79330772 | 79330888 | + | 12.0 | 32 | 159 | -902 | MER63 | AcHobo | DNA |
| 79330939 | 79331385 | + | 13.1 | 4936 | 5382 | -272 | HERVL-B3 | ERVL | LTR |
| 79331386 | 79331700 | + | 10.0 | 1 | 310 | 0 | AluSg | Alu | SINE |
| 79331701 | 79331977 | + | 13.1 | 5383 | 5651 | -3 | HERVL-B3 | ERVL | LTR |

  

|  |  |  |  |  |  |  |  |  |  |
| --- | --- | --- | --- | --- | --- | --- | --- | --- | --- |
| chr13:81255891-81262119 | | | 5952 bp | | |  |  |  |  |
| Genome Start | Genome End | Strand | Div | Repeat Start | Repeat End | Repeat Left | Name | Family | Class |
| 81255891 | 81256809 | - | 28.0 | 5117 | 6078 | -92 | L1ME3A | L1 | LINE |
| 81256839 | 81258313 | - | 27.4 | 3641 | 5116 | -1030 | L1ME3A | L1 | LINE |
| 81258311 | 81259250 | - | 9.6 | 3726 | 4699 | -1447 | L1PA8 | L1 | LINE |
| 81259250 | 81260735 | + | 8.2 | 4703 | 6172 | 0 | L1PA8 | L1 | LINE |
| 81260883 | 81261059 | - | 12.5 | 5785 | 5961 | -344 | L1MA3 | L1 | LINE |
| 81261061 | 81261387 | + | 13.9 | 5979 | 6305 | 0 | L1MA3 | L1 | LINE |
| 81261402 | 81261630 | - | 24.6 | 3275 | 3546 | -2600 | L1ME3A | L1 | LINE |
| 81261721 | 81262119 | - | 32.0 | 2890 | 3269 | -2877 | L1ME3A | L1 | LINE |

  

|  |  |  |  |  |  |  |  |  |  |
| --- | --- | --- | --- | --- | --- | --- | --- | --- | --- |
| chr13:81621956-81625210 | | | 3262 bp | | |  |  |  |  |
| Genome Start | Genome End | Strand | Div | Repeat Start | Repeat End | Repeat Left | Name | Family | Class |
| 81621956 | 81622167 | - | 17.9 | 5952 | 6165 | -3 | L1MB1 | L1 | LINE |
| 81622175 | 81622739 | - | 13.0 | 5575 | 6166 | 0 | L1PA16 | L1 | LINE |
| 81622740 | 81624438 | + | 13.6 | 3883 | 5569 | -597 | L1PA16 | L1 | LINE |
| 81624425 | 81625210 | - | 17.1 | 5166 | 5970 | -198 | L1MB1 | L1 | LINE |

  

|  |  |  |  |  |  |  |  |  |  |
| --- | --- | --- | --- | --- | --- | --- | --- | --- | --- |
| chr13:81774475-81776768 | | | 2234 bp | | |  |  |  |  |
| Genome Start | Genome End | Strand | Div | Repeat Start | Repeat End | Repeat Left | Name | Family | Class |
| 81774475 | 81774835 | + | 29.7 | 1952 | 2303 | -4097 | L1MEc | L1 | LINE |
| 81774853 | 81775259 | - | 16.6 | 5349 | 5756 | -410 | L1PA16 | L1 | LINE |
| 81775260 | 81775673 | + | 14.0 | 5754 | 6163 | -3 | L1PA16 | L1 | LINE |
| 81775692 | 81776148 | + | 29.3 | 2212 | 2672 | -3547 | L1MEc | L1 | LINE |
| 81776149 | 81776433 | + | 12.6 | 1 | 286 | -26 | AluSx | Alu | SINE |
| 81776459 | 81776768 | + | 29.3 | 2673 | 2985 | -3234 | L1MEc | L1 | LINE |

  

|  |  |  |  |  |  |  |  |  |  |
| --- | --- | --- | --- | --- | --- | --- | --- | --- | --- |
| chr13:81823617-81828417 | | | 4380 bp | | |  |  |  |  |
| Genome Start | Genome End | Strand | Div | Repeat Start | Repeat End | Repeat Left | Name | Family | Class |
| 81823617 | 81825341 | - | 24.3 | 1951 | 3852 | -2712 | L1MCb | L1 | LINE |
| 81825354 | 81825722 | - | 20.1 | 5921 | 6295 | -5 | L1MA6 | L1 | LINE |
| 81825723 | 81826368 | + | 17.1 | 5300 | 5937 | -363 | L1MA6 | L1 | LINE |
| 81826418 | 81827384 | - | 27.4 | 845 | 1886 | -4865 | L1MCb | L1 | LINE |
| 81827421 | 81827718 | - | 17.9 | 2 | 298 | -14 | AluJb | Alu | SINE |
| 81828043 | 81828417 | - | 32.2 | 41 | 422 | -5623 | L1MCb | L1 | LINE |

  

|  |  |  |  |  |  |  |  |  |  |
| --- | --- | --- | --- | --- | --- | --- | --- | --- | --- |
| chr13:82729054-82743454 | | | 13536 bp | | |  |  |  |  |
| Genome Start | Genome End | Strand | Div | Repeat Start | Repeat End | Repeat Left | Name | Family | Class |
| 82729054 | 82729110 | + | 32.2 | 37 | 100 | -6300 | L1ME1 | L1 | LINE |
| 82729135 | 82729443 | + | 32.2 | 101 | 418 | -5982 | L1ME1 | L1 | LINE |
| 82729698 | 82729790 | - | 18.3 | 4151 | 4246 | -1894 | L1MB1 | L1 | LINE |
| 82729784 | 82731374 | + | 20.5 | 4253 | 5946 | -222 | L1MB1 | L1 | LINE |
| 82731375 | 82733110 | + | 17.8 | 4563 | 6300 | 0 | L1MA6 | L1 | LINE |
| 82733276 | 82733745 | + | 33.5 | 4112 | 4611 | -1608 | L1ME1 | L1 | LINE |
| 82733748 | 82734505 | - | 23.7 | 5386 | 6177 | -1 | L1MB8 | L1 | LINE |
| 82734499 | 82734898 | - | 23.7 | 4077 | 4484 | -1662 | L1MB8 | L1 | LINE |
| 82734907 | 82735038 | - | 14.4 | 142 | 278 | -2140 | Tigger1 | MER2\_type | DNA |
| 82735043 | 82735418 | + | 12.4 | 1 | 375 | 0 | THE1C | MaLR | LTR |
| 82735420 | 82736871 | + | 14.9 | 2 | 1580 | 0 | THE1C-int | MaLR | LTR |
| 82736872 | 82737233 | + | 15.8 | 1 | 375 | 0 | THE1C | MaLR | LTR |
| 82737240 | 82737372 | - | 20.4 | 1 | 136 | -2282 | Tigger1 | MER2\_type | DNA |
| 82737379 | 82737595 | - | 25.1 | 2317 | 2551 | -3595 | L1MB8 | L1 | LINE |
| 82737596 | 82737919 | - | 22.3 | 1 | 324 | 0 | AluJb | Alu | SINE |
| 82737920 | 82738175 | - | 25.4 | 2110 | 2470 | -4004 | L1MB8 | L1 | LINE |
| 82738176 | 82738398 | - | 16.7 | 6106 | 6332 | -1 | L1MC1 | L1 | LINE |
| 82738399 | 82741373 | - | 27.2 | 13 | 3240 | -4094 | L1M4b | L1 | LINE |
| 82741432 | 82741870 | + | 30.3 | 4690 | 5163 | -1056 | L1ME1 | L1 | LINE |
| 82742129 | 82742892 | + | 16.4 | 5493 | 6300 | 0 | L1MA6 | L1 | LINE |
| 82742986 | 82743454 | + | 29.5 | 5754 | 6233 | -6 | L1ME1 | L1 | LINE |

  

|  |  |  |  |  |  |  |  |  |  |
| --- | --- | --- | --- | --- | --- | --- | --- | --- | --- |
| chr13:83084768-83087452 | | | 2649 bp | | |  |  |  |  |
| Genome Start | Genome End | Strand | Div | Repeat Start | Repeat End | Repeat Left | Name | Family | Class |
| 83084768 | 83084929 | - | 13.0 | 206 | 364 | 0 | THE1B | MaLR | LTR |
| 83084930 | 83086086 | - | 8.9 | 3 | 1254 | 0 | LTR12D | ERV1 | LTR |
| 83086087 | 83086132 | - | 13.0 | 171 | 205 | -159 | THE1B | MaLR | LTR |
| 83086136 | 83086146 | - | 5.0 | 6143 | 6154 | 0 | L1PA5 | L1 | LINE |
| 83086180 | 83086646 | - | 5.0 | 5658 | 6142 | -12 | L1PA5 | L1 | LINE |
| 83086647 | 83087268 | + | 5.3 | 5012 | 5639 | -515 | L1PA5 | L1 | LINE |
| 83087269 | 83087452 | - | 15.2 | 1 | 184 | -180 | THE1B | MaLR | LTR |

  

|  |  |  |  |  |  |  |  |  |  |
| --- | --- | --- | --- | --- | --- | --- | --- | --- | --- |
| chr13:83945641-83949931 | | | 4179 bp | | |  |  |  |  |
| Genome Start | Genome End | Strand | Div | Repeat Start | Repeat End | Repeat Left | Name | Family | Class |
| 83945641 | 83946303 | + | 21.4 | 5 | 691 | -5693 | L1M4c | L1 | LINE |
| 83946432 | 83946607 | + | 19.1 | 722 | 899 | -5485 | L1M4c | L1 | LINE |
| 83946606 | 83948429 | - | 9.2 | 3528 | 5347 | -799 | L1PA7 | L1 | LINE |
| 83948427 | 83949210 | + | 5.5 | 5363 | 6154 | 0 | L1PA7 | L1 | LINE |
| 83949212 | 83949332 | + | 19.2 | 886 | 1008 | -5376 | L1M4c | L1 | LINE |
| 83949321 | 83949931 | + | 21.2 | 2909 | 3575 | -2807 | L1M4c | L1 | LINE |

  

|  |  |  |  |  |  |  |  |  |  |
| --- | --- | --- | --- | --- | --- | --- | --- | --- | --- |
| chr13:83973578-83976346 | | | 2732 bp | | |  |  |  |  |
| Genome Start | Genome End | Strand | Div | Repeat Start | Repeat End | Repeat Left | Name | Family | Class |
| 83973578 | 83973760 | + | 13.7 | 1 | 184 | -180 | THE1B | MaLR | LTR |
| 83973761 | 83974061 | + | 13.9 | 1 | 301 | -9 | AluSg | Alu | SINE |
| 83974062 | 83974731 | - | 2.5 | 4122 | 4791 | -1355 | L1PA2 | L1 | LINE |
| 83974730 | 83976109 | + | 1.8 | 4775 | 6154 | -1 | L1PA2 | L1 | LINE |
| 83976149 | 83976346 | + | 14.0 | 171 | 345 | -19 | THE1B | MaLR | LTR |

  

|  |  |  |  |  |  |  |  |  |  |
| --- | --- | --- | --- | --- | --- | --- | --- | --- | --- |
| chr13:84724067-84726725 | | | 2514 bp | | |  |  |  |  |
| Genome Start | Genome End | Strand | Div | Repeat Start | Repeat End | Repeat Left | Name | Family | Class |
| 84724067 | 84724182 | + | 29.7 | 2968 | 3086 | -333 | L2 | L2 | LINE |
| 84724240 | 84724528 | + | 13.2 | 1 | 291 | -21 | AluSx | Alu | SINE |
| 84724530 | 84725189 | - | 9.1 | 4438 | 5121 | -1025 | L1PA7 | L1 | LINE |
| 84725185 | 84726194 | + | 5.6 | 5132 | 6154 | 0 | L1PA7 | L1 | LINE |
| 84726285 | 84726595 | - | 6.8 | 1 | 310 | -1 | AluY | Alu | SINE |
| 84726598 | 84726725 | + | 40.9 | 3250 | 3378 | 0 | L2 | L2 | LINE |

  

|  |  |  |  |  |  |  |  |  |  |
| --- | --- | --- | --- | --- | --- | --- | --- | --- | --- |
| chr13:86824722-86828193 | | | 3459 bp | | |  |  |  |  |
| Genome Start | Genome End | Strand | Div | Repeat Start | Repeat End | Repeat Left | Name | Family | Class |
| 86824722 | 86824772 | + | 22.6 | 6452 | 6493 | -1292 | L1MC3 | L1 | LINE |
| 86824773 | 86825082 | + | 7.1 | 1 | 311 | 0 | AluY | Alu | SINE |
| 86825083 | 86825958 | + | 22.6 | 6494 | 7465 | -320 | L1MC3 | L1 | LINE |
| 86825959 | 86826269 | + | 1.6 | 1 | 311 | 0 | AluY | Alu | SINE |
| 86826270 | 86826399 | + | 22.6 | 7466 | 7592 | -193 | L1MC3 | L1 | LINE |
| 86826393 | 86826763 | + | 20.0 | 5774 | 6144 | -156 | L1MA6 | L1 | LINE |
| 86826765 | 86827246 | - | 2.6 | 5694 | 6175 | 0 | L1PA4 | L1 | LINE |
| 86827247 | 86827515 | + | 2.6 | 5414 | 5681 | -474 | L1PA4 | L1 | LINE |
| 86827506 | 86827561 | + | 19.1 | 6135 | 6174 | -129 | L1MA4A | L1 | LINE |
| 86827562 | 86827850 | - | 11.1 | 1 | 291 | -22 | AluSq | Alu | SINE |
| 86827851 | 86827981 | + | 14.5 | 6164 | 6294 | -6 | L1MA6 | L1 | LINE |
| 86828011 | 86828193 | + | 18.1 | 7601 | 7784 | -1 | L1MC3 | L1 | LINE |

  

|  |  |  |  |  |  |  |  |  |  |
| --- | --- | --- | --- | --- | --- | --- | --- | --- | --- |
| chr13:86884486-86894229 | | | 9432 bp | | |  |  |  |  |
| Genome Start | Genome End | Strand | Div | Repeat Start | Repeat End | Repeat Left | Name | Family | Class |
| 86884486 | 86884542 | + | 27.0 | 403 | 449 | -6158 | L1MB4 | L1 | LINE |
| 86884543 | 86884863 | - | 6.8 | 1 | 311 | 0 | AluY | Alu | SINE |
| 86884864 | 86885263 | + | 27.0 | 450 | 855 | -5752 | L1MB4 | L1 | LINE |
| 86885278 | 86886485 | + | 20.7 | 1069 | 6757 | 0 | L1PB4 | L1 | LINE |
| 86886479 | 86887517 | + | 26.4 | 1215 | 2260 | -4489 | L1MB4 | L1 | LINE |
| 86887526 | 86888431 | - | 5.1 | 2448 | 3352 | -2794 | L1P1 | L1 | LINE |
| 86888429 | 86888525 | + | 2.1 | 3328 | 3424 | -2722 | L1P3 | L1 | LINE |
| 86888559 | 86890944 | + | 4.1 | 3755 | 6155 | 0 | L1PA4 | L1 | LINE |
| 86890949 | 86892064 | + | 25.4 | 2371 | 3702 | -3039 | L1MB4 | L1 | LINE |
| 86892103 | 86892569 | + | 19.1 | 5837 | 6310 | -2 | L1MA9 | L1 | LINE |
| 86892579 | 86892667 | + | 26.4 | 4614 | 4718 | -2029 | L1MB4 | L1 | LINE |
| 86892668 | 86892978 | + | 11.2 | 1 | 310 | 0 | AluSq | Alu | SINE |
| 86892979 | 86893052 | + | 26.4 | 4719 | 4794 | -1953 | L1MB4 | L1 | LINE |
| 86893269 | 86894229 | + | 19.1 | 5777 | 6781 | 0 | L1MB4 | L1 | LINE |

  

|  |  |  |  |  |  |  |  |  |  |
| --- | --- | --- | --- | --- | --- | --- | --- | --- | --- |
| chr13:87861520-87867276 | | | 5434 bp | | |  |  |  |  |
| Genome Start | Genome End | Strand | Div | Repeat Start | Repeat End | Repeat Left | Name | Family | Class |
| 87861520 | 87861861 | - | 24.4 | 5743 | 6096 | -4 | L1ME1 | L1 | LINE |
| 87861863 | 87862596 | - | 29.3 | 5536 | 6294 | -65 | L1MD2 | L1 | LINE |
| 87862633 | 87862782 | - | 30.1 | 5020 | 5171 | -909 | L1ME1 | L1 | LINE |
| 87862974 | 87863338 | - | 13.4 | 5306 | 5675 | -493 | L1PA13 | L1 | LINE |
| 87863342 | 87863819 | + | 9.5 | 5673 | 6162 | -1 | L1PA13 | L1 | LINE |
| 87863823 | 87863912 | - | 29.1 | 4694 | 4794 | -1286 | L1ME1 | L1 | LINE |
| 87863913 | 87864209 | - | 12.1 | 1 | 299 | -10 | AluSc | Alu | SINE |
| 87864210 | 87864484 | - | 29.1 | 4386 | 4693 | -1387 | L1ME1 | L1 | LINE |
| 87864525 | 87864852 | - | 27.9 | 3608 | 3949 | -2131 | L1ME1 | L1 | LINE |
| 87864902 | 87867276 | - | 29.9 | 333 | 2841 | -3239 | L1ME1 | L1 | LINE |

  

|  |  |  |  |  |  |  |  |  |  |
| --- | --- | --- | --- | --- | --- | --- | --- | --- | --- |
| chr13:88639148-88644651 | | | 5450 bp | | |  |  |  |  |
| Genome Start | Genome End | Strand | Div | Repeat Start | Repeat End | Repeat Left | Name | Family | Class |
| 88639148 | 88639269 | - | 27.3 | 3555 | 3679 | -2540 | L1MEc | L1 | LINE |
| 88639274 | 88639738 | - | 15.7 | 4296 | 4765 | -1378 | L1M2 | L1 | LINE |
| 88639735 | 88640396 | + | 17.2 | 4765 | 5436 | -864 | L1M2 | L1 | LINE |
| 88640416 | 88642140 | + | 18.5 | 4516 | 6289 | -11 | L1MA5 | L1 | LINE |
| 88642176 | 88642534 | - | 28.5 | 3157 | 3541 | -2678 | L1MEc | L1 | LINE |
| 88642535 | 88642814 | - | 15.1 | 1 | 288 | -24 | AluJb | Alu | SINE |
| 88642815 | 88644121 | - | 29.0 | 1913 | 3156 | -3063 | L1MEc | L1 | LINE |
| 88644122 | 88644426 | - | 12.4 | 1 | 305 | 0 | AluY | Alu | SINE |
| 88644427 | 88644651 | - | 30.5 | 1693 | 1912 | -4488 | L1MEc | L1 | LINE |

  

|  |  |  |  |  |  |  |  |  |  |
| --- | --- | --- | --- | --- | --- | --- | --- | --- | --- |
| chr13:88907827-88913447 | | | 5621 bp | | |  |  |  |  |
| Genome Start | Genome End | Strand | Div | Repeat Start | Repeat End | Repeat Left | Name | Family | Class |
| 88907827 | 88909109 | - | 27.2 | 4295 | 5653 | -14 | ERVL-F | ERVL | LTR |
| 88909110 | 88909929 | - | 28.2 | 1906 | 2735 | -2932 | ERVL-F | ERVL | LTR |
| 88909930 | 88910307 | - | 9.4 | 4390 | 4766 | -1380 | L1PREC2 | L1 | LINE |
| 88910302 | 88911690 | + | 10.6 | 4752 | 6168 | 0 | L1PA12 | L1 | LINE |
| 88911697 | 88913447 | - | 28.8 | 5 | 1900 | -3767 | ERVL-F | ERVL | LTR |

  

|  |  |  |  |  |  |  |  |  |  |
| --- | --- | --- | --- | --- | --- | --- | --- | --- | --- |
| chr13:91333727-91335075 | | | 1342 bp | | |  |  |  |  |
| Genome Start | Genome End | Strand | Div | Repeat Start | Repeat End | Repeat Left | Name | Family | Class |
| 91333727 | 91334028 | - | 28.0 | 2841 | 3142 | -277 | L2 | L2 | LINE |
| 91334035 | 91334208 | - | 13.3 | 5991 | 6163 | 0 | L1PA13 | L1 | LINE |
| 91334209 | 91334830 | + | 10.0 | 5347 | 5974 | -194 | L1PA13 | L1 | LINE |
| 91334832 | 91335075 | - | 27.5 | 2607 | 2851 | -568 | L2 | L2 | LINE |

  

|  |  |  |  |  |  |  |  |  |  |
| --- | --- | --- | --- | --- | --- | --- | --- | --- | --- |
| chr13:95356644-95364548 | | | 7843 bp | | |  |  |  |  |
| Genome Start | Genome End | Strand | Div | Repeat Start | Repeat End | Repeat Left | Name | Family | Class |
| 95356644 | 95357053 | + | 26.1 | 1140 | 1568 | -4816 | L1M4b | L1 | LINE |
| 95357065 | 95358745 | + | 7.9 | 4 | 1744 | -531 | L1PA6 | L1 | LINE |
| 95358739 | 95359479 | - | 1.6 | 4877 | 5618 | -537 | L1PA2 | L1 | LINE |
| 95359480 | 95360002 | + | 2.5 | 5615 | 6137 | -18 | L1PA2 | L1 | LINE |
| 95360062 | 95364513 | + | 6.7 | 1719 | 6153 | -1 | L1PA6 | L1 | LINE |
| 95364513 | 95364548 | + | 13.9 | 1563 | 1598 | -5112 | L1M4b | L1 | LINE |

  

|  |  |  |  |  |  |  |  |  |  |
| --- | --- | --- | --- | --- | --- | --- | --- | --- | --- |
| chr13:104370014-104376993 | | | 6948 bp | | |  |  |  |  |
| Genome Start | Genome End | Strand | Div | Repeat Start | Repeat End | Repeat Left | Name | Family | Class |
| 104370014 | 104372144 | + | 10.4 | 181 | 2364 | -4249 | L1PB1 | L1 | LINE |
| 104372141 | 104374912 | + | 8.9 | 2592 | 5352 | -1260 | L1PB1 | L1 | LINE |
| 104374934 | 104375124 | - | 4.2 | 5962 | 6152 | -3 | L1PA4 | L1 | LINE |
| 104375125 | 104375758 | + | 2.8 | 5335 | 5968 | -187 | L1PA4 | L1 | LINE |
| 104375750 | 104376619 | + | 9.8 | 5353 | 6246 | -371 | L1PB1 | L1 | LINE |
| 104376644 | 104376993 | + | 10.6 | 6247 | 6609 | -8 | L1PB1 | L1 | LINE |

  

|  |  |  |  |  |  |  |  |  |  |
| --- | --- | --- | --- | --- | --- | --- | --- | --- | --- |
| chr13:105160745-105168356 | | | 7224 bp | | |  |  |  |  |
| Genome Start | Genome End | Strand | Div | Repeat Start | Repeat End | Repeat Left | Name | Family | Class |
| 105160745 | 105162323 | - | 27.9 | 2549 | 4266 | -1953 | L1MEc | L1 | LINE |
| 105162327 | 105164445 | - | 22.8 | 4034 | 6234 | -1 | L1MB2 | L1 | LINE |
| 105164621 | 105164706 | - | 20.7 | 915 | 1002 | -5139 | L1MB2 | L1 | LINE |
| 105164786 | 105165186 | - | 34.0 | 241 | 624 | -5507 | L1MB2 | L1 | LINE |
| 105165197 | 105165405 | - | 22.2 | 2270 | 2481 | -3665 | L1MC | L1 | LINE |
| 105165439 | 105165486 | - | 16.7 | 5814 | 5861 | -436 | L1MA4 | L1 | LINE |
| 105165492 | 105165922 | + | 16.5 | 5857 | 6295 | -5 | L1MA4 | L1 | LINE |
| 105165923 | 105166148 | - | 28.7 | 2193 | 2424 | -3976 | L1MEc | L1 | LINE |
| 105166223 | 105166413 | - | 22.1 | 2003 | 2203 | -4197 | L1MEc | L1 | LINE |
| 105166423 | 105167553 | - | 24.8 | 636 | 1868 | -4532 | L1MEc | L1 | LINE |
| 105167554 | 105167852 | - | 9.7 | 1 | 299 | -14 | AluSq | Alu | SINE |
| 105167853 | 105168356 | - | 24.8 | 88 | 635 | -5765 | L1MEc | L1 | LINE |

  

|  |  |  |  |  |  |  |  |  |  |
| --- | --- | --- | --- | --- | --- | --- | --- | --- | --- |
| chr13:107029234-107031029 | | | 1446 bp | | |  |  |  |  |
| Genome Start | Genome End | Strand | Div | Repeat Start | Repeat End | Repeat Left | Name | Family | Class |
| 107029234 | 107029415 | + | 31.3 | 2542 | 2758 | -661 | L2 | L2 | LINE |
| 107029452 | 107029739 | - | 13.2 | 1 | 289 | -23 | AluSx | Alu | SINE |
| 107030025 | 107030178 | - | 2.6 | 5999 | 6152 | -3 | L1PA2 | L1 | LINE |
| 107030181 | 107030641 | + | 1.7 | 5527 | 5987 | -168 | L1PA2 | L1 | LINE |
| 107030669 | 107031029 | + | 36.5 | 2989 | 3370 | -49 | L2 | L2 | LINE |

  

|  |  |  |  |  |  |  |  |  |  |
| --- | --- | --- | --- | --- | --- | --- | --- | --- | --- |
| chr13:108651754-108653873 | | | 2021 bp | | |  |  |  |  |
| Genome Start | Genome End | Strand | Div | Repeat Start | Repeat End | Repeat Left | Name | Family | Class |
| 108651754 | 108651952 | - | 22.8 | 2313 | 2667 | -4520 | L1M4b | L1 | LINE |
| 108651954 | 108652317 | - | 9.6 | 5782 | 6151 | 0 | L1PB1 | L1 | LINE |
| 108652422 | 108652888 | - | 9.8 | 5320 | 5786 | -365 | L1PB1 | L1 | LINE |
| 108652884 | 108653723 | + | 8.5 | 4469 | 5317 | -829 | L1PB1 | L1 | LINE |
| 108653723 | 108653873 | - | 23.6 | 2135 | 2436 | -4590 | L1M4b | L1 | LINE |

  

|  |  |  |  |  |  |  |  |  |  |
| --- | --- | --- | --- | --- | --- | --- | --- | --- | --- |
| chr13:109489315-109492854 | | | 3566 bp | | |  |  |  |  |
| Genome Start | Genome End | Strand | Div | Repeat Start | Repeat End | Repeat Left | Name | Family | Class |
| 109489315 | 109489863 | + | 19.0 | 3826 | 4423 | -1717 | L1M2 | L1 | LINE |
| 109489859 | 109490144 | - | 7.7 | 3467 | 3752 | -2394 | L1PA5 | L1 | LINE |
| 109490131 | 109492516 | + | 4.5 | 3761 | 6150 | -4 | L1PA5 | L1 | LINE |
| 109492510 | 109492854 | + | 18.1 | 4353 | 4708 | -1435 | L1M2 | L1 | LINE |

  


|  |  |  |  |  |  |  |  |  |  |
| --- | --- | --- | --- | --- | --- | --- | --- | --- | --- |
| chr14:19257980-19260476 | | | 2506 bp | | |  |  |  |  |
| Genome Start | Genome End | Strand | Div | Repeat Start | Repeat End | Repeat Left | Name | Family | Class |
| 19257980 | 19258015 | - | 8.3 | 6044 | 6079 | -233 | L1MA9 | L1 | LINE |
| 19258016 | 19259719 | - | 0.7 | 4458 | 6178 | 0 | L1HS | L1 | LINE |
| 19259718 | 19260420 | + | 0.8 | 3761 | 4463 | -1683 | L1HS | L1 | LINE |
| 19260414 | 19260476 | - | 12.7 | 5996 | 6059 | -253 | L1MA9 | L1 | LINE |

  

|  |  |  |  |  |  |  |  |  |  |
| --- | --- | --- | --- | --- | --- | --- | --- | --- | --- |
| chr14:19500227-19505306 | | | 5053 bp | | |  |  |  |  |
| Genome Start | Genome End | Strand | Div | Repeat Start | Repeat End | Repeat Left | Name | Family | Class |
| 19500227 | 19500388 | + | 24.1 | 2 | 165 | -6377 | L1MB7 | L1 | LINE |
| 19500403 | 19500513 | + | 25.7 | 1984 | 2095 | -4551 | L1MB7 | L1 | LINE |
| 19500520 | 19500751 | - | 5.2 | 4331 | 4562 | -1584 | L1PA3 | L1 | LINE |
| 19500749 | 19502324 | + | 3.1 | 4579 | 6155 | 0 | L1PA3 | L1 | LINE |
| 19502340 | 19503006 | + | 23.3 | 2193 | 2876 | -3768 | L1MB7 | L1 | LINE |
| 19503007 | 19503277 | - | 4.1 | 5878 | 6148 | -7 | L1PA4 | L1 | LINE |
| 19503278 | 19503472 | + | 21.2 | 2877 | 3050 | -3594 | L1MB7 | L1 | LINE |
| 19503473 | 19503804 | - | 1.8 | 5820 | 6155 | 0 | L1PA3 | L1 | LINE |
| 19503805 | 19504215 | + | 21.2 | 3051 | 3465 | -3179 | L1MB7 | L1 | LINE |
| 19504211 | 19505306 | + | 20.4 | 5543 | 6656 | -26 | L1MB7 | L1 | LINE |

  

|  |  |  |  |  |  |  |  |  |  |
| --- | --- | --- | --- | --- | --- | --- | --- | --- | --- |
| chr14:19619267-19623992 | | | 4656 bp | | |  |  |  |  |
| Genome Start | Genome End | Strand | Div | Repeat Start | Repeat End | Repeat Left | Name | Family | Class |
| 19619267 | 19619559 | + | 18.6 | 3679 | 3958 | -2182 | L1MA8 | L1 | LINE |
| 19619560 | 19619755 | + | 18.9 | 6084 | 6289 | -2 | L1MA7 | L1 | LINE |
| 19619756 | 19620054 | + | 18.6 | 3959 | 4231 | -1909 | L1MA8 | L1 | LINE |
| 19620124 | 19620390 | + | 18.6 | 4232 | 4452 | -1688 | L1MA8 | L1 | LINE |
| 19620393 | 19620943 | - | 3.6 | 5584 | 6151 | -4 | L1PA3 | L1 | LINE |
| 19620944 | 19621192 | + | 2.4 | 5329 | 5577 | -578 | L1PA3 | L1 | LINE |
| 19621192 | 19622585 | + | 18.2 | 4448 | 5859 | -432 | L1MA8 | L1 | LINE |
| 19622586 | 19623555 | + | 7.2 | 1 | 968 | 0 | LTR5\_Hs | ERVK | LTR |
| 19623556 | 19623992 | + | 16.6 | 5860 | 6286 | -5 | L1MA8 | L1 | LINE |

  

|  |  |  |  |  |  |  |  |  |  |
| --- | --- | --- | --- | --- | --- | --- | --- | --- | --- |
| chr14:19697879-19699837 | | | 1882 bp | | |  |  |  |  |
| Genome Start | Genome End | Strand | Div | Repeat Start | Repeat End | Repeat Left | Name | Family | Class |
| 19697879 | 19698529 | - | 19.1 | 5278 | 5769 | -575 | L1MC | L1 | LINE |
| 19698541 | 19698776 | - | 10.6 | 4996 | 5235 | -911 | L1PREC2 | L1 | LINE |
| 19698768 | 19699630 | + | 11.8 | 5255 | 6152 | -11 | L1PA13 | L1 | LINE |
| 19699706 | 19699837 | - | 13.6 | 4258 | 4389 | -1757 | L1MC | L1 | LINE |

  

|  |  |  |  |  |  |  |  |  |  |
| --- | --- | --- | --- | --- | --- | --- | --- | --- | --- |
| chr14:21671632-21674239 | | | 2580 bp | | |  |  |  |  |
| Genome Start | Genome End | Strand | Div | Repeat Start | Repeat End | Repeat Left | Name | Family | Class |
| 21671632 | 21671907 | + | 26.0 | 1852 | 2125 | -4508 | L1MDa | L1 | LINE |
| 21671913 | 21672527 | - | 14.4 | 5714 | 6324 | -10 | L1MA10 | L1 | LINE |
| 21672530 | 21673268 | + | 16.2 | 4928 | 5721 | -447 | L1MA10 | L1 | LINE |
| 21673271 | 21673570 | + | 28.4 | 2117 | 2412 | -4221 | L1MDa | L1 | LINE |
| 21673582 | 21674153 | + | 21.6 | 2506 | 3065 | -3472 | L1MDa | L1 | LINE |
| 21674162 | 21674239 | + | 21.8 | 3373 | 3450 | -3087 | L1MDa | L1 | LINE |

  

|  |  |  |  |  |  |  |  |  |  |
| --- | --- | --- | --- | --- | --- | --- | --- | --- | --- |
| chr14:24183830-24192393 | | | 8575 bp | | |  |  |  |  |
| Genome Start | Genome End | Strand | Div | Repeat Start | Repeat End | Repeat Left | Name | Family | Class |
| 24183830 | 24184193 | + | 12.7 | 1 | 382 | -5822 | L1PB3 | L1 | LINE |
| 24184198 | 24185107 | + | 10.9 | 5254 | 6168 | 0 | L1PA12 | L1 | LINE |
| 24185113 | 24185890 | + | 14.2 | 363 | 1122 | -5082 | L1PB3 | L1 | LINE |
| 24185891 | 24186125 | + | 13.9 | 84 | 292 | -20 | AluSg/x | Alu | SINE |
| 24186127 | 24187064 | + | 19.1 | 1110 | 2072 | -4132 | L1PB3 | L1 | LINE |
| 24187064 | 24187365 | - | 8.6 | 4901 | 5202 | -944 | L1PA7 | L1 | LINE |
| 24187361 | 24188327 | + | 6.3 | 5181 | 6154 | 0 | L1PA7 | L1 | LINE |
| 24188313 | 24192393 | + | 15.2 | 2097 | 6211 | 0 | L1PB3 | L1 | LINE |

  

|  |  |  |  |  |  |  |  |  |  |
| --- | --- | --- | --- | --- | --- | --- | --- | --- | --- |
| chr14:26488226-26490230 | | | 1999 bp | | |  |  |  |  |
| Genome Start | Genome End | Strand | Div | Repeat Start | Repeat End | Repeat Left | Name | Family | Class |
| 26488226 | 26488480 | + | 27.7 | 5302 | 5583 | -2225 | L1MC | L1 | LINE |
| 26488497 | 26489538 | - | 3.3 | 5110 | 6155 | 0 | L1PA3 | L1 | LINE |
| 26489533 | 26489955 | + | 2.1 | 4674 | 5103 | -1043 | L1PA3 | L1 | LINE |
| 26489952 | 26490090 | + | 26.6 | 5579 | 5716 | -2092 | L1MC | L1 | LINE |
| 26490091 | 26490169 | - | 22.8 | 2 | 80 | 0 | MADE2 | Mariner | DNA |
| 26490170 | 26490230 | + | 26.6 | 5717 | 5785 | -2023 | L1MC | L1 | LINE |

  

|  |  |  |  |  |  |  |  |  |  |
| --- | --- | --- | --- | --- | --- | --- | --- | --- | --- |
| chr14:26997194-27003204 | | | 5867 bp | | |  |  |  |  |
| Genome Start | Genome End | Strand | Div | Repeat Start | Repeat End | Repeat Left | Name | Family | Class |
| 26997194 | 26997375 | + | 23.2 | 4718 | 4898 | -1248 | L1ME1 | L1 | LINE |
| 26997405 | 26997730 | + | 27.6 | 267 | 598 | -9 | MER77 | ERVL | LTR |
| 26997739 | 26998010 | + | 29.2 | 4894 | 5170 | -976 | L1ME1 | L1 | LINE |
| 26998005 | 27000575 | - | 1.6 | 3586 | 6155 | 0 | L1PA2 | L1 | LINE |
| 27000563 | 27001545 | + | 1.7 | 2607 | 3590 | -2556 | L1PA2 | L1 | LINE |
| 27001650 | 27001980 | + | 23.1 | 18 | 354 | -20 | MLT1A | MaLR | LTR |
| 27002003 | 27002792 | + | 29.5 | 5244 | 6071 | -95 | L1ME1 | L1 | LINE |
| 27002793 | 27003093 | - | 24.9 | 2 | 345 | 0 | MER2 | MER2\_type | DNA |
| 27003094 | 27003204 | + | 30.1 | 6072 | 6159 | -7 | L1ME1 | L1 | LINE |

  

|  |  |  |  |  |  |  |  |  |  |
| --- | --- | --- | --- | --- | --- | --- | --- | --- | --- |
| chr14:27186869-27190708 | | | 3602 bp | | |  |  |  |  |
| Genome Start | Genome End | Strand | Div | Repeat Start | Repeat End | Repeat Left | Name | Family | Class |
| 27186869 | 27187539 | + | 25.8 | 2671 | 3342 | -2804 | L1M5 | L1 | LINE |
| 27187648 | 27187732 | - | 17.9 | 613 | 691 | 0 | MER76 | ERVL | LTR |
| 27187787 | 27188142 | - | 9.0 | 4771 | 5126 | -1020 | L1PA8 | L1 | LINE |
| 27188141 | 27189193 | + | 7.4 | 5120 | 6172 | 0 | L1PA8 | L1 | LINE |
| 27189266 | 27189882 | - | 27.4 | 6 | 629 | -62 | MER76 | ERVL | LTR |
| 27189889 | 27190708 | + | 29.3 | 3354 | 4207 | -1939 | L1M5 | L1 | LINE |

  

|  |  |  |  |  |  |  |  |  |  |
| --- | --- | --- | --- | --- | --- | --- | --- | --- | --- |
| chr14:27503171-27510539 | | | 7377 bp | | |  |  |  |  |
| Genome Start | Genome End | Strand | Div | Repeat Start | Repeat End | Repeat Left | Name | Family | Class |
| 27503171 | 27505062 | - | 13.9 | 4408 | 6305 | 0 | L1MA3 | L1 | LINE |
| 27505064 | 27507683 | - | 8.2 | 3547 | 6154 | 0 | L1PA7 | L1 | LINE |
| 27507680 | 27509803 | + | 9.4 | 1450 | 3560 | -2586 | L1PA7 | L1 | LINE |
| 27509799 | 27510539 | - | 14.7 | 3673 | 4415 | -1731 | L1MA3 | L1 | LINE |

  

|  |  |  |  |  |  |  |  |  |  |
| --- | --- | --- | --- | --- | --- | --- | --- | --- | --- |
| chr14:27716897-27719428 | | | 2494 bp | | |  |  |  |  |
| Genome Start | Genome End | Strand | Div | Repeat Start | Repeat End | Repeat Left | Name | Family | Class |
| 27716897 | 27716995 | - | 31.3 | 3302 | 3415 | -4 | L2 | L2 | LINE |
| 27716997 | 27718496 | - | 1.7 | 4228 | 5727 | -428 | L1PA2 | L1 | LINE |
| 27718499 | 27718917 | + | 1.2 | 5737 | 6155 | 0 | L1PA2 | L1 | LINE |
| 27718953 | 27719428 | - | 34.6 | 2713 | 3191 | -228 | L2 | L2 | LINE |

  

|  |  |  |  |  |  |  |  |  |  |
| --- | --- | --- | --- | --- | --- | --- | --- | --- | --- |
| chr14:27950136-27959424 | | | 9289 bp | | |  |  |  |  |
| Genome Start | Genome End | Strand | Div | Repeat Start | Repeat End | Repeat Left | Name | Family | Class |
| 27950136 | 27951544 | + | 10.6 | 1 | 1440 | -5762 | HERVK3 | ERVK | LTR |
| 27951545 | 27951815 | + | 12.2 | 1 | 272 | -730 | LTR5B | ERVK | LTR |
| 27951833 | 27953158 | - | 2.4 | 4828 | 6155 | 0 | L1PA3 | L1 | LINE |
| 27953157 | 27954683 | + | 1.9 | 3293 | 4820 | -1326 | L1PA3 | L1 | LINE |
| 27954684 | 27955416 | + | 15.0 | 260 | 1002 | 0 | LTR5B | ERVK | LTR |
| 27955418 | 27956571 | + | 10.2 | 1437 | 2635 | -4567 | HERVK3 | ERVK | LTR |
| 27956556 | 27956826 | + | 16.9 | 3495 | 3761 | -3441 | HERVK3 | ERVK | LTR |
| 27956826 | 27958733 | + | 12.6 | 4470 | 6381 | -821 | HERVK3 | ERVK | LTR |
| 27958735 | 27959424 | + | 10.0 | 6512 | 7200 | -2 | HERVK3 | ERVK | LTR |

  

|  |  |  |  |  |  |  |  |  |  |
| --- | --- | --- | --- | --- | --- | --- | --- | --- | --- |
| chr14:28540574-28544638 | | | 4034 bp | | |  |  |  |  |
| Genome Start | Genome End | Strand | Div | Repeat Start | Repeat End | Repeat Left | Name | Family | Class |
| 28540574 | 28540777 | + | 29.7 | 5301 | 5511 | -729 | L1M5 | L1 | LINE |
| 28540791 | 28542205 | - | 3.0 | 2555 | 3970 | -2176 | L1PA3 | L1 | LINE |
| 28542206 | 28544386 | + | 3.6 | 3973 | 6152 | -3 | L1PA3 | L1 | LINE |
| 28544405 | 28544638 | + | 25.9 | 5511 | 5771 | -350 | L1M5 | L1 | LINE |

  

|  |  |  |  |  |  |  |  |  |  |
| --- | --- | --- | --- | --- | --- | --- | --- | --- | --- |
| chr14:28870156-28874772 | | | 4590 bp | | |  |  |  |  |
| Genome Start | Genome End | Strand | Div | Repeat Start | Repeat End | Repeat Left | Name | Family | Class |
| 28870156 | 28870780 | + | 28.7 | 3262 | 4007 | -2139 | L1ME3B | L1 | LINE |
| 28870781 | 28871258 | + | 18.0 | 1 | 428 | 0 | MSTC | MaLR | LTR |
| 28871259 | 28872347 | + | 28.7 | 4008 | 5155 | -991 | L1ME3B | L1 | LINE |
| 28872355 | 28872424 | - | 5.7 | 4374 | 4443 | -1703 | L1PA4 | L1 | LINE |
| 28872421 | 28874116 | + | 3.9 | 4450 | 6155 | 0 | L1PA4 | L1 | LINE |
| 28874122 | 28874232 | + | 29.7 | 5161 | 5272 | -874 | L1ME3B | L1 | LINE |
| 28874252 | 28874772 | + | 30.8 | 5299 | 5866 | -374 | L1ME3B | L1 | LINE |

  

|  |  |  |  |  |  |  |  |  |  |
| --- | --- | --- | --- | --- | --- | --- | --- | --- | --- |
| chr14:29581437-29584762 | | | 3308 bp | | |  |  |  |  |
| Genome Start | Genome End | Strand | Div | Repeat Start | Repeat End | Repeat Left | Name | Family | Class |
| 29581437 | 29581884 | + | 14.5 | 1 | 359 | -5648 | MER41A-int | ERV1 | LTR |
| 29581912 | 29582869 | - | 9.6 | 5178 | 6143 | -8 | L1PB1 | L1 | LINE |
| 29582871 | 29584363 | + | 7.4 | 3659 | 5156 | -990 | L1PB1 | L1 | LINE |
| 29584364 | 29584641 | + | 12.7 | 344 | 625 | -5321 | MER41A-int | ERV1 | LTR |
| 29584632 | 29584762 | + | 8.4 | 1702 | 1836 | -5701 | MER41A-int | ERV1 | LTR |

  

|  |  |  |  |  |  |  |  |  |  |
| --- | --- | --- | --- | --- | --- | --- | --- | --- | --- |
| chr14:31669808-31673218 | | | 3293 bp | | |  |  |  |  |
| Genome Start | Genome End | Strand | Div | Repeat Start | Repeat End | Repeat Left | Name | Family | Class |
| 31669808 | 31670212 | - | 28.6 | 7623 | 8036 | -6 | L1MC4 | L1 | LINE |
| 31670301 | 31670509 | - | 19.2 | 5959 | 6169 | -2 | L1MB2 | L1 | LINE |
| 31670542 | 31672885 | - | 3.9 | 3807 | 6154 | -1 | L1PA4 | L1 | LINE |
| 31672886 | 31673096 | + | 4.7 | 3596 | 3806 | -2340 | L1PA4 | L1 | LINE |
| 31673095 | 31673218 | - | 23.5 | 7518 | 7637 | -405 | L1MC4 | L1 | LINE |

  

|  |  |  |  |  |  |  |  |  |  |
| --- | --- | --- | --- | --- | --- | --- | --- | --- | --- |
| chr14:36146710-36152433 | | | 5377 bp | | |  |  |  |  |
| Genome Start | Genome End | Strand | Div | Repeat Start | Repeat End | Repeat Left | Name | Family | Class |
| 36146710 | 36147030 | - | 33.2 | 2566 | 2880 | -1609 | L3 | CR1 | LINE |
| 36147140 | 36147858 | - | 2.5 | 5437 | 6155 | 0 | L1PA3 | L1 | LINE |
| 36147859 | 36147974 | + | 4.3 | 5316 | 5431 | -724 | L1PA3 | L1 | LINE |
| 36148061 | 36148563 | + | 21.4 | 1 | 583 | 0 | MER119 | MER1\_type | DNA |
| 36148626 | 36149149 | + | 16.6 | 4897 | 5462 | -712 | L1MB5 | L1 | LINE |
| 36149152 | 36149362 | + | 11.6 | 89 | 305 | -6 | AluY | Alu | SINE |
| 36149381 | 36151120 | + | 6.5 | 4389 | 6163 | -5 | L1PA10 | L1 | LINE |
| 36151123 | 36151708 | + | 16.4 | 5486 | 6075 | -99 | L1MB5 | L1 | LINE |
| 36151709 | 36152155 | - | 16.4 | 1 | 457 | 0 | MSTB2 | MaLR | LTR |
| 36152156 | 36152256 | + | 16.4 | 6076 | 6167 | -7 | L1MB5 | L1 | LINE |
| 36152325 | 36152433 | - | 30.5 | 2183 | 2290 | -2199 | L3 | CR1 | LINE |

  

|  |  |  |  |  |  |  |  |  |  |
| --- | --- | --- | --- | --- | --- | --- | --- | --- | --- |
| chr14:37595140-37598646 | | | 3462 bp | | |  |  |  |  |
| Genome Start | Genome End | Strand | Div | Repeat Start | Repeat End | Repeat Left | Name | Family | Class |
| 37595140 | 37595519 | + | 26.8 | 1 | 354 | -239 | MLT1E1A | MaLR | LTR |
| 37595520 | 37595767 | + | 14.6 | 1 | 255 | -436 | LTR8 | ERV1 | LTR |
| 37595768 | 37596329 | - | 3.0 | 3970 | 4531 | -1615 | L1PA3 | L1 | LINE |
| 37596321 | 37597937 | + | 2.3 | 4537 | 6154 | -1 | L1PA3 | L1 | LINE |
| 37597979 | 37598435 | + | 13.1 | 240 | 691 | 0 | LTR8 | ERV1 | LTR |
| 37598449 | 37598646 | + | 17.9 | 484 | 691 | -7 | MLT1E1A | MaLR | LTR |

  

|  |  |  |  |  |  |  |  |  |  |
| --- | --- | --- | --- | --- | --- | --- | --- | --- | --- |
| chr14:37759320-37763736 | | | 4412 bp | | |  |  |  |  |
| Genome Start | Genome End | Strand | Div | Repeat Start | Repeat End | Repeat Left | Name | Family | Class |
| 37759320 | 37759488 | - | 17.5 | 7367 | 7534 | -3 | MER57A-int | ERV1 | LTR |
| 37759481 | 37760292 | - | 12.5 | 5005 | 5807 | -380 | MER57A-int | ERV1 | LTR |
| 37760289 | 37760803 | - | 12.1 | 1275 | 1784 | -4403 | MER57A-int | ERV1 | LTR |
| 37760810 | 37761098 | + | 3.5 | 4889 | 5177 | -969 | L1P1 | L1 | LINE |
| 37761100 | 37761169 | - | 25.7 | 285 | 356 | -229 | MER34C\_ | ERV1 | LTR |
| 37761170 | 37761410 | - | 14.0 | 968 | 1189 | -4998 | MER57A-int | ERV1 | LTR |
| 37761411 | 37761718 | - | 10.3 | 1 | 291 | -19 | AluSg | Alu | SINE |
| 37761719 | 37761800 | - | 14.0 | 892 | 967 | -5220 | MER57A-int | ERV1 | LTR |
| 37761814 | 37761864 | - | 15.7 | 295 | 347 | -265 | LTR9 | ERV1 | LTR |
| 37761865 | 37762521 | - | 6.5 | 5495 | 6154 | 0 | L1PA7 | L1 | LINE |
| 37762519 | 37762577 | + | 10.2 | 5425 | 5483 | -671 | L1PA7 | L1 | LINE |
| 37762578 | 37762983 | - | 10.0 | 513 | 899 | -5288 | MER57A-int | ERV1 | LTR |
| 37762984 | 37763280 | + | 3.7 | 14 | 310 | -1 | AluY | Alu | SINE |
| 37763281 | 37763736 | - | 13.7 | 56 | 512 | -5675 | MER57A-int | ERV1 | LTR |

  

|  |  |  |  |  |  |  |  |  |  |
| --- | --- | --- | --- | --- | --- | --- | --- | --- | --- |
| chr14:38096516-38097862 | | | 1299 bp | | |  |  |  |  |
| Genome Start | Genome End | Strand | Div | Repeat Start | Repeat End | Repeat Left | Name | Family | Class |
| 38096516 | 38096554 | - | 12.8 | 388 | 426 | -2 | MSTA | MaLR | LTR |
| 38096555 | 38096792 | - | 5.9 | 5263 | 5510 | -644 | L1PA6 | L1 | LINE |
| 38096793 | 38097421 | + | 6.5 | 5506 | 6135 | -19 | L1PA6 | L1 | LINE |
| 38097470 | 38097862 | - | 13.5 | 1 | 398 | -30 | MSTA | MaLR | LTR |

  

|  |  |  |  |  |  |  |  |  |  |
| --- | --- | --- | --- | --- | --- | --- | --- | --- | --- |
| chr14:39019799-39022840 | | | 2609 bp | | |  |  |  |  |
| Genome Start | Genome End | Strand | Div | Repeat Start | Repeat End | Repeat Left | Name | Family | Class |
| 39019799 | 39019914 | + | 30.4 | 1405 | 1521 | -4694 | L1MC4 | L1 | LINE |
| 39020184 | 39021570 | - | 3.8 | 4767 | 6155 | 0 | L1PA4 | L1 | LINE |
| 39021569 | 39022094 | + | 5.9 | 3207 | 3733 | -2413 | L1P1 | L1 | LINE |
| 39022079 | 39022539 | + | 3.0 | 4303 | 4765 | -1381 | L1P1 | L1 | LINE |
| 39022722 | 39022840 | + | 17.2 | 6820 | 6946 | -1096 | L1MC4 | L1 | LINE |

  

|  |  |  |  |  |  |  |  |  |  |
| --- | --- | --- | --- | --- | --- | --- | --- | --- | --- |
| chr14:39097452-39100534 | | | 2627 bp | | |  |  |  |  |
| Genome Start | Genome End | Strand | Div | Repeat Start | Repeat End | Repeat Left | Name | Family | Class |
| 39097452 | 39098570 | - | 18.7 | 1205 | 2424 | -4910 | L1M4b | L1 | LINE |
| 39098989 | 39099272 | - | 24.6 | 710 | 1210 | -6124 | L1M4b | L1 | LINE |
| 39099313 | 39099370 | - | 3.5 | 6097 | 6154 | -1 | L1PA3 | L1 | LINE |
| 39099371 | 39099875 | + | 2.2 | 5583 | 6089 | -66 | L1PA3 | L1 | LINE |
| 39099874 | 39100534 | - | 26.1 | 44 | 726 | -6608 | L1M4b | L1 | LINE |

  

|  |  |  |  |  |  |  |  |  |  |
| --- | --- | --- | --- | --- | --- | --- | --- | --- | --- |
| chr14:39623499-39631607 | | | 7922 bp | | |  |  |  |  |
| Genome Start | Genome End | Strand | Div | Repeat Start | Repeat End | Repeat Left | Name | Family | Class |
| 39623499 | 39623920 | - | 26.7 | 2288 | 2732 | -3487 | L1MEc | L1 | LINE |
| 39623921 | 39624237 | - | 11.9 | 1 | 303 | -7 | AluSg | Alu | SINE |
| 39624238 | 39624329 | - | 26.7 | 2195 | 2287 | -3932 | L1MEc | L1 | LINE |
| 39624403 | 39624467 | - | 21.5 | 2064 | 2133 | -4267 | L1MEc | L1 | LINE |
| 39624552 | 39627411 | - | 8.6 | 3273 | 6138 | -16 | L1PA7 | L1 | LINE |
| 39627408 | 39628192 | - | 7.9 | 2126 | 2841 | -3305 | L1PA7 | L1 | LINE |
| 39628191 | 39629600 | + | 11.3 | 705 | 2144 | -131 | L1PA7 | L1 | LINE |
| 39629598 | 39629866 | - | 28.1 | 1783 | 2069 | -4331 | L1MEc | L1 | LINE |
| 39629870 | 39630043 | + | 23.3 | 1 | 175 | 0 | FRAM | Alu | SINE |
| 39630080 | 39631607 | - | 29.4 | 42 | 1750 | -4650 | L1MEc | L1 | LINE |

  

|  |  |  |  |  |  |  |  |  |  |
| --- | --- | --- | --- | --- | --- | --- | --- | --- | --- |
| chr14:40071057-40076041 | | | 4920 bp | | |  |  |  |  |
| Genome Start | Genome End | Strand | Div | Repeat Start | Repeat End | Repeat Left | Name | Family | Class |
| 40071057 | 40071113 | + | 15.8 | 980 | 1037 | -5503 | L1MDb | L1 | LINE |
| 40071114 | 40071272 | + | 22.3 | 1536 | 1707 | -4926 | L1MDb | L1 | LINE |
| 40071297 | 40071438 | + | 23.2 | 1052 | 1201 | -5339 | L1MDb | L1 | LINE |
| 40071437 | 40071911 | - | 9.9 | 5395 | 5858 | -305 | L1PA13 | L1 | LINE |
| 40071912 | 40072204 | + | 12.3 | 5863 | 6160 | -3 | L1PA13 | L1 | LINE |
| 40072248 | 40073172 | + | 24.6 | 1262 | 2205 | -4428 | L1MDb | L1 | LINE |
| 40073168 | 40074158 | + | 23.2 | 2438 | 3500 | -3037 | L1MDb | L1 | LINE |
| 40074158 | 40074866 | - | 22.8 | 2997 | 3728 | -2418 | L1P4 | L1 | LINE |
| 40074873 | 40075548 | + | 18.5 | 4121 | 4800 | -1737 | L1MDb | L1 | LINE |
| 40075549 | 40075855 | + | 11.4 | 1 | 309 | 0 | AluSc | Alu | SINE |
| 40075856 | 40076041 | + | 18.5 | 4801 | 4986 | -1551 | L1MDb | L1 | LINE |

  

|  |  |  |  |  |  |  |  |  |  |
| --- | --- | --- | --- | --- | --- | --- | --- | --- | --- |
| chr14:40078018-40086705 | | | 8646 bp | | |  |  |  |  |
| Genome Start | Genome End | Strand | Div | Repeat Start | Repeat End | Repeat Left | Name | Family | Class |
| 40078018 | 40078198 | + | 28.0 | 3068 | 3246 | -2900 | L1MD2 | L1 | LINE |
| 40078195 | 40078715 | + | 14.5 | 3993 | 4524 | -1622 | L1PA16 | L1 | LINE |
| 40078716 | 40079087 | - | 11.3 | 1 | 375 | 0 | THE1C | MaLR | LTR |
| 40079088 | 40080764 | + | 14.9 | 4525 | 6157 | -9 | L1PA16 | L1 | LINE |
| 40080790 | 40081934 | + | 25.8 | 3296 | 4503 | -1643 | L1MD2 | L1 | LINE |
| 40081936 | 40082174 | - | 14.9 | 3706 | 3934 | -2212 | L1PA16 | L1 | LINE |
| 40082174 | 40083216 | + | 16.6 | 3950 | 5313 | -855 | L1PA16 | L1 | LINE |
| 40083217 | 40083767 | + | 2.7 | 5602 | 6152 | -3 | L1PA3 | L1 | LINE |
| 40083768 | 40084599 | + | 14.8 | 5312 | 6156 | -10 | L1PA16 | L1 | LINE |
| 40084621 | 40086051 | + | 25.0 | 4496 | 5983 | -376 | L1MD2 | L1 | LINE |
| 40086052 | 40086337 | + | 11.2 | 9 | 309 | -3 | AluSx | Alu | SINE |
| 40086338 | 40086705 | + | 25.8 | 5984 | 6345 | -14 | L1MD2 | L1 | LINE |

  

|  |  |  |  |  |  |  |  |  |  |
| --- | --- | --- | --- | --- | --- | --- | --- | --- | --- |
| chr14:40265117-40271265 | | | 6176 bp | | |  |  |  |  |
| Genome Start | Genome End | Strand | Div | Repeat Start | Repeat End | Repeat Left | Name | Family | Class |
| 40265117 | 40266148 | - | 10.6 | 6220 | 7310 | -413 | L1MA2 | L1 | LINE |
| 40266118 | 40267656 | - | 13.3 | 2715 | 4249 | -3316 | L1MA2 | L1 | LINE |
| 40267659 | 40268940 | - | 0.6 | 4874 | 6155 | 0 | L1HS | L1 | LINE |
| 40268939 | 40269486 | + | 2.2 | 4330 | 4877 | -1269 | L1HS | L1 | LINE |
| 40269491 | 40269749 | - | 18.4 | 2456 | 2709 | -4856 | L1MA2 | L1 | LINE |
| 40269750 | 40269873 | - | 8.1 | 187 | 310 | -1 | AluY | Alu | SINE |
| 40269874 | 40271265 | - | 18.4 | 1088 | 2455 | -5110 | L1MA2 | L1 | LINE |

  

|  |  |  |  |  |  |  |  |  |  |
| --- | --- | --- | --- | --- | --- | --- | --- | --- | --- |
| chr14:40522064-40534677 | | | 12228 bp | | |  |  |  |  |
| Genome Start | Genome End | Strand | Div | Repeat Start | Repeat End | Repeat Left | Name | Family | Class |
| 40522064 | 40524572 | - | 12.7 | 5198 | 7723 | 0 | L1MA2 | L1 | LINE |
| 40524573 | 40524612 | - | 13.8 | 2654 | 2706 | -4 | Charlie3 | MER1\_type | DNA |
| 40524640 | 40527216 | - | 13.8 | 2 | 2653 | -57 | Charlie3 | MER1\_type | DNA |
| 40527217 | 40529684 | - | 14.5 | 2709 | 5197 | -2368 | L1MA2 | L1 | LINE |
| 40529965 | 40531048 | - | 8.5 | 5051 | 6154 | 0 | L1PA7 | L1 | LINE |
| 40531047 | 40531851 | + | 11.7 | 2592 | 3395 | -2751 | L1P3 | L1 | LINE |
| 40531845 | 40532189 | + | 10.4 | 4681 | 5026 | -1120 | L1P3 | L1 | LINE |
| 40532278 | 40532575 | - | 21.3 | 2501 | 2754 | -4811 | L1MA2 | L1 | LINE |
| 40532576 | 40532862 | + | 15.1 | 2 | 303 | -9 | AluSx | Alu | SINE |
| 40532863 | 40534677 | - | 21.5 | 529 | 2500 | -5065 | L1MA2 | L1 | LINE |

  

|  |  |  |  |  |  |  |  |  |  |
| --- | --- | --- | --- | --- | --- | --- | --- | --- | --- |
| chr14:41105257-41106146 | | | 866 bp | | |  |  |  |  |
| Genome Start | Genome End | Strand | Div | Repeat Start | Repeat End | Repeat Left | Name | Family | Class |
| 41105257 | 41105356 | - | 25.5 | 106 | 200 | -68 | MIRb | MIR | SINE |
| 41105376 | 41105468 | - | 3.0 | 5474 | 5565 | -768 | L1PA4 | L1 | LINE |
| 41105469 | 41106075 | + | 2.7 | 5548 | 6155 | 0 | L1PA4 | L1 | LINE |
| 41106081 | 41106146 | - | 27.3 | 25 | 93 | -175 | MIRb | MIR | SINE |

  

|  |  |  |  |  |  |  |  |  |  |
| --- | --- | --- | --- | --- | --- | --- | --- | --- | --- |
| chr14:41392237-41393718 | | | 1352 bp | | |  |  |  |  |
| Genome Start | Genome End | Strand | Div | Repeat Start | Repeat End | Repeat Left | Name | Family | Class |
| 41392237 | 41392791 | - | 33.6 | 2202 | 2788 | -631 | L2 | L2 | LINE |
| 41392841 | 41393011 | - | 33.7 | 94 | 259 | -3 | MIR | MIR | SINE |
| 41393093 | 41393305 | - | 5.6 | 5764 | 5982 | -172 | L1PA5 | L1 | LINE |
| 41393306 | 41393473 | + | 6.5 | 5981 | 6153 | -1 | L1PA5 | L1 | LINE |
| 41393474 | 41393718 | - | 30.4 | 1513 | 1764 | -1655 | L2 | L2 | LINE |

  

|  |  |  |  |  |  |  |  |  |  |
| --- | --- | --- | --- | --- | --- | --- | --- | --- | --- |
| chr14:41698981-41712111 | | | 13051 bp | | |  |  |  |  |
| Genome Start | Genome End | Strand | Div | Repeat Start | Repeat End | Repeat Left | Name | Family | Class |
| 41698981 | 41699421 | - | 23.8 | 5206 | 5713 | -1 | ERVL-C1 | ERVL | LTR |
| 41699422 | 41699875 | - | 27.6 | 5 | 503 | -2 | MLT1D | MaLR | LTR |
| 41699876 | 41700319 | - | 23.8 | 4776 | 5205 | -509 | ERVL-C1 | ERVL | LTR |
| 41700320 | 41700616 | + | 13.8 | 1 | 297 | -15 | AluSx | Alu | SINE |
| 41700617 | 41701724 | - | 23.8 | 3668 | 4775 | -939 | ERVL-C1 | ERVL | LTR |
| 41701725 | 41702036 | - | 9.3 | 5852 | 6162 | -1 | L1PA13 | L1 | LINE |
| 41702037 | 41702632 | + | 8.6 | 1 | 608 | 0 | LTR14B | ERVK | LTR |
| 41702633 | 41702695 | - | 9.3 | 5789 | 5851 | -312 | L1PA13 | L1 | LINE |
| 41702696 | 41702724 | - | 23.8 | 3641 | 3667 | -2047 | ERVL-C1 | ERVL | LTR |
| 41702732 | 41702959 | - | 22.1 | 6060 | 6290 | -10 | L1MA4 | L1 | LINE |
| 41702985 | 41703106 | - | 29.5 | 3526 | 3648 | -2066 | ERVL-C1 | ERVL | LTR |
| 41703105 | 41704927 | - | 25.0 | 1029 | 2906 | -2808 | ERVL-C1 | ERVL | LTR |
| 41704981 | 41709933 | - | 9.5 | 1570 | 6469 | 0 | L1PA7 | L1 | LINE |
| 41709928 | 41711109 | + | 10.8 | 375 | 1562 | -4899 | L1PA7 | L1 | LINE |
| 41711113 | 41712111 | - | 27.1 | 1 | 1039 | -4675 | ERVL-C1 | ERVL | LTR |

  

|  |  |  |  |  |  |  |  |  |  |
| --- | --- | --- | --- | --- | --- | --- | --- | --- | --- |
| chr14:42555698-42556885 | | | 1158 bp | | |  |  |  |  |
| Genome Start | Genome End | Strand | Div | Repeat Start | Repeat End | Repeat Left | Name | Family | Class |
| 42555698 | 42555900 | + | 24.7 | 1 | 187 | -439 | MLT1E2 | MaLR | LTR |
| 42555902 | 42556075 | - | 1.7 | 5564 | 5737 | -418 | L1PA4 | L1 | LINE |
| 42556076 | 42556490 | + | 3.6 | 5729 | 6144 | -11 | L1PA4 | L1 | LINE |
| 42556520 | 42556885 | + | 31.6 | 223 | 612 | -14 | MLT1E2 | MaLR | LTR |

  

|  |  |  |  |  |  |  |  |  |  |
| --- | --- | --- | --- | --- | --- | --- | --- | --- | --- |
| chr14:42879350-42886814 | | | 7432 bp | | |  |  |  |  |
| Genome Start | Genome End | Strand | Div | Repeat Start | Repeat End | Repeat Left | Name | Family | Class |
| 42879350 | 42879648 | - | 11.4 | 5857 | 6158 | -10 | L1PA10 | L1 | LINE |
| 42879650 | 42880792 | - | 16.4 | 1246 | 2396 | -4087 | L1P3 | L1 | LINE |
| 42880817 | 42881693 | - | 17.3 | 398 | 1270 | -5191 | L1P3 | L1 | LINE |
| 42881691 | 42882699 | - | 6.9 | 2878 | 3870 | -2276 | L1P2 | L1 | LINE |
| 42882700 | 42883004 | + | 1.0 | 1 | 304 | -6 | AluYa5 | Alu | SINE |
| 42883005 | 42884078 | - | 6.3 | 1798 | 2877 | -3269 | L1P2 | L1 | LINE |
| 42884075 | 42886375 | + | 5.6 | 3856 | 6154 | 0 | L1PA5 | L1 | LINE |
| 42886391 | 42886814 | - | 22.8 | 10 | 498 | -5985 | L1PA10 | L1 | LINE |

  

|  |  |  |  |  |  |  |  |  |  |
| --- | --- | --- | --- | --- | --- | --- | --- | --- | --- |
| chr14:43583456-43587911 | | | 4155 bp | | |  |  |  |  |
| Genome Start | Genome End | Strand | Div | Repeat Start | Repeat End | Repeat Left | Name | Family | Class |
| 43583456 | 43583768 | - | 28.8 | 3109 | 3417 | -2 | L2 | L2 | LINE |
| 43583806 | 43584065 | + | 32.0 | 23 | 329 | -194 | LTR33A | ERVL | LTR |
| 43584241 | 43584516 | - | 30.4 | 2806 | 3114 | -305 | L2 | L2 | LINE |
| 43584581 | 43585779 | - | 9.1 | 3106 | 4313 | -1833 | L1PA7 | L1 | LINE |
| 43585782 | 43587611 | + | 8.6 | 4316 | 6148 | -6 | L1PA7 | L1 | LINE |
| 43587634 | 43587770 | - | 35.0 | 2585 | 2734 | -685 | L2 | L2 | LINE |
| 43587772 | 43587911 | - | 30.4 | 2316 | 2454 | -965 | L2 | L2 | LINE |

  

|  |  |  |  |  |  |  |  |  |  |
| --- | --- | --- | --- | --- | --- | --- | --- | --- | --- |
| chr14:43692644-43700069 | | | 6954 bp | | |  |  |  |  |
| Genome Start | Genome End | Strand | Div | Repeat Start | Repeat End | Repeat Left | Name | Family | Class |
| 43692644 | 43692946 | + | 33.2 | 242 | 566 | -1394 | L4 | RTE | LINE |
| 43693142 | 43693406 | + | 19.8 | 4 | 274 | -6756 | L1M3e | L1 | LINE |
| 43693408 | 43695211 | + | 27.6 | 1204 | 2697 | -4409 | L1M3e | L1 | LINE |
| 43695212 | 43695513 | + | 5.0 | 1 | 305 | -6 | AluY | Alu | SINE |
| 43695514 | 43696771 | + | 23.0 | 2698 | 3987 | -3116 | L1M3e | L1 | LINE |
| 43696784 | 43697088 | + | 15.5 | 5867 | 6177 | -135 | L1MA9 | L1 | LINE |
| 43697086 | 43697394 | - | 1.3 | 3995 | 4303 | -1843 | L1PA2 | L1 | LINE |
| 43697393 | 43699364 | + | 2.9 | 4313 | 6308 | -4 | L1PA2 | L1 | LINE |
| 43699634 | 43700069 | + | 38.0 | 1108 | 1568 | -392 | L4 | RTE | LINE |

  

|  |  |  |  |  |  |  |  |  |  |
| --- | --- | --- | --- | --- | --- | --- | --- | --- | --- |
| chr14:44297530-44299204 | | | 1675 bp | | |  |  |  |  |
| Genome Start | Genome End | Strand | Div | Repeat Start | Repeat End | Repeat Left | Name | Family | Class |
| 44297530 | 44297630 | + | 23.0 | 1 | 100 | -419 | LTR40a | ERVL | LTR |
| 44297631 | 44297666 | + | 28.1 | 5598 | 5628 | -493 | L1M5 | L1 | LINE |
| 44297667 | 44297955 | - | 14.6 | 6037 | 6312 | 0 | L1MA9 | L1 | LINE |
| 44297956 | 44298375 | + | 16.6 | 5628 | 6045 | -267 | L1MA9 | L1 | LINE |
| 44298377 | 44298571 | + | 27.2 | 81 | 287 | -232 | LTR40a | ERVL | LTR |
| 44298572 | 44299062 | + | 19.0 | 1 | 491 | -29 | MLT2B1 | ERVL | LTR |
| 44299062 | 44299204 | + | 20.9 | 379 | 519 | 0 | LTR40a | ERVL | LTR |

  

|  |  |  |  |  |  |  |  |  |  |
| --- | --- | --- | --- | --- | --- | --- | --- | --- | --- |
| chr14:44343621-44346564 | | | 2494 bp | | |  |  |  |  |
| Genome Start | Genome End | Strand | Div | Repeat Start | Repeat End | Repeat Left | Name | Family | Class |
| 44343621 | 44343774 | - | 34.0 | 3220 | 3377 | -1 | L2 | L2 | LINE |
| 44343775 | 44344169 | + | 17.1 | 1 | 390 | 0 | MLT1B | MaLR | LTR |
| 44344170 | 44344705 | - | 34.0 | 2660 | 3219 | -159 | L2 | L2 | LINE |
| 44344714 | 44345023 | - | 16.1 | 5641 | 5955 | -357 | L1MA10 | L1 | LINE |
| 44345026 | 44345389 | + | 16.2 | 5963 | 6327 | -7 | L1MA10 | L1 | LINE |
| 44345509 | 44345786 | + | 14.4 | 1 | 279 | -33 | AluSx | Alu | SINE |
| 44346108 | 44346564 | - | 31.9 | 2002 | 2513 | -906 | L2 | L2 | LINE |

  

|  |  |  |  |  |  |  |  |  |  |
| --- | --- | --- | --- | --- | --- | --- | --- | --- | --- |
| chr14:45465126-45468836 | | | 3332 bp | | |  |  |  |  |
| Genome Start | Genome End | Strand | Div | Repeat Start | Repeat End | Repeat Left | Name | Family | Class |
| 45465126 | 45465301 | - | 21.7 | 3126 | 3311 | -4023 | L1M4b | L1 | LINE |
| 45465425 | 45467054 | - | 3.0 | 4528 | 6155 | 0 | L1PA3 | L1 | LINE |
| 45467054 | 45468077 | + | 3.0 | 3510 | 4533 | -1613 | L1PA3 | L1 | LINE |
| 45468083 | 45468425 | - | 21.3 | 2728 | 3132 | -4202 | L1M4b | L1 | LINE |
| 45468678 | 45468836 | - | 35.7 | 108 | 269 | -7065 | L1M4b | L1 | LINE |

  

|  |  |  |  |  |  |  |  |  |  |
| --- | --- | --- | --- | --- | --- | --- | --- | --- | --- |
| chr14:45925388-45928306 | | | 2795 bp | | |  |  |  |  |
| Genome Start | Genome End | Strand | Div | Repeat Start | Repeat End | Repeat Left | Name | Family | Class |
| 45925388 | 45925471 | - | 19.3 | 431 | 514 | 0 | MER67D | ERV1 | LTR |
| 45925534 | 45925760 | - | 20.3 | 168 | 403 | -308 | MER67C | ERV1 | LTR |
| 45925761 | 45926708 | - | 13.5 | 4228 | 5199 | -947 | L1M1 | L1 | LINE |
| 45926714 | 45927186 | + | 15.8 | 5191 | 5729 | -571 | L1MA6 | L1 | LINE |
| 45927187 | 45927483 | + | 6.9 | 1 | 304 | 0 | AluY | Alu | SINE |
| 45927484 | 45928067 | + | 17.9 | 5730 | 6300 | 0 | L1MA6 | L1 | LINE |
| 45928087 | 45928183 | - | 17.7 | 65 | 164 | -547 | MER67C | ERV1 | LTR |
| 45928222 | 45928306 | - | 20.0 | 1 | 85 | -429 | MER67D | ERV1 | LTR |

  

|  |  |  |  |  |  |  |  |  |  |
| --- | --- | --- | --- | --- | --- | --- | --- | --- | --- |
| chr14:45966454-45968918 | | | 2410 bp | | |  |  |  |  |
| Genome Start | Genome End | Strand | Div | Repeat Start | Repeat End | Repeat Left | Name | Family | Class |
| 45966454 | 45966594 | - | 23.2 | 222 | 363 | -11 | MLT1A | MaLR | LTR |
| 45966596 | 45966766 | - | 8.3 | 137 | 305 | -6 | AluY | Alu | SINE |
| 45966808 | 45967160 | - | 2.8 | 4243 | 4595 | -1551 | L1PA3 | L1 | LINE |
| 45967159 | 45968722 | + | 3.4 | 4591 | 6155 | 0 | L1PA3 | L1 | LINE |
| 45968738 | 45968918 | - | 25.7 | 6 | 188 | -186 | MLT1A | MaLR | LTR |

  

|  |  |  |  |  |  |  |  |  |  |
| --- | --- | --- | --- | --- | --- | --- | --- | --- | --- |
| chr14:46506494-46511247 | | | 4643 bp | | |  |  |  |  |
| Genome Start | Genome End | Strand | Div | Repeat Start | Repeat End | Repeat Left | Name | Family | Class |
| 46506494 | 46506601 | + | 22.3 | 448 | 550 | -385 | MER21C | ERV1 | LTR |
| 46506619 | 46506869 | - | 16.2 | 2248 | 2491 | 0 | Tigger7 | MER2\_type | DNA |
| 46506894 | 46507090 | + | 30.8 | 223 | 423 | -6426 | L1M2 | L1 | LINE |
| 46507104 | 46507274 | + | 30.4 | 608 | 790 | -6059 | L1M2 | L1 | LINE |
| 46507274 | 46507347 | + | 16.2 | 3699 | 3776 | -2370 | L1P4 | L1 | LINE |
| 46507344 | 46507504 | + | 15.1 | 4109 | 4265 | -1878 | L1MA4A | L1 | LINE |
| 46507505 | 46507808 | + | 12.7 | 1 | 299 | -11 | AluSg | Alu | SINE |
| 46507809 | 46507963 | + | 15.1 | 4266 | 4423 | -1720 | L1MA4A | L1 | LINE |
| 46507962 | 46508653 | - | 3.7 | 5204 | 5895 | -260 | L1PA5 | L1 | LINE |
| 46508654 | 46508883 | + | 7.0 | 5909 | 6138 | -16 | L1PA5 | L1 | LINE |
| 46508931 | 46510847 | + | 16.7 | 4414 | 6296 | -7 | L1MA4A | L1 | LINE |
| 46510863 | 46510918 | - | 28.6 | 22 | 77 | -2414 | Tigger7 | MER2\_type | DNA |
| 46510921 | 46511247 | + | 21.3 | 572 | 911 | -24 | MER21C | ERV1 | LTR |

  

|  |  |  |  |  |  |  |  |  |  |
| --- | --- | --- | --- | --- | --- | --- | --- | --- | --- |
| chr14:47792920-47810643 | | | 17632 bp | | |  |  |  |  |
| Genome Start | Genome End | Strand | Div | Repeat Start | Repeat End | Repeat Left | Name | Family | Class |
| 47792920 | 47794141 | + | 22.1 | 2 | 1230 | -5278 | L1MC1 | L1 | LINE |
| 47794152 | 47794558 | - | 8.9 | 1 | 448 | -2 | LTR7 | ERV1 | LTR |
| 47794559 | 47794936 | - | 11.3 | 7321 | 7713 | 0 | HERVH | ERV1 | LTR |
| 47794934 | 47795149 | - | 13.9 | 5600 | 5819 | -1894 | HERVH | ERV1 | LTR |
| 47795151 | 47795814 | - | 15.1 | 4488 | 5149 | -2564 | HERVH | ERV1 | LTR |
| 47795817 | 47796672 | - | 7.9 | 3140 | 3993 | -3720 | HERVH | ERV1 | LTR |
| 47796659 | 47799491 | - | 10.4 | 1 | 2992 | -4721 | HERVH | ERV1 | LTR |
| 47799494 | 47799893 | - | 9.3 | 1 | 448 | -2 | LTR7 | ERV1 | LTR |
| 47799961 | 47800726 | + | 18.1 | 1273 | 2124 | -4384 | L1MC1 | L1 | LINE |
| 47800737 | 47801348 | - | 17.3 | 5665 | 6295 | -5 | L1MA4 | L1 | LINE |
| 47801349 | 47801755 | - | 9.4 | 1 | 448 | -2 | LTR7 | ERV1 | LTR |
| 47801766 | 47802133 | - | 12.4 | 7322 | 7703 | -10 | HERVH | ERV1 | LTR |
| 47802132 | 47802348 | - | 12.5 | 5600 | 5819 | -1894 | HERVH | ERV1 | LTR |
| 47802350 | 47803012 | - | 14.5 | 4488 | 5149 | -2564 | HERVH | ERV1 | LTR |
| 47803015 | 47803508 | - | 11.6 | 3500 | 3993 | -3720 | HERVH | ERV1 | LTR |
| 47803508 | 47806822 | - | 10.1 | 1 | 3290 | -4423 | HERVH | ERV1 | LTR |
| 47806825 | 47807235 | - | 10.0 | 1 | 448 | -2 | LTR7 | ERV1 | LTR |
| 47807236 | 47807671 | - | 14.9 | 5222 | 5671 | -634 | L1MA4 | L1 | LINE |
| 47807663 | 47807809 | + | 19.1 | 5082 | 5234 | -912 | L1MA4 | L1 | LINE |
| 47807824 | 47810434 | + | 17.3 | 2138 | 6012 | -496 | L1MC1 | L1 | LINE |
| 47810435 | 47810643 | + | 19.6 | 6296 | 6508 | 0 | L1MC1 | L1 | LINE |

  

|  |  |  |  |  |  |  |  |  |  |
| --- | --- | --- | --- | --- | --- | --- | --- | --- | --- |
| chr14:47948435-47955848 | | | 7427 bp | | |  |  |  |  |
| Genome Start | Genome End | Strand | Div | Repeat Start | Repeat End | Repeat Left | Name | Family | Class |
| 47948435 | 47951321 | - | 4.0 | 3252 | 6149 | -6 | L1PA4 | L1 | LINE |
| 47951320 | 47952452 | - | 3.4 | 5007 | 6155 | 0 | L1PA3 | L1 | LINE |
| 47952442 | 47953978 | + | 3.1 | 3467 | 5005 | -1141 | L1PA3 | L1 | LINE |
| 47953979 | 47955848 | - | 4.9 | 1382 | 3264 | -2882 | L1PA4 | L1 | LINE |

  

|  |  |  |  |  |  |  |  |  |  |
| --- | --- | --- | --- | --- | --- | --- | --- | --- | --- |
| chr14:48196302-48200492 | | | 3630 bp | | |  |  |  |  |
| Genome Start | Genome End | Strand | Div | Repeat Start | Repeat End | Repeat Left | Name | Family | Class |
| 48196302 | 48196402 | + | 28.3 | 99 | 198 | -5872 | L1MEa | L1 | LINE |
| 48196658 | 48197331 | + | 31.2 | 392 | 1067 | -5003 | L1MEa | L1 | LINE |
| 48197429 | 48198229 | - | 1.6 | 5338 | 6155 | 0 | L1PA3 | L1 | LINE |
| 48198231 | 48199271 | + | 2.8 | 4296 | 5341 | -805 | L1PA3 | L1 | LINE |
| 48199263 | 48199328 | + | 21.2 | 4306 | 4371 | -1769 | L1M3 | L1 | LINE |
| 48199473 | 48199542 | + | 18.6 | 4302 | 4371 | -1769 | L1M3 | L1 | LINE |
| 48199616 | 48200492 | + | 31.1 | 1086 | 2201 | -4060 | L1MEa | L1 | LINE |

  

|  |  |  |  |  |  |  |  |  |  |
| --- | --- | --- | --- | --- | --- | --- | --- | --- | --- |
| chr14:48364214-48366582 | | | 2330 bp | | |  |  |  |  |
| Genome Start | Genome End | Strand | Div | Repeat Start | Repeat End | Repeat Left | Name | Family | Class |
| 48364214 | 48364769 | + | 24.1 | 4096 | 4689 | -1639 | L1M3f | L1 | LINE |
| 48364781 | 48364837 | - | 15.8 | 6094 | 6150 | -141 | L1MA9 | L1 | LINE |
| 48364836 | 48364944 | + | 20.2 | 6153 | 6274 | -38 | L1MA9 | L1 | LINE |
| 48364975 | 48365490 | + | 29.0 | 4689 | 5256 | -1072 | L1M3f | L1 | LINE |
| 48365491 | 48365953 | + | 27.2 | 1 | 505 | 0 | MLT1D | MaLR | LTR |
| 48365954 | 48366582 | + | 31.4 | 5257 | 5921 | -431 | L1M3f | L1 | LINE |

  

|  |  |  |  |  |  |  |  |  |  |
| --- | --- | --- | --- | --- | --- | --- | --- | --- | --- |
| chr14:49343325-49350127 | | | 6769 bp | | |  |  |  |  |
| Genome Start | Genome End | Strand | Div | Repeat Start | Repeat End | Repeat Left | Name | Family | Class |
| 49343325 | 49344060 | - | 18.5 | 5438 | 6331 | -3 | L1MB7 | L1 | LINE |
| 49344061 | 49344371 | - | 11.0 | 1 | 303 | -9 | AluSx | Alu | SINE |
| 49344372 | 49344635 | - | 19.2 | 5172 | 5437 | -747 | L1MB7 | L1 | LINE |
| 49344636 | 49344942 | - | 11.1 | 1 | 307 | -6 | AluSq | Alu | SINE |
| 49344943 | 49345207 | - | 20.7 | 4900 | 5171 | -975 | L1MC1 | L1 | LINE |
| 49345201 | 49346048 | + | 14.5 | 5195 | 6128 | -205 | L1MC1 | L1 | LINE |
| 49346049 | 49346335 | + | 11.5 | 1 | 289 | -23 | AluSx | Alu | SINE |
| 49346336 | 49346550 | + | 14.5 | 6129 | 6333 | 0 | L1MC1 | L1 | LINE |
| 49346555 | 49346868 | + | 6.8 | 1 | 311 | 0 | AluY | Alu | SINE |
| 49346882 | 49347152 | - | 17.4 | 4619 | 4896 | -1250 | L1MB7 | L1 | LINE |
| 49347177 | 49347470 | + | 21.1 | 1 | 299 | -13 | AluJo | Alu | SINE |
| 49347471 | 49349267 | - | 17.4 | 2757 | 4618 | -1528 | L1MB7 | L1 | LINE |
| 49349268 | 49349542 | - | 10.9 | 1 | 291 | -19 | AluSg | Alu | SINE |
| 49349543 | 49349607 | - | 17.4 | 2692 | 2756 | -3390 | L1MB7 | L1 | LINE |
| 49349608 | 49349838 | + | 13.9 | 1 | 236 | 0 | MER46A | MER2\_type | DNA |
| 49349839 | 49350127 | - | 17.4 | 2390 | 2691 | -3455 | L1MB7 | L1 | LINE |

  

|  |  |  |  |  |  |  |  |  |  |
| --- | --- | --- | --- | --- | --- | --- | --- | --- | --- |
| chr14:49662062-49665245 | | | 2789 bp | | |  |  |  |  |
| Genome Start | Genome End | Strand | Div | Repeat Start | Repeat End | Repeat Left | Name | Family | Class |
| 49662062 | 49662365 | + | 29.9 | 201 | 530 | -1977 | HAL1 | L1 | LINE |
| 49662380 | 49662705 | + | 28.9 | 634 | 967 | -1540 | HAL1 | L1 | LINE |
| 49662713 | 49662871 | - | 13.6 | 5984 | 6195 | -105 | L1MA7 | L1 | LINE |
| 49662874 | 49662972 | + | 9.1 | 6181 | 6291 | 0 | L1MA7 | L1 | LINE |
| 49662973 | 49663460 | + | 22.2 | 958 | 1449 | -1058 | HAL1 | L1 | LINE |
| 49663471 | 49663597 | + | 25.8 | 1 | 134 | -377 | MLT1J | MaLR | LTR |
| 49663607 | 49663909 | - | 8.9 | 1 | 302 | -10 | AluSx | Alu | SINE |
| 49664114 | 49664313 | + | 32.8 | 70 | 276 | 0 | MIRm | MIR | SINE |
| 49664195 | 49664315 | + | 33.3 | 3299 | 3419 | 0 | L2 | L2 | LINE |
| 49664316 | 49664762 | + | 24.5 | 1491 | 1916 | -591 | HAL1 | L1 | LINE |
| 49665031 | 49665245 | + | 28.6 | 2250 | 2500 | -7 | HAL1 | L1 | LINE |

  

|  |  |  |  |  |  |  |  |  |  |
| --- | --- | --- | --- | --- | --- | --- | --- | --- | --- |
| chr14:50080754-50092327 | | | 11253 bp | | |  |  |  |  |
| Genome Start | Genome End | Strand | Div | Repeat Start | Repeat End | Repeat Left | Name | Family | Class |
| 50080754 | 50081116 | + | 28.4 | 26 | 412 | -203 | MLT1L | MaLR | LTR |
| 50081119 | 50081433 | + | 16.8 | 1 | 311 | -1 | AluJo | Alu | SINE |
| 50081440 | 50081837 | + | 15.8 | 1 | 411 | 0 | LTR57 | ERVL | LTR |
| 50081838 | 50082119 | + | 19.1 | 1 | 282 | -5061 | LTR57-int | ERVL | LTR |
| 50082210 | 50082833 | + | 11.1 | 274 | 875 | -4468 | LTR57-int | ERVL | LTR |
| 50082863 | 50083303 | + | 11.1 | 876 | 1324 | -4019 | LTR57-int | ERVL | LTR |
| 50083306 | 50083484 | - | 5.6 | 5976 | 6154 | 0 | L1PA7 | L1 | LINE |
| 50083485 | 50083538 | + | 5.6 | 5914 | 5967 | -205 | L1PA7 | L1 | LINE |
| 50083546 | 50084983 | + | 10.6 | 1326 | 2761 | -2582 | LTR57-int | ERVL | LTR |
| 50084984 | 50085277 | - | 8.9 | 1 | 293 | -18 | AluY | Alu | SINE |
| 50085278 | 50085741 | + | 10.6 | 2762 | 3210 | -2133 | LTR57-int | ERVL | LTR |
| 50085744 | 50087111 | + | 12.2 | 3566 | 4993 | -350 | LTR57-int | ERVL | LTR |
| 50087112 | 50089480 | + | 10.8 | 2732 | 5142 | -1004 | L1PB2 | L1 | LINE |
| 50089481 | 50089753 | + | 10.3 | 1 | 297 | -15 | AluSx | Alu | SINE |
| 50089754 | 50090567 | + | 10.5 | 5322 | 6144 | -7 | L1PB2 | L1 | LINE |
| 50090595 | 50090966 | + | 8.9 | 4969 | 5343 | 0 | LTR57-int | ERVL | LTR |
| 50090967 | 50091371 | + | 17.9 | 1 | 411 | 0 | LTR57 | ERVL | LTR |
| 50091524 | 50091730 | + | 33.6 | 157 | 368 | -143 | MLT1J | MaLR | LTR |
| 50091731 | 50092051 | - | 8.8 | 1 | 319 | 0 | AluSx | Alu | SINE |
| 50092052 | 50092191 | + | 33.6 | 369 | 511 | 0 | MLT1J | MaLR | LTR |
| 50092196 | 50092327 | + | 28.0 | 465 | 615 | 0 | MLT1L | MaLR | LTR |

  

|  |  |  |  |  |  |  |  |  |  |
| --- | --- | --- | --- | --- | --- | --- | --- | --- | --- |
| chr14:57154712-57162789 | | | 8055 bp | | |  |  |  |  |
| Genome Start | Genome End | Strand | Div | Repeat Start | Repeat End | Repeat Left | Name | Family | Class |
| 57154712 | 57157068 | + | 11.1 | 6 | 2375 | -215 | L1PA7 | L1 | LINE |
| 57157065 | 57157227 | - | 1.2 | 3919 | 4081 | -2065 | L1PA3 | L1 | LINE |
| 57157227 | 57159304 | + | 2.7 | 4079 | 6154 | -1 | L1PA3 | L1 | LINE |
| 57159333 | 57162261 | + | 8.4 | 2367 | 5295 | -1166 | L1PA7 | L1 | LINE |
| 57162262 | 57162789 | + | 4.9 | 5924 | 6454 | -15 | L1PA7 | L1 | LINE |

  

|  |  |  |  |  |  |  |  |  |  |
| --- | --- | --- | --- | --- | --- | --- | --- | --- | --- |
| chr14:60421948-60427518 | | | 5526 bp | | |  |  |  |  |
| Genome Start | Genome End | Strand | Div | Repeat Start | Repeat End | Repeat Left | Name | Family | Class |
| 60421948 | 60423636 | - | 20.2 | 3840 | 5434 | -878 | L1M2 | L1 | LINE |
| 60423676 | 60424071 | - | 4.4 | 5788 | 6189 | 0 | L1PA3 | L1 | LINE |
| 60424072 | 60426726 | + | 3.0 | 3142 | 5796 | -359 | L1PA3 | L1 | LINE |
| 60426725 | 60426875 | - | 21.4 | 3655 | 3846 | -2300 | L1M2 | L1 | LINE |
| 60426878 | 60427426 | - | 18.2 | 1 | 554 | 0 | MLT2A2 | ERVL | LTR |
| 60427433 | 60427518 | - | 16.3 | 3548 | 3633 | -2507 | L1M2 | L1 | LINE |

  

|  |  |  |  |  |  |  |  |  |  |
| --- | --- | --- | --- | --- | --- | --- | --- | --- | --- |
| chr14:61491577-61499491 | | | 7918 bp | | |  |  |  |  |
| Genome Start | Genome End | Strand | Div | Repeat Start | Repeat End | Repeat Left | Name | Family | Class |
| 61491577 | 61496309 | - | 17.9 | 1749 | 6562 | -8 | L1MA10 | L1 | LINE |
| 61496319 | 61496561 | - | 17.3 | 42 | 282 | -30 | AluJb | Alu | SINE |
| 61496566 | 61496622 | - | 21.1 | 1629 | 1689 | -4695 | L1M4c | L1 | LINE |
| 61496615 | 61497298 | - | 10.4 | 4025 | 4711 | -1435 | L1PREC2 | L1 | LINE |
| 61497298 | 61498752 | + | 12.0 | 4701 | 6162 | -3 | L1PA15 | L1 | LINE |
| 61498746 | 61499491 | - | 21.4 | 871 | 1785 | -4743 | L1MA10 | L1 | LINE |

  

|  |  |  |  |  |  |  |  |  |  |
| --- | --- | --- | --- | --- | --- | --- | --- | --- | --- |
| chr14:61985964-61987287 | | | 1203 bp | | |  |  |  |  |
| Genome Start | Genome End | Strand | Div | Repeat Start | Repeat End | Repeat Left | Name | Family | Class |
| 61985964 | 61986051 | + | 37.2 | 2618 | 2704 | -715 | L2 | L2 | LINE |
| 61986052 | 61986691 | - | 13.6 | 1 | 634 | 0 | MER41B | ERV1 | LTR |
| 61986692 | 61986772 | + | 37.2 | 2705 | 2783 | -636 | L2 | L2 | LINE |
| 61986864 | 61986943 | - | 17.5 | 6089 | 6168 | 0 | L1MB1 | L1 | LINE |
| 61986944 | 61987010 | + | 19.7 | 6011 | 6076 | -92 | L1MB1 | L1 | LINE |
| 61987041 | 61987287 | + | 37.9 | 2955 | 3197 | -212 | L2 | L2 | LINE |

  

|  |  |  |  |  |  |  |  |  |  |
| --- | --- | --- | --- | --- | --- | --- | --- | --- | --- |
| chr14:62404876-62406489 | | | 1506 bp | | |  |  |  |  |
| Genome Start | Genome End | Strand | Div | Repeat Start | Repeat End | Repeat Left | Name | Family | Class |
| 62404876 | 62404919 | + | 4.5 | 1 | 44 | -36 | MADE1 | Mariner | DNA |
| 62405028 | 62406136 | - | 6.3 | 5017 | 6151 | -3 | L1PA7 | L1 | LINE |
| 62406137 | 62406444 | + | 9.1 | 4710 | 5017 | -1129 | L1PA7 | L1 | LINE |
| 62406445 | 62406489 | + | 8.9 | 31 | 76 | -4 | MADE1 | Mariner | DNA |

  

|  |  |  |  |  |  |  |  |  |  |
| --- | --- | --- | --- | --- | --- | --- | --- | --- | --- |
| chr14:62412655-62415815 | | | 3110 bp | | |  |  |  |  |
| Genome Start | Genome End | Strand | Div | Repeat Start | Repeat End | Repeat Left | Name | Family | Class |
| 62412655 | 62413143 | + | 30.8 | 3964 | 4469 | -1677 | L1M5 | L1 | LINE |
| 62413147 | 62413453 | + | 27.4 | 4738 | 5054 | -1092 | L1M5 | L1 | LINE |
| 62413449 | 62413567 | - | 2.6 | 4788 | 4903 | -1243 | L1PA3 | L1 | LINE |
| 62413567 | 62414828 | + | 2.6 | 4893 | 6152 | -3 | L1PA3 | L1 | LINE |
| 62414842 | 62415140 | + | 27.3 | 5040 | 5351 | -795 | L1M5 | L1 | LINE |
| 62415141 | 62415493 | - | 18.1 | 24 | 387 | -3 | MLT1B | MaLR | LTR |
| 62415535 | 62415815 | + | 22.9 | 5343 | 5635 | -527 | L1M5 | L1 | LINE |

  

|  |  |  |  |  |  |  |  |  |  |
| --- | --- | --- | --- | --- | --- | --- | --- | --- | --- |
| chr14:63322227-63325882 | | | 3603 bp | | |  |  |  |  |
| Genome Start | Genome End | Strand | Div | Repeat Start | Repeat End | Repeat Left | Name | Family | Class |
| 63322227 | 63322297 | - | 27.7 | 2430 | 2499 | -8 | HAL1 | L1 | LINE |
| 63322298 | 63322439 | + | 15.6 | 4 | 143 | -169 | AluJb | Alu | SINE |
| 63322440 | 63322730 | + | 9.3 | 1 | 294 | -18 | AluSx | Alu | SINE |
| 63322731 | 63322894 | + | 15.6 | 144 | 300 | -12 | AluJb | Alu | SINE |
| 63322895 | 63323228 | - | 24.7 | 2029 | 2429 | -78 | HAL1 | L1 | LINE |
| 63323248 | 63323533 | - | 14.3 | 1 | 287 | -25 | AluJb | Alu | SINE |
| 63323576 | 63323905 | - | 13.7 | 5910 | 6240 | -60 | L1MA4 | L1 | LINE |
| 63323909 | 63325192 | - | 14.6 | 33 | 1538 | -19 | MER52D | ERV1 | LTR |
| 63325193 | 63325220 | - | 3.6 | 5862 | 5889 | -402 | L1MA4 | L1 | LINE |
| 63325214 | 63325565 | + | 11.4 | 5523 | 5875 | -425 | L1MA4 | L1 | LINE |
| 63325562 | 63325882 | - | 24.6 | 1660 | 2021 | -486 | HAL1 | L1 | LINE |

  

|  |  |  |  |  |  |  |  |  |  |
| --- | --- | --- | --- | --- | --- | --- | --- | --- | --- |
| chr14:71603349-71604151 | | | 764 bp | | |  |  |  |  |
| Genome Start | Genome End | Strand | Div | Repeat Start | Repeat End | Repeat Left | Name | Family | Class |
| 71603349 | 71603406 | - | 20.7 | 276 | 334 | -4 | MER46C | MER2\_type | DNA |
| 71603408 | 71603653 | - | 3.2 | 5633 | 5878 | -294 | L1PA8 | L1 | LINE |
| 71603654 | 71603958 | + | 7.9 | 5865 | 6168 | -4 | L1PA8 | L1 | LINE |
| 71603997 | 71604151 | - | 18.3 | 56 | 222 | -116 | MER46C | MER2\_type | DNA |

  

|  |  |  |  |  |  |  |  |  |  |
| --- | --- | --- | --- | --- | --- | --- | --- | --- | --- |
| chr14:71931637-71937143 | | | 5474 bp | | |  |  |  |  |
| Genome Start | Genome End | Strand | Div | Repeat Start | Repeat End | Repeat Left | Name | Family | Class |
| 71931637 | 71932004 | - | 24.0 | 1648 | 1986 | -23 | HAL1b | L1 | LINE |
| 71931996 | 71936274 | - | 4.1 | 1883 | 6153 | -2 | L1PA4 | L1 | LINE |
| 71936260 | 71936722 | + | 5.2 | 1398 | 1861 | -4294 | L1PA4 | L1 | LINE |
| 71936750 | 71936931 | - | 27.3 | 1433 | 1625 | -384 | HAL1b | L1 | LINE |
| 71936940 | 71937053 | - | 30.5 | 3281 | 3386 | -33 | L2 | L2 | LINE |
| 71937076 | 71937143 | - | 22.1 | 1356 | 1423 | -586 | HAL1b | L1 | LINE |

  

|  |  |  |  |  |  |  |  |  |  |
| --- | --- | --- | --- | --- | --- | --- | --- | --- | --- |
| chr14:73659555-73665977 | | | 6101 bp | | |  |  |  |  |
| Genome Start | Genome End | Strand | Div | Repeat Start | Repeat End | Repeat Left | Name | Family | Class |
| 73659555 | 73660087 | - | 36.5 | 2262 | 2863 | -556 | L2 | L2 | LINE |
| 73660094 | 73660246 | + | 17.6 | 1 | 183 | -379 | MLT1F2 | MaLR | LTR |
| 73660263 | 73660475 | + | 26.9 | 1 | 220 | -48 | MIRb | MIR | SINE |
| 73660567 | 73660977 | + | 28.7 | 4247 | 4684 | -1462 | L1M5 | L1 | LINE |
| 73660990 | 73661289 | + | 14.4 | 6 | 305 | -7 | AluJb | Alu | SINE |
| 73661325 | 73661557 | + | 15.4 | 5092 | 5328 | -818 | L1MB5 | L1 | LINE |
| 73661554 | 73662024 | - | 1.8 | 5202 | 5671 | -484 | L1PA4 | L1 | LINE |
| 73662025 | 73662505 | + | 2.7 | 5673 | 6155 | 0 | L1PA4 | L1 | LINE |
| 73662508 | 73663345 | + | 15.1 | 5320 | 6166 | -8 | L1MB5 | L1 | LINE |
| 73663413 | 73663527 | + | 26.4 | 84 | 194 | -14 | MIR3 | MIR | SINE |
| 73663552 | 73664206 | - | 32.4 | 2632 | 3374 | -4 | L2 | L2 | LINE |
| 73664207 | 73664329 | - | 13.8 | 4 | 126 | -7 | FLAM\_C | Alu | SINE |
| 73664330 | 73664415 | - | 30.2 | 2540 | 2631 | -788 | L2 | L2 | LINE |
| 73664447 | 73665021 | + | 18.8 | 34 | 638 | -5762 | L1MEc | L1 | LINE |
| 73665019 | 73665088 | + | 20.0 | 6082 | 6153 | -11 | L1ME2 | L1 | LINE |
| 73665116 | 73665423 | + | 12.8 | 1 | 308 | 0 | AluSx | Alu | SINE |
| 73665433 | 73665540 | - | 30.6 | 2382 | 2499 | -920 | L2 | L2 | LINE |
| 73665550 | 73665977 | - | 34.9 | 1550 | 1985 | -1434 | L2 | L2 | LINE |

  

|  |  |  |  |  |  |  |  |  |  |
| --- | --- | --- | --- | --- | --- | --- | --- | --- | --- |
| chr14:75879975-75881893 | | | 1883 bp | | |  |  |  |  |
| Genome Start | Genome End | Strand | Div | Repeat Start | Repeat End | Repeat Left | Name | Family | Class |
| 75879975 | 75880090 | - | 27.3 | 2824 | 2941 | -478 | L2 | L2 | LINE |
| 75880093 | 75880339 | + | 28.7 | 8 | 262 | 0 | MIR | MIR | SINE |
| 75880372 | 75880664 | - | 13.3 | 5862 | 6150 | -1 | L1PB1 | L1 | LINE |
| 75880665 | 75880964 | + | 12.4 | 1 | 303 | -9 | AluSx | Alu | SINE |
| 75880965 | 75881162 | - | 13.3 | 5660 | 5861 | -290 | L1PB1 | L1 | LINE |
| 75881163 | 75881808 | + | 6.0 | 5016 | 5661 | -490 | L1PB1 | L1 | LINE |
| 75881811 | 75881893 | - | 39.0 | 2734 | 2815 | -604 | L2 | L2 | LINE |

  

|  |  |  |  |  |  |  |  |  |  |
| --- | --- | --- | --- | --- | --- | --- | --- | --- | --- |
| chr14:79052836-79058067 | | | 4486 bp | | |  |  |  |  |
| Genome Start | Genome End | Strand | Div | Repeat Start | Repeat End | Repeat Left | Name | Family | Class |
| 79052836 | 79053430 | - | 26.4 | 5366 | 5993 | -1883 | L1MC4a | L1 | LINE |
| 79053470 | 79053992 | - | 34.3 | 4741 | 5308 | -906 | L1MC4a | L1 | LINE |
| 79053993 | 79054300 | - | 11.4 | 1 | 309 | -3 | AluSx | Alu | SINE |
| 79054301 | 79054550 | - | 34.3 | 4510 | 4740 | -1474 | L1MC4a | L1 | LINE |
| 79054645 | 79054921 | - | 30.9 | 4023 | 4315 | -1831 | L1M1 | L1 | LINE |
| 79055055 | 79055317 | - | 32.1 | 3427 | 3682 | -2532 | L1MC4a | L1 | LINE |
| 79055390 | 79056095 | - | 35.9 | 2603 | 3339 | -2875 | L1MC4a | L1 | LINE |
| 79056138 | 79056358 | - | 33.8 | 2335 | 2555 | -3660 | L1MC4a | L1 | LINE |
| 79056405 | 79056774 | - | 4.9 | 5766 | 6141 | -13 | L1PA7 | L1 | LINE |
| 79056775 | 79057144 | + | 4.6 | 5403 | 5773 | -381 | L1PA7 | L1 | LINE |
| 79057149 | 79057407 | - | 26.6 | 2074 | 2321 | -3894 | L1MC4a | L1 | LINE |
| 79057724 | 79058067 | - | 29.4 | 1580 | 1972 | -4243 | L1MC4a | L1 | LINE |

  

|  |  |  |  |  |  |  |  |  |  |
| --- | --- | --- | --- | --- | --- | --- | --- | --- | --- |
| chr14:81648003-81650253 | | | 2191 bp | | |  |  |  |  |
| Genome Start | Genome End | Strand | Div | Repeat Start | Repeat End | Repeat Left | Name | Family | Class |
| 81648003 | 81648218 | - | 32.7 | 3186 | 3419 | 0 | L2 | L2 | LINE |
| 81648220 | 81648298 | + | 19.0 | 1 | 79 | -114 | MER53 | DNA | DNA |
| 81648310 | 81648895 | - | 17.5 | 4677 | 5253 | -890 | L1MA4 | L1 | LINE |
| 81648892 | 81649897 | + | 16.7 | 5269 | 6294 | -6 | L1MA4 | L1 | LINE |
| 81649913 | 81649972 | + | 23.3 | 125 | 184 | -9 | MER53 | DNA | DNA |
| 81650010 | 81650253 | - | 37.0 | 2915 | 3158 | -220 | L2 | L2 | LINE |

  

|  |  |  |  |  |  |  |  |  |  |
| --- | --- | --- | --- | --- | --- | --- | --- | --- | --- |
| chr14:82747528-82759934 | | | 11988 bp | | |  |  |  |  |
| Genome Start | Genome End | Strand | Div | Repeat Start | Repeat End | Repeat Left | Name | Family | Class |
| 82747528 | 82748389 | - | 23.4 | 5583 | 6555 | -109 | L1ME1 | L1 | LINE |
| 82748507 | 82748719 | - | 25.5 | 5046 | 5263 | -1381 | L1ME1 | L1 | LINE |
| 82748730 | 82754334 | - | 16.0 | 16 | 5738 | -1827 | L1M1 | L1 | LINE |
| 82754336 | 82755296 | - | 27.6 | 1232 | 2315 | -4331 | L1ME1 | L1 | LINE |
| 82755300 | 82755618 | + | 23.8 | 4 | 374 | 0 | MLT1A | MaLR | LTR |
| 82755619 | 82756206 | + | 22.1 | 1 | 742 | -629 | MLT1-int | MaLR | LTR |
| 82756189 | 82756343 | + | 31.8 | 858 | 1023 | -466 | MLT1I-int | MaLR | LTR |
| 82756311 | 82756405 | + | 27.2 | 676 | 767 | -968 | MLT1I-int | MaLR | LTR |
| 82756516 | 82756725 | + | 33.5 | 1248 | 1455 | -280 | MLT1I-int | MaLR | LTR |
| 82756944 | 82757333 | - | 1.0 | 5763 | 6155 | 0 | L1PA2 | L1 | LINE |
| 82757334 | 82757687 | + | 2.3 | 5387 | 5740 | -415 | L1PA2 | L1 | LINE |
| 82757690 | 82757892 | + | 21.7 | 1444 | 1674 | -61 | MLT1I-int | MaLR | LTR |
| 82757891 | 82757964 | + | 23.9 | 713 | 783 | -952 | MLT-int | MaLR | LTR |
| 82757944 | 82758006 | + | 27.4 | 1666 | 1727 | -8 | MLT1A-int | MaLR | LTR |
| 82758018 | 82758346 | + | 23.7 | 3 | 349 | -25 | MLT1A | MaLR | LTR |
| 82758368 | 82758575 | - | 26.7 | 1055 | 1246 | -5296 | L1ME1 | L1 | LINE |
| 82758576 | 82758877 | - | 6.3 | 1 | 305 | -6 | AluY | Alu | SINE |
| 82758878 | 82759934 | - | 26.7 | 18 | 1054 | -5488 | L1ME1 | L1 | LINE |

  

|  |  |  |  |  |  |  |  |  |  |
| --- | --- | --- | --- | --- | --- | --- | --- | --- | --- |
| chr14:85777485-85783875 | | | 6178 bp | | |  |  |  |  |
| Genome Start | Genome End | Strand | Div | Repeat Start | Repeat End | Repeat Left | Name | Family | Class |
| 85777485 | 85777762 | + | 22.7 | 4260 | 4547 | -1599 | L1MC3 | L1 | LINE |
| 85777763 | 85777893 | + | 13.7 | 6163 | 6294 | -6 | L1MA5 | L1 | LINE |
| 85777963 | 85778721 | - | 12.0 | 5379 | 6149 | -2 | L1PB2 | L1 | LINE |
| 85778722 | 85779709 | + | 17.6 | 5299 | 6296 | -48 | L1MC2 | L1 | LINE |
| 85779848 | 85779883 | + | 17.6 | 6297 | 6343 | -1 | L1MC2 | L1 | LINE |
| 85779885 | 85780672 | + | 18.7 | 4549 | 5292 | -854 | L1MC3 | L1 | LINE |
| 85780675 | 85781909 | - | 2.0 | 3744 | 4975 | -1171 | L1PA3 | L1 | LINE |
| 85781909 | 85783087 | + | 2.2 | 4977 | 6155 | 0 | L1PA3 | L1 | LINE |
| 85783092 | 85783875 | + | 20.5 | 5283 | 6140 | -1645 | L1MC3 | L1 | LINE |

  

|  |  |  |  |  |  |  |  |  |  |
| --- | --- | --- | --- | --- | --- | --- | --- | --- | --- |
| chr14:85874263-85882398 | | | 8055 bp | | |  |  |  |  |
| Genome Start | Genome End | Strand | Div | Repeat Start | Repeat End | Repeat Left | Name | Family | Class |
| 85874263 | 85875252 | - | 21.9 | 5298 | 6459 | -1100 | L1M2 | L1 | LINE |
| 85875260 | 85876889 | - | 11.9 | 4503 | 6159 | -4 | L1PA13 | L1 | LINE |
| 85876862 | 85877599 | + | 15.0 | 3760 | 4501 | -1645 | L1PA13 | L1 | LINE |
| 85877593 | 85877662 | - | 21.8 | 5242 | 5312 | -2247 | L1M2 | L1 | LINE |
| 85877663 | 85878026 | + | 10.4 | 1 | 364 | 0 | THE1B | MaLR | LTR |
| 85878027 | 85881523 | - | 22.9 | 2097 | 5244 | -2318 | L1M2 | L1 | LINE |
| 85881633 | 85882398 | - | 30.0 | 403 | 1206 | -6360 | L1M2 | L1 | LINE |

  

|  |  |  |  |  |  |  |  |  |  |
| --- | --- | --- | --- | --- | --- | --- | --- | --- | --- |
| chr14:87492073-87497638 | | | 5357 bp | | |  |  |  |  |
| Genome Start | Genome End | Strand | Div | Repeat Start | Repeat End | Repeat Left | Name | Family | Class |
| 87492073 | 87492569 | + | 20.6 | 903 | 1406 | -5928 | L1M4 | L1 | LINE |
| 87492728 | 87493716 | - | 13.3 | 4794 | 5795 | -359 | L1P5 | L1 | LINE |
| 87493718 | 87494254 | + | 14.1 | 4261 | 4789 | -1357 | L1PB | L1 | LINE |
| 87494308 | 87494483 | + | 17.8 | 2956 | 3142 | -4192 | L1M4 | L1 | LINE |
| 87494481 | 87495532 | + | 14.9 | 4529 | 5607 | -693 | L1MA6 | L1 | LINE |
| 87495533 | 87495834 | + | 11.4 | 1 | 299 | -13 | AluSx | Alu | SINE |
| 87495835 | 87496304 | + | 15.2 | 5608 | 6066 | -234 | L1MA6 | L1 | LINE |
| 87496300 | 87496630 | + | 18.4 | 5940 | 6300 | 0 | L1MA6 | L1 | LINE |
| 87496636 | 87497638 | + | 17.5 | 5563 | 6585 | -459 | L1M4 | L1 | LINE |

  

|  |  |  |  |  |  |  |  |  |  |
| --- | --- | --- | --- | --- | --- | --- | --- | --- | --- |
| chr14:91212698-91215267 | | | 2569 bp | | |  |  |  |  |
| Genome Start | Genome End | Strand | Div | Repeat Start | Repeat End | Repeat Left | Name | Family | Class |
| 91212698 | 91212779 | + | 16.2 | 606 | 686 | -4657 | LTR57-int | ERVL | LTR |
| 91212780 | 91212832 | - | 11.3 | 4604 | 4656 | -1490 | L1PA8 | L1 | LINE |
| 91212831 | 91214351 | + | 8.1 | 4652 | 6170 | -2 | L1PA8 | L1 | LINE |
| 91214355 | 91215267 | + | 16.0 | 682 | 1595 | -3748 | LTR57-int | ERVL | LTR |

  

|  |  |  |  |  |  |  |  |  |  |
| --- | --- | --- | --- | --- | --- | --- | --- | --- | --- |
| chr14:94564612-94569975 | | | 5354 bp | | |  |  |  |  |
| Genome Start | Genome End | Strand | Div | Repeat Start | Repeat End | Repeat Left | Name | Family | Class |
| 94564612 | 94564674 | - | 14.3 | 6112 | 6179 | -5 | L1MB7 | L1 | LINE |
| 94564675 | 94565288 | - | 11.0 | 4005 | 4629 | -1514 | L1M2 | L1 | LINE |
| 94565282 | 94566024 | + | 12.5 | 4645 | 5411 | -901 | L1M2 | L1 | LINE |
| 94566025 | 94566102 | + | 10.4 | 5336 | 5412 | -879 | L1M | L1 | LINE |
| 94566102 | 94566638 | + | 14.6 | 5336 | 5870 | -430 | L1MA6 | L1 | LINE |
| 94566639 | 94566910 | + | 12.2 | 42 | 304 | -8 | AluSx | Alu | SINE |
| 94566911 | 94567335 | + | 14.6 | 5871 | 6298 | -2 | L1MA6 | L1 | LINE |
| 94567341 | 94569824 | - | 17.9 | 3545 | 6126 | -58 | L1MB7 | L1 | LINE |
| 94569838 | 94569975 | - | 17.1 | 2550 | 2685 | -3455 | L1MB7 | L1 | LINE |

  

|  |  |  |  |  |  |  |  |  |  |
| --- | --- | --- | --- | --- | --- | --- | --- | --- | --- |
| chr14:95873535-95876671 | | | 2807 bp | | |  |  |  |  |
| Genome Start | Genome End | Strand | Div | Repeat Start | Repeat End | Repeat Left | Name | Family | Class |
| 95873535 | 95873638 | + | 14.8 | 5500 | 5605 | -700 | L1MA4 | L1 | LINE |
| 95873640 | 95874101 | - | 12.8 | 4413 | 4881 | -1265 | L1PB | L1 | LINE |
| 95874132 | 95874611 | - | 12.8 | 3920 | 4412 | -1734 | L1PB | L1 | LINE |
| 95874611 | 95875361 | + | 15.4 | 4872 | 5635 | -516 | L1PB2 | L1 | LINE |
| 95875396 | 95875967 | + | 15.6 | 5636 | 6144 | -7 | L1PB2 | L1 | LINE |
| 95875999 | 95876133 | + | 14.8 | 5594 | 5731 | -569 | L1MA4 | L1 | LINE |
| 95876369 | 95876671 | + | 14.4 | 5723 | 6018 | -282 | L1MA4 | L1 | LINE |

  

|  |  |  |  |  |  |  |  |  |  |
| --- | --- | --- | --- | --- | --- | --- | --- | --- | --- |
| chr14:96503653-96506674 | | | 2743 bp | | |  |  |  |  |
| Genome Start | Genome End | Strand | Div | Repeat Start | Repeat End | Repeat Left | Name | Family | Class |
| 96503653 | 96503774 | - | 22.1 | 119 | 262 | 0 | MIR | MIR | SINE |
| 96503777 | 96503862 | - | 19.8 | 107 | 192 | -1 | MER53 | DNA | DNA |
| 96503863 | 96504531 | - | 17.8 | 4270 | 4962 | -1178 | L1M3 | L1 | LINE |
| 96504767 | 96504894 | - | 19.1 | 4160 | 4289 | -1851 | L1M3 | L1 | LINE |
| 96504891 | 96505722 | + | 19.0 | 4954 | 5822 | -469 | L1MA7 | L1 | LINE |
| 96505723 | 96506018 | + | 14.5 | 1 | 300 | -12 | AluSx | Alu | SINE |
| 96506019 | 96506298 | + | 20.8 | 5823 | 6162 | -129 | L1MA7 | L1 | LINE |
| 96506345 | 96506442 | + | 20.8 | 6163 | 6254 | -37 | L1MA7 | L1 | LINE |
| 96506443 | 96506574 | + | 18.0 | 58 | 193 | 0 | MER53 | DNA | DNA |
| 96506575 | 96506674 | - | 26.6 | 39 | 132 | -137 | MIR | MIR | SINE |

  

|  |  |  |  |  |  |  |  |  |  |
| --- | --- | --- | --- | --- | --- | --- | --- | --- | --- |
| chr14:98212492-98216524 | | | 3929 bp | | |  |  |  |  |
| Genome Start | Genome End | Strand | Div | Repeat Start | Repeat End | Repeat Left | Name | Family | Class |
| 98212492 | 98214007 | - | 30.9 | 2337 | 3983 | -2163 | L1M2 | L1 | LINE |
| 98214039 | 98214561 | - | 17.7 | 5744 | 6287 | -4 | L1MA7 | L1 | LINE |
| 98214557 | 98214642 | + | 20.1 | 4552 | 4639 | -1501 | L1M2 | L1 | LINE |
| 98214643 | 98214865 | + | 8.6 | 1 | 223 | -88 | AluY | Alu | SINE |
| 98214866 | 98215763 | + | 19.0 | 4640 | 5490 | -801 | L1M2 | L1 | LINE |
| 98215764 | 98216075 | + | 18.1 | 1 | 306 | -6 | AluJb | Alu | SINE |
| 98216076 | 98216334 | + | 15.8 | 5491 | 5747 | -544 | L1M2 | L1 | LINE |
| 98216413 | 98216524 | - | 29.9 | 2206 | 2313 | -3827 | L1M2 | L1 | LINE |

  

|  |  |  |  |  |  |  |  |  |  |
| --- | --- | --- | --- | --- | --- | --- | --- | --- | --- |
| chr14:100041089-100045639 | | | 4571 bp | | |  |  |  |  |
| Genome Start | Genome End | Strand | Div | Repeat Start | Repeat End | Repeat Left | Name | Family | Class |
| 100041089 | 100041193 | - | 19.1 | 6120 | 6240 | -782 | L1M | L1 | LINE |
| 100041198 | 100041990 | - | 21.9 | 5177 | 5989 | -1017 | L1M | L1 | LINE |
| 100042007 | 100044017 | - | 9.6 | 4143 | 6157 | -9 | L1PA16 | L1 | LINE |
| 100044005 | 100044529 | + | 9.9 | 3619 | 4154 | -1992 | L1PA16 | L1 | LINE |
| 100044527 | 100045507 | - | 23.5 | 4153 | 5192 | -1814 | L1M | L1 | LINE |
| 100045484 | 100045639 | - | 22.4 | 2315 | 2477 | -4857 | L1M | L1 | LINE |

  

|  |  |  |  |  |  |  |  |  |  |
| --- | --- | --- | --- | --- | --- | --- | --- | --- | --- |
| chr14:100673611-100685630 | | | 11647 bp | | |  |  |  |  |
| Genome Start | Genome End | Strand | Div | Repeat Start | Repeat End | Repeat Left | Name | Family | Class |
| 100673611 | 100673863 | - | 21.0 | 5219 | 5478 | -1166 | L1M4c | L1 | LINE |
| 100673880 | 100674329 | + | 27.0 | 12 | 467 | 0 | MLT1C | MaLR | LTR |
| 100674371 | 100674683 | + | 13.2 | 2 | 313 | 0 | AluSq | Alu | SINE |
| 100674693 | 100677199 | - | 18.3 | 2636 | 5189 | -1455 | L1M4c | L1 | LINE |
| 100677200 | 100677307 | - | 11.1 | 6062 | 6172 | -2 | L1PA11 | L1 | LINE |
| 100677308 | 100678439 | - | 28.0 | 1381 | 2737 | -4088 | L1M4c | L1 | LINE |
| 100678508 | 100678549 | + | 20.0 | 2062 | 2101 | -1318 | L2 | L2 | LINE |
| 100678562 | 100679866 | - | 5.7 | 4845 | 6150 | -4 | L1PA7 | L1 | LINE |
| 100679865 | 100681424 | + | 7.9 | 3294 | 4866 | -1280 | L1PA7 | L1 | LINE |
| 100681414 | 100681549 | - | 22.2 | 6049 | 6184 | 0 | L1MB7 | L1 | LINE |
| 100681786 | 100682140 | - | 17.6 | 5712 | 6059 | -125 | L1MB7 | L1 | LINE |
| 100682134 | 100683845 | - | 18.2 | 3552 | 5259 | -887 | L1MB7 | L1 | LINE |
| 100683849 | 100683938 | - | 21.1 | 1270 | 1361 | -5611 | L1M4c | L1 | LINE |
| 100683947 | 100684861 | - | 29.1 | 316 | 1181 | -5361 | L1M4c | L1 | LINE |
| 100684862 | 100685158 | - | 17.6 | 2 | 292 | -20 | AluJo | Alu | SINE |
| 100685159 | 100685172 | - | 29.1 | 316 | 315 | -6227 | L1M4c | L1 | LINE |
| 100685173 | 100685339 | - | 15.0 | 6131 | 6291 | -6 | L1MA5A | L1 | LINE |
| 100685340 | 100685630 | - | 29.1 | 14 | 315 | -6227 | L1M4c | L1 | LINE |

  

|  |  |  |  |  |  |  |  |  |  |
| --- | --- | --- | --- | --- | --- | --- | --- | --- | --- |
| chr14:103992500-103996322 | | | 3600 bp | | |  |  |  |  |
| Genome Start | Genome End | Strand | Div | Repeat Start | Repeat End | Repeat Left | Name | Family | Class |
| 103992500 | 103992738 | + | 34.9 | 33 | 279 | -4575 | L1ME3B | L1 | LINE |
| 103992748 | 103993042 | - | 14.1 | 5978 | 6295 | -2 | L1MA5A | L1 | LINE |
| 103993043 | 103993336 | - | 14.3 | 5796 | 6294 | -3 | L1MA5A | L1 | LINE |
| 103993379 | 103993929 | - | 14.8 | 5206 | 5795 | -502 | L1MA5A | L1 | LINE |
| 103993915 | 103994174 | + | 13.1 | 4960 | 5220 | -923 | L1MA5A | L1 | LINE |
| 103994162 | 103994418 | + | 28.8 | 254 | 513 | -4341 | L1ME3B | L1 | LINE |
| 103994463 | 103995902 | + | 29.5 | 2641 | 4284 | -644 | L1ME3B | L1 | LINE |
| 103995921 | 103995972 | - | 26.9 | 209 | 260 | -2 | MIR | MIR | SINE |
| 103996111 | 103996322 | + | 26.9 | 4446 | 4676 | -252 | L1ME3B | L1 | LINE |

  

|  |  |  |  |  |  |  |  |  |  |
| --- | --- | --- | --- | --- | --- | --- | --- | --- | --- |
| chr14:105632655-105635314 | | | 2643 bp | | |  |  |  |  |
| Genome Start | Genome End | Strand | Div | Repeat Start | Repeat End | Repeat Left | Name | Family | Class |
| 105632655 | 105633012 | + | 22.0 | 3891 | 4264 | -1882 | L1M1 | L1 | LINE |
| 105633047 | 105634274 | - | 5.7 | 4926 | 6154 | 0 | L1PA5 | L1 | LINE |
| 105634265 | 105635054 | + | 4.9 | 4130 | 4928 | -1218 | L1PA5 | L1 | LINE |
| 105635048 | 105635314 | + | 21.0 | 4257 | 4543 | -1603 | L1M1 | L1 | LINE |

  

|  |  |  |  |  |  |  |  |  |  |
| --- | --- | --- | --- | --- | --- | --- | --- | --- | --- |
| chr14:105676686-105680868 | | | 3934 bp | | |  |  |  |  |
| Genome Start | Genome End | Strand | Div | Repeat Start | Repeat End | Repeat Left | Name | Family | Class |
| 105676686 | 105676845 | + | 32.3 | 5630 | 5798 | -366 | L1ME2 | L1 | LINE |
| 105677052 | 105677219 | - | 16.1 | 5263 | 5431 | -735 | L1P4 | L1 | LINE |
| 105677220 | 105677628 | + | 15.3 | 1 | 363 | -1 | THE1B | MaLR | LTR |
| 105677630 | 105679204 | + | 14.7 | 1 | 1580 | 0 | THE1B-int | MaLR | LTR |
| 105679205 | 105679564 | + | 17.0 | 1 | 364 | 0 | THE1B | MaLR | LTR |
| 105679565 | 105679650 | - | 20.4 | 5182 | 5267 | -879 | L1P4 | L1 | LINE |
| 105679679 | 105680035 | - | 20.4 | 4833 | 5181 | -965 | L1P4 | L1 | LINE |
| 105680036 | 105680694 | + | 20.4 | 5449 | 6166 | 0 | L1PA16 | L1 | LINE |
| 105680709 | 105680868 | + | 30.1 | 5998 | 6153 | -11 | L1ME2 | L1 | LINE |

  

|  |  |  |  |  |  |  |  |  |  |
| --- | --- | --- | --- | --- | --- | --- | --- | --- | --- |
| chr14:105824152-105827786 | | | 3620 bp | | |  |  |  |  |
| Genome Start | Genome End | Strand | Div | Repeat Start | Repeat End | Repeat Left | Name | Family | Class |
| 105824152 | 105824719 | - | 22.7 | 5571 | 6152 | -4 | L1PB4 | L1 | LINE |
| 105824736 | 105826818 | - | 9.1 | 4066 | 6150 | -4 | L1PA7 | L1 | LINE |
| 105826819 | 105827077 | + | 11.2 | 3784 | 4041 | -2105 | L1PA7 | L1 | LINE |
| 105827077 | 105827786 | - | 19.6 | 4811 | 5584 | -572 | L1PB4 | L1 | LINE |

  


|  |  |  |  |  |  |  |  |  |  |
| --- | --- | --- | --- | --- | --- | --- | --- | --- | --- |
| chr15:20226608-20227936 | | | 1321 bp | | |  |  |  |  |
| Genome Start | Genome End | Strand | Div | Repeat Start | Repeat End | Repeat Left | Name | Family | Class |
| 20226608 | 20226838 | + | 26.5 | 2616 | 2849 | -570 | L2 | L2 | LINE |
| 20226841 | 20227451 | - | 10.5 | 5347 | 5974 | -189 | L1PA13 | L1 | LINE |
| 20227452 | 20227625 | + | 14.4 | 5991 | 6163 | 0 | L1PA13 | L1 | LINE |
| 20227632 | 20227936 | + | 28.9 | 2841 | 3142 | -277 | L2 | L2 | LINE |

  

|  |  |  |  |  |  |  |  |  |  |
| --- | --- | --- | --- | --- | --- | --- | --- | --- | --- |
| chr15:21779282-21791197 | | | 11478 bp | | |  |  |  |  |
| Genome Start | Genome End | Strand | Div | Repeat Start | Repeat End | Repeat Left | Name | Family | Class |
| 21779282 | 21779402 | - | 23.3 | 1607 | 1734 | -1 | MSTC-int | MaLR | LTR |
| 21779465 | 21780191 | - | 24.7 | 1 | 683 | -8 | LTR8 | ERV1 | LTR |
| 21780195 | 21780500 | - | 26.2 | 5946 | 6259 | -4 | HUERS-P1 | ERV1 | LTR |
| 21780606 | 21781944 | - | 17.2 | 5382 | 6496 | -105 | PRIMA41-int | ERV1 | LTR |
| 21781945 | 21782231 | - | 11.7 | 1 | 284 | -25 | AluSc | Alu | SINE |
| 21782232 | 21782510 | - | 16.1 | 5103 | 5381 | -1537 | PRIMA41-int | ERV1 | LTR |
| 21782511 | 21782685 | - | 18.6 | 146 | 307 | -5 | AluJb | Alu | SINE |
| 21782686 | 21782979 | - | 11.3 | 7 | 299 | -14 | AluSp | Alu | SINE |
| 21782980 | 21783119 | - | 18.6 | 4 | 145 | -167 | AluJb | Alu | SINE |
| 21783120 | 21783750 | - | 16.6 | 2677 | 5102 | -1816 | PRIMA41-int | ERV1 | LTR |
| 21783751 | 21784225 | - | 16.1 | 28 | 537 | 0 | HERV1\_LTRe | ERV1 | LTR |
| 21784239 | 21784489 | - | 20.3 | 3053 | 3292 | -1588 | PRIMA41-int | ERV1 | LTR |
| 21784490 | 21784791 | + | 8.0 | 1 | 309 | -2 | AluY | Alu | SINE |
| 21784792 | 21785551 | - | 20.3 | 1210 | 3052 | -1828 | PRIMA41-int | ERV1 | LTR |
| 21785577 | 21786094 | - | 24.8 | 366 | 2095 | -5442 | PRIMA41-int | ERV1 | LTR |
| 21786096 | 21786652 | - | 23.2 | 462 | 1021 | -5242 | PRIMA41-int | ERV1 | LTR |
| 21786807 | 21786969 | - | 29.2 | 41 | 201 | -6062 | PRIMA41-int | ERV1 | LTR |
| 21786995 | 21787756 | - | 26.2 | 1 | 691 | 0 | LTR8 | ERV1 | LTR |
| 21787759 | 21788730 | - | 29.4 | 442 | 1441 | -210 | MST-int | MaLR | LTR |
| 21788776 | 21788952 | - | 29.4 | 277 | 441 | -1210 | MST-int | MaLR | LTR |
| 21788957 | 21789968 | - | 2.1 | 5136 | 6155 | 0 | L1PA3 | L1 | LINE |
| 21789968 | 21790283 | + | 2.9 | 4816 | 5131 | -1015 | L1PA3 | L1 | LINE |
| 21790284 | 21790339 | - | 25.9 | 290 | 345 | -1390 | MLT-int | MaLR | LTR |
| 21790340 | 21790938 | - | 12.2 | 1 | 644 | 0 | LTR9B | ERV1 | LTR |
| 21790939 | 21791197 | - | 25.9 | 2 | 289 | -1446 | MSTC-int | MaLR | LTR |

  

|  |  |  |  |  |  |  |  |  |  |
| --- | --- | --- | --- | --- | --- | --- | --- | --- | --- |
| chr15:22515580-22520035 | | | 4394 bp | | |  |  |  |  |
| Genome Start | Genome End | Strand | Div | Repeat Start | Repeat End | Repeat Left | Name | Family | Class |
| 22515580 | 22515926 | - | 27.1 | 6512 | 6854 | -931 | L1MC3 | L1 | LINE |
| 22515939 | 22516129 | - | 24.9 | 6161 | 6367 | -1418 | L1MC3 | L1 | LINE |
| 22516181 | 22516456 | - | 24.9 | 5856 | 6160 | -1625 | L1MC3 | L1 | LINE |
| 22516457 | 22517419 | - | 22.0 | 4482 | 5454 | -730 | L1MC3 | L1 | LINE |
| 22517422 | 22517646 | - | 8.9 | 6066 | 6297 | -6 | L1MA4A | L1 | LINE |
| 22517647 | 22517788 | + | 14.1 | 5893 | 6043 | -260 | L1MA4A | L1 | LINE |
| 22517790 | 22517955 | - | 22.4 | 4323 | 4491 | -1655 | L1MC3 | L1 | LINE |
| 22517956 | 22519322 | + | 7.3 | 4774 | 6152 | -2 | L1PA7 | L1 | LINE |
| 22519319 | 22520035 | - | 24.5 | 3593 | 4334 | -1812 | L1MC3 | L1 | LINE |

  

|  |  |  |  |  |  |  |  |  |  |
| --- | --- | --- | --- | --- | --- | --- | --- | --- | --- |
| chr15:25162563-25164848 | | | 2221 bp | | |  |  |  |  |
| Genome Start | Genome End | Strand | Div | Repeat Start | Repeat End | Repeat Left | Name | Family | Class |
| 25162563 | 25162923 | + | 24.3 | 4909 | 5285 | -861 | L1MD2 | L1 | LINE |
| 25162926 | 25163393 | - | 21.1 | 5397 | 5828 | -346 | L1MB4 | L1 | LINE |
| 25163385 | 25163727 | + | 22.7 | 5820 | 6167 | -13 | L1MB4 | L1 | LINE |
| 25163799 | 25164245 | + | 13.5 | 1 | 446 | 0 | AluSq | Alu | SINE |
| 25164247 | 25164848 | + | 19.8 | 5320 | 5942 | -417 | L1MD2 | L1 | LINE |

  

|  |  |  |  |  |  |  |  |  |  |
| --- | --- | --- | --- | --- | --- | --- | --- | --- | --- |
| chr15:25913666-25920159 | | | 6213 bp | | |  |  |  |  |
| Genome Start | Genome End | Strand | Div | Repeat Start | Repeat End | Repeat Left | Name | Family | Class |
| 25913666 | 25914141 | + | 15.2 | 2296 | 2748 | -3392 | L1MA9 | L1 | LINE |
| 25914142 | 25914424 | - | 18.4 | 7 | 292 | -20 | AluJo | Alu | SINE |
| 25914425 | 25914554 | + | 15.2 | 2749 | 2873 | -3267 | L1MA9 | L1 | LINE |
| 25914555 | 25914851 | - | 11.4 | 1 | 297 | -13 | AluSg | Alu | SINE |
| 25914852 | 25915741 | + | 15.2 | 2874 | 3689 | -2451 | L1MA9 | L1 | LINE |
| 25915983 | 25916064 | + | 11.0 | 3673 | 3743 | -2403 | L1MA9 | L1 | LINE |
| 25916063 | 25916799 | - | 2.4 | 4273 | 5009 | -1137 | L1PA3 | L1 | LINE |
| 25916803 | 25917949 | + | 2.8 | 5005 | 6151 | -4 | L1PA3 | L1 | LINE |
| 25917945 | 25919986 | + | 14.2 | 3723 | 5792 | -520 | L1MA9 | L1 | LINE |
| 25920031 | 25920159 | + | 14.2 | 5793 | 5930 | -382 | L1MA9 | L1 | LINE |

  

|  |  |  |  |  |  |  |  |  |  |
| --- | --- | --- | --- | --- | --- | --- | --- | --- | --- |
| chr15:26220839-26228264 | | | 7434 bp | | |  |  |  |  |
| Genome Start | Genome End | Strand | Div | Repeat Start | Repeat End | Repeat Left | Name | Family | Class |
| 26220839 | 26222624 | - | 11.3 | 4323 | 6059 | -246 | L1MA3 | L1 | LINE |
| 26222625 | 26222933 | + | 10.0 | 1 | 309 | -3 | AluSx | Alu | SINE |
| 26222934 | 26223096 | - | 11.0 | 4167 | 4322 | -1824 | L1MA3 | L1 | LINE |
| 26223097 | 26223404 | - | 13.3 | 1 | 312 | 0 | AluSx | Alu | SINE |
| 26223405 | 26223443 | - | 11.0 | 4125 | 4166 | -1980 | L1MA3 | L1 | LINE |
| 26223451 | 26224058 | - | 1.6 | 5553 | 6155 | 0 | L1PA2 | L1 | LINE |
| 26224059 | 26224838 | + | 0.9 | 4781 | 5561 | -594 | L1PA2 | L1 | LINE |
| 26224835 | 26225571 | - | 12.1 | 3396 | 4127 | -2019 | L1MA3 | L1 | LINE |
| 26225572 | 26225875 | - | 13.5 | 1 | 303 | -9 | AluSx | Alu | SINE |
| 26225876 | 26226466 | - | 12.1 | 2759 | 3395 | -2751 | L1MA3 | L1 | LINE |
| 26226457 | 26227785 | - | 8.3 | 3652 | 4996 | -1150 | L1M1 | L1 | LINE |
| 26227785 | 26228264 | - | 12.8 | 2280 | 2763 | -3383 | L1MA3 | L1 | LINE |

  

|  |  |  |  |  |  |  |  |  |  |
| --- | --- | --- | --- | --- | --- | --- | --- | --- | --- |
| chr15:26343902-26345229 | | | 1320 bp | | |  |  |  |  |
| Genome Start | Genome End | Strand | Div | Repeat Start | Repeat End | Repeat Left | Name | Family | Class |
| 26343902 | 26344203 | - | 28.8 | 2841 | 3142 | -277 | L2 | L2 | LINE |
| 26344210 | 26344383 | - | 13.9 | 5991 | 6163 | 0 | L1PA13 | L1 | LINE |
| 26344384 | 26344994 | + | 11.0 | 5347 | 5974 | -189 | L1PA13 | L1 | LINE |
| 26344997 | 26345229 | - | 27.6 | 2616 | 2849 | -570 | L2 | L2 | LINE |

  

|  |  |  |  |  |  |  |  |  |  |
| --- | --- | --- | --- | --- | --- | --- | --- | --- | --- |
| chr15:26535036-26536363 | | | 1320 bp | | |  |  |  |  |
| Genome Start | Genome End | Strand | Div | Repeat Start | Repeat End | Repeat Left | Name | Family | Class |
| 26535036 | 26535268 | + | 27.6 | 2616 | 2849 | -570 | L2 | L2 | LINE |
| 26535271 | 26535881 | - | 11.1 | 5347 | 5974 | -189 | L1PA13 | L1 | LINE |
| 26535882 | 26536055 | + | 14.4 | 5991 | 6163 | 0 | L1PA13 | L1 | LINE |
| 26536062 | 26536363 | + | 27.9 | 2841 | 3142 | -277 | L2 | L2 | LINE |

  

|  |  |  |  |  |  |  |  |  |  |
| --- | --- | --- | --- | --- | --- | --- | --- | --- | --- |
| chr15:27290964-27297831 | | | 6662 bp | | |  |  |  |  |
| Genome Start | Genome End | Strand | Div | Repeat Start | Repeat End | Repeat Left | Name | Family | Class |
| 27290964 | 27292266 | - | 7.8 | 3332 | 4629 | -1983 | L1PBa1 | L1 | LINE |
| 27292267 | 27293218 | - | 4.6 | 4220 | 5173 | -973 | L1PA5 | L1 | LINE |
| 27293217 | 27294196 | + | 4.0 | 5169 | 6154 | 0 | L1PA5 | L1 | LINE |
| 27294211 | 27294680 | - | 7.9 | 2873 | 3345 | -3267 | L1PBa1 | L1 | LINE |
| 27294681 | 27295064 | + | 7.6 | 1 | 403 | -41 | MLT2A1 | ERVL | LTR |
| 27295094 | 27295133 | + | 7.6 | 512 | 551 | -1 | MLT2A1 | ERVL | LTR |
| 27295134 | 27296119 | - | 9.6 | 1882 | 2872 | -3740 | L1PBa1 | L1 | LINE |
| 27296226 | 27296318 | - | 17.8 | 6025 | 6120 | -1 | L1ME4a | L1 | LINE |
| 27296378 | 27296907 | - | 6.7 | 1 | 532 | -26 | LTR6B | ERV1 | LTR |
| 27296908 | 27297831 | - | 10.6 | 942 | 1885 | -4728 | L1PBa1 | L1 | LINE |

  

|  |  |  |  |  |  |  |  |  |  |
| --- | --- | --- | --- | --- | --- | --- | --- | --- | --- |
| chr15:27466077-27468817 | | | 2677 bp | | |  |  |  |  |
| Genome Start | Genome End | Strand | Div | Repeat Start | Repeat End | Repeat Left | Name | Family | Class |
| 27466077 | 27466275 | + | 20.4 | 1 | 209 | -165 | MLT1A | MaLR | LTR |
| 27466296 | 27466818 | - | 4.2 | 3829 | 4351 | -1795 | L1PA5 | L1 | LINE |
| 27466817 | 27468621 | + | 4.0 | 4342 | 6153 | -1 | L1PA5 | L1 | LINE |
| 27468668 | 27468817 | + | 20.1 | 237 | 390 | 0 | MLT1A | MaLR | LTR |

  

|  |  |  |  |  |  |  |  |  |  |
| --- | --- | --- | --- | --- | --- | --- | --- | --- | --- |
| chr15:28235719-28239153 | | | 3458 bp | | |  |  |  |  |
| Genome Start | Genome End | Strand | Div | Repeat Start | Repeat End | Repeat Left | Name | Family | Class |
| 28235719 | 28236116 | - | 15.4 | 5781 | 6184 | 0 | L1MB7 | L1 | LINE |
| 28236117 | 28236410 | - | 15.3 | 1 | 296 | -16 | AluJo | Alu | SINE |
| 28236411 | 28236733 | - | 15.4 | 5469 | 5780 | -404 | L1MB7 | L1 | LINE |
| 28236717 | 28238144 | - | 3.6 | 4702 | 6155 | 0 | L1PA4 | L1 | LINE |
| 28238142 | 28238844 | + | 4.5 | 4003 | 4705 | -1441 | L1PA4 | L1 | LINE |
| 28238842 | 28239153 | - | 19.2 | 5193 | 5499 | -679 | L1MB7 | L1 | LINE |

  

|  |  |  |  |  |  |  |  |  |  |
| --- | --- | --- | --- | --- | --- | --- | --- | --- | --- |
| chr15:30455832-30461622 | | | 5789 bp | | |  |  |  |  |
| Genome Start | Genome End | Strand | Div | Repeat Start | Repeat End | Repeat Left | Name | Family | Class |
| 30455832 | 30456073 | + | 16.5 | 3988 | 4255 | -1891 | L1MB7 | L1 | LINE |
| 30456099 | 30456121 | + | 16.5 | 4256 | 4275 | -1871 | L1MB7 | L1 | LINE |
| 30456122 | 30456429 | + | 13.0 | 1 | 308 | -4 | AluSx | Alu | SINE |
| 30456430 | 30456823 | + | 16.5 | 4276 | 4705 | -1441 | L1MB7 | L1 | LINE |
| 30456824 | 30457127 | + | 14.5 | 1 | 306 | -6 | AluJb | Alu | SINE |
| 30457128 | 30457457 | + | 16.5 | 4706 | 5004 | -1142 | L1MB7 | L1 | LINE |
| 30457458 | 30457649 | + | 18.8 | 96 | 288 | -24 | AluJb | Alu | SINE |
| 30457651 | 30457856 | + | 17.5 | 4993 | 5191 | -955 | L1MB7 | L1 | LINE |
| 30457857 | 30458162 | + | 11.2 | 1 | 303 | -10 | AluSq | Alu | SINE |
| 30458163 | 30458474 | + | 17.0 | 5192 | 5499 | -679 | L1MB7 | L1 | LINE |
| 30458472 | 30459174 | - | 4.7 | 4003 | 4705 | -1441 | L1PA4 | L1 | LINE |
| 30459172 | 30460624 | + | 3.9 | 4702 | 6155 | 0 | L1PA4 | L1 | LINE |
| 30460607 | 30460929 | + | 14.7 | 5469 | 5785 | -399 | L1MB7 | L1 | LINE |
| 30460930 | 30461224 | + | 15.6 | 1 | 297 | -15 | AluJo | Alu | SINE |
| 30461225 | 30461622 | + | 14.7 | 5786 | 6184 | 0 | L1MB7 | L1 | LINE |

  

|  |  |  |  |  |  |  |  |  |  |
| --- | --- | --- | --- | --- | --- | --- | --- | --- | --- |
| chr15:34027082-34030058 | | | 2826 bp | | |  |  |  |  |
| Genome Start | Genome End | Strand | Div | Repeat Start | Repeat End | Repeat Left | Name | Family | Class |
| 34027082 | 34027477 | - | 37.9 | 2953 | 3393 | -26 | L2 | L2 | LINE |
| 34027510 | 34027641 | - | 26.0 | 4 | 134 | 0 | MER94 | AcHobo | DNA |
| 34027674 | 34028281 | - | 36.3 | 2186 | 2842 | -577 | L2 | L2 | LINE |
| 34028374 | 34028506 | + | 25.2 | 340 | 473 | -18 | LTR16C | ERVL | LTR |
| 34028508 | 34028684 | - | 31.4 | 1983 | 2181 | -1238 | L2 | L2 | LINE |
| 34028687 | 34029042 | - | 4.8 | 5530 | 5887 | -267 | L1PA7 | L1 | LINE |
| 34029030 | 34029230 | + | 8.5 | 5880 | 6079 | -75 | L1PA7 | L1 | LINE |
| 34029233 | 34029472 | + | 8.3 | 58 | 298 | -13 | AluY | Alu | SINE |
| 34029474 | 34029517 | - | 20.4 | 1754 | 1797 | -1622 | L2 | L2 | LINE |
| 34029520 | 34030058 | - | 37.2 | 919 | 1504 | -1915 | L2 | L2 | LINE |

  

|  |  |  |  |  |  |  |  |  |  |
| --- | --- | --- | --- | --- | --- | --- | --- | --- | --- |
| chr15:34931594-34934596 | | | 2389 bp | | |  |  |  |  |
| Genome Start | Genome End | Strand | Div | Repeat Start | Repeat End | Repeat Left | Name | Family | Class |
| 34931594 | 34931765 | + | 23.1 | 2280 | 2479 | -3760 | L1ME3 | L1 | LINE |
| 34931777 | 34932040 | + | 14.5 | 1 | 262 | -51 | AluSq | Alu | SINE |
| 34932380 | 34932573 | + | 22.8 | 2491 | 2692 | -3527 | L1ME3 | L1 | LINE |
| 34932748 | 34933010 | + | 25.9 | 5406 | 5663 | -572 | L1ME3 | L1 | LINE |
| 34933011 | 34933325 | + | 16.8 | 4 | 339 | 0 | MER44A | MER2\_type | DNA |
| 34933326 | 34933736 | + | 25.9 | 5664 | 6085 | -150 | L1ME3 | L1 | LINE |
| 34933782 | 34934084 | - | 14.6 | 5864 | 6161 | -7 | L1MB1 | L1 | LINE |
| 34934085 | 34934418 | + | 14.4 | 5543 | 5876 | -313 | L1MB1 | L1 | LINE |
| 34934416 | 34934466 | + | 21.6 | 6091 | 6142 | -93 | L1ME3 | L1 | LINE |
| 34934515 | 34934596 | + | 25.9 | 6153 | 6234 | -1 | L1ME3 | L1 | LINE |

  

|  |  |  |  |  |  |  |  |  |  |
| --- | --- | --- | --- | --- | --- | --- | --- | --- | --- |
| chr15:35695578-35702228 | | | 6283 bp | | |  |  |  |  |
| Genome Start | Genome End | Strand | Div | Repeat Start | Repeat End | Repeat Left | Name | Family | Class |
| 35695578 | 35696216 | + | 14.9 | 1 | 664 | -2067 | Tigger4(Zombi) | MER2\_type | DNA |
| 35696217 | 35696515 | + | 14.4 | 1 | 300 | -12 | AluSx | Alu | SINE |
| 35696704 | 35697277 | + | 14.9 | 665 | 1230 | -1501 | Tigger4(Zombi) | MER2\_type | DNA |
| 35697316 | 35698300 | - | 12.6 | 5172 | 6166 | 0 | L1PA16 | L1 | LINE |
| 35698328 | 35698609 | - | 6.4 | 5868 | 6154 | 0 | L1PA6 | L1 | LINE |
| 35698610 | 35698692 | + | 2.4 | 5779 | 5861 | -293 | L1PA6 | L1 | LINE |
| 35698714 | 35698994 | - | 12.1 | 4895 | 5180 | -966 | L1PA16 | L1 | LINE |
| 35698995 | 35699783 | + | 13.8 | 1215 | 2015 | -716 | Tigger4(Zombi) | MER2\_type | DNA |
| 35699784 | 35700090 | + | 10.1 | 1 | 308 | -5 | AluSp | Alu | SINE |
| 35700091 | 35700142 | + | 13.8 | 2016 | 2071 | -660 | Tigger4(Zombi) | MER2\_type | DNA |
| 35700147 | 35700282 | - | 8.7 | 183 | 313 | 0 | AluSg | Alu | SINE |
| 35700328 | 35700526 | - | 11.1 | 1 | 201 | -109 | AluSg | Alu | SINE |
| 35700549 | 35701042 | - | 2.7 | 5075 | 5572 | -583 | L1PA3 | L1 | LINE |
| 35701044 | 35701643 | + | 3.5 | 5555 | 6154 | -1 | L1PA3 | L1 | LINE |
| 35701666 | 35702228 | + | 11.2 | 2073 | 2731 | 0 | Tigger4(Zombi) | MER2\_type | DNA |

  

|  |  |  |  |  |  |  |  |  |  |
| --- | --- | --- | --- | --- | --- | --- | --- | --- | --- |
| chr15:37046828-37049563 | | | 2303 bp | | |  |  |  |  |
| Genome Start | Genome End | Strand | Div | Repeat Start | Repeat End | Repeat Left | Name | Family | Class |
| 37046828 | 37047590 | - | 25.6 | 794 | 1685 | -4554 | L1MEc | L1 | LINE |
| 37047625 | 37048174 | - | 17.8 | 5613 | 6153 | -3 | L1PB4 | L1 | LINE |
| 37048176 | 37048878 | + | 14.7 | 4858 | 5612 | -544 | L1PB4 | L1 | LINE |
| 37049277 | 37049563 | - | 35.4 | 89 | 377 | -6023 | L1MEc | L1 | LINE |

  

|  |  |  |  |  |  |  |  |  |  |
| --- | --- | --- | --- | --- | --- | --- | --- | --- | --- |
| chr15:38217859-38221701 | | | 3797 bp | | |  |  |  |  |
| Genome Start | Genome End | Strand | Div | Repeat Start | Repeat End | Repeat Left | Name | Family | Class |
| 38217859 | 38217940 | + | 23.2 | 2353 | 2438 | -981 | L2 | L2 | LINE |
| 38217941 | 38219690 | - | 3.1 | 3581 | 5332 | -814 | L1PA4 | L1 | LINE |
| 38219680 | 38220508 | + | 4.0 | 5348 | 6175 | 0 | L1PA4 | L1 | LINE |
| 38220558 | 38220856 | + | 4.7 | 1 | 299 | -12 | AluY | Alu | SINE |
| 38220865 | 38220878 | + | 26.2 | 2476 | 2489 | -930 | L2 | L2 | LINE |
| 38220879 | 38221215 | + | 22.3 | 1 | 396 | 0 | MSTD | MaLR | LTR |
| 38221216 | 38221701 | + | 26.2 | 2490 | 3014 | -405 | L2 | L2 | LINE |

  

|  |  |  |  |  |  |  |  |  |  |
| --- | --- | --- | --- | --- | --- | --- | --- | --- | --- |
| chr15:41118652-41122221 | | | 3562 bp | | |  |  |  |  |
| Genome Start | Genome End | Strand | Div | Repeat Start | Repeat End | Repeat Left | Name | Family | Class |
| 41118652 | 41118784 | + | 18.3 | 4961 | 5076 | -1064 | L1MB3 | L1 | LINE |
| 41118780 | 41119491 | - | 5.3 | 3830 | 4541 | -1605 | L1PA5 | L1 | LINE |
| 41119494 | 41121094 | + | 5.4 | 4546 | 6154 | 0 | L1PA5 | L1 | LINE |
| 41121106 | 41122221 | + | 13.5 | 5065 | 6171 | -12 | L1MB3 | L1 | LINE |

  

|  |  |  |  |  |  |  |  |  |  |
| --- | --- | --- | --- | --- | --- | --- | --- | --- | --- |
| chr15:44278307-44283091 | | | 4727 bp | | |  |  |  |  |
| Genome Start | Genome End | Strand | Div | Repeat Start | Repeat End | Repeat Left | Name | Family | Class |
| 44278307 | 44278357 | - | 11.8 | 6236 | 6286 | -5 | L1MA8 | L1 | LINE |
| 44278429 | 44279384 | - | 18.2 | 5157 | 6110 | -181 | L1MA4 | L1 | LINE |
| 44279378 | 44279785 | + | 16.0 | 4732 | 5149 | -994 | L1MA4 | L1 | LINE |
| 44279780 | 44283091 | - | 21.5 | 2718 | 6243 | -48 | L1MA8 | L1 | LINE |

  

|  |  |  |  |  |  |  |  |  |  |
| --- | --- | --- | --- | --- | --- | --- | --- | --- | --- |
| chr15:44455128-44460093 | | | 4940 bp | | |  |  |  |  |
| Genome Start | Genome End | Strand | Div | Repeat Start | Repeat End | Repeat Left | Name | Family | Class |
| 44455128 | 44455486 | - | 34.0 | 206 | 557 | 0 | LTR67 | ERVL | LTR |
| 44455509 | 44458768 | - | 4.8 | 2876 | 6146 | -8 | L1PA5 | L1 | LINE |
| 44458755 | 44460004 | + | 5.7 | 1628 | 2881 | -3265 | L1PA5 | L1 | LINE |
| 44460023 | 44460093 | - | 21.7 | 110 | 183 | -374 | LTR67 | ERVL | LTR |

  

|  |  |  |  |  |  |  |  |  |  |
| --- | --- | --- | --- | --- | --- | --- | --- | --- | --- |
| chr15:52049217-52050667 | | | 1448 bp | | |  |  |  |  |
| Genome Start | Genome End | Strand | Div | Repeat Start | Repeat End | Repeat Left | Name | Family | Class |
| 52049217 | 52049535 | - | 18.8 | 5829 | 6152 | -19 | L1MB2 | L1 | LINE |
| 52049536 | 52049857 | - | 13.6 | 1 | 304 | -9 | AluSq | Alu | SINE |
| 52049858 | 52050024 | - | 18.8 | 5662 | 5828 | -343 | L1PA11 | L1 | LINE |
| 52050024 | 52050337 | + | 12.5 | 5848 | 6171 | -3 | L1PA11 | L1 | LINE |
| 52050342 | 52050667 | - | 15.8 | 5332 | 5660 | -511 | L1MB2 | L1 | LINE |

  

|  |  |  |  |  |  |  |  |  |  |
| --- | --- | --- | --- | --- | --- | --- | --- | --- | --- |
| chr15:52861681-52865144 | | | 3464 bp | | |  |  |  |  |
| Genome Start | Genome End | Strand | Div | Repeat Start | Repeat End | Repeat Left | Name | Family | Class |
| 52861681 | 52861816 | + | 28.1 | 4015 | 4167 | -3395 | L1M1 | L1 | LINE |
| 52861833 | 52862178 | + | 21.1 | 4557 | 4909 | -2650 | L1M1 | L1 | LINE |
| 52862175 | 52863102 | - | 10.7 | 3977 | 4915 | -1231 | L1P3 | L1 | LINE |
| 52863091 | 52864796 | + | 9.1 | 2220 | 3955 | -2191 | L1P3 | L1 | LINE |
| 52864797 | 52865106 | + | 6.8 | 1 | 310 | -1 | AluY | Alu | SINE |
| 52865107 | 52865144 | + | 9.1 | 5375 | 5412 | -2153 | L1M1 | L1 | LINE |

  

|  |  |  |  |  |  |  |  |  |  |
| --- | --- | --- | --- | --- | --- | --- | --- | --- | --- |
| chr15:53046884-53050523 | | | 3587 bp | | |  |  |  |  |
| Genome Start | Genome End | Strand | Div | Repeat Start | Repeat End | Repeat Left | Name | Family | Class |
| 53046884 | 53047315 | + | 17.1 | 4551 | 5013 | -1127 | L1MB1 | L1 | LINE |
| 53047316 | 53047688 | + | 16.7 | 5215 | 5608 | -560 | L1MB1 | L1 | LINE |
| 53047684 | 53048746 | - | 8.4 | 4472 | 5539 | -615 | L1PA7 | L1 | LINE |
| 53048748 | 53049346 | + | 6.7 | 5539 | 6143 | -11 | L1PA7 | L1 | LINE |
| 53049401 | 53049601 | + | 14.0 | 5593 | 5792 | -542 | L1MB1 | L1 | LINE |
| 53049605 | 53050155 | - | 23.2 | 2 | 547 | -7 | MLT2B3 | ERVL | LTR |
| 53050156 | 53050523 | + | 13.9 | 5787 | 6160 | -8 | L1MB1 | L1 | LINE |

  

|  |  |  |  |  |  |  |  |  |  |
| --- | --- | --- | --- | --- | --- | --- | --- | --- | --- |
| chr15:54065039-54068443 | | | 3273 bp | | |  |  |  |  |
| Genome Start | Genome End | Strand | Div | Repeat Start | Repeat End | Repeat Left | Name | Family | Class |
| 54065039 | 54065244 | + | 23.6 | 6463 | 6704 | -1338 | L1MC4 | L1 | LINE |
| 54065279 | 54066024 | + | 23.6 | 6705 | 7520 | -522 | L1MC4 | L1 | LINE |
| 54066071 | 54066384 | - | 7.2 | 1 | 308 | -3 | AluY | Alu | SINE |
| 54066425 | 54067507 | - | 0.3 | 4609 | 5691 | -464 | L1HS | L1 | LINE |
| 54067508 | 54067975 | + | 1.3 | 5686 | 6153 | -2 | L1HS | L1 | LINE |
| 54067988 | 54068443 | + | 29.4 | 7578 | 8038 | -4 | L1MC4 | L1 | LINE |

  

|  |  |  |  |  |  |  |  |  |  |
| --- | --- | --- | --- | --- | --- | --- | --- | --- | --- |
| chr15:54982606-54985392 | | | 2772 bp | | |  |  |  |  |
| Genome Start | Genome End | Strand | Div | Repeat Start | Repeat End | Repeat Left | Name | Family | Class |
| 54982606 | 54982740 | + | 11.4 | 1 | 132 | -180 | AluSx | Alu | SINE |
| 54982741 | 54983550 | - | 5.3 | 3709 | 4526 | -1620 | L1PA5 | L1 | LINE |
| 54983536 | 54985170 | + | 5.0 | 4515 | 6153 | -1 | L1PA5 | L1 | LINE |
| 54985201 | 54985392 | + | 19.3 | 121 | 312 | 0 | AluSx | Alu | SINE |

  

|  |  |  |  |  |  |  |  |  |  |
| --- | --- | --- | --- | --- | --- | --- | --- | --- | --- |
| chr15:55072410-55079392 | | | 6975 bp | | |  |  |  |  |
| Genome Start | Genome End | Strand | Div | Repeat Start | Repeat End | Repeat Left | Name | Family | Class |
| 55072410 | 55074492 | + | 25.5 | 33 | 2200 | -4440 | L1MD2 | L1 | LINE |
| 55074514 | 55074646 | + | 26.5 | 2275 | 2470 | -4255 | L1MD2 | L1 | LINE |
| 55074655 | 55075783 | - | 11.4 | 3643 | 4777 | -1369 | L1PA16 | L1 | LINE |
| 55075769 | 55077126 | + | 13.8 | 4766 | 6159 | -7 | L1PA16 | L1 | LINE |
| 55077121 | 55077666 | + | 23.9 | 2442 | 3008 | -3536 | L1MD2 | L1 | LINE |
| 55077667 | 55077967 | + | 13.0 | 3 | 302 | -10 | AluSx | Alu | SINE |
| 55077968 | 55078406 | + | 23.9 | 3009 | 3483 | -3061 | L1MD2 | L1 | LINE |
| 55078407 | 55078699 | + | 5.8 | 1 | 293 | -18 | AluY | Alu | SINE |
| 55078700 | 55079081 | + | 23.9 | 3484 | 3878 | -2666 | L1MD2 | L1 | LINE |
| 55079082 | 55079375 | + | 14.6 | 1 | 294 | -18 | AluSx | Alu | SINE |
| 55079376 | 55079392 | + | 23.9 | 3879 | 3893 | -2651 | L1MD2 | L1 | LINE |

  

|  |  |  |  |  |  |  |  |  |  |
| --- | --- | --- | --- | --- | --- | --- | --- | --- | --- |
| chr15:56175583-56184547 | | | 8867 bp | | |  |  |  |  |
| Genome Start | Genome End | Strand | Div | Repeat Start | Repeat End | Repeat Left | Name | Family | Class |
| 56175583 | 56176235 | - | 18.0 | 5963 | 6661 | -21 | L1MB7 | L1 | LINE |
| 56176236 | 56176615 | - | 11.7 | 1 | 375 | 0 | THE1C | MaLR | LTR |
| 56176616 | 56177733 | - | 17.2 | 4827 | 5962 | -720 | L1MB7 | L1 | LINE |
| 56177734 | 56178031 | - | 7.4 | 1 | 296 | -15 | AluY | Alu | SINE |
| 56178032 | 56178836 | - | 17.1 | 3993 | 4826 | -1818 | L1MB7 | L1 | LINE |
| 56178875 | 56179471 | - | 17.1 | 3363 | 3992 | -2652 | L1MB7 | L1 | LINE |
| 56179465 | 56180539 | - | 12.3 | 4855 | 5879 | -252 | L1PA16 | L1 | LINE |
| 56180540 | 56180792 | + | 15.8 | 5900 | 6158 | -8 | L1PA16 | L1 | LINE |
| 56180808 | 56181327 | - | 18.4 | 2839 | 3367 | -3277 | L1MB7 | L1 | LINE |
| 56181385 | 56181839 | - | 15.9 | 3 | 470 | -3 | MER65D | ERV1 | LTR |
| 56181840 | 56182173 | - | 19.6 | 1 | 327 | -38 | MLT1A0 | MaLR | LTR |
| 56182174 | 56184186 | - | 22.0 | 690 | 2906 | -3919 | L1MB7 | L1 | LINE |
| 56184182 | 56184547 | - | 24.5 | 6 | 374 | -6168 | L1MB7 | L1 | LINE |

  

|  |  |  |  |  |  |  |  |  |  |
| --- | --- | --- | --- | --- | --- | --- | --- | --- | --- |
| chr15:66194718-66200891 | | | 6162 bp | | |  |  |  |  |
| Genome Start | Genome End | Strand | Div | Repeat Start | Repeat End | Repeat Left | Name | Family | Class |
| 66194718 | 66196271 | - | 22.9 | 2913 | 4471 | -1748 | L1MEc | L1 | LINE |
| 66196289 | 66196465 | - | 15.8 | 6133 | 6295 | -5 | L1MA6 | L1 | LINE |
| 66196466 | 66196777 | - | 13.9 | 1 | 312 | 0 | AluSx | Alu | SINE |
| 66196778 | 66197436 | - | 15.8 | 5482 | 6132 | -168 | L1MA6 | L1 | LINE |
| 66197437 | 66197728 | + | 12.8 | 1 | 298 | -14 | AluSx | Alu | SINE |
| 66197729 | 66197746 | - | 15.8 | 5482 | 5481 | -819 | L1MA6 | L1 | LINE |
| 66197747 | 66198033 | - | 20.6 | 1 | 282 | -30 | AluJo | Alu | SINE |
| 66198034 | 66198186 | - | 15.8 | 5340 | 5481 | -819 | L1MA6 | L1 | LINE |
| 66198187 | 66198508 | + | 12.8 | 4984 | 5318 | -825 | L1MA6 | L1 | LINE |
| 66198509 | 66198844 | + | 15.6 | 1 | 292 | -19 | AluYh9 | Alu | SINE |
| 66198845 | 66198877 | + | 12.8 | 5319 | 5347 | -796 | L1M2 | L1 | LINE |
| 66198873 | 66199366 | - | 22.1 | 2404 | 2916 | -3303 | L1MEc | L1 | LINE |
| 66199367 | 66199497 | - | 17.6 | 2 | 133 | 0 | FLAM\_A | Alu | SINE |
| 66199498 | 66200061 | - | 19.2 | 1974 | 2476 | -3924 | L1MEc | L1 | LINE |
| 66200062 | 66200327 | - | 10.9 | 1 | 283 | -27 | AluSg | Alu | SINE |
| 66200328 | 66200891 | - | 18.9 | 1373 | 1973 | -4427 | L1MEc | L1 | LINE |

  

|  |  |  |  |  |  |  |  |  |  |
| --- | --- | --- | --- | --- | --- | --- | --- | --- | --- |
| chr15:67337314-67339114 | | | 1664 bp | | |  |  |  |  |
| Genome Start | Genome End | Strand | Div | Repeat Start | Repeat End | Repeat Left | Name | Family | Class |
| 67337314 | 67337374 | - | 25.1 | 5950 | 6010 | -111 | L1ME4a | L1 | LINE |
| 67337403 | 67337745 | - | 9.9 | 5800 | 6144 | -19 | L1PA13 | L1 | LINE |
| 67337746 | 67337954 | - | 25.1 | 5733 | 5949 | -172 | L1ME4a | L1 | LINE |
| 67337980 | 67338174 | - | 9.9 | 6112 | 6303 | -2 | L1MA3 | L1 | LINE |
| 67338175 | 67338221 | + | 14.9 | 6054 | 6101 | -204 | L1MA3 | L1 | LINE |
| 67338226 | 67338580 | - | 29.3 | 5357 | 5738 | -383 | L1ME4a | L1 | LINE |
| 67338661 | 67339114 | - | 36.3 | 4756 | 5240 | -906 | L1ME4a | L1 | LINE |

  

|  |  |  |  |  |  |  |  |  |  |
| --- | --- | --- | --- | --- | --- | --- | --- | --- | --- |
| chr15:74786281-74793698 | | | 7398 bp | | |  |  |  |  |
| Genome Start | Genome End | Strand | Div | Repeat Start | Repeat End | Repeat Left | Name | Family | Class |
| 74786281 | 74787421 | + | 25.3 | 1874 | 3001 | -3218 | L1ME1 | L1 | LINE |
| 74787422 | 74787742 | - | 20.3 | 1 | 344 | -1 | MER2 | MER2\_type | DNA |
| 74787743 | 74788179 | + | 23.4 | 3002 | 3371 | -2848 | L1ME1 | L1 | LINE |
| 74788180 | 74788446 | + | 14.2 | 1 | 267 | -45 | AluSx | Alu | SINE |
| 74788447 | 74788524 | + | 23.4 | 3372 | 3434 | -2785 | L1ME1 | L1 | LINE |
| 74788525 | 74788829 | - | 10.0 | 1 | 301 | -8 | AluSc | Alu | SINE |
| 74788830 | 74789735 | + | 23.4 | 3435 | 4386 | -1853 | L1ME1 | L1 | LINE |
| 74789736 | 74789808 | + | 23.3 | 2334 | 2406 | 0 | Tigger5 | MER2\_type | DNA |
| 74789809 | 74791009 | + | 23.4 | 4387 | 5649 | -590 | L1ME1 | L1 | LINE |
| 74791007 | 74792515 | - | 3.6 | 3972 | 5480 | -675 | L1PA3 | L1 | LINE |
| 74792516 | 74793185 | + | 2.1 | 5483 | 6152 | -3 | L1PA3 | L1 | LINE |
| 74793209 | 74793698 | + | 23.7 | 5645 | 6193 | -46 | L1ME1 | L1 | LINE |

  

|  |  |  |  |  |  |  |  |  |  |
| --- | --- | --- | --- | --- | --- | --- | --- | --- | --- |
| chr15:76264264-76270898 | | | 6519 bp | | |  |  |  |  |
| Genome Start | Genome End | Strand | Div | Repeat Start | Repeat End | Repeat Left | Name | Family | Class |
| 76264264 | 76264865 | + | 27.9 | 439 | 1005 | -5713 | L1MEc | L1 | LINE |
| 76264874 | 76265420 | + | 24.3 | 1473 | 2017 | -4616 | L1MEc | L1 | LINE |
| 76265418 | 76265611 | - | 5.7 | 2326 | 2519 | -3627 | L1PA3 | L1 | LINE |
| 76265598 | 76269232 | + | 2.5 | 2511 | 6155 | 0 | L1PA3 | L1 | LINE |
| 76269356 | 76269523 | + | 25.3 | 2075 | 2249 | -4469 | L1MEc | L1 | LINE |
| 76269524 | 76269659 | + | 14.8 | 1 | 135 | -177 | AluJo | Alu | SINE |
| 76269664 | 76269742 | - | 16.9 | 1922 | 1999 | -4401 | L1MEc | L1 | LINE |
| 76269743 | 76269835 | - | 18.3 | 217 | 309 | -3 | AluJ/FRAM | Alu | SINE |
| 76269836 | 76269994 | + | 12.6 | 151 | 309 | -3 | AluJb | Alu | SINE |
| 76269995 | 76270358 | + | 21.0 | 2238 | 2617 | -4101 | L1MEc | L1 | LINE |
| 76270357 | 76270898 | + | 25.5 | 2572 | 3118 | -3419 | L1MEc | L1 | LINE |

  

|  |  |  |  |  |  |  |  |  |  |
| --- | --- | --- | --- | --- | --- | --- | --- | --- | --- |
| chr15:79155774-79159301 | | | 3438 bp | | |  |  |  |  |
| Genome Start | Genome End | Strand | Div | Repeat Start | Repeat End | Repeat Left | Name | Family | Class |
| 79155774 | 79156426 | - | 32.0 | 573 | 1230 | -4889 | L1MEd | L1 | LINE |
| 79156429 | 79156715 | + | 13.7 | 1 | 301 | -11 | AluSx | Alu | SINE |
| 79156771 | 79157629 | - | 5.4 | 4228 | 5086 | -1060 | L1PA5 | L1 | LINE |
| 79157631 | 79158709 | + | 5.0 | 5084 | 6154 | 0 | L1PA5 | L1 | LINE |
| 79158712 | 79158828 | + | 11.0 | 6016 | 6166 | 0 | L1PREC2 | L1 | LINE |
| 79158855 | 79159153 | + | 11.5 | 1 | 297 | -15 | AluSx | Alu | SINE |
| 79159158 | 79159301 | - | 27.1 | 418 | 550 | -5569 | L1MEd | L1 | LINE |

  

|  |  |  |  |  |  |  |  |  |  |
| --- | --- | --- | --- | --- | --- | --- | --- | --- | --- |
| chr15:85295995-85299376 | | | 3359 bp | | |  |  |  |  |
| Genome Start | Genome End | Strand | Div | Repeat Start | Repeat End | Repeat Left | Name | Family | Class |
| 85295995 | 85296185 | - | 27.3 | 2399 | 2596 | -3623 | L1MEc | L1 | LINE |
| 85296186 | 85296478 | + | 10.9 | 1 | 298 | -15 | AluSq | Alu | SINE |
| 85296479 | 85296728 | - | 26.3 | 2193 | 2454 | -3946 | L1MEc | L1 | LINE |
| 85296736 | 85297635 | - | 14.4 | 4393 | 5339 | -807 | L1P4 | L1 | LINE |
| 85297636 | 85297923 | - | 16.3 | 3 | 299 | -13 | AluJb | Alu | SINE |
| 85297924 | 85298176 | - | 14.4 | 4134 | 4392 | -1754 | L1P4 | L1 | LINE |
| 85298179 | 85299054 | + | 15.8 | 5352 | 6164 | -2 | L1PA16 | L1 | LINE |
| 85299069 | 85299376 | - | 22.4 | 1933 | 2232 | -4168 | L1MEc | L1 | LINE |

  

|  |  |  |  |  |  |  |  |  |  |
| --- | --- | --- | --- | --- | --- | --- | --- | --- | --- |
| chr15:89506700-89508917 | | | 2212 bp | | |  |  |  |  |
| Genome Start | Genome End | Strand | Div | Repeat Start | Repeat End | Repeat Left | Name | Family | Class |
| 89506700 | 89506997 | - | 24.4 | 330 | 626 | 0 | MLT1E2 | MaLR | LTR |
| 89507005 | 89508377 | - | 5.2 | 4774 | 6154 | 0 | L1PA5 | L1 | LINE |
| 89508377 | 89508597 | + | 6.3 | 4534 | 4754 | -1392 | L1PA5 | L1 | LINE |
| 89508598 | 89508917 | - | 25.9 | 1 | 344 | -280 | MLT1E2 | MaLR | LTR |

  

|  |  |  |  |  |  |  |  |  |  |
| --- | --- | --- | --- | --- | --- | --- | --- | --- | --- |
| chr15:90355070-90356004 | | | 902 bp | | |  |  |  |  |
| Genome Start | Genome End | Strand | Div | Repeat Start | Repeat End | Repeat Left | Name | Family | Class |
| 90355070 | 90355509 | + | 29.9 | 2615 | 3139 | -280 | L2 | L2 | LINE |
| 90355516 | 90355652 | - | 14.6 | 6082 | 6219 | -81 | L1MA6 | L1 | LINE |
| 90355654 | 90355728 | + | 10.1 | 6232 | 6300 | 0 | L1MA6 | L1 | LINE |
| 90355755 | 90356004 | + | 31.9 | 3129 | 3378 | -41 | L2 | L2 | LINE |

  

|  |  |  |  |  |  |  |  |  |  |
| --- | --- | --- | --- | --- | --- | --- | --- | --- | --- |
| chr15:90829762-90833851 | | | 3971 bp | | |  |  |  |  |
| Genome Start | Genome End | Strand | Div | Repeat Start | Repeat End | Repeat Left | Name | Family | Class |
| 90829762 | 90829926 | + | 25.0 | 11 | 194 | -2313 | HAL1 | L1 | LINE |
| 90829940 | 90830396 | + | 26.4 | 275 | 766 | -1741 | HAL1 | L1 | LINE |
| 90830509 | 90830829 | + | 29.0 | 86 | 409 | 0 | MLT1I | MaLR | LTR |
| 90830833 | 90830944 | + | 25.9 | 781 | 899 | -1608 | HAL1 | L1 | LINE |
| 90830941 | 90831197 | - | 3.9 | 3694 | 3950 | -2196 | L1PA4 | L1 | LINE |
| 90831197 | 90833400 | + | 3.6 | 3950 | 6155 | 0 | L1PA4 | L1 | LINE |
| 90833396 | 90833668 | + | 25.6 | 881 | 1162 | -1345 | HAL1 | L1 | LINE |
| 90833670 | 90833851 | + | 31.4 | 1203 | 1387 | -1120 | HAL1 | L1 | LINE |

  

|  |  |  |  |  |  |  |  |  |  |
| --- | --- | --- | --- | --- | --- | --- | --- | --- | --- |
| chr15:95097386-95100497 | | | 3096 bp | | |  |  |  |  |
| Genome Start | Genome End | Strand | Div | Repeat Start | Repeat End | Repeat Left | Name | Family | Class |
| 95097386 | 95097594 | + | 30.1 | 2072 | 2307 | -4093 | L1ME1 | L1 | LINE |
| 95097596 | 95097969 | + | 9.1 | 2349 | 2725 | -4138 | L1PBa | L1 | LINE |
| 95097978 | 95098626 | - | 1.7 | 4925 | 5573 | -582 | L1PA3 | L1 | LINE |
| 95098628 | 95099211 | + | 2.7 | 5570 | 6155 | 0 | L1PA3 | L1 | LINE |
| 95099218 | 95099961 | + | 24.7 | 4986 | 5676 | -563 | L1ME1 | L1 | LINE |
| 95099962 | 95100248 | + | 7.7 | 2 | 300 | -10 | AluSg | Alu | SINE |
| 95100249 | 95100497 | + | 23.6 | 5677 | 5951 | -288 | L1ME1 | L1 | LINE |

  

|  |  |  |  |  |  |  |  |  |  |
| --- | --- | --- | --- | --- | --- | --- | --- | --- | --- |
| chr15:97884354-97893452 | | | 8797 bp | | |  |  |  |  |
| Genome Start | Genome End | Strand | Div | Repeat Start | Repeat End | Repeat Left | Name | Family | Class |
| 97884354 | 97885419 | - | 23.1 | 1637 | 2635 | -3584 | L1MEc | L1 | LINE |
| 97885406 | 97886353 | - | 16.3 | 2386 | 3354 | -2859 | L1M | L1 | LINE |
| 97886353 | 97887238 | + | 16.1 | 3362 | 4236 | -1977 | L1M | L1 | LINE |
| 97887239 | 97887612 | + | 14.8 | 1 | 381 | 0 | THE1D | MaLR | LTR |
| 97887613 | 97888346 | + | 16.1 | 4237 | 4967 | -1246 | L1M | L1 | LINE |
| 97888347 | 97888662 | - | 18.5 | 2 | 310 | -2 | AluJo | Alu | SINE |
| 97888663 | 97888755 | + | 16.1 | 4968 | 5097 | -1116 | L1M | L1 | LINE |
| 97888758 | 97888816 | + | 10.2 | 5361 | 5423 | -941 | L1M | L1 | LINE |
| 97888818 | 97889825 | - | 4.3 | 5158 | 6155 | 0 | L1PA4 | L1 | LINE |
| 97889822 | 97890179 | + | 20.5 | 5348 | 5792 | -520 | L1MA9 | L1 | LINE |
| 97890205 | 97890715 | + | 16.8 | 5793 | 6311 | -1 | L1MA9 | L1 | LINE |
| 97890816 | 97890917 | + | 15.6 | 4267 | 4366 | -1774 | L1M3 | L1 | LINE |
| 97890918 | 97891142 | + | 11.6 | 3 | 228 | -84 | AluSx | Alu | SINE |
| 97891143 | 97891216 | + | 15.6 | 4367 | 4447 | -1693 | L1M3 | L1 | LINE |
| 97891336 | 97891479 | - | 20.2 | 1461 | 1599 | -4801 | L1MEc | L1 | LINE |
| 97891506 | 97891523 | - | 20.2 | 1461 | 1460 | -4940 | L1MEc | L1 | LINE |
| 97891524 | 97891795 | - | 12.5 | 1 | 273 | -39 | AluSx | Alu | SINE |
| 97891796 | 97891862 | - | 20.2 | 1406 | 1460 | -4940 | L1MEc | L1 | LINE |
| 97891863 | 97892157 | - | 15.2 | 1 | 304 | -8 | AluJo | Alu | SINE |
| 97892158 | 97892506 | - | 20.2 | 1031 | 1405 | -4995 | L1MEc | L1 | LINE |
| 97892532 | 97892937 | - | 20.2 | 614 | 1030 | -5370 | L1MEc | L1 | LINE |
| 97892961 | 97893452 | - | 28.0 | 33 | 542 | -5858 | L1MEc | L1 | LINE |

  

|  |  |  |  |  |  |  |  |  |  |
| --- | --- | --- | --- | --- | --- | --- | --- | --- | --- |
| chr15:100245587-100248109 | | | 2483 bp | | |  |  |  |  |
| Genome Start | Genome End | Strand | Div | Repeat Start | Repeat End | Repeat Left | Name | Family | Class |
| 100245587 | 100246675 | - | 16.6 | 3657 | 4741 | -1399 | L1M3 | L1 | LINE |
| 100246699 | 100246877 | - | 4.5 | 5977 | 6155 | 0 | L1PA4 | L1 | LINE |
| 100246878 | 100247599 | + | 3.2 | 5254 | 5975 | -179 | L1PA4 | L1 | LINE |
| 100247617 | 100248109 | - | 19.3 | 3150 | 3646 | -2494 | L1M3 | L1 | LINE |

  

|  |  |  |  |  |  |  |  |  |  |
| --- | --- | --- | --- | --- | --- | --- | --- | --- | --- |
| chr15:100272225-100276173 | | | 3847 bp | | |  |  |  |  |
| Genome Start | Genome End | Strand | Div | Repeat Start | Repeat End | Repeat Left | Name | Family | Class |
| 100272225 | 100272368 | - | 30.4 | 6056 | 6201 | -1607 | L1MC4a | L1 | LINE |
| 100272470 | 100272657 | - | 24.7 | 5735 | 5942 | -1866 | L1MC4a | L1 | LINE |
| 100272658 | 100272962 | - | 13.1 | 1 | 305 | -7 | AluSx | Alu | SINE |
| 100272963 | 100273165 | - | 24.7 | 5512 | 5734 | -2074 | L1MC4a | L1 | LINE |
| 100273161 | 100274960 | - | 2.3 | 3311 | 5110 | -1036 | L1PA2 | L1 | LINE |
| 100274963 | 100276013 | + | 2.0 | 5105 | 6155 | 0 | L1PA2 | L1 | LINE |
| 100276018 | 100276173 | - | 23.5 | 5372 | 5525 | -2283 | L1MC4a | L1 | LINE |

  


|  |  |  |  |  |  |  |  |  |  |
| --- | --- | --- | --- | --- | --- | --- | --- | --- | --- |
| chr16:3320745-3322378 | | | 1596 bp | | |  |  |  |  |
| Genome Start | Genome End | Strand | Div | Repeat Start | Repeat End | Repeat Left | Name | Family | Class |
| 3320745 | 3320912 | - | 19.1 | 131 | 299 | -13 | AluJo | Alu | SINE |
| 3320931 | 3321700 | - | 2.6 | 4842 | 5611 | -544 | L1PA3 | L1 | LINE |
| 3321701 | 3322245 | + | 2.4 | 5616 | 6155 | 0 | L1PA3 | L1 | LINE |
| 3322266 | 3322378 | - | 13.3 | 19 | 135 | -177 | AluJo | Alu | SINE |

  

|  |  |  |  |  |  |  |  |  |  |
| --- | --- | --- | --- | --- | --- | --- | --- | --- | --- |
| chr16:5283458-5284392 | | | 904 bp | | |  |  |  |  |
| Genome Start | Genome End | Strand | Div | Repeat Start | Repeat End | Repeat Left | Name | Family | Class |
| 5283458 | 5283501 | - | 9.1 | 135 | 178 | 0 | MER5B | MER1\_type | DNA |
| 5283517 | 5283666 | - | 18.2 | 5712 | 5887 | -287 | L1MB5 | L1 | LINE |
| 5283665 | 5283944 | + | 19.3 | 5904 | 6174 | 0 | L1MB5 | L1 | LINE |
| 5283950 | 5284249 | + | 13.0 | 2 | 301 | -11 | AluSx | Alu | SINE |
| 5284263 | 5284392 | - | 21.3 | 5 | 132 | -46 | MER5B | MER1\_type | DNA |

  

|  |  |  |  |  |  |  |  |  |  |
| --- | --- | --- | --- | --- | --- | --- | --- | --- | --- |
| chr16:5798049-5800509 | | | 2458 bp | | |  |  |  |  |
| Genome Start | Genome End | Strand | Div | Repeat Start | Repeat End | Repeat Left | Name | Family | Class |
| 5798049 | 5798243 | + | 20.2 | 3 | 223 | -167 | MLT1B | MaLR | LTR |
| 5798244 | 5799009 | - | 4.7 | 4065 | 4830 | -1316 | L1PA3 | L1 | LINE |
| 5799013 | 5800339 | + | 3.9 | 4829 | 6154 | -1 | L1PA3 | L1 | LINE |
| 5800340 | 5800509 | + | 14.7 | 212 | 390 | 0 | MLT1B | MaLR | LTR |

  

|  |  |  |  |  |  |  |  |  |  |
| --- | --- | --- | --- | --- | --- | --- | --- | --- | --- |
| chr16:5801773-5803688 | | | 1912 bp | | |  |  |  |  |
| Genome Start | Genome End | Strand | Div | Repeat Start | Repeat End | Repeat Left | Name | Family | Class |
| 5801773 | 5802452 | - | 24.6 | 5484 | 6160 | -6 | L1ME1 | L1 | LINE |
| 5802453 | 5802766 | - | 20.4 | 1 | 308 | -4 | AluJo | Alu | SINE |
| 5802767 | 5802961 | - | 24.6 | 5290 | 5483 | -683 | L1ME1 | L1 | LINE |
| 5802962 | 5803010 | - | 16.3 | 6140 | 6179 | -1 | L1MB4 | L1 | LINE |
| 5803011 | 5803270 | - | 12.3 | 38 | 297 | -13 | AluSg | Alu | SINE |
| 5803271 | 5803435 | - | 16.3 | 5960 | 6139 | -41 | L1MB4 | L1 | LINE |
| 5803442 | 5803614 | + | 19.1 | 5776 | 5949 | -225 | L1MB4 | L1 | LINE |
| 5803613 | 5803688 | - | 16.2 | 5218 | 5299 | -847 | L1ME1 | L1 | LINE |

  

|  |  |  |  |  |  |  |  |  |  |
| --- | --- | --- | --- | --- | --- | --- | --- | --- | --- |
| chr16:6003574-6004196 | | | 621 bp | | |  |  |  |  |
| Genome Start | Genome End | Strand | Div | Repeat Start | Repeat End | Repeat Left | Name | Family | Class |
| 6003574 | 6003677 | + | 29.9 | 29 | 133 | -135 | MIR | MIR | SINE |
| 6003680 | 6003817 | - | 5.1 | 5705 | 5854 | -301 | L1PA4 | L1 | LINE |
| 6003818 | 6004129 | + | 4.5 | 5846 | 6155 | 0 | L1PA4 | L1 | LINE |
| 6004130 | 6004196 | + | 22.4 | 136 | 213 | -49 | MIR | MIR | SINE |

  

|  |  |  |  |  |  |  |  |  |  |
| --- | --- | --- | --- | --- | --- | --- | --- | --- | --- |
| chr16:7717303-7720960 | | | 3537 bp | | |  |  |  |  |
| Genome Start | Genome End | Strand | Div | Repeat Start | Repeat End | Repeat Left | Name | Family | Class |
| 7717303 | 7717418 | + | 23.3 | 5187 | 5305 | -838 | L1MA8 | L1 | LINE |
| 7717417 | 7718051 | - | 2.5 | 3645 | 4279 | -1867 | L1PA2 | L1 | LINE |
| 7718048 | 7719920 | + | 2.1 | 4269 | 6155 | 0 | L1PA2 | L1 | LINE |
| 7719893 | 7720210 | + | 13.8 | 5276 | 5618 | -673 | L1MA8 | L1 | LINE |
| 7720211 | 7720547 | - | 15.1 | 1 | 364 | 0 | THE1B | MaLR | LTR |
| 7720548 | 7720577 | + | 13.3 | 5619 | 5643 | -648 | L1MA8 | L1 | LINE |
| 7720604 | 7720639 | + | 22.1 | 5658 | 5698 | -465 | L1P4 | L1 | LINE |
| 7720769 | 7720960 | + | 19.9 | 5651 | 5852 | -439 | L1MA8 | L1 | LINE |

  

|  |  |  |  |  |  |  |  |  |  |
| --- | --- | --- | --- | --- | --- | --- | --- | --- | --- |
| chr16:8213104-8215847 | | | 2685 bp | | |  |  |  |  |
| Genome Start | Genome End | Strand | Div | Repeat Start | Repeat End | Repeat Left | Name | Family | Class |
| 8213104 | 8213141 | - | 13.2 | 1693 | 1730 | -15 | Kanga1 | Tc2 | DNA |
| 8213161 | 8215304 | - | 4.7 | 3980 | 6153 | -1 | L1PA5 | L1 | LINE |
| 8215306 | 8215715 | + | 7.1 | 3562 | 3971 | -2175 | L1PA5 | L1 | LINE |
| 8215755 | 8215847 | - | 24.7 | 1 | 90 | -1808 | Kanga1 | Tc2 | DNA |

  

|  |  |  |  |  |  |  |  |  |  |
| --- | --- | --- | --- | --- | --- | --- | --- | --- | --- |
| chr16:10478403-10481321 | | | 2921 bp | | |  |  |  |  |
| Genome Start | Genome End | Strand | Div | Repeat Start | Repeat End | Repeat Left | Name | Family | Class |
| 10478403 | 10478637 | + | 8.1 | 5087 | 5333 | -813 | L1PA8A | L1 | LINE |
| 10478636 | 10478726 | - | 0.0 | 3768 | 3858 | -2288 | L1PA3 | L1 | LINE |
| 10478727 | 10481022 | + | 2.3 | 3882 | 6155 | 0 | L1PA3 | L1 | LINE |
| 10481023 | 10481321 | + | 9.7 | 5317 | 5619 | -512 | L1PA8A | L1 | LINE |

  

|  |  |  |  |  |  |  |  |  |  |
| --- | --- | --- | --- | --- | --- | --- | --- | --- | --- |
| chr16:12950493-12953042 | | | 2368 bp | | |  |  |  |  |
| Genome Start | Genome End | Strand | Div | Repeat Start | Repeat End | Repeat Left | Name | Family | Class |
| 12950493 | 12950817 | - | 25.0 | 122 | 444 | -13 | LTR16A1 | ERVL | LTR |
| 12950836 | 12951078 | + | 17.8 | 6 | 245 | -79 | MER33 | MER1\_type | DNA |
| 12951085 | 12951751 | - | 14.3 | 5628 | 6297 | -3 | L1MA4 | L1 | LINE |
| 12951757 | 12951965 | + | 12.0 | 5414 | 5623 | -677 | L1MA4 | L1 | LINE |
| 12951972 | 12952265 | - | 10.4 | 1 | 295 | -14 | AluSc | Alu | SINE |
| 12952313 | 12952607 | + | 9.5 | 16 | 311 | -1 | AluSx | Alu | SINE |
| 12952704 | 12952926 | - | 26.0 | 1 | 223 | -1 | MER58A | MER1\_type | DNA |
| 12952931 | 12953042 | - | 28.0 | 12 | 112 | -345 | LTR16A1 | ERVL | LTR |

  

|  |  |  |  |  |  |  |  |  |  |
| --- | --- | --- | --- | --- | --- | --- | --- | --- | --- |
| chr16:13132030-13133063 | | | 1031 bp | | |  |  |  |  |
| Genome Start | Genome End | Strand | Div | Repeat Start | Repeat End | Repeat Left | Name | Family | Class |
| 13132030 | 13132192 | - | 23.9 | 171 | 335 | -6 | MER58B | MER1\_type | DNA |
| 13132193 | 13132226 | - | 2.9 | 5350 | 5383 | -785 | L1P | L1 | LINE |
| 13132226 | 13132997 | + | 9.3 | 5397 | 6174 | 0 | L1PA11 | L1 | LINE |
| 13133002 | 13133063 | - | 29.0 | 1 | 63 | -278 | MER58B | MER1\_type | DNA |

  

|  |  |  |  |  |  |  |  |  |  |
| --- | --- | --- | --- | --- | --- | --- | --- | --- | --- |
| chr16:16576427-16578487 | | | 1982 bp | | |  |  |  |  |
| Genome Start | Genome End | Strand | Div | Repeat Start | Repeat End | Repeat Left | Name | Family | Class |
| 16576427 | 16576533 | + | 18.7 | 5 | 141 | -268 | MLT1I | MaLR | LTR |
| 16576611 | 16577561 | - | 3.5 | 5200 | 6150 | -5 | L1PA4 | L1 | LINE |
| 16577560 | 16578286 | + | 4.4 | 4474 | 5200 | -946 | L1PA4 | L1 | LINE |
| 16578291 | 16578487 | + | 24.1 | 195 | 409 | 0 | MLT1I | MaLR | LTR |

  

|  |  |  |  |  |  |  |  |  |  |
| --- | --- | --- | --- | --- | --- | --- | --- | --- | --- |
| chr16:16686106-16693025 | | | 6669 bp | | |  |  |  |  |
| Genome Start | Genome End | Strand | Div | Repeat Start | Repeat End | Repeat Left | Name | Family | Class |
| 16686106 | 16686240 | - | 25.8 | 457 | 591 | 0 | MLT1K | MaLR | LTR |
| 16686283 | 16686897 | - | 24.8 | 5552 | 6160 | -6 | L1ME1 | L1 | LINE |
| 16686900 | 16687024 | + | 13.6 | 2 | 126 | -186 | AluSq/x | Alu | SINE |
| 16687052 | 16687277 | - | 24.3 | 5296 | 5516 | -650 | L1ME1 | L1 | LINE |
| 16687294 | 16690385 | - | 5.6 | 3051 | 6154 | 0 | L1PA6 | L1 | LINE |
| 16690385 | 16690895 | + | 8.8 | 2536 | 3047 | -3099 | L1PA6 | L1 | LINE |
| 16690992 | 16691352 | + | 11.0 | 5802 | 6164 | -2 | L1PA16 | L1 | LINE |
| 16691415 | 16691870 | - | 21.0 | 4874 | 5310 | -836 | L1MD | L1 | LINE |
| 16691877 | 16692172 | - | 14.9 | 1 | 296 | -16 | AluSx | Alu | SINE |
| 16692173 | 16692226 | - | 23.7 | 4822 | 4873 | -1273 | L1ME1 | L1 | LINE |
| 16692228 | 16692442 | - | 29.8 | 193 | 426 | -165 | MLT1K | MaLR | LTR |
| 16692443 | 16692852 | + | 15.4 | 1 | 428 | 0 | MSTA | MaLR | LTR |
| 16692853 | 16693025 | - | 29.8 | 17 | 192 | -399 | MLT1K | MaLR | LTR |

  

|  |  |  |  |  |  |  |  |  |  |
| --- | --- | --- | --- | --- | --- | --- | --- | --- | --- |
| chr16:16834651-16836319 | | | 1665 bp | | |  |  |  |  |
| Genome Start | Genome End | Strand | Div | Repeat Start | Repeat End | Repeat Left | Name | Family | Class |
| 16834651 | 16835098 | + | 17.5 | 2 | 450 | -70 | MLT2B1 | ERVL | LTR |
| 16835101 | 16835401 | - | 10.3 | 5863 | 6163 | 0 | L1PA13 | L1 | LINE |
| 16835404 | 16835527 | + | 6.5 | 5401 | 5524 | -639 | L1PA13 | L1 | LINE |
| 16835528 | 16835834 | + | 7.5 | 1 | 307 | -4 | AluY | Alu | SINE |
| 16835835 | 16836246 | + | 6.5 | 5525 | 5863 | -300 | L1PA13 | L1 | LINE |
| 16836247 | 16836319 | + | 13.7 | 488 | 562 | 0 | MLT2B1 | ERVL | LTR |

  

|  |  |  |  |  |  |  |  |  |  |
| --- | --- | --- | --- | --- | --- | --- | --- | --- | --- |
| chr16:18586356-18593277 | | | 6671 bp | | |  |  |  |  |
| Genome Start | Genome End | Strand | Div | Repeat Start | Repeat End | Repeat Left | Name | Family | Class |
| 18586356 | 18586528 | + | 29.8 | 16 | 191 | -400 | MLT1K | MaLR | LTR |
| 18586529 | 18586938 | - | 15.4 | 1 | 428 | 0 | MSTA | MaLR | LTR |
| 18586939 | 18587153 | + | 29.8 | 192 | 425 | -166 | MLT1K | MaLR | LTR |
| 18587155 | 18587208 | + | 23.7 | 4822 | 4873 | -1273 | L1ME1 | L1 | LINE |
| 18587209 | 18587504 | + | 15.2 | 1 | 296 | -16 | AluSx | Alu | SINE |
| 18587511 | 18587965 | + | 21.0 | 4874 | 5310 | -836 | L1MD | L1 | LINE |
| 18588028 | 18588388 | - | 11.3 | 5802 | 6164 | -2 | L1PA16 | L1 | LINE |
| 18588485 | 18588995 | - | 9.4 | 2536 | 3047 | -3099 | L1PA6 | L1 | LINE |
| 18588995 | 18592089 | + | 5.9 | 3051 | 6154 | 0 | L1PA6 | L1 | LINE |
| 18592106 | 18592331 | + | 24.5 | 5296 | 5516 | -650 | L1ME1 | L1 | LINE |
| 18592359 | 18592483 | - | 13.6 | 2 | 126 | -186 | AluSq/x | Alu | SINE |
| 18592486 | 18593100 | + | 25.0 | 5552 | 6160 | -6 | L1ME1 | L1 | LINE |
| 18593143 | 18593277 | + | 25.8 | 457 | 591 | 0 | MLT1K | MaLR | LTR |

  

|  |  |  |  |  |  |  |  |  |  |
| --- | --- | --- | --- | --- | --- | --- | --- | --- | --- |
| chr16:18695469-18697534 | | | 1985 bp | | |  |  |  |  |
| Genome Start | Genome End | Strand | Div | Repeat Start | Repeat End | Repeat Left | Name | Family | Class |
| 18695469 | 18695668 | - | 26.1 | 195 | 409 | 0 | MLT1I | MaLR | LTR |
| 18695673 | 18696399 | - | 3.4 | 4474 | 5200 | -946 | L1PA4 | L1 | LINE |
| 18696398 | 18697348 | + | 3.1 | 5200 | 6151 | -4 | L1PA4 | L1 | LINE |
| 18697428 | 18697534 | - | 18.7 | 5 | 141 | -268 | MLT1I | MaLR | LTR |

  

|  |  |  |  |  |  |  |  |  |  |
| --- | --- | --- | --- | --- | --- | --- | --- | --- | --- |
| chr16:19855283-19856806 | | | 1500 bp | | |  |  |  |  |
| Genome Start | Genome End | Strand | Div | Repeat Start | Repeat End | Repeat Left | Name | Family | Class |
| 19855283 | 19855636 | + | 22.3 | 1 | 367 | -195 | MLT1F2 | MaLR | LTR |
| 19855654 | 19855934 | - | 9.6 | 1 | 281 | -29 | AluSg | Alu | SINE |
| 19855941 | 19856269 | - | 9.4 | 5484 | 5822 | -352 | L1PA11 | L1 | LINE |
| 19856270 | 19856594 | + | 10.2 | 5844 | 6174 | 0 | L1PA11 | L1 | LINE |
| 19856596 | 19856806 | + | 22.6 | 353 | 562 | 0 | MLT1F2 | MaLR | LTR |

  

|  |  |  |  |  |  |  |  |  |  |
| --- | --- | --- | --- | --- | --- | --- | --- | --- | --- |
| chr16:20183226-20185368 | | | 2094 bp | | |  |  |  |  |
| Genome Start | Genome End | Strand | Div | Repeat Start | Repeat End | Repeat Left | Name | Family | Class |
| 20183226 | 20183616 | + | 34.7 | 4836 | 5273 | -873 | L1ME4a | L1 | LINE |
| 20183617 | 20183941 | + | 10.0 | 1 | 312 | -1 | AluSp | Alu | SINE |
| 20183942 | 20184031 | + | 34.7 | 5274 | 5371 | -775 | L1ME4a | L1 | LINE |
| 20184033 | 20184239 | - | 10.0 | 5877 | 6076 | -87 | L1PA13 | L1 | LINE |
| 20184240 | 20184310 | + | 7.0 | 6082 | 6157 | -6 | L1PA13 | L1 | LINE |
| 20184359 | 20184602 | + | 30.1 | 5401 | 5661 | -460 | L1ME4a | L1 | LINE |
| 20184603 | 20184916 | + | 12.2 | 1 | 312 | 0 | AluSx | Alu | SINE |
| 20184917 | 20185368 | + | 30.1 | 5662 | 6117 | -4 | L1ME4a | L1 | LINE |

  

|  |  |  |  |  |  |  |  |  |  |
| --- | --- | --- | --- | --- | --- | --- | --- | --- | --- |
| chr16:21225279-21226939 | | | 1274 bp | | |  |  |  |  |
| Genome Start | Genome End | Strand | Div | Repeat Start | Repeat End | Repeat Left | Name | Family | Class |
| 21225279 | 21225398 | - | 24.2 | 5316 | 5460 | -2348 | L1MC4a | L1 | LINE |
| 21225402 | 21226236 | - | 6.5 | 5310 | 6149 | -5 | L1PA7 | L1 | LINE |
| 21226234 | 21226304 | + | 7.0 | 5216 | 5290 | -856 | L1PA7 | L1 | LINE |
| 21226692 | 21226939 | - | 30.9 | 4705 | 4952 | -1194 | L1MC4a | L1 | LINE |

  

|  |  |  |  |  |  |  |  |  |  |
| --- | --- | --- | --- | --- | --- | --- | --- | --- | --- |
| chr16:22575696-22577909 | | | 2140 bp | | |  |  |  |  |
| Genome Start | Genome End | Strand | Div | Repeat Start | Repeat End | Repeat Left | Name | Family | Class |
| 22575696 | 22575797 | - | 15.7 | 162 | 282 | -30 | AluJ | Alu | SINE |
| 22575798 | 22576223 | - | 0.6 | 5184 | 5609 | -546 | L1PA2 | L1 | LINE |
| 22576224 | 22576755 | + | 0.6 | 5620 | 6151 | -4 | L1PA2 | L1 | LINE |
| 22576768 | 22576787 | - | 28.1 | 2109 | 2127 | -4273 | L1MEc | L1 | LINE |
| 22576788 | 22577149 | + | 12.8 | 1 | 379 | -2 | THE1D | MaLR | LTR |
| 22577150 | 22577359 | - | 28.1 | 1902 | 2108 | -4292 | L1MEc | L1 | LINE |
| 22577397 | 22577708 | + | 6.5 | 1 | 311 | 0 | AluY | Alu | SINE |
| 22577734 | 22577909 | - | 17.1 | 5 | 174 | -138 | AluJ | Alu | SINE |

  

|  |  |  |  |  |  |  |  |  |  |
| --- | --- | --- | --- | --- | --- | --- | --- | --- | --- |
| chr16:25258117-25260255 | | | 1929 bp | | |  |  |  |  |
| Genome Start | Genome End | Strand | Div | Repeat Start | Repeat End | Repeat Left | Name | Family | Class |
| 25258117 | 25258710 | - | 32.0 | 2811 | 3419 | 0 | L2 | L2 | LINE |
| 25258711 | 25258894 | - | 12.5 | 5988 | 6165 | -1 | L1PA16 | L1 | LINE |
| 25258895 | 25259085 | - | 9.3 | 165 | 364 | 0 | THE1B | MaLR | LTR |
| 25259086 | 25259379 | + | 10.9 | 1 | 307 | -4 | AluY | Alu | SINE |
| 25259380 | 25259542 | - | 9.3 | 1 | 164 | -200 | THE1B | MaLR | LTR |
| 25259543 | 25259592 | - | 12.5 | 5940 | 5987 | -179 | L1PA16 | L1 | LINE |
| 25259593 | 25259752 | + | 9.4 | 5780 | 5940 | -226 | L1PA16 | L1 | LINE |
| 25259936 | 25260059 | + | 28.2 | 4 | 121 | -57 | MER5B | MER1\_type | DNA |
| 25260087 | 25260255 | - | 26.2 | 2548 | 2736 | -683 | L2 | L2 | LINE |

  

|  |  |  |  |  |  |  |  |  |  |
| --- | --- | --- | --- | --- | --- | --- | --- | --- | --- |
| chr16:25277309-25278807 | | | 1455 bp | | |  |  |  |  |
| Genome Start | Genome End | Strand | Div | Repeat Start | Repeat End | Repeat Left | Name | Family | Class |
| 25277309 | 25277369 | + | 4.9 | 1 | 66 | -397 | LTR2 | ERV1 | LTR |
| 25277372 | 25277828 | - | 2.3 | 5193 | 5651 | -504 | L1PA4 | L1 | LINE |
| 25277829 | 25278347 | + | 2.5 | 5638 | 6155 | 0 | L1PA4 | L1 | LINE |
| 25278390 | 25278807 | + | 10.0 | 52 | 463 | 0 | LTR2 | ERV1 | LTR |

  

|  |  |  |  |  |  |  |  |  |  |
| --- | --- | --- | --- | --- | --- | --- | --- | --- | --- |
| chr16:26858672-26859949 | | | 1167 bp | | |  |  |  |  |
| Genome Start | Genome End | Strand | Div | Repeat Start | Repeat End | Repeat Left | Name | Family | Class |
| 26858672 | 26858821 | - | 28.0 | 110 | 252 | -16 | MIRb | MIR | SINE |
| 26858886 | 26859175 | - | 32.4 | 124 | 435 | -258 | MER115 | Tip100 | DNA |
| 26859176 | 26859611 | - | 6.7 | 5731 | 6168 | 0 | L1PA10 | L1 | LINE |
| 26859614 | 26859779 | + | 5.4 | 5565 | 5730 | -438 | L1PA10 | L1 | LINE |
| 26859780 | 26859858 | - | 24.4 | 56 | 136 | -557 | MER115 | Tip100 | DNA |
| 26859904 | 26859949 | - | 21.7 | 31 | 81 | -187 | MIRb | MIR | SINE |

  

|  |  |  |  |  |  |  |  |  |  |
| --- | --- | --- | --- | --- | --- | --- | --- | --- | --- |
| chr16:31207051-31211003 | | | 3985 bp | | |  |  |  |  |
| Genome Start | Genome End | Strand | Div | Repeat Start | Repeat End | Repeat Left | Name | Family | Class |
| 31207051 | 31207103 | + | 21.1 | 3797 | 3848 | -2298 | L1MD | L1 | LINE |
| 31207084 | 31208588 | + | 6.3 | 4598 | 6152 | -2 | L1PA5 | L1 | LINE |
| 31208589 | 31208621 | - | 15.2 | 4330 | 4360 | -1786 | L1M4 | L1 | LINE |
| 31208622 | 31208927 | + | 9.5 | 1 | 308 | -5 | AluSp | Alu | SINE |
| 31208928 | 31208960 | - | 15.2 | 4297 | 4329 | -1817 | L1M4 | L1 | LINE |
| 31208956 | 31209038 | + | 13.2 | 4676 | 4768 | -1378 | L1M4 | L1 | LINE |
| 31209022 | 31209250 | + | 20.6 | 5944 | 6171 | -13 | L1MB7 | L1 | LINE |
| 31209261 | 31210006 | + | 22.1 | 3846 | 4606 | -1540 | L1M5 | L1 | LINE |
| 31210006 | 31210404 | - | 12.1 | 5412 | 5809 | -491 | L1MA4 | L1 | LINE |
| 31210406 | 31210916 | + | 13.1 | 5788 | 6299 | -1 | L1MA4 | L1 | LINE |
| 31210917 | 31211003 | + | 20.9 | 4604 | 4696 | -1450 | L1MD | L1 | LINE |

  

|  |  |  |  |  |  |  |  |  |  |
| --- | --- | --- | --- | --- | --- | --- | --- | --- | --- |
| chr16:31537540-31541707 | | | 4176 bp | | |  |  |  |  |
| Genome Start | Genome End | Strand | Div | Repeat Start | Repeat End | Repeat Left | Name | Family | Class |
| 31537540 | 31538040 | + | 20.4 | 4501 | 5004 | -1136 | L1MB2 | L1 | LINE |
| 31538043 | 31538535 | - | 18.9 | 3888 | 4398 | -1745 | L1MA6 | L1 | LINE |
| 31538526 | 31540070 | + | 17.5 | 4386 | 5941 | -359 | L1MA6 | L1 | LINE |
| 31540071 | 31540372 | + | 10.6 | 1 | 302 | -10 | AluSx | Alu | SINE |
| 31540373 | 31540576 | + | 19.1 | 5942 | 6141 | -159 | L1MA6 | L1 | LINE |
| 31540577 | 31540862 | + | 20.5 | 22 | 306 | -6 | AluJo | Alu | SINE |
| 31540863 | 31541020 | + | 19.1 | 6142 | 6295 | -5 | L1MA6 | L1 | LINE |
| 31541009 | 31541182 | + | 19.8 | 4978 | 5145 | -995 | L1MB2 | L1 | LINE |
| 31541195 | 31541707 | + | 25.1 | 5644 | 6169 | -2 | L1MB2 | L1 | LINE |

  

|  |  |  |  |  |  |  |  |  |  |
| --- | --- | --- | --- | --- | --- | --- | --- | --- | --- |
| chr16:31652231-31657823 | | | 5513 bp | | |  |  |  |  |
| Genome Start | Genome End | Strand | Div | Repeat Start | Repeat End | Repeat Left | Name | Family | Class |
| 31652231 | 31652727 | - | 25.9 | 5880 | 6380 | -27 | L1MB2 | L1 | LINE |
| 31652740 | 31652913 | - | 19.8 | 5214 | 5381 | -995 | L1MB2 | L1 | LINE |
| 31652902 | 31653057 | - | 20.0 | 6135 | 6295 | -5 | L1MA6 | L1 | LINE |
| 31653058 | 31653373 | - | 21.8 | 1 | 312 | 0 | AluJo | Alu | SINE |
| 31653374 | 31653552 | - | 20.0 | 5948 | 6134 | -166 | L1MA6 | L1 | LINE |
| 31653557 | 31653870 | - | 12.8 | 1 | 312 | 0 | AluSx | Alu | SINE |
| 31653951 | 31655336 | - | 17.1 | 4539 | 5940 | -360 | L1MA6 | L1 | LINE |
| 31655337 | 31655641 | - | 11.6 | 1 | 303 | -10 | AluSq | Alu | SINE |
| 31655642 | 31655798 | - | 17.3 | 4386 | 4538 | -1605 | L1MA6 | L1 | LINE |
| 31655789 | 31656283 | + | 18.2 | 3888 | 4400 | -1743 | L1MA6 | L1 | LINE |
| 31656284 | 31656779 | - | 19.9 | 4749 | 5240 | -1136 | L1MB2 | L1 | LINE |
| 31656786 | 31657823 | - | 23.6 | 2037 | 3120 | -3256 | L1MB2 | L1 | LINE |

  

|  |  |  |  |  |  |  |  |  |  |
| --- | --- | --- | --- | --- | --- | --- | --- | --- | --- |
| chr16:31972362-31974058 | | | 1212 bp | | |  |  |  |  |
| Genome Start | Genome End | Strand | Div | Repeat Start | Repeat End | Repeat Left | Name | Family | Class |
| 31972362 | 31973037 | - | 31.7 | 4431 | 5133 | -1668 | MER89-int | ERV1 | LTR |
| 31973520 | 31973806 | - | 21.6 | 5563 | 5855 | -457 | L1MA9 | L1 | LINE |
| 31973810 | 31973850 | + | 17.1 | 5539 | 5579 | -755 | L1MA9 | L1 | LINE |
| 31973851 | 31974058 | - | 25.1 | 3431 | 3616 | -3185 | MER89-int | ERV1 | LTR |

  

|  |  |  |  |  |  |  |  |  |  |
| --- | --- | --- | --- | --- | --- | --- | --- | --- | --- |
| chr16:32768392-32772092 | | | 3687 bp | | |  |  |  |  |
| Genome Start | Genome End | Strand | Div | Repeat Start | Repeat End | Repeat Left | Name | Family | Class |
| 32768392 | 32769163 | + | 19.4 | 4817 | 5584 | -572 | L1PB4 | L1 | LINE |
| 32769163 | 32769419 | - | 14.0 | 3784 | 4041 | -2105 | L1PA7 | L1 | LINE |
| 32769420 | 32771506 | + | 9.5 | 4066 | 6154 | 0 | L1PA7 | L1 | LINE |
| 32771522 | 32772092 | + | 22.8 | 5570 | 6154 | -2 | L1PB4 | L1 | LINE |

  

|  |  |  |  |  |  |  |  |  |  |
| --- | --- | --- | --- | --- | --- | --- | --- | --- | --- |
| chr16:32888714-32892920 | | | 4177 bp | | |  |  |  |  |
| Genome Start | Genome End | Strand | Div | Repeat Start | Repeat End | Repeat Left | Name | Family | Class |
| 32888714 | 32888849 | - | 31.1 | 6017 | 6153 | -11 | L1ME2 | L1 | LINE |
| 32888882 | 32889538 | - | 20.9 | 5449 | 6166 | 0 | L1PA16 | L1 | LINE |
| 32889535 | 32890009 | + | 21.3 | 4829 | 5267 | -879 | L1P4 | L1 | LINE |
| 32890010 | 32890369 | - | 17.6 | 1 | 364 | 0 | THE1B | MaLR | LTR |
| 32890370 | 32891948 | - | 14.2 | 1 | 1580 | 0 | THE1B-int | MaLR | LTR |
| 32891949 | 32892356 | - | 14.7 | 1 | 364 | 0 | THE1B | MaLR | LTR |
| 32892357 | 32892524 | + | 16.7 | 5263 | 5431 | -735 | L1P4 | L1 | LINE |
| 32892527 | 32892920 | - | 32.8 | 5602 | 5975 | -187 | L1ME2 | L1 | LINE |

  

|  |  |  |  |  |  |  |  |  |  |
| --- | --- | --- | --- | --- | --- | --- | --- | --- | --- |
| chr16:33566010-33567606 | | | 1110 bp | | |  |  |  |  |
| Genome Start | Genome End | Strand | Div | Repeat Start | Repeat End | Repeat Left | Name | Family | Class |
| 33566010 | 33566117 | + | 26.4 | 3509 | 3616 | -3185 | MER89-int | ERV1 | LTR |
| 33566118 | 33566158 | - | 17.1 | 5539 | 5579 | -755 | L1MA9 | L1 | LINE |
| 33566162 | 33566446 | + | 21.1 | 5563 | 5853 | -459 | L1MA9 | L1 | LINE |
| 33566931 | 33567606 | + | 32.0 | 4431 | 5133 | -1668 | MER89-int | ERV1 | LTR |

  

|  |  |  |  |  |  |  |  |  |  |
| --- | --- | --- | --- | --- | --- | --- | --- | --- | --- |
| chr16:33589822-33594030 | | | 3983 bp | | |  |  |  |  |
| Genome Start | Genome End | Strand | Div | Repeat Start | Repeat End | Repeat Left | Name | Family | Class |
| 33589822 | 33590023 | + | 32.5 | 5602 | 5808 | -366 | L1ME2 | L1 | LINE |
| 33590218 | 33590385 | - | 16.7 | 5263 | 5431 | -735 | L1P4 | L1 | LINE |
| 33590386 | 33590793 | + | 14.7 | 1 | 364 | 0 | THE1B | MaLR | LTR |
| 33590794 | 33592374 | + | 14.2 | 1 | 1580 | 0 | THE1B-int | MaLR | LTR |
| 33592375 | 33592734 | + | 17.6 | 1 | 364 | 0 | THE1B | MaLR | LTR |
| 33592735 | 33593205 | - | 22.9 | 4833 | 5267 | -879 | L1P4 | L1 | LINE |
| 33593206 | 33593862 | + | 21.1 | 5449 | 6166 | 0 | L1PA16 | L1 | LINE |
| 33593895 | 33594030 | + | 31.1 | 6017 | 6153 | -11 | L1ME2 | L1 | LINE |

  

|  |  |  |  |  |  |  |  |  |  |
| --- | --- | --- | --- | --- | --- | --- | --- | --- | --- |
| chr16:33718237-33721941 | | | 3691 bp | | |  |  |  |  |
| Genome Start | Genome End | Strand | Div | Repeat Start | Repeat End | Repeat Left | Name | Family | Class |
| 33718237 | 33718807 | - | 22.8 | 5570 | 6154 | -2 | L1PB4 | L1 | LINE |
| 33718823 | 33720913 | - | 9.6 | 4066 | 6154 | 0 | L1PA7 | L1 | LINE |
| 33720914 | 33721170 | + | 14.0 | 3784 | 4041 | -2105 | L1PA7 | L1 | LINE |
| 33721170 | 33721941 | - | 19.7 | 4817 | 5584 | -572 | L1PB4 | L1 | LINE |

  

|  |  |  |  |  |  |  |  |  |  |
| --- | --- | --- | --- | --- | --- | --- | --- | --- | --- |
| chr16:46427225-46430152 | | | 2937 bp | | |  |  |  |  |
| Genome Start | Genome End | Strand | Div | Repeat Start | Repeat End | Repeat Left | Name | Family | Class |
| 46427225 | 46427998 | + | 19.3 | 3955 | 4754 | -1386 | L1M4 | L1 | LINE |
| 46427999 | 46428309 | + | 16.2 | 1 | 289 | -23 | AluJb | Alu | SINE |
| 46428310 | 46428720 | + | 19.3 | 4755 | 5173 | -967 | L1M4 | L1 | LINE |
| 46428711 | 46429489 | - | 3.2 | 4794 | 5572 | -583 | L1PA3 | L1 | LINE |
| 46429491 | 46430070 | + | 3.5 | 5571 | 6154 | -1 | L1PA3 | L1 | LINE |
| 46430071 | 46430152 | + | 15.8 | 5166 | 5247 | -899 | L1M4 | L1 | LINE |

  

|  |  |  |  |  |  |  |  |  |  |
| --- | --- | --- | --- | --- | --- | --- | --- | --- | --- |
| chr16:46904469-46906467 | | | 2000 bp | | |  |  |  |  |
| Genome Start | Genome End | Strand | Div | Repeat Start | Repeat End | Repeat Left | Name | Family | Class |
| 46904469 | 46904722 | - | 18.8 | 4487 | 4738 | -1408 | L1M5 | L1 | LINE |
| 46904730 | 46905997 | - | 7.4 | 4879 | 6173 | -1 | L1PA11 | L1 | LINE |
| 46905997 | 46906083 | + | 6.9 | 4774 | 4860 | -1286 | L1PA11 | L1 | LINE |
| 46906077 | 46906467 | - | 23.5 | 4090 | 4495 | -1651 | L1M5 | L1 | LINE |

  

|  |  |  |  |  |  |  |  |  |  |
| --- | --- | --- | --- | --- | --- | --- | --- | --- | --- |
| chr16:47063463-47068232 | | | 4599 bp | | |  |  |  |  |
| Genome Start | Genome End | Strand | Div | Repeat Start | Repeat End | Repeat Left | Name | Family | Class |
| 47063463 | 47063597 | - | 10.7 | 3253 | 3389 | -3255 | L1M4 | L1 | LINE |
| 47063598 | 47063900 | + | 15.9 | 1 | 301 | -11 | AluSx | Alu | SINE |
| 47063901 | 47063915 | - | 10.7 | 3237 | 3252 | -3392 | L1M4 | L1 | LINE |
| 47063927 | 47065190 | - | 4.4 | 4893 | 6154 | 0 | L1PA5 | L1 | LINE |
| 47065191 | 47065795 | + | 3.3 | 4268 | 4880 | -1266 | L1PA5 | L1 | LINE |
| 47065895 | 47066162 | - | 16.4 | 2977 | 3241 | -3403 | L1M4 | L1 | LINE |
| 47066177 | 47066367 | - | 11.5 | 2470 | 2661 | -4170 | L1M4 | L1 | LINE |
| 47066368 | 47066656 | - | 11.4 | 17 | 308 | -4 | AluSx | Alu | SINE |
| 47066662 | 47066726 | + | 12.3 | 65 | 129 | -183 | AluSq/x | Alu | SINE |
| 47066729 | 47066797 | - | 5.9 | 5761 | 5828 | -335 | L1PA13 | L1 | LINE |
| 47066808 | 47066991 | - | 15.1 | 2273 | 2459 | -4372 | L1M4 | L1 | LINE |
| 47067020 | 47067262 | - | 15.1 | 1994 | 2272 | -4559 | L1M4 | L1 | LINE |
| 47067253 | 47067353 | - | 19.1 | 683 | 776 | -6055 | L1M4 | L1 | LINE |
| 47067354 | 47067756 | + | 11.2 | 5748 | 6156 | -4 | L1PREC2 | L1 | LINE |
| 47067757 | 47067983 | - | 19.1 | 498 | 682 | -6149 | L1M4 | L1 | LINE |
| 47067996 | 47068232 | - | 26.5 | 14 | 253 | -6289 | L1M4 | L1 | LINE |

  

|  |  |  |  |  |  |  |  |  |  |
| --- | --- | --- | --- | --- | --- | --- | --- | --- | --- |
| chr16:47776372-47779299 | | | 2639 bp | | |  |  |  |  |
| Genome Start | Genome End | Strand | Div | Repeat Start | Repeat End | Repeat Left | Name | Family | Class |
| 47776372 | 47778413 | - | 24.1 | 4010 | 6153 | -11 | L1ME2 | L1 | LINE |
| 47778704 | 47779053 | - | 16.3 | 5959 | 6312 | 0 | L1MA9 | L1 | LINE |
| 47779054 | 47779110 | + | 12.3 | 5898 | 5954 | -358 | L1MA9 | L1 | LINE |
| 47779110 | 47779299 | - | 17.0 | 3804 | 4025 | -2121 | L1ME2 | L1 | LINE |

  

|  |  |  |  |  |  |  |  |  |  |
| --- | --- | --- | --- | --- | --- | --- | --- | --- | --- |
| chr16:53702306-53704213 | | | 1882 bp | | |  |  |  |  |
| Genome Start | Genome End | Strand | Div | Repeat Start | Repeat End | Repeat Left | Name | Family | Class |
| 53702306 | 53702424 | - | 33.6 | 81 | 193 | -69 | MIR | MIR | SINE |
| 53702425 | 53702910 | - | 17.9 | 5001 | 5496 | -672 | L1MA9 | L1 | LINE |
| 53702908 | 53703281 | + | 22.1 | 5479 | 5831 | -481 | L1MA9 | L1 | LINE |
| 53703282 | 53703582 | + | 9.6 | 1 | 305 | -7 | AluSx | Alu | SINE |
| 53703583 | 53704030 | + | 22.1 | 5832 | 6275 | -37 | L1MA9 | L1 | LINE |
| 53704043 | 53704137 | + | 18.7 | 7942 | 8042 | 0 | L1MC4 | L1 | LINE |
| 53704155 | 53704213 | - | 28.8 | 29 | 88 | -174 | MIR | MIR | SINE |

  

|  |  |  |  |  |  |  |  |  |  |
| --- | --- | --- | --- | --- | --- | --- | --- | --- | --- |
| chr16:58021722-58022479 | | | 745 bp | | |  |  |  |  |
| Genome Start | Genome End | Strand | Div | Repeat Start | Repeat End | Repeat Left | Name | Family | Class |
| 58021722 | 58021783 | - | 21.0 | 324 | 388 | -2 | MLT1B | MaLR | LTR |
| 58021795 | 58021946 | - | 4.6 | 5727 | 5878 | -290 | L1PA10 | L1 | LINE |
| 58021947 | 58022244 | + | 12.1 | 5870 | 6168 | 0 | L1PA10 | L1 | LINE |
| 58022247 | 58022479 | - | 23.2 | 3 | 294 | -96 | MLT1B | MaLR | LTR |

  

|  |  |  |  |  |  |  |  |  |  |
| --- | --- | --- | --- | --- | --- | --- | --- | --- | --- |
| chr16:58102805-58114820 | | | 12006 bp | | |  |  |  |  |
| Genome Start | Genome End | Strand | Div | Repeat Start | Repeat End | Repeat Left | Name | Family | Class |
| 58102805 | 58103034 | - | 17.4 | 2473 | 2708 | 0 | Tigger2 | MER2\_type | DNA |
| 58103037 | 58103450 | - | 3.4 | 4900 | 5313 | -833 | L1PA2 | L1 | LINE |
| 58103442 | 58104277 | + | 1.7 | 5314 | 6149 | -6 | L1PA2 | L1 | LINE |
| 58104278 | 58106313 | - | 14.6 | 431 | 2526 | -192 | Tigger2 | MER2\_type | DNA |
| 58106316 | 58106816 | - | 4.0 | 1 | 448 | -2 | LTR7 | ERV1 | LTR |
| 58106817 | 58113785 | - | 10.5 | 609 | 7713 | 0 | HERVH | ERV1 | LTR |
| 58113799 | 58113931 | - | 7.5 | 1 | 133 | -7580 | HERVH | ERV1 | LTR |
| 58113934 | 58114369 | - | 3.2 | 1 | 448 | -2 | LTR7 | ERV1 | LTR |
| 58114370 | 58114820 | - | 14.4 | 1 | 435 | -2283 | Tigger2 | MER2\_type | DNA |

  

|  |  |  |  |  |  |  |  |  |  |
| --- | --- | --- | --- | --- | --- | --- | --- | --- | --- |
| chr16:58389284-58392396 | | | 3059 bp | | |  |  |  |  |
| Genome Start | Genome End | Strand | Div | Repeat Start | Repeat End | Repeat Left | Name | Family | Class |
| 58389284 | 58389617 | + | 34.1 | 2933 | 3330 | -89 | L2 | L2 | LINE |
| 58389624 | 58389778 | - | 10.3 | 6014 | 6168 | 0 | L1PB1 | L1 | LINE |
| 58389779 | 58390035 | - | 22.8 | 5793 | 6012 | -156 | L1PA10 | L1 | LINE |
| 58390082 | 58390885 | - | 9.0 | 4990 | 5792 | -359 | L1PA10 | L1 | LINE |
| 58390884 | 58392306 | + | 8.2 | 3524 | 4975 | -1171 | L1PB | L1 | LINE |
| 58392311 | 58392396 | + | 24.4 | 3333 | 3419 | 0 | L2 | L2 | LINE |

  

|  |  |  |  |  |  |  |  |  |  |
| --- | --- | --- | --- | --- | --- | --- | --- | --- | --- |
| chr16:59262194-59264321 | | | 1836 bp | | |  |  |  |  |
| Genome Start | Genome End | Strand | Div | Repeat Start | Repeat End | Repeat Left | Name | Family | Class |
| 59262194 | 59262253 | - | 21.7 | 149 | 209 | 0 | MER3 | MER1\_type | DNA |
| 59262254 | 59263062 | - | 13.1 | 4635 | 5425 | -741 | L1P | L1 | LINE |
| 59263350 | 59263514 | - | 12.7 | 4470 | 4636 | -1510 | L1P | L1 | LINE |
| 59263520 | 59264238 | + | 13.9 | 5438 | 6165 | -1 | L1PA16 | L1 | LINE |
| 59264239 | 59264321 | - | 23.9 | 83 | 166 | -43 | MER3 | MER1\_type | DNA |

  

|  |  |  |  |  |  |  |  |  |  |
| --- | --- | --- | --- | --- | --- | --- | --- | --- | --- |
| chr16:60820687-60821435 | | | 706 bp | | |  |  |  |  |
| Genome Start | Genome End | Strand | Div | Repeat Start | Repeat End | Repeat Left | Name | Family | Class |
| 60820687 | 60820773 | + | 26.8 | 22 | 108 | -160 | MIRb | MIR | SINE |
| 60820787 | 60821066 | - | 24.3 | 5861 | 6149 | -17 | L1ME1 | L1 | LINE |
| 60821068 | 60821269 | + | 25.2 | 5644 | 5862 | -304 | L1ME1 | L1 | LINE |
| 60821299 | 60821435 | + | 38.2 | 128 | 268 | 0 | MIRb | MIR | SINE |

  

|  |  |  |  |  |  |  |  |  |  |
| --- | --- | --- | --- | --- | --- | --- | --- | --- | --- |
| chr16:61143099-61151166 | | | 8080 bp | | |  |  |  |  |
| Genome Start | Genome End | Strand | Div | Repeat Start | Repeat End | Repeat Left | Name | Family | Class |
| 61143099 | 61143788 | - | 14.1 | 6154 | 6850 | -874 | L1M1 | L1 | LINE |
| 61143786 | 61144976 | - | 4.2 | 4961 | 6152 | -2 | L1PA5 | L1 | LINE |
| 61144973 | 61146932 | + | 4.7 | 2983 | 4949 | -1197 | L1PA5 | L1 | LINE |
| 61146929 | 61147227 | - | 14.7 | 5872 | 6171 | -1394 | L1M1 | L1 | LINE |
| 61147227 | 61151166 | - | 15.9 | 27 | 4145 | -3420 | L1M1 | L1 | LINE |

  

|  |  |  |  |  |  |  |  |  |  |
| --- | --- | --- | --- | --- | --- | --- | --- | --- | --- |
| chr16:62456401-62461453 | | | 5055 bp | | |  |  |  |  |
| Genome Start | Genome End | Strand | Div | Repeat Start | Repeat End | Repeat Left | Name | Family | Class |
| 62456401 | 62457115 | + | 21.8 | 4692 | 5429 | -2356 | L1MC3 | L1 | LINE |
| 62457116 | 62457502 | + | 12.3 | 5914 | 6303 | -1 | L1MA2 | L1 | LINE |
| 62457503 | 62458235 | + | 22.2 | 5430 | 6299 | -1486 | L1MC3 | L1 | LINE |
| 62458236 | 62458386 | + | 15.4 | 6018 | 6168 | 0 | L1MB1 | L1 | LINE |
| 62458387 | 62459007 | + | 24.3 | 6300 | 6885 | -900 | L1MC3 | L1 | LINE |
| 62459008 | 62460491 | - | 6.9 | 4663 | 6151 | -3 | L1PA6 | L1 | LINE |
| 62460492 | 62460558 | + | 6.0 | 4597 | 4663 | -1483 | L1PA6 | L1 | LINE |
| 62460557 | 62461453 | + | 19.9 | 6868 | 7785 | 0 | L1MC3 | L1 | LINE |

  

|  |  |  |  |  |  |  |  |  |  |
| --- | --- | --- | --- | --- | --- | --- | --- | --- | --- |
| chr16:62469342-62470426 | | | 1068 bp | | |  |  |  |  |
| Genome Start | Genome End | Strand | Div | Repeat Start | Repeat End | Repeat Left | Name | Family | Class |
| 62469342 | 62469523 | + | 33.1 | 2 | 220 | -48 | MIRb | MIR | SINE |
| 62469524 | 62469806 | - | 10.9 | 5315 | 5601 | -573 | L1PA11 | L1 | LINE |
| 62469807 | 62470379 | + | 13.4 | 5591 | 6174 | 0 | L1PA11 | L1 | LINE |
| 62470397 | 62470426 | + | 16.7 | 208 | 237 | -31 | MIRb | MIR | SINE |

  

|  |  |  |  |  |  |  |  |  |  |
| --- | --- | --- | --- | --- | --- | --- | --- | --- | --- |
| chr16:62522020-62524924 | | | 2890 bp | | |  |  |  |  |
| Genome Start | Genome End | Strand | Div | Repeat Start | Repeat End | Repeat Left | Name | Family | Class |
| 62522020 | 62522223 | + | 14.2 | 5 | 206 | -1096 | HSMAR2 | Mariner | DNA |
| 62522224 | 62522543 | + | 8.7 | 1 | 311 | 0 | AluY | Alu | SINE |
| 62522544 | 62523436 | + | 14.2 | 207 | 1101 | -201 | HSMAR2 | Mariner | DNA |
| 62523437 | 62524006 | - | 15.0 | 4911 | 5484 | -681 | L1PA16 | L1 | LINE |
| 62524007 | 62524685 | + | 15.5 | 5474 | 6157 | -9 | L1PA16 | L1 | LINE |
| 62524701 | 62524924 | + | 16.0 | 1081 | 1301 | -1 | HSMAR2 | Mariner | DNA |

  

|  |  |  |  |  |  |  |  |  |  |
| --- | --- | --- | --- | --- | --- | --- | --- | --- | --- |
| chr16:70754912-70755730 | | | 781 bp | | |  |  |  |  |
| Genome Start | Genome End | Strand | Div | Repeat Start | Repeat End | Repeat Left | Name | Family | Class |
| 70754912 | 70755060 | - | 21.4 | 347 | 491 | 0 | LTR16C | ERVL | LTR |
| 70755061 | 70755105 | - | 6.7 | 5721 | 5766 | -385 | L1PB1 | L1 | LINE |
| 70755105 | 70755433 | + | 6.7 | 5776 | 6106 | -45 | L1PB1 | L1 | LINE |
| 70755473 | 70755730 | - | 26.6 | 86 | 360 | -131 | LTR16C | ERVL | LTR |

  

|  |  |  |  |  |  |  |  |  |  |
| --- | --- | --- | --- | --- | --- | --- | --- | --- | --- |
| chr16:77643100-77644242 | | | 1126 bp | | |  |  |  |  |
| Genome Start | Genome End | Strand | Div | Repeat Start | Repeat End | Repeat Left | Name | Family | Class |
| 77643100 | 77643419 | + | 2.5 | 11 | 330 | -261 | LTR10C | ERV1 | LTR |
| 77643437 | 77643813 | - | 2.9 | 5779 | 6155 | 0 | L1PA2 | L1 | LINE |
| 77643814 | 77643968 | + | 5.2 | 5626 | 5780 | -375 | L1PA2 | L1 | LINE |
| 77643969 | 77644242 | + | 6.9 | 316 | 591 | 0 | LTR10C | ERV1 | LTR |

  

|  |  |  |  |  |  |  |  |  |  |
| --- | --- | --- | --- | --- | --- | --- | --- | --- | --- |
| chr16:79405344-79407198 | | | 1837 bp | | |  |  |  |  |
| Genome Start | Genome End | Strand | Div | Repeat Start | Repeat End | Repeat Left | Name | Family | Class |
| 79405344 | 79406325 | - | 24.9 | 6909 | 7930 | -31 | L1MC5 | L1 | LINE |
| 79406330 | 79406622 | - | 8.6 | 5870 | 6165 | -3 | L1PA10 | L1 | LINE |
| 79406623 | 79406937 | + | 6.4 | 5541 | 5852 | -316 | L1PA10 | L1 | LINE |
| 79406952 | 79407198 | - | 26.0 | 6622 | 6903 | -1058 | L1MC5 | L1 | LINE |

  

|  |  |  |  |  |  |  |  |  |  |
| --- | --- | --- | --- | --- | --- | --- | --- | --- | --- |
| chr16:81154411-81157556 | | | 3076 bp | | |  |  |  |  |
| Genome Start | Genome End | Strand | Div | Repeat Start | Repeat End | Repeat Left | Name | Family | Class |
| 81154411 | 81154531 | - | 22.6 | 140 | 268 | 0 | MIRb | MIR | SINE |
| 81154591 | 81155509 | - | 13.8 | 5336 | 6287 | -4 | L1MA8 | L1 | LINE |
| 81155502 | 81156305 | + | 15.8 | 4104 | 4932 | -1211 | L1M2 | L1 | LINE |
| 81156306 | 81156597 | + | 12.7 | 1 | 311 | -2 | AluSp | Alu | SINE |
| 81156598 | 81156688 | + | 15.8 | 4933 | 5008 | -1135 | L1M2 | L1 | LINE |
| 81156693 | 81156815 | + | 8.1 | 1 | 123 | -186 | AluSg1 | Alu | SINE |
| 81156829 | 81157135 | + | 19.1 | 5027 | 5345 | -798 | L1M2 | L1 | LINE |
| 81157159 | 81157486 | - | 27.4 | 3065 | 3386 | -33 | L2 | L2 | LINE |
| 81157466 | 81157556 | - | 24.7 | 28 | 123 | -145 | MIRb | MIR | SINE |

  

|  |  |  |  |  |  |  |  |  |  |
| --- | --- | --- | --- | --- | --- | --- | --- | --- | --- |
| chr16:86670520-86673705 | | | 2948 bp | | |  |  |  |  |
| Genome Start | Genome End | Strand | Div | Repeat Start | Repeat End | Repeat Left | Name | Family | Class |
| 86670520 | 86670612 | + | 34.4 | 503 | 595 | -4259 | L1M | L1 | LINE |
| 86670635 | 86670931 | - | 7.8 | 1 | 295 | -14 | AluSc | Alu | SINE |
| 86670935 | 86671114 | - | 10.0 | 134 | 313 | 0 | AluSq | Alu | SINE |
| 86671241 | 86671938 | + | 24.8 | 1885 | 2616 | -2218 | L1M | L1 | LINE |
| 86671939 | 86672015 | + | 9.1 | 6 | 82 | -57 | MER85 | PiggyBac | DNA |
| 86672016 | 86672242 | + | 24.8 | 2617 | 2854 | -1980 | L1M | L1 | LINE |
| 86672243 | 86672531 | + | 8.7 | 1 | 304 | -9 | AluSp | Alu | SINE |
| 86672532 | 86673002 | + | 24.8 | 2857 | 3342 | -1492 | L1M | L1 | LINE |
| 86672978 | 86673114 | - | 8.8 | 5547 | 5682 | -486 | L1P4 | L1 | LINE |
| 86673111 | 86673206 | + | 6.2 | 5683 | 5779 | -384 | L1P4 | L1 | LINE |
| 86673207 | 86673491 | + | 12.5 | 45 | 329 | 0 | AluSx | Alu | SINE |
| 86673608 | 86673705 | + | 24.8 | 4000 | 4096 | -832 | L1M | L1 | LINE |

  


|  |  |  |  |  |  |  |  |  |  |
| --- | --- | --- | --- | --- | --- | --- | --- | --- | --- |
| chr17:3150629-3152733 | | | 2111 bp | | |  |  |  |  |
| Genome Start | Genome End | Strand | Div | Repeat Start | Repeat End | Repeat Left | Name | Family | Class |
| 3150629 | 3150732 | + | 26.7 | 5424 | 5528 | -642 | L1M5 | L1 | LINE |
| 3150734 | 3151931 | - | 4.3 | 4957 | 6154 | 0 | L1PA5 | L1 | LINE |
| 3151929 | 3152589 | + | 4.4 | 4306 | 4958 | -1188 | L1PA5 | L1 | LINE |
| 3152586 | 3152733 | + | 29.4 | 5511 | 5675 | -565 | L1M5 | L1 | LINE |

  

|  |  |  |  |  |  |  |  |  |  |
| --- | --- | --- | --- | --- | --- | --- | --- | --- | --- |
| chr17:5692996-5697389 | | | 4236 bp | | |  |  |  |  |
| Genome Start | Genome End | Strand | Div | Repeat Start | Repeat End | Repeat Left | Name | Family | Class |
| 5692996 | 5693232 | - | 22.1 | 4408 | 4665 | -1481 | L1MC4a | L1 | LINE |
| 5693273 | 5693496 | - | 25.6 | 2 | 224 | 0 | MER58A | MER1\_type | DNA |
| 5693601 | 5694843 | - | 1.9 | 4910 | 6155 | 0 | L1PA3 | L1 | LINE |
| 5694844 | 5696259 | + | 2.5 | 3515 | 4927 | -1219 | L1PA3 | L1 | LINE |
| 5696274 | 5697389 | - | 32.3 | 3136 | 4296 | -1850 | L1MC4a | L1 | LINE |

  

|  |  |  |  |  |  |  |  |  |  |
| --- | --- | --- | --- | --- | --- | --- | --- | --- | --- |
| chr17:6066891-6069521 | | | 2561 bp | | |  |  |  |  |
| Genome Start | Genome End | Strand | Div | Repeat Start | Repeat End | Repeat Left | Name | Family | Class |
| 6066891 | 6067022 | + | 22.1 | 722 | 852 | -5557 | L1MD2 | L1 | LINE |
| 6067017 | 6067128 | - | 7.1 | 5876 | 5987 | -167 | L1PA5 | L1 | LINE |
| 6067129 | 6067291 | + | 5.5 | 5992 | 6154 | 0 | L1PA5 | L1 | LINE |
| 6067332 | 6067653 | + | 20.6 | 836 | 1162 | -5247 | L1MD2 | L1 | LINE |
| 6067684 | 6067883 | + | 17.7 | 3085 | 3303 | -3103 | L1MD2 | L1 | LINE |
| 6067884 | 6068187 | + | 8.9 | 1 | 306 | -3 | AluSc | Alu | SINE |
| 6068188 | 6068437 | + | 17.7 | 3304 | 3562 | -2844 | L1MD2 | L1 | LINE |
| 6068439 | 6068574 | + | 14.3 | 2 | 143 | 0 | FLAM\_C | Alu | SINE |
| 6068580 | 6068734 | + | 21.4 | 3959 | 4113 | -2293 | L1MD2 | L1 | LINE |
| 6068735 | 6069031 | + | 16.9 | 1 | 308 | -4 | AluJb | Alu | SINE |
| 6069032 | 6069521 | + | 21.4 | 4114 | 4608 | -1798 | L1MD2 | L1 | LINE |

  

|  |  |  |  |  |  |  |  |  |  |
| --- | --- | --- | --- | --- | --- | --- | --- | --- | --- |
| chr17:11315814-11324409 | | | 8468 bp | | |  |  |  |  |
| Genome Start | Genome End | Strand | Div | Repeat Start | Repeat End | Repeat Left | Name | Family | Class |
| 11315814 | 11316299 | - | 15.5 | 6009 | 6508 | 0 | L1MC1 | L1 | LINE |
| 11316301 | 11316536 | - | 20.9 | 5 | 238 | -74 | AluJo | Alu | SINE |
| 11316541 | 11317366 | - | 13.4 | 5155 | 6003 | -505 | L1MC1 | L1 | LINE |
| 11317367 | 11317665 | - | 20.9 | 38 | 362 | -46 | MLT1A1 | MaLR | LTR |
| 11317787 | 11318187 | - | 7.8 | 5770 | 6168 | -4 | L1PA8 | L1 | LINE |
| 11318188 | 11318528 | - | 8.9 | 5230 | 5571 | -560 | L1PA8 | L1 | LINE |
| 11318526 | 11321492 | - | 14.3 | 2119 | 5153 | -1168 | L1MC1 | L1 | LINE |
| 11321466 | 11323016 | - | 1.5 | 4600 | 6150 | -5 | L1PA2 | L1 | LINE |
| 11323015 | 11323108 | + | 2.1 | 4514 | 4606 | -1540 | L1PA2 | L1 | LINE |
| 11323109 | 11323217 | - | 14.1 | 2044 | 2136 | -4372 | L1MC1 | L1 | LINE |
| 11323218 | 11323287 | + | 10.0 | 220 | 289 | -22 | Alu | Alu | SINE |
| 11323288 | 11323307 | - | 14.1 | 2027 | 2043 | -4465 | L1MC1 | L1 | LINE |
| 11323308 | 11323682 | + | 5.9 | 5798 | 6172 | 0 | L1PA8 | L1 | LINE |
| 11323683 | 11323780 | - | 14.1 | 1939 | 2025 | -4483 | L1MC1 | L1 | LINE |
| 11323815 | 11324409 | - | 14.1 | 1275 | 1938 | -4570 | L1MC1 | L1 | LINE |

  

|  |  |  |  |  |  |  |  |  |  |
| --- | --- | --- | --- | --- | --- | --- | --- | --- | --- |
| chr17:13013912-13020311 | | | 6282 bp | | |  |  |  |  |
| Genome Start | Genome End | Strand | Div | Repeat Start | Repeat End | Repeat Left | Name | Family | Class |
| 13013912 | 13014171 | + | 14.7 | 5193 | 5478 | -822 | L1MA6 | L1 | LINE |
| 13014209 | 13014444 | + | 14.6 | 5479 | 5732 | -568 | L1MA6 | L1 | LINE |
| 13014488 | 13016159 | + | 16.7 | 478 | 4415 | -2773 | LTR25-int | ERV1 | LTR |
| 13016144 | 13018221 | + | 11.8 | 3055 | 5206 | -11 | MER61D-int | ERV1 | LTR |
| 13018223 | 13018576 | - | 4.5 | 5802 | 6155 | 0 | L1PA4 | L1 | LINE |
| 13018577 | 13019315 | + | 3.6 | 5075 | 5813 | -342 | L1PA4 | L1 | LINE |
| 13019368 | 13019745 | + | 16.1 | 114 | 501 | 0 | MER61D | ERV1 | LTR |
| 13019747 | 13020311 | + | 15.1 | 5729 | 6300 | 0 | L1MA6 | L1 | LINE |

  

|  |  |  |  |  |  |  |  |  |  |
| --- | --- | --- | --- | --- | --- | --- | --- | --- | --- |
| chr17:13051615-13064542 | | | 12514 bp | | |  |  |  |  |
| Genome Start | Genome End | Strand | Div | Repeat Start | Repeat End | Repeat Left | Name | Family | Class |
| 13051615 | 13051845 | - | 30.1 | 1572 | 1829 | -678 | HAL1 | L1 | LINE |
| 13051858 | 13053514 | + | 23.5 | 4 | 1856 | -4849 | L1MC3 | L1 | LINE |
| 13053558 | 13055422 | - | 10.9 | 3669 | 5531 | -630 | L1PA13 | L1 | LINE |
| 13055423 | 13056042 | + | 8.8 | 5512 | 6160 | -3 | L1PA13 | L1 | LINE |
| 13056050 | 13057345 | + | 23.4 | 2046 | 3527 | -3037 | L1MC3 | L1 | LINE |
| 13057346 | 13057533 | - | 22.5 | 210 | 396 | 0 | MSTD | MaLR | LTR |
| 13057534 | 13057582 | + | 23.4 | 3528 | 3582 | -2982 | L1MC3 | L1 | LINE |
| 13057624 | 13058165 | + | 21.0 | 3586 | 4139 | -2425 | L1MC3 | L1 | LINE |
| 13058202 | 13058660 | + | 21.1 | 22 | 505 | 0 | MLT1D | MaLR | LTR |
| 13058661 | 13059935 | + | 20.8 | 4137 | 5474 | -1090 | L1MC3 | L1 | LINE |
| 13059972 | 13062505 | + | 19.4 | 5475 | 8203 | 0 | L1MC3 | L1 | LINE |
| 13062507 | 13062828 | - | 31.4 | 1234 | 1568 | -939 | HAL1 | L1 | LINE |
| 13063016 | 13063319 | - | 18.9 | 1 | 312 | 0 | AluSx | Alu | SINE |
| 13063320 | 13063336 | - | 31.4 | 1221 | 1233 | -1274 | HAL1 | L1 | LINE |
| 13063388 | 13063576 | - | 6.3 | 5966 | 6154 | 0 | L1PA5 | L1 | LINE |
| 13063577 | 13063809 | - | 31.4 | 1033 | 1212 | -1295 | HAL1 | L1 | LINE |
| 13063810 | 13064247 | - | 12.6 | 5712 | 6160 | 0 | L1PREC2 | L1 | LINE |
| 13064248 | 13064542 | - | 31.4 | 737 | 1032 | -1475 | HAL1 | L1 | LINE |

  

|  |  |  |  |  |  |  |  |  |  |
| --- | --- | --- | --- | --- | --- | --- | --- | --- | --- |
| chr17:13297809-13300078 | | | 2238 bp | | |  |  |  |  |
| Genome Start | Genome End | Strand | Div | Repeat Start | Repeat End | Repeat Left | Name | Family | Class |
| 13297809 | 13298487 | - | 18.1 | 5476 | 6165 | -9 | L1MB5 | L1 | LINE |
| 13298520 | 13298585 | - | 12.5 | 6062 | 6125 | -6 | L1PA8A | L1 | LINE |
| 13298586 | 13298800 | + | 6.5 | 5845 | 6059 | -72 | L1PA8A | L1 | LINE |
| 13298798 | 13299283 | - | 16.8 | 4961 | 5471 | -709 | L1MB5 | L1 | LINE |
| 13299287 | 13300078 | - | 22.3 | 3757 | 4530 | -1616 | L1MB5 | L1 | LINE |

  

|  |  |  |  |  |  |  |  |  |  |
| --- | --- | --- | --- | --- | --- | --- | --- | --- | --- |
| chr17:14519566-14524231 | | | 4396 bp | | |  |  |  |  |
| Genome Start | Genome End | Strand | Div | Repeat Start | Repeat End | Repeat Left | Name | Family | Class |
| 14519566 | 14521035 | + | 33.4 | 5 | 1547 | -4602 | L1MEd | L1 | LINE |
| 14521051 | 14522539 | - | 9.8 | 4660 | 6171 | -1 | L1PA8 | L1 | LINE |
| 14522540 | 14522975 | + | 7.5 | 4208 | 4647 | -1499 | L1PA8 | L1 | LINE |
| 14522963 | 14523504 | + | 29.8 | 1539 | 2094 | -4055 | L1MEd | L1 | LINE |
| 14523521 | 14523784 | - | 37.7 | 204 | 480 | -11 | LTR16C | ERVL | LTR |
| 14524037 | 14524231 | + | 31.9 | 2073 | 2280 | -3869 | L1MEd | L1 | LINE |

  

|  |  |  |  |  |  |  |  |  |  |
| --- | --- | --- | --- | --- | --- | --- | --- | --- | --- |
| chr17:14811095-14815894 | | | 4665 bp | | |  |  |  |  |
| Genome Start | Genome End | Strand | Div | Repeat Start | Repeat End | Repeat Left | Name | Family | Class |
| 14811095 | 14811236 | - | 10.6 | 537 | 669 | -65 | MER50 | ERV1 | LTR |
| 14811256 | 14811607 | - | 10.7 | 5782 | 6143 | -8 | L1PB1 | L1 | LINE |
| 14811726 | 14813001 | - | 8.1 | 4491 | 5781 | -370 | L1PB1 | L1 | LINE |
| 14813002 | 14813291 | - | 11.4 | 5 | 294 | -16 | AluSg | Alu | SINE |
| 14813292 | 14814757 | - | 7.1 | 3022 | 4490 | -1656 | L1PB1 | L1 | LINE |
| 14814753 | 14815336 | + | 7.9 | 2453 | 3035 | -3111 | L1PB1 | L1 | LINE |
| 14815340 | 14815894 | - | 17.0 | 1 | 550 | -184 | MER50 | ERV1 | LTR |

  

|  |  |  |  |  |  |  |  |  |  |
| --- | --- | --- | --- | --- | --- | --- | --- | --- | --- |
| chr17:20761202-20763811 | | | 2610 bp | | |  |  |  |  |
| Genome Start | Genome End | Strand | Div | Repeat Start | Repeat End | Repeat Left | Name | Family | Class |
| 20761202 | 20761349 | + | 31.8 | 15 | 169 | -2775 | BLACKJACK | AcHobo | DNA |
| 20761350 | 20762823 | - | 4.8 | 4686 | 6154 | 0 | L1PA5 | L1 | LINE |
| 20762824 | 20763485 | + | 4.5 | 4021 | 4682 | -1464 | L1PA5 | L1 | LINE |
| 20763486 | 20763811 | + | 31.4 | 154 | 474 | -2470 | BLACKJACK | AcHobo | DNA |

  

|  |  |  |  |  |  |  |  |  |  |
| --- | --- | --- | --- | --- | --- | --- | --- | --- | --- |
| chr17:21411721-21416719 | | | 4935 bp | | |  |  |  |  |
| Genome Start | Genome End | Strand | Div | Repeat Start | Repeat End | Repeat Left | Name | Family | Class |
| 21411721 | 21412714 | - | 16.0 | 5706 | 6758 | 0 | MER4B-int | ERV1 | LTR |
| 21412715 | 21413712 | - | 9.8 | 3697 | 4694 | -1452 | L1P3 | L1 | LINE |
| 21413755 | 21413837 | - | 9.8 | 3593 | 3696 | -2450 | L1P3 | L1 | LINE |
| 21413836 | 21415265 | + | 9.2 | 4693 | 6129 | -2 | L1PA8A | L1 | LINE |
| 21415267 | 21415456 | - | 14.5 | 5544 | 5722 | -1036 | MER4B-int | ERV1 | LTR |
| 21415457 | 21415620 | + | 16.1 | 1 | 204 | -177 | THE1D | MaLR | LTR |
| 21415648 | 21415815 | + | 16.1 | 205 | 381 | 0 | THE1D | MaLR | LTR |
| 21415816 | 21416360 | - | 14.5 | 5006 | 5543 | -1215 | MER4B-int | ERV1 | LTR |
| 21416351 | 21416507 | - | 12.6 | 4321 | 4481 | -2277 | MER4B-int | ERV1 | LTR |
| 21416514 | 21416719 | - | 20.7 | 3973 | 4184 | -2574 | MER4B-int | ERV1 | LTR |

  

|  |  |  |  |  |  |  |  |  |  |
| --- | --- | --- | --- | --- | --- | --- | --- | --- | --- |
| chr17:21836601-21841551 | | | 4921 bp | | |  |  |  |  |
| Genome Start | Genome End | Strand | Div | Repeat Start | Repeat End | Repeat Left | Name | Family | Class |
| 21836601 | 21836806 | + | 21.7 | 3975 | 4186 | -2572 | MER4B-int | ERV1 | LTR |
| 21836811 | 21836967 | + | 17.8 | 4321 | 4481 | -2277 | MER4B-int | ERV1 | LTR |
| 21836958 | 21837500 | + | 14.4 | 5004 | 5543 | -1215 | MER4B-int | ERV1 | LTR |
| 21837501 | 21837847 | - | 16.6 | 1 | 381 | 0 | THE1D | MaLR | LTR |
| 21837848 | 21838006 | + | 14.4 | 5544 | 5723 | -1035 | MER4B-int | ERV1 | LTR |
| 21838009 | 21839437 | - | 10.7 | 4693 | 6129 | -2 | L1PA8A | L1 | LINE |
| 21839436 | 21839518 | + | 10.3 | 3592 | 3695 | -2451 | L1P3 | L1 | LINE |
| 21839555 | 21840552 | + | 10.3 | 3696 | 4693 | -1453 | L1P3 | L1 | LINE |
| 21840553 | 21841551 | + | 15.9 | 5706 | 6758 | 0 | MER4B-int | ERV1 | LTR |

  

|  |  |  |  |  |  |  |  |  |  |
| --- | --- | --- | --- | --- | --- | --- | --- | --- | --- |
| chr17:22479552-22483195 | | | 3624 bp | | |  |  |  |  |
| Genome Start | Genome End | Strand | Div | Repeat Start | Repeat End | Repeat Left | Name | Family | Class |
| 22479552 | 22479625 | + | 18.9 | 5 | 79 | -435 | MER67D | ERV1 | LTR |
| 22479646 | 22482340 | - | 4.7 | 3443 | 6144 | -11 | L1PA4 | L1 | LINE |
| 22482341 | 22482791 | + | 5.3 | 2999 | 3451 | -2695 | L1PA4 | L1 | LINE |
| 22482792 | 22483195 | + | 22.2 | 71 | 509 | -5 | MER67D | ERV1 | LTR |

  

|  |  |  |  |  |  |  |  |  |  |
| --- | --- | --- | --- | --- | --- | --- | --- | --- | --- |
| chr17:28315642-28325001 | | | 9354 bp | | |  |  |  |  |
| Genome Start | Genome End | Strand | Div | Repeat Start | Repeat End | Repeat Left | Name | Family | Class |
| 28315642 | 28316666 | + | 8.6 | 1 | 1033 | -6557 | HERVP71A | ERV1 | LTR |
| 28316675 | 28316997 | - | 3.7 | 1953 | 2275 | 0 | L1PA5 | L1 | LINE |
| 28316996 | 28320901 | + | 5.2 | 2263 | 6154 | 0 | L1PA5 | L1 | LINE |
| 28320902 | 28321518 | + | 8.3 | 1022 | 1649 | -5941 | HERVP71A | ERV1 | LTR |
| 28321519 | 28321647 | + | 19.8 | 353 | 492 | -122 | LTR18B | ERVL | LTR |
| 28321648 | 28324076 | + | 7.2 | 4493 | 6954 | -636 | HERVP71A | ERV1 | LTR |
| 28324077 | 28324375 | - | 11.2 | 1 | 296 | -16 | AluSx | Alu | SINE |
| 28324376 | 28325001 | + | 7.2 | 6955 | 7590 | 0 | HERVP71A | ERV1 | LTR |

  

|  |  |  |  |  |  |  |  |  |  |
| --- | --- | --- | --- | --- | --- | --- | --- | --- | --- |
| chr17:36878940-36880558 | | | 1510 bp | | |  |  |  |  |
| Genome Start | Genome End | Strand | Div | Repeat Start | Repeat End | Repeat Left | Name | Family | Class |
| 36878940 | 36878983 | + | 27.3 | 33 | 76 | -113 | MER5A | MER1\_type | DNA |
| 36878984 | 36880122 | + | 5.0 | 5035 | 6169 | -3 | L1PA8 | L1 | LINE |
| 36880120 | 36880160 | - | 0.0 | 5940 | 5980 | -175 | L1PA3 | L1 | LINE |
| 36880161 | 36880329 | + | 3.0 | 5987 | 6155 | 0 | L1PA3 | L1 | LINE |
| 36880442 | 36880558 | + | 22.5 | 61 | 173 | -16 | MER5A | MER1\_type | DNA |

  

|  |  |  |  |  |  |  |  |  |  |
| --- | --- | --- | --- | --- | --- | --- | --- | --- | --- |
| chr17:47854011-47859890 | | | 5797 bp | | |  |  |  |  |
| Genome Start | Genome End | Strand | Div | Repeat Start | Repeat End | Repeat Left | Name | Family | Class |
| 47854011 | 47854387 | + | 25.1 | 2683 | 3082 | -3064 | L1M5 | L1 | LINE |
| 47854388 | 47855068 | + | 4.5 | 5465 | 6146 | -8 | L1PA5 | L1 | LINE |
| 47855069 | 47855504 | + | 25.1 | 3083 | 3517 | -2629 | L1M5 | L1 | LINE |
| 47855511 | 47857101 | - | 8.9 | 3133 | 4710 | -1436 | L1PA7 | L1 | LINE |
| 47857100 | 47858534 | + | 7.9 | 4718 | 6153 | -1 | L1PA7 | L1 | LINE |
| 47858546 | 47859574 | + | 28.5 | 4637 | 5762 | -402 | L1M5 | L1 | LINE |
| 47859640 | 47859716 | - | 21.5 | 1 | 75 | -85 | MER5A | MER1\_type | DNA |
| 47859720 | 47859798 | + | 27.9 | 3298 | 3384 | -35 | L2 | L2 | LINE |
| 47859799 | 47859890 | + | 26.7 | 5746 | 5837 | -327 | L1M5 | L1 | LINE |

  

|  |  |  |  |  |  |  |  |  |  |
| --- | --- | --- | --- | --- | --- | --- | --- | --- | --- |
| chr17:47902348-47907247 | | | 4785 bp | | |  |  |  |  |
| Genome Start | Genome End | Strand | Div | Repeat Start | Repeat End | Repeat Left | Name | Family | Class |
| 47902348 | 47903541 | - | 24.8 | 4390 | 5664 | -3 | ERVL-E | ERVL | LTR |
| 47903533 | 47904674 | - | 25.5 | 1469 | 2673 | -2994 | ERVL-E | ERVL | LTR |
| 47904676 | 47904849 | + | 32.0 | 37 | 216 | -447 | MLT2F | ERVL | LTR |
| 47904958 | 47905160 | + | 36.5 | 457 | 663 | 0 | MLT2F | ERVL | LTR |
| 47905162 | 47906587 | - | 27.8 | 34 | 1461 | -4206 | ERVL-E | ERVL | LTR |
| 47906588 | 47906904 | - | 11.6 | 5653 | 5971 | -192 | L1PA13 | L1 | LINE |
| 47906905 | 47906999 | - | 8.8 | 6064 | 6154 | 0 | L1PA6 | L1 | LINE |
| 47907000 | 47907025 | - | 11.6 | 5626 | 5652 | -511 | L1PA13 | L1 | LINE |
| 47907026 | 47907194 | + | 8.4 | 5995 | 6160 | -3 | L1PA13 | L1 | LINE |
| 47907209 | 47907247 | - | 17.9 | 2 | 40 | -5627 | ERVL-E | ERVL | LTR |

  

|  |  |  |  |  |  |  |  |  |  |
| --- | --- | --- | --- | --- | --- | --- | --- | --- | --- |
| chr17:48639092-48642651 | | | 3452 bp | | |  |  |  |  |
| Genome Start | Genome End | Strand | Div | Repeat Start | Repeat End | Repeat Left | Name | Family | Class |
| 48639092 | 48639225 | - | 32.1 | 97 | 244 | -18 | MIR | MIR | SINE |
| 48639229 | 48639754 | + | 7.3 | 4259 | 4767 | -1379 | L1PB1 | L1 | LINE |
| 48639756 | 48640393 | + | 9.0 | 5140 | 5785 | -366 | L1PB1 | L1 | LINE |
| 48640403 | 48640705 | + | 14.3 | 4 | 309 | -4 | AluSp | Alu | SINE |
| 48640779 | 48641149 | + | 14.4 | 5783 | 6151 | 0 | L1PB1 | L1 | LINE |
| 48641175 | 48642461 | - | 6.6 | 4866 | 6153 | -1 | L1PA7 | L1 | LINE |
| 48642459 | 48642549 | + | 16.5 | 4772 | 4862 | -1284 | L1P1 | L1 | LINE |
| 48642550 | 48642651 | - | 34.3 | 2 | 109 | -153 | MIR | MIR | SINE |

  

|  |  |  |  |  |  |  |  |  |  |
| --- | --- | --- | --- | --- | --- | --- | --- | --- | --- |
| chr17:48938311-48943394 | | | 4698 bp | | |  |  |  |  |
| Genome Start | Genome End | Strand | Div | Repeat Start | Repeat End | Repeat Left | Name | Family | Class |
| 48938311 | 48938596 | + | 36.4 | 195 | 503 | -5616 | L1MEd | L1 | LINE |
| 48938629 | 48939877 | - | 14.0 | 5038 | 6292 | -13 | L1MA3 | L1 | LINE |
| 48939876 | 48942058 | + | 13.9 | 2828 | 5045 | -1101 | L1MA3 | L1 | LINE |
| 48942415 | 48943394 | + | 30.2 | 904 | 2019 | -4130 | L1MEd | L1 | LINE |

  

|  |  |  |  |  |  |  |  |  |  |
| --- | --- | --- | --- | --- | --- | --- | --- | --- | --- |
| chr17:49452624-49454298 | | | 1452 bp | | |  |  |  |  |
| Genome Start | Genome End | Strand | Div | Repeat Start | Repeat End | Repeat Left | Name | Family | Class |
| 49452624 | 49452843 | - | 38.0 | 1513 | 1727 | -780 | HAL1 | L1 | LINE |
| 49452960 | 49453236 | - | 33.8 | 940 | 1234 | -4885 | L1MEd | L1 | LINE |
| 49453237 | 49453556 | - | 18.5 | 5968 | 6312 | 0 | L1MA9 | L1 | LINE |
| 49453554 | 49453800 | + | 11.7 | 5223 | 5483 | -808 | L1MA7 | L1 | LINE |
| 49453855 | 49454039 | + | 17.8 | 5794 | 5978 | -313 | L1MA7 | L1 | LINE |
| 49454096 | 49454298 | - | 29.9 | 817 | 1034 | -1473 | HAL1 | L1 | LINE |

  

|  |  |  |  |  |  |  |  |  |  |
| --- | --- | --- | --- | --- | --- | --- | --- | --- | --- |
| chr17:49633582-49635491 | | | 1832 bp | | |  |  |  |  |
| Genome Start | Genome End | Strand | Div | Repeat Start | Repeat End | Repeat Left | Name | Family | Class |
| 49633582 | 49633798 | + | 24.9 | 5257 | 5477 | -2331 | L1MC4a | L1 | LINE |
| 49633824 | 49633933 | + | 20.5 | 5611 | 5724 | -2084 | L1MC4a | L1 | LINE |
| 49633934 | 49634901 | + | 2.3 | 1 | 968 | 0 | LTR5\_Hs | ERVK | LTR |
| 49634917 | 49635201 | - | 3.9 | 5866 | 6151 | -4 | L1PA4 | L1 | LINE |
| 49635202 | 49635355 | + | 4.5 | 5709 | 5862 | -293 | L1PA4 | L1 | LINE |
| 49635394 | 49635491 | + | 27.6 | 5815 | 5915 | -1893 | L1MC4a | L1 | LINE |

  

|  |  |  |  |  |  |  |  |  |  |
| --- | --- | --- | --- | --- | --- | --- | --- | --- | --- |
| chr17:51486784-51498360 | | | 11462 bp | | |  |  |  |  |
| Genome Start | Genome End | Strand | Div | Repeat Start | Repeat End | Repeat Left | Name | Family | Class |
| 51486784 | 51486989 | - | 12.1 | 2216 | 2418 | 0 | Tigger1 | MER2\_type | DNA |
| 51486990 | 51487265 | + | 15.9 | 1 | 302 | -10 | AluJb | Alu | SINE |
| 51487266 | 51487417 | - | 12.1 | 2078 | 2215 | -203 | Tigger1 | MER2\_type | DNA |
| 51487420 | 51487806 | - | 16.2 | 5783 | 6166 | 0 | L1PA17 | L1 | LINE |
| 51487829 | 51487890 | + | 13.1 | 498 | 558 | -7712 | PRIMA4-int | ERV1 | LTR |
| 51487948 | 51488600 | - | 12.8 | 5140 | 5791 | -365 | L1P5 | L1 | LINE |
| 51488598 | 51489182 | + | 16.8 | 4556 | 5140 | -1006 | L1P5 | L1 | LINE |
| 51489183 | 51489863 | - | 12.1 | 1448 | 2089 | -329 | Tigger1 | MER2\_type | DNA |
| 51489864 | 51490156 | + | 12.3 | 1 | 294 | -18 | AluSx | Alu | SINE |
| 51490157 | 51490833 | - | 12.1 | 799 | 1447 | -971 | Tigger1 | MER2\_type | DNA |
| 51490838 | 51493619 | - | 8.5 | 3525 | 6298 | -18 | L1PA8A | L1 | LINE |
| 51493620 | 51493932 | - | 9.9 | 1 | 312 | -1 | AluSq | Alu | SINE |
| 51493933 | 51496291 | - | 12.5 | 1116 | 3524 | -2807 | L1PA8A | L1 | LINE |
| 51496322 | 51497528 | - | 16.4 | 11 | 1115 | -5238 | L1PA8A | L1 | LINE |
| 51497532 | 51498360 | - | 15.7 | 2 | 839 | -1579 | Tigger1 | MER2\_type | DNA |

  

|  |  |  |  |  |  |  |  |  |  |
| --- | --- | --- | --- | --- | --- | --- | --- | --- | --- |
| chr17:51786806-51788467 | | | 1649 bp | | |  |  |  |  |
| Genome Start | Genome End | Strand | Div | Repeat Start | Repeat End | Repeat Left | Name | Family | Class |
| 51786806 | 51787019 | - | 25.9 | 3163 | 3385 | -34 | L2 | L2 | LINE |
| 51787023 | 51787353 | - | 14.8 | 5595 | 6026 | -265 | L1MA6 | L1 | LINE |
| 51787355 | 51787608 | + | 16.5 | 6043 | 6295 | -5 | L1MA6 | L1 | LINE |
| 51787618 | 51788135 | - | 29.8 | 2635 | 3172 | -247 | L2 | L2 | LINE |
| 51788136 | 51788433 | - | 15.2 | 5878 | 6175 | -9 | L1MB7 | L1 | LINE |
| 51788434 | 51788467 | - | 30.4 | 2612 | 2634 | -785 | L2 | L2 | LINE |

  

|  |  |  |  |  |  |  |  |  |  |
| --- | --- | --- | --- | --- | --- | --- | --- | --- | --- |
| chr17:65275254-65282495 | | | 7198 bp | | |  |  |  |  |
| Genome Start | Genome End | Strand | Div | Repeat Start | Repeat End | Repeat Left | Name | Family | Class |
| 65275254 | 65276809 | + | 14.3 | 1 | 1588 | -830 | Tigger1 | MER2\_type | DNA |
| 65276814 | 65278458 | - | 12.9 | 1953 | 3603 | -3246 | L1PA16 | L1 | LINE |
| 65278464 | 65279057 | + | 12.3 | 3616 | 4250 | -2599 | L1PA16 | L1 | LINE |
| 65279082 | 65281670 | + | 13.2 | 4251 | 6860 | -9 | L1PA16 | L1 | LINE |
| 65281682 | 65282495 | + | 13.4 | 1577 | 2418 | 0 | Tigger1 | MER2\_type | DNA |

  


|  |  |  |  |  |  |  |  |  |  |
| --- | --- | --- | --- | --- | --- | --- | --- | --- | --- |
| chr18:1200958-1204971 | | | 3982 bp | | |  |  |  |  |
| Genome Start | Genome End | Strand | Div | Repeat Start | Repeat End | Repeat Left | Name | Family | Class |
| 1200958 | 1201380 | + | 28.4 | 1546 | 2034 | -1385 | L2 | L2 | LINE |
| 1201400 | 1203536 | - | 15.0 | 3619 | 5829 | -468 | L1MA5A | L1 | LINE |
| 1203537 | 1203663 | + | 11.8 | 6165 | 6292 | -5 | L1MA5A | L1 | LINE |
| 1203664 | 1203957 | - | 15.8 | 3377 | 3618 | -2525 | L1MA5A | L1 | LINE |
| 1203964 | 1204405 | + | 12.0 | 5845 | 6291 | -6 | L1MA5A | L1 | LINE |
| 1204413 | 1204971 | + | 31.8 | 2842 | 3415 | -4 | L2 | L2 | LINE |

  

|  |  |  |  |  |  |  |  |  |  |
| --- | --- | --- | --- | --- | --- | --- | --- | --- | --- |
| chr18:1581438-1583082 | | | 1607 bp | | |  |  |  |  |
| Genome Start | Genome End | Strand | Div | Repeat Start | Repeat End | Repeat Left | Name | Family | Class |
| 1581438 | 1582250 | + | 19.4 | 4932 | 5761 | -583 | L1MC | L1 | LINE |
| 1582273 | 1582527 | - | 21.7 | 5461 | 5705 | -478 | L1MB3 | L1 | LINE |
| 1582529 | 1583022 | + | 16.6 | 5704 | 6181 | -2 | L1MB3 | L1 | LINE |
| 1583038 | 1583082 | + | 8.9 | 5826 | 5881 | -463 | L1MC | L1 | LINE |

  

|  |  |  |  |  |  |  |  |  |  |
| --- | --- | --- | --- | --- | --- | --- | --- | --- | --- |
| chr18:1621734-1624977 | | | 3251 bp | | |  |  |  |  |
| Genome Start | Genome End | Strand | Div | Repeat Start | Repeat End | Repeat Left | Name | Family | Class |
| 1621734 | 1622164 | + | 23.2 | 4176 | 4619 | -1527 | L1M4 | L1 | LINE |
| 1622156 | 1623061 | - | 1.3 | 4134 | 5039 | -1107 | L1PA2 | L1 | LINE |
| 1623060 | 1624184 | + | 2.2 | 5037 | 6152 | -3 | L1PA2 | L1 | LINE |
| 1624183 | 1624479 | - | 15.0 | 5924 | 6222 | -83 | L1MA3 | L1 | LINE |
| 1624486 | 1624977 | + | 25.1 | 4600 | 5092 | -1054 | L1M4 | L1 | LINE |

  

|  |  |  |  |  |  |  |  |  |  |
| --- | --- | --- | --- | --- | --- | --- | --- | --- | --- |
| chr18:1745973-1751315 | | | 4927 bp | | |  |  |  |  |
| Genome Start | Genome End | Strand | Div | Repeat Start | Repeat End | Repeat Left | Name | Family | Class |
| 1745973 | 1746280 | + | 35.9 | 142 | 450 | -5950 | L1ME2 | L1 | LINE |
| 1746266 | 1746780 | - | 5.8 | 4961 | 5475 | -679 | L1P5 | L1 | LINE |
| 1746781 | 1747481 | + | 3.1 | 5455 | 6155 | 0 | L1PA4 | L1 | LINE |
| 1747903 | 1748439 | + | 26.1 | 641 | 1177 | -5223 | L1ME2 | L1 | LINE |
| 1748440 | 1748712 | + | 15.3 | 6014 | 6286 | -5 | L1MA7 | L1 | LINE |
| 1748713 | 1749855 | + | 26.1 | 1178 | 2331 | -4069 | L1ME2 | L1 | LINE |
| 1749859 | 1750096 | + | 21.6 | 6978 | 7232 | -553 | L1MC3 | L1 | LINE |
| 1750104 | 1751315 | + | 24.6 | 4719 | 6028 | -209 | L1ME2 | L1 | LINE |

  

|  |  |  |  |  |  |  |  |  |  |
| --- | --- | --- | --- | --- | --- | --- | --- | --- | --- |
| chr18:4755716-4756987 | | | 1270 bp | | |  |  |  |  |
| Genome Start | Genome End | Strand | Div | Repeat Start | Repeat End | Repeat Left | Name | Family | Class |
| 4755716 | 4756429 | - | 6.8 | 5461 | 6171 | -1 | L1PA8 | L1 | LINE |
| 4756432 | 4756607 | - | 3.4 | 5681 | 5856 | -299 | L1PA4 | L1 | LINE |
| 4756608 | 4756926 | + | 4.1 | 5835 | 6152 | -3 | L1PA4 | L1 | LINE |
| 4756927 | 4756987 | - | 8.2 | 5420 | 5480 | -651 | L1PA8 | L1 | LINE |

  

|  |  |  |  |  |  |  |  |  |  |
| --- | --- | --- | --- | --- | --- | --- | --- | --- | --- |
| chr18:7175650-7178954 | | | 3156 bp | | |  |  |  |  |
| Genome Start | Genome End | Strand | Div | Repeat Start | Repeat End | Repeat Left | Name | Family | Class |
| 7175650 | 7175705 | + | 24.6 | 661 | 718 | -4136 | L1MEe | L1 | LINE |
| 7175706 | 7176245 | + | 11.2 | 5613 | 6151 | 0 | L1PB2 | L1 | LINE |
| 7176246 | 7176471 | + | 24.6 | 719 | 926 | -3928 | L1MEe | L1 | LINE |
| 7176472 | 7176783 | - | 13.9 | 1 | 309 | -3 | AluSx | Alu | SINE |
| 7176784 | 7176908 | + | 24.6 | 927 | 1041 | -3813 | L1MEe | L1 | LINE |
| 7176909 | 7177205 | + | 6.7 | 1 | 297 | -14 | AluY | Alu | SINE |
| 7177206 | 7177237 | + | 24.6 | 1042 | 1073 | -3781 | L1MEe | L1 | LINE |
| 7177266 | 7177576 | - | 9.7 | 1 | 313 | 0 | AluSp | Alu | SINE |
| 7177603 | 7177929 | - | 9.4 | 1 | 313 | 0 | AluSq | Alu | SINE |
| 7177969 | 7178376 | - | 18.7 | 5883 | 6291 | 0 | L1MA7 | L1 | LINE |
| 7178379 | 7178668 | + | 13.9 | 5581 | 5863 | -428 | L1MA7 | L1 | LINE |
| 7178664 | 7178735 | + | 27.8 | 1091 | 1162 | -3692 | L1MEe | L1 | LINE |
| 7178795 | 7178954 | + | 30.6 | 1237 | 1402 | -3432 | L1MEe | L1 | LINE |

  

|  |  |  |  |  |  |  |  |  |  |
| --- | --- | --- | --- | --- | --- | --- | --- | --- | --- |
| chr18:9298483-9307536 | | | 8945 bp | | |  |  |  |  |
| Genome Start | Genome End | Strand | Div | Repeat Start | Repeat End | Repeat Left | Name | Family | Class |
| 9298483 | 9298677 | + | 24.6 | 752 | 976 | -3895 | L1ME1 | L1 | LINE |
| 9298678 | 9299100 | + | 16.6 | 5415 | 5831 | -502 | L1MC1 | L1 | LINE |
| 9299101 | 9299393 | + | 10.7 | 1 | 291 | -19 | AluSg | Alu | SINE |
| 9299394 | 9299889 | + | 16.6 | 5832 | 6317 | -16 | L1MC1 | L1 | LINE |
| 9299890 | 9300185 | - | 9.5 | 5 | 301 | -10 | AluY | Alu | SINE |
| 9300186 | 9300206 | + | 16.6 | 6318 | 6333 | 0 | L1MC1 | L1 | LINE |
| 9300207 | 9300493 | + | 24.3 | 940 | 1246 | -3588 | L1ME1 | L1 | LINE |
| 9300494 | 9300795 | - | 11.9 | 1 | 305 | -7 | AluSx | Alu | SINE |
| 9300796 | 9300978 | + | 24.3 | 1247 | 1424 | -3410 | L1ME1 | L1 | LINE |
| 9300979 | 9301275 | - | 4.7 | 1 | 297 | -14 | AluY | Alu | SINE |
| 9301276 | 9302208 | + | 24.3 | 1425 | 2388 | -2446 | L1ME1 | L1 | LINE |
| 9302209 | 9302517 | + | 11.0 | 1 | 309 | 0 | AluSc | Alu | SINE |
| 9302518 | 9303113 | + | 24.3 | 2389 | 2932 | -1902 | L1ME1 | L1 | LINE |
| 9303114 | 9303393 | + | 14.2 | 1 | 293 | -19 | AluSx | Alu | SINE |
| 9303394 | 9303519 | + | 24.3 | 2933 | 3029 | -1805 | L1ME1 | L1 | LINE |
| 9303565 | 9304108 | - | 6.2 | 4798 | 5349 | -797 | L1PA10 | L1 | LINE |
| 9304104 | 9304897 | + | 7.6 | 5369 | 6166 | -2 | L1PA10 | L1 | LINE |
| 9304890 | 9305008 | + | 23.9 | 3083 | 3201 | -1633 | L1ME1 | L1 | LINE |
| 9305009 | 9305285 | + | 12.3 | 22 | 297 | -13 | AluSg | Alu | SINE |
| 9305286 | 9305856 | + | 23.9 | 3202 | 3738 | -1096 | L1ME1 | L1 | LINE |
| 9305857 | 9306147 | + | 16.9 | 1 | 298 | -14 | AluSx | Alu | SINE |
| 9306225 | 9306527 | - | 10.9 | 4 | 307 | -6 | AluSp | Alu | SINE |
| 9306528 | 9307536 | + | 20.2 | 3739 | 4804 | -50 | L1ME1 | L1 | LINE |

  

|  |  |  |  |  |  |  |  |  |  |
| --- | --- | --- | --- | --- | --- | --- | --- | --- | --- |
| chr18:13332569-13343218 | | | 10624 bp | | |  |  |  |  |
| Genome Start | Genome End | Strand | Div | Repeat Start | Repeat End | Repeat Left | Name | Family | Class |
| 13332569 | 13333068 | - | 19.9 | 2155 | 2641 | -3735 | L1M4c | L1 | LINE |
| 13333089 | 13339217 | - | 5.2 | 5 | 6148 | -7 | L1PA4 | L1 | LINE |
| 13339228 | 13339618 | - | 19.2 | 1643 | 2144 | -4240 | L1M4c | L1 | LINE |
| 13339615 | 13341568 | - | 7.6 | 4212 | 6172 | 0 | L1PA8 | L1 | LINE |
| 13341572 | 13342327 | + | 7.5 | 3445 | 4204 | -1942 | L1PA8 | L1 | LINE |
| 13342325 | 13343218 | - | 23.6 | 777 | 1651 | -4733 | L1M4c | L1 | LINE |

  

|  |  |  |  |  |  |  |  |  |  |
| --- | --- | --- | --- | --- | --- | --- | --- | --- | --- |
| chr18:13794500-13797301 | | | 2781 bp | | |  |  |  |  |
| Genome Start | Genome End | Strand | Div | Repeat Start | Repeat End | Repeat Left | Name | Family | Class |
| 13794500 | 13794718 | + | 22.0 | 10 | 447 | -5828 | L1MCa | L1 | LINE |
| 13794719 | 13794993 | - | 18.1 | 269 | 622 | 0 | LTR26E | ERV1 | LTR |
| 13794994 | 13795076 | + | 19.3 | 1089 | 1175 | -4870 | L1MCa | L1 | LINE |
| 13795075 | 13795359 | - | 12.3 | 5573 | 5858 | -308 | L1PA16 | L1 | LINE |
| 13795361 | 13795614 | + | 14.1 | 5869 | 6161 | -5 | L1PA16 | L1 | LINE |
| 13795592 | 13795999 | + | 18.5 | 1242 | 1641 | -5110 | L1MCa | L1 | LINE |
| 13796000 | 13796745 | - | 5.8 | 5 | 766 | 0 | MER4E | ERV1 | LTR |
| 13796791 | 13797090 | - | 11.4 | 1 | 303 | -10 | AluSq | Alu | SINE |
| 13797091 | 13797301 | + | 18.5 | 1648 | 1870 | -4881 | L1MCa | L1 | LINE |

  

|  |  |  |  |  |  |  |  |  |  |
| --- | --- | --- | --- | --- | --- | --- | --- | --- | --- |
| chr18:18310663-18316861 | | | 6210 bp | | |  |  |  |  |
| Genome Start | Genome End | Strand | Div | Repeat Start | Repeat End | Repeat Left | Name | Family | Class |
| 18310663 | 18311547 | + | 20.2 | 2 | 891 | -5958 | L1MA4A | L1 | LINE |
| 18311542 | 18311822 | - | 11.8 | 2227 | 2511 | -4338 | L1M2 | L1 | LINE |
| 18311820 | 18311948 | + | 19.4 | 2096 | 2236 | -4613 | L1M2 | L1 | LINE |
| 18311947 | 18316065 | + | 15.3 | 2497 | 6677 | -328 | L1MA4A | L1 | LINE |
| 18316066 | 18316543 | + | 14.3 | 1 | 486 | 0 | MER66B | ERV1 | LTR |
| 18316544 | 18316861 | + | 11.1 | 6678 | 7000 | -5 | L1MA4A | L1 | LINE |

  

|  |  |  |  |  |  |  |  |  |  |
| --- | --- | --- | --- | --- | --- | --- | --- | --- | --- |
| chr18:18576105-18582257 | | | 6101 bp | | |  |  |  |  |
| Genome Start | Genome End | Strand | Div | Repeat Start | Repeat End | Repeat Left | Name | Family | Class |
| 18576105 | 18577628 | + | 17.7 | 15 | 1559 | -4769 | L1M3f | L1 | LINE |
| 18577661 | 18577695 | + | 20.6 | 5046 | 5078 | -1065 | L1M2 | L1 | LINE |
| 18577696 | 18578220 | - | 2.1 | 5631 | 6155 | 0 | L1PA3 | L1 | LINE |
| 18578221 | 18578766 | + | 1.5 | 5082 | 5627 | -528 | L1PA3 | L1 | LINE |
| 18578765 | 18580910 | + | 18.3 | 1547 | 3703 | -2622 | L1M3f | L1 | LINE |
| 18580911 | 18581205 | + | 21.6 | 6 | 298 | -14 | AluJo | Alu | SINE |
| 18581206 | 18581343 | + | 16.0 | 3704 | 3857 | -2468 | L1M3f | L1 | LINE |
| 18581366 | 18582257 | + | 16.0 | 3858 | 4792 | -1533 | L1M3f | L1 | LINE |

  

|  |  |  |  |  |  |  |  |  |  |
| --- | --- | --- | --- | --- | --- | --- | --- | --- | --- |
| chr18:24321754-24326307 | | | 4552 bp | | |  |  |  |  |
| Genome Start | Genome End | Strand | Div | Repeat Start | Repeat End | Repeat Left | Name | Family | Class |
| 24321754 | 24322727 | + | 15.2 | 2 | 1064 | -1354 | Tigger1 | MER2\_type | DNA |
| 24322731 | 24323223 | - | 6.7 | 3254 | 3745 | -2401 | L1PA5 | L1 | LINE |
| 24323223 | 24325611 | + | 4.8 | 3761 | 6145 | -9 | L1PA5 | L1 | LINE |
| 24325612 | 24325890 | + | 16.6 | 1061 | 1339 | -1079 | Tigger1 | MER2\_type | DNA |
| 24325891 | 24326277 | + | 12.1 | 1 | 375 | 0 | THE1C | MaLR | LTR |
| 24326278 | 24326307 | + | 16.6 | 1340 | 1364 | -1054 | Tigger1 | MER2\_type | DNA |

  

|  |  |  |  |  |  |  |  |  |  |
| --- | --- | --- | --- | --- | --- | --- | --- | --- | --- |
| chr18:24676485-24679429 | | | 2652 bp | | |  |  |  |  |
| Genome Start | Genome End | Strand | Div | Repeat Start | Repeat End | Repeat Left | Name | Family | Class |
| 24676485 | 24676990 | - | 33.7 | 2931 | 3419 | 0 | L2 | L2 | LINE |
| 24677056 | 24678281 | - | 11.3 | 4897 | 6115 | -36 | L1PB2 | L1 | LINE |
| 24678288 | 24678912 | + | 11.4 | 4277 | 4907 | -1239 | L1PB2 | L1 | LINE |
| 24679135 | 24679429 | - | 35.1 | 2138 | 2443 | -976 | L2 | L2 | LINE |

  

|  |  |  |  |  |  |  |  |  |  |
| --- | --- | --- | --- | --- | --- | --- | --- | --- | --- |
| chr18:25446132-25450443 | | | 4318 bp | | |  |  |  |  |
| Genome Start | Genome End | Strand | Div | Repeat Start | Repeat End | Repeat Left | Name | Family | Class |
| 25446132 | 25446224 | + | 20.6 | 4772 | 4848 | -1298 | L1MB8 | L1 | LINE |
| 25446225 | 25446552 | - | 12.8 | 1 | 313 | 0 | AluSq | Alu | SINE |
| 25446553 | 25447461 | + | 21.8 | 4849 | 5820 | -358 | L1MB8 | L1 | LINE |
| 25447459 | 25448160 | - | 12.1 | 3544 | 4242 | -1904 | L1PREC2 | L1 | LINE |
| 25448159 | 25450064 | + | 10.8 | 4249 | 6158 | -5 | L1PA13 | L1 | LINE |
| 25450064 | 25450443 | + | 22.9 | 5808 | 6177 | -1 | L1MB8 | L1 | LINE |

  

|  |  |  |  |  |  |  |  |  |  |
| --- | --- | --- | --- | --- | --- | --- | --- | --- | --- |
| chr18:29152462-29156633 | | | 4086 bp | | |  |  |  |  |
| Genome Start | Genome End | Strand | Div | Repeat Start | Repeat End | Repeat Left | Name | Family | Class |
| 29152462 | 29152611 | + | 31.3 | 1058 | 1211 | -4908 | L1MEd | L1 | LINE |
| 29152654 | 29152983 | + | 34.3 | 1308 | 1657 | -4492 | L1MEd | L1 | LINE |
| 29153011 | 29153883 | - | 4.3 | 5268 | 6147 | -7 | L1PA5 | L1 | LINE |
| 29153883 | 29155338 | + | 4.5 | 3819 | 5277 | -869 | L1PA5 | L1 | LINE |
| 29155333 | 29156192 | + | 27.1 | 1654 | 2580 | -3549 | L1MEd | L1 | LINE |
| 29156202 | 29156319 | - | 28.0 | 25 | 160 | 0 | MER5A1 | MER1\_type | DNA |
| 29156335 | 29156633 | + | 28.4 | 2602 | 2921 | -3208 | L1MEd | L1 | LINE |

  

|  |  |  |  |  |  |  |  |  |  |
| --- | --- | --- | --- | --- | --- | --- | --- | --- | --- |
| chr18:29170533-29175847 | | | 5285 bp | | |  |  |  |  |
| Genome Start | Genome End | Strand | Div | Repeat Start | Repeat End | Repeat Left | Name | Family | Class |
| 29170533 | 29170747 | + | 26.0 | 2534 | 2757 | -3423 | L1M4 | L1 | LINE |
| 29170754 | 29171491 | - | 9.7 | 4030 | 4756 | -1390 | L1PB1 | L1 | LINE |
| 29171494 | 29172859 | + | 9.9 | 4761 | 6151 | 0 | L1PB1 | L1 | LINE |
| 29172880 | 29173525 | + | 26.5 | 2800 | 3462 | -2684 | L1M4 | L1 | LINE |
| 29173526 | 29173885 | + | 9.6 | 1 | 355 | 0 | THE1A | MaLR | LTR |
| 29173888 | 29175447 | + | 31.8 | 2 | 1735 | 0 | MLT-int | MaLR | LTR |
| 29175448 | 29175807 | + | 12.1 | 1 | 355 | 0 | THE1A | MaLR | LTR |
| 29175808 | 29175847 | + | 26.5 | 3463 | 3500 | -2646 | L1M4 | L1 | LINE |

  

|  |  |  |  |  |  |  |  |  |  |
| --- | --- | --- | --- | --- | --- | --- | --- | --- | --- |
| chr18:29600055-29609920 | | | 9810 bp | | |  |  |  |  |
| Genome Start | Genome End | Strand | Div | Repeat Start | Repeat End | Repeat Left | Name | Family | Class |
| 29600055 | 29600674 | - | 20.4 | 7532 | 8179 | -24 | L1MC3 | L1 | LINE |
| 29600680 | 29600918 | - | 25.3 | 202 | 461 | -6 | MLT1C | MaLR | LTR |
| 29600939 | 29601384 | - | 4.7 | 5710 | 6155 | 0 | L1PA3 | L1 | LINE |
| 29601385 | 29602251 | + | 2.6 | 4882 | 5747 | -408 | L1PA3 | L1 | LINE |
| 29602252 | 29602417 | - | 24.4 | 18 | 194 | -311 | MLT1C | MaLR | LTR |
| 29602438 | 29605934 | - | 20.8 | 3896 | 7534 | -669 | L1MC3 | L1 | LINE |
| 29605938 | 29606130 | - | 24.7 | 725 | 917 | -5128 | L1MC3 | L1 | LINE |
| 29606122 | 29607834 | - | 4.5 | 3080 | 4791 | -1355 | L1PA4 | L1 | LINE |
| 29607831 | 29609183 | + | 3.4 | 4800 | 6155 | 0 | L1PA4 | L1 | LINE |
| 29609205 | 29609920 | - | 25.6 | 3 | 717 | -5328 | L1MC3 | L1 | LINE |

  

|  |  |  |  |  |  |  |  |  |  |
| --- | --- | --- | --- | --- | --- | --- | --- | --- | --- |
| chr18:29694241-29698669 | | | 4399 bp | | |  |  |  |  |
| Genome Start | Genome End | Strand | Div | Repeat Start | Repeat End | Repeat Left | Name | Family | Class |
| 29694241 | 29694415 | - | 13.9 | 1397 | 1579 | -1 | THE1D-int | MaLR | LTR |
| 29694449 | 29695521 | - | 3.4 | 3227 | 4299 | -1847 | L1PA3 | L1 | LINE |
| 29695519 | 29697395 | + | 3.7 | 4278 | 6155 | 0 | L1PA3 | L1 | LINE |
| 29697396 | 29698669 | - | 13.3 | 3 | 1399 | -181 | THE1D-int | MaLR | LTR |

  

|  |  |  |  |  |  |  |  |  |  |
| --- | --- | --- | --- | --- | --- | --- | --- | --- | --- |
| chr18:31699309-31703255 | | | 3906 bp | | |  |  |  |  |
| Genome Start | Genome End | Strand | Div | Repeat Start | Repeat End | Repeat Left | Name | Family | Class |
| 31699309 | 31699709 | - | 14.8 | 5772 | 6172 | -6 | L1MB8 | L1 | LINE |
| 31699716 | 31700023 | - | 17.2 | 1 | 290 | -22 | AluJb | Alu | SINE |
| 31700061 | 31700933 | - | 2.2 | 5279 | 6152 | -3 | L1PA3 | L1 | LINE |
| 31700932 | 31702917 | + | 3.3 | 3316 | 5302 | -844 | L1PA3 | L1 | LINE |
| 31702918 | 31703255 | - | 19.0 | 5417 | 5768 | -410 | L1MB8 | L1 | LINE |

  

|  |  |  |  |  |  |  |  |  |  |
| --- | --- | --- | --- | --- | --- | --- | --- | --- | --- |
| chr18:31883208-31884420 | | | 1189 bp | | |  |  |  |  |
| Genome Start | Genome End | Strand | Div | Repeat Start | Repeat End | Repeat Left | Name | Family | Class |
| 31883208 | 31883314 | - | 20.4 | 1529 | 1639 | -1122 | Charlie1 | MER1\_type | DNA |
| 31883315 | 31883411 | - | 24.2 | 354 | 444 | 0 | Tigger2a | MER2\_type | DNA |
| 31883413 | 31883481 | - | 21.7 | 20 | 1638 | 0 | Tigger2a | MER2\_type | DNA |
| 31883483 | 31884011 | - | 15.6 | 5419 | 5949 | -234 | L1MB3 | L1 | LINE |
| 31884009 | 31884216 | + | 14.6 | 5950 | 6183 | 0 | L1MB3 | L1 | LINE |
| 31884218 | 31884315 | - | 18.4 | 182 | 281 | -31 | AluJb | Alu | SINE |
| 31884340 | 31884420 | - | 21.2 | 1441 | 1529 | -1232 | Charlie1 | MER1\_type | DNA |

  

|  |  |  |  |  |  |  |  |  |  |
| --- | --- | --- | --- | --- | --- | --- | --- | --- | --- |
| chr18:32857772-32863346 | | | 5526 bp | | |  |  |  |  |
| Genome Start | Genome End | Strand | Div | Repeat Start | Repeat End | Repeat Left | Name | Family | Class |
| 32857772 | 32857960 | - | 17.1 | 5975 | 6152 | -13 | L1PA15 | L1 | LINE |
| 32857961 | 32858261 | - | 20.9 | 1 | 297 | -15 | AluJo | Alu | SINE |
| 32858262 | 32858293 | - | 17.1 | 5943 | 5974 | -191 | L1PA15 | L1 | LINE |
| 32858350 | 32859107 | - | 5.2 | 5385 | 6143 | -11 | L1PA5 | L1 | LINE |
| 32859110 | 32862074 | + | 5.4 | 2410 | 5398 | -748 | L1PA5 | L1 | LINE |
| 32862068 | 32862180 | - | 15.2 | 5845 | 5957 | -208 | L1PA15 | L1 | LINE |
| 32862179 | 32863346 | - | 16.1 | 3276 | 4471 | -1675 | L1PA15 | L1 | LINE |

  

|  |  |  |  |  |  |  |  |  |  |
| --- | --- | --- | --- | --- | --- | --- | --- | --- | --- |
| chr18:33673512-33677523 | | | 3278 bp | | |  |  |  |  |
| Genome Start | Genome End | Strand | Div | Repeat Start | Repeat End | Repeat Left | Name | Family | Class |
| 33673512 | 33673640 | - | 33.6 | 4354 | 4486 | -3 | L3 | CR1 | LINE |
| 33673641 | 33674091 | - | 19.7 | 1 | 496 | -2 | LTR54B | ERV1 | LTR |
| 33674092 | 33674679 | - | 33.6 | 3756 | 4353 | -136 | L3 | CR1 | LINE |
| 33674691 | 33674995 | - | 11.5 | 1 | 307 | -3 | AluSg | Alu | SINE |
| 33675003 | 33675075 | - | 30.6 | 3694 | 3765 | -724 | L3 | CR1 | LINE |
| 33675318 | 33675441 | - | 27.3 | 3337 | 3476 | -1013 | L3 | CR1 | LINE |
| 33675442 | 33676274 | - | 1.8 | 4789 | 5629 | -526 | L1PA3 | L1 | LINE |
| 33676275 | 33676786 | + | 1.9 | 5641 | 6152 | -3 | L1PA3 | L1 | LINE |
| 33677261 | 33677523 | - | 34.9 | 2634 | 2914 | -1575 | L3 | CR1 | LINE |

  

|  |  |  |  |  |  |  |  |  |  |
| --- | --- | --- | --- | --- | --- | --- | --- | --- | --- |
| chr18:33943431-33947828 | | | 4556 bp | | |  |  |  |  |
| Genome Start | Genome End | Strand | Div | Repeat Start | Repeat End | Repeat Left | Name | Family | Class |
| 33943431 | 33944774 | + | 26.8 | 3764 | 5184 | -962 | L1ME2 | L1 | LINE |
| 33944775 | 33945206 | - | 2.6 | 5708 | 6155 | 0 | L1PA4 | L1 | LINE |
| 33945207 | 33945412 | + | 26.8 | 5185 | 5390 | -775 | L1ME2 | L1 | LINE |
| 33945413 | 33945760 | + | 10.6 | 1 | 355 | 0 | THE1A | MaLR | LTR |
| 33945761 | 33945963 | + | 28.7 | 5390 | 5597 | -567 | L1ME2 | L1 | LINE |
| 33946076 | 33946747 | - | 15.7 | 5612 | 6290 | -1 | L1MA8 | L1 | LINE |
| 33946739 | 33947413 | + | 16.5 | 4902 | 5618 | -673 | L1MA8 | L1 | LINE |
| 33947411 | 33947733 | + | 26.3 | 5716 | 6084 | -94 | L1MB8 | L1 | LINE |
| 33947476 | 33947828 | + | 23.7 | 5814 | 6333 | -1 | L1ME2 | L1 | LINE |

  

|  |  |  |  |  |  |  |  |  |  |
| --- | --- | --- | --- | --- | --- | --- | --- | --- | --- |
| chr18:33968053-33971572 | | | 3394 bp | | |  |  |  |  |
| Genome Start | Genome End | Strand | Div | Repeat Start | Repeat End | Repeat Left | Name | Family | Class |
| 33968053 | 33968195 | - | 22.1 | 269 | 483 | -1 | MLT1H | MaLR | LTR |
| 33968297 | 33969129 | - | 10.2 | 5332 | 6158 | -2 | L1PREC2 | L1 | LINE |
| 33969159 | 33969406 | - | 8.9 | 1 | 279 | -32 | AluY | Alu | SINE |
| 33969407 | 33969745 | - | 10.2 | 4993 | 5331 | -815 | L1P | L1 | LINE |
| 33969742 | 33971390 | + | 10.7 | 3317 | 4990 | -1156 | L1P | L1 | LINE |
| 33971391 | 33971572 | - | 28.8 | 1 | 208 | -341 | MLT1H | MaLR | LTR |

  

|  |  |  |  |  |  |  |  |  |  |
| --- | --- | --- | --- | --- | --- | --- | --- | --- | --- |
| chr18:34707861-34710078 | | | 2084 bp | | |  |  |  |  |
| Genome Start | Genome End | Strand | Div | Repeat Start | Repeat End | Repeat Left | Name | Family | Class |
| 34707861 | 34707961 | + | 28.7 | 6449 | 6552 | -1490 | L1MC4 | L1 | LINE |
| 34708100 | 34709183 | + | 26.6 | 6604 | 7893 | -149 | L1MC4 | L1 | LINE |
| 34709167 | 34709237 | - | 11.5 | 6081 | 6129 | -171 | L1MA5 | L1 | LINE |
| 34709238 | 34709770 | + | 15.4 | 1 | 529 | 0 | MER1A | MER1\_type | DNA |
| 34709771 | 34709825 | - | 11.5 | 6025 | 6080 | -220 | L1MA5 | L1 | LINE |
| 34709831 | 34710016 | + | 18.8 | 6107 | 6292 | -5 | L1MA5A | L1 | LINE |
| 34710025 | 34710078 | + | 16.7 | 7984 | 8037 | -5 | L1MC4 | L1 | LINE |

  

|  |  |  |  |  |  |  |  |  |  |
| --- | --- | --- | --- | --- | --- | --- | --- | --- | --- |
| chr18:36154544-36160409 | | | 5630 bp | | |  |  |  |  |
| Genome Start | Genome End | Strand | Div | Repeat Start | Repeat End | Repeat Left | Name | Family | Class |
| 36154544 | 36155599 | + | 26.6 | 35 | 1145 | -5255 | L1ME1 | L1 | LINE |
| 36155598 | 36155824 | + | 27.6 | 3597 | 3834 | -2385 | L1ME1 | L1 | LINE |
| 36155825 | 36156198 | + | 16.1 | 3 | 428 | 0 | MSTA | MaLR | LTR |
| 36156199 | 36156924 | + | 27.6 | 3835 | 4611 | -1608 | L1ME1 | L1 | LINE |
| 36156948 | 36157258 | - | 13.3 | 1 | 312 | 0 | AluSx | Alu | SINE |
| 36157262 | 36158058 | + | 25.3 | 5142 | 5943 | -296 | L1ME1 | L1 | LINE |
| 36158102 | 36158334 | + | 16.3 | 3 | 236 | -76 | AluJb | Alu | SINE |
| 36158385 | 36159813 | - | 3.0 | 4706 | 6137 | -18 | L1PA3 | L1 | LINE |
| 36159811 | 36159993 | + | 3.8 | 4513 | 4695 | -1451 | L1PA3 | L1 | LINE |
| 36159994 | 36160067 | + | 25.7 | 226 | 299 | -3 | AluJb | Alu | SINE |
| 36160190 | 36160409 | + | 28.7 | 6002 | 6239 | 0 | L1ME1 | L1 | LINE |

  

|  |  |  |  |  |  |  |  |  |  |
| --- | --- | --- | --- | --- | --- | --- | --- | --- | --- |
| chr18:37952669-37959794 | | | 6825 bp | | |  |  |  |  |
| Genome Start | Genome End | Strand | Div | Repeat Start | Repeat End | Repeat Left | Name | Family | Class |
| 37952669 | 37952844 | - | 30.7 | 1969 | 2156 | -4083 | L1MEe | L1 | LINE |
| 37952887 | 37953316 | - | 33.6 | 1398 | 1838 | -4401 | L1MEe | L1 | LINE |
| 37953363 | 37953715 | - | 24.1 | 2780 | 3157 | -2989 | L1M4 | L1 | LINE |
| 37953708 | 37954161 | - | 15.7 | 994 | 1466 | -4934 | L1MEe | L1 | LINE |
| 37954196 | 37954353 | - | 20.4 | 2388 | 2523 | -4110 | L1MDa | L1 | LINE |
| 37954354 | 37954655 | - | 17.3 | 5 | 300 | -12 | AluJo | Alu | SINE |
| 37954656 | 37955964 | - | 20.4 | 1020 | 2387 | -4246 | L1MDa | L1 | LINE |
| 37956092 | 37956586 | - | 30.7 | 78 | 642 | -5991 | L1MDa | L1 | LINE |
| 37956588 | 37956758 | - | 21.1 | 764 | 969 | -5431 | L1MEe | L1 | LINE |
| 37956800 | 37957330 | - | 8.8 | 5599 | 6160 | -8 | L1PA10 | L1 | LINE |
| 37957330 | 37959072 | + | 7.6 | 3853 | 5622 | -546 | L1PA10 | L1 | LINE |
| 37959092 | 37959794 | - | 23.4 | 34 | 775 | -5625 | L1MEe | L1 | LINE |

  

|  |  |  |  |  |  |  |  |  |  |
| --- | --- | --- | --- | --- | --- | --- | --- | --- | --- |
| chr18:40042253-40047551 | | | 5302 bp | | |  |  |  |  |
| Genome Start | Genome End | Strand | Div | Repeat Start | Repeat End | Repeat Left | Name | Family | Class |
| 40042253 | 40043885 | + | 29.1 | 176 | 1863 | -3804 | ERVL-C1 | ERVL | LTR |
| 40043886 | 40044340 | + | 18.8 | 4153 | 4607 | -1107 | ERVL-C1 | ERVL | LTR |
| 40044341 | 40044423 | + | 22.2 | 1582 | 1677 | -283 | L4 | RTE | LINE |
| 40044427 | 40044813 | + | 20.4 | 4688 | 5122 | -592 | ERVL-C1 | ERVL | LTR |
| 40044815 | 40045142 | - | 15.8 | 4197 | 4529 | -1617 | L1PREC2 | L1 | LINE |
| 40045134 | 40045979 | + | 14.2 | 4534 | 5405 | -760 | L1PREC2 | L1 | LINE |
| 40045980 | 40046299 | + | 6.8 | 1 | 311 | 0 | AluY | Alu | SINE |
| 40046300 | 40047075 | + | 12.6 | 5406 | 6162 | -3 | L1PA15 | L1 | LINE |
| 40047078 | 40047551 | + | 22.4 | 5068 | 5593 | -61 | ERVL-C1 | ERVL | LTR |

  

|  |  |  |  |  |  |  |  |  |  |
| --- | --- | --- | --- | --- | --- | --- | --- | --- | --- |
| chr18:47752770-47759081 | | | 6252 bp | | |  |  |  |  |
| Genome Start | Genome End | Strand | Div | Repeat Start | Repeat End | Repeat Left | Name | Family | Class |
| 47752770 | 47753980 | + | 25.3 | 1 | 1394 | -5357 | L1MC3 | L1 | LINE |
| 47754001 | 47754087 | + | 14.9 | 5707 | 5803 | -2021 | L1MC | L1 | LINE |
| 47754091 | 47755040 | + | 19.4 | 4616 | 7197 | -1827 | L1MC3 | L1 | LINE |
| 47755044 | 47755646 | - | 8.3 | 4768 | 5390 | -756 | L1PB1 | L1 | LINE |
| 47755648 | 47756394 | + | 9.5 | 5406 | 6151 | 0 | L1PB1 | L1 | LINE |
| 47756400 | 47757309 | + | 16.3 | 4303 | 5261 | -879 | L1MA9 | L1 | LINE |
| 47757310 | 47757651 | - | 18.0 | 1 | 346 | 0 | MER7A | MER2\_type | DNA |
| 47757652 | 47757841 | + | 14.8 | 5262 | 5462 | -850 | L1MA9 | L1 | LINE |
| 47757844 | 47758065 | + | 13.6 | 6066 | 6307 | -5 | L1MA9 | L1 | LINE |
| 47758092 | 47759081 | + | 17.9 | 7226 | 8187 | -16 | L1MC3 | L1 | LINE |

  

|  |  |  |  |  |  |  |  |  |  |
| --- | --- | --- | --- | --- | --- | --- | --- | --- | --- |
| chr18:49399258-49405522 | | | 4700 bp | | |  |  |  |  |
| Genome Start | Genome End | Strand | Div | Repeat Start | Repeat End | Repeat Left | Name | Family | Class |
| 49399258 | 49399473 | - | 32.1 | 7816 | 8042 | 0 | L1MC4 | L1 | LINE |
| 49399600 | 49399687 | - | 21.2 | 6796 | 6899 | -1143 | L1MC4 | L1 | LINE |
| 49399688 | 49400004 | - | 6.6 | 1 | 303 | -8 | AluY | Alu | SINE |
| 49400005 | 49400075 | - | 21.2 | 6726 | 6795 | -1247 | L1MC4 | L1 | LINE |
| 49400485 | 49400710 | - | 27.7 | 5673 | 5907 | -1901 | L1MC4 | L1 | LINE |
| 49400956 | 49401171 | + | 25.6 | 5361 | 5633 | -2175 | L1MC | L1 | LINE |
| 49401208 | 49401507 | - | 29.6 | 3578 | 3905 | -2309 | L1MC4 | L1 | LINE |
| 49401527 | 49401615 | - | 9.8 | 6221 | 6302 | 0 | L1MA1 | L1 | LINE |
| 49401618 | 49401957 | + | 8.9 | 5870 | 6224 | -78 | L1MA1 | L1 | LINE |
| 49402028 | 49402222 | - | 28.9 | 3295 | 3511 | -2703 | L1MC4 | L1 | LINE |
| 49402223 | 49402570 | + | 10.9 | 1 | 355 | 0 | THE1A | MaLR | LTR |
| 49402571 | 49402665 | - | 28.9 | 3189 | 3294 | -2920 | L1MC4 | L1 | LINE |
| 49403078 | 49404627 | - | 30.7 | 960 | 2543 | -3672 | L1MC4 | L1 | LINE |
| 49404816 | 49405123 | + | 7.2 | 1 | 307 | -4 | AluY | Alu | SINE |
| 49405182 | 49405522 | - | 35.7 | 362 | 704 | -5511 | L1MC4 | L1 | LINE |

  

|  |  |  |  |  |  |  |  |  |  |
| --- | --- | --- | --- | --- | --- | --- | --- | --- | --- |
| chr18:49517060-49519517 | | | 2393 bp | | |  |  |  |  |
| Genome Start | Genome End | Strand | Div | Repeat Start | Repeat End | Repeat Left | Name | Family | Class |
| 49517060 | 49517133 | - | 17.6 | 6218 | 6294 | -3 | L1MA5A | L1 | LINE |
| 49517135 | 49517400 | + | 19.2 | 1 | 283 | -640 | MER49 | ERV1 | LTR |
| 49517460 | 49517761 | - | 12.7 | 5602 | 5904 | -262 | L1PA16 | L1 | LINE |
| 49517762 | 49518385 | + | 9.3 | 1 | 594 | 0 | PRIMA4\_LTR | ERV1 | LTR |
| 49518386 | 49518722 | - | 12.7 | 5255 | 5601 | -565 | L1PA16 | L1 | LINE |
| 49518724 | 49519036 | + | 12.9 | 4961 | 5270 | -876 | L1PA16 | L1 | LINE |
| 49519041 | 49519398 | + | 23.1 | 549 | 903 | 0 | MER4D | ERV1 | LTR |
| 49519399 | 49519517 | - | 7.3 | 6111 | 6220 | -77 | L1MA5A | L1 | LINE |

  

|  |  |  |  |  |  |  |  |  |  |
| --- | --- | --- | --- | --- | --- | --- | --- | --- | --- |
| chr18:56388877-56390812 | | | 1745 bp | | |  |  |  |  |
| Genome Start | Genome End | Strand | Div | Repeat Start | Repeat End | Repeat Left | Name | Family | Class |
| 56388877 | 56389091 | - | 29.1 | 4424 | 4654 | -1492 | L1M5 | L1 | LINE |
| 56389276 | 56389300 | - | 0.0 | 5635 | 5659 | -501 | L1PREC2 | L1 | LINE |
| 56389298 | 56389654 | - | 3.4 | 5303 | 5660 | -495 | L1PA4 | L1 | LINE |
| 56389658 | 56390160 | + | 2.6 | 5645 | 6152 | -3 | L1PA4 | L1 | LINE |
| 56390168 | 56390812 | - | 30.9 | 3277 | 3974 | -2172 | L1M5 | L1 | LINE |

  

|  |  |  |  |  |  |  |  |  |  |
| --- | --- | --- | --- | --- | --- | --- | --- | --- | --- |
| chr18:56679245-56681041 | | | 1700 bp | | |  |  |  |  |
| Genome Start | Genome End | Strand | Div | Repeat Start | Repeat End | Repeat Left | Name | Family | Class |
| 56679245 | 56679593 | - | 31.2 | 5476 | 5858 | -382 | L1ME3B | L1 | LINE |
| 56679633 | 56680143 | - | 17.9 | 5836 | 6331 | -3 | L1MA10 | L1 | LINE |
| 56680148 | 56680809 | + | 15.7 | 5160 | 5824 | -488 | L1M2 | L1 | LINE |
| 56680864 | 56681041 | - | 29.9 | 5177 | 5352 | -794 | L1ME3B | L1 | LINE |

  

|  |  |  |  |  |  |  |  |  |  |
| --- | --- | --- | --- | --- | --- | --- | --- | --- | --- |
| chr18:56775874-56778113 | | | 2200 bp | | |  |  |  |  |
| Genome Start | Genome End | Strand | Div | Repeat Start | Repeat End | Repeat Left | Name | Family | Class |
| 56775874 | 56776271 | - | 18.4 | 3951 | 4368 | -2014 | L1M4c | L1 | LINE |
| 56776285 | 56776803 | - | 3.3 | 5629 | 6147 | -7 | L1PA5 | L1 | LINE |
| 56776804 | 56777009 | + | 1.9 | 5444 | 5649 | -506 | L1PA5 | L1 | LINE |
| 56777007 | 56777995 | - | 18.2 | 2925 | 3965 | -2417 | L1M4c | L1 | LINE |
| 56778026 | 56778113 | - | 18.2 | 2396 | 2485 | -3899 | L1M4c | L1 | LINE |

  

|  |  |  |  |  |  |  |  |  |  |
| --- | --- | --- | --- | --- | --- | --- | --- | --- | --- |
| chr18:57533256-57536958 | | | 3717 bp | | |  |  |  |  |
| Genome Start | Genome End | Strand | Div | Repeat Start | Repeat End | Repeat Left | Name | Family | Class |
| 57533256 | 57533523 | + | 8.6 | 1 | 273 | -248 | MER41C | ERV1 | LTR |
| 57533524 | 57533851 | - | 2.7 | 2996 | 3323 | -2823 | L1PA2 | L1 | LINE |
| 57533833 | 57536667 | + | 1.4 | 3324 | 6155 | 0 | L1PA2 | L1 | LINE |
| 57536673 | 57536958 | + | 9.5 | 259 | 521 | 0 | MER41C | ERV1 | LTR |

  

|  |  |  |  |  |  |  |  |  |  |
| --- | --- | --- | --- | --- | --- | --- | --- | --- | --- |
| chr18:60273172-60277073 | | | 3932 bp | | |  |  |  |  |
| Genome Start | Genome End | Strand | Div | Repeat Start | Repeat End | Repeat Left | Name | Family | Class |
| 60273172 | 60273710 | + | 12.1 | 4416 | 4952 | -1194 | L1PA8 | L1 | LINE |
| 60273708 | 60273789 | + | 7.3 | 5571 | 5689 | -442 | L1PA8 | L1 | LINE |
| 60273784 | 60274497 | - | 9.5 | 3807 | 4520 | -1626 | L1PA8 | L1 | LINE |
| 60274495 | 60275591 | + | 8.2 | 4518 | 5616 | -556 | L1PA8 | L1 | LINE |
| 60275592 | 60275892 | + | 9.0 | 4 | 308 | -5 | AluSq | Alu | SINE |
| 60275893 | 60276371 | + | 6.5 | 5617 | 6082 | -90 | L1PA8 | L1 | LINE |
| 60276372 | 60276608 | + | 10.2 | 5934 | 6170 | -2 | L1PA8 | L1 | LINE |
| 60276609 | 60276631 | + | 6.5 | 6083 | 6108 | -64 | L1PA8 | L1 | LINE |
| 60276614 | 60277073 | + | 8.0 | 5685 | 6147 | -25 | L1PA8 | L1 | LINE |

  

|  |  |  |  |  |  |  |  |  |  |
| --- | --- | --- | --- | --- | --- | --- | --- | --- | --- |
| chr18:60872700-60874084 | | | 1364 bp | | |  |  |  |  |
| Genome Start | Genome End | Strand | Div | Repeat Start | Repeat End | Repeat Left | Name | Family | Class |
| 60872700 | 60872998 | + | 21.2 | 1 | 275 | -115 | MLT1B | MaLR | LTR |
| 60873019 | 60873625 | - | 6.8 | 5554 | 6161 | 0 | L1PA5 | L1 | LINE |
| 60873627 | 60874011 | + | 4.7 | 5160 | 5543 | -611 | L1PA5 | L1 | LINE |
| 60874012 | 60874084 | + | 20.8 | 309 | 385 | -5 | MLT1B | MaLR | LTR |

  

|  |  |  |  |  |  |  |  |  |  |
| --- | --- | --- | --- | --- | --- | --- | --- | --- | --- |
| chr18:61235463-61242657 | | | 7083 bp | | |  |  |  |  |
| Genome Start | Genome End | Strand | Div | Repeat Start | Repeat End | Repeat Left | Name | Family | Class |
| 61235463 | 61236083 | - | 20.2 | 2586 | 3259 | -4075 | L1M4b | L1 | LINE |
| 61236083 | 61236125 | - | 16.3 | 855 | 897 | -6437 | L1M4b | L1 | LINE |
| 61236128 | 61237594 | - | 19.6 | 4821 | 6312 | 0 | L1MA9 | L1 | LINE |
| 61237611 | 61238053 | - | 5.0 | 5710 | 6152 | -3 | L1PA3 | L1 | LINE |
| 61238054 | 61238420 | + | 0.8 | 5343 | 5709 | -446 | L1PA3 | L1 | LINE |
| 61238429 | 61238820 | - | 20.0 | 4368 | 4823 | -1317 | L1MA9 | L1 | LINE |
| 61238818 | 61238926 | + | 24.8 | 1461 | 1569 | -4572 | L1M3c | L1 | LINE |
| 61238977 | 61240582 | + | 23.0 | 2738 | 4384 | -3175 | L1M1 | L1 | LINE |
| 61240658 | 61241305 | - | 30.0 | 139 | 865 | -6469 | L1M4b | L1 | LINE |
| 61241295 | 61242030 | - | 17.8 | 5449 | 6165 | -1 | L1PA17 | L1 | LINE |
| 61242031 | 61242406 | + | 12.9 | 1 | 375 | 0 | THE1C | MaLR | LTR |
| 61242407 | 61242519 | - | 17.8 | 5335 | 5448 | -718 | L1PA17 | L1 | LINE |
| 61242496 | 61242657 | - | 25.9 | 1 | 165 | -7169 | L1M4b | L1 | LINE |

  

|  |  |  |  |  |  |  |  |  |  |
| --- | --- | --- | --- | --- | --- | --- | --- | --- | --- |
| chr18:61372252-61381093 | | | 8692 bp | | |  |  |  |  |
| Genome Start | Genome End | Strand | Div | Repeat Start | Repeat End | Repeat Left | Name | Family | Class |
| 61372252 | 61372768 | + | 29.3 | 1359 | 1929 | -5567 | L1MA10 | L1 | LINE |
| 61372814 | 61373472 | + | 28.1 | 3043 | 3714 | -3848 | L1MA10 | L1 | LINE |
| 61373473 | 61373782 | - | 10.1 | 1 | 308 | -3 | AluY | Alu | SINE |
| 61373783 | 61373851 | + | 21.4 | 3715 | 3780 | -3782 | L1MA10 | L1 | LINE |
| 61373846 | 61375312 | - | 5.9 | 3463 | 4927 | -1219 | L1PA5 | L1 | LINE |
| 61375311 | 61376506 | + | 5.2 | 4943 | 6146 | -8 | L1PA5 | L1 | LINE |
| 61376524 | 61377065 | + | 19.9 | 3765 | 4321 | -3238 | L1MA10 | L1 | LINE |
| 61377122 | 61379573 | + | 20.0 | 4322 | 6816 | -781 | L1MA10 | L1 | LINE |
| 61379607 | 61380108 | + | 18.7 | 5803 | 6312 | 0 | L1MA9 | L1 | LINE |
| 61380113 | 61380206 | + | 26.6 | 6035 | 6131 | -111 | L1MD1 | L1 | LINE |
| 61380209 | 61380349 | - | 13.7 | 1 | 140 | -241 | THE1D | MaLR | LTR |
| 61380351 | 61381093 | + | 20.6 | 6883 | 7626 | -127 | L1MA10 | L1 | LINE |

  

|  |  |  |  |  |  |  |  |  |  |
| --- | --- | --- | --- | --- | --- | --- | --- | --- | --- |
| chr18:61946444-61947970 | | | 1389 bp | | |  |  |  |  |
| Genome Start | Genome End | Strand | Div | Repeat Start | Repeat End | Repeat Left | Name | Family | Class |
| 61946444 | 61946960 | + | 28.5 | 3169 | 3724 | -1943 | ERVL-E | ERVL | LTR |
| 61947002 | 61947193 | - | 16.4 | 6104 | 6290 | -1 | L1MA8 | L1 | LINE |
| 61947193 | 61947358 | + | 15.1 | 5917 | 6094 | -197 | L1MA8 | L1 | LINE |
| 61947457 | 61947970 | + | 28.9 | 3923 | 4483 | -1171 | ERVL-E | ERVL | LTR |

  

|  |  |  |  |  |  |  |  |  |  |
| --- | --- | --- | --- | --- | --- | --- | --- | --- | --- |
| chr18:62277532-62281192 | | | 3656 bp | | |  |  |  |  |
| Genome Start | Genome End | Strand | Div | Repeat Start | Repeat End | Repeat Left | Name | Family | Class |
| 62277532 | 62278657 | + | 19.7 | 4983 | 6130 | -161 | L1MA7 | L1 | LINE |
| 62278653 | 62279978 | - | 19.4 | 3845 | 5349 | -794 | L1MA4A | L1 | LINE |
| 62279980 | 62280227 | + | 15.4 | 5352 | 5598 | -705 | L1MA4A | L1 | LINE |
| 62280231 | 62280320 | + | 6.7 | 6054 | 6143 | -11 | L1PA7 | L1 | LINE |
| 62280327 | 62281003 | + | 17.3 | 5587 | 6291 | -12 | L1MA4A | L1 | LINE |
| 62281004 | 62281192 | + | 14.5 | 6116 | 6290 | -1 | L1MA7 | L1 | LINE |

  

|  |  |  |  |  |  |  |  |  |  |
| --- | --- | --- | --- | --- | --- | --- | --- | --- | --- |
| chr18:63704293-63705290 | | | 981 bp | | |  |  |  |  |
| Genome Start | Genome End | Strand | Div | Repeat Start | Repeat End | Repeat Left | Name | Family | Class |
| 63704293 | 63704465 | - | 15.6 | 324 | 497 | 0 | LOR1a | ERV1 | LTR |
| 63704466 | 63705089 | - | 2.4 | 5402 | 6027 | -128 | L1PA4 | L1 | LINE |
| 63705090 | 63705210 | + | 3.3 | 6032 | 6152 | -3 | L1PA4 | L1 | LINE |
| 63705228 | 63705290 | - | 17.5 | 278 | 340 | -157 | LOR1a | ERV1 | LTR |

  

|  |  |  |  |  |  |  |  |  |  |
| --- | --- | --- | --- | --- | --- | --- | --- | --- | --- |
| chr18:66483149-66488754 | | | 5137 bp | | |  |  |  |  |
| Genome Start | Genome End | Strand | Div | Repeat Start | Repeat End | Repeat Left | Name | Family | Class |
| 66483149 | 66484223 | - | 15.4 | 5120 | 6198 | -2 | L1MA9 | L1 | LINE |
| 66484336 | 66485393 | - | 19.3 | 4045 | 5101 | -927 | L1MA9 | L1 | LINE |
| 66485689 | 66485810 | - | 13.9 | 24 | 145 | -31 | FRAM | Alu | SINE |
| 66485850 | 66485960 | - | 23.4 | 3919 | 4050 | -1978 | L1MA9 | L1 | LINE |
| 66485949 | 66486019 | - | 21.1 | 1071 | 1141 | -4814 | L1MA9 | L1 | LINE |
| 66486048 | 66486501 | - | 4.0 | 5698 | 6152 | -3 | L1PA3 | L1 | LINE |
| 66486502 | 66487001 | + | 1.7 | 5179 | 5678 | -477 | L1PA3 | L1 | LINE |
| 66487004 | 66487170 | - | 21.0 | 890 | 1078 | -4877 | L1MA9 | L1 | LINE |
| 66487174 | 66488497 | + | 16.0 | 4 | 1914 | -209 | MER52D | ERV1 | LTR |
| 66488500 | 66488754 | - | 22.5 | 15 | 269 | -5686 | L1MA9 | L1 | LINE |

  

|  |  |  |  |  |  |  |  |  |  |
| --- | --- | --- | --- | --- | --- | --- | --- | --- | --- |
| chr18:67473417-67478337 | | | 4866 bp | | |  |  |  |  |
| Genome Start | Genome End | Strand | Div | Repeat Start | Repeat End | Repeat Left | Name | Family | Class |
| 67473417 | 67473831 | + | 33.3 | 1075 | 1504 | -1915 | L2 | L2 | LINE |
| 67473853 | 67474865 | - | 0.8 | 5144 | 6155 | 0 | L1HS | L1 | LINE |
| 67474856 | 67476021 | + | 0.5 | 3963 | 5128 | -1018 | L1HS | L1 | LINE |
| 67476028 | 67476770 | + | 29.2 | 1513 | 2338 | -1081 | L2 | L2 | LINE |
| 67476809 | 67477332 | + | 31.3 | 2339 | 3007 | -412 | L2 | L2 | LINE |
| 67477333 | 67477642 | - | 10.1 | 1 | 308 | -3 | AluY | Alu | SINE |
| 67477643 | 67477943 | + | 33.2 | 3008 | 3348 | -71 | L2 | L2 | LINE |
| 67477944 | 67478303 | - | 11.4 | 1 | 364 | 0 | THE1B | MaLR | LTR |
| 67478304 | 67478337 | + | 33.7 | 3349 | 3378 | -41 | L2 | L2 | LINE |

  

|  |  |  |  |  |  |  |  |  |  |
| --- | --- | --- | --- | --- | --- | --- | --- | --- | --- |
| chr18:67951607-67955383 | | | 3712 bp | | |  |  |  |  |
| Genome Start | Genome End | Strand | Div | Repeat Start | Repeat End | Repeat Left | Name | Family | Class |
| 67951607 | 67951788 | + | 27.5 | 4947 | 5147 | -999 | L1ME3B | L1 | LINE |
| 67951802 | 67953775 | - | 3.5 | 4169 | 6152 | -3 | L1PA3 | L1 | LINE |
| 67953775 | 67954483 | + | 3.4 | 3454 | 4162 | -1984 | L1PA3 | L1 | LINE |
| 67954481 | 67954685 | + | 30.7 | 5137 | 5342 | -804 | L1ME3B | L1 | LINE |
| 67954742 | 67955383 | + | 31.6 | 5361 | 6067 | -345 | L1ME3B | L1 | LINE |

  

|  |  |  |  |  |  |  |  |  |  |
| --- | --- | --- | --- | --- | --- | --- | --- | --- | --- |
| chr18:68006714-68011789 | | | 5043 bp | | |  |  |  |  |
| Genome Start | Genome End | Strand | Div | Repeat Start | Repeat End | Repeat Left | Name | Family | Class |
| 68006714 | 68008338 | - | 19.2 | 4677 | 6316 | -17 | L1MC1 | L1 | LINE |
| 68008339 | 68008639 | - | 5.7 | 1 | 301 | -10 | AluY | Alu | SINE |
| 68008640 | 68008696 | - | 18.8 | 4615 | 4676 | -1470 | L1MC1 | L1 | LINE |
| 68008726 | 68009834 | - | 18.8 | 3365 | 4614 | -1532 | L1MC1 | L1 | LINE |
| 68009831 | 68010667 | - | 3.0 | 4472 | 5308 | -838 | L1PA3 | L1 | LINE |
| 68010663 | 68011498 | + | 3.1 | 5304 | 6155 | 0 | L1PA3 | L1 | LINE |
| 68011512 | 68011789 | - | 18.1 | 3086 | 3378 | -2768 | L1MC1 | L1 | LINE |

  

|  |  |  |  |  |  |  |  |  |  |
| --- | --- | --- | --- | --- | --- | --- | --- | --- | --- |
| chr18:68068936-68072006 | | | 2690 bp | | |  |  |  |  |
| Genome Start | Genome End | Strand | Div | Repeat Start | Repeat End | Repeat Left | Name | Family | Class |
| 68068936 | 68069135 | + | 39.9 | 817 | 1018 | -5354 | L1ME1 | L1 | LINE |
| 68069234 | 68069724 | - | 18.5 | 4361 | 4857 | -1286 | L1MA4A | L1 | LINE |
| 68069727 | 68070786 | + | 15.8 | 4865 | 5944 | -359 | L1MA4A | L1 | LINE |
| 68070793 | 68070867 | + | 6.8 | 6224 | 6301 | -2 | L1MA4A | L1 | LINE |
| 68071143 | 68072006 | + | 32.7 | 1297 | 2325 | -4077 | L1ME1 | L1 | LINE |

  

|  |  |  |  |  |  |  |  |  |  |
| --- | --- | --- | --- | --- | --- | --- | --- | --- | --- |
| chr18:68093472-68099857 | | | 6317 bp | | |  |  |  |  |
| Genome Start | Genome End | Strand | Div | Repeat Start | Repeat End | Repeat Left | Name | Family | Class |
| 68093472 | 68094528 | + | 25.7 | 3832 | 5021 | -1125 | L1MA7 | L1 | LINE |
| 68094535 | 68096217 | + | 10.3 | 3311 | 4998 | -1148 | L1PA7 | L1 | LINE |
| 68096207 | 68096601 | - | 4.6 | 5071 | 5465 | -689 | L1PA5 | L1 | LINE |
| 68096602 | 68097283 | + | 3.8 | 5470 | 6151 | -3 | L1PA5 | L1 | LINE |
| 68097284 | 68098467 | + | 8.8 | 4975 | 6153 | -1 | L1PA7 | L1 | LINE |
| 68098512 | 68098675 | + | 17.1 | 1 | 163 | -13 | FRAM | Alu | SINE |
| 68098706 | 68099078 | + | 17.1 | 5290 | 5668 | -623 | L1MA7 | L1 | LINE |
| 68099079 | 68099385 | + | 12.7 | 1 | 307 | -3 | AluSg | Alu | SINE |
| 68099386 | 68099857 | + | 17.1 | 5669 | 6138 | -153 | L1MA7 | L1 | LINE |

  

|  |  |  |  |  |  |  |  |  |  |
| --- | --- | --- | --- | --- | --- | --- | --- | --- | --- |
| chr18:68111438-68113366 | | | 1915 bp | | |  |  |  |  |
| Genome Start | Genome End | Strand | Div | Repeat Start | Repeat End | Repeat Left | Name | Family | Class |
| 68111438 | 68111806 | - | 22.9 | 205 | 596 | -5 | MLT2B5 | ERVL | LTR |
| 68111807 | 68112168 | - | 13.8 | 5082 | 5456 | -844 | L1MA4 | L1 | LINE |
| 68112169 | 68113010 | + | 14.8 | 5445 | 6287 | -13 | L1MA4 | L1 | LINE |
| 68113025 | 68113366 | - | 21.5 | 1 | 336 | -381 | MLT2B5 | ERVL | LTR |

  

|  |  |  |  |  |  |  |  |  |  |
| --- | --- | --- | --- | --- | --- | --- | --- | --- | --- |
| chr18:70559605-70562765 | | | 3042 bp | | |  |  |  |  |
| Genome Start | Genome End | Strand | Div | Repeat Start | Repeat End | Repeat Left | Name | Family | Class |
| 70559605 | 70559833 | - | 26.4 | 5013 | 5235 | -1771 | L1MEc | L1 | LINE |
| 70559829 | 70559937 | - | 24.8 | 3573 | 3682 | -3652 | L1MEc | L1 | LINE |
| 70559993 | 70560318 | - | 12.4 | 5969 | 6294 | -3 | L1MA5A | L1 | LINE |
| 70560362 | 70560646 | - | 5.3 | 1 | 281 | -30 | AluY | Alu | SINE |
| 70560647 | 70562176 | - | 14.3 | 4396 | 5968 | -329 | L1MA5A | L1 | LINE |
| 70562197 | 70562272 | - | 15.9 | 4320 | 4395 | -1748 | L1MA5A | L1 | LINE |
| 70562269 | 70562530 | + | 15.8 | 4069 | 4331 | -1812 | L1MA5A | L1 | LINE |
| 70562541 | 70562765 | - | 20.6 | 2978 | 3242 | -3945 | L1MEc | L1 | LINE |

  

|  |  |  |  |  |  |  |  |  |  |
| --- | --- | --- | --- | --- | --- | --- | --- | --- | --- |
| chr18:73316365-73319462 | | | 2763 bp | | |  |  |  |  |
| Genome Start | Genome End | Strand | Div | Repeat Start | Repeat End | Repeat Left | Name | Family | Class |
| 73316365 | 73316715 | + | 30.5 | 221 | 568 | -3534 | PRIMAX-int | ERV1 | LTR |
| 73316717 | 73316910 | - | 6.2 | 4594 | 4787 | -1359 | L1PB1 | L1 | LINE |
| 73316906 | 73317881 | + | 7.9 | 4795 | 5774 | -377 | L1PB1 | L1 | LINE |
| 73317924 | 73318273 | + | 10.3 | 5775 | 6142 | -9 | L1PB1 | L1 | LINE |
| 73318512 | 73318679 | + | 18.1 | 562 | 736 | -3366 | PRIMAX-int | ERV1 | LTR |
| 73318739 | 73319462 | + | 25.8 | 742 | 1469 | -2633 | PRIMAX-int | ERV1 | LTR |

  

|  |  |  |  |  |  |  |  |  |  |
| --- | --- | --- | --- | --- | --- | --- | --- | --- | --- |
| chr18:74301282-74305960 | | | 4587 bp | | |  |  |  |  |
| Genome Start | Genome End | Strand | Div | Repeat Start | Repeat End | Repeat Left | Name | Family | Class |
| 74301282 | 74301665 | + | 27.6 | 1894 | 2503 | -4571 | L1MEd | L1 | LINE |
| 74301683 | 74301855 | - | 33.3 | 73 | 261 | -1 | MIR | MIR | SINE |
| 74301856 | 74302251 | + | 30.1 | 2466 | 2866 | -4160 | L1MEd | L1 | LINE |
| 74302278 | 74303124 | + | 29.2 | 3164 | 4115 | -2891 | L1MEd | L1 | LINE |
| 74303123 | 74303414 | - | 7.2 | 4963 | 5269 | -877 | L1PA8 | L1 | LINE |
| 74303409 | 74304303 | + | 9.6 | 5272 | 6169 | -3 | L1PA8 | L1 | LINE |
| 74304331 | 74304383 | + | 28.6 | 4103 | 4157 | -2849 | L1MEd | L1 | LINE |
| 74304414 | 74305439 | + | 28.6 | 4158 | 5134 | -2257 | L1MEd | L1 | LINE |
| 74305440 | 74305742 | + | 15.4 | 1 | 285 | -27 | AluJb | Alu | SINE |
| 74305743 | 74305960 | + | 28.6 | 5135 | 5385 | -1621 | L1MEd | L1 | LINE |

  

|  |  |  |  |  |  |  |  |  |  |
| --- | --- | --- | --- | --- | --- | --- | --- | --- | --- |
| chr18:74322802-74329555 | | | 6493 bp | | |  |  |  |  |
| Genome Start | Genome End | Strand | Div | Repeat Start | Repeat End | Repeat Left | Name | Family | Class |
| 74322802 | 74323329 | - | 12.1 | 6831 | 7360 | -2 | L1MA5 | L1 | LINE |
| 74323321 | 74323741 | - | 7.4 | 5746 | 6166 | -6 | L1PA8 | L1 | LINE |
| 74323747 | 74324238 | + | 7.1 | 5254 | 5746 | -426 | L1PA8 | L1 | LINE |
| 74324234 | 74328066 | - | 17.7 | 3008 | 6851 | -511 | L1MA5 | L1 | LINE |
| 74328116 | 74328431 | - | 18.0 | 2324 | 3042 | -4167 | L1MA5 | L1 | LINE |
| 74328411 | 74328660 | - | 28.2 | 1728 | 1969 | -5240 | L1MA5 | L1 | LINE |
| 74328725 | 74328974 | - | 25.4 | 699 | 957 | -6252 | L1MA5 | L1 | LINE |
| 74329153 | 74329555 | - | 29.9 | 235 | 642 | -6567 | L1MA5 | L1 | LINE |

  

|  |  |  |  |  |  |  |  |  |  |
| --- | --- | --- | --- | --- | --- | --- | --- | --- | --- |
| chr18:75873140-75884809 | | | 11320 bp | | |  |  |  |  |
| Genome Start | Genome End | Strand | Div | Repeat Start | Repeat End | Repeat Left | Name | Family | Class |
| 75873140 | 75873816 | - | 28.8 | 7625 | 8307 | -612 | MER4-int | ERV1 | LTR |
| 75874111 | 75874408 | - | 6.8 | 1 | 297 | -14 | AluY | Alu | SINE |
| 75874419 | 75874731 | - | 10.6 | 1 | 302 | -8 | AluSg | Alu | SINE |
| 75874742 | 75874790 | - | 22.4 | 7574 | 7622 | -1297 | MER4-int | ERV1 | LTR |
| 75874794 | 75875153 | - | 12.2 | 1 | 355 | 0 | THE1A | MaLR | LTR |
| 75875159 | 75876336 | - | 10.1 | 414 | 1574 | -6 | THE1A-int | MaLR | LTR |
| 75876367 | 75879110 | - | 2.4 | 3393 | 6137 | -18 | L1PA3 | L1 | LINE |
| 75879108 | 75879740 | + | 3.6 | 2773 | 3406 | -2740 | L1PA3 | L1 | LINE |
| 75879741 | 75880166 | - | 11.0 | 1 | 431 | -1149 | THE1A-int | MaLR | LTR |
| 75880167 | 75880512 | - | 9.8 | 1 | 355 | 0 | THE1A | MaLR | LTR |
| 75880513 | 75880935 | - | 26.6 | 7104 | 7575 | -1344 | MER4-int | ERV1 | LTR |
| 75880936 | 75881177 | + | 13.2 | 1 | 236 | -128 | THE1B | MaLR | LTR |
| 75881179 | 75882170 | - | 21.3 | 3821 | 4838 | -2253 | MER4-int | ERV1 | LTR |
| 75882171 | 75882464 | - | 8.9 | 1 | 293 | -18 | AluY | Alu | SINE |
| 75882465 | 75882984 | - | 21.3 | 3311 | 3820 | -3271 | MER4-int | ERV1 | LTR |
| 75882985 | 75884809 | - | 13.3 | 539 | 2375 | -4813 | MER4-int | ERV1 | LTR |

  


|  |  |  |  |  |  |  |  |  |  |
| --- | --- | --- | --- | --- | --- | --- | --- | --- | --- |
| chr19:66282-69983 | | | 3703 bp | | |  |  |  |  |
| Genome Start | Genome End | Strand | Div | Repeat Start | Repeat End | Repeat Left | Name | Family | Class |
| 66282 | 66437 | + | 23.5 | 5372 | 5525 | -2283 | L1MC5 | L1 | LINE |
| 66440 | 67489 | - | 1.9 | 5105 | 6155 | 0 | L1PA2 | L1 | LINE |
| 67492 | 69291 | + | 2.1 | 3311 | 5110 | -1036 | L1PA2 | L1 | LINE |
| 69287 | 69486 | + | 24.1 | 5512 | 5734 | -2074 | L1MC5 | L1 | LINE |
| 69487 | 69796 | + | 12.9 | 1 | 310 | -2 | AluSx | Alu | SINE |
| 69797 | 69983 | + | 24.1 | 5735 | 5942 | -1866 | L1MC5 | L1 | LINE |

  

|  |  |  |  |  |  |  |  |  |  |
| --- | --- | --- | --- | --- | --- | --- | --- | --- | --- |
| chr19:93801-96307 | | | 2473 bp | | |  |  |  |  |
| Genome Start | Genome End | Strand | Div | Repeat Start | Repeat End | Repeat Left | Name | Family | Class |
| 93801 | 94289 | + | 19.5 | 3150 | 3646 | -2494 | L1M3 | L1 | LINE |
| 94305 | 95026 | - | 2.6 | 5254 | 5975 | -180 | L1PA4 | L1 | LINE |
| 95027 | 95204 | + | 5.1 | 5977 | 6155 | 0 | L1PA4 | L1 | LINE |
| 95224 | 96307 | + | 16.5 | 3657 | 4741 | -1399 | L1M3 | L1 | LINE |

  

|  |  |  |  |  |  |  |  |  |  |
| --- | --- | --- | --- | --- | --- | --- | --- | --- | --- |
| chr19:15567507-15573821 | | | 5916 bp | | |  |  |  |  |
| Genome Start | Genome End | Strand | Div | Repeat Start | Repeat End | Repeat Left | Name | Family | Class |
| 15567507 | 15567676 | - | 20.0 | 4792 | 4964 | -1367 | MER101-int | ERV1 | LTR |
| 15567688 | 15568709 | - | 2.9 | 2568 | 3589 | -2557 | L1PA3 | L1 | LINE |
| 15568709 | 15571246 | + | 2.8 | 3596 | 6152 | -3 | L1PA3 | L1 | LINE |
| 15571303 | 15571476 | - | 27.6 | 5040 | 5230 | -2890 | MER101-int | ERV1 | LTR |
| 15571495 | 15571793 | + | 11.7 | 1 | 306 | -6 | AluSx | Alu | SINE |
| 15571830 | 15572135 | + | 11.3 | 1 | 302 | -10 | AluSx | Alu | SINE |
| 15572410 | 15572514 | - | 21.8 | 3438 | 3530 | -3109 | MER101-int | ERV1 | LTR |
| 15572515 | 15572836 | + | 15.4 | 1 | 322 | 0 | AluJb | Alu | SINE |
| 15572837 | 15572941 | - | 21.8 | 3336 | 3437 | -3202 | MER101-int | ERV1 | LTR |
| 15572942 | 15573246 | - | 18.4 | 1 | 305 | -7 | AluJo | Alu | SINE |
| 15573247 | 15573386 | - | 21.8 | 3183 | 3335 | -3304 | MER101-int | ERV1 | LTR |
| 15573390 | 15573685 | - | 11.7 | 1 | 292 | -20 | AluSx | Alu | SINE |
| 15573688 | 15573821 | - | 22.0 | 1546 | 1680 | -6440 | MER101-int | ERV1 | LTR |

  

|  |  |  |  |  |  |  |  |  |  |
| --- | --- | --- | --- | --- | --- | --- | --- | --- | --- |
| chr19:15870958-15872430 | | | 1444 bp | | |  |  |  |  |
| Genome Start | Genome End | Strand | Div | Repeat Start | Repeat End | Repeat Left | Name | Family | Class |
| 15870958 | 15871088 | + | 5.3 | 2 | 132 | -1446 | LTR12C | ERV1 | LTR |
| 15871118 | 15871335 | - | 1.4 | 5938 | 6155 | 0 | L1PA3 | L1 | LINE |
| 15871336 | 15871424 | + | 1.1 | 5839 | 5927 | -228 | L1PA3 | L1 | LINE |
| 15871425 | 15872430 | + | 7.6 | 116 | 1457 | -121 | LTR12C | ERV1 | LTR |

  

|  |  |  |  |  |  |  |  |  |  |
| --- | --- | --- | --- | --- | --- | --- | --- | --- | --- |
| chr19:20196308-20203452 | | | 7117 bp | | |  |  |  |  |
| Genome Start | Genome End | Strand | Div | Repeat Start | Repeat End | Repeat Left | Name | Family | Class |
| 20196308 | 20196935 | + | 13.8 | 4792 | 5431 | -729 | L1PREC2 | L1 | LINE |
| 20196936 | 20197246 | + | 14.9 | 1 | 309 | -1 | AluSg | Alu | SINE |
| 20197247 | 20197588 | + | 13.6 | 5432 | 5766 | -394 | L1PREC2 | L1 | LINE |
| 20197589 | 20197897 | + | 27.2 | 1 | 302 | 0 | AluJo | Alu | SINE |
| 20197898 | 20197996 | + | 13.6 | 5767 | 5869 | -291 | L1PREC2 | L1 | LINE |
| 20198004 | 20198948 | - | 7.0 | 2206 | 3146 | -3000 | L1P2 | L1 | LINE |
| 20198949 | 20199234 | - | 10.8 | 4 | 294 | -17 | AluY | Alu | SINE |
| 20199235 | 20200290 | - | 7.3 | 1174 | 2221 | -54 | L1P2 | L1 | LINE |
| 20200289 | 20202821 | + | 6.9 | 3144 | 6154 | 0 | L1PA5 | L1 | LINE |
| 20202834 | 20202865 | + | 28.8 | 5814 | 5851 | -321 | L1PA8 | L1 | LINE |
| 20202866 | 20203171 | + | 13.4 | 6 | 312 | -1 | AluSp | Alu | SINE |
| 20203183 | 20203452 | + | 20.4 | 5875 | 6160 | 0 | L1PREC2 | L1 | LINE |

  

|  |  |  |  |  |  |  |  |  |  |
| --- | --- | --- | --- | --- | --- | --- | --- | --- | --- |
| chr19:24218280-24225409 | | | 7143 bp | | |  |  |  |  |
| Genome Start | Genome End | Strand | Div | Repeat Start | Repeat End | Repeat Left | Name | Family | Class |
| 24218280 | 24219198 | + | 14.3 | 1 | 976 | -6977 | HERVK11 | ERVK | LTR |
| 24219197 | 24219776 | + | 10.7 | 1165 | 2700 | -5253 | HERVK11 | ERVK | LTR |
| 24219769 | 24221450 | + | 9.2 | 2912 | 4601 | -3352 | HERVK11 | ERVK | LTR |
| 24221449 | 24222628 | + | 8.5 | 5525 | 6767 | -1186 | HERVK11 | ERVK | LTR |
| 24222614 | 24223132 | + | 10.6 | 6957 | 7493 | -460 | HERVK11 | ERVK | LTR |
| 24223134 | 24223609 | - | 4.0 | 4336 | 4815 | -1331 | L1PA3 | L1 | LINE |
| 24223607 | 24224928 | + | 5.0 | 4828 | 6150 | -5 | L1PA3 | L1 | LINE |
| 24224945 | 24225409 | + | 9.9 | 7486 | 7953 | 0 | HERVK11 | ERVK | LTR |

  

|  |  |  |  |  |  |  |  |  |  |
| --- | --- | --- | --- | --- | --- | --- | --- | --- | --- |
| chr19:32969553-32974324 | | | 4764 bp | | |  |  |  |  |
| Genome Start | Genome End | Strand | Div | Repeat Start | Repeat End | Repeat Left | Name | Family | Class |
| 32969553 | 32970592 | + | 19.2 | 2155 | 3270 | -3112 | L1M4c | L1 | LINE |
| 32970593 | 32970901 | + | 12.6 | 1 | 312 | 0 | AluSx | Alu | SINE |
| 32970902 | 32971188 | + | 20.7 | 3271 | 3551 | -2831 | L1M4c | L1 | LINE |
| 32971189 | 32971441 | - | 11.5 | 50 | 304 | -5 | AluSc | Alu | SINE |
| 32971442 | 32971902 | + | 20.7 | 3552 | 4028 | -2354 | L1M4c | L1 | LINE |
| 32971899 | 32972083 | - | 4.3 | 4814 | 4998 | -1148 | L1PA3 | L1 | LINE |
| 32972083 | 32973217 | + | 2.3 | 5014 | 6152 | -3 | L1PA3 | L1 | LINE |
| 32973231 | 32974324 | + | 26.8 | 4010 | 5304 | -1723 | L1M4c | L1 | LINE |

  

|  |  |  |  |  |  |  |  |  |  |
| --- | --- | --- | --- | --- | --- | --- | --- | --- | --- |
| chr19:33805565-33814821 | | | 9233 bp | | |  |  |  |  |
| Genome Start | Genome End | Strand | Div | Repeat Start | Repeat End | Repeat Left | Name | Family | Class |
| 33805565 | 33805895 | + | 27.2 | 3026 | 3374 | -4191 | L1MA5A | L1 | LINE |
| 33805931 | 33806296 | + | 24.7 | 3375 | 3734 | -3825 | L1MA5A | L1 | LINE |
| 33806291 | 33808640 | - | 10.5 | 2627 | 5098 | -1048 | L1PA6 | L1 | LINE |
| 33808634 | 33809707 | + | 5.7 | 5075 | 6153 | -1 | L1PA6 | L1 | LINE |
| 33809710 | 33809967 | + | 16.9 | 3723 | 3966 | -3593 | L1MA5A | L1 | LINE |
| 33809968 | 33810273 | - | 15.0 | 1 | 311 | -1 | AluSx | Alu | SINE |
| 33810274 | 33811627 | + | 16.9 | 3967 | 5301 | -2258 | L1MA5A | L1 | LINE |
| 33811628 | 33811937 | + | 0.7 | 1 | 310 | 0 | AluYa5 | Alu | SINE |
| 33811938 | 33813347 | + | 16.9 | 5302 | 6745 | -814 | L1MA5A | L1 | LINE |
| 33813348 | 33813637 | + | 20.1 | 1 | 283 | -29 | AluJb | Alu | SINE |
| 33813638 | 33813986 | + | 16.8 | 6737 | 7064 | -646 | L1MA5A | L1 | LINE |
| 33813987 | 33814433 | + | 16.4 | 1 | 461 | 0 | MER65C | ERV1 | LTR |
| 33814434 | 33814821 | + | 20.1 | 7065 | 7365 | -351 | L1MA5A | L1 | LINE |

  

|  |  |  |  |  |  |  |  |  |  |
| --- | --- | --- | --- | --- | --- | --- | --- | --- | --- |
| chr19:35296377-35298457 | | | 1830 bp | | |  |  |  |  |
| Genome Start | Genome End | Strand | Div | Repeat Start | Repeat End | Repeat Left | Name | Family | Class |
| 35296377 | 35296532 | - | 26.2 | 699 | 858 | -30 | Kanga2\_a | Tc2 | DNA |
| 35296533 | 35296685 | - | 20.3 | 1 | 153 | -23 | FRAM | Alu | SINE |
| 35296713 | 35297246 | - | 9.2 | 5616 | 6148 | -3 | L1PB1 | L1 | LINE |
| 35297247 | 35297610 | + | 9.1 | 5259 | 5621 | -530 | L1PB1 | L1 | LINE |
| 35297799 | 35298043 | + | 9.8 | 13 | 257 | -55 | AluSx | Alu | SINE |
| 35298080 | 35298457 | - | 27.7 | 8 | 386 | -502 | Kanga2\_a | Tc2 | DNA |

  

|  |  |  |  |  |  |  |  |  |  |
| --- | --- | --- | --- | --- | --- | --- | --- | --- | --- |
| chr19:35366409-35377879 | | | 11394 bp | | |  |  |  |  |
| Genome Start | Genome End | Strand | Div | Repeat Start | Repeat End | Repeat Left | Name | Family | Class |
| 35366409 | 35366524 | - | 21.1 | 2549 | 2663 | -4671 | L1M4 | L1 | LINE |
| 35366561 | 35367412 | - | 17.2 | 1487 | 2427 | -4907 | L1M4 | L1 | LINE |
| 35367408 | 35373505 | - | 6.9 | 4 | 6151 | -3 | L1PA5 | L1 | LINE |
| 35373549 | 35375724 | - | 4.5 | 3976 | 6151 | -4 | L1PA4 | L1 | LINE |
| 35375724 | 35376530 | + | 6.0 | 3148 | 3956 | -2190 | L1PA4 | L1 | LINE |
| 35376521 | 35377339 | - | 22.5 | 630 | 1483 | -5851 | L1M4 | L1 | LINE |
| 35377354 | 35377879 | - | 26.9 | 4 | 569 | -6765 | L1M4 | L1 | LINE |

  

|  |  |  |  |  |  |  |  |  |  |
| --- | --- | --- | --- | --- | --- | --- | --- | --- | --- |
| chr19:35943031-35956588 | | | 13241 bp | | |  |  |  |  |
| Genome Start | Genome End | Strand | Div | Repeat Start | Repeat End | Repeat Left | Name | Family | Class |
| 35943031 | 35943182 | - | 21.6 | 5121 | 5299 | -1245 | L1MD2 | L1 | LINE |
| 35943225 | 35944765 | - | 23.2 | 3291 | 4864 | -1680 | L1MD2 | L1 | LINE |
| 35944787 | 35946689 | - | 17.6 | 4504 | 6493 | -26 | L1MC2 | L1 | LINE |
| 35946693 | 35947123 | - | 17.2 | 3849 | 4332 | -1989 | L1MC2 | L1 | LINE |
| 35947122 | 35947149 | - | 17.1 | 3491 | 3518 | -2803 | L1MC2 | L1 | LINE |
| 35947150 | 35947541 | + | 19.7 | 1 | 396 | 0 | MSTD | MaLR | LTR |
| 35947542 | 35947749 | - | 17.1 | 3272 | 3490 | -2831 | L1MC2 | L1 | LINE |
| 35947750 | 35947760 | - | 8.5 | 285 | 311 | 0 | AluY | Alu | SINE |
| 35947800 | 35948088 | - | 8.5 | 2 | 284 | -27 | AluY | Alu | SINE |
| 35948089 | 35949719 | - | 18.1 | 1547 | 3271 | -3050 | L1MC2 | L1 | LINE |
| 35949720 | 35950019 | - | 14.7 | 1 | 305 | -7 | AluSx | Alu | SINE |
| 35950020 | 35950819 | - | 19.4 | 745 | 1546 | -4962 | L1MC2 | L1 | LINE |
| 35950820 | 35951347 | - | 25.2 | 2654 | 3198 | -3346 | L1MD2 | L1 | LINE |
| 35951423 | 35951623 | - | 20.4 | 2476 | 2653 | -3891 | L1MD2 | L1 | LINE |
| 35951674 | 35952573 | - | 19.7 | 5262 | 6179 | -1 | L1MB4 | L1 | LINE |
| 35952569 | 35952902 | - | 8.5 | 5156 | 5487 | -668 | L1PA3 | L1 | LINE |
| 35952903 | 35953556 | + | 2.3 | 5499 | 6152 | -3 | L1PA3 | L1 | LINE |
| 35953557 | 35954983 | - | 23.2 | 3783 | 5266 | -991 | L1MB4 | L1 | LINE |
| 35955078 | 35956588 | - | 27.6 | 25 | 1560 | -4987 | L1MD2 | L1 | LINE |

  

|  |  |  |  |  |  |  |  |  |  |
| --- | --- | --- | --- | --- | --- | --- | --- | --- | --- |
| chr19:36416571-36423622 | | | 6790 bp | | |  |  |  |  |
| Genome Start | Genome End | Strand | Div | Repeat Start | Repeat End | Repeat Left | Name | Family | Class |
| 36416571 | 36417779 | - | 24.4 | 4334 | 5594 | -1412 | L1M4 | L1 | LINE |
| 36417782 | 36418278 | - | 16.7 | 5820 | 6312 | 0 | L1MA9 | L1 | LINE |
| 36418443 | 36418747 | - | 13.8 | 1 | 310 | 0 | AluSg | Alu | SINE |
| 36418748 | 36419370 | - | 15.4 | 5164 | 5779 | -533 | L1MA9 | L1 | LINE |
| 36419371 | 36419612 | + | 13.4 | 4941 | 5185 | -955 | L1MA9 | L1 | LINE |
| 36419613 | 36419916 | + | 10.2 | 1 | 305 | -7 | AluSx | Alu | SINE |
| 36419917 | 36419939 | + | 13.4 | 5186 | 5199 | -941 | L1M3 | L1 | LINE |
| 36419937 | 36420083 | - | 31.3 | 4197 | 4344 | -2662 | L1M4 | L1 | LINE |
| 36420177 | 36421001 | - | 27.6 | 3342 | 4186 | -2820 | L1M4 | L1 | LINE |
| 36421002 | 36421301 | - | 12.7 | 1 | 300 | -12 | AluSx | Alu | SINE |
| 36421302 | 36422325 | - | 29.2 | 2419 | 3341 | -3665 | L1M4 | L1 | LINE |
| 36422327 | 36422591 | - | 21.0 | 38 | 305 | -7 | AluJo | Alu | SINE |
| 36422597 | 36422927 | - | 26.6 | 1876 | 2217 | -5117 | L1M4 | L1 | LINE |
| 36422928 | 36423400 | - | 24.4 | 2 | 523 | 0 | Charlie1b | MER1\_type | DNA |
| 36423401 | 36423622 | - | 24.4 | 1612 | 1875 | -5029 | L1M4 | L1 | LINE |

  

|  |  |  |  |  |  |  |  |  |  |
| --- | --- | --- | --- | --- | --- | --- | --- | --- | --- |
| chr19:38744042-38745311 | | | 1273 bp | | |  |  |  |  |
| Genome Start | Genome End | Strand | Div | Repeat Start | Repeat End | Repeat Left | Name | Family | Class |
| 38744042 | 38744343 | - | 15.7 | 236 | 542 | -111 | MER39B | ERV1 | LTR |
| 38744344 | 38745029 | - | 6.9 | 5404 | 6105 | -67 | L1PA8 | L1 | LINE |
| 38745027 | 38745068 | + | 7.1 | 5369 | 5410 | -762 | L1P | L1 | LINE |
| 38745069 | 38745311 | - | 9.5 | 25 | 269 | -384 | MER39B | ERV1 | LTR |

  

|  |  |  |  |  |  |  |  |  |  |
| --- | --- | --- | --- | --- | --- | --- | --- | --- | --- |
| chr19:39709170-39713249 | | | 4069 bp | | |  |  |  |  |
| Genome Start | Genome End | Strand | Div | Repeat Start | Repeat End | Repeat Left | Name | Family | Class |
| 39709170 | 39709359 | - | 15.7 | 6115 | 6300 | 0 | L1MA5 | L1 | LINE |
| 39709360 | 39709648 | + | 8.0 | 1 | 289 | -22 | AluY | Alu | SINE |
| 39709649 | 39710457 | - | 15.7 | 5331 | 6114 | -186 | L1MA5 | L1 | LINE |
| 39710458 | 39710761 | - | 9.9 | 1 | 305 | -8 | AluSq | Alu | SINE |
| 39710762 | 39711191 | - | 15.6 | 4899 | 5330 | -970 | L1M | L1 | LINE |
| 39711194 | 39711259 | - | 14.0 | 4095 | 4159 | -1984 | L1M | L1 | LINE |
| 39711260 | 39711565 | + | 8.5 | 1 | 305 | 0 | AluSg | Alu | SINE |
| 39711566 | 39711602 | - | 14.0 | 4067 | 4094 | -2049 | L1M | L1 | LINE |
| 39711597 | 39711817 | + | 15.9 | 3837 | 4061 | -2082 | L1M | L1 | LINE |
| 39711833 | 39713249 | - | 18.2 | 3472 | 4884 | -1259 | L1MA5 | L1 | LINE |

  

|  |  |  |  |  |  |  |  |  |  |
| --- | --- | --- | --- | --- | --- | --- | --- | --- | --- |
| chr19:46110453-46116672 | | | 6230 bp | | |  |  |  |  |
| Genome Start | Genome End | Strand | Div | Repeat Start | Repeat End | Repeat Left | Name | Family | Class |
| 46110453 | 46115742 | + | 7.4 | 4 | 5331 | -815 | L1PA5 | L1 | LINE |
| 46115733 | 46115892 | - | 3.8 | 5820 | 5979 | -176 | L1PA4 | L1 | LINE |
| 46115893 | 46116052 | + | 3.8 | 5990 | 6149 | -6 | L1PA4 | L1 | LINE |
| 46116053 | 46116525 | + | 3.6 | 5322 | 5795 | -359 | L1PA5 | L1 | LINE |
| 46116526 | 46116672 | + | 2.1 | 6007 | 6150 | -4 | L1PA5 | L1 | LINE |

  

|  |  |  |  |  |  |  |  |  |  |
| --- | --- | --- | --- | --- | --- | --- | --- | --- | --- |
| chr19:49088955-49090782 | | | 1813 bp | | |  |  |  |  |
| Genome Start | Genome End | Strand | Div | Repeat Start | Repeat End | Repeat Left | Name | Family | Class |
| 49088955 | 49089392 | - | 23.4 | 126 | 593 | 0 | MLT1E | MaLR | LTR |
| 49089398 | 49089530 | - | 15.6 | 5493 | 5621 | -691 | L1M2 | L1 | LINE |
| 49089531 | 49089828 | - | 4.1 | 1 | 308 | -3 | AluY | Alu | SINE |
| 49089829 | 49090022 | - | 16.2 | 5286 | 5492 | -820 | L1M2 | L1 | LINE |
| 49090033 | 49090564 | - | 7.0 | 5630 | 6164 | -8 | L1PA8 | L1 | LINE |
| 49090565 | 49090601 | + | 2.7 | 5585 | 5621 | -551 | L1PA8 | L1 | LINE |
| 49090602 | 49090782 | - | 21.1 | 1 | 160 | -433 | MLT1E | MaLR | LTR |

  

|  |  |  |  |  |  |  |  |  |  |
| --- | --- | --- | --- | --- | --- | --- | --- | --- | --- |
| chr19:49573097-49574661 | | | 1570 bp | | |  |  |  |  |
| Genome Start | Genome End | Strand | Div | Repeat Start | Repeat End | Repeat Left | Name | Family | Class |
| 49573097 | 49573812 | + | 25.6 | 4565 | 5290 | -856 | L1M5 | L1 | LINE |
| 49573807 | 49573996 | - | 1.6 | 5639 | 5828 | -327 | L1PA4 | L1 | LINE |
| 49573997 | 49574349 | + | 2.0 | 5806 | 6153 | -2 | L1PA4 | L1 | LINE |
| 49574351 | 49574661 | + | 22.9 | 5274 | 5616 | -550 | L1M5 | L1 | LINE |

  

|  |  |  |  |  |  |  |  |  |  |
| --- | --- | --- | --- | --- | --- | --- | --- | --- | --- |
| chr19:53229496-53233407 | | | 3587 bp | | |  |  |  |  |
| Genome Start | Genome End | Strand | Div | Repeat Start | Repeat End | Repeat Left | Name | Family | Class |
| 53229496 | 53229540 | - | 24.4 | 208 | 252 | -16 | MIRb | MIR | SINE |
| 53229561 | 53229604 | - | 25.1 | 6159 | 6200 | -134 | L1MA10 | L1 | LINE |
| 53229605 | 53229914 | - | 11.6 | 1 | 312 | 0 | AluSx | Alu | SINE |
| 53229920 | 53230045 | - | 20.2 | 2 | 125 | -8 | FLAM\_C | Alu | SINE |
| 53230046 | 53230384 | - | 18.8 | 5803 | 6154 | -35 | L1MA7 | L1 | LINE |
| 53230386 | 53230682 | + | 9.1 | 2 | 301 | -12 | AluSp | Alu | SINE |
| 53230736 | 53231229 | - | 34.7 | 2578 | 3066 | -353 | L2 | L2 | LINE |
| 53231357 | 53231484 | - | 15.6 | 5776 | 5908 | -281 | L1MB1 | L1 | LINE |
| 53231482 | 53231694 | + | 15.2 | 5891 | 6105 | -63 | L1MB1 | L1 | LINE |
| 53231705 | 53231749 | + | 11.1 | 35 | 80 | 0 | MADE1 | Mariner | DNA |
| 53231755 | 53231817 | + | 17.5 | 5322 | 5390 | -793 | L1M | L1 | LINE |
| 53231795 | 53231859 | + | 13.8 | 5320 | 5390 | -790 | L1M | L1 | LINE |
| 53231837 | 53232328 | + | 14.7 | 5320 | 5810 | -481 | L1M2 | L1 | LINE |
| 53232329 | 53232636 | - | 9.1 | 1 | 309 | -4 | AluSp | Alu | SINE |
| 53232637 | 53232809 | - | 16.5 | 2379 | 2542 | -70 | Charlie5 | MER1\_type | DNA |
| 53232925 | 53232981 | - | 15.8 | 2325 | 2384 | -228 | Charlie5 | MER1\_type | DNA |
| 53232985 | 53233283 | - | 11.5 | 1 | 299 | -14 | AluSq | Alu | SINE |
| 53233319 | 53233407 | - | 21.2 | 2 | 95 | -173 | MIRb | MIR | SINE |

  

|  |  |  |  |  |  |  |  |  |  |
| --- | --- | --- | --- | --- | --- | --- | --- | --- | --- |
| chr19:56549762-56551370 | | | 1630 bp | | |  |  |  |  |
| Genome Start | Genome End | Strand | Div | Repeat Start | Repeat End | Repeat Left | Name | Family | Class |
| 56549762 | 56550168 | + | 11.6 | 6 | 472 | -204 | MER61F | ERV1 | LTR |
| 56550169 | 56550202 | - | 2.9 | 5150 | 5183 | -963 | L1P4 | L1 | LINE |
| 56550173 | 56551171 | + | 3.3 | 5163 | 6155 | 0 | L1PA4 | L1 | LINE |
| 56551181 | 56551370 | + | 15.2 | 452 | 639 | -37 | MER61F | ERV1 | LTR |

  

|  |  |  |  |  |  |  |  |  |  |
| --- | --- | --- | --- | --- | --- | --- | --- | --- | --- |
| chr19:56864640-56870357 | | | 5528 bp | | |  |  |  |  |
| Genome Start | Genome End | Strand | Div | Repeat Start | Repeat End | Repeat Left | Name | Family | Class |
| 56864640 | 56864790 | - | 28.5 | 4659 | 4818 | -110 | L1ME3B | L1 | LINE |
| 56864791 | 56865101 | - | 10.3 | 2 | 312 | -1 | AluSp | Alu | SINE |
| 56865102 | 56865300 | - | 28.5 | 4451 | 4658 | -270 | L1ME3B | L1 | LINE |
| 56865304 | 56865535 | + | 7.8 | 5920 | 6160 | -3 | L1PA13 | L1 | LINE |
| 56865536 | 56865838 | - | 22.3 | 32 | 334 | -1121 | Charlie1a | MER1\_type | DNA |
| 56865905 | 56865985 | - | 22.2 | 368 | 449 | 0 | Tigger5a | MER2\_type | DNA |
| 56866022 | 56866124 | - | 22.3 | 1 | 105 | -261 | Tigger5a | MER2\_type | DNA |
| 56866136 | 56866464 | - | 26.5 | 3952 | 4302 | -626 | L1ME3B | L1 | LINE |
| 56866460 | 56867274 | - | 26.5 | 1788 | 2663 | -2171 | L1ME3B | L1 | LINE |
| 56867354 | 56867740 | - | 15.7 | 41 | 432 | 0 | MSTB1 | MaLR | LTR |
| 56867757 | 56868859 | - | 0.4 | 5053 | 6155 | 0 | L1HS | L1 | LINE |
| 56868848 | 56869326 | + | 0.4 | 4579 | 5057 | -1089 | L1HS | L1 | LINE |
| 56869323 | 56870357 | - | 25.7 | 560 | 1556 | -3278 | L1ME3B | L1 | LINE |

  

|  |  |  |  |  |  |  |  |  |  |
| --- | --- | --- | --- | --- | --- | --- | --- | --- | --- |
| chr19:60953446-60954744 | | | 1296 bp | | |  |  |  |  |
| Genome Start | Genome End | Strand | Div | Repeat Start | Repeat End | Repeat Left | Name | Family | Class |
| 60953446 | 60953542 | - | 29.9 | 7078 | 7175 | -867 | L1MC4 | L1 | LINE |
| 60953549 | 60953568 | + | 25.0 | 5585 | 5601 | -565 | L1P5 | L1 | LINE |
| 60953569 | 60953924 | - | 12.4 | 5784 | 6137 | -14 | L1PB1 | L1 | LINE |
| 60953925 | 60954109 | + | 13.5 | 5602 | 5786 | -365 | L1PB1 | L1 | LINE |
| 60954107 | 60954292 | - | 24.0 | 6915 | 7086 | -956 | L1MC4 | L1 | LINE |
| 60954293 | 60954596 | - | 5.0 | 9 | 310 | -1 | AluY | Alu | SINE |
| 60954597 | 60954744 | - | 24.0 | 6766 | 6914 | -1128 | L1MC4 | L1 | LINE |

  

|  |  |  |  |  |  |  |  |  |  |
| --- | --- | --- | --- | --- | --- | --- | --- | --- | --- |
| chr19:61581171-61583625 | | | 2323 bp | | |  |  |  |  |
| Genome Start | Genome End | Strand | Div | Repeat Start | Repeat End | Repeat Left | Name | Family | Class |
| 61581171 | 61581273 | - | 32.0 | 5423 | 5526 | -644 | L1ME2 | L1 | LINE |
| 61581362 | 61581405 | - | 4.5 | 6001 | 6044 | -111 | L1PA5 | L1 | LINE |
| 61581406 | 61581526 | + | 2.5 | 6033 | 6153 | -1 | L1PA5 | L1 | LINE |
| 61581571 | 61581675 | - | 26.6 | 5117 | 5233 | -913 | L1ME2 | L1 | LINE |
| 61581676 | 61581967 | - | 15.8 | 1 | 296 | -16 | AluJb | Alu | SINE |
| 61581968 | 61582378 | - | 26.6 | 4698 | 5116 | -1030 | L1ME2 | L1 | LINE |
| 61582379 | 61582680 | + | 17.1 | 1 | 299 | -13 | AluJo | Alu | SINE |
| 61582681 | 61583135 | - | 26.6 | 4245 | 4697 | -1449 | L1ME2 | L1 | LINE |
| 61583136 | 61583443 | - | 5.2 | 1 | 308 | -3 | AluY | Alu | SINE |
| 61583444 | 61583625 | - | 26.6 | 4055 | 4244 | -1902 | L1ME2 | L1 | LINE |

  


|  |  |  |  |  |  |  |  |  |  |
| --- | --- | --- | --- | --- | --- | --- | --- | --- | --- |
| chr20:5443208-5447678 | | | 4461 bp | | |  |  |  |  |
| Genome Start | Genome End | Strand | Div | Repeat Start | Repeat End | Repeat Left | Name | Family | Class |
| 5443208 | 5443742 | + | 6.9 | 3567 | 4101 | -2045 | L1PA5 | L1 | LINE |
| 5443732 | 5444692 | - | 4.5 | 4294 | 5254 | -892 | L1PA4 | L1 | LINE |
| 5444684 | 5445576 | + | 3.7 | 5257 | 6154 | -1 | L1PA4 | L1 | LINE |
| 5445607 | 5447678 | + | 4.9 | 4080 | 6152 | -2 | L1PA5 | L1 | LINE |

  

|  |  |  |  |  |  |  |  |  |  |
| --- | --- | --- | --- | --- | --- | --- | --- | --- | --- |
| chr20:11030242-11034480 | | | 4169 bp | | |  |  |  |  |
| Genome Start | Genome End | Strand | Div | Repeat Start | Repeat End | Repeat Left | Name | Family | Class |
| 11030242 | 11030365 | - | 29.3 | 2651 | 2783 | -636 | L2 | L2 | LINE |
| 11030366 | 11030541 | - | 18.4 | 137 | 312 | 0 | AluJb | Alu | SINE |
| 11030589 | 11030693 | - | 18.4 | 2 | 136 | -176 | AluJb | Alu | SINE |
| 11030694 | 11031239 | - | 29.3 | 2096 | 2650 | -769 | L2 | L2 | LINE |
| 11031242 | 11031968 | - | 10.0 | 4288 | 5015 | -1131 | L1PA16 | L1 | LINE |
| 11031953 | 11032234 | + | 10.9 | 5013 | 5298 | -868 | L1PA16 | L1 | LINE |
| 11032235 | 11032528 | + | 12.4 | 1 | 291 | -21 | AluSx | Alu | SINE |
| 11032529 | 11033073 | + | 12.6 | 5299 | 5828 | -338 | L1PA16 | L1 | LINE |
| 11033085 | 11033398 | + | 14.0 | 6 | 312 | 0 | AluSx | Alu | SINE |
| 11033404 | 11033638 | + | 15.4 | 5909 | 6157 | -9 | L1PA16 | L1 | LINE |
| 11033660 | 11034480 | - | 30.4 | 1199 | 2077 | -1342 | L2 | L2 | LINE |

  

|  |  |  |  |  |  |  |  |  |  |
| --- | --- | --- | --- | --- | --- | --- | --- | --- | --- |
| chr20:12109398-12122426 | | | 12629 bp | | |  |  |  |  |
| Genome Start | Genome End | Strand | Div | Repeat Start | Repeat End | Repeat Left | Name | Family | Class |
| 12109398 | 12110136 | - | 21.4 | 5820 | 6626 | -1867 | L1ME1 | L1 | LINE |
| 12110138 | 12110474 | + | 17.2 | 5584 | 5950 | -230 | L1MB4 | L1 | LINE |
| 12110475 | 12110537 | + | 12.7 | 2 | 78 | -224 | AluS | Alu | SINE |
| 12110538 | 12110565 | + | 21.4 | 5922 | 5949 | -231 | L1MB4 | L1 | LINE |
| 12110566 | 12110878 | + | 10.2 | 1 | 313 | 0 | AluSq | Alu | SINE |
| 12110879 | 12111113 | + | 21.4 | 5950 | 6178 | -2 | L1MB4 | L1 | LINE |
| 12111138 | 12112713 | - | 19.7 | 4200 | 5804 | -840 | L1ME1 | L1 | LINE |
| 12112723 | 12113605 | - | 20.0 | 2666 | 3569 | -3075 | L1ME1 | L1 | LINE |
| 12113731 | 12113852 | + | 19.6 | 58 | 167 | -300 | MLT1C | MaLR | LTR |
| 12113940 | 12114229 | + | 20.7 | 172 | 465 | -2 | MLT1C | MaLR | LTR |
| 12114237 | 12114913 | - | 29.0 | 1899 | 2759 | -4066 | L1ME1 | L1 | LINE |
| 12114994 | 12117305 | - | 14.5 | 3857 | 6157 | -9 | L1PA16 | L1 | LINE |
| 12117296 | 12118160 | + | 17.3 | 3008 | 3877 | -2269 | L1PA16 | L1 | LINE |
| 12118142 | 12118246 | - | 21.2 | 1793 | 1894 | -4752 | L1ME1 | L1 | LINE |
| 12118247 | 12118562 | - | 11.4 | 1 | 307 | -5 | AluSx | Alu | SINE |
| 12118563 | 12118785 | - | 21.2 | 1578 | 1792 | -4854 | L1ME1 | L1 | LINE |
| 12118786 | 12119705 | - | 16.2 | 2953 | 3879 | -2267 | L1MC | L1 | LINE |
| 12119748 | 12119967 | - | 28.3 | 1349 | 1569 | -4973 | L1ME1 | L1 | LINE |
| 12119976 | 12120760 | - | 4.5 | 5366 | 6154 | 0 | L1PA5 | L1 | LINE |
| 12120762 | 12120959 | - | 23.0 | 1172 | 1364 | -5178 | L1ME1 | L1 | LINE |
| 12120960 | 12121265 | + | 6.7 | 1 | 297 | -14 | AluY | Alu | SINE |
| 12121266 | 12121637 | - | 23.0 | 791 | 1171 | -5371 | L1ME1 | L1 | LINE |
| 12121683 | 12122426 | - | 23.0 | 7 | 790 | -5752 | L1ME1 | L1 | LINE |

  

|  |  |  |  |  |  |  |  |  |  |
| --- | --- | --- | --- | --- | --- | --- | --- | --- | --- |
| chr20:17181448-17187222 | | | 5701 bp | | |  |  |  |  |
| Genome Start | Genome End | Strand | Div | Repeat Start | Repeat End | Repeat Left | Name | Family | Class |
| 17181448 | 17181648 | + | 30.9 | 5318 | 5531 | -709 | L1M5 | L1 | LINE |
| 17181680 | 17181844 | + | 29.4 | 5555 | 5724 | -2084 | L1MC | L1 | LINE |
| 17181831 | 17183989 | - | 19.2 | 4473 | 6682 | 0 | L1MB7 | L1 | LINE |
| 17183990 | 17184285 | - | 8.1 | 1 | 296 | -15 | AluY | Alu | SINE |
| 17184286 | 17184780 | - | 18.1 | 3970 | 4472 | -2172 | L1MB7 | L1 | LINE |
| 17184779 | 17185242 | - | 19.1 | 949 | 1378 | -5164 | L1MB7 | L1 | LINE |
| 17185272 | 17185706 | - | 6.7 | 5715 | 6153 | -1 | L1PA7 | L1 | LINE |
| 17185707 | 17186129 | + | 7.8 | 5275 | 5703 | -451 | L1PA7 | L1 | LINE |
| 17186136 | 17187079 | - | 26.5 | 17 | 957 | -5585 | L1MB7 | L1 | LINE |
| 17187104 | 17187222 | + | 23.5 | 5703 | 5819 | -302 | L1M5 | L1 | LINE |

  

|  |  |  |  |  |  |  |  |  |  |
| --- | --- | --- | --- | --- | --- | --- | --- | --- | --- |
| chr20:18048681-18059093 | | | 10357 bp | | |  |  |  |  |
| Genome Start | Genome End | Strand | Div | Repeat Start | Repeat End | Repeat Left | Name | Family | Class |
| 18048681 | 18050343 | + | 20.6 | 2283 | 4002 | -2144 | L1MB4 | L1 | LINE |
| 18050344 | 18050709 | + | 17.8 | 1 | 408 | 0 | MLT1A1 | MaLR | LTR |
| 18050710 | 18050807 | + | 20.6 | 4003 | 4101 | -2045 | L1MB4 | L1 | LINE |
| 18050808 | 18051109 | - | 11.2 | 1 | 297 | -15 | AluSx | Alu | SINE |
| 18051110 | 18051189 | + | 20.6 | 4102 | 4166 | -1980 | L1MB4 | L1 | LINE |
| 18051190 | 18051480 | + | 11.4 | 1 | 290 | -20 | AluSg | Alu | SINE |
| 18051481 | 18051607 | + | 20.6 | 4167 | 4378 | -1768 | L1MB4 | L1 | LINE |
| 18051608 | 18051908 | + | 11.0 | 1 | 301 | -11 | AluSx | Alu | SINE |
| 18051909 | 18052199 | + | 13.4 | 1 | 291 | -21 | AluSx | Alu | SINE |
| 18052200 | 18052498 | + | 20.6 | 4379 | 4689 | -1457 | L1MB4 | L1 | LINE |
| 18052499 | 18052793 | - | 11.5 | 1 | 301 | -11 | AluSx | Alu | SINE |
| 18052794 | 18053199 | + | 20.6 | 4690 | 5092 | -1054 | L1MB4 | L1 | LINE |
| 18053194 | 18053839 | - | 6.4 | 2914 | 3559 | -2587 | L1PA5 | L1 | LINE |
| 18053837 | 18056463 | + | 5.8 | 3535 | 6154 | 0 | L1PA5 | L1 | LINE |
| 18056466 | 18056716 | + | 20.3 | 5079 | 5331 | -852 | L1MB4 | L1 | LINE |
| 18056717 | 18057015 | + | 9.7 | 1 | 299 | -14 | AluSp | Alu | SINE |
| 18057016 | 18057199 | + | 20.1 | 5332 | 5506 | -677 | L1MB4 | L1 | LINE |
| 18057200 | 18057614 | - | 10.7 | 1 | 428 | 0 | MSTA | MaLR | LTR |
| 18057615 | 18057767 | + | 20.1 | 5507 | 5654 | -529 | L1MB4 | L1 | LINE |
| 18057825 | 18058118 | - | 10.9 | 3 | 296 | -16 | AluSx | Alu | SINE |
| 18058121 | 18058408 | - | 11.5 | 1 | 298 | -14 | AluSx | Alu | SINE |
| 18058413 | 18058484 | + | 16.7 | 2332 | 2403 | 0 | Tigger5 | MER2\_type | DNA |
| 18058485 | 18058560 | - | 19.5 | 3 | 78 | -2245 | Tigger5 | MER2\_type | DNA |
| 18058561 | 18059093 | + | 15.2 | 5643 | 6179 | -1 | L1MB4 | L1 | LINE |

  

|  |  |  |  |  |  |  |  |  |  |
| --- | --- | --- | --- | --- | --- | --- | --- | --- | --- |
| chr20:18618335-18620474 | | | 2139 bp | | |  |  |  |  |
| Genome Start | Genome End | Strand | Div | Repeat Start | Repeat End | Repeat Left | Name | Family | Class |
| 18618335 | 18618494 | + | 19.4 | 1 | 166 | -43 | MER3 | MER1\_type | DNA |
| 18618495 | 18618806 | + | 15.4 | 1 | 307 | -5 | AluJo | Alu | SINE |
| 18618807 | 18618829 | + | 24.1 | 120 | 142 | -67 | MER3 | MER1\_type | DNA |
| 18618830 | 18619588 | - | 11.4 | 4546 | 5312 | -834 | L1PA16 | L1 | LINE |
| 18619590 | 18620424 | + | 13.8 | 5323 | 6163 | -3 | L1PA16 | L1 | LINE |
| 18620425 | 18620474 | + | 8.0 | 160 | 209 | 0 | MER3 | MER1\_type | DNA |

  

|  |  |  |  |  |  |  |  |  |  |
| --- | --- | --- | --- | --- | --- | --- | --- | --- | --- |
| chr20:25033740-25044526 | | | 10464 bp | | |  |  |  |  |
| Genome Start | Genome End | Strand | Div | Repeat Start | Repeat End | Repeat Left | Name | Family | Class |
| 25033740 | 25033882 | - | 24.6 | 2511 | 2675 | -107 | Charlie2b | MER1\_type | DNA |
| 25033889 | 25039673 | + | 7.0 | 4 | 5784 | -370 | L1PA5 | L1 | LINE |
| 25039673 | 25040538 | - | 4.0 | 4203 | 5068 | -1078 | L1PA3 | L1 | LINE |
| 25040534 | 25041613 | + | 2.1 | 5071 | 6155 | 0 | L1PA3 | L1 | LINE |
| 25041614 | 25042025 | + | 6.7 | 5770 | 6180 | 0 | L1PA5 | L1 | LINE |
| 25042237 | 25042436 | - | 25.4 | 2330 | 2539 | -243 | Charlie2b | MER1\_type | DNA |
| 25042437 | 25042734 | + | 12.4 | 1 | 299 | -11 | AluSg | Alu | SINE |
| 25042735 | 25043077 | - | 25.4 | 1965 | 2329 | -453 | Charlie2b | MER1\_type | DNA |
| 25043078 | 25043379 | - | 15.2 | 1 | 302 | -10 | AluJb | Alu | SINE |
| 25043380 | 25044109 | - | 24.8 | 1155 | 1964 | -818 | Charlie2b | MER1\_type | DNA |
| 25044176 | 25044351 | - | 12.5 | 132 | 307 | -5 | AluSg/x | Alu | SINE |
| 25044398 | 25044526 | - | 27.0 | 895 | 1020 | -1762 | Charlie2b | MER1\_type | DNA |

  

|  |  |  |  |  |  |  |  |  |  |
| --- | --- | --- | --- | --- | --- | --- | --- | --- | --- |
| chr20:25652217-25659146 | | | 6944 bp | | |  |  |  |  |
| Genome Start | Genome End | Strand | Div | Repeat Start | Repeat End | Repeat Left | Name | Family | Class |
| 25652217 | 25654645 | - | 20.3 | 2408 | 5024 | -2085 | L1M2 | L1 | LINE |
| 25654646 | 25655064 | + | 16.1 | 1 | 426 | -2 | MSTA | MaLR | LTR |
| 25655065 | 25655484 | - | 21.3 | 1993 | 2407 | -4709 | L1M2 | L1 | LINE |
| 25655485 | 25655895 | - | 17.6 | 1 | 428 | 0 | MSTA | MaLR | LTR |
| 25655896 | 25656034 | - | 21.3 | 1842 | 1992 | -5124 | L1M2 | L1 | LINE |
| 25656029 | 25656842 | - | 4.5 | 3322 | 4135 | -2011 | L1PA3 | L1 | LINE |
| 25656823 | 25658858 | + | 2.3 | 4117 | 6155 | 0 | L1PA3 | L1 | LINE |
| 25658871 | 25659146 | - | 12.5 | 1559 | 1840 | -5276 | L1M2 | L1 | LINE |

  

|  |  |  |  |  |  |  |  |  |  |
| --- | --- | --- | --- | --- | --- | --- | --- | --- | --- |
| chr20:31221650-31224022 | | | 2257 bp | | |  |  |  |  |
| Genome Start | Genome End | Strand | Div | Repeat Start | Repeat End | Repeat Left | Name | Family | Class |
| 31221650 | 31221856 | - | 25.8 | 1991 | 2186 | -321 | HAL1 | L1 | LINE |
| 31221884 | 31222160 | - | 14.8 | 1 | 295 | -17 | AluJb | Alu | SINE |
| 31222243 | 31222842 | - | 8.2 | 4514 | 5128 | -1018 | L1PA8 | L1 | LINE |
| 31222841 | 31223880 | + | 6.7 | 5128 | 6171 | -1 | L1PA8 | L1 | LINE |
| 31223890 | 31224022 | - | 32.3 | 1729 | 1860 | -647 | HAL1 | L1 | LINE |

  

|  |  |  |  |  |  |  |  |  |  |
| --- | --- | --- | --- | --- | --- | --- | --- | --- | --- |
| chr20:39188787-39193816 | | | 4718 bp | | |  |  |  |  |
| Genome Start | Genome End | Strand | Div | Repeat Start | Repeat End | Repeat Left | Name | Family | Class |
| 39188787 | 39189102 | + | 28.7 | 2261 | 2594 | -825 | L2 | L2 | LINE |
| 39189158 | 39189403 | + | 20.1 | 76 | 352 | -536 | Kanga2\_a | Tc2 | DNA |
| 39189480 | 39189753 | - | 8.4 | 1 | 298 | -12 | AluSg | Alu | SINE |
| 39189780 | 39189925 | + | 25.0 | 134 | 281 | -31 | AluJo | Alu | SINE |
| 39189947 | 39190132 | + | 25.3 | 7709 | 7914 | -47 | L1MC5 | L1 | LINE |
| 39190143 | 39191257 | - | 2.5 | 5037 | 6152 | -3 | L1PA3 | L1 | LINE |
| 39191243 | 39191830 | + | 1.9 | 4442 | 5029 | -1117 | L1PA3 | L1 | LINE |
| 39191831 | 39192129 | - | 12.1 | 1 | 298 | -14 | AluSx | Alu | SINE |
| 39192152 | 39192467 | - | 19.0 | 2 | 306 | -6 | AluJo | Alu | SINE |
| 39192490 | 39192668 | + | 27.0 | 346 | 523 | -365 | Kanga2\_a | Tc2 | DNA |
| 39192669 | 39192961 | + | 16.0 | 5853 | 6163 | -3 | L1ME1 | L1 | LINE |
| 39193002 | 39193296 | + | 7.1 | 1 | 297 | -16 | AluSq | Alu | SINE |
| 39193303 | 39193672 | + | 22.6 | 497 | 885 | -3 | Kanga2\_a | Tc2 | DNA |
| 39193722 | 39193816 | + | 34.0 | 3172 | 3269 | -109 | L2 | L2 | LINE |

  

|  |  |  |  |  |  |  |  |  |  |
| --- | --- | --- | --- | --- | --- | --- | --- | --- | --- |
| chr20:39926863-39933074 | | | 5925 bp | | |  |  |  |  |
| Genome Start | Genome End | Strand | Div | Repeat Start | Repeat End | Repeat Left | Name | Family | Class |
| 39926863 | 39927491 | - | 37.2 | 1057 | 1757 | -203 | L4 | RTE | LINE |
| 39927581 | 39927762 | - | 6.0 | 6121 | 6302 | 0 | L1MA1 | L1 | LINE |
| 39927763 | 39927840 | + | 14.1 | 6045 | 6122 | -180 | L1MA1 | L1 | LINE |
| 39927987 | 39928343 | - | 24.2 | 153 | 549 | 0 | MLT1H | MaLR | LTR |
| 39928352 | 39928414 | - | 15.9 | 6294 | 6356 | -3 | L1MD2 | L1 | LINE |
| 39928417 | 39928558 | - | 20.0 | 5164 | 5302 | -844 | L1MD | L1 | LINE |
| 39928566 | 39929811 | - | 9.5 | 4896 | 6161 | 0 | L1PA14 | L1 | LINE |
| 39929815 | 39930454 | + | 8.5 | 4235 | 4875 | -1271 | L1PREC2 | L1 | LINE |
| 39930434 | 39931212 | - | 18.7 | 4422 | 5195 | -951 | L1MD | L1 | LINE |
| 39931202 | 39931817 | - | 18.7 | 1484 | 2171 | -4462 | L1MDa | L1 | LINE |
| 39931844 | 39932250 | - | 18.7 | 943 | 1483 | -5150 | L1MDa | L1 | LINE |
| 39932255 | 39932451 | - | 30.1 | 20 | 225 | -324 | MLT1H | MaLR | LTR |
| 39932486 | 39933074 | - | 34.2 | 165 | 752 | -1208 | L4 | RTE | LINE |

  

|  |  |  |  |  |  |  |  |  |  |
| --- | --- | --- | --- | --- | --- | --- | --- | --- | --- |
| chr20:40266773-40267600 | | | 436 bp | | |  |  |  |  |
| Genome Start | Genome End | Strand | Div | Repeat Start | Repeat End | Repeat Left | Name | Family | Class |
| 40266773 | 40266933 | + | 34.8 | 3980 | 4139 | -350 | L3 | CR1 | LINE |
| 40267029 | 40267119 | - | 4.4 | 6064 | 6154 | 0 | L1PA5 | L1 | LINE |
| 40267122 | 40267212 | + | 0.0 | 5991 | 6081 | -74 | L1PA5 | L1 | LINE |
| 40267508 | 40267600 | + | 30.1 | 4330 | 4424 | -65 | L3 | CR1 | LINE |

  

|  |  |  |  |  |  |  |  |  |  |
| --- | --- | --- | --- | --- | --- | --- | --- | --- | --- |
| chr20:40776599-40780857 | | | 3463 bp | | |  |  |  |  |
| Genome Start | Genome End | Strand | Div | Repeat Start | Repeat End | Repeat Left | Name | Family | Class |
| 40776599 | 40776899 | + | 34.6 | 37 | 363 | -6037 | L1ME3A | L1 | LINE |
| 40777371 | 40777700 | + | 29.1 | 827 | 1158 | -5051 | L1ME3A | L1 | LINE |
| 40777720 | 40777973 | + | 26.6 | 4525 | 4772 | -1447 | L1ME3A | L1 | LINE |
| 40777991 | 40778075 | + | 17.1 | 6250 | 6333 | 0 | L1MC1 | L1 | LINE |
| 40778309 | 40778745 | - | 3.5 | 5214 | 5651 | -504 | L1PA4 | L1 | LINE |
| 40778747 | 40779235 | + | 3.1 | 5649 | 6137 | -18 | L1PA4 | L1 | LINE |
| 40779271 | 40779921 | + | 27.4 | 4775 | 5446 | -773 | L1ME3A | L1 | LINE |
| 40779942 | 40780309 | + | 23.0 | 5434 | 5893 | -350 | L1ME3A | L1 | LINE |
| 40780310 | 40780620 | + | 7.8 | 1 | 307 | -4 | AluY | Alu | SINE |
| 40780621 | 40780857 | + | 24.4 | 5894 | 6162 | -81 | L1ME3A | L1 | LINE |

  

|  |  |  |  |  |  |  |  |  |  |
| --- | --- | --- | --- | --- | --- | --- | --- | --- | --- |
| chr20:52071195-52072021 | | | 809 bp | | |  |  |  |  |
| Genome Start | Genome End | Strand | Div | Repeat Start | Repeat End | Repeat Left | Name | Family | Class |
| 52071195 | 52071379 | - | 15.1 | 2221 | 2414 | -4 | Tigger1 | MER2\_type | DNA |
| 52071384 | 52071477 | - | 3.2 | 5703 | 5796 | -372 | L1PA10 | L1 | LINE |
| 52071478 | 52071870 | + | 7.9 | 5794 | 6168 | 0 | L1PA10 | L1 | LINE |
| 52071885 | 52072021 | - | 17.6 | 21 | 157 | -2261 | Tigger1 | MER2\_type | DNA |

  

|  |  |  |  |  |  |  |  |  |  |
| --- | --- | --- | --- | --- | --- | --- | --- | --- | --- |
| chr20:52567380-52569300 | | | 1729 bp | | |  |  |  |  |
| Genome Start | Genome End | Strand | Div | Repeat Start | Repeat End | Repeat Left | Name | Family | Class |
| 52567380 | 52567523 | + | 33.3 | 32 | 173 | -89 | MIRb | MIR | SINE |
| 52567597 | 52567952 | + | 19.8 | 1 | 362 | -3 | MLT1A0 | MaLR | LTR |
| 52567957 | 52568068 | - | 28.6 | 137 | 251 | -11 | MIR | MIR | SINE |
| 52568070 | 52568689 | - | 3.2 | 5536 | 6155 | 0 | L1PA4 | L1 | LINE |
| 52568690 | 52569042 | + | 4.4 | 5181 | 5534 | -621 | L1PA4 | L1 | LINE |
| 52569043 | 52569103 | - | 24.6 | 92 | 156 | -120 | MIRm | MIR | SINE |
| 52569218 | 52569300 | + | 30.9 | 171 | 258 | -10 | MIRb | MIR | SINE |

  

|  |  |  |  |  |  |  |  |  |  |
| --- | --- | --- | --- | --- | --- | --- | --- | --- | --- |
| chr20:52773824-52775823 | | | 1989 bp | | |  |  |  |  |
| Genome Start | Genome End | Strand | Div | Repeat Start | Repeat End | Repeat Left | Name | Family | Class |
| 52773824 | 52774190 | + | 13.3 | 5545 | 5922 | -369 | L1MA8 | L1 | LINE |
| 52774203 | 52775332 | - | 6.9 | 5057 | 6170 | -2 | L1PA8 | L1 | LINE |
| 52775332 | 52775447 | + | 6.0 | 4949 | 5064 | -1082 | L1PA8 | L1 | LINE |
| 52775448 | 52775823 | + | 12.1 | 5910 | 6288 | -3 | L1MA8 | L1 | LINE |

  

|  |  |  |  |  |  |  |  |  |  |
| --- | --- | --- | --- | --- | --- | --- | --- | --- | --- |
| chr20:52802179-52813246 | | | 10728 bp | | |  |  |  |  |
| Genome Start | Genome End | Strand | Div | Repeat Start | Repeat End | Repeat Left | Name | Family | Class |
| 52802179 | 52802217 | - | 12.8 | 8002 | 8040 | -2 | L1MC4 | L1 | LINE |
| 52802220 | 52802398 | + | 25.1 | 26 | 207 | -6426 | L1MDa | L1 | LINE |
| 52802401 | 52802644 | + | 21.4 | 4876 | 5116 | -1030 | L1MC | L1 | LINE |
| 52802644 | 52803435 | - | 6.4 | 1541 | 2317 | -3829 | L1PA5 | L1 | LINE |
| 52803433 | 52807230 | + | 5.6 | 2342 | 6150 | -4 | L1PA5 | L1 | LINE |
| 52807227 | 52807596 | + | 16.6 | 5106 | 5490 | -2295 | L1MC | L1 | LINE |
| 52807590 | 52807914 | - | 24.1 | 2229 | 2580 | -4171 | L1MCb | L1 | LINE |
| 52807956 | 52809897 | - | 24.3 | 8 | 2146 | -4605 | L1MCb | L1 | LINE |
| 52809925 | 52810690 | + | 19.9 | 4845 | 5605 | -637 | L1MD1 | L1 | LINE |
| 52810691 | 52810987 | + | 17.9 | 1 | 300 | -12 | AluJo | Alu | SINE |
| 52810988 | 52811609 | + | 18.9 | 5606 | 6242 | 0 | L1MD1 | L1 | LINE |
| 52811864 | 52812173 | + | 10.3 | 1 | 309 | 0 | AluSc | Alu | SINE |
| 52812203 | 52812810 | - | 23.0 | 7365 | 8013 | -29 | L1MC4 | L1 | LINE |
| 52812811 | 52813140 | + | 9.5 | 1 | 331 | 0 | AluSq | Alu | SINE |
| 52813141 | 52813246 | - | 23.0 | 7259 | 7364 | -678 | L1MC4 | L1 | LINE |

  

|  |  |  |  |  |  |  |  |  |  |
| --- | --- | --- | --- | --- | --- | --- | --- | --- | --- |
| chr20:53541459-53552380 | | | 10971 bp | | |  |  |  |  |
| Genome Start | Genome End | Strand | Div | Repeat Start | Repeat End | Repeat Left | Name | Family | Class |
| 53541459 | 53542434 | + | 19.0 | 3604 | 4642 | -1498 | L1M3 | L1 | LINE |
| 53542448 | 53546731 | - | 9.3 | 2174 | 6464 | -5 | L1PA7 | L1 | LINE |
| 53546725 | 53547572 | - | 4.0 | 5305 | 6155 | 0 | L1PA4 | L1 | LINE |
| 53547573 | 53547890 | - | 0.9 | 1 | 318 | 0 | AluYb8 | Alu | SINE |
| 53547891 | 53548763 | - | 4.3 | 4437 | 5304 | -842 | L1P | L1 | LINE |
| 53548747 | 53548826 | + | 8.8 | 4373 | 4452 | -1694 | L1P | L1 | LINE |
| 53548813 | 53550952 | - | 12.7 | 10 | 2190 | -400 | L1PA7 | L1 | LINE |
| 53550926 | 53551664 | - | 9.3 | 4776 | 5531 | -623 | L1P3 | L1 | LINE |
| 53551668 | 53552380 | + | 17.4 | 4668 | 5424 | -744 | L1M3 | L1 | LINE |

  

|  |  |  |  |  |  |  |  |  |  |
| --- | --- | --- | --- | --- | --- | --- | --- | --- | --- |
| chr20:53821676-53824429 | | | 2622 bp | | |  |  |  |  |
| Genome Start | Genome End | Strand | Div | Repeat Start | Repeat End | Repeat Left | Name | Family | Class |
| 53821676 | 53821959 | + | 27.5 | 2816 | 3141 | -278 | L2 | L2 | LINE |
| 53821960 | 53823690 | - | 6.2 | 4414 | 6154 | 0 | L1PA5 | L1 | LINE |
| 53823689 | 53824192 | + | 5.6 | 3912 | 4417 | -1729 | L1PA5 | L1 | LINE |
| 53824327 | 53824429 | + | 29.3 | 3307 | 3418 | -1 | L2 | L2 | LINE |

  

|  |  |  |  |  |  |  |  |  |  |
| --- | --- | --- | --- | --- | --- | --- | --- | --- | --- |
| chr20:54001612-54002092 | | | 474 bp | | |  |  |  |  |
| Genome Start | Genome End | Strand | Div | Repeat Start | Repeat End | Repeat Left | Name | Family | Class |
| 54001612 | 54001677 | + | 22.7 | 1 | 67 | -93 | MER5A1 | MER1\_type | DNA |
| 54001682 | 54001821 | - | 1.4 | 5827 | 5968 | -186 | L1PA5 | L1 | LINE |
| 54001822 | 54001989 | + | 3.6 | 5986 | 6154 | 0 | L1PA5 | L1 | LINE |
| 54001993 | 54002092 | + | 24.0 | 58 | 159 | -1 | MER5A1 | MER1\_type | DNA |

  

|  |  |  |  |  |  |  |  |  |  |
| --- | --- | --- | --- | --- | --- | --- | --- | --- | --- |
| chr20:54070310-54075794 | | | 5403 bp | | |  |  |  |  |
| Genome Start | Genome End | Strand | Div | Repeat Start | Repeat End | Repeat Left | Name | Family | Class |
| 54070310 | 54070995 | + | 23.5 | 3225 | 3977 | -2169 | L1MD3 | L1 | LINE |
| 54071003 | 54071660 | - | 7.2 | 4193 | 4850 | -1296 | L1PA7 | L1 | LINE |
| 54071652 | 54072915 | + | 6.7 | 4847 | 6129 | -25 | L1PA7 | L1 | LINE |
| 54072995 | 54073336 | - | 20.2 | 5 | 363 | -2 | MLT1A0 | MaLR | LTR |
| 54073342 | 54074994 | + | 26.7 | 3977 | 5588 | -2407 | L1MD3 | L1 | LINE |
| 54074995 | 54075386 | - | 3.8 | 5763 | 6155 | 0 | L1PA4 | L1 | LINE |
| 54075387 | 54075794 | + | 29.4 | 5589 | 5999 | -1996 | L1MD3 | L1 | LINE |

  

|  |  |  |  |  |  |  |  |  |  |
| --- | --- | --- | --- | --- | --- | --- | --- | --- | --- |
| chr20:54087890-54096561 | | | 8578 bp | | |  |  |  |  |
| Genome Start | Genome End | Strand | Div | Repeat Start | Repeat End | Repeat Left | Name | Family | Class |
| 54087890 | 54089307 | - | 23.2 | 4677 | 6146 | -93 | L1ME1 | L1 | LINE |
| 54089306 | 54090436 | + | 11.7 | 5024 | 6161 | -5 | L1PA16 | L1 | LINE |
| 54090430 | 54090518 | - | 16.3 | 4608 | 4693 | -1526 | L1ME1 | L1 | LINE |
| 54090519 | 54091219 | - | 1.0 | 5450 | 6150 | -5 | L1PA2 | L1 | LINE |
| 54091220 | 54091611 | + | 1.7 | 5073 | 5464 | -691 | L1PA2 | L1 | LINE |
| 54091606 | 54093789 | - | 25.7 | 2369 | 4622 | -1597 | L1ME1 | L1 | LINE |
| 54093790 | 54093883 | + | 20.2 | 1 | 94 | -559 | MER82 | MER2\_type | DNA |
| 54093962 | 54094093 | - | 34.1 | 5 | 146 | -122 | MIRb | MIR | SINE |
| 54094125 | 54094326 | + | 25.4 | 447 | 649 | -4 | MER82 | MER2\_type | DNA |
| 54094327 | 54096561 | - | 20.3 | 36 | 2472 | -3928 | L1ME1 | L1 | LINE |

  

|  |  |  |  |  |  |  |  |  |  |
| --- | --- | --- | --- | --- | --- | --- | --- | --- | --- |
| chr20:57861711-57871709 | | | 9928 bp | | |  |  |  |  |
| Genome Start | Genome End | Strand | Div | Repeat Start | Repeat End | Repeat Left | Name | Family | Class |
| 57861711 | 57862139 | - | 33.1 | 2877 | 3418 | -1 | L2 | L2 | LINE |
| 57862146 | 57862646 | - | 14.5 | 5656 | 6173 | -1 | L1MB5 | L1 | LINE |
| 57862647 | 57863059 | + | 15.3 | 1 | 428 | 0 | MSTA | MaLR | LTR |
| 57863060 | 57863090 | - | 14.5 | 5625 | 5655 | -519 | L1MB5 | L1 | LINE |
| 57863091 | 57863183 | + | 12.9 | 32 | 130 | -1521 | MSTA-int | MaLR | LTR |
| 57863184 | 57864040 | - | 4.2 | 3795 | 4657 | -1489 | L1PA4 | L1 | LINE |
| 57864040 | 57865513 | + | 3.3 | 4662 | 6142 | -13 | L1PA4 | L1 | LINE |
| 57865514 | 57866350 | + | 18.6 | 109 | 964 | -687 | MSTA-int | MaLR | LTR |
| 57866348 | 57866708 | + | 12.9 | 1293 | 1651 | 0 | MSTA-int | MaLR | LTR |
| 57866709 | 57867120 | + | 18.8 | 1 | 426 | -2 | MSTA | MaLR | LTR |
| 57867123 | 57869087 | - | 18.9 | 3578 | 5660 | -520 | L1M3 | L1 | LINE |
| 57869088 | 57869387 | + | 20.1 | 1 | 301 | -11 | AluJo | Alu | SINE |
| 57869388 | 57870028 | - | 19.3 | 2871 | 3577 | -2569 | L1M3 | L1 | LINE |
| 57870029 | 57871564 | - | 33.1 | 911 | 2856 | -563 | L2 | L2 | LINE |
| 57871632 | 57871709 | - | 26.0 | 203 | 280 | -3139 | L2 | L2 | LINE |

  


|  |  |  |  |  |  |  |  |  |  |
| --- | --- | --- | --- | --- | --- | --- | --- | --- | --- |
| chr21:14937792-14947990 | | | 10117 bp | | |  |  |  |  |
| Genome Start | Genome End | Strand | Div | Repeat Start | Repeat End | Repeat Left | Name | Family | Class |
| 14937792 | 14941382 | + | 15.9 | 16 | 3675 | -3890 | L1MA1 | L1 | LINE |
| 14941389 | 14942391 | - | 1.8 | 3940 | 4942 | -1204 | L1PA3 | L1 | LINE |
| 14942389 | 14943628 | + | 2.4 | 4919 | 6155 | 0 | L1PA3 | L1 | LINE |
| 14943663 | 14943679 | + | 10.8 | 3672 | 3689 | -3876 | L1MA1 | L1 | LINE |
| 14943680 | 14943964 | + | 17.1 | 1 | 285 | -27 | AluSx | Alu | SINE |
| 14944010 | 14944022 | + | 17.1 | 286 | 298 | -14 | AluSx | Alu | SINE |
| 14944023 | 14947990 | + | 10.5 | 3690 | 7717 | -4 | L1MA1 | L1 | LINE |

  

|  |  |  |  |  |  |  |  |  |  |
| --- | --- | --- | --- | --- | --- | --- | --- | --- | --- |
| chr21:17197264-17203442 | | | 6015 bp | | |  |  |  |  |
| Genome Start | Genome End | Strand | Div | Repeat Start | Repeat End | Repeat Left | Name | Family | Class |
| 17197264 | 17197499 | - | 31.7 | 5414 | 5666 | -1 | ERVL-F | ERVL | LTR |
| 17197507 | 17197980 | + | 30.8 | 3 | 504 | -15 | LTR40a | ERVL | LTR |
| 17198056 | 17198279 | - | 26.8 | 5122 | 5351 | -316 | ERVL-F | ERVL | LTR |
| 17198287 | 17198442 | - | 11.5 | 6136 | 6292 | -11 | L1MA4A | L1 | LINE |
| 17198446 | 17201311 | + | 18.0 | 2627 | 5475 | -828 | L1MA4A | L1 | LINE |
| 17201312 | 17201594 | + | 13.1 | 1 | 292 | -20 | AluSx | Alu | SINE |
| 17201595 | 17202256 | + | 13.8 | 5476 | 6131 | -172 | L1MA4A | L1 | LINE |
| 17202257 | 17202918 | - | 30.1 | 4425 | 5133 | -534 | ERVL-F | ERVL | LTR |
| 17202910 | 17203139 | - | 29.6 | 4075 | 4307 | -1360 | ERVL-F | ERVL | LTR |
| 17203221 | 17203442 | - | 26.2 | 3635 | 3932 | -1735 | ERVL-F | ERVL | LTR |

  

|  |  |  |  |  |  |  |  |  |  |
| --- | --- | --- | --- | --- | --- | --- | --- | --- | --- |
| chr21:17487482-17488412 | | | 927 bp | | |  |  |  |  |
| Genome Start | Genome End | Strand | Div | Repeat Start | Repeat End | Repeat Left | Name | Family | Class |
| 17487482 | 17487893 | + | 15.1 | 1 | 435 | -51 | LTR19A | ERV1 | LTR |
| 17487895 | 17488147 | - | 5.1 | 5698 | 5950 | -204 | L1PA5 | L1 | LINE |
| 17488148 | 17488350 | + | 7.5 | 5953 | 6154 | 0 | L1PA5 | L1 | LINE |
| 17488354 | 17488412 | + | 8.5 | 425 | 484 | -2 | LTR19A | ERV1 | LTR |

  

|  |  |  |  |  |  |  |  |  |  |
| --- | --- | --- | --- | --- | --- | --- | --- | --- | --- |
| chr21:18352840-18354352 | | | 1358 bp | | |  |  |  |  |
| Genome Start | Genome End | Strand | Div | Repeat Start | Repeat End | Repeat Left | Name | Family | Class |
| 18352840 | 18353180 | + | 35.1 | 1980 | 2361 | -1058 | L2 | L2 | LINE |
| 18353181 | 18353575 | + | 25.1 | 1 | 385 | -5 | MLT1B | MaLR | LTR |
| 18353688 | 18353908 | - | 8.6 | 5931 | 6174 | 0 | L1PA11 | L1 | LINE |
| 18353911 | 18353993 | + | 12.1 | 5867 | 5949 | -219 | L1PA11 | L1 | LINE |
| 18354035 | 18354352 | + | 35.6 | 2482 | 2810 | -609 | L2 | L2 | LINE |

  

|  |  |  |  |  |  |  |  |  |  |
| --- | --- | --- | --- | --- | --- | --- | --- | --- | --- |
| chr21:18849189-18852239 | | | 2930 bp | | |  |  |  |  |
| Genome Start | Genome End | Strand | Div | Repeat Start | Repeat End | Repeat Left | Name | Family | Class |
| 18849189 | 18850109 | + | 24.9 | 3923 | 4869 | -1277 | L1M5 | L1 | LINE |
| 18850113 | 18851050 | - | 8.9 | 4102 | 5051 | -1095 | L1PA8A | L1 | LINE |
| 18851052 | 18852086 | + | 8.5 | 5059 | 6104 | -27 | L1PA8A | L1 | LINE |
| 18852204 | 18852239 | + | 16.7 | 4947 | 4982 | -1164 | L1M5 | L1 | LINE |

  

|  |  |  |  |  |  |  |  |  |  |
| --- | --- | --- | --- | --- | --- | --- | --- | --- | --- |
| chr21:20249940-20257051 | | | 7078 bp | | |  |  |  |  |
| Genome Start | Genome End | Strand | Div | Repeat Start | Repeat End | Repeat Left | Name | Family | Class |
| 20249940 | 20252826 | - | 17.5 | 3363 | 6300 | 0 | L1MA6 | L1 | LINE |
| 20252865 | 20255344 | - | 2.7 | 3671 | 6152 | -3 | L1PA4 | L1 | LINE |
| 20255343 | 20255910 | + | 3.5 | 3093 | 3660 | -2486 | L1PA4 | L1 | LINE |
| 20255909 | 20257051 | - | 18.5 | 2172 | 3321 | -2822 | L1MA6 | L1 | LINE |

  

|  |  |  |  |  |  |  |  |  |  |
| --- | --- | --- | --- | --- | --- | --- | --- | --- | --- |
| chr21:20525838-20536560 | | | 10752 bp | | |  |  |  |  |
| Genome Start | Genome End | Strand | Div | Repeat Start | Repeat End | Repeat Left | Name | Family | Class |
| 20525838 | 20529173 | - | 17.9 | 3988 | 7360 | -2 | L1MA5 | L1 | LINE |
| 20529152 | 20531983 | - | 9.2 | 3273 | 6128 | -3 | L1PA8A | L1 | LINE |
| 20531983 | 20532557 | + | 10.9 | 2679 | 3252 | -2894 | L1PA8A | L1 | LINE |
| 20532552 | 20533037 | - | 17.8 | 3576 | 4036 | -3169 | L1MA5 | L1 | LINE |
| 20533038 | 20533308 | - | 14.8 | 23 | 299 | -13 | AluSx | Alu | SINE |
| 20533309 | 20533478 | - | 22.4 | 3230 | 3410 | -3795 | L1MA5 | L1 | LINE |
| 20533479 | 20533780 | + | 14.9 | 4 | 307 | 0 | AluSx | Alu | SINE |
| 20533781 | 20534765 | - | 21.0 | 2251 | 3230 | -3975 | L1MA5 | L1 | LINE |
| 20534766 | 20535186 | - | 17.8 | 1 | 428 | 0 | MSTA | MaLR | LTR |
| 20535187 | 20536560 | - | 25.3 | 1 | 2250 | -4959 | L1MA5 | L1 | LINE |

  

|  |  |  |  |  |  |  |  |  |  |
| --- | --- | --- | --- | --- | --- | --- | --- | --- | --- |
| chr21:20671878-20680880 | | | 8963 bp | | |  |  |  |  |
| Genome Start | Genome End | Strand | Div | Repeat Start | Repeat End | Repeat Left | Name | Family | Class |
| 20671878 | 20675037 | - | 19.0 | 3174 | 6432 | -76 | L1MC1 | L1 | LINE |
| 20675038 | 20675365 | + | 19.8 | 1 | 329 | 0 | AluJb | Alu | SINE |
| 20675366 | 20675704 | - | 18.8 | 2842 | 3173 | -3148 | L1MC1 | L1 | LINE |
| 20675726 | 20675963 | - | 18.6 | 1 | 237 | -75 | AluJb | Alu | SINE |
| 20675980 | 20676406 | - | 19.4 | 2403 | 2831 | -3490 | L1MC1 | L1 | LINE |
| 20676401 | 20677553 | + | 14.4 | 4954 | 6164 | -1 | L1PA15 | L1 | LINE |
| 20677564 | 20678258 | - | 21.4 | 1723 | 2423 | -4085 | L1MC1 | L1 | LINE |
| 20678259 | 20678774 | - | 16.6 | 5804 | 6312 | 0 | L1MA9 | L1 | LINE |
| 20678776 | 20678932 | - | 15.3 | 5345 | 5508 | -804 | L1MA9 | L1 | LINE |
| 20678934 | 20679070 | + | 21.5 | 4857 | 4991 | -1149 | L1M3 | L1 | LINE |
| 20679073 | 20679189 | + | 25.7 | 5221 | 5333 | -807 | L1M3 | L1 | LINE |
| 20679185 | 20680880 | - | 20.5 | 14 | 1733 | -4775 | L1MC1 | L1 | LINE |

  

|  |  |  |  |  |  |  |  |  |  |
| --- | --- | --- | --- | --- | --- | --- | --- | --- | --- |
| chr21:21595214-21596693 | | | 1470 bp | | |  |  |  |  |
| Genome Start | Genome End | Strand | Div | Repeat Start | Repeat End | Repeat Left | Name | Family | Class |
| 21595214 | 21595563 | - | 16.4 | 5016 | 5364 | -782 | L1P4 | L1 | LINE |
| 21595576 | 21595943 | - | 0.5 | 5788 | 6155 | 0 | L1PA2 | L1 | LINE |
| 21595944 | 21596433 | + | 1.0 | 5283 | 5772 | -383 | L1PA2 | L1 | LINE |
| 21596432 | 21596693 | - | 14.3 | 4757 | 5028 | -1118 | L1P4 | L1 | LINE |

  

|  |  |  |  |  |  |  |  |  |  |
| --- | --- | --- | --- | --- | --- | --- | --- | --- | --- |
| chr21:22128361-22135372 | | | 6270 bp | | |  |  |  |  |
| Genome Start | Genome End | Strand | Div | Repeat Start | Repeat End | Repeat Left | Name | Family | Class |
| 22128361 | 22129081 | - | 24.4 | 4079 | 4773 | -1446 | L1MEc | L1 | LINE |
| 22129085 | 22129226 | - | 21.3 | 365 | 505 | 0 | MLT1D | MaLR | LTR |
| 22129223 | 22129575 | - | 20.0 | 8 | 404 | -459 | MER21B | ERV1 | LTR |
| 22129587 | 22130054 | - | 27.6 | 3424 | 3944 | -2275 | L1MEc | L1 | LINE |
| 22130073 | 22130445 | - | 18.4 | 40 | 417 | -28 | MER65A | ERV1 | LTR |
| 22130496 | 22130652 | - | 12.7 | 5994 | 6151 | 0 | L1PB1 | L1 | LINE |
| 22130719 | 22131449 | - | 27.1 | 2594 | 3374 | -2845 | L1MEc | L1 | LINE |
| 22131465 | 22132019 | - | 24.0 | 2059 | 2535 | -3684 | L1MEc | L1 | LINE |
| 22132045 | 22132095 | - | 17.6 | 813 | 865 | 0 | MER6 | MER2\_type | DNA |
| 22132096 | 22132597 | - | 2.0 | 4497 | 4999 | -1147 | L1PA3 | L1 | LINE |
| 22132596 | 22133765 | + | 3.2 | 4985 | 6153 | -2 | L1PA3 | L1 | LINE |
| 22133766 | 22133940 | + | 24.1 | 39 | 210 | 0 | MER6B | MER2\_type | DNA |
| 22133999 | 22134432 | - | 17.4 | 2 | 434 | 0 | Tigger2a | MER2\_type | DNA |
| 22134520 | 22134797 | - | 23.2 | 1677 | 1980 | -4420 | L1MEc | L1 | LINE |
| 22135213 | 22135372 | - | 29.1 | 1034 | 1198 | -5011 | L1MEc | L1 | LINE |

  

|  |  |  |  |  |  |  |  |  |  |
| --- | --- | --- | --- | --- | --- | --- | --- | --- | --- |
| chr21:22565704-22572386 | | | 6053 bp | | |  |  |  |  |
| Genome Start | Genome End | Strand | Div | Repeat Start | Repeat End | Repeat Left | Name | Family | Class |
| 22565704 | 22565981 | - | 28.1 | 5967 | 6247 | -2023 | PRIMA4-int | ERV1 | LTR |
| 22565988 | 22566800 | - | 7.2 | 3908 | 4709 | -1437 | L1PA6 | L1 | LINE |
| 22566795 | 22568222 | + | 6.9 | 4723 | 6154 | 0 | L1PA6 | L1 | LINE |
| 22568257 | 22570410 | - | 32.8 | 3734 | 5935 | -2335 | PRIMA4-int | ERV1 | LTR |
| 22570411 | 22570767 | + | 19.5 | 1 | 339 | 0 | MER1B | MER1\_type | DNA |
| 22570768 | 22571273 | - | 32.8 | 3233 | 3733 | -4537 | PRIMA4-int | ERV1 | LTR |
| 22571439 | 22571762 | - | 32.6 | 967 | 5606 | -1033 | PRIMA4-int | ERV1 | LTR |
| 22572194 | 22572386 | - | 30.2 | 1721 | 1903 | -6367 | PRIMA4-int | ERV1 | LTR |

  

|  |  |  |  |  |  |  |  |  |  |
| --- | --- | --- | --- | --- | --- | --- | --- | --- | --- |
| chr21:24580969-24582069 | | | 1003 bp | | |  |  |  |  |
| Genome Start | Genome End | Strand | Div | Repeat Start | Repeat End | Repeat Left | Name | Family | Class |
| 24580969 | 24581285 | - | 25.7 | 37 | 363 | -2 | MLT1A0 | MaLR | LTR |
| 24581287 | 24581467 | - | 14.9 | 6125 | 6305 | 0 | L1MA3 | L1 | LINE |
| 24581468 | 24581546 | + | 15.2 | 6045 | 6126 | -179 | L1MA3 | L1 | LINE |
| 24581535 | 24581918 | - | 4.4 | 3635 | 4017 | -2129 | L1P1 | L1 | LINE |
| 24582028 | 24582069 | - | 16.7 | 5 | 46 | -328 | MLT1A0 | MaLR | LTR |

  

|  |  |  |  |  |  |  |  |  |  |
| --- | --- | --- | --- | --- | --- | --- | --- | --- | --- |
| chr21:26948422-26951987 | | | 3554 bp | | |  |  |  |  |
| Genome Start | Genome End | Strand | Div | Repeat Start | Repeat End | Repeat Left | Name | Family | Class |
| 26948422 | 26948490 | + | 14.3 | 1 | 63 | -2655 | Tigger2 | MER2\_type | DNA |
| 26948503 | 26949308 | - | 3.6 | 5338 | 6147 | -7 | L1PA5 | L1 | LINE |
| 26949309 | 26949391 | + | 3.6 | 5279 | 5361 | -793 | L1P | L1 | LINE |
| 26949392 | 26951987 | + | 14.4 | 47 | 2718 | 0 | Tigger2 | MER2\_type | DNA |

  

|  |  |  |  |  |  |  |  |  |  |
| --- | --- | --- | --- | --- | --- | --- | --- | --- | --- |
| chr21:27814904-27817416 | | | 2500 bp | | |  |  |  |  |
| Genome Start | Genome End | Strand | Div | Repeat Start | Repeat End | Repeat Left | Name | Family | Class |
| 27814904 | 27815010 | + | 16.0 | 1 | 106 | -358 | MLT2C2 | ERVL | LTR |
| 27814947 | 27815020 | + | 24.6 | 65 | 139 | -485 | MLT1E3 | MaLR | LTR |
| 27815021 | 27816296 | - | 7.3 | 4315 | 5595 | -559 | L1PA6 | L1 | LINE |
| 27816297 | 27816833 | + | 7.8 | 5605 | 6141 | -13 | L1PA6 | L1 | LINE |
| 27816889 | 27816941 | + | 20.2 | 97 | 150 | -314 | MLT2C2 | ERVL | LTR |
| 27816942 | 27817211 | + | 11.2 | 1 | 280 | -29 | AluSc | Alu | SINE |
| 27817234 | 27817416 | + | 20.2 | 151 | 351 | -113 | MLT2C2 | ERVL | LTR |

  

|  |  |  |  |  |  |  |  |  |  |
| --- | --- | --- | --- | --- | --- | --- | --- | --- | --- |
| chr21:28054081-28055403 | | | 1300 bp | | |  |  |  |  |
| Genome Start | Genome End | Strand | Div | Repeat Start | Repeat End | Repeat Left | Name | Family | Class |
| 28054081 | 28054491 | + | 27.3 | 1 | 531 | -242 | LTR41 | ERVL | LTR |
| 28054513 | 28054740 | - | 9.4 | 5961 | 6174 | 0 | L1PA11 | L1 | LINE |
| 28054742 | 28055123 | + | 8.1 | 5582 | 5962 | -212 | L1PA11 | L1 | LINE |
| 28055125 | 28055403 | + | 23.7 | 526 | 795 | 0 | LTR41 | ERVL | LTR |

  

|  |  |  |  |  |  |  |  |  |  |
| --- | --- | --- | --- | --- | --- | --- | --- | --- | --- |
| chr21:29713666-29716930 | | | 3154 bp | | |  |  |  |  |
| Genome Start | Genome End | Strand | Div | Repeat Start | Repeat End | Repeat Left | Name | Family | Class |
| 29713666 | 29714209 | - | 20.4 | 5592 | 6157 | -85 | L1MD1 | L1 | LINE |
| 29714291 | 29715487 | - | 1.3 | 4959 | 6155 | 0 | L1PA2 | L1 | LINE |
| 29715482 | 29716736 | + | 0.7 | 3689 | 4943 | -1203 | L1PA2 | L1 | LINE |
| 29716773 | 29716930 | - | 19.5 | 5439 | 5593 | -766 | L1MD1 | L1 | LINE |

  

|  |  |  |  |  |  |  |  |  |  |
| --- | --- | --- | --- | --- | --- | --- | --- | --- | --- |
| chr21:31223940-31226036 | | | 2092 bp | | |  |  |  |  |
| Genome Start | Genome End | Strand | Div | Repeat Start | Repeat End | Repeat Left | Name | Family | Class |
| 31223940 | 31224538 | - | 13.0 | 582 | 1236 | -66 | HSMAR2 | Mariner | DNA |
| 31224539 | 31224768 | - | 2.1 | 5218 | 5451 | -704 | L1PA3 | L1 | LINE |
| 31224769 | 31225468 | + | 1.1 | 5455 | 6155 | 0 | L1PA3 | L1 | LINE |
| 31225474 | 31226036 | - | 14.6 | 4 | 595 | -707 | HSMAR2 | Mariner | DNA |

  

|  |  |  |  |  |  |  |  |  |  |
| --- | --- | --- | --- | --- | --- | --- | --- | --- | --- |
| chr21:32332557-32336704 | | | 4020 bp | | |  |  |  |  |
| Genome Start | Genome End | Strand | Div | Repeat Start | Repeat End | Repeat Left | Name | Family | Class |
| 32332557 | 32332742 | + | 25.1 | 2645 | 2890 | -529 | L2 | L2 | LINE |
| 32332840 | 32334919 | - | 7.0 | 4060 | 6172 | 0 | L1PA8 | L1 | LINE |
| 32334912 | 32336538 | + | 8.5 | 2403 | 4046 | -2100 | L1PA8 | L1 | LINE |
| 32336578 | 32336704 | + | 30.1 | 3251 | 3417 | -2 | L2 | L2 | LINE |

  

|  |  |  |  |  |  |  |  |  |  |
| --- | --- | --- | --- | --- | --- | --- | --- | --- | --- |
| chr21:35711900-35712573 | | | 672 bp | | |  |  |  |  |
| Genome Start | Genome End | Strand | Div | Repeat Start | Repeat End | Repeat Left | Name | Family | Class |
| 35711900 | 35711967 | + | 16.2 | 2 | 75 | -114 | MER5A | MER1\_type | DNA |
| 35711969 | 35712306 | - | 11.6 | 5627 | 5966 | -202 | L1PA13 | L1 | LINE |
| 35712307 | 35712485 | + | 11.7 | 5979 | 6163 | 0 | L1PA13 | L1 | LINE |
| 35712487 | 35712573 | + | 18.6 | 61 | 151 | -38 | MER5A | MER1\_type | DNA |

  

|  |  |  |  |  |  |  |  |  |  |
| --- | --- | --- | --- | --- | --- | --- | --- | --- | --- |
| chr21:36087348-36090188 | | | 2727 bp | | |  |  |  |  |
| Genome Start | Genome End | Strand | Div | Repeat Start | Repeat End | Repeat Left | Name | Family | Class |
| 36087348 | 36087465 | - | 30.4 | 2027 | 2151 | -4088 | L1MEd | L1 | LINE |
| 36087466 | 36087772 | - | 10.4 | 1 | 307 | -6 | AluSq | Alu | SINE |
| 36087773 | 36088300 | - | 30.4 | 1469 | 2026 | -4213 | L1MEd | L1 | LINE |
| 36088303 | 36088608 | - | 7.5 | 5864 | 6170 | -2 | L1PA8 | L1 | LINE |
| 36088610 | 36088925 | + | 4.4 | 5543 | 5858 | -314 | L1PA8 | L1 | LINE |
| 36088924 | 36089038 | - | 32.0 | 1337 | 1443 | -4872 | L1MEd | L1 | LINE |
| 36089039 | 36089322 | - | 14.8 | 1 | 291 | -21 | AluJb | Alu | SINE |
| 36089323 | 36089488 | - | 25.7 | 1162 | 1336 | -4979 | L1MEd | L1 | LINE |
| 36089509 | 36089720 | - | 27.8 | 867 | 1090 | -5119 | L1MEd | L1 | LINE |
| 36089721 | 36089944 | - | 17.6 | 84 | 306 | -6 | AluJo | Alu | SINE |
| 36090030 | 36090141 | + | 18.8 | 36 | 151 | -25 | FRAM | Alu | SINE |
| 36090150 | 36090188 | - | 15.4 | 804 | 842 | -5558 | L1MEd | L1 | LINE |

  

|  |  |  |  |  |  |  |  |  |  |
| --- | --- | --- | --- | --- | --- | --- | --- | --- | --- |
| chr21:36189354-36190045 | | | 677 bp | | |  |  |  |  |
| Genome Start | Genome End | Strand | Div | Repeat Start | Repeat End | Repeat Left | Name | Family | Class |
| 36189354 | 36189472 | + | 19.5 | 2 | 126 | -264 | MLT1C | MaLR | LTR |
| 36189474 | 36189554 | - | 6.2 | 5889 | 5969 | -182 | L1PB1 | L1 | LINE |
| 36189558 | 36189721 | + | 14.0 | 5988 | 6151 | 0 | L1PB1 | L1 | LINE |
| 36189733 | 36190045 | + | 21.6 | 112 | 463 | -4 | MLT1C | MaLR | LTR |

  

|  |  |  |  |  |  |  |  |  |  |
| --- | --- | --- | --- | --- | --- | --- | --- | --- | --- |
| chr21:40079005-40080958 | | | 1968 bp | | |  |  |  |  |
| Genome Start | Genome End | Strand | Div | Repeat Start | Repeat End | Repeat Left | Name | Family | Class |
| 40079005 | 40079092 | + | 6.8 | 5034 | 5121 | -1025 | L1P1 | L1 | LINE |
| 40079088 | 40079683 | - | 2.0 | 4324 | 4919 | -1227 | L1PA3 | L1 | LINE |
| 40079678 | 40080913 | + | 2.9 | 4915 | 6155 | 0 | L1PA3 | L1 | LINE |
| 40080911 | 40080958 | + | 2.1 | 5106 | 5153 | -993 | L1P1 | L1 | LINE |

  


|  |  |  |  |  |  |  |  |  |  |
| --- | --- | --- | --- | --- | --- | --- | --- | --- | --- |
| chr22:22918431-22922014 | | | 3368 bp | | |  |  |  |  |
| Genome Start | Genome End | Strand | Div | Repeat Start | Repeat End | Repeat Left | Name | Family | Class |
| 22918431 | 22918629 | - | 20.2 | 439 | 644 | 0 | LTR48B | ERV1 | LTR |
| 22918636 | 22918740 | + | 28.8 | 3110 | 3220 | -158 | L2 | L2 | LINE |
| 22918861 | 22919163 | - | 11.2 | 1 | 304 | -8 | AluSx | Alu | SINE |
| 22919203 | 22920020 | - | 1.6 | 4003 | 4820 | -1326 | L1PA3 | L1 | LINE |
| 22920021 | 22921356 | + | 1.8 | 4811 | 6155 | 0 | L1PA3 | L1 | LINE |
| 22921372 | 22921681 | + | 12.6 | 1 | 309 | 0 | AluSc | Alu | SINE |
| 22921718 | 22922014 | - | 23.2 | 1 | 336 | -308 | LTR48B | ERV1 | LTR |

  

|  |  |  |  |  |  |  |  |  |  |
| --- | --- | --- | --- | --- | --- | --- | --- | --- | --- |
| chr22:25596936-25604085 | | | 6837 bp | | |  |  |  |  |
| Genome Start | Genome End | Strand | Div | Repeat Start | Repeat End | Repeat Left | Name | Family | Class |
| 25596936 | 25598069 | - | 27.3 | 4842 | 6024 | -150 | L1ME3B | L1 | LINE |
| 25598081 | 25598567 | - | 24.1 | 3958 | 4426 | -1654 | L1ME3B | L1 | LINE |
| 25598568 | 25598854 | + | 8.7 | 1 | 286 | -24 | AluSg | Alu | SINE |
| 25598855 | 25600603 | - | 24.1 | 2070 | 3961 | -2119 | L1ME3B | L1 | LINE |
| 25600674 | 25602048 | - | 25.3 | 2949 | 4492 | -1654 | L1M5 | L1 | LINE |
| 25602055 | 25602616 | - | 24.7 | 5639 | 6161 | -5 | L1ME1 | L1 | LINE |
| 25602617 | 25602740 | + | 18.0 | 1 | 126 | -430 | MLT2B4 | ERVL | LTR |
| 25602741 | 25603134 | - | 16.8 | 1 | 424 | -2 | MSTB | MaLR | LTR |
| 25603135 | 25603482 | + | 19.1 | 127 | 514 | -1 | MLT2B4 | ERVL | LTR |
| 25603483 | 25603750 | - | 22.3 | 5377 | 5643 | -523 | L1ME1 | L1 | LINE |
| 25603751 | 25603794 | + | 9.3 | 5350 | 5392 | -786 | L1M | L1 | LINE |
| 25604021 | 25604085 | - | 18.5 | 46 | 116 | -5954 | L1ME3B | L1 | LINE |

  

|  |  |  |  |  |  |  |  |  |  |
| --- | --- | --- | --- | --- | --- | --- | --- | --- | --- |
| chr22:32103672-32106162 | | | 2148 bp | | |  |  |  |  |
| Genome Start | Genome End | Strand | Div | Repeat Start | Repeat End | Repeat Left | Name | Family | Class |
| 32103672 | 32104472 | - | 29.5 | 279 | 1150 | -4969 | L1MEd | L1 | LINE |
| 32104595 | 32105104 | - | 20.4 | 392 | 935 | 0 | MER21C | ERV1 | LTR |
| 32105250 | 32105467 | - | 5.5 | 5936 | 6154 | 0 | L1PA5 | L1 | LINE |
| 32105468 | 32105724 | + | 5.1 | 5671 | 5927 | -227 | L1PA5 | L1 | LINE |
| 32105725 | 32105955 | - | 14.7 | 3 | 307 | -628 | MER21C | ERV1 | LTR |
| 32106032 | 32106162 | - | 27.8 | 55 | 187 | -5932 | L1MEd | L1 | LINE |

  

|  |  |  |  |  |  |  |  |  |  |
| --- | --- | --- | --- | --- | --- | --- | --- | --- | --- |
| chr22:32124584-32125896 | | | 1293 bp | | |  |  |  |  |
| Genome Start | Genome End | Strand | Div | Repeat Start | Repeat End | Repeat Left | Name | Family | Class |
| 32124584 | 32124970 | + | 22.9 | 3 | 417 | -50 | MLT1C | MaLR | LTR |
| 32124977 | 32125508 | - | 6.2 | 5618 | 6157 | -15 | L1PA8 | L1 | LINE |
| 32125509 | 32125557 | + | 6.1 | 5563 | 5611 | -561 | L1PA8 | L1 | LINE |
| 32125572 | 32125833 | - | 5.3 | 1 | 262 | -49 | AluY | Alu | SINE |
| 32125834 | 32125896 | + | 16.1 | 402 | 465 | -2 | MLT1C | MaLR | LTR |

  

|  |  |  |  |  |  |  |  |  |  |
| --- | --- | --- | --- | --- | --- | --- | --- | --- | --- |
| chr22:33270924-33275414 | | | 4137 bp | | |  |  |  |  |
| Genome Start | Genome End | Strand | Div | Repeat Start | Repeat End | Repeat Left | Name | Family | Class |
| 33270924 | 33271218 | - | 29.1 | 5347 | 5660 | -580 | L1M5 | L1 | LINE |
| 33271248 | 33271531 | - | 10.9 | 7 | 312 | -1 | AluSq | Alu | SINE |
| 33271700 | 33271953 | + | 12.2 | 2650 | 2933 | -3213 | L1P3 | L1 | LINE |
| 33271952 | 33273089 | - | 8.2 | 4112 | 5261 | -885 | L1PB | L1 | LINE |
| 33273079 | 33273606 | + | 9.3 | 5255 | 5791 | -360 | L1P5 | L1 | LINE |
| 33273639 | 33274046 | + | 13.1 | 5746 | 6151 | 0 | L1PB1 | L1 | LINE |
| 33274092 | 33274294 | + | 9.9 | 2923 | 3131 | -3015 | L1PREC2 | L1 | LINE |
| 33274307 | 33274576 | - | 32.1 | 4990 | 5282 | -864 | L1M5 | L1 | LINE |
| 33274658 | 33275414 | - | 32.1 | 4194 | 4983 | -1163 | L1M5 | L1 | LINE |

  

|  |  |  |  |  |  |  |  |  |  |
| --- | --- | --- | --- | --- | --- | --- | --- | --- | --- |
| chr22:33295164-33304275 | | | 9073 bp | | |  |  |  |  |
| Genome Start | Genome End | Strand | Div | Repeat Start | Repeat End | Repeat Left | Name | Family | Class |
| 33295164 | 33299444 | - | 19.5 | 3631 | 8195 | -8 | L1MC3 | L1 | LINE |
| 33299461 | 33300007 | - | 12.0 | 5602 | 6146 | -5 | L1PB1 | L1 | LINE |
| 33300008 | 33300551 | + | 9.9 | 5031 | 5597 | -554 | L1PB1 | L1 | LINE |
| 33300556 | 33300866 | - | 18.2 | 1 | 309 | -3 | AluJo | Alu | SINE |
| 33300873 | 33301443 | - | 21.3 | 3027 | 3642 | -2922 | L1MC3 | L1 | LINE |
| 33301450 | 33301582 | + | 20.6 | 1 | 120 | -375 | LTR42 | ERVL | LTR |
| 33301583 | 33301874 | + | 13.8 | 1 | 297 | -15 | AluJb | Alu | SINE |
| 33301875 | 33302287 | + | 20.6 | 121 | 495 | 0 | LTR42 | ERVL | LTR |
| 33302295 | 33303205 | - | 22.9 | 2116 | 3010 | -3554 | L1MC3 | L1 | LINE |
| 33303206 | 33303516 | - | 11.6 | 1 | 312 | 0 | AluSx | Alu | SINE |
| 33303517 | 33303763 | - | 25.6 | 1837 | 2115 | -4636 | L1MC3 | L1 | LINE |
| 33303764 | 33304064 | + | 8.0 | 1 | 300 | -11 | AluY | Alu | SINE |
| 33304065 | 33304275 | - | 25.6 | 1644 | 1836 | -4915 | L1MC3 | L1 | LINE |

  

|  |  |  |  |  |  |  |  |  |  |
| --- | --- | --- | --- | --- | --- | --- | --- | --- | --- |
| chr22:33331991-33340429 | | | 7262 bp | | |  |  |  |  |
| Genome Start | Genome End | Strand | Div | Repeat Start | Repeat End | Repeat Left | Name | Family | Class |
| 33331991 | 33332118 | + | 35.8 | 3083 | 3205 | -173 | L2 | L2 | LINE |
| 33332403 | 33332498 | + | 27.9 | 2288 | 2375 | -1044 | L2 | L2 | LINE |
| 33332525 | 33332766 | + | 30.0 | 4 | 262 | 0 | MIR | MIR | SINE |
| 33332860 | 33332929 | + | 28.6 | 2485 | 2557 | -862 | L2 | L2 | LINE |
| 33333322 | 33333732 | + | 38.6 | 2989 | 3374 | -4 | L2 | L2 | LINE |
| 33333753 | 33335365 | + | 9.3 | 53 | 1725 | -985 | Charlie3 | MER1\_type | DNA |
| 33335366 | 33335672 | - | 9.2 | 1 | 305 | -8 | AluSp | Alu | SINE |
| 33335673 | 33336109 | + | 9.3 | 1726 | 2161 | -549 | Charlie3 | MER1\_type | DNA |
| 33336138 | 33336427 | + | 7.7 | 2422 | 2706 | -4 | Charlie3 | MER1\_type | DNA |
| 33336466 | 33336646 | - | 31.6 | 16 | 197 | -11 | MIR3 | MIR | SINE |
| 33336677 | 33337080 | - | 27.7 | 110 | 504 | -7 | MLT1J | MaLR | LTR |
| 33337139 | 33337406 | + | 18.0 | 12 | 285 | -27 | AluJo | Alu | SINE |
| 33337603 | 33339671 | - | 3.9 | 4077 | 6154 | -1 | L1PA4 | L1 | LINE |
| 33339650 | 33340115 | + | 5.4 | 3610 | 4086 | -2060 | L1PA4 | L1 | LINE |
| 33340116 | 33340289 | + | 35.3 | 53 | 245 | -23 | MIRb | MIR | SINE |
| 33340324 | 33340429 | + | 27.9 | 3274 | 3378 | 0 | L2 | L2 | LINE |

  

|  |  |  |  |  |  |  |  |  |  |
| --- | --- | --- | --- | --- | --- | --- | --- | --- | --- |
| chr22:46922889-46924701 | | | 1796 bp | | |  |  |  |  |
| Genome Start | Genome End | Strand | Div | Repeat Start | Repeat End | Repeat Left | Name | Family | Class |
| 46922889 | 46923167 | + | 23.3 | 1 | 289 | -178 | LTR24 | ERV1 | LTR |
| 46923170 | 46923601 | - | 7.9 | 5731 | 6161 | -11 | L1PA8 | L1 | LINE |
| 46923602 | 46923821 | + | 7.8 | 5102 | 5332 | -840 | L1P3 | L1 | LINE |
| 46923822 | 46924135 | + | 10.3 | 1 | 301 | -9 | AluSg | Alu | SINE |
| 46924136 | 46924537 | + | 6.4 | 5333 | 5722 | -450 | L1P3 | L1 | LINE |
| 46924553 | 46924701 | + | 20.9 | 313 | 467 | 0 | LTR24 | ERV1 | LTR |

  


|  |  |  |  |  |  |  |  |  |  |
| --- | --- | --- | --- | --- | --- | --- | --- | --- | --- |
| chrX:3200754-3203843 | | | 3091 bp | | |  |  |  |  |
| Genome Start | Genome End | Strand | Div | Repeat Start | Repeat End | Repeat Left | Name | Family | Class |
| 3200754 | 3201119 | - | 12.0 | 5790 | 6156 | 0 | L1PB4 | L1 | LINE |
| 3201120 | 3201289 | + | 13.5 | 3 | 173 | -191 | THE1B | MaLR | LTR |
| 3201290 | 3203147 | - | 5.9 | 4287 | 6140 | -14 | L1PA6 | L1 | LINE |
| 3203147 | 3203383 | + | 5.5 | 4043 | 4286 | -1860 | L1PA6 | L1 | LINE |
| 3203384 | 3203591 | + | 9.7 | 152 | 364 | 0 | THE1B | MaLR | LTR |
| 3203592 | 3203843 | - | 12.3 | 5550 | 5796 | -360 | L1PB4 | L1 | LINE |

  

|  |  |  |  |  |  |  |  |  |  |
| --- | --- | --- | --- | --- | --- | --- | --- | --- | --- |
| chrX:3481055-3488469 | | | 7389 bp | | |  |  |  |  |
| Genome Start | Genome End | Strand | Div | Repeat Start | Repeat End | Repeat Left | Name | Family | Class |
| 3481055 | 3481181 | + | 20.5 | 2 | 114 | -442 | MLT2D | ERVL | LTR |
| 3481183 | 3481616 | - | 7.6 | 1 | 472 | 0 | LTR7Y | ERV1 | LTR |
| 3481617 | 3481991 | - | 6.4 | 7322 | 7713 | 0 | HERVH | ERV1 | LTR |
| 3481990 | 3482207 | - | 11.2 | 5600 | 5819 | -1894 | HERVH | ERV1 | LTR |
| 3482209 | 3482865 | - | 13.7 | 4488 | 5149 | -2564 | HERVH | ERV1 | LTR |
| 3482868 | 3483372 | - | 11.2 | 3492 | 3993 | -3720 | HERVH | ERV1 | LTR |
| 3483363 | 3483510 | - | 12.8 | 3140 | 3290 | -4423 | HERVH | ERV1 | LTR |
| 3483505 | 3485587 | - | 9.1 | 899 | 2987 | -4726 | HERVH | ERV1 | LTR |
| 3485588 | 3486148 | - | 1.2 | 4986 | 5550 | -605 | L1PA2 | L1 | LINE |
| 3486149 | 3486742 | + | 1.4 | 5556 | 6150 | -5 | L1PA2 | L1 | LINE |
| 3486770 | 3487768 | - | 11.9 | 1 | 903 | -6810 | HERVH | ERV1 | LTR |
| 3487769 | 3488202 | - | 8.1 | 1 | 472 | 0 | LTR7Y | ERV1 | LTR |
| 3488216 | 3488469 | + | 23.0 | 128 | 528 | -39 | MLT2D | ERVL | LTR |

  

|  |  |  |  |  |  |  |  |  |  |
| --- | --- | --- | --- | --- | --- | --- | --- | --- | --- |
| chrX:5639281-5644187 | | | 4927 bp | | |  |  |  |  |
| Genome Start | Genome End | Strand | Div | Repeat Start | Repeat End | Repeat Left | Name | Family | Class |
| 5639281 | 5640037 | + | 26.5 | 4832 | 5604 | -562 | L1ME1 | L1 | LINE |
| 5640035 | 5641683 | - | 9.3 | 2240 | 4123 | -2023 | L1PA7 | L1 | LINE |
| 5641672 | 5643686 | + | 6.9 | 4115 | 6152 | -2 | L1PA7 | L1 | LINE |
| 5643682 | 5644187 | + | 28.4 | 5590 | 6078 | -88 | L1ME1 | L1 | LINE |

  

|  |  |  |  |  |  |  |  |  |  |
| --- | --- | --- | --- | --- | --- | --- | --- | --- | --- |
| chrX:6645569-6647592 | | | 1974 bp | | |  |  |  |  |
| Genome Start | Genome End | Strand | Div | Repeat Start | Repeat End | Repeat Left | Name | Family | Class |
| 6645569 | 6645828 | + | 26.7 | 13 | 247 | -391 | MLT1E | MaLR | LTR |
| 6645838 | 6646206 | - | 12.3 | 5185 | 5558 | -598 | L1PB4 | L1 | LINE |
| 6646207 | 6646799 | + | 12.6 | 5565 | 6154 | -2 | L1PB4 | L1 | LINE |
| 6646832 | 6647117 | + | 28.6 | 206 | 483 | -110 | MLT1E | MaLR | LTR |
| 6647123 | 6647475 | - | 13.3 | 4 | 383 | -7 | MLT1B | MaLR | LTR |
| 6647480 | 6647592 | + | 17.1 | 584 | 695 | -3 | MLT1E | MaLR | LTR |

  

|  |  |  |  |  |  |  |  |  |  |
| --- | --- | --- | --- | --- | --- | --- | --- | --- | --- |
| chrX:11015007-11019202 | | | 4178 bp | | |  |  |  |  |
| Genome Start | Genome End | Strand | Div | Repeat Start | Repeat End | Repeat Left | Name | Family | Class |
| 11015007 | 11015476 | - | 18.5 | 2072 | 2468 | -3932 | L1MEc | L1 | LINE |
| 11015477 | 11016293 | - | 8.2 | 5342 | 6161 | 0 | L1PA14 | L1 | LINE |
| 11016287 | 11017225 | + | 7.7 | 4389 | 5330 | -816 | L1PREC2 | L1 | LINE |
| 11017229 | 11017366 | - | 19.1 | 1952 | 2081 | -4319 | L1MEc | L1 | LINE |
| 11017367 | 11017662 | - | 8.2 | 5857 | 6152 | -2 | L1PA7 | L1 | LINE |
| 11017663 | 11017952 | - | 19.1 | 1645 | 1951 | -4449 | L1MEc | L1 | LINE |
| 11017980 | 11018565 | - | 19.1 | 984 | 1644 | -4756 | L1MEc | L1 | LINE |
| 11018561 | 11019202 | - | 23.3 | 36 | 709 | -5691 | L1MEc | L1 | LINE |

  

|  |  |  |  |  |  |  |  |  |  |
| --- | --- | --- | --- | --- | --- | --- | --- | --- | --- |
| chrX:14080365-14086924 | | | 6215 bp | | |  |  |  |  |
| Genome Start | Genome End | Strand | Div | Repeat Start | Repeat End | Repeat Left | Name | Family | Class |
| 14080365 | 14080761 | + | 35.7 | 20 | 444 | -5956 | L1ME2 | L1 | LINE |
| 14081029 | 14081514 | + | 29.7 | 694 | 1209 | -5000 | L1ME2 | L1 | LINE |
| 14081571 | 14082769 | - | 14.7 | 5085 | 6288 | -3 | L1MA8 | L1 | LINE |
| 14082784 | 14083364 | + | 27.2 | 1252 | 1958 | -4442 | L1ME2 | L1 | LINE |
| 14083346 | 14083427 | + | 23.8 | 4572 | 4656 | -1563 | L1ME2 | L1 | LINE |
| 14083428 | 14083857 | + | 14.4 | 1 | 428 | 0 | MSTC | MaLR | LTR |
| 14083858 | 14084278 | + | 23.8 | 4657 | 5100 | -1119 | L1ME2 | L1 | LINE |
| 14084278 | 14084995 | - | 5.8 | 4635 | 5359 | -787 | L1PA4 | L1 | LINE |
| 14084994 | 14085786 | + | 3.0 | 5364 | 6154 | -1 | L1PA4 | L1 | LINE |
| 14085817 | 14086924 | + | 24.4 | 5089 | 6237 | 0 | L1ME2 | L1 | LINE |

  

|  |  |  |  |  |  |  |  |  |  |
| --- | --- | --- | --- | --- | --- | --- | --- | --- | --- |
| chrX:14125139-14136907 | | | 11756 bp | | |  |  |  |  |
| Genome Start | Genome End | Strand | Div | Repeat Start | Repeat End | Repeat Left | Name | Family | Class |
| 14125139 | 14125815 | - | 15.3 | 5469 | 6150 | -16 | L1PA17 | L1 | LINE |
| 14125803 | 14126057 | - | 5.9 | 5896 | 6148 | -6 | L1PA5 | L1 | LINE |
| 14126068 | 14126558 | - | 3.9 | 5662 | 6152 | -3 | L1PA4 | L1 | LINE |
| 14126560 | 14127420 | - | 18.0 | 2418 | 3269 | -3580 | L1PA15-16 | L1 | LINE |
| 14127421 | 14127685 | + | 8.7 | 5898 | 6168 | 0 | L1PA10 | L1 | LINE |
| 14127686 | 14128008 | - | 17.9 | 2130 | 2417 | -4436 | L1PA15-16 | L1 | LINE |
| 14128014 | 14128238 | - | 3.6 | 5930 | 6154 | -1 | L1PA3 | L1 | LINE |
| 14128239 | 14128483 | - | 2.5 | 5685 | 5929 | -226 | L1PA3 | L1 | LINE |
| 14128484 | 14128534 | + | 0.0 | 5651 | 5701 | -454 | L1PA3 | L1 | LINE |
| 14128531 | 14128742 | - | 15.3 | 1666 | 1877 | -4700 | L1PA17 | L1 | LINE |
| 14128745 | 14133596 | - | 5.8 | 1296 | 6153 | -1 | L1PA5 | L1 | LINE |
| 14133597 | 14133931 | + | 5.7 | 734 | 1113 | -1162 | L1P2 | L1 | LINE |
| 14133933 | 14135040 | - | 10.1 | 1 | 1174 | -1101 | L1PA5 | L1 | LINE |
| 14135052 | 14136907 | - | 19.1 | 14 | 1734 | -5139 | L1PA17 | L1 | LINE |

  

|  |  |  |  |  |  |  |  |  |  |
| --- | --- | --- | --- | --- | --- | --- | --- | --- | --- |
| chrX:15207788-15209648 | | | 1788 bp | | |  |  |  |  |
| Genome Start | Genome End | Strand | Div | Repeat Start | Repeat End | Repeat Left | Name | Family | Class |
| 15207788 | 15207957 | - | 18.4 | 150 | 323 | -1 | MER33 | MER1\_type | DNA |
| 15207961 | 15208624 | - | 14.9 | 5617 | 6299 | -1 | L1MA5 | L1 | LINE |
| 15208625 | 15208947 | + | 10.3 | 5287 | 5611 | -689 | L1MA5 | L1 | LINE |
| 15209004 | 15209262 | + | 11.6 | 49 | 306 | -7 | AluSq | Alu | SINE |
| 15209270 | 15209407 | - | 18.8 | 3 | 142 | -182 | MER33 | MER1\_type | DNA |
| 15209408 | 15209501 | + | 19.6 | 211 | 302 | -11 | AluSp/q | Alu | SINE |
| 15209509 | 15209648 | - | 22.1 | 1 | 143 | -181 | MER33 | MER1\_type | DNA |

  

|  |  |  |  |  |  |  |  |  |  |
| --- | --- | --- | --- | --- | --- | --- | --- | --- | --- |
| chrX:19157692-19160035 | | | 2341 bp | | |  |  |  |  |
| Genome Start | Genome End | Strand | Div | Repeat Start | Repeat End | Repeat Left | Name | Family | Class |
| 19157692 | 19157800 | + | 21.1 | 1 | 113 | -47 | MER5A1 | MER1\_type | DNA |
| 19157807 | 19159238 | - | 5.6 | 4698 | 6141 | -13 | L1PA7 | L1 | LINE |
| 19159235 | 19159979 | + | 5.9 | 3956 | 4700 | -1446 | L1PA7 | L1 | LINE |
| 19159981 | 19160035 | + | 21.8 | 103 | 158 | -2 | MER5A1 | MER1\_type | DNA |

  

|  |  |  |  |  |  |  |  |  |  |
| --- | --- | --- | --- | --- | --- | --- | --- | --- | --- |
| chrX:20467881-20469277 | | | 1390 bp | | |  |  |  |  |
| Genome Start | Genome End | Strand | Div | Repeat Start | Repeat End | Repeat Left | Name | Family | Class |
| 20467881 | 20468047 | - | 7.8 | 6139 | 6305 | 0 | L1MA3 | L1 | LINE |
| 20468057 | 20468253 | - | 4.6 | 5955 | 6151 | -3 | L1PA6 | L1 | LINE |
| 20468254 | 20468613 | + | 2.8 | 5582 | 5942 | -212 | L1PA6 | L1 | LINE |
| 20468612 | 20469277 | - | 8.4 | 5487 | 6148 | -157 | L1MA3 | L1 | LINE |

  

|  |  |  |  |  |  |  |  |  |  |
| --- | --- | --- | --- | --- | --- | --- | --- | --- | --- |
| chrX:20479594-20484700 | | | 4638 bp | | |  |  |  |  |
| Genome Start | Genome End | Strand | Div | Repeat Start | Repeat End | Repeat Left | Name | Family | Class |
| 20479594 | 20479701 | + | 28.0 | 2248 | 2357 | -3789 | L1ME3B | L1 | LINE |
| 20479737 | 20480410 | + | 32.0 | 2423 | 3144 | -3002 | L1ME3B | L1 | LINE |
| 20480418 | 20480994 | - | 16.3 | 1 | 554 | 0 | MLT2A2 | ERVL | LTR |
| 20481088 | 20481294 | - | 11.8 | 5824 | 6034 | -278 | L1MA9 | L1 | LINE |
| 20481268 | 20481422 | + | 13.0 | 6010 | 6163 | -149 | L1MA9 | L1 | LINE |
| 20481404 | 20481650 | - | 27.8 | 5292 | 5544 | -2264 | L1MC | L1 | LINE |
| 20481661 | 20481768 | - | 32.4 | 5115 | 5226 | -920 | L1M5 | L1 | LINE |
| 20481786 | 20481919 | + | 13.4 | 6169 | 6302 | -10 | L1MA9 | L1 | LINE |
| 20481987 | 20482196 | + | 25.4 | 3513 | 3740 | -2406 | L1ME3B | L1 | LINE |
| 20482193 | 20482705 | + | 29.9 | 3869 | 4426 | -1717 | L1M2 | L1 | LINE |
| 20482777 | 20482990 | + | 23.4 | 3 | 221 | -3 | MER58A | MER1\_type | DNA |
| 20483130 | 20483200 | + | 23.9 | 211 | 281 | -60 | MER58 | MER1\_type | DNA |
| 20483228 | 20483461 | + | 5.6 | 5940 | 6172 | 0 | L1PA8 | L1 | LINE |
| 20483515 | 20484700 | + | 32.6 | 4616 | 5889 | -351 | L1ME3B | L1 | LINE |

  

|  |  |  |  |  |  |  |  |  |  |
| --- | --- | --- | --- | --- | --- | --- | --- | --- | --- |
| chrX:20542983-20544743 | | | 1748 bp | | |  |  |  |  |
| Genome Start | Genome End | Strand | Div | Repeat Start | Repeat End | Repeat Left | Name | Family | Class |
| 20542983 | 20543144 | - | 23.9 | 3264 | 3419 | 0 | L2 | L2 | LINE |
| 20543148 | 20543346 | - | 10.1 | 5506 | 5703 | -601 | L1MA3 | L1 | LINE |
| 20543347 | 20543938 | + | 12.5 | 5702 | 6305 | 0 | L1MA3 | L1 | LINE |
| 20543949 | 20544066 | - | 30.3 | 3164 | 3271 | -148 | L2 | L2 | LINE |
| 20544067 | 20544427 | - | 19.4 | 1 | 386 | -4 | MLT1B | MaLR | LTR |
| 20544428 | 20544743 | - | 28.2 | 2853 | 3163 | -256 | L2 | L2 | LINE |

  

|  |  |  |  |  |  |  |  |  |  |
| --- | --- | --- | --- | --- | --- | --- | --- | --- | --- |
| chrX:21190564-21193130 | | | 2572 bp | | |  |  |  |  |
| Genome Start | Genome End | Strand | Div | Repeat Start | Repeat End | Repeat Left | Name | Family | Class |
| 21190564 | 21191237 | - | 7.0 | 5476 | 6153 | -1 | L1PA7 | L1 | LINE |
| 21191238 | 21192333 | - | 3.1 | 5067 | 6155 | 0 | L1PA3 | L1 | LINE |
| 21192331 | 21193026 | + | 2.7 | 4384 | 5079 | -1067 | L1PA3 | L1 | LINE |
| 21193025 | 21193130 | - | 4.7 | 5386 | 5491 | -681 | L1PA7 | L1 | LINE |

  

|  |  |  |  |  |  |  |  |  |  |
| --- | --- | --- | --- | --- | --- | --- | --- | --- | --- |
| chrX:25726915-25736900 | | | 9987 bp | | |  |  |  |  |
| Genome Start | Genome End | Strand | Div | Repeat Start | Repeat End | Repeat Left | Name | Family | Class |
| 25726915 | 25731136 | - | 15.3 | 3604 | 7724 | 0 | L1MA3 | L1 | LINE |
| 25731138 | 25731731 | - | 7.0 | 5563 | 6154 | 0 | L1PA7 | L1 | LINE |
| 25731732 | 25732083 | + | 4.4 | 5199 | 5561 | -593 | L1PA7 | L1 | LINE |
| 25732082 | 25732863 | - | 14.6 | 2832 | 3609 | -3956 | L1MA3 | L1 | LINE |
| 25732864 | 25734176 | - | 7.1 | 1 | 1254 | 0 | LTR12D | ERV1 | LTR |
| 25734177 | 25736900 | - | 14.6 | 15 | 2831 | -4734 | L1MA3 | L1 | LINE |

  

|  |  |  |  |  |  |  |  |  |  |
| --- | --- | --- | --- | --- | --- | --- | --- | --- | --- |
| chrX:25914890-25919190 | | | 4297 bp | | |  |  |  |  |
| Genome Start | Genome End | Strand | Div | Repeat Start | Repeat End | Repeat Left | Name | Family | Class |
| 25914890 | 25916303 | + | 12.8 | 1 | 1450 | -968 | Tigger1 | MER2\_type | DNA |
| 25916305 | 25916750 | - | 3.4 | 5722 | 6167 | 0 | L1PA3 | L1 | LINE |
| 25916754 | 25917999 | + | 2.2 | 4480 | 5725 | -430 | L1PA3 | L1 | LINE |
| 25918000 | 25918386 | + | 12.4 | 1435 | 1827 | -591 | Tigger1 | MER2\_type | DNA |
| 25918387 | 25918627 | + | 10.0 | 65 | 305 | -4 | AluSc | Alu | SINE |
| 25918628 | 25919190 | + | 12.4 | 1828 | 2417 | -1 | Tigger1 | MER2\_type | DNA |

  

|  |  |  |  |  |  |  |  |  |  |
| --- | --- | --- | --- | --- | --- | --- | --- | --- | --- |
| chrX:26452645-26458435 | | | 5769 bp | | |  |  |  |  |
| Genome Start | Genome End | Strand | Div | Repeat Start | Repeat End | Repeat Left | Name | Family | Class |
| 26452645 | 26452899 | + | 14.1 | 1 | 259 | -2159 | Tigger1 | MER2\_type | DNA |
| 26452900 | 26453229 | - | 9.1 | 1 | 364 | 0 | THE1B | MaLR | LTR |
| 26453230 | 26453504 | + | 14.1 | 260 | 529 | -1889 | Tigger1 | MER2\_type | DNA |
| 26453505 | 26454478 | - | 13.1 | 1 | 1002 | 0 | LTR5B | ERVK | LTR |
| 26454479 | 26455338 | + | 14.1 | 530 | 1390 | -1028 | Tigger1 | MER2\_type | DNA |
| 26455367 | 26456654 | - | 9.6 | 4848 | 6163 | 0 | L1PA13 | L1 | LINE |
| 26456649 | 26457427 | + | 9.3 | 4049 | 4829 | -1317 | L1PREC2 | L1 | LINE |
| 26457428 | 26458435 | + | 15.2 | 1391 | 2418 | 0 | Tigger1 | MER2\_type | DNA |

  

|  |  |  |  |  |  |  |  |  |  |
| --- | --- | --- | --- | --- | --- | --- | --- | --- | --- |
| chrX:26950813-26952614 | | | 1771 bp | | |  |  |  |  |
| Genome Start | Genome End | Strand | Div | Repeat Start | Repeat End | Repeat Left | Name | Family | Class |
| 26950813 | 26951065 | - | 25.8 | 126 | 374 | 0 | MLT1A | MaLR | LTR |
| 26951068 | 26951702 | - | 3.1 | 4719 | 5403 | -743 | L1PA3 | L1 | LINE |
| 26951703 | 26952439 | + | 2.9 | 5419 | 6155 | 0 | L1PA3 | L1 | LINE |
| 26952469 | 26952614 | - | 16.4 | 2 | 152 | -222 | MLT1A | MaLR | LTR |

  

|  |  |  |  |  |  |  |  |  |  |
| --- | --- | --- | --- | --- | --- | --- | --- | --- | --- |
| chrX:26988246-26992532 | | | 3585 bp | | |  |  |  |  |
| Genome Start | Genome End | Strand | Div | Repeat Start | Repeat End | Repeat Left | Name | Family | Class |
| 26988246 | 26988289 | + | 31.8 | 1939 | 1982 | -1437 | L2 | L2 | LINE |
| 26988351 | 26988496 | + | 35.7 | 2489 | 2639 | -780 | L2 | L2 | LINE |
| 26988498 | 26988696 | - | 21.0 | 157 | 379 | 0 | MLT1B | MaLR | LTR |
| 26988698 | 26989291 | - | 16.2 | 5555 | 6156 | 0 | L1PB4 | L1 | LINE |
| 26989294 | 26989722 | + | 14.6 | 5099 | 5536 | -620 | L1PB4 | L1 | LINE |
| 26989728 | 26989885 | - | 21.7 | 1 | 161 | -213 | MLT1B | MaLR | LTR |
| 26990236 | 26990402 | - | 20.4 | 1 | 45 | -4 | FAM | Alu | SINE |
| 26990492 | 26990509 | + | 27.1 | 5327 | 5344 | -802 | L1MC | L1 | LINE |
| 26990510 | 26990813 | - | 14.1 | 5863 | 6168 | 0 | L1MB1 | L1 | LINE |
| 26990814 | 26991318 | + | 15.1 | 5345 | 5858 | -310 | L1MB1 | L1 | LINE |
| 26991332 | 26991422 | + | 39.7 | 2799 | 2875 | -544 | L2 | L2 | LINE |
| 26991423 | 26991745 | - | 8.6 | 1 | 292 | -18 | AluSg | Alu | SINE |
| 26991746 | 26992018 | + | 38.1 | 2876 | 3163 | -256 | L2 | L2 | LINE |
| 26992181 | 26992314 | + | 33.6 | 3268 | 3417 | -2 | L2 | L2 | LINE |
| 26992333 | 26992532 | + | 40.1 | 2481 | 2682 | -737 | L2 | L2 | LINE |

  

|  |  |  |  |  |  |  |  |  |  |
| --- | --- | --- | --- | --- | --- | --- | --- | --- | --- |
| chrX:27122066-27124716 | | | 2238 bp | | |  |  |  |  |
| Genome Start | Genome End | Strand | Div | Repeat Start | Repeat End | Repeat Left | Name | Family | Class |
| 27122066 | 27122276 | + | 27.4 | 2111 | 2332 | -3883 | L1MEe | L1 | LINE |
| 27122436 | 27123133 | - | 12.6 | 5004 | 5708 | -595 | L1MA4A | L1 | LINE |
| 27123134 | 27123722 | + | 13.1 | 5704 | 6297 | -6 | L1MA4A | L1 | LINE |
| 27123923 | 27124025 | + | 13.6 | 6056 | 6158 | -10 | L1PA10 | L1 | LINE |
| 27124047 | 27124504 | - | 33.4 | 3 | 491 | 0 | LTR16C | ERVL | LTR |
| 27124538 | 27124716 | + | 34.1 | 2758 | 2935 | -3279 | L1MEe | L1 | LINE |

  

|  |  |  |  |  |  |  |  |  |  |
| --- | --- | --- | --- | --- | --- | --- | --- | --- | --- |
| chrX:28336871-28338812 | | | 1738 bp | | |  |  |  |  |
| Genome Start | Genome End | Strand | Div | Repeat Start | Repeat End | Repeat Left | Name | Family | Class |
| 28336871 | 28336991 | - | 20.8 | 498 | 632 | -4 | MER92B | ERV1 | LTR |
| 28337184 | 28337432 | - | 20.8 | 5948 | 6290 | -22 | L1MA9 | L1 | LINE |
| 28337430 | 28337667 | + | 10.5 | 5680 | 5952 | -339 | L1MA8 | L1 | LINE |
| 28337668 | 28338088 | - | 23.3 | 92 | 494 | -142 | MER92B | ERV1 | LTR |
| 28338091 | 28338405 | + | 11.9 | 1 | 333 | -6 | MER1B | MER1\_type | DNA |
| 28338406 | 28338696 | + | 7.9 | 1 | 303 | -7 | AluSg | Alu | SINE |
| 28338710 | 28338812 | - | 14.9 | 1 | 99 | -537 | MER92B | ERV1 | LTR |

  

|  |  |  |  |  |  |  |  |  |  |
| --- | --- | --- | --- | --- | --- | --- | --- | --- | --- |
| chrX:28606825-28610662 | | | 3827 bp | | |  |  |  |  |
| Genome Start | Genome End | Strand | Div | Repeat Start | Repeat End | Repeat Left | Name | Family | Class |
| 28606825 | 28606888 | - | 17.2 | 6110 | 6173 | -1 | L1MB5 | L1 | LINE |
| 28606891 | 28609315 | - | 15.8 | 3283 | 5738 | -574 | L1MA9 | L1 | LINE |
| 28609316 | 28609864 | + | 17.1 | 5757 | 6312 | 0 | L1MA9 | L1 | LINE |
| 28609874 | 28610334 | - | 20.3 | 5670 | 6120 | -54 | L1MB5 | L1 | LINE |
| 28610335 | 28610611 | - | 12.0 | 1 | 310 | 0 | AluSg | Alu | SINE |
| 28610612 | 28610662 | - | 20.3 | 5618 | 5669 | -505 | L1MB5 | L1 | LINE |

  

|  |  |  |  |  |  |  |  |  |  |
| --- | --- | --- | --- | --- | --- | --- | --- | --- | --- |
| chrX:31998573-31999785 | | | 1217 bp | | |  |  |  |  |
| Genome Start | Genome End | Strand | Div | Repeat Start | Repeat End | Repeat Left | Name | Family | Class |
| 31998573 | 31998968 | + | 13.7 | 5735 | 6104 | -47 | L1PB1 | L1 | LINE |
| 31998965 | 31999423 | - | 3.3 | 5430 | 5892 | -262 | L1PA4 | L1 | LINE |
| 31999424 | 31999706 | + | 1.8 | 5870 | 6152 | -3 | L1PA4 | L1 | LINE |
| 31999707 | 31999785 | + | 16.9 | 6073 | 6150 | -1 | L1PB1 | L1 | LINE |

  

|  |  |  |  |  |  |  |  |  |  |
| --- | --- | --- | --- | --- | --- | --- | --- | --- | --- |
| chrX:33589022-33592903 | | | 3795 bp | | |  |  |  |  |
| Genome Start | Genome End | Strand | Div | Repeat Start | Repeat End | Repeat Left | Name | Family | Class |
| 33589022 | 33591553 | + | 15.1 | 2889 | 5480 | -832 | L1MA6 | L1 | LINE |
| 33591570 | 33591816 | - | 8.1 | 5697 | 5943 | -225 | L1PA10 | L1 | LINE |
| 33591817 | 33592024 | + | 11.1 | 5947 | 6157 | -11 | L1PA10 | L1 | LINE |
| 33592096 | 33592903 | + | 16.4 | 5484 | 6300 | 0 | L1MA6 | L1 | LINE |

  

|  |  |  |  |  |  |  |  |  |  |
| --- | --- | --- | --- | --- | --- | --- | --- | --- | --- |
| chrX:34352303-34353652 | | | 1353 bp | | |  |  |  |  |
| Genome Start | Genome End | Strand | Div | Repeat Start | Repeat End | Repeat Left | Name | Family | Class |
| 34352303 | 34352369 | - | 3.0 | 5295 | 5361 | -770 | L1P | L1 | LINE |
| 34352370 | 34352569 | - | 3.5 | 5956 | 6155 | 0 | L1PA4 | L1 | LINE |
| 34352570 | 34353158 | + | 2.2 | 5366 | 5954 | -201 | L1PA4 | L1 | LINE |
| 34353156 | 34353652 | - | 4.8 | 4812 | 5308 | -838 | L1P | L1 | LINE |

  

|  |  |  |  |  |  |  |  |  |  |
| --- | --- | --- | --- | --- | --- | --- | --- | --- | --- |
| chrX:34994277-35005610 | | | 11336 bp | | |  |  |  |  |
| Genome Start | Genome End | Strand | Div | Repeat Start | Repeat End | Repeat Left | Name | Family | Class |
| 34994277 | 34996173 | - | 10.4 | 5793 | 7720 | -3 | L1MA2 | L1 | LINE |
| 34996187 | 34997027 | - | 3.6 | 5308 | 6147 | -7 | L1PA5 | L1 | LINE |
| 34997030 | 34997649 | + | 6.0 | 4705 | 5325 | -821 | L1PA5 | L1 | LINE |
| 34997641 | 34998290 | - | 15.9 | 5158 | 5808 | -1757 | L1MA2 | L1 | LINE |
| 34998284 | 34999223 | - | 8.5 | 5177 | 6130 | -1 | L1PA8A | L1 | LINE |
| 34999224 | 35000173 | + | 8.9 | 4070 | 5022 | -1124 | L1PA8A | L1 | LINE |
| 35000173 | 35001234 | - | 12.9 | 4107 | 5170 | -2395 | L1MA2 | L1 | LINE |
| 35001235 | 35001546 | + | 9.1 | 1 | 308 | -3 | AluY | Alu | SINE |
| 35001547 | 35005610 | - | 17.1 | 23 | 4106 | -3459 | L1MA2 | L1 | LINE |

  

|  |  |  |  |  |  |  |  |  |  |
| --- | --- | --- | --- | --- | --- | --- | --- | --- | --- |
| chrX:35109191-35111443 | | | 2261 bp | | |  |  |  |  |
| Genome Start | Genome End | Strand | Div | Repeat Start | Repeat End | Repeat Left | Name | Family | Class |
| 35109191 | 35109449 | - | 20.6 | 5760 | 6022 | -152 | L1MB5 | L1 | LINE |
| 35109436 | 35110268 | - | 8.9 | 5337 | 6174 | 0 | L1PA11 | L1 | LINE |
| 35110263 | 35110829 | + | 10.1 | 4738 | 5323 | -823 | L1PA11 | L1 | LINE |
| 35110842 | 35111443 | - | 17.2 | 5156 | 5775 | -399 | L1MB5 | L1 | LINE |

  

|  |  |  |  |  |  |  |  |  |  |
| --- | --- | --- | --- | --- | --- | --- | --- | --- | --- |
| chrX:36852968-36857194 | | | 4209 bp | | |  |  |  |  |
| Genome Start | Genome End | Strand | Div | Repeat Start | Repeat End | Repeat Left | Name | Family | Class |
| 36852968 | 36853554 | - | 22.5 | 2754 | 3389 | -3255 | L1M4c | L1 | LINE |
| 36853564 | 36854195 | - | 16.5 | 5677 | 6300 | 0 | L1MA5 | L1 | LINE |
| 36854197 | 36854918 | + | 14.1 | 4966 | 5671 | -629 | L1MA5 | L1 | LINE |
| 36854927 | 36857194 | - | 24.2 | 334 | 2749 | -3991 | L1M4c | L1 | LINE |

  

|  |  |  |  |  |  |  |  |  |  |
| --- | --- | --- | --- | --- | --- | --- | --- | --- | --- |
| chrX:36954863-36959232 | | | 4279 bp | | |  |  |  |  |
| Genome Start | Genome End | Strand | Div | Repeat Start | Repeat End | Repeat Left | Name | Family | Class |
| 36954863 | 36956220 | - | 25.7 | 958 | 2394 | -113 | HAL1 | L1 | LINE |
| 36956210 | 36957181 | - | 7.1 | 5187 | 6169 | -3 | L1PA8 | L1 | LINE |
| 36957179 | 36957873 | + | 8.6 | 4503 | 5198 | -948 | L1PA8 | L1 | LINE |
| 36957979 | 36958438 | - | 28.1 | 403 | 890 | -1617 | HAL1 | L1 | LINE |
| 36958439 | 36958891 | + | 9.3 | 1 | 463 | 0 | LTR2 | ERV1 | LTR |
| 36958892 | 36959232 | - | 28.1 | 43 | 402 | -2105 | HAL1 | L1 | LINE |

  

|  |  |  |  |  |  |  |  |  |  |
| --- | --- | --- | --- | --- | --- | --- | --- | --- | --- |
| chrX:38214001-38216747 | | | 2652 bp | | |  |  |  |  |
| Genome Start | Genome End | Strand | Div | Repeat Start | Repeat End | Repeat Left | Name | Family | Class |
| 38214001 | 38214097 | + | 21.7 | 3 | 101 | -65 | MER103 | DNA | DNA |
| 38214098 | 38214171 | + | 22.2 | 1 | 75 | -103 | MER5B | MER1\_type | DNA |
| 38214245 | 38214655 | - | 16.9 | 4416 | 4827 | -1316 | L1MA8 | L1 | LINE |
| 38214643 | 38215377 | + | 13.0 | 4840 | 5599 | -692 | L1MA8 | L1 | LINE |
| 38215378 | 38215689 | + | 9.4 | 1 | 308 | -1 | AluSc | Alu | SINE |
| 38215690 | 38216349 | + | 11.9 | 5600 | 6291 | 0 | L1MA8 | L1 | LINE |
| 38216353 | 38216643 | + | 15.8 | 1 | 293 | -19 | AluJb | Alu | SINE |
| 38216676 | 38216747 | + | 25.7 | 91 | 160 | -6 | MER103 | DNA | DNA |

  

|  |  |  |  |  |  |  |  |  |  |
| --- | --- | --- | --- | --- | --- | --- | --- | --- | --- |
| chrX:42649203-42656359 | | | 7044 bp | | |  |  |  |  |
| Genome Start | Genome End | Strand | Div | Repeat Start | Repeat End | Repeat Left | Name | Family | Class |
| 42649203 | 42651036 | + | 16.4 | 3251 | 5094 | -2468 | L1MA4 | L1 | LINE |
| 42651065 | 42651147 | + | 15.6 | 5095 | 5202 | -2360 | L1MA4 | L1 | LINE |
| 42651148 | 42651473 | + | 14.8 | 5803 | 6147 | -7 | L1PB3 | L1 | LINE |
| 42651474 | 42652706 | + | 15.6 | 5203 | 6432 | -1130 | L1MA4 | L1 | LINE |
| 42652706 | 42653777 | - | 5.6 | 3882 | 4955 | -1191 | L1PA5 | L1 | LINE |
| 42653776 | 42654969 | + | 3.7 | 4955 | 6149 | -5 | L1PA5 | L1 | LINE |
| 42654947 | 42655462 | + | 14.7 | 6403 | 6905 | -814 | L1MA4 | L1 | LINE |
| 42655574 | 42656359 | + | 12.7 | 6906 | 7707 | -12 | L1MA4 | L1 | LINE |

  

|  |  |  |  |  |  |  |  |  |  |
| --- | --- | --- | --- | --- | --- | --- | --- | --- | --- |
| chrX:43172328-43175565 | | | 3196 bp | | |  |  |  |  |
| Genome Start | Genome End | Strand | Div | Repeat Start | Repeat End | Repeat Left | Name | Family | Class |
| 43172328 | 43172788 | - | 18.5 | 6034 | 6524 | -226 | L1MD2 | L1 | LINE |
| 43172786 | 43173446 | - | 10.7 | 4710 | 5382 | -764 | L1P3 | L1 | LINE |
| 43173445 | 43173494 | + | 6.0 | 4685 | 4734 | -1412 | L1P3 | L1 | LINE |
| 43173496 | 43174402 | - | 21.9 | 4435 | 5295 | -1242 | L1MD2 | L1 | LINE |
| 43174450 | 43174546 | - | 11.3 | 213 | 309 | -3 | AluSg/x | Alu | SINE |
| 43174552 | 43174925 | - | 21.3 | 5874 | 6310 | -49 | L1MD2 | L1 | LINE |
| 43174910 | 43175197 | - | 15.1 | 4153 | 4447 | -2090 | L1MD2 | L1 | LINE |
| 43175208 | 43175565 | - | 21.8 | 23 | 383 | -6250 | L1MD2 | L1 | LINE |

  

|  |  |  |  |  |  |  |  |  |  |
| --- | --- | --- | --- | --- | --- | --- | --- | --- | --- |
| chrX:43332208-43333849 | | | 1500 bp | | |  |  |  |  |
| Genome Start | Genome End | Strand | Div | Repeat Start | Repeat End | Repeat Left | Name | Family | Class |
| 43332208 | 43332589 | - | 20.9 | 245 | 667 | -2 | LTR65 | ERV1 | LTR |
| 43332590 | 43333219 | - | 1.1 | 5277 | 5906 | -249 | L1HS | L1 | LINE |
| 43333220 | 43333471 | + | 0.8 | 5904 | 6155 | 0 | L1HS | L1 | LINE |
| 43333614 | 43333849 | - | 16.8 | 3 | 253 | -416 | LTR65 | ERV1 | LTR |

  

|  |  |  |  |  |  |  |  |  |  |
| --- | --- | --- | --- | --- | --- | --- | --- | --- | --- |
| chrX:44551808-44554722 | | | 2501 bp | | |  |  |  |  |
| Genome Start | Genome End | Strand | Div | Repeat Start | Repeat End | Repeat Left | Name | Family | Class |
| 44551808 | 44552043 | - | 24.7 | 2314 | 2566 | -2268 | L1MEe | L1 | LINE |
| 44552106 | 44552461 | - | 2.5 | 5795 | 6152 | -3 | L1PA4 | L1 | LINE |
| 44552462 | 44552526 | + | 4.6 | 5744 | 5808 | -347 | L1PA4 | L1 | LINE |
| 44552638 | 44552925 | - | 11.1 | 1 | 289 | -24 | AluSp | Alu | SINE |
| 44552939 | 44553054 | - | 27.7 | 1180 | 1295 | -3539 | L1MEe | L1 | LINE |
| 44553097 | 44553308 | - | 28.4 | 767 | 988 | -3866 | L1MEe | L1 | LINE |
| 44553364 | 44553656 | - | 10.2 | 1 | 293 | -17 | AluSg | Alu | SINE |
| 44553788 | 44553835 | - | 28.1 | 337 | 381 | -4473 | L1MEe | L1 | LINE |
| 44553836 | 44554142 | - | 10.3 | 1 | 307 | 0 | AluSg | Alu | SINE |
| 44554143 | 44554160 | - | 28.1 | 326 | 336 | -4518 | L1MEe | L1 | LINE |
| 44554161 | 44554454 | + | 17.5 | 1 | 298 | -14 | AluJb | Alu | SINE |
| 44554455 | 44554722 | - | 28.1 | 27 | 325 | -4529 | L1MEe | L1 | LINE |

  

|  |  |  |  |  |  |  |  |  |  |
| --- | --- | --- | --- | --- | --- | --- | --- | --- | --- |
| chrX:45467658-45469789 | | | 2126 bp | | |  |  |  |  |
| Genome Start | Genome End | Strand | Div | Repeat Start | Repeat End | Repeat Left | Name | Family | Class |
| 45467658 | 45468887 | - | 14.8 | 4974 | 6081 | -210 | L1MA8 | L1 | LINE |
| 45468898 | 45469249 | - | 11.1 | 5802 | 6164 | -2 | L1PA16 | L1 | LINE |
| 45469250 | 45469442 | + | 12.6 | 5621 | 5813 | -353 | L1PA16 | L1 | LINE |
| 45469439 | 45469789 | - | 12.9 | 4646 | 4995 | -1148 | L1MA8 | L1 | LINE |

  

|  |  |  |  |  |  |  |  |  |  |
| --- | --- | --- | --- | --- | --- | --- | --- | --- | --- |
| chrX:45925087-45926885 | | | 1793 bp | | |  |  |  |  |
| Genome Start | Genome End | Strand | Div | Repeat Start | Repeat End | Repeat Left | Name | Family | Class |
| 45925087 | 45925766 | - | 22.1 | 4400 | 5135 | -1011 | L1MD | L1 | LINE |
| 45925775 | 45926002 | - | 10.1 | 6069 | 6300 | 0 | L1MA6 | L1 | LINE |
| 45926004 | 45926345 | + | 12.2 | 5625 | 5968 | -332 | L1MA6 | L1 | LINE |
| 45926346 | 45926638 | + | 11.3 | 1 | 295 | -18 | AluSq | Alu | SINE |
| 45926639 | 45926732 | + | 12.2 | 5969 | 6047 | -253 | L1MA6 | L1 | LINE |
| 45926730 | 45926885 | - | 24.3 | 4257 | 4414 | -1732 | L1MD | L1 | LINE |

  

|  |  |  |  |  |  |  |  |  |  |
| --- | --- | --- | --- | --- | --- | --- | --- | --- | --- |
| chrX:47414742-47419179 | | | 4433 bp | | |  |  |  |  |
| Genome Start | Genome End | Strand | Div | Repeat Start | Repeat End | Repeat Left | Name | Family | Class |
| 47414742 | 47415114 | - | 8.0 | 3880 | 4253 | -1893 | L1M1 | L1 | LINE |
| 47415117 | 47415476 | - | 9.5 | 2803 | 3300 | -4265 | L1M1 | L1 | LINE |
| 47415474 | 47417416 | - | 12.4 | 480 | 2446 | -5119 | L1M1 | L1 | LINE |
| 47417429 | 47418027 | - | 8.0 | 5528 | 6126 | -5 | L1PA8A | L1 | LINE |
| 47418028 | 47418716 | + | 6.3 | 4829 | 5524 | -607 | L1PA8A | L1 | LINE |
| 47418711 | 47419179 | - | 16.2 | 16 | 492 | -7073 | L1M1 | L1 | LINE |

  

|  |  |  |  |  |  |  |  |  |  |
| --- | --- | --- | --- | --- | --- | --- | --- | --- | --- |
| chrX:48610709-48618538 | | | 7338 bp | | |  |  |  |  |
| Genome Start | Genome End | Strand | Div | Repeat Start | Repeat End | Repeat Left | Name | Family | Class |
| 48610709 | 48610839 | - | 21.3 | 5810 | 5939 | -301 | L1ME3B | L1 | LINE |
| 48610846 | 48610907 | - | 6.5 | 394 | 457 | -5 | LTR40b | ERVL | LTR |
| 48610965 | 48611166 | + | 15.1 | 85 | 285 | -27 | AluJo | Alu | SINE |
| 48611511 | 48611578 | - | 18.2 | 77 | 147 | -372 | LTR40a | ERVL | LTR |
| 48611620 | 48611773 | - | 20.8 | 5658 | 5814 | -356 | L1ME3B | L1 | LINE |
| 48611772 | 48612453 | - | 5.7 | 746 | 1427 | -4728 | L1PA4 | L1 | LINE |
| 48612453 | 48612879 | + | 3.6 | 1433 | 1858 | -4297 | L1PA4 | L1 | LINE |
| 48612880 | 48613193 | + | 8.8 | 2 | 311 | -2 | AluSq | Alu | SINE |
| 48613194 | 48617481 | + | 4.2 | 1859 | 6153 | -2 | L1PA4 | L1 | LINE |
| 48617529 | 48617595 | - | 26.6 | 5582 | 5649 | -591 | L1ME3B | L1 | LINE |
| 48617596 | 48617907 | + | 11.5 | 1 | 312 | -1 | AluSq | Alu | SINE |
| 48617908 | 48618041 | - | 26.6 | 5474 | 5581 | -659 | L1ME3B | L1 | LINE |
| 48618042 | 48618337 | + | 9.9 | 1 | 293 | -19 | AluSx | Alu | SINE |
| 48618338 | 48618538 | - | 26.6 | 5266 | 5473 | -767 | L1ME3B | L1 | LINE |

  

|  |  |  |  |  |  |  |  |  |  |
| --- | --- | --- | --- | --- | --- | --- | --- | --- | --- |
| chrX:49680126-49682330 | | | 2172 bp | | |  |  |  |  |
| Genome Start | Genome End | Strand | Div | Repeat Start | Repeat End | Repeat Left | Name | Family | Class |
| 49680126 | 49680510 | - | 30.9 | 4811 | 5251 | -895 | L1M5 | L1 | LINE |
| 49680511 | 49680926 | - | 2.6 | 5730 | 6154 | -1 | L1PA4 | L1 | LINE |
| 49680927 | 49681315 | + | 2.3 | 5336 | 5723 | -432 | L1PA4 | L1 | LINE |
| 49681318 | 49681934 | - | 34.2 | 4097 | 4791 | -1355 | L1M5 | L1 | LINE |
| 49681966 | 49682330 | - | 29.9 | 3667 | 4034 | -2112 | L1M5 | L1 | LINE |

  

|  |  |  |  |  |  |  |  |  |  |
| --- | --- | --- | --- | --- | --- | --- | --- | --- | --- |
| chrX:49682785-49685238 | | | 2386 bp | | |  |  |  |  |
| Genome Start | Genome End | Strand | Div | Repeat Start | Repeat End | Repeat Left | Name | Family | Class |
| 49682785 | 49683004 | + | 23.6 | 7 | 2476 | -285 | Charlie1 | MER1\_type | DNA |
| 49683073 | 49684463 | - | 2.3 | 4760 | 6150 | -5 | L1PA3 | L1 | LINE |
| 49684463 | 49685115 | + | 3.7 | 4086 | 4738 | -1408 | L1PA3 | L1 | LINE |
| 49685117 | 49685238 | + | 15.7 | 2639 | 2761 | 0 | Charlie1 | MER1\_type | DNA |

  

|  |  |  |  |  |  |  |  |  |  |
| --- | --- | --- | --- | --- | --- | --- | --- | --- | --- |
| chrX:51642200-51649100 | | | 6856 bp | | |  |  |  |  |
| Genome Start | Genome End | Strand | Div | Repeat Start | Repeat End | Repeat Left | Name | Family | Class |
| 51642200 | 51642521 | + | 24.2 | 18 | 346 | -6287 | L1MD2 | L1 | LINE |
| 51642522 | 51642826 | - | 7.9 | 1 | 305 | -8 | AluSq | Alu | SINE |
| 51642827 | 51644376 | + | 24.2 | 347 | 2028 | -4605 | L1MD2 | L1 | LINE |
| 51644380 | 51644932 | + | 21.0 | 3934 | 4517 | -2020 | L1MD2 | L1 | LINE |
| 51644925 | 51645616 | - | 14.8 | 4958 | 5639 | -661 | L1MA4 | L1 | LINE |
| 51645617 | 51646298 | + | 13.0 | 5649 | 6299 | -1 | L1MA4 | L1 | LINE |
| 51646349 | 51646608 | + | 18.6 | 4504 | 4776 | -1761 | L1MD2 | L1 | LINE |
| 51646609 | 51646916 | + | 14.7 | 1 | 307 | -5 | AluJo | Alu | SINE |
| 51646917 | 51647851 | + | 18.6 | 4777 | 5707 | -830 | L1MD2 | L1 | LINE |
| 51647852 | 51648127 | + | 7.3 | 5998 | 6295 | -7 | L1MA1 | L1 | LINE |
| 51648128 | 51649100 | + | 16.8 | 5708 | 6625 | -8 | L1MD2 | L1 | LINE |

  

|  |  |  |  |  |  |  |  |  |  |
| --- | --- | --- | --- | --- | --- | --- | --- | --- | --- |
| chrX:53804487-53807748 | | | 3094 bp | | |  |  |  |  |
| Genome Start | Genome End | Strand | Div | Repeat Start | Repeat End | Repeat Left | Name | Family | Class |
| 53804487 | 53805107 | + | 28.6 | 3100 | 3703 | -1964 | ERVL-E | ERVL | LTR |
| 53805111 | 53805771 | - | 2.9 | 5199 | 5859 | -295 | L1PA5 | L1 | LINE |
| 53805772 | 53806039 | + | 4.1 | 5870 | 6136 | -18 | L1PA5 | L1 | LINE |
| 53806058 | 53806175 | + | 25.4 | 3707 | 3847 | -1820 | ERVL-E | ERVL | LTR |
| 53806187 | 53806247 | - | 22.9 | 196 | 260 | -2 | MIR | MIR | SINE |
| 53806263 | 53807371 | + | 28.6 | 3869 | 5119 | -548 | ERVL-E | ERVL | LTR |
| 53807493 | 53807748 | + | 28.3 | 5046 | 5403 | -354 | ERVL-E | ERVL | LTR |

  

|  |  |  |  |  |  |  |  |  |  |
| --- | --- | --- | --- | --- | --- | --- | --- | --- | --- |
| chrX:54308682-54311163 | | | 2444 bp | | |  |  |  |  |
| Genome Start | Genome End | Strand | Div | Repeat Start | Repeat End | Repeat Left | Name | Family | Class |
| 54308682 | 54308919 | - | 30.1 | 2709 | 2947 | -472 | L2 | L2 | LINE |
| 54308920 | 54309160 | + | 12.0 | 1 | 274 | -39 | AluSq | Alu | SINE |
| 54309161 | 54309246 | - | 30.1 | 2621 | 2708 | -711 | L2 | L2 | LINE |
| 54309252 | 54309700 | + | 9.5 | 5785 | 6255 | -50 | L1MA3 | L1 | LINE |
| 54309701 | 54309867 | - | 19.8 | 129 | 295 | -17 | AluJb | Alu | SINE |
| 54309893 | 54310210 | - | 4.4 | 5845 | 6163 | -9 | L1PA8 | L1 | LINE |
| 54310211 | 54310391 | + | 6.1 | 5640 | 5820 | -352 | L1PA8 | L1 | LINE |
| 54310392 | 54310526 | - | 13.4 | 1 | 135 | -177 | AluJb | Alu | SINE |
| 54310528 | 54310582 | + | 7.3 | 6246 | 6300 | -5 | L1MA3 | L1 | LINE |
| 54310590 | 54310663 | - | 26.1 | 2561 | 2628 | -791 | L2 | L2 | LINE |
| 54310664 | 54310981 | + | 10.7 | 3 | 312 | 0 | AluSx | Alu | SINE |
| 54310982 | 54311163 | - | 26.1 | 2386 | 2560 | -859 | L2 | L2 | LINE |

  

|  |  |  |  |  |  |  |  |  |  |
| --- | --- | --- | --- | --- | --- | --- | --- | --- | --- |
| chrX:55808522-55809005 | | | 443 bp | | |  |  |  |  |
| Genome Start | Genome End | Strand | Div | Repeat Start | Repeat End | Repeat Left | Name | Family | Class |
| 55808522 | 55808636 | + | 11.9 | 1 | 110 | -99 | MER3 | MER1\_type | DNA |
| 55808637 | 55808793 | - | 1.9 | 5939 | 6094 | -61 | L1PA2 | L1 | LINE |
| 55808794 | 55808858 | + | 3.1 | 6090 | 6155 | 0 | L1PA2 | L1 | LINE |
| 55808900 | 55809005 | + | 17.1 | 99 | 209 | 0 | MER3 | MER1\_type | DNA |

  

|  |  |  |  |  |  |  |  |  |  |
| --- | --- | --- | --- | --- | --- | --- | --- | --- | --- |
| chrX:55964051-55966676 | | | 2657 bp | | |  |  |  |  |
| Genome Start | Genome End | Strand | Div | Repeat Start | Repeat End | Repeat Left | Name | Family | Class |
| 55964051 | 55964351 | + | 10.7 | 5004 | 5318 | -828 | L1PA11 | L1 | LINE |
| 55964348 | 55964650 | - | 9.9 | 4700 | 5003 | -1143 | L1PA10 | L1 | LINE |
| 55964650 | 55965792 | + | 8.6 | 5015 | 6168 | 0 | L1PA10 | L1 | LINE |
| 55965767 | 55966676 | + | 10.5 | 5274 | 6174 | 0 | L1PA11 | L1 | LINE |

  

|  |  |  |  |  |  |  |  |  |  |
| --- | --- | --- | --- | --- | --- | --- | --- | --- | --- |
| chrX:56196115-56209698 | | | 13300 bp | | |  |  |  |  |
| Genome Start | Genome End | Strand | Div | Repeat Start | Repeat End | Repeat Left | Name | Family | Class |
| 56196115 | 56196351 | - | 19.9 | 1221 | 1455 | 0 | Charlie1a | MER1\_type | DNA |
| 56196370 | 56196525 | + | 22.4 | 1 | 160 | -16 | FRAM | Alu | SINE |
| 56196526 | 56196681 | - | 20.5 | 1050 | 1205 | -250 | Charlie1a | MER1\_type | DNA |
| 56196683 | 56196742 | - | 8.3 | 4066 | 4125 | -2021 | L1PA5 | L1 | LINE |
| 56196740 | 56198772 | + | 4.9 | 4142 | 6151 | -3 | L1PA5 | L1 | LINE |
| 56198787 | 56198813 | - | 24.7 | 1032 | 1061 | -394 | Charlie1a | MER1\_type | DNA |
| 56198814 | 56199319 | - | 17.1 | 5656 | 6164 | -2 | L1PA16 | L1 | LINE |
| 56199320 | 56199389 | - | 24.7 | 958 | 1031 | -424 | Charlie1a | MER1\_type | DNA |
| 56199406 | 56199736 | - | 8.2 | 5814 | 6144 | -19 | L1PA13 | L1 | LINE |
| 56199731 | 56200058 | - | 7.6 | 5698 | 6032 | 0 | L1PA4 | L1 | LINE |
| 56200073 | 56200552 | + | 6.7 | 5673 | 6151 | -3 | L1PA5 | L1 | LINE |
| 56200567 | 56206075 | - | 10.2 | 1 | 5512 | -520 | L1PA4 | L1 | LINE |
| 56206075 | 56207260 | - | 13.8 | 4643 | 5832 | -336 | L1PA12 | L1 | LINE |
| 56207356 | 56207613 | - | 42.0 | 3126 | 3372 | -6 | L2 | L2 | LINE |
| 56207649 | 56207739 | + | 21.4 | 80 | 171 | -90 | MER112 | MER1\_type | DNA |
| 56207772 | 56207859 | - | 24.7 | 3667 | 3755 | -734 | L3 | CR1 | LINE |
| 56207910 | 56208295 | - | 1.6 | 5770 | 6155 | 0 | L1HS | L1 | LINE |
| 56208296 | 56208333 | + | 0.0 | 5715 | 5752 | -402 | L1P2 | L1 | LINE |
| 56208329 | 56208427 | - | 12.1 | 4558 | 4657 | -1489 | L1PREC2 | L1 | LINE |
| 56208438 | 56208504 | + | 19.4 | 967 | 1033 | -5948 | L1PA12 | L1 | LINE |
| 56208505 | 56208744 | - | 23.9 | 742 | 978 | -477 | Charlie1a | MER1\_type | DNA |
| 56208745 | 56209031 | + | 20.2 | 3 | 297 | -15 | AluJo | Alu | SINE |
| 56209032 | 56209698 | - | 23.9 | 2 | 741 | -714 | Charlie1a | MER1\_type | DNA |

  

|  |  |  |  |  |  |  |  |  |  |
| --- | --- | --- | --- | --- | --- | --- | --- | --- | --- |
| chrX:56492559-56495767 | | | 3211 bp | | |  |  |  |  |
| Genome Start | Genome End | Strand | Div | Repeat Start | Repeat End | Repeat Left | Name | Family | Class |
| 56492559 | 56493190 | - | 5.1 | 5520 | 6152 | -3 | L1PA4 | L1 | LINE |
| 56493191 | 56495023 | - | 5.3 | 3272 | 5108 | -1038 | L1P1 | L1 | LINE |
| 56495025 | 56495269 | + | 4.1 | 5107 | 5351 | -795 | L1P1 | L1 | LINE |
| 56495267 | 56495767 | - | 9.8 | 7 | 507 | -5648 | L1PA4 | L1 | LINE |

  

|  |  |  |  |  |  |  |  |  |  |
| --- | --- | --- | --- | --- | --- | --- | --- | --- | --- |
| chrX:56563709-56571169 | | | 6857 bp | | |  |  |  |  |
| Genome Start | Genome End | Strand | Div | Repeat Start | Repeat End | Repeat Left | Name | Family | Class |
| 56563709 | 56564511 | - | 23.0 | 4687 | 5406 | -953 | L1M | L1 | LINE |
| 56564537 | 56567750 | - | 3.8 | 2922 | 6137 | -18 | L1PA3 | L1 | LINE |
| 56567747 | 56569565 | + | 4.7 | 1109 | 2931 | -3215 | L1PA3 | L1 | LINE |
| 56569754 | 56569903 | + | 29.3 | 33 | 183 | -79 | MIR | MIR | SINE |
| 56569991 | 56570657 | - | 17.8 | 3979 | 4691 | -1455 | L1M | L1 | LINE |
| 56570966 | 56571169 | - | 19.3 | 3777 | 3989 | -2157 | L1M | L1 | LINE |

  

|  |  |  |  |  |  |  |  |  |  |
| --- | --- | --- | --- | --- | --- | --- | --- | --- | --- |
| chrX:57398585-57403792 | | | 5115 bp | | |  |  |  |  |
| Genome Start | Genome End | Strand | Div | Repeat Start | Repeat End | Repeat Left | Name | Family | Class |
| 57398585 | 57399937 | - | 22.4 | 3365 | 4760 | -1616 | L1M3a | L1 | LINE |
| 57399949 | 57400248 | - | 10.7 | 5854 | 6153 | -1 | L1PA7 | L1 | LINE |
| 57400250 | 57401211 | + | 7.1 | 4899 | 5859 | -295 | L1PA7 | L1 | LINE |
| 57401208 | 57401329 | - | 18.0 | 3261 | 3382 | -2994 | L1M3a | L1 | LINE |
| 57401348 | 57402041 | - | 19.6 | 5492 | 6167 | -1 | L1MB1 | L1 | LINE |
| 57402048 | 57402388 | - | 11.0 | 5969 | 6305 | 0 | L1MA3 | L1 | LINE |
| 57402393 | 57402652 | - | 21.5 | 3013 | 3282 | -3094 | L1M3a | L1 | LINE |
| 57402710 | 57403792 | - | 25.8 | 911 | 2131 | -4253 | L1M3a | L1 | LINE |

  

|  |  |  |  |  |  |  |  |  |  |
| --- | --- | --- | --- | --- | --- | --- | --- | --- | --- |
| chrX:57438607-57450851 | | | 12150 bp | | |  |  |  |  |
| Genome Start | Genome End | Strand | Div | Repeat Start | Repeat End | Repeat Left | Name | Family | Class |
| 57438607 | 57440861 | - | 21.0 | 2137 | 4512 | -2061 | L1PA17 | L1 | LINE |
| 57440862 | 57441257 | - | 4.3 | 5756 | 6154 | 0 | L1PA5 | L1 | LINE |
| 57441259 | 57441423 | + | 3.0 | 5593 | 5757 | -397 | L1PA5 | L1 | LINE |
| 57441425 | 57441716 | - | 21.6 | 1858 | 2158 | -4419 | L1PA17 | L1 | LINE |
| 57441717 | 57442042 | - | 6.8 | 1 | 326 | -454 | LTR17 | ERV1 | LTR |
| 57442049 | 57442349 | - | 9.0 | 6832 | 8621 | -5 | HERV17 | ERV1 | LTR |
| 57442384 | 57443998 | - | 5.3 | 4414 | 6019 | -2607 | HERV17 | ERV1 | LTR |
| 57443999 | 57444300 | + | 11.3 | 1 | 301 | -10 | AluY | Alu | SINE |
| 57444301 | 57446303 | - | 5.3 | 2430 | 4413 | -4213 | HERV17 | ERV1 | LTR |
| 57446289 | 57448356 | - | 9.3 | 2 | 2014 | -6612 | HERV17 | ERV1 | LTR |
| 57448357 | 57448768 | - | 9.9 | 347 | 780 | 0 | LTR17 | ERV1 | LTR |
| 57448837 | 57450851 | - | 23.6 | 12 | 1857 | -4720 | L1PA17 | L1 | LINE |

  

|  |  |  |  |  |  |  |  |  |  |
| --- | --- | --- | --- | --- | --- | --- | --- | --- | --- |
| chrX:57515579-57528715 | | | 12671 bp | | |  |  |  |  |
| Genome Start | Genome End | Strand | Div | Repeat Start | Repeat End | Repeat Left | Name | Family | Class |
| 57515579 | 57517352 | - | 21.8 | 3117 | 4873 | -2441 | L1PA17 | L1 | LINE |
| 57517353 | 57520388 | - | 11.0 | 3437 | 6481 | -6 | L1PA8 | L1 | LINE |
| 57520643 | 57523022 | - | 5.3 | 3762 | 6154 | 0 | L1PA5 | L1 | LINE |
| 57523006 | 57523564 | + | 5.2 | 3225 | 3783 | -2363 | L1PA5 | L1 | LINE |
| 57523561 | 57525852 | - | 15.9 | 10 | 2299 | -4184 | L1PA8 | L1 | LINE |
| 57525887 | 57526938 | - | 21.4 | 2013 | 3100 | -4218 | L1PA17 | L1 | LINE |
| 57526939 | 57527262 | - | 7.4 | 1 | 313 | 0 | AluSp | Alu | SINE |
| 57527263 | 57527890 | - | 29.7 | 1582 | 2020 | -5594 | L1PA17 | L1 | LINE |
| 57527858 | 57528130 | - | 29.8 | 796 | 1066 | -5783 | L1M2 | L1 | LINE |
| 57528363 | 57528715 | - | 36.4 | 683 | 1034 | -6300 | L1PA17 | L1 | LINE |

  

|  |  |  |  |  |  |  |  |  |  |
| --- | --- | --- | --- | --- | --- | --- | --- | --- | --- |
| chrX:57600638-57608092 | | | 7465 bp | | |  |  |  |  |
| Genome Start | Genome End | Strand | Div | Repeat Start | Repeat End | Repeat Left | Name | Family | Class |
| 57600638 | 57601111 | + | 14.5 | 2383 | 2915 | -3951 | L1PBa | L1 | LINE |
| 57601109 | 57602441 | - | 7.7 | 619 | 1954 | -4201 | L1PA4 | L1 | LINE |
| 57602439 | 57606683 | + | 6.1 | 1931 | 6154 | -1 | L1PA4 | L1 | LINE |
| 57606680 | 57607417 | + | 11.4 | 2901 | 3641 | -3221 | L1PBa | L1 | LINE |
| 57607418 | 57607708 | + | 11.6 | 1 | 288 | -22 | AluSg | Alu | SINE |
| 57607709 | 57608092 | + | 11.4 | 3642 | 4023 | -2839 | L1PBa | L1 | LINE |

  

|  |  |  |  |  |  |  |  |  |  |
| --- | --- | --- | --- | --- | --- | --- | --- | --- | --- |
| chrX:57727334-57740141 | | | 12666 bp | | |  |  |  |  |
| Genome Start | Genome End | Strand | Div | Repeat Start | Repeat End | Repeat Left | Name | Family | Class |
| 57727334 | 57727451 | + | 22.2 | 903 | 1023 | -5022 | L1MC3 | L1 | LINE |
| 57727517 | 57733574 | + | 6.3 | 99 | 6155 | 0 | L1PA4 | L1 | LINE |
| 57733592 | 57734025 | + | 25.7 | 2469 | 2904 | -3660 | L1MC3 | L1 | LINE |
| 57734027 | 57734724 | - | 16.8 | 3534 | 4166 | -1977 | L1M2 | L1 | LINE |
| 57734725 | 57735012 | + | 12.6 | 12 | 307 | 0 | AluSx | Alu | SINE |
| 57735013 | 57735246 | - | 16.8 | 3311 | 3533 | -2610 | L1M2 | L1 | LINE |
| 57735245 | 57736533 | + | 22.5 | 4155 | 5462 | -829 | L1MA7 | L1 | LINE |
| 57736531 | 57736854 | + | 18.7 | 5970 | 6290 | -2 | L1MA7 | L1 | LINE |
| 57736931 | 57737647 | - | 5.8 | 5476 | 6152 | -2 | L1PA6 | L1 | LINE |
| 57737648 | 57738465 | - | 4.2 | 5333 | 6153 | -2 | L1PA4 | L1 | LINE |
| 57738454 | 57740141 | + | 23.0 | 4934 | 6634 | -1569 | L1MC3 | L1 | LINE |

  

|  |  |  |  |  |  |  |  |  |  |
| --- | --- | --- | --- | --- | --- | --- | --- | --- | --- |
| chrX:57823413-57830408 | | | 6987 bp | | |  |  |  |  |
| Genome Start | Genome End | Strand | Div | Repeat Start | Repeat End | Repeat Left | Name | Family | Class |
| 57823413 | 57825270 | - | 13.0 | 4828 | 6758 | 0 | MER4E-int | ERV1 | LTR |
| 57825271 | 57825338 | + | 23.8 | 471 | 534 | -1426 | L4 | RTE | LINE |
| 57825339 | 57825481 | - | 16.9 | 4616 | 4759 | -1999 | MER4E-int | ERV1 | LTR |
| 57825483 | 57825831 | - | 17.8 | 3424 | 3776 | -2982 | MER4E-int | ERV1 | LTR |
| 57825804 | 57826207 | - | 14.6 | 2727 | 3109 | -3649 | MER4E-int | ERV1 | LTR |
| 57826208 | 57826496 | + | 13.2 | 4 | 297 | -16 | AluSq | Alu | SINE |
| 57826497 | 57826806 | - | 14.6 | 2399 | 2726 | -4032 | MER4E-int | ERV1 | LTR |
| 57826809 | 57827161 | + | 8.5 | 3854 | 4208 | -1938 | L1PA7 | L1 | LINE |
| 57827147 | 57827211 | - | 12.3 | 5017 | 5082 | -1064 | L1PA6 | L1 | LINE |
| 57827200 | 57828250 | + | 6.4 | 5086 | 6141 | -13 | L1PA6 | L1 | LINE |
| 57828306 | 57828362 | + | 10.5 | 6062 | 6118 | -13 | L1PA8A | L1 | LINE |
| 57828369 | 57830305 | + | 8.9 | 4181 | 6151 | -3 | L1PA7 | L1 | LINE |
| 57830306 | 57830408 | - | 7.0 | 2282 | 2385 | -4373 | MER4E-int | ERV1 | LTR |

  

|  |  |  |  |  |  |  |  |  |  |
| --- | --- | --- | --- | --- | --- | --- | --- | --- | --- |
| chrX:57986506-57994773 | | | 8282 bp | | |  |  |  |  |
| Genome Start | Genome End | Strand | Div | Repeat Start | Repeat End | Repeat Left | Name | Family | Class |
| 57986506 | 57989660 | - | 13.7 | 2447 | 5512 | -1161 | L1PA13 | L1 | LINE |
| 57989661 | 57989966 | + | 11.5 | 5 | 309 | -2 | AluY | Alu | SINE |
| 57989967 | 57990026 | - | 13.4 | 2387 | 2446 | -4246 | L1PA13 | L1 | LINE |
| 57990015 | 57991938 | - | 6.2 | 3200 | 5133 | -1013 | L1PA5 | L1 | LINE |
| 57991930 | 57992956 | + | 6.1 | 5128 | 6154 | 0 | L1PA5 | L1 | LINE |
| 57992964 | 57994773 | - | 22.6 | 574 | 2399 | -4290 | L1PA13 | L1 | LINE |

  

|  |  |  |  |  |  |  |  |  |  |
| --- | --- | --- | --- | --- | --- | --- | --- | --- | --- |
| chrX:62090466-62094742 | | | 4272 bp | | |  |  |  |  |
| Genome Start | Genome End | Strand | Div | Repeat Start | Repeat End | Repeat Left | Name | Family | Class |
| 62090466 | 62091546 | + | 20.1 | 3945 | 5063 | -1535 | MER4C-int | ERV1 | LTR |
| 62091547 | 62091848 | - | 12.2 | 2 | 304 | -7 | AluY | Alu | SINE |
| 62091849 | 62093037 | + | 20.1 | 5064 | 6236 | -362 | MER4C-int | ERV1 | LTR |
| 62093043 | 62093808 | - | 1.7 | 5386 | 6155 | 0 | L1PA2 | L1 | LINE |
| 62093809 | 62094368 | + | 1.8 | 4816 | 5375 | -771 | L1PA2 | L1 | LINE |
| 62094369 | 62094742 | + | 18.0 | 6223 | 6593 | -5 | MER4C-int | ERV1 | LTR |

  

|  |  |  |  |  |  |  |  |  |  |
| --- | --- | --- | --- | --- | --- | --- | --- | --- | --- |
| chrX:62757660-62761538 | | | 3754 bp | | |  |  |  |  |
| Genome Start | Genome End | Strand | Div | Repeat Start | Repeat End | Repeat Left | Name | Family | Class |
| 62757660 | 62758107 | - | 19.8 | 7313 | 7764 | -21 | L1MC3 | L1 | LINE |
| 62758108 | 62758508 | + | 21.3 | 1 | 430 | -2 | MSTB1 | MaLR | LTR |
| 62758509 | 62758658 | - | 19.8 | 7162 | 7312 | -473 | L1MC3 | L1 | LINE |
| 62758657 | 62760329 | - | 5.0 | 3619 | 5296 | -850 | L1PA4 | L1 | LINE |
| 62760303 | 62761149 | + | 4.0 | 5310 | 6155 | 0 | L1PA4 | L1 | LINE |
| 62761238 | 62761381 | + | 29.6 | 67 | 210 | -58 | MIRb | MIR | SINE |
| 62761448 | 62761538 | - | 24.2 | 7080 | 7170 | -615 | L1MC3 | L1 | LINE |

  

|  |  |  |  |  |  |  |  |  |  |
| --- | --- | --- | --- | --- | --- | --- | --- | --- | --- |
| chrX:63015019-63028456 | | | 13452 bp | | |  |  |  |  |
| Genome Start | Genome End | Strand | Div | Repeat Start | Repeat End | Repeat Left | Name | Family | Class |
| 63015019 | 63016824 | + | 21.5 | 2 | 1718 | -5131 | L1MA4A | L1 | LINE |
| 63016817 | 63017167 | + | 17.4 | 4464 | 4832 | -2013 | L1MA4A | L1 | LINE |
| 63017163 | 63018514 | - | 4.0 | 3720 | 5070 | -1076 | L1PA3 | L1 | LINE |
| 63018513 | 63019591 | + | 3.2 | 5078 | 6155 | 0 | L1PA3 | L1 | LINE |
| 63019594 | 63020023 | + | 19.7 | 4813 | 5240 | -1605 | L1MA4A | L1 | LINE |
| 63020024 | 63020329 | + | 11.5 | 1 | 305 | -4 | AluSc | Alu | SINE |
| 63020330 | 63021056 | + | 19.7 | 5241 | 5978 | -867 | L1MA4A | L1 | LINE |
| 63021058 | 63025444 | + | 22.2 | 2 | 4585 | -2181 | L1PA15 | L1 | LINE |
| 63025445 | 63025749 | + | 21.1 | 1 | 295 | -17 | AluJb | Alu | SINE |
| 63025750 | 63027868 | + | 16.3 | 4586 | 6785 | 0 | L1PA15 | L1 | LINE |
| 63027867 | 63028456 | + | 11.2 | 5974 | 6569 | -436 | L1MA4A | L1 | LINE |

  

|  |  |  |  |  |  |  |  |  |  |
| --- | --- | --- | --- | --- | --- | --- | --- | --- | --- |
| chrX:63105374-63109300 | | | 3909 bp | | |  |  |  |  |
| Genome Start | Genome End | Strand | Div | Repeat Start | Repeat End | Repeat Left | Name | Family | Class |
| 63105374 | 63105974 | + | 18.0 | 1840 | 2538 | -3783 | L1MCa | L1 | LINE |
| 63105971 | 63107476 | - | 3.8 | 3225 | 4730 | -1416 | L1PA3 | L1 | LINE |
| 63107477 | 63108874 | + | 3.1 | 4747 | 6152 | -3 | L1PA3 | L1 | LINE |
| 63108897 | 63109300 | + | 16.8 | 2527 | 2942 | -3379 | L1MCa | L1 | LINE |

  

|  |  |  |  |  |  |  |  |  |  |
| --- | --- | --- | --- | --- | --- | --- | --- | --- | --- |
| chrX:63113289-63127107 | | | 13791 bp | | |  |  |  |  |
| Genome Start | Genome End | Strand | Div | Repeat Start | Repeat End | Repeat Left | Name | Family | Class |
| 63113289 | 63113360 | + | 22.9 | 8 | 78 | -7487 | L1MA1 | L1 | LINE |
| 63113361 | 63114600 | + | 20.8 | 445 | 1769 | -5796 | L1MA1 | L1 | LINE |
| 63114601 | 63114985 | - | 15.9 | 1 | 428 | 0 | MSTA | MaLR | LTR |
| 63114986 | 63115613 | - | 15.4 | 965 | 1580 | 0 | MSTA-int | MaLR | LTR |
| 63115614 | 63115977 | + | 14.3 | 1 | 364 | 0 | THE1B | MaLR | LTR |
| 63115978 | 63116313 | - | 15.4 | 633 | 964 | -616 | MSTA-int | MaLR | LTR |
| 63116327 | 63116438 | + | 22.9 | 373 | 485 | -1166 | MST-int | MaLR | LTR |
| 63116457 | 63118127 | + | 14.5 | 1778 | 3480 | -4085 | L1MA1 | L1 | LINE |
| 63118128 | 63118435 | + | 6.9 | 5 | 310 | -1 | AluY | Alu | SINE |
| 63118436 | 63118976 | + | 12.1 | 3481 | 4026 | -3539 | L1MA1 | L1 | LINE |
| 63118977 | 63119278 | + | 11.3 | 1 | 300 | -12 | AluSx | Alu | SINE |
| 63119279 | 63120124 | + | 11.8 | 4027 | 4884 | -2681 | L1MA1 | L1 | LINE |
| 63120125 | 63120539 | - | 16.8 | 1 | 427 | -1 | MSTA | MaLR | LTR |
| 63120540 | 63121586 | + | 11.8 | 4885 | 5939 | -1626 | L1MA1 | L1 | LINE |
| 63121586 | 63123081 | + | 21.3 | 162 | 1666 | -5119 | L1PA13 | L1 | LINE |
| 63123080 | 63123533 | - | 10.8 | 2533 | 2987 | -3159 | L1P1 | L1 | LINE |
| 63123534 | 63125885 | + | 11.7 | 2978 | 5303 | -843 | L1P1 | L1 | LINE |
| 63125886 | 63125942 | + | 10.9 | 6832 | 6886 | -835 | L1MA1 | L1 | LINE |
| 63125943 | 63126250 | - | 14.7 | 1 | 300 | -12 | AluSx | Alu | SINE |
| 63126251 | 63127107 | + | 10.9 | 6887 | 7721 | 0 | L1MA1 | L1 | LINE |

  

|  |  |  |  |  |  |  |  |  |  |
| --- | --- | --- | --- | --- | --- | --- | --- | --- | --- |
| chrX:63192491-63215463 | | | 22528 bp | | |  |  |  |  |
| Genome Start | Genome End | Strand | Div | Repeat Start | Repeat End | Repeat Left | Name | Family | Class |
| 63192491 | 63193886 | - | 17.3 | 4787 | 6485 | -277 | L1MC2 | L1 | LINE |
| 63194054 | 63194106 | + | 24.5 | 3886 | 3939 | -1 | Arthur1 | Tip100 | DNA |
| 63194109 | 63200022 | - | 5.9 | 4 | 6154 | -1 | L1PA4 | L1 | LINE |
| 63200023 | 63200656 | - | 19.2 | 4176 | 4801 | -1763 | L1MC2 | L1 | LINE |
| 63200656 | 63201742 | + | 22.8 | 880 | 2748 | -4855 | L1PA12 | L1 | LINE |
| 63201742 | 63202358 | - | 10.0 | 5409 | 6021 | -142 | L1PA13 | L1 | LINE |
| 63202359 | 63202880 | - | 11.0 | 1 | 511 | 0 | MER9 | ERVK | LTR |
| 63202881 | 63205137 | - | 12.5 | 3161 | 5408 | -755 | L1P | L1 | LINE |
| 63205166 | 63205471 | - | 12.7 | 2840 | 3160 | -2986 | L1P | L1 | LINE |
| 63205470 | 63205844 | - | 17.6 | 2383 | 2753 | -3811 | L1MC2 | L1 | LINE |
| 63205878 | 63205947 | + | 10.8 | 1085 | 1121 | -6444 | L1M1 | L1 | LINE |
| 63206028 | 63206272 | - | 24.2 | 29 | 269 | -43 | AluJo | Alu | SINE |
| 63206302 | 63206486 | - | 19.6 | 2181 | 2379 | -4372 | L1MC2 | L1 | LINE |
| 63206541 | 63209820 | + | 17.7 | 11 | 3346 | -4219 | L1MA2 | L1 | LINE |
| 63209865 | 63212156 | + | 11.6 | 4412 | 6743 | -822 | L1MA2 | L1 | LINE |
| 63212157 | 63212451 | + | 22.4 | 1 | 297 | -15 | AluJb | Alu | SINE |
| 63212452 | 63213428 | + | 11.7 | 6744 | 7723 | 0 | L1MA2 | L1 | LINE |
| 63213440 | 63213825 | - | 19.4 | 1689 | 2088 | -4663 | L1MC2 | L1 | LINE |
| 63213821 | 63213935 | - | 18.0 | 6056 | 6170 | -130 | L1MA5 | L1 | LINE |
| 63213934 | 63214044 | + | 10.8 | 6181 | 6294 | -6 | L1MA5 | L1 | LINE |
| 63214053 | 63215463 | - | 22.0 | 301 | 1700 | -5051 | L1MC2 | L1 | LINE |

  

|  |  |  |  |  |  |  |  |  |  |
| --- | --- | --- | --- | --- | --- | --- | --- | --- | --- |
| chrX:63446273-63450349 | | | 4069 bp | | |  |  |  |  |
| Genome Start | Genome End | Strand | Div | Repeat Start | Repeat End | Repeat Left | Name | Family | Class |
| 63446273 | 63447106 | - | 11.9 | 1577 | 2414 | -4 | Tigger1 | MER2\_type | DNA |
| 63447119 | 63449000 | - | 6.3 | 4261 | 6141 | -13 | L1PA6 | L1 | LINE |
| 63448997 | 63449190 | + | 7.2 | 4054 | 4247 | -1899 | L1PA6 | L1 | LINE |
| 63449191 | 63450349 | - | 14.5 | 353 | 1603 | -815 | Tigger1 | MER2\_type | DNA |

  

|  |  |  |  |  |  |  |  |  |  |
| --- | --- | --- | --- | --- | --- | --- | --- | --- | --- |
| chrX:63674214-63688196 | | | 13637 bp | | |  |  |  |  |
| Genome Start | Genome End | Strand | Div | Repeat Start | Repeat End | Repeat Left | Name | Family | Class |
| 63674214 | 63674340 | + | 26.2 | 1016 | 1150 | -4969 | L1MEd | L1 | LINE |
| 63674423 | 63675009 | + | 23.9 | 1319 | 1898 | -4251 | L1MEd | L1 | LINE |
| 63675044 | 63675641 | - | 4.0 | 5538 | 6137 | -18 | L1PA3 | L1 | LINE |
| 63675642 | 63675712 | + | 23.9 | 1899 | 1971 | -4178 | L1MEd | L1 | LINE |
| 63675736 | 63675770 | + | 23.9 | 1972 | 2012 | -4137 | L1MEd | L1 | LINE |
| 63675797 | 63675904 | - | 1.9 | 5122 | 5229 | -917 | L1PA3 | L1 | LINE |
| 63675906 | 63676838 | + | 3.0 | 5219 | 6152 | -3 | L1PA3 | L1 | LINE |
| 63676841 | 63677221 | + | 20.4 | 2047 | 2459 | -3690 | L1MEd | L1 | LINE |
| 63677228 | 63677341 | + | 27.1 | 2992 | 3108 | -3021 | L1MEd | L1 | LINE |
| 63677342 | 63677682 | + | 16.7 | 9 | 349 | 0 | AluJb | Alu | SINE |
| 63677683 | 63677829 | + | 27.1 | 3109 | 3245 | -2884 | L1MEd | L1 | LINE |
| 63677830 | 63677964 | + | 21.7 | 1 | 135 | -177 | AluJo | Alu | SINE |
| 63677965 | 63678276 | + | 10.7 | 1 | 309 | -3 | AluSx | Alu | SINE |
| 63678277 | 63678452 | + | 21.7 | 136 | 299 | -13 | AluJo | Alu | SINE |
| 63678453 | 63678773 | + | 27.1 | 3246 | 3611 | -2518 | L1MEd | L1 | LINE |
| 63678774 | 63679075 | - | 17.0 | 1 | 295 | -17 | AluJb | Alu | SINE |
| 63679076 | 63679393 | + | 27.1 | 3612 | 3937 | -2192 | L1MEd | L1 | LINE |
| 63679394 | 63679693 | + | 18.2 | 1 | 305 | -7 | AluJb | Alu | SINE |
| 63679694 | 63679792 | + | 27.1 | 3938 | 4031 | -2098 | L1MEd | L1 | LINE |
| 63679820 | 63680058 | - | 18.9 | 38 | 281 | -31 | AluJb | Alu | SINE |
| 63680121 | 63680381 | + | 31.9 | 4166 | 4435 | -1694 | L1MEd | L1 | LINE |
| 63680422 | 63686538 | - | 5.4 | 9 | 6152 | -3 | L1PA3 | L1 | LINE |
| 63686582 | 63686885 | + | 18.1 | 1 | 304 | -8 | AluJb | Alu | SINE |
| 63686886 | 63687618 | + | 28.6 | 4459 | 5229 | -900 | L1MEd | L1 | LINE |
| 63687619 | 63687922 | + | 22.9 | 9 | 310 | -2 | AluJo | Alu | SINE |
| 63687923 | 63688196 | + | 20.4 | 5230 | 5529 | -632 | L1MEd | L1 | LINE |

  

|  |  |  |  |  |  |  |  |  |  |
| --- | --- | --- | --- | --- | --- | --- | --- | --- | --- |
| chrX:63762598-63780628 | | | 17373 bp | | |  |  |  |  |
| Genome Start | Genome End | Strand | Div | Repeat Start | Repeat End | Repeat Left | Name | Family | Class |
| 63762598 | 63762770 | - | 18.6 | 5986 | 6160 | -6 | L1PA17 | L1 | LINE |
| 63762771 | 63763147 | + | 11.9 | 1 | 385 | -59 | MLT2A1 | ERVL | LTR |
| 63763190 | 63763234 | + | 11.9 | 494 | 551 | -1 | MLT2A1 | ERVL | LTR |
| 63763235 | 63768308 | + | 9.2 | 1 | 5104 | -550 | HERVL-A1 | ERVL | LTR |
| 63768308 | 63768661 | + | 10.7 | 5291 | 5654 | 0 | HERVL-A1 | ERVL | LTR |
| 63768662 | 63768916 | + | 11.8 | 1 | 262 | -182 | MLT2A1 | ERVL | LTR |
| 63769098 | 63769150 | + | 7.7 | 510 | 561 | 0 | MLT2A2 | ERVL | LTR |
| 63769161 | 63769327 | - | 18.6 | 5812 | 5978 | -188 | L1PA17 | L1 | LINE |
| 63769346 | 63769400 | - | 9.1 | 6246 | 6301 | -3 | L1MA2 | L1 | LINE |
| 63769395 | 63771877 | - | 5.5 | 3660 | 6154 | -1 | L1PA3 | L1 | LINE |
| 63771876 | 63772111 | + | 6.4 | 3445 | 3682 | -2464 | L1PA3 | L1 | LINE |
| 63772112 | 63773936 | - | 23.4 | 9 | 2529 | -5036 | L1M1 | L1 | LINE |
| 63773941 | 63775292 | - | 22.1 | 4437 | 5825 | -331 | L1PB4 | L1 | LINE |
| 63775293 | 63775644 | - | 14.0 | 1 | 364 | 0 | THE1B | MaLR | LTR |
| 63775645 | 63777375 | - | 24.1 | 2581 | 4436 | -1707 | L1M2 | L1 | LINE |
| 63777397 | 63777876 | - | 25.0 | 2620 | 3125 | -3790 | L1PA17 | L1 | LINE |
| 63777877 | 63778013 | - | 18.9 | 1 | 128 | -5 | FLAM\_C | Alu | SINE |
| 63778014 | 63779235 | - | 26.5 | 1618 | 2619 | -4300 | L1PA17 | L1 | LINE |
| 63779627 | 63780628 | - | 35.6 | 132 | 1111 | -5824 | L1PA17 | L1 | LINE |

  

|  |  |  |  |  |  |  |  |  |  |
| --- | --- | --- | --- | --- | --- | --- | --- | --- | --- |
| chrX:64251598-64264844 | | | 13220 bp | | |  |  |  |  |
| Genome Start | Genome End | Strand | Div | Repeat Start | Repeat End | Repeat Left | Name | Family | Class |
| 64251598 | 64251651 | - | 11.1 | 6247 | 6300 | -4 | L1MA2 | L1 | LINE |
| 64251663 | 64253176 | - | 10.7 | 4629 | 6151 | 0 | L1PB1 | L1 | LINE |
| 64253171 | 64255252 | - | 7.4 | 4068 | 6154 | 0 | L1PA5 | L1 | LINE |
| 64255256 | 64258795 | - | 5.5 | 2608 | 6155 | 0 | L1PA4 | L1 | LINE |
| 64258797 | 64259600 | + | 5.8 | 1794 | 2598 | -3548 | L1PA4 | L1 | LINE |
| 64259600 | 64260093 | - | 7.3 | 3593 | 4084 | -2062 | L1PA5 | L1 | LINE |
| 64260101 | 64264580 | - | 12.9 | 634 | 5352 | -1510 | L1PBa | L1 | LINE |
| 64264581 | 64264775 | - | 18.5 | 3 | 200 | -6663 | L1PBa | L1 | LINE |
| 64264788 | 64264844 | - | 3.5 | 6199 | 6255 | -49 | L1MA2 | L1 | LINE |

  

|  |  |  |  |  |  |  |  |  |  |
| --- | --- | --- | --- | --- | --- | --- | --- | --- | --- |
| chrX:64410480-64418502 | | | 8010 bp | | |  |  |  |  |
| Genome Start | Genome End | Strand | Div | Repeat Start | Repeat End | Repeat Left | Name | Family | Class |
| 64410480 | 64414815 | + | 21.9 | 4 | 4059 | -2726 | L1M2c | L1 | LINE |
| 64414822 | 64415146 | + | 12.0 | 219 | 546 | -88 | MER41B | ERV1 | LTR |
| 64415155 | 64415254 | + | 21.0 | 4065 | 4172 | -2610 | L1M2c | L1 | LINE |
| 64415253 | 64416043 | - | 11.2 | 5357 | 6161 | 0 | L1PA14 | L1 | LINE |
| 64416040 | 64416543 | + | 8.3 | 4856 | 5358 | -788 | L1PREC2 | L1 | LINE |
| 64416549 | 64417836 | + | 17.8 | 4169 | 5490 | -1295 | L1M2c | L1 | LINE |
| 64417837 | 64418130 | - | 15.0 | 1 | 301 | -11 | AluSx | Alu | SINE |
| 64418131 | 64418502 | + | 17.8 | 5491 | 5855 | -930 | L1M2c | L1 | LINE |

  

|  |  |  |  |  |  |  |  |  |  |
| --- | --- | --- | --- | --- | --- | --- | --- | --- | --- |
| chrX:65063677-65078719 | | | 14914 bp | | |  |  |  |  |
| Genome Start | Genome End | Strand | Div | Repeat Start | Repeat End | Repeat Left | Name | Family | Class |
| 65063677 | 65063852 | - | 23.5 | 2535 | 2705 | -2123 | L1MEe | L1 | LINE |
| 65063853 | 65064344 | - | 8.0 | 1 | 511 | 0 | LTR10F | ERV1 | LTR |
| 65064345 | 65065857 | - | 11.3 | 3583 | 5102 | 0 | HERVIP10FH | ERV1 | LTR |
| 65065919 | 65069017 | - | 14.2 | 3 | 3097 | -2005 | HERVIP10FH | ERV1 | LTR |
| 65069020 | 65069622 | - | 8.9 | 1 | 511 | 0 | LTR10F | ERV1 | LTR |
| 65069646 | 65070789 | - | 17.8 | 1 | 1231 | 0 | Tigger3b | MER2\_type | DNA |
| 65070843 | 65073174 | - | 3.8 | 3821 | 6152 | -3 | L1PA3 | L1 | LINE |
| 65073168 | 65076174 | + | 4.7 | 812 | 3819 | -2327 | L1PA3 | L1 | LINE |
| 65076172 | 65076373 | - | 21.3 | 2288 | 2480 | -2354 | L1MEe | L1 | LINE |
| 65076374 | 65076697 | - | 14.0 | 1 | 300 | -12 | AluSx | Alu | SINE |
| 65076698 | 65077259 | - | 21.3 | 1695 | 2287 | -2547 | L1MEe | L1 | LINE |
| 65077260 | 65077453 | - | 22.2 | 187 | 390 | 0 | MLT1B | MaLR | LTR |
| 65077454 | 65077888 | - | 21.3 | 1236 | 1694 | -3140 | L1MEe | L1 | LINE |
| 65077889 | 65078177 | + | 14.6 | 5 | 294 | -18 | AluSx | Alu | SINE |
| 65078178 | 65078719 | - | 22.5 | 726 | 1233 | -3601 | L1MEe | L1 | LINE |

  

|  |  |  |  |  |  |  |  |  |  |
| --- | --- | --- | --- | --- | --- | --- | --- | --- | --- |
| chrX:65347844-65350139 | | | 2246 bp | | |  |  |  |  |
| Genome Start | Genome End | Strand | Div | Repeat Start | Repeat End | Repeat Left | Name | Family | Class |
| 65347844 | 65349327 | - | 16.1 | 4703 | 6280 | -23 | L1MA4A | L1 | LINE |
| 65349353 | 65349573 | - | 18.2 | 4455 | 4702 | -1441 | L1MA4A | L1 | LINE |
| 65349598 | 65349927 | - | 3.1 | 5829 | 6155 | 0 | L1PA3 | L1 | LINE |
| 65349928 | 65350057 | + | 0.8 | 5698 | 5827 | -328 | L1PA3 | L1 | LINE |
| 65350059 | 65350139 | - | 21.0 | 4383 | 4464 | -1679 | L1MA4A | L1 | LINE |

  

|  |  |  |  |  |  |  |  |  |  |
| --- | --- | --- | --- | --- | --- | --- | --- | --- | --- |
| chrX:65440487-65447525 | | | 6968 bp | | |  |  |  |  |
| Genome Start | Genome End | Strand | Div | Repeat Start | Repeat End | Repeat Left | Name | Family | Class |
| 65440487 | 65441784 | - | 30.3 | 3203 | 4482 | -7 | L3 | CR1 | LINE |
| 65441788 | 65442022 | - | 23.4 | 135 | 342 | -3 | MER2B | MER2\_type | DNA |
| 65442064 | 65443782 | - | 8.3 | 4462 | 6170 | 0 | L1PA7 | L1 | LINE |
| 65443783 | 65444078 | - | 5.5 | 1 | 292 | -19 | AluY | Alu | SINE |
| 65444079 | 65445700 | - | 10.9 | 2825 | 4461 | -1685 | L1PA7 | L1 | LINE |
| 65445699 | 65446980 | + | 8.5 | 1468 | 2817 | -3329 | L1PA7 | L1 | LINE |
| 65446987 | 65447291 | - | 19.3 | 1 | 305 | -7 | AluSx | Alu | SINE |
| 65447315 | 65447388 | - | 27.0 | 2 | 75 | -261 | MER2B | MER2\_type | DNA |
| 65447389 | 65447525 | - | 37.6 | 3059 | 3204 | -1285 | L3 | CR1 | LINE |

  

|  |  |  |  |  |  |  |  |  |  |
| --- | --- | --- | --- | --- | --- | --- | --- | --- | --- |
| chrX:65849246-65854867 | | | 5335 bp | | |  |  |  |  |
| Genome Start | Genome End | Strand | Div | Repeat Start | Repeat End | Repeat Left | Name | Family | Class |
| 65849246 | 65849610 | - | 38.2 | 2989 | 3352 | -26 | L2 | L2 | LINE |
| 65849668 | 65849848 | - | 34.4 | 2669 | 2857 | -562 | L2 | L2 | LINE |
| 65849899 | 65850108 | + | 33.0 | 1825 | 2062 | -1357 | L2 | L2 | LINE |
| 65850114 | 65852276 | - | 2.2 | 3987 | 6153 | -2 | L1PA2 | L1 | LINE |
| 65852275 | 65852383 | + | 2.8 | 3883 | 3991 | -2155 | L1PA2 | L1 | LINE |
| 65852385 | 65852606 | + | 30.6 | 2094 | 2310 | -1109 | L2 | L2 | LINE |
| 65852631 | 65852722 | - | 20.7 | 1795 | 1881 | 0 | Kanga1 | Tc2 | DNA |
| 65852746 | 65853394 | + | 33.4 | 2477 | 3174 | -204 | L2 | L2 | LINE |
| 65853472 | 65854373 | + | 18.8 | 5409 | 6335 | -9 | L1MC2 | L1 | LINE |
| 65854441 | 65854548 | + | 32.7 | 3294 | 3419 | 0 | L2 | L2 | LINE |
| 65854534 | 65854867 | - | 32.3 | 2253 | 2630 | -789 | L2 | L2 | LINE |

  

|  |  |  |  |  |  |  |  |  |  |
| --- | --- | --- | --- | --- | --- | --- | --- | --- | --- |
| chrX:65942208-65946859 | | | 4629 bp | | |  |  |  |  |
| Genome Start | Genome End | Strand | Div | Repeat Start | Repeat End | Repeat Left | Name | Family | Class |
| 65942208 | 65942754 | + | 31.7 | 1506 | 2099 | -1320 | L2 | L2 | LINE |
| 65942762 | 65943964 | - | 17.4 | 4934 | 6165 | -3 | L1MB1 | L1 | LINE |
| 65943965 | 65944688 | - | 16.3 | 5555 | 6282 | -9 | L1MA7 | L1 | LINE |
| 65944689 | 65944922 | - | 16.1 | 4721 | 4933 | -1207 | L1MB1 | L1 | LINE |
| 65944921 | 65945987 | + | 16.7 | 3605 | 4698 | -1442 | L1MB1 | L1 | LINE |
| 65946006 | 65946355 | + | 3.2 | 5842 | 6191 | 0 | L1PA3 | L1 | LINE |
| 65946356 | 65946859 | + | 30.7 | 2106 | 2642 | -777 | L2 | L2 | LINE |

  

|  |  |  |  |  |  |  |  |  |  |
| --- | --- | --- | --- | --- | --- | --- | --- | --- | --- |
| chrX:66102902-66123091 | | | 20066 bp | | |  |  |  |  |
| Genome Start | Genome End | Strand | Div | Repeat Start | Repeat End | Repeat Left | Name | Family | Class |
| 66102902 | 66103950 | - | 18.0 | 4959 | 6019 | -1546 | L1M1 | L1 | LINE |
| 66103951 | 66104235 | + | 14.4 | 1 | 285 | -24 | AluSc | Alu | SINE |
| 66104236 | 66104716 | - | 18.0 | 4477 | 4958 | -2607 | L1M1 | L1 | LINE |
| 66104721 | 66106897 | + | 14.3 | 5 | 2189 | -401 | L1PA7 | L1 | LINE |
| 66106925 | 66109431 | - | 3.7 | 3628 | 6137 | -18 | L1PA3 | L1 | LINE |
| 66109422 | 66110581 | + | 11.9 | 2178 | 3321 | -3140 | L1PA7 | L1 | LINE |
| 66110597 | 66113468 | - | 8.9 | 3261 | 6147 | -7 | L1PA6 | L1 | LINE |
| 66113470 | 66115078 | + | 10.8 | 4195 | 5803 | -666 | L1PA7 | L1 | LINE |
| 66115079 | 66115612 | + | 8.6 | 5936 | 6469 | 0 | L1PA7 | L1 | LINE |
| 66115615 | 66116427 | - | 18.6 | 3669 | 4491 | -3074 | L1M1 | L1 | LINE |
| 66116508 | 66116726 | - | 4.1 | 5934 | 6152 | -3 | L1PA4 | L1 | LINE |
| 66116727 | 66117244 | + | 4.6 | 5412 | 5929 | -226 | L1PA4 | L1 | LINE |
| 66117250 | 66118006 | - | 16.5 | 2892 | 3675 | -3890 | L1M1 | L1 | LINE |
| 66118007 | 66118377 | - | 13.9 | 1 | 381 | 0 | THE1D | MaLR | LTR |
| 66118378 | 66119973 | - | 16.6 | 1 | 1580 | 0 | THE1D-int | MaLR | LTR |
| 66119974 | 66120343 | - | 13.9 | 1 | 381 | 0 | THE1D | MaLR | LTR |
| 66120344 | 66123091 | - | 20.5 | 20 | 2896 | -4669 | L1M1 | L1 | LINE |

  

|  |  |  |  |  |  |  |  |  |  |
| --- | --- | --- | --- | --- | --- | --- | --- | --- | --- |
| chrX:66270883-66275787 | | | 4845 bp | | |  |  |  |  |
| Genome Start | Genome End | Strand | Div | Repeat Start | Repeat End | Repeat Left | Name | Family | Class |
| 66270883 | 66270984 | - | 38.6 | 164 | 267 | -1 | MIRb | MIR | SINE |
| 66271006 | 66271057 | - | 13.5 | 6132 | 6183 | 0 | L1MB3 | L1 | LINE |
| 66271059 | 66271558 | - | 16.9 | 5676 | 6181 | -2 | L1MB3 | L1 | LINE |
| 66271545 | 66272118 | + | 17.8 | 5579 | 6148 | -8 | L1PB4 | L1 | LINE |
| 66272157 | 66272882 | - | 17.9 | 4939 | 5667 | -516 | L1MB3 | L1 | LINE |
| 66272884 | 66273942 | - | 3.3 | 5094 | 6153 | -2 | L1PA4 | L1 | LINE |
| 66273941 | 66275091 | + | 5.5 | 3939 | 5091 | -1055 | L1PA4 | L1 | LINE |
| 66275102 | 66275648 | - | 24.6 | 4385 | 4943 | -1197 | L1MB3 | L1 | LINE |
| 66275654 | 66275787 | - | 37.6 | 2 | 149 | -119 | MIRb | MIR | SINE |

  

|  |  |  |  |  |  |  |  |  |  |
| --- | --- | --- | --- | --- | --- | --- | --- | --- | --- |
| chrX:66287192-66289883 | | | 2636 bp | | |  |  |  |  |
| Genome Start | Genome End | Strand | Div | Repeat Start | Repeat End | Repeat Left | Name | Family | Class |
| 66287192 | 66287432 | - | 15.3 | 5352 | 5603 | -699 | L1M1 | L1 | LINE |
| 66287426 | 66287436 | - | 4.7 | 6141 | 6150 | -4 | L1PA5 | L1 | LINE |
| 66287503 | 66289066 | - | 5.0 | 4577 | 6140 | -14 | L1PA5 | L1 | LINE |
| 66289065 | 66289675 | + | 5.9 | 3977 | 4587 | -1559 | L1PA5 | L1 | LINE |
| 66289675 | 66289883 | - | 12.5 | 5141 | 5364 | -782 | L1M1 | L1 | LINE |

  

|  |  |  |  |  |  |  |  |  |  |
| --- | --- | --- | --- | --- | --- | --- | --- | --- | --- |
| chrX:66308709-66313057 | | | 4270 bp | | |  |  |  |  |
| Genome Start | Genome End | Strand | Div | Repeat Start | Repeat End | Repeat Left | Name | Family | Class |
| 66308709 | 66309141 | - | 16.1 | 3826 | 4257 | -2509 | L1PA13 | L1 | LINE |
| 66309176 | 66309397 | - | 16.1 | 3596 | 3825 | -2941 | L1PA13 | L1 | LINE |
| 66309388 | 66309485 | - | 2.0 | 5999 | 6096 | -59 | L1PA4 | L1 | LINE |
| 66309486 | 66309558 | + | 5.5 | 6080 | 6152 | -3 | L1PA4 | L1 | LINE |
| 66309589 | 66309665 | - | 10.4 | 3528 | 3606 | -3160 | L1PA13 | L1 | LINE |
| 66309663 | 66311232 | + | 12.4 | 4575 | 6182 | 0 | L1PB1 | L1 | LINE |
| 66311261 | 66311556 | + | 10.8 | 1 | 296 | -17 | AluSp | Alu | SINE |
| 66311557 | 66313057 | - | 14.9 | 2013 | 3543 | -3223 | L1PA13 | L1 | LINE |

  

|  |  |  |  |  |  |  |  |  |  |
| --- | --- | --- | --- | --- | --- | --- | --- | --- | --- |
| chrX:66331094-66334557 | | | 3465 bp | | |  |  |  |  |
| Genome Start | Genome End | Strand | Div | Repeat Start | Repeat End | Repeat Left | Name | Family | Class |
| 66331094 | 66331197 | + | 27.4 | 12 | 117 | -151 | MIRb | MIR | SINE |
| 66331200 | 66331873 | - | 8.8 | 5173 | 5861 | -311 | L1PA8 | L1 | LINE |
| 66331867 | 66334495 | + | 9.8 | 2544 | 5154 | -992 | L1PA8 | L1 | LINE |
| 66334500 | 66334557 | + | 29.3 | 123 | 180 | -88 | MIRb | MIR | SINE |

  

|  |  |  |  |  |  |  |  |  |  |
| --- | --- | --- | --- | --- | --- | --- | --- | --- | --- |
| chrX:66530110-66541265 | | | 11151 bp | | |  |  |  |  |
| Genome Start | Genome End | Strand | Div | Repeat Start | Repeat End | Repeat Left | Name | Family | Class |
| 66530110 | 66530581 | - | 12.5 | 6390 | 6864 | -3 | L1PB2 | L1 | LINE |
| 66530583 | 66530721 | - | 15.0 | 6039 | 6172 | 0 | L1PA8 | L1 | LINE |
| 66530724 | 66532156 | - | 15.0 | 4932 | 6396 | -471 | L1PB2 | L1 | LINE |
| 66532165 | 66534597 | - | 4.9 | 3652 | 6150 | -5 | L1PA3 | L1 | LINE |
| 66534596 | 66535536 | + | 5.8 | 2711 | 3650 | -2496 | L1PA3 | L1 | LINE |
| 66535533 | 66541265 | - | 19.4 | 3 | 4946 | -1916 | L1PB2 | L1 | LINE |

  

|  |  |  |  |  |  |  |  |  |  |
| --- | --- | --- | --- | --- | --- | --- | --- | --- | --- |
| chrX:66600508-66616870 | | | 16318 bp | | |  |  |  |  |
| Genome Start | Genome End | Strand | Div | Repeat Start | Repeat End | Repeat Left | Name | Family | Class |
| 66600508 | 66600726 | + | 24.6 | 3036 | 3251 | -2895 | L1M4 | L1 | LINE |
| 66600729 | 66600871 | - | 20.4 | 3177 | 3331 | -2815 | L1MC | L1 | LINE |
| 66600869 | 66601382 | + | 22.9 | 3624 | 4145 | -2001 | L1M4 | L1 | LINE |
| 66601417 | 66601561 | + | 22.9 | 4146 | 4303 | -1843 | L1M4 | L1 | LINE |
| 66601531 | 66603978 | + | 18.0 | 13 | 2430 | -5135 | L1M1 | L1 | LINE |
| 66603979 | 66604282 | + | 11.2 | 1 | 308 | -3 | AluY | Alu | SINE |
| 66604283 | 66605668 | + | 16.7 | 2431 | 3824 | -3741 | L1M1 | L1 | LINE |
| 66605669 | 66606157 | - | 11.4 | 1 | 454 | -234 | LTR12\_ | ERV1 | LTR |
| 66606158 | 66606835 | + | 11.5 | 3820 | 4507 | -3058 | L1M1 | L1 | LINE |
| 66606836 | 66606926 | + | 8.8 | 1 | 91 | -221 | AluSp | Alu | SINE |
| 66606927 | 66607096 | + | 10.9 | 4170 | 4347 | -3218 | L1MA1 | L1 | LINE |
| 66607098 | 66607305 | + | 14.4 | 90 | 297 | -16 | AluSp | Alu | SINE |
| 66607306 | 66607751 | + | 11.7 | 4498 | 4946 | -2619 | L1MA1 | L1 | LINE |
| 66607750 | 66609891 | - | 4.5 | 1913 | 4054 | -2092 | L1PA3 | L1 | LINE |
| 66609890 | 66611975 | + | 4.1 | 4066 | 6152 | -3 | L1PA3 | L1 | LINE |
| 66611984 | 66614692 | + | 12.8 | 4926 | 7711 | -8 | L1MA1 | L1 | LINE |
| 66614717 | 66614843 | + | 20.9 | 4249 | 4391 | -1749 | L1M4 | L1 | LINE |
| 66614844 | 66615334 | + | 4.1 | 5665 | 6155 | 0 | L1PA4 | L1 | LINE |
| 66615335 | 66615477 | + | 22.8 | 4398 | 4507 | -1639 | L1M4 | L1 | LINE |
| 66615492 | 66616354 | - | 13.2 | 5284 | 6157 | -6 | L1PA13 | L1 | LINE |
| 66616355 | 66616870 | + | 24.1 | 4531 | 5046 | -1100 | L1M4 | L1 | LINE |

  

|  |  |  |  |  |  |  |  |  |  |
| --- | --- | --- | --- | --- | --- | --- | --- | --- | --- |
| chrX:66645476-66654330 | | | 8826 bp | | |  |  |  |  |
| Genome Start | Genome End | Strand | Div | Repeat Start | Repeat End | Repeat Left | Name | Family | Class |
| 66645476 | 66647950 | + | 15.8 | 6 | 2477 | -4006 | L1P3 | L1 | LINE |
| 66647948 | 66648652 | - | 10.8 | 3279 | 3983 | -2163 | L1PA6 | L1 | LINE |
| 66648647 | 66648943 | + | 8.4 | 3984 | 4307 | -1839 | L1PA6 | L1 | LINE |
| 66648944 | 66649207 | + | 4.9 | 5873 | 6155 | 0 | L1PA4 | L1 | LINE |
| 66649208 | 66651043 | + | 7.8 | 4308 | 6154 | 0 | L1PA6 | L1 | LINE |
| 66651082 | 66654330 | + | 10.8 | 2463 | 5735 | -913 | L1P3 | L1 | LINE |

  

|  |  |  |  |  |  |  |  |  |  |
| --- | --- | --- | --- | --- | --- | --- | --- | --- | --- |
| chrX:67127021-67131107 | | | 3917 bp | | |  |  |  |  |
| Genome Start | Genome End | Strand | Div | Repeat Start | Repeat End | Repeat Left | Name | Family | Class |
| 67127021 | 67127106 | + | 11.6 | 2741 | 2826 | -3320 | L1PA8 | L1 | LINE |
| 67127107 | 67127315 | - | 8.7 | 5272 | 5480 | -692 | L1PA7 | L1 | LINE |
| 67127317 | 67127989 | + | 9.4 | 5472 | 6151 | -3 | L1PA7 | L1 | LINE |
| 67128159 | 67131107 | + | 12.1 | 2840 | 6170 | -2 | L1PA8 | L1 | LINE |

  

|  |  |  |  |  |  |  |  |  |  |
| --- | --- | --- | --- | --- | --- | --- | --- | --- | --- |
| chrX:68192450-68195288 | | | 2805 bp | | |  |  |  |  |
| Genome Start | Genome End | Strand | Div | Repeat Start | Repeat End | Repeat Left | Name | Family | Class |
| 68192450 | 68192688 | + | 13.9 | 6637 | 6892 | -1103 | L1MD3 | L1 | LINE |
| 68192724 | 68192819 | + | 13.9 | 6893 | 6993 | -1002 | L1MD3 | L1 | LINE |
| 68192818 | 68193245 | - | 16.0 | 7362 | 7785 | 0 | L1MC3 | L1 | LINE |
| 68193250 | 68194135 | - | 2.4 | 4391 | 5278 | -868 | L1PA3 | L1 | LINE |
| 68194133 | 68195011 | + | 1.9 | 5280 | 6155 | 0 | L1PA3 | L1 | LINE |
| 68195013 | 68195248 | - | 17.8 | 7102 | 7366 | -419 | L1MC3 | L1 | LINE |
| 68195248 | 68195288 | + | 23.4 | 6980 | 7031 | -964 | L1MD3 | L1 | LINE |

  

|  |  |  |  |  |  |  |  |  |  |
| --- | --- | --- | --- | --- | --- | --- | --- | --- | --- |
| chrX:68380153-68382168 | | | 1992 bp | | |  |  |  |  |
| Genome Start | Genome End | Strand | Div | Repeat Start | Repeat End | Repeat Left | Name | Family | Class |
| 68380153 | 68380299 | + | 21.5 | 5616 | 5768 | -2040 | L1MC4a | L1 | LINE |
| 68380324 | 68381482 | - | 5.9 | 4434 | 5606 | -548 | L1PA7 | L1 | LINE |
| 68381483 | 68382037 | + | 6.3 | 5599 | 6153 | -1 | L1PA7 | L1 | LINE |
| 68382038 | 68382168 | + | 28.2 | 5821 | 5963 | -1845 | L1MC4a | L1 | LINE |

  

|  |  |  |  |  |  |  |  |  |  |
| --- | --- | --- | --- | --- | --- | --- | --- | --- | --- |
| chrX:69883914-69888869 | | | 4909 bp | | |  |  |  |  |
| Genome Start | Genome End | Strand | Div | Repeat Start | Repeat End | Repeat Left | Name | Family | Class |
| 69883914 | 69884331 | + | 18.6 | 1205 | 1614 | -5720 | L1M4b | L1 | LINE |
| 69884332 | 69884474 | - | 3.6 | 6017 | 6154 | 0 | L1PA5 | L1 | LINE |
| 69884475 | 69885015 | + | 18.6 | 1615 | 2213 | -5121 | L1M4b | L1 | LINE |
| 69885027 | 69886497 | - | 2.0 | 3651 | 5123 | -1023 | L1PA2 | L1 | LINE |
| 69886492 | 69887508 | + | 2.2 | 5136 | 6154 | -1 | L1PA2 | L1 | LINE |
| 69887542 | 69888220 | + | 3.4 | 5478 | 6154 | -1 | L1PA4 | L1 | LINE |
| 69888230 | 69888869 | + | 19.6 | 2274 | 3020 | -4314 | L1M4b | L1 | LINE |

  

|  |  |  |  |  |  |  |  |  |  |
| --- | --- | --- | --- | --- | --- | --- | --- | --- | --- |
| chrX:70468227-70476163 | | | 7886 bp | | |  |  |  |  |
| Genome Start | Genome End | Strand | Div | Repeat Start | Repeat End | Repeat Left | Name | Family | Class |
| 70468227 | 70468351 | - | 17.2 | 5708 | 5830 | -1176 | L1M4c | L1 | LINE |
| 70468346 | 70468566 | - | 15.8 | 5702 | 5922 | -369 | L1MA7 | L1 | LINE |
| 70468564 | 70468738 | + | 14.9 | 5901 | 6062 | -229 | L1MA7 | L1 | LINE |
| 70468739 | 70469035 | + | 7.4 | 5 | 301 | -12 | AluSq | Alu | SINE |
| 70469036 | 70469108 | + | 14.9 | 6063 | 6130 | -161 | L1MA7 | L1 | LINE |
| 70469109 | 70469251 | + | 16.5 | 1 | 133 | -10 | FLAM\_C | Alu | SINE |
| 70469269 | 70469431 | + | 17.2 | 6116 | 6281 | -10 | L1MA7 | L1 | LINE |
| 70469458 | 70469671 | - | 18.3 | 5519 | 5719 | -1287 | L1M4c | L1 | LINE |
| 70469672 | 70469982 | - | 10.2 | 1 | 295 | -15 | AluSg | Alu | SINE |
| 70469983 | 70470083 | - | 18.3 | 5424 | 5518 | -1488 | L1M4c | L1 | LINE |
| 70470084 | 70470377 | - | 12.2 | 3 | 297 | -15 | AluSx | Alu | SINE |
| 70470378 | 70470765 | - | 18.3 | 4957 | 5423 | -1583 | L1M4c | L1 | LINE |
| 70470766 | 70471064 | + | 9.4 | 1 | 301 | -12 | AluSp | Alu | SINE |
| 70471065 | 70471590 | - | 18.3 | 4444 | 4956 | -2050 | L1M4c | L1 | LINE |
| 70471591 | 70471888 | + | 10.8 | 1 | 298 | -12 | AluSg | Alu | SINE |
| 70471889 | 70472183 | + | 7.1 | 1 | 294 | -17 | AluY | Alu | SINE |
| 70472184 | 70472448 | - | 18.3 | 4169 | 4443 | -2563 | L1M4c | L1 | LINE |
| 70472449 | 70472739 | - | 13.8 | 1 | 291 | -21 | AluSx | Alu | SINE |
| 70472740 | 70472760 | - | 18.3 | 4141 | 4168 | -2838 | L1M4c | L1 | LINE |
| 70472761 | 70473481 | - | 13.7 | 1 | 691 | 0 | LTR8 | ERV1 | LTR |
| 70473482 | 70473605 | - | 18.3 | 3974 | 4140 | -2866 | L1M4c | L1 | LINE |
| 70473606 | 70473719 | - | 18.9 | 33 | 143 | 0 | FLAM\_C | Alu | SINE |
| 70473720 | 70473769 | - | 18.3 | 3907 | 3973 | -3033 | L1M4c | L1 | LINE |
| 70473770 | 70474066 | - | 12.0 | 1 | 293 | -19 | AluSx | Alu | SINE |
| 70474067 | 70474631 | - | 18.3 | 3317 | 3903 | -3103 | L1M4c | L1 | LINE |
| 70474635 | 70475067 | + | 14.3 | 15 | 457 | 0 | MSTB2 | MaLR | LTR |
| 70475068 | 70475414 | - | 20.3 | 2966 | 3319 | -3687 | L1M4c | L1 | LINE |
| 70475415 | 70475716 | - | 6.0 | 1 | 302 | -9 | AluY | Alu | SINE |
| 70475717 | 70475866 | - | 23.1 | 2814 | 2978 | -4048 | L1M4c | L1 | LINE |
| 70475879 | 70476004 | + | 12.3 | 12 | 134 | -9 | FLAM\_C | Alu | SINE |
| 70476007 | 70476163 | - | 18.5 | 1924 | 2087 | -4921 | L1M4c | L1 | LINE |

  

|  |  |  |  |  |  |  |  |  |  |
| --- | --- | --- | --- | --- | --- | --- | --- | --- | --- |
| chrX:70932669-70946102 | | | 13308 bp | | |  |  |  |  |
| Genome Start | Genome End | Strand | Div | Repeat Start | Repeat End | Repeat Left | Name | Family | Class |
| 70932669 | 70933786 | + | 24.8 | 1200 | 2352 | -5137 | L1M3e | L1 | LINE |
| 70933787 | 70934057 | - | 10.0 | 1 | 270 | -43 | AluSq | Alu | SINE |
| 70934058 | 70934286 | + | 25.9 | 2353 | 2590 | -4976 | L1M3e | L1 | LINE |
| 70934321 | 70934639 | - | 17.1 | 1 | 309 | -3 | AluJb | Alu | SINE |
| 70934640 | 70935039 | + | 26.2 | 3008 | 3429 | -4136 | L1M3e | L1 | LINE |
| 70935033 | 70938407 | - | 3.8 | 895 | 4274 | -1749 | L1HS | L1 | LINE |
| 70938407 | 70940163 | + | 3.7 | 4395 | 6151 | -4 | L1PA4 | L1 | LINE |
| 70940154 | 70940358 | + | 15.6 | 3555 | 3770 | -2370 | L1M3 | L1 | LINE |
| 70940410 | 70941770 | + | 16.4 | 297 | 1678 | -5887 | L1M1 | L1 | LINE |
| 70941768 | 70942638 | + | 10.7 | 3586 | 4467 | -3098 | L1M3e | L1 | LINE |
| 70942627 | 70942856 | + | 11.8 | 4972 | 5193 | -2372 | L1M3e | L1 | LINE |
| 70942934 | 70944190 | - | 5.9 | 4884 | 6140 | -14 | L1PA6 | L1 | LINE |
| 70944190 | 70945499 | + | 7.4 | 3550 | 4861 | -1285 | L1PA6 | L1 | LINE |
| 70945496 | 70945622 | - | 11.8 | 4050 | 4177 | -1969 | L1M1 | L1 | LINE |
| 70945625 | 70946102 | + | 12.0 | 5205 | 5835 | -1730 | L1M3e | L1 | LINE |

  

|  |  |  |  |  |  |  |  |  |  |
| --- | --- | --- | --- | --- | --- | --- | --- | --- | --- |
| chrX:71805298-71808955 | | | 3606 bp | | |  |  |  |  |
| Genome Start | Genome End | Strand | Div | Repeat Start | Repeat End | Repeat Left | Name | Family | Class |
| 71805298 | 71805912 | - | 22.8 | 966 | 1651 | 0 | MST-int | MaLR | LTR |
| 71805958 | 71807016 | - | 3.5 | 5088 | 6152 | -3 | L1PA4 | L1 | LINE |
| 71807018 | 71808597 | + | 3.4 | 3497 | 5076 | -1070 | L1PA4 | L1 | LINE |
| 71808604 | 71808955 | - | 25.5 | 591 | 959 | -692 | MST-int | MaLR | LTR |

  

|  |  |  |  |  |  |  |  |  |  |
| --- | --- | --- | --- | --- | --- | --- | --- | --- | --- |
| chrX:72145270-72150074 | | | 4751 bp | | |  |  |  |  |
| Genome Start | Genome End | Strand | Div | Repeat Start | Repeat End | Repeat Left | Name | Family | Class |
| 72145270 | 72145857 | - | 11.8 | 3018 | 3614 | -2143 | ERVL | ERVL | LTR |
| 72145859 | 72147496 | - | 1.3 | 3441 | 5078 | -1068 | L1PA2 | L1 | LINE |
| 72147496 | 72148573 | + | 1.5 | 5078 | 6155 | 0 | L1PA2 | L1 | LINE |
| 72148628 | 72150074 | - | 15.7 | 1562 | 3027 | -2730 | ERVL | ERVL | LTR |

  

|  |  |  |  |  |  |  |  |  |  |
| --- | --- | --- | --- | --- | --- | --- | --- | --- | --- |
| chrX:72557788-72576008 | | | 18208 bp | | |  |  |  |  |
| Genome Start | Genome End | Strand | Div | Repeat Start | Repeat End | Repeat Left | Name | Family | Class |
| 72557788 | 72558029 | + | 21.9 | 78 | 325 | -6183 | L1MCa | L1 | LINE |
| 72558026 | 72558090 | + | 21.9 | 966 | 1029 | -5479 | L1MCa | L1 | LINE |
| 72558089 | 72559311 | - | 13.4 | 1672 | 2900 | -4081 | L1PA14 | L1 | LINE |
| 72559310 | 72563853 | + | 11.2 | 2898 | 7452 | -144 | L1PA14 | L1 | LINE |
| 72563850 | 72564171 | - | 26.4 | 2423 | 2766 | -3453 | L1MEc | L1 | LINE |
| 72564180 | 72564331 | - | 21.7 | 5816 | 5968 | -210 | L1MB8 | L1 | LINE |
| 72564325 | 72565180 | - | 11.7 | 5293 | 6159 | -2 | L1PA14 | L1 | LINE |
| 72565189 | 72571312 | - | 5.9 | 4 | 6147 | -8 | L1PA4 | L1 | LINE |
| 72571312 | 72571937 | - | 21.4 | 1879 | 2471 | -3929 | L1MEc | L1 | LINE |
| 72571924 | 72572745 | + | 20.8 | 1015 | 1897 | -4611 | L1MCa | L1 | LINE |
| 72572766 | 72572952 | + | 20.8 | 1898 | 2137 | -4371 | L1MCa | L1 | LINE |
| 72572964 | 72573321 | - | 16.9 | 1 | 364 | 0 | THE1B | MaLR | LTR |
| 72573322 | 72574844 | - | 11.7 | 1 | 1580 | 0 | THE1B-int | MaLR | LTR |
| 72574845 | 72575198 | - | 14.5 | 1 | 364 | 0 | THE1B | MaLR | LTR |
| 72575199 | 72575279 | + | 14.4 | 2152 | 2226 | -4282 | L1MCa | L1 | LINE |
| 72575280 | 72575627 | - | 22.5 | 1 | 363 | -2 | MLT1A0 | MaLR | LTR |
| 72575628 | 72576008 | + | 14.7 | 2227 | 2614 | -3707 | L1MCa | L1 | LINE |

  

|  |  |  |  |  |  |  |  |  |  |
| --- | --- | --- | --- | --- | --- | --- | --- | --- | --- |
| chrX:72731191-72775090 | | | 43645 bp | | |  |  |  |  |
| Genome Start | Genome End | Strand | Div | Repeat Start | Repeat End | Repeat Left | Name | Family | Class |
| 72731191 | 72731733 | - | 22.2 | 5579 | 6129 | -51 | L1MB4 | L1 | LINE |
| 72731742 | 72732705 | - | 7.7 | 5181 | 6148 | -6 | L1PA7 | L1 | LINE |
| 72732706 | 72733626 | + | 9.9 | 4258 | 5178 | -968 | L1PA7 | L1 | LINE |
| 72733619 | 72734105 | - | 22.1 | 5088 | 5585 | -598 | L1MB4 | L1 | LINE |
| 72734200 | 72734891 | - | 6.7 | 5413 | 6136 | -18 | L1PA7 | L1 | LINE |
| 72734892 | 72737351 | - | 2.2 | 3696 | 6155 | 0 | L1PA2 | L1 | LINE |
| 72737351 | 72738809 | + | 4.0 | 2220 | 3670 | -2476 | L1PA2 | L1 | LINE |
| 72738807 | 72744511 | - | 10.8 | 2 | 5743 | -744 | L1P2 | L1 | LINE |
| 72744523 | 72745430 | - | 22.0 | 4875 | 5797 | -1045 | L1M2 | L1 | LINE |
| 72745431 | 72745732 | + | 11.4 | 1 | 299 | -13 | AluSx | Alu | SINE |
| 72745733 | 72745955 | - | 22.0 | 4657 | 4874 | -1968 | L1M2 | L1 | LINE |
| 72745963 | 72746225 | - | 20.7 | 44 | 300 | -12 | AluJo | Alu | SINE |
| 72746228 | 72746779 | - | 14.2 | 5817 | 6301 | -2 | L1MA4A | L1 | LINE |
| 72746814 | 72747083 | - | 14.5 | 10 | 293 | -6168 | L1P3 | L1 | LINE |
| 72747096 | 72747584 | - | 16.9 | 3897 | 4393 | -2452 | L1M2 | L1 | LINE |
| 72747580 | 72748809 | - | 3.2 | 4925 | 6155 | 0 | L1PA4 | L1 | LINE |
| 72748810 | 72749794 | - | 19.5 | 2917 | 3912 | -2933 | L1M2 | L1 | LINE |
| 72749795 | 72750214 | + | 18.5 | 3 | 432 | 0 | MSTB1 | MaLR | LTR |
| 72750215 | 72752883 | - | 20.2 | 237 | 2916 | -3929 | L1M2 | L1 | LINE |
| 72752884 | 72754041 | - | 10.5 | 1 | 1176 | -404 | THE1B-int | MaLR | LTR |
| 72754042 | 72754399 | - | 12.6 | 1 | 364 | 0 | THE1B | MaLR | LTR |
| 72754400 | 72755889 | - | 21.2 | 10 | 1468 | -5144 | L1PBa1 | L1 | LINE |
| 72755890 | 72756269 | - | 14.2 | 3238 | 3628 | -3937 | L1M1 | L1 | LINE |
| 72756266 | 72760127 | - | 8.8 | 2265 | 6154 | 0 | L1PA7 | L1 | LINE |
| 72760128 | 72760329 | - | 13.4 | 3300 | 3505 | -2441 | MER41B-int | ERV1 | LTR |
| 72760317 | 72760932 | - | 12.9 | 2258 | 2946 | -3000 | MER41B-int | ERV1 | LTR |
| 72760918 | 72761066 | - | 10.9 | 1846 | 2006 | -3940 | MER41B-int | ERV1 | LTR |
| 72761055 | 72762586 | - | 12.9 | 1 | 1607 | -4339 | MER41B-int | ERV1 | LTR |
| 72762587 | 72762734 | - | 9.5 | 481 | 634 | 0 | MER41B | ERV1 | LTR |
| 72762737 | 72765433 | - | 18.3 | 22 | 2772 | -4793 | L1M1 | L1 | LINE |
| 72765441 | 72767904 | - | 16.7 | 3117 | 5669 | -707 | L1M2 | L1 | LINE |
| 72767905 | 72768200 | + | 12.4 | 1 | 295 | -17 | AluSx | Alu | SINE |
| 72768201 | 72769320 | - | 19.7 | 1740 | 3116 | -3094 | L1M2 | L1 | LINE |
| 72769321 | 72769626 | - | 6.9 | 1 | 307 | -4 | AluY | Alu | SINE |
| 72769627 | 72771660 | - | 27.2 | 1 | 2040 | -4171 | L1M2 | L1 | LINE |
| 72771661 | 72772371 | - | 20.3 | 3792 | 4531 | -2210 | L1MB4 | L1 | LINE |
| 72772358 | 72772829 | - | 21.0 | 2824 | 3335 | -3412 | L1MB4 | L1 | LINE |
| 72772846 | 72773008 | - | 25.8 | 1 | 164 | -21 | FAM | Alu | SINE |
| 72773018 | 72774727 | - | 23.8 | 580 | 2807 | -3942 | L1MB4 | L1 | LINE |
| 72774856 | 72775090 | - | 24.8 | 158 | 432 | -6175 | L1MB4 | L1 | LINE |

  

|  |  |  |  |  |  |  |  |  |  |
| --- | --- | --- | --- | --- | --- | --- | --- | --- | --- |
| chrX:73793704-73798602 | | | 4885 bp | | |  |  |  |  |
| Genome Start | Genome End | Strand | Div | Repeat Start | Repeat End | Repeat Left | Name | Family | Class |
| 73793704 | 73793840 | - | 18.4 | 2193 | 2329 | -5236 | L1M1 | L1 | LINE |
| 73793841 | 73794130 | - | 11.8 | 1 | 288 | -24 | AluSx | Alu | SINE |
| 73794131 | 73795524 | - | 18.4 | 731 | 2192 | -5373 | L1M1 | L1 | LINE |
| 73795525 | 73795970 | - | 9.6 | 1 | 608 | 0 | LTR14B | ERVK | LTR |
| 73795972 | 73796521 | - | 23.0 | 197 | 738 | -6827 | L1M1 | L1 | LINE |
| 73796515 | 73797255 | - | 3.4 | 4291 | 5029 | -1117 | L1PA3 | L1 | LINE |
| 73797247 | 73798382 | + | 1.8 | 5019 | 6154 | -1 | L1PA3 | L1 | LINE |
| 73798412 | 73798602 | - | 21.1 | 16 | 208 | -7357 | L1M1 | L1 | LINE |

  

|  |  |  |  |  |  |  |  |  |  |
| --- | --- | --- | --- | --- | --- | --- | --- | --- | --- |
| chrX:73811989-73814490 | | | 2038 bp | | |  |  |  |  |
| Genome Start | Genome End | Strand | Div | Repeat Start | Repeat End | Repeat Left | Name | Family | Class |
| 73811989 | 73812159 | - | 23.8 | 5998 | 6166 | -4 | L1ME3A | L1 | LINE |
| 73812239 | 73812493 | - | 9.8 | 5528 | 5783 | -385 | L1PA13 | L1 | LINE |
| 73812494 | 73812851 | + | 7.0 | 5796 | 6152 | -11 | L1PA13 | L1 | LINE |
| 73812876 | 73813050 | - | 30.4 | 4785 | 4968 | -1178 | L1M5 | L1 | LINE |
| 73813053 | 73813264 | - | 28.6 | 2634 | 2846 | -573 | L2 | L2 | LINE |
| 73813286 | 73813556 | - | 28.4 | 4508 | 4775 | -1371 | L1M5 | L1 | LINE |
| 73813895 | 73814012 | - | 28.7 | 5866 | 5983 | -257 | L1ME3A | L1 | LINE |
| 73814013 | 73814297 | + | 14.4 | 1 | 292 | -20 | AluSx | Alu | SINE |
| 73814298 | 73814490 | - | 26.7 | 5678 | 5865 | -375 | L1ME3A | L1 | LINE |

  

|  |  |  |  |  |  |  |  |  |  |
| --- | --- | --- | --- | --- | --- | --- | --- | --- | --- |
| chrX:74675298-74681848 | | | 6523 bp | | |  |  |  |  |
| Genome Start | Genome End | Strand | Div | Repeat Start | Repeat End | Repeat Left | Name | Family | Class |
| 74675298 | 74676449 | - | 13.1 | 2559 | 3760 | -3102 | L1PB1 | L1 | LINE |
| 74676451 | 74676807 | - | 14.5 | 2 | 380 | -1 | THE1D | MaLR | LTR |
| 74676837 | 74677337 | - | 2.6 | 4846 | 5345 | -801 | L1PA3 | L1 | LINE |
| 74677326 | 74678144 | + | 1.6 | 5337 | 6155 | 0 | L1PA3 | L1 | LINE |
| 74678155 | 74678294 | - | 17.1 | 1491 | 1632 | -19 | MST-int | MaLR | LTR |
| 74678287 | 74678947 | - | 16.5 | 9 | 635 | -945 | THE1D-int | MaLR | LTR |
| 74678956 | 74679319 | - | 14.9 | 1 | 381 | 0 | THE1D | MaLR | LTR |
| 74679320 | 74681848 | - | 14.8 | 3 | 2560 | -4303 | L1PB1 | L1 | LINE |

  

|  |  |  |  |  |  |  |  |  |  |
| --- | --- | --- | --- | --- | --- | --- | --- | --- | --- |
| chrX:74836788-74841250 | | | 4441 bp | | |  |  |  |  |
| Genome Start | Genome End | Strand | Div | Repeat Start | Repeat End | Repeat Left | Name | Family | Class |
| 74836788 | 74837263 | + | 25.3 | 819 | 1291 | -5690 | L1PA12 | L1 | LINE |
| 74837265 | 74838393 | - | 3.5 | 5027 | 6155 | 0 | L1PA3 | L1 | LINE |
| 74838393 | 74839578 | + | 3.6 | 3814 | 5006 | -1140 | L1PA3 | L1 | LINE |
| 74839601 | 74841250 | + | 21.6 | 1793 | 3471 | -4132 | L1PA12 | L1 | LINE |

  

|  |  |  |  |  |  |  |  |  |  |
| --- | --- | --- | --- | --- | --- | --- | --- | --- | --- |
| chrX:74844859-74850549 | | | 5655 bp | | |  |  |  |  |
| Genome Start | Genome End | Strand | Div | Repeat Start | Repeat End | Repeat Left | Name | Family | Class |
| 74844859 | 74846424 | - | 16.4 | 3212 | 4876 | -1733 | L1PBa1 | L1 | LINE |
| 74846459 | 74847355 | - | 3.9 | 5254 | 6151 | -4 | L1PA4 | L1 | LINE |
| 74847362 | 74847598 | + | 6.8 | 5014 | 5250 | -896 | L1PA4 | L1 | LINE |
| 74847595 | 74850549 | - | 18.7 | 2 | 3227 | -3385 | L1PBa1 | L1 | LINE |

  

|  |  |  |  |  |  |  |  |  |  |
| --- | --- | --- | --- | --- | --- | --- | --- | --- | --- |
| chrX:74959760-74988588 | | | 28521 bp | | |  |  |  |  |
| Genome Start | Genome End | Strand | Div | Repeat Start | Repeat End | Repeat Left | Name | Family | Class |
| 74959760 | 74960345 | - | 20.6 | 4468 | 5076 | -1461 | L1MDa | L1 | LINE |
| 74960362 | 74960436 | - | 18.9 | 6076 | 6153 | -12 | L1PA15 | L1 | LINE |
| 74960543 | 74961283 | - | 26.6 | 2 | 750 | -5541 | L1P4e | L1 | LINE |
| 74961293 | 74961502 | - | 23.8 | 4251 | 4472 | -2065 | L1MDa | L1 | LINE |
| 74961506 | 74961859 | - | 14.1 | 1 | 364 | 0 | THE1B | MaLR | LTR |
| 74961860 | 74961992 | - | 13.5 | 1448 | 1580 | 0 | THE1B-int | MaLR | LTR |
| 74961989 | 74963322 | - | 11.3 | 2 | 1339 | -241 | THE1B-int | MaLR | LTR |
| 74963324 | 74963680 | - | 13.8 | 1 | 364 | 0 | THE1B | MaLR | LTR |
| 74963682 | 74963882 | - | 20.1 | 3653 | 3860 | -2286 | L1MC | L1 | LINE |
| 74963887 | 74964091 | + | 22.4 | 1 | 215 | -217 | MSTB1 | MaLR | LTR |
| 74964122 | 74964308 | + | 22.4 | 216 | 418 | -14 | MSTB1 | MaLR | LTR |
| 74964312 | 74964754 | - | 23.3 | 3600 | 4036 | -2501 | L1MDa | L1 | LINE |
| 74964755 | 74965056 | - | 21.3 | 3 | 293 | -19 | AluJb | Alu | SINE |
| 74965057 | 74965623 | - | 23.6 | 3006 | 3599 | -2938 | L1MDa | L1 | LINE |
| 74965624 | 74965690 | + | 13.9 | 1 | 72 | -34 | MADE1 | Mariner | DNA |
| 74965691 | 74966282 | - | 23.6 | 2385 | 3005 | -3532 | L1MDa | L1 | LINE |
| 74966303 | 74969752 | - | 10.6 | 2736 | 6172 | 0 | L1PA8 | L1 | LINE |
| 74969838 | 74976263 | - | 10.2 | 10 | 6466 | -4 | L1PA3 | L1 | LINE |
| 74976283 | 74979860 | - | 14.6 | 2568 | 6160 | 0 | L1PREC2 | L1 | LINE |
| 74979867 | 74979928 | - | 5.2 | 6111 | 6154 | 0 | L1PA5 | L1 | LINE |
| 74979929 | 74980239 | - | 10.3 | 1 | 311 | 0 | AluY | Alu | SINE |
| 74980240 | 74981227 | - | 5.4 | 5122 | 6110 | -44 | L1PA5 | L1 | LINE |
| 74981221 | 74981808 | - | 7.9 | 4169 | 4753 | -1393 | L1PA5 | L1 | LINE |
| 74981807 | 74982857 | + | 6.8 | 3097 | 4148 | -1998 | L1PA5 | L1 | LINE |
| 74982854 | 74984895 | - | 17.6 | 1123 | 3201 | -3565 | L1PA13 | L1 | LINE |
| 74984906 | 74985258 | - | 9.8 | 1 | 355 | 0 | THE1A | MaLR | LTR |
| 74985259 | 74986707 | - | 10.3 | 1 | 1580 | 0 | THE1A-int | MaLR | LTR |
| 74986708 | 74987090 | - | 11.1 | 1 | 355 | 0 | THE1A | MaLR | LTR |
| 74987094 | 74988213 | - | 23.7 | 11 | 1114 | -5671 | L1PA13 | L1 | LINE |
| 74988223 | 74988588 | - | 29.1 | 2025 | 2391 | -4242 | L1MDa | L1 | LINE |

  

|  |  |  |  |  |  |  |  |  |  |
| --- | --- | --- | --- | --- | --- | --- | --- | --- | --- |
| chrX:75108590-75112176 | | | 3594 bp | | |  |  |  |  |
| Genome Start | Genome End | Strand | Div | Repeat Start | Repeat End | Repeat Left | Name | Family | Class |
| 75108590 | 75108657 | + | 17.8 | 4021 | 4088 | -2052 | L1MB1 | L1 | LINE |
| 75108658 | 75108705 | + | 0.0 | 6106 | 6153 | -2 | L1PA4 | L1 | LINE |
| 75108706 | 75109836 | + | 17.8 | 4089 | 5260 | -880 | L1MB1 | L1 | LINE |
| 75109837 | 75110070 | + | 4.7 | 5935 | 6169 | 0 | L1PA3 | L1 | LINE |
| 75110071 | 75110885 | + | 19.5 | 5274 | 6113 | -55 | L1MB1 | L1 | LINE |
| 75110882 | 75111485 | - | 8.9 | 5363 | 5966 | -206 | L1PA8 | L1 | LINE |
| 75111484 | 75112136 | + | 11.0 | 4705 | 5357 | -789 | L1PA8 | L1 | LINE |
| 75112136 | 75112176 | + | 17.1 | 6127 | 6167 | -1 | L1MB1 | L1 | LINE |

  

|  |  |  |  |  |  |  |  |  |  |
| --- | --- | --- | --- | --- | --- | --- | --- | --- | --- |
| chrX:75192620-75196674 | | | 4044 bp | | |  |  |  |  |
| Genome Start | Genome End | Strand | Div | Repeat Start | Repeat End | Repeat Left | Name | Family | Class |
| 75192620 | 75193107 | + | 13.1 | 4857 | 5344 | -802 | L1PREC2 | L1 | LINE |
| 75193108 | 75193340 | - | 6.4 | 5940 | 6172 | 0 | L1PA8 | L1 | LINE |
| 75193341 | 75193480 | + | 11.7 | 5345 | 5472 | -688 | L1PREC2 | L1 | LINE |
| 75193480 | 75194565 | - | 2.1 | 5068 | 6155 | 0 | L1PA2 | L1 | LINE |
| 75194565 | 75195576 | + | 2.7 | 4058 | 5069 | -1077 | L1PA2 | L1 | LINE |
| 75195572 | 75196262 | + | 19.5 | 5459 | 6132 | -19 | L1PB2 | L1 | LINE |
| 75196281 | 75196674 | + | 16.5 | 5766 | 6159 | -1 | L1PREC2 | L1 | LINE |

  

|  |  |  |  |  |  |  |  |  |  |
| --- | --- | --- | --- | --- | --- | --- | --- | --- | --- |
| chrX:75416797-75424039 | | | 7250 bp | | |  |  |  |  |
| Genome Start | Genome End | Strand | Div | Repeat Start | Repeat End | Repeat Left | Name | Family | Class |
| 75416797 | 75417329 | - | 14.3 | 7185 | 7723 | 0 | L1MA2 | L1 | LINE |
| 75417330 | 75418495 | - | 10.6 | 4995 | 6168 | -4 | L1PA8 | L1 | LINE |
| 75418493 | 75419384 | + | 8.7 | 4084 | 4980 | -1166 | L1PA8 | L1 | LINE |
| 75419381 | 75422379 | - | 14.4 | 4188 | 7199 | -524 | L1MA2 | L1 | LINE |
| 75422380 | 75422670 | - | 14.8 | 1 | 290 | -22 | AluSx | Alu | SINE |
| 75422671 | 75423595 | - | 14.7 | 3276 | 4187 | -3378 | L1MA2 | L1 | LINE |
| 75423596 | 75423903 | + | 19.2 | 1 | 309 | -3 | AluSx | Alu | SINE |
| 75423904 | 75424039 | - | 15.4 | 3140 | 3275 | -4290 | L1MA2 | L1 | LINE |

  

|  |  |  |  |  |  |  |  |  |  |
| --- | --- | --- | --- | --- | --- | --- | --- | --- | --- |
| chrX:75498993-75507473 | | | 8387 bp | | |  |  |  |  |
| Genome Start | Genome End | Strand | Div | Repeat Start | Repeat End | Repeat Left | Name | Family | Class |
| 75498993 | 75499724 | - | 17.1 | 4314 | 5074 | -1692 | L1PA13 | L1 | LINE |
| 75499772 | 75500593 | - | 17.1 | 3447 | 4313 | -2453 | L1PA13 | L1 | LINE |
| 75500595 | 75501147 | - | 15.9 | 2468 | 3025 | -3741 | L1PA13 | L1 | LINE |
| 75501162 | 75502433 | - | 8.5 | 4872 | 6152 | -2 | L1PA7 | L1 | LINE |
| 75502423 | 75504340 | - | 24.7 | 525 | 2474 | -4312 | L1PA13 | L1 | LINE |
| 75504384 | 75505277 | - | 4.0 | 5262 | 6155 | 0 | L1PA4 | L1 | LINE |
| 75505278 | 75506346 | + | 5.2 | 3927 | 4995 | -1151 | L1P1 | L1 | LINE |
| 75506347 | 75506641 | + | 7.1 | 5858 | 6152 | -3 | L1PA3 | L1 | LINE |
| 75506642 | 75507028 | + | 5.2 | 4996 | 5237 | -909 | L1P1 | L1 | LINE |
| 75507029 | 75507473 | - | 21.6 | 87 | 531 | -6254 | L1PA13 | L1 | LINE |

  

|  |  |  |  |  |  |  |  |  |  |
| --- | --- | --- | --- | --- | --- | --- | --- | --- | --- |
| chrX:75785690-75790403 | | | 4673 bp | | |  |  |  |  |
| Genome Start | Genome End | Strand | Div | Repeat Start | Repeat End | Repeat Left | Name | Family | Class |
| 75785690 | 75786163 | + | 22.9 | 2456 | 2928 | -3218 | L1M4 | L1 | LINE |
| 75786163 | 75786383 | - | 12.3 | 3426 | 3671 | -2475 | L1P3 | L1 | LINE |
| 75786399 | 75786813 | - | 5.3 | 5741 | 6155 | 0 | L1PA4 | L1 | LINE |
| 75786814 | 75787050 | + | 2.5 | 5499 | 5737 | -418 | L1PA4 | L1 | LINE |
| 75787049 | 75787145 | - | 13.4 | 3370 | 3439 | -2707 | L1P3 | L1 | LINE |
| 75787146 | 75787436 | - | 4.8 | 5851 | 6143 | -11 | L1PA5 | L1 | LINE |
| 75787437 | 75787775 | - | 13.4 | 3030 | 3369 | -2777 | L1P3 | L1 | LINE |
| 75787775 | 75787866 | + | 19.6 | 2936 | 3027 | -3119 | L1M4 | L1 | LINE |
| 75787900 | 75788040 | - | 5.7 | 6008 | 6148 | -7 | L1PA4 | L1 | LINE |
| 75788041 | 75789718 | - | 5.3 | 4076 | 5764 | -391 | L1PA4 | L1 | LINE |
| 75789716 | 75790236 | + | 23.1 | 3012 | 3556 | -2590 | L1M4 | L1 | LINE |
| 75790237 | 75790403 | + | 22.3 | 3831 | 3998 | -2148 | L1M4 | L1 | LINE |

  

|  |  |  |  |  |  |  |  |  |  |
| --- | --- | --- | --- | --- | --- | --- | --- | --- | --- |
| chrX:75919003-75925964 | | | 6740 bp | | |  |  |  |  |
| Genome Start | Genome End | Strand | Div | Repeat Start | Repeat End | Repeat Left | Name | Family | Class |
| 75919003 | 75919093 | + | 26.6 | 2785 | 2863 | -556 | L2 | L2 | LINE |
| 75919098 | 75919401 | - | 14.8 | 1 | 305 | -7 | AluSx | Alu | SINE |
| 75919415 | 75919522 | - | 18.5 | 7663 | 7777 | -8 | L1MC3 | L1 | LINE |
| 75919530 | 75920101 | + | 18.9 | 6 | 588 | -1830 | Tigger1 | MER2\_type | DNA |
| 75920102 | 75920917 | - | 4.2 | 3567 | 4382 | -1764 | L1PA3 | L1 | LINE |
| 75920919 | 75922701 | + | 4.2 | 4376 | 6152 | -3 | L1PA3 | L1 | LINE |
| 75922736 | 75923787 | + | 16.7 | 578 | 1753 | -665 | Tigger1 | MER2\_type | DNA |
| 75923788 | 75925686 | + | 15.7 | 4173 | 6162 | -3 | L1PA15 | L1 | LINE |
| 75925850 | 75925964 | + | 29.5 | 3244 | 3366 | -12 | L2 | L2 | LINE |

  

|  |  |  |  |  |  |  |  |  |  |
| --- | --- | --- | --- | --- | --- | --- | --- | --- | --- |
| chrX:76390346-76399284 | | | 8500 bp | | |  |  |  |  |
| Genome Start | Genome End | Strand | Div | Repeat Start | Repeat End | Repeat Left | Name | Family | Class |
| 76390346 | 76390475 | - | 28.3 | 3239 | 3366 | -12 | L2 | L2 | LINE |
| 76390616 | 76391158 | + | 15.3 | 10 | 572 | -5889 | L1PA8 | L1 | LINE |
| 76391160 | 76391434 | + | 15.3 | 1 | 275 | -37 | AluSx | Alu | SINE |
| 76391731 | 76391872 | + | 18.3 | 560 | 704 | -5757 | L1PA8 | L1 | LINE |
| 76391872 | 76393244 | + | 14.3 | 1310 | 2676 | -3785 | L1PA8 | L1 | LINE |
| 76393253 | 76394568 | - | 5.0 | 4836 | 6154 | 0 | L1PA5 | L1 | LINE |
| 76394566 | 76395307 | + | 6.7 | 4090 | 4831 | -1315 | L1PA5 | L1 | LINE |
| 76395303 | 76399116 | + | 9.9 | 2662 | 6487 | 0 | L1PA8 | L1 | LINE |
| 76399120 | 76399284 | - | 33.1 | 2648 | 2805 | -614 | L2 | L2 | LINE |

  

|  |  |  |  |  |  |  |  |  |  |
| --- | --- | --- | --- | --- | --- | --- | --- | --- | --- |
| chrX:77199023-77201671 | | | 2629 bp | | |  |  |  |  |
| Genome Start | Genome End | Strand | Div | Repeat Start | Repeat End | Repeat Left | Name | Family | Class |
| 77199023 | 77199456 | - | 25.2 | 5646 | 6079 | -87 | L1ME1 | L1 | LINE |
| 77199459 | 77199595 | + | 15.6 | 3 | 153 | -66 | MER20 | MER1\_type | DNA |
| 77199600 | 77199906 | - | 12.4 | 115 | 381 | 0 | THE1D | MaLR | LTR |
| 77199907 | 77200313 | - | 7.9 | 63 | 472 | 0 | MER4A1 | ERV1 | LTR |
| 77200324 | 77200562 | - | 0.4 | 5917 | 6155 | 0 | L1HS | L1 | LINE |
| 77200563 | 77201264 | + | 0.8 | 5212 | 5913 | -242 | L1HS | L1 | LINE |
| 77201265 | 77201334 | - | 12.9 | 1 | 79 | -393 | MER4A1 | ERV1 | LTR |
| 77201335 | 77201460 | - | 11.6 | 1 | 118 | -263 | THE1D | MaLR | LTR |
| 77201465 | 77201527 | + | 19.1 | 157 | 219 | 0 | MER20 | MER1\_type | DNA |
| 77201528 | 77201671 | - | 18.8 | 5494 | 5647 | -519 | L1ME1 | L1 | LINE |

  

|  |  |  |  |  |  |  |  |  |  |
| --- | --- | --- | --- | --- | --- | --- | --- | --- | --- |
| chrX:77361985-77368062 | | | 6129 bp | | |  |  |  |  |
| Genome Start | Genome End | Strand | Div | Repeat Start | Repeat End | Repeat Left | Name | Family | Class |
| 77361985 | 77362824 | + | 25.1 | 923 | 1762 | -4622 | L1M4c | L1 | LINE |
| 77362801 | 77363379 | + | 22.8 | 2594 | 3182 | -3194 | L1M4c | L1 | LINE |
| 77363378 | 77364509 | - | 12.0 | 3079 | 4220 | -1926 | L1PREC2 | L1 | LINE |
| 77364510 | 77364806 | - | 10.8 | 1 | 297 | -12 | AluSc | Alu | SINE |
| 77364807 | 77364965 | - | 12.0 | 2920 | 3078 | -3068 | L1PREC2 | L1 | LINE |
| 77364965 | 77366920 | + | 11.9 | 4226 | 6158 | -2 | L1PREC2 | L1 | LINE |
| 77366897 | 77367151 | + | 19.5 | 3138 | 3397 | -2979 | L1M4c | L1 | LINE |
| 77367152 | 77367455 | + | 20.1 | 1 | 306 | -6 | AluJo | Alu | SINE |
| 77367456 | 77367843 | + | 19.5 | 3398 | 3750 | -2626 | L1M4c | L1 | LINE |
| 77367844 | 77367961 | - | 15.2 | 1 | 118 | -15 | FLAM\_C | Alu | SINE |
| 77367962 | 77368062 | + | 19.5 | 3751 | 3854 | -2522 | L1M4c | L1 | LINE |

  

|  |  |  |  |  |  |  |  |  |  |
| --- | --- | --- | --- | --- | --- | --- | --- | --- | --- |
| chrX:77428339-77430368 | | | 1930 bp | | |  |  |  |  |
| Genome Start | Genome End | Strand | Div | Repeat Start | Repeat End | Repeat Left | Name | Family | Class |
| 77428339 | 77428445 | + | 17.8 | 24 | 133 | -179 | AluJ | Alu | SINE |
| 77428449 | 77428649 | - | 22.9 | 5598 | 5809 | -357 | L1M5 | L1 | LINE |
| 77428650 | 77428896 | - | 4.5 | 5902 | 6155 | 0 | L1PA3 | L1 | LINE |
| 77428897 | 77429404 | - | 22.8 | 5061 | 5597 | -569 | L1M5 | L1 | LINE |
| 77429403 | 77429723 | + | 22.3 | 5824 | 6159 | -19 | L1MB8 | L1 | LINE |
| 77429745 | 77430110 | + | 24.0 | 5810 | 6159 | -7 | L1ME1 | L1 | LINE |
| 77430189 | 77430368 | + | 18.0 | 120 | 292 | -20 | AluJ | Alu | SINE |

  

|  |  |  |  |  |  |  |  |  |  |
| --- | --- | --- | --- | --- | --- | --- | --- | --- | --- |
| chrX:77707590-77715743 | | | 8148 bp | | |  |  |  |  |
| Genome Start | Genome End | Strand | Div | Repeat Start | Repeat End | Repeat Left | Name | Family | Class |
| 77707590 | 77708047 | - | 16.4 | 4807 | 5277 | -869 | L1M1 | L1 | LINE |
| 77708056 | 77709670 | - | 13.5 | 4685 | 6293 | -9 | L1PB2 | L1 | LINE |
| 77709672 | 77710905 | + | 13.4 | 3451 | 4697 | -1449 | L1PB2 | L1 | LINE |
| 77710903 | 77711117 | - | 14.6 | 5935 | 6155 | -147 | L1MA1 | L1 | LINE |
| 77711116 | 77711346 | + | 6.1 | 4262 | 4505 | -1641 | L1M1 | L1 | LINE |
| 77711345 | 77713269 | - | 19.0 | 2656 | 4821 | -1322 | L1M1 | L1 | LINE |
| 77713274 | 77715743 | - | 18.7 | 12 | 2563 | -5002 | L1M1 | L1 | LINE |

  

|  |  |  |  |  |  |  |  |  |  |
| --- | --- | --- | --- | --- | --- | --- | --- | --- | --- |
| chrX:77943229-77948746 | | | 5494 bp | | |  |  |  |  |
| Genome Start | Genome End | Strand | Div | Repeat Start | Repeat End | Repeat Left | Name | Family | Class |
| 77943229 | 77943668 | + | 13.0 | 1 | 499 | -1081 | THE1C-int | MaLR | LTR |
| 77943692 | 77944524 | + | 13.0 | 500 | 1327 | -253 | THE1C-int | MaLR | LTR |
| 77944531 | 77947308 | - | 3.8 | 3376 | 6152 | -3 | L1PA3 | L1 | LINE |
| 77947304 | 77948478 | + | 4.0 | 2208 | 3381 | -2765 | L1PA3 | L1 | LINE |
| 77948479 | 77948746 | + | 12.0 | 1314 | 1580 | 0 | THE1C-int | MaLR | LTR |

  

|  |  |  |  |  |  |  |  |  |  |
| --- | --- | --- | --- | --- | --- | --- | --- | --- | --- |
| chrX:78015626-78027947 | | | 12289 bp | | |  |  |  |  |
| Genome Start | Genome End | Strand | Div | Repeat Start | Repeat End | Repeat Left | Name | Family | Class |
| 78015626 | 78016360 | + | 15.2 | 2 | 762 | -419 | Ricksha\_a | MuDR | DNA |
| 78016373 | 78016849 | + | 25.8 | 10 | 479 | -5982 | L1PA10 | L1 | LINE |
| 78016850 | 78017153 | + | 5.9 | 1 | 304 | -7 | AluY | Alu | SINE |
| 78017154 | 78022203 | + | 11.5 | 480 | 5496 | -965 | L1PA10 | L1 | LINE |
| 78022227 | 78023202 | + | 11.5 | 5497 | 6480 | -3 | L1PA10 | L1 | LINE |
| 78023203 | 78023491 | + | 16.3 | 759 | 1057 | -129 | Ricksha\_a | MuDR | DNA |
| 78023492 | 78025592 | - | 10.6 | 1884 | 3933 | -2213 | L1PA7 | L1 | LINE |
| 78025590 | 78027810 | + | 7.8 | 3928 | 6148 | -6 | L1PA7 | L1 | LINE |
| 78027812 | 78027947 | + | 17.9 | 1042 | 1175 | -1 | Ricksha\_a | MuDR | DNA |

  

|  |  |  |  |  |  |  |  |  |  |
| --- | --- | --- | --- | --- | --- | --- | --- | --- | --- |
| chrX:78196578-78202607 | | | 6000 bp | | |  |  |  |  |
| Genome Start | Genome End | Strand | Div | Repeat Start | Repeat End | Repeat Left | Name | Family | Class |
| 78196578 | 78197946 | - | 22.9 | 2746 | 4155 | -2227 | L1M4c | L1 | LINE |
| 78197951 | 78198089 | - | 7.2 | 6014 | 6155 | 0 | L1PA4 | L1 | LINE |
| 78198104 | 78198606 | + | 4.4 | 1356 | 1858 | -4297 | L1P1 | L1 | LINE |
| 78198605 | 78199658 | - | 3.7 | 4034 | 5087 | -1059 | L1PA3 | L1 | LINE |
| 78199658 | 78200713 | + | 3.6 | 5098 | 6152 | -3 | L1PA3 | L1 | LINE |
| 78200732 | 78201572 | + | 3.8 | 1859 | 2682 | -3464 | L1P1 | L1 | LINE |
| 78201570 | 78202607 | - | 24.8 | 1607 | 2753 | -3629 | L1M4c | L1 | LINE |

  

|  |  |  |  |  |  |  |  |  |  |
| --- | --- | --- | --- | --- | --- | --- | --- | --- | --- |
| chrX:78449859-78462875 | | | 12894 bp | | |  |  |  |  |
| Genome Start | Genome End | Strand | Div | Repeat Start | Repeat End | Repeat Left | Name | Family | Class |
| 78449859 | 78449884 | + | 20.3 | 10 | 34 | -7531 | L1MA3 | L1 | LINE |
| 78449885 | 78450254 | + | 16.3 | 1 | 364 | 0 | THE1B | MaLR | LTR |
| 78450255 | 78450324 | + | 20.3 | 35 | 100 | -7465 | L1MA3 | L1 | LINE |
| 78450352 | 78452249 | + | 20.3 | 101 | 2026 | -5539 | L1MA3 | L1 | LINE |
| 78452284 | 78453087 | + | 20.3 | 2027 | 2847 | -4718 | L1MA3 | L1 | LINE |
| 78453092 | 78453331 | - | 2.5 | 5814 | 6056 | -99 | L1PA3 | L1 | LINE |
| 78453332 | 78453421 | + | 0.0 | 6068 | 6154 | -1 | L1PA3 | L1 | LINE |
| 78453422 | 78457632 | + | 15.8 | 2849 | 7345 | -379 | L1MA3 | L1 | LINE |
| 78457657 | 78458272 | - | 11.4 | 5989 | 6609 | -8 | L1PB1 | L1 | LINE |
| 78458273 | 78459420 | - | 8.9 | 3122 | 4262 | -2350 | L1PB1 | L1 | LINE |
| 78459444 | 78462485 | - | 10.7 | 2 | 3121 | -3491 | L1PB1 | L1 | LINE |
| 78462497 | 78462875 | + | 11.5 | 7335 | 7722 | -2 | L1MA3 | L1 | LINE |

  

|  |  |  |  |  |  |  |  |  |  |
| --- | --- | --- | --- | --- | --- | --- | --- | --- | --- |
| chrX:78568478-78580601 | | | 11950 bp | | |  |  |  |  |
| Genome Start | Genome End | Strand | Div | Repeat Start | Repeat End | Repeat Left | Name | Family | Class |
| 78568478 | 78568626 | - | 26.4 | 5976 | 6139 | -25 | L1ME2 | L1 | LINE |
| 78568638 | 78569005 | - | 28.7 | 5548 | 5925 | -245 | L1ME2 | L1 | LINE |
| 78569072 | 78569433 | - | 22.7 | 5984 | 6334 | 0 | L1MA10 | L1 | LINE |
| 78569434 | 78569653 | + | 19.4 | 2 | 181 | -251 | MSTB1 | MaLR | LTR |
| 78569654 | 78569964 | - | 13.3 | 1 | 310 | 0 | AluSg | Alu | SINE |
| 78569965 | 78570201 | + | 19.4 | 182 | 432 | 0 | MSTB1 | MaLR | LTR |
| 78570202 | 78570403 | - | 22.7 | 5781 | 5983 | -206 | L1MB1 | L1 | LINE |
| 78570427 | 78570439 | - | 22.7 | 5752 | 5780 | -554 | L1MB1 | L1 | LINE |
| 78570440 | 78570733 | + | 11.3 | 1 | 293 | -17 | AluSg | Alu | SINE |
| 78570734 | 78572244 | - | 19.5 | 4119 | 5751 | -583 | L1MB1 | L1 | LINE |
| 78572246 | 78572852 | + | 20.0 | 3448 | 4107 | -2033 | L1MB1 | L1 | LINE |
| 78572853 | 78573627 | - | 27.9 | 4485 | 5302 | -844 | L1ME2 | L1 | LINE |
| 78573648 | 78573814 | - | 19.8 | 6167 | 6333 | -1 | L1MA10 | L1 | LINE |
| 78573838 | 78573855 | - | 19.8 | 6145 | 6166 | -23 | L1MB1 | L1 | LINE |
| 78573856 | 78574168 | + | 13.1 | 1 | 312 | 0 | AluSx | Alu | SINE |
| 78574169 | 78574492 | - | 22.7 | 5743 | 6144 | -45 | L1MB1 | L1 | LINE |
| 78574496 | 78574688 | - | 26.6 | 4253 | 4457 | -1689 | L1ME2 | L1 | LINE |
| 78574689 | 78574994 | - | 13.6 | 1 | 302 | -10 | AluSx | Alu | SINE |
| 78574995 | 78575287 | - | 26.6 | 3970 | 4252 | -1894 | L1ME2 | L1 | LINE |
| 78575288 | 78575587 | - | 5.7 | 1 | 299 | -12 | AluY | Alu | SINE |
| 78575588 | 78576799 | - | 26.6 | 2716 | 3969 | -2177 | L1ME2 | L1 | LINE |
| 78576802 | 78577534 | + | 15.8 | 2953 | 3695 | -2451 | L1PA16 | L1 | LINE |
| 78577558 | 78579320 | + | 15.6 | 3696 | 5499 | -667 | L1PA16 | L1 | LINE |
| 78579321 | 78579620 | + | 9.0 | 1 | 299 | -12 | AluY | Alu | SINE |
| 78579621 | 78580299 | + | 14.1 | 5500 | 6166 | 0 | L1PA16 | L1 | LINE |
| 78580302 | 78580601 | - | 24.4 | 2381 | 2724 | -3422 | L1ME2 | L1 | LINE |

  

|  |  |  |  |  |  |  |  |  |  |
| --- | --- | --- | --- | --- | --- | --- | --- | --- | --- |
| chrX:78595707-78597977 | | | 2245 bp | | |  |  |  |  |
| Genome Start | Genome End | Strand | Div | Repeat Start | Repeat End | Repeat Left | Name | Family | Class |
| 78595707 | 78596073 | + | 13.3 | 1 | 345 | -319 | MER4A | ERV1 | LTR |
| 78596074 | 78596318 | - | 8.2 | 4893 | 5137 | -1009 | L1PA7 | L1 | LINE |
| 78596321 | 78597322 | + | 6.4 | 5140 | 6149 | -5 | L1PA7 | L1 | LINE |
| 78597329 | 78597407 | + | 24.4 | 360 | 442 | -152 | PRIMA4\_LTR | ERV1 | LTR |
| 78597426 | 78597477 | + | 15.2 | 363 | 414 | -250 | MER4A | ERV1 | LTR |
| 78597478 | 78597735 | - | 2.7 | 39 | 296 | -15 | AluY | Alu | SINE |
| 78597736 | 78597977 | + | 15.2 | 415 | 664 | 0 | MER4A | ERV1 | LTR |

  

|  |  |  |  |  |  |  |  |  |  |
| --- | --- | --- | --- | --- | --- | --- | --- | --- | --- |
| chrX:78884920-78889509 | | | 4234 bp | | |  |  |  |  |
| Genome Start | Genome End | Strand | Div | Repeat Start | Repeat End | Repeat Left | Name | Family | Class |
| 78884920 | 78885216 | - | 26.2 | 2596 | 2910 | -4062 | L1M4b | L1 | LINE |
| 78885236 | 78886167 | - | 1.6 | 5215 | 6146 | -9 | L1PA3 | L1 | LINE |
| 78886164 | 78887115 | + | 1.9 | 4254 | 5205 | -941 | L1PA3 | L1 | LINE |
| 78887376 | 78887574 | - | 24.7 | 1517 | 1723 | -4923 | L1M4b | L1 | LINE |
| 78887584 | 78888295 | - | 17.7 | 5386 | 6157 | -9 | L1PA16 | L1 | LINE |
| 78888300 | 78889312 | - | 23.3 | 515 | 1531 | -5011 | L1M4b | L1 | LINE |
| 78889381 | 78889509 | - | 19.4 | 3 | 137 | -6405 | L1M4b | L1 | LINE |

  

|  |  |  |  |  |  |  |  |  |  |
| --- | --- | --- | --- | --- | --- | --- | --- | --- | --- |
| chrX:79078013-79087444 | | | 9238 bp | | |  |  |  |  |
| Genome Start | Genome End | Strand | Div | Repeat Start | Repeat End | Repeat Left | Name | Family | Class |
| 79078013 | 79078121 | - | 10.1 | 6047 | 6155 | -8 | L1PA13 | L1 | LINE |
| 79078122 | 79078795 | - | 15.8 | 5602 | 6289 | -11 | L1MA6 | L1 | LINE |
| 79078796 | 79078940 | - | 19.3 | 5323 | 5469 | -890 | L1MD | L1 | LINE |
| 79078999 | 79080253 | - | 10.5 | 4873 | 6152 | -9 | L1PA14 | L1 | LINE |
| 79080246 | 79080416 | - | 19.4 | 3002 | 3188 | -2958 | L1MD | L1 | LINE |
| 79080431 | 79081553 | - | 20.8 | 5192 | 6262 | -50 | L1MA9 | L1 | LINE |
| 79081570 | 79081841 | - | 27.3 | 6010 | 6274 | -38 | L1MA9 | L1 | LINE |
| 79081799 | 79082021 | + | 24.1 | 5802 | 6025 | -287 | L1MA9 | L1 | LINE |
| 79082028 | 79082669 | - | 26.8 | 3247 | 3897 | -937 | L1MEe | L1 | LINE |
| 79082670 | 79083018 | + | 21.5 | 1 | 233 | -3 | MER46B | MER2\_type | DNA |
| 79083019 | 79085449 | - | 26.8 | 949 | 3246 | -1588 | L1MEe | L1 | LINE |
| 79085453 | 79086755 | - | 24.9 | 1209 | 2537 | -4248 | L1PA13 | L1 | LINE |
| 79086904 | 79087444 | - | 28.3 | 744 | 1245 | -5541 | L1PA13 | L1 | LINE |

  

|  |  |  |  |  |  |  |  |  |  |
| --- | --- | --- | --- | --- | --- | --- | --- | --- | --- |
| chrX:79306429-79313535 | | | 7361 bp | | |  |  |  |  |
| Genome Start | Genome End | Strand | Div | Repeat Start | Repeat End | Repeat Left | Name | Family | Class |
| 79306429 | 79306566 | + | 21.2 | 6154 | 6291 | -1494 | L1MC3 | L1 | LINE |
| 79306619 | 79307161 | - | 18.7 | 1 | 554 | 0 | MLT2A2 | ERVL | LTR |
| 79307165 | 79307854 | + | 24.3 | 6366 | 7109 | -886 | L1MD3 | L1 | LINE |
| 79307419 | 79308096 | + | 26.1 | 6660 | 7323 | -462 | L1MC3 | L1 | LINE |
| 79308100 | 79308140 | - | 21.0 | 527 | 567 | 0 | MLT2B1 | ERVL | LTR |
| 79308206 | 79308441 | - | 23.9 | 172 | 526 | -41 | MLT2B1 | ERVL | LTR |
| 79308442 | 79308670 | - | 21.5 | 5415 | 5642 | -72 | ERVL-D | ERVL | LTR |
| 79308674 | 79310510 | - | 11.7 | 2951 | 4853 | -1293 | L1PREC2 | L1 | LINE |
| 79310512 | 79310946 | + | 13.8 | 4850 | 5284 | -862 | L1PREC2 | L1 | LINE |
| 79310934 | 79311118 | + | 5.4 | 2508 | 2692 | -3454 | L1P1 | L1 | LINE |
| 79311135 | 79311957 | + | 3.3 | 5330 | 6152 | -3 | L1PA4 | L1 | LINE |
| 79311971 | 79312179 | - | 19.6 | 2311 | 2522 | -3192 | ERVL-D | ERVL | LTR |
| 79312172 | 79312752 | - | 20.3 | 2 | 596 | -5118 | ERVL-D | ERVL | LTR |
| 79312768 | 79313075 | - | 22.6 | 82 | 400 | -14 | MLT2D | ERVL | LTR |
| 79313108 | 79313535 | + | 17.5 | 7326 | 7777 | -8 | L1MC3 | L1 | LINE |

  

|  |  |  |  |  |  |  |  |  |  |
| --- | --- | --- | --- | --- | --- | --- | --- | --- | --- |
| chrX:79361405-79366928 | | | 5458 bp | | |  |  |  |  |
| Genome Start | Genome End | Strand | Div | Repeat Start | Repeat End | Repeat Left | Name | Family | Class |
| 79361405 | 79362703 | - | 20.0 | 2045 | 3412 | -4153 | L1M1 | L1 | LINE |
| 79362705 | 79362891 | + | 14.5 | 1 | 203 | -178 | THE1D | MaLR | LTR |
| 79362916 | 79363085 | + | 14.5 | 204 | 380 | -1 | THE1D | MaLR | LTR |
| 79363087 | 79363570 | + | 16.1 | 2 | 488 | -1154 | THE1D-int | MaLR | LTR |
| 79363571 | 79363806 | - | 8.3 | 5152 | 5418 | -750 | L1P | L1 | LINE |
| 79363807 | 79364524 | + | 9.6 | 5436 | 6166 | -2 | L1PA10 | L1 | LINE |
| 79364551 | 79364615 | + | 20.0 | 471 | 535 | -1116 | MST-int | MaLR | LTR |
| 79364630 | 79365207 | + | 16.1 | 1012 | 1580 | 0 | THE1D-int | MaLR | LTR |
| 79365208 | 79365560 | + | 15.8 | 1 | 381 | 0 | THE1D | MaLR | LTR |
| 79365561 | 79366928 | - | 27.7 | 16 | 2048 | -5517 | L1M1 | L1 | LINE |

  

|  |  |  |  |  |  |  |  |  |  |
| --- | --- | --- | --- | --- | --- | --- | --- | --- | --- |
| chrX:79435330-79439220 | | | 3730 bp | | |  |  |  |  |
| Genome Start | Genome End | Strand | Div | Repeat Start | Repeat End | Repeat Left | Name | Family | Class |
| 79435330 | 79436381 | - | 29.4 | 2333 | 3419 | 0 | L2 | L2 | LINE |
| 79436438 | 79436558 | - | 33.3 | 1869 | 1986 | -1433 | L2 | L2 | LINE |
| 79436563 | 79437201 | - | 3.6 | 4326 | 4964 | -1182 | L1PA4 | L1 | LINE |
| 79437201 | 79438403 | + | 3.0 | 4968 | 6172 | 0 | L1PA4 | L1 | LINE |
| 79438460 | 79438771 | - | 8.8 | 6 | 313 | 0 | AluSp | Alu | SINE |
| 79438812 | 79438854 | + | 11.6 | 6109 | 6151 | -3 | L1PA6 | L1 | LINE |
| 79438861 | 79439220 | - | 24.0 | 1378 | 1784 | -1635 | L2 | L2 | LINE |

  

|  |  |  |  |  |  |  |  |  |  |
| --- | --- | --- | --- | --- | --- | --- | --- | --- | --- |
| chrX:79664966-79669884 | | | 4868 bp | | |  |  |  |  |
| Genome Start | Genome End | Strand | Div | Repeat Start | Repeat End | Repeat Left | Name | Family | Class |
| 79664966 | 79667387 | + | 8.9 | 2978 | 5374 | -280 | HERVL-A2 | ERVL | LTR |
| 79667446 | 79668520 | - | 8.8 | 5047 | 6128 | -3 | L1PA8A | L1 | LINE |
| 79668514 | 79669587 | + | 9.7 | 3967 | 5047 | -1099 | L1PA8A | L1 | LINE |
| 79669588 | 79669884 | + | 11.1 | 5355 | 5654 | 0 | HERVL-A2 | ERVL | LTR |

  

|  |  |  |  |  |  |  |  |  |  |
| --- | --- | --- | --- | --- | --- | --- | --- | --- | --- |
| chrX:79675292-79682080 | | | 6763 bp | | |  |  |  |  |
| Genome Start | Genome End | Strand | Div | Repeat Start | Repeat End | Repeat Left | Name | Family | Class |
| 79675292 | 79675837 | + | 23.1 | 1 | 556 | -1041 | Tigger6b | MER2\_type | DNA |
| 79675842 | 79676362 | + | 14.2 | 3261 | 3789 | -2357 | L1PA13 | L1 | LINE |
| 79676359 | 79678156 | - | 0.5 | 3420 | 5217 | -929 | L1HS | L1 | LINE |
| 79678150 | 79679090 | + | 1.0 | 5215 | 6155 | 0 | L1HS | L1 | LINE |
| 79679129 | 79681498 | + | 12.0 | 3775 | 6144 | -19 | L1PA13 | L1 | LINE |
| 79681506 | 79681867 | + | 17.7 | 546 | 913 | -684 | Tigger6b | MER2\_type | DNA |
| 79681856 | 79682080 | + | 21.8 | 1080 | 1312 | -285 | Tigger6b | MER2\_type | DNA |

  

|  |  |  |  |  |  |  |  |  |  |
| --- | --- | --- | --- | --- | --- | --- | --- | --- | --- |
| chrX:80281576-80297225 | | | 15489 bp | | |  |  |  |  |
| Genome Start | Genome End | Strand | Div | Repeat Start | Repeat End | Repeat Left | Name | Family | Class |
| 80281576 | 80282433 | + | 20.6 | 17 | 883 | -6682 | L1M1 | L1 | LINE |
| 80282434 | 80282739 | + | 4.6 | 1 | 306 | -5 | AluY | Alu | SINE |
| 80282740 | 80283617 | + | 16.5 | 884 | 1755 | -5810 | L1M1 | L1 | LINE |
| 80283665 | 80285520 | + | 16.5 | 1756 | 3635 | -3930 | L1M1 | L1 | LINE |
| 80285534 | 80291784 | + | 10.4 | 8 | 6150 | -4 | L1PA7 | L1 | LINE |
| 80291777 | 80292437 | + | 12.6 | 3619 | 4278 | -3287 | L1M1 | L1 | LINE |
| 80292438 | 80292753 | + | 9.9 | 2 | 313 | 0 | AluSq | Alu | SINE |
| 80292754 | 80292767 | + | 12.6 | 4279 | 4292 | -3273 | L1M1 | L1 | LINE |
| 80292769 | 80293329 | + | 12.3 | 4544 | 5124 | -2441 | L1M1 | L1 | LINE |
| 80293324 | 80294093 | - | 2.9 | 4163 | 4932 | -1214 | L1PA3 | L1 | LINE |
| 80294094 | 80295313 | + | 3.1 | 4936 | 6155 | 0 | L1PA3 | L1 | LINE |
| 80295325 | 80295425 | + | 11.9 | 5103 | 5203 | -2362 | L1M1 | L1 | LINE |
| 80295426 | 80295704 | + | 14.0 | 1 | 278 | -34 | AluSx | Alu | SINE |
| 80295808 | 80297225 | + | 9.4 | 5644 | 7092 | -631 | L1M1 | L1 | LINE |

  

|  |  |  |  |  |  |  |  |  |  |
| --- | --- | --- | --- | --- | --- | --- | --- | --- | --- |
| chrX:80606213-80608731 | | | 2505 bp | | |  |  |  |  |
| Genome Start | Genome End | Strand | Div | Repeat Start | Repeat End | Repeat Left | Name | Family | Class |
| 80606213 | 80607045 | - | 18.8 | 2203 | 3262 | -2812 | L1MEa | L1 | LINE |
| 80607060 | 80607261 | - | 13.4 | 6080 | 6296 | -4 | L1MA6 | L1 | LINE |
| 80607263 | 80607317 | + | 20.0 | 6011 | 6065 | -235 | L1MA6 | L1 | LINE |
| 80607317 | 80608731 | - | 30.4 | 715 | 2225 | -3875 | L1MEa | L1 | LINE |

  

|  |  |  |  |  |  |  |  |  |  |
| --- | --- | --- | --- | --- | --- | --- | --- | --- | --- |
| chrX:80773183-80794454 | | | 20807 bp | | |  |  |  |  |
| Genome Start | Genome End | Strand | Div | Repeat Start | Repeat End | Repeat Left | Name | Family | Class |
| 80773183 | 80773235 | - | 26.3 | 4840 | 4888 | -1433 | L1MCa | L1 | LINE |
| 80773264 | 80773471 | - | 26.3 | 4625 | 4839 | -1482 | L1MCa | L1 | LINE |
| 80773472 | 80773812 | + | 16.1 | 2 | 366 | 0 | Tigger5a | MER2\_type | DNA |
| 80773813 | 80775735 | - | 26.3 | 2581 | 4624 | -1697 | L1MCa | L1 | LINE |
| 80775736 | 80776170 | - | 10.6 | 5724 | 6159 | -9 | L1PA10 | L1 | LINE |
| 80776171 | 80776855 | - | 23.6 | 1841 | 2580 | -3741 | L1MCa | L1 | LINE |
| 80776856 | 80776942 | + | 9.2 | 6065 | 6155 | -8 | L1PA13 | L1 | LINE |
| 80776943 | 80777175 | - | 22.9 | 1636 | 1840 | -4668 | L1MCa | L1 | LINE |
| 80777178 | 80778053 | - | 21.1 | 5288 | 6168 | 0 | L1MB1 | L1 | LINE |
| 80778351 | 80778726 | + | 37.3 | 1545 | 1928 | -5240 | L1M2a | L1 | LINE |
| 80778738 | 80778847 | + | 19.1 | 20 | 133 | -7432 | L1M1 | L1 | LINE |
| 80778842 | 80781450 | + | 14.1 | 1844 | 4492 | -3073 | L1M1 | L1 | LINE |
| 80781451 | 80782991 | - | 11.3 | 4523 | 6143 | -17 | L1PREC2 | L1 | LINE |
| 80782992 | 80783360 | - | 13.3 | 1 | 381 | 0 | THE1D | MaLR | LTR |
| 80783361 | 80786308 | - | 14.0 | 2186 | 5142 | -1624 | L1PA13 | L1 | LINE |
| 80786309 | 80786671 | + | 17.9 | 1 | 364 | 0 | THE1B | MaLR | LTR |
| 80786672 | 80788224 | + | 10.1 | 1 | 1580 | 0 | THE1B-int | MaLR | LTR |
| 80788225 | 80788587 | + | 17.6 | 2 | 364 | 0 | THE1B | MaLR | LTR |
| 80788590 | 80789778 | - | 19.5 | 978 | 2188 | -4597 | L1PA13 | L1 | LINE |
| 80789801 | 80791265 | + | 17.7 | 3059 | 4551 | -1595 | L1M1 | L1 | LINE |
| 80791262 | 80791312 | - | 5.9 | 5651 | 5701 | -453 | L1P2 | L1 | LINE |
| 80791309 | 80791425 | + | 4.3 | 5698 | 5814 | -340 | L1P2 | L1 | LINE |
| 80791516 | 80792295 | + | 25.7 | 816 | 1722 | -13 | MSTD-int | MaLR | LTR |
| 80792307 | 80792679 | + | 25.1 | 1 | 384 | -12 | MSTD | MaLR | LTR |
| 80792696 | 80794081 | - | 20.7 | 106 | 1539 | -4969 | L1MCa | L1 | LINE |
| 80794082 | 80794358 | + | 6.9 | 5873 | 6155 | -8 | L1PA13 | L1 | LINE |
| 80794359 | 80794454 | - | 20.7 | 4 | 105 | -6403 | L1MCa | L1 | LINE |

  

|  |  |  |  |  |  |  |  |  |  |
| --- | --- | --- | --- | --- | --- | --- | --- | --- | --- |
| chrX:80811584-80813509 | | | 1945 bp | | |  |  |  |  |
| Genome Start | Genome End | Strand | Div | Repeat Start | Repeat End | Repeat Left | Name | Family | Class |
| 80811584 | 80811804 | - | 18.2 | 5936 | 6164 | -7 | L1MB2 | L1 | LINE |
| 80811805 | 80812606 | - | 13.8 | 5336 | 6149 | -2 | L1PB1 | L1 | LINE |
| 80812592 | 80813044 | + | 6.9 | 4892 | 5348 | -798 | L1PB1 | L1 | LINE |
| 80813041 | 80813509 | - | 20.4 | 5477 | 5949 | -234 | L1MB2 | L1 | LINE |

  

|  |  |  |  |  |  |  |  |  |  |
| --- | --- | --- | --- | --- | --- | --- | --- | --- | --- |
| chrX:80852454-80859058 | | | 6307 bp | | |  |  |  |  |
| Genome Start | Genome End | Strand | Div | Repeat Start | Repeat End | Repeat Left | Name | Family | Class |
| 80852454 | 80852837 | - | 25.1 | 3102 | 3515 | -1319 | L1MEe | L1 | LINE |
| 80852971 | 80853232 | - | 16.5 | 5879 | 6162 | -129 | L1MA7 | L1 | LINE |
| 80853356 | 80853689 | - | 26.5 | 2745 | 3100 | -1734 | L1MEe | L1 | LINE |
| 80853690 | 80853765 | - | 11.8 | 306 | 381 | 0 | THE1D | MaLR | LTR |
| 80853767 | 80853886 | - | 5.8 | 3893 | 4012 | -2134 | L1PA3 | L1 | LINE |
| 80853870 | 80856017 | + | 3.8 | 4006 | 6154 | -1 | L1PA3 | L1 | LINE |
| 80856042 | 80856335 | - | 13.0 | 1 | 325 | -56 | THE1D | MaLR | LTR |
| 80856336 | 80857587 | - | 26.3 | 1404 | 2749 | -2085 | L1MEe | L1 | LINE |
| 80857589 | 80857650 | - | 22.8 | 987 | 1045 | -3789 | L1MEe | L1 | LINE |
| 80857684 | 80857960 | - | 14.4 | 4 | 281 | -31 | AluJb | Alu | SINE |
| 80857961 | 80858250 | - | 26.3 | 695 | 999 | -3855 | L1MEe | L1 | LINE |
| 80858251 | 80858554 | - | 5.9 | 1 | 304 | -7 | AluY | Alu | SINE |
| 80858555 | 80859058 | - | 30.6 | 161 | 694 | -4160 | L1MEe | L1 | LINE |

  

|  |  |  |  |  |  |  |  |  |  |
| --- | --- | --- | --- | --- | --- | --- | --- | --- | --- |
| chrX:80880358-80882140 | | | 1752 bp | | |  |  |  |  |
| Genome Start | Genome End | Strand | Div | Repeat Start | Repeat End | Repeat Left | Name | Family | Class |
| 80880358 | 80880584 | - | 17.4 | 5077 | 5330 | -982 | L1M | L1 | LINE |
| 80880576 | 80880812 | - | 13.2 | 5367 | 5601 | -562 | L1PA13 | L1 | LINE |
| 80880813 | 80881387 | + | 11.0 | 5591 | 6163 | 0 | L1PA13 | L1 | LINE |
| 80881428 | 80882140 | - | 20.4 | 4406 | 5046 | -1094 | L1M | L1 | LINE |

  

|  |  |  |  |  |  |  |  |  |  |
| --- | --- | --- | --- | --- | --- | --- | --- | --- | --- |
| chrX:81105581-81108866 | | | 3253 bp | | |  |  |  |  |
| Genome Start | Genome End | Strand | Div | Repeat Start | Repeat End | Repeat Left | Name | Family | Class |
| 81105581 | 81105683 | + | 26.5 | 5555 | 5659 | -462 | L1ME4a | L1 | LINE |
| 81105688 | 81107184 | - | 8.1 | 3030 | 4536 | -1610 | L1PA7 | L1 | LINE |
| 81107185 | 81108173 | + | 9.0 | 4536 | 5525 | -629 | L1PA7 | L1 | LINE |
| 81108174 | 81108649 | + | 5.9 | 5677 | 6152 | -2 | L1PA7 | L1 | LINE |
| 81108679 | 81108866 | + | 24.2 | 5701 | 5904 | -217 | L1ME4a | L1 | LINE |

  

|  |  |  |  |  |  |  |  |  |  |
| --- | --- | --- | --- | --- | --- | --- | --- | --- | --- |
| chrX:81449631-81457629 | | | 7681 bp | | |  |  |  |  |
| Genome Start | Genome End | Strand | Div | Repeat Start | Repeat End | Repeat Left | Name | Family | Class |
| 81449631 | 81450503 | - | 24.5 | 5272 | 6166 | -12 | L1MB8 | L1 | LINE |
| 81450498 | 81451244 | - | 20.9 | 5561 | 6312 | 0 | L1MA9 | L1 | LINE |
| 81451246 | 81451599 | - | 11.0 | 1 | 354 | -1 | THE1A | MaLR | LTR |
| 81451600 | 81452736 | - | 12.8 | 443 | 1580 | 0 | THE1A-int | MaLR | LTR |
| 81453028 | 81455260 | - | 22.6 | 2963 | 5286 | -860 | L1MB8 | L1 | LINE |
| 81455285 | 81456804 | - | 19.0 | 4763 | 6294 | -6 | L1MA4 | L1 | LINE |
| 81456807 | 81457503 | + | 16.1 | 4048 | 4756 | -1387 | L1MA4 | L1 | LINE |
| 81457510 | 81457629 | - | 20.0 | 2842 | 2967 | -3179 | L1MB8 | L1 | LINE |

  

|  |  |  |  |  |  |  |  |  |  |
| --- | --- | --- | --- | --- | --- | --- | --- | --- | --- |
| chrX:81629792-81635760 | | | 5831 bp | | |  |  |  |  |
| Genome Start | Genome End | Strand | Div | Repeat Start | Repeat End | Repeat Left | Name | Family | Class |
| 81629792 | 81630002 | + | 25.2 | 3294 | 3508 | -2638 | L1M4 | L1 | LINE |
| 81630004 | 81631373 | + | 11.1 | 3014 | 4398 | -1748 | L1PB1 | L1 | LINE |
| 81631402 | 81632708 | + | 10.1 | 4399 | 5792 | -359 | L1PB1 | L1 | LINE |
| 81632832 | 81633153 | + | 9.3 | 5785 | 6107 | -44 | L1PB1 | L1 | LINE |
| 81633153 | 81633305 | + | 19.6 | 3460 | 3619 | -2521 | L1M4 | L1 | LINE |
| 81633306 | 81634271 | - | 0.9 | 5189 | 6154 | -1 | L1HS | L1 | LINE |
| 81634265 | 81634806 | + | 1.1 | 4639 | 5180 | -966 | L1HS | L1 | LINE |
| 81634801 | 81635760 | + | 24.3 | 3611 | 4592 | -1554 | L1M4 | L1 | LINE |

  

|  |  |  |  |  |  |  |  |  |  |
| --- | --- | --- | --- | --- | --- | --- | --- | --- | --- |
| chrX:81645577-81647870 | | | 2280 bp | | |  |  |  |  |
| Genome Start | Genome End | Strand | Div | Repeat Start | Repeat End | Repeat Left | Name | Family | Class |
| 81645577 | 81645697 | - | 21.5 | 4518 | 4641 | -1735 | L1M4c | L1 | LINE |
| 81645702 | 81645994 | + | 16.8 | 1341 | 1651 | 0 | MSTA-int | MaLR | LTR |
| 81645995 | 81646413 | + | 22.4 | 1 | 428 | 0 | MSTA | MaLR | LTR |
| 81646423 | 81647035 | - | 8.5 | 5338 | 5949 | -223 | L1PA8 | L1 | LINE |
| 81647037 | 81647635 | + | 9.8 | 4739 | 5337 | -809 | L1PA8 | L1 | LINE |
| 81647636 | 81647870 | - | 23.0 | 3504 | 3748 | -2628 | L1M4c | L1 | LINE |

  

|  |  |  |  |  |  |  |  |  |  |
| --- | --- | --- | --- | --- | --- | --- | --- | --- | --- |
| chrX:82094749-82099644 | | | 4899 bp | | |  |  |  |  |
| Genome Start | Genome End | Strand | Div | Repeat Start | Repeat End | Repeat Left | Name | Family | Class |
| 82094749 | 82096003 | + | 27.5 | 382 | 1790 | -5059 | L1M2 | L1 | LINE |
| 82096001 | 82096107 | - | 3.7 | 5236 | 5342 | -804 | L1PA3 | L1 | LINE |
| 82096107 | 82096921 | + | 2.3 | 5338 | 6152 | -3 | L1PA3 | L1 | LINE |
| 82096923 | 82099644 | + | 25.8 | 1778 | 4858 | -1990 | L1M2 | L1 | LINE |

  

|  |  |  |  |  |  |  |  |  |  |
| --- | --- | --- | --- | --- | --- | --- | --- | --- | --- |
| chrX:82159233-82174092 | | | 14748 bp | | |  |  |  |  |
| Genome Start | Genome End | Strand | Div | Repeat Start | Repeat End | Repeat Left | Name | Family | Class |
| 82159233 | 82159608 | + | 28.0 | 1258 | 1648 | -4998 | L1MB4 | L1 | LINE |
| 82159609 | 82159712 | + | 2.9 | 6049 | 6152 | -3 | L1PA3 | L1 | LINE |
| 82159713 | 82163288 | + | 26.8 | 1649 | 5393 | -1245 | L1MB4 | L1 | LINE |
| 82163294 | 82165673 | + | 20.0 | 1 | 2457 | -5108 | L1MA2 | L1 | LINE |
| 82165700 | 82166348 | + | 20.0 | 2458 | 3122 | -4443 | L1MA2 | L1 | LINE |
| 82166346 | 82170503 | + | 18.4 | 3373 | 7720 | -3 | L1MA2 | L1 | LINE |
| 82170544 | 82170867 | + | 26.1 | 5413 | 5749 | -895 | L1MB4 | L1 | LINE |
| 82170868 | 82171727 | - | 16.8 | 223 | 1071 | 0 | MER11C | ERVK | LTR |
| 82171728 | 82172012 | - | 2.1 | 5282 | 5565 | -590 | L1PA4 | L1 | LINE |
| 82172013 | 82172596 | + | 2.9 | 5569 | 6152 | -3 | L1PA4 | L1 | LINE |
| 82172641 | 82172875 | - | 8.9 | 1 | 237 | -999 | MER11B | ERVK | LTR |
| 82172876 | 82173072 | + | 24.7 | 5744 | 5940 | -738 | L1MB4 | L1 | LINE |
| 82173073 | 82173347 | - | 15.6 | 1 | 298 | -14 | AluSx | Alu | SINE |
| 82173348 | 82174092 | + | 24.7 | 5941 | 6678 | 0 | L1MB4 | L1 | LINE |

  

|  |  |  |  |  |  |  |  |  |  |
| --- | --- | --- | --- | --- | --- | --- | --- | --- | --- |
| chrX:82450387-82458591 | | | 8100 bp | | |  |  |  |  |
| Genome Start | Genome End | Strand | Div | Repeat Start | Repeat End | Repeat Left | Name | Family | Class |
| 82450387 | 82451480 | - | 25.5 | 2868 | 4000 | -2146 | L1M4 | L1 | LINE |
| 82451486 | 82451939 | + | 13.5 | 3841 | 4313 | -1830 | L1M2 | L1 | LINE |
| 82451981 | 82453727 | + | 17.9 | 13 | 1755 | -5810 | L1MA2 | L1 | LINE |
| 82453765 | 82455370 | + | 17.9 | 1756 | 3302 | -4263 | L1MA2 | L1 | LINE |
| 82455377 | 82456598 | + | 14.4 | 6423 | 7718 | -1 | L1MA2 | L1 | LINE |
| 82456615 | 82456788 | + | 13.9 | 4292 | 4469 | -1674 | L1M2 | L1 | LINE |
| 82456778 | 82456844 | - | 13.4 | 6011 | 6077 | -88 | L1PA15 | L1 | LINE |
| 82456842 | 82456916 | + | 21.3 | 6087 | 6161 | -4 | L1PA15 | L1 | LINE |
| 82456919 | 82457085 | + | 16.8 | 6117 | 6291 | 0 | L1MA8 | L1 | LINE |
| 82457095 | 82457527 | - | 23.4 | 2890 | 3377 | -3267 | L1M4 | L1 | LINE |
| 82457531 | 82457646 | - | 21.1 | 1433 | 1545 | -4997 | L1M4 | L1 | LINE |
| 82457647 | 82457964 | + | 1.9 | 1 | 318 | 0 | AluYb8 | Alu | SINE |
| 82457965 | 82458591 | - | 21.1 | 836 | 1432 | -5110 | L1M4 | L1 | LINE |

  

|  |  |  |  |  |  |  |  |  |  |
| --- | --- | --- | --- | --- | --- | --- | --- | --- | --- |
| chrX:82511027-82515215 | | | 4107 bp | | |  |  |  |  |
| Genome Start | Genome End | Strand | Div | Repeat Start | Repeat End | Repeat Left | Name | Family | Class |
| 82511027 | 82511909 | + | 24.0 | 4500 | 5426 | -2359 | L1M3 | L1 | LINE |
| 82511943 | 82513019 | - | 19.5 | 4557 | 5633 | -679 | L1M2 | L1 | LINE |
| 82513020 | 82513476 | - | 22.9 | 1 | 408 | 0 | MLT1A1 | MaLR | LTR |
| 82513477 | 82514152 | - | 20.6 | 3850 | 4556 | -1584 | L1M2 | L1 | LINE |
| 82514155 | 82514662 | + | 21.1 | 5627 | 6113 | -199 | L1MA9 | L1 | LINE |
| 82514700 | 82514908 | + | 19.5 | 6067 | 6272 | -40 | L1MA9 | L1 | LINE |
| 82514919 | 82515000 | + | 20.5 | 5486 | 5566 | -605 | L1M3 | L1 | LINE |
| 82515001 | 82515104 | + | 4.8 | 6051 | 6154 | 0 | L1PA7 | L1 | LINE |
| 82515105 | 82515215 | + | 20.5 | 5567 | 5681 | -490 | L1M3 | L1 | LINE |

  

|  |  |  |  |  |  |  |  |  |  |
| --- | --- | --- | --- | --- | --- | --- | --- | --- | --- |
| chrX:82703413-82711191 | | | 7635 bp | | |  |  |  |  |
| Genome Start | Genome End | Strand | Div | Repeat Start | Repeat End | Repeat Left | Name | Family | Class |
| 82703413 | 82709306 | - | 11.7 | 503 | 6486 | -1 | L1PA8 | L1 | LINE |
| 82709328 | 82709604 | - | 1.4 | 4871 | 5147 | -999 | L1PA2 | L1 | LINE |
| 82709601 | 82710589 | + | 1.6 | 5164 | 6153 | -2 | L1PA2 | L1 | LINE |
| 82710717 | 82711191 | - | 18.1 | 3 | 478 | -5397 | L1PA8 | L1 | LINE |

  

|  |  |  |  |  |  |  |  |  |  |
| --- | --- | --- | --- | --- | --- | --- | --- | --- | --- |
| chrX:82915973-82927551 | | | 11274 bp | | |  |  |  |  |
| Genome Start | Genome End | Strand | Div | Repeat Start | Repeat End | Repeat Left | Name | Family | Class |
| 82915973 | 82917996 | + | 19.6 | 2 | 2083 | -4425 | L1MCa | L1 | LINE |
| 82918256 | 82920558 | - | 8.5 | 3840 | 6154 | 0 | L1PA7 | L1 | LINE |
| 82920552 | 82922259 | + | 10.0 | 2321 | 4010 | -2311 | L1MCa | L1 | LINE |
| 82922275 | 82923198 | + | 18.3 | 2090 | 3055 | -3266 | L1MCa | L1 | LINE |
| 82923198 | 82923282 | - | 15.8 | 5767 | 5848 | -452 | L1MA6 | L1 | LINE |
| 82923286 | 82923704 | + | 21.9 | 5864 | 6291 | -9 | L1MA6 | L1 | LINE |
| 82923721 | 82924039 | + | 17.2 | 3038 | 3377 | -2944 | L1MCa | L1 | LINE |
| 82924044 | 82925055 | + | 6.2 | 1 | 1262 | -4 | MER11A | ERVK | LTR |
| 82925072 | 82927551 | + | 19.5 | 3387 | 5923 | -585 | L1MCa | L1 | LINE |

  

|  |  |  |  |  |  |  |  |  |  |
| --- | --- | --- | --- | --- | --- | --- | --- | --- | --- |
| chrX:83571556-83578704 | | | 7095 bp | | |  |  |  |  |
| Genome Start | Genome End | Strand | Div | Repeat Start | Repeat End | Repeat Left | Name | Family | Class |
| 83571556 | 83572476 | - | 14.4 | 5366 | 6291 | -6 | L1MA5A | L1 | LINE |
| 83572535 | 83574616 | - | 13.7 | 4024 | 6155 | -10 | L1PA15 | L1 | LINE |
| 83574617 | 83576900 | - | 19.4 | 3070 | 5379 | -764 | L1MA5A | L1 | LINE |
| 83576904 | 83577419 | - | 2.5 | 4960 | 5475 | -680 | L1PA2 | L1 | LINE |
| 83577420 | 83578069 | + | 1.2 | 5470 | 6155 | 0 | L1PA2 | L1 | LINE |
| 83578070 | 83578084 | - | 2.4 | 4945 | 4959 | -1187 | L1P1 | L1 | LINE |
| 83578078 | 83578704 | - | 18.9 | 2444 | 3061 | -3082 | L1MA5A | L1 | LINE |

  

|  |  |  |  |  |  |  |  |  |  |
| --- | --- | --- | --- | --- | --- | --- | --- | --- | --- |
| chrX:84538291-84551175 | | | 12838 bp | | |  |  |  |  |
| Genome Start | Genome End | Strand | Div | Repeat Start | Repeat End | Repeat Left | Name | Family | Class |
| 84538291 | 84538485 | - | 10.1 | 7536 | 7721 | 0 | L1MA1 | L1 | LINE |
| 84538486 | 84538741 | + | 14.9 | 2 | 295 | -17 | AluSx | Alu | SINE |
| 84538742 | 84538842 | - | 10.1 | 7426 | 7535 | -186 | L1MA1 | L1 | LINE |
| 84538850 | 84540027 | - | 10.7 | 5745 | 6892 | -831 | L1MA1 | L1 | LINE |
| 84540028 | 84540337 | + | 11.0 | 2 | 311 | -1 | AluSx | Alu | SINE |
| 84540338 | 84543644 | - | 11.6 | 2447 | 5744 | -1821 | L1MA1 | L1 | LINE |
| 84543680 | 84545676 | - | 8.3 | 4168 | 6149 | -5 | L1PA7 | L1 | LINE |
| 84545677 | 84545984 | - | 7.8 | 1 | 310 | -1 | AluY | Alu | SINE |
| 84545985 | 84547214 | - | 9.0 | 2945 | 4167 | -1979 | L1PA7 | L1 | LINE |
| 84547214 | 84548763 | + | 6.7 | 1414 | 2945 | -3201 | L1PA7 | L1 | LINE |
| 84548770 | 84551175 | - | 17.6 | 13 | 2453 | -5112 | L1MA1 | L1 | LINE |

  

|  |  |  |  |  |  |  |  |  |  |
| --- | --- | --- | --- | --- | --- | --- | --- | --- | --- |
| chrX:84805467-84807056 | | | 1533 bp | | |  |  |  |  |
| Genome Start | Genome End | Strand | Div | Repeat Start | Repeat End | Repeat Left | Name | Family | Class |
| 84805467 | 84805755 | - | 21.7 | 247 | 505 | 0 | MLT1D | MaLR | LTR |
| 84805803 | 84806624 | - | 2.7 | 5078 | 5899 | -256 | L1PA4 | L1 | LINE |
| 84806626 | 84806874 | + | 5.2 | 5899 | 6155 | 0 | L1PA4 | L1 | LINE |
| 84806884 | 84807056 | - | 19.6 | 5 | 164 | -462 | MLT1D | MaLR | LTR |

  

|  |  |  |  |  |  |  |  |  |  |
| --- | --- | --- | --- | --- | --- | --- | --- | --- | --- |
| chrX:85206189-85207081 | | | 878 bp | | |  |  |  |  |
| Genome Start | Genome End | Strand | Div | Repeat Start | Repeat End | Repeat Left | Name | Family | Class |
| 85206189 | 85206296 | + | 11.3 | 38 | 159 | -165 | MER5C | MER1\_type | DNA |
| 85206305 | 85206719 | - | 15.3 | 5654 | 6029 | -292 | L1MA9 | L1 | LINE |
| 85206722 | 85207011 | + | 17.0 | 6032 | 6309 | -3 | L1MA9 | L1 | LINE |
| 85207017 | 85207081 | + | 12.3 | 157 | 234 | -90 | MER5C | MER1\_type | DNA |

  

|  |  |  |  |  |  |  |  |  |  |
| --- | --- | --- | --- | --- | --- | --- | --- | --- | --- |
| chrX:85747846-85753703 | | | 5679 bp | | |  |  |  |  |
| Genome Start | Genome End | Strand | Div | Repeat Start | Repeat End | Repeat Left | Name | Family | Class |
| 85747846 | 85749256 | - | 13.3 | 3161 | 4566 | -2999 | L1M1 | L1 | LINE |
| 85749258 | 85749776 | - | 1.5 | 5632 | 6150 | -5 | L1PA3 | L1 | LINE |
| 85749777 | 85750079 | + | 1.3 | 5343 | 5645 | -510 | L1PA3 | L1 | LINE |
| 85750073 | 85751958 | - | 18.8 | 1286 | 3178 | -4387 | L1M1 | L1 | LINE |
| 85752116 | 85752405 | - | 14.8 | 1 | 280 | -32 | AluJb | Alu | SINE |
| 85752406 | 85753616 | - | 18.8 | 73 | 1285 | -6280 | L1M1 | L1 | LINE |
| 85753645 | 85753703 | - | 18.8 | 10 | 72 | -7493 | L1M1 | L1 | LINE |

  

|  |  |  |  |  |  |  |  |  |  |
| --- | --- | --- | --- | --- | --- | --- | --- | --- | --- |
| chrX:85896115-85903749 | | | 7609 bp | | |  |  |  |  |
| Genome Start | Genome End | Strand | Div | Repeat Start | Repeat End | Repeat Left | Name | Family | Class |
| 85896115 | 85897252 | - | 15.1 | 5684 | 6827 | -178 | L1MA4A | L1 | LINE |
| 85897253 | 85897555 | - | 5.9 | 1 | 303 | -8 | AluY | Alu | SINE |
| 85897556 | 85899192 | - | 16.4 | 4190 | 5683 | -1162 | L1MA4A | L1 | LINE |
| 85899193 | 85899579 | + | 14.1 | 1 | 428 | 0 | MSTA | MaLR | LTR |
| 85899580 | 85900434 | - | 17.3 | 3324 | 4189 | -2656 | L1MA4A | L1 | LINE |
| 85900435 | 85900796 | - | 16.0 | 5922 | 6301 | -3 | L1MA2 | L1 | LINE |
| 85900797 | 85902662 | - | 17.9 | 1472 | 3323 | -3522 | L1MA4A | L1 | LINE |
| 85902686 | 85903042 | - | 3.9 | 5816 | 6171 | -1 | L1PA8 | L1 | LINE |
| 85903043 | 85903355 | + | 8.6 | 5479 | 5791 | -381 | L1PA8 | L1 | LINE |
| 85903357 | 85903648 | - | 25.0 | 1176 | 1465 | -5384 | L1MA4A | L1 | LINE |
| 85903651 | 85903749 | - | 18.2 | 3 | 101 | -6748 | L1MA4A | L1 | LINE |

  

|  |  |  |  |  |  |  |  |  |  |
| --- | --- | --- | --- | --- | --- | --- | --- | --- | --- |
| chrX:86095793-86099150 | | | 3346 bp | | |  |  |  |  |
| Genome Start | Genome End | Strand | Div | Repeat Start | Repeat End | Repeat Left | Name | Family | Class |
| 86095793 | 86096000 | - | 15.4 | 6085 | 6298 | -2 | L1MA5 | L1 | LINE |
| 86096001 | 86096264 | - | 8.7 | 4874 | 5138 | -1008 | L1PREC2 | L1 | LINE |
| 86096266 | 86097272 | + | 9.3 | 5141 | 6158 | -3 | L1PA14 | L1 | LINE |
| 86097284 | 86099150 | - | 16.1 | 4197 | 6097 | -203 | L1MA5 | L1 | LINE |

  

|  |  |  |  |  |  |  |  |  |  |
| --- | --- | --- | --- | --- | --- | --- | --- | --- | --- |
| chrX:86641088-86643524 | | | 2438 bp | | |  |  |  |  |
| Genome Start | Genome End | Strand | Div | Repeat Start | Repeat End | Repeat Left | Name | Family | Class |
| 86641088 | 86641205 | + | 13.5 | 1 | 112 | -278 | MLT1B | MaLR | LTR |
| 86641206 | 86642163 | - | 2.0 | 4109 | 5067 | -1079 | L1PA3 | L1 | LINE |
| 86642158 | 86643225 | + | 2.8 | 5078 | 6145 | -10 | L1PA3 | L1 | LINE |
| 86643231 | 86643524 | + | 20.4 | 96 | 390 | 0 | MLT1B | MaLR | LTR |

  

|  |  |  |  |  |  |  |  |  |  |
| --- | --- | --- | --- | --- | --- | --- | --- | --- | --- |
| chrX:86854964-86857224 | | | 2067 bp | | |  |  |  |  |
| Genome Start | Genome End | Strand | Div | Repeat Start | Repeat End | Repeat Left | Name | Family | Class |
| 86854964 | 86855243 | + | 22.2 | 1678 | 1951 | -993 | BLACKJACK | AcHobo | DNA |
| 86855244 | 86855661 | + | 18.4 | 1 | 426 | 0 | MSTB | MaLR | LTR |
| 86855662 | 86856449 | + | 22.2 | 1952 | 2779 | -165 | BLACKJACK | AcHobo | DNA |
| 86856642 | 86857009 | - | 16.7 | 5910 | 6295 | -5 | L1MA6 | L1 | LINE |
| 86857012 | 86857071 | + | 13.3 | 5852 | 5914 | -386 | L1MA6 | L1 | LINE |
| 86857072 | 86857224 | + | 24.5 | 2769 | 2921 | -23 | BLACKJACK | AcHobo | DNA |

  

|  |  |  |  |  |  |  |  |  |  |
| --- | --- | --- | --- | --- | --- | --- | --- | --- | --- |
| chrX:87952763-87955953 | | | 3188 bp | | |  |  |  |  |
| Genome Start | Genome End | Strand | Div | Repeat Start | Repeat End | Repeat Left | Name | Family | Class |
| 87952763 | 87952907 | - | 21.7 | 6007 | 6162 | -4 | L1ME1 | L1 | LINE |
| 87952912 | 87953097 | - | 21.6 | 303 | 488 | -75 | MER68 | ERVL | LTR |
| 87953102 | 87954791 | - | 15.6 | 4477 | 6160 | -6 | L1PA16 | L1 | LINE |
| 87954786 | 87954915 | + | 14.6 | 3623 | 3749 | -2397 | L1P4 | L1 | LINE |
| 87954916 | 87954999 | + | 9.1 | 3 | 80 | 0 | MADE1 | Mariner | DNA |
| 87955000 | 87955711 | + | 14.6 | 3750 | 4460 | -1686 | L1P4 | L1 | LINE |
| 87955713 | 87955953 | - | 23.7 | 5773 | 6022 | -144 | L1ME1 | L1 | LINE |

  

|  |  |  |  |  |  |  |  |  |  |
| --- | --- | --- | --- | --- | --- | --- | --- | --- | --- |
| chrX:88755159-88758861 | | | 3709 bp | | |  |  |  |  |
| Genome Start | Genome End | Strand | Div | Repeat Start | Repeat End | Repeat Left | Name | Family | Class |
| 88755159 | 88755446 | + | 15.6 | 2639 | 2941 | -3205 | L1MC | L1 | LINE |
| 88755440 | 88755965 | - | 10.3 | 5145 | 5680 | -481 | L1PA14 | L1 | LINE |
| 88755966 | 88756454 | + | 10.3 | 5671 | 6159 | -2 | L1PA14 | L1 | LINE |
| 88756456 | 88756553 | + | 18.3 | 2928 | 3025 | -3121 | L1MC | L1 | LINE |
| 88756554 | 88756771 | - | 21.1 | 1 | 236 | 0 | MER46A | MER2\_type | DNA |
| 88756772 | 88757232 | + | 18.3 | 3026 | 3467 | -2679 | L1MC | L1 | LINE |
| 88757233 | 88757587 | - | 14.3 | 1 | 375 | 0 | THE1C | MaLR | LTR |
| 88757588 | 88758861 | + | 18.3 | 3468 | 4676 | -1470 | L1MC | L1 | LINE |

  

|  |  |  |  |  |  |  |  |  |  |
| --- | --- | --- | --- | --- | --- | --- | --- | --- | --- |
| chrX:88777404-88786602 | | | 9205 bp | | |  |  |  |  |
| Genome Start | Genome End | Strand | Div | Repeat Start | Repeat End | Repeat Left | Name | Family | Class |
| 88777404 | 88778790 | + | 17.9 | 16 | 1458 | -6107 | L1M1 | L1 | LINE |
| 88778793 | 88779620 | - | 5.1 | 5326 | 6154 | 0 | L1PA5 | L1 | LINE |
| 88779617 | 88780212 | + | 6.9 | 4718 | 5317 | -829 | L1PA5 | L1 | LINE |
| 88780209 | 88782622 | + | 13.8 | 1444 | 3930 | -3635 | L1M1 | L1 | LINE |
| 88782623 | 88783154 | + | 11.5 | 1 | 453 | 0 | MLT2A2 | ERVL | LTR |
| 88783155 | 88784410 | + | 13.1 | 3931 | 5190 | -2375 | L1M1 | L1 | LINE |
| 88784411 | 88784714 | + | 8.2 | 5851 | 6154 | 0 | L1PA7 | L1 | LINE |
| 88784715 | 88786602 | + | 17.1 | 5191 | 6889 | -696 | L1M1 | L1 | LINE |

  

|  |  |  |  |  |  |  |  |  |  |
| --- | --- | --- | --- | --- | --- | --- | --- | --- | --- |
| chrX:90080253-90083288 | | | 3029 bp | | |  |  |  |  |
| Genome Start | Genome End | Strand | Div | Repeat Start | Repeat End | Repeat Left | Name | Family | Class |
| 90080253 | 90080564 | - | 9.9 | 1275 | 1579 | -1 | THE1D-int | MaLR | LTR |
| 90080573 | 90082029 | - | 12.0 | 4703 | 6173 | -1 | L1PA11 | L1 | LINE |
| 90082028 | 90082880 | + | 10.6 | 3835 | 4689 | -1457 | L1PA11 | L1 | LINE |
| 90082882 | 90083288 | - | 15.5 | 867 | 1277 | -303 | THE1D-int | MaLR | LTR |

  

|  |  |  |  |  |  |  |  |  |  |
| --- | --- | --- | --- | --- | --- | --- | --- | --- | --- |
| chrX:90494668-90505947 | | | 11124 bp | | |  |  |  |  |
| Genome Start | Genome End | Strand | Div | Repeat Start | Repeat End | Repeat Left | Name | Family | Class |
| 90494668 | 90496054 | - | 26.4 | 4771 | 6158 | -12 | L1ME3A | L1 | LINE |
| 90496065 | 90497948 | + | 17.4 | 4331 | 6089 | -244 | L1MC1 | L1 | LINE |
| 90497949 | 90499853 | - | 12.9 | 3805 | 5776 | -822 | MER4A1\_-int | ERV1 | LTR |
| 90499854 | 90501609 | - | 2.3 | 3211 | 4969 | -1177 | L1PA3 | L1 | LINE |
| 90501604 | 90502798 | + | 1.8 | 4961 | 6155 | 0 | L1PA3 | L1 | LINE |
| 90502804 | 90502958 | - | 13.2 | 1269 | 1429 | -5169 | MER4A1\_-int | ERV1 | LTR |
| 90503018 | 90503226 | - | 14.8 | 524 | 1033 | -5758 | MER4A1\_-int | ERV1 | LTR |
| 90503233 | 90503519 | - | 18.0 | 435 | 734 | 0 | MER50 | ERV1 | LTR |
| 90503523 | 90503741 | - | 14.7 | 1 | 222 | -512 | MER50 | ERV1 | LTR |
| 90503775 | 90504180 | - | 11.6 | 1 | 429 | -6169 | MER4A1\_-int | ERV1 | LTR |
| 90504184 | 90504780 | - | 13.1 | 3 | 599 | -1 | MER4A1\_ | ERV1 | LTR |
| 90504783 | 90504992 | + | 19.6 | 6093 | 6297 | -36 | L1MC1 | L1 | LINE |
| 90505034 | 90505552 | - | 25.8 | 4222 | 4756 | -1390 | L1ME3A | L1 | LINE |
| 90505553 | 90505815 | - | 17.9 | 6028 | 6291 | -9 | L1MA4 | L1 | LINE |
| 90505816 | 90505947 | - | 25.8 | 4093 | 4221 | -1925 | L1ME3A | L1 | LINE |

  

|  |  |  |  |  |  |  |  |  |  |
| --- | --- | --- | --- | --- | --- | --- | --- | --- | --- |
| chrX:90638298-90639419 | | | 1115 bp | | |  |  |  |  |
| Genome Start | Genome End | Strand | Div | Repeat Start | Repeat End | Repeat Left | Name | Family | Class |
| 90638298 | 90638407 | + | 22.2 | 1 | 116 | -349 | MER4C | ERV1 | LTR |
| 90638392 | 90638741 | + | 28.7 | 83 | 455 | -244 | LTR34 | ERV1 | LTR |
| 90638742 | 90638852 | + | 16.4 | 278 | 496 | -203 | LTR34 | ERV1 | LTR |
| 90638853 | 90638920 | - | 8.8 | 5768 | 5838 | -330 | L1PA13 | L1 | LINE |
| 90638925 | 90639217 | + | 10.2 | 5860 | 6152 | -11 | L1PA13 | L1 | LINE |
| 90639237 | 90639419 | + | 25.1 | 275 | 461 | -4 | MER4C | ERV1 | LTR |

  

|  |  |  |  |  |  |  |  |  |  |
| --- | --- | --- | --- | --- | --- | --- | --- | --- | --- |
| chrX:91113548-91115595 | | | 2039 bp | | |  |  |  |  |
| Genome Start | Genome End | Strand | Div | Repeat Start | Repeat End | Repeat Left | Name | Family | Class |
| 91113548 | 91113696 | + | 15.4 | 5968 | 6124 | -167 | L1MA8 | L1 | LINE |
| 91113690 | 91114505 | - | 1.5 | 4741 | 5567 | -588 | L1PA2 | L1 | LINE |
| 91114506 | 91115105 | + | 1.7 | 5555 | 6154 | -1 | L1PA2 | L1 | LINE |
| 91115122 | 91115268 | + | 10.8 | 6115 | 6265 | -26 | L1MA8 | L1 | LINE |
| 91115269 | 91115569 | - | 8.3 | 1 | 301 | -11 | AluSx | Alu | SINE |
| 91115570 | 91115595 | + | 10.8 | 6266 | 6286 | -5 | L1MA8 | L1 | LINE |

  

|  |  |  |  |  |  |  |  |  |  |
| --- | --- | --- | --- | --- | --- | --- | --- | --- | --- |
| chrX:92295049-92302954 | | | 7910 bp | | |  |  |  |  |
| Genome Start | Genome End | Strand | Div | Repeat Start | Repeat End | Repeat Left | Name | Family | Class |
| 92295049 | 92300268 | + | 18.1 | 11 | 5410 | -1435 | L1MA4A | L1 | LINE |
| 92300267 | 92301081 | - | 15.9 | 4643 | 5484 | -682 | L1PA16 | L1 | LINE |
| 92301082 | 92301760 | + | 15.9 | 5498 | 6166 | 0 | L1PA16 | L1 | LINE |
| 92301761 | 92301801 | + | 18.0 | 5406 | 5446 | -1399 | L1MA4A | L1 | LINE |
| 92301802 | 92302096 | + | 13.6 | 1 | 295 | -15 | AluSg | Alu | SINE |
| 92302097 | 92302540 | + | 18.0 | 5447 | 5890 | -955 | L1MA4A | L1 | LINE |
| 92302539 | 92302954 | + | 17.2 | 6235 | 6665 | -340 | L1MA4A | L1 | LINE |

  

|  |  |  |  |  |  |  |  |  |  |
| --- | --- | --- | --- | --- | --- | --- | --- | --- | --- |
| chrX:93703087-93710413 | | | 7274 bp | | |  |  |  |  |
| Genome Start | Genome End | Strand | Div | Repeat Start | Repeat End | Repeat Left | Name | Family | Class |
| 93703087 | 93706567 | - | 15.9 | 3191 | 6785 | -780 | L1M1 | L1 | LINE |
| 93706593 | 93706720 | - | 13.3 | 3578 | 3705 | -2441 | L1M1 | L1 | LINE |
| 93706727 | 93706974 | - | 6.0 | 5867 | 6114 | -40 | L1PA7 | L1 | LINE |
| 93706972 | 93707712 | + | 13.1 | 5156 | 5844 | -322 | L1PA7 | L1 | LINE |
| 93707695 | 93707994 | - | 20.7 | 2900 | 3219 | -4346 | L1M1 | L1 | LINE |
| 93708007 | 93708319 | - | 12.8 | 1 | 305 | -5 | AluSg | Alu | SINE |
| 93708351 | 93710413 | - | 22.6 | 10 | 2777 | -4788 | L1M1 | L1 | LINE |

  

|  |  |  |  |  |  |  |  |  |  |
| --- | --- | --- | --- | --- | --- | --- | --- | --- | --- |
| chrX:93960666-93970187 | | | 9345 bp | | |  |  |  |  |
| Genome Start | Genome End | Strand | Div | Repeat Start | Repeat End | Repeat Left | Name | Family | Class |
| 93960666 | 93960711 | - | 10.9 | 512 | 561 | 0 | MLT2A1 | ERVL | LTR |
| 93960868 | 93961259 | - | 6.9 | 15 | 404 | -40 | MLT2A1 | ERVL | LTR |
| 93961260 | 93961565 | - | 13.1 | 1 | 306 | -4 | AluSg | Alu | SINE |
| 93961580 | 93967020 | - | 9.3 | 181 | 5654 | 0 | HERVL-A1 | ERVL | LTR |
| 93967025 | 93969348 | - | 3.5 | 3839 | 6154 | -1 | L1PA4 | L1 | LINE |
| 93969347 | 93969520 | + | 8.1 | 3671 | 3844 | -2302 | L1PA4 | L1 | LINE |
| 93969526 | 93969699 | - | 10.3 | 1 | 178 | -5476 | HERVL-A1 | ERVL | LTR |
| 93969700 | 93970187 | - | 8.4 | 1 | 444 | 0 | MLT2A1 | ERVL | LTR |

  

|  |  |  |  |  |  |  |  |  |  |
| --- | --- | --- | --- | --- | --- | --- | --- | --- | --- |
| chrX:94510476-94518751 | | | 8151 bp | | |  |  |  |  |
| Genome Start | Genome End | Strand | Div | Repeat Start | Repeat End | Repeat Left | Name | Family | Class |
| 94510476 | 94511411 | + | 20.6 | 1 | 940 | -5264 | L1PB3 | L1 | LINE |
| 94511438 | 94511543 | + | 20.6 | 941 | 1050 | -5154 | L1PB3 | L1 | LINE |
| 94511579 | 94512277 | - | 3.0 | 5458 | 6152 | -3 | L1PA3 | L1 | LINE |
| 94512278 | 94513274 | + | 2.8 | 4453 | 5449 | -706 | L1PA3 | L1 | LINE |
| 94513274 | 94514796 | + | 18.6 | 1038 | 2653 | -3550 | L1PB3 | L1 | LINE |
| 94514797 | 94515164 | - | 14.3 | 3 | 373 | -2 | THE1C | MaLR | LTR |
| 94515199 | 94516987 | + | 16.8 | 2661 | 4469 | -1734 | L1PB3 | L1 | LINE |
| 94517019 | 94518751 | + | 14.7 | 4470 | 6209 | -2 | L1PB3 | L1 | LINE |

  

|  |  |  |  |  |  |  |  |  |  |
| --- | --- | --- | --- | --- | --- | --- | --- | --- | --- |
| chrX:94737920-94746210 | | | 8154 bp | | |  |  |  |  |
| Genome Start | Genome End | Strand | Div | Repeat Start | Repeat End | Repeat Left | Name | Family | Class |
| 94737920 | 94738023 | + | 19.6 | 16 | 123 | -7442 | L1MA1 | L1 | LINE |
| 94738070 | 94742247 | + | 13.3 | 1109 | 5329 | -2236 | L1MA1 | L1 | LINE |
| 94742225 | 94743132 | - | 0.5 | 4568 | 5475 | -680 | L1HS | L1 | LINE |
| 94743133 | 94743788 | + | 0.6 | 5500 | 6155 | 0 | L1HS | L1 | LINE |
| 94743903 | 94746210 | + | 10.3 | 5287 | 7616 | -105 | L1MA1 | L1 | LINE |

  

|  |  |  |  |  |  |  |  |  |  |
| --- | --- | --- | --- | --- | --- | --- | --- | --- | --- |
| chrX:94951044-94955316 | | | 4254 bp | | |  |  |  |  |
| Genome Start | Genome End | Strand | Div | Repeat Start | Repeat End | Repeat Left | Name | Family | Class |
| 94951044 | 94951492 | + | 19.4 | 694 | 1189 | -5211 | L1MEc | L1 | LINE |
| 94951503 | 94953014 | - | 1.1 | 4641 | 6153 | -2 | L1HS | L1 | LINE |
| 94953010 | 94954245 | + | 0.3 | 3430 | 4665 | -1481 | L1HS | L1 | LINE |
| 94954260 | 94955316 | + | 22.4 | 1206 | 2352 | -4048 | L1MEc | L1 | LINE |

  

|  |  |  |  |  |  |  |  |  |  |
| --- | --- | --- | --- | --- | --- | --- | --- | --- | --- |
| chrX:95215994-95217875 | | | 1877 bp | | |  |  |  |  |
| Genome Start | Genome End | Strand | Div | Repeat Start | Repeat End | Repeat Left | Name | Family | Class |
| 95215994 | 95216958 | + | 19.5 | 1253 | 2199 | -4366 | L1M3f | L1 | LINE |
| 95216964 | 95217466 | - | 6.8 | 5647 | 6148 | -6 | L1PA7 | L1 | LINE |
| 95217467 | 95217601 | + | 5.2 | 5520 | 5654 | -500 | L1PA7 | L1 | LINE |
| 95217602 | 95217875 | + | 12.1 | 2190 | 2470 | -4095 | L1M3f | L1 | LINE |

  

|  |  |  |  |  |  |  |  |  |  |
| --- | --- | --- | --- | --- | --- | --- | --- | --- | --- |
| chrX:95430661-95437913 | | | 6895 bp | | |  |  |  |  |
| Genome Start | Genome End | Strand | Div | Repeat Start | Repeat End | Repeat Left | Name | Family | Class |
| 95430661 | 95430935 | + | 30.3 | 242 | 528 | -4326 | L1MEe | L1 | LINE |
| 95431012 | 95431249 | - | 18.1 | 5870 | 6109 | -59 | L1MB1 | L1 | LINE |
| 95431251 | 95431310 | + | 13.3 | 6104 | 6167 | -1 | L1MB1 | L1 | LINE |
| 95431354 | 95431502 | + | 30.9 | 5670 | 5819 | -302 | L1M5 | L1 | LINE |
| 95431554 | 95431829 | + | 22.8 | 637 | 913 | -3941 | L1MEe | L1 | LINE |
| 95431835 | 95431894 | + | 26.7 | 5533 | 5592 | -572 | L1M5 | L1 | LINE |
| 95431941 | 95432209 | - | 26.8 | 258 | 519 | 0 | LTR53 | ERVL | LTR |
| 95432331 | 95433044 | + | 24.2 | 1047 | 1783 | -4648 | L1M3de | L1 | LINE |
| 95433058 | 95433507 | + | 13.6 | 2 | 453 | -6008 | L1PA7 | L1 | LINE |
| 95433505 | 95436589 | + | 9.1 | 3376 | 6466 | -3 | L1PA7 | L1 | LINE |
| 95436591 | 95436710 | + | 18.3 | 892 | 1018 | -3816 | L1MEe | L1 | LINE |
| 95436715 | 95437031 | - | 14.8 | 1 | 317 | 0 | AluSx | Alu | SINE |
| 95437032 | 95437913 | + | 11.3 | 1014 | 1895 | -2939 | L1MEe | L1 | LINE |

  

|  |  |  |  |  |  |  |  |  |  |
| --- | --- | --- | --- | --- | --- | --- | --- | --- | --- |
| chrX:97234524-97235547 | | | 816 bp | | |  |  |  |  |
| Genome Start | Genome End | Strand | Div | Repeat Start | Repeat End | Repeat Left | Name | Family | Class |
| 97234524 | 97234723 | + | 35.0 | 5617 | 5823 | -298 | L1ME4a | L1 | LINE |
| 97234722 | 97234784 | - | 4.8 | 5650 | 5713 | -441 | L1PA7 | L1 | LINE |
| 97234785 | 97235216 | + | 4.4 | 5715 | 6153 | -1 | L1PA7 | L1 | LINE |
| 97235427 | 97235547 | + | 19.0 | 5950 | 6074 | -47 | L1ME4a | L1 | LINE |

  

|  |  |  |  |  |  |  |  |  |  |
| --- | --- | --- | --- | --- | --- | --- | --- | --- | --- |
| chrX:98486539-98490758 | | | 4225 bp | | |  |  |  |  |
| Genome Start | Genome End | Strand | Div | Repeat Start | Repeat End | Repeat Left | Name | Family | Class |
| 98486539 | 98487837 | - | 18.9 | 2250 | 3575 | -3098 | L1PA14 | L1 | LINE |
| 98487838 | 98488081 | - | 2.9 | 5903 | 6154 | 0 | L1PA5 | L1 | LINE |
| 98488082 | 98488459 | + | 3.4 | 5540 | 5918 | -236 | L1PA5 | L1 | LINE |
| 98488455 | 98490758 | - | 25.7 | 9 | 2266 | -4427 | L1PA14 | L1 | LINE |

  

|  |  |  |  |  |  |  |  |  |  |
| --- | --- | --- | --- | --- | --- | --- | --- | --- | --- |
| chrX:98622184-98630107 | | | 7715 bp | | |  |  |  |  |
| Genome Start | Genome End | Strand | Div | Repeat Start | Repeat End | Repeat Left | Name | Family | Class |
| 98622184 | 98627062 | - | 25.3 | 2887 | 8048 | -20 | L1MD3 | L1 | LINE |
| 98627141 | 98627658 | - | 1.7 | 5639 | 6155 | 0 | L1PA3 | L1 | LINE |
| 98627659 | 98628933 | + | 2.2 | 4365 | 5636 | -519 | L1PA3 | L1 | LINE |
| 98629028 | 98629208 | + | 23.3 | 2794 | 2971 | -3175 | L1MD | L1 | LINE |
| 98629246 | 98630107 | - | 27.4 | 1962 | 2792 | -3427 | L1MD3 | L1 | LINE |

  

|  |  |  |  |  |  |  |  |  |  |
| --- | --- | --- | --- | --- | --- | --- | --- | --- | --- |
| chrX:98692479-98694520 | | | 2021 bp | | |  |  |  |  |
| Genome Start | Genome End | Strand | Div | Repeat Start | Repeat End | Repeat Left | Name | Family | Class |
| 98692479 | 98692735 | - | 29.8 | 5901 | 6155 | -9 | L1ME2 | L1 | LINE |
| 98692736 | 98692919 | - | 8.2 | 5969 | 6150 | -4 | L1PA7 | L1 | LINE |
| 98692920 | 98693104 | + | 7.0 | 5801 | 5972 | -182 | L1PA7 | L1 | LINE |
| 98693100 | 98693174 | - | 22.7 | 5830 | 5908 | -254 | L1ME2 | L1 | LINE |
| 98693205 | 98694062 | - | 15.5 | 5279 | 6165 | -1 | L1PA16 | L1 | LINE |
| 98694061 | 98694239 | + | 14.5 | 5108 | 5295 | -851 | L1PA16 | L1 | LINE |
| 98694238 | 98694520 | - | 21.4 | 5555 | 5825 | -341 | L1ME2 | L1 | LINE |

  

|  |  |  |  |  |  |  |  |  |  |
| --- | --- | --- | --- | --- | --- | --- | --- | --- | --- |
| chrX:100931086-100933508 | | | 2112 bp | | |  |  |  |  |
| Genome Start | Genome End | Strand | Div | Repeat Start | Repeat End | Repeat Left | Name | Family | Class |
| 100931086 | 100931620 | - | 27.0 | 2308 | 3018 | -4169 | L1MEb | L1 | LINE |
| 100931653 | 100931996 | + | 10.4 | 1 | 339 | 0 | MER1B | MER1\_type | DNA |
| 100932048 | 100932468 | - | 28.6 | 1022 | 1431 | -5565 | L1MEb | L1 | LINE |
| 100932698 | 100932914 | - | 15.2 | 6074 | 6293 | -7 | L1MA4 | L1 | LINE |
| 100932915 | 100933408 | + | 13.6 | 5575 | 6076 | -224 | L1MA4 | L1 | LINE |
| 100933408 | 100933508 | - | 31.0 | 938 | 1041 | -5955 | L1MEb | L1 | LINE |

  

|  |  |  |  |  |  |  |  |  |  |
| --- | --- | --- | --- | --- | --- | --- | --- | --- | --- |
| chrX:101079590-101084892 | | | 5283 bp | | |  |  |  |  |
| Genome Start | Genome End | Strand | Div | Repeat Start | Repeat End | Repeat Left | Name | Family | Class |
| 101079590 | 101080074 | + | 32.3 | 1794 | 2285 | -2711 | HERV16 | ERVL | LTR |
| 101080075 | 101080405 | + | 14.1 | 1 | 315 | 0 | AluSx | Alu | SINE |
| 101080406 | 101081514 | + | 33.1 | 2286 | 3464 | -1905 | HERV16 | ERVL | LTR |
| 101081520 | 101082312 | - | 2.9 | 5360 | 6153 | -2 | L1PA3 | L1 | LINE |
| 101082312 | 101084150 | + | 3.2 | 3547 | 5378 | -787 | L1PA3 | L1 | LINE |
| 101084167 | 101084892 | + | 34.7 | 3391 | 4111 | -1556 | HERV16 | ERVL | LTR |

  

|  |  |  |  |  |  |  |  |  |  |
| --- | --- | --- | --- | --- | --- | --- | --- | --- | --- |
| chrX:101429801-101433178 | | | 3364 bp | | |  |  |  |  |
| Genome Start | Genome End | Strand | Div | Repeat Start | Repeat End | Repeat Left | Name | Family | Class |
| 101429801 | 101430878 | - | 10.3 | 5100 | 6150 | -1 | L1PB2 | L1 | LINE |
| 101430890 | 101431634 | - | 4.6 | 4553 | 5297 | -849 | L1PA4 | L1 | LINE |
| 101431635 | 101432476 | + | 1.8 | 5304 | 6153 | -2 | L1PA4 | L1 | LINE |
| 101432480 | 101433178 | - | 11.3 | 4398 | 5093 | -1053 | L1PB2 | L1 | LINE |

  

|  |  |  |  |  |  |  |  |  |  |
| --- | --- | --- | --- | --- | --- | --- | --- | --- | --- |
| chrX:101537129-101540506 | | | 3364 bp | | |  |  |  |  |
| Genome Start | Genome End | Strand | Div | Repeat Start | Repeat End | Repeat Left | Name | Family | Class |
| 101537129 | 101537827 | + | 11.3 | 4398 | 5093 | -1053 | L1PB2 | L1 | LINE |
| 101537831 | 101538672 | - | 1.7 | 5304 | 6153 | -2 | L1PA4 | L1 | LINE |
| 101538673 | 101539417 | + | 4.7 | 4553 | 5297 | -849 | L1PA4 | L1 | LINE |
| 101539429 | 101540506 | + | 10.3 | 5100 | 6150 | -1 | L1PB2 | L1 | LINE |

  

|  |  |  |  |  |  |  |  |  |  |
| --- | --- | --- | --- | --- | --- | --- | --- | --- | --- |
| chrX:101693397-101697037 | | | 3241 bp | | |  |  |  |  |
| Genome Start | Genome End | Strand | Div | Repeat Start | Repeat End | Repeat Left | Name | Family | Class |
| 101693397 | 101693485 | - | 28.7 | 2629 | 2739 | -3390 | L1MEe | L1 | LINE |
| 101693486 | 101693800 | - | 4.9 | 1 | 308 | -3 | AluY | Alu | SINE |
| 101693801 | 101694401 | - | 26.8 | 2059 | 2628 | -3501 | L1MEe | L1 | LINE |
| 101694667 | 101694871 | - | 7.8 | 5959 | 6165 | -3 | L1PA10 | L1 | LINE |
| 101694872 | 101694950 | + | 6.3 | 5874 | 5952 | -216 | L1PA10 | L1 | LINE |
| 101694966 | 101695159 | - | 25.8 | 1568 | 1774 | -4375 | L1MEe | L1 | LINE |
| 101695168 | 101695468 | + | 12.0 | 1 | 301 | -12 | AluSq | Alu | SINE |
| 101695478 | 101695683 | + | 13.8 | 87 | 296 | -16 | AluJb | Alu | SINE |
| 101695711 | 101696505 | - | 19.6 | 1 | 848 | -15 | MER21B | ERV1 | LTR |
| 101696565 | 101696774 | - | 34.8 | 1315 | 1523 | -4626 | L1MEe | L1 | LINE |
| 101696792 | 101697037 | - | 34.7 | 1009 | 1250 | -4869 | L1MEe | L1 | LINE |

  

|  |  |  |  |  |  |  |  |  |  |
| --- | --- | --- | --- | --- | --- | --- | --- | --- | --- |
| chrX:101747345-101754234 | | | 6856 bp | | |  |  |  |  |
| Genome Start | Genome End | Strand | Div | Repeat Start | Repeat End | Repeat Left | Name | Family | Class |
| 101747345 | 101748070 | - | 13.5 | 5022 | 5761 | -419 | L1M3 | L1 | LINE |
| 101748075 | 101749057 | - | 15.1 | 5271 | 6214 | -89 | L1MA4A | L1 | LINE |
| 101749058 | 101749458 | + | 16.5 | 2 | 345 | -1 | MER7A | MER2\_type | DNA |
| 101749459 | 101749725 | - | 8.3 | 4983 | 5268 | -875 | L1M2 | L1 | LINE |
| 101749725 | 101749790 | + | 16.7 | 4911 | 4976 | -1167 | L1M | L1 | LINE |
| 101749786 | 101751969 | - | 19.9 | 2484 | 4777 | -1369 | L1M | L1 | LINE |
| 101752004 | 101752249 | + | 34.7 | 1723 | 1985 | -1434 | L2 | L2 | LINE |
| 101752252 | 101753141 | + | 12.3 | 699 | 1580 | 0 | THE1D-int | MaLR | LTR |
| 101753142 | 101753518 | + | 10.7 | 1 | 381 | 0 | THE1D | MaLR | LTR |
| 101753519 | 101754234 | - | 25.7 | 3462 | 4207 | -2351 | L1M3 | L1 | LINE |

  

|  |  |  |  |  |  |  |  |  |  |
| --- | --- | --- | --- | --- | --- | --- | --- | --- | --- |
| chrX:102706897-102711282 | | | 4106 bp | | |  |  |  |  |
| Genome Start | Genome End | Strand | Div | Repeat Start | Repeat End | Repeat Left | Name | Family | Class |
| 102706897 | 102707022 | + | 27.4 | 3161 | 3288 | -1648 | MER31-int | ERV1 | LTR |
| 102707077 | 102707137 | - | 21.3 | 455 | 515 | -47 | LTR75 | ERVL | LTR |
| 102707178 | 102707264 | - | 28.7 | 292 | 380 | -182 | LTR75 | ERVL | LTR |
| 102707276 | 102707378 | - | 16.5 | 6198 | 6300 | 0 | L1MA6 | L1 | LINE |
| 102707379 | 102707518 | + | 18.4 | 6041 | 6177 | -123 | L1MA6 | L1 | LINE |
| 102707519 | 102707819 | - | 27.5 | 4 | 308 | -254 | LTR75 | ERVL | LTR |
| 102707885 | 102708063 | - | 14.8 | 134 | 309 | -3 | AluJo | Alu | SINE |
| 102708093 | 102708230 | - | 14.8 | 1 | 133 | -179 | AluJo | Alu | SINE |
| 102708262 | 102708965 | + | 24.4 | 3337 | 4055 | -881 | MER31-int | ERV1 | LTR |
| 102708995 | 102711107 | + | 15.1 | 3993 | 6164 | -19 | L1MB3 | L1 | LINE |
| 102711129 | 102711282 | + | 28.8 | 4068 | 4224 | -712 | MER31-int | ERV1 | LTR |

  

|  |  |  |  |  |  |  |  |  |  |
| --- | --- | --- | --- | --- | --- | --- | --- | --- | --- |
| chrX:103785606-103790067 | | | 4444 bp | | |  |  |  |  |
| Genome Start | Genome End | Strand | Div | Repeat Start | Repeat End | Repeat Left | Name | Family | Class |
| 103785606 | 103785919 | - | 32.1 | 2897 | 3250 | -169 | L2 | L2 | LINE |
| 103785922 | 103788554 | - | 7.7 | 2110 | 4741 | -1405 | L1PA6 | L1 | LINE |
| 103788559 | 103789967 | + | 7.4 | 4740 | 6154 | 0 | L1PA6 | L1 | LINE |
| 103789980 | 103790067 | - | 28.7 | 2815 | 2901 | -518 | L2 | L2 | LINE |

  

|  |  |  |  |  |  |  |  |  |  |
| --- | --- | --- | --- | --- | --- | --- | --- | --- | --- |
| chrX:104712849-104717827 | | | 4780 bp | | |  |  |  |  |
| Genome Start | Genome End | Strand | Div | Repeat Start | Repeat End | Repeat Left | Name | Family | Class |
| 104712849 | 104713225 | - | 19.7 | 4456 | 4850 | -1369 | L1MEc | L1 | LINE |
| 104713232 | 104713779 | - | 25.1 | 3565 | 4144 | -2075 | L1MEc | L1 | LINE |
| 104713780 | 104714082 | - | 8.2 | 1 | 303 | -8 | AluY | Alu | SINE |
| 104714083 | 104715187 | - | 25.1 | 2276 | 3564 | -2655 | L1MEc | L1 | LINE |
| 104715298 | 104715385 | - | 27.3 | 1500 | 1592 | -4723 | L1MEc | L1 | LINE |
| 104715459 | 104715999 | - | 27.2 | 727 | 1338 | -5062 | L1MEc | L1 | LINE |
| 104716010 | 104716549 | - | 7.8 | 5621 | 6166 | -2 | L1PA10 | L1 | LINE |
| 104716550 | 104717115 | + | 9.2 | 5043 | 5609 | -559 | L1PA10 | L1 | LINE |
| 104717116 | 104717827 | - | 32.0 | 29 | 737 | -5663 | L1MEc | L1 | LINE |

  

|  |  |  |  |  |  |  |  |  |  |
| --- | --- | --- | --- | --- | --- | --- | --- | --- | --- |
| chrX:105118418-105125243 | | | 6553 bp | | |  |  |  |  |
| Genome Start | Genome End | Strand | Div | Repeat Start | Repeat End | Repeat Left | Name | Family | Class |
| 105118418 | 105118732 | - | 17.0 | 3708 | 4034 | -2530 | L1MCb | L1 | LINE |
| 105118797 | 105119184 | - | 17.0 | 3301 | 3707 | -2857 | L1MCb | L1 | LINE |
| 105119240 | 105119531 | - | 13.0 | 1 | 302 | -10 | AluSx | Alu | SINE |
| 105119535 | 105120019 | - | 17.3 | 2438 | 2957 | -3607 | L1MCb | L1 | LINE |
| 105120023 | 105120189 | - | 21.8 | 1 | 166 | -10 | FRAM | Alu | SINE |
| 105120194 | 105121056 | - | 23.6 | 1476 | 2539 | -4206 | L1MCb | L1 | LINE |
| 105121057 | 105121503 | + | 10.1 | 5 | 472 | 0 | MER4A1 | ERV1 | LTR |
| 105121504 | 105122114 | - | 21.0 | 786 | 1475 | -5276 | L1MCb | L1 | LINE |
| 105122133 | 105122682 | - | 19.6 | 4173 | 4708 | -1438 | L1MD | L1 | LINE |
| 105122734 | 105122814 | - | 22.8 | 4117 | 4195 | -1951 | L1M4 | L1 | LINE |
| 105122816 | 105123419 | - | 0.3 | 5571 | 6174 | 0 | L1PA2 | L1 | LINE |
| 105123422 | 105123850 | + | 1.7 | 5154 | 5582 | -573 | L1PA2 | L1 | LINE |
| 105123848 | 105124010 | - | 27.6 | 636 | 799 | -5246 | L1MCb | L1 | LINE |
| 105124049 | 105124158 | - | 15.4 | 6177 | 6291 | 0 | L1MA7 | L1 | LINE |
| 105124174 | 105124566 | - | 17.6 | 5648 | 6052 | -239 | L1MA7 | L1 | LINE |
| 105124589 | 105124940 | + | 14.8 | 5254 | 5608 | -683 | L1M2 | L1 | LINE |
| 105124941 | 105125243 | - | 22.0 | 308 | 647 | -5398 | L1MCb | L1 | LINE |

  

|  |  |  |  |  |  |  |  |  |  |
| --- | --- | --- | --- | --- | --- | --- | --- | --- | --- |
| chrX:105209139-105219189 | | | 9774 bp | | |  |  |  |  |
| Genome Start | Genome End | Strand | Div | Repeat Start | Repeat End | Repeat Left | Name | Family | Class |
| 105209139 | 105210071 | - | 9.5 | 6771 | 7713 | -8 | L1MA1 | L1 | LINE |
| 105210301 | 105212152 | - | 1.5 | 4304 | 6155 | 0 | L1PA2 | L1 | LINE |
| 105212150 | 105212246 | + | 3.1 | 4207 | 4303 | -1843 | L1PA2 | L1 | LINE |
| 105212247 | 105213456 | - | 11.9 | 5542 | 6783 | -782 | L1MA1 | L1 | LINE |
| 105213472 | 105214910 | - | 10.3 | 1861 | 3306 | -4259 | L1MA1 | L1 | LINE |
| 105214911 | 105217490 | + | 4.5 | 3547 | 6141 | -13 | L1PA5 | L1 | LINE |
| 105217527 | 105219189 | - | 17.9 | 11 | 1843 | -5722 | L1MA1 | L1 | LINE |

  

|  |  |  |  |  |  |  |  |  |  |
| --- | --- | --- | --- | --- | --- | --- | --- | --- | --- |
| chrX:105262328-105267868 | | | 5551 bp | | |  |  |  |  |
| Genome Start | Genome End | Strand | Div | Repeat Start | Repeat End | Repeat Left | Name | Family | Class |
| 105262328 | 105262747 | + | 24.4 | 1007 | 1443 | -5190 | L1MD1 | L1 | LINE |
| 105262748 | 105263058 | + | 9.9 | 1 | 312 | 0 | AluSx | Alu | SINE |
| 105263059 | 105263577 | + | 24.4 | 1444 | 2005 | -4628 | L1MD1 | L1 | LINE |
| 105263578 | 105263646 | + | 10.6 | 6089 | 6154 | -12 | L1PA16 | L1 | LINE |
| 105263647 | 105264334 | + | 23.1 | 2006 | 2717 | -3820 | L1MD1 | L1 | LINE |
| 105264333 | 105265108 | - | 2.7 | 4068 | 4843 | -1303 | L1PA3 | L1 | LINE |
| 105265107 | 105266420 | + | 2.6 | 4841 | 6155 | 0 | L1PA3 | L1 | LINE |
| 105266415 | 105266859 | + | 21.7 | 2702 | 3163 | -3374 | L1MD1 | L1 | LINE |
| 105266860 | 105267152 | + | 20.3 | 1 | 298 | -14 | AluJo | Alu | SINE |
| 105267153 | 105267868 | + | 21.7 | 3164 | 3886 | -2651 | L1MD1 | L1 | LINE |

  

|  |  |  |  |  |  |  |  |  |  |
| --- | --- | --- | --- | --- | --- | --- | --- | --- | --- |
| chrX:105280495-105286631 | | | 6080 bp | | |  |  |  |  |
| Genome Start | Genome End | Strand | Div | Repeat Start | Repeat End | Repeat Left | Name | Family | Class |
| 105280495 | 105280958 | + | 26.9 | 7 | 486 | -5914 | L1ME1 | L1 | LINE |
| 105280959 | 105281258 | + | 18.0 | 3 | 311 | -1 | AluJb | Alu | SINE |
| 105281259 | 105281602 | + | 26.9 | 487 | 791 | -5609 | L1ME1 | L1 | LINE |
| 105281603 | 105281838 | + | 20.3 | 186 | 426 | -2 | MSTC | MaLR | LTR |
| 105281882 | 105282055 | + | 19.3 | 1003 | 1182 | -5218 | L1ME1 | L1 | LINE |
| 105282092 | 105282779 | + | 20.3 | 1414 | 2272 | -4286 | L1ME1 | L1 | LINE |
| 105282779 | 105282920 | - | 3.6 | 4081 | 4220 | -1926 | L1PA8 | L1 | LINE |
| 105282909 | 105284845 | + | 7.2 | 4238 | 6172 | 0 | L1PA8 | L1 | LINE |
| 105284827 | 105285094 | + | 21.2 | 2183 | 2476 | -3781 | L1ME1 | L1 | LINE |
| 105285105 | 105286631 | + | 24.1 | 4592 | 6407 | 0 | L1ME1 | L1 | LINE |

  

|  |  |  |  |  |  |  |  |  |  |
| --- | --- | --- | --- | --- | --- | --- | --- | --- | --- |
| chrX:105626258-105628153 | | | 1907 bp | | |  |  |  |  |
| Genome Start | Genome End | Strand | Div | Repeat Start | Repeat End | Repeat Left | Name | Family | Class |
| 105626258 | 105626353 | - | 20.9 | 6071 | 6165 | -13 | L1MB8 | L1 | LINE |
| 105626354 | 105626815 | + | 18.4 | 1 | 457 | 0 | MSTB2 | MaLR | LTR |
| 105626816 | 105627684 | - | 20.5 | 5188 | 6070 | -108 | L1MB8 | L1 | LINE |
| 105627692 | 105627957 | - | 1.1 | 5887 | 6152 | -3 | L1PA2 | L1 | LINE |
| 105627958 | 105627992 | + | 0.0 | 5851 | 5885 | -270 | L1PA2 | L1 | LINE |
| 105627975 | 105628153 | - | 17.8 | 5033 | 5216 | -930 | L1MB8 | L1 | LINE |

  

|  |  |  |  |  |  |  |  |  |  |
| --- | --- | --- | --- | --- | --- | --- | --- | --- | --- |
| chrX:105782615-105788118 | | | 5334 bp | | |  |  |  |  |
| Genome Start | Genome End | Strand | Div | Repeat Start | Repeat End | Repeat Left | Name | Family | Class |
| 105782615 | 105782733 | + | 20.5 | 1437 | 1552 | -4848 | L1MEc | L1 | LINE |
| 105782738 | 105782887 | + | 13.3 | 149 | 300 | -12 | AluSg/x | Alu | SINE |
| 105782889 | 105783349 | + | 24.2 | 1649 | 2142 | -4258 | L1MEc | L1 | LINE |
| 105783391 | 105784298 | - | 5.3 | 5277 | 6186 | 0 | L1PA7 | L1 | LINE |
| 105784299 | 105784773 | + | 7.6 | 4798 | 5275 | -871 | L1PA7 | L1 | LINE |
| 105784898 | 105785228 | - | 12.8 | 2097 | 2418 | 0 | Tigger1 | MER2\_type | DNA |
| 105785229 | 105785798 | - | 4.9 | 5584 | 6153 | -1 | L1PA5 | L1 | LINE |
| 105785799 | 105787921 | - | 12.8 | 4 | 2096 | -322 | Tigger1 | MER2\_type | DNA |
| 105787922 | 105788118 | + | 24.4 | 2214 | 2433 | -3786 | L1MEc | L1 | LINE |

  

|  |  |  |  |  |  |  |  |  |  |
| --- | --- | --- | --- | --- | --- | --- | --- | --- | --- |
| chrX:107167594-107169766 | | | 2021 bp | | |  |  |  |  |
| Genome Start | Genome End | Strand | Div | Repeat Start | Repeat End | Repeat Left | Name | Family | Class |
| 107167594 | 107167961 | - | 30.4 | 3036 | 3419 | 0 | L2 | L2 | LINE |
| 107167970 | 107168320 | - | 6.3 | 5821 | 6171 | -1 | L1PA8 | L1 | LINE |
| 107168321 | 107168563 | + | 4.1 | 5568 | 5810 | -362 | L1PA8 | L1 | LINE |
| 107168566 | 107168729 | - | 27.6 | 2891 | 3044 | -375 | L2 | L2 | LINE |
| 107168738 | 107168923 | - | 16.7 | 2 | 228 | -96 | MER33 | MER1\_type | DNA |
| 107169054 | 107169380 | - | 12.5 | 1 | 312 | 0 | AluSx | Alu | SINE |
| 107169385 | 107169766 | - | 26.2 | 2508 | 2899 | -520 | L2 | L2 | LINE |

  

|  |  |  |  |  |  |  |  |  |  |
| --- | --- | --- | --- | --- | --- | --- | --- | --- | --- |
| chrX:107446121-107450521 | | | 4178 bp | | |  |  |  |  |
| Genome Start | Genome End | Strand | Div | Repeat Start | Repeat End | Repeat Left | Name | Family | Class |
| 107446121 | 107446417 | - | 27.2 | 3119 | 3416 | -3 | L2 | L2 | LINE |
| 107446418 | 107446598 | + | 20.0 | 1 | 189 | 0 | MER5A | MER1\_type | DNA |
| 107446599 | 107447306 | - | 26.6 | 2361 | 3118 | -301 | L2 | L2 | LINE |
| 107447307 | 107447400 | - | 24.2 | 320 | 428 | -4 | MLT1I | MaLR | LTR |
| 107447401 | 107447901 | - | 3.0 | 4566 | 5066 | -1080 | L1P1 | L1 | LINE |
| 107447902 | 107448705 | + | 3.4 | 3782 | 4585 | -1561 | L1P1 | L1 | LINE |
| 107448929 | 107448974 | - | 27.6 | 2304 | 2344 | -1075 | L2 | L2 | LINE |
| 107448975 | 107449254 | + | 13.2 | 1 | 294 | -18 | AluJo | Alu | SINE |
| 107449255 | 107450521 | - | 27.6 | 975 | 2303 | -1116 | L2 | L2 | LINE |

  

|  |  |  |  |  |  |  |  |  |  |
| --- | --- | --- | --- | --- | --- | --- | --- | --- | --- |
| chrX:107757912-107763372 | | | 5346 bp | | |  |  |  |  |
| Genome Start | Genome End | Strand | Div | Repeat Start | Repeat End | Repeat Left | Name | Family | Class |
| 107757912 | 107758426 | - | 33.9 | 5381 | 5910 | -1898 | L1MC4a | L1 | LINE |
| 107758428 | 107758651 | - | 32.9 | 5138 | 5358 | -788 | L1MC4a | L1 | LINE |
| 107758664 | 107759525 | - | 26.3 | 2463 | 3419 | 0 | L2 | L2 | LINE |
| 107759562 | 107761374 | - | 5.7 | 4320 | 6142 | -12 | L1PA6 | L1 | LINE |
| 107761375 | 107763025 | + | 7.0 | 2645 | 4299 | -1847 | L1PA6 | L1 | LINE |
| 107763032 | 107763117 | - | 29.1 | 1442 | 1532 | -1887 | L2 | L2 | LINE |
| 107763178 | 107763372 | - | 33.2 | 4882 | 5079 | -1067 | L1MC4a | L1 | LINE |

  

|  |  |  |  |  |  |  |  |  |  |
| --- | --- | --- | --- | --- | --- | --- | --- | --- | --- |
| chrX:109902984-109906481 | | | 3490 bp | | |  |  |  |  |
| Genome Start | Genome End | Strand | Div | Repeat Start | Repeat End | Repeat Left | Name | Family | Class |
| 109902984 | 109903330 | - | 16.0 | 2955 | 3303 | -3018 | L1MCa | L1 | LINE |
| 109903331 | 109903629 | + | 5.3 | 1 | 303 | -8 | AluY | Alu | SINE |
| 109903630 | 109904167 | - | 16.0 | 2409 | 2954 | -3367 | L1MCa | L1 | LINE |
| 109904160 | 109904260 | - | 19.8 | 5785 | 5887 | -302 | L1MA9 | L1 | LINE |
| 109904258 | 109904537 | + | 24.4 | 5865 | 6136 | -176 | L1MA9 | L1 | LINE |
| 109904545 | 109904754 | - | 5.5 | 1010 | 1172 | -3 | Merlin1\_HS | Merlin | DNA |
| 109904744 | 109905330 | - | 12.6 | 1 | 615 | -560 | Merlin1\_HS | Merlin | DNA |
| 109905352 | 109905460 | + | 23.4 | 6162 | 6274 | -38 | L1MA9 | L1 | LINE |
| 109905463 | 109906481 | - | 22.8 | 1512 | 2445 | -3876 | L1MCa | L1 | LINE |

  

|  |  |  |  |  |  |  |  |  |  |
| --- | --- | --- | --- | --- | --- | --- | --- | --- | --- |
| chrX:110365381-110367449 | | | 2046 bp | | |  |  |  |  |
| Genome Start | Genome End | Strand | Div | Repeat Start | Repeat End | Repeat Left | Name | Family | Class |
| 110365381 | 110366283 | + | 17.8 | 4539 | 5439 | -735 | L1MB8 | L1 | LINE |
| 110366289 | 110366472 | - | 15.2 | 5866 | 6053 | -259 | L1MA9 | L1 | LINE |
| 110366457 | 110366670 | + | 16.8 | 6032 | 6257 | -55 | L1MA9 | L1 | LINE |
| 110366705 | 110367449 | + | 20.0 | 5423 | 6171 | -7 | L1MB8 | L1 | LINE |

  

|  |  |  |  |  |  |  |  |  |  |
| --- | --- | --- | --- | --- | --- | --- | --- | --- | --- |
| chrX:110594029-110595186 | | | 1152 bp | | |  |  |  |  |
| Genome Start | Genome End | Strand | Div | Repeat Start | Repeat End | Repeat Left | Name | Family | Class |
| 110594029 | 110594202 | - | 18.4 | 131 | 306 | -6 | AluJ | Alu | SINE |
| 110594205 | 110594268 | - | 4.6 | 5328 | 5390 | -765 | L1P | L1 | LINE |
| 110594270 | 110595059 | + | 3.0 | 5386 | 6175 | 0 | L1PA4 | L1 | LINE |
| 110595063 | 110595186 | - | 13.7 | 1 | 124 | -178 | AluJ | Alu | SINE |

  

|  |  |  |  |  |  |  |  |  |  |
| --- | --- | --- | --- | --- | --- | --- | --- | --- | --- |
| chrX:110699658-110706544 | | | 6779 bp | | |  |  |  |  |
| Genome Start | Genome End | Strand | Div | Repeat Start | Repeat End | Repeat Left | Name | Family | Class |
| 110699658 | 110700181 | + | 22.5 | 2186 | 2763 | -3568 | LTR49-int | ERV1 | LTR |
| 110700220 | 110701175 | + | 20.7 | 2764 | 3711 | -2620 | MER83B-int | ERV1 | LTR |
| 110701212 | 110701389 | + | 21.7 | 3168 | 3344 | -1536 | MER83B-int | ERV1 | LTR |
| 110701401 | 110701517 | + | 10.3 | 1 | 117 | -193 | AluSg | Alu | SINE |
| 110701549 | 110701775 | + | 15.2 | 3889 | 4097 | -2234 | MER83B-int | ERV1 | LTR |
| 110701776 | 110702062 | - | 17.8 | 1 | 288 | -24 | AluSx | Alu | SINE |
| 110702063 | 110702426 | + | 15.2 | 4098 | 4451 | -1880 | MER83B-int | ERV1 | LTR |
| 110702439 | 110705531 | - | 2.6 | 3058 | 6155 | 0 | L1PA3 | L1 | LINE |
| 110705509 | 110705660 | + | 4.6 | 2924 | 3072 | -3074 | L1PA3 | L1 | LINE |
| 110705664 | 110706544 | + | 17.7 | 4904 | 5959 | -921 | LTR49-int | ERV1 | LTR |

  

|  |  |  |  |  |  |  |  |  |  |
| --- | --- | --- | --- | --- | --- | --- | --- | --- | --- |
| chrX:110744096-110745429 | | | 1297 bp | | |  |  |  |  |
| Genome Start | Genome End | Strand | Div | Repeat Start | Repeat End | Repeat Left | Name | Family | Class |
| 110744096 | 110744221 | + | 15.9 | 1 | 127 | -175 | AluJo | Alu | SINE |
| 110744246 | 110744914 | - | 1.9 | 5475 | 6143 | -12 | L1PA4 | L1 | LINE |
| 110744915 | 110745243 | + | 4.9 | 5160 | 5492 | -662 | L1PA4 | L1 | LINE |
| 110745257 | 110745429 | + | 19.9 | 134 | 305 | -7 | AluJo | Alu | SINE |

  

|  |  |  |  |  |  |  |  |  |  |
| --- | --- | --- | --- | --- | --- | --- | --- | --- | --- |
| chrX:111227044-111230186 | | | 2641 bp | | |  |  |  |  |
| Genome Start | Genome End | Strand | Div | Repeat Start | Repeat End | Repeat Left | Name | Family | Class |
| 111227044 | 111227104 | - | 23.3 | 3296 | 3355 | -64 | L2 | L2 | LINE |
| 111227238 | 111227510 | + | 19.1 | 1 | 273 | -39 | AluJb | Alu | SINE |
| 111227845 | 111228651 | - | 2.7 | 5347 | 6153 | -2 | L1PA4 | L1 | LINE |
| 111228646 | 111229119 | + | 4.4 | 4869 | 5342 | -804 | L1PA4 | L1 | LINE |
| 111229120 | 111229981 | - | 35.0 | 2370 | 3284 | -94 | L2 | L2 | LINE |
| 111230023 | 111230186 | - | 28.8 | 1903 | 2080 | -1339 | L2 | L2 | LINE |

  

|  |  |  |  |  |  |  |  |  |  |
| --- | --- | --- | --- | --- | --- | --- | --- | --- | --- |
| chrX:112595982-112599975 | | | 3968 bp | | |  |  |  |  |
| Genome Start | Genome End | Strand | Div | Repeat Start | Repeat End | Repeat Left | Name | Family | Class |
| 112595982 | 112597536 | + | 21.2 | 1725 | 3488 | -2699 | MER57A-int | ERV1 | LTR |
| 112597537 | 112598060 | - | 7.5 | 4745 | 5269 | -877 | L1PA8 | L1 | LINE |
| 112598060 | 112598958 | + | 4.6 | 5267 | 6172 | 0 | L1PA8 | L1 | LINE |
| 112598986 | 112599975 | + | 19.0 | 4625 | 5634 | -697 | MER57A-int | ERV1 | LTR |

  

|  |  |  |  |  |  |  |  |  |  |
| --- | --- | --- | --- | --- | --- | --- | --- | --- | --- |
| chrX:112940749-112945314 | | | 4496 bp | | |  |  |  |  |
| Genome Start | Genome End | Strand | Div | Repeat Start | Repeat End | Repeat Left | Name | Family | Class |
| 112940749 | 112941138 | + | 25.1 | 4265 | 4670 | -1476 | L1ME1 | L1 | LINE |
| 112941143 | 112941228 | + | 12.2 | 2 | 85 | -480 | MER34B | ERV1 | LTR |
| 112941229 | 112941517 | + | 7.0 | 1 | 301 | -10 | AluY | Alu | SINE |
| 112941518 | 112941651 | + | 12.2 | 86 | 224 | -341 | MER34B | ERV1 | LTR |
| 112941652 | 112942210 | + | 15.6 | 1 | 603 | 0 | LTR26 | ERV1 | LTR |
| 112942211 | 112942536 | + | 12.2 | 225 | 565 | 0 | MER34B | ERV1 | LTR |
| 112942558 | 112943037 | + | 27.1 | 4698 | 5203 | -943 | L1ME1 | L1 | LINE |
| 112943043 | 112943390 | - | 1.1 | 5227 | 5574 | -581 | L1PA4 | L1 | LINE |
| 112943391 | 112943963 | + | 4.0 | 5572 | 6144 | -11 | L1PA4 | L1 | LINE |
| 112943981 | 112944231 | + | 19.9 | 5188 | 5389 | -777 | L1ME1 | L1 | LINE |
| 112944232 | 112944534 | + | 11.3 | 1 | 305 | -5 | AluSg | Alu | SINE |
| 112944535 | 112944589 | + | 25.1 | 5390 | 5444 | -722 | L1ME1 | L1 | LINE |
| 112944613 | 112945314 | + | 25.1 | 5445 | 6161 | -5 | L1ME1 | L1 | LINE |

  

|  |  |  |  |  |  |  |  |  |  |
| --- | --- | --- | --- | --- | --- | --- | --- | --- | --- |
| chrX:113698315-113702391 | | | 4042 bp | | |  |  |  |  |
| Genome Start | Genome End | Strand | Div | Repeat Start | Repeat End | Repeat Left | Name | Family | Class |
| 113698315 | 113699293 | + | 14.7 | 13 | 1029 | -5355 | L1M4c | L1 | LINE |
| 113699293 | 113699871 | - | 1.4 | 5284 | 5862 | -293 | L1PA3 | L1 | LINE |
| 113699872 | 113700156 | + | 1.4 | 5867 | 6155 | 0 | L1PA3 | L1 | LINE |
| 113700159 | 113700526 | + | 15.2 | 1021 | 1395 | -4989 | L1M4c | L1 | LINE |
| 113700527 | 113700824 | + | 6.7 | 1 | 299 | -12 | AluY | Alu | SINE |
| 113700825 | 113701199 | + | 15.2 | 1396 | 1755 | -4629 | L1M4c | L1 | LINE |
| 113701234 | 113702391 | + | 17.6 | 1756 | 2910 | -3472 | L1M4c | L1 | LINE |

  

|  |  |  |  |  |  |  |  |  |  |
| --- | --- | --- | --- | --- | --- | --- | --- | --- | --- |
| chrX:115437870-115444714 | | | 6675 bp | | |  |  |  |  |
| Genome Start | Genome End | Strand | Div | Repeat Start | Repeat End | Repeat Left | Name | Family | Class |
| 115437870 | 115438399 | - | 24.8 | 1454 | 2010 | -2824 | L1MEe | L1 | LINE |
| 115438434 | 115438618 | + | 11.3 | 126 | 311 | 0 | AluY | Alu | SINE |
| 115438647 | 115439597 | - | 15.7 | 5311 | 6334 | 0 | L1MA10 | L1 | LINE |
| 115439627 | 115439922 | - | 8.8 | 1 | 298 | -14 | AluSx | Alu | SINE |
| 115439923 | 115440400 | - | 16.7 | 4814 | 5296 | -1038 | L1M | L1 | LINE |
| 115440423 | 115440872 | - | 16.8 | 4349 | 4813 | -1327 | L1M | L1 | LINE |
| 115440872 | 115441034 | + | 21.5 | 4190 | 4352 | -1788 | L1M | L1 | LINE |
| 115441032 | 115441199 | - | 26.3 | 1267 | 1464 | -3370 | L1MEe | L1 | LINE |
| 115441200 | 115441504 | - | 10.2 | 1 | 309 | 0 | AluSc | Alu | SINE |
| 115441505 | 115441799 | - | 26.3 | 952 | 1266 | -3568 | L1MEe | L1 | LINE |
| 115441780 | 115442655 | - | 13.6 | 5311 | 6166 | 0 | L1PA16 | L1 | LINE |
| 115442680 | 115443474 | - | 12.2 | 4519 | 5310 | -836 | L1P | L1 | LINE |
| 115443514 | 115443739 | - | 25.3 | 662 | 904 | -3950 | L1MEe | L1 | LINE |
| 115443755 | 115444123 | + | 22.1 | 6 | 365 | -158 | Charlie1b | MER1\_type | DNA |
| 115444124 | 115444288 | - | 18.1 | 5 | 164 | -2 | FRAM | Alu | SINE |
| 115444289 | 115444454 | + | 22.1 | 366 | 521 | -2 | Charlie1b | MER1\_type | DNA |
| 115444458 | 115444714 | - | 32.3 | 388 | 658 | -4196 | L1MEe | L1 | LINE |

  

|  |  |  |  |  |  |  |  |  |  |
| --- | --- | --- | --- | --- | --- | --- | --- | --- | --- |
| chrX:115661416-115662273 | | | 838 bp | | |  |  |  |  |
| Genome Start | Genome End | Strand | Div | Repeat Start | Repeat End | Repeat Left | Name | Family | Class |
| 115661416 | 115661542 | + | 28.4 | 2064 | 2193 | -1226 | L2 | L2 | LINE |
| 115661543 | 115661793 | - | 11.3 | 5900 | 6147 | -153 | L1MA2 | L1 | LINE |
| 115661795 | 115661933 | + | 10.1 | 6163 | 6304 | 0 | L1MA2 | L1 | LINE |
| 115661953 | 115662273 | + | 29.8 | 2220 | 2554 | -865 | L2 | L2 | LINE |

  

|  |  |  |  |  |  |  |  |  |  |
| --- | --- | --- | --- | --- | --- | --- | --- | --- | --- |
| chrX:115698807-115702359 | | | 3518 bp | | |  |  |  |  |
| Genome Start | Genome End | Strand | Div | Repeat Start | Repeat End | Repeat Left | Name | Family | Class |
| 115698807 | 115699425 | - | 18.3 | 5531 | 6165 | -19 | L1MB7 | L1 | LINE |
| 115699454 | 115699570 | + | 13.9 | 5544 | 5664 | -520 | L1M4 | L1 | LINE |
| 115699578 | 115700542 | + | 6.3 | 5182 | 6153 | -1 | L1PA7 | L1 | LINE |
| 115700548 | 115700728 | - | 12.8 | 5341 | 5533 | -645 | L1MB7 | L1 | LINE |
| 115700728 | 115701867 | - | 9.4 | 4989 | 6163 | 0 | L1PA13 | L1 | LINE |
| 115701867 | 115701912 | + | 8.7 | 4950 | 4995 | -1151 | L1PA13 | L1 | LINE |
| 115701910 | 115702359 | - | 19.6 | 4890 | 5350 | -796 | L1MB7 | L1 | LINE |

  

|  |  |  |  |  |  |  |  |  |  |
| --- | --- | --- | --- | --- | --- | --- | --- | --- | --- |
| chrX:115873962-115881778 | | | 7782 bp | | |  |  |  |  |
| Genome Start | Genome End | Strand | Div | Repeat Start | Repeat End | Repeat Left | Name | Family | Class |
| 115873962 | 115874260 | - | 23.3 | 6153 | 6416 | -1143 | L1M1 | L1 | LINE |
| 115874275 | 115874426 | - | 12.1 | 6156 | 6304 | 0 | L1MA2 | L1 | LINE |
| 115874427 | 115874839 | + | 12.7 | 4937 | 5318 | -986 | L1MA2 | L1 | LINE |
| 115874840 | 115875134 | + | 13.2 | 3 | 299 | -13 | AluSx | Alu | SINE |
| 115875135 | 115875974 | + | 14.5 | 5319 | 6145 | -159 | L1MA2 | L1 | LINE |
| 115875975 | 115876438 | - | 14.8 | 5221 | 5671 | -1894 | L1M1 | L1 | LINE |
| 115876439 | 115876554 | + | 17.0 | 1 | 119 | -193 | AluJo | Alu | SINE |
| 115876576 | 115876760 | + | 17.0 | 120 | 309 | -3 | AluJo | Alu | SINE |
| 115876761 | 115881778 | - | 18.4 | 64 | 5220 | -2345 | L1M1 | L1 | LINE |

  

|  |  |  |  |  |  |  |  |  |  |
| --- | --- | --- | --- | --- | --- | --- | --- | --- | --- |
| chrX:116323822-116327601 | | | 3793 bp | | |  |  |  |  |
| Genome Start | Genome End | Strand | Div | Repeat Start | Repeat End | Repeat Left | Name | Family | Class |
| 116323822 | 116324424 | + | 14.1 | 5258 | 5838 | -465 | L1MA4A | L1 | LINE |
| 116324417 | 116325266 | - | 7.5 | 5232 | 6154 | 0 | L1PA7 | L1 | LINE |
| 116325264 | 116325371 | - | 1.9 | 4071 | 4178 | -1968 | L1P3 | L1 | LINE |
| 116325370 | 116327119 | + | 10.5 | 2330 | 4066 | -2080 | L1P3 | L1 | LINE |
| 116327120 | 116327601 | + | 13.9 | 5823 | 6301 | -2 | L1MA4A | L1 | LINE |

  

|  |  |  |  |  |  |  |  |  |  |
| --- | --- | --- | --- | --- | --- | --- | --- | --- | --- |
| chrX:116710236-116721187 | | | 10918 bp | | |  |  |  |  |
| Genome Start | Genome End | Strand | Div | Repeat Start | Repeat End | Repeat Left | Name | Family | Class |
| 116710236 | 116712181 | - | 17.4 | 4373 | 6344 | 0 | L1MC2 | L1 | LINE |
| 116712187 | 116712732 | - | 15.0 | 5739 | 6291 | 0 | L1MA7 | L1 | LINE |
| 116712737 | 116712777 | + | 7.3 | 5406 | 5446 | -854 | L1M2 | L1 | LINE |
| 116712778 | 116720336 | + | 13.6 | 11 | 7712 | -9 | L1MA1 | L1 | LINE |
| 116720363 | 116720666 | + | 16.9 | 5428 | 5728 | -563 | L1M2 | L1 | LINE |
| 116720666 | 116721187 | - | 15.7 | 3843 | 4386 | -1760 | L1MC2 | L1 | LINE |

  

|  |  |  |  |  |  |  |  |  |  |
| --- | --- | --- | --- | --- | --- | --- | --- | --- | --- |
| chrX:116816198-116820003 | | | 3802 bp | | |  |  |  |  |
| Genome Start | Genome End | Strand | Div | Repeat Start | Repeat End | Repeat Left | Name | Family | Class |
| 116816198 | 116816315 | - | 13.6 | 2301 | 2418 | 0 | Tigger1 | MER2\_type | DNA |
| 116816317 | 116816788 | - | 9.9 | 4708 | 5173 | -973 | L1PA7 | L1 | LINE |
| 116816785 | 116817753 | + | 5.2 | 5171 | 6150 | -4 | L1PA7 | L1 | LINE |
| 116817761 | 116820003 | - | 13.5 | 48 | 2312 | -106 | Tigger1 | MER2\_type | DNA |

  

|  |  |  |  |  |  |  |  |  |  |
| --- | --- | --- | --- | --- | --- | --- | --- | --- | --- |
| chrX:117026597-117029825 | | | 2996 bp | | |  |  |  |  |
| Genome Start | Genome End | Strand | Div | Repeat Start | Repeat End | Repeat Left | Name | Family | Class |
| 117026597 | 117026704 | - | 30.1 | 3288 | 3392 | -27 | L2 | L2 | LINE |
| 117026710 | 117026959 | + | 24.8 | 7 | 261 | -1 | MIR | MIR | SINE |
| 117027039 | 117027949 | - | 1.2 | 4446 | 5356 | -790 | L1PA2 | L1 | LINE |
| 117027944 | 117028744 | + | 0.9 | 5354 | 6155 | 0 | L1PA2 | L1 | LINE |
| 117028782 | 117029094 | + | 13.8 | 1 | 313 | 0 | AluJb | Alu | SINE |
| 117029095 | 117029225 | - | 40.8 | 3056 | 3188 | -190 | L2 | L2 | LINE |
| 117029344 | 117029825 | - | 39.7 | 2383 | 2860 | -559 | L2 | L2 | LINE |

  

|  |  |  |  |  |  |  |  |  |  |
| --- | --- | --- | --- | --- | --- | --- | --- | --- | --- |
| chrX:117088230-117090788 | | | 2538 bp | | |  |  |  |  |
| Genome Start | Genome End | Strand | Div | Repeat Start | Repeat End | Repeat Left | Name | Family | Class |
| 117088230 | 117088722 | - | 24.7 | 2505 | 3020 | -3126 | L1M5 | L1 | LINE |
| 117088726 | 117088737 | - | 2.6 | 6143 | 6155 | 0 | L1PA3 | L1 | LINE |
| 117088760 | 117089562 | - | 2.6 | 5341 | 6142 | -13 | L1PA3 | L1 | LINE |
| 117089560 | 117090673 | + | 2.0 | 4230 | 5343 | -803 | L1PA3 | L1 | LINE |
| 117090673 | 117090788 | - | 28.4 | 2400 | 2516 | -3630 | L1M5 | L1 | LINE |

  

|  |  |  |  |  |  |  |  |  |  |
| --- | --- | --- | --- | --- | --- | --- | --- | --- | --- |
| chrX:119652289-119656558 | | | 4248 bp | | |  |  |  |  |
| Genome Start | Genome End | Strand | Div | Repeat Start | Repeat End | Repeat Left | Name | Family | Class |
| 119652289 | 119652369 | + | 18.5 | 5572 | 5661 | -460 | L1M5 | L1 | LINE |
| 119652402 | 119652701 | - | 15.7 | 1 | 311 | -1 | AluJo | Alu | SINE |
| 119652710 | 119652960 | - | 12.1 | 5918 | 6165 | 0 | L1PA15 | L1 | LINE |
| 119652961 | 119653257 | + | 9.8 | 4 | 301 | -12 | AluSq | Alu | SINE |
| 119653263 | 119653553 | + | 9.0 | 6 | 295 | -17 | AluSx | Alu | SINE |
| 119653554 | 119654137 | - | 12.1 | 5336 | 5917 | -248 | L1PA15 | L1 | LINE |
| 119654138 | 119654225 | - | 13.5 | 308 | 381 | 0 | THE1D | MaLR | LTR |
| 119654226 | 119654530 | - | 12.8 | 6 | 312 | 0 | AluSx | Alu | SINE |
| 119654531 | 119654829 | - | 13.5 | 1 | 307 | -74 | THE1D | MaLR | LTR |
| 119654830 | 119655174 | - | 9.2 | 4994 | 5335 | -811 | L1P | L1 | LINE |
| 119655175 | 119655459 | - | 8.8 | 24 | 308 | -2 | AluSg | Alu | SINE |
| 119655460 | 119655494 | - | 9.4 | 4961 | 4993 | -1153 | L1P | L1 | LINE |
| 119655492 | 119656489 | + | 9.1 | 3978 | 4985 | -1161 | L1P | L1 | LINE |
| 119656470 | 119656558 | + | 15.7 | 5710 | 5808 | -313 | L1M5 | L1 | LINE |

  

|  |  |  |  |  |  |  |  |  |  |
| --- | --- | --- | --- | --- | --- | --- | --- | --- | --- |
| chrX:120472465-120475107 | | | 2584 bp | | |  |  |  |  |
| Genome Start | Genome End | Strand | Div | Repeat Start | Repeat End | Repeat Left | Name | Family | Class |
| 120472465 | 120472692 | - | 23.4 | 273 | 505 | 0 | MLT1D | MaLR | LTR |
| 120472702 | 120474625 | - | 4.2 | 4217 | 6151 | -3 | L1PA5 | L1 | LINE |
| 120474626 | 120474834 | + | 5.7 | 3997 | 4205 | -1941 | L1PA5 | L1 | LINE |
| 120474885 | 120475107 | - | 22.5 | 1 | 230 | -275 | MLT1D | MaLR | LTR |

  

|  |  |  |  |  |  |  |  |  |  |
| --- | --- | --- | --- | --- | --- | --- | --- | --- | --- |
| chrX:120648231-120656263 | | | 7967 bp | | |  |  |  |  |
| Genome Start | Genome End | Strand | Div | Repeat Start | Repeat End | Repeat Left | Name | Family | Class |
| 120648231 | 120649150 | - | 20.2 | 5513 | 6402 | -8 | L1MB5 | L1 | LINE |
| 120649182 | 120649624 | - | 21.3 | 5047 | 5512 | -870 | L1MB5 | L1 | LINE |
| 120649625 | 120650374 | - | 16.4 | 5596 | 6299 | -1 | L1MA5 | L1 | LINE |
| 120650375 | 120650877 | - | 21.3 | 4494 | 5046 | -1336 | L1MB5 | L1 | LINE |
| 120650861 | 120651343 | - | 17.0 | 5658 | 6132 | -36 | L1MB1 | L1 | LINE |
| 120651401 | 120651896 | - | 20.2 | 3953 | 4461 | -1921 | L1MB5 | L1 | LINE |
| 120651898 | 120653238 | - | 3.0 | 4815 | 6155 | 0 | L1PA3 | L1 | LINE |
| 120653236 | 120654282 | + | 1.8 | 3773 | 4819 | -1327 | L1PA3 | L1 | LINE |
| 120654280 | 120656263 | - | 21.5 | 1945 | 3965 | -2417 | L1MB5 | L1 | LINE |

  

|  |  |  |  |  |  |  |  |  |  |
| --- | --- | --- | --- | --- | --- | --- | --- | --- | --- |
| chrX:120656267-120666807 | | | 10480 bp | | |  |  |  |  |
| Genome Start | Genome End | Strand | Div | Repeat Start | Repeat End | Repeat Left | Name | Family | Class |
| 120656267 | 120657028 | - | 18.9 | 5214 | 5976 | -753 | L1M3de | L1 | LINE |
| 120657022 | 120659433 | - | 1.4 | 2967 | 5379 | -767 | L1PA2 | L1 | LINE |
| 120659427 | 120660207 | + | 2.0 | 5372 | 6152 | -3 | L1PA2 | L1 | LINE |
| 120660203 | 120661554 | - | 16.5 | 3835 | 5202 | -1527 | L1M3de | L1 | LINE |
| 120661583 | 120663556 | - | 17.0 | 4311 | 6286 | -14 | L1MA4 | L1 | LINE |
| 120663558 | 120666676 | - | 23.9 | 945 | 3843 | -2886 | L1M3de | L1 | LINE |
| 120666728 | 120666807 | - | 35.4 | 278 | 357 | -6296 | L1M3de | L1 | LINE |

  

|  |  |  |  |  |  |  |  |  |  |
| --- | --- | --- | --- | --- | --- | --- | --- | --- | --- |
| chrX:120860517-120860986 | | | 464 bp | | |  |  |  |  |
| Genome Start | Genome End | Strand | Div | Repeat Start | Repeat End | Repeat Left | Name | Family | Class |
| 120860517 | 120860576 | - | 28.3 | 101 | 160 | 0 | MER5A1 | MER1\_type | DNA |
| 120860582 | 120860746 | - | 12.0 | 5860 | 6018 | -142 | L1PREC2 | L1 | LINE |
| 120860747 | 120860873 | + | 7.9 | 6034 | 6160 | 0 | L1PREC2 | L1 | LINE |
| 120860875 | 120860986 | - | 19.4 | 1 | 108 | -52 | MER5A1 | MER1\_type | DNA |

  

|  |  |  |  |  |  |  |  |  |  |
| --- | --- | --- | --- | --- | --- | --- | --- | --- | --- |
| chrX:121349593-121353815 | | | 4193 bp | | |  |  |  |  |
| Genome Start | Genome End | Strand | Div | Repeat Start | Repeat End | Repeat Left | Name | Family | Class |
| 121349593 | 121349680 | - | 14.8 | 6088 | 6176 | -7 | L1MB3 | L1 | LINE |
| 121349681 | 121349712 | - | 16.9 | 5685 | 5715 | -576 | L1M2 | L1 | LINE |
| 121349713 | 121350948 | - | 9.4 | 1 | 1253 | -1 | LTR12D | ERV1 | LTR |
| 121350949 | 121352716 | - | 18.0 | 3871 | 5684 | -607 | L1MA7 | L1 | LINE |
| 121352719 | 121353293 | + | 18.5 | 5707 | 6283 | -8 | L1MA7 | L1 | LINE |
| 121353322 | 121353815 | - | 18.3 | 5605 | 6104 | -79 | L1MB3 | L1 | LINE |

  

|  |  |  |  |  |  |  |  |  |  |
| --- | --- | --- | --- | --- | --- | --- | --- | --- | --- |
| chrX:121453991-121460721 | | | 6736 bp | | |  |  |  |  |
| Genome Start | Genome End | Strand | Div | Repeat Start | Repeat End | Repeat Left | Name | Family | Class |
| 121453991 | 121458207 | - | 8.7 | 1859 | 6171 | -1 | L1PA8 | L1 | LINE |
| 121458220 | 121458548 | - | 4.6 | 5826 | 6155 | 0 | L1PA3 | L1 | LINE |
| 121458549 | 121460328 | + | 3.0 | 4054 | 5838 | -317 | L1PA3 | L1 | LINE |
| 121460312 | 121460721 | - | 9.3 | 1466 | 1875 | -400 | L1PA8 | L1 | LINE |

  

|  |  |  |  |  |  |  |  |  |  |
| --- | --- | --- | --- | --- | --- | --- | --- | --- | --- |
| chrX:121971210-121972899 | | | 1687 bp | | |  |  |  |  |
| Genome Start | Genome End | Strand | Div | Repeat Start | Repeat End | Repeat Left | Name | Family | Class |
| 121971210 | 121971249 | + | 7.5 | 1 | 40 | -40 | MADE1 | Mariner | DNA |
| 121971250 | 121971942 | - | 6.9 | 5465 | 6158 | -10 | L1PA10 | L1 | LINE |
| 121971946 | 121972848 | + | 7.8 | 4562 | 5473 | -701 | L1PA10 | L1 | LINE |
| 121972849 | 121972899 | + | 9.8 | 29 | 80 | 0 | MADE1 | Mariner | DNA |

  

|  |  |  |  |  |  |  |  |  |  |
| --- | --- | --- | --- | --- | --- | --- | --- | --- | --- |
| chrX:122121930-122127717 | | | 5795 bp | | |  |  |  |  |
| Genome Start | Genome End | Strand | Div | Repeat Start | Repeat End | Repeat Left | Name | Family | Class |
| 122121930 | 122122903 | - | 16.4 | 5504 | 6502 | -6 | L1MC1 | L1 | LINE |
| 122122901 | 122123565 | - | 13.2 | 1522 | 2206 | -4302 | L1MC1 | L1 | LINE |
| 122123539 | 122123908 | - | 9.5 | 5789 | 6149 | -2 | L1PB1 | L1 | LINE |
| 122123930 | 122125675 | - | 7.8 | 4018 | 5788 | -363 | L1PB1 | L1 | LINE |
| 122125679 | 122126196 | + | 7.9 | 3475 | 3998 | -2148 | L1PB1 | L1 | LINE |
| 122126196 | 122127717 | - | 16.1 | 2 | 1581 | -4927 | L1MC1 | L1 | LINE |

  

|  |  |  |  |  |  |  |  |  |  |
| --- | --- | --- | --- | --- | --- | --- | --- | --- | --- |
| chrX:123312764-123314857 | | | 1539 bp | | |  |  |  |  |
| Genome Start | Genome End | Strand | Div | Repeat Start | Repeat End | Repeat Left | Name | Family | Class |
| 123312764 | 123313113 | - | 29.8 | 5483 | 5828 | -293 | L1ME4a | L1 | LINE |
| 123313545 | 123313904 | - | 32.6 | 4770 | 5147 | -999 | L1ME4a | L1 | LINE |
| 123314008 | 123314329 | - | 16.1 | 5625 | 5955 | -348 | L1MA6 | L1 | LINE |
| 123314332 | 123314670 | + | 15.1 | 5946 | 6288 | -12 | L1MA6 | L1 | LINE |
| 123314690 | 123314857 | - | 24.4 | 4427 | 4598 | -1548 | L1ME4a | L1 | LINE |

  

|  |  |  |  |  |  |  |  |  |  |
| --- | --- | --- | --- | --- | --- | --- | --- | --- | --- |
| chrX:124020755-124021711 | | | 920 bp | | |  |  |  |  |
| Genome Start | Genome End | Strand | Div | Repeat Start | Repeat End | Repeat Left | Name | Family | Class |
| 124020755 | 124020842 | + | 12.5 | 1 | 91 | -323 | MLT2D | ERVL | LTR |
| 124020873 | 124021249 | - | 22.9 | 5894 | 6266 | -46 | L1MA9 | L1 | LINE |
| 124021248 | 124021403 | + | 19.2 | 5741 | 5900 | -405 | L1MA9 | L1 | LINE |
| 124021407 | 124021688 | + | 20.4 | 73 | 394 | -20 | MLT2D | ERVL | LTR |
| 124021695 | 124021711 | + | 10.7 | 504 | 520 | -40 | MLT2D | ERVL | LTR |

  

|  |  |  |  |  |  |  |  |  |  |
| --- | --- | --- | --- | --- | --- | --- | --- | --- | --- |
| chrX:124560263-124565330 | | | 5033 bp | | |  |  |  |  |
| Genome Start | Genome End | Strand | Div | Repeat Start | Repeat End | Repeat Left | Name | Family | Class |
| 124560263 | 124560416 | + | 18.8 | 5012 | 5165 | -978 | L1MA5 | L1 | LINE |
| 124560415 | 124560939 | - | 14.8 | 4756 | 5293 | -853 | L1P4 | L1 | LINE |
| 124560940 | 124561306 | + | 14.2 | 1 | 381 | 0 | THE1D | MaLR | LTR |
| 124561307 | 124562783 | + | 14.2 | 1 | 1580 | 0 | THE1D-int | MaLR | LTR |
| 124562784 | 124563159 | + | 15.2 | 1 | 381 | 0 | THE1D | MaLR | LTR |
| 124563160 | 124563369 | - | 15.3 | 4549 | 4760 | -1386 | L1P4 | L1 | LINE |
| 124563375 | 124564228 | + | 15.0 | 5307 | 6166 | 0 | L1PA17 | L1 | LINE |
| 124564223 | 124564836 | + | 15.1 | 5154 | 5780 | -520 | L1MA5 | L1 | LINE |
| 124564875 | 124565330 | + | 15.5 | 5781 | 6300 | 0 | L1MA5 | L1 | LINE |

  

|  |  |  |  |  |  |  |  |  |  |
| --- | --- | --- | --- | --- | --- | --- | --- | --- | --- |
| chrX:124742720-124756420 | | | 13631 bp | | |  |  |  |  |
| Genome Start | Genome End | Strand | Div | Repeat Start | Repeat End | Repeat Left | Name | Family | Class |
| 124742720 | 124743314 | - | 10.5 | 4558 | 5150 | -996 | L1PB | L1 | LINE |
| 124743315 | 124743952 | - | 13.9 | 1 | 676 | 0 | MER61F | ERV1 | LTR |
| 124743954 | 124746934 | - | 11.3 | 4192 | 7187 | -1 | MER61F-int | ERV1 | LTR |
| 124746947 | 124747241 | - | 6.8 | 1 | 296 | -15 | AluY | Alu | SINE |
| 124747273 | 124747386 | - | 7.9 | 1 | 115 | -196 | AluY | Alu | SINE |
| 124747393 | 124750359 | - | 16.9 | 755 | 4142 | -3046 | MER61F-int | ERV1 | LTR |
| 124750360 | 124751386 | - | 5.5 | 3231 | 4254 | -1892 | L1PA5 | L1 | LINE |
| 124751382 | 124753275 | + | 4.4 | 4259 | 6151 | -3 | L1PA5 | L1 | LINE |
| 124753292 | 124753982 | - | 17.6 | 262 | 931 | -6257 | MER61F-int | ERV1 | LTR |
| 124753987 | 124754079 | - | 13.8 | 5 | 91 | -7097 | MER61F-int | ERV1 | LTR |
| 124754085 | 124754714 | - | 13.9 | 1 | 676 | 0 | MER61F | ERV1 | LTR |
| 124754715 | 124755383 | - | 12.0 | 3890 | 4561 | -1585 | L1PB | L1 | LINE |
| 124755384 | 124755680 | - | 11.8 | 1 | 297 | -16 | AluSq | Alu | SINE |
| 124755681 | 124756420 | - | 12.0 | 3129 | 3889 | -2257 | L1PB | L1 | LINE |

  

|  |  |  |  |  |  |  |  |  |  |
| --- | --- | --- | --- | --- | --- | --- | --- | --- | --- |
| chrX:124997573-125004216 | | | 6617 bp | | |  |  |  |  |
| Genome Start | Genome End | Strand | Div | Repeat Start | Repeat End | Repeat Left | Name | Family | Class |
| 124997573 | 125001268 | + | 8.3 | 1 | 3696 | -1958 | HERVL-A1 | ERVL | LTR |
| 125001283 | 125001821 | + | 9.5 | 3971 | 4511 | -1143 | HERVL-A1 | ERVL | LTR |
| 125001827 | 125002712 | - | 7.6 | 4893 | 5778 | -376 | L1PA7 | L1 | LINE |
| 125002713 | 125003066 | + | 4.2 | 5788 | 6141 | -13 | L1PA7 | L1 | LINE |
| 125003075 | 125004216 | + | 7.6 | 4506 | 5654 | 0 | HERVL-A1 | ERVL | LTR |

  

|  |  |  |  |  |  |  |  |  |  |
| --- | --- | --- | --- | --- | --- | --- | --- | --- | --- |
| chrX:125100107-125102475 | | | 2052 bp | | |  |  |  |  |
| Genome Start | Genome End | Strand | Div | Repeat Start | Repeat End | Repeat Left | Name | Family | Class |
| 125100107 | 125101574 | - | 24.1 | 279 | 1825 | -5324 | L1PA15-16 | L1 | LINE |
| 125101556 | 125101597 | - | 14.3 | 5955 | 5996 | -176 | L1PA8A | L1 | LINE |
| 125101600 | 125101736 | + | 12.8 | 5999 | 6131 | 0 | L1PA8A | L1 | LINE |
| 125102039 | 125102146 | + | 27.8 | 5317 | 5429 | -722 | L1P | L1 | LINE |
| 125102179 | 125102475 | - | 19.9 | 5 | 302 | -6551 | L1PA15-16 | L1 | LINE |

  

|  |  |  |  |  |  |  |  |  |  |
| --- | --- | --- | --- | --- | --- | --- | --- | --- | --- |
| chrX:125238281-125239358 | | | 1063 bp | | |  |  |  |  |
| Genome Start | Genome End | Strand | Div | Repeat Start | Repeat End | Repeat Left | Name | Family | Class |
| 125238281 | 125238476 | - | 28.5 | 2542 | 2738 | -681 | L2 | L2 | LINE |
| 125238483 | 125238701 | - | 16.0 | 5415 | 5636 | -676 | L1MA9 | L1 | LINE |
| 125238702 | 125239296 | + | 19.3 | 5646 | 6274 | -38 | L1MA9 | L1 | LINE |
| 125239306 | 125239358 | - | 26.4 | 2481 | 2533 | -886 | L2 | L2 | LINE |

  

|  |  |  |  |  |  |  |  |  |  |
| --- | --- | --- | --- | --- | --- | --- | --- | --- | --- |
| chrX:125402752-125411076 | | | 8176 bp | | |  |  |  |  |
| Genome Start | Genome End | Strand | Div | Repeat Start | Repeat End | Repeat Left | Name | Family | Class |
| 125402752 | 125406007 | - | 17.7 | 3868 | 7158 | -552 | L1M2 | L1 | LINE |
| 125406010 | 125406785 | - | 25.8 | 2914 | 3724 | -3838 | L1M2 | L1 | LINE |
| 125406796 | 125407995 | - | 10.3 | 4964 | 6156 | -5 | L1PA14 | L1 | LINE |
| 125407989 | 125408317 | + | 12.7 | 4083 | 4401 | -1745 | L1PREC2 | L1 | LINE |
| 125408354 | 125408935 | + | 12.7 | 4402 | 4984 | -1162 | L1PREC2 | L1 | LINE |
| 125408934 | 125409380 | - | 13.0 | 5689 | 6126 | -34 | L1PREC2 | L1 | LINE |
| 125409384 | 125410434 | - | 30.1 | 1898 | 2929 | -4636 | L1M2 | L1 | LINE |
| 125410542 | 125411076 | - | 26.2 | 466 | 992 | -6497 | L1M2 | L1 | LINE |

  

|  |  |  |  |  |  |  |  |  |  |
| --- | --- | --- | --- | --- | --- | --- | --- | --- | --- |
| chrX:125760053-125770405 | | | 10365 bp | | |  |  |  |  |
| Genome Start | Genome End | Strand | Div | Repeat Start | Repeat End | Repeat Left | Name | Family | Class |
| 125760053 | 125761591 | + | 22.2 | 17 | 1632 | -5933 | L1M1 | L1 | LINE |
| 125761590 | 125763061 | + | 14.6 | 3237 | 4705 | -1441 | L1M1 | L1 | LINE |
| 125763060 | 125765023 | - | 6.8 | 2796 | 4758 | -1388 | L1P2 | L1 | LINE |
| 125765013 | 125765306 | - | 4.4 | 1764 | 2062 | -213 | L1P2 | L1 | LINE |
| 125765303 | 125765491 | + | 6.5 | 4768 | 4956 | -1190 | L1PA5 | L1 | LINE |
| 125765492 | 125765805 | + | 9.0 | 1 | 311 | 0 | AluY | Alu | SINE |
| 125765806 | 125767014 | + | 5.8 | 4957 | 6153 | -1 | L1PA5 | L1 | LINE |
| 125767013 | 125767602 | + | 12.8 | 4692 | 5283 | -863 | L1M1 | L1 | LINE |
| 125767603 | 125767968 | + | 11.0 | 1 | 364 | 0 | THE1B | MaLR | LTR |
| 125767971 | 125769545 | + | 30.6 | 2 | 1731 | -4 | MSTD-int | MaLR | LTR |
| 125769553 | 125769914 | + | 11.4 | 4 | 364 | 0 | THE1B | MaLR | LTR |
| 125769915 | 125770405 | + | 11.9 | 5284 | 5770 | -534 | L1M1 | L1 | LINE |

  

|  |  |  |  |  |  |  |  |  |  |
| --- | --- | --- | --- | --- | --- | --- | --- | --- | --- |
| chrX:126027477-126037396 | | | 9379 bp | | |  |  |  |  |
| Genome Start | Genome End | Strand | Div | Repeat Start | Repeat End | Repeat Left | Name | Family | Class |
| 126027477 | 126029357 | - | 14.0 | 5820 | 7724 | 0 | L1MA3 | L1 | LINE |
| 126029480 | 126029565 | - | 19.0 | 6219 | 6297 | 0 | L1MA5A | L1 | LINE |
| 126029846 | 126030101 | + | 1.9 | 5081 | 5336 | -810 | L1P1 | L1 | LINE |
| 126030187 | 126030531 | - | 15.7 | 5859 | 6205 | -99 | L1MA2 | L1 | LINE |
| 126030531 | 126030962 | + | 11.3 | 5404 | 5838 | -467 | L1MA2 | L1 | LINE |
| 126030965 | 126031265 | - | 13.3 | 5508 | 5817 | -1748 | L1MA3 | L1 | LINE |
| 126031272 | 126032588 | - | 17.6 | 3006 | 4314 | -3251 | L1MA3 | L1 | LINE |
| 126032633 | 126034156 | - | 12.7 | 4612 | 6123 | -28 | L1PB2 | L1 | LINE |
| 126034160 | 126035734 | - | 19.9 | 1397 | 3017 | -4548 | L1MA3 | L1 | LINE |
| 126035735 | 126036030 | - | 10.8 | 1 | 296 | -14 | AluSg | Alu | SINE |
| 126036031 | 126037396 | - | 19.9 | 36 | 1396 | -6169 | L1MA3 | L1 | LINE |

  

|  |  |  |  |  |  |  |  |  |  |
| --- | --- | --- | --- | --- | --- | --- | --- | --- | --- |
| chrX:126158044-126166160 | | | 7649 bp | | |  |  |  |  |
| Genome Start | Genome End | Strand | Div | Repeat Start | Repeat End | Repeat Left | Name | Family | Class |
| 126158044 | 126159001 | + | 22.1 | 15 | 1010 | -4318 | L1M3f | L1 | LINE |
| 126159002 | 126161670 | + | 17.5 | 3532 | 6300 | 0 | L1MA4 | L1 | LINE |
| 126161677 | 126161753 | + | 20.8 | 2880 | 2956 | -3372 | L1M3f | L1 | LINE |
| 126161755 | 126162396 | - | 9.3 | 4336 | 4988 | -1158 | L1PREC2 | L1 | LINE |
| 126162396 | 126162435 | + | 10.0 | 6117 | 6156 | -9 | L1PA15 | L1 | LINE |
| 126162506 | 126162717 | + | 30.7 | 2995 | 3202 | -3126 | L1M3f | L1 | LINE |
| 126163114 | 126163536 | - | 4.3 | 5714 | 6136 | -18 | L1PA5 | L1 | LINE |
| 126163537 | 126165929 | + | 4.7 | 3323 | 5722 | -433 | L1PA5 | L1 | LINE |
| 126165926 | 126166160 | + | 21.7 | 3189 | 3620 | -2708 | L1M3f | L1 | LINE |

  

|  |  |  |  |  |  |  |  |  |  |
| --- | --- | --- | --- | --- | --- | --- | --- | --- | --- |
| chrX:126441977-126454615 | | | 12403 bp | | |  |  |  |  |
| Genome Start | Genome End | Strand | Div | Repeat Start | Repeat End | Repeat Left | Name | Family | Class |
| 126441977 | 126445183 | - | 14.1 | 4550 | 7723 | 0 | L1MA2 | L1 | LINE |
| 126445153 | 126446781 | - | 1.8 | 4525 | 6155 | 0 | L1PA3 | L1 | LINE |
| 126446781 | 126447566 | + | 2.4 | 3730 | 4515 | -1631 | L1PA3 | L1 | LINE |
| 126447792 | 126448145 | - | 28.5 | 2992 | 3414 | -5 | L2 | L2 | LINE |
| 126448152 | 126448358 | + | 24.8 | 7209 | 7443 | -552 | L1MD3 | L1 | LINE |
| 126448396 | 126452099 | - | 16.2 | 772 | 4584 | -2981 | L1MA2 | L1 | LINE |
| 126452100 | 126452170 | - | 13.4 | 365 | 428 | 0 | MSTA | MaLR | LTR |
| 126452171 | 126452479 | + | 14.9 | 1 | 302 | -8 | AluSg | Alu | SINE |
| 126452480 | 126452834 | - | 13.4 | 1 | 364 | -64 | MSTA | MaLR | LTR |
| 126452835 | 126453344 | - | 17.0 | 265 | 771 | -6794 | L1MA2 | L1 | LINE |
| 126453345 | 126454366 | - | 6.5 | 1 | 1266 | 0 | MER11A | ERVK | LTR |
| 126454367 | 126454615 | - | 23.2 | 14 | 265 | -7300 | L1MA2 | L1 | LINE |

  

|  |  |  |  |  |  |  |  |  |  |
| --- | --- | --- | --- | --- | --- | --- | --- | --- | --- |
| chrX:127060379-127070297 | | | 9799 bp | | |  |  |  |  |
| Genome Start | Genome End | Strand | Div | Repeat Start | Repeat End | Repeat Left | Name | Family | Class |
| 127060379 | 127060901 | - | 25.1 | 3435 | 3967 | -2179 | L1M4 | L1 | LINE |
| 127060952 | 127061084 | - | 7.5 | 223 | 355 | 0 | THE1A | MaLR | LTR |
| 127061092 | 127062075 | - | 6.2 | 5171 | 6159 | -13 | L1PA8 | L1 | LINE |
| 127062075 | 127062384 | + | 10.3 | 4861 | 5172 | -974 | L1PA8 | L1 | LINE |
| 127062385 | 127062620 | - | 11.9 | 1 | 238 | -117 | THE1A | MaLR | LTR |
| 127062621 | 127064189 | - | 29.2 | 2 | 1735 | 0 | MLT1A1-int | MaLR | LTR |
| 127064192 | 127064545 | - | 11.0 | 1 | 355 | 0 | THE1A | MaLR | LTR |
| 127064546 | 127065047 | - | 18.4 | 6879 | 7646 | -73 | L1MA4 | L1 | LINE |
| 127065036 | 127065916 | - | 21.8 | 5521 | 6349 | -1213 | L1MA4 | L1 | LINE |
| 127065924 | 127066112 | - | 18.7 | 3673 | 3864 | -3698 | L1MA4 | L1 | LINE |
| 127066120 | 127066437 | - | 15.4 | 5822 | 6157 | -9 | L1PA16 | L1 | LINE |
| 127066438 | 127066701 | + | 11.0 | 5477 | 5742 | -424 | L1PA16 | L1 | LINE |
| 127066711 | 127069335 | - | 26.4 | 256 | 3675 | -3890 | L1MA4 | L1 | LINE |
| 127069387 | 127070297 | - | 23.7 | 2386 | 3360 | -2786 | L1M4 | L1 | LINE |

  

|  |  |  |  |  |  |  |  |  |  |
| --- | --- | --- | --- | --- | --- | --- | --- | --- | --- |
| chrX:127310976-127314302 | | | 3290 bp | | |  |  |  |  |
| Genome Start | Genome End | Strand | Div | Repeat Start | Repeat End | Repeat Left | Name | Family | Class |
| 127310976 | 127311039 | + | 27.0 | 34 | 96 | -332 | MSTD | MaLR | LTR |
| 127311040 | 127312324 | - | 9.1 | 3190 | 4463 | -1683 | L1PA7 | L1 | LINE |
| 127312324 | 127313998 | + | 7.3 | 4471 | 6151 | -3 | L1PA7 | L1 | LINE |
| 127313999 | 127314088 | + | 27.8 | 85 | 175 | -221 | MSTD | MaLR | LTR |
| 127314127 | 127314302 | + | 25.4 | 220 | 395 | -1 | MSTD | MaLR | LTR |

  

|  |  |  |  |  |  |  |  |  |  |
| --- | --- | --- | --- | --- | --- | --- | --- | --- | --- |
| chrX:127751611-127758389 | | | 6750 bp | | |  |  |  |  |
| Genome Start | Genome End | Strand | Div | Repeat Start | Repeat End | Repeat Left | Name | Family | Class |
| 127751611 | 127751685 | + | 12.0 | 1144 | 1218 | -5135 | L1PA8 | L1 | LINE |
| 127751710 | 127752681 | + | 12.0 | 1219 | 2244 | -4109 | L1PA8 | L1 | LINE |
| 127752680 | 127753165 | - | 9.5 | 4809 | 5295 | -851 | L1PA7 | L1 | LINE |
| 127753166 | 127754006 | + | 4.8 | 5309 | 6152 | -2 | L1PA7 | L1 | LINE |
| 127754014 | 127755144 | + | 9.0 | 2246 | 3376 | -2955 | L1PA8 | L1 | LINE |
| 127755145 | 127755419 | + | 8.8 | 27 | 304 | -7 | AluY | Alu | SINE |
| 127755420 | 127758389 | + | 9.1 | 3377 | 6356 | -1 | L1PA8 | L1 | LINE |

  

|  |  |  |  |  |  |  |  |  |  |
| --- | --- | --- | --- | --- | --- | --- | --- | --- | --- |
| chrX:127878205-127880441 | | | 2234 bp | | |  |  |  |  |
| Genome Start | Genome End | Strand | Div | Repeat Start | Repeat End | Repeat Left | Name | Family | Class |
| 127878205 | 127879513 | + | 2.6 | 4470 | 5783 | -372 | L1PA5 | L1 | LINE |
| 127879509 | 127879544 | - | 2.8 | 5594 | 5629 | -525 | L1PA2 | L1 | LINE |
| 127879545 | 127880053 | + | 1.6 | 5645 | 6153 | -2 | L1PA2 | L1 | LINE |
| 127880062 | 127880441 | + | 5.0 | 5770 | 6151 | -3 | L1PA5 | L1 | LINE |

  

|  |  |  |  |  |  |  |  |  |  |
| --- | --- | --- | --- | --- | --- | --- | --- | --- | --- |
| chrX:128045946-128047787 | | | 1844 bp | | |  |  |  |  |
| Genome Start | Genome End | Strand | Div | Repeat Start | Repeat End | Repeat Left | Name | Family | Class |
| 128045946 | 128046160 | + | 10.2 | 26 | 244 | -351 | LTR49 | ERV1 | LTR |
| 128046161 | 128046653 | - | 2.8 | 4755 | 5247 | -899 | L1PA3 | L1 | LINE |
| 128046652 | 128047551 | + | 1.9 | 5254 | 6153 | -2 | L1PA3 | L1 | LINE |
| 128047552 | 128047787 | + | 12.8 | 227 | 508 | -87 | LTR49 | ERV1 | LTR |

  

|  |  |  |  |  |  |  |  |  |  |
| --- | --- | --- | --- | --- | --- | --- | --- | --- | --- |
| chrX:129964383-129968526 | | | 4108 bp | | |  |  |  |  |
| Genome Start | Genome End | Strand | Div | Repeat Start | Repeat End | Repeat Left | Name | Family | Class |
| 129964383 | 129964630 | - | 39.5 | 2782 | 3034 | -385 | L2 | L2 | LINE |
| 129964635 | 129964698 | - | 14.1 | 115 | 178 | 0 | MER5B | MER1\_type | DNA |
| 129964699 | 129966970 | - | 0.4 | 2684 | 4955 | -1191 | L1HS | L1 | LINE |
| 129966962 | 129968175 | + | 0.8 | 4942 | 6155 | 0 | L1HS | L1 | LINE |
| 129968213 | 129968283 | - | 25.4 | 60 | 130 | -48 | MER5B | MER1\_type | DNA |
| 129968288 | 129968526 | - | 29.6 | 2432 | 2675 | -744 | L2 | L2 | LINE |

  

|  |  |  |  |  |  |  |  |  |  |
| --- | --- | --- | --- | --- | --- | --- | --- | --- | --- |
| chrX:130102998-130107371 | | | 3580 bp | | |  |  |  |  |
| Genome Start | Genome End | Strand | Div | Repeat Start | Repeat End | Repeat Left | Name | Family | Class |
| 130102998 | 130103282 | + | 30.5 | 2298 | 2602 | -817 | L2 | L2 | LINE |
| 130103298 | 130103681 | - | 9.1 | 3908 | 4291 | -1855 | L1PB1 | L1 | LINE |
| 130103684 | 130105173 | + | 10.0 | 4286 | 5780 | -371 | L1PB1 | L1 | LINE |
| 130105174 | 130105291 | + | 11.0 | 6056 | 6173 | -1 | L1PA11 | L1 | LINE |
| 130105289 | 130105497 | + | 8.2 | 5769 | 5967 | -184 | L1PB1 | L1 | LINE |
| 130105498 | 130105801 | + | 15.1 | 1 | 306 | -6 | AluSx | Alu | SINE |
| 130105802 | 130105991 | + | 8.2 | 5968 | 6146 | -5 | L1PB1 | L1 | LINE |
| 130106023 | 130106255 | + | 36.6 | 2612 | 2863 | -556 | L2 | L2 | LINE |
| 130106602 | 130106676 | + | 37.3 | 3344 | 3419 | 0 | L2 | L2 | LINE |
| 130107042 | 130107284 | + | 31.5 | 2458 | 2704 | -715 | L2 | L2 | LINE |
| 130107323 | 130107371 | + | 24.5 | 3244 | 3293 | -85 | L2 | L2 | LINE |

  

|  |  |  |  |  |  |  |  |  |  |
| --- | --- | --- | --- | --- | --- | --- | --- | --- | --- |
| chrX:130294397-130295486 | | | 1058 bp | | |  |  |  |  |
| Genome Start | Genome End | Strand | Div | Repeat Start | Repeat End | Repeat Left | Name | Family | Class |
| 130294397 | 130294633 | - | 36.1 | 2806 | 3123 | -255 | L2 | L2 | LINE |
| 130294662 | 130295300 | - | 8.9 | 5650 | 6296 | -6 | L1MA1 | L1 | LINE |
| 130295304 | 130295362 | + | 5.1 | 5587 | 5645 | -659 | L1MA1 | L1 | LINE |
| 130295364 | 130295486 | - | 32.8 | 2693 | 2815 | -604 | L2 | L2 | LINE |

  

|  |  |  |  |  |  |  |  |  |  |
| --- | --- | --- | --- | --- | --- | --- | --- | --- | --- |
| chrX:130440592-130451876 | | | 11181 bp | | |  |  |  |  |
| Genome Start | Genome End | Strand | Div | Repeat Start | Repeat End | Repeat Left | Name | Family | Class |
| 130440592 | 130441328 | + | 17.8 | 3 | 794 | -6069 | L1PB1 | L1 | LINE |
| 130441322 | 130442896 | + | 11.4 | 1101 | 2717 | -4146 | L1PB1 | L1 | LINE |
| 130442897 | 130443241 | + | 13.9 | 1 | 364 | 0 | THE1B | MaLR | LTR |
| 130443242 | 130444829 | + | 11.3 | 1 | 1580 | 0 | THE1B-int | MaLR | LTR |
| 130444830 | 130444997 | + | 13.7 | 1 | 183 | -181 | THE1B | MaLR | LTR |
| 130445001 | 130445193 | + | 15.7 | 137 | 308 | -5 | AluSq | Alu | SINE |
| 130445197 | 130445384 | + | 11.8 | 177 | 364 | 0 | THE1B | MaLR | LTR |
| 130445402 | 130447900 | + | 7.6 | 2780 | 5278 | -1584 | L1PB1 | L1 | LINE |
| 130447898 | 130448495 | - | 3.0 | 3837 | 4434 | -1712 | L1PA3 | L1 | LINE |
| 130448493 | 130450194 | + | 3.1 | 4452 | 6152 | -3 | L1PA3 | L1 | LINE |
| 130450229 | 130451458 | + | 8.1 | 5264 | 6509 | -358 | L1PB1 | L1 | LINE |
| 130451519 | 130451876 | + | 10.8 | 6510 | 6867 | 0 | L1PB1 | L1 | LINE |

  

|  |  |  |  |  |  |  |  |  |  |
| --- | --- | --- | --- | --- | --- | --- | --- | --- | --- |
| chrX:131276930-131283852 | | | 6837 bp | | |  |  |  |  |
| Genome Start | Genome End | Strand | Div | Repeat Start | Repeat End | Repeat Left | Name | Family | Class |
| 131276930 | 131277202 | - | 25.5 | 1932 | 2214 | -4186 | L1MEc | L1 | LINE |
| 131277203 | 131277491 | - | 10.4 | 10 | 298 | -12 | AluSg | Alu | SINE |
| 131277492 | 131277570 | - | 25.5 | 1877 | 1931 | -4469 | L1MEc | L1 | LINE |
| 131277571 | 131277854 | + | 10.9 | 5864 | 6160 | -3 | L1PA13 | L1 | LINE |
| 131277855 | 131278000 | - | 25.5 | 1725 | 1876 | -4524 | L1MEc | L1 | LINE |
| 131278014 | 131279848 | - | 4.8 | 1885 | 3723 | -2423 | L1PA4 | L1 | LINE |
| 131279848 | 131282285 | + | 4.4 | 3714 | 6155 | 0 | L1PA4 | L1 | LINE |
| 131282360 | 131283852 | - | 29.2 | 1 | 1558 | -4842 | L1MEc | L1 | LINE |

  

|  |  |  |  |  |  |  |  |  |  |
| --- | --- | --- | --- | --- | --- | --- | --- | --- | --- |
| chrX:131299827-131304710 | | | 4716 bp | | |  |  |  |  |
| Genome Start | Genome End | Strand | Div | Repeat Start | Repeat End | Repeat Left | Name | Family | Class |
| 131299827 | 131300004 | + | 25.3 | 4885 | 5095 | -1052 | L1MD3 | L1 | LINE |
| 131300013 | 131300271 | - | 17.0 | 5017 | 5283 | -863 | L1P4 | L1 | LINE |
| 131300247 | 131301072 | - | 2.5 | 5321 | 6149 | -5 | L1PA5 | L1 | LINE |
| 131301072 | 131302257 | - | 17.7 | 3793 | 5031 | -1115 | L1P4 | L1 | LINE |
| 131302258 | 131302747 | + | 11.6 | 5299 | 5792 | -364 | L1PB4 | L1 | LINE |
| 131302778 | 131303136 | + | 11.6 | 5793 | 6155 | -1 | L1PB4 | L1 | LINE |
| 131303195 | 131303379 | - | 14.1 | 816 | 1011 | -50 | MER63 | AcHobo | DNA |
| 131303478 | 131303958 | + | 25.0 | 5083 | 5629 | -2366 | L1MD3 | L1 | LINE |
| 131303959 | 131304274 | + | 11.5 | 1 | 328 | 0 | AluSx | Alu | SINE |
| 131304275 | 131304710 | + | 25.4 | 5630 | 6074 | -90 | L1MD3 | L1 | LINE |

  

|  |  |  |  |  |  |  |  |  |  |
| --- | --- | --- | --- | --- | --- | --- | --- | --- | --- |
| chrX:133877230-133880555 | | | 2842 bp | | |  |  |  |  |
| Genome Start | Genome End | Strand | Div | Repeat Start | Repeat End | Repeat Left | Name | Family | Class |
| 133877230 | 133877537 | + | 34.2 | 2555 | 2853 | -566 | L2 | L2 | LINE |
| 133877579 | 133877669 | + | 12.1 | 45 | 135 | -177 | AluSq/x | Alu | SINE |
| 133877670 | 133877978 | + | 14.1 | 1 | 308 | -4 | AluSx | Alu | SINE |
| 133877984 | 133878092 | - | 15.3 | 6181 | 6275 | -25 | L1MA4 | L1 | LINE |
| 133878093 | 133878386 | + | 6.5 | 1 | 298 | -13 | AluY | Alu | SINE |
| 133878387 | 133878515 | - | 15.3 | 6054 | 6180 | -120 | L1MA4 | L1 | LINE |
| 133878514 | 133878716 | + | 13.3 | 5018 | 5227 | -916 | L1MA4 | L1 | LINE |
| 133878718 | 133878950 | - | 15.0 | 1 | 237 | -75 | AluSx | Alu | SINE |
| 133878951 | 133879135 | + | 13.0 | 5858 | 6043 | -257 | L1MA4 | L1 | LINE |
| 133879130 | 133879301 | - | 11.1 | 5046 | 5226 | -917 | L1M2 | L1 | LINE |
| 133879302 | 133879608 | - | 9.6 | 1 | 302 | -10 | AluSx | Alu | SINE |
| 133879609 | 133879979 | - | 11.1 | 4684 | 5045 | -1098 | L1M2 | L1 | LINE |
| 133880425 | 133880555 | + | 34.6 | 3026 | 3166 | -212 | L2 | L2 | LINE |

  

|  |  |  |  |  |  |  |  |  |  |
| --- | --- | --- | --- | --- | --- | --- | --- | --- | --- |
| chrX:133987761-133990673 | | | 2881 bp | | |  |  |  |  |
| Genome Start | Genome End | Strand | Div | Repeat Start | Repeat End | Repeat Left | Name | Family | Class |
| 133987761 | 133987836 | + | 18.4 | 1 | 77 | -268 | MER2 | MER2\_type | DNA |
| 133987847 | 133987982 | - | 16.2 | 4188 | 4323 | -1823 | L1PB | L1 | LINE |
| 133987989 | 133988134 | - | 13.7 | 3713 | 3860 | -2286 | L1PB | L1 | LINE |
| 133988122 | 133989183 | + | 11.8 | 4330 | 5441 | -713 | L1PB2 | L1 | LINE |
| 133989179 | 133989254 | - | 1.3 | 5642 | 5717 | -438 | L1HS | L1 | LINE |
| 133989255 | 133989705 | + | 0.9 | 5705 | 6155 | 0 | L1HS | L1 | LINE |
| 133989716 | 133990439 | + | 8.8 | 5423 | 6148 | -3 | L1PB2 | L1 | LINE |
| 133990464 | 133990673 | + | 16.2 | 134 | 345 | 0 | MER2 | MER2\_type | DNA |

  

|  |  |  |  |  |  |  |  |  |  |
| --- | --- | --- | --- | --- | --- | --- | --- | --- | --- |
| chrX:134684743-134689537 | | | 4469 bp | | |  |  |  |  |
| Genome Start | Genome End | Strand | Div | Repeat Start | Repeat End | Repeat Left | Name | Family | Class |
| 134684743 | 134685050 | - | 26.0 | 4962 | 5280 | -387 | ERVL-F | ERVL | LTR |
| 134685279 | 134685366 | - | 22.6 | 535 | 620 | -43 | MLT2F | ERVL | LTR |
| 134685393 | 134685694 | - | 17.1 | 1 | 297 | -15 | AluJb | Alu | SINE |
| 134685763 | 134687259 | - | 3.7 | 4654 | 6150 | -5 | L1PA4 | L1 | LINE |
| 134687258 | 134687615 | + | 4.8 | 4302 | 4659 | -1487 | L1PA4 | L1 | LINE |
| 134687616 | 134688429 | - | 25.6 | 4118 | 4918 | -796 | ERVL-F | ERVL | LTR |
| 134688431 | 134688783 | + | 30.1 | 259 | 624 | -2 | MLT1E2 | MaLR | LTR |
| 134688787 | 134688899 | - | 24.1 | 3502 | 3613 | -2054 | ERVL-F | ERVL | LTR |
| 134688902 | 134689537 | - | 29.3 | 2420 | 3080 | -2634 | ERVL-F | ERVL | LTR |

  

|  |  |  |  |  |  |  |  |  |  |
| --- | --- | --- | --- | --- | --- | --- | --- | --- | --- |
| chrX:134702003-134706812 | | | 4472 bp | | |  |  |  |  |
| Genome Start | Genome End | Strand | Div | Repeat Start | Repeat End | Repeat Left | Name | Family | Class |
| 134702003 | 134702310 | - | 25.6 | 4962 | 5280 | -387 | ERVL-F | ERVL | LTR |
| 134702551 | 134702638 | - | 22.6 | 535 | 620 | -43 | MLT2F | ERVL | LTR |
| 134702665 | 134702966 | - | 17.1 | 1 | 297 | -15 | AluJb | Alu | SINE |
| 134703035 | 134704534 | - | 3.8 | 4654 | 6150 | -5 | L1PA4 | L1 | LINE |
| 134704533 | 134704890 | + | 4.2 | 4302 | 4659 | -1487 | L1PA4 | L1 | LINE |
| 134704891 | 134705704 | - | 25.5 | 4118 | 4918 | -796 | ERVL-F | ERVL | LTR |
| 134705706 | 134706058 | + | 30.7 | 259 | 624 | -2 | MLT1E2 | MaLR | LTR |
| 134706062 | 134706174 | - | 24.1 | 3502 | 3613 | -2054 | ERVL-F | ERVL | LTR |
| 134706177 | 134706812 | - | 29.2 | 2420 | 3080 | -2634 | ERVL-F | ERVL | LTR |

  

|  |  |  |  |  |  |  |  |  |  |
| --- | --- | --- | --- | --- | --- | --- | --- | --- | --- |
| chrX:134768743-134773541 | | | 4469 bp | | |  |  |  |  |
| Genome Start | Genome End | Strand | Div | Repeat Start | Repeat End | Repeat Left | Name | Family | Class |
| 134768743 | 134769378 | + | 29.2 | 2420 | 3080 | -2634 | ERVL-B4 | ERVL | LTR |
| 134769381 | 134769493 | + | 24.1 | 3502 | 3613 | -2054 | ERVL-B4 | ERVL | LTR |
| 134769497 | 134769849 | - | 30.1 | 259 | 624 | -2 | MLT1E2 | MaLR | LTR |
| 134769851 | 134770664 | + | 25.5 | 4118 | 4918 | -796 | ERVL-B4 | ERVL | LTR |
| 134770665 | 134771022 | - | 3.7 | 4302 | 4659 | -1487 | L1PA4 | L1 | LINE |
| 134771021 | 134772517 | + | 3.7 | 4654 | 6150 | -5 | L1PA4 | L1 | LINE |
| 134772586 | 134772887 | + | 17.1 | 1 | 297 | -15 | AluJb | Alu | SINE |
| 134772914 | 134773001 | + | 22.6 | 535 | 620 | -43 | MLT2F | ERVL | LTR |
| 134773234 | 134773541 | + | 25.6 | 4962 | 5280 | -387 | ERVL-B4 | ERVL | LTR |

  

|  |  |  |  |  |  |  |  |  |  |
| --- | --- | --- | --- | --- | --- | --- | --- | --- | --- |
| chrX:134785998-134790792 | | | 4469 bp | | |  |  |  |  |
| Genome Start | Genome End | Strand | Div | Repeat Start | Repeat End | Repeat Left | Name | Family | Class |
| 134785998 | 134786633 | + | 29.5 | 2420 | 3080 | -2634 | ERVL-B4 | ERVL | LTR |
| 134786636 | 134786748 | + | 24.1 | 3502 | 3613 | -2054 | ERVL-B4 | ERVL | LTR |
| 134786752 | 134787104 | - | 30.4 | 259 | 624 | -2 | MLT1E2 | MaLR | LTR |
| 134787106 | 134787919 | + | 25.6 | 4118 | 4918 | -796 | ERVL-B4 | ERVL | LTR |
| 134787920 | 134788277 | - | 4.5 | 4302 | 4659 | -1487 | L1PA4 | L1 | LINE |
| 134788276 | 134789772 | + | 3.8 | 4654 | 6151 | -4 | L1PA4 | L1 | LINE |
| 134789841 | 134790142 | + | 17.1 | 1 | 297 | -15 | AluJb | Alu | SINE |
| 134790169 | 134790256 | + | 22.6 | 535 | 620 | -43 | MLT2F | ERVL | LTR |
| 134790485 | 134790792 | + | 26.0 | 4962 | 5280 | -387 | ERVL-B4 | ERVL | LTR |

  

|  |  |  |  |  |  |  |  |  |  |
| --- | --- | --- | --- | --- | --- | --- | --- | --- | --- |
| chrX:135239756-135249651 | | | 9680 bp | | |  |  |  |  |
| Genome Start | Genome End | Strand | Div | Repeat Start | Repeat End | Repeat Left | Name | Family | Class |
| 135239756 | 135240503 | - | 19.0 | 5855 | 6619 | 0 | L1MD2 | L1 | LINE |
| 135240527 | 135241618 | - | 14.4 | 5285 | 6300 | -4 | L1MA2 | L1 | LINE |
| 135241644 | 135241822 | - | 6.7 | 119 | 297 | -14 | AluY | Alu | SINE |
| 135241830 | 135242894 | - | 1.9 | 5090 | 6155 | 0 | L1PA2 | L1 | LINE |
| 135242889 | 135243118 | + | 1.3 | 4866 | 5095 | -1051 | L1PA2 | L1 | LINE |
| 135243124 | 135243246 | - | 7.3 | 1 | 123 | -188 | AluY | Alu | SINE |
| 135243251 | 135243318 | - | 20.3 | 5457 | 5523 | -883 | L1MD2 | L1 | LINE |
| 135243361 | 135243641 | - | 14.2 | 1 | 282 | -30 | AluSx | Alu | SINE |
| 135243642 | 135243895 | - | 20.3 | 5203 | 5456 | -950 | L1MD2 | L1 | LINE |
| 135243985 | 135246791 | - | 20.5 | 2094 | 5202 | -1204 | L1MD2 | L1 | LINE |
| 135246792 | 135247093 | - | 18.2 | 1 | 290 | -22 | AluJo | Alu | SINE |
| 135247094 | 135247985 | - | 22.7 | 1178 | 2093 | -4409 | L1MD2 | L1 | LINE |
| 135247987 | 135248272 | - | 12.9 | 1 | 296 | -16 | AluSx | Alu | SINE |
| 135248300 | 135248381 | - | 8.8 | 6058 | 6145 | -20 | L1PA10 | L1 | LINE |
| 135248381 | 135249651 | - | 25.3 | 7 | 1175 | -5234 | L1MD2 | L1 | LINE |

  

|  |  |  |  |  |  |  |  |  |  |
| --- | --- | --- | --- | --- | --- | --- | --- | --- | --- |
| chrX:137320526-137330737 | | | 10146 bp | | |  |  |  |  |
| Genome Start | Genome End | Strand | Div | Repeat Start | Repeat End | Repeat Left | Name | Family | Class |
| 137320526 | 137325608 | + | 13.3 | 13 | 5213 | -2352 | L1MA3 | L1 | LINE |
| 137325668 | 137326336 | - | 13.2 | 5486 | 6158 | -7 | L1PA15 | L1 | LINE |
| 137326337 | 137326643 | - | 11.1 | 1 | 308 | -1 | AluSc | Alu | SINE |
| 137326644 | 137327045 | - | 14.5 | 5086 | 5485 | -680 | L1PA15 | L1 | LINE |
| 137327051 | 137327414 | + | 10.4 | 1 | 364 | 0 | THE1B | MaLR | LTR |
| 137327415 | 137327905 | + | 11.6 | 1 | 537 | -1043 | THE1B-int | MaLR | LTR |
| 137327908 | 137328489 | + | 11.1 | 1001 | 1580 | 0 | THE1B-int | MaLR | LTR |
| 137328495 | 137328857 | + | 13.2 | 1 | 364 | 0 | THE1B | MaLR | LTR |
| 137328858 | 137329101 | - | 11.2 | 4849 | 5090 | -1056 | L1PREC2 | L1 | LINE |
| 137329101 | 137329168 | + | 8.8 | 4768 | 4835 | -1311 | L1PREC2 | L1 | LINE |
| 137329165 | 137330737 | + | 10.9 | 6143 | 7714 | -10 | L1MA3 | L1 | LINE |

  

|  |  |  |  |  |  |  |  |  |  |
| --- | --- | --- | --- | --- | --- | --- | --- | --- | --- |
| chrX:137412454-137427880 | | | 15353 bp | | |  |  |  |  |
| Genome Start | Genome End | Strand | Div | Repeat Start | Repeat End | Repeat Left | Name | Family | Class |
| 137412454 | 137412618 | - | 29.3 | 4308 | 4485 | -4 | L3 | CR1 | LINE |
| 137412775 | 137413307 | + | 20.1 | 1 | 563 | -4 | MLT1F1 | MaLR | LTR |
| 137413309 | 137413387 | - | 32.9 | 4006 | 4088 | -401 | L3 | CR1 | LINE |
| 137413408 | 137413480 | - | 26.0 | 6047 | 6121 | -238 | L1MD2 | L1 | LINE |
| 137413579 | 137414742 | + | 23.7 | 15 | 1281 | -5364 | L1MB4 | L1 | LINE |
| 137414753 | 137416233 | - | 1.7 | 4671 | 6151 | -4 | L1PA3 | L1 | LINE |
| 137416233 | 137417438 | + | 24.0 | 911 | 2258 | -4126 | L1M4c | L1 | LINE |
| 137417439 | 137417815 | - | 14.7 | 1 | 428 | 0 | MSTA | MaLR | LTR |
| 137417816 | 137417956 | + | 24.0 | 2259 | 2429 | -3955 | L1M4c | L1 | LINE |
| 137417955 | 137418194 | - | 9.2 | 4853 | 5094 | -1052 | L1PA8A | L1 | LINE |
| 137418186 | 137419208 | + | 7.0 | 5104 | 6131 | 0 | L1PA8A | L1 | LINE |
| 137419237 | 137419707 | - | 20.6 | 4312 | 4742 | -1398 | L1MA9 | L1 | LINE |
| 137419708 | 137420076 | + | 17.2 | 4757 | 5127 | -1013 | L1MA9 | L1 | LINE |
| 137420077 | 137420381 | + | 17.4 | 1 | 305 | -7 | AluSx | Alu | SINE |
| 137420382 | 137421335 | + | 17.3 | 5128 | 6041 | -271 | L1MA9 | L1 | LINE |
| 137421336 | 137421624 | + | 12.1 | 1 | 289 | -23 | AluSx | Alu | SINE |
| 137421625 | 137421870 | + | 17.3 | 6042 | 6281 | -31 | L1MA9 | L1 | LINE |
| 137421886 | 137422009 | + | 18.2 | 2172 | 2294 | -3852 | L1M3 | L1 | LINE |
| 137422011 | 137424417 | - | 13.1 | 32 | 2418 | 0 | Tigger1 | MER2\_type | DNA |
| 137424436 | 137424595 | + | 22.1 | 2726 | 2879 | -3261 | L1M3 | L1 | LINE |
| 137424630 | 137425438 | + | 19.1 | 2726 | 3553 | -2593 | L1MC4 | L1 | LINE |
| 137425437 | 137426684 | + | 20.1 | 4960 | 6126 | -1916 | L1MC4 | L1 | LINE |
| 137426324 | 137426734 | + | 17.1 | 5759 | 6166 | -8 | L1MB5 | L1 | LINE |
| 137426742 | 137426829 | - | 12.5 | 5845 | 5932 | -427 | L1MD2 | L1 | LINE |
| 137426834 | 137427044 | - | 34.9 | 3361 | 3593 | -896 | L3 | CR1 | LINE |
| 137427060 | 137427339 | - | 18.6 | 206 | 502 | -3 | MLT1D | MaLR | LTR |
| 137427374 | 137427491 | - | 5.1 | 6031 | 6149 | -5 | L1PA7 | L1 | LINE |
| 137427492 | 137427764 | + | 7.1 | 3561 | 3833 | -2313 | L1P1 | L1 | LINE |
| 137427773 | 137427880 | - | 27.2 | 3248 | 3357 | -1132 | L3 | CR1 | LINE |

  

|  |  |  |  |  |  |  |  |  |  |
| --- | --- | --- | --- | --- | --- | --- | --- | --- | --- |
| chrX:137787250-137792241 | | | 4978 bp | | |  |  |  |  |
| Genome Start | Genome End | Strand | Div | Repeat Start | Repeat End | Repeat Left | Name | Family | Class |
| 137787250 | 137787984 | - | 20.3 | 5423 | 6180 | 0 | L1MB4 | L1 | LINE |
| 137787982 | 137788945 | - | 2.8 | 5185 | 6152 | -3 | L1PA4 | L1 | LINE |
| 137788949 | 137789330 | - | 17.9 | 5006 | 5403 | -743 | L1MB4 | L1 | LINE |
| 137789350 | 137789565 | - | 3.7 | 5926 | 6151 | -3 | L1PA7 | L1 | LINE |
| 137789566 | 137790430 | + | 7.5 | 5048 | 5913 | -241 | L1PA7 | L1 | LINE |
| 137790429 | 137791227 | - | 19.9 | 4175 | 4992 | -1154 | L1MB4 | L1 | LINE |
| 137791225 | 137792241 | - | 17.7 | 2613 | 3665 | -2481 | L1MB4 | L1 | LINE |

  

|  |  |  |  |  |  |  |  |  |  |
| --- | --- | --- | --- | --- | --- | --- | --- | --- | --- |
| chrX:137974935-137980968 | | | 6055 bp | | |  |  |  |  |
| Genome Start | Genome End | Strand | Div | Repeat Start | Repeat End | Repeat Left | Name | Family | Class |
| 137974935 | 137977615 | + | 9.0 | 1956 | 4644 | -1502 | L1PA7 | L1 | LINE |
| 137977608 | 137978666 | - | 2.5 | 4301 | 5359 | -787 | L1PA2 | L1 | LINE |
| 137978662 | 137979454 | + | 1.9 | 5363 | 6155 | 0 | L1PA2 | L1 | LINE |
| 137979447 | 137980968 | + | 7.4 | 4629 | 6153 | -1 | L1PA7 | L1 | LINE |

  

|  |  |  |  |  |  |  |  |  |  |
| --- | --- | --- | --- | --- | --- | --- | --- | --- | --- |
| chrX:138280406-138289300 | | | 8730 bp | | |  |  |  |  |
| Genome Start | Genome End | Strand | Div | Repeat Start | Repeat End | Repeat Left | Name | Family | Class |
| 138280406 | 138281949 | - | 19.3 | 5167 | 6746 | -4 | L1MD2 | L1 | LINE |
| 138281950 | 138282000 | - | 9.8 | 236 | 286 | -14 | AluYc3 | Alu | SINE |
| 138282001 | 138282734 | - | 19.3 | 4416 | 5166 | -1584 | L1MD2 | L1 | LINE |
| 138282727 | 138283026 | - | 8.6 | 5020 | 5308 | -838 | L1P3 | L1 | LINE |
| 138283027 | 138283368 | - | 1.8 | 5812 | 6154 | -1 | L1PA4 | L1 | LINE |
| 138283369 | 138286105 | - | 8.6 | 2571 | 5019 | -1127 | L1P3 | L1 | LINE |
| 138286105 | 138286918 | + | 4.4 | 5325 | 6136 | -18 | L1PA7 | L1 | LINE |
| 138287071 | 138288844 | - | 20.2 | 2639 | 4429 | -2108 | L1MD2 | L1 | LINE |
| 138288859 | 138288970 | + | 21.7 | 2983 | 3088 | -3058 | L1MD | L1 | LINE |
| 138288979 | 138289300 | - | 26.9 | 2298 | 2629 | -4004 | L1MD2 | L1 | LINE |

  

|  |  |  |  |  |  |  |  |  |  |
| --- | --- | --- | --- | --- | --- | --- | --- | --- | --- |
| chrX:138399125-138401062 | | | 1860 bp | | |  |  |  |  |
| Genome Start | Genome End | Strand | Div | Repeat Start | Repeat End | Repeat Left | Name | Family | Class |
| 138399125 | 138399215 | - | 15.4 | 334 | 428 | 0 | MSTA | MaLR | LTR |
| 138399216 | 138399336 | - | 3.3 | 4696 | 4816 | -1330 | L1PA4 | L1 | LINE |
| 138399336 | 138400630 | + | 4.2 | 4836 | 6139 | -16 | L1PA4 | L1 | LINE |
| 138400710 | 138401062 | - | 13.3 | 1 | 361 | -67 | MSTA | MaLR | LTR |

  

|  |  |  |  |  |  |  |  |  |  |
| --- | --- | --- | --- | --- | --- | --- | --- | --- | --- |
| chrX:141822478-141829463 | | | 6954 bp | | |  |  |  |  |
| Genome Start | Genome End | Strand | Div | Repeat Start | Repeat End | Repeat Left | Name | Family | Class |
| 141822478 | 141823353 | + | 15.9 | 1 | 860 | -1558 | Tigger1 | MER2\_type | DNA |
| 141823354 | 141826089 | - | 2.7 | 2262 | 5004 | -1142 | L1PA3 | L1 | LINE |
| 141826090 | 141827231 | + | 2.9 | 5021 | 6152 | -3 | L1PA3 | L1 | LINE |
| 141827264 | 141827406 | + | 14.8 | 842 | 991 | -1427 | Tigger1 | MER2\_type | DNA |
| 141827407 | 141827723 | + | 11.9 | 1 | 314 | 0 | AluSp | Alu | SINE |
| 141827724 | 141827752 | + | 11.9 | 283 | 310 | -3 | AluSp | Alu | SINE |
| 141827753 | 141828960 | + | 14.8 | 992 | 2203 | -215 | Tigger1 | MER2\_type | DNA |
| 141828961 | 141829266 | - | 12.9 | 1 | 302 | -11 | AluSq | Alu | SINE |
| 141829267 | 141829463 | + | 14.8 | 2204 | 2416 | -2 | Tigger1 | MER2\_type | DNA |

  

|  |  |  |  |  |  |  |  |  |  |
| --- | --- | --- | --- | --- | --- | --- | --- | --- | --- |
| chrX:142126042-142128957 | | | 2903 bp | | |  |  |  |  |
| Genome Start | Genome End | Strand | Div | Repeat Start | Repeat End | Repeat Left | Name | Family | Class |
| 142126042 | 142126177 | - | 11.0 | 6016 | 6151 | 0 | L1PB1 | L1 | LINE |
| 142126180 | 142126736 | - | 3.8 | 5591 | 6146 | -8 | L1PA5 | L1 | LINE |
| 142126738 | 142126784 | + | 6.4 | 5548 | 5594 | -560 | L1PA5 | L1 | LINE |
| 142126780 | 142127200 | - | 14.5 | 5693 | 6035 | -116 | L1PB1 | L1 | LINE |
| 142127216 | 142128957 | - | 9.7 | 3039 | 4784 | -1362 | L1PB1 | L1 | LINE |

  

|  |  |  |  |  |  |  |  |  |  |
| --- | --- | --- | --- | --- | --- | --- | --- | --- | --- |
| chrX:142614915-142617794 | | | 2842 bp | | |  |  |  |  |
| Genome Start | Genome End | Strand | Div | Repeat Start | Repeat End | Repeat Left | Name | Family | Class |
| 142614915 | 142614980 | - | 21.2 | 4995 | 5048 | -1092 | L1M3 | L1 | LINE |
| 142614978 | 142615200 | - | 10.8 | 5593 | 5822 | -346 | L1PA12 | L1 | LINE |
| 142615201 | 142615528 | + | 13.6 | 5826 | 6150 | -18 | L1PA12 | L1 | LINE |
| 142615539 | 142615746 | - | 19.6 | 4799 | 5006 | -1137 | L1M3 | L1 | LINE |
| 142615776 | 142615924 | - | 19.6 | 4634 | 4798 | -1345 | L1M3 | L1 | LINE |
| 142615925 | 142616318 | + | 28.0 | 1 | 805 | -1318 | MER52D | ERV1 | LTR |
| 142616327 | 142617316 | + | 21.1 | 634 | 1538 | -585 | MER52D | ERV1 | LTR |
| 142617307 | 142617380 | + | 31.1 | 1682 | 1755 | 0 | MER52A | ERV1 | LTR |
| 142617385 | 142617794 | - | 20.5 | 4201 | 4629 | -1511 | L1M3 | L1 | LINE |

  

|  |  |  |  |  |  |  |  |  |  |
| --- | --- | --- | --- | --- | --- | --- | --- | --- | --- |
| chrX:142746578-142749658 | | | 3082 bp | | |  |  |  |  |
| Genome Start | Genome End | Strand | Div | Repeat Start | Repeat End | Repeat Left | Name | Family | Class |
| 142746578 | 142746918 | - | 21.2 | 4986 | 5332 | -808 | L1M3 | L1 | LINE |
| 142746910 | 142747143 | - | 9.6 | 5593 | 5823 | -345 | L1PA12 | L1 | LINE |
| 142747144 | 142747470 | + | 11.9 | 5826 | 6168 | 0 | L1PA12 | L1 | LINE |
| 142747475 | 142747840 | - | 16.9 | 4630 | 5003 | -1140 | L1M3 | L1 | LINE |
| 142747841 | 142749235 | + | 20.9 | 1 | 1538 | 0 | MER52D | ERV1 | LTR |
| 142749240 | 142749658 | - | 21.2 | 4188 | 4628 | -1512 | L1M3 | L1 | LINE |

  

|  |  |  |  |  |  |  |  |  |  |
| --- | --- | --- | --- | --- | --- | --- | --- | --- | --- |
| chrX:143366952-143369299 | | | 1973 bp | | |  |  |  |  |
| Genome Start | Genome End | Strand | Div | Repeat Start | Repeat End | Repeat Left | Name | Family | Class |
| 143366952 | 143367058 | - | 25.5 | 4943 | 5055 | -1091 | L1M5 | L1 | LINE |
| 143367265 | 143367349 | - | 24.7 | 2322 | 2406 | 0 | Tigger5 | MER2\_type | DNA |
| 143367364 | 143367447 | + | 22.6 | 2 | 88 | -2235 | Tigger5 | MER2\_type | DNA |
| 143367469 | 143367530 | - | 19.4 | 2338 | 2400 | 0 | Tigger5 | MER2\_type | DNA |
| 143367531 | 143367944 | - | 6.5 | 5355 | 5768 | -406 | L1PA11 | L1 | LINE |
| 143367945 | 143368345 | + | 10.8 | 5768 | 6173 | -1 | L1PA11 | L1 | LINE |
| 143368428 | 143368591 | - | 25.8 | 2 | 187 | -75 | MIR | MIR | SINE |
| 143368644 | 143369299 | - | 26.7 | 3548 | 4163 | -1983 | L1M5 | L1 | LINE |

  

|  |  |  |  |  |  |  |  |  |  |
| --- | --- | --- | --- | --- | --- | --- | --- | --- | --- |
| chrX:143445868-143448162 | | | 2249 bp | | |  |  |  |  |
| Genome Start | Genome End | Strand | Div | Repeat Start | Repeat End | Repeat Left | Name | Family | Class |
| 143445868 | 143446243 | - | 18.4 | 5912 | 6296 | -4 | L1MA5 | L1 | LINE |
| 143446278 | 143446474 | - | 12.7 | 5388 | 5585 | -715 | L1MA5 | L1 | LINE |
| 143446483 | 143446761 | - | 20.8 | 49 | 584 | -42 | MLT1A0 | MaLR | LTR |
| 143446763 | 143447490 | - | 13.5 | 5430 | 6163 | 0 | L1PA13 | L1 | LINE |
| 143447491 | 143448019 | + | 13.5 | 4889 | 5427 | -733 | L1PREC2 | L1 | LINE |
| 143448023 | 143448162 | - | 20.7 | 5239 | 5388 | -755 | L1MA5 | L1 | LINE |

  

|  |  |  |  |  |  |  |  |  |  |
| --- | --- | --- | --- | --- | --- | --- | --- | --- | --- |
| chrX:144210092-144214220 | | | 4069 bp | | |  |  |  |  |
| Genome Start | Genome End | Strand | Div | Repeat Start | Repeat End | Repeat Left | Name | Family | Class |
| 144210092 | 144210320 | - | 27.5 | 1629 | 1862 | -4399 | L1MEb | L1 | LINE |
| 144210380 | 144212562 | - | 5.3 | 3948 | 6136 | -18 | L1PA5 | L1 | LINE |
| 144212562 | 144212777 | + | 5.1 | 3736 | 3951 | -2195 | L1PA5 | L1 | LINE |
| 144212780 | 144214220 | - | 28.9 | 21 | 1521 | -4655 | L1MEb | L1 | LINE |

  

|  |  |  |  |  |  |  |  |  |  |
| --- | --- | --- | --- | --- | --- | --- | --- | --- | --- |
| chrX:144998981-145004009 | | | 4579 bp | | |  |  |  |  |
| Genome Start | Genome End | Strand | Div | Repeat Start | Repeat End | Repeat Left | Name | Family | Class |
| 144998981 | 144999242 | - | 16.2 | 6117 | 6392 | -973 | L1M | L1 | LINE |
| 144999243 | 144999548 | - | 7.3 | 1 | 303 | -8 | AluY | Alu | SINE |
| 144999549 | 145000105 | - | 17.6 | 5567 | 6116 | -1089 | L1M | L1 | LINE |
| 145000528 | 145001485 | - | 1.0 | 5198 | 6155 | 0 | L1PA2 | L1 | LINE |
| 145001486 | 145002020 | + | 0.6 | 4668 | 5202 | -944 | L1PA2 | L1 | LINE |
| 145002013 | 145002963 | - | 19.3 | 4600 | 5583 | -1622 | L1M | L1 | LINE |
| 145002989 | 145003204 | - | 31.6 | 1153 | 1367 | -5842 | L1M | L1 | LINE |
| 145003216 | 145004009 | - | 23.8 | 1 | 835 | -6374 | L1M | L1 | LINE |

  

|  |  |  |  |  |  |  |  |  |  |
| --- | --- | --- | --- | --- | --- | --- | --- | --- | --- |
| chrX:146294199-146302723 | | | 8496 bp | | |  |  |  |  |
| Genome Start | Genome End | Strand | Div | Repeat Start | Repeat End | Repeat Left | Name | Family | Class |
| 146294199 | 146298042 | + | 11.6 | 10 | 5717 | -744 | L1PA8 | L1 | LINE |
| 146298022 | 146300185 | - | 1.2 | 2292 | 4453 | -1693 | L1PA2 | L1 | LINE |
| 146300186 | 146301887 | + | 1.2 | 4455 | 6155 | 0 | L1PA2 | L1 | LINE |
| 146301938 | 146302723 | + | 7.1 | 5701 | 6490 | 0 | L1PA8 | L1 | LINE |

  

|  |  |  |  |  |  |  |  |  |  |
| --- | --- | --- | --- | --- | --- | --- | --- | --- | --- |
| chrX:146343934-146351552 | | | 7477 bp | | |  |  |  |  |
| Genome Start | Genome End | Strand | Div | Repeat Start | Repeat End | Repeat Left | Name | Family | Class |
| 146343934 | 146344283 | + | 22.5 | 1950 | 2312 | -4088 | L1ME1 | L1 | LINE |
| 146344284 | 146344568 | - | 12.6 | 2 | 288 | -25 | AluSq | Alu | SINE |
| 146344569 | 146344810 | + | 24.0 | 2209 | 2481 | -3756 | L1MEa | L1 | LINE |
| 146344811 | 146345256 | - | 8.7 | 5726 | 6172 | 0 | L1PA8 | L1 | LINE |
| 146345257 | 146345888 | + | 26.7 | 2464 | 3206 | -3013 | L1ME1 | L1 | LINE |
| 146345864 | 146346314 | + | 27.0 | 2577 | 3040 | -3106 | L1MD | L1 | LINE |
| 146346312 | 146347566 | + | 14.4 | 4909 | 6157 | -9 | L1PA16 | L1 | LINE |
| 146347732 | 146349307 | + | 25.8 | 3103 | 4817 | -1402 | L1ME1 | L1 | LINE |
| 146349324 | 146350109 | - | 3.4 | 5362 | 6146 | -8 | L1PA5 | L1 | LINE |
| 146350101 | 146350487 | + | 4.9 | 4977 | 5367 | -779 | L1PA5 | L1 | LINE |
| 146350486 | 146351552 | + | 27.4 | 4803 | 6121 | -118 | L1ME1 | L1 | LINE |

  

|  |  |  |  |  |  |  |  |  |  |
| --- | --- | --- | --- | --- | --- | --- | --- | --- | --- |
| chrX:146622190-146622991 | | | 712 bp | | |  |  |  |  |
| Genome Start | Genome End | Strand | Div | Repeat Start | Repeat End | Repeat Left | Name | Family | Class |
| 146622190 | 146622338 | - | 34.9 | 263 | 412 | -38 | MLT1J2 | MaLR | LTR |
| 146622391 | 146622652 | - | 18.0 | 5881 | 6138 | -51 | L1MB1 | L1 | LINE |
| 146622654 | 146622857 | + | 15.3 | 6122 | 6333 | -1 | L1MA10 | L1 | LINE |
| 146622895 | 146622991 | - | 23.6 | 59 | 156 | -294 | MLT1J2 | MaLR | LTR |

  

|  |  |  |  |  |  |  |  |  |  |
| --- | --- | --- | --- | --- | --- | --- | --- | --- | --- |
| chrX:146752206-146753971 | | | 1761 bp | | |  |  |  |  |
| Genome Start | Genome End | Strand | Div | Repeat Start | Repeat End | Repeat Left | Name | Family | Class |
| 146752206 | 146752802 | + | 28.3 | 3 | 705 | -1802 | HAL1 | L1 | LINE |
| 146752803 | 146753179 | + | 16.0 | 1 | 390 | 0 | MLT1B | MaLR | LTR |
| 146753180 | 146753318 | + | 28.3 | 706 | 843 | -1664 | HAL1 | L1 | LINE |
| 146753316 | 146753671 | - | 2.9 | 5628 | 5968 | -187 | L1PA4 | L1 | LINE |
| 146753673 | 146753839 | + | 3.6 | 5986 | 6152 | -3 | L1PA4 | L1 | LINE |
| 146753847 | 146753971 | + | 26.4 | 826 | 954 | -1553 | HAL1 | L1 | LINE |

  

|  |  |  |  |  |  |  |  |  |  |
| --- | --- | --- | --- | --- | --- | --- | --- | --- | --- |
| chrX:146905433-146910719 | | | 5267 bp | | |  |  |  |  |
| Genome Start | Genome End | Strand | Div | Repeat Start | Repeat End | Repeat Left | Name | Family | Class |
| 146905433 | 146906741 | - | 12.0 | 2486 | 3795 | -3770 | L1M1 | L1 | LINE |
| 146906770 | 146907575 | - | 1.5 | 5332 | 6152 | -3 | L1PA3 | L1 | LINE |
| 146907573 | 146908245 | + | 3.1 | 4678 | 5350 | -796 | L1PA3 | L1 | LINE |
| 146908241 | 146910719 | - | 15.9 | 17 | 2504 | -5061 | L1M1 | L1 | LINE |

  

|  |  |  |  |  |  |  |  |  |  |
| --- | --- | --- | --- | --- | --- | --- | --- | --- | --- |
| chrX:147027722-147031801 | | | 4047 bp | | |  |  |  |  |
| Genome Start | Genome End | Strand | Div | Repeat Start | Repeat End | Repeat Left | Name | Family | Class |
| 147027722 | 147027829 | + | 18.5 | 1 | 126 | -2486 | Charlie5 | MER1\_type | DNA |
| 147027828 | 147027987 | + | 14.4 | 2234 | 2402 | -210 | Charlie5 | MER1\_type | DNA |
| 147027994 | 147028705 | + | 7.7 | 4780 | 5488 | -686 | L1PA11 | L1 | LINE |
| 147028702 | 147030052 | - | 7.7 | 3988 | 5350 | -796 | L1PA7 | L1 | LINE |
| 147030051 | 147030837 | + | 5.8 | 5355 | 6141 | -13 | L1PA7 | L1 | LINE |
| 147030842 | 147031148 | + | 10.3 | 5476 | 5781 | -393 | L1PA11 | L1 | LINE |
| 147031179 | 147031564 | + | 10.3 | 5782 | 6170 | -4 | L1PA11 | L1 | LINE |
| 147031566 | 147031801 | + | 24.6 | 2353 | 2585 | -27 | Charlie5 | MER1\_type | DNA |

  

|  |  |  |  |  |  |  |  |  |  |
| --- | --- | --- | --- | --- | --- | --- | --- | --- | --- |
| chrX:147448395-147449982 | | | 1577 bp | | |  |  |  |  |
| Genome Start | Genome End | Strand | Div | Repeat Start | Repeat End | Repeat Left | Name | Family | Class |
| 147448395 | 147448482 | - | 18.4 | 266 | 352 | -65 | MER96B | MER1\_type? | DNA |
| 147448491 | 147449352 | - | 4.7 | 5293 | 6151 | -3 | L1PA7 | L1 | LINE |
| 147449350 | 147449895 | + | 8.1 | 4731 | 5290 | -856 | L1PA7 | L1 | LINE |
| 147449902 | 147449982 | - | 10.4 | 191 | 267 | -150 | MER96B | MER1\_type? | DNA |

  

|  |  |  |  |  |  |  |  |  |  |
| --- | --- | --- | --- | --- | --- | --- | --- | --- | --- |
| chrX:148940671-148942192 | | | 1486 bp | | |  |  |  |  |
| Genome Start | Genome End | Strand | Div | Repeat Start | Repeat End | Repeat Left | Name | Family | Class |
| 148940671 | 148940757 | + | 24.7 | 1 | 85 | -868 | MER45C | Tip100 | DNA |
| 148940791 | 148940955 | - | 4.2 | 5970 | 6137 | -18 | L1PA2 | L1 | LINE |
| 148940956 | 148941502 | + | 0.9 | 5445 | 5991 | -164 | L1PA2 | L1 | LINE |
| 148941506 | 148942192 | + | 31.1 | 77 | 778 | -175 | MER45C | Tip100 | DNA |

  

|  |  |  |  |  |  |  |  |  |  |
| --- | --- | --- | --- | --- | --- | --- | --- | --- | --- |
| chrX:149049123-149054859 | | | 5376 bp | | |  |  |  |  |
| Genome Start | Genome End | Strand | Div | Repeat Start | Repeat End | Repeat Left | Name | Family | Class |
| 149049123 | 149049192 | + | 27.4 | 326 | 395 | -6238 | L1MDa | L1 | LINE |
| 149049222 | 149050221 | + | 27.4 | 396 | 1446 | -5187 | L1MDa | L1 | LINE |
| 149050297 | 149051993 | - | 1.9 | 4459 | 6155 | 0 | L1PA2 | L1 | LINE |
| 149051989 | 149053703 | + | 1.8 | 2729 | 4443 | -1703 | L1PA2 | L1 | LINE |
| 149053944 | 149054412 | + | 17.4 | 1449 | 1972 | -4661 | L1MDa | L1 | LINE |
| 149054435 | 149054859 | + | 17.4 | 1973 | 2409 | -4224 | L1MDa | L1 | LINE |

  

|  |  |  |  |  |  |  |  |  |  |
| --- | --- | --- | --- | --- | --- | --- | --- | --- | --- |
| chrX:150109934-150128770 | | | 18739 bp | | |  |  |  |  |
| Genome Start | Genome End | Strand | Div | Repeat Start | Repeat End | Repeat Left | Name | Family | Class |
| 150109934 | 150112312 | + | 15.9 | 14 | 2657 | -4908 | L1M1 | L1 | LINE |
| 150112313 | 150112446 | - | 15.0 | 1 | 133 | -10 | FLAM\_C | Alu | SINE |
| 150112447 | 150112744 | + | 15.9 | 2658 | 2988 | -4577 | L1M1 | L1 | LINE |
| 150112745 | 150113575 | + | 11.4 | 5472 | 6305 | 0 | L1MA3 | L1 | LINE |
| 150113576 | 150113968 | + | 15.9 | 2991 | 3349 | -4216 | L1M1 | L1 | LINE |
| 150113964 | 150114260 | + | 12.3 | 4799 | 5090 | -1056 | L1PREC2 | L1 | LINE |
| 150114313 | 150115606 | - | 15.0 | 4990 | 6293 | -7 | L1MA4 | L1 | LINE |
| 150115619 | 150116229 | - | 8.4 | 5688 | 6305 | 0 | L1MA3 | L1 | LINE |
| 150116232 | 150116579 | + | 12.9 | 5333 | 5682 | -623 | L1MA3 | L1 | LINE |
| 150116571 | 150117620 | + | 9.4 | 5070 | 6152 | -11 | L1PA13 | L1 | LINE |
| 150117680 | 150118953 | + | 10.9 | 3331 | 4596 | -2969 | L1M1 | L1 | LINE |
| 150118957 | 150125125 | + | 6.5 | 1 | 6154 | 0 | L1PA5 | L1 | LINE |
| 150125110 | 150125504 | + | 15.4 | 4563 | 4973 | -2592 | L1M1 | L1 | LINE |
| 150125506 | 150126331 | - | 13.9 | 5332 | 6163 | -3 | L1PA16 | L1 | LINE |
| 150126332 | 150126630 | - | 9.7 | 1 | 299 | -13 | AluSx | Alu | SINE |
| 150126631 | 150128313 | - | 11.5 | 3663 | 5331 | -815 | L1P | L1 | LINE |
| 150128313 | 150128770 | + | 13.7 | 4966 | 5426 | -2139 | L1M1 | L1 | LINE |

  

|  |  |  |  |  |  |  |  |  |  |
| --- | --- | --- | --- | --- | --- | --- | --- | --- | --- |
| chrX:150976707-150979978 | | | 3260 bp | | |  |  |  |  |
| Genome Start | Genome End | Strand | Div | Repeat Start | Repeat End | Repeat Left | Name | Family | Class |
| 150976707 | 150976740 | - | 8.8 | 5317 | 5350 | -816 | L1P | L1 | LINE |
| 150976753 | 150977484 | - | 2.7 | 5415 | 6147 | -8 | L1PA4 | L1 | LINE |
| 150977486 | 150979150 | + | 3.5 | 3730 | 5394 | -752 | L1PA4 | L1 | LINE |
| 150979150 | 150979978 | - | 9.2 | 4491 | 5331 | -815 | L1P | L1 | LINE |

  

|  |  |  |  |  |  |  |  |  |  |
| --- | --- | --- | --- | --- | --- | --- | --- | --- | --- |
| chrX:151286636-151289011 | | | 2248 bp | | |  |  |  |  |
| Genome Start | Genome End | Strand | Div | Repeat Start | Repeat End | Repeat Left | Name | Family | Class |
| 151286636 | 151286710 | + | 25.3 | 2313 | 2389 | -1030 | L2 | L2 | LINE |
| 151286812 | 151287279 | + | 29.1 | 2410 | 2867 | -552 | L2 | L2 | LINE |
| 151287290 | 151287821 | - | 13.6 | 5599 | 6130 | -161 | L1MA8 | L1 | LINE |
| 151287822 | 151288132 | - | 5.5 | 1 | 311 | 0 | AluY | Alu | SINE |
| 151288133 | 151288356 | - | 13.6 | 5360 | 5598 | -693 | L1MA8 | L1 | LINE |
| 151288358 | 151288509 | + | 18.0 | 6148 | 6301 | 0 | L1MA8 | L1 | LINE |
| 151288526 | 151289011 | + | 28.0 | 2910 | 3419 | 0 | L2 | L2 | LINE |

  

|  |  |  |  |  |  |  |  |  |  |
| --- | --- | --- | --- | --- | --- | --- | --- | --- | --- |
| chrX:151302547-151306673 | | | 4137 bp | | |  |  |  |  |
| Genome Start | Genome End | Strand | Div | Repeat Start | Repeat End | Repeat Left | Name | Family | Class |
| 151302547 | 151304640 | + | 23.3 | 3684 | 5898 | -342 | L1ME3B | L1 | LINE |
| 151304638 | 151305507 | - | 2.3 | 4367 | 5236 | -910 | L1PA3 | L1 | LINE |
| 151305507 | 151306437 | + | 2.1 | 5223 | 6154 | -1 | L1PA3 | L1 | LINE |
| 151306432 | 151306673 | + | 27.7 | 5879 | 6169 | -71 | L1ME3B | L1 | LINE |

  

|  |  |  |  |  |  |  |  |  |  |
| --- | --- | --- | --- | --- | --- | --- | --- | --- | --- |
| chrX:151377799-151382988 | | | 5185 bp | | |  |  |  |  |
| Genome Start | Genome End | Strand | Div | Repeat Start | Repeat End | Repeat Left | Name | Family | Class |
| 151377799 | 151378475 | + | 28.3 | 1674 | 2370 | -4014 | L1MCa | L1 | LINE |
| 151378476 | 151378589 | - | 4.4 | 242 | 355 | 0 | THE1A | MaLR | LTR |
| 151378566 | 151378644 | + | 15.2 | 85 | 163 | -192 | THE1A | MaLR | LTR |
| 151378645 | 151379202 | + | 16.3 | 3214 | 3789 | -2587 | L1MCa | L1 | LINE |
| 151379203 | 151379500 | - | 9.5 | 1 | 295 | -16 | AluY | Alu | SINE |
| 151379501 | 151379736 | + | 16.3 | 3790 | 4018 | -2358 | L1MCa | L1 | LINE |
| 151379737 | 151380032 | + | 12.8 | 1 | 296 | -16 | AluSx | Alu | SINE |
| 151380071 | 151380284 | - | 3.7 | 5077 | 5290 | -856 | L1PA10 | L1 | LINE |
| 151380280 | 151381130 | + | 8.2 | 5311 | 6165 | -3 | L1PA10 | L1 | LINE |
| 151381127 | 151381288 | + | 17.9 | 4005 | 4186 | -2190 | L1MCa | L1 | LINE |
| 151381289 | 151381582 | - | 4.8 | 5860 | 6153 | -1 | L1PA7 | L1 | LINE |
| 151381583 | 151382988 | + | 17.6 | 4187 | 5625 | -902 | L1MCa | L1 | LINE |

  

|  |  |  |  |  |  |  |  |  |  |
| --- | --- | --- | --- | --- | --- | --- | --- | --- | --- |
| chrX:151407216-151417091 | | | 9721 bp | | |  |  |  |  |
| Genome Start | Genome End | Strand | Div | Repeat Start | Repeat End | Repeat Left | Name | Family | Class |
| 151407216 | 151408037 | + | 25.5 | 1049 | 1921 | -1498 | L2 | L2 | LINE |
| 151408042 | 151408693 | - | 17.4 | 5613 | 6311 | -1 | L1MA9 | L1 | LINE |
| 151408690 | 151408838 | - | 20.1 | 1020 | 1189 | -4776 | L1M3c | L1 | LINE |
| 151408841 | 151409050 | + | 13.2 | 1 | 210 | -165 | THE1C | MaLR | LTR |
| 151409078 | 151410006 | + | 2.2 | 5217 | 6144 | -11 | L1PA4 | L1 | LINE |
| 151410007 | 151410160 | + | 15.6 | 221 | 375 | 0 | THE1C | MaLR | LTR |
| 151410161 | 151410589 | + | 9.8 | 1 | 375 | -1205 | THE1C-int | MaLR | LTR |
| 151410599 | 151412050 | - | 8.1 | 4705 | 6151 | 0 | L1PB1 | L1 | LINE |
| 151412044 | 151412756 | + | 7.4 | 3983 | 4691 | -1455 | L1PB1 | L1 | LINE |
| 151412757 | 151413938 | + | 11.4 | 359 | 1578 | -2 | THE1C-int | MaLR | LTR |
| 151413941 | 151414315 | + | 11.9 | 1 | 375 | 0 | THE1C | MaLR | LTR |
| 151414316 | 151414887 | - | 28.2 | 450 | 1015 | -4940 | L1M3c | L1 | LINE |
| 151414888 | 151415241 | - | 11.0 | 1 | 364 | 0 | THE1B | MaLR | LTR |
| 151415242 | 151415785 | - | 29.2 | 12 | 771 | -5506 | L1M3a | L1 | LINE |
| 151415908 | 151417091 | + | 29.6 | 2057 | 3402 | -17 | L2 | L2 | LINE |

  

|  |  |  |  |  |  |  |  |  |  |
| --- | --- | --- | --- | --- | --- | --- | --- | --- | --- |
| chrX:151420529-151423427 | | | 2870 bp | | |  |  |  |  |
| Genome Start | Genome End | Strand | Div | Repeat Start | Repeat End | Repeat Left | Name | Family | Class |
| 151420529 | 151420662 | + | 32.3 | 17 | 149 | -113 | MIR | MIR | SINE |
| 151420666 | 151420730 | - | 7.7 | 364 | 428 | 0 | MSTB2 | MaLR | LTR |
| 151420734 | 151421050 | - | 4.4 | 4687 | 5003 | -1143 | L1PA5 | L1 | LINE |
| 151421051 | 151422235 | + | 3.7 | 5003 | 6152 | -2 | L1PA5 | L1 | LINE |
| 151422236 | 151422636 | - | 15.6 | 1 | 402 | -55 | MSTB2 | MaLR | LTR |
| 151422637 | 151423315 | - | 20.6 | 920 | 1651 | 0 | MST-int | MaLR | LTR |
| 151423339 | 151423427 | + | 24.1 | 175 | 262 | 0 | MIR | MIR | SINE |

  

|  |  |  |  |  |  |  |  |  |  |
| --- | --- | --- | --- | --- | --- | --- | --- | --- | --- |
| chrX:154412130-154415129 | | | 2781 bp | | |  |  |  |  |
| Genome Start | Genome End | Strand | Div | Repeat Start | Repeat End | Repeat Left | Name | Family | Class |
| 154412130 | 154412249 | + | 21.4 | 30 | 157 | -7177 | L1M4b | L1 | LINE |
| 154412203 | 154412917 | - | 14.4 | 4934 | 5656 | -703 | L1MA9 | L1 | LINE |
| 154412918 | 154413542 | + | 19.9 | 5635 | 6274 | -38 | L1MA9 | L1 | LINE |
| 154413561 | 154413714 | + | 30.8 | 146 | 293 | -7041 | L1M4b | L1 | LINE |
| 154413816 | 154414072 | + | 29.8 | 879 | 1103 | -5801 | L1M4b | L1 | LINE |
| 154414073 | 154414467 | - | 15.5 | 1 | 423 | -3 | MSTB | MaLR | LTR |
| 154414468 | 154414598 | + | 29.8 | 1104 | 1237 | -5667 | L1M4b | L1 | LINE |
| 154414675 | 154414779 | - | 17.1 | 1 | 106 | -113 | MER20 | MER1\_type | DNA |
| 154414851 | 154415129 | + | 21.4 | 1206 | 1460 | -5874 | L1M4b | L1 | LINE |

  

|  |  |  |  |  |  |  |  |  |  |
| --- | --- | --- | --- | --- | --- | --- | --- | --- | --- |
| chrX:154425503-154427645 | | | 2023 bp | | |  |  |  |  |
| Genome Start | Genome End | Strand | Div | Repeat Start | Repeat End | Repeat Left | Name | Family | Class |
| 154425503 | 154425785 | - | 20.2 | 1625 | 1908 | -4843 | L1MCa | L1 | LINE |
| 154425777 | 154425790 | - | 21.1 | 4821 | 4833 | -1313 | L1PB | L1 | LINE |
| 154425811 | 154425963 | - | 12.1 | 4662 | 4820 | -1326 | L1M2 | L1 | LINE |
| 154425964 | 154426536 | + | 15.2 | 4045 | 4646 | -1497 | L1M2 | L1 | LINE |
| 154426532 | 154427036 | - | 25.4 | 558 | 1063 | -4982 | L1MCa | L1 | LINE |
| 154427042 | 154427468 | + | 20.3 | 1 | 467 | 0 | MLT1C | MaLR | LTR |
| 154427578 | 154427645 | - | 16.7 | 498 | 565 | -5480 | L1MCa | L1 | LINE |

  

|  |  |  |  |  |  |  |  |  |  |
| --- | --- | --- | --- | --- | --- | --- | --- | --- | --- |
| chrX:154509803-154512857 | | | 3063 bp | | |  |  |  |  |
| Genome Start | Genome End | Strand | Div | Repeat Start | Repeat End | Repeat Left | Name | Family | Class |
| 154509803 | 154510512 | - | 14.2 | 5593 | 6289 | -2 | L1MA8 | L1 | LINE |
| 154510507 | 154511156 | - | 7.7 | 5511 | 6167 | -1 | L1PA10 | L1 | LINE |
| 154511157 | 154511544 | + | 6.7 | 5109 | 5509 | -622 | L1PA10 | L1 | LINE |
| 154511543 | 154511871 | - | 12.6 | 5296 | 5608 | -683 | L1MA8 | L1 | LINE |
| 154511872 | 154512607 | - | 4.3 | 5409 | 6154 | 0 | L1PA5 | L1 | LINE |
| 154512608 | 154512857 | - | 10.6 | 5020 | 5328 | -815 | L1MA8 | L1 | LINE |

  


|  |  |  |  |  |  |  |  |  |  |
| --- | --- | --- | --- | --- | --- | --- | --- | --- | --- |
| chrY:3197689-3201427 | | | 3745 bp | | |  |  |  |  |
| Genome Start | Genome End | Strand | Div | Repeat Start | Repeat End | Repeat Left | Name | Family | Class |
| 3197689 | 3197976 | + | 16.3 | 2639 | 2941 | -3205 | L1MC | L1 | LINE |
| 3197970 | 3198495 | - | 10.6 | 5145 | 5680 | -481 | L1PA14 | L1 | LINE |
| 3198496 | 3198984 | + | 10.3 | 5671 | 6159 | -2 | L1PA14 | L1 | LINE |
| 3198986 | 3199083 | + | 18.4 | 2928 | 3025 | -3121 | L1MC | L1 | LINE |
| 3199084 | 3199339 | - | 21.6 | 1 | 236 | 0 | MER46A | MER2\_type | DNA |
| 3199340 | 3199800 | + | 18.4 | 3026 | 3467 | -2679 | L1MC | L1 | LINE |
| 3199801 | 3200152 | - | 14.2 | 4 | 375 | 0 | THE1C | MaLR | LTR |
| 3200153 | 3201427 | + | 18.4 | 3468 | 4677 | -1469 | L1MC | L1 | LINE |

  

|  |  |  |  |  |  |  |  |  |  |
| --- | --- | --- | --- | --- | --- | --- | --- | --- | --- |
| chrY:3225158-3234363 | | | 9213 bp | | |  |  |  |  |
| Genome Start | Genome End | Strand | Div | Repeat Start | Repeat End | Repeat Left | Name | Family | Class |
| 3225158 | 3226548 | + | 18.1 | 16 | 1458 | -6107 | L1M1 | L1 | LINE |
| 3226550 | 3227382 | - | 4.7 | 5326 | 6154 | 0 | L1PA5 | L1 | LINE |
| 3227379 | 3227981 | + | 7.0 | 4718 | 5317 | -829 | L1PA5 | L1 | LINE |
| 3227978 | 3230387 | + | 15.0 | 1444 | 3930 | -3635 | L1M1 | L1 | LINE |
| 3230388 | 3230921 | + | 10.8 | 1 | 453 | 0 | MLT2A2 | ERVL | LTR |
| 3230922 | 3232166 | + | 17.3 | 3931 | 5190 | -2375 | L1M1 | L1 | LINE |
| 3232167 | 3232469 | + | 8.2 | 5851 | 6154 | 0 | L1PA7 | L1 | LINE |
| 3232470 | 3234363 | + | 17.1 | 5191 | 6889 | -696 | L1M1 | L1 | LINE |

  

|  |  |  |  |  |  |  |  |  |  |
| --- | --- | --- | --- | --- | --- | --- | --- | --- | --- |
| chrY:4443056-4446090 | | | 3028 bp | | |  |  |  |  |
| Genome Start | Genome End | Strand | Div | Repeat Start | Repeat End | Repeat Left | Name | Family | Class |
| 4443056 | 4443367 | - | 10.6 | 1275 | 1579 | -1 | THE1D-int | MaLR | LTR |
| 4443376 | 4444831 | - | 12.8 | 4703 | 6173 | -1 | L1PA11 | L1 | LINE |
| 4444830 | 4445682 | + | 10.7 | 3835 | 4689 | -1457 | L1PA11 | L1 | LINE |
| 4445684 | 4446090 | - | 16.0 | 867 | 1277 | -303 | THE1D-int | MaLR | LTR |

  

|  |  |  |  |  |  |  |  |  |  |
| --- | --- | --- | --- | --- | --- | --- | --- | --- | --- |
| chrY:4665994-4667114 | | | 1028 bp | | |  |  |  |  |
| Genome Start | Genome End | Strand | Div | Repeat Start | Repeat End | Repeat Left | Name | Family | Class |
| 4665994 | 4666103 | + | 22.2 | 1 | 116 | -349 | MER4C | ERV1 | LTR |
| 4666174 | 4666437 | + | 24.2 | 136 | 455 | -244 | LTR34 | ERV1 | LTR |
| 4666438 | 4666548 | + | 16.4 | 278 | 496 | -203 | LTR34 | ERV1 | LTR |
| 4666549 | 4666616 | - | 8.8 | 5768 | 5838 | -330 | L1PA13 | L1 | LINE |
| 4666621 | 4666912 | + | 10.6 | 5860 | 6152 | -11 | L1PA13 | L1 | LINE |
| 4666932 | 4667114 | + | 25.1 | 275 | 461 | -4 | MER4C | ERV1 | LTR |

  

|  |  |  |  |  |  |  |  |  |  |
| --- | --- | --- | --- | --- | --- | --- | --- | --- | --- |
| chrY:5124464-5126514 | | | 2050 bp | | |  |  |  |  |
| Genome Start | Genome End | Strand | Div | Repeat Start | Repeat End | Repeat Left | Name | Family | Class |
| 5124464 | 5124612 | + | 16.1 | 5968 | 6124 | -167 | L1MA8 | L1 | LINE |
| 5124606 | 5125432 | - | 0.9 | 4741 | 5567 | -588 | L1PA2 | L1 | LINE |
| 5125433 | 5126030 | + | 1.2 | 5555 | 6152 | -3 | L1PA2 | L1 | LINE |
| 5126039 | 5126184 | + | 11.5 | 6116 | 6265 | -26 | L1MA8 | L1 | LINE |
| 5126185 | 5126488 | - | 8.9 | 1 | 304 | -8 | AluSx | Alu | SINE |
| 5126489 | 5126514 | + | 11.5 | 6266 | 6286 | -5 | L1MA8 | L1 | LINE |

  

|  |  |  |  |  |  |  |  |  |  |
| --- | --- | --- | --- | --- | --- | --- | --- | --- | --- |
| chrY:7320982-7328691 | | | 7635 bp | | |  |  |  |  |
| Genome Start | Genome End | Strand | Div | Repeat Start | Repeat End | Repeat Left | Name | Family | Class |
| 7320982 | 7322460 | + | 27.8 | 384 | 1928 | -5809 | HERVIP10FH | ERV1 | LTR |
| 7322547 | 7323590 | + | 26.6 | 2083 | 3111 | -4626 | HERVIP10FH | ERV1 | LTR |
| 7323589 | 7323701 | + | 24.8 | 1213 | 1325 | -3777 | HERVIP10FH | ERV1 | LTR |
| 7323706 | 7324492 | - | 2.8 | 5375 | 6153 | -2 | L1PA3 | L1 | LINE |
| 7324493 | 7325009 | + | 4.5 | 4863 | 5387 | -796 | L1PA3 | L1 | LINE |
| 7324997 | 7328691 | + | 27.5 | 2719 | 5905 | -20 | HERVIP10FH | ERV1 | LTR |

  

|  |  |  |  |  |  |  |  |  |  |
| --- | --- | --- | --- | --- | --- | --- | --- | --- | --- |
| chrY:11660865-11662535 | | | 1675 bp | | |  |  |  |  |
| Genome Start | Genome End | Strand | Div | Repeat Start | Repeat End | Repeat Left | Name | Family | Class |
| 11660865 | 11661047 | + | 17.7 | 1 | 190 | -277 | MLT1C | MaLR | LTR |
| 11661048 | 11661361 | - | 8.0 | 4939 | 5257 | -889 | L1PA6 | L1 | LINE |
| 11661356 | 11662243 | + | 6.5 | 5267 | 6154 | 0 | L1PA6 | L1 | LINE |
| 11662246 | 11662535 | + | 19.6 | 176 | 465 | -2 | MLT1C | MaLR | LTR |

  

|  |  |  |  |  |  |  |  |  |  |
| --- | --- | --- | --- | --- | --- | --- | --- | --- | --- |
| chrY:12005835-12009999 | | | 3806 bp | | |  |  |  |  |
| Genome Start | Genome End | Strand | Div | Repeat Start | Repeat End | Repeat Left | Name | Family | Class |
| 12005835 | 12005979 | + | 35.2 | 1250 | 1411 | -5585 | L1MEa | L1 | LINE |
| 12006260 | 12006966 | + | 21.2 | 1331 | 2135 | -5199 | L1MEa | L1 | LINE |
| 12007017 | 12008846 | - | 1.2 | 4322 | 6152 | -3 | L1HS | L1 | LINE |
| 12008846 | 12009604 | + | 1.7 | 3574 | 4336 | -1810 | L1HS | L1 | LINE |
| 12009598 | 12009878 | + | 27.6 | 2281 | 2673 | -4514 | L1MEa | L1 | LINE |
| 12009916 | 12009999 | + | 27.5 | 2674 | 2764 | -4423 | L1MEa | L1 | LINE |

  

|  |  |  |  |  |  |  |  |  |  |
| --- | --- | --- | --- | --- | --- | --- | --- | --- | --- |
| chrY:12663066-12668315 | | | 5214 bp | | |  |  |  |  |
| Genome Start | Genome End | Strand | Div | Repeat Start | Repeat End | Repeat Left | Name | Family | Class |
| 12663066 | 12663196 | + | 12.6 | 5331 | 5452 | -714 | L1PA15 | L1 | LINE |
| 12663194 | 12665731 | - | 7.8 | 1746 | 4273 | -1873 | L1PA4 | L1 | LINE |
| 12665725 | 12667571 | + | 6.0 | 4292 | 6142 | -13 | L1PA4 | L1 | LINE |
| 12667618 | 12668315 | + | 16.7 | 5442 | 6150 | -15 | L1PA15 | L1 | LINE |

  

|  |  |  |  |  |  |  |  |  |  |
| --- | --- | --- | --- | --- | --- | --- | --- | --- | --- |
| chrY:13730912-13745897 | | | 14958 bp | | |  |  |  |  |
| Genome Start | Genome End | Strand | Div | Repeat Start | Repeat End | Repeat Left | Name | Family | Class |
| 13730912 | 13730938 | - | 18.4 | 6012 | 6023 | -982 | L1M | L1 | LINE |
| 13730939 | 13731233 | - | 15.2 | 1 | 289 | -21 | AluSg | Alu | SINE |
| 13731234 | 13732351 | - | 19.5 | 4896 | 6011 | -994 | L1M | L1 | LINE |
| 13732362 | 13732841 | + | 10.8 | 1 | 486 | -7 | LTR15 | ERV1 | LTR |
| 13732849 | 13740230 | + | 4.0 | 1 | 7373 | -1362 | HERV15 | ERV1 | LTR |
| 13740240 | 13741367 | - | 4.2 | 5033 | 6155 | 0 | L1PA4 | L1 | LINE |
| 13741362 | 13741769 | + | 5.4 | 4611 | 5030 | -1116 | L1PA4 | L1 | LINE |
| 13741770 | 13743134 | + | 2.3 | 7371 | 8735 | 0 | HERV15 | ERV1 | LTR |
| 13743135 | 13743611 | + | 10.5 | 1 | 486 | -7 | LTR15 | ERV1 | LTR |
| 13743618 | 13744563 | - | 23.5 | 3939 | 4892 | -1953 | L1M | L1 | LINE |
| 13744564 | 13744686 | - | 16.3 | 1 | 123 | -189 | AluSq/x | Alu | SINE |
| 13744689 | 13745008 | - | 12.9 | 1 | 310 | -2 | AluSx | Alu | SINE |
| 13745009 | 13745480 | - | 23.5 | 3459 | 3936 | -2909 | L1M | L1 | LINE |
| 13745481 | 13745767 | - | 11.2 | 1 | 291 | -18 | AluSc | Alu | SINE |
| 13745768 | 13745897 | - | 23.5 | 3329 | 3458 | -3387 | L1M | L1 | LINE |

  

|  |  |  |  |  |  |  |  |  |  |
| --- | --- | --- | --- | --- | --- | --- | --- | --- | --- |
| chrY:14225823-14228156 | | | 2324 bp | | |  |  |  |  |
| Genome Start | Genome End | Strand | Div | Repeat Start | Repeat End | Repeat Left | Name | Family | Class |
| 14225823 | 14226439 | + | 35.0 | 1053 | 1668 | -5181 | L1M2 | L1 | LINE |
| 14226442 | 14226780 | - | 12.1 | 5050 | 5403 | -743 | L1PA8 | L1 | LINE |
| 14226781 | 14227556 | + | 11.5 | 5416 | 6164 | -8 | L1PA8 | L1 | LINE |
| 14227565 | 14228156 | + | 22.7 | 1664 | 2267 | -4582 | L1M2 | L1 | LINE |

  

|  |  |  |  |  |  |  |  |  |  |
| --- | --- | --- | --- | --- | --- | --- | --- | --- | --- |
| chrY:15662516-15667695 | | | 5092 bp | | |  |  |  |  |
| Genome Start | Genome End | Strand | Div | Repeat Start | Repeat End | Repeat Left | Name | Family | Class |
| 15662516 | 15662725 | - | 23.9 | 5866 | 6062 | -177 | L1ME1 | L1 | LINE |
| 15662722 | 15664794 | - | 6.3 | 4069 | 6155 | 0 | L1PA4 | L1 | LINE |
| 15664798 | 15665396 | + | 6.3 | 3450 | 4049 | -2097 | L1PA4 | L1 | LINE |
| 15665395 | 15666165 | - | 30.4 | 4361 | 5166 | -1053 | L1ME1 | L1 | LINE |
| 15666242 | 15666630 | - | 29.2 | 437 | 807 | -5593 | L1ME1 | L1 | LINE |
| 15666646 | 15666710 | + | 14.7 | 3 | 58 | -306 | THE1B | MaLR | LTR |
| 15666711 | 15666978 | + | 5.6 | 5882 | 6155 | 0 | L1PA4 | L1 | LINE |
| 15666979 | 15667278 | + | 14.7 | 59 | 364 | 0 | THE1B | MaLR | LTR |
| 15667279 | 15667695 | - | 30.5 | 40 | 485 | -5915 | L1ME1 | L1 | LINE |

  

|  |  |  |  |  |  |  |  |  |  |
| --- | --- | --- | --- | --- | --- | --- | --- | --- | --- |
| chrY:16060622-16061717 | | | 1088 bp | | |  |  |  |  |
| Genome Start | Genome End | Strand | Div | Repeat Start | Repeat End | Repeat Left | Name | Family | Class |
| 16060622 | 16060727 | - | 19.8 | 537 | 644 | 0 | LTR9B | ERV1 | LTR |
| 16060728 | 16060799 | - | 1.4 | 5714 | 5785 | -369 | L1PA5 | L1 | LINE |
| 16060800 | 16061203 | + | 5.5 | 5785 | 6189 | 0 | L1PA5 | L1 | LINE |
| 16061212 | 16061717 | - | 16.1 | 1 | 548 | -96 | LTR9B | ERV1 | LTR |

  

|  |  |  |  |  |  |  |  |  |  |
| --- | --- | --- | --- | --- | --- | --- | --- | --- | --- |
| chrY:18067676-18072138 | | | 4459 bp | | |  |  |  |  |
| Genome Start | Genome End | Strand | Div | Repeat Start | Repeat End | Repeat Left | Name | Family | Class |
| 18067676 | 18068062 | - | 14.8 | 5769 | 6158 | -10 | L1PA12 | L1 | LINE |
| 18068073 | 18068821 | - | 6.1 | 5404 | 6152 | -3 | L1PA4 | L1 | LINE |
| 18068822 | 18072096 | + | 5.8 | 1823 | 5403 | -743 | L1PA4 | L1 | LINE |
| 18072091 | 18072138 | - | 12.5 | 5737 | 5784 | -347 | L1PA12 | L1 | LINE |

  

|  |  |  |  |  |  |  |  |  |  |
| --- | --- | --- | --- | --- | --- | --- | --- | --- | --- |
| chrY:18310872-18317479 | | | 6611 bp | | |  |  |  |  |
| Genome Start | Genome End | Strand | Div | Repeat Start | Repeat End | Repeat Left | Name | Family | Class |
| 18310872 | 18311142 | - | 6.6 | 5867 | 6137 | -18 | L1PA4 | L1 | LINE |
| 18311143 | 18312826 | - | 7.4 | 3955 | 5638 | -517 | L1PA4 | L1 | LINE |
| 18312827 | 18313024 | - | 2.5 | 5958 | 6155 | 0 | L1PA2 | L1 | LINE |
| 18313025 | 18313550 | + | 2.5 | 5422 | 5947 | -208 | L1PA2 | L1 | LINE |
| 18313548 | 18317479 | - | 9.0 | 4 | 3964 | -2182 | L1PA4 | L1 | LINE |

  

|  |  |  |  |  |  |  |  |  |  |
| --- | --- | --- | --- | --- | --- | --- | --- | --- | --- |
| chrY:18831083-18837690 | | | 6611 bp | | |  |  |  |  |
| Genome Start | Genome End | Strand | Div | Repeat Start | Repeat End | Repeat Left | Name | Family | Class |
| 18831083 | 18835014 | + | 9.0 | 4 | 3964 | -2182 | L1PA4 | L1 | LINE |
| 18835012 | 18835537 | - | 2.3 | 5422 | 5947 | -208 | L1PA2 | L1 | LINE |
| 18835538 | 18835735 | + | 2.5 | 5958 | 6155 | 0 | L1PA2 | L1 | LINE |
| 18835736 | 18837419 | + | 7.3 | 3955 | 5638 | -517 | L1PA4 | L1 | LINE |
| 18837420 | 18837690 | + | 6.6 | 5867 | 6137 | -18 | L1PA4 | L1 | LINE |

  

|  |  |  |  |  |  |  |  |  |  |
| --- | --- | --- | --- | --- | --- | --- | --- | --- | --- |
| chrY:19627215-19632859 | | | 5618 bp | | |  |  |  |  |
| Genome Start | Genome End | Strand | Div | Repeat Start | Repeat End | Repeat Left | Name | Family | Class |
| 19627215 | 19629602 | + | 30.1 | 16 | 2603 | -3616 | L1MEc | L1 | LINE |
| 19629623 | 19630804 | - | 2.3 | 4213 | 5391 | -755 | L1PA2 | L1 | LINE |
| 19630800 | 19631565 | + | 1.6 | 5395 | 6155 | 0 | L1PA2 | L1 | LINE |
| 19631578 | 19632859 | + | 27.4 | 2629 | 4064 | -2155 | L1MEc | L1 | LINE |

  

|  |  |  |  |  |  |  |  |  |  |
| --- | --- | --- | --- | --- | --- | --- | --- | --- | --- |
| chrY:20286131-20294060 | | | 7783 bp | | |  |  |  |  |
| Genome Start | Genome End | Strand | Div | Repeat Start | Repeat End | Repeat Left | Name | Family | Class |
| 20286131 | 20286664 | + | 27.0 | 94 | 620 | -245 | MER6 | MER2\_type | DNA |
| 20286684 | 20287010 | + | 13.5 | 1313 | 1648 | -4705 | L1PA10 | L1 | LINE |
| 20286999 | 20287941 | + | 13.8 | 1494 | 2444 | -3909 | L1PA10 | L1 | LINE |
| 20287947 | 20288816 | - | 2.3 | 4560 | 5429 | -726 | L1P | L1 | LINE |
| 20288819 | 20289543 | + | 1.8 | 5430 | 6154 | -1 | L1PA3 | L1 | LINE |
| 20289616 | 20293474 | + | 12.6 | 2439 | 6348 | -5 | L1PA10 | L1 | LINE |
| 20293536 | 20293569 | + | 23.6 | 640 | 670 | -195 | MER6 | MER2\_type | DNA |
| 20293570 | 20293864 | - | 14.9 | 12 | 304 | 0 | AluSc | Alu | SINE |
| 20293865 | 20294060 | + | 23.6 | 671 | 865 | 0 | MER6 | MER2\_type | DNA |

  

|  |  |  |  |  |  |  |  |  |  |
| --- | --- | --- | --- | --- | --- | --- | --- | --- | --- |
| chrY:20525454-20531119 | | | 5616 bp | | |  |  |  |  |
| Genome Start | Genome End | Strand | Div | Repeat Start | Repeat End | Repeat Left | Name | Family | Class |
| 20525454 | 20526341 | - | 28.1 | 4855 | 5742 | -1820 | L1M1 | L1 | LINE |
| 20526342 | 20527204 | - | 19.4 | 3403 | 6172 | 0 | L1PREC2 | L1 | LINE |
| 20527205 | 20527491 | - | 30.2 | 1 | 291 | -11 | AluJb | Alu | SINE |
| 20527492 | 20527751 | - | 16.1 | 5103 | 5365 | -781 | L1P | L1 | LINE |
| 20527749 | 20528610 | + | 18.6 | 4213 | 5100 | -1046 | L1P | L1 | LINE |
| 20528613 | 20529288 | - | 26.7 | 4140 | 4867 | -2695 | L1M1 | L1 | LINE |
| 20529314 | 20529683 | - | 22.5 | 1 | 381 | 0 | THE1D | MaLR | LTR |
| 20529690 | 20530530 | - | 22.9 | 693 | 1573 | -7 | THE1D-int | MaLR | LTR |
| 20530551 | 20531119 | - | 24.3 | 2006 | 2597 | -4968 | L1M1 | L1 | LINE |

  

|  |  |  |  |  |  |  |  |  |  |
| --- | --- | --- | --- | --- | --- | --- | --- | --- | --- |
| chrY:20547416-20552402 | | | 4932 bp | | |  |  |  |  |
| Genome Start | Genome End | Strand | Div | Repeat Start | Repeat End | Repeat Left | Name | Family | Class |
| 20547416 | 20547670 | + | 19.2 | 5680 | 5935 | -228 | L1PA13 | L1 | LINE |
| 20547671 | 20547950 | + | 26.1 | 3802 | 4065 | -3123 | LTR25-int | ERV1 | LTR |
| 20547951 | 20548232 | + | 14.9 | 1 | 282 | -30 | AluSx | Alu | SINE |
| 20548250 | 20548537 | + | 17.4 | 1 | 297 | -16 | AluSq | Alu | SINE |
| 20548538 | 20549499 | + | 26.1 | 4066 | 5000 | -2188 | LTR25-int | ERV1 | LTR |
| 20549504 | 20549723 | - | 6.4 | 5132 | 5352 | -794 | L1PA4 | L1 | LINE |
| 20549714 | 20550528 | + | 3.2 | 5344 | 6155 | 0 | L1PA4 | L1 | LINE |
| 20550529 | 20551247 | + | 20.3 | 4135 | 5672 | -1468 | MER52C-int | ERV1 | LTR |
| 20551249 | 20551388 | + | 26.6 | 4549 | 4689 | -1257 | MER52C-int | ERV1 | LTR |
| 20551395 | 20551455 | + | 14.8 | 5976 | 6037 | -1168 | MER52C-int | ERV1 | LTR |
| 20551446 | 20551642 | + | 20.6 | 6892 | 7090 | -1 | MER52C-int | ERV1 | LTR |
| 20551670 | 20551977 | + | 26.4 | 12 | 316 | -962 | MER52C | ERV1 | LTR |
| 20551990 | 20552319 | - | 20.4 | 6 | 335 | 0 | AluSx | Alu | SINE |
| 20552328 | 20552402 | + | 17.6 | 6082 | 6157 | -6 | L1PA13 | L1 | LINE |

  

|  |  |  |  |  |  |  |  |  |  |
| --- | --- | --- | --- | --- | --- | --- | --- | --- | --- |
| chrY:20999905-21009443 | | | 9006 bp | | |  |  |  |  |
| Genome Start | Genome End | Strand | Div | Repeat Start | Repeat End | Repeat Left | Name | Family | Class |
| 20999905 | 21000069 | + | 20.0 | 3163 | 3340 | -2806 | L1MA2 | L1 | LINE |
| 21000067 | 21000153 | - | 6.9 | 5160 | 5246 | -900 | L1PA4 | L1 | LINE |
| 21000152 | 21001074 | + | 4.0 | 5228 | 6152 | -3 | L1PA4 | L1 | LINE |
| 21001084 | 21001238 | + | 21.3 | 3324 | 3501 | -2645 | L1MA2 | L1 | LINE |
| 21001246 | 21002006 | - | 7.5 | 3948 | 4717 | -1429 | L1P1 | L1 | LINE |
| 21002094 | 21003473 | + | 6.6 | 4751 | 6140 | -14 | L1PA5 | L1 | LINE |
| 21003477 | 21004122 | + | 8.2 | 5476 | 6141 | -13 | L1PA5 | L1 | LINE |
| 21004187 | 21004805 | + | 20.1 | 3511 | 4135 | -2011 | L1MA2 | L1 | LINE |
| 21004809 | 21006367 | - | 25.8 | 471 | 2120 | -3 | MER52D | ERV1 | LTR |
| 21006729 | 21007135 | - | 31.2 | 5 | 471 | -1652 | MER52D | ERV1 | LTR |
| 21007136 | 21008001 | + | 20.3 | 4137 | 4988 | -1158 | L1MA2 | L1 | LINE |
| 21008006 | 21008148 | + | 10.5 | 154 | 296 | -15 | AluY | Alu | SINE |
| 21008149 | 21009443 | + | 18.8 | 4990 | 6304 | 0 | L1MA2 | L1 | LINE |

  

|  |  |  |  |  |  |  |  |  |  |
| --- | --- | --- | --- | --- | --- | --- | --- | --- | --- |
| chrY:21456285-21466741 | | | 10009 bp | | |  |  |  |  |
| Genome Start | Genome End | Strand | Div | Repeat Start | Repeat End | Repeat Left | Name | Family | Class |
| 21456285 | 21456734 | + | 25.8 | 2 | 438 | -6980 | HUERS-P3b | ERV1 | LTR |
| 21456735 | 21458434 | + | 11.3 | 3152 | 4984 | -1162 | L1PA6 | L1 | LINE |
| 21458434 | 21458804 | + | 7.8 | 5413 | 5783 | -371 | L1PA6 | L1 | LINE |
| 21458806 | 21459032 | + | 12.8 | 5907 | 6136 | -18 | L1PA6 | L1 | LINE |
| 21459288 | 21459340 | + | 17.0 | 451 | 504 | -6914 | HUERS-P3b | ERV1 | LTR |
| 21459502 | 21459721 | - | 6.4 | 3240 | 3462 | -2684 | L1PA4 | L1 | LINE |
| 21459723 | 21462101 | + | 6.6 | 3458 | 6155 | 0 | L1PA4 | L1 | LINE |
| 21462114 | 21462321 | + | 25.0 | 878 | 1097 | -7822 | HUERS-P3b | ERV1 | LTR |
| 21462322 | 21462622 | - | 17.3 | 1 | 302 | -11 | AluSq | Alu | SINE |
| 21462642 | 21462768 | + | 18.1 | 2506 | 2642 | -4776 | HUERS-P3b | ERV1 | LTR |
| 21462769 | 21463198 | - | 22.1 | 1 | 441 | -250 | LTR8 | ERV1 | LTR |
| 21463199 | 21466741 | + | 14.6 | 1275 | 6780 | -638 | HUERS-P3b | ERV1 | LTR |

  

|  |  |  |  |  |  |  |  |  |  |
| --- | --- | --- | --- | --- | --- | --- | --- | --- | --- |
| chrY:23328791-23337336 | | | 8530 bp | | |  |  |  |  |
| Genome Start | Genome End | Strand | Div | Repeat Start | Repeat End | Repeat Left | Name | Family | Class |
| 23328791 | 23333525 | - | 7.5 | 1446 | 6152 | -2 | L1PA5 | L1 | LINE |
| 23333526 | 23334262 | - | 5.8 | 5408 | 6155 | 0 | L1PA3 | L1 | LINE |
| 23334263 | 23335864 | + | 5.2 | 3803 | 5403 | -743 | L1PA3 | L1 | LINE |
| 23335881 | 23337336 | - | 11.7 | 1 | 1460 | -4695 | L1PA5 | L1 | LINE |

  

|  |  |  |  |  |  |  |  |  |  |
| --- | --- | --- | --- | --- | --- | --- | --- | --- | --- |
| chrY:23426571-23432131 | | | 5397 bp | | |  |  |  |  |
| Genome Start | Genome End | Strand | Div | Repeat Start | Repeat End | Repeat Left | Name | Family | Class |
| 23426571 | 23426632 | + | 25.8 | 4519 | 4580 | -1563 | L1M3 | L1 | LINE |
| 23426649 | 23426958 | - | 12.6 | 1 | 310 | -1 | AluY | Alu | SINE |
| 23427067 | 23427194 | - | 7.0 | 1111 | 1242 | -4913 | L1PA4 | L1 | LINE |
| 23427195 | 23431952 | + | 5.8 | 1230 | 6014 | -141 | L1PA4 | L1 | LINE |
| 23431993 | 23432131 | + | 29.5 | 4687 | 4827 | -1313 | L1M3 | L1 | LINE |

  

|  |  |  |  |  |  |  |  |  |  |
| --- | --- | --- | --- | --- | --- | --- | --- | --- | --- |
| chrY:24962534-24971080 | | | 8531 bp | | |  |  |  |  |
| Genome Start | Genome End | Strand | Div | Repeat Start | Repeat End | Repeat Left | Name | Family | Class |
| 24962534 | 24967269 | - | 7.4 | 1446 | 6152 | -2 | L1PA5 | L1 | LINE |
| 24967270 | 24968006 | - | 5.7 | 5408 | 6155 | 0 | L1PA3 | L1 | LINE |
| 24968007 | 24969608 | + | 5.1 | 3803 | 5403 | -743 | L1PA3 | L1 | LINE |
| 24969625 | 24971080 | - | 11.7 | 1 | 1460 | -4695 | L1PA5 | L1 | LINE |

  

|  |  |  |  |  |  |  |  |  |  |
| --- | --- | --- | --- | --- | --- | --- | --- | --- | --- |
| chrY:25715294-25720854 | | | 5397 bp | | |  |  |  |  |
| Genome Start | Genome End | Strand | Div | Repeat Start | Repeat End | Repeat Left | Name | Family | Class |
| 25715294 | 25715432 | - | 29.5 | 4687 | 4827 | -1313 | L1M3 | L1 | LINE |
| 25715473 | 25720230 | - | 5.8 | 1230 | 6014 | -141 | L1PA4 | L1 | LINE |
| 25720231 | 25720358 | + | 7.0 | 1111 | 1242 | -4913 | L1PA4 | L1 | LINE |
| 25720467 | 25720776 | + | 12.6 | 1 | 310 | -1 | AluY | Alu | SINE |
| 25720793 | 25720854 | - | 25.8 | 4519 | 4580 | -1563 | L1M3 | L1 | LINE |

  

|  |  |  |  |  |  |  |  |  |  |
| --- | --- | --- | --- | --- | --- | --- | --- | --- | --- |
| chrY:25810104-25818650 | | | 8531 bp | | |  |  |  |  |
| Genome Start | Genome End | Strand | Div | Repeat Start | Repeat End | Repeat Left | Name | Family | Class |
| 25810104 | 25811559 | + | 11.7 | 1 | 1460 | -4695 | L1PA5 | L1 | LINE |
| 25811576 | 25813177 | - | 5.1 | 3803 | 5403 | -743 | L1PA3 | L1 | LINE |
| 25813178 | 25813914 | + | 5.7 | 5408 | 6155 | 0 | L1PA3 | L1 | LINE |
| 25813915 | 25818650 | + | 7.4 | 1446 | 6152 | -2 | L1PA5 | L1 | LINE |
