# Supplementary material for: Evolutionary History of Mammalian Transposons Determined by Genome-Wide Defragmentation
Source: PLoS Comput Biol. 2007 Jul 13;3(7):e137. doi: 10.1371/journal.pcbi.0030137 (PMC1914374; doi:10.1371/journal.pcbi.0030137)
Supplement: Supplementary file 6 [file pcbi.0030137.sd003.htm]

Clusters with Tandem Repeats

**Supplementary Table S1c: Clusters with Tandem Repeats**  
  
To load the custom track into the UCSC Genome Browser, click here. The custom track must be loaded in order to view the clusters in Supplementary Table S1. It may take a minute to load, so please be patient.  
  
Jump to:  
chr1  
chr2  
chr4  
chr5  
chr6  
chr7  
chr8  
chr9  
chr10  
chr12  
chr13  
chr17  
chr18  
chr19  
chr20  
chr21  
chrX  
  


|  |  |  |  |  |  |  |  |  |  |
| --- | --- | --- | --- | --- | --- | --- | --- | --- | --- |
| chr1:18009084-18017953 | | |  |  |  |  |  |  |  |
| Genome Start | Genome End | Strand | Div | Repeat Start | Repeat End | Repeat Left | Name | Family | Class |
| 18009084 | 18009282 | + | 22.5 | 1735 | 1939 | -1480 | L2 | L2 | LINE |
| 18009303 | 18009606 | - | 28.3 | 7684 | 8013 | -29 | L1MC4 | L1 | LINE |
| 18009623 | 18010943 | - | 22.2 | 4754 | 6142 | -24 | L1ME1 | L1 | LINE |
| 18010944 | 18011239 | - | 15.3 | 1 | 306 | -6 | AluJb | Alu | SINE |
| 18011240 | 18011499 | - | 22.5 | 4509 | 4753 | -1393 | L1MD | L1 | LINE |
| 18011500 | 18011746 | - | 11.0 | 76 | 312 | 0 | AluSg/x | Alu | SINE |
| 18011747 | 18011874 | - | 13.3 | 1 | 129 | -183 | AluSq/x | Alu | SINE |
| 18011875 | 18013169 | - | 22.5 | 3154 | 4508 | -1638 | L1MD | L1 | LINE |
| 18013175 | 18013295 | + | 14.1 | 1 | 132 | -214 | MER7A | MER2\_type | DNA |
| 18013298 | 18013427 | + | 23.9 | 2 | 132 | -11 | FLAM\_C | Alu | SINE |
| 18013429 | 18013514 | + | 17.4 | 2806 | 2894 | -135 | Tigger3(Golem) | MER2\_type | DNA |
| 18013532 | 18013601 | + | 17.1 | 2806 | 2877 | -152 | Tigger3(Golem) | MER2\_type | DNA |
| 18013633 | 18013704 | + | 16.7 | 2806 | 2877 | -152 | Tigger3(Golem) | MER2\_type | DNA |
| 18013736 | 18013807 | + | 15.3 | 2806 | 2877 | -152 | Tigger3(Golem) | MER2\_type | DNA |
| 18013839 | 18013910 | + | 9.7 | 2806 | 2877 | -152 | Tigger3(Golem) | MER2\_type | DNA |
| 18013942 | 18014013 | + | 12.5 | 2806 | 2877 | -152 | Tigger3(Golem) | MER2\_type | DNA |
| 18014035 | 18014106 | + | 15.3 | 2806 | 2877 | -152 | Tigger3(Golem) | MER2\_type | DNA |
| 18014138 | 18014258 | + | 12.4 | 2806 | 2926 | -103 | Tigger3(Golem) | MER2\_type | DNA |
| 18014259 | 18015014 | - | 23.7 | 1389 | 2171 | -4469 | L1MEa | L1 | LINE |
| 18015044 | 18015272 | - | 21.8 | 6057 | 6291 | 0 | L1MA7 | L1 | LINE |
| 18015283 | 18015585 | - | 16.2 | 1 | 298 | -14 | AluJb | Alu | SINE |
| 18015625 | 18015834 | - | 24.1 | 851 | 1067 | -5480 | L1MEa | L1 | LINE |
| 18015837 | 18016165 | - | 21.8 | 521 | 853 | -5681 | L1MEa | L1 | LINE |
| 18016285 | 18016424 | - | 27.3 | 7518 | 7678 | -364 | L1MC4 | L1 | LINE |
| 18016425 | 18016772 | + | 29.6 | 1927 | 2304 | -1115 | L2 | L2 | LINE |
| 18016773 | 18017083 | - | 10.6 | 1 | 310 | -2 | AluSx | Alu | SINE |
| 18017084 | 18017953 | + | 30.2 | 2305 | 3325 | -94 | L2 | L2 | LINE |

|  |  |  |  |  |  |  |  |  |  |
| --- | --- | --- | --- | --- | --- | --- | --- | --- | --- |
| chr1:91900472-91910675 | | |  |  |  |  |  |  |  |
| Genome Start | Genome End | Strand | Div | Repeat Start | Repeat End | Repeat Left | Name | Family | Class |
| 91900472 | 91900588 | - | 26.3 | 3297 | 3414 | -5 | L2 | L2 | LINE |
| 91900596 | 91901056 | - | 5.9 | 1 | 463 | 0 | LTR2 | ERV1 | LTR |
| 91901057 | 91906145 | - | 6.6 | 1806 | 6896 | 0 | Harlequin | ERV1 | LTR |
| 91906127 | 91906217 | - | 5.9 | 1333 | 1422 | -5474 | Harlequin | ERV1 | LTR |
| 91906244 | 91906788 | - | 5.9 | 770 | 1332 | -5564 | Harlequin | ERV1 | LTR |
| 91906821 | 91906860 | - | 10.0 | 769 | 808 | -6088 | Harlequin | ERV1 | LTR |
| 91906857 | 91906896 | - | 10.0 | 769 | 808 | -6088 | Harlequin | ERV1 | LTR |
| 91906893 | 91906932 | - | 10.0 | 769 | 808 | -6088 | Harlequin | ERV1 | LTR |
| 91906929 | 91906968 | - | 10.0 | 769 | 808 | -6088 | Harlequin | ERV1 | LTR |
| 91906965 | 91907005 | - | 14.6 | 765 | 805 | -7008 | Harlequin | ERV1 | LTR |
| 91907001 | 91907040 | - | 10.0 | 769 | 808 | -6088 | Harlequin | ERV1 | LTR |
| 91907037 | 91907076 | - | 10.0 | 769 | 808 | -6088 | Harlequin | ERV1 | LTR |
| 91907073 | 91907112 | - | 7.5 | 769 | 808 | -6088 | Harlequin | ERV1 | LTR |
| 91907109 | 91907148 | - | 7.5 | 769 | 808 | -6088 | Harlequin | ERV1 | LTR |
| 91907145 | 91907180 | - | 8.3 | 773 | 808 | -6088 | Harlequin | ERV1 | LTR |
| 91907181 | 91907220 | - | 10.0 | 769 | 808 | -6088 | Harlequin | ERV1 | LTR |
| 91907217 | 91907256 | - | 7.5 | 769 | 808 | -6088 | Harlequin | ERV1 | LTR |
| 91907253 | 91907292 | - | 7.5 | 769 | 808 | -6088 | Harlequin | ERV1 | LTR |
| 91907289 | 91907328 | - | 10.0 | 769 | 808 | -6088 | Harlequin | ERV1 | LTR |
| 91907325 | 91907364 | - | 10.0 | 769 | 808 | -6088 | Harlequin | ERV1 | LTR |
| 91907361 | 91907400 | - | 10.0 | 769 | 808 | -6088 | Harlequin | ERV1 | LTR |
| 91907397 | 91907436 | - | 10.0 | 769 | 808 | -6088 | Harlequin | ERV1 | LTR |
| 91907433 | 91907472 | - | 10.0 | 769 | 808 | -6088 | Harlequin | ERV1 | LTR |
| 91907469 | 91907508 | - | 7.5 | 769 | 808 | -6088 | Harlequin | ERV1 | LTR |
| 91907505 | 91908049 | - | 6.3 | 263 | 808 | -6088 | Harlequin | ERV1 | LTR |
| 91908043 | 91908432 | - | 12.7 | 1 | 387 | -6509 | Harlequin | ERV1 | LTR |
| 91908433 | 91908892 | - | 8.3 | 1 | 463 | 0 | LTR2 | ERV1 | LTR |
| 91908903 | 91909218 | - | 28.8 | 2959 | 3289 | -130 | L2 | L2 | LINE |
| 91909266 | 91909766 | + | 25.6 | 5254 | 5762 | -2046 | L1MC4a | L1 | LINE |
| 91909767 | 91910076 | + | 12.9 | 1 | 310 | 0 | AluSg | Alu | SINE |
| 91910077 | 91910329 | + | 25.6 | 5763 | 6030 | -1778 | L1MC4a | L1 | LINE |
| 91910392 | 91910675 | - | 30.3 | 2572 | 2885 | -534 | L2 | L2 | LINE |

|  |  |  |  |  |  |  |  |  |  |
| --- | --- | --- | --- | --- | --- | --- | --- | --- | --- |
| chr1:245878999-245880437 | | |  |  |  |  |  |  |  |
| Genome Start | Genome End | Strand | Div | Repeat Start | Repeat End | Repeat Left | Name | Family | Class |
| 245878999 | 245879154 | - | 11.5 | 745 | 903 | 0 | MER4D | ERV1 | LTR |
| 245879151 | 245879256 | - | 10.4 | 714 | 800 | -103 | MER4D | ERV1 | LTR |
| 245879252 | 245879306 | - | 12.7 | 509 | 564 | -100 | MER4A | ERV1 | LTR |
| 245879304 | 245879358 | - | 10.9 | 714 | 769 | -103 | MER4D0 | ERV1 | LTR |
| 245879359 | 245879405 | - | 10.6 | 718 | 765 | -107 | MER4D0 | ERV1 | LTR |
| 245879406 | 245879460 | - | 9.1 | 714 | 769 | -103 | MER4D0 | ERV1 | LTR |
| 245879461 | 245879562 | - | 12.3 | 714 | 796 | -107 | MER4D0 | ERV1 | LTR |
| 245879559 | 245879613 | - | 12.7 | 714 | 769 | -103 | MER4D0 | ERV1 | LTR |
| 245879617 | 245879661 | - | 15.6 | 718 | 762 | -110 | MER4D0 | ERV1 | LTR |
| 245879662 | 245880437 | - | 13.5 | 1 | 800 | -103 | MER4D | ERV1 | LTR |

|  |  |  |  |  |  |  |  |  |  |
| --- | --- | --- | --- | --- | --- | --- | --- | --- | --- |
| chr2:95860533-95865476 | | |  |  |  |  |  |  |  |
| Genome Start | Genome End | Strand | Div | Repeat Start | Repeat End | Repeat Left | Name | Family | Class |
| 95860533 | 95861233 | + | 20.5 | 5 | 1166 | -5815 | L1PA12 | L1 | LINE |
| 95861238 | 95861516 | - | 23.6 | 802 | 1090 | -5891 | L1PA10 | L1 | LINE |
| 95861520 | 95861636 | - | 25.5 | 793 | 900 | -6081 | L1PA10 | L1 | LINE |
| 95861749 | 95861838 | - | 17.8 | 814 | 904 | -6077 | L1PA10 | L1 | LINE |
| 95861852 | 95861939 | - | 23.9 | 815 | 903 | -6078 | L1PA10 | L1 | LINE |
| 95861953 | 95862071 | - | 22.9 | 793 | 903 | -6078 | L1PA10 | L1 | LINE |
| 95862067 | 95862155 | - | 23.9 | 814 | 901 | -6080 | L1PA10 | L1 | LINE |
| 95862169 | 95862662 | - | 22.7 | 799 | 1174 | -5807 | L1PA12 | L1 | LINE |
| 95862682 | 95862764 | - | 15.7 | 815 | 897 | -6084 | L1PA12 | L1 | LINE |
| 95862779 | 95862864 | - | 15.1 | 814 | 903 | -6078 | L1PA12 | L1 | LINE |
| 95862889 | 95862980 | - | 25.0 | 799 | 892 | -6089 | L1PA12 | L1 | LINE |
| 95862981 | 95863083 | - | 22.6 | 798 | 904 | -6077 | L1PA12 | L1 | LINE |
| 95863087 | 95863398 | - | 25.7 | 799 | 1169 | -5812 | L1PA12 | L1 | LINE |
| 95863399 | 95863512 | - | 25.0 | 798 | 903 | -6078 | L1PA12 | L1 | LINE |
| 95863513 | 95863601 | - | 21.6 | 814 | 901 | -6080 | L1PA12 | L1 | LINE |
| 95863615 | 95863914 | - | 25.3 | 814 | 1174 | -5807 | L1PA12 | L1 | LINE |
| 95863931 | 95864031 | - | 22.0 | 799 | 900 | -6081 | L1PA12 | L1 | LINE |
| 95864032 | 95864434 | - | 24.4 | 810 | 1174 | -5807 | L1PA12 | L1 | LINE |
| 95864444 | 95864507 | - | 20.3 | 838 | 904 | -6077 | L1PA12 | L1 | LINE |
| 95864506 | 95865476 | + | 24.8 | 734 | 1627 | -5890 | L1PA12 | L1 | LINE |

|  |  |  |  |  |  |  |  |  |  |
| --- | --- | --- | --- | --- | --- | --- | --- | --- | --- |
| chr2:114082492-114085741 | | |  |  |  |  |  |  |  |
| Genome Start | Genome End | Strand | Div | Repeat Start | Repeat End | Repeat Left | Name | Family | Class |
| 114082492 | 114082603 | + | 23.6 | 500 | 623 | -4733 | MER31-int | ERV1 | LTR |
| 114082645 | 114082984 | + | 25.9 | 173 | 515 | -4421 | MER31-int | ERV1 | LTR |
| 114082960 | 114083323 | + | 26.7 | 620 | 1063 | -3873 | MER31-int | ERV1 | LTR |
| 114083330 | 114083680 | - | 18.8 | 167 | 551 | -3 | MLT2B3 | ERVL | LTR |
| 114083681 | 114084042 | - | 15.0 | 1 | 375 | 0 | THE1C | MaLR | LTR |
| 114084043 | 114084269 | - | 20.4 | 3 | 274 | -388 | MLT2B3 | ERVL | LTR |
| 114084295 | 114084362 | - | 20.6 | 3 | 72 | -482 | MLT2B3 | ERVL | LTR |
| 114084363 | 114084455 | - | 21.6 | 3 | 96 | -501 | MLT2B3 | ERVL | LTR |
| 114084456 | 114084548 | - | 21.6 | 3 | 96 | -501 | MLT2B3 | ERVL | LTR |
| 114084574 | 114084641 | - | 19.1 | 3 | 72 | -482 | MLT2B3 | ERVL | LTR |
| 114084667 | 114084734 | - | 20.6 | 3 | 72 | -482 | MLT2B3 | ERVL | LTR |
| 114084760 | 114084827 | - | 19.1 | 3 | 72 | -482 | MLT2B3 | ERVL | LTR |
| 114084853 | 114084920 | - | 19.1 | 3 | 72 | -482 | MLT2B3 | ERVL | LTR |
| 114084946 | 114085013 | - | 22.1 | 3 | 72 | -482 | MLT2B3 | ERVL | LTR |
| 114085039 | 114085106 | - | 17.6 | 3 | 72 | -482 | MLT2B3 | ERVL | LTR |
| 114085107 | 114085199 | - | 16.1 | 3 | 97 | -483 | MLT2B3 | ERVL | LTR |
| 114085225 | 114085291 | - | 22.4 | 3 | 72 | -482 | MLT2B3 | ERVL | LTR |
| 114085295 | 114085741 | + | 21.6 | 1063 | 1506 | -3430 | MER31-int | ERV1 | LTR |

|  |  |  |  |  |  |  |  |  |  |
| --- | --- | --- | --- | --- | --- | --- | --- | --- | --- |
| chr2:128696761-128704217 | | |  |  |  |  |  |  |  |
| Genome Start | Genome End | Strand | Div | Repeat Start | Repeat End | Repeat Left | Name | Family | Class |
| 128696761 | 128696830 | - | 18.8 | 5749 | 5816 | -817 | L1M | L1 | LINE |
| 128696836 | 128696861 | - | 15.5 | 401 | 423 | -5 | MSTC | MaLR | LTR |
| 128696862 | 128697276 | - | 17.4 | 136 | 594 | -1 | LTR49 | ERV1 | LTR |
| 128697277 | 128697585 | - | 10.9 | 1 | 309 | 0 | AluSq | Alu | SINE |
| 128697586 | 128697724 | - | 17.4 | 2 | 135 | -460 | LTR49 | ERV1 | LTR |
| 128697725 | 128697844 | - | 15.5 | 284 | 400 | -28 | MSTC | MaLR | LTR |
| 128697848 | 128698064 | - | 16.6 | 3907 | 4123 | -2414 | L1M | L1 | LINE |
| 128698065 | 128698366 | - | 16.6 | 1 | 302 | -10 | AluJb | Alu | SINE |
| 128698393 | 128698528 | - | 18.8 | 2 | 142 | -1 | FLAM\_C | Alu | SINE |
| 128698530 | 128698679 | - | 18.2 | 3394 | 3537 | -3000 | L1M | L1 | LINE |
| 128698680 | 128698979 | + | 10.0 | 1 | 299 | -13 | AluSx | Alu | SINE |
| 128698980 | 128699052 | - | 18.2 | 3322 | 3393 | -3144 | L1M | L1 | LINE |
| 128699053 | 128699353 | - | 9.3 | 1 | 302 | -10 | AluSx | Alu | SINE |
| 128699354 | 128699679 | - | 18.2 | 2990 | 3321 | -3216 | L1M | L1 | LINE |
| 128699680 | 128699853 | + | 16.6 | 1 | 178 | -250 | MSTB | MaLR | LTR |
| 128699840 | 128699871 | + | 6.2 | 102 | 133 | -324 | MSTB2 | MaLR | LTR |
| 128699877 | 128699908 | + | 6.2 | 102 | 133 | -324 | MSTB2 | MaLR | LTR |
| 128699914 | 128699945 | + | 6.2 | 102 | 133 | -324 | MSTB2 | MaLR | LTR |
| 128699951 | 128699982 | + | 6.2 | 102 | 133 | -324 | MSTB2 | MaLR | LTR |
| 128699988 | 128700019 | + | 6.2 | 102 | 133 | -324 | MSTB2 | MaLR | LTR |
| 128700025 | 128700056 | + | 6.2 | 102 | 133 | -324 | MSTB2 | MaLR | LTR |
| 128700062 | 128700093 | + | 6.2 | 102 | 133 | -324 | MSTB2 | MaLR | LTR |
| 128700099 | 128700130 | + | 6.2 | 102 | 133 | -324 | MSTB2 | MaLR | LTR |
| 128700136 | 128700167 | + | 6.2 | 102 | 133 | -324 | MSTB | MaLR | LTR |
| 128700173 | 128700283 | + | 16.3 | 86 | 190 | -236 | MSTB | MaLR | LTR |
| 128700284 | 128700590 | + | 14.4 | 1 | 306 | -6 | AluJo | Alu | SINE |
| 128700591 | 128700824 | + | 16.3 | 191 | 426 | 0 | MSTB | MaLR | LTR |
| 128700825 | 128701093 | - | 13.9 | 2690 | 2994 | -3543 | L1M | L1 | LINE |
| 128701095 | 128701505 | + | 15.3 | 1 | 426 | -2 | MSTC | MaLR | LTR |
| 128701506 | 128701665 | - | 15.2 | 2521 | 2689 | -3848 | L1M | L1 | LINE |
| 128701668 | 128701954 | - | 10.1 | 24 | 310 | -2 | AluSx | Alu | SINE |
| 128701956 | 128702133 | + | 16.7 | 170 | 347 | 0 | AluSg/x | Alu | SINE |
| 128702238 | 128702347 | - | 21.7 | 2383 | 2498 | -4135 | L1M | L1 | LINE |
| 128702424 | 128702734 | + | 9.5 | 1 | 305 | -6 | AluY | Alu | SINE |
| 128702776 | 128702957 | - | 21.7 | 2211 | 2387 | -4246 | L1M | L1 | LINE |
| 128702958 | 128703197 | + | 15.2 | 8 | 336 | -1 | MER7A | MER2\_type | DNA |
| 128703198 | 128703317 | - | 22.4 | 2075 | 2209 | -4424 | L1M | L1 | LINE |
| 128703318 | 128703623 | - | 18.6 | 5 | 310 | -2 | AluSx | Alu | SINE |
| 128703624 | 128703691 | - | 22.4 | 2013 | 2074 | -4559 | L1M | L1 | LINE |
| 128703692 | 128703981 | - | 10.7 | 10 | 298 | -12 | AluSg | Alu | SINE |
| 128703982 | 128704217 | - | 22.4 | 1789 | 2012 | -4621 | L1M | L1 | LINE |

|  |  |  |  |  |  |  |  |  |  |
| --- | --- | --- | --- | --- | --- | --- | --- | --- | --- |
| chr2:241429813-241435302 | | |  |  |  |  |  |  |  |
| Genome Start | Genome End | Strand | Div | Repeat Start | Repeat End | Repeat Left | Name | Family | Class |
| 241429813 | 241429951 | - | 16.8 | 476 | 653 | 0 | LTR49 | ERV1 | LTR |
| 241429952 | 241430257 | + | 15.3 | 1 | 309 | -3 | AluJo | Alu | SINE |
| 241430258 | 241430282 | - | 23.9 | 481 | 507 | -146 | MER39B | ERV1 | LTR |
| 241430295 | 241430345 | + | 14.0 | 1 | 50 | -850 | MER4D1 | ERV1 | LTR |
| 241430350 | 241430743 | - | 10.7 | 104 | 508 | -3 | LTR10F | ERV1 | LTR |
| 241430744 | 241430794 | - | 11.8 | 107 | 159 | -352 | LTR10F | ERV1 | LTR |
| 241430795 | 241430878 | - | 8.3 | 104 | 197 | -314 | LTR10F | ERV1 | LTR |
| 241430902 | 241430987 | - | 8.1 | 104 | 197 | -314 | LTR10F | ERV1 | LTR |
| 241431011 | 241431096 | - | 8.1 | 104 | 197 | -314 | LTR10F | ERV1 | LTR |
| 241431120 | 241431204 | - | 5.9 | 104 | 197 | -314 | LTR10F | ERV1 | LTR |
| 241431228 | 241431310 | - | 3.6 | 104 | 197 | -314 | LTR10F | ERV1 | LTR |
| 241431311 | 241431361 | - | 11.8 | 107 | 159 | -352 | LTR10F | ERV1 | LTR |
| 241431362 | 241431447 | - | 8.1 | 104 | 197 | -314 | LTR10F | ERV1 | LTR |
| 241431471 | 241431551 | - | 6.2 | 108 | 197 | -314 | LTR10F | ERV1 | LTR |
| 241431553 | 241431738 | - | 8.6 | 1 | 197 | -314 | LTR10F | ERV1 | LTR |
| 241431740 | 241432595 | + | 16.0 | 49 | 895 | -8 | MER4D | ERV1 | LTR |
| 241432602 | 241432761 | - | 16.2 | 205 | 415 | -180 | LTR49 | ERV1 | LTR |
| 241432762 | 241433122 | + | 14.6 | 1 | 373 | -2 | THE1C | MaLR | LTR |
| 241433125 | 241434727 | + | 10.9 | 1 | 1580 | 0 | THE1C-int | MaLR | LTR |
| 241434728 | 241435098 | + | 11.2 | 1 | 375 | 0 | THE1C | MaLR | LTR |
| 241435099 | 241435302 | - | 10.3 | 3 | 207 | -388 | LTR49 | ERV1 | LTR |

|  |  |  |  |  |  |  |  |  |  |
| --- | --- | --- | --- | --- | --- | --- | --- | --- | --- |
| chr4:183278667-183284028 | | |  |  |  |  |  |  |  |
| Genome Start | Genome End | Strand | Div | Repeat Start | Repeat End | Repeat Left | Name | Family | Class |
| 183278667 | 183279134 | - | 31.1 | 2814 | 3278 | -141 | L2 | L2 | LINE |
| 183279135 | 183279444 | - | 11.3 | 1 | 311 | -1 | AluSx | Alu | SINE |
| 183279445 | 183279640 | - | 30.3 | 2621 | 2813 | -606 | L2 | L2 | LINE |
| 183279656 | 183281293 | - | 13.3 | 706 | 2383 | -35 | Tigger1 | MER2\_type | DNA |
| 183281320 | 183282002 | - | 13.3 | 19 | 705 | -1713 | Tigger1 | MER2\_type | DNA |
| 183282007 | 183282184 | - | 25.8 | 2439 | 2616 | -803 | L2 | L2 | LINE |
| 183282185 | 183282498 | - | 12.5 | 1 | 312 | 0 | AluSx | Alu | SINE |
| 183282499 | 183282876 | - | 25.8 | 2046 | 2438 | -981 | L2 | L2 | LINE |
| 183282881 | 183283093 | + | 20.4 | 7 | 230 | -2261 | Tigger7 | MER2\_type | DNA |
| 183283089 | 183283154 | + | 20.0 | 166 | 230 | -475 | Tigger7 | MER2\_type | DNA |
| 183283132 | 183283215 | + | 18.8 | 150 | 230 | -321 | Tigger7 | MER2\_type | DNA |
| 183283219 | 183283276 | + | 15.8 | 174 | 230 | -2261 | Tigger7 | MER2\_type | DNA |
| 183283272 | 183283337 | + | 20.0 | 166 | 230 | -475 | Tigger7 | MER2\_type | DNA |
| 183283333 | 183283550 | + | 18.9 | 166 | 395 | -2096 | Tigger7 | MER2\_type | DNA |
| 183283561 | 183283632 | + | 19.4 | 2410 | 2482 | -9 | Tigger7 | MER2\_type | DNA |
| 183283643 | 183284028 | - | 27.0 | 1699 | 2055 | -1364 | L2 | L2 | LINE |

|  |  |  |  |  |  |  |  |  |  |
| --- | --- | --- | --- | --- | --- | --- | --- | --- | --- |
| chr4:185113171-185116769 | | |  |  |  |  |  |  |  |
| Genome Start | Genome End | Strand | Div | Repeat Start | Repeat End | Repeat Left | Name | Family | Class |
| 185113171 | 185113842 | - | 28.1 | 1683 | 2397 | -110 | HAL1 | L1 | LINE |
| 185113945 | 185114349 | - | 27.5 | 313 | 819 | -1688 | HAL1 | L1 | LINE |
| 185114425 | 185114509 | - | 26.3 | 7931 | 8015 | -27 | L1MC4 | L1 | LINE |
| 185114631 | 185115118 | - | 26.3 | 7434 | 7930 | -112 | L1MC4 | L1 | LINE |
| 185115119 | 185115295 | + | 3.4 | 5952 | 6136 | -18 | L1PA5 | L1 | LINE |
| 185115333 | 185115979 | - | 26.3 | 6777 | 7433 | -609 | L1MC4 | L1 | LINE |
| 185115986 | 185116040 | - | 14.6 | 6778 | 6825 | -1217 | L1MC4 | L1 | LINE |
| 185116049 | 185116100 | - | 13.3 | 6781 | 6825 | -1217 | L1MC4 | L1 | LINE |
| 185116113 | 185116168 | - | 10.2 | 6776 | 6824 | -1218 | L1MC4 | L1 | LINE |
| 185116176 | 185116231 | - | 14.3 | 6776 | 6824 | -1218 | L1MC4 | L1 | LINE |
| 185116238 | 185116294 | - | 14.0 | 6776 | 6825 | -1217 | L1MC4 | L1 | LINE |
| 185116301 | 185116466 | - | 19.6 | 6570 | 6740 | -1068 | L1MC4a | L1 | LINE |
| 185116621 | 185116769 | - | 27.1 | 51 | 207 | -2300 | HAL1 | L1 | LINE |

|  |  |  |  |  |  |  |  |  |  |
| --- | --- | --- | --- | --- | --- | --- | --- | --- | --- |
| chr4:189354756-189356151 | | |  |  |  |  |  |  |  |
| Genome Start | Genome End | Strand | Div | Repeat Start | Repeat End | Repeat Left | Name | Family | Class |
| 189354756 | 189355200 | + | 15.2 | 16 | 429 | -356 | LTR1 | ERV1 | LTR |
| 189355200 | 189355260 | + | 7.3 | 374 | 429 | -356 | LTR1 | ERV1 | LTR |
| 189355320 | 189355380 | + | 16.4 | 362 | 424 | -402 | LTR1B | ERV1 | LTR |
| 189355380 | 189355440 | + | 13.1 | 362 | 424 | -402 | LTR1B | ERV1 | LTR |
| 189355440 | 189355500 | + | 16.4 | 362 | 424 | -402 | LTR1B | ERV1 | LTR |
| 189355500 | 189355560 | + | 14.8 | 362 | 424 | -402 | LTR1B | ERV1 | LTR |
| 189355560 | 189355620 | + | 13.1 | 362 | 424 | -402 | LTR1B | ERV1 | LTR |
| 189355621 | 189355680 | + | 16.7 | 363 | 424 | -402 | LTR1B | ERV1 | LTR |
| 189355680 | 189355739 | + | 15.0 | 362 | 423 | -403 | LTR1B | ERV1 | LTR |
| 189355740 | 189356151 | + | 12.4 | 374 | 785 | 0 | LTR1 | ERV1 | LTR |

|  |  |  |  |  |  |  |  |  |  |
| --- | --- | --- | --- | --- | --- | --- | --- | --- | --- |
| chr5:2132993-2142793 | | |  |  |  |  |  |  |  |
| Genome Start | Genome End | Strand | Div | Repeat Start | Repeat End | Repeat Left | Name | Family | Class |
| 2132993 | 2134322 | + | 27.4 | 310 | 1641 | -4759 | L1MEc | L1 | LINE |
| 2134348 | 2135069 | + | 20.3 | 6219 | 6925 | -860 | L1MC3 | L1 | LINE |
| 2135070 | 2135131 | + | 14.5 | 6863 | 6924 | -861 | L1MC3 | L1 | LINE |
| 2135132 | 2135195 | + | 14.1 | 6862 | 6925 | -860 | L1MC3 | L1 | LINE |
| 2135195 | 2135258 | + | 12.5 | 6862 | 6925 | -860 | L1MC3 | L1 | LINE |
| 2135258 | 2135321 | + | 14.1 | 6862 | 6925 | -860 | L1MC3 | L1 | LINE |
| 2135304 | 2135384 | + | 12.8 | 6848 | 6925 | -860 | L1MC3 | L1 | LINE |
| 2135384 | 2135428 | + | 11.1 | 6862 | 6906 | -879 | L1MC3 | L1 | LINE |
| 2135430 | 2135510 | + | 16.7 | 6848 | 6925 | -860 | L1MC3 | L1 | LINE |
| 2135511 | 2135573 | + | 14.3 | 6863 | 6925 | -860 | L1MC3 | L1 | LINE |
| 2135573 | 2135636 | + | 12.5 | 6862 | 6925 | -860 | L1MC3 | L1 | LINE |
| 2135636 | 2135699 | + | 15.6 | 6862 | 6925 | -860 | L1MC3 | L1 | LINE |
| 2135682 | 2135762 | + | 14.1 | 6848 | 6925 | -860 | L1MC3 | L1 | LINE |
| 2135763 | 2135825 | + | 12.7 | 6863 | 6925 | -860 | L1MC3 | L1 | LINE |
| 2135825 | 2135888 | + | 15.6 | 6862 | 6925 | -860 | L1MC3 | L1 | LINE |
| 2135889 | 2135954 | + | 22.7 | 6863 | 6928 | -857 | L1MC3 | L1 | LINE |
| 2136202 | 2136407 | + | 21.0 | 7003 | 7207 | -578 | L1MC3 | L1 | LINE |
| 2136408 | 2136669 | + | 11.1 | 1 | 286 | -25 | AluY | Alu | SINE |
| 2136726 | 2137276 | + | 21.0 | 7208 | 7785 | 0 | L1MC3 | L1 | LINE |
| 2137270 | 2138793 | + | 23.2 | 20 | 1654 | -4746 | L1MEc | L1 | LINE |
| 2138794 | 2138926 | + | 17.6 | 2 | 133 | -10 | FLAM\_C | Alu | SINE |
| 2138927 | 2139500 | + | 23.2 | 1655 | 2270 | -4130 | L1MEc | L1 | LINE |
| 2139507 | 2140476 | + | 13.7 | 5 | 973 | -329 | HSMAR2 | Mariner | DNA |
| 2140477 | 2141070 | - | 11.6 | 1 | 602 | 0 | LTR43 | ERV1 | LTR |
| 2141071 | 2141409 | + | 16.1 | 974 | 1301 | -1 | HSMAR2 | Mariner | DNA |
| 2141410 | 2141512 | + | 20.4 | 2277 | 2379 | -4021 | L1MEc | L1 | LINE |
| 2141570 | 2141597 | + | 20.7 | 2369 | 2388 | -4012 | L1MEc | L1 | LINE |
| 2141598 | 2141903 | + | 17.0 | 4 | 307 | -5 | AluJb | Alu | SINE |
| 2141904 | 2142793 | + | 25.8 | 2285 | 3261 | -2958 | L1MEc | L1 | LINE |

|  |  |  |  |  |  |  |  |  |  |
| --- | --- | --- | --- | --- | --- | --- | --- | --- | --- |
| chr5:133841124-133852948 | | |  |  |  |  |  |  |  |
| Genome Start | Genome End | Strand | Div | Repeat Start | Repeat End | Repeat Left | Name | Family | Class |
| 133841124 | 133841163 | - | 5.0 | 5616 | 5660 | -461 | L1M5 | L1 | LINE |
| 133841164 | 133841577 | + | 19.5 | 1 | 412 | -74 | MER66B | ERV1 | LTR |
| 133841596 | 133841731 | + | 12.1 | 1 | 132 | -178 | AluSg | Alu | SINE |
| 133841732 | 133841848 | + | 11.1 | 1 | 118 | -191 | AluSc | Alu | SINE |
| 133841873 | 133842057 | + | 11.1 | 119 | 304 | -5 | AluSc | Alu | SINE |
| 133842058 | 133842161 | + | 13.5 | 383 | 486 | 0 | MER66B | ERV1 | LTR |
| 133842266 | 133842547 | + | 19.5 | 1 | 296 | -16 | AluJb | Alu | SINE |
| 133842585 | 133842866 | - | 12.2 | 1 | 280 | -32 | AluSx | Alu | SINE |
| 133842876 | 133843043 | - | 20.2 | 1 | 166 | -10 | FRAM | Alu | SINE |
| 133843050 | 133843291 | + | 25.1 | 5031 | 5309 | -837 | L1M5 | L1 | LINE |
| 133843292 | 133843838 | - | 18.9 | 2 | 550 | 0 | MER44B | MER2\_type | DNA |
| 133843839 | 133843852 | + | 25.1 | 5310 | 5322 | -824 | L1M5 | L1 | LINE |
| 133843842 | 133844056 | + | 27.0 | 2942 | 3181 | -2965 | L1M5 | L1 | LINE |
| 133844059 | 133844347 | - | 11.1 | 6 | 294 | -16 | AluSg | Alu | SINE |
| 133844348 | 133844638 | + | 25.6 | 3184 | 3478 | -2668 | L1M5 | L1 | LINE |
| 133844639 | 133844929 | + | 12.4 | 1 | 291 | -22 | AluSq | Alu | SINE |
| 133844930 | 133845287 | + | 25.6 | 3479 | 3844 | -2302 | L1M5 | L1 | LINE |
| 133845288 | 133845586 | + | 8.8 | 1 | 311 | -2 | AluSp | Alu | SINE |
| 133845587 | 133845688 | + | 25.6 | 3845 | 3944 | -2202 | L1M5 | L1 | LINE |
| 133845689 | 133845986 | - | 8.1 | 1 | 301 | -12 | AluSq | Alu | SINE |
| 133846012 | 133846322 | - | 4.8 | 1 | 311 | 0 | AluY | Alu | SINE |
| 133846323 | 133846494 | + | 25.6 | 3945 | 4103 | -2043 | L1M5 | L1 | LINE |
| 133846495 | 133846599 | - | 9.6 | 202 | 305 | -6 | AluY | Alu | SINE |
| 133846600 | 133846635 | - | 13.9 | 205 | 240 | -60 | Alu | Alu | SINE |
| 133846636 | 133846693 | - | 17.2 | 191 | 248 | -52 | Alu | Alu | SINE |
| 133846677 | 133846731 | - | 23.6 | 215 | 269 | -33 | AluSg/x | Alu | SINE |
| 133846730 | 133846787 | - | 15.5 | 191 | 248 | -52 | Alu | Alu | SINE |
| 133846771 | 133846825 | - | 21.8 | 203 | 257 | -43 | Alu | Alu | SINE |
| 133846824 | 133846864 | - | 12.2 | 208 | 248 | -52 | Alu | Alu | SINE |
| 133846865 | 133847132 | - | 9.0 | 1 | 268 | -43 | AluY | Alu | SINE |
| 133847153 | 133847299 | + | 35.4 | 3955 | 4102 | -2044 | L1M5 | L1 | LINE |
| 133847301 | 133847400 | - | 10.1 | 202 | 300 | -11 | AluY | Alu | SINE |
| 133847401 | 133847436 | - | 13.9 | 205 | 240 | -60 | Alu | Alu | SINE |
| 133847437 | 133847494 | - | 15.5 | 191 | 248 | -52 | Alu | Alu | SINE |
| 133847495 | 133847532 | - | 13.2 | 203 | 240 | -60 | Alu | Alu | SINE |
| 133847531 | 133847588 | - | 15.5 | 191 | 248 | -52 | Alu | Alu | SINE |
| 133847572 | 133847626 | - | 21.8 | 203 | 257 | -43 | Alu | Alu | SINE |
| 133847625 | 133847665 | - | 12.2 | 208 | 248 | -52 | Alu | Alu | SINE |
| 133847666 | 133847933 | - | 9.3 | 1 | 268 | -43 | AluY | Alu | SINE |
| 133847996 | 133848017 | + | 26.5 | 4160 | 4178 | -1968 | L1M5 | L1 | LINE |
| 133848018 | 133848322 | + | 12.0 | 2 | 312 | 0 | AluSx | Alu | SINE |
| 133848323 | 133848717 | + | 26.5 | 4179 | 4609 | -1537 | L1M5 | L1 | LINE |
| 133848718 | 133849002 | + | 16.2 | 1 | 273 | -39 | AluJo | Alu | SINE |
| 133849003 | 133850059 | + | 26.3 | 4610 | 5724 | -516 | L1M5 | L1 | LINE |
| 133850061 | 133850133 | - | 33.8 | 368 | 450 | 0 | LTR16A | ERVL | LTR |
| 133850134 | 133850518 | + | 17.9 | 1 | 361 | -65 | MSTB | MaLR | LTR |
| 133850519 | 133850831 | - | 13.4 | 1 | 313 | 0 | AluSq | Alu | SINE |
| 133850832 | 133850896 | + | 17.9 | 362 | 426 | 0 | MSTB | MaLR | LTR |
| 133850897 | 133851249 | - | 33.8 | 4 | 367 | -83 | LTR16A | ERVL | LTR |
| 133851359 | 133851823 | - | 19.4 | 5478 | 5957 | -209 | L1ME1 | L1 | LINE |
| 133851824 | 133852134 | - | 11.6 | 1 | 311 | -1 | AluSx | Alu | SINE |
| 133852135 | 133852696 | - | 20.0 | 4832 | 5477 | -689 | L1ME1 | L1 | LINE |
| 133852720 | 133852948 | - | 33.2 | 4791 | 5016 | -1130 | L1M5 | L1 | LINE |

|  |  |  |  |  |  |  |  |  |  |
| --- | --- | --- | --- | --- | --- | --- | --- | --- | --- |
| chr5:175565061-175571358 | | |  |  |  |  |  |  |  |
| Genome Start | Genome End | Strand | Div | Repeat Start | Repeat End | Repeat Left | Name | Family | Class |
| 175565061 | 175565292 | - | 17.6 | 4472 | 4716 | -1430 | L1M4 | L1 | LINE |
| 175565293 | 175565601 | + | 9.1 | 1 | 310 | 0 | AluSg | Alu | SINE |
| 175565602 | 175565694 | - | 17.6 | 4379 | 4471 | -1675 | L1M4 | L1 | LINE |
| 175565695 | 175565927 | - | 16.0 | 192 | 428 | 0 | MSTA | MaLR | LTR |
| 175565928 | 175566221 | - | 13.9 | 1 | 294 | -18 | AluSx | Alu | SINE |
| 175566229 | 175566525 | - | 12.8 | 1 | 302 | -10 | AluSx | Alu | SINE |
| 175566526 | 175566668 | - | 16.0 | 58 | 191 | -237 | MSTA | MaLR | LTR |
| 175566669 | 175566720 | - | 13.5 | 58 | 110 | -318 | MSTA | MaLR | LTR |
| 175566721 | 175566772 | - | 13.5 | 58 | 110 | -318 | MSTA | MaLR | LTR |
| 175566773 | 175566824 | - | 11.5 | 58 | 110 | -318 | MSTA | MaLR | LTR |
| 175566877 | 175566928 | - | 11.5 | 58 | 110 | -318 | MSTA | MaLR | LTR |
| 175566929 | 175566980 | - | 15.4 | 58 | 110 | -318 | MSTA | MaLR | LTR |
| 175567085 | 175567136 | - | 13.5 | 58 | 110 | -318 | MSTA | MaLR | LTR |
| 175567137 | 175567188 | - | 11.5 | 58 | 110 | -318 | MSTA | MaLR | LTR |
| 175567189 | 175567240 | - | 9.6 | 58 | 110 | -318 | MSTA | MaLR | LTR |
| 175567241 | 175567312 | - | 16.7 | 38 | 110 | -318 | MSTA | MaLR | LTR |
| 175567315 | 175568929 | - | 14.3 | 1 | 1635 | -16 | MSTA-int | MaLR | LTR |
| 175568930 | 175568986 | - | 15.1 | 390 | 428 | 0 | MSTA | MaLR | LTR |
| 175568987 | 175569298 | - | 14.0 | 2 | 310 | -2 | AluSx | Alu | SINE |
| 175569299 | 175569672 | - | 15.1 | 1 | 389 | -39 | MSTA | MaLR | LTR |
| 175569673 | 175569769 | - | 15.9 | 4294 | 4381 | -1765 | L1M4 | L1 | LINE |
| 175569770 | 175570059 | - | 12.4 | 1 | 291 | -21 | AluSx | Alu | SINE |
| 175570060 | 175571358 | - | 15.9 | 2987 | 4293 | -1853 | L1M4 | L1 | LINE |

|  |  |  |  |  |  |  |  |  |  |
| --- | --- | --- | --- | --- | --- | --- | --- | --- | --- |
| chr5:177058798-177065789 | | |  |  |  |  |  |  |  |
| Genome Start | Genome End | Strand | Div | Repeat Start | Repeat End | Repeat Left | Name | Family | Class |
| 177058798 | 177060127 | + | 15.0 | 2987 | 4293 | -1853 | L1M4 | L1 | LINE |
| 177060128 | 177060417 | + | 11.0 | 1 | 291 | -21 | AluSx | Alu | SINE |
| 177060418 | 177060514 | + | 15.0 | 4294 | 4381 | -1765 | L1M4 | L1 | LINE |
| 177060515 | 177060889 | + | 14.4 | 1 | 389 | -39 | MSTA | MaLR | LTR |
| 177060890 | 177061178 | + | 15.6 | 2 | 291 | -21 | AluSx | Alu | SINE |
| 177061179 | 177061256 | + | 14.4 | 390 | 428 | 0 | MSTA | MaLR | LTR |
| 177061257 | 177061518 | + | 14.8 | 1 | 263 | -1388 | MSTA-int | MaLR | LTR |
| 177061519 | 177061814 | - | 3.7 | 1 | 295 | -16 | AluY | Alu | SINE |
| 177061815 | 177063176 | + | 14.8 | 264 | 1635 | -16 | MSTA-int | MaLR | LTR |
| 177063179 | 177063250 | + | 15.3 | 38 | 110 | -318 | MSTA | MaLR | LTR |
| 177063251 | 177063302 | + | 15.4 | 58 | 110 | -318 | MSTA | MaLR | LTR |
| 177063303 | 177063354 | + | 13.5 | 58 | 110 | -318 | MSTA | MaLR | LTR |
| 177063355 | 177063406 | + | 13.5 | 58 | 110 | -318 | MSTA | MaLR | LTR |
| 177063407 | 177063458 | + | 13.5 | 58 | 110 | -318 | MSTA | MaLR | LTR |
| 177063459 | 177063510 | + | 11.5 | 58 | 110 | -318 | MSTA | MaLR | LTR |
| 177063511 | 177063562 | + | 11.5 | 58 | 110 | -318 | MSTA | MaLR | LTR |
| 177063563 | 177063614 | + | 13.5 | 58 | 110 | -318 | MSTA | MaLR | LTR |
| 177063615 | 177063666 | + | 13.5 | 58 | 110 | -318 | MSTA | MaLR | LTR |
| 177063667 | 177063718 | + | 15.4 | 58 | 110 | -318 | MSTA | MaLR | LTR |
| 177063719 | 177063770 | + | 15.4 | 58 | 110 | -318 | MSTA | MaLR | LTR |
| 177063771 | 177063822 | + | 13.5 | 58 | 110 | -318 | MSTA | MaLR | LTR |
| 177063823 | 177063874 | + | 13.5 | 58 | 110 | -318 | MSTA | MaLR | LTR |
| 177063875 | 177063926 | + | 11.5 | 58 | 110 | -318 | MSTA | MaLR | LTR |
| 177063927 | 177063978 | + | 13.5 | 58 | 110 | -318 | MSTA | MaLR | LTR |
| 177063979 | 177064030 | + | 13.5 | 58 | 110 | -318 | MSTA | MaLR | LTR |
| 177064031 | 177064082 | + | 13.5 | 58 | 110 | -318 | MSTA | MaLR | LTR |
| 177064083 | 177064134 | + | 13.5 | 58 | 110 | -318 | MSTA | MaLR | LTR |
| 177064135 | 177064186 | + | 11.5 | 58 | 110 | -318 | MSTA | MaLR | LTR |
| 177064187 | 177064329 | + | 14.9 | 58 | 194 | -234 | MSTA | MaLR | LTR |
| 177064330 | 177064621 | + | 13.8 | 1 | 296 | -16 | AluSx | Alu | SINE |
| 177064629 | 177064922 | + | 13.9 | 1 | 294 | -18 | AluSx | Alu | SINE |
| 177064923 | 177065155 | + | 14.9 | 195 | 428 | 0 | MSTA | MaLR | LTR |
| 177065156 | 177065255 | + | 16.7 | 4379 | 4475 | -1671 | L1M4 | L1 | LINE |
| 177065256 | 177065564 | - | 8.4 | 1 | 310 | 0 | AluSg | Alu | SINE |
| 177065565 | 177065789 | + | 16.7 | 4476 | 4716 | -1430 | L1M4 | L1 | LINE |

|  |  |  |  |  |  |  |  |  |  |
| --- | --- | --- | --- | --- | --- | --- | --- | --- | --- |
| chr5:180273505-180283529 | | |  |  |  |  |  |  |  |
| Genome Start | Genome End | Strand | Div | Repeat Start | Repeat End | Repeat Left | Name | Family | Class |
| 180273505 | 180273682 | + | 30.2 | 2589 | 2768 | -3378 | L1M5 | L1 | LINE |
| 180273683 | 180274070 | + | 17.8 | 4 | 407 | -1 | MLT1A1 | MaLR | LTR |
| 180274071 | 180274263 | + | 30.2 | 2769 | 2965 | -3181 | L1M5 | L1 | LINE |
| 180274290 | 180275233 | + | 26.2 | 2 | 1006 | -5135 | L1MB1 | L1 | LINE |
| 180275249 | 180275545 | + | 14.7 | 3737 | 4040 | -2103 | L1M2 | L1 | LINE |
| 180275547 | 180275888 | - | 18.7 | 3 | 396 | 0 | MSTD | MaLR | LTR |
| 180275907 | 180276288 | + | 13.9 | 3013 | 3408 | -2796 | L1MB1 | L1 | LINE |
| 180276291 | 180276383 | - | 17.2 | 2397 | 2489 | -2 | Tigger7 | MER2\_type | DNA |
| 180276382 | 180276563 | - | 23.1 | 3 | 185 | -520 | Tigger7 | MER2\_type | DNA |
| 180276567 | 180276611 | + | 15.6 | 3408 | 3452 | -2752 | L1MB1 | L1 | LINE |
| 180276636 | 180276680 | + | 15.6 | 3408 | 3452 | -2752 | L1MB1 | L1 | LINE |
| 180276695 | 180276749 | + | 13.8 | 3406 | 3455 | -2752 | L1MB1 | L1 | LINE |
| 180276774 | 180276818 | + | 11.1 | 3408 | 3452 | -2752 | L1MB1 | L1 | LINE |
| 180276843 | 180276887 | + | 11.1 | 3408 | 3452 | -2752 | L1MB1 | L1 | LINE |
| 180276912 | 180276956 | + | 11.1 | 3408 | 3452 | -2752 | L1MB1 | L1 | LINE |
| 180276981 | 180277025 | + | 11.1 | 3408 | 3452 | -2752 | L1MB1 | L1 | LINE |
| 180277050 | 180277094 | + | 11.1 | 3408 | 3452 | -2752 | L1MB1 | L1 | LINE |
| 180277089 | 180277347 | + | 17.4 | 3384 | 3641 | -2566 | L1MB1 | L1 | LINE |
| 180277353 | 180277664 | + | 7.7 | 1 | 311 | 0 | AluY | Alu | SINE |
| 180277665 | 180278137 | + | 17.7 | 3633 | 4070 | -2134 | L1MB1 | L1 | LINE |
| 180278138 | 180279036 | - | 14.6 | 1 | 900 | 0 | MER4D1 | ERV1 | LTR |
| 180279037 | 180279734 | + | 17.7 | 4071 | 4811 | -1393 | L1MB1 | L1 | LINE |
| 180279735 | 180279814 | - | 7.5 | 1 | 80 | 0 | MADE1 | Mariner | DNA |
| 180279815 | 180281221 | + | 19.8 | 4748 | 6119 | -181 | L1MA6 | L1 | LINE |
| 180280761 | 180281266 | + | 21.3 | 5617 | 6158 | -31 | L1MB1 | L1 | LINE |
| 180281314 | 180281480 | + | 21.3 | 6159 | 6313 | -21 | L1MA10 | L1 | LINE |
| 180281549 | 180282786 | - | 11.6 | 2156 | 3404 | -2742 | L1PREC2 | L1 | LINE |
| 180282819 | 180283529 | + | 30.3 | 3713 | 4437 | -1709 | L1M5 | L1 | LINE |

|  |  |  |  |  |  |  |  |  |  |
| --- | --- | --- | --- | --- | --- | --- | --- | --- | --- |
| chr5:180832122-180835373 | | |  |  |  |  |  |  |  |
| Genome Start | Genome End | Strand | Div | Repeat Start | Repeat End | Repeat Left | Name | Family | Class |
| 180832122 | 180832568 | - | 21.1 | 1063 | 1506 | -3430 | MER34B-int | ERV1 | LTR |
| 180832572 | 180832639 | + | 25.0 | 3 | 72 | -482 | MLT2B3 | ERVL | LTR |
| 180832665 | 180832757 | + | 20.3 | 3 | 96 | -501 | MLT2B3 | ERVL | LTR |
| 180832758 | 180832825 | + | 19.1 | 3 | 72 | -482 | MLT2B3 | ERVL | LTR |
| 180832851 | 180832943 | + | 21.6 | 3 | 96 | -501 | MLT2B3 | ERVL | LTR |
| 180832944 | 180833011 | + | 19.1 | 3 | 72 | -482 | MLT2B3 | ERVL | LTR |
| 180833037 | 180833104 | + | 16.2 | 3 | 72 | -482 | MLT2B3 | ERVL | LTR |
| 180833130 | 180833197 | + | 19.1 | 3 | 72 | -482 | MLT2B3 | ERVL | LTR |
| 180833223 | 180833290 | + | 19.1 | 3 | 72 | -482 | MLT2B3 | ERVL | LTR |
| 180833316 | 180833383 | + | 20.6 | 3 | 72 | -482 | MLT2B3 | ERVL | LTR |
| 180833409 | 180833476 | + | 16.2 | 3 | 72 | -482 | MLT2B3 | ERVL | LTR |
| 180833502 | 180833569 | + | 17.6 | 3 | 72 | -482 | MLT2B5 | ERVL | LTR |
| 180833595 | 180833821 | + | 20.4 | 3 | 274 | -388 | MLT2B3 | ERVL | LTR |
| 180833822 | 180834183 | + | 13.9 | 1 | 375 | 0 | THE1C | MaLR | LTR |
| 180834184 | 180834533 | + | 23.1 | 159 | 597 | -4 | MLT2B5 | ERVL | LTR |
| 180834541 | 180834905 | - | 27.3 | 620 | 1063 | -3873 | MER34B-int | ERV1 | LTR |
| 180834881 | 180835220 | - | 26.5 | 173 | 515 | -4421 | MER34B-int | ERV1 | LTR |
| 180835262 | 180835373 | - | 19.2 | 500 | 623 | -4733 | MER34B-int | ERV1 | LTR |

|  |  |  |  |  |  |  |  |  |  |
| --- | --- | --- | --- | --- | --- | --- | --- | --- | --- |
| chr6:161650260-161659077 | | |  |  |  |  |  |  |  |
| Genome Start | Genome End | Strand | Div | Repeat Start | Repeat End | Repeat Left | Name | Family | Class |
| 161650260 | 161650372 | - | 27.7 | 7930 | 8042 | 0 | L1MC4 | L1 | LINE |
| 161650380 | 161650675 | - | 11.7 | 19 | 313 | 0 | AluSp | Alu | SINE |
| 161650676 | 161651180 | - | 9.0 | 1 | 508 | -3 | LTR10F | ERV1 | LTR |
| 161651181 | 161656136 | - | 9.6 | 1 | 5102 | 0 | HERVIP10FH | ERV1 | LTR |
| 161656137 | 161656537 | - | 9.3 | 104 | 511 | 0 | LTR10F | ERV1 | LTR |
| 161656543 | 161656649 | - | 3.0 | 104 | 210 | -301 | LTR10F | ERV1 | LTR |
| 161656655 | 161656684 | - | 0.0 | 127 | 156 | -355 | LTR10F | ERV1 | LTR |
| 161656685 | 161656896 | - | 5.4 | 1 | 210 | -301 | LTR10F | ERV1 | LTR |
| 161656993 | 161657875 | + | 14.7 | 45 | 977 | -254 | Tigger3b | MER2\_type | DNA |
| 161657876 | 161658167 | - | 13.0 | 1 | 295 | -18 | AluSq | Alu | SINE |
| 161658168 | 161658355 | + | 14.7 | 978 | 1170 | -61 | Tigger3b | MER2\_type | DNA |
| 161658356 | 161658644 | - | 8.0 | 10 | 298 | -14 | AluSx | Alu | SINE |
| 161658645 | 161658725 | + | 14.7 | 1171 | 1231 | 0 | Tigger3b | MER2\_type | DNA |
| 161658920 | 161659077 | - | 27.4 | 7742 | 7912 | -130 | L1MC4 | L1 | LINE |

|  |  |  |  |  |  |  |  |  |  |
| --- | --- | --- | --- | --- | --- | --- | --- | --- | --- |
| chr6:170475891-170479754 | | |  |  |  |  |  |  |  |
| Genome Start | Genome End | Strand | Div | Repeat Start | Repeat End | Repeat Left | Name | Family | Class |
| 170475891 | 170476548 | - | 24.7 | 2154 | 2780 | -3439 | L1MEc | L1 | LINE |
| 170476572 | 170476881 | + | 9.0 | 1 | 300 | -13 | AluSq | Alu | SINE |
| 170476902 | 170476947 | + | 6.5 | 1 | 46 | -34 | MADE1 | Mariner | DNA |
| 170476948 | 170477266 | - | 25.0 | 1968 | 8008 | -4290 | L1MC4 | L1 | LINE |
| 170477080 | 170477510 | - | 23.3 | 5961 | 6416 | -16 | L1MD2 | L1 | LINE |
| 170477541 | 170477740 | - | 19.4 | 163 | 340 | -1 | MER58B | MER1\_type | DNA |
| 170477741 | 170478056 | - | 16.4 | 1 | 311 | -1 | AluJo | Alu | SINE |
| 170478067 | 170478358 | - | 5.8 | 1 | 292 | -19 | AluY | Alu | SINE |
| 170478359 | 170478508 | - | 19.4 | 7 | 162 | -179 | MER58B | MER1\_type | DNA |
| 170478548 | 170478650 | - | 20.4 | 2587 | 2693 | -336 | Tigger3(Golem) | MER2\_type | DNA |
| 170478644 | 170478746 | - | 19.4 | 2587 | 2691 | -338 | Tigger3(Golem) | MER2\_type | DNA |
| 170478737 | 170478842 | - | 21.7 | 2587 | 2694 | -335 | Tigger3(Golem) | MER2\_type | DNA |
| 170478836 | 170478939 | - | 22.1 | 2584 | 2691 | -338 | Tigger3(Golem) | MER2\_type | DNA |
| 170478930 | 170479356 | - | 12.7 | 2284 | 2691 | -338 | Tigger3(Golem) | MER2\_type | DNA |
| 170479488 | 170479754 | - | 28.7 | 1266 | 1561 | -4839 | L1MEc | L1 | LINE |

|  |  |  |  |  |  |  |  |  |  |
| --- | --- | --- | --- | --- | --- | --- | --- | --- | --- |
| chr7:1964627-1969750 | | |  |  |  |  |  |  |  |
| Genome Start | Genome End | Strand | Div | Repeat Start | Repeat End | Repeat Left | Name | Family | Class |
| 1964627 | 1965130 | + | 27.0 | 624 | 1174 | -5226 | L1ME1 | L1 | LINE |
| 1965131 | 1965434 | + | 13.9 | 1 | 307 | -5 | AluSx | Alu | SINE |
| 1965435 | 1967829 | + | 27.5 | 1175 | 5177 | -1042 | L1ME1 | L1 | LINE |
| 1967831 | 1967961 | + | 11.4 | 1 | 133 | -242 | THE1C | MaLR | LTR |
| 1967945 | 1968027 | + | 18.5 | 51 | 133 | -242 | THE1C | MaLR | LTR |
| 1968011 | 1968093 | + | 17.3 | 51 | 133 | -242 | THE1C | MaLR | LTR |
| 1968077 | 1968159 | + | 19.8 | 51 | 133 | -242 | THE1C | MaLR | LTR |
| 1968143 | 1968222 | + | 20.5 | 51 | 130 | -245 | THE1C | MaLR | LTR |
| 1968209 | 1968290 | + | 18.8 | 51 | 132 | -243 | THE1C | MaLR | LTR |
| 1968269 | 1968581 | + | 15.9 | 45 | 353 | -2 | THE1A | MaLR | LTR |
| 1968585 | 1968947 | + | 19.3 | 5180 | 5550 | -689 | L1ME1 | L1 | LINE |
| 1968954 | 1969135 | + | 13.4 | 1 | 171 | -5 | FRAM | Alu | SINE |
| 1969187 | 1969750 | + | 34.3 | 5633 | 6234 | -5 | L1ME1 | L1 | LINE |

|  |  |  |  |  |  |  |  |  |  |
| --- | --- | --- | --- | --- | --- | --- | --- | --- | --- |
| chr8:10920764-10922384 | | |  |  |  |  |  |  |  |
| Genome Start | Genome End | Strand | Div | Repeat Start | Repeat End | Repeat Left | Name | Family | Class |
| 10920764 | 10920948 | + | 20.9 | 9 | 191 | -133 | MER33 | MER1\_type | DNA |
| 10920952 | 10921105 | - | 15.6 | 2880 | 3028 | -1 | Tigger3(Golem) | MER2\_type | DNA |
| 10921129 | 10921180 | - | 13.5 | 2880 | 2936 | -93 | Tigger3(Golem) | MER2\_type | DNA |
| 10921195 | 10921252 | - | 10.5 | 2882 | 2943 | -86 | Tigger3(Golem) | MER2\_type | DNA |
| 10921253 | 10921302 | - | 20.0 | 2882 | 2936 | -93 | Tigger3(Golem) | MER2\_type | DNA |
| 10921303 | 10921354 | - | 11.5 | 2880 | 2936 | -93 | Tigger3(Golem) | MER2\_type | DNA |
| 10921378 | 10921429 | - | 9.6 | 2880 | 2936 | -93 | Tigger3(Golem) | MER2\_type | DNA |
| 10921432 | 10921486 | - | 11.3 | 2875 | 2932 | -97 | Tigger3(Golem) | MER2\_type | DNA |
| 10921494 | 10921553 | - | 13.6 | 2880 | 2943 | -86 | Tigger3(Golem) | MER2\_type | DNA |
| 10921593 | 10921652 | - | 11.9 | 2880 | 2943 | -86 | Tigger3(Golem) | MER2\_type | DNA |
| 10921655 | 10921685 | - | 9.7 | 2902 | 2932 | -97 | Tigger3(Golem) | MER2\_type | DNA |
| 10921694 | 10921750 | - | 14.3 | 2882 | 2942 | -87 | Tigger3(Golem) | MER2\_type | DNA |
| 10921751 | 10921805 | - | 16.4 | 2877 | 2936 | -93 | Tigger3(Golem) | MER2\_type | DNA |
| 10921826 | 10921877 | - | 11.5 | 2880 | 2936 | -93 | Tigger3(Golem) | MER2\_type | DNA |
| 10921893 | 10921952 | - | 18.6 | 2880 | 2943 | -86 | Tigger3(Golem) | MER2\_type | DNA |
| 10921976 | 10922229 | - | 14.2 | 2 | 254 | -92 | MER7A | MER2\_type | DNA |
| 10922233 | 10922384 | + | 14.1 | 195 | 322 | -2 | MER33 | MER1\_type | DNA |

|  |  |  |  |  |  |  |  |  |  |
| --- | --- | --- | --- | --- | --- | --- | --- | --- | --- |
| chr8:122738771-122740537 | | |  |  |  |  |  |  |  |
| Genome Start | Genome End | Strand | Div | Repeat Start | Repeat End | Repeat Left | Name | Family | Class |
| 122738771 | 122738955 | - | 16.3 | 4168 | 4362 | -1778 | L1M3 | L1 | LINE |
| 122738938 | 122738957 | - | 13.3 | 6146 | 6164 | -1 | L1PA15 | L1 | LINE |
| 122738958 | 122739009 | - | 1.9 | 6105 | 6161 | -4 | L1PA15 | L1 | LINE |
| 122739010 | 122739050 | - | 13.3 | 6105 | 6145 | -20 | L1PA15 | L1 | LINE |
| 122739051 | 122739091 | - | 4.9 | 6105 | 6145 | -20 | L1PA15 | L1 | LINE |
| 122739092 | 122739132 | - | 4.9 | 6105 | 6145 | -20 | L1PA15 | L1 | LINE |
| 122739133 | 122739173 | - | 4.9 | 6105 | 6145 | -15 | L1PREC2 | L1 | LINE |
| 122739174 | 122739214 | - | 2.4 | 6105 | 6145 | -15 | L1PREC2 | L1 | LINE |
| 122739215 | 122739255 | - | 2.4 | 6105 | 6145 | -20 | L1PA15 | L1 | LINE |
| 122739256 | 122739296 | - | 4.9 | 6105 | 6145 | -15 | L1PREC2 | L1 | LINE |
| 122739297 | 122739337 | - | 2.4 | 6105 | 6145 | -15 | L1PREC2 | L1 | LINE |
| 122739338 | 122739564 | - | 8.6 | 5925 | 6145 | -15 | L1PREC2 | L1 | LINE |
| 122739570 | 122739996 | - | 18.3 | 3743 | 4150 | -1990 | L1M3 | L1 | LINE |
| 122739997 | 122740290 | - | 10.9 | 18 | 311 | -1 | AluSx | Alu | SINE |
| 122740291 | 122740537 | - | 18.3 | 3508 | 3742 | -2398 | L1M3 | L1 | LINE |

|  |  |  |  |  |  |  |  |  |  |
| --- | --- | --- | --- | --- | --- | --- | --- | --- | --- |
| chr8:122861964-122864322 | | |  |  |  |  |  |  |  |
| Genome Start | Genome End | Strand | Div | Repeat Start | Repeat End | Repeat Left | Name | Family | Class |
| 122861964 | 122862049 | + | 28.1 | 2552 | 2630 | -789 | L2 | L2 | LINE |
| 122862050 | 122862344 | - | 6.1 | 1 | 295 | -16 | AluY | Alu | SINE |
| 122862345 | 122862872 | + | 28.9 | 2631 | 3192 | -227 | L2 | L2 | LINE |
| 122862887 | 122863202 | - | 8.9 | 141 | 464 | 0 | LTR7B | ERV1 | LTR |
| 122863203 | 122863255 | - | 7.5 | 185 | 238 | -226 | LTR7B | ERV1 | LTR |
| 122863256 | 122863382 | - | 11.8 | 141 | 278 | -186 | LTR7B | ERV1 | LTR |
| 122863406 | 122863532 | - | 12.6 | 141 | 278 | -186 | LTR7B | ERV1 | LTR |
| 122863533 | 122863636 | - | 13.5 | 163 | 269 | -195 | LTR7B | ERV1 | LTR |
| 122863646 | 122863918 | - | 6.2 | 1 | 278 | -186 | LTR7B | ERV1 | LTR |
| 122864058 | 122864190 | + | 18.8 | 1 | 133 | 0 | FLAM\_A | Alu | SINE |
| 122864219 | 122864322 | + | 31.1 | 3305 | 3415 | -4 | L2 | L2 | LINE |

|  |  |  |  |  |  |  |  |  |  |
| --- | --- | --- | --- | --- | --- | --- | --- | --- | --- |
| chr8:145804715-145811191 | | |  |  |  |  |  |  |  |
| Genome Start | Genome End | Strand | Div | Repeat Start | Repeat End | Repeat Left | Name | Family | Class |
| 145804715 | 145805590 | - | 27.1 | 3860 | 4804 | -1415 | L1MEc | L1 | LINE |
| 145805591 | 145805894 | - | 10.0 | 1 | 302 | -8 | AluSg | Alu | SINE |
| 145805895 | 145806622 | - | 27.1 | 3109 | 3859 | -2360 | L1MEc | L1 | LINE |
| 145806623 | 145806916 | + | 7.5 | 1 | 293 | -18 | AluY | Alu | SINE |
| 145806917 | 145807023 | - | 27.1 | 3003 | 3108 | -3111 | L1MEc | L1 | LINE |
| 145807024 | 145807311 | - | 11.2 | 1 | 286 | -26 | AluSx | Alu | SINE |
| 145807312 | 145807926 | - | 27.1 | 2312 | 3002 | -3217 | L1MEc | L1 | LINE |
| 145807927 | 145808219 | + | 17.1 | 1 | 300 | -13 | AluSq | Alu | SINE |
| 145808220 | 145808314 | + | 22.1 | 177 | 271 | -41 | AluJb | Alu | SINE |
| 145808315 | 145808363 | + | 16.3 | 224 | 272 | -30 | AluJ/FRAM | Alu | SINE |
| 145808364 | 145808411 | + | 16.7 | 225 | 272 | -30 | AluJ/FRAM | Alu | SINE |
| 145808412 | 145808507 | + | 18.5 | 89 | 272 | -30 | AluJ/FRAM | Alu | SINE |
| 145808508 | 145808555 | + | 16.7 | 225 | 272 | -30 | AluJ/FRAM | Alu | SINE |
| 145808556 | 145808651 | + | 18.5 | 89 | 272 | -30 | AluJ/FRAM | Alu | SINE |
| 145808652 | 145808699 | + | 16.7 | 225 | 272 | -30 | AluJ/FRAM | Alu | SINE |
| 145808700 | 145808747 | + | 16.7 | 225 | 272 | -30 | AluJ/FRAM | Alu | SINE |
| 145808748 | 145808843 | + | 18.5 | 89 | 272 | -30 | AluJ/FRAM | Alu | SINE |
| 145808844 | 145808891 | + | 16.7 | 225 | 272 | -30 | AluJ/FRAM | Alu | SINE |
| 145808892 | 145808939 | + | 16.7 | 225 | 272 | -30 | AluJ/FRAM | Alu | SINE |
| 145808962 | 145809054 | + | 19.4 | 197 | 291 | -21 | AluJb | Alu | SINE |
| 145809062 | 145810547 | - | 21.2 | 762 | 2340 | -4060 | L1MEc | L1 | LINE |
| 145810548 | 145810845 | - | 9.1 | 1 | 298 | -14 | AluSx | Alu | SINE |
| 145810846 | 145811191 | - | 21.2 | 400 | 761 | -5639 | L1MEc | L1 | LINE |

|  |  |  |  |  |  |  |  |  |  |
| --- | --- | --- | --- | --- | --- | --- | --- | --- | --- |
| chr9:135147896-135150343 | | |  |  |  |  |  |  |  |
| Genome Start | Genome End | Strand | Div | Repeat Start | Repeat End | Repeat Left | Name | Family | Class |
| 135147896 | 135148139 | - | 13.4 | 217 | 480 | -2 | MER51E | ERV1 | LTR |
| 135148261 | 135148525 | - | 15.0 | 49 | 309 | -3 | AluJo | Alu | SINE |
| 135148552 | 135148673 | - | 16.4 | 209 | 330 | -131 | MER65C | ERV1 | LTR |
| 135148729 | 135148765 | - | 10.8 | 209 | 245 | -216 | MER65C | ERV1 | LTR |
| 135148792 | 135149788 | + | 15.1 | 1 | 1002 | 0 | LTR5B | ERVK | LTR |
| 135149796 | 135149832 | - | 13.5 | 209 | 245 | -216 | MER65C | ERV1 | LTR |
| 135149925 | 135149961 | - | 16.2 | 209 | 245 | -216 | MER65C | ERV1 | LTR |
| 135149957 | 135149993 | - | 13.5 | 209 | 245 | -216 | MER65C | ERV1 | LTR |
| 135150081 | 135150198 | - | 16.2 | 156 | 266 | -195 | MER65C | ERV1 | LTR |
| 135150198 | 135150343 | - | 10.3 | 86 | 231 | -251 | MER51E | ERV1 | LTR |

|  |  |  |  |  |  |  |  |  |  |
| --- | --- | --- | --- | --- | --- | --- | --- | --- | --- |
| chr10:1491080-1493171 | | |  |  |  |  |  |  |  |
| Genome Start | Genome End | Strand | Div | Repeat Start | Repeat End | Repeat Left | Name | Family | Class |
| 1491080 | 1491675 | + | 12.2 | 5336 | 5951 | -212 | L1PA13 | L1 | LINE |
| 1491676 | 1491968 | + | 13.0 | 1 | 295 | -18 | AluSq | Alu | SINE |
| 1491969 | 1492149 | + | 12.2 | 5952 | 6122 | -41 | L1PA13 | L1 | LINE |
| 1492165 | 1492217 | + | 19.6 | 6072 | 6122 | -38 | L1PREC2 | L1 | LINE |
| 1492199 | 1492251 | + | 19.6 | 6072 | 6122 | -38 | L1PREC2 | L1 | LINE |
| 1492335 | 1492403 | + | 22.4 | 6072 | 6141 | -19 | L1PREC2 | L1 | LINE |
| 1492390 | 1492455 | + | 21.0 | 6061 | 6122 | -38 | L1PREC2 | L1 | LINE |
| 1492437 | 1492489 | + | 21.6 | 6072 | 6122 | -41 | L1PA13 | L1 | LINE |
| 1492471 | 1492523 | + | 17.6 | 6072 | 6122 | -38 | L1PREC2 | L1 | LINE |
| 1492508 | 1492555 | + | 17.4 | 6075 | 6120 | -41 | L1PA14 | L1 | LINE |
| 1492539 | 1492625 | + | 20.7 | 6072 | 6141 | -38 | L1PREC2 | L1 | LINE |
| 1492811 | 1492863 | + | 21.6 | 6072 | 6122 | -38 | L1PREC2 | L1 | LINE |
| 1492845 | 1492913 | + | 22.4 | 6072 | 6141 | -19 | L1PREC2 | L1 | LINE |
| 1492900 | 1492965 | + | 19.4 | 6061 | 6122 | -38 | L1PREC2 | L1 | LINE |
| 1492947 | 1492998 | + | 20.0 | 6072 | 6121 | -39 | L1PREC2 | L1 | LINE |
| 1492981 | 1493033 | + | 21.6 | 6072 | 6122 | -38 | L1PREC2 | L1 | LINE |
| 1493015 | 1493067 | + | 19.6 | 6072 | 6122 | -38 | L1PREC2 | L1 | LINE |
| 1493092 | 1493171 | + | 12.8 | 6081 | 6163 | 0 | L1PA13 | L1 | LINE |

|  |  |  |  |  |  |  |  |  |  |
| --- | --- | --- | --- | --- | --- | --- | --- | --- | --- |
| chr10:116684136-116685757 | | |  |  |  |  |  |  |  |
| Genome Start | Genome End | Strand | Div | Repeat Start | Repeat End | Repeat Left | Name | Family | Class |
| 116684136 | 116684160 | - | 23.4 | 3394 | 3419 | 0 | L2 | L2 | LINE |
| 116684161 | 116684479 | + | 9.4 | 1 | 313 | 0 | AluSq | Alu | SINE |
| 116684480 | 116684620 | - | 23.4 | 3255 | 3393 | -26 | L2 | L2 | LINE |
| 116684622 | 116684987 | - | 15.2 | 52 | 425 | -1 | MSTB | MaLR | LTR |
| 116684989 | 116685060 | - | 13.9 | 53 | 124 | -304 | MSTA | MaLR | LTR |
| 116685059 | 116685133 | - | 14.7 | 53 | 127 | -301 | MSTA | MaLR | LTR |
| 116685132 | 116685206 | - | 14.7 | 53 | 127 | -301 | MSTA | MaLR | LTR |
| 116685205 | 116685279 | - | 14.7 | 53 | 127 | -301 | MSTA | MaLR | LTR |
| 116685278 | 116685352 | - | 13.3 | 53 | 127 | -301 | MSTA | MaLR | LTR |
| 116685351 | 116685425 | - | 13.3 | 53 | 127 | -301 | MSTA | MaLR | LTR |
| 116685424 | 116685498 | - | 12.0 | 53 | 127 | -301 | MSTA | MaLR | LTR |
| 116685497 | 116685618 | - | 13.9 | 5 | 126 | -300 | MSTB | MaLR | LTR |
| 116685623 | 116685757 | - | 28.6 | 3136 | 3260 | -159 | L2 | L2 | LINE |

|  |  |  |  |  |  |  |  |  |  |
| --- | --- | --- | --- | --- | --- | --- | --- | --- | --- |
| chr12:131975201-131985541 | | |  |  |  |  |  |  |  |
| Genome Start | Genome End | Strand | Div | Repeat Start | Repeat End | Repeat Left | Name | Family | Class |
| 131975201 | 131975682 | + | 12.7 | 1 | 533 | -367 | MER4D1 | ERV1 | LTR |
| 131975683 | 131975967 | + | 15.8 | 1 | 306 | -6 | AluJb | Alu | SINE |
| 131975968 | 131976083 | + | 12.7 | 534 | 659 | -241 | MER4D1 | ERV1 | LTR |
| 131976107 | 131976169 | + | 9.5 | 1 | 63 | -255 | AluSg1 | Alu | SINE |
| 131976170 | 131976628 | + | 7.8 | 1 | 448 | -2 | LTR7 | ERV1 | LTR |
| 131976631 | 131980865 | + | 18.3 | 1 | 4017 | -2512 | HERVH | ERV1 | LTR |
| 131980866 | 131981174 | - | 8.1 | 2 | 311 | -2 | AluSp | Alu | SINE |
| 131981175 | 131981342 | + | 7.9 | 4015 | 4181 | -2372 | HERVH | ERV1 | LTR |
| 131981337 | 131981734 | + | 7.8 | 6283 | 6680 | -1033 | HERVH | ERV1 | LTR |
| 131981735 | 131981812 | + | 19.2 | 6600 | 6678 | -1035 | HERVH | ERV1 | LTR |
| 131981815 | 131981895 | + | 13.6 | 6600 | 6680 | -1033 | HERVH | ERV1 | LTR |
| 131981896 | 131981976 | + | 13.6 | 6600 | 6680 | -1033 | HERVH | ERV1 | LTR |
| 131981977 | 131982057 | + | 13.6 | 6600 | 6680 | -1033 | HERVH | ERV1 | LTR |
| 131982058 | 131982138 | + | 13.6 | 6600 | 6680 | -1033 | HERVH | ERV1 | LTR |
| 131982139 | 131982216 | + | 19.2 | 6600 | 6678 | -1035 | HERVH | ERV1 | LTR |
| 131982219 | 131982299 | + | 13.6 | 6600 | 6680 | -1033 | HERVH | ERV1 | LTR |
| 131982300 | 131982380 | + | 14.8 | 6600 | 6680 | -1033 | HERVH | ERV1 | LTR |
| 131982381 | 131982461 | + | 16.1 | 6600 | 6680 | -1033 | HERVH | ERV1 | LTR |
| 131982462 | 131982541 | + | 18.8 | 6600 | 6680 | -1033 | HERVH | ERV1 | LTR |
| 131982542 | 131982622 | + | 13.6 | 6600 | 6680 | -1033 | HERVH | ERV1 | LTR |
| 131982623 | 131982703 | + | 16.1 | 6600 | 6680 | -1033 | HERVH | ERV1 | LTR |
| 131982704 | 131982784 | + | 16.1 | 6600 | 6680 | -1033 | HERVH | ERV1 | LTR |
| 131982785 | 131982865 | + | 13.6 | 6600 | 6680 | -1033 | HERVH | ERV1 | LTR |
| 131982866 | 131982945 | + | 17.5 | 6600 | 6680 | -1033 | HERVH | ERV1 | LTR |
| 131982946 | 131983026 | + | 14.8 | 6600 | 6680 | -1033 | HERVH | ERV1 | LTR |
| 131983027 | 131983107 | + | 12.3 | 6600 | 6680 | -1033 | HERVH | ERV1 | LTR |
| 131983108 | 131983187 | + | 15.0 | 6600 | 6680 | -1033 | HERVH | ERV1 | LTR |
| 131983188 | 131983268 | + | 14.8 | 6600 | 6680 | -1033 | HERVH | ERV1 | LTR |
| 131983269 | 131983349 | + | 16.1 | 6600 | 6680 | -1033 | HERVH | ERV1 | LTR |
| 131983350 | 131983430 | + | 14.8 | 6600 | 6680 | -1033 | HERVH | ERV1 | LTR |
| 131983431 | 131983510 | + | 11.2 | 6600 | 6680 | -1033 | HERVH | ERV1 | LTR |
| 131983511 | 131983590 | + | 12.5 | 6600 | 6680 | -1033 | HERVH | ERV1 | LTR |
| 131983591 | 131984707 | + | 8.5 | 6600 | 7713 | 0 | HERVH | ERV1 | LTR |
| 131984708 | 131985158 | + | 6.1 | 1 | 448 | -2 | LTR7 | ERV1 | LTR |
| 131985161 | 131985407 | + | 9.9 | 59 | 301 | -8 | AluSg1 | Alu | SINE |
| 131985408 | 131985541 | + | 17.2 | 765 | 900 | 0 | MER4D1 | ERV1 | LTR |

|  |  |  |  |  |  |  |  |  |  |
| --- | --- | --- | --- | --- | --- | --- | --- | --- | --- |
| chr13:113224343-113230575 | | |  |  |  |  |  |  |  |
| Genome Start | Genome End | Strand | Div | Repeat Start | Repeat End | Repeat Left | Name | Family | Class |
| 113224343 | 113224640 | - | 17.9 | 4279 | 4589 | -1948 | L1MDa | L1 | LINE |
| 113224674 | 113224955 | - | 7.6 | 2 | 283 | -28 | AluY | Alu | SINE |
| 113224957 | 113225245 | - | 11.8 | 1 | 291 | -19 | AluSg | Alu | SINE |
| 113225246 | 113226657 | - | 17.9 | 2793 | 4278 | -2259 | L1MDa | L1 | LINE |
| 113226658 | 113226951 | - | 12.3 | 1 | 303 | -9 | AluSx | Alu | SINE |
| 113226952 | 113227479 | - | 20.5 | 2230 | 2792 | -3745 | L1MDa | L1 | LINE |
| 113227487 | 113227905 | + | 19.9 | 2 | 434 | -797 | Tigger3b | MER2\_type | DNA |
| 113227902 | 113227952 | + | 19.6 | 2177 | 2227 | -802 | Tigger3(Golem) | MER2\_type | DNA |
| 113227953 | 113228007 | + | 16.4 | 2177 | 2231 | -798 | Tigger3(Golem) | MER2\_type | DNA |
| 113228004 | 113228058 | + | 20.0 | 2177 | 2231 | -798 | Tigger3(Golem) | MER2\_type | DNA |
| 113228106 | 113228160 | + | 20.0 | 2177 | 2231 | -798 | Tigger3(Golem) | MER2\_type | DNA |
| 113228208 | 113228262 | + | 23.6 | 2177 | 2231 | -798 | Tigger3(Golem) | MER2\_type | DNA |
| 113228263 | 113228313 | + | 25.5 | 2182 | 2232 | -797 | Tigger3(Golem) | MER2\_type | DNA |
| 113228310 | 113228356 | + | 23.4 | 2177 | 2223 | -806 | Tigger3(Golem) | MER2\_type | DNA |
| 113228361 | 113228753 | + | 15.9 | 2177 | 2580 | -449 | Tigger3(Golem) | MER2\_type | DNA |
| 113228754 | 113229035 | + | 16.0 | 1 | 283 | -29 | AluJo | Alu | SINE |
| 113229036 | 113229439 | + | 15.9 | 2581 | 3028 | -1 | Tigger3(Golem) | MER2\_type | DNA |
| 113229440 | 113230575 | - | 27.6 | 1027 | 2223 | -4410 | L1MDa | L1 | LINE |

|  |  |  |  |  |  |  |  |  |  |
| --- | --- | --- | --- | --- | --- | --- | --- | --- | --- |
| chr17:245915-249940 | | |  |  |  |  |  |  |  |
| Genome Start | Genome End | Strand | Div | Repeat Start | Repeat End | Repeat Left | Name | Family | Class |
| 245915 | 246042 | - | 23.6 | 317 | 468 | 0 | LTR37B | ERV1 | LTR |
| 246183 | 246493 | + | 4.9 | 1 | 308 | -3 | AluY | Alu | SINE |
| 246516 | 247079 | - | 18.1 | 475 | 1068 | 0 | Tigger2b\_Pri | MER2\_type | DNA |
| 247080 | 247368 | - | 17.2 | 9 | 312 | 0 | AluJo | Alu | SINE |
| 247369 | 247528 | - | 18.1 | 331 | 474 | -594 | Tigger2b\_Pri | MER2\_type | DNA |
| 247524 | 247588 | - | 18.5 | 336 | 404 | -664 | Tigger2b\_Pri | MER2\_type | DNA |
| 247589 | 247662 | - | 18.9 | 331 | 404 | -664 | Tigger2b\_Pri | MER2\_type | DNA |
| 247658 | 247726 | - | 18.8 | 336 | 404 | -664 | Tigger2b\_Pri | MER2\_type | DNA |
| 247728 | 247795 | - | 19.1 | 336 | 403 | -665 | Tigger2b\_Pri | MER2\_type | DNA |
| 247796 | 247869 | - | 18.9 | 331 | 404 | -664 | Tigger2b\_Pri | MER2\_type | DNA |
| 247865 | 247938 | - | 17.6 | 331 | 404 | -664 | Tigger2b\_Pri | MER2\_type | DNA |
| 247934 | 248007 | - | 18.9 | 331 | 404 | -664 | Tigger2b\_Pri | MER2\_type | DNA |
| 248003 | 248071 | - | 17.4 | 336 | 404 | -664 | Tigger2b\_Pri | MER2\_type | DNA |
| 248072 | 248145 | - | 17.6 | 331 | 404 | -664 | Tigger2b\_Pri | MER2\_type | DNA |
| 248141 | 248214 | - | 18.9 | 331 | 404 | -664 | Tigger2b\_Pri | MER2\_type | DNA |
| 248210 | 248283 | - | 17.6 | 331 | 404 | -664 | Tigger2b\_Pri | MER2\_type | DNA |
| 248279 | 248352 | - | 18.9 | 331 | 404 | -664 | Tigger2b\_Pri | MER2\_type | DNA |
| 248348 | 248421 | - | 18.9 | 331 | 404 | -664 | Tigger2b\_Pri | MER2\_type | DNA |
| 248417 | 248448 | - | 18.9 | 377 | 404 | -664 | Tigger2b\_Pri | MER2\_type | DNA |
| 248449 | 248756 | - | 10.8 | 1 | 305 | -8 | AluSq | Alu | SINE |
| 248757 | 248802 | - | 18.9 | 331 | 376 | -692 | Tigger2b\_Pri | MER2\_type | DNA |
| 248798 | 248995 | - | 13.8 | 197 | 404 | -664 | Tigger2b\_Pri | MER2\_type | DNA |
| 248999 | 249125 | + | 22.1 | 2 | 128 | -15 | FLAM\_C | Alu | SINE |
| 249283 | 249492 | + | 16.0 | 95 | 291 | -21 | AluJo | Alu | SINE |
| 249493 | 249686 | - | 11.9 | 9 | 207 | -861 | Tigger2b\_Pri | MER2\_type | DNA |
| 249698 | 249940 | - | 21.1 | 94 | 304 | -164 | LTR37B | ERV1 | LTR |

|  |  |  |  |  |  |  |  |  |  |
| --- | --- | --- | --- | --- | --- | --- | --- | --- | --- |
| chr17:5083597-5086174 | | |  |  |  |  |  |  |  |
| Genome Start | Genome End | Strand | Div | Repeat Start | Repeat End | Repeat Left | Name | Family | Class |
| 5083597 | 5083760 | + | 24.7 | 10 | 177 | -413 | MLT1H1 | MaLR | LTR |
| 5083764 | 5083856 | + | 8.6 | 2 | 94 | -215 | AluSg1 | Alu | SINE |
| 5083892 | 5084089 | + | 21.9 | 25 | 190 | -274 | LTR7B | ERV1 | LTR |
| 5084187 | 5084255 | + | 23.5 | 123 | 190 | -282 | LTR7Y | ERV1 | LTR |
| 5084229 | 5084295 | + | 23.9 | 123 | 190 | -282 | LTR7Y | ERV1 | LTR |
| 5084310 | 5084378 | + | 20.6 | 123 | 190 | -282 | LTR7Y | ERV1 | LTR |
| 5084394 | 5084462 | + | 23.5 | 123 | 190 | -282 | LTR7Y | ERV1 | LTR |
| 5084477 | 5084549 | + | 19.4 | 123 | 189 | -283 | LTR7Y | ERV1 | LTR |
| 5084519 | 5084586 | + | 20.9 | 123 | 190 | -282 | LTR7Y | ERV1 | LTR |
| 5084628 | 5084710 | + | 21.9 | 107 | 190 | -282 | LTR7Y | ERV1 | LTR |
| 5084752 | 5084834 | + | 21.9 | 107 | 190 | -282 | LTR7Y | ERV1 | LTR |
| 5084891 | 5084959 | + | 23.5 | 123 | 190 | -282 | LTR7Y | ERV1 | LTR |
| 5084933 | 5084999 | + | 23.9 | 123 | 190 | -282 | LTR7Y | ERV1 | LTR |
| 5085014 | 5085085 | + | 18.2 | 123 | 189 | -283 | LTR7Y | ERV1 | LTR |
| 5085082 | 5085205 | + | 19.3 | 107 | 190 | -282 | LTR7Y | ERV1 | LTR |
| 5085220 | 5085600 | + | 20.5 | 107 | 464 | 0 | LTR7B | ERV1 | LTR |
| 5085601 | 5085803 | + | 8.4 | 93 | 296 | -16 | AluSg1 | Alu | SINE |
| 5085807 | 5086174 | + | 27.1 | 168 | 555 | 0 | MLT1H1 | MaLR | LTR |

|  |  |  |  |  |  |  |  |  |  |
| --- | --- | --- | --- | --- | --- | --- | --- | --- | --- |
| chr18:65578567-65580563 | | |  |  |  |  |  |  |  |
| Genome Start | Genome End | Strand | Div | Repeat Start | Repeat End | Repeat Left | Name | Family | Class |
| 65578567 | 65578609 | + | 13.9 | 3 | 45 | -257 | AluJb | Alu | SINE |
| 65578629 | 65578733 | - | 16.2 | 502 | 600 | -134 | MER50 | ERV1 | LTR |
| 65578724 | 65579161 | - | 25.2 | 122 | 563 | -171 | MER50 | ERV1 | LTR |
| 65579164 | 65579261 | - | 7.1 | 122 | 222 | -512 | MER50 | ERV1 | LTR |
| 65579264 | 65579361 | - | 9.2 | 122 | 222 | -512 | MER50 | ERV1 | LTR |
| 65579364 | 65579461 | - | 8.2 | 122 | 222 | -512 | MER50 | ERV1 | LTR |
| 65579464 | 65579687 | - | 14.7 | 1 | 222 | -512 | MER50 | ERV1 | LTR |
| 65579688 | 65580015 | - | 13.1 | 6882 | 7197 | -8 | MER50-int | ERV1 | LTR |
| 65580050 | 65580294 | - | 17.1 | 513 | 734 | 0 | MER50 | ERV1 | LTR |
| 65580295 | 65580361 | + | 18.9 | 51 | 133 | -179 | AluJb | Alu | SINE |
| 65580410 | 65580563 | + | 18.9 | 134 | 288 | -24 | AluJb | Alu | SINE |

|  |  |  |  |  |  |  |  |  |  |
| --- | --- | --- | --- | --- | --- | --- | --- | --- | --- |
| chr19:34325264-34326765 | | |  |  |  |  |  |  |  |
| Genome Start | Genome End | Strand | Div | Repeat Start | Repeat End | Repeat Left | Name | Family | Class |
| 34325264 | 34325380 | - | 23.4 | 1348 | 1469 | -3 | MLT1H-int | MaLR | LTR |
| 34325586 | 34325865 | + | 16.8 | 5854 | 6136 | -25 | L1PA17 | L1 | LINE |
| 34325840 | 34325880 | + | 12.2 | 6084 | 6124 | -42 | L1PA17 | L1 | LINE |
| 34325880 | 34325917 | + | 13.2 | 6086 | 6123 | -43 | L1PA17 | L1 | LINE |
| 34325918 | 34325956 | + | 7.7 | 6086 | 6124 | -42 | L1PA17 | L1 | LINE |
| 34325986 | 34326034 | + | 12.2 | 6076 | 6124 | -42 | L1PA17 | L1 | LINE |
| 34326034 | 34326071 | + | 7.9 | 6086 | 6123 | -43 | L1PA17 | L1 | LINE |
| 34326100 | 34326152 | + | 17.5 | 6074 | 6126 | -40 | L1PA16 | L1 | LINE |
| 34326187 | 34326225 | + | 7.7 | 6086 | 6124 | -42 | L1PA17 | L1 | LINE |
| 34326225 | 34326263 | + | 7.7 | 6086 | 6124 | -42 | L1PA17 | L1 | LINE |
| 34326263 | 34326301 | + | 7.7 | 6086 | 6124 | -42 | L1PA17 | L1 | LINE |
| 34326329 | 34326377 | + | 18.4 | 6076 | 6124 | -42 | L1PA17 | L1 | LINE |
| 34326407 | 34326483 | + | 16.9 | 6076 | 6152 | -14 | L1PA17 | L1 | LINE |
| 34326613 | 34326765 | - | 32.2 | 768 | 923 | -549 | MLT1H-int | MaLR | LTR |

|  |  |  |  |  |  |  |  |  |  |
| --- | --- | --- | --- | --- | --- | --- | --- | --- | --- |
| chr19:63802699-63805611 | | |  |  |  |  |  |  |  |
| Genome Start | Genome End | Strand | Div | Repeat Start | Repeat End | Repeat Left | Name | Family | Class |
| 63802699 | 63803145 | - | 21.6 | 1063 | 1506 | -3430 | MER72-int | ERV1 | LTR |
| 63803149 | 63803216 | + | 22.1 | 3 | 72 | -482 | MLT2B3 | ERVL | LTR |
| 63803242 | 63803309 | + | 16.2 | 3 | 72 | -482 | MLT2B3 | ERVL | LTR |
| 63803335 | 63803402 | + | 17.6 | 3 | 72 | -482 | MLT2B3 | ERVL | LTR |
| 63803428 | 63803495 | + | 22.1 | 3 | 72 | -482 | MLT2B3 | ERVL | LTR |
| 63803521 | 63803588 | + | 20.6 | 3 | 72 | -482 | MLT2B3 | ERVL | LTR |
| 63803614 | 63803681 | + | 20.6 | 3 | 72 | -482 | MLT2B3 | ERVL | LTR |
| 63803707 | 63803774 | + | 17.6 | 3 | 72 | -482 | MLT2B3 | ERVL | LTR |
| 63803800 | 63803867 | + | 19.1 | 3 | 72 | -482 | MLT2B3 | ERVL | LTR |
| 63803893 | 63803960 | + | 22.1 | 3 | 72 | -482 | MLT2B5 | ERVL | LTR |
| 63803986 | 63804210 | + | 21.1 | 3 | 274 | -388 | MLT2B3 | ERVL | LTR |
| 63804211 | 63804572 | + | 14.2 | 1 | 375 | 0 | THE1C | MaLR | LTR |
| 63804573 | 63804922 | + | 24.0 | 159 | 597 | -4 | MLT2B5 | ERVL | LTR |
| 63804930 | 63805296 | - | 27.0 | 620 | 1063 | -3873 | MER72-int | ERV1 | LTR |
| 63805272 | 63805611 | - | 26.5 | 173 | 515 | -4421 | MER72-int | ERV1 | LTR |

|  |  |  |  |  |  |  |  |  |  |
| --- | --- | --- | --- | --- | --- | --- | --- | --- | --- |
| chr20:2370303-2373113 | | |  |  |  |  |  |  |  |
| Genome Start | Genome End | Strand | Div | Repeat Start | Repeat End | Repeat Left | Name | Family | Class |
| 2370303 | 2371100 | + | 22.8 | 4070 | 4952 | -1194 | L1M5 | L1 | LINE |
| 2371105 | 2371403 | + | 11.0 | 1 | 304 | -8 | AluSx | Alu | SINE |
| 2371423 | 2371701 | + | 22.6 | 1 | 258 | -194 | LTR47A | ERVL | LTR |
| 2371704 | 2371762 | + | 19.6 | 200 | 258 | -185 | LTR47B | ERVL | LTR |
| 2371765 | 2371823 | + | 19.6 | 200 | 258 | -185 | LTR47B | ERVL | LTR |
| 2371826 | 2371884 | + | 19.6 | 200 | 258 | -185 | LTR47B | ERVL | LTR |
| 2371887 | 2371945 | + | 27.1 | 200 | 258 | -194 | LTR47A | ERVL | LTR |
| 2371948 | 2372006 | + | 19.6 | 200 | 258 | -185 | LTR47B | ERVL | LTR |
| 2372009 | 2372067 | + | 19.6 | 200 | 258 | -185 | LTR47B | ERVL | LTR |
| 2372070 | 2372128 | + | 19.6 | 200 | 258 | -185 | LTR47B | ERVL | LTR |
| 2372131 | 2372189 | + | 19.6 | 200 | 258 | -185 | LTR47B | ERVL | LTR |
| 2372216 | 2372250 | + | 17.1 | 224 | 258 | -194 | LTR47A | ERVL | LTR |
| 2372253 | 2372311 | + | 19.6 | 200 | 258 | -185 | LTR47B | ERVL | LTR |
| 2372314 | 2372372 | + | 19.6 | 200 | 258 | -185 | LTR47B | ERVL | LTR |
| 2372375 | 2372433 | + | 19.6 | 200 | 258 | -185 | LTR47B | ERVL | LTR |
| 2372436 | 2372494 | + | 19.6 | 200 | 258 | -185 | LTR47B | ERVL | LTR |
| 2372505 | 2372740 | + | 20.5 | 210 | 443 | 0 | LTR47B | ERVL | LTR |
| 2372742 | 2372780 | + | 17.9 | 405 | 443 | 0 | LTR47B | ERVL | LTR |
| 2372781 | 2373113 | + | 24.3 | 4962 | 5298 | -848 | L1M5 | L1 | LINE |

|  |  |  |  |  |  |  |  |  |  |
| --- | --- | --- | --- | --- | --- | --- | --- | --- | --- |
| chr20:55192488-55194895 | | |  |  |  |  |  |  |  |
| Genome Start | Genome End | Strand | Div | Repeat Start | Repeat End | Repeat Left | Name | Family | Class |
| 55192488 | 55192620 | - | 32.6 | 3286 | 3419 | 0 | L2 | L2 | LINE |
| 55192697 | 55192812 | - | 26.1 | 6 | 133 | -276 | MLT1I | MaLR | LTR |
| 55192814 | 55193006 | - | 29.3 | 2938 | 3139 | -280 | L2 | L2 | LINE |
| 55193062 | 55193427 | + | 24.0 | 22 | 478 | -27 | MLT1C | MaLR | LTR |
| 55193503 | 55193682 | - | 30.3 | 2629 | 2809 | -610 | L2 | L2 | LINE |
| 55193692 | 55193847 | - | 27.6 | 6204 | 6359 | 0 | L1MD2 | L1 | LINE |
| 55193856 | 55194089 | - | 30.6 | 2396 | 2621 | -798 | L2 | L2 | LINE |
| 55194199 | 55194262 | + | 26.6 | 198 | 261 | -7 | MIRb | MIR | SINE |
| 55194267 | 55194307 | + | 21.9 | 221 | 261 | -7 | MIRb | MIR | SINE |
| 55194329 | 55194397 | + | 24.5 | 185 | 261 | -6 | MIRb | MIR | SINE |
| 55194402 | 55194442 | + | 19.5 | 221 | 261 | -7 | MIRb | MIR | SINE |
| 55194464 | 55194532 | + | 22.6 | 185 | 261 | -6 | MIRb | MIR | SINE |
| 55194528 | 55194895 | - | 29.6 | 1999 | 2377 | -1042 | L2 | L2 | LINE |

|  |  |  |  |  |  |  |  |  |  |
| --- | --- | --- | --- | --- | --- | --- | --- | --- | --- |
| chr21:46935652-46938527 | | |  |  |  |  |  |  |  |
| Genome Start | Genome End | Strand | Div | Repeat Start | Repeat End | Repeat Left | Name | Family | Class |
| 46935652 | 46936098 | - | 21.6 | 1063 | 1506 | -3430 | MER34B-int | ERV1 | LTR |
| 46936102 | 46936169 | + | 22.1 | 3 | 72 | -482 | MLT2B3 | ERVL | LTR |
| 46936195 | 46936262 | + | 14.7 | 3 | 72 | -482 | MLT2B3 | ERVL | LTR |
| 46936288 | 46936355 | + | 16.2 | 3 | 72 | -482 | MLT2B3 | ERVL | LTR |
| 46936381 | 46936448 | + | 22.1 | 3 | 72 | -482 | MLT2B3 | ERVL | LTR |
| 46936474 | 46936541 | + | 19.1 | 3 | 72 | -482 | MLT2B3 | ERVL | LTR |
| 46936567 | 46936632 | + | 16.7 | 3 | 72 | -482 | MLT2B3 | ERVL | LTR |
| 46936658 | 46936725 | + | 14.7 | 3 | 72 | -482 | MLT2B5 | ERVL | LTR |
| 46936751 | 46936977 | + | 19.9 | 3 | 279 | -388 | MLT2B3 | ERVL | LTR |
| 46936978 | 46937339 | + | 14.4 | 1 | 375 | 0 | THE1C | MaLR | LTR |
| 46937340 | 46937689 | + | 21.8 | 164 | 597 | -4 | MLT2B5 | ERVL | LTR |
| 46937697 | 46938061 | - | 27.3 | 620 | 1063 | -3873 | MER34B-int | ERV1 | LTR |
| 46938037 | 46938374 | - | 26.9 | 173 | 515 | -4421 | MER34B-int | ERV1 | LTR |
| 46938416 | 46938527 | - | 19.2 | 500 | 623 | -4733 | MER34B-int | ERV1 | LTR |

|  |  |  |  |  |  |  |  |  |  |
| --- | --- | --- | --- | --- | --- | --- | --- | --- | --- |
| chrX:1770724-1778476 | | |  |  |  |  |  |  |  |
| Genome Start | Genome End | Strand | Div | Repeat Start | Repeat End | Repeat Left | Name | Family | Class |
| 1770724 | 1770900 | - | 16.9 | 6101 | 6283 | -8 | L1MA8 | L1 | LINE |
| 1770905 | 1771205 | + | 12.0 | 1 | 301 | -11 | AluSx | Alu | SINE |
| 1771206 | 1771448 | + | 6.2 | 5900 | 6154 | 0 | L1PA7 | L1 | LINE |
| 1771487 | 1771722 | - | 12.3 | 1 | 236 | -77 | AluSq | Alu | SINE |
| 1771727 | 1772028 | - | 7.3 | 1 | 302 | -9 | AluY | Alu | SINE |
| 1772057 | 1772186 | + | 17.6 | 12 | 137 | -6 | FLAM\_C | Alu | SINE |
| 1772288 | 1772591 | - | 5.6 | 1 | 303 | -8 | AluY | Alu | SINE |
| 1772593 | 1772762 | - | 21.2 | 130 | 299 | -13 | AluJb | Alu | SINE |
| 1772887 | 1772943 | - | 27.8 | 6098 | 6178 | -5 | L1MB3 | L1 | LINE |
| 1772944 | 1773078 | + | 10.7 | 1 | 135 | -177 | AluSx | Alu | SINE |
| 1773079 | 1773392 | + | 14.5 | 1 | 312 | 0 | AluSx | Alu | SINE |
| 1773393 | 1773558 | + | 10.7 | 136 | 298 | -14 | AluSx | Alu | SINE |
| 1773560 | 1773593 | - | 22.5 | 6061 | 6094 | -80 | L1MB5 | L1 | LINE |
| 1773594 | 1773879 | - | 29.9 | 5767 | 6057 | -1938 | L1MD3 | L1 | LINE |
| 1773925 | 1774226 | - | 12.9 | 1 | 303 | -9 | AluSx | Alu | SINE |
| 1774242 | 1774425 | - | 16.3 | 6098 | 6286 | -5 | L1MA8 | L1 | LINE |
| 1774426 | 1774714 | + | 12.2 | 1 | 286 | -26 | AluSx | Alu | SINE |
| 1774736 | 1774811 | - | 16.3 | 6030 | 6097 | -194 | L1MA8 | L1 | LINE |
| 1774812 | 1775112 | - | 10.0 | 1 | 301 | -10 | AluY | Alu | SINE |
| 1775113 | 1775221 | - | 16.3 | 5921 | 6029 | -262 | L1MA8 | L1 | LINE |
| 1775222 | 1775457 | - | 26.8 | 5703 | 5938 | -228 | L1PA16 | L1 | LINE |
| 1775458 | 1775577 | - | 21.2 | 5648 | 5829 | -355 | L1MB7 | L1 | LINE |
| 1775585 | 1775706 | - | 20.0 | 5707 | 5828 | -346 | L1MB5 | L1 | LINE |
| 1775711 | 1775831 | - | 25.0 | 5711 | 5831 | -469 | L1MA5 | L1 | LINE |
| 1775821 | 1775966 | - | 20.5 | 5702 | 5847 | -309 | L1MB5 | L1 | LINE |
| 1775966 | 1776090 | - | 20.2 | 5707 | 5831 | -353 | L1MB7 | L1 | LINE |
| 1776086 | 1776207 | - | 25.8 | 5707 | 5837 | -463 | L1MA5 | L1 | LINE |
| 1776212 | 1776332 | - | 25.0 | 5711 | 5831 | -469 | L1MA5 | L1 | LINE |
| 1776320 | 1776467 | - | 21.8 | 5699 | 5846 | -445 | L1MB5 | L1 | LINE |
| 1776467 | 1776587 | - | 24.2 | 5711 | 5831 | -469 | L1MA5 | L1 | LINE |
| 1776577 | 1776722 | - | 20.5 | 5702 | 5847 | -309 | L1MB5 | L1 | LINE |
| 1776722 | 1776846 | - | 19.4 | 5707 | 5831 | -353 | L1MB7 | L1 | LINE |
| 1776842 | 1776963 | - | 25.8 | 5707 | 5837 | -463 | L1MA5 | L1 | LINE |
| 1776968 | 1777088 | - | 25.0 | 5711 | 5831 | -469 | L1MA5 | L1 | LINE |
| 1777076 | 1777223 | - | 20.9 | 5699 | 5846 | -445 | L1MB5 | L1 | LINE |
| 1777223 | 1777343 | - | 24.2 | 5711 | 5831 | -469 | L1MA5 | L1 | LINE |
| 1777333 | 1777478 | - | 20.5 | 5702 | 5847 | -309 | L1MB5 | L1 | LINE |
| 1777478 | 1777602 | - | 20.2 | 5707 | 5831 | -353 | L1MB7 | L1 | LINE |
| 1777598 | 1777719 | - | 25.8 | 5707 | 5837 | -463 | L1MA5 | L1 | LINE |
| 1777724 | 1777844 | - | 25.0 | 5711 | 5831 | -469 | L1MA5 | L1 | LINE |
| 1777832 | 1777974 | - | 20.8 | 5707 | 5846 | -454 | L1MB5 | L1 | LINE |
| 1777979 | 1778099 | - | 25.0 | 5711 | 5831 | -343 | L1MB5 | L1 | LINE |
| 1778089 | 1778224 | - | 24.6 | 5711 | 5844 | -461 | L1MB5 | L1 | LINE |
| 1778212 | 1778351 | - | 25.9 | 5711 | 5846 | -454 | L1MA4 | L1 | LINE |
| 1778339 | 1778476 | - | 26.7 | 5707 | 5846 | -445 | L1MA8 | L1 | LINE |

|  |  |  |  |  |  |  |  |  |  |
| --- | --- | --- | --- | --- | --- | --- | --- | --- | --- |
| chrX:1902319-1906638 | | |  |  |  |  |  |  |  |
| Genome Start | Genome End | Strand | Div | Repeat Start | Repeat End | Repeat Left | Name | Family | Class |
| 1902319 | 1902488 | + | 21.3 | 1 | 161 | -306 | MLT1C | MaLR | LTR |
| 1902489 | 1902669 | - | 24.3 | 179 | 365 | 0 | MLT1A0 | MaLR | LTR |
| 1902707 | 1902766 | - | 15.0 | 32 | 92 | -334 | MLT1A0 | MaLR | LTR |
| 1902765 | 1902823 | - | 16.9 | 32 | 92 | -334 | MSTB | MaLR | LTR |
| 1902817 | 1902880 | - | 14.5 | 32 | 95 | -331 | MSTB | MaLR | LTR |
| 1902881 | 1902937 | - | 10.5 | 32 | 89 | -337 | MSTB | MaLR | LTR |
| 1902940 | 1902995 | - | 19.6 | 32 | 88 | -338 | MSTB | MaLR | LTR |
| 1902994 | 1903053 | - | 18.3 | 32 | 92 | -334 | MSTB | MaLR | LTR |
| 1903047 | 1903105 | - | 14.0 | 37 | 95 | -331 | MSTB | MaLR | LTR |
| 1903109 | 1903167 | - | 11.9 | 32 | 92 | -334 | MSTB | MaLR | LTR |
| 1903166 | 1903224 | - | 16.9 | 33 | 92 | -340 | MSTB1 | MaLR | LTR |
| 1903228 | 1903283 | - | 14.3 | 32 | 88 | -338 | MSTB | MaLR | LTR |
| 1903282 | 1903340 | - | 16.9 | 33 | 92 | -340 | MSTB1 | MaLR | LTR |
| 1903340 | 1903399 | - | 15.0 | 32 | 92 | -334 | MSTB | MaLR | LTR |
| 1903398 | 1903456 | - | 20.3 | 33 | 92 | -340 | MSTB1 | MaLR | LTR |
| 1903456 | 1903515 | - | 16.7 | 32 | 92 | -334 | MSTB | MaLR | LTR |
| 1903514 | 1903571 | - | 17.2 | 34 | 92 | -340 | MSTB1 | MaLR | LTR |
| 1903567 | 1903631 | - | 12.9 | 31 | 95 | -331 | MSTB | MaLR | LTR |
| 1903624 | 1903688 | - | 15.9 | 32 | 95 | -331 | MSTB | MaLR | LTR |
| 1903687 | 1903746 | - | 15.0 | 32 | 92 | -334 | MSTB | MaLR | LTR |
| 1903745 | 1903804 | - | 18.3 | 32 | 92 | -334 | MSTB | MaLR | LTR |
| 1903861 | 1903920 | - | 16.7 | 32 | 92 | -340 | MSTB1 | MaLR | LTR |
| 1903924 | 1903975 | - | 15.4 | 35 | 87 | -339 | MSTB | MaLR | LTR |
| 1903969 | 1904035 | - | 21.5 | 33 | 98 | -334 | MSTB1 | MaLR | LTR |
| 1904035 | 1904094 | - | 21.7 | 32 | 92 | -334 | MSTB | MaLR | LTR |
| 1904093 | 1904152 | - | 13.3 | 32 | 92 | -334 | MSTB | MaLR | LTR |
| 1904151 | 1904210 | - | 11.7 | 32 | 92 | -334 | MSTB | MaLR | LTR |
| 1904213 | 1904268 | - | 17.9 | 32 | 88 | -338 | MSTB | MaLR | LTR |
| 1904267 | 1904326 | - | 20.0 | 32 | 92 | -334 | MSTB | MaLR | LTR |
| 1904325 | 1904383 | - | 18.6 | 33 | 92 | -340 | MSTB1 | MaLR | LTR |
| 1904383 | 1904442 | - | 16.7 | 32 | 92 | -334 | MSTB | MaLR | LTR |
| 1904441 | 1904500 | - | 18.3 | 32 | 92 | -334 | MSTB | MaLR | LTR |
| 1904499 | 1904558 | - | 15.0 | 32 | 92 | -334 | MSTB | MaLR | LTR |
| 1904557 | 1904616 | - | 20.0 | 32 | 92 | -334 | MSTB | MaLR | LTR |
| 1904615 | 1904674 | - | 13.3 | 32 | 92 | -340 | MSTB1 | MaLR | LTR |
| 1904668 | 1904732 | - | 17.5 | 32 | 95 | -337 | MSTB1 | MaLR | LTR |
| 1904726 | 1904790 | - | 11.3 | 32 | 95 | -331 | MSTB | MaLR | LTR |
| 1904789 | 1904848 | - | 16.7 | 32 | 92 | -334 | MSTB | MaLR | LTR |
| 1904847 | 1904906 | - | 15.0 | 32 | 92 | -334 | MSTB | MaLR | LTR |
| 1904905 | 1904962 | - | 17.2 | 34 | 92 | -334 | MSTB | MaLR | LTR |
| 1904958 | 1905022 | - | 19.1 | 32 | 95 | -331 | MSTB | MaLR | LTR |
| 1905021 | 1905080 | - | 18.3 | 32 | 92 | -334 | MSTB | MaLR | LTR |
| 1905083 | 1905138 | - | 14.3 | 32 | 88 | -338 | MSTB | MaLR | LTR |
| 1905137 | 1905196 | - | 16.7 | 32 | 92 | -334 | MSTB | MaLR | LTR |
| 1905195 | 1905254 | - | 18.3 | 32 | 92 | -334 | MSTB | MaLR | LTR |
| 1905253 | 1905312 | - | 13.3 | 32 | 92 | -334 | MSTB | MaLR | LTR |
| 1905311 | 1905371 | - | 15.0 | 32 | 92 | -334 | MSTB | MaLR | LTR |
| 1905370 | 1905424 | - | 16.4 | 33 | 92 | -340 | MSTB1 | MaLR | LTR |
| 1905424 | 1905483 | - | 16.7 | 32 | 92 | -340 | MSTB1 | MaLR | LTR |
| 1905477 | 1905541 | - | 15.9 | 32 | 95 | -331 | MSTB | MaLR | LTR |
| 1905540 | 1905598 | - | 15.2 | 33 | 92 | -340 | MSTB1 | MaLR | LTR |
| 1905598 | 1905657 | - | 16.7 | 32 | 92 | -334 | MSTB | MaLR | LTR |
| 1905656 | 1905715 | - | 20.0 | 32 | 92 | -334 | MSTB | MaLR | LTR |
| 1905714 | 1905773 | - | 16.7 | 32 | 92 | -334 | MSTB | MaLR | LTR |
| 1905772 | 1905832 | - | 16.4 | 31 | 92 | -334 | MSTB | MaLR | LTR |
| 1905830 | 1905893 | - | 16.4 | 32 | 92 | -334 | MSTB | MaLR | LTR |
| 1905892 | 1905955 | - | 16.4 | 32 | 92 | -334 | MSTB | MaLR | LTR |
| 1905954 | 1906012 | - | 16.9 | 32 | 92 | -334 | MSTB | MaLR | LTR |
| 1906016 | 1906072 | - | 17.5 | 30 | 87 | -339 | MSTB | MaLR | LTR |
| 1906069 | 1906129 | - | 17.2 | 34 | 92 | -340 | MSTB1 | MaLR | LTR |
| 1906125 | 1906186 | - | 11.7 | 34 | 95 | -331 | MSTB | MaLR | LTR |
| 1906240 | 1906335 | - | 13.8 | 1 | 95 | -331 | MSTB | MaLR | LTR |
| 1906373 | 1906638 | + | 25.7 | 157 | 465 | -2 | MLT1C | MaLR | LTR |

|  |  |  |  |  |  |  |  |  |  |
| --- | --- | --- | --- | --- | --- | --- | --- | --- | --- |
| chrX:1957407-1958538 | | |  |  |  |  |  |  |  |
| Genome Start | Genome End | Strand | Div | Repeat Start | Repeat End | Repeat Left | Name | Family | Class |
| 1957407 | 1957484 | + | 7.7 | 6073 | 6151 | -153 | L1MA2 | L1 | LINE |
| 1957485 | 1957526 | - | 4.8 | 314 | 355 | 0 | THE1B | MaLR | LTR |
| 1957527 | 1957836 | + | 10.3 | 1 | 310 | -2 | AluSx | Alu | SINE |
| 1957837 | 1958188 | - | 14.3 | 16 | 334 | -41 | THE1B | MaLR | LTR |
| 1958189 | 1958245 | - | 10.5 | 16 | 72 | -292 | THE1B | MaLR | LTR |
| 1958245 | 1958302 | - | 10.3 | 16 | 73 | -291 | THE1B | MaLR | LTR |
| 1958302 | 1958358 | - | 10.5 | 17 | 73 | -291 | THE1B | MaLR | LTR |
| 1958359 | 1958431 | - | 12.3 | 1 | 73 | -291 | THE1B | MaLR | LTR |
| 1958432 | 1958538 | + | 9.3 | 6147 | 6253 | -51 | L1MA2 | L1 | LINE |

|  |  |  |  |  |  |  |  |  |  |
| --- | --- | --- | --- | --- | --- | --- | --- | --- | --- |
| chrX:82837600-82852732 | | |  |  |  |  |  |  |  |
| Genome Start | Genome End | Strand | Div | Repeat Start | Repeat End | Repeat Left | Name | Family | Class |
| 82837600 | 82838654 | - | 20.4 | 3197 | 4481 | -2364 | L1M2 | L1 | LINE |
| 82838698 | 82839189 | - | 21.8 | 2691 | 3196 | -3649 | L1M2 | L1 | LINE |
| 82839301 | 82839586 | - | 13.4 | 5851 | 6149 | -2 | L1PB1 | L1 | LINE |
| 82839622 | 82839651 | - | 10.0 | 5851 | 5880 | -276 | L1PB1 | L1 | LINE |
| 82839636 | 82839683 | - | 8.3 | 5851 | 5902 | -249 | L1PB1 | L1 | LINE |
| 82839668 | 82839715 | - | 8.3 | 5851 | 5902 | -249 | L1PB1 | L1 | LINE |
| 82839700 | 82839747 | - | 8.3 | 5851 | 5902 | -249 | L1PB1 | L1 | LINE |
| 82839732 | 82839779 | - | 8.3 | 5851 | 5902 | -249 | L1PB1 | L1 | LINE |
| 82839764 | 82839811 | - | 8.3 | 5851 | 5902 | -249 | L1PB1 | L1 | LINE |
| 82839796 | 82839936 | - | 12.9 | 5783 | 5902 | -249 | L1PB1 | L1 | LINE |
| 82840027 | 82841089 | - | 11.8 | 4706 | 5782 | -369 | L1PB1 | L1 | LINE |
| 82841221 | 82841409 | - | 27.5 | 1672 | 1937 | -4445 | L1M4c | L1 | LINE |
| 82841398 | 82843795 | - | 16.5 | 3790 | 6164 | -1 | L1PA15 | L1 | LINE |
| 82843796 | 82844050 | - | 9.8 | 1 | 301 | -10 | AluY | Alu | SINE |
| 82844051 | 82844637 | - | 17.2 | 3359 | 3993 | -2357 | L1M2 | L1 | LINE |
| 82844623 | 82844989 | - | 29.0 | 1685 | 2053 | -4298 | L1M2 | L1 | LINE |
| 82844997 | 82846132 | - | 17.4 | 5740 | 6865 | -5 | L1PB3 | L1 | LINE |
| 82846133 | 82846513 | - | 17.1 | 1 | 381 | 0 | THE1D | MaLR | LTR |
| 82846514 | 82850279 | - | 19.6 | 2013 | 5739 | -1123 | L1PB3 | L1 | LINE |
| 82850298 | 82850740 | - | 33.6 | 142 | 595 | -6268 | L1PB3 | L1 | LINE |
| 82850789 | 82851902 | - | 29.2 | 602 | 1709 | -4572 | L1M2 | L1 | LINE |
| 82851878 | 82852732 | - | 30.8 | 3 | 759 | -5537 | L1M2 | L1 | LINE |
