# Supplementary material for: Evolutionary History of Mammalian Transposons Determined by Genome-Wide Defragmentation
Source: PLoS Comput Biol. 2007 Jul 13;3(7):e137. doi: 10.1371/journal.pcbi.0030137 (PMC1914374; doi:10.1371/journal.pcbi.0030137)
Supplement: Supplementary file 7 [file pcbi.0030137.sd004.htm]

Tandem Arrays

**Supplementary Table S1d: Large Tandem Arrays**
  
To load the custom track into the UCSC Genome Browser, click here. The custom track must be loaded in order to view the clusters in Supplementary Table S1. It may take a minute to load, so please be patient.  
  
Tandem Arrays  
chr1:141614596-141629292  
chr1:142011066-142025761  
chr1:142245576-142259928  
chr2:94793805-94815544  
chr2:130527955-130533249  
chr2:130918665-130923963  
chr2:131145648-131150946  
chr2:131752314-131757592  
chr3:199259377-199263452  
chr4:48911896-48929687  
chr4:49259299-49274079  
chr8:43489114-43506146  
chr8:86744493-86911178  
chr9:33567377-33587718  
chr9:38536498-38557002  
chr9:42429334-42456540  
chr9:43025353-43052560  
chr9:43964543-43986620  
chr9:66111804-66133881  
chr9:68742783-68763792  
chr9:89731067-89735730  
chr9:89926704-89931368  
chr9:98936138-98953601  
chr10:124329561-124349273  
chr13:18218665-18279040  
chr18:14244666-14324176  
chr19:55280783-55336942  
chr21:14145755-14197329  
chrX:49059954-49271622  
chrY:9226249-10017916  
  
Clusters excluded from Tandem Arrays  
Jump to:  
chr1  
chr2  
chr3  
chr4  
chr8  
chr9  
chr10  
chr13  
chr18  
chr19  
chr21  
chrX  
chrY  
  


|  |  |  |  |  |  |  |  |  |  |
| --- | --- | --- | --- | --- | --- | --- | --- | --- | --- |
| chr1:141614596-141625495 | | | 9970 bp | | |  |  |  |  |
| Genome Start | Genome End | Strand | Div | Repeat Start | Repeat End | Repeat Left | Name | Family | Class |
| 141614596 | 141614738 | - | 28.2 | 129 | 274 | -154 | MSTA | MaLR | LTR |
| 141614785 | 141615018 | + | 21.9 | 907 | 1178 | -402 | THE1-int | MaLR | LTR |
| 141615017 | 141615062 | - | 19.6 | 921 | 967 | -613 | THE1-int | MaLR | LTR |
| 141615138 | 141615238 | - | 19.6 | 1 | 106 | -322 | MSTA | MaLR | LTR |
| 141615239 | 141615551 | - | 22.4 | 1317 | 1651 | 0 | MST-int | MaLR | LTR |
| 141615544 | 141615649 | - | 26.7 | 60 | 176 | -252 | MSTA | MaLR | LTR |
| 141615699 | 141615796 | - | 18.6 | 1 | 99 | -329 | MSTA | MaLR | LTR |
| 141615797 | 141615906 | - | 19.1 | 1541 | 1651 | 0 | MSTA-int | MaLR | LTR |
| 141615909 | 141617203 | - | 25.1 | 32 | 1389 | -262 | MSTA-int | MaLR | LTR |
| 141617206 | 141617338 | + | 15.9 | 507 | 661 | -990 | MST-int | MaLR | LTR |
| 141617365 | 141617669 | - | 25.1 | 76 | 385 | -43 | MSTA | MaLR | LTR |
| 141617674 | 141617907 | + | 23.7 | 907 | 1178 | -402 | THE1-int | MaLR | LTR |
| 141618050 | 141618155 | - | 19.6 | 1 | 113 | -262 | MSTA | MaLR | LTR |
| 141618156 | 141618473 | - | 21.3 | 1341 | 1651 | 0 | MST-int | MaLR | LTR |
| 141618480 | 141618591 | - | 22.3 | 61 | 171 | -257 | MSTA | MaLR | LTR |
| 141618627 | 141618722 | - | 24.0 | 1 | 97 | -331 | MSTA | MaLR | LTR |
| 141618723 | 141618834 | - | 13.4 | 1538 | 1651 | 0 | MSTA-int | MaLR | LTR |
| 141618950 | 141619056 | - | 19.4 | 1 | 104 | -324 | MSTA | MaLR | LTR |
| 141619057 | 141620636 | - | 23.7 | 28 | 1651 | 0 | MSTA-int | MaLR | LTR |
| 141620632 | 141620774 | + | 23.2 | 504 | 670 | -981 | MST-int | MaLR | LTR |
| 141620804 | 141621069 | - | 25.8 | 98 | 374 | -54 | MSTA | MaLR | LTR |
| 141621097 | 141621328 | + | 22.9 | 907 | 1183 | -397 | THE1-int | MaLR | LTR |
| 141621336 | 141621392 | - | 22.8 | 910 | 967 | -613 | MSTA-int | MaLR | LTR |
| 141621477 | 141621572 | - | 15.8 | 1 | 97 | -331 | MSTA | MaLR | LTR |
| 141621573 | 141621920 | - | 20.7 | 1317 | 1651 | 0 | MSTA-int | MaLR | LTR |
| 141621919 | 141622021 | - | 25.2 | 58 | 171 | -257 | MSTA | MaLR | LTR |
| 141622067 | 141622165 | - | 25.2 | 1 | 100 | -328 | MSTA | MaLR | LTR |
| 141622166 | 141622242 | - | 16.9 | 1574 | 1651 | 0 | MSTA-int | MaLR | LTR |
| 141622245 | 141622306 | + | 18.3 | 317 | 376 | -52 | MSTA | MaLR | LTR |
| 141622341 | 141622379 | - | 12.8 | 623 | 661 | -990 | MSTA-int | MaLR | LTR |
| 141622380 | 141623910 | - | 23.5 | 28 | 1583 | -68 | MSTA-int | MaLR | LTR |
| 141623906 | 141624059 | + | 25.3 | 463 | 617 | -963 | THE1-int | MaLR | LTR |
| 141624077 | 141624343 | - | 22.9 | 96 | 376 | -52 | MSTA | MaLR | LTR |
| 141624368 | 141624608 | + | 23.4 | 907 | 1188 | -392 | THE1-int | MaLR | LTR |
| 141624600 | 141624655 | - | 18.2 | 911 | 967 | -613 | THE1-int | MaLR | LTR |
| 141624753 | 141624848 | - | 19.8 | 1 | 97 | -331 | MSTA | MaLR | LTR |
| 141624851 | 141625195 | - | 23.6 | 1318 | 1649 | -2 | MST-int | MaLR | LTR |
| 141625203 | 141625312 | - | 20.6 | 56 | 159 | -269 | MSTA | MaLR | LTR |
| 141625397 | 141625495 | - | 23.2 | 1 | 100 | -328 | MSTA | MaLR | LTR |

  

|  |  |  |  |  |  |  |  |  |  |
| --- | --- | --- | --- | --- | --- | --- | --- | --- | --- |
| chr1:141627690-141628929 | | | 1120 bp | | |  |  |  |  |
| Genome Start | Genome End | Strand | Div | Repeat Start | Repeat End | Repeat Left | Name | Family | Class |
| 141627690 | 141627745 | - | 25.0 | 911 | 967 | -613 | MSTA-int | MaLR | LTR |
| 141627857 | 141627962 | - | 27.4 | 28 | 134 | -1517 | MSTA-int | MaLR | LTR |
| 141627960 | 141628110 | + | 28.0 | 465 | 621 | -959 | THE1-int | MaLR | LTR |
| 141628126 | 141628479 | - | 25.3 | 1 | 376 | -52 | MSTA | MaLR | LTR |
| 141628480 | 141628698 | - | 14.0 | 1401 | 1651 | 0 | MSTA-int | MaLR | LTR |
| 141628696 | 141628929 | - | 17.1 | 13 | 250 | -1401 | MSTA-int | MaLR | LTR |

  

|  |  |  |  |  |  |  |  |  |  |
| --- | --- | --- | --- | --- | --- | --- | --- | --- | --- |
| chr1:142011066-142021963 | | | 9954 bp | | |  |  |  |  |
| Genome Start | Genome End | Strand | Div | Repeat Start | Repeat End | Repeat Left | Name | Family | Class |
| 142011066 | 142011208 | - | 27.5 | 129 | 274 | -154 | MSTA | MaLR | LTR |
| 142011255 | 142011488 | + | 22.8 | 907 | 1178 | -402 | THE1-int | MaLR | LTR |
| 142011608 | 142011708 | - | 21.6 | 1 | 106 | -322 | MSTA | MaLR | LTR |
| 142011709 | 142012021 | - | 23.7 | 1317 | 1651 | 0 | MSTA-int | MaLR | LTR |
| 142012014 | 142012107 | - | 26.9 | 76 | 176 | -252 | MSTA | MaLR | LTR |
| 142012169 | 142012266 | - | 19.6 | 1 | 99 | -329 | MSTA | MaLR | LTR |
| 142012267 | 142012382 | - | 19.8 | 1535 | 1651 | 0 | MSTA-int | MaLR | LTR |
| 142012386 | 142013674 | - | 25.2 | 32 | 1382 | -269 | MSTA-int | MaLR | LTR |
| 142013677 | 142013809 | + | 15.9 | 507 | 661 | -990 | MST-int | MaLR | LTR |
| 142013836 | 142014142 | - | 25.2 | 76 | 385 | -43 | MSTA | MaLR | LTR |
| 142014147 | 142014386 | + | 24.7 | 907 | 1188 | -392 | THE1-int | MaLR | LTR |
| 142014378 | 142014433 | - | 21.4 | 979 | 1035 | -616 | MST-int | MaLR | LTR |
| 142014535 | 142014627 | - | 20.4 | 1 | 94 | -334 | MSTA | MaLR | LTR |
| 142014628 | 142014945 | - | 21.3 | 1341 | 1651 | 0 | MST-int | MaLR | LTR |
| 142014952 | 142015063 | - | 23.3 | 61 | 171 | -257 | MSTA | MaLR | LTR |
| 142015099 | 142015194 | - | 22.9 | 1 | 97 | -331 | MSTA | MaLR | LTR |
| 142015195 | 142015306 | - | 13.4 | 1538 | 1651 | 0 | MSTA-int | MaLR | LTR |
| 142015434 | 142015526 | - | 21.5 | 1 | 96 | -332 | MSTA | MaLR | LTR |
| 142015527 | 142017106 | - | 23.7 | 28 | 1651 | 0 | MSTA-int | MaLR | LTR |
| 142017102 | 142017255 | + | 27.5 | 463 | 617 | -963 | THE1-int | MaLR | LTR |
| 142017274 | 142017539 | - | 25.4 | 98 | 374 | -54 | MSTA | MaLR | LTR |
| 142017567 | 142017798 | + | 22.9 | 907 | 1183 | -397 | THE1-int | MaLR | LTR |
| 142017806 | 142017862 | - | 22.8 | 910 | 967 | -613 | THE1-int | MaLR | LTR |
| 142017947 | 142018042 | - | 15.8 | 1 | 97 | -331 | MSTA | MaLR | LTR |
| 142018043 | 142018385 | - | 22.2 | 1322 | 1651 | 0 | MSTA-int | MaLR | LTR |
| 142018389 | 142018491 | - | 22.6 | 58 | 171 | -257 | MSTA | MaLR | LTR |
| 142018537 | 142018635 | - | 25.2 | 1 | 100 | -328 | MSTA | MaLR | LTR |
| 142018636 | 142018712 | - | 15.6 | 1574 | 1651 | 0 | MSTA-int | MaLR | LTR |
| 142018715 | 142018775 | + | 18.3 | 317 | 376 | -52 | MSTA | MaLR | LTR |
| 142018810 | 142018848 | - | 15.4 | 623 | 661 | -990 | MSTA-int | MaLR | LTR |
| 142018849 | 142020379 | - | 23.7 | 28 | 1583 | -68 | MSTA-int | MaLR | LTR |
| 142020375 | 142020528 | + | 24.0 | 463 | 617 | -963 | THE1-int | MaLR | LTR |
| 142020546 | 142020812 | - | 21.4 | 96 | 376 | -52 | MSTA | MaLR | LTR |
| 142020837 | 142021077 | + | 23.0 | 907 | 1188 | -392 | THE1-int | MaLR | LTR |
| 142021069 | 142021124 | - | 20.0 | 911 | 967 | -613 | THE1-int | MaLR | LTR |
| 142021221 | 142021316 | - | 19.8 | 1 | 97 | -331 | MSTA | MaLR | LTR |
| 142021319 | 142021663 | - | 23.3 | 1318 | 1649 | -2 | MST-int | MaLR | LTR |
| 142021671 | 142021780 | - | 21.6 | 56 | 159 | -269 | MSTA | MaLR | LTR |
| 142021865 | 142021963 | - | 23.2 | 1 | 100 | -328 | MSTA | MaLR | LTR |

  

|  |  |  |  |  |  |  |  |  |  |
| --- | --- | --- | --- | --- | --- | --- | --- | --- | --- |
| chr1:142245939-142247179 | | | 1119 bp | | |  |  |  |  |
| Genome Start | Genome End | Strand | Div | Repeat Start | Repeat End | Repeat Left | Name | Family | Class |
| 142245939 | 142246172 | + | 16.7 | 13 | 250 | -1401 | MSTA-int | MaLR | LTR |
| 142246170 | 142246388 | + | 14.1 | 1401 | 1651 | 0 | MSTA-int | MaLR | LTR |
| 142246389 | 142246742 | + | 25.6 | 1 | 376 | -52 | MSTA | MaLR | LTR |
| 142246758 | 142246909 | - | 21.9 | 465 | 661 | -959 | MST-int | MaLR | LTR |
| 142246907 | 142247010 | + | 25.2 | 28 | 130 | -1521 | MSTA-int | MaLR | LTR |
| 142247124 | 142247179 | + | 25.0 | 911 | 967 | -613 | MSTA-int | MaLR | LTR |

  

|  |  |  |  |  |  |  |  |  |  |
| --- | --- | --- | --- | --- | --- | --- | --- | --- | --- |
| chr1:142250021-142254064 | | | 3750 bp | | |  |  |  |  |
| Genome Start | Genome End | Strand | Div | Repeat Start | Repeat End | Repeat Left | Name | Family | Class |
| 142250021 | 142250116 | + | 20.8 | 1 | 97 | -331 | MSTA | MaLR | LTR |
| 142250213 | 142250268 | + | 18.2 | 911 | 967 | -613 | THE1-int | MaLR | LTR |
| 142250260 | 142250500 | - | 23.8 | 907 | 1188 | -392 | THE1-int | MaLR | LTR |
| 142250525 | 142250789 | + | 22.0 | 96 | 374 | -54 | MSTA | MaLR | LTR |
| 142250809 | 142250962 | - | 24.7 | 463 | 617 | -963 | THE1-int | MaLR | LTR |
| 142250958 | 142252488 | + | 23.8 | 28 | 1583 | -68 | MST-int | MaLR | LTR |
| 142252489 | 142252527 | + | 15.4 | 623 | 661 | -990 | MST-int | MaLR | LTR |
| 142252562 | 142252624 | - | 18.3 | 317 | 376 | -52 | MSTA | MaLR | LTR |
| 142252627 | 142252703 | + | 16.9 | 1574 | 1651 | 0 | MST-int | MaLR | LTR |
| 142252704 | 142252802 | + | 25.2 | 1 | 100 | -328 | MSTA | MaLR | LTR |
| 142252848 | 142252955 | + | 24.1 | 58 | 180 | -248 | MSTA | MaLR | LTR |
| 142252949 | 142253295 | + | 21.3 | 1317 | 1651 | 0 | MSTA-int | MaLR | LTR |
| 142253296 | 142253391 | + | 16.9 | 1 | 97 | -331 | MSTA | MaLR | LTR |
| 142253476 | 142253532 | + | 22.8 | 910 | 967 | -613 | THE1-int | MaLR | LTR |
| 142253540 | 142253771 | - | 22.5 | 907 | 1183 | -397 | THE1-int | MaLR | LTR |
| 142253776 | 142254064 | + | 24.2 | 76 | 374 | -54 | MSTA | MaLR | LTR |

  


|  |  |  |  |  |  |  |  |  |  |
| --- | --- | --- | --- | --- | --- | --- | --- | --- | --- |
| chr2:94793805-94802160 | | | 7719 bp | | |  |  |  |  |
| Genome Start | Genome End | Strand | Div | Repeat Start | Repeat End | Repeat Left | Name | Family | Class |
| 94793805 | 94794002 | - | 25.4 | 75 | 285 | -141 | MSTA | MaLR | LTR |
| 94794007 | 94794283 | + | 21.5 | 907 | 1218 | -362 | THE1-int | MaLR | LTR |
| 94794375 | 94794482 | - | 20.2 | 1 | 106 | -322 | MSTA | MaLR | LTR |
| 94794483 | 94794795 | - | 21.5 | 1317 | 1651 | 0 | MST-int | MaLR | LTR |
| 94794794 | 94794882 | - | 23.0 | 75 | 171 | -257 | MSTA | MaLR | LTR |
| 94794934 | 94795031 | - | 22.7 | 1 | 99 | -329 | MSTA | MaLR | LTR |
| 94795032 | 94796650 | - | 22.6 | 28 | 1651 | 0 | MSTA-int | MaLR | LTR |
| 94796648 | 94796780 | + | 18.9 | 506 | 661 | -990 | MST-int | MaLR | LTR |
| 94796823 | 94797079 | - | 23.2 | 95 | 368 | -60 | MSTA | MaLR | LTR |
| 94797094 | 94797337 | + | 21.3 | 907 | 1191 | -389 | THE1-int | MaLR | LTR |
| 94797326 | 94797385 | - | 19.3 | 910 | 967 | -613 | THE1-int | MaLR | LTR |
| 94797476 | 94797570 | - | 20.0 | 1 | 97 | -331 | MSTA | MaLR | LTR |
| 94797571 | 94797918 | - | 22.4 | 1318 | 1651 | 0 | MST-int | MaLR | LTR |
| 94797922 | 94798012 | - | 28.6 | 65 | 167 | -261 | MSTA | MaLR | LTR |
| 94798060 | 94798157 | - | 21.4 | 1 | 100 | -328 | MSTA | MaLR | LTR |
| 94798158 | 94799775 | - | 24.1 | 34 | 1651 | 0 | MST-int | MaLR | LTR |
| 94799779 | 94799911 | + | 22.0 | 506 | 659 | -992 | MST-int | MaLR | LTR |
| 94799948 | 94800232 | - | 24.5 | 76 | 376 | -52 | MSTA | MaLR | LTR |
| 94800237 | 94800480 | + | 20.5 | 907 | 1191 | -389 | THE1-int | MaLR | LTR |
| 94800469 | 94800524 | - | 23.2 | 911 | 967 | -613 | THE1-int | MaLR | LTR |
| 94800621 | 94800715 | - | 17.9 | 1 | 97 | -331 | MSTA | MaLR | LTR |
| 94800716 | 94800788 | - | 20.6 | 1579 | 1651 | 0 | MST-int | MaLR | LTR |
| 94800825 | 94801107 | - | 24.7 | 76 | 376 | -52 | MSTA | MaLR | LTR |
| 94801113 | 94801353 | + | 26.6 | 976 | 1256 | -395 | MST-int | MaLR | LTR |
| 94801342 | 94801397 | - | 18.2 | 911 | 967 | -613 | THE1-int | MaLR | LTR |
| 94801501 | 94801576 | - | 19.0 | 1 | 76 | -356 | MSTA | MaLR | LTR |
| 94801577 | 94801909 | - | 22.9 | 1322 | 1651 | 0 | MST-int | MaLR | LTR |
| 94801914 | 94802014 | - | 27.7 | 60 | 171 | -257 | MSTA | MaLR | LTR |
| 94802064 | 94802160 | - | 23.7 | 1 | 99 | -329 | MSTA | MaLR | LTR |

  

|  |  |  |  |  |  |  |  |  |  |
| --- | --- | --- | --- | --- | --- | --- | --- | --- | --- |
| chr2:94803900-94811612 | | | 7020 bp | | |  |  |  |  |
| Genome Start | Genome End | Strand | Div | Repeat Start | Repeat End | Repeat Left | Name | Family | Class |
| 94803900 | 94804195 | - | 24.8 | 76 | 385 | -43 | MSTA | MaLR | LTR |
| 94804200 | 94804465 | + | 29.1 | 907 | 1191 | -389 | THE1-int | MaLR | LTR |
| 94804454 | 94804509 | - | 19.6 | 911 | 967 | -613 | THE1-int | MaLR | LTR |
| 94804595 | 94804702 | - | 18.4 | 1 | 105 | -323 | MSTA | MaLR | LTR |
| 94804703 | 94805020 | - | 22.6 | 1341 | 1651 | 0 | MST-int | MaLR | LTR |
| 94805027 | 94805205 | - | 23.5 | 1 | 171 | -257 | MSTA | MaLR | LTR |
| 94805206 | 94806761 | - | 21.9 | 28 | 1651 | 0 | MSTA-int | MaLR | LTR |
| 94806757 | 94806892 | + | 21.5 | 504 | 661 | -990 | MST-int | MaLR | LTR |
| 94806929 | 94807196 | - | 22.9 | 96 | 374 | -54 | MSTA | MaLR | LTR |
| 94807222 | 94807464 | + | 21.4 | 907 | 1191 | -389 | THE1-int | MaLR | LTR |
| 94807592 | 94807687 | - | 15.6 | 1 | 97 | -331 | MSTA | MaLR | LTR |
| 94807688 | 94808028 | - | 23.1 | 1317 | 1651 | 0 | MSTA-int | MaLR | LTR |
| 94808022 | 94808127 | - | 17.0 | 58 | 180 | -248 | MSTA | MaLR | LTR |
| 94808169 | 94808270 | - | 19.8 | 1 | 103 | -325 | MSTA | MaLR | LTR |
| 94808271 | 94808348 | - | 16.9 | 1574 | 1651 | 0 | MSTA-int | MaLR | LTR |
| 94808351 | 94808410 | + | 16.7 | 317 | 376 | -52 | MSTA | MaLR | LTR |
| 94808445 | 94808483 | - | 15.4 | 623 | 661 | -990 | MSTA-int | MaLR | LTR |
| 94808484 | 94810013 | - | 22.2 | 15 | 1583 | -68 | MSTA-int | MaLR | LTR |
| 94810087 | 94810199 | + | 18.5 | 506 | 661 | -963 | MST-int | MaLR | LTR |
| 94810218 | 94810483 | - | 23.9 | 97 | 374 | -54 | MSTA | MaLR | LTR |
| 94810510 | 94810743 | + | 24.9 | 907 | 1178 | -402 | THE1-int | MaLR | LTR |
| 94810894 | 94810987 | - | 20.2 | 1 | 97 | -331 | MSTA | MaLR | LTR |
| 94810990 | 94811333 | - | 21.0 | 1318 | 1649 | -2 | MSTA-int | MaLR | LTR |
| 94811337 | 94811420 | - | 21.4 | 75 | 167 | -261 | MSTA | MaLR | LTR |
| 94811506 | 94811612 | - | 19.4 | 1 | 104 | -324 | MSTA | MaLR | LTR |

  

|  |  |  |  |  |  |  |  |  |  |
| --- | --- | --- | --- | --- | --- | --- | --- | --- | --- |
| chr2:130527955-130528613 | | | 552 bp | | |  |  |  |  |
| Genome Start | Genome End | Strand | Div | Repeat Start | Repeat End | Repeat Left | Name | Family | Class |
| 130527955 | 130528118 | - | 29.4 | 99 | 274 | -154 | MSTA | MaLR | LTR |
| 130528141 | 130528377 | + | 18.6 | 907 | 1191 | -389 | THE1-int | MaLR | LTR |
| 130528366 | 130528421 | - | 19.6 | 911 | 967 | -613 | THE1-int | MaLR | LTR |
| 130528519 | 130528613 | - | 17.9 | 1 | 97 | -331 | MSTA | MaLR | LTR |

  

|  |  |  |  |  |  |  |  |  |  |
| --- | --- | --- | --- | --- | --- | --- | --- | --- | --- |
| chr2:130529946-130530521 | | | 455 bp | | |  |  |  |  |
| Genome Start | Genome End | Strand | Div | Repeat Start | Repeat End | Repeat Left | Name | Family | Class |
| 130529946 | 130530018 | - | 19.2 | 95 | 171 | -257 | MSTA | MaLR | LTR |
| 130530042 | 130530274 | + | 21.9 | 907 | 1178 | -402 | THE1-int | MaLR | LTR |
| 130530273 | 130530325 | - | 20.8 | 914 | 967 | -613 | MSTA-int | MaLR | LTR |
| 130530426 | 130530521 | - | 16.7 | 1 | 97 | -331 | MSTA | MaLR | LTR |

  

|  |  |  |  |  |  |  |  |  |  |
| --- | --- | --- | --- | --- | --- | --- | --- | --- | --- |
| chr2:130918665-130919323 | | | 551 bp | | |  |  |  |  |
| Genome Start | Genome End | Strand | Div | Repeat Start | Repeat End | Repeat Left | Name | Family | Class |
| 130918665 | 130918828 | - | 29.4 | 99 | 274 | -154 | MSTA | MaLR | LTR |
| 130918852 | 130919087 | + | 21.7 | 976 | 1256 | -395 | MST-int | MaLR | LTR |
| 130919076 | 130919131 | - | 19.6 | 911 | 967 | -613 | THE1-int | MaLR | LTR |
| 130919229 | 130919323 | - | 17.9 | 1 | 97 | -331 | MSTA | MaLR | LTR |

  

|  |  |  |  |  |  |  |  |  |  |
| --- | --- | --- | --- | --- | --- | --- | --- | --- | --- |
| chr2:130920660-130921236 | | | 456 bp | | |  |  |  |  |
| Genome Start | Genome End | Strand | Div | Repeat Start | Repeat End | Repeat Left | Name | Family | Class |
| 130920660 | 130920732 | - | 19.2 | 95 | 171 | -257 | MSTA | MaLR | LTR |
| 130920756 | 130920989 | + | 21.8 | 907 | 1178 | -402 | THE1-int | MaLR | LTR |
| 130920988 | 130921040 | - | 20.8 | 914 | 967 | -613 | MSTA-int | MaLR | LTR |
| 130921141 | 130921236 | - | 15.6 | 1 | 97 | -331 | MSTA | MaLR | LTR |

  

|  |  |  |  |  |  |  |  |  |  |
| --- | --- | --- | --- | --- | --- | --- | --- | --- | --- |
| chr2:131148375-131148951 | | | 456 bp | | |  |  |  |  |
| Genome Start | Genome End | Strand | Div | Repeat Start | Repeat End | Repeat Left | Name | Family | Class |
| 131148375 | 131148470 | + | 15.6 | 1 | 97 | -331 | MSTA | MaLR | LTR |
| 131148571 | 131148623 | + | 22.6 | 914 | 967 | -613 | THE1-int | MaLR | LTR |
| 131148622 | 131148855 | - | 22.3 | 907 | 1178 | -402 | THE1-int | MaLR | LTR |
| 131148879 | 131148951 | + | 19.2 | 95 | 171 | -257 | MSTA | MaLR | LTR |

  

|  |  |  |  |  |  |  |  |  |  |
| --- | --- | --- | --- | --- | --- | --- | --- | --- | --- |
| chr2:131755024-131755600 | | | 416 bp | | |  |  |  |  |
| Genome Start | Genome End | Strand | Div | Repeat Start | Repeat End | Repeat Left | Name | Family | Class |
| 131755024 | 131755132 | + | 16.4 | 1 | 105 | -323 | MSTA | MaLR | LTR |
| 131755271 | 131755504 | - | 21.8 | 907 | 1178 | -402 | THE1-int | MaLR | LTR |
| 131755528 | 131755600 | + | 17.8 | 95 | 171 | -257 | MSTA | MaLR | LTR |

  


|  |  |  |  |  |  |  |  |  |  |
| --- | --- | --- | --- | --- | --- | --- | --- | --- | --- |
| chr3:199259377-199260041 | | | 569 bp | | |  |  |  |  |
| Genome Start | Genome End | Strand | Div | Repeat Start | Repeat End | Repeat Left | Name | Family | Class |
| 199259377 | 199259561 | - | 26.2 | 76 | 274 | -154 | MSTA | MaLR | LTR |
| 199259566 | 199259809 | + | 19.7 | 907 | 1191 | -389 | THE1-int | MaLR | LTR |
| 199259798 | 199259843 | - | 15.2 | 921 | 967 | -613 | THE1-int | MaLR | LTR |
| 199259948 | 199260041 | - | 11.9 | 1 | 96 | -332 | MSTA | MaLR | LTR |

  

|  |  |  |  |  |  |  |  |  |  |
| --- | --- | --- | --- | --- | --- | --- | --- | --- | --- |
| chr3:199260631-199263234 | | | 2604 bp | | |  |  |  |  |
| Genome Start | Genome End | Strand | Div | Repeat Start | Repeat End | Repeat Left | Name | Family | Class |
| 199260631 | 199261939 | - | 22.4 | 323 | 1651 | 0 | MSTA-int | MaLR | LTR |
| 199261940 | 199262941 | + | 6.5 | 1 | 1007 | 0 | LTR13 | ERVK | LTR |
| 199262942 | 199263234 | - | 22.4 | 28 | 322 | -1329 | MSTA-int | MaLR | LTR |

  


|  |  |  |  |  |  |  |  |  |  |
| --- | --- | --- | --- | --- | --- | --- | --- | --- | --- |
| chr4:48911896-48925136 | | | 12330 bp | | |  |  |  |  |
| Genome Start | Genome End | Strand | Div | Repeat Start | Repeat End | Repeat Left | Name | Family | Class |
| 48911896 | 48912080 | - | 27.9 | 76 | 274 | -154 | MSTA | MaLR | LTR |
| 48912085 | 48912328 | + | 22.6 | 907 | 1191 | -389 | THE1-int | MaLR | LTR |
| 48912317 | 48912400 | - | 17.1 | 921 | 1035 | -613 | THE1-int | MaLR | LTR |
| 48912437 | 48912537 | - | 20.6 | 1 | 106 | -322 | MSTA | MaLR | LTR |
| 48912538 | 48912851 | - | 21.8 | 1317 | 1651 | 0 | MST-int | MaLR | LTR |
| 48912850 | 48912950 | - | 24.8 | 60 | 171 | -257 | MSTA | MaLR | LTR |
| 48913000 | 48913097 | - | 18.6 | 1 | 99 | -329 | MSTA | MaLR | LTR |
| 48913100 | 48913213 | - | 19.3 | 1535 | 1643 | -8 | MSTA-int | MaLR | LTR |
| 48913210 | 48914468 | - | 25.2 | 28 | 1389 | -262 | MSTA-int | MaLR | LTR |
| 48914467 | 48914606 | + | 24.5 | 507 | 667 | -984 | MST-int | MaLR | LTR |
| 48914635 | 48914912 | - | 21.6 | 95 | 376 | -52 | MSTA | MaLR | LTR |
| 48914925 | 48915157 | + | 22.0 | 907 | 1178 | -402 | THE1-int | MaLR | LTR |
| 48915156 | 48915211 | - | 21.4 | 911 | 967 | -613 | THE1-int | MaLR | LTR |
| 48915315 | 48915388 | - | 19.5 | 3 | 76 | -356 | MSTA | MaLR | LTR |
| 48915391 | 48915727 | - | 20.7 | 1317 | 1651 | 0 | MSTA-int | MaLR | LTR |
| 48915720 | 48915826 | - | 26.4 | 60 | 176 | -252 | MSTA | MaLR | LTR |
| 48915876 | 48915972 | - | 19.6 | 1 | 99 | -329 | MSTA | MaLR | LTR |
| 48915973 | 48917538 | - | 23.1 | 29 | 1651 | 0 | MSTA-int | MaLR | LTR |
| 48917536 | 48917666 | + | 19.2 | 508 | 661 | -990 | MST-int | MaLR | LTR |
| 48917705 | 48917997 | - | 25.5 | 76 | 372 | -56 | MSTA | MaLR | LTR |
| 48918003 | 48918238 | + | 25.5 | 976 | 1248 | -403 | MST-int | MaLR | LTR |
| 48918232 | 48918287 | - | 23.2 | 911 | 967 | -613 | THE1-int | MaLR | LTR |
| 48918389 | 48918481 | - | 24.7 | 1 | 94 | -334 | MSTA | MaLR | LTR |
| 48918482 | 48918797 | - | 25.5 | 1341 | 1651 | 0 | MST-int | MaLR | LTR |
| 48918804 | 48918905 | - | 22.6 | 74 | 171 | -257 | MSTA | MaLR | LTR |
| 48918951 | 48919046 | - | 19.8 | 1 | 97 | -331 | MSTA | MaLR | LTR |
| 48919047 | 48919158 | - | 16.1 | 1538 | 1651 | 0 | MSTA-int | MaLR | LTR |
| 48919275 | 48919381 | - | 21.4 | 1 | 104 | -324 | MSTA | MaLR | LTR |
| 48919382 | 48920955 | - | 24.5 | 34 | 1651 | 0 | MSTA-int | MaLR | LTR |
| 48920958 | 48921099 | + | 22.0 | 505 | 670 | -981 | MST-int | MaLR | LTR |
| 48921129 | 48921391 | - | 22.9 | 98 | 374 | -54 | MSTA | MaLR | LTR |
| 48921419 | 48921650 | + | 22.5 | 907 | 1187 | -393 | THE1-int | MaLR | LTR |
| 48921643 | 48921698 | - | 23.2 | 911 | 967 | -613 | MSTA-int | MaLR | LTR |
| 48921784 | 48921876 | - | 19.6 | 4 | 97 | -331 | MSTA | MaLR | LTR |
| 48921880 | 48922222 | - | 20.4 | 1322 | 1651 | 0 | MSTA-int | MaLR | LTR |
| 48922226 | 48922328 | - | 25.2 | 58 | 171 | -257 | MSTA | MaLR | LTR |
| 48922374 | 48922470 | - | 23.7 | 3 | 100 | -328 | MSTA | MaLR | LTR |
| 48922473 | 48922549 | - | 16.9 | 1574 | 1651 | 0 | MSTA-int | MaLR | LTR |
| 48922552 | 48922612 | + | 18.3 | 317 | 376 | -52 | MSTA | MaLR | LTR |
| 48922647 | 48922685 | - | 17.9 | 623 | 661 | -990 | MSTA-int | MaLR | LTR |
| 48922689 | 48924214 | - | 23.3 | 28 | 1580 | -71 | MSTA-int | MaLR | LTR |
| 48924210 | 48924344 | + | 22.2 | 504 | 661 | -990 | MST-int | MaLR | LTR |
| 48924380 | 48924645 | - | 23.0 | 96 | 376 | -52 | MSTA | MaLR | LTR |
| 48924670 | 48924910 | + | 23.0 | 907 | 1188 | -392 | THE1-int | MaLR | LTR |
| 48924901 | 48924956 | - | 19.6 | 911 | 968 | -612 | THE1-int | MaLR | LTR |
| 48925041 | 48925136 | - | 18.9 | 1 | 97 | -331 | MSTA | MaLR | LTR |

  

|  |  |  |  |  |  |  |  |  |  |
| --- | --- | --- | --- | --- | --- | --- | --- | --- | --- |
| chr4:49263851-49264616 | | | 591 bp | | |  |  |  |  |
| Genome Start | Genome End | Strand | Div | Repeat Start | Repeat End | Repeat Left | Name | Family | Class |
| 49263851 | 49263943 | + | 22.6 | 4 | 97 | -331 | MSTA | MaLR | LTR |
| 49264095 | 49264325 | - | 22.7 | 907 | 1175 | -405 | THE1-int | MaLR | LTR |
| 49264350 | 49264616 | + | 22.2 | 96 | 376 | -52 | MSTA | MaLR | LTR |

  

|  |  |  |  |  |  |  |  |  |  |
| --- | --- | --- | --- | --- | --- | --- | --- | --- | --- |
| chr4:49264782-49266522 | | | 1663 bp | | |  |  |  |  |
| Genome Start | Genome End | Strand | Div | Repeat Start | Repeat End | Repeat Left | Name | Family | Class |
| 49264782 | 49266306 | + | 23.1 | 28 | 1580 | -71 | MST-int | MaLR | LTR |
| 49266383 | 49266443 | - | 20.0 | 317 | 376 | -52 | MSTA | MaLR | LTR |
| 49266446 | 49266522 | + | 16.9 | 1574 | 1651 | 0 | MST-int | MaLR | LTR |

  

|  |  |  |  |  |  |  |  |  |  |
| --- | --- | --- | --- | --- | --- | --- | --- | --- | --- |
| chr4:49270520-49271308 | | | 637 bp | | |  |  |  |  |
| Genome Start | Genome End | Strand | Div | Repeat Start | Repeat End | Repeat Left | Name | Family | Class |
| 49270520 | 49270609 | + | 18.9 | 4 | 94 | -334 | MSTA | MaLR | LTR |
| 49270758 | 49270997 | - | 23.4 | 907 | 1188 | -392 | THE1-int | MaLR | LTR |
| 49271002 | 49271308 | + | 26.6 | 76 | 385 | -43 | MSTA | MaLR | LTR |

  

|  |  |  |  |  |  |  |  |  |  |
| --- | --- | --- | --- | --- | --- | --- | --- | --- | --- |
| chr4:49272879-49274079 | | | 1028 bp | | |  |  |  |  |
| Genome Start | Genome End | Strand | Div | Repeat Start | Repeat End | Repeat Left | Name | Family | Class |
| 49272879 | 49272976 | + | 17.5 | 1 | 99 | -329 | MSTA | MaLR | LTR |
| 49273026 | 49273131 | + | 27.6 | 60 | 176 | -252 | MSTA | MaLR | LTR |
| 49273124 | 49273436 | + | 22.4 | 1317 | 1651 | 0 | MSTA-int | MaLR | LTR |
| 49273437 | 49273524 | + | 21.6 | 1 | 97 | -331 | MSTA | MaLR | LTR |
| 49273613 | 49273658 | + | 19.6 | 921 | 967 | -613 | THE1-int | MaLR | LTR |
| 49273657 | 49273890 | - | 21.5 | 907 | 1178 | -402 | THE1-int | MaLR | LTR |
| 49273937 | 49274079 | + | 27.5 | 129 | 274 | -154 | MSTA | MaLR | LTR |

  


|  |  |  |  |  |  |  |  |  |  |
| --- | --- | --- | --- | --- | --- | --- | --- | --- | --- |
| chr8:43489490-43490778 | | | 1153 bp | | |  |  |  |  |
| Genome Start | Genome End | Strand | Div | Repeat Start | Repeat End | Repeat Left | Name | Family | Class |
| 43489490 | 43489718 | + | 17.3 | 13 | 239 | -1412 | MSTA-int | MaLR | LTR |
| 43489726 | 43489973 | + | 19.2 | 1401 | 1651 | 0 | MSTA-int | MaLR | LTR |
| 43489974 | 43490334 | + | 24.1 | 1 | 374 | -54 | MSTA | MaLR | LTR |
| 43490351 | 43490503 | - | 22.6 | 466 | 661 | -958 | MST-int | MaLR | LTR |
| 43490494 | 43490599 | + | 18.9 | 18 | 129 | -1522 | MSTA-int | MaLR | LTR |
| 43490723 | 43490778 | + | 23.2 | 911 | 967 | -613 | MSTA-int | MaLR | LTR |

  

|  |  |  |  |  |  |  |  |  |  |
| --- | --- | --- | --- | --- | --- | --- | --- | --- | --- |
| chr8:43490777-43501444 | | | 10122 bp | | |  |  |  |  |
| Genome Start | Genome End | Strand | Div | Repeat Start | Repeat End | Repeat Left | Name | Family | Class |
| 43490777 | 43491011 | - | 20.5 | 907 | 1178 | -402 | THE1-int | MaLR | LTR |
| 43491032 | 43491299 | + | 21.1 | 96 | 374 | -54 | MSTA | MaLR | LTR |
| 43491320 | 43491470 | - | 23.1 | 507 | 675 | -976 | MST-int | MaLR | LTR |
| 43491483 | 43493036 | + | 24.7 | 47 | 1651 | 0 | MST-int | MaLR | LTR |
| 43493037 | 43493132 | + | 20.8 | 1 | 97 | -331 | MSTA | MaLR | LTR |
| 43493213 | 43493288 | + | 20.0 | 97 | 176 | -252 | MSTA | MaLR | LTR |
| 43493281 | 43493617 | + | 23.1 | 1317 | 1651 | 0 | MST-int | MaLR | LTR |
| 43493618 | 43493716 | + | 21.4 | 1 | 100 | -328 | MSTA | MaLR | LTR |
| 43493808 | 43493865 | + | 14.3 | 978 | 1034 | -617 | MST-int | MaLR | LTR |
| 43493855 | 43494096 | - | 21.1 | 907 | 1191 | -389 | THE1-int | MaLR | LTR |
| 43494103 | 43494389 | + | 24.6 | 82 | 385 | -43 | MSTA | MaLR | LTR |
| 43494398 | 43494548 | - | 21.9 | 469 | 661 | -963 | MST-int | MaLR | LTR |
| 43494556 | 43496116 | + | 26.4 | 35 | 1649 | -2 | MST-int | MaLR | LTR |
| 43496119 | 43496214 | + | 22.9 | 1 | 97 | -331 | MSTA | MaLR | LTR |
| 43496313 | 43496368 | + | 19.6 | 911 | 967 | -613 | THE1-int | MaLR | LTR |
| 43496357 | 43496597 | - | 24.8 | 907 | 1191 | -389 | THE1-int | MaLR | LTR |
| 43496618 | 43496882 | + | 25.5 | 96 | 371 | -57 | MSTA | MaLR | LTR |
| 43496883 | 43497208 | + | 15.1 | 1 | 304 | -5 | AluSc | Alu | SINE |
| 43497254 | 43497391 | - | 23.4 | 466 | 608 | -972 | THE1-int | MaLR | LTR |
| 43497389 | 43497429 | + | 23.7 | 27 | 67 | -1584 | MST-int | MaLR | LTR |
| 43497430 | 43497934 | - | 11.9 | 1 | 513 | 0 | LTR10D | ERV1 | LTR |
| 43497935 | 43499134 | + | 23.7 | 68 | 1335 | -316 | MST-int | MaLR | LTR |
| 43499135 | 43499576 | - | 9.1 | 1 | 463 | 0 | LTR2 | ERV1 | LTR |
| 43499577 | 43499908 | + | 23.7 | 1336 | 1651 | 0 | MST-int | MaLR | LTR |
| 43499909 | 43500003 | + | 21.1 | 1 | 96 | -332 | MSTA | MaLR | LTR |
| 43500052 | 43500166 | + | 25.0 | 68 | 176 | -252 | MSTA | MaLR | LTR |
| 43500159 | 43500506 | + | 23.1 | 1317 | 1651 | 0 | MSTA-int | MaLR | LTR |
| 43500507 | 43500612 | + | 19.2 | 1 | 105 | -323 | MSTA | MaLR | LTR |
| 43500707 | 43500756 | + | 18.0 | 917 | 967 | -613 | THE1-int | MaLR | LTR |
| 43500745 | 43500987 | - | 23.5 | 907 | 1191 | -389 | THE1-int | MaLR | LTR |
| 43501004 | 43501267 | + | 21.4 | 94 | 368 | -60 | MSTA | MaLR | LTR |
| 43501301 | 43501444 | - | 26.6 | 464 | 613 | -967 | THE1-int | MaLR | LTR |

  

|  |  |  |  |  |  |  |  |  |  |
| --- | --- | --- | --- | --- | --- | --- | --- | --- | --- |
| chr8:43503847-43505812 | | | 1966 bp | | |  |  |  |  |
| Genome Start | Genome End | Strand | Div | Repeat Start | Repeat End | Repeat Left | Name | Family | Class |
| 43503847 | 43504710 | + | 23.6 | 1 | 873 | -778 | MSTA-int | MaLR | LTR |
| 43504711 | 43505013 | - | 11.3 | 1 | 302 | -9 | AluY | Alu | SINE |
| 43505014 | 43505812 | + | 23.6 | 874 | 1651 | 0 | MSTA-int | MaLR | LTR |

  


|  |  |  |  |  |  |  |  |  |  |
| --- | --- | --- | --- | --- | --- | --- | --- | --- | --- |
| chr9:33567605-33587040 | | | 18290 bp | | |  |  |  |  |
| Genome Start | Genome End | Strand | Div | Repeat Start | Repeat End | Repeat Left | Name | Family | Class |
| 33567605 | 33567836 | + | 12.1 | 13 | 250 | -1401 | MSTA-int | MaLR | LTR |
| 33567834 | 33568078 | + | 19.0 | 1401 | 1651 | 0 | MSTA-int | MaLR | LTR |
| 33568079 | 33568452 | + | 22.9 | 1 | 373 | -55 | MSTA | MaLR | LTR |
| 33568481 | 33568623 | - | 23.8 | 506 | 670 | -981 | MST-int | MaLR | LTR |
| 33568621 | 33568722 | + | 22.6 | 28 | 129 | -1522 | MSTA-int | MaLR | LTR |
| 33568854 | 33568899 | + | 13.0 | 921 | 967 | -613 | MSTA-int | MaLR | LTR |
| 33568888 | 33569016 | - | 19.5 | 1062 | 1191 | -389 | THE1-int | MaLR | LTR |
| 33569004 | 33569045 | - | 12.8 | 976 | 1014 | -637 | MST-int | MaLR | LTR |
| 33569051 | 33569342 | + | 21.0 | 66 | 376 | -52 | MSTA | MaLR | LTR |
| 33569359 | 33569510 | - | 22.3 | 466 | 661 | -963 | MST-int | MaLR | LTR |
| 33569513 | 33571044 | + | 23.2 | 32 | 1651 | 0 | MSTA-int | MaLR | LTR |
| 33571047 | 33571145 | + | 21.3 | 3 | 104 | -324 | MSTA | MaLR | LTR |
| 33571186 | 33571274 | + | 25.8 | 57 | 155 | -273 | MSTA | MaLR | LTR |
| 33571282 | 33571622 | + | 23.6 | 1318 | 1644 | -7 | MST-int | MaLR | LTR |
| 33571633 | 33571733 | + | 19.2 | 4 | 104 | -324 | MSTA | MaLR | LTR |
| 33571812 | 33571867 | + | 19.6 | 911 | 967 | -613 | THE1-int | MaLR | LTR |
| 33571856 | 33572103 | - | 22.3 | 907 | 1191 | -389 | THE1-int | MaLR | LTR |
| 33572108 | 33572401 | + | 20.2 | 66 | 376 | -52 | MSTA | MaLR | LTR |
| 33572437 | 33572567 | - | 24.4 | 507 | 659 | -992 | MST-int | MaLR | LTR |
| 33572565 | 33574132 | + | 21.8 | 27 | 1651 | 0 | MST-int | MaLR | LTR |
| 33574132 | 33574228 | + | 23.7 | 3 | 100 | -328 | MSTA | MaLR | LTR |
| 33574274 | 33574384 | + | 20.2 | 58 | 178 | -250 | MSTA | MaLR | LTR |
| 33574374 | 33574720 | + | 21.9 | 1317 | 1651 | 0 | MSTA-int | MaLR | LTR |
| 33574721 | 33574807 | + | 18.4 | 1 | 88 | -340 | MSTA | MaLR | LTR |
| 33574914 | 33574969 | + | 19.6 | 911 | 967 | -613 | THE1-int | MaLR | LTR |
| 33574958 | 33575201 | - | 20.5 | 907 | 1191 | -389 | THE1-int | MaLR | LTR |
| 33575207 | 33575488 | + | 23.8 | 77 | 374 | -54 | MSTA | MaLR | LTR |
| 33575507 | 33575657 | - | 19.1 | 466 | 661 | -963 | MST-int | MaLR | LTR |
| 33575660 | 33577238 | + | 21.6 | 32 | 1651 | 0 | MST-int | MaLR | LTR |
| 33577239 | 33577337 | + | 23.2 | 1 | 100 | -328 | MSTA | MaLR | LTR |
| 33577360 | 33577482 | + | 22.7 | 57 | 171 | -257 | MSTA | MaLR | LTR |
| 33577492 | 33577805 | + | 26.2 | 1345 | 1651 | 0 | MST-int | MaLR | LTR |
| 33577810 | 33577899 | + | 20.0 | 5 | 95 | -333 | MSTA | MaLR | LTR |
| 33578053 | 33578284 | - | 20.3 | 907 | 1178 | -402 | THE1-int | MaLR | LTR |
| 33578289 | 33578576 | + | 24.6 | 76 | 376 | -52 | MSTA | MaLR | LTR |
| 33578611 | 33578747 | - | 22.1 | 504 | 661 | -990 | MST-int | MaLR | LTR |
| 33578743 | 33580325 | + | 22.2 | 28 | 1651 | 0 | MST-int | MaLR | LTR |
| 33580328 | 33580424 | + | 24.7 | 3 | 100 | -328 | MSTA | MaLR | LTR |
| 33580470 | 33580572 | + | 26.2 | 58 | 171 | -257 | MSTA | MaLR | LTR |
| 33580585 | 33580898 | + | 26.6 | 1347 | 1650 | -1 | MST-int | MaLR | LTR |
| 33580900 | 33580995 | + | 19.8 | 1 | 97 | -331 | MSTA | MaLR | LTR |
| 33581087 | 33581148 | + | 17.7 | 902 | 967 | -613 | THE1-int | MaLR | LTR |
| 33581147 | 33581379 | - | 18.0 | 907 | 1178 | -402 | THE1-int | MaLR | LTR |
| 33581386 | 33581675 | + | 22.6 | 65 | 376 | -52 | MSTA | MaLR | LTR |
| 33581710 | 33581847 | - | 22.8 | 504 | 661 | -990 | MST-int | MaLR | LTR |
| 33581843 | 33583436 | + | 23.9 | 28 | 1651 | 0 | MST-int | MaLR | LTR |
| 33583437 | 33583532 | + | 22.9 | 1 | 97 | -331 | MSTA | MaLR | LTR |
| 33583584 | 33583708 | + | 23.6 | 60 | 195 | -233 | MSTA | MaLR | LTR |
| 33583681 | 33583999 | + | 19.3 | 1317 | 1651 | 0 | MSTA-int | MaLR | LTR |
| 33584000 | 33584093 | + | 19.1 | 1 | 97 | -331 | MSTA | MaLR | LTR |
| 33584191 | 33584246 | + | 15.4 | 921 | 1035 | -613 | THE1-int | MaLR | LTR |
| 33584245 | 33584478 | - | 22.7 | 907 | 1178 | -402 | THE1-int | MaLR | LTR |
| 33584499 | 33584736 | + | 23.4 | 96 | 351 | -77 | MSTA | MaLR | LTR |
| 33584777 | 33584909 | - | 24.2 | 504 | 657 | -994 | MST-int | MaLR | LTR |
| 33584905 | 33586495 | + | 23.9 | 28 | 1651 | 0 | MST-int | MaLR | LTR |
| 33586590 | 33586685 | + | 23.4 | 58 | 159 | -269 | MSTA | MaLR | LTR |
| 33586696 | 33587040 | + | 20.9 | 1321 | 1651 | 0 | MSTA-int | MaLR | LTR |

  

|  |  |  |  |  |  |  |  |  |  |
| --- | --- | --- | --- | --- | --- | --- | --- | --- | --- |
| chr9:38536498-38546427 | | | 9219 bp | | |  |  |  |  |
| Genome Start | Genome End | Strand | Div | Repeat Start | Repeat End | Repeat Left | Name | Family | Class |
| 38536498 | 38536703 | - | 25.3 | 95 | 296 | -130 | MSTA | MaLR | LTR |
| 38536721 | 38536962 | + | 22.8 | 909 | 1191 | -389 | THE1-int | MaLR | LTR |
| 38536951 | 38537006 | - | 17.9 | 911 | 967 | -613 | THE1-int | MaLR | LTR |
| 38537088 | 38537194 | - | 19.1 | 1 | 107 | -321 | MSTA | MaLR | LTR |
| 38537195 | 38537539 | - | 19.9 | 1321 | 1651 | 0 | MST-int | MaLR | LTR |
| 38537550 | 38537644 | - | 24.5 | 58 | 159 | -269 | MSTA | MaLR | LTR |
| 38537739 | 38539328 | - | 23.8 | 32 | 1651 | 0 | MSTA-int | MaLR | LTR |
| 38539331 | 38539470 | + | 24.9 | 507 | 667 | -984 | MST-int | MaLR | LTR |
| 38539510 | 38539747 | - | 23.8 | 96 | 351 | -77 | MSTA | MaLR | LTR |
| 38539768 | 38540011 | + | 23.4 | 907 | 1191 | -389 | THE1-int | MaLR | LTR |
| 38540000 | 38540045 | - | 13.3 | 921 | 967 | -613 | THE1-int | MaLR | LTR |
| 38540153 | 38540246 | - | 18.1 | 1 | 97 | -331 | MSTA | MaLR | LTR |
| 38540247 | 38540664 | - | 22.2 | 60 | 1651 | 0 | MST-int | MaLR | LTR |
| 38540716 | 38540811 | - | 21.9 | 1 | 97 | -331 | MSTA | MaLR | LTR |
| 38540812 | 38542391 | - | 23.4 | 28 | 1651 | 0 | MSTA-int | MaLR | LTR |
| 38542387 | 38542524 | + | 23.5 | 504 | 661 | -990 | MST-int | MaLR | LTR |
| 38542559 | 38542843 | - | 23.4 | 75 | 376 | -52 | MSTA | MaLR | LTR |
| 38542855 | 38543087 | + | 18.0 | 907 | 1178 | -402 | THE1-int | MaLR | LTR |
| 38543086 | 38543147 | - | 17.7 | 902 | 967 | -613 | THE1-int | MaLR | LTR |
| 38543240 | 38543335 | - | 19.8 | 1 | 97 | -331 | MSTA | MaLR | LTR |
| 38543337 | 38543651 | - | 26.6 | 1347 | 1650 | -1 | MST-int | MaLR | LTR |
| 38543664 | 38543766 | - | 26.2 | 58 | 171 | -257 | MSTA | MaLR | LTR |
| 38543812 | 38543908 | - | 25.8 | 3 | 100 | -328 | MSTA | MaLR | LTR |
| 38543911 | 38545493 | - | 22.4 | 28 | 1651 | 0 | MSTA-int | MaLR | LTR |
| 38545489 | 38545625 | + | 22.8 | 504 | 661 | -990 | MST-int | MaLR | LTR |
| 38545660 | 38545947 | - | 23.4 | 66 | 376 | -52 | MSTA | MaLR | LTR |
| 38545952 | 38546190 | + | 21.4 | 907 | 1188 | -392 | THE1-int | MaLR | LTR |
| 38546182 | 38546237 | - | 21.4 | 911 | 967 | -613 | THE1-int | MaLR | LTR |
| 38546338 | 38546427 | - | 20.0 | 5 | 95 | -333 | MSTA | MaLR | LTR |

  

|  |  |  |  |  |  |  |  |  |  |
| --- | --- | --- | --- | --- | --- | --- | --- | --- | --- |
| chr9:38548738-38550094 | | | 1224 bp | | |  |  |  |  |
| Genome Start | Genome End | Strand | Div | Repeat Start | Repeat End | Repeat Left | Name | Family | Class |
| 38548738 | 38549019 | - | 21.8 | 77 | 374 | -54 | MSTA | MaLR | LTR |
| 38549025 | 38549268 | + | 19.7 | 907 | 1191 | -389 | THE1-int | MaLR | LTR |
| 38549257 | 38549312 | - | 19.6 | 911 | 967 | -613 | THE1-int | MaLR | LTR |
| 38549419 | 38549505 | - | 17.2 | 1 | 88 | -340 | MSTA | MaLR | LTR |
| 38549506 | 38549852 | - | 22.5 | 1317 | 1651 | 0 | MST-int | MaLR | LTR |
| 38549842 | 38549952 | - | 20.2 | 58 | 178 | -250 | MSTA | MaLR | LTR |
| 38549998 | 38550094 | - | 24.7 | 3 | 100 | -328 | MSTA | MaLR | LTR |

  

|  |  |  |  |  |  |  |  |  |  |
| --- | --- | --- | --- | --- | --- | --- | --- | --- | --- |
| chr9:38551658-38555221 | | | 3370 bp | | |  |  |  |  |
| Genome Start | Genome End | Strand | Div | Repeat Start | Repeat End | Repeat Left | Name | Family | Class |
| 38551658 | 38551790 | + | 24.1 | 507 | 661 | -990 | MST-int | MaLR | LTR |
| 38551825 | 38552118 | - | 19.5 | 66 | 376 | -52 | MSTA | MaLR | LTR |
| 38552124 | 38552365 | + | 20.2 | 908 | 1191 | -389 | THE1-int | MaLR | LTR |
| 38552353 | 38552409 | - | 19.3 | 979 | 1036 | -615 | MST-int | MaLR | LTR |
| 38552497 | 38552589 | - | 19.4 | 4 | 97 | -331 | MSTA | MaLR | LTR |
| 38552600 | 38552940 | - | 23.0 | 1318 | 1644 | -7 | MST-int | MaLR | LTR |
| 38552948 | 38553035 | - | 27.3 | 58 | 155 | -273 | MSTA | MaLR | LTR |
| 38553077 | 38553177 | - | 24.0 | 1 | 104 | -324 | MSTA | MaLR | LTR |
| 38553178 | 38554712 | - | 22.9 | 32 | 1651 | 0 | MSTA-int | MaLR | LTR |
| 38554715 | 38554866 | + | 26.4 | 466 | 617 | -963 | THE1-int | MaLR | LTR |
| 38554883 | 38555174 | - | 21.0 | 66 | 376 | -52 | MSTA | MaLR | LTR |
| 38555180 | 38555221 | + | 12.8 | 976 | 1014 | -637 | MST-int | MaLR | LTR |

  

|  |  |  |  |  |  |  |  |  |  |
| --- | --- | --- | --- | --- | --- | --- | --- | --- | --- |
| chr9:42429712-42432898 | | | 3042 bp | | |  |  |  |  |
| Genome Start | Genome End | Strand | Div | Repeat Start | Repeat End | Repeat Left | Name | Family | Class |
| 42429712 | 42429942 | + | 16.9 | 13 | 247 | -1404 | MSTA-int | MaLR | LTR |
| 42429946 | 42430187 | + | 17.6 | 1404 | 1651 | 0 | MSTA-int | MaLR | LTR |
| 42430188 | 42430561 | + | 23.6 | 1 | 376 | -52 | MSTA | MaLR | LTR |
| 42430577 | 42430728 | - | 22.2 | 465 | 657 | -959 | MST-int | MaLR | LTR |
| 42430726 | 42430831 | + | 23.6 | 28 | 133 | -1518 | MSTA-int | MaLR | LTR |
| 42430951 | 42431006 | + | 21.4 | 911 | 967 | -613 | MSTA-int | MaLR | LTR |
| 42430995 | 42431235 | - | 22.0 | 907 | 1191 | -389 | THE1-int | MaLR | LTR |
| 42431240 | 42431525 | + | 22.5 | 76 | 376 | -52 | MSTA | MaLR | LTR |
| 42431542 | 42431692 | - | 22.9 | 466 | 661 | -963 | MST-int | MaLR | LTR |
| 42431690 | 42432328 | + | 24.5 | 27 | 696 | -955 | MSTA-int | MaLR | LTR |
| 42432335 | 42432898 | + | 21.3 | 1107 | 1650 | -1 | MSTA-int | MaLR | LTR |

  

|  |  |  |  |  |  |  |  |  |  |
| --- | --- | --- | --- | --- | --- | --- | --- | --- | --- |
| chr9:42433522-42437621 | | | 3707 bp | | |  |  |  |  |
| Genome Start | Genome End | Strand | Div | Repeat Start | Repeat End | Repeat Left | Name | Family | Class |
| 42433522 | 42433617 | + | 19.8 | 1 | 97 | -331 | MSTA | MaLR | LTR |
| 42433714 | 42433769 | + | 21.4 | 911 | 967 | -613 | THE1-int | MaLR | LTR |
| 42433723 | 42434001 | - | 24.9 | 907 | 1218 | -362 | THE1-int | MaLR | LTR |
| 42434006 | 42434295 | + | 21.9 | 66 | 376 | -52 | MSTA | MaLR | LTR |
| 42434312 | 42434424 | - | 19.1 | 506 | 661 | -963 | MST-int | MaLR | LTR |
| 42434528 | 42436054 | + | 22.9 | 15 | 1583 | -68 | MST-int | MaLR | LTR |
| 42436127 | 42436187 | - | 18.0 | 317 | 377 | -51 | MSTA | MaLR | LTR |
| 42436190 | 42436267 | + | 18.7 | 1574 | 1651 | 0 | MST-int | MaLR | LTR |
| 42436268 | 42436363 | + | 28.1 | 1 | 100 | -328 | MSTA | MaLR | LTR |
| 42436409 | 42436516 | + | 22.4 | 58 | 180 | -248 | MSTA | MaLR | LTR |
| 42436510 | 42436857 | + | 22.1 | 1317 | 1651 | 0 | MSTA-int | MaLR | LTR |
| 42436858 | 42436953 | + | 15.6 | 1 | 97 | -331 | MSTA | MaLR | LTR |
| 42437050 | 42437105 | + | 19.6 | 911 | 967 | -613 | THE1-int | MaLR | LTR |
| 42437094 | 42437328 | - | 20.4 | 907 | 1191 | -389 | THE1-int | MaLR | LTR |
| 42437354 | 42437621 | + | 26.0 | 96 | 374 | -54 | MSTA | MaLR | LTR |

  

|  |  |  |  |  |  |  |  |  |  |
| --- | --- | --- | --- | --- | --- | --- | --- | --- | --- |
| chr9:42437909-42453756 | | | 14633 bp | | |  |  |  |  |
| Genome Start | Genome End | Strand | Div | Repeat Start | Repeat End | Repeat Left | Name | Family | Class |
| 42437909 | 42437978 | - | 30.0 | 907 | 977 | -603 | THE1-int | MaLR | LTR |
| 42437983 | 42438286 | + | 25.1 | 75 | 385 | -43 | MSTA | MaLR | LTR |
| 42438295 | 42438448 | - | 20.1 | 463 | 661 | -963 | MST-int | MaLR | LTR |
| 42438444 | 42440021 | + | 24.8 | 28 | 1651 | 0 | MSTA-int | MaLR | LTR |
| 42440022 | 42440118 | + | 23.7 | 1 | 99 | -329 | MSTA | MaLR | LTR |
| 42440168 | 42440268 | + | 26.7 | 60 | 171 | -257 | MSTA | MaLR | LTR |
| 42440267 | 42440606 | + | 22.2 | 1317 | 1651 | 0 | MSTA-int | MaLR | LTR |
| 42440607 | 42440669 | + | 19.1 | 1 | 65 | -363 | MSTA | MaLR | LTR |
| 42440789 | 42440842 | + | 11.8 | 982 | 1035 | -616 | MST-int | MaLR | LTR |
| 42440831 | 42441070 | - | 24.9 | 976 | 1256 | -395 | MST-int | MaLR | LTR |
| 42441076 | 42441259 | + | 25.3 | 76 | 266 | -162 | MSTA | MaLR | LTR |
| 42441264 | 42441399 | - | 28.9 | 466 | 605 | -975 | THE1-int | MaLR | LTR |
| 42441397 | 42442972 | + | 24.6 | 27 | 1651 | 0 | MST-int | MaLR | LTR |
| 42442973 | 42443078 | + | 19.6 | 1 | 105 | -323 | MSTA | MaLR | LTR |
| 42443196 | 42443260 | + | 23.1 | 95 | 159 | -269 | MSTA | MaLR | LTR |
| 42443268 | 42443606 | + | 22.7 | 1318 | 1649 | -2 | MSTA-int | MaLR | LTR |
| 42443609 | 42443704 | + | 19.8 | 1 | 97 | -331 | MSTA | MaLR | LTR |
| 42443801 | 42443856 | + | 19.6 | 911 | 967 | -613 | THE1-int | MaLR | LTR |
| 42443848 | 42444088 | - | 25.0 | 907 | 1188 | -392 | THE1-int | MaLR | LTR |
| 42444114 | 42444382 | + | 22.5 | 96 | 376 | -52 | MSTA | MaLR | LTR |
| 42444417 | 42444511 | - | 21.2 | 506 | 661 | -990 | MST-int | MaLR | LTR |
| 42444637 | 42446227 | + | 22.4 | 28 | 1651 | 0 | MST-int | MaLR | LTR |
| 42446228 | 42446324 | + | 23.7 | 1 | 99 | -329 | MSTA | MaLR | LTR |
| 42446374 | 42446473 | + | 28.0 | 60 | 171 | -257 | MSTA | MaLR | LTR |
| 42446472 | 42446811 | + | 23.1 | 1317 | 1651 | 0 | MST-int | MaLR | LTR |
| 42446812 | 42446874 | + | 19.1 | 1 | 65 | -363 | MSTA | MaLR | LTR |
| 42446994 | 42447047 | + | 18.9 | 914 | 967 | -613 | THE1-int | MaLR | LTR |
| 42447036 | 42447276 | - | 19.9 | 907 | 1191 | -389 | THE1-int | MaLR | LTR |
| 42447281 | 42447560 | + | 23.6 | 76 | 372 | -56 | MSTA | MaLR | LTR |
| 42447590 | 42447730 | - | 24.1 | 508 | 670 | -981 | MST-int | MaLR | LTR |
| 42447734 | 42449051 | + | 25.2 | 32 | 1389 | -262 | MST-int | MaLR | LTR |
| 42449048 | 42449163 | + | 15.7 | 1535 | 1651 | 0 | MST-int | MaLR | LTR |
| 42449164 | 42449261 | + | 20.6 | 1 | 99 | -329 | MSTA | MaLR | LTR |
| 42449311 | 42449411 | + | 19.8 | 60 | 171 | -257 | MSTA | MaLR | LTR |
| 42449410 | 42449746 | + | 20.2 | 1317 | 1651 | 0 | MST-int | MaLR | LTR |
| 42449747 | 42449841 | + | 17.9 | 1 | 97 | -331 | MSTA | MaLR | LTR |
| 42449993 | 42450226 | - | 17.9 | 904 | 1178 | -402 | THE1-int | MaLR | LTR |
| 42450226 | 42450510 | + | 23.4 | 74 | 376 | -52 | MSTA | MaLR | LTR |
| 42450547 | 42450680 | - | 23.3 | 505 | 659 | -992 | MST-int | MaLR | LTR |
| 42450683 | 42452252 | + | 23.7 | 34 | 1651 | 0 | MST-int | MaLR | LTR |
| 42452253 | 42452347 | + | 22.1 | 1 | 97 | -331 | MSTA | MaLR | LTR |
| 42452398 | 42452491 | + | 28.6 | 65 | 159 | -269 | MSTA | MaLR | LTR |
| 42452499 | 42452838 | + | 22.1 | 1318 | 1644 | -7 | MSTA-int | MaLR | LTR |
| 42452845 | 42452939 | + | 17.9 | 1 | 97 | -331 | MSTA | MaLR | LTR |
| 42453067 | 42453310 | - | 20.1 | 907 | 1191 | -389 | THE1-int | MaLR | LTR |
| 42453315 | 42453580 | + | 21.2 | 76 | 368 | -60 | MSTA | MaLR | LTR |
| 42453617 | 42453756 | - | 25.9 | 466 | 608 | -972 | THE1-int | MaLR | LTR |

  

|  |  |  |  |  |  |  |  |  |  |
| --- | --- | --- | --- | --- | --- | --- | --- | --- | --- |
| chr9:42455336-42456540 | | | 1059 bp | | |  |  |  |  |
| Genome Start | Genome End | Strand | Div | Repeat Start | Repeat End | Repeat Left | Name | Family | Class |
| 42455336 | 42455433 | + | 21.6 | 1 | 99 | -329 | MSTA | MaLR | LTR |
| 42455483 | 42455579 | + | 24.7 | 60 | 171 | -257 | MSTA | MaLR | LTR |
| 42455578 | 42455897 | + | 24.8 | 1317 | 1650 | -1 | MSTA-int | MaLR | LTR |
| 42455898 | 42455999 | + | 22.4 | 1 | 106 | -322 | MSTA | MaLR | LTR |
| 42456083 | 42456138 | + | 19.6 | 911 | 967 | -613 | THE1-int | MaLR | LTR |
| 42456127 | 42456368 | - | 23.2 | 976 | 1256 | -395 | MST-int | MaLR | LTR |
| 42456397 | 42456540 | + | 25.9 | 128 | 274 | -154 | MSTA | MaLR | LTR |

  

|  |  |  |  |  |  |  |  |  |  |
| --- | --- | --- | --- | --- | --- | --- | --- | --- | --- |
| chr9:43025353-43048994 | | | 21887 bp | | |  |  |  |  |
| Genome Start | Genome End | Strand | Div | Repeat Start | Repeat End | Repeat Left | Name | Family | Class |
| 43025353 | 43025496 | - | 25.2 | 128 | 274 | -154 | MSTA | MaLR | LTR |
| 43025525 | 43025766 | + | 23.2 | 976 | 1256 | -395 | MST-int | MaLR | LTR |
| 43025755 | 43025810 | - | 19.6 | 911 | 967 | -613 | THE1-int | MaLR | LTR |
| 43025894 | 43025995 | - | 22.4 | 1 | 106 | -322 | MSTA | MaLR | LTR |
| 43025996 | 43026315 | - | 24.8 | 1317 | 1650 | -1 | MST-int | MaLR | LTR |
| 43026314 | 43026410 | - | 24.7 | 60 | 171 | -257 | MSTA | MaLR | LTR |
| 43026460 | 43026557 | - | 21.6 | 1 | 99 | -329 | MSTA | MaLR | LTR |
| 43026558 | 43028138 | - | 23.2 | 28 | 1651 | 0 | MSTA-int | MaLR | LTR |
| 43028137 | 43028276 | + | 25.9 | 466 | 608 | -972 | THE1-int | MaLR | LTR |
| 43028313 | 43028578 | - | 20.8 | 76 | 368 | -60 | MSTA | MaLR | LTR |
| 43028583 | 43028826 | + | 18.6 | 907 | 1191 | -389 | THE1-int | MaLR | LTR |
| 43028954 | 43029048 | - | 17.9 | 1 | 97 | -331 | MSTA | MaLR | LTR |
| 43029049 | 43029394 | - | 21.7 | 1318 | 1651 | 0 | MSTA-int | MaLR | LTR |
| 43029402 | 43029495 | - | 28.6 | 65 | 159 | -269 | MSTA | MaLR | LTR |
| 43029546 | 43029640 | - | 21.1 | 1 | 97 | -331 | MSTA | MaLR | LTR |
| 43029641 | 43031211 | - | 23.4 | 34 | 1651 | 0 | MST-int | MaLR | LTR |
| 43031214 | 43031367 | + | 28.0 | 464 | 617 | -963 | THE1-int | MaLR | LTR |
| 43031384 | 43031668 | - | 23.4 | 74 | 376 | -52 | MSTA | MaLR | LTR |
| 43031668 | 43031911 | + | 18.4 | 904 | 1191 | -389 | THE1-int | MaLR | LTR |
| 43031900 | 43031956 | - | 21.4 | 911 | 967 | -613 | THE1-int | MaLR | LTR |
| 43032053 | 43032147 | - | 17.9 | 1 | 97 | -331 | MSTA | MaLR | LTR |
| 43032148 | 43032484 | - | 20.5 | 1317 | 1651 | 0 | MST-int | MaLR | LTR |
| 43032483 | 43032583 | - | 19.8 | 60 | 171 | -257 | MSTA | MaLR | LTR |
| 43032633 | 43032730 | - | 20.6 | 1 | 99 | -329 | MSTA | MaLR | LTR |
| 43032731 | 43032846 | - | 15.7 | 1535 | 1651 | 0 | MSTA-int | MaLR | LTR |
| 43032843 | 43034160 | - | 25.1 | 32 | 1389 | -262 | MSTA-int | MaLR | LTR |
| 43034164 | 43034304 | + | 24.1 | 508 | 670 | -981 | MST-int | MaLR | LTR |
| 43034334 | 43034613 | - | 24.0 | 76 | 372 | -56 | MSTA | MaLR | LTR |
| 43034618 | 43034858 | + | 19.5 | 907 | 1191 | -389 | THE1-int | MaLR | LTR |
| 43034847 | 43034900 | - | 18.9 | 914 | 967 | -613 | THE1-int | MaLR | LTR |
| 43034980 | 43035082 | - | 20.0 | 1 | 104 | -324 | MSTA | MaLR | LTR |
| 43035083 | 43035422 | - | 23.1 | 1317 | 1651 | 0 | MST-int | MaLR | LTR |
| 43035421 | 43035520 | - | 28.0 | 60 | 171 | -257 | MSTA | MaLR | LTR |
| 43035570 | 43035666 | - | 23.7 | 1 | 99 | -329 | MSTA | MaLR | LTR |
| 43035667 | 43037257 | - | 22.0 | 28 | 1651 | 0 | MSTA-int | MaLR | LTR |
| 43037383 | 43037495 | + | 22.7 | 506 | 661 | -963 | MST-int | MaLR | LTR |
| 43037512 | 43037801 | - | 20.1 | 66 | 376 | -52 | MSTA | MaLR | LTR |
| 43037806 | 43038045 | + | 24.2 | 907 | 1187 | -393 | THE1-int | MaLR | LTR |
| 43038038 | 43038093 | - | 21.4 | 911 | 967 | -613 | THE1-int | MaLR | LTR |
| 43038190 | 43038285 | - | 19.8 | 1 | 97 | -331 | MSTA | MaLR | LTR |
| 43038288 | 43038626 | - | 22.7 | 1318 | 1649 | -2 | MST-int | MaLR | LTR |
| 43038634 | 43038725 | - | 26.7 | 65 | 159 | -269 | MSTA | MaLR | LTR |
| 43038816 | 43038921 | - | 19.6 | 1 | 105 | -323 | MSTA | MaLR | LTR |
| 43038922 | 43040498 | - | 24.3 | 27 | 1651 | 0 | MSTA-int | MaLR | LTR |
| 43040496 | 43040631 | + | 28.9 | 466 | 605 | -975 | THE1-int | MaLR | LTR |
| 43040636 | 43040819 | - | 25.3 | 76 | 266 | -162 | MSTA | MaLR | LTR |
| 43040825 | 43041064 | + | 24.5 | 976 | 1256 | -395 | MST-int | MaLR | LTR |
| 43041053 | 43041106 | - | 18.9 | 914 | 967 | -613 | THE1-int | MaLR | LTR |
| 43041226 | 43041288 | - | 19.1 | 1 | 65 | -363 | MSTA | MaLR | LTR |
| 43041289 | 43041628 | - | 21.5 | 1317 | 1651 | 0 | MST-int | MaLR | LTR |
| 43041627 | 43041727 | - | 27.7 | 60 | 171 | -257 | MSTA | MaLR | LTR |
| 43041777 | 43041873 | - | 23.7 | 1 | 99 | -329 | MSTA | MaLR | LTR |
| 43041874 | 43043451 | - | 24.1 | 28 | 1651 | 0 | MSTA-int | MaLR | LTR |
| 43043447 | 43043582 | + | 19.3 | 504 | 661 | -990 | MST-int | MaLR | LTR |
| 43043609 | 43043912 | - | 24.7 | 75 | 385 | -43 | MSTA | MaLR | LTR |
| 43043917 | 43043986 | + | 30.0 | 907 | 977 | -603 | THE1-int | MaLR | LTR |
| 43044035 | 43044100 | - | 19.7 | 36 | 102 | -1549 | MSTA-int | MaLR | LTR |
| 43044107 | 43044244 | + | 21.2 | 507 | 670 | -981 | MST-int | MaLR | LTR |
| 43044274 | 43044541 | - | 27.2 | 96 | 374 | -54 | MSTA | MaLR | LTR |
| 43044567 | 43044801 | + | 20.0 | 907 | 1191 | -389 | THE1-int | MaLR | LTR |
| 43044790 | 43044845 | - | 19.6 | 911 | 967 | -613 | THE1-int | MaLR | LTR |
| 43044942 | 43045037 | - | 15.6 | 1 | 97 | -331 | MSTA | MaLR | LTR |
| 43045038 | 43045385 | - | 22.1 | 1317 | 1651 | 0 | MST-int | MaLR | LTR |
| 43045379 | 43045486 | - | 22.4 | 58 | 180 | -248 | MSTA | MaLR | LTR |
| 43045532 | 43045627 | - | 28.1 | 1 | 100 | -328 | MSTA | MaLR | LTR |
| 43045628 | 43045705 | - | 18.7 | 1574 | 1651 | 0 | MSTA-int | MaLR | LTR |
| 43045708 | 43045768 | + | 18.0 | 317 | 377 | -51 | MSTA | MaLR | LTR |
| 43045841 | 43047367 | - | 22.9 | 15 | 1583 | -68 | MSTA-int | MaLR | LTR |
| 43047471 | 43047583 | + | 19.1 | 506 | 661 | -963 | MST-int | MaLR | LTR |
| 43047600 | 43047889 | - | 20.1 | 66 | 376 | -52 | MSTA | MaLR | LTR |
| 43047894 | 43048172 | + | 24.9 | 907 | 1218 | -362 | THE1-int | MaLR | LTR |
| 43048126 | 43048181 | - | 21.4 | 911 | 967 | -613 | THE1-int | MaLR | LTR |
| 43048278 | 43048373 | - | 20.8 | 1 | 97 | -331 | MSTA | MaLR | LTR |
| 43048376 | 43048721 | - | 22.1 | 1318 | 1649 | -2 | MST-int | MaLR | LTR |
| 43048726 | 43048793 | - | 20.6 | 95 | 166 | -262 | MSTA | MaLR | LTR |
| 43048888 | 43048994 | - | 20.4 | 1 | 104 | -324 | MSTA | MaLR | LTR |

  

|  |  |  |  |  |  |  |  |  |  |
| --- | --- | --- | --- | --- | --- | --- | --- | --- | --- |
| chr9:43967503-43968239 | | | 652 bp | | |  |  |  |  |
| Genome Start | Genome End | Strand | Div | Repeat Start | Repeat End | Repeat Left | Name | Family | Class |
| 43967503 | 43967769 | - | 21.1 | 76 | 368 | -60 | MSTA | MaLR | LTR |
| 43967774 | 43968017 | + | 19.2 | 907 | 1191 | -389 | THE1-int | MaLR | LTR |
| 43968006 | 43968051 | - | 21.7 | 921 | 967 | -613 | THE1-int | MaLR | LTR |
| 43968145 | 43968239 | - | 17.9 | 1 | 97 | -331 | MSTA | MaLR | LTR |

  

|  |  |  |  |  |  |  |  |  |  |
| --- | --- | --- | --- | --- | --- | --- | --- | --- | --- |
| chr9:43970572-43971335 | | | 681 bp | | |  |  |  |  |
| Genome Start | Genome End | Strand | Div | Repeat Start | Repeat End | Repeat Left | Name | Family | Class |
| 43970572 | 43970856 | - | 23.4 | 74 | 376 | -52 | MSTA | MaLR | LTR |
| 43970856 | 43971099 | + | 20.1 | 904 | 1191 | -389 | THE1-int | MaLR | LTR |
| 43971088 | 43971144 | - | 21.4 | 911 | 967 | -613 | THE1-int | MaLR | LTR |
| 43971241 | 43971335 | - | 18.9 | 1 | 97 | -331 | MSTA | MaLR | LTR |

  

|  |  |  |  |  |  |  |  |  |  |
| --- | --- | --- | --- | --- | --- | --- | --- | --- | --- |
| chr9:43971671-43974267 | | | 2407 bp | | |  |  |  |  |
| Genome Start | Genome End | Strand | Div | Repeat Start | Repeat End | Repeat Left | Name | Family | Class |
| 43971671 | 43971771 | - | 21.8 | 60 | 171 | -257 | MSTA | MaLR | LTR |
| 43971821 | 43971918 | - | 18.6 | 1 | 99 | -329 | MSTA | MaLR | LTR |
| 43971919 | 43972030 | - | 15.3 | 1539 | 1651 | 0 | MSTA-int | MaLR | LTR |
| 43972031 | 43973348 | - | 25.3 | 32 | 1389 | -262 | MSTA-int | MaLR | LTR |
| 43973352 | 43973492 | + | 23.4 | 508 | 670 | -981 | MST-int | MaLR | LTR |
| 43973522 | 43973801 | - | 24.4 | 76 | 372 | -56 | MSTA | MaLR | LTR |
| 43973806 | 43974043 | + | 21.5 | 907 | 1191 | -389 | THE1-int | MaLR | LTR |
| 43974032 | 43974087 | - | 20.0 | 912 | 967 | -613 | THE1-int | MaLR | LTR |
| 43974205 | 43974267 | - | 17.5 | 1 | 65 | -363 | MSTA | MaLR | LTR |

  

|  |  |  |  |  |  |  |  |  |  |
| --- | --- | --- | --- | --- | --- | --- | --- | --- | --- |
| chr9:43979071-43982077 | | | 2716 bp | | |  |  |  |  |
| Genome Start | Genome End | Strand | Div | Repeat Start | Repeat End | Repeat Left | Name | Family | Class |
| 43979071 | 43979178 | - | 22.4 | 58 | 180 | -248 | MSTA | MaLR | LTR |
| 43979224 | 43979322 | - | 28.3 | 1 | 100 | -328 | MSTA | MaLR | LTR |
| 43979323 | 43979400 | - | 17.3 | 1574 | 1651 | 0 | MSTA-int | MaLR | LTR |
| 43979403 | 43979463 | + | 18.0 | 317 | 377 | -51 | MSTA | MaLR | LTR |
| 43979536 | 43981070 | - | 22.4 | 15 | 1583 | -68 | MSTA-int | MaLR | LTR |
| 43981174 | 43981286 | + | 20.3 | 506 | 661 | -963 | MST-int | MaLR | LTR |
| 43981303 | 43981593 | - | 23.9 | 66 | 376 | -52 | MSTA | MaLR | LTR |
| 43981598 | 43981876 | + | 23.4 | 907 | 1218 | -362 | THE1-int | MaLR | LTR |
| 43981830 | 43981885 | - | 21.4 | 911 | 967 | -613 | THE1-int | MaLR | LTR |
| 43981982 | 43982077 | - | 20.8 | 1 | 97 | -331 | MSTA | MaLR | LTR |

  

|  |  |  |  |  |  |  |  |  |  |
| --- | --- | --- | --- | --- | --- | --- | --- | --- | --- |
| chr9:66111804-66112446 | | | 544 bp | | |  |  |  |  |
| Genome Start | Genome End | Strand | Div | Repeat Start | Repeat End | Repeat Left | Name | Family | Class |
| 66111804 | 66111947 | - | 25.2 | 128 | 274 | -154 | MSTA | MaLR | LTR |
| 66111976 | 66112217 | + | 23.5 | 976 | 1256 | -395 | MST-int | MaLR | LTR |
| 66112206 | 66112261 | - | 19.6 | 911 | 967 | -613 | THE1-int | MaLR | LTR |
| 66112345 | 66112446 | - | 21.4 | 1 | 106 | -322 | MSTA | MaLR | LTR |

  

|  |  |  |  |  |  |  |  |  |  |
| --- | --- | --- | --- | --- | --- | --- | --- | --- | --- |
| chr9:66114764-66115500 | | | 652 bp | | |  |  |  |  |
| Genome Start | Genome End | Strand | Div | Repeat Start | Repeat End | Repeat Left | Name | Family | Class |
| 66114764 | 66115030 | - | 21.1 | 76 | 368 | -60 | MSTA | MaLR | LTR |
| 66115035 | 66115278 | + | 19.2 | 907 | 1191 | -389 | THE1-int | MaLR | LTR |
| 66115267 | 66115312 | - | 21.7 | 921 | 967 | -613 | THE1-int | MaLR | LTR |
| 66115406 | 66115500 | - | 17.9 | 1 | 97 | -331 | MSTA | MaLR | LTR |

  

|  |  |  |  |  |  |  |  |  |  |
| --- | --- | --- | --- | --- | --- | --- | --- | --- | --- |
| chr9:66117833-66118596 | | | 681 bp | | |  |  |  |  |
| Genome Start | Genome End | Strand | Div | Repeat Start | Repeat End | Repeat Left | Name | Family | Class |
| 66117833 | 66118117 | - | 23.4 | 74 | 376 | -52 | MSTA | MaLR | LTR |
| 66118117 | 66118360 | + | 19.7 | 904 | 1191 | -389 | THE1-int | MaLR | LTR |
| 66118349 | 66118405 | - | 21.4 | 911 | 967 | -613 | THE1-int | MaLR | LTR |
| 66118502 | 66118596 | - | 18.9 | 1 | 97 | -331 | MSTA | MaLR | LTR |

  

|  |  |  |  |  |  |  |  |  |  |
| --- | --- | --- | --- | --- | --- | --- | --- | --- | --- |
| chr9:66118932-66121528 | | | 2407 bp | | |  |  |  |  |
| Genome Start | Genome End | Strand | Div | Repeat Start | Repeat End | Repeat Left | Name | Family | Class |
| 66118932 | 66119032 | - | 21.8 | 60 | 171 | -257 | MSTA | MaLR | LTR |
| 66119082 | 66119179 | - | 18.6 | 1 | 99 | -329 | MSTA | MaLR | LTR |
| 66119180 | 66119291 | - | 16.2 | 1539 | 1651 | 0 | MSTA-int | MaLR | LTR |
| 66119292 | 66120609 | - | 25.3 | 32 | 1389 | -262 | MSTA-int | MaLR | LTR |
| 66120613 | 66120753 | + | 23.4 | 508 | 670 | -981 | MST-int | MaLR | LTR |
| 66120783 | 66121062 | - | 24.4 | 76 | 372 | -56 | MSTA | MaLR | LTR |
| 66121067 | 66121304 | + | 21.5 | 907 | 1191 | -389 | THE1-int | MaLR | LTR |
| 66121293 | 66121348 | - | 20.0 | 912 | 967 | -613 | THE1-int | MaLR | LTR |
| 66121466 | 66121528 | - | 17.5 | 1 | 65 | -363 | MSTA | MaLR | LTR |

  

|  |  |  |  |  |  |  |  |  |  |
| --- | --- | --- | --- | --- | --- | --- | --- | --- | --- |
| chr9:66126332-66129338 | | | 2716 bp | | |  |  |  |  |
| Genome Start | Genome End | Strand | Div | Repeat Start | Repeat End | Repeat Left | Name | Family | Class |
| 66126332 | 66126439 | - | 21.5 | 58 | 180 | -248 | MSTA | MaLR | LTR |
| 66126485 | 66126583 | - | 28.3 | 1 | 100 | -328 | MSTA | MaLR | LTR |
| 66126584 | 66126661 | - | 17.3 | 1574 | 1651 | 0 | MSTA-int | MaLR | LTR |
| 66126664 | 66126724 | + | 18.0 | 317 | 377 | -51 | MSTA | MaLR | LTR |
| 66126797 | 66128331 | - | 22.4 | 15 | 1583 | -68 | MSTA-int | MaLR | LTR |
| 66128435 | 66128547 | + | 20.3 | 506 | 661 | -963 | MST-int | MaLR | LTR |
| 66128564 | 66128854 | - | 22.0 | 66 | 376 | -52 | MSTA | MaLR | LTR |
| 66128859 | 66129137 | + | 23.4 | 907 | 1218 | -362 | THE1-int | MaLR | LTR |
| 66129091 | 66129146 | - | 21.4 | 911 | 967 | -613 | THE1-int | MaLR | LTR |
| 66129243 | 66129338 | - | 20.8 | 1 | 97 | -331 | MSTA | MaLR | LTR |

  

|  |  |  |  |  |  |  |  |  |  |
| --- | --- | --- | --- | --- | --- | --- | --- | --- | --- |
| chr9:66131523-66132220 | | | 678 bp | | |  |  |  |  |
| Genome Start | Genome End | Strand | Div | Repeat Start | Repeat End | Repeat Left | Name | Family | Class |
| 66131523 | 66131673 | + | 27.9 | 466 | 617 | -963 | THE1-int | MaLR | LTR |
| 66131690 | 66131975 | - | 21.5 | 76 | 376 | -52 | MSTA | MaLR | LTR |
| 66131980 | 66132220 | + | 21.2 | 907 | 1191 | -389 | THE1-int | MaLR | LTR |

  

|  |  |  |  |  |  |  |  |  |  |
| --- | --- | --- | --- | --- | --- | --- | --- | --- | --- |
| chr9:89927665-89928379 | | | 618 bp | | |  |  |  |  |
| Genome Start | Genome End | Strand | Div | Repeat Start | Repeat End | Repeat Left | Name | Family | Class |
| 89927665 | 89927760 | + | 20.8 | 1 | 97 | -331 | MSTA | MaLR | LTR |
| 89927850 | 89927905 | + | 21.4 | 911 | 967 | -613 | THE1-int | MaLR | LTR |
| 89927894 | 89928115 | - | 18.5 | 907 | 1191 | -389 | THE1-int | MaLR | LTR |
| 89928136 | 89928379 | + | 22.4 | 96 | 351 | -77 | MSTA | MaLR | LTR |

  

|  |  |  |  |  |  |  |  |  |  |
| --- | --- | --- | --- | --- | --- | --- | --- | --- | --- |
| chr9:89930692-89931368 | | | 578 bp | | |  |  |  |  |
| Genome Start | Genome End | Strand | Div | Repeat Start | Repeat End | Repeat Left | Name | Family | Class |
| 89930692 | 89930785 | + | 18.1 | 1 | 97 | -331 | MSTA | MaLR | LTR |
| 89930878 | 89930933 | + | 25.0 | 910 | 966 | -614 | THE1-int | MaLR | LTR |
| 89930936 | 89931165 | - | 20.4 | 908 | 1175 | -405 | THE1-int | MaLR | LTR |
| 89931171 | 89931368 | + | 26.5 | 76 | 287 | -141 | MSTA | MaLR | LTR |

  

|  |  |  |  |  |  |  |  |  |  |
| --- | --- | --- | --- | --- | --- | --- | --- | --- | --- |
| chr9:98936138-98936815 | | | 573 bp | | |  |  |  |  |
| Genome Start | Genome End | Strand | Div | Repeat Start | Repeat End | Repeat Left | Name | Family | Class |
| 98936138 | 98936335 | - | 25.5 | 76 | 287 | -141 | MSTA | MaLR | LTR |
| 98936340 | 98936573 | + | 20.1 | 907 | 1178 | -402 | THE1-int | MaLR | LTR |
| 98936572 | 98936617 | - | 21.7 | 921 | 967 | -613 | THE1-int | MaLR | LTR |
| 98936721 | 98936815 | - | 18.9 | 1 | 97 | -331 | MSTA | MaLR | LTR |

  

|  |  |  |  |  |  |  |  |  |  |
| --- | --- | --- | --- | --- | --- | --- | --- | --- | --- |
| chr9:98939127-98940456 | | | 1190 bp | | |  |  |  |  |
| Genome Start | Genome End | Strand | Div | Repeat Start | Repeat End | Repeat Left | Name | Family | Class |
| 98939127 | 98939391 | - | 21.4 | 96 | 372 | -56 | MSTA | MaLR | LTR |
| 98939412 | 98939655 | + | 23.0 | 907 | 1191 | -389 | THE1-int | MaLR | LTR |
| 98939644 | 98939689 | - | 13.3 | 921 | 967 | -613 | THE1-int | MaLR | LTR |
| 98939798 | 98939891 | - | 16.0 | 1 | 97 | -331 | MSTA | MaLR | LTR |
| 98939892 | 98940211 | - | 20.9 | 1317 | 1651 | 0 | MST-int | MaLR | LTR |
| 98940184 | 98940309 | - | 25.0 | 60 | 195 | -233 | MSTA | MaLR | LTR |
| 98940362 | 98940456 | - | 22.1 | 1 | 96 | -332 | MSTA | MaLR | LTR |

  

|  |  |  |  |  |  |  |  |  |  |
| --- | --- | --- | --- | --- | --- | --- | --- | --- | --- |
| chr9:98942225-98943000 | | | 681 bp | | |  |  |  |  |
| Genome Start | Genome End | Strand | Div | Repeat Start | Repeat End | Repeat Left | Name | Family | Class |
| 98942225 | 98942510 | - | 21.3 | 75 | 376 | -52 | MSTA | MaLR | LTR |
| 98942522 | 98942759 | + | 19.3 | 907 | 1187 | -393 | THE1-int | MaLR | LTR |
| 98942752 | 98942813 | - | 17.7 | 902 | 967 | -613 | THE1-int | MaLR | LTR |
| 98942906 | 98943000 | - | 15.8 | 1 | 97 | -331 | MSTA | MaLR | LTR |

  

|  |  |  |  |  |  |  |  |  |  |
| --- | --- | --- | --- | --- | --- | --- | --- | --- | --- |
| chr9:98948420-98949777 | | | 1157 bp | | |  |  |  |  |
| Genome Start | Genome End | Strand | Div | Repeat Start | Repeat End | Repeat Left | Name | Family | Class |
| 98948420 | 98948692 | - | 21.0 | 96 | 376 | -52 | MSTA | MaLR | LTR |
| 98948718 | 98948960 | + | 20.6 | 907 | 1191 | -389 | THE1-int | MaLR | LTR |
| 98948949 | 98948977 | - | 10.3 | 939 | 967 | -613 | THE1-int | MaLR | LTR |
| 98949093 | 98949205 | - | 19.2 | 4 | 112 | -324 | MSTA | MaLR | LTR |
| 98949206 | 98949543 | - | 21.8 | 1318 | 1727 | -8 | MST-int | MaLR | LTR |
| 98949551 | 98949611 | - | 26.2 | 95 | 155 | -273 | MSTA | MaLR | LTR |
| 98949678 | 98949777 | - | 22.9 | 1 | 104 | -324 | MSTA | MaLR | LTR |

  

|  |  |  |  |  |  |  |  |  |  |
| --- | --- | --- | --- | --- | --- | --- | --- | --- | --- |
| chr9:98951306-98951929 | | | 574 bp | | |  |  |  |  |
| Genome Start | Genome End | Strand | Div | Repeat Start | Repeat End | Repeat Left | Name | Family | Class |
| 98951306 | 98951457 | + | 27.9 | 466 | 617 | -963 | THE1-int | MaLR | LTR |
| 98951476 | 98951769 | - | 16.8 | 55 | 374 | -54 | MSTA | MaLR | LTR |
| 98951802 | 98951929 | + | 18.9 | 1062 | 1191 | -389 | THE1-int | MaLR | LTR |

  

|  |  |  |  |  |  |  |  |  |  |
| --- | --- | --- | --- | --- | --- | --- | --- | --- | --- |
| chr9:98951918-98953220 | | | 1140 bp | | |  |  |  |  |
| Genome Start | Genome End | Strand | Div | Repeat Start | Repeat End | Repeat Left | Name | Family | Class |
| 98951918 | 98951965 | - | 14.6 | 987 | 1035 | -616 | MSTA-int | MaLR | LTR |
| 98952096 | 98952197 | - | 25.5 | 28 | 129 | -1522 | MSTA-int | MaLR | LTR |
| 98952195 | 98952328 | + | 21.6 | 506 | 661 | -990 | MST-int | MaLR | LTR |
| 98952368 | 98952739 | - | 22.3 | 1 | 371 | -57 | MSTA | MaLR | LTR |
| 98952740 | 98952984 | - | 17.1 | 1401 | 1651 | 0 | MSTA-int | MaLR | LTR |
| 98952982 | 98953220 | - | 16.8 | 13 | 250 | -1401 | MSTA-int | MaLR | LTR |

  


|  |  |  |  |  |  |  |  |  |  |
| --- | --- | --- | --- | --- | --- | --- | --- | --- | --- |
| chr10:124332589-124333533 | | | 932 bp | | |  |  |  |  |
| Genome Start | Genome End | Strand | Div | Repeat Start | Repeat End | Repeat Left | Name | Family | Class |
| 124332589 | 124332699 | - | 28.8 | 539 | 653 | -38 | MER76 | ERVL | LTR |
| 124332700 | 124333123 | - | 24.0 | 1 | 431 | -1 | MSTB1 | MaLR | LTR |
| 124333124 | 124333252 | - | 28.8 | 421 | 538 | -153 | MER76 | ERVL | LTR |
| 124333266 | 124333533 | - | 26.4 | 1 | 269 | -422 | MER76 | ERVL | LTR |

  

|  |  |  |  |  |  |  |  |  |  |
| --- | --- | --- | --- | --- | --- | --- | --- | --- | --- |
| chr10:124336050-124337028 | | | 974 bp | | |  |  |  |  |
| Genome Start | Genome End | Strand | Div | Repeat Start | Repeat End | Repeat Left | Name | Family | Class |
| 124336050 | 124336160 | - | 30.2 | 537 | 653 | -38 | MER76 | ERVL | LTR |
| 124336161 | 124336648 | - | 21.5 | 1 | 431 | -1 | MSTB1 | MaLR | LTR |
| 124336649 | 124336777 | - | 30.2 | 421 | 536 | -155 | MER76 | ERVL | LTR |
| 124336783 | 124337028 | - | 28.5 | 33 | 276 | -415 | MER76 | ERVL | LTR |

  

|  |  |  |  |  |  |  |  |  |  |
| --- | --- | --- | --- | --- | --- | --- | --- | --- | --- |
| chr10:124338935-124339875 | | | 934 bp | | |  |  |  |  |
| Genome Start | Genome End | Strand | Div | Repeat Start | Repeat End | Repeat Left | Name | Family | Class |
| 124338935 | 124339045 | - | 31.2 | 543 | 653 | -38 | MER76 | ERVL | LTR |
| 124339046 | 124339465 | - | 23.3 | 1 | 431 | -1 | MSTB1 | MaLR | LTR |
| 124339466 | 124339594 | - | 31.2 | 421 | 542 | -149 | MER76 | ERVL | LTR |
| 124339602 | 124339875 | - | 27.3 | 1 | 275 | -416 | MER76 | ERVL | LTR |

  

|  |  |  |  |  |  |  |  |  |  |
| --- | --- | --- | --- | --- | --- | --- | --- | --- | --- |
| chr10:124345292-124346236 | | | 932 bp | | |  |  |  |  |
| Genome Start | Genome End | Strand | Div | Repeat Start | Repeat End | Repeat Left | Name | Family | Class |
| 124345292 | 124345402 | - | 28.8 | 539 | 653 | -38 | MER76 | ERVL | LTR |
| 124345403 | 124345826 | - | 24.0 | 1 | 431 | -1 | MSTB1 | MaLR | LTR |
| 124345827 | 124345955 | - | 28.8 | 421 | 538 | -153 | MER76 | ERVL | LTR |
| 124345969 | 124346236 | - | 26.4 | 1 | 269 | -422 | MER76 | ERVL | LTR |

  


|  |  |  |  |  |  |  |  |  |  |
| --- | --- | --- | --- | --- | --- | --- | --- | --- | --- |
| chr13:18218666-18219318 | | | 543 bp | | |  |  |  |  |
| Genome Start | Genome End | Strand | Div | Repeat Start | Repeat End | Repeat Left | Name | Family | Class |
| 18218666 | 18218817 | - | 27.5 | 133 | 285 | -141 | MSTA | MaLR | LTR |
| 18218865 | 18219108 | + | 24.7 | 907 | 1191 | -389 | THE1-int | MaLR | LTR |
| 18219097 | 18219142 | - | 17.4 | 921 | 967 | -613 | THE1-int | MaLR | LTR |
| 18219218 | 18219318 | - | 18.6 | 1 | 106 | -322 | MSTA | MaLR | LTR |

  

|  |  |  |  |  |  |  |  |  |  |
| --- | --- | --- | --- | --- | --- | --- | --- | --- | --- |
| chr13:18221661-18222406 | | | 623 bp | | |  |  |  |  |
| Genome Start | Genome End | Strand | Div | Repeat Start | Repeat End | Repeat Left | Name | Family | Class |
| 18221661 | 18221888 | - | 20.0 | 128 | 368 | -60 | MSTA | MaLR | LTR |
| 18221930 | 18222173 | + | 19.7 | 907 | 1191 | -389 | THE1-int | MaLR | LTR |
| 18222162 | 18222217 | - | 21.4 | 911 | 967 | -613 | THE1-int | MaLR | LTR |
| 18222312 | 18222406 | - | 18.9 | 1 | 97 | -331 | MSTA | MaLR | LTR |

  

|  |  |  |  |  |  |  |  |  |  |
| --- | --- | --- | --- | --- | --- | --- | --- | --- | --- |
| chr13:18226449-18254644 | | | 26196 bp | | |  |  |  |  |
| Genome Start | Genome End | Strand | Div | Repeat Start | Repeat End | Repeat Left | Name | Family | Class |
| 18226449 | 18226550 | - | 24.7 | 75 | 176 | -252 | MSTA | MaLR | LTR |
| 18226605 | 18226702 | - | 21.6 | 1 | 99 | -329 | MSTA | MaLR | LTR |
| 18226703 | 18226812 | - | 20.9 | 1541 | 1651 | 0 | MSTA-int | MaLR | LTR |
| 18226816 | 18228123 | - | 23.8 | 26 | 1388 | -263 | MSTA-int | MaLR | LTR |
| 18228120 | 18228251 | + | 22.9 | 507 | 659 | -992 | MST-int | MaLR | LTR |
| 18228288 | 18228571 | - | 23.8 | 76 | 376 | -52 | MSTA | MaLR | LTR |
| 18228576 | 18228808 | + | 22.1 | 907 | 1178 | -402 | THE1-int | MaLR | LTR |
| 18228807 | 18228862 | - | 23.2 | 911 | 967 | -613 | THE1-int | MaLR | LTR |
| 18228939 | 18229039 | - | 20.4 | 3 | 103 | -323 | MSTA | MaLR | LTR |
| 18229042 | 18229377 | - | 23.0 | 1317 | 1651 | 0 | MST-int | MaLR | LTR |
| 18229370 | 18229449 | - | 26.9 | 95 | 176 | -252 | MSTA | MaLR | LTR |
| 18229518 | 18229620 | - | 21.4 | 1 | 105 | -323 | MSTA | MaLR | LTR |
| 18229621 | 18230788 | - | 23.4 | 427 | 1651 | 0 | MSTA-int | MaLR | LTR |
| 18230787 | 18230990 | - | 23.7 | 28 | 230 | -1270 | MSTA-int | MaLR | LTR |
| 18230987 | 18231119 | + | 18.2 | 507 | 661 | -990 | MST-int | MaLR | LTR |
| 18231146 | 18231452 | - | 25.9 | 76 | 385 | -43 | MSTA | MaLR | LTR |
| 18231457 | 18231697 | + | 24.1 | 907 | 1188 | -392 | THE1-int | MaLR | LTR |
| 18231689 | 18231745 | - | 19.3 | 910 | 967 | -613 | THE1-int | MaLR | LTR |
| 18231833 | 18231936 | - | 16.8 | 3 | 113 | -262 | MSTA | MaLR | LTR |
| 18231939 | 18232256 | - | 20.5 | 1341 | 1651 | 0 | MST-int | MaLR | LTR |
| 18232263 | 18232384 | - | 25.7 | 58 | 171 | -257 | MSTA | MaLR | LTR |
| 18232410 | 18232505 | - | 20.8 | 1 | 97 | -331 | MSTA | MaLR | LTR |
| 18232506 | 18232616 | - | 19.8 | 1538 | 1651 | 0 | MSTA-int | MaLR | LTR |
| 18232740 | 18232834 | - | 23.2 | 5 | 100 | -328 | MSTA | MaLR | LTR |
| 18232837 | 18234406 | - | 23.9 | 34 | 1651 | 0 | MSTA-int | MaLR | LTR |
| 18234407 | 18234543 | + | 25.0 | 503 | 661 | -990 | MST-int | MaLR | LTR |
| 18234580 | 18234868 | - | 22.8 | 76 | 374 | -54 | MSTA | MaLR | LTR |
| 18234873 | 18235151 | + | 26.0 | 907 | 1218 | -362 | THE1-int | MaLR | LTR |
| 18235105 | 18235160 | - | 21.4 | 911 | 967 | -613 | MSTA-int | MaLR | LTR |
| 18235246 | 18235341 | - | 13.7 | 1 | 97 | -331 | MSTA | MaLR | LTR |
| 18235342 | 18235689 | - | 22.8 | 1317 | 1651 | 0 | MST-int | MaLR | LTR |
| 18235683 | 18235788 | - | 21.7 | 58 | 180 | -248 | MSTA | MaLR | LTR |
| 18235834 | 18235932 | - | 22.2 | 1 | 100 | -328 | MSTA | MaLR | LTR |
| 18235933 | 18236008 | - | 17.1 | 1574 | 1651 | 0 | MSTA-int | MaLR | LTR |
| 18236021 | 18236072 | + | 15.4 | 323 | 374 | -52 | MSTB | MaLR | LTR |
| 18236146 | 18237676 | - | 23.7 | 28 | 1583 | -68 | MSTA-int | MaLR | LTR |
| 18237672 | 18237823 | + | 24.3 | 463 | 615 | -965 | THE1-int | MaLR | LTR |
| 18237843 | 18238109 | - | 24.4 | 96 | 376 | -52 | MSTA | MaLR | LTR |
| 18238134 | 18238364 | + | 23.1 | 907 | 1175 | -405 | THE1-int | MaLR | LTR |
| 18238367 | 18238421 | - | 16.7 | 911 | 966 | -614 | THE1-int | MaLR | LTR |
| 18238518 | 18238613 | - | 21.9 | 1 | 97 | -331 | MSTA | MaLR | LTR |
| 18238616 | 18238960 | - | 23.0 | 1318 | 1649 | -2 | MST-int | MaLR | LTR |
| 18238968 | 18239077 | - | 23.5 | 56 | 159 | -269 | MSTA | MaLR | LTR |
| 18239136 | 18239234 | - | 25.2 | 1 | 100 | -328 | MSTA | MaLR | LTR |
| 18239235 | 18240851 | - | 23.1 | 30 | 1651 | 0 | MSTA-int | MaLR | LTR |
| 18240853 | 18240997 | + | 27.3 | 467 | 608 | -972 | THE1-int | MaLR | LTR |
| 18241035 | 18241289 | - | 24.1 | 95 | 367 | -61 | MSTA | MaLR | LTR |
| 18241305 | 18241548 | + | 19.2 | 907 | 1191 | -389 | THE1-int | MaLR | LTR |
| 18241537 | 18241592 | - | 23.2 | 911 | 967 | -613 | THE1-int | MaLR | LTR |
| 18241687 | 18241781 | - | 17.9 | 1 | 97 | -331 | MSTA | MaLR | LTR |
| 18241782 | 18242128 | - | 22.0 | 1318 | 1651 | 0 | MST-int | MaLR | LTR |
| 18242136 | 18242211 | - | 25.3 | 75 | 155 | -273 | MSTA | MaLR | LTR |
| 18242255 | 18242361 | - | 22.3 | 1 | 105 | -323 | MSTA | MaLR | LTR |
| 18242362 | 18243953 | - | 23.3 | 34 | 1651 | 0 | MST-int | MaLR | LTR |
| 18243958 | 18244066 | + | 29.6 | 466 | 577 | -1003 | THE1-int | MaLR | LTR |
| 18244109 | 18244392 | - | 23.1 | 76 | 376 | -52 | MSTA | MaLR | LTR |
| 18244397 | 18244647 | + | 19.4 | 907 | 1191 | -389 | THE1-int | MaLR | LTR |
| 18244636 | 18244691 | - | 17.9 | 911 | 967 | -613 | THE1-int | MaLR | LTR |
| 18244763 | 18245312 | + | 9.3 | 1 | 563 | -1 | LTR6A | ERV1 | LTR |
| 18245344 | 18245438 | - | 20.0 | 1 | 97 | -331 | MSTA | MaLR | LTR |
| 18245439 | 18245771 | - | 20.9 | 1317 | 1651 | 0 | MST-int | MaLR | LTR |
| 18245764 | 18245857 | - | 26.9 | 76 | 176 | -252 | MSTA | MaLR | LTR |
| 18245915 | 18246012 | - | 22.7 | 1 | 99 | -329 | MSTA | MaLR | LTR |
| 18246013 | 18246127 | - | 24.4 | 1535 | 1651 | 0 | MSTA-int | MaLR | LTR |
| 18246124 | 18247429 | - | 24.6 | 28 | 1389 | -262 | MSTA-int | MaLR | LTR |
| 18247428 | 18247567 | + | 21.0 | 507 | 667 | -984 | MST-int | MaLR | LTR |
| 18247596 | 18247866 | - | 25.0 | 95 | 376 | -52 | MSTA | MaLR | LTR |
| 18247885 | 18248117 | + | 23.4 | 907 | 1178 | -402 | THE1-int | MaLR | LTR |
| 18248116 | 18248168 | - | 22.6 | 914 | 967 | -613 | THE1-int | MaLR | LTR |
| 18248275 | 18248348 | - | 19.5 | 3 | 76 | -356 | MSTA | MaLR | LTR |
| 18248351 | 18248684 | - | 23.7 | 1321 | 1651 | 0 | MST-int | MaLR | LTR |
| 18248831 | 18248927 | - | 19.6 | 1 | 99 | -329 | MSTA | MaLR | LTR |
| 18248928 | 18250088 | - | 24.3 | 427 | 1651 | 0 | MSTA-int | MaLR | LTR |
| 18250087 | 18250290 | - | 23.7 | 28 | 230 | -1269 | MSTA-int | MaLR | LTR |
| 18250287 | 18250419 | + | 16.7 | 507 | 661 | -990 | MST-int | MaLR | LTR |
| 18250446 | 18250752 | - | 25.9 | 76 | 385 | -43 | MSTA | MaLR | LTR |
| 18250757 | 18250997 | + | 23.3 | 907 | 1188 | -392 | THE1-int | MaLR | LTR |
| 18250989 | 18251045 | - | 19.3 | 910 | 967 | -613 | THE1-int | MaLR | LTR |
| 18251133 | 18251236 | - | 16.8 | 3 | 113 | -262 | MSTA | MaLR | LTR |
| 18251239 | 18251556 | - | 22.2 | 1341 | 1651 | 0 | MST-int | MaLR | LTR |
| 18251564 | 18251657 | - | 21.8 | 75 | 170 | -258 | MSTA | MaLR | LTR |
| 18251710 | 18251804 | - | 20.0 | 1 | 97 | -331 | MSTA | MaLR | LTR |
| 18251805 | 18251915 | - | 16.2 | 1538 | 1651 | 0 | MSTA-int | MaLR | LTR |
| 18252042 | 18252137 | - | 22.9 | 1 | 97 | -331 | MSTA | MaLR | LTR |
| 18252138 | 18253709 | - | 23.5 | 34 | 1651 | 0 | MSTA-int | MaLR | LTR |
| 18253710 | 18253844 | + | 24.6 | 503 | 659 | -992 | MST-int | MaLR | LTR |
| 18253883 | 18254148 | - | 26.9 | 98 | 374 | -54 | MSTA | MaLR | LTR |
| 18254176 | 18254406 | + | 24.8 | 907 | 1175 | -405 | THE1-int | MaLR | LTR |
| 18254409 | 18254463 | - | 23.6 | 911 | 966 | -614 | MSTA-int | MaLR | LTR |
| 18254549 | 18254644 | - | 13.7 | 1 | 97 | -331 | MSTA | MaLR | LTR |

  

|  |  |  |  |  |  |  |  |  |  |
| --- | --- | --- | --- | --- | --- | --- | --- | --- | --- |
| chr13:18255237-18256995 | | | 1685 bp | | |  |  |  |  |
| Genome Start | Genome End | Strand | Div | Repeat Start | Repeat End | Repeat Left | Name | Family | Class |
| 18255237 | 18255312 | - | 15.8 | 1574 | 1651 | 0 | MSTA-int | MaLR | LTR |
| 18255315 | 18255377 | + | 16.7 | 317 | 376 | -52 | MSTA | MaLR | LTR |
| 18255450 | 18256995 | - | 22.7 | 28 | 1583 | -68 | MSTA-int | MaLR | LTR |

  

|  |  |  |  |  |  |  |  |  |  |
| --- | --- | --- | --- | --- | --- | --- | --- | --- | --- |
| chr13:18258286-18261098 | | | 2595 bp | | |  |  |  |  |
| Genome Start | Genome End | Strand | Div | Repeat Start | Repeat End | Repeat Left | Name | Family | Class |
| 18258286 | 18258349 | - | 23.4 | 96 | 159 | -269 | MSTA | MaLR | LTR |
| 18258431 | 18258552 | - | 25.6 | 1 | 122 | -304 | MSTA | MaLR | LTR |
| 18258553 | 18260169 | - | 23.7 | 28 | 1651 | 0 | MSTA-int | MaLR | LTR |
| 18260167 | 18260308 | + | 19.3 | 506 | 661 | -990 | MST-int | MaLR | LTR |
| 18260352 | 18260606 | - | 23.4 | 95 | 367 | -61 | MSTA | MaLR | LTR |
| 18260622 | 18260865 | + | 19.2 | 907 | 1191 | -389 | THE1-int | MaLR | LTR |
| 18260854 | 18260909 | - | 23.2 | 911 | 967 | -613 | THE1-int | MaLR | LTR |
| 18261004 | 18261098 | - | 16.8 | 1 | 97 | -331 | MSTA | MaLR | LTR |

  

|  |  |  |  |  |  |  |  |  |  |
| --- | --- | --- | --- | --- | --- | --- | --- | --- | --- |
| chr13:18270468-18271222 | | | 618 bp | | |  |  |  |  |
| Genome Start | Genome End | Strand | Div | Repeat Start | Repeat End | Repeat Left | Name | Family | Class |
| 18270468 | 18270756 | - | 23.8 | 76 | 374 | -54 | MSTA | MaLR | LTR |
| 18270761 | 18270993 | + | 21.1 | 907 | 1187 | -393 | THE1-int | MaLR | LTR |
| 18271127 | 18271222 | - | 15.8 | 1 | 97 | -331 | MSTA | MaLR | LTR |

  

|  |  |  |  |  |  |  |  |  |  |
| --- | --- | --- | --- | --- | --- | --- | --- | --- | --- |
| chr13:18271564-18274521 | | | 2745 bp | | |  |  |  |  |
| Genome Start | Genome End | Strand | Div | Repeat Start | Repeat End | Repeat Left | Name | Family | Class |
| 18271564 | 18271671 | - | 21.5 | 58 | 180 | -248 | MSTA | MaLR | LTR |
| 18271717 | 18271815 | - | 25.2 | 1 | 100 | -328 | MSTA | MaLR | LTR |
| 18271816 | 18271890 | - | 16.0 | 1574 | 1651 | 0 | MSTA-int | MaLR | LTR |
| 18271893 | 18271955 | + | 16.7 | 317 | 376 | -52 | MSTA | MaLR | LTR |
| 18271990 | 18272028 | - | 12.8 | 623 | 661 | -990 | MSTA-int | MaLR | LTR |
| 18272029 | 18273584 | - | 23.6 | 28 | 1583 | -68 | MSTA-int | MaLR | LTR |
| 18273580 | 18273733 | + | 24.7 | 463 | 617 | -963 | THE1-int | MaLR | LTR |
| 18273751 | 18274019 | - | 23.2 | 95 | 376 | -52 | MSTA | MaLR | LTR |
| 18274042 | 18274272 | + | 24.4 | 907 | 1175 | -405 | THE1-int | MaLR | LTR |
| 18274275 | 18274329 | - | 18.5 | 911 | 966 | -614 | THE1-int | MaLR | LTR |
| 18274426 | 18274521 | - | 20.8 | 1 | 97 | -331 | MSTA | MaLR | LTR |

  


|  |  |  |  |  |  |  |  |  |  |
| --- | --- | --- | --- | --- | --- | --- | --- | --- | --- |
| chr18:14245033-14307552 | | | 57758 bp | | |  |  |  |  |
| Genome Start | Genome End | Strand | Div | Repeat Start | Repeat End | Repeat Left | Name | Family | Class |
| 14245033 | 14245269 | + | 18.6 | 13 | 250 | -1401 | MSTA-int | MaLR | LTR |
| 14245267 | 14245518 | + | 17.3 | 1401 | 1651 | 0 | MSTA-int | MaLR | LTR |
| 14245519 | 14245902 | + | 25.4 | 1 | 376 | -52 | MSTA | MaLR | LTR |
| 14245928 | 14246074 | - | 21.2 | 502 | 670 | -981 | MST-int | MaLR | LTR |
| 14246066 | 14246166 | + | 29.7 | 28 | 131 | -1449 | MSTA-int | MaLR | LTR |
| 14246284 | 14246338 | + | 16.4 | 979 | 1035 | -616 | MSTA-int | MaLR | LTR |
| 14246327 | 14246568 | - | 21.5 | 907 | 1191 | -389 | THE1-int | MaLR | LTR |
| 14246574 | 14246857 | + | 20.6 | 77 | 376 | -52 | MSTA | MaLR | LTR |
| 14246874 | 14247024 | - | 22.5 | 466 | 661 | -963 | MST-int | MaLR | LTR |
| 14247027 | 14248582 | + | 24.8 | 32 | 1651 | 0 | MSTA-int | MaLR | LTR |
| 14248583 | 14248681 | + | 24.2 | 1 | 100 | -328 | MSTA | MaLR | LTR |
| 14248784 | 14248846 | + | 23.8 | 97 | 159 | -269 | MSTA | MaLR | LTR |
| 14248854 | 14249198 | + | 22.7 | 1318 | 1649 | -2 | MSTA-int | MaLR | LTR |
| 14249201 | 14249297 | + | 12.6 | 1 | 98 | -330 | MSTA | MaLR | LTR |
| 14249393 | 14249449 | + | 16.1 | 911 | 968 | -612 | THE1-int | MaLR | LTR |
| 14249440 | 14249680 | - | 24.2 | 907 | 1188 | -392 | THE1-int | MaLR | LTR |
| 14249682 | 14249844 | + | 19.2 | 64 | 229 | -155 | MSTA | MaLR | LTR |
| 14249874 | 14249993 | - | 20.8 | 463 | 588 | -992 | THE1-int | MaLR | LTR |
| 14249989 | 14251513 | + | 23.8 | 28 | 1579 | -72 | MST-int | MaLR | LTR |
| 14251518 | 14251556 | + | 15.4 | 623 | 661 | -990 | MST-int | MaLR | LTR |
| 14251591 | 14251642 | - | 17.3 | 323 | 374 | -52 | MSTB | MaLR | LTR |
| 14251656 | 14251731 | + | 15.8 | 1574 | 1651 | 0 | MST-int | MaLR | LTR |
| 14251732 | 14251830 | + | 23.2 | 1 | 100 | -328 | MSTA | MaLR | LTR |
| 14251867 | 14251978 | + | 22.6 | 65 | 171 | -257 | MSTA | MaLR | LTR |
| 14251979 | 14252325 | + | 22.6 | 1320 | 1651 | 0 | MSTA-int | MaLR | LTR |
| 14252326 | 14252421 | + | 13.7 | 1 | 97 | -331 | MSTA | MaLR | LTR |
| 14252507 | 14252562 | + | 23.2 | 979 | 1035 | -616 | MST-int | MaLR | LTR |
| 14252561 | 14252794 | - | 25.8 | 907 | 1178 | -402 | THE1-int | MaLR | LTR |
| 14252790 | 14253087 | + | 23.2 | 60 | 374 | -54 | MSTA | MaLR | LTR |
| 14253106 | 14253259 | - | 26.4 | 462 | 617 | -963 | THE1-int | MaLR | LTR |
| 14253254 | 14254829 | + | 23.4 | 28 | 1651 | 0 | MST-int | MaLR | LTR |
| 14254830 | 14254928 | + | 23.2 | 1 | 100 | -328 | MSTA | MaLR | LTR |
| 14255052 | 14255161 | + | 15.4 | 1538 | 1651 | 0 | MSTA-int | MaLR | LTR |
| 14255162 | 14255256 | + | 20.0 | 1 | 97 | -331 | MSTA | MaLR | LTR |
| 14255329 | 14255403 | + | 28.4 | 94 | 171 | -257 | MSTA | MaLR | LTR |
| 14255410 | 14255726 | + | 23.5 | 1341 | 1651 | 0 | MSTA-int | MaLR | LTR |
| 14255729 | 14255831 | + | 18.6 | 3 | 104 | -324 | MSTA | MaLR | LTR |
| 14255907 | 14255963 | + | 17.5 | 910 | 967 | -613 | THE1-int | MaLR | LTR |
| 14255955 | 14256195 | - | 22.9 | 907 | 1188 | -392 | THE1-int | MaLR | LTR |
| 14256219 | 14256506 | + | 27.9 | 96 | 385 | -43 | MSTA | MaLR | LTR |
| 14256515 | 14256665 | - | 19.0 | 466 | 661 | -963 | MST-int | MaLR | LTR |
| 14256662 | 14256865 | + | 20.6 | 28 | 230 | -1269 | MSTA-int | MaLR | LTR |
| 14256864 | 14258025 | + | 24.1 | 427 | 1651 | 0 | MSTA-int | MaLR | LTR |
| 14258026 | 14258122 | + | 18.6 | 1 | 99 | -329 | MSTA | MaLR | LTR |
| 14258176 | 14258262 | + | 25.3 | 65 | 159 | -269 | MSTA | MaLR | LTR |
| 14258269 | 14258604 | + | 22.4 | 1317 | 1650 | -1 | MSTA-int | MaLR | LTR |
| 14258608 | 14258681 | + | 19.5 | 3 | 76 | -356 | MSTA | MaLR | LTR |
| 14258788 | 14258840 | + | 24.5 | 914 | 967 | -613 | THE1-int | MaLR | LTR |
| 14258839 | 14259071 | - | 26.3 | 907 | 1178 | -402 | THE1-int | MaLR | LTR |
| 14259092 | 14259360 | + | 23.6 | 96 | 376 | -52 | MSTA | MaLR | LTR |
| 14259397 | 14259528 | - | 21.4 | 507 | 659 | -992 | MST-int | MaLR | LTR |
| 14259531 | 14260832 | + | 24.7 | 32 | 1389 | -262 | MST-int | MaLR | LTR |
| 14260829 | 14260944 | + | 19.8 | 1535 | 1651 | 0 | MST-int | MaLR | LTR |
| 14260945 | 14261042 | + | 19.4 | 1 | 99 | -329 | MSTA | MaLR | LTR |
| 14261116 | 14261193 | + | 24.7 | 96 | 176 | -252 | MSTA | MaLR | LTR |
| 14261186 | 14261522 | + | 22.1 | 1317 | 1651 | 0 | MST-int | MaLR | LTR |
| 14261523 | 14261630 | + | 19.7 | 1 | 113 | -268 | MSTA | MaLR | LTR |
| 14261649 | 14262199 | - | 9.7 | 1 | 563 | -1 | LTR6A | ERV1 | LTR |
| 14262315 | 14262565 | - | 19.1 | 907 | 1191 | -389 | THE1-int | MaLR | LTR |
| 14262578 | 14262853 | + | 20.7 | 95 | 376 | -52 | MSTA | MaLR | LTR |
| 14262896 | 14263004 | - | 28.7 | 466 | 577 | -1003 | THE1-int | MaLR | LTR |
| 14263009 | 14264600 | + | 24.4 | 34 | 1651 | 0 | MST-int | MaLR | LTR |
| 14264601 | 14264706 | + | 21.6 | 1 | 104 | -324 | MSTA | MaLR | LTR |
| 14264744 | 14264837 | + | 26.6 | 58 | 159 | -269 | MSTA | MaLR | LTR |
| 14264845 | 14265191 | + | 23.1 | 1318 | 1651 | 0 | MSTA-int | MaLR | LTR |
| 14265192 | 14265286 | + | 16.8 | 1 | 97 | -331 | MSTA | MaLR | LTR |
| 14265370 | 14265425 | + | 23.2 | 979 | 1035 | -616 | MST-int | MaLR | LTR |
| 14265414 | 14265657 | - | 22.2 | 907 | 1191 | -389 | THE1-int | MaLR | LTR |
| 14265673 | 14265927 | + | 24.5 | 95 | 368 | -60 | MSTA | MaLR | LTR |
| 14265970 | 14266113 | - | 20.7 | 465 | 661 | -990 | THE1-int | MaLR | LTR |
| 14266111 | 14267722 | + | 23.4 | 28 | 1651 | 0 | MST-int | MaLR | LTR |
| 14267723 | 14267821 | + | 25.2 | 1 | 100 | -328 | MSTA | MaLR | LTR |
| 14267983 | 14268047 | + | 23.1 | 95 | 159 | -269 | MSTA | MaLR | LTR |
| 14268055 | 14268387 | + | 22.9 | 1318 | 1637 | -14 | MSTA-int | MaLR | LTR |
| 14268403 | 14268498 | + | 15.8 | 1 | 97 | -331 | MSTA | MaLR | LTR |
| 14268592 | 14268651 | + | 14.0 | 910 | 968 | -612 | THE1-int | MaLR | LTR |
| 14268642 | 14268882 | - | 24.2 | 907 | 1188 | -392 | THE1-int | MaLR | LTR |
| 14268878 | 14269176 | + | 23.9 | 60 | 376 | -52 | MSTA | MaLR | LTR |
| 14269194 | 14269346 | - | 23.5 | 463 | 617 | -963 | THE1-int | MaLR | LTR |
| 14269342 | 14270870 | + | 23.9 | 28 | 1583 | -68 | MSTA-int | MaLR | LTR |
| 14270944 | 14271005 | - | 16.7 | 317 | 376 | -52 | MSTA | MaLR | LTR |
| 14271023 | 14271082 | + | 10.0 | 1590 | 1651 | 0 | MSTA-int | MaLR | LTR |
| 14271083 | 14271181 | + | 26.3 | 1 | 100 | -328 | MSTA | MaLR | LTR |
| 14271227 | 14271335 | + | 24.1 | 58 | 176 | -252 | MSTA | MaLR | LTR |
| 14271328 | 14271675 | + | 23.4 | 1317 | 1651 | 0 | MSTA-int | MaLR | LTR |
| 14271676 | 14271771 | + | 14.7 | 1 | 97 | -331 | MSTA | MaLR | LTR |
| 14271904 | 14272136 | - | 21.5 | 907 | 1187 | -393 | THE1-int | MaLR | LTR |
| 14272141 | 14272429 | + | 25.0 | 76 | 374 | -54 | MSTA | MaLR | LTR |
| 14272448 | 14272602 | - | 25.0 | 463 | 661 | -963 | MST-int | MaLR | LTR |
| 14272598 | 14274164 | + | 22.7 | 28 | 1651 | 0 | MST-int | MaLR | LTR |
| 14274165 | 14274263 | + | 21.2 | 1 | 100 | -328 | MSTA | MaLR | LTR |
| 14274437 | 14274548 | + | 14.3 | 1538 | 1651 | 0 | MST-int | MaLR | LTR |
| 14274549 | 14274644 | + | 21.9 | 1 | 97 | -331 | MSTA | MaLR | LTR |
| 14274717 | 14274791 | + | 24.3 | 94 | 171 | -257 | MSTA | MaLR | LTR |
| 14274798 | 14275114 | + | 24.6 | 1341 | 1650 | -1 | MSTA-int | MaLR | LTR |
| 14275116 | 14275218 | + | 17.9 | 1 | 103 | -323 | MSTA | MaLR | LTR |
| 14275356 | 14275596 | - | 23.3 | 907 | 1188 | -392 | THE1-int | MaLR | LTR |
| 14275601 | 14275907 | + | 26.6 | 76 | 385 | -43 | MSTA | MaLR | LTR |
| 14275916 | 14276074 | - | 19.9 | 461 | 661 | -963 | MST-int | MaLR | LTR |
| 14276069 | 14277612 | + | 23.7 | 32 | 1651 | 0 | MSTA-int | MaLR | LTR |
| 14277613 | 14277709 | + | 23.7 | 1 | 99 | -329 | MSTA | MaLR | LTR |
| 14277759 | 14277864 | + | 25.7 | 60 | 176 | -252 | MSTA | MaLR | LTR |
| 14277857 | 14278191 | + | 23.7 | 1317 | 1651 | 0 | MSTA-int | MaLR | LTR |
| 14278194 | 14278254 | + | 21.3 | 3 | 65 | -363 | MSTA | MaLR | LTR |
| 14278371 | 14278426 | + | 18.4 | 921 | 1035 | -613 | THE1-int | MaLR | LTR |
| 14278415 | 14278657 | - | 23.5 | 907 | 1191 | -389 | THE1-int | MaLR | LTR |
| 14278678 | 14278946 | + | 23.8 | 96 | 376 | -52 | MSTA | MaLR | LTR |
| 14278983 | 14279110 | - | 19.7 | 510 | 659 | -992 | MST-int | MaLR | LTR |
| 14279118 | 14280425 | + | 24.4 | 34 | 1389 | -262 | MST-int | MaLR | LTR |
| 14280426 | 14280537 | + | 18.8 | 1539 | 1651 | 0 | MST-int | MaLR | LTR |
| 14280538 | 14280648 | + | 22.6 | 1 | 116 | -248 | MSTA | MaLR | LTR |
| 14280685 | 14280777 | + | 31.2 | 60 | 159 | -269 | MSTA | MaLR | LTR |
| 14280784 | 14281116 | + | 20.4 | 1317 | 1651 | 0 | MST-int | MaLR | LTR |
| 14281117 | 14281211 | + | 16.8 | 1 | 97 | -331 | MSTA | MaLR | LTR |
| 14281243 | 14281792 | - | 10.2 | 1 | 563 | -1 | LTR6A | ERV1 | LTR |
| 14281864 | 14281919 | + | 17.9 | 911 | 967 | -613 | THE1-int | MaLR | LTR |
| 14281908 | 14282149 | - | 20.7 | 907 | 1191 | -389 | THE1-int | MaLR | LTR |
| 14282154 | 14282437 | + | 21.5 | 76 | 376 | -52 | MSTA | MaLR | LTR |
| 14282480 | 14282588 | - | 28.7 | 466 | 577 | -1003 | THE1-int | MaLR | LTR |
| 14282593 | 14284187 | + | 24.5 | 34 | 1651 | 0 | MST-int | MaLR | LTR |
| 14284188 | 14284286 | + | 24.2 | 1 | 104 | -324 | MSTA | MaLR | LTR |
| 14284362 | 14284423 | + | 24.2 | 96 | 159 | -269 | MSTA | MaLR | LTR |
| 14284431 | 14284776 | + | 22.9 | 1318 | 1651 | 0 | MSTA-int | MaLR | LTR |
| 14284777 | 14284871 | + | 15.8 | 1 | 97 | -331 | MSTA | MaLR | LTR |
| 14285022 | 14285251 | - | 23.3 | 976 | 1240 | -411 | MST-int | MaLR | LTR |
| 14285268 | 14285523 | + | 22.5 | 95 | 368 | -60 | MSTA | MaLR | LTR |
| 14285560 | 14285702 | - | 26.2 | 465 | 608 | -972 | THE1-int | MaLR | LTR |
| 14285700 | 14287314 | + | 23.0 | 28 | 1651 | 0 | MST-int | MaLR | LTR |
| 14287315 | 14287409 | + | 23.2 | 1 | 96 | -332 | MSTA | MaLR | LTR |
| 14287516 | 14287580 | + | 24.6 | 95 | 159 | -269 | MSTA | MaLR | LTR |
| 14287588 | 14287920 | + | 20.8 | 1318 | 1637 | -14 | MST-int | MaLR | LTR |
| 14287937 | 14288033 | + | 18.6 | 1 | 98 | -330 | MSTA | MaLR | LTR |
| 14288129 | 14288185 | + | 17.9 | 911 | 968 | -612 | THE1-int | MaLR | LTR |
| 14288176 | 14288416 | - | 24.2 | 907 | 1188 | -392 | THE1-int | MaLR | LTR |
| 14288441 | 14288707 | + | 19.5 | 96 | 376 | -52 | MSTA | MaLR | LTR |
| 14288725 | 14288878 | - | 24.7 | 463 | 617 | -963 | THE1-int | MaLR | LTR |
| 14288874 | 14290404 | + | 25.4 | 28 | 1583 | -68 | MST-int | MaLR | LTR |
| 14290478 | 14290539 | - | 16.7 | 317 | 376 | -52 | MSTA | MaLR | LTR |
| 14290542 | 14290617 | + | 17.1 | 1574 | 1651 | 0 | MST-int | MaLR | LTR |
| 14290618 | 14290716 | + | 26.3 | 1 | 100 | -328 | MSTA | MaLR | LTR |
| 14290793 | 14290870 | + | 22.1 | 96 | 176 | -252 | MSTA | MaLR | LTR |
| 14290863 | 14291210 | + | 20.5 | 1317 | 1651 | 0 | MSTA-int | MaLR | LTR |
| 14291211 | 14291306 | + | 14.7 | 1 | 97 | -331 | MSTA | MaLR | LTR |
| 14291446 | 14291672 | - | 20.8 | 907 | 1178 | -402 | THE1-int | MaLR | LTR |
| 14291677 | 14291964 | + | 26.9 | 76 | 374 | -54 | MSTA | MaLR | LTR |
| 14291983 | 14292137 | - | 26.4 | 463 | 661 | -963 | MST-int | MaLR | LTR |
| 14292133 | 14293703 | + | 22.9 | 28 | 1651 | 0 | MST-int | MaLR | LTR |
| 14293704 | 14293802 | + | 21.2 | 1 | 100 | -328 | MSTA | MaLR | LTR |
| 14293949 | 14294059 | + | 18.0 | 1538 | 1651 | 0 | MST-int | MaLR | LTR |
| 14294060 | 14294155 | + | 20.8 | 1 | 97 | -331 | MSTA | MaLR | LTR |
| 14294205 | 14294302 | + | 25.3 | 76 | 171 | -257 | MSTA | MaLR | LTR |
| 14294309 | 14294626 | + | 23.5 | 1341 | 1651 | 0 | MST-int | MaLR | LTR |
| 14294627 | 14294719 | + | 19.4 | 1 | 94 | -334 | MSTA | MaLR | LTR |
| 14294870 | 14295110 | - | 22.9 | 907 | 1188 | -392 | THE1-int | MaLR | LTR |
| 14295134 | 14295409 | + | 25.1 | 96 | 372 | -56 | MSTA | MaLR | LTR |
| 14295430 | 14295588 | - | 18.9 | 461 | 661 | -963 | MST-int | MaLR | LTR |
| 14295583 | 14296848 | + | 26.0 | 32 | 1389 | -262 | MST-int | MaLR | LTR |
| 14296845 | 14296960 | + | 20.7 | 1535 | 1651 | 0 | MST-int | MaLR | LTR |
| 14296961 | 14297070 | + | 21.7 | 1 | 107 | -321 | MSTA | MaLR | LTR |
| 14297108 | 14297214 | + | 26.4 | 60 | 176 | -252 | MSTA | MaLR | LTR |
| 14297207 | 14297460 | + | 27.3 | 1317 | 1651 | 0 | MST-int | MaLR | LTR |
| 14297461 | 14297555 | + | 17.9 | 1 | 97 | -331 | MSTA | MaLR | LTR |
| 14297587 | 14298137 | - | 12.2 | 1 | 563 | -1 | LTR6A | ERV1 | LTR |
| 14298209 | 14298264 | + | 25.0 | 911 | 967 | -613 | THE1-int | MaLR | LTR |
| 14298263 | 14298494 | - | 20.4 | 907 | 1178 | -402 | THE1-int | MaLR | LTR |
| 14298507 | 14298782 | + | 23.2 | 95 | 376 | -52 | MSTA | MaLR | LTR |
| 14298825 | 14298933 | - | 28.7 | 466 | 577 | -1003 | THE1-int | MaLR | LTR |
| 14298938 | 14300529 | + | 23.9 | 34 | 1651 | 0 | MST-int | MaLR | LTR |
| 14300530 | 14300628 | + | 24.2 | 1 | 104 | -324 | MSTA | MaLR | LTR |
| 14300705 | 14300766 | + | 22.6 | 96 | 159 | -269 | MSTA | MaLR | LTR |
| 14300774 | 14301120 | + | 21.9 | 1318 | 1651 | 0 | MSTA-int | MaLR | LTR |
| 14301121 | 14301215 | + | 16.8 | 1 | 97 | -331 | MSTA | MaLR | LTR |
| 14301310 | 14301365 | + | 21.4 | 911 | 967 | -613 | THE1-int | MaLR | LTR |
| 14301354 | 14301597 | - | 18.0 | 907 | 1191 | -389 | THE1-int | MaLR | LTR |
| 14301613 | 14301868 | + | 23.7 | 95 | 368 | -60 | MSTA | MaLR | LTR |
| 14301911 | 14302051 | - | 22.3 | 465 | 661 | -990 | THE1-int | MaLR | LTR |
| 14302049 | 14303662 | + | 23.8 | 28 | 1651 | 0 | MST-int | MaLR | LTR |
| 14303663 | 14303757 | + | 24.2 | 1 | 96 | -332 | MSTA | MaLR | LTR |
| 14303866 | 14303928 | + | 22.2 | 97 | 159 | -269 | MSTA | MaLR | LTR |
| 14303936 | 14304280 | + | 23.1 | 1318 | 1649 | -2 | MST-int | MaLR | LTR |
| 14304283 | 14304379 | + | 17.5 | 1 | 98 | -330 | MSTA | MaLR | LTR |
| 14304475 | 14304530 | + | 23.2 | 911 | 967 | -613 | THE1-int | MaLR | LTR |
| 14304529 | 14304762 | - | 24.4 | 907 | 1178 | -402 | THE1-int | MaLR | LTR |
| 14304787 | 14305053 | + | 23.3 | 96 | 376 | -52 | MSTA | MaLR | LTR |
| 14305071 | 14305223 | - | 22.8 | 463 | 617 | -963 | THE1-int | MaLR | LTR |
| 14305219 | 14306747 | + | 23.7 | 28 | 1583 | -68 | MST-int | MaLR | LTR |
| 14306748 | 14306786 | + | 15.4 | 623 | 661 | -990 | MST-int | MaLR | LTR |
| 14306829 | 14306882 | - | 17.3 | 317 | 368 | -60 | MSTA | MaLR | LTR |
| 14306885 | 14306960 | + | 15.8 | 1574 | 1651 | 0 | MST-int | MaLR | LTR |
| 14306961 | 14307059 | + | 23.2 | 1 | 100 | -328 | MSTA | MaLR | LTR |
| 14307105 | 14307211 | + | 23.4 | 58 | 180 | -248 | MSTA | MaLR | LTR |
| 14307205 | 14307552 | + | 22.1 | 1317 | 1651 | 0 | MSTA-int | MaLR | LTR |

  

|  |  |  |  |  |  |  |  |  |  |
| --- | --- | --- | --- | --- | --- | --- | --- | --- | --- |
| chr18:14307553-14308310 | | | 619 bp | | |  |  |  |  |
| Genome Start | Genome End | Strand | Div | Repeat Start | Repeat End | Repeat Left | Name | Family | Class |
| 14307553 | 14307646 | + | 14.0 | 1 | 97 | -331 | MSTA | MaLR | LTR |
| 14307780 | 14307921 | - | 22.6 | 1045 | 1187 | -393 | THE1-int | MaLR | LTR |
| 14307920 | 14308018 | - | 31.3 | 976 | 1075 | -576 | MST-int | MaLR | LTR |
| 14308027 | 14308310 | + | 26.6 | 75 | 374 | -54 | MSTA | MaLR | LTR |

  

|  |  |  |  |  |  |  |  |  |  |
| --- | --- | --- | --- | --- | --- | --- | --- | --- | --- |
| chr18:14310390-14311733 | | | 1220 bp | | |  |  |  |  |
| Genome Start | Genome End | Strand | Div | Repeat Start | Repeat End | Repeat Left | Name | Family | Class |
| 14310390 | 14310482 | + | 22.6 | 4 | 97 | -331 | MSTA | MaLR | LTR |
| 14310518 | 14310629 | + | 27.6 | 61 | 171 | -257 | MSTA | MaLR | LTR |
| 14310636 | 14310953 | + | 19.9 | 1341 | 1651 | 0 | MSTA-int | MaLR | LTR |
| 14310956 | 14311059 | + | 18.0 | 3 | 113 | -262 | MSTA | MaLR | LTR |
| 14311147 | 14311204 | + | 19.0 | 910 | 968 | -612 | THE1-int | MaLR | LTR |
| 14311195 | 14311435 | - | 23.3 | 907 | 1188 | -392 | THE1-int | MaLR | LTR |
| 14311440 | 14311733 | + | 25.5 | 66 | 372 | -56 | MSTA | MaLR | LTR |

  

|  |  |  |  |  |  |  |  |  |  |
| --- | --- | --- | --- | --- | --- | --- | --- | --- | --- |
| chr18:14313269-14320072 | | | 6350 bp | | |  |  |  |  |
| Genome Start | Genome End | Strand | Div | Repeat Start | Repeat End | Repeat Left | Name | Family | Class |
| 14313269 | 14313365 | + | 17.7 | 1 | 99 | -329 | MSTA | MaLR | LTR |
| 14313415 | 14313505 | + | 27.5 | 60 | 159 | -269 | MSTA | MaLR | LTR |
| 14313512 | 14313848 | + | 22.9 | 1317 | 1651 | 0 | MST-int | MaLR | LTR |
| 14313851 | 14313911 | + | 19.7 | 3 | 65 | -363 | MSTA | MaLR | LTR |
| 14314033 | 14314082 | + | 20.0 | 985 | 1035 | -616 | MST-int | MaLR | LTR |
| 14314081 | 14314311 | - | 24.3 | 908 | 1178 | -402 | THE1-int | MaLR | LTR |
| 14314317 | 14314601 | + | 25.1 | 76 | 376 | -52 | MSTA | MaLR | LTR |
| 14314638 | 14314769 | - | 21.4 | 507 | 659 | -992 | MST-int | MaLR | LTR |
| 14314768 | 14316073 | + | 24.4 | 28 | 1389 | -262 | MST-int | MaLR | LTR |
| 14316070 | 14316185 | + | 20.7 | 1535 | 1651 | 0 | MST-int | MaLR | LTR |
| 14316186 | 14316283 | + | 20.6 | 1 | 99 | -329 | MSTA | MaLR | LTR |
| 14316333 | 14316439 | + | 28.3 | 60 | 176 | -252 | MSTA | MaLR | LTR |
| 14316432 | 14316764 | + | 21.0 | 1317 | 1651 | 0 | MSTA-int | MaLR | LTR |
| 14316765 | 14316872 | + | 18.7 | 1 | 113 | -268 | MSTA | MaLR | LTR |
| 14316891 | 14317439 | - | 10.8 | 1 | 563 | -1 | LTR6A | ERV1 | LTR |
| 14317511 | 14317566 | + | 19.6 | 911 | 967 | -613 | THE1-int | MaLR | LTR |
| 14317555 | 14317805 | - | 20.2 | 907 | 1191 | -389 | THE1-int | MaLR | LTR |
| 14317810 | 14318091 | + | 21.6 | 76 | 376 | -52 | MSTA | MaLR | LTR |
| 14318134 | 14318242 | - | 26.9 | 466 | 577 | -1003 | THE1-int | MaLR | LTR |
| 14318247 | 14319836 | + | 23.8 | 34 | 1651 | 0 | MST-int | MaLR | LTR |
| 14319837 | 14319935 | + | 23.2 | 1 | 104 | -324 | MSTA | MaLR | LTR |
| 14320011 | 14320072 | + | 21.0 | 96 | 159 | -269 | MSTA | MaLR | LTR |

  


|  |  |  |  |  |  |  |  |  |  |
| --- | --- | --- | --- | --- | --- | --- | --- | --- | --- |
| chr19:55282513-55283105 | | | 593 bp | | |  |  |  |  |
| Genome Start | Genome End | Strand | Div | Repeat Start | Repeat End | Repeat Left | Name | Family | Class |
| 55282513 | 55282644 | + | 17.5 | 6 | 131 | -181 | AluJo | Alu | SINE |
| 55282645 | 55282957 | + | 19.2 | 1 | 312 | 0 | AluJb | Alu | SINE |
| 55282958 | 55283105 | + | 17.5 | 132 | 282 | -30 | AluJo | Alu | SINE |

  

|  |  |  |  |  |  |  |  |  |  |
| --- | --- | --- | --- | --- | --- | --- | --- | --- | --- |
| chr19:55287848-55288715 | | | 850 bp | | |  |  |  |  |
| Genome Start | Genome End | Strand | Div | Repeat Start | Repeat End | Repeat Left | Name | Family | Class |
| 55287848 | 55288049 | - | 29.2 | 3058 | 3274 | -145 | L2 | L2 | LINE |
| 55288061 | 55288377 | + | 10.0 | 1 | 310 | 0 | AluSg | Alu | SINE |
| 55288378 | 55288509 | - | 19.9 | 104 | 239 | -85 | MER33 | MER1\_type | DNA |
| 55288507 | 55288582 | - | 21.1 | 1 | 77 | -247 | MER33 | MER1\_type | DNA |
| 55288593 | 55288715 | - | 20.3 | 2945 | 3076 | -343 | L2 | L2 | LINE |

  

|  |  |  |  |  |  |  |  |  |  |
| --- | --- | --- | --- | --- | --- | --- | --- | --- | --- |
| chr19:55289285-55291358 | | | 1853 bp | | |  |  |  |  |
| Genome Start | Genome End | Strand | Div | Repeat Start | Repeat End | Repeat Left | Name | Family | Class |
| 55289285 | 55289765 | - | 16.5 | 3279 | 3745 | -2469 | L1MC4 | L1 | LINE |
| 55289766 | 55290086 | + | 14.0 | 5822 | 6150 | -1 | L1PB1 | L1 | LINE |
| 55290087 | 55290459 | - | 16.5 | 2923 | 3278 | -2936 | L1MC4 | L1 | LINE |
| 55290470 | 55290637 | + | 10.1 | 137 | 304 | -8 | AluSg/x | Alu | SINE |
| 55290639 | 55290837 | - | 24.6 | 891 | 1117 | -5098 | L1MC4 | L1 | LINE |
| 55290960 | 55291091 | - | 10.6 | 1 | 132 | -178 | Alu | Alu | SINE |
| 55291131 | 55291253 | - | 30.4 | 582 | 710 | -5505 | L1MC4 | L1 | LINE |
| 55291303 | 55291358 | - | 30.4 | 524 | 581 | -5634 | L1MC4 | L1 | LINE |

  

|  |  |  |  |  |  |  |  |  |  |
| --- | --- | --- | --- | --- | --- | --- | --- | --- | --- |
| chr19:55291526-55291973 | | | 448 bp | | |  |  |  |  |
| Genome Start | Genome End | Strand | Div | Repeat Start | Repeat End | Repeat Left | Name | Family | Class |
| 55291526 | 55291603 | - | 17.5 | 417 | 486 | 0 | MER66B | ERV1 | LTR |
| 55291604 | 55291901 | + | 9.7 | 1 | 298 | -14 | AluSx | Alu | SINE |
| 55291902 | 55291973 | - | 17.5 | 345 | 416 | -70 | MER66B | ERV1 | LTR |

  

|  |  |  |  |  |  |  |  |  |  |
| --- | --- | --- | --- | --- | --- | --- | --- | --- | --- |
| chr19:55293703-55294423 | | | 511 bp | | |  |  |  |  |
| Genome Start | Genome End | Strand | Div | Repeat Start | Repeat End | Repeat Left | Name | Family | Class |
| 55293703 | 55293902 | - | 25.0 | 891 | 1117 | -5098 | L1MC4 | L1 | LINE |
| 55294025 | 55294156 | - | 12.1 | 1 | 132 | -178 | Alu | Alu | SINE |
| 55294196 | 55294318 | - | 29.9 | 582 | 710 | -5505 | L1MC4 | L1 | LINE |
| 55294368 | 55294423 | - | 29.9 | 524 | 581 | -5634 | L1MC4 | L1 | LINE |

  

|  |  |  |  |  |  |  |  |  |  |
| --- | --- | --- | --- | --- | --- | --- | --- | --- | --- |
| chr19:55294591-55295038 | | | 448 bp | | |  |  |  |  |
| Genome Start | Genome End | Strand | Div | Repeat Start | Repeat End | Repeat Left | Name | Family | Class |
| 55294591 | 55294668 | - | 17.5 | 417 | 486 | 0 | MER66B | ERV1 | LTR |
| 55294669 | 55294966 | + | 9.7 | 1 | 298 | -14 | AluSx | Alu | SINE |
| 55294967 | 55295038 | - | 17.5 | 345 | 416 | -70 | MER66B | ERV1 | LTR |

  

|  |  |  |  |  |  |  |  |  |  |
| --- | --- | --- | --- | --- | --- | --- | --- | --- | --- |
| chr19:55296768-55297488 | | | 511 bp | | |  |  |  |  |
| Genome Start | Genome End | Strand | Div | Repeat Start | Repeat End | Repeat Left | Name | Family | Class |
| 55296768 | 55296967 | - | 25.0 | 891 | 1117 | -5098 | L1MC4 | L1 | LINE |
| 55297090 | 55297221 | - | 10.6 | 1 | 132 | -178 | Alu | Alu | SINE |
| 55297261 | 55297383 | - | 29.9 | 582 | 710 | -5505 | L1MC4 | L1 | LINE |
| 55297433 | 55297488 | - | 29.9 | 524 | 581 | -5634 | L1MC4 | L1 | LINE |

  


|  |  |  |  |  |  |  |  |  |  |
| --- | --- | --- | --- | --- | --- | --- | --- | --- | --- |
| chr21:14145756-14146398 | | | 526 bp | | |  |  |  |  |
| Genome Start | Genome End | Strand | Div | Repeat Start | Repeat End | Repeat Left | Name | Family | Class |
| 14145756 | 14145894 | - | 28.3 | 133 | 274 | -154 | MSTA | MaLR | LTR |
| 14145945 | 14146174 | + | 23.6 | 907 | 1174 | -406 | THE1-int | MaLR | LTR |
| 14146177 | 14146232 | - | 21.4 | 911 | 967 | -613 | THE1-int | MaLR | LTR |
| 14146298 | 14146398 | - | 21.6 | 1 | 106 | -322 | MSTA | MaLR | LTR |

  

|  |  |  |  |  |  |  |  |  |  |
| --- | --- | --- | --- | --- | --- | --- | --- | --- | --- |
| chr21:14146717-14166312 | | | 18147 bp | | |  |  |  |  |
| Genome Start | Genome End | Strand | Div | Repeat Start | Repeat End | Repeat Left | Name | Family | Class |
| 14146717 | 14146790 | - | 20.6 | 95 | 171 | -257 | MSTA | MaLR | LTR |
| 14146868 | 14146963 | - | 21.1 | 1 | 97 | -331 | MSTA | MaLR | LTR |
| 14146964 | 14148583 | - | 23.6 | 28 | 1651 | 0 | MSTA-int | MaLR | LTR |
| 14148581 | 14148719 | + | 21.1 | 465 | 661 | -990 | THE1-int | MaLR | LTR |
| 14148762 | 14149017 | - | 24.1 | 95 | 368 | -60 | MSTA | MaLR | LTR |
| 14149033 | 14149276 | + | 19.2 | 907 | 1191 | -389 | THE1-int | MaLR | LTR |
| 14149265 | 14149320 | - | 21.4 | 911 | 967 | -613 | THE1-int | MaLR | LTR |
| 14149415 | 14149509 | - | 17.9 | 1 | 97 | -331 | MSTA | MaLR | LTR |
| 14149510 | 14149856 | - | 21.0 | 1318 | 1651 | 0 | MST-int | MaLR | LTR |
| 14149864 | 14149925 | - | 22.6 | 96 | 159 | -269 | MSTA | MaLR | LTR |
| 14149997 | 14150099 | - | 24.3 | 1 | 105 | -323 | MSTA | MaLR | LTR |
| 14150100 | 14151710 | - | 24.2 | 34 | 1651 | 0 | MST-int | MaLR | LTR |
| 14151715 | 14151823 | + | 27.8 | 466 | 577 | -1003 | THE1-int | MaLR | LTR |
| 14151866 | 14152147 | - | 22.3 | 76 | 376 | -52 | MSTA | MaLR | LTR |
| 14152152 | 14152402 | + | 20.2 | 907 | 1191 | -389 | THE1-int | MaLR | LTR |
| 14152393 | 14152446 | - | 22.2 | 911 | 965 | -615 | THE1-int | MaLR | LTR |
| 14152518 | 14153067 | + | 11.1 | 1 | 563 | -1 | LTR6A | ERV1 | LTR |
| 14153101 | 14153195 | - | 20.0 | 1 | 97 | -331 | MSTA | MaLR | LTR |
| 14153196 | 14153531 | - | 22.1 | 1318 | 1651 | 0 | MST-int | MaLR | LTR |
| 14153539 | 14153602 | - | 34.4 | 96 | 159 | -269 | MSTA | MaLR | LTR |
| 14153681 | 14153778 | - | 23.5 | 1 | 99 | -329 | MSTA | MaLR | LTR |
| 14153779 | 14153894 | - | 19.8 | 1535 | 1651 | 0 | MSTA-int | MaLR | LTR |
| 14153891 | 14155204 | - | 24.8 | 26 | 1389 | -262 | MSTA-int | MaLR | LTR |
| 14155201 | 14155339 | + | 23.2 | 507 | 667 | -984 | MST-int | MaLR | LTR |
| 14155368 | 14155635 | - | 23.3 | 97 | 376 | -52 | MSTA | MaLR | LTR |
| 14155657 | 14155899 | + | 26.1 | 907 | 1191 | -389 | THE1-int | MaLR | LTR |
| 14155888 | 14155943 | - | 19.6 | 911 | 967 | -613 | THE1-int | MaLR | LTR |
| 14156060 | 14156120 | - | 19.7 | 3 | 65 | -363 | MSTA | MaLR | LTR |
| 14156123 | 14156458 | - | 22.1 | 1317 | 1651 | 0 | MST-int | MaLR | LTR |
| 14156451 | 14156556 | - | 24.8 | 60 | 176 | -252 | MSTA | MaLR | LTR |
| 14156606 | 14156702 | - | 22.7 | 1 | 99 | -329 | MSTA | MaLR | LTR |
| 14156703 | 14157866 | - | 25.2 | 427 | 1651 | 0 | MSTA-int | MaLR | LTR |
| 14157865 | 14158068 | - | 22.5 | 28 | 230 | -1269 | MSTA-int | MaLR | LTR |
| 14158065 | 14158197 | + | 17.4 | 507 | 661 | -990 | MST-int | MaLR | LTR |
| 14158224 | 14158528 | - | 25.0 | 76 | 385 | -43 | MSTA | MaLR | LTR |
| 14158533 | 14158766 | + | 22.7 | 907 | 1188 | -392 | THE1-int | MaLR | LTR |
| 14158758 | 14158814 | - | 19.3 | 910 | 967 | -613 | THE1-int | MaLR | LTR |
| 14158915 | 14159007 | - | 19.4 | 1 | 94 | -334 | MSTA | MaLR | LTR |
| 14159008 | 14159325 | - | 21.6 | 1341 | 1651 | 0 | MST-int | MaLR | LTR |
| 14159332 | 14159453 | - | 23.9 | 58 | 171 | -257 | MSTA | MaLR | LTR |
| 14159479 | 14159574 | - | 21.9 | 1 | 97 | -331 | MSTA | MaLR | LTR |
| 14159575 | 14159685 | - | 18.9 | 1538 | 1651 | 0 | MSTA-int | MaLR | LTR |
| 14159809 | 14159907 | - | 20.2 | 1 | 100 | -328 | MSTA | MaLR | LTR |
| 14159908 | 14161485 | - | 24.6 | 28 | 1651 | 0 | MSTA-int | MaLR | LTR |
| 14161480 | 14161634 | + | 31.1 | 462 | 617 | -963 | THE1-int | MaLR | LTR |
| 14161653 | 14161917 | - | 24.8 | 98 | 374 | -54 | MSTA | MaLR | LTR |
| 14161945 | 14162184 | + | 27.2 | 907 | 1187 | -393 | THE1-int | MaLR | LTR |
| 14162177 | 14162232 | - | 23.2 | 911 | 967 | -613 | MSTA-int | MaLR | LTR |
| 14162318 | 14162413 | - | 14.7 | 1 | 97 | -331 | MSTA | MaLR | LTR |
| 14162414 | 14162761 | - | 20.9 | 1317 | 1651 | 0 | MST-int | MaLR | LTR |
| 14162760 | 14162862 | - | 21.6 | 58 | 171 | -257 | MSTA | MaLR | LTR |
| 14162908 | 14163006 | - | 23.2 | 1 | 100 | -328 | MSTA | MaLR | LTR |
| 14163007 | 14163082 | - | 15.8 | 1574 | 1651 | 0 | MSTA-int | MaLR | LTR |
| 14163085 | 14163146 | + | 18.3 | 317 | 376 | -52 | MSTA | MaLR | LTR |
| 14163220 | 14164754 | - | 23.9 | 28 | 1583 | -68 | MSTA-int | MaLR | LTR |
| 14164750 | 14164904 | + | 25.3 | 463 | 617 | -963 | THE1-int | MaLR | LTR |
| 14164922 | 14165188 | - | 24.4 | 96 | 376 | -52 | MSTA | MaLR | LTR |
| 14165213 | 14165453 | + | 23.8 | 907 | 1188 | -392 | THE1-int | MaLR | LTR |
| 14165444 | 14165500 | - | 19.6 | 911 | 968 | -612 | THE1-int | MaLR | LTR |
| 14165597 | 14165692 | - | 20.8 | 1 | 97 | -331 | MSTA | MaLR | LTR |
| 14165695 | 14166038 | - | 23.9 | 1318 | 1649 | -2 | MST-int | MaLR | LTR |
| 14166046 | 14166110 | - | 21.5 | 95 | 159 | -269 | MSTA | MaLR | LTR |
| 14166218 | 14166312 | - | 24.2 | 1 | 96 | -332 | MSTA | MaLR | LTR |

  

|  |  |  |  |  |  |  |  |  |  |
| --- | --- | --- | --- | --- | --- | --- | --- | --- | --- |
| chr21:14167928-14171718 | | | 3508 bp | | |  |  |  |  |
| Genome Start | Genome End | Strand | Div | Repeat Start | Repeat End | Repeat Left | Name | Family | Class |
| 14167928 | 14168070 | + | 23.7 | 506 | 661 | -990 | MST-int | MaLR | LTR |
| 14168113 | 14168368 | - | 23.6 | 95 | 368 | -60 | MSTA | MaLR | LTR |
| 14168384 | 14168623 | + | 19.1 | 907 | 1191 | -389 | THE1-int | MaLR | LTR |
| 14168612 | 14168667 | - | 21.4 | 979 | 1035 | -616 | MST-int | MaLR | LTR |
| 14168762 | 14168855 | - | 20.2 | 1 | 97 | -331 | MSTA | MaLR | LTR |
| 14168856 | 14169202 | - | 22.4 | 1318 | 1651 | 0 | MST-int | MaLR | LTR |
| 14169210 | 14169270 | - | 22.9 | 97 | 159 | -269 | MSTA | MaLR | LTR |
| 14169349 | 14169446 | - | 22.4 | 1 | 100 | -328 | MSTA | MaLR | LTR |
| 14169447 | 14171044 | - | 23.7 | 34 | 1651 | 0 | MST-int | MaLR | LTR |
| 14171049 | 14171156 | + | 27.1 | 466 | 577 | -1003 | THE1-int | MaLR | LTR |
| 14171199 | 14171474 | - | 22.2 | 95 | 376 | -52 | MSTA | MaLR | LTR |
| 14171488 | 14171718 | + | 23.9 | 976 | 1243 | -408 | MST-int | MaLR | LTR |

  

|  |  |  |  |  |  |  |  |  |  |
| --- | --- | --- | --- | --- | --- | --- | --- | --- | --- |
| chr21:14172844-14195630 | | | 20995 bp | | |  |  |  |  |
| Genome Start | Genome End | Strand | Div | Repeat Start | Repeat End | Repeat Left | Name | Family | Class |
| 14172844 | 14172945 | - | 21.5 | 75 | 176 | -252 | MSTA | MaLR | LTR |
| 14172990 | 14173097 | - | 23.1 | 1 | 105 | -323 | MSTA | MaLR | LTR |
| 14173098 | 14173213 | - | 18.1 | 1535 | 1651 | 0 | MSTA-int | MaLR | LTR |
| 14173210 | 14174518 | - | 24.7 | 32 | 1389 | -262 | MSTA-int | MaLR | LTR |
| 14174524 | 14174660 | + | 22.8 | 510 | 667 | -984 | MST-int | MaLR | LTR |
| 14174689 | 14174973 | - | 23.8 | 76 | 376 | -52 | MSTA | MaLR | LTR |
| 14174978 | 14175220 | + | 23.5 | 907 | 1191 | -389 | THE1-int | MaLR | LTR |
| 14175209 | 14175264 | - | 21.4 | 911 | 967 | -613 | THE1-int | MaLR | LTR |
| 14175381 | 14175441 | - | 19.7 | 3 | 65 | -363 | MSTA | MaLR | LTR |
| 14175444 | 14175779 | - | 22.1 | 1317 | 1651 | 0 | MST-int | MaLR | LTR |
| 14175772 | 14175877 | - | 24.8 | 60 | 176 | -252 | MSTA | MaLR | LTR |
| 14175927 | 14176023 | - | 22.7 | 1 | 99 | -329 | MSTA | MaLR | LTR |
| 14176024 | 14177574 | - | 24.0 | 28 | 1651 | 0 | MSTA-int | MaLR | LTR |
| 14177571 | 14177703 | + | 20.4 | 507 | 661 | -990 | MST-int | MaLR | LTR |
| 14177730 | 14178017 | - | 26.2 | 96 | 385 | -43 | MSTA | MaLR | LTR |
| 14178040 | 14178280 | + | 22.4 | 907 | 1188 | -392 | THE1-int | MaLR | LTR |
| 14178272 | 14178329 | - | 17.9 | 911 | 967 | -613 | THE1-int | MaLR | LTR |
| 14178430 | 14178522 | - | 20.4 | 1 | 94 | -334 | MSTA | MaLR | LTR |
| 14178523 | 14178841 | - | 23.4 | 1341 | 1651 | 0 | MST-int | MaLR | LTR |
| 14178848 | 14178929 | - | 24.0 | 93 | 171 | -257 | MSTA | MaLR | LTR |
| 14179002 | 14179097 | - | 20.8 | 1 | 97 | -331 | MSTA | MaLR | LTR |
| 14179098 | 14179210 | - | 15.9 | 1538 | 1651 | 0 | MSTA-int | MaLR | LTR |
| 14179372 | 14179470 | - | 21.2 | 1 | 100 | -328 | MSTA | MaLR | LTR |
| 14179471 | 14181047 | - | 23.5 | 28 | 1651 | 0 | MSTA-int | MaLR | LTR |
| 14181042 | 14181179 | + | 23.4 | 503 | 661 | -990 | MST-int | MaLR | LTR |
| 14181216 | 14181481 | - | 25.4 | 98 | 374 | -54 | MSTA | MaLR | LTR |
| 14181509 | 14181748 | + | 23.8 | 907 | 1187 | -393 | THE1-int | MaLR | LTR |
| 14181883 | 14181977 | - | 17.0 | 1 | 96 | -332 | MSTA | MaLR | LTR |
| 14181978 | 14182325 | - | 23.9 | 1317 | 1651 | 0 | MSTA-int | MaLR | LTR |
| 14182319 | 14182425 | - | 23.4 | 58 | 180 | -248 | MSTA | MaLR | LTR |
| 14182471 | 14182569 | - | 26.3 | 1 | 100 | -328 | MSTA | MaLR | LTR |
| 14182570 | 14182645 | - | 14.5 | 1574 | 1651 | 0 | MST-int | MaLR | LTR |
| 14182658 | 14182700 | + | 16.3 | 323 | 365 | -61 | MSTB | MaLR | LTR |
| 14182781 | 14184312 | - | 22.9 | 28 | 1583 | -68 | MST-int | MaLR | LTR |
| 14184308 | 14184461 | + | 24.7 | 463 | 617 | -963 | THE1-int | MaLR | LTR |
| 14184479 | 14184767 | - | 24.5 | 75 | 376 | -52 | MSTA | MaLR | LTR |
| 14184770 | 14185010 | + | 23.4 | 907 | 1188 | -392 | THE1-int | MaLR | LTR |
| 14185001 | 14185057 | - | 19.6 | 911 | 968 | -612 | THE1-int | MaLR | LTR |
| 14185154 | 14185249 | - | 20.8 | 1 | 97 | -331 | MSTA | MaLR | LTR |
| 14185252 | 14185595 | - | 21.6 | 1318 | 1649 | -2 | MST-int | MaLR | LTR |
| 14185603 | 14185667 | - | 23.1 | 95 | 159 | -269 | MSTA | MaLR | LTR |
| 14185771 | 14185869 | - | 26.3 | 1 | 100 | -328 | MSTA | MaLR | LTR |
| 14185871 | 14187481 | - | 23.3 | 28 | 1650 | -1 | MSTA-int | MaLR | LTR |
| 14187479 | 14187620 | + | 17.7 | 465 | 661 | -990 | THE1-int | MaLR | LTR |
| 14187664 | 14187918 | - | 23.8 | 95 | 367 | -61 | MSTA | MaLR | LTR |
| 14187934 | 14188177 | + | 18.0 | 907 | 1191 | -389 | THE1-int | MaLR | LTR |
| 14188166 | 14188221 | - | 21.4 | 911 | 967 | -613 | THE1-int | MaLR | LTR |
| 14188316 | 14188410 | - | 18.9 | 1 | 97 | -331 | MSTA | MaLR | LTR |
| 14188411 | 14188757 | - | 22.2 | 1318 | 1651 | 0 | MST-int | MaLR | LTR |
| 14188765 | 14188826 | - | 25.8 | 96 | 159 | -269 | MSTA | MaLR | LTR |
| 14188904 | 14188999 | - | 22.3 | 3 | 100 | -328 | MSTA | MaLR | LTR |
| 14189002 | 14190587 | - | 24.0 | 34 | 1651 | 0 | MST-int | MaLR | LTR |
| 14190592 | 14190700 | + | 24.8 | 466 | 577 | -1003 | THE1-int | MaLR | LTR |
| 14190743 | 14191018 | - | 21.8 | 95 | 376 | -52 | MSTA | MaLR | LTR |
| 14191031 | 14191262 | + | 21.6 | 907 | 1178 | -402 | THE1-int | MaLR | LTR |
| 14191386 | 14191935 | + | 10.4 | 1 | 563 | -1 | LTR6A | ERV1 | LTR |
| 14191967 | 14192061 | - | 20.0 | 1 | 97 | -331 | MSTA | MaLR | LTR |
| 14192062 | 14192393 | - | 22.0 | 1317 | 1651 | 0 | MST-int | MaLR | LTR |
| 14192386 | 14192492 | - | 26.4 | 60 | 176 | -252 | MSTA | MaLR | LTR |
| 14192542 | 14192639 | - | 22.4 | 1 | 99 | -329 | MSTA | MaLR | LTR |
| 14192640 | 14192755 | - | 19.0 | 1535 | 1651 | 0 | MSTA-int | MaLR | LTR |
| 14192752 | 14194071 | - | 22.7 | 28 | 1389 | -262 | MSTA-int | MaLR | LTR |
| 14194067 | 14194220 | + | 24.7 | 463 | 617 | -963 | THE1-int | MaLR | LTR |
| 14194241 | 14194504 | - | 24.3 | 96 | 373 | -55 | MSTA | MaLR | LTR |
| 14194529 | 14194769 | + | 23.4 | 907 | 1188 | -392 | THE1-int | MaLR | LTR |
| 14194760 | 14194810 | - | 14.0 | 917 | 968 | -612 | THE1-int | MaLR | LTR |
| 14194902 | 14195008 | - | 21.6 | 1 | 113 | -251 | MSTA | MaLR | LTR |
| 14195011 | 14195355 | - | 22.1 | 1318 | 1649 | -2 | MST-int | MaLR | LTR |
| 14195363 | 14195472 | - | 25.5 | 56 | 159 | -269 | MSTA | MaLR | LTR |
| 14195532 | 14195630 | - | 26.6 | 1 | 99 | -327 | MSTA | MaLR | LTR |

  


|  |  |  |  |  |  |  |  |  |  |
| --- | --- | --- | --- | --- | --- | --- | --- | --- | --- |
| chrX:49063226-49064306 | | | 1037 bp | | |  |  |  |  |
| Genome Start | Genome End | Strand | Div | Repeat Start | Repeat End | Repeat Left | Name | Family | Class |
| 49063226 | 49063383 | - | 30.4 | 4324 | 4488 | -1658 | L1M5 | L1 | LINE |
| 49063384 | 49063689 | - | 11.1 | 3 | 309 | 0 | AluSg1 | Alu | SINE |
| 49063690 | 49063831 | - | 30.4 | 4189 | 4323 | -1823 | L1M5 | L1 | LINE |
| 49063854 | 49063984 | - | 18.5 | 2 | 132 | -180 | AluJb | Alu | SINE |
| 49064007 | 49064306 | - | 26.9 | 3841 | 4167 | -1979 | L1M5 | L1 | LINE |

  

|  |  |  |  |  |  |  |  |  |  |
| --- | --- | --- | --- | --- | --- | --- | --- | --- | --- |
| chrX:49068866-49069691 | | | 791 bp | | |  |  |  |  |
| Genome Start | Genome End | Strand | Div | Repeat Start | Repeat End | Repeat Left | Name | Family | Class |
| 49068866 | 49068990 | - | 26.5 | 7905 | 8019 | -23 | L1MC4 | L1 | LINE |
| 49069026 | 49069246 | - | 26.5 | 7690 | 7904 | -138 | L1MC4 | L1 | LINE |
| 49069247 | 49069488 | - | 16.1 | 1 | 299 | -3 | AluSx | Alu | SINE |
| 49069489 | 49069691 | - | 26.5 | 7503 | 7689 | -353 | L1MC4 | L1 | LINE |

  


|  |  |  |  |  |  |  |  |  |  |
| --- | --- | --- | --- | --- | --- | --- | --- | --- | --- |
| chrY:9246493-9246979 | | | 487 bp | | |  |  |  |  |
| Genome Start | Genome End | Strand | Div | Repeat Start | Repeat End | Repeat Left | Name | Family | Class |
| 9246493 | 9246628 | + | 31.3 | 2384 | 2518 | -901 | L2 | L2 | LINE |
| 9246629 | 9246935 | + | 20.3 | 1 | 306 | -6 | AluSx | Alu | SINE |
| 9246936 | 9246979 | + | 31.3 | 2519 | 2562 | -857 | L2 | L2 | LINE |
