# Supplementary material for: Evolutionary History of Mammalian Transposons Determined by Genome-Wide Defragmentation
Source: PLoS Comput Biol. 2007 Jul 13;3(7):e137. doi: 10.1371/journal.pcbi.0030137 (PMC1914374; doi:10.1371/journal.pcbi.0030137)
Supplement: Supplementary file 8 [file pcbi.0030137.sd005.htm]

Clusters excluded by Segmental Duplications

**Supplementary Table S1e: Clusters Excluded by Segmental Duplications**  
  
To load the custom track into the UCSC Genome Browser, click here. The custom track must be loaded in order to view the clusters in Supplementary Table S1. It may take a minute to load, so please be patient.  
  
Jump to:  
chr1  
chr2  
chr3  
chr4  
chr5  
chr6  
chr7  
chr8  
chr9  
chr10  
chr11  
chr12  
chr13  
chr14  
chr15  
chr16  
chr17  
chr18  
chr19  
chr20  
chr21  
chr22  
chrX  
chrY  
  


|  |  |  |  |  |  |  |  |  |  |
| --- | --- | --- | --- | --- | --- | --- | --- | --- | --- |
| chr1:13688-14311 | | | 546 bp | | |  |  |  |  |
| Genome Start | Genome End | Strand | Div | Repeat Start | Repeat End | Repeat Left | Name | Family | Class |
| 13688 | 13869 | + | 28.3 | 2957 | 3178 | -200 | L2 | L2 | LINE |
| 13935 | 14113 | + | 27.1 | 33 | 260 | -2 | MIR | MIR | SINE |
| 14127 | 14311 | + | 34.6 | 3232 | 3418 | -1 | L2 | L2 | LINE |

  

|  |  |  |  |  |  |  |  |  |  |
| --- | --- | --- | --- | --- | --- | --- | --- | --- | --- |
| chr1:27922-30491 | | | 2468 bp | | |  |  |  |  |
| Genome Start | Genome End | Strand | Div | Repeat Start | Repeat End | Repeat Left | Name | Family | Class |
| 27922 | 28054 | + | 17.4 | 2705 | 2846 | -573 | L2 | L2 | LINE |
| 28119 | 29327 | + | 22.9 | 131 | 1489 | 0 | MLT1E-int | MaLR | LTR |
| 29328 | 29486 | + | 24.3 | 1 | 168 | -425 | MLT1E | MaLR | LTR |
| 29487 | 29787 | + | 12.0 | 1 | 301 | -11 | AluSx | Alu | SINE |
| 29788 | 30157 | + | 24.3 | 169 | 579 | -14 | MLT1E | MaLR | LTR |
| 30196 | 30491 | + | 31.9 | 2924 | 3228 | -191 | L2 | L2 | LINE |

  

|  |  |  |  |  |  |  |  |  |  |
| --- | --- | --- | --- | --- | --- | --- | --- | --- | --- |
| chr1:63551-67251 | | | 3703 bp | | |  |  |  |  |
| Genome Start | Genome End | Strand | Div | Repeat Start | Repeat End | Repeat Left | Name | Family | Class |
| 63551 | 63706 | + | 24.2 | 5372 | 5525 | -2283 | L1MC5 | L1 | LINE |
| 63708 | 64758 | - | 1.6 | 5105 | 6155 | 0 | L1PA2 | L1 | LINE |
| 64761 | 66560 | + | 2.1 | 3311 | 5110 | -1036 | L1PA2 | L1 | LINE |
| 66556 | 66755 | + | 23.9 | 5512 | 5734 | -2074 | L1MC5 | L1 | LINE |
| 66756 | 67064 | + | 13.3 | 1 | 309 | -3 | AluSx | Alu | SINE |
| 67065 | 67251 | + | 23.9 | 5735 | 5942 | -1866 | L1MC5 | L1 | LINE |

  

|  |  |  |  |  |  |  |  |  |  |
| --- | --- | --- | --- | --- | --- | --- | --- | --- | --- |
| chr1:70575-71554 | | | 980 bp | | |  |  |  |  |
| Genome Start | Genome End | Strand | Div | Repeat Start | Repeat End | Repeat Left | Name | Family | Class |
| 70575 | 70667 | + | 23.8 | 1 | 87 | -2236 | Tigger5 | MER2\_type | DNA |
| 70668 | 70959 | + | 7.9 | 5 | 295 | -14 | AluSc | Alu | SINE |
| 70960 | 71094 | + | 23.8 | 88 | 218 | -2105 | Tigger5 | MER2\_type | DNA |
| 71095 | 71554 | + | 17.3 | 1919 | 2406 | 0 | Tigger5 | MER2\_type | DNA |

  

|  |  |  |  |  |  |  |  |  |  |
| --- | --- | --- | --- | --- | --- | --- | --- | --- | --- |
| chr1:92646-94939 | | | 2137 bp | | |  |  |  |  |
| Genome Start | Genome End | Strand | Div | Repeat Start | Repeat End | Repeat Left | Name | Family | Class |
| 92646 | 92838 | + | 30.8 | 3573 | 3761 | -728 | L3 | CR1 | LINE |
| 92839 | 93143 | + | 11.1 | 1 | 306 | 0 | AluSg | Alu | SINE |
| 93151 | 93220 | + | 30.8 | 3762 | 3831 | -658 | L3 | CR1 | LINE |
| 93238 | 93330 | - | 21.9 | 415 | 519 | -2 | LTR33 | ERVL | LTR |
| 93331 | 93479 | + | 15.1 | 1 | 149 | -283 | MSTB1 | MaLR | LTR |
| 93480 | 93772 | - | 10.6 | 6 | 297 | -16 | AluSq | Alu | SINE |
| 93773 | 94023 | + | 15.1 | 150 | 415 | -17 | MSTB1 | MaLR | LTR |
| 94063 | 94078 | + | 15.1 | 416 | 431 | -1 | MSTB1 | MaLR | LTR |
| 94079 | 94117 | - | 21.9 | 380 | 414 | -107 | LTR33 | ERVL | LTR |
| 94201 | 94369 | - | 28.5 | 1 | 173 | -348 | LTR33 | ERVL | LTR |
| 94381 | 94939 | + | 34.0 | 3856 | 4479 | -10 | L3 | CR1 | LINE |

  

|  |  |  |  |  |  |  |  |  |  |
| --- | --- | --- | --- | --- | --- | --- | --- | --- | --- |
| chr1:106895-108999 | | | 2110 bp | | |  |  |  |  |
| Genome Start | Genome End | Strand | Div | Repeat Start | Repeat End | Repeat Left | Name | Family | Class |
| 106895 | 107091 | + | 23.9 | 3777 | 3965 | -2164 | L1ME3B | L1 | LINE |
| 107085 | 107471 | + | 31.6 | 4488 | 4909 | -1220 | L1ME3B | L1 | LINE |
| 107474 | 107963 | + | 22.9 | 1 | 495 | -10 | MLT1D | MaLR | LTR |
| 107964 | 108999 | + | 27.0 | 4906 | 6042 | -181 | L1ME3B | L1 | LINE |

  

|  |  |  |  |  |  |  |  |  |  |
| --- | --- | --- | --- | --- | --- | --- | --- | --- | --- |
| chr1:118351-118850 | | | 500 bp | | |  |  |  |  |
| Genome Start | Genome End | Strand | Div | Repeat Start | Repeat End | Repeat Left | Name | Family | Class |
| 118351 | 118458 | - | 31.7 | 3313 | 3414 | -5 | L2 | L2 | LINE |
| 118459 | 118757 | - | 12.4 | 1 | 299 | -14 | AluSq | Alu | SINE |
| 118758 | 118850 | - | 31.7 | 3223 | 3312 | -107 | L2 | L2 | LINE |

  

|  |  |  |  |  |  |  |  |  |  |
| --- | --- | --- | --- | --- | --- | --- | --- | --- | --- |
| chr1:130648-132134 | | | 1487 bp | | |  |  |  |  |
| Genome Start | Genome End | Strand | Div | Repeat Start | Repeat End | Repeat Left | Name | Family | Class |
| 130648 | 131153 | + | 20.6 | 18 | 525 | -410 | MER21C | ERV1 | LTR |
| 131154 | 131460 | - | 19.2 | 2 | 294 | -18 | AluJb | Alu | SINE |
| 131461 | 131530 | + | 20.6 | 526 | 603 | -332 | MER21C | ERV1 | LTR |
| 131531 | 131833 | + | 20.5 | 1 | 302 | -10 | AluJo | Alu | SINE |
| 131834 | 132134 | + | 20.6 | 604 | 935 | 0 | MER21C | ERV1 | LTR |

  

|  |  |  |  |  |  |  |  |  |  |
| --- | --- | --- | --- | --- | --- | --- | --- | --- | --- |
| chr1:137458-138633 | | | 1128 bp | | |  |  |  |  |
| Genome Start | Genome End | Strand | Div | Repeat Start | Repeat End | Repeat Left | Name | Family | Class |
| 137458 | 137488 | + | 18.9 | 5476 | 5506 | -678 | L1MB8 | L1 | LINE |
| 137489 | 137780 | + | 19.0 | 1 | 293 | -19 | AluJb | Alu | SINE |
| 137781 | 137854 | + | 18.9 | 5507 | 5570 | -614 | L1MB8 | L1 | LINE |
| 137903 | 138013 | - | 18.0 | 4 | 114 | -19 | FLAM\_A | Alu | SINE |
| 138014 | 138633 | + | 20.2 | 5552 | 6173 | -5 | L1MB8 | L1 | LINE |

  

|  |  |  |  |  |  |  |  |  |  |
| --- | --- | --- | --- | --- | --- | --- | --- | --- | --- |
| chr1:141180-141757 | | | 578 bp | | |  |  |  |  |
| Genome Start | Genome End | Strand | Div | Repeat Start | Repeat End | Repeat Left | Name | Family | Class |
| 141180 | 141342 | - | 19.9 | 137 | 299 | -13 | AluJb | Alu | SINE |
| 141343 | 141629 | - | 10.1 | 1 | 297 | -12 | AluSg1 | Alu | SINE |
| 141630 | 141757 | - | 19.9 | 12 | 136 | -176 | AluJb | Alu | SINE |

  

|  |  |  |  |  |  |  |  |  |  |
| --- | --- | --- | --- | --- | --- | --- | --- | --- | --- |
| chr1:147259-147604 | | | 346 bp | | |  |  |  |  |
| Genome Start | Genome End | Strand | Div | Repeat Start | Repeat End | Repeat Left | Name | Family | Class |
| 147259 | 147358 | + | 31.0 | 5483 | 5575 | -2233 | L1MC4 | L1 | LINE |
| 147359 | 147481 | - | 16.5 | 4 | 126 | -7 | FLAM\_C | Alu | SINE |
| 147482 | 147604 | + | 31.0 | 5576 | 5690 | -2118 | L1MC4 | L1 | LINE |

  

|  |  |  |  |  |  |  |  |  |  |
| --- | --- | --- | --- | --- | --- | --- | --- | --- | --- |
| chr1:220151-222360 | | | 2210 bp | | |  |  |  |  |
| Genome Start | Genome End | Strand | Div | Repeat Start | Repeat End | Repeat Left | Name | Family | Class |
| 220151 | 220559 | + | 23.4 | 5762 | 6232 | -1553 | L1MC3 | L1 | LINE |
| 220560 | 220999 | + | 19.1 | 5 | 432 | 0 | MSTB1 | MaLR | LTR |
| 221000 | 222360 | + | 23.4 | 6233 | 7783 | -2 | L1MC3 | L1 | LINE |

  

|  |  |  |  |  |  |  |  |  |  |
| --- | --- | --- | --- | --- | --- | --- | --- | --- | --- |
| chr1:237898-238373 | | | 476 bp | | |  |  |  |  |
| Genome Start | Genome End | Strand | Div | Repeat Start | Repeat End | Repeat Left | Name | Family | Class |
| 237898 | 238000 | + | 30.5 | 3044 | 3144 | -3002 | L1M5 | L1 | LINE |
| 238001 | 238301 | + | 14.3 | 1 | 301 | -11 | AluSx | Alu | SINE |
| 238302 | 238373 | + | 30.5 | 3145 | 3217 | -2929 | L1M5 | L1 | LINE |

  

|  |  |  |  |  |  |  |  |  |  |
| --- | --- | --- | --- | --- | --- | --- | --- | --- | --- |
| chr1:238962-241254 | | | 2145 bp | | |  |  |  |  |
| Genome Start | Genome End | Strand | Div | Repeat Start | Repeat End | Repeat Left | Name | Family | Class |
| 238962 | 239155 | + | 28.9 | 3573 | 3757 | -732 | L3 | CR1 | LINE |
| 239156 | 239460 | + | 11.0 | 1 | 306 | 0 | AluSg | Alu | SINE |
| 239461 | 239537 | + | 28.9 | 3758 | 3834 | -655 | L3 | CR1 | LINE |
| 239555 | 239647 | - | 21.9 | 415 | 519 | -2 | LTR33 | ERVL | LTR |
| 239648 | 239796 | + | 15.3 | 1 | 149 | -283 | MSTB1 | MaLR | LTR |
| 239797 | 240089 | - | 9.9 | 6 | 297 | -16 | AluSq | Alu | SINE |
| 240090 | 240340 | + | 15.3 | 150 | 415 | -17 | MSTB1 | MaLR | LTR |
| 240378 | 240393 | + | 15.3 | 416 | 431 | -1 | MSTB1 | MaLR | LTR |
| 240394 | 240432 | - | 21.9 | 380 | 414 | -107 | LTR33 | ERVL | LTR |
| 240516 | 240684 | - | 28.5 | 1 | 173 | -348 | LTR33 | ERVL | LTR |
| 240696 | 241254 | + | 33.8 | 3856 | 4479 | -10 | L3 | CR1 | LINE |

  

|  |  |  |  |  |  |  |  |  |  |
| --- | --- | --- | --- | --- | --- | --- | --- | --- | --- |
| chr1:244046-244864 | | | 819 bp | | |  |  |  |  |
| Genome Start | Genome End | Strand | Div | Repeat Start | Repeat End | Repeat Left | Name | Family | Class |
| 244046 | 244507 | + | 34.1 | 2753 | 3307 | -112 | L2 | L2 | LINE |
| 244508 | 244745 | + | 19.1 | 1 | 302 | 0 | AluJb | Alu | SINE |
| 244746 | 244864 | + | 34.1 | 3308 | 3413 | -6 | L2 | L2 | LINE |

  

|  |  |  |  |  |  |  |  |  |  |
| --- | --- | --- | --- | --- | --- | --- | --- | --- | --- |
| chr1:308320-309806 | | | 1487 bp | | |  |  |  |  |
| Genome Start | Genome End | Strand | Div | Repeat Start | Repeat End | Repeat Left | Name | Family | Class |
| 308320 | 308620 | - | 20.7 | 604 | 935 | 0 | MER21C | ERV1 | LTR |
| 308621 | 308923 | - | 20.5 | 1 | 302 | -10 | AluJo | Alu | SINE |
| 308924 | 308993 | - | 20.7 | 526 | 603 | -332 | MER21C | ERV1 | LTR |
| 308994 | 309300 | + | 19.2 | 2 | 294 | -18 | AluJb | Alu | SINE |
| 309301 | 309806 | - | 20.7 | 18 | 525 | -410 | MER21C | ERV1 | LTR |

  

|  |  |  |  |  |  |  |  |  |  |
| --- | --- | --- | --- | --- | --- | --- | --- | --- | --- |
| chr1:322465-323306 | | | 842 bp | | |  |  |  |  |
| Genome Start | Genome End | Strand | Div | Repeat Start | Repeat End | Repeat Left | Name | Family | Class |
| 322465 | 322899 | - | 23.8 | 2025 | 2563 | -4226 | L1M2 | L1 | LINE |
| 322900 | 323214 | - | 12.7 | 1 | 309 | -1 | AluSg | Alu | SINE |
| 323215 | 323306 | - | 25.7 | 1946 | 2024 | -4765 | L1M2 | L1 | LINE |

  

|  |  |  |  |  |  |  |  |  |  |
| --- | --- | --- | --- | --- | --- | --- | --- | --- | --- |
| chr1:324228-324728 | | | 501 bp | | |  |  |  |  |
| Genome Start | Genome End | Strand | Div | Repeat Start | Repeat End | Repeat Left | Name | Family | Class |
| 324228 | 324320 | + | 31.7 | 3223 | 3312 | -107 | L2 | L2 | LINE |
| 324321 | 324620 | + | 11.3 | 1 | 300 | -13 | AluSq | Alu | SINE |
| 324621 | 324728 | + | 31.7 | 3313 | 3414 | -5 | L2 | L2 | LINE |

  

|  |  |  |  |  |  |  |  |  |  |
| --- | --- | --- | --- | --- | --- | --- | --- | --- | --- |
| chr1:343159-345974 | | | 2812 bp | | |  |  |  |  |
| Genome Start | Genome End | Strand | Div | Repeat Start | Repeat End | Repeat Left | Name | Family | Class |
| 343159 | 343452 | - | 29.0 | 252 | 542 | -13 | MLT1H1 | MaLR | LTR |
| 343453 | 343812 | + | 15.0 | 3 | 364 | 0 | THE1B | MaLR | LTR |
| 343817 | 345376 | + | 30.2 | 4 | 1735 | 0 | MLT-int | MaLR | LTR |
| 345377 | 345734 | + | 18.6 | 1 | 364 | 0 | THE1B | MaLR | LTR |
| 345735 | 345974 | - | 29.0 | 3 | 251 | -304 | MLT1H1 | MaLR | LTR |

  

|  |  |  |  |  |  |  |  |  |  |
| --- | --- | --- | --- | --- | --- | --- | --- | --- | --- |
| chr1:347476-348046 | | | 423 bp | | |  |  |  |  |
| Genome Start | Genome End | Strand | Div | Repeat Start | Repeat End | Repeat Left | Name | Family | Class |
| 347476 | 347624 | - | 28.9 | 1398 | 1552 | -457 | HAL1b | L1 | LINE |
| 347696 | 347840 | - | 14.3 | 1 | 133 | 0 | FLAM\_C | Alu | SINE |
| 347918 | 348046 | - | 29.1 | 866 | 995 | -1014 | HAL1b | L1 | LINE |

  

|  |  |  |  |  |  |  |  |  |  |
| --- | --- | --- | --- | --- | --- | --- | --- | --- | --- |
| chr1:352935-353582 | | | 575 bp | | |  |  |  |  |
| Genome Start | Genome End | Strand | Div | Repeat Start | Repeat End | Repeat Left | Name | Family | Class |
| 352935 | 353071 | - | 24.8 | 143 | 267 | -1 | MIRb | MIR | SINE |
| 353121 | 353367 | - | 14.6 | 6049 | 6295 | -5 | L1MA4 | L1 | LINE |
| 353384 | 353432 | - | 18.4 | 6055 | 6103 | -197 | L1MA4 | L1 | LINE |
| 353441 | 353582 | - | 34.1 | 21 | 155 | -113 | MIRb | MIR | SINE |

  

|  |  |  |  |  |  |  |  |  |  |
| --- | --- | --- | --- | --- | --- | --- | --- | --- | --- |
| chr1:369434-371928 | | | 2460 bp | | |  |  |  |  |
| Genome Start | Genome End | Strand | Div | Repeat Start | Repeat End | Repeat Left | Name | Family | Class |
| 369434 | 370518 | - | 16.4 | 3657 | 4741 | -1399 | L1M3 | L1 | LINE |
| 370538 | 370715 | - | 4.5 | 5977 | 6155 | 0 | L1PA4 | L1 | LINE |
| 370716 | 371437 | + | 2.2 | 5254 | 5975 | -180 | L1PA4 | L1 | LINE |
| 371454 | 371928 | - | 18.7 | 3167 | 3646 | -2494 | L1M3 | L1 | LINE |

  

|  |  |  |  |  |  |  |  |  |  |
| --- | --- | --- | --- | --- | --- | --- | --- | --- | --- |
| chr1:379316-385047 | | | 5561 bp | | |  |  |  |  |
| Genome Start | Genome End | Strand | Div | Repeat Start | Repeat End | Repeat Left | Name | Family | Class |
| 379316 | 379454 | + | 26.1 | 3224 | 3368 | -2778 | L1ME3B | L1 | LINE |
| 379453 | 381434 | + | 15.6 | 4080 | 6108 | -183 | L1MA8 | L1 | LINE |
| 381435 | 382170 | - | 10.3 | 5556 | 6303 | -1 | L1MA2 | L1 | LINE |
| 382171 | 382294 | + | 15.8 | 6109 | 6238 | -53 | L1MA8 | L1 | LINE |
| 382329 | 383069 | + | 25.0 | 3352 | 4119 | -2027 | L1ME3B | L1 | LINE |
| 383120 | 384133 | + | 17.4 | 3687 | 4697 | -1449 | L1P4 | L1 | LINE |
| 384192 | 384776 | + | 30.5 | 5508 | 6122 | -118 | L1ME3B | L1 | LINE |
| 384803 | 384964 | - | 18.7 | 123 | 298 | -15 | AluSp | Alu | SINE |
| 384970 | 385047 | + | 26.0 | 6128 | 6204 | -36 | L1ME3B | L1 | LINE |

  

|  |  |  |  |  |  |  |  |  |  |
| --- | --- | --- | --- | --- | --- | --- | --- | --- | --- |
| chr1:385214-386217 | | | 799 bp | | |  |  |  |  |
| Genome Start | Genome End | Strand | Div | Repeat Start | Repeat End | Repeat Left | Name | Family | Class |
| 385214 | 385434 | + | 38.9 | 2572 | 2801 | -618 | L2 | L2 | LINE |
| 385440 | 385715 | + | 4.3 | 5861 | 6150 | -4 | L1PA5 | L1 | LINE |
| 385916 | 386217 | + | 37.6 | 3030 | 3355 | -23 | L2 | L2 | LINE |

  

|  |  |  |  |  |  |  |  |  |  |
| --- | --- | --- | --- | --- | --- | --- | --- | --- | --- |
| chr1:393938-395948 | | | 1820 bp | | |  |  |  |  |
| Genome Start | Genome End | Strand | Div | Repeat Start | Repeat End | Repeat Left | Name | Family | Class |
| 393938 | 394203 | - | 32.3 | 3856 | 4151 | -338 | L3 | CR1 | LINE |
| 394215 | 394383 | + | 27.3 | 1 | 173 | -348 | LTR33 | ERVL | LTR |
| 394467 | 394505 | + | 21.9 | 378 | 414 | -107 | LTR33 | ERVL | LTR |
| 394506 | 394521 | - | 16.1 | 417 | 432 | 0 | MSTB1 | MaLR | LTR |
| 394571 | 394821 | - | 16.1 | 151 | 416 | -16 | MSTB1 | MaLR | LTR |
| 394822 | 395113 | + | 10.7 | 6 | 296 | -17 | AluSq | Alu | SINE |
| 395114 | 395262 | - | 16.1 | 2 | 150 | -282 | MSTB1 | MaLR | LTR |
| 395263 | 395355 | + | 21.9 | 415 | 517 | -4 | LTR33 | ERVL | LTR |
| 395373 | 395442 | - | 31.2 | 3763 | 3834 | -655 | L3 | CR1 | LINE |
| 395474 | 395754 | - | 11.8 | 2 | 283 | -27 | AluSg | Alu | SINE |
| 395755 | 395948 | - | 31.2 | 3576 | 3762 | -727 | L3 | CR1 | LINE |

  

|  |  |  |  |  |  |  |  |  |  |
| --- | --- | --- | --- | --- | --- | --- | --- | --- | --- |
| chr1:396598-397073 | | | 476 bp | | |  |  |  |  |
| Genome Start | Genome End | Strand | Div | Repeat Start | Repeat End | Repeat Left | Name | Family | Class |
| 396598 | 396669 | - | 31.1 | 3145 | 3217 | -2929 | L1M5 | L1 | LINE |
| 396670 | 396970 | - | 13.6 | 1 | 301 | -11 | AluSx | Alu | SINE |
| 396971 | 397073 | - | 31.1 | 3044 | 3144 | -3002 | L1M5 | L1 | LINE |

  

|  |  |  |  |  |  |  |  |  |  |
| --- | --- | --- | --- | --- | --- | --- | --- | --- | --- |
| chr1:403685-404341 | | | 657 bp | | |  |  |  |  |
| Genome Start | Genome End | Strand | Div | Repeat Start | Repeat End | Repeat Left | Name | Family | Class |
| 403685 | 403730 | + | 28.3 | 62 | 107 | -442 | MLT1H | MaLR | LTR |
| 403731 | 404050 | + | 9.3 | 1 | 310 | -1 | AluY | Alu | SINE |
| 404051 | 404341 | + | 26.7 | 93 | 375 | -167 | MLT1H | MaLR | LTR |

  

|  |  |  |  |  |  |  |  |  |  |
| --- | --- | --- | --- | --- | --- | --- | --- | --- | --- |
| chr1:404478-405316 | | | 839 bp | | |  |  |  |  |
| Genome Start | Genome End | Strand | Div | Repeat Start | Repeat End | Repeat Left | Name | Family | Class |
| 404478 | 404829 | - | 33.7 | 2624 | 2980 | -439 | L2 | L2 | LINE |
| 404830 | 405119 | + | 13.8 | 1 | 290 | -19 | AluSc | Alu | SINE |
| 405120 | 405316 | - | 33.7 | 2383 | 2623 | -796 | L2 | L2 | LINE |

  

|  |  |  |  |  |  |  |  |  |  |
| --- | --- | --- | --- | --- | --- | --- | --- | --- | --- |
| chr1:408231-409409 | | | 1179 bp | | |  |  |  |  |
| Genome Start | Genome End | Strand | Div | Repeat Start | Repeat End | Repeat Left | Name | Family | Class |
| 408231 | 408374 | + | 18.3 | 51 | 190 | -593 | LTR39 | ERV1 | LTR |
| 408375 | 408494 | + | 3.3 | 6034 | 6155 | 0 | L1PA4 | L1 | LINE |
| 408495 | 408796 | + | 18.3 | 191 | 459 | -324 | LTR39 | ERV1 | LTR |
| 408797 | 409095 | - | 15.4 | 1 | 299 | -13 | AluSx | Alu | SINE |
| 409096 | 409409 | + | 18.3 | 460 | 782 | -1 | LTR39 | ERV1 | LTR |

  

|  |  |  |  |  |  |  |  |  |  |
| --- | --- | --- | --- | --- | --- | --- | --- | --- | --- |
| chr1:420046-421115 | | | 1070 bp | | |  |  |  |  |
| Genome Start | Genome End | Strand | Div | Repeat Start | Repeat End | Repeat Left | Name | Family | Class |
| 420046 | 420467 | - | 6.6 | 334 | 781 | 0 | MER4E1 | ERV1 | LTR |
| 420468 | 420766 | + | 10.4 | 1 | 297 | -13 | AluSg | Alu | SINE |
| 420767 | 421115 | - | 6.6 | 1 | 333 | -448 | MER4E1 | ERV1 | LTR |

  

|  |  |  |  |  |  |  |  |  |  |
| --- | --- | --- | --- | --- | --- | --- | --- | --- | --- |
| chr1:422128-423151 | | | 917 bp | | |  |  |  |  |
| Genome Start | Genome End | Strand | Div | Repeat Start | Repeat End | Repeat Left | Name | Family | Class |
| 422128 | 422298 | - | 24.2 | 398 | 575 | -16 | MLT1K | MaLR | LTR |
| 422400 | 423057 | - | 11.2 | 1 | 644 | 0 | LTR9B | ERV1 | LTR |
| 423064 | 423151 | - | 26.2 | 4 | 89 | -502 | MLT1K | MaLR | LTR |

  

|  |  |  |  |  |  |  |  |  |  |
| --- | --- | --- | --- | --- | --- | --- | --- | --- | --- |
| chr1:435706-439136 | | | 2499 bp | | |  |  |  |  |
| Genome Start | Genome End | Strand | Div | Repeat Start | Repeat End | Repeat Left | Name | Family | Class |
| 435706 | 436280 | + | 31.2 | 5936 | 6537 | -1458 | L1MD3 | L1 | LINE |
| 436634 | 436760 | + | 32.5 | 6539 | 6668 | -1327 | L1MD3 | L1 | LINE |
| 437054 | 437136 | + | 32.9 | 6539 | 6620 | -1375 | L1MD3 | L1 | LINE |
| 437297 | 437373 | + | 30.3 | 6545 | 6620 | -1375 | L1MD3 | L1 | LINE |
| 437449 | 437885 | + | 24.7 | 6503 | 6945 | -840 | L1MC3 | L1 | LINE |
| 437926 | 438008 | + | 24.7 | 6946 | 7047 | -738 | L1MC3 | L1 | LINE |
| 438014 | 438191 | + | 8.7 | 134 | 311 | 0 | AluY | Alu | SINE |
| 438196 | 438266 | + | 24.2 | 7068 | 7148 | -847 | L1MD3 | L1 | LINE |
| 438267 | 438573 | + | 11.8 | 1 | 313 | 0 | AluSq | Alu | SINE |
| 438574 | 438714 | + | 24.2 | 7149 | 7285 | -710 | L1MD3 | L1 | LINE |
| 438717 | 438796 | + | 22.5 | 25 | 125 | -442 | MLT1F1 | MaLR | LTR |
| 438797 | 439136 | + | 25.3 | 7406 | 7753 | -242 | L1MD3 | L1 | LINE |

  

|  |  |  |  |  |  |  |  |  |  |
| --- | --- | --- | --- | --- | --- | --- | --- | --- | --- |
| chr1:450341-451634 | | | 1298 bp | | |  |  |  |  |
| Genome Start | Genome End | Strand | Div | Repeat Start | Repeat End | Repeat Left | Name | Family | Class |
| 450341 | 451136 | + | 15.6 | 2 | 836 | -64 | MER4D1 | ERV1 | LTR |
| 451133 | 451564 | - | 22.2 | 1 | 589 | -5 | MER4A | ERV1 | LTR |
| 451565 | 451634 | + | 15.3 | 824 | 900 | 0 | MER4D1 | ERV1 | LTR |

  

|  |  |  |  |  |  |  |  |  |  |
| --- | --- | --- | --- | --- | --- | --- | --- | --- | --- |
| chr1:457052-459258 | | | 2207 bp | | |  |  |  |  |
| Genome Start | Genome End | Strand | Div | Repeat Start | Repeat End | Repeat Left | Name | Family | Class |
| 457052 | 458409 | - | 23.5 | 6234 | 7784 | -1 | L1MC3 | L1 | LINE |
| 458410 | 458849 | - | 18.1 | 5 | 432 | 0 | MSTB1 | MaLR | LTR |
| 458850 | 459258 | - | 23.5 | 5763 | 6233 | -1552 | L1MC3 | L1 | LINE |

  

|  |  |  |  |  |  |  |  |  |  |
| --- | --- | --- | --- | --- | --- | --- | --- | --- | --- |
| chr1:514805-517227 | | | 2420 bp | | |  |  |  |  |
| Genome Start | Genome End | Strand | Div | Repeat Start | Repeat End | Repeat Left | Name | Family | Class |
| 514805 | 515617 | + | 16.8 | 4763 | 5605 | -695 | L1MA4 | L1 | LINE |
| 515618 | 515917 | + | 12.7 | 1 | 299 | -11 | AluSg | Alu | SINE |
| 515921 | 516233 | + | 8.9 | 1 | 304 | -5 | AluSc | Alu | SINE |
| 516234 | 516695 | + | 18.1 | 5606 | 6067 | -233 | L1MA4 | L1 | LINE |
| 516696 | 517006 | + | 5.1 | 1 | 311 | 0 | AluY | Alu | SINE |
| 517007 | 517227 | + | 18.1 | 6068 | 6300 | 0 | L1MA4 | L1 | LINE |

  

|  |  |  |  |  |  |  |  |  |  |
| --- | --- | --- | --- | --- | --- | --- | --- | --- | --- |
| chr1:543217-544287 | | | 1071 bp | | |  |  |  |  |
| Genome Start | Genome End | Strand | Div | Repeat Start | Repeat End | Repeat Left | Name | Family | Class |
| 543217 | 543565 | + | 6.6 | 1 | 333 | -448 | MER4E1 | ERV1 | LTR |
| 543566 | 543865 | - | 10.7 | 1 | 298 | -12 | AluSg | Alu | SINE |
| 543866 | 544287 | + | 6.6 | 334 | 781 | 0 | MER4E1 | ERV1 | LTR |

  

|  |  |  |  |  |  |  |  |  |  |
| --- | --- | --- | --- | --- | --- | --- | --- | --- | --- |
| chr1:551830-552248 | | | 379 bp | | |  |  |  |  |
| Genome Start | Genome End | Strand | Div | Repeat Start | Repeat End | Repeat Left | Name | Family | Class |
| 551830 | 551875 | + | 13.5 | 319 | 361 | -67 | MSTA | MaLR | LTR |
| 551876 | 552024 | - | 8.9 | 156 | 304 | -6 | AluSg | Alu | SINE |
| 552065 | 552184 | - | 8.9 | 2 | 155 | -155 | AluSg | Alu | SINE |
| 552185 | 552248 | + | 13.5 | 362 | 428 | 0 | MSTA | MaLR | LTR |

  

|  |  |  |  |  |  |  |  |  |  |
| --- | --- | --- | --- | --- | --- | --- | --- | --- | --- |
| chr1:560786-561963 | | | 1178 bp | | |  |  |  |  |
| Genome Start | Genome End | Strand | Div | Repeat Start | Repeat End | Repeat Left | Name | Family | Class |
| 560786 | 561099 | - | 18.1 | 460 | 782 | -1 | LTR39 | ERV1 | LTR |
| 561100 | 561398 | + | 15.1 | 1 | 299 | -13 | AluSx | Alu | SINE |
| 561399 | 561701 | - | 18.1 | 191 | 459 | -324 | LTR39 | ERV1 | LTR |
| 561702 | 561821 | - | 3.3 | 6034 | 6155 | 0 | L1PA4 | L1 | LINE |
| 561822 | 561963 | - | 18.1 | 53 | 190 | -593 | LTR39 | ERV1 | LTR |

  

|  |  |  |  |  |  |  |  |  |  |
| --- | --- | --- | --- | --- | --- | --- | --- | --- | --- |
| chr1:565852-566508 | | | 657 bp | | |  |  |  |  |
| Genome Start | Genome End | Strand | Div | Repeat Start | Repeat End | Repeat Left | Name | Family | Class |
| 565852 | 566141 | - | 27.0 | 93 | 375 | -167 | MLT1H | MaLR | LTR |
| 566142 | 566462 | - | 10.0 | 1 | 311 | 0 | AluY | Alu | SINE |
| 566463 | 566508 | - | 27.6 | 62 | 107 | -442 | MLT1H | MaLR | LTR |

  

|  |  |  |  |  |  |  |  |  |  |
| --- | --- | --- | --- | --- | --- | --- | --- | --- | --- |
| chr1:573125-573600 | | | 476 bp | | |  |  |  |  |
| Genome Start | Genome End | Strand | Div | Repeat Start | Repeat End | Repeat Left | Name | Family | Class |
| 573125 | 573227 | + | 31.1 | 3044 | 3144 | -3002 | L1M5 | L1 | LINE |
| 573228 | 573528 | + | 13.6 | 1 | 301 | -11 | AluSx | Alu | SINE |
| 573529 | 573600 | + | 31.1 | 3145 | 3217 | -2929 | L1M5 | L1 | LINE |

  

|  |  |  |  |  |  |  |  |  |  |
| --- | --- | --- | --- | --- | --- | --- | --- | --- | --- |
| chr1:574250-576253 | | | 1843 bp | | |  |  |  |  |
| Genome Start | Genome End | Strand | Div | Repeat Start | Repeat End | Repeat Left | Name | Family | Class |
| 574250 | 574442 | + | 31.2 | 3573 | 3761 | -728 | L3 | CR1 | LINE |
| 574443 | 574747 | + | 11.8 | 1 | 306 | 0 | AluSg | Alu | SINE |
| 574755 | 574824 | + | 31.2 | 3762 | 3831 | -658 | L3 | CR1 | LINE |
| 574842 | 574934 | - | 22.6 | 415 | 519 | -2 | LTR33 | ERVL | LTR |
| 574935 | 575083 | + | 16.1 | 1 | 149 | -283 | MSTB1 | MaLR | LTR |
| 575084 | 575375 | - | 10.3 | 6 | 296 | -17 | AluSq | Alu | SINE |
| 575376 | 575626 | + | 16.1 | 150 | 415 | -17 | MSTB1 | MaLR | LTR |
| 575670 | 575685 | + | 16.1 | 416 | 431 | -1 | MSTB1 | MaLR | LTR |
| 575686 | 575724 | - | 22.6 | 380 | 414 | -107 | LTR33 | ERVL | LTR |
| 575808 | 575976 | - | 28.5 | 1 | 173 | -348 | LTR33 | ERVL | LTR |
| 575988 | 576253 | + | 30.2 | 3856 | 4151 | -338 | L3 | CR1 | LINE |

  

|  |  |  |  |  |  |  |  |  |  |
| --- | --- | --- | --- | --- | --- | --- | --- | --- | --- |
| chr1:583967-584970 | | | 801 bp | | |  |  |  |  |
| Genome Start | Genome End | Strand | Div | Repeat Start | Repeat End | Repeat Left | Name | Family | Class |
| 583967 | 584268 | - | 37.3 | 3030 | 3355 | -23 | L2 | L2 | LINE |
| 584469 | 584734 | - | 3.8 | 5881 | 6150 | -4 | L1PA5 | L1 | LINE |
| 584738 | 584970 | - | 37.7 | 2572 | 2813 | -606 | L2 | L2 | LINE |

  

|  |  |  |  |  |  |  |  |  |  |
| --- | --- | --- | --- | --- | --- | --- | --- | --- | --- |
| chr1:585408-590870 | | | 5321 bp | | |  |  |  |  |
| Genome Start | Genome End | Strand | Div | Repeat Start | Repeat End | Repeat Left | Name | Family | Class |
| 585408 | 585992 | - | 30.2 | 5508 | 6122 | -118 | L1ME3B | L1 | LINE |
| 586051 | 587064 | - | 17.5 | 3687 | 4697 | -1449 | L1P4 | L1 | LINE |
| 587115 | 587855 | - | 25.5 | 3352 | 4119 | -2027 | L1ME3B | L1 | LINE |
| 587890 | 588013 | - | 12.7 | 6109 | 6238 | -53 | L1MA8 | L1 | LINE |
| 588014 | 588749 | + | 10.5 | 5556 | 6303 | -1 | L1MA2 | L1 | LINE |
| 588750 | 590731 | - | 15.2 | 4080 | 6108 | -183 | L1MA8 | L1 | LINE |
| 590732 | 590870 | - | 26.8 | 3222 | 3366 | -2780 | L1ME3B | L1 | LINE |

  

|  |  |  |  |  |  |  |  |  |  |
| --- | --- | --- | --- | --- | --- | --- | --- | --- | --- |
| chr1:597494-599988 | | | 2460 bp | | |  |  |  |  |
| Genome Start | Genome End | Strand | Div | Repeat Start | Repeat End | Repeat Left | Name | Family | Class |
| 597494 | 597968 | + | 18.7 | 3167 | 3646 | -2494 | L1M3 | L1 | LINE |
| 597985 | 598706 | - | 2.4 | 5254 | 5975 | -180 | L1PA4 | L1 | LINE |
| 598707 | 598884 | + | 3.9 | 5977 | 6155 | 0 | L1PA4 | L1 | LINE |
| 598904 | 599988 | + | 16.5 | 3657 | 4741 | -1399 | L1M3 | L1 | LINE |

  

|  |  |  |  |  |  |  |  |  |  |
| --- | --- | --- | --- | --- | --- | --- | --- | --- | --- |
| chr1:615834-616481 | | | 575 bp | | |  |  |  |  |
| Genome Start | Genome End | Strand | Div | Repeat Start | Repeat End | Repeat Left | Name | Family | Class |
| 615834 | 615975 | + | 34.1 | 21 | 155 | -113 | MIRb | MIR | SINE |
| 615984 | 616032 | + | 18.4 | 6055 | 6103 | -197 | L1MA4 | L1 | LINE |
| 616049 | 616295 | + | 15.8 | 6049 | 6295 | -5 | L1MA4 | L1 | LINE |
| 616345 | 616481 | + | 24.0 | 143 | 267 | -1 | MIRb | MIR | SINE |

  

|  |  |  |  |  |  |  |  |  |  |
| --- | --- | --- | --- | --- | --- | --- | --- | --- | --- |
| chr1:621368-621938 | | | 423 bp | | |  |  |  |  |
| Genome Start | Genome End | Strand | Div | Repeat Start | Repeat End | Repeat Left | Name | Family | Class |
| 621368 | 621496 | + | 29.1 | 866 | 995 | -1014 | HAL1b | L1 | LINE |
| 621574 | 621718 | + | 14.3 | 1 | 133 | 0 | FLAM\_C | Alu | SINE |
| 621790 | 621938 | + | 28.9 | 1398 | 1552 | -457 | HAL1b | L1 | LINE |

  

|  |  |  |  |  |  |  |  |  |  |
| --- | --- | --- | --- | --- | --- | --- | --- | --- | --- |
| chr1:623446-626243 | | | 2794 bp | | |  |  |  |  |
| Genome Start | Genome End | Strand | Div | Repeat Start | Repeat End | Repeat Left | Name | Family | Class |
| 623446 | 623684 | + | 29.8 | 2 | 249 | -306 | MLT1H1 | MaLR | LTR |
| 623685 | 624042 | - | 18.1 | 1 | 364 | 0 | THE1B | MaLR | LTR |
| 624043 | 625602 | - | 31.2 | 4 | 1735 | 0 | MLT-int | MaLR | LTR |
| 625607 | 625966 | - | 16.1 | 3 | 364 | 0 | THE1B | MaLR | LTR |
| 625967 | 626243 | + | 29.8 | 250 | 524 | -31 | MLT1H1 | MaLR | LTR |

  

|  |  |  |  |  |  |  |  |  |  |
| --- | --- | --- | --- | --- | --- | --- | --- | --- | --- |
| chr1:633260-635368 | | | 2114 bp | | |  |  |  |  |
| Genome Start | Genome End | Strand | Div | Repeat Start | Repeat End | Repeat Left | Name | Family | Class |
| 633260 | 633456 | + | 24.5 | 3777 | 3965 | -2164 | L1ME3B | L1 | LINE |
| 633450 | 633836 | + | 31.4 | 4488 | 4909 | -1220 | L1ME3B | L1 | LINE |
| 633839 | 634328 | + | 23.1 | 1 | 495 | -10 | MLT1D | MaLR | LTR |
| 634329 | 635368 | + | 27.2 | 4906 | 6042 | -181 | L1ME3B | L1 | LINE |

  

|  |  |  |  |  |  |  |  |  |  |
| --- | --- | --- | --- | --- | --- | --- | --- | --- | --- |
| chr1:646128-646969 | | | 842 bp | | |  |  |  |  |
| Genome Start | Genome End | Strand | Div | Repeat Start | Repeat End | Repeat Left | Name | Family | Class |
| 646128 | 646219 | + | 25.7 | 1946 | 2024 | -4765 | L1M2c | L1 | LINE |
| 646220 | 646534 | + | 12.7 | 1 | 309 | -1 | AluSg | Alu | SINE |
| 646535 | 646969 | + | 21.8 | 2025 | 2563 | -4226 | L1M2c | L1 | LINE |

  

|  |  |  |  |  |  |  |  |  |  |
| --- | --- | --- | --- | --- | --- | --- | --- | --- | --- |
| chr1:666367-667853 | | | 1487 bp | | |  |  |  |  |
| Genome Start | Genome End | Strand | Div | Repeat Start | Repeat End | Repeat Left | Name | Family | Class |
| 666367 | 666872 | + | 20.7 | 18 | 525 | -410 | MER21C | ERV1 | LTR |
| 666873 | 667179 | - | 19.5 | 2 | 294 | -18 | AluJb | Alu | SINE |
| 667180 | 667249 | + | 20.7 | 526 | 603 | -332 | MER21C | ERV1 | LTR |
| 667250 | 667552 | + | 20.5 | 1 | 302 | -10 | AluJo | Alu | SINE |
| 667553 | 667853 | + | 20.7 | 604 | 935 | 0 | MER21C | ERV1 | LTR |

  

|  |  |  |  |  |  |  |  |  |  |
| --- | --- | --- | --- | --- | --- | --- | --- | --- | --- |
| chr1:672609-673087 | | | 432 bp | | |  |  |  |  |
| Genome Start | Genome End | Strand | Div | Repeat Start | Repeat End | Repeat Left | Name | Family | Class |
| 672609 | 672710 | - | 22.2 | 72 | 167 | -145 | AluJo | Alu | SINE |
| 672711 | 672980 | + | 13.8 | 1 | 275 | -37 | AluSx | Alu | SINE |
| 673028 | 673087 | - | 22.2 | 24 | 71 | -241 | AluJo | Alu | SINE |

  

|  |  |  |  |  |  |  |  |  |  |
| --- | --- | --- | --- | --- | --- | --- | --- | --- | --- |
| chr1:673147-674322 | | | 1128 bp | | |  |  |  |  |
| Genome Start | Genome End | Strand | Div | Repeat Start | Repeat End | Repeat Left | Name | Family | Class |
| 673147 | 673177 | + | 17.9 | 5476 | 5506 | -678 | L1MB8 | L1 | LINE |
| 673178 | 673469 | + | 19.0 | 1 | 293 | -19 | AluJb | Alu | SINE |
| 673470 | 673543 | + | 17.9 | 5507 | 5570 | -614 | L1MB8 | L1 | LINE |
| 673592 | 673702 | - | 18.0 | 4 | 114 | -19 | FLAM\_A | Alu | SINE |
| 673703 | 674322 | + | 20.2 | 5552 | 6173 | -5 | L1MB8 | L1 | LINE |

  

|  |  |  |  |  |  |  |  |  |  |
| --- | --- | --- | --- | --- | --- | --- | --- | --- | --- |
| chr1:676870-677445 | | | 576 bp | | |  |  |  |  |
| Genome Start | Genome End | Strand | Div | Repeat Start | Repeat End | Repeat Left | Name | Family | Class |
| 676870 | 677032 | - | 19.9 | 137 | 299 | -13 | AluJb | Alu | SINE |
| 677033 | 677318 | - | 10.1 | 1 | 296 | -13 | AluSg1 | Alu | SINE |
| 677319 | 677445 | - | 19.9 | 12 | 136 | -176 | AluJb | Alu | SINE |

  

|  |  |  |  |  |  |  |  |  |  |
| --- | --- | --- | --- | --- | --- | --- | --- | --- | --- |
| chr1:678119-679588 | | | 1470 bp | | |  |  |  |  |
| Genome Start | Genome End | Strand | Div | Repeat Start | Repeat End | Repeat Left | Name | Family | Class |
| 678119 | 678377 | + | 20.2 | 18 | 269 | -666 | MER21C | ERV1 | LTR |
| 678378 | 678671 | - | 14.3 | 1 | 293 | -20 | AluSq | Alu | SINE |
| 678672 | 678981 | + | 20.2 | 270 | 603 | -332 | MER21C | ERV1 | LTR |
| 678982 | 679288 | + | 17.4 | 1 | 304 | -8 | AluJo | Alu | SINE |
| 679289 | 679588 | + | 20.2 | 604 | 934 | -1 | MER21C | ERV1 | LTR |

  

|  |  |  |  |  |  |  |  |  |  |
| --- | --- | --- | --- | --- | --- | --- | --- | --- | --- |
| chr1:683088-683433 | | | 346 bp | | |  |  |  |  |
| Genome Start | Genome End | Strand | Div | Repeat Start | Repeat End | Repeat Left | Name | Family | Class |
| 683088 | 683187 | + | 31.0 | 5483 | 5575 | -2233 | L1MC4 | L1 | LINE |
| 683188 | 683310 | - | 16.5 | 4 | 126 | -7 | FLAM\_C | Alu | SINE |
| 683311 | 683433 | + | 31.0 | 5576 | 5690 | -2118 | L1MC4 | L1 | LINE |

  

|  |  |  |  |  |  |  |  |  |  |
| --- | --- | --- | --- | --- | --- | --- | --- | --- | --- |
| chr1:700034-700572 | | | 539 bp | | |  |  |  |  |
| Genome Start | Genome End | Strand | Div | Repeat Start | Repeat End | Repeat Left | Name | Family | Class |
| 700034 | 700169 | + | 14.7 | 1 | 136 | -166 | AluSx | Alu | SINE |
| 700170 | 700473 | + | 11.5 | 1 | 305 | -8 | AluSp | Alu | SINE |
| 700474 | 700572 | + | 14.7 | 137 | 302 | 0 | AluSx | Alu | SINE |

  

|  |  |  |  |  |  |  |  |  |  |
| --- | --- | --- | --- | --- | --- | --- | --- | --- | --- |
| chr1:720605-721343 | | | 739 bp | | |  |  |  |  |
| Genome Start | Genome End | Strand | Div | Repeat Start | Repeat End | Repeat Left | Name | Family | Class |
| 720605 | 720852 | + | 25.4 | 4 | 315 | -208 | Charlie1b | MER1\_type | DNA |
| 720853 | 721175 | - | 9.6 | 1 | 304 | 0 | AluSq | Alu | SINE |
| 721176 | 721343 | + | 25.4 | 316 | 523 | 0 | Charlie1b | MER1\_type | DNA |

  

|  |  |  |  |  |  |  |  |  |  |
| --- | --- | --- | --- | --- | --- | --- | --- | --- | --- |
| chr1:740416-741376 | | | 587 bp | | |  |  |  |  |
| Genome Start | Genome End | Strand | Div | Repeat Start | Repeat End | Repeat Left | Name | Family | Class |
| 740416 | 740600 | + | 33.0 | 1555 | 1749 | -4466 | L1MC4 | L1 | LINE |
| 740959 | 741058 | + | 26.0 | 2223 | 2322 | -3893 | L1M5 | L1 | LINE |
| 741075 | 741376 | + | 30.3 | 3031 | 3349 | -2865 | L1MC4 | L1 | LINE |

  

|  |  |  |  |  |  |  |  |  |  |
| --- | --- | --- | --- | --- | --- | --- | --- | --- | --- |
| chr1:796291-797035 | | | 745 bp | | |  |  |  |  |
| Genome Start | Genome End | Strand | Div | Repeat Start | Repeat End | Repeat Left | Name | Family | Class |
| 796291 | 796503 | - | 22.1 | 3783 | 4016 | -2130 | L1M4 | L1 | LINE |
| 796504 | 796668 | - | 20.0 | 1 | 165 | -1 | FRAM | Alu | SINE |
| 796669 | 797035 | - | 22.1 | 3398 | 3782 | -2364 | L1M4 | L1 | LINE |

  

|  |  |  |  |  |  |  |  |  |  |
| --- | --- | --- | --- | --- | --- | --- | --- | --- | --- |
| chr1:1371265-1371868 | | | 604 bp | | |  |  |  |  |
| Genome Start | Genome End | Strand | Div | Repeat Start | Repeat End | Repeat Left | Name | Family | Class |
| 1371265 | 1371403 | + | 17.9 | 1 | 133 | -179 | AluSx | Alu | SINE |
| 1371404 | 1371700 | + | 10.5 | 3 | 299 | -14 | AluSq | Alu | SINE |
| 1371701 | 1371868 | + | 17.9 | 134 | 309 | -3 | AluSx | Alu | SINE |

  

|  |  |  |  |  |  |  |  |  |  |
| --- | --- | --- | --- | --- | --- | --- | --- | --- | --- |
| chr1:1564044-1564650 | | | 607 bp | | |  |  |  |  |
| Genome Start | Genome End | Strand | Div | Repeat Start | Repeat End | Repeat Left | Name | Family | Class |
| 1564044 | 1564155 | + | 16.6 | 1 | 112 | -200 | AluJo | Alu | SINE |
| 1564156 | 1564464 | - | 7.8 | 1 | 307 | -3 | AluSg | Alu | SINE |
| 1564465 | 1564650 | + | 16.6 | 113 | 292 | -20 | AluJo | Alu | SINE |

  

|  |  |  |  |  |  |  |  |  |  |
| --- | --- | --- | --- | --- | --- | --- | --- | --- | --- |
| chr1:1610816-1611753 | | | 929 bp | | |  |  |  |  |
| Genome Start | Genome End | Strand | Div | Repeat Start | Repeat End | Repeat Left | Name | Family | Class |
| 1610816 | 1611003 | + | 23.8 | 4999 | 5190 | -956 | L1M5 | L1 | LINE |
| 1611004 | 1611301 | + | 10.7 | 1 | 298 | -14 | AluSx | Alu | SINE |
| 1611311 | 1611607 | + | 10.8 | 13 | 311 | -2 | AluSp | Alu | SINE |
| 1611608 | 1611753 | + | 23.8 | 5191 | 5324 | -822 | L1M5 | L1 | LINE |

  

|  |  |  |  |  |  |  |  |  |  |
| --- | --- | --- | --- | --- | --- | --- | --- | --- | --- |
| chr1:1631202-1633145 | | | 1942 bp | | |  |  |  |  |
| Genome Start | Genome End | Strand | Div | Repeat Start | Repeat End | Repeat Left | Name | Family | Class |
| 1631202 | 1632649 | + | 20.4 | 17 | 1610 | -4774 | L1M4c | L1 | LINE |
| 1632651 | 1632900 | + | 15.6 | 50 | 300 | -12 | AluJo | Alu | SINE |
| 1632902 | 1633145 | + | 19.3 | 1604 | 1842 | -4542 | L1M4c | L1 | LINE |

  

|  |  |  |  |  |  |  |  |  |  |
| --- | --- | --- | --- | --- | --- | --- | --- | --- | --- |
| chr1:12783471-12784402 | | | 932 bp | | |  |  |  |  |
| Genome Start | Genome End | Strand | Div | Repeat Start | Repeat End | Repeat Left | Name | Family | Class |
| 12783471 | 12783769 | - | 18.1 | 572 | 885 | 0 | MER41G | ERV1 | LTR |
| 12783770 | 12784087 | - | 11.0 | 1 | 324 | 0 | AluSp | Alu | SINE |
| 12784088 | 12784402 | - | 18.1 | 264 | 571 | -314 | MER41G | ERV1 | LTR |

  

|  |  |  |  |  |  |  |  |  |  |
| --- | --- | --- | --- | --- | --- | --- | --- | --- | --- |
| chr1:12812960-12813576 | | | 617 bp | | |  |  |  |  |
| Genome Start | Genome End | Strand | Div | Repeat Start | Repeat End | Repeat Left | Name | Family | Class |
| 12812960 | 12813147 | - | 20.4 | 126 | 299 | -13 | AluJb | Alu | SINE |
| 12813148 | 12813455 | + | 15.9 | 1 | 296 | -16 | AluSx | Alu | SINE |
| 12813456 | 12813576 | - | 20.4 | 5 | 125 | -187 | AluJb | Alu | SINE |

  

|  |  |  |  |  |  |  |  |  |  |
| --- | --- | --- | --- | --- | --- | --- | --- | --- | --- |
| chr1:12818747-12819623 | | | 877 bp | | |  |  |  |  |
| Genome Start | Genome End | Strand | Div | Repeat Start | Repeat End | Repeat Left | Name | Family | Class |
| 12818747 | 12818967 | + | 21.9 | 5473 | 5691 | -609 | L1MA4 | L1 | LINE |
| 12818968 | 12819069 | - | 15.7 | 2 | 103 | -209 | AluSq/x | Alu | SINE |
| 12819070 | 12819623 | + | 18.9 | 5685 | 6250 | -50 | L1MA4 | L1 | LINE |

  

|  |  |  |  |  |  |  |  |  |  |
| --- | --- | --- | --- | --- | --- | --- | --- | --- | --- |
| chr1:12825353-12825973 | | | 621 bp | | |  |  |  |  |
| Genome Start | Genome End | Strand | Div | Repeat Start | Repeat End | Repeat Left | Name | Family | Class |
| 12825353 | 12825570 | - | 20.2 | 126 | 311 | -1 | AluJb | Alu | SINE |
| 12825571 | 12825856 | + | 17.5 | 13 | 292 | -20 | AluSx | Alu | SINE |
| 12825857 | 12825973 | - | 20.2 | 9 | 125 | -187 | AluJb | Alu | SINE |

  

|  |  |  |  |  |  |  |  |  |  |
| --- | --- | --- | --- | --- | --- | --- | --- | --- | --- |
| chr1:12848940-12849865 | | | 926 bp | | |  |  |  |  |
| Genome Start | Genome End | Strand | Div | Repeat Start | Repeat End | Repeat Left | Name | Family | Class |
| 12848940 | 12849233 | - | 18.5 | 577 | 885 | 0 | MER41G | ERV1 | LTR |
| 12849234 | 12849550 | - | 12.6 | 1 | 318 | 0 | AluSp | Alu | SINE |
| 12849551 | 12849865 | - | 18.5 | 264 | 576 | -309 | MER41G | ERV1 | LTR |

  

|  |  |  |  |  |  |  |  |  |  |
| --- | --- | --- | --- | --- | --- | --- | --- | --- | --- |
| chr1:12867768-12868330 | | | 563 bp | | |  |  |  |  |
| Genome Start | Genome End | Strand | Div | Repeat Start | Repeat End | Repeat Left | Name | Family | Class |
| 12867768 | 12867912 | - | 20.1 | 156 | 298 | -14 | AluJb | Alu | SINE |
| 12867913 | 12868209 | + | 16.8 | 7 | 292 | -20 | AluSx | Alu | SINE |
| 12868210 | 12868330 | - | 20.1 | 6 | 155 | -157 | AluJb | Alu | SINE |

  

|  |  |  |  |  |  |  |  |  |  |
| --- | --- | --- | --- | --- | --- | --- | --- | --- | --- |
| chr1:12873500-12874375 | | | 876 bp | | |  |  |  |  |
| Genome Start | Genome End | Strand | Div | Repeat Start | Repeat End | Repeat Left | Name | Family | Class |
| 12873500 | 12873720 | + | 22.4 | 5473 | 5691 | -609 | L1MA4 | L1 | LINE |
| 12873721 | 12873823 | - | 12.6 | 1 | 103 | -209 | AluSq/x | Alu | SINE |
| 12873824 | 12874375 | + | 19.3 | 5686 | 6250 | -50 | L1MA4 | L1 | LINE |

  

|  |  |  |  |  |  |  |  |  |  |
| --- | --- | --- | --- | --- | --- | --- | --- | --- | --- |
| chr1:12879805-12880410 | | | 606 bp | | |  |  |  |  |
| Genome Start | Genome End | Strand | Div | Repeat Start | Repeat End | Repeat Left | Name | Family | Class |
| 12879805 | 12879991 | - | 21.5 | 126 | 292 | -20 | AluJb | Alu | SINE |
| 12879992 | 12880274 | + | 16.3 | 1 | 282 | -30 | AluSx | Alu | SINE |
| 12880275 | 12880410 | - | 21.5 | 5 | 125 | -187 | AluJb | Alu | SINE |

  

|  |  |  |  |  |  |  |  |  |  |
| --- | --- | --- | --- | --- | --- | --- | --- | --- | --- |
| chr1:12887566-12892958 | | | 5386 bp | | |  |  |  |  |
| Genome Start | Genome End | Strand | Div | Repeat Start | Repeat End | Repeat Left | Name | Family | Class |
| 12887566 | 12890050 | + | 5.4 | 13 | 2483 | -3663 | L1PA4 | L1 | LINE |
| 12890051 | 12890128 | - | 5.1 | 1162 | 1239 | -339 | LTR12C | ERV1 | LTR |
| 12890129 | 12892707 | + | 5.4 | 2484 | 5047 | -1099 | L1PA4 | L1 | LINE |
| 12892715 | 12892958 | + | 5.3 | 5912 | 6155 | 0 | L1PA4 | L1 | LINE |

  

|  |  |  |  |  |  |  |  |  |  |
| --- | --- | --- | --- | --- | --- | --- | --- | --- | --- |
| chr1:12917372-12917949 | | | 578 bp | | |  |  |  |  |
| Genome Start | Genome End | Strand | Div | Repeat Start | Repeat End | Repeat Left | Name | Family | Class |
| 12917372 | 12917542 | - | 14.0 | 126 | 300 | -12 | AluSx | Alu | SINE |
| 12917543 | 12917767 | - | 5.3 | 5923 | 6151 | -4 | L1PA4 | L1 | LINE |
| 12917768 | 12917814 | + | 6.4 | 5885 | 5931 | -223 | L1PA4 | L1 | LINE |
| 12917815 | 12917949 | - | 14.1 | 1 | 135 | -177 | AluSx | Alu | SINE |

  

|  |  |  |  |  |  |  |  |  |  |
| --- | --- | --- | --- | --- | --- | --- | --- | --- | --- |
| chr1:12926935-12927549 | | | 615 bp | | |  |  |  |  |
| Genome Start | Genome End | Strand | Div | Repeat Start | Repeat End | Repeat Left | Name | Family | Class |
| 12926935 | 12927122 | - | 21.1 | 126 | 299 | -13 | AluJb | Alu | SINE |
| 12927123 | 12927428 | + | 16.0 | 1 | 294 | -18 | AluSx | Alu | SINE |
| 12927429 | 12927549 | - | 21.1 | 5 | 125 | -187 | AluJb | Alu | SINE |

  

|  |  |  |  |  |  |  |  |  |  |
| --- | --- | --- | --- | --- | --- | --- | --- | --- | --- |
| chr1:12929098-12929711 | | | 614 bp | | |  |  |  |  |
| Genome Start | Genome End | Strand | Div | Repeat Start | Repeat End | Repeat Left | Name | Family | Class |
| 12929098 | 12929283 | - | 20.2 | 126 | 297 | -15 | AluJb | Alu | SINE |
| 12929284 | 12929590 | + | 15.6 | 1 | 295 | -17 | AluSx | Alu | SINE |
| 12929591 | 12929711 | - | 20.2 | 5 | 125 | -187 | AluJb | Alu | SINE |

  

|  |  |  |  |  |  |  |  |  |  |
| --- | --- | --- | --- | --- | --- | --- | --- | --- | --- |
| chr1:12934862-12935429 | | | 568 bp | | |  |  |  |  |
| Genome Start | Genome End | Strand | Div | Repeat Start | Repeat End | Repeat Left | Name | Family | Class |
| 12934862 | 12934998 | + | 15.8 | 1 | 136 | -176 | AluSx | Alu | SINE |
| 12934999 | 12935271 | + | 8.4 | 35 | 307 | -4 | AluY | Alu | SINE |
| 12935272 | 12935429 | + | 15.8 | 137 | 293 | -19 | AluSx | Alu | SINE |

  

|  |  |  |  |  |  |  |  |  |  |
| --- | --- | --- | --- | --- | --- | --- | --- | --- | --- |
| chr1:12943709-12945564 | | | 1742 bp | | |  |  |  |  |
| Genome Start | Genome End | Strand | Div | Repeat Start | Repeat End | Repeat Left | Name | Family | Class |
| 12943709 | 12944285 | + | 10.2 | 1 | 599 | -182 | MER4E1 | ERV1 | LTR |
| 12944368 | 12944449 | - | 25.6 | 218 | 299 | -3 | AluSg/x | Alu | SINE |
| 12944451 | 12944722 | - | 13.5 | 1 | 266 | -46 | AluSx | Alu | SINE |
| 12944735 | 12945035 | - | 13.9 | 12 | 312 | 0 | AluSx | Alu | SINE |
| 12945055 | 12945156 | + | 10.4 | 589 | 683 | -98 | MER4E1 | ERV1 | LTR |
| 12945157 | 12945460 | - | 5.7 | 1 | 301 | -10 | AluY | Alu | SINE |
| 12945461 | 12945564 | + | 10.4 | 684 | 781 | 0 | MER4E1 | ERV1 | LTR |

  

|  |  |  |  |  |  |  |  |  |  |
| --- | --- | --- | --- | --- | --- | --- | --- | --- | --- |
| chr1:12956288-12956926 | | | 639 bp | | |  |  |  |  |
| Genome Start | Genome End | Strand | Div | Repeat Start | Repeat End | Repeat Left | Name | Family | Class |
| 12956288 | 12956479 | - | 25.4 | 126 | 301 | -1 | AluJo | Alu | SINE |
| 12956480 | 12956791 | - | 15.4 | 1 | 312 | 0 | AluSx | Alu | SINE |
| 12956792 | 12956926 | - | 25.4 | 1 | 125 | -177 | AluJo | Alu | SINE |

  

|  |  |  |  |  |  |  |  |  |  |
| --- | --- | --- | --- | --- | --- | --- | --- | --- | --- |
| chr1:13037267-13037881 | | | 615 bp | | |  |  |  |  |
| Genome Start | Genome End | Strand | Div | Repeat Start | Repeat End | Repeat Left | Name | Family | Class |
| 13037267 | 13037454 | - | 21.1 | 126 | 299 | -13 | AluJb | Alu | SINE |
| 13037455 | 13037760 | + | 16.0 | 1 | 294 | -18 | AluSx | Alu | SINE |
| 13037761 | 13037881 | - | 21.1 | 5 | 125 | -187 | AluJb | Alu | SINE |

  

|  |  |  |  |  |  |  |  |  |  |
| --- | --- | --- | --- | --- | --- | --- | --- | --- | --- |
| chr1:13039429-13040042 | | | 614 bp | | |  |  |  |  |
| Genome Start | Genome End | Strand | Div | Repeat Start | Repeat End | Repeat Left | Name | Family | Class |
| 13039429 | 13039614 | - | 20.2 | 126 | 297 | -15 | AluJb | Alu | SINE |
| 13039615 | 13039921 | + | 15.9 | 1 | 295 | -17 | AluSx | Alu | SINE |
| 13039922 | 13040042 | - | 20.2 | 5 | 125 | -187 | AluJb | Alu | SINE |

  

|  |  |  |  |  |  |  |  |  |  |
| --- | --- | --- | --- | --- | --- | --- | --- | --- | --- |
| chr1:13045193-13045760 | | | 568 bp | | |  |  |  |  |
| Genome Start | Genome End | Strand | Div | Repeat Start | Repeat End | Repeat Left | Name | Family | Class |
| 13045193 | 13045329 | + | 15.5 | 1 | 136 | -176 | AluSx | Alu | SINE |
| 13045330 | 13045602 | + | 8.4 | 35 | 307 | -4 | AluY | Alu | SINE |
| 13045603 | 13045760 | + | 15.5 | 137 | 293 | -19 | AluSx | Alu | SINE |

  

|  |  |  |  |  |  |  |  |  |  |
| --- | --- | --- | --- | --- | --- | --- | --- | --- | --- |
| chr1:13061190-13061831 | | | 642 bp | | |  |  |  |  |
| Genome Start | Genome End | Strand | Div | Repeat Start | Repeat End | Repeat Left | Name | Family | Class |
| 13061190 | 13061385 | - | 25.7 | 126 | 302 | 0 | AluJo | Alu | SINE |
| 13061386 | 13061696 | - | 15.1 | 1 | 311 | -1 | AluSx | Alu | SINE |
| 13061697 | 13061831 | - | 25.7 | 1 | 125 | -177 | AluJo | Alu | SINE |

  

|  |  |  |  |  |  |  |  |  |  |
| --- | --- | --- | --- | --- | --- | --- | --- | --- | --- |
| chr1:13097292-13097917 | | | 626 bp | | |  |  |  |  |
| Genome Start | Genome End | Strand | Div | Repeat Start | Repeat End | Repeat Left | Name | Family | Class |
| 13097292 | 13097510 | - | 20.5 | 126 | 312 | 0 | AluJb | Alu | SINE |
| 13097511 | 13097796 | + | 18.2 | 13 | 292 | -20 | AluSx | Alu | SINE |
| 13097797 | 13097917 | - | 20.5 | 5 | 125 | -187 | AluJb | Alu | SINE |

  

|  |  |  |  |  |  |  |  |  |  |
| --- | --- | --- | --- | --- | --- | --- | --- | --- | --- |
| chr1:13216090-13216723 | | | 652 bp | | |  |  |  |  |
| Genome Start | Genome End | Strand | Div | Repeat Start | Repeat End | Repeat Left | Name | Family | Class |
| 13216090 | 13216343 | + | 17.7 | 1 | 248 | -474 | MER72 | ERV1 | LTR |
| 13216326 | 13216375 | + | 15.5 | 608 | 655 | -67 | MER72 | ERV1 | LTR |
| 13216376 | 13216679 | - | 12.2 | 1 | 309 | -3 | AluSx | Alu | SINE |
| 13216680 | 13216723 | + | 15.5 | 656 | 696 | -26 | MER72 | ERV1 | LTR |

  

|  |  |  |  |  |  |  |  |  |  |
| --- | --- | --- | --- | --- | --- | --- | --- | --- | --- |
| chr1:13220424-13221556 | | | 1097 bp | | |  |  |  |  |
| Genome Start | Genome End | Strand | Div | Repeat Start | Repeat End | Repeat Left | Name | Family | Class |
| 13220424 | 13220604 | - | 14.9 | 121 | 304 | -8 | AluSx | Alu | SINE |
| 13220637 | 13220707 | - | 15.5 | 226 | 298 | -2 | AluSg | Alu | SINE |
| 13220708 | 13221179 | - | 2.5 | 1 | 472 | 0 | LTR7Y | ERV1 | LTR |
| 13221180 | 13221418 | - | 9.2 | 1 | 240 | -70 | AluSg | Alu | SINE |
| 13221423 | 13221556 | - | 14.4 | 1 | 132 | -180 | AluSx | Alu | SINE |

  

|  |  |  |  |  |  |  |  |  |  |
| --- | --- | --- | --- | --- | --- | --- | --- | --- | --- |
| chr1:13226983-13227551 | | | 569 bp | | |  |  |  |  |
| Genome Start | Genome End | Strand | Div | Repeat Start | Repeat End | Repeat Left | Name | Family | Class |
| 13226983 | 13227141 | - | 15.8 | 137 | 294 | -18 | AluSx | Alu | SINE |
| 13227142 | 13227414 | - | 8.4 | 35 | 307 | -4 | AluY | Alu | SINE |
| 13227415 | 13227551 | - | 15.8 | 1 | 136 | -176 | AluSx | Alu | SINE |

  

|  |  |  |  |  |  |  |  |  |  |
| --- | --- | --- | --- | --- | --- | --- | --- | --- | --- |
| chr1:13234864-13235478 | | | 615 bp | | |  |  |  |  |
| Genome Start | Genome End | Strand | Div | Repeat Start | Repeat End | Repeat Left | Name | Family | Class |
| 13234864 | 13234984 | + | 21.1 | 5 | 125 | -187 | AluJb | Alu | SINE |
| 13234985 | 13235290 | - | 16.0 | 1 | 294 | -18 | AluSx | Alu | SINE |
| 13235291 | 13235478 | + | 21.1 | 126 | 299 | -13 | AluJb | Alu | SINE |

  

|  |  |  |  |  |  |  |  |  |  |
| --- | --- | --- | --- | --- | --- | --- | --- | --- | --- |
| chr1:13244463-13245044 | | | 582 bp | | |  |  |  |  |
| Genome Start | Genome End | Strand | Div | Repeat Start | Repeat End | Repeat Left | Name | Family | Class |
| 13244463 | 13244597 | + | 14.1 | 1 | 135 | -177 | AluSx | Alu | SINE |
| 13244598 | 13244644 | - | 4.3 | 5885 | 5931 | -223 | L1PA4 | L1 | LINE |
| 13244645 | 13244869 | + | 5.3 | 5923 | 6151 | -4 | L1PA4 | L1 | LINE |
| 13244870 | 13245044 | + | 13.7 | 126 | 304 | -8 | AluSx | Alu | SINE |

  

|  |  |  |  |  |  |  |  |  |  |
| --- | --- | --- | --- | --- | --- | --- | --- | --- | --- |
| chr1:13254109-13254562 | | | 405 bp | | |  |  |  |  |
| Genome Start | Genome End | Strand | Div | Repeat Start | Repeat End | Repeat Left | Name | Family | Class |
| 13254109 | 13254135 | - | 18.2 | 128 | 146 | -635 | MER4E1 | ERV1 | LTR |
| 13254185 | 13254470 | - | 13.3 | 1 | 287 | -26 | AluSp | Alu | SINE |
| 13254471 | 13254562 | - | 18.2 | 37 | 127 | -654 | MER4E1 | ERV1 | LTR |

  

|  |  |  |  |  |  |  |  |  |  |
| --- | --- | --- | --- | --- | --- | --- | --- | --- | --- |
| chr1:13269439-13274839 | | | 5394 bp | | |  |  |  |  |
| Genome Start | Genome End | Strand | Div | Repeat Start | Repeat End | Repeat Left | Name | Family | Class |
| 13269439 | 13269682 | - | 4.9 | 5912 | 6155 | 0 | L1PA4 | L1 | LINE |
| 13269690 | 13272268 | - | 5.4 | 2490 | 5049 | -1097 | L1PA4 | L1 | LINE |
| 13272269 | 13272346 | + | 3.9 | 1162 | 1239 | -339 | LTR12C | ERV1 | LTR |
| 13272347 | 13274839 | - | 5.4 | 15 | 2489 | -3657 | L1PA4 | L1 | LINE |

  

|  |  |  |  |  |  |  |  |  |  |
| --- | --- | --- | --- | --- | --- | --- | --- | --- | --- |
| chr1:13277777-13278100 | | | 324 bp | | |  |  |  |  |
| Genome Start | Genome End | Strand | Div | Repeat Start | Repeat End | Repeat Left | Name | Family | Class |
| 13277777 | 13277794 | + | 26.3 | 398 | 411 | -78 | LTR45B | ERV1 | LTR |
| 13277795 | 13278086 | - | 16.8 | 1 | 292 | -18 | AluSg | Alu | SINE |
| 13278087 | 13278100 | + | 26.3 | 412 | 421 | -68 | LTR45B | ERV1 | LTR |

  

|  |  |  |  |  |  |  |  |  |  |
| --- | --- | --- | --- | --- | --- | --- | --- | --- | --- |
| chr1:13281993-13282598 | | | 606 bp | | |  |  |  |  |
| Genome Start | Genome End | Strand | Div | Repeat Start | Repeat End | Repeat Left | Name | Family | Class |
| 13281993 | 13282130 | + | 21.5 | 5 | 126 | -176 | AluJb | Alu | SINE |
| 13282131 | 13282411 | - | 15.7 | 1 | 280 | -32 | AluSx | Alu | SINE |
| 13282412 | 13282598 | + | 21.5 | 127 | 294 | -8 | AluJb | Alu | SINE |

  

|  |  |  |  |  |  |  |  |  |  |
| --- | --- | --- | --- | --- | --- | --- | --- | --- | --- |
| chr1:13294064-13294624 | | | 561 bp | | |  |  |  |  |
| Genome Start | Genome End | Strand | Div | Repeat Start | Repeat End | Repeat Left | Name | Family | Class |
| 13294064 | 13294184 | + | 20.4 | 5 | 154 | -148 | AluJb | Alu | SINE |
| 13294185 | 13294479 | - | 17.1 | 7 | 292 | -20 | AluSx | Alu | SINE |
| 13294480 | 13294624 | + | 20.4 | 155 | 297 | -5 | AluJb | Alu | SINE |

  

|  |  |  |  |  |  |  |  |  |  |
| --- | --- | --- | --- | --- | --- | --- | --- | --- | --- |
| chr1:13351750-13352362 | | | 613 bp | | |  |  |  |  |
| Genome Start | Genome End | Strand | Div | Repeat Start | Repeat End | Repeat Left | Name | Family | Class |
| 13351750 | 13351885 | + | 14.9 | 1 | 136 | -176 | AluSx | Alu | SINE |
| 13351886 | 13352187 | + | 6.0 | 1 | 302 | -9 | AluY | Alu | SINE |
| 13352188 | 13352362 | + | 14.9 | 137 | 309 | -3 | AluSx | Alu | SINE |

  

|  |  |  |  |  |  |  |  |  |  |
| --- | --- | --- | --- | --- | --- | --- | --- | --- | --- |
| chr1:13360855-13361472 | | | 637 bp | | |  |  |  |  |
| Genome Start | Genome End | Strand | Div | Repeat Start | Repeat End | Repeat Left | Name | Family | Class |
| 13360855 | 13361101 | + | 17.7 | 8 | 249 | -473 | MER72 | ERV1 | LTR |
| 13361083 | 13361132 | + | 19.1 | 608 | 655 | -67 | MER72 | ERV1 | LTR |
| 13361133 | 13361428 | - | 10.1 | 1 | 301 | -11 | AluSx | Alu | SINE |
| 13361429 | 13361472 | + | 19.1 | 656 | 696 | -26 | MER72 | ERV1 | LTR |

  

|  |  |  |  |  |  |  |  |  |  |
| --- | --- | --- | --- | --- | --- | --- | --- | --- | --- |
| chr1:13361573-13362992 | | | 1420 bp | | |  |  |  |  |
| Genome Start | Genome End | Strand | Div | Repeat Start | Repeat End | Repeat Left | Name | Family | Class |
| 13361573 | 13361774 | - | 10.8 | 594 | 781 | 0 | MER4E1 | ERV1 | LTR |
| 13361775 | 13362084 | + | 11.0 | 2 | 311 | -1 | AluSx | Alu | SINE |
| 13362085 | 13362095 | - | 10.8 | 594 | 593 | -188 | MER4E1 | ERV1 | LTR |
| 13362096 | 13362411 | + | 14.8 | 1 | 310 | -2 | AluSx | Alu | SINE |
| 13362412 | 13362992 | - | 10.8 | 1 | 593 | -188 | MER4E1 | ERV1 | LTR |

  

|  |  |  |  |  |  |  |  |  |  |
| --- | --- | --- | --- | --- | --- | --- | --- | --- | --- |
| chr1:13373943-13375723 | | | 1757 bp | | |  |  |  |  |
| Genome Start | Genome End | Strand | Div | Repeat Start | Repeat End | Repeat Left | Name | Family | Class |
| 13373943 | 13373991 | - | 18.1 | 6251 | 6294 | -6 | L1MA4 | L1 | LINE |
| 13374016 | 13374298 | - | 13.1 | 1 | 283 | -29 | AluSx | Alu | SINE |
| 13374299 | 13374546 | - | 18.1 | 6014 | 6250 | -50 | L1MA4 | L1 | LINE |
| 13374547 | 13374855 | - | 8.8 | 5 | 311 | 0 | AluY | Alu | SINE |
| 13374856 | 13375186 | - | 18.1 | 5692 | 6013 | -287 | L1MA4 | L1 | LINE |
| 13375187 | 13375499 | + | 13.5 | 1 | 313 | 0 | AluSq | Alu | SINE |
| 13375500 | 13375723 | - | 18.1 | 5476 | 5691 | -609 | L1MA4 | L1 | LINE |

  

|  |  |  |  |  |  |  |  |  |  |
| --- | --- | --- | --- | --- | --- | --- | --- | --- | --- |
| chr1:13382022-13383169 | | | 1148 bp | | |  |  |  |  |
| Genome Start | Genome End | Strand | Div | Repeat Start | Repeat End | Repeat Left | Name | Family | Class |
| 13382022 | 13382097 | + | 32.0 | 3491 | 3568 | -2578 | L1M5 | L1 | LINE |
| 13382098 | 13382410 | - | 14.8 | 1 | 312 | 0 | AluSx | Alu | SINE |
| 13382411 | 13382559 | + | 32.0 | 3569 | 3723 | -2423 | L1M5 | L1 | LINE |
| 13382560 | 13382857 | - | 9.7 | 1 | 298 | -14 | AluSx | Alu | SINE |
| 13382858 | 13383169 | + | 32.0 | 3724 | 4086 | -2060 | L1M5 | L1 | LINE |

  

|  |  |  |  |  |  |  |  |  |  |
| --- | --- | --- | --- | --- | --- | --- | --- | --- | --- |
| chr1:13388944-13389530 | | | 587 bp | | |  |  |  |  |
| Genome Start | Genome End | Strand | Div | Repeat Start | Repeat End | Repeat Left | Name | Family | Class |
| 13388944 | 13389064 | + | 17.1 | 5 | 123 | -189 | AluJb | Alu | SINE |
| 13389065 | 13389361 | - | 13.2 | 1 | 296 | -16 | AluSx | Alu | SINE |
| 13389362 | 13389530 | + | 17.1 | 124 | 278 | -34 | AluJb | Alu | SINE |

  

|  |  |  |  |  |  |  |  |  |  |
| --- | --- | --- | --- | --- | --- | --- | --- | --- | --- |
| chr1:13395095-13395819 | | | 724 bp | | |  |  |  |  |
| Genome Start | Genome End | Strand | Div | Repeat Start | Repeat End | Repeat Left | Name | Family | Class |
| 13395095 | 13395198 | + | 10.6 | 1 | 104 | -208 | AluSx | Alu | SINE |
| 13395199 | 13395619 | - | 12.3 | 3 | 432 | -235 | LTR12B | ERV1 | LTR |
| 13395621 | 13395819 | + | 9.6 | 94 | 292 | -18 | AluSx | Alu | SINE |

  

|  |  |  |  |  |  |  |  |  |  |
| --- | --- | --- | --- | --- | --- | --- | --- | --- | --- |
| chr1:13402607-13403599 | | | 993 bp | | |  |  |  |  |
| Genome Start | Genome End | Strand | Div | Repeat Start | Repeat End | Repeat Left | Name | Family | Class |
| 13402607 | 13403079 | - | 20.3 | 5839 | 6320 | -13 | L1MC1 | L1 | LINE |
| 13403080 | 13403386 | - | 10.4 | 1 | 307 | -2 | AluSg1 | Alu | SINE |
| 13403387 | 13403599 | - | 20.3 | 5602 | 5838 | -495 | L1MC1 | L1 | LINE |

  

|  |  |  |  |  |  |  |  |  |  |
| --- | --- | --- | --- | --- | --- | --- | --- | --- | --- |
| chr1:13415656-13416406 | | | 751 bp | | |  |  |  |  |
| Genome Start | Genome End | Strand | Div | Repeat Start | Repeat End | Repeat Left | Name | Family | Class |
| 13415656 | 13416020 | - | 18.9 | 89 | 461 | 0 | LOR1b | ERV1 | LTR |
| 13416021 | 13416323 | + | 10.6 | 1 | 313 | 0 | AluSp | Alu | SINE |
| 13416324 | 13416406 | - | 18.9 | 1 | 88 | -373 | LOR1b | ERV1 | LTR |

  

|  |  |  |  |  |  |  |  |  |  |
| --- | --- | --- | --- | --- | --- | --- | --- | --- | --- |
| chr1:13416784-13419360 | | | 2515 bp | | |  |  |  |  |
| Genome Start | Genome End | Strand | Div | Repeat Start | Repeat End | Repeat Left | Name | Family | Class |
| 13416784 | 13416853 | + | 27.1 | 3 | 72 | -269 | MER58B | MER1\_type | DNA |
| 13416854 | 13417096 | + | 18.1 | 5884 | 6126 | -186 | L1MA9 | L1 | LINE |
| 13417101 | 13417295 | + | 16.7 | 1 | 187 | -125 | AluJo | Alu | SINE |
| 13417296 | 13418593 | + | 5.3 | 1 | 1578 | 0 | LTR12C | ERV1 | LTR |
| 13418603 | 13418845 | + | 14.1 | 64 | 305 | -7 | AluSx | Alu | SINE |
| 13418846 | 13419007 | + | 16.7 | 193 | 301 | -11 | AluJo | Alu | SINE |
| 13419052 | 13419242 | + | 25.4 | 6119 | 6321 | -13 | L1MA10 | L1 | LINE |
| 13419248 | 13419360 | + | 22.2 | 98 | 330 | -11 | MER58B | MER1\_type | DNA |

  

|  |  |  |  |  |  |  |  |  |  |
| --- | --- | --- | --- | --- | --- | --- | --- | --- | --- |
| chr1:13422215-13422703 | | | 489 bp | | |  |  |  |  |
| Genome Start | Genome End | Strand | Div | Repeat Start | Repeat End | Repeat Left | Name | Family | Class |
| 13422215 | 13422360 | + | 26.0 | 9 | 168 | -100 | MIRb | MIR | SINE |
| 13422361 | 13422653 | + | 4.8 | 1 | 293 | -18 | AluY | Alu | SINE |
| 13422654 | 13422703 | + | 26.0 | 169 | 208 | -60 | MIRb | MIR | SINE |

  

|  |  |  |  |  |  |  |  |  |  |
| --- | --- | --- | --- | --- | --- | --- | --- | --- | --- |
| chr1:13422734-13423540 | | | 807 bp | | |  |  |  |  |
| Genome Start | Genome End | Strand | Div | Repeat Start | Repeat End | Repeat Left | Name | Family | Class |
| 13422734 | 13422764 | + | 17.1 | 1 | 22 | -631 | MER82 | MER2\_type | DNA |
| 13422765 | 13423068 | - | 9.3 | 1 | 301 | -10 | AluY | Alu | SINE |
| 13423069 | 13423325 | + | 17.1 | 23 | 259 | -394 | MER82 | MER2\_type | DNA |
| 13423326 | 13423455 | - | 15.4 | 1 | 133 | 0 | FLAM\_C | Alu | SINE |
| 13423456 | 13423540 | + | 17.1 | 260 | 339 | -314 | MER82 | MER2\_type | DNA |

  

|  |  |  |  |  |  |  |  |  |  |
| --- | --- | --- | --- | --- | --- | --- | --- | --- | --- |
| chr1:13502790-13503395 | | | 606 bp | | |  |  |  |  |
| Genome Start | Genome End | Strand | Div | Repeat Start | Repeat End | Repeat Left | Name | Family | Class |
| 13502790 | 13502927 | + | 21.5 | 5 | 126 | -176 | AluJb | Alu | SINE |
| 13502928 | 13503208 | - | 15.7 | 1 | 280 | -32 | AluSx | Alu | SINE |
| 13503209 | 13503395 | + | 21.5 | 127 | 294 | -8 | AluJb | Alu | SINE |

  

|  |  |  |  |  |  |  |  |  |  |
| --- | --- | --- | --- | --- | --- | --- | --- | --- | --- |
| chr1:13508825-13509700 | | | 876 bp | | |  |  |  |  |
| Genome Start | Genome End | Strand | Div | Repeat Start | Repeat End | Repeat Left | Name | Family | Class |
| 13508825 | 13509377 | - | 19.1 | 5686 | 6250 | -50 | L1MA4 | L1 | LINE |
| 13509378 | 13509480 | + | 12.6 | 1 | 103 | -209 | AluSq/x | Alu | SINE |
| 13509481 | 13509700 | - | 22.0 | 5473 | 5691 | -609 | L1MA4 | L1 | LINE |

  

|  |  |  |  |  |  |  |  |  |  |
| --- | --- | --- | --- | --- | --- | --- | --- | --- | --- |
| chr1:13514861-13515421 | | | 561 bp | | |  |  |  |  |
| Genome Start | Genome End | Strand | Div | Repeat Start | Repeat End | Repeat Left | Name | Family | Class |
| 13514861 | 13514981 | + | 20.4 | 5 | 154 | -148 | AluJb | Alu | SINE |
| 13514982 | 13515276 | - | 17.1 | 7 | 292 | -20 | AluSx | Alu | SINE |
| 13515277 | 13515421 | + | 20.4 | 155 | 297 | -5 | AluJb | Alu | SINE |

  

|  |  |  |  |  |  |  |  |  |  |
| --- | --- | --- | --- | --- | --- | --- | --- | --- | --- |
| chr1:13529109-13535728 | | | 6620 bp | | |  |  |  |  |
| Genome Start | Genome End | Strand | Div | Repeat Start | Repeat End | Repeat Left | Name | Family | Class |
| 13529109 | 13532121 | + | 10.4 | 1 | 2840 | -3306 | L1PA7 | L1 | LINE |
| 13532122 | 13532264 | + | 7.7 | 6013 | 6155 | 0 | L1PA4 | L1 | LINE |
| 13532265 | 13535728 | + | 12.0 | 2841 | 6298 | 0 | L1PA7 | L1 | LINE |

  

|  |  |  |  |  |  |  |  |  |  |
| --- | --- | --- | --- | --- | --- | --- | --- | --- | --- |
| chr1:13535804-13536741 | | | 938 bp | | |  |  |  |  |
| Genome Start | Genome End | Strand | Div | Repeat Start | Repeat End | Repeat Left | Name | Family | Class |
| 13535804 | 13536118 | + | 17.6 | 264 | 574 | -311 | MER41G | ERV1 | LTR |
| 13536119 | 13536445 | + | 12.2 | 1 | 328 | 0 | AluSp | Alu | SINE |
| 13536446 | 13536741 | + | 17.6 | 575 | 885 | 0 | MER41G | ERV1 | LTR |

  

|  |  |  |  |  |  |  |  |  |  |
| --- | --- | --- | --- | --- | --- | --- | --- | --- | --- |
| chr1:13562543-13564324 | | | 1782 bp | | |  |  |  |  |
| Genome Start | Genome End | Strand | Div | Repeat Start | Repeat End | Repeat Left | Name | Family | Class |
| 13562543 | 13562858 | + | 19.5 | 1 | 300 | -623 | MER49 | ERV1 | LTR |
| 13562859 | 13563155 | - | 10.8 | 1 | 297 | -13 | AluSg | Alu | SINE |
| 13563156 | 13563483 | + | 19.5 | 301 | 613 | -310 | MER49 | ERV1 | LTR |
| 13563484 | 13563622 | + | 13.6 | 7 | 133 | -179 | AluSx | Alu | SINE |
| 13563623 | 13563918 | + | 7.1 | 1 | 297 | -14 | AluY | Alu | SINE |
| 13563919 | 13564078 | + | 13.6 | 134 | 297 | -15 | AluSx | Alu | SINE |
| 13564079 | 13564324 | + | 19.5 | 614 | 859 | -64 | MER49 | ERV1 | LTR |

  

|  |  |  |  |  |  |  |  |  |  |
| --- | --- | --- | --- | --- | --- | --- | --- | --- | --- |
| chr1:13564927-13565712 | | | 786 bp | | |  |  |  |  |
| Genome Start | Genome End | Strand | Div | Repeat Start | Repeat End | Repeat Left | Name | Family | Class |
| 13564927 | 13565333 | + | 19.5 | 5656 | 6036 | -129 | L1PA15 | L1 | LINE |
| 13565334 | 13565623 | + | 14.9 | 1 | 306 | -6 | AluSx | Alu | SINE |
| 13565624 | 13565712 | + | 18.8 | 6037 | 6158 | -7 | L1PA15 | L1 | LINE |

  

|  |  |  |  |  |  |  |  |  |  |
| --- | --- | --- | --- | --- | --- | --- | --- | --- | --- |
| chr1:13585571-13586229 | | | 659 bp | | |  |  |  |  |
| Genome Start | Genome End | Strand | Div | Repeat Start | Repeat End | Repeat Left | Name | Family | Class |
| 13585571 | 13585785 | - | 15.3 | 133 | 301 | -1 | AluSx | Alu | SINE |
| 13585786 | 13586091 | - | 11.4 | 1 | 308 | -2 | AluSg | Alu | SINE |
| 13586092 | 13586229 | - | 15.3 | 1 | 132 | -170 | AluSx | Alu | SINE |

  

|  |  |  |  |  |  |  |  |  |  |
| --- | --- | --- | --- | --- | --- | --- | --- | --- | --- |
| chr1:13609785-13610371 | | | 587 bp | | |  |  |  |  |
| Genome Start | Genome End | Strand | Div | Repeat Start | Repeat End | Repeat Left | Name | Family | Class |
| 13609785 | 13609905 | + | 17.9 | 5 | 123 | -189 | AluJb | Alu | SINE |
| 13609906 | 13610202 | - | 13.2 | 1 | 296 | -16 | AluSx | Alu | SINE |
| 13610203 | 13610371 | + | 17.9 | 124 | 278 | -34 | AluJb | Alu | SINE |

  

|  |  |  |  |  |  |  |  |  |  |
| --- | --- | --- | --- | --- | --- | --- | --- | --- | --- |
| chr1:13630599-13631194 | | | 596 bp | | |  |  |  |  |
| Genome Start | Genome End | Strand | Div | Repeat Start | Repeat End | Repeat Left | Name | Family | Class |
| 13630599 | 13630733 | + | 12.8 | 1 | 133 | -180 | AluSq | Alu | SINE |
| 13630734 | 13631030 | + | 9.8 | 1 | 295 | -14 | AluSg1 | Alu | SINE |
| 13631031 | 13631194 | + | 12.8 | 134 | 297 | -16 | AluSq | Alu | SINE |

  

|  |  |  |  |  |  |  |  |  |  |
| --- | --- | --- | --- | --- | --- | --- | --- | --- | --- |
| chr1:13642058-13642439 | | | 343 bp | | |  |  |  |  |
| Genome Start | Genome End | Strand | Div | Repeat Start | Repeat End | Repeat Left | Name | Family | Class |
| 13642058 | 13642115 | + | 26.8 | 4292 | 4354 | -135 | L3 | CR1 | LINE |
| 13642116 | 13642352 | + | 13.1 | 40 | 283 | -29 | AluSx | Alu | SINE |
| 13642392 | 13642439 | + | 26.8 | 4355 | 4396 | -93 | L3 | CR1 | LINE |

  

|  |  |  |  |  |  |  |  |  |  |
| --- | --- | --- | --- | --- | --- | --- | --- | --- | --- |
| chr1:13645027-13645785 | | | 759 bp | | |  |  |  |  |
| Genome Start | Genome End | Strand | Div | Repeat Start | Repeat End | Repeat Left | Name | Family | Class |
| 13645027 | 13645099 | - | 33.1 | 3305 | 3378 | 0 | L2 | L2 | LINE |
| 13645100 | 13645396 | + | 15.9 | 1 | 296 | -16 | AluSx | Alu | SINE |
| 13645397 | 13645705 | + | 11.0 | 1 | 308 | -4 | AluSx | Alu | SINE |
| 13645706 | 13645785 | - | 33.1 | 3227 | 3304 | -74 | L2 | L2 | LINE |

  

|  |  |  |  |  |  |  |  |  |  |
| --- | --- | --- | --- | --- | --- | --- | --- | --- | --- |
| chr1:13652209-13652708 | | | 500 bp | | |  |  |  |  |
| Genome Start | Genome End | Strand | Div | Repeat Start | Repeat End | Repeat Left | Name | Family | Class |
| 13652209 | 13652258 | + | 27.2 | 21 | 64 | -198 | MIR | MIR | SINE |
| 13652259 | 13652555 | + | 11.8 | 1 | 298 | -14 | AluSx | Alu | SINE |
| 13652556 | 13652708 | + | 27.2 | 65 | 221 | -41 | MIR | MIR | SINE |

  

|  |  |  |  |  |  |  |  |  |  |
| --- | --- | --- | --- | --- | --- | --- | --- | --- | --- |
| chr1:15662742-15663209 | | | 468 bp | | |  |  |  |  |
| Genome Start | Genome End | Strand | Div | Repeat Start | Repeat End | Repeat Left | Name | Family | Class |
| 15662742 | 15662849 | + | 23.6 | 44 | 142 | -120 | MIR | MIR | SINE |
| 15662850 | 15663143 | + | 13.3 | 1 | 292 | -20 | AluSx | Alu | SINE |
| 15663144 | 15663209 | + | 23.6 | 143 | 208 | -54 | MIR | MIR | SINE |

  

|  |  |  |  |  |  |  |  |  |  |
| --- | --- | --- | --- | --- | --- | --- | --- | --- | --- |
| chr1:16676310-16676722 | | | 413 bp | | |  |  |  |  |
| Genome Start | Genome End | Strand | Div | Repeat Start | Repeat End | Repeat Left | Name | Family | Class |
| 16676310 | 16676374 | + | 19.4 | 104 | 171 | -62 | MIR | MIR | SINE |
| 16676375 | 16676672 | - | 8.7 | 1 | 298 | -14 | AluSx | Alu | SINE |
| 16676673 | 16676722 | + | 20.9 | 172 | 230 | -32 | MIR | MIR | SINE |

  

|  |  |  |  |  |  |  |  |  |  |
| --- | --- | --- | --- | --- | --- | --- | --- | --- | --- |
| chr1:16694189-16694807 | | | 619 bp | | |  |  |  |  |
| Genome Start | Genome End | Strand | Div | Repeat Start | Repeat End | Repeat Left | Name | Family | Class |
| 16694189 | 16694367 | + | 23.9 | 42 | 233 | -29 | MIR | MIR | SINE |
| 16694368 | 16694771 | + | 27.8 | 1 | 449 | -1 | MLT1J2 | MaLR | LTR |
| 16694772 | 16694807 | + | 23.9 | 234 | 261 | -1 | MIR | MIR | SINE |

  

|  |  |  |  |  |  |  |  |  |  |
| --- | --- | --- | --- | --- | --- | --- | --- | --- | --- |
| chr1:16701655-16702207 | | | 553 bp | | |  |  |  |  |
| Genome Start | Genome End | Strand | Div | Repeat Start | Repeat End | Repeat Left | Name | Family | Class |
| 16701655 | 16701756 | - | 8.9 | 157 | 248 | -259 | MER84 | ERV1 | LTR |
| 16701757 | 16702053 | + | 10.4 | 1 | 298 | -11 | AluSg1 | Alu | SINE |
| 16702054 | 16702207 | - | 8.9 | 8 | 156 | -351 | MER84 | ERV1 | LTR |

  

|  |  |  |  |  |  |  |  |  |  |
| --- | --- | --- | --- | --- | --- | --- | --- | --- | --- |
| chr1:16715238-16716309 | | | 1072 bp | | |  |  |  |  |
| Genome Start | Genome End | Strand | Div | Repeat Start | Repeat End | Repeat Left | Name | Family | Class |
| 16715238 | 16715819 | - | 16.6 | 163 | 727 | 0 | LTR8A | ERV1 | LTR |
| 16715820 | 16716159 | - | 15.9 | 1 | 337 | -2 | MER1B | MER1\_type | DNA |
| 16716160 | 16716309 | - | 16.6 | 2 | 162 | -565 | LTR8A | ERV1 | LTR |

  

|  |  |  |  |  |  |  |  |  |  |
| --- | --- | --- | --- | --- | --- | --- | --- | --- | --- |
| chr1:16717358-16718811 | | | 1454 bp | | |  |  |  |  |
| Genome Start | Genome End | Strand | Div | Repeat Start | Repeat End | Repeat Left | Name | Family | Class |
| 16717358 | 16717514 | + | 21.1 | 1 | 160 | -562 | MER72 | ERV1 | LTR |
| 16717515 | 16717674 | + | 10.3 | 1 | 163 | -494 | MER51D | ERV1 | LTR |
| 16717675 | 16717980 | + | 8.5 | 1 | 305 | -7 | AluSx | Alu | SINE |
| 16717981 | 16718458 | + | 10.3 | 164 | 655 | -2 | MER51D | ERV1 | LTR |
| 16718459 | 16718811 | + | 21.1 | 161 | 504 | -218 | MER72 | ERV1 | LTR |

  

|  |  |  |  |  |  |  |  |  |  |
| --- | --- | --- | --- | --- | --- | --- | --- | --- | --- |
| chr1:16748964-16749462 | | | 499 bp | | |  |  |  |  |
| Genome Start | Genome End | Strand | Div | Repeat Start | Repeat End | Repeat Left | Name | Family | Class |
| 16748964 | 16749011 | - | 24.5 | 205 | 245 | -17 | MIR | MIR | SINE |
| 16749012 | 16749313 | - | 10.9 | 1 | 302 | -10 | AluSx | Alu | SINE |
| 16749314 | 16749462 | - | 24.5 | 33 | 204 | -58 | MIR | MIR | SINE |

  

|  |  |  |  |  |  |  |  |  |  |
| --- | --- | --- | --- | --- | --- | --- | --- | --- | --- |
| chr1:16768886-16771988 | | | 3103 bp | | |  |  |  |  |
| Genome Start | Genome End | Strand | Div | Repeat Start | Repeat End | Repeat Left | Name | Family | Class |
| 16768886 | 16769956 | + | 5.9 | 857 | 1849 | -426 | L1P2 | L1 | LINE |
| 16769957 | 16770601 | - | 4.7 | 5494 | 6139 | -16 | L1PA4 | L1 | LINE |
| 16770602 | 16771988 | + | 6.8 | 1850 | 3226 | -2920 | L1P2 | L1 | LINE |

  

|  |  |  |  |  |  |  |  |  |  |
| --- | --- | --- | --- | --- | --- | --- | --- | --- | --- |
| chr1:16811018-16811414 | | | 397 bp | | |  |  |  |  |
| Genome Start | Genome End | Strand | Div | Repeat Start | Repeat End | Repeat Left | Name | Family | Class |
| 16811018 | 16811072 | + | 15.8 | 6037 | 6103 | -67 | L1ME3A | L1 | LINE |
| 16811073 | 16811369 | - | 15.2 | 3 | 300 | -12 | AluJb | Alu | SINE |
| 16811370 | 16811414 | + | 15.8 | 6104 | 6159 | -11 | L1ME3A | L1 | LINE |

  

|  |  |  |  |  |  |  |  |  |  |
| --- | --- | --- | --- | --- | --- | --- | --- | --- | --- |
| chr1:16839202-16841012 | | | 1811 bp | | |  |  |  |  |
| Genome Start | Genome End | Strand | Div | Repeat Start | Repeat End | Repeat Left | Name | Family | Class |
| 16839202 | 16839660 | + | 9.4 | 5147 | 5609 | -695 | L1MA2 | L1 | LINE |
| 16839661 | 16839968 | + | 11.0 | 4 | 311 | -2 | AluSq | Alu | SINE |
| 16839969 | 16840281 | + | 8.8 | 1 | 306 | -5 | AluY | Alu | SINE |
| 16840282 | 16841012 | + | 9.3 | 5610 | 6304 | 0 | L1MA2 | L1 | LINE |

  

|  |  |  |  |  |  |  |  |  |  |
| --- | --- | --- | --- | --- | --- | --- | --- | --- | --- |
| chr1:16976746-16978387 | | | 1642 bp | | |  |  |  |  |
| Genome Start | Genome End | Strand | Div | Repeat Start | Repeat End | Repeat Left | Name | Family | Class |
| 16976746 | 16977555 | - | 27.8 | 2520 | 3419 | 0 | L2 | L2 | LINE |
| 16977556 | 16977981 | - | 10.6 | 1 | 428 | 0 | MSTA | MaLR | LTR |
| 16977982 | 16978387 | - | 27.6 | 2090 | 2519 | -900 | L2 | L2 | LINE |

  

|  |  |  |  |  |  |  |  |  |  |
| --- | --- | --- | --- | --- | --- | --- | --- | --- | --- |
| chr1:17063670-17066989 | | | 3229 bp | | |  |  |  |  |
| Genome Start | Genome End | Strand | Div | Repeat Start | Repeat End | Repeat Left | Name | Family | Class |
| 17063670 | 17063874 | - | 23.7 | 5264 | 5471 | -703 | L1M4 | L1 | LINE |
| 17063890 | 17065840 | - | 5.9 | 4293 | 6154 | 0 | L1PA6 | L1 | LINE |
| 17065908 | 17066818 | - | 18.3 | 5 | 923 | 0 | MER49 | ERV1 | LTR |
| 17066828 | 17066989 | - | 33.3 | 4987 | 5154 | -992 | L1M4 | L1 | LINE |

  

|  |  |  |  |  |  |  |  |  |  |
| --- | --- | --- | --- | --- | --- | --- | --- | --- | --- |
| chr1:17089694-17091149 | | | 1456 bp | | |  |  |  |  |
| Genome Start | Genome End | Strand | Div | Repeat Start | Repeat End | Repeat Left | Name | Family | Class |
| 17089694 | 17090044 | - | 19.6 | 161 | 504 | -218 | MER72 | ERV1 | LTR |
| 17090045 | 17090523 | - | 10.6 | 156 | 655 | -2 | MER51D | ERV1 | LTR |
| 17090524 | 17090835 | - | 8.0 | 1 | 311 | -1 | AluSx | Alu | SINE |
| 17090836 | 17090995 | - | 10.6 | 1 | 155 | -502 | MER51D | ERV1 | LTR |
| 17090996 | 17091149 | - | 19.6 | 3 | 160 | -562 | MER72 | ERV1 | LTR |

  

|  |  |  |  |  |  |  |  |  |  |
| --- | --- | --- | --- | --- | --- | --- | --- | --- | --- |
| chr1:17091314-17091818 | | | 505 bp | | |  |  |  |  |
| Genome Start | Genome End | Strand | Div | Repeat Start | Repeat End | Repeat Left | Name | Family | Class |
| 17091314 | 17091367 | + | 23.9 | 350 | 404 | -155 | MER89 | ERV1 | LTR |
| 17091368 | 17091663 | - | 10.2 | 1 | 311 | 0 | AluY | Alu | SINE |
| 17091664 | 17091818 | + | 23.9 | 405 | 556 | -3 | MER89 | ERV1 | LTR |

  

|  |  |  |  |  |  |  |  |  |  |
| --- | --- | --- | --- | --- | --- | --- | --- | --- | --- |
| chr1:17092144-17093210 | | | 1067 bp | | |  |  |  |  |
| Genome Start | Genome End | Strand | Div | Repeat Start | Repeat End | Repeat Left | Name | Family | Class |
| 17092144 | 17092279 | + | 17.8 | 34 | 162 | -565 | LTR8A | ERV1 | LTR |
| 17092280 | 17092628 | + | 14.4 | 1 | 337 | -2 | MER1B | MER1\_type | DNA |
| 17092629 | 17093210 | + | 17.8 | 163 | 727 | 0 | LTR8A | ERV1 | LTR |

  

|  |  |  |  |  |  |  |  |  |  |
| --- | --- | --- | --- | --- | --- | --- | --- | --- | --- |
| chr1:17114786-17115507 | | | 722 bp | | |  |  |  |  |
| Genome Start | Genome End | Strand | Div | Repeat Start | Repeat End | Repeat Left | Name | Family | Class |
| 17114786 | 17114978 | - | 24.8 | 2977 | 3186 | -192 | L2 | L2 | LINE |
| 17114979 | 17115274 | + | 11.5 | 1 | 291 | -21 | AluSx | Alu | SINE |
| 17115275 | 17115507 | - | 25.7 | 2710 | 2981 | -397 | L2 | L2 | LINE |

  

|  |  |  |  |  |  |  |  |  |  |
| --- | --- | --- | --- | --- | --- | --- | --- | --- | --- |
| chr1:17139303-17139787 | | | 485 bp | | |  |  |  |  |
| Genome Start | Genome End | Strand | Div | Repeat Start | Repeat End | Repeat Left | Name | Family | Class |
| 17139303 | 17139472 | - | 10.2 | 134 | 302 | -9 | AluY | Alu | SINE |
| 17139473 | 17139769 | - | 10.2 | 1 | 295 | -14 | AluSg1 | Alu | SINE |
| 17139770 | 17139787 | - | 10.2 | 115 | 133 | -178 | AluY | Alu | SINE |

  

|  |  |  |  |  |  |  |  |  |  |
| --- | --- | --- | --- | --- | --- | --- | --- | --- | --- |
| chr1:17143983-17144382 | | | 400 bp | | |  |  |  |  |
| Genome Start | Genome End | Strand | Div | Repeat Start | Repeat End | Repeat Left | Name | Family | Class |
| 17143983 | 17144032 | - | 23.8 | 173 | 231 | -31 | MIR | MIR | SINE |
| 17144033 | 17144328 | + | 10.8 | 1 | 296 | -16 | AluSx | Alu | SINE |
| 17144329 | 17144382 | - | 23.8 | 112 | 172 | -90 | MIR | MIR | SINE |

  

|  |  |  |  |  |  |  |  |  |  |
| --- | --- | --- | --- | --- | --- | --- | --- | --- | --- |
| chr1:19482170-19482605 | | | 307 bp | | |  |  |  |  |
| Genome Start | Genome End | Strand | Div | Repeat Start | Repeat End | Repeat Left | Name | Family | Class |
| 19482170 | 19482290 | + | 33.8 | 46 | 165 | -111 | MIRb | MIR | SINE |
| 19482317 | 19482396 | - | 15.4 | 3333 | 3400 | -19 | L2 | L2 | LINE |
| 19482500 | 19482605 | + | 28.6 | 121 | 237 | -31 | MIRb | MIR | SINE |

  

|  |  |  |  |  |  |  |  |  |  |
| --- | --- | --- | --- | --- | --- | --- | --- | --- | --- |
| chr1:21659580-21661531 | | | 1952 bp | | |  |  |  |  |
| Genome Start | Genome End | Strand | Div | Repeat Start | Repeat End | Repeat Left | Name | Family | Class |
| 21659580 | 21660553 | + | 30.4 | 4935 | 5983 | -252 | L1ME3 | L1 | LINE |
| 21660554 | 21660842 | - | 12.6 | 1 | 298 | -14 | AluSx | Alu | SINE |
| 21660843 | 21660897 | + | 28.6 | 5984 | 6033 | -202 | L1ME3 | L1 | LINE |
| 21660898 | 21661327 | + | 17.0 | 1 | 431 | -1 | MSTB1 | MaLR | LTR |
| 21661328 | 21661531 | + | 28.6 | 6034 | 6234 | -1 | L1ME3 | L1 | LINE |

  

|  |  |  |  |  |  |  |  |  |  |
| --- | --- | --- | --- | --- | --- | --- | --- | --- | --- |
| chr1:22176618-22177137 | | | 520 bp | | |  |  |  |  |
| Genome Start | Genome End | Strand | Div | Repeat Start | Repeat End | Repeat Left | Name | Family | Class |
| 22176618 | 22176807 | - | 30.1 | 70 | 260 | -2 | MIR | MIR | SINE |
| 22176808 | 22177088 | - | 17.7 | 1 | 302 | 0 | AluJo | Alu | SINE |
| 22177089 | 22177137 | - | 30.1 | 21 | 69 | -193 | MIR | MIR | SINE |

  

|  |  |  |  |  |  |  |  |  |  |
| --- | --- | --- | --- | --- | --- | --- | --- | --- | --- |
| chr1:22178453-22178938 | | | 486 bp | | |  |  |  |  |
| Genome Start | Genome End | Strand | Div | Repeat Start | Repeat End | Repeat Left | Name | Family | Class |
| 22178453 | 22178520 | - | 17.9 | 112 | 159 | -50 | MER3 | MER1\_type | DNA |
| 22178521 | 22178833 | - | 15.7 | 1 | 312 | 0 | AluJb | Alu | SINE |
| 22178834 | 22178938 | - | 17.9 | 9 | 111 | -98 | MER3 | MER1\_type | DNA |

  

|  |  |  |  |  |  |  |  |  |  |
| --- | --- | --- | --- | --- | --- | --- | --- | --- | --- |
| chr1:22206952-22207557 | | | 606 bp | | |  |  |  |  |
| Genome Start | Genome End | Strand | Div | Repeat Start | Repeat End | Repeat Left | Name | Family | Class |
| 22206952 | 22207225 | + | 18.9 | 3 | 274 | -28 | AluJo | Alu | SINE |
| 22207226 | 22207519 | - | 18.0 | 12 | 305 | -7 | AluJb | Alu | SINE |
| 22207520 | 22207557 | + | 18.9 | 275 | 297 | -5 | AluJo | Alu | SINE |

  

|  |  |  |  |  |  |  |  |  |  |
| --- | --- | --- | --- | --- | --- | --- | --- | --- | --- |
| chr1:25467171-25468534 | | | 1364 bp | | |  |  |  |  |
| Genome Start | Genome End | Strand | Div | Repeat Start | Repeat End | Repeat Left | Name | Family | Class |
| 25467171 | 25467314 | - | 24.4 | 3272 | 3417 | -2 | L2 | L2 | LINE |
| 25467315 | 25467602 | + | 12.2 | 1 | 291 | -21 | AluSx | Alu | SINE |
| 25467603 | 25467743 | - | 24.4 | 3169 | 3271 | -148 | L2 | L2 | LINE |
| 25467744 | 25468043 | + | 11.3 | 1 | 300 | -13 | AluSq | Alu | SINE |
| 25468044 | 25468120 | - | 24.4 | 3088 | 3168 | -251 | L2 | L2 | LINE |
| 25468121 | 25468486 | - | 14.5 | 1 | 366 | 0 | Tigger5a | MER2\_type | DNA |
| 25468487 | 25468534 | - | 24.4 | 3041 | 3087 | -332 | L2 | L2 | LINE |

  

|  |  |  |  |  |  |  |  |  |  |
| --- | --- | --- | --- | --- | --- | --- | --- | --- | --- |
| chr1:25469342-25470555 | | | 1185 bp | | |  |  |  |  |
| Genome Start | Genome End | Strand | Div | Repeat Start | Repeat End | Repeat Left | Name | Family | Class |
| 25469342 | 25469384 | - | 27.9 | 4440 | 4482 | -7 | L3 | CR1 | LINE |
| 25469389 | 25469710 | + | 9.2 | 3 | 339 | 0 | MER1B | MER1\_type | DNA |
| 25469727 | 25469948 | + | 24.3 | 13 | 256 | -6 | MIR | MIR | SINE |
| 25469965 | 25470267 | - | 26.0 | 5696 | 6015 | -106 | L1ME4a | L1 | LINE |
| 25470276 | 25470428 | + | 30.1 | 68 | 219 | -18 | MIR3 | MIR | SINE |
| 25470414 | 25470555 | - | 29.1 | 4200 | 4370 | -119 | L3 | CR1 | LINE |

  

|  |  |  |  |  |  |  |  |  |  |
| --- | --- | --- | --- | --- | --- | --- | --- | --- | --- |
| chr1:25474631-25477180 | | | 2172 bp | | |  |  |  |  |
| Genome Start | Genome End | Strand | Div | Repeat Start | Repeat End | Repeat Left | Name | Family | Class |
| 25474631 | 25474775 | - | 22.7 | 2415 | 2565 | -47 | Charlie7 | MER1\_type | DNA |
| 25474822 | 25475141 | - | 22.0 | 303 | 634 | -1978 | Charlie7 | MER1\_type | DNA |
| 25475156 | 25475505 | + | 28.6 | 7581 | 7931 | -30 | L1MC5 | L1 | LINE |
| 25475521 | 25475805 | + | 18.2 | 33 | 296 | -16 | AluJo | Alu | SINE |
| 25475863 | 25476173 | + | 9.3 | 1 | 310 | -1 | AluY | Alu | SINE |
| 25476401 | 25476585 | + | 17.8 | 4 | 189 | 0 | MER5A | MER1\_type | DNA |
| 25476586 | 25476745 | + | 24.5 | 36 | 214 | -48 | MIR | MIR | SINE |
| 25476763 | 25477051 | - | 6.6 | 1 | 292 | -21 | AluSp | Alu | SINE |
| 25477054 | 25477180 | - | 28.0 | 1 | 131 | -2481 | Charlie7 | MER1\_type | DNA |

  

|  |  |  |  |  |  |  |  |  |  |
| --- | --- | --- | --- | --- | --- | --- | --- | --- | --- |
| chr1:25488115-25488642 | | | 528 bp | | |  |  |  |  |
| Genome Start | Genome End | Strand | Div | Repeat Start | Repeat End | Repeat Left | Name | Family | Class |
| 25488115 | 25488274 | + | 29.3 | 18 | 174 | -88 | MIR | MIR | SINE |
| 25488275 | 25488572 | - | 15.8 | 1 | 298 | -14 | AluSx | Alu | SINE |
| 25488573 | 25488642 | + | 29.3 | 175 | 249 | -13 | MIR | MIR | SINE |

  

|  |  |  |  |  |  |  |  |  |  |
| --- | --- | --- | --- | --- | --- | --- | --- | --- | --- |
| chr1:25493735-25494915 | | | 1112 bp | | |  |  |  |  |
| Genome Start | Genome End | Strand | Div | Repeat Start | Repeat End | Repeat Left | Name | Family | Class |
| 25493735 | 25494235 | - | 31.6 | 1103 | 1629 | -1790 | L2 | L2 | LINE |
| 25494252 | 25494478 | + | 23.6 | 37 | 241 | -21 | MIR | MIR | SINE |
| 25494532 | 25494915 | - | 28.1 | 593 | 1032 | -2387 | L2 | L2 | LINE |

  

|  |  |  |  |  |  |  |  |  |  |
| --- | --- | --- | --- | --- | --- | --- | --- | --- | --- |
| chr1:25514908-25515838 | | | 508 bp | | |  |  |  |  |
| Genome Start | Genome End | Strand | Div | Repeat Start | Repeat End | Repeat Left | Name | Family | Class |
| 25514908 | 25515002 | - | 28.9 | 104 | 196 | -66 | MIR | MIR | SINE |
| 25515416 | 25515712 | + | 11.4 | 1 | 312 | 0 | AluSx | Alu | SINE |
| 25515723 | 25515838 | - | 22.4 | 15 | 131 | -77 | MIR | MIR | SINE |

  

|  |  |  |  |  |  |  |  |  |  |
| --- | --- | --- | --- | --- | --- | --- | --- | --- | --- |
| chr1:25523735-25524259 | | | 525 bp | | |  |  |  |  |
| Genome Start | Genome End | Strand | Div | Repeat Start | Repeat End | Repeat Left | Name | Family | Class |
| 25523735 | 25523841 | - | 28.4 | 158 | 262 | -6 | MIRb | MIR | SINE |
| 25523842 | 25524141 | + | 13.2 | 3 | 298 | -14 | AluSx | Alu | SINE |
| 25524142 | 25524259 | - | 28.4 | 37 | 157 | -111 | MIRb | MIR | SINE |

  

|  |  |  |  |  |  |  |  |  |  |
| --- | --- | --- | --- | --- | --- | --- | --- | --- | --- |
| chr1:25527203-25527866 | | | 664 bp | | |  |  |  |  |
| Genome Start | Genome End | Strand | Div | Repeat Start | Repeat End | Repeat Left | Name | Family | Class |
| 25527203 | 25527287 | + | 28.2 | 4106 | 4183 | -306 | L3 | CR1 | LINE |
| 25527288 | 25527607 | + | 10.1 | 1 | 313 | 0 | AluSq | Alu | SINE |
| 25527608 | 25527866 | + | 28.2 | 4184 | 4482 | -7 | L3 | CR1 | LINE |

  

|  |  |  |  |  |  |  |  |  |  |
| --- | --- | --- | --- | --- | --- | --- | --- | --- | --- |
| chr1:25529805-25530512 | | | 708 bp | | |  |  |  |  |
| Genome Start | Genome End | Strand | Div | Repeat Start | Repeat End | Repeat Left | Name | Family | Class |
| 25529805 | 25530031 | - | 17.5 | 174 | 390 | 0 | MLT1B | MaLR | LTR |
| 25530032 | 25530343 | - | 7.1 | 1 | 308 | -2 | AluSg | Alu | SINE |
| 25530344 | 25530512 | - | 17.5 | 1 | 173 | -217 | MLT1B | MaLR | LTR |

  

|  |  |  |  |  |  |  |  |  |  |
| --- | --- | --- | --- | --- | --- | --- | --- | --- | --- |
| chr1:25531682-25534859 | | | 3160 bp | | |  |  |  |  |
| Genome Start | Genome End | Strand | Div | Repeat Start | Repeat End | Repeat Left | Name | Family | Class |
| 25531682 | 25531856 | - | 24.0 | 3219 | 3419 | 0 | L2 | L2 | LINE |
| 25531865 | 25532398 | - | 19.5 | 1 | 549 | -5 | MLT2B3 | ERVL | LTR |
| 25532409 | 25532718 | - | 27.3 | 2858 | 3210 | -209 | L2 | L2 | LINE |
| 25532719 | 25533057 | - | 10.0 | 1 | 364 | 0 | THE1B | MaLR | LTR |
| 25533058 | 25534628 | - | 29.5 | 2 | 1735 | 0 | MLT-int | MaLR | LTR |
| 25534629 | 25534859 | - | 30.9 | 2618 | 2857 | -562 | L2 | L2 | LINE |

  

|  |  |  |  |  |  |  |  |  |  |
| --- | --- | --- | --- | --- | --- | --- | --- | --- | --- |
| chr1:25594782-25596238 | | | 1432 bp | | |  |  |  |  |
| Genome Start | Genome End | Strand | Div | Repeat Start | Repeat End | Repeat Left | Name | Family | Class |
| 25594782 | 25595523 | + | 17.5 | 7046 | 7830 | -212 | L1MC4 | L1 | LINE |
| 25595524 | 25595695 | - | 15.1 | 211 | 381 | 0 | THE1D | MaLR | LTR |
| 25595721 | 25595895 | - | 15.1 | 1 | 210 | -171 | THE1D | MaLR | LTR |
| 25595896 | 25595909 | + | 17.5 | 7831 | 7834 | -208 | L1MC4 | L1 | LINE |
| 25595910 | 25596215 | + | 12.4 | 1 | 308 | -4 | AluSx | Alu | SINE |
| 25596216 | 25596238 | + | 17.5 | 7835 | 7852 | -190 | L1MC4 | L1 | LINE |

  

|  |  |  |  |  |  |  |  |  |  |
| --- | --- | --- | --- | --- | --- | --- | --- | --- | --- |
| chr1:47308540-47310215 | | | 1622 bp | | |  |  |  |  |
| Genome Start | Genome End | Strand | Div | Repeat Start | Repeat End | Repeat Left | Name | Family | Class |
| 47308540 | 47309228 | - | 14.0 | 5483 | 6179 | -4 | L1MB3 | L1 | LINE |
| 47309257 | 47309374 | + | 16.1 | 10 | 134 | -179 | AluSp | Alu | SINE |
| 47309376 | 47309682 | + | 13.5 | 1 | 304 | -8 | AluSx | Alu | SINE |
| 47309708 | 47310215 | - | 16.8 | 4964 | 5476 | -695 | L1MB3 | L1 | LINE |

  

|  |  |  |  |  |  |  |  |  |  |
| --- | --- | --- | --- | --- | --- | --- | --- | --- | --- |
| chr1:47311219-47313037 | | | 1728 bp | | |  |  |  |  |
| Genome Start | Genome End | Strand | Div | Repeat Start | Repeat End | Repeat Left | Name | Family | Class |
| 47311219 | 47311636 | - | 18.1 | 6306 | 6739 | -36 | L1MB5 | L1 | LINE |
| 47311682 | 47311900 | - | 12.3 | 1 | 223 | -89 | AluSx | Alu | SINE |
| 47311914 | 47312365 | - | 18.8 | 5593 | 6032 | -743 | L1MB5 | L1 | LINE |
| 47312394 | 47312754 | - | 14.7 | 5814 | 6173 | 0 | L1PA16 | L1 | LINE |
| 47312760 | 47313037 | - | 19.1 | 5305 | 5606 | -1141 | L1MB5 | L1 | LINE |

  

|  |  |  |  |  |  |  |  |  |  |
| --- | --- | --- | --- | --- | --- | --- | --- | --- | --- |
| chr1:83414424-83416975 | | | 2552 bp | | |  |  |  |  |
| Genome Start | Genome End | Strand | Div | Repeat Start | Repeat End | Repeat Left | Name | Family | Class |
| 83414424 | 83415098 | - | 13.8 | 1749 | 2418 | 0 | Tigger1 | MER2\_type | DNA |
| 83415099 | 83415312 | - | 13.1 | 5960 | 6172 | 0 | L1PA8 | L1 | LINE |
| 83415313 | 83416975 | - | 14.2 | 1 | 1748 | -670 | Tigger1 | MER2\_type | DNA |

  

|  |  |  |  |  |  |  |  |  |  |
| --- | --- | --- | --- | --- | --- | --- | --- | --- | --- |
| chr1:83443207-83443714 | | | 477 bp | | |  |  |  |  |
| Genome Start | Genome End | Strand | Div | Repeat Start | Repeat End | Repeat Left | Name | Family | Class |
| 83443207 | 83443486 | - | 25.0 | 476 | 782 | -1 | MER20B | MER1\_type | DNA |
| 83443490 | 83443607 | + | 31.6 | 3302 | 3418 | -1 | L2 | L2 | LINE |
| 83443636 | 83443714 | - | 26.6 | 369 | 447 | -336 | MER20B | MER1\_type | DNA |

  

|  |  |  |  |  |  |  |  |  |  |
| --- | --- | --- | --- | --- | --- | --- | --- | --- | --- |
| chr1:83458585-83459389 | | | 800 bp | | |  |  |  |  |
| Genome Start | Genome End | Strand | Div | Repeat Start | Repeat End | Repeat Left | Name | Family | Class |
| 83458585 | 83458874 | - | 19.0 | 81 | 377 | -13 | MLT1B | MaLR | LTR |
| 83458880 | 83459306 | - | 12.2 | 1 | 432 | -6 | MER93B | ERV1 | LTR |
| 83459307 | 83459389 | - | 13.2 | 1 | 84 | -306 | MLT1B | MaLR | LTR |

  

|  |  |  |  |  |  |  |  |  |  |
| --- | --- | --- | --- | --- | --- | --- | --- | --- | --- |
| chr1:83488970-83492666 | | | 3697 bp | | |  |  |  |  |
| Genome Start | Genome End | Strand | Div | Repeat Start | Repeat End | Repeat Left | Name | Family | Class |
| 83488970 | 83489623 | - | 27.9 | 4007 | 4672 | -1547 | L1MEc | L1 | LINE |
| 83489624 | 83490722 | - | 6.6 | 1 | 1266 | 0 | MER11A | ERVK | LTR |
| 83490723 | 83492666 | - | 27.1 | 1990 | 4006 | -2213 | L1MEc | L1 | LINE |

  

|  |  |  |  |  |  |  |  |  |  |
| --- | --- | --- | --- | --- | --- | --- | --- | --- | --- |
| chr1:83517414-83518820 | | | 1324 bp | | |  |  |  |  |
| Genome Start | Genome End | Strand | Div | Repeat Start | Repeat End | Repeat Left | Name | Family | Class |
| 83517414 | 83517790 | + | 27.6 | 2105 | 2489 | -3781 | L1MC4 | L1 | LINE |
| 83517865 | 83518673 | - | 18.4 | 1 | 931 | 0 | MER21A | ERV1 | LTR |
| 83518683 | 83518820 | + | 31.1 | 2567 | 2704 | -3510 | L1MC4 | L1 | LINE |

  

|  |  |  |  |  |  |  |  |  |  |
| --- | --- | --- | --- | --- | --- | --- | --- | --- | --- |
| chr1:83535222-83536411 | | | 1190 bp | | |  |  |  |  |
| Genome Start | Genome End | Strand | Div | Repeat Start | Repeat End | Repeat Left | Name | Family | Class |
| 83535222 | 83535732 | - | 31.6 | 2743 | 3322 | -97 | L2 | L2 | LINE |
| 83535733 | 83535993 | + | 7.3 | 44 | 303 | -8 | AluY | Alu | SINE |
| 83535994 | 83536411 | - | 33.4 | 2280 | 2742 | -677 | L2 | L2 | LINE |

  

|  |  |  |  |  |  |  |  |  |  |
| --- | --- | --- | --- | --- | --- | --- | --- | --- | --- |
| chr1:83553538-83554278 | | | 741 bp | | |  |  |  |  |
| Genome Start | Genome End | Strand | Div | Repeat Start | Repeat End | Repeat Left | Name | Family | Class |
| 83553538 | 83553698 | - | 30.4 | 2125 | 2316 | -1103 | L2 | L2 | LINE |
| 83553699 | 83554078 | + | 18.5 | 2 | 390 | 0 | MLT1B | MaLR | LTR |
| 83554079 | 83554278 | - | 30.4 | 1923 | 2124 | -1295 | L2 | L2 | LINE |

  

|  |  |  |  |  |  |  |  |  |  |
| --- | --- | --- | --- | --- | --- | --- | --- | --- | --- |
| chr1:83555304-83557061 | | | 1606 bp | | |  |  |  |  |
| Genome Start | Genome End | Strand | Div | Repeat Start | Repeat End | Repeat Left | Name | Family | Class |
| 83555304 | 83555617 | - | 19.8 | 5796 | 6161 | -5 | L1ME1 | L1 | LINE |
| 83555726 | 83556216 | - | 19.1 | 1 | 499 | -6 | MLT1D | MaLR | LTR |
| 83556226 | 83556648 | - | 25.4 | 5269 | 5724 | -440 | L1ME1 | L1 | LINE |
| 83556684 | 83557061 | - | 30.4 | 4853 | 5268 | -878 | L1ME1 | L1 | LINE |

  

|  |  |  |  |  |  |  |  |  |  |
| --- | --- | --- | --- | --- | --- | --- | --- | --- | --- |
| chr1:83561815-83563226 | | | 1212 bp | | |  |  |  |  |
| Genome Start | Genome End | Strand | Div | Repeat Start | Repeat End | Repeat Left | Name | Family | Class |
| 83561815 | 83561921 | - | 24.3 | 1691 | 1814 | -693 | HAL1 | L1 | LINE |
| 83561977 | 83562358 | + | 28.2 | 15 | 397 | -114 | MLT1J | MaLR | LTR |
| 83562504 | 83563226 | - | 31.9 | 968 | 1672 | -835 | HAL1 | L1 | LINE |

  

|  |  |  |  |  |  |  |  |  |  |
| --- | --- | --- | --- | --- | --- | --- | --- | --- | --- |
| chr1:83585521-83586632 | | | 888 bp | | |  |  |  |  |
| Genome Start | Genome End | Strand | Div | Repeat Start | Repeat End | Repeat Left | Name | Family | Class |
| 83585521 | 83585614 | - | 28.8 | 3284 | 3419 | 0 | L2 | L2 | LINE |
| 83585654 | 83585973 | + | 24.5 | 3 | 331 | -622 | MER45C | Tip100 | DNA |
| 83586016 | 83586295 | + | 32.0 | 318 | 618 | -335 | MER45C | Tip100 | DNA |
| 83586439 | 83586632 | - | 31.8 | 2950 | 3134 | -244 | L2 | L2 | LINE |

  

|  |  |  |  |  |  |  |  |  |  |
| --- | --- | --- | --- | --- | --- | --- | --- | --- | --- |
| chr1:83622253-83626379 | | | 4145 bp | | |  |  |  |  |
| Genome Start | Genome End | Strand | Div | Repeat Start | Repeat End | Repeat Left | Name | Family | Class |
| 83622253 | 83623049 | + | 20.7 | 1 | 763 | -5745 | L1MC1 | L1 | LINE |
| 83623032 | 83623181 | + | 15.8 | 3368 | 3520 | -2801 | L1MC1 | L1 | LINE |
| 83623182 | 83623491 | + | 12.3 | 1 | 308 | -1 | AluSc | Alu | SINE |
| 83623492 | 83626379 | + | 15.4 | 3521 | 6493 | -15 | L1MC1 | L1 | LINE |

  

|  |  |  |  |  |  |  |  |  |  |
| --- | --- | --- | --- | --- | --- | --- | --- | --- | --- |
| chr1:83657674-83659443 | | | 1359 bp | | |  |  |  |  |
| Genome Start | Genome End | Strand | Div | Repeat Start | Repeat End | Repeat Left | Name | Family | Class |
| 83657674 | 83658012 | + | 28.2 | 2870 | 3261 | -158 | L2 | L2 | LINE |
| 83658035 | 83658262 | - | 32.4 | 253 | 475 | -16 | LTR16C | ERVL | LTR |
| 83658619 | 83659338 | - | 34.1 | 4517 | 5245 | -422 | ERVL-E | ERVL | LTR |
| 83659372 | 83659443 | + | 29.2 | 3340 | 3419 | 0 | L2 | L2 | LINE |

  

|  |  |  |  |  |  |  |  |  |  |
| --- | --- | --- | --- | --- | --- | --- | --- | --- | --- |
| chr1:83668711-83670314 | | | 1604 bp | | |  |  |  |  |
| Genome Start | Genome End | Strand | Div | Repeat Start | Repeat End | Repeat Left | Name | Family | Class |
| 83668711 | 83669366 | - | 26.1 | 497 | 1162 | -2778 | Arthur1 | Tip100 | DNA |
| 83669367 | 83669815 | - | 18.3 | 5778 | 6242 | 0 | L1MD1 | L1 | LINE |
| 83669816 | 83670314 | - | 26.1 | 3 | 496 | -3444 | Arthur1 | Tip100 | DNA |

  

|  |  |  |  |  |  |  |  |  |  |
| --- | --- | --- | --- | --- | --- | --- | --- | --- | --- |
| chr1:83716805-83717346 | | | 542 bp | | |  |  |  |  |
| Genome Start | Genome End | Strand | Div | Repeat Start | Repeat End | Repeat Left | Name | Family | Class |
| 83716805 | 83716888 | - | 26.4 | 170 | 254 | -8 | MIR | MIR | SINE |
| 83716889 | 83717263 | - | 17.8 | 1 | 381 | 0 | THE1D | MaLR | LTR |
| 83717264 | 83717346 | - | 26.4 | 88 | 169 | -93 | MIR | MIR | SINE |

  

|  |  |  |  |  |  |  |  |  |  |
| --- | --- | --- | --- | --- | --- | --- | --- | --- | --- |
| chr1:87654918-87655749 | | | 832 bp | | |  |  |  |  |
| Genome Start | Genome End | Strand | Div | Repeat Start | Repeat End | Repeat Left | Name | Family | Class |
| 87654918 | 87655300 | + | 26.5 | 5527 | 5898 | -1910 | L1MC4a | L1 | LINE |
| 87655301 | 87655732 | - | 12.3 | 1 | 434 | 0 | Tigger2a | MER2\_type | DNA |
| 87655733 | 87655749 | + | 26.5 | 5899 | 5915 | -1893 | L1MC4a | L1 | LINE |

  

|  |  |  |  |  |  |  |  |  |  |
| --- | --- | --- | --- | --- | --- | --- | --- | --- | --- |
| chr1:87660195-87661264 | | | 1070 bp | | |  |  |  |  |
| Genome Start | Genome End | Strand | Div | Repeat Start | Repeat End | Repeat Left | Name | Family | Class |
| 87660195 | 87660425 | - | 14.5 | 156 | 593 | 0 | MLT1B | MaLR | LTR |
| 87660426 | 87660679 | - | 11.9 | 120 | 364 | 0 | THE1B | MaLR | LTR |
| 87660680 | 87660982 | + | 15.8 | 1 | 304 | -8 | AluSx | Alu | SINE |
| 87660983 | 87661105 | - | 11.9 | 3 | 119 | -245 | THE1B | MaLR | LTR |
| 87661106 | 87661264 | - | 13.6 | 6 | 155 | -235 | MLT1B | MaLR | LTR |

  

|  |  |  |  |  |  |  |  |  |  |
| --- | --- | --- | --- | --- | --- | --- | --- | --- | --- |
| chr1:87681849-87684458 | | | 2604 bp | | |  |  |  |  |
| Genome Start | Genome End | Strand | Div | Repeat Start | Repeat End | Repeat Left | Name | Family | Class |
| 87681849 | 87681936 | + | 17.1 | 1 | 89 | -301 | MLT1B | MaLR | LTR |
| 87681939 | 87682309 | - | 14.1 | 5 | 428 | 0 | MSTA | MaLR | LTR |
| 87682314 | 87683784 | - | 19.6 | 1 | 1580 | 0 | MSTA-int | MaLR | LTR |
| 87683785 | 87684163 | - | 13.3 | 1 | 428 | 0 | MSTA | MaLR | LTR |
| 87684164 | 87684458 | + | 16.1 | 88 | 379 | -11 | MLT1B | MaLR | LTR |

  

|  |  |  |  |  |  |  |  |  |  |
| --- | --- | --- | --- | --- | --- | --- | --- | --- | --- |
| chr1:87740627-87743454 | | | 2630 bp | | |  |  |  |  |
| Genome Start | Genome End | Strand | Div | Repeat Start | Repeat End | Repeat Left | Name | Family | Class |
| 87740627 | 87740681 | - | 14.0 | 143 | 192 | -76 | MIRb | MIR | SINE |
| 87740769 | 87741013 | + | 33.6 | 16 | 258 | -10 | MIRb | MIR | SINE |
| 87741031 | 87741439 | - | 11.1 | 1 | 381 | 0 | THE1D | MaLR | LTR |
| 87741460 | 87741559 | + | 26.0 | 4 | 109 | -25 | MER94 | AcHobo | DNA |
| 87741562 | 87741946 | + | 21.8 | 5944 | 6334 | -25 | L1MD2 | L1 | LINE |
| 87742020 | 87742135 | + | 37.1 | 3156 | 3279 | -140 | L2 | L2 | LINE |
| 87742129 | 87742313 | - | 29.8 | 3213 | 3418 | -1 | L2 | L2 | LINE |
| 87742314 | 87742806 | - | 19.9 | 4 | 556 | 0 | MLT2B4 | ERVL | LTR |
| 87742807 | 87742927 | - | 29.8 | 3094 | 3212 | -207 | L2 | L2 | LINE |
| 87742927 | 87743057 | + | 20.8 | 3268 | 3416 | -3 | L2 | L2 | LINE |
| 87743068 | 87743163 | + | 30.5 | 3274 | 3375 | -3 | L2 | L2 | LINE |
| 87743151 | 87743310 | + | 30.2 | 3220 | 3409 | -10 | L2 | L2 | LINE |
| 87743321 | 87743454 | - | 36.1 | 8 | 143 | -125 | MIRb | MIR | SINE |

  

|  |  |  |  |  |  |  |  |  |  |
| --- | --- | --- | --- | --- | --- | --- | --- | --- | --- |
| chr1:89667978-89669353 | | | 1376 bp | | |  |  |  |  |
| Genome Start | Genome End | Strand | Div | Repeat Start | Repeat End | Repeat Left | Name | Family | Class |
| 89667978 | 89668262 | - | 17.0 | 6314 | 6622 | -17 | MER101-int | ERV1 | LTR |
| 89668263 | 89668575 | + | 9.9 | 1 | 313 | 0 | AluSp | Alu | SINE |
| 89668576 | 89669353 | - | 17.0 | 5553 | 6313 | -326 | MER101-int | ERV1 | LTR |

  

|  |  |  |  |  |  |  |  |  |  |
| --- | --- | --- | --- | --- | --- | --- | --- | --- | --- |
| chr1:103566541-103572771 | | | 6202 bp | | |  |  |  |  |
| Genome Start | Genome End | Strand | Div | Repeat Start | Repeat End | Repeat Left | Name | Family | Class |
| 103566541 | 103568862 | - | 11.4 | 5321 | 7598 | -5 | L1PA12 | L1 | LINE |
| 103568863 | 103569159 | - | 18.2 | 1 | 297 | -15 | AluSx | Alu | SINE |
| 103569160 | 103572375 | - | 16.5 | 2042 | 5320 | -2261 | L1PA12 | L1 | LINE |
| 103572405 | 103572771 | - | 21.8 | 905 | 2041 | -5562 | L1PA12 | L1 | LINE |

  

|  |  |  |  |  |  |  |  |  |  |
| --- | --- | --- | --- | --- | --- | --- | --- | --- | --- |
| chr1:103574037-103575684 | | | 1475 bp | | |  |  |  |  |
| Genome Start | Genome End | Strand | Div | Repeat Start | Repeat End | Repeat Left | Name | Family | Class |
| 103574037 | 103574219 | - | 29.3 | 1787 | 1968 | -4247 | L1MC4 | L1 | LINE |
| 103574220 | 103574287 | + | 14.9 | 3 | 84 | -485 | MER61E | ERV1 | LTR |
| 103574415 | 103574490 | + | 27.6 | 141 | 216 | -420 | LTR77 | ERV1 | LTR |
| 103574485 | 103574549 | + | 23.1 | 208 | 281 | -353 | MER51A | ERV1 | LTR |
| 103574528 | 103574582 | + | 25.4 | 139 | 193 | -289 | MER51E | ERV1 | LTR |
| 103574576 | 103574858 | + | 23.3 | 211 | 781 | 0 | LTR77 | ERV1 | LTR |
| 103574940 | 103575684 | - | 32.0 | 983 | 1747 | -4468 | L1MC4 | L1 | LINE |

  

|  |  |  |  |  |  |  |  |  |  |
| --- | --- | --- | --- | --- | --- | --- | --- | --- | --- |
| chr1:103576546-103579207 | | | 2662 bp | | |  |  |  |  |
| Genome Start | Genome End | Strand | Div | Repeat Start | Repeat End | Repeat Left | Name | Family | Class |
| 103576546 | 103578037 | - | 10.9 | 86 | 1580 | 0 | THE1B-int | MaLR | LTR |
| 103578038 | 103579122 | + | 7.3 | 1 | 1236 | 0 | MER11B | ERVK | LTR |
| 103579123 | 103579207 | - | 10.9 | 1 | 85 | -1495 | THE1B-int | MaLR | LTR |

  

|  |  |  |  |  |  |  |  |  |  |
| --- | --- | --- | --- | --- | --- | --- | --- | --- | --- |
| chr1:103581032-103582896 | | | 1360 bp | | |  |  |  |  |
| Genome Start | Genome End | Strand | Div | Repeat Start | Repeat End | Repeat Left | Name | Family | Class |
| 103581032 | 103581549 | + | 34.9 | 10 | 553 | -4443 | HERV16 | ERVL | LTR |
| 103581808 | 103581932 | + | 25.8 | 920 | 1056 | -3940 | HERV16 | ERVL | LTR |
| 103581933 | 103582270 | + | 15.0 | 3 | 339 | 0 | MER1B | MER1\_type | DNA |
| 103582271 | 103582389 | + | 25.8 | 1057 | 1172 | -3824 | HERV16 | ERVL | LTR |
| 103582637 | 103582896 | + | 27.7 | 1621 | 1888 | -3108 | HERV16 | ERVL | LTR |

  

|  |  |  |  |  |  |  |  |  |  |
| --- | --- | --- | --- | --- | --- | --- | --- | --- | --- |
| chr1:103582909-103584444 | | | 1363 bp | | |  |  |  |  |
| Genome Start | Genome End | Strand | Div | Repeat Start | Repeat End | Repeat Left | Name | Family | Class |
| 103582909 | 103583222 | + | 27.2 | 722 | 1042 | -3806 | L1MEe | L1 | LINE |
| 103583223 | 103583306 | + | 9.0 | 2 | 80 | 0 | MADE1 | Mariner | DNA |
| 103583307 | 103583826 | + | 29.3 | 1029 | 1592 | -3242 | L1MEe | L1 | LINE |
| 103583990 | 103584148 | + | 25.5 | 2114 | 2288 | -2546 | L1MEe | L1 | LINE |
| 103584149 | 103584337 | + | 27.9 | 64 | 262 | 0 | MIR | MIR | SINE |
| 103584348 | 103584444 | + | 27.8 | 2339 | 2436 | -2398 | L1MEe | L1 | LINE |

  

|  |  |  |  |  |  |  |  |  |  |
| --- | --- | --- | --- | --- | --- | --- | --- | --- | --- |
| chr1:103597695-103598459 | | | 750 bp | | |  |  |  |  |
| Genome Start | Genome End | Strand | Div | Repeat Start | Repeat End | Repeat Left | Name | Family | Class |
| 103597695 | 103597837 | + | 12.1 | 1 | 136 | -229 | MLT1A0 | MaLR | LTR |
| 103597840 | 103598232 | - | 15.9 | 10 | 426 | 0 | MSTB | MaLR | LTR |
| 103598246 | 103598459 | + | 21.5 | 152 | 365 | 0 | MLT1A0 | MaLR | LTR |

  

|  |  |  |  |  |  |  |  |  |  |
| --- | --- | --- | --- | --- | --- | --- | --- | --- | --- |
| chr1:103618658-103620357 | | | 1700 bp | | |  |  |  |  |
| Genome Start | Genome End | Strand | Div | Repeat Start | Repeat End | Repeat Left | Name | Family | Class |
| 103618658 | 103619989 | + | 21.1 | 6114 | 7475 | -310 | L1MC3 | L1 | LINE |
| 103619990 | 103620278 | + | 21.4 | 3 | 302 | -10 | AluJb | Alu | SINE |
| 103620279 | 103620357 | + | 21.1 | 7476 | 7552 | -233 | L1MC3 | L1 | LINE |

  

|  |  |  |  |  |  |  |  |  |  |
| --- | --- | --- | --- | --- | --- | --- | --- | --- | --- |
| chr1:103750875-103752873 | | | 1999 bp | | |  |  |  |  |
| Genome Start | Genome End | Strand | Div | Repeat Start | Repeat End | Repeat Left | Name | Family | Class |
| 103750875 | 103751135 | + | 26.2 | 5218 | 5482 | -682 | L1ME2 | L1 | LINE |
| 103751136 | 103751429 | + | 11.9 | 3 | 296 | -14 | AluSg | Alu | SINE |
| 103751430 | 103751602 | + | 25.8 | 5483 | 5646 | -518 | L1ME2 | L1 | LINE |
| 103751603 | 103752659 | - | 7.6 | 1 | 1232 | -4 | MER11B | ERVK | LTR |
| 103752660 | 103752873 | + | 25.8 | 5647 | 5861 | -303 | L1ME2 | L1 | LINE |

  

|  |  |  |  |  |  |  |  |  |  |
| --- | --- | --- | --- | --- | --- | --- | --- | --- | --- |
| chr1:103760345-103764740 | | | 4341 bp | | |  |  |  |  |
| Genome Start | Genome End | Strand | Div | Repeat Start | Repeat End | Repeat Left | Name | Family | Class |
| 103760345 | 103760560 | - | 21.3 | 2198 | 2418 | 0 | Tigger1 | MER2\_type | DNA |
| 103760561 | 103760655 | + | 8.5 | 4777 | 4874 | -1272 | L1PA11 | L1 | LINE |
| 103760680 | 103761949 | + | 9.1 | 4875 | 6166 | -8 | L1PA11 | L1 | LINE |
| 103761950 | 103761997 | - | 20.8 | 2167 | 2214 | -204 | Tigger1 | MER2\_type | DNA |
| 103761999 | 103762192 | - | 15.3 | 124 | 321 | 0 | AluJb | Alu | SINE |
| 103762222 | 103762292 | - | 15.3 | 64 | 123 | -189 | AluJb | Alu | SINE |
| 103762294 | 103762991 | - | 14.0 | 1488 | 2186 | -232 | Tigger1 | MER2\_type | DNA |
| 103762992 | 103763303 | - | 20.8 | 1 | 303 | -9 | AluJo | Alu | SINE |
| 103763304 | 103764740 | - | 14.0 | 25 | 1487 | -931 | Tigger1 | MER2\_type | DNA |

  

|  |  |  |  |  |  |  |  |  |  |
| --- | --- | --- | --- | --- | --- | --- | --- | --- | --- |
| chr1:103980048-103984547 | | | 4030 bp | | |  |  |  |  |
| Genome Start | Genome End | Strand | Div | Repeat Start | Repeat End | Repeat Left | Name | Family | Class |
| 103980048 | 103981590 | + | 22.3 | 3025 | 4600 | -2962 | L1MA9 | L1 | LINE |
| 103981588 | 103982256 | + | 24.1 | 5914 | 6610 | -949 | L1MA9 | L1 | LINE |
| 103982284 | 103982729 | + | 20.3 | 6611 | 7065 | -666 | L1MA9 | L1 | LINE |
| 103982730 | 103982962 | + | 14.3 | 5 | 235 | -78 | AluSq | Alu | SINE |
| 103982963 | 103983124 | + | 19.7 | 7066 | 7210 | -521 | L1MA9 | L1 | LINE |
| 103983178 | 103983296 | + | 19.7 | 7211 | 7330 | -401 | L1MA9 | L1 | LINE |
| 103983309 | 103983492 | + | 21.7 | 7446 | 7641 | -90 | L1MA9 | L1 | LINE |
| 103983498 | 103983677 | + | 16.8 | 5987 | 6166 | 0 | L1PA16 | L1 | LINE |
| 103983865 | 103984285 | + | 26.5 | 5727 | 6162 | 0 | L1ME3 | L1 | LINE |
| 103984475 | 103984547 | + | 17.8 | 7657 | 7729 | -2 | L1MA9 | L1 | LINE |

  

|  |  |  |  |  |  |  |  |  |  |
| --- | --- | --- | --- | --- | --- | --- | --- | --- | --- |
| chr1:103990930-103991589 | | | 660 bp | | |  |  |  |  |
| Genome Start | Genome End | Strand | Div | Repeat Start | Repeat End | Repeat Left | Name | Family | Class |
| 103990930 | 103991029 | + | 30.2 | 5321 | 5433 | -807 | L1M5 | L1 | LINE |
| 103991030 | 103991336 | - | 11.8 | 1 | 304 | -7 | AluY | Alu | SINE |
| 103991337 | 103991589 | + | 30.8 | 5433 | 5726 | -514 | L1M5 | L1 | LINE |

  

|  |  |  |  |  |  |  |  |  |  |
| --- | --- | --- | --- | --- | --- | --- | --- | --- | --- |
| chr1:104085016-104085727 | | | 712 bp | | |  |  |  |  |
| Genome Start | Genome End | Strand | Div | Repeat Start | Repeat End | Repeat Left | Name | Family | Class |
| 104085016 | 104085167 | + | 32.1 | 5273 | 5436 | -807 | L1M5 | L1 | LINE |
| 104085168 | 104085474 | - | 11.5 | 1 | 304 | -7 | AluY | Alu | SINE |
| 104085475 | 104085727 | + | 31.4 | 5434 | 5726 | -514 | L1M5 | L1 | LINE |

  

|  |  |  |  |  |  |  |  |  |  |
| --- | --- | --- | --- | --- | --- | --- | --- | --- | --- |
| chr1:108594864-108596151 | | | 1288 bp | | |  |  |  |  |
| Genome Start | Genome End | Strand | Div | Repeat Start | Repeat End | Repeat Left | Name | Family | Class |
| 108594864 | 108594965 | - | 27.5 | 6058 | 6159 | -7 | L1ME1 | L1 | LINE |
| 108594966 | 108595267 | + | 15.2 | 1 | 303 | -9 | AluJo | Alu | SINE |
| 108595268 | 108596151 | - | 27.4 | 5120 | 6057 | -109 | L1ME1 | L1 | LINE |

  

|  |  |  |  |  |  |  |  |  |  |
| --- | --- | --- | --- | --- | --- | --- | --- | --- | --- |
| chr1:108605692-108610394 | | | 4707 bp | | |  |  |  |  |
| Genome Start | Genome End | Strand | Div | Repeat Start | Repeat End | Repeat Left | Name | Family | Class |
| 108605692 | 108609189 | + | 12.9 | 10 | 3456 | -3005 | L1P3 | L1 | LINE |
| 108609187 | 108610201 | + | 3.2 | 5142 | 6155 | 0 | L1PA3 | L1 | LINE |
| 108610201 | 108610394 | + | 14.8 | 3430 | 3618 | -2843 | L1P3 | L1 | LINE |

  

|  |  |  |  |  |  |  |  |  |  |
| --- | --- | --- | --- | --- | --- | --- | --- | --- | --- |
| chr1:108626410-108627387 | | | 668 bp | | |  |  |  |  |
| Genome Start | Genome End | Strand | Div | Repeat Start | Repeat End | Repeat Left | Name | Family | Class |
| 108626410 | 108626504 | - | 18.1 | 1861 | 1955 | -4678 | L1MEc | L1 | LINE |
| 108626534 | 108626835 | + | 9.4 | 1 | 297 | -16 | AluSq | Alu | SINE |
| 108627117 | 108627387 | - | 36.1 | 355 | 638 | -6080 | L1MEc | L1 | LINE |

  

|  |  |  |  |  |  |  |  |  |  |
| --- | --- | --- | --- | --- | --- | --- | --- | --- | --- |
| chr1:108711198-108713566 | | | 2072 bp | | |  |  |  |  |
| Genome Start | Genome End | Strand | Div | Repeat Start | Repeat End | Repeat Left | Name | Family | Class |
| 108711198 | 108711983 | + | 28.6 | 4329 | 5175 | -971 | L1ME3 | L1 | LINE |
| 108712281 | 108712927 | + | 27.2 | 5234 | 5958 | -204 | L1ME3 | L1 | LINE |
| 108712928 | 108713365 | + | 16.3 | 1 | 432 | 0 | MSTB1 | MaLR | LTR |
| 108713366 | 108713566 | + | 28.4 | 5959 | 6158 | -4 | L1ME3 | L1 | LINE |

  

|  |  |  |  |  |  |  |  |  |  |
| --- | --- | --- | --- | --- | --- | --- | --- | --- | --- |
| chr1:108781331-108785028 | | | 3698 bp | | |  |  |  |  |
| Genome Start | Genome End | Strand | Div | Repeat Start | Repeat End | Repeat Left | Name | Family | Class |
| 108781331 | 108781601 | + | 16.7 | 2752 | 3027 | -3113 | L1M2 | L1 | LINE |
| 108781602 | 108781881 | + | 14.4 | 1 | 278 | -34 | AluSx | Alu | SINE |
| 108781882 | 108784139 | + | 16.7 | 3028 | 5140 | -1000 | L1M2 | L1 | LINE |
| 108784140 | 108784441 | + | 10.7 | 1 | 302 | -11 | AluSq | Alu | SINE |
| 108784442 | 108785028 | + | 14.7 | 5141 | 5761 | -551 | L1M2 | L1 | LINE |

  

|  |  |  |  |  |  |  |  |  |  |
| --- | --- | --- | --- | --- | --- | --- | --- | --- | --- |
| chr1:110038390-110038929 | | | 540 bp | | |  |  |  |  |
| Genome Start | Genome End | Strand | Div | Repeat Start | Repeat End | Repeat Left | Name | Family | Class |
| 110038390 | 110038471 | - | 28.8 | 1600 | 1681 | -826 | HAL1 | L1 | LINE |
| 110038472 | 110038824 | - | 13.9 | 1 | 360 | -4 | THE1B | MaLR | LTR |
| 110038825 | 110038929 | - | 28.8 | 1488 | 1599 | -908 | HAL1 | L1 | LINE |

  

|  |  |  |  |  |  |  |  |  |  |
| --- | --- | --- | --- | --- | --- | --- | --- | --- | --- |
| chr1:116964647-116965779 | | | 1114 bp | | |  |  |  |  |
| Genome Start | Genome End | Strand | Div | Repeat Start | Repeat End | Repeat Left | Name | Family | Class |
| 116964647 | 116964884 | + | 16.7 | 78 | 378 | -164 | MLT1F1 | MaLR | LTR |
| 116964890 | 116965078 | - | 16.0 | 111 | 296 | -16 | AluJo | Alu | SINE |
| 116965079 | 116965494 | + | 14.0 | 1 | 428 | 0 | MSTA | MaLR | LTR |
| 116965495 | 116965597 | - | 16.0 | 10 | 110 | -202 | AluJo | Alu | SINE |
| 116965612 | 116965779 | + | 20.6 | 398 | 564 | -3 | MLT1F1 | MaLR | LTR |

  

|  |  |  |  |  |  |  |  |  |  |
| --- | --- | --- | --- | --- | --- | --- | --- | --- | --- |
| chr1:116981387-116982066 | | | 617 bp | | |  |  |  |  |
| Genome Start | Genome End | Strand | Div | Repeat Start | Repeat End | Repeat Left | Name | Family | Class |
| 116981387 | 116981429 | - | 23.3 | 4260 | 4302 | -187 | L3 | CR1 | LINE |
| 116981462 | 116981581 | - | 36.2 | 3298 | 3417 | -2 | L2 | L2 | LINE |
| 116981613 | 116982066 | - | 32.5 | 3508 | 4041 | -448 | L3 | CR1 | LINE |

  

|  |  |  |  |  |  |  |  |  |  |
| --- | --- | --- | --- | --- | --- | --- | --- | --- | --- |
| chr1:116995740-116995947 | | | 208 bp | | |  |  |  |  |
| Genome Start | Genome End | Strand | Div | Repeat Start | Repeat End | Repeat Left | Name | Family | Class |
| 116995740 | 116995773 | - | 35.8 | 3349 | 3378 | 0 | L2 | L2 | LINE |
| 116995774 | 116995848 | + | 22.7 | 1 | 80 | 0 | MADE2 | Mariner | DNA |
| 116995849 | 116995947 | - | 35.8 | 3255 | 3348 | -30 | L2 | L2 | LINE |

  

|  |  |  |  |  |  |  |  |  |  |
| --- | --- | --- | --- | --- | --- | --- | --- | --- | --- |
| chr1:120198637-120201548 | | | 2707 bp | | |  |  |  |  |
| Genome Start | Genome End | Strand | Div | Repeat Start | Repeat End | Repeat Left | Name | Family | Class |
| 120198637 | 120199093 | - | 27.1 | 5764 | 6234 | -5 | L1ME1 | L1 | LINE |
| 120199094 | 120199659 | + | 6.3 | 1 | 660 | 0 | PABL\_A | ERV1 | LTR |
| 120199660 | 120200030 | - | 27.1 | 5367 | 5763 | -476 | L1ME1 | L1 | LINE |
| 120200236 | 120200665 | - | 28.9 | 4505 | 4929 | -1290 | L1ME1 | L1 | LINE |
| 120200666 | 120200965 | - | 14.3 | 1 | 301 | -10 | AluY | Alu | SINE |
| 120200966 | 120201548 | - | 28.4 | 3937 | 4504 | -1715 | L1ME1 | L1 | LINE |

  

|  |  |  |  |  |  |  |  |  |  |
| --- | --- | --- | --- | --- | --- | --- | --- | --- | --- |
| chr1:120354469-120357044 | | | 2163 bp | | |  |  |  |  |
| Genome Start | Genome End | Strand | Div | Repeat Start | Repeat End | Repeat Left | Name | Family | Class |
| 120354469 | 120354539 | - | 25.4 | 4364 | 4435 | -54 | L3 | CR1 | LINE |
| 120354610 | 120354799 | + | 22.1 | 18 | 241 | -21 | MIR | MIR | SINE |
| 120354914 | 120355427 | + | 14.8 | 5631 | 6164 | -20 | L1MB7 | L1 | LINE |
| 120355442 | 120355651 | - | 33.6 | 4043 | 4270 | -219 | L3 | CR1 | LINE |
| 120355732 | 120355880 | - | 29.8 | 86 | 251 | -11 | MIR | MIR | SINE |
| 120355881 | 120356182 | + | 6.0 | 1 | 302 | -9 | AluY | Alu | SINE |
| 120356183 | 120356264 | - | 29.8 | 2 | 85 | -177 | MIR | MIR | SINE |
| 120356265 | 120356720 | - | 31.9 | 3666 | 4056 | -433 | L3 | CR1 | LINE |
| 120356731 | 120356830 | - | 28.0 | 89 | 205 | -3 | MIR3 | MIR | SINE |
| 120356956 | 120357044 | - | 25.0 | 3350 | 3455 | -1034 | L3 | CR1 | LINE |

  

|  |  |  |  |  |  |  |  |  |  |
| --- | --- | --- | --- | --- | --- | --- | --- | --- | --- |
| chr1:120377042-120378061 | | | 1020 bp | | |  |  |  |  |
| Genome Start | Genome End | Strand | Div | Repeat Start | Repeat End | Repeat Left | Name | Family | Class |
| 120377042 | 120377289 | + | 17.9 | 5505 | 5776 | -515 | L1MA7 | L1 | LINE |
| 120377290 | 120377586 | + | 9.1 | 1 | 310 | 0 | AluSg | Alu | SINE |
| 120377587 | 120378061 | + | 17.9 | 5777 | 6281 | -10 | L1MA7 | L1 | LINE |

  

|  |  |  |  |  |  |  |  |  |  |
| --- | --- | --- | --- | --- | --- | --- | --- | --- | --- |
| chr1:120392547-120393067 | | | 521 bp | | |  |  |  |  |
| Genome Start | Genome End | Strand | Div | Repeat Start | Repeat End | Repeat Left | Name | Family | Class |
| 120392547 | 120392591 | + | 24.4 | 17 | 67 | -201 | MIRb | MIR | SINE |
| 120392592 | 120392892 | - | 15.3 | 1 | 302 | -10 | AluJo | Alu | SINE |
| 120392893 | 120393067 | + | 24.4 | 68 | 256 | -12 | MIRb | MIR | SINE |

  

|  |  |  |  |  |  |  |  |  |  |
| --- | --- | --- | --- | --- | --- | --- | --- | --- | --- |
| chr1:120424326-120425055 | | | 730 bp | | |  |  |  |  |
| Genome Start | Genome End | Strand | Div | Repeat Start | Repeat End | Repeat Left | Name | Family | Class |
| 120424326 | 120424674 | + | 14.4 | 3 | 353 | -73 | MSTB | MaLR | LTR |
| 120424675 | 120424981 | - | 17.9 | 1 | 307 | -5 | AluSx | Alu | SINE |
| 120424982 | 120425055 | + | 14.4 | 354 | 425 | -1 | MSTB | MaLR | LTR |

  

|  |  |  |  |  |  |  |  |  |  |
| --- | --- | --- | --- | --- | --- | --- | --- | --- | --- |
| chr1:120428173-120428911 | | | 739 bp | | |  |  |  |  |
| Genome Start | Genome End | Strand | Div | Repeat Start | Repeat End | Repeat Left | Name | Family | Class |
| 120428173 | 120428272 | - | 23.1 | 962 | 1059 | 0 | MER106B | MER1\_type | DNA |
| 120428273 | 120428799 | + | 15.9 | 1 | 520 | 0 | MLT2B1 | ERVL | LTR |
| 120428800 | 120428911 | - | 20.0 | 30 | 962 | -97 | MER106B | MER1\_type | DNA |

  

|  |  |  |  |  |  |  |  |  |  |
| --- | --- | --- | --- | --- | --- | --- | --- | --- | --- |
| chr1:120464958-120465689 | | | 732 bp | | |  |  |  |  |
| Genome Start | Genome End | Strand | Div | Repeat Start | Repeat End | Repeat Left | Name | Family | Class |
| 120464958 | 120465027 | + | 29.5 | 2 | 83 | -185 | MIRb | MIR | SINE |
| 120465028 | 120465540 | - | 15.0 | 5814 | 6333 | 0 | L1MC1 | L1 | LINE |
| 120465541 | 120465689 | + | 29.3 | 84 | 245 | -23 | MIRb | MIR | SINE |

  

|  |  |  |  |  |  |  |  |  |  |
| --- | --- | --- | --- | --- | --- | --- | --- | --- | --- |
| chr1:120470683-120471723 | | | 941 bp | | |  |  |  |  |
| Genome Start | Genome End | Strand | Div | Repeat Start | Repeat End | Repeat Left | Name | Family | Class |
| 120470683 | 120471085 | + | 25.3 | 5274 | 5668 | -2140 | L1MC4a | L1 | LINE |
| 120471186 | 120471340 | + | 27.1 | 5669 | 5824 | -1984 | L1MC4a | L1 | LINE |
| 120471341 | 120471643 | + | 16.5 | 1 | 306 | -6 | AluJo | Alu | SINE |
| 120471644 | 120471723 | + | 27.1 | 5825 | 5906 | -1902 | L1MC4a | L1 | LINE |

  

|  |  |  |  |  |  |  |  |  |  |
| --- | --- | --- | --- | --- | --- | --- | --- | --- | --- |
| chr1:120554348-120556981 | | | 2571 bp | | |  |  |  |  |
| Genome Start | Genome End | Strand | Div | Repeat Start | Repeat End | Repeat Left | Name | Family | Class |
| 120554348 | 120555286 | + | 25.8 | 859 | 1850 | -159 | HAL1b | L1 | LINE |
| 120555340 | 120556868 | + | 25.8 | 1596 | 3419 | 0 | L2 | L2 | LINE |
| 120556879 | 120556981 | + | 19.4 | 1905 | 2007 | -2 | HAL1b | L1 | LINE |

  

|  |  |  |  |  |  |  |  |  |  |
| --- | --- | --- | --- | --- | --- | --- | --- | --- | --- |
| chr1:120564411-120565259 | | | 849 bp | | |  |  |  |  |
| Genome Start | Genome End | Strand | Div | Repeat Start | Repeat End | Repeat Left | Name | Family | Class |
| 120564411 | 120564693 | + | 25.5 | 2933 | 3185 | -234 | L2 | L2 | LINE |
| 120564694 | 120565017 | + | 14.1 | 1 | 306 | -6 | AluJo | Alu | SINE |
| 120565018 | 120565259 | + | 25.5 | 3186 | 3408 | -11 | L2 | L2 | LINE |

  

|  |  |  |  |  |  |  |  |  |  |
| --- | --- | --- | --- | --- | --- | --- | --- | --- | --- |
| chr1:120584368-120585812 | | | 972 bp | | |  |  |  |  |
| Genome Start | Genome End | Strand | Div | Repeat Start | Repeat End | Repeat Left | Name | Family | Class |
| 120584368 | 120584551 | + | 29.9 | 2328 | 2522 | -897 | L2 | L2 | LINE |
| 120584902 | 120584977 | + | 31.1 | 2754 | 2831 | -588 | L2 | L2 | LINE |
| 120584978 | 120585155 | - | 16.3 | 1 | 224 | 0 | MER58A | MER1\_type | DNA |
| 120585156 | 120585544 | + | 28.1 | 2832 | 3271 | -148 | L2 | L2 | LINE |
| 120585668 | 120585812 | + | 27.4 | 3272 | 3407 | -12 | L2 | L2 | LINE |

  

|  |  |  |  |  |  |  |  |  |  |
| --- | --- | --- | --- | --- | --- | --- | --- | --- | --- |
| chr1:120593441-120594658 | | | 1218 bp | | |  |  |  |  |
| Genome Start | Genome End | Strand | Div | Repeat Start | Repeat End | Repeat Left | Name | Family | Class |
| 120593441 | 120593645 | - | 26.0 | 3157 | 3419 | 0 | L2 | L2 | LINE |
| 120593646 | 120594153 | - | 6.3 | 5649 | 6154 | 0 | L1PA7 | L1 | LINE |
| 120594154 | 120594658 | - | 25.0 | 2597 | 3156 | -263 | L2 | L2 | LINE |

  

|  |  |  |  |  |  |  |  |  |  |
| --- | --- | --- | --- | --- | --- | --- | --- | --- | --- |
| chr1:120619474-120620251 | | | 778 bp | | |  |  |  |  |
| Genome Start | Genome End | Strand | Div | Repeat Start | Repeat End | Repeat Left | Name | Family | Class |
| 120619474 | 120619781 | + | 27.6 | 4833 | 5159 | -987 | L1M5 | L1 | LINE |
| 120619782 | 120620090 | - | 16.9 | 3 | 309 | -3 | AluJb | Alu | SINE |
| 120620091 | 120620251 | + | 27.6 | 5160 | 5318 | -828 | L1M5 | L1 | LINE |

  

|  |  |  |  |  |  |  |  |  |  |
| --- | --- | --- | --- | --- | --- | --- | --- | --- | --- |
| chr1:120622036-120622442 | | | 407 bp | | |  |  |  |  |
| Genome Start | Genome End | Strand | Div | Repeat Start | Repeat End | Repeat Left | Name | Family | Class |
| 120622036 | 120622119 | + | 29.7 | 3293 | 3383 | -36 | L2 | L2 | LINE |
| 120622120 | 120622414 | + | 9.8 | 1 | 295 | -15 | AluSg | Alu | SINE |
| 120622415 | 120622442 | + | 29.7 | 3384 | 3400 | -19 | L2 | L2 | LINE |

  

|  |  |  |  |  |  |  |  |  |  |
| --- | --- | --- | --- | --- | --- | --- | --- | --- | --- |
| chr1:120643074-120643448 | | | 370 bp | | |  |  |  |  |
| Genome Start | Genome End | Strand | Div | Repeat Start | Repeat End | Repeat Left | Name | Family | Class |
| 120643074 | 120643280 | + | 14.0 | 5566 | 5777 | -394 | L1MB2 | L1 | LINE |
| 120643281 | 120643349 | + | 24.6 | 8 | 76 | -90 | FRAM/FAM | Alu | SINE |
| 120643355 | 120643448 | + | 17.0 | 6069 | 6167 | -4 | L1MB2 | L1 | LINE |

  

|  |  |  |  |  |  |  |  |  |  |
| --- | --- | --- | --- | --- | --- | --- | --- | --- | --- |
| chr1:120648175-120651434 | | | 3251 bp | | |  |  |  |  |
| Genome Start | Genome End | Strand | Div | Repeat Start | Repeat End | Repeat Left | Name | Family | Class |
| 120648175 | 120648378 | - | 11.4 | 2225 | 2418 | 0 | Tigger1 | MER2\_type | DNA |
| 120648379 | 120648678 | + | 15.5 | 1 | 298 | -14 | AluJb | Alu | SINE |
| 120648679 | 120649903 | - | 11.4 | 973 | 2224 | -194 | Tigger1 | MER2\_type | DNA |
| 120649904 | 120650174 | + | 14.8 | 2 | 295 | -18 | AluSq | Alu | SINE |
| 120650184 | 120650495 | + | 6.7 | 1 | 313 | 0 | AluSp | Alu | SINE |
| 120650496 | 120651434 | - | 11.4 | 2 | 972 | -1446 | Tigger1 | MER2\_type | DNA |

  

|  |  |  |  |  |  |  |  |  |  |
| --- | --- | --- | --- | --- | --- | --- | --- | --- | --- |
| chr1:120670573-120671102 | | | 530 bp | | |  |  |  |  |
| Genome Start | Genome End | Strand | Div | Repeat Start | Repeat End | Repeat Left | Name | Family | Class |
| 120670573 | 120670816 | - | 25.1 | 5540 | 5805 | -316 | L1M5 | L1 | LINE |
| 120670817 | 120671074 | + | 9.7 | 43 | 302 | -11 | AluSq | Alu | SINE |
| 120671075 | 120671102 | - | 25.1 | 5510 | 5539 | -582 | L1M5 | L1 | LINE |

  

|  |  |  |  |  |  |  |  |  |  |
| --- | --- | --- | --- | --- | --- | --- | --- | --- | --- |
| chr1:120672404-120673053 | | | 650 bp | | |  |  |  |  |
| Genome Start | Genome End | Strand | Div | Repeat Start | Repeat End | Repeat Left | Name | Family | Class |
| 120672404 | 120672604 | - | 19.7 | 163 | 349 | -25 | MLT1A | MaLR | LTR |
| 120672605 | 120672885 | - | 14.2 | 1 | 298 | -14 | AluJo | Alu | SINE |
| 120672886 | 120673053 | - | 19.7 | 2 | 162 | -212 | MLT1A | MaLR | LTR |

  

|  |  |  |  |  |  |  |  |  |  |
| --- | --- | --- | --- | --- | --- | --- | --- | --- | --- |
| chr1:120679393-120681761 | | | 2369 bp | | |  |  |  |  |
| Genome Start | Genome End | Strand | Div | Repeat Start | Repeat End | Repeat Left | Name | Family | Class |
| 120679393 | 120679864 | - | 20.5 | 5790 | 6276 | -36 | L1MA9 | L1 | LINE |
| 120679865 | 120681116 | - | 11.5 | 47 | 1301 | -1 | HSMAR2 | Mariner | DNA |
| 120681117 | 120681430 | - | 6.1 | 1 | 311 | 0 | AluY | Alu | SINE |
| 120681431 | 120681493 | - | 11.5 | 1 | 46 | -1256 | HSMAR2 | Mariner | DNA |
| 120681494 | 120681761 | - | 20.5 | 5532 | 5789 | -523 | L1MA9 | L1 | LINE |

  

|  |  |  |  |  |  |  |  |  |  |
| --- | --- | --- | --- | --- | --- | --- | --- | --- | --- |
| chr1:120693725-120694524 | | | 800 bp | | |  |  |  |  |
| Genome Start | Genome End | Strand | Div | Repeat Start | Repeat End | Repeat Left | Name | Family | Class |
| 120693725 | 120694161 | + | 20.6 | 1 | 447 | -58 | MLT1D | MaLR | LTR |
| 120694162 | 120694456 | - | 9.8 | 2 | 296 | -17 | AluSq | Alu | SINE |
| 120694457 | 120694524 | + | 20.6 | 448 | 505 | 0 | MLT1D | MaLR | LTR |

  

|  |  |  |  |  |  |  |  |  |  |
| --- | --- | --- | --- | --- | --- | --- | --- | --- | --- |
| chr1:120694980-120695936 | | | 961 bp | | |  |  |  |  |
| Genome Start | Genome End | Strand | Div | Repeat Start | Repeat End | Repeat Left | Name | Family | Class |
| 120694980 | 120695418 | + | 14.8 | 1 | 459 | -2272 | Tigger4(Zombi) | MER2\_type | DNA |
| 120695419 | 120695711 | + | 9.6 | 1 | 299 | -13 | AluSx | Alu | SINE |
| 120695712 | 120695788 | + | 14.8 | 460 | 531 | -2200 | Tigger4(Zombi) | MER2\_type | DNA |
| 120695785 | 120695936 | + | 9.9 | 2568 | 2729 | -2 | Tigger4(Zombi) | MER2\_type | DNA |

  

|  |  |  |  |  |  |  |  |  |  |
| --- | --- | --- | --- | --- | --- | --- | --- | --- | --- |
| chr1:120697390-120697821 | | | 432 bp | | |  |  |  |  |
| Genome Start | Genome End | Strand | Div | Repeat Start | Repeat End | Repeat Left | Name | Family | Class |
| 120697390 | 120697432 | - | 20.0 | 2612 | 2646 | -13 | Charlie9 | MER1\_type | DNA |
| 120697433 | 120697747 | + | 5.0 | 1 | 300 | -11 | AluY | Alu | SINE |
| 120697748 | 120697821 | - | 20.0 | 2536 | 2611 | -48 | Charlie9 | MER1\_type | DNA |

  

|  |  |  |  |  |  |  |  |  |  |
| --- | --- | --- | --- | --- | --- | --- | --- | --- | --- |
| chr1:120713000-120713950 | | | 951 bp | | |  |  |  |  |
| Genome Start | Genome End | Strand | Div | Repeat Start | Repeat End | Repeat Left | Name | Family | Class |
| 120713000 | 120713584 | + | 26.7 | 2580 | 3217 | -202 | L2 | L2 | LINE |
| 120713585 | 120713882 | - | 10.3 | 1 | 295 | -16 | AluY | Alu | SINE |
| 120713883 | 120713950 | + | 31.1 | 3218 | 3286 | -133 | L2 | L2 | LINE |

  

|  |  |  |  |  |  |  |  |  |  |
| --- | --- | --- | --- | --- | --- | --- | --- | --- | --- |
| chr1:120813203-120814420 | | | 1218 bp | | |  |  |  |  |
| Genome Start | Genome End | Strand | Div | Repeat Start | Repeat End | Repeat Left | Name | Family | Class |
| 120813203 | 120813823 | - | 25.2 | 359 | 1079 | -1428 | HAL1 | L1 | LINE |
| 120813824 | 120814127 | - | 6.3 | 1 | 301 | -10 | AluY | Alu | SINE |
| 120814128 | 120814420 | - | 25.2 | 23 | 358 | -2149 | HAL1 | L1 | LINE |

  

|  |  |  |  |  |  |  |  |  |  |
| --- | --- | --- | --- | --- | --- | --- | --- | --- | --- |
| chr1:120855238-120857335 | | | 2029 bp | | |  |  |  |  |
| Genome Start | Genome End | Strand | Div | Repeat Start | Repeat End | Repeat Left | Name | Family | Class |
| 120855238 | 120855775 | - | 35.0 | 2799 | 3378 | 0 | L2 | L2 | LINE |
| 120855834 | 120855905 | + | 11.6 | 2 | 70 | -10 | MADE1 | Mariner | DNA |
| 120855917 | 120856519 | - | 35.7 | 2067 | 2694 | -725 | L2 | L2 | LINE |
| 120856520 | 120856810 | + | 11.3 | 1 | 299 | -11 | AluSg | Alu | SINE |
| 120856811 | 120857335 | - | 35.7 | 1486 | 2066 | -1353 | L2 | L2 | LINE |

  

|  |  |  |  |  |  |  |  |  |  |
| --- | --- | --- | --- | --- | --- | --- | --- | --- | --- |
| chr1:120958308-120959528 | | | 1221 bp | | |  |  |  |  |
| Genome Start | Genome End | Strand | Div | Repeat Start | Repeat End | Repeat Left | Name | Family | Class |
| 120958308 | 120958635 | + | 16.1 | 4436 | 4747 | -1396 | L1M2 | L1 | LINE |
| 120958636 | 120959081 | + | 10.0 | 5709 | 6173 | -1 | L1PA11 | L1 | LINE |
| 120959082 | 120959528 | + | 16.1 | 4748 | 5188 | -955 | L1M2 | L1 | LINE |

  

|  |  |  |  |  |  |  |  |  |  |
| --- | --- | --- | --- | --- | --- | --- | --- | --- | --- |
| chr1:141581194-141582417 | | | 1224 bp | | |  |  |  |  |
| Genome Start | Genome End | Strand | Div | Repeat Start | Repeat End | Repeat Left | Name | Family | Class |
| 141581194 | 141581999 | - | 27.2 | 2501 | 3343 | -3194 | L1MDa | L1 | LINE |
| 141582000 | 141582306 | - | 19.2 | 1 | 309 | -3 | AluJb | Alu | SINE |
| 141582307 | 141582417 | - | 25.1 | 2366 | 2501 | -4056 | L1MDa | L1 | LINE |

  

|  |  |  |  |  |  |  |  |  |  |
| --- | --- | --- | --- | --- | --- | --- | --- | --- | --- |
| chr1:141588768-141589260 | | | 444 bp | | |  |  |  |  |
| Genome Start | Genome End | Strand | Div | Repeat Start | Repeat End | Repeat Left | Name | Family | Class |
| 141588768 | 141588813 | - | 29.8 | 6270 | 6315 | -19 | L1MA10 | L1 | LINE |
| 141588863 | 141589136 | - | 10.3 | 10 | 280 | -33 | AluSq | Alu | SINE |
| 141589137 | 141589260 | - | 29.8 | 6160 | 6269 | -65 | L1MA10 | L1 | LINE |

  

|  |  |  |  |  |  |  |  |  |  |
| --- | --- | --- | --- | --- | --- | --- | --- | --- | --- |
| chr1:141589620-141592874 | | | 3255 bp | | |  |  |  |  |
| Genome Start | Genome End | Strand | Div | Repeat Start | Repeat End | Repeat Left | Name | Family | Class |
| 141589620 | 141589837 | - | 16.0 | 2174 | 2414 | -4 | Tigger1 | MER2\_type | DNA |
| 141589838 | 141590103 | - | 19.1 | 1 | 295 | -7 | AluJo | Alu | SINE |
| 141590104 | 141590696 | - | 16.0 | 1633 | 2173 | -245 | Tigger1 | MER2\_type | DNA |
| 141590697 | 141590990 | - | 12.8 | 1 | 298 | -12 | AluSg | Alu | SINE |
| 141590991 | 141591843 | - | 16.0 | 757 | 1632 | -786 | Tigger1 | MER2\_type | DNA |
| 141591844 | 141592154 | + | 11.7 | 1 | 308 | -5 | AluSp | Alu | SINE |
| 141592155 | 141592874 | - | 16.0 | 1 | 756 | -1662 | Tigger1 | MER2\_type | DNA |

  

|  |  |  |  |  |  |  |  |  |  |
| --- | --- | --- | --- | --- | --- | --- | --- | --- | --- |
| chr1:141606598-141607586 | | | 848 bp | | |  |  |  |  |
| Genome Start | Genome End | Strand | Div | Repeat Start | Repeat End | Repeat Left | Name | Family | Class |
| 141606598 | 141606758 | - | 22.9 | 935 | 1097 | -5501 | MER4-int | ERV1 | LTR |
| 141606806 | 141606849 | - | 11.9 | 273 | 314 | -281 | LTR49 | ERV1 | LTR |
| 141606939 | 141607018 | - | 19.2 | 949 | 1028 | -7242 | MER4-int | ERV1 | LTR |
| 141607024 | 141607586 | - | 19.8 | 29 | 653 | -5945 | MER4-int | ERV1 | LTR |

  

|  |  |  |  |  |  |  |  |  |  |
| --- | --- | --- | --- | --- | --- | --- | --- | --- | --- |
| chr1:141642702-141644423 | | | 1656 bp | | |  |  |  |  |
| Genome Start | Genome End | Strand | Div | Repeat Start | Repeat End | Repeat Left | Name | Family | Class |
| 141642702 | 141643822 | + | 21.1 | 4805 | 5946 | -354 | L1MA5 | L1 | LINE |
| 141643824 | 141644082 | + | 25.3 | 1 | 264 | -48 | AluJo | Alu | SINE |
| 141644148 | 141644423 | + | 16.3 | 6001 | 6294 | -6 | L1MA5 | L1 | LINE |

  

|  |  |  |  |  |  |  |  |  |  |
| --- | --- | --- | --- | --- | --- | --- | --- | --- | --- |
| chr1:141646180-141647965 | | | 1777 bp | | |  |  |  |  |
| Genome Start | Genome End | Strand | Div | Repeat Start | Repeat End | Repeat Left | Name | Family | Class |
| 141646180 | 141647228 | + | 19.2 | 4777 | 5836 | -342 | L1MB8 | L1 | LINE |
| 141647238 | 141647642 | + | 13.2 | 11 | 428 | 0 | MER61C | ERV1 | LTR |
| 141647643 | 141647965 | + | 21.9 | 5833 | 6173 | -5 | L1MB8 | L1 | LINE |

  

|  |  |  |  |  |  |  |  |  |  |
| --- | --- | --- | --- | --- | --- | --- | --- | --- | --- |
| chr1:141722609-141724273 | | | 1663 bp | | |  |  |  |  |
| Genome Start | Genome End | Strand | Div | Repeat Start | Repeat End | Repeat Left | Name | Family | Class |
| 141722609 | 141723320 | + | 25.2 | 2810 | 3568 | -2578 | L1M5 | L1 | LINE |
| 141723323 | 141723667 | + | 17.4 | 1 | 2405 | 0 | Tigger5 | MER2\_type | DNA |
| 141723668 | 141723713 | + | 27.5 | 3571 | 3613 | -2533 | L1M5 | L1 | LINE |
| 141723714 | 141724026 | + | 12.5 | 1 | 313 | 0 | AluSp | Alu | SINE |
| 141724027 | 141724273 | + | 27.5 | 3614 | 3862 | -2284 | L1M5 | L1 | LINE |

  

|  |  |  |  |  |  |  |  |  |  |
| --- | --- | --- | --- | --- | --- | --- | --- | --- | --- |
| chr1:141725855-141727424 | | | 1570 bp | | |  |  |  |  |
| Genome Start | Genome End | Strand | Div | Repeat Start | Repeat End | Repeat Left | Name | Family | Class |
| 141725855 | 141726481 | - | 13.7 | 5545 | 6165 | 0 | L1PA15 | L1 | LINE |
| 141726482 | 141727236 | - | 15.1 | 1 | 826 | 0 | LTR1B | ERV1 | LTR |
| 141727237 | 141727424 | - | 13.7 | 5347 | 5544 | -621 | L1PA15 | L1 | LINE |

  

|  |  |  |  |  |  |  |  |  |  |
| --- | --- | --- | --- | --- | --- | --- | --- | --- | --- |
| chr1:141778785-141780511 | | | 1727 bp | | |  |  |  |  |
| Genome Start | Genome End | Strand | Div | Repeat Start | Repeat End | Repeat Left | Name | Family | Class |
| 141778785 | 141779072 | - | 21.5 | 5948 | 6296 | -1 | L1MA5A | L1 | LINE |
| 141779073 | 141779389 | - | 23.9 | 1 | 296 | -6 | AluJo | Alu | SINE |
| 141779390 | 141780511 | - | 21.5 | 4805 | 5947 | -350 | L1MA5A | L1 | LINE |

  

|  |  |  |  |  |  |  |  |  |  |
| --- | --- | --- | --- | --- | --- | --- | --- | --- | --- |
| chr1:141809764-141810976 | | | 1213 bp | | |  |  |  |  |
| Genome Start | Genome End | Strand | Div | Repeat Start | Repeat End | Repeat Left | Name | Family | Class |
| 141809764 | 141809874 | + | 26.9 | 2366 | 2500 | -4057 | L1MDa | L1 | LINE |
| 141809875 | 141810180 | + | 18.3 | 1 | 309 | -3 | AluJb | Alu | SINE |
| 141810181 | 141810976 | + | 27.4 | 2487 | 3343 | -3194 | L1MDa | L1 | LINE |

  

|  |  |  |  |  |  |  |  |  |  |
| --- | --- | --- | --- | --- | --- | --- | --- | --- | --- |
| chr1:141898906-141900608 | | | 1685 bp | | |  |  |  |  |
| Genome Start | Genome End | Strand | Div | Repeat Start | Repeat End | Repeat Left | Name | Family | Class |
| 141898906 | 141899059 | + | 27.0 | 724 | 874 | -2545 | L2 | L2 | LINE |
| 141899069 | 141899508 | - | 20.5 | 458 | 929 | -6 | MER21C | ERV1 | LTR |
| 141899494 | 141899750 | - | 23.2 | 22 | 335 | -600 | MER21C | ERV1 | LTR |
| 141899766 | 141899953 | - | 27.0 | 1 | 189 | 0 | MER5A | MER1\_type | DNA |
| 141899963 | 141900608 | + | 30.6 | 916 | 1640 | -1779 | L2 | L2 | LINE |

  

|  |  |  |  |  |  |  |  |  |  |
| --- | --- | --- | --- | --- | --- | --- | --- | --- | --- |
| chr1:141962506-141963287 | | | 782 bp | | |  |  |  |  |
| Genome Start | Genome End | Strand | Div | Repeat Start | Repeat End | Repeat Left | Name | Family | Class |
| 141962506 | 141962885 | - | 11.4 | 7132 | 7537 | 0 | MER57B-int | ERV1 | LTR |
| 141962886 | 141963177 | + | 10.3 | 2 | 293 | -18 | AluY | Alu | SINE |
| 141963178 | 141963287 | - | 11.4 | 7032 | 7131 | -406 | MER57B-int | ERV1 | LTR |

  

|  |  |  |  |  |  |  |  |  |  |
| --- | --- | --- | --- | --- | --- | --- | --- | --- | --- |
| chr1:142003060-142004050 | | | 847 bp | | |  |  |  |  |
| Genome Start | Genome End | Strand | Div | Repeat Start | Repeat End | Repeat Left | Name | Family | Class |
| 142003060 | 142003220 | - | 20.5 | 935 | 1097 | -5501 | MER4-int | ERV1 | LTR |
| 142003268 | 142003311 | - | 9.5 | 273 | 314 | -281 | LTR49 | ERV1 | LTR |
| 142003403 | 142003482 | - | 19.2 | 949 | 1028 | -7242 | MER4-int | ERV1 | LTR |
| 142003489 | 142004050 | - | 19.6 | 29 | 652 | -5946 | MER4-int | ERV1 | LTR |

  

|  |  |  |  |  |  |  |  |  |  |
| --- | --- | --- | --- | --- | --- | --- | --- | --- | --- |
| chr1:142004052-142006859 | | | 2771 bp | | |  |  |  |  |
| Genome Start | Genome End | Strand | Div | Repeat Start | Repeat End | Repeat Left | Name | Family | Class |
| 142004052 | 142006583 | - | 18.7 | 486 | 3021 | -3745 | L1PA13 | L1 | LINE |
| 142006604 | 142006673 | + | 18.6 | 1261 | 1330 | -5651 | L1PA12 | L1 | LINE |
| 142006683 | 142006757 | + | 21.3 | 1288 | 1362 | -6238 | L1PA12 | L1 | LINE |
| 142006766 | 142006859 | - | 24.5 | 344 | 471 | -6314 | L1PA13 | L1 | LINE |

  

|  |  |  |  |  |  |  |  |  |  |
| --- | --- | --- | --- | --- | --- | --- | --- | --- | --- |
| chr1:142042652-142044437 | | | 1777 bp | | |  |  |  |  |
| Genome Start | Genome End | Strand | Div | Repeat Start | Repeat End | Repeat Left | Name | Family | Class |
| 142042652 | 142043700 | + | 19.1 | 4777 | 5836 | -342 | L1MB8 | L1 | LINE |
| 142043710 | 142044114 | + | 13.6 | 11 | 428 | 0 | MER61C | ERV1 | LTR |
| 142044115 | 142044437 | + | 21.3 | 5833 | 6173 | -5 | L1MB8 | L1 | LINE |

  

|  |  |  |  |  |  |  |  |  |  |
| --- | --- | --- | --- | --- | --- | --- | --- | --- | --- |
| chr1:142091975-142093540 | | | 1566 bp | | |  |  |  |  |
| Genome Start | Genome End | Strand | Div | Repeat Start | Repeat End | Repeat Left | Name | Family | Class |
| 142091975 | 142092162 | + | 14.2 | 5347 | 5544 | -621 | L1PA15 | L1 | LINE |
| 142092163 | 142092915 | + | 15.8 | 1 | 826 | 0 | LTR1B | ERV1 | LTR |
| 142092916 | 142093540 | + | 14.2 | 5545 | 6165 | 0 | L1PA15 | L1 | LINE |

  

|  |  |  |  |  |  |  |  |  |  |
| --- | --- | --- | --- | --- | --- | --- | --- | --- | --- |
| chr1:142095126-142096789 | | | 1662 bp | | |  |  |  |  |
| Genome Start | Genome End | Strand | Div | Repeat Start | Repeat End | Repeat Left | Name | Family | Class |
| 142095126 | 142095372 | - | 28.6 | 3614 | 3862 | -2284 | L1M5 | L1 | LINE |
| 142095373 | 142095684 | - | 11.9 | 1 | 312 | -1 | AluSp | Alu | SINE |
| 142095685 | 142095730 | - | 28.6 | 3571 | 3613 | -2533 | L1M5 | L1 | LINE |
| 142095731 | 142096075 | - | 16.7 | 1 | 2405 | 0 | Tigger5 | MER2\_type | DNA |
| 142096078 | 142096789 | - | 25.9 | 2810 | 3568 | -2578 | L1M5 | L1 | LINE |

  

|  |  |  |  |  |  |  |  |  |  |
| --- | --- | --- | --- | --- | --- | --- | --- | --- | --- |
| chr1:142096783-142098072 | | | 1290 bp | | |  |  |  |  |
| Genome Start | Genome End | Strand | Div | Repeat Start | Repeat End | Repeat Left | Name | Family | Class |
| 142096783 | 142097342 | - | 23.1 | 7057 | 7678 | -364 | L1MC4 | L1 | LINE |
| 142097343 | 142097632 | - | 12.8 | 2 | 291 | -21 | AluSx | Alu | SINE |
| 142097633 | 142098072 | - | 23.1 | 6565 | 7056 | -986 | L1MC4 | L1 | LINE |

  

|  |  |  |  |  |  |  |  |  |  |
| --- | --- | --- | --- | --- | --- | --- | --- | --- | --- |
| chr1:142105293-142106800 | | | 1508 bp | | |  |  |  |  |
| Genome Start | Genome End | Strand | Div | Repeat Start | Repeat End | Repeat Left | Name | Family | Class |
| 142105293 | 142105810 | + | 24.2 | 18 | 528 | -407 | MER21C | ERV1 | LTR |
| 142105811 | 142106140 | - | 21.3 | 2 | 297 | -5 | AluJb | Alu | SINE |
| 142106141 | 142106218 | + | 24.2 | 529 | 601 | -334 | MER21C | ERV1 | LTR |
| 142106219 | 142106512 | + | 18.6 | 9 | 300 | -12 | AluJo | Alu | SINE |
| 142106513 | 142106800 | + | 24.2 | 602 | 916 | -19 | MER21C | ERV1 | LTR |

  

|  |  |  |  |  |  |  |  |  |  |
| --- | --- | --- | --- | --- | --- | --- | --- | --- | --- |
| chr1:142230500-142232231 | | | 1732 bp | | |  |  |  |  |
| Genome Start | Genome End | Strand | Div | Repeat Start | Repeat End | Repeat Left | Name | Family | Class |
| 142230500 | 142230787 | - | 21.2 | 5948 | 6296 | -1 | L1MA5A | L1 | LINE |
| 142230788 | 142231103 | - | 25.0 | 1 | 295 | -7 | AluJo | Alu | SINE |
| 142231104 | 142232231 | - | 21.2 | 4805 | 5947 | -350 | L1MA5A | L1 | LINE |

  

|  |  |  |  |  |  |  |  |  |  |
| --- | --- | --- | --- | --- | --- | --- | --- | --- | --- |
| chr1:142250021-142254064 | | | 3750 bp | | |  |  |  |  |
| Genome Start | Genome End | Strand | Div | Repeat Start | Repeat End | Repeat Left | Name | Family | Class |
| 142250021 | 142250116 | + | 20.8 | 1 | 97 | -331 | MSTA | MaLR | LTR |
| 142250213 | 142250268 | + | 18.2 | 911 | 967 | -613 | THE1-int | MaLR | LTR |
| 142250260 | 142250500 | - | 23.8 | 907 | 1188 | -392 | THE1-int | MaLR | LTR |
| 142250525 | 142250789 | + | 22.0 | 96 | 374 | -54 | MSTA | MaLR | LTR |
| 142250809 | 142250962 | - | 24.7 | 463 | 617 | -963 | THE1-int | MaLR | LTR |
| 142250958 | 142252488 | + | 23.8 | 28 | 1583 | -68 | MST-int | MaLR | LTR |
| 142252489 | 142252527 | + | 15.4 | 623 | 661 | -990 | MST-int | MaLR | LTR |
| 142252562 | 142252624 | - | 18.3 | 317 | 376 | -52 | MSTA | MaLR | LTR |
| 142252627 | 142252703 | + | 16.9 | 1574 | 1651 | 0 | MST-int | MaLR | LTR |
| 142252704 | 142252802 | + | 25.2 | 1 | 100 | -328 | MSTA | MaLR | LTR |
| 142252848 | 142252955 | + | 24.1 | 58 | 180 | -248 | MSTA | MaLR | LTR |
| 142252949 | 142253295 | + | 21.3 | 1317 | 1651 | 0 | MSTA-int | MaLR | LTR |
| 142253296 | 142253391 | + | 16.9 | 1 | 97 | -331 | MSTA | MaLR | LTR |
| 142253476 | 142253532 | + | 22.8 | 910 | 967 | -613 | THE1-int | MaLR | LTR |
| 142253540 | 142253771 | - | 22.5 | 907 | 1183 | -397 | THE1-int | MaLR | LTR |
| 142253776 | 142254064 | + | 24.2 | 76 | 374 | -54 | MSTA | MaLR | LTR |

  

|  |  |  |  |  |  |  |  |  |  |
| --- | --- | --- | --- | --- | --- | --- | --- | --- | --- |
| chr1:142286106-142286580 | | | 439 bp | | |  |  |  |  |
| Genome Start | Genome End | Strand | Div | Repeat Start | Repeat End | Repeat Left | Name | Family | Class |
| 142286106 | 142286229 | + | 29.8 | 6160 | 6269 | -65 | L1MA10 | L1 | LINE |
| 142286230 | 142286497 | + | 9.0 | 10 | 275 | -38 | AluSq | Alu | SINE |
| 142286534 | 142286580 | + | 29.8 | 6270 | 6315 | -19 | L1MA10 | L1 | LINE |

  

|  |  |  |  |  |  |  |  |  |  |
| --- | --- | --- | --- | --- | --- | --- | --- | --- | --- |
| chr1:142292986-142294207 | | | 1222 bp | | |  |  |  |  |
| Genome Start | Genome End | Strand | Div | Repeat Start | Repeat End | Repeat Left | Name | Family | Class |
| 142292986 | 142293096 | + | 25.5 | 2366 | 2500 | -4057 | L1MDa | L1 | LINE |
| 142293097 | 142293406 | + | 18.4 | 1 | 312 | 0 | AluJb | Alu | SINE |
| 142293407 | 142294207 | + | 26.7 | 2487 | 3352 | -3185 | L1MDa | L1 | LINE |

  

|  |  |  |  |  |  |  |  |  |  |
| --- | --- | --- | --- | --- | --- | --- | --- | --- | --- |
| chr1:142312776-142315793 | | | 3018 bp | | |  |  |  |  |
| Genome Start | Genome End | Strand | Div | Repeat Start | Repeat End | Repeat Left | Name | Family | Class |
| 142312776 | 142314168 | + | 15.0 | 3396 | 4937 | -1209 | L1MA3 | L1 | LINE |
| 142314169 | 142314428 | - | 10.9 | 36 | 294 | -17 | AluY | Alu | SINE |
| 142314429 | 142314854 | + | 13.2 | 4938 | 5359 | -787 | L1MA3 | L1 | LINE |
| 142314855 | 142315147 | + | 21.6 | 1 | 308 | -4 | AluJb | Alu | SINE |
| 142315148 | 142315793 | + | 11.3 | 5360 | 6008 | -297 | L1MA3 | L1 | LINE |

  

|  |  |  |  |  |  |  |  |  |  |
| --- | --- | --- | --- | --- | --- | --- | --- | --- | --- |
| chr1:142443220-142447755 | | | 3917 bp | | |  |  |  |  |
| Genome Start | Genome End | Strand | Div | Repeat Start | Repeat End | Repeat Left | Name | Family | Class |
| 142443220 | 142443426 | - | 29.9 | 6178 | 6387 | -1883 | PRIMA4-int | ERV1 | LTR |
| 142443427 | 142443579 | + | 24.2 | 1 | 153 | -13 | FRAM | Alu | SINE |
| 142443580 | 142444121 | - | 29.9 | 5630 | 6177 | -2093 | PRIMA4-int | ERV1 | LTR |
| 142444122 | 142444421 | - | 9.0 | 1 | 300 | -9 | AluSc | Alu | SINE |
| 142444422 | 142444894 | - | 29.9 | 5161 | 5629 | -2641 | PRIMA4-int | ERV1 | LTR |
| 142444895 | 142445187 | - | 14.3 | 1 | 296 | -16 | AluSx | Alu | SINE |
| 142445188 | 142445517 | - | 29.9 | 4838 | 5160 | -3110 | PRIMA4-int | ERV1 | LTR |
| 142445518 | 142445778 | + | 18.4 | 44 | 288 | -24 | AluJo | Alu | SINE |
| 142445982 | 142446113 | - | 28.0 | 1764 | 1898 | -6372 | PRIMA4-int | ERV1 | LTR |
| 142446141 | 142446374 | + | 9.1 | 1 | 232 | -77 | AluSg1 | Alu | SINE |
| 142446435 | 142446738 | - | 13.2 | 1 | 305 | -5 | AluSg | Alu | SINE |
| 142447068 | 142447155 | - | 28.9 | 1220 | 1297 | -6973 | PRIMA4-int | ERV1 | LTR |
| 142447156 | 142447461 | + | 11.2 | 1 | 307 | -2 | AluSc | Alu | SINE |
| 142447462 | 142447755 | - | 28.9 | 922 | 1219 | -7051 | PRIMA4-int | ERV1 | LTR |

  

|  |  |  |  |  |  |  |  |  |  |
| --- | --- | --- | --- | --- | --- | --- | --- | --- | --- |
| chr1:142449235-142449992 | | | 758 bp | | |  |  |  |  |
| Genome Start | Genome End | Strand | Div | Repeat Start | Repeat End | Repeat Left | Name | Family | Class |
| 142449235 | 142449571 | - | 21.9 | 98 | 443 | -2 | LTR56 | ERV1 | LTR |
| 142449572 | 142449865 | + | 14.6 | 1 | 298 | -14 | AluSx | Alu | SINE |
| 142449866 | 142449992 | - | 21.9 | 9 | 97 | -348 | LTR56 | ERV1 | LTR |

  

|  |  |  |  |  |  |  |  |  |  |
| --- | --- | --- | --- | --- | --- | --- | --- | --- | --- |
| chr1:142456645-142457532 | | | 888 bp | | |  |  |  |  |
| Genome Start | Genome End | Strand | Div | Repeat Start | Repeat End | Repeat Left | Name | Family | Class |
| 142456645 | 142457077 | - | 21.3 | 1846 | 2309 | -4193 | L1MCa | L1 | LINE |
| 142457078 | 142457393 | - | 12.2 | 1 | 302 | -8 | AluSg | Alu | SINE |
| 142457394 | 142457532 | - | 23.2 | 1544 | 1845 | -4657 | L1MCa | L1 | LINE |

  

|  |  |  |  |  |  |  |  |  |  |
| --- | --- | --- | --- | --- | --- | --- | --- | --- | --- |
| chr1:142466682-142467754 | | | 1073 bp | | |  |  |  |  |
| Genome Start | Genome End | Strand | Div | Repeat Start | Repeat End | Repeat Left | Name | Family | Class |
| 142466682 | 142467263 | - | 16.6 | 163 | 727 | 0 | LTR8A | ERV1 | LTR |
| 142467264 | 142467603 | - | 16.5 | 1 | 337 | -2 | MER1B | MER1\_type | DNA |
| 142467604 | 142467754 | - | 17.2 | 1 | 162 | -545 | LTR8A | ERV1 | LTR |

  

|  |  |  |  |  |  |  |  |  |  |
| --- | --- | --- | --- | --- | --- | --- | --- | --- | --- |
| chr1:142468464-142469887 | | | 1424 bp | | |  |  |  |  |
| Genome Start | Genome End | Strand | Div | Repeat Start | Repeat End | Repeat Left | Name | Family | Class |
| 142468464 | 142468620 | + | 22.3 | 1 | 155 | -567 | MER72 | ERV1 | LTR |
| 142468621 | 142468780 | + | 11.9 | 1 | 154 | -503 | MER51D | ERV1 | LTR |
| 142468781 | 142469086 | + | 13.4 | 1 | 299 | -13 | AluSx | Alu | SINE |
| 142469087 | 142469356 | + | 11.9 | 155 | 445 | -212 | MER51D | ERV1 | LTR |
| 142469357 | 142469654 | + | 8.4 | 1 | 297 | -14 | AluY | Alu | SINE |
| 142469655 | 142469872 | + | 11.9 | 446 | 655 | -2 | MER51D | ERV1 | LTR |
| 142469873 | 142469887 | + | 22.3 | 156 | 166 | -556 | MER72 | ERV1 | LTR |

  

|  |  |  |  |  |  |  |  |  |  |
| --- | --- | --- | --- | --- | --- | --- | --- | --- | --- |
| chr1:142470328-142470806 | | | 479 bp | | |  |  |  |  |
| Genome Start | Genome End | Strand | Div | Repeat Start | Repeat End | Repeat Left | Name | Family | Class |
| 142470328 | 142470386 | - | 30.1 | 465 | 518 | -3 | LTR33 | ERVL | LTR |
| 142470387 | 142470681 | - | 6.8 | 1 | 295 | -16 | AluY | Alu | SINE |
| 142470682 | 142470806 | - | 30.1 | 344 | 464 | -57 | LTR33 | ERVL | LTR |

  

|  |  |  |  |  |  |  |  |  |  |
| --- | --- | --- | --- | --- | --- | --- | --- | --- | --- |
| chr1:142471321-142473212 | | | 1892 bp | | |  |  |  |  |
| Genome Start | Genome End | Strand | Div | Repeat Start | Repeat End | Repeat Left | Name | Family | Class |
| 142471321 | 142471336 | + | 16.3 | 1 | 13 | -453 | MER31B | ERV1 | LTR |
| 142471337 | 142471411 | + | 13.4 | 1 | 66 | -569 | LTR24C | ERV1 | LTR |
| 142471412 | 142471723 | + | 10.6 | 1 | 312 | -1 | AluSp | Alu | SINE |
| 142471724 | 142471822 | + | 13.4 | 67 | 165 | -470 | LTR24C | ERV1 | LTR |
| 142471823 | 142472120 | - | 9.4 | 1 | 299 | -12 | AluY | Alu | SINE |
| 142472121 | 142472355 | + | 13.4 | 166 | 390 | -245 | LTR24C | ERV1 | LTR |
| 142472356 | 142472669 | + | 15.1 | 1 | 311 | -1 | AluSx | Alu | SINE |
| 142472670 | 142472963 | + | 13.4 | 391 | 635 | 0 | LTR24C | ERV1 | LTR |
| 142472964 | 142473212 | + | 16.3 | 14 | 243 | -223 | MER31B | ERV1 | LTR |

  

|  |  |  |  |  |  |  |  |  |  |
| --- | --- | --- | --- | --- | --- | --- | --- | --- | --- |
| chr1:142473215-142474843 | | | 1487 bp | | |  |  |  |  |
| Genome Start | Genome End | Strand | Div | Repeat Start | Repeat End | Repeat Left | Name | Family | Class |
| 142473215 | 142473293 | + | 15.3 | 11 | 121 | -464 | LTR49 | ERV1 | LTR |
| 142473313 | 142473867 | - | 23.2 | 1 | 551 | -21 | LTR73 | ERV1 | LTR |
| 142473923 | 142474106 | + | 23.9 | 78 | 236 | -359 | LTR49 | ERV1 | LTR |
| 142474107 | 142474267 | - | 12.5 | 135 | 293 | -19 | AluSx | Alu | SINE |
| 142474336 | 142474616 | - | 8.6 | 1 | 280 | -31 | AluY | Alu | SINE |
| 142474617 | 142474742 | - | 12.5 | 6 | 134 | -178 | AluSx | Alu | SINE |
| 142474743 | 142474843 | + | 23.9 | 237 | 335 | -260 | LTR49 | ERV1 | LTR |

  

|  |  |  |  |  |  |  |  |  |  |
| --- | --- | --- | --- | --- | --- | --- | --- | --- | --- |
| chr1:142476442-142477024 | | | 530 bp | | |  |  |  |  |
| Genome Start | Genome End | Strand | Div | Repeat Start | Repeat End | Repeat Left | Name | Family | Class |
| 142476442 | 142476521 | - | 19.7 | 7518 | 7589 | -1 | HERVP71A | ERV1 | LTR |
| 142476522 | 142476805 | - | 9.0 | 1 | 292 | -20 | AluSx | Alu | SINE |
| 142476806 | 142476929 | - | 19.7 | 7393 | 7517 | -73 | HERVP71A | ERV1 | LTR |
| 142476983 | 142477024 | - | 19.7 | 7361 | 7392 | -198 | HERVP71A | ERV1 | LTR |

  

|  |  |  |  |  |  |  |  |  |  |
| --- | --- | --- | --- | --- | --- | --- | --- | --- | --- |
| chr1:142483570-142484482 | | | 892 bp | | |  |  |  |  |
| Genome Start | Genome End | Strand | Div | Repeat Start | Repeat End | Repeat Left | Name | Family | Class |
| 142483570 | 142483780 | + | 24.6 | 1 | 268 | -346 | MER90a | ERV1 | LTR |
| 142483806 | 142484109 | - | 11.4 | 1 | 299 | -11 | AluSg | Alu | SINE |
| 142484111 | 142484284 | - | 12.6 | 124 | 303 | -9 | AluSg/x | Alu | SINE |
| 142484285 | 142484381 | + | 28.1 | 290 | 386 | -228 | MER90a | ERV1 | LTR |
| 142484377 | 142484482 | + | 26.5 | 505 | 609 | -5 | MER90a | ERV1 | LTR |

  

|  |  |  |  |  |  |  |  |  |  |
| --- | --- | --- | --- | --- | --- | --- | --- | --- | --- |
| chr1:142487951-142488929 | | | 979 bp | | |  |  |  |  |
| Genome Start | Genome End | Strand | Div | Repeat Start | Repeat End | Repeat Left | Name | Family | Class |
| 142487951 | 142487991 | + | 15.2 | 2297 | 2334 | -3812 | L1M4 | L1 | LINE |
| 142487992 | 142488488 | + | 14.6 | 3 | 497 | 0 | LOR1a | ERV1 | LTR |
| 142488489 | 142488929 | + | 15.2 | 2335 | 2781 | -3365 | L1M4 | L1 | LINE |

  

|  |  |  |  |  |  |  |  |  |  |
| --- | --- | --- | --- | --- | --- | --- | --- | --- | --- |
| chr1:142496825-142497341 | | | 517 bp | | |  |  |  |  |
| Genome Start | Genome End | Strand | Div | Repeat Start | Repeat End | Repeat Left | Name | Family | Class |
| 142496825 | 142496958 | - | 31.0 | 90 | 221 | -6326 | L1MD2 | L1 | LINE |
| 142496959 | 142497256 | - | 17.8 | 1 | 298 | -14 | AluJb | Alu | SINE |
| 142497257 | 142497341 | - | 31.0 | 10 | 89 | -6458 | L1MD2 | L1 | LINE |

  

|  |  |  |  |  |  |  |  |  |  |
| --- | --- | --- | --- | --- | --- | --- | --- | --- | --- |
| chr1:142513945-142516481 | | | 2535 bp | | |  |  |  |  |
| Genome Start | Genome End | Strand | Div | Repeat Start | Repeat End | Repeat Left | Name | Family | Class |
| 142513945 | 142514182 | + | 22.1 | 1 | 242 | -70 | AluJo | Alu | SINE |
| 142514183 | 142514524 | + | 13.9 | 1 | 364 | 0 | THE1B | MaLR | LTR |
| 142514527 | 142516099 | + | 29.5 | 2 | 1735 | 0 | MLT-int | MaLR | LTR |
| 142516100 | 142516424 | + | 12.2 | 5 | 364 | 0 | THE1B | MaLR | LTR |
| 142516425 | 142516481 | + | 22.1 | 243 | 299 | -13 | AluJo | Alu | SINE |

  

|  |  |  |  |  |  |  |  |  |  |
| --- | --- | --- | --- | --- | --- | --- | --- | --- | --- |
| chr1:142551473-142552489 | | | 1017 bp | | |  |  |  |  |
| Genome Start | Genome End | Strand | Div | Repeat Start | Repeat End | Repeat Left | Name | Family | Class |
| 142551473 | 142551711 | + | 32.5 | 5348 | 5596 | -2212 | L1MC4a | L1 | LINE |
| 142551712 | 142552157 | + | 12.4 | 5704 | 6157 | -9 | L1PA16 | L1 | LINE |
| 142552158 | 142552489 | + | 32.5 | 5597 | 5933 | -1875 | L1MC4a | L1 | LINE |

  

|  |  |  |  |  |  |  |  |  |  |
| --- | --- | --- | --- | --- | --- | --- | --- | --- | --- |
| chr1:142560151-142561891 | | | 1546 bp | | |  |  |  |  |
| Genome Start | Genome End | Strand | Div | Repeat Start | Repeat End | Repeat Left | Name | Family | Class |
| 142560151 | 142561122 | - | 33.5 | 3819 | 4811 | -856 | ERVL-E | ERVL | LTR |
| 142561123 | 142561434 | - | 14.7 | 1 | 312 | 0 | AluSx | Alu | SINE |
| 142561435 | 142561538 | - | 33.5 | 3698 | 3818 | -1849 | ERVL-E | ERVL | LTR |
| 142561734 | 142561891 | - | 30.8 | 3230 | 3397 | -2317 | ERVL-E | ERVL | LTR |

  

|  |  |  |  |  |  |  |  |  |  |
| --- | --- | --- | --- | --- | --- | --- | --- | --- | --- |
| chr1:142613568-142616824 | | | 3248 bp | | |  |  |  |  |
| Genome Start | Genome End | Strand | Div | Repeat Start | Repeat End | Repeat Left | Name | Family | Class |
| 142613568 | 142614505 | + | 11.5 | 2 | 971 | -1447 | Tigger1 | MER2\_type | DNA |
| 142614506 | 142614816 | - | 6.4 | 1 | 312 | -1 | AluSp | Alu | SINE |
| 142614826 | 142615095 | - | 13.7 | 2 | 294 | -19 | AluSq | Alu | SINE |
| 142615096 | 142616320 | + | 11.5 | 972 | 2224 | -194 | Tigger1 | MER2\_type | DNA |
| 142616321 | 142616620 | - | 15.1 | 1 | 299 | -13 | AluJb | Alu | SINE |
| 142616621 | 142616824 | + | 11.5 | 2225 | 2418 | 0 | Tigger1 | MER2\_type | DNA |

  

|  |  |  |  |  |  |  |  |  |  |
| --- | --- | --- | --- | --- | --- | --- | --- | --- | --- |
| chr1:142634374-142634948 | | | 575 bp | | |  |  |  |  |
| Genome Start | Genome End | Strand | Div | Repeat Start | Repeat End | Repeat Left | Name | Family | Class |
| 142634374 | 142634520 | + | 17.9 | 1 | 133 | -179 | AluJo | Alu | SINE |
| 142634521 | 142634806 | + | 11.2 | 1 | 292 | -21 | AluSq | Alu | SINE |
| 142634807 | 142634948 | + | 17.9 | 134 | 269 | -43 | AluJo | Alu | SINE |

  

|  |  |  |  |  |  |  |  |  |  |
| --- | --- | --- | --- | --- | --- | --- | --- | --- | --- |
| chr1:142642521-142642926 | | | 406 bp | | |  |  |  |  |
| Genome Start | Genome End | Strand | Div | Repeat Start | Repeat End | Repeat Left | Name | Family | Class |
| 142642521 | 142642548 | - | 29.7 | 3384 | 3400 | -19 | L2 | L2 | LINE |
| 142642549 | 142642842 | - | 10.2 | 1 | 294 | -16 | AluSg | Alu | SINE |
| 142642843 | 142642926 | - | 29.7 | 3293 | 3383 | -36 | L2 | L2 | LINE |

  

|  |  |  |  |  |  |  |  |  |  |
| --- | --- | --- | --- | --- | --- | --- | --- | --- | --- |
| chr1:142644711-142645486 | | | 776 bp | | |  |  |  |  |
| Genome Start | Genome End | Strand | Div | Repeat Start | Repeat End | Repeat Left | Name | Family | Class |
| 142644711 | 142644871 | - | 27.9 | 5160 | 5318 | -828 | L1M5 | L1 | LINE |
| 142644872 | 142645178 | + | 17.1 | 3 | 307 | -5 | AluJb | Alu | SINE |
| 142645179 | 142645486 | - | 27.9 | 4833 | 5159 | -987 | L1M5 | L1 | LINE |

  

|  |  |  |  |  |  |  |  |  |  |
| --- | --- | --- | --- | --- | --- | --- | --- | --- | --- |
| chr1:142674925-142675469 | | | 543 bp | | |  |  |  |  |
| Genome Start | Genome End | Strand | Div | Repeat Start | Repeat End | Repeat Left | Name | Family | Class |
| 142674925 | 142675091 | + | 16.2 | 176 | 341 | -55 | MSTD | MaLR | LTR |
| 142675092 | 142675401 | - | 6.8 | 75 | 369 | 0 | AluY | Alu | SINE |
| 142675404 | 142675469 | + | 22.7 | 330 | 395 | -1 | MSTD | MaLR | LTR |

  

|  |  |  |  |  |  |  |  |  |  |
| --- | --- | --- | --- | --- | --- | --- | --- | --- | --- |
| chr1:142679212-142680558 | | | 951 bp | | |  |  |  |  |
| Genome Start | Genome End | Strand | Div | Repeat Start | Repeat End | Repeat Left | Name | Family | Class |
| 142679212 | 142679356 | - | 27.4 | 3272 | 3407 | -12 | L2 | L2 | LINE |
| 142679403 | 142679791 | - | 28.0 | 2833 | 3271 | -148 | L2 | L2 | LINE |
| 142679792 | 142680010 | + | 20.6 | 1 | 224 | 0 | MER58A | MER1\_type | DNA |
| 142680011 | 142680086 | - | 30.8 | 2755 | 2832 | -587 | L2 | L2 | LINE |
| 142680437 | 142680558 | - | 27.4 | 2391 | 2522 | -897 | L2 | L2 | LINE |

  

|  |  |  |  |  |  |  |  |  |  |
| --- | --- | --- | --- | --- | --- | --- | --- | --- | --- |
| chr1:142742684-142745235 | | | 2552 bp | | |  |  |  |  |
| Genome Start | Genome End | Strand | Div | Repeat Start | Repeat End | Repeat Left | Name | Family | Class |
| 142742684 | 142744284 | - | 20.1 | 6423 | 8037 | -5 | L1MC4 | L1 | LINE |
| 142744285 | 142744597 | + | 7.2 | 1 | 306 | -5 | AluY | Alu | SINE |
| 142744598 | 142744727 | - | 20.1 | 6297 | 6422 | -1620 | L1MC4 | L1 | LINE |
| 142744728 | 142745158 | - | 22.5 | 1 | 457 | 0 | MSTB2 | MaLR | LTR |
| 142745159 | 142745235 | - | 20.1 | 6224 | 6296 | -1746 | L1MC4 | L1 | LINE |

  

|  |  |  |  |  |  |  |  |  |  |
| --- | --- | --- | --- | --- | --- | --- | --- | --- | --- |
| chr1:142775026-142776409 | | | 1276 bp | | |  |  |  |  |
| Genome Start | Genome End | Strand | Div | Repeat Start | Repeat End | Repeat Left | Name | Family | Class |
| 142775026 | 142775390 | - | 24.9 | 1935 | 2275 | -4912 | L1MEc | L1 | LINE |
| 142775405 | 142775638 | + | 11.5 | 1 | 234 | -78 | AluSx | Alu | SINE |
| 142775639 | 142775784 | + | 10.1 | 187 | 332 | 0 | AluSg/x | Alu | SINE |
| 142775855 | 142776113 | - | 19.7 | 42 | 309 | -3 | AluJo | Alu | SINE |
| 142776138 | 142776409 | - | 30.5 | 1376 | 1693 | -5303 | L1MEc | L1 | LINE |

  

|  |  |  |  |  |  |  |  |  |  |
| --- | --- | --- | --- | --- | --- | --- | --- | --- | --- |
| chr1:142784611-142785892 | | | 961 bp | | |  |  |  |  |
| Genome Start | Genome End | Strand | Div | Repeat Start | Repeat End | Repeat Left | Name | Family | Class |
| 142784611 | 142784727 | - | 38.3 | 3206 | 3329 | -49 | L2 | L2 | LINE |
| 142785047 | 142785743 | - | 21.6 | 7093 | 7808 | 0 | L1MC4a | L1 | LINE |
| 142785746 | 142785892 | - | 34.0 | 2658 | 2832 | -587 | L2 | L2 | LINE |

  

|  |  |  |  |  |  |  |  |  |  |
| --- | --- | --- | --- | --- | --- | --- | --- | --- | --- |
| chr1:142795112-142796057 | | | 946 bp | | |  |  |  |  |
| Genome Start | Genome End | Strand | Div | Repeat Start | Repeat End | Repeat Left | Name | Family | Class |
| 142795112 | 142795170 | + | 23.4 | 2328 | 2387 | -3742 | L1MEd | L1 | LINE |
| 142795171 | 142795462 | + | 12.1 | 1 | 291 | -21 | AluSx | Alu | SINE |
| 142795463 | 142795720 | + | 23.4 | 2388 | 2660 | -3469 | L1MEd | L1 | LINE |
| 142795721 | 142796016 | - | 3.1 | 1 | 304 | -7 | AluY | Alu | SINE |
| 142796017 | 142796057 | + | 23.4 | 2661 | 2699 | -3430 | L1MEd | L1 | LINE |

  

|  |  |  |  |  |  |  |  |  |  |
| --- | --- | --- | --- | --- | --- | --- | --- | --- | --- |
| chr1:142864657-142867761 | | | 3071 bp | | |  |  |  |  |
| Genome Start | Genome End | Strand | Div | Repeat Start | Repeat End | Repeat Left | Name | Family | Class |
| 142864657 | 142866040 | - | 6.8 | 1850 | 3226 | -2920 | L1P2 | L1 | LINE |
| 142866041 | 142866685 | + | 5.0 | 5494 | 6139 | -16 | L1PA4 | L1 | LINE |
| 142866720 | 142866743 | + | 5.0 | 6140 | 6154 | -1 | L1PA4 | L1 | LINE |
| 142866744 | 142867761 | - | 5.7 | 857 | 1849 | -426 | L1P2 | L1 | LINE |

  

|  |  |  |  |  |  |  |  |  |  |
| --- | --- | --- | --- | --- | --- | --- | --- | --- | --- |
| chr1:142992915-142995418 | | | 2501 bp | | |  |  |  |  |
| Genome Start | Genome End | Strand | Div | Repeat Start | Repeat End | Repeat Left | Name | Family | Class |
| 142992915 | 142993408 | + | 22.7 | 22 | 603 | -5797 | L1MEc | L1 | LINE |
| 142993409 | 142993760 | + | 13.2 | 1 | 339 | 0 | MER1B | MER1\_type | DNA |
| 142993764 | 142993990 | + | 13.8 | 6100 | 6328 | -5 | L1MC1 | L1 | LINE |
| 142993991 | 142994410 | + | 22.7 | 604 | 990 | -5410 | L1MEc | L1 | LINE |
| 142994411 | 142994712 | + | 16.9 | 1 | 307 | 0 | AluSx | Alu | SINE |
| 142994713 | 142995418 | + | 22.7 | 991 | 1698 | -4702 | L1MEc | L1 | LINE |

  

|  |  |  |  |  |  |  |  |  |  |
| --- | --- | --- | --- | --- | --- | --- | --- | --- | --- |
| chr1:142995407-142995923 | | | 517 bp | | |  |  |  |  |
| Genome Start | Genome End | Strand | Div | Repeat Start | Repeat End | Repeat Left | Name | Family | Class |
| 142995407 | 142995491 | + | 31.5 | 10 | 89 | -6458 | L1MD2 | L1 | LINE |
| 142995492 | 142995789 | + | 16.8 | 1 | 298 | -14 | AluJb | Alu | SINE |
| 142995790 | 142995923 | + | 31.5 | 90 | 221 | -6326 | L1MD2 | L1 | LINE |

  

|  |  |  |  |  |  |  |  |  |  |
| --- | --- | --- | --- | --- | --- | --- | --- | --- | --- |
| chr1:143002160-143002657 | | | 498 bp | | |  |  |  |  |
| Genome Start | Genome End | Strand | Div | Repeat Start | Repeat End | Repeat Left | Name | Family | Class |
| 143002160 | 143002308 | + | 18.3 | 1 | 147 | -155 | AluJb | Alu | SINE |
| 143002309 | 143002562 | + | 10.2 | 1 | 259 | -52 | AluY | Alu | SINE |
| 143002563 | 143002657 | + | 18.3 | 148 | 299 | -3 | AluJb | Alu | SINE |

  

|  |  |  |  |  |  |  |  |  |  |
| --- | --- | --- | --- | --- | --- | --- | --- | --- | --- |
| chr1:143003802-143004780 | | | 979 bp | | |  |  |  |  |
| Genome Start | Genome End | Strand | Div | Repeat Start | Repeat End | Repeat Left | Name | Family | Class |
| 143003802 | 143004242 | - | 15.6 | 2335 | 2781 | -3365 | L1M4 | L1 | LINE |
| 143004243 | 143004739 | - | 14.6 | 3 | 497 | 0 | LOR1a | ERV1 | LTR |
| 143004740 | 143004780 | - | 15.6 | 2297 | 2334 | -3812 | L1M4 | L1 | LINE |

  

|  |  |  |  |  |  |  |  |  |  |
| --- | --- | --- | --- | --- | --- | --- | --- | --- | --- |
| chr1:143005931-143007397 | | | 1350 bp | | |  |  |  |  |
| Genome Start | Genome End | Strand | Div | Repeat Start | Repeat End | Repeat Left | Name | Family | Class |
| 143005931 | 143006385 | + | 10.0 | 1 | 474 | -6124 | MER4C-int | ERV1 | LTR |
| 143006386 | 143006519 | + | 4.5 | 178 | 311 | 0 | AluY | Alu | SINE |
| 143006520 | 143006771 | + | 16.4 | 506 | 837 | -5761 | MER4C-int | ERV1 | LTR |
| 143006862 | 143007177 | + | 12.2 | 5 | 308 | -3 | AluY | Alu | SINE |
| 143007205 | 143007255 | + | 19.6 | 263 | 313 | -282 | LTR49 | ERV1 | LTR |
| 143007256 | 143007397 | + | 13.5 | 865 | 1003 | -5595 | MER4C-int | ERV1 | LTR |

  

|  |  |  |  |  |  |  |  |  |  |
| --- | --- | --- | --- | --- | --- | --- | --- | --- | --- |
| chr1:143016158-143016878 | | | 721 bp | | |  |  |  |  |
| Genome Start | Genome End | Strand | Div | Repeat Start | Repeat End | Repeat Left | Name | Family | Class |
| 143016158 | 143016529 | + | 26.0 | 71 | 482 | -67 | MLT1H | MaLR | LTR |
| 143016530 | 143016828 | - | 9.1 | 1 | 298 | -13 | AluY | Alu | SINE |
| 143016829 | 143016878 | + | 26.0 | 483 | 533 | -16 | MLT1H | MaLR | LTR |

  

|  |  |  |  |  |  |  |  |  |  |
| --- | --- | --- | --- | --- | --- | --- | --- | --- | --- |
| chr1:143018101-143018859 | | | 755 bp | | |  |  |  |  |
| Genome Start | Genome End | Strand | Div | Repeat Start | Repeat End | Repeat Left | Name | Family | Class |
| 143018101 | 143018185 | + | 15.3 | 1 | 85 | -227 | AluSx | Alu | SINE |
| 143018186 | 143018634 | - | 19.5 | 11 | 463 | 0 | LTR2 | ERV1 | LTR |
| 143018639 | 143018859 | + | 11.2 | 79 | 293 | -19 | AluSx | Alu | SINE |

  

|  |  |  |  |  |  |  |  |  |  |
| --- | --- | --- | --- | --- | --- | --- | --- | --- | --- |
| chr1:143024601-143025184 | | | 533 bp | | |  |  |  |  |
| Genome Start | Genome End | Strand | Div | Repeat Start | Repeat End | Repeat Left | Name | Family | Class |
| 143024601 | 143024642 | + | 19.2 | 7360 | 7391 | -199 | HERVP71A | ERV1 | LTR |
| 143024694 | 143024817 | + | 19.2 | 7392 | 7516 | -74 | HERVP71A | ERV1 | LTR |
| 143024818 | 143025103 | + | 7.8 | 1 | 294 | -18 | AluSx | Alu | SINE |
| 143025104 | 143025184 | + | 19.2 | 7517 | 7589 | -1 | HERVP71A | ERV1 | LTR |

  

|  |  |  |  |  |  |  |  |  |  |
| --- | --- | --- | --- | --- | --- | --- | --- | --- | --- |
| chr1:143026770-143028358 | | | 1514 bp | | |  |  |  |  |
| Genome Start | Genome End | Strand | Div | Repeat Start | Repeat End | Repeat Left | Name | Family | Class |
| 143026770 | 143026870 | - | 21.6 | 236 | 340 | -255 | LTR49 | ERV1 | LTR |
| 143026871 | 143026996 | + | 11.7 | 3 | 129 | -183 | AluSx | Alu | SINE |
| 143026997 | 143027297 | + | 9.0 | 1 | 300 | -11 | AluY | Alu | SINE |
| 143027298 | 143027478 | + | 11.7 | 130 | 293 | -19 | AluSx | Alu | SINE |
| 143027479 | 143027661 | - | 21.6 | 81 | 235 | -360 | LTR49 | ERV1 | LTR |
| 143027717 | 143028272 | + | 23.3 | 1 | 552 | -20 | LTR73 | ERV1 | LTR |
| 143028293 | 143028358 | - | 13.6 | 11 | 76 | -519 | LTR49 | ERV1 | LTR |

  

|  |  |  |  |  |  |  |  |  |  |
| --- | --- | --- | --- | --- | --- | --- | --- | --- | --- |
| chr1:143030777-143031256 | | | 480 bp | | |  |  |  |  |
| Genome Start | Genome End | Strand | Div | Repeat Start | Repeat End | Repeat Left | Name | Family | Class |
| 143030777 | 143030901 | + | 30.1 | 344 | 464 | -57 | LTR33 | ERVL | LTR |
| 143030902 | 143031196 | + | 7.8 | 1 | 295 | -16 | AluY | Alu | SINE |
| 143031197 | 143031256 | + | 30.1 | 465 | 518 | -3 | LTR33 | ERVL | LTR |

  

|  |  |  |  |  |  |  |  |  |  |
| --- | --- | --- | --- | --- | --- | --- | --- | --- | --- |
| chr1:143067624-143068647 | | | 889 bp | | |  |  |  |  |
| Genome Start | Genome End | Strand | Div | Repeat Start | Repeat End | Repeat Left | Name | Family | Class |
| 143067624 | 143067798 | + | 31.8 | 5348 | 5531 | -2277 | L1MC4a | L1 | LINE |
| 143067864 | 143068316 | + | 13.2 | 5704 | 6157 | -9 | L1PA16 | L1 | LINE |
| 143068387 | 143068647 | + | 25.9 | 5675 | 5933 | -1875 | L1MC4a | L1 | LINE |

  

|  |  |  |  |  |  |  |  |  |  |
| --- | --- | --- | --- | --- | --- | --- | --- | --- | --- |
| chr1:143076302-143078042 | | | 1546 bp | | |  |  |  |  |
| Genome Start | Genome End | Strand | Div | Repeat Start | Repeat End | Repeat Left | Name | Family | Class |
| 143076302 | 143077274 | - | 33.7 | 3819 | 4811 | -856 | ERVL-E | ERVL | LTR |
| 143077275 | 143077586 | - | 15.4 | 1 | 312 | 0 | AluSx | Alu | SINE |
| 143077587 | 143077690 | - | 33.7 | 3698 | 3818 | -1849 | ERVL-E | ERVL | LTR |
| 143077886 | 143078042 | - | 30.3 | 3230 | 3397 | -2317 | ERVL-E | ERVL | LTR |

  

|  |  |  |  |  |  |  |  |  |  |
| --- | --- | --- | --- | --- | --- | --- | --- | --- | --- |
| chr1:143095012-143097876 | | | 2853 bp | | |  |  |  |  |
| Genome Start | Genome End | Strand | Div | Repeat Start | Repeat End | Repeat Left | Name | Family | Class |
| 143095012 | 143097262 | - | 13.1 | 386 | 2716 | -2 | Tigger2 | MER2\_type | DNA |
| 143097268 | 143097472 | + | 16.7 | 5965 | 6153 | -3 | L1PB4 | L1 | LINE |
| 143097480 | 143097876 | - | 15.1 | 2 | 403 | -2315 | Tigger2 | MER2\_type | DNA |

  

|  |  |  |  |  |  |  |  |  |  |
| --- | --- | --- | --- | --- | --- | --- | --- | --- | --- |
| chr1:143175649-143176165 | | | 517 bp | | |  |  |  |  |
| Genome Start | Genome End | Strand | Div | Repeat Start | Repeat End | Repeat Left | Name | Family | Class |
| 143175649 | 143175733 | + | 31.5 | 10 | 89 | -6458 | L1MD2 | L1 | LINE |
| 143175734 | 143176031 | + | 16.8 | 1 | 298 | -14 | AluJb | Alu | SINE |
| 143176032 | 143176165 | + | 31.5 | 90 | 221 | -6326 | L1MD2 | L1 | LINE |

  

|  |  |  |  |  |  |  |  |  |  |
| --- | --- | --- | --- | --- | --- | --- | --- | --- | --- |
| chr1:143182396-143182893 | | | 498 bp | | |  |  |  |  |
| Genome Start | Genome End | Strand | Div | Repeat Start | Repeat End | Repeat Left | Name | Family | Class |
| 143182396 | 143182544 | + | 18.3 | 1 | 147 | -155 | AluJb | Alu | SINE |
| 143182545 | 143182798 | + | 10.6 | 1 | 259 | -52 | AluY | Alu | SINE |
| 143182799 | 143182893 | + | 18.3 | 148 | 299 | -3 | AluJb | Alu | SINE |

  

|  |  |  |  |  |  |  |  |  |  |
| --- | --- | --- | --- | --- | --- | --- | --- | --- | --- |
| chr1:143184041-143185019 | | | 979 bp | | |  |  |  |  |
| Genome Start | Genome End | Strand | Div | Repeat Start | Repeat End | Repeat Left | Name | Family | Class |
| 143184041 | 143184481 | - | 15.8 | 2335 | 2781 | -3365 | L1M4 | L1 | LINE |
| 143184482 | 143184978 | - | 14.3 | 3 | 497 | 0 | LOR1a | ERV1 | LTR |
| 143184979 | 143185019 | - | 15.8 | 2297 | 2334 | -3812 | L1M4 | L1 | LINE |

  

|  |  |  |  |  |  |  |  |  |  |
| --- | --- | --- | --- | --- | --- | --- | --- | --- | --- |
| chr1:143186170-143187638 | | | 1350 bp | | |  |  |  |  |
| Genome Start | Genome End | Strand | Div | Repeat Start | Repeat End | Repeat Left | Name | Family | Class |
| 143186170 | 143186624 | + | 9.8 | 1 | 474 | -6124 | MER4C-int | ERV1 | LTR |
| 143186625 | 143186758 | + | 4.5 | 178 | 311 | 0 | AluY | Alu | SINE |
| 143186759 | 143187010 | + | 16.8 | 506 | 837 | -5761 | MER4C-int | ERV1 | LTR |
| 143187101 | 143187406 | + | 12.2 | 5 | 298 | -13 | AluY | Alu | SINE |
| 143187436 | 143187486 | + | 19.6 | 263 | 313 | -282 | LTR49 | ERV1 | LTR |
| 143187487 | 143187638 | + | 14.1 | 865 | 1030 | -5595 | MER4C-int | ERV1 | LTR |

  

|  |  |  |  |  |  |  |  |  |  |
| --- | --- | --- | --- | --- | --- | --- | --- | --- | --- |
| chr1:143196386-143197106 | | | 721 bp | | |  |  |  |  |
| Genome Start | Genome End | Strand | Div | Repeat Start | Repeat End | Repeat Left | Name | Family | Class |
| 143196386 | 143196757 | + | 26.0 | 71 | 482 | -67 | MLT1H | MaLR | LTR |
| 143196758 | 143197056 | - | 9.4 | 1 | 298 | -13 | AluY | Alu | SINE |
| 143197057 | 143197106 | + | 26.0 | 483 | 533 | -16 | MLT1H | MaLR | LTR |

  

|  |  |  |  |  |  |  |  |  |  |
| --- | --- | --- | --- | --- | --- | --- | --- | --- | --- |
| chr1:143197383-143198298 | | | 894 bp | | |  |  |  |  |
| Genome Start | Genome End | Strand | Div | Repeat Start | Repeat End | Repeat Left | Name | Family | Class |
| 143197383 | 143197488 | - | 24.5 | 505 | 609 | -5 | MER90a | ERV1 | LTR |
| 143197484 | 143197580 | - | 27.1 | 290 | 386 | -228 | MER90a | ERV1 | LTR |
| 143197581 | 143197756 | + | 12.5 | 124 | 305 | -7 | AluSg/x | Alu | SINE |
| 143197759 | 143198062 | + | 11.2 | 1 | 299 | -11 | AluSg | Alu | SINE |
| 143198088 | 143198298 | - | 23.4 | 1 | 268 | -346 | MER90a | ERV1 | LTR |

  

|  |  |  |  |  |  |  |  |  |  |
| --- | --- | --- | --- | --- | --- | --- | --- | --- | --- |
| chr1:143198330-143199088 | | | 755 bp | | |  |  |  |  |
| Genome Start | Genome End | Strand | Div | Repeat Start | Repeat End | Repeat Left | Name | Family | Class |
| 143198330 | 143198414 | + | 16.5 | 1 | 85 | -227 | AluSx | Alu | SINE |
| 143198415 | 143198863 | - | 21.5 | 11 | 463 | 0 | LTR2 | ERV1 | LTR |
| 143198868 | 143199088 | + | 11.2 | 79 | 293 | -19 | AluSx | Alu | SINE |

  

|  |  |  |  |  |  |  |  |  |  |
| --- | --- | --- | --- | --- | --- | --- | --- | --- | --- |
| chr1:143204831-143205414 | | | 533 bp | | |  |  |  |  |
| Genome Start | Genome End | Strand | Div | Repeat Start | Repeat End | Repeat Left | Name | Family | Class |
| 143204831 | 143204872 | + | 19.6 | 7360 | 7391 | -199 | HERVP71A | ERV1 | LTR |
| 143204924 | 143205047 | + | 19.6 | 7392 | 7516 | -74 | HERVP71A | ERV1 | LTR |
| 143205048 | 143205333 | + | 7.8 | 1 | 294 | -18 | AluSx | Alu | SINE |
| 143205334 | 143205414 | + | 19.6 | 7517 | 7589 | -1 | HERVP71A | ERV1 | LTR |

  

|  |  |  |  |  |  |  |  |  |  |
| --- | --- | --- | --- | --- | --- | --- | --- | --- | --- |
| chr1:143207013-143208601 | | | 1514 bp | | |  |  |  |  |
| Genome Start | Genome End | Strand | Div | Repeat Start | Repeat End | Repeat Left | Name | Family | Class |
| 143207013 | 143207113 | - | 21.6 | 236 | 340 | -255 | LTR49 | ERV1 | LTR |
| 143207114 | 143207239 | + | 11.7 | 3 | 129 | -183 | AluSx | Alu | SINE |
| 143207240 | 143207540 | + | 9.0 | 1 | 300 | -11 | AluY | Alu | SINE |
| 143207541 | 143207721 | + | 11.7 | 130 | 293 | -19 | AluSx | Alu | SINE |
| 143207722 | 143207904 | - | 21.6 | 81 | 235 | -360 | LTR49 | ERV1 | LTR |
| 143207960 | 143208515 | + | 23.3 | 1 | 552 | -20 | LTR73 | ERV1 | LTR |
| 143208536 | 143208601 | - | 13.6 | 11 | 76 | -519 | LTR49 | ERV1 | LTR |

  

|  |  |  |  |  |  |  |  |  |  |
| --- | --- | --- | --- | --- | --- | --- | --- | --- | --- |
| chr1:143208862-143210493 | | | 1599 bp | | |  |  |  |  |
| Genome Start | Genome End | Strand | Div | Repeat Start | Repeat End | Repeat Left | Name | Family | Class |
| 143208862 | 143208915 | - | 13.0 | 582 | 635 | 0 | LTR24C | ERV1 | LTR |
| 143208949 | 143209138 | - | 13.6 | 395 | 595 | -40 | LTR24C | ERV1 | LTR |
| 143209139 | 143209460 | - | 14.1 | 1 | 312 | 0 | AluSx | Alu | SINE |
| 143209461 | 143209695 | - | 13.6 | 172 | 394 | -241 | LTR24C | ERV1 | LTR |
| 143209696 | 143210005 | + | 10.7 | 1 | 311 | 0 | AluY | Alu | SINE |
| 143210006 | 143210113 | - | 13.6 | 77 | 171 | -464 | LTR24C | ERV1 | LTR |
| 143210114 | 143210418 | - | 11.2 | 1 | 305 | -8 | AluSp | Alu | SINE |
| 143210419 | 143210493 | - | 13.6 | 2 | 76 | -559 | LTR24C | ERV1 | LTR |

  

|  |  |  |  |  |  |  |  |  |  |
| --- | --- | --- | --- | --- | --- | --- | --- | --- | --- |
| chr1:143211024-143211503 | | | 480 bp | | |  |  |  |  |
| Genome Start | Genome End | Strand | Div | Repeat Start | Repeat End | Repeat Left | Name | Family | Class |
| 143211024 | 143211148 | + | 30.1 | 344 | 464 | -57 | LTR33 | ERVL | LTR |
| 143211149 | 143211443 | + | 7.5 | 1 | 295 | -16 | AluY | Alu | SINE |
| 143211444 | 143211503 | + | 30.1 | 465 | 518 | -3 | LTR33 | ERVL | LTR |

  

|  |  |  |  |  |  |  |  |  |  |
| --- | --- | --- | --- | --- | --- | --- | --- | --- | --- |
| chr1:143302157-143303474 | | | 1306 bp | | |  |  |  |  |
| Genome Start | Genome End | Strand | Div | Repeat Start | Repeat End | Repeat Left | Name | Family | Class |
| 143302157 | 143302212 | - | 12.5 | 3899 | 3955 | -2191 | L1MD | L1 | LINE |
| 143302225 | 143302329 | - | 18.6 | 3568 | 3672 | -2474 | L1MD | L1 | LINE |
| 143302330 | 143302632 | + | 7.6 | 1 | 303 | -8 | AluY | Alu | SINE |
| 143302633 | 143303086 | - | 18.6 | 3112 | 3567 | -2579 | L1MD | L1 | LINE |
| 143303087 | 143303389 | - | 12.4 | 1 | 299 | -13 | AluSx | Alu | SINE |
| 143303390 | 143303474 | - | 18.6 | 3028 | 3111 | -3035 | L1MD | L1 | LINE |

  

|  |  |  |  |  |  |  |  |  |  |
| --- | --- | --- | --- | --- | --- | --- | --- | --- | --- |
| chr1:143306296-143306708 | | | 413 bp | | |  |  |  |  |
| Genome Start | Genome End | Strand | Div | Repeat Start | Repeat End | Repeat Left | Name | Family | Class |
| 143306296 | 143306342 | - | 15.5 | 6104 | 6159 | -11 | L1ME3A | L1 | LINE |
| 143306343 | 143306647 | + | 14.8 | 3 | 307 | -5 | AluJb | Alu | SINE |
| 143306648 | 143306708 | - | 15.5 | 6037 | 6103 | -67 | L1ME3A | L1 | LINE |

  

|  |  |  |  |  |  |  |  |  |  |
| --- | --- | --- | --- | --- | --- | --- | --- | --- | --- |
| chr1:143306710-143308075 | | | 1366 bp | | |  |  |  |  |
| Genome Start | Genome End | Strand | Div | Repeat Start | Repeat End | Repeat Left | Name | Family | Class |
| 143306710 | 143307167 | - | 23.3 | 5165 | 5654 | -586 | L1M5 | L1 | LINE |
| 143307168 | 143307466 | - | 16.8 | 1 | 298 | -14 | AluJb | Alu | SINE |
| 143307467 | 143307620 | - | 21.6 | 4978 | 5164 | -982 | L1M5 | L1 | LINE |
| 143307621 | 143307919 | - | 15.4 | 1 | 299 | -13 | AluJb | Alu | SINE |
| 143307920 | 143308075 | - | 21.6 | 4823 | 4977 | -1169 | L1M5 | L1 | LINE |

  

|  |  |  |  |  |  |  |  |  |  |
| --- | --- | --- | --- | --- | --- | --- | --- | --- | --- |
| chr1:143319918-143322875 | | | 2782 bp | | |  |  |  |  |
| Genome Start | Genome End | Strand | Div | Repeat Start | Repeat End | Repeat Left | Name | Family | Class |
| 143319918 | 143320492 | + | 29.6 | 3863 | 4431 | -1715 | L1ME1 | L1 | LINE |
| 143320493 | 143320799 | + | 11.7 | 1 | 308 | -3 | AluY | Alu | SINE |
| 143320800 | 143321232 | + | 29.6 | 4432 | 4859 | -1287 | L1ME1 | L1 | LINE |
| 143321409 | 143321775 | + | 25.2 | 5294 | 5690 | -476 | L1ME1 | L1 | LINE |
| 143321776 | 143322439 | - | 8.3 | 2 | 660 | 0 | PABL\_A | ERV1 | LTR |
| 143322440 | 143322875 | + | 25.2 | 5691 | 6161 | -5 | L1ME1 | L1 | LINE |

  

|  |  |  |  |  |  |  |  |  |  |
| --- | --- | --- | --- | --- | --- | --- | --- | --- | --- |
| chr1:143324589-143325879 | | | 1291 bp | | |  |  |  |  |
| Genome Start | Genome End | Strand | Div | Repeat Start | Repeat End | Repeat Left | Name | Family | Class |
| 143324589 | 143325526 | + | 29.4 | 4857 | 5887 | -277 | L1ME2 | L1 | LINE |
| 143325527 | 143325844 | + | 5.4 | 1 | 295 | -16 | AluY | Alu | SINE |
| 143325845 | 143325879 | + | 27.7 | 5888 | 5921 | -243 | L1ME2 | L1 | LINE |

  

|  |  |  |  |  |  |  |  |  |  |
| --- | --- | --- | --- | --- | --- | --- | --- | --- | --- |
| chr1:143387175-143388001 | | | 827 bp | | |  |  |  |  |
| Genome Start | Genome End | Strand | Div | Repeat Start | Repeat End | Repeat Left | Name | Family | Class |
| 143387175 | 143387388 | + | 31.2 | 2950 | 3158 | -220 | L2 | L2 | LINE |
| 143387389 | 143387793 | - | 16.8 | 1 | 365 | 0 | MLT1A0 | MaLR | LTR |
| 143387794 | 143388001 | + | 31.2 | 3159 | 3378 | 0 | L2 | L2 | LINE |

  

|  |  |  |  |  |  |  |  |  |  |
| --- | --- | --- | --- | --- | --- | --- | --- | --- | --- |
| chr1:143405428-143407271 | | | 1844 bp | | |  |  |  |  |
| Genome Start | Genome End | Strand | Div | Repeat Start | Repeat End | Repeat Left | Name | Family | Class |
| 143405428 | 143405905 | + | 26.8 | 1542 | 2030 | -1389 | L2 | L2 | LINE |
| 143405906 | 143406195 | - | 10.0 | 2 | 291 | -21 | AluSx | Alu | SINE |
| 143406196 | 143407271 | + | 27.1 | 2031 | 3142 | -277 | L2 | L2 | LINE |

  

|  |  |  |  |  |  |  |  |  |  |
| --- | --- | --- | --- | --- | --- | --- | --- | --- | --- |
| chr1:143410317-143410573 | | | 252 bp | | |  |  |  |  |
| Genome Start | Genome End | Strand | Div | Repeat Start | Repeat End | Repeat Left | Name | Family | Class |
| 143410317 | 143410415 | + | 28.1 | 3236 | 3335 | -84 | L2 | L2 | LINE |
| 143410421 | 143410496 | + | 18.4 | 5607 | 5695 | -471 | L1M5 | L1 | LINE |
| 143410497 | 143410573 | + | 26.7 | 3343 | 3418 | -1 | L2 | L2 | LINE |

  

|  |  |  |  |  |  |  |  |  |  |
| --- | --- | --- | --- | --- | --- | --- | --- | --- | --- |
| chr1:143529610-143532710 | | | 3101 bp | | |  |  |  |  |
| Genome Start | Genome End | Strand | Div | Repeat Start | Repeat End | Repeat Left | Name | Family | Class |
| 143529610 | 143530996 | - | 6.8 | 1850 | 3226 | -2920 | L1P2 | L1 | LINE |
| 143530997 | 143531650 | + | 4.3 | 5494 | 6153 | -2 | L1PA4 | L1 | LINE |
| 143531651 | 143532710 | - | 5.8 | 857 | 1849 | -426 | L1P2 | L1 | LINE |

  

|  |  |  |  |  |  |  |  |  |  |
| --- | --- | --- | --- | --- | --- | --- | --- | --- | --- |
| chr1:143589630-143590146 | | | 517 bp | | |  |  |  |  |
| Genome Start | Genome End | Strand | Div | Repeat Start | Repeat End | Repeat Left | Name | Family | Class |
| 143589630 | 143589677 | - | 21.3 | 203 | 245 | -17 | MIR | MIR | SINE |
| 143589678 | 143589978 | - | 11.0 | 1 | 301 | -11 | AluSx | Alu | SINE |
| 143589979 | 143590146 | - | 21.3 | 4 | 202 | -60 | MIR | MIR | SINE |

  

|  |  |  |  |  |  |  |  |  |  |
| --- | --- | --- | --- | --- | --- | --- | --- | --- | --- |
| chr1:143618777-143619517 | | | 776 bp | | |  |  |  |  |
| Genome Start | Genome End | Strand | Div | Repeat Start | Repeat End | Repeat Left | Name | Family | Class |
| 143618777 | 143618894 | - | 32.8 | 136 | 261 | -1 | MIR | MIR | SINE |
| 143618863 | 143619318 | + | 29.9 | 2915 | 3419 | 0 | L2 | L2 | LINE |
| 143619316 | 143619517 | - | 24.7 | 1 | 185 | -77 | MIR | MIR | SINE |

  

|  |  |  |  |  |  |  |  |  |  |
| --- | --- | --- | --- | --- | --- | --- | --- | --- | --- |
| chr1:143852578-143854879 | | | 2290 bp | | |  |  |  |  |
| Genome Start | Genome End | Strand | Div | Repeat Start | Repeat End | Repeat Left | Name | Family | Class |
| 143852578 | 143852773 | + | 25.9 | 2622 | 2817 | -602 | L2 | L2 | LINE |
| 143852775 | 143854279 | + | 13.6 | 4643 | 6152 | -2 | L1PB3 | L1 | LINE |
| 143854291 | 143854879 | + | 26.6 | 2804 | 3419 | 0 | L2 | L2 | LINE |

  

|  |  |  |  |  |  |  |  |  |  |
| --- | --- | --- | --- | --- | --- | --- | --- | --- | --- |
| chr1:143908663-143910144 | | | 1131 bp | | |  |  |  |  |
| Genome Start | Genome End | Strand | Div | Repeat Start | Repeat End | Repeat Left | Name | Family | Class |
| 143908663 | 143909005 | - | 35.4 | 2719 | 3077 | -342 | L2 | L2 | LINE |
| 143909009 | 143909141 | - | 32.1 | 1951 | 2085 | -1334 | L2 | L2 | LINE |
| 143909149 | 143909329 | + | 23.2 | 1 | 188 | -1 | MER5A | MER1\_type | DNA |
| 143909496 | 143909730 | + | 34.7 | 3105 | 3361 | -17 | L2 | L2 | LINE |
| 143909906 | 143910144 | - | 29.9 | 1532 | 1771 | -1648 | L2 | L2 | LINE |

  

|  |  |  |  |  |  |  |  |  |  |
| --- | --- | --- | --- | --- | --- | --- | --- | --- | --- |
| chr1:143942416-143942813 | | | 398 bp | | |  |  |  |  |
| Genome Start | Genome End | Strand | Div | Repeat Start | Repeat End | Repeat Left | Name | Family | Class |
| 143942416 | 143942479 | - | 27.5 | 58 | 117 | -151 | MIRb | MIR | SINE |
| 143942480 | 143942780 | - | 13.1 | 1 | 298 | -14 | AluSx | Alu | SINE |
| 143942781 | 143942813 | - | 27.5 | 19 | 57 | -211 | MIRb | MIR | SINE |

  

|  |  |  |  |  |  |  |  |  |  |
| --- | --- | --- | --- | --- | --- | --- | --- | --- | --- |
| chr1:144017589-144020689 | | | 3101 bp | | |  |  |  |  |
| Genome Start | Genome End | Strand | Div | Repeat Start | Repeat End | Repeat Left | Name | Family | Class |
| 144017589 | 144018975 | - | 6.4 | 1850 | 3226 | -2920 | L1P2 | L1 | LINE |
| 144018976 | 144019629 | + | 4.1 | 5494 | 6153 | -2 | L1PA4 | L1 | LINE |
| 144019630 | 144020689 | - | 5.7 | 857 | 1849 | -426 | L1P2 | L1 | LINE |

  

|  |  |  |  |  |  |  |  |  |  |
| --- | --- | --- | --- | --- | --- | --- | --- | --- | --- |
| chr1:144326280-144328032 | | | 1753 bp | | |  |  |  |  |
| Genome Start | Genome End | Strand | Div | Repeat Start | Repeat End | Repeat Left | Name | Family | Class |
| 144326280 | 144326403 | - | 11.9 | 3789 | 3898 | -2248 | L1P4 | L1 | LINE |
| 144326404 | 144326700 | - | 15.2 | 7 | 303 | -9 | AluSx | Alu | SINE |
| 144326701 | 144326987 | - | 11.9 | 3504 | 3788 | -2358 | L1P4 | L1 | LINE |
| 144326988 | 144327294 | - | 18.1 | 1 | 305 | -7 | AluJb | Alu | SINE |
| 144327295 | 144328032 | - | 11.9 | 2760 | 3503 | -2643 | L1P4 | L1 | LINE |

  

|  |  |  |  |  |  |  |  |  |  |
| --- | --- | --- | --- | --- | --- | --- | --- | --- | --- |
| chr1:144332110-144332628 | | | 519 bp | | |  |  |  |  |
| Genome Start | Genome End | Strand | Div | Repeat Start | Repeat End | Repeat Left | Name | Family | Class |
| 144332110 | 144332172 | + | 20.1 | 1 | 62 | -157 | MER20 | MER1\_type | DNA |
| 144332173 | 144332455 | - | 15.9 | 1 | 300 | -12 | AluJb | Alu | SINE |
| 144332456 | 144332628 | + | 20.1 | 63 | 219 | 0 | MER20 | MER1\_type | DNA |

  

|  |  |  |  |  |  |  |  |  |  |
| --- | --- | --- | --- | --- | --- | --- | --- | --- | --- |
| chr1:144461191-144462824 | | | 1626 bp | | |  |  |  |  |
| Genome Start | Genome End | Strand | Div | Repeat Start | Repeat End | Repeat Left | Name | Family | Class |
| 144461191 | 144461488 | + | 16.9 | 5 | 315 | -2176 | Tigger7 | MER2\_type | DNA |
| 144461489 | 144461903 | + | 11.4 | 5880 | 6298 | -5 | L1MA4A | L1 | LINE |
| 144461914 | 144462213 | + | 5.3 | 1 | 300 | -11 | AluY | Alu | SINE |
| 144462214 | 144462274 | + | 16.9 | 324 | 383 | -2108 | Tigger7 | MER2\_type | DNA |
| 144462275 | 144462575 | + | 11.7 | 1 | 299 | -13 | AluSx | Alu | SINE |
| 144462576 | 144462657 | + | 16.9 | 384 | 460 | -2031 | Tigger7 | MER2\_type | DNA |
| 144462656 | 144462824 | + | 12.8 | 2320 | 2490 | -1 | Tigger7 | MER2\_type | DNA |

  

|  |  |  |  |  |  |  |  |  |  |
| --- | --- | --- | --- | --- | --- | --- | --- | --- | --- |
| chr1:144613727-144622423 | | | 8628 bp | | |  |  |  |  |
| Genome Start | Genome End | Strand | Div | Repeat Start | Repeat End | Repeat Left | Name | Family | Class |
| 144613727 | 144614545 | - | 22.8 | 5314 | 6115 | -51 | L1ME1 | L1 | LINE |
| 144614541 | 144618836 | - | 6.0 | 2027 | 6353 | 0 | L1PA10 | L1 | LINE |
| 144618837 | 144619145 | + | 7.1 | 1 | 309 | -2 | AluY | Alu | SINE |
| 144619146 | 144621190 | - | 9.8 | 3 | 2026 | -4327 | L1PA10 | L1 | LINE |
| 144621265 | 144621691 | - | 21.5 | 3726 | 4160 | -1986 | L1ME1 | L1 | LINE |
| 144621692 | 144621982 | + | 16.8 | 3 | 296 | -16 | AluJo | Alu | SINE |
| 144621983 | 144622423 | - | 21.5 | 3293 | 3725 | -2421 | L1ME1 | L1 | LINE |

  

|  |  |  |  |  |  |  |  |  |  |
| --- | --- | --- | --- | --- | --- | --- | --- | --- | --- |
| chr1:144641095-144642269 | | | 1033 bp | | |  |  |  |  |
| Genome Start | Genome End | Strand | Div | Repeat Start | Repeat End | Repeat Left | Name | Family | Class |
| 144641095 | 144641210 | - | 29.4 | 4205 | 4335 | -154 | L3 | CR1 | LINE |
| 144641211 | 144641495 | + | 8.8 | 3 | 295 | -14 | AluSc | Alu | SINE |
| 144641496 | 144641661 | - | 29.4 | 3981 | 4204 | -285 | L3 | CR1 | LINE |
| 144641672 | 144641808 | - | 31.4 | 98 | 245 | -17 | MIR | MIR | SINE |
| 144641941 | 144642269 | - | 32.8 | 3442 | 3821 | -668 | L3 | CR1 | LINE |

  

|  |  |  |  |  |  |  |  |  |  |
| --- | --- | --- | --- | --- | --- | --- | --- | --- | --- |
| chr1:144657720-144658868 | | | 1149 bp | | |  |  |  |  |
| Genome Start | Genome End | Strand | Div | Repeat Start | Repeat End | Repeat Left | Name | Family | Class |
| 144657720 | 144658502 | - | 21.2 | 1332 | 2204 | -4196 | L1MEc | L1 | LINE |
| 144658503 | 144658615 | + | 22.5 | 1 | 132 | -1 | FLAM\_C | Alu | SINE |
| 144658616 | 144658868 | - | 21.2 | 1050 | 1331 | -5069 | L1MEc | L1 | LINE |

  

|  |  |  |  |  |  |  |  |  |  |
| --- | --- | --- | --- | --- | --- | --- | --- | --- | --- |
| chr1:144669482-144672839 | | | 3368 bp | | |  |  |  |  |
| Genome Start | Genome End | Strand | Div | Repeat Start | Repeat End | Repeat Left | Name | Family | Class |
| 144669482 | 144669892 | + | 10.0 | 3008 | 3407 | -4713 | LOR1b-int | ERV1 | LTR |
| 144669893 | 144670180 | + | 16.3 | 1 | 299 | -13 | AluSx | Alu | SINE |
| 144670181 | 144670380 | + | 10.0 | 3408 | 3605 | -4515 | LOR1b-int | ERV1 | LTR |
| 144670373 | 144670933 | + | 14.0 | 3529 | 4062 | -4058 | LOR1b-int | ERV1 | LTR |
| 144670944 | 144671114 | - | 18.9 | 38 | 206 | 0 | FLAM\_C | Alu | SINE |
| 144671115 | 144672507 | + | 13.5 | 3570 | 5538 | -1050 | LOR1b-int | ERV1 | LTR |
| 144672497 | 144672749 | + | 7.9 | 5811 | 6064 | -2056 | LOR1b-int | ERV1 | LTR |
| 144672749 | 144672839 | + | 17.6 | 8030 | 8120 | 0 | LOR1b-int | ERV1 | LTR |

  

|  |  |  |  |  |  |  |  |  |  |
| --- | --- | --- | --- | --- | --- | --- | --- | --- | --- |
| chr1:144687073-144688420 | | | 1348 bp | | |  |  |  |  |
| Genome Start | Genome End | Strand | Div | Repeat Start | Repeat End | Repeat Left | Name | Family | Class |
| 144687073 | 144687287 | - | 31.0 | 6162 | 6387 | -1883 | MER4B-int | ERV1 | LTR |
| 144687288 | 144687444 | + | 19.8 | 1 | 157 | -9 | FRAM | Alu | SINE |
| 144687445 | 144688420 | - | 31.0 | 5189 | 6161 | -2109 | MER4B-int | ERV1 | LTR |

  

|  |  |  |  |  |  |  |  |  |  |
| --- | --- | --- | --- | --- | --- | --- | --- | --- | --- |
| chr1:144707218-144709123 | | | 1634 bp | | |  |  |  |  |
| Genome Start | Genome End | Strand | Div | Repeat Start | Repeat End | Repeat Left | Name | Family | Class |
| 144707218 | 144707306 | + | 30.0 | 7 | 89 | -694 | MER20B | MER1\_type | DNA |
| 144707307 | 144707742 | + | 9.5 | 5708 | 6174 | 0 | L1PA11 | L1 | LINE |
| 144707743 | 144707927 | + | 30.0 | 90 | 301 | -482 | MER20B | MER1\_type | DNA |
| 144707928 | 144708238 | - | 12.3 | 1 | 307 | 0 | AluJb | Alu | SINE |
| 144708239 | 144708308 | + | 30.0 | 302 | 366 | -417 | MER20B | MER1\_type | DNA |
| 144708309 | 144708439 | + | 17.1 | 1 | 133 | 0 | FLAM\_C | Alu | SINE |
| 144708651 | 144708957 | + | 11.0 | 1 | 299 | -13 | AluSx | Alu | SINE |
| 144709019 | 144709123 | + | 24.0 | 669 | 780 | -3 | MER20B | MER1\_type | DNA |

  

|  |  |  |  |  |  |  |  |  |  |
| --- | --- | --- | --- | --- | --- | --- | --- | --- | --- |
| chr1:144710891-144711409 | | | 519 bp | | |  |  |  |  |
| Genome Start | Genome End | Strand | Div | Repeat Start | Repeat End | Repeat Left | Name | Family | Class |
| 144710891 | 144711063 | - | 19.6 | 63 | 219 | 0 | MER20 | MER1\_type | DNA |
| 144711064 | 144711346 | + | 15.2 | 1 | 300 | -12 | AluJb | Alu | SINE |
| 144711347 | 144711409 | - | 19.6 | 1 | 62 | -157 | MER20 | MER1\_type | DNA |

  

|  |  |  |  |  |  |  |  |  |  |
| --- | --- | --- | --- | --- | --- | --- | --- | --- | --- |
| chr1:144715491-144717248 | | | 1758 bp | | |  |  |  |  |
| Genome Start | Genome End | Strand | Div | Repeat Start | Repeat End | Repeat Left | Name | Family | Class |
| 144715491 | 144716228 | + | 12.1 | 2760 | 3503 | -2643 | L1P4 | L1 | LINE |
| 144716229 | 144716540 | + | 17.8 | 1 | 305 | -7 | AluJb | Alu | SINE |
| 144716541 | 144716822 | + | 12.1 | 3504 | 3788 | -2358 | L1P4 | L1 | LINE |
| 144716823 | 144717124 | + | 15.6 | 1 | 303 | -9 | AluSx | Alu | SINE |
| 144717125 | 144717248 | + | 12.1 | 3789 | 3898 | -2248 | L1P4 | L1 | LINE |

  

|  |  |  |  |  |  |  |  |  |  |
| --- | --- | --- | --- | --- | --- | --- | --- | --- | --- |
| chr1:144752978-144756070 | | | 3093 bp | | |  |  |  |  |
| Genome Start | Genome End | Strand | Div | Repeat Start | Repeat End | Repeat Left | Name | Family | Class |
| 144752978 | 144754031 | + | 6.0 | 857 | 1849 | -426 | L1P2 | L1 | LINE |
| 144754032 | 144754683 | - | 4.6 | 5494 | 6146 | -9 | L1PA4 | L1 | LINE |
| 144754684 | 144756070 | + | 6.9 | 1850 | 3226 | -2920 | L1P2 | L1 | LINE |

  

|  |  |  |  |  |  |  |  |  |  |
| --- | --- | --- | --- | --- | --- | --- | --- | --- | --- |
| chr1:144769409-144770689 | | | 1281 bp | | |  |  |  |  |
| Genome Start | Genome End | Strand | Div | Repeat Start | Repeat End | Repeat Left | Name | Family | Class |
| 144769409 | 144769443 | - | 27.5 | 5889 | 5922 | -242 | L1ME2 | L1 | LINE |
| 144769444 | 144769757 | - | 4.7 | 1 | 301 | -10 | AluY | Alu | SINE |
| 144769758 | 144770689 | - | 29.2 | 4862 | 5888 | -276 | L1ME2 | L1 | LINE |

  

|  |  |  |  |  |  |  |  |  |  |
| --- | --- | --- | --- | --- | --- | --- | --- | --- | --- |
| chr1:144772452-144773915 | | | 1464 bp | | |  |  |  |  |
| Genome Start | Genome End | Strand | Div | Repeat Start | Repeat End | Repeat Left | Name | Family | Class |
| 144772452 | 144772880 | - | 26.0 | 5691 | 6151 | -13 | L1ME2 | L1 | LINE |
| 144772881 | 144773546 | + | 8.9 | 2 | 660 | 0 | PABL\_A | ERV1 | LTR |
| 144773547 | 144773915 | - | 26.0 | 5299 | 5690 | -474 | L1ME2 | L1 | LINE |

  

|  |  |  |  |  |  |  |  |  |  |
| --- | --- | --- | --- | --- | --- | --- | --- | --- | --- |
| chr1:144842685-144845321 | | | 2633 bp | | |  |  |  |  |
| Genome Start | Genome End | Strand | Div | Repeat Start | Repeat End | Repeat Left | Name | Family | Class |
| 144842685 | 144842991 | - | 15.9 | 88 | 390 | 0 | MLT1B | MaLR | LTR |
| 144842992 | 144843372 | + | 13.6 | 1 | 428 | 0 | MSTA | MaLR | LTR |
| 144843373 | 144844852 | + | 17.2 | 1 | 1651 | 0 | MSTA-int | MaLR | LTR |
| 144844857 | 144845230 | + | 14.6 | 5 | 428 | 0 | MSTA | MaLR | LTR |
| 144845231 | 144845321 | - | 15.8 | 5 | 96 | -294 | MLT1B | MaLR | LTR |

  

|  |  |  |  |  |  |  |  |  |  |
| --- | --- | --- | --- | --- | --- | --- | --- | --- | --- |
| chr1:144872695-144874712 | | | 1948 bp | | |  |  |  |  |
| Genome Start | Genome End | Strand | Div | Repeat Start | Repeat End | Repeat Left | Name | Family | Class |
| 144872695 | 144873371 | + | 29.9 | 534 | 1205 | -4914 | L1MEd | L1 | LINE |
| 144873407 | 144873891 | + | 29.5 | 1310 | 1873 | -4276 | L1MEd | L1 | LINE |
| 144873926 | 144874126 | + | 10.9 | 108 | 308 | -5 | AluSp | Alu | SINE |
| 144874128 | 144874191 | + | 18.4 | 1860 | 1914 | -4235 | L1MEd | L1 | LINE |
| 144874192 | 144874500 | + | 17.0 | 1 | 300 | -12 | AluJo | Alu | SINE |
| 144874501 | 144874712 | + | 18.4 | 1915 | 2111 | -4038 | L1MEd | L1 | LINE |

  

|  |  |  |  |  |  |  |  |  |  |
| --- | --- | --- | --- | --- | --- | --- | --- | --- | --- |
| chr1:145866093-145866754 | | | 662 bp | | |  |  |  |  |
| Genome Start | Genome End | Strand | Div | Repeat Start | Repeat End | Repeat Left | Name | Family | Class |
| 145866093 | 145866342 | - | 28.7 | 4029 | 4343 | -146 | L3 | CR1 | LINE |
| 145866343 | 145866647 | - | 7.2 | 1 | 311 | -2 | AluSp | Alu | SINE |
| 145866648 | 145866754 | - | 28.7 | 3911 | 4028 | -461 | L3 | CR1 | LINE |

  

|  |  |  |  |  |  |  |  |  |  |
| --- | --- | --- | --- | --- | --- | --- | --- | --- | --- |
| chr1:145871236-145873621 | | | 1860 bp | | |  |  |  |  |
| Genome Start | Genome End | Strand | Div | Repeat Start | Repeat End | Repeat Left | Name | Family | Class |
| 145871236 | 145871519 | + | 34.0 | 1384 | 1662 | -1757 | L2 | L2 | LINE |
| 145871608 | 145871816 | + | 28.8 | 25 | 260 | -8 | MIRb | MIR | SINE |
| 145872056 | 145872873 | + | 33.9 | 2191 | 3082 | -337 | L2 | L2 | LINE |
| 145872909 | 145873201 | - | 17.1 | 1 | 306 | -6 | AluJb | Alu | SINE |
| 145873366 | 145873621 | + | 34.1 | 3121 | 3371 | -7 | L2 | L2 | LINE |

  

|  |  |  |  |  |  |  |  |  |  |
| --- | --- | --- | --- | --- | --- | --- | --- | --- | --- |
| chr1:145878013-145880706 | | | 2542 bp | | |  |  |  |  |
| Genome Start | Genome End | Strand | Div | Repeat Start | Repeat End | Repeat Left | Name | Family | Class |
| 145878013 | 145878368 | + | 30.0 | 13 | 431 | -1529 | L4 | RTE | LINE |
| 145878454 | 145878732 | + | 17.2 | 1 | 290 | -22 | AluJo | Alu | SINE |
| 145878746 | 145878859 | + | 35.6 | 421 | 527 | -1433 | L4 | RTE | LINE |
| 145878860 | 145879162 | + | 5.9 | 1 | 303 | -8 | AluY | Alu | SINE |
| 145879163 | 145879266 | + | 35.6 | 528 | 636 | -1324 | L4 | RTE | LINE |
| 145879290 | 145879492 | - | 15.9 | 9 | 224 | 0 | MER58A | MER1\_type | DNA |
| 145879524 | 145880706 | + | 38.3 | 701 | 1942 | -18 | L4 | RTE | LINE |

  

|  |  |  |  |  |  |  |  |  |  |
| --- | --- | --- | --- | --- | --- | --- | --- | --- | --- |
| chr1:145910658-145919396 | | | 8670 bp | | |  |  |  |  |
| Genome Start | Genome End | Strand | Div | Repeat Start | Repeat End | Repeat Left | Name | Family | Class |
| 145910658 | 145911517 | - | 22.8 | 5314 | 6160 | -6 | L1ME1 | L1 | LINE |
| 145911513 | 145915808 | - | 6.0 | 2027 | 6353 | 0 | L1PA10 | L1 | LINE |
| 145915809 | 145916118 | + | 7.1 | 1 | 310 | -1 | AluY | Alu | SINE |
| 145916119 | 145918163 | - | 9.8 | 3 | 2026 | -4327 | L1PA10 | L1 | LINE |
| 145918238 | 145918664 | - | 21.6 | 3726 | 4160 | -1986 | L1ME1 | L1 | LINE |
| 145918665 | 145918955 | + | 16.8 | 3 | 296 | -16 | AluJo | Alu | SINE |
| 145918956 | 145919396 | - | 21.6 | 3293 | 3725 | -2421 | L1ME1 | L1 | LINE |

  

|  |  |  |  |  |  |  |  |  |  |
| --- | --- | --- | --- | --- | --- | --- | --- | --- | --- |
| chr1:145923238-145924697 | | | 1444 bp | | |  |  |  |  |
| Genome Start | Genome End | Strand | Div | Repeat Start | Repeat End | Repeat Left | Name | Family | Class |
| 145923238 | 145924302 | - | 22.9 | 2232 | 3385 | -2834 | L1MEc | L1 | LINE |
| 145924303 | 145924439 | + | 22.6 | 1 | 138 | 0 | MER58A | MER1\_type | DNA |
| 145924456 | 145924697 | - | 18.2 | 2123 | 2337 | -4063 | L1MEc | L1 | LINE |

  

|  |  |  |  |  |  |  |  |  |  |
| --- | --- | --- | --- | --- | --- | --- | --- | --- | --- |
| chr1:145945326-145946953 | | | 1620 bp | | |  |  |  |  |
| Genome Start | Genome End | Strand | Div | Repeat Start | Repeat End | Repeat Left | Name | Family | Class |
| 145945326 | 145945494 | - | 12.2 | 2320 | 2490 | -1 | Tigger7 | MER2\_type | DNA |
| 145945493 | 145945574 | - | 16.9 | 385 | 461 | -2030 | Tigger7 | MER2\_type | DNA |
| 145945575 | 145945872 | - | 12.8 | 1 | 296 | -16 | AluSx | Alu | SINE |
| 145945873 | 145945933 | - | 16.9 | 325 | 384 | -2107 | Tigger7 | MER2\_type | DNA |
| 145945934 | 145946230 | - | 6.4 | 1 | 297 | -14 | AluY | Alu | SINE |
| 145946241 | 145946655 | - | 11.7 | 5880 | 6298 | -5 | L1MA4A | L1 | LINE |
| 145946656 | 145946953 | - | 16.9 | 7 | 317 | -2174 | Tigger7 | MER2\_type | DNA |

  

|  |  |  |  |  |  |  |  |  |  |
| --- | --- | --- | --- | --- | --- | --- | --- | --- | --- |
| chr1:145979106-145980039 | | | 934 bp | | |  |  |  |  |
| Genome Start | Genome End | Strand | Div | Repeat Start | Repeat End | Repeat Left | Name | Family | Class |
| 145979106 | 145979170 | - | 23.5 | 5893 | 5949 | -215 | L1ME2 | L1 | LINE |
| 145979171 | 145979895 | - | 14.7 | 1 | 691 | 0 | LTR8 | ERV1 | LTR |
| 145979896 | 145980039 | - | 20.4 | 5746 | 5892 | -272 | L1ME2 | L1 | LINE |

  

|  |  |  |  |  |  |  |  |  |  |
| --- | --- | --- | --- | --- | --- | --- | --- | --- | --- |
| chr1:145989127-145989860 | | | 712 bp | | |  |  |  |  |
| Genome Start | Genome End | Strand | Div | Repeat Start | Repeat End | Repeat Left | Name | Family | Class |
| 145989127 | 145989321 | - | 16.1 | 1961 | 2204 | -4196 | L1MEc | L1 | LINE |
| 145989344 | 145989471 | - | 22.4 | 1727 | 1858 | -4542 | L1MEc | L1 | LINE |
| 145989472 | 145989772 | - | 12.0 | 7 | 307 | -5 | AluSx | Alu | SINE |
| 145989773 | 145989860 | - | 22.4 | 1640 | 1726 | -4674 | L1MEc | L1 | LINE |

  

|  |  |  |  |  |  |  |  |  |  |
| --- | --- | --- | --- | --- | --- | --- | --- | --- | --- |
| chr1:146004278-146006180 | | | 1646 bp | | |  |  |  |  |
| Genome Start | Genome End | Strand | Div | Repeat Start | Repeat End | Repeat Left | Name | Family | Class |
| 146004278 | 146004366 | + | 30.0 | 7 | 89 | -694 | MER20B | MER1\_type | DNA |
| 146004367 | 146004802 | + | 9.5 | 5708 | 6174 | 0 | L1PA11 | L1 | LINE |
| 146004803 | 146004987 | + | 30.0 | 90 | 301 | -482 | MER20B | MER1\_type | DNA |
| 146004988 | 146005298 | - | 12.3 | 1 | 307 | 0 | AluJb | Alu | SINE |
| 146005299 | 146005368 | + | 30.0 | 302 | 366 | -417 | MER20B | MER1\_type | DNA |
| 146005369 | 146005499 | + | 16.3 | 1 | 133 | 0 | FLAM\_C | Alu | SINE |
| 146005710 | 146006028 | + | 10.8 | 1 | 311 | -1 | AluSx | Alu | SINE |
| 146006076 | 146006180 | + | 24.0 | 669 | 780 | -3 | MER20B | MER1\_type | DNA |

  

|  |  |  |  |  |  |  |  |  |  |
| --- | --- | --- | --- | --- | --- | --- | --- | --- | --- |
| chr1:146071192-146072527 | | | 1336 bp | | |  |  |  |  |
| Genome Start | Genome End | Strand | Div | Repeat Start | Repeat End | Repeat Left | Name | Family | Class |
| 146071192 | 146071642 | - | 29.0 | 4432 | 4859 | -1287 | L1M4 | L1 | LINE |
| 146071643 | 146071952 | - | 11.9 | 1 | 311 | 0 | AluY | Alu | SINE |
| 146071953 | 146072527 | - | 29.0 | 3863 | 4431 | -1715 | L1M4 | L1 | LINE |

  

|  |  |  |  |  |  |  |  |  |  |
| --- | --- | --- | --- | --- | --- | --- | --- | --- | --- |
| chr1:146084413-146084966 | | | 554 bp | | |  |  |  |  |
| Genome Start | Genome End | Strand | Div | Repeat Start | Repeat End | Repeat Left | Name | Family | Class |
| 146084413 | 146084501 | + | 20.6 | 5079 | 5164 | -982 | L1ME2 | L1 | LINE |
| 146084502 | 146084796 | + | 17.1 | 1 | 296 | -16 | AluJb | Alu | SINE |
| 146084797 | 146084966 | + | 20.6 | 5165 | 5336 | -810 | L1ME2 | L1 | LINE |

  

|  |  |  |  |  |  |  |  |  |  |
| --- | --- | --- | --- | --- | --- | --- | --- | --- | --- |
| chr1:146162485-146163569 | | | 1085 bp | | |  |  |  |  |
| Genome Start | Genome End | Strand | Div | Repeat Start | Repeat End | Repeat Left | Name | Family | Class |
| 146162485 | 146162644 | + | 13.7 | 1 | 154 | -236 | MLT1B | MaLR | LTR |
| 146162645 | 146162769 | + | 12.0 | 1 | 117 | -247 | THE1B | MaLR | LTR |
| 146162770 | 146163077 | - | 14.6 | 1 | 309 | -3 | AluSx | Alu | SINE |
| 146163078 | 146163337 | + | 12.0 | 118 | 364 | 0 | THE1B | MaLR | LTR |
| 146163338 | 146163569 | + | 14.2 | 155 | 624 | 0 | MLT1B | MaLR | LTR |

  

|  |  |  |  |  |  |  |  |  |  |
| --- | --- | --- | --- | --- | --- | --- | --- | --- | --- |
| chr1:146168339-146169156 | | | 818 bp | | |  |  |  |  |
| Genome Start | Genome End | Strand | Div | Repeat Start | Repeat End | Repeat Left | Name | Family | Class |
| 146168339 | 146168355 | - | 28.8 | 5899 | 5915 | -1893 | L1MC4a | L1 | LINE |
| 146168356 | 146168789 | + | 13.9 | 1 | 434 | 0 | Tigger2a | MER2\_type | DNA |
| 146168790 | 146169156 | - | 28.8 | 5538 | 5898 | -1910 | L1MC4a | L1 | LINE |

  

|  |  |  |  |  |  |  |  |  |  |
| --- | --- | --- | --- | --- | --- | --- | --- | --- | --- |
| chr1:146200579-146201515 | | | 937 bp | | |  |  |  |  |
| Genome Start | Genome End | Strand | Div | Repeat Start | Repeat End | Repeat Left | Name | Family | Class |
| 146200579 | 146200722 | + | 23.5 | 5746 | 5891 | -273 | L1ME2 | L1 | LINE |
| 146200723 | 146201450 | + | 15.2 | 1 | 691 | 0 | LTR8 | ERV1 | LTR |
| 146201451 | 146201515 | + | 23.5 | 5892 | 5949 | -215 | L1ME2 | L1 | LINE |

  

|  |  |  |  |  |  |  |  |  |  |
| --- | --- | --- | --- | --- | --- | --- | --- | --- | --- |
| chr1:146237675-146239281 | | | 1560 bp | | |  |  |  |  |
| Genome Start | Genome End | Strand | Div | Repeat Start | Repeat End | Repeat Left | Name | Family | Class |
| 146237675 | 146237834 | + | 30.4 | 4691 | 4851 | -1295 | L1ME2 | L1 | LINE |
| 146237835 | 146238123 | - | 16.6 | 1 | 312 | 0 | AluJb | Alu | SINE |
| 146238124 | 146238544 | + | 30.4 | 4852 | 5298 | -848 | L1ME2 | L1 | LINE |
| 146238592 | 146239281 | + | 26.1 | 5299 | 5982 | -182 | L1ME2 | L1 | LINE |

  

|  |  |  |  |  |  |  |  |  |  |
| --- | --- | --- | --- | --- | --- | --- | --- | --- | --- |
| chr1:146332942-146333420 | | | 479 bp | | |  |  |  |  |
| Genome Start | Genome End | Strand | Div | Repeat Start | Repeat End | Repeat Left | Name | Family | Class |
| 146332942 | 146333000 | - | 30.1 | 465 | 518 | -3 | LTR33 | ERVL | LTR |
| 146333001 | 146333295 | - | 6.8 | 1 | 295 | -16 | AluY | Alu | SINE |
| 146333296 | 146333420 | - | 30.1 | 344 | 464 | -57 | LTR33 | ERVL | LTR |

  

|  |  |  |  |  |  |  |  |  |  |
| --- | --- | --- | --- | --- | --- | --- | --- | --- | --- |
| chr1:146335829-146337461 | | | 1495 bp | | |  |  |  |  |
| Genome Start | Genome End | Strand | Div | Repeat Start | Repeat End | Repeat Left | Name | Family | Class |
| 146335829 | 146335907 | + | 15.3 | 11 | 121 | -464 | LTR49 | ERV1 | LTR |
| 146335927 | 146336481 | - | 23.2 | 1 | 551 | -21 | LTR73 | ERV1 | LTR |
| 146336537 | 146336720 | + | 23.9 | 78 | 236 | -359 | LTR49 | ERV1 | LTR |
| 146336721 | 146336881 | - | 12.5 | 135 | 293 | -19 | AluSx | Alu | SINE |
| 146336946 | 146337226 | - | 8.6 | 1 | 280 | -31 | AluY | Alu | SINE |
| 146337227 | 146337352 | - | 12.5 | 6 | 134 | -178 | AluSx | Alu | SINE |
| 146337353 | 146337461 | + | 23.9 | 237 | 345 | -250 | LTR49 | ERV1 | LTR |

  

|  |  |  |  |  |  |  |  |  |  |
| --- | --- | --- | --- | --- | --- | --- | --- | --- | --- |
| chr1:146339052-146339634 | | | 530 bp | | |  |  |  |  |
| Genome Start | Genome End | Strand | Div | Repeat Start | Repeat End | Repeat Left | Name | Family | Class |
| 146339052 | 146339131 | - | 19.7 | 7518 | 7589 | -1 | HERVP71A | ERV1 | LTR |
| 146339132 | 146339415 | - | 9.0 | 1 | 292 | -20 | AluSx | Alu | SINE |
| 146339416 | 146339539 | - | 19.7 | 7393 | 7517 | -73 | HERVP71A | ERV1 | LTR |
| 146339593 | 146339634 | - | 19.7 | 7361 | 7392 | -198 | HERVP71A | ERV1 | LTR |

  

|  |  |  |  |  |  |  |  |  |  |
| --- | --- | --- | --- | --- | --- | --- | --- | --- | --- |
| chr1:146345383-146346131 | | | 745 bp | | |  |  |  |  |
| Genome Start | Genome End | Strand | Div | Repeat Start | Repeat End | Repeat Left | Name | Family | Class |
| 146345383 | 146345602 | - | 11.1 | 79 | 295 | -17 | AluSx | Alu | SINE |
| 146345607 | 146346057 | + | 22.0 | 11 | 463 | 0 | LTR2 | ERV1 | LTR |
| 146346058 | 146346131 | - | 13.5 | 12 | 85 | -227 | AluSx | Alu | SINE |

  

|  |  |  |  |  |  |  |  |  |  |
| --- | --- | --- | --- | --- | --- | --- | --- | --- | --- |
| chr1:146346180-146347092 | | | 892 bp | | |  |  |  |  |
| Genome Start | Genome End | Strand | Div | Repeat Start | Repeat End | Repeat Left | Name | Family | Class |
| 146346180 | 146346390 | + | 24.6 | 1 | 268 | -346 | MER90a | ERV1 | LTR |
| 146346416 | 146346719 | - | 11.7 | 1 | 299 | -11 | AluSg | Alu | SINE |
| 146346721 | 146346894 | - | 12.6 | 124 | 303 | -9 | AluSg/x | Alu | SINE |
| 146346895 | 146346991 | + | 28.1 | 290 | 386 | -228 | MER90a | ERV1 | LTR |
| 146346987 | 146347092 | + | 26.5 | 505 | 609 | -5 | MER90a | ERV1 | LTR |

  

|  |  |  |  |  |  |  |  |  |  |
| --- | --- | --- | --- | --- | --- | --- | --- | --- | --- |
| chr1:146347369-146348118 | | | 750 bp | | |  |  |  |  |
| Genome Start | Genome End | Strand | Div | Repeat Start | Repeat End | Repeat Left | Name | Family | Class |
| 146347369 | 146347418 | - | 25.5 | 494 | 533 | -16 | MLT1H | MaLR | LTR |
| 146347419 | 146347717 | + | 8.4 | 1 | 300 | -11 | AluY | Alu | SINE |
| 146347718 | 146348118 | - | 25.5 | 1 | 493 | -56 | MLT1H | MaLR | LTR |

  

|  |  |  |  |  |  |  |  |  |  |
| --- | --- | --- | --- | --- | --- | --- | --- | --- | --- |
| chr1:146350564-146351539 | | | 976 bp | | |  |  |  |  |
| Genome Start | Genome End | Strand | Div | Repeat Start | Repeat End | Repeat Left | Name | Family | Class |
| 146350564 | 146350601 | + | 15.3 | 2300 | 2334 | -3812 | L1M4 | L1 | LINE |
| 146350602 | 146351098 | + | 14.3 | 3 | 497 | 0 | LOR1a | ERV1 | LTR |
| 146351099 | 146351539 | + | 15.3 | 2335 | 2781 | -3365 | L1M4 | L1 | LINE |

  

|  |  |  |  |  |  |  |  |  |  |
| --- | --- | --- | --- | --- | --- | --- | --- | --- | --- |
| chr1:146352681-146353178 | | | 498 bp | | |  |  |  |  |
| Genome Start | Genome End | Strand | Div | Repeat Start | Repeat End | Repeat Left | Name | Family | Class |
| 146352681 | 146352775 | - | 19.4 | 165 | 299 | -3 | AluJb | Alu | SINE |
| 146352776 | 146353029 | - | 10.6 | 1 | 259 | -52 | AluY | Alu | SINE |
| 146353030 | 146353178 | - | 19.4 | 2 | 164 | -138 | AluJb | Alu | SINE |

  

|  |  |  |  |  |  |  |  |  |  |
| --- | --- | --- | --- | --- | --- | --- | --- | --- | --- |
| chr1:146359435-146359924 | | | 490 bp | | |  |  |  |  |
| Genome Start | Genome End | Strand | Div | Repeat Start | Repeat End | Repeat Left | Name | Family | Class |
| 146359435 | 146359568 | - | 29.5 | 90 | 221 | -6326 | L1MD2 | L1 | LINE |
| 146359569 | 146359866 | - | 17.8 | 1 | 298 | -14 | AluJb | Alu | SINE |
| 146359867 | 146359924 | - | 29.5 | 41 | 89 | -6458 | L1MD2 | L1 | LINE |

  

|  |  |  |  |  |  |  |  |  |  |
| --- | --- | --- | --- | --- | --- | --- | --- | --- | --- |
| chr1:146372536-146373029 | | | 494 bp | | |  |  |  |  |
| Genome Start | Genome End | Strand | Div | Repeat Start | Repeat End | Repeat Left | Name | Family | Class |
| 146372536 | 146372722 | - | 23.0 | 4749 | 4948 | -1728 | MER66-int | ERV1 | LTR |
| 146372723 | 146373017 | + | 7.2 | 1 | 294 | -17 | AluY | Alu | SINE |
| 146373018 | 146373029 | - | 23.0 | 4737 | 4748 | -1928 | MER66-int | ERV1 | LTR |

  

|  |  |  |  |  |  |  |  |  |  |
| --- | --- | --- | --- | --- | --- | --- | --- | --- | --- |
| chr1:146414033-146415085 | | | 1053 bp | | |  |  |  |  |
| Genome Start | Genome End | Strand | Div | Repeat Start | Repeat End | Repeat Left | Name | Family | Class |
| 146414033 | 146414308 | + | 32.2 | 5316 | 5598 | -2210 | L1MC4a | L1 | LINE |
| 146414309 | 146414754 | + | 13.5 | 5704 | 6157 | -9 | L1PA16 | L1 | LINE |
| 146414755 | 146415085 | + | 34.3 | 5599 | 5933 | -1875 | L1MC4a | L1 | LINE |

  

|  |  |  |  |  |  |  |  |  |  |
| --- | --- | --- | --- | --- | --- | --- | --- | --- | --- |
| chr1:146441439-146444306 | | | 2856 bp | | |  |  |  |  |
| Genome Start | Genome End | Strand | Div | Repeat Start | Repeat End | Repeat Left | Name | Family | Class |
| 146441439 | 146443692 | - | 13.2 | 386 | 2716 | -2 | Tigger2 | MER2\_type | DNA |
| 146443698 | 146443902 | + | 16.7 | 5965 | 6153 | -3 | L1PB4 | L1 | LINE |
| 146443910 | 146444306 | - | 15.1 | 2 | 403 | -2315 | Tigger2 | MER2\_type | DNA |

  

|  |  |  |  |  |  |  |  |  |  |
| --- | --- | --- | --- | --- | --- | --- | --- | --- | --- |
| chr1:146462462-146462929 | | | 468 bp | | |  |  |  |  |
| Genome Start | Genome End | Strand | Div | Repeat Start | Repeat End | Repeat Left | Name | Family | Class |
| 146462462 | 146462493 | + | 27.9 | 2452 | 2484 | -935 | L2 | L2 | LINE |
| 146462494 | 146462796 | - | 13.2 | 1 | 303 | -9 | AluSx | Alu | SINE |
| 146462797 | 146462929 | + | 27.9 | 2485 | 2627 | -792 | L2 | L2 | LINE |

  

|  |  |  |  |  |  |  |  |  |  |
| --- | --- | --- | --- | --- | --- | --- | --- | --- | --- |
| chr1:146484857-146487965 | | | 3084 bp | | |  |  |  |  |
| Genome Start | Genome End | Strand | Div | Repeat Start | Repeat End | Repeat Left | Name | Family | Class |
| 146484857 | 146485899 | + | 5.6 | 857 | 1849 | -426 | L1P2 | L1 | LINE |
| 146485925 | 146486577 | - | 4.1 | 5494 | 6152 | -3 | L1PA4 | L1 | LINE |
| 146486578 | 146487965 | + | 6.8 | 1850 | 3226 | -2920 | L1P2 | L1 | LINE |

  

|  |  |  |  |  |  |  |  |  |  |
| --- | --- | --- | --- | --- | --- | --- | --- | --- | --- |
| chr1:146545106-146549505 | | | 4259 bp | | |  |  |  |  |
| Genome Start | Genome End | Strand | Div | Repeat Start | Repeat End | Repeat Left | Name | Family | Class |
| 146545106 | 146545928 | - | 17.1 | 4081 | 4910 | -26 | MER31A-int | ERV1 | LTR |
| 146545929 | 146546227 | - | 11.4 | 1 | 298 | -12 | AluSg | Alu | SINE |
| 146546228 | 146547937 | - | 19.5 | 2669 | 4365 | -856 | MER31A-int | ERV1 | LTR |
| 146547976 | 146548401 | - | 19.6 | 1080 | 1514 | -3422 | MER31A-int | ERV1 | LTR |
| 146548402 | 146548690 | + | 10.5 | 1 | 306 | -6 | AluSx | Alu | SINE |
| 146548691 | 146548874 | - | 19.6 | 887 | 1079 | -3857 | MER31A-int | ERV1 | LTR |
| 146549011 | 146549241 | - | 22.1 | 201 | 842 | -4514 | MER31A-int | ERV1 | LTR |
| 146549209 | 146549505 | - | 22.1 | 15 | 290 | -4646 | MER31A-int | ERV1 | LTR |

  

|  |  |  |  |  |  |  |  |  |  |
| --- | --- | --- | --- | --- | --- | --- | --- | --- | --- |
| chr1:146558341-146559370 | | | 1030 bp | | |  |  |  |  |
| Genome Start | Genome End | Strand | Div | Repeat Start | Repeat End | Repeat Left | Name | Family | Class |
| 146558341 | 146558592 | + | 32.8 | 5327 | 5596 | -2212 | L1MC4a | L1 | LINE |
| 146558593 | 146559038 | + | 11.7 | 5704 | 6157 | -9 | L1PA16 | L1 | LINE |
| 146559039 | 146559370 | + | 32.8 | 5597 | 5933 | -1875 | L1MC4a | L1 | LINE |

  

|  |  |  |  |  |  |  |  |  |  |
| --- | --- | --- | --- | --- | --- | --- | --- | --- | --- |
| chr1:146582916-146583539 | | | 624 bp | | |  |  |  |  |
| Genome Start | Genome End | Strand | Div | Repeat Start | Repeat End | Repeat Left | Name | Family | Class |
| 146582916 | 146583278 | + | 31.1 | 5554 | 5942 | -179 | L1ME4a | L1 | LINE |
| 146583279 | 146583367 | - | 12.4 | 6210 | 6300 | 0 | L1MA6 | L1 | LINE |
| 146583368 | 146583539 | + | 31.1 | 5943 | 6121 | 0 | L1ME4a | L1 | LINE |

  

|  |  |  |  |  |  |  |  |  |  |
| --- | --- | --- | --- | --- | --- | --- | --- | --- | --- |
| chr1:146605200-146605668 | | | 469 bp | | |  |  |  |  |
| Genome Start | Genome End | Strand | Div | Repeat Start | Repeat End | Repeat Left | Name | Family | Class |
| 146605200 | 146605325 | + | 30.4 | 346 | 468 | -53 | LTR33 | ERVL | LTR |
| 146605326 | 146605605 | + | 4.6 | 1 | 302 | -9 | AluY | Alu | SINE |
| 146605606 | 146605668 | + | 30.4 | 469 | 517 | -4 | LTR33 | ERVL | LTR |

  

|  |  |  |  |  |  |  |  |  |  |
| --- | --- | --- | --- | --- | --- | --- | --- | --- | --- |
| chr1:146697479-146700583 | | | 3071 bp | | |  |  |  |  |
| Genome Start | Genome End | Strand | Div | Repeat Start | Repeat End | Repeat Left | Name | Family | Class |
| 146697479 | 146698496 | + | 5.8 | 857 | 1849 | -426 | L1P2 | L1 | LINE |
| 146698497 | 146698520 | - | 5.0 | 6140 | 6154 | -1 | L1PA4 | L1 | LINE |
| 146698555 | 146699199 | - | 5.0 | 5494 | 6139 | -16 | L1PA4 | L1 | LINE |
| 146699200 | 146700583 | + | 6.8 | 1850 | 3226 | -2920 | L1P2 | L1 | LINE |

  

|  |  |  |  |  |  |  |  |  |  |
| --- | --- | --- | --- | --- | --- | --- | --- | --- | --- |
| chr1:146713990-146715273 | | | 1284 bp | | |  |  |  |  |
| Genome Start | Genome End | Strand | Div | Repeat Start | Repeat End | Repeat Left | Name | Family | Class |
| 146713990 | 146714024 | - | 27.5 | 5889 | 5922 | -242 | L1ME2 | L1 | LINE |
| 146714025 | 146714341 | - | 5.7 | 1 | 301 | -10 | AluY | Alu | SINE |
| 146714342 | 146715273 | - | 29.1 | 4862 | 5888 | -276 | L1ME2 | L1 | LINE |

  

|  |  |  |  |  |  |  |  |  |  |
| --- | --- | --- | --- | --- | --- | --- | --- | --- | --- |
| chr1:146717028-146718422 | | | 1395 bp | | |  |  |  |  |
| Genome Start | Genome End | Strand | Div | Repeat Start | Repeat End | Repeat Left | Name | Family | Class |
| 146717028 | 146717456 | - | 25.8 | 5691 | 6151 | -13 | L1ME2 | L1 | LINE |
| 146717457 | 146718114 | + | 8.7 | 2 | 660 | 0 | PABL\_A | ERV1 | LTR |
| 146718115 | 146718422 | - | 25.8 | 5363 | 5690 | -474 | L1ME2 | L1 | LINE |

  

|  |  |  |  |  |  |  |  |  |  |
| --- | --- | --- | --- | --- | --- | --- | --- | --- | --- |
| chr1:146806733-146808030 | | | 1269 bp | | |  |  |  |  |
| Genome Start | Genome End | Strand | Div | Repeat Start | Repeat End | Repeat Left | Name | Family | Class |
| 146806733 | 146807179 | + | 19.6 | 2748 | 3198 | -2942 | L1M3 | L1 | LINE |
| 146807209 | 146807223 | + | 19.6 | 3199 | 3210 | -2930 | L1M3 | L1 | LINE |
| 146807224 | 146807319 | - | 31.2 | 41 | 133 | 0 | FLAM | Alu | SINE |
| 146807320 | 146807481 | + | 19.6 | 3211 | 3346 | -2794 | L1M3 | L1 | LINE |
| 146807482 | 146807819 | + | 4.5 | 1 | 308 | -3 | AluY | Alu | SINE |
| 146807820 | 146808030 | + | 19.6 | 3349 | 3564 | -2576 | L1M3 | L1 | LINE |

  

|  |  |  |  |  |  |  |  |  |  |
| --- | --- | --- | --- | --- | --- | --- | --- | --- | --- |
| chr1:146823890-146824310 | | | 421 bp | | |  |  |  |  |
| Genome Start | Genome End | Strand | Div | Repeat Start | Repeat End | Repeat Left | Name | Family | Class |
| 146823890 | 146823936 | - | 15.5 | 6104 | 6159 | -11 | L1ME3A | L1 | LINE |
| 146823937 | 146824246 | + | 14.5 | 3 | 312 | 0 | AluJb | Alu | SINE |
| 146824247 | 146824310 | - | 15.5 | 6037 | 6103 | -67 | L1ME3A | L1 | LINE |

  

|  |  |  |  |  |  |  |  |  |  |
| --- | --- | --- | --- | --- | --- | --- | --- | --- | --- |
| chr1:146824312-146825674 | | | 1363 bp | | |  |  |  |  |
| Genome Start | Genome End | Strand | Div | Repeat Start | Repeat End | Repeat Left | Name | Family | Class |
| 146824312 | 146824769 | - | 23.0 | 5165 | 5654 | -586 | L1M5 | L1 | LINE |
| 146824770 | 146825067 | - | 15.9 | 1 | 297 | -15 | AluJb | Alu | SINE |
| 146825068 | 146825202 | - | 21.5 | 4955 | 5164 | -982 | L1M5 | L1 | LINE |
| 146825203 | 146825518 | - | 15.5 | 1 | 311 | -1 | AluJb | Alu | SINE |
| 146825519 | 146825674 | - | 21.5 | 4823 | 4954 | -1192 | L1M5 | L1 | LINE |

  

|  |  |  |  |  |  |  |  |  |  |
| --- | --- | --- | --- | --- | --- | --- | --- | --- | --- |
| chr1:146842379-146843664 | | | 1286 bp | | |  |  |  |  |
| Genome Start | Genome End | Strand | Div | Repeat Start | Repeat End | Repeat Left | Name | Family | Class |
| 146842379 | 146843309 | + | 29.6 | 4862 | 5887 | -277 | L1ME2 | L1 | LINE |
| 146843310 | 146843629 | + | 5.3 | 1 | 304 | -7 | AluY | Alu | SINE |
| 146843630 | 146843664 | + | 28.0 | 5888 | 5921 | -243 | L1ME2 | L1 | LINE |

  

|  |  |  |  |  |  |  |  |  |  |
| --- | --- | --- | --- | --- | --- | --- | --- | --- | --- |
| chr1:146850737-146853844 | | | 3075 bp | | |  |  |  |  |
| Genome Start | Genome End | Strand | Div | Repeat Start | Repeat End | Repeat Left | Name | Family | Class |
| 146850737 | 146852120 | - | 6.4 | 1859 | 3226 | -2920 | L1P2 | L1 | LINE |
| 146852121 | 146852769 | + | 5.2 | 5494 | 6139 | -16 | L1PA4 | L1 | LINE |
| 146852803 | 146852826 | + | 5.2 | 6140 | 6154 | -1 | L1PA4 | L1 | LINE |
| 146852827 | 146853844 | - | 5.5 | 857 | 1858 | -417 | L1P2 | L1 | LINE |

  

|  |  |  |  |  |  |  |  |  |  |
| --- | --- | --- | --- | --- | --- | --- | --- | --- | --- |
| chr1:146866301-146867102 | | | 780 bp | | |  |  |  |  |
| Genome Start | Genome End | Strand | Div | Repeat Start | Repeat End | Repeat Left | Name | Family | Class |
| 146866301 | 146866322 | - | 18.9 | 833 | 856 | -4159 | L1MEe | L1 | LINE |
| 146866323 | 146866638 | - | 9.0 | 2 | 311 | -1 | AluSx | Alu | SINE |
| 146866639 | 146866848 | - | 19.4 | 576 | 832 | -4183 | L1MEe | L1 | LINE |
| 146866871 | 146867102 | - | 23.0 | 176 | 473 | -4542 | L1MEe | L1 | LINE |

  

|  |  |  |  |  |  |  |  |  |  |
| --- | --- | --- | --- | --- | --- | --- | --- | --- | --- |
| chr1:146868776-146869493 | | | 718 bp | | |  |  |  |  |
| Genome Start | Genome End | Strand | Div | Repeat Start | Repeat End | Repeat Left | Name | Family | Class |
| 146868776 | 146868958 | - | 16.6 | 258 | 430 | -31 | LOR1b | ERV1 | LTR |
| 146868959 | 146869270 | + | 4.5 | 1 | 309 | -2 | AluY | Alu | SINE |
| 146869271 | 146869493 | - | 16.6 | 1 | 257 | -204 | LOR1b | ERV1 | LTR |

  

|  |  |  |  |  |  |  |  |  |  |
| --- | --- | --- | --- | --- | --- | --- | --- | --- | --- |
| chr1:146869536-146870007 | | | 472 bp | | |  |  |  |  |
| Genome Start | Genome End | Strand | Div | Repeat Start | Repeat End | Repeat Left | Name | Family | Class |
| 146869536 | 146869668 | - | 30.9 | 2489 | 2628 | -791 | L2 | L2 | LINE |
| 146869669 | 146869971 | + | 12.9 | 1 | 303 | -9 | AluSx | Alu | SINE |
| 146869972 | 146870007 | - | 30.9 | 2456 | 2488 | -931 | L2 | L2 | LINE |

  

|  |  |  |  |  |  |  |  |  |  |
| --- | --- | --- | --- | --- | --- | --- | --- | --- | --- |
| chr1:146888833-146891700 | | | 2856 bp | | |  |  |  |  |
| Genome Start | Genome End | Strand | Div | Repeat Start | Repeat End | Repeat Left | Name | Family | Class |
| 146888833 | 146889230 | + | 15.1 | 2 | 403 | -2315 | Tigger2 | MER2\_type | DNA |
| 146889238 | 146889442 | - | 16.7 | 5965 | 6153 | -3 | L1PB4 | L1 | LINE |
| 146889448 | 146891700 | + | 13.6 | 386 | 2716 | -2 | Tigger2 | MER2\_type | DNA |

  

|  |  |  |  |  |  |  |  |  |  |
| --- | --- | --- | --- | --- | --- | --- | --- | --- | --- |
| chr1:146917468-146918484 | | | 1017 bp | | |  |  |  |  |
| Genome Start | Genome End | Strand | Div | Repeat Start | Repeat End | Repeat Left | Name | Family | Class |
| 146917468 | 146917799 | - | 32.5 | 5597 | 5933 | -1875 | L1MC4a | L1 | LINE |
| 146917800 | 146918245 | - | 11.9 | 5704 | 6157 | -9 | L1PA16 | L1 | LINE |
| 146918246 | 146918484 | - | 32.5 | 5348 | 5596 | -2212 | L1MC4a | L1 | LINE |

  

|  |  |  |  |  |  |  |  |  |  |
| --- | --- | --- | --- | --- | --- | --- | --- | --- | --- |
| chr1:146932332-146933024 | | | 693 bp | | |  |  |  |  |
| Genome Start | Genome End | Strand | Div | Repeat Start | Repeat End | Repeat Left | Name | Family | Class |
| 146932332 | 146932366 | + | 18.4 | 1 | 36 | -559 | LTR49 | ERV1 | LTR |
| 146932367 | 146932642 | - | 14.5 | 14 | 290 | -23 | AluSq | Alu | SINE |
| 146932643 | 146933024 | + | 18.4 | 37 | 427 | -168 | LTR49 | ERV1 | LTR |

  

|  |  |  |  |  |  |  |  |  |  |
| --- | --- | --- | --- | --- | --- | --- | --- | --- | --- |
| chr1:147004466-147005750 | | | 1285 bp | | |  |  |  |  |
| Genome Start | Genome End | Strand | Div | Repeat Start | Repeat End | Repeat Left | Name | Family | Class |
| 147004466 | 147005396 | + | 29.4 | 4862 | 5887 | -277 | L1ME2 | L1 | LINE |
| 147005397 | 147005715 | + | 5.3 | 1 | 303 | -8 | AluY | Alu | SINE |
| 147005716 | 147005750 | + | 27.9 | 5888 | 5921 | -243 | L1ME2 | L1 | LINE |

  

|  |  |  |  |  |  |  |  |  |  |
| --- | --- | --- | --- | --- | --- | --- | --- | --- | --- |
| chr1:147030805-147031523 | | | 719 bp | | |  |  |  |  |
| Genome Start | Genome End | Strand | Div | Repeat Start | Repeat End | Repeat Left | Name | Family | Class |
| 147030805 | 147030987 | - | 17.1 | 258 | 430 | -31 | LOR1b | ERV1 | LTR |
| 147030988 | 147031300 | + | 4.5 | 1 | 310 | -1 | AluY | Alu | SINE |
| 147031301 | 147031523 | - | 17.1 | 1 | 257 | -204 | LOR1b | ERV1 | LTR |

  

|  |  |  |  |  |  |  |  |  |  |
| --- | --- | --- | --- | --- | --- | --- | --- | --- | --- |
| chr1:147031566-147032037 | | | 472 bp | | |  |  |  |  |
| Genome Start | Genome End | Strand | Div | Repeat Start | Repeat End | Repeat Left | Name | Family | Class |
| 147031566 | 147031698 | - | 30.9 | 2489 | 2628 | -791 | L2 | L2 | LINE |
| 147031699 | 147032001 | + | 13.5 | 1 | 303 | -9 | AluSx | Alu | SINE |
| 147032002 | 147032037 | - | 30.9 | 2456 | 2488 | -931 | L2 | L2 | LINE |

  

|  |  |  |  |  |  |  |  |  |  |
| --- | --- | --- | --- | --- | --- | --- | --- | --- | --- |
| chr1:147036134-147036607 | | | 474 bp | | |  |  |  |  |
| Genome Start | Genome End | Strand | Div | Repeat Start | Repeat End | Repeat Left | Name | Family | Class |
| 147036134 | 147036198 | - | 30.6 | 469 | 517 | -4 | LTR33 | ERVL | LTR |
| 147036199 | 147036479 | - | 4.3 | 1 | 303 | -8 | AluY | Alu | SINE |
| 147036480 | 147036607 | - | 30.6 | 344 | 468 | -53 | LTR33 | ERVL | LTR |

  

|  |  |  |  |  |  |  |  |  |  |
| --- | --- | --- | --- | --- | --- | --- | --- | --- | --- |
| chr1:147058289-147058907 | | | 619 bp | | |  |  |  |  |
| Genome Start | Genome End | Strand | Div | Repeat Start | Repeat End | Repeat Left | Name | Family | Class |
| 147058289 | 147058454 | - | 30.2 | 5943 | 6115 | -6 | L1ME4a | L1 | LINE |
| 147058455 | 147058543 | + | 13.5 | 6210 | 6300 | 0 | L1MA6 | L1 | LINE |
| 147058544 | 147058907 | - | 30.2 | 5554 | 5942 | -179 | L1ME4a | L1 | LINE |

  

|  |  |  |  |  |  |  |  |  |  |
| --- | --- | --- | --- | --- | --- | --- | --- | --- | --- |
| chr1:147070198-147071935 | | | 1698 bp | | |  |  |  |  |
| Genome Start | Genome End | Strand | Div | Repeat Start | Repeat End | Repeat Left | Name | Family | Class |
| 147070198 | 147070352 | + | 27.6 | 3230 | 3397 | -2317 | ERVL-B4 | ERVL | LTR |
| 147070393 | 147070651 | + | 31.4 | 3508 | 3818 | -1849 | ERVL-B4 | ERVL | LTR |
| 147070652 | 147070963 | + | 14.4 | 1 | 312 | 0 | AluSx | Alu | SINE |
| 147070964 | 147071935 | + | 33.8 | 3819 | 4811 | -856 | ERVL-B4 | ERVL | LTR |

  

|  |  |  |  |  |  |  |  |  |  |
| --- | --- | --- | --- | --- | --- | --- | --- | --- | --- |
| chr1:147079472-147080488 | | | 1017 bp | | |  |  |  |  |
| Genome Start | Genome End | Strand | Div | Repeat Start | Repeat End | Repeat Left | Name | Family | Class |
| 147079472 | 147079803 | - | 32.5 | 5597 | 5933 | -1875 | L1MC4a | L1 | LINE |
| 147079804 | 147080249 | - | 11.7 | 5704 | 6157 | -9 | L1PA16 | L1 | LINE |
| 147080250 | 147080488 | - | 32.5 | 5348 | 5596 | -2212 | L1MC4a | L1 | LINE |

  

|  |  |  |  |  |  |  |  |  |  |
| --- | --- | --- | --- | --- | --- | --- | --- | --- | --- |
| chr1:147094244-147094936 | | | 693 bp | | |  |  |  |  |
| Genome Start | Genome End | Strand | Div | Repeat Start | Repeat End | Repeat Left | Name | Family | Class |
| 147094244 | 147094278 | + | 18.4 | 1 | 36 | -559 | LTR49 | ERV1 | LTR |
| 147094279 | 147094554 | - | 14.2 | 14 | 290 | -23 | AluSq | Alu | SINE |
| 147094555 | 147094936 | + | 18.4 | 37 | 427 | -168 | LTR49 | ERV1 | LTR |

  

|  |  |  |  |  |  |  |  |  |  |
| --- | --- | --- | --- | --- | --- | --- | --- | --- | --- |
| chr1:147144240-147145427 | | | 1188 bp | | |  |  |  |  |
| Genome Start | Genome End | Strand | Div | Repeat Start | Repeat End | Repeat Left | Name | Family | Class |
| 147144240 | 147144449 | - | 24.7 | 3502 | 3703 | -2443 | L1M5 | L1 | LINE |
| 147144450 | 147144774 | - | 11.7 | 3 | 311 | -2 | AluSq | Alu | SINE |
| 147144775 | 147145019 | - | 12.7 | 1 | 255 | -57 | AluSx | Alu | SINE |
| 147145020 | 147145427 | - | 24.7 | 3081 | 3501 | -2645 | L1M5 | L1 | LINE |

  

|  |  |  |  |  |  |  |  |  |  |
| --- | --- | --- | --- | --- | --- | --- | --- | --- | --- |
| chr1:147152285-147153596 | | | 1312 bp | | |  |  |  |  |
| Genome Start | Genome End | Strand | Div | Repeat Start | Repeat End | Repeat Left | Name | Family | Class |
| 147152285 | 147152341 | - | 27.9 | 2439 | 2497 | -10 | HAL1 | L1 | LINE |
| 147152342 | 147152646 | + | 13.4 | 1 | 306 | -6 | AluSx | Alu | SINE |
| 147152647 | 147153596 | - | 27.9 | 1337 | 2438 | -69 | HAL1 | L1 | LINE |

  

|  |  |  |  |  |  |  |  |  |  |
| --- | --- | --- | --- | --- | --- | --- | --- | --- | --- |
| chr1:147159446-147160432 | | | 987 bp | | |  |  |  |  |
| Genome Start | Genome End | Strand | Div | Repeat Start | Repeat End | Repeat Left | Name | Family | Class |
| 147159446 | 147159637 | - | 30.9 | 3192 | 3408 | -11 | L2 | L2 | LINE |
| 147159638 | 147159945 | + | 12.0 | 1 | 308 | -4 | AluSx | Alu | SINE |
| 147159946 | 147160432 | - | 31.7 | 2684 | 3191 | -228 | L2 | L2 | LINE |

  

|  |  |  |  |  |  |  |  |  |  |
| --- | --- | --- | --- | --- | --- | --- | --- | --- | --- |
| chr1:147163834-147166562 | | | 2729 bp | | |  |  |  |  |
| Genome Start | Genome End | Strand | Div | Repeat Start | Repeat End | Repeat Left | Name | Family | Class |
| 147163834 | 147164300 | - | 17.2 | 5789 | 6274 | -38 | L1MA9 | L1 | LINE |
| 147164301 | 147164503 | - | 21.0 | 4 | 206 | -4 | MER6B | MER2\_type | DNA |
| 147164504 | 147165827 | - | 17.3 | 4485 | 5788 | -524 | L1MA9 | L1 | LINE |
| 147165828 | 147166131 | - | 10.8 | 1 | 297 | -15 | AluSx | Alu | SINE |
| 147166132 | 147166562 | - | 17.3 | 4032 | 4484 | -1656 | L1MA9 | L1 | LINE |

  

|  |  |  |  |  |  |  |  |  |  |
| --- | --- | --- | --- | --- | --- | --- | --- | --- | --- |
| chr1:147173533-147173958 | | | 426 bp | | |  |  |  |  |
| Genome Start | Genome End | Strand | Div | Repeat Start | Repeat End | Repeat Left | Name | Family | Class |
| 147173533 | 147173701 | - | 27.8 | 53 | 222 | -46 | MIRb | MIR | SINE |
| 147173702 | 147173906 | + | 19.8 | 1 | 219 | 0 | MER20 | MER1\_type | DNA |
| 147173907 | 147173958 | - | 27.8 | 7 | 52 | -216 | MIRb | MIR | SINE |

  

|  |  |  |  |  |  |  |  |  |  |
| --- | --- | --- | --- | --- | --- | --- | --- | --- | --- |
| chr1:147174298-147176079 | | | 1610 bp | | |  |  |  |  |
| Genome Start | Genome End | Strand | Div | Repeat Start | Repeat End | Repeat Left | Name | Family | Class |
| 147174298 | 147175129 | - | 22.0 | 7043 | 8039 | 0 | L1MC4 | L1 | LINE |
| 147175130 | 147175435 | - | 16.2 | 1 | 298 | -14 | AluJo | Alu | SINE |
| 147175436 | 147175474 | - | 21.0 | 7014 | 7042 | -1000 | L1MC4 | L1 | LINE |
| 147175647 | 147176079 | - | 22.7 | 6586 | 7014 | -1028 | L1MC4 | L1 | LINE |

  

|  |  |  |  |  |  |  |  |  |  |
| --- | --- | --- | --- | --- | --- | --- | --- | --- | --- |
| chr1:147182088-147182553 | | | 466 bp | | |  |  |  |  |
| Genome Start | Genome End | Strand | Div | Repeat Start | Repeat End | Repeat Left | Name | Family | Class |
| 147182088 | 147182163 | - | 25.2 | 200 | 261 | -1 | MIR | MIR | SINE |
| 147182164 | 147182475 | - | 9.8 | 1 | 308 | -1 | AluSc | Alu | SINE |
| 147182476 | 147182553 | - | 25.2 | 122 | 199 | -63 | MIR | MIR | SINE |

  

|  |  |  |  |  |  |  |  |  |  |
| --- | --- | --- | --- | --- | --- | --- | --- | --- | --- |
| chr1:147285865-147286557 | | | 693 bp | | |  |  |  |  |
| Genome Start | Genome End | Strand | Div | Repeat Start | Repeat End | Repeat Left | Name | Family | Class |
| 147285865 | 147286246 | - | 18.4 | 37 | 427 | -168 | LTR49 | ERV1 | LTR |
| 147286247 | 147286522 | + | 15.6 | 14 | 290 | -23 | AluSq | Alu | SINE |
| 147286523 | 147286557 | - | 18.4 | 1 | 36 | -559 | LTR49 | ERV1 | LTR |

  

|  |  |  |  |  |  |  |  |  |  |
| --- | --- | --- | --- | --- | --- | --- | --- | --- | --- |
| chr1:147290896-147292088 | | | 1146 bp | | |  |  |  |  |
| Genome Start | Genome End | Strand | Div | Repeat Start | Repeat End | Repeat Left | Name | Family | Class |
| 147290896 | 147291310 | + | 25.6 | 1 | 430 | -471 | MER54A | ERVL | LTR |
| 147291349 | 147291686 | - | 11.7 | 7 | 364 | 0 | THE1B | MaLR | LTR |
| 147291696 | 147292088 | + | 20.9 | 490 | 901 | 0 | MER54A | ERVL | LTR |

  

|  |  |  |  |  |  |  |  |  |  |
| --- | --- | --- | --- | --- | --- | --- | --- | --- | --- |
| chr1:147294562-147295371 | | | 810 bp | | |  |  |  |  |
| Genome Start | Genome End | Strand | Div | Repeat Start | Repeat End | Repeat Left | Name | Family | Class |
| 147294562 | 147294803 | - | 29.6 | 275 | 503 | -2 | MLT1D | MaLR | LTR |
| 147294804 | 147295100 | - | 14.1 | 1 | 299 | -12 | AluY | Alu | SINE |
| 147295101 | 147295371 | - | 29.6 | 5 | 274 | -231 | MLT1D | MaLR | LTR |

  

|  |  |  |  |  |  |  |  |  |  |
| --- | --- | --- | --- | --- | --- | --- | --- | --- | --- |
| chr1:147382555-147385869 | | | 2826 bp | | |  |  |  |  |
| Genome Start | Genome End | Strand | Div | Repeat Start | Repeat End | Repeat Left | Name | Family | Class |
| 147382555 | 147383008 | - | 26.6 | 5763 | 6234 | -5 | L1ME1 | L1 | LINE |
| 147383009 | 147383669 | + | 8.1 | 2 | 660 | 0 | PABL\_A | ERV1 | LTR |
| 147383670 | 147384033 | - | 26.6 | 5367 | 5762 | -477 | L1ME1 | L1 | LINE |
| 147384523 | 147384953 | - | 28.2 | 4505 | 4929 | -1290 | L1ME1 | L1 | LINE |
| 147384954 | 147385259 | - | 14.4 | 1 | 306 | -5 | AluY | Alu | SINE |
| 147385260 | 147385869 | - | 28.2 | 3902 | 4504 | -1715 | L1ME1 | L1 | LINE |

  

|  |  |  |  |  |  |  |  |  |  |
| --- | --- | --- | --- | --- | --- | --- | --- | --- | --- |
| chr1:147472519-147473316 | | | 798 bp | | |  |  |  |  |
| Genome Start | Genome End | Strand | Div | Repeat Start | Repeat End | Repeat Left | Name | Family | Class |
| 147472519 | 147472884 | - | 21.4 | 102 | 436 | -1 | LTR56 | ERV1 | LTR |
| 147472885 | 147473182 | + | 17.2 | 1 | 284 | -28 | AluSx | Alu | SINE |
| 147473183 | 147473316 | - | 20.9 | 9 | 101 | -344 | LTR56 | ERV1 | LTR |

  

|  |  |  |  |  |  |  |  |  |  |
| --- | --- | --- | --- | --- | --- | --- | --- | --- | --- |
| chr1:147492771-147493840 | | | 1070 bp | | |  |  |  |  |
| Genome Start | Genome End | Strand | Div | Repeat Start | Repeat End | Repeat Left | Name | Family | Class |
| 147492771 | 147493350 | - | 16.2 | 163 | 727 | 0 | LTR8A | ERV1 | LTR |
| 147493351 | 147493690 | - | 11.7 | 1 | 337 | -2 | MER1B | MER1\_type | DNA |
| 147493691 | 147493840 | - | 16.2 | 2 | 162 | -565 | LTR8A | ERV1 | LTR |

  

|  |  |  |  |  |  |  |  |  |  |
| --- | --- | --- | --- | --- | --- | --- | --- | --- | --- |
| chr1:147511965-147512480 | | | 516 bp | | |  |  |  |  |
| Genome Start | Genome End | Strand | Div | Repeat Start | Repeat End | Repeat Left | Name | Family | Class |
| 147511965 | 147512098 | - | 31.5 | 98 | 221 | -6326 | L1MD2 | L1 | LINE |
| 147512099 | 147512395 | - | 16.8 | 1 | 298 | -14 | AluJb | Alu | SINE |
| 147512396 | 147512480 | - | 31.5 | 10 | 97 | -6450 | L1MD2 | L1 | LINE |

  

|  |  |  |  |  |  |  |  |  |  |
| --- | --- | --- | --- | --- | --- | --- | --- | --- | --- |
| chr1:147533105-147539188 | | | 5906 bp | | |  |  |  |  |
| Genome Start | Genome End | Strand | Div | Repeat Start | Repeat End | Repeat Left | Name | Family | Class |
| 147533105 | 147534154 | - | 34.3 | 4756 | 5845 | -1911 | MER66-int | ERV1 | LTR |
| 147534155 | 147534443 | - | 11.1 | 11 | 299 | -13 | AluSx | Alu | SINE |
| 147534444 | 147535996 | - | 34.3 | 3229 | 4755 | -3001 | MER66-int | ERV1 | LTR |
| 147535997 | 147536289 | - | 17.1 | 7 | 303 | -9 | AluSx | Alu | SINE |
| 147536290 | 147538499 | - | 22.1 | 1211 | 3228 | -3822 | MER66-int | ERV1 | LTR |
| 147538605 | 147538759 | - | 26.1 | 2947 | 3102 | -3574 | MER66-int | ERV1 | LTR |
| 147538833 | 147539188 | - | 19.3 | 732 | 1114 | -5562 | MER66-int | ERV1 | LTR |

  

|  |  |  |  |  |  |  |  |  |  |
| --- | --- | --- | --- | --- | --- | --- | --- | --- | --- |
| chr1:147566134-147572158 | | | 5989 bp | | |  |  |  |  |
| Genome Start | Genome End | Strand | Div | Repeat Start | Repeat End | Repeat Left | Name | Family | Class |
| 147566134 | 147568297 | + | 19.9 | 637 | 2616 | -3957 | L1PA17 | L1 | LINE |
| 147568298 | 147568577 | + | 10.4 | 3 | 299 | -14 | AluSq | Alu | SINE |
| 147568578 | 147570763 | + | 13.9 | 2617 | 4821 | -1752 | L1PA17 | L1 | LINE |
| 147570800 | 147571056 | + | 13.9 | 4822 | 5078 | -1495 | L1PA17 | L1 | LINE |
| 147571057 | 147571357 | + | 14.7 | 1 | 301 | -11 | AluSx | Alu | SINE |
| 147571358 | 147572158 | + | 12.4 | 5079 | 5898 | -695 | L1PA17 | L1 | LINE |

  

|  |  |  |  |  |  |  |  |  |  |
| --- | --- | --- | --- | --- | --- | --- | --- | --- | --- |
| chr1:147589206-147589753 | | | 515 bp | | |  |  |  |  |
| Genome Start | Genome End | Strand | Div | Repeat Start | Repeat End | Repeat Left | Name | Family | Class |
| 147589206 | 147589296 | - | 24.1 | 159 | 242 | -20 | MIR | MIR | SINE |
| 147589297 | 147589311 | - | 12.7 | 284 | 294 | -18 | AluSx | Alu | SINE |
| 147589345 | 147589627 | - | 12.7 | 1 | 283 | -29 | AluSx | Alu | SINE |
| 147589628 | 147589753 | - | 24.1 | 36 | 158 | -104 | MIR | MIR | SINE |

  

|  |  |  |  |  |  |  |  |  |  |
| --- | --- | --- | --- | --- | --- | --- | --- | --- | --- |
| chr1:147589868-147590515 | | | 648 bp | | |  |  |  |  |
| Genome Start | Genome End | Strand | Div | Repeat Start | Repeat End | Repeat Left | Name | Family | Class |
| 147589868 | 147589964 | + | 14.6 | 5461 | 5551 | -632 | L1MB3 | L1 | LINE |
| 147589965 | 147590044 | + | 3.8 | 1 | 80 | 0 | MADE1 | Mariner | DNA |
| 147590045 | 147590515 | + | 14.6 | 5552 | 6034 | -149 | L1MB3 | L1 | LINE |

  

|  |  |  |  |  |  |  |  |  |  |
| --- | --- | --- | --- | --- | --- | --- | --- | --- | --- |
| chr1:147611881-147613583 | | | 1692 bp | | |  |  |  |  |
| Genome Start | Genome End | Strand | Div | Repeat Start | Repeat End | Repeat Left | Name | Family | Class |
| 147611881 | 147612339 | - | 27.2 | 1935 | 2375 | -3774 | L1MEe | L1 | LINE |
| 147612340 | 147612624 | - | 8.5 | 1 | 297 | -12 | AluSc | Alu | SINE |
| 147612625 | 147613246 | - | 27.0 | 1303 | 1934 | -4215 | L1MEe | L1 | LINE |
| 147613258 | 147613583 | - | 34.3 | 903 | 1235 | -4884 | L1MEe | L1 | LINE |

  

|  |  |  |  |  |  |  |  |  |  |
| --- | --- | --- | --- | --- | --- | --- | --- | --- | --- |
| chr1:147675517-147675948 | | | 432 bp | | |  |  |  |  |
| Genome Start | Genome End | Strand | Div | Repeat Start | Repeat End | Repeat Left | Name | Family | Class |
| 147675517 | 147675590 | + | 20.0 | 2536 | 2611 | -48 | Charlie9 | MER1\_type | DNA |
| 147675591 | 147675905 | - | 5.7 | 1 | 300 | -11 | AluY | Alu | SINE |
| 147675906 | 147675948 | + | 20.0 | 2612 | 2646 | -13 | Charlie9 | MER1\_type | DNA |

  

|  |  |  |  |  |  |  |  |  |  |
| --- | --- | --- | --- | --- | --- | --- | --- | --- | --- |
| chr1:147679137-147679939 | | | 803 bp | | |  |  |  |  |
| Genome Start | Genome End | Strand | Div | Repeat Start | Repeat End | Repeat Left | Name | Family | Class |
| 147679137 | 147679204 | - | 20.1 | 448 | 505 | 0 | MLT1D | MaLR | LTR |
| 147679205 | 147679502 | + | 8.7 | 2 | 299 | -14 | AluSq | Alu | SINE |
| 147679503 | 147679939 | - | 20.1 | 1 | 447 | -58 | MLT1D | MaLR | LTR |

  

|  |  |  |  |  |  |  |  |  |  |
| --- | --- | --- | --- | --- | --- | --- | --- | --- | --- |
| chr1:147682218-147683601 | | | 1384 bp | | |  |  |  |  |
| Genome Start | Genome End | Strand | Div | Repeat Start | Repeat End | Repeat Left | Name | Family | Class |
| 147682218 | 147682941 | + | 25.8 | 123 | 830 | -1952 | Charlie2b | MER1\_type | DNA |
| 147682942 | 147683073 | - | 16.3 | 1 | 131 | -2 | FLAM\_C | Alu | SINE |
| 147683074 | 147683601 | + | 25.8 | 831 | 1387 | -1395 | Charlie2b | MER1\_type | DNA |

  

|  |  |  |  |  |  |  |  |  |  |
| --- | --- | --- | --- | --- | --- | --- | --- | --- | --- |
| chr1:147688424-147690594 | | | 2122 bp | | |  |  |  |  |
| Genome Start | Genome End | Strand | Div | Repeat Start | Repeat End | Repeat Left | Name | Family | Class |
| 147688424 | 147688693 | - | 15.6 | 956 | 1231 | 0 | Tigger3b | MER2\_type | DNA |
| 147688694 | 147689641 | + | 17.6 | 1 | 911 | -20 | MER21A | ERV1 | LTR |
| 147689658 | 147689732 | - | 11.0 | 886 | 960 | -271 | Tigger3b | MER2\_type | DNA |
| 147689766 | 147690594 | - | 11.0 | 4 | 885 | -346 | Tigger3b | MER2\_type | DNA |

  

|  |  |  |  |  |  |  |  |  |  |
| --- | --- | --- | --- | --- | --- | --- | --- | --- | --- |
| chr1:147691598-147692218 | | | 621 bp | | |  |  |  |  |
| Genome Start | Genome End | Strand | Div | Repeat Start | Repeat End | Repeat Left | Name | Family | Class |
| 147691598 | 147691855 | - | 13.9 | 5084 | 5330 | -810 | L1M3 | L1 | LINE |
| 147691856 | 147692145 | + | 8.7 | 1 | 288 | -24 | AluSx | Alu | SINE |
| 147692146 | 147692218 | - | 13.9 | 5012 | 5083 | -1057 | L1M3 | L1 | LINE |

  

|  |  |  |  |  |  |  |  |  |  |
| --- | --- | --- | --- | --- | --- | --- | --- | --- | --- |
| chr1:147692223-147694597 | | | 2375 bp | | |  |  |  |  |
| Genome Start | Genome End | Strand | Div | Repeat Start | Repeat End | Repeat Left | Name | Family | Class |
| 147692223 | 147692490 | + | 20.6 | 5530 | 5789 | -523 | L1MA9 | L1 | LINE |
| 147692491 | 147692553 | + | 11.5 | 1 | 39 | -1263 | HSMAR2 | Mariner | DNA |
| 147692554 | 147692865 | + | 5.5 | 1 | 308 | -3 | AluY | Alu | SINE |
| 147692866 | 147694126 | + | 11.5 | 40 | 1301 | -1 | HSMAR2 | Mariner | DNA |
| 147694127 | 147694597 | + | 20.6 | 5790 | 6275 | -37 | L1MA9 | L1 | LINE |

  

|  |  |  |  |  |  |  |  |  |  |
| --- | --- | --- | --- | --- | --- | --- | --- | --- | --- |
| chr1:147700615-147701264 | | | 650 bp | | |  |  |  |  |
| Genome Start | Genome End | Strand | Div | Repeat Start | Repeat End | Repeat Left | Name | Family | Class |
| 147700615 | 147700782 | + | 19.7 | 2 | 162 | -212 | MLT1A | MaLR | LTR |
| 147700783 | 147701063 | + | 14.5 | 1 | 298 | -14 | AluJo | Alu | SINE |
| 147701064 | 147701264 | + | 19.7 | 163 | 349 | -25 | MLT1A | MaLR | LTR |

  

|  |  |  |  |  |  |  |  |  |  |
| --- | --- | --- | --- | --- | --- | --- | --- | --- | --- |
| chr1:147777963-147778679 | | | 717 bp | | |  |  |  |  |
| Genome Start | Genome End | Strand | Div | Repeat Start | Repeat End | Repeat Left | Name | Family | Class |
| 147777963 | 147778145 | - | 17.1 | 258 | 430 | -31 | LOR1b | ERV1 | LTR |
| 147778146 | 147778459 | + | 5.2 | 1 | 311 | 0 | AluY | Alu | SINE |
| 147778460 | 147778679 | - | 17.1 | 1 | 257 | -204 | LOR1b | ERV1 | LTR |

  

|  |  |  |  |  |  |  |  |  |  |
| --- | --- | --- | --- | --- | --- | --- | --- | --- | --- |
| chr1:147778722-147779191 | | | 470 bp | | |  |  |  |  |
| Genome Start | Genome End | Strand | Div | Repeat Start | Repeat End | Repeat Left | Name | Family | Class |
| 147778722 | 147778854 | - | 30.0 | 2489 | 2628 | -791 | L2 | L2 | LINE |
| 147778855 | 147779157 | + | 13.9 | 1 | 303 | -9 | AluSx | Alu | SINE |
| 147779158 | 147779191 | - | 30.0 | 2456 | 2488 | -931 | L2 | L2 | LINE |

  

|  |  |  |  |  |  |  |  |  |  |
| --- | --- | --- | --- | --- | --- | --- | --- | --- | --- |
| chr1:147864004-147864484 | | | 481 bp | | |  |  |  |  |
| Genome Start | Genome End | Strand | Div | Repeat Start | Repeat End | Repeat Left | Name | Family | Class |
| 147864004 | 147864063 | - | 30.1 | 465 | 518 | -3 | LTR33 | ERVL | LTR |
| 147864064 | 147864359 | - | 7.8 | 1 | 296 | -15 | AluY | Alu | SINE |
| 147864360 | 147864484 | - | 30.1 | 344 | 464 | -57 | LTR33 | ERVL | LTR |

  

|  |  |  |  |  |  |  |  |  |  |
| --- | --- | --- | --- | --- | --- | --- | --- | --- | --- |
| chr1:147865015-147866648 | | | 1601 bp | | |  |  |  |  |
| Genome Start | Genome End | Strand | Div | Repeat Start | Repeat End | Repeat Left | Name | Family | Class |
| 147865015 | 147865089 | + | 13.4 | 1 | 75 | -560 | LTR24C | ERV1 | LTR |
| 147865090 | 147865397 | + | 10.7 | 1 | 308 | -5 | AluSp | Alu | SINE |
| 147865398 | 147865504 | + | 13.4 | 76 | 170 | -465 | LTR24C | ERV1 | LTR |
| 147865505 | 147865813 | - | 10.4 | 1 | 310 | -1 | AluY | Alu | SINE |
| 147865814 | 147866048 | + | 13.4 | 171 | 391 | -244 | LTR24C | ERV1 | LTR |
| 147866049 | 147866370 | + | 14.1 | 1 | 312 | 0 | AluSx | Alu | SINE |
| 147866371 | 147866561 | + | 13.4 | 392 | 594 | -41 | LTR24C | ERV1 | LTR |
| 147866595 | 147866648 | + | 13.0 | 582 | 635 | 0 | LTR24C | ERV1 | LTR |

  

|  |  |  |  |  |  |  |  |  |  |
| --- | --- | --- | --- | --- | --- | --- | --- | --- | --- |
| chr1:147876418-147877174 | | | 753 bp | | |  |  |  |  |
| Genome Start | Genome End | Strand | Div | Repeat Start | Repeat End | Repeat Left | Name | Family | Class |
| 147876418 | 147876637 | - | 11.2 | 79 | 293 | -19 | AluSx | Alu | SINE |
| 147876642 | 147877090 | + | 21.8 | 11 | 463 | 0 | LTR2 | ERV1 | LTR |
| 147877091 | 147877174 | - | 15.5 | 2 | 85 | -227 | AluSx | Alu | SINE |

  

|  |  |  |  |  |  |  |  |  |  |
| --- | --- | --- | --- | --- | --- | --- | --- | --- | --- |
| chr1:147878399-147879119 | | | 721 bp | | |  |  |  |  |
| Genome Start | Genome End | Strand | Div | Repeat Start | Repeat End | Repeat Left | Name | Family | Class |
| 147878399 | 147878448 | - | 26.2 | 483 | 533 | -16 | MLT1H | MaLR | LTR |
| 147878449 | 147878747 | + | 8.1 | 1 | 298 | -13 | AluY | Alu | SINE |
| 147878748 | 147879119 | - | 26.2 | 71 | 482 | -67 | MLT1H | MaLR | LTR |

  

|  |  |  |  |  |  |  |  |  |  |
| --- | --- | --- | --- | --- | --- | --- | --- | --- | --- |
| chr1:147892639-147893136 | | | 498 bp | | |  |  |  |  |
| Genome Start | Genome End | Strand | Div | Repeat Start | Repeat End | Repeat Left | Name | Family | Class |
| 147892639 | 147892733 | - | 18.3 | 148 | 299 | -3 | AluJb | Alu | SINE |
| 147892734 | 147892987 | - | 10.2 | 1 | 259 | -52 | AluY | Alu | SINE |
| 147892988 | 147893136 | - | 18.3 | 1 | 147 | -155 | AluJb | Alu | SINE |

  

|  |  |  |  |  |  |  |  |  |  |
| --- | --- | --- | --- | --- | --- | --- | --- | --- | --- |
| chr1:147899876-147902379 | | | 2501 bp | | |  |  |  |  |
| Genome Start | Genome End | Strand | Div | Repeat Start | Repeat End | Repeat Left | Name | Family | Class |
| 147899876 | 147900581 | - | 22.7 | 995 | 1702 | -4698 | L1MEc | L1 | LINE |
| 147900582 | 147900883 | - | 16.9 | 1 | 307 | 0 | AluSx | Alu | SINE |
| 147900884 | 147901303 | - | 22.7 | 608 | 994 | -5406 | L1MEc | L1 | LINE |
| 147901304 | 147901530 | - | 13.8 | 6100 | 6328 | -5 | L1MC1 | L1 | LINE |
| 147901534 | 147901885 | - | 13.2 | 1 | 339 | 0 | MER1B | MER1\_type | DNA |
| 147901886 | 147902379 | - | 22.7 | 26 | 607 | -5793 | L1MEc | L1 | LINE |

  

|  |  |  |  |  |  |  |  |  |  |
| --- | --- | --- | --- | --- | --- | --- | --- | --- | --- |
| chr1:147929370-147930608 | | | 1239 bp | | |  |  |  |  |
| Genome Start | Genome End | Strand | Div | Repeat Start | Repeat End | Repeat Left | Name | Family | Class |
| 147929370 | 147929517 | - | 30.2 | 216 | 352 | -5718 | L1MEb | L1 | LINE |
| 147929518 | 147930416 | + | 17.1 | 1 | 923 | 0 | MER49 | ERV1 | LTR |
| 147930417 | 147930608 | - | 30.2 | 8 | 215 | -5855 | L1MEb | L1 | LINE |

  

|  |  |  |  |  |  |  |  |  |  |
| --- | --- | --- | --- | --- | --- | --- | --- | --- | --- |
| chr1:147935357-147936109 | | | 666 bp | | |  |  |  |  |
| Genome Start | Genome End | Strand | Div | Repeat Start | Repeat End | Repeat Left | Name | Family | Class |
| 147935357 | 147935539 | + | 21.9 | 943 | 1131 | -5269 | L1MEc | L1 | LINE |
| 147935550 | 147935687 | + | 9.5 | 2 | 132 | -179 | AluY | Alu | SINE |
| 147935733 | 147935899 | + | 9.5 | 133 | 295 | -16 | AluY | Alu | SINE |
| 147935932 | 147936109 | + | 24.2 | 1723 | 1889 | -4511 | L1MEc | L1 | LINE |

  

|  |  |  |  |  |  |  |  |  |  |
| --- | --- | --- | --- | --- | --- | --- | --- | --- | --- |
| chr1:148007775-148008845 | | | 1071 bp | | |  |  |  |  |
| Genome Start | Genome End | Strand | Div | Repeat Start | Repeat End | Repeat Left | Name | Family | Class |
| 148007775 | 148008277 | - | 16.6 | 223 | 787 | 0 | LTR48 | ERV1 | LTR |
| 148008278 | 148008648 | - | 13.4 | 2 | 371 | 0 | AluSx | Alu | SINE |
| 148008649 | 148008845 | - | 16.6 | 1 | 222 | -565 | LTR48 | ERV1 | LTR |

  

|  |  |  |  |  |  |  |  |  |  |
| --- | --- | --- | --- | --- | --- | --- | --- | --- | --- |
| chr1:148013557-148016232 | | | 2625 bp | | |  |  |  |  |
| Genome Start | Genome End | Strand | Div | Repeat Start | Repeat End | Repeat Left | Name | Family | Class |
| 148013557 | 148013614 | - | 15.4 | 1291 | 1349 | -5051 | L1MEc | L1 | LINE |
| 148013615 | 148014243 | - | 9.1 | 1 | 634 | 0 | MER41B | ERV1 | LTR |
| 148014244 | 148014276 | - | 15.4 | 1260 | 1290 | -5110 | L1MEc | L1 | LINE |
| 148014277 | 148014978 | - | 9.8 | 1 | 722 | 0 | LTR30 | ERV1 | LTR |
| 148014979 | 148015198 | - | 15.4 | 1000 | 1259 | -5141 | L1MEc | L1 | LINE |
| 148015201 | 148015361 | - | 21.1 | 443 | 627 | -5773 | L1MEc | L1 | LINE |
| 148015387 | 148015809 | + | 23.2 | 9 | 445 | 0 | LTR56 | ERV1 | LTR |
| 148015834 | 148016232 | - | 19.4 | 45 | 440 | -5960 | L1MEc | L1 | LINE |

  

|  |  |  |  |  |  |  |  |  |  |
| --- | --- | --- | --- | --- | --- | --- | --- | --- | --- |
| chr1:148060255-148060691 | | | 437 bp | | |  |  |  |  |
| Genome Start | Genome End | Strand | Div | Repeat Start | Repeat End | Repeat Left | Name | Family | Class |
| 148060255 | 148060328 | + | 20.0 | 2536 | 2611 | -48 | Charlie9 | MER1\_type | DNA |
| 148060329 | 148060648 | - | 5.3 | 1 | 300 | -11 | AluY | Alu | SINE |
| 148060649 | 148060691 | + | 20.0 | 2612 | 2646 | -13 | Charlie9 | MER1\_type | DNA |

  

|  |  |  |  |  |  |  |  |  |  |
| --- | --- | --- | --- | --- | --- | --- | --- | --- | --- |
| chr1:148063555-148064361 | | | 807 bp | | |  |  |  |  |
| Genome Start | Genome End | Strand | Div | Repeat Start | Repeat End | Repeat Left | Name | Family | Class |
| 148063555 | 148063622 | - | 19.5 | 442 | 505 | 0 | MLT1D | MaLR | LTR |
| 148063623 | 148063923 | + | 8.0 | 2 | 302 | -11 | AluSq | Alu | SINE |
| 148063924 | 148064361 | - | 19.5 | 1 | 441 | -64 | MLT1D | MaLR | LTR |

  

|  |  |  |  |  |  |  |  |  |  |
| --- | --- | --- | --- | --- | --- | --- | --- | --- | --- |
| chr1:148065206-148065955 | | | 750 bp | | |  |  |  |  |
| Genome Start | Genome End | Strand | Div | Repeat Start | Repeat End | Repeat Left | Name | Family | Class |
| 148065206 | 148065577 | + | 32.9 | 2096 | 2486 | -933 | L2 | L2 | LINE |
| 148065578 | 148065882 | + | 9.5 | 1 | 306 | -6 | AluSx | Alu | SINE |
| 148065883 | 148065955 | + | 32.9 | 2487 | 2555 | -864 | L2 | L2 | LINE |

  

|  |  |  |  |  |  |  |  |  |  |
| --- | --- | --- | --- | --- | --- | --- | --- | --- | --- |
| chr1:148074468-148075903 | | | 1215 bp | | |  |  |  |  |
| Genome Start | Genome End | Strand | Div | Repeat Start | Repeat End | Repeat Left | Name | Family | Class |
| 148074468 | 148074547 | - | 32.5 | 3040 | 3127 | -251 | L2 | L2 | LINE |
| 148074694 | 148074747 | - | 22.0 | 216 | 266 | -2 | MIRb | MIR | SINE |
| 148074764 | 148074914 | - | 16.7 | 8 | 158 | -18 | FRAM | Alu | SINE |
| 148074917 | 148075090 | - | 33.5 | 29 | 210 | -58 | MIRb | MIR | SINE |
| 148075126 | 148075209 | - | 33.0 | 2817 | 2891 | -528 | L2 | L2 | LINE |
| 148075232 | 148075263 | - | 33.0 | 2793 | 2816 | -603 | L2 | L2 | LINE |
| 148075264 | 148075529 | + | 9.4 | 1 | 304 | -7 | AluY | Alu | SINE |
| 148075530 | 148075903 | - | 33.0 | 2391 | 2792 | -627 | L2 | L2 | LINE |

  

|  |  |  |  |  |  |  |  |  |  |
| --- | --- | --- | --- | --- | --- | --- | --- | --- | --- |
| chr1:148107090-148108045 | | | 960 bp | | |  |  |  |  |
| Genome Start | Genome End | Strand | Div | Repeat Start | Repeat End | Repeat Left | Name | Family | Class |
| 148107090 | 148107528 | + | 15.4 | 1 | 464 | -2267 | Tigger4(Zombi) | MER2\_type | DNA |
| 148107529 | 148107831 | + | 10.2 | 1 | 310 | -2 | AluSx | Alu | SINE |
| 148107832 | 148107897 | + | 15.4 | 465 | 530 | -2201 | Tigger4(Zombi) | MER2\_type | DNA |
| 148107894 | 148108045 | + | 9.9 | 2568 | 2729 | -2 | Tigger4(Zombi) | MER2\_type | DNA |

  

|  |  |  |  |  |  |  |  |  |  |
| --- | --- | --- | --- | --- | --- | --- | --- | --- | --- |
| chr1:159788748-159789901 | | | 935 bp | | |  |  |  |  |
| Genome Start | Genome End | Strand | Div | Repeat Start | Repeat End | Repeat Left | Name | Family | Class |
| 159788748 | 159788826 | - | 29.5 | 3002 | 3082 | -296 | L2 | L2 | LINE |
| 159788918 | 159789075 | + | 25.6 | 36 | 233 | -35 | MIRb | MIR | SINE |
| 159789167 | 159789214 | + | 32.7 | 113 | 265 | -11 | MIRm | MIR | SINE |
| 159789215 | 159789323 | + | 31.2 | 3257 | 3365 | -13 | L2 | L2 | LINE |
| 159789361 | 159789901 | - | 37.2 | 2327 | 2879 | -540 | L2 | L2 | LINE |

  

|  |  |  |  |  |  |  |  |  |  |
| --- | --- | --- | --- | --- | --- | --- | --- | --- | --- |
| chr1:159790661-159791256 | | | 596 bp | | |  |  |  |  |
| Genome Start | Genome End | Strand | Div | Repeat Start | Repeat End | Repeat Left | Name | Family | Class |
| 159790661 | 159790907 | + | 12.4 | 1 | 275 | -115 | MLT1B | MaLR | LTR |
| 159790908 | 159791119 | + | 10.5 | 5951 | 6160 | 0 | L1PREC2 | L1 | LINE |
| 159791120 | 159791256 | + | 12.4 | 276 | 390 | 0 | MLT1B | MaLR | LTR |

  

|  |  |  |  |  |  |  |  |  |  |
| --- | --- | --- | --- | --- | --- | --- | --- | --- | --- |
| chr1:159793031-159794334 | | | 1276 bp | | |  |  |  |  |
| Genome Start | Genome End | Strand | Div | Repeat Start | Repeat End | Repeat Left | Name | Family | Class |
| 159793031 | 159793076 | - | 19.3 | 766 | 811 | -52 | MER21B | ERV1 | LTR |
| 159793105 | 159793139 | - | 19.3 | 720 | 765 | -98 | MER21B | ERV1 | LTR |
| 159793140 | 159793663 | - | 13.1 | 1 | 529 | 0 | MER1A | MER1\_type | DNA |
| 159793664 | 159794334 | - | 19.3 | 28 | 719 | -144 | MER21B | ERV1 | LTR |

  

|  |  |  |  |  |  |  |  |  |  |
| --- | --- | --- | --- | --- | --- | --- | --- | --- | --- |
| chr1:159806575-159809451 | | | 2428 bp | | |  |  |  |  |
| Genome Start | Genome End | Strand | Div | Repeat Start | Repeat End | Repeat Left | Name | Family | Class |
| 159806575 | 159806617 | - | 26.5 | 2021 | 2066 | -5062 | L1MCc | L1 | LINE |
| 159806656 | 159807641 | - | 22.5 | 899 | 2020 | -5108 | L1MCc | L1 | LINE |
| 159807689 | 159808000 | + | 12.0 | 1 | 310 | -2 | AluSx | Alu | SINE |
| 159808020 | 159808137 | + | 23.1 | 11 | 148 | -393 | MER87 | ERV1 | LTR |
| 159808285 | 159808769 | + | 26.8 | 107 | 727 | 0 | LTR8A | ERV1 | LTR |
| 159808770 | 159808920 | - | 32.7 | 752 | 903 | -5520 | L1MCc | L1 | LINE |
| 159809119 | 159809451 | - | 26.1 | 80 | 431 | -5992 | L1MCc | L1 | LINE |

  

|  |  |  |  |  |  |  |  |  |  |
| --- | --- | --- | --- | --- | --- | --- | --- | --- | --- |
| chr1:159815197-159816233 | | | 827 bp | | |  |  |  |  |
| Genome Start | Genome End | Strand | Div | Repeat Start | Repeat End | Repeat Left | Name | Family | Class |
| 159815197 | 159815345 | + | 12.8 | 1 | 146 | -319 | MER4C | ERV1 | LTR |
| 159815346 | 159815650 | + | 14.7 | 1 | 278 | -34 | AluSx | Alu | SINE |
| 159815683 | 159815727 | + | 12.8 | 147 | 155 | -310 | MER4C | ERV1 | LTR |
| 159815906 | 159816233 | + | 12.8 | 156 | 465 | 0 | MER4C | ERV1 | LTR |

  

|  |  |  |  |  |  |  |  |  |  |
| --- | --- | --- | --- | --- | --- | --- | --- | --- | --- |
| chr1:159819494-159822326 | | | 2530 bp | | |  |  |  |  |
| Genome Start | Genome End | Strand | Div | Repeat Start | Repeat End | Repeat Left | Name | Family | Class |
| 159819494 | 159819560 | - | 25.4 | 3317 | 3384 | -35 | L2 | L2 | LINE |
| 159819716 | 159820013 | + | 36.8 | 2253 | 2552 | -867 | L2 | L2 | LINE |
| 159820159 | 159820975 | - | 32.2 | 2492 | 3348 | -71 | L2 | L2 | LINE |
| 159820979 | 159821162 | - | 32.5 | 1865 | 2040 | -1379 | L2 | L2 | LINE |
| 159821163 | 159821453 | + | 12.6 | 1 | 263 | -49 | AluSx | Alu | SINE |
| 159821454 | 159822326 | - | 32.5 | 982 | 1864 | -1555 | L2 | L2 | LINE |

  

|  |  |  |  |  |  |  |  |  |  |
| --- | --- | --- | --- | --- | --- | --- | --- | --- | --- |
| chr1:159880321-159881044 | | | 724 bp | | |  |  |  |  |
| Genome Start | Genome End | Strand | Div | Repeat Start | Repeat End | Repeat Left | Name | Family | Class |
| 159880321 | 159880459 | + | 25.5 | 18 | 153 | -462 | MLT1L | MaLR | LTR |
| 159880460 | 159880640 | - | 15.4 | 1 | 189 | 0 | MER5A | MER1\_type | DNA |
| 159880641 | 159881044 | + | 25.5 | 154 | 611 | -4 | MLT1L | MaLR | LTR |

  

|  |  |  |  |  |  |  |  |  |  |
| --- | --- | --- | --- | --- | --- | --- | --- | --- | --- |
| chr1:159895433-159895924 | | | 492 bp | | |  |  |  |  |
| Genome Start | Genome End | Strand | Div | Repeat Start | Repeat End | Repeat Left | Name | Family | Class |
| 159895433 | 159895557 | - | 33.2 | 110 | 232 | -30 | MIR | MIR | SINE |
| 159895558 | 159895851 | + | 8.8 | 1 | 297 | -15 | AluSx | Alu | SINE |
| 159895852 | 159895924 | - | 33.2 | 34 | 109 | -153 | MIR | MIR | SINE |

  

|  |  |  |  |  |  |  |  |  |  |
| --- | --- | --- | --- | --- | --- | --- | --- | --- | --- |
| chr1:159898485-159898873 | | | 389 bp | | |  |  |  |  |
| Genome Start | Genome End | Strand | Div | Repeat Start | Repeat End | Repeat Left | Name | Family | Class |
| 159898485 | 159898575 | - | 29.6 | 170 | 265 | -3 | MIRb | MIR | SINE |
| 159898576 | 159898779 | + | 20.6 | 3 | 219 | 0 | MER20 | MER1\_type | DNA |
| 159898780 | 159898873 | - | 29.6 | 86 | 169 | -99 | MIRb | MIR | SINE |

  

|  |  |  |  |  |  |  |  |  |  |
| --- | --- | --- | --- | --- | --- | --- | --- | --- | --- |
| chr1:161034536-161036084 | | | 1549 bp | | |  |  |  |  |
| Genome Start | Genome End | Strand | Div | Repeat Start | Repeat End | Repeat Left | Name | Family | Class |
| 161034536 | 161035127 | - | 16.4 | 7169 | 7758 | -27 | L1MC3 | L1 | LINE |
| 161035128 | 161035436 | + | 10.4 | 1 | 311 | -2 | AluSp | Alu | SINE |
| 161035437 | 161036084 | - | 16.4 | 6516 | 7168 | -617 | L1MC3 | L1 | LINE |

  

|  |  |  |  |  |  |  |  |  |  |
| --- | --- | --- | --- | --- | --- | --- | --- | --- | --- |
| chr1:176241907-176242572 | | | 666 bp | | |  |  |  |  |
| Genome Start | Genome End | Strand | Div | Repeat Start | Repeat End | Repeat Left | Name | Family | Class |
| 176241907 | 176242124 | - | 12.5 | 5967 | 6182 | -1 | L1MB3 | L1 | LINE |
| 176242125 | 176242343 | - | 13.7 | 1 | 232 | -4 | MER46A | MER2\_type | DNA |
| 176242344 | 176242572 | - | 12.5 | 5731 | 5966 | -217 | L1MB3 | L1 | LINE |

  

|  |  |  |  |  |  |  |  |  |  |
| --- | --- | --- | --- | --- | --- | --- | --- | --- | --- |
| chr1:176242626-176243822 | | | 753 bp | | |  |  |  |  |
| Genome Start | Genome End | Strand | Div | Repeat Start | Repeat End | Repeat Left | Name | Family | Class |
| 176242626 | 176242745 | - | 32.8 | 3249 | 3374 | -4 | L2 | L2 | LINE |
| 176242768 | 176242984 | - | 26.4 | 1 | 257 | -5 | MIR | MIR | SINE |
| 176243280 | 176243461 | - | 34.3 | 2707 | 2891 | -528 | L2 | L2 | LINE |
| 176243563 | 176243668 | + | 16.0 | 1 | 129 | -5 | MER94 | AcHobo | DNA |
| 176243695 | 176243822 | - | 27.6 | 2438 | 2575 | -844 | L2 | L2 | LINE |

  

|  |  |  |  |  |  |  |  |  |  |
| --- | --- | --- | --- | --- | --- | --- | --- | --- | --- |
| chr1:195059858-195060501 | | | 644 bp | | |  |  |  |  |
| Genome Start | Genome End | Strand | Div | Repeat Start | Repeat End | Repeat Left | Name | Family | Class |
| 195059858 | 195059998 | - | 21.9 | 218 | 345 | 0 | MER2 | MER2\_type | DNA |
| 195059999 | 195060310 | - | 12.3 | 1 | 308 | -4 | AluSx | Alu | SINE |
| 195060311 | 195060501 | - | 21.9 | 1 | 217 | -128 | MER2 | MER2\_type | DNA |

  

|  |  |  |  |  |  |  |  |  |  |
| --- | --- | --- | --- | --- | --- | --- | --- | --- | --- |
| chr1:200609864-200611802 | | | 1919 bp | | |  |  |  |  |
| Genome Start | Genome End | Strand | Div | Repeat Start | Repeat End | Repeat Left | Name | Family | Class |
| 200609864 | 200610059 | + | 30.6 | 2365 | 2589 | -830 | L2 | L2 | LINE |
| 200610057 | 200610153 | - | 26.0 | 3312 | 3417 | -2 | L2 | L2 | LINE |
| 200610177 | 200610244 | + | 32.3 | 2608 | 2675 | -744 | L2 | L2 | LINE |
| 200610245 | 200610594 | - | 7.6 | 1 | 304 | -8 | AluSx | Alu | SINE |
| 200610595 | 200610688 | + | 32.3 | 2676 | 2767 | -652 | L2 | L2 | LINE |
| 200610689 | 200610994 | - | 11.5 | 1 | 305 | -7 | AluSx | Alu | SINE |
| 200610995 | 200611325 | + | 37.6 | 2768 | 3185 | -193 | L2 | L2 | LINE |
| 200611326 | 200611595 | - | 19.6 | 1 | 300 | -2 | AluJo | Alu | SINE |
| 200611596 | 200611802 | + | 39.1 | 3186 | 3377 | -1 | L2 | L2 | LINE |

  

|  |  |  |  |  |  |  |  |  |  |
| --- | --- | --- | --- | --- | --- | --- | --- | --- | --- |
| chr1:200614133-200614986 | | | 854 bp | | |  |  |  |  |
| Genome Start | Genome End | Strand | Div | Repeat Start | Repeat End | Repeat Left | Name | Family | Class |
| 200614133 | 200614227 | + | 36.3 | 1939 | 2036 | -1383 | L2 | L2 | LINE |
| 200614228 | 200614528 | - | 10.3 | 1 | 301 | -9 | AluSg | Alu | SINE |
| 200614529 | 200614986 | + | 36.3 | 2037 | 2506 | -913 | L2 | L2 | LINE |

  

|  |  |  |  |  |  |  |  |  |  |
| --- | --- | --- | --- | --- | --- | --- | --- | --- | --- |
| chr1:200621578-200622018 | | | 441 bp | | |  |  |  |  |
| Genome Start | Genome End | Strand | Div | Repeat Start | Repeat End | Repeat Left | Name | Family | Class |
| 200621578 | 200621677 | - | 26.6 | 505 | 605 | -31 | MER92B | ERV1 | LTR |
| 200621678 | 200621978 | - | 7.0 | 1 | 306 | -5 | AluY | Alu | SINE |
| 200621979 | 200622018 | - | 26.6 | 460 | 504 | -132 | MER92B | ERV1 | LTR |

  

|  |  |  |  |  |  |  |  |  |  |
| --- | --- | --- | --- | --- | --- | --- | --- | --- | --- |
| chr1:200690167-200691728 | | | 1562 bp | | |  |  |  |  |
| Genome Start | Genome End | Strand | Div | Repeat Start | Repeat End | Repeat Left | Name | Family | Class |
| 200690167 | 200690402 | - | 35.3 | 3172 | 3419 | 0 | L2 | L2 | LINE |
| 200690403 | 200690685 | + | 19.8 | 1 | 295 | -17 | AluJo | Alu | SINE |
| 200690686 | 200690965 | - | 34.3 | 2863 | 3171 | -248 | L2 | L2 | LINE |
| 200690966 | 200691511 | - | 16.3 | 5612 | 6160 | -18 | L1MB8 | L1 | LINE |
| 200691512 | 200691728 | - | 33.8 | 2658 | 2862 | -557 | L2 | L2 | LINE |

  

|  |  |  |  |  |  |  |  |  |  |
| --- | --- | --- | --- | --- | --- | --- | --- | --- | --- |
| chr1:200715053-200716176 | | | 716 bp | | |  |  |  |  |
| Genome Start | Genome End | Strand | Div | Repeat Start | Repeat End | Repeat Left | Name | Family | Class |
| 200715053 | 200715170 | + | 24.4 | 976 | 1095 | -5901 | L1MEb | L1 | LINE |
| 200715469 | 200715647 | + | 19.3 | 4 | 188 | -1 | MER5A | MER1\_type | DNA |
| 200715758 | 200716176 | + | 26.4 | 1378 | 2025 | -5077 | L1MEb | L1 | LINE |

  

|  |  |  |  |  |  |  |  |  |  |
| --- | --- | --- | --- | --- | --- | --- | --- | --- | --- |
| chr1:200722128-200723296 | | | 1169 bp | | |  |  |  |  |
| Genome Start | Genome End | Strand | Div | Repeat Start | Repeat End | Repeat Left | Name | Family | Class |
| 200722128 | 200722274 | + | 21.8 | 3345 | 3501 | -2645 | L1M5 | L1 | LINE |
| 200722275 | 200722569 | + | 15.8 | 1 | 299 | -13 | AluJo | Alu | SINE |
| 200722570 | 200723296 | + | 21.8 | 3502 | 4228 | -1918 | L1M5 | L1 | LINE |

  

|  |  |  |  |  |  |  |  |  |  |
| --- | --- | --- | --- | --- | --- | --- | --- | --- | --- |
| chr1:204254097-204254627 | | | 524 bp | | |  |  |  |  |
| Genome Start | Genome End | Strand | Div | Repeat Start | Repeat End | Repeat Left | Name | Family | Class |
| 204254097 | 204254161 | - | 20.0 | 330 | 395 | -1 | MSTD | MaLR | LTR |
| 204254167 | 204254464 | + | 7.2 | 75 | 364 | 0 | AluY | Alu | SINE |
| 204254467 | 204254627 | - | 13.6 | 176 | 340 | -56 | MSTD | MaLR | LTR |

  

|  |  |  |  |  |  |  |  |  |  |
| --- | --- | --- | --- | --- | --- | --- | --- | --- | --- |
| chr1:204258014-204259231 | | | 1218 bp | | |  |  |  |  |
| Genome Start | Genome End | Strand | Div | Repeat Start | Repeat End | Repeat Left | Name | Family | Class |
| 204258014 | 204258223 | - | 25.0 | 3159 | 3419 | 0 | L2 | L2 | LINE |
| 204258224 | 204258726 | - | 6.6 | 5649 | 6152 | -2 | L1PA7 | L1 | LINE |
| 204258727 | 204259231 | - | 25.0 | 2597 | 3158 | -261 | L2 | L2 | LINE |

  

|  |  |  |  |  |  |  |  |  |  |
| --- | --- | --- | --- | --- | --- | --- | --- | --- | --- |
| chr1:204294611-204295185 | | | 575 bp | | |  |  |  |  |
| Genome Start | Genome End | Strand | Div | Repeat Start | Repeat End | Repeat Left | Name | Family | Class |
| 204294611 | 204294752 | - | 17.9 | 134 | 269 | -43 | AluJo | Alu | SINE |
| 204294753 | 204295038 | - | 10.1 | 1 | 292 | -21 | AluSq | Alu | SINE |
| 204295039 | 204295185 | - | 17.9 | 1 | 133 | -179 | AluJo | Alu | SINE |

  

|  |  |  |  |  |  |  |  |  |  |
| --- | --- | --- | --- | --- | --- | --- | --- | --- | --- |
| chr1:204307645-204308019 | | | 370 bp | | |  |  |  |  |
| Genome Start | Genome End | Strand | Div | Repeat Start | Repeat End | Repeat Left | Name | Family | Class |
| 204307645 | 204307851 | + | 14.6 | 5566 | 5777 | -394 | L1MB2 | L1 | LINE |
| 204307852 | 204307920 | + | 23.2 | 8 | 76 | -90 | FRAM/FAM | Alu | SINE |
| 204307926 | 204308019 | + | 18.1 | 6069 | 6167 | -4 | L1MB2 | L1 | LINE |

  

|  |  |  |  |  |  |  |  |  |  |
| --- | --- | --- | --- | --- | --- | --- | --- | --- | --- |
| chr1:204312734-204315994 | | | 3257 bp | | |  |  |  |  |
| Genome Start | Genome End | Strand | Div | Repeat Start | Repeat End | Repeat Left | Name | Family | Class |
| 204312734 | 204312937 | - | 10.2 | 2225 | 2418 | 0 | Tigger1 | MER2\_type | DNA |
| 204312938 | 204313236 | + | 15.2 | 1 | 298 | -14 | AluJb | Alu | SINE |
| 204313232 | 204314461 | - | 11.2 | 977 | 2231 | -187 | Tigger1 | MER2\_type | DNA |
| 204314462 | 204314731 | + | 13.3 | 2 | 294 | -19 | AluSq | Alu | SINE |
| 204314741 | 204315050 | + | 6.5 | 1 | 311 | -2 | AluSp | Alu | SINE |
| 204315051 | 204315994 | - | 11.2 | 2 | 976 | -1442 | Tigger1 | MER2\_type | DNA |

  

|  |  |  |  |  |  |  |  |  |  |
| --- | --- | --- | --- | --- | --- | --- | --- | --- | --- |
| chr1:204335168-204335695 | | | 528 bp | | |  |  |  |  |
| Genome Start | Genome End | Strand | Div | Repeat Start | Repeat End | Repeat Left | Name | Family | Class |
| 204335168 | 204335411 | - | 24.3 | 5540 | 5805 | -316 | L1M5 | L1 | LINE |
| 204335412 | 204335667 | + | 9.4 | 43 | 300 | -13 | AluSq | Alu | SINE |
| 204335668 | 204335695 | - | 24.3 | 5510 | 5539 | -582 | L1M5 | L1 | LINE |

  

|  |  |  |  |  |  |  |  |  |  |
| --- | --- | --- | --- | --- | --- | --- | --- | --- | --- |
| chr1:204346037-204346657 | | | 621 bp | | |  |  |  |  |
| Genome Start | Genome End | Strand | Div | Repeat Start | Repeat End | Repeat Left | Name | Family | Class |
| 204346037 | 204346109 | + | 14.9 | 5012 | 5083 | -1057 | L1M3 | L1 | LINE |
| 204346110 | 204346399 | - | 9.7 | 1 | 288 | -24 | AluSx | Alu | SINE |
| 204346400 | 204346657 | + | 14.9 | 5084 | 5330 | -810 | L1M3 | L1 | LINE |

  

|  |  |  |  |  |  |  |  |  |  |
| --- | --- | --- | --- | --- | --- | --- | --- | --- | --- |
| chr1:204347661-204349828 | | | 2119 bp | | |  |  |  |  |
| Genome Start | Genome End | Strand | Div | Repeat Start | Repeat End | Repeat Left | Name | Family | Class |
| 204347661 | 204348486 | + | 11.1 | 2 | 852 | -379 | Tigger3b | MER2\_type | DNA |
| 204348520 | 204348594 | + | 11.1 | 853 | 959 | -272 | Tigger3b | MER2\_type | DNA |
| 204348611 | 204349558 | - | 17.1 | 1 | 911 | -20 | MER21A | ERV1 | LTR |
| 204349559 | 204349828 | + | 15.6 | 956 | 1231 | 0 | Tigger3b | MER2\_type | DNA |

  

|  |  |  |  |  |  |  |  |  |  |
| --- | --- | --- | --- | --- | --- | --- | --- | --- | --- |
| chr1:204565157-204565741 | | | 585 bp | | |  |  |  |  |
| Genome Start | Genome End | Strand | Div | Repeat Start | Repeat End | Repeat Left | Name | Family | Class |
| 204565157 | 204565304 | - | 23.0 | 106 | 261 | 0 | MER112 | MER1\_type | DNA |
| 204565305 | 204565612 | - | 17.6 | 3 | 309 | -3 | AluSx | Alu | SINE |
| 204565613 | 204565741 | - | 23.0 | 1 | 105 | -155 | MER112 | MER1\_type | DNA |

  

|  |  |  |  |  |  |  |  |  |  |
| --- | --- | --- | --- | --- | --- | --- | --- | --- | --- |
| chr1:204588821-204590450 | | | 1628 bp | | |  |  |  |  |
| Genome Start | Genome End | Strand | Div | Repeat Start | Repeat End | Repeat Left | Name | Family | Class |
| 204588821 | 204589460 | + | 21.6 | 5058 | 5708 | -458 | L1ME1 | L1 | LINE |
| 204589461 | 204589971 | + | 19.5 | 1 | 494 | -11 | MLT1D | MaLR | LTR |
| 204589974 | 204590450 | + | 21.4 | 5705 | 6161 | -5 | L1ME1 | L1 | LINE |

  

|  |  |  |  |  |  |  |  |  |  |
| --- | --- | --- | --- | --- | --- | --- | --- | --- | --- |
| chr1:204608935-204612579 | | | 3650 bp | | |  |  |  |  |
| Genome Start | Genome End | Strand | Div | Repeat Start | Repeat End | Repeat Left | Name | Family | Class |
| 204608935 | 204609213 | + | 32.3 | 598 | 885 | -5234 | L1ME1 | L1 | LINE |
| 204609215 | 204609956 | + | 16.0 | 4980 | 5725 | -459 | L1MB7 | L1 | LINE |
| 204609957 | 204610269 | + | 7.5 | 1 | 312 | 0 | AluY | Alu | SINE |
| 204610270 | 204610744 | + | 17.0 | 5726 | 6184 | 0 | L1MB7 | L1 | LINE |
| 204610738 | 204611088 | + | 29.2 | 893 | 1255 | -4864 | L1ME1 | L1 | LINE |
| 204611090 | 204611421 | + | 25.7 | 1358 | 1729 | -4420 | L1ME1 | L1 | LINE |
| 204611422 | 204611728 | + | 8.5 | 1 | 306 | -5 | AluY | Alu | SINE |
| 204611729 | 204611814 | + | 25.7 | 1730 | 1779 | -4370 | L1ME1 | L1 | LINE |
| 204611815 | 204612096 | - | 16.6 | 1 | 280 | -32 | AluJo | Alu | SINE |
| 204612097 | 204612579 | + | 25.1 | 1780 | 6126 | -23 | L1ME1 | L1 | LINE |

  

|  |  |  |  |  |  |  |  |  |  |
| --- | --- | --- | --- | --- | --- | --- | --- | --- | --- |
| chr1:204640285-204640625 | | | 228 bp | | |  |  |  |  |
| Genome Start | Genome End | Strand | Div | Repeat Start | Repeat End | Repeat Left | Name | Family | Class |
| 204640285 | 204640343 | - | 22.8 | 150 | 206 | -2 | MIR3 | MIR | SINE |
| 204640288 | 204640385 | - | 31.4 | 4403 | 4488 | -1 | L3 | CR1 | LINE |
| 204640555 | 204640625 | - | 29.6 | 86 | 158 | -50 | MIR3 | MIR | SINE |

  

|  |  |  |  |  |  |  |  |  |  |
| --- | --- | --- | --- | --- | --- | --- | --- | --- | --- |
| chr1:205753765-205754898 | | | 1134 bp | | |  |  |  |  |
| Genome Start | Genome End | Strand | Div | Repeat Start | Repeat End | Repeat Left | Name | Family | Class |
| 205753765 | 205754226 | - | 25.2 | 3731 | 4192 | -1954 | L1M5 | L1 | LINE |
| 205754227 | 205754310 | - | 17.2 | 6084 | 6166 | -18 | L1MB7 | L1 | LINE |
| 205754311 | 205754627 | + | 16.4 | 1 | 365 | 0 | MLT1A0 | MaLR | LTR |
| 205754628 | 205754676 | - | 17.2 | 6039 | 6083 | -101 | L1MB7 | L1 | LINE |
| 205754677 | 205754898 | - | 25.2 | 3498 | 3730 | -2416 | L1M5 | L1 | LINE |

  

|  |  |  |  |  |  |  |  |  |  |
| --- | --- | --- | --- | --- | --- | --- | --- | --- | --- |
| chr1:205782823-205783333 | | | 511 bp | | |  |  |  |  |
| Genome Start | Genome End | Strand | Div | Repeat Start | Repeat End | Repeat Left | Name | Family | Class |
| 205782823 | 205782924 | - | 32.0 | 152 | 247 | -21 | MIRb | MIR | SINE |
| 205782925 | 205783222 | - | 18.4 | 1 | 295 | -17 | AluJb | Alu | SINE |
| 205783223 | 205783333 | - | 32.0 | 27 | 151 | -117 | MIRb | MIR | SINE |

  

|  |  |  |  |  |  |  |  |  |  |
| --- | --- | --- | --- | --- | --- | --- | --- | --- | --- |
| chr1:205801380-205801890 | | | 511 bp | | |  |  |  |  |
| Genome Start | Genome End | Strand | Div | Repeat Start | Repeat End | Repeat Left | Name | Family | Class |
| 205801380 | 205801481 | - | 32.0 | 152 | 247 | -21 | MIRb | MIR | SINE |
| 205801482 | 205801779 | - | 18.4 | 1 | 295 | -17 | AluJb | Alu | SINE |
| 205801780 | 205801890 | - | 32.0 | 27 | 151 | -117 | MIRb | MIR | SINE |

  

|  |  |  |  |  |  |  |  |  |  |
| --- | --- | --- | --- | --- | --- | --- | --- | --- | --- |
| chr1:205935012-205935510 | | | 499 bp | | |  |  |  |  |
| Genome Start | Genome End | Strand | Div | Repeat Start | Repeat End | Repeat Left | Name | Family | Class |
| 205935012 | 205935098 | - | 34.8 | 148 | 231 | -37 | MIRb | MIR | SINE |
| 205935099 | 205935397 | - | 18.3 | 1 | 295 | -17 | AluJb | Alu | SINE |
| 205935398 | 205935510 | - | 34.8 | 27 | 147 | -121 | MIRb | MIR | SINE |

  

|  |  |  |  |  |  |  |  |  |  |
| --- | --- | --- | --- | --- | --- | --- | --- | --- | --- |
| chr1:220722337-220722951 | | | 615 bp | | |  |  |  |  |
| Genome Start | Genome End | Strand | Div | Repeat Start | Repeat End | Repeat Left | Name | Family | Class |
| 220722337 | 220722510 | - | 18.0 | 136 | 312 | 0 | AluJo | Alu | SINE |
| 220722511 | 220722817 | - | 11.4 | 1 | 307 | -6 | AluSq | Alu | SINE |
| 220722818 | 220722951 | - | 18.0 | 1 | 135 | -177 | AluJo | Alu | SINE |

  

|  |  |  |  |  |  |  |  |  |  |
| --- | --- | --- | --- | --- | --- | --- | --- | --- | --- |
| chr1:220723863-220725349 | | | 1487 bp | | |  |  |  |  |
| Genome Start | Genome End | Strand | Div | Repeat Start | Repeat End | Repeat Left | Name | Family | Class |
| 220723863 | 220724366 | + | 21.3 | 18 | 525 | -410 | MER21C | ERV1 | LTR |
| 220724367 | 220724675 | - | 20.1 | 2 | 296 | -16 | AluJb | Alu | SINE |
| 220724676 | 220724745 | + | 21.3 | 526 | 603 | -332 | MER21C | ERV1 | LTR |
| 220724746 | 220725048 | + | 20.5 | 1 | 302 | -10 | AluJo | Alu | SINE |
| 220725049 | 220725349 | + | 21.3 | 604 | 935 | 0 | MER21C | ERV1 | LTR |

  

|  |  |  |  |  |  |  |  |  |  |
| --- | --- | --- | --- | --- | --- | --- | --- | --- | --- |
| chr1:220732717-220733196 | | | 416 bp | | |  |  |  |  |
| Genome Start | Genome End | Strand | Div | Repeat Start | Repeat End | Repeat Left | Name | Family | Class |
| 220732717 | 220732813 | - | 22.7 | 71 | 167 | -145 | AluJo | Alu | SINE |
| 220732819 | 220733081 | + | 14.9 | 1 | 268 | -44 | AluSx | Alu | SINE |
| 220733141 | 220733196 | - | 23.2 | 24 | 79 | -223 | AluJo | Alu | SINE |

  

|  |  |  |  |  |  |  |  |  |  |
| --- | --- | --- | --- | --- | --- | --- | --- | --- | --- |
| chr1:220733255-220734431 | | | 1169 bp | | |  |  |  |  |
| Genome Start | Genome End | Strand | Div | Repeat Start | Repeat End | Repeat Left | Name | Family | Class |
| 220733255 | 220733285 | + | 17.9 | 5476 | 5505 | -679 | L1MB8 | L1 | LINE |
| 220733286 | 220733578 | + | 18.2 | 1 | 294 | -18 | AluJb | Alu | SINE |
| 220733579 | 220733652 | + | 17.9 | 5506 | 5570 | -614 | L1MB8 | L1 | LINE |
| 220733661 | 220733814 | - | 21.1 | 1 | 142 | 0 | FLAM\_A | Alu | SINE |
| 220733815 | 220734431 | + | 20.3 | 5555 | 6173 | -5 | L1MB8 | L1 | LINE |

  

|  |  |  |  |  |  |  |  |  |  |
| --- | --- | --- | --- | --- | --- | --- | --- | --- | --- |
| chr1:220736964-220737552 | | | 589 bp | | |  |  |  |  |
| Genome Start | Genome End | Strand | Div | Repeat Start | Repeat End | Repeat Left | Name | Family | Class |
| 220736964 | 220737130 | - | 20.0 | 137 | 303 | -9 | AluJb | Alu | SINE |
| 220737131 | 220737425 | - | 10.2 | 1 | 305 | -4 | AluSg1 | Alu | SINE |
| 220737426 | 220737552 | - | 20.0 | 12 | 136 | -176 | AluJb | Alu | SINE |

  

|  |  |  |  |  |  |  |  |  |  |
| --- | --- | --- | --- | --- | --- | --- | --- | --- | --- |
| chr1:220738231-220739698 | | | 1468 bp | | |  |  |  |  |
| Genome Start | Genome End | Strand | Div | Repeat Start | Repeat End | Repeat Left | Name | Family | Class |
| 220738231 | 220738489 | + | 20.6 | 18 | 275 | -660 | MER21C | ERV1 | LTR |
| 220738490 | 220738783 | - | 14.0 | 1 | 293 | -20 | AluSq | Alu | SINE |
| 220738784 | 220739093 | + | 20.6 | 276 | 603 | -332 | MER21C | ERV1 | LTR |
| 220739094 | 220739398 | + | 17.6 | 1 | 302 | -10 | AluJo | Alu | SINE |
| 220739399 | 220739698 | + | 20.6 | 604 | 934 | -1 | MER21C | ERV1 | LTR |

  

|  |  |  |  |  |  |  |  |  |  |
| --- | --- | --- | --- | --- | --- | --- | --- | --- | --- |
| chr1:220743209-220743554 | | | 346 bp | | |  |  |  |  |
| Genome Start | Genome End | Strand | Div | Repeat Start | Repeat End | Repeat Left | Name | Family | Class |
| 220743209 | 220743308 | + | 31.5 | 5483 | 5575 | -2233 | L1MC4 | L1 | LINE |
| 220743309 | 220743431 | - | 16.5 | 4 | 126 | -7 | FLAM\_C | Alu | SINE |
| 220743432 | 220743554 | + | 31.5 | 5576 | 5690 | -2118 | L1MC4 | L1 | LINE |

  

|  |  |  |  |  |  |  |  |  |  |
| --- | --- | --- | --- | --- | --- | --- | --- | --- | --- |
| chr1:222144525-222145551 | | | 976 bp | | |  |  |  |  |
| Genome Start | Genome End | Strand | Div | Repeat Start | Repeat End | Repeat Left | Name | Family | Class |
| 222144525 | 222144618 | + | 27.1 | 4 | 98 | -493 | MLT1K | MaLR | LTR |
| 222144619 | 222145279 | + | 10.7 | 1 | 644 | 0 | LTR9B | ERV1 | LTR |
| 222145280 | 222145329 | + | 27.1 | 99 | 145 | -446 | MLT1K | MaLR | LTR |
| 222145381 | 222145551 | + | 24.2 | 398 | 575 | -16 | MLT1K | MaLR | LTR |

  

|  |  |  |  |  |  |  |  |  |  |
| --- | --- | --- | --- | --- | --- | --- | --- | --- | --- |
| chr1:222146564-222147634 | | | 1071 bp | | |  |  |  |  |
| Genome Start | Genome End | Strand | Div | Repeat Start | Repeat End | Repeat Left | Name | Family | Class |
| 222146564 | 222146914 | + | 6.8 | 1 | 333 | -448 | MER4E1 | ERV1 | LTR |
| 222146915 | 222147212 | - | 10.8 | 1 | 296 | -14 | AluSg | Alu | SINE |
| 222147213 | 222147634 | + | 6.8 | 334 | 781 | 0 | MER4E1 | ERV1 | LTR |

  

|  |  |  |  |  |  |  |  |  |  |
| --- | --- | --- | --- | --- | --- | --- | --- | --- | --- |
| chr1:222156203-222157154 | | | 925 bp | | |  |  |  |  |
| Genome Start | Genome End | Strand | Div | Repeat Start | Repeat End | Repeat Left | Name | Family | Class |
| 222156203 | 222156289 | + | 26.4 | 41 | 113 | -155 | MIRb | MIR | SINE |
| 222156290 | 222156620 | + | 11.6 | 1 | 361 | -67 | MSTA | MaLR | LTR |
| 222156621 | 222156795 | - | 12.2 | 130 | 306 | -4 | AluSg | Alu | SINE |
| 222156823 | 222156942 | - | 12.2 | 2 | 129 | -181 | AluSg | Alu | SINE |
| 222156943 | 222157006 | + | 11.6 | 362 | 428 | 0 | MSTA | MaLR | LTR |
| 222157007 | 222157154 | + | 26.4 | 114 | 258 | -10 | MIRb | MIR | SINE |

  

|  |  |  |  |  |  |  |  |  |  |
| --- | --- | --- | --- | --- | --- | --- | --- | --- | --- |
| chr1:222159673-222160841 | | | 1169 bp | | |  |  |  |  |
| Genome Start | Genome End | Strand | Div | Repeat Start | Repeat End | Repeat Left | Name | Family | Class |
| 222159673 | 222159986 | - | 18.2 | 460 | 782 | -1 | LTR39 | ERV1 | LTR |
| 222159987 | 222160285 | + | 14.1 | 1 | 299 | -13 | AluSx | Alu | SINE |
| 222160286 | 222160587 | - | 18.2 | 191 | 459 | -324 | LTR39 | ERV1 | LTR |
| 222160588 | 222160706 | - | 3.4 | 6034 | 6154 | -1 | L1PA4 | L1 | LINE |
| 222160707 | 222160841 | - | 18.2 | 59 | 190 | -593 | LTR39 | ERV1 | LTR |

  

|  |  |  |  |  |  |  |  |  |  |
| --- | --- | --- | --- | --- | --- | --- | --- | --- | --- |
| chr1:222172050-222172525 | | | 476 bp | | |  |  |  |  |
| Genome Start | Genome End | Strand | Div | Repeat Start | Repeat End | Repeat Left | Name | Family | Class |
| 222172050 | 222172152 | + | 30.5 | 3044 | 3144 | -3002 | L1M5 | L1 | LINE |
| 222172153 | 222172453 | + | 13.6 | 1 | 301 | -11 | AluSx | Alu | SINE |
| 222172454 | 222172525 | + | 30.5 | 3145 | 3217 | -2929 | L1M5 | L1 | LINE |

  

|  |  |  |  |  |  |  |  |  |  |
| --- | --- | --- | --- | --- | --- | --- | --- | --- | --- |
| chr1:222178200-222179022 | | | 823 bp | | |  |  |  |  |
| Genome Start | Genome End | Strand | Div | Repeat Start | Repeat End | Repeat Left | Name | Family | Class |
| 222178200 | 222178661 | + | 35.1 | 2753 | 3307 | -112 | L2 | L2 | LINE |
| 222178662 | 222178899 | + | 20.0 | 1 | 302 | 0 | AluJb | Alu | SINE |
| 222178900 | 222179022 | + | 33.8 | 3308 | 3413 | -6 | L2 | L2 | LINE |

  

|  |  |  |  |  |  |  |  |  |  |
| --- | --- | --- | --- | --- | --- | --- | --- | --- | --- |
| chr1:222187367-222189473 | | | 1840 bp | | |  |  |  |  |
| Genome Start | Genome End | Strand | Div | Repeat Start | Repeat End | Repeat Left | Name | Family | Class |
| 222187367 | 222187563 | + | 23.4 | 3777 | 3965 | -2164 | L1ME3B | L1 | LINE |
| 222187617 | 222187748 | + | 26.4 | 4579 | 4716 | -1430 | L1MC | L1 | LINE |
| 222187946 | 222188435 | + | 22.6 | 1 | 495 | -10 | MLT1D | MaLR | LTR |
| 222188436 | 222188700 | + | 28.1 | 4906 | 5224 | -905 | L1ME3B | L1 | LINE |
| 222188718 | 222189473 | + | 27.9 | 5242 | 6040 | -183 | L1ME3B | L1 | LINE |

  

|  |  |  |  |  |  |  |  |  |  |
| --- | --- | --- | --- | --- | --- | --- | --- | --- | --- |
| chr1:222199285-222199751 | | | 467 bp | | |  |  |  |  |
| Genome Start | Genome End | Strand | Div | Repeat Start | Repeat End | Repeat Left | Name | Family | Class |
| 222199285 | 222199399 | - | 28.3 | 3313 | 3414 | -5 | L2 | L2 | LINE |
| 222199400 | 222199703 | - | 11.5 | 1 | 304 | -9 | AluSq | Alu | SINE |
| 222199704 | 222199751 | - | 28.3 | 3268 | 3312 | -107 | L2 | L2 | LINE |

  

|  |  |  |  |  |  |  |  |  |  |
| --- | --- | --- | --- | --- | --- | --- | --- | --- | --- |
| chr1:222211813-222212432 | | | 591 bp | | |  |  |  |  |
| Genome Start | Genome End | Strand | Div | Repeat Start | Repeat End | Repeat Left | Name | Family | Class |
| 222211813 | 222211986 | - | 18.4 | 136 | 312 | 0 | AluJo | Alu | SINE |
| 222212016 | 222212298 | - | 10.4 | 1 | 283 | -30 | AluSq | Alu | SINE |
| 222212299 | 222212432 | - | 18.4 | 3 | 135 | -177 | AluJo | Alu | SINE |

  

|  |  |  |  |  |  |  |  |  |  |
| --- | --- | --- | --- | --- | --- | --- | --- | --- | --- |
| chr1:222213345-222214819 | | | 1475 bp | | |  |  |  |  |
| Genome Start | Genome End | Strand | Div | Repeat Start | Repeat End | Repeat Left | Name | Family | Class |
| 222213345 | 222213850 | + | 20.3 | 18 | 525 | -410 | MER21C | ERV1 | LTR |
| 222213851 | 222214144 | - | 20.4 | 2 | 294 | -18 | AluJb | Alu | SINE |
| 222214145 | 222214214 | + | 20.3 | 526 | 603 | -332 | MER21C | ERV1 | LTR |
| 222214215 | 222214518 | + | 21.4 | 1 | 303 | -9 | AluJo | Alu | SINE |
| 222214519 | 222214819 | + | 20.3 | 604 | 935 | 0 | MER21C | ERV1 | LTR |

  

|  |  |  |  |  |  |  |  |  |  |
| --- | --- | --- | --- | --- | --- | --- | --- | --- | --- |
| chr1:222219590-222220070 | | | 436 bp | | |  |  |  |  |
| Genome Start | Genome End | Strand | Div | Repeat Start | Repeat End | Repeat Left | Name | Family | Class |
| 222219590 | 222219691 | - | 23.6 | 72 | 167 | -145 | AluJo | Alu | SINE |
| 222219692 | 222219965 | + | 13.6 | 1 | 279 | -33 | AluSx | Alu | SINE |
| 222220011 | 222220070 | - | 23.6 | 24 | 71 | -241 | AluJo | Alu | SINE |

  

|  |  |  |  |  |  |  |  |  |  |
| --- | --- | --- | --- | --- | --- | --- | --- | --- | --- |
| chr1:222220129-222221306 | | | 1130 bp | | |  |  |  |  |
| Genome Start | Genome End | Strand | Div | Repeat Start | Repeat End | Repeat Left | Name | Family | Class |
| 222220129 | 222220159 | + | 17.9 | 5476 | 5505 | -679 | L1MB8 | L1 | LINE |
| 222220160 | 222220453 | + | 18.8 | 1 | 295 | -17 | AluJb | Alu | SINE |
| 222220454 | 222220527 | + | 17.9 | 5506 | 5570 | -614 | L1MB8 | L1 | LINE |
| 222220576 | 222220686 | - | 18.0 | 4 | 114 | -19 | FLAM\_A | Alu | SINE |
| 222220687 | 222221306 | + | 20.4 | 5552 | 6173 | -5 | L1MB8 | L1 | LINE |

  

|  |  |  |  |  |  |  |  |  |  |
| --- | --- | --- | --- | --- | --- | --- | --- | --- | --- |
| chr1:222223855-222224433 | | | 579 bp | | |  |  |  |  |
| Genome Start | Genome End | Strand | Div | Repeat Start | Repeat End | Repeat Left | Name | Family | Class |
| 222223855 | 222224017 | - | 19.9 | 137 | 299 | -13 | AluJb | Alu | SINE |
| 222224018 | 222224306 | - | 10.7 | 1 | 299 | -10 | AluSg1 | Alu | SINE |
| 222224307 | 222224433 | - | 19.9 | 12 | 136 | -176 | AluJb | Alu | SINE |

  

|  |  |  |  |  |  |  |  |  |  |
| --- | --- | --- | --- | --- | --- | --- | --- | --- | --- |
| chr1:222225112-222226580 | | | 1469 bp | | |  |  |  |  |
| Genome Start | Genome End | Strand | Div | Repeat Start | Repeat End | Repeat Left | Name | Family | Class |
| 222225112 | 222225370 | + | 20.0 | 18 | 269 | -666 | MER21C | ERV1 | LTR |
| 222225371 | 222225664 | - | 15.4 | 1 | 293 | -20 | AluSq | Alu | SINE |
| 222225665 | 222225974 | + | 20.0 | 270 | 603 | -332 | MER21C | ERV1 | LTR |
| 222225975 | 222226280 | + | 18.1 | 1 | 303 | -9 | AluJo | Alu | SINE |
| 222226281 | 222226580 | + | 20.0 | 604 | 934 | -1 | MER21C | ERV1 | LTR |

  

|  |  |  |  |  |  |  |  |  |  |
| --- | --- | --- | --- | --- | --- | --- | --- | --- | --- |
| chr1:222230083-222230428 | | | 346 bp | | |  |  |  |  |
| Genome Start | Genome End | Strand | Div | Repeat Start | Repeat End | Repeat Left | Name | Family | Class |
| 222230083 | 222230182 | + | 32.0 | 5483 | 5575 | -2233 | L1MC4 | L1 | LINE |
| 222230183 | 222230305 | - | 16.5 | 4 | 126 | -7 | FLAM\_C | Alu | SINE |
| 222230306 | 222230428 | + | 32.0 | 5576 | 5690 | -2118 | L1MC4 | L1 | LINE |

  

|  |  |  |  |  |  |  |  |  |  |
| --- | --- | --- | --- | --- | --- | --- | --- | --- | --- |
| chr1:222242999-222243613 | | | 615 bp | | |  |  |  |  |
| Genome Start | Genome End | Strand | Div | Repeat Start | Repeat End | Repeat Left | Name | Family | Class |
| 222242999 | 222243134 | + | 11.7 | 1 | 136 | -176 | AluSx | Alu | SINE |
| 222243135 | 222243437 | + | 10.6 | 1 | 304 | -9 | AluSp | Alu | SINE |
| 222243438 | 222243613 | + | 11.7 | 137 | 309 | -3 | AluSx | Alu | SINE |

  

|  |  |  |  |  |  |  |  |  |  |
| --- | --- | --- | --- | --- | --- | --- | --- | --- | --- |
| chr1:222263569-222264595 | | | 1003 bp | | |  |  |  |  |
| Genome Start | Genome End | Strand | Div | Repeat Start | Repeat End | Repeat Left | Name | Family | Class |
| 222263569 | 222263628 | - | 17.4 | 5166 | 5216 | -924 | L1M3 | L1 | LINE |
| 222263629 | 222263759 | - | 19.2 | 1 | 131 | -2 | FLAM\_C | Alu | SINE |
| 222263760 | 222264236 | - | 17.4 | 4683 | 5165 | -975 | L1M3 | L1 | LINE |
| 222264261 | 222264595 | - | 17.4 | 4338 | 4682 | -1458 | L1M3 | L1 | LINE |

  

|  |  |  |  |  |  |  |  |  |  |
| --- | --- | --- | --- | --- | --- | --- | --- | --- | --- |
| chr1:225768902-225770729 | | | 1828 bp | | |  |  |  |  |
| Genome Start | Genome End | Strand | Div | Repeat Start | Repeat End | Repeat Left | Name | Family | Class |
| 225768902 | 225768954 | + | 29.0 | 1 | 57 | -510 | MLT1F1 | MaLR | LTR |
| 225768955 | 225770249 | + | 15.8 | 2 | 1301 | -1 | HSMAR2 | Mariner | DNA |
| 225770250 | 225770729 | + | 29.0 | 58 | 560 | -7 | MLT1F1 | MaLR | LTR |

  

|  |  |  |  |  |  |  |  |  |  |
| --- | --- | --- | --- | --- | --- | --- | --- | --- | --- |
| chr1:225773051-225774448 | | | 1128 bp | | |  |  |  |  |
| Genome Start | Genome End | Strand | Div | Repeat Start | Repeat End | Repeat Left | Name | Family | Class |
| 225773051 | 225773337 | - | 33.1 | 2942 | 3240 | -179 | L2 | L2 | LINE |
| 225773390 | 225773677 | - | 25.7 | 228 | 533 | -29 | MLT1F2 | MaLR | LTR |
| 225773857 | 225774192 | + | 25.0 | 1 | 348 | -17 | MLT1A0 | MaLR | LTR |
| 225774194 | 225774264 | - | 23.7 | 90 | 161 | -406 | MLT1F2 | MaLR | LTR |
| 225774303 | 225774448 | - | 31.2 | 2749 | 2902 | -517 | L2 | L2 | LINE |

  

|  |  |  |  |  |  |  |  |  |  |
| --- | --- | --- | --- | --- | --- | --- | --- | --- | --- |
| chr1:225791197-225791929 | | | 733 bp | | |  |  |  |  |
| Genome Start | Genome End | Strand | Div | Repeat Start | Repeat End | Repeat Left | Name | Family | Class |
| 225791197 | 225791548 | + | 19.8 | 3414 | 3778 | -2368 | L1M4 | L1 | LINE |
| 225791549 | 225791710 | + | 24.7 | 1 | 165 | -1 | FRAM | Alu | SINE |
| 225791711 | 225791929 | + | 19.8 | 3779 | 4016 | -2130 | L1M4 | L1 | LINE |

  

|  |  |  |  |  |  |  |  |  |  |
| --- | --- | --- | --- | --- | --- | --- | --- | --- | --- |
| chr1:226234612-226235120 | | | 509 bp | | |  |  |  |  |
| Genome Start | Genome End | Strand | Div | Repeat Start | Repeat End | Repeat Left | Name | Family | Class |
| 226234612 | 226234703 | + | 31.8 | 3224 | 3312 | -107 | L2 | L2 | LINE |
| 226234704 | 226235010 | + | 11.4 | 1 | 307 | -6 | AluSq | Alu | SINE |
| 226235011 | 226235120 | + | 31.8 | 3313 | 3418 | -1 | L2 | L2 | LINE |

  

|  |  |  |  |  |  |  |  |  |  |
| --- | --- | --- | --- | --- | --- | --- | --- | --- | --- |
| chr1:240478086-240478650 | | | 538 bp | | |  |  |  |  |
| Genome Start | Genome End | Strand | Div | Repeat Start | Repeat End | Repeat Left | Name | Family | Class |
| 240478086 | 240478305 | + | 14.2 | 9 | 229 | -216 | LTR56 | ERV1 | LTR |
| 240478306 | 240478380 | + | 6.7 | 6077 | 6153 | -1 | L1PA7 | L1 | LINE |
| 240478408 | 240478650 | + | 19.9 | 200 | 445 | 0 | LTR56 | ERV1 | LTR |

  

|  |  |  |  |  |  |  |  |  |  |
| --- | --- | --- | --- | --- | --- | --- | --- | --- | --- |
| chr1:240478737-240479406 | | | 670 bp | | |  |  |  |  |
| Genome Start | Genome End | Strand | Div | Repeat Start | Repeat End | Repeat Left | Name | Family | Class |
| 240478737 | 240478790 | + | 17.0 | 1 | 56 | -352 | MLT1A1 | MaLR | LTR |
| 240478791 | 240479049 | + | 12.0 | 1 | 291 | -21 | AluSx | Alu | SINE |
| 240479050 | 240479406 | + | 17.0 | 57 | 408 | 0 | MLT1A1 | MaLR | LTR |

  

|  |  |  |  |  |  |  |  |  |  |
| --- | --- | --- | --- | --- | --- | --- | --- | --- | --- |
| chr1:240550970-240551819 | | | 850 bp | | |  |  |  |  |
| Genome Start | Genome End | Strand | Div | Repeat Start | Repeat End | Repeat Left | Name | Family | Class |
| 240550970 | 240551218 | + | 16.9 | 2 | 262 | -333 | LTR49 | ERV1 | LTR |
| 240551219 | 240551529 | - | 11.9 | 1 | 311 | -2 | AluSq | Alu | SINE |
| 240551530 | 240551819 | + | 16.9 | 263 | 594 | -1 | LTR49 | ERV1 | LTR |

  

|  |  |  |  |  |  |  |  |  |  |
| --- | --- | --- | --- | --- | --- | --- | --- | --- | --- |
| chr1:240555720-240556198 | | | 479 bp | | |  |  |  |  |
| Genome Start | Genome End | Strand | Div | Repeat Start | Repeat End | Repeat Left | Name | Family | Class |
| 240555720 | 240555805 | + | 25.7 | 145 | 239 | -347 | MARNA | Mariner | DNA |
| 240555806 | 240556098 | - | 19.1 | 1 | 307 | -5 | AluJo | Alu | SINE |
| 240556099 | 240556198 | + | 25.7 | 240 | 334 | -252 | MARNA | Mariner | DNA |

  

|  |  |  |  |  |  |  |  |  |  |
| --- | --- | --- | --- | --- | --- | --- | --- | --- | --- |
| chr1:240556624-240557226 | | | 603 bp | | |  |  |  |  |
| Genome Start | Genome End | Strand | Div | Repeat Start | Repeat End | Repeat Left | Name | Family | Class |
| 240556624 | 240556795 | - | 17.2 | 139 | 308 | -4 | AluJb | Alu | SINE |
| 240556796 | 240557088 | - | 9.9 | 2 | 294 | -17 | AluY | Alu | SINE |
| 240557089 | 240557226 | - | 17.2 | 6 | 138 | -174 | AluJb | Alu | SINE |

  

|  |  |  |  |  |  |  |  |  |  |
| --- | --- | --- | --- | --- | --- | --- | --- | --- | --- |
| chr1:241039988-241042564 | | | 2510 bp | | |  |  |  |  |
| Genome Start | Genome End | Strand | Div | Repeat Start | Repeat End | Repeat Left | Name | Family | Class |
| 241039988 | 241040061 | - | 18.8 | 6276 | 6346 | -13 | L1MD2 | L1 | LINE |
| 241040109 | 241040418 | - | 8.4 | 1 | 312 | 0 | AluSx | Alu | SINE |
| 241040419 | 241040544 | - | 18.4 | 6158 | 6281 | -78 | L1MD2 | L1 | LINE |
| 241040545 | 241041384 | - | 16.9 | 1 | 865 | 0 | MER6 | MER2\_type | DNA |
| 241041385 | 241041396 | - | 18.4 | 6146 | 6157 | -202 | L1MD2 | L1 | LINE |
| 241041417 | 241042564 | - | 16.9 | 4942 | 6145 | -214 | L1MD2 | L1 | LINE |

  

|  |  |  |  |  |  |  |  |  |  |
| --- | --- | --- | --- | --- | --- | --- | --- | --- | --- |
| chr1:241047367-241048133 | | | 758 bp | | |  |  |  |  |
| Genome Start | Genome End | Strand | Div | Repeat Start | Repeat End | Repeat Left | Name | Family | Class |
| 241047367 | 241047459 | + | 32.6 | 2 | 90 | -178 | MIRb | MIR | SINE |
| 241047459 | 241047526 | - | 32.3 | 198 | 259 | -9 | MIRb | MIR | SINE |
| 241047527 | 241047806 | - | 17.5 | 3 | 295 | -17 | AluJo | Alu | SINE |
| 241047807 | 241047949 | - | 32.3 | 16 | 197 | -71 | MIR | MIR | SINE |
| 241047960 | 241048133 | + | 31.0 | 73 | 265 | -3 | MIRb | MIR | SINE |

  

|  |  |  |  |  |  |  |  |  |  |
| --- | --- | --- | --- | --- | --- | --- | --- | --- | --- |
| chr1:241117527-241119819 | | | 2257 bp | | |  |  |  |  |
| Genome Start | Genome End | Strand | Div | Repeat Start | Repeat End | Repeat Left | Name | Family | Class |
| 241117527 | 241118061 | + | 27.4 | 1 | 556 | -6245 | MER89-int | ERV1 | LTR |
| 241118084 | 241118570 | + | 27.4 | 557 | 1005 | -5796 | MER89-int | ERV1 | LTR |
| 241118571 | 241118626 | - | 5.4 | 1 | 80 | 0 | MADE1 | Mariner | DNA |
| 241118627 | 241119013 | + | 27.4 | 1006 | 1357 | -5444 | MER89-int | ERV1 | LTR |
| 241119014 | 241119236 | + | 23.3 | 1 | 203 | -162 | MLT1A0 | MaLR | LTR |
| 241119237 | 241119536 | + | 11.3 | 1 | 300 | -11 | AluY | Alu | SINE |
| 241119537 | 241119670 | + | 23.3 | 204 | 350 | -15 | MLT1A0 | MaLR | LTR |
| 241119685 | 241119819 | + | 24.4 | 1352 | 1488 | -5313 | MER89-int | ERV1 | LTR |

  

|  |  |  |  |  |  |  |  |  |  |
| --- | --- | --- | --- | --- | --- | --- | --- | --- | --- |
| chr1:241123593-241124284 | | | 692 bp | | |  |  |  |  |
| Genome Start | Genome End | Strand | Div | Repeat Start | Repeat End | Repeat Left | Name | Family | Class |
| 241123593 | 241123693 | + | 18.8 | 1 | 109 | -281 | MLT1B | MaLR | LTR |
| 241123694 | 241124000 | + | 11.4 | 1 | 299 | -13 | AluSx | Alu | SINE |
| 241124001 | 241124284 | + | 18.8 | 110 | 384 | -6 | MLT1B | MaLR | LTR |

  

|  |  |  |  |  |  |  |  |  |  |
| --- | --- | --- | --- | --- | --- | --- | --- | --- | --- |
| chr1:241126073-241128165 | | | 2093 bp | | |  |  |  |  |
| Genome Start | Genome End | Strand | Div | Repeat Start | Repeat End | Repeat Left | Name | Family | Class |
| 241126073 | 241127796 | - | 12.4 | 4408 | 6148 | -13 | L1PA14 | L1 | LINE |
| 241127797 | 241127872 | - | 9.2 | 5 | 80 | 0 | MADE1 | Mariner | DNA |
| 241127873 | 241128165 | - | 13.7 | 4093 | 4407 | -1739 | L1PA14 | L1 | LINE |

  

|  |  |  |  |  |  |  |  |  |  |
| --- | --- | --- | --- | --- | --- | --- | --- | --- | --- |
| chr1:241131175-241131927 | | | 753 bp | | |  |  |  |  |
| Genome Start | Genome End | Strand | Div | Repeat Start | Repeat End | Repeat Left | Name | Family | Class |
| 241131175 | 241131492 | + | 21.1 | 250 | 540 | -167 | MER39 | ERV1 | LTR |
| 241131493 | 241131797 | + | 12.0 | 1 | 301 | -11 | AluSx | Alu | SINE |
| 241131798 | 241131927 | + | 21.1 | 541 | 666 | -41 | MER39 | ERV1 | LTR |

  

|  |  |  |  |  |  |  |  |  |  |
| --- | --- | --- | --- | --- | --- | --- | --- | --- | --- |
| chr1:241142436-241142769 | | | 334 bp | | |  |  |  |  |
| Genome Start | Genome End | Strand | Div | Repeat Start | Repeat End | Repeat Left | Name | Family | Class |
| 241142436 | 241142603 | - | 32.6 | 40 | 208 | -60 | MIRb | MIR | SINE |
| 241142604 | 241142734 | - | 12.4 | 5 | 133 | 0 | FLAM\_A | Alu | SINE |
| 241142735 | 241142769 | - | 32.6 | 8 | 39 | -229 | MIRb | MIR | SINE |

  

|  |  |  |  |  |  |  |  |  |  |
| --- | --- | --- | --- | --- | --- | --- | --- | --- | --- |
| chr1:241160120-241160897 | | | 778 bp | | |  |  |  |  |
| Genome Start | Genome End | Strand | Div | Repeat Start | Repeat End | Repeat Left | Name | Family | Class |
| 241160120 | 241160396 | - | 20.3 | 789 | 1062 | 0 | MER63 | AcHobo | DNA |
| 241160397 | 241160694 | + | 12.5 | 1 | 297 | -15 | AluSx | Alu | SINE |
| 241160695 | 241160897 | - | 20.3 | 6 | 788 | -274 | MER63 | AcHobo | DNA |

  

|  |  |  |  |  |  |  |  |  |  |
| --- | --- | --- | --- | --- | --- | --- | --- | --- | --- |
| chr1:241171189-241173535 | | | 2318 bp | | |  |  |  |  |
| Genome Start | Genome End | Strand | Div | Repeat Start | Repeat End | Repeat Left | Name | Family | Class |
| 241171189 | 241171525 | - | 28.8 | 2125 | 2497 | -10 | HAL1 | L1 | LINE |
| 241171527 | 241171706 | + | 24.9 | 1 | 172 | -611 | MER20B | MER1\_type | DNA |
| 241171739 | 241171845 | - | 20.0 | 2024 | 2129 | -378 | HAL1 | L1 | LINE |
| 241171842 | 241172123 | - | 26.6 | 1165 | 1463 | -1044 | HAL1 | L1 | LINE |
| 241172124 | 241172426 | - | 13.2 | 1 | 306 | -6 | AluSx | Alu | SINE |
| 241172427 | 241173535 | - | 26.6 | 47 | 1164 | -1343 | HAL1 | L1 | LINE |

  

|  |  |  |  |  |  |  |  |  |  |
| --- | --- | --- | --- | --- | --- | --- | --- | --- | --- |
| chr1:241173578-241174449 | | | 872 bp | | |  |  |  |  |
| Genome Start | Genome End | Strand | Div | Repeat Start | Repeat End | Repeat Left | Name | Family | Class |
| 241173578 | 241173924 | + | 28.9 | 2765 | 3095 | -324 | L2 | L2 | LINE |
| 241173925 | 241174188 | - | 12.5 | 1 | 282 | -29 | AluY | Alu | SINE |
| 241174189 | 241174449 | + | 30.0 | 3096 | 3370 | -49 | L2 | L2 | LINE |

  

|  |  |  |  |  |  |  |  |  |  |
| --- | --- | --- | --- | --- | --- | --- | --- | --- | --- |
| chr1:241175490-241182976 | | | 7487 bp | | |  |  |  |  |
| Genome Start | Genome End | Strand | Div | Repeat Start | Repeat End | Repeat Left | Name | Family | Class |
| 241175490 | 241179720 | + | 15.1 | 1 | 4197 | -2569 | L1PA13 | L1 | LINE |
| 241179721 | 241180029 | - | 12.6 | 1 | 309 | -3 | AluSx | Alu | SINE |
| 241180030 | 241181307 | + | 16.9 | 4198 | 5451 | -1315 | L1PA13 | L1 | LINE |
| 241181308 | 241181577 | + | 10.4 | 3 | 274 | -39 | AluSp | Alu | SINE |
| 241181578 | 241182976 | + | 11.9 | 5452 | 6781 | -2 | L1PA13 | L1 | LINE |

  

|  |  |  |  |  |  |  |  |  |  |
| --- | --- | --- | --- | --- | --- | --- | --- | --- | --- |
| chr1:241188890-241191649 | | | 2760 bp | | |  |  |  |  |
| Genome Start | Genome End | Strand | Div | Repeat Start | Repeat End | Repeat Left | Name | Family | Class |
| 241188890 | 241189859 | - | 18.2 | 5205 | 6178 | 0 | L1MB8 | L1 | LINE |
| 241189860 | 241190156 | - | 12.5 | 1 | 297 | -13 | AluSg | Alu | SINE |
| 241190157 | 241191649 | - | 18.5 | 3549 | 5204 | -942 | L1MB8 | L1 | LINE |

  

|  |  |  |  |  |  |  |  |  |  |
| --- | --- | --- | --- | --- | --- | --- | --- | --- | --- |
| chr1:241207738-241208578 | | | 841 bp | | |  |  |  |  |
| Genome Start | Genome End | Strand | Div | Repeat Start | Repeat End | Repeat Left | Name | Family | Class |
| 241207738 | 241208140 | - | 27.5 | 7619 | 8042 | 0 | L1MC4 | L1 | LINE |
| 241208141 | 241208441 | - | 12.0 | 1 | 300 | -12 | AluSx | Alu | SINE |
| 241208442 | 241208578 | - | 27.5 | 7492 | 7618 | -424 | L1MC4 | L1 | LINE |

  

|  |  |  |  |  |  |  |  |  |  |
| --- | --- | --- | --- | --- | --- | --- | --- | --- | --- |
| chr1:241208689-241209623 | | | 935 bp | | |  |  |  |  |
| Genome Start | Genome End | Strand | Div | Repeat Start | Repeat End | Repeat Left | Name | Family | Class |
| 241208689 | 241208928 | + | 18.0 | 5546 | 5772 | -406 | L1MB8 | L1 | LINE |
| 241208929 | 241209230 | + | 11.3 | 6 | 307 | -5 | AluSx | Alu | SINE |
| 241209231 | 241209623 | + | 18.0 | 5773 | 6178 | 0 | L1MB8 | L1 | LINE |

  

|  |  |  |  |  |  |  |  |  |  |
| --- | --- | --- | --- | --- | --- | --- | --- | --- | --- |
| chr1:241222994-241224016 | | | 956 bp | | |  |  |  |  |
| Genome Start | Genome End | Strand | Div | Repeat Start | Repeat End | Repeat Left | Name | Family | Class |
| 241222994 | 241223087 | + | 28.1 | 4 | 98 | -493 | MLT1K | MaLR | LTR |
| 241223088 | 241223744 | + | 11.2 | 1 | 644 | 0 | LTR9B | ERV1 | LTR |
| 241223745 | 241223778 | + | 28.1 | 99 | 133 | -458 | MLT1K | MaLR | LTR |
| 241223846 | 241224016 | + | 23.6 | 398 | 575 | -16 | MLT1K | MaLR | LTR |

  

|  |  |  |  |  |  |  |  |  |  |
| --- | --- | --- | --- | --- | --- | --- | --- | --- | --- |
| chr1:241238053-241239220 | | | 1168 bp | | |  |  |  |  |
| Genome Start | Genome End | Strand | Div | Repeat Start | Repeat End | Repeat Left | Name | Family | Class |
| 241238053 | 241238366 | - | 18.5 | 460 | 782 | -1 | LTR39 | ERV1 | LTR |
| 241238367 | 241238665 | + | 15.1 | 1 | 299 | -13 | AluSx | Alu | SINE |
| 241238666 | 241238967 | - | 18.5 | 194 | 459 | -324 | LTR39 | ERV1 | LTR |
| 241238968 | 241239085 | - | 3.4 | 6034 | 6154 | -1 | L1PA4 | L1 | LINE |
| 241239086 | 241239220 | - | 18.5 | 59 | 193 | -590 | LTR39 | ERV1 | LTR |

  

|  |  |  |  |  |  |  |  |  |  |
| --- | --- | --- | --- | --- | --- | --- | --- | --- | --- |
| chr1:241243118-241243770 | | | 653 bp | | |  |  |  |  |
| Genome Start | Genome End | Strand | Div | Repeat Start | Repeat End | Repeat Left | Name | Family | Class |
| 241243118 | 241243405 | - | 26.9 | 93 | 375 | -167 | MLT1H | MaLR | LTR |
| 241243406 | 241243724 | - | 8.7 | 1 | 311 | 0 | AluY | Alu | SINE |
| 241243725 | 241243770 | - | 27.2 | 62 | 107 | -442 | MLT1H | MaLR | LTR |

  

|  |  |  |  |  |  |  |  |  |  |
| --- | --- | --- | --- | --- | --- | --- | --- | --- | --- |
| chr1:241250399-241250874 | | | 476 bp | | |  |  |  |  |
| Genome Start | Genome End | Strand | Div | Repeat Start | Repeat End | Repeat Left | Name | Family | Class |
| 241250399 | 241250501 | + | 29.3 | 3044 | 3141 | -3005 | L1M5 | L1 | LINE |
| 241250502 | 241250802 | + | 13.0 | 1 | 301 | -11 | AluSx | Alu | SINE |
| 241250803 | 241250874 | + | 29.3 | 3142 | 3217 | -2929 | L1M5 | L1 | LINE |

  

|  |  |  |  |  |  |  |  |  |  |
| --- | --- | --- | --- | --- | --- | --- | --- | --- | --- |
| chr1:241251463-241253758 | | | 2120 bp | | |  |  |  |  |
| Genome Start | Genome End | Strand | Div | Repeat Start | Repeat End | Repeat Left | Name | Family | Class |
| 241251463 | 241251655 | + | 28.5 | 3573 | 3757 | -732 | L3 | CR1 | LINE |
| 241251656 | 241251960 | + | 9.6 | 1 | 306 | 0 | AluSg | Alu | SINE |
| 241251961 | 241252037 | + | 28.5 | 3758 | 3834 | -655 | L3 | CR1 | LINE |
| 241252055 | 241252146 | - | 21.7 | 414 | 519 | -2 | LTR33 | ERVL | LTR |
| 241252148 | 241252298 | + | 15.6 | 1 | 149 | -283 | MSTB1 | MaLR | LTR |
| 241252299 | 241252592 | - | 9.9 | 5 | 297 | -16 | AluSq | Alu | SINE |
| 241252593 | 241252842 | + | 15.6 | 150 | 412 | -20 | MSTB1 | MaLR | LTR |
| 241252868 | 241252897 | + | 19.1 | 377 | 396 | 0 | MSTB1 | MaLR | LTR |
| 241253020 | 241253188 | - | 28.5 | 1 | 173 | -348 | LTR33 | ERVL | LTR |
| 241253200 | 241253758 | + | 34.0 | 3856 | 4479 | -10 | L3 | CR1 | LINE |

  

|  |  |  |  |  |  |  |  |  |  |
| --- | --- | --- | --- | --- | --- | --- | --- | --- | --- |
| chr1:241256547-241257363 | | | 817 bp | | |  |  |  |  |
| Genome Start | Genome End | Strand | Div | Repeat Start | Repeat End | Repeat Left | Name | Family | Class |
| 241256547 | 241257007 | + | 34.1 | 2753 | 3307 | -112 | L2 | L2 | LINE |
| 241257008 | 241257243 | + | 19.7 | 1 | 302 | 0 | AluJb | Alu | SINE |
| 241257244 | 241257363 | + | 34.3 | 3308 | 3413 | -6 | L2 | L2 | LINE |

  

|  |  |  |  |  |  |  |  |  |  |
| --- | --- | --- | --- | --- | --- | --- | --- | --- | --- |
| chr1:241277068-241277568 | | | 501 bp | | |  |  |  |  |
| Genome Start | Genome End | Strand | Div | Repeat Start | Repeat End | Repeat Left | Name | Family | Class |
| 241277068 | 241277177 | - | 30.3 | 3313 | 3418 | -1 | L2 | L2 | LINE |
| 241277178 | 241277476 | - | 11.4 | 1 | 299 | -14 | AluSq | Alu | SINE |
| 241277477 | 241277568 | - | 30.3 | 3224 | 3312 | -107 | L2 | L2 | LINE |

  

|  |  |  |  |  |  |  |  |  |  |
| --- | --- | --- | --- | --- | --- | --- | --- | --- | --- |
| chr1:241278470-241279342 | | | 873 bp | | |  |  |  |  |
| Genome Start | Genome End | Strand | Div | Repeat Start | Repeat End | Repeat Left | Name | Family | Class |
| 241278470 | 241278576 | + | 24.9 | 1930 | 2031 | -4758 | L1M2c | L1 | LINE |
| 241278577 | 241278894 | + | 9.8 | 1 | 306 | -4 | AluSg | Alu | SINE |
| 241278895 | 241279342 | + | 22.3 | 2032 | 2563 | -4226 | L1M2c | L1 | LINE |

  

|  |  |  |  |  |  |  |  |  |  |
| --- | --- | --- | --- | --- | --- | --- | --- | --- | --- |
| chr1:241292414-241293033 | | | 591 bp | | |  |  |  |  |
| Genome Start | Genome End | Strand | Div | Repeat Start | Repeat End | Repeat Left | Name | Family | Class |
| 241292414 | 241292587 | - | 17.7 | 136 | 312 | 0 | AluJo | Alu | SINE |
| 241292617 | 241292899 | - | 10.0 | 1 | 283 | -30 | AluSq | Alu | SINE |
| 241292900 | 241293033 | - | 17.7 | 3 | 135 | -177 | AluJo | Alu | SINE |

  

|  |  |  |  |  |  |  |  |  |  |
| --- | --- | --- | --- | --- | --- | --- | --- | --- | --- |
| chr1:241293946-241295429 | | | 1484 bp | | |  |  |  |  |
| Genome Start | Genome End | Strand | Div | Repeat Start | Repeat End | Repeat Left | Name | Family | Class |
| 241293946 | 241294451 | + | 20.4 | 18 | 525 | -410 | MER21C | ERV1 | LTR |
| 241294452 | 241294755 | - | 20.2 | 2 | 294 | -18 | AluJb | Alu | SINE |
| 241294756 | 241294825 | + | 20.4 | 526 | 603 | -332 | MER21C | ERV1 | LTR |
| 241294826 | 241295128 | + | 20.9 | 1 | 302 | -10 | AluJo | Alu | SINE |
| 241295129 | 241295429 | + | 20.4 | 604 | 935 | 0 | MER21C | ERV1 | LTR |

  

|  |  |  |  |  |  |  |  |  |  |
| --- | --- | --- | --- | --- | --- | --- | --- | --- | --- |
| chr1:241300184-241300667 | | | 436 bp | | |  |  |  |  |
| Genome Start | Genome End | Strand | Div | Repeat Start | Repeat End | Repeat Left | Name | Family | Class |
| 241300184 | 241300285 | - | 22.9 | 72 | 167 | -145 | AluJo | Alu | SINE |
| 241300286 | 241300559 | + | 14.7 | 1 | 279 | -33 | AluSx | Alu | SINE |
| 241300608 | 241300667 | - | 22.9 | 24 | 71 | -241 | AluJo | Alu | SINE |

  

|  |  |  |  |  |  |  |  |  |  |
| --- | --- | --- | --- | --- | --- | --- | --- | --- | --- |
| chr1:241300727-241301902 | | | 1126 bp | | |  |  |  |  |
| Genome Start | Genome End | Strand | Div | Repeat Start | Repeat End | Repeat Left | Name | Family | Class |
| 241300727 | 241300757 | + | 17.9 | 5476 | 5505 | -679 | L1MB8 | L1 | LINE |
| 241300758 | 241301049 | + | 17.9 | 1 | 293 | -19 | AluJb | Alu | SINE |
| 241301050 | 241301123 | + | 17.9 | 5506 | 5570 | -614 | L1MB8 | L1 | LINE |
| 241301174 | 241301282 | - | 18.4 | 4 | 112 | -30 | FLAM\_A | Alu | SINE |
| 241301283 | 241301902 | + | 20.4 | 5552 | 6173 | -5 | L1MB8 | L1 | LINE |

  

|  |  |  |  |  |  |  |  |  |  |
| --- | --- | --- | --- | --- | --- | --- | --- | --- | --- |
| chr1:241305706-241307176 | | | 1471 bp | | |  |  |  |  |
| Genome Start | Genome End | Strand | Div | Repeat Start | Repeat End | Repeat Left | Name | Family | Class |
| 241305706 | 241305964 | + | 20.7 | 18 | 269 | -666 | MER21C | ERV1 | LTR |
| 241305965 | 241306260 | - | 13.9 | 1 | 295 | -18 | AluSq | Alu | SINE |
| 241306261 | 241306571 | + | 20.7 | 270 | 603 | -332 | MER21C | ERV1 | LTR |
| 241306572 | 241306876 | + | 16.9 | 1 | 302 | -10 | AluJo | Alu | SINE |
| 241306877 | 241307176 | + | 20.7 | 604 | 934 | -1 | MER21C | ERV1 | LTR |

  

|  |  |  |  |  |  |  |  |  |  |
| --- | --- | --- | --- | --- | --- | --- | --- | --- | --- |
| chr1:241310693-241311038 | | | 346 bp | | |  |  |  |  |
| Genome Start | Genome End | Strand | Div | Repeat Start | Repeat End | Repeat Left | Name | Family | Class |
| 241310693 | 241310792 | + | 31.0 | 5483 | 5575 | -2233 | L1MC4 | L1 | LINE |
| 241310793 | 241310915 | - | 16.5 | 4 | 126 | -7 | FLAM\_C | Alu | SINE |
| 241310916 | 241311038 | + | 31.0 | 5576 | 5690 | -2118 | L1MC4 | L1 | LINE |

  

|  |  |  |  |  |  |  |  |  |  |
| --- | --- | --- | --- | --- | --- | --- | --- | --- | --- |
| chr1:241327740-241328346 | | | 607 bp | | |  |  |  |  |
| Genome Start | Genome End | Strand | Div | Repeat Start | Repeat End | Repeat Left | Name | Family | Class |
| 241327740 | 241327875 | + | 11.6 | 1 | 136 | -176 | AluSx | Alu | SINE |
| 241327876 | 241328176 | + | 10.0 | 1 | 302 | -11 | AluSp | Alu | SINE |
| 241328177 | 241328346 | + | 11.6 | 137 | 303 | -9 | AluSx | Alu | SINE |

  

|  |  |  |  |  |  |  |  |  |  |
| --- | --- | --- | --- | --- | --- | --- | --- | --- | --- |
| chr1:241341016-241344020 | | | 2722 bp | | |  |  |  |  |
| Genome Start | Genome End | Strand | Div | Repeat Start | Repeat End | Repeat Left | Name | Family | Class |
| 241341016 | 241341419 | + | 23.0 | 547 | 2392 | -248 | MER69B | Tip100 | DNA |
| 241341421 | 241342038 | + | 12.1 | 5342 | 5928 | -363 | L1MA8 | L1 | LINE |
| 241342105 | 241342407 | + | 10.2 | 1 | 294 | -19 | AluSq | Alu | SINE |
| 241342431 | 241342650 | - | 21.5 | 232 | 458 | -83 | MER87 | ERV1 | LTR |
| 241342651 | 241342939 | - | 16.3 | 5 | 307 | -5 | AluJo | Alu | SINE |
| 241343103 | 241343292 | + | 13.7 | 5915 | 6107 | -184 | L1MA8 | L1 | LINE |
| 241343293 | 241343611 | + | 13.1 | 1 | 312 | 0 | AluSx | Alu | SINE |
| 241343612 | 241343809 | + | 13.7 | 6108 | 6291 | 0 | L1MA8 | L1 | LINE |
| 241343840 | 241344020 | + | 17.8 | 2441 | 2624 | -16 | MER69B | Tip100 | DNA |

  

|  |  |  |  |  |  |  |  |  |  |
| --- | --- | --- | --- | --- | --- | --- | --- | --- | --- |
| chr1:241347722-241348443 | | | 722 bp | | |  |  |  |  |
| Genome Start | Genome End | Strand | Div | Repeat Start | Repeat End | Repeat Left | Name | Family | Class |
| 241347722 | 241347811 | - | 19.8 | 6116 | 6178 | 0 | L1MB8 | L1 | LINE |
| 241347812 | 241348107 | - | 8.4 | 1 | 296 | -14 | AluSg | Alu | SINE |
| 241348108 | 241348443 | - | 19.8 | 5787 | 6115 | -63 | L1MB8 | L1 | LINE |

  

|  |  |  |  |  |  |  |  |  |  |
| --- | --- | --- | --- | --- | --- | --- | --- | --- | --- |
| chr1:241411619-241413163 | | | 1110 bp | | |  |  |  |  |
| Genome Start | Genome End | Strand | Div | Repeat Start | Repeat End | Repeat Left | Name | Family | Class |
| 241411619 | 241411878 | + | 30.2 | 2036 | 2318 | -1101 | L2 | L2 | LINE |
| 241411879 | 241412297 | + | 17.4 | 1 | 457 | 0 | MSTB2 | MaLR | LTR |
| 241412298 | 241412391 | + | 30.2 | 2319 | 2410 | -1009 | L2 | L2 | LINE |
| 241412472 | 241412669 | + | 36.4 | 2820 | 3079 | -340 | L2 | L2 | LINE |
| 241413025 | 241413163 | + | 30.4 | 3273 | 3419 | 0 | L2 | L2 | LINE |

  

|  |  |  |  |  |  |  |  |  |  |
| --- | --- | --- | --- | --- | --- | --- | --- | --- | --- |
| chr1:246661344-246664534 | | | 2780 bp | | |  |  |  |  |
| Genome Start | Genome End | Strand | Div | Repeat Start | Repeat End | Repeat Left | Name | Family | Class |
| 246661344 | 246661597 | + | 17.4 | 5651 | 5933 | -247 | L1MB4 | L1 | LINE |
| 246661599 | 246662049 | + | 13.1 | 1 | 450 | -49 | LTR3B\_ | ERVK | LTR |
| 246662257 | 246662456 | + | 24.8 | 15 | 232 | -6970 | HERVK3 | ERVK | LTR |
| 246662591 | 246663854 | + | 21.5 | 1280 | 5473 | -623 | HERVK3 | ERVK | LTR |
| 246663924 | 246664387 | + | 19.9 | 2 | 499 | 0 | LTR3B\_ | ERVK | LTR |
| 246664388 | 246664534 | + | 20.7 | 5929 | 6082 | -98 | L1MB4 | L1 | LINE |

  

|  |  |  |  |  |  |  |  |  |  |
| --- | --- | --- | --- | --- | --- | --- | --- | --- | --- |
| chr1:247192703-247194906 | | | 2204 bp | | |  |  |  |  |
| Genome Start | Genome End | Strand | Div | Repeat Start | Repeat End | Repeat Left | Name | Family | Class |
| 247192703 | 247194058 | - | 22.5 | 6229 | 7784 | -1 | L1MC3 | L1 | LINE |
| 247194059 | 247194502 | - | 19.4 | 1 | 432 | 0 | MSTB1 | MaLR | LTR |
| 247194503 | 247194906 | - | 22.5 | 5763 | 6228 | -1557 | L1MC3 | L1 | LINE |

  


|  |  |  |  |  |  |  |  |  |  |
| --- | --- | --- | --- | --- | --- | --- | --- | --- | --- |
| chr2:5684921-5685298 | | | 377 bp | | |  |  |  |  |
| Genome Start | Genome End | Strand | Div | Repeat Start | Repeat End | Repeat Left | Name | Family | Class |
| 5684921 | 5685001 | + | 15.9 | 5862 | 5924 | -247 | L1MB3 | L1 | LINE |
| 5685003 | 5685047 | - | 6.7 | 35 | 80 | 0 | MADE1 | Mariner | DNA |
| 5685048 | 5685298 | + | 20.6 | 5925 | 6181 | -2 | L1MB3 | L1 | LINE |

  

|  |  |  |  |  |  |  |  |  |  |
| --- | --- | --- | --- | --- | --- | --- | --- | --- | --- |
| chr2:13471136-13471916 | | | 781 bp | | |  |  |  |  |
| Genome Start | Genome End | Strand | Div | Repeat Start | Repeat End | Repeat Left | Name | Family | Class |
| 13471136 | 13471199 | - | 23.2 | 438 | 505 | 0 | MLT1D | MaLR | LTR |
| 13471200 | 13471473 | + | 9.6 | 1 | 291 | -18 | AluSc | Alu | SINE |
| 13471474 | 13471916 | - | 23.2 | 1 | 437 | -68 | MLT1D | MaLR | LTR |

  

|  |  |  |  |  |  |  |  |  |  |
| --- | --- | --- | --- | --- | --- | --- | --- | --- | --- |
| chr2:19071965-19072593 | | | 629 bp | | |  |  |  |  |
| Genome Start | Genome End | Strand | Div | Repeat Start | Repeat End | Repeat Left | Name | Family | Class |
| 19071965 | 19072020 | + | 24.0 | 8 | 61 | -201 | MIR | MIR | SINE |
| 19072021 | 19072423 | + | 17.8 | 1 | 426 | 0 | MSTB | MaLR | LTR |
| 19072424 | 19072593 | + | 24.0 | 62 | 254 | -8 | MIR | MIR | SINE |

  

|  |  |  |  |  |  |  |  |  |  |
| --- | --- | --- | --- | --- | --- | --- | --- | --- | --- |
| chr2:38780318-38781676 | | | 1359 bp | | |  |  |  |  |
| Genome Start | Genome End | Strand | Div | Repeat Start | Repeat End | Repeat Left | Name | Family | Class |
| 38780318 | 38780602 | - | 14.6 | 3328 | 3612 | -3233 | L1M2 | L1 | LINE |
| 38780603 | 38780897 | - | 12.9 | 1 | 296 | -16 | AluSx | Alu | SINE |
| 38780898 | 38781676 | - | 13.4 | 2536 | 3327 | -3518 | L1M2 | L1 | LINE |

  

|  |  |  |  |  |  |  |  |  |  |
| --- | --- | --- | --- | --- | --- | --- | --- | --- | --- |
| chr2:38794222-38794740 | | | 519 bp | | |  |  |  |  |
| Genome Start | Genome End | Strand | Div | Repeat Start | Repeat End | Repeat Left | Name | Family | Class |
| 38794222 | 38794268 | + | 23.4 | 11 | 66 | -196 | MIR | MIR | SINE |
| 38794269 | 38794567 | + | 15.8 | 2 | 299 | -13 | AluJo | Alu | SINE |
| 38794568 | 38794740 | + | 23.4 | 67 | 261 | -1 | MIR | MIR | SINE |

  

|  |  |  |  |  |  |  |  |  |  |
| --- | --- | --- | --- | --- | --- | --- | --- | --- | --- |
| chr2:71111963-71112633 | | | 671 bp | | |  |  |  |  |
| Genome Start | Genome End | Strand | Div | Repeat Start | Repeat End | Repeat Left | Name | Family | Class |
| 71111963 | 71112145 | + | 22.6 | 5771 | 5940 | -360 | L1MA4 | L1 | LINE |
| 71112146 | 71112426 | + | 14.6 | 1 | 280 | -32 | AluSx | Alu | SINE |
| 71112427 | 71112633 | + | 22.6 | 5941 | 6130 | -170 | L1MA4 | L1 | LINE |

  

|  |  |  |  |  |  |  |  |  |  |
| --- | --- | --- | --- | --- | --- | --- | --- | --- | --- |
| chr2:71113067-71115041 | | | 1927 bp | | |  |  |  |  |
| Genome Start | Genome End | Strand | Div | Repeat Start | Repeat End | Repeat Left | Name | Family | Class |
| 71113067 | 71114058 | + | 23.8 | 4818 | 5824 | -359 | L1MB3 | L1 | LINE |
| 71114059 | 71114336 | + | 11.6 | 1 | 279 | -33 | AluSx | Alu | SINE |
| 71114385 | 71114485 | + | 22.7 | 5825 | 5907 | -276 | L1MB3 | L1 | LINE |
| 71114486 | 71114789 | + | 15.1 | 1 | 304 | -8 | AluSx | Alu | SINE |
| 71114790 | 71115041 | + | 22.7 | 5908 | 6153 | -30 | L1MB3 | L1 | LINE |

  

|  |  |  |  |  |  |  |  |  |  |
| --- | --- | --- | --- | --- | --- | --- | --- | --- | --- |
| chr2:73778704-73780156 | | | 1453 bp | | |  |  |  |  |
| Genome Start | Genome End | Strand | Div | Repeat Start | Repeat End | Repeat Left | Name | Family | Class |
| 73778704 | 73779097 | + | 18.1 | 459 | 882 | -769 | MSTB-int | MaLR | LTR |
| 73779098 | 73779402 | - | 14.4 | 1 | 306 | -6 | AluSx | Alu | SINE |
| 73779403 | 73780156 | + | 18.1 | 883 | 1650 | -1 | MSTB-int | MaLR | LTR |

  

|  |  |  |  |  |  |  |  |  |  |
| --- | --- | --- | --- | --- | --- | --- | --- | --- | --- |
| chr2:73793182-73794076 | | | 895 bp | | |  |  |  |  |
| Genome Start | Genome End | Strand | Div | Repeat Start | Repeat End | Repeat Left | Name | Family | Class |
| 73793182 | 73793372 | + | 33.3 | 5394 | 5588 | -533 | L1ME4a | L1 | LINE |
| 73793373 | 73793656 | - | 22.8 | 1 | 291 | -11 | AluJo | Alu | SINE |
| 73793657 | 73794076 | + | 33.3 | 5589 | 6038 | -83 | L1ME4a | L1 | LINE |

  

|  |  |  |  |  |  |  |  |  |  |
| --- | --- | --- | --- | --- | --- | --- | --- | --- | --- |
| chr2:77759923-77760710 | | | 788 bp | | |  |  |  |  |
| Genome Start | Genome End | Strand | Div | Repeat Start | Repeat End | Repeat Left | Name | Family | Class |
| 77759923 | 77760238 | - | 17.8 | 203 | 501 | 0 | LTR27 | ERV1 | LTR |
| 77760239 | 77760536 | + | 14.1 | 1 | 297 | -15 | AluSx | Alu | SINE |
| 77760537 | 77760710 | - | 22.7 | 4 | 202 | -434 | LTR27 | ERV1 | LTR |

  

|  |  |  |  |  |  |  |  |  |  |
| --- | --- | --- | --- | --- | --- | --- | --- | --- | --- |
| chr2:77764736-77765954 | | | 954 bp | | |  |  |  |  |
| Genome Start | Genome End | Strand | Div | Repeat Start | Repeat End | Repeat Left | Name | Family | Class |
| 77764736 | 77764959 | + | 26.0 | 1 | 224 | -338 | MLT1F2 | MaLR | LTR |
| 77765052 | 77765465 | + | 18.5 | 3 | 399 | -502 | MER54A | ERVL | LTR |
| 77765639 | 77765954 | + | 30.1 | 241 | 558 | -4 | MLT1F2 | MaLR | LTR |

  

|  |  |  |  |  |  |  |  |  |  |
| --- | --- | --- | --- | --- | --- | --- | --- | --- | --- |
| chr2:83298126-83298550 | | | 425 bp | | |  |  |  |  |
| Genome Start | Genome End | Strand | Div | Repeat Start | Repeat End | Repeat Left | Name | Family | Class |
| 83298126 | 83298207 | + | 10.7 | 1 | 80 | -110 | MER30B | MER1\_type | DNA |
| 83298208 | 83298452 | - | 9.0 | 58 | 303 | -10 | AluSp | Alu | SINE |
| 83298453 | 83298550 | + | 10.7 | 81 | 189 | -1 | MER30B | MER1\_type | DNA |

  

|  |  |  |  |  |  |  |  |  |  |
| --- | --- | --- | --- | --- | --- | --- | --- | --- | --- |
| chr2:86792985-86794327 | | | 1335 bp | | |  |  |  |  |
| Genome Start | Genome End | Strand | Div | Repeat Start | Repeat End | Repeat Left | Name | Family | Class |
| 86792985 | 86793062 | + | 14.1 | 1 | 82 | -6426 | L1MCa | L1 | LINE |
| 86793072 | 86793266 | - | 17.6 | 84 | 291 | -21 | AluJb | Alu | SINE |
| 86793268 | 86793642 | + | 21.4 | 99 | 468 | -6040 | L1MCa | L1 | LINE |
| 86793641 | 86794327 | + | 12.5 | 1436 | 2188 | -4320 | L1MCa | L1 | LINE |

  

|  |  |  |  |  |  |  |  |  |  |
| --- | --- | --- | --- | --- | --- | --- | --- | --- | --- |
| chr2:86794654-86795237 | | | 584 bp | | |  |  |  |  |
| Genome Start | Genome End | Strand | Div | Repeat Start | Repeat End | Repeat Left | Name | Family | Class |
| 86794654 | 86794792 | + | 24.4 | 32 | 160 | -108 | MIRb | MIR | SINE |
| 86794793 | 86795135 | - | 12.4 | 2 | 345 | 0 | AluSp | Alu | SINE |
| 86795136 | 86795237 | + | 24.4 | 161 | 268 | 0 | MIRb | MIR | SINE |

  

|  |  |  |  |  |  |  |  |  |  |
| --- | --- | --- | --- | --- | --- | --- | --- | --- | --- |
| chr2:86798003-86798527 | | | 525 bp | | |  |  |  |  |
| Genome Start | Genome End | Strand | Div | Repeat Start | Repeat End | Repeat Left | Name | Family | Class |
| 86798003 | 86798171 | + | 19.3 | 1 | 129 | -80 | MER3 | MER1\_type | DNA |
| 86798172 | 86798489 | + | 10.3 | 1 | 317 | 0 | AluSg | Alu | SINE |
| 86798490 | 86798527 | + | 19.3 | 177 | 209 | 0 | MER3 | MER1\_type | DNA |

  

|  |  |  |  |  |  |  |  |  |  |
| --- | --- | --- | --- | --- | --- | --- | --- | --- | --- |
| chr2:86820144-86821097 | | | 912 bp | | |  |  |  |  |
| Genome Start | Genome End | Strand | Div | Repeat Start | Repeat End | Repeat Left | Name | Family | Class |
| 86820144 | 86820304 | - | 19.5 | 405 | 563 | 0 | MER68 | ERVL | LTR |
| 86820326 | 86820490 | + | 13.9 | 225 | 392 | -171 | MER68 | ERVL | LTR |
| 86820512 | 86820629 | - | 17.7 | 122 | 242 | -321 | MER68 | ERVL | LTR |
| 86820630 | 86820970 | + | 12.6 | 1 | 339 | 0 | MER1B | MER1\_type | DNA |
| 86820971 | 86821097 | - | 17.7 | 1 | 121 | -442 | MER68 | ERVL | LTR |

  

|  |  |  |  |  |  |  |  |  |  |
| --- | --- | --- | --- | --- | --- | --- | --- | --- | --- |
| chr2:86931395-86931930 | | | 536 bp | | |  |  |  |  |
| Genome Start | Genome End | Strand | Div | Repeat Start | Repeat End | Repeat Left | Name | Family | Class |
| 86931395 | 86931442 | + | 31.0 | 1478 | 1516 | -444 | L4 | RTE | LINE |
| 86931443 | 86931752 | + | 10.3 | 1 | 310 | -2 | AluSx | Alu | SINE |
| 86931753 | 86931930 | + | 31.0 | 1517 | 1695 | -265 | L4 | RTE | LINE |

  

|  |  |  |  |  |  |  |  |  |  |
| --- | --- | --- | --- | --- | --- | --- | --- | --- | --- |
| chr2:86950435-86951030 | | | 596 bp | | |  |  |  |  |
| Genome Start | Genome End | Strand | Div | Repeat Start | Repeat End | Repeat Left | Name | Family | Class |
| 86950435 | 86950588 | - | 23.2 | 471 | 622 | 0 | LTR26E | ERV1 | LTR |
| 86950589 | 86950888 | - | 7.3 | 1 | 300 | -11 | AluY | Alu | SINE |
| 86950889 | 86951030 | - | 23.2 | 316 | 470 | -152 | LTR26E | ERV1 | LTR |

  

|  |  |  |  |  |  |  |  |  |  |
| --- | --- | --- | --- | --- | --- | --- | --- | --- | --- |
| chr2:86960327-86963169 | | | 2839 bp | | |  |  |  |  |
| Genome Start | Genome End | Strand | Div | Repeat Start | Repeat End | Repeat Left | Name | Family | Class |
| 86960327 | 86960456 | + | 16.9 | 1 | 137 | -99 | MER46A | MER2\_type | DNA |
| 86960460 | 86961058 | + | 13.4 | 3772 | 4377 | -1769 | L1PB3 | L1 | LINE |
| 86961059 | 86961351 | - | 8.9 | 1 | 292 | -21 | AluSp | Alu | SINE |
| 86961352 | 86963080 | + | 13.9 | 4378 | 6136 | -18 | L1PB3 | L1 | LINE |
| 86963082 | 86963169 | + | 13.6 | 124 | 236 | 0 | MER46A | MER2\_type | DNA |

  

|  |  |  |  |  |  |  |  |  |  |
| --- | --- | --- | --- | --- | --- | --- | --- | --- | --- |
| chr2:86964715-86965226 | | | 512 bp | | |  |  |  |  |
| Genome Start | Genome End | Strand | Div | Repeat Start | Repeat End | Repeat Left | Name | Family | Class |
| 86964715 | 86964800 | + | 22.2 | 7 | 83 | -132 | MER58C | MER1\_type | DNA |
| 86964801 | 86965106 | - | 11.8 | 5 | 313 | 0 | AluSq | Alu | SINE |
| 86965107 | 86965226 | + | 22.2 | 84 | 203 | -12 | MER58C | MER1\_type | DNA |

  

|  |  |  |  |  |  |  |  |  |  |
| --- | --- | --- | --- | --- | --- | --- | --- | --- | --- |
| chr2:86976841-86978952 | | | 2112 bp | | |  |  |  |  |
| Genome Start | Genome End | Strand | Div | Repeat Start | Repeat End | Repeat Left | Name | Family | Class |
| 86976841 | 86978322 | + | 11.7 | 5 | 1548 | -870 | Tigger1 | MER2\_type | DNA |
| 86978323 | 86978618 | + | 7.8 | 1 | 296 | -15 | AluY | Alu | SINE |
| 86978619 | 86978952 | + | 11.7 | 1549 | 1831 | -587 | Tigger1 | MER2\_type | DNA |

  

|  |  |  |  |  |  |  |  |  |  |
| --- | --- | --- | --- | --- | --- | --- | --- | --- | --- |
| chr2:86978953-86979806 | | | 825 bp | | |  |  |  |  |
| Genome Start | Genome End | Strand | Div | Repeat Start | Repeat End | Repeat Left | Name | Family | Class |
| 86978953 | 86979252 | + | 20.8 | 2183 | 2507 | -3712 | L1MEc | L1 | LINE |
| 86979258 | 86979315 | - | 20.7 | 2604 | 2661 | -3479 | L1M3 | L1 | LINE |
| 86979329 | 86979493 | - | 20.2 | 137 | 300 | -12 | AluJb | Alu | SINE |
| 86979496 | 86979687 | - | 26.0 | 5 | 207 | -8 | MER58C | MER1\_type | DNA |
| 86979697 | 86979806 | + | 20.2 | 3976 | 4088 | -2131 | L1MEc | L1 | LINE |

  

|  |  |  |  |  |  |  |  |  |  |
| --- | --- | --- | --- | --- | --- | --- | --- | --- | --- |
| chr2:87035004-87035393 | | | 390 bp | | |  |  |  |  |
| Genome Start | Genome End | Strand | Div | Repeat Start | Repeat End | Repeat Left | Name | Family | Class |
| 87035004 | 87035111 | - | 21.4 | 7886 | 7993 | -2 | L1MD3 | L1 | LINE |
| 87035112 | 87035339 | + | 12.8 | 1 | 236 | 0 | MER46A | MER2\_type | DNA |
| 87035340 | 87035393 | - | 21.4 | 7832 | 7885 | -110 | L1MD3 | L1 | LINE |

  

|  |  |  |  |  |  |  |  |  |  |
| --- | --- | --- | --- | --- | --- | --- | --- | --- | --- |
| chr2:87035393-87036325 | | | 932 bp | | |  |  |  |  |
| Genome Start | Genome End | Strand | Div | Repeat Start | Repeat End | Repeat Left | Name | Family | Class |
| 87035393 | 87035625 | - | 17.5 | 5909 | 6145 | -214 | L1MD2 | L1 | LINE |
| 87035626 | 87035933 | - | 12.0 | 1 | 309 | -3 | AluSx | Alu | SINE |
| 87035934 | 87036067 | - | 17.5 | 5759 | 5908 | -451 | L1MD2 | L1 | LINE |
| 87036068 | 87036236 | - | 17.4 | 137 | 303 | -9 | AluSg/x | Alu | SINE |
| 87036238 | 87036325 | - | 19.3 | 5678 | 5770 | -589 | L1MD2 | L1 | LINE |

  

|  |  |  |  |  |  |  |  |  |  |
| --- | --- | --- | --- | --- | --- | --- | --- | --- | --- |
| chr2:87037047-87038101 | | | 545 bp | | |  |  |  |  |
| Genome Start | Genome End | Strand | Div | Repeat Start | Repeat End | Repeat Left | Name | Family | Class |
| 87037047 | 87037257 | - | 28.8 | 1301 | 1527 | -1892 | L2 | L2 | LINE |
| 87037597 | 87037660 | - | 22.2 | 832 | 894 | -2525 | L2 | L2 | LINE |
| 87037665 | 87037781 | + | 19.3 | 86 | 199 | -113 | AluJo | Alu | SINE |
| 87037949 | 87038101 | - | 27.2 | 192 | 354 | -3065 | L2 | L2 | LINE |

  

|  |  |  |  |  |  |  |  |  |  |
| --- | --- | --- | --- | --- | --- | --- | --- | --- | --- |
| chr2:87040059-87042710 | | | 2365 bp | | |  |  |  |  |
| Genome Start | Genome End | Strand | Div | Repeat Start | Repeat End | Repeat Left | Name | Family | Class |
| 87040059 | 87040128 | - | 20.0 | 436 | 515 | -8 | Charlie1b | MER1\_type | DNA |
| 87040133 | 87040150 | - | 16.6 | 293 | 309 | 0 | AluSc | Alu | SINE |
| 87040151 | 87040374 | - | 8.0 | 72 | 308 | -1 | AluSc | Alu | SINE |
| 87040375 | 87040575 | - | 16.6 | 88 | 292 | -17 | AluSc | Alu | SINE |
| 87040577 | 87040664 | - | 14.3 | 201 | 242 | -58 | AluY | Alu | SINE |
| 87040946 | 87041158 | - | 6.1 | 1 | 212 | -99 | AluY | Alu | SINE |
| 87041159 | 87041228 | - | 11.2 | 85 | 144 | -165 | AluSc | Alu | SINE |
| 87041229 | 87041522 | - | 6.8 | 1 | 294 | -17 | AluY | Alu | SINE |
| 87041523 | 87041578 | - | 11.2 | 29 | 84 | -225 | AluSc | Alu | SINE |
| 87041579 | 87041662 | - | 13.1 | 1 | 84 | -225 | AluSc | Alu | SINE |
| 87041664 | 87041770 | - | 21.6 | 333 | 445 | -78 | Charlie1b | MER1\_type | DNA |
| 87041771 | 87042082 | - | 8.7 | 1 | 312 | -1 | AluSq | Alu | SINE |
| 87042083 | 87042274 | - | 21.6 | 144 | 332 | -191 | Charlie1b | MER1\_type | DNA |
| 87042275 | 87042575 | + | 11.7 | 1 | 299 | -10 | AluSc | Alu | SINE |
| 87042576 | 87042710 | - | 21.6 | 13 | 143 | -380 | Charlie1b | MER1\_type | DNA |

  

|  |  |  |  |  |  |  |  |  |  |
| --- | --- | --- | --- | --- | --- | --- | --- | --- | --- |
| chr2:87047161-87047628 | | | 468 bp | | |  |  |  |  |
| Genome Start | Genome End | Strand | Div | Repeat Start | Repeat End | Repeat Left | Name | Family | Class |
| 87047161 | 87047282 | + | 31.2 | 3286 | 3407 | -1082 | L3 | CR1 | LINE |
| 87047283 | 87047583 | + | 11.8 | 1 | 299 | -13 | AluSx | Alu | SINE |
| 87047584 | 87047628 | + | 31.2 | 3408 | 3452 | -1037 | L3 | CR1 | LINE |

  

|  |  |  |  |  |  |  |  |  |  |
| --- | --- | --- | --- | --- | --- | --- | --- | --- | --- |
| chr2:87079574-87080187 | | | 614 bp | | |  |  |  |  |
| Genome Start | Genome End | Strand | Div | Repeat Start | Repeat End | Repeat Left | Name | Family | Class |
| 87079574 | 87079644 | - | 28.9 | 7514 | 7588 | -373 | L1MC5 | L1 | LINE |
| 87079645 | 87079945 | - | 10.7 | 1 | 309 | 0 | AluSc | Alu | SINE |
| 87079946 | 87080187 | - | 28.9 | 7250 | 7513 | -448 | L1MC5 | L1 | LINE |

  

|  |  |  |  |  |  |  |  |  |  |
| --- | --- | --- | --- | --- | --- | --- | --- | --- | --- |
| chr2:87108341-87110155 | | | 1654 bp | | |  |  |  |  |
| Genome Start | Genome End | Strand | Div | Repeat Start | Repeat End | Repeat Left | Name | Family | Class |
| 87108341 | 87108601 | - | 32.8 | 3072 | 3339 | -39 | L2 | L2 | LINE |
| 87108602 | 87109043 | + | 12.1 | 1 | 448 | 0 | MER83 | ERV1 | LTR |
| 87109044 | 87109095 | - | 32.8 | 3020 | 3071 | -307 | L2 | L2 | LINE |
| 87109257 | 87109572 | - | 31.7 | 2384 | 2739 | -680 | L2 | L2 | LINE |
| 87109573 | 87109786 | + | 21.1 | 1 | 224 | 0 | MER58A | MER1\_type | DNA |
| 87109787 | 87110155 | - | 31.7 | 1975 | 2383 | -1036 | L2 | L2 | LINE |

  

|  |  |  |  |  |  |  |  |  |  |
| --- | --- | --- | --- | --- | --- | --- | --- | --- | --- |
| chr2:87110190-87111260 | | | 1003 bp | | |  |  |  |  |
| Genome Start | Genome End | Strand | Div | Repeat Start | Repeat End | Repeat Left | Name | Family | Class |
| 87110190 | 87110507 | - | 21.3 | 3424 | 3772 | -168 | Arthur1 | Tip100 | DNA |
| 87110564 | 87110898 | - | 8.1 | 5829 | 6163 | 0 | L1PA13 | L1 | LINE |
| 87110911 | 87111260 | - | 24.1 | 59 | 461 | -765 | Arthur1 | Tip100 | DNA |

  

|  |  |  |  |  |  |  |  |  |  |
| --- | --- | --- | --- | --- | --- | --- | --- | --- | --- |
| chr2:87138225-87140161 | | | 1912 bp | | |  |  |  |  |
| Genome Start | Genome End | Strand | Div | Repeat Start | Repeat End | Repeat Left | Name | Family | Class |
| 87138225 | 87138807 | - | 29.5 | 2802 | 3413 | -6 | L2 | L2 | LINE |
| 87138808 | 87139099 | - | 13.7 | 1 | 293 | -19 | AluJb | Alu | SINE |
| 87139100 | 87139319 | - | 29.1 | 2509 | 2801 | -618 | L2 | L2 | LINE |
| 87139320 | 87139563 | + | 16.1 | 27 | 275 | -37 | AluJb | Alu | SINE |
| 87139564 | 87139768 | - | 29.1 | 2326 | 2508 | -911 | L2 | L2 | LINE |
| 87139794 | 87140161 | - | 29.1 | 1955 | 2325 | -1094 | L2 | L2 | LINE |

  

|  |  |  |  |  |  |  |  |  |  |
| --- | --- | --- | --- | --- | --- | --- | --- | --- | --- |
| chr2:87148680-87149115 | | | 436 bp | | |  |  |  |  |
| Genome Start | Genome End | Strand | Div | Repeat Start | Repeat End | Repeat Left | Name | Family | Class |
| 87148680 | 87148765 | - | 30.9 | 99 | 210 | -58 | MIRb | MIR | SINE |
| 87148766 | 87149052 | + | 13.0 | 1 | 295 | -17 | AluSx | Alu | SINE |
| 87149053 | 87149115 | - | 30.9 | 36 | 98 | -170 | MIRb | MIR | SINE |

  

|  |  |  |  |  |  |  |  |  |  |
| --- | --- | --- | --- | --- | --- | --- | --- | --- | --- |
| chr2:87212933-87214311 | | | 1372 bp | | |  |  |  |  |
| Genome Start | Genome End | Strand | Div | Repeat Start | Repeat End | Repeat Left | Name | Family | Class |
| 87212933 | 87213392 | - | 21.8 | 5397 | 5871 | -371 | L1MD1 | L1 | LINE |
| 87213400 | 87213947 | - | 21.3 | 4649 | 5266 | -880 | L1MD1 | L1 | LINE |
| 87213948 | 87214252 | - | 11.5 | 1 | 304 | -8 | AluSx | Alu | SINE |
| 87214253 | 87214311 | - | 21.3 | 4600 | 4648 | -1498 | L1MD1 | L1 | LINE |

  

|  |  |  |  |  |  |  |  |  |  |
| --- | --- | --- | --- | --- | --- | --- | --- | --- | --- |
| chr2:87216889-87217835 | | | 947 bp | | |  |  |  |  |
| Genome Start | Genome End | Strand | Div | Repeat Start | Repeat End | Repeat Left | Name | Family | Class |
| 87216889 | 87217109 | + | 20.4 | 5478 | 5701 | -465 | L1ME1 | L1 | LINE |
| 87217110 | 87217407 | + | 8.7 | 1 | 306 | -3 | AluSc | Alu | SINE |
| 87217408 | 87217835 | + | 20.4 | 5702 | 6143 | -23 | L1ME1 | L1 | LINE |

  

|  |  |  |  |  |  |  |  |  |  |
| --- | --- | --- | --- | --- | --- | --- | --- | --- | --- |
| chr2:87220074-87221317 | | | 1244 bp | | |  |  |  |  |
| Genome Start | Genome End | Strand | Div | Repeat Start | Repeat End | Repeat Left | Name | Family | Class |
| 87220074 | 87220756 | - | 26.6 | 5520 | 6229 | -11 | L1ME3B | L1 | LINE |
| 87220757 | 87221072 | + | 7.1 | 1 | 309 | -2 | AluY | Alu | SINE |
| 87221073 | 87221317 | - | 24.9 | 5251 | 5519 | -721 | L1ME3B | L1 | LINE |

  

|  |  |  |  |  |  |  |  |  |  |
| --- | --- | --- | --- | --- | --- | --- | --- | --- | --- |
| chr2:87246089-87248518 | | | 2351 bp | | |  |  |  |  |
| Genome Start | Genome End | Strand | Div | Repeat Start | Repeat End | Repeat Left | Name | Family | Class |
| 87246089 | 87246397 | - | 24.8 | 5737 | 6088 | -10 | L1ME2 | L1 | LINE |
| 87246398 | 87246617 | - | 14.2 | 1 | 236 | 0 | MER46A | MER2\_type | DNA |
| 87246618 | 87246628 | - | 24.8 | 5727 | 5736 | -362 | L1ME2 | L1 | LINE |
| 87246650 | 87247040 | - | 26.0 | 5177 | 5568 | -530 | L1ME2 | L1 | LINE |
| 87247041 | 87247340 | - | 5.3 | 1 | 300 | -11 | AluY | Alu | SINE |
| 87247341 | 87247617 | - | 24.8 | 4897 | 5176 | -904 | L1ME2 | L1 | LINE |
| 87247627 | 87247790 | - | 18.2 | 138 | 307 | -256 | MER68 | ERVL | LTR |
| 87247804 | 87248244 | - | 28.5 | 99 | 550 | -5520 | L1ME2 | L1 | LINE |
| 87248265 | 87248416 | + | 17.9 | 43 | 224 | 0 | MER58A | MER1\_type | DNA |
| 87248433 | 87248518 | - | 27.7 | 3 | 91 | -5979 | L1ME2 | L1 | LINE |

  

|  |  |  |  |  |  |  |  |  |  |
| --- | --- | --- | --- | --- | --- | --- | --- | --- | --- |
| chr2:87276438-87276971 | | | 534 bp | | |  |  |  |  |
| Genome Start | Genome End | Strand | Div | Repeat Start | Repeat End | Repeat Left | Name | Family | Class |
| 87276438 | 87276573 | + | 17.9 | 1 | 143 | -93 | MER46A | MER2\_type | DNA |
| 87276574 | 87276870 | + | 8.1 | 1 | 296 | -15 | AluY | Alu | SINE |
| 87276871 | 87276971 | + | 17.9 | 144 | 232 | -4 | MER46A | MER2\_type | DNA |

  

|  |  |  |  |  |  |  |  |  |  |
| --- | --- | --- | --- | --- | --- | --- | --- | --- | --- |
| chr2:87310646-87312860 | | | 2215 bp | | |  |  |  |  |
| Genome Start | Genome End | Strand | Div | Repeat Start | Repeat End | Repeat Left | Name | Family | Class |
| 87310646 | 87310878 | - | 14.6 | 5934 | 6168 | -3 | L1MB2 | L1 | LINE |
| 87310879 | 87311494 | - | 12.9 | 2 | 766 | 0 | MER4E | ERV1 | LTR |
| 87311495 | 87311640 | - | 14.1 | 5793 | 5938 | -245 | L1MB2 | L1 | LINE |
| 87311641 | 87311849 | + | 16.3 | 2 | 203 | -312 | MLT2B2 | ERVL | LTR |
| 87311850 | 87312142 | + | 6.5 | 1 | 291 | -20 | AluY | Alu | SINE |
| 87312143 | 87312502 | + | 16.3 | 204 | 513 | -2 | MLT2B2 | ERVL | LTR |
| 87312503 | 87312860 | - | 14.1 | 5442 | 5792 | -391 | L1MB2 | L1 | LINE |

  

|  |  |  |  |  |  |  |  |  |  |
| --- | --- | --- | --- | --- | --- | --- | --- | --- | --- |
| chr2:87317215-87318106 | | | 892 bp | | |  |  |  |  |
| Genome Start | Genome End | Strand | Div | Repeat Start | Repeat End | Repeat Left | Name | Family | Class |
| 87317215 | 87317248 | - | 21.0 | 6136 | 6159 | -7 | L1ME1 | L1 | LINE |
| 87317249 | 87317555 | + | 8.9 | 1 | 310 | -3 | AluSp | Alu | SINE |
| 87317556 | 87318106 | - | 21.0 | 5549 | 6135 | -31 | L1ME1 | L1 | LINE |

  

|  |  |  |  |  |  |  |  |  |  |
| --- | --- | --- | --- | --- | --- | --- | --- | --- | --- |
| chr2:87330648-87331425 | | | 778 bp | | |  |  |  |  |
| Genome Start | Genome End | Strand | Div | Repeat Start | Repeat End | Repeat Left | Name | Family | Class |
| 87330648 | 87330769 | - | 35.6 | 3242 | 3377 | -1 | L2 | L2 | LINE |
| 87330770 | 87331232 | + | 20.6 | 1 | 505 | 0 | MLT1D | MaLR | LTR |
| 87331233 | 87331425 | - | 35.6 | 3039 | 3241 | -137 | L2 | L2 | LINE |

  

|  |  |  |  |  |  |  |  |  |  |
| --- | --- | --- | --- | --- | --- | --- | --- | --- | --- |
| chr2:87372020-87373326 | | | 1189 bp | | |  |  |  |  |
| Genome Start | Genome End | Strand | Div | Repeat Start | Repeat End | Repeat Left | Name | Family | Class |
| 87372020 | 87372555 | - | 28.7 | 5472 | 6038 | -1770 | L1MC4a | L1 | LINE |
| 87372556 | 87372597 | + | 12.2 | 1 | 41 | -258 | Alu | Alu | SINE |
| 87372627 | 87372659 | - | 22.4 | 401 | 431 | -5 | MER63B | AcHobo | DNA |
| 87372684 | 87373072 | - | 22.4 | 1 | 400 | -36 | MER63B | AcHobo | DNA |
| 87373138 | 87373326 | - | 28.9 | 5197 | 5388 | -758 | L1MC4a | L1 | LINE |

  

|  |  |  |  |  |  |  |  |  |  |
| --- | --- | --- | --- | --- | --- | --- | --- | --- | --- |
| chr2:87415777-87416165 | | | 389 bp | | |  |  |  |  |
| Genome Start | Genome End | Strand | Div | Repeat Start | Repeat End | Repeat Left | Name | Family | Class |
| 87415777 | 87415889 | - | 24.0 | 3270 | 3383 | -2763 | L1M5 | L1 | LINE |
| 87415890 | 87416149 | - | 11.6 | 38 | 307 | -4 | AluY | Alu | SINE |
| 87416150 | 87416165 | - | 24.0 | 3257 | 3269 | -2877 | L1M5 | L1 | LINE |

  

|  |  |  |  |  |  |  |  |  |  |
| --- | --- | --- | --- | --- | --- | --- | --- | --- | --- |
| chr2:87426955-87428848 | | | 1797 bp | | |  |  |  |  |
| Genome Start | Genome End | Strand | Div | Repeat Start | Repeat End | Repeat Left | Name | Family | Class |
| 87426955 | 87427808 | + | 23.1 | 1892 | 2784 | -3753 | L1MDa | L1 | LINE |
| 87427822 | 87428325 | + | 27.0 | 20 | 519 | 0 | LTR53 | ERVL | LTR |
| 87428410 | 87428677 | + | 21.3 | 1 | 274 | -38 | AluJo | Alu | SINE |
| 87428678 | 87428848 | + | 26.6 | 2971 | 3151 | -3386 | L1MDa | L1 | LINE |

  

|  |  |  |  |  |  |  |  |  |  |
| --- | --- | --- | --- | --- | --- | --- | --- | --- | --- |
| chr2:87465369-87466225 | | | 857 bp | | |  |  |  |  |
| Genome Start | Genome End | Strand | Div | Repeat Start | Repeat End | Repeat Left | Name | Family | Class |
| 87465369 | 87465506 | + | 18.5 | 5769 | 5884 | -282 | L1PA16 | L1 | LINE |
| 87465507 | 87465953 | - | 10.8 | 5708 | 6154 | 0 | L1PA7 | L1 | LINE |
| 87465954 | 87466225 | + | 18.5 | 5885 | 6144 | -22 | L1PA16 | L1 | LINE |

  

|  |  |  |  |  |  |  |  |  |  |
| --- | --- | --- | --- | --- | --- | --- | --- | --- | --- |
| chr2:87466223-87466835 | | | 613 bp | | |  |  |  |  |
| Genome Start | Genome End | Strand | Div | Repeat Start | Repeat End | Repeat Left | Name | Family | Class |
| 87466223 | 87466317 | + | 16.8 | 5603 | 5689 | -477 | L1PA16 | L1 | LINE |
| 87466318 | 87466598 | - | 10.7 | 23 | 305 | -7 | AluSx | Alu | SINE |
| 87466599 | 87466835 | + | 16.8 | 5690 | 5917 | -249 | L1PA16 | L1 | LINE |

  

|  |  |  |  |  |  |  |  |  |  |
| --- | --- | --- | --- | --- | --- | --- | --- | --- | --- |
| chr2:87518891-87519427 | | | 537 bp | | |  |  |  |  |
| Genome Start | Genome End | Strand | Div | Repeat Start | Repeat End | Repeat Left | Name | Family | Class |
| 87518891 | 87519061 | + | 26.5 | 5838 | 6051 | -70 | L1ME4a | L1 | LINE |
| 87519062 | 87519357 | + | 9.1 | 1 | 299 | -11 | AluSg | Alu | SINE |
| 87519358 | 87519427 | + | 27.3 | 6052 | 6120 | -1 | L1ME4a | L1 | LINE |

  

|  |  |  |  |  |  |  |  |  |  |
| --- | --- | --- | --- | --- | --- | --- | --- | --- | --- |
| chr2:87525093-87526646 | | | 1554 bp | | |  |  |  |  |
| Genome Start | Genome End | Strand | Div | Repeat Start | Repeat End | Repeat Left | Name | Family | Class |
| 87525093 | 87525797 | + | 30.5 | 1942 | 2783 | -636 | L2 | L2 | LINE |
| 87525798 | 87526105 | + | 15.6 | 1 | 311 | -1 | AluJb | Alu | SINE |
| 87526106 | 87526646 | + | 32.2 | 2784 | 3419 | 0 | L2 | L2 | LINE |

  

|  |  |  |  |  |  |  |  |  |  |
| --- | --- | --- | --- | --- | --- | --- | --- | --- | --- |
| chr2:87605872-87606826 | | | 682 bp | | |  |  |  |  |
| Genome Start | Genome End | Strand | Div | Repeat Start | Repeat End | Repeat Left | Name | Family | Class |
| 87605872 | 87605939 | - | 17.8 | 3571 | 3632 | -857 | L3 | CR1 | LINE |
| 87605942 | 87606049 | - | 7.4 | 1 | 108 | -201 | AluSc | Alu | SINE |
| 87606065 | 87606111 | - | 23.4 | 3514 | 3560 | -929 | L3 | CR1 | LINE |
| 87606368 | 87606826 | - | 38.8 | 2984 | 3466 | -1023 | L3 | CR1 | LINE |

  

|  |  |  |  |  |  |  |  |  |  |
| --- | --- | --- | --- | --- | --- | --- | --- | --- | --- |
| chr2:87631410-87632383 | | | 954 bp | | |  |  |  |  |
| Genome Start | Genome End | Strand | Div | Repeat Start | Repeat End | Repeat Left | Name | Family | Class |
| 87631410 | 87631526 | + | 28.4 | 28 | 148 | -114 | MIR | MIR | SINE |
| 87631527 | 87631820 | + | 9.2 | 1 | 312 | 0 | AluSx | Alu | SINE |
| 87631841 | 87632284 | + | 9.0 | 5716 | 6160 | -3 | L1PA13 | L1 | LINE |
| 87632285 | 87632383 | + | 28.4 | 149 | 250 | -12 | MIR | MIR | SINE |

  

|  |  |  |  |  |  |  |  |  |  |
| --- | --- | --- | --- | --- | --- | --- | --- | --- | --- |
| chr2:87642232-87646299 | | | 4068 bp | | |  |  |  |  |
| Genome Start | Genome End | Strand | Div | Repeat Start | Repeat End | Repeat Left | Name | Family | Class |
| 87642232 | 87642870 | + | 20.7 | 2983 | 3648 | -2498 | L1MC3 | L1 | LINE |
| 87642871 | 87643257 | - | 17.0 | 1 | 365 | 0 | MLT1A0 | MaLR | LTR |
| 87643258 | 87646299 | + | 20.6 | 3649 | 6742 | -1043 | L1MC3 | L1 | LINE |

  

|  |  |  |  |  |  |  |  |  |  |
| --- | --- | --- | --- | --- | --- | --- | --- | --- | --- |
| chr2:87665849-87668304 | | | 2357 bp | | |  |  |  |  |
| Genome Start | Genome End | Strand | Div | Repeat Start | Repeat End | Repeat Left | Name | Family | Class |
| 87665849 | 87666376 | - | 22.7 | 4992 | 5545 | -625 | L1M5 | L1 | LINE |
| 87666377 | 87666682 | - | 11.6 | 1 | 306 | 0 | AluSx | Alu | SINE |
| 87666683 | 87666856 | - | 22.1 | 4799 | 4991 | -1155 | L1M5 | L1 | LINE |
| 87666882 | 87667370 | - | 26.2 | 45 | 506 | -49 | MLT1H1 | MaLR | LTR |
| 87667445 | 87668304 | - | 22.0 | 3899 | 4770 | -1376 | L1M5 | L1 | LINE |

  

|  |  |  |  |  |  |  |  |  |  |
| --- | --- | --- | --- | --- | --- | --- | --- | --- | --- |
| chr2:87697599-87698086 | | | 488 bp | | |  |  |  |  |
| Genome Start | Genome End | Strand | Div | Repeat Start | Repeat End | Repeat Left | Name | Family | Class |
| 87697599 | 87697661 | + | 27.5 | 1 | 67 | -201 | MIRb | MIR | SINE |
| 87697662 | 87697960 | - | 8.7 | 1 | 303 | -8 | AluY | Alu | SINE |
| 87697961 | 87698086 | + | 27.5 | 68 | 212 | -56 | MIRb | MIR | SINE |

  

|  |  |  |  |  |  |  |  |  |  |
| --- | --- | --- | --- | --- | --- | --- | --- | --- | --- |
| chr2:87729923-87730693 | | | 768 bp | | |  |  |  |  |
| Genome Start | Genome End | Strand | Div | Repeat Start | Repeat End | Repeat Left | Name | Family | Class |
| 87729923 | 87730223 | - | 17.4 | 253 | 559 | 0 | MER89 | ERV1 | LTR |
| 87730227 | 87730534 | + | 11.4 | 1 | 307 | 0 | AluSx | Alu | SINE |
| 87730535 | 87730693 | - | 17.9 | 1 | 259 | -300 | MER89 | ERV1 | LTR |

  

|  |  |  |  |  |  |  |  |  |  |
| --- | --- | --- | --- | --- | --- | --- | --- | --- | --- |
| chr2:87782366-87784338 | | | 1950 bp | | |  |  |  |  |
| Genome Start | Genome End | Strand | Div | Repeat Start | Repeat End | Repeat Left | Name | Family | Class |
| 87782366 | 87782512 | - | 21.4 | 6014 | 6164 | 0 | L1ME2 | L1 | LINE |
| 87782513 | 87782801 | - | 10.7 | 1 | 298 | -12 | AluSg | Alu | SINE |
| 87782802 | 87783167 | - | 21.4 | 5649 | 6013 | -151 | L1ME2 | L1 | LINE |
| 87783177 | 87783455 | - | 15.4 | 1 | 280 | -32 | AluSx | Alu | SINE |
| 87783468 | 87783652 | - | 18.6 | 129 | 312 | 0 | AluJo/FRAM | Alu | SINE |
| 87783654 | 87783751 | + | 22.1 | 101 | 196 | -116 | AluJo | Alu | SINE |
| 87783753 | 87783976 | - | 25.9 | 5135 | 5398 | -748 | L1ME2 | L1 | LINE |
| 87783977 | 87784287 | - | 7.5 | 1 | 306 | 0 | AluY | Alu | SINE |
| 87784288 | 87784338 | - | 25.9 | 5084 | 5134 | -1012 | L1ME2 | L1 | LINE |

  

|  |  |  |  |  |  |  |  |  |  |
| --- | --- | --- | --- | --- | --- | --- | --- | --- | --- |
| chr2:87790551-87791820 | | | 1270 bp | | |  |  |  |  |
| Genome Start | Genome End | Strand | Div | Repeat Start | Repeat End | Repeat Left | Name | Family | Class |
| 87790551 | 87790613 | - | 16.9 | 6136 | 6180 | 0 | L1MB4 | L1 | LINE |
| 87790614 | 87790898 | - | 8.8 | 1 | 288 | -24 | AluSx | Alu | SINE |
| 87790899 | 87791820 | - | 17.0 | 5204 | 6135 | -45 | L1MB4 | L1 | LINE |

  

|  |  |  |  |  |  |  |  |  |  |
| --- | --- | --- | --- | --- | --- | --- | --- | --- | --- |
| chr2:87792680-87793627 | | | 948 bp | | |  |  |  |  |
| Genome Start | Genome End | Strand | Div | Repeat Start | Repeat End | Repeat Left | Name | Family | Class |
| 87792680 | 87792900 | + | 20.2 | 5478 | 5701 | -465 | L1ME1 | L1 | LINE |
| 87792901 | 87793198 | + | 8.7 | 1 | 306 | -3 | AluSc | Alu | SINE |
| 87793199 | 87793627 | + | 20.2 | 5702 | 6143 | -23 | L1ME1 | L1 | LINE |

  

|  |  |  |  |  |  |  |  |  |  |
| --- | --- | --- | --- | --- | --- | --- | --- | --- | --- |
| chr2:87821042-87822887 | | | 1685 bp | | |  |  |  |  |
| Genome Start | Genome End | Strand | Div | Repeat Start | Repeat End | Repeat Left | Name | Family | Class |
| 87821042 | 87821410 | + | 31.7 | 1975 | 2383 | -1036 | L2 | L2 | LINE |
| 87821411 | 87821624 | - | 21.1 | 1 | 224 | 0 | MER58A | MER1\_type | DNA |
| 87821625 | 87821940 | + | 31.7 | 2384 | 2739 | -680 | L2 | L2 | LINE |
| 87822102 | 87822153 | + | 32.8 | 3020 | 3071 | -307 | L2 | L2 | LINE |
| 87822154 | 87822595 | - | 12.3 | 1 | 448 | 0 | MER83 | ERV1 | LTR |
| 87822596 | 87822887 | + | 32.8 | 3072 | 3413 | -6 | L2 | L2 | LINE |

  

|  |  |  |  |  |  |  |  |  |  |
| --- | --- | --- | --- | --- | --- | --- | --- | --- | --- |
| chr2:87840793-87842257 | | | 1465 bp | | |  |  |  |  |
| Genome Start | Genome End | Strand | Div | Repeat Start | Repeat End | Repeat Left | Name | Family | Class |
| 87840793 | 87840834 | - | 16.7 | 1567 | 1607 | -5144 | L1MCb | L1 | LINE |
| 87840835 | 87841182 | - | 19.6 | 2 | 362 | -3 | MLT1A0 | MaLR | LTR |
| 87841183 | 87841241 | - | 16.7 | 1515 | 1566 | -5185 | L1MCb | L1 | LINE |
| 87841242 | 87841550 | - | 8.1 | 1 | 309 | -2 | AluY | Alu | SINE |
| 87841551 | 87842257 | - | 16.7 | 721 | 1514 | -5237 | L1MCb | L1 | LINE |

  

|  |  |  |  |  |  |  |  |  |  |
| --- | --- | --- | --- | --- | --- | --- | --- | --- | --- |
| chr2:87848326-87848804 | | | 479 bp | | |  |  |  |  |
| Genome Start | Genome End | Strand | Div | Repeat Start | Repeat End | Repeat Left | Name | Family | Class |
| 87848326 | 87848389 | - | 23.9 | 96 | 161 | -100 | MER112 | MER1\_type | DNA |
| 87848390 | 87848709 | - | 23.4 | 1 | 305 | -7 | AluJo | Alu | SINE |
| 87848710 | 87848804 | - | 23.9 | 2 | 95 | -165 | MER112 | MER1\_type | DNA |

  

|  |  |  |  |  |  |  |  |  |  |
| --- | --- | --- | --- | --- | --- | --- | --- | --- | --- |
| chr2:87849045-87849978 | | | 922 bp | | |  |  |  |  |
| Genome Start | Genome End | Strand | Div | Repeat Start | Repeat End | Repeat Left | Name | Family | Class |
| 87849045 | 87849177 | - | 27.1 | 1566 | 1713 | -4436 | L1MEd | L1 | LINE |
| 87849190 | 87849592 | - | 24.7 | 1002 | 1517 | -4632 | L1MEd | L1 | LINE |
| 87849593 | 87849900 | - | 9.4 | 1 | 308 | -5 | AluSq | Alu | SINE |
| 87849901 | 87849978 | - | 23.7 | 936 | 1001 | -5118 | L1MEd | L1 | LINE |

  

|  |  |  |  |  |  |  |  |  |  |
| --- | --- | --- | --- | --- | --- | --- | --- | --- | --- |
| chr2:87851029-87851642 | | | 614 bp | | |  |  |  |  |
| Genome Start | Genome End | Strand | Div | Repeat Start | Repeat End | Repeat Left | Name | Family | Class |
| 87851029 | 87851270 | + | 28.9 | 7250 | 7513 | -448 | L1MC5 | L1 | LINE |
| 87851271 | 87851571 | + | 10.7 | 1 | 309 | 0 | AluSc | Alu | SINE |
| 87851572 | 87851642 | + | 28.9 | 7514 | 7588 | -373 | L1MC5 | L1 | LINE |

  

|  |  |  |  |  |  |  |  |  |  |
| --- | --- | --- | --- | --- | --- | --- | --- | --- | --- |
| chr2:87877177-87878552 | | | 1226 bp | | |  |  |  |  |
| Genome Start | Genome End | Strand | Div | Repeat Start | Repeat End | Repeat Left | Name | Family | Class |
| 87877177 | 87877448 | + | 29.1 | 5534 | 5827 | -294 | L1ME4a | L1 | LINE |
| 87877535 | 87877571 | + | 24.3 | 1 | 37 | -96 | AluJo | Alu | SINE |
| 87877572 | 87877860 | - | 9.7 | 1 | 292 | -20 | AluSx | Alu | SINE |
| 87877861 | 87878122 | + | 15.8 | 26 | 286 | -26 | AluJo | Alu | SINE |
| 87878131 | 87878437 | + | 10.1 | 1 | 307 | -6 | AluSq | Alu | SINE |
| 87878494 | 87878552 | + | 22.0 | 5978 | 6038 | -83 | L1ME4a | L1 | LINE |

  

|  |  |  |  |  |  |  |  |  |  |
| --- | --- | --- | --- | --- | --- | --- | --- | --- | --- |
| chr2:87879153-87880301 | | | 1149 bp | | |  |  |  |  |
| Genome Start | Genome End | Strand | Div | Repeat Start | Repeat End | Repeat Left | Name | Family | Class |
| 87879153 | 87879207 | - | 36.5 | 122 | 172 | -6 | MER5B | MER1\_type | DNA |
| 87879208 | 87879518 | + | 7.4 | 1 | 300 | -11 | AluY | Alu | SINE |
| 87879519 | 87879592 | - | 36.5 | 55 | 121 | -57 | MER5B | MER1\_type | DNA |
| 87879593 | 87879720 | + | 17.4 | 5820 | 5949 | -229 | L1MB8 | L1 | LINE |
| 87879721 | 87880019 | + | 1.0 | 1 | 299 | -11 | AluYa5 | Alu | SINE |
| 87880020 | 87880237 | + | 17.4 | 5950 | 6163 | -15 | L1MB8 | L1 | LINE |
| 87880238 | 87880301 | - | 36.5 | 2 | 54 | -124 | MER5B | MER1\_type | DNA |

  

|  |  |  |  |  |  |  |  |  |  |
| --- | --- | --- | --- | --- | --- | --- | --- | --- | --- |
| chr2:87883560-87884027 | | | 468 bp | | |  |  |  |  |
| Genome Start | Genome End | Strand | Div | Repeat Start | Repeat End | Repeat Left | Name | Family | Class |
| 87883560 | 87883604 | - | 31.2 | 3408 | 3452 | -1037 | L3 | CR1 | LINE |
| 87883605 | 87883905 | - | 11.8 | 1 | 299 | -13 | AluSx | Alu | SINE |
| 87883906 | 87884027 | - | 31.2 | 3286 | 3407 | -1082 | L3 | CR1 | LINE |

  

|  |  |  |  |  |  |  |  |  |  |
| --- | --- | --- | --- | --- | --- | --- | --- | --- | --- |
| chr2:87902827-87903943 | | | 1069 bp | | |  |  |  |  |
| Genome Start | Genome End | Strand | Div | Repeat Start | Repeat End | Repeat Left | Name | Family | Class |
| 87902827 | 87903010 | + | 18.4 | 1113 | 1344 | -1163 | HAL1 | L1 | LINE |
| 87903031 | 87903187 | + | 27.1 | 1480 | 1667 | -840 | HAL1 | L1 | LINE |
| 87903188 | 87903414 | + | 11.9 | 78 | 304 | -8 | AluSg/x | Alu | SINE |
| 87903415 | 87903647 | + | 27.1 | 1668 | 1903 | -604 | HAL1 | L1 | LINE |
| 87903676 | 87903943 | + | 27.1 | 1904 | 2217 | -290 | HAL1 | L1 | LINE |

  

|  |  |  |  |  |  |  |  |  |  |
| --- | --- | --- | --- | --- | --- | --- | --- | --- | --- |
| chr2:87935268-87936017 | | | 722 bp | | |  |  |  |  |
| Genome Start | Genome End | Strand | Div | Repeat Start | Repeat End | Repeat Left | Name | Family | Class |
| 87935268 | 87935482 | - | 15.0 | 5688 | 5903 | -441 | L1MC2 | L1 | LINE |
| 87935483 | 87935631 | - | 15.3 | 6155 | 6311 | -1 | L1MA9 | L1 | LINE |
| 87935660 | 87935822 | - | 15.3 | 5997 | 6154 | -158 | L1MA9 | L1 | LINE |
| 87935823 | 87936017 | - | 15.0 | 5503 | 5687 | -657 | L1MC2 | L1 | LINE |

  

|  |  |  |  |  |  |  |  |  |  |
| --- | --- | --- | --- | --- | --- | --- | --- | --- | --- |
| chr2:87950469-87951755 | | | 1114 bp | | |  |  |  |  |
| Genome Start | Genome End | Strand | Div | Repeat Start | Repeat End | Repeat Left | Name | Family | Class |
| 87950469 | 87950684 | + | 36.8 | 2059 | 2284 | -1135 | L2 | L2 | LINE |
| 87950770 | 87951268 | + | 28.3 | 58 | 539 | -83 | MER74B | ERVL | LTR |
| 87951357 | 87951755 | + | 36.8 | 2421 | 2828 | -591 | L2 | L2 | LINE |

  

|  |  |  |  |  |  |  |  |  |  |
| --- | --- | --- | --- | --- | --- | --- | --- | --- | --- |
| chr2:87953145-87959104 | | | 5916 bp | | |  |  |  |  |
| Genome Start | Genome End | Strand | Div | Repeat Start | Repeat End | Repeat Left | Name | Family | Class |
| 87953145 | 87953403 | - | 9.7 | 6123 | 6356 | -3 | L1PA11 | L1 | LINE |
| 87953404 | 87953691 | - | 12.8 | 1 | 288 | -22 | AluSg | Alu | SINE |
| 87953692 | 87954206 | - | 9.7 | 5631 | 6122 | -237 | L1PA11 | L1 | LINE |
| 87954207 | 87954574 | - | 15.2 | 1 | 364 | 0 | THE1B | MaLR | LTR |
| 87954575 | 87956299 | - | 12.1 | 3882 | 5630 | -729 | L1PA11 | L1 | LINE |
| 87956324 | 87956761 | - | 12.4 | 3420 | 3881 | -2450 | L1PA11 | L1 | LINE |
| 87956782 | 87958155 | - | 12.0 | 2027 | 3419 | -2912 | L1PA11 | L1 | LINE |
| 87958156 | 87958468 | + | 8.0 | 1 | 311 | 0 | AluY | Alu | SINE |
| 87958469 | 87959104 | - | 11.2 | 1390 | 2026 | -4327 | L1PA11 | L1 | LINE |

  

|  |  |  |  |  |  |  |  |  |  |
| --- | --- | --- | --- | --- | --- | --- | --- | --- | --- |
| chr2:87963641-87966204 | | | 2564 bp | | |  |  |  |  |
| Genome Start | Genome End | Strand | Div | Repeat Start | Repeat End | Repeat Left | Name | Family | Class |
| 87963641 | 87965681 | + | 21.6 | 3069 | 5147 | -2415 | L1M1 | L1 | LINE |
| 87965682 | 87965736 | + | 25.4 | 1 | 55 | -2268 | Tigger5 | MER2\_type | DNA |
| 87965737 | 87966204 | + | 21.6 | 5148 | 5623 | -1939 | L1M1 | L1 | LINE |

  

|  |  |  |  |  |  |  |  |  |  |
| --- | --- | --- | --- | --- | --- | --- | --- | --- | --- |
| chr2:88008682-88013989 | | | 5263 bp | | |  |  |  |  |
| Genome Start | Genome End | Strand | Div | Repeat Start | Repeat End | Repeat Left | Name | Family | Class |
| 88008682 | 88010109 | - | 11.5 | 5319 | 6780 | -3 | L1PA13 | L1 | LINE |
| 88010155 | 88012179 | - | 13.1 | 3303 | 5318 | -1448 | L1PA13 | L1 | LINE |
| 88012180 | 88012552 | + | 9.2 | 1 | 373 | -2 | THE1C | MaLR | LTR |
| 88012553 | 88013989 | - | 13.0 | 1863 | 3302 | -3464 | L1PA13 | L1 | LINE |

  

|  |  |  |  |  |  |  |  |  |  |
| --- | --- | --- | --- | --- | --- | --- | --- | --- | --- |
| chr2:88017575-88018773 | | | 1031 bp | | |  |  |  |  |
| Genome Start | Genome End | Strand | Div | Repeat Start | Repeat End | Repeat Left | Name | Family | Class |
| 88017575 | 88017741 | - | 29.4 | 3233 | 3419 | 0 | L2 | L2 | LINE |
| 88017910 | 88017977 | - | 34.5 | 2218 | 2278 | -1141 | L2 | L2 | LINE |
| 88017978 | 88018369 | - | 18.6 | 1 | 426 | 0 | MSTB | MaLR | LTR |
| 88018370 | 88018773 | - | 34.5 | 1796 | 2217 | -1202 | L2 | L2 | LINE |

  

|  |  |  |  |  |  |  |  |  |  |
| --- | --- | --- | --- | --- | --- | --- | --- | --- | --- |
| chr2:88022383-88025969 | | | 3392 bp | | |  |  |  |  |
| Genome Start | Genome End | Strand | Div | Repeat Start | Repeat End | Repeat Left | Name | Family | Class |
| 88022383 | 88022552 | + | 31.4 | 5464 | 5650 | -590 | L1ME3B | L1 | LINE |
| 88022558 | 88023075 | + | 26.4 | 1 | 510 | -945 | Charlie1a | MER1\_type | DNA |
| 88023103 | 88024657 | - | 17.6 | 4742 | 6300 | 0 | L1MA5 | L1 | LINE |
| 88024658 | 88025584 | + | 20.8 | 1816 | 2761 | 0 | Charlie1 | MER1\_type | DNA |
| 88025599 | 88025696 | - | 13.3 | 6067 | 6169 | -2 | L1MB2 | L1 | LINE |
| 88025846 | 88025969 | + | 32.0 | 5771 | 5893 | -347 | L1ME3B | L1 | LINE |

  

|  |  |  |  |  |  |  |  |  |  |
| --- | --- | --- | --- | --- | --- | --- | --- | --- | --- |
| chr2:88043880-88044389 | | | 510 bp | | |  |  |  |  |
| Genome Start | Genome End | Strand | Div | Repeat Start | Repeat End | Repeat Left | Name | Family | Class |
| 88043880 | 88044026 | - | 27.2 | 115 | 261 | -1 | MIR | MIR | SINE |
| 88044027 | 88044339 | + | 16.3 | 1 | 308 | -4 | AluJo | Alu | SINE |
| 88044340 | 88044389 | - | 27.2 | 74 | 114 | -148 | MIR | MIR | SINE |

  

|  |  |  |  |  |  |  |  |  |  |
| --- | --- | --- | --- | --- | --- | --- | --- | --- | --- |
| chr2:88908210-88908731 | | | 522 bp | | |  |  |  |  |
| Genome Start | Genome End | Strand | Div | Repeat Start | Repeat End | Repeat Left | Name | Family | Class |
| 88908210 | 88908287 | + | 31.0 | 11 | 91 | -169 | MER112 | MER1\_type | DNA |
| 88908288 | 88908581 | + | 20.0 | 3 | 307 | -5 | AluJo | Alu | SINE |
| 88908582 | 88908731 | + | 31.0 | 92 | 233 | -28 | MER112 | MER1\_type | DNA |

  

|  |  |  |  |  |  |  |  |  |  |
| --- | --- | --- | --- | --- | --- | --- | --- | --- | --- |
| chr2:89026644-89027573 | | | 930 bp | | |  |  |  |  |
| Genome Start | Genome End | Strand | Div | Repeat Start | Repeat End | Repeat Left | Name | Family | Class |
| 89026644 | 89026751 | + | 21.4 | 1 | 115 | -346 | MER65C | ERV1 | LTR |
| 89026752 | 89027237 | + | 11.7 | 1 | 497 | 0 | LOR1a | ERV1 | LTR |
| 89027238 | 89027573 | + | 21.4 | 116 | 457 | -4 | MER65C | ERV1 | LTR |

  

|  |  |  |  |  |  |  |  |  |  |
| --- | --- | --- | --- | --- | --- | --- | --- | --- | --- |
| chr2:89071747-89072682 | | | 936 bp | | |  |  |  |  |
| Genome Start | Genome End | Strand | Div | Repeat Start | Repeat End | Repeat Left | Name | Family | Class |
| 89071747 | 89071853 | + | 21.8 | 3 | 112 | -349 | MER65C | ERV1 | LTR |
| 89071854 | 89072330 | + | 14.7 | 1 | 495 | -2 | LOR1a | ERV1 | LTR |
| 89072331 | 89072682 | + | 21.8 | 113 | 457 | -4 | MER65C | ERV1 | LTR |

  

|  |  |  |  |  |  |  |  |  |  |
| --- | --- | --- | --- | --- | --- | --- | --- | --- | --- |
| chr2:89075297-89076403 | | | 1107 bp | | |  |  |  |  |
| Genome Start | Genome End | Strand | Div | Repeat Start | Repeat End | Repeat Left | Name | Family | Class |
| 89075297 | 89075360 | + | 23.0 | 3 | 58 | -447 | MLT1D | MaLR | LTR |
| 89075361 | 89075998 | + | 14.7 | 3 | 644 | 0 | LTR9B | ERV1 | LTR |
| 89075999 | 89076403 | + | 23.0 | 59 | 505 | 0 | MLT1D | MaLR | LTR |

  

|  |  |  |  |  |  |  |  |  |  |
| --- | --- | --- | --- | --- | --- | --- | --- | --- | --- |
| chr2:89089310-89090233 | | | 924 bp | | |  |  |  |  |
| Genome Start | Genome End | Strand | Div | Repeat Start | Repeat End | Repeat Left | Name | Family | Class |
| 89089310 | 89089416 | + | 23.1 | 1 | 115 | -346 | MER65C | ERV1 | LTR |
| 89089417 | 89089899 | + | 14.1 | 1 | 497 | 0 | LOR1a | ERV1 | LTR |
| 89089900 | 89090233 | + | 23.1 | 116 | 457 | -4 | MER65C | ERV1 | LTR |

  

|  |  |  |  |  |  |  |  |  |  |
| --- | --- | --- | --- | --- | --- | --- | --- | --- | --- |
| chr2:89119537-89120476 | | | 940 bp | | |  |  |  |  |
| Genome Start | Genome End | Strand | Div | Repeat Start | Repeat End | Repeat Left | Name | Family | Class |
| 89119537 | 89119644 | + | 21.9 | 1 | 112 | -349 | MER65C | ERV1 | LTR |
| 89119645 | 89120143 | + | 13.2 | 1 | 497 | 0 | LOR1a | ERV1 | LTR |
| 89120144 | 89120476 | + | 21.9 | 113 | 456 | -5 | MER65C | ERV1 | LTR |

  

|  |  |  |  |  |  |  |  |  |  |
| --- | --- | --- | --- | --- | --- | --- | --- | --- | --- |
| chr2:89125337-89126244 | | | 908 bp | | |  |  |  |  |
| Genome Start | Genome End | Strand | Div | Repeat Start | Repeat End | Repeat Left | Name | Family | Class |
| 89125337 | 89125444 | + | 22.6 | 1 | 112 | -349 | MER65C | ERV1 | LTR |
| 89125445 | 89125924 | + | 12.9 | 1 | 497 | 0 | LOR1a | ERV1 | LTR |
| 89125925 | 89126244 | + | 22.6 | 113 | 461 | 0 | MER65C | ERV1 | LTR |

  

|  |  |  |  |  |  |  |  |  |  |
| --- | --- | --- | --- | --- | --- | --- | --- | --- | --- |
| chr2:89129011-89129582 | | | 572 bp | | |  |  |  |  |
| Genome Start | Genome End | Strand | Div | Repeat Start | Repeat End | Repeat Left | Name | Family | Class |
| 89129011 | 89129066 | + | 30.7 | 134 | 189 | -302 | LTR16C | ERVL | LTR |
| 89129067 | 89129456 | + | 26.1 | 1 | 406 | -2 | MLT1A1 | MaLR | LTR |
| 89129457 | 89129582 | + | 30.7 | 190 | 325 | -166 | LTR16C | ERVL | LTR |

  

|  |  |  |  |  |  |  |  |  |  |
| --- | --- | --- | --- | --- | --- | --- | --- | --- | --- |
| chr2:89168547-89170439 | | | 1893 bp | | |  |  |  |  |
| Genome Start | Genome End | Strand | Div | Repeat Start | Repeat End | Repeat Left | Name | Family | Class |
| 89168547 | 89168997 | - | 22.8 | 1182 | 1618 | -5058 | MER66A-int | ERV1 | LTR |
| 89168998 | 89169308 | - | 15.6 | 7 | 312 | 0 | AluSx | Alu | SINE |
| 89169309 | 89170439 | - | 22.8 | 30 | 1181 | -5495 | MER66A-int | ERV1 | LTR |

  

|  |  |  |  |  |  |  |  |  |  |
| --- | --- | --- | --- | --- | --- | --- | --- | --- | --- |
| chr2:89179183-89180114 | | | 932 bp | | |  |  |  |  |
| Genome Start | Genome End | Strand | Div | Repeat Start | Repeat End | Repeat Left | Name | Family | Class |
| 89179183 | 89179289 | + | 22.0 | 3 | 112 | -349 | MER65C | ERV1 | LTR |
| 89179290 | 89179774 | + | 15.2 | 1 | 495 | -2 | LOR1a | ERV1 | LTR |
| 89179775 | 89180114 | + | 22.0 | 113 | 461 | 0 | MER65C | ERV1 | LTR |

  

|  |  |  |  |  |  |  |  |  |  |
| --- | --- | --- | --- | --- | --- | --- | --- | --- | --- |
| chr2:89185148-89185980 | | | 833 bp | | |  |  |  |  |
| Genome Start | Genome End | Strand | Div | Repeat Start | Repeat End | Repeat Left | Name | Family | Class |
| 89185148 | 89185206 | + | 23.2 | 6 | 58 | -447 | MLT1D | MaLR | LTR |
| 89185207 | 89185830 | + | 14.7 | 1 | 644 | 0 | LTR9B | ERV1 | LTR |
| 89185831 | 89185980 | + | 23.2 | 59 | 228 | -277 | MLT1D | MaLR | LTR |

  

|  |  |  |  |  |  |  |  |  |  |
| --- | --- | --- | --- | --- | --- | --- | --- | --- | --- |
| chr2:89196666-89197589 | | | 924 bp | | |  |  |  |  |
| Genome Start | Genome End | Strand | Div | Repeat Start | Repeat End | Repeat Left | Name | Family | Class |
| 89196666 | 89196773 | + | 23.4 | 1 | 115 | -346 | MER65C | ERV1 | LTR |
| 89196774 | 89197254 | + | 13.3 | 1 | 497 | 0 | LOR1a | ERV1 | LTR |
| 89197255 | 89197589 | + | 23.4 | 116 | 457 | -4 | MER65C | ERV1 | LTR |

  

|  |  |  |  |  |  |  |  |  |  |
| --- | --- | --- | --- | --- | --- | --- | --- | --- | --- |
| chr2:89217694-89218569 | | | 832 bp | | |  |  |  |  |
| Genome Start | Genome End | Strand | Div | Repeat Start | Repeat End | Repeat Left | Name | Family | Class |
| 89217694 | 89217825 | + | 19.7 | 5931 | 6085 | -206 | L1MA7 | L1 | LINE |
| 89217868 | 89218375 | - | 22.9 | 3 | 501 | -30 | LTR26B | ERV1 | LTR |
| 89218378 | 89218569 | + | 15.6 | 6088 | 6291 | 0 | L1MA7 | L1 | LINE |

  

|  |  |  |  |  |  |  |  |  |  |
| --- | --- | --- | --- | --- | --- | --- | --- | --- | --- |
| chr2:89230535-89231320 | | | 786 bp | | |  |  |  |  |
| Genome Start | Genome End | Strand | Div | Repeat Start | Repeat End | Repeat Left | Name | Family | Class |
| 89230535 | 89230820 | + | 20.5 | 32 | 300 | -205 | MLT1D | MaLR | LTR |
| 89230821 | 89231130 | - | 6.1 | 1 | 310 | -1 | AluY | Alu | SINE |
| 89231131 | 89231320 | + | 20.5 | 301 | 505 | 0 | MLT1D | MaLR | LTR |

  

|  |  |  |  |  |  |  |  |  |  |
| --- | --- | --- | --- | --- | --- | --- | --- | --- | --- |
| chr2:89305555-89306653 | | | 1099 bp | | |  |  |  |  |
| Genome Start | Genome End | Strand | Div | Repeat Start | Repeat End | Repeat Left | Name | Family | Class |
| 89305555 | 89305773 | - | 25.8 | 301 | 537 | -11 | LTR55 | ERV | LTR |
| 89305774 | 89306358 | - | 7.9 | 4 | 608 | 0 | LTR14B | ERVK | LTR |
| 89306359 | 89306653 | - | 25.8 | 1 | 300 | -248 | LTR55 | ERV | LTR |

  

|  |  |  |  |  |  |  |  |  |  |
| --- | --- | --- | --- | --- | --- | --- | --- | --- | --- |
| chr2:89317783-89318615 | | | 833 bp | | |  |  |  |  |
| Genome Start | Genome End | Strand | Div | Repeat Start | Repeat End | Repeat Left | Name | Family | Class |
| 89317783 | 89318178 | - | 26.2 | 150 | 543 | -5 | LTR55 | ERV | LTR |
| 89318179 | 89318477 | + | 10.1 | 1 | 298 | -13 | AluY | Alu | SINE |
| 89318478 | 89318615 | - | 26.2 | 1 | 149 | -399 | LTR55 | ERV | LTR |

  

|  |  |  |  |  |  |  |  |  |  |
| --- | --- | --- | --- | --- | --- | --- | --- | --- | --- |
| chr2:89346251-89347973 | | | 1723 bp | | |  |  |  |  |
| Genome Start | Genome End | Strand | Div | Repeat Start | Repeat End | Repeat Left | Name | Family | Class |
| 89346251 | 89346923 | - | 10.7 | 5493 | 6168 | 0 | L1PA10 | L1 | LINE |
| 89346924 | 89347217 | - | 17.5 | 12 | 303 | -9 | AluSx | Alu | SINE |
| 89347218 | 89347973 | - | 9.9 | 4742 | 5492 | -676 | L1PA10 | L1 | LINE |

  

|  |  |  |  |  |  |  |  |  |  |
| --- | --- | --- | --- | --- | --- | --- | --- | --- | --- |
| chr2:89588101-89589293 | | | 1143 bp | | |  |  |  |  |
| Genome Start | Genome End | Strand | Div | Repeat Start | Repeat End | Repeat Left | Name | Family | Class |
| 89588101 | 89588344 | - | 24.3 | 154 | 406 | -2 | MLT1A1 | MaLR | LTR |
| 89588346 | 89589029 | + | 20.1 | 1 | 687 | -96 | LTR39 | ERV1 | LTR |
| 89589060 | 89589136 | + | 20.1 | 688 | 775 | -8 | LTR39 | ERV1 | LTR |
| 89589156 | 89589293 | - | 20.4 | 1 | 139 | -257 | MLT1A1 | MaLR | LTR |

  

|  |  |  |  |  |  |  |  |  |  |
| --- | --- | --- | --- | --- | --- | --- | --- | --- | --- |
| chr2:89596117-89599196 | | | 3080 bp | | |  |  |  |  |
| Genome Start | Genome End | Strand | Div | Repeat Start | Repeat End | Repeat Left | Name | Family | Class |
| 89596117 | 89597692 | - | 10.0 | 4648 | 6157 | -11 | L1PA10 | L1 | LINE |
| 89597693 | 89597996 | - | 7.9 | 1 | 304 | -7 | AluY | Alu | SINE |
| 89597997 | 89599196 | - | 10.4 | 3425 | 4647 | -1499 | L1PA10 | L1 | LINE |

  

|  |  |  |  |  |  |  |  |  |  |
| --- | --- | --- | --- | --- | --- | --- | --- | --- | --- |
| chr2:89606459-89608575 | | | 1690 bp | | |  |  |  |  |
| Genome Start | Genome End | Strand | Div | Repeat Start | Repeat End | Repeat Left | Name | Family | Class |
| 89606459 | 89606631 | + | 23.2 | 4471 | 4656 | -1490 | L1M5 | L1 | LINE |
| 89606867 | 89607177 | + | 13.5 | 1 | 312 | 0 | AluSx | Alu | SINE |
| 89607200 | 89607511 | + | 14.9 | 1 | 312 | 0 | AluSx | Alu | SINE |
| 89607627 | 89607932 | + | 10.5 | 1 | 310 | 0 | AluSg | Alu | SINE |
| 89607967 | 89608300 | - | 20.7 | 1 | 380 | -28 | MLT1A1 | MaLR | LTR |
| 89608322 | 89608575 | + | 28.8 | 4906 | 5151 | -995 | L1M5 | L1 | LINE |

  

|  |  |  |  |  |  |  |  |  |  |
| --- | --- | --- | --- | --- | --- | --- | --- | --- | --- |
| chr2:89658439-89659780 | | | 1342 bp | | |  |  |  |  |
| Genome Start | Genome End | Strand | Div | Repeat Start | Repeat End | Repeat Left | Name | Family | Class |
| 89658439 | 89658494 | - | 19.0 | 675 | 727 | 0 | LTR8A | ERV1 | LTR |
| 89658495 | 89659176 | - | 9.0 | 1 | 691 | 0 | LTR8 | ERV1 | LTR |
| 89659177 | 89659780 | - | 18.3 | 1 | 674 | -53 | LTR8A | ERV1 | LTR |

  

|  |  |  |  |  |  |  |  |  |  |
| --- | --- | --- | --- | --- | --- | --- | --- | --- | --- |
| chr2:89659781-89661365 | | | 1585 bp | | |  |  |  |  |
| Genome Start | Genome End | Strand | Div | Repeat Start | Repeat End | Repeat Left | Name | Family | Class |
| 89659781 | 89660425 | + | 27.2 | 1581 | 2341 | -4993 | L1M4b | L1 | LINE |
| 89660426 | 89661207 | - | 8.2 | 1 | 781 | 0 | MER4E1 | ERV1 | LTR |
| 89661208 | 89661365 | + | 27.2 | 2342 | 2495 | -4839 | L1M4b | L1 | LINE |

  

|  |  |  |  |  |  |  |  |  |  |
| --- | --- | --- | --- | --- | --- | --- | --- | --- | --- |
| chr2:89666640-89668369 | | | 1733 bp | | |  |  |  |  |
| Genome Start | Genome End | Strand | Div | Repeat Start | Repeat End | Repeat Left | Name | Family | Class |
| 89666640 | 89667344 | + | 29.9 | 4123 | 4897 | -1249 | L1ME3A | L1 | LINE |
| 89667342 | 89667613 | + | 31.9 | 5383 | 5700 | -470 | L1ME3A | L1 | LINE |
| 89667614 | 89667942 | + | 8.9 | 1 | 329 | 0 | AluSg | Alu | SINE |
| 89667943 | 89668369 | + | 28.9 | 5701 | 6127 | -43 | L1ME3A | L1 | LINE |

  

|  |  |  |  |  |  |  |  |  |  |
| --- | --- | --- | --- | --- | --- | --- | --- | --- | --- |
| chr2:89759797-89760718 | | | 922 bp | | |  |  |  |  |
| Genome Start | Genome End | Strand | Div | Repeat Start | Repeat End | Repeat Left | Name | Family | Class |
| 89759797 | 89760131 | - | 23.4 | 116 | 457 | -4 | MER65C | ERV1 | LTR |
| 89760132 | 89760610 | - | 13.8 | 1 | 497 | 0 | LOR1a | ERV1 | LTR |
| 89760611 | 89760718 | - | 23.4 | 1 | 115 | -346 | MER65C | ERV1 | LTR |

  

|  |  |  |  |  |  |  |  |  |  |
| --- | --- | --- | --- | --- | --- | --- | --- | --- | --- |
| chr2:89777242-89778168 | | | 927 bp | | |  |  |  |  |
| Genome Start | Genome End | Strand | Div | Repeat Start | Repeat End | Repeat Left | Name | Family | Class |
| 89777242 | 89777577 | - | 21.5 | 111 | 457 | -4 | MER65C | ERV1 | LTR |
| 89777578 | 89778061 | - | 15.4 | 1 | 495 | -2 | LOR1a | ERV1 | LTR |
| 89778062 | 89778168 | - | 21.5 | 3 | 115 | -346 | MER65C | ERV1 | LTR |

  

|  |  |  |  |  |  |  |  |  |  |
| --- | --- | --- | --- | --- | --- | --- | --- | --- | --- |
| chr2:89820204-89826600 | | | 6397 bp | | |  |  |  |  |
| Genome Start | Genome End | Strand | Div | Repeat Start | Repeat End | Repeat Left | Name | Family | Class |
| 89820204 | 89825295 | - | 18.2 | 2670 | 7713 | 0 | HERVH | ERV1 | LTR |
| 89825296 | 89825765 | - | 7.0 | 1 | 483 | 0 | LTR2B | ERV1 | LTR |
| 89825766 | 89826600 | - | 18.2 | 1793 | 2669 | -5044 | HERVH | ERV1 | LTR |

  

|  |  |  |  |  |  |  |  |  |  |
| --- | --- | --- | --- | --- | --- | --- | --- | --- | --- |
| chr2:89831087-89831994 | | | 908 bp | | |  |  |  |  |
| Genome Start | Genome End | Strand | Div | Repeat Start | Repeat End | Repeat Left | Name | Family | Class |
| 89831087 | 89831406 | - | 22.8 | 112 | 461 | 0 | MER65C | ERV1 | LTR |
| 89831407 | 89831886 | - | 12.9 | 1 | 497 | 0 | LOR1a | ERV1 | LTR |
| 89831887 | 89831994 | - | 22.8 | 2 | 111 | -350 | MER65C | ERV1 | LTR |

  

|  |  |  |  |  |  |  |  |  |  |
| --- | --- | --- | --- | --- | --- | --- | --- | --- | --- |
| chr2:89836855-89837794 | | | 940 bp | | |  |  |  |  |
| Genome Start | Genome End | Strand | Div | Repeat Start | Repeat End | Repeat Left | Name | Family | Class |
| 89836855 | 89837187 | - | 21.9 | 113 | 456 | -5 | MER65C | ERV1 | LTR |
| 89837188 | 89837686 | - | 13.2 | 1 | 497 | 0 | LOR1a | ERV1 | LTR |
| 89837687 | 89837794 | - | 21.9 | 1 | 112 | -349 | MER65C | ERV1 | LTR |

  

|  |  |  |  |  |  |  |  |  |  |
| --- | --- | --- | --- | --- | --- | --- | --- | --- | --- |
| chr2:89854049-89855314 | | | 1261 bp | | |  |  |  |  |
| Genome Start | Genome End | Strand | Div | Repeat Start | Repeat End | Repeat Left | Name | Family | Class |
| 89854049 | 89854410 | + | 29.6 | 1512 | 1928 | -4976 | L1M4b | L1 | LINE |
| 89854415 | 89854634 | - | 12.7 | 5754 | 5971 | -195 | L1PA16 | L1 | LINE |
| 89854635 | 89854840 | + | 19.5 | 5962 | 6166 | 0 | L1PA16 | L1 | LINE |
| 89854842 | 89855314 | + | 28.7 | 1930 | 2473 | -4861 | L1M4b | L1 | LINE |

  

|  |  |  |  |  |  |  |  |  |  |
| --- | --- | --- | --- | --- | --- | --- | --- | --- | --- |
| chr2:89887061-89887985 | | | 925 bp | | |  |  |  |  |
| Genome Start | Genome End | Strand | Div | Repeat Start | Repeat End | Repeat Left | Name | Family | Class |
| 89887061 | 89887394 | - | 22.6 | 116 | 457 | -4 | MER65C | ERV1 | LTR |
| 89887395 | 89887877 | - | 15.3 | 1 | 497 | 0 | LOR1a | ERV1 | LTR |
| 89887878 | 89887985 | - | 22.6 | 1 | 115 | -346 | MER65C | ERV1 | LTR |

  

|  |  |  |  |  |  |  |  |  |  |
| --- | --- | --- | --- | --- | --- | --- | --- | --- | --- |
| chr2:89897914-89898836 | | | 923 bp | | |  |  |  |  |
| Genome Start | Genome End | Strand | Div | Repeat Start | Repeat End | Repeat Left | Name | Family | Class |
| 89897914 | 89898250 | - | 20.8 | 113 | 457 | -4 | MER65C | ERV1 | LTR |
| 89898251 | 89898729 | - | 14.5 | 1 | 495 | -2 | LOR1a | ERV1 | LTR |
| 89898730 | 89898836 | - | 20.8 | 3 | 112 | -349 | MER65C | ERV1 | LTR |

  

|  |  |  |  |  |  |  |  |  |  |
| --- | --- | --- | --- | --- | --- | --- | --- | --- | --- |
| chr2:89907624-89908012 | | | 389 bp | | |  |  |  |  |
| Genome Start | Genome End | Strand | Div | Repeat Start | Repeat End | Repeat Left | Name | Family | Class |
| 89907624 | 89907736 | - | 25.6 | 3270 | 3383 | -2763 | L1M5 | L1 | LINE |
| 89907737 | 89907996 | - | 12.4 | 38 | 307 | -4 | AluY | Alu | SINE |
| 89907997 | 89908012 | - | 25.6 | 3257 | 3269 | -2877 | L1M5 | L1 | LINE |

  

|  |  |  |  |  |  |  |  |  |  |
| --- | --- | --- | --- | --- | --- | --- | --- | --- | --- |
| chr2:89951330-89951959 | | | 630 bp | | |  |  |  |  |
| Genome Start | Genome End | Strand | Div | Repeat Start | Repeat End | Repeat Left | Name | Family | Class |
| 89951330 | 89951506 | - | 10.6 | 137 | 308 | -2 | AluSg | Alu | SINE |
| 89951507 | 89951817 | - | 9.5 | 1 | 304 | -7 | AluY | Alu | SINE |
| 89951818 | 89951959 | - | 10.6 | 1 | 136 | -174 | AluSg | Alu | SINE |

  

|  |  |  |  |  |  |  |  |  |  |
| --- | --- | --- | --- | --- | --- | --- | --- | --- | --- |
| chr2:89953678-89954282 | | | 605 bp | | |  |  |  |  |
| Genome Start | Genome End | Strand | Div | Repeat Start | Repeat End | Repeat Left | Name | Family | Class |
| 89953678 | 89953768 | + | 15.6 | 5611 | 5689 | -477 | L1PA16 | L1 | LINE |
| 89953769 | 89954045 | - | 10.5 | 23 | 301 | -11 | AluSx | Alu | SINE |
| 89954046 | 89954282 | + | 15.6 | 5690 | 5917 | -249 | L1PA16 | L1 | LINE |

  

|  |  |  |  |  |  |  |  |  |  |
| --- | --- | --- | --- | --- | --- | --- | --- | --- | --- |
| chr2:89954598-89955681 | | | 841 bp | | |  |  |  |  |
| Genome Start | Genome End | Strand | Div | Repeat Start | Repeat End | Repeat Left | Name | Family | Class |
| 89954598 | 89955034 | + | 17.8 | 5718 | 6033 | -133 | L1PA16 | L1 | LINE |
| 89955144 | 89955445 | - | 9.9 | 1 | 302 | -10 | AluSx | Alu | SINE |
| 89955580 | 89955681 | + | 17.6 | 6040 | 6141 | -25 | L1PA16 | L1 | LINE |

  

|  |  |  |  |  |  |  |  |  |  |
| --- | --- | --- | --- | --- | --- | --- | --- | --- | --- |
| chr2:91018300-91019491 | | | 1145 bp | | |  |  |  |  |
| Genome Start | Genome End | Strand | Div | Repeat Start | Repeat End | Repeat Left | Name | Family | Class |
| 91018300 | 91018713 | + | 25.1 | 1 | 430 | -471 | MER54A | ERVL | LTR |
| 91018752 | 91019089 | - | 12.3 | 7 | 364 | 0 | THE1B | MaLR | LTR |
| 91019099 | 91019491 | + | 23.7 | 490 | 901 | 0 | MER54A | ERVL | LTR |

  

|  |  |  |  |  |  |  |  |  |  |
| --- | --- | --- | --- | --- | --- | --- | --- | --- | --- |
| chr2:91021977-91022799 | | | 823 bp | | |  |  |  |  |
| Genome Start | Genome End | Strand | Div | Repeat Start | Repeat End | Repeat Left | Name | Family | Class |
| 91021977 | 91022219 | - | 28.4 | 275 | 504 | -1 | MLT1D | MaLR | LTR |
| 91022220 | 91022528 | - | 12.6 | 1 | 311 | 0 | AluY | Alu | SINE |
| 91022529 | 91022799 | - | 28.4 | 5 | 274 | -231 | MLT1D | MaLR | LTR |

  

|  |  |  |  |  |  |  |  |  |  |
| --- | --- | --- | --- | --- | --- | --- | --- | --- | --- |
| chr2:91044440-91045602 | | | 1056 bp | | |  |  |  |  |
| Genome Start | Genome End | Strand | Div | Repeat Start | Repeat End | Repeat Left | Name | Family | Class |
| 91044440 | 91044526 | + | 32.3 | 1803 | 1901 | -1518 | L2 | L2 | LINE |
| 91044527 | 91044810 | + | 9.9 | 2 | 285 | -24 | AluSc | Alu | SINE |
| 91044831 | 91045225 | + | 32.3 | 1902 | 2353 | -1066 | L2 | L2 | LINE |
| 91045313 | 91045602 | + | 34.8 | 2537 | 2854 | -565 | L2 | L2 | LINE |

  

|  |  |  |  |  |  |  |  |  |  |
| --- | --- | --- | --- | --- | --- | --- | --- | --- | --- |
| chr2:91079773-91080856 | | | 841 bp | | |  |  |  |  |
| Genome Start | Genome End | Strand | Div | Repeat Start | Repeat End | Repeat Left | Name | Family | Class |
| 91079773 | 91079874 | - | 17.6 | 6040 | 6141 | -25 | L1PA16 | L1 | LINE |
| 91080009 | 91080310 | + | 9.9 | 1 | 302 | -10 | AluSx | Alu | SINE |
| 91080420 | 91080856 | - | 18.0 | 5718 | 6033 | -133 | L1PA16 | L1 | LINE |

  

|  |  |  |  |  |  |  |  |  |  |
| --- | --- | --- | --- | --- | --- | --- | --- | --- | --- |
| chr2:91081940-91082794 | | | 855 bp | | |  |  |  |  |
| Genome Start | Genome End | Strand | Div | Repeat Start | Repeat End | Repeat Left | Name | Family | Class |
| 91081940 | 91082211 | - | 18.0 | 5885 | 6144 | -22 | L1PA16 | L1 | LINE |
| 91082212 | 91082655 | + | 10.4 | 5708 | 6151 | -3 | L1PA7 | L1 | LINE |
| 91082656 | 91082794 | - | 18.0 | 5770 | 5884 | -282 | L1PA16 | L1 | LINE |

  

|  |  |  |  |  |  |  |  |  |  |
| --- | --- | --- | --- | --- | --- | --- | --- | --- | --- |
| chr2:91083630-91084256 | | | 627 bp | | |  |  |  |  |
| Genome Start | Genome End | Strand | Div | Repeat Start | Repeat End | Repeat Left | Name | Family | Class |
| 91083630 | 91083771 | + | 10.1 | 1 | 136 | -174 | AluSg | Alu | SINE |
| 91083772 | 91084082 | + | 9.5 | 1 | 304 | -7 | AluY | Alu | SINE |
| 91084083 | 91084256 | + | 10.1 | 137 | 305 | -5 | AluSg | Alu | SINE |

  

|  |  |  |  |  |  |  |  |  |  |
| --- | --- | --- | --- | --- | --- | --- | --- | --- | --- |
| chr2:91115209-91117102 | | | 1797 bp | | |  |  |  |  |
| Genome Start | Genome End | Strand | Div | Repeat Start | Repeat End | Repeat Left | Name | Family | Class |
| 91115209 | 91115379 | - | 27.2 | 2971 | 3151 | -3386 | L1MDa | L1 | LINE |
| 91115380 | 91115647 | - | 22.4 | 1 | 274 | -38 | AluJo | Alu | SINE |
| 91115732 | 91116235 | - | 27.4 | 20 | 519 | 0 | LTR53 | ERVL | LTR |
| 91116249 | 91117102 | - | 23.0 | 1892 | 2784 | -3753 | L1MDa | L1 | LINE |

  

|  |  |  |  |  |  |  |  |  |  |
| --- | --- | --- | --- | --- | --- | --- | --- | --- | --- |
| chr2:91146355-91147204 | | | 635 bp | | |  |  |  |  |
| Genome Start | Genome End | Strand | Div | Repeat Start | Repeat End | Repeat Left | Name | Family | Class |
| 91146355 | 91146501 | + | 7.5 | 1 | 150 | -162 | AluSx | Alu | SINE |
| 91146519 | 91146619 | - | 28.3 | 3275 | 3385 | -34 | L2 | L2 | LINE |
| 91146815 | 91147040 | - | 14.9 | 97 | 311 | -1 | AluJo | Alu | SINE |
| 91147044 | 91147204 | + | 11.9 | 154 | 312 | 0 | AluSx | Alu | SINE |

  

|  |  |  |  |  |  |  |  |  |  |
| --- | --- | --- | --- | --- | --- | --- | --- | --- | --- |
| chr2:91164851-91165881 | | | 1031 bp | | |  |  |  |  |
| Genome Start | Genome End | Strand | Div | Repeat Start | Repeat End | Repeat Left | Name | Family | Class |
| 91164851 | 91165223 | - | 15.2 | 5780 | 6153 | -13 | L1PA16 | L1 | LINE |
| 91165224 | 91165403 | - | 17.5 | 134 | 303 | -9 | AluJo | Alu | SINE |
| 91165404 | 91165695 | - | 13.2 | 1 | 296 | -17 | AluSq | Alu | SINE |
| 91165696 | 91165834 | - | 17.5 | 1 | 133 | -179 | AluJo | Alu | SINE |
| 91165835 | 91165881 | - | 15.2 | 5738 | 5779 | -387 | L1PA16 | L1 | LINE |

  

|  |  |  |  |  |  |  |  |  |  |
| --- | --- | --- | --- | --- | --- | --- | --- | --- | --- |
| chr2:91183176-91183935 | | | 760 bp | | |  |  |  |  |
| Genome Start | Genome End | Strand | Div | Repeat Start | Repeat End | Repeat Left | Name | Family | Class |
| 91183176 | 91183494 | + | 12.6 | 5847 | 6184 | -120 | L1MA2 | L1 | LINE |
| 91183495 | 91183805 | + | 11.0 | 2 | 309 | -2 | AluY | Alu | SINE |
| 91183806 | 91183935 | + | 12.6 | 6185 | 6304 | 0 | L1MA2 | L1 | LINE |

  

|  |  |  |  |  |  |  |  |  |  |
| --- | --- | --- | --- | --- | --- | --- | --- | --- | --- |
| chr2:91199400-91200017 | | | 618 bp | | |  |  |  |  |
| Genome Start | Genome End | Strand | Div | Repeat Start | Repeat End | Repeat Left | Name | Family | Class |
| 91199400 | 91199562 | - | 21.8 | 134 | 294 | -8 | AluJo | Alu | SINE |
| 91199563 | 91199874 | - | 13.0 | 1 | 309 | -1 | AluSg | Alu | SINE |
| 91199875 | 91200017 | - | 21.8 | 1 | 133 | -169 | AluJo | Alu | SINE |

  

|  |  |  |  |  |  |  |  |  |  |
| --- | --- | --- | --- | --- | --- | --- | --- | --- | --- |
| chr2:91224859-91225382 | | | 524 bp | | |  |  |  |  |
| Genome Start | Genome End | Strand | Div | Repeat Start | Repeat End | Repeat Left | Name | Family | Class |
| 91224859 | 91225010 | + | 32.4 | 43 | 195 | -73 | MIRb | MIR | SINE |
| 91225011 | 91225314 | + | 8.9 | 1 | 296 | -17 | AluSq | Alu | SINE |
| 91225315 | 91225382 | + | 32.4 | 196 | 253 | -15 | MIRb | MIR | SINE |

  

|  |  |  |  |  |  |  |  |  |  |
| --- | --- | --- | --- | --- | --- | --- | --- | --- | --- |
| chr2:91246158-91247137 | | | 980 bp | | |  |  |  |  |
| Genome Start | Genome End | Strand | Div | Repeat Start | Repeat End | Repeat Left | Name | Family | Class |
| 91246158 | 91246644 | + | 31.9 | 2684 | 3191 | -228 | L2 | L2 | LINE |
| 91246645 | 91246945 | - | 11.6 | 1 | 301 | -11 | AluSx | Alu | SINE |
| 91246946 | 91247137 | + | 30.7 | 3192 | 3408 | -11 | L2 | L2 | LINE |

  

|  |  |  |  |  |  |  |  |  |  |
| --- | --- | --- | --- | --- | --- | --- | --- | --- | --- |
| chr2:91258329-91258804 | | | 476 bp | | |  |  |  |  |
| Genome Start | Genome End | Strand | Div | Repeat Start | Repeat End | Repeat Left | Name | Family | Class |
| 91258329 | 91258473 | + | 17.5 | 51 | 246 | -78 | MER33 | MER1\_type | DNA |
| 91258474 | 91258714 | - | 12.1 | 56 | 296 | -17 | AluSq | Alu | SINE |
| 91258715 | 91258804 | + | 15.6 | 247 | 323 | -1 | MER33 | MER1\_type | DNA |

  

|  |  |  |  |  |  |  |  |  |  |
| --- | --- | --- | --- | --- | --- | --- | --- | --- | --- |
| chr2:91261112-91262293 | | | 1182 bp | | |  |  |  |  |
| Genome Start | Genome End | Strand | Div | Repeat Start | Repeat End | Repeat Left | Name | Family | Class |
| 91261112 | 91261519 | + | 24.7 | 3081 | 3501 | -2645 | L1M5 | L1 | LINE |
| 91261520 | 91261764 | + | 13.9 | 1 | 255 | -57 | AluSx | Alu | SINE |
| 91261765 | 91262083 | + | 11.0 | 3 | 313 | 0 | AluSq | Alu | SINE |
| 91262084 | 91262293 | + | 24.7 | 3502 | 3703 | -2443 | L1M5 | L1 | LINE |

  

|  |  |  |  |  |  |  |  |  |  |
| --- | --- | --- | --- | --- | --- | --- | --- | --- | --- |
| chr2:91340843-91341533 | | | 691 bp | | |  |  |  |  |
| Genome Start | Genome End | Strand | Div | Repeat Start | Repeat End | Repeat Left | Name | Family | Class |
| 91340843 | 91341223 | - | 18.9 | 36 | 427 | -168 | LTR49 | ERV1 | LTR |
| 91341224 | 91341501 | + | 13.7 | 14 | 292 | -21 | AluSq | Alu | SINE |
| 91341502 | 91341533 | - | 18.9 | 2 | 35 | -560 | LTR49 | ERV1 | LTR |

  

|  |  |  |  |  |  |  |  |  |  |
| --- | --- | --- | --- | --- | --- | --- | --- | --- | --- |
| chr2:91345877-91347070 | | | 1147 bp | | |  |  |  |  |
| Genome Start | Genome End | Strand | Div | Repeat Start | Repeat End | Repeat Left | Name | Family | Class |
| 91345877 | 91346292 | + | 25.1 | 1 | 430 | -471 | MER54A | ERVL | LTR |
| 91346331 | 91346668 | - | 12.0 | 7 | 364 | 0 | THE1B | MaLR | LTR |
| 91346678 | 91347070 | + | 21.7 | 490 | 901 | 0 | MER54A | ERVL | LTR |

  

|  |  |  |  |  |  |  |  |  |  |
| --- | --- | --- | --- | --- | --- | --- | --- | --- | --- |
| chr2:91349547-91350371 | | | 825 bp | | |  |  |  |  |
| Genome Start | Genome End | Strand | Div | Repeat Start | Repeat End | Repeat Left | Name | Family | Class |
| 91349547 | 91349791 | - | 28.6 | 275 | 503 | -2 | MLT1D | MaLR | LTR |
| 91349792 | 91350100 | - | 12.0 | 1 | 311 | 0 | AluY | Alu | SINE |
| 91350101 | 91350371 | - | 28.6 | 5 | 274 | -231 | MLT1D | MaLR | LTR |

  

|  |  |  |  |  |  |  |  |  |  |
| --- | --- | --- | --- | --- | --- | --- | --- | --- | --- |
| chr2:91378991-91381240 | | | 2249 bp | | |  |  |  |  |
| Genome Start | Genome End | Strand | Div | Repeat Start | Repeat End | Repeat Left | Name | Family | Class |
| 91378991 | 91379732 | - | 12.5 | 5512 | 6293 | -4 | L1MA5A | L1 | LINE |
| 91379733 | 91379851 | + | 14.7 | 168 | 294 | -18 | AluSg/x | Alu | SINE |
| 91379853 | 91381240 | - | 15.0 | 4106 | 5523 | -774 | L1MA5A | L1 | LINE |

  

|  |  |  |  |  |  |  |  |  |  |
| --- | --- | --- | --- | --- | --- | --- | --- | --- | --- |
| chr2:91382464-91383173 | | | 705 bp | | |  |  |  |  |
| Genome Start | Genome End | Strand | Div | Repeat Start | Repeat End | Repeat Left | Name | Family | Class |
| 91382464 | 91382654 | + | 19.1 | 4834 | 5024 | -1122 | L1MD | L1 | LINE |
| 91382658 | 91382844 | + | 22.1 | 126 | 315 | 0 | AluJo | Alu | SINE |
| 91382847 | 91383173 | + | 20.8 | 5029 | 5328 | -818 | L1MD | L1 | LINE |

  

|  |  |  |  |  |  |  |  |  |  |
| --- | --- | --- | --- | --- | --- | --- | --- | --- | --- |
| chr2:91387228-91389233 | | | 2005 bp | | |  |  |  |  |
| Genome Start | Genome End | Strand | Div | Repeat Start | Repeat End | Repeat Left | Name | Family | Class |
| 91387228 | 91387520 | + | 15.4 | 4774 | 5048 | -1098 | L1MA4 | L1 | LINE |
| 91387521 | 91387731 | + | 5.6 | 5 | 201 | -110 | AluY | Alu | SINE |
| 91387732 | 91388035 | + | 15.4 | 5049 | 5325 | -821 | L1MA4 | L1 | LINE |
| 91388036 | 91388062 | + | 21.3 | 33 | 59 | -117 | FRAM/FAM | Alu | SINE |
| 91388063 | 91388370 | - | 5.9 | 1 | 308 | -3 | AluY | Alu | SINE |
| 91388371 | 91388472 | + | 21.3 | 196 | 290 | -22 | AluJo/FRAM | Alu | SINE |
| 91388474 | 91389233 | + | 17.8 | 5290 | 6022 | -278 | L1MA4 | L1 | LINE |

  

|  |  |  |  |  |  |  |  |  |  |
| --- | --- | --- | --- | --- | --- | --- | --- | --- | --- |
| chr2:91438274-91438891 | | | 618 bp | | |  |  |  |  |
| Genome Start | Genome End | Strand | Div | Repeat Start | Repeat End | Repeat Left | Name | Family | Class |
| 91438274 | 91438411 | + | 20.6 | 1 | 133 | -179 | AluJo | Alu | SINE |
| 91438412 | 91438718 | + | 11.6 | 1 | 305 | -7 | AluSx | Alu | SINE |
| 91438719 | 91438891 | + | 20.6 | 134 | 306 | -6 | AluJo | Alu | SINE |

  

|  |  |  |  |  |  |  |  |  |  |
| --- | --- | --- | --- | --- | --- | --- | --- | --- | --- |
| chr2:91452097-91452600 | | | 504 bp | | |  |  |  |  |
| Genome Start | Genome End | Strand | Div | Repeat Start | Repeat End | Repeat Left | Name | Family | Class |
| 91452097 | 91452191 | + | 17.4 | 5226 | 5325 | -815 | L1M3 | L1 | LINE |
| 91452192 | 91452544 | + | 21.4 | 1 | 365 | 0 | MLT1A0 | MaLR | LTR |
| 91452545 | 91452600 | + | 17.4 | 5326 | 5381 | -759 | L1M3 | L1 | LINE |

  

|  |  |  |  |  |  |  |  |  |  |
| --- | --- | --- | --- | --- | --- | --- | --- | --- | --- |
| chr2:91467730-91469310 | | | 1317 bp | | |  |  |  |  |
| Genome Start | Genome End | Strand | Div | Repeat Start | Repeat End | Repeat Left | Name | Family | Class |
| 91467730 | 91468172 | + | 30.2 | 4533 | 5043 | -1103 | L1M5 | L1 | LINE |
| 91468173 | 91468429 | + | 15.2 | 1 | 294 | -16 | AluSg | Alu | SINE |
| 91468430 | 91468547 | + | 30.2 | 5044 | 5166 | -980 | L1M5 | L1 | LINE |
| 91468741 | 91469113 | + | 27.9 | 5224 | 5626 | -495 | L1M5 | L1 | LINE |
| 91469185 | 91469310 | + | 27.6 | 5768 | 5896 | -344 | L1M5 | L1 | LINE |

  

|  |  |  |  |  |  |  |  |  |  |
| --- | --- | --- | --- | --- | --- | --- | --- | --- | --- |
| chr2:91499026-91504712 | | | 5690 bp | | |  |  |  |  |
| Genome Start | Genome End | Strand | Div | Repeat Start | Repeat End | Repeat Left | Name | Family | Class |
| 91499026 | 91500507 | + | 10.1 | 5 | 1557 | -718 | L1P2 | L1 | LINE |
| 91500505 | 91502470 | - | 4.7 | 3055 | 5103 | -1043 | L1P1 | L1 | LINE |
| 91502471 | 91504712 | + | 7.4 | 1646 | 3859 | -2287 | L1P2 | L1 | LINE |

  

|  |  |  |  |  |  |  |  |  |  |
| --- | --- | --- | --- | --- | --- | --- | --- | --- | --- |
| chr2:91512506-91513087 | | | 582 bp | | |  |  |  |  |
| Genome Start | Genome End | Strand | Div | Repeat Start | Repeat End | Repeat Left | Name | Family | Class |
| 91512506 | 91512646 | - | 18.6 | 137 | 278 | -34 | AluJo | Alu | SINE |
| 91512647 | 91512948 | - | 12.2 | 2 | 304 | -8 | AluSx | Alu | SINE |
| 91512949 | 91513087 | - | 18.6 | 1 | 136 | -176 | AluJo | Alu | SINE |

  

|  |  |  |  |  |  |  |  |  |  |
| --- | --- | --- | --- | --- | --- | --- | --- | --- | --- |
| chr2:91517441-91518605 | | | 1165 bp | | |  |  |  |  |
| Genome Start | Genome End | Strand | Div | Repeat Start | Repeat End | Repeat Left | Name | Family | Class |
| 91517441 | 91517604 | + | 20.6 | 3 | 171 | -334 | MLT1D | MaLR | LTR |
| 91517605 | 91517901 | - | 10.6 | 1 | 298 | -15 | AluSq | Alu | SINE |
| 91517902 | 91517963 | + | 20.6 | 172 | 229 | -276 | MLT1D | MaLR | LTR |
| 91517964 | 91518338 | - | 16.4 | 1 | 396 | 0 | MSTD | MaLR | LTR |
| 91518339 | 91518605 | + | 20.6 | 230 | 502 | -3 | MLT1D | MaLR | LTR |

  

|  |  |  |  |  |  |  |  |  |  |
| --- | --- | --- | --- | --- | --- | --- | --- | --- | --- |
| chr2:91564006-91564894 | | | 889 bp | | |  |  |  |  |
| Genome Start | Genome End | Strand | Div | Repeat Start | Repeat End | Repeat Left | Name | Family | Class |
| 91564006 | 91564407 | + | 22.2 | 5188 | 5602 | -564 | L1M5 | L1 | LINE |
| 91564408 | 91564702 | + | 5.4 | 1 | 296 | -15 | AluY | Alu | SINE |
| 91564703 | 91564894 | + | 22.1 | 5603 | 5796 | -370 | L1M5 | L1 | LINE |

  

|  |  |  |  |  |  |  |  |  |  |
| --- | --- | --- | --- | --- | --- | --- | --- | --- | --- |
| chr2:91573097-91573806 | | | 702 bp | | |  |  |  |  |
| Genome Start | Genome End | Strand | Div | Repeat Start | Repeat End | Repeat Left | Name | Family | Class |
| 91573097 | 91573420 | - | 20.7 | 5032 | 5328 | -818 | L1MD | L1 | LINE |
| 91573426 | 91573612 | - | 22.1 | 126 | 315 | 0 | AluJo | Alu | SINE |
| 91573616 | 91573806 | - | 19.1 | 4834 | 5024 | -1122 | L1MD | L1 | LINE |

  

|  |  |  |  |  |  |  |  |  |  |
| --- | --- | --- | --- | --- | --- | --- | --- | --- | --- |
| chr2:91575050-91577279 | | | 2229 bp | | |  |  |  |  |
| Genome Start | Genome End | Strand | Div | Repeat Start | Repeat End | Repeat Left | Name | Family | Class |
| 91575050 | 91576417 | + | 15.0 | 4123 | 5523 | -774 | L1MA5A | L1 | LINE |
| 91576419 | 91576537 | - | 14.7 | 168 | 294 | -18 | AluSg/x | Alu | SINE |
| 91576538 | 91577279 | + | 12.5 | 5512 | 6293 | -4 | L1MA5A | L1 | LINE |

  

|  |  |  |  |  |  |  |  |  |  |
| --- | --- | --- | --- | --- | --- | --- | --- | --- | --- |
| chr2:91601215-91602689 | | | 1475 bp | | |  |  |  |  |
| Genome Start | Genome End | Strand | Div | Repeat Start | Repeat End | Repeat Left | Name | Family | Class |
| 91601215 | 91602027 | - | 22.9 | 2624 | 3354 | -3688 | L1M4b | L1 | LINE |
| 91602028 | 91602310 | - | 10.2 | 1 | 283 | -28 | AluY | Alu | SINE |
| 91602311 | 91602689 | - | 21.3 | 2146 | 2623 | -4711 | L1M4b | L1 | LINE |

  

|  |  |  |  |  |  |  |  |  |  |
| --- | --- | --- | --- | --- | --- | --- | --- | --- | --- |
| chr2:91605914-91606736 | | | 823 bp | | |  |  |  |  |
| Genome Start | Genome End | Strand | Div | Repeat Start | Repeat End | Repeat Left | Name | Family | Class |
| 91605914 | 91606184 | + | 28.4 | 5 | 274 | -231 | MLT1D | MaLR | LTR |
| 91606185 | 91606493 | + | 12.0 | 1 | 311 | 0 | AluY | Alu | SINE |
| 91606494 | 91606736 | + | 28.4 | 275 | 503 | -2 | MLT1D | MaLR | LTR |

  

|  |  |  |  |  |  |  |  |  |  |
| --- | --- | --- | --- | --- | --- | --- | --- | --- | --- |
| chr2:91609214-91610407 | | | 1147 bp | | |  |  |  |  |
| Genome Start | Genome End | Strand | Div | Repeat Start | Repeat End | Repeat Left | Name | Family | Class |
| 91609214 | 91609606 | - | 20.9 | 490 | 901 | 0 | MER54A | ERVL | LTR |
| 91609616 | 91609953 | + | 12.0 | 7 | 364 | 0 | THE1B | MaLR | LTR |
| 91609992 | 91610407 | - | 24.6 | 1 | 430 | -471 | MER54A | ERVL | LTR |

  

|  |  |  |  |  |  |  |  |  |  |
| --- | --- | --- | --- | --- | --- | --- | --- | --- | --- |
| chr2:91614761-91615451 | | | 691 bp | | |  |  |  |  |
| Genome Start | Genome End | Strand | Div | Repeat Start | Repeat End | Repeat Left | Name | Family | Class |
| 91614761 | 91614792 | + | 19.4 | 1 | 34 | -561 | LTR49 | ERV1 | LTR |
| 91614793 | 91615069 | - | 13.4 | 14 | 292 | -21 | AluSq | Alu | SINE |
| 91615070 | 91615451 | + | 19.4 | 35 | 427 | -168 | LTR49 | ERV1 | LTR |

  

|  |  |  |  |  |  |  |  |  |  |
| --- | --- | --- | --- | --- | --- | --- | --- | --- | --- |
| chr2:94692799-94694534 | | | 1736 bp | | |  |  |  |  |
| Genome Start | Genome End | Strand | Div | Repeat Start | Repeat End | Repeat Left | Name | Family | Class |
| 94692799 | 94693967 | - | 12.2 | 408 | 1574 | -6 | THE1B-int | MaLR | LTR |
| 94693968 | 94694128 | - | 12.6 | 134 | 301 | -11 | AluSg/x | Alu | SINE |
| 94694129 | 94694534 | - | 12.2 | 3 | 407 | -1173 | THE1B-int | MaLR | LTR |

  

|  |  |  |  |  |  |  |  |  |  |
| --- | --- | --- | --- | --- | --- | --- | --- | --- | --- |
| chr2:94700986-94701478 | | | 493 bp | | |  |  |  |  |
| Genome Start | Genome End | Strand | Div | Repeat Start | Repeat End | Repeat Left | Name | Family | Class |
| 94700986 | 94701103 | + | 28.1 | 3 | 119 | -59 | MER5B | MER1\_type | DNA |
| 94701104 | 94701422 | - | 13.5 | 1 | 312 | 0 | AluSx | Alu | SINE |
| 94701423 | 94701478 | + | 28.1 | 120 | 172 | -6 | MER5B | MER1\_type | DNA |

  

|  |  |  |  |  |  |  |  |  |  |
| --- | --- | --- | --- | --- | --- | --- | --- | --- | --- |
| chr2:94702018-94702542 | | | 466 bp | | |  |  |  |  |
| Genome Start | Genome End | Strand | Div | Repeat Start | Repeat End | Repeat Left | Name | Family | Class |
| 94702018 | 94702061 | + | 27.3 | 1 | 44 | -134 | MER5B | MER1\_type | DNA |
| 94702062 | 94702355 | + | 5.1 | 1 | 294 | -17 | AluY | Alu | SINE |
| 94702415 | 94702542 | + | 27.2 | 50 | 177 | -1 | MER5B | MER1\_type | DNA |

  

|  |  |  |  |  |  |  |  |  |  |
| --- | --- | --- | --- | --- | --- | --- | --- | --- | --- |
| chr2:94727225-94728899 | | | 1641 bp | | |  |  |  |  |
| Genome Start | Genome End | Strand | Div | Repeat Start | Repeat End | Repeat Left | Name | Family | Class |
| 94727225 | 94727588 | - | 10.9 | 5783 | 6145 | -6 | L1PB1 | L1 | LINE |
| 94727623 | 94728384 | - | 10.2 | 5016 | 5782 | -369 | L1PB1 | L1 | LINE |
| 94728385 | 94728698 | - | 14.3 | 1 | 284 | -28 | AluSx | Alu | SINE |
| 94728699 | 94728899 | - | 9.4 | 4815 | 5015 | -1131 | L1PB1 | L1 | LINE |

  

|  |  |  |  |  |  |  |  |  |  |
| --- | --- | --- | --- | --- | --- | --- | --- | --- | --- |
| chr2:94777097-94777734 | | | 612 bp | | |  |  |  |  |
| Genome Start | Genome End | Strand | Div | Repeat Start | Repeat End | Repeat Left | Name | Family | Class |
| 94777097 | 94777398 | - | 18.9 | 117 | 426 | 0 | MSTB | MaLR | LTR |
| 94777399 | 94777490 | + | 12.7 | 1 | 100 | -130 | MER30 | MER1\_type | DNA |
| 94777517 | 94777612 | + | 12.7 | 101 | 229 | -1 | MER30 | MER1\_type | DNA |
| 94777613 | 94777734 | - | 12.2 | 1 | 124 | -302 | MSTB | MaLR | LTR |

  

|  |  |  |  |  |  |  |  |  |  |
| --- | --- | --- | --- | --- | --- | --- | --- | --- | --- |
| chr2:94829345-94831062 | | | 1643 bp | | |  |  |  |  |
| Genome Start | Genome End | Strand | Div | Repeat Start | Repeat End | Repeat Left | Name | Family | Class |
| 94829345 | 94830455 | + | 19.8 | 4805 | 5941 | -359 | L1MA5 | L1 | LINE |
| 94830464 | 94830721 | + | 22.7 | 1 | 264 | -48 | AluJo | Alu | SINE |
| 94830789 | 94831062 | + | 17.5 | 5997 | 6294 | -6 | L1MA5 | L1 | LINE |

  

|  |  |  |  |  |  |  |  |  |  |
| --- | --- | --- | --- | --- | --- | --- | --- | --- | --- |
| chr2:94832802-94834613 | | | 1803 bp | | |  |  |  |  |
| Genome Start | Genome End | Strand | Div | Repeat Start | Repeat End | Repeat Left | Name | Family | Class |
| 94832802 | 94833869 | + | 20.1 | 4766 | 5836 | -342 | L1MB8 | L1 | LINE |
| 94833879 | 94834290 | + | 14.0 | 11 | 428 | 0 | MER61C | ERV1 | LTR |
| 94834291 | 94834613 | + | 20.4 | 5833 | 6173 | -5 | L1MB8 | L1 | LINE |

  

|  |  |  |  |  |  |  |  |  |  |
| --- | --- | --- | --- | --- | --- | --- | --- | --- | --- |
| chr2:94873764-94874421 | | | 658 bp | | |  |  |  |  |
| Genome Start | Genome End | Strand | Div | Repeat Start | Repeat End | Repeat Left | Name | Family | Class |
| 94873764 | 94873980 | + | 17.8 | 284 | 518 | -3006 | Zaphod2 | Tip100 | DNA |
| 94873981 | 94874404 | + | 18.4 | 1 | 434 | 0 | Tigger2a | MER2\_type | DNA |
| 94874405 | 94874421 | + | 17.8 | 519 | 536 | -2988 | Zaphod2 | Tip100 | DNA |

  

|  |  |  |  |  |  |  |  |  |  |
| --- | --- | --- | --- | --- | --- | --- | --- | --- | --- |
| chr2:94940518-94941894 | | | 1377 bp | | |  |  |  |  |
| Genome Start | Genome End | Strand | Div | Repeat Start | Repeat End | Repeat Left | Name | Family | Class |
| 94940518 | 94941093 | - | 22.7 | 7053 | 7678 | -364 | L1MC4 | L1 | LINE |
| 94941094 | 94941390 | - | 12.2 | 1 | 297 | -15 | AluSx | Alu | SINE |
| 94941391 | 94941894 | - | 22.7 | 6490 | 7052 | -990 | L1MC4 | L1 | LINE |

  

|  |  |  |  |  |  |  |  |  |  |
| --- | --- | --- | --- | --- | --- | --- | --- | --- | --- |
| chr2:94955920-94957404 | | | 1485 bp | | |  |  |  |  |
| Genome Start | Genome End | Strand | Div | Repeat Start | Repeat End | Repeat Left | Name | Family | Class |
| 94955920 | 94956428 | + | 24.5 | 18 | 528 | -407 | MER21C | ERV1 | LTR |
| 94956429 | 94956748 | - | 21.1 | 2 | 304 | -8 | AluJb | Alu | SINE |
| 94956749 | 94956826 | + | 24.5 | 529 | 603 | -332 | MER21C | ERV1 | LTR |
| 94956827 | 94957120 | + | 17.2 | 9 | 300 | -12 | AluJo | Alu | SINE |
| 94957121 | 94957404 | + | 24.5 | 604 | 916 | -19 | MER21C | ERV1 | LTR |

  

|  |  |  |  |  |  |  |  |  |  |
| --- | --- | --- | --- | --- | --- | --- | --- | --- | --- |
| chr2:94966414-94966913 | | | 446 bp | | |  |  |  |  |
| Genome Start | Genome End | Strand | Div | Repeat Start | Repeat End | Repeat Left | Name | Family | Class |
| 94966414 | 94966514 | + | 25.2 | 4 | 104 | -1794 | Kanga1 | Tc2 | DNA |
| 94966527 | 94966787 | - | 31.1 | 353 | 626 | -5493 | L1MEd | L1 | LINE |
| 94966830 | 94966913 | + | 20.0 | 1656 | 1735 | -10 | Kanga1 | Tc2 | DNA |

  

|  |  |  |  |  |  |  |  |  |  |
| --- | --- | --- | --- | --- | --- | --- | --- | --- | --- |
| chr2:95448983-95449998 | | | 937 bp | | |  |  |  |  |
| Genome Start | Genome End | Strand | Div | Repeat Start | Repeat End | Repeat Left | Name | Family | Class |
| 95448983 | 95449164 | - | 27.1 | 468 | 655 | 0 | LTR16D | ERVL | LTR |
| 95449168 | 95449247 | - | 19.2 | 478 | 555 | 0 | MLT2B1 | ERVL | LTR |
| 95449324 | 95449759 | - | 18.1 | 1 | 472 | -48 | MLT2B1 | ERVL | LTR |
| 95449760 | 95449998 | - | 29.0 | 217 | 469 | -186 | LTR16D | ERVL | LTR |

  

|  |  |  |  |  |  |  |  |  |  |
| --- | --- | --- | --- | --- | --- | --- | --- | --- | --- |
| chr2:95500861-95501328 | | | 468 bp | | |  |  |  |  |
| Genome Start | Genome End | Strand | Div | Repeat Start | Repeat End | Repeat Left | Name | Family | Class |
| 95500861 | 95500890 | + | 18.6 | 2405 | 2428 | -184 | Charlie5 | MER1\_type | DNA |
| 95500891 | 95501192 | + | 22.1 | 1 | 305 | -7 | AluJb | Alu | SINE |
| 95501193 | 95501328 | + | 18.6 | 2429 | 2579 | -33 | Charlie5 | MER1\_type | DNA |

  

|  |  |  |  |  |  |  |  |  |  |
| --- | --- | --- | --- | --- | --- | --- | --- | --- | --- |
| chr2:95501732-95503472 | | | 1730 bp | | |  |  |  |  |
| Genome Start | Genome End | Strand | Div | Repeat Start | Repeat End | Repeat Left | Name | Family | Class |
| 95501732 | 95502002 | + | 28.2 | 2888 | 3193 | -2953 | L1M5 | L1 | LINE |
| 95502003 | 95502297 | - | 12.2 | 5 | 299 | -13 | AluSx | Alu | SINE |
| 95502298 | 95502725 | + | 28.2 | 3194 | 3631 | -2515 | L1M5 | L1 | LINE |
| 95502737 | 95502912 | - | 25.7 | 1 | 174 | -2 | FRAM | Alu | SINE |
| 95502913 | 95503472 | + | 29.1 | 3644 | 4239 | -1907 | L1M5 | L1 | LINE |

  

|  |  |  |  |  |  |  |  |  |  |
| --- | --- | --- | --- | --- | --- | --- | --- | --- | --- |
| chr2:95521407-95522309 | | | 903 bp | | |  |  |  |  |
| Genome Start | Genome End | Strand | Div | Repeat Start | Repeat End | Repeat Left | Name | Family | Class |
| 95521407 | 95521961 | - | 30.8 | 847 | 1426 | -4997 | L1MCc | L1 | LINE |
| 95521962 | 95522257 | - | 12.4 | 1 | 304 | -7 | AluY | Alu | SINE |
| 95522258 | 95522309 | - | 30.8 | 797 | 846 | -5577 | L1MCc | L1 | LINE |

  

|  |  |  |  |  |  |  |  |  |  |
| --- | --- | --- | --- | --- | --- | --- | --- | --- | --- |
| chr2:95543488-95544118 | | | 631 bp | | |  |  |  |  |
| Genome Start | Genome End | Strand | Div | Repeat Start | Repeat End | Repeat Left | Name | Family | Class |
| 95543488 | 95543593 | + | 26.2 | 1 | 108 | -237 | MER2 | MER2\_type | DNA |
| 95543594 | 95543875 | - | 12.5 | 1 | 279 | -32 | AluY | Alu | SINE |
| 95543876 | 95544118 | + | 26.2 | 109 | 344 | -1 | MER2 | MER2\_type | DNA |

  

|  |  |  |  |  |  |  |  |  |  |
| --- | --- | --- | --- | --- | --- | --- | --- | --- | --- |
| chr2:95578457-95579154 | | | 698 bp | | |  |  |  |  |
| Genome Start | Genome End | Strand | Div | Repeat Start | Repeat End | Repeat Left | Name | Family | Class |
| 95578457 | 95578636 | + | 23.9 | 5770 | 5938 | -362 | L1MA4 | L1 | LINE |
| 95578637 | 95578917 | + | 15.4 | 1 | 280 | -32 | AluSx | Alu | SINE |
| 95578918 | 95579154 | + | 23.9 | 5939 | 6160 | -140 | L1MA4 | L1 | LINE |

  

|  |  |  |  |  |  |  |  |  |  |
| --- | --- | --- | --- | --- | --- | --- | --- | --- | --- |
| chr2:95634358-95634825 | | | 468 bp | | |  |  |  |  |
| Genome Start | Genome End | Strand | Div | Repeat Start | Repeat End | Repeat Left | Name | Family | Class |
| 95634358 | 95634493 | - | 18.6 | 2429 | 2579 | -33 | Charlie5 | MER1\_type | DNA |
| 95634494 | 95634795 | - | 22.1 | 1 | 305 | -7 | AluJb | Alu | SINE |
| 95634796 | 95634825 | - | 18.6 | 2405 | 2428 | -184 | Charlie5 | MER1\_type | DNA |

  

|  |  |  |  |  |  |  |  |  |  |
| --- | --- | --- | --- | --- | --- | --- | --- | --- | --- |
| chr2:95685683-95686696 | | | 937 bp | | |  |  |  |  |
| Genome Start | Genome End | Strand | Div | Repeat Start | Repeat End | Repeat Left | Name | Family | Class |
| 95685683 | 95685921 | + | 29.4 | 217 | 469 | -186 | LTR16D | ERVL | LTR |
| 95685922 | 95686357 | + | 17.9 | 1 | 472 | -48 | MLT2B1 | ERVL | LTR |
| 95686432 | 95686511 | + | 17.9 | 478 | 555 | 0 | MLT2B1 | ERVL | LTR |
| 95686515 | 95686696 | + | 28.1 | 468 | 655 | 0 | LTR16D | ERVL | LTR |

  

|  |  |  |  |  |  |  |  |  |  |
| --- | --- | --- | --- | --- | --- | --- | --- | --- | --- |
| chr2:95759292-95759787 | | | 489 bp | | |  |  |  |  |
| Genome Start | Genome End | Strand | Div | Repeat Start | Repeat End | Repeat Left | Name | Family | Class |
| 95759292 | 95759402 | + | 25.2 | 20 | 130 | -48 | MER5B | MER1\_type | DNA |
| 95759410 | 95759722 | - | 8.4 | 1 | 310 | -1 | AluY | Alu | SINE |
| 95759723 | 95759787 | + | 35.9 | 114 | 177 | -1 | MER5B | MER1\_type | DNA |

  

|  |  |  |  |  |  |  |  |  |  |
| --- | --- | --- | --- | --- | --- | --- | --- | --- | --- |
| chr2:95799783-95800406 | | | 624 bp | | |  |  |  |  |
| Genome Start | Genome End | Strand | Div | Repeat Start | Repeat End | Repeat Left | Name | Family | Class |
| 95799783 | 95800015 | + | 20.8 | 1 | 242 | -82 | MER33 | MER1\_type | DNA |
| 95800016 | 95800325 | - | 13.6 | 1 | 312 | 0 | AluSx | Alu | SINE |
| 95800326 | 95800406 | + | 20.8 | 243 | 324 | 0 | MER33 | MER1\_type | DNA |

  

|  |  |  |  |  |  |  |  |  |  |
| --- | --- | --- | --- | --- | --- | --- | --- | --- | --- |
| chr2:95997977-95999503 | | | 1527 bp | | |  |  |  |  |
| Genome Start | Genome End | Strand | Div | Repeat Start | Repeat End | Repeat Left | Name | Family | Class |
| 95997977 | 95998007 | + | 22.3 | 1 | 21 | -684 | MER44D | MER2\_type | DNA |
| 95998008 | 95998835 | - | 19.2 | 1 | 859 | -4 | MER21B | ERV1 | LTR |
| 95998836 | 95999503 | + | 22.3 | 22 | 705 | 0 | MER44D | MER2\_type | DNA |

  

|  |  |  |  |  |  |  |  |  |  |
| --- | --- | --- | --- | --- | --- | --- | --- | --- | --- |
| chr2:96025976-96026786 | | | 811 bp | | |  |  |  |  |
| Genome Start | Genome End | Strand | Div | Repeat Start | Repeat End | Repeat Left | Name | Family | Class |
| 96025976 | 96026066 | + | 28.2 | 73 | 162 | -1847 | HAL1b | L1 | LINE |
| 96026067 | 96026709 | - | 21.8 | 1 | 614 | 0 | MER90a | ERV1 | LTR |
| 96026710 | 96026786 | + | 28.2 | 163 | 239 | -1770 | HAL1b | L1 | LINE |

  

|  |  |  |  |  |  |  |  |  |  |
| --- | --- | --- | --- | --- | --- | --- | --- | --- | --- |
| chr2:96037154-96038476 | | | 1323 bp | | |  |  |  |  |
| Genome Start | Genome End | Strand | Div | Repeat Start | Repeat End | Repeat Left | Name | Family | Class |
| 96037154 | 96037430 | - | 22.8 | 5643 | 5924 | -242 | L1ME1 | L1 | LINE |
| 96037431 | 96038032 | - | 22.7 | 1 | 636 | 0 | MER92B | ERV1 | LTR |
| 96038033 | 96038476 | - | 22.9 | 5195 | 5642 | -524 | L1ME1 | L1 | LINE |

  

|  |  |  |  |  |  |  |  |  |  |
| --- | --- | --- | --- | --- | --- | --- | --- | --- | --- |
| chr2:97050492-97052247 | | | 1745 bp | | |  |  |  |  |
| Genome Start | Genome End | Strand | Div | Repeat Start | Repeat End | Repeat Left | Name | Family | Class |
| 97050492 | 97050756 | + | 27.6 | 2888 | 3193 | -2953 | L1M5 | L1 | LINE |
| 97050757 | 97051055 | - | 12.1 | 5 | 301 | -11 | AluSx | Alu | SINE |
| 97051056 | 97051482 | + | 27.6 | 3194 | 3631 | -2515 | L1M5 | L1 | LINE |
| 97051494 | 97051670 | - | 25.6 | 1 | 175 | -1 | FRAM | Alu | SINE |
| 97051671 | 97052247 | + | 28.9 | 3644 | 4239 | -1907 | L1M5 | L1 | LINE |

  

|  |  |  |  |  |  |  |  |  |  |
| --- | --- | --- | --- | --- | --- | --- | --- | --- | --- |
| chr2:97071654-97072723 | | | 1070 bp | | |  |  |  |  |
| Genome Start | Genome End | Strand | Div | Repeat Start | Repeat End | Repeat Left | Name | Family | Class |
| 97071654 | 97071934 | - | 27.9 | 4881 | 5156 | -990 | L1M5 | L1 | LINE |
| 97071935 | 97072302 | + | 22.5 | 1 | 408 | 0 | MLT1A1 | MaLR | LTR |
| 97072303 | 97072723 | - | 27.9 | 4462 | 4880 | -1266 | L1M5 | L1 | LINE |

  

|  |  |  |  |  |  |  |  |  |  |
| --- | --- | --- | --- | --- | --- | --- | --- | --- | --- |
| chr2:97083895-97084601 | | | 684 bp | | |  |  |  |  |
| Genome Start | Genome End | Strand | Div | Repeat Start | Repeat End | Repeat Left | Name | Family | Class |
| 97083895 | 97084021 | + | 25.5 | 1 | 146 | -228 | MLT1A | MaLR | LTR |
| 97084022 | 97084041 | - | 7.6 | 285 | 311 | 0 | AluY | Alu | SINE |
| 97084065 | 97084346 | - | 7.6 | 3 | 284 | -27 | AluY | Alu | SINE |
| 97084347 | 97084601 | + | 25.5 | 147 | 368 | -6 | MLT1A | MaLR | LTR |

  

|  |  |  |  |  |  |  |  |  |  |
| --- | --- | --- | --- | --- | --- | --- | --- | --- | --- |
| chr2:97122888-97123407 | | | 520 bp | | |  |  |  |  |
| Genome Start | Genome End | Strand | Div | Repeat Start | Repeat End | Repeat Left | Name | Family | Class |
| 97122888 | 97122932 | - | 29.7 | 189 | 237 | -31 | MIRb | MIR | SINE |
| 97122933 | 97123236 | - | 6.9 | 1 | 305 | -6 | AluY | Alu | SINE |
| 97123237 | 97123407 | - | 29.7 | 31 | 188 | -80 | MIRb | MIR | SINE |

  

|  |  |  |  |  |  |  |  |  |  |
| --- | --- | --- | --- | --- | --- | --- | --- | --- | --- |
| chr2:97137601-97138409 | | | 809 bp | | |  |  |  |  |
| Genome Start | Genome End | Strand | Div | Repeat Start | Repeat End | Repeat Left | Name | Family | Class |
| 97137601 | 97137677 | - | 28.9 | 163 | 239 | -1770 | HAL1b | L1 | LINE |
| 97137678 | 97138318 | + | 21.7 | 1 | 614 | 0 | MER90a | ERV1 | LTR |
| 97138319 | 97138409 | - | 28.9 | 73 | 162 | -1847 | HAL1b | L1 | LINE |

  

|  |  |  |  |  |  |  |  |  |  |
| --- | --- | --- | --- | --- | --- | --- | --- | --- | --- |
| chr2:97358754-97359460 | | | 707 bp | | |  |  |  |  |
| Genome Start | Genome End | Strand | Div | Repeat Start | Repeat End | Repeat Left | Name | Family | Class |
| 97358754 | 97359008 | - | 25.2 | 148 | 368 | -6 | MLT1A | MaLR | LTR |
| 97359009 | 97359313 | + | 7.6 | 2 | 306 | 0 | AluY | Alu | SINE |
| 97359314 | 97359333 | + | 7.6 | 284 | 303 | -8 | AluY | Alu | SINE |
| 97359334 | 97359460 | - | 25.2 | 3 | 147 | -227 | MLT1A | MaLR | LTR |

  

|  |  |  |  |  |  |  |  |  |  |
| --- | --- | --- | --- | --- | --- | --- | --- | --- | --- |
| chr2:97370605-97371724 | | | 1120 bp | | |  |  |  |  |
| Genome Start | Genome End | Strand | Div | Repeat Start | Repeat End | Repeat Left | Name | Family | Class |
| 97370605 | 97371072 | + | 28.7 | 4428 | 4880 | -1266 | L1M5 | L1 | LINE |
| 97371073 | 97371443 | - | 22.6 | 1 | 408 | 0 | MLT1A1 | MaLR | LTR |
| 97371444 | 97371724 | + | 28.7 | 4881 | 5156 | -990 | L1M5 | L1 | LINE |

  

|  |  |  |  |  |  |  |  |  |  |
| --- | --- | --- | --- | --- | --- | --- | --- | --- | --- |
| chr2:97375142-97376192 | | | 1051 bp | | |  |  |  |  |
| Genome Start | Genome End | Strand | Div | Repeat Start | Repeat End | Repeat Left | Name | Family | Class |
| 97375142 | 97375651 | + | 23.4 | 5553 | 6070 | -108 | L1MB8 | L1 | LINE |
| 97375652 | 97376094 | - | 13.1 | 1 | 472 | 0 | MER4A1 | ERV1 | LTR |
| 97376095 | 97376192 | + | 23.4 | 6071 | 6167 | -11 | L1MB8 | L1 | LINE |

  

|  |  |  |  |  |  |  |  |  |  |
| --- | --- | --- | --- | --- | --- | --- | --- | --- | --- |
| chr2:97474649-97475496 | | | 848 bp | | |  |  |  |  |
| Genome Start | Genome End | Strand | Div | Repeat Start | Repeat End | Repeat Left | Name | Family | Class |
| 97474649 | 97475024 | - | 21.0 | 774 | 1166 | -5815 | L1PA12 | L1 | LINE |
| 97475025 | 97475336 | + | 13.5 | 1 | 312 | 0 | AluSx | Alu | SINE |
| 97475337 | 97475496 | - | 21.0 | 610 | 773 | -6208 | L1PA12 | L1 | LINE |

  

|  |  |  |  |  |  |  |  |  |  |
| --- | --- | --- | --- | --- | --- | --- | --- | --- | --- |
| chr2:97549628-97551151 | | | 1524 bp | | |  |  |  |  |
| Genome Start | Genome End | Strand | Div | Repeat Start | Repeat End | Repeat Left | Name | Family | Class |
| 97549628 | 97549658 | + | 21.9 | 1 | 21 | -684 | MER44D | MER2\_type | DNA |
| 97549659 | 97550485 | - | 19.5 | 1 | 859 | -4 | MER21B | ERV1 | LTR |
| 97550486 | 97551151 | + | 21.9 | 22 | 705 | 0 | MER44D | MER2\_type | DNA |

  

|  |  |  |  |  |  |  |  |  |  |
| --- | --- | --- | --- | --- | --- | --- | --- | --- | --- |
| chr2:97577403-97578213 | | | 811 bp | | |  |  |  |  |
| Genome Start | Genome End | Strand | Div | Repeat Start | Repeat End | Repeat Left | Name | Family | Class |
| 97577403 | 97577493 | + | 26.3 | 73 | 162 | -1847 | HAL1b | L1 | LINE |
| 97577494 | 97578136 | - | 21.8 | 1 | 614 | 0 | MER90a | ERV1 | LTR |
| 97578137 | 97578213 | + | 26.3 | 163 | 239 | -1770 | HAL1b | L1 | LINE |

  

|  |  |  |  |  |  |  |  |  |  |
| --- | --- | --- | --- | --- | --- | --- | --- | --- | --- |
| chr2:97592637-97593256 | | | 620 bp | | |  |  |  |  |
| Genome Start | Genome End | Strand | Div | Repeat Start | Repeat End | Repeat Left | Name | Family | Class |
| 97592637 | 97592717 | - | 20.8 | 242 | 324 | 0 | MER33 | MER1\_type | DNA |
| 97592718 | 97593026 | + | 13.6 | 1 | 312 | 0 | AluSx | Alu | SINE |
| 97593027 | 97593256 | - | 20.8 | 2 | 241 | -83 | MER33 | MER1\_type | DNA |

  

|  |  |  |  |  |  |  |  |  |  |
| --- | --- | --- | --- | --- | --- | --- | --- | --- | --- |
| chr2:106233195-106235784 | | | 2583 bp | | |  |  |  |  |
| Genome Start | Genome End | Strand | Div | Repeat Start | Repeat End | Repeat Left | Name | Family | Class |
| 106233195 | 106233863 | - | 21.1 | 4405 | 5095 | -1287 | L1M4c | L1 | LINE |
| 106233864 | 106234155 | - | 14.7 | 1 | 293 | -19 | AluSx | Alu | SINE |
| 106234163 | 106234295 | - | 12.8 | 1 | 133 | -10 | FLAM\_C | Alu | SINE |
| 106234296 | 106234513 | - | 21.1 | 4199 | 4398 | -1984 | L1M4c | L1 | LINE |
| 106234514 | 106234816 | - | 11.6 | 1 | 301 | -11 | AluSx | Alu | SINE |
| 106234817 | 106235026 | - | 21.1 | 3991 | 4196 | -2186 | L1M4c | L1 | LINE |
| 106235027 | 106235332 | - | 9.2 | 1 | 307 | -5 | AluSx | Alu | SINE |
| 106235333 | 106235784 | - | 21.1 | 3525 | 3990 | -2392 | L1M4c | L1 | LINE |

  

|  |  |  |  |  |  |  |  |  |  |
| --- | --- | --- | --- | --- | --- | --- | --- | --- | --- |
| chr2:106259859-106260575 | | | 717 bp | | |  |  |  |  |
| Genome Start | Genome End | Strand | Div | Repeat Start | Repeat End | Repeat Left | Name | Family | Class |
| 106259859 | 106260038 | - | 22.7 | 463 | 631 | -13 | LTR48B | ERV1 | LTR |
| 106260039 | 106260334 | + | 12.5 | 1 | 295 | -17 | AluSx | Alu | SINE |
| 106260335 | 106260575 | - | 22.7 | 243 | 462 | -182 | LTR48B | ERV1 | LTR |

  

|  |  |  |  |  |  |  |  |  |  |
| --- | --- | --- | --- | --- | --- | --- | --- | --- | --- |
| chr2:106268365-106269471 | | | 1094 bp | | |  |  |  |  |
| Genome Start | Genome End | Strand | Div | Repeat Start | Repeat End | Repeat Left | Name | Family | Class |
| 106268365 | 106268447 | + | 16.9 | 1170 | 1263 | -746 | HAL1b | L1 | LINE |
| 106268456 | 106268660 | + | 14.1 | 96 | 295 | -17 | AluJo | Alu | SINE |
| 106268666 | 106269030 | + | 25.1 | 1271 | 1653 | -356 | HAL1b | L1 | LINE |
| 106269031 | 106269322 | - | 19.0 | 29 | 312 | 0 | AluJo | Alu | SINE |
| 106269323 | 106269471 | + | 25.1 | 1654 | 1777 | -232 | HAL1b | L1 | LINE |

  

|  |  |  |  |  |  |  |  |  |  |
| --- | --- | --- | --- | --- | --- | --- | --- | --- | --- |
| chr2:106377140-106378611 | | | 1472 bp | | |  |  |  |  |
| Genome Start | Genome End | Strand | Div | Repeat Start | Repeat End | Repeat Left | Name | Family | Class |
| 106377140 | 106377181 | - | 16.6 | 1566 | 1607 | -5144 | L1MCb | L1 | LINE |
| 106377182 | 106377529 | - | 19.0 | 2 | 362 | -3 | MLT1A0 | MaLR | LTR |
| 106377530 | 106377589 | - | 16.6 | 1514 | 1565 | -5186 | L1MCb | L1 | LINE |
| 106377590 | 106377900 | - | 8.4 | 1 | 311 | 0 | AluY | Alu | SINE |
| 106377901 | 106378611 | - | 16.6 | 721 | 1513 | -5238 | L1MCb | L1 | LINE |

  

|  |  |  |  |  |  |  |  |  |  |
| --- | --- | --- | --- | --- | --- | --- | --- | --- | --- |
| chr2:106383270-106383785 | | | 516 bp | | |  |  |  |  |
| Genome Start | Genome End | Strand | Div | Repeat Start | Repeat End | Repeat Left | Name | Family | Class |
| 106383270 | 106383286 | - | 25.4 | 226 | 242 | -26 | MIRb | MIR | SINE |
| 106383287 | 106383604 | - | 9.0 | 1 | 310 | -1 | AluY | Alu | SINE |
| 106383605 | 106383785 | - | 27.1 | 3 | 225 | -41 | MIRb | MIR | SINE |

  

|  |  |  |  |  |  |  |  |  |  |
| --- | --- | --- | --- | --- | --- | --- | --- | --- | --- |
| chr2:106391512-106391967 | | | 456 bp | | |  |  |  |  |
| Genome Start | Genome End | Strand | Div | Repeat Start | Repeat End | Repeat Left | Name | Family | Class |
| 106391512 | 106391554 | - | 24.6 | 96 | 141 | -120 | MER112 | MER1\_type | DNA |
| 106391555 | 106391873 | - | 21.5 | 1 | 304 | -8 | AluJo | Alu | SINE |
| 106391874 | 106391967 | - | 24.6 | 3 | 95 | -165 | MER112 | MER1\_type | DNA |

  

|  |  |  |  |  |  |  |  |  |  |
| --- | --- | --- | --- | --- | --- | --- | --- | --- | --- |
| chr2:106392214-106393140 | | | 834 bp | | |  |  |  |  |
| Genome Start | Genome End | Strand | Div | Repeat Start | Repeat End | Repeat Left | Name | Family | Class |
| 106392214 | 106392309 | - | 22.9 | 1614 | 1713 | -4436 | L1MEd | L1 | LINE |
| 106392403 | 106392759 | - | 24.5 | 1002 | 1407 | -4903 | L1MEd | L1 | LINE |
| 106392760 | 106393062 | - | 9.9 | 1 | 303 | -10 | AluSq | Alu | SINE |
| 106393063 | 106393140 | - | 23.6 | 936 | 1001 | -5118 | L1MEd | L1 | LINE |

  

|  |  |  |  |  |  |  |  |  |  |
| --- | --- | --- | --- | --- | --- | --- | --- | --- | --- |
| chr2:106394191-106394806 | | | 616 bp | | |  |  |  |  |
| Genome Start | Genome End | Strand | Div | Repeat Start | Repeat End | Repeat Left | Name | Family | Class |
| 106394191 | 106394434 | + | 28.2 | 7250 | 7514 | -447 | L1MC5 | L1 | LINE |
| 106394435 | 106394735 | + | 12.0 | 1 | 309 | 0 | AluSc | Alu | SINE |
| 106394736 | 106394806 | + | 28.2 | 7515 | 7588 | -373 | L1MC5 | L1 | LINE |

  

|  |  |  |  |  |  |  |  |  |  |
| --- | --- | --- | --- | --- | --- | --- | --- | --- | --- |
| chr2:106420441-106421812 | | | 1264 bp | | |  |  |  |  |
| Genome Start | Genome End | Strand | Div | Repeat Start | Repeat End | Repeat Left | Name | Family | Class |
| 106420441 | 106420745 | + | 27.7 | 5539 | 5864 | -257 | L1ME4a | L1 | LINE |
| 106420790 | 106420826 | + | 21.6 | 1 | 37 | -96 | AluJo | Alu | SINE |
| 106420827 | 106421115 | - | 10.0 | 1 | 293 | -19 | AluSx | Alu | SINE |
| 106421116 | 106421379 | + | 15.7 | 26 | 286 | -26 | AluJo | Alu | SINE |
| 106421388 | 106421694 | + | 11.1 | 1 | 307 | -6 | AluSq | Alu | SINE |
| 106421751 | 106421812 | + | 22.6 | 5978 | 6041 | -80 | L1ME4a | L1 | LINE |

  

|  |  |  |  |  |  |  |  |  |  |
| --- | --- | --- | --- | --- | --- | --- | --- | --- | --- |
| chr2:106431445-106434091 | | | 2318 bp | | |  |  |  |  |
| Genome Start | Genome End | Strand | Div | Repeat Start | Repeat End | Repeat Left | Name | Family | Class |
| 106431445 | 106431577 | + | 20.9 | 13 | 143 | -380 | Charlie1b | MER1\_type | DNA |
| 106431578 | 106431878 | - | 12.0 | 1 | 299 | -10 | AluSc | Alu | SINE |
| 106431879 | 106432070 | + | 20.9 | 144 | 332 | -191 | Charlie1b | MER1\_type | DNA |
| 106432071 | 106432375 | + | 9.5 | 1 | 304 | -9 | AluSq | Alu | SINE |
| 106432376 | 106432482 | + | 20.9 | 333 | 445 | -78 | Charlie1b | MER1\_type | DNA |
| 106432485 | 106432567 | + | 13.2 | 2 | 84 | -225 | AluSc | Alu | SINE |
| 106432568 | 106432623 | + | 11.2 | 29 | 84 | -225 | AluSc | Alu | SINE |
| 106432624 | 106432915 | + | 6.2 | 1 | 292 | -19 | AluY | Alu | SINE |
| 106432916 | 106432985 | + | 11.2 | 85 | 144 | -165 | AluSc | Alu | SINE |
| 106432986 | 106433198 | + | 7.5 | 1 | 212 | -99 | AluY | Alu | SINE |
| 106433524 | 106433610 | + | 8.1 | 212 | 298 | -13 | AluY | Alu | SINE |
| 106433611 | 106433780 | + | 15.8 | 119 | 289 | -20 | AluSc | Alu | SINE |
| 106433781 | 106434003 | + | 8.1 | 72 | 307 | -2 | AluSc | Alu | SINE |
| 106434004 | 106434019 | + | 15.8 | 290 | 304 | -5 | AluSc | Alu | SINE |
| 106434022 | 106434091 | + | 20.0 | 436 | 515 | -8 | Charlie1b | MER1\_type | DNA |

  

|  |  |  |  |  |  |  |  |  |  |
| --- | --- | --- | --- | --- | --- | --- | --- | --- | --- |
| chr2:106436053-106437101 | | | 545 bp | | |  |  |  |  |
| Genome Start | Genome End | Strand | Div | Repeat Start | Repeat End | Repeat Left | Name | Family | Class |
| 106436053 | 106436205 | + | 27.8 | 192 | 354 | -3065 | L2 | L2 | LINE |
| 106436373 | 106436489 | - | 19.3 | 86 | 199 | -113 | AluJo | Alu | SINE |
| 106436494 | 106436557 | + | 19.1 | 832 | 894 | -2525 | L2 | L2 | LINE |
| 106436891 | 106437101 | + | 28.3 | 1301 | 1527 | -1892 | L2 | L2 | LINE |

  

|  |  |  |  |  |  |  |  |  |  |
| --- | --- | --- | --- | --- | --- | --- | --- | --- | --- |
| chr2:106437819-106438750 | | | 931 bp | | |  |  |  |  |
| Genome Start | Genome End | Strand | Div | Repeat Start | Repeat End | Repeat Left | Name | Family | Class |
| 106437819 | 106437906 | + | 18.2 | 5678 | 5770 | -589 | L1MD2 | L1 | LINE |
| 106437908 | 106438076 | + | 18.6 | 137 | 303 | -9 | AluSg/x | Alu | SINE |
| 106438077 | 106438210 | + | 17.6 | 5759 | 5908 | -451 | L1MD2 | L1 | LINE |
| 106438211 | 106438518 | + | 11.7 | 1 | 308 | -4 | AluSx | Alu | SINE |
| 106438519 | 106438750 | + | 17.6 | 5909 | 6145 | -214 | L1MD2 | L1 | LINE |

  

|  |  |  |  |  |  |  |  |  |  |
| --- | --- | --- | --- | --- | --- | --- | --- | --- | --- |
| chr2:106445797-106446886 | | | 1050 bp | | |  |  |  |  |
| Genome Start | Genome End | Strand | Div | Repeat Start | Repeat End | Repeat Left | Name | Family | Class |
| 106445797 | 106445980 | + | 19.0 | 1113 | 1344 | -1163 | HAL1 | L1 | LINE |
| 106446001 | 106446157 | + | 24.8 | 1480 | 1667 | -840 | HAL1 | L1 | LINE |
| 106446158 | 106446384 | + | 11.1 | 78 | 304 | -8 | AluSg/x | Alu | SINE |
| 106446385 | 106446614 | + | 24.8 | 1668 | 1901 | -606 | HAL1 | L1 | LINE |
| 106446635 | 106446886 | + | 24.8 | 1902 | 2204 | -303 | HAL1 | L1 | LINE |

  

|  |  |  |  |  |  |  |  |  |  |
| --- | --- | --- | --- | --- | --- | --- | --- | --- | --- |
| chr2:106482121-106482774 | | | 654 bp | | |  |  |  |  |
| Genome Start | Genome End | Strand | Div | Repeat Start | Repeat End | Repeat Left | Name | Family | Class |
| 106482121 | 106482138 | - | 23.0 | 6170 | 6184 | 0 | L1MB7 | L1 | LINE |
| 106482139 | 106482439 | + | 11.3 | 1 | 302 | -10 | AluSx | Alu | SINE |
| 106482440 | 106482774 | - | 23.3 | 5829 | 6169 | -15 | L1MB7 | L1 | LINE |

  

|  |  |  |  |  |  |  |  |  |  |
| --- | --- | --- | --- | --- | --- | --- | --- | --- | --- |
| chr2:106484723-106485346 | | | 624 bp | | |  |  |  |  |
| Genome Start | Genome End | Strand | Div | Repeat Start | Repeat End | Repeat Left | Name | Family | Class |
| 106484723 | 106484901 | + | 30.3 | 5790 | 5971 | -150 | L1ME4a | L1 | LINE |
| 106484902 | 106485207 | + | 11.8 | 1 | 307 | -5 | AluSx | Alu | SINE |
| 106485208 | 106485346 | + | 30.3 | 5972 | 6101 | -20 | L1ME4a | L1 | LINE |

  

|  |  |  |  |  |  |  |  |  |  |
| --- | --- | --- | --- | --- | --- | --- | --- | --- | --- |
| chr2:107822572-107822961 | | | 390 bp | | |  |  |  |  |
| Genome Start | Genome End | Strand | Div | Repeat Start | Repeat End | Repeat Left | Name | Family | Class |
| 107822572 | 107822679 | - | 21.4 | 7886 | 7993 | -2 | L1MD3 | L1 | LINE |
| 107822680 | 107822907 | + | 12.8 | 1 | 236 | 0 | MER46A | MER2\_type | DNA |
| 107822908 | 107822961 | - | 21.4 | 7832 | 7885 | -110 | L1MD3 | L1 | LINE |

  

|  |  |  |  |  |  |  |  |  |  |
| --- | --- | --- | --- | --- | --- | --- | --- | --- | --- |
| chr2:107822961-107823892 | | | 931 bp | | |  |  |  |  |
| Genome Start | Genome End | Strand | Div | Repeat Start | Repeat End | Repeat Left | Name | Family | Class |
| 107822961 | 107823192 | - | 17.8 | 5909 | 6145 | -214 | L1MD2 | L1 | LINE |
| 107823193 | 107823500 | - | 11.4 | 1 | 308 | -4 | AluSx | Alu | SINE |
| 107823501 | 107823634 | - | 17.8 | 5759 | 5908 | -451 | L1MD2 | L1 | LINE |
| 107823635 | 107823803 | - | 19.8 | 137 | 303 | -9 | AluSg/x | Alu | SINE |
| 107823805 | 107823892 | - | 18.2 | 5678 | 5770 | -589 | L1MD2 | L1 | LINE |

  

|  |  |  |  |  |  |  |  |  |  |
| --- | --- | --- | --- | --- | --- | --- | --- | --- | --- |
| chr2:107824610-107825658 | | | 545 bp | | |  |  |  |  |
| Genome Start | Genome End | Strand | Div | Repeat Start | Repeat End | Repeat Left | Name | Family | Class |
| 107824610 | 107824820 | - | 28.3 | 1301 | 1527 | -1892 | L2 | L2 | LINE |
| 107825154 | 107825217 | - | 19.1 | 832 | 894 | -2525 | L2 | L2 | LINE |
| 107825222 | 107825338 | + | 19.3 | 86 | 199 | -113 | AluJo | Alu | SINE |
| 107825506 | 107825658 | - | 27.8 | 192 | 354 | -3065 | L2 | L2 | LINE |

  

|  |  |  |  |  |  |  |  |  |  |
| --- | --- | --- | --- | --- | --- | --- | --- | --- | --- |
| chr2:107834725-107835203 | | | 479 bp | | |  |  |  |  |
| Genome Start | Genome End | Strand | Div | Repeat Start | Repeat End | Repeat Left | Name | Family | Class |
| 107834725 | 107834846 | + | 30.6 | 3286 | 3407 | -1082 | L3 | CR1 | LINE |
| 107834847 | 107835158 | + | 11.0 | 1 | 302 | -10 | AluSx | Alu | SINE |
| 107835159 | 107835203 | + | 30.6 | 3408 | 3452 | -1037 | L3 | CR1 | LINE |

  

|  |  |  |  |  |  |  |  |  |  |
| --- | --- | --- | --- | --- | --- | --- | --- | --- | --- |
| chr2:107839904-107841279 | | | 1230 bp | | |  |  |  |  |
| Genome Start | Genome End | Strand | Div | Repeat Start | Repeat End | Repeat Left | Name | Family | Class |
| 107839904 | 107839962 | - | 22.0 | 5978 | 6038 | -83 | L1ME4a | L1 | LINE |
| 107840019 | 107840326 | - | 11.1 | 1 | 307 | -6 | AluSq | Alu | SINE |
| 107840335 | 107840597 | - | 15.7 | 26 | 286 | -26 | AluJo | Alu | SINE |
| 107840598 | 107840887 | + | 10.3 | 1 | 293 | -19 | AluSx | Alu | SINE |
| 107840888 | 107840924 | - | 21.6 | 1 | 37 | -96 | AluJo | Alu | SINE |
| 107841007 | 107841279 | - | 29.4 | 5533 | 5827 | -294 | L1ME4a | L1 | LINE |

  

|  |  |  |  |  |  |  |  |  |  |
| --- | --- | --- | --- | --- | --- | --- | --- | --- | --- |
| chr2:107866916-107867531 | | | 616 bp | | |  |  |  |  |
| Genome Start | Genome End | Strand | Div | Repeat Start | Repeat End | Repeat Left | Name | Family | Class |
| 107866916 | 107866986 | - | 27.9 | 7515 | 7588 | -373 | L1MC5 | L1 | LINE |
| 107866987 | 107867287 | - | 11.3 | 1 | 309 | 0 | AluSc | Alu | SINE |
| 107867288 | 107867531 | - | 27.9 | 7250 | 7514 | -447 | L1MC5 | L1 | LINE |

  

|  |  |  |  |  |  |  |  |  |  |
| --- | --- | --- | --- | --- | --- | --- | --- | --- | --- |
| chr2:107868582-107869506 | | | 843 bp | | |  |  |  |  |
| Genome Start | Genome End | Strand | Div | Repeat Start | Repeat End | Repeat Left | Name | Family | Class |
| 107868582 | 107868659 | + | 23.6 | 936 | 1001 | -5118 | L1MEd | L1 | LINE |
| 107868660 | 107868958 | + | 10.1 | 1 | 299 | -14 | AluSq | Alu | SINE |
| 107868959 | 107869291 | + | 24.4 | 1002 | 1407 | -4903 | L1MEd | L1 | LINE |
| 107869374 | 107869506 | + | 26.3 | 1566 | 1713 | -4436 | L1MEd | L1 | LINE |

  

|  |  |  |  |  |  |  |  |  |  |
| --- | --- | --- | --- | --- | --- | --- | --- | --- | --- |
| chr2:107869755-107870209 | | | 455 bp | | |  |  |  |  |
| Genome Start | Genome End | Strand | Div | Repeat Start | Repeat End | Repeat Left | Name | Family | Class |
| 107869755 | 107869848 | + | 24.6 | 2 | 94 | -166 | MER112 | MER1\_type | DNA |
| 107869849 | 107870166 | + | 20.9 | 1 | 303 | -9 | AluJo | Alu | SINE |
| 107870167 | 107870209 | + | 24.6 | 95 | 140 | -121 | MER112 | MER1\_type | DNA |

  

|  |  |  |  |  |  |  |  |  |  |
| --- | --- | --- | --- | --- | --- | --- | --- | --- | --- |
| chr2:107877936-107878445 | | | 510 bp | | |  |  |  |  |
| Genome Start | Genome End | Strand | Div | Repeat Start | Repeat End | Repeat Left | Name | Family | Class |
| 107877936 | 107878116 | + | 27.1 | 3 | 225 | -41 | MIRb | MIR | SINE |
| 107878117 | 107878428 | + | 6.9 | 1 | 304 | -7 | AluY | Alu | SINE |
| 107878429 | 107878445 | + | 25.4 | 226 | 242 | -26 | MIRb | MIR | SINE |

  

|  |  |  |  |  |  |  |  |  |  |
| --- | --- | --- | --- | --- | --- | --- | --- | --- | --- |
| chr2:107883115-107884586 | | | 1472 bp | | |  |  |  |  |
| Genome Start | Genome End | Strand | Div | Repeat Start | Repeat End | Repeat Left | Name | Family | Class |
| 107883115 | 107883825 | + | 16.8 | 721 | 1513 | -5238 | L1MCb | L1 | LINE |
| 107883826 | 107884136 | + | 8.4 | 1 | 311 | 0 | AluY | Alu | SINE |
| 107884137 | 107884196 | + | 16.7 | 1514 | 1565 | -5186 | L1MCb | L1 | LINE |
| 107884197 | 107884544 | + | 19.6 | 2 | 362 | -3 | MLT1A0 | MaLR | LTR |
| 107884545 | 107884586 | + | 16.7 | 1566 | 1607 | -5144 | L1MCb | L1 | LINE |

  

|  |  |  |  |  |  |  |  |  |  |
| --- | --- | --- | --- | --- | --- | --- | --- | --- | --- |
| chr2:108486620-108487097 | | | 478 bp | | |  |  |  |  |
| Genome Start | Genome End | Strand | Div | Repeat Start | Repeat End | Repeat Left | Name | Family | Class |
| 108486620 | 108486714 | + | 22.6 | 1 | 94 | -166 | MER112 | MER1\_type | DNA |
| 108486715 | 108487033 | + | 22.5 | 1 | 304 | -8 | AluJo | Alu | SINE |
| 108487034 | 108487097 | + | 22.6 | 95 | 160 | -101 | MER112 | MER1\_type | DNA |

  

|  |  |  |  |  |  |  |  |  |  |
| --- | --- | --- | --- | --- | --- | --- | --- | --- | --- |
| chr2:108494840-108495286 | | | 447 bp | | |  |  |  |  |
| Genome Start | Genome End | Strand | Div | Repeat Start | Repeat End | Repeat Left | Name | Family | Class |
| 108494840 | 108494966 | + | 27.7 | 80 | 225 | -43 | MIRb | MIR | SINE |
| 108494967 | 108495269 | + | 10.6 | 1 | 303 | -8 | AluY | Alu | SINE |
| 108495270 | 108495286 | + | 27.7 | 226 | 242 | -26 | MIRb | MIR | SINE |

  

|  |  |  |  |  |  |  |  |  |  |
| --- | --- | --- | --- | --- | --- | --- | --- | --- | --- |
| chr2:108505720-108506584 | | | 865 bp | | |  |  |  |  |
| Genome Start | Genome End | Strand | Div | Repeat Start | Repeat End | Repeat Left | Name | Family | Class |
| 108505720 | 108505811 | - | 15.1 | 5823 | 5909 | -262 | L1MB2 | L1 | LINE |
| 108505812 | 108506118 | - | 10.4 | 1 | 307 | -6 | AluSq | Alu | SINE |
| 108506119 | 108506584 | - | 15.1 | 5362 | 5822 | -349 | L1MB2 | L1 | LINE |

  

|  |  |  |  |  |  |  |  |  |  |
| --- | --- | --- | --- | --- | --- | --- | --- | --- | --- |
| chr2:108661896-108662505 | | | 610 bp | | |  |  |  |  |
| Genome Start | Genome End | Strand | Div | Repeat Start | Repeat End | Repeat Left | Name | Family | Class |
| 108661896 | 108662084 | + | 17.5 | 2 | 225 | -120 | MER2 | MER2\_type | DNA |
| 108662085 | 108662385 | + | 11.7 | 1 | 299 | -14 | AluSq | Alu | SINE |
| 108662386 | 108662505 | + | 17.5 | 226 | 345 | 0 | MER2 | MER2\_type | DNA |

  

|  |  |  |  |  |  |  |  |  |  |
| --- | --- | --- | --- | --- | --- | --- | --- | --- | --- |
| chr2:108699181-108699686 | | | 506 bp | | |  |  |  |  |
| Genome Start | Genome End | Strand | Div | Repeat Start | Repeat End | Repeat Left | Name | Family | Class |
| 108699181 | 108699230 | + | 25.1 | 74 | 114 | -148 | MIR | MIR | SINE |
| 108699231 | 108699542 | - | 15.7 | 1 | 307 | -5 | AluJo | Alu | SINE |
| 108699543 | 108699686 | + | 25.1 | 115 | 262 | 0 | MIR | MIR | SINE |

  

|  |  |  |  |  |  |  |  |  |  |
| --- | --- | --- | --- | --- | --- | --- | --- | --- | --- |
| chr2:108706528-108707637 | | | 1060 bp | | |  |  |  |  |
| Genome Start | Genome End | Strand | Div | Repeat Start | Repeat End | Repeat Left | Name | Family | Class |
| 108706528 | 108706780 | - | 26.5 | 1905 | 2204 | -303 | HAL1 | L1 | LINE |
| 108706809 | 108707042 | - | 26.5 | 1669 | 1904 | -603 | HAL1 | L1 | LINE |
| 108707043 | 108707273 | - | 10.0 | 78 | 308 | -4 | AluSg/x | Alu | SINE |
| 108707274 | 108707432 | - | 26.5 | 1481 | 1668 | -839 | HAL1 | L1 | LINE |
| 108707455 | 108707637 | - | 22.9 | 1113 | 1344 | -1163 | HAL1 | L1 | LINE |

  

|  |  |  |  |  |  |  |  |  |  |
| --- | --- | --- | --- | --- | --- | --- | --- | --- | --- |
| chr2:108715082-108715472 | | | 391 bp | | |  |  |  |  |
| Genome Start | Genome End | Strand | Div | Repeat Start | Repeat End | Repeat Left | Name | Family | Class |
| 108715082 | 108715189 | - | 20.8 | 7886 | 7993 | -2 | L1MD3 | L1 | LINE |
| 108715190 | 108715418 | + | 11.8 | 1 | 236 | 0 | MER46A | MER2\_type | DNA |
| 108715419 | 108715472 | - | 20.8 | 7832 | 7885 | -110 | L1MD3 | L1 | LINE |

  

|  |  |  |  |  |  |  |  |  |  |
| --- | --- | --- | --- | --- | --- | --- | --- | --- | --- |
| chr2:108715472-108716408 | | | 934 bp | | |  |  |  |  |
| Genome Start | Genome End | Strand | Div | Repeat Start | Repeat End | Repeat Left | Name | Family | Class |
| 108715472 | 108715705 | - | 17.9 | 5899 | 6145 | -214 | L1MD2 | L1 | LINE |
| 108715706 | 108716011 | - | 11.4 | 1 | 306 | -6 | AluSx | Alu | SINE |
| 108716012 | 108716143 | - | 17.9 | 5761 | 5898 | -461 | L1MD2 | L1 | LINE |
| 108716146 | 108716319 | - | 17.4 | 137 | 308 | -4 | AluSg/x | Alu | SINE |
| 108716321 | 108716408 | - | 17.1 | 5678 | 5770 | -589 | L1MD2 | L1 | LINE |

  

|  |  |  |  |  |  |  |  |  |  |
| --- | --- | --- | --- | --- | --- | --- | --- | --- | --- |
| chr2:108726888-108727356 | | | 469 bp | | |  |  |  |  |
| Genome Start | Genome End | Strand | Div | Repeat Start | Repeat End | Repeat Left | Name | Family | Class |
| 108726888 | 108727009 | + | 31.2 | 3286 | 3407 | -1082 | L3 | CR1 | LINE |
| 108727010 | 108727311 | + | 10.7 | 1 | 300 | -12 | AluSx | Alu | SINE |
| 108727312 | 108727356 | + | 31.2 | 3408 | 3452 | -1037 | L3 | CR1 | LINE |

  

|  |  |  |  |  |  |  |  |  |  |
| --- | --- | --- | --- | --- | --- | --- | --- | --- | --- |
| chr2:108732064-108733438 | | | 1225 bp | | |  |  |  |  |
| Genome Start | Genome End | Strand | Div | Repeat Start | Repeat End | Repeat Left | Name | Family | Class |
| 108732064 | 108732122 | - | 22.0 | 5978 | 6038 | -83 | L1ME4a | L1 | LINE |
| 108732179 | 108732485 | - | 10.8 | 1 | 307 | -6 | AluSq | Alu | SINE |
| 108732494 | 108732755 | - | 16.9 | 26 | 286 | -26 | AluJo | Alu | SINE |
| 108732756 | 108733044 | + | 9.7 | 1 | 293 | -19 | AluSx | Alu | SINE |
| 108733045 | 108733081 | - | 21.6 | 1 | 37 | -96 | AluJo | Alu | SINE |
| 108733168 | 108733438 | - | 28.5 | 5533 | 5827 | -294 | L1ME4a | L1 | LINE |

  

|  |  |  |  |  |  |  |  |  |  |
| --- | --- | --- | --- | --- | --- | --- | --- | --- | --- |
| chr2:109832917-109833963 | | | 1047 bp | | |  |  |  |  |
| Genome Start | Genome End | Strand | Div | Repeat Start | Repeat End | Repeat Left | Name | Family | Class |
| 109832917 | 109833563 | + | 17.1 | 5473 | 6117 | -61 | L1MB8 | L1 | LINE |
| 109833564 | 109833904 | + | 10.0 | 1 | 309 | 0 | AluSc | Alu | SINE |
| 109833905 | 109833963 | + | 17.1 | 6118 | 6158 | -20 | L1MB8 | L1 | LINE |

  

|  |  |  |  |  |  |  |  |  |  |
| --- | --- | --- | --- | --- | --- | --- | --- | --- | --- |
| chr2:109848084-109849721 | | | 1634 bp | | |  |  |  |  |
| Genome Start | Genome End | Strand | Div | Repeat Start | Repeat End | Repeat Left | Name | Family | Class |
| 109848084 | 109848443 | + | 21.1 | 3777 | 4169 | -1977 | L1M4 | L1 | LINE |
| 109848444 | 109848751 | + | 18.0 | 8 | 300 | -12 | AluJb | Alu | SINE |
| 109848756 | 109849059 | + | 12.1 | 1 | 297 | -15 | AluSx | Alu | SINE |
| 109849060 | 109849721 | + | 21.1 | 4170 | 4857 | -1289 | L1M4 | L1 | LINE |

  

|  |  |  |  |  |  |  |  |  |  |
| --- | --- | --- | --- | --- | --- | --- | --- | --- | --- |
| chr2:109855153-109861102 | | | 5914 bp | | |  |  |  |  |
| Genome Start | Genome End | Strand | Div | Repeat Start | Repeat End | Repeat Left | Name | Family | Class |
| 109855153 | 109855472 | + | 15.0 | 2426 | 2773 | -3373 | L1MC2 | L1 | LINE |
| 109855503 | 109856302 | + | 18.4 | 3205 | 4024 | -2122 | L1MC2 | L1 | LINE |
| 109856303 | 109856605 | + | 12.9 | 1 | 303 | -9 | AluSx | Alu | SINE |
| 109856606 | 109857005 | + | 18.4 | 4025 | 4422 | -1724 | L1MC2 | L1 | LINE |
| 109857006 | 109857314 | + | 11.0 | 1 | 310 | -3 | AluSq | Alu | SINE |
| 109857315 | 109857854 | + | 18.4 | 4423 | 4951 | -1195 | L1MC2 | L1 | LINE |
| 109857855 | 109858151 | + | 17.9 | 1 | 297 | -15 | AluJb | Alu | SINE |
| 109858152 | 109858476 | + | 18.4 | 4952 | 5281 | -865 | L1MC2 | L1 | LINE |
| 109858485 | 109859406 | - | 12.9 | 5192 | 6166 | 0 | L1PA16 | L1 | LINE |
| 109859405 | 109859971 | + | 16.9 | 5260 | 5822 | -522 | L1MC2 | L1 | LINE |
| 109859972 | 109860279 | - | 21.9 | 7 | 312 | 0 | AluJb | Alu | SINE |
| 109860280 | 109860592 | + | 16.9 | 5823 | 6123 | -221 | L1MC2 | L1 | LINE |
| 109860593 | 109860886 | + | 12.2 | 1 | 295 | -17 | AluSx | Alu | SINE |
| 109860887 | 109861102 | + | 16.9 | 6124 | 6343 | -1 | L1MC2 | L1 | LINE |

  

|  |  |  |  |  |  |  |  |  |  |
| --- | --- | --- | --- | --- | --- | --- | --- | --- | --- |
| chr2:109899669-109901958 | | | 2223 bp | | |  |  |  |  |
| Genome Start | Genome End | Strand | Div | Repeat Start | Repeat End | Repeat Left | Name | Family | Class |
| 109899669 | 109900702 | + | 21.7 | 4304 | 5444 | -900 | L1MC3 | L1 | LINE |
| 109900706 | 109901048 | + | 20.2 | 76 | 459 | -56 | MLT2B2 | ERVL | LTR |
| 109901049 | 109901331 | - | 16.6 | 2 | 299 | -13 | AluJb | Alu | SINE |
| 109901332 | 109901351 | + | 20.2 | 507 | 522 | -40 | MLT2B2 | ERVL | LTR |
| 109901416 | 109901958 | + | 22.8 | 5456 | 6018 | -1767 | L1MC3 | L1 | LINE |

  

|  |  |  |  |  |  |  |  |  |  |
| --- | --- | --- | --- | --- | --- | --- | --- | --- | --- |
| chr2:109911396-109913071 | | | 1654 bp | | |  |  |  |  |
| Genome Start | Genome End | Strand | Div | Repeat Start | Repeat End | Repeat Left | Name | Family | Class |
| 109911396 | 109911498 | - | 25.6 | 2287 | 2372 | -135 | HAL1 | L1 | LINE |
| 109911499 | 109911806 | - | 5.4 | 1 | 312 | 0 | AluY | Alu | SINE |
| 109911807 | 109911987 | - | 25.6 | 2061 | 2286 | -221 | HAL1 | L1 | LINE |
| 109911988 | 109912088 | - | 2.0 | 6055 | 6155 | 0 | L1PA2 | L1 | LINE |
| 109912089 | 109912481 | - | 25.6 | 1668 | 2060 | -447 | HAL1 | L1 | LINE |
| 109912482 | 109912708 | - | 11.5 | 78 | 304 | -8 | AluSg/x | Alu | SINE |
| 109912709 | 109912865 | - | 25.6 | 1480 | 1667 | -840 | HAL1 | L1 | LINE |
| 109912888 | 109913071 | - | 19.6 | 1113 | 1344 | -1163 | HAL1 | L1 | LINE |

  

|  |  |  |  |  |  |  |  |  |  |
| --- | --- | --- | --- | --- | --- | --- | --- | --- | --- |
| chr2:109919716-109920105 | | | 390 bp | | |  |  |  |  |
| Genome Start | Genome End | Strand | Div | Repeat Start | Repeat End | Repeat Left | Name | Family | Class |
| 109919716 | 109919823 | - | 21.4 | 7886 | 7993 | -2 | L1MD3 | L1 | LINE |
| 109919824 | 109920051 | + | 12.8 | 1 | 236 | 0 | MER46A | MER2\_type | DNA |
| 109920052 | 109920105 | - | 21.4 | 7832 | 7885 | -110 | L1MD3 | L1 | LINE |

  

|  |  |  |  |  |  |  |  |  |  |
| --- | --- | --- | --- | --- | --- | --- | --- | --- | --- |
| chr2:109931852-109932322 | | | 471 bp | | |  |  |  |  |
| Genome Start | Genome End | Strand | Div | Repeat Start | Repeat End | Repeat Left | Name | Family | Class |
| 109931852 | 109931973 | + | 31.2 | 3286 | 3407 | -1082 | L3 | CR1 | LINE |
| 109931974 | 109932277 | + | 11.3 | 1 | 302 | -10 | AluSx | Alu | SINE |
| 109932278 | 109932322 | + | 31.2 | 3408 | 3452 | -1037 | L3 | CR1 | LINE |

  

|  |  |  |  |  |  |  |  |  |  |
| --- | --- | --- | --- | --- | --- | --- | --- | --- | --- |
| chr2:109935586-109936426 | | | 841 bp | | |  |  |  |  |
| Genome Start | Genome End | Strand | Div | Repeat Start | Repeat End | Repeat Left | Name | Family | Class |
| 109935586 | 109935649 | + | 35.9 | 2 | 57 | -121 | MER5B | MER1\_type | DNA |
| 109935650 | 109935983 | - | 15.7 | 5820 | 6163 | -15 | L1MB8 | L1 | LINE |
| 109935984 | 109936057 | + | 35.9 | 58 | 121 | -57 | MER5B | MER1\_type | DNA |
| 109936058 | 109936371 | - | 7.6 | 1 | 305 | -6 | AluY | Alu | SINE |
| 109936372 | 109936426 | + | 35.9 | 122 | 172 | -6 | MER5B | MER1\_type | DNA |

  

|  |  |  |  |  |  |  |  |  |  |
| --- | --- | --- | --- | --- | --- | --- | --- | --- | --- |
| chr2:109937027-109938400 | | | 1196 bp | | |  |  |  |  |
| Genome Start | Genome End | Strand | Div | Repeat Start | Repeat End | Repeat Left | Name | Family | Class |
| 109937027 | 109937085 | - | 22.0 | 5978 | 6038 | -83 | L1ME4a | L1 | LINE |
| 109937170 | 109937451 | - | 9.8 | 6 | 287 | -26 | AluSq | Alu | SINE |
| 109937460 | 109937721 | - | 17.7 | 26 | 286 | -26 | AluJo | Alu | SINE |
| 109937722 | 109938010 | + | 9.3 | 1 | 293 | -19 | AluSx | Alu | SINE |
| 109938011 | 109938047 | - | 21.6 | 1 | 37 | -96 | AluJo | Alu | SINE |
| 109938134 | 109938400 | - | 28.6 | 5539 | 5827 | -294 | L1ME4a | L1 | LINE |

  

|  |  |  |  |  |  |  |  |  |  |
| --- | --- | --- | --- | --- | --- | --- | --- | --- | --- |
| chr2:109964024-109964639 | | | 616 bp | | |  |  |  |  |
| Genome Start | Genome End | Strand | Div | Repeat Start | Repeat End | Repeat Left | Name | Family | Class |
| 109964024 | 109964094 | - | 28.8 | 7520 | 7588 | -373 | L1MC5 | L1 | LINE |
| 109964095 | 109964395 | - | 10.7 | 1 | 309 | 0 | AluSc | Alu | SINE |
| 109964396 | 109964639 | - | 28.8 | 7250 | 7519 | -442 | L1MC5 | L1 | LINE |

  

|  |  |  |  |  |  |  |  |  |  |
| --- | --- | --- | --- | --- | --- | --- | --- | --- | --- |
| chr2:109964662-109964959 | | | 298 bp | | |  |  |  |  |
| Genome Start | Genome End | Strand | Div | Repeat Start | Repeat End | Repeat Left | Name | Family | Class |
| 109964662 | 109964743 | - | 20.1 | 7342 | 7413 | -548 | L1MC5 | L1 | LINE |
| 109964744 | 109964891 | - | 21.6 | 5 | 188 | -1 | MER5A | MER1\_type | DNA |
| 109964892 | 109964959 | - | 20.1 | 7269 | 7341 | -620 | L1MC5 | L1 | LINE |

  

|  |  |  |  |  |  |  |  |  |  |
| --- | --- | --- | --- | --- | --- | --- | --- | --- | --- |
| chr2:109966891-109967368 | | | 478 bp | | |  |  |  |  |
| Genome Start | Genome End | Strand | Div | Repeat Start | Repeat End | Repeat Left | Name | Family | Class |
| 109966891 | 109966987 | + | 25.5 | 1 | 94 | -166 | MER112 | MER1\_type | DNA |
| 109966988 | 109967306 | + | 20.0 | 1 | 304 | -8 | AluJo | Alu | SINE |
| 109967307 | 109967368 | + | 25.5 | 95 | 158 | -103 | MER112 | MER1\_type | DNA |

  

|  |  |  |  |  |  |  |  |  |  |
| --- | --- | --- | --- | --- | --- | --- | --- | --- | --- |
| chr2:109974955-109975461 | | | 507 bp | | |  |  |  |  |
| Genome Start | Genome End | Strand | Div | Repeat Start | Repeat End | Repeat Left | Name | Family | Class |
| 109974955 | 109975138 | + | 25.6 | 3 | 225 | -41 | MIRb | MIR | SINE |
| 109975139 | 109975444 | + | 8.2 | 1 | 306 | -5 | AluY | Alu | SINE |
| 109975445 | 109975461 | + | 23.4 | 226 | 242 | -26 | MIRb | MIR | SINE |

  

|  |  |  |  |  |  |  |  |  |  |
| --- | --- | --- | --- | --- | --- | --- | --- | --- | --- |
| chr2:109980151-109982737 | | | 2492 bp | | |  |  |  |  |
| Genome Start | Genome End | Strand | Div | Repeat Start | Repeat End | Repeat Left | Name | Family | Class |
| 109980151 | 109980861 | + | 16.4 | 721 | 1513 | -5238 | L1MC2 | L1 | LINE |
| 109980862 | 109981172 | + | 8.0 | 1 | 311 | 0 | AluY | Alu | SINE |
| 109981173 | 109981233 | + | 16.0 | 1514 | 1564 | -5187 | L1MC2 | L1 | LINE |
| 109981234 | 109981579 | + | 19.1 | 4 | 362 | -3 | MLT1A0 | MaLR | LTR |
| 109981580 | 109981641 | + | 16.0 | 1565 | 1626 | -5125 | L1MC2 | L1 | LINE |
| 109981661 | 109981956 | - | 10.8 | 4 | 298 | -15 | AluSq | Alu | SINE |
| 109981971 | 109982255 | + | 17.2 | 5370 | 5655 | -909 | L1MC2 | L1 | LINE |
| 109982318 | 109982737 | + | 16.7 | 6350 | 6762 | 0 | L1MC2 | L1 | LINE |

  

|  |  |  |  |  |  |  |  |  |  |
| --- | --- | --- | --- | --- | --- | --- | --- | --- | --- |
| chr2:109982739-109983774 | | | 1033 bp | | |  |  |  |  |
| Genome Start | Genome End | Strand | Div | Repeat Start | Repeat End | Repeat Left | Name | Family | Class |
| 109982739 | 109982807 | + | 19.1 | 1221 | 1289 | -5120 | L1MDb | L1 | LINE |
| 109982811 | 109982980 | + | 19.9 | 2370 | 2535 | -3871 | L1MDb | L1 | LINE |
| 109982981 | 109983266 | + | 12.2 | 1 | 286 | -25 | AluY | Alu | SINE |
| 109983267 | 109983327 | + | 19.9 | 2536 | 2585 | -3821 | L1MDb | L1 | LINE |
| 109983328 | 109983649 | + | 19.0 | 1 | 310 | -2 | AluJo | Alu | SINE |
| 109983650 | 109983774 | + | 19.9 | 2586 | 2713 | -3693 | L1MDb | L1 | LINE |

  

|  |  |  |  |  |  |  |  |  |  |
| --- | --- | --- | --- | --- | --- | --- | --- | --- | --- |
| chr2:109990470-109991063 | | | 594 bp | | |  |  |  |  |
| Genome Start | Genome End | Strand | Div | Repeat Start | Repeat End | Repeat Left | Name | Family | Class |
| 109990470 | 109990668 | + | 16.2 | 1 | 180 | -132 | AluJb | Alu | SINE |
| 109990669 | 109990956 | + | 8.3 | 3 | 290 | -21 | AluY | Alu | SINE |
| 109990957 | 109991063 | + | 16.2 | 181 | 284 | -28 | AluJb | Alu | SINE |

  

|  |  |  |  |  |  |  |  |  |  |
| --- | --- | --- | --- | --- | --- | --- | --- | --- | --- |
| chr2:109996325-109997414 | | | 1090 bp | | |  |  |  |  |
| Genome Start | Genome End | Strand | Div | Repeat Start | Repeat End | Repeat Left | Name | Family | Class |
| 109996325 | 109996682 | - | 13.2 | 5738 | 6085 | -71 | L1PB4 | L1 | LINE |
| 109996683 | 109996984 | - | 12.9 | 1 | 298 | -14 | AluSx | Alu | SINE |
| 109996985 | 109997414 | - | 13.2 | 5287 | 5737 | -419 | L1PB4 | L1 | LINE |

  

|  |  |  |  |  |  |  |  |  |  |
| --- | --- | --- | --- | --- | --- | --- | --- | --- | --- |
| chr2:109998317-110000720 | | | 2404 bp | | |  |  |  |  |
| Genome Start | Genome End | Strand | Div | Repeat Start | Repeat End | Repeat Left | Name | Family | Class |
| 109998317 | 109999799 | - | 17.0 | 2914 | 4396 | -1747 | L1M2 | L1 | LINE |
| 109999800 | 110000097 | + | 14.2 | 1 | 298 | -15 | AluSp | Alu | SINE |
| 110000098 | 110000273 | - | 17.0 | 2750 | 2913 | -3230 | L1M2 | L1 | LINE |
| 110000274 | 110000578 | + | 9.5 | 1 | 305 | -8 | AluSq | Alu | SINE |
| 110000579 | 110000720 | - | 17.0 | 2591 | 2749 | -3394 | L1M2 | L1 | LINE |

  

|  |  |  |  |  |  |  |  |  |  |
| --- | --- | --- | --- | --- | --- | --- | --- | --- | --- |
| chr2:110008361-110009256 | | | 896 bp | | |  |  |  |  |
| Genome Start | Genome End | Strand | Div | Repeat Start | Repeat End | Repeat Left | Name | Family | Class |
| 110008361 | 110008525 | - | 12.4 | 147 | 312 | 0 | AluSx | Alu | SINE |
| 110008526 | 110009099 | - | 7.5 | 1 | 610 | 0 | LTR66 | ERVL | LTR |
| 110009100 | 110009256 | - | 12.4 | 1 | 146 | -166 | AluSx | Alu | SINE |

  

|  |  |  |  |  |  |  |  |  |  |
| --- | --- | --- | --- | --- | --- | --- | --- | --- | --- |
| chr2:110023440-110028916 | | | 5244 bp | | |  |  |  |  |
| Genome Start | Genome End | Strand | Div | Repeat Start | Repeat End | Repeat Left | Name | Family | Class |
| 110023440 | 110023642 | + | 18.3 | 187 | 478 | -2383 | Charlie2a | MER1\_type | DNA |
| 110023651 | 110023968 | - | 16.7 | 1 | 338 | -8 | MER7A | MER2\_type | DNA |
| 110024026 | 110024333 | - | 11.4 | 1 | 309 | -1 | AluSg | Alu | SINE |
| 110024335 | 110024455 | + | 24.2 | 506 | 627 | -2234 | Charlie2a | MER1\_type | DNA |
| 110024466 | 110024823 | - | 18.5 | 474 | 859 | -4 | MER21B | ERV1 | LTR |
| 110024834 | 110024996 | - | 20.9 | 136 | 298 | -14 | AluJb | Alu | SINE |
| 110025025 | 110025312 | + | 17.4 | 1 | 291 | -21 | AluJo | Alu | SINE |
| 110025339 | 110025806 | - | 17.9 | 1 | 480 | -383 | MER21B | ERV1 | LTR |
| 110025868 | 110025972 | - | 23.8 | 12 | 121 | -141 | MIR | MIR | SINE |
| 110025993 | 110026196 | + | 18.8 | 932 | 1136 | -1725 | Charlie2a | MER1\_type | DNA |
| 110026197 | 110026557 | - | 17.5 | 5 | 345 | 0 | MER2 | MER2\_type | DNA |
| 110026577 | 110027274 | - | 12.3 | 5604 | 6300 | 0 | L1MA6 | L1 | LINE |
| 110027275 | 110027454 | + | 26.3 | 1151 | 1361 | -1500 | Charlie2a | MER1\_type | DNA |
| 110027455 | 110027578 | + | 21.8 | 2 | 127 | -6 | FLAM\_A | Alu | SINE |
| 110027579 | 110027625 | + | 24.9 | 1362 | 1502 | -1359 | Charlie2a | MER1\_type | DNA |
| 110027626 | 110027914 | + | 11.5 | 1 | 292 | -20 | AluSx | Alu | SINE |
| 110027915 | 110028040 | + | 22.8 | 1503 | 1644 | -1217 | Charlie2a | MER1\_type | DNA |
| 110028034 | 110028698 | + | 22.5 | 1945 | 2714 | -148 | Charlie2a | MER1\_type | DNA |
| 110028699 | 110028830 | + | 22.1 | 1 | 132 | -1 | FLAM\_C | Alu | SINE |
| 110028831 | 110028916 | + | 25.0 | 2715 | 2800 | -62 | Charlie2a | MER1\_type | DNA |

  

|  |  |  |  |  |  |  |  |  |  |
| --- | --- | --- | --- | --- | --- | --- | --- | --- | --- |
| chr2:110037536-110038145 | | | 610 bp | | |  |  |  |  |
| Genome Start | Genome End | Strand | Div | Repeat Start | Repeat End | Repeat Left | Name | Family | Class |
| 110037536 | 110037724 | + | 19.4 | 2 | 225 | -120 | MER2 | MER2\_type | DNA |
| 110037725 | 110038025 | + | 11.0 | 1 | 300 | -13 | AluSq | Alu | SINE |
| 110038026 | 110038145 | + | 19.4 | 226 | 345 | 0 | MER2 | MER2\_type | DNA |

  

|  |  |  |  |  |  |  |  |  |  |
| --- | --- | --- | --- | --- | --- | --- | --- | --- | --- |
| chr2:110038477-110039083 | | | 607 bp | | |  |  |  |  |
| Genome Start | Genome End | Strand | Div | Repeat Start | Repeat End | Repeat Left | Name | Family | Class |
| 110038477 | 110038622 | + | 15.9 | 1 | 142 | -170 | AluJb | Alu | SINE |
| 110038623 | 110038920 | + | 8.1 | 1 | 300 | -9 | AluSc | Alu | SINE |
| 110038921 | 110039083 | + | 15.9 | 143 | 291 | -21 | AluJb | Alu | SINE |

  

|  |  |  |  |  |  |  |  |  |  |
| --- | --- | --- | --- | --- | --- | --- | --- | --- | --- |
| chr2:110048241-110050042 | | | 1740 bp | | |  |  |  |  |
| Genome Start | Genome End | Strand | Div | Repeat Start | Repeat End | Repeat Left | Name | Family | Class |
| 110048241 | 110048618 | - | 24.5 | 7505 | 7919 | -42 | L1MC5 | L1 | LINE |
| 110048619 | 110048897 | + | 14.5 | 13 | 288 | -24 | AluJo | Alu | SINE |
| 110048898 | 110048942 | - | 24.5 | 7459 | 7504 | -457 | L1MC5 | L1 | LINE |
| 110048965 | 110049762 | - | 24.5 | 6683 | 7458 | -503 | L1MC5 | L1 | LINE |
| 110049803 | 110050042 | - | 28.0 | 5746 | 6006 | -1802 | L1MC5 | L1 | LINE |

  

|  |  |  |  |  |  |  |  |  |  |
| --- | --- | --- | --- | --- | --- | --- | --- | --- | --- |
| chr2:110050752-110051305 | | | 554 bp | | |  |  |  |  |
| Genome Start | Genome End | Strand | Div | Repeat Start | Repeat End | Repeat Left | Name | Family | Class |
| 110050752 | 110050910 | + | 29.0 | 8 | 160 | -56 | MIR | MIR | SINE |
| 110050911 | 110051277 | + | 17.4 | 3 | 386 | -4 | MLT1B | MaLR | LTR |
| 110051278 | 110051305 | + | 28.5 | 161 | 182 | -80 | MIR | MIR | SINE |

  

|  |  |  |  |  |  |  |  |  |  |
| --- | --- | --- | --- | --- | --- | --- | --- | --- | --- |
| chr2:110080495-110081179 | | | 694 bp | | |  |  |  |  |
| Genome Start | Genome End | Strand | Div | Repeat Start | Repeat End | Repeat Left | Name | Family | Class |
| 110080495 | 110080591 | + | 24.0 | 1 | 98 | -243 | MER58B | MER1\_type | DNA |
| 110080583 | 110080924 | + | 14.6 | 12 | 365 | 0 | MLT1A0 | MaLR | LTR |
| 110080925 | 110081179 | + | 23.0 | 80 | 340 | -1 | MER58B | MER1\_type | DNA |

  

|  |  |  |  |  |  |  |  |  |  |
| --- | --- | --- | --- | --- | --- | --- | --- | --- | --- |
| chr2:110103347-110104958 | | | 1586 bp | | |  |  |  |  |
| Genome Start | Genome End | Strand | Div | Repeat Start | Repeat End | Repeat Left | Name | Family | Class |
| 110103347 | 110104217 | - | 21.5 | 5275 | 6177 | -1 | L1MB8 | L1 | LINE |
| 110104244 | 110104543 | - | 8.7 | 1 | 299 | -10 | AluSc | Alu | SINE |
| 110104544 | 110104958 | - | 21.4 | 4853 | 5274 | -872 | L1MB8 | L1 | LINE |

  

|  |  |  |  |  |  |  |  |  |  |
| --- | --- | --- | --- | --- | --- | --- | --- | --- | --- |
| chr2:110159245-110161178 | | | 1934 bp | | |  |  |  |  |
| Genome Start | Genome End | Strand | Div | Repeat Start | Repeat End | Repeat Left | Name | Family | Class |
| 110159245 | 110160605 | + | 26.3 | 2724 | 4194 | -1952 | L1M5 | L1 | LINE |
| 110160606 | 110160921 | + | 12.2 | 1 | 313 | 0 | AluSp | Alu | SINE |
| 110160922 | 110161178 | + | 25.3 | 4195 | 4466 | -1680 | L1M5 | L1 | LINE |

  

|  |  |  |  |  |  |  |  |  |  |
| --- | --- | --- | --- | --- | --- | --- | --- | --- | --- |
| chr2:110170646-110172053 | | | 1396 bp | | |  |  |  |  |
| Genome Start | Genome End | Strand | Div | Repeat Start | Repeat End | Repeat Left | Name | Family | Class |
| 110170646 | 110170935 | - | 15.9 | 145 | 443 | -2 | LTR56 | ERV1 | LTR |
| 110170936 | 110171894 | + | 12.2 | 1 | 954 | -14 | LTR5\_Hs | ERVK | LTR |
| 110171907 | 110172053 | - | 18.5 | 3 | 150 | -295 | LTR56 | ERV1 | LTR |

  

|  |  |  |  |  |  |  |  |  |  |
| --- | --- | --- | --- | --- | --- | --- | --- | --- | --- |
| chr2:110173296-110177805 | | | 4509 bp | | |  |  |  |  |
| Genome Start | Genome End | Strand | Div | Repeat Start | Repeat End | Repeat Left | Name | Family | Class |
| 110173296 | 110173402 | + | 17.8 | 3570 | 3678 | -4529 | MER34-int | ERV1 | LTR |
| 110173404 | 110174091 | + | 17.1 | 3943 | 4617 | -3590 | MER34-int | ERV1 | LTR |
| 110174092 | 110174784 | - | 12.5 | 1 | 697 | 0 | MER50B | ERV1 | LTR |
| 110174785 | 110175691 | + | 17.9 | 4618 | 5646 | -2561 | MER34-int | ERV1 | LTR |
| 110175692 | 110176001 | - | 12.6 | 1 | 310 | -2 | AluSx | Alu | SINE |
| 110176002 | 110177805 | + | 19.1 | 5647 | 7548 | -659 | MER34-int | ERV1 | LTR |

  

|  |  |  |  |  |  |  |  |  |  |
| --- | --- | --- | --- | --- | --- | --- | --- | --- | --- |
| chr2:110346197-110347987 | | | 1695 bp | | |  |  |  |  |
| Genome Start | Genome End | Strand | Div | Repeat Start | Repeat End | Repeat Left | Name | Family | Class |
| 110346197 | 110346563 | - | 24.2 | 7505 | 7909 | -52 | L1MC5 | L1 | LINE |
| 110346564 | 110346842 | + | 14.5 | 13 | 288 | -24 | AluJo | Alu | SINE |
| 110346843 | 110346887 | - | 24.2 | 7459 | 7504 | -457 | L1MC5 | L1 | LINE |
| 110346910 | 110347707 | - | 24.2 | 6683 | 7458 | -503 | L1MC5 | L1 | LINE |
| 110347782 | 110347987 | - | 27.2 | 5746 | 5967 | -1841 | L1MC5 | L1 | LINE |

  

|  |  |  |  |  |  |  |  |  |  |
| --- | --- | --- | --- | --- | --- | --- | --- | --- | --- |
| chr2:110604379-110610714 | | | 6320 bp | | |  |  |  |  |
| Genome Start | Genome End | Strand | Div | Repeat Start | Repeat End | Repeat Left | Name | Family | Class |
| 110604379 | 110604507 | + | 26.8 | 3 | 127 | -51 | MER5B | MER1\_type | DNA |
| 110604510 | 110610646 | - | 6.4 | 1 | 6143 | -11 | L1PA6 | L1 | LINE |
| 110610661 | 110610714 | + | 24.1 | 124 | 178 | 0 | MER5B | MER1\_type | DNA |

  

|  |  |  |  |  |  |  |  |  |  |
| --- | --- | --- | --- | --- | --- | --- | --- | --- | --- |
| chr2:110616768-110617514 | | | 746 bp | | |  |  |  |  |
| Genome Start | Genome End | Strand | Div | Repeat Start | Repeat End | Repeat Left | Name | Family | Class |
| 110616768 | 110616824 | + | 12.3 | 1 | 58 | -244 | AluJo | Alu | SINE |
| 110616825 | 110617277 | + | 14.8 | 1 | 436 | -231 | LTR12B | ERV1 | LTR |
| 110617279 | 110617514 | + | 21.9 | 56 | 304 | -8 | AluJo | Alu | SINE |

  

|  |  |  |  |  |  |  |  |  |  |
| --- | --- | --- | --- | --- | --- | --- | --- | --- | --- |
| chr2:110618450-110625746 | | | 7297 bp | | |  |  |  |  |
| Genome Start | Genome End | Strand | Div | Repeat Start | Repeat End | Repeat Left | Name | Family | Class |
| 110618450 | 110619614 | - | 11.4 | 5733 | 6858 | -9 | L1PB2 | L1 | LINE |
| 110619615 | 110619914 | - | 14.1 | 1 | 310 | -2 | AluSx | Alu | SINE |
| 110619915 | 110623630 | - | 13.1 | 1861 | 5732 | -1130 | L1PB2 | L1 | LINE |
| 110623631 | 110623931 | - | 10.6 | 1 | 310 | -1 | AluY | Alu | SINE |
| 110623932 | 110625746 | - | 15.2 | 1 | 1860 | -5003 | L1PB2 | L1 | LINE |

  

|  |  |  |  |  |  |  |  |  |  |
| --- | --- | --- | --- | --- | --- | --- | --- | --- | --- |
| chr2:110639405-110640010 | | | 606 bp | | |  |  |  |  |
| Genome Start | Genome End | Strand | Div | Repeat Start | Repeat End | Repeat Left | Name | Family | Class |
| 110639405 | 110639567 | - | 15.9 | 143 | 291 | -21 | AluJb | Alu | SINE |
| 110639568 | 110639864 | - | 8.1 | 1 | 299 | -10 | AluSc | Alu | SINE |
| 110639865 | 110640010 | - | 15.9 | 1 | 142 | -170 | AluJb | Alu | SINE |

  

|  |  |  |  |  |  |  |  |  |  |
| --- | --- | --- | --- | --- | --- | --- | --- | --- | --- |
| chr2:110670131-110672033 | | | 1871 bp | | |  |  |  |  |
| Genome Start | Genome End | Strand | Div | Repeat Start | Repeat End | Repeat Left | Name | Family | Class |
| 110670131 | 110670674 | + | 8.7 | 4629 | 5170 | -976 | L1MA2 | L1 | LINE |
| 110670675 | 110670962 | + | 9.9 | 1 | 283 | -29 | AluSx | Alu | SINE |
| 110670995 | 110672033 | + | 8.6 | 5171 | 6202 | -102 | L1MA2 | L1 | LINE |

  

|  |  |  |  |  |  |  |  |  |  |
| --- | --- | --- | --- | --- | --- | --- | --- | --- | --- |
| chr2:110682987-110684471 | | | 1358 bp | | |  |  |  |  |
| Genome Start | Genome End | Strand | Div | Repeat Start | Repeat End | Repeat Left | Name | Family | Class |
| 110682987 | 110683366 | + | 18.3 | 6755 | 7153 | -632 | L1MC3 | L1 | LINE |
| 110683377 | 110683574 | - | 12.2 | 313 | 509 | -12 | MER41C | ERV1 | LTR |
| 110683688 | 110683989 | - | 8.6 | 1 | 308 | -213 | MER41C | ERV1 | LTR |
| 110683994 | 110684471 | + | 18.8 | 7162 | 7785 | 0 | L1MC3 | L1 | LINE |

  

|  |  |  |  |  |  |  |  |  |  |
| --- | --- | --- | --- | --- | --- | --- | --- | --- | --- |
| chr2:110687424-110688017 | | | 594 bp | | |  |  |  |  |
| Genome Start | Genome End | Strand | Div | Repeat Start | Repeat End | Repeat Left | Name | Family | Class |
| 110687424 | 110687530 | - | 16.2 | 181 | 284 | -28 | AluJb | Alu | SINE |
| 110687531 | 110687818 | - | 8.3 | 3 | 290 | -21 | AluY | Alu | SINE |
| 110687819 | 110688017 | - | 16.2 | 1 | 180 | -132 | AluJb | Alu | SINE |

  

|  |  |  |  |  |  |  |  |  |  |
| --- | --- | --- | --- | --- | --- | --- | --- | --- | --- |
| chr2:110690901-110691720 | | | 811 bp | | |  |  |  |  |
| Genome Start | Genome End | Strand | Div | Repeat Start | Repeat End | Repeat Left | Name | Family | Class |
| 110690901 | 110691301 | + | 16.7 | 5654 | 6072 | -112 | L1MB7 | L1 | LINE |
| 110691302 | 110691609 | + | 11.4 | 1 | 308 | 0 | AluSg | Alu | SINE |
| 110691619 | 110691720 | + | 16.7 | 6073 | 6175 | -9 | L1MB7 | L1 | LINE |

  

|  |  |  |  |  |  |  |  |  |  |
| --- | --- | --- | --- | --- | --- | --- | --- | --- | --- |
| chr2:110693149-110694714 | | | 1273 bp | | |  |  |  |  |
| Genome Start | Genome End | Strand | Div | Repeat Start | Repeat End | Repeat Left | Name | Family | Class |
| 110693149 | 110693557 | - | 28.4 | 7349 | 7852 | -190 | L1MC4 | L1 | LINE |
| 110693708 | 110693983 | - | 9.8 | 1 | 276 | -37 | AluSq | Alu | SINE |
| 110694127 | 110694714 | - | 26.6 | 6709 | 7233 | -728 | L1MC4 | L1 | LINE |

  

|  |  |  |  |  |  |  |  |  |  |
| --- | --- | --- | --- | --- | --- | --- | --- | --- | --- |
| chr2:110694713-110695748 | | | 1033 bp | | |  |  |  |  |
| Genome Start | Genome End | Strand | Div | Repeat Start | Repeat End | Repeat Left | Name | Family | Class |
| 110694713 | 110694837 | - | 19.9 | 2586 | 2713 | -3693 | L1MDb | L1 | LINE |
| 110694838 | 110695159 | - | 19.0 | 1 | 310 | -2 | AluJo | Alu | SINE |
| 110695160 | 110695220 | - | 19.9 | 2536 | 2585 | -3821 | L1MDb | L1 | LINE |
| 110695221 | 110695506 | - | 12.2 | 1 | 286 | -25 | AluY | Alu | SINE |
| 110695507 | 110695676 | - | 19.9 | 2370 | 2535 | -3871 | L1MDb | L1 | LINE |
| 110695680 | 110695748 | - | 19.1 | 1221 | 1289 | -5120 | L1MDb | L1 | LINE |

  

|  |  |  |  |  |  |  |  |  |  |
| --- | --- | --- | --- | --- | --- | --- | --- | --- | --- |
| chr2:110695750-110698336 | | | 2492 bp | | |  |  |  |  |
| Genome Start | Genome End | Strand | Div | Repeat Start | Repeat End | Repeat Left | Name | Family | Class |
| 110695750 | 110696169 | - | 16.7 | 6350 | 6762 | 0 | L1MC2 | L1 | LINE |
| 110696232 | 110696516 | - | 17.2 | 5370 | 5655 | -909 | L1MC2 | L1 | LINE |
| 110696531 | 110696826 | + | 10.8 | 4 | 298 | -15 | AluSq | Alu | SINE |
| 110696846 | 110696907 | - | 16.0 | 1566 | 1627 | -5124 | L1MC2 | L1 | LINE |
| 110696908 | 110697253 | - | 19.1 | 4 | 362 | -3 | MLT1A0 | MaLR | LTR |
| 110697254 | 110697314 | - | 16.0 | 1515 | 1565 | -5186 | L1MC2 | L1 | LINE |
| 110697315 | 110697625 | - | 8.0 | 1 | 311 | 0 | AluY | Alu | SINE |
| 110697626 | 110698336 | - | 16.4 | 721 | 1514 | -5237 | L1MC2 | L1 | LINE |

  

|  |  |  |  |  |  |  |  |  |  |
| --- | --- | --- | --- | --- | --- | --- | --- | --- | --- |
| chr2:110703026-110703532 | | | 507 bp | | |  |  |  |  |
| Genome Start | Genome End | Strand | Div | Repeat Start | Repeat End | Repeat Left | Name | Family | Class |
| 110703026 | 110703042 | - | 23.4 | 226 | 242 | -26 | MIRb | MIR | SINE |
| 110703043 | 110703348 | - | 8.2 | 1 | 306 | -5 | AluY | Alu | SINE |
| 110703349 | 110703532 | - | 25.6 | 3 | 225 | -41 | MIRb | MIR | SINE |

  

|  |  |  |  |  |  |  |  |  |  |
| --- | --- | --- | --- | --- | --- | --- | --- | --- | --- |
| chr2:110711119-110711596 | | | 478 bp | | |  |  |  |  |
| Genome Start | Genome End | Strand | Div | Repeat Start | Repeat End | Repeat Left | Name | Family | Class |
| 110711119 | 110711180 | - | 25.5 | 96 | 159 | -102 | MER112 | MER1\_type | DNA |
| 110711181 | 110711499 | - | 20.0 | 1 | 304 | -8 | AluJo | Alu | SINE |
| 110711500 | 110711596 | - | 25.5 | 2 | 95 | -165 | MER112 | MER1\_type | DNA |

  

|  |  |  |  |  |  |  |  |  |  |
| --- | --- | --- | --- | --- | --- | --- | --- | --- | --- |
| chr2:110713528-110713825 | | | 298 bp | | |  |  |  |  |
| Genome Start | Genome End | Strand | Div | Repeat Start | Repeat End | Repeat Left | Name | Family | Class |
| 110713528 | 110713595 | + | 20.1 | 7269 | 7341 | -620 | L1MC5 | L1 | LINE |
| 110713596 | 110713743 | + | 21.6 | 5 | 188 | -1 | MER5A | MER1\_type | DNA |
| 110713744 | 110713825 | + | 20.1 | 7342 | 7413 | -548 | L1MC5 | L1 | LINE |

  

|  |  |  |  |  |  |  |  |  |  |
| --- | --- | --- | --- | --- | --- | --- | --- | --- | --- |
| chr2:110742111-110742952 | | | 842 bp | | |  |  |  |  |
| Genome Start | Genome End | Strand | Div | Repeat Start | Repeat End | Repeat Left | Name | Family | Class |
| 110742111 | 110742165 | - | 35.9 | 122 | 172 | -6 | MER5B | MER1\_type | DNA |
| 110742166 | 110742480 | + | 7.6 | 1 | 305 | -6 | AluY | Alu | SINE |
| 110742481 | 110742554 | - | 35.9 | 58 | 121 | -57 | MER5B | MER1\_type | DNA |
| 110742555 | 110742888 | + | 15.4 | 5820 | 6163 | -15 | L1MB8 | L1 | LINE |
| 110742889 | 110742952 | - | 35.9 | 2 | 57 | -121 | MER5B | MER1\_type | DNA |

  

|  |  |  |  |  |  |  |  |  |  |
| --- | --- | --- | --- | --- | --- | --- | --- | --- | --- |
| chr2:110746221-110746690 | | | 470 bp | | |  |  |  |  |
| Genome Start | Genome End | Strand | Div | Repeat Start | Repeat End | Repeat Left | Name | Family | Class |
| 110746221 | 110746265 | - | 31.2 | 3408 | 3452 | -1037 | L3 | CR1 | LINE |
| 110746266 | 110746568 | - | 11.0 | 1 | 301 | -11 | AluSx | Alu | SINE |
| 110746569 | 110746690 | - | 31.2 | 3286 | 3407 | -1082 | L3 | CR1 | LINE |

  

|  |  |  |  |  |  |  |  |  |  |
| --- | --- | --- | --- | --- | --- | --- | --- | --- | --- |
| chr2:110751162-110753794 | | | 2347 bp | | |  |  |  |  |
| Genome Start | Genome End | Strand | Div | Repeat Start | Repeat End | Repeat Left | Name | Family | Class |
| 110751162 | 110751295 | + | 21.1 | 13 | 143 | -380 | Charlie1b | MER1\_type | DNA |
| 110751296 | 110751596 | - | 11.0 | 1 | 299 | -10 | AluSc | Alu | SINE |
| 110751597 | 110751788 | + | 21.1 | 144 | 332 | -191 | Charlie1b | MER1\_type | DNA |
| 110751789 | 110752091 | + | 8.6 | 1 | 302 | -11 | AluSq | Alu | SINE |
| 110752092 | 110752194 | + | 21.1 | 333 | 445 | -78 | Charlie1b | MER1\_type | DNA |
| 110752197 | 110752279 | + | 12.1 | 2 | 84 | -225 | AluSc | Alu | SINE |
| 110752280 | 110752335 | + | 11.2 | 29 | 76 | -233 | AluSc | Alu | SINE |
| 110752336 | 110752627 | + | 6.5 | 1 | 292 | -19 | AluY | Alu | SINE |
| 110752628 | 110752697 | + | 11.2 | 77 | 144 | -165 | AluSc | Alu | SINE |
| 110752698 | 110752911 | + | 6.1 | 1 | 212 | -99 | AluY | Alu | SINE |
| 110753193 | 110753280 | + | 14.3 | 201 | 242 | -58 | Alu | Alu | SINE |
| 110753282 | 110753486 | + | 17.8 | 88 | 292 | -17 | AluSc | Alu | SINE |
| 110753487 | 110753710 | + | 7.6 | 72 | 308 | -1 | AluSc | Alu | SINE |
| 110753711 | 110753722 | + | 17.8 | 293 | 303 | -6 | AluSc | Alu | SINE |
| 110753725 | 110753794 | + | 18.6 | 436 | 515 | -8 | Charlie1b | MER1\_type | DNA |

  

|  |  |  |  |  |  |  |  |  |  |
| --- | --- | --- | --- | --- | --- | --- | --- | --- | --- |
| chr2:110755748-110756809 | | | 545 bp | | |  |  |  |  |
| Genome Start | Genome End | Strand | Div | Repeat Start | Repeat End | Repeat Left | Name | Family | Class |
| 110755748 | 110755900 | + | 27.8 | 192 | 354 | -3065 | L2 | L2 | LINE |
| 110756068 | 110756184 | - | 20.2 | 86 | 199 | -113 | AluJo | Alu | SINE |
| 110756189 | 110756252 | + | 20.6 | 832 | 894 | -2525 | L2 | L2 | LINE |
| 110756599 | 110756809 | + | 28.3 | 1301 | 1527 | -1892 | L2 | L2 | LINE |

  

|  |  |  |  |  |  |  |  |  |  |
| --- | --- | --- | --- | --- | --- | --- | --- | --- | --- |
| chr2:110757528-110758441 | | | 913 bp | | |  |  |  |  |
| Genome Start | Genome End | Strand | Div | Repeat Start | Repeat End | Repeat Left | Name | Family | Class |
| 110757528 | 110757615 | + | 17.1 | 5678 | 5770 | -589 | L1MD2 | L1 | LINE |
| 110757617 | 110757786 | + | 17.3 | 137 | 304 | -8 | AluSg/x | Alu | SINE |
| 110757787 | 110757920 | + | 17.5 | 5759 | 5908 | -451 | L1MD2 | L1 | LINE |
| 110757921 | 110758208 | + | 12.2 | 1 | 308 | -4 | AluSx | Alu | SINE |
| 110758209 | 110758441 | + | 17.5 | 5909 | 6145 | -214 | L1MD2 | L1 | LINE |

  

|  |  |  |  |  |  |  |  |  |  |
| --- | --- | --- | --- | --- | --- | --- | --- | --- | --- |
| chr2:110758441-110758830 | | | 390 bp | | |  |  |  |  |
| Genome Start | Genome End | Strand | Div | Repeat Start | Repeat End | Repeat Left | Name | Family | Class |
| 110758441 | 110758494 | + | 21.4 | 7832 | 7885 | -110 | L1MD3 | L1 | LINE |
| 110758495 | 110758722 | - | 12.3 | 1 | 236 | 0 | MER46A | MER2\_type | DNA |
| 110758723 | 110758830 | + | 21.4 | 7886 | 7993 | -2 | L1MD3 | L1 | LINE |

  

|  |  |  |  |  |  |  |  |  |  |
| --- | --- | --- | --- | --- | --- | --- | --- | --- | --- |
| chr2:110769860-110770999 | | | 1051 bp | | |  |  |  |  |
| Genome Start | Genome End | Strand | Div | Repeat Start | Repeat End | Repeat Left | Name | Family | Class |
| 110769860 | 110770185 | + | 20.0 | 4215 | 4533 | -1613 | L1MB7 | L1 | LINE |
| 110770218 | 110770350 | + | 14.4 | 1 | 132 | -1 | FLAM\_C | Alu | SINE |
| 110770357 | 110770465 | + | 18.4 | 5674 | 5784 | -390 | L1MB7 | L1 | LINE |
| 110770481 | 110770556 | + | 14.5 | 15 | 90 | -86 | FAM | Alu | SINE |
| 110770593 | 110770667 | + | 16.2 | 93 | 168 | -17 | FAM | Alu | SINE |
| 110770668 | 110770999 | + | 19.8 | 5777 | 6107 | -77 | L1MB7 | L1 | LINE |

  

|  |  |  |  |  |  |  |  |  |  |
| --- | --- | --- | --- | --- | --- | --- | --- | --- | --- |
| chr2:110817857-110823800 | | | 5906 bp | | |  |  |  |  |
| Genome Start | Genome End | Strand | Div | Repeat Start | Repeat End | Repeat Left | Name | Family | Class |
| 110817857 | 110818072 | - | 16.9 | 6124 | 6343 | -1 | L1MC2 | L1 | LINE |
| 110818073 | 110818366 | - | 12.2 | 1 | 295 | -17 | AluSx | Alu | SINE |
| 110818367 | 110818679 | - | 16.9 | 5823 | 6123 | -221 | L1MC2 | L1 | LINE |
| 110818680 | 110818987 | + | 22.2 | 7 | 312 | 0 | AluJb | Alu | SINE |
| 110818988 | 110819554 | - | 16.9 | 5260 | 5822 | -522 | L1MC2 | L1 | LINE |
| 110819553 | 110820474 | + | 12.9 | 5192 | 6166 | 0 | L1PA16 | L1 | LINE |
| 110820483 | 110820805 | - | 17.5 | 4953 | 5282 | -864 | L1MC2 | L1 | LINE |
| 110820806 | 110821102 | - | 17.9 | 1 | 297 | -15 | AluJb | Alu | SINE |
| 110821103 | 110821638 | - | 17.5 | 4424 | 4952 | -1194 | L1MC2 | L1 | LINE |
| 110821639 | 110821947 | - | 11.3 | 1 | 310 | -3 | AluSq | Alu | SINE |
| 110821948 | 110822345 | - | 17.5 | 4020 | 4423 | -1723 | L1MC2 | L1 | LINE |
| 110822348 | 110822650 | - | 13.2 | 1 | 303 | -9 | AluSx | Alu | SINE |
| 110822651 | 110823450 | - | 18.6 | 3205 | 4024 | -2122 | L1MC2 | L1 | LINE |
| 110823481 | 110823800 | - | 15.0 | 2426 | 2773 | -3373 | L1MC2 | L1 | LINE |

  

|  |  |  |  |  |  |  |  |  |  |
| --- | --- | --- | --- | --- | --- | --- | --- | --- | --- |
| chr2:110832874-110833521 | | | 648 bp | | |  |  |  |  |
| Genome Start | Genome End | Strand | Div | Repeat Start | Repeat End | Repeat Left | Name | Family | Class |
| 110832874 | 110832997 | + | 21.1 | 10 | 141 | -224 | MLT1A0 | MaLR | LTR |
| 110832998 | 110833307 | + | 9.3 | 1 | 311 | -2 | AluSp | Alu | SINE |
| 110833308 | 110833521 | + | 21.1 | 142 | 365 | 0 | MLT1A0 | MaLR | LTR |

  

|  |  |  |  |  |  |  |  |  |  |
| --- | --- | --- | --- | --- | --- | --- | --- | --- | --- |
| chr2:110839611-110841614 | | | 2004 bp | | |  |  |  |  |
| Genome Start | Genome End | Strand | Div | Repeat Start | Repeat End | Repeat Left | Name | Family | Class |
| 110839611 | 110840638 | + | 6.4 | 4434 | 5475 | -693 | L1PA10 | L1 | LINE |
| 110840639 | 110840945 | + | 12.8 | 1 | 309 | -3 | AluSx | Alu | SINE |
| 110840946 | 110841614 | + | 9.3 | 5476 | 6168 | 0 | L1PA10 | L1 | LINE |

  

|  |  |  |  |  |  |  |  |  |  |
| --- | --- | --- | --- | --- | --- | --- | --- | --- | --- |
| chr2:110846019-110850392 | | | 4171 bp | | |  |  |  |  |
| Genome Start | Genome End | Strand | Div | Repeat Start | Repeat End | Repeat Left | Name | Family | Class |
| 110846019 | 110846146 | - | 24.2 | 4381 | 4509 | -1637 | L1M5 | L1 | LINE |
| 110846147 | 110846454 | - | 12.3 | 1 | 309 | -4 | AluSq | Alu | SINE |
| 110846455 | 110846465 | - | 24.2 | 4379 | 4380 | -1766 | L1M5 | L1 | LINE |
| 110846497 | 110846779 | - | 8.8 | 5 | 287 | -26 | AluSq | Alu | SINE |
| 110846780 | 110847047 | - | 24.2 | 4147 | 4378 | -1768 | L1M5 | L1 | LINE |
| 110847048 | 110847234 | + | 9.0 | 1 | 187 | -278 | MER4C | ERV1 | LTR |
| 110847235 | 110847530 | + | 15.7 | 1 | 293 | -19 | AluJb | Alu | SINE |
| 110847534 | 110847847 | + | 9.8 | 1 | 307 | -3 | AluSg | Alu | SINE |
| 110847848 | 110848122 | + | 9.0 | 188 | 465 | 0 | MER4C | ERV1 | LTR |
| 110848123 | 110848193 | - | 16.3 | 4080 | 4144 | -2002 | L1M5 | L1 | LINE |
| 110848205 | 110848693 | + | 19.5 | 1 | 556 | -307 | MER21B | ERV1 | LTR |
| 110848708 | 110848811 | + | 13.5 | 33 | 136 | -176 | AluJb | Alu | SINE |
| 110848817 | 110849091 | + | 12.0 | 1 | 278 | -35 | AluSq | Alu | SINE |
| 110849176 | 110849483 | + | 14.3 | 3 | 312 | 0 | AluSx | Alu | SINE |
| 110849487 | 110849785 | + | 11.0 | 4 | 302 | -7 | AluSc | Alu | SINE |
| 110849791 | 110849803 | + | 20.5 | 120 | 131 | -181 | AluJb | Alu | SINE |
| 110849829 | 110850006 | + | 20.5 | 132 | 302 | -10 | AluJb | Alu | SINE |
| 110850026 | 110850240 | + | 18.6 | 604 | 844 | -19 | MER21B | ERV1 | LTR |
| 110850244 | 110850392 | - | 20.0 | 3944 | 4074 | -2072 | L1M5 | L1 | LINE |

  

|  |  |  |  |  |  |  |  |  |  |
| --- | --- | --- | --- | --- | --- | --- | --- | --- | --- |
| chr2:110851181-110851726 | | | 546 bp | | |  |  |  |  |
| Genome Start | Genome End | Strand | Div | Repeat Start | Repeat End | Repeat Left | Name | Family | Class |
| 110851181 | 110851351 | + | 19.7 | 31 | 202 | -73 | ORSL | Tip100 | DNA |
| 110851352 | 110851654 | + | 10.6 | 1 | 301 | -9 | AluSg | Alu | SINE |
| 110851655 | 110851726 | + | 19.7 | 203 | 275 | 0 | ORSL | Tip100 | DNA |

  

|  |  |  |  |  |  |  |  |  |  |
| --- | --- | --- | --- | --- | --- | --- | --- | --- | --- |
| chr2:111021613-111022986 | | | 1196 bp | | |  |  |  |  |
| Genome Start | Genome End | Strand | Div | Repeat Start | Repeat End | Repeat Left | Name | Family | Class |
| 111021613 | 111021879 | + | 28.6 | 5539 | 5827 | -294 | L1ME4a | L1 | LINE |
| 111021966 | 111022002 | + | 21.6 | 1 | 37 | -96 | AluJo | Alu | SINE |
| 111022003 | 111022291 | - | 9.3 | 1 | 293 | -19 | AluSx | Alu | SINE |
| 111022292 | 111022553 | + | 17.7 | 26 | 286 | -26 | AluJo | Alu | SINE |
| 111022562 | 111022843 | + | 9.8 | 1 | 282 | -31 | AluSq | Alu | SINE |
| 111022928 | 111022986 | + | 22.0 | 5978 | 6038 | -83 | L1ME4a | L1 | LINE |

  

|  |  |  |  |  |  |  |  |  |  |
| --- | --- | --- | --- | --- | --- | --- | --- | --- | --- |
| chr2:111027691-111028161 | | | 471 bp | | |  |  |  |  |
| Genome Start | Genome End | Strand | Div | Repeat Start | Repeat End | Repeat Left | Name | Family | Class |
| 111027691 | 111027735 | - | 31.2 | 3408 | 3452 | -1037 | L3 | CR1 | LINE |
| 111027736 | 111028039 | - | 11.3 | 1 | 302 | -10 | AluSx | Alu | SINE |
| 111028040 | 111028161 | - | 31.2 | 3286 | 3407 | -1082 | L3 | CR1 | LINE |

  

|  |  |  |  |  |  |  |  |  |  |
| --- | --- | --- | --- | --- | --- | --- | --- | --- | --- |
| chr2:111032630-111035264 | | | 2296 bp | | |  |  |  |  |
| Genome Start | Genome End | Strand | Div | Repeat Start | Repeat End | Repeat Left | Name | Family | Class |
| 111032630 | 111032763 | + | 21.1 | 13 | 143 | -380 | Charlie1b | MER1\_type | DNA |
| 111032764 | 111033064 | - | 11.0 | 1 | 299 | -10 | AluSc | Alu | SINE |
| 111033065 | 111033256 | + | 21.1 | 144 | 332 | -191 | Charlie1b | MER1\_type | DNA |
| 111033257 | 111033558 | + | 8.3 | 1 | 301 | -12 | AluSq | Alu | SINE |
| 111033559 | 111033661 | + | 21.1 | 333 | 445 | -78 | Charlie1b | MER1\_type | DNA |
| 111033663 | 111033746 | + | 11.9 | 1 | 84 | -225 | AluSc | Alu | SINE |
| 111033747 | 111033802 | + | 11.2 | 29 | 76 | -233 | AluSc | Alu | SINE |
| 111033803 | 111034096 | + | 6.5 | 1 | 294 | -17 | AluY | Alu | SINE |
| 111034097 | 111034166 | + | 11.2 | 77 | 144 | -165 | AluSc | Alu | SINE |
| 111034167 | 111034384 | + | 5.7 | 1 | 212 | -99 | AluY | Alu | SINE |
| 111034712 | 111034786 | + | 12.0 | 212 | 286 | -25 | AluY | Alu | SINE |
| 111034787 | 111034958 | + | 15.4 | 119 | 292 | -17 | AluSc | Alu | SINE |
| 111034959 | 111035183 | + | 8.4 | 72 | 309 | 0 | AluSc | Alu | SINE |
| 111035195 | 111035264 | + | 20.0 | 436 | 515 | -8 | Charlie1b | MER1\_type | DNA |

  

|  |  |  |  |  |  |  |  |  |  |
| --- | --- | --- | --- | --- | --- | --- | --- | --- | --- |
| chr2:111037212-111038325 | | | 1109 bp | | |  |  |  |  |
| Genome Start | Genome End | Strand | Div | Repeat Start | Repeat End | Repeat Left | Name | Family | Class |
| 111037212 | 111037530 | + | 31.2 | 192 | 588 | -2831 | L2 | L2 | LINE |
| 111037532 | 111037648 | - | 19.3 | 86 | 199 | -113 | AluJo | Alu | SINE |
| 111037653 | 111038325 | + | 32.7 | 832 | 1595 | -1824 | L2 | L2 | LINE |

  

|  |  |  |  |  |  |  |  |  |  |
| --- | --- | --- | --- | --- | --- | --- | --- | --- | --- |
| chr2:111038993-111039908 | | | 915 bp | | |  |  |  |  |
| Genome Start | Genome End | Strand | Div | Repeat Start | Repeat End | Repeat Left | Name | Family | Class |
| 111038993 | 111039080 | + | 18.2 | 5678 | 5770 | -589 | L1MD2 | L1 | LINE |
| 111039082 | 111039253 | + | 17.1 | 137 | 306 | -6 | AluSg/x | Alu | SINE |
| 111039254 | 111039387 | + | 18.5 | 5759 | 5908 | -451 | L1MD2 | L1 | LINE |
| 111039388 | 111039675 | + | 12.2 | 1 | 308 | -4 | AluSx | Alu | SINE |
| 111039676 | 111039908 | + | 18.5 | 5909 | 6145 | -214 | L1MD2 | L1 | LINE |

  

|  |  |  |  |  |  |  |  |  |  |
| --- | --- | --- | --- | --- | --- | --- | --- | --- | --- |
| chr2:111039908-111040297 | | | 390 bp | | |  |  |  |  |
| Genome Start | Genome End | Strand | Div | Repeat Start | Repeat End | Repeat Left | Name | Family | Class |
| 111039908 | 111039961 | + | 21.4 | 7832 | 7885 | -110 | L1MD3 | L1 | LINE |
| 111039962 | 111040189 | - | 12.8 | 1 | 236 | 0 | MER46A | MER2\_type | DNA |
| 111040190 | 111040297 | + | 21.4 | 7886 | 7993 | -2 | L1MD3 | L1 | LINE |

  

|  |  |  |  |  |  |  |  |  |  |
| --- | --- | --- | --- | --- | --- | --- | --- | --- | --- |
| chr2:111046943-111048618 | | | 1653 bp | | |  |  |  |  |
| Genome Start | Genome End | Strand | Div | Repeat Start | Repeat End | Repeat Left | Name | Family | Class |
| 111046943 | 111047126 | + | 19.6 | 1113 | 1344 | -1163 | HAL1 | L1 | LINE |
| 111047149 | 111047305 | + | 25.6 | 1480 | 1667 | -840 | HAL1 | L1 | LINE |
| 111047306 | 111047532 | + | 11.5 | 78 | 304 | -8 | AluSg/x | Alu | SINE |
| 111047533 | 111047924 | + | 25.3 | 1668 | 2059 | -448 | HAL1 | L1 | LINE |
| 111047926 | 111048026 | + | 2.0 | 6055 | 6155 | 0 | L1PA2 | L1 | LINE |
| 111048027 | 111048207 | + | 25.6 | 2061 | 2286 | -221 | HAL1 | L1 | LINE |
| 111048208 | 111048515 | + | 5.4 | 1 | 312 | 0 | AluY | Alu | SINE |
| 111048516 | 111048618 | + | 25.6 | 2287 | 2372 | -135 | HAL1 | L1 | LINE |

  

|  |  |  |  |  |  |  |  |  |  |
| --- | --- | --- | --- | --- | --- | --- | --- | --- | --- |
| chr2:111051336-111052475 | | | 1051 bp | | |  |  |  |  |
| Genome Start | Genome End | Strand | Div | Repeat Start | Repeat End | Repeat Left | Name | Family | Class |
| 111051336 | 111051661 | + | 20.0 | 4215 | 4533 | -1613 | L1MB7 | L1 | LINE |
| 111051694 | 111051826 | + | 14.4 | 1 | 132 | -1 | FLAM\_C | Alu | SINE |
| 111051833 | 111051941 | + | 18.4 | 5674 | 5784 | -390 | L1MB7 | L1 | LINE |
| 111051957 | 111052032 | + | 14.5 | 15 | 90 | -86 | FAM | Alu | SINE |
| 111052069 | 111052143 | + | 16.2 | 93 | 168 | -17 | FAM | Alu | SINE |
| 111052144 | 111052475 | + | 20.1 | 5777 | 6107 | -77 | L1MB7 | L1 | LINE |

  

|  |  |  |  |  |  |  |  |  |  |
| --- | --- | --- | --- | --- | --- | --- | --- | --- | --- |
| chr2:111058444-111060733 | | | 2223 bp | | |  |  |  |  |
| Genome Start | Genome End | Strand | Div | Repeat Start | Repeat End | Repeat Left | Name | Family | Class |
| 111058444 | 111058986 | - | 22.8 | 5456 | 6018 | -1767 | L1MC3 | L1 | LINE |
| 111059051 | 111059070 | - | 20.2 | 507 | 522 | -40 | MLT2B2 | ERVL | LTR |
| 111059071 | 111059353 | + | 16.6 | 2 | 299 | -13 | AluJb | Alu | SINE |
| 111059354 | 111059696 | - | 20.2 | 76 | 459 | -56 | MLT2B2 | ERVL | LTR |
| 111059700 | 111060733 | - | 21.7 | 4304 | 5444 | -900 | L1MC3 | L1 | LINE |

  

|  |  |  |  |  |  |  |  |  |  |
| --- | --- | --- | --- | --- | --- | --- | --- | --- | --- |
| chr2:111735706-111736637 | | | 932 bp | | |  |  |  |  |
| Genome Start | Genome End | Strand | Div | Repeat Start | Repeat End | Repeat Left | Name | Family | Class |
| 111735706 | 111736033 | + | 18.0 | 1 | 316 | -110 | MSTB | MaLR | LTR |
| 111736034 | 111736339 | - | 12.1 | 2 | 309 | 0 | AluSg1 | Alu | SINE |
| 111736340 | 111736411 | + | 18.0 | 317 | 379 | -47 | MSTB | MaLR | LTR |
| 111736412 | 111736592 | + | 7.2 | 130 | 310 | -1 | AluY | Alu | SINE |
| 111736593 | 111736637 | + | 18.0 | 380 | 426 | 0 | MSTB | MaLR | LTR |

  

|  |  |  |  |  |  |  |  |  |  |
| --- | --- | --- | --- | --- | --- | --- | --- | --- | --- |
| chr2:111741652-111743054 | | | 1169 bp | | |  |  |  |  |
| Genome Start | Genome End | Strand | Div | Repeat Start | Repeat End | Repeat Left | Name | Family | Class |
| 111741652 | 111742196 | - | 16.2 | 390 | 929 | -2 | MER21A | ERV1 | LTR |
| 111742429 | 111742691 | - | 15.0 | 1 | 273 | -39 | AluJb | Alu | SINE |
| 111742694 | 111743054 | - | 15.0 | 1 | 400 | -531 | MER21A | ERV1 | LTR |

  

|  |  |  |  |  |  |  |  |  |  |
| --- | --- | --- | --- | --- | --- | --- | --- | --- | --- |
| chr2:111761162-111761603 | | | 442 bp | | |  |  |  |  |
| Genome Start | Genome End | Strand | Div | Repeat Start | Repeat End | Repeat Left | Name | Family | Class |
| 111761162 | 111761274 | + | 26.7 | 150 | 264 | -2153 | Charlie8 | MER1\_type | DNA |
| 111761275 | 111761435 | - | 24.8 | 1 | 175 | 0 | MER96 | MER1\_type? | DNA |
| 111761436 | 111761603 | + | 26.7 | 265 | 409 | -2008 | Charlie8 | MER1\_type | DNA |

  

|  |  |  |  |  |  |  |  |  |  |
| --- | --- | --- | --- | --- | --- | --- | --- | --- | --- |
| chr2:111773529-111774212 | | | 684 bp | | |  |  |  |  |
| Genome Start | Genome End | Strand | Div | Repeat Start | Repeat End | Repeat Left | Name | Family | Class |
| 111773529 | 111773560 | - | 28.0 | 292 | 316 | -14 | MER102a | MER1\_type | DNA |
| 111773561 | 111773911 | - | 21.3 | 2 | 362 | -3 | MLT1A0 | MaLR | LTR |
| 111773912 | 111774212 | - | 28.0 | 1 | 291 | -39 | MER102a | MER1\_type | DNA |

  

|  |  |  |  |  |  |  |  |  |  |
| --- | --- | --- | --- | --- | --- | --- | --- | --- | --- |
| chr2:111779044-111779832 | | | 712 bp | | |  |  |  |  |
| Genome Start | Genome End | Strand | Div | Repeat Start | Repeat End | Repeat Left | Name | Family | Class |
| 111779044 | 111779299 | - | 24.9 | 208 | 464 | -3 | MLT1C | MaLR | LTR |
| 111779377 | 111779397 | - | 24.9 | 183 | 207 | -260 | MLT1C | MaLR | LTR |
| 111779398 | 111779700 | - | 19.1 | 1 | 305 | 0 | AluJo | Alu | SINE |
| 111779701 | 111779832 | - | 24.9 | 54 | 182 | -285 | MLT1C | MaLR | LTR |

  

|  |  |  |  |  |  |  |  |  |  |
| --- | --- | --- | --- | --- | --- | --- | --- | --- | --- |
| chr2:111781454-111782252 | | | 771 bp | | |  |  |  |  |
| Genome Start | Genome End | Strand | Div | Repeat Start | Repeat End | Repeat Left | Name | Family | Class |
| 111781454 | 111781867 | - | 19.3 | 86 | 552 | -4 | MLT2B4 | ERVL | LTR |
| 111781896 | 111782177 | - | 10.6 | 1 | 282 | -28 | AluSg | Alu | SINE |
| 111782178 | 111782252 | - | 19.3 | 6 | 85 | -471 | MLT2B4 | ERVL | LTR |

  

|  |  |  |  |  |  |  |  |  |  |
| --- | --- | --- | --- | --- | --- | --- | --- | --- | --- |
| chr2:111823608-111825817 | | | 1426 bp | | |  |  |  |  |
| Genome Start | Genome End | Strand | Div | Repeat Start | Repeat End | Repeat Left | Name | Family | Class |
| 111823608 | 111823997 | + | 35.6 | 2483 | 2871 | -1618 | L3 | CR1 | LINE |
| 111824178 | 111824473 | + | 34.8 | 3155 | 3476 | -1013 | L3 | CR1 | LINE |
| 111824714 | 111824816 | + | 28.2 | 34 | 140 | -68 | MIR | MIR | SINE |
| 111824796 | 111824991 | + | 37.1 | 3551 | 3737 | -752 | L3 | CR1 | LINE |
| 111825014 | 111825350 | + | 11.6 | 1 | 317 | 0 | AluSq | Alu | SINE |
| 111825714 | 111825817 | + | 34.6 | 3813 | 3924 | -565 | L3 | CR1 | LINE |

  

|  |  |  |  |  |  |  |  |  |  |
| --- | --- | --- | --- | --- | --- | --- | --- | --- | --- |
| chr2:111844612-111845748 | | | 1137 bp | | |  |  |  |  |
| Genome Start | Genome End | Strand | Div | Repeat Start | Repeat End | Repeat Left | Name | Family | Class |
| 111844612 | 111844760 | - | 17.6 | 5977 | 6132 | -39 | L1MB2 | L1 | LINE |
| 111844761 | 111845045 | - | 11.6 | 2 | 291 | -21 | AluSx | Alu | SINE |
| 111845046 | 111845748 | - | 17.6 | 5271 | 5976 | -195 | L1MB2 | L1 | LINE |

  

|  |  |  |  |  |  |  |  |  |  |
| --- | --- | --- | --- | --- | --- | --- | --- | --- | --- |
| chr2:111879206-111880378 | | | 1141 bp | | |  |  |  |  |
| Genome Start | Genome End | Strand | Div | Repeat Start | Repeat End | Repeat Left | Name | Family | Class |
| 111879206 | 111879904 | + | 24.7 | 5001 | 5721 | -443 | L1M5 | L1 | LINE |
| 111879905 | 111880172 | + | 21.0 | 1 | 268 | -2223 | Tigger7 | MER2\_type | DNA |
| 111880199 | 111880287 | + | 13.8 | 2403 | 2491 | 0 | Tigger7 | MER2\_type | DNA |
| 111880294 | 111880378 | + | 15.3 | 5726 | 5810 | -354 | L1M5 | L1 | LINE |

  

|  |  |  |  |  |  |  |  |  |  |
| --- | --- | --- | --- | --- | --- | --- | --- | --- | --- |
| chr2:111934167-111934655 | | | 489 bp | | |  |  |  |  |
| Genome Start | Genome End | Strand | Div | Repeat Start | Repeat End | Repeat Left | Name | Family | Class |
| 111934167 | 111934385 | - | 14.6 | 168 | 388 | -2 | MLT1B | MaLR | LTR |
| 111934386 | 111934478 | - | 5.4 | 6062 | 6154 | 0 | L1PA5 | L1 | LINE |
| 111934479 | 111934655 | - | 14.6 | 1 | 167 | -223 | MLT1B | MaLR | LTR |

  

|  |  |  |  |  |  |  |  |  |  |
| --- | --- | --- | --- | --- | --- | --- | --- | --- | --- |
| chr2:111974422-111975993 | | | 1572 bp | | |  |  |  |  |
| Genome Start | Genome End | Strand | Div | Repeat Start | Repeat End | Repeat Left | Name | Family | Class |
| 111974422 | 111975346 | - | 20.9 | 4856 | 5826 | -533 | L1MD2 | L1 | LINE |
| 111975347 | 111975641 | + | 15.9 | 1 | 295 | -17 | AluSx | Alu | SINE |
| 111975642 | 111975993 | - | 20.3 | 4495 | 4855 | -1291 | L1MD2 | L1 | LINE |

  

|  |  |  |  |  |  |  |  |  |  |
| --- | --- | --- | --- | --- | --- | --- | --- | --- | --- |
| chr2:112046212-112046836 | | | 625 bp | | |  |  |  |  |
| Genome Start | Genome End | Strand | Div | Repeat Start | Repeat End | Repeat Left | Name | Family | Class |
| 112046212 | 112046404 | - | 23.6 | 7758 | 7953 | -89 | L1MC4 | L1 | LINE |
| 112046405 | 112046693 | - | 12.5 | 1 | 298 | -14 | AluSx | Alu | SINE |
| 112046694 | 112046836 | - | 23.6 | 7636 | 7757 | -285 | L1MC4 | L1 | LINE |

  

|  |  |  |  |  |  |  |  |  |  |
| --- | --- | --- | --- | --- | --- | --- | --- | --- | --- |
| chr2:112060919-112066924 | | | 5991 bp | | |  |  |  |  |
| Genome Start | Genome End | Strand | Div | Repeat Start | Repeat End | Repeat Left | Name | Family | Class |
| 112060919 | 112061740 | + | 11.7 | 24 | 881 | -1537 | Tigger1 | MER2\_type | DNA |
| 112061741 | 112062043 | + | 10.7 | 1 | 303 | -10 | AluSq | Alu | SINE |
| 112062044 | 112062323 | + | 11.7 | 882 | 1173 | -1245 | Tigger1 | MER2\_type | DNA |
| 112062331 | 112062895 | - | 3.7 | 2798 | 3359 | -2787 | L1PA4 | L1 | LINE |
| 112062896 | 112065680 | + | 2.9 | 3357 | 6172 | 0 | L1PA4 | L1 | LINE |
| 112065689 | 112066924 | + | 12.3 | 1183 | 2418 | 0 | Tigger1 | MER2\_type | DNA |

  

|  |  |  |  |  |  |  |  |  |  |
| --- | --- | --- | --- | --- | --- | --- | --- | --- | --- |
| chr2:112077429-112079025 | | | 1597 bp | | |  |  |  |  |
| Genome Start | Genome End | Strand | Div | Repeat Start | Repeat End | Repeat Left | Name | Family | Class |
| 112077429 | 112078365 | - | 12.7 | 343 | 1302 | 0 | HSMAR2 | Mariner | DNA |
| 112078366 | 112078675 | - | 11.1 | 1 | 299 | -13 | AluSx | Alu | SINE |
| 112078676 | 112079025 | - | 12.7 | 2 | 342 | -960 | HSMAR2 | Mariner | DNA |

  

|  |  |  |  |  |  |  |  |  |  |
| --- | --- | --- | --- | --- | --- | --- | --- | --- | --- |
| chr2:112087285-112088060 | | | 776 bp | | |  |  |  |  |
| Genome Start | Genome End | Strand | Div | Repeat Start | Repeat End | Repeat Left | Name | Family | Class |
| 112087285 | 112087435 | - | 25.8 | 555 | 707 | 0 | MER39 | ERV1 | LTR |
| 112087436 | 112087746 | + | 15.1 | 1 | 312 | 0 | AluSx | Alu | SINE |
| 112087747 | 112088060 | - | 25.8 | 260 | 554 | -153 | MER39 | ERV1 | LTR |

  

|  |  |  |  |  |  |  |  |  |  |
| --- | --- | --- | --- | --- | --- | --- | --- | --- | --- |
| chr2:112225843-112230250 | | | 4382 bp | | |  |  |  |  |
| Genome Start | Genome End | Strand | Div | Repeat Start | Repeat End | Repeat Left | Name | Family | Class |
| 112225843 | 112226025 | + | 25.5 | 19 | 204 | -6429 | L1MDa | L1 | LINE |
| 112226026 | 112226324 | - | 13.3 | 1 | 292 | -20 | AluSx | Alu | SINE |
| 112226325 | 112226858 | + | 25.5 | 205 | 741 | -5892 | L1MDa | L1 | LINE |
| 112226885 | 112229184 | + | 18.8 | 1194 | 3502 | -3035 | L1MDa | L1 | LINE |
| 112229185 | 112229489 | + | 20.4 | 1 | 311 | -1 | AluJo | Alu | SINE |
| 112229490 | 112230250 | + | 15.9 | 3503 | 4320 | -2217 | L1MDa | L1 | LINE |

  

|  |  |  |  |  |  |  |  |  |  |
| --- | --- | --- | --- | --- | --- | --- | --- | --- | --- |
| chr2:112231018-112231594 | | | 397 bp | | |  |  |  |  |
| Genome Start | Genome End | Strand | Div | Repeat Start | Repeat End | Repeat Left | Name | Family | Class |
| 112231018 | 112231045 | + | 24.3 | 5166 | 5194 | -946 | L1M4 | L1 | LINE |
| 112231226 | 112231525 | + | 11.0 | 1 | 300 | -12 | AluSx | Alu | SINE |
| 112231526 | 112231594 | + | 16.3 | 5200 | 5276 | -870 | L1M4 | L1 | LINE |

  

|  |  |  |  |  |  |  |  |  |  |
| --- | --- | --- | --- | --- | --- | --- | --- | --- | --- |
| chr2:112231731-112233448 | | | 1690 bp | | |  |  |  |  |
| Genome Start | Genome End | Strand | Div | Repeat Start | Repeat End | Repeat Left | Name | Family | Class |
| 112231731 | 112232040 | + | 22.7 | 5456 | 5932 | -427 | L1MD2 | L1 | LINE |
| 112232054 | 112232116 | - | 18.3 | 238 | 302 | 0 | AluJb | Alu | SINE |
| 112232117 | 112232817 | + | 15.1 | 1 | 710 | -24 | MER50 | ERV1 | LTR |
| 112232833 | 112233060 | - | 16.2 | 1 | 228 | -84 | AluJb | Alu | SINE |
| 112233061 | 112233448 | + | 21.8 | 5927 | 6352 | -7 | L1MD2 | L1 | LINE |

  

|  |  |  |  |  |  |  |  |  |  |
| --- | --- | --- | --- | --- | --- | --- | --- | --- | --- |
| chr2:112264199-112265952 | | | 1754 bp | | |  |  |  |  |
| Genome Start | Genome End | Strand | Div | Repeat Start | Repeat End | Repeat Left | Name | Family | Class |
| 112264199 | 112264908 | + | 22.7 | 4495 | 5298 | -848 | L1ME3A | L1 | LINE |
| 112264909 | 112265130 | + | 13.5 | 5937 | 6169 | -2 | L1MB2 | L1 | LINE |
| 112265131 | 112265952 | + | 25.1 | 5299 | 6139 | -31 | L1ME3A | L1 | LINE |

  

|  |  |  |  |  |  |  |  |  |  |
| --- | --- | --- | --- | --- | --- | --- | --- | --- | --- |
| chr2:112275064-112275751 | | | 688 bp | | |  |  |  |  |
| Genome Start | Genome End | Strand | Div | Repeat Start | Repeat End | Repeat Left | Name | Family | Class |
| 112275064 | 112275122 | - | 20.1 | 344 | 394 | -24 | Tigger5b | MER2\_type | DNA |
| 112275123 | 112275431 | + | 12.9 | 1 | 309 | -3 | AluSx | Alu | SINE |
| 112275432 | 112275751 | - | 20.1 | 17 | 343 | -75 | Tigger5b | MER2\_type | DNA |

  

|  |  |  |  |  |  |  |  |  |  |
| --- | --- | --- | --- | --- | --- | --- | --- | --- | --- |
| chr2:112296790-112298045 | | | 1256 bp | | |  |  |  |  |
| Genome Start | Genome End | Strand | Div | Repeat Start | Repeat End | Repeat Left | Name | Family | Class |
| 112296790 | 112297045 | + | 26.5 | 5249 | 5529 | -711 | L1ME3B | L1 | LINE |
| 112297046 | 112297364 | - | 3.9 | 1 | 311 | 0 | AluY | Alu | SINE |
| 112297365 | 112298045 | + | 27.3 | 5530 | 6229 | -11 | L1ME3B | L1 | LINE |

  

|  |  |  |  |  |  |  |  |  |  |
| --- | --- | --- | --- | --- | --- | --- | --- | --- | --- |
| chr2:112302055-112303328 | | | 1274 bp | | |  |  |  |  |
| Genome Start | Genome End | Strand | Div | Repeat Start | Repeat End | Repeat Left | Name | Family | Class |
| 112302055 | 112302980 | + | 17.2 | 5204 | 6135 | -45 | L1MB4 | L1 | LINE |
| 112302981 | 112303265 | + | 8.8 | 1 | 288 | -24 | AluSx | Alu | SINE |
| 112303266 | 112303328 | + | 16.9 | 6136 | 6180 | 0 | L1MB4 | L1 | LINE |

  

|  |  |  |  |  |  |  |  |  |  |
| --- | --- | --- | --- | --- | --- | --- | --- | --- | --- |
| chr2:112303777-112305145 | | | 1362 bp | | |  |  |  |  |
| Genome Start | Genome End | Strand | Div | Repeat Start | Repeat End | Repeat Left | Name | Family | Class |
| 112303777 | 112303835 | + | 21.6 | 4600 | 4652 | -1494 | L1MD1 | L1 | LINE |
| 112303836 | 112304139 | + | 11.2 | 1 | 304 | -8 | AluSx | Alu | SINE |
| 112304140 | 112304687 | + | 21.6 | 4653 | 5266 | -880 | L1MD1 | L1 | LINE |
| 112304695 | 112305145 | + | 22.5 | 5397 | 5859 | -383 | L1MD1 | L1 | LINE |

  

|  |  |  |  |  |  |  |  |  |  |
| --- | --- | --- | --- | --- | --- | --- | --- | --- | --- |
| chr2:112326218-112327129 | | | 878 bp | | |  |  |  |  |
| Genome Start | Genome End | Strand | Div | Repeat Start | Repeat End | Repeat Left | Name | Family | Class |
| 112326218 | 112326572 | + | 30.2 | 82 | 437 | -6196 | L1MDa | L1 | LINE |
| 112326577 | 112326686 | - | 22.7 | 3903 | 4015 | -2131 | L1MC | L1 | LINE |
| 112326696 | 112326858 | + | 23.3 | 5 | 176 | -39 | MER58C | MER1\_type | DNA |
| 112326876 | 112327058 | + | 18.8 | 129 | 305 | -7 | AluJb | Alu | SINE |
| 112327063 | 112327129 | + | 23.9 | 2986 | 3052 | -3485 | L1MDa | L1 | LINE |

  

|  |  |  |  |  |  |  |  |  |  |
| --- | --- | --- | --- | --- | --- | --- | --- | --- | --- |
| chr2:112348288-112349345 | | | 1048 bp | | |  |  |  |  |
| Genome Start | Genome End | Strand | Div | Repeat Start | Repeat End | Repeat Left | Name | Family | Class |
| 112348288 | 112348877 | - | 16.9 | 5594 | 6180 | 0 | L1MB4 | L1 | LINE |
| 112348885 | 112349032 | - | 21.8 | 1 | 146 | -30 | FRAM | Alu | SINE |
| 112349036 | 112349345 | - | 14.6 | 5273 | 5604 | -576 | L1MB4 | L1 | LINE |

  

|  |  |  |  |  |  |  |  |  |  |
| --- | --- | --- | --- | --- | --- | --- | --- | --- | --- |
| chr2:112350955-112352337 | | | 1231 bp | | |  |  |  |  |
| Genome Start | Genome End | Strand | Div | Repeat Start | Repeat End | Repeat Left | Name | Family | Class |
| 112350955 | 112351431 | - | 24.0 | 7555 | 8042 | 0 | L1MC4 | L1 | LINE |
| 112351454 | 112351538 | - | 15.5 | 7832 | 7925 | -36 | L1MC5 | L1 | LINE |
| 112351543 | 112351853 | + | 9.9 | 1 | 303 | -8 | AluY | Alu | SINE |
| 112351980 | 112352337 | - | 31.8 | 7098 | 7475 | -567 | L1MC4 | L1 | LINE |

  

|  |  |  |  |  |  |  |  |  |  |
| --- | --- | --- | --- | --- | --- | --- | --- | --- | --- |
| chr2:112831213-112832248 | | | 1036 bp | | |  |  |  |  |
| Genome Start | Genome End | Strand | Div | Repeat Start | Repeat End | Repeat Left | Name | Family | Class |
| 112831213 | 112831337 | - | 19.0 | 2586 | 2713 | -3693 | L1MDb | L1 | LINE |
| 112831338 | 112831659 | - | 19.0 | 1 | 310 | -2 | AluJo | Alu | SINE |
| 112831660 | 112831720 | - | 19.0 | 2546 | 2585 | -3821 | L1MDb | L1 | LINE |
| 112831721 | 112832006 | - | 11.5 | 1 | 286 | -25 | AluY | Alu | SINE |
| 112832007 | 112832176 | - | 19.0 | 2370 | 2545 | -3861 | L1MDb | L1 | LINE |
| 112832177 | 112832248 | - | 15.5 | 1221 | 1295 | -5114 | L1MDb | L1 | LINE |

  

|  |  |  |  |  |  |  |  |  |  |
| --- | --- | --- | --- | --- | --- | --- | --- | --- | --- |
| chr2:112847631-112848110 | | | 480 bp | | |  |  |  |  |
| Genome Start | Genome End | Strand | Div | Repeat Start | Repeat End | Repeat Left | Name | Family | Class |
| 112847631 | 112847694 | - | 25.8 | 96 | 161 | -100 | MER112 | MER1\_type | DNA |
| 112847695 | 112848013 | - | 20.0 | 1 | 304 | -8 | AluJo | Alu | SINE |
| 112848014 | 112848110 | - | 25.8 | 2 | 95 | -165 | MER112 | MER1\_type | DNA |

  

|  |  |  |  |  |  |  |  |  |  |
| --- | --- | --- | --- | --- | --- | --- | --- | --- | --- |
| chr2:112848356-112849287 | | | 920 bp | | |  |  |  |  |
| Genome Start | Genome End | Strand | Div | Repeat Start | Repeat End | Repeat Left | Name | Family | Class |
| 112848356 | 112848488 | - | 25.6 | 1566 | 1713 | -4436 | L1MEd | L1 | LINE |
| 112848501 | 112848903 | - | 24.3 | 1002 | 1517 | -4632 | L1MEd | L1 | LINE |
| 112848904 | 112849209 | - | 9.5 | 1 | 306 | -7 | AluSq | Alu | SINE |
| 112849210 | 112849287 | - | 23.4 | 936 | 1001 | -5118 | L1MEd | L1 | LINE |

  

|  |  |  |  |  |  |  |  |  |  |
| --- | --- | --- | --- | --- | --- | --- | --- | --- | --- |
| chr2:112850018-112850315 | | | 298 bp | | |  |  |  |  |
| Genome Start | Genome End | Strand | Div | Repeat Start | Repeat End | Repeat Left | Name | Family | Class |
| 112850018 | 112850085 | + | 20.1 | 7269 | 7341 | -620 | L1MC5 | L1 | LINE |
| 112850086 | 112850233 | + | 22.3 | 5 | 188 | -1 | MER5A | MER1\_type | DNA |
| 112850234 | 112850315 | + | 20.1 | 7342 | 7413 | -548 | L1MC5 | L1 | LINE |

  

|  |  |  |  |  |  |  |  |  |  |
| --- | --- | --- | --- | --- | --- | --- | --- | --- | --- |
| chr2:112878568-112879408 | | | 841 bp | | |  |  |  |  |
| Genome Start | Genome End | Strand | Div | Repeat Start | Repeat End | Repeat Left | Name | Family | Class |
| 112878568 | 112878622 | - | 35.9 | 122 | 172 | -6 | MER5B | MER1\_type | DNA |
| 112878623 | 112878936 | + | 7.6 | 1 | 305 | -6 | AluY | Alu | SINE |
| 112878937 | 112879010 | - | 35.9 | 58 | 121 | -57 | MER5B | MER1\_type | DNA |
| 112879011 | 112879344 | + | 16.0 | 5820 | 6163 | -15 | L1MB8 | L1 | LINE |
| 112879345 | 112879408 | - | 35.9 | 2 | 57 | -121 | MER5B | MER1\_type | DNA |

  

|  |  |  |  |  |  |  |  |  |  |
| --- | --- | --- | --- | --- | --- | --- | --- | --- | --- |
| chr2:112882672-112883141 | | | 470 bp | | |  |  |  |  |
| Genome Start | Genome End | Strand | Div | Repeat Start | Repeat End | Repeat Left | Name | Family | Class |
| 112882672 | 112882716 | - | 31.2 | 3408 | 3452 | -1037 | L3 | CR1 | LINE |
| 112882717 | 112883019 | - | 10.7 | 1 | 301 | -11 | AluSx | Alu | SINE |
| 112883020 | 112883141 | - | 31.2 | 3286 | 3407 | -1082 | L3 | CR1 | LINE |

  

|  |  |  |  |  |  |  |  |  |  |
| --- | --- | --- | --- | --- | --- | --- | --- | --- | --- |
| chr2:112887618-112890247 | | | 2291 bp | | |  |  |  |  |
| Genome Start | Genome End | Strand | Div | Repeat Start | Repeat End | Repeat Left | Name | Family | Class |
| 112887618 | 112887748 | + | 21.5 | 13 | 143 | -380 | Charlie1b | MER1\_type | DNA |
| 112887749 | 112888049 | - | 11.0 | 1 | 299 | -10 | AluSc | Alu | SINE |
| 112888050 | 112888241 | + | 21.5 | 144 | 332 | -191 | Charlie1b | MER1\_type | DNA |
| 112888242 | 112888542 | + | 8.7 | 1 | 300 | -13 | AluSq | Alu | SINE |
| 112888543 | 112888645 | + | 21.5 | 333 | 445 | -78 | Charlie1b | MER1\_type | DNA |
| 112888647 | 112888730 | + | 11.9 | 1 | 84 | -225 | AluSc | Alu | SINE |
| 112888731 | 112888786 | + | 11.2 | 29 | 76 | -233 | AluSc | Alu | SINE |
| 112888787 | 112889079 | + | 6.5 | 1 | 293 | -18 | AluY | Alu | SINE |
| 112889080 | 112889149 | + | 11.2 | 77 | 144 | -165 | AluSc | Alu | SINE |
| 112889150 | 112889363 | + | 6.6 | 1 | 212 | -99 | AluY | Alu | SINE |
| 112889691 | 112889768 | + | 10.3 | 212 | 289 | -22 | AluY | Alu | SINE |
| 112889769 | 112889941 | + | 15.3 | 119 | 293 | -16 | AluSc | Alu | SINE |
| 112889942 | 112890166 | + | 8.0 | 72 | 309 | 0 | AluSc | Alu | SINE |
| 112890178 | 112890247 | + | 20.0 | 436 | 515 | -8 | Charlie1b | MER1\_type | DNA |

  

|  |  |  |  |  |  |  |  |  |  |
| --- | --- | --- | --- | --- | --- | --- | --- | --- | --- |
| chr2:112892201-112893314 | | | 1109 bp | | |  |  |  |  |
| Genome Start | Genome End | Strand | Div | Repeat Start | Repeat End | Repeat Left | Name | Family | Class |
| 112892201 | 112892519 | + | 31.2 | 192 | 588 | -2831 | L2 | L2 | LINE |
| 112892521 | 112892637 | - | 19.3 | 86 | 199 | -113 | AluJo | Alu | SINE |
| 112892642 | 112893314 | + | 30.5 | 832 | 1595 | -1824 | L2 | L2 | LINE |

  

|  |  |  |  |  |  |  |  |  |  |
| --- | --- | --- | --- | --- | --- | --- | --- | --- | --- |
| chr2:112893981-112894900 | | | 919 bp | | |  |  |  |  |
| Genome Start | Genome End | Strand | Div | Repeat Start | Repeat End | Repeat Left | Name | Family | Class |
| 112893981 | 112894068 | + | 17.1 | 5678 | 5770 | -589 | L1MD2 | L1 | LINE |
| 112894070 | 112894245 | + | 17.2 | 137 | 310 | -2 | AluSg/x | Alu | SINE |
| 112894246 | 112894379 | + | 17.5 | 5759 | 5908 | -451 | L1MD2 | L1 | LINE |
| 112894380 | 112894667 | + | 12.2 | 1 | 308 | -4 | AluSx | Alu | SINE |
| 112894668 | 112894900 | + | 17.5 | 5909 | 6145 | -214 | L1MD2 | L1 | LINE |

  

|  |  |  |  |  |  |  |  |  |  |
| --- | --- | --- | --- | --- | --- | --- | --- | --- | --- |
| chr2:112894900-112895289 | | | 390 bp | | |  |  |  |  |
| Genome Start | Genome End | Strand | Div | Repeat Start | Repeat End | Repeat Left | Name | Family | Class |
| 112894900 | 112894953 | + | 21.4 | 7832 | 7885 | -110 | L1MD3 | L1 | LINE |
| 112894954 | 112895181 | - | 12.3 | 1 | 236 | 0 | MER46A | MER2\_type | DNA |
| 112895182 | 112895289 | + | 21.4 | 7886 | 7993 | -2 | L1MD3 | L1 | LINE |

  

|  |  |  |  |  |  |  |  |  |  |
| --- | --- | --- | --- | --- | --- | --- | --- | --- | --- |
| chr2:112973939-112974757 | | | 810 bp | | |  |  |  |  |
| Genome Start | Genome End | Strand | Div | Repeat Start | Repeat End | Repeat Left | Name | Family | Class |
| 112973939 | 112974338 | + | 16.3 | 5654 | 6072 | -112 | L1MB7 | L1 | LINE |
| 112974339 | 112974646 | + | 10.0 | 1 | 308 | 0 | AluSg | Alu | SINE |
| 112974656 | 112974757 | + | 16.3 | 6073 | 6175 | -9 | L1MB7 | L1 | LINE |

  

|  |  |  |  |  |  |  |  |  |  |
| --- | --- | --- | --- | --- | --- | --- | --- | --- | --- |
| chr2:113781105-113781578 | | | 276 bp | | |  |  |  |  |
| Genome Start | Genome End | Strand | Div | Repeat Start | Repeat End | Repeat Left | Name | Family | Class |
| 113781105 | 113781194 | - | 20.0 | 68 | 177 | -1 | MER5B | MER1\_type | DNA |
| 113781391 | 113781530 | - | 22.1 | 6021 | 6162 | -4 | L1ME1 | L1 | LINE |
| 113781533 | 113781578 | - | 8.7 | 19 | 66 | -112 | MER5B | MER1\_type | DNA |

  

|  |  |  |  |  |  |  |  |  |  |
| --- | --- | --- | --- | --- | --- | --- | --- | --- | --- |
| chr2:113823615-113824433 | | | 692 bp | | |  |  |  |  |
| Genome Start | Genome End | Strand | Div | Repeat Start | Repeat End | Repeat Left | Name | Family | Class |
| 113823615 | 113823925 | + | 37.1 | 3945 | 4268 | -221 | L3 | CR1 | LINE |
| 113823965 | 113824191 | + | 26.0 | 29 | 258 | -4 | MIR | MIR | SINE |
| 113824280 | 113824433 | + | 33.8 | 4299 | 4464 | -25 | L3 | CR1 | LINE |

  

|  |  |  |  |  |  |  |  |  |  |
| --- | --- | --- | --- | --- | --- | --- | --- | --- | --- |
| chr2:113837277-113837657 | | | 381 bp | | |  |  |  |  |
| Genome Start | Genome End | Strand | Div | Repeat Start | Repeat End | Repeat Left | Name | Family | Class |
| 113837277 | 113837327 | - | 33.3 | 156 | 203 | -5 | MIR3 | MIR | SINE |
| 113837328 | 113837515 | + | 18.2 | 1 | 189 | 0 | MER5A | MER1\_type | DNA |
| 113837516 | 113837657 | - | 33.3 | 16 | 155 | -53 | MIR3 | MIR | SINE |

  

|  |  |  |  |  |  |  |  |  |  |
| --- | --- | --- | --- | --- | --- | --- | --- | --- | --- |
| chr2:113855087-113855823 | | | 737 bp | | |  |  |  |  |
| Genome Start | Genome End | Strand | Div | Repeat Start | Repeat End | Repeat Left | Name | Family | Class |
| 113855087 | 113855468 | + | 15.8 | 1 | 385 | -43 | MSTA | MaLR | LTR |
| 113855469 | 113855780 | - | 8.4 | 1 | 310 | 0 | AluSg | Alu | SINE |
| 113855781 | 113855823 | + | 15.8 | 386 | 426 | -2 | MSTA | MaLR | LTR |

  

|  |  |  |  |  |  |  |  |  |  |
| --- | --- | --- | --- | --- | --- | --- | --- | --- | --- |
| chr2:113892054-113896493 | | | 4156 bp | | |  |  |  |  |
| Genome Start | Genome End | Strand | Div | Repeat Start | Repeat End | Repeat Left | Name | Family | Class |
| 113892054 | 113892968 | + | 28.3 | 1 | 962 | -1545 | HAL1 | L1 | LINE |
| 113892969 | 113893657 | + | 10.7 | 5555 | 6251 | -54 | L1MA3 | L1 | LINE |
| 113893658 | 113893955 | + | 7.0 | 2 | 302 | -11 | AluSp | Alu | SINE |
| 113893956 | 113894008 | + | 10.7 | 6252 | 6304 | -1 | L1MA3 | L1 | LINE |
| 113894009 | 113894490 | + | 26.4 | 963 | 1419 | -1088 | HAL1 | L1 | LINE |
| 113894491 | 113894895 | - | 17.2 | 3 | 390 | 0 | MLT1B | MaLR | LTR |
| 113894896 | 113895056 | + | 26.4 | 1420 | 1572 | -935 | HAL1 | L1 | LINE |
| 113895057 | 113895188 | + | 9.1 | 1 | 133 | -177 | AluSg | Alu | SINE |
| 113895189 | 113895498 | + | 11.6 | 1 | 310 | 0 | AluSg | Alu | SINE |
| 113895775 | 113895876 | + | 26.9 | 324 | 426 | -6 | MLT1I | MaLR | LTR |
| 113895885 | 113896493 | + | 25.4 | 1597 | 2211 | -296 | HAL1 | L1 | LINE |

  

|  |  |  |  |  |  |  |  |  |  |
| --- | --- | --- | --- | --- | --- | --- | --- | --- | --- |
| chr2:113906649-113907971 | | | 1301 bp | | |  |  |  |  |
| Genome Start | Genome End | Strand | Div | Repeat Start | Repeat End | Repeat Left | Name | Family | Class |
| 113906649 | 113906760 | + | 22.1 | 32 | 144 | -197 | MER58 | MER1\_type | DNA |
| 113906761 | 113907071 | - | 16.8 | 1 | 403 | 0 | MER57A | ERV1 | LTR |
| 113907073 | 113907200 | - | 19.8 | 6074 | 6185 | -2 | MER57A-int | ERV1 | LTR |
| 113907201 | 113907292 | - | 21.2 | 280 | 377 | -121 | LTR54B | ERV1 | LTR |
| 113907293 | 113907558 | - | 13.9 | 1 | 267 | -45 | AluSx | Alu | SINE |
| 113907559 | 113907828 | - | 21.2 | 1 | 279 | -219 | LTR54B | ERV1 | LTR |
| 113907850 | 113907971 | + | 19.2 | 2294 | 2416 | -4 | MER58 | MER1\_type | DNA |

  

|  |  |  |  |  |  |  |  |  |  |
| --- | --- | --- | --- | --- | --- | --- | --- | --- | --- |
| chr2:113921074-113923538 | | | 2461 bp | | |  |  |  |  |
| Genome Start | Genome End | Strand | Div | Repeat Start | Repeat End | Repeat Left | Name | Family | Class |
| 113921074 | 113921188 | - | 18.3 | 2304 | 2418 | 0 | Tigger1 | MER2\_type | DNA |
| 113921193 | 113921346 | + | 14.5 | 1 | 162 | -14 | FRAM | Alu | SINE |
| 113921347 | 113922669 | - | 9.6 | 972 | 2309 | -109 | Tigger1 | MER2\_type | DNA |
| 113922670 | 113922979 | + | 11.1 | 1 | 310 | 0 | AluSg | Alu | SINE |
| 113922980 | 113923538 | - | 9.6 | 388 | 971 | -1447 | Tigger1 | MER2\_type | DNA |

  

|  |  |  |  |  |  |  |  |  |  |
| --- | --- | --- | --- | --- | --- | --- | --- | --- | --- |
| chr2:113979263-113979803 | | | 541 bp | | |  |  |  |  |
| Genome Start | Genome End | Strand | Div | Repeat Start | Repeat End | Repeat Left | Name | Family | Class |
| 113979263 | 113979394 | - | 26.1 | 7702 | 7826 | -135 | L1MC5 | L1 | LINE |
| 113979395 | 113979520 | + | 17.5 | 1 | 126 | -7 | FLAM\_C | Alu | SINE |
| 113979521 | 113979803 | - | 26.1 | 7409 | 7701 | -260 | L1MC5 | L1 | LINE |

  

|  |  |  |  |  |  |  |  |  |  |
| --- | --- | --- | --- | --- | --- | --- | --- | --- | --- |
| chr2:113986246-113987031 | | | 786 bp | | |  |  |  |  |
| Genome Start | Genome End | Strand | Div | Repeat Start | Repeat End | Repeat Left | Name | Family | Class |
| 113986246 | 113986280 | - | 23.9 | 459 | 493 | -5907 | L1MEc | L1 | LINE |
| 113986281 | 113986581 | - | 13.3 | 1 | 309 | 0 | AluSc | Alu | SINE |
| 113986582 | 113987031 | - | 23.9 | 38 | 458 | -5942 | L1MEc | L1 | LINE |

  

|  |  |  |  |  |  |  |  |  |  |
| --- | --- | --- | --- | --- | --- | --- | --- | --- | --- |
| chr2:113990770-113991281 | | | 512 bp | | |  |  |  |  |
| Genome Start | Genome End | Strand | Div | Repeat Start | Repeat End | Repeat Left | Name | Family | Class |
| 113990770 | 113990850 | + | 18.1 | 1 | 67 | -152 | MER20 | MER1\_type | DNA |
| 113990851 | 113991120 | - | 13.8 | 5906 | 6176 | -7 | L1MB3 | L1 | LINE |
| 113991121 | 113991281 | + | 18.1 | 68 | 219 | 0 | MER20 | MER1\_type | DNA |

  

|  |  |  |  |  |  |  |  |  |  |
| --- | --- | --- | --- | --- | --- | --- | --- | --- | --- |
| chr2:114003675-114004926 | | | 1252 bp | | |  |  |  |  |
| Genome Start | Genome End | Strand | Div | Repeat Start | Repeat End | Repeat Left | Name | Family | Class |
| 114003675 | 114003758 | - | 16.2 | 4269 | 4315 | -1828 | L1M2 | L1 | LINE |
| 114003759 | 114003982 | - | 12.3 | 5955 | 6166 | 0 | L1PA17 | L1 | LINE |
| 114003983 | 114004926 | - | 16.2 | 3278 | 4268 | -1875 | L1M2 | L1 | LINE |

  

|  |  |  |  |  |  |  |  |  |  |
| --- | --- | --- | --- | --- | --- | --- | --- | --- | --- |
| chr2:114005648-114006153 | | | 506 bp | | |  |  |  |  |
| Genome Start | Genome End | Strand | Div | Repeat Start | Repeat End | Repeat Left | Name | Family | Class |
| 114005648 | 114005742 | + | 32.2 | 60 | 155 | -113 | MIRb | MIR | SINE |
| 114005743 | 114006093 | + | 14.1 | 1 | 366 | 0 | Tigger5a | MER2\_type | DNA |
| 114006094 | 114006153 | + | 32.2 | 156 | 211 | -57 | MIRb | MIR | SINE |

  

|  |  |  |  |  |  |  |  |  |  |
| --- | --- | --- | --- | --- | --- | --- | --- | --- | --- |
| chr2:114020064-114021155 | | | 1244 bp | | |  |  |  |  |
| Genome Start | Genome End | Strand | Div | Repeat Start | Repeat End | Repeat Left | Name | Family | Class |
| 114020064 | 114020290 | - | 25.7 | 7333 | 7560 | -401 | L1MC5 | L1 | LINE |
| 114020292 | 114020650 | - | 20.1 | 1 | 359 | -6 | MLT1A0 | MaLR | LTR |
| 114020651 | 114020722 | - | 26.4 | 7257 | 7331 | -630 | L1MC5 | L1 | LINE |
| 114020700 | 114021009 | - | 27.3 | 7096 | 7416 | -626 | L1MC4 | L1 | LINE |
| 114020880 | 114021155 | - | 28.2 | 6960 | 7228 | -733 | L1MC5 | L1 | LINE |

  

|  |  |  |  |  |  |  |  |  |  |
| --- | --- | --- | --- | --- | --- | --- | --- | --- | --- |
| chr2:114033346-114035182 | | | 1678 bp | | |  |  |  |  |
| Genome Start | Genome End | Strand | Div | Repeat Start | Repeat End | Repeat Left | Name | Family | Class |
| 114033346 | 114033428 | + | 31.3 | 2500 | 2586 | -833 | L2 | L2 | LINE |
| 114033438 | 114033489 | - | 13.5 | 490 | 541 | -1 | MLT1F | MaLR | LTR |
| 114033495 | 114033569 | - | 10.7 | 282 | 357 | -185 | MLT1F | MaLR | LTR |
| 114033658 | 114033812 | - | 23.9 | 3236 | 3419 | 0 | L2 | L2 | LINE |
| 114033818 | 114034264 | - | 23.2 | 1 | 509 | -58 | MLT1F | MaLR | LTR |
| 114034269 | 114034469 | + | 32.8 | 2584 | 2793 | -626 | L2 | L2 | LINE |
| 114034494 | 114035086 | - | 27.5 | 2786 | 3419 | 0 | L2 | L2 | LINE |
| 114035111 | 114035182 | + | 29.6 | 2821 | 2891 | -528 | L2 | L2 | LINE |

  

|  |  |  |  |  |  |  |  |  |  |
| --- | --- | --- | --- | --- | --- | --- | --- | --- | --- |
| chr2:114035620-114036264 | | | 463 bp | | |  |  |  |  |
| Genome Start | Genome End | Strand | Div | Repeat Start | Repeat End | Repeat Left | Name | Family | Class |
| 114035620 | 114035687 | - | 17.6 | 58 | 125 | -35 | MER5A1 | MER1\_type | DNA |
| 114035870 | 114036202 | - | 14.5 | 1 | 334 | 0 | AluSx | Alu | SINE |
| 114036203 | 114036264 | - | 17.7 | 1 | 73 | -87 | MER5A1 | MER1\_type | DNA |

  

|  |  |  |  |  |  |  |  |  |  |
| --- | --- | --- | --- | --- | --- | --- | --- | --- | --- |
| chr2:114063040-114063663 | | | 530 bp | | |  |  |  |  |
| Genome Start | Genome End | Strand | Div | Repeat Start | Repeat End | Repeat Left | Name | Family | Class |
| 114063040 | 114063224 | - | 35.2 | 3232 | 3418 | -1 | L2 | L2 | LINE |
| 114063238 | 114063400 | - | 24.7 | 49 | 260 | -2 | MIR | MIR | SINE |
| 114063482 | 114063663 | - | 28.9 | 2957 | 3178 | -200 | L2 | L2 | LINE |

  

|  |  |  |  |  |  |  |  |  |  |
| --- | --- | --- | --- | --- | --- | --- | --- | --- | --- |
| chr2:114079670-114081034 | | | 1349 bp | | |  |  |  |  |
| Genome Start | Genome End | Strand | Div | Repeat Start | Repeat End | Repeat Left | Name | Family | Class |
| 114079670 | 114080280 | + | 22.4 | 4898 | 5546 | -2239 | L1MC | L1 | LINE |
| 114080292 | 114080841 | + | 17.8 | 7 | 583 | -2 | MER34C\_ | ERV1 | LTR |
| 114080847 | 114081034 | + | 27.0 | 5577 | 5765 | -2020 | L1MC | L1 | LINE |

  

|  |  |  |  |  |  |  |  |  |  |
| --- | --- | --- | --- | --- | --- | --- | --- | --- | --- |
| chr2:114081244-114082244 | | | 1001 bp | | |  |  |  |  |
| Genome Start | Genome End | Strand | Div | Repeat Start | Repeat End | Repeat Left | Name | Family | Class |
| 114081244 | 114081890 | + | 14.2 | 1 | 668 | -54 | MER72 | ERV1 | LTR |
| 114081891 | 114082182 | + | 9.6 | 11 | 301 | -12 | AluSq | Alu | SINE |
| 114082183 | 114082244 | + | 14.2 | 669 | 722 | 0 | MER72 | ERV1 | LTR |

  

|  |  |  |  |  |  |  |  |  |  |
| --- | --- | --- | --- | --- | --- | --- | --- | --- | --- |
| chr2:114087344-114089459 | | | 1959 bp | | |  |  |  |  |
| Genome Start | Genome End | Strand | Div | Repeat Start | Repeat End | Repeat Left | Name | Family | Class |
| 114087344 | 114088134 | + | 22.8 | 3121 | 3873 | -1063 | MER31-int | ERV1 | LTR |
| 114088254 | 114088615 | + | 26.2 | 188 | 562 | -29 | LTR10C | ERV1 | LTR |
| 114088654 | 114089081 | + | 19.7 | 3859 | 4297 | -639 | MER31-int | ERV1 | LTR |
| 114089082 | 114089346 | + | 14.7 | 2 | 290 | -12 | AluJb | Alu | SINE |
| 114089347 | 114089459 | + | 19.7 | 4298 | 4375 | -561 | MER31-int | ERV1 | LTR |

  

|  |  |  |  |  |  |  |  |  |  |
| --- | --- | --- | --- | --- | --- | --- | --- | --- | --- |
| chr2:114119877-114120737 | | | 861 bp | | |  |  |  |  |
| Genome Start | Genome End | Strand | Div | Repeat Start | Repeat End | Repeat Left | Name | Family | Class |
| 114119877 | 114119960 | + | 19.4 | 5748 | 5822 | -512 | L1MA10 | L1 | LINE |
| 114119961 | 114120282 | + | 6.5 | 1 | 310 | 0 | AluY | Alu | SINE |
| 114120283 | 114120737 | + | 19.4 | 5823 | 6289 | -45 | L1MA10 | L1 | LINE |

  

|  |  |  |  |  |  |  |  |  |  |
| --- | --- | --- | --- | --- | --- | --- | --- | --- | --- |
| chr2:130403072-130403555 | | | 484 bp | | |  |  |  |  |
| Genome Start | Genome End | Strand | Div | Repeat Start | Repeat End | Repeat Left | Name | Family | Class |
| 130403072 | 130403167 | + | 29.3 | 3968 | 4074 | -415 | L3 | CR1 | LINE |
| 130403168 | 130403460 | - | 15.4 | 1 | 296 | -16 | AluJb | Alu | SINE |
| 130403461 | 130403555 | + | 29.3 | 4075 | 4166 | -323 | L3 | CR1 | LINE |

  

|  |  |  |  |  |  |  |  |  |  |
| --- | --- | --- | --- | --- | --- | --- | --- | --- | --- |
| chr2:130433653-130434400 | | | 703 bp | | |  |  |  |  |
| Genome Start | Genome End | Strand | Div | Repeat Start | Repeat End | Repeat Left | Name | Family | Class |
| 130433653 | 130433703 | - | 14.3 | 4612 | 4652 | -1491 | L1M2 | L1 | LINE |
| 130433749 | 130434032 | - | 8.8 | 1 | 285 | -28 | AluSp | Alu | SINE |
| 130434033 | 130434400 | - | 14.3 | 4247 | 4611 | -1532 | L1M2 | L1 | LINE |

  

|  |  |  |  |  |  |  |  |  |  |
| --- | --- | --- | --- | --- | --- | --- | --- | --- | --- |
| chr2:130439601-130440282 | | | 682 bp | | |  |  |  |  |
| Genome Start | Genome End | Strand | Div | Repeat Start | Repeat End | Repeat Left | Name | Family | Class |
| 130439601 | 130439710 | + | 17.8 | 1 | 109 | -237 | MER7A | MER2\_type | DNA |
| 130439711 | 130440013 | - | 10.6 | 1 | 305 | -7 | AluSx | Alu | SINE |
| 130440014 | 130440282 | + | 17.8 | 110 | 341 | -5 | MER7A | MER2\_type | DNA |

  

|  |  |  |  |  |  |  |  |  |  |
| --- | --- | --- | --- | --- | --- | --- | --- | --- | --- |
| chr2:130459721-130460746 | | | 1014 bp | | |  |  |  |  |
| Genome Start | Genome End | Strand | Div | Repeat Start | Repeat End | Repeat Left | Name | Family | Class |
| 130459721 | 130459864 | - | 35.7 | 1678 | 1817 | -1602 | L2 | L2 | LINE |
| 130459865 | 130460395 | + | 17.8 | 1 | 554 | 0 | MLT2A2 | ERVL | LTR |
| 130460408 | 130460746 | - | 29.8 | 1299 | 1670 | -1749 | L2 | L2 | LINE |

  

|  |  |  |  |  |  |  |  |  |  |
| --- | --- | --- | --- | --- | --- | --- | --- | --- | --- |
| chr2:130555642-130557763 | | | 1764 bp | | |  |  |  |  |
| Genome Start | Genome End | Strand | Div | Repeat Start | Repeat End | Repeat Left | Name | Family | Class |
| 130555642 | 130555901 | - | 32.8 | 3137 | 3419 | 0 | L2 | L2 | LINE |
| 130556260 | 130557096 | - | 31.4 | 2301 | 3146 | -273 | L2 | L2 | LINE |
| 130557097 | 130557390 | + | 12.6 | 1 | 295 | -17 | AluSx | Alu | SINE |
| 130557391 | 130557763 | - | 31.3 | 1882 | 2300 | -1119 | L2 | L2 | LINE |

  

|  |  |  |  |  |  |  |  |  |  |
| --- | --- | --- | --- | --- | --- | --- | --- | --- | --- |
| chr2:130558348-130558687 | | | 318 bp | | |  |  |  |  |
| Genome Start | Genome End | Strand | Div | Repeat Start | Repeat End | Repeat Left | Name | Family | Class |
| 130558348 | 130558445 | + | 21.6 | 1 | 98 | -9 | MER58A | MER1\_type | DNA |
| 130558461 | 130558614 | + | 19.5 | 134 | 289 | -23 | AluJb | Alu | SINE |
| 130558622 | 130558687 | + | 16.7 | 159 | 224 | 0 | MER58A | MER1\_type | DNA |

  

|  |  |  |  |  |  |  |  |  |  |
| --- | --- | --- | --- | --- | --- | --- | --- | --- | --- |
| chr2:130559019-130559757 | | | 739 bp | | |  |  |  |  |
| Genome Start | Genome End | Strand | Div | Repeat Start | Repeat End | Repeat Left | Name | Family | Class |
| 130559019 | 130559285 | - | 31.4 | 5523 | 5825 | -1983 | L1MC | L1 | LINE |
| 130559286 | 130559631 | + | 16.7 | 5821 | 6165 | 0 | L1PA15 | L1 | LINE |
| 130559632 | 130559757 | - | 31.4 | 5385 | 5522 | -2286 | L1MC | L1 | LINE |

  

|  |  |  |  |  |  |  |  |  |  |
| --- | --- | --- | --- | --- | --- | --- | --- | --- | --- |
| chr2:130569414-130571375 | | | 1684 bp | | |  |  |  |  |
| Genome Start | Genome End | Strand | Div | Repeat Start | Repeat End | Repeat Left | Name | Family | Class |
| 130569414 | 130569673 | - | 30.4 | 3137 | 3419 | 0 | L2 | L2 | LINE |
| 130569952 | 130570702 | - | 30.6 | 2281 | 3150 | -269 | L2 | L2 | LINE |
| 130570703 | 130571003 | + | 13.0 | 1 | 301 | -11 | AluSx | Alu | SINE |
| 130571004 | 130571375 | - | 30.5 | 1883 | 2280 | -1139 | L2 | L2 | LINE |

  

|  |  |  |  |  |  |  |  |  |  |
| --- | --- | --- | --- | --- | --- | --- | --- | --- | --- |
| chr2:130571978-130572320 | | | 322 bp | | |  |  |  |  |
| Genome Start | Genome End | Strand | Div | Repeat Start | Repeat End | Repeat Left | Name | Family | Class |
| 130571978 | 130572074 | + | 15.8 | 1 | 98 | -9 | MER58A | MER1\_type | DNA |
| 130572088 | 130572247 | + | 20.0 | 132 | 291 | -21 | AluJb | Alu | SINE |
| 130572256 | 130572320 | + | 15.4 | 160 | 224 | 0 | MER58A | MER1\_type | DNA |

  

|  |  |  |  |  |  |  |  |  |  |
| --- | --- | --- | --- | --- | --- | --- | --- | --- | --- |
| chr2:130605058-130608845 | | | 3788 bp | | |  |  |  |  |
| Genome Start | Genome End | Strand | Div | Repeat Start | Repeat End | Repeat Left | Name | Family | Class |
| 130605058 | 130607616 | - | 17.1 | 4188 | 6738 | -824 | L1M1 | L1 | LINE |
| 130607617 | 130607919 | - | 7.2 | 1 | 305 | 0 | AluY | Alu | SINE |
| 130607920 | 130608845 | - | 16.5 | 3306 | 4187 | -3375 | L1M1 | L1 | LINE |

  

|  |  |  |  |  |  |  |  |  |  |
| --- | --- | --- | --- | --- | --- | --- | --- | --- | --- |
| chr2:130651829-130652427 | | | 585 bp | | |  |  |  |  |
| Genome Start | Genome End | Strand | Div | Repeat Start | Repeat End | Repeat Left | Name | Family | Class |
| 130651829 | 130651903 | - | 34.7 | 178 | 252 | -10 | MIR | MIR | SINE |
| 130651904 | 130652043 | + | 16.7 | 2 | 160 | -464 | MLT1E2 | MaLR | LTR |
| 130652040 | 130652273 | + | 19.7 | 330 | 594 | -32 | MLT1E2 | MaLR | LTR |
| 130652292 | 130652427 | - | 23.8 | 45 | 182 | -80 | MIR | MIR | SINE |

  

|  |  |  |  |  |  |  |  |  |  |
| --- | --- | --- | --- | --- | --- | --- | --- | --- | --- |
| chr2:130658932-130659544 | | | 613 bp | | |  |  |  |  |
| Genome Start | Genome End | Strand | Div | Repeat Start | Repeat End | Repeat Left | Name | Family | Class |
| 130658932 | 130659070 | + | 12.9 | 7 | 143 | -169 | AluJo | Alu | SINE |
| 130659071 | 130659368 | + | 5.1 | 1 | 296 | -15 | AluY | Alu | SINE |
| 130659369 | 130659544 | + | 12.9 | 144 | 304 | -8 | AluJo | Alu | SINE |

  

|  |  |  |  |  |  |  |  |  |  |
| --- | --- | --- | --- | --- | --- | --- | --- | --- | --- |
| chr2:130681589-130682125 | | | 537 bp | | |  |  |  |  |
| Genome Start | Genome End | Strand | Div | Repeat Start | Repeat End | Repeat Left | Name | Family | Class |
| 130681589 | 130681629 | - | 8.5 | 402 | 428 | 0 | MSTA | MaLR | LTR |
| 130681630 | 130681894 | + | 10.6 | 1 | 267 | -45 | AluSx | Alu | SINE |
| 130681895 | 130682125 | - | 8.5 | 166 | 401 | -27 | MSTA | MaLR | LTR |

  

|  |  |  |  |  |  |  |  |  |  |
| --- | --- | --- | --- | --- | --- | --- | --- | --- | --- |
| chr2:130701216-130702131 | | | 916 bp | | |  |  |  |  |
| Genome Start | Genome End | Strand | Div | Repeat Start | Repeat End | Repeat Left | Name | Family | Class |
| 130701216 | 130701559 | + | 26.8 | 1386 | 1793 | -1626 | L2 | L2 | LINE |
| 130701560 | 130702019 | + | 21.6 | 1 | 464 | 0 | MER88 | ERVL | LTR |
| 130702020 | 130702131 | + | 28.9 | 1794 | 1908 | -1511 | L2 | L2 | LINE |

  

|  |  |  |  |  |  |  |  |  |  |
| --- | --- | --- | --- | --- | --- | --- | --- | --- | --- |
| chr2:130895559-130895932 | | | 374 bp | | |  |  |  |  |
| Genome Start | Genome End | Strand | Div | Repeat Start | Repeat End | Repeat Left | Name | Family | Class |
| 130895559 | 130895746 | - | 35.7 | 63 | 260 | -2 | MIR | MIR | SINE |
| 130895767 | 130895867 | + | 28.0 | 3296 | 3417 | -2 | L2 | L2 | LINE |
| 130895848 | 130895932 | - | 33.7 | 1 | 86 | -176 | MIR | MIR | SINE |

  

|  |  |  |  |  |  |  |  |  |  |
| --- | --- | --- | --- | --- | --- | --- | --- | --- | --- |
| chr2:130944209-130946322 | | | 1757 bp | | |  |  |  |  |
| Genome Start | Genome End | Strand | Div | Repeat Start | Repeat End | Repeat Left | Name | Family | Class |
| 130944209 | 130944468 | - | 32.4 | 3137 | 3419 | 0 | L2 | L2 | LINE |
| 130944826 | 130945640 | - | 31.7 | 2301 | 3146 | -273 | L2 | L2 | LINE |
| 130945641 | 130945949 | + | 11.3 | 1 | 310 | -2 | AluSx | Alu | SINE |
| 130945950 | 130946322 | - | 31.5 | 1882 | 2300 | -1119 | L2 | L2 | LINE |

  

|  |  |  |  |  |  |  |  |  |  |
| --- | --- | --- | --- | --- | --- | --- | --- | --- | --- |
| chr2:130946908-130947247 | | | 318 bp | | |  |  |  |  |
| Genome Start | Genome End | Strand | Div | Repeat Start | Repeat End | Repeat Left | Name | Family | Class |
| 130946908 | 130947005 | + | 21.6 | 1 | 98 | -9 | MER58A | MER1\_type | DNA |
| 130947021 | 130947174 | + | 18.2 | 134 | 289 | -23 | AluJb | Alu | SINE |
| 130947182 | 130947247 | + | 16.7 | 159 | 224 | 0 | MER58A | MER1\_type | DNA |

  

|  |  |  |  |  |  |  |  |  |  |
| --- | --- | --- | --- | --- | --- | --- | --- | --- | --- |
| chr2:130957981-130959941 | | | 1687 bp | | |  |  |  |  |
| Genome Start | Genome End | Strand | Div | Repeat Start | Repeat End | Repeat Left | Name | Family | Class |
| 130957981 | 130958240 | - | 30.0 | 3137 | 3419 | 0 | L2 | L2 | LINE |
| 130958515 | 130959266 | - | 30.6 | 2281 | 3150 | -269 | L2 | L2 | LINE |
| 130959267 | 130959567 | + | 13.3 | 1 | 301 | -11 | AluSx | Alu | SINE |
| 130959568 | 130959941 | - | 30.5 | 1883 | 2280 | -1139 | L2 | L2 | LINE |

  

|  |  |  |  |  |  |  |  |  |  |
| --- | --- | --- | --- | --- | --- | --- | --- | --- | --- |
| chr2:130960544-130960886 | | | 322 bp | | |  |  |  |  |
| Genome Start | Genome End | Strand | Div | Repeat Start | Repeat End | Repeat Left | Name | Family | Class |
| 130960544 | 130960640 | + | 14.7 | 1 | 98 | -9 | MER58A | MER1\_type | DNA |
| 130960654 | 130960813 | + | 19.4 | 132 | 291 | -21 | AluJb | Alu | SINE |
| 130960822 | 130960886 | + | 15.4 | 160 | 224 | 0 | MER58A | MER1\_type | DNA |

  

|  |  |  |  |  |  |  |  |  |  |
| --- | --- | --- | --- | --- | --- | --- | --- | --- | --- |
| chr2:130961306-130961975 | | | 670 bp | | |  |  |  |  |
| Genome Start | Genome End | Strand | Div | Repeat Start | Repeat End | Repeat Left | Name | Family | Class |
| 130961306 | 130961505 | - | 29.3 | 5524 | 5762 | -2046 | L1MC | L1 | LINE |
| 130961506 | 130961851 | + | 18.1 | 5821 | 6165 | 0 | L1PA15 | L1 | LINE |
| 130961852 | 130961975 | - | 29.3 | 5385 | 5523 | -2285 | L1MC | L1 | LINE |

  

|  |  |  |  |  |  |  |  |  |  |
| --- | --- | --- | --- | --- | --- | --- | --- | --- | --- |
| chr2:130984523-130986284 | | | 1547 bp | | |  |  |  |  |
| Genome Start | Genome End | Strand | Div | Repeat Start | Repeat End | Repeat Left | Name | Family | Class |
| 130984523 | 130984661 | + | 30.6 | 1397 | 1547 | -1872 | L2 | L2 | LINE |
| 130984662 | 130984992 | + | 7.8 | 1 | 332 | 0 | AluSp | Alu | SINE |
| 130984993 | 130985815 | + | 30.6 | 1548 | 2628 | -791 | L2 | L2 | LINE |
| 130986031 | 130986284 | + | 24.7 | 3137 | 3419 | 0 | L2 | L2 | LINE |

  

|  |  |  |  |  |  |  |  |  |  |
| --- | --- | --- | --- | --- | --- | --- | --- | --- | --- |
| chr2:131025837-131027840 | | | 2004 bp | | |  |  |  |  |
| Genome Start | Genome End | Strand | Div | Repeat Start | Repeat End | Repeat Left | Name | Family | Class |
| 131025837 | 131026769 | - | 25.1 | 319 | 1401 | -88 | MLT1E1-int | MaLR | LTR |
| 131026770 | 131027512 | + | 21.2 | 1 | 723 | -4 | LTR8A | ERV1 | LTR |
| 131027513 | 131027840 | - | 25.1 | 2 | 318 | -1171 | MLT1E1-int | MaLR | LTR |

  

|  |  |  |  |  |  |  |  |  |  |
| --- | --- | --- | --- | --- | --- | --- | --- | --- | --- |
| chr2:131043002-131044374 | | | 1373 bp | | |  |  |  |  |
| Genome Start | Genome End | Strand | Div | Repeat Start | Repeat End | Repeat Left | Name | Family | Class |
| 131043002 | 131043049 | + | 21.9 | 1359 | 1402 | -87 | MLT1E1-int | MaLR | LTR |
| 131043050 | 131043145 | + | 10.4 | 1 | 97 | -537 | MER41B | ERV1 | LTR |
| 131043146 | 131043459 | - | 7.4 | 1 | 309 | -2 | AluY | Alu | SINE |
| 131043460 | 131043757 | - | 8.4 | 1 | 297 | -15 | AluSx | Alu | SINE |
| 131043758 | 131044293 | + | 10.4 | 98 | 634 | 0 | MER41B | ERV1 | LTR |
| 131044294 | 131044374 | + | 21.9 | 1403 | 1480 | -9 | MLT1E1-int | MaLR | LTR |

  

|  |  |  |  |  |  |  |  |  |  |
| --- | --- | --- | --- | --- | --- | --- | --- | --- | --- |
| chr2:131063567-131064327 | | | 761 bp | | |  |  |  |  |
| Genome Start | Genome End | Strand | Div | Repeat Start | Repeat End | Repeat Left | Name | Family | Class |
| 131063567 | 131063934 | + | 21.9 | 537 | 993 | -68 | MER63 | AcHobo | DNA |
| 131063935 | 131064250 | + | 8.0 | 1 | 311 | 0 | AluY | Alu | SINE |
| 131064251 | 131064327 | + | 21.9 | 994 | 1057 | -4 | MER63 | AcHobo | DNA |

  

|  |  |  |  |  |  |  |  |  |  |
| --- | --- | --- | --- | --- | --- | --- | --- | --- | --- |
| chr2:131106802-131107295 | | | 494 bp | | |  |  |  |  |
| Genome Start | Genome End | Strand | Div | Repeat Start | Repeat End | Repeat Left | Name | Family | Class |
| 131106802 | 131106922 | + | 26.5 | 5388 | 5523 | -598 | L1M5 | L1 | LINE |
| 131106923 | 131107268 | - | 17.8 | 5821 | 6165 | 0 | L1PA15 | L1 | LINE |
| 131107269 | 131107295 | + | 26.5 | 5524 | 5539 | -582 | L1M5 | L1 | LINE |

  

|  |  |  |  |  |  |  |  |  |  |
| --- | --- | --- | --- | --- | --- | --- | --- | --- | --- |
| chr2:131107891-131108233 | | | 322 bp | | |  |  |  |  |
| Genome Start | Genome End | Strand | Div | Repeat Start | Repeat End | Repeat Left | Name | Family | Class |
| 131107891 | 131107955 | - | 15.4 | 160 | 224 | 0 | MER58A | MER1\_type | DNA |
| 131107964 | 131108123 | - | 18.8 | 132 | 291 | -21 | AluJb | Alu | SINE |
| 131108137 | 131108233 | - | 15.8 | 1 | 98 | -9 | MER58A | MER1\_type | DNA |

  

|  |  |  |  |  |  |  |  |  |  |
| --- | --- | --- | --- | --- | --- | --- | --- | --- | --- |
| chr2:131108836-131110796 | | | 1686 bp | | |  |  |  |  |
| Genome Start | Genome End | Strand | Div | Repeat Start | Repeat End | Repeat Left | Name | Family | Class |
| 131108836 | 131109208 | + | 30.4 | 1882 | 2279 | -1140 | L2 | L2 | LINE |
| 131109209 | 131109509 | - | 13.3 | 1 | 301 | -11 | AluSx | Alu | SINE |
| 131109510 | 131110261 | + | 30.5 | 2280 | 3150 | -269 | L2 | L2 | LINE |
| 131110537 | 131110796 | + | 28.9 | 3137 | 3419 | 0 | L2 | L2 | LINE |

  

|  |  |  |  |  |  |  |  |  |  |
| --- | --- | --- | --- | --- | --- | --- | --- | --- | --- |
| chr2:131120453-131121196 | | | 744 bp | | |  |  |  |  |
| Genome Start | Genome End | Strand | Div | Repeat Start | Repeat End | Repeat Left | Name | Family | Class |
| 131120453 | 131120578 | + | 30.9 | 5385 | 5522 | -2286 | L1MC4a | L1 | LINE |
| 131120579 | 131120924 | - | 16.4 | 5821 | 6165 | 0 | L1PA15 | L1 | LINE |
| 131120925 | 131121196 | + | 30.9 | 5523 | 5825 | -1983 | L1MC4a | L1 | LINE |

  

|  |  |  |  |  |  |  |  |  |  |
| --- | --- | --- | --- | --- | --- | --- | --- | --- | --- |
| chr2:131121528-131121867 | | | 322 bp | | |  |  |  |  |
| Genome Start | Genome End | Strand | Div | Repeat Start | Repeat End | Repeat Left | Name | Family | Class |
| 131121528 | 131121593 | - | 16.7 | 159 | 224 | 0 | MER58A | MER1\_type | DNA |
| 131121597 | 131121754 | - | 18.4 | 134 | 293 | -19 | AluJb | Alu | SINE |
| 131121770 | 131121867 | - | 21.6 | 1 | 98 | -9 | MER58A | MER1\_type | DNA |

  

|  |  |  |  |  |  |  |  |  |  |
| --- | --- | --- | --- | --- | --- | --- | --- | --- | --- |
| chr2:131122452-131124566 | | | 1758 bp | | |  |  |  |  |
| Genome Start | Genome End | Strand | Div | Repeat Start | Repeat End | Repeat Left | Name | Family | Class |
| 131122452 | 131122824 | + | 31.0 | 1882 | 2300 | -1119 | L2 | L2 | LINE |
| 131122825 | 131123134 | - | 11.3 | 1 | 311 | -1 | AluSx | Alu | SINE |
| 131123135 | 131123949 | + | 31.2 | 2301 | 3146 | -273 | L2 | L2 | LINE |
| 131124307 | 131124566 | + | 32.0 | 3137 | 3419 | 0 | L2 | L2 | LINE |

  

|  |  |  |  |  |  |  |  |  |  |
| --- | --- | --- | --- | --- | --- | --- | --- | --- | --- |
| chr2:131150288-131150946 | | | 551 bp | | |  |  |  |  |
| Genome Start | Genome End | Strand | Div | Repeat Start | Repeat End | Repeat Left | Name | Family | Class |
| 131150288 | 131150382 | + | 17.9 | 1 | 97 | -331 | MSTA | MaLR | LTR |
| 131150480 | 131150535 | + | 21.4 | 911 | 967 | -613 | THE1-int | MaLR | LTR |
| 131150524 | 131150759 | - | 21.7 | 976 | 1256 | -395 | MST-int | MaLR | LTR |
| 131150783 | 131150946 | + | 29.4 | 99 | 274 | -154 | MSTA | MaLR | LTR |

  

|  |  |  |  |  |  |  |  |  |  |
| --- | --- | --- | --- | --- | --- | --- | --- | --- | --- |
| chr2:131171748-131173610 | | | 1867 bp | | |  |  |  |  |
| Genome Start | Genome End | Strand | Div | Repeat Start | Repeat End | Repeat Left | Name | Family | Class |
| 131171748 | 131172127 | - | 27.1 | 4149 | 4524 | -1622 | L1M5 | L1 | LINE |
| 131172128 | 131172368 | - | 10.0 | 1 | 242 | -69 | AluY | Alu | SINE |
| 131172369 | 131172462 | - | 27.1 | 4048 | 4148 | -1998 | L1M5 | L1 | LINE |
| 131172453 | 131172548 | - | 26.3 | 3747 | 3841 | -2305 | L1M5 | L1 | LINE |
| 131172555 | 131172975 | - | 26.9 | 3182 | 3620 | -2526 | L1M5 | L1 | LINE |
| 131172976 | 131173291 | - | 11.6 | 1 | 313 | 0 | AluSp | Alu | SINE |
| 131173292 | 131173610 | - | 26.9 | 2850 | 3181 | -2965 | L1M5 | L1 | LINE |

  

|  |  |  |  |  |  |  |  |  |  |
| --- | --- | --- | --- | --- | --- | --- | --- | --- | --- |
| chr2:131185490-131185927 | | | 438 bp | | |  |  |  |  |
| Genome Start | Genome End | Strand | Div | Repeat Start | Repeat End | Repeat Left | Name | Family | Class |
| 131185490 | 131185537 | + | 29.7 | 31 | 76 | -6043 | L1MEd | L1 | LINE |
| 131185538 | 131185846 | + | 14.3 | 1 | 301 | -12 | AluSq | Alu | SINE |
| 131185847 | 131185927 | + | 29.7 | 77 | 163 | -5956 | L1MEd | L1 | LINE |

  

|  |  |  |  |  |  |  |  |  |  |
| --- | --- | --- | --- | --- | --- | --- | --- | --- | --- |
| chr2:131694659-131695190 | | | 532 bp | | |  |  |  |  |
| Genome Start | Genome End | Strand | Div | Repeat Start | Repeat End | Repeat Left | Name | Family | Class |
| 131694659 | 131694722 | + | 36.5 | 5411 | 5480 | -641 | L1M5 | L1 | LINE |
| 131694723 | 131695028 | + | 12.5 | 1 | 305 | -5 | AluSg | Alu | SINE |
| 131695029 | 131695190 | + | 36.5 | 5481 | 5636 | -485 | L1M5 | L1 | LINE |

  

|  |  |  |  |  |  |  |  |  |  |
| --- | --- | --- | --- | --- | --- | --- | --- | --- | --- |
| chr2:131713589-131714083 | | | 495 bp | | |  |  |  |  |
| Genome Start | Genome End | Strand | Div | Repeat Start | Repeat End | Repeat Left | Name | Family | Class |
| 131713589 | 131713710 | + | 23.1 | 5387 | 5523 | -2285 | L1MC | L1 | LINE |
| 131713711 | 131714056 | - | 17.8 | 5821 | 6165 | 0 | L1PA15 | L1 | LINE |
| 131714057 | 131714083 | + | 23.1 | 5524 | 5539 | -2269 | L1MC | L1 | LINE |

  

|  |  |  |  |  |  |  |  |  |  |
| --- | --- | --- | --- | --- | --- | --- | --- | --- | --- |
| chr2:131714679-131715021 | | | 322 bp | | |  |  |  |  |
| Genome Start | Genome End | Strand | Div | Repeat Start | Repeat End | Repeat Left | Name | Family | Class |
| 131714679 | 131714743 | - | 15.4 | 160 | 224 | 0 | MER58A | MER1\_type | DNA |
| 131714752 | 131714911 | - | 20.0 | 132 | 291 | -21 | AluJb | Alu | SINE |
| 131714925 | 131715021 | - | 15.8 | 1 | 98 | -9 | MER58A | MER1\_type | DNA |

  

|  |  |  |  |  |  |  |  |  |  |
| --- | --- | --- | --- | --- | --- | --- | --- | --- | --- |
| chr2:131715624-131717585 | | | 1684 bp | | |  |  |  |  |
| Genome Start | Genome End | Strand | Div | Repeat Start | Repeat End | Repeat Left | Name | Family | Class |
| 131715624 | 131715995 | + | 30.5 | 1882 | 2279 | -1140 | L2 | L2 | LINE |
| 131715996 | 131716296 | - | 13.0 | 1 | 301 | -11 | AluSx | Alu | SINE |
| 131716297 | 131717047 | + | 30.6 | 2280 | 3150 | -269 | L2 | L2 | LINE |
| 131717326 | 131717585 | + | 30.4 | 3137 | 3419 | 0 | L2 | L2 | LINE |

  

|  |  |  |  |  |  |  |  |  |  |
| --- | --- | --- | --- | --- | --- | --- | --- | --- | --- |
| chr2:131728312-131728651 | | | 318 bp | | |  |  |  |  |
| Genome Start | Genome End | Strand | Div | Repeat Start | Repeat End | Repeat Left | Name | Family | Class |
| 131728312 | 131728377 | - | 16.7 | 159 | 224 | 0 | MER58A | MER1\_type | DNA |
| 131728385 | 131728538 | - | 19.5 | 134 | 289 | -23 | AluJb | Alu | SINE |
| 131728554 | 131728651 | - | 21.6 | 1 | 98 | -9 | MER58A | MER1\_type | DNA |

  

|  |  |  |  |  |  |  |  |  |  |
| --- | --- | --- | --- | --- | --- | --- | --- | --- | --- |
| chr2:131729236-131731365 | | | 1773 bp | | |  |  |  |  |
| Genome Start | Genome End | Strand | Div | Repeat Start | Repeat End | Repeat Left | Name | Family | Class |
| 131729236 | 131729608 | + | 31.2 | 1882 | 2300 | -1119 | L2 | L2 | LINE |
| 131729609 | 131729911 | - | 11.9 | 1 | 304 | -8 | AluSx | Alu | SINE |
| 131729912 | 131730748 | + | 31.3 | 2301 | 3146 | -273 | L2 | L2 | LINE |
| 131731106 | 131731365 | + | 32.8 | 3137 | 3419 | 0 | L2 | L2 | LINE |

  

|  |  |  |  |  |  |  |  |  |  |
| --- | --- | --- | --- | --- | --- | --- | --- | --- | --- |
| chr2:131756937-131757592 | | | 548 bp | | |  |  |  |  |
| Genome Start | Genome End | Strand | Div | Repeat Start | Repeat End | Repeat Left | Name | Family | Class |
| 131756937 | 131757031 | + | 17.9 | 1 | 97 | -331 | MSTA | MaLR | LTR |
| 131757129 | 131757184 | + | 19.6 | 911 | 967 | -613 | THE1-int | MaLR | LTR |
| 131757173 | 131757408 | - | 22.1 | 976 | 1256 | -395 | MST-int | MaLR | LTR |
| 131757432 | 131757592 | + | 29.4 | 99 | 274 | -154 | MSTA | MaLR | LTR |

  

|  |  |  |  |  |  |  |  |  |  |
| --- | --- | --- | --- | --- | --- | --- | --- | --- | --- |
| chr2:131778449-131780213 | | | 1769 bp | | |  |  |  |  |
| Genome Start | Genome End | Strand | Div | Repeat Start | Repeat End | Repeat Left | Name | Family | Class |
| 131778449 | 131778816 | - | 26.4 | 4142 | 4524 | -1622 | L1M5 | L1 | LINE |
| 131778817 | 131779057 | - | 11.2 | 1 | 242 | -69 | AluY | Alu | SINE |
| 131779058 | 131779151 | - | 26.4 | 4047 | 4141 | -2005 | L1M5 | L1 | LINE |
| 131779142 | 131779237 | - | 27.4 | 3747 | 3841 | -2305 | L1M5 | L1 | LINE |
| 131779244 | 131779656 | - | 27.1 | 3182 | 3620 | -2526 | L1M5 | L1 | LINE |
| 131779657 | 131779932 | - | 12.7 | 44 | 313 | 0 | AluSp | Alu | SINE |
| 131779933 | 131780213 | - | 27.1 | 2886 | 3181 | -2965 | L1M5 | L1 | LINE |

  

|  |  |  |  |  |  |  |  |  |  |
| --- | --- | --- | --- | --- | --- | --- | --- | --- | --- |
| chr2:131792071-131792513 | | | 443 bp | | |  |  |  |  |
| Genome Start | Genome End | Strand | Div | Repeat Start | Repeat End | Repeat Left | Name | Family | Class |
| 131792071 | 131792120 | + | 32.6 | 31 | 78 | -6041 | L1MEd | L1 | LINE |
| 131792121 | 131792429 | + | 16.0 | 3 | 303 | -10 | AluSq | Alu | SINE |
| 131792430 | 131792513 | + | 32.6 | 79 | 164 | -5955 | L1MEd | L1 | LINE |

  

|  |  |  |  |  |  |  |  |  |  |
| --- | --- | --- | --- | --- | --- | --- | --- | --- | --- |
| chr2:131925785-131927676 | | | 1862 bp | | |  |  |  |  |
| Genome Start | Genome End | Strand | Div | Repeat Start | Repeat End | Repeat Left | Name | Family | Class |
| 131925785 | 131926213 | - | 26.3 | 1139 | 1596 | -1 | Tigger6b | MER2\_type | DNA |
| 131926244 | 131927111 | - | 26.3 | 263 | 1138 | -459 | Tigger6b | MER2\_type | DNA |
| 131927112 | 131927414 | + | 8.4 | 1 | 296 | -13 | AluSc | Alu | SINE |
| 131927415 | 131927676 | - | 26.3 | 3 | 262 | -1335 | Tigger6b | MER2\_type | DNA |

  

|  |  |  |  |  |  |  |  |  |  |
| --- | --- | --- | --- | --- | --- | --- | --- | --- | --- |
| chr2:132027984-132028690 | | | 658 bp | | |  |  |  |  |
| Genome Start | Genome End | Strand | Div | Repeat Start | Repeat End | Repeat Left | Name | Family | Class |
| 132027984 | 132028205 | - | 29.4 | 5123 | 5356 | -784 | L1M3 | L1 | LINE |
| 132028208 | 132028480 | + | 17.2 | 1 | 268 | -44 | AluJb | Alu | SINE |
| 132028526 | 132028584 | - | 20.3 | 230 | 289 | -13 | AluJ/FRAM | Alu | SINE |
| 132028587 | 132028690 | - | 24.3 | 4612 | 4716 | -1424 | L1M3 | L1 | LINE |

  

|  |  |  |  |  |  |  |  |  |  |
| --- | --- | --- | --- | --- | --- | --- | --- | --- | --- |
| chr2:132043886-132046071 | | | 2186 bp | | |  |  |  |  |
| Genome Start | Genome End | Strand | Div | Repeat Start | Repeat End | Repeat Left | Name | Family | Class |
| 132043886 | 132043938 | + | 21.7 | 4130 | 4182 | -1961 | L1M2 | L1 | LINE |
| 132043939 | 132044195 | - | 11.8 | 1 | 255 | -55 | AluSg | Alu | SINE |
| 132044196 | 132045465 | + | 21.8 | 4183 | 5480 | -811 | L1M2 | L1 | LINE |
| 132045466 | 132045778 | + | 10.0 | 1 | 310 | -2 | AluSx | Alu | SINE |
| 132045779 | 132046071 | + | 22.6 | 5481 | 5780 | -511 | L1M2 | L1 | LINE |

  

|  |  |  |  |  |  |  |  |  |  |
| --- | --- | --- | --- | --- | --- | --- | --- | --- | --- |
| chr2:132085856-132086350 | | | 495 bp | | |  |  |  |  |
| Genome Start | Genome End | Strand | Div | Repeat Start | Repeat End | Repeat Left | Name | Family | Class |
| 132085856 | 132085977 | + | 28.5 | 5388 | 5522 | -599 | L1M5 | L1 | LINE |
| 132085978 | 132086323 | - | 15.0 | 5821 | 6165 | 0 | L1PA15 | L1 | LINE |
| 132086324 | 132086350 | + | 28.5 | 5523 | 5539 | -582 | L1M5 | L1 | LINE |

  

|  |  |  |  |  |  |  |  |  |  |
| --- | --- | --- | --- | --- | --- | --- | --- | --- | --- |
| chr2:132086922-132087260 | | | 319 bp | | |  |  |  |  |
| Genome Start | Genome End | Strand | Div | Repeat Start | Repeat End | Repeat Left | Name | Family | Class |
| 132086922 | 132086987 | - | 19.7 | 159 | 224 | 0 | MER58A | MER1\_type | DNA |
| 132086995 | 132087152 | - | 18.4 | 132 | 289 | -23 | AluJb | Alu | SINE |
| 132087166 | 132087260 | - | 22.3 | 4 | 98 | -9 | MER58A | MER1\_type | DNA |

  

|  |  |  |  |  |  |  |  |  |  |
| --- | --- | --- | --- | --- | --- | --- | --- | --- | --- |
| chr2:132128072-132130849 | | | 2780 bp | | |  |  |  |  |
| Genome Start | Genome End | Strand | Div | Repeat Start | Repeat End | Repeat Left | Name | Family | Class |
| 132128072 | 132128574 | - | 20.2 | 5769 | 6291 | 0 | L1MA7 | L1 | LINE |
| 132128579 | 132129221 | - | 21.9 | 5508 | 6176 | -2 | L1MB8 | L1 | LINE |
| 132129216 | 132130849 | - | 20.9 | 4128 | 5781 | -510 | L1MA7 | L1 | LINE |

  

|  |  |  |  |  |  |  |  |  |  |
| --- | --- | --- | --- | --- | --- | --- | --- | --- | --- |
| chr2:132161792-132163174 | | | 1383 bp | | |  |  |  |  |
| Genome Start | Genome End | Strand | Div | Repeat Start | Repeat End | Repeat Left | Name | Family | Class |
| 132161792 | 132162542 | - | 26.2 | 5562 | 6295 | -5 | L1MA6 | L1 | LINE |
| 132162543 | 132162843 | - | 6.6 | 1 | 302 | -9 | AluY | Alu | SINE |
| 132162844 | 132163174 | - | 25.5 | 5216 | 5561 | -739 | L1MA6 | L1 | LINE |

  

|  |  |  |  |  |  |  |  |  |  |
| --- | --- | --- | --- | --- | --- | --- | --- | --- | --- |
| chr2:132174462-132176207 | | | 1746 bp | | |  |  |  |  |
| Genome Start | Genome End | Strand | Div | Repeat Start | Repeat End | Repeat Left | Name | Family | Class |
| 132174462 | 132174862 | - | 17.6 | 5781 | 6165 | 0 | L1PA15 | L1 | LINE |
| 132174863 | 132175156 | - | 17.0 | 3 | 296 | -16 | AluSx | Alu | SINE |
| 132175157 | 132176207 | - | 17.6 | 4736 | 5780 | -385 | L1PA15 | L1 | LINE |

  

|  |  |  |  |  |  |  |  |  |  |
| --- | --- | --- | --- | --- | --- | --- | --- | --- | --- |
| chr2:132220071-132222107 | | | 2030 bp | | |  |  |  |  |
| Genome Start | Genome End | Strand | Div | Repeat Start | Repeat End | Repeat Left | Name | Family | Class |
| 132220071 | 132220211 | - | 25.0 | 4750 | 4895 | -2111 | L1M4b | L1 | LINE |
| 132220219 | 132220753 | - | 18.6 | 3564 | 4114 | -2892 | L1M4b | L1 | LINE |
| 132220754 | 132221040 | - | 18.2 | 1 | 289 | -23 | AluJo | Alu | SINE |
| 132221041 | 132222107 | - | 17.7 | 2694 | 3563 | -3443 | L1M4b | L1 | LINE |

  

|  |  |  |  |  |  |  |  |  |  |
| --- | --- | --- | --- | --- | --- | --- | --- | --- | --- |
| chr2:132241453-132242453 | | | 1000 bp | | |  |  |  |  |
| Genome Start | Genome End | Strand | Div | Repeat Start | Repeat End | Repeat Left | Name | Family | Class |
| 132241453 | 132241783 | - | 25.6 | 1340 | 1659 | -5142 | MER89-int | ERV1 | LTR |
| 132241785 | 132242119 | - | 22.6 | 1 | 340 | -25 | MLT1A0 | MaLR | LTR |
| 132242120 | 132242453 | - | 24.5 | 1030 | 1357 | -5444 | MER89-int | ERV1 | LTR |

  

|  |  |  |  |  |  |  |  |  |  |
| --- | --- | --- | --- | --- | --- | --- | --- | --- | --- |
| chr2:132255178-132257555 | | | 2244 bp | | |  |  |  |  |
| Genome Start | Genome End | Strand | Div | Repeat Start | Repeat End | Repeat Left | Name | Family | Class |
| 132255178 | 132255320 | - | 21.2 | 2286 | 2416 | -4519 | L1PA17 | L1 | LINE |
| 132255321 | 132255614 | + | 15.3 | 1 | 294 | -18 | AluSx | Alu | SINE |
| 132255615 | 132256383 | - | 21.4 | 1504 | 2285 | -4650 | L1PA17 | L1 | LINE |
| 132256461 | 132256744 | - | 28.9 | 920 | 1197 | -6018 | L1PA17 | L1 | LINE |
| 132256802 | 132257555 | - | 19.8 | 68 | 799 | -6120 | L1PA17 | L1 | LINE |

  

|  |  |  |  |  |  |  |  |  |  |
| --- | --- | --- | --- | --- | --- | --- | --- | --- | --- |
| chr2:132259067-132261396 | | | 2293 bp | | |  |  |  |  |
| Genome Start | Genome End | Strand | Div | Repeat Start | Repeat End | Repeat Left | Name | Family | Class |
| 132259067 | 132259940 | - | 18.0 | 725 | 1651 | 0 | MSTA-int | MaLR | LTR |
| 132259943 | 132260434 | + | 8.8 | 1 | 487 | -6 | LTR15 | ERV1 | LTR |
| 132260443 | 132260737 | + | 10.2 | 3 | 297 | -14 | AluY | Alu | SINE |
| 132260765 | 132261396 | - | 24.3 | 3 | 667 | -913 | MSTA-int | MaLR | LTR |

  

|  |  |  |  |  |  |  |  |  |  |
| --- | --- | --- | --- | --- | --- | --- | --- | --- | --- |
| chr2:132268005-132268998 | | | 993 bp | | |  |  |  |  |
| Genome Start | Genome End | Strand | Div | Repeat Start | Repeat End | Repeat Left | Name | Family | Class |
| 132268005 | 132268338 | + | 25.1 | 1030 | 1357 | -5444 | MER89-int | ERV1 | LTR |
| 132268339 | 132268673 | + | 22.3 | 1 | 340 | -25 | MLT1A0 | MaLR | LTR |
| 132268675 | 132268998 | + | 25.6 | 1340 | 1659 | -5142 | MER89-int | ERV1 | LTR |

  

|  |  |  |  |  |  |  |  |  |  |
| --- | --- | --- | --- | --- | --- | --- | --- | --- | --- |
| chr2:132279523-132280275 | | | 753 bp | | |  |  |  |  |
| Genome Start | Genome End | Strand | Div | Repeat Start | Repeat End | Repeat Left | Name | Family | Class |
| 132279523 | 132279738 | - | 21.7 | 3782 | 4016 | -2130 | L1M4 | L1 | LINE |
| 132279739 | 132279903 | - | 23.0 | 1 | 165 | -1 | FRAM | Alu | SINE |
| 132279904 | 132280275 | - | 21.7 | 3398 | 3781 | -2365 | L1M4 | L1 | LINE |

  

|  |  |  |  |  |  |  |  |  |  |
| --- | --- | --- | --- | --- | --- | --- | --- | --- | --- |
| chr2:132297469-132298274 | | | 806 bp | | |  |  |  |  |
| Genome Start | Genome End | Strand | Div | Repeat Start | Repeat End | Repeat Left | Name | Family | Class |
| 132297469 | 132297709 | + | 12.3 | 2495 | 2741 | -288 | Tigger3(Golem) | MER2\_type | DNA |
| 132297710 | 132298000 | - | 11.7 | 1 | 307 | -5 | AluSx | Alu | SINE |
| 132298001 | 132298274 | + | 12.3 | 2742 | 3028 | -1 | Tigger3(Golem) | MER2\_type | DNA |

  

|  |  |  |  |  |  |  |  |  |  |
| --- | --- | --- | --- | --- | --- | --- | --- | --- | --- |
| chr2:132311737-132312627 | | | 794 bp | | |  |  |  |  |
| Genome Start | Genome End | Strand | Div | Repeat Start | Repeat End | Repeat Left | Name | Family | Class |
| 132311737 | 132311975 | + | 36.7 | 5133 | 5392 | -754 | L1MC4a | L1 | LINE |
| 132312024 | 132312331 | + | 30.8 | 4 | 406 | -3 | MLT1I | MaLR | LTR |
| 132312381 | 132312627 | + | 33.3 | 5609 | 5863 | -1945 | L1MC4a | L1 | LINE |

  

|  |  |  |  |  |  |  |  |  |  |
| --- | --- | --- | --- | --- | --- | --- | --- | --- | --- |
| chr2:132324484-132326200 | | | 1717 bp | | |  |  |  |  |
| Genome Start | Genome End | Strand | Div | Repeat Start | Repeat End | Repeat Left | Name | Family | Class |
| 132324484 | 132325629 | - | 10.4 | 275 | 1574 | -6 | THE1B-int | MaLR | LTR |
| 132325630 | 132325921 | + | 7.5 | 1 | 303 | -8 | AluY | Alu | SINE |
| 132325922 | 132326200 | - | 10.4 | 1 | 274 | -1306 | THE1B-int | MaLR | LTR |

  

|  |  |  |  |  |  |  |  |  |  |
| --- | --- | --- | --- | --- | --- | --- | --- | --- | --- |
| chr2:132362122-132364105 | | | 1984 bp | | |  |  |  |  |
| Genome Start | Genome End | Strand | Div | Repeat Start | Repeat End | Repeat Left | Name | Family | Class |
| 132362122 | 132362969 | + | 8.2 | 4472 | 5333 | -813 | L1PA10 | L1 | LINE |
| 132362970 | 132363263 | + | 10.9 | 1 | 294 | -15 | AluSg1 | Alu | SINE |
| 132363264 | 132364105 | + | 9.8 | 5323 | 6168 | 0 | L1PA10 | L1 | LINE |

  

|  |  |  |  |  |  |  |  |  |  |
| --- | --- | --- | --- | --- | --- | --- | --- | --- | --- |
| chr2:132411988-132412688 | | | 669 bp | | |  |  |  |  |
| Genome Start | Genome End | Strand | Div | Repeat Start | Repeat End | Repeat Left | Name | Family | Class |
| 132411988 | 132412148 | - | 14.4 | 5599 | 5773 | -527 | L1M1 | L1 | LINE |
| 132412149 | 132412431 | - | 11.7 | 21 | 303 | -9 | AluSx | Alu | SINE |
| 132412464 | 132412688 | - | 14.4 | 5372 | 5598 | -702 | L1M1 | L1 | LINE |

  

|  |  |  |  |  |  |  |  |  |  |
| --- | --- | --- | --- | --- | --- | --- | --- | --- | --- |
| chr2:132449185-132458111 | | | 8876 bp | | |  |  |  |  |
| Genome Start | Genome End | Strand | Div | Repeat Start | Repeat End | Repeat Left | Name | Family | Class |
| 132449185 | 132452039 | + | 25.5 | 15 | 2991 | -3653 | L1M4 | L1 | LINE |
| 132452095 | 132452258 | + | 17.7 | 4026 | 4211 | -2433 | L1M4 | L1 | LINE |
| 132452260 | 132453740 | + | 22.4 | 1 | 1257 | -5705 | L1PA15-16 | L1 | LINE |
| 132453742 | 132454112 | - | 9.9 | 1 | 364 | 0 | THE1B | MaLR | LTR |
| 132454113 | 132455577 | - | 12.1 | 133 | 1580 | 0 | THE1B-int | MaLR | LTR |
| 132455578 | 132457145 | + | 15.6 | 4605 | 6161 | -5 | L1PA16 | L1 | LINE |
| 132457139 | 132457425 | + | 23.0 | 4187 | 4465 | -2179 | L1M4 | L1 | LINE |
| 132457427 | 132458111 | + | 19.8 | 5594 | 6277 | -405 | L1M4 | L1 | LINE |

  

|  |  |  |  |  |  |  |  |  |  |
| --- | --- | --- | --- | --- | --- | --- | --- | --- | --- |
| chr2:132458491-132459587 | | | 1097 bp | | |  |  |  |  |
| Genome Start | Genome End | Strand | Div | Repeat Start | Repeat End | Repeat Left | Name | Family | Class |
| 132458491 | 132458710 | + | 9.9 | 5414 | 5639 | -524 | L1PA13 | L1 | LINE |
| 132458711 | 132459084 | - | 10.7 | 1 | 364 | 0 | THE1B | MaLR | LTR |
| 132459085 | 132459587 | + | 9.9 | 5640 | 6160 | -3 | L1PA13 | L1 | LINE |

  

|  |  |  |  |  |  |  |  |  |  |
| --- | --- | --- | --- | --- | --- | --- | --- | --- | --- |
| chr2:132523332-132524137 | | | 806 bp | | |  |  |  |  |
| Genome Start | Genome End | Strand | Div | Repeat Start | Repeat End | Repeat Left | Name | Family | Class |
| 132523332 | 132523602 | + | 26.8 | 5 | 274 | -231 | MLT1D | MaLR | LTR |
| 132523603 | 132523895 | + | 10.7 | 1 | 293 | -16 | AluSc | Alu | SINE |
| 132523896 | 132524137 | + | 26.8 | 275 | 505 | 0 | MLT1D | MaLR | LTR |

  

|  |  |  |  |  |  |  |  |  |  |
| --- | --- | --- | --- | --- | --- | --- | --- | --- | --- |
| chr2:132526606-132527798 | | | 1144 bp | | |  |  |  |  |
| Genome Start | Genome End | Strand | Div | Repeat Start | Repeat End | Repeat Left | Name | Family | Class |
| 132526606 | 132526998 | - | 20.9 | 490 | 901 | 0 | MER54A | ERVL | LTR |
| 132527008 | 132527345 | + | 12.9 | 7 | 364 | 0 | THE1B | MaLR | LTR |
| 132527386 | 132527798 | - | 25.7 | 1 | 428 | -473 | MER54A | ERVL | LTR |

  

|  |  |  |  |  |  |  |  |  |  |
| --- | --- | --- | --- | --- | --- | --- | --- | --- | --- |
| chr2:132532138-132532829 | | | 692 bp | | |  |  |  |  |
| Genome Start | Genome End | Strand | Div | Repeat Start | Repeat End | Repeat Left | Name | Family | Class |
| 132532138 | 132532168 | + | 18.7 | 1 | 33 | -562 | LTR49 | ERV1 | LTR |
| 132532169 | 132532452 | - | 12.0 | 1 | 293 | -20 | AluSq | Alu | SINE |
| 132532453 | 132532829 | + | 18.7 | 34 | 427 | -168 | LTR49 | ERV1 | LTR |

  

|  |  |  |  |  |  |  |  |  |  |
| --- | --- | --- | --- | --- | --- | --- | --- | --- | --- |
| chr2:132583051-132585527 | | | 2467 bp | | |  |  |  |  |
| Genome Start | Genome End | Strand | Div | Repeat Start | Repeat End | Repeat Left | Name | Family | Class |
| 132583051 | 132583273 | - | 22.5 | 6025 | 6287 | -4 | L1MA8 | L1 | LINE |
| 132583285 | 132585035 | - | 24.8 | 4102 | 5941 | -359 | L1MA8 | L1 | LINE |
| 132585036 | 132585331 | + | 9.1 | 1 | 297 | -14 | AluY | Alu | SINE |
| 132585331 | 132585527 | - | 23.3 | 3894 | 4106 | -2037 | L1MA8 | L1 | LINE |

  

|  |  |  |  |  |  |  |  |  |  |
| --- | --- | --- | --- | --- | --- | --- | --- | --- | --- |
| chr2:132603052-132604319 | | | 1268 bp | | |  |  |  |  |
| Genome Start | Genome End | Strand | Div | Repeat Start | Repeat End | Repeat Left | Name | Family | Class |
| 132603052 | 132603524 | + | 10.8 | 2808 | 3303 | -2843 | L1P3 | L1 | LINE |
| 132603525 | 132603820 | + | 12.8 | 1 | 296 | -14 | AluSg | Alu | SINE |
| 132603821 | 132604319 | + | 10.8 | 3304 | 3793 | -2353 | L1P3 | L1 | LINE |

  

|  |  |  |  |  |  |  |  |  |  |
| --- | --- | --- | --- | --- | --- | --- | --- | --- | --- |
| chr2:132604433-132605194 | | | 713 bp | | |  |  |  |  |
| Genome Start | Genome End | Strand | Div | Repeat Start | Repeat End | Repeat Left | Name | Family | Class |
| 132604433 | 132604563 | - | 33.9 | 3271 | 3418 | -1 | L2 | L2 | LINE |
| 132604601 | 132604767 | + | 18.6 | 120 | 290 | -22 | AluJo | Alu | SINE |
| 132604780 | 132605194 | - | 31.4 | 2766 | 3230 | -189 | L2 | L2 | LINE |

  

|  |  |  |  |  |  |  |  |  |  |
| --- | --- | --- | --- | --- | --- | --- | --- | --- | --- |
| chr2:132605870-132606799 | | | 930 bp | | |  |  |  |  |
| Genome Start | Genome End | Strand | Div | Repeat Start | Repeat End | Repeat Left | Name | Family | Class |
| 132605870 | 132606323 | + | 21.5 | 5023 | 5497 | -687 | L1M4 | L1 | LINE |
| 132606324 | 132606582 | + | 8.5 | 1 | 258 | -53 | AluY | Alu | SINE |
| 132606583 | 132606799 | + | 21.1 | 5498 | 5719 | -465 | L1M4 | L1 | LINE |

  

|  |  |  |  |  |  |  |  |  |  |
| --- | --- | --- | --- | --- | --- | --- | --- | --- | --- |
| chr2:132614085-132621579 | | | 7514 bp | | |  |  |  |  |
| Genome Start | Genome End | Strand | Div | Repeat Start | Repeat End | Repeat Left | Name | Family | Class |
| 132614085 | 132616013 | - | 15.9 | 5804 | 7730 | -7 | HERVIP10F | ERV1 | LTR |
| 132615995 | 132619366 | - | 14.2 | 1778 | 5115 | -2622 | HERVIP10F | ERV1 | LTR |
| 132619367 | 132619664 | + | 10.7 | 1 | 298 | -13 | AluY | Alu | SINE |
| 132619665 | 132621579 | - | 15.6 | 1 | 1941 | -5844 | HERVIP10F | ERV1 | LTR |

  

|  |  |  |  |  |  |  |  |  |  |
| --- | --- | --- | --- | --- | --- | --- | --- | --- | --- |
| chr2:132650155-132650684 | | | 530 bp | | |  |  |  |  |
| Genome Start | Genome End | Strand | Div | Repeat Start | Repeat End | Repeat Left | Name | Family | Class |
| 132650155 | 132650290 | + | 14.4 | 1 | 133 | -179 | AluSx | Alu | SINE |
| 132650291 | 132650504 | + | 11.7 | 1 | 214 | -96 | AluSg | Alu | SINE |
| 132650505 | 132650684 | + | 14.4 | 134 | 312 | 0 | AluSx | Alu | SINE |

  

|  |  |  |  |  |  |  |  |  |  |
| --- | --- | --- | --- | --- | --- | --- | --- | --- | --- |
| chr2:132653051-132654532 | | | 1482 bp | | |  |  |  |  |
| Genome Start | Genome End | Strand | Div | Repeat Start | Repeat End | Repeat Left | Name | Family | Class |
| 132653051 | 132653323 | + | 16.3 | 1 | 290 | -941 | Tigger3b | MER2\_type | DNA |
| 132653324 | 132653631 | - | 15.0 | 1 | 307 | -5 | AluSx | Alu | SINE |
| 132653632 | 132654532 | + | 16.3 | 291 | 1231 | 0 | Tigger3b | MER2\_type | DNA |

  

|  |  |  |  |  |  |  |  |  |  |
| --- | --- | --- | --- | --- | --- | --- | --- | --- | --- |
| chr2:132666262-132667905 | | | 1483 bp | | |  |  |  |  |
| Genome Start | Genome End | Strand | Div | Repeat Start | Repeat End | Repeat Left | Name | Family | Class |
| 132666262 | 132667311 | + | 34.9 | 2980 | 3900 | -1096 | HERV16 | ERVL | LTR |
| 132667472 | 132667524 | + | 17.3 | 431 | 484 | -83 | MLT1F1 | MaLR | LTR |
| 132667526 | 132667905 | + | 35.6 | 4389 | 4779 | -888 | HERV16 | ERVL | LTR |

  

|  |  |  |  |  |  |  |  |  |  |
| --- | --- | --- | --- | --- | --- | --- | --- | --- | --- |
| chr2:132747692-132748259 | | | 568 bp | | |  |  |  |  |
| Genome Start | Genome End | Strand | Div | Repeat Start | Repeat End | Repeat Left | Name | Family | Class |
| 132747692 | 132747799 | + | 23.8 | 3 | 137 | -165 | AluJo | Alu | SINE |
| 132747800 | 132748104 | + | 10.8 | 1 | 306 | -7 | AluSp | Alu | SINE |
| 132748105 | 132748259 | + | 23.8 | 138 | 286 | -16 | AluJo | Alu | SINE |

  

|  |  |  |  |  |  |  |  |  |  |
| --- | --- | --- | --- | --- | --- | --- | --- | --- | --- |
| chr2:132784205-132785345 | | | 968 bp | | |  |  |  |  |
| Genome Start | Genome End | Strand | Div | Repeat Start | Repeat End | Repeat Left | Name | Family | Class |
| 132784205 | 132784373 | - | 27.4 | 7768 | 7929 | -32 | L1MC5 | L1 | LINE |
| 132784389 | 132784619 | - | 23.7 | 198 | 432 | -1040 | MLT1H-int | MaLR | LTR |
| 132784681 | 132784869 | - | 33.1 | 84 | 273 | -276 | MLT1H | MaLR | LTR |
| 132784967 | 132785345 | - | 25.2 | 7273 | 7660 | -301 | L1MC5 | L1 | LINE |

  

|  |  |  |  |  |  |  |  |  |  |
| --- | --- | --- | --- | --- | --- | --- | --- | --- | --- |
| chr2:132788035-132788695 | | | 661 bp | | |  |  |  |  |
| Genome Start | Genome End | Strand | Div | Repeat Start | Repeat End | Repeat Left | Name | Family | Class |
| 132788035 | 132788222 | - | 12.2 | 184 | 363 | -1 | THE1B | MaLR | LTR |
| 132788223 | 132788509 | - | 14.0 | 5 | 294 | -15 | AluSc | Alu | SINE |
| 132788510 | 132788695 | - | 12.2 | 1 | 183 | -181 | THE1B | MaLR | LTR |

  

|  |  |  |  |  |  |  |  |  |  |
| --- | --- | --- | --- | --- | --- | --- | --- | --- | --- |
| chr2:132813862-132815048 | | | 1181 bp | | |  |  |  |  |
| Genome Start | Genome End | Strand | Div | Repeat Start | Repeat End | Repeat Left | Name | Family | Class |
| 132813862 | 132814000 | + | 26.5 | 274 | 411 | -5659 | L1ME2 | L1 | LINE |
| 132814007 | 132814231 | + | 26.1 | 5195 | 5422 | -676 | L1ME2 | L1 | LINE |
| 132814232 | 132814524 | + | 9.6 | 1 | 293 | -20 | AluSq | Alu | SINE |
| 132814525 | 132815048 | + | 26.1 | 5423 | 5982 | -116 | L1ME2 | L1 | LINE |

  

|  |  |  |  |  |  |  |  |  |  |
| --- | --- | --- | --- | --- | --- | --- | --- | --- | --- |
| chr2:152159315-152159526 | | | 211 bp | | |  |  |  |  |
| Genome Start | Genome End | Strand | Div | Repeat Start | Repeat End | Repeat Left | Name | Family | Class |
| 152159315 | 152159379 | - | 32.3 | 198 | 262 | 0 | MIR | MIR | SINE |
| 152159381 | 152159460 | - | 25.6 | 3 | 80 | 0 | MADE2 | Mariner | DNA |
| 152159461 | 152159526 | - | 22.7 | 113 | 179 | -83 | MIR | MIR | SINE |

  

|  |  |  |  |  |  |  |  |  |  |
| --- | --- | --- | --- | --- | --- | --- | --- | --- | --- |
| chr2:156828160-156829298 | | | 1139 bp | | |  |  |  |  |
| Genome Start | Genome End | Strand | Div | Repeat Start | Repeat End | Repeat Left | Name | Family | Class |
| 156828160 | 156828463 | + | 28.3 | 2529 | 2890 | -529 | L2 | L2 | LINE |
| 156828464 | 156828806 | - | 13.4 | 3 | 339 | 0 | MER1B | MER1\_type | DNA |
| 156828807 | 156829298 | + | 34.7 | 2891 | 3412 | -7 | L2 | L2 | LINE |

  

|  |  |  |  |  |  |  |  |  |  |
| --- | --- | --- | --- | --- | --- | --- | --- | --- | --- |
| chr2:159421829-159423801 | | | 1973 bp | | |  |  |  |  |
| Genome Start | Genome End | Strand | Div | Repeat Start | Repeat End | Repeat Left | Name | Family | Class |
| 159421829 | 159422820 | + | 24.2 | 4818 | 5818 | -365 | L1MB3 | L1 | LINE |
| 159422821 | 159423129 | + | 13.0 | 1 | 310 | -2 | AluSx | Alu | SINE |
| 159423130 | 159423263 | + | 23.0 | 5819 | 5908 | -275 | L1MB3 | L1 | LINE |
| 159423264 | 159423554 | + | 13.8 | 12 | 304 | -8 | AluSx | Alu | SINE |
| 159423555 | 159423801 | + | 23.0 | 5909 | 6151 | -32 | L1MB3 | L1 | LINE |

  

|  |  |  |  |  |  |  |  |  |  |
| --- | --- | --- | --- | --- | --- | --- | --- | --- | --- |
| chr2:165547154-165548095 | | | 942 bp | | |  |  |  |  |
| Genome Start | Genome End | Strand | Div | Repeat Start | Repeat End | Repeat Left | Name | Family | Class |
| 165547154 | 165547354 | - | 8.0 | 450 | 639 | -28 | PABL\_B | ERV1 | LTR |
| 165547355 | 165547658 | - | 9.9 | 1 | 305 | -8 | AluSp | Alu | SINE |
| 165547659 | 165548095 | - | 8.0 | 1 | 449 | -218 | PABL\_B | ERV1 | LTR |

  

|  |  |  |  |  |  |  |  |  |  |
| --- | --- | --- | --- | --- | --- | --- | --- | --- | --- |
| chr2:174114252-174114779 | | | 492 bp | | |  |  |  |  |
| Genome Start | Genome End | Strand | Div | Repeat Start | Repeat End | Repeat Left | Name | Family | Class |
| 174114252 | 174114384 | + | 21.2 | 1 | 134 | -44 | MER5B | MER1\_type | DNA |
| 174114399 | 174114706 | + | 15.0 | 1 | 309 | -3 | AluJb | Alu | SINE |
| 174114729 | 174114779 | + | 25.0 | 131 | 178 | 0 | MER5B | MER1\_type | DNA |

  

|  |  |  |  |  |  |  |  |  |  |
| --- | --- | --- | --- | --- | --- | --- | --- | --- | --- |
| chr2:234203874-234209014 | | | 5059 bp | | |  |  |  |  |
| Genome Start | Genome End | Strand | Div | Repeat Start | Repeat End | Repeat Left | Name | Family | Class |
| 234203874 | 234204105 | - | 15.1 | 186 | 426 | 0 | MSTB | MaLR | LTR |
| 234204106 | 234204189 | - | 11.9 | 6085 | 6168 | 0 | L1PA10 | L1 | LINE |
| 234204224 | 234208783 | - | 8.5 | 1569 | 6151 | -3 | L1PA7 | L1 | LINE |
| 234208832 | 234209014 | - | 21.3 | 1 | 198 | -228 | MSTB | MaLR | LTR |

  

|  |  |  |  |  |  |  |  |  |  |
| --- | --- | --- | --- | --- | --- | --- | --- | --- | --- |
| chr2:241284080-241285127 | | | 834 bp | | |  |  |  |  |
| Genome Start | Genome End | Strand | Div | Repeat Start | Repeat End | Repeat Left | Name | Family | Class |
| 241284080 | 241284321 | - | 30.1 | 1547 | 1795 | -712 | HAL1 | L1 | LINE |
| 241284360 | 241284725 | - | 30.4 | 53 | 440 | -27 | MLT1C | MaLR | LTR |
| 241284902 | 241285127 | - | 32.0 | 223 | 468 | -2039 | HAL1 | L1 | LINE |

  

|  |  |  |  |  |  |  |  |  |  |
| --- | --- | --- | --- | --- | --- | --- | --- | --- | --- |
| chr2:242656512-242657073 | | | 562 bp | | |  |  |  |  |
| Genome Start | Genome End | Strand | Div | Repeat Start | Repeat End | Repeat Left | Name | Family | Class |
| 242656512 | 242656704 | + | 26.1 | 1120 | 1358 | -651 | HAL1b | L1 | LINE |
| 242656705 | 242657000 | + | 8.1 | 1 | 298 | -15 | AluSp | Alu | SINE |
| 242657001 | 242657073 | + | 26.1 | 1359 | 1436 | -573 | HAL1b | L1 | LINE |

  

|  |  |  |  |  |  |  |  |  |  |
| --- | --- | --- | --- | --- | --- | --- | --- | --- | --- |
| chr2:242683810-242685474 | | | 1490 bp | | |  |  |  |  |
| Genome Start | Genome End | Strand | Div | Repeat Start | Repeat End | Repeat Left | Name | Family | Class |
| 242683810 | 242684197 | + | 36.4 | 1971 | 2394 | -1025 | L2 | L2 | LINE |
| 242684197 | 242684451 | - | 32.3 | 3128 | 3378 | 0 | L2 | L2 | LINE |
| 242684505 | 242684661 | - | 33.8 | 1 | 159 | -19 | MER5B | MER1\_type | DNA |
| 242684785 | 242685474 | + | 36.5 | 2415 | 3158 | -220 | L2 | L2 | LINE |

  

|  |  |  |  |  |  |  |  |  |  |
| --- | --- | --- | --- | --- | --- | --- | --- | --- | --- |
| chr2:242697333-242698923 | | | 1591 bp | | |  |  |  |  |
| Genome Start | Genome End | Strand | Div | Repeat Start | Repeat End | Repeat Left | Name | Family | Class |
| 242697333 | 242698153 | - | 13.0 | 5323 | 6162 | -4 | L1PA16 | L1 | LINE |
| 242698154 | 242698226 | - | 6.8 | 223 | 295 | -16 | Alu | Alu | SINE |
| 242698227 | 242698923 | - | 13.8 | 4623 | 5332 | -814 | L1PA16 | L1 | LINE |

  

|  |  |  |  |  |  |  |  |  |  |
| --- | --- | --- | --- | --- | --- | --- | --- | --- | --- |
| chr2:242699364-242700382 | | | 651 bp | | |  |  |  |  |
| Genome Start | Genome End | Strand | Div | Repeat Start | Repeat End | Repeat Left | Name | Family | Class |
| 242699364 | 242699729 | - | 30.1 | 3023 | 3418 | -2796 | L1MC4 | L1 | LINE |
| 242699737 | 242699836 | - | 26.0 | 2223 | 2322 | -3893 | L1M5 | L1 | LINE |
| 242700198 | 242700382 | - | 32.4 | 1555 | 1749 | -4466 | L1MC4 | L1 | LINE |

  

|  |  |  |  |  |  |  |  |  |  |
| --- | --- | --- | --- | --- | --- | --- | --- | --- | --- |
| chr2:242713393-242713837 | | | 445 bp | | |  |  |  |  |
| Genome Start | Genome End | Strand | Div | Repeat Start | Repeat End | Repeat Left | Name | Family | Class |
| 242713393 | 242713443 | + | 29.1 | 3268 | 3312 | -107 | L2 | L2 | LINE |
| 242713444 | 242713731 | + | 11.5 | 5 | 292 | -21 | AluSq | Alu | SINE |
| 242713732 | 242713837 | + | 29.1 | 3313 | 3414 | -5 | L2 | L2 | LINE |

  

|  |  |  |  |  |  |  |  |  |  |
| --- | --- | --- | --- | --- | --- | --- | --- | --- | --- |
| chr2:242723274-242725380 | | | 2112 bp | | |  |  |  |  |
| Genome Start | Genome End | Strand | Div | Repeat Start | Repeat End | Repeat Left | Name | Family | Class |
| 242723274 | 242724311 | - | 27.1 | 4906 | 6040 | -183 | L1ME3B | L1 | LINE |
| 242724312 | 242724801 | - | 23.1 | 1 | 495 | -10 | MLT1D | MaLR | LTR |
| 242724804 | 242725190 | - | 33.2 | 4488 | 4909 | -1220 | L1ME3B | L1 | LINE |
| 242725184 | 242725380 | - | 23.4 | 3777 | 3965 | -2164 | L1ME3B | L1 | LINE |

  

|  |  |  |  |  |  |  |  |  |  |
| --- | --- | --- | --- | --- | --- | --- | --- | --- | --- |
| chr2:242740225-242740699 | | | 475 bp | | |  |  |  |  |
| Genome Start | Genome End | Strand | Div | Repeat Start | Repeat End | Repeat Left | Name | Family | Class |
| 242740225 | 242740296 | - | 30.1 | 3145 | 3217 | -2929 | L1M5 | L1 | LINE |
| 242740297 | 242740597 | - | 14.3 | 1 | 301 | -11 | AluSx | Alu | SINE |
| 242740598 | 242740699 | - | 30.1 | 3044 | 3144 | -3002 | L1M5 | L1 | LINE |

  


|  |  |  |  |  |  |  |  |  |  |
| --- | --- | --- | --- | --- | --- | --- | --- | --- | --- |
| chr3:8707240-8707910 | | | 671 bp | | |  |  |  |  |
| Genome Start | Genome End | Strand | Div | Repeat Start | Repeat End | Repeat Left | Name | Family | Class |
| 8707240 | 8707422 | + | 22.0 | 5771 | 5938 | -362 | L1MA4 | L1 | LINE |
| 8707423 | 8707703 | + | 16.8 | 1 | 280 | -32 | AluSx | Alu | SINE |
| 8707704 | 8707910 | + | 22.0 | 5939 | 6130 | -170 | L1MA4 | L1 | LINE |

  

|  |  |  |  |  |  |  |  |  |  |
| --- | --- | --- | --- | --- | --- | --- | --- | --- | --- |
| chr3:8708337-8710310 | | | 1974 bp | | |  |  |  |  |
| Genome Start | Genome End | Strand | Div | Repeat Start | Repeat End | Repeat Left | Name | Family | Class |
| 8708337 | 8709328 | + | 23.9 | 4818 | 5824 | -359 | L1MB3 | L1 | LINE |
| 8709329 | 8709650 | + | 12.9 | 1 | 312 | 0 | AluSx | Alu | SINE |
| 8709651 | 8709756 | + | 22.9 | 5825 | 5907 | -276 | L1MB3 | L1 | LINE |
| 8709757 | 8710060 | + | 14.5 | 1 | 304 | -8 | AluSx | Alu | SINE |
| 8710061 | 8710310 | + | 22.9 | 5908 | 6153 | -30 | L1MB3 | L1 | LINE |

  

|  |  |  |  |  |  |  |  |  |  |
| --- | --- | --- | --- | --- | --- | --- | --- | --- | --- |
| chr3:11893577-11894095 | | | 519 bp | | |  |  |  |  |
| Genome Start | Genome End | Strand | Div | Repeat Start | Repeat End | Repeat Left | Name | Family | Class |
| 11893577 | 11893766 | - | 12.7 | 5134 | 5331 | -815 | L1M | L1 | LINE |
| 11893767 | 11894065 | - | 10.7 | 1 | 299 | -10 | AluSc | Alu | SINE |
| 11894066 | 11894095 | - | 12.7 | 5106 | 5133 | -1013 | L1M | L1 | LINE |

  

|  |  |  |  |  |  |  |  |  |  |
| --- | --- | --- | --- | --- | --- | --- | --- | --- | --- |
| chr3:15154157-15154663 | | | 507 bp | | |  |  |  |  |
| Genome Start | Genome End | Strand | Div | Repeat Start | Repeat End | Repeat Left | Name | Family | Class |
| 15154157 | 15154255 | + | 22.8 | 5996 | 6078 | -92 | L1ME3A | L1 | LINE |
| 15154256 | 15154575 | - | 15.8 | 1 | 312 | 0 | AluJo | Alu | SINE |
| 15154576 | 15154663 | + | 22.8 | 6079 | 6170 | 0 | L1ME3A | L1 | LINE |

  

|  |  |  |  |  |  |  |  |  |  |
| --- | --- | --- | --- | --- | --- | --- | --- | --- | --- |
| chr3:16569304-16570060 | | | 567 bp | | |  |  |  |  |
| Genome Start | Genome End | Strand | Div | Repeat Start | Repeat End | Repeat Left | Name | Family | Class |
| 16569304 | 16569413 | - | 23.1 | 3289 | 3415 | -4 | L2 | L2 | LINE |
| 16569594 | 16569703 | + | 23.6 | 941 | 1062 | 0 | MER63 | AcHobo | DNA |
| 16569720 | 16569993 | - | 32.7 | 2577 | 2862 | -557 | L2 | L2 | LINE |
| 16569988 | 16570060 | - | 27.8 | 3243 | 3316 | -62 | L2 | L2 | LINE |

  

|  |  |  |  |  |  |  |  |  |  |
| --- | --- | --- | --- | --- | --- | --- | --- | --- | --- |
| chr3:16587376-16589545 | | | 1692 bp | | |  |  |  |  |
| Genome Start | Genome End | Strand | Div | Repeat Start | Repeat End | Repeat Left | Name | Family | Class |
| 16587376 | 16587506 | + | 21.1 | 5455 | 5577 | -587 | L1ME2 | L1 | LINE |
| 16587507 | 16587821 | + | 9.4 | 1 | 310 | 0 | AluSg | Alu | SINE |
| 16587822 | 16588349 | + | 21.1 | 5578 | 6141 | -23 | L1ME2 | L1 | LINE |
| 16588350 | 16588505 | + | 25.2 | 31 | 178 | 0 | MER5B | MER1\_type | DNA |
| 16588984 | 16589545 | + | 22.2 | 5571 | 6155 | -9 | L1ME2 | L1 | LINE |

  

|  |  |  |  |  |  |  |  |  |  |
| --- | --- | --- | --- | --- | --- | --- | --- | --- | --- |
| chr3:33855611-33856981 | | | 1371 bp | | |  |  |  |  |
| Genome Start | Genome End | Strand | Div | Repeat Start | Repeat End | Repeat Left | Name | Family | Class |
| 33855611 | 33856517 | - | 23.3 | 5224 | 6101 | -65 | L1ME1 | L1 | LINE |
| 33856518 | 33856944 | - | 13.1 | 1 | 433 | -1 | Tigger2a | MER2\_type | DNA |
| 33856945 | 33856981 | - | 22.7 | 5188 | 5223 | -923 | L1ME1 | L1 | LINE |

  

|  |  |  |  |  |  |  |  |  |  |
| --- | --- | --- | --- | --- | --- | --- | --- | --- | --- |
| chr3:33858975-33859474 | | | 479 bp | | |  |  |  |  |
| Genome Start | Genome End | Strand | Div | Repeat Start | Repeat End | Repeat Left | Name | Family | Class |
| 33858975 | 33859115 | - | 24.8 | 699 | 840 | -5279 | L1MEd | L1 | LINE |
| 33859139 | 33859364 | - | 21.8 | 7729 | 7971 | -24 | L1MD3 | L1 | LINE |
| 33859363 | 33859474 | - | 24.1 | 598 | 714 | -5405 | L1MEd | L1 | LINE |

  

|  |  |  |  |  |  |  |  |  |  |
| --- | --- | --- | --- | --- | --- | --- | --- | --- | --- |
| chr3:41769013-41769844 | | | 832 bp | | |  |  |  |  |
| Genome Start | Genome End | Strand | Div | Repeat Start | Repeat End | Repeat Left | Name | Family | Class |
| 41769013 | 41769426 | + | 18.7 | 1882 | 2298 | -122 | Cheshire | MER1\_type | DNA |
| 41769427 | 41769720 | + | 18.1 | 1 | 288 | -24 | AluJb | Alu | SINE |
| 41769721 | 41769844 | + | 18.7 | 2299 | 2419 | -1 | Cheshire | MER1\_type | DNA |

  

|  |  |  |  |  |  |  |  |  |  |
| --- | --- | --- | --- | --- | --- | --- | --- | --- | --- |
| chr3:41797932-41798551 | | | 620 bp | | |  |  |  |  |
| Genome Start | Genome End | Strand | Div | Repeat Start | Repeat End | Repeat Left | Name | Family | Class |
| 41797932 | 41798152 | + | 21.4 | 7616 | 7820 | -141 | L1MC5 | L1 | LINE |
| 41798153 | 41798449 | + | 10.4 | 1 | 297 | -16 | AluSq | Alu | SINE |
| 41798450 | 41798551 | + | 21.4 | 7821 | 7912 | -49 | L1MC5 | L1 | LINE |

  

|  |  |  |  |  |  |  |  |  |  |
| --- | --- | --- | --- | --- | --- | --- | --- | --- | --- |
| chr3:51873082-51873875 | | | 778 bp | | |  |  |  |  |
| Genome Start | Genome End | Strand | Div | Repeat Start | Repeat End | Repeat Left | Name | Family | Class |
| 51873082 | 51873220 | + | 29.5 | 748 | 908 | -2511 | L2 | L2 | LINE |
| 51873225 | 51873377 | + | 18.9 | 1 | 157 | -52 | MER3 | MER1\_type | DNA |
| 51873390 | 51873875 | + | 32.9 | 904 | 1440 | -1979 | L2 | L2 | LINE |

  

|  |  |  |  |  |  |  |  |  |  |
| --- | --- | --- | --- | --- | --- | --- | --- | --- | --- |
| chr3:73935370-73936127 | | | 758 bp | | |  |  |  |  |
| Genome Start | Genome End | Strand | Div | Repeat Start | Repeat End | Repeat Left | Name | Family | Class |
| 73935370 | 73935422 | - | 25.0 | 367 | 409 | 0 | MLT1I | MaLR | LTR |
| 73935423 | 73935806 | + | 12.2 | 1 | 381 | 0 | THE1D | MaLR | LTR |
| 73935807 | 73936127 | - | 25.0 | 7 | 366 | -43 | MLT1I | MaLR | LTR |

  

|  |  |  |  |  |  |  |  |  |  |
| --- | --- | --- | --- | --- | --- | --- | --- | --- | --- |
| chr3:73946777-73948761 | | | 1429 bp | | |  |  |  |  |
| Genome Start | Genome End | Strand | Div | Repeat Start | Repeat End | Repeat Left | Name | Family | Class |
| 73946777 | 73946928 | - | 37.6 | 3202 | 3389 | -30 | L2 | L2 | LINE |
| 73947126 | 73947490 | + | 10.4 | 1 | 364 | 0 | THE1B | MaLR | LTR |
| 73947756 | 73947976 | - | 26.8 | 3217 | 3419 | 0 | L2 | L2 | LINE |
| 73947988 | 73948093 | + | 30.2 | 3 | 108 | -26 | MER94 | AcHobo | DNA |
| 73948177 | 73948761 | - | 27.9 | 2586 | 3173 | -246 | L2 | L2 | LINE |

  

|  |  |  |  |  |  |  |  |  |  |
| --- | --- | --- | --- | --- | --- | --- | --- | --- | --- |
| chr3:75388192-75389304 | | | 1113 bp | | |  |  |  |  |
| Genome Start | Genome End | Strand | Div | Repeat Start | Repeat End | Repeat Left | Name | Family | Class |
| 75388192 | 75388555 | + | 24.1 | 5 | 389 | -176 | MER34B | ERV1 | LTR |
| 75388556 | 75388782 | - | 15.4 | 84 | 312 | 0 | AluJo | Alu | SINE |
| 75388783 | 75388925 | + | 24.1 | 390 | 531 | -34 | MER34B | ERV1 | LTR |
| 75388926 | 75389269 | - | 20.9 | 1 | 365 | 0 | MLT1A0 | MaLR | LTR |
| 75389270 | 75389304 | + | 24.1 | 532 | 565 | 0 | MER34B | ERV1 | LTR |

  

|  |  |  |  |  |  |  |  |  |  |
| --- | --- | --- | --- | --- | --- | --- | --- | --- | --- |
| chr3:75417688-75419323 | | | 1633 bp | | |  |  |  |  |
| Genome Start | Genome End | Strand | Div | Repeat Start | Repeat End | Repeat Left | Name | Family | Class |
| 75417688 | 75418222 | - | 21.6 | 5579 | 6130 | -36 | L1ME1 | L1 | LINE |
| 75418223 | 75418535 | + | 13.1 | 1 | 313 | 0 | AluSq | Alu | SINE |
| 75418536 | 75418919 | - | 20.9 | 5215 | 5578 | -588 | L1ME1 | L1 | LINE |
| 75418921 | 75419135 | - | 19.6 | 1 | 194 | -118 | AluJo | Alu | SINE |
| 75419138 | 75419323 | - | 25.1 | 5009 | 5201 | -945 | L1ME1 | L1 | LINE |

  

|  |  |  |  |  |  |  |  |  |  |
| --- | --- | --- | --- | --- | --- | --- | --- | --- | --- |
| chr3:75429059-75429304 | | | 246 bp | | |  |  |  |  |
| Genome Start | Genome End | Strand | Div | Repeat Start | Repeat End | Repeat Left | Name | Family | Class |
| 75429059 | 75429145 | + | 33.3 | 3252 | 3329 | -49 | L2 | L2 | LINE |
| 75429146 | 75429254 | + | 27.8 | 3 | 111 | -3 | MER81 | AcHobo | DNA |
| 75429255 | 75429304 | + | 33.3 | 3330 | 3377 | -1 | L2 | L2 | LINE |

  

|  |  |  |  |  |  |  |  |  |  |
| --- | --- | --- | --- | --- | --- | --- | --- | --- | --- |
| chr3:75483897-75485861 | | | 1965 bp | | |  |  |  |  |
| Genome Start | Genome End | Strand | Div | Repeat Start | Repeat End | Repeat Left | Name | Family | Class |
| 75483897 | 75484144 | - | 22.6 | 5903 | 6152 | -31 | L1MB3 | L1 | LINE |
| 75484145 | 75484449 | - | 14.4 | 1 | 305 | -7 | AluSx | Alu | SINE |
| 75484450 | 75484551 | - | 22.6 | 5825 | 5902 | -281 | L1MB3 | L1 | LINE |
| 75484552 | 75484865 | - | 15.4 | 7 | 312 | 0 | AluSx | Alu | SINE |
| 75484866 | 75485861 | - | 24.0 | 4818 | 5824 | -359 | L1MB3 | L1 | LINE |

  

|  |  |  |  |  |  |  |  |  |  |
| --- | --- | --- | --- | --- | --- | --- | --- | --- | --- |
| chr3:75486286-75486956 | | | 671 bp | | |  |  |  |  |
| Genome Start | Genome End | Strand | Div | Repeat Start | Repeat End | Repeat Left | Name | Family | Class |
| 75486286 | 75486492 | - | 24.0 | 5941 | 6130 | -170 | L1MA4 | L1 | LINE |
| 75486493 | 75486773 | - | 18.6 | 1 | 280 | -32 | AluSx | Alu | SINE |
| 75486774 | 75486956 | - | 24.0 | 5767 | 5940 | -360 | L1MA4 | L1 | LINE |

  

|  |  |  |  |  |  |  |  |  |  |
| --- | --- | --- | --- | --- | --- | --- | --- | --- | --- |
| chr3:75507530-75508159 | | | 630 bp | | |  |  |  |  |
| Genome Start | Genome End | Strand | Div | Repeat Start | Repeat End | Repeat Left | Name | Family | Class |
| 75507530 | 75507776 | - | 22.9 | 5907 | 6153 | -30 | L1MB3 | L1 | LINE |
| 75507777 | 75508071 | - | 12.9 | 3 | 298 | -14 | AluSx | Alu | SINE |
| 75508072 | 75508159 | - | 22.9 | 5822 | 5906 | -277 | L1MB3 | L1 | LINE |

  

|  |  |  |  |  |  |  |  |  |  |
| --- | --- | --- | --- | --- | --- | --- | --- | --- | --- |
| chr3:75509279-75510221 | | | 943 bp | | |  |  |  |  |
| Genome Start | Genome End | Strand | Div | Repeat Start | Repeat End | Repeat Left | Name | Family | Class |
| 75509279 | 75509358 | + | 38.7 | 2797 | 2873 | -546 | L2 | L2 | LINE |
| 75509359 | 75509411 | - | 6.5 | 31 | 80 | 0 | MADE1 | Mariner | DNA |
| 75509412 | 75509714 | + | 12.8 | 1 | 301 | -11 | AluSx | Alu | SINE |
| 75509715 | 75509753 | - | 6.5 | 1 | 30 | -50 | MADE1 | Mariner | DNA |
| 75509754 | 75510221 | + | 36.5 | 2874 | 3377 | -1 | L2 | L2 | LINE |

  

|  |  |  |  |  |  |  |  |  |  |
| --- | --- | --- | --- | --- | --- | --- | --- | --- | --- |
| chr3:75524991-75526375 | | | 1385 bp | | |  |  |  |  |
| Genome Start | Genome End | Strand | Div | Repeat Start | Repeat End | Repeat Left | Name | Family | Class |
| 75524991 | 75525033 | + | 21.7 | 1 | 51 | -416 | MLT1C | MaLR | LTR |
| 75525034 | 75525993 | + | 11.4 | 1 | 968 | 0 | LTR5\_Hs | ERVK | LTR |
| 75525994 | 75526375 | + | 20.3 | 52 | 505 | 0 | MLT1C | MaLR | LTR |

  

|  |  |  |  |  |  |  |  |  |  |
| --- | --- | --- | --- | --- | --- | --- | --- | --- | --- |
| chr3:75560109-75560697 | | | 589 bp | | |  |  |  |  |
| Genome Start | Genome End | Strand | Div | Repeat Start | Repeat End | Repeat Left | Name | Family | Class |
| 75560109 | 75560264 | - | 9.8 | 137 | 301 | -11 | AluSx | Alu | SINE |
| 75560265 | 75560564 | - | 13.0 | 1 | 300 | -13 | AluSq | Alu | SINE |
| 75560565 | 75560697 | - | 9.8 | 1 | 136 | -176 | AluSx | Alu | SINE |

  

|  |  |  |  |  |  |  |  |  |  |
| --- | --- | --- | --- | --- | --- | --- | --- | --- | --- |
| chr3:75573726-75574317 | | | 592 bp | | |  |  |  |  |
| Genome Start | Genome End | Strand | Div | Repeat Start | Repeat End | Repeat Left | Name | Family | Class |
| 75573726 | 75573901 | - | 19.4 | 138 | 305 | -7 | AluJb | Alu | SINE |
| 75573902 | 75574193 | - | 12.0 | 6 | 309 | -3 | AluSx | Alu | SINE |
| 75574194 | 75574317 | - | 19.4 | 18 | 137 | -175 | AluJb | Alu | SINE |

  

|  |  |  |  |  |  |  |  |  |  |
| --- | --- | --- | --- | --- | --- | --- | --- | --- | --- |
| chr3:75574345-75575194 | | | 805 bp | | |  |  |  |  |
| Genome Start | Genome End | Strand | Div | Repeat Start | Repeat End | Repeat Left | Name | Family | Class |
| 75574345 | 75574414 | - | 17.0 | 6116 | 6184 | 0 | L1MB7 | L1 | LINE |
| 75574415 | 75574710 | + | 17.7 | 1 | 294 | -18 | AluJb | Alu | SINE |
| 75574711 | 75574913 | - | 17.0 | 5932 | 6115 | -69 | L1MB7 | L1 | LINE |
| 75574959 | 75575194 | - | 17.0 | 5672 | 5931 | -253 | L1MB7 | L1 | LINE |

  

|  |  |  |  |  |  |  |  |  |  |
| --- | --- | --- | --- | --- | --- | --- | --- | --- | --- |
| chr3:75579717-75580313 | | | 564 bp | | |  |  |  |  |
| Genome Start | Genome End | Strand | Div | Repeat Start | Repeat End | Repeat Left | Name | Family | Class |
| 75579717 | 75579819 | - | 17.3 | 146 | 248 | -64 | AluJo | Alu | SINE |
| 75579853 | 75580164 | - | 15.7 | 1 | 312 | 0 | AluSx | Alu | SINE |
| 75580165 | 75580313 | - | 17.3 | 5 | 145 | -167 | AluJo | Alu | SINE |

  

|  |  |  |  |  |  |  |  |  |  |
| --- | --- | --- | --- | --- | --- | --- | --- | --- | --- |
| chr3:75586153-75586666 | | | 514 bp | | |  |  |  |  |
| Genome Start | Genome End | Strand | Div | Repeat Start | Repeat End | Repeat Left | Name | Family | Class |
| 75586153 | 75586220 | - | 20.5 | 158 | 209 | 0 | MER3 | MER1\_type | DNA |
| 75586221 | 75586515 | + | 12.9 | 1 | 296 | -16 | AluSx | Alu | SINE |
| 75586516 | 75586666 | - | 20.5 | 9 | 157 | -52 | MER3 | MER1\_type | DNA |

  

|  |  |  |  |  |  |  |  |  |  |
| --- | --- | --- | --- | --- | --- | --- | --- | --- | --- |
| chr3:75590301-75591649 | | | 1309 bp | | |  |  |  |  |
| Genome Start | Genome End | Strand | Div | Repeat Start | Repeat End | Repeat Left | Name | Family | Class |
| 75590301 | 75590584 | - | 15.8 | 41 | 299 | -13 | AluJo | Alu | SINE |
| 75590588 | 75590884 | - | 14.1 | 474 | 785 | 0 | LTR1 | ERV1 | LTR |
| 75590885 | 75591114 | - | 5.7 | 16 | 244 | -67 | AluY | Alu | SINE |
| 75591115 | 75591560 | - | 14.1 | 3 | 473 | -312 | LTR1 | ERV1 | LTR |
| 75591598 | 75591649 | - | 23.1 | 1 | 52 | -248 | AluJo | Alu | SINE |

  

|  |  |  |  |  |  |  |  |  |  |
| --- | --- | --- | --- | --- | --- | --- | --- | --- | --- |
| chr3:75592706-75593424 | | | 719 bp | | |  |  |  |  |
| Genome Start | Genome End | Strand | Div | Repeat Start | Repeat End | Repeat Left | Name | Family | Class |
| 75592706 | 75593056 | - | 23.0 | 91 | 464 | -3 | MLT1C | MaLR | LTR |
| 75593057 | 75593348 | - | 15.1 | 5 | 297 | -15 | AluSx | Alu | SINE |
| 75593349 | 75593424 | - | 23.0 | 6 | 90 | -377 | MLT1C | MaLR | LTR |

  

|  |  |  |  |  |  |  |  |  |  |
| --- | --- | --- | --- | --- | --- | --- | --- | --- | --- |
| chr3:75594019-75594475 | | | 457 bp | | |  |  |  |  |
| Genome Start | Genome End | Strand | Div | Repeat Start | Repeat End | Repeat Left | Name | Family | Class |
| 75594019 | 75594042 | - | 25.9 | 116 | 135 | -43 | MER5B | MER1\_type | DNA |
| 75594043 | 75594353 | + | 17.4 | 1 | 311 | -1 | AluJo | Alu | SINE |
| 75594354 | 75594475 | - | 25.9 | 3 | 115 | -63 | MER5B | MER1\_type | DNA |

  

|  |  |  |  |  |  |  |  |  |  |
| --- | --- | --- | --- | --- | --- | --- | --- | --- | --- |
| chr3:75601245-75608107 | | | 6803 bp | | |  |  |  |  |
| Genome Start | Genome End | Strand | Div | Repeat Start | Repeat End | Repeat Left | Name | Family | Class |
| 75601245 | 75601425 | + | 22.9 | 1 | 185 | -320 | MLT1D | MaLR | LTR |
| 75601436 | 75601734 | + | 10.0 | 3 | 302 | -11 | AluSp | Alu | SINE |
| 75601747 | 75602145 | - | 9.1 | 3 | 448 | -2 | LTR7 | ERV1 | LTR |
| 75602148 | 75602524 | - | 10.8 | 7324 | 7713 | 0 | HERVH | ERV1 | LTR |
| 75602525 | 75602736 | - | 11.8 | 5600 | 5819 | -1894 | HERVH | ERV1 | LTR |
| 75602741 | 75603399 | - | 14.7 | 4488 | 5146 | -2567 | HERVH | ERV1 | LTR |
| 75603402 | 75604251 | - | 10.0 | 3140 | 3993 | -3720 | HERVH | ERV1 | LTR |
| 75604238 | 75607173 | - | 9.9 | 1 | 2992 | -4721 | HERVH | ERV1 | LTR |
| 75607176 | 75607524 | - | 9.5 | 1 | 448 | -2 | LTR7 | ERV1 | LTR |
| 75607567 | 75607738 | + | 22.9 | 206 | 425 | -79 | MLT1D | MaLR | LTR |
| 75607739 | 75608042 | - | 15.2 | 1 | 303 | -9 | AluSx | Alu | SINE |
| 75608043 | 75608107 | + | 23.4 | 389 | 484 | 0 | MLT1D | MaLR | LTR |

  

|  |  |  |  |  |  |  |  |  |  |
| --- | --- | --- | --- | --- | --- | --- | --- | --- | --- |
| chr3:75612307-75613093 | | | 787 bp | | |  |  |  |  |
| Genome Start | Genome End | Strand | Div | Repeat Start | Repeat End | Repeat Left | Name | Family | Class |
| 75612307 | 75612397 | + | 21.4 | 3 | 87 | -380 | MLT1C | MaLR | LTR |
| 75612398 | 75612709 | + | 10.3 | 1 | 313 | 0 | AluSp | Alu | SINE |
| 75612710 | 75613093 | + | 21.4 | 88 | 467 | 0 | MLT1C | MaLR | LTR |

  

|  |  |  |  |  |  |  |  |  |  |
| --- | --- | --- | --- | --- | --- | --- | --- | --- | --- |
| chr3:75613688-75614533 | | | 846 bp | | |  |  |  |  |
| Genome Start | Genome End | Strand | Div | Repeat Start | Repeat End | Repeat Left | Name | Family | Class |
| 75613688 | 75613954 | + | 19.9 | 3 | 320 | -334 | MLT1E1 | MaLR | LTR |
| 75613955 | 75614266 | - | 10.9 | 1 | 312 | 0 | AluSx | Alu | SINE |
| 75614267 | 75614533 | + | 19.9 | 321 | 653 | -1 | MLT1E1 | MaLR | LTR |

  

|  |  |  |  |  |  |  |  |  |  |
| --- | --- | --- | --- | --- | --- | --- | --- | --- | --- |
| chr3:75627540-75628672 | | | 948 bp | | |  |  |  |  |
| Genome Start | Genome End | Strand | Div | Repeat Start | Repeat End | Repeat Left | Name | Family | Class |
| 75627540 | 75627605 | - | 21.5 | 521 | 590 | -1 | MLT1K | MaLR | LTR |
| 75627637 | 75627940 | - | 29.6 | 164 | 474 | -17 | LTR16C | ERVL | LTR |
| 75627945 | 75628255 | - | 13.6 | 1 | 310 | 0 | AluSg | Alu | SINE |
| 75628406 | 75628672 | - | 27.7 | 183 | 442 | -149 | MLT1K | MaLR | LTR |

  

|  |  |  |  |  |  |  |  |  |  |
| --- | --- | --- | --- | --- | --- | --- | --- | --- | --- |
| chr3:75629710-75630543 | | | 832 bp | | |  |  |  |  |
| Genome Start | Genome End | Strand | Div | Repeat Start | Repeat End | Repeat Left | Name | Family | Class |
| 75629710 | 75629752 | + | 7.0 | 2 | 44 | -428 | MER4A1 | ERV1 | LTR |
| 75629754 | 75629830 | + | 5.2 | 2 | 78 | -234 | AluS | Alu | SINE |
| 75629831 | 75630101 | + | 10.2 | 29 | 295 | -17 | AluSx | Alu | SINE |
| 75630103 | 75630543 | + | 11.9 | 34 | 472 | 0 | MER4A1 | ERV1 | LTR |

  

|  |  |  |  |  |  |  |  |  |  |
| --- | --- | --- | --- | --- | --- | --- | --- | --- | --- |
| chr3:75639316-75640780 | | | 1375 bp | | |  |  |  |  |
| Genome Start | Genome End | Strand | Div | Repeat Start | Repeat End | Repeat Left | Name | Family | Class |
| 75639316 | 75639494 | - | 31.6 | 4092 | 4289 | -200 | L3 | CR1 | LINE |
| 75639518 | 75639616 | - | 19.9 | 116 | 207 | -17 | MER58A | MER1\_type | DNA |
| 75639617 | 75640013 | + | 14.4 | 1 | 428 | 0 | MSTA | MaLR | LTR |
| 75640014 | 75640129 | - | 19.9 | 1 | 115 | 0 | MER58A | MER1\_type | DNA |
| 75640173 | 75640483 | - | 12.9 | 1 | 313 | 0 | AluSq | Alu | SINE |
| 75640484 | 75640610 | - | 11.8 | 9 | 135 | -177 | AluSq/x | Alu | SINE |
| 75640635 | 75640780 | - | 38.3 | 3965 | 4108 | -381 | L3 | CR1 | LINE |

  

|  |  |  |  |  |  |  |  |  |  |
| --- | --- | --- | --- | --- | --- | --- | --- | --- | --- |
| chr3:75641442-75643916 | | | 2429 bp | | |  |  |  |  |
| Genome Start | Genome End | Strand | Div | Repeat Start | Repeat End | Repeat Left | Name | Family | Class |
| 75641442 | 75641697 | + | 33.4 | 2230 | 2473 | -946 | L2 | L2 | LINE |
| 75641698 | 75641986 | + | 14.9 | 23 | 290 | -22 | AluJo | Alu | SINE |
| 75641987 | 75642667 | + | 32.5 | 2474 | 3196 | -223 | L2 | L2 | LINE |
| 75642687 | 75643750 | + | 9.6 | 1 | 1266 | 0 | MER11A | ERVK | LTR |
| 75643778 | 75643916 | + | 31.6 | 3242 | 3378 | 0 | L2 | L2 | LINE |

  

|  |  |  |  |  |  |  |  |  |  |
| --- | --- | --- | --- | --- | --- | --- | --- | --- | --- |
| chr3:75644250-75644870 | | | 621 bp | | |  |  |  |  |
| Genome Start | Genome End | Strand | Div | Repeat Start | Repeat End | Repeat Left | Name | Family | Class |
| 75644250 | 75644319 | - | 26.2 | 340 | 402 | -7 | MLT1I | MaLR | LTR |
| 75644320 | 75644614 | - | 16.7 | 1 | 303 | -9 | AluSx | Alu | SINE |
| 75644615 | 75644870 | - | 26.2 | 87 | 339 | -70 | MLT1I | MaLR | LTR |

  

|  |  |  |  |  |  |  |  |  |  |
| --- | --- | --- | --- | --- | --- | --- | --- | --- | --- |
| chr3:75649985-75650923 | | | 931 bp | | |  |  |  |  |
| Genome Start | Genome End | Strand | Div | Repeat Start | Repeat End | Repeat Left | Name | Family | Class |
| 75649985 | 75650110 | + | 19.5 | 5 | 128 | -50 | MER5B | MER1\_type | DNA |
| 75650129 | 75650431 | - | 14.5 | 2 | 304 | -8 | AluSx | Alu | SINE |
| 75650437 | 75650718 | - | 18.0 | 5902 | 6174 | 0 | L1MB5 | L1 | LINE |
| 75650689 | 75650864 | + | 19.4 | 5684 | 5887 | -297 | L1MB5 | L1 | LINE |
| 75650880 | 75650923 | + | 11.4 | 135 | 178 | 0 | MER5B | MER1\_type | DNA |

  

|  |  |  |  |  |  |  |  |  |  |
| --- | --- | --- | --- | --- | --- | --- | --- | --- | --- |
| chr3:75652486-75653242 | | | 752 bp | | |  |  |  |  |
| Genome Start | Genome End | Strand | Div | Repeat Start | Repeat End | Repeat Left | Name | Family | Class |
| 75652486 | 75652688 | - | 22.3 | 5976 | 6184 | 0 | L1MB7 | L1 | LINE |
| 75652694 | 75652866 | - | 9.8 | 104 | 289 | -23 | AluSg/x | Alu | SINE |
| 75652867 | 75653242 | - | 18.9 | 5575 | 5959 | -219 | L1MB7 | L1 | LINE |

  

|  |  |  |  |  |  |  |  |  |  |
| --- | --- | --- | --- | --- | --- | --- | --- | --- | --- |
| chr3:75675568-75676201 | | | 634 bp | | |  |  |  |  |
| Genome Start | Genome End | Strand | Div | Repeat Start | Repeat End | Repeat Left | Name | Family | Class |
| 75675568 | 75675729 | + | 21.0 | 5333 | 5490 | -684 | L1M4 | L1 | LINE |
| 75675730 | 75676038 | + | 12.7 | 1 | 307 | -5 | AluSx | Alu | SINE |
| 75676039 | 75676201 | + | 20.9 | 5491 | 5653 | -521 | L1M4 | L1 | LINE |

  

|  |  |  |  |  |  |  |  |  |  |
| --- | --- | --- | --- | --- | --- | --- | --- | --- | --- |
| chr3:75677997-75678836 | | | 808 bp | | |  |  |  |  |
| Genome Start | Genome End | Strand | Div | Repeat Start | Repeat End | Repeat Left | Name | Family | Class |
| 75677997 | 75678086 | - | 27.4 | 6089 | 6170 | 0 | L1ME3A | L1 | LINE |
| 75678087 | 75678378 | + | 16.0 | 3 | 284 | -28 | AluJo | Alu | SINE |
| 75678411 | 75678560 | - | 27.4 | 5925 | 6088 | -82 | L1ME3A | L1 | LINE |
| 75678561 | 75678781 | + | 23.8 | 1 | 219 | 0 | MER20 | MER1\_type | DNA |
| 75678782 | 75678836 | - | 27.4 | 5874 | 5924 | -246 | L1ME3A | L1 | LINE |

  

|  |  |  |  |  |  |  |  |  |  |
| --- | --- | --- | --- | --- | --- | --- | --- | --- | --- |
| chr3:75695838-75696327 | | | 409 bp | | |  |  |  |  |
| Genome Start | Genome End | Strand | Div | Repeat Start | Repeat End | Repeat Left | Name | Family | Class |
| 75695838 | 75695953 | + | 32.5 | 3096 | 3216 | -162 | L2 | L2 | LINE |
| 75696053 | 75696267 | - | 27.7 | 12 | 260 | -2 | MIR | MIR | SINE |
| 75696250 | 75696327 | + | 33.3 | 3347 | 3418 | -1 | L2 | L2 | LINE |

  

|  |  |  |  |  |  |  |  |  |  |
| --- | --- | --- | --- | --- | --- | --- | --- | --- | --- |
| chr3:75704777-75705590 | | | 814 bp | | |  |  |  |  |
| Genome Start | Genome End | Strand | Div | Repeat Start | Repeat End | Repeat Left | Name | Family | Class |
| 75704777 | 75704952 | + | 12.8 | 5775 | 5950 | -341 | L1MA8 | L1 | LINE |
| 75704953 | 75705263 | + | 18.1 | 1 | 312 | 0 | AluJo | Alu | SINE |
| 75705264 | 75705590 | + | 12.8 | 5951 | 6288 | -3 | L1MA8 | L1 | LINE |

  

|  |  |  |  |  |  |  |  |  |  |
| --- | --- | --- | --- | --- | --- | --- | --- | --- | --- |
| chr3:75716986-75718153 | | | 1168 bp | | |  |  |  |  |
| Genome Start | Genome End | Strand | Div | Repeat Start | Repeat End | Repeat Left | Name | Family | Class |
| 75716986 | 75717167 | - | 35.3 | 3172 | 3335 | -43 | L2 | L2 | LINE |
| 75717168 | 75717466 | + | 13.1 | 1 | 305 | -8 | AluSq | Alu | SINE |
| 75717467 | 75718153 | - | 31.3 | 2348 | 3171 | -207 | L2 | L2 | LINE |

  

|  |  |  |  |  |  |  |  |  |  |
| --- | --- | --- | --- | --- | --- | --- | --- | --- | --- |
| chr3:75726590-75728007 | | | 1418 bp | | |  |  |  |  |
| Genome Start | Genome End | Strand | Div | Repeat Start | Repeat End | Repeat Left | Name | Family | Class |
| 75726590 | 75726716 | - | 23.5 | 5822 | 5910 | -274 | L1MB3 | L1 | LINE |
| 75726717 | 75727007 | - | 15.2 | 11 | 300 | -12 | AluSx | Alu | SINE |
| 75727008 | 75728007 | - | 24.8 | 4818 | 5834 | -349 | L1MB3 | L1 | LINE |

  

|  |  |  |  |  |  |  |  |  |  |
| --- | --- | --- | --- | --- | --- | --- | --- | --- | --- |
| chr3:75728443-75729108 | | | 666 bp | | |  |  |  |  |
| Genome Start | Genome End | Strand | Div | Repeat Start | Repeat End | Repeat Left | Name | Family | Class |
| 75728443 | 75728641 | - | 21.8 | 5934 | 6125 | -175 | L1MA4 | L1 | LINE |
| 75728642 | 75728925 | - | 15.5 | 1 | 283 | -29 | AluSx | Alu | SINE |
| 75728926 | 75729108 | - | 21.8 | 5767 | 5933 | -367 | L1MA4 | L1 | LINE |

  

|  |  |  |  |  |  |  |  |  |  |
| --- | --- | --- | --- | --- | --- | --- | --- | --- | --- |
| chr3:75747112-75748446 | | | 1232 bp | | |  |  |  |  |
| Genome Start | Genome End | Strand | Div | Repeat Start | Repeat End | Repeat Left | Name | Family | Class |
| 75747112 | 75747328 | - | 28.3 | 565 | 793 | -5326 | L1MEd | L1 | LINE |
| 75747329 | 75747617 | - | 9.1 | 1 | 292 | -21 | AluSq | Alu | SINE |
| 75747618 | 75747854 | - | 28.3 | 329 | 564 | -5555 | L1MEd | L1 | LINE |
| 75747916 | 75748028 | - | 24.5 | 108 | 220 | -5899 | L1MEd | L1 | LINE |
| 75748071 | 75748356 | - | 10.5 | 1 | 289 | -24 | AluSq | Alu | SINE |
| 75748357 | 75748446 | - | 24.5 | 23 | 107 | -6012 | L1MEd | L1 | LINE |

  

|  |  |  |  |  |  |  |  |  |  |
| --- | --- | --- | --- | --- | --- | --- | --- | --- | --- |
| chr3:75757048-75760153 | | | 2946 bp | | |  |  |  |  |
| Genome Start | Genome End | Strand | Div | Repeat Start | Repeat End | Repeat Left | Name | Family | Class |
| 75757048 | 75757558 | + | 23.0 | 1699 | 2231 | -2705 | MER31-int | ERV1 | LTR |
| 75757573 | 75757742 | + | 22.5 | 2939 | 3112 | -1824 | MER31-int | ERV1 | LTR |
| 75757743 | 75758038 | + | 10.1 | 2 | 297 | -14 | AluY | Alu | SINE |
| 75758039 | 75758839 | + | 22.5 | 3113 | 3873 | -1063 | MER31-int | ERV1 | LTR |
| 75758949 | 75759310 | + | 27.3 | 188 | 562 | -29 | LTR10C | ERV1 | LTR |
| 75759348 | 75759774 | + | 20.1 | 3859 | 4296 | -640 | MER31-int | ERV1 | LTR |
| 75759775 | 75760040 | + | 15.8 | 1 | 290 | -22 | AluJb | Alu | SINE |
| 75760041 | 75760153 | + | 20.1 | 4297 | 4375 | -561 | MER31-int | ERV1 | LTR |

  

|  |  |  |  |  |  |  |  |  |  |
| --- | --- | --- | --- | --- | --- | --- | --- | --- | --- |
| chr3:75807863-75809836 | | | 1947 bp | | |  |  |  |  |
| Genome Start | Genome End | Strand | Div | Repeat Start | Repeat End | Repeat Left | Name | Family | Class |
| 75807863 | 75808407 | - | 12.9 | 3307 | 3880 | -2307 | MER57A-int | ERV1 | LTR |
| 75808435 | 75808681 | - | 11.6 | 2433 | 2663 | -3524 | MER57A-int | ERV1 | LTR |
| 75808682 | 75808954 | + | 12.1 | 22 | 294 | -18 | AluSx | Alu | SINE |
| 75808955 | 75809836 | - | 11.6 | 1495 | 2432 | -3755 | MER57A-int | ERV1 | LTR |

  

|  |  |  |  |  |  |  |  |  |  |
| --- | --- | --- | --- | --- | --- | --- | --- | --- | --- |
| chr3:75897580-75898791 | | | 1212 bp | | |  |  |  |  |
| Genome Start | Genome End | Strand | Div | Repeat Start | Repeat End | Repeat Left | Name | Family | Class |
| 75897580 | 75897667 | + | 26.7 | 2394 | 2500 | -4057 | L1MDa | L1 | LINE |
| 75897668 | 75897975 | + | 17.9 | 1 | 310 | -2 | AluJb | Alu | SINE |
| 75897976 | 75898791 | + | 27.1 | 2487 | 3343 | -3194 | L1MDa | L1 | LINE |

  

|  |  |  |  |  |  |  |  |  |  |
| --- | --- | --- | --- | --- | --- | --- | --- | --- | --- |
| chr3:75912140-75912914 | | | 775 bp | | |  |  |  |  |
| Genome Start | Genome End | Strand | Div | Repeat Start | Repeat End | Repeat Left | Name | Family | Class |
| 75912140 | 75912253 | + | 12.4 | 7032 | 7145 | -392 | MER57B-int | ERV1 | LTR |
| 75912254 | 75912540 | - | 8.7 | 1 | 291 | -20 | AluY | Alu | SINE |
| 75912541 | 75912914 | + | 12.4 | 7146 | 7537 | 0 | MER57B-int | ERV1 | LTR |

  

|  |  |  |  |  |  |  |  |  |  |
| --- | --- | --- | --- | --- | --- | --- | --- | --- | --- |
| chr3:90343301-90345419 | | | 2119 bp | | |  |  |  |  |
| Genome Start | Genome End | Strand | Div | Repeat Start | Repeat End | Repeat Left | Name | Family | Class |
| 90343301 | 90344229 | + | 29.9 | 26 | 1060 | -5363 | L1MCc | L1 | LINE |
| 90344230 | 90344537 | + | 13.7 | 1 | 312 | 0 | AluSx | Alu | SINE |
| 90344538 | 90344619 | + | 29.9 | 1061 | 1123 | -5300 | L1MCc | L1 | LINE |
| 90344620 | 90344902 | + | 13.8 | 1 | 300 | -10 | AluSg | Alu | SINE |
| 90344903 | 90345419 | + | 29.1 | 1124 | 1764 | -5364 | L1MCc | L1 | LINE |

  

|  |  |  |  |  |  |  |  |  |  |
| --- | --- | --- | --- | --- | --- | --- | --- | --- | --- |
| chr3:90348389-90350179 | | | 1699 bp | | |  |  |  |  |
| Genome Start | Genome End | Strand | Div | Repeat Start | Repeat End | Repeat Left | Name | Family | Class |
| 90348389 | 90348564 | + | 25.1 | 2022 | 2208 | -1211 | L2 | L2 | LINE |
| 90348637 | 90349362 | + | 25.5 | 37 | 783 | 0 | MER20B | MER1\_type | DNA |
| 90349374 | 90349422 | + | 22.4 | 2298 | 2346 | -1073 | L2 | L2 | LINE |
| 90349432 | 90350179 | + | 29.7 | 2624 | 3415 | -4 | L2 | L2 | LINE |

  

|  |  |  |  |  |  |  |  |  |  |
| --- | --- | --- | --- | --- | --- | --- | --- | --- | --- |
| chr3:90389573-90390055 | | | 469 bp | | |  |  |  |  |
| Genome Start | Genome End | Strand | Div | Repeat Start | Repeat End | Repeat Left | Name | Family | Class |
| 90389573 | 90389705 | + | 18.1 | 1 | 133 | -169 | AluJo | Alu | SINE |
| 90389720 | 90389878 | - | 12.6 | 144 | 304 | -8 | AluSg/x | Alu | SINE |
| 90389879 | 90390055 | + | 19.9 | 125 | 310 | -2 | AluJo | Alu | SINE |

  

|  |  |  |  |  |  |  |  |  |  |
| --- | --- | --- | --- | --- | --- | --- | --- | --- | --- |
| chr3:95023539-95024277 | | | 739 bp | | |  |  |  |  |
| Genome Start | Genome End | Strand | Div | Repeat Start | Repeat End | Repeat Left | Name | Family | Class |
| 95023539 | 95023750 | + | 11.7 | 5874 | 6080 | -217 | L1MA5A | L1 | LINE |
| 95023751 | 95024075 | - | 13.5 | 1 | 339 | 0 | MER1B | MER1\_type | DNA |
| 95024076 | 95024277 | + | 11.7 | 6081 | 6287 | -10 | L1MA5A | L1 | LINE |

  

|  |  |  |  |  |  |  |  |  |  |
| --- | --- | --- | --- | --- | --- | --- | --- | --- | --- |
| chr3:95114527-95115267 | | | 741 bp | | |  |  |  |  |
| Genome Start | Genome End | Strand | Div | Repeat Start | Repeat End | Repeat Left | Name | Family | Class |
| 95114527 | 95114766 | + | 13.6 | 1 | 242 | -139 | THE1D | MaLR | LTR |
| 95114767 | 95115122 | + | 14.1 | 1 | 363 | -1 | THE1B | MaLR | LTR |
| 95115123 | 95115267 | + | 13.6 | 243 | 381 | 0 | THE1D | MaLR | LTR |

  

|  |  |  |  |  |  |  |  |  |  |
| --- | --- | --- | --- | --- | --- | --- | --- | --- | --- |
| chr3:95115268-95117485 | | | 2218 bp | | |  |  |  |  |
| Genome Start | Genome End | Strand | Div | Repeat Start | Repeat End | Repeat Left | Name | Family | Class |
| 95115268 | 95116208 | - | 23.6 | 670 | 1668 | -67 | MSTD-int | MaLR | LTR |
| 95116209 | 95116496 | - | 11.3 | 5867 | 6149 | -2 | L1PB1 | L1 | LINE |
| 95116497 | 95116718 | - | 23.6 | 462 | 669 | -1066 | MSTD-int | MaLR | LTR |
| 95116719 | 95117028 | + | 16.4 | 1 | 311 | 0 | AluSx | Alu | SINE |
| 95117029 | 95117485 | - | 23.6 | 4 | 461 | -1274 | MSTD-int | MaLR | LTR |

  

|  |  |  |  |  |  |  |  |  |  |
| --- | --- | --- | --- | --- | --- | --- | --- | --- | --- |
| chr3:107587533-107588085 | | | 553 bp | | |  |  |  |  |
| Genome Start | Genome End | Strand | Div | Repeat Start | Repeat End | Repeat Left | Name | Family | Class |
| 107587533 | 107587583 | + | 16.7 | 6244 | 6291 | -21 | L1MA9 | L1 | LINE |
| 107587584 | 107588067 | + | 18.1 | 1 | 497 | 0 | LOR1a | ERV1 | LTR |
| 107588068 | 107588085 | + | 16.7 | 6292 | 6309 | -3 | L1MA9 | L1 | LINE |

  

|  |  |  |  |  |  |  |  |  |  |
| --- | --- | --- | --- | --- | --- | --- | --- | --- | --- |
| chr3:113723657-113724315 | | | 659 bp | | |  |  |  |  |
| Genome Start | Genome End | Strand | Div | Repeat Start | Repeat End | Repeat Left | Name | Family | Class |
| 113723657 | 113723863 | - | 23.6 | 5941 | 6130 | -170 | L1MA4 | L1 | LINE |
| 113723864 | 113724144 | - | 13.6 | 1 | 280 | -32 | AluSx | Alu | SINE |
| 113724145 | 113724315 | - | 23.6 | 5779 | 5940 | -360 | L1MA4 | L1 | LINE |

  

|  |  |  |  |  |  |  |  |  |  |
| --- | --- | --- | --- | --- | --- | --- | --- | --- | --- |
| chr3:124912070-124912916 | | | 845 bp | | |  |  |  |  |
| Genome Start | Genome End | Strand | Div | Repeat Start | Repeat End | Repeat Left | Name | Family | Class |
| 124912070 | 124912500 | + | 19.6 | 5443 | 5885 | -281 | L1ME1 | L1 | LINE |
| 124912503 | 124912638 | + | 10.3 | 158 | 293 | -19 | AluJb | Alu | SINE |
| 124912639 | 124912916 | + | 25.9 | 5875 | 6163 | -3 | L1ME1 | L1 | LINE |

  

|  |  |  |  |  |  |  |  |  |  |
| --- | --- | --- | --- | --- | --- | --- | --- | --- | --- |
| chr3:126907282-126907952 | | | 671 bp | | |  |  |  |  |
| Genome Start | Genome End | Strand | Div | Repeat Start | Repeat End | Repeat Left | Name | Family | Class |
| 126907282 | 126907464 | + | 21.7 | 5771 | 5940 | -360 | L1MA4 | L1 | LINE |
| 126907465 | 126907745 | + | 14.6 | 1 | 280 | -32 | AluSx | Alu | SINE |
| 126907746 | 126907952 | + | 21.7 | 5941 | 6130 | -170 | L1MA4 | L1 | LINE |

  

|  |  |  |  |  |  |  |  |  |  |
| --- | --- | --- | --- | --- | --- | --- | --- | --- | --- |
| chr3:126908385-126910363 | | | 1979 bp | | |  |  |  |  |
| Genome Start | Genome End | Strand | Div | Repeat Start | Repeat End | Repeat Left | Name | Family | Class |
| 126908385 | 126909376 | + | 24.1 | 4818 | 5824 | -359 | L1MB3 | L1 | LINE |
| 126909377 | 126909695 | + | 11.6 | 1 | 312 | 0 | AluSx | Alu | SINE |
| 126909696 | 126909807 | + | 22.6 | 5825 | 5904 | -279 | L1MB3 | L1 | LINE |
| 126909808 | 126910111 | + | 15.1 | 1 | 304 | -8 | AluSx | Alu | SINE |
| 126910112 | 126910363 | + | 22.6 | 5905 | 6153 | -30 | L1MB3 | L1 | LINE |

  

|  |  |  |  |  |  |  |  |  |  |
| --- | --- | --- | --- | --- | --- | --- | --- | --- | --- |
| chr3:126928433-126929103 | | | 671 bp | | |  |  |  |  |
| Genome Start | Genome End | Strand | Div | Repeat Start | Repeat End | Repeat Left | Name | Family | Class |
| 126928433 | 126928615 | + | 25.3 | 5767 | 5933 | -367 | L1MA4 | L1 | LINE |
| 126928616 | 126928896 | + | 15.7 | 1 | 280 | -32 | AluSx | Alu | SINE |
| 126928897 | 126929103 | + | 25.3 | 5934 | 6130 | -170 | L1MA4 | L1 | LINE |

  

|  |  |  |  |  |  |  |  |  |  |
| --- | --- | --- | --- | --- | --- | --- | --- | --- | --- |
| chr3:126929529-126931475 | | | 1947 bp | | |  |  |  |  |
| Genome Start | Genome End | Strand | Div | Repeat Start | Repeat End | Repeat Left | Name | Family | Class |
| 126929529 | 126930521 | + | 25.5 | 4818 | 5824 | -359 | L1MB3 | L1 | LINE |
| 126930522 | 126930822 | + | 14.4 | 1 | 300 | -12 | AluSx | Alu | SINE |
| 126930823 | 126930940 | + | 24.7 | 5825 | 5908 | -275 | L1MB3 | L1 | LINE |
| 126930941 | 126931223 | + | 14.1 | 3 | 295 | -17 | AluSx | Alu | SINE |
| 126931224 | 126931475 | + | 24.7 | 5909 | 6153 | -30 | L1MB3 | L1 | LINE |

  

|  |  |  |  |  |  |  |  |  |  |
| --- | --- | --- | --- | --- | --- | --- | --- | --- | --- |
| chr3:126951011-126951694 | | | 684 bp | | |  |  |  |  |
| Genome Start | Genome End | Strand | Div | Repeat Start | Repeat End | Repeat Left | Name | Family | Class |
| 126951011 | 126951193 | + | 25.8 | 5767 | 5933 | -367 | L1MA4 | L1 | LINE |
| 126951194 | 126951474 | + | 17.1 | 1 | 280 | -32 | AluSx | Alu | SINE |
| 126951475 | 126951694 | + | 25.8 | 5934 | 6143 | -157 | L1MA4 | L1 | LINE |

  

|  |  |  |  |  |  |  |  |  |  |
| --- | --- | --- | --- | --- | --- | --- | --- | --- | --- |
| chr3:126952106-126954052 | | | 1947 bp | | |  |  |  |  |
| Genome Start | Genome End | Strand | Div | Repeat Start | Repeat End | Repeat Left | Name | Family | Class |
| 126952106 | 126953089 | + | 24.9 | 4818 | 5820 | -363 | L1MB3 | L1 | LINE |
| 126953090 | 126953409 | + | 14.5 | 1 | 312 | 0 | AluSx | Alu | SINE |
| 126953410 | 126953521 | + | 23.7 | 5821 | 5906 | -277 | L1MB3 | L1 | LINE |
| 126953522 | 126953807 | + | 13.3 | 10 | 295 | -17 | AluSx | Alu | SINE |
| 126953808 | 126954052 | + | 23.7 | 5907 | 6148 | -35 | L1MB3 | L1 | LINE |

  

|  |  |  |  |  |  |  |  |  |  |
| --- | --- | --- | --- | --- | --- | --- | --- | --- | --- |
| chr3:126958120-126959007 | | | 888 bp | | |  |  |  |  |
| Genome Start | Genome End | Strand | Div | Repeat Start | Repeat End | Repeat Left | Name | Family | Class |
| 126958120 | 126958491 | - | 28.8 | 2558 | 2939 | -3207 | L1M5 | L1 | LINE |
| 126958492 | 126958873 | - | 24.1 | 1 | 448 | -2 | MLT1J2 | MaLR | LTR |
| 126958874 | 126959007 | - | 28.8 | 2424 | 2557 | -3589 | L1M5 | L1 | LINE |

  

|  |  |  |  |  |  |  |  |  |  |
| --- | --- | --- | --- | --- | --- | --- | --- | --- | --- |
| chr3:126961963-126963144 | | | 1151 bp | | |  |  |  |  |
| Genome Start | Genome End | Strand | Div | Repeat Start | Repeat End | Repeat Left | Name | Family | Class |
| 126961963 | 126962650 | + | 30.2 | 2348 | 3193 | -226 | L2 | L2 | LINE |
| 126962682 | 126962965 | - | 14.6 | 1 | 281 | -32 | AluSq | Alu | SINE |
| 126962966 | 126963144 | + | 32.6 | 3194 | 3376 | -43 | L2 | L2 | LINE |

  

|  |  |  |  |  |  |  |  |  |  |
| --- | --- | --- | --- | --- | --- | --- | --- | --- | --- |
| chr3:126965684-126966491 | | | 803 bp | | |  |  |  |  |
| Genome Start | Genome End | Strand | Div | Repeat Start | Repeat End | Repeat Left | Name | Family | Class |
| 126965684 | 126965743 | + | 36.7 | 15 | 75 | -187 | MIR | MIR | SINE |
| 126965749 | 126966328 | - | 19.5 | 1493 | 2104 | -3610 | ERVL-B4 | ERVL | LTR |
| 126966329 | 126966491 | + | 21.9 | 71 | 224 | -38 | MIR | MIR | SINE |

  

|  |  |  |  |  |  |  |  |  |  |
| --- | --- | --- | --- | --- | --- | --- | --- | --- | --- |
| chr3:126974889-126975704 | | | 816 bp | | |  |  |  |  |
| Genome Start | Genome End | Strand | Div | Repeat Start | Repeat End | Repeat Left | Name | Family | Class |
| 126974889 | 126975217 | - | 13.8 | 5951 | 6288 | -3 | L1MA8 | L1 | LINE |
| 126975218 | 126975528 | - | 15.5 | 1 | 312 | 0 | AluJo | Alu | SINE |
| 126975529 | 126975704 | - | 13.8 | 5775 | 5950 | -341 | L1MA8 | L1 | LINE |

  

|  |  |  |  |  |  |  |  |  |  |
| --- | --- | --- | --- | --- | --- | --- | --- | --- | --- |
| chr3:127018215-127018970 | | | 753 bp | | |  |  |  |  |
| Genome Start | Genome End | Strand | Div | Repeat Start | Repeat End | Repeat Left | Name | Family | Class |
| 127018215 | 127018590 | + | 18.4 | 5575 | 5959 | -219 | L1MB7 | L1 | LINE |
| 127018591 | 127018763 | + | 9.8 | 104 | 289 | -23 | AluSg/x | Alu | SINE |
| 127018767 | 127018970 | + | 22.1 | 5974 | 6184 | 0 | L1MB7 | L1 | LINE |

  

|  |  |  |  |  |  |  |  |  |  |
| --- | --- | --- | --- | --- | --- | --- | --- | --- | --- |
| chr3:127018971-127020297 | | | 1181 bp | | |  |  |  |  |
| Genome Start | Genome End | Strand | Div | Repeat Start | Repeat End | Repeat Left | Name | Family | Class |
| 127018971 | 127019127 | - | 33.9 | 3013 | 3157 | -221 | L2 | L2 | LINE |
| 127019128 | 127019426 | - | 10.4 | 1 | 299 | -14 | AluSq | Alu | SINE |
| 127019427 | 127019528 | - | 33.9 | 2910 | 3012 | -366 | L2 | L2 | LINE |
| 127019570 | 127019627 | + | 29.3 | 95 | 152 | -124 | MIRm | MIR | SINE |
| 127019685 | 127019987 | - | 6.5 | 1 | 303 | 0 | AluY | Alu | SINE |
| 127020036 | 127020297 | - | 32.6 | 2563 | 2832 | -587 | L2 | L2 | LINE |

  

|  |  |  |  |  |  |  |  |  |  |
| --- | --- | --- | --- | --- | --- | --- | --- | --- | --- |
| chr3:127021894-127022823 | | | 907 bp | | |  |  |  |  |
| Genome Start | Genome End | Strand | Div | Repeat Start | Repeat End | Repeat Left | Name | Family | Class |
| 127021894 | 127021937 | - | 6.8 | 135 | 178 | 0 | MER5B | MER1\_type | DNA |
| 127021953 | 127022128 | - | 24.4 | 5684 | 5887 | -287 | L1MB5 | L1 | LINE |
| 127022099 | 127022381 | + | 19.1 | 5902 | 6174 | 0 | L1MB5 | L1 | LINE |
| 127022387 | 127022665 | + | 12.9 | 2 | 286 | -26 | AluSx | Alu | SINE |
| 127022699 | 127022823 | - | 20.5 | 5 | 127 | -51 | MER5B | MER1\_type | DNA |

  

|  |  |  |  |  |  |  |  |  |  |
| --- | --- | --- | --- | --- | --- | --- | --- | --- | --- |
| chr3:127027923-127028535 | | | 613 bp | | |  |  |  |  |
| Genome Start | Genome End | Strand | Div | Repeat Start | Repeat End | Repeat Left | Name | Family | Class |
| 127027923 | 127028178 | + | 25.5 | 87 | 339 | -70 | MLT1I | MaLR | LTR |
| 127028179 | 127028457 | + | 14.4 | 1 | 287 | -25 | AluSx | Alu | SINE |
| 127028458 | 127028535 | + | 25.5 | 340 | 402 | -7 | MLT1I | MaLR | LTR |

  

|  |  |  |  |  |  |  |  |  |  |
| --- | --- | --- | --- | --- | --- | --- | --- | --- | --- |
| chr3:127028873-127031357 | | | 2449 bp | | |  |  |  |  |
| Genome Start | Genome End | Strand | Div | Repeat Start | Repeat End | Repeat Left | Name | Family | Class |
| 127028873 | 127029011 | - | 30.9 | 3242 | 3378 | 0 | L2 | L2 | LINE |
| 127029039 | 127030110 | - | 12.3 | 1 | 1236 | 0 | MER11A | ERVK | LTR |
| 127030120 | 127030812 | - | 32.0 | 2466 | 3207 | -212 | L2 | L2 | LINE |
| 127030813 | 127031123 | - | 16.5 | 1 | 291 | -21 | AluJo | Alu | SINE |
| 127031124 | 127031357 | - | 33.1 | 2230 | 2465 | -954 | L2 | L2 | LINE |

  

|  |  |  |  |  |  |  |  |  |  |
| --- | --- | --- | --- | --- | --- | --- | --- | --- | --- |
| chr3:127042074-127043346 | | | 1273 bp | | |  |  |  |  |
| Genome Start | Genome End | Strand | Div | Repeat Start | Repeat End | Repeat Left | Name | Family | Class |
| 127042074 | 127042429 | - | 29.4 | 194 | 545 | -4 | MLT1H | MaLR | LTR |
| 127042430 | 127042868 | - | 10.1 | 45 | 472 | 0 | MER4A1 | ERV1 | LTR |
| 127042869 | 127043171 | - | 9.0 | 1 | 300 | -12 | AluSx | Alu | SINE |
| 127043172 | 127043214 | - | 10.1 | 2 | 44 | -428 | MER4A1 | ERV1 | LTR |
| 127043215 | 127043346 | - | 29.4 | 60 | 193 | -356 | MLT1H | MaLR | LTR |

  

|  |  |  |  |  |  |  |  |  |  |
| --- | --- | --- | --- | --- | --- | --- | --- | --- | --- |
| chr3:127044155-127045272 | | | 930 bp | | |  |  |  |  |
| Genome Start | Genome End | Strand | Div | Repeat Start | Repeat End | Repeat Left | Name | Family | Class |
| 127044155 | 127044413 | + | 28.8 | 183 | 432 | -159 | MLT1K | MaLR | LTR |
| 127044571 | 127044872 | + | 15.2 | 1 | 304 | -6 | AluSg | Alu | SINE |
| 127044873 | 127045175 | + | 29.4 | 164 | 474 | -17 | LTR16C | ERVL | LTR |
| 127045207 | 127045272 | + | 18.5 | 521 | 590 | -1 | MLT1K | MaLR | LTR |

  

|  |  |  |  |  |  |  |  |  |  |
| --- | --- | --- | --- | --- | --- | --- | --- | --- | --- |
| chr3:127058279-127059124 | | | 846 bp | | |  |  |  |  |
| Genome Start | Genome End | Strand | Div | Repeat Start | Repeat End | Repeat Left | Name | Family | Class |
| 127058279 | 127058545 | - | 19.1 | 322 | 654 | 0 | MLT1E1 | MaLR | LTR |
| 127058546 | 127058857 | + | 11.5 | 1 | 312 | 0 | AluSx | Alu | SINE |
| 127058858 | 127059124 | - | 19.1 | 4 | 321 | -333 | MLT1E1 | MaLR | LTR |

  

|  |  |  |  |  |  |  |  |  |  |
| --- | --- | --- | --- | --- | --- | --- | --- | --- | --- |
| chr3:127078623-127079079 | | | 457 bp | | |  |  |  |  |
| Genome Start | Genome End | Strand | Div | Repeat Start | Repeat End | Repeat Left | Name | Family | Class |
| 127078623 | 127078744 | + | 26.7 | 3 | 115 | -63 | MER5B | MER1\_type | DNA |
| 127078745 | 127079055 | - | 17.0 | 1 | 311 | -1 | AluJo | Alu | SINE |
| 127079056 | 127079079 | + | 26.7 | 116 | 135 | -43 | MER5B | MER1\_type | DNA |

  

|  |  |  |  |  |  |  |  |  |  |
| --- | --- | --- | --- | --- | --- | --- | --- | --- | --- |
| chr3:127079669-127080388 | | | 720 bp | | |  |  |  |  |
| Genome Start | Genome End | Strand | Div | Repeat Start | Repeat End | Repeat Left | Name | Family | Class |
| 127079669 | 127079744 | + | 23.9 | 6 | 90 | -377 | MLT1C | MaLR | LTR |
| 127079745 | 127080038 | + | 15.7 | 5 | 299 | -13 | AluSx | Alu | SINE |
| 127080039 | 127080388 | + | 23.9 | 91 | 464 | -3 | MLT1C | MaLR | LTR |

  

|  |  |  |  |  |  |  |  |  |  |
| --- | --- | --- | --- | --- | --- | --- | --- | --- | --- |
| chr3:127081464-127082816 | | | 1324 bp | | |  |  |  |  |
| Genome Start | Genome End | Strand | Div | Repeat Start | Repeat End | Repeat Left | Name | Family | Class |
| 127081464 | 127081526 | + | 19.4 | 1 | 63 | -239 | AluJo | Alu | SINE |
| 127081553 | 127081996 | + | 13.7 | 1 | 442 | -343 | LTR1 | ERV1 | LTR |
| 127081997 | 127082226 | + | 6.1 | 16 | 244 | -67 | AluY | Alu | SINE |
| 127082227 | 127082523 | + | 13.7 | 443 | 784 | -1 | LTR1 | ERV1 | LTR |
| 127082527 | 127082816 | + | 15.7 | 43 | 309 | -3 | AluJo | Alu | SINE |

  

|  |  |  |  |  |  |  |  |  |  |
| --- | --- | --- | --- | --- | --- | --- | --- | --- | --- |
| chr3:127086457-127086976 | | | 520 bp | | |  |  |  |  |
| Genome Start | Genome End | Strand | Div | Repeat Start | Repeat End | Repeat Left | Name | Family | Class |
| 127086457 | 127086616 | + | 20.6 | 4 | 152 | -57 | MER3 | MER1\_type | DNA |
| 127086617 | 127086911 | - | 11.9 | 1 | 296 | -16 | AluSx | Alu | SINE |
| 127086912 | 127086976 | + | 20.6 | 153 | 209 | 0 | MER3 | MER1\_type | DNA |

  

|  |  |  |  |  |  |  |  |  |  |
| --- | --- | --- | --- | --- | --- | --- | --- | --- | --- |
| chr3:127101938-127102490 | | | 553 bp | | |  |  |  |  |
| Genome Start | Genome End | Strand | Div | Repeat Start | Repeat End | Repeat Left | Name | Family | Class |
| 127101938 | 127102081 | + | 16.4 | 1 | 137 | -175 | AluJo | Alu | SINE |
| 127102082 | 127102381 | + | 16.7 | 1 | 300 | -12 | AluSx | Alu | SINE |
| 127102382 | 127102490 | + | 16.4 | 138 | 248 | -64 | AluJo | Alu | SINE |

  

|  |  |  |  |  |  |  |  |  |  |
| --- | --- | --- | --- | --- | --- | --- | --- | --- | --- |
| chr3:127106770-127107653 | | | 847 bp | | |  |  |  |  |
| Genome Start | Genome End | Strand | Div | Repeat Start | Repeat End | Repeat Left | Name | Family | Class |
| 127106770 | 127107035 | + | 20.6 | 5651 | 5915 | -269 | L1MB7 | L1 | LINE |
| 127107073 | 127107284 | + | 20.6 | 5916 | 6117 | -67 | L1MB7 | L1 | LINE |
| 127107285 | 127107583 | - | 16.5 | 1 | 298 | -14 | AluJb | Alu | SINE |
| 127107584 | 127107653 | + | 20.6 | 6118 | 6184 | 0 | L1MB7 | L1 | LINE |

  

|  |  |  |  |  |  |  |  |  |  |
| --- | --- | --- | --- | --- | --- | --- | --- | --- | --- |
| chr3:127107665-127108240 | | | 576 bp | | |  |  |  |  |
| Genome Start | Genome End | Strand | Div | Repeat Start | Repeat End | Repeat Left | Name | Family | Class |
| 127107665 | 127107799 | + | 18.9 | 1 | 136 | -176 | AluJb | Alu | SINE |
| 127107800 | 127108087 | + | 11.1 | 1 | 299 | -13 | AluSx | Alu | SINE |
| 127108088 | 127108240 | + | 18.9 | 137 | 286 | -26 | AluJb | Alu | SINE |

  

|  |  |  |  |  |  |  |  |  |  |
| --- | --- | --- | --- | --- | --- | --- | --- | --- | --- |
| chr3:127113352-127114048 | | | 697 bp | | |  |  |  |  |
| Genome Start | Genome End | Strand | Div | Repeat Start | Repeat End | Repeat Left | Name | Family | Class |
| 127113352 | 127113587 | - | 21.3 | 184 | 422 | -45 | MLT1C | MaLR | LTR |
| 127113588 | 127113895 | - | 13.4 | 1 | 307 | -5 | AluSx | Alu | SINE |
| 127113896 | 127114048 | - | 21.3 | 41 | 183 | -284 | MLT1C | MaLR | LTR |

  

|  |  |  |  |  |  |  |  |  |  |
| --- | --- | --- | --- | --- | --- | --- | --- | --- | --- |
| chr3:130539480-130539974 | | | 495 bp | | |  |  |  |  |
| Genome Start | Genome End | Strand | Div | Repeat Start | Repeat End | Repeat Left | Name | Family | Class |
| 130539480 | 130539538 | + | 29.4 | 3045 | 3110 | -309 | L2 | L2 | LINE |
| 130539539 | 130539882 | + | 8.7 | 1 | 355 | 0 | THE1A | MaLR | LTR |
| 130539883 | 130539974 | + | 29.4 | 3111 | 3211 | -208 | L2 | L2 | LINE |

  

|  |  |  |  |  |  |  |  |  |  |
| --- | --- | --- | --- | --- | --- | --- | --- | --- | --- |
| chr3:131209498-131210672 | | | 1175 bp | | |  |  |  |  |
| Genome Start | Genome End | Strand | Div | Repeat Start | Repeat End | Repeat Left | Name | Family | Class |
| 131209498 | 131209662 | - | 32.5 | 3194 | 3359 | -60 | L2 | L2 | LINE |
| 131209663 | 131209977 | + | 12.8 | 1 | 313 | 0 | AluSq | Alu | SINE |
| 131209978 | 131210672 | - | 29.2 | 2348 | 3193 | -226 | L2 | L2 | LINE |

  

|  |  |  |  |  |  |  |  |  |  |
| --- | --- | --- | --- | --- | --- | --- | --- | --- | --- |
| chr3:131213665-131214586 | | | 717 bp | | |  |  |  |  |
| Genome Start | Genome End | Strand | Div | Repeat Start | Repeat End | Repeat Left | Name | Family | Class |
| 131213665 | 131213790 | + | 29.0 | 2424 | 2552 | -3594 | L1M5 | L1 | LINE |
| 131213800 | 131213987 | + | 21.3 | 2 | 211 | -198 | MLT1J1 | MaLR | LTR |
| 131214000 | 131214181 | + | 28.8 | 240 | 448 | -2 | MLT1J2 | MaLR | LTR |
| 131214366 | 131214586 | + | 25.5 | 2718 | 2945 | -3201 | L1M5 | L1 | LINE |

  

|  |  |  |  |  |  |  |  |  |  |
| --- | --- | --- | --- | --- | --- | --- | --- | --- | --- |
| chr3:131218634-131220593 | | | 1960 bp | | |  |  |  |  |
| Genome Start | Genome End | Strand | Div | Repeat Start | Repeat End | Repeat Left | Name | Family | Class |
| 131218634 | 131218885 | - | 23.6 | 5909 | 6153 | -30 | L1MB3 | L1 | LINE |
| 131218886 | 131219168 | - | 13.8 | 3 | 295 | -17 | AluSx | Alu | SINE |
| 131219169 | 131219285 | - | 23.6 | 5825 | 5908 | -275 | L1MB3 | L1 | LINE |
| 131219286 | 131219605 | - | 14.5 | 1 | 312 | 0 | AluSx | Alu | SINE |
| 131219606 | 131220593 | - | 24.4 | 4818 | 5824 | -359 | L1MB3 | L1 | LINE |

  

|  |  |  |  |  |  |  |  |  |  |
| --- | --- | --- | --- | --- | --- | --- | --- | --- | --- |
| chr3:131221025-131221695 | | | 671 bp | | |  |  |  |  |
| Genome Start | Genome End | Strand | Div | Repeat Start | Repeat End | Repeat Left | Name | Family | Class |
| 131221025 | 131221231 | - | 26.3 | 5934 | 6130 | -170 | L1MA4 | L1 | LINE |
| 131221232 | 131221512 | - | 15.7 | 1 | 280 | -32 | AluSx | Alu | SINE |
| 131221513 | 131221695 | - | 26.3 | 5767 | 5933 | -367 | L1MA4 | L1 | LINE |

  

|  |  |  |  |  |  |  |  |  |  |
| --- | --- | --- | --- | --- | --- | --- | --- | --- | --- |
| chr3:131241347-131241984 | | | 638 bp | | |  |  |  |  |
| Genome Start | Genome End | Strand | Div | Repeat Start | Repeat End | Repeat Left | Name | Family | Class |
| 131241347 | 131241598 | - | 22.9 | 5907 | 6153 | -30 | L1MB3 | L1 | LINE |
| 131241599 | 131241892 | - | 14.6 | 7 | 300 | -12 | AluSx | Alu | SINE |
| 131241893 | 131241984 | - | 22.9 | 5822 | 5906 | -277 | L1MB3 | L1 | LINE |

  

|  |  |  |  |  |  |  |  |  |  |
| --- | --- | --- | --- | --- | --- | --- | --- | --- | --- |
| chr3:131252774-131253488 | | | 715 bp | | |  |  |  |  |
| Genome Start | Genome End | Strand | Div | Repeat Start | Repeat End | Repeat Left | Name | Family | Class |
| 131252774 | 131252831 | + | 21.6 | 5769 | 5826 | -352 | L1MB8 | L1 | LINE |
| 131252832 | 131253127 | + | 12.6 | 1 | 295 | -17 | AluSx | Alu | SINE |
| 131253128 | 131253488 | + | 21.6 | 5827 | 6174 | -4 | L1MB8 | L1 | LINE |

  

|  |  |  |  |  |  |  |  |  |  |
| --- | --- | --- | --- | --- | --- | --- | --- | --- | --- |
| chr3:131258779-131260180 | | | 1402 bp | | |  |  |  |  |
| Genome Start | Genome End | Strand | Div | Repeat Start | Repeat End | Repeat Left | Name | Family | Class |
| 131258779 | 131258821 | + | 22.7 | 1 | 50 | -417 | MLT1C | MaLR | LTR |
| 131258822 | 131259798 | + | 10.4 | 1 | 968 | 0 | LTR5\_Hs | ERVK | LTR |
| 131259799 | 131260180 | + | 21.3 | 51 | 505 | 0 | MLT1C | MaLR | LTR |

  

|  |  |  |  |  |  |  |  |  |  |
| --- | --- | --- | --- | --- | --- | --- | --- | --- | --- |
| chr3:131267971-131268947 | | | 874 bp | | |  |  |  |  |
| Genome Start | Genome End | Strand | Div | Repeat Start | Repeat End | Repeat Left | Name | Family | Class |
| 131267971 | 131268485 | + | 14.7 | 5783 | 6291 | -42 | L1MC1 | L1 | LINE |
| 131268589 | 131268890 | - | 14.3 | 1 | 310 | 0 | AluSg | Alu | SINE |
| 131268891 | 131268947 | + | 17.5 | 6276 | 6332 | -1 | L1MC1 | L1 | LINE |

  

|  |  |  |  |  |  |  |  |  |  |
| --- | --- | --- | --- | --- | --- | --- | --- | --- | --- |
| chr3:131283758-131285381 | | | 1624 bp | | |  |  |  |  |
| Genome Start | Genome End | Strand | Div | Repeat Start | Repeat End | Repeat Left | Name | Family | Class |
| 131283758 | 131283878 | + | 21.6 | 11 | 127 | -913 | MER45B | Tip100 | DNA |
| 131283879 | 131284170 | - | 13.0 | 1 | 296 | -16 | AluSx | Alu | SINE |
| 131284171 | 131284696 | + | 21.6 | 128 | 658 | -382 | MER45B | Tip100 | DNA |
| 131284697 | 131285008 | + | 12.3 | 1 | 310 | 0 | AluSg | Alu | SINE |
| 131285009 | 131285381 | + | 21.6 | 659 | 1040 | 0 | MER45B | Tip100 | DNA |

  

|  |  |  |  |  |  |  |  |  |  |
| --- | --- | --- | --- | --- | --- | --- | --- | --- | --- |
| chr3:131289796-131290461 | | | 666 bp | | |  |  |  |  |
| Genome Start | Genome End | Strand | Div | Repeat Start | Repeat End | Repeat Left | Name | Family | Class |
| 131289796 | 131289950 | + | 11.2 | 1 | 161 | -203 | THE1B | MaLR | LTR |
| 131289951 | 131290250 | - | 16.0 | 1 | 300 | -12 | AluSx | Alu | SINE |
| 131290251 | 131290461 | + | 11.2 | 162 | 364 | 0 | THE1B | MaLR | LTR |

  

|  |  |  |  |  |  |  |  |  |  |
| --- | --- | --- | --- | --- | --- | --- | --- | --- | --- |
| chr3:131322286-131322788 | | | 503 bp | | |  |  |  |  |
| Genome Start | Genome End | Strand | Div | Repeat Start | Repeat End | Repeat Left | Name | Family | Class |
| 131322286 | 131322353 | - | 19.8 | 149 | 209 | 0 | MER3 | MER1\_type | DNA |
| 131322354 | 131322641 | + | 13.5 | 1 | 291 | -21 | AluSx | Alu | SINE |
| 131322642 | 131322788 | - | 19.8 | 8 | 148 | -61 | MER3 | MER1\_type | DNA |

  

|  |  |  |  |  |  |  |  |  |  |
| --- | --- | --- | --- | --- | --- | --- | --- | --- | --- |
| chr3:131327441-131328802 | | | 1336 bp | | |  |  |  |  |
| Genome Start | Genome End | Strand | Div | Repeat Start | Repeat End | Repeat Left | Name | Family | Class |
| 131327441 | 131327740 | - | 19.3 | 38 | 312 | 0 | AluJo | Alu | SINE |
| 131327741 | 131328037 | - | 13.4 | 474 | 785 | 0 | LTR1 | ERV1 | LTR |
| 131328038 | 131328267 | - | 6.5 | 16 | 244 | -67 | AluY | Alu | SINE |
| 131328268 | 131328713 | - | 13.4 | 3 | 473 | -312 | LTR1 | ERV1 | LTR |
| 131328740 | 131328802 | - | 25.4 | 1 | 63 | -239 | AluJo | Alu | SINE |

  

|  |  |  |  |  |  |  |  |  |  |
| --- | --- | --- | --- | --- | --- | --- | --- | --- | --- |
| chr3:131329876-131330582 | | | 707 bp | | |  |  |  |  |
| Genome Start | Genome End | Strand | Div | Repeat Start | Repeat End | Repeat Left | Name | Family | Class |
| 131329876 | 131330211 | - | 23.2 | 90 | 451 | -16 | MLT1C | MaLR | LTR |
| 131330212 | 131330510 | - | 14.7 | 1 | 301 | -11 | AluSx | Alu | SINE |
| 131330511 | 131330582 | - | 23.2 | 6 | 89 | -378 | MLT1C | MaLR | LTR |

  

|  |  |  |  |  |  |  |  |  |  |
| --- | --- | --- | --- | --- | --- | --- | --- | --- | --- |
| chr3:131331173-131331614 | | | 442 bp | | |  |  |  |  |
| Genome Start | Genome End | Strand | Div | Repeat Start | Repeat End | Repeat Left | Name | Family | Class |
| 131331173 | 131331198 | - | 24.2 | 116 | 135 | -43 | MER5B | MER1\_type | DNA |
| 131331199 | 131331495 | + | 15.8 | 3 | 299 | -13 | AluJo | Alu | SINE |
| 131331496 | 131331614 | - | 24.2 | 3 | 115 | -63 | MER5B | MER1\_type | DNA |

  

|  |  |  |  |  |  |  |  |  |  |
| --- | --- | --- | --- | --- | --- | --- | --- | --- | --- |
| chr3:131332357-131332978 | | | 622 bp | | |  |  |  |  |
| Genome Start | Genome End | Strand | Div | Repeat Start | Repeat End | Repeat Left | Name | Family | Class |
| 131332357 | 131332502 | + | 15.1 | 1 | 144 | -168 | AluSx | Alu | SINE |
| 131332503 | 131332800 | + | 7.8 | 1 | 297 | -14 | AluY | Alu | SINE |
| 131332801 | 131332978 | + | 15.1 | 145 | 312 | 0 | AluSx | Alu | SINE |

  

|  |  |  |  |  |  |  |  |  |  |
| --- | --- | --- | --- | --- | --- | --- | --- | --- | --- |
| chr3:131333804-131334036 | | | 233 bp | | |  |  |  |  |
| Genome Start | Genome End | Strand | Div | Repeat Start | Repeat End | Repeat Left | Name | Family | Class |
| 131333804 | 131333846 | + | 29.8 | 4 | 41 | -137 | MER5B | MER1\_type | DNA |
| 131333847 | 131333979 | - | 16.5 | 1 | 133 | 0 | FLAM\_A | Alu | SINE |
| 131333980 | 131334036 | + | 29.8 | 42 | 97 | -81 | MER5B | MER1\_type | DNA |

  

|  |  |  |  |  |  |  |  |  |  |
| --- | --- | --- | --- | --- | --- | --- | --- | --- | --- |
| chr3:131338681-131354804 | | | 16093 bp | | |  |  |  |  |
| Genome Start | Genome End | Strand | Div | Repeat Start | Repeat End | Repeat Left | Name | Family | Class |
| 131338681 | 131338861 | + | 24.0 | 1 | 185 | -320 | MLT1D | MaLR | LTR |
| 131338872 | 131339161 | + | 10.7 | 3 | 293 | -20 | AluSp | Alu | SINE |
| 131339174 | 131339571 | - | 10.3 | 2 | 448 | -2 | LTR7 | ERV1 | LTR |
| 131339573 | 131339948 | - | 8.9 | 7323 | 7713 | 0 | HERVH | ERV1 | LTR |
| 131339949 | 131340154 | - | 11.7 | 5600 | 5818 | -1895 | HERVH | ERV1 | LTR |
| 131340156 | 131340221 | - | 15.0 | 5090 | 5149 | -2564 | HERVH | ERV1 | LTR |
| 131340222 | 131341289 | - | 4.3 | 1 | 1071 | 0 | MER11C | ERVK | LTR |
| 131341290 | 131341892 | - | 15.0 | 4488 | 5089 | -2624 | HERVH | ERV1 | LTR |
| 131341895 | 131342444 | - | 9.1 | 3442 | 3993 | -3720 | HERVH | ERV1 | LTR |
| 131342445 | 131342941 | - | 8.9 | 1 | 501 | 0 | LTR2C | ERV1 | LTR |
| 131342942 | 131350763 | - | 6.6 | 3 | 7811 | -2 | HERVE | ERV1 | LTR |
| 131350764 | 131351067 | - | 11.4 | 3140 | 3441 | -4272 | HERVH | ERV1 | LTR |
| 131351054 | 131353871 | - | 10.2 | 1 | 2992 | -4721 | HERVH | ERV1 | LTR |
| 131353874 | 131354223 | - | 9.8 | 1 | 448 | -2 | LTR7 | ERV1 | LTR |
| 131354241 | 131354434 | + | 25.1 | 193 | 430 | -74 | MLT1D | MaLR | LTR |
| 131354435 | 131354739 | - | 15.5 | 1 | 303 | -9 | AluSx | Alu | SINE |
| 131354740 | 131354804 | + | 25.2 | 394 | 484 | 0 | MLT1D | MaLR | LTR |

  

|  |  |  |  |  |  |  |  |  |  |
| --- | --- | --- | --- | --- | --- | --- | --- | --- | --- |
| chr3:131358963-131359772 | | | 810 bp | | |  |  |  |  |
| Genome Start | Genome End | Strand | Div | Repeat Start | Repeat End | Repeat Left | Name | Family | Class |
| 131358963 | 131359055 | + | 21.5 | 3 | 88 | -379 | MLT1C | MaLR | LTR |
| 131359056 | 131359355 | + | 9.7 | 1 | 301 | -12 | AluSp | Alu | SINE |
| 131359356 | 131359772 | + | 21.5 | 89 | 467 | 0 | MLT1C | MaLR | LTR |

  

|  |  |  |  |  |  |  |  |  |  |
| --- | --- | --- | --- | --- | --- | --- | --- | --- | --- |
| chr3:131360365-131361205 | | | 841 bp | | |  |  |  |  |
| Genome Start | Genome End | Strand | Div | Repeat Start | Repeat End | Repeat Left | Name | Family | Class |
| 131360365 | 131360631 | + | 20.3 | 3 | 320 | -334 | MLT1E1 | MaLR | LTR |
| 131360632 | 131360934 | - | 11.2 | 1 | 312 | 0 | AluSx | Alu | SINE |
| 131360935 | 131361205 | + | 20.3 | 321 | 653 | -1 | MLT1E1 | MaLR | LTR |

  

|  |  |  |  |  |  |  |  |  |  |
| --- | --- | --- | --- | --- | --- | --- | --- | --- | --- |
| chr3:131373905-131375000 | | | 937 bp | | |  |  |  |  |
| Genome Start | Genome End | Strand | Div | Repeat Start | Repeat End | Repeat Left | Name | Family | Class |
| 131373905 | 131373966 | - | 19.7 | 521 | 585 | -6 | MLT1K | MaLR | LTR |
| 131373998 | 131374301 | - | 28.9 | 164 | 474 | -17 | LTR16C | ERVL | LTR |
| 131374307 | 131374612 | - | 13.1 | 1 | 308 | -2 | AluSg | Alu | SINE |
| 131374736 | 131375000 | - | 28.4 | 176 | 429 | -162 | MLT1K | MaLR | LTR |

  

|  |  |  |  |  |  |  |  |  |  |
| --- | --- | --- | --- | --- | --- | --- | --- | --- | --- |
| chr3:131375740-131377043 | | | 1304 bp | | |  |  |  |  |
| Genome Start | Genome End | Strand | Div | Repeat Start | Repeat End | Repeat Left | Name | Family | Class |
| 131375740 | 131375911 | + | 26.7 | 1 | 193 | -356 | MLT1H | MaLR | LTR |
| 131375912 | 131375954 | + | 11.3 | 2 | 44 | -428 | MER4A1 | ERV1 | LTR |
| 131375955 | 131376237 | + | 10.3 | 1 | 282 | -30 | AluSx | Alu | SINE |
| 131376238 | 131376682 | + | 11.3 | 45 | 470 | -2 | MER4A1 | ERV1 | LTR |
| 131376683 | 131377043 | + | 26.7 | 194 | 549 | 0 | MLT1H | MaLR | LTR |

  

|  |  |  |  |  |  |  |  |  |  |
| --- | --- | --- | --- | --- | --- | --- | --- | --- | --- |
| chr3:131385845-131386471 | | | 627 bp | | |  |  |  |  |
| Genome Start | Genome End | Strand | Div | Repeat Start | Repeat End | Repeat Left | Name | Family | Class |
| 131385845 | 131385957 | - | 20.0 | 116 | 221 | -3 | MER58A | MER1\_type | DNA |
| 131385958 | 131386354 | + | 12.3 | 1 | 428 | 0 | MSTA | MaLR | LTR |
| 131386355 | 131386471 | - | 20.0 | 1 | 115 | 0 | MER58A | MER1\_type | DNA |

  

|  |  |  |  |  |  |  |  |  |  |
| --- | --- | --- | --- | --- | --- | --- | --- | --- | --- |
| chr3:131387783-131390245 | | | 2439 bp | | |  |  |  |  |
| Genome Start | Genome End | Strand | Div | Repeat Start | Repeat End | Repeat Left | Name | Family | Class |
| 131387783 | 131388013 | + | 30.8 | 2233 | 2467 | -952 | L2 | L2 | LINE |
| 131388014 | 131388334 | + | 20.1 | 1 | 304 | -8 | AluJo | Alu | SINE |
| 131388335 | 131388999 | + | 28.7 | 2468 | 3210 | -209 | L2 | L2 | LINE |
| 131389000 | 131390083 | + | 6.5 | 1 | 1266 | 0 | MER11A | ERVK | LTR |
| 131390108 | 131390245 | + | 27.4 | 3243 | 3378 | 0 | L2 | L2 | LINE |

  

|  |  |  |  |  |  |  |  |  |  |
| --- | --- | --- | --- | --- | --- | --- | --- | --- | --- |
| chr3:131390578-131391210 | | | 633 bp | | |  |  |  |  |
| Genome Start | Genome End | Strand | Div | Repeat Start | Repeat End | Repeat Left | Name | Family | Class |
| 131390578 | 131390652 | - | 25.0 | 340 | 407 | -2 | MLT1I | MaLR | LTR |
| 131390653 | 131390954 | - | 14.0 | 1 | 308 | 0 | AluSx | Alu | SINE |
| 131390955 | 131391210 | - | 25.0 | 87 | 339 | -70 | MLT1I | MaLR | LTR |

  

|  |  |  |  |  |  |  |  |  |  |
| --- | --- | --- | --- | --- | --- | --- | --- | --- | --- |
| chr3:131398770-131400082 | | | 1224 bp | | |  |  |  |  |
| Genome Start | Genome End | Strand | Div | Repeat Start | Repeat End | Repeat Left | Name | Family | Class |
| 131398770 | 131399051 | + | 28.7 | 2563 | 2860 | -559 | L2 | L2 | LINE |
| 131399084 | 131399391 | + | 8.2 | 1 | 307 | -4 | AluY | Alu | SINE |
| 131399408 | 131399498 | - | 26.4 | 95 | 206 | -62 | MIRm | MIR | SINE |
| 131399540 | 131399641 | + | 34.0 | 2910 | 3012 | -366 | L2 | L2 | LINE |
| 131399642 | 131399932 | + | 13.1 | 1 | 291 | -22 | AluSq | Alu | SINE |
| 131399933 | 131400082 | + | 34.0 | 3013 | 3153 | -225 | L2 | L2 | LINE |

  

|  |  |  |  |  |  |  |  |  |  |
| --- | --- | --- | --- | --- | --- | --- | --- | --- | --- |
| chr3:131400086-131400840 | | | 751 bp | | |  |  |  |  |
| Genome Start | Genome End | Strand | Div | Repeat Start | Repeat End | Repeat Left | Name | Family | Class |
| 131400086 | 131400289 | - | 22.7 | 5974 | 6178 | 0 | L1MB8 | L1 | LINE |
| 131400293 | 131400463 | - | 8.8 | 106 | 289 | -23 | AluSg/x | Alu | SINE |
| 131400465 | 131400840 | - | 17.6 | 5575 | 5960 | -218 | L1MB8 | L1 | LINE |

  

|  |  |  |  |  |  |  |  |  |  |
| --- | --- | --- | --- | --- | --- | --- | --- | --- | --- |
| chr3:157911993-157912704 | | | 712 bp | | |  |  |  |  |
| Genome Start | Genome End | Strand | Div | Repeat Start | Repeat End | Repeat Left | Name | Family | Class |
| 157911993 | 157912248 | - | 18.4 | 179 | 466 | -1 | MLT1C | MaLR | LTR |
| 157912249 | 157912537 | - | 12.2 | 1 | 306 | -6 | AluSx | Alu | SINE |
| 157912538 | 157912704 | - | 18.4 | 6 | 178 | -289 | MLT1C | MaLR | LTR |

  

|  |  |  |  |  |  |  |  |  |  |
| --- | --- | --- | --- | --- | --- | --- | --- | --- | --- |
| chr3:180415733-180417310 | | | 1561 bp | | |  |  |  |  |
| Genome Start | Genome End | Strand | Div | Repeat Start | Repeat End | Repeat Left | Name | Family | Class |
| 180415733 | 180416349 | + | 16.7 | 46 | 660 | -898 | Looper | PiggyBac | DNA |
| 180416350 | 180416576 | - | 17.1 | 2 | 219 | 0 | MER20 | MER1\_type | DNA |
| 180416577 | 180417143 | + | 16.7 | 661 | 1187 | -371 | Looper | PiggyBac | DNA |
| 180417161 | 180417310 | + | 13.4 | 1340 | 1500 | -58 | Looper | PiggyBac | DNA |

  

|  |  |  |  |  |  |  |  |  |  |
| --- | --- | --- | --- | --- | --- | --- | --- | --- | --- |
| chr3:196834330-196835376 | | | 857 bp | | |  |  |  |  |
| Genome Start | Genome End | Strand | Div | Repeat Start | Repeat End | Repeat Left | Name | Family | Class |
| 196834330 | 196834460 | - | 27.4 | 1075 | 1207 | -4912 | L1MEd | L1 | LINE |
| 196834471 | 196834629 | - | 22.6 | 192 | 377 | -13 | MLT1B | MaLR | LTR |
| 196834737 | 196834834 | - | 21.6 | 76 | 173 | -217 | MLT1B | MaLR | LTR |
| 196834865 | 196835191 | + | 18.1 | 7 | 354 | -11 | MLT1A0 | MaLR | LTR |
| 196835235 | 196835376 | - | 28.8 | 945 | 1089 | -5030 | L1MEd | L1 | LINE |

  

|  |  |  |  |  |  |  |  |  |  |
| --- | --- | --- | --- | --- | --- | --- | --- | --- | --- |
| chr3:197168803-197169249 | | | 447 bp | | |  |  |  |  |
| Genome Start | Genome End | Strand | Div | Repeat Start | Repeat End | Repeat Left | Name | Family | Class |
| 197168803 | 197168825 | - | 27.3 | 7167 | 7189 | -772 | L1MC5 | L1 | LINE |
| 197168826 | 197169046 | - | 10.9 | 86 | 308 | -5 | AluSq | Alu | SINE |
| 197169047 | 197169249 | - | 27.3 | 6960 | 7166 | -795 | L1MC5 | L1 | LINE |

  

|  |  |  |  |  |  |  |  |  |  |
| --- | --- | --- | --- | --- | --- | --- | --- | --- | --- |
| chr3:197174757-197175379 | | | 623 bp | | |  |  |  |  |
| Genome Start | Genome End | Strand | Div | Repeat Start | Repeat End | Repeat Left | Name | Family | Class |
| 197174757 | 197175051 | + | 33.8 | 5656 | 5947 | -174 | L1ME4a | L1 | LINE |
| 197175052 | 197175264 | + | 18.3 | 2 | 232 | -4 | MER46A | MER2\_type | DNA |
| 197175265 | 197175379 | + | 33.8 | 5948 | 6065 | -56 | L1ME4a | L1 | LINE |

  

|  |  |  |  |  |  |  |  |  |  |
| --- | --- | --- | --- | --- | --- | --- | --- | --- | --- |
| chr3:197206428-197207385 | | | 766 bp | | |  |  |  |  |
| Genome Start | Genome End | Strand | Div | Repeat Start | Repeat End | Repeat Left | Name | Family | Class |
| 197206428 | 197206644 | + | 25.7 | 5296 | 5539 | -623 | L1ME3B | L1 | LINE |
| 197206764 | 197206983 | + | 33.5 | 5650 | 5896 | -274 | L1ME3B | L1 | LINE |
| 197207052 | 197207102 | + | 17.6 | 248 | 298 | -4 | Alu | Alu | SINE |
| 197207108 | 197207385 | + | 32.5 | 5944 | 6219 | -21 | L1ME3B | L1 | LINE |

  

|  |  |  |  |  |  |  |  |  |  |
| --- | --- | --- | --- | --- | --- | --- | --- | --- | --- |
| chr3:198859220-198859775 | | | 556 bp | | |  |  |  |  |
| Genome Start | Genome End | Strand | Div | Repeat Start | Repeat End | Repeat Left | Name | Family | Class |
| 198859220 | 198859252 | + | 28.9 | 3183 | 3214 | -205 | L2 | L2 | LINE |
| 198859253 | 198859567 | - | 15.4 | 1 | 312 | 0 | AluJb | Alu | SINE |
| 198859568 | 198859775 | + | 28.9 | 3215 | 3418 | -1 | L2 | L2 | LINE |

  

|  |  |  |  |  |  |  |  |  |  |
| --- | --- | --- | --- | --- | --- | --- | --- | --- | --- |
| chr3:199341175-199343812 | | | 2638 bp | | |  |  |  |  |
| Genome Start | Genome End | Strand | Div | Repeat Start | Repeat End | Repeat Left | Name | Family | Class |
| 199341175 | 199341433 | - | 5.8 | 5895 | 6149 | -5 | L1PA7 | L1 | LINE |
| 199341434 | 199341746 | - | 14.4 | 1 | 312 | -1 | AluSp | Alu | SINE |
| 199341747 | 199343812 | - | 7.8 | 3784 | 5894 | -260 | L1PA7 | L1 | LINE |

  

|  |  |  |  |  |  |  |  |  |  |
| --- | --- | --- | --- | --- | --- | --- | --- | --- | --- |
| chr3:199369909-199370830 | | | 925 bp | | |  |  |  |  |
| Genome Start | Genome End | Strand | Div | Repeat Start | Repeat End | Repeat Left | Name | Family | Class |
| 199369909 | 199370132 | - | 19.3 | 2799 | 3028 | -1 | Tigger3(Golem) | MER2\_type | DNA |
| 199370133 | 199370713 | - | 7.9 | 3 | 600 | 0 | MER4A1\_ | ERV1 | LTR |
| 199370714 | 199370737 | - | 19.3 | 2772 | 2798 | -231 | Tigger3(Golem) | MER2\_type | DNA |
| 199370735 | 199370830 | - | 15.9 | 40 | 142 | -2887 | Tigger3(Golem) | MER2\_type | DNA |

  

|  |  |  |  |  |  |  |  |  |  |
| --- | --- | --- | --- | --- | --- | --- | --- | --- | --- |
| chr3:199373435-199375643 | | | 2190 bp | | |  |  |  |  |
| Genome Start | Genome End | Strand | Div | Repeat Start | Repeat End | Repeat Left | Name | Family | Class |
| 199373435 | 199373572 | - | 19.6 | 7658 | 7785 | 0 | L1MC3 | L1 | LINE |
| 199373573 | 199373870 | - | 8.1 | 1 | 307 | -6 | AluSq | Alu | SINE |
| 199373871 | 199374192 | - | 19.6 | 7324 | 7657 | -128 | L1MC3 | L1 | LINE |
| 199374193 | 199374808 | - | 11.0 | 1 | 900 | 0 | MER4B | ERV1 | LTR |
| 199374828 | 199375643 | - | 22.4 | 6432 | 7295 | -490 | L1MC3 | L1 | LINE |

  

|  |  |  |  |  |  |  |  |  |  |
| --- | --- | --- | --- | --- | --- | --- | --- | --- | --- |
| chr3:199381348-199383917 | | | 2497 bp | | |  |  |  |  |
| Genome Start | Genome End | Strand | Div | Repeat Start | Repeat End | Repeat Left | Name | Family | Class |
| 199381348 | 199381786 | - | 19.9 | 5202 | 5689 | -485 | L1M4 | L1 | LINE |
| 199381806 | 199382402 | + | 26.3 | 19 | 647 | -216 | MER21B | ERV1 | LTR |
| 199382403 | 199382907 | + | 20.7 | 1 | 586 | -25 | LTR36 | ERV1 | LTR |
| 199382926 | 199383086 | + | 21.1 | 648 | 826 | -37 | MER21B | ERV1 | LTR |
| 199383123 | 199383917 | - | 26.0 | 4315 | 5205 | -941 | L1M4 | L1 | LINE |

  

|  |  |  |  |  |  |  |  |  |  |
| --- | --- | --- | --- | --- | --- | --- | --- | --- | --- |
| chr3:199390963-199391503 | | | 541 bp | | |  |  |  |  |
| Genome Start | Genome End | Strand | Div | Repeat Start | Repeat End | Repeat Left | Name | Family | Class |
| 199390963 | 199391064 | - | 13.6 | 137 | 302 | 0 | AluSx | Alu | SINE |
| 199391065 | 199391367 | - | 10.9 | 1 | 304 | -9 | AluSp | Alu | SINE |
| 199391368 | 199391503 | - | 13.6 | 1 | 136 | -166 | AluSx | Alu | SINE |

  

|  |  |  |  |  |  |  |  |  |  |
| --- | --- | --- | --- | --- | --- | --- | --- | --- | --- |
| chr3:199408160-199408505 | | | 346 bp | | |  |  |  |  |
| Genome Start | Genome End | Strand | Div | Repeat Start | Repeat End | Repeat Left | Name | Family | Class |
| 199408160 | 199408282 | - | 31.0 | 5576 | 5690 | -2118 | L1MC4 | L1 | LINE |
| 199408283 | 199408405 | + | 16.5 | 4 | 126 | -7 | FLAM\_C | Alu | SINE |
| 199408406 | 199408505 | - | 31.0 | 5483 | 5575 | -2233 | L1MC4 | L1 | LINE |

  

|  |  |  |  |  |  |  |  |  |  |
| --- | --- | --- | --- | --- | --- | --- | --- | --- | --- |
| chr3:199412010-199413478 | | | 1469 bp | | |  |  |  |  |
| Genome Start | Genome End | Strand | Div | Repeat Start | Repeat End | Repeat Left | Name | Family | Class |
| 199412010 | 199412309 | - | 20.4 | 604 | 934 | -1 | MER21C | ERV1 | LTR |
| 199412310 | 199412615 | - | 17.8 | 1 | 303 | -9 | AluJo | Alu | SINE |
| 199412616 | 199412925 | - | 20.4 | 270 | 603 | -332 | MER21C | ERV1 | LTR |
| 199412926 | 199413219 | + | 14.7 | 1 | 293 | -20 | AluSq | Alu | SINE |
| 199413220 | 199413478 | - | 20.4 | 18 | 269 | -666 | MER21C | ERV1 | LTR |

  

|  |  |  |  |  |  |  |  |  |  |
| --- | --- | --- | --- | --- | --- | --- | --- | --- | --- |
| chr3:199414151-199414728 | | | 578 bp | | |  |  |  |  |
| Genome Start | Genome End | Strand | Div | Repeat Start | Repeat End | Repeat Left | Name | Family | Class |
| 199414151 | 199414278 | + | 19.9 | 10 | 131 | -181 | AluJb | Alu | SINE |
| 199414279 | 199414565 | + | 10.1 | 1 | 297 | -12 | AluSg1 | Alu | SINE |
| 199414566 | 199414728 | + | 19.9 | 132 | 298 | -14 | AluJb | Alu | SINE |

  

|  |  |  |  |  |  |  |  |  |  |
| --- | --- | --- | --- | --- | --- | --- | --- | --- | --- |
| chr3:199418037-199418446 | | | 410 bp | | |  |  |  |  |
| Genome Start | Genome End | Strand | Div | Repeat Start | Repeat End | Repeat Left | Name | Family | Class |
| 199418037 | 199418124 | - | 18.4 | 5503 | 5589 | -577 | L1M5 | L1 | LINE |
| 199418125 | 199418415 | - | 18.7 | 1 | 292 | -20 | AluJb | Alu | SINE |
| 199418416 | 199418446 | - | 17.9 | 5476 | 5506 | -678 | L1M5 | L1 | LINE |

  

|  |  |  |  |  |  |  |  |  |  |
| --- | --- | --- | --- | --- | --- | --- | --- | --- | --- |
| chr3:199418506-199418981 | | | 432 bp | | |  |  |  |  |
| Genome Start | Genome End | Strand | Div | Repeat Start | Repeat End | Repeat Left | Name | Family | Class |
| 199418506 | 199418565 | + | 22.2 | 24 | 71 | -241 | AluJo | Alu | SINE |
| 199418610 | 199418879 | - | 13.8 | 1 | 275 | -37 | AluSx | Alu | SINE |
| 199418880 | 199418981 | + | 22.3 | 75 | 167 | -145 | AluJo | Alu | SINE |

  

|  |  |  |  |  |  |  |  |  |  |
| --- | --- | --- | --- | --- | --- | --- | --- | --- | --- |
| chr3:199423754-199425240 | | | 1487 bp | | |  |  |  |  |
| Genome Start | Genome End | Strand | Div | Repeat Start | Repeat End | Repeat Left | Name | Family | Class |
| 199423754 | 199424054 | - | 21.0 | 604 | 935 | 0 | MER21C | ERV1 | LTR |
| 199424055 | 199424357 | - | 20.5 | 1 | 302 | -10 | AluJo | Alu | SINE |
| 199424358 | 199424427 | - | 21.0 | 526 | 603 | -332 | MER21C | ERV1 | LTR |
| 199424428 | 199424734 | + | 19.5 | 2 | 294 | -18 | AluJb | Alu | SINE |
| 199424735 | 199425240 | - | 21.0 | 18 | 525 | -410 | MER21C | ERV1 | LTR |

  

|  |  |  |  |  |  |  |  |  |  |
| --- | --- | --- | --- | --- | --- | --- | --- | --- | --- |
| chr3:199439666-199440201 | | | 536 bp | | |  |  |  |  |
| Genome Start | Genome End | Strand | Div | Repeat Start | Repeat End | Repeat Left | Name | Family | Class |
| 199439666 | 199439790 | + | 32.3 | 3190 | 3312 | -107 | L2 | L2 | LINE |
| 199439791 | 199440089 | + | 11.4 | 1 | 299 | -14 | AluSq | Alu | SINE |
| 199440090 | 199440201 | + | 32.3 | 3313 | 3418 | -1 | L2 | L2 | LINE |

  


|  |  |  |  |  |  |  |  |  |  |
| --- | --- | --- | --- | --- | --- | --- | --- | --- | --- |
| chr4:1783334-1783915 | | | 582 bp | | |  |  |  |  |
| Genome Start | Genome End | Strand | Div | Repeat Start | Repeat End | Repeat Left | Name | Family | Class |
| 1783334 | 1783461 | + | 8.9 | 1 | 128 | -184 | AluSx | Alu | SINE |
| 1783462 | 1783740 | + | 8.6 | 21 | 298 | -13 | AluY | Alu | SINE |
| 1783741 | 1783915 | + | 8.9 | 129 | 307 | -5 | AluSx | Alu | SINE |

  

|  |  |  |  |  |  |  |  |  |  |
| --- | --- | --- | --- | --- | --- | --- | --- | --- | --- |
| chr4:3519327-3520213 | | | 887 bp | | |  |  |  |  |
| Genome Start | Genome End | Strand | Div | Repeat Start | Repeat End | Repeat Left | Name | Family | Class |
| 3519327 | 3519493 | + | 25.1 | 520 | 699 | -527 | MER69B | Tip100 | DNA |
| 3519494 | 3519778 | - | 17.0 | 1 | 297 | -15 | AluJb | Alu | SINE |
| 3519779 | 3520213 | + | 25.1 | 700 | 2598 | -42 | MER69B | Tip100 | DNA |

  

|  |  |  |  |  |  |  |  |  |  |
| --- | --- | --- | --- | --- | --- | --- | --- | --- | --- |
| chr4:3528707-3529581 | | | 428 bp | | |  |  |  |  |
| Genome Start | Genome End | Strand | Div | Repeat Start | Repeat End | Repeat Left | Name | Family | Class |
| 3528707 | 3528849 | + | 22.5 | 49 | 192 | -181 | MER83C | ERV1 | LTR |
| 3528908 | 3528969 | + | 22.6 | 134 | 197 | -181 | MER83B | ERV1 | LTR |
| 3529359 | 3529581 | + | 24.7 | 130 | 373 | 0 | MER83C | ERV1 | LTR |

  

|  |  |  |  |  |  |  |  |  |  |
| --- | --- | --- | --- | --- | --- | --- | --- | --- | --- |
| chr4:3532706-3533821 | | | 1116 bp | | |  |  |  |  |
| Genome Start | Genome End | Strand | Div | Repeat Start | Repeat End | Repeat Left | Name | Family | Class |
| 3532706 | 3532728 | + | 20.6 | 1 | 22 | -253 | ORSL | Tip100 | DNA |
| 3532729 | 3532865 | - | 12.2 | 468 | 600 | 0 | MER4A1\_ | ERV1 | LTR |
| 3532866 | 3533169 | - | 11.6 | 7 | 309 | 0 | AluSg1 | Alu | SINE |
| 3533170 | 3533644 | - | 12.2 | 1 | 467 | -133 | MER4A1\_ | ERV1 | LTR |
| 3533645 | 3533821 | + | 23.0 | 23 | 235 | -40 | ORSL | Tip100 | DNA |

  

|  |  |  |  |  |  |  |  |  |  |
| --- | --- | --- | --- | --- | --- | --- | --- | --- | --- |
| chr4:3856393-3858342 | | | 1920 bp | | |  |  |  |  |
| Genome Start | Genome End | Strand | Div | Repeat Start | Repeat End | Repeat Left | Name | Family | Class |
| 3856393 | 3856644 | - | 25.2 | 5909 | 6153 | -30 | L1MB3 | L1 | LINE |
| 3856645 | 3856927 | - | 13.1 | 3 | 295 | -17 | AluSx | Alu | SINE |
| 3856928 | 3857017 | - | 25.2 | 5821 | 5908 | -275 | L1MB3 | L1 | LINE |
| 3857048 | 3857342 | - | 17.4 | 7 | 300 | -12 | AluSx | Alu | SINE |
| 3857343 | 3858342 | - | 25.1 | 4818 | 5820 | -363 | L1MB3 | L1 | LINE |

  

|  |  |  |  |  |  |  |  |  |  |
| --- | --- | --- | --- | --- | --- | --- | --- | --- | --- |
| chr4:3858775-3859445 | | | 671 bp | | |  |  |  |  |
| Genome Start | Genome End | Strand | Div | Repeat Start | Repeat End | Repeat Left | Name | Family | Class |
| 3858775 | 3858978 | - | 23.8 | 5934 | 6130 | -170 | L1MA4 | L1 | LINE |
| 3858979 | 3859262 | - | 17.0 | 1 | 283 | -29 | AluSx | Alu | SINE |
| 3859263 | 3859445 | - | 23.8 | 5767 | 5933 | -367 | L1MA4 | L1 | LINE |

  

|  |  |  |  |  |  |  |  |  |  |
| --- | --- | --- | --- | --- | --- | --- | --- | --- | --- |
| chr4:3878374-3879007 | | | 634 bp | | |  |  |  |  |
| Genome Start | Genome End | Strand | Div | Repeat Start | Repeat End | Repeat Left | Name | Family | Class |
| 3878374 | 3878624 | - | 23.9 | 5907 | 6152 | -31 | L1MB3 | L1 | LINE |
| 3878625 | 3878919 | - | 14.6 | 3 | 297 | -15 | AluSx | Alu | SINE |
| 3878920 | 3879007 | - | 23.9 | 5822 | 5906 | -277 | L1MB3 | L1 | LINE |

  

|  |  |  |  |  |  |  |  |  |  |
| --- | --- | --- | --- | --- | --- | --- | --- | --- | --- |
| chr4:3970772-3971511 | | | 740 bp | | |  |  |  |  |
| Genome Start | Genome End | Strand | Div | Repeat Start | Repeat End | Repeat Left | Name | Family | Class |
| 3970772 | 3970857 | + | 23.3 | 5744 | 5827 | -351 | L1MB8 | L1 | LINE |
| 3970858 | 3971150 | + | 13.1 | 6 | 297 | -15 | AluSx | Alu | SINE |
| 3971151 | 3971511 | + | 23.3 | 5828 | 6174 | -4 | L1MB8 | L1 | LINE |

  

|  |  |  |  |  |  |  |  |  |  |
| --- | --- | --- | --- | --- | --- | --- | --- | --- | --- |
| chr4:3978254-3979645 | | | 1392 bp | | |  |  |  |  |
| Genome Start | Genome End | Strand | Div | Repeat Start | Repeat End | Repeat Left | Name | Family | Class |
| 3978254 | 3978296 | + | 23.4 | 1 | 51 | -416 | MLT1C | MaLR | LTR |
| 3978297 | 3979263 | + | 11.3 | 1 | 968 | 0 | LTR5\_Hs | ERVK | LTR |
| 3979264 | 3979645 | + | 23.4 | 52 | 448 | 0 | MLT1C | MaLR | LTR |

  

|  |  |  |  |  |  |  |  |  |  |
| --- | --- | --- | --- | --- | --- | --- | --- | --- | --- |
| chr4:4001250-4001817 | | | 568 bp | | |  |  |  |  |
| Genome Start | Genome End | Strand | Div | Repeat Start | Repeat End | Repeat Left | Name | Family | Class |
| 4001250 | 4001384 | - | 10.2 | 137 | 269 | -43 | AluSx | Alu | SINE |
| 4001385 | 4001684 | - | 11.7 | 1 | 300 | -13 | AluSq | Alu | SINE |
| 4001685 | 4001817 | - | 10.2 | 1 | 136 | -176 | AluSx | Alu | SINE |

  

|  |  |  |  |  |  |  |  |  |  |
| --- | --- | --- | --- | --- | --- | --- | --- | --- | --- |
| chr4:4014873-4015458 | | | 586 bp | | |  |  |  |  |
| Genome Start | Genome End | Strand | Div | Repeat Start | Repeat End | Repeat Left | Name | Family | Class |
| 4014873 | 4015025 | - | 18.9 | 138 | 286 | -26 | AluJb | Alu | SINE |
| 4015026 | 4015318 | - | 12.3 | 6 | 309 | -3 | AluSx | Alu | SINE |
| 4015319 | 4015458 | - | 18.9 | 1 | 137 | -175 | AluJb | Alu | SINE |

  

|  |  |  |  |  |  |  |  |  |  |
| --- | --- | --- | --- | --- | --- | --- | --- | --- | --- |
| chr4:4016423-4018589 | | | 2167 bp | | |  |  |  |  |
| Genome Start | Genome End | Strand | Div | Repeat Start | Repeat End | Repeat Left | Name | Family | Class |
| 4016423 | 4016480 | - | 19.7 | 244 | 298 | -4 | AluJb | Alu | SINE |
| 4016481 | 4016516 | - | 15.2 | 453 | 486 | 0 | MER66B | ERV1 | LTR |
| 4016517 | 4017903 | - | 8.2 | 2 | 1578 | 0 | LTR12C | ERV1 | LTR |
| 4017904 | 4018341 | - | 15.2 | 1 | 452 | -34 | MER66B | ERV1 | LTR |
| 4018342 | 4018589 | - | 16.7 | 1 | 246 | -66 | AluJb | Alu | SINE |

  

|  |  |  |  |  |  |  |  |  |  |
| --- | --- | --- | --- | --- | --- | --- | --- | --- | --- |
| chr4:4020807-4021356 | | | 550 bp | | |  |  |  |  |
| Genome Start | Genome End | Strand | Div | Repeat Start | Repeat End | Repeat Left | Name | Family | Class |
| 4020807 | 4020917 | - | 18.9 | 139 | 249 | -63 | AluJo | Alu | SINE |
| 4020918 | 4021212 | - | 16.9 | 1 | 295 | -17 | AluSx | Alu | SINE |
| 4021213 | 4021356 | - | 18.9 | 2 | 138 | -174 | AluJo | Alu | SINE |

  

|  |  |  |  |  |  |  |  |  |  |
| --- | --- | --- | --- | --- | --- | --- | --- | --- | --- |
| chr4:4027340-4027849 | | | 510 bp | | |  |  |  |  |
| Genome Start | Genome End | Strand | Div | Repeat Start | Repeat End | Repeat Left | Name | Family | Class |
| 4027340 | 4027407 | - | 19.3 | 149 | 209 | 0 | MER3 | MER1\_type | DNA |
| 4027408 | 4027703 | + | 11.5 | 1 | 298 | -14 | AluSx | Alu | SINE |
| 4027704 | 4027849 | - | 19.3 | 8 | 148 | -61 | MER3 | MER1\_type | DNA |

  

|  |  |  |  |  |  |  |  |  |  |
| --- | --- | --- | --- | --- | --- | --- | --- | --- | --- |
| chr4:4041308-4042217 | | | 910 bp | | |  |  |  |  |
| Genome Start | Genome End | Strand | Div | Repeat Start | Repeat End | Repeat Left | Name | Family | Class |
| 4041308 | 4041544 | - | 12.1 | 474 | 725 | -60 | LTR1 | ERV1 | LTR |
| 4041545 | 4041774 | - | 6.1 | 16 | 244 | -67 | AluY | Alu | SINE |
| 4041775 | 4042217 | - | 12.1 | 3 | 473 | -312 | LTR1 | ERV1 | LTR |

  

|  |  |  |  |  |  |  |  |  |  |
| --- | --- | --- | --- | --- | --- | --- | --- | --- | --- |
| chr4:4043370-4044085 | | | 716 bp | | |  |  |  |  |
| Genome Start | Genome End | Strand | Div | Repeat Start | Repeat End | Repeat Left | Name | Family | Class |
| 4043370 | 4043718 | - | 22.1 | 90 | 464 | -3 | MLT1C | MaLR | LTR |
| 4043719 | 4044018 | - | 16.0 | 1 | 301 | -11 | AluSx | Alu | SINE |
| 4044019 | 4044085 | - | 22.1 | 11 | 89 | -378 | MLT1C | MaLR | LTR |

  

|  |  |  |  |  |  |  |  |  |  |
| --- | --- | --- | --- | --- | --- | --- | --- | --- | --- |
| chr4:4044683-4045130 | | | 448 bp | | |  |  |  |  |
| Genome Start | Genome End | Strand | Div | Repeat Start | Repeat End | Repeat Left | Name | Family | Class |
| 4044683 | 4044708 | - | 27.3 | 116 | 135 | -43 | MER5B | MER1\_type | DNA |
| 4044709 | 4045010 | + | 15.2 | 3 | 304 | -8 | AluJo | Alu | SINE |
| 4045011 | 4045130 | - | 27.3 | 3 | 115 | -63 | MER5B | MER1\_type | DNA |

  

|  |  |  |  |  |  |  |  |  |  |
| --- | --- | --- | --- | --- | --- | --- | --- | --- | --- |
| chr4:4047336-4047572 | | | 237 bp | | |  |  |  |  |
| Genome Start | Genome End | Strand | Div | Repeat Start | Repeat End | Repeat Left | Name | Family | Class |
| 4047336 | 4047383 | + | 29.8 | 4 | 49 | -129 | MER5B | MER1\_type | DNA |
| 4047384 | 4047515 | - | 15.2 | 1 | 133 | 0 | FLAM\_A | Alu | SINE |
| 4047516 | 4047572 | + | 29.8 | 50 | 97 | -81 | MER5B | MER1\_type | DNA |

  

|  |  |  |  |  |  |  |  |  |  |
| --- | --- | --- | --- | --- | --- | --- | --- | --- | --- |
| chr4:4052210-4058947 | | | 6685 bp | | |  |  |  |  |
| Genome Start | Genome End | Strand | Div | Repeat Start | Repeat End | Repeat Left | Name | Family | Class |
| 4052210 | 4052395 | + | 23.3 | 1 | 185 | -320 | MLT1D | MaLR | LTR |
| 4052408 | 4052705 | + | 10.7 | 5 | 303 | -10 | AluSp | Alu | SINE |
| 4052715 | 4053113 | - | 10.4 | 2 | 448 | -2 | LTR7 | ERV1 | LTR |
| 4053115 | 4053491 | - | 8.9 | 7324 | 7713 | 0 | HERVH | ERV1 | LTR |
| 4053492 | 4053701 | - | 12.4 | 5600 | 5819 | -1894 | HERVH | ERV1 | LTR |
| 4053703 | 4054364 | - | 15.4 | 4488 | 5149 | -2564 | HERVH | ERV1 | LTR |
| 4054367 | 4055221 | - | 10.3 | 3140 | 3993 | -3720 | HERVH | ERV1 | LTR |
| 4055207 | 4058038 | - | 10.0 | 1 | 2993 | -4720 | HERVH | ERV1 | LTR |
| 4058044 | 4058389 | - | 9.9 | 1 | 447 | -3 | LTR7 | ERV1 | LTR |
| 4058428 | 4058600 | + | 23.0 | 206 | 435 | -69 | MLT1D | MaLR | LTR |
| 4058601 | 4058903 | - | 14.6 | 1 | 302 | -10 | AluSx | Alu | SINE |
| 4058904 | 4058947 | + | 22.2 | 436 | 483 | -22 | MLT1D | MaLR | LTR |

  

|  |  |  |  |  |  |  |  |  |  |
| --- | --- | --- | --- | --- | --- | --- | --- | --- | --- |
| chr4:4063145-4063915 | | | 771 bp | | |  |  |  |  |
| Genome Start | Genome End | Strand | Div | Repeat Start | Repeat End | Repeat Left | Name | Family | Class |
| 4063145 | 4063237 | + | 20.6 | 3 | 88 | -379 | MLT1C | MaLR | LTR |
| 4063238 | 4063539 | + | 8.6 | 1 | 303 | -10 | AluSp | Alu | SINE |
| 4063540 | 4063915 | + | 20.6 | 89 | 467 | 0 | MLT1C | MaLR | LTR |

  

|  |  |  |  |  |  |  |  |  |  |
| --- | --- | --- | --- | --- | --- | --- | --- | --- | --- |
| chr4:4064508-4065359 | | | 852 bp | | |  |  |  |  |
| Genome Start | Genome End | Strand | Div | Repeat Start | Repeat End | Repeat Left | Name | Family | Class |
| 4064508 | 4064782 | + | 20.8 | 3 | 321 | -333 | MLT1E1 | MaLR | LTR |
| 4064783 | 4065088 | - | 9.2 | 1 | 307 | 0 | AluSx | Alu | SINE |
| 4065089 | 4065359 | + | 20.8 | 322 | 654 | 0 | MLT1E1 | MaLR | LTR |

  

|  |  |  |  |  |  |  |  |  |  |
| --- | --- | --- | --- | --- | --- | --- | --- | --- | --- |
| chr4:4078461-4079572 | | | 893 bp | | |  |  |  |  |
| Genome Start | Genome End | Strand | Div | Repeat Start | Repeat End | Repeat Left | Name | Family | Class |
| 4078461 | 4078499 | - | 12.8 | 549 | 590 | -1 | MLT1K | MaLR | LTR |
| 4078562 | 4078855 | - | 28.6 | 170 | 468 | -23 | LTR16C | ERVL | LTR |
| 4078860 | 4079161 | - | 13.6 | 1 | 305 | -5 | AluSg | Alu | SINE |
| 4079315 | 4079572 | - | 29.4 | 183 | 429 | -162 | MLT1K | MaLR | LTR |

  

|  |  |  |  |  |  |  |  |  |  |
| --- | --- | --- | --- | --- | --- | --- | --- | --- | --- |
| chr4:4102186-4102803 | | | 618 bp | | |  |  |  |  |
| Genome Start | Genome End | Strand | Div | Repeat Start | Repeat End | Repeat Left | Name | Family | Class |
| 4102186 | 4102255 | - | 26.1 | 340 | 402 | -7 | MLT1I | MaLR | LTR |
| 4102256 | 4102547 | - | 15.1 | 1 | 299 | -13 | AluSx | Alu | SINE |
| 4102548 | 4102803 | - | 26.1 | 87 | 339 | -70 | MLT1I | MaLR | LTR |

  

|  |  |  |  |  |  |  |  |  |  |
| --- | --- | --- | --- | --- | --- | --- | --- | --- | --- |
| chr4:4108790-4109335 | | | 546 bp | | |  |  |  |  |
| Genome Start | Genome End | Strand | Div | Repeat Start | Repeat End | Repeat Left | Name | Family | Class |
| 4108790 | 4108891 | + | 32.2 | 2910 | 3012 | -407 | L2 | L2 | LINE |
| 4108892 | 4109180 | + | 13.5 | 1 | 289 | -24 | AluSq | Alu | SINE |
| 4109181 | 4109335 | + | 32.2 | 3013 | 3158 | -261 | L2 | L2 | LINE |

  

|  |  |  |  |  |  |  |  |  |  |
| --- | --- | --- | --- | --- | --- | --- | --- | --- | --- |
| chr4:4109336-4110091 | | | 753 bp | | |  |  |  |  |
| Genome Start | Genome End | Strand | Div | Repeat Start | Repeat End | Repeat Left | Name | Family | Class |
| 4109336 | 4109539 | - | 22.6 | 5974 | 6184 | 0 | L1MB7 | L1 | LINE |
| 4109543 | 4109715 | - | 8.7 | 104 | 289 | -23 | AluSg/x | Alu | SINE |
| 4109716 | 4110091 | - | 19.1 | 5575 | 5959 | -219 | L1MB7 | L1 | LINE |

  

|  |  |  |  |  |  |  |  |  |  |
| --- | --- | --- | --- | --- | --- | --- | --- | --- | --- |
| chr4:4130914-4131563 | | | 641 bp | | |  |  |  |  |
| Genome Start | Genome End | Strand | Div | Repeat Start | Repeat End | Repeat Left | Name | Family | Class |
| 4130914 | 4131077 | + | 19.2 | 5331 | 5490 | -688 | L1M4 | L1 | LINE |
| 4131078 | 4131391 | + | 13.1 | 1 | 312 | 0 | AluSx | Alu | SINE |
| 4131401 | 4131563 | + | 19.0 | 5491 | 5652 | -526 | L1M4 | L1 | LINE |

  

|  |  |  |  |  |  |  |  |  |  |
| --- | --- | --- | --- | --- | --- | --- | --- | --- | --- |
| chr4:4133351-4134174 | | | 824 bp | | |  |  |  |  |
| Genome Start | Genome End | Strand | Div | Repeat Start | Repeat End | Repeat Left | Name | Family | Class |
| 4133351 | 4133438 | - | 26.0 | 6079 | 6170 | 0 | L1ME3A | L1 | LINE |
| 4133439 | 4133750 | + | 15.8 | 1 | 303 | -9 | AluJo | Alu | SINE |
| 4133751 | 4133898 | - | 26.0 | 5925 | 6078 | -92 | L1ME3A | L1 | LINE |
| 4133899 | 4134119 | + | 23.4 | 1 | 219 | 0 | MER20 | MER1\_type | DNA |
| 4134120 | 4134174 | - | 26.0 | 5874 | 5924 | -246 | L1ME3A | L1 | LINE |

  

|  |  |  |  |  |  |  |  |  |  |
| --- | --- | --- | --- | --- | --- | --- | --- | --- | --- |
| chr4:4152875-4153699 | | | 825 bp | | |  |  |  |  |
| Genome Start | Genome End | Strand | Div | Repeat Start | Repeat End | Repeat Left | Name | Family | Class |
| 4152875 | 4153050 | + | 12.1 | 5775 | 5950 | -341 | L1MA8 | L1 | LINE |
| 4153051 | 4153361 | + | 14.2 | 1 | 312 | 0 | AluJo | Alu | SINE |
| 4153362 | 4153699 | + | 12.1 | 5951 | 6288 | -3 | L1MA8 | L1 | LINE |

  

|  |  |  |  |  |  |  |  |  |  |
| --- | --- | --- | --- | --- | --- | --- | --- | --- | --- |
| chr4:4161765-4162570 | | | 781 bp | | |  |  |  |  |
| Genome Start | Genome End | Strand | Div | Repeat Start | Repeat End | Repeat Left | Name | Family | Class |
| 4161765 | 4161882 | - | 27.1 | 104 | 224 | -38 | MIR | MIR | SINE |
| 4161903 | 4162505 | + | 19.4 | 1473 | 2104 | -3610 | ERVL-B4 | ERVL | LTR |
| 4162511 | 4162570 | - | 33.3 | 15 | 75 | -187 | MIR | MIR | SINE |

  

|  |  |  |  |  |  |  |  |  |  |
| --- | --- | --- | --- | --- | --- | --- | --- | --- | --- |
| chr4:4165082-4166250 | | | 1169 bp | | |  |  |  |  |
| Genome Start | Genome End | Strand | Div | Repeat Start | Repeat End | Repeat Left | Name | Family | Class |
| 4165082 | 4165264 | - | 33.9 | 3171 | 3335 | -43 | L2 | L2 | LINE |
| 4165265 | 4165563 | + | 13.1 | 1 | 299 | -14 | AluSq | Alu | SINE |
| 4165564 | 4166250 | - | 30.6 | 2348 | 3170 | -208 | L2 | L2 | LINE |

  

|  |  |  |  |  |  |  |  |  |  |
| --- | --- | --- | --- | --- | --- | --- | --- | --- | --- |
| chr4:4207067-4207731 | | | 665 bp | | |  |  |  |  |
| Genome Start | Genome End | Strand | Div | Repeat Start | Repeat End | Repeat Left | Name | Family | Class |
| 4207067 | 4207265 | - | 23.0 | 5934 | 6130 | -170 | L1MA4 | L1 | LINE |
| 4207266 | 4207548 | - | 16.3 | 1 | 283 | -29 | AluSx | Alu | SINE |
| 4207549 | 4207731 | - | 23.0 | 5767 | 5933 | -367 | L1MA4 | L1 | LINE |

  

|  |  |  |  |  |  |  |  |  |  |
| --- | --- | --- | --- | --- | --- | --- | --- | --- | --- |
| chr4:4230974-4232946 | | | 1973 bp | | |  |  |  |  |
| Genome Start | Genome End | Strand | Div | Repeat Start | Repeat End | Repeat Left | Name | Family | Class |
| 4230974 | 4231220 | - | 24.3 | 5908 | 6151 | -32 | L1MB3 | L1 | LINE |
| 4231221 | 4231525 | - | 16.1 | 1 | 305 | -7 | AluSx | Alu | SINE |
| 4231526 | 4231627 | - | 24.3 | 5825 | 5907 | -276 | L1MB3 | L1 | LINE |
| 4231628 | 4231954 | - | 13.0 | 1 | 327 | 0 | AluSx | Alu | SINE |
| 4231955 | 4232946 | - | 24.5 | 4818 | 5824 | -359 | L1MB3 | L1 | LINE |

  

|  |  |  |  |  |  |  |  |  |  |
| --- | --- | --- | --- | --- | --- | --- | --- | --- | --- |
| chr4:4233380-4234069 | | | 690 bp | | |  |  |  |  |
| Genome Start | Genome End | Strand | Div | Repeat Start | Repeat End | Repeat Left | Name | Family | Class |
| 4233380 | 4233605 | - | 21.8 | 5938 | 6130 | -170 | L1MA4 | L1 | LINE |
| 4233606 | 4233886 | - | 18.6 | 1 | 280 | -32 | AluSx | Alu | SINE |
| 4233887 | 4234069 | - | 21.8 | 5767 | 5937 | -363 | L1MA4 | L1 | LINE |

  

|  |  |  |  |  |  |  |  |  |  |
| --- | --- | --- | --- | --- | --- | --- | --- | --- | --- |
| chr4:4239621-4240045 | | | 425 bp | | |  |  |  |  |
| Genome Start | Genome End | Strand | Div | Repeat Start | Repeat End | Repeat Left | Name | Family | Class |
| 4239621 | 4239729 | - | 29.3 | 101 | 207 | -12 | MER20 | MER1\_type | DNA |
| 4239730 | 4239943 | + | 23.4 | 6071 | 6285 | -15 | L1MA4 | L1 | LINE |
| 4239944 | 4240045 | - | 29.3 | 6 | 100 | -119 | MER20 | MER1\_type | DNA |

  

|  |  |  |  |  |  |  |  |  |  |
| --- | --- | --- | --- | --- | --- | --- | --- | --- | --- |
| chr4:4262959-4263632 | | | 674 bp | | |  |  |  |  |
| Genome Start | Genome End | Strand | Div | Repeat Start | Repeat End | Repeat Left | Name | Family | Class |
| 4262959 | 4263176 | - | 18.1 | 159 | 379 | -11 | MLT1B | MaLR | LTR |
| 4263177 | 4263475 | + | 13.0 | 1 | 301 | -12 | AluSq | Alu | SINE |
| 4263476 | 4263632 | - | 18.1 | 1 | 158 | -232 | MLT1B | MaLR | LTR |

  

|  |  |  |  |  |  |  |  |  |  |
| --- | --- | --- | --- | --- | --- | --- | --- | --- | --- |
| chr4:4284786-4285897 | | | 1112 bp | | |  |  |  |  |
| Genome Start | Genome End | Strand | Div | Repeat Start | Repeat End | Repeat Left | Name | Family | Class |
| 4284786 | 4284910 | + | 15.0 | 5719 | 5828 | -463 | L1MA8 | L1 | LINE |
| 4284911 | 4285052 | + | 16.6 | 1 | 133 | -179 | AluJb | Alu | SINE |
| 4285053 | 4285332 | + | 7.5 | 2 | 309 | -4 | AluSp | Alu | SINE |
| 4285333 | 4285502 | + | 16.6 | 134 | 303 | -9 | AluJb | Alu | SINE |
| 4285503 | 4285897 | + | 15.0 | 5829 | 6220 | -71 | L1MA8 | L1 | LINE |

  

|  |  |  |  |  |  |  |  |  |  |
| --- | --- | --- | --- | --- | --- | --- | --- | --- | --- |
| chr4:4287572-4287911 | | | 340 bp | | |  |  |  |  |
| Genome Start | Genome End | Strand | Div | Repeat Start | Repeat End | Repeat Left | Name | Family | Class |
| 4287572 | 4287696 | - | 24.3 | 112 | 257 | -11 | MIRb | MIR | SINE |
| 4287697 | 4287862 | - | 12.7 | 1 | 236 | 0 | MER46A | MER2\_type | DNA |
| 4287863 | 4287911 | - | 24.3 | 58 | 111 | -157 | MIRb | MIR | SINE |

  

|  |  |  |  |  |  |  |  |  |  |
| --- | --- | --- | --- | --- | --- | --- | --- | --- | --- |
| chr4:8680809-8681288 | | | 480 bp | | |  |  |  |  |
| Genome Start | Genome End | Strand | Div | Repeat Start | Repeat End | Repeat Left | Name | Family | Class |
| 8680809 | 8680940 | - | 28.6 | 6590 | 6721 | -1087 | L1MC4a | L1 | LINE |
| 8680941 | 8681250 | + | 12.4 | 1 | 306 | -6 | AluSx | Alu | SINE |
| 8681251 | 8681288 | - | 28.6 | 6555 | 6589 | -1219 | L1MC4a | L1 | LINE |

  

|  |  |  |  |  |  |  |  |  |  |
| --- | --- | --- | --- | --- | --- | --- | --- | --- | --- |
| chr4:8980830-8983329 | | | 2471 bp | | |  |  |  |  |
| Genome Start | Genome End | Strand | Div | Repeat Start | Repeat End | Repeat Left | Name | Family | Class |
| 8980830 | 8982156 | + | 11.5 | 3693 | 5035 | -1111 | L1PA10 | L1 | LINE |
| 8982184 | 8982472 | + | 19.1 | 1 | 288 | -24 | AluSx | Alu | SINE |
| 8982475 | 8983329 | + | 9.0 | 5298 | 6168 | 0 | L1PA10 | L1 | LINE |

  

|  |  |  |  |  |  |  |  |  |  |
| --- | --- | --- | --- | --- | --- | --- | --- | --- | --- |
| chr4:9016348-9018571 | | | 2221 bp | | |  |  |  |  |
| Genome Start | Genome End | Strand | Div | Repeat Start | Repeat End | Repeat Left | Name | Family | Class |
| 9016348 | 9017656 | + | 27.4 | 6 | 1262 | -5587 | L1M2 | L1 | LINE |
| 9017657 | 9017964 | + | 8.4 | 13 | 311 | -2 | AluSp | Alu | SINE |
| 9017965 | 9018461 | + | 27.4 | 1263 | 1777 | -5072 | L1M2 | L1 | LINE |
| 9018465 | 9018571 | + | 18.9 | 1917 | 2022 | -4827 | L1M2 | L1 | LINE |

  

|  |  |  |  |  |  |  |  |  |  |
| --- | --- | --- | --- | --- | --- | --- | --- | --- | --- |
| chr4:9018991-9021073 | | | 2083 bp | | |  |  |  |  |
| Genome Start | Genome End | Strand | Div | Repeat Start | Repeat End | Repeat Left | Name | Family | Class |
| 9018991 | 9019979 | + | 11.4 | 2 | 1005 | -5607 | L1PBa1 | L1 | LINE |
| 9019980 | 9020986 | - | 12.0 | 1 | 1130 | -124 | LTR12D | ERV1 | LTR |
| 9020987 | 9021073 | + | 9.3 | 1003 | 1088 | -5524 | L1PBa1 | L1 | LINE |

  

|  |  |  |  |  |  |  |  |  |  |
| --- | --- | --- | --- | --- | --- | --- | --- | --- | --- |
| chr4:9042951-9043923 | | | 973 bp | | |  |  |  |  |
| Genome Start | Genome End | Strand | Div | Repeat Start | Repeat End | Repeat Left | Name | Family | Class |
| 9042951 | 9043376 | - | 30.5 | 2470 | 2887 | -1760 | MER110-int | ERV1 | LTR |
| 9043377 | 9043663 | + | 11.5 | 1 | 303 | -7 | AluSg | Alu | SINE |
| 9043664 | 9043923 | - | 30.5 | 2202 | 2469 | -2178 | MER110-int | ERV1 | LTR |

  

|  |  |  |  |  |  |  |  |  |  |
| --- | --- | --- | --- | --- | --- | --- | --- | --- | --- |
| chr4:9044428-9047332 | | | 2905 bp | | |  |  |  |  |
| Genome Start | Genome End | Strand | Div | Repeat Start | Repeat End | Repeat Left | Name | Family | Class |
| 9044428 | 9045931 | - | 14.6 | 1494 | 3018 | -3253 | L1P4e | L1 | LINE |
| 9045932 | 9046401 | - | 7.9 | 5685 | 6154 | 0 | L1PA5 | L1 | LINE |
| 9046402 | 9046776 | - | 14.0 | 1117 | 1493 | -4794 | L1P4e | L1 | LINE |
| 9046777 | 9047109 | - | 14.4 | 1 | 364 | 0 | THE1B | MaLR | LTR |
| 9047110 | 9047332 | - | 15.1 | 901 | 1124 | -5167 | L1P4e | L1 | LINE |

  

|  |  |  |  |  |  |  |  |  |  |
| --- | --- | --- | --- | --- | --- | --- | --- | --- | --- |
| chr4:9058646-9059551 | | | 906 bp | | |  |  |  |  |
| Genome Start | Genome End | Strand | Div | Repeat Start | Repeat End | Repeat Left | Name | Family | Class |
| 9058646 | 9059185 | + | 20.6 | 1066 | 1627 | -4881 | L1MCa | L1 | LINE |
| 9059186 | 9059447 | - | 13.0 | 1 | 289 | -23 | AluSx | Alu | SINE |
| 9059448 | 9059551 | + | 20.6 | 1628 | 1731 | -4777 | L1MCa | L1 | LINE |

  

|  |  |  |  |  |  |  |  |  |  |
| --- | --- | --- | --- | --- | --- | --- | --- | --- | --- |
| chr4:9072452-9073116 | | | 665 bp | | |  |  |  |  |
| Genome Start | Genome End | Strand | Div | Repeat Start | Repeat End | Repeat Left | Name | Family | Class |
| 9072452 | 9072634 | + | 24.1 | 5767 | 5933 | -367 | L1MA4 | L1 | LINE |
| 9072635 | 9072915 | + | 16.4 | 1 | 280 | -32 | AluSx | Alu | SINE |
| 9072916 | 9073116 | + | 24.1 | 5934 | 6124 | -176 | L1MA4 | L1 | LINE |

  

|  |  |  |  |  |  |  |  |  |  |
| --- | --- | --- | --- | --- | --- | --- | --- | --- | --- |
| chr4:9073561-9075521 | | | 1961 bp | | |  |  |  |  |
| Genome Start | Genome End | Strand | Div | Repeat Start | Repeat End | Repeat Left | Name | Family | Class |
| 9073561 | 9074554 | + | 24.6 | 4818 | 5824 | -359 | L1MB3 | L1 | LINE |
| 9074555 | 9074874 | + | 15.4 | 1 | 312 | 0 | AluSx | Alu | SINE |
| 9074875 | 9074988 | + | 23.6 | 5825 | 5907 | -276 | L1MB3 | L1 | LINE |
| 9074989 | 9075271 | + | 14.5 | 3 | 295 | -17 | AluSx | Alu | SINE |
| 9075272 | 9075521 | + | 23.6 | 5908 | 6153 | -30 | L1MB3 | L1 | LINE |

  

|  |  |  |  |  |  |  |  |  |  |
| --- | --- | --- | --- | --- | --- | --- | --- | --- | --- |
| chr4:9096856-9097556 | | | 701 bp | | |  |  |  |  |
| Genome Start | Genome End | Strand | Div | Repeat Start | Repeat End | Repeat Left | Name | Family | Class |
| 9096856 | 9097038 | + | 25.7 | 5767 | 5933 | -367 | L1MA4 | L1 | LINE |
| 9097039 | 9097322 | + | 18.4 | 1 | 283 | -29 | AluSx | Alu | SINE |
| 9097323 | 9097556 | + | 25.7 | 5934 | 6160 | -140 | L1MA4 | L1 | LINE |

  

|  |  |  |  |  |  |  |  |  |  |
| --- | --- | --- | --- | --- | --- | --- | --- | --- | --- |
| chr4:9097956-9099922 | | | 1967 bp | | |  |  |  |  |
| Genome Start | Genome End | Strand | Div | Repeat Start | Repeat End | Repeat Left | Name | Family | Class |
| 9097956 | 9098949 | + | 24.5 | 4818 | 5824 | -359 | L1MB3 | L1 | LINE |
| 9098950 | 9099268 | + | 15.5 | 1 | 311 | -1 | AluSx | Alu | SINE |
| 9099269 | 9099395 | + | 23.9 | 5825 | 5902 | -281 | L1MB3 | L1 | LINE |
| 9099396 | 9099670 | + | 14.9 | 10 | 295 | -17 | AluSx | Alu | SINE |
| 9099671 | 9099922 | + | 23.9 | 5903 | 6153 | -30 | L1MB3 | L1 | LINE |

  

|  |  |  |  |  |  |  |  |  |  |
| --- | --- | --- | --- | --- | --- | --- | --- | --- | --- |
| chr4:9111664-9112978 | | | 1222 bp | | |  |  |  |  |
| Genome Start | Genome End | Strand | Div | Repeat Start | Repeat End | Repeat Left | Name | Family | Class |
| 9111664 | 9111753 | + | 26.0 | 23 | 108 | -6011 | L1MEd | L1 | LINE |
| 9111754 | 9112039 | + | 10.1 | 1 | 289 | -24 | AluSq | Alu | SINE |
| 9112072 | 9112184 | + | 26.0 | 109 | 220 | -5899 | L1MEd | L1 | LINE |
| 9112246 | 9112482 | + | 28.3 | 329 | 564 | -5555 | L1MEd | L1 | LINE |
| 9112483 | 9112771 | + | 10.4 | 1 | 293 | -20 | AluSq | Alu | SINE |
| 9112772 | 9112978 | + | 28.3 | 565 | 778 | -5341 | L1MEd | L1 | LINE |

  

|  |  |  |  |  |  |  |  |  |  |
| --- | --- | --- | --- | --- | --- | --- | --- | --- | --- |
| chr4:9126018-9126688 | | | 671 bp | | |  |  |  |  |
| Genome Start | Genome End | Strand | Div | Repeat Start | Repeat End | Repeat Left | Name | Family | Class |
| 9126018 | 9126200 | + | 25.6 | 5767 | 5940 | -360 | L1MA4 | L1 | LINE |
| 9126201 | 9126484 | + | 18.0 | 1 | 283 | -29 | AluSx | Alu | SINE |
| 9126485 | 9126688 | + | 25.6 | 5941 | 6130 | -170 | L1MA4 | L1 | LINE |

  

|  |  |  |  |  |  |  |  |  |  |
| --- | --- | --- | --- | --- | --- | --- | --- | --- | --- |
| chr4:9133143-9134030 | | | 888 bp | | |  |  |  |  |
| Genome Start | Genome End | Strand | Div | Repeat Start | Repeat End | Repeat Left | Name | Family | Class |
| 9133143 | 9133514 | - | 29.6 | 2559 | 2945 | -3201 | L1M5 | L1 | LINE |
| 9133515 | 9133896 | - | 25.1 | 1 | 448 | -2 | MLT1J2 | MaLR | LTR |
| 9133897 | 9134030 | - | 29.6 | 2424 | 2558 | -3588 | L1M5 | L1 | LINE |

  

|  |  |  |  |  |  |  |  |  |  |
| --- | --- | --- | --- | --- | --- | --- | --- | --- | --- |
| chr4:9136993-9138169 | | | 1177 bp | | |  |  |  |  |
| Genome Start | Genome End | Strand | Div | Repeat Start | Repeat End | Repeat Left | Name | Family | Class |
| 9136993 | 9137679 | + | 30.6 | 2348 | 3193 | -226 | L2 | L2 | LINE |
| 9137680 | 9137986 | - | 14.4 | 1 | 307 | -6 | AluSq | Alu | SINE |
| 9137987 | 9138169 | + | 32.7 | 3194 | 3374 | -45 | L2 | L2 | LINE |

  

|  |  |  |  |  |  |  |  |  |  |
| --- | --- | --- | --- | --- | --- | --- | --- | --- | --- |
| chr4:9140671-9141505 | | | 814 bp | | |  |  |  |  |
| Genome Start | Genome End | Strand | Div | Repeat Start | Repeat End | Repeat Left | Name | Family | Class |
| 9140671 | 9140745 | + | 34.5 | 2 | 75 | -133 | MIR | MIR | SINE |
| 9140751 | 9141366 | - | 20.6 | 1473 | 2104 | -3610 | ERVL-B4 | ERVL | LTR |
| 9141383 | 9141505 | + | 26.8 | 99 | 223 | -39 | MIR | MIR | SINE |

  

|  |  |  |  |  |  |  |  |  |  |
| --- | --- | --- | --- | --- | --- | --- | --- | --- | --- |
| chr4:9149579-9150406 | | | 828 bp | | |  |  |  |  |
| Genome Start | Genome End | Strand | Div | Repeat Start | Repeat End | Repeat Left | Name | Family | Class |
| 9149579 | 9149920 | - | 13.4 | 5936 | 6288 | -3 | L1MA8 | L1 | LINE |
| 9149921 | 9150230 | - | 15.9 | 1 | 312 | 0 | AluJo | Alu | SINE |
| 9150231 | 9150406 | - | 13.4 | 5775 | 5935 | -356 | L1MA8 | L1 | LINE |

  

|  |  |  |  |  |  |  |  |  |  |
| --- | --- | --- | --- | --- | --- | --- | --- | --- | --- |
| chr4:9172466-9173094 | | | 629 bp | | |  |  |  |  |
| Genome Start | Genome End | Strand | Div | Repeat Start | Repeat End | Repeat Left | Name | Family | Class |
| 9172466 | 9172628 | - | 17.9 | 5491 | 5653 | -521 | L1M4 | L1 | LINE |
| 9172629 | 9172930 | - | 11.7 | 1 | 300 | -12 | AluSx | Alu | SINE |
| 9172931 | 9173094 | - | 18.0 | 5331 | 5490 | -684 | L1M4 | L1 | LINE |

  

|  |  |  |  |  |  |  |  |  |  |
| --- | --- | --- | --- | --- | --- | --- | --- | --- | --- |
| chr4:9195275-9196023 | | | 746 bp | | |  |  |  |  |
| Genome Start | Genome End | Strand | Div | Repeat Start | Repeat End | Repeat Left | Name | Family | Class |
| 9195275 | 9195643 | + | 17.9 | 5575 | 5959 | -219 | L1MB7 | L1 | LINE |
| 9195644 | 9195816 | + | 10.4 | 104 | 289 | -23 | AluSg/x | Alu | SINE |
| 9195820 | 9196023 | + | 20.6 | 5974 | 6184 | 0 | L1MB7 | L1 | LINE |

  

|  |  |  |  |  |  |  |  |  |  |
| --- | --- | --- | --- | --- | --- | --- | --- | --- | --- |
| chr4:9196024-9197348 | | | 1184 bp | | |  |  |  |  |
| Genome Start | Genome End | Strand | Div | Repeat Start | Repeat End | Repeat Left | Name | Family | Class |
| 9196024 | 9196175 | - | 32.5 | 3015 | 3157 | -221 | L2 | L2 | LINE |
| 9196176 | 9196473 | - | 14.1 | 1 | 298 | -15 | AluSq | Alu | SINE |
| 9196474 | 9196575 | - | 32.5 | 2910 | 3014 | -364 | L2 | L2 | LINE |
| 9196617 | 9196707 | + | 25.3 | 95 | 206 | -62 | MIRm | MIR | SINE |
| 9196738 | 9197034 | - | 5.4 | 1 | 300 | -11 | AluY | Alu | SINE |
| 9197105 | 9197348 | - | 28.4 | 2563 | 2810 | -609 | L2 | L2 | LINE |

  

|  |  |  |  |  |  |  |  |  |  |
| --- | --- | --- | --- | --- | --- | --- | --- | --- | --- |
| chr4:9198938-9199870 | | | 903 bp | | |  |  |  |  |
| Genome Start | Genome End | Strand | Div | Repeat Start | Repeat End | Repeat Left | Name | Family | Class |
| 9198938 | 9198981 | - | 9.1 | 135 | 178 | 0 | MER5B | MER1\_type | DNA |
| 9198998 | 9199147 | - | 21.5 | 5711 | 5887 | -287 | L1MB5 | L1 | LINE |
| 9199143 | 9199425 | + | 20.7 | 5902 | 6174 | 0 | L1MB5 | L1 | LINE |
| 9199430 | 9199729 | + | 15.0 | 2 | 301 | -11 | AluSx | Alu | SINE |
| 9199745 | 9199870 | - | 20.3 | 5 | 128 | -50 | MER5B | MER1\_type | DNA |

  

|  |  |  |  |  |  |  |  |  |  |
| --- | --- | --- | --- | --- | --- | --- | --- | --- | --- |
| chr4:9204989-9205607 | | | 619 bp | | |  |  |  |  |
| Genome Start | Genome End | Strand | Div | Repeat Start | Repeat End | Repeat Left | Name | Family | Class |
| 9204989 | 9205244 | + | 27.8 | 87 | 339 | -70 | MLT1I | MaLR | LTR |
| 9205245 | 9205537 | + | 14.4 | 1 | 299 | -13 | AluSx | Alu | SINE |
| 9205538 | 9205607 | + | 27.8 | 340 | 402 | -7 | MLT1I | MaLR | LTR |

  

|  |  |  |  |  |  |  |  |  |  |
| --- | --- | --- | --- | --- | --- | --- | --- | --- | --- |
| chr4:9205956-9208446 | | | 2455 bp | | |  |  |  |  |
| Genome Start | Genome End | Strand | Div | Repeat Start | Repeat End | Repeat Left | Name | Family | Class |
| 9205956 | 9206094 | - | 29.4 | 3242 | 3378 | 0 | L2 | L2 | LINE |
| 9206122 | 9207202 | - | 6.6 | 1 | 1266 | 0 | MER11A | ERVK | LTR |
| 9207212 | 9207903 | - | 31.9 | 2466 | 3207 | -212 | L2 | L2 | LINE |
| 9207904 | 9208212 | - | 16.8 | 1 | 292 | -20 | AluJo | Alu | SINE |
| 9208213 | 9208446 | - | 32.9 | 2230 | 2465 | -954 | L2 | L2 | LINE |

  

|  |  |  |  |  |  |  |  |  |  |
| --- | --- | --- | --- | --- | --- | --- | --- | --- | --- |
| chr4:9209081-9210543 | | | 1347 bp | | |  |  |  |  |
| Genome Start | Genome End | Strand | Div | Repeat Start | Repeat End | Repeat Left | Name | Family | Class |
| 9209081 | 9209254 | + | 39.1 | 3945 | 4108 | -381 | L3 | CR1 | LINE |
| 9209277 | 9209403 | + | 12.6 | 9 | 135 | -177 | AluSq/x | Alu | SINE |
| 9209404 | 9209706 | + | 13.6 | 1 | 305 | -8 | AluSq | Alu | SINE |
| 9209747 | 9209863 | + | 19.1 | 1 | 110 | 0 | MER58A | MER1\_type | DNA |
| 9209864 | 9210240 | - | 15.1 | 1 | 428 | 0 | MSTA | MaLR | LTR |
| 9210241 | 9210353 | + | 19.1 | 111 | 221 | -3 | MER58A | MER1\_type | DNA |
| 9210408 | 9210543 | + | 31.9 | 4142 | 4289 | -200 | L3 | CR1 | LINE |

  

|  |  |  |  |  |  |  |  |  |  |
| --- | --- | --- | --- | --- | --- | --- | --- | --- | --- |
| chr4:9242684-9243540 | | | 857 bp | | |  |  |  |  |
| Genome Start | Genome End | Strand | Div | Repeat Start | Repeat End | Repeat Left | Name | Family | Class |
| 9242684 | 9242955 | - | 19.4 | 322 | 654 | 0 | MLT1E1 | MaLR | LTR |
| 9242956 | 9243264 | + | 9.2 | 1 | 310 | 0 | AluSx | Alu | SINE |
| 9243265 | 9243540 | - | 19.4 | 3 | 321 | -333 | MLT1E1 | MaLR | LTR |

  

|  |  |  |  |  |  |  |  |  |  |
| --- | --- | --- | --- | --- | --- | --- | --- | --- | --- |
| chr4:9244138-9244927 | | | 790 bp | | |  |  |  |  |
| Genome Start | Genome End | Strand | Div | Repeat Start | Repeat End | Repeat Left | Name | Family | Class |
| 9244138 | 9244522 | - | 22.1 | 83 | 467 | 0 | MLT1C | MaLR | LTR |
| 9244523 | 9244834 | - | 10.3 | 1 | 313 | 0 | AluSp | Alu | SINE |
| 9244835 | 9244927 | - | 22.1 | 3 | 82 | -385 | MLT1C | MaLR | LTR |

  

|  |  |  |  |  |  |  |  |  |  |
| --- | --- | --- | --- | --- | --- | --- | --- | --- | --- |
| chr4:9249116-9255979 | | | 6809 bp | | |  |  |  |  |
| Genome Start | Genome End | Strand | Div | Repeat Start | Repeat End | Repeat Left | Name | Family | Class |
| 9249116 | 9249180 | - | 23.0 | 389 | 484 | 0 | MLT1D | MaLR | LTR |
| 9249181 | 9249482 | + | 14.7 | 1 | 301 | -11 | AluSx | Alu | SINE |
| 9249483 | 9249655 | - | 22.5 | 206 | 430 | -75 | MLT1D | MaLR | LTR |
| 9249695 | 9250041 | + | 9.3 | 1 | 447 | -3 | LTR7 | ERV1 | LTR |
| 9250047 | 9252988 | + | 9.5 | 1 | 2993 | -4720 | HERVH | ERV1 | LTR |
| 9252974 | 9253825 | + | 10.1 | 3140 | 3993 | -3720 | HERVH | ERV1 | LTR |
| 9253828 | 9254490 | + | 15.9 | 4488 | 5149 | -2564 | HERVH | ERV1 | LTR |
| 9254492 | 9254702 | + | 13.3 | 5600 | 5819 | -1894 | HERVH | ERV1 | LTR |
| 9254703 | 9255079 | + | 9.9 | 7324 | 7713 | 0 | HERVH | ERV1 | LTR |
| 9255081 | 9255475 | + | 10.4 | 2 | 448 | -2 | LTR7 | ERV1 | LTR |
| 9255486 | 9255781 | - | 9.8 | 5 | 301 | -12 | AluSp | Alu | SINE |
| 9255794 | 9255979 | - | 23.9 | 1 | 185 | -320 | MLT1D | MaLR | LTR |

  

|  |  |  |  |  |  |  |  |  |  |
| --- | --- | --- | --- | --- | --- | --- | --- | --- | --- |
| chr4:9261714-9262314 | | | 601 bp | | |  |  |  |  |
| Genome Start | Genome End | Strand | Div | Repeat Start | Repeat End | Repeat Left | Name | Family | Class |
| 9261714 | 9261859 | - | 15.8 | 146 | 280 | -32 | AluSx | Alu | SINE |
| 9261860 | 9262168 | - | 9.2 | 1 | 295 | -16 | AluY | Alu | SINE |
| 9262169 | 9262314 | - | 15.8 | 1 | 145 | -167 | AluSx | Alu | SINE |

  

|  |  |  |  |  |  |  |  |  |  |
| --- | --- | --- | --- | --- | --- | --- | --- | --- | --- |
| chr4:9263035-9263521 | | | 487 bp | | |  |  |  |  |
| Genome Start | Genome End | Strand | Div | Repeat Start | Repeat End | Repeat Left | Name | Family | Class |
| 9263035 | 9263170 | + | 28.1 | 2 | 133 | -56 | MER5B | MER1\_type | DNA |
| 9263171 | 9263472 | - | 16.6 | 3 | 304 | -8 | AluJo | Alu | SINE |
| 9263473 | 9263521 | + | 29.6 | 116 | 188 | -1 | MER5B | MER1\_type | DNA |

  

|  |  |  |  |  |  |  |  |  |  |
| --- | --- | --- | --- | --- | --- | --- | --- | --- | --- |
| chr4:9264092-9264795 | | | 704 bp | | |  |  |  |  |
| Genome Start | Genome End | Strand | Div | Repeat Start | Repeat End | Repeat Left | Name | Family | Class |
| 9264092 | 9264158 | + | 22.6 | 11 | 89 | -378 | MLT1C | MaLR | LTR |
| 9264159 | 9264459 | + | 16.3 | 1 | 302 | -10 | AluSx | Alu | SINE |
| 9264460 | 9264795 | + | 22.6 | 90 | 451 | -16 | MLT1C | MaLR | LTR |

  

|  |  |  |  |  |  |  |  |  |  |
| --- | --- | --- | --- | --- | --- | --- | --- | --- | --- |
| chr4:9265960-9266872 | | | 913 bp | | |  |  |  |  |
| Genome Start | Genome End | Strand | Div | Repeat Start | Repeat End | Repeat Left | Name | Family | Class |
| 9265960 | 9266405 | + | 12.1 | 1 | 442 | -343 | LTR1 | ERV1 | LTR |
| 9266406 | 9266635 | + | 7.0 | 16 | 244 | -67 | AluY | Alu | SINE |
| 9266636 | 9266872 | + | 12.1 | 443 | 724 | -61 | LTR1 | ERV1 | LTR |

  

|  |  |  |  |  |  |  |  |  |  |
| --- | --- | --- | --- | --- | --- | --- | --- | --- | --- |
| chr4:9280361-9280876 | | | 516 bp | | |  |  |  |  |
| Genome Start | Genome End | Strand | Div | Repeat Start | Repeat End | Repeat Left | Name | Family | Class |
| 9280361 | 9280509 | + | 19.4 | 4 | 148 | -61 | MER3 | MER1\_type | DNA |
| 9280510 | 9280808 | - | 11.4 | 1 | 300 | -12 | AluSx | Alu | SINE |
| 9280809 | 9280876 | + | 19.4 | 149 | 209 | 0 | MER3 | MER1\_type | DNA |

  

|  |  |  |  |  |  |  |  |  |  |
| --- | --- | --- | --- | --- | --- | --- | --- | --- | --- |
| chr4:9286931-9287481 | | | 551 bp | | |  |  |  |  |
| Genome Start | Genome End | Strand | Div | Repeat Start | Repeat End | Repeat Left | Name | Family | Class |
| 9286931 | 9287073 | + | 19.7 | 1 | 137 | -175 | AluJo | Alu | SINE |
| 9287074 | 9287370 | + | 18.5 | 1 | 297 | -15 | AluSx | Alu | SINE |
| 9287371 | 9287481 | + | 19.7 | 138 | 248 | -64 | AluJo | Alu | SINE |

  

|  |  |  |  |  |  |  |  |  |  |
| --- | --- | --- | --- | --- | --- | --- | --- | --- | --- |
| chr4:9289722-9291788 | | | 2067 bp | | |  |  |  |  |
| Genome Start | Genome End | Strand | Div | Repeat Start | Repeat End | Repeat Left | Name | Family | Class |
| 9289722 | 9289970 | + | 18.3 | 1 | 246 | -66 | AluJb | Alu | SINE |
| 9289971 | 9290408 | + | 15.0 | 1 | 451 | -35 | MER66B | ERV1 | LTR |
| 9290409 | 9291694 | + | 7.8 | 2 | 1578 | 0 | LTR12C | ERV1 | LTR |
| 9291695 | 9291730 | + | 15.0 | 452 | 486 | 0 | MER66B | ERV1 | LTR |
| 9291731 | 9291788 | + | 16.4 | 244 | 298 | -4 | AluJb | Alu | SINE |

  

|  |  |  |  |  |  |  |  |  |  |
| --- | --- | --- | --- | --- | --- | --- | --- | --- | --- |
| chr4:9291922-9292763 | | | 819 bp | | |  |  |  |  |
| Genome Start | Genome End | Strand | Div | Repeat Start | Repeat End | Repeat Left | Name | Family | Class |
| 9291922 | 9292157 | + | 17.2 | 5670 | 5915 | -269 | L1MB7 | L1 | LINE |
| 9292181 | 9292393 | + | 17.2 | 5916 | 6114 | -70 | L1MB7 | L1 | LINE |
| 9292394 | 9292693 | - | 16.1 | 1 | 298 | -14 | AluJb | Alu | SINE |
| 9292694 | 9292763 | + | 17.2 | 6115 | 6184 | 0 | L1MB7 | L1 | LINE |

  

|  |  |  |  |  |  |  |  |  |  |
| --- | --- | --- | --- | --- | --- | --- | --- | --- | --- |
| chr4:9292791-9293394 | | | 604 bp | | |  |  |  |  |
| Genome Start | Genome End | Strand | Div | Repeat Start | Repeat End | Repeat Left | Name | Family | Class |
| 9292791 | 9292909 | + | 16.3 | 18 | 136 | -176 | AluJb | Alu | SINE |
| 9292910 | 9293206 | + | 14.2 | 1 | 308 | -4 | AluSx | Alu | SINE |
| 9293207 | 9293394 | + | 16.3 | 137 | 312 | 0 | AluJb | Alu | SINE |

  

|  |  |  |  |  |  |  |  |  |  |
| --- | --- | --- | --- | --- | --- | --- | --- | --- | --- |
| chr4:9298391-9299086 | | | 696 bp | | |  |  |  |  |
| Genome Start | Genome End | Strand | Div | Repeat Start | Repeat End | Repeat Left | Name | Family | Class |
| 9298391 | 9298634 | - | 21.4 | 175 | 422 | -45 | MLT1C | MaLR | LTR |
| 9298635 | 9298932 | - | 11.8 | 2 | 298 | -14 | AluSx | Alu | SINE |
| 9298933 | 9299086 | - | 21.4 | 41 | 174 | -293 | MLT1C | MaLR | LTR |

  

|  |  |  |  |  |  |  |  |  |  |
| --- | --- | --- | --- | --- | --- | --- | --- | --- | --- |
| chr4:9307794-9308380 | | | 587 bp | | |  |  |  |  |
| Genome Start | Genome End | Strand | Div | Repeat Start | Repeat End | Repeat Left | Name | Family | Class |
| 9307794 | 9307926 | + | 9.5 | 1 | 136 | -176 | AluSx | Alu | SINE |
| 9307927 | 9308225 | + | 10.7 | 1 | 299 | -14 | AluSq | Alu | SINE |
| 9308226 | 9308380 | + | 9.5 | 137 | 300 | -12 | AluSx | Alu | SINE |

  

|  |  |  |  |  |  |  |  |  |  |
| --- | --- | --- | --- | --- | --- | --- | --- | --- | --- |
| chr4:9324106-9324768 | | | 663 bp | | |  |  |  |  |
| Genome Start | Genome End | Strand | Div | Repeat Start | Repeat End | Repeat Left | Name | Family | Class |
| 9324106 | 9324314 | - | 12.1 | 162 | 362 | -2 | THE1B | MaLR | LTR |
| 9324315 | 9324614 | + | 14.0 | 1 | 300 | -12 | AluSx | Alu | SINE |
| 9324615 | 9324768 | - | 12.1 | 2 | 161 | -203 | THE1B | MaLR | LTR |

  

|  |  |  |  |  |  |  |  |  |  |
| --- | --- | --- | --- | --- | --- | --- | --- | --- | --- |
| chr4:9329188-9330822 | | | 1635 bp | | |  |  |  |  |
| Genome Start | Genome End | Strand | Div | Repeat Start | Repeat End | Repeat Left | Name | Family | Class |
| 9329188 | 9329563 | - | 22.1 | 659 | 1040 | 0 | MER45B | Tip100 | DNA |
| 9329564 | 9329878 | - | 13.4 | 4 | 317 | 0 | AluSg | Alu | SINE |
| 9329879 | 9330405 | - | 22.1 | 125 | 658 | -382 | MER45B | Tip100 | DNA |
| 9330406 | 9330701 | + | 13.8 | 1 | 296 | -16 | AluSx | Alu | SINE |
| 9330702 | 9330822 | - | 22.1 | 11 | 124 | -916 | MER45B | Tip100 | DNA |

  

|  |  |  |  |  |  |  |  |  |  |
| --- | --- | --- | --- | --- | --- | --- | --- | --- | --- |
| chr4:9334592-9337294 | | | 2591 bp | | |  |  |  |  |
| Genome Start | Genome End | Strand | Div | Repeat Start | Repeat End | Repeat Left | Name | Family | Class |
| 9334592 | 9334728 | - | 20.6 | 3273 | 3402 | -789 | MER21-int | ERV1 | LTR |
| 9334729 | 9335012 | - | 15.0 | 195 | 428 | 0 | MSTA | MaLR | LTR |
| 9335013 | 9335327 | - | 14.0 | 1 | 300 | -12 | AluSx | Alu | SINE |
| 9335328 | 9335523 | - | 15.0 | 1 | 194 | -234 | MSTA | MaLR | LTR |
| 9335524 | 9335717 | - | 20.6 | 3078 | 3272 | -919 | MER21-int | ERV1 | LTR |
| 9335718 | 9336004 | - | 10.5 | 1 | 308 | -4 | AluSx | Alu | SINE |
| 9336005 | 9336224 | - | 20.6 | 2875 | 3077 | -1114 | MER21-int | ERV1 | LTR |
| 9336225 | 9336508 | - | 11.3 | 2 | 298 | -15 | AluSp | Alu | SINE |
| 9336509 | 9336879 | - | 20.6 | 2495 | 2874 | -1317 | MER21-int | ERV1 | LTR |
| 9336992 | 9337294 | - | 18.3 | 2178 | 2480 | -1711 | MER21-int | ERV1 | LTR |

  

|  |  |  |  |  |  |  |  |  |  |
| --- | --- | --- | --- | --- | --- | --- | --- | --- | --- |
| chr4:9337834-9338298 | | | 465 bp | | |  |  |  |  |
| Genome Start | Genome End | Strand | Div | Repeat Start | Repeat End | Repeat Left | Name | Family | Class |
| 9337834 | 9337851 | + | 27.3 | 1902 | 1922 | -1497 | L2 | L2 | LINE |
| 9337852 | 9338161 | - | 12.9 | 1 | 311 | -2 | AluSp | Alu | SINE |
| 9338162 | 9338298 | + | 27.3 | 1923 | 2061 | -1358 | L2 | L2 | LINE |

  

|  |  |  |  |  |  |  |  |  |  |
| --- | --- | --- | --- | --- | --- | --- | --- | --- | --- |
| chr4:9349374-9350089 | | | 716 bp | | |  |  |  |  |
| Genome Start | Genome End | Strand | Div | Repeat Start | Repeat End | Repeat Left | Name | Family | Class |
| 9349374 | 9349734 | - | 21.1 | 5827 | 6175 | -9 | L1MB7 | L1 | LINE |
| 9349735 | 9350025 | - | 12.8 | 6 | 295 | -17 | AluSx | Alu | SINE |
| 9350026 | 9350089 | - | 21.1 | 5769 | 5826 | -358 | L1MB7 | L1 | LINE |

  

|  |  |  |  |  |  |  |  |  |  |
| --- | --- | --- | --- | --- | --- | --- | --- | --- | --- |
| chr4:9358593-9359531 | | | 939 bp | | |  |  |  |  |
| Genome Start | Genome End | Strand | Div | Repeat Start | Repeat End | Repeat Left | Name | Family | Class |
| 9358593 | 9359057 | - | 34.0 | 2875 | 3377 | -1 | L2 | L2 | LINE |
| 9359058 | 9359096 | + | 6.5 | 1 | 30 | -50 | MADE1 | Mariner | DNA |
| 9359097 | 9359398 | - | 13.9 | 1 | 300 | -12 | AluSx | Alu | SINE |
| 9359399 | 9359451 | + | 6.5 | 31 | 80 | 0 | MADE1 | Mariner | DNA |
| 9359452 | 9359531 | - | 36.2 | 2799 | 2874 | -545 | L2 | L2 | LINE |

  

|  |  |  |  |  |  |  |  |  |  |
| --- | --- | --- | --- | --- | --- | --- | --- | --- | --- |
| chr4:9377540-9379284 | | | 1745 bp | | |  |  |  |  |
| Genome Start | Genome End | Strand | Div | Repeat Start | Repeat End | Repeat Left | Name | Family | Class |
| 9377540 | 9377943 | + | 30.9 | 2120 | 2590 | -829 | L2 | L2 | LINE |
| 9377944 | 9378573 | + | 16.4 | 1 | 707 | 0 | MER39 | ERV1 | LTR |
| 9378574 | 9379284 | + | 30.1 | 2591 | 3319 | -59 | L2 | L2 | LINE |

  

|  |  |  |  |  |  |  |  |  |  |
| --- | --- | --- | --- | --- | --- | --- | --- | --- | --- |
| chr4:9398087-9399852 | | | 1594 bp | | |  |  |  |  |
| Genome Start | Genome End | Strand | Div | Repeat Start | Repeat End | Repeat Left | Name | Family | Class |
| 9398087 | 9398902 | - | 20.7 | 7052 | 8039 | -3 | L1MC4 | L1 | LINE |
| 9398903 | 9399213 | - | 17.8 | 1 | 310 | -2 | AluJo | Alu | SINE |
| 9399214 | 9399252 | - | 19.8 | 7014 | 7051 | -991 | L1MC4 | L1 | LINE |
| 9399425 | 9399852 | - | 24.1 | 6586 | 7014 | -1028 | L1MC4 | L1 | LINE |

  

|  |  |  |  |  |  |  |  |  |  |
| --- | --- | --- | --- | --- | --- | --- | --- | --- | --- |
| chr4:22200567-22201253 | | | 687 bp | | |  |  |  |  |
| Genome Start | Genome End | Strand | Div | Repeat Start | Repeat End | Repeat Left | Name | Family | Class |
| 22200567 | 22201072 | + | 16.0 | 4872 | 5374 | -917 | L1M | L1 | LINE |
| 22201073 | 22201239 | + | 16.8 | 6119 | 6289 | -2 | L1MA8 | L1 | LINE |
| 22201240 | 22201253 | + | 19.9 | 5375 | 5388 | -903 | L1M | L1 | LINE |

  

|  |  |  |  |  |  |  |  |  |  |
| --- | --- | --- | --- | --- | --- | --- | --- | --- | --- |
| chr4:34133061-34133804 | | | 705 bp | | |  |  |  |  |
| Genome Start | Genome End | Strand | Div | Repeat Start | Repeat End | Repeat Left | Name | Family | Class |
| 34133061 | 34133352 | + | 24.4 | 7262 | 7555 | -230 | L1MC3 | L1 | LINE |
| 34133391 | 34133564 | - | 18.6 | 5923 | 6097 | -215 | L1MA9 | L1 | LINE |
| 34133565 | 34133690 | + | 16.1 | 6179 | 6310 | -2 | L1MA9 | L1 | LINE |
| 34133692 | 34133804 | + | 22.3 | 7616 | 7734 | -51 | L1MC3 | L1 | LINE |

  

|  |  |  |  |  |  |  |  |  |  |
| --- | --- | --- | --- | --- | --- | --- | --- | --- | --- |
| chr4:34135794-34137135 | | | 1335 bp | | |  |  |  |  |
| Genome Start | Genome End | Strand | Div | Repeat Start | Repeat End | Repeat Left | Name | Family | Class |
| 34135794 | 34136081 | + | 30.7 | 2 | 284 | -1205 | MLT1F1-int | MaLR | LTR |
| 34136089 | 34136472 | + | 19.4 | 8 | 390 | 0 | MLT1B | MaLR | LTR |
| 34136473 | 34136602 | + | 23.3 | 280 | 397 | -1092 | MLT1F1-int | MaLR | LTR |
| 34136603 | 34136915 | + | 12.9 | 1 | 311 | -2 | AluSp | Alu | SINE |
| 34136916 | 34137135 | + | 23.3 | 398 | 616 | -873 | MLT1F1-int | MaLR | LTR |

  

|  |  |  |  |  |  |  |  |  |  |
| --- | --- | --- | --- | --- | --- | --- | --- | --- | --- |
| chr4:48905060-48907694 | | | 2598 bp | | |  |  |  |  |
| Genome Start | Genome End | Strand | Div | Repeat Start | Repeat End | Repeat Left | Name | Family | Class |
| 48905060 | 48907444 | - | 19.5 | 486 | 3017 | -3749 | L1PA13 | L1 | LINE |
| 48907465 | 48907534 | + | 15.7 | 1261 | 1330 | -5651 | L1PA12 | L1 | LINE |
| 48907544 | 48907618 | + | 22.7 | 1288 | 1362 | -6238 | L1PA12 | L1 | LINE |
| 48907627 | 48907694 | - | 24.6 | 407 | 471 | -6314 | L1PA13 | L1 | LINE |

  

|  |  |  |  |  |  |  |  |  |  |
| --- | --- | --- | --- | --- | --- | --- | --- | --- | --- |
| chr4:48947879-48949446 | | | 1568 bp | | |  |  |  |  |
| Genome Start | Genome End | Strand | Div | Repeat Start | Repeat End | Repeat Left | Name | Family | Class |
| 48947879 | 48948066 | + | 13.8 | 5347 | 5544 | -621 | L1PA15 | L1 | LINE |
| 48948067 | 48948820 | + | 17.3 | 1 | 826 | 0 | LTR1B | ERV1 | LTR |
| 48948821 | 48949446 | + | 13.8 | 5545 | 6165 | 0 | L1PA15 | L1 | LINE |

  

|  |  |  |  |  |  |  |  |  |  |
| --- | --- | --- | --- | --- | --- | --- | --- | --- | --- |
| chr4:48951031-48952699 | | | 1667 bp | | |  |  |  |  |
| Genome Start | Genome End | Strand | Div | Repeat Start | Repeat End | Repeat Left | Name | Family | Class |
| 48951031 | 48951278 | - | 27.9 | 3614 | 3862 | -2284 | L1M5 | L1 | LINE |
| 48951279 | 48951591 | - | 12.2 | 1 | 313 | 0 | AluSp | Alu | SINE |
| 48951592 | 48951637 | - | 27.9 | 3571 | 3613 | -2533 | L1M5 | L1 | LINE |
| 48951638 | 48951982 | - | 16.5 | 1 | 2405 | 0 | Tigger5 | MER2\_type | DNA |
| 48951985 | 48952699 | - | 25.0 | 2808 | 3568 | -2578 | L1M5 | L1 | LINE |

  

|  |  |  |  |  |  |  |  |  |  |
| --- | --- | --- | --- | --- | --- | --- | --- | --- | --- |
| chr4:48952691-48953981 | | | 1291 bp | | |  |  |  |  |
| Genome Start | Genome End | Strand | Div | Repeat Start | Repeat End | Repeat Left | Name | Family | Class |
| 48952691 | 48953250 | - | 23.3 | 7057 | 7678 | -364 | L1MC4 | L1 | LINE |
| 48953251 | 48953542 | - | 14.1 | 1 | 292 | -20 | AluSx | Alu | SINE |
| 48953543 | 48953981 | - | 23.3 | 6565 | 7056 | -986 | L1MC4 | L1 | LINE |

  

|  |  |  |  |  |  |  |  |  |  |
| --- | --- | --- | --- | --- | --- | --- | --- | --- | --- |
| chr4:48960930-48962433 | | | 1504 bp | | |  |  |  |  |
| Genome Start | Genome End | Strand | Div | Repeat Start | Repeat End | Repeat Left | Name | Family | Class |
| 48960930 | 48961443 | + | 23.9 | 18 | 528 | -407 | MER21C | ERV1 | LTR |
| 48961444 | 48961774 | - | 20.2 | 2 | 298 | -14 | AluJb | Alu | SINE |
| 48961775 | 48961852 | + | 23.9 | 529 | 601 | -334 | MER21C | ERV1 | LTR |
| 48961853 | 48962148 | + | 19.3 | 9 | 299 | -13 | AluJo | Alu | SINE |
| 48962149 | 48962433 | + | 23.9 | 602 | 916 | -19 | MER21C | ERV1 | LTR |

  

|  |  |  |  |  |  |  |  |  |  |
| --- | --- | --- | --- | --- | --- | --- | --- | --- | --- |
| chr4:49187186-49188913 | | | 1663 bp | | |  |  |  |  |
| Genome Start | Genome End | Strand | Div | Repeat Start | Repeat End | Repeat Left | Name | Family | Class |
| 49187186 | 49188313 | + | 21.0 | 4805 | 5946 | -354 | L1MA5 | L1 | LINE |
| 49188315 | 49188573 | + | 26.1 | 1 | 264 | -48 | AluJo | Alu | SINE |
| 49188638 | 49188913 | + | 17.4 | 6001 | 6294 | -6 | L1MA5 | L1 | LINE |

  

|  |  |  |  |  |  |  |  |  |  |
| --- | --- | --- | --- | --- | --- | --- | --- | --- | --- |
| chr4:49190674-49192458 | | | 1776 bp | | |  |  |  |  |
| Genome Start | Genome End | Strand | Div | Repeat Start | Repeat End | Repeat Left | Name | Family | Class |
| 49190674 | 49191722 | + | 19.7 | 4777 | 5836 | -342 | L1MB8 | L1 | LINE |
| 49191732 | 49192135 | + | 13.0 | 11 | 428 | 0 | MER61C | ERV1 | LTR |
| 49192136 | 49192458 | + | 21.9 | 5833 | 6173 | -5 | L1MB8 | L1 | LINE |

  

|  |  |  |  |  |  |  |  |  |  |
| --- | --- | --- | --- | --- | --- | --- | --- | --- | --- |
| chr4:49217559-49220116 | | | 2558 bp | | |  |  |  |  |
| Genome Start | Genome End | Strand | Div | Repeat Start | Repeat End | Repeat Left | Name | Family | Class |
| 49217559 | 49218284 | - | 10.3 | 4505 | 5219 | -927 | L1PB | L1 | LINE |
| 49218285 | 49218462 | - | 15.2 | 136 | 313 | 0 | AluSp | Alu | SINE |
| 49218463 | 49220116 | - | 10.3 | 2875 | 4504 | -1642 | L1PB | L1 | LINE |

  

|  |  |  |  |  |  |  |  |  |  |
| --- | --- | --- | --- | --- | --- | --- | --- | --- | --- |
| chr4:49226200-49227700 | | | 1501 bp | | |  |  |  |  |
| Genome Start | Genome End | Strand | Div | Repeat Start | Repeat End | Repeat Left | Name | Family | Class |
| 49226200 | 49226487 | - | 24.6 | 602 | 916 | -19 | MER21C | ERV1 | LTR |
| 49226488 | 49226778 | - | 19.4 | 9 | 297 | -15 | AluJo | Alu | SINE |
| 49226779 | 49226856 | - | 24.6 | 529 | 601 | -334 | MER21C | ERV1 | LTR |
| 49226857 | 49227188 | + | 19.8 | 2 | 299 | -13 | AluJb | Alu | SINE |
| 49227189 | 49227700 | - | 24.6 | 18 | 528 | -407 | MER21C | ERV1 | LTR |

  

|  |  |  |  |  |  |  |  |  |  |
| --- | --- | --- | --- | --- | --- | --- | --- | --- | --- |
| chr4:49231579-49232503 | | | 925 bp | | |  |  |  |  |
| Genome Start | Genome End | Strand | Div | Repeat Start | Repeat End | Repeat Left | Name | Family | Class |
| 49231579 | 49231641 | - | 24.1 | 6122 | 6173 | -5 | L1MB8 | L1 | LINE |
| 49231642 | 49231945 | + | 22.8 | 1 | 305 | -7 | AluJo | Alu | SINE |
| 49231946 | 49232503 | - | 24.1 | 5557 | 6121 | -57 | L1MB8 | L1 | LINE |

  

|  |  |  |  |  |  |  |  |  |  |
| --- | --- | --- | --- | --- | --- | --- | --- | --- | --- |
| chr4:49236190-49237851 | | | 1660 bp | | |  |  |  |  |
| Genome Start | Genome End | Strand | Div | Repeat Start | Repeat End | Repeat Left | Name | Family | Class |
| 49236190 | 49236901 | + | 26.0 | 2810 | 3568 | -2578 | L1M5 | L1 | LINE |
| 49236904 | 49237248 | + | 16.8 | 1 | 2405 | 0 | Tigger5 | MER2\_type | DNA |
| 49237249 | 49237298 | + | 27.9 | 3571 | 3613 | -2533 | L1M5 | L1 | LINE |
| 49237299 | 49237604 | + | 12.1 | 5 | 310 | -3 | AluSp | Alu | SINE |
| 49237605 | 49237851 | + | 27.9 | 3614 | 3862 | -2284 | L1M5 | L1 | LINE |

  

|  |  |  |  |  |  |  |  |  |  |
| --- | --- | --- | --- | --- | --- | --- | --- | --- | --- |
| chr4:49239435-49241002 | | | 1568 bp | | |  |  |  |  |
| Genome Start | Genome End | Strand | Div | Repeat Start | Repeat End | Repeat Left | Name | Family | Class |
| 49239435 | 49240060 | - | 14.2 | 5545 | 6165 | 0 | L1PA15 | L1 | LINE |
| 49240061 | 49240814 | - | 16.0 | 1 | 826 | 0 | LTR1B | ERV1 | LTR |
| 49240815 | 49241002 | - | 14.2 | 5347 | 5544 | -621 | L1PA15 | L1 | LINE |

  

|  |  |  |  |  |  |  |  |  |  |
| --- | --- | --- | --- | --- | --- | --- | --- | --- | --- |
| chr4:49309412-49310930 | | | 1519 bp | | |  |  |  |  |
| Genome Start | Genome End | Strand | Div | Repeat Start | Repeat End | Repeat Left | Name | Family | Class |
| 49309412 | 49309423 | + | 26.2 | 2355 | 2365 | -4268 | L1MDa | L1 | LINE |
| 49309424 | 49309713 | - | 18.2 | 1 | 288 | -24 | AluJb | Alu | SINE |
| 49309714 | 49309824 | + | 26.2 | 2366 | 2500 | -4057 | L1MDa | L1 | LINE |
| 49309825 | 49310130 | + | 19.6 | 1 | 308 | -4 | AluJb | Alu | SINE |
| 49310131 | 49310930 | + | 27.1 | 2487 | 3343 | -3194 | L1MDa | L1 | LINE |

  

|  |  |  |  |  |  |  |  |  |  |
| --- | --- | --- | --- | --- | --- | --- | --- | --- | --- |
| chr4:60334708-60335283 | | | 576 bp | | |  |  |  |  |
| Genome Start | Genome End | Strand | Div | Repeat Start | Repeat End | Repeat Left | Name | Family | Class |
| 60334708 | 60334785 | + | 31.4 | 29 | 104 | -158 | MIR | MIR | SINE |
| 60334786 | 60335201 | - | 16.8 | 1 | 428 | 0 | MSTA | MaLR | LTR |
| 60335202 | 60335283 | + | 31.4 | 105 | 196 | -66 | MIR | MIR | SINE |

  

|  |  |  |  |  |  |  |  |  |  |
| --- | --- | --- | --- | --- | --- | --- | --- | --- | --- |
| chr4:68912105-68915704 | | | 3597 bp | | |  |  |  |  |
| Genome Start | Genome End | Strand | Div | Repeat Start | Repeat End | Repeat Left | Name | Family | Class |
| 68912105 | 68912187 | + | 15.7 | 1 | 78 | -533 | LTR36 | ERV1 | LTR |
| 68912188 | 68912570 | + | 13.6 | 1 | 381 | 0 | THE1D | MaLR | LTR |
| 68912574 | 68912941 | + | 32.5 | 3 | 432 | -1303 | MLT-int | MaLR | LTR |
| 68912942 | 68913238 | - | 9.8 | 1 | 295 | -16 | AluY | Alu | SINE |
| 68913239 | 68914519 | + | 32.5 | 433 | 1735 | 0 | MLT1H-int | MaLR | LTR |
| 68914520 | 68914887 | + | 11.3 | 1 | 381 | 0 | THE1D | MaLR | LTR |
| 68914888 | 68915265 | + | 15.7 | 79 | 438 | -173 | LTR36 | ERV1 | LTR |
| 68915266 | 68915485 | + | 11.0 | 5913 | 6131 | 0 | L1PA8A | L1 | LINE |
| 68915486 | 68915704 | + | 15.7 | 439 | 611 | 0 | LTR36 | ERV1 | LTR |

  

|  |  |  |  |  |  |  |  |  |  |
| --- | --- | --- | --- | --- | --- | --- | --- | --- | --- |
| chr4:68915963-68916769 | | | 807 bp | | |  |  |  |  |
| Genome Start | Genome End | Strand | Div | Repeat Start | Repeat End | Repeat Left | Name | Family | Class |
| 68915963 | 68916076 | + | 17.9 | 1 | 113 | -199 | AluJb | Alu | SINE |
| 68916077 | 68916556 | + | 10.0 | 1 | 492 | 0 | LTR22B | ERVK | LTR |
| 68916557 | 68916769 | + | 17.9 | 114 | 312 | 0 | AluJb | Alu | SINE |

  

|  |  |  |  |  |  |  |  |  |  |
| --- | --- | --- | --- | --- | --- | --- | --- | --- | --- |
| chr4:68942043-68942855 | | | 813 bp | | |  |  |  |  |
| Genome Start | Genome End | Strand | Div | Repeat Start | Repeat End | Repeat Left | Name | Family | Class |
| 68942043 | 68942171 | + | 17.7 | 415 | 553 | -378 | MER21A | ERV1 | LTR |
| 68942172 | 68942476 | - | 14.8 | 1 | 304 | -8 | AluSx | Alu | SINE |
| 68942477 | 68942855 | + | 17.7 | 554 | 924 | -7 | MER21A | ERV1 | LTR |

  

|  |  |  |  |  |  |  |  |  |  |
| --- | --- | --- | --- | --- | --- | --- | --- | --- | --- |
| chr4:68951845-68953014 | | | 1170 bp | | |  |  |  |  |
| Genome Start | Genome End | Strand | Div | Repeat Start | Repeat End | Repeat Left | Name | Family | Class |
| 68951845 | 68952041 | + | 10.9 | 1 | 197 | -703 | MER4D1 | ERV1 | LTR |
| 68952042 | 68952349 | + | 6.4 | 1 | 296 | -13 | AluSg1 | Alu | SINE |
| 68952350 | 68953014 | + | 11.3 | 195 | 827 | -4 | MER4D1 | ERV1 | LTR |

  

|  |  |  |  |  |  |  |  |  |  |
| --- | --- | --- | --- | --- | --- | --- | --- | --- | --- |
| chr4:68953493-68958398 | | | 4785 bp | | |  |  |  |  |
| Genome Start | Genome End | Strand | Div | Repeat Start | Repeat End | Repeat Left | Name | Family | Class |
| 68953493 | 68953811 | + | 11.1 | 1 | 348 | -6250 | MER4A1-int | ERV1 | LTR |
| 68953812 | 68954120 | - | 13.6 | 1 | 310 | -2 | AluSx | Alu | SINE |
| 68954121 | 68954206 | + | 11.1 | 349 | 419 | -6179 | MER4A1-int | ERV1 | LTR |
| 68954207 | 68954608 | + | 8.5 | 1 | 448 | -2 | LTR7 | ERV1 | LTR |
| 68954609 | 68954882 | + | 10.0 | 420 | 816 | -5782 | MER4A1-int | ERV1 | LTR |
| 68955010 | 68955064 | + | 16.4 | 838 | 892 | -5706 | MER4A1-int | ERV1 | LTR |
| 68955059 | 68955516 | + | 11.6 | 994 | 1484 | -5114 | MER4A1-int | ERV1 | LTR |
| 68955517 | 68955916 | - | 8.0 | 1 | 448 | -2 | LTR7 | ERV1 | LTR |
| 68955917 | 68956910 | + | 11.6 | 1485 | 2473 | -4125 | MER4A1-int | ERV1 | LTR |
| 68956911 | 68957236 | - | 13.9 | 1 | 311 | 0 | AluY | Alu | SINE |
| 68957237 | 68957534 | - | 16.4 | 1 | 298 | -14 | AluSx | Alu | SINE |
| 68957535 | 68957557 | + | 11.6 | 2474 | 2495 | -4103 | MER4A1-int | ERV1 | LTR |
| 68957558 | 68957687 | - | 16.9 | 111 | 257 | -434 | LTR8 | ERV1 | LTR |
| 68957688 | 68958398 | + | 12.7 | 5860 | 6596 | -2 | MER4A1-int | ERV1 | LTR |

  

|  |  |  |  |  |  |  |  |  |  |
| --- | --- | --- | --- | --- | --- | --- | --- | --- | --- |
| chr4:69023834-69024411 | | | 381 bp | | |  |  |  |  |
| Genome Start | Genome End | Strand | Div | Repeat Start | Repeat End | Repeat Left | Name | Family | Class |
| 69023834 | 69023941 | - | 32.4 | 3294 | 3406 | -13 | L2 | L2 | LINE |
| 69023991 | 69024191 | + | 26.4 | 7 | 219 | 0 | MER20 | MER1\_type | DNA |
| 69024340 | 69024411 | - | 34.7 | 3093 | 3165 | -213 | L2 | L2 | LINE |

  

|  |  |  |  |  |  |  |  |  |  |
| --- | --- | --- | --- | --- | --- | --- | --- | --- | --- |
| chr4:69027819-69039817 | | | 11884 bp | | |  |  |  |  |
| Genome Start | Genome End | Strand | Div | Repeat Start | Repeat End | Repeat Left | Name | Family | Class |
| 69027819 | 69029045 | - | 20.4 | 1040 | 2332 | -4301 | L1MDa | L1 | LINE |
| 69029109 | 69029940 | - | 13.5 | 5457 | 6289 | -1 | L1PA15 | L1 | LINE |
| 69029941 | 69029980 | - | 18.4 | 389 | 428 | 0 | MSTA | MaLR | LTR |
| 69029981 | 69030270 | + | 17.3 | 1 | 300 | -12 | AluJb | Alu | SINE |
| 69030271 | 69030665 | - | 18.4 | 1 | 388 | -40 | MSTA | MaLR | LTR |
| 69030666 | 69030784 | - | 12.1 | 1543 | 1651 | 0 | MSTA-int | MaLR | LTR |
| 69030785 | 69031099 | + | 21.9 | 1 | 297 | -15 | AluJb | Alu | SINE |
| 69031100 | 69031157 | - | 12.1 | 1486 | 1542 | -109 | MSTA-int | MaLR | LTR |
| 69031144 | 69031353 | - | 18.1 | 1 | 217 | -1434 | MSTA-int | MaLR | LTR |
| 69031354 | 69031758 | - | 19.2 | 2 | 428 | 0 | MSTA | MaLR | LTR |
| 69031765 | 69032618 | - | 15.4 | 4615 | 5458 | -813 | L1PA15 | L1 | LINE |
| 69032619 | 69032920 | - | 17.3 | 1 | 301 | -11 | AluJo | Alu | SINE |
| 69032921 | 69033455 | - | 15.4 | 4066 | 4614 | -1657 | L1PA15 | L1 | LINE |
| 69033456 | 69033759 | - | 11.5 | 1 | 304 | -8 | AluSx | Alu | SINE |
| 69033760 | 69035567 | - | 15.5 | 2145 | 4065 | -2206 | L1PA15 | L1 | LINE |
| 69035568 | 69035864 | - | 14.5 | 1 | 298 | -15 | AluSq | Alu | SINE |
| 69035865 | 69036054 | - | 17.6 | 1957 | 2144 | -4143 | L1PA15 | L1 | LINE |
| 69036055 | 69036281 | + | 12.8 | 84 | 310 | -3 | AluSq | Alu | SINE |
| 69036282 | 69036843 | - | 17.6 | 1416 | 1956 | -4331 | L1PA15 | L1 | LINE |
| 69036844 | 69037152 | - | 9.4 | 1 | 311 | 0 | AluY | Alu | SINE |
| 69037153 | 69038633 | - | 21.5 | 5 | 1415 | -4872 | L1PA15 | L1 | LINE |
| 69038684 | 69038765 | - | 30.5 | 968 | 1050 | -5583 | L1MDa | L1 | LINE |
| 69038776 | 69039061 | - | 29.5 | 523 | 857 | -5776 | L1MDa | L1 | LINE |
| 69039062 | 69039369 | - | 14.3 | 3 | 311 | -1 | AluSx | Alu | SINE |
| 69039370 | 69039817 | - | 29.5 | 34 | 522 | -6111 | L1MDa | L1 | LINE |

  

|  |  |  |  |  |  |  |  |  |  |
| --- | --- | --- | --- | --- | --- | --- | --- | --- | --- |
| chr4:69048121-69049176 | | | 956 bp | | |  |  |  |  |
| Genome Start | Genome End | Strand | Div | Repeat Start | Repeat End | Repeat Left | Name | Family | Class |
| 69048121 | 69048198 | + | 32.0 | 3123 | 3197 | -181 | L2 | L2 | LINE |
| 69048212 | 69048968 | - | 20.8 | 5571 | 6343 | -16 | L1MD2 | L1 | LINE |
| 69049056 | 69049176 | + | 32.2 | 3296 | 3414 | -5 | L2 | L2 | LINE |

  

|  |  |  |  |  |  |  |  |  |  |
| --- | --- | --- | --- | --- | --- | --- | --- | --- | --- |
| chr4:69177408-69180090 | | | 2630 bp | | |  |  |  |  |
| Genome Start | Genome End | Strand | Div | Repeat Start | Repeat End | Repeat Left | Name | Family | Class |
| 69177408 | 69178249 | - | 22.8 | 4685 | 5591 | -587 | L1M4 | L1 | LINE |
| 69178250 | 69178378 | - | 28.1 | 1 | 128 | -5 | FLAM\_A | Alu | SINE |
| 69178379 | 69178607 | - | 23.1 | 4457 | 4684 | -1462 | L1M4 | L1 | LINE |
| 69178608 | 69179289 | + | 18.6 | 1 | 711 | -23 | MER50 | ERV1 | LTR |
| 69179317 | 69179648 | - | 23.6 | 4095 | 4447 | -1699 | L1M4 | L1 | LINE |
| 69179675 | 69179818 | - | 0.7 | 168 | 311 | -7 | AluYb8 | Alu | SINE |
| 69179819 | 69180090 | - | 25.2 | 3748 | 4026 | -2120 | L1M4 | L1 | LINE |

  

|  |  |  |  |  |  |  |  |  |  |
| --- | --- | --- | --- | --- | --- | --- | --- | --- | --- |
| chr4:69187287-69188511 | | | 1225 bp | | |  |  |  |  |
| Genome Start | Genome End | Strand | Div | Repeat Start | Repeat End | Repeat Left | Name | Family | Class |
| 69187287 | 69187475 | + | 23.4 | 1 | 179 | -433 | LTR9 | ERV1 | LTR |
| 69187476 | 69188046 | + | 8.2 | 5583 | 6154 | 0 | L1PA7 | L1 | LINE |
| 69188047 | 69188511 | + | 23.4 | 180 | 610 | -2 | LTR9 | ERV1 | LTR |

  

|  |  |  |  |  |  |  |  |  |  |
| --- | --- | --- | --- | --- | --- | --- | --- | --- | --- |
| chr4:69204220-69209469 | | | 5097 bp | | |  |  |  |  |
| Genome Start | Genome End | Strand | Div | Repeat Start | Repeat End | Repeat Left | Name | Family | Class |
| 69204220 | 69204715 | + | 12.6 | 1 | 585 | -6013 | MER4A1-int | ERV1 | LTR |
| 69204761 | 69204832 | + | 22.9 | 950 | 1019 | -7251 | MER4A1-int | ERV1 | LTR |
| 69204924 | 69205279 | + | 25.5 | 1845 | 2200 | -4131 | MER4A1-int | ERV1 | LTR |
| 69205295 | 69206210 | + | 12.7 | 200 | 1185 | -5573 | MER4B-int | ERV1 | LTR |
| 69206209 | 69206951 | + | 9.8 | 1748 | 2495 | -4103 | MER4B-int | ERV1 | LTR |
| 69206952 | 69207056 | - | 22.9 | 136 | 257 | -434 | LTR8 | ERV1 | LTR |
| 69207058 | 69207155 | + | 22.9 | 3533 | 3632 | -2966 | MER4A1-int | ERV1 | LTR |
| 69207159 | 69209469 | + | 11.8 | 4173 | 6584 | -14 | MER4A1-int | ERV1 | LTR |

  

|  |  |  |  |  |  |  |  |  |  |
| --- | --- | --- | --- | --- | --- | --- | --- | --- | --- |
| chr4:69245740-69249082 | | | 3343 bp | | |  |  |  |  |
| Genome Start | Genome End | Strand | Div | Repeat Start | Repeat End | Repeat Left | Name | Family | Class |
| 69245740 | 69246782 | + | 18.8 | 24 | 1130 | -1288 | Tigger1 | MER2\_type | DNA |
| 69246783 | 69247064 | - | 8.9 | 3 | 296 | -15 | AluY | Alu | SINE |
| 69247065 | 69247956 | + | 18.8 | 1131 | 2014 | -404 | Tigger1 | MER2\_type | DNA |
| 69247957 | 69248707 | + | 12.5 | 5413 | 6166 | -8 | L1PA11 | L1 | LINE |
| 69248708 | 69249082 | + | 18.8 | 2015 | 2416 | -2 | Tigger1 | MER2\_type | DNA |

  

|  |  |  |  |  |  |  |  |  |  |
| --- | --- | --- | --- | --- | --- | --- | --- | --- | --- |
| chr4:69268623-69271277 | | | 2693 bp | | |  |  |  |  |
| Genome Start | Genome End | Strand | Div | Repeat Start | Repeat End | Repeat Left | Name | Family | Class |
| 69268623 | 69268802 | + | 22.5 | 1673 | 1854 | -2337 | MER21-int | ERV1 | LTR |
| 69268808 | 69269341 | + | 14.7 | 3 | 552 | -5603 | L1P1 | L1 | LINE |
| 69269260 | 69269375 | - | 9.5 | 467 | 582 | -5573 | L1P1 | L1 | LINE |
| 69269368 | 69270075 | + | 7.3 | 2834 | 3542 | -2604 | L1P1 | L1 | LINE |
| 69270074 | 69271138 | + | 3.9 | 5081 | 6155 | 0 | L1PA4 | L1 | LINE |
| 69271188 | 69271277 | + | 30.3 | 1840 | 1928 | -2263 | MER21-int | ERV1 | LTR |

  

|  |  |  |  |  |  |  |  |  |  |
| --- | --- | --- | --- | --- | --- | --- | --- | --- | --- |
| chr4:69412186-69416864 | | | 4680 bp | | |  |  |  |  |
| Genome Start | Genome End | Strand | Div | Repeat Start | Repeat End | Repeat Left | Name | Family | Class |
| 69412186 | 69412692 | + | 16.0 | 1 | 509 | -3682 | MER21A-int | ERV1 | LTR |
| 69412693 | 69413156 | + | 15.8 | 1 | 471 | -263 | MER50 | ERV1 | LTR |
| 69413155 | 69413217 | + | 15.9 | 646 | 711 | -23 | MER50 | ERV1 | LTR |
| 69413219 | 69416864 | + | 17.6 | 506 | 4191 | 0 | MER21A-int | ERV1 | LTR |

  

|  |  |  |  |  |  |  |  |  |  |
| --- | --- | --- | --- | --- | --- | --- | --- | --- | --- |
| chr4:69430680-69431486 | | | 807 bp | | |  |  |  |  |
| Genome Start | Genome End | Strand | Div | Repeat Start | Repeat End | Repeat Left | Name | Family | Class |
| 69430680 | 69430960 | - | 8.7 | 229 | 513 | 0 | LTR10D | ERV1 | LTR |
| 69430961 | 69431258 | + | 6.1 | 1 | 309 | -2 | AluY | Alu | SINE |
| 69431259 | 69431486 | - | 8.7 | 1 | 228 | -285 | LTR10D | ERV1 | LTR |

  

|  |  |  |  |  |  |  |  |  |  |
| --- | --- | --- | --- | --- | --- | --- | --- | --- | --- |
| chr4:69539309-69540533 | | | 1225 bp | | |  |  |  |  |
| Genome Start | Genome End | Strand | Div | Repeat Start | Repeat End | Repeat Left | Name | Family | Class |
| 69539309 | 69539497 | + | 23.4 | 1 | 179 | -433 | LTR9 | ERV1 | LTR |
| 69539498 | 69540068 | + | 8.2 | 5583 | 6154 | 0 | L1PA7 | L1 | LINE |
| 69540069 | 69540533 | + | 23.4 | 180 | 610 | -2 | LTR9 | ERV1 | LTR |

  

|  |  |  |  |  |  |  |  |  |  |
| --- | --- | --- | --- | --- | --- | --- | --- | --- | --- |
| chr4:69593040-69597139 | | | 4056 bp | | |  |  |  |  |
| Genome Start | Genome End | Strand | Div | Repeat Start | Repeat End | Repeat Left | Name | Family | Class |
| 69593040 | 69593779 | - | 22.2 | 6300 | 7022 | -16 | L1MB8 | L1 | LINE |
| 69593780 | 69594732 | + | 14.2 | 1 | 954 | -14 | LTR5\_Hs | ERVK | LTR |
| 69594745 | 69595452 | - | 21.1 | 5567 | 6305 | -729 | L1MB8 | L1 | LINE |
| 69595479 | 69595731 | - | 21.4 | 34 | 281 | -31 | AluJo | Alu | SINE |
| 69595732 | 69596949 | - | 23.8 | 4287 | 5570 | -1436 | L1MB8 | L1 | LINE |
| 69596956 | 69597139 | - | 30.8 | 103 | 289 | -7045 | L1MB8 | L1 | LINE |

  

|  |  |  |  |  |  |  |  |  |  |
| --- | --- | --- | --- | --- | --- | --- | --- | --- | --- |
| chr4:69656545-69657290 | | | 746 bp | | |  |  |  |  |
| Genome Start | Genome End | Strand | Div | Repeat Start | Repeat End | Repeat Left | Name | Family | Class |
| 69656545 | 69656823 | + | 32.0 | 19 | 305 | -145 | LTR16A | ERVL | LTR |
| 69656824 | 69657169 | - | 26.4 | 1 | 365 | 0 | MLT1A0 | MaLR | LTR |
| 69657170 | 69657290 | + | 32.0 | 306 | 428 | -22 | LTR16A | ERVL | LTR |

  

|  |  |  |  |  |  |  |  |  |  |
| --- | --- | --- | --- | --- | --- | --- | --- | --- | --- |
| chr4:69816979-69818234 | | | 1248 bp | | |  |  |  |  |
| Genome Start | Genome End | Strand | Div | Repeat Start | Repeat End | Repeat Left | Name | Family | Class |
| 69816979 | 69817240 | - | 36.5 | 5881 | 6147 | -23 | L1ME3A | L1 | LINE |
| 69817249 | 69817399 | - | 32.0 | 5690 | 5838 | -402 | L1ME3A | L1 | LINE |
| 69817400 | 69818149 | - | 17.5 | 1 | 826 | 0 | LTR1B | ERV1 | LTR |
| 69818150 | 69818234 | - | 32.0 | 5606 | 5689 | -551 | L1ME3A | L1 | LINE |

  

|  |  |  |  |  |  |  |  |  |  |
| --- | --- | --- | --- | --- | --- | --- | --- | --- | --- |
| chr4:70117770-70118534 | | | 765 bp | | |  |  |  |  |
| Genome Start | Genome End | Strand | Div | Repeat Start | Repeat End | Repeat Left | Name | Family | Class |
| 70117770 | 70118050 | + | 31.6 | 18 | 305 | -145 | LTR16A | ERVL | LTR |
| 70118051 | 70118392 | - | 26.4 | 5 | 365 | 0 | MLT1A0 | MaLR | LTR |
| 70118393 | 70118534 | + | 31.6 | 306 | 447 | -3 | LTR16A | ERVL | LTR |

  

|  |  |  |  |  |  |  |  |  |  |
| --- | --- | --- | --- | --- | --- | --- | --- | --- | --- |
| chr4:70177319-70178072 | | | 754 bp | | |  |  |  |  |
| Genome Start | Genome End | Strand | Div | Repeat Start | Repeat End | Repeat Left | Name | Family | Class |
| 70177319 | 70177444 | - | 35.1 | 306 | 432 | -18 | LTR16A | ERVL | LTR |
| 70177445 | 70177790 | + | 27.0 | 1 | 365 | 0 | MLT1A0 | MaLR | LTR |
| 70177791 | 70178072 | - | 35.1 | 18 | 305 | -145 | LTR16A | ERVL | LTR |

  

|  |  |  |  |  |  |  |  |  |  |
| --- | --- | --- | --- | --- | --- | --- | --- | --- | --- |
| chr4:70195893-70198495 | | | 2500 bp | | |  |  |  |  |
| Genome Start | Genome End | Strand | Div | Repeat Start | Repeat End | Repeat Left | Name | Family | Class |
| 70195893 | 70196251 | - | 19.6 | 5792 | 6151 | 0 | L1PB1 | L1 | LINE |
| 70196339 | 70197370 | - | 7.4 | 1 | 1266 | 0 | MER11A | ERVK | LTR |
| 70197387 | 70198495 | - | 15.5 | 4668 | 5791 | -360 | L1PB1 | L1 | LINE |

  

|  |  |  |  |  |  |  |  |  |  |
| --- | --- | --- | --- | --- | --- | --- | --- | --- | --- |
| chr4:70229557-70230941 | | | 1385 bp | | |  |  |  |  |
| Genome Start | Genome End | Strand | Div | Repeat Start | Repeat End | Repeat Left | Name | Family | Class |
| 70229557 | 70229686 | + | 27.2 | 3135 | 3235 | -956 | MER21-int | ERV1 | LTR |
| 70229687 | 70230048 | + | 21.0 | 1 | 408 | 0 | MLT1A1 | MaLR | LTR |
| 70230049 | 70230941 | + | 27.2 | 3236 | 4135 | -56 | MER21-int | ERV1 | LTR |

  

|  |  |  |  |  |  |  |  |  |  |
| --- | --- | --- | --- | --- | --- | --- | --- | --- | --- |
| chr4:70286294-70287059 | | | 766 bp | | |  |  |  |  |
| Genome Start | Genome End | Strand | Div | Repeat Start | Repeat End | Repeat Left | Name | Family | Class |
| 70286294 | 70286431 | - | 32.8 | 306 | 447 | -3 | LTR16A | ERVL | LTR |
| 70286432 | 70286777 | + | 26.7 | 1 | 365 | 0 | MLT1A0 | MaLR | LTR |
| 70286778 | 70287059 | - | 32.8 | 18 | 305 | -145 | LTR16A | ERVL | LTR |

  

|  |  |  |  |  |  |  |  |  |  |
| --- | --- | --- | --- | --- | --- | --- | --- | --- | --- |
| chr4:70305643-70306403 | | | 761 bp | | |  |  |  |  |
| Genome Start | Genome End | Strand | Div | Repeat Start | Repeat End | Repeat Left | Name | Family | Class |
| 70305643 | 70305780 | - | 32.7 | 306 | 447 | -3 | LTR16A | ERVL | LTR |
| 70305781 | 70306126 | + | 25.7 | 1 | 365 | 0 | MLT1A0 | MaLR | LTR |
| 70306127 | 70306403 | - | 32.7 | 25 | 305 | -145 | LTR16A | ERVL | LTR |

  

|  |  |  |  |  |  |  |  |  |  |
| --- | --- | --- | --- | --- | --- | --- | --- | --- | --- |
| chr4:75533925-75534450 | | | 526 bp | | |  |  |  |  |
| Genome Start | Genome End | Strand | Div | Repeat Start | Repeat End | Repeat Left | Name | Family | Class |
| 75533925 | 75534048 | - | 20.2 | 88 | 215 | 0 | MER58C | MER1\_type | DNA |
| 75534049 | 75534354 | + | 14.8 | 5 | 301 | 0 | AluJb | Alu | SINE |
| 75534355 | 75534450 | - | 20.2 | 1 | 87 | -128 | MER58C | MER1\_type | DNA |

  

|  |  |  |  |  |  |  |  |  |  |
| --- | --- | --- | --- | --- | --- | --- | --- | --- | --- |
| chr4:104045168-104045789 | | | 622 bp | | |  |  |  |  |
| Genome Start | Genome End | Strand | Div | Repeat Start | Repeat End | Repeat Left | Name | Family | Class |
| 104045168 | 104045305 | + | 19.6 | 1 | 133 | -179 | AluJo | Alu | SINE |
| 104045306 | 104045610 | + | 9.9 | 1 | 303 | -9 | AluSx | Alu | SINE |
| 104045611 | 104045789 | + | 19.6 | 134 | 312 | 0 | AluJo | Alu | SINE |

  

|  |  |  |  |  |  |  |  |  |  |
| --- | --- | --- | --- | --- | --- | --- | --- | --- | --- |
| chr4:104059021-104059531 | | | 511 bp | | |  |  |  |  |
| Genome Start | Genome End | Strand | Div | Repeat Start | Repeat End | Repeat Left | Name | Family | Class |
| 104059021 | 104059116 | + | 15.9 | 5224 | 5325 | -815 | L1M3 | L1 | LINE |
| 104059117 | 104059475 | + | 20.5 | 1 | 365 | 0 | MLT1A0 | MaLR | LTR |
| 104059476 | 104059531 | + | 15.9 | 5326 | 5381 | -759 | L1M3 | L1 | LINE |

  

|  |  |  |  |  |  |  |  |  |  |
| --- | --- | --- | --- | --- | --- | --- | --- | --- | --- |
| chr4:104095544-104096052 | | | 509 bp | | |  |  |  |  |
| Genome Start | Genome End | Strand | Div | Repeat Start | Repeat End | Repeat Left | Name | Family | Class |
| 104095544 | 104095680 | + | 28.6 | 91 | 219 | -7115 | L1M4b | L1 | LINE |
| 104095681 | 104096020 | + | 7.4 | 1 | 355 | 0 | THE1A | MaLR | LTR |
| 104096021 | 104096052 | + | 28.6 | 220 | 248 | -7086 | L1M4b | L1 | LINE |

  

|  |  |  |  |  |  |  |  |  |  |
| --- | --- | --- | --- | --- | --- | --- | --- | --- | --- |
| chr4:104104377-104109135 | | | 4747 bp | | |  |  |  |  |
| Genome Start | Genome End | Strand | Div | Repeat Start | Repeat End | Repeat Left | Name | Family | Class |
| 104104377 | 104105784 | + | 22.6 | 3808 | 5265 | -1741 | L1M4b | L1 | LINE |
| 104105786 | 104106213 | - | 18.8 | 16 | 457 | 0 | MSTB2 | MaLR | LTR |
| 104106218 | 104107977 | - | 27.4 | 2 | 1735 | 0 | MLT-int | MaLR | LTR |
| 104107980 | 104108392 | - | 19.9 | 15 | 457 | 0 | MSTB2 | MaLR | LTR |
| 104108398 | 104109135 | + | 22.1 | 5269 | 6037 | -969 | L1M4b | L1 | LINE |

  

|  |  |  |  |  |  |  |  |  |  |
| --- | --- | --- | --- | --- | --- | --- | --- | --- | --- |
| chr4:104377268-104378398 | | | 1090 bp | | |  |  |  |  |
| Genome Start | Genome End | Strand | Div | Repeat Start | Repeat End | Repeat Left | Name | Family | Class |
| 104377268 | 104377654 | + | 16.8 | 5513 | 5910 | -449 | L1MD2 | L1 | LINE |
| 104377655 | 104377948 | + | 10.9 | 1 | 294 | -16 | AluSg | Alu | SINE |
| 104377949 | 104378157 | + | 16.8 | 5911 | 6154 | -205 | L1MD2 | L1 | LINE |
| 104378199 | 104378398 | + | 16.8 | 6155 | 6346 | -13 | L1MD2 | L1 | LINE |

  

|  |  |  |  |  |  |  |  |  |  |
| --- | --- | --- | --- | --- | --- | --- | --- | --- | --- |
| chr4:111553777-111554555 | | | 779 bp | | |  |  |  |  |
| Genome Start | Genome End | Strand | Div | Repeat Start | Repeat End | Repeat Left | Name | Family | Class |
| 111553777 | 111554193 | - | 19.1 | 86 | 467 | 0 | MLT1C | MaLR | LTR |
| 111554194 | 111554501 | - | 13.6 | 1 | 312 | 0 | AluSx | Alu | SINE |
| 111554502 | 111554555 | - | 19.1 | 36 | 85 | -382 | MLT1C | MaLR | LTR |

  

|  |  |  |  |  |  |  |  |  |  |
| --- | --- | --- | --- | --- | --- | --- | --- | --- | --- |
| chr4:111579644-111580313 | | | 670 bp | | |  |  |  |  |
| Genome Start | Genome End | Strand | Div | Repeat Start | Repeat End | Repeat Left | Name | Family | Class |
| 111579644 | 111579694 | - | 16.6 | 6111 | 6156 | 0 | L1PB4 | L1 | LINE |
| 111579695 | 111580140 | - | 8.1 | 3 | 453 | 0 | MLT2A2 | ERVL | LTR |
| 111580141 | 111580313 | - | 16.6 | 5936 | 6110 | -46 | L1PB4 | L1 | LINE |

  

|  |  |  |  |  |  |  |  |  |  |
| --- | --- | --- | --- | --- | --- | --- | --- | --- | --- |
| chr4:111583803-111584971 | | | 1169 bp | | |  |  |  |  |
| Genome Start | Genome End | Strand | Div | Repeat Start | Repeat End | Repeat Left | Name | Family | Class |
| 111583803 | 111584148 | + | 32.7 | 22 | 399 | -122 | LTR33 | ERVL | LTR |
| 111584149 | 111584322 | + | 13.8 | 1 | 197 | -231 | MSTA | MaLR | LTR |
| 111584323 | 111584629 | + | 9.3 | 1 | 307 | 0 | AluSc | Alu | SINE |
| 111584630 | 111584854 | + | 13.8 | 198 | 428 | 0 | MSTA | MaLR | LTR |
| 111584855 | 111584971 | + | 32.7 | 400 | 510 | -11 | LTR33 | ERVL | LTR |

  

|  |  |  |  |  |  |  |  |  |  |
| --- | --- | --- | --- | --- | --- | --- | --- | --- | --- |
| chr4:111585325-111585809 | | | 485 bp | | |  |  |  |  |
| Genome Start | Genome End | Strand | Div | Repeat Start | Repeat End | Repeat Left | Name | Family | Class |
| 111585325 | 111585397 | - | 18.4 | 463 | 524 | -7 | MER65B | ERV1 | LTR |
| 111585398 | 111585633 | + | 9.3 | 1 | 236 | -77 | AluSq | Alu | SINE |
| 111585634 | 111585809 | - | 18.4 | 296 | 462 | -69 | MER65B | ERV1 | LTR |

  

|  |  |  |  |  |  |  |  |  |  |
| --- | --- | --- | --- | --- | --- | --- | --- | --- | --- |
| chr4:119562009-119562654 | | | 646 bp | | |  |  |  |  |
| Genome Start | Genome End | Strand | Div | Repeat Start | Repeat End | Repeat Left | Name | Family | Class |
| 119562009 | 119562054 | + | 27.5 | 62 | 107 | -442 | MLT1H | MaLR | LTR |
| 119562055 | 119562371 | + | 8.4 | 1 | 308 | -3 | AluY | Alu | SINE |
| 119562372 | 119562654 | + | 26.9 | 93 | 375 | -167 | MLT1H | MaLR | LTR |

  

|  |  |  |  |  |  |  |  |  |  |
| --- | --- | --- | --- | --- | --- | --- | --- | --- | --- |
| chr4:119566568-119567736 | | | 1169 bp | | |  |  |  |  |
| Genome Start | Genome End | Strand | Div | Repeat Start | Repeat End | Repeat Left | Name | Family | Class |
| 119566568 | 119566702 | + | 18.4 | 59 | 190 | -593 | LTR39 | ERV1 | LTR |
| 119566703 | 119566826 | + | 5.7 | 6034 | 6155 | 0 | L1PA4 | L1 | LINE |
| 119566827 | 119567123 | + | 18.4 | 191 | 459 | -324 | LTR39 | ERV1 | LTR |
| 119567124 | 119567422 | - | 14.4 | 1 | 299 | -13 | AluSx | Alu | SINE |
| 119567423 | 119567736 | + | 18.4 | 460 | 782 | -1 | LTR39 | ERV1 | LTR |

  

|  |  |  |  |  |  |  |  |  |  |
| --- | --- | --- | --- | --- | --- | --- | --- | --- | --- |
| chr4:119579764-119580825 | | | 1062 bp | | |  |  |  |  |
| Genome Start | Genome End | Strand | Div | Repeat Start | Repeat End | Repeat Left | Name | Family | Class |
| 119579764 | 119580175 | - | 6.6 | 334 | 771 | -10 | MER4E1 | ERV1 | LTR |
| 119580176 | 119580473 | + | 10.1 | 1 | 296 | -14 | AluSg | Alu | SINE |
| 119580474 | 119580825 | - | 6.6 | 1 | 333 | -448 | MER4E1 | ERV1 | LTR |

  

|  |  |  |  |  |  |  |  |  |  |
| --- | --- | --- | --- | --- | --- | --- | --- | --- | --- |
| chr4:119591881-119592545 | | | 646 bp | | |  |  |  |  |
| Genome Start | Genome End | Strand | Div | Repeat Start | Repeat End | Repeat Left | Name | Family | Class |
| 119591881 | 119592288 | - | 21.2 | 116 | 567 | 0 | MLT1F1 | MaLR | LTR |
| 119592307 | 119592433 | - | 18.1 | 3 | 134 | -178 | AluJo | Alu | SINE |
| 119592435 | 119592545 | - | 22.5 | 23 | 133 | -434 | MLT1F1 | MaLR | LTR |

  

|  |  |  |  |  |  |  |  |  |  |
| --- | --- | --- | --- | --- | --- | --- | --- | --- | --- |
| chr4:119596209-119597140 | | | 932 bp | | |  |  |  |  |
| Genome Start | Genome End | Strand | Div | Repeat Start | Repeat End | Repeat Left | Name | Family | Class |
| 119596209 | 119596593 | - | 17.4 | 5776 | 6176 | -2 | L1MB8 | L1 | LINE |
| 119596594 | 119596900 | - | 12.7 | 6 | 312 | 0 | AluSx | Alu | SINE |
| 119596901 | 119597140 | - | 17.4 | 5546 | 5775 | -403 | L1MB8 | L1 | LINE |

  

|  |  |  |  |  |  |  |  |  |  |
| --- | --- | --- | --- | --- | --- | --- | --- | --- | --- |
| chr4:119599610-119600080 | | | 471 bp | | |  |  |  |  |
| Genome Start | Genome End | Strand | Div | Repeat Start | Repeat End | Repeat Left | Name | Family | Class |
| 119599610 | 119599710 | - | 24.0 | 73 | 160 | -6 | MER103 | DNA | DNA |
| 119599711 | 119600016 | + | 4.9 | 1 | 306 | -5 | AluY | Alu | SINE |
| 119600017 | 119600080 | - | 24.0 | 9 | 72 | -94 | MER103 | DNA | DNA |

  

|  |  |  |  |  |  |  |  |  |  |
| --- | --- | --- | --- | --- | --- | --- | --- | --- | --- |
| chr4:119641574-119642368 | | | 795 bp | | |  |  |  |  |
| Genome Start | Genome End | Strand | Div | Repeat Start | Repeat End | Repeat Left | Name | Family | Class |
| 119641574 | 119641928 | - | 18.1 | 5825 | 6164 | -10 | L1MB5 | L1 | LINE |
| 119641929 | 119642215 | - | 10.1 | 1 | 307 | -5 | AluSx | Alu | SINE |
| 119642216 | 119642368 | - | 18.1 | 5668 | 5824 | -350 | L1MB5 | L1 | LINE |

  

|  |  |  |  |  |  |  |  |  |  |
| --- | --- | --- | --- | --- | --- | --- | --- | --- | --- |
| chr4:119666450-119668915 | | | 2465 bp | | |  |  |  |  |
| Genome Start | Genome End | Strand | Div | Repeat Start | Repeat End | Repeat Left | Name | Family | Class |
| 119666450 | 119667431 | - | 12.0 | 1447 | 2418 | 0 | Tigger1 | MER2\_type | DNA |
| 119667432 | 119667734 | + | 7.9 | 1 | 303 | -8 | AluY | Alu | SINE |
| 119667736 | 119668029 | + | 7.5 | 1 | 294 | -17 | AluY | Alu | SINE |
| 119668030 | 119668915 | - | 12.0 | 536 | 1446 | -972 | Tigger1 | MER2\_type | DNA |

  

|  |  |  |  |  |  |  |  |  |  |
| --- | --- | --- | --- | --- | --- | --- | --- | --- | --- |
| chr4:119714307-119718386 | | | 3626 bp | | |  |  |  |  |
| Genome Start | Genome End | Strand | Div | Repeat Start | Repeat End | Repeat Left | Name | Family | Class |
| 119714307 | 119714432 | + | 31.4 | 2412 | 2534 | -885 | L2 | L2 | LINE |
| 119714469 | 119714606 | - | 20.3 | 6169 | 6307 | -5 | L1MA9 | L1 | LINE |
| 119714607 | 119714795 | + | 17.4 | 1 | 193 | -471 | MER4A | ERV1 | LTR |
| 119714796 | 119715149 | - | 12.7 | 1 | 364 | 0 | THE1B | MaLR | LTR |
| 119715150 | 119715613 | + | 19.6 | 189 | 581 | 0 | MER4C | ERV1 | LTR |
| 119715631 | 119716442 | - | 16.2 | 5319 | 6161 | -151 | L1MA9 | L1 | LINE |
| 119716482 | 119716717 | + | 29.0 | 2549 | 2783 | -636 | L2 | L2 | LINE |
| 119716718 | 119717010 | - | 15.2 | 1 | 291 | -21 | AluJb | Alu | SINE |
| 119717019 | 119717350 | - | 10.8 | 1 | 329 | 0 | AluSc | Alu | SINE |
| 119717351 | 119717675 | + | 28.9 | 2791 | 3122 | -297 | L2 | L2 | LINE |
| 119718003 | 119718134 | - | 21.4 | 5764 | 5898 | -266 | L1ME2 | L1 | LINE |
| 119718137 | 119718269 | + | 27.7 | 227 | 360 | -49 | MLT1I | MaLR | LTR |
| 119718295 | 119718386 | + | 32.6 | 3327 | 3419 | 0 | L2 | L2 | LINE |

  

|  |  |  |  |  |  |  |  |  |  |
| --- | --- | --- | --- | --- | --- | --- | --- | --- | --- |
| chr4:119735395-119735996 | | | 602 bp | | |  |  |  |  |
| Genome Start | Genome End | Strand | Div | Repeat Start | Repeat End | Repeat Left | Name | Family | Class |
| 119735395 | 119735555 | - | 11.9 | 137 | 294 | -18 | AluSx | Alu | SINE |
| 119735556 | 119735860 | - | 10.8 | 1 | 306 | -7 | AluSp | Alu | SINE |
| 119735861 | 119735996 | - | 11.9 | 1 | 136 | -176 | AluSx | Alu | SINE |

  

|  |  |  |  |  |  |  |  |  |  |
| --- | --- | --- | --- | --- | --- | --- | --- | --- | --- |
| chr4:119747198-119747543 | | | 346 bp | | |  |  |  |  |
| Genome Start | Genome End | Strand | Div | Repeat Start | Repeat End | Repeat Left | Name | Family | Class |
| 119747198 | 119747321 | - | 30.7 | 5574 | 5693 | -428 | L1M5 | L1 | LINE |
| 119747322 | 119747444 | + | 16.5 | 4 | 126 | -7 | FLAM\_C | Alu | SINE |
| 119747445 | 119747543 | - | 30.7 | 5484 | 5573 | -548 | L1M5 | L1 | LINE |

  

|  |  |  |  |  |  |  |  |  |  |
| --- | --- | --- | --- | --- | --- | --- | --- | --- | --- |
| chr4:119751061-119752528 | | | 1468 bp | | |  |  |  |  |
| Genome Start | Genome End | Strand | Div | Repeat Start | Repeat End | Repeat Left | Name | Family | Class |
| 119751061 | 119751360 | - | 20.0 | 604 | 934 | -1 | MER21C | ERV1 | LTR |
| 119751361 | 119751665 | - | 17.6 | 1 | 302 | -10 | AluJo | Alu | SINE |
| 119751666 | 119751975 | - | 20.0 | 270 | 603 | -332 | MER21C | ERV1 | LTR |
| 119751976 | 119752269 | + | 15.0 | 1 | 293 | -20 | AluSq | Alu | SINE |
| 119752270 | 119752528 | - | 20.0 | 18 | 269 | -666 | MER21C | ERV1 | LTR |

  

|  |  |  |  |  |  |  |  |  |  |
| --- | --- | --- | --- | --- | --- | --- | --- | --- | --- |
| chr4:119753207-119753787 | | | 581 bp | | |  |  |  |  |
| Genome Start | Genome End | Strand | Div | Repeat Start | Repeat End | Repeat Left | Name | Family | Class |
| 119753207 | 119753333 | + | 20.5 | 10 | 132 | -180 | AluJb | Alu | SINE |
| 119753334 | 119753628 | + | 10.5 | 1 | 305 | -4 | AluSg1 | Alu | SINE |
| 119753629 | 119753787 | + | 20.5 | 133 | 294 | -18 | AluJb | Alu | SINE |

  

|  |  |  |  |  |  |  |  |  |  |
| --- | --- | --- | --- | --- | --- | --- | --- | --- | --- |
| chr4:119757095-119757505 | | | 411 bp | | |  |  |  |  |
| Genome Start | Genome End | Strand | Div | Repeat Start | Repeat End | Repeat Left | Name | Family | Class |
| 119757095 | 119757182 | - | 18.4 | 5503 | 5589 | -577 | L1M5 | L1 | LINE |
| 119757183 | 119757474 | - | 19.0 | 1 | 292 | -20 | AluJb | Alu | SINE |
| 119757475 | 119757505 | - | 17.9 | 5476 | 5505 | -679 | L1M5 | L1 | LINE |

  

|  |  |  |  |  |  |  |  |  |  |
| --- | --- | --- | --- | --- | --- | --- | --- | --- | --- |
| chr4:119757565-119758034 | | | 422 bp | | |  |  |  |  |
| Genome Start | Genome End | Strand | Div | Repeat Start | Repeat End | Repeat Left | Name | Family | Class |
| 119757565 | 119757624 | + | 21.5 | 24 | 71 | -241 | AluJo | Alu | SINE |
| 119757673 | 119757946 | - | 14.7 | 1 | 279 | -33 | AluSx | Alu | SINE |
| 119757947 | 119758034 | + | 21.5 | 72 | 153 | -159 | AluJo | Alu | SINE |

  

|  |  |  |  |  |  |  |  |  |  |
| --- | --- | --- | --- | --- | --- | --- | --- | --- | --- |
| chr4:119762816-119764289 | | | 1474 bp | | |  |  |  |  |
| Genome Start | Genome End | Strand | Div | Repeat Start | Repeat End | Repeat Left | Name | Family | Class |
| 119762816 | 119763116 | - | 20.6 | 604 | 935 | 0 | MER21C | ERV1 | LTR |
| 119763117 | 119763406 | - | 19.7 | 1 | 300 | -12 | AluJo | Alu | SINE |
| 119763407 | 119763476 | - | 20.6 | 526 | 603 | -332 | MER21C | ERV1 | LTR |
| 119763477 | 119763783 | + | 19.9 | 2 | 294 | -18 | AluJb | Alu | SINE |
| 119763784 | 119764289 | - | 20.6 | 18 | 525 | -410 | MER21C | ERV1 | LTR |

  

|  |  |  |  |  |  |  |  |  |  |
| --- | --- | --- | --- | --- | --- | --- | --- | --- | --- |
| chr4:119779750-119780589 | | | 840 bp | | |  |  |  |  |
| Genome Start | Genome End | Strand | Div | Repeat Start | Repeat End | Repeat Left | Name | Family | Class |
| 119779750 | 119780197 | - | 24.5 | 2032 | 2563 | -4226 | L1M2 | L1 | LINE |
| 119780198 | 119780507 | - | 10.6 | 1 | 304 | -6 | AluSg | Alu | SINE |
| 119780508 | 119780589 | - | 26.4 | 1956 | 2031 | -4758 | L1M2 | L1 | LINE |

  

|  |  |  |  |  |  |  |  |  |  |
| --- | --- | --- | --- | --- | --- | --- | --- | --- | --- |
| chr4:119781516-119782014 | | | 499 bp | | |  |  |  |  |
| Genome Start | Genome End | Strand | Div | Repeat Start | Repeat End | Repeat Left | Name | Family | Class |
| 119781516 | 119781607 | + | 31.7 | 3224 | 3312 | -107 | L2 | L2 | LINE |
| 119781608 | 119781905 | + | 11.7 | 1 | 298 | -15 | AluSq | Alu | SINE |
| 119781906 | 119782014 | + | 31.7 | 3313 | 3418 | -1 | L2 | L2 | LINE |

  

|  |  |  |  |  |  |  |  |  |  |
| --- | --- | --- | --- | --- | --- | --- | --- | --- | --- |
| chr4:119791300-119793398 | | | 2108 bp | | |  |  |  |  |
| Genome Start | Genome End | Strand | Div | Repeat Start | Repeat End | Repeat Left | Name | Family | Class |
| 119791300 | 119792329 | - | 27.8 | 4906 | 6040 | -183 | L1ME3B | L1 | LINE |
| 119792330 | 119792819 | - | 22.6 | 1 | 495 | -10 | MLT1D | MaLR | LTR |
| 119792822 | 119793212 | - | 33.4 | 4484 | 4909 | -1220 | L1ME3B | L1 | LINE |
| 119793202 | 119793398 | - | 22.9 | 3777 | 3965 | -2164 | L1ME3B | L1 | LINE |

  

|  |  |  |  |  |  |  |  |  |  |
| --- | --- | --- | --- | --- | --- | --- | --- | --- | --- |
| chr4:119801738-119802564 | | | 827 bp | | |  |  |  |  |
| Genome Start | Genome End | Strand | Div | Repeat Start | Repeat End | Repeat Left | Name | Family | Class |
| 119801738 | 119801856 | - | 34.6 | 3308 | 3413 | -6 | L2 | L2 | LINE |
| 119801857 | 119802101 | - | 18.9 | 1 | 300 | -2 | AluJb | Alu | SINE |
| 119802102 | 119802564 | - | 33.7 | 2753 | 3307 | -112 | L2 | L2 | LINE |

  

|  |  |  |  |  |  |  |  |  |  |
| --- | --- | --- | --- | --- | --- | --- | --- | --- | --- |
| chr4:119805362-119807663 | | | 2147 bp | | |  |  |  |  |
| Genome Start | Genome End | Strand | Div | Repeat Start | Repeat End | Repeat Left | Name | Family | Class |
| 119805362 | 119805920 | - | 34.6 | 3856 | 4479 | -10 | L3 | CR1 | LINE |
| 119805933 | 119806100 | + | 30.1 | 2 | 173 | -348 | LTR33 | ERVL | LTR |
| 119806184 | 119806222 | + | 21.9 | 378 | 414 | -107 | LTR33 | ERVL | LTR |
| 119806223 | 119806238 | - | 15.6 | 417 | 432 | 0 | MSTB1 | MaLR | LTR |
| 119806282 | 119806531 | - | 15.6 | 151 | 416 | -16 | MSTB1 | MaLR | LTR |
| 119806532 | 119806826 | + | 9.9 | 5 | 298 | -15 | AluSq | Alu | SINE |
| 119806827 | 119806975 | - | 15.6 | 2 | 150 | -282 | MSTB1 | MaLR | LTR |
| 119806976 | 119807068 | + | 21.9 | 415 | 517 | -4 | LTR33 | ERVL | LTR |
| 119807086 | 119807162 | - | 28.5 | 3758 | 3834 | -655 | L3 | CR1 | LINE |
| 119807163 | 119807470 | - | 10.0 | 1 | 306 | 0 | AluSg | Alu | SINE |
| 119807471 | 119807663 | - | 28.5 | 3573 | 3757 | -732 | L3 | CR1 | LINE |

  

|  |  |  |  |  |  |  |  |  |  |
| --- | --- | --- | --- | --- | --- | --- | --- | --- | --- |
| chr4:119808252-119808727 | | | 476 bp | | |  |  |  |  |
| Genome Start | Genome End | Strand | Div | Repeat Start | Repeat End | Repeat Left | Name | Family | Class |
| 119808252 | 119808323 | - | 29.3 | 3142 | 3217 | -2929 | L1M5 | L1 | LINE |
| 119808324 | 119808624 | - | 13.6 | 1 | 301 | -11 | AluSx | Alu | SINE |
| 119808625 | 119808727 | - | 29.3 | 3044 | 3141 | -3005 | L1M5 | L1 | LINE |

  

|  |  |  |  |  |  |  |  |  |  |
| --- | --- | --- | --- | --- | --- | --- | --- | --- | --- |
| chr4:120475274-120475681 | | | 408 bp | | |  |  |  |  |
| Genome Start | Genome End | Strand | Div | Repeat Start | Repeat End | Repeat Left | Name | Family | Class |
| 120475274 | 120475434 | + | 17.8 | 55 | 208 | -116 | MER33 | MER1\_type | DNA |
| 120475435 | 120475552 | + | 16.1 | 1 | 118 | -15 | FLAM\_C | Alu | SINE |
| 120475553 | 120475681 | + | 17.8 | 209 | 324 | 0 | MER33 | MER1\_type | DNA |

  

|  |  |  |  |  |  |  |  |  |  |
| --- | --- | --- | --- | --- | --- | --- | --- | --- | --- |
| chr4:120487074-120487494 | | | 421 bp | | |  |  |  |  |
| Genome Start | Genome End | Strand | Div | Repeat Start | Repeat End | Repeat Left | Name | Family | Class |
| 120487074 | 120487129 | - | 17.1 | 6124 | 6178 | 0 | L1MB8 | L1 | LINE |
| 120487130 | 120487397 | + | 16.9 | 1 | 273 | -39 | AluJb | Alu | SINE |
| 120487398 | 120487494 | - | 17.1 | 6049 | 6123 | -55 | L1MB8 | L1 | LINE |

  

|  |  |  |  |  |  |  |  |  |  |
| --- | --- | --- | --- | --- | --- | --- | --- | --- | --- |
| chr4:120541593-120542061 | | | 469 bp | | |  |  |  |  |
| Genome Start | Genome End | Strand | Div | Repeat Start | Repeat End | Repeat Left | Name | Family | Class |
| 120541593 | 120541685 | + | 16.1 | 5987 | 6091 | -80 | L1MB2 | L1 | LINE |
| 120541686 | 120541975 | - | 10.0 | 1 | 290 | -23 | AluSq | Alu | SINE |
| 120541976 | 120542061 | + | 16.1 | 6092 | 6171 | 0 | L1MB2 | L1 | LINE |

  

|  |  |  |  |  |  |  |  |  |  |
| --- | --- | --- | --- | --- | --- | --- | --- | --- | --- |
| chr4:120555961-120556591 | | | 592 bp | | |  |  |  |  |
| Genome Start | Genome End | Strand | Div | Repeat Start | Repeat End | Repeat Left | Name | Family | Class |
| 120555961 | 120556134 | - | 18.0 | 136 | 312 | 0 | AluJo | Alu | SINE |
| 120556174 | 120556457 | - | 10.0 | 1 | 283 | -30 | AluSq | Alu | SINE |
| 120556458 | 120556591 | - | 18.0 | 3 | 135 | -177 | AluJo | Alu | SINE |

  

|  |  |  |  |  |  |  |  |  |  |
| --- | --- | --- | --- | --- | --- | --- | --- | --- | --- |
| chr4:120557503-120558974 | | | 1472 bp | | |  |  |  |  |
| Genome Start | Genome End | Strand | Div | Repeat Start | Repeat End | Repeat Left | Name | Family | Class |
| 120557503 | 120558006 | + | 20.4 | 18 | 525 | -410 | MER21C | ERV1 | LTR |
| 120558007 | 120558313 | - | 19.9 | 2 | 294 | -18 | AluJb | Alu | SINE |
| 120558314 | 120558383 | + | 20.4 | 526 | 603 | -332 | MER21C | ERV1 | LTR |
| 120558384 | 120558673 | + | 20.1 | 1 | 300 | -12 | AluJo | Alu | SINE |
| 120558674 | 120558974 | + | 20.4 | 604 | 935 | 0 | MER21C | ERV1 | LTR |

  

|  |  |  |  |  |  |  |  |  |  |
| --- | --- | --- | --- | --- | --- | --- | --- | --- | --- |
| chr4:120563726-120564210 | | | 437 bp | | |  |  |  |  |
| Genome Start | Genome End | Strand | Div | Repeat Start | Repeat End | Repeat Left | Name | Family | Class |
| 120563726 | 120563827 | - | 22.9 | 72 | 167 | -145 | AluJo | Alu | SINE |
| 120563828 | 120564101 | + | 14.7 | 1 | 279 | -33 | AluSx | Alu | SINE |
| 120564150 | 120564210 | - | 22.9 | 24 | 71 | -241 | AluJo | Alu | SINE |

  

|  |  |  |  |  |  |  |  |  |  |
| --- | --- | --- | --- | --- | --- | --- | --- | --- | --- |
| chr4:120564269-120565443 | | | 1125 bp | | |  |  |  |  |
| Genome Start | Genome End | Strand | Div | Repeat Start | Repeat End | Repeat Left | Name | Family | Class |
| 120564269 | 120564299 | + | 17.9 | 5476 | 5505 | -679 | L1MB8 | L1 | LINE |
| 120564300 | 120564591 | + | 18.0 | 1 | 292 | -20 | AluJb | Alu | SINE |
| 120564592 | 120564665 | + | 17.9 | 5506 | 5570 | -614 | L1MB8 | L1 | LINE |
| 120564716 | 120564824 | - | 18.4 | 4 | 112 | -30 | FLAM\_A | Alu | SINE |
| 120564825 | 120565443 | + | 19.6 | 5552 | 6173 | -5 | L1MB8 | L1 | LINE |

  

|  |  |  |  |  |  |  |  |  |  |
| --- | --- | --- | --- | --- | --- | --- | --- | --- | --- |
| chr4:120567986-120568558 | | | 573 bp | | |  |  |  |  |
| Genome Start | Genome End | Strand | Div | Repeat Start | Repeat End | Repeat Left | Name | Family | Class |
| 120567986 | 120568143 | - | 19.4 | 138 | 295 | -17 | AluJo | Alu | SINE |
| 120568144 | 120568434 | - | 10.0 | 1 | 301 | -8 | AluSg1 | Alu | SINE |
| 120568435 | 120568558 | - | 19.4 | 12 | 137 | -175 | AluJo | Alu | SINE |

  

|  |  |  |  |  |  |  |  |  |  |
| --- | --- | --- | --- | --- | --- | --- | --- | --- | --- |
| chr4:120569237-120570703 | | | 1467 bp | | |  |  |  |  |
| Genome Start | Genome End | Strand | Div | Repeat Start | Repeat End | Repeat Left | Name | Family | Class |
| 120569237 | 120569495 | + | 20.0 | 18 | 269 | -666 | MER21C | ERV1 | LTR |
| 120569496 | 120569788 | - | 15.1 | 1 | 292 | -21 | AluSq | Alu | SINE |
| 120569789 | 120570098 | + | 20.0 | 270 | 603 | -332 | MER21C | ERV1 | LTR |
| 120570099 | 120570403 | + | 17.2 | 1 | 302 | -10 | AluJo | Alu | SINE |
| 120570404 | 120570703 | + | 20.0 | 604 | 934 | -1 | MER21C | ERV1 | LTR |

  

|  |  |  |  |  |  |  |  |  |  |
| --- | --- | --- | --- | --- | --- | --- | --- | --- | --- |
| chr4:120574230-120574565 | | | 336 bp | | |  |  |  |  |
| Genome Start | Genome End | Strand | Div | Repeat Start | Repeat End | Repeat Left | Name | Family | Class |
| 120574230 | 120574318 | + | 30.2 | 5495 | 5575 | -2233 | L1MC4 | L1 | LINE |
| 120574319 | 120574441 | - | 16.5 | 4 | 126 | -7 | FLAM\_C | Alu | SINE |
| 120574442 | 120574565 | + | 30.2 | 5576 | 5690 | -2118 | L1MC4 | L1 | LINE |

  

|  |  |  |  |  |  |  |  |  |  |
| --- | --- | --- | --- | --- | --- | --- | --- | --- | --- |
| chr4:132816767-132819085 | | | 2286 bp | | |  |  |  |  |
| Genome Start | Genome End | Strand | Div | Repeat Start | Repeat End | Repeat Left | Name | Family | Class |
| 132816767 | 132816862 | + | 20.4 | 1 | 94 | -1137 | Tigger3b | MER2\_type | DNA |
| 132816886 | 132817129 | + | 14.8 | 336 | 586 | -645 | Tigger3b | MER2\_type | DNA |
| 132817140 | 132818489 | - | 3.0 | 4801 | 6152 | -3 | L1PA3 | L1 | LINE |
| 132818490 | 132819085 | + | 16.1 | 588 | 1231 | 0 | Tigger3b | MER2\_type | DNA |

  

|  |  |  |  |  |  |  |  |  |  |
| --- | --- | --- | --- | --- | --- | --- | --- | --- | --- |
| chr4:132822153-132824688 | | | 2499 bp | | |  |  |  |  |
| Genome Start | Genome End | Strand | Div | Repeat Start | Repeat End | Repeat Left | Name | Family | Class |
| 132822153 | 132823090 | - | 16.1 | 1423 | 2351 | -4434 | L1PA13 | L1 | LINE |
| 132823093 | 132823535 | - | 23.2 | 19 | 509 | 0 | LTR22C | ERVK | LTR |
| 132823536 | 132824188 | + | 13.1 | 1 | 675 | 0 | LTR76 | ERV1 | LTR |
| 132824189 | 132824207 | - | 23.2 | 1 | 18 | -491 | LTR22C | ERVK | LTR |
| 132824243 | 132824688 | - | 31.8 | 745 | 1255 | -5530 | L1PA13 | L1 | LINE |

  

|  |  |  |  |  |  |  |  |  |  |
| --- | --- | --- | --- | --- | --- | --- | --- | --- | --- |
| chr4:132846714-132849372 | | | 2659 bp | | |  |  |  |  |
| Genome Start | Genome End | Strand | Div | Repeat Start | Repeat End | Repeat Left | Name | Family | Class |
| 132846714 | 132847039 | - | 15.7 | 2080 | 2417 | -1 | Tigger1 | MER2\_type | DNA |
| 132847040 | 132847346 | - | 14.0 | 3 | 312 | 0 | AluSx | Alu | SINE |
| 132847347 | 132849372 | - | 15.7 | 24 | 2079 | -339 | Tigger1 | MER2\_type | DNA |

  

|  |  |  |  |  |  |  |  |  |  |
| --- | --- | --- | --- | --- | --- | --- | --- | --- | --- |
| chr4:132915635-132916935 | | | 880 bp | | |  |  |  |  |
| Genome Start | Genome End | Strand | Div | Repeat Start | Repeat End | Repeat Left | Name | Family | Class |
| 132915635 | 132915986 | - | 29.0 | 1359 | 1728 | -4421 | L1MEe | L1 | LINE |
| 132916021 | 132916245 | - | 34.2 | 3179 | 3415 | -4 | L2 | L2 | LINE |
| 132916633 | 132916935 | - | 34.7 | 500 | 807 | -5312 | L1MEe | L1 | LINE |

  

|  |  |  |  |  |  |  |  |  |  |
| --- | --- | --- | --- | --- | --- | --- | --- | --- | --- |
| chr4:132942717-132943615 | | | 899 bp | | |  |  |  |  |
| Genome Start | Genome End | Strand | Div | Repeat Start | Repeat End | Repeat Left | Name | Family | Class |
| 132942717 | 132942803 | - | 17.9 | 255 | 337 | -2 | MER1B | MER1\_type | DNA |
| 132942804 | 132943373 | + | 25.7 | 1 | 554 | 0 | MLT2B3 | ERVL | LTR |
| 132943374 | 132943615 | - | 17.9 | 1 | 254 | -85 | MER1B | MER1\_type | DNA |

  

|  |  |  |  |  |  |  |  |  |  |
| --- | --- | --- | --- | --- | --- | --- | --- | --- | --- |
| chr4:132944300-132945093 | | | 794 bp | | |  |  |  |  |
| Genome Start | Genome End | Strand | Div | Repeat Start | Repeat End | Repeat Left | Name | Family | Class |
| 132944300 | 132944477 | - | 16.7 | 309 | 501 | 0 | MER61D | ERV1 | LTR |
| 132944478 | 132944773 | - | 14.0 | 2 | 295 | -15 | AluSg | Alu | SINE |
| 132944774 | 132945093 | - | 16.7 | 1 | 308 | -193 | MER61D | ERV1 | LTR |

  

|  |  |  |  |  |  |  |  |  |  |
| --- | --- | --- | --- | --- | --- | --- | --- | --- | --- |
| chr4:132972681-132973886 | | | 1206 bp | | |  |  |  |  |
| Genome Start | Genome End | Strand | Div | Repeat Start | Repeat End | Repeat Left | Name | Family | Class |
| 132972681 | 132972789 | - | 17.9 | 194 | 298 | -14 | AluJb | Alu | SINE |
| 132972790 | 132973694 | - | 18.7 | 1 | 983 | 0 | LTR1D | ERV1 | LTR |
| 132973695 | 132973886 | - | 17.9 | 1 | 193 | -119 | AluJb | Alu | SINE |

  

|  |  |  |  |  |  |  |  |  |  |
| --- | --- | --- | --- | --- | --- | --- | --- | --- | --- |
| chr4:133000978-133002058 | | | 1081 bp | | |  |  |  |  |
| Genome Start | Genome End | Strand | Div | Repeat Start | Repeat End | Repeat Left | Name | Family | Class |
| 133000978 | 133001211 | - | 26.6 | 244 | 477 | -28 | MLT1D | MaLR | LTR |
| 133001212 | 133001418 | + | 15.5 | 6087 | 6294 | -6 | L1MA6 | L1 | LINE |
| 133001419 | 133001674 | - | 26.6 | 30 | 243 | -262 | MLT1D | MaLR | LTR |
| 133001675 | 133002027 | - | 15.9 | 1 | 364 | 0 | THE1B | MaLR | LTR |
| 133002028 | 133002058 | - | 26.6 | 1 | 29 | -476 | MLT1D | MaLR | LTR |

  

|  |  |  |  |  |  |  |  |  |  |
| --- | --- | --- | --- | --- | --- | --- | --- | --- | --- |
| chr4:133005863-133009904 | | | 3942 bp | | |  |  |  |  |
| Genome Start | Genome End | Strand | Div | Repeat Start | Repeat End | Repeat Left | Name | Family | Class |
| 133005863 | 133006272 | + | 29.9 | 4755 | 5173 | -973 | L1ME1 | L1 | LINE |
| 133006269 | 133008317 | - | 7.9 | 4089 | 6152 | -2 | L1PA7 | L1 | LINE |
| 133008422 | 133008998 | + | 24.1 | 62 | 607 | 0 | MER77 | ERVL | LTR |
| 133008999 | 133009904 | + | 26.4 | 5243 | 6161 | -5 | L1ME1 | L1 | LINE |

  

|  |  |  |  |  |  |  |  |  |  |
| --- | --- | --- | --- | --- | --- | --- | --- | --- | --- |
| chr4:133025497-133027441 | | | 1655 bp | | |  |  |  |  |
| Genome Start | Genome End | Strand | Div | Repeat Start | Repeat End | Repeat Left | Name | Family | Class |
| 133025497 | 133025872 | + | 30.7 | 4326 | 4700 | -1446 | L1ME2 | L1 | LINE |
| 133026086 | 133026210 | - | 20.2 | 1 | 130 | -46 | FRAM | Alu | SINE |
| 133026288 | 133027441 | + | 29.2 | 5004 | 6132 | -38 | L1ME2 | L1 | LINE |

  

|  |  |  |  |  |  |  |  |  |  |
| --- | --- | --- | --- | --- | --- | --- | --- | --- | --- |
| chr4:133038495-133039925 | | | 1431 bp | | |  |  |  |  |
| Genome Start | Genome End | Strand | Div | Repeat Start | Repeat End | Repeat Left | Name | Family | Class |
| 133038495 | 133038760 | - | 22.6 | 7518 | 7808 | 0 | L1MC4a | L1 | LINE |
| 133038761 | 133039065 | + | 7.7 | 4 | 303 | -8 | AluY | Alu | SINE |
| 133039066 | 133039925 | - | 22.6 | 6607 | 7517 | -291 | L1MC4a | L1 | LINE |

  

|  |  |  |  |  |  |  |  |  |  |
| --- | --- | --- | --- | --- | --- | --- | --- | --- | --- |
| chr4:133046087-133046676 | | | 590 bp | | |  |  |  |  |
| Genome Start | Genome End | Strand | Div | Repeat Start | Repeat End | Repeat Left | Name | Family | Class |
| 133046087 | 133046201 | - | 32.3 | 2683 | 2789 | -630 | L2 | L2 | LINE |
| 133046202 | 133046572 | + | 26.3 | 1 | 365 | 0 | MLT1A0 | MaLR | LTR |
| 133046573 | 133046676 | - | 32.3 | 2581 | 2682 | -737 | L2 | L2 | LINE |

  

|  |  |  |  |  |  |  |  |  |  |
| --- | --- | --- | --- | --- | --- | --- | --- | --- | --- |
| chr4:133054625-133055396 | | | 772 bp | | |  |  |  |  |
| Genome Start | Genome End | Strand | Div | Repeat Start | Repeat End | Repeat Left | Name | Family | Class |
| 133054625 | 133054798 | - | 32.5 | 91 | 258 | -2603 | Charlie2 | MER1\_type | DNA |
| 133054799 | 133055306 | + | 26.1 | 1 | 519 | 0 | LTR40a | ERVL | LTR |
| 133055307 | 133055396 | - | 32.5 | 3 | 90 | -2771 | Charlie2 | MER1\_type | DNA |

  

|  |  |  |  |  |  |  |  |  |  |
| --- | --- | --- | --- | --- | --- | --- | --- | --- | --- |
| chr4:133080852-133081661 | | | 810 bp | | |  |  |  |  |
| Genome Start | Genome End | Strand | Div | Repeat Start | Repeat End | Repeat Left | Name | Family | Class |
| 133080852 | 133081022 | - | 37.3 | 85 | 251 | -17 | MIRb | MIR | SINE |
| 133081023 | 133081611 | + | 18.2 | 2 | 604 | -1 | MER6A | MER2\_type | DNA |
| 133081612 | 133081661 | - | 37.3 | 38 | 84 | -184 | MIRb | MIR | SINE |

  

|  |  |  |  |  |  |  |  |  |  |
| --- | --- | --- | --- | --- | --- | --- | --- | --- | --- |
| chr4:133086312-133087545 | | | 1234 bp | | |  |  |  |  |
| Genome Start | Genome End | Strand | Div | Repeat Start | Repeat End | Repeat Left | Name | Family | Class |
| 133086312 | 133086427 | + | 18.6 | 5661 | 5784 | -372 | L1PB4 | L1 | LINE |
| 133086428 | 133087134 | + | 6.9 | 5441 | 6147 | -7 | L1PA7 | L1 | LINE |
| 133087135 | 133087545 | + | 18.6 | 5785 | 6153 | -3 | L1PB4 | L1 | LINE |

  

|  |  |  |  |  |  |  |  |  |  |
| --- | --- | --- | --- | --- | --- | --- | --- | --- | --- |
| chr4:133095322-133097725 | | | 2404 bp | | |  |  |  |  |
| Genome Start | Genome End | Strand | Div | Repeat Start | Repeat End | Repeat Left | Name | Family | Class |
| 133095322 | 133095729 | - | 27.7 | 5556 | 5973 | -191 | L1ME2 | L1 | LINE |
| 133095730 | 133096040 | - | 8.3 | 1 | 300 | -11 | AluY | Alu | SINE |
| 133096041 | 133096964 | - | 27.7 | 4583 | 5555 | -609 | L1ME2 | L1 | LINE |
| 133096965 | 133097271 | - | 11.9 | 1 | 303 | -8 | AluY | Alu | SINE |
| 133097272 | 133097725 | - | 27.8 | 4157 | 4582 | -1564 | L1ME2 | L1 | LINE |

  

|  |  |  |  |  |  |  |  |  |  |
| --- | --- | --- | --- | --- | --- | --- | --- | --- | --- |
| chr4:133107409-133108225 | | | 817 bp | | |  |  |  |  |
| Genome Start | Genome End | Strand | Div | Repeat Start | Repeat End | Repeat Left | Name | Family | Class |
| 133107409 | 133107595 | + | 26.4 | 3 | 163 | -342 | MLT1D | MaLR | LTR |
| 133107596 | 133107907 | - | 12.9 | 1 | 310 | -2 | AluSx | Alu | SINE |
| 133107908 | 133108225 | + | 26.4 | 164 | 505 | 0 | MLT1D | MaLR | LTR |

  

|  |  |  |  |  |  |  |  |  |  |
| --- | --- | --- | --- | --- | --- | --- | --- | --- | --- |
| chr4:144985302-144986030 | | | 729 bp | | |  |  |  |  |
| Genome Start | Genome End | Strand | Div | Repeat Start | Repeat End | Repeat Left | Name | Family | Class |
| 144985302 | 144985676 | + | 30.4 | 71 | 449 | -42 | LTR16C | ERVL | LTR |
| 144985677 | 144985989 | - | 9.6 | 1 | 313 | 0 | AluSp | Alu | SINE |
| 144985990 | 144986030 | + | 30.4 | 450 | 487 | -4 | LTR16C | ERVL | LTR |

  

|  |  |  |  |  |  |  |  |  |  |
| --- | --- | --- | --- | --- | --- | --- | --- | --- | --- |
| chr4:144994330-144996030 | | | 1694 bp | | |  |  |  |  |
| Genome Start | Genome End | Strand | Div | Repeat Start | Repeat End | Repeat Left | Name | Family | Class |
| 144994330 | 144994422 | + | 12.9 | 6010 | 6104 | -208 | L1MA9 | L1 | LINE |
| 144994423 | 144994491 | - | 15.9 | 300 | 366 | 0 | Tigger5a | MER2\_type | DNA |
| 144994492 | 144994710 | - | 13.7 | 1 | 230 | 0 | MER30 | MER1\_type | DNA |
| 144994711 | 144994982 | - | 15.9 | 26 | 299 | -67 | Tigger5a | MER2\_type | DNA |
| 144994990 | 144995124 | + | 16.4 | 6100 | 6246 | -66 | L1MA9 | L1 | LINE |
| 144995125 | 144995729 | - | 15.8 | 1 | 611 | 0 | LTR36 | ERV1 | LTR |
| 144995730 | 144995746 | + | 16.4 | 6247 | 6250 | -62 | L1MA9 | L1 | LINE |
| 144995747 | 144995973 | + | 12.8 | 2 | 230 | 0 | MER30 | MER1\_type | DNA |
| 144995974 | 144996030 | + | 16.4 | 6251 | 6312 | 0 | L1MA9 | L1 | LINE |

  

|  |  |  |  |  |  |  |  |  |  |
| --- | --- | --- | --- | --- | --- | --- | --- | --- | --- |
| chr4:145003123-145003909 | | | 640 bp | | |  |  |  |  |
| Genome Start | Genome End | Strand | Div | Repeat Start | Repeat End | Repeat Left | Name | Family | Class |
| 145003123 | 145003310 | - | 25.3 | 3231 | 3418 | -1 | L2 | L2 | LINE |
| 145003397 | 145003732 | - | 25.8 | 73 | 431 | -19 | LTR16A | ERVL | LTR |
| 145003794 | 145003909 | - | 27.7 | 3072 | 3193 | -226 | L2 | L2 | LINE |

  

|  |  |  |  |  |  |  |  |  |  |
| --- | --- | --- | --- | --- | --- | --- | --- | --- | --- |
| chr4:145006393-145008021 | | | 1531 bp | | |  |  |  |  |
| Genome Start | Genome End | Strand | Div | Repeat Start | Repeat End | Repeat Left | Name | Family | Class |
| 145006393 | 145006515 | - | 25.4 | 3038 | 3168 | -2978 | L1MEe | L1 | LINE |
| 145006611 | 145006807 | - | 10.8 | 355 | 554 | 0 | MER41A | ERV1 | LTR |
| 145006808 | 145007560 | + | 16.1 | 1 | 826 | 0 | LTR1B | ERV1 | LTR |
| 145007561 | 145007911 | - | 10.8 | 1 | 354 | -200 | MER41A | ERV1 | LTR |
| 145007915 | 145008021 | - | 19.4 | 2318 | 2431 | -3715 | L1MEe | L1 | LINE |

  

|  |  |  |  |  |  |  |  |  |  |
| --- | --- | --- | --- | --- | --- | --- | --- | --- | --- |
| chr4:145031672-145032338 | | | 667 bp | | |  |  |  |  |
| Genome Start | Genome End | Strand | Div | Repeat Start | Repeat End | Repeat Left | Name | Family | Class |
| 145031672 | 145031919 | - | 24.4 | 111 | 341 | 0 | MER58B | MER1\_type | DNA |
| 145031920 | 145032230 | - | 10.3 | 1 | 311 | 0 | AluY | Alu | SINE |
| 145032231 | 145032338 | - | 24.4 | 1 | 110 | -231 | MER58B | MER1\_type | DNA |

  

|  |  |  |  |  |  |  |  |  |  |
| --- | --- | --- | --- | --- | --- | --- | --- | --- | --- |
| chr4:145039863-145041679 | | | 1814 bp | | |  |  |  |  |
| Genome Start | Genome End | Strand | Div | Repeat Start | Repeat End | Repeat Left | Name | Family | Class |
| 145039863 | 145040049 | + | 33.7 | 2 | 205 | -57 | MIR | MIR | SINE |
| 145040053 | 145041608 | + | 18.8 | 4615 | 6174 | 0 | L1MB5 | L1 | LINE |
| 145041609 | 145041679 | + | 17.1 | 193 | 262 | 0 | MIR | MIR | SINE |

  

|  |  |  |  |  |  |  |  |  |  |
| --- | --- | --- | --- | --- | --- | --- | --- | --- | --- |
| chr4:145078761-145079375 | | | 615 bp | | |  |  |  |  |
| Genome Start | Genome End | Strand | Div | Repeat Start | Repeat End | Repeat Left | Name | Family | Class |
| 145078761 | 145078888 | - | 14.3 | 217 | 339 | 0 | MER1B | MER1\_type | DNA |
| 145078889 | 145079196 | - | 8.4 | 1 | 308 | -3 | AluY | Alu | SINE |
| 145079197 | 145079375 | - | 14.3 | 39 | 216 | -81 | MER1B | MER1\_type | DNA |

  

|  |  |  |  |  |  |  |  |  |  |
| --- | --- | --- | --- | --- | --- | --- | --- | --- | --- |
| chr4:145113497-145114862 | | | 1365 bp | | |  |  |  |  |
| Genome Start | Genome End | Strand | Div | Repeat Start | Repeat End | Repeat Left | Name | Family | Class |
| 145113497 | 145114209 | - | 12.4 | 5440 | 6154 | 0 | L1PB3 | L1 | LINE |
| 145114210 | 145114311 | - | 10.8 | 5558 | 5659 | -495 | L1P5 | L1 | LINE |
| 145114313 | 145114862 | - | 12.9 | 3638 | 4191 | -1955 | L1PB3 | L1 | LINE |

  

|  |  |  |  |  |  |  |  |  |  |
| --- | --- | --- | --- | --- | --- | --- | --- | --- | --- |
| chr4:145115234-145116936 | | | 1696 bp | | |  |  |  |  |
| Genome Start | Genome End | Strand | Div | Repeat Start | Repeat End | Repeat Left | Name | Family | Class |
| 145115234 | 145115326 | + | 11.8 | 6010 | 6104 | -208 | L1MA9 | L1 | LINE |
| 145115327 | 145115395 | - | 15.9 | 300 | 366 | 0 | Tigger5a | MER2\_type | DNA |
| 145115396 | 145115613 | - | 11.1 | 1 | 230 | 0 | MER30 | MER1\_type | DNA |
| 145115614 | 145115885 | - | 15.9 | 26 | 299 | -67 | Tigger5a | MER2\_type | DNA |
| 145115893 | 145116027 | + | 17.2 | 6100 | 6246 | -66 | L1MA9 | L1 | LINE |
| 145116028 | 145116633 | - | 15.8 | 1 | 611 | 0 | LTR36 | ERV1 | LTR |
| 145116634 | 145116650 | + | 17.2 | 6247 | 6250 | -62 | L1MA9 | L1 | LINE |
| 145116651 | 145116877 | + | 13.8 | 2 | 230 | 0 | MER30 | MER1\_type | DNA |
| 145116878 | 145116936 | + | 17.2 | 6251 | 6312 | 0 | L1MA9 | L1 | LINE |

  

|  |  |  |  |  |  |  |  |  |  |
| --- | --- | --- | --- | --- | --- | --- | --- | --- | --- |
| chr4:145149946-145150623 | | | 678 bp | | |  |  |  |  |
| Genome Start | Genome End | Strand | Div | Repeat Start | Repeat End | Repeat Left | Name | Family | Class |
| 145149946 | 145150204 | - | 22.6 | 107 | 341 | 0 | MER58B | MER1\_type | DNA |
| 145150205 | 145150515 | - | 8.0 | 1 | 311 | 0 | AluY | Alu | SINE |
| 145150516 | 145150623 | - | 22.6 | 1 | 106 | -235 | MER58B | MER1\_type | DNA |

  

|  |  |  |  |  |  |  |  |  |  |
| --- | --- | --- | --- | --- | --- | --- | --- | --- | --- |
| chr4:145150723-145151192 | | | 470 bp | | |  |  |  |  |
| Genome Start | Genome End | Strand | Div | Repeat Start | Repeat End | Repeat Left | Name | Family | Class |
| 145150723 | 145150789 | + | 24.9 | 29 | 93 | -167 | MER112 | MER1\_type | DNA |
| 145150790 | 145151080 | + | 19.2 | 6 | 291 | -21 | AluJo | Alu | SINE |
| 145151081 | 145151192 | + | 24.9 | 94 | 199 | -62 | MER112 | MER1\_type | DNA |

  

|  |  |  |  |  |  |  |  |  |  |
| --- | --- | --- | --- | --- | --- | --- | --- | --- | --- |
| chr4:145227165-145227943 | | | 635 bp | | |  |  |  |  |
| Genome Start | Genome End | Strand | Div | Repeat Start | Repeat End | Repeat Left | Name | Family | Class |
| 145227165 | 145227352 | - | 25.3 | 3231 | 3418 | -1 | L2 | L2 | LINE |
| 145227440 | 145227774 | - | 26.4 | 73 | 431 | -19 | LTR16A | ERVL | LTR |
| 145227832 | 145227943 | - | 29.6 | 3077 | 3193 | -226 | L2 | L2 | LINE |

  

|  |  |  |  |  |  |  |  |  |  |
| --- | --- | --- | --- | --- | --- | --- | --- | --- | --- |
| chr4:145273843-145274470 | | | 628 bp | | |  |  |  |  |
| Genome Start | Genome End | Strand | Div | Repeat Start | Repeat End | Repeat Left | Name | Family | Class |
| 145273843 | 145273888 | + | 32.5 | 2251 | 2295 | -3851 | L1M5 | L1 | LINE |
| 145273889 | 145274187 | + | 12.1 | 1 | 298 | -14 | AluSx | Alu | SINE |
| 145274188 | 145274470 | + | 32.5 | 2296 | 2615 | -3531 | L1M5 | L1 | LINE |

  

|  |  |  |  |  |  |  |  |  |  |
| --- | --- | --- | --- | --- | --- | --- | --- | --- | --- |
| chr4:165411162-165413268 | | | 2112 bp | | |  |  |  |  |
| Genome Start | Genome End | Strand | Div | Repeat Start | Repeat End | Repeat Left | Name | Family | Class |
| 165411162 | 165411359 | + | 25.4 | 3777 | 3965 | -2164 | L1ME3B | L1 | LINE |
| 165411353 | 165411739 | + | 31.9 | 4488 | 4909 | -1220 | L1ME3B | L1 | LINE |
| 165411742 | 165412232 | + | 22.0 | 1 | 495 | -10 | MLT1D | MaLR | LTR |
| 165412233 | 165413268 | + | 28.1 | 4906 | 6042 | -181 | L1ME3B | L1 | LINE |

  

|  |  |  |  |  |  |  |  |  |  |
| --- | --- | --- | --- | --- | --- | --- | --- | --- | --- |
| chr4:166393493-166394123 | | | 631 bp | | |  |  |  |  |
| Genome Start | Genome End | Strand | Div | Repeat Start | Repeat End | Repeat Left | Name | Family | Class |
| 166393493 | 166393630 | - | 17.6 | 226 | 345 | 0 | MER2 | MER2\_type | DNA |
| 166393631 | 166393935 | - | 10.3 | 1 | 303 | -9 | AluSx | Alu | SINE |
| 166393936 | 166394123 | - | 17.6 | 32 | 225 | -120 | MER2 | MER2\_type | DNA |

  

|  |  |  |  |  |  |  |  |  |  |
| --- | --- | --- | --- | --- | --- | --- | --- | --- | --- |
| chr4:170820980-170821485 | | | 469 bp | | |  |  |  |  |
| Genome Start | Genome End | Strand | Div | Repeat Start | Repeat End | Repeat Left | Name | Family | Class |
| 170820980 | 170821035 | - | 25.9 | 219 | 262 | 0 | MIR | MIR | SINE |
| 170821073 | 170821365 | - | 18.2 | 2 | 288 | -24 | AluJo | Alu | SINE |
| 170821366 | 170821485 | - | 25.9 | 102 | 218 | -44 | MIR | MIR | SINE |

  

|  |  |  |  |  |  |  |  |  |  |
| --- | --- | --- | --- | --- | --- | --- | --- | --- | --- |
| chr4:189898260-189899186 | | | 578 bp | | |  |  |  |  |
| Genome Start | Genome End | Strand | Div | Repeat Start | Repeat End | Repeat Left | Name | Family | Class |
| 189898260 | 189898368 | - | 15.2 | 660 | 760 | -13 | LTR41 | ERVL | LTR |
| 189898654 | 189898967 | - | 25.7 | 55 | 364 | -50 | MLT2D | ERVL | LTR |
| 189899015 | 189899085 | - | 18.3 | 1 | 72 | -484 | MLT2D | ERVL | LTR |
| 189899103 | 189899186 | - | 25.0 | 83 | 169 | -604 | LTR41 | ERVL | LTR |

  

|  |  |  |  |  |  |  |  |  |  |
| --- | --- | --- | --- | --- | --- | --- | --- | --- | --- |
| chr4:191100273-191101160 | | | 861 bp | | |  |  |  |  |
| Genome Start | Genome End | Strand | Div | Repeat Start | Repeat End | Repeat Left | Name | Family | Class |
| 191100273 | 191100348 | - | 30.7 | 186 | 262 | 0 | MIR | MIR | SINE |
| 191100375 | 191101007 | - | 15.8 | 5497 | 6168 | -16 | L1MB7 | L1 | LINE |
| 191101009 | 191101160 | - | 28.5 | 17 | 171 | -91 | MIR | MIR | SINE |

  

|  |  |  |  |  |  |  |  |  |  |
| --- | --- | --- | --- | --- | --- | --- | --- | --- | --- |
| chr4:191102103-191105564 | | | 3466 bp | | |  |  |  |  |
| Genome Start | Genome End | Strand | Div | Repeat Start | Repeat End | Repeat Left | Name | Family | Class |
| 191102103 | 191102278 | - | 16.8 | 5477 | 5655 | -509 | L1M5 | L1 | LINE |
| 191102275 | 191102335 | - | 25.9 | 5168 | 5224 | -922 | L1M5 | L1 | LINE |
| 191102336 | 191102544 | - | 11.0 | 81 | 289 | -22 | AluY | Alu | SINE |
| 191102545 | 191104362 | - | 25.9 | 3194 | 5167 | -979 | L1M5 | L1 | LINE |
| 191104363 | 191104577 | - | 11.6 | 1 | 230 | 0 | MER30 | MER1\_type | DNA |
| 191104578 | 191104990 | - | 25.9 | 2666 | 3193 | -2953 | L1M5 | L1 | LINE |
| 191104991 | 191105290 | - | 9.0 | 1 | 311 | 0 | AluSq | Alu | SINE |
| 191105291 | 191105564 | - | 25.9 | 2366 | 2665 | -3481 | L1M5 | L1 | LINE |

  

|  |  |  |  |  |  |  |  |  |  |
| --- | --- | --- | --- | --- | --- | --- | --- | --- | --- |
| chr4:191112699-191113092 | | | 394 bp | | |  |  |  |  |
| Genome Start | Genome End | Strand | Div | Repeat Start | Repeat End | Repeat Left | Name | Family | Class |
| 191112699 | 191112737 | + | 22.5 | 1 | 39 | -285 | MER33 | MER1\_type | DNA |
| 191112738 | 191113034 | - | 17.7 | 9 | 304 | -8 | AluJo | Alu | SINE |
| 191113035 | 191113092 | + | 22.5 | 40 | 80 | -244 | MER33 | MER1\_type | DNA |

  

|  |  |  |  |  |  |  |  |  |  |
| --- | --- | --- | --- | --- | --- | --- | --- | --- | --- |
| chr4:191167712-191169675 | | | 1937 bp | | |  |  |  |  |
| Genome Start | Genome End | Strand | Div | Repeat Start | Repeat End | Repeat Left | Name | Family | Class |
| 191167712 | 191168587 | + | 12.4 | 1495 | 2432 | -3755 | MER57A-int | ERV1 | LTR |
| 191168588 | 191168869 | - | 10.6 | 18 | 299 | -13 | AluSx | Alu | SINE |
| 191168870 | 191169104 | + | 12.4 | 2433 | 2663 | -3524 | MER57A-int | ERV1 | LTR |
| 191169132 | 191169675 | + | 12.9 | 3307 | 3880 | -2307 | MER57A-int | ERV1 | LTR |

  


|  |  |  |  |  |  |  |  |  |  |
| --- | --- | --- | --- | --- | --- | --- | --- | --- | --- |
| chr5:327875-328647 | | | 336 bp | | |  |  |  |  |
| Genome Start | Genome End | Strand | Div | Repeat Start | Repeat End | Repeat Left | Name | Family | Class |
| 327875 | 328034 | + | 39.0 | 1136 | 1317 | -643 | L4 | RTE | LINE |
| 328255 | 328366 | + | 14.3 | 2 | 113 | -30 | FLAM\_C | Alu | SINE |
| 328584 | 328647 | + | 37.5 | 1703 | 1766 | -194 | L4 | RTE | LINE |

  

|  |  |  |  |  |  |  |  |  |  |
| --- | --- | --- | --- | --- | --- | --- | --- | --- | --- |
| chr5:331341-331892 | | | 552 bp | | |  |  |  |  |
| Genome Start | Genome End | Strand | Div | Repeat Start | Repeat End | Repeat Left | Name | Family | Class |
| 331341 | 331542 | - | 28.6 | 3217 | 3419 | 0 | L2 | L2 | LINE |
| 331543 | 331854 | + | 16.2 | 1 | 309 | -3 | AluJb | Alu | SINE |
| 331855 | 331892 | - | 28.6 | 3183 | 3216 | -203 | L2 | L2 | LINE |

  

|  |  |  |  |  |  |  |  |  |  |
| --- | --- | --- | --- | --- | --- | --- | --- | --- | --- |
| chr5:344093-345355 | | | 802 bp | | |  |  |  |  |
| Genome Start | Genome End | Strand | Div | Repeat Start | Repeat End | Repeat Left | Name | Family | Class |
| 344093 | 344336 | - | 29.1 | 5944 | 6180 | -60 | L1ME3B | L1 | LINE |
| 344342 | 344392 | - | 17.6 | 248 | 298 | -4 | Alu | Alu | SINE |
| 344849 | 345355 | - | 31.1 | 5296 | 5823 | -339 | L1ME3B | L1 | LINE |

  

|  |  |  |  |  |  |  |  |  |  |
| --- | --- | --- | --- | --- | --- | --- | --- | --- | --- |
| chr5:772136-774559 | | | 2412 bp | | |  |  |  |  |
| Genome Start | Genome End | Strand | Div | Repeat Start | Repeat End | Repeat Left | Name | Family | Class |
| 772136 | 772871 | + | 24.3 | 816 | 1571 | -4971 | L1M4 | L1 | LINE |
| 772872 | 773102 | + | 18.3 | 1 | 235 | -315 | Tigger7 | MER2\_type | DNA |
| 773101 | 773201 | + | 17.8 | 2385 | 2485 | -6 | Tigger7 | MER2\_type | DNA |
| 773216 | 773943 | + | 23.0 | 1570 | 2394 | -4252 | L1M4 | L1 | LINE |
| 773944 | 774227 | + | 20.2 | 1 | 291 | -21 | AluJo | Alu | SINE |
| 774228 | 774559 | + | 24.4 | 2395 | 2707 | -3939 | L1M4 | L1 | LINE |

  

|  |  |  |  |  |  |  |  |  |  |
| --- | --- | --- | --- | --- | --- | --- | --- | --- | --- |
| chr5:794892-797658 | | | 2591 bp | | |  |  |  |  |
| Genome Start | Genome End | Strand | Div | Repeat Start | Repeat End | Repeat Left | Name | Family | Class |
| 794892 | 795266 | - | 24.2 | 5313 | 5677 | -489 | L1M5 | L1 | LINE |
| 795280 | 795397 | - | 18.8 | 1 | 134 | -178 | AluJo | Alu | SINE |
| 795561 | 795702 | + | 19.0 | 6 | 149 | -2269 | Tigger1 | MER2\_type | DNA |
| 795701 | 795897 | + | 20.8 | 2202 | 2418 | 0 | Tigger1 | MER2\_type | DNA |
| 795900 | 797658 | - | 29.8 | 3413 | 5200 | -946 | L1M5 | L1 | LINE |

  

|  |  |  |  |  |  |  |  |  |  |
| --- | --- | --- | --- | --- | --- | --- | --- | --- | --- |
| chr5:831756-832433 | | | 678 bp | | |  |  |  |  |
| Genome Start | Genome End | Strand | Div | Repeat Start | Repeat End | Repeat Left | Name | Family | Class |
| 831756 | 831924 | + | 27.1 | 25 | 234 | -257 | MLT1C | MaLR | LTR |
| 831925 | 832249 | + | 26.3 | 1 | 365 | 0 | MLT1A0 | MaLR | LTR |
| 832250 | 832433 | + | 28.8 | 211 | 420 | -47 | MLT1C | MaLR | LTR |

  

|  |  |  |  |  |  |  |  |  |  |
| --- | --- | --- | --- | --- | --- | --- | --- | --- | --- |
| chr5:875582-876586 | | | 1005 bp | | |  |  |  |  |
| Genome Start | Genome End | Strand | Div | Repeat Start | Repeat End | Repeat Left | Name | Family | Class |
| 875582 | 875785 | - | 23.7 | 5482 | 5679 | -487 | L1M5 | L1 | LINE |
| 875786 | 875922 | - | 18.3 | 3 | 133 | 0 | FLAM\_C | Alu | SINE |
| 875923 | 876586 | - | 28.4 | 4796 | 5481 | -685 | L1M5 | L1 | LINE |

  

|  |  |  |  |  |  |  |  |  |  |
| --- | --- | --- | --- | --- | --- | --- | --- | --- | --- |
| chr5:1683062-1684012 | | | 628 bp | | |  |  |  |  |
| Genome Start | Genome End | Strand | Div | Repeat Start | Repeat End | Repeat Left | Name | Family | Class |
| 1683062 | 1683350 | - | 39.6 | 1645 | 1958 | -2 | L4 | RTE | LINE |
| 1683508 | 1683624 | - | 16.2 | 1 | 117 | -16 | FLAM\_C | Alu | SINE |
| 1683791 | 1684012 | - | 33.8 | 1136 | 1394 | -566 | L4 | RTE | LINE |

  

|  |  |  |  |  |  |  |  |  |  |
| --- | --- | --- | --- | --- | --- | --- | --- | --- | --- |
| chr5:5445018-5445897 | | | 880 bp | | |  |  |  |  |
| Genome Start | Genome End | Strand | Div | Repeat Start | Repeat End | Repeat Left | Name | Family | Class |
| 5445018 | 5445283 | + | 27.1 | 25 | 305 | -288 | MLT1E | MaLR | LTR |
| 5445284 | 5445655 | + | 14.0 | 1 | 364 | 0 | THE1B | MaLR | LTR |
| 5445656 | 5445897 | + | 27.1 | 306 | 593 | 0 | MLT1E | MaLR | LTR |

  

|  |  |  |  |  |  |  |  |  |  |
| --- | --- | --- | --- | --- | --- | --- | --- | --- | --- |
| chr5:7345349-7345839 | | | 491 bp | | |  |  |  |  |
| Genome Start | Genome End | Strand | Div | Repeat Start | Repeat End | Repeat Left | Name | Family | Class |
| 7345349 | 7345502 | - | 12.8 | 147 | 300 | -12 | AluSx | Alu | SINE |
| 7345503 | 7345713 | - | 9.9 | 12 | 225 | -85 | AluSg | Alu | SINE |
| 7345714 | 7345839 | - | 12.8 | 2 | 146 | -166 | AluSx | Alu | SINE |

  

|  |  |  |  |  |  |  |  |  |  |
| --- | --- | --- | --- | --- | --- | --- | --- | --- | --- |
| chr5:7346380-7347002 | | | 597 bp | | |  |  |  |  |
| Genome Start | Genome End | Strand | Div | Repeat Start | Repeat End | Repeat Left | Name | Family | Class |
| 7346380 | 7346638 | - | 24.0 | 80 | 340 | -1 | MER58B | MER1\_type | DNA |
| 7346645 | 7346854 | + | 7.6 | 1 | 212 | -100 | AluSx | Alu | SINE |
| 7346855 | 7346896 | + | 14.3 | 194 | 235 | -68 | AluSp/q | Alu | SINE |
| 7346917 | 7347002 | - | 27.9 | 3 | 91 | -250 | MER58B | MER1\_type | DNA |

  

|  |  |  |  |  |  |  |  |  |  |
| --- | --- | --- | --- | --- | --- | --- | --- | --- | --- |
| chr5:20755911-20756445 | | | 535 bp | | |  |  |  |  |
| Genome Start | Genome End | Strand | Div | Repeat Start | Repeat End | Repeat Left | Name | Family | Class |
| 20755911 | 20756003 | + | 19.3 | 261 | 351 | -116 | MLT1C | MaLR | LTR |
| 20756004 | 20756311 | + | 12.7 | 1 | 310 | -1 | AluY | Alu | SINE |
| 20756312 | 20756445 | + | 19.3 | 352 | 467 | 0 | MLT1C | MaLR | LTR |

  

|  |  |  |  |  |  |  |  |  |  |
| --- | --- | --- | --- | --- | --- | --- | --- | --- | --- |
| chr5:20778376-20781381 | | | 2977 bp | | |  |  |  |  |
| Genome Start | Genome End | Strand | Div | Repeat Start | Repeat End | Repeat Left | Name | Family | Class |
| 20778376 | 20778957 | + | 25.5 | 1828 | 2476 | -4858 | L1M4b | L1 | LINE |
| 20778958 | 20779248 | + | 15.5 | 1 | 294 | -18 | AluSx | Alu | SINE |
| 20779249 | 20779536 | + | 25.5 | 2477 | 2749 | -4585 | L1M4b | L1 | LINE |
| 20779537 | 20779817 | - | 11.5 | 1 | 304 | -8 | AluSx | Alu | SINE |
| 20779818 | 20779875 | + | 25.5 | 2750 | 2806 | -4528 | L1M4b | L1 | LINE |
| 20779905 | 20780446 | + | 24.2 | 2962 | 3330 | -3789 | L1M4b | L1 | LINE |
| 20780447 | 20780745 | + | 14.1 | 1 | 299 | -13 | AluSx | Alu | SINE |
| 20780746 | 20781381 | + | 23.7 | 3218 | 3920 | -3086 | L1M4b | L1 | LINE |

  

|  |  |  |  |  |  |  |  |  |  |
| --- | --- | --- | --- | --- | --- | --- | --- | --- | --- |
| chr5:20811760-20812707 | | | 948 bp | | |  |  |  |  |
| Genome Start | Genome End | Strand | Div | Repeat Start | Repeat End | Repeat Left | Name | Family | Class |
| 20811760 | 20812069 | + | 12.5 | 4705 | 5015 | -1131 | L1P3 | L1 | LINE |
| 20812070 | 20812381 | + | 6.5 | 1 | 308 | -3 | AluY | Alu | SINE |
| 20812382 | 20812707 | + | 12.5 | 5016 | 5304 | -842 | L1P3 | L1 | LINE |

  

|  |  |  |  |  |  |  |  |  |  |
| --- | --- | --- | --- | --- | --- | --- | --- | --- | --- |
| chr5:20829951-20831435 | | | 1317 bp | | |  |  |  |  |
| Genome Start | Genome End | Strand | Div | Repeat Start | Repeat End | Repeat Left | Name | Family | Class |
| 20829951 | 20830118 | - | 25.6 | 7751 | 7922 | -39 | L1MC5 | L1 | LINE |
| 20830121 | 20830476 | + | 10.7 | 1 | 364 | 0 | THE1B | MaLR | LTR |
| 20830477 | 20831137 | + | 11.4 | 1 | 664 | -916 | THE1B-int | MaLR | LTR |
| 20831304 | 20831435 | - | 19.4 | 7460 | 7591 | -370 | L1MC5 | L1 | LINE |

  

|  |  |  |  |  |  |  |  |  |  |
| --- | --- | --- | --- | --- | --- | --- | --- | --- | --- |
| chr5:20938415-20939671 | | | 1257 bp | | |  |  |  |  |
| Genome Start | Genome End | Strand | Div | Repeat Start | Repeat End | Repeat Left | Name | Family | Class |
| 20938415 | 20938941 | - | 26.7 | 5609 | 6153 | -11 | L1ME2 | L1 | LINE |
| 20938942 | 20939364 | - | 22.0 | 1 | 431 | -1 | MSTB1 | MaLR | LTR |
| 20939365 | 20939671 | - | 26.7 | 5298 | 5608 | -556 | L1ME2 | L1 | LINE |

  

|  |  |  |  |  |  |  |  |  |  |
| --- | --- | --- | --- | --- | --- | --- | --- | --- | --- |
| chr5:20968171-20969454 | | | 1172 bp | | |  |  |  |  |
| Genome Start | Genome End | Strand | Div | Repeat Start | Repeat End | Repeat Left | Name | Family | Class |
| 20968171 | 20968320 | + | 23.9 | 3 | 164 | -356 | MLT2B1 | ERVL | LTR |
| 20968321 | 20968617 | - | 10.1 | 1 | 297 | -16 | AluSq | Alu | SINE |
| 20968618 | 20968867 | + | 23.9 | 165 | 453 | -67 | MLT2B1 | ERVL | LTR |
| 20968868 | 20969306 | - | 16.7 | 1 | 434 | 0 | Tigger2a | MER2\_type | DNA |
| 20969419 | 20969454 | + | 11.1 | 525 | 560 | 0 | MLT2B1 | ERVL | LTR |

  

|  |  |  |  |  |  |  |  |  |  |
| --- | --- | --- | --- | --- | --- | --- | --- | --- | --- |
| chr5:21333396-21333772 | | | 377 bp | | |  |  |  |  |
| Genome Start | Genome End | Strand | Div | Repeat Start | Repeat End | Repeat Left | Name | Family | Class |
| 21333396 | 21333426 | + | 33.3 | 79 | 106 | -162 | MIRb | MIR | SINE |
| 21333427 | 21333718 | - | 13.7 | 1 | 294 | -18 | AluSx | Alu | SINE |
| 21333719 | 21333772 | + | 33.3 | 107 | 160 | -108 | MIRb | MIR | SINE |

  

|  |  |  |  |  |  |  |  |  |  |
| --- | --- | --- | --- | --- | --- | --- | --- | --- | --- |
| chr5:21335237-21335967 | | | 731 bp | | |  |  |  |  |
| Genome Start | Genome End | Strand | Div | Repeat Start | Repeat End | Repeat Left | Name | Family | Class |
| 21335237 | 21335286 | + | 34.6 | 861 | 910 | -2509 | L2 | L2 | LINE |
| 21335287 | 21335804 | - | 27.4 | 3 | 560 | -2 | MLT1F2 | MaLR | LTR |
| 21335805 | 21335967 | + | 34.6 | 911 | 1075 | -2344 | L2 | L2 | LINE |

  

|  |  |  |  |  |  |  |  |  |  |
| --- | --- | --- | --- | --- | --- | --- | --- | --- | --- |
| chr5:21340360-21341150 | | | 791 bp | | |  |  |  |  |
| Genome Start | Genome End | Strand | Div | Repeat Start | Repeat End | Repeat Left | Name | Family | Class |
| 21340360 | 21340651 | + | 26.9 | 1 | 324 | -121 | MER65A | ERV1 | LTR |
| 21340652 | 21341025 | + | 13.4 | 1 | 428 | 0 | MSTA | MaLR | LTR |
| 21341026 | 21341150 | + | 26.9 | 325 | 445 | 0 | MER65A | ERV1 | LTR |

  

|  |  |  |  |  |  |  |  |  |  |
| --- | --- | --- | --- | --- | --- | --- | --- | --- | --- |
| chr5:21365842-21368465 | | | 2391 bp | | |  |  |  |  |
| Genome Start | Genome End | Strand | Div | Repeat Start | Repeat End | Repeat Left | Name | Family | Class |
| 21365842 | 21366084 | - | 25.6 | 5765 | 6005 | -161 | L1ME2 | L1 | LINE |
| 21366098 | 21366283 | + | 22.0 | 1 | 213 | -6 | MER20 | MER1\_type | DNA |
| 21366289 | 21366662 | - | 24.6 | 5329 | 5758 | -406 | L1ME2 | L1 | LINE |
| 21366878 | 21367416 | - | 30.7 | 4523 | 5064 | -1082 | L1ME2 | L1 | LINE |
| 21367417 | 21367835 | - | 13.8 | 5 | 428 | 0 | MSTA | MaLR | LTR |
| 21367836 | 21368465 | - | 30.7 | 3855 | 4522 | -1624 | L1ME2 | L1 | LINE |

  

|  |  |  |  |  |  |  |  |  |  |
| --- | --- | --- | --- | --- | --- | --- | --- | --- | --- |
| chr5:21397011-21397963 | | | 806 bp | | |  |  |  |  |
| Genome Start | Genome End | Strand | Div | Repeat Start | Repeat End | Repeat Left | Name | Family | Class |
| 21397011 | 21397239 | - | 27.4 | 275 | 505 | 0 | MLT1D | MaLR | LTR |
| 21397240 | 21397450 | - | 19.1 | 182 | 379 | 0 | MLT1A | MaLR | LTR |
| 21397503 | 21397605 | - | 31.1 | 58 | 160 | -214 | MLT1A | MaLR | LTR |
| 21397660 | 21397706 | - | 17.0 | 1 | 47 | -327 | MLT1A | MaLR | LTR |
| 21397748 | 21397963 | - | 25.7 | 1 | 225 | -280 | MLT1D | MaLR | LTR |

  

|  |  |  |  |  |  |  |  |  |  |
| --- | --- | --- | --- | --- | --- | --- | --- | --- | --- |
| chr5:21417722-21419034 | | | 1313 bp | | |  |  |  |  |
| Genome Start | Genome End | Strand | Div | Repeat Start | Repeat End | Repeat Left | Name | Family | Class |
| 21417722 | 21418222 | - | 23.1 | 6587 | 7121 | -664 | L1MC3 | L1 | LINE |
| 21418223 | 21418606 | - | 17.3 | 1 | 428 | 0 | MSTA | MaLR | LTR |
| 21418607 | 21419034 | - | 23.1 | 6150 | 6586 | -1199 | L1MC3 | L1 | LINE |

  

|  |  |  |  |  |  |  |  |  |  |
| --- | --- | --- | --- | --- | --- | --- | --- | --- | --- |
| chr5:21419717-21420970 | | | 1254 bp | | |  |  |  |  |
| Genome Start | Genome End | Strand | Div | Repeat Start | Repeat End | Repeat Left | Name | Family | Class |
| 21419717 | 21420281 | - | 20.2 | 2897 | 3392 | -3453 | L1M2 | L1 | LINE |
| 21420282 | 21420580 | - | 13.0 | 1 | 299 | -11 | AluSg | Alu | SINE |
| 21420581 | 21420970 | - | 21.7 | 2528 | 2896 | -3949 | L1M2 | L1 | LINE |

  

|  |  |  |  |  |  |  |  |  |  |
| --- | --- | --- | --- | --- | --- | --- | --- | --- | --- |
| chr5:21425595-21426231 | | | 637 bp | | |  |  |  |  |
| Genome Start | Genome End | Strand | Div | Repeat Start | Repeat End | Repeat Left | Name | Family | Class |
| 21425595 | 21425689 | + | 19.5 | 43 | 126 | -239 | MLT1A0 | MaLR | LTR |
| 21425690 | 21425999 | - | 12.6 | 1 | 310 | 0 | AluSg | Alu | SINE |
| 21426000 | 21426231 | + | 19.5 | 127 | 365 | 0 | MLT1A0 | MaLR | LTR |

  

|  |  |  |  |  |  |  |  |  |  |
| --- | --- | --- | --- | --- | --- | --- | --- | --- | --- |
| chr5:21507631-21508517 | | | 887 bp | | |  |  |  |  |
| Genome Start | Genome End | Strand | Div | Repeat Start | Repeat End | Repeat Left | Name | Family | Class |
| 21507631 | 21507872 | - | 26.4 | 303 | 593 | 0 | MLT1E | MaLR | LTR |
| 21507873 | 21508234 | - | 14.4 | 1 | 364 | 0 | THE1B | MaLR | LTR |
| 21508235 | 21508517 | - | 26.4 | 1 | 302 | -291 | MLT1E | MaLR | LTR |

  

|  |  |  |  |  |  |  |  |  |  |
| --- | --- | --- | --- | --- | --- | --- | --- | --- | --- |
| chr5:21513793-21514297 | | | 505 bp | | |  |  |  |  |
| Genome Start | Genome End | Strand | Div | Repeat Start | Repeat End | Repeat Left | Name | Family | Class |
| 21513793 | 21513857 | - | 21.1 | 134 | 209 | 0 | MER3 | MER1\_type | DNA |
| 21513858 | 21514169 | + | 6.8 | 1 | 310 | -1 | AluY | Alu | SINE |
| 21514170 | 21514297 | - | 21.1 | 3 | 133 | -76 | MER3 | MER1\_type | DNA |

  

|  |  |  |  |  |  |  |  |  |  |
| --- | --- | --- | --- | --- | --- | --- | --- | --- | --- |
| chr5:21514318-21515326 | | | 1009 bp | | |  |  |  |  |
| Genome Start | Genome End | Strand | Div | Repeat Start | Repeat End | Repeat Left | Name | Family | Class |
| 21514318 | 21514385 | - | 23.3 | 309 | 374 | 0 | MLT1A | MaLR | LTR |
| 21514386 | 21514688 | + | 15.5 | 1 | 312 | 0 | AluSx | Alu | SINE |
| 21514689 | 21514968 | - | 23.3 | 45 | 308 | -66 | MLT1A | MaLR | LTR |
| 21514969 | 21515282 | - | 6.2 | 1 | 310 | -1 | AluY | Alu | SINE |
| 21515283 | 21515326 | - | 23.3 | 1 | 44 | -330 | MLT1A | MaLR | LTR |

  

|  |  |  |  |  |  |  |  |  |  |
| --- | --- | --- | --- | --- | --- | --- | --- | --- | --- |
| chr5:21524223-21525143 | | | 891 bp | | |  |  |  |  |
| Genome Start | Genome End | Strand | Div | Repeat Start | Repeat End | Repeat Left | Name | Family | Class |
| 21524223 | 21524677 | - | 21.9 | 179 | 653 | 0 | MER82 | MER2\_type | DNA |
| 21524678 | 21524833 | - | 12.8 | 135 | 290 | -22 | AluSx | Alu | SINE |
| 21524864 | 21524994 | - | 12.8 | 2 | 134 | -178 | AluSx | Alu | SINE |
| 21524995 | 21525143 | - | 21.9 | 3 | 178 | -475 | MER82 | MER2\_type | DNA |

  

|  |  |  |  |  |  |  |  |  |  |
| --- | --- | --- | --- | --- | --- | --- | --- | --- | --- |
| chr5:21527581-21528150 | | | 570 bp | | |  |  |  |  |
| Genome Start | Genome End | Strand | Div | Repeat Start | Repeat End | Repeat Left | Name | Family | Class |
| 21527581 | 21527731 | - | 16.3 | 126 | 275 | -37 | AluJo | Alu | SINE |
| 21527732 | 21528022 | + | 11.3 | 1 | 294 | -18 | AluSx | Alu | SINE |
| 21528023 | 21528150 | - | 16.3 | 1 | 125 | -187 | AluJo | Alu | SINE |

  

|  |  |  |  |  |  |  |  |  |  |
| --- | --- | --- | --- | --- | --- | --- | --- | --- | --- |
| chr5:21529512-21530108 | | | 597 bp | | |  |  |  |  |
| Genome Start | Genome End | Strand | Div | Repeat Start | Repeat End | Repeat Left | Name | Family | Class |
| 21529512 | 21529692 | - | 17.2 | 138 | 305 | -7 | AluJo | Alu | SINE |
| 21529693 | 21529985 | - | 12.7 | 1 | 288 | -22 | AluSg | Alu | SINE |
| 21529986 | 21530108 | - | 17.2 | 2 | 137 | -175 | AluJo | Alu | SINE |

  

|  |  |  |  |  |  |  |  |  |  |
| --- | --- | --- | --- | --- | --- | --- | --- | --- | --- |
| chr5:21535588-21536181 | | | 594 bp | | |  |  |  |  |
| Genome Start | Genome End | Strand | Div | Repeat Start | Repeat End | Repeat Left | Name | Family | Class |
| 21535588 | 21535722 | + | 19.2 | 2 | 136 | -176 | AluJb | Alu | SINE |
| 21535723 | 21536024 | + | 9.3 | 1 | 300 | -10 | AluSg | Alu | SINE |
| 21536025 | 21536181 | + | 19.2 | 137 | 291 | -21 | AluJb | Alu | SINE |

  

|  |  |  |  |  |  |  |  |  |  |
| --- | --- | --- | --- | --- | --- | --- | --- | --- | --- |
| chr5:21557142-21558659 | | | 1292 bp | | |  |  |  |  |
| Genome Start | Genome End | Strand | Div | Repeat Start | Repeat End | Repeat Left | Name | Family | Class |
| 21557142 | 21557550 | - | 20.2 | 1326 | 1735 | 0 | MLT1A0-int | MaLR | LTR |
| 21557791 | 21557971 | - | 33.1 | 858 | 1029 | -460 | MLT1F-int | MaLR | LTR |
| 21557958 | 21558659 | - | 24.9 | 1 | 738 | -633 | MLT1A0-int | MaLR | LTR |

  

|  |  |  |  |  |  |  |  |  |  |
| --- | --- | --- | --- | --- | --- | --- | --- | --- | --- |
| chr5:21980880-21986563 | | | 5581 bp | | |  |  |  |  |
| Genome Start | Genome End | Strand | Div | Repeat Start | Repeat End | Repeat Left | Name | Family | Class |
| 21980880 | 21981008 | + | 27.4 | 1032 | 1167 | -5466 | L1MDa | L1 | LINE |
| 21981009 | 21981319 | + | 12.3 | 1 | 312 | 0 | AluSx | Alu | SINE |
| 21981320 | 21982468 | + | 27.4 | 1168 | 2359 | -4274 | L1MDa | L1 | LINE |
| 21982486 | 21982712 | + | 19.4 | 1 | 229 | -1002 | Tigger3b | MER2\_type | DNA |
| 21982713 | 21983083 | + | 15.2 | 1 | 379 | -2 | THE1D | MaLR | LTR |
| 21983086 | 21984584 | + | 14.4 | 1 | 1580 | 0 | THE1D-int | MaLR | LTR |
| 21984585 | 21984963 | + | 14.5 | 1 | 381 | 0 | THE1D | MaLR | LTR |
| 21984964 | 21985080 | + | 16.8 | 225 | 339 | -892 | Tigger3b | MER2\_type | DNA |
| 21985081 | 21985337 | + | 5.8 | 47 | 303 | -8 | AluY | Alu | SINE |
| 21985338 | 21986197 | + | 16.8 | 340 | 1225 | -6 | Tigger3b | MER2\_type | DNA |
| 21986205 | 21986419 | + | 20.1 | 2379 | 2601 | -4032 | L1MDa | L1 | LINE |
| 21986497 | 21986563 | + | 22.4 | 2614 | 2683 | -3854 | L1MDa | L1 | LINE |

  

|  |  |  |  |  |  |  |  |  |  |
| --- | --- | --- | --- | --- | --- | --- | --- | --- | --- |
| chr5:21995220-21996149 | | | 930 bp | | |  |  |  |  |
| Genome Start | Genome End | Strand | Div | Repeat Start | Repeat End | Repeat Left | Name | Family | Class |
| 21995220 | 21995493 | + | 8.8 | 2118 | 2393 | -3753 | L1PREC2 | L1 | LINE |
| 21995494 | 21995811 | + | 9.3 | 1 | 300 | -9 | AluSc | Alu | SINE |
| 21995812 | 21996149 | + | 8.8 | 2394 | 2732 | -3414 | L1PREC2 | L1 | LINE |

  

|  |  |  |  |  |  |  |  |  |  |
| --- | --- | --- | --- | --- | --- | --- | --- | --- | --- |
| chr5:22040242-22041648 | | | 1224 bp | | |  |  |  |  |
| Genome Start | Genome End | Strand | Div | Repeat Start | Repeat End | Repeat Left | Name | Family | Class |
| 22040242 | 22040489 | + | 25.4 | 2857 | 3136 | -283 | L2 | L2 | LINE |
| 22040539 | 22040918 | - | 18.7 | 5788 | 6134 | -20 | L1PB3 | L1 | LINE |
| 22040919 | 22041208 | - | 11.5 | 1 | 288 | -24 | AluSx | Alu | SINE |
| 22041209 | 22041369 | - | 18.7 | 5631 | 5787 | -367 | L1PB3 | L1 | LINE |
| 22041504 | 22041648 | + | 29.6 | 3267 | 3419 | 0 | L2 | L2 | LINE |

  

|  |  |  |  |  |  |  |  |  |  |
| --- | --- | --- | --- | --- | --- | --- | --- | --- | --- |
| chr5:22184347-22185158 | | | 812 bp | | |  |  |  |  |
| Genome Start | Genome End | Strand | Div | Repeat Start | Repeat End | Repeat Left | Name | Family | Class |
| 22184347 | 22184704 | - | 26.8 | 155 | 467 | 0 | MLT1C | MaLR | LTR |
| 22184705 | 22185001 | + | 10.4 | 1 | 299 | -11 | AluSg | Alu | SINE |
| 22185002 | 22185158 | - | 26.8 | 6 | 154 | -313 | MLT1C | MaLR | LTR |

  

|  |  |  |  |  |  |  |  |  |  |
| --- | --- | --- | --- | --- | --- | --- | --- | --- | --- |
| chr5:22198698-22199546 | | | 849 bp | | |  |  |  |  |
| Genome Start | Genome End | Strand | Div | Repeat Start | Repeat End | Repeat Left | Name | Family | Class |
| 22198698 | 22198757 | + | 32.2 | 2155 | 2214 | -1205 | L2 | L2 | LINE |
| 22198758 | 22199046 | - | 7.3 | 1 | 292 | -7 | AluYd8 | Alu | SINE |
| 22199047 | 22199546 | + | 32.2 | 2215 | 2726 | -693 | L2 | L2 | LINE |

  

|  |  |  |  |  |  |  |  |  |  |
| --- | --- | --- | --- | --- | --- | --- | --- | --- | --- |
| chr5:22200919-22202188 | | | 1209 bp | | |  |  |  |  |
| Genome Start | Genome End | Strand | Div | Repeat Start | Repeat End | Repeat Left | Name | Family | Class |
| 22200919 | 22201341 | + | 31.6 | 2729 | 3191 | -228 | L2 | L2 | LINE |
| 22201403 | 22201455 | + | 34.0 | 3192 | 3232 | -187 | L2 | L2 | LINE |
| 22201456 | 22202013 | + | 10.0 | 1 | 554 | 0 | MER41A | ERV1 | LTR |
| 22202014 | 22202188 | + | 34.0 | 3233 | 3415 | -4 | L2 | L2 | LINE |

  

|  |  |  |  |  |  |  |  |  |  |
| --- | --- | --- | --- | --- | --- | --- | --- | --- | --- |
| chr5:26258134-26259073 | | | 738 bp | | |  |  |  |  |
| Genome Start | Genome End | Strand | Div | Repeat Start | Repeat End | Repeat Left | Name | Family | Class |
| 26258134 | 26258335 | + | 27.9 | 5296 | 5525 | -715 | L1ME3B | L1 | LINE |
| 26258466 | 26258685 | + | 31.9 | 5650 | 5896 | -344 | L1ME3B | L1 | LINE |
| 26258754 | 26258804 | + | 15.7 | 248 | 298 | -4 | Alu | Alu | SINE |
| 26258809 | 26259073 | + | 31.5 | 5943 | 6219 | -21 | L1ME3B | L1 | LINE |

  

|  |  |  |  |  |  |  |  |  |  |
| --- | --- | --- | --- | --- | --- | --- | --- | --- | --- |
| chr5:34213184-34213783 | | | 600 bp | | |  |  |  |  |
| Genome Start | Genome End | Strand | Div | Repeat Start | Repeat End | Repeat Left | Name | Family | Class |
| 34213184 | 34213342 | - | 18.9 | 137 | 291 | -21 | AluJb | Alu | SINE |
| 34213343 | 34213648 | - | 9.9 | 1 | 304 | -6 | AluSg | Alu | SINE |
| 34213649 | 34213783 | - | 18.9 | 2 | 136 | -176 | AluJb | Alu | SINE |

  

|  |  |  |  |  |  |  |  |  |  |
| --- | --- | --- | --- | --- | --- | --- | --- | --- | --- |
| chr5:34215804-34216401 | | | 598 bp | | |  |  |  |  |
| Genome Start | Genome End | Strand | Div | Repeat Start | Repeat End | Repeat Left | Name | Family | Class |
| 34215804 | 34215926 | + | 17.9 | 2 | 137 | -175 | AluJo | Alu | SINE |
| 34215927 | 34216205 | + | 13.4 | 1 | 280 | -30 | AluSg | Alu | SINE |
| 34216206 | 34216401 | + | 17.9 | 138 | 305 | -7 | AluJo | Alu | SINE |

  

|  |  |  |  |  |  |  |  |  |  |
| --- | --- | --- | --- | --- | --- | --- | --- | --- | --- |
| chr5:34217763-34218331 | | | 569 bp | | |  |  |  |  |
| Genome Start | Genome End | Strand | Div | Repeat Start | Repeat End | Repeat Left | Name | Family | Class |
| 34217763 | 34217890 | + | 16.7 | 1 | 125 | -187 | AluJo | Alu | SINE |
| 34217891 | 34218180 | - | 12.4 | 1 | 293 | -19 | AluSx | Alu | SINE |
| 34218181 | 34218331 | + | 16.7 | 126 | 275 | -37 | AluJo | Alu | SINE |

  

|  |  |  |  |  |  |  |  |  |  |
| --- | --- | --- | --- | --- | --- | --- | --- | --- | --- |
| chr5:34220778-34221684 | | | 907 bp | | |  |  |  |  |
| Genome Start | Genome End | Strand | Div | Repeat Start | Repeat End | Repeat Left | Name | Family | Class |
| 34220778 | 34220926 | + | 21.8 | 1 | 178 | -475 | MER82 | MER2\_type | DNA |
| 34220927 | 34221229 | + | 11.7 | 1 | 290 | -22 | AluSx | Alu | SINE |
| 34221230 | 34221684 | + | 21.8 | 179 | 653 | 0 | MER82 | MER2\_type | DNA |

  

|  |  |  |  |  |  |  |  |  |  |
| --- | --- | --- | --- | --- | --- | --- | --- | --- | --- |
| chr5:34231378-34232367 | | | 990 bp | | |  |  |  |  |
| Genome Start | Genome End | Strand | Div | Repeat Start | Repeat End | Repeat Left | Name | Family | Class |
| 34231378 | 34231421 | + | 23.0 | 1 | 41 | -333 | MLT1A | MaLR | LTR |
| 34231422 | 34231718 | + | 5.7 | 1 | 298 | -13 | AluY | Alu | SINE |
| 34231719 | 34231996 | + | 23.0 | 42 | 308 | -66 | MLT1A | MaLR | LTR |
| 34231997 | 34232299 | - | 16.2 | 1 | 312 | 0 | AluSx | Alu | SINE |
| 34232300 | 34232367 | + | 23.0 | 309 | 374 | 0 | MLT1A | MaLR | LTR |

  

|  |  |  |  |  |  |  |  |  |  |
| --- | --- | --- | --- | --- | --- | --- | --- | --- | --- |
| chr5:34232388-34232886 | | | 499 bp | | |  |  |  |  |
| Genome Start | Genome End | Strand | Div | Repeat Start | Repeat End | Repeat Left | Name | Family | Class |
| 34232388 | 34232515 | + | 19.1 | 1 | 129 | -80 | MER3 | MER1\_type | DNA |
| 34232516 | 34232821 | - | 5.9 | 1 | 303 | -8 | AluY | Alu | SINE |
| 34232822 | 34232886 | + | 19.1 | 130 | 208 | -1 | MER3 | MER1\_type | DNA |

  

|  |  |  |  |  |  |  |  |  |  |
| --- | --- | --- | --- | --- | --- | --- | --- | --- | --- |
| chr5:34233584-34234238 | | | 655 bp | | |  |  |  |  |
| Genome Start | Genome End | Strand | Div | Repeat Start | Repeat End | Repeat Left | Name | Family | Class |
| 34233584 | 34233815 | - | 20.8 | 127 | 365 | 0 | MLT1A0 | MaLR | LTR |
| 34233816 | 34234130 | + | 13.4 | 1 | 307 | -3 | AluSg | Alu | SINE |
| 34234131 | 34234238 | - | 20.8 | 43 | 126 | -239 | MLT1A0 | MaLR | LTR |

  

|  |  |  |  |  |  |  |  |  |  |
| --- | --- | --- | --- | --- | --- | --- | --- | --- | --- |
| chr5:34240773-34242083 | | | 1311 bp | | |  |  |  |  |
| Genome Start | Genome End | Strand | Div | Repeat Start | Repeat End | Repeat Left | Name | Family | Class |
| 34240773 | 34241198 | + | 23.8 | 6152 | 6586 | -1199 | L1MC3 | L1 | LINE |
| 34241199 | 34241581 | + | 17.1 | 1 | 428 | 0 | MSTA | MaLR | LTR |
| 34241582 | 34242083 | + | 23.8 | 6587 | 7119 | -666 | L1MC3 | L1 | LINE |

  

|  |  |  |  |  |  |  |  |  |  |
| --- | --- | --- | --- | --- | --- | --- | --- | --- | --- |
| chr5:34265319-34266265 | | | 850 bp | | |  |  |  |  |
| Genome Start | Genome End | Strand | Div | Repeat Start | Repeat End | Repeat Left | Name | Family | Class |
| 34265319 | 34265571 | + | 27.5 | 1 | 277 | -228 | MLT1D | MaLR | LTR |
| 34265575 | 34265621 | + | 17.0 | 1 | 47 | -327 | MLT1A | MaLR | LTR |
| 34265664 | 34265773 | + | 30.0 | 51 | 160 | -214 | MLT1A | MaLR | LTR |
| 34265826 | 34266036 | + | 19.7 | 182 | 379 | 0 | MLT1A | MaLR | LTR |
| 34266037 | 34266265 | + | 27.4 | 275 | 505 | 0 | MLT1D | MaLR | LTR |

  

|  |  |  |  |  |  |  |  |  |  |
| --- | --- | --- | --- | --- | --- | --- | --- | --- | --- |
| chr5:34321196-34323102 | | | 1854 bp | | |  |  |  |  |
| Genome Start | Genome End | Strand | Div | Repeat Start | Repeat End | Repeat Left | Name | Family | Class |
| 34321196 | 34321361 | - | 31.9 | 5982 | 6151 | -15 | L1ME1 | L1 | LINE |
| 34321404 | 34321568 | + | 19.6 | 1 | 163 | -202 | MLT1A0 | MaLR | LTR |
| 34321569 | 34322135 | + | 22.9 | 1 | 554 | 0 | MLT2B3 | ERVL | LTR |
| 34322136 | 34322338 | + | 19.6 | 164 | 364 | -1 | MLT1A0 | MaLR | LTR |
| 34322339 | 34322912 | - | 29.8 | 5324 | 5941 | -225 | L1ME1 | L1 | LINE |
| 34322924 | 34323102 | - | 29.6 | 5099 | 5293 | -853 | L1ME1 | L1 | LINE |

  

|  |  |  |  |  |  |  |  |  |  |
| --- | --- | --- | --- | --- | --- | --- | --- | --- | --- |
| chr5:34356509-34357237 | | | 701 bp | | |  |  |  |  |
| Genome Start | Genome End | Strand | Div | Repeat Start | Repeat End | Repeat Left | Name | Family | Class |
| 34356509 | 34356841 | - | 18.8 | 73 | 396 | -1 | MLT2C1 | ERVL | LTR |
| 34356870 | 34357164 | - | 12.9 | 1 | 294 | -19 | AluSq | Alu | SINE |
| 34357165 | 34357237 | - | 18.8 | 1 | 72 | -325 | MLT2C1 | ERVL | LTR |

  

|  |  |  |  |  |  |  |  |  |  |
| --- | --- | --- | --- | --- | --- | --- | --- | --- | --- |
| chr5:34393679-34394610 | | | 893 bp | | |  |  |  |  |
| Genome Start | Genome End | Strand | Div | Repeat Start | Repeat End | Repeat Left | Name | Family | Class |
| 34393679 | 34393903 | + | 13.8 | 5550 | 5775 | -516 | L1MA6 | L1 | LINE |
| 34393943 | 34394123 | + | 18.1 | 5931 | 6111 | -189 | L1MA6 | L1 | LINE |
| 34394124 | 34394423 | - | 11.1 | 1 | 302 | -10 | AluSx | Alu | SINE |
| 34394424 | 34394610 | + | 18.1 | 6112 | 6295 | -5 | L1MA6 | L1 | LINE |

  

|  |  |  |  |  |  |  |  |  |  |
| --- | --- | --- | --- | --- | --- | --- | --- | --- | --- |
| chr5:34394679-34395787 | | | 1079 bp | | |  |  |  |  |
| Genome Start | Genome End | Strand | Div | Repeat Start | Repeat End | Repeat Left | Name | Family | Class |
| 34394679 | 34394833 | + | 41.5 | 2 | 156 | -112 | MIR | MIR | SINE |
| 34394846 | 34395175 | - | 34.8 | 250 | 627 | -1 | MLT2E | ERVL | LTR |
| 34395176 | 34395481 | + | 8.8 | 6 | 310 | -3 | AluSp | Alu | SINE |
| 34395482 | 34395738 | - | 34.8 | 2 | 249 | -379 | MLT2E | ERVL | LTR |
| 34395757 | 34395787 | + | 12.9 | 176 | 206 | -56 | MIR | MIR | SINE |

  

|  |  |  |  |  |  |  |  |  |  |
| --- | --- | --- | --- | --- | --- | --- | --- | --- | --- |
| chr5:34432250-34435534 | | | 3285 bp | | |  |  |  |  |
| Genome Start | Genome End | Strand | Div | Repeat Start | Repeat End | Repeat Left | Name | Family | Class |
| 34432250 | 34432310 | - | 15.3 | 2360 | 2417 | -1 | Tigger1 | MER2\_type | DNA |
| 34432311 | 34432618 | - | 5.2 | 2 | 309 | -2 | AluY | Alu | SINE |
| 34432619 | 34432646 | - | 15.3 | 2349 | 2359 | -59 | Tigger1 | MER2\_type | DNA |
| 34432647 | 34433089 | + | 19.3 | 1 | 432 | 0 | MSTB1 | MaLR | LTR |
| 34433090 | 34433879 | - | 15.3 | 1385 | 2348 | -70 | Tigger1 | MER2\_type | DNA |
| 34433880 | 34434162 | + | 11.7 | 1 | 286 | -26 | AluSx | Alu | SINE |
| 34434163 | 34435534 | - | 15.3 | 2 | 1384 | -1034 | Tigger1 | MER2\_type | DNA |

  

|  |  |  |  |  |  |  |  |  |  |
| --- | --- | --- | --- | --- | --- | --- | --- | --- | --- |
| chr5:37420033-37420641 | | | 609 bp | | |  |  |  |  |
| Genome Start | Genome End | Strand | Div | Repeat Start | Repeat End | Repeat Left | Name | Family | Class |
| 37420033 | 37420245 | - | 18.3 | 108 | 307 | -5 | AluJb | Alu | SINE |
| 37420246 | 37420546 | + | 5.0 | 1 | 308 | -3 | AluY | Alu | SINE |
| 37420547 | 37420641 | - | 18.3 | 13 | 107 | -205 | AluJb | Alu | SINE |

  

|  |  |  |  |  |  |  |  |  |  |
| --- | --- | --- | --- | --- | --- | --- | --- | --- | --- |
| chr5:37420649-37421669 | | | 1021 bp | | |  |  |  |  |
| Genome Start | Genome End | Strand | Div | Repeat Start | Repeat End | Repeat Left | Name | Family | Class |
| 37420649 | 37421096 | - | 21.2 | 291 | 803 | -60 | MER21B | ERV1 | LTR |
| 37421097 | 37421402 | + | 15.2 | 1 | 307 | -5 | AluJo | Alu | SINE |
| 37421403 | 37421669 | - | 21.2 | 15 | 290 | -573 | MER21B | ERV1 | LTR |

  

|  |  |  |  |  |  |  |  |  |  |
| --- | --- | --- | --- | --- | --- | --- | --- | --- | --- |
| chr5:37481001-37484354 | | | 3365 bp | | |  |  |  |  |
| Genome Start | Genome End | Strand | Div | Repeat Start | Repeat End | Repeat Left | Name | Family | Class |
| 37481001 | 37481331 | - | 31.6 | 7099 | 7430 | -612 | L1MC4 | L1 | LINE |
| 37481326 | 37481388 | - | 9.5 | 3670 | 3732 | -2414 | L1PA5 | L1 | LINE |
| 37481385 | 37483808 | + | 5.3 | 3726 | 6154 | 0 | L1PA5 | L1 | LINE |
| 37483808 | 37484354 | - | 23.3 | 6625 | 7118 | -843 | L1MC4 | L1 | LINE |

  

|  |  |  |  |  |  |  |  |  |  |
| --- | --- | --- | --- | --- | --- | --- | --- | --- | --- |
| chr5:37502219-37502724 | | | 506 bp | | |  |  |  |  |
| Genome Start | Genome End | Strand | Div | Repeat Start | Repeat End | Repeat Left | Name | Family | Class |
| 37502219 | 37502283 | - | 18.9 | 138 | 209 | 0 | MER3 | MER1\_type | DNA |
| 37502284 | 37502594 | + | 6.1 | 1 | 311 | 0 | AluY | Alu | SINE |
| 37502595 | 37502724 | - | 18.9 | 1 | 137 | -72 | MER3 | MER1\_type | DNA |

  

|  |  |  |  |  |  |  |  |  |  |
| --- | --- | --- | --- | --- | --- | --- | --- | --- | --- |
| chr5:37521106-37522022 | | | 917 bp | | |  |  |  |  |
| Genome Start | Genome End | Strand | Div | Repeat Start | Repeat End | Repeat Left | Name | Family | Class |
| 37521106 | 37521563 | - | 21.2 | 179 | 653 | 0 | MER82 | MER2\_type | DNA |
| 37521564 | 37521873 | - | 11.2 | 1 | 287 | -25 | AluSx | Alu | SINE |
| 37521874 | 37522022 | - | 21.2 | 1 | 178 | -475 | MER82 | MER2\_type | DNA |

  

|  |  |  |  |  |  |  |  |  |  |
| --- | --- | --- | --- | --- | --- | --- | --- | --- | --- |
| chr5:49776631-49777193 | | | 517 bp | | |  |  |  |  |
| Genome Start | Genome End | Strand | Div | Repeat Start | Repeat End | Repeat Left | Name | Family | Class |
| 49776631 | 49776713 | + | 25.6 | 100 | 183 | -412 | MLT1G1 | MaLR | LTR |
| 49776718 | 49777028 | + | 12.1 | 1 | 292 | -18 | AluSg | Alu | SINE |
| 49777071 | 49777193 | + | 29.3 | 173 | 292 | -298 | MLT1G1 | MaLR | LTR |

  

|  |  |  |  |  |  |  |  |  |  |
| --- | --- | --- | --- | --- | --- | --- | --- | --- | --- |
| chr5:49942421-49945298 | | | 2866 bp | | |  |  |  |  |
| Genome Start | Genome End | Strand | Div | Repeat Start | Repeat End | Repeat Left | Name | Family | Class |
| 49942421 | 49942816 | + | 14.5 | 2 | 403 | -2315 | Tigger2 | MER2\_type | DNA |
| 49942824 | 49943028 | - | 18.3 | 5965 | 6153 | -3 | L1PB4 | L1 | LINE |
| 49943034 | 49945298 | + | 13.7 | 386 | 2716 | -2 | Tigger2 | MER2\_type | DNA |

  

|  |  |  |  |  |  |  |  |  |  |
| --- | --- | --- | --- | --- | --- | --- | --- | --- | --- |
| chr5:49971592-49972621 | | | 1030 bp | | |  |  |  |  |
| Genome Start | Genome End | Strand | Div | Repeat Start | Repeat End | Repeat Left | Name | Family | Class |
| 49971592 | 49971923 | - | 33.0 | 5597 | 5933 | -1875 | L1MC4a | L1 | LINE |
| 49971924 | 49972369 | - | 12.4 | 5704 | 6157 | -9 | L1PA16 | L1 | LINE |
| 49972370 | 49972621 | - | 33.0 | 5327 | 5596 | -2212 | L1MC4a | L1 | LINE |

  

|  |  |  |  |  |  |  |  |  |  |
| --- | --- | --- | --- | --- | --- | --- | --- | --- | --- |
| chr5:61552116-61552858 | | | 743 bp | | |  |  |  |  |
| Genome Start | Genome End | Strand | Div | Repeat Start | Repeat End | Repeat Left | Name | Family | Class |
| 61552116 | 61552303 | + | 14.1 | 5442 | 5623 | -548 | L1M3 | L1 | LINE |
| 61552304 | 61552701 | + | 10.7 | 1 | 428 | 0 | MSTA | MaLR | LTR |
| 61552702 | 61552858 | + | 14.1 | 5624 | 5776 | -395 | L1M3 | L1 | LINE |

  

|  |  |  |  |  |  |  |  |  |  |
| --- | --- | --- | --- | --- | --- | --- | --- | --- | --- |
| chr5:61568956-61569544 | | | 551 bp | | |  |  |  |  |
| Genome Start | Genome End | Strand | Div | Repeat Start | Repeat End | Repeat Left | Name | Family | Class |
| 61568956 | 61569156 | + | 34.4 | 3 | 188 | -80 | MIRb | MIR | SINE |
| 61569157 | 61569279 | + | 16.7 | 1 | 126 | -186 | AluJb | Alu | SINE |
| 61569318 | 61569469 | + | 16.7 | 127 | 284 | -28 | AluJb | Alu | SINE |
| 61569470 | 61569544 | + | 34.4 | 189 | 265 | -3 | MIRb | MIR | SINE |

  

|  |  |  |  |  |  |  |  |  |  |
| --- | --- | --- | --- | --- | --- | --- | --- | --- | --- |
| chr5:61583233-61583819 | | | 587 bp | | |  |  |  |  |
| Genome Start | Genome End | Strand | Div | Repeat Start | Repeat End | Repeat Left | Name | Family | Class |
| 61583233 | 61583396 | + | 26.1 | 5698 | 5877 | -244 | L1ME4a | L1 | LINE |
| 61583397 | 61583705 | + | 15.6 | 2 | 312 | 0 | AluJb | Alu | SINE |
| 61583706 | 61583819 | + | 26.1 | 5878 | 5999 | -122 | L1ME4a | L1 | LINE |

  

|  |  |  |  |  |  |  |  |  |  |
| --- | --- | --- | --- | --- | --- | --- | --- | --- | --- |
| chr5:65065311-65065882 | | | 572 bp | | |  |  |  |  |
| Genome Start | Genome End | Strand | Div | Repeat Start | Repeat End | Repeat Left | Name | Family | Class |
| 65065311 | 65065469 | - | 14.4 | 137 | 294 | -18 | AluSx | Alu | SINE |
| 65065470 | 65065745 | - | 9.1 | 35 | 310 | -1 | AluY | Alu | SINE |
| 65065746 | 65065882 | - | 14.4 | 1 | 136 | -176 | AluSx | Alu | SINE |

  

|  |  |  |  |  |  |  |  |  |  |
| --- | --- | --- | --- | --- | --- | --- | --- | --- | --- |
| chr5:68883286-68884518 | | | 1233 bp | | |  |  |  |  |
| Genome Start | Genome End | Strand | Div | Repeat Start | Repeat End | Repeat Left | Name | Family | Class |
| 68883286 | 68883626 | - | 23.8 | 5831 | 6169 | -1 | L1ME3A | L1 | LINE |
| 68883627 | 68883908 | - | 11.3 | 1 | 289 | -23 | AluSx | Alu | SINE |
| 68883909 | 68883920 | - | 23.8 | 5824 | 5830 | -340 | L1ME3A | L1 | LINE |
| 68883921 | 68884218 | - | 10.7 | 1 | 298 | -14 | AluSx | Alu | SINE |
| 68884219 | 68884518 | - | 23.8 | 5472 | 5823 | -347 | L1ME3A | L1 | LINE |

  

|  |  |  |  |  |  |  |  |  |  |
| --- | --- | --- | --- | --- | --- | --- | --- | --- | --- |
| chr5:68895117-68895949 | | | 833 bp | | |  |  |  |  |
| Genome Start | Genome End | Strand | Div | Repeat Start | Repeat End | Repeat Left | Name | Family | Class |
| 68895117 | 68895398 | - | 15.4 | 5888 | 6166 | 0 | L1PA16 | L1 | LINE |
| 68895399 | 68895658 | - | 10.5 | 1 | 257 | -56 | AluSq | Alu | SINE |
| 68895659 | 68895949 | - | 15.4 | 5618 | 5887 | -279 | L1PA16 | L1 | LINE |

  

|  |  |  |  |  |  |  |  |  |  |
| --- | --- | --- | --- | --- | --- | --- | --- | --- | --- |
| chr5:68896822-68897663 | | | 842 bp | | |  |  |  |  |
| Genome Start | Genome End | Strand | Div | Repeat Start | Repeat End | Repeat Left | Name | Family | Class |
| 68896822 | 68897097 | - | 24.1 | 5911 | 6178 | 0 | L1MB8 | L1 | LINE |
| 68897098 | 68897411 | + | 14.1 | 1 | 345 | -1 | MER7A | MER2\_type | DNA |
| 68897412 | 68897663 | - | 24.1 | 5649 | 5910 | -268 | L1MB8 | L1 | LINE |

  

|  |  |  |  |  |  |  |  |  |  |
| --- | --- | --- | --- | --- | --- | --- | --- | --- | --- |
| chr5:68902967-68903772 | | | 806 bp | | |  |  |  |  |
| Genome Start | Genome End | Strand | Div | Repeat Start | Repeat End | Repeat Left | Name | Family | Class |
| 68902967 | 68903251 | - | 25.3 | 247 | 547 | -1351 | Kanga1 | Tc2 | DNA |
| 68903252 | 68903553 | - | 14.6 | 1 | 301 | -12 | AluSq | Alu | SINE |
| 68903554 | 68903772 | - | 25.3 | 1 | 246 | -1652 | Kanga1 | Tc2 | DNA |

  

|  |  |  |  |  |  |  |  |  |  |
| --- | --- | --- | --- | --- | --- | --- | --- | --- | --- |
| chr5:68905801-68909405 | | | 3598 bp | | |  |  |  |  |
| Genome Start | Genome End | Strand | Div | Repeat Start | Repeat End | Repeat Left | Name | Family | Class |
| 68905801 | 68906410 | + | 13.0 | 2 | 617 | -1801 | Tigger1 | MER2\_type | DNA |
| 68906411 | 68906717 | + | 17.8 | 1 | 297 | -15 | AluJo | Alu | SINE |
| 68906718 | 68907001 | + | 13.0 | 618 | 874 | -1544 | Tigger1 | MER2\_type | DNA |
| 68907002 | 68907300 | + | 9.7 | 1 | 298 | -12 | AluSg | Alu | SINE |
| 68907301 | 68907400 | + | 13.0 | 875 | 967 | -1451 | Tigger1 | MER2\_type | DNA |
| 68907401 | 68907704 | - | 14.4 | 1 | 305 | -7 | AluJo | Alu | SINE |
| 68907705 | 68908319 | + | 13.0 | 968 | 1618 | -800 | Tigger1 | MER2\_type | DNA |
| 68908322 | 68908617 | + | 11.2 | 1 | 297 | -15 | AluSx | Alu | SINE |
| 68908623 | 68908998 | + | 14.5 | 1909 | 2309 | -109 | Tigger1 | MER2\_type | DNA |
| 68908999 | 68909271 | - | 7.4 | 1 | 309 | -2 | AluY | Alu | SINE |
| 68909272 | 68909405 | + | 14.5 | 2310 | 2418 | 0 | Tigger1 | MER2\_type | DNA |

  

|  |  |  |  |  |  |  |  |  |  |
| --- | --- | --- | --- | --- | --- | --- | --- | --- | --- |
| chr5:68909395-68909828 | | | 434 bp | | |  |  |  |  |
| Genome Start | Genome End | Strand | Div | Repeat Start | Repeat End | Repeat Left | Name | Family | Class |
| 68909395 | 68909425 | + | 12.3 | 2377 | 2407 | -84 | Tigger7 | MER2\_type | DNA |
| 68909426 | 68909742 | + | 17.0 | 2 | 345 | 0 | MER2 | MER2\_type | DNA |
| 68909743 | 68909828 | + | 12.3 | 2408 | 2490 | -1 | Tigger7 | MER2\_type | DNA |

  

|  |  |  |  |  |  |  |  |  |  |
| --- | --- | --- | --- | --- | --- | --- | --- | --- | --- |
| chr5:68919733-68921019 | | | 1283 bp | | |  |  |  |  |
| Genome Start | Genome End | Strand | Div | Repeat Start | Repeat End | Repeat Left | Name | Family | Class |
| 68919733 | 68919982 | + | 23.5 | 1084 | 1345 | -1162 | HAL1 | L1 | LINE |
| 68919987 | 68920118 | + | 23.5 | 1 | 132 | -11 | FLAM\_C | Alu | SINE |
| 68920119 | 68921019 | + | 23.9 | 1337 | 2316 | -191 | HAL1 | L1 | LINE |

  

|  |  |  |  |  |  |  |  |  |  |
| --- | --- | --- | --- | --- | --- | --- | --- | --- | --- |
| chr5:68928695-68929400 | | | 521 bp | | |  |  |  |  |
| Genome Start | Genome End | Strand | Div | Repeat Start | Repeat End | Repeat Left | Name | Family | Class |
| 68928695 | 68928802 | - | 25.0 | 225 | 328 | -13 | MER102b | MER1\_type | DNA |
| 68928985 | 68929301 | - | 19.0 | 1 | 311 | -1 | AluJb | Alu | SINE |
| 68929305 | 68929400 | - | 24.2 | 24 | 129 | -212 | MER102b | MER1\_type | DNA |

  

|  |  |  |  |  |  |  |  |  |  |
| --- | --- | --- | --- | --- | --- | --- | --- | --- | --- |
| chr5:68930227-68930721 | | | 495 bp | | |  |  |  |  |
| Genome Start | Genome End | Strand | Div | Repeat Start | Repeat End | Repeat Left | Name | Family | Class |
| 68930227 | 68930304 | + | 24.4 | 1 | 75 | -114 | MER5A | MER1\_type | DNA |
| 68930305 | 68930601 | + | 9.8 | 1 | 297 | -13 | AluSg | Alu | SINE |
| 68930602 | 68930721 | + | 24.4 | 76 | 189 | 0 | MER5A | MER1\_type | DNA |

  

|  |  |  |  |  |  |  |  |  |  |
| --- | --- | --- | --- | --- | --- | --- | --- | --- | --- |
| chr5:68934301-68936354 | | | 2027 bp | | |  |  |  |  |
| Genome Start | Genome End | Strand | Div | Repeat Start | Repeat End | Repeat Left | Name | Family | Class |
| 68934301 | 68934922 | + | 20.2 | 1 | 650 | -285 | MER21C | ERV1 | LTR |
| 68934925 | 68935094 | + | 15.4 | 443 | 612 | 0 | LTR9 | ERV1 | LTR |
| 68935097 | 68935218 | + | 14.9 | 3 | 128 | -8791 | HUERS-P3 | ERV1 | LTR |
| 68935231 | 68935273 | + | 16.3 | 885 | 927 | -6491 | HUERS-P3 | ERV1 | LTR |
| 68935270 | 68935817 | + | 17.4 | 142 | 692 | -8227 | HUERS-P3 | ERV1 | LTR |
| 68935818 | 68936062 | + | 16.9 | 284 | 532 | -80 | LTR9 | ERV1 | LTR |
| 68936078 | 68936354 | + | 19.8 | 634 | 931 | -4 | MER21C | ERV1 | LTR |

  

|  |  |  |  |  |  |  |  |  |  |
| --- | --- | --- | --- | --- | --- | --- | --- | --- | --- |
| chr5:68937453-68938241 | | | 789 bp | | |  |  |  |  |
| Genome Start | Genome End | Strand | Div | Repeat Start | Repeat End | Repeat Left | Name | Family | Class |
| 68937453 | 68937756 | + | 27.7 | 798 | 1137 | -1370 | HAL1 | L1 | LINE |
| 68937757 | 68938036 | + | 14.6 | 1 | 277 | -35 | AluJb | Alu | SINE |
| 68938037 | 68938241 | + | 27.7 | 1138 | 1369 | -1138 | HAL1 | L1 | LINE |

  

|  |  |  |  |  |  |  |  |  |  |
| --- | --- | --- | --- | --- | --- | --- | --- | --- | --- |
| chr5:68946985-68947747 | | | 599 bp | | |  |  |  |  |
| Genome Start | Genome End | Strand | Div | Repeat Start | Repeat End | Repeat Left | Name | Family | Class |
| 68946985 | 68947176 | - | 18.0 | 277 | 473 | 0 | MER65D | ERV1 | LTR |
| 68947177 | 68947294 | + | 21.4 | 1 | 117 | -195 | AluJb | Alu | SINE |
| 68947375 | 68947519 | - | 18.9 | 157 | 288 | -185 | MER65D | ERV1 | LTR |
| 68947604 | 68947747 | - | 19.4 | 1 | 147 | -326 | MER65D | ERV1 | LTR |

  

|  |  |  |  |  |  |  |  |  |  |
| --- | --- | --- | --- | --- | --- | --- | --- | --- | --- |
| chr5:68950923-68951558 | | | 636 bp | | |  |  |  |  |
| Genome Start | Genome End | Strand | Div | Repeat Start | Repeat End | Repeat Left | Name | Family | Class |
| 68950923 | 68951029 | + | 10.5 | 1 | 101 | -211 | AluSx | Alu | SINE |
| 68951030 | 68951345 | + | 7.8 | 1 | 308 | -3 | AluY | Alu | SINE |
| 68951346 | 68951558 | + | 10.5 | 102 | 303 | -9 | AluSx | Alu | SINE |

  

|  |  |  |  |  |  |  |  |  |  |
| --- | --- | --- | --- | --- | --- | --- | --- | --- | --- |
| chr5:68952826-68953376 | | | 509 bp | | |  |  |  |  |
| Genome Start | Genome End | Strand | Div | Repeat Start | Repeat End | Repeat Left | Name | Family | Class |
| 68952826 | 68952886 | - | 24.6 | 152 | 200 | -311 | MLT1J | MaLR | LTR |
| 68952887 | 68953173 | + | 8.7 | 1 | 286 | -27 | AluSq | Alu | SINE |
| 68953216 | 68953376 | - | 24.6 | 1 | 151 | -360 | MLT1J | MaLR | LTR |

  

|  |  |  |  |  |  |  |  |  |  |
| --- | --- | --- | --- | --- | --- | --- | --- | --- | --- |
| chr5:68958899-68960806 | | | 1745 bp | | |  |  |  |  |
| Genome Start | Genome End | Strand | Div | Repeat Start | Repeat End | Repeat Left | Name | Family | Class |
| 68958899 | 68959013 | - | 24.7 | 6161 | 6276 | -37 | L1ME3B | L1 | LINE |
| 68959014 | 68959320 | + | 12.1 | 1 | 309 | -3 | AluSx | Alu | SINE |
| 68959321 | 68959932 | - | 24.7 | 5536 | 6160 | -153 | L1ME3B | L1 | LINE |
| 68959966 | 68960275 | - | 6.1 | 2 | 312 | -1 | AluSp | Alu | SINE |
| 68960399 | 68960690 | + | 20.6 | 4 | 295 | -17 | AluJo | Alu | SINE |
| 68960698 | 68960806 | - | 24.1 | 2407 | 2526 | -3874 | L1ME3B | L1 | LINE |

  

|  |  |  |  |  |  |  |  |  |  |
| --- | --- | --- | --- | --- | --- | --- | --- | --- | --- |
| chr5:68969874-68970874 | | | 1001 bp | | |  |  |  |  |
| Genome Start | Genome End | Strand | Div | Repeat Start | Repeat End | Repeat Left | Name | Family | Class |
| 68969874 | 68969917 | + | 22.5 | 1 | 44 | -330 | MLT1A | MaLR | LTR |
| 68969918 | 68970224 | + | 6.9 | 1 | 304 | -7 | AluY | Alu | SINE |
| 68970225 | 68970503 | + | 22.5 | 45 | 308 | -66 | MLT1A | MaLR | LTR |
| 68970504 | 68970806 | - | 15.2 | 1 | 312 | 0 | AluSx | Alu | SINE |
| 68970807 | 68970874 | + | 22.5 | 309 | 374 | 0 | MLT1A | MaLR | LTR |

  

|  |  |  |  |  |  |  |  |  |  |
| --- | --- | --- | --- | --- | --- | --- | --- | --- | --- |
| chr5:68970895-68971391 | | | 497 bp | | |  |  |  |  |
| Genome Start | Genome End | Strand | Div | Repeat Start | Repeat End | Repeat Left | Name | Family | Class |
| 68970895 | 68971022 | + | 19.1 | 1 | 129 | -80 | MER3 | MER1\_type | DNA |
| 68971023 | 68971326 | - | 7.2 | 1 | 304 | -7 | AluY | Alu | SINE |
| 68971327 | 68971391 | + | 19.1 | 130 | 208 | -1 | MER3 | MER1\_type | DNA |

  

|  |  |  |  |  |  |  |  |  |  |
| --- | --- | --- | --- | --- | --- | --- | --- | --- | --- |
| chr5:68974729-68975326 | | | 598 bp | | |  |  |  |  |
| Genome Start | Genome End | Strand | Div | Repeat Start | Repeat End | Repeat Left | Name | Family | Class |
| 68974729 | 68974851 | + | 17.9 | 2 | 137 | -175 | AluJo | Alu | SINE |
| 68974852 | 68975145 | + | 14.8 | 1 | 295 | -15 | AluSg | Alu | SINE |
| 68975146 | 68975326 | + | 17.9 | 138 | 305 | -7 | AluJo | Alu | SINE |

  

|  |  |  |  |  |  |  |  |  |  |
| --- | --- | --- | --- | --- | --- | --- | --- | --- | --- |
| chr5:68976688-68977258 | | | 571 bp | | |  |  |  |  |
| Genome Start | Genome End | Strand | Div | Repeat Start | Repeat End | Repeat Left | Name | Family | Class |
| 68976688 | 68976815 | + | 15.9 | 1 | 125 | -187 | AluJo | Alu | SINE |
| 68976816 | 68977107 | - | 12.7 | 1 | 295 | -17 | AluSx | Alu | SINE |
| 68977108 | 68977258 | + | 15.9 | 126 | 275 | -37 | AluJo | Alu | SINE |

  

|  |  |  |  |  |  |  |  |  |  |
| --- | --- | --- | --- | --- | --- | --- | --- | --- | --- |
| chr5:68979698-68980607 | | | 891 bp | | |  |  |  |  |
| Genome Start | Genome End | Strand | Div | Repeat Start | Repeat End | Repeat Left | Name | Family | Class |
| 68979698 | 68979827 | + | 21.3 | 1 | 193 | -460 | MER82 | MER2\_type | DNA |
| 68979847 | 68980161 | + | 11.8 | 1 | 302 | -10 | AluSx | Alu | SINE |
| 68980162 | 68980607 | + | 21.3 | 194 | 653 | 0 | MER82 | MER2\_type | DNA |

  

|  |  |  |  |  |  |  |  |  |  |
| --- | --- | --- | --- | --- | --- | --- | --- | --- | --- |
| chr5:68987907-68988906 | | | 1000 bp | | |  |  |  |  |
| Genome Start | Genome End | Strand | Div | Repeat Start | Repeat End | Repeat Left | Name | Family | Class |
| 68987907 | 68987950 | + | 22.9 | 1 | 44 | -330 | MLT1A | MaLR | LTR |
| 68987951 | 68988259 | + | 6.5 | 1 | 306 | -5 | AluY | Alu | SINE |
| 68988260 | 68988535 | + | 22.9 | 45 | 308 | -66 | MLT1A | MaLR | LTR |
| 68988536 | 68988838 | - | 15.2 | 1 | 312 | 0 | AluSx | Alu | SINE |
| 68988839 | 68988906 | + | 22.9 | 309 | 374 | 0 | MLT1A | MaLR | LTR |

  

|  |  |  |  |  |  |  |  |  |  |
| --- | --- | --- | --- | --- | --- | --- | --- | --- | --- |
| chr5:68988927-68989423 | | | 497 bp | | |  |  |  |  |
| Genome Start | Genome End | Strand | Div | Repeat Start | Repeat End | Repeat Left | Name | Family | Class |
| 68988927 | 68989061 | + | 18.3 | 1 | 136 | -73 | MER3 | MER1\_type | DNA |
| 68989062 | 68989358 | - | 7.1 | 1 | 297 | -14 | AluY | Alu | SINE |
| 68989359 | 68989423 | + | 18.3 | 137 | 209 | 0 | MER3 | MER1\_type | DNA |

  

|  |  |  |  |  |  |  |  |  |  |
| --- | --- | --- | --- | --- | --- | --- | --- | --- | --- |
| chr5:68990121-68990781 | | | 661 bp | | |  |  |  |  |
| Genome Start | Genome End | Strand | Div | Repeat Start | Repeat End | Repeat Left | Name | Family | Class |
| 68990121 | 68990352 | - | 19.8 | 127 | 365 | 0 | MLT1A0 | MaLR | LTR |
| 68990353 | 68990667 | + | 12.7 | 1 | 307 | -3 | AluSg | Alu | SINE |
| 68990668 | 68990781 | - | 19.8 | 43 | 126 | -239 | MLT1A0 | MaLR | LTR |

  

|  |  |  |  |  |  |  |  |  |  |
| --- | --- | --- | --- | --- | --- | --- | --- | --- | --- |
| chr5:68995400-68996619 | | | 1220 bp | | |  |  |  |  |
| Genome Start | Genome End | Strand | Div | Repeat Start | Repeat End | Repeat Left | Name | Family | Class |
| 68995400 | 68995800 | + | 23.0 | 2528 | 2897 | -3948 | L1M2 | L1 | LINE |
| 68995801 | 68996103 | + | 12.9 | 1 | 303 | -7 | AluSg | Alu | SINE |
| 68996104 | 68996619 | + | 21.9 | 2898 | 3392 | -3453 | L1M2 | L1 | LINE |

  

|  |  |  |  |  |  |  |  |  |  |
| --- | --- | --- | --- | --- | --- | --- | --- | --- | --- |
| chr5:68997308-68998618 | | | 1311 bp | | |  |  |  |  |
| Genome Start | Genome End | Strand | Div | Repeat Start | Repeat End | Repeat Left | Name | Family | Class |
| 68997308 | 68997733 | + | 23.4 | 6152 | 6586 | -1199 | L1MC3 | L1 | LINE |
| 68997734 | 68998116 | + | 17.6 | 1 | 428 | 0 | MSTA | MaLR | LTR |
| 68998117 | 68998618 | + | 23.4 | 6587 | 7119 | -666 | L1MC3 | L1 | LINE |

  

|  |  |  |  |  |  |  |  |  |  |
| --- | --- | --- | --- | --- | --- | --- | --- | --- | --- |
| chr5:69021881-69022833 | | | 791 bp | | |  |  |  |  |
| Genome Start | Genome End | Strand | Div | Repeat Start | Repeat End | Repeat Left | Name | Family | Class |
| 69021881 | 69022096 | + | 23.9 | 1 | 225 | -280 | MLT1D | MaLR | LTR |
| 69022138 | 69022184 | + | 17.0 | 1 | 47 | -327 | MLT1A | MaLR | LTR |
| 69022232 | 69022341 | + | 30.9 | 51 | 160 | -214 | MLT1A | MaLR | LTR |
| 69022416 | 69022604 | + | 18.5 | 189 | 374 | 0 | MLT1A | MaLR | LTR |
| 69022605 | 69022833 | + | 27.0 | 275 | 505 | 0 | MLT1D | MaLR | LTR |

  

|  |  |  |  |  |  |  |  |  |  |
| --- | --- | --- | --- | --- | --- | --- | --- | --- | --- |
| chr5:69024124-69026815 | | | 2681 bp | | |  |  |  |  |
| Genome Start | Genome End | Strand | Div | Repeat Start | Repeat End | Repeat Left | Name | Family | Class |
| 69024124 | 69024346 | + | 18.4 | 1 | 238 | -128 | Tigger5a | MER2\_type | DNA |
| 69024357 | 69026744 | - | 6.8 | 3742 | 6150 | -4 | L1PA6 | L1 | LINE |
| 69026746 | 69026815 | + | 20.0 | 356 | 434 | -15 | Tigger5a | MER2\_type | DNA |

  

|  |  |  |  |  |  |  |  |  |  |
| --- | --- | --- | --- | --- | --- | --- | --- | --- | --- |
| chr5:69034840-69041191 | | | 6197 bp | | |  |  |  |  |
| Genome Start | Genome End | Strand | Div | Repeat Start | Repeat End | Repeat Left | Name | Family | Class |
| 69034840 | 69035046 | + | 20.8 | 13 | 235 | -6268 | L1PA16 | L1 | LINE |
| 69035046 | 69038901 | + | 16.9 | 947 | 4819 | -1664 | L1PA16 | L1 | LINE |
| 69038902 | 69039163 | + | 18.4 | 2 | 286 | -16 | AluJo | Alu | SINE |
| 69039320 | 69040146 | + | 16.8 | 4820 | 5660 | -823 | L1PA16 | L1 | LINE |
| 69040147 | 69040460 | + | 8.7 | 5876 | 6167 | -1 | L1PA10 | L1 | LINE |
| 69040461 | 69041191 | + | 14.2 | 5661 | 6398 | -105 | L1PA16 | L1 | LINE |

  

|  |  |  |  |  |  |  |  |  |  |
| --- | --- | --- | --- | --- | --- | --- | --- | --- | --- |
| chr5:69051097-69053742 | | | 2413 bp | | |  |  |  |  |
| Genome Start | Genome End | Strand | Div | Repeat Start | Repeat End | Repeat Left | Name | Family | Class |
| 69051097 | 69051726 | + | 30.7 | 3855 | 4522 | -1624 | L1ME2 | L1 | LINE |
| 69051727 | 69052145 | + | 14.2 | 5 | 428 | 0 | MSTA | MaLR | LTR |
| 69052146 | 69052684 | + | 30.7 | 4523 | 5064 | -1082 | L1ME2 | L1 | LINE |
| 69052900 | 69053273 | + | 24.7 | 5329 | 5758 | -406 | L1ME2 | L1 | LINE |
| 69053279 | 69053463 | - | 22.1 | 1 | 213 | -6 | MER20 | MER1\_type | DNA |
| 69053477 | 69053742 | + | 26.4 | 5768 | 6039 | -125 | L1ME2 | L1 | LINE |

  

|  |  |  |  |  |  |  |  |  |  |
| --- | --- | --- | --- | --- | --- | --- | --- | --- | --- |
| chr5:69071462-69073809 | | | 2326 bp | | |  |  |  |  |
| Genome Start | Genome End | Strand | Div | Repeat Start | Repeat End | Repeat Left | Name | Family | Class |
| 69071462 | 69072065 | - | 23.1 | 3317 | 3934 | -2705 | HERV35I | ERV1 | LTR |
| 69072076 | 69072945 | - | 10.8 | 5275 | 6165 | 0 | L1PA15 | L1 | LINE |
| 69072948 | 69073683 | - | 27.0 | 8 | 767 | -6567 | L1P4b | L1 | LINE |
| 69073694 | 69073809 | - | 18.3 | 3206 | 3321 | -3318 | HERV35I | ERV1 | LTR |

  

|  |  |  |  |  |  |  |  |  |  |
| --- | --- | --- | --- | --- | --- | --- | --- | --- | --- |
| chr5:69074988-69075514 | | | 515 bp | | |  |  |  |  |
| Genome Start | Genome End | Strand | Div | Repeat Start | Repeat End | Repeat Left | Name | Family | Class |
| 69074988 | 69075260 | - | 26.9 | 1151 | 3596 | -3322 | HERV35I | ERV1 | LTR |
| 69075271 | 69075413 | - | 20.7 | 124 | 292 | -266 | MER34C\_ | ERV1 | LTR |
| 69075416 | 69075514 | - | 19.8 | 891 | 990 | -4133 | HERV35I | ERV1 | LTR |

  

|  |  |  |  |  |  |  |  |  |  |
| --- | --- | --- | --- | --- | --- | --- | --- | --- | --- |
| chr5:69075847-69076642 | | | 796 bp | | |  |  |  |  |
| Genome Start | Genome End | Strand | Div | Repeat Start | Repeat End | Repeat Left | Name | Family | Class |
| 69075847 | 69075976 | - | 30.3 | 325 | 445 | 0 | MER65A | ERV1 | LTR |
| 69075977 | 69076350 | - | 13.2 | 1 | 428 | 0 | MSTA | MaLR | LTR |
| 69076351 | 69076642 | - | 30.3 | 1 | 324 | -121 | MER65A | ERV1 | LTR |

  

|  |  |  |  |  |  |  |  |  |  |
| --- | --- | --- | --- | --- | --- | --- | --- | --- | --- |
| chr5:69083221-69083627 | | | 407 bp | | |  |  |  |  |
| Genome Start | Genome End | Strand | Div | Repeat Start | Repeat End | Repeat Left | Name | Family | Class |
| 69083221 | 69083297 | - | 32.0 | 107 | 184 | -84 | MIRb | MIR | SINE |
| 69083298 | 69083596 | + | 14.1 | 1 | 301 | -11 | AluSx | Alu | SINE |
| 69083597 | 69083627 | - | 32.0 | 79 | 106 | -162 | MIRb | MIR | SINE |

  

|  |  |  |  |  |  |  |  |  |  |
| --- | --- | --- | --- | --- | --- | --- | --- | --- | --- |
| chr5:69088021-69090064 | | | 1742 bp | | |  |  |  |  |
| Genome Start | Genome End | Strand | Div | Repeat Start | Repeat End | Repeat Left | Name | Family | Class |
| 69088021 | 69088412 | - | 29.4 | 5751 | 6161 | -5 | L1ME1 | L1 | LINE |
| 69088544 | 69088900 | - | 25.7 | 4340 | 4721 | -1425 | L1ME1 | L1 | LINE |
| 69088901 | 69089107 | + | 20.0 | 1 | 211 | -200 | LTR57 | ERVL | LTR |
| 69089108 | 69089386 | + | 5.0 | 1 | 279 | -32 | AluY | Alu | SINE |
| 69089558 | 69089757 | + | 20.0 | 212 | 411 | 0 | LTR57 | ERVL | LTR |
| 69089758 | 69090064 | - | 25.7 | 4110 | 4339 | -1807 | L1ME1 | L1 | LINE |

  

|  |  |  |  |  |  |  |  |  |  |
| --- | --- | --- | --- | --- | --- | --- | --- | --- | --- |
| chr5:69134803-69135209 | | | 407 bp | | |  |  |  |  |
| Genome Start | Genome End | Strand | Div | Repeat Start | Repeat End | Repeat Left | Name | Family | Class |
| 69134803 | 69134833 | + | 32.0 | 79 | 106 | -162 | MIRb | MIR | SINE |
| 69134834 | 69135132 | - | 14.1 | 1 | 301 | -11 | AluSx | Alu | SINE |
| 69135133 | 69135209 | + | 32.0 | 107 | 184 | -84 | MIRb | MIR | SINE |

  

|  |  |  |  |  |  |  |  |  |  |
| --- | --- | --- | --- | --- | --- | --- | --- | --- | --- |
| chr5:69136650-69137381 | | | 732 bp | | |  |  |  |  |
| Genome Start | Genome End | Strand | Div | Repeat Start | Repeat End | Repeat Left | Name | Family | Class |
| 69136650 | 69136699 | + | 34.0 | 861 | 910 | -2509 | L2 | L2 | LINE |
| 69136700 | 69137217 | - | 27.0 | 3 | 560 | -2 | MLT1F2 | MaLR | LTR |
| 69137218 | 69137381 | + | 34.0 | 911 | 1076 | -2343 | L2 | L2 | LINE |

  

|  |  |  |  |  |  |  |  |  |  |
| --- | --- | --- | --- | --- | --- | --- | --- | --- | --- |
| chr5:69141787-69142582 | | | 796 bp | | |  |  |  |  |
| Genome Start | Genome End | Strand | Div | Repeat Start | Repeat End | Repeat Left | Name | Family | Class |
| 69141787 | 69142078 | + | 30.3 | 1 | 324 | -121 | MER65A | ERV1 | LTR |
| 69142079 | 69142452 | + | 13.2 | 1 | 428 | 0 | MSTA | MaLR | LTR |
| 69142453 | 69142582 | + | 30.3 | 325 | 445 | 0 | MER65A | ERV1 | LTR |

  

|  |  |  |  |  |  |  |  |  |  |
| --- | --- | --- | --- | --- | --- | --- | --- | --- | --- |
| chr5:69164679-69167324 | | | 2413 bp | | |  |  |  |  |
| Genome Start | Genome End | Strand | Div | Repeat Start | Repeat End | Repeat Left | Name | Family | Class |
| 69164679 | 69164944 | - | 26.4 | 5768 | 6039 | -125 | L1ME2 | L1 | LINE |
| 69164958 | 69165142 | + | 22.1 | 1 | 213 | -6 | MER20 | MER1\_type | DNA |
| 69165148 | 69165521 | - | 24.7 | 5329 | 5758 | -406 | L1ME2 | L1 | LINE |
| 69165737 | 69166275 | - | 30.7 | 4523 | 5064 | -1082 | L1ME2 | L1 | LINE |
| 69166276 | 69166694 | - | 14.5 | 5 | 428 | 0 | MSTA | MaLR | LTR |
| 69166695 | 69167324 | - | 30.7 | 3855 | 4522 | -1624 | L1ME2 | L1 | LINE |

  

|  |  |  |  |  |  |  |  |  |  |
| --- | --- | --- | --- | --- | --- | --- | --- | --- | --- |
| chr5:69191581-69194272 | | | 2681 bp | | |  |  |  |  |
| Genome Start | Genome End | Strand | Div | Repeat Start | Repeat End | Repeat Left | Name | Family | Class |
| 69191581 | 69191650 | - | 20.0 | 356 | 434 | -15 | Tigger5a | MER2\_type | DNA |
| 69191652 | 69194039 | + | 6.8 | 3742 | 6150 | -4 | L1PA6 | L1 | LINE |
| 69194050 | 69194272 | - | 18.8 | 1 | 238 | -128 | Tigger5a | MER2\_type | DNA |

  

|  |  |  |  |  |  |  |  |  |  |
| --- | --- | --- | --- | --- | --- | --- | --- | --- | --- |
| chr5:69195563-69196515 | | | 784 bp | | |  |  |  |  |
| Genome Start | Genome End | Strand | Div | Repeat Start | Repeat End | Repeat Left | Name | Family | Class |
| 69195563 | 69195791 | - | 27.0 | 275 | 505 | 0 | MLT1D | MaLR | LTR |
| 69195792 | 69195980 | - | 18.5 | 189 | 374 | 0 | MLT1A | MaLR | LTR |
| 69196055 | 69196157 | - | 30.1 | 58 | 160 | -214 | MLT1A | MaLR | LTR |
| 69196212 | 69196258 | - | 17.0 | 1 | 47 | -327 | MLT1A | MaLR | LTR |
| 69196300 | 69196515 | - | 24.4 | 1 | 225 | -280 | MLT1D | MaLR | LTR |

  

|  |  |  |  |  |  |  |  |  |  |
| --- | --- | --- | --- | --- | --- | --- | --- | --- | --- |
| chr5:69217737-69219055 | | | 1319 bp | | |  |  |  |  |
| Genome Start | Genome End | Strand | Div | Repeat Start | Repeat End | Repeat Left | Name | Family | Class |
| 69217737 | 69218238 | - | 23.6 | 6587 | 7119 | -666 | L1MC3 | L1 | LINE |
| 69218239 | 69218621 | - | 17.3 | 1 | 428 | 0 | MSTA | MaLR | LTR |
| 69218622 | 69219055 | - | 23.6 | 6147 | 6586 | -1199 | L1MC3 | L1 | LINE |

  

|  |  |  |  |  |  |  |  |  |  |
| --- | --- | --- | --- | --- | --- | --- | --- | --- | --- |
| chr5:69219736-69220958 | | | 1223 bp | | |  |  |  |  |
| Genome Start | Genome End | Strand | Div | Repeat Start | Repeat End | Repeat Left | Name | Family | Class |
| 69219736 | 69220251 | - | 21.9 | 2898 | 3392 | -3453 | L1M2 | L1 | LINE |
| 69220252 | 69220557 | - | 12.8 | 1 | 306 | -4 | AluSg | Alu | SINE |
| 69220558 | 69220958 | - | 23.0 | 2528 | 2897 | -3948 | L1M2 | L1 | LINE |

  

|  |  |  |  |  |  |  |  |  |  |
| --- | --- | --- | --- | --- | --- | --- | --- | --- | --- |
| chr5:69225589-69226244 | | | 656 bp | | |  |  |  |  |
| Genome Start | Genome End | Strand | Div | Repeat Start | Repeat End | Repeat Left | Name | Family | Class |
| 69225589 | 69225696 | + | 19.8 | 43 | 126 | -239 | MLT1A0 | MaLR | LTR |
| 69225697 | 69226012 | - | 13.0 | 1 | 308 | -2 | AluSg | Alu | SINE |
| 69226013 | 69226244 | + | 19.8 | 127 | 365 | 0 | MLT1A0 | MaLR | LTR |

  

|  |  |  |  |  |  |  |  |  |  |
| --- | --- | --- | --- | --- | --- | --- | --- | --- | --- |
| chr5:69226943-69227440 | | | 498 bp | | |  |  |  |  |
| Genome Start | Genome End | Strand | Div | Repeat Start | Repeat End | Repeat Left | Name | Family | Class |
| 69226943 | 69227007 | - | 19.1 | 134 | 209 | 0 | MER3 | MER1\_type | DNA |
| 69227008 | 69227312 | + | 6.6 | 1 | 303 | -8 | AluY | Alu | SINE |
| 69227313 | 69227440 | - | 19.1 | 2 | 133 | -76 | MER3 | MER1\_type | DNA |

  

|  |  |  |  |  |  |  |  |  |  |
| --- | --- | --- | --- | --- | --- | --- | --- | --- | --- |
| chr5:69227461-69228460 | | | 1000 bp | | |  |  |  |  |
| Genome Start | Genome End | Strand | Div | Repeat Start | Repeat End | Repeat Left | Name | Family | Class |
| 69227461 | 69227528 | - | 22.5 | 309 | 374 | 0 | MLT1A | MaLR | LTR |
| 69227529 | 69227831 | + | 16.2 | 1 | 312 | 0 | AluSx | Alu | SINE |
| 69227832 | 69228108 | - | 22.5 | 45 | 308 | -66 | MLT1A | MaLR | LTR |
| 69228109 | 69228416 | - | 6.6 | 1 | 305 | -6 | AluY | Alu | SINE |
| 69228417 | 69228460 | - | 22.5 | 1 | 44 | -330 | MLT1A | MaLR | LTR |

  

|  |  |  |  |  |  |  |  |  |  |
| --- | --- | --- | --- | --- | --- | --- | --- | --- | --- |
| chr5:69239003-69239917 | | | 915 bp | | |  |  |  |  |
| Genome Start | Genome End | Strand | Div | Repeat Start | Repeat End | Repeat Left | Name | Family | Class |
| 69239003 | 69239460 | - | 21.2 | 179 | 653 | 0 | MER82 | MER2\_type | DNA |
| 69239461 | 69239768 | - | 11.7 | 1 | 290 | -22 | AluSx | Alu | SINE |
| 69239769 | 69239917 | - | 21.2 | 1 | 178 | -475 | MER82 | MER2\_type | DNA |

  

|  |  |  |  |  |  |  |  |  |  |
| --- | --- | --- | --- | --- | --- | --- | --- | --- | --- |
| chr5:69242294-69242924 | | | 631 bp | | |  |  |  |  |
| Genome Start | Genome End | Strand | Div | Repeat Start | Repeat End | Repeat Left | Name | Family | Class |
| 69242294 | 69242506 | - | 17.1 | 126 | 339 | 0 | AluJo | Alu | SINE |
| 69242507 | 69242796 | + | 13.4 | 1 | 293 | -19 | AluSx | Alu | SINE |
| 69242797 | 69242924 | - | 17.1 | 1 | 125 | -187 | AluJo | Alu | SINE |

  

|  |  |  |  |  |  |  |  |  |  |
| --- | --- | --- | --- | --- | --- | --- | --- | --- | --- |
| chr5:69244286-69244884 | | | 599 bp | | |  |  |  |  |
| Genome Start | Genome End | Strand | Div | Repeat Start | Repeat End | Repeat Left | Name | Family | Class |
| 69244286 | 69244466 | - | 17.5 | 138 | 305 | -7 | AluJo | Alu | SINE |
| 69244467 | 69244761 | - | 13.0 | 1 | 290 | -20 | AluSg | Alu | SINE |
| 69244762 | 69244884 | - | 17.5 | 2 | 137 | -175 | AluJo | Alu | SINE |

  

|  |  |  |  |  |  |  |  |  |  |
| --- | --- | --- | --- | --- | --- | --- | --- | --- | --- |
| chr5:69250365-69250959 | | | 595 bp | | |  |  |  |  |
| Genome Start | Genome End | Strand | Div | Repeat Start | Repeat End | Repeat Left | Name | Family | Class |
| 69250365 | 69250499 | + | 19.2 | 2 | 135 | -177 | AluJb | Alu | SINE |
| 69250500 | 69250802 | + | 10.0 | 1 | 301 | -9 | AluSg | Alu | SINE |
| 69250803 | 69250959 | + | 19.2 | 136 | 291 | -21 | AluJb | Alu | SINE |

  

|  |  |  |  |  |  |  |  |  |  |
| --- | --- | --- | --- | --- | --- | --- | --- | --- | --- |
| chr5:69272012-69273523 | | | 1286 bp | | |  |  |  |  |
| Genome Start | Genome End | Strand | Div | Repeat Start | Repeat End | Repeat Left | Name | Family | Class |
| 69272012 | 69272420 | - | 20.4 | 1326 | 1735 | 0 | MLT1A0-int | MaLR | LTR |
| 69272661 | 69272835 | - | 30.5 | 858 | 1029 | -460 | MLT1F-int | MaLR | LTR |
| 69272822 | 69273523 | - | 24.0 | 1 | 738 | -633 | MLT1A0-int | MaLR | LTR |

  

|  |  |  |  |  |  |  |  |  |  |
| --- | --- | --- | --- | --- | --- | --- | --- | --- | --- |
| chr5:69301067-69301990 | | | 924 bp | | |  |  |  |  |
| Genome Start | Genome End | Strand | Div | Repeat Start | Repeat End | Repeat Left | Name | Family | Class |
| 69301067 | 69301340 | + | 9.9 | 2118 | 2393 | -3753 | L1PREC2 | L1 | LINE |
| 69301341 | 69301652 | + | 9.3 | 1 | 300 | -9 | AluSc | Alu | SINE |
| 69301653 | 69301990 | + | 9.9 | 2394 | 2732 | -3414 | L1PREC2 | L1 | LINE |

  

|  |  |  |  |  |  |  |  |  |  |
| --- | --- | --- | --- | --- | --- | --- | --- | --- | --- |
| chr5:69310167-69311043 | | | 868 bp | | |  |  |  |  |
| Genome Start | Genome End | Strand | Div | Repeat Start | Repeat End | Repeat Left | Name | Family | Class |
| 69310167 | 69310458 | + | 26.3 | 982 | 1276 | -213 | MLT1F2-int | MaLR | LTR |
| 69310462 | 69310870 | + | 9.9 | 5763 | 6168 | 0 | L1PA10 | L1 | LINE |
| 69310877 | 69311043 | + | 21.6 | 1319 | 1488 | -1 | MLT1F2-int | MaLR | LTR |

  

|  |  |  |  |  |  |  |  |  |  |
| --- | --- | --- | --- | --- | --- | --- | --- | --- | --- |
| chr5:69370821-69371289 | | | 469 bp | | |  |  |  |  |
| Genome Start | Genome End | Strand | Div | Repeat Start | Repeat End | Repeat Left | Name | Family | Class |
| 69370821 | 69370841 | - | 25.0 | 83 | 99 | -492 | MLT1K | MaLR | LTR |
| 69370842 | 69371217 | - | 8.3 | 1 | 375 | 0 | THE1C | MaLR | LTR |
| 69371218 | 69371289 | - | 25.0 | 11 | 82 | -509 | MLT1K | MaLR | LTR |

  

|  |  |  |  |  |  |  |  |  |  |
| --- | --- | --- | --- | --- | --- | --- | --- | --- | --- |
| chr5:69375412-69377641 | | | 2073 bp | | |  |  |  |  |
| Genome Start | Genome End | Strand | Div | Repeat Start | Repeat End | Repeat Left | Name | Family | Class |
| 69375412 | 69375588 | - | 30.1 | 5616 | 5792 | -329 | L1M5 | L1 | LINE |
| 69375589 | 69375720 | - | 19.8 | 327 | 461 | 0 | MER65C | ERV1 | LTR |
| 69375721 | 69376018 | + | 9.7 | 1 | 300 | -13 | AluSp | Alu | SINE |
| 69376019 | 69376347 | - | 19.8 | 1 | 326 | -135 | MER65C | ERV1 | LTR |
| 69376348 | 69376379 | - | 30.1 | 5584 | 5615 | -506 | L1M5 | L1 | LINE |
| 69376385 | 69376688 | - | 10.0 | 2 | 301 | -11 | AluSx | Alu | SINE |
| 69376689 | 69376710 | - | 33.0 | 5559 | 5583 | -2225 | L1MC | L1 | LINE |
| 69376711 | 69376844 | - | 18.2 | 2 | 133 | 0 | FLAM\_C | Alu | SINE |
| 69376845 | 69376960 | - | 33.0 | 5437 | 5558 | -2250 | L1MC | L1 | LINE |
| 69377065 | 69377178 | - | 13.2 | 3 | 116 | -17 | FLAM\_C | Alu | SINE |
| 69377226 | 69377530 | - | 12.1 | 1 | 307 | -5 | AluSx | Alu | SINE |
| 69377532 | 69377641 | - | 20.4 | 5337 | 5452 | -788 | L1M5 | L1 | LINE |

  

|  |  |  |  |  |  |  |  |  |  |
| --- | --- | --- | --- | --- | --- | --- | --- | --- | --- |
| chr5:69379416-69379841 | | | 426 bp | | |  |  |  |  |
| Genome Start | Genome End | Strand | Div | Repeat Start | Repeat End | Repeat Left | Name | Family | Class |
| 69379416 | 69379448 | - | 12.7 | 6280 | 6308 | -4 | L1MA9 | L1 | LINE |
| 69379449 | 69379741 | - | 12.3 | 1 | 293 | -19 | AluSx | Alu | SINE |
| 69379742 | 69379841 | - | 12.7 | 6161 | 6279 | -33 | L1MA9 | L1 | LINE |

  

|  |  |  |  |  |  |  |  |  |  |
| --- | --- | --- | --- | --- | --- | --- | --- | --- | --- |
| chr5:69381608-69386116 | | | 4353 bp | | |  |  |  |  |
| Genome Start | Genome End | Strand | Div | Repeat Start | Repeat End | Repeat Left | Name | Family | Class |
| 69381608 | 69381890 | - | 24.6 | 2448 | 2796 | -623 | L2 | L2 | LINE |
| 69381960 | 69382157 | + | 15.8 | 83 | 295 | -17 | AluJb | Alu | SINE |
| 69382159 | 69382469 | + | 3.5 | 1 | 311 | 0 | AluY | Alu | SINE |
| 69382516 | 69382653 | - | 20.3 | 7 | 154 | -22 | FRAM | Alu | SINE |
| 69382655 | 69382725 | - | 21.4 | 1811 | 1882 | -1537 | L2 | L2 | LINE |
| 69382754 | 69382893 | - | 17.0 | 6 | 141 | -35 | FRAM | Alu | SINE |
| 69382895 | 69383489 | - | 25.9 | 1104 | 1801 | -1618 | L2 | L2 | LINE |
| 69383490 | 69383792 | + | 11.6 | 1 | 301 | -11 | AluSx | Alu | SINE |
| 69383793 | 69383843 | - | 25.9 | 1073 | 1103 | -2316 | L2 | L2 | LINE |
| 69383844 | 69384154 | + | 11.6 | 1 | 313 | 0 | AluSq | Alu | SINE |
| 69384158 | 69384484 | + | 16.9 | 1 | 302 | -10 | AluJb | Alu | SINE |
| 69384485 | 69384684 | - | 25.9 | 873 | 1072 | -2347 | L2 | L2 | LINE |
| 69384685 | 69385074 | - | 14.5 | 1 | 428 | 0 | MSTA | MaLR | LTR |
| 69385075 | 69385296 | - | 25.9 | 644 | 872 | -2547 | L2 | L2 | LINE |
| 69385297 | 69385479 | - | 17.3 | 127 | 302 | -10 | AluJb | Alu | SINE |
| 69385487 | 69385557 | - | 25.4 | 583 | 641 | -2778 | L2 | L2 | LINE |
| 69385558 | 69385867 | - | 5.6 | 6 | 310 | -1 | AluY | Alu | SINE |
| 69385868 | 69386116 | - | 25.4 | 330 | 582 | -2837 | L2 | L2 | LINE |

  

|  |  |  |  |  |  |  |  |  |  |
| --- | --- | --- | --- | --- | --- | --- | --- | --- | --- |
| chr5:69387683-69390618 | | | 2746 bp | | |  |  |  |  |
| Genome Start | Genome End | Strand | Div | Repeat Start | Repeat End | Repeat Left | Name | Family | Class |
| 69387683 | 69387891 | - | 22.6 | 5185 | 5394 | -820 | L1M | L1 | LINE |
| 69387901 | 69388662 | + | 23.3 | 7 | 779 | 0 | LTR19C | ERV1 | LTR |
| 69388684 | 69389018 | - | 26.8 | 4808 | 5146 | -1068 | L1M | L1 | LINE |
| 69389019 | 69389627 | + | 9.5 | 1 | 610 | 0 | MER4B | ERV1 | LTR |
| 69389628 | 69389718 | - | 26.8 | 4693 | 4807 | -1407 | L1M | L1 | LINE |
| 69389734 | 69389775 | - | 19.4 | 1769 | 1808 | -4407 | L1M | L1 | LINE |
| 69389776 | 69390084 | - | 8.1 | 1 | 309 | -4 | AluSq | Alu | SINE |
| 69390085 | 69390287 | - | 19.4 | 1562 | 1768 | -4447 | L1M | L1 | LINE |
| 69390433 | 69390618 | - | 21.4 | 1065 | 1283 | -4932 | L1M | L1 | LINE |

  

|  |  |  |  |  |  |  |  |  |  |
| --- | --- | --- | --- | --- | --- | --- | --- | --- | --- |
| chr5:69392778-69393395 | | | 618 bp | | |  |  |  |  |
| Genome Start | Genome End | Strand | Div | Repeat Start | Repeat End | Repeat Left | Name | Family | Class |
| 69392778 | 69392963 | - | 17.6 | 136 | 312 | 0 | AluJb | Alu | SINE |
| 69392964 | 69393263 | - | 10.7 | 1 | 301 | -9 | AluSg | Alu | SINE |
| 69393264 | 69393395 | - | 17.6 | 1 | 135 | -177 | AluJb | Alu | SINE |

  

|  |  |  |  |  |  |  |  |  |  |
| --- | --- | --- | --- | --- | --- | --- | --- | --- | --- |
| chr5:69404358-69404878 | | | 521 bp | | |  |  |  |  |
| Genome Start | Genome End | Strand | Div | Repeat Start | Repeat End | Repeat Left | Name | Family | Class |
| 69404358 | 69404423 | - | 15.3 | 6122 | 6177 | -1 | L1MB8 | L1 | LINE |
| 69404424 | 69404729 | + | 8.6 | 1 | 303 | -8 | AluY | Alu | SINE |
| 69404730 | 69404878 | - | 15.3 | 5973 | 6121 | -57 | L1MB8 | L1 | LINE |

  

|  |  |  |  |  |  |  |  |  |  |
| --- | --- | --- | --- | --- | --- | --- | --- | --- | --- |
| chr5:69406616-69407235 | | | 620 bp | | |  |  |  |  |
| Genome Start | Genome End | Strand | Div | Repeat Start | Repeat End | Repeat Left | Name | Family | Class |
| 69406616 | 69406785 | - | 12.6 | 131 | 297 | -16 | AluSp | Alu | SINE |
| 69406786 | 69407096 | - | 8.7 | 1 | 310 | -1 | AluY | Alu | SINE |
| 69407097 | 69407235 | - | 12.6 | 2 | 130 | -183 | AluSp | Alu | SINE |

  

|  |  |  |  |  |  |  |  |  |  |
| --- | --- | --- | --- | --- | --- | --- | --- | --- | --- |
| chr5:69407263-69407883 | | | 621 bp | | |  |  |  |  |
| Genome Start | Genome End | Strand | Div | Repeat Start | Repeat End | Repeat Left | Name | Family | Class |
| 69407263 | 69407424 | - | 16.8 | 152 | 294 | -18 | AluJb | Alu | SINE |
| 69407425 | 69407737 | - | 12.2 | 1 | 311 | -1 | AluSx | Alu | SINE |
| 69407738 | 69407883 | - | 16.8 | 9 | 151 | -161 | AluJb | Alu | SINE |

  

|  |  |  |  |  |  |  |  |  |  |
| --- | --- | --- | --- | --- | --- | --- | --- | --- | --- |
| chr5:69412034-69416359 | | | 4290 bp | | |  |  |  |  |
| Genome Start | Genome End | Strand | Div | Repeat Start | Repeat End | Repeat Left | Name | Family | Class |
| 69412034 | 69412083 | - | 16.0 | 5816 | 5869 | -1939 | L1MC | L1 | LINE |
| 69412082 | 69414939 | - | 2.5 | 2655 | 5524 | -631 | L1PA3 | L1 | LINE |
| 69414940 | 69415547 | + | 3.3 | 5547 | 6154 | -1 | L1PA3 | L1 | LINE |
| 69415586 | 69415771 | - | 28.8 | 5599 | 5824 | -1984 | L1MC | L1 | LINE |
| 69415772 | 69416069 | - | 8.4 | 1 | 296 | -14 | AluSg | Alu | SINE |
| 69416070 | 69416359 | - | 28.8 | 5273 | 5598 | -2210 | L1MC | L1 | LINE |

  

|  |  |  |  |  |  |  |  |  |  |
| --- | --- | --- | --- | --- | --- | --- | --- | --- | --- |
| chr5:69417334-69418096 | | | 763 bp | | |  |  |  |  |
| Genome Start | Genome End | Strand | Div | Repeat Start | Repeat End | Repeat Left | Name | Family | Class |
| 69417334 | 69417650 | - | 16.6 | 1164 | 1485 | -73 | Looper | PiggyBac | DNA |
| 69417651 | 69417773 | - | 20.5 | 3 | 130 | -3 | FLAM\_A | Alu | SINE |
| 69417774 | 69418096 | - | 16.6 | 870 | 1163 | -395 | Looper | PiggyBac | DNA |

  

|  |  |  |  |  |  |  |  |  |  |
| --- | --- | --- | --- | --- | --- | --- | --- | --- | --- |
| chr5:69423108-69424502 | | | 1392 bp | | |  |  |  |  |
| Genome Start | Genome End | Strand | Div | Repeat Start | Repeat End | Repeat Left | Name | Family | Class |
| 69423108 | 69423739 | + | 26.4 | 7 | 701 | -72 | LTR41 | ERVL | LTR |
| 69423740 | 69424101 | + | 14.9 | 1 | 365 | -1 | Tigger5a | MER2\_type | DNA |
| 69424105 | 69424429 | - | 9.6 | 1 | 305 | 0 | AluSx | Alu | SINE |
| 69424430 | 69424502 | + | 26.4 | 702 | 770 | -3 | LTR41 | ERVL | LTR |

  

|  |  |  |  |  |  |  |  |  |  |
| --- | --- | --- | --- | --- | --- | --- | --- | --- | --- |
| chr5:69426731-69427194 | | | 464 bp | | |  |  |  |  |
| Genome Start | Genome End | Strand | Div | Repeat Start | Repeat End | Repeat Left | Name | Family | Class |
| 69426731 | 69426788 | - | 17.5 | 92 | 126 | -7 | FLAM\_A | Alu | SINE |
| 69426789 | 69427099 | - | 5.8 | 1 | 311 | 0 | AluY | Alu | SINE |
| 69427100 | 69427194 | - | 17.5 | 1 | 91 | -42 | FLAM\_A | Alu | SINE |

  

|  |  |  |  |  |  |  |  |  |  |
| --- | --- | --- | --- | --- | --- | --- | --- | --- | --- |
| chr5:69430914-69431653 | | | 740 bp | | |  |  |  |  |
| Genome Start | Genome End | Strand | Div | Repeat Start | Repeat End | Repeat Left | Name | Family | Class |
| 69430914 | 69431308 | - | 23.0 | 6482 | 6902 | -1140 | L1MC4 | L1 | LINE |
| 69431309 | 69431613 | - | 11.8 | 1 | 304 | -8 | AluSx | Alu | SINE |
| 69431614 | 69431653 | - | 23.0 | 6449 | 6481 | -1561 | L1MC4 | L1 | LINE |

  

|  |  |  |  |  |  |  |  |  |  |
| --- | --- | --- | --- | --- | --- | --- | --- | --- | --- |
| chr5:69446407-69447722 | | | 1316 bp | | |  |  |  |  |
| Genome Start | Genome End | Strand | Div | Repeat Start | Repeat End | Repeat Left | Name | Family | Class |
| 69446407 | 69446491 | + | 22.4 | 1696 | 1782 | -4618 | L1MEc | L1 | LINE |
| 69446492 | 69446796 | + | 10.8 | 1 | 305 | -5 | AluSg | Alu | SINE |
| 69446797 | 69447087 | + | 22.4 | 1783 | 2074 | -4326 | L1MEc | L1 | LINE |
| 69447088 | 69447378 | - | 13.5 | 1 | 289 | -23 | AluSx | Alu | SINE |
| 69447379 | 69447722 | + | 22.4 | 2075 | 2389 | -4011 | L1MEc | L1 | LINE |

  

|  |  |  |  |  |  |  |  |  |  |
| --- | --- | --- | --- | --- | --- | --- | --- | --- | --- |
| chr5:69451612-69454753 | | | 2933 bp | | |  |  |  |  |
| Genome Start | Genome End | Strand | Div | Repeat Start | Repeat End | Repeat Left | Name | Family | Class |
| 69451612 | 69452111 | + | 26.3 | 1437 | 1934 | -573 | HAL1 | L1 | LINE |
| 69452130 | 69452439 | + | 12.0 | 1 | 309 | -3 | AluSx | Alu | SINE |
| 69452444 | 69452540 | + | 30.9 | 1902 | 2001 | -506 | HAL1 | L1 | LINE |
| 69452644 | 69452961 | + | 6.1 | 1 | 311 | 0 | AluY | Alu | SINE |
| 69453043 | 69453240 | + | 20.5 | 5903 | 6094 | -80 | L1MB5 | L1 | LINE |
| 69453240 | 69453313 | + | 19.2 | 5535 | 5603 | -571 | L1MB5 | L1 | LINE |
| 69453314 | 69453611 | + | 11.8 | 1 | 306 | -6 | AluSx | Alu | SINE |
| 69453612 | 69454156 | + | 19.2 | 5604 | 6140 | -34 | L1MB5 | L1 | LINE |
| 69454157 | 69454463 | + | 18.0 | 2 | 308 | -4 | AluJo | Alu | SINE |
| 69454464 | 69454488 | + | 19.2 | 6141 | 6164 | -10 | L1MB5 | L1 | LINE |
| 69454493 | 69454753 | + | 30.8 | 2012 | 2268 | -239 | HAL1 | L1 | LINE |

  

|  |  |  |  |  |  |  |  |  |  |
| --- | --- | --- | --- | --- | --- | --- | --- | --- | --- |
| chr5:69456530-69457083 | | | 488 bp | | |  |  |  |  |
| Genome Start | Genome End | Strand | Div | Repeat Start | Repeat End | Repeat Left | Name | Family | Class |
| 69456530 | 69456673 | + | 21.8 | 1 | 148 | -363 | MLT1J | MaLR | LTR |
| 69456734 | 69457022 | - | 8.7 | 1 | 288 | -25 | AluSq | Alu | SINE |
| 69457029 | 69457083 | + | 25.9 | 147 | 200 | -311 | MLT1J | MaLR | LTR |

  

|  |  |  |  |  |  |  |  |  |  |
| --- | --- | --- | --- | --- | --- | --- | --- | --- | --- |
| chr5:69458351-69458986 | | | 636 bp | | |  |  |  |  |
| Genome Start | Genome End | Strand | Div | Repeat Start | Repeat End | Repeat Left | Name | Family | Class |
| 69458351 | 69458563 | - | 10.5 | 102 | 303 | -9 | AluSx | Alu | SINE |
| 69458564 | 69458879 | - | 7.8 | 1 | 308 | -3 | AluY | Alu | SINE |
| 69458880 | 69458986 | - | 10.5 | 1 | 101 | -211 | AluSx | Alu | SINE |

  

|  |  |  |  |  |  |  |  |  |  |
| --- | --- | --- | --- | --- | --- | --- | --- | --- | --- |
| chr5:69470307-69471183 | | | 868 bp | | |  |  |  |  |
| Genome Start | Genome End | Strand | Div | Repeat Start | Repeat End | Repeat Left | Name | Family | Class |
| 69470307 | 69470473 | - | 21.6 | 1319 | 1488 | -1 | MLT1F2-int | MaLR | LTR |
| 69470480 | 69470888 | - | 9.9 | 5763 | 6168 | 0 | L1PA10 | L1 | LINE |
| 69470892 | 69471183 | - | 26.0 | 982 | 1276 | -213 | MLT1F2-int | MaLR | LTR |

  

|  |  |  |  |  |  |  |  |  |  |
| --- | --- | --- | --- | --- | --- | --- | --- | --- | --- |
| chr5:69530329-69530923 | | | 595 bp | | |  |  |  |  |
| Genome Start | Genome End | Strand | Div | Repeat Start | Repeat End | Repeat Left | Name | Family | Class |
| 69530329 | 69530485 | - | 19.2 | 136 | 291 | -21 | AluJb | Alu | SINE |
| 69530486 | 69530788 | - | 10.0 | 1 | 301 | -9 | AluSg | Alu | SINE |
| 69530789 | 69530923 | - | 19.2 | 2 | 135 | -177 | AluJb | Alu | SINE |

  

|  |  |  |  |  |  |  |  |  |  |
| --- | --- | --- | --- | --- | --- | --- | --- | --- | --- |
| chr5:69538364-69538934 | | | 571 bp | | |  |  |  |  |
| Genome Start | Genome End | Strand | Div | Repeat Start | Repeat End | Repeat Left | Name | Family | Class |
| 69538364 | 69538491 | + | 15.9 | 1 | 125 | -187 | AluJo | Alu | SINE |
| 69538492 | 69538783 | - | 12.7 | 1 | 295 | -17 | AluSx | Alu | SINE |
| 69538784 | 69538934 | + | 15.9 | 126 | 275 | -37 | AluJo | Alu | SINE |

  

|  |  |  |  |  |  |  |  |  |  |
| --- | --- | --- | --- | --- | --- | --- | --- | --- | --- |
| chr5:69541388-69542297 | | | 910 bp | | |  |  |  |  |
| Genome Start | Genome End | Strand | Div | Repeat Start | Repeat End | Repeat Left | Name | Family | Class |
| 69541388 | 69541536 | + | 21.4 | 1 | 178 | -475 | MER82 | MER2\_type | DNA |
| 69541537 | 69541839 | + | 11.7 | 1 | 290 | -22 | AluSx | Alu | SINE |
| 69541840 | 69542297 | + | 21.4 | 179 | 653 | 0 | MER82 | MER2\_type | DNA |

  

|  |  |  |  |  |  |  |  |  |  |
| --- | --- | --- | --- | --- | --- | --- | --- | --- | --- |
| chr5:69549598-69550600 | | | 1003 bp | | |  |  |  |  |
| Genome Start | Genome End | Strand | Div | Repeat Start | Repeat End | Repeat Left | Name | Family | Class |
| 69549598 | 69549641 | + | 22.5 | 1 | 44 | -330 | MLT1A | MaLR | LTR |
| 69549642 | 69549950 | + | 6.5 | 1 | 306 | -5 | AluY | Alu | SINE |
| 69549951 | 69550229 | + | 22.5 | 45 | 308 | -66 | MLT1A | MaLR | LTR |
| 69550230 | 69550532 | - | 15.2 | 1 | 312 | 0 | AluSx | Alu | SINE |
| 69550533 | 69550600 | + | 22.5 | 309 | 374 | 0 | MLT1A | MaLR | LTR |

  

|  |  |  |  |  |  |  |  |  |  |
| --- | --- | --- | --- | --- | --- | --- | --- | --- | --- |
| chr5:69550621-69551117 | | | 497 bp | | |  |  |  |  |
| Genome Start | Genome End | Strand | Div | Repeat Start | Repeat End | Repeat Left | Name | Family | Class |
| 69550621 | 69550748 | + | 19.6 | 1 | 129 | -80 | MER3 | MER1\_type | DNA |
| 69550749 | 69551052 | - | 7.6 | 1 | 304 | -7 | AluY | Alu | SINE |
| 69551053 | 69551117 | + | 19.6 | 130 | 208 | -1 | MER3 | MER1\_type | DNA |

  

|  |  |  |  |  |  |  |  |  |  |
| --- | --- | --- | --- | --- | --- | --- | --- | --- | --- |
| chr5:69554455-69555052 | | | 598 bp | | |  |  |  |  |
| Genome Start | Genome End | Strand | Div | Repeat Start | Repeat End | Repeat Left | Name | Family | Class |
| 69554455 | 69554577 | + | 17.9 | 2 | 137 | -175 | AluJo | Alu | SINE |
| 69554578 | 69554871 | + | 13.0 | 1 | 289 | -21 | AluSg | Alu | SINE |
| 69554872 | 69555052 | + | 17.9 | 138 | 305 | -7 | AluJo | Alu | SINE |

  

|  |  |  |  |  |  |  |  |  |  |
| --- | --- | --- | --- | --- | --- | --- | --- | --- | --- |
| chr5:69556414-69556984 | | | 571 bp | | |  |  |  |  |
| Genome Start | Genome End | Strand | Div | Repeat Start | Repeat End | Repeat Left | Name | Family | Class |
| 69556414 | 69556541 | + | 15.9 | 1 | 125 | -187 | AluJo | Alu | SINE |
| 69556542 | 69556833 | - | 12.7 | 1 | 286 | -26 | AluSx | Alu | SINE |
| 69556834 | 69556984 | + | 15.9 | 126 | 275 | -37 | AluJo | Alu | SINE |

  

|  |  |  |  |  |  |  |  |  |  |
| --- | --- | --- | --- | --- | --- | --- | --- | --- | --- |
| chr5:69559438-69560347 | | | 910 bp | | |  |  |  |  |
| Genome Start | Genome End | Strand | Div | Repeat Start | Repeat End | Repeat Left | Name | Family | Class |
| 69559438 | 69559586 | + | 21.4 | 1 | 178 | -475 | MER82 | MER2\_type | DNA |
| 69559587 | 69559889 | + | 11.7 | 1 | 290 | -22 | AluSx | Alu | SINE |
| 69559890 | 69560347 | + | 21.4 | 179 | 653 | 0 | MER82 | MER2\_type | DNA |

  

|  |  |  |  |  |  |  |  |  |  |
| --- | --- | --- | --- | --- | --- | --- | --- | --- | --- |
| chr5:69567648-69568647 | | | 1000 bp | | |  |  |  |  |
| Genome Start | Genome End | Strand | Div | Repeat Start | Repeat End | Repeat Left | Name | Family | Class |
| 69567648 | 69567691 | + | 22.9 | 1 | 44 | -330 | MLT1A | MaLR | LTR |
| 69567692 | 69568000 | + | 6.5 | 1 | 306 | -5 | AluY | Alu | SINE |
| 69568001 | 69568276 | + | 22.9 | 45 | 308 | -66 | MLT1A | MaLR | LTR |
| 69568277 | 69568579 | - | 15.2 | 1 | 312 | 0 | AluSx | Alu | SINE |
| 69568580 | 69568647 | + | 22.9 | 309 | 374 | 0 | MLT1A | MaLR | LTR |

  

|  |  |  |  |  |  |  |  |  |  |
| --- | --- | --- | --- | --- | --- | --- | --- | --- | --- |
| chr5:69568668-69569164 | | | 497 bp | | |  |  |  |  |
| Genome Start | Genome End | Strand | Div | Repeat Start | Repeat End | Repeat Left | Name | Family | Class |
| 69568668 | 69568802 | + | 18.3 | 1 | 136 | -73 | MER3 | MER1\_type | DNA |
| 69568803 | 69569099 | - | 7.1 | 1 | 297 | -14 | AluY | Alu | SINE |
| 69569100 | 69569164 | + | 18.3 | 137 | 209 | 0 | MER3 | MER1\_type | DNA |

  

|  |  |  |  |  |  |  |  |  |  |
| --- | --- | --- | --- | --- | --- | --- | --- | --- | --- |
| chr5:69569862-69570522 | | | 661 bp | | |  |  |  |  |
| Genome Start | Genome End | Strand | Div | Repeat Start | Repeat End | Repeat Left | Name | Family | Class |
| 69569862 | 69570093 | - | 19.8 | 127 | 365 | 0 | MLT1A0 | MaLR | LTR |
| 69570094 | 69570408 | + | 12.7 | 1 | 307 | -3 | AluSg | Alu | SINE |
| 69570409 | 69570522 | - | 19.8 | 43 | 126 | -239 | MLT1A0 | MaLR | LTR |

  

|  |  |  |  |  |  |  |  |  |  |
| --- | --- | --- | --- | --- | --- | --- | --- | --- | --- |
| chr5:69575141-69576360 | | | 1220 bp | | |  |  |  |  |
| Genome Start | Genome End | Strand | Div | Repeat Start | Repeat End | Repeat Left | Name | Family | Class |
| 69575141 | 69575541 | + | 23.0 | 2528 | 2897 | -3948 | L1M2 | L1 | LINE |
| 69575542 | 69575844 | + | 12.9 | 1 | 303 | -7 | AluSg | Alu | SINE |
| 69575845 | 69576360 | + | 21.9 | 2898 | 3392 | -3453 | L1M2 | L1 | LINE |

  

|  |  |  |  |  |  |  |  |  |  |
| --- | --- | --- | --- | --- | --- | --- | --- | --- | --- |
| chr5:69577049-69578359 | | | 1311 bp | | |  |  |  |  |
| Genome Start | Genome End | Strand | Div | Repeat Start | Repeat End | Repeat Left | Name | Family | Class |
| 69577049 | 69577474 | + | 23.4 | 6152 | 6586 | -1199 | L1MC3 | L1 | LINE |
| 69577475 | 69577857 | + | 17.6 | 1 | 428 | 0 | MSTA | MaLR | LTR |
| 69577858 | 69578359 | + | 23.4 | 6587 | 7119 | -666 | L1MC3 | L1 | LINE |

  

|  |  |  |  |  |  |  |  |  |  |
| --- | --- | --- | --- | --- | --- | --- | --- | --- | --- |
| chr5:69601621-69602573 | | | 784 bp | | |  |  |  |  |
| Genome Start | Genome End | Strand | Div | Repeat Start | Repeat End | Repeat Left | Name | Family | Class |
| 69601621 | 69601836 | + | 23.9 | 1 | 225 | -280 | MLT1D | MaLR | LTR |
| 69601878 | 69601924 | + | 17.0 | 1 | 47 | -327 | MLT1A | MaLR | LTR |
| 69601979 | 69602081 | + | 30.1 | 58 | 160 | -214 | MLT1A | MaLR | LTR |
| 69602156 | 69602344 | + | 18.5 | 189 | 374 | 0 | MLT1A | MaLR | LTR |
| 69602345 | 69602573 | + | 27.0 | 275 | 505 | 0 | MLT1D | MaLR | LTR |

  

|  |  |  |  |  |  |  |  |  |  |
| --- | --- | --- | --- | --- | --- | --- | --- | --- | --- |
| chr5:69603864-69606555 | | | 2681 bp | | |  |  |  |  |
| Genome Start | Genome End | Strand | Div | Repeat Start | Repeat End | Repeat Left | Name | Family | Class |
| 69603864 | 69604086 | + | 18.4 | 1 | 238 | -128 | Tigger5a | MER2\_type | DNA |
| 69604097 | 69606484 | - | 6.8 | 3742 | 6150 | -4 | L1PA6 | L1 | LINE |
| 69606486 | 69606555 | + | 20.0 | 356 | 434 | -15 | Tigger5a | MER2\_type | DNA |

  

|  |  |  |  |  |  |  |  |  |  |
| --- | --- | --- | --- | --- | --- | --- | --- | --- | --- |
| chr5:69614576-69620923 | | | 6197 bp | | |  |  |  |  |
| Genome Start | Genome End | Strand | Div | Repeat Start | Repeat End | Repeat Left | Name | Family | Class |
| 69614576 | 69614782 | + | 20.8 | 13 | 235 | -6268 | L1PA16 | L1 | LINE |
| 69614782 | 69618637 | + | 16.9 | 947 | 4819 | -1664 | L1PA16 | L1 | LINE |
| 69618638 | 69618899 | + | 18.4 | 2 | 286 | -16 | AluJo | Alu | SINE |
| 69619052 | 69619878 | + | 16.8 | 4820 | 5660 | -823 | L1PA16 | L1 | LINE |
| 69619879 | 69620192 | + | 8.7 | 5876 | 6167 | -1 | L1PA10 | L1 | LINE |
| 69620193 | 69620923 | + | 14.2 | 5661 | 6398 | -105 | L1PA16 | L1 | LINE |

  

|  |  |  |  |  |  |  |  |  |  |
| --- | --- | --- | --- | --- | --- | --- | --- | --- | --- |
| chr5:69630821-69633466 | | | 2413 bp | | |  |  |  |  |
| Genome Start | Genome End | Strand | Div | Repeat Start | Repeat End | Repeat Left | Name | Family | Class |
| 69630821 | 69631450 | + | 30.7 | 3855 | 4522 | -1624 | L1ME2 | L1 | LINE |
| 69631451 | 69631869 | + | 14.2 | 5 | 428 | 0 | MSTA | MaLR | LTR |
| 69631870 | 69632408 | + | 30.7 | 4523 | 5064 | -1082 | L1ME2 | L1 | LINE |
| 69632624 | 69632997 | + | 24.4 | 5329 | 5758 | -406 | L1ME2 | L1 | LINE |
| 69633003 | 69633187 | - | 22.1 | 1 | 213 | -6 | MER20 | MER1\_type | DNA |
| 69633201 | 69633466 | + | 26.4 | 5768 | 6039 | -125 | L1ME2 | L1 | LINE |

  

|  |  |  |  |  |  |  |  |  |  |
| --- | --- | --- | --- | --- | --- | --- | --- | --- | --- |
| chr5:69655800-69656595 | | | 796 bp | | |  |  |  |  |
| Genome Start | Genome End | Strand | Div | Repeat Start | Repeat End | Repeat Left | Name | Family | Class |
| 69655800 | 69655929 | - | 30.3 | 325 | 445 | 0 | MER65A | ERV1 | LTR |
| 69655930 | 69656303 | - | 13.2 | 1 | 428 | 0 | MSTA | MaLR | LTR |
| 69656304 | 69656595 | - | 30.3 | 1 | 324 | -121 | MER65A | ERV1 | LTR |

  

|  |  |  |  |  |  |  |  |  |  |
| --- | --- | --- | --- | --- | --- | --- | --- | --- | --- |
| chr5:69660992-69661723 | | | 732 bp | | |  |  |  |  |
| Genome Start | Genome End | Strand | Div | Repeat Start | Repeat End | Repeat Left | Name | Family | Class |
| 69660992 | 69661155 | - | 34.0 | 911 | 1076 | -2343 | L2 | L2 | LINE |
| 69661156 | 69661673 | + | 27.2 | 3 | 560 | -2 | MLT1F2 | MaLR | LTR |
| 69661674 | 69661723 | - | 34.0 | 861 | 910 | -2509 | L2 | L2 | LINE |

  

|  |  |  |  |  |  |  |  |  |  |
| --- | --- | --- | --- | --- | --- | --- | --- | --- | --- |
| chr5:69663166-69663569 | | | 404 bp | | |  |  |  |  |
| Genome Start | Genome End | Strand | Div | Repeat Start | Repeat End | Repeat Left | Name | Family | Class |
| 69663166 | 69663242 | - | 32.0 | 107 | 184 | -84 | MIRb | MIR | SINE |
| 69663243 | 69663538 | + | 13.8 | 1 | 298 | -14 | AluSx | Alu | SINE |
| 69663539 | 69663569 | - | 32.0 | 79 | 106 | -162 | MIRb | MIR | SINE |

  

|  |  |  |  |  |  |  |  |  |  |
| --- | --- | --- | --- | --- | --- | --- | --- | --- | --- |
| chr5:69667960-69669920 | | | 1816 bp | | |  |  |  |  |
| Genome Start | Genome End | Strand | Div | Repeat Start | Repeat End | Repeat Left | Name | Family | Class |
| 69667960 | 69668351 | - | 29.4 | 5751 | 6161 | -5 | L1ME1 | L1 | LINE |
| 69668497 | 69668853 | - | 26.1 | 4340 | 4721 | -1425 | L1ME1 | L1 | LINE |
| 69668854 | 69669060 | + | 20.3 | 1 | 211 | -200 | LTR57 | ERVL | LTR |
| 69669061 | 69669369 | + | 6.2 | 1 | 311 | 0 | AluY | Alu | SINE |
| 69669370 | 69669613 | + | 20.3 | 212 | 411 | 0 | LTR57 | ERVL | LTR |
| 69669614 | 69669920 | - | 26.1 | 4108 | 4339 | -1807 | L1ME1 | L1 | LINE |

  

|  |  |  |  |  |  |  |  |  |  |
| --- | --- | --- | --- | --- | --- | --- | --- | --- | --- |
| chr5:69721406-69724051 | | | 2413 bp | | |  |  |  |  |
| Genome Start | Genome End | Strand | Div | Repeat Start | Repeat End | Repeat Left | Name | Family | Class |
| 69721406 | 69721671 | - | 26.4 | 5768 | 6039 | -125 | L1ME2 | L1 | LINE |
| 69721685 | 69721869 | + | 22.1 | 1 | 213 | -6 | MER20 | MER1\_type | DNA |
| 69721875 | 69722248 | - | 24.4 | 5329 | 5758 | -406 | L1ME2 | L1 | LINE |
| 69722464 | 69723002 | - | 30.7 | 4523 | 5064 | -1082 | L1ME2 | L1 | LINE |
| 69723003 | 69723421 | - | 14.5 | 5 | 428 | 0 | MSTA | MaLR | LTR |
| 69723422 | 69724051 | - | 30.7 | 3855 | 4522 | -1624 | L1ME2 | L1 | LINE |

  

|  |  |  |  |  |  |  |  |  |  |
| --- | --- | --- | --- | --- | --- | --- | --- | --- | --- |
| chr5:69745575-69746558 | | | 1007 bp | | |  |  |  |  |
| Genome Start | Genome End | Strand | Div | Repeat Start | Repeat End | Repeat Left | Name | Family | Class |
| 69745575 | 69745846 | + | 22.8 | 2412 | 2767 | -652 | L2 | L2 | LINE |
| 69745824 | 69746046 | + | 27.8 | 2918 | 3168 | -251 | L2 | L2 | LINE |
| 69746047 | 69746335 | - | 9.3 | 1 | 293 | -19 | AluSx | Alu | SINE |
| 69746336 | 69746558 | + | 27.8 | 3169 | 3413 | -6 | L2 | L2 | LINE |

  

|  |  |  |  |  |  |  |  |  |  |
| --- | --- | --- | --- | --- | --- | --- | --- | --- | --- |
| chr5:69747825-69748339 | | | 515 bp | | |  |  |  |  |
| Genome Start | Genome End | Strand | Div | Repeat Start | Repeat End | Repeat Left | Name | Family | Class |
| 69747825 | 69747997 | - | 28.6 | 4288 | 4463 | -26 | L3 | CR1 | LINE |
| 69747998 | 69748305 | + | 11.0 | 1 | 304 | -8 | AluSx | Alu | SINE |
| 69748306 | 69748339 | - | 28.6 | 4255 | 4287 | -202 | L3 | CR1 | LINE |

  

|  |  |  |  |  |  |  |  |  |  |
| --- | --- | --- | --- | --- | --- | --- | --- | --- | --- |
| chr5:69750266-69751097 | | | 832 bp | | |  |  |  |  |
| Genome Start | Genome End | Strand | Div | Repeat Start | Repeat End | Repeat Left | Name | Family | Class |
| 69750266 | 69750530 | - | 14.6 | 5897 | 6163 | -3 | L1PA16 | L1 | LINE |
| 69750531 | 69750805 | - | 12.5 | 1 | 272 | -41 | AluSq | Alu | SINE |
| 69750806 | 69751097 | - | 14.6 | 5618 | 5896 | -270 | L1PA16 | L1 | LINE |

  

|  |  |  |  |  |  |  |  |  |  |
| --- | --- | --- | --- | --- | --- | --- | --- | --- | --- |
| chr5:69760941-69764550 | | | 3603 bp | | |  |  |  |  |
| Genome Start | Genome End | Strand | Div | Repeat Start | Repeat End | Repeat Left | Name | Family | Class |
| 69760941 | 69761551 | + | 13.0 | 2 | 617 | -1801 | Tigger1 | MER2\_type | DNA |
| 69761552 | 69761860 | + | 17.8 | 1 | 297 | -15 | AluJo | Alu | SINE |
| 69761861 | 69762144 | + | 13.0 | 618 | 874 | -1544 | Tigger1 | MER2\_type | DNA |
| 69762145 | 69762443 | + | 9.4 | 1 | 298 | -12 | AluSg | Alu | SINE |
| 69762444 | 69762543 | + | 13.0 | 875 | 967 | -1451 | Tigger1 | MER2\_type | DNA |
| 69762544 | 69762847 | - | 14.4 | 1 | 305 | -7 | AluJo | Alu | SINE |
| 69762848 | 69763462 | + | 13.0 | 968 | 1618 | -800 | Tigger1 | MER2\_type | DNA |
| 69763465 | 69763760 | + | 11.2 | 1 | 297 | -15 | AluSx | Alu | SINE |
| 69763766 | 69764142 | + | 14.5 | 1909 | 2309 | -109 | Tigger1 | MER2\_type | DNA |
| 69764143 | 69764416 | - | 7.3 | 1 | 310 | -1 | AluY | Alu | SINE |
| 69764417 | 69764550 | + | 14.5 | 2310 | 2418 | 0 | Tigger1 | MER2\_type | DNA |

  

|  |  |  |  |  |  |  |  |  |  |
| --- | --- | --- | --- | --- | --- | --- | --- | --- | --- |
| chr5:69769948-69771407 | | | 1436 bp | | |  |  |  |  |
| Genome Start | Genome End | Strand | Div | Repeat Start | Repeat End | Repeat Left | Name | Family | Class |
| 69769948 | 69770524 | + | 23.1 | 35 | 644 | -5756 | L1MEc | L1 | LINE |
| 69770549 | 69770866 | + | 23.1 | 645 | 932 | -5468 | L1MEc | L1 | LINE |
| 69770867 | 69771164 | + | 15.8 | 1 | 300 | -12 | AluSx | Alu | SINE |
| 69771165 | 69771407 | + | 23.1 | 933 | 1167 | -5233 | L1MEc | L1 | LINE |

  

|  |  |  |  |  |  |  |  |  |  |
| --- | --- | --- | --- | --- | --- | --- | --- | --- | --- |
| chr5:69774893-69776179 | | | 1283 bp | | |  |  |  |  |
| Genome Start | Genome End | Strand | Div | Repeat Start | Repeat End | Repeat Left | Name | Family | Class |
| 69774893 | 69775141 | + | 23.6 | 1083 | 1345 | -1162 | HAL1 | L1 | LINE |
| 69775146 | 69775277 | + | 22.0 | 1 | 132 | -11 | FLAM\_C | Alu | SINE |
| 69775278 | 69776179 | + | 23.9 | 1337 | 2316 | -191 | HAL1 | L1 | LINE |

  

|  |  |  |  |  |  |  |  |  |  |
| --- | --- | --- | --- | --- | --- | --- | --- | --- | --- |
| chr5:69777529-69778674 | | | 1146 bp | | |  |  |  |  |
| Genome Start | Genome End | Strand | Div | Repeat Start | Repeat End | Repeat Left | Name | Family | Class |
| 69777529 | 69777837 | - | 23.6 | 552 | 871 | -17 | Kanga2\_a | Tc2 | DNA |
| 69777838 | 69778136 | - | 13.7 | 1 | 301 | -11 | AluSx | Alu | SINE |
| 69778137 | 69778674 | - | 23.6 | 4 | 551 | -337 | Kanga2\_a | Tc2 | DNA |

  

|  |  |  |  |  |  |  |  |  |  |
| --- | --- | --- | --- | --- | --- | --- | --- | --- | --- |
| chr5:69787378-69787996 | | | 615 bp | | |  |  |  |  |
| Genome Start | Genome End | Strand | Div | Repeat Start | Repeat End | Repeat Left | Name | Family | Class |
| 69787378 | 69787643 | + | 23.8 | 5060 | 5338 | -808 | L1M4 | L1 | LINE |
| 69787648 | 69787946 | + | 7.4 | 1 | 299 | -11 | AluSg | Alu | SINE |
| 69787947 | 69787996 | + | 23.9 | 5342 | 5388 | -758 | L1M4 | L1 | LINE |

  

|  |  |  |  |  |  |  |  |  |  |
| --- | --- | --- | --- | --- | --- | --- | --- | --- | --- |
| chr5:69802126-69802882 | | | 593 bp | | |  |  |  |  |
| Genome Start | Genome End | Strand | Div | Repeat Start | Repeat End | Repeat Left | Name | Family | Class |
| 69802126 | 69802317 | - | 18.0 | 277 | 473 | 0 | MER65D | ERV1 | LTR |
| 69802318 | 69802435 | + | 21.4 | 1 | 117 | -195 | AluJb | Alu | SINE |
| 69802516 | 69802654 | - | 16.7 | 157 | 288 | -185 | MER65D | ERV1 | LTR |
| 69802739 | 69802882 | - | 19.4 | 1 | 147 | -326 | MER65D | ERV1 | LTR |

  

|  |  |  |  |  |  |  |  |  |  |
| --- | --- | --- | --- | --- | --- | --- | --- | --- | --- |
| chr5:69806064-69806699 | | | 636 bp | | |  |  |  |  |
| Genome Start | Genome End | Strand | Div | Repeat Start | Repeat End | Repeat Left | Name | Family | Class |
| 69806064 | 69806170 | + | 10.5 | 1 | 101 | -211 | AluSx | Alu | SINE |
| 69806171 | 69806486 | + | 7.8 | 1 | 308 | -3 | AluY | Alu | SINE |
| 69806487 | 69806699 | + | 10.5 | 102 | 303 | -9 | AluSx | Alu | SINE |

  

|  |  |  |  |  |  |  |  |  |  |
| --- | --- | --- | --- | --- | --- | --- | --- | --- | --- |
| chr5:69816622-69818349 | | | 1702 bp | | |  |  |  |  |
| Genome Start | Genome End | Strand | Div | Repeat Start | Repeat End | Repeat Left | Name | Family | Class |
| 69816622 | 69816799 | - | 15.7 | 129 | 308 | -4 | AluJ | Alu | SINE |
| 69816810 | 69818000 | + | 10.9 | 310 | 1574 | -6 | THE1B-int | MaLR | LTR |
| 69818008 | 69818210 | + | 12.9 | 3 | 214 | -150 | THE1B | MaLR | LTR |
| 69818220 | 69818349 | - | 19.2 | 1 | 132 | -170 | AluJ | Alu | SINE |

  

|  |  |  |  |  |  |  |  |  |  |
| --- | --- | --- | --- | --- | --- | --- | --- | --- | --- |
| chr5:69824703-69825706 | | | 1004 bp | | |  |  |  |  |
| Genome Start | Genome End | Strand | Div | Repeat Start | Repeat End | Repeat Left | Name | Family | Class |
| 69824703 | 69824746 | + | 22.8 | 1 | 44 | -330 | MLT1A | MaLR | LTR |
| 69824747 | 69825056 | + | 6.5 | 1 | 307 | -4 | AluY | Alu | SINE |
| 69825057 | 69825335 | + | 22.8 | 45 | 308 | -66 | MLT1A | MaLR | LTR |
| 69825336 | 69825638 | - | 15.2 | 1 | 312 | 0 | AluSx | Alu | SINE |
| 69825639 | 69825706 | + | 22.8 | 309 | 374 | 0 | MLT1A | MaLR | LTR |

  

|  |  |  |  |  |  |  |  |  |  |
| --- | --- | --- | --- | --- | --- | --- | --- | --- | --- |
| chr5:69825727-69826223 | | | 497 bp | | |  |  |  |  |
| Genome Start | Genome End | Strand | Div | Repeat Start | Repeat End | Repeat Left | Name | Family | Class |
| 69825727 | 69825854 | + | 19.6 | 1 | 129 | -80 | MER3 | MER1\_type | DNA |
| 69825855 | 69826158 | - | 7.9 | 1 | 304 | -7 | AluY | Alu | SINE |
| 69826159 | 69826223 | + | 19.6 | 130 | 208 | -1 | MER3 | MER1\_type | DNA |

  

|  |  |  |  |  |  |  |  |  |  |
| --- | --- | --- | --- | --- | --- | --- | --- | --- | --- |
| chr5:69829565-69830162 | | | 598 bp | | |  |  |  |  |
| Genome Start | Genome End | Strand | Div | Repeat Start | Repeat End | Repeat Left | Name | Family | Class |
| 69829565 | 69829687 | + | 18.2 | 2 | 137 | -175 | AluJo | Alu | SINE |
| 69829688 | 69829981 | + | 14.1 | 1 | 295 | -15 | AluSg | Alu | SINE |
| 69829982 | 69830162 | + | 18.2 | 138 | 305 | -7 | AluJo | Alu | SINE |

  

|  |  |  |  |  |  |  |  |  |  |
| --- | --- | --- | --- | --- | --- | --- | --- | --- | --- |
| chr5:69831524-69832093 | | | 570 bp | | |  |  |  |  |
| Genome Start | Genome End | Strand | Div | Repeat Start | Repeat End | Repeat Left | Name | Family | Class |
| 69831524 | 69831651 | + | 15.9 | 1 | 125 | -187 | AluJo | Alu | SINE |
| 69831652 | 69831942 | - | 12.7 | 1 | 294 | -18 | AluSx | Alu | SINE |
| 69831943 | 69832093 | + | 15.9 | 126 | 275 | -37 | AluJo | Alu | SINE |

  

|  |  |  |  |  |  |  |  |  |  |
| --- | --- | --- | --- | --- | --- | --- | --- | --- | --- |
| chr5:69834535-69835444 | | | 910 bp | | |  |  |  |  |
| Genome Start | Genome End | Strand | Div | Repeat Start | Repeat End | Repeat Left | Name | Family | Class |
| 69834535 | 69834683 | + | 21.3 | 1 | 193 | -460 | MER82 | MER2\_type | DNA |
| 69834684 | 69834998 | + | 12.6 | 1 | 302 | -10 | AluSx | Alu | SINE |
| 69834999 | 69835444 | + | 21.3 | 194 | 653 | 0 | MER82 | MER2\_type | DNA |

  

|  |  |  |  |  |  |  |  |  |  |
| --- | --- | --- | --- | --- | --- | --- | --- | --- | --- |
| chr5:69845984-69846989 | | | 1006 bp | | |  |  |  |  |
| Genome Start | Genome End | Strand | Div | Repeat Start | Repeat End | Repeat Left | Name | Family | Class |
| 69845984 | 69846027 | + | 22.8 | 1 | 44 | -330 | MLT1A | MaLR | LTR |
| 69846028 | 69846339 | + | 6.5 | 1 | 309 | -2 | AluY | Alu | SINE |
| 69846340 | 69846618 | + | 22.8 | 45 | 308 | -66 | MLT1A | MaLR | LTR |
| 69846619 | 69846921 | - | 16.2 | 1 | 312 | 0 | AluSx | Alu | SINE |
| 69846922 | 69846989 | + | 22.8 | 309 | 374 | 0 | MLT1A | MaLR | LTR |

  

|  |  |  |  |  |  |  |  |  |  |
| --- | --- | --- | --- | --- | --- | --- | --- | --- | --- |
| chr5:69850857-69851454 | | | 598 bp | | |  |  |  |  |
| Genome Start | Genome End | Strand | Div | Repeat Start | Repeat End | Repeat Left | Name | Family | Class |
| 69850857 | 69850979 | + | 17.9 | 2 | 137 | -175 | AluJo | Alu | SINE |
| 69850980 | 69851273 | + | 14.1 | 1 | 295 | -15 | AluSg | Alu | SINE |
| 69851274 | 69851454 | + | 17.9 | 138 | 305 | -7 | AluJo | Alu | SINE |

  

|  |  |  |  |  |  |  |  |  |  |
| --- | --- | --- | --- | --- | --- | --- | --- | --- | --- |
| chr5:69855814-69856722 | | | 909 bp | | |  |  |  |  |
| Genome Start | Genome End | Strand | Div | Repeat Start | Repeat End | Repeat Left | Name | Family | Class |
| 69855814 | 69855962 | + | 21.3 | 1 | 193 | -460 | MER82 | MER2\_type | DNA |
| 69855963 | 69856276 | + | 12.3 | 1 | 301 | -11 | AluSx | Alu | SINE |
| 69856277 | 69856722 | + | 21.3 | 194 | 653 | 0 | MER82 | MER2\_type | DNA |

  

|  |  |  |  |  |  |  |  |  |  |
| --- | --- | --- | --- | --- | --- | --- | --- | --- | --- |
| chr5:69864025-69865030 | | | 1006 bp | | |  |  |  |  |
| Genome Start | Genome End | Strand | Div | Repeat Start | Repeat End | Repeat Left | Name | Family | Class |
| 69864025 | 69864068 | + | 22.5 | 1 | 41 | -333 | MLT1A | MaLR | LTR |
| 69864069 | 69864380 | + | 6.5 | 1 | 309 | -2 | AluY | Alu | SINE |
| 69864381 | 69864659 | + | 22.5 | 42 | 308 | -66 | MLT1A | MaLR | LTR |
| 69864660 | 69864962 | - | 14.8 | 1 | 312 | 0 | AluSx | Alu | SINE |
| 69864963 | 69865030 | + | 22.5 | 309 | 374 | 0 | MLT1A | MaLR | LTR |

  

|  |  |  |  |  |  |  |  |  |  |
| --- | --- | --- | --- | --- | --- | --- | --- | --- | --- |
| chr5:69865052-69865548 | | | 497 bp | | |  |  |  |  |
| Genome Start | Genome End | Strand | Div | Repeat Start | Repeat End | Repeat Left | Name | Family | Class |
| 69865052 | 69865186 | + | 17.8 | 1 | 136 | -73 | MER3 | MER1\_type | DNA |
| 69865187 | 69865483 | - | 7.1 | 1 | 297 | -14 | AluY | Alu | SINE |
| 69865484 | 69865548 | + | 17.8 | 137 | 209 | 0 | MER3 | MER1\_type | DNA |

  

|  |  |  |  |  |  |  |  |  |  |
| --- | --- | --- | --- | --- | --- | --- | --- | --- | --- |
| chr5:69871517-69872736 | | | 1220 bp | | |  |  |  |  |
| Genome Start | Genome End | Strand | Div | Repeat Start | Repeat End | Repeat Left | Name | Family | Class |
| 69871517 | 69871917 | + | 23.4 | 2528 | 2892 | -3953 | L1M2 | L1 | LINE |
| 69871918 | 69872220 | + | 12.9 | 1 | 303 | -7 | AluSg | Alu | SINE |
| 69872221 | 69872736 | + | 21.9 | 2893 | 3392 | -3453 | L1M2 | L1 | LINE |

  

|  |  |  |  |  |  |  |  |  |  |
| --- | --- | --- | --- | --- | --- | --- | --- | --- | --- |
| chr5:69897185-69898137 | | | 791 bp | | |  |  |  |  |
| Genome Start | Genome End | Strand | Div | Repeat Start | Repeat End | Repeat Left | Name | Family | Class |
| 69897185 | 69897400 | + | 25.2 | 1 | 225 | -280 | MLT1D | MaLR | LTR |
| 69897442 | 69897488 | + | 17.0 | 1 | 47 | -327 | MLT1A | MaLR | LTR |
| 69897536 | 69897645 | + | 30.9 | 51 | 160 | -214 | MLT1A | MaLR | LTR |
| 69897720 | 69897908 | + | 18.5 | 189 | 374 | 0 | MLT1A | MaLR | LTR |
| 69897909 | 69898137 | + | 27.4 | 275 | 505 | 0 | MLT1D | MaLR | LTR |

  

|  |  |  |  |  |  |  |  |  |  |
| --- | --- | --- | --- | --- | --- | --- | --- | --- | --- |
| chr5:69899428-69902118 | | | 2680 bp | | |  |  |  |  |
| Genome Start | Genome End | Strand | Div | Repeat Start | Repeat End | Repeat Left | Name | Family | Class |
| 69899428 | 69899650 | + | 18.8 | 1 | 238 | -128 | Tigger5a | MER2\_type | DNA |
| 69899661 | 69902047 | - | 6.9 | 3742 | 6150 | -4 | L1PA6 | L1 | LINE |
| 69902049 | 69902118 | + | 20.0 | 356 | 434 | -15 | Tigger5a | MER2\_type | DNA |

  

|  |  |  |  |  |  |  |  |  |  |
| --- | --- | --- | --- | --- | --- | --- | --- | --- | --- |
| chr5:69910151-69916469 | | | 6320 bp | | |  |  |  |  |
| Genome Start | Genome End | Strand | Div | Repeat Start | Repeat End | Repeat Left | Name | Family | Class |
| 69910151 | 69910357 | + | 20.8 | 13 | 235 | -6268 | L1PA16 | L1 | LINE |
| 69910357 | 69914212 | + | 19.2 | 947 | 4816 | -1667 | L1PA16 | L1 | LINE |
| 69914213 | 69914484 | + | 19.1 | 2 | 297 | -5 | AluJo | Alu | SINE |
| 69914485 | 69915424 | + | 20.6 | 4817 | 5660 | -823 | L1PA16 | L1 | LINE |
| 69915425 | 69915738 | + | 8.7 | 5876 | 6167 | -1 | L1PA10 | L1 | LINE |
| 69915739 | 69916469 | + | 14.3 | 5661 | 6398 | -105 | L1PA16 | L1 | LINE |

  

|  |  |  |  |  |  |  |  |  |  |
| --- | --- | --- | --- | --- | --- | --- | --- | --- | --- |
| chr5:69926411-69929056 | | | 2413 bp | | |  |  |  |  |
| Genome Start | Genome End | Strand | Div | Repeat Start | Repeat End | Repeat Left | Name | Family | Class |
| 69926411 | 69927040 | + | 30.7 | 3855 | 4522 | -1624 | L1ME2 | L1 | LINE |
| 69927041 | 69927459 | + | 14.5 | 5 | 428 | 0 | MSTA | MaLR | LTR |
| 69927460 | 69927998 | + | 30.7 | 4523 | 5064 | -1082 | L1ME2 | L1 | LINE |
| 69928214 | 69928587 | + | 24.7 | 5329 | 5758 | -406 | L1ME2 | L1 | LINE |
| 69928593 | 69928777 | - | 22.1 | 1 | 213 | -6 | MER20 | MER1\_type | DNA |
| 69928791 | 69929056 | + | 26.4 | 5768 | 6039 | -125 | L1ME2 | L1 | LINE |

  

|  |  |  |  |  |  |  |  |  |  |
| --- | --- | --- | --- | --- | --- | --- | --- | --- | --- |
| chr5:69951161-69951956 | | | 796 bp | | |  |  |  |  |
| Genome Start | Genome End | Strand | Div | Repeat Start | Repeat End | Repeat Left | Name | Family | Class |
| 69951161 | 69951290 | - | 30.3 | 325 | 445 | 0 | MER65A | ERV1 | LTR |
| 69951291 | 69951664 | - | 13.2 | 1 | 428 | 0 | MSTA | MaLR | LTR |
| 69951665 | 69951956 | - | 30.3 | 1 | 324 | -121 | MER65A | ERV1 | LTR |

  

|  |  |  |  |  |  |  |  |  |  |
| --- | --- | --- | --- | --- | --- | --- | --- | --- | --- |
| chr5:69958534-69958939 | | | 406 bp | | |  |  |  |  |
| Genome Start | Genome End | Strand | Div | Repeat Start | Repeat End | Repeat Left | Name | Family | Class |
| 69958534 | 69958610 | - | 32.0 | 107 | 184 | -84 | MIRb | MIR | SINE |
| 69958611 | 69958908 | + | 13.8 | 1 | 300 | -12 | AluSx | Alu | SINE |
| 69958909 | 69958939 | - | 32.0 | 79 | 106 | -162 | MIRb | MIR | SINE |

  

|  |  |  |  |  |  |  |  |  |  |
| --- | --- | --- | --- | --- | --- | --- | --- | --- | --- |
| chr5:69963340-69965282 | | | 1737 bp | | |  |  |  |  |
| Genome Start | Genome End | Strand | Div | Repeat Start | Repeat End | Repeat Left | Name | Family | Class |
| 69963340 | 69963731 | - | 29.2 | 5751 | 6161 | -5 | L1ME1 | L1 | LINE |
| 69963818 | 69964175 | - | 27.9 | 4340 | 4721 | -1425 | L1ME1 | L1 | LINE |
| 69964176 | 69964382 | + | 20.2 | 1 | 211 | -200 | LTR57 | ERVL | LTR |
| 69964383 | 69964661 | + | 5.0 | 1 | 279 | -32 | AluY | Alu | SINE |
| 69964782 | 69964975 | + | 20.2 | 212 | 411 | 0 | LTR57 | ERVL | LTR |
| 69964976 | 69965282 | - | 27.9 | 4108 | 4339 | -1807 | L1ME1 | L1 | LINE |

  

|  |  |  |  |  |  |  |  |  |  |
| --- | --- | --- | --- | --- | --- | --- | --- | --- | --- |
| chr5:70002864-70005309 | | | 2086 bp | | |  |  |  |  |
| Genome Start | Genome End | Strand | Div | Repeat Start | Repeat End | Repeat Left | Name | Family | Class |
| 70002864 | 70003211 | + | 30.8 | 1662 | 2097 | -5096 | L1ME1 | L1 | LINE |
| 70003301 | 70003607 | + | 25.8 | 4968 | 5199 | -1807 | L1ME1 | L1 | LINE |
| 70003608 | 70003803 | - | 20.3 | 212 | 411 | 0 | LTR57 | ERVL | LTR |
| 70003930 | 70004208 | - | 5.0 | 1 | 279 | -32 | AluY | Alu | SINE |
| 70004209 | 70004415 | - | 20.3 | 1 | 211 | -200 | LTR57 | ERVL | LTR |
| 70004416 | 70004772 | + | 25.8 | 5200 | 5581 | -1425 | L1ME1 | L1 | LINE |
| 70004918 | 70005309 | + | 29.4 | 6611 | 7021 | -5 | L1ME1 | L1 | LINE |

  

|  |  |  |  |  |  |  |  |  |  |
| --- | --- | --- | --- | --- | --- | --- | --- | --- | --- |
| chr5:70011545-70012276 | | | 732 bp | | |  |  |  |  |
| Genome Start | Genome End | Strand | Div | Repeat Start | Repeat End | Repeat Left | Name | Family | Class |
| 70011545 | 70011594 | + | 34.0 | 861 | 910 | -2509 | L2 | L2 | LINE |
| 70011595 | 70012112 | - | 27.2 | 3 | 560 | -2 | MLT1F2 | MaLR | LTR |
| 70012113 | 70012276 | + | 34.0 | 911 | 1076 | -2343 | L2 | L2 | LINE |

  

|  |  |  |  |  |  |  |  |  |  |
| --- | --- | --- | --- | --- | --- | --- | --- | --- | --- |
| chr5:70019513-70021860 | | | 2326 bp | | |  |  |  |  |
| Genome Start | Genome End | Strand | Div | Repeat Start | Repeat End | Repeat Left | Name | Family | Class |
| 70019513 | 70019628 | + | 18.3 | 3206 | 3321 | -3318 | MER65D-int | ERV1 | LTR |
| 70019639 | 70020374 | + | 26.8 | 8 | 767 | -6567 | L1P4b | L1 | LINE |
| 70020377 | 70021246 | + | 10.8 | 5275 | 6165 | 0 | L1PA15 | L1 | LINE |
| 70021257 | 70021860 | + | 22.9 | 3317 | 3934 | -2705 | MER65D-int | ERV1 | LTR |

  

|  |  |  |  |  |  |  |  |  |  |
| --- | --- | --- | --- | --- | --- | --- | --- | --- | --- |
| chr5:70039798-70042443 | | | 2413 bp | | |  |  |  |  |
| Genome Start | Genome End | Strand | Div | Repeat Start | Repeat End | Repeat Left | Name | Family | Class |
| 70039798 | 70040063 | - | 26.4 | 5768 | 6039 | -125 | L1ME2 | L1 | LINE |
| 70040077 | 70040261 | + | 22.1 | 1 | 213 | -6 | MER20 | MER1\_type | DNA |
| 70040267 | 70040640 | - | 24.7 | 5329 | 5758 | -406 | L1ME2 | L1 | LINE |
| 70040856 | 70041394 | - | 30.8 | 4523 | 5064 | -1082 | L1ME2 | L1 | LINE |
| 70041395 | 70041813 | - | 14.2 | 5 | 428 | 0 | MSTA | MaLR | LTR |
| 70041814 | 70042443 | - | 30.8 | 3855 | 4522 | -1624 | L1ME2 | L1 | LINE |

  

|  |  |  |  |  |  |  |  |  |  |
| --- | --- | --- | --- | --- | --- | --- | --- | --- | --- |
| chr5:70066729-70069420 | | | 2681 bp | | |  |  |  |  |
| Genome Start | Genome End | Strand | Div | Repeat Start | Repeat End | Repeat Left | Name | Family | Class |
| 70066729 | 70066798 | - | 20.0 | 356 | 434 | -15 | Tigger5a | MER2\_type | DNA |
| 70066800 | 70069187 | + | 6.8 | 3742 | 6150 | -4 | L1PA6 | L1 | LINE |
| 70069198 | 70069420 | - | 18.8 | 1 | 238 | -128 | Tigger5a | MER2\_type | DNA |

  

|  |  |  |  |  |  |  |  |  |  |
| --- | --- | --- | --- | --- | --- | --- | --- | --- | --- |
| chr5:70093619-70094929 | | | 1311 bp | | |  |  |  |  |
| Genome Start | Genome End | Strand | Div | Repeat Start | Repeat End | Repeat Left | Name | Family | Class |
| 70093619 | 70094120 | - | 23.7 | 6587 | 7119 | -666 | L1MC3 | L1 | LINE |
| 70094121 | 70094503 | - | 17.3 | 1 | 428 | 0 | MSTA | MaLR | LTR |
| 70094504 | 70094929 | - | 23.7 | 6152 | 6586 | -1199 | L1MC3 | L1 | LINE |

  

|  |  |  |  |  |  |  |  |  |  |
| --- | --- | --- | --- | --- | --- | --- | --- | --- | --- |
| chr5:70095618-70096840 | | | 1223 bp | | |  |  |  |  |
| Genome Start | Genome End | Strand | Div | Repeat Start | Repeat End | Repeat Left | Name | Family | Class |
| 70095618 | 70096133 | - | 21.1 | 2898 | 3392 | -3453 | L1M2 | L1 | LINE |
| 70096134 | 70096439 | - | 12.8 | 1 | 306 | -4 | AluSg | Alu | SINE |
| 70096440 | 70096840 | - | 23.0 | 2528 | 2897 | -3948 | L1M2 | L1 | LINE |

  

|  |  |  |  |  |  |  |  |  |  |
| --- | --- | --- | --- | --- | --- | --- | --- | --- | --- |
| chr5:70102825-70103322 | | | 498 bp | | |  |  |  |  |
| Genome Start | Genome End | Strand | Div | Repeat Start | Repeat End | Repeat Left | Name | Family | Class |
| 70102825 | 70102889 | - | 19.1 | 134 | 209 | 0 | MER3 | MER1\_type | DNA |
| 70102890 | 70103194 | + | 6.6 | 1 | 303 | -8 | AluY | Alu | SINE |
| 70103195 | 70103322 | - | 19.1 | 2 | 133 | -76 | MER3 | MER1\_type | DNA |

  

|  |  |  |  |  |  |  |  |  |  |
| --- | --- | --- | --- | --- | --- | --- | --- | --- | --- |
| chr5:70103343-70104342 | | | 1000 bp | | |  |  |  |  |
| Genome Start | Genome End | Strand | Div | Repeat Start | Repeat End | Repeat Left | Name | Family | Class |
| 70103343 | 70103410 | - | 22.5 | 309 | 374 | 0 | MLT1A | MaLR | LTR |
| 70103411 | 70103713 | + | 16.2 | 1 | 312 | 0 | AluSx | Alu | SINE |
| 70103714 | 70103990 | - | 22.5 | 45 | 308 | -66 | MLT1A | MaLR | LTR |
| 70103991 | 70104298 | - | 6.6 | 1 | 305 | -6 | AluY | Alu | SINE |
| 70104299 | 70104342 | - | 22.5 | 1 | 44 | -330 | MLT1A | MaLR | LTR |

  

|  |  |  |  |  |  |  |  |  |  |
| --- | --- | --- | --- | --- | --- | --- | --- | --- | --- |
| chr5:70114069-70114984 | | | 916 bp | | |  |  |  |  |
| Genome Start | Genome End | Strand | Div | Repeat Start | Repeat End | Repeat Left | Name | Family | Class |
| 70114069 | 70114526 | - | 21.5 | 179 | 653 | 0 | MER82 | MER2\_type | DNA |
| 70114527 | 70114835 | - | 11.4 | 1 | 290 | -22 | AluSx | Alu | SINE |
| 70114836 | 70114984 | - | 21.5 | 1 | 178 | -475 | MER82 | MER2\_type | DNA |

  

|  |  |  |  |  |  |  |  |  |  |
| --- | --- | --- | --- | --- | --- | --- | --- | --- | --- |
| chr5:70117373-70117975 | | | 603 bp | | |  |  |  |  |
| Genome Start | Genome End | Strand | Div | Repeat Start | Repeat End | Repeat Left | Name | Family | Class |
| 70117373 | 70117557 | - | 17.1 | 126 | 311 | -1 | AluJo | Alu | SINE |
| 70117558 | 70117847 | + | 13.4 | 1 | 293 | -19 | AluSx | Alu | SINE |
| 70117848 | 70117975 | - | 17.1 | 1 | 125 | -187 | AluJo | Alu | SINE |

  

|  |  |  |  |  |  |  |  |  |  |
| --- | --- | --- | --- | --- | --- | --- | --- | --- | --- |
| chr5:70119337-70119935 | | | 599 bp | | |  |  |  |  |
| Genome Start | Genome End | Strand | Div | Repeat Start | Repeat End | Repeat Left | Name | Family | Class |
| 70119337 | 70119517 | - | 17.5 | 138 | 305 | -7 | AluJo | Alu | SINE |
| 70119518 | 70119812 | - | 12.3 | 1 | 290 | -20 | AluSg | Alu | SINE |
| 70119813 | 70119935 | - | 17.5 | 2 | 137 | -175 | AluJo | Alu | SINE |

  

|  |  |  |  |  |  |  |  |  |  |
| --- | --- | --- | --- | --- | --- | --- | --- | --- | --- |
| chr5:70125416-70126007 | | | 592 bp | | |  |  |  |  |
| Genome Start | Genome End | Strand | Div | Repeat Start | Repeat End | Repeat Left | Name | Family | Class |
| 70125416 | 70125550 | + | 19.2 | 2 | 135 | -177 | AluJb | Alu | SINE |
| 70125551 | 70125850 | + | 10.1 | 1 | 298 | -12 | AluSg | Alu | SINE |
| 70125851 | 70126007 | + | 19.2 | 136 | 291 | -21 | AluJb | Alu | SINE |

  

|  |  |  |  |  |  |  |  |  |  |
| --- | --- | --- | --- | --- | --- | --- | --- | --- | --- |
| chr5:70161811-70167483 | | | 5566 bp | | |  |  |  |  |
| Genome Start | Genome End | Strand | Div | Repeat Start | Repeat End | Repeat Left | Name | Family | Class |
| 70161811 | 70161935 | + | 27.4 | 1036 | 1160 | -5473 | L1MDa | L1 | LINE |
| 70161936 | 70162246 | + | 11.9 | 1 | 312 | 0 | AluSx | Alu | SINE |
| 70162247 | 70163396 | + | 27.4 | 1161 | 2359 | -4274 | L1MDa | L1 | LINE |
| 70163414 | 70163640 | + | 19.4 | 1 | 229 | -1002 | Tigger3b | MER2\_type | DNA |
| 70163641 | 70164011 | + | 15.4 | 1 | 379 | -2 | THE1D | MaLR | LTR |
| 70164014 | 70165510 | + | 14.6 | 1 | 1580 | 0 | THE1D-int | MaLR | LTR |
| 70165511 | 70165889 | + | 15.6 | 1 | 381 | 0 | THE1D | MaLR | LTR |
| 70165890 | 70166006 | + | 16.9 | 225 | 338 | -893 | Tigger3b | MER2\_type | DNA |
| 70166007 | 70166261 | + | 5.9 | 47 | 301 | -10 | AluY | Alu | SINE |
| 70166262 | 70167122 | + | 16.9 | 339 | 1225 | -6 | Tigger3b | MER2\_type | DNA |
| 70167134 | 70167343 | + | 19.1 | 2384 | 2601 | -4032 | L1MDa | L1 | LINE |
| 70167421 | 70167483 | + | 22.2 | 2614 | 2679 | -3858 | L1MDa | L1 | LINE |

  

|  |  |  |  |  |  |  |  |  |  |
| --- | --- | --- | --- | --- | --- | --- | --- | --- | --- |
| chr5:70176138-70177061 | | | 924 bp | | |  |  |  |  |
| Genome Start | Genome End | Strand | Div | Repeat Start | Repeat End | Repeat Left | Name | Family | Class |
| 70176138 | 70176411 | + | 9.8 | 2118 | 2393 | -3753 | L1PREC2 | L1 | LINE |
| 70176412 | 70176723 | + | 9.3 | 1 | 300 | -9 | AluSc | Alu | SINE |
| 70176724 | 70177061 | + | 9.8 | 2394 | 2732 | -3414 | L1PREC2 | L1 | LINE |

  

|  |  |  |  |  |  |  |  |  |  |
| --- | --- | --- | --- | --- | --- | --- | --- | --- | --- |
| chr5:70177693-70179196 | | | 1514 bp | | |  |  |  |  |
| Genome Start | Genome End | Strand | Div | Repeat Start | Repeat End | Repeat Left | Name | Family | Class |
| 70177693 | 70178246 | - | 27.5 | 2666 | 3271 | -3373 | L1M4 | L1 | LINE |
| 70178247 | 70178643 | + | 20.0 | 1 | 467 | 0 | MLT1C | MaLR | LTR |
| 70178644 | 70178829 | - | 26.5 | 2506 | 2768 | -4057 | L1M4 | L1 | LINE |
| 70178820 | 70179196 | - | 28.9 | 1309 | 1861 | -4964 | L1M4 | L1 | LINE |

  

|  |  |  |  |  |  |  |  |  |  |
| --- | --- | --- | --- | --- | --- | --- | --- | --- | --- |
| chr5:70245302-70245854 | | | 553 bp | | |  |  |  |  |
| Genome Start | Genome End | Strand | Div | Repeat Start | Repeat End | Repeat Left | Name | Family | Class |
| 70245302 | 70245328 | - | 7.3 | 219 | 243 | 0 | MER75B | PiggyBac | DNA |
| 70245329 | 70245648 | + | 6.4 | 1 | 311 | 0 | AluY | Alu | SINE |
| 70245649 | 70245854 | - | 7.3 | 1 | 218 | -25 | MER75B | PiggyBac | DNA |

  

|  |  |  |  |  |  |  |  |  |  |
| --- | --- | --- | --- | --- | --- | --- | --- | --- | --- |
| chr5:70262337-70262799 | | | 460 bp | | |  |  |  |  |
| Genome Start | Genome End | Strand | Div | Repeat Start | Repeat End | Repeat Left | Name | Family | Class |
| 70262337 | 70262486 | - | 25.9 | 5549 | 5711 | -453 | L1M5 | L1 | LINE |
| 70262489 | 70262571 | - | 22.9 | 295 | 378 | -25 | MER57A | ERV1 | LTR |
| 70262573 | 70262799 | - | 19.6 | 5324 | 5554 | -612 | L1M5 | L1 | LINE |

  

|  |  |  |  |  |  |  |  |  |  |
| --- | --- | --- | --- | --- | --- | --- | --- | --- | --- |
| chr5:70267569-70268118 | | | 550 bp | | |  |  |  |  |
| Genome Start | Genome End | Strand | Div | Repeat Start | Repeat End | Repeat Left | Name | Family | Class |
| 70267569 | 70267731 | - | 21.2 | 5970 | 6139 | -32 | L1MB2 | L1 | LINE |
| 70267732 | 70268026 | - | 7.1 | 1 | 302 | -11 | AluSp | Alu | SINE |
| 70268027 | 70268118 | - | 23.9 | 5879 | 5969 | -205 | L1MB2 | L1 | LINE |

  

|  |  |  |  |  |  |  |  |  |  |
| --- | --- | --- | --- | --- | --- | --- | --- | --- | --- |
| chr5:70291833-70292781 | | | 949 bp | | |  |  |  |  |
| Genome Start | Genome End | Strand | Div | Repeat Start | Repeat End | Repeat Left | Name | Family | Class |
| 70291833 | 70292290 | - | 19.2 | 204 | 653 | 0 | MER82 | MER2\_type | DNA |
| 70292291 | 70292579 | - | 14.2 | 1 | 288 | -24 | AluSx | Alu | SINE |
| 70292580 | 70292781 | - | 19.2 | 1 | 203 | -450 | MER82 | MER2\_type | DNA |

  

|  |  |  |  |  |  |  |  |  |  |
| --- | --- | --- | --- | --- | --- | --- | --- | --- | --- |
| chr5:70310729-70311280 | | | 521 bp | | |  |  |  |  |
| Genome Start | Genome End | Strand | Div | Repeat Start | Repeat End | Repeat Left | Name | Family | Class |
| 70310729 | 70310878 | + | 28.8 | 12 | 164 | -98 | MIR | MIR | SINE |
| 70310879 | 70311167 | + | 5.9 | 1 | 293 | -18 | AluY | Alu | SINE |
| 70311199 | 70311280 | + | 28.8 | 165 | 247 | -15 | MIR | MIR | SINE |

  

|  |  |  |  |  |  |  |  |  |  |
| --- | --- | --- | --- | --- | --- | --- | --- | --- | --- |
| chr5:70312165-70314661 | | | 2495 bp | | |  |  |  |  |
| Genome Start | Genome End | Strand | Div | Repeat Start | Repeat End | Repeat Left | Name | Family | Class |
| 70312165 | 70312739 | + | 16.2 | 4886 | 5489 | -691 | L1MB4 | L1 | LINE |
| 70312740 | 70312833 | + | 16.1 | 1 | 88 | -224 | AluJo | Alu | SINE |
| 70312834 | 70313144 | + | 8.4 | 1 | 311 | -1 | AluSx | Alu | SINE |
| 70313145 | 70313366 | + | 16.1 | 89 | 305 | -7 | AluJo | Alu | SINE |
| 70313369 | 70313664 | + | 14.2 | 1 | 296 | -16 | AluSx | Alu | SINE |
| 70313665 | 70314205 | + | 15.0 | 5490 | 5999 | -181 | L1MB4 | L1 | LINE |
| 70314206 | 70314511 | - | 9.8 | 1 | 306 | -6 | AluSx | Alu | SINE |
| 70314512 | 70314661 | + | 15.0 | 6000 | 6177 | -3 | L1MB4 | L1 | LINE |

  

|  |  |  |  |  |  |  |  |  |  |
| --- | --- | --- | --- | --- | --- | --- | --- | --- | --- |
| chr5:70324909-70325945 | | | 1037 bp | | |  |  |  |  |
| Genome Start | Genome End | Strand | Div | Repeat Start | Repeat End | Repeat Left | Name | Family | Class |
| 70324909 | 70325523 | + | 24.9 | 5463 | 6085 | -155 | L1ME3B | L1 | LINE |
| 70325524 | 70325830 | - | 11.8 | 1 | 309 | -3 | AluSx | Alu | SINE |
| 70325831 | 70325945 | + | 24.9 | 6086 | 6202 | -38 | L1ME3B | L1 | LINE |

  

|  |  |  |  |  |  |  |  |  |  |
| --- | --- | --- | --- | --- | --- | --- | --- | --- | --- |
| chr5:70327085-70330183 | | | 2995 bp | | |  |  |  |  |
| Genome Start | Genome End | Strand | Div | Repeat Start | Repeat End | Repeat Left | Name | Family | Class |
| 70327085 | 70327573 | + | 24.8 | 1437 | 1901 | -606 | HAL1 | L1 | LINE |
| 70327574 | 70327883 | + | 12.0 | 1 | 309 | -3 | AluSx | Alu | SINE |
| 70327884 | 70327984 | + | 24.8 | 1902 | 2001 | -506 | HAL1 | L1 | LINE |
| 70328088 | 70328396 | + | 6.6 | 1 | 302 | -9 | AluY | Alu | SINE |
| 70328404 | 70328480 | + | 16.2 | 5463 | 5551 | -629 | L1MB5 | L1 | LINE |
| 70328472 | 70328669 | + | 20.5 | 5903 | 6094 | -80 | L1MB5 | L1 | LINE |
| 70328669 | 70328742 | + | 19.2 | 5535 | 5603 | -571 | L1MB5 | L1 | LINE |
| 70328743 | 70329039 | + | 11.5 | 1 | 305 | -7 | AluSx | Alu | SINE |
| 70329040 | 70329584 | + | 19.2 | 5604 | 6140 | -34 | L1MB5 | L1 | LINE |
| 70329585 | 70329893 | + | 17.7 | 2 | 308 | -4 | AluJo | Alu | SINE |
| 70329894 | 70329918 | + | 19.2 | 6141 | 6164 | -10 | L1MB5 | L1 | LINE |
| 70329923 | 70330183 | + | 30.8 | 2012 | 2268 | -239 | HAL1 | L1 | LINE |

  

|  |  |  |  |  |  |  |  |  |  |
| --- | --- | --- | --- | --- | --- | --- | --- | --- | --- |
| chr5:70331959-70332497 | | | 494 bp | | |  |  |  |  |
| Genome Start | Genome End | Strand | Div | Repeat Start | Repeat End | Repeat Left | Name | Family | Class |
| 70331959 | 70332102 | + | 23.2 | 1 | 148 | -363 | MLT1J | MaLR | LTR |
| 70332148 | 70332436 | - | 8.7 | 1 | 288 | -25 | AluSq | Alu | SINE |
| 70332437 | 70332497 | + | 23.2 | 149 | 200 | -311 | MLT1J | MaLR | LTR |

  

|  |  |  |  |  |  |  |  |  |  |
| --- | --- | --- | --- | --- | --- | --- | --- | --- | --- |
| chr5:70333765-70334405 | | | 641 bp | | |  |  |  |  |
| Genome Start | Genome End | Strand | Div | Repeat Start | Repeat End | Repeat Left | Name | Family | Class |
| 70333765 | 70333984 | - | 10.6 | 102 | 308 | -4 | AluSx | Alu | SINE |
| 70333985 | 70334298 | - | 7.5 | 1 | 306 | -5 | AluY | Alu | SINE |
| 70334299 | 70334405 | - | 10.6 | 1 | 101 | -211 | AluSx | Alu | SINE |

  

|  |  |  |  |  |  |  |  |  |  |
| --- | --- | --- | --- | --- | --- | --- | --- | --- | --- |
| chr5:70352779-70353587 | | | 809 bp | | |  |  |  |  |
| Genome Start | Genome End | Strand | Div | Repeat Start | Repeat End | Repeat Left | Name | Family | Class |
| 70352779 | 70353003 | - | 28.3 | 1139 | 1393 | -1114 | HAL1 | L1 | LINE |
| 70353004 | 70353283 | - | 14.6 | 1 | 277 | -35 | AluJb | Alu | SINE |
| 70353284 | 70353587 | - | 28.3 | 799 | 1138 | -1369 | HAL1 | L1 | LINE |

  

|  |  |  |  |  |  |  |  |  |  |
| --- | --- | --- | --- | --- | --- | --- | --- | --- | --- |
| chr5:70358188-70358806 | | | 619 bp | | |  |  |  |  |
| Genome Start | Genome End | Strand | Div | Repeat Start | Repeat End | Repeat Left | Name | Family | Class |
| 70358188 | 70358239 | - | 22.6 | 5344 | 5388 | -758 | L1M4 | L1 | LINE |
| 70358240 | 70358536 | - | 7.5 | 1 | 295 | -15 | AluSg | Alu | SINE |
| 70358537 | 70358806 | - | 22.6 | 5061 | 5343 | -803 | L1M4 | L1 | LINE |

  

|  |  |  |  |  |  |  |  |  |  |
| --- | --- | --- | --- | --- | --- | --- | --- | --- | --- |
| chr5:70360325-70360818 | | | 494 bp | | |  |  |  |  |
| Genome Start | Genome End | Strand | Div | Repeat Start | Repeat End | Repeat Left | Name | Family | Class |
| 70360325 | 70360444 | - | 24.4 | 76 | 189 | 0 | MER5A | MER1\_type | DNA |
| 70360445 | 70360740 | - | 9.8 | 1 | 296 | -14 | AluSg | Alu | SINE |
| 70360741 | 70360818 | - | 24.4 | 1 | 75 | -114 | MER5A | MER1\_type | DNA |

  

|  |  |  |  |  |  |  |  |  |  |
| --- | --- | --- | --- | --- | --- | --- | --- | --- | --- |
| chr5:70361629-70362362 | | | 540 bp | | |  |  |  |  |
| Genome Start | Genome End | Strand | Div | Repeat Start | Repeat End | Repeat Left | Name | Family | Class |
| 70361629 | 70361740 | + | 25.2 | 1 | 129 | -212 | MER102b | MER1\_type | DNA |
| 70361744 | 70362061 | + | 18.9 | 1 | 312 | 0 | AluJb | Alu | SINE |
| 70362253 | 70362362 | + | 25.5 | 225 | 330 | -11 | MER102b | MER1\_type | DNA |

  

|  |  |  |  |  |  |  |  |  |  |
| --- | --- | --- | --- | --- | --- | --- | --- | --- | --- |
| chr5:70367522-70368666 | | | 1145 bp | | |  |  |  |  |
| Genome Start | Genome End | Strand | Div | Repeat Start | Repeat End | Repeat Left | Name | Family | Class |
| 70367522 | 70368058 | + | 23.9 | 4 | 551 | -337 | Kanga2\_a | Tc2 | DNA |
| 70368059 | 70368357 | + | 14.1 | 1 | 301 | -11 | AluSx | Alu | SINE |
| 70368358 | 70368666 | + | 23.9 | 552 | 871 | -17 | Kanga2\_a | Tc2 | DNA |

  

|  |  |  |  |  |  |  |  |  |  |
| --- | --- | --- | --- | --- | --- | --- | --- | --- | --- |
| chr5:70370021-70371311 | | | 1287 bp | | |  |  |  |  |
| Genome Start | Genome End | Strand | Div | Repeat Start | Repeat End | Repeat Left | Name | Family | Class |
| 70370021 | 70370921 | - | 23.6 | 1337 | 2316 | -191 | HAL1 | L1 | LINE |
| 70370922 | 70371053 | - | 22.7 | 1 | 132 | -11 | FLAM\_C | Alu | SINE |
| 70371058 | 70371311 | - | 23.1 | 1084 | 1345 | -1162 | HAL1 | L1 | LINE |

  

|  |  |  |  |  |  |  |  |  |  |
| --- | --- | --- | --- | --- | --- | --- | --- | --- | --- |
| chr5:70381208-70381643 | | | 436 bp | | |  |  |  |  |
| Genome Start | Genome End | Strand | Div | Repeat Start | Repeat End | Repeat Left | Name | Family | Class |
| 70381208 | 70381293 | - | 12.3 | 2408 | 2490 | -1 | Tigger7 | MER2\_type | DNA |
| 70381294 | 70381623 | - | 17.3 | 2 | 345 | 0 | MER2 | MER2\_type | DNA |
| 70381624 | 70381643 | - | 12.3 | 2388 | 2407 | -84 | Tigger7 | MER2\_type | DNA |

  

|  |  |  |  |  |  |  |  |  |  |
| --- | --- | --- | --- | --- | --- | --- | --- | --- | --- |
| chr5:70387296-70388101 | | | 806 bp | | |  |  |  |  |
| Genome Start | Genome End | Strand | Div | Repeat Start | Repeat End | Repeat Left | Name | Family | Class |
| 70387296 | 70387514 | + | 24.9 | 1 | 246 | -1652 | Kanga1 | Tc2 | DNA |
| 70387515 | 70387816 | + | 14.3 | 1 | 301 | -12 | AluSq | Alu | SINE |
| 70387817 | 70388101 | + | 24.9 | 247 | 547 | -1351 | Kanga1 | Tc2 | DNA |

  

|  |  |  |  |  |  |  |  |  |  |
| --- | --- | --- | --- | --- | --- | --- | --- | --- | --- |
| chr5:70393399-70394240 | | | 842 bp | | |  |  |  |  |
| Genome Start | Genome End | Strand | Div | Repeat Start | Repeat End | Repeat Left | Name | Family | Class |
| 70393399 | 70393650 | + | 24.3 | 5649 | 5910 | -268 | L1MB8 | L1 | LINE |
| 70393651 | 70393964 | - | 13.7 | 1 | 345 | -1 | MER7A | MER2\_type | DNA |
| 70393965 | 70394240 | + | 24.3 | 5911 | 6178 | 0 | L1MB8 | L1 | LINE |

  

|  |  |  |  |  |  |  |  |  |  |
| --- | --- | --- | --- | --- | --- | --- | --- | --- | --- |
| chr5:70397871-70398385 | | | 515 bp | | |  |  |  |  |
| Genome Start | Genome End | Strand | Div | Repeat Start | Repeat End | Repeat Left | Name | Family | Class |
| 70397871 | 70397904 | + | 28.6 | 4255 | 4287 | -202 | L3 | CR1 | LINE |
| 70397905 | 70398212 | - | 11.0 | 1 | 304 | -8 | AluSx | Alu | SINE |
| 70398213 | 70398385 | + | 28.6 | 4288 | 4463 | -26 | L3 | CR1 | LINE |

  

|  |  |  |  |  |  |  |  |  |  |
| --- | --- | --- | --- | --- | --- | --- | --- | --- | --- |
| chr5:70399652-70400635 | | | 1007 bp | | |  |  |  |  |
| Genome Start | Genome End | Strand | Div | Repeat Start | Repeat End | Repeat Left | Name | Family | Class |
| 70399652 | 70399874 | - | 27.8 | 3176 | 3414 | -5 | L2 | L2 | LINE |
| 70399875 | 70400163 | + | 9.3 | 1 | 293 | -19 | AluSx | Alu | SINE |
| 70400164 | 70400386 | - | 27.8 | 2920 | 3175 | -244 | L2 | L2 | LINE |
| 70400364 | 70400635 | - | 22.8 | 2412 | 2767 | -652 | L2 | L2 | LINE |

  

|  |  |  |  |  |  |  |  |  |  |
| --- | --- | --- | --- | --- | --- | --- | --- | --- | --- |
| chr5:70408882-70409420 | | | 539 bp | | |  |  |  |  |
| Genome Start | Genome End | Strand | Div | Repeat Start | Repeat End | Repeat Left | Name | Family | Class |
| 70408882 | 70408987 | + | 29.8 | 5549 | 5662 | -459 | L1ME3 | L1 | LINE |
| 70408988 | 70409292 | + | 9.2 | 1 | 305 | -7 | AluSx | Alu | SINE |
| 70409293 | 70409420 | + | 29.8 | 5663 | 5791 | -330 | L1ME3 | L1 | LINE |

  

|  |  |  |  |  |  |  |  |  |  |
| --- | --- | --- | --- | --- | --- | --- | --- | --- | --- |
| chr5:70418844-70419417 | | | 574 bp | | |  |  |  |  |
| Genome Start | Genome End | Strand | Div | Repeat Start | Repeat End | Repeat Left | Name | Family | Class |
| 70418844 | 70419065 | + | 30.0 | 5541 | 5778 | -2030 | L1MC | L1 | LINE |
| 70419066 | 70419374 | + | 12.6 | 1 | 310 | -2 | AluSx | Alu | SINE |
| 70419375 | 70419417 | + | 30.0 | 5779 | 5821 | -1987 | L1MC | L1 | LINE |

  

|  |  |  |  |  |  |  |  |  |  |
| --- | --- | --- | --- | --- | --- | --- | --- | --- | --- |
| chr5:70431328-70432720 | | | 1390 bp | | |  |  |  |  |
| Genome Start | Genome End | Strand | Div | Repeat Start | Repeat End | Repeat Left | Name | Family | Class |
| 70431328 | 70431959 | + | 26.4 | 7 | 701 | -72 | LTR41 | ERVL | LTR |
| 70431960 | 70432321 | + | 15.2 | 1 | 365 | -1 | Tigger5a | MER2\_type | DNA |
| 70432325 | 70432647 | - | 9.6 | 1 | 305 | 0 | AluSx | Alu | SINE |
| 70432648 | 70432720 | + | 26.4 | 702 | 770 | -3 | LTR41 | ERVL | LTR |

  

|  |  |  |  |  |  |  |  |  |  |
| --- | --- | --- | --- | --- | --- | --- | --- | --- | --- |
| chr5:70435598-70436155 | | | 558 bp | | |  |  |  |  |
| Genome Start | Genome End | Strand | Div | Repeat Start | Repeat End | Repeat Left | Name | Family | Class |
| 70435598 | 70435748 | + | 27.9 | 12 | 145 | -117 | MIR | MIR | SINE |
| 70435749 | 70436048 | + | 5.3 | 2 | 305 | -6 | AluY | Alu | SINE |
| 70436049 | 70436155 | + | 27.9 | 146 | 247 | -15 | MIR | MIR | SINE |

  

|  |  |  |  |  |  |  |  |  |  |
| --- | --- | --- | --- | --- | --- | --- | --- | --- | --- |
| chr5:70437041-70439546 | | | 2504 bp | | |  |  |  |  |
| Genome Start | Genome End | Strand | Div | Repeat Start | Repeat End | Repeat Left | Name | Family | Class |
| 70437041 | 70437617 | + | 16.2 | 4886 | 5489 | -691 | L1MB4 | L1 | LINE |
| 70437618 | 70437711 | + | 15.8 | 1 | 88 | -224 | AluJo | Alu | SINE |
| 70437712 | 70438023 | + | 8.3 | 1 | 312 | 0 | AluSx | Alu | SINE |
| 70438024 | 70438245 | + | 15.8 | 89 | 305 | -7 | AluJo | Alu | SINE |
| 70438248 | 70438543 | + | 14.2 | 1 | 296 | -16 | AluSx | Alu | SINE |
| 70438544 | 70439084 | + | 15.2 | 5490 | 5999 | -181 | L1MB4 | L1 | LINE |
| 70439085 | 70439396 | - | 10.0 | 1 | 312 | 0 | AluSx | Alu | SINE |
| 70439397 | 70439546 | + | 15.2 | 6000 | 6177 | -3 | L1MB4 | L1 | LINE |

  

|  |  |  |  |  |  |  |  |  |  |
| --- | --- | --- | --- | --- | --- | --- | --- | --- | --- |
| chr5:70449795-70450829 | | | 1035 bp | | |  |  |  |  |
| Genome Start | Genome End | Strand | Div | Repeat Start | Repeat End | Repeat Left | Name | Family | Class |
| 70449795 | 70450407 | + | 25.1 | 5465 | 6085 | -155 | L1ME3B | L1 | LINE |
| 70450408 | 70450714 | - | 11.8 | 1 | 309 | -3 | AluSx | Alu | SINE |
| 70450715 | 70450829 | + | 25.1 | 6086 | 6202 | -38 | L1ME3B | L1 | LINE |

  

|  |  |  |  |  |  |  |  |  |  |
| --- | --- | --- | --- | --- | --- | --- | --- | --- | --- |
| chr5:70456853-70457394 | | | 494 bp | | |  |  |  |  |
| Genome Start | Genome End | Strand | Div | Repeat Start | Repeat End | Repeat Left | Name | Family | Class |
| 70456853 | 70456996 | + | 23.2 | 1 | 148 | -363 | MLT1J | MaLR | LTR |
| 70457045 | 70457333 | - | 8.7 | 1 | 288 | -25 | AluSq | Alu | SINE |
| 70457334 | 70457394 | + | 23.2 | 149 | 200 | -311 | MLT1J | MaLR | LTR |

  

|  |  |  |  |  |  |  |  |  |  |
| --- | --- | --- | --- | --- | --- | --- | --- | --- | --- |
| chr5:70470628-70471497 | | | 861 bp | | |  |  |  |  |
| Genome Start | Genome End | Strand | Div | Repeat Start | Repeat End | Repeat Left | Name | Family | Class |
| 70470628 | 70470787 | - | 21.2 | 1319 | 1481 | -8 | MLT1F2-int | MaLR | LTR |
| 70470794 | 70471202 | - | 10.1 | 5763 | 6168 | 0 | L1PA10 | L1 | LINE |
| 70471206 | 70471497 | - | 26.0 | 982 | 1276 | -213 | MLT1F2-int | MaLR | LTR |

  

|  |  |  |  |  |  |  |  |  |  |
| --- | --- | --- | --- | --- | --- | --- | --- | --- | --- |
| chr5:70479667-70480589 | | | 923 bp | | |  |  |  |  |
| Genome Start | Genome End | Strand | Div | Repeat Start | Repeat End | Repeat Left | Name | Family | Class |
| 70479667 | 70480004 | - | 9.8 | 2394 | 2732 | -3414 | L1PREC2 | L1 | LINE |
| 70480005 | 70480315 | - | 9.3 | 1 | 300 | -9 | AluSc | Alu | SINE |
| 70480316 | 70480589 | - | 9.8 | 2118 | 2393 | -3753 | L1PREC2 | L1 | LINE |

  

|  |  |  |  |  |  |  |  |  |  |
| --- | --- | --- | --- | --- | --- | --- | --- | --- | --- |
| chr5:70489404-70494918 | | | 5485 bp | | |  |  |  |  |
| Genome Start | Genome End | Strand | Div | Repeat Start | Repeat End | Repeat Left | Name | Family | Class |
| 70489404 | 70489603 | - | 19.5 | 2384 | 2601 | -4032 | L1MDa | L1 | LINE |
| 70489615 | 70490472 | - | 16.9 | 339 | 1225 | -6 | Tigger3b | MER2\_type | DNA |
| 70490473 | 70490727 | - | 5.9 | 47 | 301 | -10 | AluY | Alu | SINE |
| 70490728 | 70490844 | - | 16.9 | 225 | 338 | -893 | Tigger3b | MER2\_type | DNA |
| 70490845 | 70491223 | - | 14.7 | 1 | 381 | 0 | THE1D | MaLR | LTR |
| 70491224 | 70492720 | - | 14.3 | 1 | 1580 | 0 | THE1D-int | MaLR | LTR |
| 70492723 | 70493093 | - | 15.4 | 1 | 379 | -2 | THE1D | MaLR | LTR |
| 70493094 | 70493320 | - | 18.5 | 1 | 229 | -1002 | Tigger3b | MER2\_type | DNA |
| 70493338 | 70494482 | - | 27.1 | 1161 | 2359 | -4274 | L1MDa | L1 | LINE |
| 70494483 | 70494793 | - | 11.9 | 1 | 312 | 0 | AluSx | Alu | SINE |
| 70494794 | 70494918 | - | 27.1 | 1036 | 1160 | -5473 | L1MDa | L1 | LINE |

  

|  |  |  |  |  |  |  |  |  |  |
| --- | --- | --- | --- | --- | --- | --- | --- | --- | --- |
| chr5:70530720-70531311 | | | 592 bp | | |  |  |  |  |
| Genome Start | Genome End | Strand | Div | Repeat Start | Repeat End | Repeat Left | Name | Family | Class |
| 70530720 | 70530876 | - | 19.2 | 136 | 291 | -21 | AluJb | Alu | SINE |
| 70530877 | 70531176 | - | 10.1 | 1 | 298 | -12 | AluSg | Alu | SINE |
| 70531177 | 70531311 | - | 19.2 | 2 | 135 | -177 | AluJb | Alu | SINE |

  

|  |  |  |  |  |  |  |  |  |  |
| --- | --- | --- | --- | --- | --- | --- | --- | --- | --- |
| chr5:70536795-70537392 | | | 598 bp | | |  |  |  |  |
| Genome Start | Genome End | Strand | Div | Repeat Start | Repeat End | Repeat Left | Name | Family | Class |
| 70536795 | 70536917 | + | 17.9 | 2 | 137 | -175 | AluJo | Alu | SINE |
| 70536918 | 70537211 | + | 12.3 | 1 | 289 | -21 | AluSg | Alu | SINE |
| 70537212 | 70537392 | + | 17.9 | 138 | 305 | -7 | AluJo | Alu | SINE |

  

|  |  |  |  |  |  |  |  |  |  |
| --- | --- | --- | --- | --- | --- | --- | --- | --- | --- |
| chr5:70538754-70539323 | | | 570 bp | | |  |  |  |  |
| Genome Start | Genome End | Strand | Div | Repeat Start | Repeat End | Repeat Left | Name | Family | Class |
| 70538754 | 70538881 | + | 15.9 | 1 | 125 | -187 | AluJo | Alu | SINE |
| 70538882 | 70539172 | - | 12.7 | 1 | 294 | -18 | AluSx | Alu | SINE |
| 70539173 | 70539323 | + | 15.9 | 126 | 275 | -37 | AluJo | Alu | SINE |

  

|  |  |  |  |  |  |  |  |  |  |
| --- | --- | --- | --- | --- | --- | --- | --- | --- | --- |
| chr5:70541755-70542664 | | | 910 bp | | |  |  |  |  |
| Genome Start | Genome End | Strand | Div | Repeat Start | Repeat End | Repeat Left | Name | Family | Class |
| 70541755 | 70541903 | + | 21.4 | 1 | 178 | -475 | MER82 | MER2\_type | DNA |
| 70541904 | 70542209 | + | 11.6 | 1 | 293 | -19 | AluSx | Alu | SINE |
| 70542210 | 70542664 | + | 21.4 | 179 | 653 | 0 | MER82 | MER2\_type | DNA |

  

|  |  |  |  |  |  |  |  |  |  |
| --- | --- | --- | --- | --- | --- | --- | --- | --- | --- |
| chr5:70549962-70550964 | | | 1003 bp | | |  |  |  |  |
| Genome Start | Genome End | Strand | Div | Repeat Start | Repeat End | Repeat Left | Name | Family | Class |
| 70549962 | 70550005 | + | 22.8 | 1 | 44 | -330 | MLT1A | MaLR | LTR |
| 70550006 | 70550314 | + | 6.5 | 1 | 306 | -5 | AluY | Alu | SINE |
| 70550315 | 70550593 | + | 22.8 | 45 | 308 | -66 | MLT1A | MaLR | LTR |
| 70550594 | 70550896 | - | 15.2 | 1 | 312 | 0 | AluSx | Alu | SINE |
| 70550897 | 70550964 | + | 22.8 | 309 | 374 | 0 | MLT1A | MaLR | LTR |

  

|  |  |  |  |  |  |  |  |  |  |
| --- | --- | --- | --- | --- | --- | --- | --- | --- | --- |
| chr5:70550985-70551481 | | | 497 bp | | |  |  |  |  |
| Genome Start | Genome End | Strand | Div | Repeat Start | Repeat End | Repeat Left | Name | Family | Class |
| 70550985 | 70551112 | + | 19.6 | 1 | 129 | -80 | MER3 | MER1\_type | DNA |
| 70551113 | 70551416 | - | 7.6 | 1 | 304 | -7 | AluY | Alu | SINE |
| 70551417 | 70551481 | + | 19.6 | 130 | 208 | -1 | MER3 | MER1\_type | DNA |

  

|  |  |  |  |  |  |  |  |  |  |
| --- | --- | --- | --- | --- | --- | --- | --- | --- | --- |
| chr5:70554824-70555421 | | | 598 bp | | |  |  |  |  |
| Genome Start | Genome End | Strand | Div | Repeat Start | Repeat End | Repeat Left | Name | Family | Class |
| 70554824 | 70554946 | + | 17.9 | 2 | 137 | -175 | AluJo | Alu | SINE |
| 70554947 | 70555240 | + | 12.3 | 1 | 289 | -21 | AluSg | Alu | SINE |
| 70555241 | 70555421 | + | 17.9 | 138 | 305 | -7 | AluJo | Alu | SINE |

  

|  |  |  |  |  |  |  |  |  |  |
| --- | --- | --- | --- | --- | --- | --- | --- | --- | --- |
| chr5:70556783-70557385 | | | 603 bp | | |  |  |  |  |
| Genome Start | Genome End | Strand | Div | Repeat Start | Repeat End | Repeat Left | Name | Family | Class |
| 70556783 | 70556910 | + | 17.1 | 1 | 125 | -187 | AluJo | Alu | SINE |
| 70556911 | 70557200 | - | 13.4 | 1 | 293 | -19 | AluSx | Alu | SINE |
| 70557201 | 70557385 | + | 17.1 | 126 | 311 | -1 | AluJo | Alu | SINE |

  

|  |  |  |  |  |  |  |  |  |  |
| --- | --- | --- | --- | --- | --- | --- | --- | --- | --- |
| chr5:70559780-70560690 | | | 911 bp | | |  |  |  |  |
| Genome Start | Genome End | Strand | Div | Repeat Start | Repeat End | Repeat Left | Name | Family | Class |
| 70559780 | 70559928 | + | 21.4 | 1 | 178 | -475 | MER82 | MER2\_type | DNA |
| 70559929 | 70560235 | + | 11.6 | 1 | 293 | -19 | AluSx | Alu | SINE |
| 70560236 | 70560690 | + | 21.4 | 179 | 653 | 0 | MER82 | MER2\_type | DNA |

  

|  |  |  |  |  |  |  |  |  |  |
| --- | --- | --- | --- | --- | --- | --- | --- | --- | --- |
| chr5:70567983-70568988 | | | 1006 bp | | |  |  |  |  |
| Genome Start | Genome End | Strand | Div | Repeat Start | Repeat End | Repeat Left | Name | Family | Class |
| 70567983 | 70568026 | + | 22.5 | 1 | 41 | -333 | MLT1A | MaLR | LTR |
| 70568027 | 70568338 | + | 6.5 | 1 | 309 | -2 | AluY | Alu | SINE |
| 70568339 | 70568617 | + | 22.5 | 42 | 308 | -66 | MLT1A | MaLR | LTR |
| 70568618 | 70568920 | - | 15.2 | 1 | 312 | 0 | AluSx | Alu | SINE |
| 70568921 | 70568988 | + | 22.5 | 309 | 374 | 0 | MLT1A | MaLR | LTR |

  

|  |  |  |  |  |  |  |  |  |  |
| --- | --- | --- | --- | --- | --- | --- | --- | --- | --- |
| chr5:70569010-70569506 | | | 497 bp | | |  |  |  |  |
| Genome Start | Genome End | Strand | Div | Repeat Start | Repeat End | Repeat Left | Name | Family | Class |
| 70569010 | 70569144 | + | 18.3 | 1 | 136 | -73 | MER3 | MER1\_type | DNA |
| 70569145 | 70569441 | - | 7.1 | 1 | 297 | -14 | AluY | Alu | SINE |
| 70569442 | 70569506 | + | 18.3 | 137 | 209 | 0 | MER3 | MER1\_type | DNA |

  

|  |  |  |  |  |  |  |  |  |  |
| --- | --- | --- | --- | --- | --- | --- | --- | --- | --- |
| chr5:70570204-70570864 | | | 661 bp | | |  |  |  |  |
| Genome Start | Genome End | Strand | Div | Repeat Start | Repeat End | Repeat Left | Name | Family | Class |
| 70570204 | 70570435 | - | 18.4 | 127 | 365 | 0 | MLT1A0 | MaLR | LTR |
| 70570436 | 70570750 | + | 13.0 | 1 | 307 | -3 | AluSg | Alu | SINE |
| 70570751 | 70570864 | - | 18.4 | 43 | 126 | -239 | MLT1A0 | MaLR | LTR |

  

|  |  |  |  |  |  |  |  |  |  |
| --- | --- | --- | --- | --- | --- | --- | --- | --- | --- |
| chr5:70575479-70576698 | | | 1220 bp | | |  |  |  |  |
| Genome Start | Genome End | Strand | Div | Repeat Start | Repeat End | Repeat Left | Name | Family | Class |
| 70575479 | 70575879 | + | 23.0 | 2528 | 2897 | -3948 | L1M2 | L1 | LINE |
| 70575880 | 70576182 | + | 12.9 | 1 | 303 | -7 | AluSg | Alu | SINE |
| 70576183 | 70576698 | + | 21.9 | 2898 | 3392 | -3453 | L1M2 | L1 | LINE |

  

|  |  |  |  |  |  |  |  |  |  |
| --- | --- | --- | --- | --- | --- | --- | --- | --- | --- |
| chr5:70577387-70578685 | | | 1299 bp | | |  |  |  |  |
| Genome Start | Genome End | Strand | Div | Repeat Start | Repeat End | Repeat Left | Name | Family | Class |
| 70577387 | 70577812 | + | 23.0 | 6152 | 6586 | -1199 | L1MC3 | L1 | LINE |
| 70577813 | 70578195 | + | 17.3 | 1 | 428 | 0 | MSTA | MaLR | LTR |
| 70578196 | 70578685 | + | 23.0 | 6587 | 7119 | -666 | L1MC3 | L1 | LINE |

  

|  |  |  |  |  |  |  |  |  |  |
| --- | --- | --- | --- | --- | --- | --- | --- | --- | --- |
| chr5:70601153-70602105 | | | 784 bp | | |  |  |  |  |
| Genome Start | Genome End | Strand | Div | Repeat Start | Repeat End | Repeat Left | Name | Family | Class |
| 70601153 | 70601368 | + | 25.2 | 1 | 225 | -280 | MLT1D | MaLR | LTR |
| 70601410 | 70601456 | + | 17.0 | 1 | 47 | -327 | MLT1A | MaLR | LTR |
| 70601511 | 70601613 | + | 30.1 | 58 | 160 | -214 | MLT1A | MaLR | LTR |
| 70601688 | 70601876 | + | 18.5 | 189 | 374 | 0 | MLT1A | MaLR | LTR |
| 70601877 | 70602105 | + | 27.8 | 275 | 505 | 0 | MLT1D | MaLR | LTR |

  

|  |  |  |  |  |  |  |  |  |  |
| --- | --- | --- | --- | --- | --- | --- | --- | --- | --- |
| chr5:70603396-70606087 | | | 2681 bp | | |  |  |  |  |
| Genome Start | Genome End | Strand | Div | Repeat Start | Repeat End | Repeat Left | Name | Family | Class |
| 70603396 | 70603618 | + | 18.4 | 1 | 238 | -128 | Tigger5a | MER2\_type | DNA |
| 70603629 | 70606016 | - | 7.0 | 3742 | 6150 | -4 | L1PA6 | L1 | LINE |
| 70606018 | 70606087 | + | 20.0 | 356 | 434 | -15 | Tigger5a | MER2\_type | DNA |

  

|  |  |  |  |  |  |  |  |  |  |
| --- | --- | --- | --- | --- | --- | --- | --- | --- | --- |
| chr5:70614114-70620445 | | | 6198 bp | | |  |  |  |  |
| Genome Start | Genome End | Strand | Div | Repeat Start | Repeat End | Repeat Left | Name | Family | Class |
| 70614114 | 70614320 | + | 20.8 | 13 | 235 | -6268 | L1PA16 | L1 | LINE |
| 70614320 | 70618176 | + | 16.9 | 947 | 4819 | -1664 | L1PA16 | L1 | LINE |
| 70618177 | 70618437 | + | 18.4 | 2 | 286 | -16 | AluJo | Alu | SINE |
| 70618573 | 70619400 | + | 16.7 | 4820 | 5660 | -823 | L1PA16 | L1 | LINE |
| 70619401 | 70619714 | + | 8.7 | 5876 | 6167 | -1 | L1PA10 | L1 | LINE |
| 70619715 | 70620445 | + | 14.3 | 5661 | 6398 | -105 | L1PA16 | L1 | LINE |

  

|  |  |  |  |  |  |  |  |  |  |
| --- | --- | --- | --- | --- | --- | --- | --- | --- | --- |
| chr5:70630381-70633025 | | | 2412 bp | | |  |  |  |  |
| Genome Start | Genome End | Strand | Div | Repeat Start | Repeat End | Repeat Left | Name | Family | Class |
| 70630381 | 70631010 | + | 30.7 | 3855 | 4522 | -1624 | L1ME2 | L1 | LINE |
| 70631011 | 70631429 | + | 14.5 | 5 | 428 | 0 | MSTA | MaLR | LTR |
| 70631430 | 70631968 | + | 30.7 | 4523 | 5064 | -1082 | L1ME2 | L1 | LINE |
| 70632184 | 70632557 | + | 24.2 | 5329 | 5758 | -406 | L1ME2 | L1 | LINE |
| 70632563 | 70632748 | - | 22.5 | 1 | 213 | -6 | MER20 | MER1\_type | DNA |
| 70632762 | 70633025 | + | 27.1 | 5768 | 6039 | -125 | L1ME2 | L1 | LINE |

  

|  |  |  |  |  |  |  |  |  |  |
| --- | --- | --- | --- | --- | --- | --- | --- | --- | --- |
| chr5:70650747-70654791 | | | 4013 bp | | |  |  |  |  |
| Genome Start | Genome End | Strand | Div | Repeat Start | Repeat End | Repeat Left | Name | Family | Class |
| 70650747 | 70651344 | - | 22.1 | 3317 | 3934 | -2705 | MER57A-int | ERV1 | LTR |
| 70651355 | 70652224 | - | 10.8 | 5275 | 6165 | 0 | L1PA15 | L1 | LINE |
| 70652227 | 70652962 | - | 27.1 | 8 | 767 | -6567 | L1P4b | L1 | LINE |
| 70652973 | 70653088 | - | 18.3 | 3206 | 3321 | -3318 | MER57A-int | ERV1 | LTR |
| 70653087 | 70654537 | - | 25.6 | 1150 | 2573 | -2550 | MER57A-int | ERV1 | LTR |
| 70654548 | 70654690 | - | 20.7 | 124 | 292 | -266 | MER34C\_ | ERV1 | LTR |
| 70654693 | 70654791 | - | 19.8 | 891 | 990 | -4133 | MER57A-int | ERV1 | LTR |

  

|  |  |  |  |  |  |  |  |  |  |
| --- | --- | --- | --- | --- | --- | --- | --- | --- | --- |
| chr5:70655124-70655914 | | | 791 bp | | |  |  |  |  |
| Genome Start | Genome End | Strand | Div | Repeat Start | Repeat End | Repeat Left | Name | Family | Class |
| 70655124 | 70655248 | - | 30.0 | 325 | 445 | 0 | MER65A | ERV1 | LTR |
| 70655249 | 70655622 | - | 13.2 | 1 | 428 | 0 | MSTA | MaLR | LTR |
| 70655623 | 70655914 | - | 30.0 | 1 | 324 | -121 | MER65A | ERV1 | LTR |

  

|  |  |  |  |  |  |  |  |  |  |
| --- | --- | --- | --- | --- | --- | --- | --- | --- | --- |
| chr5:70662481-70662892 | | | 412 bp | | |  |  |  |  |
| Genome Start | Genome End | Strand | Div | Repeat Start | Repeat End | Repeat Left | Name | Family | Class |
| 70662481 | 70662557 | - | 32.0 | 107 | 184 | -84 | MIRb | MIR | SINE |
| 70662558 | 70662861 | + | 13.8 | 1 | 306 | -6 | AluSx | Alu | SINE |
| 70662862 | 70662892 | - | 32.0 | 79 | 106 | -162 | MIRb | MIR | SINE |

  

|  |  |  |  |  |  |  |  |  |  |
| --- | --- | --- | --- | --- | --- | --- | --- | --- | --- |
| chr5:70700522-70701339 | | | 818 bp | | |  |  |  |  |
| Genome Start | Genome End | Strand | Div | Repeat Start | Repeat End | Repeat Left | Name | Family | Class |
| 70700522 | 70700552 | - | 37.0 | 3392 | 3419 | 0 | L2 | L2 | LINE |
| 70700553 | 70701010 | - | 20.8 | 1 | 461 | 0 | MER65C | ERV1 | LTR |
| 70701011 | 70701339 | - | 37.0 | 3056 | 3391 | -28 | L2 | L2 | LINE |

  

|  |  |  |  |  |  |  |  |  |  |
| --- | --- | --- | --- | --- | --- | --- | --- | --- | --- |
| chr5:74593252-74594048 | | | 734 bp | | |  |  |  |  |
| Genome Start | Genome End | Strand | Div | Repeat Start | Repeat End | Repeat Left | Name | Family | Class |
| 74593252 | 74593440 | + | 20.1 | 1 | 200 | -861 | MER63 | AcHobo | DNA |
| 74593504 | 74593547 | + | 17.3 | 966 | 1017 | -45 | MER63 | AcHobo | DNA |
| 74593548 | 74594009 | + | 11.5 | 1 | 485 | 0 | MER31A | ERV1 | LTR |
| 74594010 | 74594048 | + | 17.3 | 1018 | 1062 | 0 | MER63 | AcHobo | DNA |

  

|  |  |  |  |  |  |  |  |  |  |
| --- | --- | --- | --- | --- | --- | --- | --- | --- | --- |
| chr5:79958012-79958934 | | | 794 bp | | |  |  |  |  |
| Genome Start | Genome End | Strand | Div | Repeat Start | Repeat End | Repeat Left | Name | Family | Class |
| 79958012 | 79958440 | - | 26.4 | 2012 | 2507 | 0 | HAL1 | L1 | LINE |
| 79958512 | 79958632 | - | 19.8 | 1 | 122 | -11 | FLAM\_A | Alu | SINE |
| 79958691 | 79958934 | - | 21.3 | 1522 | 1781 | -726 | HAL1 | L1 | LINE |

  

|  |  |  |  |  |  |  |  |  |  |
| --- | --- | --- | --- | --- | --- | --- | --- | --- | --- |
| chr5:85603783-85605337 | | | 1493 bp | | |  |  |  |  |
| Genome Start | Genome End | Strand | Div | Repeat Start | Repeat End | Repeat Left | Name | Family | Class |
| 85603783 | 85604183 | + | 24.3 | 5184 | 5610 | -554 | L1ME1 | L1 | LINE |
| 85604240 | 85604970 | - | 6.7 | 22 | 773 | -7 | LTR17 | ERV1 | LTR |
| 85604977 | 85605337 | + | 31.3 | 5776 | 6160 | -6 | L1ME1 | L1 | LINE |

  

|  |  |  |  |  |  |  |  |  |  |
| --- | --- | --- | --- | --- | --- | --- | --- | --- | --- |
| chr5:98755925-98756676 | | | 752 bp | | |  |  |  |  |
| Genome Start | Genome End | Strand | Div | Repeat Start | Repeat End | Repeat Left | Name | Family | Class |
| 98755925 | 98756088 | - | 24.8 | 7739 | 7907 | -54 | L1MC5 | L1 | LINE |
| 98756089 | 98756447 | - | 11.2 | 1 | 364 | 0 | THE1B | MaLR | LTR |
| 98756448 | 98756676 | - | 24.8 | 7458 | 7738 | -223 | L1MC5 | L1 | LINE |

  

|  |  |  |  |  |  |  |  |  |  |
| --- | --- | --- | --- | --- | --- | --- | --- | --- | --- |
| chr5:98765455-98766885 | | | 1383 bp | | |  |  |  |  |
| Genome Start | Genome End | Strand | Div | Repeat Start | Repeat End | Repeat Left | Name | Family | Class |
| 98765455 | 98765815 | - | 28.0 | 409 | 772 | -1 | LTR41 | ERVL | LTR |
| 98765833 | 98766495 | - | 15.3 | 9 | 705 | 0 | MER44D | MER2\_type | DNA |
| 98766527 | 98766885 | - | 29.9 | 3 | 440 | -333 | LTR41 | ERVL | LTR |

  

|  |  |  |  |  |  |  |  |  |  |
| --- | --- | --- | --- | --- | --- | --- | --- | --- | --- |
| chr5:98773103-98775372 | | | 2232 bp | | |  |  |  |  |
| Genome Start | Genome End | Strand | Div | Repeat Start | Repeat End | Repeat Left | Name | Family | Class |
| 98773103 | 98773441 | - | 23.9 | 5825 | 6147 | -19 | L1ME1 | L1 | LINE |
| 98773442 | 98774433 | - | 9.0 | 1 | 1254 | 0 | LTR12D | ERV1 | LTR |
| 98774434 | 98774856 | - | 23.9 | 5385 | 5824 | -342 | L1ME1 | L1 | LINE |
| 98774895 | 98775001 | - | 20.4 | 4610 | 4710 | -1436 | L1ME1 | L1 | LINE |
| 98775002 | 98775309 | - | 17.2 | 1 | 308 | 0 | AluJb | Alu | SINE |
| 98775310 | 98775372 | - | 20.4 | 4553 | 4609 | -1537 | L1ME1 | L1 | LINE |

  

|  |  |  |  |  |  |  |  |  |  |
| --- | --- | --- | --- | --- | --- | --- | --- | --- | --- |
| chr5:98778028-98779130 | | | 1103 bp | | |  |  |  |  |
| Genome Start | Genome End | Strand | Div | Repeat Start | Repeat End | Repeat Left | Name | Family | Class |
| 98778028 | 98778155 | - | 27.4 | 6967 | 7097 | -864 | L1MC5 | L1 | LINE |
| 98778156 | 98778844 | - | 8.7 | 5476 | 6162 | -1 | L1PA13 | L1 | LINE |
| 98778845 | 98779130 | - | 27.4 | 6694 | 6966 | -995 | L1MC5 | L1 | LINE |

  

|  |  |  |  |  |  |  |  |  |  |
| --- | --- | --- | --- | --- | --- | --- | --- | --- | --- |
| chr5:98794367-98795366 | | | 1010 bp | | |  |  |  |  |
| Genome Start | Genome End | Strand | Div | Repeat Start | Repeat End | Repeat Left | Name | Family | Class |
| 98794367 | 98794718 | - | 17.2 | 5936 | 6294 | -6 | L1MA4 | L1 | LINE |
| 98794715 | 98795256 | - | 15.6 | 5604 | 6159 | -6 | L1PA15 | L1 | LINE |
| 98795258 | 98795320 | - | 17.5 | 6070 | 6132 | -33 | L1PA15 | L1 | LINE |
| 98795314 | 98795366 | - | 9.8 | 5652 | 5702 | -602 | L1MA4 | L1 | LINE |

  

|  |  |  |  |  |  |  |  |  |  |
| --- | --- | --- | --- | --- | --- | --- | --- | --- | --- |
| chr5:98864567-98867928 | | | 3376 bp | | |  |  |  |  |
| Genome Start | Genome End | Strand | Div | Repeat Start | Repeat End | Repeat Left | Name | Family | Class |
| 98864567 | 98864916 | - | 29.5 | 7096 | 7439 | -603 | L1MC4 | L1 | LINE |
| 98864908 | 98864970 | - | 7.9 | 3670 | 3732 | -2414 | L1PA5 | L1 | LINE |
| 98864967 | 98867382 | + | 5.6 | 3726 | 6154 | 0 | L1PA5 | L1 | LINE |
| 98867382 | 98867928 | - | 23.9 | 6625 | 7118 | -843 | L1MC4 | L1 | LINE |

  

|  |  |  |  |  |  |  |  |  |  |
| --- | --- | --- | --- | --- | --- | --- | --- | --- | --- |
| chr5:98871469-98872171 | | | 703 bp | | |  |  |  |  |
| Genome Start | Genome End | Strand | Div | Repeat Start | Repeat End | Repeat Left | Name | Family | Class |
| 98871469 | 98871693 | - | 35.1 | 3485 | 3736 | -753 | L3 | CR1 | LINE |
| 98871694 | 98871985 | - | 10.3 | 5 | 296 | -17 | AluSq | Alu | SINE |
| 98871986 | 98872171 | - | 35.1 | 3333 | 3484 | -1005 | L3 | CR1 | LINE |

  

|  |  |  |  |  |  |  |  |  |  |
| --- | --- | --- | --- | --- | --- | --- | --- | --- | --- |
| chr5:98879097-98879556 | | | 460 bp | | |  |  |  |  |
| Genome Start | Genome End | Strand | Div | Repeat Start | Repeat End | Repeat Left | Name | Family | Class |
| 98879097 | 98879140 | - | 28.0 | 123 | 161 | -47 | MIR3 | MIR | SINE |
| 98879141 | 98879437 | + | 8.1 | 1 | 297 | -13 | AluSg | Alu | SINE |
| 98879438 | 98879556 | - | 28.0 | 12 | 122 | -86 | MIR3 | MIR | SINE |

  

|  |  |  |  |  |  |  |  |  |  |
| --- | --- | --- | --- | --- | --- | --- | --- | --- | --- |
| chr5:98882502-98883020 | | | 519 bp | | |  |  |  |  |
| Genome Start | Genome End | Strand | Div | Repeat Start | Repeat End | Repeat Left | Name | Family | Class |
| 98882502 | 98882566 | - | 18.9 | 146 | 209 | 0 | MER3 | MER1\_type | DNA |
| 98882567 | 98882876 | + | 7.4 | 1 | 310 | -1 | AluY | Alu | SINE |
| 98882877 | 98883020 | - | 18.9 | 1 | 145 | -64 | MER3 | MER1\_type | DNA |

  

|  |  |  |  |  |  |  |  |  |  |
| --- | --- | --- | --- | --- | --- | --- | --- | --- | --- |
| chr5:98883041-98884026 | | | 986 bp | | |  |  |  |  |
| Genome Start | Genome End | Strand | Div | Repeat Start | Repeat End | Repeat Left | Name | Family | Class |
| 98883041 | 98883108 | - | 22.0 | 309 | 374 | 0 | MLT1A | MaLR | LTR |
| 98883109 | 98883406 | + | 14.8 | 1 | 307 | -5 | AluJb | Alu | SINE |
| 98883407 | 98883683 | - | 22.0 | 42 | 308 | -66 | MLT1A | MaLR | LTR |
| 98883684 | 98883982 | - | 9.2 | 1 | 296 | -15 | AluY | Alu | SINE |
| 98883983 | 98884026 | - | 22.0 | 1 | 41 | -333 | MLT1A | MaLR | LTR |

  

|  |  |  |  |  |  |  |  |  |  |
| --- | --- | --- | --- | --- | --- | --- | --- | --- | --- |
| chr5:98893292-98894201 | | | 910 bp | | |  |  |  |  |
| Genome Start | Genome End | Strand | Div | Repeat Start | Repeat End | Repeat Left | Name | Family | Class |
| 98893292 | 98893748 | - | 21.9 | 179 | 653 | 0 | MER82 | MER2\_type | DNA |
| 98893749 | 98894052 | - | 11.9 | 1 | 293 | -19 | AluSx | Alu | SINE |
| 98894053 | 98894201 | - | 21.9 | 1 | 178 | -475 | MER82 | MER2\_type | DNA |

  

|  |  |  |  |  |  |  |  |  |  |
| --- | --- | --- | --- | --- | --- | --- | --- | --- | --- |
| chr5:98896641-98897206 | | | 566 bp | | |  |  |  |  |
| Genome Start | Genome End | Strand | Div | Repeat Start | Repeat End | Repeat Left | Name | Family | Class |
| 98896641 | 98896791 | - | 16.7 | 126 | 275 | -37 | AluJo | Alu | SINE |
| 98896792 | 98897076 | + | 12.6 | 1 | 288 | -24 | AluSx | Alu | SINE |
| 98897077 | 98897206 | - | 16.7 | 1 | 125 | -187 | AluJo | Alu | SINE |

  

|  |  |  |  |  |  |  |  |  |  |
| --- | --- | --- | --- | --- | --- | --- | --- | --- | --- |
| chr5:99430603-99431168 | | | 566 bp | | |  |  |  |  |
| Genome Start | Genome End | Strand | Div | Repeat Start | Repeat End | Repeat Left | Name | Family | Class |
| 99430603 | 99430732 | + | 16.7 | 1 | 125 | -187 | AluJo | Alu | SINE |
| 99430733 | 99431017 | - | 13.0 | 1 | 288 | -24 | AluSx | Alu | SINE |
| 99431018 | 99431168 | + | 16.7 | 126 | 275 | -37 | AluJo | Alu | SINE |

  

|  |  |  |  |  |  |  |  |  |  |
| --- | --- | --- | --- | --- | --- | --- | --- | --- | --- |
| chr5:99721585-99722427 | | | 843 bp | | |  |  |  |  |
| Genome Start | Genome End | Strand | Div | Repeat Start | Repeat End | Repeat Left | Name | Family | Class |
| 99721585 | 99721770 | + | 21.8 | 1 | 191 | -330 | MER41C | ERV1 | LTR |
| 99721771 | 99722079 | - | 9.7 | 3 | 311 | 0 | AluY | Alu | SINE |
| 99722080 | 99722427 | + | 21.8 | 192 | 520 | -1 | MER41C | ERV1 | LTR |

  

|  |  |  |  |  |  |  |  |  |  |
| --- | --- | --- | --- | --- | --- | --- | --- | --- | --- |
| chr5:99740231-99740819 | | | 589 bp | | |  |  |  |  |
| Genome Start | Genome End | Strand | Div | Repeat Start | Repeat End | Repeat Left | Name | Family | Class |
| 99740231 | 99740349 | + | 18.1 | 2 | 137 | -175 | AluJo | Alu | SINE |
| 99740350 | 99740625 | + | 14.0 | 1 | 280 | -30 | AluSg | Alu | SINE |
| 99740626 | 99740819 | + | 18.1 | 138 | 305 | -7 | AluJo | Alu | SINE |

  

|  |  |  |  |  |  |  |  |  |  |
| --- | --- | --- | --- | --- | --- | --- | --- | --- | --- |
| chr5:99744031-99744596 | | | 566 bp | | |  |  |  |  |
| Genome Start | Genome End | Strand | Div | Repeat Start | Repeat End | Repeat Left | Name | Family | Class |
| 99744031 | 99744160 | + | 16.3 | 1 | 125 | -187 | AluJo | Alu | SINE |
| 99744161 | 99744445 | - | 13.0 | 1 | 288 | -24 | AluSx | Alu | SINE |
| 99744446 | 99744596 | + | 16.3 | 126 | 275 | -37 | AluJo | Alu | SINE |

  

|  |  |  |  |  |  |  |  |  |  |
| --- | --- | --- | --- | --- | --- | --- | --- | --- | --- |
| chr5:99759354-99759863 | | | 510 bp | | |  |  |  |  |
| Genome Start | Genome End | Strand | Div | Repeat Start | Repeat End | Repeat Left | Name | Family | Class |
| 99759354 | 99759487 | + | 18.8 | 1 | 135 | -74 | MER3 | MER1\_type | DNA |
| 99759488 | 99759798 | - | 7.1 | 1 | 309 | -2 | AluY | Alu | SINE |
| 99759799 | 99759863 | + | 18.8 | 136 | 209 | 0 | MER3 | MER1\_type | DNA |

  

|  |  |  |  |  |  |  |  |  |  |
| --- | --- | --- | --- | --- | --- | --- | --- | --- | --- |
| chr5:99762813-99763269 | | | 457 bp | | |  |  |  |  |
| Genome Start | Genome End | Strand | Div | Repeat Start | Repeat End | Repeat Left | Name | Family | Class |
| 99762813 | 99762931 | + | 27.3 | 12 | 122 | -86 | MIR3 | MIR | SINE |
| 99762932 | 99763225 | - | 9.5 | 1 | 294 | -16 | AluSg | Alu | SINE |
| 99763226 | 99763269 | + | 27.3 | 123 | 161 | -47 | MIR3 | MIR | SINE |

  

|  |  |  |  |  |  |  |  |  |  |
| --- | --- | --- | --- | --- | --- | --- | --- | --- | --- |
| chr5:122040644-122041250 | | | 607 bp | | |  |  |  |  |
| Genome Start | Genome End | Strand | Div | Repeat Start | Repeat End | Repeat Left | Name | Family | Class |
| 122040644 | 122040803 | - | 17.5 | 136 | 291 | -21 | AluJb | Alu | SINE |
| 122040804 | 122041114 | - | 12.9 | 1 | 311 | -1 | AluSx | Alu | SINE |
| 122041115 | 122041250 | - | 17.5 | 1 | 135 | -177 | AluJb | Alu | SINE |

  

|  |  |  |  |  |  |  |  |  |  |
| --- | --- | --- | --- | --- | --- | --- | --- | --- | --- |
| chr5:170189631-170190575 | | | 945 bp | | |  |  |  |  |
| Genome Start | Genome End | Strand | Div | Repeat Start | Repeat End | Repeat Left | Name | Family | Class |
| 170189631 | 170190154 | + | 12.8 | 1 | 521 | -113 | MER41B | ERV1 | LTR |
| 170190155 | 170190456 | - | 12.2 | 1 | 302 | -10 | AluSx | Alu | SINE |
| 170190457 | 170190575 | + | 12.8 | 522 | 634 | 0 | MER41B | ERV1 | LTR |

  

|  |  |  |  |  |  |  |  |  |  |
| --- | --- | --- | --- | --- | --- | --- | --- | --- | --- |
| chr5:175295532-175296465 | | | 934 bp | | |  |  |  |  |
| Genome Start | Genome End | Strand | Div | Repeat Start | Repeat End | Repeat Left | Name | Family | Class |
| 175295532 | 175295762 | + | 18.6 | 7357 | 7619 | -342 | L1MC5 | L1 | LINE |
| 175295763 | 175296056 | + | 14.1 | 2 | 301 | -11 | AluSx | Alu | SINE |
| 175296057 | 175296141 | + | 18.6 | 7620 | 7694 | -267 | L1MC5 | L1 | LINE |
| 175296142 | 175296442 | - | 15.2 | 1 | 298 | -14 | AluJb | Alu | SINE |
| 175296443 | 175296465 | + | 18.6 | 7695 | 7720 | -241 | L1MC5 | L1 | LINE |

  

|  |  |  |  |  |  |  |  |  |  |
| --- | --- | --- | --- | --- | --- | --- | --- | --- | --- |
| chr5:175311877-175312312 | | | 436 bp | | |  |  |  |  |
| Genome Start | Genome End | Strand | Div | Repeat Start | Repeat End | Repeat Left | Name | Family | Class |
| 175311877 | 175311926 | + | 23.9 | 30 | 98 | -91 | MER5A | MER1\_type | DNA |
| 175311927 | 175312233 | + | 9.9 | 14 | 308 | -5 | AluSq | Alu | SINE |
| 175312234 | 175312312 | + | 23.9 | 99 | 188 | -1 | MER5A | MER1\_type | DNA |

  

|  |  |  |  |  |  |  |  |  |  |
| --- | --- | --- | --- | --- | --- | --- | --- | --- | --- |
| chr5:175330747-175332188 | | | 1442 bp | | |  |  |  |  |
| Genome Start | Genome End | Strand | Div | Repeat Start | Repeat End | Repeat Left | Name | Family | Class |
| 175330747 | 175330867 | - | 23.5 | 7926 | 8042 | 0 | L1MC4 | L1 | LINE |
| 175330868 | 175331166 | - | 12.1 | 1 | 308 | -4 | AluSx | Alu | SINE |
| 175331167 | 175331228 | - | 23.5 | 7879 | 7925 | -117 | L1MC4 | L1 | LINE |
| 175331229 | 175331304 | - | 13.4 | 365 | 428 | 0 | MSTA | MaLR | LTR |
| 175331305 | 175331608 | + | 9.2 | 1 | 303 | -8 | AluY | Alu | SINE |
| 175331609 | 175331858 | - | 13.4 | 1 | 364 | -64 | MSTA | MaLR | LTR |
| 175331859 | 175332188 | - | 23.5 | 7549 | 7878 | -164 | L1MC4 | L1 | LINE |

  

|  |  |  |  |  |  |  |  |  |  |
| --- | --- | --- | --- | --- | --- | --- | --- | --- | --- |
| chr5:175338055-175339578 | | | 1524 bp | | |  |  |  |  |
| Genome Start | Genome End | Strand | Div | Repeat Start | Repeat End | Repeat Left | Name | Family | Class |
| 175338055 | 175338574 | + | 13.9 | 1 | 507 | -103 | MER4B | ERV1 | LTR |
| 175338575 | 175339491 | + | 17.6 | 1 | 1020 | 0 | LTR28 | ERV1 | LTR |
| 175339492 | 175339578 | + | 13.9 | 508 | 610 | 0 | MER4B | ERV1 | LTR |

  

|  |  |  |  |  |  |  |  |  |  |
| --- | --- | --- | --- | --- | --- | --- | --- | --- | --- |
| chr5:175342975-175344490 | | | 1378 bp | | |  |  |  |  |
| Genome Start | Genome End | Strand | Div | Repeat Start | Repeat End | Repeat Left | Name | Family | Class |
| 175342975 | 175343508 | - | 21.7 | 395 | 935 | 0 | MER21C | ERV1 | LTR |
| 175343519 | 175344007 | - | 22.2 | 109 | 604 | -15 | LTR31 | ERV1 | LTR |
| 175344136 | 175344490 | - | 17.0 | 1 | 398 | -537 | MER21C | ERV1 | LTR |

  

|  |  |  |  |  |  |  |  |  |  |
| --- | --- | --- | --- | --- | --- | --- | --- | --- | --- |
| chr5:175349143-175349654 | | | 512 bp | | |  |  |  |  |
| Genome Start | Genome End | Strand | Div | Repeat Start | Repeat End | Repeat Left | Name | Family | Class |
| 175349143 | 175349313 | + | 20.5 | 5039 | 5198 | -948 | L1MD | L1 | LINE |
| 175349314 | 175349543 | + | 11.0 | 6060 | 6297 | 0 | L1MA5A | L1 | LINE |
| 175349544 | 175349654 | + | 20.5 | 5199 | 5299 | -847 | L1MD | L1 | LINE |

  

|  |  |  |  |  |  |  |  |  |  |
| --- | --- | --- | --- | --- | --- | --- | --- | --- | --- |
| chr5:175371691-175372566 | | | 816 bp | | |  |  |  |  |
| Genome Start | Genome End | Strand | Div | Repeat Start | Repeat End | Repeat Left | Name | Family | Class |
| 175371691 | 175372026 | + | 25.2 | 4845 | 5187 | -959 | L1ME3 | L1 | LINE |
| 175372027 | 175372341 | + | 10.5 | 1 | 306 | -7 | AluSq | Alu | SINE |
| 175372342 | 175372401 | + | 25.2 | 5188 | 5226 | -920 | L1ME3 | L1 | LINE |
| 175372462 | 175372566 | + | 25.2 | 5227 | 5335 | -811 | L1ME3 | L1 | LINE |

  

|  |  |  |  |  |  |  |  |  |  |
| --- | --- | --- | --- | --- | --- | --- | --- | --- | --- |
| chr5:175374499-175375662 | | | 976 bp | | |  |  |  |  |
| Genome Start | Genome End | Strand | Div | Repeat Start | Repeat End | Repeat Left | Name | Family | Class |
| 175374499 | 175374603 | - | 27.6 | 2508 | 2615 | -804 | L2 | L2 | LINE |
| 175374628 | 175374938 | + | 11.1 | 4 | 309 | 0 | AluSc | Alu | SINE |
| 175374947 | 175375037 | + | 17.8 | 6109 | 6202 | -131 | L1MC1 | L1 | LINE |
| 175375191 | 175375446 | - | 20.0 | 1 | 302 | 0 | AluJb | Alu | SINE |
| 175375450 | 175375590 | + | 17.9 | 6192 | 6342 | -2 | L1MC2 | L1 | LINE |
| 175375591 | 175375662 | - | 34.3 | 2429 | 2498 | -921 | L2 | L2 | LINE |

  

|  |  |  |  |  |  |  |  |  |  |
| --- | --- | --- | --- | --- | --- | --- | --- | --- | --- |
| chr5:175379870-175382124 | | | 2239 bp | | |  |  |  |  |
| Genome Start | Genome End | Strand | Div | Repeat Start | Repeat End | Repeat Left | Name | Family | Class |
| 175379870 | 175380146 | + | 19.1 | 5326 | 5614 | -550 | L1ME3 | L1 | LINE |
| 175380147 | 175380338 | + | 21.7 | 1 | 189 | -185 | MLT1A | MaLR | LTR |
| 175380339 | 175381233 | - | 18.1 | 41 | 901 | 0 | MER54A | ERVL | LTR |
| 175381234 | 175381533 | + | 13.7 | 1 | 303 | -9 | AluSx | Alu | SINE |
| 175381534 | 175381573 | - | 18.1 | 1 | 40 | -861 | MER54A | ERVL | LTR |
| 175381574 | 175381772 | + | 21.7 | 190 | 368 | -6 | MLT1A | MaLR | LTR |
| 175381789 | 175382124 | + | 26.5 | 5623 | 5971 | -191 | L1ME3 | L1 | LINE |

  

|  |  |  |  |  |  |  |  |  |  |
| --- | --- | --- | --- | --- | --- | --- | --- | --- | --- |
| chr5:175397719-175399907 | | | 2189 bp | | |  |  |  |  |
| Genome Start | Genome End | Strand | Div | Repeat Start | Repeat End | Repeat Left | Name | Family | Class |
| 175397719 | 175398956 | - | 19.1 | 4082 | 5329 | -814 | L1M2 | L1 | LINE |
| 175398957 | 175399256 | + | 8.6 | 1 | 291 | -20 | AluY | Alu | SINE |
| 175399257 | 175399907 | - | 19.1 | 3425 | 4081 | -2062 | L1M2 | L1 | LINE |

  

|  |  |  |  |  |  |  |  |  |  |
| --- | --- | --- | --- | --- | --- | --- | --- | --- | --- |
| chr5:175423717-175424462 | | | 746 bp | | |  |  |  |  |
| Genome Start | Genome End | Strand | Div | Repeat Start | Repeat End | Repeat Left | Name | Family | Class |
| 175423717 | 175423799 | - | 22.4 | 519 | 590 | -2934 | Zaphod2 | Tip100 | DNA |
| 175423800 | 175424227 | - | 18.6 | 1 | 434 | 0 | Tigger2a | MER2\_type | DNA |
| 175424228 | 175424462 | - | 22.4 | 265 | 518 | -3006 | Zaphod2 | Tip100 | DNA |

  

|  |  |  |  |  |  |  |  |  |  |
| --- | --- | --- | --- | --- | --- | --- | --- | --- | --- |
| chr5:175571393-175571600 | | | 208 bp | | |  |  |  |  |
| Genome Start | Genome End | Strand | Div | Repeat Start | Repeat End | Repeat Left | Name | Family | Class |
| 175571393 | 175571449 | + | 20.3 | 2636 | 2691 | -171 | Charlie2 | MER1\_type | DNA |
| 175571450 | 175571573 | + | 18.7 | 1 | 123 | -10 | FLAM\_A | Alu | SINE |
| 175571574 | 175571600 | + | 20.3 | 2692 | 2708 | -154 | Charlie2 | MER1\_type | DNA |

  

|  |  |  |  |  |  |  |  |  |  |
| --- | --- | --- | --- | --- | --- | --- | --- | --- | --- |
| chr5:175580480-175583112 | | | 2633 bp | | |  |  |  |  |
| Genome Start | Genome End | Strand | Div | Repeat Start | Repeat End | Repeat Left | Name | Family | Class |
| 175580480 | 175580532 | + | 20.3 | 5492 | 5541 | -637 | L1MB8 | L1 | LINE |
| 175580533 | 175582197 | - | 6.5 | 2 | 1578 | 0 | LTR12C | ERV1 | LTR |
| 175582198 | 175582449 | + | 20.3 | 5542 | 5818 | -360 | L1MB8 | L1 | LINE |
| 175582450 | 175582751 | + | 10.0 | 1 | 301 | -10 | AluY | Alu | SINE |
| 175582752 | 175583112 | + | 20.3 | 5819 | 6177 | -1 | L1MB8 | L1 | LINE |

  

|  |  |  |  |  |  |  |  |  |  |
| --- | --- | --- | --- | --- | --- | --- | --- | --- | --- |
| chr5:175591256-175592163 | | | 908 bp | | |  |  |  |  |
| Genome Start | Genome End | Strand | Div | Repeat Start | Repeat End | Repeat Left | Name | Family | Class |
| 175591256 | 175591571 | + | 30.0 | 5315 | 5647 | -2161 | L1MC4a | L1 | LINE |
| 175591572 | 175591875 | + | 8.6 | 1 | 301 | -10 | AluY | Alu | SINE |
| 175591876 | 175592163 | + | 30.0 | 5648 | 5929 | -1879 | L1MC4a | L1 | LINE |

  

|  |  |  |  |  |  |  |  |  |  |
| --- | --- | --- | --- | --- | --- | --- | --- | --- | --- |
| chr5:175595996-175596902 | | | 907 bp | | |  |  |  |  |
| Genome Start | Genome End | Strand | Div | Repeat Start | Repeat End | Repeat Left | Name | Family | Class |
| 175595996 | 175596122 | + | 17.5 | 1 | 119 | -70 | MER5A | MER1\_type | DNA |
| 175596123 | 175596520 | - | 9.6 | 1 | 426 | -2 | MSTA | MaLR | LTR |
| 175596521 | 175596548 | + | 17.5 | 120 | 139 | -50 | MER5A | MER1\_type | DNA |
| 175596549 | 175596840 | - | 8.6 | 1 | 302 | -10 | AluSx | Alu | SINE |
| 175596841 | 175596902 | + | 17.5 | 140 | 189 | 0 | MER5A | MER1\_type | DNA |

  

|  |  |  |  |  |  |  |  |  |  |
| --- | --- | --- | --- | --- | --- | --- | --- | --- | --- |
| chr5:177008587-177010129 | | | 1543 bp | | |  |  |  |  |
| Genome Start | Genome End | Strand | Div | Repeat Start | Repeat End | Repeat Left | Name | Family | Class |
| 177008587 | 177009097 | - | 14.8 | 4169 | 4679 | -1467 | L1MC | L1 | LINE |
| 177009098 | 177009392 | - | 16.6 | 1 | 295 | -17 | AluSx | Alu | SINE |
| 177009393 | 177010129 | - | 14.8 | 3444 | 4168 | -1978 | L1MC | L1 | LINE |

  

|  |  |  |  |  |  |  |  |  |  |
| --- | --- | --- | --- | --- | --- | --- | --- | --- | --- |
| chr5:177014201-177014675 | | | 473 bp | | |  |  |  |  |
| Genome Start | Genome End | Strand | Div | Repeat Start | Repeat End | Repeat Left | Name | Family | Class |
| 177014201 | 177014548 | + | 15.8 | 14 | 401 | -27 | MSTA | MaLR | LTR |
| 177014551 | 177014632 | - | 16.9 | 4958 | 5034 | -1112 | L1PB | L1 | LINE |
| 177014633 | 177014675 | + | 16.3 | 386 | 428 | 0 | MSTA | MaLR | LTR |

  

|  |  |  |  |  |  |  |  |  |  |
| --- | --- | --- | --- | --- | --- | --- | --- | --- | --- |
| chr5:177023905-177024610 | | | 706 bp | | |  |  |  |  |
| Genome Start | Genome End | Strand | Div | Repeat Start | Repeat End | Repeat Left | Name | Family | Class |
| 177023905 | 177023936 | + | 13.4 | 1 | 34 | -411 | MER65A | ERV1 | LTR |
| 177023937 | 177024238 | + | 8.0 | 1 | 301 | -9 | AluSg | Alu | SINE |
| 177024239 | 177024610 | + | 13.4 | 35 | 444 | -1 | MER65A | ERV1 | LTR |

  

|  |  |  |  |  |  |  |  |  |  |
| --- | --- | --- | --- | --- | --- | --- | --- | --- | --- |
| chr5:177040833-177041373 | | | 538 bp | | |  |  |  |  |
| Genome Start | Genome End | Strand | Div | Repeat Start | Repeat End | Repeat Left | Name | Family | Class |
| 177040833 | 177040946 | + | 20.0 | 7505 | 7622 | -339 | L1MC5 | L1 | LINE |
| 177040950 | 177041105 | + | 14.3 | 1 | 155 | -21 | FRAM | Alu | SINE |
| 177041106 | 177041373 | + | 22.2 | 7614 | 7925 | -36 | L1MC5 | L1 | LINE |

  

|  |  |  |  |  |  |  |  |  |  |
| --- | --- | --- | --- | --- | --- | --- | --- | --- | --- |
| chr5:177056054-177056937 | | | 884 bp | | |  |  |  |  |
| Genome Start | Genome End | Strand | Div | Repeat Start | Repeat End | Repeat Left | Name | Family | Class |
| 177056054 | 177056275 | - | 17.6 | 4769 | 4995 | -1151 | L1M5 | L1 | LINE |
| 177056276 | 177056437 | - | 17.9 | 1 | 164 | -2 | FRAM | Alu | SINE |
| 177056438 | 177056937 | - | 17.6 | 4273 | 4768 | -1378 | L1M5 | L1 | LINE |

  

|  |  |  |  |  |  |  |  |  |  |
| --- | --- | --- | --- | --- | --- | --- | --- | --- | --- |
| chr5:177108830-177109456 | | | 626 bp | | |  |  |  |  |
| Genome Start | Genome End | Strand | Div | Repeat Start | Repeat End | Repeat Left | Name | Family | Class |
| 177108830 | 177109156 | + | 25.2 | 3042 | 3369 | -2777 | L1MC | L1 | LINE |
| 177109158 | 177109270 | - | 20.9 | 500 | 609 | -125 | MER50 | ERV1 | LTR |
| 177109271 | 177109456 | + | 20.0 | 3362 | 3558 | -2588 | L1MC | L1 | LINE |

  

|  |  |  |  |  |  |  |  |  |  |
| --- | --- | --- | --- | --- | --- | --- | --- | --- | --- |
| chr5:177138969-177139714 | | | 746 bp | | |  |  |  |  |
| Genome Start | Genome End | Strand | Div | Repeat Start | Repeat End | Repeat Left | Name | Family | Class |
| 177138969 | 177139203 | + | 21.7 | 265 | 518 | -3006 | Zaphod2 | Tip100 | DNA |
| 177139204 | 177139631 | + | 18.6 | 1 | 434 | 0 | Tigger2a | MER2\_type | DNA |
| 177139632 | 177139714 | + | 21.7 | 519 | 590 | -2934 | Zaphod2 | Tip100 | DNA |

  

|  |  |  |  |  |  |  |  |  |  |
| --- | --- | --- | --- | --- | --- | --- | --- | --- | --- |
| chr5:177176339-177177160 | | | 822 bp | | |  |  |  |  |
| Genome Start | Genome End | Strand | Div | Repeat Start | Repeat End | Repeat Left | Name | Family | Class |
| 177176339 | 177176435 | + | 16.3 | 2209 | 2315 | -3831 | L1P4 | L1 | LINE |
| 177176436 | 177176731 | - | 15.9 | 1 | 309 | -3 | AluJb | Alu | SINE |
| 177176732 | 177177160 | + | 16.3 | 2316 | 2766 | -3380 | L1P4 | L1 | LINE |

  

|  |  |  |  |  |  |  |  |  |  |
| --- | --- | --- | --- | --- | --- | --- | --- | --- | --- |
| chr5:177177798-177179640 | | | 1835 bp | | |  |  |  |  |
| Genome Start | Genome End | Strand | Div | Repeat Start | Repeat End | Repeat Left | Name | Family | Class |
| 177177798 | 177178138 | - | 16.7 | 3415 | 3752 | -3254 | L1M4b | L1 | LINE |
| 177178139 | 177178450 | + | 9.1 | 1 | 307 | -4 | AluY | Alu | SINE |
| 177178451 | 177178637 | - | 16.7 | 3229 | 3414 | -3592 | L1M4b | L1 | LINE |
| 177178638 | 177178961 | - | 11.9 | 1 | 323 | 0 | AluSx | Alu | SINE |
| 177178962 | 177179367 | - | 20.2 | 2833 | 3242 | -3784 | L1M4b | L1 | LINE |
| 177179376 | 177179640 | - | 22.0 | 108 | 374 | -6960 | L1M4b | L1 | LINE |

  

|  |  |  |  |  |  |  |  |  |  |
| --- | --- | --- | --- | --- | --- | --- | --- | --- | --- |
| chr5:177201398-177205428 | | | 4031 bp | | |  |  |  |  |
| Genome Start | Genome End | Strand | Div | Repeat Start | Repeat End | Repeat Left | Name | Family | Class |
| 177201398 | 177201660 | - | 23.2 | 2533 | 2794 | -3425 | L1MEc | L1 | LINE |
| 177201661 | 177202105 | - | 13.6 | 1 | 461 | 0 | MER65C | ERV1 | LTR |
| 177202106 | 177202372 | - | 22.2 | 2264 | 2532 | -3687 | L1MEc | L1 | LINE |
| 177202373 | 177202676 | - | 10.4 | 1 | 299 | -13 | AluSx | Alu | SINE |
| 177202677 | 177203121 | - | 20.9 | 1922 | 2365 | -4035 | L1MEc | L1 | LINE |
| 177203122 | 177203415 | - | 11.3 | 1 | 294 | -19 | AluSq | Alu | SINE |
| 177203416 | 177203911 | - | 20.9 | 1349 | 1921 | -4479 | L1MEc | L1 | LINE |
| 177203912 | 177204210 | - | 8.0 | 1 | 301 | -9 | AluSg | Alu | SINE |
| 177204211 | 177205428 | - | 20.9 | 33 | 1348 | -5052 | L1MEc | L1 | LINE |

  

|  |  |  |  |  |  |  |  |  |  |
| --- | --- | --- | --- | --- | --- | --- | --- | --- | --- |
| chr5:177237629-177238078 | | | 450 bp | | |  |  |  |  |
| Genome Start | Genome End | Strand | Div | Repeat Start | Repeat End | Repeat Left | Name | Family | Class |
| 177237629 | 177237681 | + | 28.2 | 16 | 74 | -194 | MIRb | MIR | SINE |
| 177237682 | 177237929 | + | 4.9 | 1 | 247 | -64 | AluY | Alu | SINE |
| 177237930 | 177238078 | + | 28.2 | 75 | 210 | -58 | MIRb | MIR | SINE |

  

|  |  |  |  |  |  |  |  |  |  |
| --- | --- | --- | --- | --- | --- | --- | --- | --- | --- |
| chr5:177256714-177262983 | | | 6163 bp | | |  |  |  |  |
| Genome Start | Genome End | Strand | Div | Repeat Start | Repeat End | Repeat Left | Name | Family | Class |
| 177256714 | 177257002 | - | 28.3 | 5964 | 6238 | -1 | L1ME1 | L1 | LINE |
| 177257021 | 177257101 | - | 24.1 | 6080 | 6164 | -7 | L1MB2 | L1 | LINE |
| 177257102 | 177257394 | + | 11.0 | 1 | 290 | -22 | AluSx | Alu | SINE |
| 177257395 | 177257676 | - | 15.1 | 5787 | 6079 | -92 | L1MA4 | L1 | LINE |
| 177257676 | 177257803 | - | 12.7 | 4128 | 4253 | -1887 | L1M3 | L1 | LINE |
| 177257804 | 177258981 | - | 21.2 | 4764 | 5979 | -260 | L1ME1 | L1 | LINE |
| 177259021 | 177259169 | - | 23.3 | 4605 | 4763 | -1456 | L1ME1 | L1 | LINE |
| 177259178 | 177259225 | - | 8.3 | 6245 | 6292 | -11 | L1MA4A | L1 | LINE |
| 177259226 | 177260036 | + | 16.0 | 1 | 983 | 0 | LTR1D | ERV1 | LTR |
| 177260037 | 177261444 | - | 12.6 | 4805 | 6248 | -55 | L1MA4A | L1 | LINE |
| 177261488 | 177262983 | - | 20.6 | 37 | 1598 | -4802 | L1ME1 | L1 | LINE |

  

|  |  |  |  |  |  |  |  |  |  |
| --- | --- | --- | --- | --- | --- | --- | --- | --- | --- |
| chr5:177266212-177266423 | | | 149 bp | | |  |  |  |  |
| Genome Start | Genome End | Strand | Div | Repeat Start | Repeat End | Repeat Left | Name | Family | Class |
| 177266212 | 177266257 | + | 8.7 | 1 | 46 | -34 | MADE1 | Mariner | DNA |
| 177266321 | 177266370 | + | 12.0 | 2 | 51 | -251 | Alu | Alu | SINE |
| 177266371 | 177266423 | + | 9.4 | 28 | 80 | 0 | MADE1 | Mariner | DNA |

  

|  |  |  |  |  |  |  |  |  |  |
| --- | --- | --- | --- | --- | --- | --- | --- | --- | --- |
| chr5:177285060-177285899 | | | 840 bp | | |  |  |  |  |
| Genome Start | Genome End | Strand | Div | Repeat Start | Repeat End | Repeat Left | Name | Family | Class |
| 177285060 | 177285211 | + | 21.7 | 2874 | 3041 | -378 | L2 | L2 | LINE |
| 177285212 | 177285558 | + | 17.2 | 1 | 362 | -3 | MLT1A0 | MaLR | LTR |
| 177285559 | 177285899 | + | 26.0 | 3042 | 3419 | 0 | L2 | L2 | LINE |

  

|  |  |  |  |  |  |  |  |  |  |
| --- | --- | --- | --- | --- | --- | --- | --- | --- | --- |
| chr5:177373900-177374779 | | | 880 bp | | |  |  |  |  |
| Genome Start | Genome End | Strand | Div | Repeat Start | Repeat End | Repeat Left | Name | Family | Class |
| 177373900 | 177374132 | - | 20.3 | 452 | 705 | 0 | MER44D | MER2\_type | DNA |
| 177374133 | 177374435 | - | 7.1 | 1 | 295 | -16 | AluY | Alu | SINE |
| 177374436 | 177374779 | - | 20.3 | 16 | 451 | -254 | MER44D | MER2\_type | DNA |

  

|  |  |  |  |  |  |  |  |  |  |
| --- | --- | --- | --- | --- | --- | --- | --- | --- | --- |
| chr5:177393552-177394178 | | | 626 bp | | |  |  |  |  |
| Genome Start | Genome End | Strand | Div | Repeat Start | Repeat End | Repeat Left | Name | Family | Class |
| 177393552 | 177393737 | - | 20.0 | 3362 | 3558 | -2588 | L1MC | L1 | LINE |
| 177393738 | 177393850 | + | 19.1 | 500 | 609 | -125 | MER50 | ERV1 | LTR |
| 177393852 | 177394178 | - | 24.8 | 3042 | 3369 | -2777 | L1MC | L1 | LINE |

  

|  |  |  |  |  |  |  |  |  |  |
| --- | --- | --- | --- | --- | --- | --- | --- | --- | --- |
| chr5:178806750-178807379 | | | 630 bp | | |  |  |  |  |
| Genome Start | Genome End | Strand | Div | Repeat Start | Repeat End | Repeat Left | Name | Family | Class |
| 178806750 | 178806985 | - | 27.2 | 90 | 332 | -9 | MER102b | MER1\_type | DNA |
| 178806986 | 178807286 | - | 5.7 | 1 | 300 | -11 | AluY | Alu | SINE |
| 178807287 | 178807379 | - | 27.2 | 1 | 89 | -252 | MER102b | MER1\_type | DNA |

  

|  |  |  |  |  |  |  |  |  |  |
| --- | --- | --- | --- | --- | --- | --- | --- | --- | --- |
| chr5:179015931-179016538 | | | 608 bp | | |  |  |  |  |
| Genome Start | Genome End | Strand | Div | Repeat Start | Repeat End | Repeat Left | Name | Family | Class |
| 179015931 | 179016148 | - | 22.8 | 95 | 292 | -20 | AluJo | Alu | SINE |
| 179016149 | 179016450 | - | 9.9 | 1 | 309 | 0 | AluSc | Alu | SINE |
| 179016451 | 179016538 | - | 22.8 | 7 | 94 | -218 | AluJo | Alu | SINE |

  

|  |  |  |  |  |  |  |  |  |  |
| --- | --- | --- | --- | --- | --- | --- | --- | --- | --- |
| chr5:180635143-180639121 | | | 3974 bp | | |  |  |  |  |
| Genome Start | Genome End | Strand | Div | Repeat Start | Repeat End | Repeat Left | Name | Family | Class |
| 180635143 | 180635753 | + | 21.1 | 1954 | 2564 | -4289 | L1PA15-16 | L1 | LINE |
| 180635754 | 180636048 | - | 11.9 | 1 | 295 | -17 | AluSx | Alu | SINE |
| 180636049 | 180637633 | + | 17.9 | 2565 | 4154 | -2695 | L1PA15-16 | L1 | LINE |
| 180637635 | 180638254 | + | 19.7 | 1020 | 1651 | 0 | MSTB-int | MaLR | LTR |
| 180638255 | 180638625 | + | 23.2 | 1 | 377 | -49 | MSTB | MaLR | LTR |
| 180638630 | 180639121 | + | 19.4 | 4152 | 4646 | -2203 | L1PA15-16 | L1 | LINE |

  

|  |  |  |  |  |  |  |  |  |  |
| --- | --- | --- | --- | --- | --- | --- | --- | --- | --- |
| chr5:180665578-180667046 | | | 1469 bp | | |  |  |  |  |
| Genome Start | Genome End | Strand | Div | Repeat Start | Repeat End | Repeat Left | Name | Family | Class |
| 180665578 | 180665877 | - | 20.7 | 604 | 934 | -1 | MER21C | ERV1 | LTR |
| 180665878 | 180666183 | - | 17.5 | 1 | 303 | -9 | AluJo | Alu | SINE |
| 180666184 | 180666493 | - | 20.7 | 270 | 603 | -332 | MER21C | ERV1 | LTR |
| 180666494 | 180666787 | + | 14.3 | 1 | 293 | -20 | AluSq | Alu | SINE |
| 180666788 | 180667046 | - | 20.7 | 18 | 269 | -666 | MER21C | ERV1 | LTR |

  

|  |  |  |  |  |  |  |  |  |  |
| --- | --- | --- | --- | --- | --- | --- | --- | --- | --- |
| chr5:180667719-180668294 | | | 576 bp | | |  |  |  |  |
| Genome Start | Genome End | Strand | Div | Repeat Start | Repeat End | Repeat Left | Name | Family | Class |
| 180667719 | 180667845 | + | 19.9 | 10 | 131 | -181 | AluJb | Alu | SINE |
| 180667846 | 180668131 | + | 10.1 | 1 | 296 | -13 | AluSg1 | Alu | SINE |
| 180668132 | 180668294 | + | 19.9 | 132 | 298 | -14 | AluJb | Alu | SINE |

  

|  |  |  |  |  |  |  |  |  |  |
| --- | --- | --- | --- | --- | --- | --- | --- | --- | --- |
| chr5:180672407-180672885 | | | 432 bp | | |  |  |  |  |
| Genome Start | Genome End | Strand | Div | Repeat Start | Repeat End | Repeat Left | Name | Family | Class |
| 180672407 | 180672466 | + | 22.2 | 24 | 71 | -241 | AluJo | Alu | SINE |
| 180672514 | 180672783 | - | 14.1 | 1 | 275 | -37 | AluSx | Alu | SINE |
| 180672784 | 180672885 | + | 22.2 | 72 | 167 | -145 | AluJo | Alu | SINE |

  

|  |  |  |  |  |  |  |  |  |  |
| --- | --- | --- | --- | --- | --- | --- | --- | --- | --- |
| chr5:180677678-180679164 | | | 1487 bp | | |  |  |  |  |
| Genome Start | Genome End | Strand | Div | Repeat Start | Repeat End | Repeat Left | Name | Family | Class |
| 180677678 | 180677978 | - | 21.1 | 604 | 935 | 0 | MER21C | ERV1 | LTR |
| 180677979 | 180678281 | - | 20.5 | 1 | 302 | -10 | AluJo | Alu | SINE |
| 180678282 | 180678351 | - | 21.1 | 526 | 603 | -332 | MER21C | ERV1 | LTR |
| 180678352 | 180678658 | + | 19.5 | 2 | 294 | -18 | AluJb | Alu | SINE |
| 180678659 | 180679164 | - | 21.1 | 18 | 525 | -410 | MER21C | ERV1 | LTR |

  

|  |  |  |  |  |  |  |  |  |  |
| --- | --- | --- | --- | --- | --- | --- | --- | --- | --- |
| chr5:180693554-180694090 | | | 537 bp | | |  |  |  |  |
| Genome Start | Genome End | Strand | Div | Repeat Start | Repeat End | Repeat Left | Name | Family | Class |
| 180693554 | 180693678 | + | 32.3 | 3190 | 3312 | -107 | L2 | L2 | LINE |
| 180693679 | 180693978 | + | 11.3 | 1 | 300 | -13 | AluSq | Alu | SINE |
| 180693979 | 180694090 | + | 32.3 | 3313 | 3418 | -1 | L2 | L2 | LINE |

  

|  |  |  |  |  |  |  |  |  |  |
| --- | --- | --- | --- | --- | --- | --- | --- | --- | --- |
| chr5:180712516-180715330 | | | 2811 bp | | |  |  |  |  |
| Genome Start | Genome End | Strand | Div | Repeat Start | Repeat End | Repeat Left | Name | Family | Class |
| 180712516 | 180712809 | - | 29.1 | 252 | 542 | -13 | MLT1H1 | MaLR | LTR |
| 180712810 | 180713169 | + | 15.0 | 3 | 364 | 0 | THE1B | MaLR | LTR |
| 180713174 | 180714731 | + | 31.0 | 4 | 1735 | 0 | MLT-int | MaLR | LTR |
| 180714732 | 180715089 | + | 18.3 | 1 | 364 | 0 | THE1B | MaLR | LTR |
| 180715090 | 180715330 | - | 29.1 | 3 | 251 | -304 | MLT1H1 | MaLR | LTR |

  

|  |  |  |  |  |  |  |  |  |  |
| --- | --- | --- | --- | --- | --- | --- | --- | --- | --- |
| chr5:180716837-180717410 | | | 423 bp | | |  |  |  |  |
| Genome Start | Genome End | Strand | Div | Repeat Start | Repeat End | Repeat Left | Name | Family | Class |
| 180716837 | 180716985 | - | 28.9 | 1398 | 1552 | -457 | HAL1b | L1 | LINE |
| 180717057 | 180717201 | - | 14.3 | 1 | 133 | 0 | FLAM\_C | Alu | SINE |
| 180717282 | 180717410 | - | 28.4 | 866 | 995 | -1014 | HAL1b | L1 | LINE |

  

|  |  |  |  |  |  |  |  |  |  |
| --- | --- | --- | --- | --- | --- | --- | --- | --- | --- |
| chr5:180722309-180722956 | | | 575 bp | | |  |  |  |  |
| Genome Start | Genome End | Strand | Div | Repeat Start | Repeat End | Repeat Left | Name | Family | Class |
| 180722309 | 180722445 | - | 24.8 | 143 | 267 | -1 | MIRb | MIR | SINE |
| 180722495 | 180722741 | - | 14.6 | 6049 | 6295 | -5 | L1MA4 | L1 | LINE |
| 180722758 | 180722806 | - | 18.4 | 6055 | 6103 | -197 | L1MA4 | L1 | LINE |
| 180722815 | 180722956 | - | 34.1 | 21 | 155 | -113 | MIRb | MIR | SINE |

  

|  |  |  |  |  |  |  |  |  |  |
| --- | --- | --- | --- | --- | --- | --- | --- | --- | --- |
| chr5:180738806-180741300 | | | 2460 bp | | |  |  |  |  |
| Genome Start | Genome End | Strand | Div | Repeat Start | Repeat End | Repeat Left | Name | Family | Class |
| 180738806 | 180739890 | - | 16.4 | 3657 | 4741 | -1399 | L1M3 | L1 | LINE |
| 180739910 | 180740087 | - | 4.5 | 5977 | 6155 | 0 | L1PA4 | L1 | LINE |
| 180740088 | 180740809 | + | 2.2 | 5254 | 5975 | -180 | L1PA4 | L1 | LINE |
| 180740826 | 180741300 | - | 18.7 | 3167 | 3646 | -2494 | L1M3 | L1 | LINE |

  

|  |  |  |  |  |  |  |  |  |  |
| --- | --- | --- | --- | --- | --- | --- | --- | --- | --- |
| chr5:180748611-180754342 | | | 5561 bp | | |  |  |  |  |
| Genome Start | Genome End | Strand | Div | Repeat Start | Repeat End | Repeat Left | Name | Family | Class |
| 180748611 | 180748749 | + | 26.1 | 3224 | 3368 | -2778 | L1ME3B | L1 | LINE |
| 180748748 | 180750729 | + | 15.6 | 4080 | 6108 | -183 | L1MA8 | L1 | LINE |
| 180750730 | 180751465 | - | 10.3 | 5556 | 6303 | -1 | L1MA2 | L1 | LINE |
| 180751466 | 180751589 | + | 15.8 | 6109 | 6238 | -53 | L1MA8 | L1 | LINE |
| 180751624 | 180752364 | + | 25.0 | 3352 | 4119 | -2027 | L1ME3B | L1 | LINE |
| 180752415 | 180753428 | + | 17.4 | 3687 | 4697 | -1449 | L1P4 | L1 | LINE |
| 180753487 | 180754071 | + | 30.5 | 5508 | 6122 | -118 | L1ME3B | L1 | LINE |
| 180754098 | 180754259 | - | 19.3 | 123 | 298 | -15 | AluSp | Alu | SINE |
| 180754265 | 180754342 | + | 26.0 | 6128 | 6204 | -36 | L1ME3B | L1 | LINE |

  

|  |  |  |  |  |  |  |  |  |  |
| --- | --- | --- | --- | --- | --- | --- | --- | --- | --- |
| chr5:180772978-180773641 | | | 664 bp | | |  |  |  |  |
| Genome Start | Genome End | Strand | Div | Repeat Start | Repeat End | Repeat Left | Name | Family | Class |
| 180772978 | 180773023 | + | 28.3 | 62 | 107 | -442 | MLT1H | MaLR | LTR |
| 180773024 | 180773345 | + | 9.3 | 1 | 311 | 0 | AluY | Alu | SINE |
| 180773346 | 180773641 | + | 26.7 | 93 | 375 | -167 | MLT1H | MaLR | LTR |

  

|  |  |  |  |  |  |  |  |  |  |
| --- | --- | --- | --- | --- | --- | --- | --- | --- | --- |
| chr5:180781377-180781800 | | | 386 bp | | |  |  |  |  |
| Genome Start | Genome End | Strand | Div | Repeat Start | Repeat End | Repeat Left | Name | Family | Class |
| 180781377 | 180781440 | - | 13.5 | 362 | 428 | 0 | MSTA | MaLR | LTR |
| 180781441 | 180781558 | + | 8.4 | 1 | 118 | -192 | AluSg | Alu | SINE |
| 180781597 | 180781750 | + | 8.4 | 119 | 307 | -3 | AluSg | Alu | SINE |
| 180781751 | 180781800 | - | 13.5 | 319 | 361 | -67 | MSTA | MaLR | LTR |

  

|  |  |  |  |  |  |  |  |  |  |
| --- | --- | --- | --- | --- | --- | --- | --- | --- | --- |
| chr5:180789343-180790412 | | | 1070 bp | | |  |  |  |  |
| Genome Start | Genome End | Strand | Div | Repeat Start | Repeat End | Repeat Left | Name | Family | Class |
| 180789343 | 180789764 | - | 6.6 | 334 | 781 | 0 | MER4E1 | ERV1 | LTR |
| 180789765 | 180790063 | + | 10.4 | 1 | 297 | -13 | AluSg | Alu | SINE |
| 180790064 | 180790412 | - | 6.6 | 1 | 333 | -448 | MER4E1 | ERV1 | LTR |

  

|  |  |  |  |  |  |  |  |  |  |
| --- | --- | --- | --- | --- | --- | --- | --- | --- | --- |
| chr5:180791425-180792448 | | | 973 bp | | |  |  |  |  |
| Genome Start | Genome End | Strand | Div | Repeat Start | Repeat End | Repeat Left | Name | Family | Class |
| 180791425 | 180791595 | - | 24.2 | 398 | 575 | -16 | MLT1K | MaLR | LTR |
| 180791647 | 180791696 | - | 27.1 | 99 | 145 | -446 | MLT1K | MaLR | LTR |
| 180791697 | 180792354 | - | 11.2 | 1 | 644 | 0 | LTR9B | ERV1 | LTR |
| 180792355 | 180792448 | - | 27.1 | 4 | 98 | -493 | MLT1K | MaLR | LTR |

  

|  |  |  |  |  |  |  |  |  |  |
| --- | --- | --- | --- | --- | --- | --- | --- | --- | --- |
| chr5:180817081-180819201 | | | 2118 bp | | |  |  |  |  |
| Genome Start | Genome End | Strand | Div | Repeat Start | Repeat End | Repeat Left | Name | Family | Class |
| 180817081 | 180817762 | - | 18.6 | 5600 | 6300 | 0 | L1MA4 | L1 | LINE |
| 180817763 | 180818075 | - | 8.2 | 1 | 304 | -5 | AluSc | Alu | SINE |
| 180818079 | 180818382 | - | 12.5 | 1 | 303 | -7 | AluSg | Alu | SINE |
| 180818383 | 180819201 | - | 17.3 | 4760 | 5599 | -701 | L1MA4 | L1 | LINE |

  

|  |  |  |  |  |  |  |  |  |  |
| --- | --- | --- | --- | --- | --- | --- | --- | --- | --- |
| chr5:180821141-180822434 | | | 1298 bp | | |  |  |  |  |
| Genome Start | Genome End | Strand | Div | Repeat Start | Repeat End | Repeat Left | Name | Family | Class |
| 180821141 | 180821936 | + | 15.4 | 2 | 836 | -64 | MER4D1 | ERV1 | LTR |
| 180821933 | 180822364 | - | 22.2 | 1 | 589 | -5 | MER4A | ERV1 | LTR |
| 180822365 | 180822434 | + | 15.3 | 824 | 900 | 0 | MER4D1 | ERV1 | LTR |

  

|  |  |  |  |  |  |  |  |  |  |
| --- | --- | --- | --- | --- | --- | --- | --- | --- | --- |
| chr5:180827852-180830058 | | | 2207 bp | | |  |  |  |  |
| Genome Start | Genome End | Strand | Div | Repeat Start | Repeat End | Repeat Left | Name | Family | Class |
| 180827852 | 180829209 | - | 23.4 | 6234 | 7784 | -1 | L1MC3 | L1 | LINE |
| 180829210 | 180829649 | - | 18.1 | 5 | 432 | 0 | MSTB1 | MaLR | LTR |
| 180829650 | 180830058 | - | 23.4 | 5763 | 6233 | -1552 | L1MC3 | L1 | LINE |

  

|  |  |  |  |  |  |  |  |  |  |
| --- | --- | --- | --- | --- | --- | --- | --- | --- | --- |
| chr5:180835621-180836621 | | | 1001 bp | | |  |  |  |  |
| Genome Start | Genome End | Strand | Div | Repeat Start | Repeat End | Repeat Left | Name | Family | Class |
| 180835621 | 180835673 | - | 14.6 | 684 | 722 | 0 | MER72 | ERV1 | LTR |
| 180835674 | 180835981 | - | 11.7 | 3 | 310 | -3 | AluSq | Alu | SINE |
| 180835982 | 180836621 | - | 14.6 | 1 | 683 | -39 | MER72 | ERV1 | LTR |

  


|  |  |  |  |  |  |  |  |  |  |
| --- | --- | --- | --- | --- | --- | --- | --- | --- | --- |
| chr6:15711-16690 | | | 980 bp | | |  |  |  |  |
| Genome Start | Genome End | Strand | Div | Repeat Start | Repeat End | Repeat Left | Name | Family | Class |
| 15711 | 15803 | + | 22.9 | 1 | 87 | -2236 | Tigger5 | MER2\_type | DNA |
| 15804 | 16095 | + | 7.9 | 5 | 295 | -14 | AluSc | Alu | SINE |
| 16096 | 16230 | + | 22.9 | 88 | 218 | -2105 | Tigger5 | MER2\_type | DNA |
| 16231 | 16690 | + | 17.7 | 1919 | 2406 | 0 | Tigger5 | MER2\_type | DNA |

  

|  |  |  |  |  |  |  |  |  |  |
| --- | --- | --- | --- | --- | --- | --- | --- | --- | --- |
| chr6:23939-24943 | | | 802 bp | | |  |  |  |  |
| Genome Start | Genome End | Strand | Div | Repeat Start | Repeat End | Repeat Left | Name | Family | Class |
| 23939 | 24240 | - | 37.3 | 3030 | 3355 | -23 | L2 | L2 | LINE |
| 24441 | 24706 | - | 4.5 | 5881 | 6150 | -4 | L1PA5 | L1 | LINE |
| 24710 | 24943 | - | 39.0 | 2572 | 2813 | -606 | L2 | L2 | LINE |

  

|  |  |  |  |  |  |  |  |  |  |
| --- | --- | --- | --- | --- | --- | --- | --- | --- | --- |
| chr6:25110-30842 | | | 5562 bp | | |  |  |  |  |
| Genome Start | Genome End | Strand | Div | Repeat Start | Repeat End | Repeat Left | Name | Family | Class |
| 25110 | 25187 | - | 26.0 | 6128 | 6204 | -36 | L1ME3B | L1 | LINE |
| 25193 | 25355 | + | 19.1 | 123 | 299 | -14 | AluSp | Alu | SINE |
| 25382 | 25966 | - | 30.5 | 5508 | 6122 | -118 | L1ME3B | L1 | LINE |
| 26025 | 27038 | - | 17.7 | 3687 | 4697 | -1449 | L1P4 | L1 | LINE |
| 27089 | 27829 | - | 25.4 | 3352 | 4119 | -2027 | L1ME3B | L1 | LINE |
| 27864 | 27987 | - | 15.8 | 6109 | 6238 | -53 | L1MA8 | L1 | LINE |
| 27988 | 28723 | + | 10.8 | 5556 | 6303 | -1 | L1MA2 | L1 | LINE |
| 28724 | 30705 | - | 15.6 | 4080 | 6108 | -183 | L1MA8 | L1 | LINE |
| 30704 | 30842 | - | 26.1 | 3224 | 3368 | -2778 | L1ME3B | L1 | LINE |

  

|  |  |  |  |  |  |  |  |  |  |
| --- | --- | --- | --- | --- | --- | --- | --- | --- | --- |
| chr6:37439-39945 | | | 2472 bp | | |  |  |  |  |
| Genome Start | Genome End | Strand | Div | Repeat Start | Repeat End | Repeat Left | Name | Family | Class |
| 37439 | 37927 | + | 19.5 | 3150 | 3646 | -2494 | L1M3 | L1 | LINE |
| 37944 | 38664 | - | 2.6 | 5254 | 5975 | -180 | L1PA4 | L1 | LINE |
| 38665 | 38842 | + | 5.1 | 5977 | 6155 | 0 | L1PA4 | L1 | LINE |
| 38862 | 39945 | + | 16.4 | 3657 | 4741 | -1399 | L1M3 | L1 | LINE |

  

|  |  |  |  |  |  |  |  |  |  |
| --- | --- | --- | --- | --- | --- | --- | --- | --- | --- |
| chr6:55793-56440 | | | 575 bp | | |  |  |  |  |
| Genome Start | Genome End | Strand | Div | Repeat Start | Repeat End | Repeat Left | Name | Family | Class |
| 55793 | 55934 | + | 34.1 | 21 | 155 | -113 | MIRb | MIR | SINE |
| 55943 | 55991 | + | 18.4 | 6055 | 6103 | -197 | L1MA4 | L1 | LINE |
| 56008 | 56254 | + | 15.0 | 6049 | 6295 | -5 | L1MA4 | L1 | LINE |
| 56304 | 56440 | + | 24.8 | 143 | 267 | -1 | MIRb | MIR | SINE |

  

|  |  |  |  |  |  |  |  |  |  |
| --- | --- | --- | --- | --- | --- | --- | --- | --- | --- |
| chr6:61327-61897 | | | 423 bp | | |  |  |  |  |
| Genome Start | Genome End | Strand | Div | Repeat Start | Repeat End | Repeat Left | Name | Family | Class |
| 61327 | 61455 | + | 29.1 | 866 | 995 | -1014 | HAL1b | L1 | LINE |
| 61533 | 61677 | + | 14.3 | 1 | 133 | 0 | FLAM\_C | Alu | SINE |
| 61749 | 61897 | + | 28.9 | 1398 | 1552 | -457 | HAL1b | L1 | LINE |

  

|  |  |  |  |  |  |  |  |  |  |
| --- | --- | --- | --- | --- | --- | --- | --- | --- | --- |
| chr6:84638-85097 | | | 460 bp | | |  |  |  |  |
| Genome Start | Genome End | Strand | Div | Repeat Start | Repeat End | Repeat Left | Name | Family | Class |
| 84638 | 84742 | - | 28.4 | 3313 | 3414 | -5 | L2 | L2 | LINE |
| 84743 | 85050 | - | 10.7 | 1 | 308 | -5 | AluSq | Alu | SINE |
| 85051 | 85097 | - | 28.4 | 3268 | 3312 | -107 | L2 | L2 | LINE |

  

|  |  |  |  |  |  |  |  |  |  |
| --- | --- | --- | --- | --- | --- | --- | --- | --- | --- |
| chr6:86047-86898 | | | 852 bp | | |  |  |  |  |
| Genome Start | Genome End | Strand | Div | Repeat Start | Repeat End | Repeat Left | Name | Family | Class |
| 86047 | 86153 | + | 25.1 | 1930 | 2031 | -4758 | L1M2c | L1 | LINE |
| 86154 | 86462 | + | 9.9 | 1 | 303 | -7 | AluSg | Alu | SINE |
| 86463 | 86898 | + | 20.6 | 2032 | 2551 | -4238 | L1M2c | L1 | LINE |

  

|  |  |  |  |  |  |  |  |  |  |
| --- | --- | --- | --- | --- | --- | --- | --- | --- | --- |
| chr6:94841-96216 | | | 1366 bp | | |  |  |  |  |
| Genome Start | Genome End | Strand | Div | Repeat Start | Repeat End | Repeat Left | Name | Family | Class |
| 94841 | 95452 | + | 22.1 | 4898 | 5546 | -2239 | L1MC | L1 | LINE |
| 95458 | 96023 | + | 15.4 | 1 | 583 | -2 | MER34C\_ | ERV1 | LTR |
| 96029 | 96216 | + | 26.9 | 5577 | 5765 | -2020 | L1MC | L1 | LINE |

  

|  |  |  |  |  |  |  |  |  |  |
| --- | --- | --- | --- | --- | --- | --- | --- | --- | --- |
| chr6:5644616-5644988 | | | 373 bp | | |  |  |  |  |
| Genome Start | Genome End | Strand | Div | Repeat Start | Repeat End | Repeat Left | Name | Family | Class |
| 5644616 | 5644742 | + | 17.1 | 1 | 131 | -29 | MER5A1 | MER1\_type | DNA |
| 5644743 | 5644958 | - | 8.8 | 83 | 297 | -15 | AluSg/x | Alu | SINE |
| 5644959 | 5644988 | + | 17.1 | 132 | 160 | 0 | MER5A1 | MER1\_type | DNA |

  

|  |  |  |  |  |  |  |  |  |  |
| --- | --- | --- | --- | --- | --- | --- | --- | --- | --- |
| chr6:5661334-5663027 | | | 1443 bp | | |  |  |  |  |
| Genome Start | Genome End | Strand | Div | Repeat Start | Repeat End | Repeat Left | Name | Family | Class |
| 5661334 | 5661611 | - | 26.5 | 7483 | 7779 | -182 | L1MC5 | L1 | LINE |
| 5661669 | 5662577 | - | 18.6 | 16 | 905 | -26 | MER21A | ERV1 | LTR |
| 5662664 | 5662877 | - | 16.3 | 7258 | 7467 | -494 | L1MC5 | L1 | LINE |
| 5662986 | 5663027 | - | 14.3 | 7182 | 7224 | -737 | L1MC5 | L1 | LINE |

  

|  |  |  |  |  |  |  |  |  |  |
| --- | --- | --- | --- | --- | --- | --- | --- | --- | --- |
| chr6:5764849-5765430 | | | 582 bp | | |  |  |  |  |
| Genome Start | Genome End | Strand | Div | Repeat Start | Repeat End | Repeat Left | Name | Family | Class |
| 5764849 | 5764995 | - | 14.3 | 143 | 272 | -40 | AluJo | Alu | SINE |
| 5764996 | 5765285 | - | 11.1 | 20 | 298 | -12 | AluSg | Alu | SINE |
| 5765286 | 5765430 | - | 14.3 | 1 | 142 | -170 | AluJo | Alu | SINE |

  

|  |  |  |  |  |  |  |  |  |  |
| --- | --- | --- | --- | --- | --- | --- | --- | --- | --- |
| chr6:5770645-5771325 | | | 681 bp | | |  |  |  |  |
| Genome Start | Genome End | Strand | Div | Repeat Start | Repeat End | Repeat Left | Name | Family | Class |
| 5770645 | 5770933 | + | 14.1 | 1 | 291 | -75 | Tigger5a | MER2\_type | DNA |
| 5770934 | 5771242 | - | 14.9 | 1 | 309 | -3 | AluJb | Alu | SINE |
| 5771243 | 5771325 | + | 14.1 | 292 | 366 | 0 | Tigger5a | MER2\_type | DNA |

  

|  |  |  |  |  |  |  |  |  |  |
| --- | --- | --- | --- | --- | --- | --- | --- | --- | --- |
| chr6:5776087-5776585 | | | 499 bp | | |  |  |  |  |
| Genome Start | Genome End | Strand | Div | Repeat Start | Repeat End | Repeat Left | Name | Family | Class |
| 5776087 | 5776226 | - | 28.8 | 82 | 223 | -39 | MIRb | MIR | SINE |
| 5776227 | 5776520 | + | 12.2 | 1 | 306 | -6 | AluSx | Alu | SINE |
| 5776521 | 5776585 | - | 28.7 | 7 | 88 | -180 | MIRb | MIR | SINE |

  

|  |  |  |  |  |  |  |  |  |  |
| --- | --- | --- | --- | --- | --- | --- | --- | --- | --- |
| chr6:9100650-9101378 | | | 623 bp | | |  |  |  |  |
| Genome Start | Genome End | Strand | Div | Repeat Start | Repeat End | Repeat Left | Name | Family | Class |
| 9100650 | 9100716 | - | 16.1 | 404 | 462 | 0 | LTR40b | ERVL | LTR |
| 9100725 | 9100926 | + | 23.0 | 1625 | 1894 | -4696 | L1MEc | L1 | LINE |
| 9101025 | 9101378 | - | 29.8 | 1 | 357 | -105 | LTR40b | ERVL | LTR |

  

|  |  |  |  |  |  |  |  |  |  |
| --- | --- | --- | --- | --- | --- | --- | --- | --- | --- |
| chr6:9124744-9126619 | | | 1571 bp | | |  |  |  |  |
| Genome Start | Genome End | Strand | Div | Repeat Start | Repeat End | Repeat Left | Name | Family | Class |
| 9124744 | 9124851 | + | 21.1 | 2 | 210 | -725 | MER21C | ERV1 | LTR |
| 9124855 | 9124938 | - | 13.7 | 476 | 555 | 0 | MLT2A1 | ERVL | LTR |
| 9125071 | 9125117 | - | 20.9 | 499 | 520 | -35 | MLT2A1 | ERVL | LTR |
| 9125175 | 9125562 | - | 7.8 | 1 | 477 | -54 | MLT2A1 | ERVL | LTR |
| 9125676 | 9126002 | + | 24.2 | 280 | 640 | -295 | MER21C | ERV1 | LTR |
| 9126003 | 9126421 | - | 12.1 | 1 | 427 | -1 | MSTA | MaLR | LTR |
| 9126422 | 9126619 | + | 24.2 | 641 | 839 | -96 | MER21C | ERV1 | LTR |

  

|  |  |  |  |  |  |  |  |  |  |
| --- | --- | --- | --- | --- | --- | --- | --- | --- | --- |
| chr6:9187418-9188890 | | | 1144 bp | | |  |  |  |  |
| Genome Start | Genome End | Strand | Div | Repeat Start | Repeat End | Repeat Left | Name | Family | Class |
| 9187418 | 9187648 | - | 29.5 | 3187 | 3411 | -8 | L2 | L2 | LINE |
| 9187621 | 9187895 | + | 22.9 | 5 | 241 | -21 | MIR | MIR | SINE |
| 9188152 | 9188470 | - | 15.2 | 2 | 324 | 0 | MER33 | MER1\_type | DNA |
| 9188572 | 9188890 | - | 34.8 | 2528 | 2839 | -580 | L2 | L2 | LINE |

  

|  |  |  |  |  |  |  |  |  |  |
| --- | --- | --- | --- | --- | --- | --- | --- | --- | --- |
| chr6:9251321-9253242 | | | 1905 bp | | |  |  |  |  |
| Genome Start | Genome End | Strand | Div | Repeat Start | Repeat End | Repeat Left | Name | Family | Class |
| 9251321 | 9251649 | - | 25.6 | 7467 | 7832 | -210 | L1MC4 | L1 | LINE |
| 9251650 | 9252032 | + | 16.4 | 1 | 397 | 0 | MLT2C1 | ERVL | LTR |
| 9252033 | 9252471 | - | 25.6 | 7022 | 7466 | -576 | L1MC4 | L1 | LINE |
| 9252484 | 9252665 | + | 25.0 | 16 | 1685 | -3 | MER63 | AcHobo | DNA |
| 9252671 | 9252691 | - | 23.2 | 7009 | 7027 | -1015 | L1MC4 | L1 | LINE |
| 9252692 | 9252991 | - | 10.5 | 1 | 298 | -11 | AluSg1 | Alu | SINE |
| 9252992 | 9253242 | - | 23.2 | 6766 | 7008 | -1034 | L1MC4 | L1 | LINE |

  

|  |  |  |  |  |  |  |  |  |  |
| --- | --- | --- | --- | --- | --- | --- | --- | --- | --- |
| chr6:9269465-9274309 | | | 4199 bp | | |  |  |  |  |
| Genome Start | Genome End | Strand | Div | Repeat Start | Repeat End | Repeat Left | Name | Family | Class |
| 9269465 | 9269525 | + | 30.4 | 24 | 89 | -6334 | L1MCc | L1 | LINE |
| 9269526 | 9269628 | + | 16.3 | 1 | 118 | -194 | AluJo | Alu | SINE |
| 9269655 | 9269807 | + | 16.3 | 119 | 290 | -22 | AluJo | Alu | SINE |
| 9269808 | 9270268 | + | 30.4 | 90 | 580 | -5843 | L1MCc | L1 | LINE |
| 9270467 | 9270750 | + | 35.0 | 868 | 1197 | -5226 | L1MCc | L1 | LINE |
| 9271178 | 9273870 | + | 26.6 | 1246 | 4196 | -2745 | L1MCc | L1 | LINE |
| 9273866 | 9274309 | + | 25.8 | 4720 | 5186 | -1755 | L1MCc | L1 | LINE |

  

|  |  |  |  |  |  |  |  |  |  |
| --- | --- | --- | --- | --- | --- | --- | --- | --- | --- |
| chr6:9293846-9294857 | | | 819 bp | | |  |  |  |  |
| Genome Start | Genome End | Strand | Div | Repeat Start | Repeat End | Repeat Left | Name | Family | Class |
| 9293846 | 9293989 | + | 20.8 | 59 | 203 | -421 | MLT1E2 | MaLR | LTR |
| 9294002 | 9294169 | + | 24.6 | 1 | 167 | -18 | FAM | Alu | SINE |
| 9294197 | 9294330 | - | 28.4 | 867 | 1034 | -455 | MLT1F-int | MaLR | LTR |
| 9294485 | 9294857 | + | 22.2 | 215 | 626 | 0 | MLT1E2 | MaLR | LTR |

  

|  |  |  |  |  |  |  |  |  |  |
| --- | --- | --- | --- | --- | --- | --- | --- | --- | --- |
| chr6:9301416-9302984 | | | 1347 bp | | |  |  |  |  |
| Genome Start | Genome End | Strand | Div | Repeat Start | Repeat End | Repeat Left | Name | Family | Class |
| 9301416 | 9301900 | + | 24.4 | 2635 | 3146 | -3043 | L1ME3A | L1 | LINE |
| 9302028 | 9302337 | + | 26.9 | 5535 | 5857 | -313 | L1ME3A | L1 | LINE |
| 9302338 | 9302631 | - | 5.4 | 1 | 294 | -17 | AluY | Alu | SINE |
| 9302632 | 9302701 | + | 26.9 | 5858 | 5928 | -242 | L1ME3A | L1 | LINE |
| 9302797 | 9302984 | + | 31.6 | 5976 | 6162 | -8 | L1ME3A | L1 | LINE |

  

|  |  |  |  |  |  |  |  |  |  |
| --- | --- | --- | --- | --- | --- | --- | --- | --- | --- |
| chr6:9321715-9328829 | | | 7115 bp | | |  |  |  |  |
| Genome Start | Genome End | Strand | Div | Repeat Start | Repeat End | Repeat Left | Name | Family | Class |
| 9321715 | 9324568 | + | 6.9 | 9 | 2865 | -3281 | L1PA6 | L1 | LINE |
| 9324569 | 9325220 | - | 4.9 | 5493 | 6146 | -8 | L1PA5 | L1 | LINE |
| 9325221 | 9325493 | + | 7.1 | 2866 | 3138 | -3008 | L1PA6 | L1 | LINE |
| 9325494 | 9325797 | + | 1.6 | 1 | 304 | -6 | AluYa5 | Alu | SINE |
| 9325798 | 9328829 | + | 6.9 | 3139 | 6148 | -6 | L1PA6 | L1 | LINE |

  

|  |  |  |  |  |  |  |  |  |  |
| --- | --- | --- | --- | --- | --- | --- | --- | --- | --- |
| chr6:9339985-9342963 | | | 2979 bp | | |  |  |  |  |
| Genome Start | Genome End | Strand | Div | Repeat Start | Repeat End | Repeat Left | Name | Family | Class |
| 9339985 | 9341128 | + | 13.8 | 1 | 411 | -1570 | Tigger2 | MER2\_type | DNA |
| 9341129 | 9341432 | + | 13.5 | 1 | 304 | -6 | AluSg | Alu | SINE |
| 9341433 | 9342963 | + | 13.8 | 412 | 2680 | -38 | Tigger2 | MER2\_type | DNA |

  

|  |  |  |  |  |  |  |  |  |  |
| --- | --- | --- | --- | --- | --- | --- | --- | --- | --- |
| chr6:26822172-26824841 | | | 2670 bp | | |  |  |  |  |
| Genome Start | Genome End | Strand | Div | Repeat Start | Repeat End | Repeat Left | Name | Family | Class |
| 26822172 | 26824020 | + | 26.5 | 515 | 2519 | -4121 | L1MD2 | L1 | LINE |
| 26824021 | 26824315 | + | 12.2 | 1 | 294 | -19 | AluSq | Alu | SINE |
| 26824316 | 26824841 | + | 24.6 | 2520 | 3043 | -3501 | L1MD2 | L1 | LINE |

  

|  |  |  |  |  |  |  |  |  |  |
| --- | --- | --- | --- | --- | --- | --- | --- | --- | --- |
| chr6:26840001-26840581 | | | 581 bp | | |  |  |  |  |
| Genome Start | Genome End | Strand | Div | Repeat Start | Repeat End | Repeat Left | Name | Family | Class |
| 26840001 | 26840214 | + | 29.6 | 7258 | 7471 | -490 | L1MC5 | L1 | LINE |
| 26840215 | 26840519 | + | 16.5 | 6048 | 6344 | 0 | L1MC2 | L1 | LINE |
| 26840520 | 26840581 | + | 29.6 | 7472 | 7527 | -434 | L1MC5 | L1 | LINE |

  

|  |  |  |  |  |  |  |  |  |  |
| --- | --- | --- | --- | --- | --- | --- | --- | --- | --- |
| chr6:26877467-26878047 | | | 581 bp | | |  |  |  |  |
| Genome Start | Genome End | Strand | Div | Repeat Start | Repeat End | Repeat Left | Name | Family | Class |
| 26877467 | 26877528 | - | 29.6 | 7472 | 7527 | -434 | L1MC5 | L1 | LINE |
| 26877529 | 26877831 | - | 16.3 | 6050 | 6344 | 0 | L1MC2 | L1 | LINE |
| 26877832 | 26878047 | - | 29.6 | 7258 | 7471 | -490 | L1MC5 | L1 | LINE |

  

|  |  |  |  |  |  |  |  |  |  |
| --- | --- | --- | --- | --- | --- | --- | --- | --- | --- |
| chr6:26893243-26895912 | | | 2670 bp | | |  |  |  |  |
| Genome Start | Genome End | Strand | Div | Repeat Start | Repeat End | Repeat Left | Name | Family | Class |
| 26893243 | 26893768 | - | 24.7 | 2520 | 3043 | -3501 | L1MD2 | L1 | LINE |
| 26893769 | 26894063 | - | 12.2 | 1 | 294 | -19 | AluSq | Alu | SINE |
| 26894064 | 26895912 | - | 26.4 | 515 | 2519 | -4121 | L1MD2 | L1 | LINE |

  

|  |  |  |  |  |  |  |  |  |  |
| --- | --- | --- | --- | --- | --- | --- | --- | --- | --- |
| chr6:26907770-26909164 | | | 1366 bp | | |  |  |  |  |
| Genome Start | Genome End | Strand | Div | Repeat Start | Repeat End | Repeat Left | Name | Family | Class |
| 26907770 | 26908078 | - | 31.1 | 5633 | 5944 | -296 | L1ME3B | L1 | LINE |
| 26908079 | 26908482 | + | 22.7 | 1 | 376 | -315 | LTR8 | ERV1 | LTR |
| 26908512 | 26908722 | + | 9.0 | 85 | 303 | -9 | AluSg/x | Alu | SINE |
| 26908723 | 26909050 | + | 22.7 | 377 | 691 | 0 | LTR8 | ERV1 | LTR |
| 26909051 | 26909164 | - | 31.1 | 5514 | 5632 | -608 | L1ME3B | L1 | LINE |

  

|  |  |  |  |  |  |  |  |  |  |
| --- | --- | --- | --- | --- | --- | --- | --- | --- | --- |
| chr6:26924976-26925501 | | | 526 bp | | |  |  |  |  |
| Genome Start | Genome End | Strand | Div | Repeat Start | Repeat End | Repeat Left | Name | Family | Class |
| 26924976 | 26925111 | - | 19.3 | 352 | 467 | 0 | MLT1C | MaLR | LTR |
| 26925112 | 26925408 | - | 10.1 | 1 | 299 | -12 | AluY | Alu | SINE |
| 26925409 | 26925501 | - | 19.3 | 261 | 351 | -116 | MLT1C | MaLR | LTR |

  

|  |  |  |  |  |  |  |  |  |  |
| --- | --- | --- | --- | --- | --- | --- | --- | --- | --- |
| chr6:26925610-26931995 | | | 6374 bp | | |  |  |  |  |
| Genome Start | Genome End | Strand | Div | Repeat Start | Repeat End | Repeat Left | Name | Family | Class |
| 26925610 | 26925769 | + | 23.1 | 5881 | 6041 | -271 | L1MA9 | L1 | LINE |
| 26925789 | 26926140 | + | 18.4 | 634 | 997 | -5506 | L1PA16 | L1 | LINE |
| 26926141 | 26926444 | + | 9.6 | 1 | 304 | -8 | AluSx | Alu | SINE |
| 26926445 | 26928858 | + | 18.0 | 998 | 3470 | -3013 | L1PA16 | L1 | LINE |
| 26928859 | 26929152 | + | 10.0 | 14 | 306 | 0 | AluSg | Alu | SINE |
| 26929153 | 26931838 | + | 17.5 | 3471 | 6503 | 0 | L1PA16 | L1 | LINE |
| 26931832 | 26931995 | + | 23.7 | 6163 | 6323 | -1 | L1MA9 | L1 | LINE |

  

|  |  |  |  |  |  |  |  |  |  |
| --- | --- | --- | --- | --- | --- | --- | --- | --- | --- |
| chr6:26955962-26956555 | | | 594 bp | | |  |  |  |  |
| Genome Start | Genome End | Strand | Div | Repeat Start | Repeat End | Repeat Left | Name | Family | Class |
| 26955962 | 26956118 | - | 18.9 | 136 | 291 | -21 | AluJb | Alu | SINE |
| 26956119 | 26956420 | - | 10.7 | 1 | 300 | -10 | AluSg | Alu | SINE |
| 26956421 | 26956555 | - | 18.9 | 2 | 135 | -177 | AluJb | Alu | SINE |

  

|  |  |  |  |  |  |  |  |  |  |
| --- | --- | --- | --- | --- | --- | --- | --- | --- | --- |
| chr6:26962050-26962643 | | | 594 bp | | |  |  |  |  |
| Genome Start | Genome End | Strand | Div | Repeat Start | Repeat End | Repeat Left | Name | Family | Class |
| 26962050 | 26962172 | + | 18.2 | 2 | 137 | -175 | AluJo | Alu | SINE |
| 26962173 | 26962462 | + | 12.5 | 1 | 288 | -22 | AluSg | Alu | SINE |
| 26962463 | 26962643 | + | 18.2 | 138 | 305 | -7 | AluJo | Alu | SINE |

  

|  |  |  |  |  |  |  |  |  |  |
| --- | --- | --- | --- | --- | --- | --- | --- | --- | --- |
| chr6:26964002-26964585 | | | 584 bp | | |  |  |  |  |
| Genome Start | Genome End | Strand | Div | Repeat Start | Repeat End | Repeat Left | Name | Family | Class |
| 26964002 | 26964128 | + | 16.4 | 1 | 125 | -187 | AluJo | Alu | SINE |
| 26964129 | 26964417 | - | 12.8 | 1 | 292 | -20 | AluSx | Alu | SINE |
| 26964418 | 26964585 | + | 16.4 | 126 | 292 | -20 | AluJo | Alu | SINE |

  

|  |  |  |  |  |  |  |  |  |  |
| --- | --- | --- | --- | --- | --- | --- | --- | --- | --- |
| chr6:26966980-26967885 | | | 906 bp | | |  |  |  |  |
| Genome Start | Genome End | Strand | Div | Repeat Start | Repeat End | Repeat Left | Name | Family | Class |
| 26966980 | 26967128 | + | 21.9 | 1 | 178 | -475 | MER82 | MER2\_type | DNA |
| 26967129 | 26967426 | + | 11.8 | 1 | 288 | -24 | AluSx | Alu | SINE |
| 26967427 | 26967885 | + | 21.9 | 179 | 653 | 0 | MER82 | MER2\_type | DNA |

  

|  |  |  |  |  |  |  |  |  |  |
| --- | --- | --- | --- | --- | --- | --- | --- | --- | --- |
| chr6:26979001-26979495 | | | 495 bp | | |  |  |  |  |
| Genome Start | Genome End | Strand | Div | Repeat Start | Repeat End | Repeat Left | Name | Family | Class |
| 26979001 | 26979131 | + | 16.6 | 1 | 136 | -73 | MER3 | MER1\_type | DNA |
| 26979132 | 26979430 | - | 5.3 | 1 | 299 | -12 | AluY | Alu | SINE |
| 26979431 | 26979495 | + | 16.6 | 137 | 209 | 0 | MER3 | MER1\_type | DNA |

  

|  |  |  |  |  |  |  |  |  |  |
| --- | --- | --- | --- | --- | --- | --- | --- | --- | --- |
| chr6:26985488-26986700 | | | 1213 bp | | |  |  |  |  |
| Genome Start | Genome End | Strand | Div | Repeat Start | Repeat End | Repeat Left | Name | Family | Class |
| 26985488 | 26985880 | + | 22.2 | 2528 | 2896 | -3949 | L1M2 | L1 | LINE |
| 26985881 | 26986184 | + | 12.5 | 1 | 304 | -6 | AluSg | Alu | SINE |
| 26986185 | 26986700 | + | 21.3 | 2897 | 3392 | -3453 | L1M2 | L1 | LINE |

  

|  |  |  |  |  |  |  |  |  |  |
| --- | --- | --- | --- | --- | --- | --- | --- | --- | --- |
| chr6:26987387-26988699 | | | 1313 bp | | |  |  |  |  |
| Genome Start | Genome End | Strand | Div | Repeat Start | Repeat End | Repeat Left | Name | Family | Class |
| 26987387 | 26987814 | + | 23.2 | 6150 | 6586 | -1199 | L1MC3 | L1 | LINE |
| 26987815 | 26988197 | + | 16.5 | 1 | 428 | 0 | MSTA | MaLR | LTR |
| 26988198 | 26988699 | + | 23.2 | 6587 | 7119 | -666 | L1MC3 | L1 | LINE |

  

|  |  |  |  |  |  |  |  |  |  |
| --- | --- | --- | --- | --- | --- | --- | --- | --- | --- |
| chr6:27014310-27016990 | | | 2670 bp | | |  |  |  |  |
| Genome Start | Genome End | Strand | Div | Repeat Start | Repeat End | Repeat Left | Name | Family | Class |
| 27014310 | 27014533 | + | 18.4 | 1 | 238 | -128 | Tigger5a | MER2\_type | DNA |
| 27014544 | 27016919 | - | 6.8 | 3742 | 6150 | -4 | L1PA6 | L1 | LINE |
| 27016921 | 27016990 | + | 20.0 | 356 | 434 | -15 | Tigger5a | MER2\_type | DNA |

  

|  |  |  |  |  |  |  |  |  |  |
| --- | --- | --- | --- | --- | --- | --- | --- | --- | --- |
| chr6:27025034-27031409 | | | 6213 bp | | |  |  |  |  |
| Genome Start | Genome End | Strand | Div | Repeat Start | Repeat End | Repeat Left | Name | Family | Class |
| 27025034 | 27025248 | + | 20.2 | 7 | 235 | -6268 | L1PA16 | L1 | LINE |
| 27025248 | 27029101 | + | 16.9 | 947 | 4819 | -1664 | L1PA16 | L1 | LINE |
| 27029102 | 27029366 | + | 18.0 | 2 | 286 | -16 | AluJo | Alu | SINE |
| 27029531 | 27030356 | + | 17.1 | 4820 | 5660 | -823 | L1PA16 | L1 | LINE |
| 27030357 | 27030677 | + | 8.7 | 5876 | 6167 | -1 | L1PA10 | L1 | LINE |
| 27030678 | 27031409 | + | 13.8 | 5661 | 6398 | -105 | L1PA16 | L1 | LINE |

  

|  |  |  |  |  |  |  |  |  |  |
| --- | --- | --- | --- | --- | --- | --- | --- | --- | --- |
| chr6:27070416-27071206 | | | 791 bp | | |  |  |  |  |
| Genome Start | Genome End | Strand | Div | Repeat Start | Repeat End | Repeat Left | Name | Family | Class |
| 27070416 | 27070540 | - | 29.0 | 325 | 445 | 0 | MER65A | ERV1 | LTR |
| 27070541 | 27070914 | - | 13.4 | 1 | 428 | 0 | MSTA | MaLR | LTR |
| 27070915 | 27071206 | - | 29.0 | 1 | 324 | -121 | MER65A | ERV1 | LTR |

  

|  |  |  |  |  |  |  |  |  |  |
| --- | --- | --- | --- | --- | --- | --- | --- | --- | --- |
| chr6:27074548-27076345 | | | 1382 bp | | |  |  |  |  |
| Genome Start | Genome End | Strand | Div | Repeat Start | Repeat End | Repeat Left | Name | Family | Class |
| 27074548 | 27074890 | - | 30.9 | 1415 | 1768 | -1651 | L2 | L2 | LINE |
| 27075148 | 27075347 | - | 24.8 | 4 | 212 | -12 | MER58A | MER1\_type | DNA |
| 27075454 | 27075563 | - | 32.0 | 1 | 103 | -75 | MER5B | MER1\_type | DNA |
| 27075617 | 27075779 | - | 34.6 | 911 | 1075 | -2344 | L2 | L2 | LINE |
| 27075780 | 27076295 | + | 26.2 | 3 | 560 | -2 | MLT1F2 | MaLR | LTR |
| 27076296 | 27076345 | - | 34.6 | 861 | 910 | -2509 | L2 | L2 | LINE |

  

|  |  |  |  |  |  |  |  |  |  |
| --- | --- | --- | --- | --- | --- | --- | --- | --- | --- |
| chr6:31439457-31440363 | | | 756 bp | | |  |  |  |  |
| Genome Start | Genome End | Strand | Div | Repeat Start | Repeat End | Repeat Left | Name | Family | Class |
| 31439457 | 31439845 | - | 25.1 | 2036 | 2490 | -17 | HAL1 | L1 | LINE |
| 31439942 | 31440064 | - | 22.1 | 1 | 122 | -11 | FLAM\_A | Alu | SINE |
| 31440120 | 31440363 | - | 22.2 | 1530 | 1784 | -723 | HAL1 | L1 | LINE |

  

|  |  |  |  |  |  |  |  |  |  |
| --- | --- | --- | --- | --- | --- | --- | --- | --- | --- |
| chr6:57849127-57851043 | | | 1877 bp | | |  |  |  |  |
| Genome Start | Genome End | Strand | Div | Repeat Start | Repeat End | Repeat Left | Name | Family | Class |
| 57849127 | 57849167 | - | 19.2 | 475 | 505 | 0 | MLT1D | MaLR | LTR |
| 57849168 | 57849470 | + | 18.8 | 3 | 306 | -6 | AluJo | Alu | SINE |
| 57849471 | 57849786 | - | 18.8 | 74 | 474 | -31 | MLT1D | MaLR | LTR |
| 57849827 | 57850452 | - | 4.2 | 5527 | 6154 | 0 | L1PA5 | L1 | LINE |
| 57850453 | 57850991 | - | 22.5 | 1 | 545 | -18 | MER68 | ERVL | LTR |
| 57850992 | 57851043 | - | 24.1 | 28 | 79 | -426 | MLT1D | MaLR | LTR |

  

|  |  |  |  |  |  |  |  |  |  |
| --- | --- | --- | --- | --- | --- | --- | --- | --- | --- |
| chr6:57922635-57924267 | | | 1619 bp | | |  |  |  |  |
| Genome Start | Genome End | Strand | Div | Repeat Start | Repeat End | Repeat Left | Name | Family | Class |
| 57922635 | 57923188 | - | 23.7 | 5581 | 6161 | -5 | L1ME1 | L1 | LINE |
| 57923196 | 57923734 | - | 22.0 | 1 | 554 | 0 | MLT2B3 | ERVL | LTR |
| 57923742 | 57924267 | - | 23.7 | 5048 | 5556 | -610 | L1ME1 | L1 | LINE |

  

|  |  |  |  |  |  |  |  |  |  |
| --- | --- | --- | --- | --- | --- | --- | --- | --- | --- |
| chr6:57945125-57946073 | | | 876 bp | | |  |  |  |  |
| Genome Start | Genome End | Strand | Div | Repeat Start | Repeat End | Repeat Left | Name | Family | Class |
| 57945125 | 57945276 | - | 34.4 | 3268 | 3414 | -5 | L2 | L2 | LINE |
| 57945277 | 57945593 | + | 16.0 | 1 | 312 | 0 | AluSx | Alu | SINE |
| 57945594 | 57945870 | - | 34.4 | 2985 | 3267 | -152 | L2 | L2 | LINE |
| 57945944 | 57946073 | - | 26.1 | 2692 | 2859 | -560 | L2 | L2 | LINE |

  

|  |  |  |  |  |  |  |  |  |  |
| --- | --- | --- | --- | --- | --- | --- | --- | --- | --- |
| chr6:57959164-57963347 | | | 4192 bp | | |  |  |  |  |
| Genome Start | Genome End | Strand | Div | Repeat Start | Repeat End | Repeat Left | Name | Family | Class |
| 57959164 | 57959244 | + | 18.5 | 5 | 88 | -426 | MER67D | ERV1 | LTR |
| 57959245 | 57961617 | - | 7.3 | 3824 | 6201 | 0 | L1PA8 | L1 | LINE |
| 57961609 | 57962978 | + | 8.9 | 2430 | 3812 | -2334 | L1PA8 | L1 | LINE |
| 57962980 | 57963347 | + | 21.2 | 63 | 436 | -78 | MER67D | ERV1 | LTR |

  

|  |  |  |  |  |  |  |  |  |  |
| --- | --- | --- | --- | --- | --- | --- | --- | --- | --- |
| chr6:57981911-57983001 | | | 1091 bp | | |  |  |  |  |
| Genome Start | Genome End | Strand | Div | Repeat Start | Repeat End | Repeat Left | Name | Family | Class |
| 57981911 | 57982575 | - | 14.4 | 5581 | 6305 | 0 | L1MA3 | L1 | LINE |
| 57982576 | 57982827 | - | 11.9 | 56 | 307 | -2 | AluSc | Alu | SINE |
| 57982828 | 57983001 | - | 14.4 | 5407 | 5580 | -725 | L1MA3 | L1 | LINE |

  

|  |  |  |  |  |  |  |  |  |  |
| --- | --- | --- | --- | --- | --- | --- | --- | --- | --- |
| chr6:58015205-58016278 | | | 844 bp | | |  |  |  |  |
| Genome Start | Genome End | Strand | Div | Repeat Start | Repeat End | Repeat Left | Name | Family | Class |
| 58015205 | 58015761 | - | 35.1 | 2806 | 3378 | 0 | L2 | L2 | LINE |
| 58015984 | 58016151 | - | 16.3 | 6002 | 6167 | -1 | L1MB1 | L1 | LINE |
| 58016160 | 58016278 | - | 28.8 | 2716 | 2837 | -582 | L2 | L2 | LINE |

  

|  |  |  |  |  |  |  |  |  |  |
| --- | --- | --- | --- | --- | --- | --- | --- | --- | --- |
| chr6:58046057-58047241 | | | 1185 bp | | |  |  |  |  |
| Genome Start | Genome End | Strand | Div | Repeat Start | Repeat End | Repeat Left | Name | Family | Class |
| 58046057 | 58046144 | + | 26.2 | 65 | 152 | -110 | MIR | MIR | SINE |
| 58046145 | 58046746 | + | 17.7 | 1 | 554 | 0 | MLT2B3 | ERVL | LTR |
| 58046747 | 58046813 | + | 26.2 | 153 | 214 | -48 | MIR | MIR | SINE |
| 58046814 | 58047196 | - | 15.7 | 1 | 428 | 0 | MSTA | MaLR | LTR |
| 58047197 | 58047241 | + | 26.2 | 215 | 259 | -3 | MIR | MIR | SINE |

  

|  |  |  |  |  |  |  |  |  |  |
| --- | --- | --- | --- | --- | --- | --- | --- | --- | --- |
| chr6:58055550-58067950 | | | 12394 bp | | |  |  |  |  |
| Genome Start | Genome End | Strand | Div | Repeat Start | Repeat End | Repeat Left | Name | Family | Class |
| 58055550 | 58056655 | - | 14.1 | 5344 | 6494 | -9 | L1PA16 | L1 | LINE |
| 58056660 | 58062780 | - | 5.3 | 7 | 6147 | -8 | L1PA4 | L1 | LINE |
| 58062784 | 58067950 | - | 17.8 | 218 | 5365 | -1118 | L1PA16 | L1 | LINE |

  

|  |  |  |  |  |  |  |  |  |  |
| --- | --- | --- | --- | --- | --- | --- | --- | --- | --- |
| chr6:58130260-58132732 | | | 2447 bp | | |  |  |  |  |
| Genome Start | Genome End | Strand | Div | Repeat Start | Repeat End | Repeat Left | Name | Family | Class |
| 58130260 | 58130584 | - | 17.3 | 127 | 449 | -3 | LTR47A | ERVL | LTR |
| 58130609 | 58132131 | - | 13.1 | 4625 | 6160 | -6 | L1PA16 | L1 | LINE |
| 58132133 | 58132634 | + | 13.7 | 4126 | 4627 | -1519 | L1PA16 | L1 | LINE |
| 58132636 | 58132732 | - | 19.6 | 1 | 97 | -355 | LTR47A | ERVL | LTR |

  

|  |  |  |  |  |  |  |  |  |  |
| --- | --- | --- | --- | --- | --- | --- | --- | --- | --- |
| chr6:58133058-58136153 | | | 3096 bp | | |  |  |  |  |
| Genome Start | Genome End | Strand | Div | Repeat Start | Repeat End | Repeat Left | Name | Family | Class |
| 58133058 | 58134058 | - | 15.1 | 1767 | 2769 | -3692 | L1P3 | L1 | LINE |
| 58134059 | 58134360 | - | 14.3 | 1 | 304 | -8 | AluSx | Alu | SINE |
| 58134361 | 58136153 | - | 20.1 | 2 | 1766 | -4717 | L1P3 | L1 | LINE |

  

|  |  |  |  |  |  |  |  |  |  |
| --- | --- | --- | --- | --- | --- | --- | --- | --- | --- |
| chr6:58145496-58155361 | | | 9836 bp | | |  |  |  |  |
| Genome Start | Genome End | Strand | Div | Repeat Start | Repeat End | Repeat Left | Name | Family | Class |
| 58145496 | 58146230 | - | 16.8 | 6656 | 7395 | -315 | L1MA8 | L1 | LINE |
| 58146231 | 58146653 | + | 17.3 | 1 | 432 | 0 | MSTB1 | MaLR | LTR |
| 58146657 | 58148182 | + | 27.0 | 2 | 1735 | 0 | MLT-int | MaLR | LTR |
| 58148187 | 58148609 | + | 18.7 | 5 | 432 | 0 | MSTB1 | MaLR | LTR |
| 58148610 | 58149096 | - | 17.0 | 6157 | 6655 | -907 | L1MA8 | L1 | LINE |
| 58149097 | 58149529 | + | 15.7 | 1 | 434 | 0 | Tigger2a | MER2\_type | DNA |
| 58149530 | 58150435 | - | 17.0 | 5274 | 6156 | -1406 | L1MA8 | L1 | LINE |
| 58150436 | 58150797 | + | 17.9 | 1 | 364 | 0 | THE1B | MaLR | LTR |
| 58150798 | 58150958 | - | 17.0 | 5123 | 5273 | -2289 | L1MA8 | L1 | LINE |
| 58150959 | 58151249 | - | 18.1 | 1 | 299 | -13 | AluJb | Alu | SINE |
| 58151250 | 58152567 | - | 17.0 | 3924 | 5122 | -2440 | L1MA8 | L1 | LINE |
| 58152568 | 58152875 | - | 10.1 | 1 | 309 | -2 | AluY | Alu | SINE |
| 58152876 | 58154770 | - | 24.6 | 2008 | 3923 | -3639 | L1MA8 | L1 | LINE |
| 58154794 | 58155361 | - | 25.1 | 466 | 1050 | -6439 | L1MA8 | L1 | LINE |

  

|  |  |  |  |  |  |  |  |  |  |
| --- | --- | --- | --- | --- | --- | --- | --- | --- | --- |
| chr6:58160638-58162920 | | | 2283 bp | | |  |  |  |  |
| Genome Start | Genome End | Strand | Div | Repeat Start | Repeat End | Repeat Left | Name | Family | Class |
| 58160638 | 58160982 | - | 18.6 | 4852 | 5183 | -963 | L1M4 | L1 | LINE |
| 58160983 | 58161261 | + | 15.8 | 21 | 300 | -12 | AluSx | Alu | SINE |
| 58161262 | 58161691 | - | 18.6 | 4425 | 4851 | -1295 | L1M4 | L1 | LINE |
| 58161692 | 58162887 | - | 14.8 | 1 | 1174 | -1 | Merlin1\_HS | Merlin | DNA |
| 58162888 | 58162920 | - | 18.6 | 4392 | 4424 | -1722 | L1M4 | L1 | LINE |

  

|  |  |  |  |  |  |  |  |  |  |
| --- | --- | --- | --- | --- | --- | --- | --- | --- | --- |
| chr6:58167334-58167958 | | | 533 bp | | |  |  |  |  |
| Genome Start | Genome End | Strand | Div | Repeat Start | Repeat End | Repeat Left | Name | Family | Class |
| 58167334 | 58167403 | + | 31.4 | 2 | 74 | -447 | LTR33 | ERVL | LTR |
| 58167404 | 58167582 | + | 27.4 | 4 | 178 | 0 | MER45A | Tip100 | DNA |
| 58167583 | 58167716 | + | 31.4 | 75 | 202 | -319 | LTR33 | ERVL | LTR |
| 58167809 | 58167958 | + | 25.7 | 367 | 519 | -2 | LTR33 | ERVL | LTR |

  

|  |  |  |  |  |  |  |  |  |  |
| --- | --- | --- | --- | --- | --- | --- | --- | --- | --- |
| chr6:58247757-58248333 | | | 577 bp | | |  |  |  |  |
| Genome Start | Genome End | Strand | Div | Repeat Start | Repeat End | Repeat Left | Name | Family | Class |
| 58247757 | 58247817 | - | 30.7 | 7478 | 7527 | -434 | L1MC5 | L1 | LINE |
| 58247818 | 58248117 | - | 15.9 | 6050 | 6344 | 0 | L1MC2 | L1 | LINE |
| 58248118 | 58248333 | - | 30.7 | 7258 | 7477 | -484 | L1MC5 | L1 | LINE |

  

|  |  |  |  |  |  |  |  |  |  |
| --- | --- | --- | --- | --- | --- | --- | --- | --- | --- |
| chr6:58308420-58309824 | | | 1368 bp | | |  |  |  |  |
| Genome Start | Genome End | Strand | Div | Repeat Start | Repeat End | Repeat Left | Name | Family | Class |
| 58308420 | 58308728 | - | 31.6 | 5633 | 5944 | -296 | L1ME3B | L1 | LINE |
| 58308729 | 58309134 | + | 22.5 | 1 | 380 | -311 | LTR8 | ERV1 | LTR |
| 58309172 | 58309393 | + | 10.0 | 85 | 312 | 0 | AluSg/x | Alu | SINE |
| 58309394 | 58309710 | + | 22.5 | 381 | 691 | 0 | LTR8 | ERV1 | LTR |
| 58309711 | 58309824 | - | 31.6 | 5514 | 5632 | -608 | L1ME3B | L1 | LINE |

  

|  |  |  |  |  |  |  |  |  |  |
| --- | --- | --- | --- | --- | --- | --- | --- | --- | --- |
| chr6:58325082-58325608 | | | 527 bp | | |  |  |  |  |
| Genome Start | Genome End | Strand | Div | Repeat Start | Repeat End | Repeat Left | Name | Family | Class |
| 58325082 | 58325208 | - | 20.3 | 352 | 467 | 0 | MLT1C | MaLR | LTR |
| 58325209 | 58325515 | - | 12.1 | 1 | 309 | -2 | AluY | Alu | SINE |
| 58325516 | 58325608 | - | 20.3 | 261 | 351 | -116 | MLT1C | MaLR | LTR |

  

|  |  |  |  |  |  |  |  |  |  |
| --- | --- | --- | --- | --- | --- | --- | --- | --- | --- |
| chr6:58355527-58356118 | | | 592 bp | | |  |  |  |  |
| Genome Start | Genome End | Strand | Div | Repeat Start | Repeat End | Repeat Left | Name | Family | Class |
| 58355527 | 58355681 | - | 19.9 | 137 | 291 | -21 | AluJb | Alu | SINE |
| 58355682 | 58355983 | - | 10.0 | 1 | 299 | -11 | AluSg | Alu | SINE |
| 58355984 | 58356118 | - | 19.9 | 2 | 136 | -176 | AluJb | Alu | SINE |

  

|  |  |  |  |  |  |  |  |  |  |
| --- | --- | --- | --- | --- | --- | --- | --- | --- | --- |
| chr6:58361600-58362196 | | | 597 bp | | |  |  |  |  |
| Genome Start | Genome End | Strand | Div | Repeat Start | Repeat End | Repeat Left | Name | Family | Class |
| 58361600 | 58361722 | + | 18.6 | 2 | 137 | -175 | AluJo | Alu | SINE |
| 58361723 | 58362015 | + | 12.7 | 1 | 288 | -22 | AluSg | Alu | SINE |
| 58362016 | 58362196 | + | 18.6 | 138 | 305 | -7 | AluJo | Alu | SINE |

  

|  |  |  |  |  |  |  |  |  |  |
| --- | --- | --- | --- | --- | --- | --- | --- | --- | --- |
| chr6:58363562-58364127 | | | 566 bp | | |  |  |  |  |
| Genome Start | Genome End | Strand | Div | Repeat Start | Repeat End | Repeat Left | Name | Family | Class |
| 58363562 | 58363688 | + | 17.4 | 1 | 125 | -187 | AluJo | Alu | SINE |
| 58363689 | 58363976 | - | 12.2 | 1 | 291 | -21 | AluSx | Alu | SINE |
| 58363977 | 58364127 | + | 17.4 | 126 | 275 | -37 | AluJo | Alu | SINE |

  

|  |  |  |  |  |  |  |  |  |  |
| --- | --- | --- | --- | --- | --- | --- | --- | --- | --- |
| chr6:58366570-58367474 | | | 905 bp | | |  |  |  |  |
| Genome Start | Genome End | Strand | Div | Repeat Start | Repeat End | Repeat Left | Name | Family | Class |
| 58366570 | 58366718 | + | 21.9 | 1 | 178 | -475 | MER82 | MER2\_type | DNA |
| 58366719 | 58367022 | + | 12.8 | 1 | 290 | -22 | AluSx | Alu | SINE |
| 58367023 | 58367474 | + | 21.9 | 179 | 653 | 0 | MER82 | MER2\_type | DNA |

  

|  |  |  |  |  |  |  |  |  |  |
| --- | --- | --- | --- | --- | --- | --- | --- | --- | --- |
| chr6:58375342-58376350 | | | 1009 bp | | |  |  |  |  |
| Genome Start | Genome End | Strand | Div | Repeat Start | Repeat End | Repeat Left | Name | Family | Class |
| 58375342 | 58375385 | + | 22.8 | 1 | 44 | -330 | MLT1A | MaLR | LTR |
| 58375386 | 58375695 | + | 5.9 | 1 | 307 | -4 | AluY | Alu | SINE |
| 58375696 | 58375979 | + | 22.8 | 45 | 308 | -66 | MLT1A | MaLR | LTR |
| 58375980 | 58376282 | - | 15.5 | 1 | 312 | 0 | AluSx | Alu | SINE |
| 58376283 | 58376350 | + | 22.8 | 309 | 374 | 0 | MLT1A | MaLR | LTR |

  

|  |  |  |  |  |  |  |  |  |  |
| --- | --- | --- | --- | --- | --- | --- | --- | --- | --- |
| chr6:58376371-58376865 | | | 495 bp | | |  |  |  |  |
| Genome Start | Genome End | Strand | Div | Repeat Start | Repeat End | Repeat Left | Name | Family | Class |
| 58376371 | 58376501 | + | 17.6 | 1 | 136 | -73 | MER3 | MER1\_type | DNA |
| 58376502 | 58376800 | - | 6.3 | 1 | 299 | -12 | AluY | Alu | SINE |
| 58376801 | 58376865 | + | 17.6 | 137 | 209 | 0 | MER3 | MER1\_type | DNA |

  

|  |  |  |  |  |  |  |  |  |  |
| --- | --- | --- | --- | --- | --- | --- | --- | --- | --- |
| chr6:58389148-58390165 | | | 1018 bp | | |  |  |  |  |
| Genome Start | Genome End | Strand | Div | Repeat Start | Repeat End | Repeat Left | Name | Family | Class |
| 58389148 | 58389414 | + | 22.2 | 15 | 268 | -595 | MER21B | ERV1 | LTR |
| 58389415 | 58389717 | - | 15.4 | 1 | 304 | -8 | AluJo | Alu | SINE |
| 58389718 | 58390165 | + | 22.2 | 269 | 802 | -61 | MER21B | ERV1 | LTR |

  

|  |  |  |  |  |  |  |  |  |  |
| --- | --- | --- | --- | --- | --- | --- | --- | --- | --- |
| chr6:58438393-58439426 | | | 914 bp | | |  |  |  |  |
| Genome Start | Genome End | Strand | Div | Repeat Start | Repeat End | Repeat Left | Name | Family | Class |
| 58438393 | 58438656 | + | 28.5 | 993 | 1261 | -1246 | HAL1 | L1 | LINE |
| 58438691 | 58439000 | + | 11.6 | 1 | 312 | 0 | AluSx | Alu | SINE |
| 58439087 | 58439426 | + | 26.9 | 1491 | 1888 | -619 | HAL1 | L1 | LINE |

  

|  |  |  |  |  |  |  |  |  |  |
| --- | --- | --- | --- | --- | --- | --- | --- | --- | --- |
| chr6:58497769-58504288 | | | 6415 bp | | |  |  |  |  |
| Genome Start | Genome End | Strand | Div | Repeat Start | Repeat End | Repeat Left | Name | Family | Class |
| 58497769 | 58499028 | - | 17.8 | 4985 | 6166 | -165 | MER39B-int | ERV1 | LTR |
| 58499029 | 58499321 | - | 11.7 | 1 | 296 | -17 | AluSq | Alu | SINE |
| 58499322 | 58501194 | - | 17.8 | 3147 | 4984 | -1347 | MER39B-int | ERV1 | LTR |
| 58501195 | 58501499 | - | 9.4 | 1 | 298 | -13 | AluY | Alu | SINE |
| 58501500 | 58503309 | - | 17.8 | 1236 | 3146 | -3185 | MER39B-int | ERV1 | LTR |
| 58503383 | 58503729 | - | 17.8 | 870 | 1235 | -5096 | MER39B-int | ERV1 | LTR |
| 58503762 | 58504288 | - | 17.6 | 1 | 569 | -5762 | MER39B-int | ERV1 | LTR |

  

|  |  |  |  |  |  |  |  |  |  |
| --- | --- | --- | --- | --- | --- | --- | --- | --- | --- |
| chr6:58558625-58559633 | | | 1009 bp | | |  |  |  |  |
| Genome Start | Genome End | Strand | Div | Repeat Start | Repeat End | Repeat Left | Name | Family | Class |
| 58558625 | 58559001 | - | 19.5 | 322 | 787 | 0 | LTR48 | ERV1 | LTR |
| 58559002 | 58559325 | - | 16.0 | 1 | 312 | 0 | AluSx | Alu | SINE |
| 58559326 | 58559633 | - | 19.5 | 2 | 321 | -466 | LTR48 | ERV1 | LTR |

  

|  |  |  |  |  |  |  |  |  |  |
| --- | --- | --- | --- | --- | --- | --- | --- | --- | --- |
| chr6:58600192-58601053 | | | 834 bp | | |  |  |  |  |
| Genome Start | Genome End | Strand | Div | Repeat Start | Repeat End | Repeat Left | Name | Family | Class |
| 58600192 | 58600348 | + | 24.0 | 2 | 155 | -107 | MIR | MIR | SINE |
| 58600364 | 58600698 | - | 2.1 | 5539 | 5873 | -282 | L1PA5 | L1 | LINE |
| 58600699 | 58600983 | + | 3.2 | 5866 | 6150 | -4 | L1PA5 | L1 | LINE |
| 58600997 | 58601053 | + | 23.2 | 196 | 257 | -10 | MIR | MIR | SINE |

  

|  |  |  |  |  |  |  |  |  |  |
| --- | --- | --- | --- | --- | --- | --- | --- | --- | --- |
| chr6:58627802-58629089 | | | 1288 bp | | |  |  |  |  |
| Genome Start | Genome End | Strand | Div | Repeat Start | Repeat End | Repeat Left | Name | Family | Class |
| 58627802 | 58628141 | - | 9.6 | 5830 | 6161 | -2 | L1PA13 | L1 | LINE |
| 58628142 | 58628441 | - | 11.0 | 1 | 301 | -12 | AluSp | Alu | SINE |
| 58628442 | 58629089 | - | 9.1 | 5179 | 5829 | -334 | L1PA13 | L1 | LINE |

  

|  |  |  |  |  |  |  |  |  |  |
| --- | --- | --- | --- | --- | --- | --- | --- | --- | --- |
| chr6:58654906-58655817 | | | 912 bp | | |  |  |  |  |
| Genome Start | Genome End | Strand | Div | Repeat Start | Repeat End | Repeat Left | Name | Family | Class |
| 58654906 | 58655143 | - | 32.2 | 345 | 591 | 0 | MLT1K | MaLR | LTR |
| 58655144 | 58655534 | + | 16.7 | 1 | 497 | 0 | LOR1a | ERV1 | LTR |
| 58655535 | 58655817 | - | 32.2 | 41 | 344 | -247 | MLT1K | MaLR | LTR |

  

|  |  |  |  |  |  |  |  |  |  |
| --- | --- | --- | --- | --- | --- | --- | --- | --- | --- |
| chr6:58782097-58782898 | | | 685 bp | | |  |  |  |  |
| Genome Start | Genome End | Strand | Div | Repeat Start | Repeat End | Repeat Left | Name | Family | Class |
| 58782097 | 58782324 | + | 37.0 | 2599 | 2830 | -589 | L2 | L2 | LINE |
| 58782418 | 58782730 | - | 6.4 | 1 | 296 | -15 | AluY | Alu | SINE |
| 58782755 | 58782898 | + | 31.7 | 3033 | 3184 | -194 | L2 | L2 | LINE |

  

|  |  |  |  |  |  |  |  |  |  |
| --- | --- | --- | --- | --- | --- | --- | --- | --- | --- |
| chr6:58796487-58798565 | | | 2002 bp | | |  |  |  |  |
| Genome Start | Genome End | Strand | Div | Repeat Start | Repeat End | Repeat Left | Name | Family | Class |
| 58796487 | 58796606 | - | 15.0 | 1333 | 1455 | 0 | Charlie1a | MER1\_type | DNA |
| 58796627 | 58796721 | - | 15.8 | 1043 | 1137 | -38 | Merlin1\_HS | Merlin | DNA |
| 58796779 | 58797054 | - | 19.9 | 1048 | 1322 | -133 | Charlie1a | MER1\_type | DNA |
| 58797055 | 58797251 | + | 13.7 | 6104 | 6302 | 0 | L1MA1 | L1 | LINE |
| 58797252 | 58798170 | - | 19.9 | 100 | 1047 | -408 | Charlie1a | MER1\_type | DNA |
| 58798171 | 58798464 | - | 12.6 | 1 | 295 | -18 | AluSp | Alu | SINE |
| 58798465 | 58798565 | - | 19.9 | 1 | 99 | -1356 | Charlie1a | MER1\_type | DNA |

  

|  |  |  |  |  |  |  |  |  |  |
| --- | --- | --- | --- | --- | --- | --- | --- | --- | --- |
| chr6:58818997-58820027 | | | 1031 bp | | |  |  |  |  |
| Genome Start | Genome End | Strand | Div | Repeat Start | Repeat End | Repeat Left | Name | Family | Class |
| 58818997 | 58819166 | - | 35.3 | 3177 | 3345 | -74 | L2 | L2 | LINE |
| 58819167 | 58819635 | - | 18.8 | 1 | 505 | 0 | MLT1D | MaLR | LTR |
| 58819636 | 58820027 | - | 30.1 | 2730 | 3176 | -243 | L2 | L2 | LINE |

  

|  |  |  |  |  |  |  |  |  |  |
| --- | --- | --- | --- | --- | --- | --- | --- | --- | --- |
| chr6:66076322-66077643 | | | 1219 bp | | |  |  |  |  |
| Genome Start | Genome End | Strand | Div | Repeat Start | Repeat End | Repeat Left | Name | Family | Class |
| 66076322 | 66076658 | + | 32.9 | 2503 | 2869 | -550 | L2 | L2 | LINE |
| 66076676 | 66076956 | + | 11.4 | 1 | 281 | -31 | AluSx | Alu | SINE |
| 66077008 | 66077120 | + | 20.4 | 2 | 106 | -436 | MLT1F | MaLR | LTR |
| 66077156 | 66077643 | + | 28.1 | 2876 | 3415 | -4 | L2 | L2 | LINE |

  

|  |  |  |  |  |  |  |  |  |  |
| --- | --- | --- | --- | --- | --- | --- | --- | --- | --- |
| chr6:89827654-89828272 | | | 619 bp | | |  |  |  |  |
| Genome Start | Genome End | Strand | Div | Repeat Start | Repeat End | Repeat Left | Name | Family | Class |
| 89827654 | 89827785 | + | 19.7 | 3 | 127 | -185 | AluJo | Alu | SINE |
| 89827786 | 89828088 | + | 11.3 | 1 | 300 | -12 | AluSx | Alu | SINE |
| 89828089 | 89828272 | + | 19.7 | 128 | 307 | -5 | AluJo | Alu | SINE |

  

|  |  |  |  |  |  |  |  |  |  |
| --- | --- | --- | --- | --- | --- | --- | --- | --- | --- |
| chr6:90402570-90403563 | | | 982 bp | | |  |  |  |  |
| Genome Start | Genome End | Strand | Div | Repeat Start | Repeat End | Repeat Left | Name | Family | Class |
| 90402570 | 90402909 | - | 13.4 | 5801 | 6178 | 0 | L1MB8 | L1 | LINE |
| 90402922 | 90403120 | - | 20.3 | 5442 | 5638 | -540 | L1MB8 | L1 | LINE |
| 90403121 | 90403420 | - | 12.7 | 1 | 301 | -11 | AluSx | Alu | SINE |
| 90403421 | 90403563 | - | 23.2 | 5287 | 5441 | -737 | L1MB8 | L1 | LINE |

  

|  |  |  |  |  |  |  |  |  |  |
| --- | --- | --- | --- | --- | --- | --- | --- | --- | --- |
| chr6:132075754-132076226 | | | 473 bp | | |  |  |  |  |
| Genome Start | Genome End | Strand | Div | Repeat Start | Repeat End | Repeat Left | Name | Family | Class |
| 132075754 | 132075767 | - | 28.2 | 562 | 573 | -5954 | L1MEa | L1 | LINE |
| 132075768 | 132076149 | + | 12.6 | 1 | 364 | 0 | THE1B | MaLR | LTR |
| 132076150 | 132076226 | - | 28.2 | 488 | 561 | -5966 | L1MEa | L1 | LINE |

  

|  |  |  |  |  |  |  |  |  |  |
| --- | --- | --- | --- | --- | --- | --- | --- | --- | --- |
| chr6:132076529-132077076 | | | 523 bp | | |  |  |  |  |
| Genome Start | Genome End | Strand | Div | Repeat Start | Repeat End | Repeat Left | Name | Family | Class |
| 132076529 | 132076609 | - | 23.5 | 368 | 448 | -1 | Tigger5a | MER2\_type | DNA |
| 132076635 | 132076779 | - | 22.1 | 1 | 140 | -45 | FAM | Alu | SINE |
| 132076780 | 132077076 | - | 15.2 | 1 | 298 | -68 | Tigger5a | MER2\_type | DNA |

  

|  |  |  |  |  |  |  |  |  |  |
| --- | --- | --- | --- | --- | --- | --- | --- | --- | --- |
| chr6:150371985-150372580 | | | 596 bp | | |  |  |  |  |
| Genome Start | Genome End | Strand | Div | Repeat Start | Repeat End | Repeat Left | Name | Family | Class |
| 150371985 | 150372143 | - | 35.1 | 2193 | 2364 | -3870 | L1MC4 | L1 | LINE |
| 150372144 | 150372446 | - | 12.6 | 3 | 307 | -5 | AluJo | Alu | SINE |
| 150372447 | 150372580 | - | 35.1 | 2037 | 2192 | -4042 | L1MC4 | L1 | LINE |

  

|  |  |  |  |  |  |  |  |  |  |
| --- | --- | --- | --- | --- | --- | --- | --- | --- | --- |
| chr6:151411624-151412587 | | | 964 bp | | |  |  |  |  |
| Genome Start | Genome End | Strand | Div | Repeat Start | Repeat End | Repeat Left | Name | Family | Class |
| 151411624 | 151411902 | - | 21.9 | 569 | 859 | -4 | MER21B | ERV1 | LTR |
| 151411903 | 151412038 | + | 10.7 | 1 | 136 | -176 | AluSx | Alu | SINE |
| 151412039 | 151412341 | + | 6.3 | 1 | 302 | -9 | AluY | Alu | SINE |
| 151412342 | 151412508 | + | 10.7 | 137 | 300 | -12 | AluSx | Alu | SINE |
| 151412509 | 151412587 | - | 21.9 | 492 | 568 | -295 | MER21B | ERV1 | LTR |

  

|  |  |  |  |  |  |  |  |  |  |
| --- | --- | --- | --- | --- | --- | --- | --- | --- | --- |
| chr6:153351180-153352104 | | | 577 bp | | |  |  |  |  |
| Genome Start | Genome End | Strand | Div | Repeat Start | Repeat End | Repeat Left | Name | Family | Class |
| 153351180 | 153351263 | + | 25.0 | 83 | 169 | -604 | LTR41 | ERVL | LTR |
| 153351281 | 153351351 | + | 18.3 | 1 | 72 | -484 | MLT2D | ERVL | LTR |
| 153351398 | 153351711 | + | 26.0 | 55 | 364 | -50 | MLT2D | ERVL | LTR |
| 153351997 | 153352104 | + | 15.2 | 660 | 760 | -13 | LTR41 | ERVL | LTR |

  

|  |  |  |  |  |  |  |  |  |  |
| --- | --- | --- | --- | --- | --- | --- | --- | --- | --- |
| chr6:167693333-167693845 | | | 513 bp | | |  |  |  |  |
| Genome Start | Genome End | Strand | Div | Repeat Start | Repeat End | Repeat Left | Name | Family | Class |
| 167693333 | 167693396 | - | 18.2 | 131 | 193 | 0 | MER53 | DNA | DNA |
| 167693397 | 167693704 | - | 12.7 | 1 | 308 | -4 | AluSx | Alu | SINE |
| 167693705 | 167693845 | - | 18.2 | 1 | 130 | -63 | MER53 | DNA | DNA |

  

|  |  |  |  |  |  |  |  |  |  |
| --- | --- | --- | --- | --- | --- | --- | --- | --- | --- |
| chr6:170756313-170757334 | | | 1022 bp | | |  |  |  |  |
| Genome Start | Genome End | Strand | Div | Repeat Start | Repeat End | Repeat Left | Name | Family | Class |
| 170756313 | 170756365 | - | 14.8 | 684 | 722 | 0 | MER72 | ERV1 | LTR |
| 170756366 | 170756673 | - | 14.0 | 5 | 311 | -2 | AluSq | Alu | SINE |
| 170756674 | 170757334 | - | 14.8 | 1 | 683 | -39 | MER72 | ERV1 | LTR |

  

|  |  |  |  |  |  |  |  |  |  |
| --- | --- | --- | --- | --- | --- | --- | --- | --- | --- |
| chr6:170767170-170769238 | | | 1782 bp | | |  |  |  |  |
| Genome Start | Genome End | Strand | Div | Repeat Start | Repeat End | Repeat Left | Name | Family | Class |
| 170767170 | 170767887 | - | 27.8 | 5242 | 5996 | -227 | L1ME3B | L1 | LINE |
| 170767925 | 170768169 | - | 28.4 | 4906 | 5199 | -930 | L1ME3B | L1 | LINE |
| 170768170 | 170768659 | - | 22.9 | 1 | 495 | -10 | MLT1D | MaLR | LTR |
| 170768857 | 170768988 | - | 26.4 | 4579 | 4716 | -1430 | L1MC | L1 | LINE |
| 170769042 | 170769238 | - | 23.9 | 3777 | 3965 | -2164 | L1ME3B | L1 | LINE |

  

|  |  |  |  |  |  |  |  |  |  |
| --- | --- | --- | --- | --- | --- | --- | --- | --- | --- |
| chr6:170776257-170779053 | | | 2793 bp | | |  |  |  |  |
| Genome Start | Genome End | Strand | Div | Repeat Start | Repeat End | Repeat Left | Name | Family | Class |
| 170776257 | 170776533 | - | 29.4 | 252 | 526 | -29 | MLT1H1 | MaLR | LTR |
| 170776534 | 170776893 | + | 14.7 | 3 | 364 | 0 | THE1B | MaLR | LTR |
| 170776898 | 170778455 | + | 31.1 | 4 | 1735 | 0 | MLT-int | MaLR | LTR |
| 170778456 | 170778813 | + | 18.1 | 1 | 364 | 0 | THE1B | MaLR | LTR |
| 170778814 | 170779053 | - | 29.4 | 3 | 251 | -304 | MLT1H1 | MaLR | LTR |

  

|  |  |  |  |  |  |  |  |  |  |
| --- | --- | --- | --- | --- | --- | --- | --- | --- | --- |
| chr6:170786035-170786682 | | | 575 bp | | |  |  |  |  |
| Genome Start | Genome End | Strand | Div | Repeat Start | Repeat End | Repeat Left | Name | Family | Class |
| 170786035 | 170786171 | - | 25.6 | 143 | 267 | -1 | MIRb | MIR | SINE |
| 170786221 | 170786467 | - | 15.0 | 6049 | 6295 | -5 | L1MA4 | L1 | LINE |
| 170786484 | 170786532 | - | 18.4 | 6055 | 6103 | -197 | L1MA4 | L1 | LINE |
| 170786541 | 170786682 | - | 34.8 | 21 | 155 | -113 | MIRb | MIR | SINE |

  

|  |  |  |  |  |  |  |  |  |  |
| --- | --- | --- | --- | --- | --- | --- | --- | --- | --- |
| chr6:170802529-170805035 | | | 2473 bp | | |  |  |  |  |
| Genome Start | Genome End | Strand | Div | Repeat Start | Repeat End | Repeat Left | Name | Family | Class |
| 170802529 | 170803612 | - | 16.7 | 3657 | 4741 | -1399 | L1M3 | L1 | LINE |
| 170803632 | 170803809 | - | 4.5 | 5977 | 6155 | 0 | L1PA4 | L1 | LINE |
| 170803810 | 170804531 | + | 2.8 | 5254 | 5975 | -180 | L1PA4 | L1 | LINE |
| 170804547 | 170805035 | - | 19.7 | 3150 | 3646 | -2494 | L1M3 | L1 | LINE |

  

|  |  |  |  |  |  |  |  |  |  |
| --- | --- | --- | --- | --- | --- | --- | --- | --- | --- |
| chr6:170821444-170822447 | | | 895 bp | | |  |  |  |  |
| Genome Start | Genome End | Strand | Div | Repeat Start | Repeat End | Repeat Left | Name | Family | Class |
| 170821444 | 170821664 | + | 39.4 | 2572 | 2801 | -618 | L2 | L2 | LINE |
| 170821670 | 170821945 | + | 4.3 | 5861 | 6150 | -4 | L1PA5 | L1 | LINE |
| 170822050 | 170822447 | + | 37.6 | 2934 | 3355 | -23 | L2 | L2 | LINE |

  

|  |  |  |  |  |  |  |  |  |  |
| --- | --- | --- | --- | --- | --- | --- | --- | --- | --- |
| chr6:170830201-170832202 | | | 1819 bp | | |  |  |  |  |
| Genome Start | Genome End | Strand | Div | Repeat Start | Repeat End | Repeat Left | Name | Family | Class |
| 170830201 | 170830466 | - | 31.9 | 3856 | 4151 | -338 | L3 | CR1 | LINE |
| 170830478 | 170830646 | + | 27.9 | 1 | 173 | -348 | LTR33 | ERVL | LTR |
| 170830730 | 170830768 | + | 21.9 | 378 | 414 | -107 | LTR33 | ERVL | LTR |
| 170830769 | 170830784 | - | 16.1 | 417 | 432 | 0 | MSTB1 | MaLR | LTR |
| 170830826 | 170831076 | - | 16.1 | 151 | 416 | -16 | MSTB1 | MaLR | LTR |
| 170831077 | 170831368 | + | 10.3 | 6 | 296 | -17 | AluSq | Alu | SINE |
| 170831369 | 170831517 | - | 16.1 | 2 | 150 | -282 | MSTB1 | MaLR | LTR |
| 170831518 | 170831610 | + | 21.9 | 415 | 517 | -4 | LTR33 | ERVL | LTR |
| 170831628 | 170831697 | - | 31.2 | 3763 | 3834 | -655 | L3 | CR1 | LINE |
| 170831729 | 170832009 | - | 12.1 | 2 | 283 | -27 | AluSg | Alu | SINE |
| 170832010 | 170832202 | - | 31.2 | 3576 | 3762 | -727 | L3 | CR1 | LINE |

  

|  |  |  |  |  |  |  |  |  |  |
| --- | --- | --- | --- | --- | --- | --- | --- | --- | --- |
| chr6:170832852-170833327 | | | 476 bp | | |  |  |  |  |
| Genome Start | Genome End | Strand | Div | Repeat Start | Repeat End | Repeat Left | Name | Family | Class |
| 170832852 | 170832923 | - | 31.1 | 3145 | 3217 | -2929 | L1M5 | L1 | LINE |
| 170832924 | 170833224 | - | 13.9 | 1 | 301 | -11 | AluSx | Alu | SINE |
| 170833225 | 170833327 | - | 31.1 | 3044 | 3144 | -3002 | L1M5 | L1 | LINE |

  

|  |  |  |  |  |  |  |  |  |  |
| --- | --- | --- | --- | --- | --- | --- | --- | --- | --- |
| chr6:170839943-170840599 | | | 657 bp | | |  |  |  |  |
| Genome Start | Genome End | Strand | Div | Repeat Start | Repeat End | Repeat Left | Name | Family | Class |
| 170839943 | 170839988 | + | 27.6 | 62 | 107 | -442 | MLT1H | MaLR | LTR |
| 170839989 | 170840309 | + | 9.7 | 1 | 311 | 0 | AluY | Alu | SINE |
| 170840310 | 170840599 | + | 27.0 | 93 | 375 | -167 | MLT1H | MaLR | LTR |

  

|  |  |  |  |  |  |  |  |  |  |
| --- | --- | --- | --- | --- | --- | --- | --- | --- | --- |
| chr6:170840736-170841575 | | | 840 bp | | |  |  |  |  |
| Genome Start | Genome End | Strand | Div | Repeat Start | Repeat End | Repeat Left | Name | Family | Class |
| 170840736 | 170841087 | - | 33.7 | 2624 | 2980 | -439 | L2 | L2 | LINE |
| 170841088 | 170841378 | + | 13.4 | 1 | 291 | -18 | AluSc | Alu | SINE |
| 170841379 | 170841575 | - | 33.7 | 2383 | 2623 | -796 | L2 | L2 | LINE |

  

|  |  |  |  |  |  |  |  |  |  |
| --- | --- | --- | --- | --- | --- | --- | --- | --- | --- |
| chr6:170844489-170845667 | | | 1179 bp | | |  |  |  |  |
| Genome Start | Genome End | Strand | Div | Repeat Start | Repeat End | Repeat Left | Name | Family | Class |
| 170844489 | 170844632 | + | 18.4 | 51 | 190 | -593 | LTR39 | ERV1 | LTR |
| 170844633 | 170844752 | + | 3.3 | 6034 | 6155 | 0 | L1PA4 | L1 | LINE |
| 170844753 | 170845054 | + | 18.4 | 191 | 459 | -324 | LTR39 | ERV1 | LTR |
| 170845055 | 170845353 | - | 15.4 | 1 | 299 | -13 | AluSx | Alu | SINE |
| 170845354 | 170845667 | + | 18.4 | 460 | 782 | -1 | LTR39 | ERV1 | LTR |

  

|  |  |  |  |  |  |  |  |  |  |
| --- | --- | --- | --- | --- | --- | --- | --- | --- | --- |
| chr6:170848335-170848766 | | | 384 bp | | |  |  |  |  |
| Genome Start | Genome End | Strand | Div | Repeat Start | Repeat End | Repeat Left | Name | Family | Class |
| 170848335 | 170848398 | - | 13.5 | 362 | 428 | 0 | MSTA | MaLR | LTR |
| 170848399 | 170848518 | + | 9.1 | 1 | 120 | -190 | AluSg | Alu | SINE |
| 170848567 | 170848720 | + | 9.1 | 121 | 307 | -3 | AluSg | Alu | SINE |
| 170848721 | 170848766 | - | 13.5 | 319 | 361 | -67 | MSTA | MaLR | LTR |

  

|  |  |  |  |  |  |  |  |  |  |
| --- | --- | --- | --- | --- | --- | --- | --- | --- | --- |
| chr6:170856349-170857422 | | | 1074 bp | | |  |  |  |  |
| Genome Start | Genome End | Strand | Div | Repeat Start | Repeat End | Repeat Left | Name | Family | Class |
| 170856349 | 170856770 | - | 6.4 | 334 | 781 | 0 | MER4E1 | ERV1 | LTR |
| 170856771 | 170857070 | + | 10.7 | 1 | 298 | -12 | AluSg | Alu | SINE |
| 170857071 | 170857422 | - | 6.4 | 1 | 333 | -448 | MER4E1 | ERV1 | LTR |

  

|  |  |  |  |  |  |  |  |  |  |
| --- | --- | --- | --- | --- | --- | --- | --- | --- | --- |
| chr6:170858431-170859450 | | | 969 bp | | |  |  |  |  |
| Genome Start | Genome End | Strand | Div | Repeat Start | Repeat End | Repeat Left | Name | Family | Class |
| 170858431 | 170858602 | - | 24.2 | 398 | 575 | -16 | MLT1K | MaLR | LTR |
| 170858654 | 170858703 | - | 27.1 | 99 | 145 | -446 | MLT1K | MaLR | LTR |
| 170858704 | 170859356 | - | 11.3 | 1 | 644 | 0 | LTR9B | ERV1 | LTR |
| 170859357 | 170859450 | - | 27.1 | 4 | 98 | -493 | MLT1K | MaLR | LTR |

  

|  |  |  |  |  |  |  |  |  |  |
| --- | --- | --- | --- | --- | --- | --- | --- | --- | --- |
| chr6:170872148-170875226 | | | 2465 bp | | |  |  |  |  |
| Genome Start | Genome End | Strand | Div | Repeat Start | Repeat End | Repeat Left | Name | Family | Class |
| 170872148 | 170872722 | + | 31.0 | 5936 | 6537 | -1458 | L1MD3 | L1 | LINE |
| 170872762 | 170872888 | + | 32.5 | 6539 | 6668 | -1327 | L1MD3 | L1 | LINE |
| 170873182 | 170873264 | + | 32.9 | 6539 | 6620 | -1375 | L1MD3 | L1 | LINE |
| 170873425 | 170873501 | + | 30.3 | 6545 | 6620 | -1375 | L1MD3 | L1 | LINE |
| 170873577 | 170874013 | + | 24.5 | 6503 | 6945 | -840 | L1MC3 | L1 | LINE |
| 170874052 | 170874134 | + | 24.5 | 6946 | 7047 | -738 | L1MC3 | L1 | LINE |
| 170874140 | 170874309 | + | 10.0 | 134 | 303 | -8 | AluY | Alu | SINE |
| 170874312 | 170874382 | + | 23.3 | 7068 | 7148 | -847 | L1MD3 | L1 | LINE |
| 170874383 | 170874689 | + | 11.4 | 1 | 313 | 0 | AluSq | Alu | SINE |
| 170874690 | 170874830 | + | 23.3 | 7149 | 7285 | -710 | L1MD3 | L1 | LINE |
| 170874833 | 170874912 | + | 22.5 | 25 | 125 | -442 | MLT1F1 | MaLR | LTR |
| 170874913 | 170875226 | + | 25.2 | 7406 | 7726 | -269 | L1MD3 | L1 | LINE |

  

|  |  |  |  |  |  |  |  |  |  |
| --- | --- | --- | --- | --- | --- | --- | --- | --- | --- |
| chr6:170886088-170887381 | | | 1298 bp | | |  |  |  |  |
| Genome Start | Genome End | Strand | Div | Repeat Start | Repeat End | Repeat Left | Name | Family | Class |
| 170886088 | 170886883 | + | 15.1 | 2 | 836 | -64 | MER4D1 | ERV1 | LTR |
| 170886880 | 170887311 | - | 21.7 | 1 | 589 | -5 | MER4A | ERV1 | LTR |
| 170887312 | 170887381 | + | 15.3 | 824 | 900 | 0 | MER4D1 | ERV1 | LTR |

  

|  |  |  |  |  |  |  |  |  |  |
| --- | --- | --- | --- | --- | --- | --- | --- | --- | --- |
| chr6:170892806-170895019 | | | 2214 bp | | |  |  |  |  |
| Genome Start | Genome End | Strand | Div | Repeat Start | Repeat End | Repeat Left | Name | Family | Class |
| 170892806 | 170894170 | - | 23.1 | 6234 | 7784 | -1 | L1MC3 | L1 | LINE |
| 170894171 | 170894610 | - | 18.4 | 5 | 432 | 0 | MSTB1 | MaLR | LTR |
| 170894611 | 170895019 | - | 23.1 | 5763 | 6233 | -1552 | L1MC3 | L1 | LINE |

  

|  |  |  |  |  |  |  |  |  |  |
| --- | --- | --- | --- | --- | --- | --- | --- | --- | --- |
| chr6:170895147-170896477 | | | 1288 bp | | |  |  |  |  |
| Genome Start | Genome End | Strand | Div | Repeat Start | Repeat End | Repeat Left | Name | Family | Class |
| 170895147 | 170895256 | - | 19.0 | 4275 | 4375 | -561 | MER31-int | ERV1 | LTR |
| 170895259 | 170895451 | - | 14.6 | 100 | 291 | -21 | AluJb | Alu | SINE |
| 170895456 | 170895481 | - | 23.2 | 3115 | 3140 | -1796 | MER31-int | ERV1 | LTR |
| 170895482 | 170895782 | - | 9.6 | 2 | 302 | 0 | AluY | Alu | SINE |
| 170895783 | 170895952 | - | 23.2 | 2939 | 3114 | -1822 | MER31-int | ERV1 | LTR |
| 170895990 | 170896477 | - | 22.1 | 1699 | 2195 | -2741 | MER31-int | ERV1 | LTR |

  


|  |  |  |  |  |  |  |  |  |  |
| --- | --- | --- | --- | --- | --- | --- | --- | --- | --- |
| chr7:42448-44552 | | | 2110 bp | | |  |  |  |  |
| Genome Start | Genome End | Strand | Div | Repeat Start | Repeat End | Repeat Left | Name | Family | Class |
| 42448 | 42644 | + | 23.9 | 3777 | 3965 | -2164 | L1ME3B | L1 | LINE |
| 42638 | 43024 | + | 31.6 | 4488 | 4909 | -1220 | L1ME3B | L1 | LINE |
| 43027 | 43516 | + | 22.9 | 1 | 495 | -10 | MLT1D | MaLR | LTR |
| 43517 | 44552 | + | 27.0 | 4906 | 6042 | -181 | L1ME3B | L1 | LINE |

  

|  |  |  |  |  |  |  |  |  |  |
| --- | --- | --- | --- | --- | --- | --- | --- | --- | --- |
| chr7:53906-54404 | | | 499 bp | | |  |  |  |  |
| Genome Start | Genome End | Strand | Div | Repeat Start | Repeat End | Repeat Left | Name | Family | Class |
| 53906 | 54013 | - | 31.7 | 3313 | 3414 | -5 | L2 | L2 | LINE |
| 54014 | 54311 | - | 12.1 | 1 | 298 | -15 | AluSq | Alu | SINE |
| 54312 | 54404 | - | 31.7 | 3223 | 3312 | -107 | L2 | L2 | LINE |

  

|  |  |  |  |  |  |  |  |  |  |
| --- | --- | --- | --- | --- | --- | --- | --- | --- | --- |
| chr7:55321-56162 | | | 842 bp | | |  |  |  |  |
| Genome Start | Genome End | Strand | Div | Repeat Start | Repeat End | Repeat Left | Name | Family | Class |
| 55321 | 55412 | + | 25.7 | 1946 | 2024 | -4765 | L1M2c | L1 | LINE |
| 55413 | 55727 | + | 12.7 | 1 | 309 | -1 | AluSg | Alu | SINE |
| 55728 | 56162 | + | 21.8 | 2025 | 2563 | -4226 | L1M2c | L1 | LINE |

  

|  |  |  |  |  |  |  |  |  |  |
| --- | --- | --- | --- | --- | --- | --- | --- | --- | --- |
| chr7:69857-71343 | | | 1487 bp | | |  |  |  |  |
| Genome Start | Genome End | Strand | Div | Repeat Start | Repeat End | Repeat Left | Name | Family | Class |
| 69857 | 70362 | + | 20.5 | 18 | 525 | -410 | MER21C | ERV1 | LTR |
| 70363 | 70669 | - | 19.2 | 2 | 294 | -18 | AluJb | Alu | SINE |
| 70670 | 70739 | + | 20.5 | 526 | 603 | -332 | MER21C | ERV1 | LTR |
| 70740 | 71042 | + | 20.5 | 1 | 302 | -10 | AluJo | Alu | SINE |
| 71043 | 71343 | + | 20.5 | 604 | 935 | 0 | MER21C | ERV1 | LTR |

  

|  |  |  |  |  |  |  |  |  |  |
| --- | --- | --- | --- | --- | --- | --- | --- | --- | --- |
| chr7:76136-76614 | | | 432 bp | | |  |  |  |  |
| Genome Start | Genome End | Strand | Div | Repeat Start | Repeat End | Repeat Left | Name | Family | Class |
| 76136 | 76237 | - | 22.9 | 72 | 167 | -145 | AluJo | Alu | SINE |
| 76238 | 76507 | + | 13.8 | 1 | 275 | -37 | AluSx | Alu | SINE |
| 76555 | 76614 | - | 22.9 | 24 | 71 | -241 | AluJo | Alu | SINE |

  

|  |  |  |  |  |  |  |  |  |  |
| --- | --- | --- | --- | --- | --- | --- | --- | --- | --- |
| chr7:76674-77848 | | | 1127 bp | | |  |  |  |  |
| Genome Start | Genome End | Strand | Div | Repeat Start | Repeat End | Repeat Left | Name | Family | Class |
| 76674 | 76704 | + | 18.9 | 5476 | 5505 | -679 | L1MB8 | L1 | LINE |
| 76705 | 76996 | + | 19.0 | 1 | 293 | -19 | AluJb | Alu | SINE |
| 76997 | 77070 | + | 18.9 | 5506 | 5570 | -614 | L1MB8 | L1 | LINE |
| 77119 | 77229 | - | 18.0 | 4 | 114 | -19 | FLAM\_A | Alu | SINE |
| 77230 | 77848 | + | 20.6 | 5552 | 6173 | -5 | L1MB8 | L1 | LINE |

  

|  |  |  |  |  |  |  |  |  |  |
| --- | --- | --- | --- | --- | --- | --- | --- | --- | --- |
| chr7:80396-80967 | | | 572 bp | | |  |  |  |  |
| Genome Start | Genome End | Strand | Div | Repeat Start | Repeat End | Repeat Left | Name | Family | Class |
| 80396 | 80554 | - | 19.9 | 137 | 295 | -17 | AluJb | Alu | SINE |
| 80555 | 80840 | - | 9.8 | 1 | 296 | -13 | AluSg1 | Alu | SINE |
| 80841 | 80967 | - | 19.9 | 12 | 136 | -176 | AluJb | Alu | SINE |

  

|  |  |  |  |  |  |  |  |  |  |
| --- | --- | --- | --- | --- | --- | --- | --- | --- | --- |
| chr7:81640-83109 | | | 1470 bp | | |  |  |  |  |
| Genome Start | Genome End | Strand | Div | Repeat Start | Repeat End | Repeat Left | Name | Family | Class |
| 81640 | 81898 | + | 20.2 | 18 | 269 | -666 | MER21C | ERV1 | LTR |
| 81899 | 82193 | - | 15.0 | 1 | 294 | -19 | AluSq | Alu | SINE |
| 82194 | 82503 | + | 20.2 | 270 | 603 | -332 | MER21C | ERV1 | LTR |
| 82504 | 82809 | + | 16.9 | 1 | 301 | -11 | AluJo | Alu | SINE |
| 82810 | 83109 | + | 20.2 | 604 | 934 | -1 | MER21C | ERV1 | LTR |

  

|  |  |  |  |  |  |  |  |  |  |
| --- | --- | --- | --- | --- | --- | --- | --- | --- | --- |
| chr7:86470-86815 | | | 346 bp | | |  |  |  |  |
| Genome Start | Genome End | Strand | Div | Repeat Start | Repeat End | Repeat Left | Name | Family | Class |
| 86470 | 86569 | + | 31.0 | 5483 | 5575 | -2233 | L1MC4 | L1 | LINE |
| 86570 | 86692 | - | 16.5 | 4 | 126 | -7 | FLAM\_C | Alu | SINE |
| 86693 | 86815 | + | 31.0 | 5576 | 5690 | -2118 | L1MC4 | L1 | LINE |

  

|  |  |  |  |  |  |  |  |  |  |
| --- | --- | --- | --- | --- | --- | --- | --- | --- | --- |
| chr7:103448-103988 | | | 541 bp | | |  |  |  |  |
| Genome Start | Genome End | Strand | Div | Repeat Start | Repeat End | Repeat Left | Name | Family | Class |
| 103448 | 103583 | + | 13.6 | 1 | 136 | -166 | AluSx | Alu | SINE |
| 103584 | 103886 | + | 10.6 | 1 | 304 | -9 | AluSp | Alu | SINE |
| 103887 | 103988 | + | 13.6 | 137 | 302 | 0 | AluSx | Alu | SINE |

  

|  |  |  |  |  |  |  |  |  |  |
| --- | --- | --- | --- | --- | --- | --- | --- | --- | --- |
| chr7:124235-125156 | | | 925 bp | | |  |  |  |  |
| Genome Start | Genome End | Strand | Div | Repeat Start | Repeat End | Repeat Left | Name | Family | Class |
| 124235 | 124330 | + | 15.9 | 40 | 142 | -2887 | Tigger3(Golem) | MER2\_type | DNA |
| 124328 | 124351 | + | 19.3 | 2771 | 2797 | -232 | Tigger3(Golem) | MER2\_type | DNA |
| 124352 | 124932 | + | 7.9 | 3 | 600 | 0 | MER4A1\_ | ERV1 | LTR |
| 124933 | 125156 | + | 19.3 | 2798 | 3027 | -2 | Tigger3(Golem) | MER2\_type | DNA |

  

|  |  |  |  |  |  |  |  |  |  |
| --- | --- | --- | --- | --- | --- | --- | --- | --- | --- |
| chr7:169362-171609 | | | 2134 bp | | |  |  |  |  |
| Genome Start | Genome End | Strand | Div | Repeat Start | Repeat End | Repeat Left | Name | Family | Class |
| 169362 | 170200 | + | 27.7 | 4019 | 4906 | -1240 | L1M5 | L1 | LINE |
| 170201 | 170463 | + | 18.7 | 1 | 302 | 0 | AluJo | Alu | SINE |
| 170464 | 170525 | + | 30.8 | 4907 | 4970 | -1176 | L1M5 | L1 | LINE |
| 170601 | 171186 | + | 25.2 | 7175 | 7785 | 0 | L1MC3 | L1 | LINE |
| 171226 | 171609 | + | 31.2 | 5186 | 5593 | -571 | L1M5 | L1 | LINE |

  

|  |  |  |  |  |  |  |  |  |  |
| --- | --- | --- | --- | --- | --- | --- | --- | --- | --- |
| chr7:5038333-5038933 | | | 576 bp | | |  |  |  |  |
| Genome Start | Genome End | Strand | Div | Repeat Start | Repeat End | Repeat Left | Name | Family | Class |
| 5038333 | 5038470 | + | 17.9 | 1 | 136 | -176 | AluJo | Alu | SINE |
| 5038471 | 5038743 | + | 17.3 | 2 | 280 | -32 | AluSx | Alu | SINE |
| 5038769 | 5038933 | + | 17.9 | 137 | 298 | -14 | AluJo | Alu | SINE |

  

|  |  |  |  |  |  |  |  |  |  |
| --- | --- | --- | --- | --- | --- | --- | --- | --- | --- |
| chr7:5045684-5046382 | | | 699 bp | | |  |  |  |  |
| Genome Start | Genome End | Strand | Div | Repeat Start | Repeat End | Repeat Left | Name | Family | Class |
| 5045684 | 5046026 | - | 35.7 | 3987 | 4333 | -156 | L3 | CR1 | LINE |
| 5046027 | 5046323 | + | 12.2 | 4 | 298 | -14 | AluSx | Alu | SINE |
| 5046324 | 5046382 | - | 35.7 | 3933 | 3986 | -503 | L3 | CR1 | LINE |

  

|  |  |  |  |  |  |  |  |  |  |
| --- | --- | --- | --- | --- | --- | --- | --- | --- | --- |
| chr7:5047916-5049492 | | | 1577 bp | | |  |  |  |  |
| Genome Start | Genome End | Strand | Div | Repeat Start | Repeat End | Repeat Left | Name | Family | Class |
| 5047916 | 5048094 | + | 24.1 | 21 | 201 | -510 | MER67C | ERV1 | LTR |
| 5048095 | 5048396 | - | 11.9 | 1 | 305 | -7 | AluSx | Alu | SINE |
| 5048397 | 5048644 | + | 24.1 | 202 | 454 | -257 | MER67C | ERV1 | LTR |
| 5048645 | 5048960 | + | 14.5 | 1 | 298 | -14 | AluSx | Alu | SINE |
| 5048961 | 5049057 | + | 24.1 | 455 | 543 | -168 | MER67C | ERV1 | LTR |
| 5049058 | 5049352 | + | 12.9 | 1 | 294 | -16 | AluSg | Alu | SINE |
| 5049353 | 5049492 | + | 24.1 | 544 | 683 | -28 | MER67C | ERV1 | LTR |

  

|  |  |  |  |  |  |  |  |  |  |
| --- | --- | --- | --- | --- | --- | --- | --- | --- | --- |
| chr7:5049945-5051151 | | | 1207 bp | | |  |  |  |  |
| Genome Start | Genome End | Strand | Div | Repeat Start | Repeat End | Repeat Left | Name | Family | Class |
| 5049945 | 5050584 | + | 12.5 | 1 | 575 | -328 | MER4D | ERV1 | LTR |
| 5050585 | 5050842 | - | 14.8 | 9 | 268 | -44 | AluJo | Alu | SINE |
| 5050843 | 5051151 | + | 12.5 | 576 | 903 | 0 | MER4D | ERV1 | LTR |

  

|  |  |  |  |  |  |  |  |  |  |
| --- | --- | --- | --- | --- | --- | --- | --- | --- | --- |
| chr7:5056429-5057279 | | | 851 bp | | |  |  |  |  |
| Genome Start | Genome End | Strand | Div | Repeat Start | Repeat End | Repeat Left | Name | Family | Class |
| 5056429 | 5056737 | - | 25.1 | 7133 | 7430 | -531 | L1MC5 | L1 | LINE |
| 5056738 | 5057027 | - | 17.9 | 1 | 304 | -8 | AluJo | Alu | SINE |
| 5057028 | 5057279 | - | 25.1 | 6852 | 7132 | -829 | L1MC5 | L1 | LINE |

  

|  |  |  |  |  |  |  |  |  |  |
| --- | --- | --- | --- | --- | --- | --- | --- | --- | --- |
| chr7:5144527-5145420 | | | 894 bp | | |  |  |  |  |
| Genome Start | Genome End | Strand | Div | Repeat Start | Repeat End | Repeat Left | Name | Family | Class |
| 5144527 | 5144737 | - | 31.5 | 3332 | 3547 | -2599 | L1M5 | L1 | LINE |
| 5144738 | 5145053 | + | 10.7 | 1 | 309 | -3 | AluSx | Alu | SINE |
| 5145054 | 5145365 | + | 12.3 | 1 | 310 | -2 | AluSx | Alu | SINE |
| 5145366 | 5145420 | - | 30.9 | 3274 | 3331 | -2815 | L1M5 | L1 | LINE |

  

|  |  |  |  |  |  |  |  |  |  |
| --- | --- | --- | --- | --- | --- | --- | --- | --- | --- |
| chr7:5154109-5154738 | | | 630 bp | | |  |  |  |  |
| Genome Start | Genome End | Strand | Div | Repeat Start | Repeat End | Repeat Left | Name | Family | Class |
| 5154109 | 5154251 | + | 9.2 | 1 | 139 | -173 | AluSx | Alu | SINE |
| 5154252 | 5154548 | + | 10.2 | 15 | 308 | -5 | AluSp | Alu | SINE |
| 5154549 | 5154738 | + | 9.2 | 140 | 307 | -5 | AluSx | Alu | SINE |

  

|  |  |  |  |  |  |  |  |  |  |
| --- | --- | --- | --- | --- | --- | --- | --- | --- | --- |
| chr7:5156808-5157407 | | | 600 bp | | |  |  |  |  |
| Genome Start | Genome End | Strand | Div | Repeat Start | Repeat End | Repeat Left | Name | Family | Class |
| 5156808 | 5156944 | + | 15.6 | 1 | 135 | -177 | AluSx | Alu | SINE |
| 5156945 | 5157241 | + | 10.4 | 2 | 299 | -10 | AluSc | Alu | SINE |
| 5157242 | 5157407 | + | 15.6 | 136 | 297 | -15 | AluSx | Alu | SINE |

  

|  |  |  |  |  |  |  |  |  |  |
| --- | --- | --- | --- | --- | --- | --- | --- | --- | --- |
| chr7:5258253-5259370 | | | 1112 bp | | |  |  |  |  |
| Genome Start | Genome End | Strand | Div | Repeat Start | Repeat End | Repeat Left | Name | Family | Class |
| 5258253 | 5258512 | + | 12.9 | 220 | 472 | -311 | LTR39 | ERV1 | LTR |
| 5258513 | 5258799 | + | 8.4 | 1 | 289 | -20 | AluSc | Alu | SINE |
| 5258800 | 5258851 | + | 12.9 | 473 | 522 | -261 | LTR39 | ERV1 | LTR |
| 5258853 | 5259172 | - | 12.8 | 3 | 317 | 0 | AluSx | Alu | SINE |
| 5259178 | 5259370 | + | 24.4 | 601 | 783 | 0 | LTR39 | ERV1 | LTR |

  

|  |  |  |  |  |  |  |  |  |  |
| --- | --- | --- | --- | --- | --- | --- | --- | --- | --- |
| chr7:5262300-5262976 | | | 677 bp | | |  |  |  |  |
| Genome Start | Genome End | Strand | Div | Repeat Start | Repeat End | Repeat Left | Name | Family | Class |
| 5262300 | 5262402 | + | 26.3 | 7604 | 7686 | -275 | L1MC5 | L1 | LINE |
| 5262403 | 5262722 | - | 11.9 | 1 | 312 | 0 | AluSx | Alu | SINE |
| 5262723 | 5262976 | + | 26.3 | 7687 | 7927 | -34 | L1MC5 | L1 | LINE |

  

|  |  |  |  |  |  |  |  |  |  |
| --- | --- | --- | --- | --- | --- | --- | --- | --- | --- |
| chr7:5265689-5266316 | | | 628 bp | | |  |  |  |  |
| Genome Start | Genome End | Strand | Div | Repeat Start | Repeat End | Repeat Left | Name | Family | Class |
| 5265689 | 5265823 | + | 11.0 | 3 | 133 | -179 | AluSx | Alu | SINE |
| 5265824 | 5266139 | + | 6.8 | 1 | 309 | -2 | AluY | Alu | SINE |
| 5266140 | 5266316 | + | 11.0 | 134 | 310 | -2 | AluSx | Alu | SINE |

  

|  |  |  |  |  |  |  |  |  |  |
| --- | --- | --- | --- | --- | --- | --- | --- | --- | --- |
| chr7:5278276-5278908 | | | 587 bp | | |  |  |  |  |
| Genome Start | Genome End | Strand | Div | Repeat Start | Repeat End | Repeat Left | Name | Family | Class |
| 5278276 | 5278496 | - | 23.7 | 92 | 336 | -725 | MER63D | AcHobo | DNA |
| 5278497 | 5278773 | + | 6.1 | 1 | 277 | -32 | AluSc | Alu | SINE |
| 5278820 | 5278908 | - | 23.7 | 6 | 91 | -970 | MER63D | AcHobo | DNA |

  

|  |  |  |  |  |  |  |  |  |  |
| --- | --- | --- | --- | --- | --- | --- | --- | --- | --- |
| chr7:5283749-5284280 | | | 532 bp | | |  |  |  |  |
| Genome Start | Genome End | Strand | Div | Repeat Start | Repeat End | Repeat Left | Name | Family | Class |
| 5283749 | 5283880 | + | 21.6 | 1 | 134 | -178 | AluJo | Alu | SINE |
| 5283881 | 5284186 | + | 13.1 | 1 | 306 | -6 | AluSx | Alu | SINE |
| 5284187 | 5284280 | + | 21.6 | 135 | 219 | -93 | AluJo | Alu | SINE |

  

|  |  |  |  |  |  |  |  |  |  |
| --- | --- | --- | --- | --- | --- | --- | --- | --- | --- |
| chr7:5296043-5296639 | | | 597 bp | | |  |  |  |  |
| Genome Start | Genome End | Strand | Div | Repeat Start | Repeat End | Repeat Left | Name | Family | Class |
| 5296043 | 5296209 | - | 11.6 | 134 | 301 | -11 | AluSx | Alu | SINE |
| 5296210 | 5296519 | - | 5.3 | 1 | 300 | -11 | AluY | Alu | SINE |
| 5296520 | 5296639 | - | 11.6 | 15 | 133 | -179 | AluSx | Alu | SINE |

  

|  |  |  |  |  |  |  |  |  |  |
| --- | --- | --- | --- | --- | --- | --- | --- | --- | --- |
| chr7:5298582-5299303 | | | 658 bp | | |  |  |  |  |
| Genome Start | Genome End | Strand | Div | Repeat Start | Repeat End | Repeat Left | Name | Family | Class |
| 5298582 | 5298845 | - | 30.3 | 7804 | 8042 | 0 | L1MC4 | L1 | LINE |
| 5298848 | 5299155 | - | 7.8 | 1 | 310 | -2 | AluSx | Alu | SINE |
| 5299218 | 5299303 | - | 27.9 | 7532 | 7620 | -422 | L1MC4 | L1 | LINE |

  

|  |  |  |  |  |  |  |  |  |  |
| --- | --- | --- | --- | --- | --- | --- | --- | --- | --- |
| chr7:5307001-5308431 | | | 1423 bp | | |  |  |  |  |
| Genome Start | Genome End | Strand | Div | Repeat Start | Repeat End | Repeat Left | Name | Family | Class |
| 5307001 | 5307133 | - | 29.9 | 3281 | 3418 | -1 | L2 | L2 | LINE |
| 5307134 | 5307435 | + | 13.9 | 1 | 301 | -11 | AluJo | Alu | SINE |
| 5307436 | 5307687 | - | 29.9 | 3030 | 3280 | -139 | L2 | L2 | LINE |
| 5307696 | 5308111 | - | 28.4 | 2365 | 2848 | -571 | L2 | L2 | LINE |
| 5308112 | 5308407 | - | 13.8 | 1 | 296 | -16 | AluSx | Alu | SINE |
| 5308408 | 5308431 | - | 28.4 | 2337 | 2364 | -1055 | L2 | L2 | LINE |

  

|  |  |  |  |  |  |  |  |  |  |
| --- | --- | --- | --- | --- | --- | --- | --- | --- | --- |
| chr7:5320065-5320460 | | | 396 bp | | |  |  |  |  |
| Genome Start | Genome End | Strand | Div | Repeat Start | Repeat End | Repeat Left | Name | Family | Class |
| 5320065 | 5320109 | + | 24.4 | 1 | 44 | -232 | MIRm | MIR | SINE |
| 5320110 | 5320425 | - | 19.5 | 2 | 309 | -3 | AluJo | Alu | SINE |
| 5320426 | 5320460 | + | 24.4 | 45 | 81 | -195 | MIRm | MIR | SINE |

  

|  |  |  |  |  |  |  |  |  |  |
| --- | --- | --- | --- | --- | --- | --- | --- | --- | --- |
| chr7:5325634-5326432 | | | 660 bp | | |  |  |  |  |
| Genome Start | Genome End | Strand | Div | Repeat Start | Repeat End | Repeat Left | Name | Family | Class |
| 5325634 | 5325666 | + | 30.2 | 7 | 39 | -552 | MLT1K | MaLR | LTR |
| 5325667 | 5325966 | - | 10.7 | 1 | 299 | -13 | AluSx | Alu | SINE |
| 5325967 | 5326106 | + | 30.2 | 40 | 180 | -411 | MLT1K | MaLR | LTR |
| 5326246 | 5326432 | + | 19.7 | 386 | 587 | -4 | MLT1K | MaLR | LTR |

  

|  |  |  |  |  |  |  |  |  |  |
| --- | --- | --- | --- | --- | --- | --- | --- | --- | --- |
| chr7:5334543-5335166 | | | 624 bp | | |  |  |  |  |
| Genome Start | Genome End | Strand | Div | Repeat Start | Repeat End | Repeat Left | Name | Family | Class |
| 5334543 | 5334669 | + | 19.7 | 1 | 109 | -203 | AluJo | Alu | SINE |
| 5334670 | 5334966 | - | 8.2 | 1 | 294 | -16 | AluSg | Alu | SINE |
| 5334967 | 5335166 | + | 19.7 | 110 | 304 | -8 | AluJo | Alu | SINE |

  

|  |  |  |  |  |  |  |  |  |  |
| --- | --- | --- | --- | --- | --- | --- | --- | --- | --- |
| chr7:5839737-5840930 | | | 1194 bp | | |  |  |  |  |
| Genome Start | Genome End | Strand | Div | Repeat Start | Repeat End | Repeat Left | Name | Family | Class |
| 5839737 | 5839799 | - | 40.4 | 1890 | 1957 | -3 | L4 | RTE | LINE |
| 5839800 | 5840096 | + | 12.8 | 1 | 298 | -14 | AluSx | Alu | SINE |
| 5840097 | 5840220 | - | 40.4 | 1764 | 1889 | -71 | L4 | RTE | LINE |
| 5840221 | 5840512 | - | 13.8 | 1 | 291 | -21 | AluSx | Alu | SINE |
| 5840513 | 5840930 | - | 40.4 | 1326 | 1763 | -197 | L4 | RTE | LINE |

  

|  |  |  |  |  |  |  |  |  |  |
| --- | --- | --- | --- | --- | --- | --- | --- | --- | --- |
| chr7:5869534-5870209 | | | 676 bp | | |  |  |  |  |
| Genome Start | Genome End | Strand | Div | Repeat Start | Repeat End | Repeat Left | Name | Family | Class |
| 5869534 | 5869688 | - | 28.6 | 1619 | 1774 | -1645 | L2 | L2 | LINE |
| 5869689 | 5869988 | - | 11.0 | 1 | 299 | -11 | AluSg | Alu | SINE |
| 5869989 | 5870209 | - | 28.6 | 1394 | 1618 | -1801 | L2 | L2 | LINE |

  

|  |  |  |  |  |  |  |  |  |  |
| --- | --- | --- | --- | --- | --- | --- | --- | --- | --- |
| chr7:5873286-5874282 | | | 997 bp | | |  |  |  |  |
| Genome Start | Genome End | Strand | Div | Repeat Start | Repeat End | Repeat Left | Name | Family | Class |
| 5873286 | 5873370 | - | 18.5 | 1519 | 1602 | -4798 | L1MEc | L1 | LINE |
| 5873371 | 5873485 | + | 13.9 | 5 | 132 | -1 | FLAM\_A | Alu | SINE |
| 5873486 | 5873925 | - | 18.5 | 1158 | 1518 | -4882 | L1MEc | L1 | LINE |
| 5873926 | 5874193 | - | 13.5 | 2 | 293 | -19 | AluJb | Alu | SINE |
| 5874194 | 5874282 | - | 18.5 | 1063 | 1157 | -5243 | L1MEc | L1 | LINE |

  

|  |  |  |  |  |  |  |  |  |  |
| --- | --- | --- | --- | --- | --- | --- | --- | --- | --- |
| chr7:5903536-5904363 | | | 828 bp | | |  |  |  |  |
| Genome Start | Genome End | Strand | Div | Repeat Start | Repeat End | Repeat Left | Name | Family | Class |
| 5903536 | 5903576 | + | 17.1 | 11 | 46 | -266 | AluJo | Alu | SINE |
| 5903577 | 5904087 | + | 11.6 | 1 | 482 | 0 | MER51E | ERV1 | LTR |
| 5904088 | 5904363 | + | 17.1 | 47 | 286 | -26 | AluJo | Alu | SINE |

  

|  |  |  |  |  |  |  |  |  |  |
| --- | --- | --- | --- | --- | --- | --- | --- | --- | --- |
| chr7:5942831-5943446 | | | 616 bp | | |  |  |  |  |
| Genome Start | Genome End | Strand | Div | Repeat Start | Repeat End | Repeat Left | Name | Family | Class |
| 5942831 | 5942964 | + | 15.5 | 1 | 133 | -179 | AluJb | Alu | SINE |
| 5942965 | 5943265 | + | 10.0 | 1 | 304 | -8 | AluSx | Alu | SINE |
| 5943266 | 5943446 | + | 15.5 | 134 | 310 | -2 | AluJb | Alu | SINE |

  

|  |  |  |  |  |  |  |  |  |  |
| --- | --- | --- | --- | --- | --- | --- | --- | --- | --- |
| chr7:5945943-5946390 | | | 448 bp | | |  |  |  |  |
| Genome Start | Genome End | Strand | Div | Repeat Start | Repeat End | Repeat Left | Name | Family | Class |
| 5945943 | 5946071 | - | 21.6 | 20 | 147 | -55 | MER6C | MER2\_type | DNA |
| 5946072 | 5946370 | + | 10.1 | 1 | 298 | -11 | AluSc | Alu | SINE |
| 5946371 | 5946390 | - | 21.6 | 1 | 19 | -183 | MER6C | MER2\_type | DNA |

  

|  |  |  |  |  |  |  |  |  |  |
| --- | --- | --- | --- | --- | --- | --- | --- | --- | --- |
| chr7:5955475-5956432 | | | 958 bp | | |  |  |  |  |
| Genome Start | Genome End | Strand | Div | Repeat Start | Repeat End | Repeat Left | Name | Family | Class |
| 5955475 | 5955558 | + | 22.0 | 3696 | 3782 | -2364 | L1MD | L1 | LINE |
| 5955559 | 5955705 | + | 17.7 | 1 | 138 | -174 | AluJo | Alu | SINE |
| 5955706 | 5955993 | + | 6.9 | 1 | 299 | -12 | AluY | Alu | SINE |
| 5955994 | 5956177 | + | 17.7 | 139 | 311 | -1 | AluJo | Alu | SINE |
| 5956178 | 5956432 | + | 22.0 | 3783 | 4055 | -2091 | L1MD | L1 | LINE |

  

|  |  |  |  |  |  |  |  |  |  |
| --- | --- | --- | --- | --- | --- | --- | --- | --- | --- |
| chr7:5958192-5958780 | | | 589 bp | | |  |  |  |  |
| Genome Start | Genome End | Strand | Div | Repeat Start | Repeat End | Repeat Left | Name | Family | Class |
| 5958192 | 5958335 | + | 19.6 | 1 | 140 | -172 | AluJo | Alu | SINE |
| 5958336 | 5958631 | + | 11.5 | 1 | 296 | -14 | AluSg | Alu | SINE |
| 5958632 | 5958780 | + | 19.6 | 141 | 285 | -27 | AluJo | Alu | SINE |

  

|  |  |  |  |  |  |  |  |  |  |
| --- | --- | --- | --- | --- | --- | --- | --- | --- | --- |
| chr7:5969844-5970309 | | | 466 bp | | |  |  |  |  |
| Genome Start | Genome End | Strand | Div | Repeat Start | Repeat End | Repeat Left | Name | Family | Class |
| 5969844 | 5969940 | + | 28.5 | 5758 | 5866 | -1942 | L1MC4a | L1 | LINE |
| 5969941 | 5970237 | + | 13.5 | 1 | 283 | -29 | AluJo | Alu | SINE |
| 5970238 | 5970309 | + | 28.5 | 5867 | 5934 | -1874 | L1MC4a | L1 | LINE |

  

|  |  |  |  |  |  |  |  |  |  |
| --- | --- | --- | --- | --- | --- | --- | --- | --- | --- |
| chr7:5981273-5982604 | | | 1078 bp | | |  |  |  |  |
| Genome Start | Genome End | Strand | Div | Repeat Start | Repeat End | Repeat Left | Name | Family | Class |
| 5981273 | 5981356 | + | 25.3 | 2234 | 2323 | -1096 | L2 | L2 | LINE |
| 5981611 | 5981693 | + | 32.1 | 3017 | 3101 | -318 | L2 | L2 | LINE |
| 5981694 | 5981994 | - | 15.0 | 1 | 300 | -12 | AluJo | Alu | SINE |
| 5981995 | 5982115 | + | 32.1 | 3102 | 3212 | -207 | L2 | L2 | LINE |
| 5982116 | 5982410 | + | 10.2 | 1 | 298 | -11 | AluSc | Alu | SINE |
| 5982411 | 5982604 | + | 32.1 | 3213 | 3418 | -1 | L2 | L2 | LINE |

  

|  |  |  |  |  |  |  |  |  |  |
| --- | --- | --- | --- | --- | --- | --- | --- | --- | --- |
| chr7:5990571-5990994 | | | 424 bp | | |  |  |  |  |
| Genome Start | Genome End | Strand | Div | Repeat Start | Repeat End | Repeat Left | Name | Family | Class |
| 5990571 | 5990682 | - | 29.3 | 329 | 440 | -5227 | ERVL-E | ERVL | LTR |
| 5990683 | 5990956 | + | 12.8 | 24 | 296 | -16 | AluSx | Alu | SINE |
| 5990957 | 5990994 | - | 29.3 | 296 | 328 | -5339 | ERVL-E | ERVL | LTR |

  

|  |  |  |  |  |  |  |  |  |  |
| --- | --- | --- | --- | --- | --- | --- | --- | --- | --- |
| chr7:6729891-6730594 | | | 704 bp | | |  |  |  |  |
| Genome Start | Genome End | Strand | Div | Repeat Start | Repeat End | Repeat Left | Name | Family | Class |
| 6729891 | 6730231 | - | 36.0 | 3989 | 4333 | -156 | L3 | CR1 | LINE |
| 6730232 | 6730535 | + | 13.0 | 4 | 305 | -7 | AluSx | Alu | SINE |
| 6730536 | 6730594 | - | 36.0 | 3933 | 3988 | -501 | L3 | CR1 | LINE |

  

|  |  |  |  |  |  |  |  |  |  |
| --- | --- | --- | --- | --- | --- | --- | --- | --- | --- |
| chr7:6733849-6735067 | | | 1219 bp | | |  |  |  |  |
| Genome Start | Genome End | Strand | Div | Repeat Start | Repeat End | Repeat Left | Name | Family | Class |
| 6733849 | 6734494 | + | 13.6 | 1 | 576 | -327 | MER4D | ERV1 | LTR |
| 6734495 | 6734758 | - | 16.2 | 9 | 268 | -44 | AluJo | Alu | SINE |
| 6734759 | 6735067 | + | 13.6 | 577 | 903 | 0 | MER4D | ERV1 | LTR |

  

|  |  |  |  |  |  |  |  |  |  |
| --- | --- | --- | --- | --- | --- | --- | --- | --- | --- |
| chr7:6748196-6748809 | | | 614 bp | | |  |  |  |  |
| Genome Start | Genome End | Strand | Div | Repeat Start | Repeat End | Repeat Left | Name | Family | Class |
| 6748196 | 6748328 | + | 9.2 | 1 | 133 | -179 | AluSx | Alu | SINE |
| 6748329 | 6748639 | + | 9.3 | 1 | 313 | 0 | AluSq | Alu | SINE |
| 6748640 | 6748809 | + | 9.2 | 134 | 293 | -19 | AluSx | Alu | SINE |

  

|  |  |  |  |  |  |  |  |  |  |
| --- | --- | --- | --- | --- | --- | --- | --- | --- | --- |
| chr7:6774991-6775531 | | | 541 bp | | |  |  |  |  |
| Genome Start | Genome End | Strand | Div | Repeat Start | Repeat End | Repeat Left | Name | Family | Class |
| 6774991 | 6775157 | - | 17.7 | 2384 | 2549 | -3597 | L1MD | L1 | LINE |
| 6775158 | 6775460 | - | 10.9 | 1 | 303 | -9 | AluSx | Alu | SINE |
| 6775461 | 6775531 | - | 17.7 | 2315 | 2383 | -3763 | L1MD | L1 | LINE |

  

|  |  |  |  |  |  |  |  |  |  |
| --- | --- | --- | --- | --- | --- | --- | --- | --- | --- |
| chr7:6783089-6783950 | | | 862 bp | | |  |  |  |  |
| Genome Start | Genome End | Strand | Div | Repeat Start | Repeat End | Repeat Left | Name | Family | Class |
| 6783089 | 6783200 | + | 13.3 | 1 | 100 | -427 | MER1A | MER1\_type | DNA |
| 6783201 | 6783512 | - | 9.9 | 1 | 311 | 0 | AluSp | Alu | SINE |
| 6783513 | 6783950 | + | 13.3 | 101 | 529 | 0 | MER1A | MER1\_type | DNA |

  

|  |  |  |  |  |  |  |  |  |  |
| --- | --- | --- | --- | --- | --- | --- | --- | --- | --- |
| chr7:6795952-6796809 | | | 693 bp | | |  |  |  |  |
| Genome Start | Genome End | Strand | Div | Repeat Start | Repeat End | Repeat Left | Name | Family | Class |
| 6795952 | 6796057 | - | 26.9 | 3286 | 3397 | -22 | L2 | L2 | LINE |
| 6796082 | 6796298 | - | 32.6 | 3 | 245 | -23 | MIR | MIR | SINE |
| 6796440 | 6796809 | - | 24.4 | 2305 | 2743 | -676 | L2 | L2 | LINE |

  

|  |  |  |  |  |  |  |  |  |  |
| --- | --- | --- | --- | --- | --- | --- | --- | --- | --- |
| chr7:6803046-6803732 | | | 687 bp | | |  |  |  |  |
| Genome Start | Genome End | Strand | Div | Repeat Start | Repeat End | Repeat Left | Name | Family | Class |
| 6803046 | 6803168 | + | 18.4 | 3 | 135 | -800 | MER21C | ERV1 | LTR |
| 6803169 | 6803456 | - | 10.4 | 9 | 298 | -14 | AluSx | Alu | SINE |
| 6803457 | 6803732 | + | 18.4 | 136 | 417 | -518 | MER21C | ERV1 | LTR |

  

|  |  |  |  |  |  |  |  |  |  |
| --- | --- | --- | --- | --- | --- | --- | --- | --- | --- |
| chr7:6813115-6814270 | | | 1156 bp | | |  |  |  |  |
| Genome Start | Genome End | Strand | Div | Repeat Start | Repeat End | Repeat Left | Name | Family | Class |
| 6813115 | 6813200 | + | 16.8 | 3030 | 3113 | -3033 | L1M4 | L1 | LINE |
| 6813201 | 6813510 | + | 10.4 | 1 | 310 | -2 | AluSx | Alu | SINE |
| 6813511 | 6813943 | + | 16.8 | 3114 | 3485 | -2661 | L1M4 | L1 | LINE |
| 6813944 | 6814068 | - | 9.6 | 1 | 139 | 0 | MER85 | PiggyBac | DNA |
| 6814069 | 6814270 | + | 16.8 | 3486 | 3714 | -2432 | L1M4 | L1 | LINE |

  

|  |  |  |  |  |  |  |  |  |  |
| --- | --- | --- | --- | --- | --- | --- | --- | --- | --- |
| chr7:6814298-6815392 | | | 933 bp | | |  |  |  |  |
| Genome Start | Genome End | Strand | Div | Repeat Start | Repeat End | Repeat Left | Name | Family | Class |
| 6814298 | 6814507 | + | 25.2 | 225 | 441 | -166 | MER77 | ERVL | LTR |
| 6814616 | 6814724 | + | 21.8 | 376 | 529 | -77 | MER77 | ERVL | LTR |
| 6814771 | 6814877 | + | 22.4 | 2225 | 2330 | -161 | Tigger7 | MER2\_type | DNA |
| 6814878 | 6815164 | - | 13.2 | 1 | 289 | -23 | AluSx | Alu | SINE |
| 6815165 | 6815319 | + | 22.4 | 2331 | 2491 | 0 | Tigger7 | MER2\_type | DNA |
| 6815328 | 6815392 | + | 21.5 | 537 | 603 | -4 | MER77 | ERVL | LTR |

  

|  |  |  |  |  |  |  |  |  |  |
| --- | --- | --- | --- | --- | --- | --- | --- | --- | --- |
| chr7:6816000-6816593 | | | 594 bp | | |  |  |  |  |
| Genome Start | Genome End | Strand | Div | Repeat Start | Repeat End | Repeat Left | Name | Family | Class |
| 6816000 | 6816168 | - | 29.7 | 1639 | 1810 | -697 | HAL1 | L1 | LINE |
| 6816169 | 6816464 | - | 10.2 | 5 | 299 | -13 | AluSx | Alu | SINE |
| 6816465 | 6816593 | - | 29.7 | 1515 | 1638 | -869 | HAL1 | L1 | LINE |

  

|  |  |  |  |  |  |  |  |  |  |
| --- | --- | --- | --- | --- | --- | --- | --- | --- | --- |
| chr7:6857468-6858784 | | | 1285 bp | | |  |  |  |  |
| Genome Start | Genome End | Strand | Div | Repeat Start | Repeat End | Repeat Left | Name | Family | Class |
| 6857468 | 6857676 | - | 27.9 | 572 | 778 | -5341 | L1MEd | L1 | LINE |
| 6857677 | 6857965 | - | 8.7 | 1 | 293 | -20 | AluSq | Alu | SINE |
| 6857966 | 6858376 | - | 27.9 | 109 | 571 | -5548 | L1MEd | L1 | LINE |
| 6858409 | 6858694 | - | 10.8 | 1 | 289 | -24 | AluSq | Alu | SINE |
| 6858695 | 6858784 | - | 27.9 | 23 | 108 | -6011 | L1MEd | L1 | LINE |

  

|  |  |  |  |  |  |  |  |  |  |
| --- | --- | --- | --- | --- | --- | --- | --- | --- | --- |
| chr7:6870758-6871428 | | | 671 bp | | |  |  |  |  |
| Genome Start | Genome End | Strand | Div | Repeat Start | Repeat End | Repeat Left | Name | Family | Class |
| 6870758 | 6870961 | - | 24.2 | 5934 | 6130 | -170 | L1MA4 | L1 | LINE |
| 6870962 | 6871245 | - | 17.3 | 1 | 283 | -29 | AluSx | Alu | SINE |
| 6871246 | 6871428 | - | 24.2 | 5767 | 5933 | -367 | L1MA4 | L1 | LINE |

  

|  |  |  |  |  |  |  |  |  |  |
| --- | --- | --- | --- | --- | --- | --- | --- | --- | --- |
| chr7:6892829-6893769 | | | 941 bp | | |  |  |  |  |
| Genome Start | Genome End | Strand | Div | Repeat Start | Repeat End | Repeat Left | Name | Family | Class |
| 6892829 | 6892909 | + | 37.8 | 2796 | 2873 | -546 | L2 | L2 | LINE |
| 6892910 | 6892962 | - | 6.5 | 31 | 80 | 0 | MADE1 | Mariner | DNA |
| 6892963 | 6893261 | + | 15.4 | 1 | 297 | -15 | AluSx | Alu | SINE |
| 6893262 | 6893300 | - | 6.5 | 1 | 30 | -50 | MADE1 | Mariner | DNA |
| 6893301 | 6893769 | + | 33.8 | 2874 | 3377 | -1 | L2 | L2 | LINE |

  

|  |  |  |  |  |  |  |  |  |  |
| --- | --- | --- | --- | --- | --- | --- | --- | --- | --- |
| chr7:6902083-6902797 | | | 715 bp | | |  |  |  |  |
| Genome Start | Genome End | Strand | Div | Repeat Start | Repeat End | Repeat Left | Name | Family | Class |
| 6902083 | 6902146 | + | 22.5 | 5769 | 5826 | -352 | L1MB8 | L1 | LINE |
| 6902147 | 6902435 | + | 12.6 | 6 | 293 | -19 | AluSx | Alu | SINE |
| 6902436 | 6902797 | + | 22.5 | 5827 | 6174 | -4 | L1MB8 | L1 | LINE |

  

|  |  |  |  |  |  |  |  |  |  |
| --- | --- | --- | --- | --- | --- | --- | --- | --- | --- |
| chr7:6908110-6909494 | | | 1385 bp | | |  |  |  |  |
| Genome Start | Genome End | Strand | Div | Repeat Start | Repeat End | Repeat Left | Name | Family | Class |
| 6908110 | 6908152 | + | 21.0 | 1 | 50 | -417 | MLT1C | MaLR | LTR |
| 6908153 | 6909119 | + | 11.9 | 1 | 968 | 0 | LTR5\_Hs | ERVK | LTR |
| 6909120 | 6909494 | + | 20.6 | 51 | 505 | 0 | MLT1C | MaLR | LTR |

  

|  |  |  |  |  |  |  |  |  |  |
| --- | --- | --- | --- | --- | --- | --- | --- | --- | --- |
| chr7:6926430-6927093 | | | 664 bp | | |  |  |  |  |
| Genome Start | Genome End | Strand | Div | Repeat Start | Repeat End | Repeat Left | Name | Family | Class |
| 6926430 | 6926584 | + | 11.3 | 1 | 161 | -203 | THE1B | MaLR | LTR |
| 6926585 | 6926884 | - | 14.7 | 1 | 300 | -12 | AluSx | Alu | SINE |
| 6926885 | 6927093 | + | 11.3 | 162 | 364 | 0 | THE1B | MaLR | LTR |

  

|  |  |  |  |  |  |  |  |  |  |
| --- | --- | --- | --- | --- | --- | --- | --- | --- | --- |
| chr7:6943155-6943737 | | | 583 bp | | |  |  |  |  |
| Genome Start | Genome End | Strand | Div | Repeat Start | Repeat End | Repeat Left | Name | Family | Class |
| 6943155 | 6943309 | - | 10.9 | 135 | 298 | -12 | AluSg | Alu | SINE |
| 6943310 | 6943606 | - | 9.4 | 1 | 300 | -13 | AluSq | Alu | SINE |
| 6943607 | 6943737 | - | 10.9 | 3 | 134 | -176 | AluSg | Alu | SINE |

  

|  |  |  |  |  |  |  |  |  |  |
| --- | --- | --- | --- | --- | --- | --- | --- | --- | --- |
| chr7:6948989-6949221 | | | 233 bp | | |  |  |  |  |
| Genome Start | Genome End | Strand | Div | Repeat Start | Repeat End | Repeat Left | Name | Family | Class |
| 6948989 | 6949031 | + | 28.7 | 4 | 42 | -136 | MER5B | MER1\_type | DNA |
| 6949032 | 6949164 | - | 12.8 | 1 | 133 | 0 | FLAM\_A | Alu | SINE |
| 6949165 | 6949221 | + | 28.7 | 43 | 97 | -81 | MER5B | MER1\_type | DNA |

  

|  |  |  |  |  |  |  |  |  |  |
| --- | --- | --- | --- | --- | --- | --- | --- | --- | --- |
| chr7:6973160-6973933 | | | 774 bp | | |  |  |  |  |
| Genome Start | Genome End | Strand | Div | Repeat Start | Repeat End | Repeat Left | Name | Family | Class |
| 6973160 | 6973252 | + | 21.9 | 3 | 88 | -379 | MLT1C | MaLR | LTR |
| 6973253 | 6973558 | + | 9.9 | 1 | 307 | 0 | AluSp | Alu | SINE |
| 6973559 | 6973933 | + | 21.9 | 89 | 467 | 0 | MLT1C | MaLR | LTR |

  

|  |  |  |  |  |  |  |  |  |  |
| --- | --- | --- | --- | --- | --- | --- | --- | --- | --- |
| chr7:6974527-6975369 | | | 843 bp | | |  |  |  |  |
| Genome Start | Genome End | Strand | Div | Repeat Start | Repeat End | Repeat Left | Name | Family | Class |
| 6974527 | 6974793 | + | 19.8 | 3 | 320 | -334 | MLT1E1 | MaLR | LTR |
| 6974794 | 6975098 | - | 9.8 | 1 | 305 | -7 | AluSx | Alu | SINE |
| 6975099 | 6975369 | + | 19.8 | 321 | 653 | -1 | MLT1E1 | MaLR | LTR |

  

|  |  |  |  |  |  |  |  |  |  |
| --- | --- | --- | --- | --- | --- | --- | --- | --- | --- |
| chr7:6990139-6991216 | | | 951 bp | | |  |  |  |  |
| Genome Start | Genome End | Strand | Div | Repeat Start | Repeat End | Repeat Left | Name | Family | Class |
| 6990139 | 6990204 | - | 18.5 | 522 | 590 | -1 | MLT1K | MaLR | LTR |
| 6990209 | 6990534 | - | 29.7 | 164 | 491 | 0 | LTR16C | ERVL | LTR |
| 6990535 | 6990835 | - | 14.9 | 1 | 303 | -7 | AluSg | Alu | SINE |
| 6990959 | 6991216 | - | 28.8 | 183 | 429 | -162 | MLT1K | MaLR | LTR |

  

|  |  |  |  |  |  |  |  |  |  |
| --- | --- | --- | --- | --- | --- | --- | --- | --- | --- |
| chr7:6992160-6992981 | | | 820 bp | | |  |  |  |  |
| Genome Start | Genome End | Strand | Div | Repeat Start | Repeat End | Repeat Left | Name | Family | Class |
| 6992160 | 6992202 | + | 7.0 | 2 | 44 | -428 | MER4A1 | ERV1 | LTR |
| 6992204 | 6992281 | + | 7.8 | 2 | 78 | -234 | AluS | Alu | SINE |
| 6992282 | 6992551 | + | 11.3 | 29 | 293 | -19 | AluSx | Alu | SINE |
| 6992553 | 6992981 | + | 11.7 | 34 | 472 | 0 | MER4A1 | ERV1 | LTR |

  

|  |  |  |  |  |  |  |  |  |  |
| --- | --- | --- | --- | --- | --- | --- | --- | --- | --- |
| chr7:22503829-22504516 | | | 683 bp | | |  |  |  |  |
| Genome Start | Genome End | Strand | Div | Repeat Start | Repeat End | Repeat Left | Name | Family | Class |
| 22503829 | 22504016 | - | 14.0 | 235 | 428 | 0 | MSTA | MaLR | LTR |
| 22504022 | 22504362 | - | 12.5 | 1 | 339 | 0 | MER1B | MER1\_type | DNA |
| 22504363 | 22504516 | - | 13.9 | 1 | 156 | -272 | MSTA | MaLR | LTR |

  

|  |  |  |  |  |  |  |  |  |  |
| --- | --- | --- | --- | --- | --- | --- | --- | --- | --- |
| chr7:29636047-29637053 | | | 1007 bp | | |  |  |  |  |
| Genome Start | Genome End | Strand | Div | Repeat Start | Repeat End | Repeat Left | Name | Family | Class |
| 29636047 | 29636384 | - | 21.2 | 120 | 467 | 0 | MLT1C | MaLR | LTR |
| 29636385 | 29636635 | - | 13.9 | 1 | 251 | -61 | AluSx | Alu | SINE |
| 29636636 | 29636732 | - | 21.2 | 34 | 119 | -348 | MLT1C | MaLR | LTR |
| 29636733 | 29637021 | + | 12.5 | 1 | 291 | -21 | AluSx | Alu | SINE |
| 29637022 | 29637053 | - | 21.2 | 1 | 33 | -434 | MLT1C | MaLR | LTR |

  

|  |  |  |  |  |  |  |  |  |  |
| --- | --- | --- | --- | --- | --- | --- | --- | --- | --- |
| chr7:29638473-29638988 | | | 516 bp | | |  |  |  |  |
| Genome Start | Genome End | Strand | Div | Repeat Start | Repeat End | Repeat Left | Name | Family | Class |
| 29638473 | 29638533 | + | 23.3 | 1 | 54 | -124 | MER5B | MER1\_type | DNA |
| 29638534 | 29638866 | + | 10.5 | 1 | 333 | 0 | AluSx | Alu | SINE |
| 29638867 | 29638988 | + | 23.3 | 55 | 178 | 0 | MER5B | MER1\_type | DNA |

  

|  |  |  |  |  |  |  |  |  |  |
| --- | --- | --- | --- | --- | --- | --- | --- | --- | --- |
| chr7:29653581-29655313 | | | 1263 bp | | |  |  |  |  |
| Genome Start | Genome End | Strand | Div | Repeat Start | Repeat End | Repeat Left | Name | Family | Class |
| 29653581 | 29653741 | + | 32.1 | 2828 | 2991 | -428 | L2 | L2 | LINE |
| 29653793 | 29653977 | - | 11.7 | 1 | 174 | -19 | MER53 | DNA | DNA |
| 29654034 | 29654255 | + | 33.3 | 3087 | 3288 | -90 | L2 | L2 | LINE |
| 29654305 | 29654530 | - | 27.5 | 21 | 251 | -11 | MIR | MIR | SINE |
| 29654809 | 29655109 | + | 11.0 | 1 | 300 | -12 | AluSx | Alu | SINE |
| 29655148 | 29655276 | + | 36.3 | 8 | 153 | -115 | MIR | MIR | SINE |
| 29655275 | 29655313 | + | 23.1 | 3323 | 3361 | -17 | L2 | L2 | LINE |

  

|  |  |  |  |  |  |  |  |  |  |
| --- | --- | --- | --- | --- | --- | --- | --- | --- | --- |
| chr7:29682143-29683273 | | | 781 bp | | |  |  |  |  |
| Genome Start | Genome End | Strand | Div | Repeat Start | Repeat End | Repeat Left | Name | Family | Class |
| 29682143 | 29682433 | + | 27.9 | 2919 | 3236 | -2910 | L1M5 | L1 | LINE |
| 29682784 | 29682881 | + | 33.5 | 5562 | 5670 | -451 | L1M5 | L1 | LINE |
| 29682882 | 29683179 | + | 10.4 | 1 | 305 | -7 | AluSx | Alu | SINE |
| 29683180 | 29683273 | + | 33.5 | 5671 | 5774 | -347 | L1M5 | L1 | LINE |

  

|  |  |  |  |  |  |  |  |  |  |
| --- | --- | --- | --- | --- | --- | --- | --- | --- | --- |
| chr7:29719441-29721540 | | | 2098 bp | | |  |  |  |  |
| Genome Start | Genome End | Strand | Div | Repeat Start | Repeat End | Repeat Left | Name | Family | Class |
| 29719441 | 29720785 | + | 23.1 | 494 | 1888 | -4614 | L1MDb | L1 | LINE |
| 29720783 | 29721001 | - | 9.1 | 4658 | 4876 | -1270 | L1P3 | L1 | LINE |
| 29721006 | 29721431 | + | 5.4 | 5731 | 6165 | -3 | L1PA10 | L1 | LINE |
| 29721433 | 29721540 | + | 23.3 | 1883 | 1990 | -4512 | L1MDb | L1 | LINE |

  

|  |  |  |  |  |  |  |  |  |  |
| --- | --- | --- | --- | --- | --- | --- | --- | --- | --- |
| chr7:29740269-29743142 | | | 2830 bp | | |  |  |  |  |
| Genome Start | Genome End | Strand | Div | Repeat Start | Repeat End | Repeat Left | Name | Family | Class |
| 29740269 | 29741626 | + | 27.9 | 1332 | 2945 | -4242 | L1MEb | L1 | LINE |
| 29741659 | 29742107 | + | 14.7 | 5845 | 6300 | 0 | L1MA5 | L1 | LINE |
| 29742113 | 29742738 | + | 18.6 | 5593 | 6242 | 0 | L1MD1 | L1 | LINE |
| 29742746 | 29743142 | + | 28.5 | 2970 | 3382 | -3624 | L1MEb | L1 | LINE |

  

|  |  |  |  |  |  |  |  |  |  |
| --- | --- | --- | --- | --- | --- | --- | --- | --- | --- |
| chr7:32632674-32634982 | | | 2290 bp | | |  |  |  |  |
| Genome Start | Genome End | Strand | Div | Repeat Start | Repeat End | Repeat Left | Name | Family | Class |
| 32632674 | 32634286 | + | 14.8 | 4404 | 6099 | -234 | L1MC1 | L1 | LINE |
| 32634288 | 32634449 | + | 22.8 | 137 | 298 | -14 | AluJb | Alu | SINE |
| 32634468 | 32634648 | + | 13.3 | 6116 | 6291 | -42 | L1MC1 | L1 | LINE |
| 32634649 | 32634942 | + | 9.9 | 1 | 295 | -17 | AluSx | Alu | SINE |
| 32634943 | 32634982 | + | 13.3 | 6292 | 6321 | -12 | L1MC1 | L1 | LINE |

  

|  |  |  |  |  |  |  |  |  |  |
| --- | --- | --- | --- | --- | --- | --- | --- | --- | --- |
| chr7:32653481-32654729 | | | 1249 bp | | |  |  |  |  |
| Genome Start | Genome End | Strand | Div | Repeat Start | Repeat End | Repeat Left | Name | Family | Class |
| 32653481 | 32653968 | + | 25.6 | 6956 | 7478 | -564 | L1MC4 | L1 | LINE |
| 32653969 | 32654270 | + | 16.3 | 1 | 302 | 0 | AluSx | Alu | SINE |
| 32654271 | 32654729 | + | 25.6 | 7479 | 7951 | -91 | L1MC4 | L1 | LINE |

  

|  |  |  |  |  |  |  |  |  |  |
| --- | --- | --- | --- | --- | --- | --- | --- | --- | --- |
| chr7:32664657-32665300 | | | 605 bp | | |  |  |  |  |
| Genome Start | Genome End | Strand | Div | Repeat Start | Repeat End | Repeat Left | Name | Family | Class |
| 32664657 | 32664739 | + | 20.7 | 70 | 153 | -423 | LTR24B | ERV1 | LTR |
| 32664777 | 32664924 | - | 18.2 | 13 | 160 | -16 | FRAM | Alu | SINE |
| 32664927 | 32665300 | + | 26.9 | 187 | 569 | -7 | LTR24B | ERV1 | LTR |

  

|  |  |  |  |  |  |  |  |  |  |
| --- | --- | --- | --- | --- | --- | --- | --- | --- | --- |
| chr7:32750190-32753419 | | | 3208 bp | | |  |  |  |  |
| Genome Start | Genome End | Strand | Div | Repeat Start | Repeat End | Repeat Left | Name | Family | Class |
| 32750190 | 32750382 | + | 16.8 | 2983 | 3185 | -2961 | L1MD | L1 | LINE |
| 32750383 | 32750832 | - | 17.3 | 3 | 426 | 0 | MSTB | MaLR | LTR |
| 32750833 | 32752375 | + | 16.8 | 3186 | 4768 | -1378 | L1MD | L1 | LINE |
| 32752376 | 32752679 | + | 15.5 | 1 | 305 | -7 | AluSx | Alu | SINE |
| 32752680 | 32752865 | + | 16.8 | 4769 | 4940 | -1206 | L1MD | L1 | LINE |
| 32752888 | 32753419 | + | 17.0 | 4941 | 5519 | -723 | L1MD | L1 | LINE |

  

|  |  |  |  |  |  |  |  |  |  |
| --- | --- | --- | --- | --- | --- | --- | --- | --- | --- |
| chr7:32787451-32789380 | | | 1930 bp | | |  |  |  |  |
| Genome Start | Genome End | Strand | Div | Repeat Start | Repeat End | Repeat Left | Name | Family | Class |
| 32787451 | 32788535 | - | 16.1 | 516 | 1645 | -6 | MSTB-int | MaLR | LTR |
| 32788536 | 32788880 | - | 15.6 | 1 | 364 | 0 | THE1B | MaLR | LTR |
| 32788881 | 32789380 | - | 16.1 | 3 | 515 | -1136 | MSTB-int | MaLR | LTR |

  

|  |  |  |  |  |  |  |  |  |  |
| --- | --- | --- | --- | --- | --- | --- | --- | --- | --- |
| chr7:32798101-32798625 | | | 427 bp | | |  |  |  |  |
| Genome Start | Genome End | Strand | Div | Repeat Start | Repeat End | Repeat Left | Name | Family | Class |
| 32798101 | 32798210 | + | 16.4 | 7672 | 7785 | -176 | L1MC5 | L1 | LINE |
| 32798214 | 32798431 | + | 27.4 | 5909 | 6151 | -19 | L1ME3A | L1 | LINE |
| 32798527 | 32798625 | + | 26.8 | 7813 | 7913 | -48 | L1MC5 | L1 | LINE |

  

|  |  |  |  |  |  |  |  |  |  |
| --- | --- | --- | --- | --- | --- | --- | --- | --- | --- |
| chr7:32999332-33000184 | | | 853 bp | | |  |  |  |  |
| Genome Start | Genome End | Strand | Div | Repeat Start | Repeat End | Repeat Left | Name | Family | Class |
| 32999332 | 32999679 | - | 18.0 | 5827 | 6181 | -2 | L1MB3 | L1 | LINE |
| 32999680 | 32999961 | + | 10.3 | 1 | 294 | -18 | AluSx | Alu | SINE |
| 32999962 | 33000184 | - | 18.0 | 5639 | 5826 | -357 | L1MB3 | L1 | LINE |

  

|  |  |  |  |  |  |  |  |  |  |
| --- | --- | --- | --- | --- | --- | --- | --- | --- | --- |
| chr7:34970589-34971758 | | | 1128 bp | | |  |  |  |  |
| Genome Start | Genome End | Strand | Div | Repeat Start | Repeat End | Repeat Left | Name | Family | Class |
| 34970589 | 34970820 | + | 26.3 | 3694 | 3947 | -2199 | L1ME3B | L1 | LINE |
| 34970831 | 34970962 | + | 20.4 | 6906 | 7044 | -998 | L1MC4 | L1 | LINE |
| 34970995 | 34971758 | + | 26.2 | 5325 | 6160 | -80 | L1ME3B | L1 | LINE |

  

|  |  |  |  |  |  |  |  |  |  |
| --- | --- | --- | --- | --- | --- | --- | --- | --- | --- |
| chr7:35017837-35018797 | | | 766 bp | | |  |  |  |  |
| Genome Start | Genome End | Strand | Div | Repeat Start | Repeat End | Repeat Left | Name | Family | Class |
| 35017837 | 35017983 | + | 30.6 | 4457 | 4608 | -1538 | L1M5 | L1 | LINE |
| 35017998 | 35018111 | + | 33.3 | 4687 | 4804 | -1342 | L1M5 | L1 | LINE |
| 35018140 | 35018572 | + | 27.9 | 25 | 450 | 0 | LTR16A | ERVL | LTR |
| 35018726 | 35018797 | + | 25.0 | 4957 | 5032 | -1114 | L1M5 | L1 | LINE |

  

|  |  |  |  |  |  |  |  |  |  |
| --- | --- | --- | --- | --- | --- | --- | --- | --- | --- |
| chr7:35074702-35076613 | | | 1912 bp | | |  |  |  |  |
| Genome Start | Genome End | Strand | Div | Repeat Start | Repeat End | Repeat Left | Name | Family | Class |
| 35074702 | 35076111 | + | 30.6 | 3 | 1490 | -245 | MLT-int | MaLR | LTR |
| 35076112 | 35076408 | - | 12.5 | 1 | 297 | -15 | AluSx | Alu | SINE |
| 35076409 | 35076613 | + | 30.6 | 1491 | 1735 | 0 | MLT-int | MaLR | LTR |

  

|  |  |  |  |  |  |  |  |  |  |
| --- | --- | --- | --- | --- | --- | --- | --- | --- | --- |
| chr7:35112753-35113585 | | | 816 bp | | |  |  |  |  |
| Genome Start | Genome End | Strand | Div | Repeat Start | Repeat End | Repeat Left | Name | Family | Class |
| 35112753 | 35112792 | - | 22.5 | 3365 | 3404 | -15 | L2 | L2 | LINE |
| 35112810 | 35113523 | + | 15.8 | 5 | 733 | 0 | MER44C | MER2\_type | DNA |
| 35113524 | 35113585 | - | 29.0 | 3285 | 3351 | -68 | L2 | L2 | LINE |

  

|  |  |  |  |  |  |  |  |  |  |
| --- | --- | --- | --- | --- | --- | --- | --- | --- | --- |
| chr7:35131373-35133120 | | | 1643 bp | | |  |  |  |  |
| Genome Start | Genome End | Strand | Div | Repeat Start | Repeat End | Repeat Left | Name | Family | Class |
| 35131373 | 35131620 | + | 35.0 | 233 | 506 | -5709 | L1MC4 | L1 | LINE |
| 35131624 | 35132708 | + | 24.2 | 549 | 1678 | -4537 | L1MC4 | L1 | LINE |
| 35132804 | 35132927 | + | 25.2 | 2251 | 2375 | -3840 | L1M5 | L1 | LINE |
| 35132935 | 35133120 | + | 31.7 | 3025 | 3213 | -3001 | L1MC4 | L1 | LINE |

  

|  |  |  |  |  |  |  |  |  |  |
| --- | --- | --- | --- | --- | --- | --- | --- | --- | --- |
| chr7:35133110-35135199 | | | 2091 bp | | |  |  |  |  |
| Genome Start | Genome End | Strand | Div | Repeat Start | Repeat End | Repeat Left | Name | Family | Class |
| 35133110 | 35133218 | - | 27.4 | 1882 | 1990 | -4512 | L1MDb | L1 | LINE |
| 35133219 | 35133647 | - | 6.3 | 5729 | 6165 | -3 | L1PA10 | L1 | LINE |
| 35133650 | 35133868 | + | 11.0 | 4658 | 4876 | -1270 | L1P3 | L1 | LINE |
| 35133866 | 35135199 | - | 22.8 | 494 | 1888 | -4614 | L1MDb | L1 | LINE |

  

|  |  |  |  |  |  |  |  |  |  |
| --- | --- | --- | --- | --- | --- | --- | --- | --- | --- |
| chr7:35165221-35167038 | | | 1811 bp | | |  |  |  |  |
| Genome Start | Genome End | Strand | Div | Repeat Start | Repeat End | Repeat Left | Name | Family | Class |
| 35165221 | 35165845 | - | 26.7 | 5299 | 5984 | -256 | L1ME3B | L1 | LINE |
| 35165848 | 35165969 | + | 23.3 | 3285 | 3419 | 0 | L2 | L2 | LINE |
| 35165975 | 35167038 | - | 29.0 | 4109 | 5266 | -880 | L1ME3B | L1 | LINE |

  

|  |  |  |  |  |  |  |  |  |  |
| --- | --- | --- | --- | --- | --- | --- | --- | --- | --- |
| chr7:35171583-35172742 | | | 1153 bp | | |  |  |  |  |
| Genome Start | Genome End | Strand | Div | Repeat Start | Repeat End | Repeat Left | Name | Family | Class |
| 35171583 | 35171674 | + | 21.6 | 180 | 272 | -6361 | L1MD | L1 | LINE |
| 35171675 | 35172059 | + | 15.8 | 1 | 408 | 0 | MLT1A1 | MaLR | LTR |
| 35172060 | 35172085 | + | 21.6 | 273 | 298 | -6335 | L1MD | L1 | LINE |
| 35172093 | 35172166 | + | 20.8 | 392 | 947 | -5686 | L1MD | L1 | LINE |
| 35172167 | 35172409 | - | 11.6 | 58 | 299 | -14 | AluSq | Alu | SINE |
| 35172410 | 35172742 | + | 19.7 | 948 | 1297 | -5336 | L1MD | L1 | LINE |

  

|  |  |  |  |  |  |  |  |  |  |
| --- | --- | --- | --- | --- | --- | --- | --- | --- | --- |
| chr7:35182889-35184081 | | | 1189 bp | | |  |  |  |  |
| Genome Start | Genome End | Strand | Div | Repeat Start | Repeat End | Repeat Left | Name | Family | Class |
| 35182889 | 35183526 | + | 23.0 | 4168 | 4894 | -1252 | L1M5 | L1 | LINE |
| 35183517 | 35183682 | + | 28.5 | 5989 | 6161 | -5 | L1ME1 | L1 | LINE |
| 35183697 | 35184081 | + | 30.5 | 5329 | 5739 | -501 | L1M5 | L1 | LINE |

  

|  |  |  |  |  |  |  |  |  |  |
| --- | --- | --- | --- | --- | --- | --- | --- | --- | --- |
| chr7:35212346-35219394 | | | 7049 bp | | |  |  |  |  |
| Genome Start | Genome End | Strand | Div | Repeat Start | Repeat End | Repeat Left | Name | Family | Class |
| 35212346 | 35212402 | - | 26.8 | 6190 | 6232 | -7 | L1ME1 | L1 | LINE |
| 35212403 | 35212706 | + | 13.2 | 1 | 306 | -7 | AluSq | Alu | SINE |
| 35212707 | 35214597 | - | 25.8 | 4174 | 6189 | -50 | L1ME1 | L1 | LINE |
| 35214598 | 35214909 | + | 8.7 | 1 | 313 | 0 | AluSp | Alu | SINE |
| 35214910 | 35216246 | - | 25.2 | 2736 | 4173 | -2046 | L1ME1 | L1 | LINE |
| 35216247 | 35216551 | - | 7.9 | 1 | 304 | -7 | AluY | Alu | SINE |
| 35216552 | 35217675 | - | 20.5 | 1610 | 2735 | -3484 | L1ME1 | L1 | LINE |
| 35217676 | 35217754 | + | 20.2 | 1 | 79 | -2244 | Tigger5 | MER2\_type | DNA |
| 35217755 | 35217793 | - | 20.5 | 1571 | 1609 | -4610 | L1ME1 | L1 | LINE |
| 35217794 | 35217870 | + | 19.5 | 2330 | 2406 | 0 | Tigger5 | MER2\_type | DNA |
| 35217871 | 35219394 | - | 20.5 | 45 | 1570 | -4649 | L1ME1 | L1 | LINE |

  

|  |  |  |  |  |  |  |  |  |  |
| --- | --- | --- | --- | --- | --- | --- | --- | --- | --- |
| chr7:35860005-35860404 | | | 400 bp | | |  |  |  |  |
| Genome Start | Genome End | Strand | Div | Repeat Start | Repeat End | Repeat Left | Name | Family | Class |
| 35860005 | 35860114 | + | 30.4 | 40 | 153 | -109 | MIR | MIR | SINE |
| 35860115 | 35860348 | + | 15.9 | 1 | 236 | 0 | MER46A | MER2\_type | DNA |
| 35860349 | 35860404 | + | 30.4 | 154 | 211 | -51 | MIR | MIR | SINE |

  

|  |  |  |  |  |  |  |  |  |  |
| --- | --- | --- | --- | --- | --- | --- | --- | --- | --- |
| chr7:35882691-35883262 | | | 572 bp | | |  |  |  |  |
| Genome Start | Genome End | Strand | Div | Repeat Start | Repeat End | Repeat Left | Name | Family | Class |
| 35882691 | 35882821 | + | 16.9 | 1 | 133 | -169 | AluJo | Alu | SINE |
| 35882822 | 35883114 | + | 9.2 | 1 | 300 | -13 | AluSq | Alu | SINE |
| 35883115 | 35883262 | + | 16.9 | 134 | 302 | 0 | AluJo | Alu | SINE |

  

|  |  |  |  |  |  |  |  |  |  |
| --- | --- | --- | --- | --- | --- | --- | --- | --- | --- |
| chr7:35926463-35927079 | | | 617 bp | | |  |  |  |  |
| Genome Start | Genome End | Strand | Div | Repeat Start | Repeat End | Repeat Left | Name | Family | Class |
| 35926463 | 35926606 | + | 14.5 | 1 | 138 | -174 | AluJo | Alu | SINE |
| 35926607 | 35926912 | + | 10.8 | 3 | 307 | -5 | AluSx | Alu | SINE |
| 35926913 | 35927079 | + | 14.5 | 139 | 300 | -12 | AluJo | Alu | SINE |

  

|  |  |  |  |  |  |  |  |  |  |
| --- | --- | --- | --- | --- | --- | --- | --- | --- | --- |
| chr7:35935562-35936175 | | | 614 bp | | |  |  |  |  |
| Genome Start | Genome End | Strand | Div | Repeat Start | Repeat End | Repeat Left | Name | Family | Class |
| 35935562 | 35935736 | - | 11.7 | 137 | 308 | -4 | AluSx | Alu | SINE |
| 35935737 | 35936038 | - | 10.3 | 1 | 303 | -8 | AluY | Alu | SINE |
| 35936039 | 35936175 | - | 11.7 | 1 | 136 | -176 | AluSx | Alu | SINE |

  

|  |  |  |  |  |  |  |  |  |  |
| --- | --- | --- | --- | --- | --- | --- | --- | --- | --- |
| chr7:39775010-39775803 | | | 768 bp | | |  |  |  |  |
| Genome Start | Genome End | Strand | Div | Repeat Start | Repeat End | Repeat Left | Name | Family | Class |
| 39775010 | 39775175 | - | 9.1 | 136 | 298 | -12 | AluSg | Alu | SINE |
| 39775202 | 39775670 | - | 3.6 | 5666 | 6137 | -18 | L1PA4 | L1 | LINE |
| 39775671 | 39775803 | - | 9.1 | 1 | 135 | -175 | AluSg | Alu | SINE |

  

|  |  |  |  |  |  |  |  |  |  |
| --- | --- | --- | --- | --- | --- | --- | --- | --- | --- |
| chr7:39777877-39779329 | | | 1453 bp | | |  |  |  |  |
| Genome Start | Genome End | Strand | Div | Repeat Start | Repeat End | Repeat Left | Name | Family | Class |
| 39777877 | 39778177 | - | 19.7 | 604 | 935 | 0 | MER21C | ERV1 | LTR |
| 39778178 | 39778478 | - | 16.2 | 1 | 299 | -13 | AluJo | Alu | SINE |
| 39778479 | 39778779 | - | 19.7 | 276 | 603 | -332 | MER21C | ERV1 | LTR |
| 39778780 | 39779070 | + | 13.8 | 1 | 290 | -23 | AluSq | Alu | SINE |
| 39779071 | 39779329 | - | 19.7 | 18 | 275 | -660 | MER21C | ERV1 | LTR |

  

|  |  |  |  |  |  |  |  |  |  |
| --- | --- | --- | --- | --- | --- | --- | --- | --- | --- |
| chr7:39780021-39780602 | | | 582 bp | | |  |  |  |  |
| Genome Start | Genome End | Strand | Div | Repeat Start | Repeat End | Repeat Left | Name | Family | Class |
| 39780021 | 39780147 | + | 19.4 | 10 | 133 | -179 | AluJo | Alu | SINE |
| 39780148 | 39780436 | + | 11.8 | 1 | 297 | -12 | AluSg1 | Alu | SINE |
| 39780437 | 39780602 | + | 19.4 | 134 | 299 | -13 | AluJo | Alu | SINE |

  

|  |  |  |  |  |  |  |  |  |  |
| --- | --- | --- | --- | --- | --- | --- | --- | --- | --- |
| chr7:39783147-39784316 | | | 1136 bp | | |  |  |  |  |
| Genome Start | Genome End | Strand | Div | Repeat Start | Repeat End | Repeat Left | Name | Family | Class |
| 39783147 | 39783765 | - | 20.3 | 5552 | 6173 | -5 | L1MB8 | L1 | LINE |
| 39783766 | 39783891 | + | 17.9 | 4 | 129 | -4 | FLAM\_A | Alu | SINE |
| 39783926 | 39783999 | - | 23.1 | 5507 | 5570 | -608 | L1MB8 | L1 | LINE |
| 39784000 | 39784285 | - | 18.9 | 1 | 297 | -15 | AluJb | Alu | SINE |
| 39784286 | 39784316 | - | 23.1 | 5476 | 5506 | -672 | L1MB8 | L1 | LINE |

  

|  |  |  |  |  |  |  |  |  |  |
| --- | --- | --- | --- | --- | --- | --- | --- | --- | --- |
| chr7:39784370-39784826 | | | 457 bp | | |  |  |  |  |
| Genome Start | Genome End | Strand | Div | Repeat Start | Repeat End | Repeat Left | Name | Family | Class |
| 39784370 | 39784433 | + | 22.9 | 24 | 71 | -241 | AluJo | Alu | SINE |
| 39784434 | 39784724 | - | 12.9 | 1 | 312 | 0 | AluSx | Alu | SINE |
| 39784725 | 39784826 | + | 22.9 | 75 | 167 | -145 | AluJo | Alu | SINE |

  

|  |  |  |  |  |  |  |  |  |  |
| --- | --- | --- | --- | --- | --- | --- | --- | --- | --- |
| chr7:39789600-39791094 | | | 1495 bp | | |  |  |  |  |
| Genome Start | Genome End | Strand | Div | Repeat Start | Repeat End | Repeat Left | Name | Family | Class |
| 39789600 | 39789901 | - | 20.6 | 604 | 935 | 0 | MER21C | ERV1 | LTR |
| 39789902 | 39790213 | - | 21.5 | 1 | 302 | -10 | AluJo | Alu | SINE |
| 39790214 | 39790283 | - | 20.6 | 526 | 603 | -332 | MER21C | ERV1 | LTR |
| 39790284 | 39790590 | + | 21.6 | 2 | 294 | -18 | AluJb | Alu | SINE |
| 39790591 | 39791094 | - | 20.6 | 18 | 525 | -410 | MER21C | ERV1 | LTR |

  

|  |  |  |  |  |  |  |  |  |  |
| --- | --- | --- | --- | --- | --- | --- | --- | --- | --- |
| chr7:39877470-39879053 | | | 1584 bp | | |  |  |  |  |
| Genome Start | Genome End | Strand | Div | Repeat Start | Repeat End | Repeat Left | Name | Family | Class |
| 39877470 | 39878191 | - | 18.1 | 5451 | 6178 | 0 | L1MB8 | L1 | LINE |
| 39878192 | 39878486 | - | 9.9 | 2 | 297 | -14 | AluY | Alu | SINE |
| 39878487 | 39879053 | - | 18.3 | 4772 | 5450 | -728 | L1MB8 | L1 | LINE |

  

|  |  |  |  |  |  |  |  |  |  |
| --- | --- | --- | --- | --- | --- | --- | --- | --- | --- |
| chr7:43969254-43970107 | | | 853 bp | | |  |  |  |  |
| Genome Start | Genome End | Strand | Div | Repeat Start | Repeat End | Repeat Left | Name | Family | Class |
| 43969254 | 43969313 | + | 16.5 | 4 | 59 | -134 | MER53 | DNA | DNA |
| 43969314 | 43969445 | - | 10.6 | 1 | 132 | -1 | FLAM\_C | Alu | SINE |
| 43969446 | 43969497 | + | 16.5 | 60 | 108 | -85 | MER53 | DNA | DNA |
| 43969498 | 43969800 | - | 12.2 | 1 | 309 | -1 | AluSg | Alu | SINE |
| 43969802 | 43970080 | - | 11.1 | 2 | 290 | -23 | AluSq | Alu | SINE |
| 43970081 | 43970107 | + | 16.5 | 109 | 134 | -59 | MER53 | DNA | DNA |

  

|  |  |  |  |  |  |  |  |  |  |
| --- | --- | --- | --- | --- | --- | --- | --- | --- | --- |
| chr7:43980253-43980785 | | | 499 bp | | |  |  |  |  |
| Genome Start | Genome End | Strand | Div | Repeat Start | Repeat End | Repeat Left | Name | Family | Class |
| 43980253 | 43980293 | + | 31.8 | 3144 | 3194 | -184 | L2 | L2 | LINE |
| 43980294 | 43980480 | - | 12.0 | 126 | 312 | 0 | AluSx | Alu | SINE |
| 43980515 | 43980634 | - | 12.0 | 4 | 125 | -187 | AluSx | Alu | SINE |
| 43980635 | 43980785 | + | 31.8 | 3195 | 3346 | -32 | L2 | L2 | LINE |

  

|  |  |  |  |  |  |  |  |  |  |
| --- | --- | --- | --- | --- | --- | --- | --- | --- | --- |
| chr7:43988218-43988839 | | | 622 bp | | |  |  |  |  |
| Genome Start | Genome End | Strand | Div | Repeat Start | Repeat End | Repeat Left | Name | Family | Class |
| 43988218 | 43988376 | - | 14.0 | 151 | 292 | -20 | AluJo | Alu | SINE |
| 43988377 | 43988678 | - | 13.0 | 1 | 302 | -10 | AluSx | Alu | SINE |
| 43988679 | 43988839 | - | 14.0 | 1 | 150 | -162 | AluJo | Alu | SINE |

  

|  |  |  |  |  |  |  |  |  |  |
| --- | --- | --- | --- | --- | --- | --- | --- | --- | --- |
| chr7:43998436-43999061 | | | 603 bp | | |  |  |  |  |
| Genome Start | Genome End | Strand | Div | Repeat Start | Repeat End | Repeat Left | Name | Family | Class |
| 43998436 | 43998615 | - | 17.5 | 144 | 311 | -1 | AluJb | Alu | SINE |
| 43998639 | 43998920 | - | 12.1 | 1 | 283 | -29 | AluSx | Alu | SINE |
| 43998921 | 43999061 | - | 17.5 | 1 | 143 | -169 | AluJb | Alu | SINE |

  

|  |  |  |  |  |  |  |  |  |  |
| --- | --- | --- | --- | --- | --- | --- | --- | --- | --- |
| chr7:44002632-44003245 | | | 614 bp | | |  |  |  |  |
| Genome Start | Genome End | Strand | Div | Repeat Start | Repeat End | Repeat Left | Name | Family | Class |
| 44002632 | 44002800 | - | 14.7 | 136 | 303 | -9 | AluJo | Alu | SINE |
| 44002801 | 44003112 | - | 10.4 | 1 | 310 | -3 | AluSq | Alu | SINE |
| 44003113 | 44003245 | - | 14.7 | 1 | 135 | -177 | AluJo | Alu | SINE |

  

|  |  |  |  |  |  |  |  |  |  |
| --- | --- | --- | --- | --- | --- | --- | --- | --- | --- |
| chr7:44012572-44013174 | | | 580 bp | | |  |  |  |  |
| Genome Start | Genome End | Strand | Div | Repeat Start | Repeat End | Repeat Left | Name | Family | Class |
| 44012572 | 44012733 | - | 16.6 | 137 | 298 | -14 | AluJo | Alu | SINE |
| 44012757 | 44013036 | - | 17.6 | 2 | 282 | -30 | AluSx | Alu | SINE |
| 44013037 | 44013174 | - | 16.6 | 1 | 136 | -176 | AluJo | Alu | SINE |

  

|  |  |  |  |  |  |  |  |  |  |
| --- | --- | --- | --- | --- | --- | --- | --- | --- | --- |
| chr7:44024121-44024756 | | | 628 bp | | |  |  |  |  |
| Genome Start | Genome End | Strand | Div | Repeat Start | Repeat End | Repeat Left | Name | Family | Class |
| 44024121 | 44024197 | - | 33.1 | 184 | 262 | 0 | MIR | MIR | SINE |
| 44024198 | 44024356 | - | 18.2 | 1 | 159 | -7 | FRAM | Alu | SINE |
| 44024365 | 44024660 | - | 8.4 | 1 | 297 | -16 | AluSp | Alu | SINE |
| 44024661 | 44024756 | - | 33.1 | 79 | 177 | -85 | MIR | MIR | SINE |

  

|  |  |  |  |  |  |  |  |  |  |
| --- | --- | --- | --- | --- | --- | --- | --- | --- | --- |
| chr7:44025902-44027815 | | | 1560 bp | | |  |  |  |  |
| Genome Start | Genome End | Strand | Div | Repeat Start | Repeat End | Repeat Left | Name | Family | Class |
| 44025902 | 44026180 | - | 16.1 | 34 | 310 | -2 | AluJo | Alu | SINE |
| 44026191 | 44027163 | + | 3.6 | 1 | 1007 | 0 | LTR13 | ERVK | LTR |
| 44027508 | 44027772 | + | 7.0 | 1 | 257 | -56 | AluSq | Alu | SINE |
| 44027773 | 44027815 | - | 13.9 | 1 | 43 | -259 | AluJo | Alu | SINE |

  

|  |  |  |  |  |  |  |  |  |  |
| --- | --- | --- | --- | --- | --- | --- | --- | --- | --- |
| chr7:44028360-44029332 | | | 973 bp | | |  |  |  |  |
| Genome Start | Genome End | Strand | Div | Repeat Start | Repeat End | Repeat Left | Name | Family | Class |
| 44028360 | 44028472 | + | 19.1 | 113 | 226 | -242 | LTR37B | ERV1 | LTR |
| 44028473 | 44028782 | + | 9.0 | 1 | 310 | 0 | AluSg | Alu | SINE |
| 44028783 | 44028866 | + | 19.1 | 227 | 309 | -159 | LTR37B | ERV1 | LTR |
| 44028867 | 44029171 | + | 17.1 | 1 | 307 | 0 | AluJo | Alu | SINE |
| 44029172 | 44029332 | + | 19.1 | 310 | 466 | -2 | LTR37B | ERV1 | LTR |

  

|  |  |  |  |  |  |  |  |  |  |
| --- | --- | --- | --- | --- | --- | --- | --- | --- | --- |
| chr7:44032467-44032844 | | | 378 bp | | |  |  |  |  |
| Genome Start | Genome End | Strand | Div | Repeat Start | Repeat End | Repeat Left | Name | Family | Class |
| 44032467 | 44032630 | - | 21.1 | 7677 | 7820 | -141 | L1MC5 | L1 | LINE |
| 44032631 | 44032762 | - | 15.3 | 1 | 131 | -2 | FLAM\_A | Alu | SINE |
| 44032763 | 44032844 | - | 21.1 | 7604 | 7676 | -285 | L1MC5 | L1 | LINE |

  

|  |  |  |  |  |  |  |  |  |  |
| --- | --- | --- | --- | --- | --- | --- | --- | --- | --- |
| chr7:44038439-44039238 | | | 800 bp | | |  |  |  |  |
| Genome Start | Genome End | Strand | Div | Repeat Start | Repeat End | Repeat Left | Name | Family | Class |
| 44038439 | 44038518 | - | 25.2 | 151 | 208 | -1 | MER3 | MER1\_type | DNA |
| 44038519 | 44038833 | - | 11.6 | 1 | 312 | 0 | AluSx | Alu | SINE |
| 44038834 | 44038863 | - | 25.2 | 126 | 150 | -59 | MER3 | MER1\_type | DNA |
| 44038864 | 44039167 | + | 15.0 | 1 | 302 | -10 | AluJo | Alu | SINE |
| 44039168 | 44039238 | - | 25.2 | 54 | 125 | -84 | MER3 | MER1\_type | DNA |

  

|  |  |  |  |  |  |  |  |  |  |
| --- | --- | --- | --- | --- | --- | --- | --- | --- | --- |
| chr7:44040534-44041060 | | | 393 bp | | |  |  |  |  |
| Genome Start | Genome End | Strand | Div | Repeat Start | Repeat End | Repeat Left | Name | Family | Class |
| 44040534 | 44040641 | - | 29.0 | 81 | 191 | -5 | MER91A | Tip100 | DNA |
| 44040676 | 44040800 | - | 16.8 | 23 | 151 | -161 | AluJo | Alu | SINE |
| 44040806 | 44040884 | + | 16.5 | 36 | 114 | -29 | FLAM\_C | Alu | SINE |
| 44040980 | 44041060 | - | 22.2 | 1 | 85 | -111 | MER91A | Tip100 | DNA |

  

|  |  |  |  |  |  |  |  |  |  |
| --- | --- | --- | --- | --- | --- | --- | --- | --- | --- |
| chr7:45759999-45760564 | | | 566 bp | | |  |  |  |  |
| Genome Start | Genome End | Strand | Div | Repeat Start | Repeat End | Repeat Left | Name | Family | Class |
| 45759999 | 45760142 | - | 17.9 | 142 | 297 | -5 | AluJo | Alu | SINE |
| 45760143 | 45760434 | - | 7.9 | 1 | 298 | -15 | AluSq | Alu | SINE |
| 45760435 | 45760564 | - | 17.9 | 1 | 141 | -161 | AluJo | Alu | SINE |

  

|  |  |  |  |  |  |  |  |  |  |
| --- | --- | --- | --- | --- | --- | --- | --- | --- | --- |
| chr7:45778562-45779168 | | | 607 bp | | |  |  |  |  |
| Genome Start | Genome End | Strand | Div | Repeat Start | Repeat End | Repeat Left | Name | Family | Class |
| 45778562 | 45778731 | - | 10.2 | 137 | 303 | -9 | AluSx | Alu | SINE |
| 45778732 | 45779032 | - | 11.0 | 1 | 302 | -11 | AluSp | Alu | SINE |
| 45779033 | 45779168 | - | 10.2 | 1 | 136 | -176 | AluSx | Alu | SINE |

  

|  |  |  |  |  |  |  |  |  |  |
| --- | --- | --- | --- | --- | --- | --- | --- | --- | --- |
| chr7:45795859-45796201 | | | 343 bp | | |  |  |  |  |
| Genome Start | Genome End | Strand | Div | Repeat Start | Repeat End | Repeat Left | Name | Family | Class |
| 45795859 | 45795978 | - | 30.4 | 5574 | 5691 | -430 | L1M5 | L1 | LINE |
| 45795979 | 45796101 | + | 18.3 | 4 | 126 | -7 | FLAM\_C | Alu | SINE |
| 45796102 | 45796201 | - | 30.4 | 5483 | 5573 | -548 | L1M5 | L1 | LINE |

  

|  |  |  |  |  |  |  |  |  |  |
| --- | --- | --- | --- | --- | --- | --- | --- | --- | --- |
| chr7:45800160-45800648 | | | 435 bp | | |  |  |  |  |
| Genome Start | Genome End | Strand | Div | Repeat Start | Repeat End | Repeat Left | Name | Family | Class |
| 45800160 | 45800219 | + | 22.9 | 24 | 71 | -241 | AluJo | Alu | SINE |
| 45800274 | 45800546 | - | 12.9 | 1 | 279 | -33 | AluSx | Alu | SINE |
| 45800547 | 45800648 | + | 22.9 | 72 | 167 | -145 | AluJo | Alu | SINE |

  

|  |  |  |  |  |  |  |  |  |  |
| --- | --- | --- | --- | --- | --- | --- | --- | --- | --- |
| chr7:45805400-45806885 | | | 1486 bp | | |  |  |  |  |
| Genome Start | Genome End | Strand | Div | Repeat Start | Repeat End | Repeat Left | Name | Family | Class |
| 45805400 | 45805701 | - | 18.9 | 604 | 935 | 0 | MER21C | ERV1 | LTR |
| 45805702 | 45805999 | - | 19.8 | 1 | 302 | -10 | AluJo | Alu | SINE |
| 45806000 | 45806069 | - | 18.9 | 526 | 603 | -332 | MER21C | ERV1 | LTR |
| 45806070 | 45806374 | + | 20.5 | 2 | 294 | -18 | AluJb | Alu | SINE |
| 45806375 | 45806885 | - | 18.9 | 18 | 525 | -410 | MER21C | ERV1 | LTR |

  

|  |  |  |  |  |  |  |  |  |  |
| --- | --- | --- | --- | --- | --- | --- | --- | --- | --- |
| chr7:45827283-45827739 | | | 457 bp | | |  |  |  |  |
| Genome Start | Genome End | Strand | Div | Repeat Start | Repeat End | Repeat Left | Name | Family | Class |
| 45827283 | 45827374 | + | 30.6 | 3224 | 3303 | -116 | L2 | L2 | LINE |
| 45827375 | 45827673 | + | 10.4 | 1 | 299 | -14 | AluSq | Alu | SINE |
| 45827674 | 45827739 | + | 22.0 | 3263 | 3369 | -50 | L2 | L2 | LINE |

  

|  |  |  |  |  |  |  |  |  |  |
| --- | --- | --- | --- | --- | --- | --- | --- | --- | --- |
| chr7:45832134-45834792 | | | 2071 bp | | |  |  |  |  |
| Genome Start | Genome End | Strand | Div | Repeat Start | Repeat End | Repeat Left | Name | Family | Class |
| 45832134 | 45832302 | + | 33.3 | 2818 | 2996 | -423 | L2 | L2 | LINE |
| 45832389 | 45832697 | - | 11.7 | 1 | 309 | -1 | AluSg | Alu | SINE |
| 45832718 | 45832949 | + | 32.6 | 3132 | 3370 | -8 | L2 | L2 | LINE |
| 45832952 | 45833243 | + | 18.5 | 1 | 292 | -20 | AluJo | Alu | SINE |
| 45833460 | 45833739 | + | 12.6 | 1 | 298 | -14 | AluJo | Alu | SINE |
| 45833742 | 45833901 | + | 11.9 | 1 | 159 | -154 | AluSq | Alu | SINE |
| 45833960 | 45834069 | + | 13.6 | 2 | 111 | -201 | AluSq/x | Alu | SINE |
| 45834097 | 45834402 | + | 12.5 | 6 | 309 | -1 | AluSg | Alu | SINE |
| 45834405 | 45834535 | + | 22.3 | 25 | 184 | -84 | MIRb | MIR | SINE |
| 45834711 | 45834792 | + | 35.4 | 3337 | 3419 | 0 | L2 | L2 | LINE |

  

|  |  |  |  |  |  |  |  |  |  |
| --- | --- | --- | --- | --- | --- | --- | --- | --- | --- |
| chr7:45853977-45854880 | | | 880 bp | | |  |  |  |  |
| Genome Start | Genome End | Strand | Div | Repeat Start | Repeat End | Repeat Left | Name | Family | Class |
| 45853977 | 45854123 | - | 17.3 | 130 | 273 | -39 | AluJb | Alu | SINE |
| 45854124 | 45854136 | - | 9.9 | 286 | 311 | 0 | AluY | Alu | SINE |
| 45854161 | 45854418 | - | 9.9 | 3 | 285 | -26 | AluY | Alu | SINE |
| 45854419 | 45854451 | - | 17.3 | 109 | 129 | -183 | AluJb | Alu | SINE |
| 45854452 | 45854759 | + | 14.6 | 1 | 313 | 0 | AluSq | Alu | SINE |
| 45854760 | 45854880 | - | 17.3 | 1 | 108 | -204 | AluJb | Alu | SINE |

  

|  |  |  |  |  |  |  |  |  |  |
| --- | --- | --- | --- | --- | --- | --- | --- | --- | --- |
| chr7:48928499-48929090 | | | 592 bp | | |  |  |  |  |
| Genome Start | Genome End | Strand | Div | Repeat Start | Repeat End | Repeat Left | Name | Family | Class |
| 48928499 | 48928691 | + | 27.4 | 2090 | 2304 | -203 | HAL1 | L1 | LINE |
| 48928692 | 48928994 | + | 13.9 | 1 | 303 | -9 | AluSx | Alu | SINE |
| 48928995 | 48929090 | + | 27.4 | 2305 | 2394 | -113 | HAL1 | L1 | LINE |

  

|  |  |  |  |  |  |  |  |  |  |
| --- | --- | --- | --- | --- | --- | --- | --- | --- | --- |
| chr7:51434632-51436115 | | | 1484 bp | | |  |  |  |  |
| Genome Start | Genome End | Strand | Div | Repeat Start | Repeat End | Repeat Left | Name | Family | Class |
| 51434632 | 51435142 | + | 19.6 | 18 | 525 | -410 | MER21C | ERV1 | LTR |
| 51435143 | 51435446 | - | 19.8 | 2 | 295 | -17 | AluJb | Alu | SINE |
| 51435447 | 51435516 | + | 19.6 | 526 | 603 | -332 | MER21C | ERV1 | LTR |
| 51435517 | 51435814 | + | 19.8 | 1 | 302 | -10 | AluJo | Alu | SINE |
| 51435815 | 51436115 | + | 19.6 | 604 | 935 | 0 | MER21C | ERV1 | LTR |

  

|  |  |  |  |  |  |  |  |  |  |
| --- | --- | --- | --- | --- | --- | --- | --- | --- | --- |
| chr7:51441208-51441660 | | | 453 bp | | |  |  |  |  |
| Genome Start | Genome End | Strand | Div | Repeat Start | Repeat End | Repeat Left | Name | Family | Class |
| 51441208 | 51441309 | - | 23.6 | 72 | 167 | -145 | AluJo | Alu | SINE |
| 51441310 | 51441596 | + | 13.3 | 1 | 312 | 0 | AluSx | Alu | SINE |
| 51441597 | 51441660 | - | 23.6 | 24 | 71 | -241 | AluJo | Alu | SINE |

  

|  |  |  |  |  |  |  |  |  |  |
| --- | --- | --- | --- | --- | --- | --- | --- | --- | --- |
| chr7:51441716-51442876 | | | 1132 bp | | |  |  |  |  |
| Genome Start | Genome End | Strand | Div | Repeat Start | Repeat End | Repeat Left | Name | Family | Class |
| 51441716 | 51441745 | + | 20.0 | 5476 | 5506 | -668 | L1MB8 | L1 | LINE |
| 51441746 | 51442027 | + | 18.1 | 1 | 293 | -19 | AluJb | Alu | SINE |
| 51442028 | 51442102 | + | 20.0 | 5507 | 5571 | -603 | L1MB8 | L1 | LINE |
| 51442132 | 51442261 | - | 19.8 | 1 | 129 | -4 | FLAM\_A | Alu | SINE |
| 51442262 | 51442876 | + | 20.1 | 5555 | 6173 | -5 | L1MB8 | L1 | LINE |

  

|  |  |  |  |  |  |  |  |  |  |
| --- | --- | --- | --- | --- | --- | --- | --- | --- | --- |
| chr7:53165240-53166755 | | | 1133 bp | | |  |  |  |  |
| Genome Start | Genome End | Strand | Div | Repeat Start | Repeat End | Repeat Left | Name | Family | Class |
| 53165240 | 53165552 | + | 28.9 | 39 | 388 | -6035 | L1MCc | L1 | LINE |
| 53165639 | 53165916 | + | 24.6 | 88 | 365 | 0 | MLT1A0 | MaLR | LTR |
| 53166156 | 53166300 | + | 30.3 | 1851 | 1995 | -5133 | L1MCc | L1 | LINE |
| 53166359 | 53166755 | + | 26.4 | 3035 | 3438 | -3503 | L1MCc | L1 | LINE |

  

|  |  |  |  |  |  |  |  |  |  |
| --- | --- | --- | --- | --- | --- | --- | --- | --- | --- |
| chr7:53167553-53168364 | | | 812 bp | | |  |  |  |  |
| Genome Start | Genome End | Strand | Div | Repeat Start | Repeat End | Repeat Left | Name | Family | Class |
| 53167553 | 53167823 | + | 25.0 | 5 | 274 | -231 | MLT1D | MaLR | LTR |
| 53167824 | 53168118 | + | 12.2 | 1 | 299 | -11 | AluSg | Alu | SINE |
| 53168119 | 53168364 | + | 25.0 | 275 | 505 | 0 | MLT1D | MaLR | LTR |

  

|  |  |  |  |  |  |  |  |  |  |
| --- | --- | --- | --- | --- | --- | --- | --- | --- | --- |
| chr7:53176198-53176889 | | | 692 bp | | |  |  |  |  |
| Genome Start | Genome End | Strand | Div | Repeat Start | Repeat End | Repeat Left | Name | Family | Class |
| 53176198 | 53176229 | + | 16.2 | 1 | 34 | -561 | LTR49 | ERV1 | LTR |
| 53176230 | 53176512 | - | 13.5 | 1 | 292 | -21 | AluSq | Alu | SINE |
| 53176513 | 53176889 | + | 16.2 | 35 | 427 | -168 | LTR49 | ERV1 | LTR |

  

|  |  |  |  |  |  |  |  |  |  |
| --- | --- | --- | --- | --- | --- | --- | --- | --- | --- |
| chr7:55706309-55707898 | | | 1590 bp | | |  |  |  |  |
| Genome Start | Genome End | Strand | Div | Repeat Start | Repeat End | Repeat Left | Name | Family | Class |
| 55706309 | 55706876 | + | 18.0 | 4772 | 5434 | -744 | L1MB8 | L1 | LINE |
| 55706877 | 55707174 | + | 9.4 | 2 | 299 | -12 | AluY | Alu | SINE |
| 55707175 | 55707898 | + | 18.6 | 5435 | 6178 | 0 | L1MB8 | L1 | LINE |

  

|  |  |  |  |  |  |  |  |  |  |
| --- | --- | --- | --- | --- | --- | --- | --- | --- | --- |
| chr7:55732329-55733100 | | | 772 bp | | |  |  |  |  |
| Genome Start | Genome End | Strand | Div | Repeat Start | Repeat End | Repeat Left | Name | Family | Class |
| 55732329 | 55732710 | - | 21.9 | 76 | 505 | 0 | MLT1D | MaLR | LTR |
| 55732711 | 55733027 | + | 17.6 | 1 | 295 | -17 | AluJo | Alu | SINE |
| 55733028 | 55733100 | - | 21.9 | 3 | 75 | -430 | MLT1D | MaLR | LTR |

  

|  |  |  |  |  |  |  |  |  |  |
| --- | --- | --- | --- | --- | --- | --- | --- | --- | --- |
| chr7:55737883-55740107 | | | 1990 bp | | |  |  |  |  |
| Genome Start | Genome End | Strand | Div | Repeat Start | Repeat End | Repeat Left | Name | Family | Class |
| 55737883 | 55738164 | + | 32.2 | 4742 | 5020 | -1126 | L1MC4a | L1 | LINE |
| 55738313 | 55738630 | + | 13.3 | 1 | 294 | -18 | AluSx | Alu | SINE |
| 55738681 | 55739266 | + | 28.5 | 5016 | 5615 | -506 | L1MC4a | L1 | LINE |
| 55739267 | 55739555 | + | 11.8 | 3 | 291 | -21 | AluSx | Alu | SINE |
| 55739556 | 55739841 | + | 11.2 | 1 | 286 | -27 | AluSq | Alu | SINE |
| 55739879 | 55740107 | + | 29.4 | 5613 | 5856 | -1952 | L1MC4a | L1 | LINE |

  

|  |  |  |  |  |  |  |  |  |  |
| --- | --- | --- | --- | --- | --- | --- | --- | --- | --- |
| chr7:55740242-55740911 | | | 670 bp | | |  |  |  |  |
| Genome Start | Genome End | Strand | Div | Repeat Start | Repeat End | Repeat Left | Name | Family | Class |
| 55740242 | 55740542 | + | 25.3 | 5679 | 5979 | -1829 | L1MC4a | L1 | LINE |
| 55740543 | 55740838 | + | 11.5 | 1 | 296 | -16 | AluSx | Alu | SINE |
| 55740839 | 55740911 | + | 25.3 | 5980 | 6048 | -1760 | L1MC4a | L1 | LINE |

  

|  |  |  |  |  |  |  |  |  |  |
| --- | --- | --- | --- | --- | --- | --- | --- | --- | --- |
| chr7:55788771-55789395 | | | 599 bp | | |  |  |  |  |
| Genome Start | Genome End | Strand | Div | Repeat Start | Repeat End | Repeat Left | Name | Family | Class |
| 55788771 | 55788952 | - | 18.4 | 134 | 312 | 0 | AluJo | Alu | SINE |
| 55788979 | 55789261 | - | 12.4 | 1 | 283 | -30 | AluSq | Alu | SINE |
| 55789262 | 55789395 | - | 18.4 | 1 | 133 | -179 | AluJo | Alu | SINE |

  

|  |  |  |  |  |  |  |  |  |  |
| --- | --- | --- | --- | --- | --- | --- | --- | --- | --- |
| chr7:55790293-55791697 | | | 1405 bp | | |  |  |  |  |
| Genome Start | Genome End | Strand | Div | Repeat Start | Repeat End | Repeat Left | Name | Family | Class |
| 55790293 | 55790720 | + | 20.1 | 115 | 525 | -410 | MER21C | ERV1 | LTR |
| 55790721 | 55791027 | - | 19.3 | 2 | 296 | -16 | AluJb | Alu | SINE |
| 55791028 | 55791097 | + | 20.1 | 526 | 603 | -332 | MER21C | ERV1 | LTR |
| 55791098 | 55791395 | + | 20.8 | 1 | 302 | -10 | AluJo | Alu | SINE |
| 55791396 | 55791697 | + | 20.1 | 604 | 935 | 0 | MER21C | ERV1 | LTR |

  

|  |  |  |  |  |  |  |  |  |  |
| --- | --- | --- | --- | --- | --- | --- | --- | --- | --- |
| chr7:55796787-55797274 | | | 443 bp | | |  |  |  |  |
| Genome Start | Genome End | Strand | Div | Repeat Start | Repeat End | Repeat Left | Name | Family | Class |
| 55796787 | 55796888 | - | 20.1 | 72 | 167 | -145 | AluJo | Alu | SINE |
| 55796889 | 55797168 | + | 12.0 | 1 | 275 | -37 | AluSx | Alu | SINE |
| 55797214 | 55797274 | - | 20.1 | 24 | 71 | -241 | AluJo | Alu | SINE |

  

|  |  |  |  |  |  |  |  |  |  |
| --- | --- | --- | --- | --- | --- | --- | --- | --- | --- |
| chr7:55830533-55831042 | | | 510 bp | | |  |  |  |  |
| Genome Start | Genome End | Strand | Div | Repeat Start | Repeat End | Repeat Left | Name | Family | Class |
| 55830533 | 55830649 | - | 28.9 | 3313 | 3418 | -1 | L2 | L2 | LINE |
| 55830650 | 55830954 | - | 11.2 | 1 | 305 | -8 | AluSq | Alu | SINE |
| 55830955 | 55831042 | - | 28.9 | 3224 | 3312 | -107 | L2 | L2 | LINE |

  

|  |  |  |  |  |  |  |  |  |  |
| --- | --- | --- | --- | --- | --- | --- | --- | --- | --- |
| chr7:55831924-55832872 | | | 949 bp | | |  |  |  |  |
| Genome Start | Genome End | Strand | Div | Repeat Start | Repeat End | Repeat Left | Name | Family | Class |
| 55831924 | 55832029 | + | 25.2 | 1967 | 2075 | -4774 | L1M2 | L1 | LINE |
| 55832030 | 55832339 | + | 11.6 | 1 | 310 | 0 | AluSg | Alu | SINE |
| 55832340 | 55832872 | + | 25.2 | 2076 | 2629 | -4220 | L1M2 | L1 | LINE |

  

|  |  |  |  |  |  |  |  |  |  |
| --- | --- | --- | --- | --- | --- | --- | --- | --- | --- |
| chr7:56079057-56080409 | | | 1269 bp | | |  |  |  |  |
| Genome Start | Genome End | Strand | Div | Repeat Start | Repeat End | Repeat Left | Name | Family | Class |
| 56079057 | 56079151 | + | 33.9 | 1601 | 1692 | -4523 | L1MC4 | L1 | LINE |
| 56079152 | 56079428 | - | 10.5 | 1 | 293 | -20 | AluSp | Alu | SINE |
| 56079429 | 56079723 | + | 33.9 | 1693 | 1971 | -4244 | L1MC4 | L1 | LINE |
| 56079810 | 56079873 | + | 9.4 | 11 | 74 | -238 | AluJ/FLAM | Alu | SINE |
| 56079870 | 56079995 | + | 14.9 | 166 | 292 | -20 | AluJo/FRAM | Alu | SINE |
| 56079997 | 56080278 | + | 9.9 | 1 | 302 | -11 | AluSp | Alu | SINE |
| 56080280 | 56080409 | + | 20.1 | 7017 | 7164 | -854 | L1MC4 | L1 | LINE |

  

|  |  |  |  |  |  |  |  |  |  |
| --- | --- | --- | --- | --- | --- | --- | --- | --- | --- |
| chr7:56102764-56103179 | | | 416 bp | | |  |  |  |  |
| Genome Start | Genome End | Strand | Div | Repeat Start | Repeat End | Repeat Left | Name | Family | Class |
| 56102764 | 56102797 | - | 22.0 | 122 | 155 | -113 | MIRb | MIR | SINE |
| 56102798 | 56103107 | + | 15.3 | 1 | 309 | -3 | AluJb | Alu | SINE |
| 56103108 | 56103179 | - | 22.0 | 56 | 121 | -147 | MIRb | MIR | SINE |

  

|  |  |  |  |  |  |  |  |  |  |
| --- | --- | --- | --- | --- | --- | --- | --- | --- | --- |
| chr7:56195245-56195977 | | | 733 bp | | |  |  |  |  |
| Genome Start | Genome End | Strand | Div | Repeat Start | Repeat End | Repeat Left | Name | Family | Class |
| 56195245 | 56195357 | - | 18.9 | 351 | 457 | 0 | MSTB2 | MaLR | LTR |
| 56195358 | 56195640 | + | 12.1 | 1 | 299 | -13 | AluSx | Alu | SINE |
| 56195641 | 56195977 | - | 18.9 | 1 | 350 | -107 | MSTB2 | MaLR | LTR |

  

|  |  |  |  |  |  |  |  |  |  |
| --- | --- | --- | --- | --- | --- | --- | --- | --- | --- |
| chr7:56229809-56230217 | | | 409 bp | | |  |  |  |  |
| Genome Start | Genome End | Strand | Div | Repeat Start | Repeat End | Repeat Left | Name | Family | Class |
| 56229809 | 56229861 | - | 6.2 | 51 | 80 | 0 | MADE1 | Mariner | DNA |
| 56229862 | 56230163 | + | 11.6 | 1 | 302 | -10 | AluSx | Alu | SINE |
| 56230164 | 56230217 | - | 6.2 | 1 | 50 | -30 | MADE1 | Mariner | DNA |

  

|  |  |  |  |  |  |  |  |  |  |
| --- | --- | --- | --- | --- | --- | --- | --- | --- | --- |
| chr7:56238201-56239922 | | | 1722 bp | | |  |  |  |  |
| Genome Start | Genome End | Strand | Div | Repeat Start | Repeat End | Repeat Left | Name | Family | Class |
| 56238201 | 56238464 | - | 34.3 | 3125 | 3417 | -2 | L2 | L2 | LINE |
| 56238465 | 56238671 | - | 19.4 | 157 | 365 | 0 | MLT1A0 | MaLR | LTR |
| 56238672 | 56238972 | - | 13.6 | 1 | 301 | -11 | AluSx | Alu | SINE |
| 56238973 | 56239134 | - | 19.4 | 1 | 156 | -209 | MLT1A0 | MaLR | LTR |
| 56239135 | 56239922 | - | 31.0 | 2260 | 3124 | -295 | L2 | L2 | LINE |

  

|  |  |  |  |  |  |  |  |  |  |
| --- | --- | --- | --- | --- | --- | --- | --- | --- | --- |
| chr7:56255072-56255914 | | | 454 bp | | |  |  |  |  |
| Genome Start | Genome End | Strand | Div | Repeat Start | Repeat End | Repeat Left | Name | Family | Class |
| 56255072 | 56255229 | + | 37.0 | 12 | 177 | -91 | MIRb | MIR | SINE |
| 56255614 | 56255842 | + | 28.3 | 189 | 440 | -10 | MLT1J2 | MaLR | LTR |
| 56255848 | 56255914 | + | 29.9 | 184 | 254 | -14 | MIRb | MIR | SINE |

  

|  |  |  |  |  |  |  |  |  |  |
| --- | --- | --- | --- | --- | --- | --- | --- | --- | --- |
| chr7:56365609-56366229 | | | 621 bp | | |  |  |  |  |
| Genome Start | Genome End | Strand | Div | Repeat Start | Repeat End | Repeat Left | Name | Family | Class |
| 56365609 | 56365752 | + | 15.3 | 1 | 142 | -170 | AluJo | Alu | SINE |
| 56365753 | 56366058 | + | 12.5 | 3 | 307 | -5 | AluSx | Alu | SINE |
| 56366059 | 56366229 | + | 15.3 | 143 | 304 | -8 | AluJo | Alu | SINE |

  

|  |  |  |  |  |  |  |  |  |  |
| --- | --- | --- | --- | --- | --- | --- | --- | --- | --- |
| chr7:56394983-56395488 | | | 506 bp | | |  |  |  |  |
| Genome Start | Genome End | Strand | Div | Repeat Start | Repeat End | Repeat Left | Name | Family | Class |
| 56394983 | 56395095 | - | 32.3 | 3304 | 3418 | -1 | L2 | L2 | LINE |
| 56395096 | 56395396 | - | 11.6 | 1 | 301 | -12 | AluSq | Alu | SINE |
| 56395397 | 56395488 | - | 32.3 | 3224 | 3303 | -116 | L2 | L2 | LINE |

  

|  |  |  |  |  |  |  |  |  |  |
| --- | --- | --- | --- | --- | --- | --- | --- | --- | --- |
| chr7:56396395-56397285 | | | 891 bp | | |  |  |  |  |
| Genome Start | Genome End | Strand | Div | Repeat Start | Repeat End | Repeat Left | Name | Family | Class |
| 56396395 | 56396504 | + | 26.2 | 1967 | 2070 | -4779 | L1M2 | L1 | LINE |
| 56396505 | 56396820 | + | 13.3 | 1 | 310 | 0 | AluSg | Alu | SINE |
| 56396821 | 56397285 | + | 22.3 | 2071 | 2627 | -4222 | L1M2 | L1 | LINE |

  

|  |  |  |  |  |  |  |  |  |  |
| --- | --- | --- | --- | --- | --- | --- | --- | --- | --- |
| chr7:56418923-56419542 | | | 591 bp | | |  |  |  |  |
| Genome Start | Genome End | Strand | Div | Repeat Start | Repeat End | Repeat Left | Name | Family | Class |
| 56418923 | 56419096 | - | 17.1 | 136 | 312 | 0 | AluJo | Alu | SINE |
| 56419126 | 56419408 | - | 11.8 | 1 | 283 | -30 | AluSq | Alu | SINE |
| 56419409 | 56419542 | - | 17.1 | 3 | 135 | -177 | AluJo | Alu | SINE |

  

|  |  |  |  |  |  |  |  |  |  |
| --- | --- | --- | --- | --- | --- | --- | --- | --- | --- |
| chr7:56427516-56427908 | | | 393 bp | | |  |  |  |  |
| Genome Start | Genome End | Strand | Div | Repeat Start | Repeat End | Repeat Left | Name | Family | Class |
| 56427516 | 56427549 | + | 22.9 | 5476 | 5505 | -673 | L1M5 | L1 | LINE |
| 56427550 | 56427833 | + | 19.1 | 1 | 295 | -17 | AluJb | Alu | SINE |
| 56427834 | 56427908 | + | 22.7 | 5494 | 5568 | -598 | L1M5 | L1 | LINE |

  

|  |  |  |  |  |  |  |  |  |  |
| --- | --- | --- | --- | --- | --- | --- | --- | --- | --- |
| chr7:56432494-56433950 | | | 1457 bp | | |  |  |  |  |
| Genome Start | Genome End | Strand | Div | Repeat Start | Repeat End | Repeat Left | Name | Family | Class |
| 56432494 | 56432752 | + | 20.6 | 18 | 275 | -660 | MER21C | ERV1 | LTR |
| 56432753 | 56433043 | - | 12.1 | 1 | 290 | -23 | AluSq | Alu | SINE |
| 56433044 | 56433345 | + | 20.6 | 276 | 603 | -332 | MER21C | ERV1 | LTR |
| 56433346 | 56433649 | + | 16.4 | 1 | 299 | -13 | AluJo | Alu | SINE |
| 56433650 | 56433950 | + | 20.6 | 604 | 935 | 0 | MER21C | ERV1 | LTR |

  

|  |  |  |  |  |  |  |  |  |  |
| --- | --- | --- | --- | --- | --- | --- | --- | --- | --- |
| chr7:56550689-56551391 | | | 689 bp | | |  |  |  |  |
| Genome Start | Genome End | Strand | Div | Repeat Start | Repeat End | Repeat Left | Name | Family | Class |
| 56550689 | 56551024 | - | 28.0 | 219 | 595 | 0 | MLT1G1 | MaLR | LTR |
| 56551030 | 56551211 | - | 20.3 | 1 | 203 | -7 | MER63A | AcHobo | DNA |
| 56551221 | 56551391 | - | 25.9 | 11 | 201 | -394 | MLT1G1 | MaLR | LTR |

  

|  |  |  |  |  |  |  |  |  |  |
| --- | --- | --- | --- | --- | --- | --- | --- | --- | --- |
| chr7:56604625-56605986 | | | 1362 bp | | |  |  |  |  |
| Genome Start | Genome End | Strand | Div | Repeat Start | Repeat End | Repeat Left | Name | Family | Class |
| 56604625 | 56605608 | + | 23.6 | 5083 | 6106 | -72 | L1MB8 | L1 | LINE |
| 56605609 | 56605918 | + | 14.9 | 1 | 307 | 0 | AluSx | Alu | SINE |
| 56605919 | 56605986 | + | 25.9 | 6107 | 6177 | -1 | L1MB8 | L1 | LINE |

  

|  |  |  |  |  |  |  |  |  |  |
| --- | --- | --- | --- | --- | --- | --- | --- | --- | --- |
| chr7:56736268-56738087 | | | 1646 bp | | |  |  |  |  |
| Genome Start | Genome End | Strand | Div | Repeat Start | Repeat End | Repeat Left | Name | Family | Class |
| 56736268 | 56736563 | + | 13.8 | 1483 | 1784 | -6336 | PABL\_B-int | ERV1 | LTR |
| 56736635 | 56736970 | + | 17.3 | 53 | 392 | -531 | MER49 | ERV1 | LTR |
| 56736975 | 56737288 | + | 14.4 | 1 | 312 | 0 | AluSx | Alu | SINE |
| 56737295 | 56737498 | + | 11.4 | 488 | 746 | -172 | MER49 | ERV1 | LTR |
| 56737582 | 56737876 | + | 12.0 | 1 | 292 | -20 | AluSx | Alu | SINE |
| 56737887 | 56738087 | + | 34.0 | 6655 | 6869 | -253 | PABL\_B-int | ERV1 | LTR |

  

|  |  |  |  |  |  |  |  |  |  |
| --- | --- | --- | --- | --- | --- | --- | --- | --- | --- |
| chr7:56778498-56779104 | | | 607 bp | | |  |  |  |  |
| Genome Start | Genome End | Strand | Div | Repeat Start | Repeat End | Repeat Left | Name | Family | Class |
| 56778498 | 56778677 | - | 10.1 | 134 | 313 | 0 | AluSp | Alu | SINE |
| 56778678 | 56778972 | - | 6.8 | 1 | 295 | -16 | AluY | Alu | SINE |
| 56778973 | 56779104 | - | 10.1 | 6 | 133 | -180 | AluSp | Alu | SINE |

  

|  |  |  |  |  |  |  |  |  |  |
| --- | --- | --- | --- | --- | --- | --- | --- | --- | --- |
| chr7:56781894-56782300 | | | 407 bp | | |  |  |  |  |
| Genome Start | Genome End | Strand | Div | Repeat Start | Repeat End | Repeat Left | Name | Family | Class |
| 56781894 | 56781957 | + | 34.0 | 3316 | 3379 | -40 | L2 | L2 | LINE |
| 56781958 | 56782254 | - | 11.8 | 1 | 300 | -12 | AluSx | Alu | SINE |
| 56782255 | 56782300 | + | 34.0 | 3380 | 3419 | 0 | L2 | L2 | LINE |

  

|  |  |  |  |  |  |  |  |  |  |
| --- | --- | --- | --- | --- | --- | --- | --- | --- | --- |
| chr7:56782508-56783378 | | | 871 bp | | |  |  |  |  |
| Genome Start | Genome End | Strand | Div | Repeat Start | Repeat End | Repeat Left | Name | Family | Class |
| 56782508 | 56782971 | - | 12.1 | 95 | 521 | 0 | MER41C | ERV1 | LTR |
| 56782972 | 56783275 | - | 11.5 | 1 | 305 | -5 | AluSg | Alu | SINE |
| 56783276 | 56783378 | - | 12.1 | 1 | 94 | -427 | MER41C | ERV1 | LTR |

  

|  |  |  |  |  |  |  |  |  |  |
| --- | --- | --- | --- | --- | --- | --- | --- | --- | --- |
| chr7:56784183-56785818 | | | 1554 bp | | |  |  |  |  |
| Genome Start | Genome End | Strand | Div | Repeat Start | Repeat End | Repeat Left | Name | Family | Class |
| 56784183 | 56784250 | + | 29.9 | 1864 | 1930 | -1489 | L2 | L2 | LINE |
| 56784261 | 56784628 | + | 15.0 | 4 | 364 | 0 | THE1B | MaLR | LTR |
| 56784630 | 56785216 | + | 25.5 | 1943 | 2682 | -737 | L2 | L2 | LINE |
| 56785288 | 56785357 | + | 26.1 | 3074 | 3131 | -288 | L2 | L2 | LINE |
| 56785358 | 56785660 | - | 18.2 | 1 | 302 | -10 | AluJo | Alu | SINE |
| 56785661 | 56785818 | + | 26.1 | 3132 | 3295 | -124 | L2 | L2 | LINE |

  

|  |  |  |  |  |  |  |  |  |  |
| --- | --- | --- | --- | --- | --- | --- | --- | --- | --- |
| chr7:56789810-56790942 | | | 1126 bp | | |  |  |  |  |
| Genome Start | Genome End | Strand | Div | Repeat Start | Repeat End | Repeat Left | Name | Family | Class |
| 56789810 | 56789845 | - | 27.7 | 534 | 566 | -29 | MLT1G1 | MaLR | LTR |
| 56789846 | 56790134 | + | 11.1 | 1 | 290 | -19 | AluSc | Alu | SINE |
| 56790142 | 56790437 | + | 10.5 | 1 | 296 | -14 | AluSg | Alu | SINE |
| 56790438 | 56790942 | - | 27.7 | 1 | 528 | -67 | MLT1G1 | MaLR | LTR |

  

|  |  |  |  |  |  |  |  |  |  |
| --- | --- | --- | --- | --- | --- | --- | --- | --- | --- |
| chr7:56797415-56798499 | | | 887 bp | | |  |  |  |  |
| Genome Start | Genome End | Strand | Div | Repeat Start | Repeat End | Repeat Left | Name | Family | Class |
| 56797415 | 56797645 | - | 30.4 | 5685 | 5925 | -1883 | L1MC4a | L1 | LINE |
| 56797845 | 56798204 | - | 25.5 | 5758 | 6155 | -7 | L1ME3 | L1 | LINE |
| 56798204 | 56798499 | - | 29.7 | 5296 | 5617 | -2191 | L1MC4a | L1 | LINE |

  

|  |  |  |  |  |  |  |  |  |  |
| --- | --- | --- | --- | --- | --- | --- | --- | --- | --- |
| chr7:56809595-56811248 | | | 1356 bp | | |  |  |  |  |
| Genome Start | Genome End | Strand | Div | Repeat Start | Repeat End | Repeat Left | Name | Family | Class |
| 56809595 | 56810340 | + | 30.3 | 4509 | 5357 | -789 | L1ME4a | L1 | LINE |
| 56810667 | 56810880 | + | 29.7 | 5348 | 5580 | -660 | L1ME4a | L1 | LINE |
| 56810853 | 56810888 | + | 19.4 | 5833 | 5868 | -253 | L1ME4a | L1 | LINE |
| 56810889 | 56811184 | - | 13.8 | 1 | 303 | -9 | AluSx | Alu | SINE |
| 56811185 | 56811248 | + | 19.4 | 5869 | 5933 | -188 | L1ME4a | L1 | LINE |

  

|  |  |  |  |  |  |  |  |  |  |
| --- | --- | --- | --- | --- | --- | --- | --- | --- | --- |
| chr7:56824526-56825119 | | | 594 bp | | |  |  |  |  |
| Genome Start | Genome End | Strand | Div | Repeat Start | Repeat End | Repeat Left | Name | Family | Class |
| 56824526 | 56824685 | - | 11.9 | 134 | 296 | -17 | AluSq | Alu | SINE |
| 56824686 | 56824983 | - | 13.1 | 1 | 297 | -15 | AluSx | Alu | SINE |
| 56824984 | 56825119 | - | 11.9 | 1 | 133 | -180 | AluSq | Alu | SINE |

  

|  |  |  |  |  |  |  |  |  |  |
| --- | --- | --- | --- | --- | --- | --- | --- | --- | --- |
| chr7:56831734-56833149 | | | 1362 bp | | |  |  |  |  |
| Genome Start | Genome End | Strand | Div | Repeat Start | Repeat End | Repeat Left | Name | Family | Class |
| 56831734 | 56831966 | + | 31.9 | 936 | 1169 | -840 | HAL1b | L1 | LINE |
| 56831967 | 56832241 | + | 10.9 | 1 | 284 | -28 | AluSx | Alu | SINE |
| 56832263 | 56832340 | + | 31.9 | 1170 | 1247 | -762 | HAL1b | L1 | LINE |
| 56832343 | 56832552 | + | 25.7 | 1560 | 1804 | -205 | HAL1b | L1 | LINE |
| 56832553 | 56832843 | - | 12.7 | 2 | 294 | -15 | AluSc | Alu | SINE |
| 56832844 | 56832914 | + | 25.7 | 1805 | 1877 | -132 | HAL1b | L1 | LINE |
| 56832919 | 56833040 | - | 15.1 | 180 | 298 | -15 | AluSp/q | Alu | SINE |
| 56833068 | 56833149 | + | 13.9 | 1882 | 1967 | -42 | HAL1b | L1 | LINE |

  

|  |  |  |  |  |  |  |  |  |  |
| --- | --- | --- | --- | --- | --- | --- | --- | --- | --- |
| chr7:56880946-56882342 | | | 1397 bp | | |  |  |  |  |
| Genome Start | Genome End | Strand | Div | Repeat Start | Repeat End | Repeat Left | Name | Family | Class |
| 56880946 | 56881354 | + | 20.1 | 115 | 532 | -403 | MER21C | ERV1 | LTR |
| 56881355 | 56881664 | - | 18.1 | 3 | 295 | -17 | AluJb | Alu | SINE |
| 56881665 | 56881735 | + | 20.1 | 533 | 603 | -332 | MER21C | ERV1 | LTR |
| 56881736 | 56882040 | + | 18.1 | 1 | 299 | -13 | AluJo | Alu | SINE |
| 56882041 | 56882342 | + | 20.1 | 604 | 935 | 0 | MER21C | ERV1 | LTR |

  

|  |  |  |  |  |  |  |  |  |  |
| --- | --- | --- | --- | --- | --- | --- | --- | --- | --- |
| chr7:56899267-56899692 | | | 426 bp | | |  |  |  |  |
| Genome Start | Genome End | Strand | Div | Repeat Start | Repeat End | Repeat Left | Name | Family | Class |
| 56899267 | 56899378 | + | 20.8 | 1 | 111 | -22 | FLAM\_A | Alu | SINE |
| 56899379 | 56899669 | - | 11.3 | 1 | 302 | -11 | AluSp | Alu | SINE |
| 56899670 | 56899692 | + | 20.8 | 112 | 125 | -8 | FLAM\_A | Alu | SINE |

  

|  |  |  |  |  |  |  |  |  |  |
| --- | --- | --- | --- | --- | --- | --- | --- | --- | --- |
| chr7:56947709-56948356 | | | 648 bp | | |  |  |  |  |
| Genome Start | Genome End | Strand | Div | Repeat Start | Repeat End | Repeat Left | Name | Family | Class |
| 56947709 | 56947773 | - | 30.2 | 7985 | 8042 | 0 | L1MC4 | L1 | LINE |
| 56947774 | 56948059 | - | 9.4 | 1 | 298 | -15 | AluSq | Alu | SINE |
| 56948060 | 56948356 | - | 30.2 | 7689 | 7984 | -58 | L1MC4 | L1 | LINE |

  

|  |  |  |  |  |  |  |  |  |  |
| --- | --- | --- | --- | --- | --- | --- | --- | --- | --- |
| chr7:56949783-56952996 | | | 3136 bp | | |  |  |  |  |
| Genome Start | Genome End | Strand | Div | Repeat Start | Repeat End | Repeat Left | Name | Family | Class |
| 56949783 | 56949900 | + | 23.9 | 4281 | 4401 | -1745 | L1M5 | L1 | LINE |
| 56949913 | 56950042 | + | 27.7 | 4670 | 4823 | -1323 | L1M5 | L1 | LINE |
| 56950043 | 56950345 | + | 16.5 | 1 | 311 | -1 | AluSx | Alu | SINE |
| 56950346 | 56950484 | + | 27.7 | 4824 | 4945 | -1201 | L1M5 | L1 | LINE |
| 56950485 | 56950791 | + | 19.4 | 1 | 304 | -8 | AluJo | Alu | SINE |
| 56950792 | 56950919 | + | 27.7 | 4946 | 5072 | -1074 | L1M5 | L1 | LINE |
| 56950920 | 56951231 | - | 9.9 | 1 | 313 | 0 | AluSp | Alu | SINE |
| 56951232 | 56951357 | + | 27.7 | 5073 | 5201 | -945 | L1M5 | L1 | LINE |
| 56951360 | 56951652 | + | 13.7 | 1 | 293 | -19 | AluSx | Alu | SINE |
| 56951673 | 56951854 | - | 26.1 | 29 | 184 | -6 | MER91B | Tip100 | DNA |
| 56951884 | 56952138 | + | 31.4 | 5217 | 5482 | -758 | L1M5 | L1 | LINE |
| 56952154 | 56952459 | + | 7.4 | 1 | 299 | -12 | AluY | Alu | SINE |
| 56952460 | 56952592 | + | 31.8 | 5539 | 5671 | -569 | L1M5 | L1 | LINE |
| 56952593 | 56952901 | + | 17.2 | 5 | 308 | -4 | AluJo | Alu | SINE |
| 56952902 | 56952996 | + | 31.8 | 5672 | 5769 | -471 | L1M5 | L1 | LINE |

  

|  |  |  |  |  |  |  |  |  |  |
| --- | --- | --- | --- | --- | --- | --- | --- | --- | --- |
| chr7:57009605-57010193 | | | 589 bp | | |  |  |  |  |
| Genome Start | Genome End | Strand | Div | Repeat Start | Repeat End | Repeat Left | Name | Family | Class |
| 57009605 | 57009725 | + | 18.6 | 1 | 121 | -191 | AluJb | Alu | SINE |
| 57009726 | 57010012 | - | 13.6 | 7 | 292 | -18 | AluSg | Alu | SINE |
| 57010013 | 57010193 | + | 18.6 | 122 | 295 | -17 | AluJb | Alu | SINE |

  

|  |  |  |  |  |  |  |  |  |  |
| --- | --- | --- | --- | --- | --- | --- | --- | --- | --- |
| chr7:57013325-57013937 | | | 613 bp | | |  |  |  |  |
| Genome Start | Genome End | Strand | Div | Repeat Start | Repeat End | Repeat Left | Name | Family | Class |
| 57013325 | 57013444 | + | 11.5 | 15 | 133 | -179 | AluSx | Alu | SINE |
| 57013445 | 57013768 | + | 5.7 | 1 | 298 | -13 | AluY | Alu | SINE |
| 57013769 | 57013937 | + | 11.5 | 134 | 302 | -10 | AluSx | Alu | SINE |

  

|  |  |  |  |  |  |  |  |  |  |
| --- | --- | --- | --- | --- | --- | --- | --- | --- | --- |
| chr7:57045914-57046477 | | | 564 bp | | |  |  |  |  |
| Genome Start | Genome End | Strand | Div | Repeat Start | Repeat End | Repeat Left | Name | Family | Class |
| 57045914 | 57046081 | - | 15.4 | 121 | 279 | -33 | AluJb | Alu | SINE |
| 57046082 | 57046376 | + | 8.5 | 1 | 295 | -16 | AluY | Alu | SINE |
| 57046377 | 57046477 | - | 15.4 | 21 | 120 | -192 | AluJb | Alu | SINE |

  

|  |  |  |  |  |  |  |  |  |  |
| --- | --- | --- | --- | --- | --- | --- | --- | --- | --- |
| chr7:57048406-57049089 | | | 684 bp | | |  |  |  |  |
| Genome Start | Genome End | Strand | Div | Repeat Start | Repeat End | Repeat Left | Name | Family | Class |
| 57048406 | 57048652 | - | 27.5 | 7694 | 7927 | -34 | L1MC5 | L1 | LINE |
| 57048653 | 57048976 | + | 12.5 | 1 | 312 | 0 | AluSx | Alu | SINE |
| 57048977 | 57049089 | - | 27.5 | 7604 | 7693 | -268 | L1MC5 | L1 | LINE |

  

|  |  |  |  |  |  |  |  |  |  |
| --- | --- | --- | --- | --- | --- | --- | --- | --- | --- |
| chr7:57052013-57053116 | | | 1098 bp | | |  |  |  |  |
| Genome Start | Genome End | Strand | Div | Repeat Start | Repeat End | Repeat Left | Name | Family | Class |
| 57052013 | 57052205 | - | 23.3 | 601 | 783 | 0 | LTR39 | ERV1 | LTR |
| 57052211 | 57052518 | + | 12.8 | 3 | 305 | 0 | AluSx | Alu | SINE |
| 57052520 | 57052572 | - | 13.8 | 476 | 522 | -261 | LTR39 | ERV1 | LTR |
| 57052573 | 57052859 | - | 10.1 | 1 | 289 | -20 | AluSc | Alu | SINE |
| 57052860 | 57053116 | - | 13.8 | 220 | 475 | -308 | LTR39 | ERV1 | LTR |

  

|  |  |  |  |  |  |  |  |  |  |
| --- | --- | --- | --- | --- | --- | --- | --- | --- | --- |
| chr7:57059804-57060427 | | | 624 bp | | |  |  |  |  |
| Genome Start | Genome End | Strand | Div | Repeat Start | Repeat End | Repeat Left | Name | Family | Class |
| 57059804 | 57060004 | - | 19.1 | 110 | 304 | -8 | AluJo | Alu | SINE |
| 57060005 | 57060301 | + | 8.2 | 1 | 294 | -16 | AluSg | Alu | SINE |
| 57060302 | 57060427 | - | 19.1 | 1 | 109 | -203 | AluJo | Alu | SINE |

  

|  |  |  |  |  |  |  |  |  |  |
| --- | --- | --- | --- | --- | --- | --- | --- | --- | --- |
| chr7:57064679-57065431 | | | 753 bp | | |  |  |  |  |
| Genome Start | Genome End | Strand | Div | Repeat Start | Repeat End | Repeat Left | Name | Family | Class |
| 57064679 | 57064915 | - | 32.5 | 2792 | 3060 | -359 | L2 | L2 | LINE |
| 57064916 | 57065216 | + | 11.0 | 1 | 301 | -12 | AluSq | Alu | SINE |
| 57065217 | 57065431 | - | 32.5 | 2563 | 2791 | -628 | L2 | L2 | LINE |

  

|  |  |  |  |  |  |  |  |  |  |
| --- | --- | --- | --- | --- | --- | --- | --- | --- | --- |
| chr7:57115242-57116039 | | | 798 bp | | |  |  |  |  |
| Genome Start | Genome End | Strand | Div | Repeat Start | Repeat End | Repeat Left | Name | Family | Class |
| 57115242 | 57115569 | + | 26.6 | 2 | 359 | -149 | Charlie4a | MER1\_type | DNA |
| 57115570 | 57115869 | + | 16.1 | 3 | 308 | -4 | AluSx | Alu | SINE |
| 57115870 | 57116039 | + | 26.6 | 360 | 504 | -4 | Charlie4a | MER1\_type | DNA |

  

|  |  |  |  |  |  |  |  |  |  |
| --- | --- | --- | --- | --- | --- | --- | --- | --- | --- |
| chr7:57148215-57149091 | | | 877 bp | | |  |  |  |  |
| Genome Start | Genome End | Strand | Div | Repeat Start | Repeat End | Repeat Left | Name | Family | Class |
| 57148215 | 57148330 | + | 17.2 | 3064 | 3170 | -2976 | L1M4 | L1 | LINE |
| 57148331 | 57148641 | + | 10.0 | 1 | 312 | -1 | AluSq | Alu | SINE |
| 57148642 | 57148661 | + | 17.2 | 3171 | 3181 | -2965 | L1M4 | L1 | LINE |
| 57148662 | 57148973 | + | 9.1 | 1 | 308 | -1 | AluSc | Alu | SINE |
| 57148974 | 57149091 | + | 17.2 | 3182 | 3303 | -2843 | L1M4 | L1 | LINE |

  

|  |  |  |  |  |  |  |  |  |  |
| --- | --- | --- | --- | --- | --- | --- | --- | --- | --- |
| chr7:57153088-57153470 | | | 383 bp | | |  |  |  |  |
| Genome Start | Genome End | Strand | Div | Repeat Start | Repeat End | Repeat Left | Name | Family | Class |
| 57153088 | 57153143 | - | 31.5 | 153 | 211 | -51 | MIR | MIR | SINE |
| 57153144 | 57153377 | - | 15.9 | 1 | 236 | 0 | MER46A | MER2\_type | DNA |
| 57153378 | 57153470 | - | 31.5 | 45 | 152 | -110 | MIR | MIR | SINE |

  

|  |  |  |  |  |  |  |  |  |  |
| --- | --- | --- | --- | --- | --- | --- | --- | --- | --- |
| chr7:57170168-57170555 | | | 324 bp | | |  |  |  |  |
| Genome Start | Genome End | Strand | Div | Repeat Start | Repeat End | Repeat Left | Name | Family | Class |
| 57170168 | 57170336 | + | 18.3 | 3 | 175 | -137 | AluJ | Alu | SINE |
| 57170353 | 57170385 | + | 3.0 | 5219 | 5251 | -895 | L1M4 | L1 | LINE |
| 57170434 | 57170555 | + | 19.8 | 176 | 297 | -15 | AluJ | Alu | SINE |

  

|  |  |  |  |  |  |  |  |  |  |
| --- | --- | --- | --- | --- | --- | --- | --- | --- | --- |
| chr7:57194213-57197063 | | | 2759 bp | | |  |  |  |  |
| Genome Start | Genome End | Strand | Div | Repeat Start | Repeat End | Repeat Left | Name | Family | Class |
| 57194213 | 57194408 | + | 25.3 | 2029 | 2233 | -4151 | L1M3 | L1 | LINE |
| 57194423 | 57194672 | + | 23.2 | 2617 | 2861 | -3515 | L1M3 | L1 | LINE |
| 57194676 | 57195073 | - | 22.3 | 1 | 413 | 0 | MSTB | MaLR | LTR |
| 57195075 | 57195200 | + | 27.2 | 2861 | 3005 | -3371 | L1M3 | L1 | LINE |
| 57195201 | 57195507 | + | 23.3 | 1 | 306 | -6 | AluJb | Alu | SINE |
| 57195508 | 57195604 | + | 27.2 | 3006 | 3114 | -3262 | L1M3 | L1 | LINE |
| 57195679 | 57196441 | + | 28.2 | 4762 | 5562 | -814 | L1M3 | L1 | LINE |
| 57196442 | 57196742 | + | 14.3 | 1 | 304 | -9 | AluSp | Alu | SINE |
| 57196743 | 57197063 | + | 26.9 | 5563 | 5891 | -516 | L1M3 | L1 | LINE |

  

|  |  |  |  |  |  |  |  |  |  |
| --- | --- | --- | --- | --- | --- | --- | --- | --- | --- |
| chr7:57294130-57294938 | | | 732 bp | | |  |  |  |  |
| Genome Start | Genome End | Strand | Div | Repeat Start | Repeat End | Repeat Left | Name | Family | Class |
| 57294130 | 57294229 | + | 32.6 | 2896 | 2991 | -428 | L2 | L2 | LINE |
| 57294260 | 57294621 | - | 29.8 | 39 | 461 | -50 | MLT1J | MaLR | LTR |
| 57294673 | 57294767 | - | 30.5 | 20 | 114 | 0 | MER81 | AcHobo | DNA |
| 57294763 | 57294859 | - | 20.6 | 8 | 104 | -10 | MER81 | AcHobo | DNA |
| 57294861 | 57294938 | + | 34.6 | 3028 | 3105 | -314 | L2 | L2 | LINE |

  

|  |  |  |  |  |  |  |  |  |  |
| --- | --- | --- | --- | --- | --- | --- | --- | --- | --- |
| chr7:57295579-57296634 | | | 1056 bp | | |  |  |  |  |
| Genome Start | Genome End | Strand | Div | Repeat Start | Repeat End | Repeat Left | Name | Family | Class |
| 57295579 | 57295707 | + | 19.4 | 51 | 202 | -309 | MLT1J | MaLR | LTR |
| 57295708 | 57296118 | + | 13.9 | 1 | 426 | 0 | MSTB | MaLR | LTR |
| 57296119 | 57296293 | + | 26.8 | 203 | 413 | -98 | MLT1J | MaLR | LTR |
| 57296294 | 57296599 | - | 16.0 | 1 | 307 | -5 | AluSx | Alu | SINE |
| 57296600 | 57296634 | + | 26.8 | 414 | 448 | -63 | MLT1J | MaLR | LTR |

  

|  |  |  |  |  |  |  |  |  |  |
| --- | --- | --- | --- | --- | --- | --- | --- | --- | --- |
| chr7:57306977-57307900 | | | 922 bp | | |  |  |  |  |
| Genome Start | Genome End | Strand | Div | Repeat Start | Repeat End | Repeat Left | Name | Family | Class |
| 57306977 | 57307051 | - | 12.0 | 222 | 296 | -15 | AluSx | Alu | SINE |
| 57307052 | 57307672 | - | 11.5 | 1 | 644 | 0 | LTR9B | ERV1 | LTR |
| 57307675 | 57307900 | - | 12.1 | 1 | 224 | -88 | AluSx | Alu | SINE |

  

|  |  |  |  |  |  |  |  |  |  |
| --- | --- | --- | --- | --- | --- | --- | --- | --- | --- |
| chr7:57397066-57398957 | | | 1772 bp | | |  |  |  |  |
| Genome Start | Genome End | Strand | Div | Repeat Start | Repeat End | Repeat Left | Name | Family | Class |
| 57397066 | 57397162 | + | 30.5 | 2695 | 2789 | -630 | L2 | L2 | LINE |
| 57397173 | 57397642 | - | 21.3 | 3 | 496 | -9 | MLT1D | MaLR | LTR |
| 57397644 | 57397912 | + | 24.9 | 2786 | 3078 | -341 | L2 | L2 | LINE |
| 57397924 | 57398230 | - | 10.4 | 1 | 307 | -3 | AluSg | Alu | SINE |
| 57398326 | 57398631 | - | 12.4 | 7 | 312 | 0 | AluSx | Alu | SINE |
| 57398635 | 57398957 | + | 29.4 | 3057 | 3415 | -4 | L2 | L2 | LINE |

  

|  |  |  |  |  |  |  |  |  |  |
| --- | --- | --- | --- | --- | --- | --- | --- | --- | --- |
| chr7:57413943-57415073 | | | 1131 bp | | |  |  |  |  |
| Genome Start | Genome End | Strand | Div | Repeat Start | Repeat End | Repeat Left | Name | Family | Class |
| 57413943 | 57413994 | + | 27.0 | 2523 | 2574 | -845 | L2 | L2 | LINE |
| 57413995 | 57414276 | - | 9.2 | 5882 | 6168 | 0 | L1PA10 | L1 | LINE |
| 57414277 | 57415073 | + | 26.1 | 2575 | 3354 | -65 | L2 | L2 | LINE |

  

|  |  |  |  |  |  |  |  |  |  |
| --- | --- | --- | --- | --- | --- | --- | --- | --- | --- |
| chr7:57443681-57443995 | | | 315 bp | | |  |  |  |  |
| Genome Start | Genome End | Strand | Div | Repeat Start | Repeat End | Repeat Left | Name | Family | Class |
| 57443681 | 57443716 | - | 23.5 | 6123 | 6151 | -15 | L1ME1 | L1 | LINE |
| 57443717 | 57443925 | - | 17.7 | 1 | 219 | 0 | MER20 | MER1\_type | DNA |
| 57443926 | 57443995 | - | 23.5 | 6054 | 6122 | -44 | L1ME1 | L1 | LINE |

  

|  |  |  |  |  |  |  |  |  |  |
| --- | --- | --- | --- | --- | --- | --- | --- | --- | --- |
| chr7:57448313-57449613 | | | 1301 bp | | |  |  |  |  |
| Genome Start | Genome End | Strand | Div | Repeat Start | Repeat End | Repeat Left | Name | Family | Class |
| 57448313 | 57448828 | + | 32.0 | 5338 | 5868 | -253 | L1ME4a | L1 | LINE |
| 57448829 | 57449138 | + | 15.9 | 1 | 310 | 0 | AluSg | Alu | SINE |
| 57449139 | 57449323 | + | 32.5 | 5869 | 6050 | -71 | L1ME4a | L1 | LINE |
| 57449324 | 57449539 | - | 16.7 | 2 | 219 | 0 | MER20 | MER1\_type | DNA |
| 57449540 | 57449613 | + | 32.5 | 6051 | 6121 | 0 | L1ME4a | L1 | LINE |

  

|  |  |  |  |  |  |  |  |  |  |
| --- | --- | --- | --- | --- | --- | --- | --- | --- | --- |
| chr7:57453898-57454633 | | | 736 bp | | |  |  |  |  |
| Genome Start | Genome End | Strand | Div | Repeat Start | Repeat End | Repeat Left | Name | Family | Class |
| 57453898 | 57454318 | + | 29.4 | 2584 | 3077 | -309 | L2 | L2 | LINE |
| 57454319 | 57454409 | - | 13.3 | 6079 | 6168 | -6 | L1MB5 | L1 | LINE |
| 57454410 | 57454633 | + | 33.0 | 3070 | 3307 | -71 | L2 | L2 | LINE |

  

|  |  |  |  |  |  |  |  |  |  |
| --- | --- | --- | --- | --- | --- | --- | --- | --- | --- |
| chr7:57605163-57606198 | | | 1036 bp | | |  |  |  |  |
| Genome Start | Genome End | Strand | Div | Repeat Start | Repeat End | Repeat Left | Name | Family | Class |
| 57605163 | 57605440 | - | 35.4 | 115 | 395 | -14 | MLT1I | MaLR | LTR |
| 57605441 | 57605599 | + | 20.9 | 1 | 157 | -233 | MLT1B | MaLR | LTR |
| 57605600 | 57605897 | - | 8.8 | 1 | 309 | -2 | AluY | Alu | SINE |
| 57605898 | 57606142 | + | 20.9 | 158 | 390 | 0 | MLT1B | MaLR | LTR |
| 57606143 | 57606198 | - | 35.4 | 59 | 114 | -295 | MLT1I | MaLR | LTR |

  

|  |  |  |  |  |  |  |  |  |  |
| --- | --- | --- | --- | --- | --- | --- | --- | --- | --- |
| chr7:57633687-57637049 | | | 3364 bp | | |  |  |  |  |
| Genome Start | Genome End | Strand | Div | Repeat Start | Repeat End | Repeat Left | Name | Family | Class |
| 57633687 | 57634593 | + | 30.9 | 2 | 955 | -6379 | L1M4b | L1 | LINE |
| 57634593 | 57634702 | + | 20.3 | 2767 | 2877 | -4457 | L1M4b | L1 | LINE |
| 57634703 | 57635077 | - | 21.1 | 1 | 408 | 0 | MLT1A1 | MaLR | LTR |
| 57635078 | 57637049 | + | 22.1 | 2878 | 4683 | -2323 | L1M4b | L1 | LINE |

  

|  |  |  |  |  |  |  |  |  |  |
| --- | --- | --- | --- | --- | --- | --- | --- | --- | --- |
| chr7:57641484-57641967 | | | 484 bp | | |  |  |  |  |
| Genome Start | Genome End | Strand | Div | Repeat Start | Repeat End | Repeat Left | Name | Family | Class |
| 57641484 | 57641526 | + | 19.9 | 5159 | 5195 | -948 | L1M2 | L1 | LINE |
| 57641527 | 57641815 | + | 9.1 | 1 | 290 | -21 | AluY | Alu | SINE |
| 57641816 | 57641967 | + | 19.9 | 5196 | 5319 | -824 | L1M2 | L1 | LINE |

  

|  |  |  |  |  |  |  |  |  |  |
| --- | --- | --- | --- | --- | --- | --- | --- | --- | --- |
| chr7:57652763-57654230 | | | 1042 bp | | |  |  |  |  |
| Genome Start | Genome End | Strand | Div | Repeat Start | Repeat End | Repeat Left | Name | Family | Class |
| 57652763 | 57652853 | - | 31.0 | 5265 | 5364 | -2444 | L1M | L1 | LINE |
| 57652857 | 57653198 | - | 11.2 | 1 | 343 | 0 | AluSp | Alu | SINE |
| 57653258 | 57653414 | + | 26.1 | 34 | 203 | -59 | MIR | MIR | SINE |
| 57653779 | 57654230 | - | 26.6 | 4547 | 5006 | -1140 | L1M | L1 | LINE |

  

|  |  |  |  |  |  |  |  |  |  |
| --- | --- | --- | --- | --- | --- | --- | --- | --- | --- |
| chr7:57655686-57656296 | | | 611 bp | | |  |  |  |  |
| Genome Start | Genome End | Strand | Div | Repeat Start | Repeat End | Repeat Left | Name | Family | Class |
| 57655686 | 57655860 | - | 14.3 | 134 | 308 | -4 | AluSx | Alu | SINE |
| 57655861 | 57656159 | - | 11.7 | 1 | 301 | -9 | AluSg | Alu | SINE |
| 57656160 | 57656296 | - | 14.3 | 1 | 133 | -179 | AluSx | Alu | SINE |

  

|  |  |  |  |  |  |  |  |  |  |
| --- | --- | --- | --- | --- | --- | --- | --- | --- | --- |
| chr7:57661026-57661647 | | | 622 bp | | |  |  |  |  |
| Genome Start | Genome End | Strand | Div | Repeat Start | Repeat End | Repeat Left | Name | Family | Class |
| 57661026 | 57661192 | - | 18.3 | 144 | 300 | -12 | AluJb | Alu | SINE |
| 57661193 | 57661491 | - | 8.4 | 1 | 301 | -10 | AluY | Alu | SINE |
| 57661492 | 57661647 | - | 18.3 | 1 | 143 | -169 | AluJb | Alu | SINE |

  

|  |  |  |  |  |  |  |  |  |  |
| --- | --- | --- | --- | --- | --- | --- | --- | --- | --- |
| chr7:57667803-57668355 | | | 553 bp | | |  |  |  |  |
| Genome Start | Genome End | Strand | Div | Repeat Start | Repeat End | Repeat Left | Name | Family | Class |
| 57667803 | 57667961 | - | 17.3 | 134 | 294 | -18 | AluSx | Alu | SINE |
| 57667962 | 57668262 | - | 10.3 | 1 | 302 | -11 | AluSp | Alu | SINE |
| 57668263 | 57668355 | - | 17.3 | 50 | 133 | -179 | AluSx | Alu | SINE |

  

|  |  |  |  |  |  |  |  |  |  |
| --- | --- | --- | --- | --- | --- | --- | --- | --- | --- |
| chr7:57680747-57681082 | | | 336 bp | | |  |  |  |  |
| Genome Start | Genome End | Strand | Div | Repeat Start | Repeat End | Repeat Left | Name | Family | Class |
| 57680747 | 57680789 | + | 26.2 | 5575 | 5628 | -493 | L1M5 | L1 | LINE |
| 57680790 | 57680915 | - | 23.8 | 1 | 126 | -7 | FLAM\_A | Alu | SINE |
| 57680916 | 57681082 | + | 26.2 | 5629 | 5796 | -325 | L1M5 | L1 | LINE |

  

|  |  |  |  |  |  |  |  |  |  |
| --- | --- | --- | --- | --- | --- | --- | --- | --- | --- |
| chr7:57701000-57701742 | | | 743 bp | | |  |  |  |  |
| Genome Start | Genome End | Strand | Div | Repeat Start | Repeat End | Repeat Left | Name | Family | Class |
| 57701000 | 57701118 | - | 30.0 | 153 | 260 | -8 | MIRb | MIR | SINE |
| 57701119 | 57701607 | - | 17.4 | 5670 | 6164 | -7 | L1MB2 | L1 | LINE |
| 57701608 | 57701742 | - | 30.0 | 2 | 152 | -116 | MIRb | MIR | SINE |

  

|  |  |  |  |  |  |  |  |  |  |
| --- | --- | --- | --- | --- | --- | --- | --- | --- | --- |
| chr7:57728943-57730527 | | | 1597 bp | | |  |  |  |  |
| Genome Start | Genome End | Strand | Div | Repeat Start | Repeat End | Repeat Left | Name | Family | Class |
| 57728943 | 57729059 | + | 9.7 | 1066 | 1208 | -1210 | Tigger1 | MER2\_type | DNA |
| 57729048 | 57729277 | + | 14.1 | 1545 | 1773 | -645 | Tigger1 | MER2\_type | DNA |
| 57729278 | 57729577 | - | 13.3 | 1 | 301 | -12 | AluSp | Alu | SINE |
| 57729578 | 57730112 | + | 14.1 | 1774 | 2308 | -110 | Tigger1 | MER2\_type | DNA |
| 57730113 | 57730389 | - | 12.6 | 2 | 296 | -16 | AluSx | Alu | SINE |
| 57730390 | 57730527 | + | 14.1 | 2309 | 2417 | -1 | Tigger1 | MER2\_type | DNA |

  

|  |  |  |  |  |  |  |  |  |  |
| --- | --- | --- | --- | --- | --- | --- | --- | --- | --- |
| chr7:57746768-57747470 | | | 703 bp | | |  |  |  |  |
| Genome Start | Genome End | Strand | Div | Repeat Start | Repeat End | Repeat Left | Name | Family | Class |
| 57746768 | 57747055 | - | 21.1 | 85 | 365 | 0 | MLT1A0 | MaLR | LTR |
| 57747056 | 57747385 | - | 11.7 | 1 | 330 | 0 | AluSg | Alu | SINE |
| 57747386 | 57747470 | - | 21.1 | 1 | 84 | -281 | MLT1A0 | MaLR | LTR |

  

|  |  |  |  |  |  |  |  |  |  |
| --- | --- | --- | --- | --- | --- | --- | --- | --- | --- |
| chr7:57748521-57750037 | | | 1522 bp | | |  |  |  |  |
| Genome Start | Genome End | Strand | Div | Repeat Start | Repeat End | Repeat Left | Name | Family | Class |
| 57748521 | 57748763 | + | 21.3 | 1 | 240 | -72 | AluJo | Alu | SINE |
| 57748769 | 57749905 | + | 21.2 | 25 | 1196 | -559 | MER52A | ERV1 | LTR |
| 57749896 | 57749967 | + | 22.2 | 1682 | 1755 | 0 | MER52A | ERV1 | LTR |
| 57749968 | 57750037 | + | 16.2 | 235 | 302 | 0 | AluJo | Alu | SINE |

  

|  |  |  |  |  |  |  |  |  |  |
| --- | --- | --- | --- | --- | --- | --- | --- | --- | --- |
| chr7:57753074-57758177 | | | 4634 bp | | |  |  |  |  |
| Genome Start | Genome End | Strand | Div | Repeat Start | Repeat End | Repeat Left | Name | Family | Class |
| 57753074 | 57753435 | - | 21.3 | 5611 | 5979 | -13 | L1MB1 | L1 | LINE |
| 57753573 | 57754554 | - | 17.6 | 4643 | 5617 | -375 | L1MB1 | L1 | LINE |
| 57754555 | 57754888 | - | 14.7 | 40 | 372 | 0 | AluSx | Alu | SINE |
| 57754889 | 57756955 | - | 18.0 | 2531 | 4642 | -1322 | L1MB1 | L1 | LINE |
| 57756970 | 57757207 | - | 32.9 | 691 | 929 | -4962 | L1MB1 | L1 | LINE |
| 57757386 | 57757724 | - | 35.6 | 249 | 613 | -5278 | L1MB1 | L1 | LINE |
| 57757866 | 57758177 | - | 33.1 | 5 | 320 | -5571 | L1MB1 | L1 | LINE |

  

|  |  |  |  |  |  |  |  |  |  |
| --- | --- | --- | --- | --- | --- | --- | --- | --- | --- |
| chr7:57770451-57771485 | | | 772 bp | | |  |  |  |  |
| Genome Start | Genome End | Strand | Div | Repeat Start | Repeat End | Repeat Left | Name | Family | Class |
| 57770451 | 57770642 | + | 31.9 | 3677 | 3874 | -2272 | L1ME4a | L1 | LINE |
| 57770747 | 57771054 | + | 20.3 | 60 | 374 | 0 | MLT1A | MaLR | LTR |
| 57771214 | 57771485 | + | 33.8 | 5644 | 5940 | -181 | L1ME4a | L1 | LINE |

  

|  |  |  |  |  |  |  |  |  |  |
| --- | --- | --- | --- | --- | --- | --- | --- | --- | --- |
| chr7:57775804-57776580 | | | 777 bp | | |  |  |  |  |
| Genome Start | Genome End | Strand | Div | Repeat Start | Repeat End | Repeat Left | Name | Family | Class |
| 57775804 | 57775907 | + | 23.5 | 1 | 109 | -396 | MLT1D | MaLR | LTR |
| 57775908 | 57776223 | + | 12.5 | 1 | 312 | 0 | AluSx | Alu | SINE |
| 57776224 | 57776580 | + | 23.5 | 110 | 505 | 0 | MLT1D | MaLR | LTR |

  

|  |  |  |  |  |  |  |  |  |  |
| --- | --- | --- | --- | --- | --- | --- | --- | --- | --- |
| chr7:57777883-57778548 | | | 666 bp | | |  |  |  |  |
| Genome Start | Genome End | Strand | Div | Repeat Start | Repeat End | Repeat Left | Name | Family | Class |
| 57777883 | 57778077 | - | 13.7 | 178 | 381 | 0 | THE1D | MaLR | LTR |
| 57778078 | 57778367 | - | 14.2 | 7 | 295 | -17 | AluSx | Alu | SINE |
| 57778368 | 57778548 | - | 13.7 | 1 | 177 | -204 | THE1D | MaLR | LTR |

  

|  |  |  |  |  |  |  |  |  |  |
| --- | --- | --- | --- | --- | --- | --- | --- | --- | --- |
| chr7:57797518-57800694 | | | 3147 bp | | |  |  |  |  |
| Genome Start | Genome End | Strand | Div | Repeat Start | Repeat End | Repeat Left | Name | Family | Class |
| 57797518 | 57798657 | - | 27.8 | 4931 | 6166 | -4 | L1ME3A | L1 | LINE |
| 57798664 | 57799561 | - | 27.6 | 3832 | 4817 | -1329 | L1ME3A | L1 | LINE |
| 57799592 | 57799767 | - | 24.0 | 7854 | 8026 | -16 | L1MC4 | L1 | LINE |
| 57799768 | 57800111 | + | 14.8 | 1 | 364 | 0 | THE1B | MaLR | LTR |
| 57800112 | 57800205 | - | 24.0 | 7759 | 7853 | -189 | L1MC4 | L1 | LINE |
| 57800200 | 57800694 | - | 26.3 | 3345 | 3848 | -2298 | L1ME3A | L1 | LINE |

  

|  |  |  |  |  |  |  |  |  |  |
| --- | --- | --- | --- | --- | --- | --- | --- | --- | --- |
| chr7:57805987-57806856 | | | 870 bp | | |  |  |  |  |
| Genome Start | Genome End | Strand | Div | Repeat Start | Repeat End | Repeat Left | Name | Family | Class |
| 57805987 | 57806291 | + | 17.1 | 1 | 288 | -7249 | MER57B-int | ERV1 | LTR |
| 57806292 | 57806601 | - | 14.8 | 1 | 310 | 0 | AluSx | Alu | SINE |
| 57806602 | 57806856 | + | 17.1 | 289 | 539 | -6998 | MER57B-int | ERV1 | LTR |

  

|  |  |  |  |  |  |  |  |  |  |
| --- | --- | --- | --- | --- | --- | --- | --- | --- | --- |
| chr7:57809758-57811649 | | | 1892 bp | | |  |  |  |  |
| Genome Start | Genome End | Strand | Div | Repeat Start | Repeat End | Repeat Left | Name | Family | Class |
| 57809758 | 57810115 | + | 29.9 | 5421 | 5795 | -445 | L1ME3B | L1 | LINE |
| 57810116 | 57810418 | - | 14.5 | 1 | 304 | -8 | AluSx | Alu | SINE |
| 57810419 | 57810586 | - | 14.4 | 138 | 300 | -12 | AluSx | Alu | SINE |
| 57810587 | 57810867 | - | 15.3 | 9 | 291 | -20 | AluY | Alu | SINE |
| 57810868 | 57811009 | - | 14.4 | 1 | 137 | -175 | AluSx | Alu | SINE |
| 57811010 | 57811189 | + | 29.9 | 5796 | 5978 | -262 | L1ME3B | L1 | LINE |
| 57811190 | 57811444 | + | 11.4 | 65 | 311 | -1 | AluSg/x | Alu | SINE |
| 57811445 | 57811649 | + | 29.9 | 5979 | 6162 | -78 | L1ME3B | L1 | LINE |

  

|  |  |  |  |  |  |  |  |  |  |
| --- | --- | --- | --- | --- | --- | --- | --- | --- | --- |
| chr7:57843216-57843903 | | | 688 bp | | |  |  |  |  |
| Genome Start | Genome End | Strand | Div | Repeat Start | Repeat End | Repeat Left | Name | Family | Class |
| 57843216 | 57843354 | + | 17.1 | 1 | 93 | -209 | AluSx | Alu | SINE |
| 57843355 | 57843661 | + | 17.2 | 1 | 291 | -21 | AluJb | Alu | SINE |
| 57843662 | 57843903 | + | 17.1 | 94 | 302 | 0 | AluSx | Alu | SINE |

  

|  |  |  |  |  |  |  |  |  |  |
| --- | --- | --- | --- | --- | --- | --- | --- | --- | --- |
| chr7:57861704-57863470 | | | 1765 bp | | |  |  |  |  |
| Genome Start | Genome End | Strand | Div | Repeat Start | Repeat End | Repeat Left | Name | Family | Class |
| 57861704 | 57861740 | - | 29.5 | 5981 | 6013 | -227 | L1ME3B | L1 | LINE |
| 57861741 | 57861990 | - | 14.0 | 65 | 301 | -11 | AluSg/x | Alu | SINE |
| 57861991 | 57862170 | - | 29.5 | 5834 | 5980 | -260 | L1ME3B | L1 | LINE |
| 57862171 | 57862312 | + | 14.2 | 1 | 137 | -175 | AluSx | Alu | SINE |
| 57862313 | 57862596 | + | 15.2 | 9 | 290 | -19 | AluSc | Alu | SINE |
| 57862597 | 57862769 | + | 14.2 | 138 | 307 | -5 | AluSx | Alu | SINE |
| 57862772 | 57863062 | + | 14.7 | 3 | 288 | -24 | AluSx | Alu | SINE |
| 57863063 | 57863470 | - | 29.5 | 5425 | 5833 | -407 | L1ME3B | L1 | LINE |

  

|  |  |  |  |  |  |  |  |  |  |
| --- | --- | --- | --- | --- | --- | --- | --- | --- | --- |
| chr7:57870812-57873334 | | | 2103 bp | | |  |  |  |  |
| Genome Start | Genome End | Strand | Div | Repeat Start | Repeat End | Repeat Left | Name | Family | Class |
| 57870812 | 57870949 | - | 14.6 | 2303 | 2418 | 0 | Tigger1 | MER2\_type | DNA |
| 57870950 | 57871234 | + | 12.6 | 2 | 297 | -15 | AluSx | Alu | SINE |
| 57871235 | 57871743 | - | 14.6 | 1801 | 2302 | -116 | Tigger1 | MER2\_type | DNA |
| 57872108 | 57872303 | - | 24.5 | 445 | 643 | -50 | MER115 | Tip100 | DNA |
| 57872360 | 57872653 | + | 13.6 | 1 | 296 | -17 | AluSp | Alu | SINE |
| 57872654 | 57873334 | - | 13.7 | 1066 | 1773 | -645 | Tigger1 | MER2\_type | DNA |

  

|  |  |  |  |  |  |  |  |  |  |
| --- | --- | --- | --- | --- | --- | --- | --- | --- | --- |
| chr7:57876658-57877701 | | | 1044 bp | | |  |  |  |  |
| Genome Start | Genome End | Strand | Div | Repeat Start | Repeat End | Repeat Left | Name | Family | Class |
| 57876658 | 57877132 | + | 25.4 | 4657 | 5186 | -960 | L1M5 | L1 | LINE |
| 57877133 | 57877416 | + | 15.0 | 1 | 310 | -2 | AluSx | Alu | SINE |
| 57877417 | 57877701 | + | 25.3 | 5187 | 5492 | -748 | L1M5 | L1 | LINE |

  

|  |  |  |  |  |  |  |  |  |  |
| --- | --- | --- | --- | --- | --- | --- | --- | --- | --- |
| chr7:57905130-57907343 | | | 1985 bp | | |  |  |  |  |
| Genome Start | Genome End | Strand | Div | Repeat Start | Repeat End | Repeat Left | Name | Family | Class |
| 57905130 | 57905271 | - | 31.7 | 3245 | 3392 | -27 | L2 | L2 | LINE |
| 57905358 | 57905588 | - | 30.3 | 1410 | 1654 | -1765 | L2 | L2 | LINE |
| 57905628 | 57905750 | + | 26.4 | 93 | 215 | -47 | MIR | MIR | SINE |
| 57905799 | 57906403 | - | 28.3 | 704 | 1394 | -2025 | L2 | L2 | LINE |
| 57906457 | 57907065 | - | 18.7 | 1 | 560 | -42 | MER7C | MER2\_type | DNA |
| 57907069 | 57907343 | - | 28.2 | 318 | 626 | -2793 | L2 | L2 | LINE |

  

|  |  |  |  |  |  |  |  |  |  |
| --- | --- | --- | --- | --- | --- | --- | --- | --- | --- |
| chr7:57938757-57940222 | | | 1466 bp | | |  |  |  |  |
| Genome Start | Genome End | Strand | Div | Repeat Start | Repeat End | Repeat Left | Name | Family | Class |
| 57938757 | 57939566 | - | 23.3 | 2624 | 3651 | -3688 | L1M4b | L1 | LINE |
| 57939567 | 57939849 | - | 10.9 | 1 | 283 | -28 | AluY | Alu | SINE |
| 57939850 | 57940222 | - | 23.3 | 2142 | 2623 | -4711 | L1M4b | L1 | LINE |

  

|  |  |  |  |  |  |  |  |  |  |
| --- | --- | --- | --- | --- | --- | --- | --- | --- | --- |
| chr7:57941135-57942629 | | | 1135 bp | | |  |  |  |  |
| Genome Start | Genome End | Strand | Div | Repeat Start | Repeat End | Repeat Left | Name | Family | Class |
| 57941135 | 57941431 | + | 26.6 | 54 | 388 | -6035 | L1MCc | L1 | LINE |
| 57941502 | 57941795 | + | 25.0 | 73 | 365 | 0 | MLT1A0 | MaLR | LTR |
| 57942029 | 57942175 | + | 28.9 | 1851 | 1995 | -5133 | L1MCc | L1 | LINE |
| 57942233 | 57942629 | + | 26.1 | 3035 | 3438 | -3503 | L1MCc | L1 | LINE |

  

|  |  |  |  |  |  |  |  |  |  |
| --- | --- | --- | --- | --- | --- | --- | --- | --- | --- |
| chr7:61064319-61065134 | | | 816 bp | | |  |  |  |  |
| Genome Start | Genome End | Strand | Div | Repeat Start | Repeat End | Repeat Left | Name | Family | Class |
| 61064319 | 61064595 | + | 28.1 | 5 | 282 | -223 | MLT1D | MaLR | LTR |
| 61064596 | 61064892 | + | 13.2 | 7 | 304 | -7 | AluY | Alu | SINE |
| 61064893 | 61065134 | + | 28.1 | 283 | 503 | -2 | MLT1D | MaLR | LTR |

  

|  |  |  |  |  |  |  |  |  |  |
| --- | --- | --- | --- | --- | --- | --- | --- | --- | --- |
| chr7:61067601-61068790 | | | 1181 bp | | |  |  |  |  |
| Genome Start | Genome End | Strand | Div | Repeat Start | Repeat End | Repeat Left | Name | Family | Class |
| 61067601 | 61067993 | - | 21.9 | 490 | 901 | 0 | MER54A | ERVL | LTR |
| 61068003 | 61068337 | + | 13.0 | 7 | 362 | -2 | THE1B | MaLR | LTR |
| 61068338 | 61068790 | - | 27.8 | 1 | 496 | -405 | MER54A | ERVL | LTR |

  

|  |  |  |  |  |  |  |  |  |  |
| --- | --- | --- | --- | --- | --- | --- | --- | --- | --- |
| chr7:61401295-61402102 | | | 808 bp | | |  |  |  |  |
| Genome Start | Genome End | Strand | Div | Repeat Start | Repeat End | Repeat Left | Name | Family | Class |
| 61401295 | 61401565 | + | 28.5 | 5 | 274 | -231 | MLT1D | MaLR | LTR |
| 61401566 | 61401865 | + | 15.7 | 1 | 302 | -9 | AluY | Alu | SINE |
| 61401866 | 61402102 | + | 28.5 | 275 | 503 | -2 | MLT1D | MaLR | LTR |

  

|  |  |  |  |  |  |  |  |  |  |
| --- | --- | --- | --- | --- | --- | --- | --- | --- | --- |
| chr7:61405848-61407039 | | | 1145 bp | | |  |  |  |  |
| Genome Start | Genome End | Strand | Div | Repeat Start | Repeat End | Repeat Left | Name | Family | Class |
| 61405848 | 61406240 | - | 22.4 | 490 | 901 | 0 | MER54A | ERVL | LTR |
| 61406250 | 61406586 | + | 15.1 | 7 | 364 | 0 | THE1B | MaLR | LTR |
| 61406625 | 61407039 | - | 25.1 | 1 | 430 | -471 | MER54A | ERVL | LTR |

  

|  |  |  |  |  |  |  |  |  |  |
| --- | --- | --- | --- | --- | --- | --- | --- | --- | --- |
| chr7:61411770-61412450 | | | 681 bp | | |  |  |  |  |
| Genome Start | Genome End | Strand | Div | Repeat Start | Repeat End | Repeat Left | Name | Family | Class |
| 61411770 | 61411792 | + | 19.9 | 10 | 33 | -562 | LTR49 | ERV1 | LTR |
| 61411793 | 61412068 | - | 15.9 | 14 | 291 | -22 | AluSq | Alu | SINE |
| 61412069 | 61412450 | + | 19.9 | 34 | 427 | -168 | LTR49 | ERV1 | LTR |

  

|  |  |  |  |  |  |  |  |  |  |
| --- | --- | --- | --- | --- | --- | --- | --- | --- | --- |
| chr7:61473324-61474003 | | | 680 bp | | |  |  |  |  |
| Genome Start | Genome End | Strand | Div | Repeat Start | Repeat End | Repeat Left | Name | Family | Class |
| 61473324 | 61473705 | - | 18.8 | 35 | 427 | -168 | LTR49 | ERV1 | LTR |
| 61473706 | 61473980 | + | 14.2 | 14 | 291 | -22 | AluSq | Alu | SINE |
| 61473981 | 61474003 | - | 18.8 | 10 | 34 | -561 | LTR49 | ERV1 | LTR |

  

|  |  |  |  |  |  |  |  |  |  |
| --- | --- | --- | --- | --- | --- | --- | --- | --- | --- |
| chr7:61478361-61479544 | | | 1137 bp | | |  |  |  |  |
| Genome Start | Genome End | Strand | Div | Repeat Start | Repeat End | Repeat Left | Name | Family | Class |
| 61478361 | 61478775 | + | 24.8 | 1 | 430 | -471 | MER54A | ERVL | LTR |
| 61478814 | 61479150 | - | 12.7 | 7 | 364 | 0 | THE1B | MaLR | LTR |
| 61479160 | 61479544 | + | 21.9 | 490 | 894 | -7 | MER54A | ERVL | LTR |

  

|  |  |  |  |  |  |  |  |  |  |
| --- | --- | --- | --- | --- | --- | --- | --- | --- | --- |
| chr7:61482020-61482830 | | | 811 bp | | |  |  |  |  |
| Genome Start | Genome End | Strand | Div | Repeat Start | Repeat End | Repeat Left | Name | Family | Class |
| 61482020 | 61482263 | - | 28.1 | 275 | 503 | -2 | MLT1D | MaLR | LTR |
| 61482264 | 61482559 | - | 16.2 | 1 | 298 | -13 | AluY | Alu | SINE |
| 61482560 | 61482830 | - | 28.1 | 5 | 274 | -231 | MLT1D | MaLR | LTR |

  

|  |  |  |  |  |  |  |  |  |  |
| --- | --- | --- | --- | --- | --- | --- | --- | --- | --- |
| chr7:62238916-62239485 | | | 570 bp | | |  |  |  |  |
| Genome Start | Genome End | Strand | Div | Repeat Start | Repeat End | Repeat Left | Name | Family | Class |
| 62238916 | 62239102 | - | 22.3 | 5584 | 5770 | -413 | L1M3 | L1 | LINE |
| 62239103 | 62239456 | - | 10.8 | 1 | 355 | 0 | THE1A | MaLR | LTR |
| 62239457 | 62239485 | - | 22.3 | 5558 | 5583 | -600 | L1M3 | L1 | LINE |

  

|  |  |  |  |  |  |  |  |  |  |
| --- | --- | --- | --- | --- | --- | --- | --- | --- | --- |
| chr7:62280452-62281024 | | | 573 bp | | |  |  |  |  |
| Genome Start | Genome End | Strand | Div | Repeat Start | Repeat End | Repeat Left | Name | Family | Class |
| 62280452 | 62280560 | - | 26.6 | 152 | 262 | 0 | MIR | MIR | SINE |
| 62280561 | 62280872 | - | 8.3 | 1 | 313 | 0 | AluSp | Alu | SINE |
| 62280873 | 62281024 | - | 26.6 | 15 | 151 | -111 | MIR | MIR | SINE |

  

|  |  |  |  |  |  |  |  |  |  |
| --- | --- | --- | --- | --- | --- | --- | --- | --- | --- |
| chr7:62321447-62322146 | | | 700 bp | | |  |  |  |  |
| Genome Start | Genome End | Strand | Div | Repeat Start | Repeat End | Repeat Left | Name | Family | Class |
| 62321447 | 62321596 | + | 14.9 | 14 | 174 | -252 | MSTB | MaLR | LTR |
| 62321597 | 62321892 | - | 13.9 | 17 | 312 | 0 | AluY | Alu | SINE |
| 62321893 | 62322146 | + | 14.9 | 175 | 426 | 0 | MSTB | MaLR | LTR |

  

|  |  |  |  |  |  |  |  |  |  |
| --- | --- | --- | --- | --- | --- | --- | --- | --- | --- |
| chr7:62352871-62353251 | | | 381 bp | | |  |  |  |  |
| Genome Start | Genome End | Strand | Div | Repeat Start | Repeat End | Repeat Left | Name | Family | Class |
| 62352871 | 62352926 | - | 29.6 | 154 | 211 | -51 | MIR | MIR | SINE |
| 62352927 | 62353158 | - | 16.5 | 1 | 236 | 0 | MER46A | MER2\_type | DNA |
| 62353159 | 62353251 | - | 29.6 | 44 | 153 | -109 | MIR | MIR | SINE |

  

|  |  |  |  |  |  |  |  |  |  |
| --- | --- | --- | --- | --- | --- | --- | --- | --- | --- |
| chr7:62369213-62369944 | | | 553 bp | | |  |  |  |  |
| Genome Start | Genome End | Strand | Div | Repeat Start | Repeat End | Repeat Left | Name | Family | Class |
| 62369213 | 62369333 | + | 20.2 | 643 | 783 | -4071 | L1MEe | L1 | LINE |
| 62369334 | 62369516 | + | 12.2 | 2281 | 2487 | 0 | Tigger7 | MER2\_type | DNA |
| 62369640 | 62369740 | - | 22.8 | 189 | 296 | -118 | MLT2D | ERVL | LTR |
| 62369797 | 62369944 | + | 26.4 | 849 | 999 | -3835 | L1MEe | L1 | LINE |

  

|  |  |  |  |  |  |  |  |  |  |
| --- | --- | --- | --- | --- | --- | --- | --- | --- | --- |
| chr7:62375570-62376223 | | | 613 bp | | |  |  |  |  |
| Genome Start | Genome End | Strand | Div | Repeat Start | Repeat End | Repeat Left | Name | Family | Class |
| 62375570 | 62375714 | + | 18.5 | 1 | 126 | -186 | AluJo | Alu | SINE |
| 62375715 | 62375728 | - | 11.9 | 284 | 313 | 0 | AluSq | Alu | SINE |
| 62375770 | 62376052 | - | 11.9 | 1 | 283 | -30 | AluSq | Alu | SINE |
| 62376053 | 62376223 | + | 18.5 | 127 | 287 | -25 | AluJo | Alu | SINE |

  

|  |  |  |  |  |  |  |  |  |  |
| --- | --- | --- | --- | --- | --- | --- | --- | --- | --- |
| chr7:62402278-62402948 | | | 671 bp | | |  |  |  |  |
| Genome Start | Genome End | Strand | Div | Repeat Start | Repeat End | Repeat Left | Name | Family | Class |
| 62402278 | 62402338 | + | 19.9 | 1 | 56 | -325 | THE1D | MaLR | LTR |
| 62402339 | 62402625 | + | 12.2 | 7 | 294 | -16 | AluSg | Alu | SINE |
| 62402626 | 62402948 | + | 19.9 | 57 | 381 | 0 | THE1D | MaLR | LTR |

  

|  |  |  |  |  |  |  |  |  |  |
| --- | --- | --- | --- | --- | --- | --- | --- | --- | --- |
| chr7:62403565-62404511 | | | 947 bp | | |  |  |  |  |
| Genome Start | Genome End | Strand | Div | Repeat Start | Repeat End | Repeat Left | Name | Family | Class |
| 62403565 | 62404135 | + | 17.9 | 1 | 564 | -70 | MER41B | ERV1 | LTR |
| 62404136 | 62404444 | - | 12.9 | 3 | 312 | 0 | AluSx | Alu | SINE |
| 62404445 | 62404511 | + | 17.9 | 565 | 629 | -5 | MER41B | ERV1 | LTR |

  

|  |  |  |  |  |  |  |  |  |  |
| --- | --- | --- | --- | --- | --- | --- | --- | --- | --- |
| chr7:62428899-62429765 | | | 867 bp | | |  |  |  |  |
| Genome Start | Genome End | Strand | Div | Repeat Start | Repeat End | Repeat Left | Name | Family | Class |
| 62428899 | 62429211 | + | 22.3 | 3398 | 3732 | -2414 | L1M4 | L1 | LINE |
| 62429212 | 62429506 | - | 10.0 | 1 | 293 | -20 | AluSq | Alu | SINE |
| 62429507 | 62429765 | + | 22.3 | 3733 | 4014 | -2132 | L1M4 | L1 | LINE |

  

|  |  |  |  |  |  |  |  |  |  |
| --- | --- | --- | --- | --- | --- | --- | --- | --- | --- |
| chr7:62453919-62456184 | | | 2266 bp | | |  |  |  |  |
| Genome Start | Genome End | Strand | Div | Repeat Start | Repeat End | Repeat Left | Name | Family | Class |
| 62453919 | 62455014 | - | 19.1 | 4024 | 5111 | -1035 | L1MC | L1 | LINE |
| 62455015 | 62455308 | - | 10.6 | 1 | 295 | -14 | AluSc | Alu | SINE |
| 62455309 | 62455475 | - | 19.1 | 3852 | 4023 | -2123 | L1MC | L1 | LINE |
| 62455476 | 62455600 | - | 23.2 | 1 | 125 | -8 | FLAM\_C | Alu | SINE |
| 62455601 | 62456184 | - | 19.1 | 3259 | 3851 | -2295 | L1MC | L1 | LINE |

  

|  |  |  |  |  |  |  |  |  |  |
| --- | --- | --- | --- | --- | --- | --- | --- | --- | --- |
| chr7:62521639-62522417 | | | 779 bp | | |  |  |  |  |
| Genome Start | Genome End | Strand | Div | Repeat Start | Repeat End | Repeat Left | Name | Family | Class |
| 62521639 | 62521993 | + | 29.0 | 2 | 365 | -37 | MER93a | ERV1 | LTR |
| 62521994 | 62522386 | - | 17.3 | 1 | 426 | -2 | MSTA | MaLR | LTR |
| 62522387 | 62522417 | + | 29.0 | 366 | 392 | -10 | MER93a | ERV1 | LTR |

  

|  |  |  |  |  |  |  |  |  |  |
| --- | --- | --- | --- | --- | --- | --- | --- | --- | --- |
| chr7:62539303-62540250 | | | 948 bp | | |  |  |  |  |
| Genome Start | Genome End | Strand | Div | Repeat Start | Repeat End | Repeat Left | Name | Family | Class |
| 62539303 | 62539369 | - | 18.2 | 565 | 629 | -5 | MER41B | ERV1 | LTR |
| 62539370 | 62539669 | + | 12.0 | 3 | 303 | -9 | AluSx | Alu | SINE |
| 62539670 | 62540250 | - | 18.2 | 1 | 564 | -70 | MER41B | ERV1 | LTR |

  

|  |  |  |  |  |  |  |  |  |  |
| --- | --- | --- | --- | --- | --- | --- | --- | --- | --- |
| chr7:62540265-62540826 | | | 562 bp | | |  |  |  |  |
| Genome Start | Genome End | Strand | Div | Repeat Start | Repeat End | Repeat Left | Name | Family | Class |
| 62540265 | 62540453 | + | 21.3 | 51 | 241 | -83 | MER33 | MER1\_type | DNA |
| 62540454 | 62540750 | - | 12.1 | 1 | 301 | -11 | AluSx | Alu | SINE |
| 62540751 | 62540826 | + | 21.3 | 242 | 307 | -17 | MER33 | MER1\_type | DNA |

  

|  |  |  |  |  |  |  |  |  |  |
| --- | --- | --- | --- | --- | --- | --- | --- | --- | --- |
| chr7:62540868-62541538 | | | 671 bp | | |  |  |  |  |
| Genome Start | Genome End | Strand | Div | Repeat Start | Repeat End | Repeat Left | Name | Family | Class |
| 62540868 | 62541190 | - | 20.4 | 57 | 381 | 0 | THE1D | MaLR | LTR |
| 62541191 | 62541477 | - | 12.2 | 7 | 294 | -16 | AluSg | Alu | SINE |
| 62541478 | 62541538 | - | 20.4 | 1 | 56 | -325 | THE1D | MaLR | LTR |

  

|  |  |  |  |  |  |  |  |  |  |
| --- | --- | --- | --- | --- | --- | --- | --- | --- | --- |
| chr7:62543735-62544498 | | | 764 bp | | |  |  |  |  |
| Genome Start | Genome End | Strand | Div | Repeat Start | Repeat End | Repeat Left | Name | Family | Class |
| 62543735 | 62543835 | - | 20.8 | 3194 | 3292 | -2854 | L1PREC2 | L1 | LINE |
| 62543836 | 62544119 | - | 12.3 | 7 | 290 | -20 | AluSg | Alu | SINE |
| 62544120 | 62544498 | - | 20.8 | 2816 | 3193 | -2953 | L1PREC2 | L1 | LINE |

  

|  |  |  |  |  |  |  |  |  |  |
| --- | --- | --- | --- | --- | --- | --- | --- | --- | --- |
| chr7:62573985-62574376 | | | 327 bp | | |  |  |  |  |
| Genome Start | Genome End | Strand | Div | Repeat Start | Repeat End | Repeat Left | Name | Family | Class |
| 62573985 | 62574109 | - | 22.6 | 177 | 291 | -21 | AluJ | Alu | SINE |
| 62574159 | 62574191 | - | 6.1 | 5219 | 5251 | -895 | L1M4 | L1 | LINE |
| 62574208 | 62574376 | - | 18.9 | 3 | 175 | -137 | AluJ | Alu | SINE |

  

|  |  |  |  |  |  |  |  |  |  |
| --- | --- | --- | --- | --- | --- | --- | --- | --- | --- |
| chr7:62616631-62617326 | | | 696 bp | | |  |  |  |  |
| Genome Start | Genome End | Strand | Div | Repeat Start | Repeat End | Repeat Left | Name | Family | Class |
| 62616631 | 62616880 | - | 13.9 | 175 | 426 | 0 | MSTB | MaLR | LTR |
| 62616881 | 62617176 | + | 14.3 | 17 | 312 | 0 | AluY | Alu | SINE |
| 62617177 | 62617326 | - | 13.9 | 14 | 174 | -252 | MSTB | MaLR | LTR |

  

|  |  |  |  |  |  |  |  |  |  |
| --- | --- | --- | --- | --- | --- | --- | --- | --- | --- |
| chr7:62626761-62627444 | | | 681 bp | | |  |  |  |  |
| Genome Start | Genome End | Strand | Div | Repeat Start | Repeat End | Repeat Left | Name | Family | Class |
| 62626761 | 62626798 | + | 8.8 | 1 | 30 | -50 | MADE1 | Mariner | DNA |
| 62626799 | 62627092 | - | 10.6 | 1 | 293 | -20 | AluSq | Alu | SINE |
| 62627096 | 62627394 | - | 14.1 | 1 | 299 | -14 | AluSq | Alu | SINE |
| 62627395 | 62627444 | + | 8.8 | 31 | 80 | 0 | MADE1 | Mariner | DNA |

  

|  |  |  |  |  |  |  |  |  |  |
| --- | --- | --- | --- | --- | --- | --- | --- | --- | --- |
| chr7:62652313-62652883 | | | 534 bp | | |  |  |  |  |
| Genome Start | Genome End | Strand | Div | Repeat Start | Repeat End | Repeat Left | Name | Family | Class |
| 62652313 | 62652437 | + | 33.6 | 5 | 119 | -149 | MIRb | MIR | SINE |
| 62652438 | 62652734 | + | 13.2 | 1 | 296 | -16 | AluSx | Alu | SINE |
| 62652772 | 62652883 | + | 33.6 | 120 | 230 | -38 | MIRb | MIR | SINE |

  

|  |  |  |  |  |  |  |  |  |  |
| --- | --- | --- | --- | --- | --- | --- | --- | --- | --- |
| chr7:62677057-62677849 | | | 654 bp | | |  |  |  |  |
| Genome Start | Genome End | Strand | Div | Repeat Start | Repeat End | Repeat Left | Name | Family | Class |
| 62677057 | 62677085 | + | 29.7 | 11 | 39 | -552 | MLT1K | MaLR | LTR |
| 62677086 | 62677384 | - | 13.0 | 1 | 299 | -13 | AluSx | Alu | SINE |
| 62677385 | 62677524 | + | 29.7 | 40 | 180 | -411 | MLT1K | MaLR | LTR |
| 62677664 | 62677849 | + | 18.7 | 386 | 587 | -4 | MLT1K | MaLR | LTR |

  

|  |  |  |  |  |  |  |  |  |  |
| --- | --- | --- | --- | --- | --- | --- | --- | --- | --- |
| chr7:62680992-62681743 | | | 752 bp | | |  |  |  |  |
| Genome Start | Genome End | Strand | Div | Repeat Start | Repeat End | Repeat Left | Name | Family | Class |
| 62680992 | 62681206 | + | 33.3 | 2563 | 2791 | -628 | L2 | L2 | LINE |
| 62681207 | 62681506 | - | 11.0 | 1 | 300 | -13 | AluSq | Alu | SINE |
| 62681507 | 62681743 | + | 33.3 | 2792 | 3060 | -359 | L2 | L2 | LINE |

  

|  |  |  |  |  |  |  |  |  |  |
| --- | --- | --- | --- | --- | --- | --- | --- | --- | --- |
| chr7:62690156-62691915 | | | 1681 bp | | |  |  |  |  |
| Genome Start | Genome End | Strand | Div | Repeat Start | Repeat End | Repeat Left | Name | Family | Class |
| 62690156 | 62690197 | - | 21.1 | 415 | 442 | -19 | MER65C | ERV1 | LTR |
| 62690198 | 62690495 | + | 10.1 | 5 | 302 | -10 | AluSx | Alu | SINE |
| 62690496 | 62690588 | - | 21.1 | 317 | 414 | -47 | MER65C | ERV1 | LTR |
| 62690628 | 62690659 | - | 21.1 | 287 | 316 | -145 | MER65C | ERV1 | LTR |
| 62690660 | 62690819 | - | 13.3 | 142 | 294 | -18 | AluJo | Alu | SINE |
| 62690820 | 62691127 | - | 11.7 | 1 | 307 | -6 | AluSp | Alu | SINE |
| 62691128 | 62691139 | - | 13.3 | 135 | 141 | -171 | AluJo | Alu | SINE |
| 62691180 | 62691481 | - | 7.4 | 2 | 302 | -11 | AluSq | Alu | SINE |
| 62691482 | 62691617 | - | 13.3 | 3 | 134 | -178 | AluJo | Alu | SINE |
| 62691618 | 62691915 | - | 21.1 | 2 | 286 | -175 | MER65C | ERV1 | LTR |

  

|  |  |  |  |  |  |  |  |  |  |
| --- | --- | --- | --- | --- | --- | --- | --- | --- | --- |
| chr7:62698030-62698878 | | | 849 bp | | |  |  |  |  |
| Genome Start | Genome End | Strand | Div | Repeat Start | Repeat End | Repeat Left | Name | Family | Class |
| 62698030 | 62698202 | + | 12.7 | 4204 | 4366 | -1780 | L1MC | L1 | LINE |
| 62698203 | 62698498 | + | 10.5 | 1 | 296 | -13 | AluSc | Alu | SINE |
| 62698499 | 62698878 | + | 12.7 | 4367 | 4768 | -1378 | L1MC | L1 | LINE |

  

|  |  |  |  |  |  |  |  |  |  |
| --- | --- | --- | --- | --- | --- | --- | --- | --- | --- |
| chr7:62699934-62700496 | | | 563 bp | | |  |  |  |  |
| Genome Start | Genome End | Strand | Div | Repeat Start | Repeat End | Repeat Left | Name | Family | Class |
| 62699934 | 62700034 | + | 15.4 | 21 | 120 | -192 | AluJb | Alu | SINE |
| 62700035 | 62700328 | - | 8.8 | 1 | 294 | -17 | AluY | Alu | SINE |
| 62700329 | 62700496 | + | 15.4 | 121 | 278 | -34 | AluJb | Alu | SINE |

  

|  |  |  |  |  |  |  |  |  |  |
| --- | --- | --- | --- | --- | --- | --- | --- | --- | --- |
| chr7:62700710-62701330 | | | 621 bp | | |  |  |  |  |
| Genome Start | Genome End | Strand | Div | Repeat Start | Repeat End | Repeat Left | Name | Family | Class |
| 62700710 | 62700844 | + | 12.3 | 3 | 136 | -176 | AluSx | Alu | SINE |
| 62700845 | 62701145 | + | 6.8 | 1 | 293 | -18 | AluY | Alu | SINE |
| 62701146 | 62701330 | + | 12.3 | 137 | 312 | 0 | AluSx | Alu | SINE |

  

|  |  |  |  |  |  |  |  |  |  |
| --- | --- | --- | --- | --- | --- | --- | --- | --- | --- |
| chr7:62711879-62712594 | | | 716 bp | | |  |  |  |  |
| Genome Start | Genome End | Strand | Div | Repeat Start | Repeat End | Repeat Left | Name | Family | Class |
| 62711879 | 62711942 | - | 11.3 | 364 | 428 | 0 | MSTC | MaLR | LTR |
| 62711943 | 62712249 | + | 5.9 | 1 | 307 | -4 | AluY | Alu | SINE |
| 62712250 | 62712594 | - | 11.3 | 1 | 363 | -65 | MSTC | MaLR | LTR |

  

|  |  |  |  |  |  |  |  |  |  |
| --- | --- | --- | --- | --- | --- | --- | --- | --- | --- |
| chr7:62714692-62715312 | | | 590 bp | | |  |  |  |  |
| Genome Start | Genome End | Strand | Div | Repeat Start | Repeat End | Repeat Left | Name | Family | Class |
| 62714692 | 62714912 | - | 23.8 | 92 | 336 | -725 | MER63D | AcHobo | DNA |
| 62714913 | 62715189 | + | 7.9 | 1 | 277 | -32 | AluSc | Alu | SINE |
| 62715221 | 62715312 | - | 23.8 | 3 | 91 | -970 | MER63D | AcHobo | DNA |

  

|  |  |  |  |  |  |  |  |  |  |
| --- | --- | --- | --- | --- | --- | --- | --- | --- | --- |
| chr7:62720154-62720679 | | | 526 bp | | |  |  |  |  |
| Genome Start | Genome End | Strand | Div | Repeat Start | Repeat End | Repeat Left | Name | Family | Class |
| 62720154 | 62720285 | + | 20.9 | 1 | 126 | -186 | AluJo | Alu | SINE |
| 62720286 | 62720585 | + | 11.3 | 1 | 300 | -12 | AluSx | Alu | SINE |
| 62720586 | 62720679 | + | 20.9 | 127 | 220 | -92 | AluJo | Alu | SINE |

  

|  |  |  |  |  |  |  |  |  |  |
| --- | --- | --- | --- | --- | --- | --- | --- | --- | --- |
| chr7:62732486-62733096 | | | 611 bp | | |  |  |  |  |
| Genome Start | Genome End | Strand | Div | Repeat Start | Repeat End | Repeat Left | Name | Family | Class |
| 62732486 | 62732654 | - | 11.5 | 134 | 302 | -10 | AluSx | Alu | SINE |
| 62732655 | 62732976 | - | 5.4 | 1 | 298 | -13 | AluY | Alu | SINE |
| 62732977 | 62733096 | - | 11.5 | 15 | 133 | -179 | AluSx | Alu | SINE |

  

|  |  |  |  |  |  |  |  |  |  |
| --- | --- | --- | --- | --- | --- | --- | --- | --- | --- |
| chr7:62735028-62735736 | | | 646 bp | | |  |  |  |  |
| Genome Start | Genome End | Strand | Div | Repeat Start | Repeat End | Repeat Left | Name | Family | Class |
| 62735028 | 62735283 | - | 29.6 | 7812 | 8042 | 0 | L1MC4 | L1 | LINE |
| 62735285 | 62735588 | - | 9.5 | 1 | 306 | -6 | AluSx | Alu | SINE |
| 62735651 | 62735736 | - | 27.9 | 7532 | 7620 | -422 | L1MC4 | L1 | LINE |

  

|  |  |  |  |  |  |  |  |  |  |
| --- | --- | --- | --- | --- | --- | --- | --- | --- | --- |
| chr7:62752046-62753306 | | | 1207 bp | | |  |  |  |  |
| Genome Start | Genome End | Strand | Div | Repeat Start | Repeat End | Repeat Left | Name | Family | Class |
| 62752046 | 62752440 | + | 16.2 | 5360 | 5793 | -519 | L1MA9 | L1 | LINE |
| 62752441 | 62752524 | - | 18.3 | 158 | 239 | 0 | MER8 | MER2\_type | DNA |
| 62752525 | 62752656 | + | 21.0 | 6019 | 6150 | -162 | L1MA9 | L1 | LINE |
| 62752694 | 62752780 | + | 21.0 | 6151 | 6252 | -60 | L1MA9 | L1 | LINE |
| 62752781 | 62753081 | + | 18.7 | 1 | 305 | -7 | AluSx | Alu | SINE |
| 62753095 | 62753235 | + | 13.5 | 154 | 294 | -18 | AluJb | Alu | SINE |
| 62753240 | 62753306 | + | 19.4 | 6240 | 6306 | -6 | L1MA9 | L1 | LINE |

  

|  |  |  |  |  |  |  |  |  |  |
| --- | --- | --- | --- | --- | --- | --- | --- | --- | --- |
| chr7:62810237-62810651 | | | 415 bp | | |  |  |  |  |
| Genome Start | Genome End | Strand | Div | Repeat Start | Repeat End | Repeat Left | Name | Family | Class |
| 62810237 | 62810259 | - | 20.8 | 112 | 125 | -8 | FLAM\_A | Alu | SINE |
| 62810260 | 62810539 | + | 11.4 | 1 | 291 | -22 | AluSp | Alu | SINE |
| 62810540 | 62810651 | - | 20.8 | 1 | 111 | -22 | FLAM\_A | Alu | SINE |

  

|  |  |  |  |  |  |  |  |  |  |
| --- | --- | --- | --- | --- | --- | --- | --- | --- | --- |
| chr7:62816289-62817190 | | | 902 bp | | |  |  |  |  |
| Genome Start | Genome End | Strand | Div | Repeat Start | Repeat End | Repeat Left | Name | Family | Class |
| 62816289 | 62816469 | + | 29.3 | 104 | 301 | -5366 | ERVL-E | ERVL | LTR |
| 62816470 | 62816968 | + | 13.0 | 1 | 529 | 0 | MER1A | MER1\_type | DNA |
| 62816969 | 62817190 | + | 29.3 | 302 | 532 | -5173 | ERVL-E | ERVL | LTR |

  

|  |  |  |  |  |  |  |  |  |  |
| --- | --- | --- | --- | --- | --- | --- | --- | --- | --- |
| chr7:62827648-62829044 | | | 1397 bp | | |  |  |  |  |
| Genome Start | Genome End | Strand | Div | Repeat Start | Repeat End | Repeat Left | Name | Family | Class |
| 62827648 | 62827949 | - | 20.8 | 604 | 935 | 0 | MER21C | ERV1 | LTR |
| 62827950 | 62828254 | - | 18.4 | 1 | 299 | -13 | AluJo | Alu | SINE |
| 62828255 | 62828325 | - | 20.8 | 533 | 603 | -332 | MER21C | ERV1 | LTR |
| 62828326 | 62828635 | + | 18.4 | 3 | 295 | -17 | AluJb | Alu | SINE |
| 62828636 | 62829044 | - | 20.8 | 115 | 532 | -403 | MER21C | ERV1 | LTR |

  

|  |  |  |  |  |  |  |  |  |  |
| --- | --- | --- | --- | --- | --- | --- | --- | --- | --- |
| chr7:62878439-62879028 | | | 590 bp | | |  |  |  |  |
| Genome Start | Genome End | Strand | Div | Repeat Start | Repeat End | Repeat Left | Name | Family | Class |
| 62878439 | 62878590 | + | 19.3 | 1 | 145 | -167 | AluJo | Alu | SINE |
| 62878591 | 62878866 | + | 14.1 | 2 | 281 | -32 | AluSq | Alu | SINE |
| 62878867 | 62879028 | + | 19.3 | 146 | 301 | -11 | AluJo | Alu | SINE |

  

|  |  |  |  |  |  |  |  |  |  |
| --- | --- | --- | --- | --- | --- | --- | --- | --- | --- |
| chr7:62881879-62883269 | | | 1391 bp | | |  |  |  |  |
| Genome Start | Genome End | Strand | Div | Repeat Start | Repeat End | Repeat Left | Name | Family | Class |
| 62881879 | 62882010 | + | 15.9 | 11 | 144 | -168 | AluJo | Alu | SINE |
| 62882011 | 62882339 | + | 9.8 | 3 | 348 | -262 | LTR71B | ERV1 | LTR |
| 62882340 | 62882906 | + | 18.1 | 1 | 633 | 0 | LTR10E | ERV1 | LTR |
| 62882907 | 62883169 | + | 9.8 | 349 | 610 | 0 | LTR71B | ERV1 | LTR |
| 62883170 | 62883269 | + | 17.0 | 138 | 237 | -75 | AluJo | Alu | SINE |

  

|  |  |  |  |  |  |  |  |  |  |
| --- | --- | --- | --- | --- | --- | --- | --- | --- | --- |
| chr7:62926840-62927727 | | | 888 bp | | |  |  |  |  |
| Genome Start | Genome End | Strand | Div | Repeat Start | Repeat End | Repeat Left | Name | Family | Class |
| 62926840 | 62927002 | + | 17.6 | 2432 | 2599 | -3783 | L1M4c | L1 | LINE |
| 62927003 | 62927313 | + | 9.9 | 1 | 304 | -8 | AluSx | Alu | SINE |
| 62927314 | 62927727 | + | 18.4 | 2600 | 3036 | -3346 | L1M4c | L1 | LINE |

  

|  |  |  |  |  |  |  |  |  |  |
| --- | --- | --- | --- | --- | --- | --- | --- | --- | --- |
| chr7:62955993-62956727 | | | 735 bp | | |  |  |  |  |
| Genome Start | Genome End | Strand | Div | Repeat Start | Repeat End | Repeat Left | Name | Family | Class |
| 62955993 | 62956387 | - | 30.0 | 168 | 556 | 0 | LTR38 | ERV1 | LTR |
| 62956388 | 62956688 | - | 13.7 | 1 | 300 | -12 | AluJo | Alu | SINE |
| 62956689 | 62956727 | - | 30.0 | 126 | 167 | -389 | LTR38 | ERV1 | LTR |

  

|  |  |  |  |  |  |  |  |  |  |
| --- | --- | --- | --- | --- | --- | --- | --- | --- | --- |
| chr7:63177170-63181730 | | | 4561 bp | | |  |  |  |  |
| Genome Start | Genome End | Strand | Div | Repeat Start | Repeat End | Repeat Left | Name | Family | Class |
| 63177170 | 63180406 | - | 24.7 | 1035 | 4506 | -3635 | L1PREC2 | L1 | LINE |
| 63180407 | 63180712 | + | 12.4 | 1 | 301 | -11 | AluSx | Alu | SINE |
| 63180713 | 63181730 | - | 26.1 | 24 | 1034 | -7121 | L1PREC2 | L1 | LINE |

  

|  |  |  |  |  |  |  |  |  |  |
| --- | --- | --- | --- | --- | --- | --- | --- | --- | --- |
| chr7:63233219-63234228 | | | 1010 bp | | |  |  |  |  |
| Genome Start | Genome End | Strand | Div | Repeat Start | Repeat End | Repeat Left | Name | Family | Class |
| 63233219 | 63233562 | + | 21.8 | 1 | 362 | -365 | LTR8A | ERV1 | LTR |
| 63233563 | 63233852 | - | 14.5 | 1 | 296 | -16 | AluSx | Alu | SINE |
| 63233853 | 63234228 | + | 23.1 | 363 | 726 | -1 | LTR8A | ERV1 | LTR |

  

|  |  |  |  |  |  |  |  |  |  |
| --- | --- | --- | --- | --- | --- | --- | --- | --- | --- |
| chr7:63303233-63303806 | | | 574 bp | | |  |  |  |  |
| Genome Start | Genome End | Strand | Div | Repeat Start | Repeat End | Repeat Left | Name | Family | Class |
| 63303233 | 63303435 | + | 23.8 | 36 | 241 | -83 | MER33 | MER1\_type | DNA |
| 63303436 | 63303732 | - | 16.2 | 1 | 301 | -11 | AluSx | Alu | SINE |
| 63303733 | 63303806 | + | 23.8 | 242 | 307 | -17 | MER33 | MER1\_type | DNA |

  

|  |  |  |  |  |  |  |  |  |  |
| --- | --- | --- | --- | --- | --- | --- | --- | --- | --- |
| chr7:63303848-63304531 | | | 684 bp | | |  |  |  |  |
| Genome Start | Genome End | Strand | Div | Repeat Start | Repeat End | Repeat Left | Name | Family | Class |
| 63303848 | 63304182 | - | 19.9 | 57 | 381 | 0 | THE1D | MaLR | LTR |
| 63304183 | 63304474 | - | 8.6 | 3 | 294 | -16 | AluSg | Alu | SINE |
| 63304475 | 63304531 | - | 19.9 | 1 | 56 | -325 | THE1D | MaLR | LTR |

  

|  |  |  |  |  |  |  |  |  |  |
| --- | --- | --- | --- | --- | --- | --- | --- | --- | --- |
| chr7:64109879-64111239 | | | 1361 bp | | |  |  |  |  |
| Genome Start | Genome End | Strand | Div | Repeat Start | Repeat End | Repeat Left | Name | Family | Class |
| 64109879 | 64110775 | + | 12.5 | 5254 | 6144 | -161 | L1MA3 | L1 | LINE |
| 64110776 | 64111077 | + | 14.9 | 1 | 302 | -10 | AluSx | Alu | SINE |
| 64111078 | 64111239 | + | 12.5 | 6145 | 6304 | -1 | L1MA3 | L1 | LINE |

  

|  |  |  |  |  |  |  |  |  |  |
| --- | --- | --- | --- | --- | --- | --- | --- | --- | --- |
| chr7:64116109-64116760 | | | 649 bp | | |  |  |  |  |
| Genome Start | Genome End | Strand | Div | Repeat Start | Repeat End | Repeat Left | Name | Family | Class |
| 64116109 | 64116199 | + | 5.5 | 1 | 91 | -519 | LTR71B | ERV1 | LTR |
| 64116202 | 64116506 | + | 13.0 | 1 | 301 | -10 | AluY | Alu | SINE |
| 64116508 | 64116760 | + | 9.6 | 360 | 610 | 0 | LTR71B | ERV1 | LTR |

  

|  |  |  |  |  |  |  |  |  |  |
| --- | --- | --- | --- | --- | --- | --- | --- | --- | --- |
| chr7:64119423-64120032 | | | 610 bp | | |  |  |  |  |
| Genome Start | Genome End | Strand | Div | Repeat Start | Repeat End | Repeat Left | Name | Family | Class |
| 64119423 | 64119583 | - | 17.3 | 142 | 297 | -15 | AluJo | Alu | SINE |
| 64119584 | 64119887 | - | 5.4 | 1 | 296 | -15 | AluY | Alu | SINE |
| 64119888 | 64120032 | - | 17.3 | 1 | 141 | -171 | AluJo | Alu | SINE |

  

|  |  |  |  |  |  |  |  |  |  |
| --- | --- | --- | --- | --- | --- | --- | --- | --- | --- |
| chr7:64133981-64134819 | | | 839 bp | | |  |  |  |  |
| Genome Start | Genome End | Strand | Div | Repeat Start | Repeat End | Repeat Left | Name | Family | Class |
| 64133981 | 64134056 | + | 21.1 | 40 | 99 | -463 | MLT1F2 | MaLR | LTR |
| 64134057 | 64134367 | - | 15.1 | 1 | 312 | 0 | AluSx | Alu | SINE |
| 64134368 | 64134819 | + | 21.1 | 100 | 562 | 0 | MLT1F2 | MaLR | LTR |

  

|  |  |  |  |  |  |  |  |  |  |
| --- | --- | --- | --- | --- | --- | --- | --- | --- | --- |
| chr7:64140726-64141152 | | | 427 bp | | |  |  |  |  |
| Genome Start | Genome End | Strand | Div | Repeat Start | Repeat End | Repeat Left | Name | Family | Class |
| 64140726 | 64140783 | + | 30.2 | 103 | 158 | -104 | MIR | MIR | SINE |
| 64140784 | 64141088 | + | 16.2 | 1 | 303 | -9 | AluJb | Alu | SINE |
| 64141089 | 64141152 | + | 30.2 | 159 | 225 | -37 | MIR | MIR | SINE |

  

|  |  |  |  |  |  |  |  |  |  |
| --- | --- | --- | --- | --- | --- | --- | --- | --- | --- |
| chr7:64202498-64203472 | | | 975 bp | | |  |  |  |  |
| Genome Start | Genome End | Strand | Div | Repeat Start | Repeat End | Repeat Left | Name | Family | Class |
| 64202498 | 64202677 | + | 24.1 | 2597 | 2779 | -640 | L2 | L2 | LINE |
| 64202678 | 64202980 | - | 11.6 | 1 | 301 | -11 | AluSx | Alu | SINE |
| 64202981 | 64203472 | + | 25.3 | 2780 | 3417 | -2 | L2 | L2 | LINE |

  

|  |  |  |  |  |  |  |  |  |  |
| --- | --- | --- | --- | --- | --- | --- | --- | --- | --- |
| chr7:64206089-64207278 | | | 1143 bp | | |  |  |  |  |
| Genome Start | Genome End | Strand | Div | Repeat Start | Repeat End | Repeat Left | Name | Family | Class |
| 64206089 | 64206503 | + | 24.3 | 1 | 430 | -471 | MER54A | ERVL | LTR |
| 64206542 | 64206876 | - | 10.9 | 7 | 364 | 0 | THE1B | MaLR | LTR |
| 64206886 | 64207278 | + | 20.9 | 490 | 901 | 0 | MER54A | ERVL | LTR |

  

|  |  |  |  |  |  |  |  |  |  |
| --- | --- | --- | --- | --- | --- | --- | --- | --- | --- |
| chr7:64209737-64210540 | | | 804 bp | | |  |  |  |  |
| Genome Start | Genome End | Strand | Div | Repeat Start | Repeat End | Repeat Left | Name | Family | Class |
| 64209737 | 64209977 | - | 24.2 | 275 | 505 | 0 | MLT1D | MaLR | LTR |
| 64209978 | 64210269 | - | 12.8 | 1 | 292 | -17 | AluSc | Alu | SINE |
| 64210270 | 64210540 | - | 24.2 | 5 | 274 | -231 | MLT1D | MaLR | LTR |

  

|  |  |  |  |  |  |  |  |  |  |
| --- | --- | --- | --- | --- | --- | --- | --- | --- | --- |
| chr7:64211275-64212786 | | | 1141 bp | | |  |  |  |  |
| Genome Start | Genome End | Strand | Div | Repeat Start | Repeat End | Repeat Left | Name | Family | Class |
| 64211275 | 64211671 | - | 26.9 | 3035 | 3438 | -3503 | L1MCa | L1 | LINE |
| 64211729 | 64211872 | - | 28.8 | 1854 | 1995 | -5133 | L1MCa | L1 | LINE |
| 64212104 | 64212383 | - | 21.9 | 86 | 365 | 0 | MLT1A0 | MaLR | LTR |
| 64212467 | 64212786 | - | 30.0 | 32 | 388 | -6035 | L1MCa | L1 | LINE |

  

|  |  |  |  |  |  |  |  |  |  |
| --- | --- | --- | --- | --- | --- | --- | --- | --- | --- |
| chr7:64213673-64215166 | | | 1494 bp | | |  |  |  |  |
| Genome Start | Genome End | Strand | Div | Repeat Start | Repeat End | Repeat Left | Name | Family | Class |
| 64213673 | 64214061 | + | 21.8 | 2146 | 2623 | -4711 | L1M4b | L1 | LINE |
| 64214062 | 64214361 | + | 8.8 | 1 | 283 | -28 | AluY | Alu | SINE |
| 64214362 | 64215166 | + | 22.6 | 2624 | 3354 | -3688 | L1M4b | L1 | LINE |

  

|  |  |  |  |  |  |  |  |  |  |
| --- | --- | --- | --- | --- | --- | --- | --- | --- | --- |
| chr7:64250992-64251433 | | | 442 bp | | |  |  |  |  |
| Genome Start | Genome End | Strand | Div | Repeat Start | Repeat End | Repeat Left | Name | Family | Class |
| 64250992 | 64251102 | - | 20.9 | 36 | 138 | -5 | FLAM\_C | Alu | SINE |
| 64251103 | 64251397 | + | 9.6 | 1 | 293 | -16 | AluSc | Alu | SINE |
| 64251398 | 64251433 | - | 20.9 | 1 | 35 | -108 | FLAM\_C | Alu | SINE |

  

|  |  |  |  |  |  |  |  |  |  |
| --- | --- | --- | --- | --- | --- | --- | --- | --- | --- |
| chr7:64251988-64252730 | | | 743 bp | | |  |  |  |  |
| Genome Start | Genome End | Strand | Div | Repeat Start | Repeat End | Repeat Left | Name | Family | Class |
| 64251988 | 64252018 | - | 28.7 | 225 | 255 | -13 | MIRb | MIR | SINE |
| 64252019 | 64252350 | + | 17.9 | 1 | 338 | -1 | MER1B | MER1\_type | DNA |
| 64252351 | 64252440 | - | 28.7 | 151 | 224 | -44 | MIRb | MIR | SINE |
| 64252441 | 64252571 | + | 13.6 | 5 | 129 | -4 | FLAM\_C | Alu | SINE |
| 64252572 | 64252730 | - | 29.0 | 27 | 150 | -118 | MIRb | MIR | SINE |

  

|  |  |  |  |  |  |  |  |  |  |
| --- | --- | --- | --- | --- | --- | --- | --- | --- | --- |
| chr7:64256187-64257768 | | | 1618 bp | | |  |  |  |  |
| Genome Start | Genome End | Strand | Div | Repeat Start | Repeat End | Repeat Left | Name | Family | Class |
| 64256187 | 64256538 | - | 20.4 | 5936 | 6290 | -10 | L1MA6 | L1 | LINE |
| 64256532 | 64257686 | - | 6.7 | 4998 | 6151 | -3 | L1PA7 | L1 | LINE |
| 64257658 | 64257768 | - | 18.4 | 5828 | 5939 | -352 | L1MA6 | L1 | LINE |

  

|  |  |  |  |  |  |  |  |  |  |
| --- | --- | --- | --- | --- | --- | --- | --- | --- | --- |
| chr7:64264257-64265387 | | | 707 bp | | |  |  |  |  |
| Genome Start | Genome End | Strand | Div | Repeat Start | Repeat End | Repeat Left | Name | Family | Class |
| 64264257 | 64264395 | + | 35.0 | 99 | 250 | -18 | MIRb | MIR | SINE |
| 64264820 | 64265047 | + | 24.5 | 3 | 238 | -28 | MIR | MIR | SINE |
| 64265048 | 64265356 | + | 9.8 | 1 | 308 | -5 | AluSq | Alu | SINE |
| 64265357 | 64265387 | + | 29.3 | 239 | 265 | -3 | MIRb | MIR | SINE |

  

|  |  |  |  |  |  |  |  |  |  |
| --- | --- | --- | --- | --- | --- | --- | --- | --- | --- |
| chr7:64268625-64271653 | | | 3026 bp | | |  |  |  |  |
| Genome Start | Genome End | Strand | Div | Repeat Start | Repeat End | Repeat Left | Name | Family | Class |
| 64268625 | 64268682 | - | 17.3 | 1670 | 1732 | -4668 | L1MEc | L1 | LINE |
| 64268683 | 64268999 | + | 13.7 | 1 | 315 | 0 | AluSq | Alu | SINE |
| 64269000 | 64269060 | - | 17.3 | 1596 | 1669 | -4731 | L1MEc | L1 | LINE |
| 64269061 | 64269355 | - | 18.0 | 1 | 295 | -17 | AluJo | Alu | SINE |
| 64269356 | 64269660 | - | 17.3 | 1293 | 1595 | -4805 | L1MEc | L1 | LINE |
| 64269661 | 64269923 | - | 16.0 | 1 | 263 | -50 | AluSq | Alu | SINE |
| 64269924 | 64270218 | - | 17.3 | 1019 | 1292 | -5108 | L1MEc | L1 | LINE |
| 64270219 | 64270433 | + | 14.9 | 85 | 299 | -13 | AluJo | Alu | SINE |
| 64270437 | 64270892 | - | 22.7 | 559 | 1028 | -5372 | L1MEc | L1 | LINE |
| 64270893 | 64271192 | - | 18.0 | 1 | 301 | -11 | AluJb | Alu | SINE |
| 64271193 | 64271653 | - | 22.7 | 52 | 558 | -5842 | L1MEc | L1 | LINE |

  

|  |  |  |  |  |  |  |  |  |  |
| --- | --- | --- | --- | --- | --- | --- | --- | --- | --- |
| chr7:64278418-64279236 | | | 732 bp | | |  |  |  |  |
| Genome Start | Genome End | Strand | Div | Repeat Start | Repeat End | Repeat Left | Name | Family | Class |
| 64278418 | 64278758 | + | 38.1 | 2911 | 3277 | -101 | L2 | L2 | LINE |
| 64278841 | 64279153 | - | 12.1 | 1 | 312 | 0 | AluSx | Alu | SINE |
| 64279159 | 64279236 | + | 29.9 | 3299 | 3376 | -2 | L2 | L2 | LINE |

  

|  |  |  |  |  |  |  |  |  |  |
| --- | --- | --- | --- | --- | --- | --- | --- | --- | --- |
| chr7:64282258-64282991 | | | 734 bp | | |  |  |  |  |
| Genome Start | Genome End | Strand | Div | Repeat Start | Repeat End | Repeat Left | Name | Family | Class |
| 64282258 | 64282339 | - | 25.9 | 402 | 522 | -93 | MLT1L | MaLR | LTR |
| 64282340 | 64282636 | - | 10.4 | 1 | 299 | -10 | AluSc | Alu | SINE |
| 64282637 | 64282991 | - | 25.9 | 17 | 401 | -214 | MLT1L | MaLR | LTR |

  

|  |  |  |  |  |  |  |  |  |  |
| --- | --- | --- | --- | --- | --- | --- | --- | --- | --- |
| chr7:64287226-64287990 | | | 765 bp | | |  |  |  |  |
| Genome Start | Genome End | Strand | Div | Repeat Start | Repeat End | Repeat Left | Name | Family | Class |
| 64287226 | 64287331 | - | 26.4 | 358 | 482 | -29 | MLT1J | MaLR | LTR |
| 64287332 | 64287640 | - | 13.9 | 1 | 301 | -11 | AluJb | Alu | SINE |
| 64287641 | 64287990 | - | 26.4 | 3 | 357 | -154 | MLT1J | MaLR | LTR |

  

|  |  |  |  |  |  |  |  |  |  |
| --- | --- | --- | --- | --- | --- | --- | --- | --- | --- |
| chr7:64295909-64297319 | | | 1411 bp | | |  |  |  |  |
| Genome Start | Genome End | Strand | Div | Repeat Start | Repeat End | Repeat Left | Name | Family | Class |
| 64295909 | 64296573 | - | 14.5 | 566 | 1242 | -13 | Ricksha\_b | MuDR | DNA |
| 64296574 | 64296881 | - | 12.8 | 1 | 306 | -6 | AluSx | Alu | SINE |
| 64296882 | 64297319 | - | 13.3 | 135 | 565 | -690 | Ricksha\_b | MuDR | DNA |

  

|  |  |  |  |  |  |  |  |  |  |
| --- | --- | --- | --- | --- | --- | --- | --- | --- | --- |
| chr7:64302610-64303202 | | | 593 bp | | |  |  |  |  |
| Genome Start | Genome End | Strand | Div | Repeat Start | Repeat End | Repeat Left | Name | Family | Class |
| 64302610 | 64302822 | + | 25.3 | 2 | 189 | -73 | MIR | MIR | SINE |
| 64302823 | 64303125 | - | 14.2 | 1 | 304 | -8 | AluSx | Alu | SINE |
| 64303126 | 64303202 | + | 25.3 | 190 | 262 | 0 | MIR | MIR | SINE |

  

|  |  |  |  |  |  |  |  |  |  |
| --- | --- | --- | --- | --- | --- | --- | --- | --- | --- |
| chr7:64537279-64539179 | | | 1696 bp | | |  |  |  |  |
| Genome Start | Genome End | Strand | Div | Repeat Start | Repeat End | Repeat Left | Name | Family | Class |
| 64537279 | 64537433 | + | 36.8 | 2394 | 2552 | -867 | L2 | L2 | LINE |
| 64537434 | 64537738 | + | 16.7 | 1 | 299 | -13 | AluJo | Alu | SINE |
| 64537739 | 64538050 | + | 9.7 | 14 | 311 | -2 | AluSq | Alu | SINE |
| 64538051 | 64538461 | + | 36.8 | 2553 | 2963 | -456 | L2 | L2 | LINE |
| 64538482 | 64538672 | + | 18.7 | 7 | 194 | -25 | MER20 | MER1\_type | DNA |
| 64538858 | 64539179 | + | 29.7 | 3061 | 3375 | -3 | L2 | L2 | LINE |

  

|  |  |  |  |  |  |  |  |  |  |
| --- | --- | --- | --- | --- | --- | --- | --- | --- | --- |
| chr7:64562548-64563177 | | | 630 bp | | |  |  |  |  |
| Genome Start | Genome End | Strand | Div | Repeat Start | Repeat End | Repeat Left | Name | Family | Class |
| 64562548 | 64562780 | - | 24.6 | 107 | 324 | 0 | MER5C | MER1\_type | DNA |
| 64562781 | 64563076 | + | 17.5 | 1 | 294 | -18 | AluJb | Alu | SINE |
| 64563077 | 64563177 | - | 24.6 | 1 | 106 | -218 | MER5C | MER1\_type | DNA |

  

|  |  |  |  |  |  |  |  |  |  |
| --- | --- | --- | --- | --- | --- | --- | --- | --- | --- |
| chr7:64618099-64619595 | | | 1497 bp | | |  |  |  |  |
| Genome Start | Genome End | Strand | Div | Repeat Start | Repeat End | Repeat Left | Name | Family | Class |
| 64618099 | 64618905 | - | 20.3 | 2624 | 3651 | -3688 | L1M4b | L1 | LINE |
| 64618906 | 64619205 | - | 8.1 | 1 | 283 | -28 | AluY | Alu | SINE |
| 64619206 | 64619595 | - | 21.7 | 2146 | 2623 | -4711 | L1M4b | L1 | LINE |

  

|  |  |  |  |  |  |  |  |  |  |
| --- | --- | --- | --- | --- | --- | --- | --- | --- | --- |
| chr7:64620480-64621988 | | | 1134 bp | | |  |  |  |  |
| Genome Start | Genome End | Strand | Div | Repeat Start | Repeat End | Repeat Left | Name | Family | Class |
| 64620480 | 64620799 | + | 30.8 | 32 | 388 | -6035 | L1MCc | L1 | LINE |
| 64620883 | 64621162 | + | 23.7 | 86 | 365 | 0 | MLT1A0 | MaLR | LTR |
| 64621398 | 64621537 | + | 28.9 | 1858 | 1995 | -5133 | L1MCc | L1 | LINE |
| 64621595 | 64621988 | + | 26.8 | 3035 | 3438 | -3503 | L1MCc | L1 | LINE |

  

|  |  |  |  |  |  |  |  |  |  |
| --- | --- | --- | --- | --- | --- | --- | --- | --- | --- |
| chr7:64624497-64627526 | | | 3027 bp | | |  |  |  |  |
| Genome Start | Genome End | Strand | Div | Repeat Start | Repeat End | Repeat Left | Name | Family | Class |
| 64624497 | 64624957 | + | 23.7 | 52 | 558 | -5842 | L1MEc | L1 | LINE |
| 64624958 | 64625259 | + | 18.2 | 1 | 303 | -9 | AluJb | Alu | SINE |
| 64625260 | 64625714 | + | 23.7 | 559 | 1028 | -5372 | L1MEc | L1 | LINE |
| 64625718 | 64625931 | - | 15.5 | 85 | 298 | -14 | AluJo | Alu | SINE |
| 64625932 | 64626230 | + | 17.5 | 1017 | 1290 | -5110 | L1MEc | L1 | LINE |
| 64626231 | 64626493 | + | 15.6 | 1 | 263 | -50 | AluSq | Alu | SINE |
| 64626494 | 64626798 | + | 17.5 | 1291 | 1593 | -4807 | L1MEc | L1 | LINE |
| 64626799 | 64627113 | + | 17.3 | 1 | 306 | -6 | AluJo | Alu | SINE |
| 64627114 | 64627155 | + | 17.5 | 1594 | 1666 | -4734 | L1MEc | L1 | LINE |
| 64627156 | 64627468 | - | 13.7 | 1 | 311 | 0 | AluSq | Alu | SINE |
| 64627469 | 64627526 | + | 17.5 | 1667 | 1729 | -4671 | L1MEc | L1 | LINE |

  

|  |  |  |  |  |  |  |  |  |  |
| --- | --- | --- | --- | --- | --- | --- | --- | --- | --- |
| chr7:64659957-64660716 | | | 491 bp | | |  |  |  |  |
| Genome Start | Genome End | Strand | Div | Repeat Start | Repeat End | Repeat Left | Name | Family | Class |
| 64659957 | 64660064 | + | 38.0 | 1960 | 2072 | -1347 | L2 | L2 | LINE |
| 64660309 | 64660486 | - | 19.7 | 2 | 187 | -22 | MER3 | MER1\_type | DNA |
| 64660512 | 64660716 | + | 38.9 | 3024 | 3218 | -160 | L2 | L2 | LINE |

  

|  |  |  |  |  |  |  |  |  |  |
| --- | --- | --- | --- | --- | --- | --- | --- | --- | --- |
| chr7:64683392-64684881 | | | 1133 bp | | |  |  |  |  |
| Genome Start | Genome End | Strand | Div | Repeat Start | Repeat End | Repeat Left | Name | Family | Class |
| 64683392 | 64683689 | + | 26.1 | 54 | 388 | -6035 | L1MCc | L1 | LINE |
| 64683759 | 64684052 | + | 23.3 | 73 | 365 | 0 | MLT1A0 | MaLR | LTR |
| 64684284 | 64684427 | + | 28.8 | 1854 | 1995 | -5133 | L1MCc | L1 | LINE |
| 64684485 | 64684881 | + | 26.9 | 3035 | 3438 | -3503 | L1MCc | L1 | LINE |

  

|  |  |  |  |  |  |  |  |  |  |
| --- | --- | --- | --- | --- | --- | --- | --- | --- | --- |
| chr7:64685634-64686442 | | | 809 bp | | |  |  |  |  |
| Genome Start | Genome End | Strand | Div | Repeat Start | Repeat End | Repeat Left | Name | Family | Class |
| 64685634 | 64685904 | + | 24.0 | 5 | 274 | -231 | MLT1D | MaLR | LTR |
| 64685905 | 64686201 | + | 12.5 | 1 | 299 | -12 | AluY | Alu | SINE |
| 64686202 | 64686442 | + | 24.0 | 275 | 505 | 0 | MLT1D | MaLR | LTR |

  

|  |  |  |  |  |  |  |  |  |  |
| --- | --- | --- | --- | --- | --- | --- | --- | --- | --- |
| chr7:64688903-64690092 | | | 1143 bp | | |  |  |  |  |
| Genome Start | Genome End | Strand | Div | Repeat Start | Repeat End | Repeat Left | Name | Family | Class |
| 64688903 | 64689295 | - | 21.2 | 490 | 901 | 0 | MER54A | ERVL | LTR |
| 64689305 | 64689639 | + | 10.9 | 7 | 364 | 0 | THE1B | MaLR | LTR |
| 64689678 | 64690092 | - | 23.9 | 1 | 430 | -471 | MER54A | ERVL | LTR |

  

|  |  |  |  |  |  |  |  |  |  |
| --- | --- | --- | --- | --- | --- | --- | --- | --- | --- |
| chr7:64694446-64695137 | | | 692 bp | | |  |  |  |  |
| Genome Start | Genome End | Strand | Div | Repeat Start | Repeat End | Repeat Left | Name | Family | Class |
| 64694446 | 64694477 | + | 18.1 | 1 | 34 | -561 | LTR49 | ERV1 | LTR |
| 64694478 | 64694755 | - | 12.6 | 14 | 292 | -21 | AluSq | Alu | SINE |
| 64694756 | 64695137 | + | 18.1 | 35 | 426 | -169 | LTR49 | ERV1 | LTR |

  

|  |  |  |  |  |  |  |  |  |  |
| --- | --- | --- | --- | --- | --- | --- | --- | --- | --- |
| chr7:64746607-64747366 | | | 491 bp | | |  |  |  |  |
| Genome Start | Genome End | Strand | Div | Repeat Start | Repeat End | Repeat Left | Name | Family | Class |
| 64746607 | 64746811 | - | 38.9 | 3024 | 3218 | -160 | L2 | L2 | LINE |
| 64746837 | 64747014 | + | 19.7 | 2 | 187 | -22 | MER3 | MER1\_type | DNA |
| 64747259 | 64747366 | - | 38.0 | 1960 | 2072 | -1347 | L2 | L2 | LINE |

  

|  |  |  |  |  |  |  |  |  |  |
| --- | --- | --- | --- | --- | --- | --- | --- | --- | --- |
| chr7:64762191-64762632 | | | 442 bp | | |  |  |  |  |
| Genome Start | Genome End | Strand | Div | Repeat Start | Repeat End | Repeat Left | Name | Family | Class |
| 64762191 | 64762301 | - | 20.3 | 36 | 138 | -5 | FLAM\_C | Alu | SINE |
| 64762302 | 64762597 | + | 9.6 | 1 | 294 | -15 | AluSc | Alu | SINE |
| 64762598 | 64762632 | - | 20.3 | 2 | 35 | -108 | FLAM\_C | Alu | SINE |

  

|  |  |  |  |  |  |  |  |  |  |
| --- | --- | --- | --- | --- | --- | --- | --- | --- | --- |
| chr7:64763188-64763920 | | | 708 bp | | |  |  |  |  |
| Genome Start | Genome End | Strand | Div | Repeat Start | Repeat End | Repeat Left | Name | Family | Class |
| 64763188 | 64763218 | - | 28.6 | 225 | 255 | -13 | MIRb | MIR | SINE |
| 64763219 | 64763550 | + | 17.3 | 1 | 338 | -1 | MER1B | MER1\_type | DNA |
| 64763551 | 64763635 | - | 28.6 | 151 | 224 | -44 | MIRb | MIR | SINE |
| 64763641 | 64763768 | + | 13.8 | 5 | 127 | -16 | FLAM\_C | Alu | SINE |
| 64763789 | 64763920 | - | 30.5 | 27 | 165 | -103 | MIRb | MIR | SINE |

  

|  |  |  |  |  |  |  |  |  |  |
| --- | --- | --- | --- | --- | --- | --- | --- | --- | --- |
| chr7:64767389-64768972 | | | 1620 bp | | |  |  |  |  |
| Genome Start | Genome End | Strand | Div | Repeat Start | Repeat End | Repeat Left | Name | Family | Class |
| 64767389 | 64767740 | - | 20.6 | 5936 | 6290 | -10 | L1MA6 | L1 | LINE |
| 64767734 | 64768890 | - | 6.8 | 4998 | 6151 | -3 | L1PA7 | L1 | LINE |
| 64768862 | 64768972 | - | 17.4 | 5828 | 5939 | -352 | L1MA6 | L1 | LINE |

  

|  |  |  |  |  |  |  |  |  |  |
| --- | --- | --- | --- | --- | --- | --- | --- | --- | --- |
| chr7:64813103-64813696 | | | 594 bp | | |  |  |  |  |
| Genome Start | Genome End | Strand | Div | Repeat Start | Repeat End | Repeat Left | Name | Family | Class |
| 64813103 | 64813315 | + | 25.7 | 2 | 189 | -73 | MIR | MIR | SINE |
| 64813316 | 64813619 | - | 14.8 | 1 | 304 | -8 | AluSx | Alu | SINE |
| 64813620 | 64813696 | + | 25.7 | 190 | 262 | 0 | MIR | MIR | SINE |

  

|  |  |  |  |  |  |  |  |  |  |
| --- | --- | --- | --- | --- | --- | --- | --- | --- | --- |
| chr7:64869990-64870402 | | | 413 bp | | |  |  |  |  |
| Genome Start | Genome End | Strand | Div | Repeat Start | Repeat End | Repeat Left | Name | Family | Class |
| 64869990 | 64870023 | - | 24.0 | 122 | 155 | -113 | MIRb | MIR | SINE |
| 64870024 | 64870334 | + | 14.2 | 1 | 310 | -2 | AluJb | Alu | SINE |
| 64870335 | 64870402 | - | 24.0 | 60 | 121 | -147 | MIRb | MIR | SINE |

  

|  |  |  |  |  |  |  |  |  |  |
| --- | --- | --- | --- | --- | --- | --- | --- | --- | --- |
| chr7:64874942-64875524 | | | 583 bp | | |  |  |  |  |
| Genome Start | Genome End | Strand | Div | Repeat Start | Repeat End | Repeat Left | Name | Family | Class |
| 64874942 | 64875098 | - | 14.0 | 137 | 290 | -22 | AluSx | Alu | SINE |
| 64875099 | 64875388 | - | 7.9 | 1 | 300 | -13 | AluSp | Alu | SINE |
| 64875389 | 64875524 | - | 14.0 | 1 | 136 | -176 | AluSx | Alu | SINE |

  

|  |  |  |  |  |  |  |  |  |  |
| --- | --- | --- | --- | --- | --- | --- | --- | --- | --- |
| chr7:64912864-64913502 | | | 639 bp | | |  |  |  |  |
| Genome Start | Genome End | Strand | Div | Repeat Start | Repeat End | Repeat Left | Name | Family | Class |
| 64912864 | 64912997 | + | 18.3 | 1 | 128 | -184 | AluJo | Alu | SINE |
| 64912998 | 64913308 | + | 13.5 | 1 | 311 | -1 | AluSx | Alu | SINE |
| 64913309 | 64913502 | + | 18.3 | 129 | 312 | 0 | AluJo | Alu | SINE |

  

|  |  |  |  |  |  |  |  |  |  |
| --- | --- | --- | --- | --- | --- | --- | --- | --- | --- |
| chr7:64922190-64922781 | | | 592 bp | | |  |  |  |  |
| Genome Start | Genome End | Strand | Div | Repeat Start | Repeat End | Repeat Left | Name | Family | Class |
| 64922190 | 64922325 | + | 17.6 | 1 | 133 | -179 | AluJo | Alu | SINE |
| 64922326 | 64922619 | + | 11.6 | 1 | 294 | -15 | AluSg1 | Alu | SINE |
| 64922620 | 64922781 | + | 17.6 | 134 | 295 | -17 | AluJo | Alu | SINE |

  

|  |  |  |  |  |  |  |  |  |  |
| --- | --- | --- | --- | --- | --- | --- | --- | --- | --- |
| chr7:64925279-64926449 | | | 1133 bp | | |  |  |  |  |
| Genome Start | Genome End | Strand | Div | Repeat Start | Repeat End | Repeat Left | Name | Family | Class |
| 64925279 | 64925903 | - | 21.3 | 5552 | 6173 | -5 | L1MB8 | L1 | LINE |
| 64925904 | 64926014 | + | 16.2 | 4 | 114 | -19 | FLAM\_A | Alu | SINE |
| 64926053 | 64926121 | - | 15.4 | 5503 | 5566 | -608 | L1MB8 | L1 | LINE |
| 64926122 | 64926418 | - | 17.3 | 1 | 296 | -16 | AluJb | Alu | SINE |
| 64926419 | 64926449 | - | 15.4 | 5476 | 5502 | -672 | L1MB8 | L1 | LINE |

  

|  |  |  |  |  |  |  |  |  |  |
| --- | --- | --- | --- | --- | --- | --- | --- | --- | --- |
| chr7:64926508-64926997 | | | 490 bp | | |  |  |  |  |
| Genome Start | Genome End | Strand | Div | Repeat Start | Repeat End | Repeat Left | Name | Family | Class |
| 64926508 | 64926568 | + | 20.1 | 24 | 71 | -241 | AluJo | Alu | SINE |
| 64926569 | 64926895 | - | 12.4 | 1 | 322 | 0 | AluSx | Alu | SINE |
| 64926896 | 64926997 | + | 20.1 | 72 | 167 | -145 | AluJo | Alu | SINE |

  

|  |  |  |  |  |  |  |  |  |  |
| --- | --- | --- | --- | --- | --- | --- | --- | --- | --- |
| chr7:64931780-64933267 | | | 1488 bp | | |  |  |  |  |
| Genome Start | Genome End | Strand | Div | Repeat Start | Repeat End | Repeat Left | Name | Family | Class |
| 64931780 | 64932079 | - | 18.8 | 604 | 935 | 0 | MER21C | ERV1 | LTR |
| 64932080 | 64932382 | - | 17.6 | 1 | 302 | -10 | AluJo | Alu | SINE |
| 64932383 | 64932452 | - | 18.8 | 526 | 603 | -332 | MER21C | ERV1 | LTR |
| 64932453 | 64932755 | + | 18.6 | 2 | 299 | -13 | AluJb | Alu | SINE |
| 64932756 | 64933267 | - | 18.8 | 18 | 525 | -410 | MER21C | ERV1 | LTR |

  

|  |  |  |  |  |  |  |  |  |  |
| --- | --- | --- | --- | --- | --- | --- | --- | --- | --- |
| chr7:64950938-64952308 | | | 1371 bp | | |  |  |  |  |
| Genome Start | Genome End | Strand | Div | Repeat Start | Repeat End | Repeat Left | Name | Family | Class |
| 64950938 | 64951850 | + | 12.5 | 5237 | 6144 | -161 | L1MA3 | L1 | LINE |
| 64951851 | 64952151 | + | 12.6 | 1 | 301 | -11 | AluSx | Alu | SINE |
| 64952152 | 64952308 | + | 12.5 | 6145 | 6304 | -1 | L1MA3 | L1 | LINE |

  

|  |  |  |  |  |  |  |  |  |  |
| --- | --- | --- | --- | --- | --- | --- | --- | --- | --- |
| chr7:64953565-64954484 | | | 920 bp | | |  |  |  |  |
| Genome Start | Genome End | Strand | Div | Repeat Start | Repeat End | Repeat Left | Name | Family | Class |
| 64953565 | 64953693 | + | 17.4 | 1 | 121 | -191 | AluJo | Alu | SINE |
| 64953694 | 64953984 | - | 10.7 | 1 | 294 | -19 | AluSp | Alu | SINE |
| 64953985 | 64954016 | + | 17.4 | 122 | 133 | -179 | AluJo | Alu | SINE |
| 64954017 | 64954307 | + | 11.7 | 3 | 293 | -20 | AluSq | Alu | SINE |
| 64954308 | 64954484 | + | 17.4 | 134 | 311 | -1 | AluJo | Alu | SINE |

  

|  |  |  |  |  |  |  |  |  |  |
| --- | --- | --- | --- | --- | --- | --- | --- | --- | --- |
| chr7:64964246-64964745 | | | 500 bp | | |  |  |  |  |
| Genome Start | Genome End | Strand | Div | Repeat Start | Repeat End | Repeat Left | Name | Family | Class |
| 64964246 | 64964376 | + | 23.5 | 863 | 991 | -744 | MLT-int | MaLR | LTR |
| 64964377 | 64964689 | - | 7.1 | 1 | 311 | 0 | AluY | Alu | SINE |
| 64964690 | 64964745 | + | 23.5 | 992 | 1040 | -695 | MLT-int | MaLR | LTR |

  

|  |  |  |  |  |  |  |  |  |  |
| --- | --- | --- | --- | --- | --- | --- | --- | --- | --- |
| chr7:64992008-64993231 | | | 1224 bp | | |  |  |  |  |
| Genome Start | Genome End | Strand | Div | Repeat Start | Repeat End | Repeat Left | Name | Family | Class |
| 64992008 | 64992367 | - | 21.2 | 5768 | 6158 | -82 | L1ME3B | L1 | LINE |
| 64992368 | 64992546 | - | 15.1 | 138 | 312 | -1 | AluSq | Alu | SINE |
| 64992547 | 64992852 | - | 8.2 | 1 | 305 | -6 | AluY | Alu | SINE |
| 64992853 | 64992990 | - | 15.1 | 1 | 137 | -176 | AluSq | Alu | SINE |
| 64992991 | 64993231 | - | 22.8 | 5447 | 5767 | -473 | L1ME3B | L1 | LINE |

  

|  |  |  |  |  |  |  |  |  |  |
| --- | --- | --- | --- | --- | --- | --- | --- | --- | --- |
| chr7:65103631-65104454 | | | 598 bp | | |  |  |  |  |
| Genome Start | Genome End | Strand | Div | Repeat Start | Repeat End | Repeat Left | Name | Family | Class |
| 65103631 | 65103928 | + | 31.1 | 2738 | 3056 | -363 | L2 | L2 | LINE |
| 65104050 | 65104096 | + | 25.5 | 4 | 50 | -139 | MER5A | MER1\_type | DNA |
| 65104127 | 65104226 | + | 20.2 | 57 | 160 | 0 | MER5A1 | MER1\_type | DNA |
| 65104302 | 65104454 | + | 21.2 | 3268 | 3419 | 0 | L2 | L2 | LINE |

  

|  |  |  |  |  |  |  |  |  |  |
| --- | --- | --- | --- | --- | --- | --- | --- | --- | --- |
| chr7:65218545-65218991 | | | 447 bp | | |  |  |  |  |
| Genome Start | Genome End | Strand | Div | Repeat Start | Repeat End | Repeat Left | Name | Family | Class |
| 65218545 | 65218634 | - | 24.5 | 64 | 183 | -78 | MER112 | MER1\_type | DNA |
| 65218635 | 65218931 | + | 18.9 | 1 | 303 | -9 | AluJo | Alu | SINE |
| 65218932 | 65218991 | - | 24.5 | 5 | 63 | -197 | MER112 | MER1\_type | DNA |

  

|  |  |  |  |  |  |  |  |  |  |
| --- | --- | --- | --- | --- | --- | --- | --- | --- | --- |
| chr7:65488395-65489002 | | | 608 bp | | |  |  |  |  |
| Genome Start | Genome End | Strand | Div | Repeat Start | Repeat End | Repeat Left | Name | Family | Class |
| 65488395 | 65488559 | - | 15.2 | 134 | 298 | -14 | AluJo | Alu | SINE |
| 65488560 | 65488862 | - | 16.8 | 7 | 309 | -4 | AluSq | Alu | SINE |
| 65488863 | 65489002 | - | 15.2 | 1 | 133 | -179 | AluJo | Alu | SINE |

  

|  |  |  |  |  |  |  |  |  |  |
| --- | --- | --- | --- | --- | --- | --- | --- | --- | --- |
| chr7:65494155-65494622 | | | 468 bp | | |  |  |  |  |
| Genome Start | Genome End | Strand | Div | Repeat Start | Repeat End | Repeat Left | Name | Family | Class |
| 65494155 | 65494212 | - | 22.6 | 719 | 773 | 0 | LTR41 | ERVL | LTR |
| 65494213 | 65494512 | + | 16.1 | 5 | 308 | -4 | AluJb | Alu | SINE |
| 65494513 | 65494622 | - | 22.6 | 637 | 718 | -55 | LTR41 | ERVL | LTR |

  

|  |  |  |  |  |  |  |  |  |  |
| --- | --- | --- | --- | --- | --- | --- | --- | --- | --- |
| chr7:65562782-65564720 | | | 1918 bp | | |  |  |  |  |
| Genome Start | Genome End | Strand | Div | Repeat Start | Repeat End | Repeat Left | Name | Family | Class |
| 65562782 | 65562914 | + | 22.0 | 53 | 184 | -442 | MLT1E2 | MaLR | LTR |
| 65562922 | 65564381 | + | 18.2 | 4664 | 6184 | 0 | L1MB7 | L1 | LINE |
| 65564396 | 65564720 | + | 25.6 | 246 | 571 | -55 | MLT1E2 | MaLR | LTR |

  

|  |  |  |  |  |  |  |  |  |  |
| --- | --- | --- | --- | --- | --- | --- | --- | --- | --- |
| chr7:65564940-65565708 | | | 668 bp | | |  |  |  |  |
| Genome Start | Genome End | Strand | Div | Repeat Start | Repeat End | Repeat Left | Name | Family | Class |
| 65564940 | 65565102 | + | 30.6 | 3036 | 3210 | -209 | L2 | L2 | LINE |
| 65565164 | 65565240 | - | 22.5 | 73 | 119 | -14 | FLAM | Alu | SINE |
| 65565253 | 65565564 | - | 14.9 | 1 | 311 | -1 | AluJb | Alu | SINE |
| 65565593 | 65565708 | + | 33.0 | 3262 | 3374 | -45 | L2 | L2 | LINE |

  

|  |  |  |  |  |  |  |  |  |  |
| --- | --- | --- | --- | --- | --- | --- | --- | --- | --- |
| chr7:65565711-65566786 | | | 1069 bp | | |  |  |  |  |
| Genome Start | Genome End | Strand | Div | Repeat Start | Repeat End | Repeat Left | Name | Family | Class |
| 65565711 | 65565784 | + | 16.9 | 1 | 70 | -861 | MER21A | ERV1 | LTR |
| 65565785 | 65566079 | - | 10.8 | 1 | 296 | -16 | AluSx | Alu | SINE |
| 65566080 | 65566255 | + | 15.1 | 71 | 264 | -667 | MER21A | ERV1 | LTR |
| 65566263 | 65566786 | + | 18.1 | 378 | 925 | -6 | MER21A | ERV1 | LTR |

  

|  |  |  |  |  |  |  |  |  |  |
| --- | --- | --- | --- | --- | --- | --- | --- | --- | --- |
| chr7:65577701-65578581 | | | 878 bp | | |  |  |  |  |
| Genome Start | Genome End | Strand | Div | Repeat Start | Repeat End | Repeat Left | Name | Family | Class |
| 65577701 | 65577864 | + | 28.3 | 2158 | 2403 | -4699 | L1M4b | L1 | LINE |
| 65577865 | 65578171 | + | 13.4 | 1 | 307 | -3 | AluSg | Alu | SINE |
| 65578175 | 65578481 | + | 12.7 | 1 | 310 | 0 | AluSg | Alu | SINE |
| 65578482 | 65578581 | + | 31.3 | 2404 | 2502 | -4600 | L1M4b | L1 | LINE |

  

|  |  |  |  |  |  |  |  |  |  |
| --- | --- | --- | --- | --- | --- | --- | --- | --- | --- |
| chr7:65635865-65636449 | | | 585 bp | | |  |  |  |  |
| Genome Start | Genome End | Strand | Div | Repeat Start | Repeat End | Repeat Left | Name | Family | Class |
| 65635865 | 65635926 | - | 30.4 | 5938 | 6000 | -240 | L1ME3B | L1 | LINE |
| 65635927 | 65636224 | - | 11.1 | 1 | 303 | -8 | AluY | Alu | SINE |
| 65636225 | 65636449 | - | 30.4 | 5707 | 5937 | -303 | L1ME3B | L1 | LINE |

  

|  |  |  |  |  |  |  |  |  |  |
| --- | --- | --- | --- | --- | --- | --- | --- | --- | --- |
| chr7:65652897-65653924 | | | 917 bp | | |  |  |  |  |
| Genome Start | Genome End | Strand | Div | Repeat Start | Repeat End | Repeat Left | Name | Family | Class |
| 65652897 | 65653020 | + | 15.3 | 1 | 124 | -188 | AluJo | Alu | SINE |
| 65653132 | 65653285 | + | 19.0 | 129 | 282 | -30 | AluJo | Alu | SINE |
| 65653286 | 65653582 | - | 13.1 | 1 | 297 | -13 | AluSg | Alu | SINE |
| 65653583 | 65653879 | - | 14.5 | 1 | 297 | -15 | AluSx | Alu | SINE |
| 65653880 | 65653924 | + | 19.0 | 283 | 312 | 0 | AluJo | Alu | SINE |

  

|  |  |  |  |  |  |  |  |  |  |
| --- | --- | --- | --- | --- | --- | --- | --- | --- | --- |
| chr7:65685062-65686600 | | | 1521 bp | | |  |  |  |  |
| Genome Start | Genome End | Strand | Div | Repeat Start | Repeat End | Repeat Left | Name | Family | Class |
| 65685062 | 65685307 | + | 28.0 | 5761 | 6016 | -343 | L1MD2 | L1 | LINE |
| 65685326 | 65685607 | + | 26.6 | 13 | 245 | -318 | MER68 | ERVL | LTR |
| 65685608 | 65685996 | + | 13.4 | 1 | 428 | 0 | MSTA | MaLR | LTR |
| 65685997 | 65686313 | + | 26.6 | 246 | 563 | 0 | MER68 | ERVL | LTR |
| 65686314 | 65686600 | + | 20.9 | 6018 | 6356 | -3 | L1MD2 | L1 | LINE |

  

|  |  |  |  |  |  |  |  |  |  |
| --- | --- | --- | --- | --- | --- | --- | --- | --- | --- |
| chr7:65739341-65740309 | | | 969 bp | | |  |  |  |  |
| Genome Start | Genome End | Strand | Div | Repeat Start | Repeat End | Repeat Left | Name | Family | Class |
| 65739341 | 65739503 | + | 21.6 | 5486 | 5680 | -482 | L1ME3 | L1 | LINE |
| 65739504 | 65739807 | - | 14.1 | 1 | 304 | -8 | AluSx | Alu | SINE |
| 65739808 | 65740309 | + | 21.1 | 5681 | 6162 | 0 | L1ME3 | L1 | LINE |

  

|  |  |  |  |  |  |  |  |  |  |
| --- | --- | --- | --- | --- | --- | --- | --- | --- | --- |
| chr7:65845774-65846352 | | | 579 bp | | |  |  |  |  |
| Genome Start | Genome End | Strand | Div | Repeat Start | Repeat End | Repeat Left | Name | Family | Class |
| 65845774 | 65845924 | - | 19.6 | 133 | 273 | -39 | AluJb | Alu | SINE |
| 65845925 | 65846220 | - | 8.8 | 3 | 298 | -13 | AluY | Alu | SINE |
| 65846221 | 65846352 | - | 19.6 | 2 | 132 | -180 | AluJb | Alu | SINE |

  

|  |  |  |  |  |  |  |  |  |  |
| --- | --- | --- | --- | --- | --- | --- | --- | --- | --- |
| chr7:65857314-65857919 | | | 606 bp | | |  |  |  |  |
| Genome Start | Genome End | Strand | Div | Repeat Start | Repeat End | Repeat Left | Name | Family | Class |
| 65857314 | 65857434 | + | 20.1 | 1 | 126 | -186 | AluJo | Alu | SINE |
| 65857435 | 65857745 | + | 9.3 | 1 | 311 | 0 | AluY | Alu | SINE |
| 65857746 | 65857919 | + | 20.1 | 127 | 304 | -8 | AluJo | Alu | SINE |

  

|  |  |  |  |  |  |  |  |  |  |
| --- | --- | --- | --- | --- | --- | --- | --- | --- | --- |
| chr7:65861301-65862823 | | | 1516 bp | | |  |  |  |  |
| Genome Start | Genome End | Strand | Div | Repeat Start | Repeat End | Repeat Left | Name | Family | Class |
| 65861301 | 65861588 | - | 22.2 | 6018 | 6356 | -3 | L1MD2 | L1 | LINE |
| 65861589 | 65861904 | - | 27.1 | 246 | 563 | 0 | MER68 | ERVL | LTR |
| 65861905 | 65862297 | - | 14.0 | 1 | 428 | 0 | MSTA | MaLR | LTR |
| 65862298 | 65862579 | - | 27.1 | 13 | 245 | -318 | MER68 | ERVL | LTR |
| 65862587 | 65862823 | - | 29.0 | 5761 | 6019 | -340 | L1MD2 | L1 | LINE |

  

|  |  |  |  |  |  |  |  |  |  |
| --- | --- | --- | --- | --- | --- | --- | --- | --- | --- |
| chr7:65863507-65864584 | | | 1071 bp | | |  |  |  |  |
| Genome Start | Genome End | Strand | Div | Repeat Start | Repeat End | Repeat Left | Name | Family | Class |
| 65863507 | 65864026 | - | 16.4 | 378 | 922 | -9 | MER21A | ERV1 | LTR |
| 65864034 | 65864195 | - | 12.1 | 71 | 264 | -667 | MER21A | ERV1 | LTR |
| 65864196 | 65864504 | + | 13.4 | 5 | 311 | -1 | AluSx | Alu | SINE |
| 65864505 | 65864584 | - | 12.1 | 1 | 70 | -861 | MER21A | ERV1 | LTR |

  

|  |  |  |  |  |  |  |  |  |  |
| --- | --- | --- | --- | --- | --- | --- | --- | --- | --- |
| chr7:65865586-65867533 | | | 1942 bp | | |  |  |  |  |
| Genome Start | Genome End | Strand | Div | Repeat Start | Repeat End | Repeat Left | Name | Family | Class |
| 65865586 | 65865927 | - | 27.2 | 215 | 571 | -55 | MLT1E2 | MaLR | LTR |
| 65865928 | 65867367 | - | 17.8 | 4662 | 6183 | -1 | L1MB7 | L1 | LINE |
| 65867374 | 65867533 | - | 19.7 | 32 | 184 | -442 | MLT1E2 | MaLR | LTR |

  

|  |  |  |  |  |  |  |  |  |  |
| --- | --- | --- | --- | --- | --- | --- | --- | --- | --- |
| chr7:65904255-65904775 | | | 490 bp | | |  |  |  |  |
| Genome Start | Genome End | Strand | Div | Repeat Start | Repeat End | Repeat Left | Name | Family | Class |
| 65904255 | 65904313 | + | 23.1 | 1 | 61 | -158 | MER20 | MER1\_type | DNA |
| 65904345 | 65904615 | - | 13.5 | 1 | 287 | -25 | AluJb | Alu | SINE |
| 65904616 | 65904775 | + | 23.1 | 62 | 219 | 0 | MER20 | MER1\_type | DNA |

  

|  |  |  |  |  |  |  |  |  |  |
| --- | --- | --- | --- | --- | --- | --- | --- | --- | --- |
| chr7:65921873-65922477 | | | 605 bp | | |  |  |  |  |
| Genome Start | Genome End | Strand | Div | Repeat Start | Repeat End | Repeat Left | Name | Family | Class |
| 65921873 | 65922014 | + | 33.7 | 2925 | 3070 | -349 | L2 | L2 | LINE |
| 65922015 | 65922310 | - | 11.2 | 1 | 296 | -14 | AluSg | Alu | SINE |
| 65922311 | 65922477 | + | 33.7 | 3071 | 3276 | -143 | L2 | L2 | LINE |

  

|  |  |  |  |  |  |  |  |  |  |
| --- | --- | --- | --- | --- | --- | --- | --- | --- | --- |
| chr7:65927057-65927515 | | | 459 bp | | |  |  |  |  |
| Genome Start | Genome End | Strand | Div | Repeat Start | Repeat End | Repeat Left | Name | Family | Class |
| 65927057 | 65927186 | + | 27.4 | 67 | 206 | -62 | MIRb | MIR | SINE |
| 65927187 | 65927485 | + | 13.0 | 1 | 301 | -11 | AluSx | Alu | SINE |
| 65927486 | 65927515 | + | 27.4 | 207 | 232 | -36 | MIRb | MIR | SINE |

  

|  |  |  |  |  |  |  |  |  |  |
| --- | --- | --- | --- | --- | --- | --- | --- | --- | --- |
| chr7:65931407-65932309 | | | 859 bp | | |  |  |  |  |
| Genome Start | Genome End | Strand | Div | Repeat Start | Repeat End | Repeat Left | Name | Family | Class |
| 65931407 | 65931572 | - | 17.8 | 130 | 295 | -17 | AluJo | Alu | SINE |
| 65931614 | 65931898 | - | 13.0 | 1 | 286 | -26 | AluSx | Alu | SINE |
| 65931902 | 65932166 | - | 16.2 | 9 | 293 | -19 | AluJb | Alu | SINE |
| 65932167 | 65932309 | - | 17.8 | 2 | 127 | -185 | AluJo | Alu | SINE |

  

|  |  |  |  |  |  |  |  |  |  |
| --- | --- | --- | --- | --- | --- | --- | --- | --- | --- |
| chr7:65938477-65938971 | | | 495 bp | | |  |  |  |  |
| Genome Start | Genome End | Strand | Div | Repeat Start | Repeat End | Repeat Left | Name | Family | Class |
| 65938477 | 65938618 | + | 25.6 | 60 | 207 | -61 | MIRb | MIR | SINE |
| 65938619 | 65938927 | + | 12.1 | 1 | 309 | 0 | AluSx | Alu | SINE |
| 65938928 | 65938971 | + | 25.6 | 208 | 241 | -27 | MIRb | MIR | SINE |

  

|  |  |  |  |  |  |  |  |  |  |
| --- | --- | --- | --- | --- | --- | --- | --- | --- | --- |
| chr7:65958515-65959122 | | | 608 bp | | |  |  |  |  |
| Genome Start | Genome End | Strand | Div | Repeat Start | Repeat End | Repeat Left | Name | Family | Class |
| 65958515 | 65958720 | - | 14.5 | 114 | 310 | 0 | AluSg | Alu | SINE |
| 65958721 | 65959013 | - | 6.1 | 1 | 293 | -18 | AluY | Alu | SINE |
| 65959014 | 65959122 | - | 14.5 | 5 | 113 | -197 | AluSg | Alu | SINE |

  

|  |  |  |  |  |  |  |  |  |  |
| --- | --- | --- | --- | --- | --- | --- | --- | --- | --- |
| chr7:65990077-65990701 | | | 625 bp | | |  |  |  |  |
| Genome Start | Genome End | Strand | Div | Repeat Start | Repeat End | Repeat Left | Name | Family | Class |
| 65990077 | 65990202 | + | 23.0 | 9 | 127 | -185 | AluJo | Alu | SINE |
| 65990203 | 65990513 | + | 9.4 | 1 | 307 | -5 | AluSx | Alu | SINE |
| 65990514 | 65990701 | + | 23.0 | 128 | 312 | 0 | AluJo | Alu | SINE |

  

|  |  |  |  |  |  |  |  |  |  |
| --- | --- | --- | --- | --- | --- | --- | --- | --- | --- |
| chr7:66157484-66158120 | | | 637 bp | | |  |  |  |  |
| Genome Start | Genome End | Strand | Div | Repeat Start | Repeat End | Repeat Left | Name | Family | Class |
| 66157484 | 66157740 | - | 29.6 | 2501 | 2767 | -3379 | L1M5 | L1 | LINE |
| 66157741 | 66158034 | + | 15.1 | 5 | 298 | -14 | AluSx | Alu | SINE |
| 66158035 | 66158120 | - | 29.6 | 2411 | 2500 | -3646 | L1M5 | L1 | LINE |

  

|  |  |  |  |  |  |  |  |  |  |
| --- | --- | --- | --- | --- | --- | --- | --- | --- | --- |
| chr7:66177872-66178902 | | | 1031 bp | | |  |  |  |  |
| Genome Start | Genome End | Strand | Div | Repeat Start | Repeat End | Repeat Left | Name | Family | Class |
| 66177872 | 66178235 | + | 20.4 | 5711 | 6078 | -100 | L1MB8 | L1 | LINE |
| 66178236 | 66178828 | + | 6.9 | 1 | 591 | 0 | LTR10C | ERV1 | LTR |
| 66178829 | 66178902 | + | 20.4 | 6079 | 6151 | -27 | L1MB8 | L1 | LINE |

  

|  |  |  |  |  |  |  |  |  |  |
| --- | --- | --- | --- | --- | --- | --- | --- | --- | --- |
| chr7:66186826-66187302 | | | 477 bp | | |  |  |  |  |
| Genome Start | Genome End | Strand | Div | Repeat Start | Repeat End | Repeat Left | Name | Family | Class |
| 66186826 | 66186931 | - | 24.0 | 79 | 186 | -3 | MER5A | MER1\_type | DNA |
| 66186932 | 66187226 | - | 16.9 | 11 | 310 | -2 | AluJb | Alu | SINE |
| 66187227 | 66187302 | - | 24.0 | 3 | 78 | -111 | MER5A | MER1\_type | DNA |

  

|  |  |  |  |  |  |  |  |  |  |
| --- | --- | --- | --- | --- | --- | --- | --- | --- | --- |
| chr7:66199702-66200467 | | | 764 bp | | |  |  |  |  |
| Genome Start | Genome End | Strand | Div | Repeat Start | Repeat End | Repeat Left | Name | Family | Class |
| 66199702 | 66199823 | + | 26.3 | 1 | 124 | -54 | MER5B | MER1\_type | DNA |
| 66199824 | 66200119 | - | 10.1 | 1 | 297 | -15 | AluSx | Alu | SINE |
| 66200122 | 66200422 | - | 16.7 | 1 | 294 | -18 | AluJo | Alu | SINE |
| 66200423 | 66200467 | + | 26.3 | 125 | 159 | -19 | MER5B | MER1\_type | DNA |

  

|  |  |  |  |  |  |  |  |  |  |
| --- | --- | --- | --- | --- | --- | --- | --- | --- | --- |
| chr7:66220378-66221003 | | | 626 bp | | |  |  |  |  |
| Genome Start | Genome End | Strand | Div | Repeat Start | Repeat End | Repeat Left | Name | Family | Class |
| 66220378 | 66220460 | - | 20.3 | 263 | 336 | -9 | MER2 | MER2\_type | DNA |
| 66220461 | 66220775 | + | 14.8 | 1 | 309 | -3 | AluSx | Alu | SINE |
| 66220776 | 66221003 | - | 20.3 | 2 | 262 | -83 | MER2 | MER2\_type | DNA |

  

|  |  |  |  |  |  |  |  |  |  |
| --- | --- | --- | --- | --- | --- | --- | --- | --- | --- |
| chr7:66235564-66236099 | | | 497 bp | | |  |  |  |  |
| Genome Start | Genome End | Strand | Div | Repeat Start | Repeat End | Repeat Left | Name | Family | Class |
| 66235564 | 66235838 | - | 16.8 | 27 | 297 | -15 | AluJb | Alu | SINE |
| 66235841 | 66236022 | + | 9.9 | 3 | 184 | -125 | AluSc | Alu | SINE |
| 66236060 | 66236099 | - | 17.5 | 1 | 40 | -262 | AluJb | Alu | SINE |

  

|  |  |  |  |  |  |  |  |  |  |
| --- | --- | --- | --- | --- | --- | --- | --- | --- | --- |
| chr7:66244039-66244530 | | | 492 bp | | |  |  |  |  |
| Genome Start | Genome End | Strand | Div | Repeat Start | Repeat End | Repeat Left | Name | Family | Class |
| 66244039 | 66244175 | + | 27.1 | 59 | 195 | -73 | MIRb | MIR | SINE |
| 66244176 | 66244464 | + | 9.7 | 1 | 290 | -23 | AluSq | Alu | SINE |
| 66244465 | 66244530 | + | 27.1 | 196 | 268 | 0 | MIRb | MIR | SINE |

  

|  |  |  |  |  |  |  |  |  |  |
| --- | --- | --- | --- | --- | --- | --- | --- | --- | --- |
| chr7:66260389-66261547 | | | 1154 bp | | |  |  |  |  |
| Genome Start | Genome End | Strand | Div | Repeat Start | Repeat End | Repeat Left | Name | Family | Class |
| 66260389 | 66260504 | - | 19.1 | 3958 | 4071 | -2075 | L1MC | L1 | LINE |
| 66260505 | 66260809 | - | 12.2 | 1 | 304 | -6 | AluSg | Alu | SINE |
| 66260810 | 66260997 | - | 19.1 | 3772 | 3957 | -2189 | L1MC | L1 | LINE |
| 66261000 | 66261292 | - | 6.9 | 1 | 291 | -20 | AluY | Alu | SINE |
| 66261296 | 66261547 | - | 18.4 | 3046 | 3298 | -2848 | L1MC | L1 | LINE |

  

|  |  |  |  |  |  |  |  |  |  |
| --- | --- | --- | --- | --- | --- | --- | --- | --- | --- |
| chr7:66299641-66300173 | | | 533 bp | | |  |  |  |  |
| Genome Start | Genome End | Strand | Div | Repeat Start | Repeat End | Repeat Left | Name | Family | Class |
| 66299641 | 66299748 | - | 22.7 | 148 | 261 | -1 | MIR | MIR | SINE |
| 66299749 | 66300047 | - | 12.7 | 1 | 299 | -13 | AluSx | Alu | SINE |
| 66300048 | 66300173 | - | 22.7 | 22 | 147 | -115 | MIR | MIR | SINE |

  

|  |  |  |  |  |  |  |  |  |  |
| --- | --- | --- | --- | --- | --- | --- | --- | --- | --- |
| chr7:66300417-66302137 | | | 1624 bp | | |  |  |  |  |
| Genome Start | Genome End | Strand | Div | Repeat Start | Repeat End | Repeat Left | Name | Family | Class |
| 66300417 | 66300621 | - | 25.6 | 7688 | 7899 | -143 | L1MC4 | L1 | LINE |
| 66300687 | 66300988 | + | 11.1 | 1 | 298 | -14 | AluSx | Alu | SINE |
| 66301021 | 66301185 | - | 28.2 | 2 | 179 | -36 | MER58C | MER1\_type | DNA |
| 66301186 | 66301340 | - | 28.6 | 7521 | 7663 | -379 | L1MC4 | L1 | LINE |
| 66301341 | 66301645 | + | 19.7 | 6 | 311 | -1 | AluJb | Alu | SINE |
| 66301646 | 66302137 | - | 28.6 | 6997 | 7520 | -522 | L1MC4 | L1 | LINE |

  

|  |  |  |  |  |  |  |  |  |  |
| --- | --- | --- | --- | --- | --- | --- | --- | --- | --- |
| chr7:66327209-66327730 | | | 522 bp | | |  |  |  |  |
| Genome Start | Genome End | Strand | Div | Repeat Start | Repeat End | Repeat Left | Name | Family | Class |
| 66327209 | 66327330 | - | 27.2 | 109 | 224 | -44 | MIRb | MIR | SINE |
| 66327331 | 66327636 | + | 11.4 | 1 | 306 | -6 | AluSx | Alu | SINE |
| 66327637 | 66327730 | - | 27.8 | 4 | 108 | -160 | MIRb | MIR | SINE |

  

|  |  |  |  |  |  |  |  |  |  |
| --- | --- | --- | --- | --- | --- | --- | --- | --- | --- |
| chr7:66329555-66330422 | | | 868 bp | | |  |  |  |  |
| Genome Start | Genome End | Strand | Div | Repeat Start | Repeat End | Repeat Left | Name | Family | Class |
| 66329555 | 66329688 | + | 23.8 | 54 | 173 | -434 | MER77 | ERVL | LTR |
| 66329689 | 66329978 | - | 9.7 | 1 | 289 | -23 | AluSx | Alu | SINE |
| 66329979 | 66330422 | + | 23.8 | 174 | 603 | -4 | MER77 | ERVL | LTR |

  

|  |  |  |  |  |  |  |  |  |  |
| --- | --- | --- | --- | --- | --- | --- | --- | --- | --- |
| chr7:66332822-66335714 | | | 2849 bp | | |  |  |  |  |
| Genome Start | Genome End | Strand | Div | Repeat Start | Repeat End | Repeat Left | Name | Family | Class |
| 66332822 | 66333074 | - | 26.9 | 2290 | 2507 | 0 | HAL1 | L1 | LINE |
| 66333075 | 66333361 | - | 9.4 | 1 | 300 | -12 | AluSx | Alu | SINE |
| 66333362 | 66334352 | - | 26.9 | 1192 | 2289 | -218 | HAL1 | L1 | LINE |
| 66334371 | 66334640 | + | 11.2 | 2 | 270 | -42 | AluSx | Alu | SINE |
| 66334667 | 66334760 | - | 28.3 | 810 | 905 | -1602 | HAL1 | L1 | LINE |
| 66334761 | 66334992 | + | 10.4 | 82 | 312 | 0 | AluSg/x | Alu | SINE |
| 66334993 | 66335714 | - | 28.3 | 11 | 809 | -1698 | HAL1 | L1 | LINE |

  

|  |  |  |  |  |  |  |  |  |  |
| --- | --- | --- | --- | --- | --- | --- | --- | --- | --- |
| chr7:66336310-66337028 | | | 719 bp | | |  |  |  |  |
| Genome Start | Genome End | Strand | Div | Repeat Start | Repeat End | Repeat Left | Name | Family | Class |
| 66336310 | 66336486 | + | 22.6 | 3 | 171 | -219 | MLT1B | MaLR | LTR |
| 66336487 | 66336795 | + | 18.8 | 1 | 309 | -3 | AluJo | Alu | SINE |
| 66336796 | 66337028 | + | 22.6 | 172 | 389 | -1 | MLT1B | MaLR | LTR |

  

|  |  |  |  |  |  |  |  |  |  |
| --- | --- | --- | --- | --- | --- | --- | --- | --- | --- |
| chr7:66360992-66362831 | | | 1836 bp | | |  |  |  |  |
| Genome Start | Genome End | Strand | Div | Repeat Start | Repeat End | Repeat Left | Name | Family | Class |
| 66360992 | 66361014 | + | 10.8 | 3 | 15 | -381 | MSTD | MaLR | LTR |
| 66361015 | 66361307 | + | 14.3 | 1 | 293 | -19 | AluSx | Alu | SINE |
| 66361308 | 66361387 | + | 10.8 | 16 | 96 | -300 | MSTD | MaLR | LTR |
| 66361387 | 66361475 | + | 16.3 | 112 | 190 | -444 | MER41B | ERV1 | LTR |
| 66361476 | 66361779 | + | 15.4 | 1 | 299 | -13 | AluJo | Alu | SINE |
| 66361780 | 66362212 | + | 17.6 | 108 | 595 | -5 | MER41B | ERV1 | LTR |
| 66362218 | 66362321 | + | 15.6 | 93 | 212 | -184 | MSTD | MaLR | LTR |
| 66362322 | 66362612 | - | 13.2 | 13 | 299 | -13 | AluJb | Alu | SINE |
| 66362613 | 66362831 | + | 15.6 | 213 | 395 | -1 | MSTD | MaLR | LTR |

  

|  |  |  |  |  |  |  |  |  |  |
| --- | --- | --- | --- | --- | --- | --- | --- | --- | --- |
| chr7:66367454-66367916 | | | 463 bp | | |  |  |  |  |
| Genome Start | Genome End | Strand | Div | Repeat Start | Repeat End | Repeat Left | Name | Family | Class |
| 66367454 | 66367514 | - | 29.9 | 3124 | 3177 | -201 | L2 | L2 | LINE |
| 66367515 | 66367819 | - | 10.6 | 3 | 305 | -7 | AluSx | Alu | SINE |
| 66367820 | 66367916 | - | 29.9 | 3040 | 3123 | -255 | L2 | L2 | LINE |

  

|  |  |  |  |  |  |  |  |  |  |
| --- | --- | --- | --- | --- | --- | --- | --- | --- | --- |
| chr7:66375870-66377428 | | | 1559 bp | | |  |  |  |  |
| Genome Start | Genome End | Strand | Div | Repeat Start | Repeat End | Repeat Left | Name | Family | Class |
| 66375870 | 66376000 | - | 21.8 | 544 | 674 | -37 | MER67C | ERV1 | LTR |
| 66376001 | 66376302 | - | 10.3 | 1 | 301 | -9 | AluSg | Alu | SINE |
| 66376303 | 66376402 | - | 21.8 | 453 | 543 | -168 | MER67C | ERV1 | LTR |
| 66376403 | 66376725 | - | 14.9 | 1 | 304 | -8 | AluSx | Alu | SINE |
| 66376726 | 66376973 | - | 21.8 | 201 | 452 | -259 | MER67C | ERV1 | LTR |
| 66376974 | 66377283 | + | 11.9 | 1 | 312 | 0 | AluSx | Alu | SINE |
| 66377284 | 66377428 | - | 21.8 | 66 | 200 | -511 | MER67C | ERV1 | LTR |

  

|  |  |  |  |  |  |  |  |  |  |
| --- | --- | --- | --- | --- | --- | --- | --- | --- | --- |
| chr7:66378691-66379399 | | | 709 bp | | |  |  |  |  |
| Genome Start | Genome End | Strand | Div | Repeat Start | Repeat End | Repeat Left | Name | Family | Class |
| 66378691 | 66378750 | + | 35.7 | 3933 | 3989 | -500 | L3 | CR1 | LINE |
| 66378751 | 66379055 | - | 11.9 | 3 | 305 | -7 | AluSx | Alu | SINE |
| 66379056 | 66379399 | + | 35.7 | 3990 | 4333 | -156 | L3 | CR1 | LINE |

  

|  |  |  |  |  |  |  |  |  |  |
| --- | --- | --- | --- | --- | --- | --- | --- | --- | --- |
| chr7:66387646-66388248 | | | 576 bp | | |  |  |  |  |
| Genome Start | Genome End | Strand | Div | Repeat Start | Repeat End | Repeat Left | Name | Family | Class |
| 66387646 | 66387803 | - | 16.8 | 135 | 294 | -18 | AluJo | Alu | SINE |
| 66387831 | 66388110 | - | 18.6 | 2 | 282 | -30 | AluSx | Alu | SINE |
| 66388111 | 66388248 | - | 16.8 | 1 | 134 | -178 | AluJo | Alu | SINE |

  

|  |  |  |  |  |  |  |  |  |  |
| --- | --- | --- | --- | --- | --- | --- | --- | --- | --- |
| chr7:66395890-66396596 | | | 707 bp | | |  |  |  |  |
| Genome Start | Genome End | Strand | Div | Repeat Start | Repeat End | Repeat Left | Name | Family | Class |
| 66395890 | 66395920 | + | 16.5 | 3 | 32 | -457 | LTR45B | ERV1 | LTR |
| 66395921 | 66396209 | - | 12.1 | 1 | 300 | -13 | AluSq | Alu | SINE |
| 66396210 | 66396596 | + | 16.5 | 33 | 414 | -75 | LTR45B | ERV1 | LTR |

  

|  |  |  |  |  |  |  |  |  |  |
| --- | --- | --- | --- | --- | --- | --- | --- | --- | --- |
| chr7:66425075-66425688 | | | 614 bp | | |  |  |  |  |
| Genome Start | Genome End | Strand | Div | Repeat Start | Repeat End | Repeat Left | Name | Family | Class |
| 66425075 | 66425178 | + | 20.1 | 11 | 109 | -215 | MER33 | MER1\_type | DNA |
| 66425179 | 66425486 | - | 9.4 | 1 | 298 | -13 | AluY | Alu | SINE |
| 66425487 | 66425688 | + | 20.1 | 110 | 322 | -2 | MER33 | MER1\_type | DNA |

  

|  |  |  |  |  |  |  |  |  |  |
| --- | --- | --- | --- | --- | --- | --- | --- | --- | --- |
| chr7:66969280-66969973 | | | 619 bp | | |  |  |  |  |
| Genome Start | Genome End | Strand | Div | Repeat Start | Repeat End | Repeat Left | Name | Family | Class |
| 66969280 | 66969447 | + | 18.2 | 28 | 183 | -231 | MLT2D | ERVL | LTR |
| 66969451 | 66969689 | - | 17.7 | 44 | 284 | -28 | AluJo | Alu | SINE |
| 66969701 | 66969869 | + | 21.4 | 195 | 366 | -48 | MLT2D | ERVL | LTR |
| 66969931 | 66969973 | + | 13.8 | 520 | 563 | 0 | MLT2D | ERVL | LTR |

  

|  |  |  |  |  |  |  |  |  |  |
| --- | --- | --- | --- | --- | --- | --- | --- | --- | --- |
| chr7:67212066-67212852 | | | 787 bp | | |  |  |  |  |
| Genome Start | Genome End | Strand | Div | Repeat Start | Repeat End | Repeat Left | Name | Family | Class |
| 67212066 | 67212156 | + | 16.0 | 3 | 88 | -338 | MSTB | MaLR | LTR |
| 67212157 | 67212518 | - | 10.9 | 1 | 381 | 0 | THE1D | MaLR | LTR |
| 67212519 | 67212852 | + | 16.0 | 89 | 426 | 0 | MSTB | MaLR | LTR |

  

|  |  |  |  |  |  |  |  |  |  |
| --- | --- | --- | --- | --- | --- | --- | --- | --- | --- |
| chr7:71629418-71631000 | | | 1573 bp | | |  |  |  |  |
| Genome Start | Genome End | Strand | Div | Repeat Start | Repeat End | Repeat Left | Name | Family | Class |
| 71629418 | 71629643 | + | 25.3 | 2276 | 2513 | -3706 | L1MEc | L1 | LINE |
| 71629644 | 71629940 | - | 10.4 | 1 | 309 | -1 | AluSg | Alu | SINE |
| 71629941 | 71630280 | + | 25.3 | 2514 | 2850 | -3369 | L1MEc | L1 | LINE |
| 71630291 | 71631000 | + | 23.7 | 3358 | 4099 | -2120 | L1MEc | L1 | LINE |

  

|  |  |  |  |  |  |  |  |  |  |
| --- | --- | --- | --- | --- | --- | --- | --- | --- | --- |
| chr7:71633286-71634288 | | | 1003 bp | | |  |  |  |  |
| Genome Start | Genome End | Strand | Div | Repeat Start | Repeat End | Repeat Left | Name | Family | Class |
| 71633286 | 71633368 | + | 11.1 | 2665 | 2744 | -285 | Tigger3(Golem) | MER2\_type | DNA |
| 71633369 | 71633652 | - | 17.9 | 1 | 304 | -8 | AluJb | Alu | SINE |
| 71633653 | 71633738 | + | 11.1 | 2745 | 2773 | -256 | Tigger3(Golem) | MER2\_type | DNA |
| 71633739 | 71634027 | - | 12.5 | 3 | 295 | -18 | AluSq | Alu | SINE |
| 71634028 | 71634288 | + | 11.1 | 2774 | 3026 | -3 | Tigger3(Golem) | MER2\_type | DNA |

  

|  |  |  |  |  |  |  |  |  |  |
| --- | --- | --- | --- | --- | --- | --- | --- | --- | --- |
| chr7:71650208-71651733 | | | 1526 bp | | |  |  |  |  |
| Genome Start | Genome End | Strand | Div | Repeat Start | Repeat End | Repeat Left | Name | Family | Class |
| 71650208 | 71650525 | - | 11.2 | 596 | 900 | 0 | MER4D1 | ERV1 | LTR |
| 71650526 | 71650803 | + | 4.3 | 1 | 278 | -33 | AluY | Alu | SINE |
| 71650804 | 71650975 | - | 11.2 | 453 | 595 | -305 | MER4D1 | ERV1 | LTR |
| 71650976 | 71651279 | + | 10.5 | 1 | 304 | -5 | AluSc | Alu | SINE |
| 71651280 | 71651733 | - | 11.2 | 1 | 452 | -448 | MER4D1 | ERV1 | LTR |

  

|  |  |  |  |  |  |  |  |  |  |
| --- | --- | --- | --- | --- | --- | --- | --- | --- | --- |
| chr7:71655463-71656045 | | | 583 bp | | |  |  |  |  |
| Genome Start | Genome End | Strand | Div | Repeat Start | Repeat End | Repeat Left | Name | Family | Class |
| 71655463 | 71655589 | + | 18.9 | 1 | 125 | -187 | AluJo | Alu | SINE |
| 71655590 | 71655873 | + | 8.8 | 13 | 297 | -13 | AluSg | Alu | SINE |
| 71655874 | 71656045 | + | 18.9 | 126 | 307 | -5 | AluJo | Alu | SINE |

  

|  |  |  |  |  |  |  |  |  |  |
| --- | --- | --- | --- | --- | --- | --- | --- | --- | --- |
| chr7:71666037-71667356 | | | 1320 bp | | |  |  |  |  |
| Genome Start | Genome End | Strand | Div | Repeat Start | Repeat End | Repeat Left | Name | Family | Class |
| 71666037 | 71666214 | - | 16.6 | 552 | 727 | 0 | LTR8A | ERV1 | LTR |
| 71666215 | 71666507 | + | 10.9 | 1 | 298 | -14 | AluSx | Alu | SINE |
| 71666508 | 71666592 | - | 16.6 | 485 | 551 | -176 | LTR8A | ERV1 | LTR |
| 71666593 | 71666890 | + | 10.1 | 1 | 299 | -12 | AluY | Alu | SINE |
| 71666891 | 71667356 | - | 16.6 | 1 | 484 | -243 | LTR8A | ERV1 | LTR |

  

|  |  |  |  |  |  |  |  |  |  |
| --- | --- | --- | --- | --- | --- | --- | --- | --- | --- |
| chr7:71674627-71676087 | | | 1461 bp | | |  |  |  |  |
| Genome Start | Genome End | Strand | Div | Repeat Start | Repeat End | Repeat Left | Name | Family | Class |
| 71674627 | 71675141 | + | 19.7 | 1 | 573 | -362 | MER21C | ERV1 | LTR |
| 71675142 | 71675434 | + | 12.6 | 5 | 299 | -13 | AluSx | Alu | SINE |
| 71675435 | 71675642 | + | 19.7 | 574 | 766 | -169 | MER21C | ERV1 | LTR |
| 71675643 | 71675949 | - | 8.8 | 2 | 308 | -4 | AluSx | Alu | SINE |
| 71675950 | 71676087 | + | 19.7 | 767 | 914 | -21 | MER21C | ERV1 | LTR |

  

|  |  |  |  |  |  |  |  |  |  |
| --- | --- | --- | --- | --- | --- | --- | --- | --- | --- |
| chr7:71713697-71714689 | | | 993 bp | | |  |  |  |  |
| Genome Start | Genome End | Strand | Div | Repeat Start | Repeat End | Repeat Left | Name | Family | Class |
| 71713697 | 71714236 | + | 34.9 | 2718 | 3285 | -93 | L2 | L2 | LINE |
| 71714237 | 71714589 | + | 10.8 | 1 | 364 | 0 | THE1B | MaLR | LTR |
| 71714590 | 71714689 | + | 31.4 | 3286 | 3402 | -17 | L2 | L2 | LINE |

  

|  |  |  |  |  |  |  |  |  |  |
| --- | --- | --- | --- | --- | --- | --- | --- | --- | --- |
| chr7:71757158-71757957 | | | 794 bp | | |  |  |  |  |
| Genome Start | Genome End | Strand | Div | Repeat Start | Repeat End | Repeat Left | Name | Family | Class |
| 71757158 | 71757213 | + | 14.3 | 6095 | 6151 | -1634 | L1MC3 | L1 | LINE |
| 71757220 | 71757519 | + | 15.3 | 7332 | 7628 | -157 | L1MC3 | L1 | LINE |
| 71757520 | 71757821 | + | 11.3 | 1 | 302 | -10 | AluSx | Alu | SINE |
| 71757822 | 71757957 | + | 15.3 | 7629 | 7754 | -31 | L1MC3 | L1 | LINE |

  

|  |  |  |  |  |  |  |  |  |  |
| --- | --- | --- | --- | --- | --- | --- | --- | --- | --- |
| chr7:71762861-71763416 | | | 556 bp | | |  |  |  |  |
| Genome Start | Genome End | Strand | Div | Repeat Start | Repeat End | Repeat Left | Name | Family | Class |
| 71762861 | 71763008 | - | 18.8 | 87 | 224 | 0 | MER58A | MER1\_type | DNA |
| 71763009 | 71763326 | + | 16.7 | 1 | 314 | 0 | AluJo | Alu | SINE |
| 71763327 | 71763416 | - | 18.8 | 2 | 86 | -21 | MER58A | MER1\_type | DNA |

  

|  |  |  |  |  |  |  |  |  |  |
| --- | --- | --- | --- | --- | --- | --- | --- | --- | --- |
| chr7:71802426-71803021 | | | 596 bp | | |  |  |  |  |
| Genome Start | Genome End | Strand | Div | Repeat Start | Repeat End | Repeat Left | Name | Family | Class |
| 71802426 | 71802549 | + | 26.6 | 8 | 136 | -202 | MER46C | MER2\_type | DNA |
| 71802550 | 71802855 | - | 11.8 | 1 | 306 | -6 | AluSx | Alu | SINE |
| 71802856 | 71803021 | + | 26.6 | 137 | 338 | 0 | MER46C | MER2\_type | DNA |

  

|  |  |  |  |  |  |  |  |  |  |
| --- | --- | --- | --- | --- | --- | --- | --- | --- | --- |
| chr7:71892808-71893298 | | | 491 bp | | |  |  |  |  |
| Genome Start | Genome End | Strand | Div | Repeat Start | Repeat End | Repeat Left | Name | Family | Class |
| 71892808 | 71892892 | + | 26.4 | 56 | 128 | -479 | MER77 | ERVL | LTR |
| 71892893 | 71893197 | - | 10.3 | 1 | 307 | -6 | AluSq | Alu | SINE |
| 71893198 | 71893298 | + | 26.4 | 129 | 220 | -387 | MER77 | ERVL | LTR |

  

|  |  |  |  |  |  |  |  |  |  |
| --- | --- | --- | --- | --- | --- | --- | --- | --- | --- |
| chr7:71921165-71921668 | | | 504 bp | | |  |  |  |  |
| Genome Start | Genome End | Strand | Div | Repeat Start | Repeat End | Repeat Left | Name | Family | Class |
| 71921165 | 71921216 | + | 22.4 | 4 | 51 | -290 | MER58B | MER1\_type | DNA |
| 71921217 | 71921508 | - | 14.5 | 1 | 298 | -14 | AluSx | Alu | SINE |
| 71921509 | 71921668 | + | 22.4 | 169 | 339 | -2 | MER58B | MER1\_type | DNA |

  

|  |  |  |  |  |  |  |  |  |  |
| --- | --- | --- | --- | --- | --- | --- | --- | --- | --- |
| chr7:71922202-71922614 | | | 413 bp | | |  |  |  |  |
| Genome Start | Genome End | Strand | Div | Repeat Start | Repeat End | Repeat Left | Name | Family | Class |
| 71922202 | 71922249 | - | 23.8 | 106 | 148 | -120 | MIRb | MIR | SINE |
| 71922250 | 71922544 | + | 13.2 | 3 | 299 | -14 | AluSq | Alu | SINE |
| 71922545 | 71922614 | - | 23.8 | 25 | 105 | -163 | MIRb | MIR | SINE |

  

|  |  |  |  |  |  |  |  |  |  |
| --- | --- | --- | --- | --- | --- | --- | --- | --- | --- |
| chr7:71922727-71923290 | | | 564 bp | | |  |  |  |  |
| Genome Start | Genome End | Strand | Div | Repeat Start | Repeat End | Repeat Left | Name | Family | Class |
| 71922727 | 71922817 | + | 16.7 | 23 | 129 | -183 | AluJb | Alu | SINE |
| 71922818 | 71923117 | + | 4.3 | 1 | 300 | -11 | AluY | Alu | SINE |
| 71923118 | 71923290 | + | 16.7 | 130 | 307 | -5 | AluJb | Alu | SINE |

  

|  |  |  |  |  |  |  |  |  |  |
| --- | --- | --- | --- | --- | --- | --- | --- | --- | --- |
| chr7:71925984-71927557 | | | 1574 bp | | |  |  |  |  |
| Genome Start | Genome End | Strand | Div | Repeat Start | Repeat End | Repeat Left | Name | Family | Class |
| 71925984 | 71926101 | + | 17.8 | 1 | 120 | -306 | MSTB | MaLR | LTR |
| 71926102 | 71926533 | - | 15.1 | 1 | 640 | 0 | MER51E | ERV1 | LTR |
| 71926534 | 71926776 | + | 17.9 | 117 | 376 | -50 | MSTB | MaLR | LTR |
| 71926777 | 71926814 | - | 12.2 | 400 | 427 | -1 | MSTA | MaLR | LTR |
| 71926815 | 71927123 | + | 12.0 | 1 | 312 | 0 | AluSx | Alu | SINE |
| 71927124 | 71927506 | - | 12.2 | 1 | 399 | -29 | MSTA | MaLR | LTR |
| 71927507 | 71927557 | + | 17.9 | 377 | 426 | 0 | MSTB | MaLR | LTR |

  

|  |  |  |  |  |  |  |  |  |  |
| --- | --- | --- | --- | --- | --- | --- | --- | --- | --- |
| chr7:71973046-71973652 | | | 607 bp | | |  |  |  |  |
| Genome Start | Genome End | Strand | Div | Repeat Start | Repeat End | Repeat Left | Name | Family | Class |
| 71973046 | 71973183 | + | 16.6 | 1 | 134 | -178 | AluJo | Alu | SINE |
| 71973184 | 71973490 | + | 17.5 | 2 | 309 | 0 | AluSx | Alu | SINE |
| 71973491 | 71973652 | + | 16.6 | 135 | 298 | -14 | AluJo | Alu | SINE |

  

|  |  |  |  |  |  |  |  |  |  |
| --- | --- | --- | --- | --- | --- | --- | --- | --- | --- |
| chr7:71983723-71985321 | | | 1599 bp | | |  |  |  |  |
| Genome Start | Genome End | Strand | Div | Repeat Start | Repeat End | Repeat Left | Name | Family | Class |
| 71983723 | 71983905 | + | 21.7 | 21 | 201 | -510 | MER67C | ERV1 | LTR |
| 71983906 | 71984213 | - | 11.7 | 1 | 310 | -2 | AluSx | Alu | SINE |
| 71984214 | 71984461 | + | 21.7 | 202 | 452 | -259 | MER67C | ERV1 | LTR |
| 71984462 | 71984786 | + | 15.2 | 1 | 304 | -8 | AluSx | Alu | SINE |
| 71984787 | 71984886 | + | 21.7 | 453 | 543 | -168 | MER67C | ERV1 | LTR |
| 71984887 | 71985190 | + | 12.2 | 1 | 303 | -7 | AluSg | Alu | SINE |
| 71985191 | 71985321 | + | 21.7 | 544 | 674 | -37 | MER67C | ERV1 | LTR |

  

|  |  |  |  |  |  |  |  |  |  |
| --- | --- | --- | --- | --- | --- | --- | --- | --- | --- |
| chr7:71992099-71992955 | | | 857 bp | | |  |  |  |  |
| Genome Start | Genome End | Strand | Div | Repeat Start | Repeat End | Repeat Left | Name | Family | Class |
| 71992099 | 71992421 | - | 24.3 | 7133 | 7430 | -531 | L1MC5 | L1 | LINE |
| 71992422 | 71992704 | - | 17.7 | 1 | 300 | -12 | AluJo | Alu | SINE |
| 71992705 | 71992955 | - | 24.3 | 6852 | 7132 | -829 | L1MC5 | L1 | LINE |

  

|  |  |  |  |  |  |  |  |  |  |
| --- | --- | --- | --- | --- | --- | --- | --- | --- | --- |
| chr7:71993690-71996470 | | | 2692 bp | | |  |  |  |  |
| Genome Start | Genome End | Strand | Div | Repeat Start | Repeat End | Repeat Left | Name | Family | Class |
| 71993690 | 71994628 | - | 22.6 | 1929 | 2815 | -3404 | L1MEc | L1 | LINE |
| 71994629 | 71994948 | - | 12.0 | 1 | 320 | 0 | AluSx | Alu | SINE |
| 71994949 | 71995844 | - | 20.6 | 847 | 1928 | -4472 | L1MEc | L1 | LINE |
| 71995879 | 71996016 | - | 21.7 | 119 | 273 | -6127 | L1MEc | L1 | LINE |
| 71996019 | 71996332 | + | 13.3 | 3 | 310 | 0 | AluSg | Alu | SINE |
| 71996386 | 71996470 | - | 25.3 | 36 | 115 | -6285 | L1MEc | L1 | LINE |

  

|  |  |  |  |  |  |  |  |  |  |
| --- | --- | --- | --- | --- | --- | --- | --- | --- | --- |
| chr7:71996666-71997583 | | | 626 bp | | |  |  |  |  |
| Genome Start | Genome End | Strand | Div | Repeat Start | Repeat End | Repeat Left | Name | Family | Class |
| 71996666 | 71996819 | - | 27.4 | 4536 | 4700 | -1446 | L1M5 | L1 | LINE |
| 71996852 | 71996980 | - | 8.5 | 5 | 133 | -179 | AluSq/x | Alu | SINE |
| 71997060 | 71997115 | - | 25.0 | 4030 | 4085 | -2061 | L1MD | L1 | LINE |
| 71997297 | 71997583 | - | 32.3 | 3692 | 3992 | -2154 | L1M5 | L1 | LINE |

  

|  |  |  |  |  |  |  |  |  |  |
| --- | --- | --- | --- | --- | --- | --- | --- | --- | --- |
| chr7:72030253-72031229 | | | 977 bp | | |  |  |  |  |
| Genome Start | Genome End | Strand | Div | Repeat Start | Repeat End | Repeat Left | Name | Family | Class |
| 72030253 | 72030867 | - | 27.9 | 5169 | 5823 | -417 | L1M5 | L1 | LINE |
| 72030868 | 72031161 | - | 14.8 | 1 | 292 | -20 | AluSx | Alu | SINE |
| 72031162 | 72031229 | - | 25.5 | 5101 | 5168 | -978 | L1M5 | L1 | LINE |

  

|  |  |  |  |  |  |  |  |  |  |
| --- | --- | --- | --- | --- | --- | --- | --- | --- | --- |
| chr7:72039121-72039478 | | | 358 bp | | |  |  |  |  |
| Genome Start | Genome End | Strand | Div | Repeat Start | Repeat End | Repeat Left | Name | Family | Class |
| 72039121 | 72039185 | + | 32.5 | 11 | 77 | -191 | MIRb | MIR | SINE |
| 72039186 | 72039318 | + | 18.1 | 1 | 133 | 0 | FLAM\_C | Alu | SINE |
| 72039319 | 72039478 | + | 32.5 | 78 | 259 | -9 | MIRb | MIR | SINE |

  

|  |  |  |  |  |  |  |  |  |  |
| --- | --- | --- | --- | --- | --- | --- | --- | --- | --- |
| chr7:72044661-72045185 | | | 525 bp | | |  |  |  |  |
| Genome Start | Genome End | Strand | Div | Repeat Start | Repeat End | Repeat Left | Name | Family | Class |
| 72044661 | 72044839 | - | 19.1 | 134 | 302 | 0 | AluSx | Alu | SINE |
| 72044840 | 72045050 | - | 7.6 | 1 | 218 | -93 | AluY | Alu | SINE |
| 72045051 | 72045185 | - | 19.1 | 1 | 133 | -169 | AluSx | Alu | SINE |

  

|  |  |  |  |  |  |  |  |  |  |
| --- | --- | --- | --- | --- | --- | --- | --- | --- | --- |
| chr7:72052190-72053078 | | | 855 bp | | |  |  |  |  |
| Genome Start | Genome End | Strand | Div | Repeat Start | Repeat End | Repeat Left | Name | Family | Class |
| 72052190 | 72052230 | - | 28.9 | 3122 | 3163 | -256 | L2 | L2 | LINE |
| 72052231 | 72052524 | + | 13.7 | 1 | 292 | -20 | AluSx | Alu | SINE |
| 72052525 | 72052786 | - | 29.7 | 2821 | 3121 | -298 | L2 | L2 | LINE |
| 72052821 | 72053078 | - | 29.2 | 1236 | 1525 | -1894 | L2 | L2 | LINE |

  

|  |  |  |  |  |  |  |  |  |  |
| --- | --- | --- | --- | --- | --- | --- | --- | --- | --- |
| chr7:72059524-72060819 | | | 1296 bp | | |  |  |  |  |
| Genome Start | Genome End | Strand | Div | Repeat Start | Repeat End | Repeat Left | Name | Family | Class |
| 72059524 | 72059688 | + | 8.9 | 1 | 160 | -153 | AluSp | Alu | SINE |
| 72059689 | 72060671 | + | 6.9 | 1 | 1002 | 0 | LTR5B | ERVK | LTR |
| 72060672 | 72060819 | + | 8.9 | 161 | 306 | -7 | AluSp | Alu | SINE |

  

|  |  |  |  |  |  |  |  |  |  |
| --- | --- | --- | --- | --- | --- | --- | --- | --- | --- |
| chr7:72086037-72086638 | | | 602 bp | | |  |  |  |  |
| Genome Start | Genome End | Strand | Div | Repeat Start | Repeat End | Repeat Left | Name | Family | Class |
| 72086037 | 72086166 | + | 14.9 | 1 | 128 | -184 | AluJo | Alu | SINE |
| 72086167 | 72086475 | + | 11.3 | 2 | 312 | 0 | AluSx | Alu | SINE |
| 72086476 | 72086638 | + | 14.9 | 129 | 289 | -23 | AluJo | Alu | SINE |

  

|  |  |  |  |  |  |  |  |  |  |
| --- | --- | --- | --- | --- | --- | --- | --- | --- | --- |
| chr7:72087433-72088369 | | | 937 bp | | |  |  |  |  |
| Genome Start | Genome End | Strand | Div | Repeat Start | Repeat End | Repeat Left | Name | Family | Class |
| 72087433 | 72087908 | + | 14.1 | 91 | 560 | -174 | MER50 | ERV1 | LTR |
| 72087909 | 72088223 | - | 11.9 | 2 | 312 | 0 | AluSx | Alu | SINE |
| 72088224 | 72088369 | + | 14.1 | 561 | 711 | -23 | MER50 | ERV1 | LTR |

  

|  |  |  |  |  |  |  |  |  |  |
| --- | --- | --- | --- | --- | --- | --- | --- | --- | --- |
| chr7:72091196-72091809 | | | 614 bp | | |  |  |  |  |
| Genome Start | Genome End | Strand | Div | Repeat Start | Repeat End | Repeat Left | Name | Family | Class |
| 72091196 | 72091262 | - | 14.7 | 214 | 267 | -45 | AluJb | Alu | SINE |
| 72091263 | 72091583 | + | 9.3 | 1 | 313 | 0 | AluSp | Alu | SINE |
| 72091584 | 72091809 | - | 14.7 | 1 | 213 | -99 | AluJb | Alu | SINE |

  

|  |  |  |  |  |  |  |  |  |  |
| --- | --- | --- | --- | --- | --- | --- | --- | --- | --- |
| chr7:72096758-72097378 | | | 621 bp | | |  |  |  |  |
| Genome Start | Genome End | Strand | Div | Repeat Start | Repeat End | Repeat Left | Name | Family | Class |
| 72096758 | 72096891 | + | 14.1 | 2 | 135 | -177 | AluSx | Alu | SINE |
| 72096892 | 72097199 | + | 6.8 | 1 | 310 | -1 | AluY | Alu | SINE |
| 72097200 | 72097378 | + | 14.1 | 136 | 307 | -5 | AluSx | Alu | SINE |

  

|  |  |  |  |  |  |  |  |  |  |
| --- | --- | --- | --- | --- | --- | --- | --- | --- | --- |
| chr7:72102646-72103404 | | | 759 bp | | |  |  |  |  |
| Genome Start | Genome End | Strand | Div | Repeat Start | Repeat End | Repeat Left | Name | Family | Class |
| 72102646 | 72102707 | - | 25.5 | 6170 | 6235 | -5 | L1ME3B | L1 | LINE |
| 72102708 | 72103043 | - | 15.7 | 1 | 294 | -18 | AluJo | Alu | SINE |
| 72103044 | 72103404 | - | 25.5 | 5765 | 6169 | -71 | L1ME3B | L1 | LINE |

  

|  |  |  |  |  |  |  |  |  |  |
| --- | --- | --- | --- | --- | --- | --- | --- | --- | --- |
| chr7:72103405-72103967 | | | 563 bp | | |  |  |  |  |
| Genome Start | Genome End | Strand | Div | Repeat Start | Repeat End | Repeat Left | Name | Family | Class |
| 72103405 | 72103488 | + | 30.9 | 1010 | 1087 | -1420 | HAL1 | L1 | LINE |
| 72103489 | 72103797 | + | 14.7 | 1 | 312 | 0 | AluSx | Alu | SINE |
| 72103798 | 72103967 | + | 30.9 | 1088 | 1261 | -1246 | HAL1 | L1 | LINE |

  

|  |  |  |  |  |  |  |  |  |  |
| --- | --- | --- | --- | --- | --- | --- | --- | --- | --- |
| chr7:72104781-72105585 | | | 796 bp | | |  |  |  |  |
| Genome Start | Genome End | Strand | Div | Repeat Start | Repeat End | Repeat Left | Name | Family | Class |
| 72104781 | 72104885 | + | 15.1 | 82 | 189 | -86 | ORSL | Tip100 | DNA |
| 72104886 | 72105198 | - | 17.4 | 1 | 302 | -10 | AluJo | Alu | SINE |
| 72105208 | 72105506 | - | 10.4 | 1 | 300 | -13 | AluSp | Alu | SINE |
| 72105507 | 72105585 | + | 15.1 | 196 | 267 | -8 | ORSL | Tip100 | DNA |

  

|  |  |  |  |  |  |  |  |  |  |
| --- | --- | --- | --- | --- | --- | --- | --- | --- | --- |
| chr7:72118304-72119079 | | | 775 bp | | |  |  |  |  |
| Genome Start | Genome End | Strand | Div | Repeat Start | Repeat End | Repeat Left | Name | Family | Class |
| 72118304 | 72118436 | + | 16.8 | 1 | 130 | -13 | FLAM\_C | Alu | SINE |
| 72118437 | 72118747 | + | 11.7 | 1 | 313 | 0 | AluSx | Alu | SINE |
| 72118749 | 72119065 | + | 15.1 | 3 | 313 | 0 | AluSq | Alu | SINE |
| 72119066 | 72119079 | + | 16.8 | 131 | 143 | 0 | FLAM\_C | Alu | SINE |

  

|  |  |  |  |  |  |  |  |  |  |
| --- | --- | --- | --- | --- | --- | --- | --- | --- | --- |
| chr7:72119102-72119577 | | | 476 bp | | |  |  |  |  |
| Genome Start | Genome End | Strand | Div | Repeat Start | Repeat End | Repeat Left | Name | Family | Class |
| 72119102 | 72119241 | - | 27.9 | 31 | 187 | -75 | MIR | MIR | SINE |
| 72119242 | 72119552 | - | 11.2 | 1 | 312 | -1 | AluSp | Alu | SINE |
| 72119553 | 72119577 | - | 27.9 | 7 | 30 | -232 | MIR | MIR | SINE |

  

|  |  |  |  |  |  |  |  |  |  |
| --- | --- | --- | --- | --- | --- | --- | --- | --- | --- |
| chr7:72123170-72123881 | | | 712 bp | | |  |  |  |  |
| Genome Start | Genome End | Strand | Div | Repeat Start | Repeat End | Repeat Left | Name | Family | Class |
| 72123170 | 72123559 | - | 17.2 | 33 | 414 | -75 | LTR45B | ERV1 | LTR |
| 72123560 | 72123850 | + | 10.0 | 4 | 296 | -17 | AluSq | Alu | SINE |
| 72123851 | 72123881 | - | 17.2 | 3 | 32 | -457 | LTR45B | ERV1 | LTR |

  

|  |  |  |  |  |  |  |  |  |  |
| --- | --- | --- | --- | --- | --- | --- | --- | --- | --- |
| chr7:72125132-72126326 | | | 915 bp | | |  |  |  |  |
| Genome Start | Genome End | Strand | Div | Repeat Start | Repeat End | Repeat Left | Name | Family | Class |
| 72125132 | 72125259 | - | 29.0 | 5398 | 5522 | -2914 | HERV9 | ERV1 | LTR |
| 72125307 | 72125610 | + | 13.0 | 1 | 300 | -13 | AluSq | Alu | SINE |
| 72125613 | 72125913 | + | 14.9 | 3 | 303 | -7 | AluSg | Alu | SINE |
| 72126145 | 72126326 | - | 25.8 | 4474 | 4650 | -1889 | HERV9 | ERV1 | LTR |

  

|  |  |  |  |  |  |  |  |  |  |
| --- | --- | --- | --- | --- | --- | --- | --- | --- | --- |
| chr7:72131207-72131812 | | | 574 bp | | |  |  |  |  |
| Genome Start | Genome End | Strand | Div | Repeat Start | Repeat End | Repeat Left | Name | Family | Class |
| 72131207 | 72131344 | + | 16.6 | 1 | 136 | -176 | AluJo | Alu | SINE |
| 72131345 | 72131618 | + | 18.6 | 2 | 276 | -36 | AluSx | Alu | SINE |
| 72131651 | 72131812 | + | 16.6 | 144 | 295 | -17 | AluJo | Alu | SINE |

  

|  |  |  |  |  |  |  |  |  |  |
| --- | --- | --- | --- | --- | --- | --- | --- | --- | --- |
| chr7:72138222-72138920 | | | 699 bp | | |  |  |  |  |
| Genome Start | Genome End | Strand | Div | Repeat Start | Repeat End | Repeat Left | Name | Family | Class |
| 72138222 | 72138554 | - | 37.5 | 3989 | 4324 | -165 | L3 | CR1 | LINE |
| 72138555 | 72138860 | + | 15.1 | 3 | 301 | -11 | AluSx | Alu | SINE |
| 72138861 | 72138920 | - | 37.5 | 3933 | 3988 | -501 | L3 | CR1 | LINE |

  

|  |  |  |  |  |  |  |  |  |  |
| --- | --- | --- | --- | --- | --- | --- | --- | --- | --- |
| chr7:72142940-72143533 | | | 594 bp | | |  |  |  |  |
| Genome Start | Genome End | Strand | Div | Repeat Start | Repeat End | Repeat Left | Name | Family | Class |
| 72142940 | 72143072 | + | 16.7 | 1 | 131 | -181 | AluJo | Alu | SINE |
| 72143073 | 72143364 | + | 12.0 | 1 | 292 | -20 | AluSx | Alu | SINE |
| 72143365 | 72143533 | + | 16.7 | 132 | 294 | -18 | AluJo | Alu | SINE |

  

|  |  |  |  |  |  |  |  |  |  |
| --- | --- | --- | --- | --- | --- | --- | --- | --- | --- |
| chr7:72146280-72147055 | | | 775 bp | | |  |  |  |  |
| Genome Start | Genome End | Strand | Div | Repeat Start | Repeat End | Repeat Left | Name | Family | Class |
| 72146280 | 72146412 | + | 16.8 | 1 | 130 | -13 | FLAM\_C | Alu | SINE |
| 72146413 | 72146723 | + | 11.7 | 1 | 313 | 0 | AluSx | Alu | SINE |
| 72146725 | 72147041 | + | 15.1 | 3 | 313 | 0 | AluSq | Alu | SINE |
| 72147042 | 72147055 | + | 16.8 | 131 | 143 | 0 | FLAM\_C | Alu | SINE |

  

|  |  |  |  |  |  |  |  |  |  |
| --- | --- | --- | --- | --- | --- | --- | --- | --- | --- |
| chr7:72151232-72151943 | | | 712 bp | | |  |  |  |  |
| Genome Start | Genome End | Strand | Div | Repeat Start | Repeat End | Repeat Left | Name | Family | Class |
| 72151232 | 72151621 | - | 17.2 | 33 | 414 | -75 | LTR45B | ERV1 | LTR |
| 72151622 | 72151912 | + | 10.0 | 4 | 296 | -17 | AluSq | Alu | SINE |
| 72151913 | 72151943 | - | 17.2 | 3 | 32 | -457 | LTR45B | ERV1 | LTR |

  

|  |  |  |  |  |  |  |  |  |  |
| --- | --- | --- | --- | --- | --- | --- | --- | --- | --- |
| chr7:72159269-72159875 | | | 575 bp | | |  |  |  |  |
| Genome Start | Genome End | Strand | Div | Repeat Start | Repeat End | Repeat Left | Name | Family | Class |
| 72159269 | 72159406 | + | 16.6 | 1 | 136 | -176 | AluJo | Alu | SINE |
| 72159407 | 72159680 | + | 18.6 | 2 | 276 | -36 | AluSx | Alu | SINE |
| 72159713 | 72159875 | + | 16.6 | 144 | 296 | -16 | AluJo | Alu | SINE |

  

|  |  |  |  |  |  |  |  |  |  |
| --- | --- | --- | --- | --- | --- | --- | --- | --- | --- |
| chr7:72165871-72166476 | | | 606 bp | | |  |  |  |  |
| Genome Start | Genome End | Strand | Div | Repeat Start | Repeat End | Repeat Left | Name | Family | Class |
| 72165871 | 72166012 | + | 11.7 | 1 | 137 | -175 | AluSx | Alu | SINE |
| 72166013 | 72166301 | + | 10.4 | 1 | 296 | -15 | AluY | Alu | SINE |
| 72166302 | 72166476 | + | 11.7 | 138 | 309 | -3 | AluSx | Alu | SINE |

  

|  |  |  |  |  |  |  |  |  |  |
| --- | --- | --- | --- | --- | --- | --- | --- | --- | --- |
| chr7:72169153-72169773 | | | 621 bp | | |  |  |  |  |
| Genome Start | Genome End | Strand | Div | Repeat Start | Repeat End | Repeat Left | Name | Family | Class |
| 72169153 | 72169328 | - | 11.6 | 137 | 302 | -10 | AluSx | Alu | SINE |
| 72169329 | 72169636 | - | 5.5 | 1 | 308 | -3 | AluY | Alu | SINE |
| 72169637 | 72169773 | - | 11.6 | 1 | 136 | -176 | AluSx | Alu | SINE |

  

|  |  |  |  |  |  |  |  |  |  |
| --- | --- | --- | --- | --- | --- | --- | --- | --- | --- |
| chr7:72170719-72172681 | | | 1924 bp | | |  |  |  |  |
| Genome Start | Genome End | Strand | Div | Repeat Start | Repeat End | Repeat Left | Name | Family | Class |
| 72170719 | 72170786 | - | 16.2 | 142 | 209 | 0 | MER3 | MER1\_type | DNA |
| 72170791 | 72171101 | + | 12.7 | 1 | 309 | -3 | AluSx | Alu | SINE |
| 72171114 | 72171304 | + | 23.2 | 1 | 188 | -186 | MLT1A | MaLR | LTR |
| 72171305 | 72171374 | - | 13.8 | 361 | 428 | 0 | MSTA | MaLR | LTR |
| 72171398 | 72171679 | - | 11.7 | 1 | 283 | -30 | AluSq | Alu | SINE |
| 72171680 | 72172020 | - | 13.8 | 40 | 360 | -68 | MSTA | MaLR | LTR |
| 72172021 | 72172331 | - | 10.6 | 1 | 312 | 0 | AluSx | Alu | SINE |
| 72172332 | 72172375 | - | 13.8 | 1 | 39 | -389 | MSTA | MaLR | LTR |
| 72172376 | 72172551 | + | 23.2 | 189 | 373 | -1 | MLT1A | MaLR | LTR |
| 72172552 | 72172681 | - | 25.6 | 1 | 121 | -88 | MER3 | MER1\_type | DNA |

  

|  |  |  |  |  |  |  |  |  |  |
| --- | --- | --- | --- | --- | --- | --- | --- | --- | --- |
| chr7:72175461-72176432 | | | 898 bp | | |  |  |  |  |
| Genome Start | Genome End | Strand | Div | Repeat Start | Repeat End | Repeat Left | Name | Family | Class |
| 72175461 | 72175711 | + | 26.3 | 85 | 350 | -141 | LTR16C | ERVL | LTR |
| 72175715 | 72176017 | - | 13.2 | 1 | 312 | -1 | AluSq | Alu | SINE |
| 72176089 | 72176342 | - | 17.9 | 1 | 256 | -56 | AluJb | Alu | SINE |
| 72176343 | 72176432 | + | 21.1 | 346 | 453 | -38 | LTR16C | ERVL | LTR |

  

|  |  |  |  |  |  |  |  |  |  |
| --- | --- | --- | --- | --- | --- | --- | --- | --- | --- |
| chr7:72190383-72190842 | | | 460 bp | | |  |  |  |  |
| Genome Start | Genome End | Strand | Div | Repeat Start | Repeat End | Repeat Left | Name | Family | Class |
| 72190383 | 72190419 | - | 35.1 | 224 | 258 | -4 | MIR | MIR | SINE |
| 72190420 | 72190722 | - | 7.7 | 1 | 298 | -11 | AluSc | Alu | SINE |
| 72190723 | 72190842 | - | 35.1 | 93 | 223 | -39 | MIR | MIR | SINE |

  

|  |  |  |  |  |  |  |  |  |  |
| --- | --- | --- | --- | --- | --- | --- | --- | --- | --- |
| chr7:72193554-72194182 | | | 629 bp | | |  |  |  |  |
| Genome Start | Genome End | Strand | Div | Repeat Start | Repeat End | Repeat Left | Name | Family | Class |
| 72193554 | 72193644 | + | 19.8 | 7481 | 7571 | -214 | L1MC3 | L1 | LINE |
| 72193645 | 72193956 | + | 6.4 | 1 | 313 | 0 | AluSp | Alu | SINE |
| 72193957 | 72194182 | + | 19.8 | 7572 | 7783 | -2 | L1MC3 | L1 | LINE |

  

|  |  |  |  |  |  |  |  |  |  |
| --- | --- | --- | --- | --- | --- | --- | --- | --- | --- |
| chr7:72195138-72195714 | | | 577 bp | | |  |  |  |  |
| Genome Start | Genome End | Strand | Div | Repeat Start | Repeat End | Repeat Left | Name | Family | Class |
| 72195138 | 72195243 | + | 23.3 | 3 | 108 | -487 | MLT1G1 | MaLR | LTR |
| 72195244 | 72195649 | - | 15.0 | 1 | 432 | 0 | MSTB1 | MaLR | LTR |
| 72195650 | 72195714 | + | 23.9 | 68 | 162 | -433 | MLT1G1 | MaLR | LTR |

  

|  |  |  |  |  |  |  |  |  |  |
| --- | --- | --- | --- | --- | --- | --- | --- | --- | --- |
| chr7:72198254-72199052 | | | 796 bp | | |  |  |  |  |
| Genome Start | Genome End | Strand | Div | Repeat Start | Repeat End | Repeat Left | Name | Family | Class |
| 72198254 | 72198271 | - | 19.0 | 2480 | 2493 | -119 | Charlie5 | MER1\_type | DNA |
| 72198272 | 72198572 | + | 11.6 | 1 | 301 | -11 | AluSx | Alu | SINE |
| 72198576 | 72198865 | + | 10.7 | 6 | 298 | -14 | AluSx | Alu | SINE |
| 72198866 | 72199052 | - | 19.0 | 2292 | 2479 | -133 | Charlie5 | MER1\_type | DNA |

  

|  |  |  |  |  |  |  |  |  |  |
| --- | --- | --- | --- | --- | --- | --- | --- | --- | --- |
| chr7:72222212-72222595 | | | 384 bp | | |  |  |  |  |
| Genome Start | Genome End | Strand | Div | Repeat Start | Repeat End | Repeat Left | Name | Family | Class |
| 72222212 | 72222286 | + | 25.4 | 46 | 123 | -145 | MIR | MIR | SINE |
| 72222287 | 72222563 | - | 20.2 | 6 | 277 | -35 | AluJo | Alu | SINE |
| 72222564 | 72222595 | + | 23.3 | 116 | 149 | -113 | MIR | MIR | SINE |

  

|  |  |  |  |  |  |  |  |  |  |
| --- | --- | --- | --- | --- | --- | --- | --- | --- | --- |
| chr7:72252805-72253303 | | | 499 bp | | |  |  |  |  |
| Genome Start | Genome End | Strand | Div | Repeat Start | Repeat End | Repeat Left | Name | Family | Class |
| 72252805 | 72252994 | + | 23.4 | 2 | 210 | -14 | MER58A | MER1\_type | DNA |
| 72252995 | 72253288 | + | 11.6 | 1 | 295 | -17 | AluSx | Alu | SINE |
| 72253289 | 72253303 | + | 23.4 | 211 | 224 | 0 | MER58A | MER1\_type | DNA |

  

|  |  |  |  |  |  |  |  |  |  |
| --- | --- | --- | --- | --- | --- | --- | --- | --- | --- |
| chr7:72266860-72267368 | | | 509 bp | | |  |  |  |  |
| Genome Start | Genome End | Strand | Div | Repeat Start | Repeat End | Repeat Left | Name | Family | Class |
| 72266860 | 72266917 | + | 30.9 | 8 | 68 | -194 | MIR | MIR | SINE |
| 72266918 | 72267215 | + | 9.4 | 1 | 301 | -11 | AluSx | Alu | SINE |
| 72267216 | 72267368 | + | 30.9 | 69 | 230 | -32 | MIR | MIR | SINE |

  

|  |  |  |  |  |  |  |  |  |  |
| --- | --- | --- | --- | --- | --- | --- | --- | --- | --- |
| chr7:72276698-72277229 | | | 532 bp | | |  |  |  |  |
| Genome Start | Genome End | Strand | Div | Repeat Start | Repeat End | Repeat Left | Name | Family | Class |
| 72276698 | 72276713 | - | 27.5 | 2818 | 2834 | -585 | L2 | L2 | LINE |
| 72276714 | 72277021 | - | 16.5 | 1 | 306 | -6 | AluJb | Alu | SINE |
| 72277022 | 72277229 | - | 27.5 | 2583 | 2817 | -602 | L2 | L2 | LINE |

  

|  |  |  |  |  |  |  |  |  |  |
| --- | --- | --- | --- | --- | --- | --- | --- | --- | --- |
| chr7:72285563-72286154 | | | 592 bp | | |  |  |  |  |
| Genome Start | Genome End | Strand | Div | Repeat Start | Repeat End | Repeat Left | Name | Family | Class |
| 72285563 | 72285769 | + | 23.9 | 4868 | 5036 | -1116 | L1M5 | L1 | LINE |
| 72285770 | 72286039 | + | 13.1 | 15 | 282 | -31 | AluSq | Alu | SINE |
| 72286040 | 72286154 | + | 23.4 | 5031 | 5140 | -1006 | L1M5 | L1 | LINE |

  

|  |  |  |  |  |  |  |  |  |  |
| --- | --- | --- | --- | --- | --- | --- | --- | --- | --- |
| chr7:72290475-72290991 | | | 517 bp | | |  |  |  |  |
| Genome Start | Genome End | Strand | Div | Repeat Start | Repeat End | Repeat Left | Name | Family | Class |
| 72290475 | 72290655 | - | 30.9 | 58 | 257 | -5 | MIRb | MIR | SINE |
| 72290656 | 72290964 | - | 10.1 | 1 | 311 | -2 | AluSq | Alu | SINE |
| 72290965 | 72290991 | - | 30.9 | 35 | 57 | -211 | MIRb | MIR | SINE |

  

|  |  |  |  |  |  |  |  |  |  |
| --- | --- | --- | --- | --- | --- | --- | --- | --- | --- |
| chr7:72294634-72297395 | | | 2762 bp | | |  |  |  |  |
| Genome Start | Genome End | Strand | Div | Repeat Start | Repeat End | Repeat Left | Name | Family | Class |
| 72294634 | 72294857 | - | 20.2 | 2195 | 2420 | 0 | Charlie8 | MER1\_type | DNA |
| 72294858 | 72295152 | + | 9.5 | 1 | 298 | -14 | AluSx | Alu | SINE |
| 72295153 | 72297081 | - | 20.2 | 124 | 2194 | -223 | Charlie8 | MER1\_type | DNA |
| 72297082 | 72297383 | - | 10.9 | 1 | 302 | -10 | AluSx | Alu | SINE |
| 72297384 | 72297395 | - | 20.2 | 112 | 123 | -2294 | Charlie8 | MER1\_type | DNA |

  

|  |  |  |  |  |  |  |  |  |  |
| --- | --- | --- | --- | --- | --- | --- | --- | --- | --- |
| chr7:72305672-72306270 | | | 599 bp | | |  |  |  |  |
| Genome Start | Genome End | Strand | Div | Repeat Start | Repeat End | Repeat Left | Name | Family | Class |
| 72305672 | 72305844 | - | 15.2 | 138 | 306 | -6 | AluJb | Alu | SINE |
| 72305845 | 72306143 | - | 11.6 | 1 | 300 | -12 | AluSx | Alu | SINE |
| 72306144 | 72306270 | - | 15.2 | 11 | 137 | -175 | AluJb | Alu | SINE |

  

|  |  |  |  |  |  |  |  |  |  |
| --- | --- | --- | --- | --- | --- | --- | --- | --- | --- |
| chr7:72312512-72313189 | | | 678 bp | | |  |  |  |  |
| Genome Start | Genome End | Strand | Div | Repeat Start | Repeat End | Repeat Left | Name | Family | Class |
| 72312512 | 72312621 | + | 28.4 | 8 | 113 | -436 | MLT1H | MaLR | LTR |
| 72312622 | 72312916 | - | 15.2 | 1 | 299 | -13 | AluJb | Alu | SINE |
| 72312917 | 72313189 | + | 28.4 | 114 | 406 | -143 | MLT1H | MaLR | LTR |

  

|  |  |  |  |  |  |  |  |  |  |
| --- | --- | --- | --- | --- | --- | --- | --- | --- | --- |
| chr7:72318972-72319810 | | | 839 bp | | |  |  |  |  |
| Genome Start | Genome End | Strand | Div | Repeat Start | Repeat End | Repeat Left | Name | Family | Class |
| 72318972 | 72319373 | + | 16.9 | 7 | 405 | -145 | MER44B | MER2\_type | DNA |
| 72319374 | 72319664 | - | 9.3 | 1 | 292 | -20 | AluSx | Alu | SINE |
| 72319665 | 72319810 | + | 16.9 | 406 | 550 | 0 | MER44B | MER2\_type | DNA |

  

|  |  |  |  |  |  |  |  |  |  |
| --- | --- | --- | --- | --- | --- | --- | --- | --- | --- |
| chr7:72324236-72326068 | | | 1833 bp | | |  |  |  |  |
| Genome Start | Genome End | Strand | Div | Repeat Start | Repeat End | Repeat Left | Name | Family | Class |
| 72324236 | 72324283 | - | 29.1 | 719 | 761 | -12 | LTR41 | ERVL | LTR |
| 72324284 | 72324321 | - | 14.2 | 6127 | 6156 | 0 | L1PB4 | L1 | LINE |
| 72324322 | 72324630 | - | 7.8 | 1 | 307 | -4 | AluY | Alu | SINE |
| 72324631 | 72324805 | - | 14.2 | 5960 | 6126 | -30 | L1PB4 | L1 | LINE |
| 72324806 | 72325105 | - | 13.0 | 1 | 300 | -12 | AluSx | Alu | SINE |
| 72325106 | 72325242 | - | 14.2 | 5829 | 5959 | -197 | L1PB4 | L1 | LINE |
| 72325243 | 72325549 | - | 11.7 | 1 | 310 | -2 | AluSx | Alu | SINE |
| 72325550 | 72325834 | - | 14.2 | 5535 | 5828 | -328 | L1PB4 | L1 | LINE |
| 72325835 | 72326068 | - | 29.1 | 506 | 718 | -55 | LTR41 | ERVL | LTR |

  

|  |  |  |  |  |  |  |  |  |  |
| --- | --- | --- | --- | --- | --- | --- | --- | --- | --- |
| chr7:72327242-72329215 | | | 1974 bp | | |  |  |  |  |
| Genome Start | Genome End | Strand | Div | Repeat Start | Repeat End | Repeat Left | Name | Family | Class |
| 72327242 | 72327523 | + | 17.0 | 3789 | 4075 | -2065 | L1M3 | L1 | LINE |
| 72327524 | 72327829 | + | 11.2 | 1 | 304 | -8 | AluSx | Alu | SINE |
| 72327830 | 72328239 | + | 17.0 | 4076 | 4473 | -1667 | L1M3 | L1 | LINE |
| 72328240 | 72328560 | + | 10.9 | 1 | 311 | -1 | AluSx | Alu | SINE |
| 72328561 | 72328737 | + | 17.0 | 4474 | 4645 | -1495 | L1M3 | L1 | LINE |
| 72328738 | 72329040 | + | 10.7 | 1 | 299 | -11 | AluSg | Alu | SINE |
| 72329041 | 72329215 | + | 17.0 | 4646 | 4829 | -1311 | L1M3 | L1 | LINE |

  

|  |  |  |  |  |  |  |  |  |  |
| --- | --- | --- | --- | --- | --- | --- | --- | --- | --- |
| chr7:72342679-72343220 | | | 542 bp | | |  |  |  |  |
| Genome Start | Genome End | Strand | Div | Repeat Start | Repeat End | Repeat Left | Name | Family | Class |
| 72342679 | 72342874 | - | 18.4 | 134 | 302 | 0 | AluSx | Alu | SINE |
| 72342875 | 72343085 | - | 9.0 | 1 | 218 | -93 | AluY | Alu | SINE |
| 72343086 | 72343220 | - | 18.4 | 1 | 133 | -169 | AluSx | Alu | SINE |

  

|  |  |  |  |  |  |  |  |  |  |
| --- | --- | --- | --- | --- | --- | --- | --- | --- | --- |
| chr7:72362557-72363348 | | | 792 bp | | |  |  |  |  |
| Genome Start | Genome End | Strand | Div | Repeat Start | Repeat End | Repeat Left | Name | Family | Class |
| 72362557 | 72362750 | + | 26.4 | 2 | 201 | -304 | MLT1D | MaLR | LTR |
| 72362751 | 72363056 | + | 10.5 | 7 | 312 | 0 | AluSx | Alu | SINE |
| 72363057 | 72363348 | + | 26.4 | 202 | 500 | -5 | MLT1D | MaLR | LTR |

  

|  |  |  |  |  |  |  |  |  |  |
| --- | --- | --- | --- | --- | --- | --- | --- | --- | --- |
| chr7:72425277-72425743 | | | 467 bp | | |  |  |  |  |
| Genome Start | Genome End | Strand | Div | Repeat Start | Repeat End | Repeat Left | Name | Family | Class |
| 72425277 | 72425424 | - | 11.3 | 4461 | 4612 | -1534 | L1MC | L1 | LINE |
| 72425425 | 72425721 | - | 9.8 | 1 | 297 | -15 | AluSx | Alu | SINE |
| 72425722 | 72425743 | - | 18.5 | 4445 | 4460 | -1686 | L1MC | L1 | LINE |

  

|  |  |  |  |  |  |  |  |  |  |
| --- | --- | --- | --- | --- | --- | --- | --- | --- | --- |
| chr7:72431312-72431910 | | | 599 bp | | |  |  |  |  |
| Genome Start | Genome End | Strand | Div | Repeat Start | Repeat End | Repeat Left | Name | Family | Class |
| 72431312 | 72431441 | + | 14.8 | 1 | 134 | -178 | AluJo | Alu | SINE |
| 72431442 | 72431749 | + | 10.3 | 2 | 312 | 0 | AluSx | Alu | SINE |
| 72431750 | 72431910 | + | 14.8 | 135 | 289 | -23 | AluJo | Alu | SINE |

  

|  |  |  |  |  |  |  |  |  |  |
| --- | --- | --- | --- | --- | --- | --- | --- | --- | --- |
| chr7:72455388-72456034 | | | 647 bp | | |  |  |  |  |
| Genome Start | Genome End | Strand | Div | Repeat Start | Repeat End | Repeat Left | Name | Family | Class |
| 72455388 | 72455458 | - | 15.5 | 201 | 271 | -41 | AluJb | Alu | SINE |
| 72455459 | 72455804 | + | 11.2 | 1 | 347 | 0 | AluSp | Alu | SINE |
| 72455805 | 72456034 | - | 15.4 | 1 | 214 | -98 | AluJb | Alu | SINE |

  

|  |  |  |  |  |  |  |  |  |  |
| --- | --- | --- | --- | --- | --- | --- | --- | --- | --- |
| chr7:72467113-72467870 | | | 758 bp | | |  |  |  |  |
| Genome Start | Genome End | Strand | Div | Repeat Start | Repeat End | Repeat Left | Name | Family | Class |
| 72467113 | 72467169 | - | 25.8 | 6175 | 6235 | -5 | L1ME3B | L1 | LINE |
| 72467170 | 72467509 | - | 16.2 | 1 | 298 | -14 | AluJo | Alu | SINE |
| 72467510 | 72467870 | - | 25.8 | 5765 | 6174 | -66 | L1ME3B | L1 | LINE |

  

|  |  |  |  |  |  |  |  |  |  |
| --- | --- | --- | --- | --- | --- | --- | --- | --- | --- |
| chr7:72467871-72468456 | | | 586 bp | | |  |  |  |  |
| Genome Start | Genome End | Strand | Div | Repeat Start | Repeat End | Repeat Left | Name | Family | Class |
| 72467871 | 72467952 | + | 28.2 | 1010 | 1087 | -1420 | HAL1 | L1 | LINE |
| 72467953 | 72468249 | + | 13.9 | 1 | 304 | -8 | AluSx | Alu | SINE |
| 72468250 | 72468456 | + | 28.2 | 1088 | 1261 | -1246 | HAL1 | L1 | LINE |

  

|  |  |  |  |  |  |  |  |  |  |
| --- | --- | --- | --- | --- | --- | --- | --- | --- | --- |
| chr7:73018142-73018606 | | | 465 bp | | |  |  |  |  |
| Genome Start | Genome End | Strand | Div | Repeat Start | Repeat End | Repeat Left | Name | Family | Class |
| 73018142 | 73018185 | + | 11.1 | 6028 | 6070 | -108 | L1MB8 | L1 | LINE |
| 73018186 | 73018495 | + | 11.8 | 1 | 310 | 0 | AluSx | Alu | SINE |
| 73018496 | 73018606 | + | 11.1 | 6071 | 6172 | -6 | L1MB8 | L1 | LINE |

  

|  |  |  |  |  |  |  |  |  |  |
| --- | --- | --- | --- | --- | --- | --- | --- | --- | --- |
| chr7:73601922-73602473 | | | 552 bp | | |  |  |  |  |
| Genome Start | Genome End | Strand | Div | Repeat Start | Repeat End | Repeat Left | Name | Family | Class |
| 73601922 | 73602019 | + | 13.1 | 1 | 136 | -176 | AluSx | Alu | SINE |
| 73602020 | 73602314 | + | 9.2 | 1 | 294 | -19 | AluSp | Alu | SINE |
| 73602315 | 73602473 | + | 13.1 | 137 | 295 | -17 | AluSx | Alu | SINE |

  

|  |  |  |  |  |  |  |  |  |  |
| --- | --- | --- | --- | --- | --- | --- | --- | --- | --- |
| chr7:73636277-73637056 | | | 780 bp | | |  |  |  |  |
| Genome Start | Genome End | Strand | Div | Repeat Start | Repeat End | Repeat Left | Name | Family | Class |
| 73636277 | 73636440 | - | 24.6 | 295 | 465 | 0 | MER4C | ERV1 | LTR |
| 73636441 | 73636725 | + | 13.0 | 3 | 301 | -12 | AluSq | Alu | SINE |
| 73636726 | 73637056 | - | 19.1 | 5 | 359 | -171 | MER4C | ERV1 | LTR |

  

|  |  |  |  |  |  |  |  |  |  |
| --- | --- | --- | --- | --- | --- | --- | --- | --- | --- |
| chr7:73825067-73826044 | | | 977 bp | | |  |  |  |  |
| Genome Start | Genome End | Strand | Div | Repeat Start | Repeat End | Repeat Left | Name | Family | Class |
| 73825067 | 73825197 | - | 27.3 | 3271 | 3405 | -14 | L2 | L2 | LINE |
| 73825198 | 73825493 | + | 10.5 | 1 | 295 | -18 | AluSp | Alu | SINE |
| 73825495 | 73825771 | + | 15.8 | 40 | 312 | 0 | AluJo | Alu | SINE |
| 73825772 | 73826044 | - | 27.3 | 3018 | 3270 | -149 | L2 | L2 | LINE |

  

|  |  |  |  |  |  |  |  |  |  |
| --- | --- | --- | --- | --- | --- | --- | --- | --- | --- |
| chr7:73827729-73828661 | | | 911 bp | | |  |  |  |  |
| Genome Start | Genome End | Strand | Div | Repeat Start | Repeat End | Repeat Left | Name | Family | Class |
| 73827729 | 73828007 | - | 23.6 | 7600 | 7897 | -145 | L1MC4 | L1 | LINE |
| 73828009 | 73828217 | - | 12.5 | 93 | 306 | -3 | AluSc | Alu | SINE |
| 73828232 | 73828523 | - | 8.9 | 6 | 297 | -15 | AluSx | Alu | SINE |
| 73828531 | 73828661 | - | 23.7 | 7465 | 7610 | -432 | L1MC4 | L1 | LINE |

  

|  |  |  |  |  |  |  |  |  |  |
| --- | --- | --- | --- | --- | --- | --- | --- | --- | --- |
| chr7:73830380-73830911 | | | 532 bp | | |  |  |  |  |
| Genome Start | Genome End | Strand | Div | Repeat Start | Repeat End | Repeat Left | Name | Family | Class |
| 73830380 | 73830395 | - | 27.5 | 2818 | 2834 | -585 | L2 | L2 | LINE |
| 73830396 | 73830703 | - | 15.8 | 1 | 306 | -6 | AluJb | Alu | SINE |
| 73830704 | 73830911 | - | 27.5 | 2583 | 2817 | -602 | L2 | L2 | LINE |

  

|  |  |  |  |  |  |  |  |  |  |
| --- | --- | --- | --- | --- | --- | --- | --- | --- | --- |
| chr7:73844174-73844672 | | | 499 bp | | |  |  |  |  |
| Genome Start | Genome End | Strand | Div | Repeat Start | Repeat End | Repeat Left | Name | Family | Class |
| 73844174 | 73844334 | - | 30.3 | 58 | 231 | -37 | MIRb | MIR | SINE |
| 73844335 | 73844645 | - | 9.3 | 1 | 313 | 0 | AluSq | Alu | SINE |
| 73844646 | 73844672 | - | 30.3 | 35 | 57 | -211 | MIRb | MIR | SINE |

  

|  |  |  |  |  |  |  |  |  |  |
| --- | --- | --- | --- | --- | --- | --- | --- | --- | --- |
| chr7:73859386-73859983 | | | 598 bp | | |  |  |  |  |
| Genome Start | Genome End | Strand | Div | Repeat Start | Repeat End | Repeat Left | Name | Family | Class |
| 73859386 | 73859558 | - | 15.2 | 138 | 306 | -6 | AluJb | Alu | SINE |
| 73859559 | 73859856 | - | 10.9 | 1 | 299 | -13 | AluSx | Alu | SINE |
| 73859857 | 73859983 | - | 15.2 | 11 | 137 | -175 | AluJb | Alu | SINE |

  

|  |  |  |  |  |  |  |  |  |  |
| --- | --- | --- | --- | --- | --- | --- | --- | --- | --- |
| chr7:73860253-73860864 | | | 612 bp | | |  |  |  |  |
| Genome Start | Genome End | Strand | Div | Repeat Start | Repeat End | Repeat Left | Name | Family | Class |
| 73860253 | 73860426 | - | 18.8 | 137 | 305 | -7 | AluJo | Alu | SINE |
| 73860427 | 73860726 | - | 17.2 | 1 | 300 | -12 | AluJb | Alu | SINE |
| 73860727 | 73860864 | - | 18.8 | 6 | 136 | -176 | AluJo | Alu | SINE |

  

|  |  |  |  |  |  |  |  |  |  |
| --- | --- | --- | --- | --- | --- | --- | --- | --- | --- |
| chr7:73863882-73864542 | | | 568 bp | | |  |  |  |  |
| Genome Start | Genome End | Strand | Div | Repeat Start | Repeat End | Repeat Left | Name | Family | Class |
| 73863882 | 73864007 | - | 20.7 | 7308 | 7432 | -529 | L1MC5 | L1 | LINE |
| 73864008 | 73864317 | - | 14.2 | 1 | 310 | 0 | AluJb | Alu | SINE |
| 73864318 | 73864377 | - | 20.7 | 7260 | 7307 | -654 | L1MC5 | L1 | LINE |
| 73864471 | 73864542 | - | 29.2 | 7162 | 7233 | -728 | L1MC5 | L1 | LINE |

  

|  |  |  |  |  |  |  |  |  |  |
| --- | --- | --- | --- | --- | --- | --- | --- | --- | --- |
| chr7:73866432-73867044 | | | 613 bp | | |  |  |  |  |
| Genome Start | Genome End | Strand | Div | Repeat Start | Repeat End | Repeat Left | Name | Family | Class |
| 73866432 | 73866541 | + | 30.5 | 8 | 113 | -436 | MLT1H | MaLR | LTR |
| 73866542 | 73866836 | - | 15.2 | 1 | 299 | -13 | AluJb | Alu | SINE |
| 73866837 | 73867044 | + | 30.5 | 114 | 327 | -222 | MLT1H | MaLR | LTR |

  

|  |  |  |  |  |  |  |  |  |  |
| --- | --- | --- | --- | --- | --- | --- | --- | --- | --- |
| chr7:73872900-73873738 | | | 839 bp | | |  |  |  |  |
| Genome Start | Genome End | Strand | Div | Repeat Start | Repeat End | Repeat Left | Name | Family | Class |
| 73872900 | 73873301 | + | 17.1 | 7 | 405 | -145 | MER44B | MER2\_type | DNA |
| 73873302 | 73873592 | - | 10.0 | 1 | 292 | -20 | AluSx | Alu | SINE |
| 73873593 | 73873738 | + | 17.1 | 406 | 550 | 0 | MER44B | MER2\_type | DNA |

  

|  |  |  |  |  |  |  |  |  |  |
| --- | --- | --- | --- | --- | --- | --- | --- | --- | --- |
| chr7:73878161-73879985 | | | 1825 bp | | |  |  |  |  |
| Genome Start | Genome End | Strand | Div | Repeat Start | Repeat End | Repeat Left | Name | Family | Class |
| 73878161 | 73878213 | - | 29.1 | 713 | 761 | -12 | LTR41 | ERVL | LTR |
| 73878214 | 73878252 | - | 14.6 | 6127 | 6156 | 0 | L1PB4 | L1 | LINE |
| 73878253 | 73878548 | - | 8.1 | 1 | 311 | 0 | AluY | Alu | SINE |
| 73878549 | 73878723 | - | 14.6 | 5960 | 6126 | -30 | L1PB4 | L1 | LINE |
| 73878724 | 73879023 | - | 12.7 | 1 | 300 | -12 | AluSx | Alu | SINE |
| 73879024 | 73879160 | - | 14.6 | 5829 | 5959 | -197 | L1PB4 | L1 | LINE |
| 73879161 | 73879466 | - | 12.8 | 1 | 309 | -3 | AluSx | Alu | SINE |
| 73879467 | 73879751 | - | 14.6 | 5535 | 5828 | -328 | L1PB4 | L1 | LINE |
| 73879752 | 73879985 | - | 29.1 | 505 | 712 | -61 | LTR41 | ERVL | LTR |

  

|  |  |  |  |  |  |  |  |  |  |
| --- | --- | --- | --- | --- | --- | --- | --- | --- | --- |
| chr7:73881151-73883134 | | | 1984 bp | | |  |  |  |  |
| Genome Start | Genome End | Strand | Div | Repeat Start | Repeat End | Repeat Left | Name | Family | Class |
| 73881151 | 73881433 | + | 17.5 | 3789 | 4075 | -2065 | L1M3 | L1 | LINE |
| 73881434 | 73881740 | + | 11.1 | 1 | 305 | -7 | AluSx | Alu | SINE |
| 73881741 | 73882149 | + | 17.5 | 4076 | 4489 | -1651 | L1M3 | L1 | LINE |
| 73882150 | 73882471 | + | 11.2 | 1 | 312 | 0 | AluSx | Alu | SINE |
| 73882472 | 73882644 | + | 17.5 | 4490 | 4645 | -1495 | L1M3 | L1 | LINE |
| 73882645 | 73882959 | + | 10.5 | 1 | 307 | -3 | AluSg | Alu | SINE |
| 73882960 | 73883134 | + | 17.5 | 4646 | 4829 | -1311 | L1M3 | L1 | LINE |

  

|  |  |  |  |  |  |  |  |  |  |
| --- | --- | --- | --- | --- | --- | --- | --- | --- | --- |
| chr7:73895951-73897173 | | | 1216 bp | | |  |  |  |  |
| Genome Start | Genome End | Strand | Div | Repeat Start | Repeat End | Repeat Left | Name | Family | Class |
| 73895951 | 73896104 | + | 17.7 | 5893 | 6063 | -115 | L1MB8 | L1 | LINE |
| 73896105 | 73896409 | + | 5.9 | 1 | 304 | -5 | AluSc | Alu | SINE |
| 73896410 | 73896476 | + | 17.7 | 6064 | 6114 | -64 | L1MB8 | L1 | LINE |
| 73896477 | 73896786 | - | 11.1 | 5 | 311 | -1 | AluSx | Alu | SINE |
| 73896794 | 73897113 | - | 15.7 | 1 | 304 | -8 | AluJo | Alu | SINE |
| 73897114 | 73897173 | + | 17.7 | 6115 | 6168 | -10 | L1MB8 | L1 | LINE |

  

|  |  |  |  |  |  |  |  |  |  |
| --- | --- | --- | --- | --- | --- | --- | --- | --- | --- |
| chr7:73902282-73904130 | | | 1822 bp | | |  |  |  |  |
| Genome Start | Genome End | Strand | Div | Repeat Start | Repeat End | Repeat Left | Name | Family | Class |
| 73902282 | 73902365 | - | 21.4 | 2400 | 2483 | -8 | Tigger7 | MER2\_type | DNA |
| 73902366 | 73902579 | - | 9.9 | 85 | 297 | -15 | AluSg/x | Alu | SINE |
| 73902592 | 73902805 | + | 19.1 | 83 | 299 | -13 | AluJo | Alu | SINE |
| 73902811 | 73903103 | + | 11.6 | 2 | 296 | -16 | AluSx | Alu | SINE |
| 73903115 | 73903218 | - | 16.8 | 1 | 104 | -2627 | Tigger4(Zombi) | MER2\_type | DNA |
| 73903219 | 73903397 | - | 9.2 | 2206 | 2389 | -102 | Tigger7 | MER2\_type | DNA |
| 73903397 | 73904130 | - | 9.8 | 1 | 773 | -1718 | Tigger7 | MER2\_type | DNA |

  

|  |  |  |  |  |  |  |  |  |  |
| --- | --- | --- | --- | --- | --- | --- | --- | --- | --- |
| chr7:73911485-73914237 | | | 2648 bp | | |  |  |  |  |
| Genome Start | Genome End | Strand | Div | Repeat Start | Repeat End | Repeat Left | Name | Family | Class |
| 73911485 | 73911877 | + | 17.5 | 4652 | 5026 | -1120 | L1MB4 | L1 | LINE |
| 73911878 | 73912190 | - | 17.1 | 3 | 311 | -1 | AluJb | Alu | SINE |
| 73912191 | 73912451 | + | 17.5 | 5027 | 5332 | -814 | L1MB4 | L1 | LINE |
| 73912452 | 73912760 | + | 9.8 | 1 | 302 | 0 | AluSx | Alu | SINE |
| 73912768 | 73913048 | + | 17.4 | 1 | 236 | -76 | AluSx | Alu | SINE |
| 73913059 | 73913154 | + | 18.8 | 5813 | 5908 | -272 | L1MB4 | L1 | LINE |
| 73913155 | 73913237 | + | 16.9 | 37 | 119 | -24 | FLAM\_C | Alu | SINE |
| 73913306 | 73913600 | - | 7.5 | 1 | 295 | -16 | AluY | Alu | SINE |
| 73913605 | 73913903 | - | 14.1 | 1 | 298 | -15 | AluSq | Alu | SINE |
| 73913920 | 73914090 | + | 15.4 | 1 | 165 | -11 | FRAM | Alu | SINE |
| 73914091 | 73914237 | + | 23.1 | 5908 | 6060 | -120 | L1MB4 | L1 | LINE |

  

|  |  |  |  |  |  |  |  |  |  |
| --- | --- | --- | --- | --- | --- | --- | --- | --- | --- |
| chr7:73927026-73927648 | | | 603 bp | | |  |  |  |  |
| Genome Start | Genome End | Strand | Div | Repeat Start | Repeat End | Repeat Left | Name | Family | Class |
| 73927026 | 73927159 | + | 14.1 | 2 | 135 | -177 | AluSx | Alu | SINE |
| 73927160 | 73927453 | + | 5.4 | 1 | 296 | -15 | AluY | Alu | SINE |
| 73927474 | 73927648 | + | 14.1 | 136 | 306 | -6 | AluSx | Alu | SINE |

  

|  |  |  |  |  |  |  |  |  |  |
| --- | --- | --- | --- | --- | --- | --- | --- | --- | --- |
| chr7:73935053-73935859 | | | 798 bp | | |  |  |  |  |
| Genome Start | Genome End | Strand | Div | Repeat Start | Repeat End | Repeat Left | Name | Family | Class |
| 73935053 | 73935157 | + | 14.5 | 82 | 189 | -86 | ORSL | Tip100 | DNA |
| 73935158 | 73935470 | - | 17.1 | 1 | 302 | -10 | AluJo | Alu | SINE |
| 73935480 | 73935780 | - | 10.0 | 1 | 302 | -11 | AluSp | Alu | SINE |
| 73935781 | 73935859 | + | 14.5 | 196 | 267 | -8 | ORSL | Tip100 | DNA |

  

|  |  |  |  |  |  |  |  |  |  |
| --- | --- | --- | --- | --- | --- | --- | --- | --- | --- |
| chr7:73940217-73940657 | | | 441 bp | | |  |  |  |  |
| Genome Start | Genome End | Strand | Div | Repeat Start | Repeat End | Repeat Left | Name | Family | Class |
| 73940217 | 73940251 | + | 25.2 | 2728 | 2761 | -3385 | L1M5 | L1 | LINE |
| 73940252 | 73940555 | - | 12.5 | 1 | 304 | -8 | AluSx | Alu | SINE |
| 73940556 | 73940657 | + | 25.2 | 2762 | 2864 | -3282 | L1M5 | L1 | LINE |

  

|  |  |  |  |  |  |  |  |  |  |
| --- | --- | --- | --- | --- | --- | --- | --- | --- | --- |
| chr7:73949373-73949848 | | | 476 bp | | |  |  |  |  |
| Genome Start | Genome End | Strand | Div | Repeat Start | Repeat End | Repeat Left | Name | Family | Class |
| 73949373 | 73949512 | - | 27.9 | 31 | 187 | -75 | MIR | MIR | SINE |
| 73949513 | 73949823 | - | 11.2 | 1 | 312 | -1 | AluSp | Alu | SINE |
| 73949824 | 73949848 | - | 27.9 | 7 | 30 | -232 | MIR | MIR | SINE |

  

|  |  |  |  |  |  |  |  |  |  |
| --- | --- | --- | --- | --- | --- | --- | --- | --- | --- |
| chr7:73953315-73954027 | | | 713 bp | | |  |  |  |  |
| Genome Start | Genome End | Strand | Div | Repeat Start | Repeat End | Repeat Left | Name | Family | Class |
| 73953315 | 73953705 | - | 17.4 | 33 | 414 | -75 | LTR45B | ERV1 | LTR |
| 73953706 | 73953996 | + | 10.7 | 5 | 297 | -16 | AluSq | Alu | SINE |
| 73953997 | 73954027 | - | 17.4 | 3 | 32 | -457 | LTR45B | ERV1 | LTR |

  

|  |  |  |  |  |  |  |  |  |  |
| --- | --- | --- | --- | --- | --- | --- | --- | --- | --- |
| chr7:73961354-73961973 | | | 572 bp | | |  |  |  |  |
| Genome Start | Genome End | Strand | Div | Repeat Start | Repeat End | Repeat Left | Name | Family | Class |
| 73961354 | 73961492 | + | 17.4 | 1 | 136 | -176 | AluJo | Alu | SINE |
| 73961493 | 73961765 | + | 18.7 | 3 | 276 | -36 | AluSx | Alu | SINE |
| 73961814 | 73961973 | + | 17.4 | 137 | 295 | -17 | AluJo | Alu | SINE |

  

|  |  |  |  |  |  |  |  |  |  |
| --- | --- | --- | --- | --- | --- | --- | --- | --- | --- |
| chr7:73969076-73969776 | | | 701 bp | | |  |  |  |  |
| Genome Start | Genome End | Strand | Div | Repeat Start | Repeat End | Repeat Left | Name | Family | Class |
| 73969076 | 73969412 | - | 36.0 | 3989 | 4324 | -165 | L3 | CR1 | LINE |
| 73969413 | 73969716 | + | 14.8 | 3 | 299 | -13 | AluSx | Alu | SINE |
| 73969717 | 73969776 | - | 36.0 | 3933 | 3988 | -501 | L3 | CR1 | LINE |

  

|  |  |  |  |  |  |  |  |  |  |
| --- | --- | --- | --- | --- | --- | --- | --- | --- | --- |
| chr7:73972713-73973309 | | | 597 bp | | |  |  |  |  |
| Genome Start | Genome End | Strand | Div | Repeat Start | Repeat End | Repeat Left | Name | Family | Class |
| 73972713 | 73972845 | + | 16.6 | 1 | 131 | -181 | AluJo | Alu | SINE |
| 73972846 | 73973137 | + | 11.7 | 1 | 292 | -20 | AluSx | Alu | SINE |
| 73973138 | 73973309 | + | 16.6 | 132 | 297 | -15 | AluJo | Alu | SINE |

  

|  |  |  |  |  |  |  |  |  |  |
| --- | --- | --- | --- | --- | --- | --- | --- | --- | --- |
| chr7:73973914-73974839 | | | 926 bp | | |  |  |  |  |
| Genome Start | Genome End | Strand | Div | Repeat Start | Repeat End | Repeat Left | Name | Family | Class |
| 73973914 | 73974082 | - | 11.9 | 6110 | 6283 | -17 | L1MA5 | L1 | LINE |
| 73974083 | 73974382 | - | 11.3 | 1 | 300 | -12 | AluSx | Alu | SINE |
| 73974383 | 73974444 | - | 16.1 | 5906 | 5968 | -332 | L1MA5 | L1 | LINE |
| 73974445 | 73974750 | - | 17.3 | 1 | 306 | -6 | AluJo | Alu | SINE |
| 73974751 | 73974839 | - | 16.1 | 5818 | 5905 | -395 | L1MA5 | L1 | LINE |

  

|  |  |  |  |  |  |  |  |  |  |
| --- | --- | --- | --- | --- | --- | --- | --- | --- | --- |
| chr7:73991147-73991947 | | | 801 bp | | |  |  |  |  |
| Genome Start | Genome End | Strand | Div | Repeat Start | Repeat End | Repeat Left | Name | Family | Class |
| 73991147 | 73991573 | - | 25.7 | 4920 | 5333 | -813 | L1MC | L1 | LINE |
| 73991574 | 73991874 | - | 17.9 | 1 | 306 | -6 | AluJo | Alu | SINE |
| 73991875 | 73991947 | - | 25.7 | 4847 | 4919 | -1227 | L1MC | L1 | LINE |

  

|  |  |  |  |  |  |  |  |  |  |
| --- | --- | --- | --- | --- | --- | --- | --- | --- | --- |
| chr7:73992580-73993133 | | | 554 bp | | |  |  |  |  |
| Genome Start | Genome End | Strand | Div | Repeat Start | Repeat End | Repeat Left | Name | Family | Class |
| 73992580 | 73992652 | - | 30.6 | 371 | 445 | -5 | LTR16A | ERVL | LTR |
| 73992653 | 73992957 | + | 12.6 | 1 | 304 | -8 | AluJo | Alu | SINE |
| 73992958 | 73993133 | - | 30.6 | 170 | 370 | -80 | LTR16A | ERVL | LTR |

  

|  |  |  |  |  |  |  |  |  |  |
| --- | --- | --- | --- | --- | --- | --- | --- | --- | --- |
| chr7:74000941-74001399 | | | 459 bp | | |  |  |  |  |
| Genome Start | Genome End | Strand | Div | Repeat Start | Repeat End | Repeat Left | Name | Family | Class |
| 74000941 | 74000977 | - | 35.1 | 224 | 258 | -4 | MIR | MIR | SINE |
| 74000978 | 74001279 | - | 7.8 | 1 | 297 | -12 | AluSc | Alu | SINE |
| 74001280 | 74001399 | - | 35.1 | 93 | 223 | -39 | MIR | MIR | SINE |

  

|  |  |  |  |  |  |  |  |  |  |
| --- | --- | --- | --- | --- | --- | --- | --- | --- | --- |
| chr7:74004119-74004751 | | | 633 bp | | |  |  |  |  |
| Genome Start | Genome End | Strand | Div | Repeat Start | Repeat End | Repeat Left | Name | Family | Class |
| 74004119 | 74004209 | + | 20.5 | 7481 | 7571 | -214 | L1MC3 | L1 | LINE |
| 74004210 | 74004521 | + | 6.4 | 1 | 313 | 0 | AluSp | Alu | SINE |
| 74004522 | 74004751 | + | 20.5 | 7572 | 7783 | -2 | L1MC3 | L1 | LINE |

  

|  |  |  |  |  |  |  |  |  |  |
| --- | --- | --- | --- | --- | --- | --- | --- | --- | --- |
| chr7:74008653-74009437 | | | 782 bp | | |  |  |  |  |
| Genome Start | Genome End | Strand | Div | Repeat Start | Repeat End | Repeat Left | Name | Family | Class |
| 74008653 | 74008670 | - | 17.3 | 2480 | 2493 | -119 | Charlie5 | MER1\_type | DNA |
| 74008671 | 74008972 | + | 11.3 | 1 | 302 | -10 | AluSx | Alu | SINE |
| 74008976 | 74009266 | + | 10.7 | 6 | 299 | -13 | AluSx | Alu | SINE |
| 74009267 | 74009437 | - | 17.3 | 2310 | 2479 | -133 | Charlie5 | MER1\_type | DNA |

  

|  |  |  |  |  |  |  |  |  |  |
| --- | --- | --- | --- | --- | --- | --- | --- | --- | --- |
| chr7:74010541-74011198 | | | 658 bp | | |  |  |  |  |
| Genome Start | Genome End | Strand | Div | Repeat Start | Repeat End | Repeat Left | Name | Family | Class |
| 74010541 | 74010641 | - | 16.0 | 302 | 397 | -74 | LTR32 | ERVL | LTR |
| 74010642 | 74010936 | + | 7.1 | 1 | 295 | -16 | AluY | Alu | SINE |
| 74010937 | 74011198 | - | 16.0 | 15 | 301 | -170 | LTR32 | ERVL | LTR |

  

|  |  |  |  |  |  |  |  |  |  |
| --- | --- | --- | --- | --- | --- | --- | --- | --- | --- |
| chr7:74011707-74012395 | | | 693 bp | | |  |  |  |  |
| Genome Start | Genome End | Strand | Div | Repeat Start | Repeat End | Repeat Left | Name | Family | Class |
| 74011707 | 74011725 | - | 25.0 | 519 | 537 | -58 | MLT1H2 | MaLR | LTR |
| 74011737 | 74012015 | - | 8.6 | 1 | 279 | -32 | AluY | Alu | SINE |
| 74012016 | 74012285 | - | 26.5 | 149 | 430 | -54 | MLT1H2 | MaLR | LTR |
| 74012271 | 74012395 | - | 19.5 | 55 | 189 | -295 | MLT1H2 | MaLR | LTR |

  

|  |  |  |  |  |  |  |  |  |  |
| --- | --- | --- | --- | --- | --- | --- | --- | --- | --- |
| chr7:74032021-74032404 | | | 384 bp | | |  |  |  |  |
| Genome Start | Genome End | Strand | Div | Repeat Start | Repeat End | Repeat Left | Name | Family | Class |
| 74032021 | 74032095 | + | 25.4 | 46 | 123 | -145 | MIR | MIR | SINE |
| 74032096 | 74032372 | - | 20.2 | 6 | 277 | -35 | AluJo | Alu | SINE |
| 74032373 | 74032404 | + | 23.3 | 116 | 149 | -113 | MIR | MIR | SINE |

  

|  |  |  |  |  |  |  |  |  |  |
| --- | --- | --- | --- | --- | --- | --- | --- | --- | --- |
| chr7:74036719-74037251 | | | 533 bp | | |  |  |  |  |
| Genome Start | Genome End | Strand | Div | Repeat Start | Repeat End | Repeat Left | Name | Family | Class |
| 74036719 | 74036931 | - | 34.6 | 26 | 267 | -1 | MIRb | MIR | SINE |
| 74036932 | 74037226 | + | 13.4 | 5 | 294 | -18 | AluSx | Alu | SINE |
| 74037227 | 74037251 | - | 34.6 | 3 | 25 | -243 | MIRb | MIR | SINE |

  

|  |  |  |  |  |  |  |  |  |  |
| --- | --- | --- | --- | --- | --- | --- | --- | --- | --- |
| chr7:74042519-74043171 | | | 653 bp | | |  |  |  |  |
| Genome Start | Genome End | Strand | Div | Repeat Start | Repeat End | Repeat Left | Name | Family | Class |
| 74042519 | 74042653 | + | 15.5 | 1 | 136 | -166 | AluJo | Alu | SINE |
| 74042654 | 74042959 | + | 13.1 | 1 | 306 | -6 | AluSx | Alu | SINE |
| 74042960 | 74043171 | + | 15.5 | 137 | 299 | -3 | AluJo | Alu | SINE |

  

|  |  |  |  |  |  |  |  |  |  |
| --- | --- | --- | --- | --- | --- | --- | --- | --- | --- |
| chr7:74044413-74045024 | | | 612 bp | | |  |  |  |  |
| Genome Start | Genome End | Strand | Div | Repeat Start | Repeat End | Repeat Left | Name | Family | Class |
| 74044413 | 74044530 | + | 15.0 | 11 | 127 | -185 | AluJo | Alu | SINE |
| 74044531 | 74044844 | - | 9.0 | 1 | 290 | -20 | AluSg | Alu | SINE |
| 74044845 | 74045024 | + | 15.0 | 128 | 297 | -15 | AluJo | Alu | SINE |

  

|  |  |  |  |  |  |  |  |  |  |
| --- | --- | --- | --- | --- | --- | --- | --- | --- | --- |
| chr7:74047490-74048099 | | | 610 bp | | |  |  |  |  |
| Genome Start | Genome End | Strand | Div | Repeat Start | Repeat End | Repeat Left | Name | Family | Class |
| 74047490 | 74047635 | + | 14.8 | 1 | 133 | -179 | AluJo | Alu | SINE |
| 74047636 | 74047935 | + | 7.0 | 1 | 299 | -12 | AluY | Alu | SINE |
| 74047936 | 74048099 | + | 14.8 | 134 | 294 | -18 | AluJo | Alu | SINE |

  

|  |  |  |  |  |  |  |  |  |  |
| --- | --- | --- | --- | --- | --- | --- | --- | --- | --- |
| chr7:74108874-74109459 | | | 586 bp | | |  |  |  |  |
| Genome Start | Genome End | Strand | Div | Repeat Start | Repeat End | Repeat Left | Name | Family | Class |
| 74108874 | 74109020 | - | 12.9 | 142 | 288 | -24 | AluJo | Alu | SINE |
| 74109021 | 74109315 | - | 13.9 | 1 | 306 | -6 | AluSx | Alu | SINE |
| 74109316 | 74109459 | - | 12.9 | 1 | 141 | -171 | AluJo | Alu | SINE |

  

|  |  |  |  |  |  |  |  |  |  |
| --- | --- | --- | --- | --- | --- | --- | --- | --- | --- |
| chr7:74133351-74136060 | | | 2692 bp | | |  |  |  |  |
| Genome Start | Genome End | Strand | Div | Repeat Start | Repeat End | Repeat Left | Name | Family | Class |
| 74133351 | 74133472 | - | 33.2 | 5490 | 5623 | -498 | L1M5 | L1 | LINE |
| 74133473 | 74133776 | - | 17.4 | 1 | 294 | -18 | AluJo | Alu | SINE |
| 74133777 | 74133939 | - | 33.2 | 5311 | 5489 | -632 | L1M5 | L1 | LINE |
| 74133940 | 74134243 | - | 11.4 | 9 | 305 | -7 | AluSx | Alu | SINE |
| 74134252 | 74134330 | - | 25.3 | 5225 | 5316 | -830 | L1M5 | L1 | LINE |
| 74134331 | 74134651 | - | 11.5 | 1 | 310 | -3 | AluSp | Alu | SINE |
| 74134659 | 74134969 | - | 12.0 | 1 | 308 | -4 | AluSx | Alu | SINE |
| 74134970 | 74135106 | - | 25.3 | 5061 | 5217 | -929 | L1M5 | L1 | LINE |
| 74135107 | 74135235 | - | 16.3 | 1 | 136 | -176 | AluJo | Alu | SINE |
| 74135239 | 74135259 | - | 31.9 | 5007 | 5026 | -1120 | L1M5 | L1 | LINE |
| 74135260 | 74135560 | - | 10.6 | 1 | 301 | -9 | AluSg | Alu | SINE |
| 74135561 | 74136060 | - | 31.9 | 4513 | 5006 | -1140 | L1M5 | L1 | LINE |

  

|  |  |  |  |  |  |  |  |  |  |
| --- | --- | --- | --- | --- | --- | --- | --- | --- | --- |
| chr7:74140919-74144274 | | | 3324 bp | | |  |  |  |  |
| Genome Start | Genome End | Strand | Div | Repeat Start | Repeat End | Repeat Left | Name | Family | Class |
| 74140919 | 74141580 | - | 23.9 | 2888 | 3583 | -2636 | L1MEc | L1 | LINE |
| 74141581 | 74141888 | - | 12.5 | 1 | 305 | -7 | AluSx | Alu | SINE |
| 74141921 | 74142144 | - | 19.6 | 1060 | 1283 | -5117 | L1MEc | L1 | LINE |
| 74142145 | 74142436 | + | 12.4 | 1 | 292 | -20 | AluSx | Alu | SINE |
| 74142437 | 74142473 | - | 19.6 | 1030 | 1059 | -5341 | L1MEc | L1 | LINE |
| 74142474 | 74142771 | + | 6.5 | 1 | 295 | -16 | AluY | Alu | SINE |
| 74142772 | 74143076 | - | 19.6 | 715 | 1029 | -5371 | L1MEc | L1 | LINE |
| 74143077 | 74143218 | + | 11.6 | 2 | 133 | -179 | AluSx | Alu | SINE |
| 74143219 | 74143474 | + | 9.4 | 37 | 291 | -19 | AluSg | Alu | SINE |
| 74143475 | 74143644 | + | 11.6 | 134 | 303 | -9 | AluSx | Alu | SINE |
| 74143645 | 74144274 | - | 19.6 | 37 | 714 | -5686 | L1MEc | L1 | LINE |

  

|  |  |  |  |  |  |  |  |  |  |
| --- | --- | --- | --- | --- | --- | --- | --- | --- | --- |
| chr7:74144294-74145739 | | | 1446 bp | | |  |  |  |  |
| Genome Start | Genome End | Strand | Div | Repeat Start | Repeat End | Repeat Left | Name | Family | Class |
| 74144294 | 74144460 | - | 24.1 | 2664 | 2851 | -568 | L2 | L2 | LINE |
| 74144461 | 74144588 | + | 17.4 | 1 | 125 | -187 | AluJo | Alu | SINE |
| 74144589 | 74144889 | - | 9.8 | 1 | 303 | -9 | AluSx | Alu | SINE |
| 74144890 | 74144900 | + | 17.4 | 126 | 131 | -181 | AluJo | Alu | SINE |
| 74144901 | 74145197 | - | 8.8 | 1 | 297 | -13 | AluSg | Alu | SINE |
| 74145198 | 74145390 | + | 17.4 | 132 | 308 | -4 | AluJo | Alu | SINE |
| 74145391 | 74145739 | - | 24.1 | 2322 | 2663 | -756 | L2 | L2 | LINE |

  

|  |  |  |  |  |  |  |  |  |  |
| --- | --- | --- | --- | --- | --- | --- | --- | --- | --- |
| chr7:74164401-74165025 | | | 625 bp | | |  |  |  |  |
| Genome Start | Genome End | Strand | Div | Repeat Start | Repeat End | Repeat Left | Name | Family | Class |
| 74164401 | 74164530 | + | 17.9 | 6 | 127 | -185 | AluJo | Alu | SINE |
| 74164531 | 74164848 | + | 9.7 | 1 | 309 | -1 | AluSg | Alu | SINE |
| 74164849 | 74165025 | + | 17.9 | 128 | 301 | -11 | AluJo | Alu | SINE |

  

|  |  |  |  |  |  |  |  |  |  |
| --- | --- | --- | --- | --- | --- | --- | --- | --- | --- |
| chr7:74165505-74166093 | | | 552 bp | | |  |  |  |  |
| Genome Start | Genome End | Strand | Div | Repeat Start | Repeat End | Repeat Left | Name | Family | Class |
| 74165505 | 74165687 | + | 13.9 | 3647 | 3836 | -104 | Arthur1 | Tip100 | DNA |
| 74165688 | 74165811 | + | 5.4 | 1 | 124 | -187 | AluY | Alu | SINE |
| 74165849 | 74166022 | + | 5.4 | 125 | 302 | 0 | AluY | Alu | SINE |
| 74166023 | 74166093 | + | 13.9 | 3837 | 3940 | 0 | Arthur1 | Tip100 | DNA |

  

|  |  |  |  |  |  |  |  |  |  |
| --- | --- | --- | --- | --- | --- | --- | --- | --- | --- |
| chr7:74187624-74188287 | | | 571 bp | | |  |  |  |  |
| Genome Start | Genome End | Strand | Div | Repeat Start | Repeat End | Repeat Left | Name | Family | Class |
| 74187624 | 74187695 | + | 29.2 | 7162 | 7233 | -728 | L1MC5 | L1 | LINE |
| 74187789 | 74187851 | + | 21.3 | 7260 | 7307 | -654 | L1MC5 | L1 | LINE |
| 74187852 | 74188161 | + | 14.6 | 1 | 310 | 0 | AluJb | Alu | SINE |
| 74188162 | 74188287 | + | 21.3 | 7308 | 7432 | -529 | L1MC5 | L1 | LINE |

  

|  |  |  |  |  |  |  |  |  |  |
| --- | --- | --- | --- | --- | --- | --- | --- | --- | --- |
| chr7:74191115-74191726 | | | 612 bp | | |  |  |  |  |
| Genome Start | Genome End | Strand | Div | Repeat Start | Repeat End | Repeat Left | Name | Family | Class |
| 74191115 | 74191252 | + | 18.8 | 6 | 136 | -176 | AluJo | Alu | SINE |
| 74191253 | 74191552 | + | 17.5 | 1 | 300 | -12 | AluJb | Alu | SINE |
| 74191553 | 74191726 | + | 18.8 | 137 | 305 | -7 | AluJo | Alu | SINE |

  

|  |  |  |  |  |  |  |  |  |  |
| --- | --- | --- | --- | --- | --- | --- | --- | --- | --- |
| chr7:74204338-74205084 | | | 604 bp | | |  |  |  |  |
| Genome Start | Genome End | Strand | Div | Repeat Start | Repeat End | Repeat Left | Name | Family | Class |
| 74204338 | 74204352 | - | 25.0 | 8003 | 8017 | -25 | L1MC4 | L1 | LINE |
| 74204353 | 74204667 | + | 10.3 | 1 | 311 | -1 | AluSx | Alu | SINE |
| 74204668 | 74204739 | - | 25.0 | 7924 | 8002 | -40 | L1MC4 | L1 | LINE |
| 74204883 | 74205084 | - | 27.4 | 7695 | 7901 | -141 | L1MC4 | L1 | LINE |

  

|  |  |  |  |  |  |  |  |  |  |
| --- | --- | --- | --- | --- | --- | --- | --- | --- | --- |
| chr7:74212142-74212733 | | | 592 bp | | |  |  |  |  |
| Genome Start | Genome End | Strand | Div | Repeat Start | Repeat End | Repeat Left | Name | Family | Class |
| 74212142 | 74212256 | - | 23.4 | 5031 | 5140 | -1006 | L1M5 | L1 | LINE |
| 74212257 | 74212526 | - | 13.1 | 15 | 282 | -31 | AluSq | Alu | SINE |
| 74212527 | 74212733 | - | 23.9 | 4868 | 5036 | -1116 | L1M5 | L1 | LINE |

  

|  |  |  |  |  |  |  |  |  |  |
| --- | --- | --- | --- | --- | --- | --- | --- | --- | --- |
| chr7:74223340-74224269 | | | 909 bp | | |  |  |  |  |
| Genome Start | Genome End | Strand | Div | Repeat Start | Repeat End | Repeat Left | Name | Family | Class |
| 74223340 | 74223470 | + | 23.7 | 7465 | 7610 | -432 | L1MC4 | L1 | LINE |
| 74223478 | 74223773 | + | 8.4 | 6 | 301 | -11 | AluSx | Alu | SINE |
| 74223788 | 74223991 | + | 11.8 | 97 | 308 | -1 | AluSc | Alu | SINE |
| 74223992 | 74224269 | + | 23.3 | 7601 | 7897 | -145 | L1MC4 | L1 | LINE |

  

|  |  |  |  |  |  |  |  |  |  |
| --- | --- | --- | --- | --- | --- | --- | --- | --- | --- |
| chr7:74225954-74226936 | | | 982 bp | | |  |  |  |  |
| Genome Start | Genome End | Strand | Div | Repeat Start | Repeat End | Repeat Left | Name | Family | Class |
| 74225954 | 74226226 | + | 27.6 | 3018 | 3270 | -149 | L2 | L2 | LINE |
| 74226227 | 74226503 | - | 16.1 | 40 | 312 | 0 | AluJo | Alu | SINE |
| 74226505 | 74226805 | - | 10.1 | 1 | 298 | -15 | AluSp | Alu | SINE |
| 74226806 | 74226936 | + | 27.6 | 3271 | 3405 | -14 | L2 | L2 | LINE |

  

|  |  |  |  |  |  |  |  |  |  |
| --- | --- | --- | --- | --- | --- | --- | --- | --- | --- |
| chr7:74230927-74231435 | | | 509 bp | | |  |  |  |  |
| Genome Start | Genome End | Strand | Div | Repeat Start | Repeat End | Repeat Left | Name | Family | Class |
| 74230927 | 74231079 | - | 30.9 | 69 | 230 | -32 | MIR | MIR | SINE |
| 74231080 | 74231377 | - | 9.1 | 1 | 301 | -11 | AluSx | Alu | SINE |
| 74231378 | 74231435 | - | 30.9 | 8 | 68 | -194 | MIR | MIR | SINE |

  

|  |  |  |  |  |  |  |  |  |  |
| --- | --- | --- | --- | --- | --- | --- | --- | --- | --- |
| chr7:74245007-74245505 | | | 499 bp | | |  |  |  |  |
| Genome Start | Genome End | Strand | Div | Repeat Start | Repeat End | Repeat Left | Name | Family | Class |
| 74245007 | 74245021 | - | 23.4 | 211 | 224 | 0 | MER58A | MER1\_type | DNA |
| 74245022 | 74245315 | - | 11.6 | 1 | 295 | -17 | AluSx | Alu | SINE |
| 74245316 | 74245505 | - | 23.4 | 2 | 210 | -14 | MER58A | MER1\_type | DNA |

  

|  |  |  |  |  |  |  |  |  |  |
| --- | --- | --- | --- | --- | --- | --- | --- | --- | --- |
| chr7:74297546-74298203 | | | 658 bp | | |  |  |  |  |
| Genome Start | Genome End | Strand | Div | Repeat Start | Repeat End | Repeat Left | Name | Family | Class |
| 74297546 | 74297807 | + | 16.0 | 15 | 301 | -170 | LTR32 | ERVL | LTR |
| 74297808 | 74298102 | - | 7.1 | 1 | 295 | -16 | AluY | Alu | SINE |
| 74298103 | 74298203 | + | 16.0 | 302 | 397 | -74 | LTR32 | ERVL | LTR |

  

|  |  |  |  |  |  |  |  |  |  |
| --- | --- | --- | --- | --- | --- | --- | --- | --- | --- |
| chr7:74299311-74300093 | | | 780 bp | | |  |  |  |  |
| Genome Start | Genome End | Strand | Div | Repeat Start | Repeat End | Repeat Left | Name | Family | Class |
| 74299311 | 74299481 | + | 17.3 | 2310 | 2479 | -133 | Charlie5 | MER1\_type | DNA |
| 74299482 | 74299771 | - | 10.7 | 6 | 298 | -14 | AluSx | Alu | SINE |
| 74299775 | 74300075 | - | 11.3 | 1 | 301 | -11 | AluSx | Alu | SINE |
| 74300076 | 74300093 | + | 17.3 | 2480 | 2493 | -119 | Charlie5 | MER1\_type | DNA |

  

|  |  |  |  |  |  |  |  |  |  |
| --- | --- | --- | --- | --- | --- | --- | --- | --- | --- |
| chr7:74302633-74303209 | | | 577 bp | | |  |  |  |  |
| Genome Start | Genome End | Strand | Div | Repeat Start | Repeat End | Repeat Left | Name | Family | Class |
| 74302633 | 74302697 | - | 23.9 | 68 | 162 | -433 | MLT1G1 | MaLR | LTR |
| 74302698 | 74303103 | + | 15.0 | 1 | 432 | 0 | MSTB1 | MaLR | LTR |
| 74303104 | 74303209 | - | 23.3 | 3 | 108 | -487 | MLT1G1 | MaLR | LTR |

  

|  |  |  |  |  |  |  |  |  |  |
| --- | --- | --- | --- | --- | --- | --- | --- | --- | --- |
| chr7:74304181-74304809 | | | 629 bp | | |  |  |  |  |
| Genome Start | Genome End | Strand | Div | Repeat Start | Repeat End | Repeat Left | Name | Family | Class |
| 74304181 | 74304406 | - | 20.1 | 7572 | 7783 | -2 | L1MC3 | L1 | LINE |
| 74304407 | 74304718 | - | 6.4 | 1 | 313 | 0 | AluSp | Alu | SINE |
| 74304719 | 74304809 | - | 20.1 | 7481 | 7571 | -214 | L1MC3 | L1 | LINE |

  

|  |  |  |  |  |  |  |  |  |  |
| --- | --- | --- | --- | --- | --- | --- | --- | --- | --- |
| chr7:74307521-74307980 | | | 460 bp | | |  |  |  |  |
| Genome Start | Genome End | Strand | Div | Repeat Start | Repeat End | Repeat Left | Name | Family | Class |
| 74307521 | 74307640 | + | 35.1 | 92 | 222 | -40 | MIR | MIR | SINE |
| 74307641 | 74307943 | + | 7.7 | 1 | 298 | -11 | AluSc | Alu | SINE |
| 74307944 | 74307980 | + | 35.1 | 223 | 257 | -5 | MIR | MIR | SINE |

  

|  |  |  |  |  |  |  |  |  |  |
| --- | --- | --- | --- | --- | --- | --- | --- | --- | --- |
| chr7:74319575-74320171 | | | 597 bp | | |  |  |  |  |
| Genome Start | Genome End | Strand | Div | Repeat Start | Repeat End | Repeat Left | Name | Family | Class |
| 74319575 | 74319704 | - | 32.6 | 885 | 1005 | -1004 | HAL1b | L1 | LINE |
| 74319705 | 74320015 | - | 11.6 | 1 | 311 | -1 | AluSx | Alu | SINE |
| 74320016 | 74320171 | - | 32.6 | 723 | 884 | -1125 | HAL1b | L1 | LINE |

  

|  |  |  |  |  |  |  |  |  |  |
| --- | --- | --- | --- | --- | --- | --- | --- | --- | --- |
| chr7:74322068-74323047 | | | 898 bp | | |  |  |  |  |
| Genome Start | Genome End | Strand | Div | Repeat Start | Repeat End | Repeat Left | Name | Family | Class |
| 74322068 | 74322157 | - | 21.1 | 346 | 453 | -38 | LTR16C | ERVL | LTR |
| 74322158 | 74322411 | + | 17.9 | 1 | 256 | -56 | AluJb | Alu | SINE |
| 74322491 | 74322793 | + | 13.2 | 1 | 312 | -1 | AluSq | Alu | SINE |
| 74322797 | 74323047 | - | 26.3 | 85 | 350 | -141 | LTR16C | ERVL | LTR |

  

|  |  |  |  |  |  |  |  |  |  |
| --- | --- | --- | --- | --- | --- | --- | --- | --- | --- |
| chr7:74325827-74327789 | | | 1947 bp | | |  |  |  |  |
| Genome Start | Genome End | Strand | Div | Repeat Start | Repeat End | Repeat Left | Name | Family | Class |
| 74325827 | 74325956 | + | 25.6 | 1 | 121 | -88 | MER3 | MER1\_type | DNA |
| 74325957 | 74326132 | - | 23.2 | 189 | 374 | 0 | MLT1A | MaLR | LTR |
| 74326133 | 74326176 | + | 13.8 | 1 | 39 | -389 | MSTA | MaLR | LTR |
| 74326177 | 74326487 | + | 10.9 | 1 | 312 | 0 | AluSx | Alu | SINE |
| 74326488 | 74326828 | + | 13.8 | 40 | 360 | -68 | MSTA | MaLR | LTR |
| 74326829 | 74327133 | + | 11.7 | 1 | 306 | 0 | AluSq | Alu | SINE |
| 74327134 | 74327203 | + | 13.8 | 361 | 428 | 0 | MSTA | MaLR | LTR |
| 74327204 | 74327394 | - | 23.2 | 7 | 188 | -186 | MLT1A | MaLR | LTR |
| 74327407 | 74327717 | - | 12.7 | 1 | 309 | -3 | AluSx | Alu | SINE |
| 74327722 | 74327789 | + | 16.2 | 142 | 209 | 0 | MER3 | MER1\_type | DNA |

  

|  |  |  |  |  |  |  |  |  |  |
| --- | --- | --- | --- | --- | --- | --- | --- | --- | --- |
| chr7:74328735-74329355 | | | 621 bp | | |  |  |  |  |
| Genome Start | Genome End | Strand | Div | Repeat Start | Repeat End | Repeat Left | Name | Family | Class |
| 74328735 | 74328871 | + | 11.6 | 1 | 136 | -176 | AluSx | Alu | SINE |
| 74328872 | 74329179 | + | 5.8 | 1 | 308 | -3 | AluY | Alu | SINE |
| 74329180 | 74329355 | + | 11.6 | 137 | 302 | -10 | AluSx | Alu | SINE |

  

|  |  |  |  |  |  |  |  |  |  |
| --- | --- | --- | --- | --- | --- | --- | --- | --- | --- |
| chr7:74332032-74332638 | | | 607 bp | | |  |  |  |  |
| Genome Start | Genome End | Strand | Div | Repeat Start | Repeat End | Repeat Left | Name | Family | Class |
| 74332032 | 74332207 | - | 11.9 | 138 | 310 | -2 | AluSx | Alu | SINE |
| 74332208 | 74332496 | - | 10.4 | 1 | 296 | -15 | AluY | Alu | SINE |
| 74332497 | 74332638 | - | 11.9 | 1 | 137 | -175 | AluSx | Alu | SINE |

  

|  |  |  |  |  |  |  |  |  |  |
| --- | --- | --- | --- | --- | --- | --- | --- | --- | --- |
| chr7:74338620-74339225 | | | 574 bp | | |  |  |  |  |
| Genome Start | Genome End | Strand | Div | Repeat Start | Repeat End | Repeat Left | Name | Family | Class |
| 74338620 | 74338781 | - | 16.6 | 137 | 297 | -15 | AluJo | Alu | SINE |
| 74338814 | 74339087 | - | 18.6 | 2 | 276 | -36 | AluSx | Alu | SINE |
| 74339088 | 74339225 | - | 16.6 | 1 | 136 | -176 | AluJo | Alu | SINE |

  

|  |  |  |  |  |  |  |  |  |  |
| --- | --- | --- | --- | --- | --- | --- | --- | --- | --- |
| chr7:74344106-74345300 | | | 915 bp | | |  |  |  |  |
| Genome Start | Genome End | Strand | Div | Repeat Start | Repeat End | Repeat Left | Name | Family | Class |
| 74344106 | 74344287 | + | 25.8 | 4474 | 4650 | -1889 | MER84-int | ERV1 | LTR |
| 74344519 | 74344819 | - | 14.9 | 3 | 303 | -7 | AluSg | Alu | SINE |
| 74344822 | 74345125 | - | 13.0 | 1 | 300 | -13 | AluSq | Alu | SINE |
| 74345173 | 74345300 | + | 29.0 | 5398 | 5522 | -2914 | MER84-int | ERV1 | LTR |

  

|  |  |  |  |  |  |  |  |  |  |
| --- | --- | --- | --- | --- | --- | --- | --- | --- | --- |
| chr7:74346551-74347262 | | | 712 bp | | |  |  |  |  |
| Genome Start | Genome End | Strand | Div | Repeat Start | Repeat End | Repeat Left | Name | Family | Class |
| 74346551 | 74346581 | + | 17.2 | 3 | 32 | -457 | LTR45B | ERV1 | LTR |
| 74346582 | 74346872 | - | 10.0 | 4 | 296 | -17 | AluSq | Alu | SINE |
| 74346873 | 74347262 | + | 17.2 | 33 | 414 | -75 | LTR45B | ERV1 | LTR |

  

|  |  |  |  |  |  |  |  |  |  |
| --- | --- | --- | --- | --- | --- | --- | --- | --- | --- |
| chr7:74350908-74351383 | | | 476 bp | | |  |  |  |  |
| Genome Start | Genome End | Strand | Div | Repeat Start | Repeat End | Repeat Left | Name | Family | Class |
| 74350908 | 74350932 | + | 27.9 | 7 | 30 | -232 | MIR | MIR | SINE |
| 74350933 | 74351243 | + | 11.2 | 1 | 312 | -1 | AluSp | Alu | SINE |
| 74351244 | 74351383 | + | 27.9 | 31 | 187 | -75 | MIR | MIR | SINE |

  

|  |  |  |  |  |  |  |  |  |  |
| --- | --- | --- | --- | --- | --- | --- | --- | --- | --- |
| chr7:74351406-74352180 | | | 746 bp | | |  |  |  |  |
| Genome Start | Genome End | Strand | Div | Repeat Start | Repeat End | Repeat Left | Name | Family | Class |
| 74351406 | 74351419 | - | 16.8 | 131 | 143 | 0 | FLAM\_C | Alu | SINE |
| 74351420 | 74351735 | - | 15.2 | 3 | 312 | -1 | AluSq | Alu | SINE |
| 74351765 | 74352047 | - | 11.7 | 1 | 285 | -27 | AluSx | Alu | SINE |
| 74352048 | 74352180 | - | 16.8 | 1 | 130 | -13 | FLAM\_C | Alu | SINE |

  

|  |  |  |  |  |  |  |  |  |  |
| --- | --- | --- | --- | --- | --- | --- | --- | --- | --- |
| chr7:74611060-74611542 | | | 469 bp | | |  |  |  |  |
| Genome Start | Genome End | Strand | Div | Repeat Start | Repeat End | Repeat Left | Name | Family | Class |
| 74611060 | 74611235 | - | 17.8 | 130 | 312 | 0 | AluJo | Alu | SINE |
| 74611250 | 74611422 | + | 9.9 | 124 | 295 | -16 | AluY | Alu | SINE |
| 74611423 | 74611542 | - | 14.2 | 9 | 129 | -183 | AluJo | Alu | SINE |

  

|  |  |  |  |  |  |  |  |  |  |
| --- | --- | --- | --- | --- | --- | --- | --- | --- | --- |
| chr7:74612828-74613417 | | | 590 bp | | |  |  |  |  |
| Genome Start | Genome End | Strand | Div | Repeat Start | Repeat End | Repeat Left | Name | Family | Class |
| 74612828 | 74612971 | + | 13.4 | 1 | 141 | -171 | AluJo | Alu | SINE |
| 74612972 | 74613266 | + | 13.9 | 1 | 306 | -6 | AluSx | Alu | SINE |
| 74613267 | 74613417 | + | 13.4 | 142 | 292 | -20 | AluJo | Alu | SINE |

  

|  |  |  |  |  |  |  |  |  |  |
| --- | --- | --- | --- | --- | --- | --- | --- | --- | --- |
| chr7:74625067-74625521 | | | 455 bp | | |  |  |  |  |
| Genome Start | Genome End | Strand | Div | Repeat Start | Repeat End | Repeat Left | Name | Family | Class |
| 74625067 | 74625206 | + | 15.9 | 134 | 274 | -38 | AluJb | Alu | SINE |
| 74625207 | 74625487 | - | 11.0 | 1 | 294 | -16 | AluSg | Alu | SINE |
| 74625488 | 74625521 | + | 15.9 | 275 | 303 | -9 | AluJb | Alu | SINE |

  

|  |  |  |  |  |  |  |  |  |  |
| --- | --- | --- | --- | --- | --- | --- | --- | --- | --- |
| chr7:74628785-74629407 | | | 592 bp | | |  |  |  |  |
| Genome Start | Genome End | Strand | Div | Repeat Start | Repeat End | Repeat Left | Name | Family | Class |
| 74628785 | 74628991 | - | 33.2 | 3187 | 3419 | 0 | L2 | L2 | LINE |
| 74629023 | 74629301 | - | 7.2 | 1 | 283 | -29 | AluSx | Alu | SINE |
| 74629302 | 74629407 | - | 33.2 | 3066 | 3186 | -233 | L2 | L2 | LINE |

  

|  |  |  |  |  |  |  |  |  |  |
| --- | --- | --- | --- | --- | --- | --- | --- | --- | --- |
| chr7:74663791-74665414 | | | 1624 bp | | |  |  |  |  |
| Genome Start | Genome End | Strand | Div | Repeat Start | Repeat End | Repeat Left | Name | Family | Class |
| 74663791 | 74663882 | + | 25.7 | 5491 | 5583 | -583 | L1ME1 | L1 | LINE |
| 74663883 | 74664049 | - | 19.9 | 134 | 299 | -13 | AluJo | Alu | SINE |
| 74664050 | 74664333 | - | 12.7 | 3 | 288 | -24 | AluSx | Alu | SINE |
| 74664334 | 74664475 | - | 19.9 | 1 | 133 | -179 | AluJo | Alu | SINE |
| 74664476 | 74665016 | + | 25.7 | 5584 | 6091 | -75 | L1ME1 | L1 | LINE |
| 74665017 | 74665315 | + | 12.1 | 1 | 298 | -15 | AluSq | Alu | SINE |
| 74665316 | 74665414 | + | 25.7 | 6092 | 6161 | -5 | L1ME1 | L1 | LINE |

  

|  |  |  |  |  |  |  |  |  |  |
| --- | --- | --- | --- | --- | --- | --- | --- | --- | --- |
| chr7:74689865-74690248 | | | 384 bp | | |  |  |  |  |
| Genome Start | Genome End | Strand | Div | Repeat Start | Repeat End | Repeat Left | Name | Family | Class |
| 74689865 | 74689896 | - | 23.3 | 116 | 149 | -113 | MIR | MIR | SINE |
| 74689897 | 74690173 | + | 20.2 | 6 | 277 | -35 | AluJo | Alu | SINE |
| 74690174 | 74690248 | - | 25.4 | 46 | 123 | -145 | MIR | MIR | SINE |

  

|  |  |  |  |  |  |  |  |  |  |
| --- | --- | --- | --- | --- | --- | --- | --- | --- | --- |
| chr7:74706433-74710305 | | | 3445 bp | | |  |  |  |  |
| Genome Start | Genome End | Strand | Div | Repeat Start | Repeat End | Repeat Left | Name | Family | Class |
| 74706433 | 74706689 | + | 33.9 | 932 | 1215 | -745 | L4 | RTE | LINE |
| 74706709 | 74706876 | - | 15.7 | 134 | 310 | -2 | AluJo | Alu | SINE |
| 74706877 | 74707174 | - | 15.2 | 1 | 312 | 0 | AluSx | Alu | SINE |
| 74707175 | 74707481 | - | 6.9 | 1 | 306 | -5 | AluY | Alu | SINE |
| 74707482 | 74707572 | - | 15.7 | 46 | 133 | -179 | AluJo | Alu | SINE |
| 74707573 | 74707724 | + | 29.5 | 3264 | 3419 | 0 | L2 | L2 | LINE |
| 74707757 | 74707817 | - | 26.2 | 1368 | 1428 | -989 | Charlie8 | MER1\_type | DNA |
| 74707843 | 74708117 | + | 9.9 | 1 | 295 | -17 | AluSx | Alu | SINE |
| 74708194 | 74708501 | + | 13.3 | 1 | 308 | -2 | AluSg | Alu | SINE |
| 74708593 | 74708681 | + | 21.4 | 2 | 85 | -2574 | Charlie9 | MER1\_type | DNA |
| 74708689 | 74708944 | + | 19.1 | 1 | 238 | -64 | AluJo | Alu | SINE |
| 74708945 | 74709260 | - | 10.6 | 1 | 310 | 0 | AluSg | Alu | SINE |
| 74709261 | 74709337 | + | 19.1 | 239 | 298 | -4 | AluJo | Alu | SINE |
| 74709473 | 74709551 | + | 25.3 | 2667 | 2742 | 0 | Charlie9 | MER1\_type | DNA |
| 74709583 | 74709750 | - | 9.6 | 136 | 301 | -11 | AluSx | Alu | SINE |
| 74709751 | 74710053 | - | 12.9 | 1 | 304 | -5 | AluSc | Alu | SINE |
| 74710054 | 74710188 | - | 9.6 | 1 | 135 | -177 | AluSx | Alu | SINE |
| 74710201 | 74710305 | + | 35.6 | 1366 | 1472 | -488 | L4 | RTE | LINE |

  

|  |  |  |  |  |  |  |  |  |  |
| --- | --- | --- | --- | --- | --- | --- | --- | --- | --- |
| chr7:74710530-74711218 | | | 704 bp | | |  |  |  |  |
| Genome Start | Genome End | Strand | Div | Repeat Start | Repeat End | Repeat Left | Name | Family | Class |
| 74710530 | 74710654 | + | 19.5 | 55 | 189 | -295 | MLT1H2 | MaLR | LTR |
| 74710640 | 74710910 | + | 28.0 | 149 | 482 | -113 | MLT1H2 | MaLR | LTR |
| 74710911 | 74711189 | + | 8.6 | 1 | 279 | -32 | AluY | Alu | SINE |
| 74711190 | 74711218 | + | 28.7 | 483 | 534 | -61 | MLT1H2 | MaLR | LTR |

  

|  |  |  |  |  |  |  |  |  |  |
| --- | --- | --- | --- | --- | --- | --- | --- | --- | --- |
| chr7:74711729-74712386 | | | 658 bp | | |  |  |  |  |
| Genome Start | Genome End | Strand | Div | Repeat Start | Repeat End | Repeat Left | Name | Family | Class |
| 74711729 | 74711990 | + | 16.0 | 15 | 301 | -170 | LTR32 | ERVL | LTR |
| 74711991 | 74712285 | - | 7.1 | 1 | 295 | -16 | AluY | Alu | SINE |
| 74712286 | 74712386 | + | 16.0 | 302 | 397 | -74 | LTR32 | ERVL | LTR |

  

|  |  |  |  |  |  |  |  |  |  |
| --- | --- | --- | --- | --- | --- | --- | --- | --- | --- |
| chr7:74716808-74717384 | | | 577 bp | | |  |  |  |  |
| Genome Start | Genome End | Strand | Div | Repeat Start | Repeat End | Repeat Left | Name | Family | Class |
| 74716808 | 74716872 | - | 23.9 | 68 | 162 | -433 | MLT1G1 | MaLR | LTR |
| 74716873 | 74717278 | + | 15.0 | 1 | 432 | 0 | MSTB1 | MaLR | LTR |
| 74717279 | 74717384 | - | 23.3 | 3 | 108 | -487 | MLT1G1 | MaLR | LTR |

  

|  |  |  |  |  |  |  |  |  |  |
| --- | --- | --- | --- | --- | --- | --- | --- | --- | --- |
| chr7:74729212-74729639 | | | 428 bp | | |  |  |  |  |
| Genome Start | Genome End | Strand | Div | Repeat Start | Repeat End | Repeat Left | Name | Family | Class |
| 74729212 | 74729265 | + | 26.1 | 316 | 370 | -80 | LTR16A | ERVL | LTR |
| 74729266 | 74729574 | - | 12.8 | 1 | 308 | -4 | AluJo | Alu | SINE |
| 74729575 | 74729639 | + | 26.1 | 371 | 437 | -13 | LTR16A | ERVL | LTR |

  

|  |  |  |  |  |  |  |  |  |  |
| --- | --- | --- | --- | --- | --- | --- | --- | --- | --- |
| chr7:74730279-74731078 | | | 800 bp | | |  |  |  |  |
| Genome Start | Genome End | Strand | Div | Repeat Start | Repeat End | Repeat Left | Name | Family | Class |
| 74730279 | 74730351 | + | 25.7 | 4847 | 4919 | -1227 | L1MC | L1 | LINE |
| 74730352 | 74730651 | + | 17.9 | 1 | 305 | -7 | AluJo | Alu | SINE |
| 74730652 | 74731078 | + | 25.7 | 4920 | 5333 | -813 | L1MC | L1 | LINE |

  

|  |  |  |  |  |  |  |  |  |  |
| --- | --- | --- | --- | --- | --- | --- | --- | --- | --- |
| chr7:74736321-74737304 | | | 898 bp | | |  |  |  |  |
| Genome Start | Genome End | Strand | Div | Repeat Start | Repeat End | Repeat Left | Name | Family | Class |
| 74736321 | 74736410 | - | 21.1 | 346 | 453 | -38 | LTR16C | ERVL | LTR |
| 74736411 | 74736664 | + | 17.5 | 1 | 256 | -56 | AluJb | Alu | SINE |
| 74736748 | 74737050 | + | 13.5 | 1 | 312 | -1 | AluSq | Alu | SINE |
| 74737054 | 74737304 | - | 26.3 | 85 | 350 | -141 | LTR16C | ERVL | LTR |

  

|  |  |  |  |  |  |  |  |  |  |
| --- | --- | --- | --- | --- | --- | --- | --- | --- | --- |
| chr7:74740084-74742046 | | | 1947 bp | | |  |  |  |  |
| Genome Start | Genome End | Strand | Div | Repeat Start | Repeat End | Repeat Left | Name | Family | Class |
| 74740084 | 74740213 | + | 25.6 | 1 | 121 | -88 | MER3 | MER1\_type | DNA |
| 74740214 | 74740389 | - | 23.2 | 189 | 374 | 0 | MLT1A | MaLR | LTR |
| 74740390 | 74740433 | + | 13.8 | 1 | 39 | -389 | MSTA | MaLR | LTR |
| 74740434 | 74740744 | + | 10.6 | 1 | 312 | 0 | AluSx | Alu | SINE |
| 74740745 | 74741085 | + | 13.8 | 40 | 360 | -68 | MSTA | MaLR | LTR |
| 74741086 | 74741390 | + | 11.7 | 1 | 306 | 0 | AluSq | Alu | SINE |
| 74741391 | 74741460 | + | 13.8 | 361 | 428 | 0 | MSTA | MaLR | LTR |
| 74741461 | 74741651 | - | 23.2 | 7 | 188 | -186 | MLT1A | MaLR | LTR |
| 74741664 | 74741974 | - | 12.7 | 1 | 309 | -3 | AluSx | Alu | SINE |
| 74741979 | 74742046 | + | 16.2 | 142 | 209 | 0 | MER3 | MER1\_type | DNA |

  

|  |  |  |  |  |  |  |  |  |  |
| --- | --- | --- | --- | --- | --- | --- | --- | --- | --- |
| chr7:74742986-74743605 | | | 620 bp | | |  |  |  |  |
| Genome Start | Genome End | Strand | Div | Repeat Start | Repeat End | Repeat Left | Name | Family | Class |
| 74742986 | 74743122 | + | 11.6 | 1 | 136 | -176 | AluSx | Alu | SINE |
| 74743123 | 74743429 | + | 5.2 | 1 | 307 | -4 | AluY | Alu | SINE |
| 74743430 | 74743605 | + | 11.6 | 137 | 302 | -10 | AluSx | Alu | SINE |

  

|  |  |  |  |  |  |  |  |  |  |
| --- | --- | --- | --- | --- | --- | --- | --- | --- | --- |
| chr7:74746281-74746887 | | | 607 bp | | |  |  |  |  |
| Genome Start | Genome End | Strand | Div | Repeat Start | Repeat End | Repeat Left | Name | Family | Class |
| 74746281 | 74746456 | - | 11.6 | 138 | 310 | -2 | AluSx | Alu | SINE |
| 74746457 | 74746745 | - | 10.8 | 1 | 296 | -15 | AluY | Alu | SINE |
| 74746746 | 74746887 | - | 11.6 | 1 | 137 | -175 | AluSx | Alu | SINE |

  

|  |  |  |  |  |  |  |  |  |  |
| --- | --- | --- | --- | --- | --- | --- | --- | --- | --- |
| chr7:74752884-74753489 | | | 574 bp | | |  |  |  |  |
| Genome Start | Genome End | Strand | Div | Repeat Start | Repeat End | Repeat Left | Name | Family | Class |
| 74752884 | 74753045 | - | 16.6 | 137 | 297 | -15 | AluJo | Alu | SINE |
| 74753078 | 74753351 | - | 18.6 | 2 | 276 | -36 | AluSx | Alu | SINE |
| 74753352 | 74753489 | - | 16.6 | 1 | 136 | -176 | AluJo | Alu | SINE |

  

|  |  |  |  |  |  |  |  |  |  |
| --- | --- | --- | --- | --- | --- | --- | --- | --- | --- |
| chr7:74758371-74759565 | | | 915 bp | | |  |  |  |  |
| Genome Start | Genome End | Strand | Div | Repeat Start | Repeat End | Repeat Left | Name | Family | Class |
| 74758371 | 74758552 | + | 25.8 | 4474 | 4650 | -1889 | MER84-int | ERV1 | LTR |
| 74758784 | 74759084 | - | 14.9 | 3 | 303 | -7 | AluSg | Alu | SINE |
| 74759087 | 74759390 | - | 13.0 | 1 | 300 | -13 | AluSq | Alu | SINE |
| 74759438 | 74759565 | + | 29.0 | 5398 | 5522 | -2914 | MER84-int | ERV1 | LTR |

  

|  |  |  |  |  |  |  |  |  |  |
| --- | --- | --- | --- | --- | --- | --- | --- | --- | --- |
| chr7:74773794-74774492 | | | 699 bp | | |  |  |  |  |
| Genome Start | Genome End | Strand | Div | Repeat Start | Repeat End | Repeat Left | Name | Family | Class |
| 74773794 | 74773853 | + | 34.5 | 3933 | 3988 | -501 | L3 | CR1 | LINE |
| 74773854 | 74774159 | - | 15.1 | 3 | 301 | -11 | AluSx | Alu | SINE |
| 74774160 | 74774492 | + | 34.5 | 3989 | 4324 | -165 | L3 | CR1 | LINE |

  

|  |  |  |  |  |  |  |  |  |  |
| --- | --- | --- | --- | --- | --- | --- | --- | --- | --- |
| chr7:74780907-74781512 | | | 574 bp | | |  |  |  |  |
| Genome Start | Genome End | Strand | Div | Repeat Start | Repeat End | Repeat Left | Name | Family | Class |
| 74780907 | 74781068 | - | 16.3 | 137 | 297 | -15 | AluJo | Alu | SINE |
| 74781101 | 74781374 | - | 18.6 | 2 | 276 | -36 | AluSx | Alu | SINE |
| 74781375 | 74781512 | - | 16.3 | 1 | 136 | -176 | AluJo | Alu | SINE |

  

|  |  |  |  |  |  |  |  |  |  |
| --- | --- | --- | --- | --- | --- | --- | --- | --- | --- |
| chr7:74788838-74789549 | | | 712 bp | | |  |  |  |  |
| Genome Start | Genome End | Strand | Div | Repeat Start | Repeat End | Repeat Left | Name | Family | Class |
| 74788838 | 74788868 | + | 16.9 | 3 | 32 | -457 | LTR45B | ERV1 | LTR |
| 74788869 | 74789159 | - | 10.3 | 4 | 296 | -17 | AluSp | Alu | SINE |
| 74789160 | 74789549 | + | 16.9 | 33 | 414 | -75 | LTR45B | ERV1 | LTR |

  

|  |  |  |  |  |  |  |  |  |  |
| --- | --- | --- | --- | --- | --- | --- | --- | --- | --- |
| chr7:74797210-74797803 | | | 594 bp | | |  |  |  |  |
| Genome Start | Genome End | Strand | Div | Repeat Start | Repeat End | Repeat Left | Name | Family | Class |
| 74797210 | 74797378 | - | 16.7 | 132 | 294 | -18 | AluJo | Alu | SINE |
| 74797379 | 74797670 | - | 12.0 | 1 | 292 | -20 | AluSx | Alu | SINE |
| 74797671 | 74797803 | - | 16.7 | 1 | 131 | -181 | AluJo | Alu | SINE |

  

|  |  |  |  |  |  |  |  |  |  |
| --- | --- | --- | --- | --- | --- | --- | --- | --- | --- |
| chr7:74814414-74815608 | | | 915 bp | | |  |  |  |  |
| Genome Start | Genome End | Strand | Div | Repeat Start | Repeat End | Repeat Left | Name | Family | Class |
| 74814414 | 74814595 | + | 25.8 | 4474 | 4650 | -1889 | MER84-int | ERV1 | LTR |
| 74814827 | 74815127 | - | 14.9 | 3 | 303 | -7 | AluSg | Alu | SINE |
| 74815130 | 74815433 | - | 13.3 | 1 | 300 | -13 | AluSq | Alu | SINE |
| 74815481 | 74815608 | + | 29.0 | 5398 | 5522 | -2914 | MER84-int | ERV1 | LTR |

  

|  |  |  |  |  |  |  |  |  |  |
| --- | --- | --- | --- | --- | --- | --- | --- | --- | --- |
| chr7:74816855-74817566 | | | 712 bp | | |  |  |  |  |
| Genome Start | Genome End | Strand | Div | Repeat Start | Repeat End | Repeat Left | Name | Family | Class |
| 74816855 | 74816885 | + | 17.2 | 3 | 32 | -457 | LTR45B | ERV1 | LTR |
| 74816886 | 74817176 | - | 10.0 | 4 | 296 | -17 | AluSq | Alu | SINE |
| 74817177 | 74817566 | + | 17.2 | 33 | 414 | -75 | LTR45B | ERV1 | LTR |

  

|  |  |  |  |  |  |  |  |  |  |
| --- | --- | --- | --- | --- | --- | --- | --- | --- | --- |
| chr7:74830376-74830817 | | | 442 bp | | |  |  |  |  |
| Genome Start | Genome End | Strand | Div | Repeat Start | Repeat End | Repeat Left | Name | Family | Class |
| 74830376 | 74830477 | - | 25.2 | 2762 | 2864 | -3282 | L1M5 | L1 | LINE |
| 74830478 | 74830782 | + | 12.5 | 1 | 305 | -7 | AluSx | Alu | SINE |
| 74830783 | 74830817 | - | 25.2 | 2728 | 2761 | -3385 | L1M5 | L1 | LINE |

  

|  |  |  |  |  |  |  |  |  |  |
| --- | --- | --- | --- | --- | --- | --- | --- | --- | --- |
| chr7:74836809-74837371 | | | 563 bp | | |  |  |  |  |
| Genome Start | Genome End | Strand | Div | Repeat Start | Repeat End | Repeat Left | Name | Family | Class |
| 74836809 | 74836978 | - | 30.9 | 1088 | 1261 | -1246 | HAL1 | L1 | LINE |
| 74836979 | 74837287 | - | 14.7 | 1 | 312 | 0 | AluSx | Alu | SINE |
| 74837288 | 74837371 | - | 30.9 | 1010 | 1087 | -1420 | HAL1 | L1 | LINE |

  

|  |  |  |  |  |  |  |  |  |  |
| --- | --- | --- | --- | --- | --- | --- | --- | --- | --- |
| chr7:74837372-74838130 | | | 759 bp | | |  |  |  |  |
| Genome Start | Genome End | Strand | Div | Repeat Start | Repeat End | Repeat Left | Name | Family | Class |
| 74837372 | 74837732 | + | 25.5 | 5765 | 6169 | -71 | L1ME3B | L1 | LINE |
| 74837733 | 74838068 | + | 15.7 | 1 | 294 | -18 | AluJo | Alu | SINE |
| 74838069 | 74838130 | + | 25.5 | 6170 | 6235 | -5 | L1ME3B | L1 | LINE |

  

|  |  |  |  |  |  |  |  |  |  |
| --- | --- | --- | --- | --- | --- | --- | --- | --- | --- |
| chr7:74843397-74844018 | | | 602 bp | | |  |  |  |  |
| Genome Start | Genome End | Strand | Div | Repeat Start | Repeat End | Repeat Left | Name | Family | Class |
| 74843397 | 74843573 | - | 14.0 | 136 | 308 | -4 | AluSx | Alu | SINE |
| 74843594 | 74843884 | - | 5.8 | 1 | 293 | -18 | AluY | Alu | SINE |
| 74843885 | 74844018 | - | 14.0 | 2 | 135 | -177 | AluSx | Alu | SINE |

  

|  |  |  |  |  |  |  |  |  |  |
| --- | --- | --- | --- | --- | --- | --- | --- | --- | --- |
| chr7:74854135-74854736 | | | 602 bp | | |  |  |  |  |
| Genome Start | Genome End | Strand | Div | Repeat Start | Repeat End | Repeat Left | Name | Family | Class |
| 74854135 | 74854296 | - | 15.6 | 135 | 289 | -23 | AluJo | Alu | SINE |
| 74854297 | 74854606 | - | 11.6 | 2 | 312 | 0 | AluSx | Alu | SINE |
| 74854607 | 74854736 | - | 15.6 | 1 | 134 | -178 | AluJo | Alu | SINE |

  

|  |  |  |  |  |  |  |  |  |  |
| --- | --- | --- | --- | --- | --- | --- | --- | --- | --- |
| chr7:74879312-74879894 | | | 583 bp | | |  |  |  |  |
| Genome Start | Genome End | Strand | Div | Repeat Start | Repeat End | Repeat Left | Name | Family | Class |
| 74879312 | 74879476 | + | 31.4 | 2469 | 2654 | -765 | L2 | L2 | LINE |
| 74879477 | 74879773 | - | 13.1 | 1 | 300 | -12 | AluSx | Alu | SINE |
| 74879774 | 74879894 | + | 31.4 | 2655 | 2774 | -645 | L2 | L2 | LINE |

  

|  |  |  |  |  |  |  |  |  |  |
| --- | --- | --- | --- | --- | --- | --- | --- | --- | --- |
| chr7:74939020-74939419 | | | 400 bp | | |  |  |  |  |
| Genome Start | Genome End | Strand | Div | Repeat Start | Repeat End | Repeat Left | Name | Family | Class |
| 74939020 | 74939093 | + | 11.5 | 6069 | 6147 | -24 | L1MB2 | L1 | LINE |
| 74939094 | 74939387 | - | 6.1 | 1 | 293 | -18 | AluY | Alu | SINE |
| 74939388 | 74939419 | + | 11.5 | 6148 | 6169 | -2 | L1MB2 | L1 | LINE |

  

|  |  |  |  |  |  |  |  |  |  |
| --- | --- | --- | --- | --- | --- | --- | --- | --- | --- |
| chr7:74943910-74944832 | | | 647 bp | | |  |  |  |  |
| Genome Start | Genome End | Strand | Div | Repeat Start | Repeat End | Repeat Left | Name | Family | Class |
| 74943910 | 74944217 | + | 31.7 | 3692 | 4016 | -2130 | L1M5 | L1 | LINE |
| 74944383 | 74944438 | + | 25.0 | 4030 | 4085 | -2061 | L1MD | L1 | LINE |
| 74944518 | 74944646 | + | 7.8 | 5 | 133 | -179 | AluSq/x | Alu | SINE |
| 74944679 | 74944832 | + | 26.8 | 4536 | 4700 | -1446 | L1M5 | L1 | LINE |

  

|  |  |  |  |  |  |  |  |  |  |
| --- | --- | --- | --- | --- | --- | --- | --- | --- | --- |
| chr7:74945028-74947805 | | | 2688 bp | | |  |  |  |  |
| Genome Start | Genome End | Strand | Div | Repeat Start | Repeat End | Repeat Left | Name | Family | Class |
| 74945028 | 74945112 | + | 24.1 | 36 | 115 | -6285 | L1MEc | L1 | LINE |
| 74945167 | 74945477 | - | 12.7 | 3 | 310 | 0 | AluSg | Alu | SINE |
| 74945480 | 74945617 | + | 21.0 | 119 | 273 | -6127 | L1MEc | L1 | LINE |
| 74945652 | 74946546 | + | 20.6 | 846 | 1927 | -4473 | L1MEc | L1 | LINE |
| 74946547 | 74946866 | + | 11.3 | 1 | 320 | 0 | AluSx | Alu | SINE |
| 74946867 | 74947805 | + | 22.7 | 1928 | 2815 | -3404 | L1MEc | L1 | LINE |

  

|  |  |  |  |  |  |  |  |  |  |
| --- | --- | --- | --- | --- | --- | --- | --- | --- | --- |
| chr7:74954381-74955589 | | | 1209 bp | | |  |  |  |  |
| Genome Start | Genome End | Strand | Div | Repeat Start | Repeat End | Repeat Left | Name | Family | Class |
| 74954381 | 74954689 | - | 14.4 | 567 | 903 | 0 | MER4D | ERV1 | LTR |
| 74954690 | 74954950 | + | 16.5 | 9 | 268 | -44 | AluJo | Alu | SINE |
| 74954951 | 74955589 | - | 14.4 | 1 | 566 | -337 | MER4D | ERV1 | LTR |

  

|  |  |  |  |  |  |  |  |  |  |
| --- | --- | --- | --- | --- | --- | --- | --- | --- | --- |
| chr7:74956033-74957632 | | | 1600 bp | | |  |  |  |  |
| Genome Start | Genome End | Strand | Div | Repeat Start | Repeat End | Repeat Left | Name | Family | Class |
| 74956033 | 74956170 | - | 22.6 | 544 | 681 | -30 | MER67C | ERV1 | LTR |
| 74956171 | 74956474 | - | 11.2 | 1 | 303 | -7 | AluSg | Alu | SINE |
| 74956475 | 74956574 | - | 22.6 | 453 | 543 | -168 | MER67C | ERV1 | LTR |
| 74956575 | 74956899 | - | 13.9 | 1 | 304 | -8 | AluSx | Alu | SINE |
| 74956900 | 74957147 | - | 22.6 | 202 | 452 | -259 | MER67C | ERV1 | LTR |
| 74957148 | 74957449 | + | 12.2 | 1 | 305 | -7 | AluSx | Alu | SINE |
| 74957450 | 74957632 | - | 22.6 | 21 | 201 | -510 | MER67C | ERV1 | LTR |

  

|  |  |  |  |  |  |  |  |  |  |
| --- | --- | --- | --- | --- | --- | --- | --- | --- | --- |
| chr7:74958856-74959570 | | | 715 bp | | |  |  |  |  |
| Genome Start | Genome End | Strand | Div | Repeat Start | Repeat End | Repeat Left | Name | Family | Class |
| 74958856 | 74958915 | + | 36.5 | 3933 | 3988 | -501 | L3 | CR1 | LINE |
| 74958916 | 74959226 | - | 12.0 | 3 | 311 | -1 | AluSx | Alu | SINE |
| 74959227 | 74959570 | + | 36.5 | 3989 | 4333 | -156 | L3 | CR1 | LINE |

  

|  |  |  |  |  |  |  |  |  |  |
| --- | --- | --- | --- | --- | --- | --- | --- | --- | --- |
| chr7:74967798-74968404 | | | 581 bp | | |  |  |  |  |
| Genome Start | Genome End | Strand | Div | Repeat Start | Repeat End | Repeat Left | Name | Family | Class |
| 74967798 | 74967958 | - | 16.9 | 138 | 298 | -14 | AluJo | Alu | SINE |
| 74967985 | 74968266 | - | 17.7 | 2 | 284 | -28 | AluSx | Alu | SINE |
| 74968267 | 74968404 | - | 16.9 | 4 | 137 | -175 | AluJo | Alu | SINE |

  

|  |  |  |  |  |  |  |  |  |  |
| --- | --- | --- | --- | --- | --- | --- | --- | --- | --- |
| chr7:74976041-74976746 | | | 706 bp | | |  |  |  |  |
| Genome Start | Genome End | Strand | Div | Repeat Start | Repeat End | Repeat Left | Name | Family | Class |
| 74976041 | 74976071 | + | 16.0 | 3 | 32 | -457 | LTR45B | ERV1 | LTR |
| 74976072 | 74976359 | - | 11.8 | 1 | 296 | -14 | AluSg | Alu | SINE |
| 74976360 | 74976746 | + | 16.0 | 33 | 414 | -75 | LTR45B | ERV1 | LTR |

  

|  |  |  |  |  |  |  |  |  |  |
| --- | --- | --- | --- | --- | --- | --- | --- | --- | --- |
| chr7:74989873-74990655 | | | 783 bp | | |  |  |  |  |
| Genome Start | Genome End | Strand | Div | Repeat Start | Repeat End | Repeat Left | Name | Family | Class |
| 74989873 | 74990124 | + | 25.1 | 6852 | 7132 | -829 | L1MC5 | L1 | LINE |
| 74990125 | 74990411 | + | 17.8 | 1 | 304 | -8 | AluJo | Alu | SINE |
| 74990412 | 74990655 | + | 25.1 | 7133 | 7361 | -600 | L1MC5 | L1 | LINE |

  

|  |  |  |  |  |  |  |  |  |  |
| --- | --- | --- | --- | --- | --- | --- | --- | --- | --- |
| chr7:75557468-75558124 | | | 657 bp | | |  |  |  |  |
| Genome Start | Genome End | Strand | Div | Repeat Start | Repeat End | Repeat Left | Name | Family | Class |
| 75557468 | 75557679 | - | 19.4 | 129 | 309 | -3 | AluJo | Alu | SINE |
| 75557680 | 75557990 | - | 10.9 | 1 | 311 | -1 | AluSx | Alu | SINE |
| 75557991 | 75558124 | - | 19.4 | 1 | 128 | -184 | AluJo | Alu | SINE |

  

|  |  |  |  |  |  |  |  |  |  |
| --- | --- | --- | --- | --- | --- | --- | --- | --- | --- |
| chr7:75561145-75562154 | | | 1010 bp | | |  |  |  |  |
| Genome Start | Genome End | Strand | Div | Repeat Start | Repeat End | Repeat Left | Name | Family | Class |
| 75561145 | 75561298 | - | 26.0 | 639 | 772 | -1 | LTR41 | ERVL | LTR |
| 75561299 | 75561605 | + | 19.5 | 1 | 292 | -20 | AluJb | Alu | SINE |
| 75561606 | 75562154 | - | 26.0 | 1 | 638 | -135 | LTR41 | ERVL | LTR |

  

|  |  |  |  |  |  |  |  |  |  |
| --- | --- | --- | --- | --- | --- | --- | --- | --- | --- |
| chr7:75591255-75592812 | | | 1537 bp | | |  |  |  |  |
| Genome Start | Genome End | Strand | Div | Repeat Start | Repeat End | Repeat Left | Name | Family | Class |
| 75591255 | 75591366 | - | 25.7 | 7702 | 7810 | -232 | L1MC4 | L1 | LINE |
| 75591388 | 75591626 | - | 25.2 | 7314 | 7587 | -455 | L1MC4 | L1 | LINE |
| 75591627 | 75591755 | + | 13.0 | 1 | 133 | 0 | FLAM\_C | Alu | SINE |
| 75591756 | 75591916 | - | 25.2 | 7158 | 7313 | -729 | L1MC4 | L1 | LINE |
| 75591917 | 75592223 | - | 11.1 | 1 | 311 | -2 | AluSp | Alu | SINE |
| 75592224 | 75592305 | - | 25.2 | 7019 | 7157 | -885 | L1MC4 | L1 | LINE |
| 75592306 | 75592603 | - | 11.7 | 1 | 299 | -13 | AluSx | Alu | SINE |
| 75592604 | 75592812 | - | 25.2 | 6767 | 7018 | -1024 | L1MC4 | L1 | LINE |

  

|  |  |  |  |  |  |  |  |  |  |
| --- | --- | --- | --- | --- | --- | --- | --- | --- | --- |
| chr7:75921799-75922689 | | | 891 bp | | |  |  |  |  |
| Genome Start | Genome End | Strand | Div | Repeat Start | Repeat End | Repeat Left | Name | Family | Class |
| 75921799 | 75922183 | - | 6.2 | 179 | 554 | 0 | MER41A | ERV1 | LTR |
| 75922184 | 75922510 | - | 4.9 | 1 | 327 | 0 | AluY | Alu | SINE |
| 75922511 | 75922689 | - | 6.2 | 1 | 178 | -376 | MER41A | ERV1 | LTR |

  

|  |  |  |  |  |  |  |  |  |  |
| --- | --- | --- | --- | --- | --- | --- | --- | --- | --- |
| chr7:75927380-75928045 | | | 645 bp | | |  |  |  |  |
| Genome Start | Genome End | Strand | Div | Repeat Start | Repeat End | Repeat Left | Name | Family | Class |
| 75927380 | 75927588 | - | 9.4 | 146 | 340 | 0 | AluSc | Alu | SINE |
| 75927610 | 75927900 | - | 3.9 | 1 | 294 | -17 | AluY | Alu | SINE |
| 75927901 | 75928045 | - | 9.4 | 2 | 145 | -164 | AluSc | Alu | SINE |

  

|  |  |  |  |  |  |  |  |  |  |
| --- | --- | --- | --- | --- | --- | --- | --- | --- | --- |
| chr7:75935654-75936411 | | | 757 bp | | |  |  |  |  |
| Genome Start | Genome End | Strand | Div | Repeat Start | Repeat End | Repeat Left | Name | Family | Class |
| 75935654 | 75935701 | + | 27.1 | 1 | 47 | -177 | MER58A | MER1\_type | DNA |
| 75935702 | 75936010 | - | 7.8 | 2 | 310 | -2 | AluSx | Alu | SINE |
| 75936012 | 75936317 | - | 13.2 | 1 | 304 | -8 | AluSx | Alu | SINE |
| 75936318 | 75936411 | + | 27.1 | 48 | 137 | 0 | MER58A | MER1\_type | DNA |

  

|  |  |  |  |  |  |  |  |  |  |
| --- | --- | --- | --- | --- | --- | --- | --- | --- | --- |
| chr7:75944675-75945537 | | | 639 bp | | |  |  |  |  |
| Genome Start | Genome End | Strand | Div | Repeat Start | Repeat End | Repeat Left | Name | Family | Class |
| 75944675 | 75944814 | - | 35.0 | 95 | 239 | -29 | MIRb | MIR | SINE |
| 75944816 | 75944856 | + | 9.8 | 123 | 167 | -132 | AluSc | Alu | SINE |
| 75944857 | 75945094 | + | 18.1 | 90 | 323 | 0 | AluJo | Alu | SINE |
| 75945127 | 75945223 | - | 30.4 | 15 | 107 | -155 | MIR | MIR | SINE |
| 75945415 | 75945537 | - | 23.4 | 25 | 161 | -62 | MIRb | MIR | SINE |

  

|  |  |  |  |  |  |  |  |  |  |
| --- | --- | --- | --- | --- | --- | --- | --- | --- | --- |
| chr7:75973600-75974194 | | | 595 bp | | |  |  |  |  |
| Genome Start | Genome End | Strand | Div | Repeat Start | Repeat End | Repeat Left | Name | Family | Class |
| 75973600 | 75973727 | + | 19.7 | 3 | 136 | -176 | AluJo | Alu | SINE |
| 75973728 | 75974028 | + | 13.0 | 1 | 300 | -12 | AluSx | Alu | SINE |
| 75974029 | 75974194 | + | 19.7 | 137 | 303 | -9 | AluJo | Alu | SINE |

  

|  |  |  |  |  |  |  |  |  |  |
| --- | --- | --- | --- | --- | --- | --- | --- | --- | --- |
| chr7:75987773-75988331 | | | 559 bp | | |  |  |  |  |
| Genome Start | Genome End | Strand | Div | Repeat Start | Repeat End | Repeat Left | Name | Family | Class |
| 75987773 | 75987946 | + | 39.6 | 2624 | 2804 | -615 | L2 | L2 | LINE |
| 75987947 | 75988250 | - | 13.6 | 1 | 305 | -7 | AluSx | Alu | SINE |
| 75988251 | 75988331 | + | 39.6 | 2805 | 2882 | -537 | L2 | L2 | LINE |

  

|  |  |  |  |  |  |  |  |  |  |
| --- | --- | --- | --- | --- | --- | --- | --- | --- | --- |
| chr7:75993396-75994099 | | | 704 bp | | |  |  |  |  |
| Genome Start | Genome End | Strand | Div | Repeat Start | Repeat End | Repeat Left | Name | Family | Class |
| 75993396 | 75993782 | - | 15.8 | 33 | 414 | -75 | LTR45B | ERV1 | LTR |
| 75993783 | 75994068 | + | 12.6 | 1 | 294 | -16 | AluSg | Alu | SINE |
| 75994069 | 75994099 | - | 15.8 | 3 | 32 | -457 | LTR45B | ERV1 | LTR |

  

|  |  |  |  |  |  |  |  |  |  |
| --- | --- | --- | --- | --- | --- | --- | --- | --- | --- |
| chr7:76001590-76002196 | | | 607 bp | | |  |  |  |  |
| Genome Start | Genome End | Strand | Div | Repeat Start | Repeat End | Repeat Left | Name | Family | Class |
| 76001590 | 76001727 | + | 16.6 | 1 | 134 | -178 | AluJo | Alu | SINE |
| 76001728 | 76002034 | + | 17.5 | 2 | 309 | 0 | AluSx | Alu | SINE |
| 76002035 | 76002196 | + | 16.6 | 135 | 298 | -14 | AluJo | Alu | SINE |

  

|  |  |  |  |  |  |  |  |  |  |
| --- | --- | --- | --- | --- | --- | --- | --- | --- | --- |
| chr7:76010514-76011230 | | | 717 bp | | |  |  |  |  |
| Genome Start | Genome End | Strand | Div | Repeat Start | Repeat End | Repeat Left | Name | Family | Class |
| 76010514 | 76010857 | - | 36.2 | 3992 | 4333 | -156 | L3 | CR1 | LINE |
| 76010858 | 76011169 | + | 12.6 | 3 | 312 | 0 | AluSx | Alu | SINE |
| 76011170 | 76011230 | - | 36.2 | 3933 | 3991 | -498 | L3 | CR1 | LINE |

  

|  |  |  |  |  |  |  |  |  |  |
| --- | --- | --- | --- | --- | --- | --- | --- | --- | --- |
| chr7:76012454-76014053 | | | 1600 bp | | |  |  |  |  |
| Genome Start | Genome End | Strand | Div | Repeat Start | Repeat End | Repeat Left | Name | Family | Class |
| 76012454 | 76012637 | + | 22.5 | 20 | 201 | -510 | MER67C | ERV1 | LTR |
| 76012638 | 76012943 | - | 11.8 | 1 | 308 | -4 | AluSx | Alu | SINE |
| 76012944 | 76013191 | + | 22.5 | 202 | 452 | -259 | MER67C | ERV1 | LTR |
| 76013192 | 76013516 | + | 14.2 | 1 | 304 | -8 | AluSx | Alu | SINE |
| 76013517 | 76013616 | + | 22.5 | 453 | 543 | -168 | MER67C | ERV1 | LTR |
| 76013617 | 76013915 | + | 10.4 | 1 | 298 | -12 | AluSg | Alu | SINE |
| 76013916 | 76014053 | + | 22.5 | 544 | 681 | -30 | MER67C | ERV1 | LTR |

  

|  |  |  |  |  |  |  |  |  |  |
| --- | --- | --- | --- | --- | --- | --- | --- | --- | --- |
| chr7:76014497-76015704 | | | 1208 bp | | |  |  |  |  |
| Genome Start | Genome End | Strand | Div | Repeat Start | Repeat End | Repeat Left | Name | Family | Class |
| 76014497 | 76015135 | + | 14.0 | 1 | 576 | -327 | MER4D | ERV1 | LTR |
| 76015136 | 76015395 | - | 17.0 | 9 | 268 | -44 | AluJo | Alu | SINE |
| 76015396 | 76015704 | + | 14.0 | 577 | 903 | 0 | MER4D | ERV1 | LTR |

  

|  |  |  |  |  |  |  |  |  |  |
| --- | --- | --- | --- | --- | --- | --- | --- | --- | --- |
| chr7:76082837-76083746 | | | 738 bp | | |  |  |  |  |
| Genome Start | Genome End | Strand | Div | Repeat Start | Repeat End | Repeat Left | Name | Family | Class |
| 76082837 | 76082882 | + | 19.6 | 1 | 47 | -131 | MER5B | MER1\_type | DNA |
| 76082956 | 76083242 | + | 15.7 | 1 | 298 | -14 | AluSx | Alu | SINE |
| 76083263 | 76083551 | + | 11.5 | 8 | 298 | -12 | AluSg | Alu | SINE |
| 76083631 | 76083746 | + | 25.1 | 57 | 177 | -1 | MER5B | MER1\_type | DNA |

  

|  |  |  |  |  |  |  |  |  |  |
| --- | --- | --- | --- | --- | --- | --- | --- | --- | --- |
| chr7:76088733-76089613 | | | 881 bp | | |  |  |  |  |
| Genome Start | Genome End | Strand | Div | Repeat Start | Repeat End | Repeat Left | Name | Family | Class |
| 76088733 | 76088911 | + | 5.8 | 1 | 178 | -376 | MER41A | ERV1 | LTR |
| 76088912 | 76089228 | + | 5.0 | 1 | 319 | 0 | AluY | Alu | SINE |
| 76089229 | 76089613 | + | 5.8 | 179 | 554 | 0 | MER41A | ERV1 | LTR |

  

|  |  |  |  |  |  |  |  |  |  |
| --- | --- | --- | --- | --- | --- | --- | --- | --- | --- |
| chr7:76096411-76097373 | | | 963 bp | | |  |  |  |  |
| Genome Start | Genome End | Strand | Div | Repeat Start | Repeat End | Repeat Left | Name | Family | Class |
| 76096411 | 76096477 | + | 26.8 | 5101 | 5168 | -978 | L1M5 | L1 | LINE |
| 76096478 | 76096747 | + | 14.3 | 1 | 273 | -39 | AluSx | Alu | SINE |
| 76096748 | 76097373 | + | 27.0 | 5169 | 5823 | -417 | L1M5 | L1 | LINE |

  

|  |  |  |  |  |  |  |  |  |  |
| --- | --- | --- | --- | --- | --- | --- | --- | --- | --- |
| chr7:76097538-76100228 | | | 2624 bp | | |  |  |  |  |
| Genome Start | Genome End | Strand | Div | Repeat Start | Repeat End | Repeat Left | Name | Family | Class |
| 76097538 | 76097815 | + | 31.4 | 131 | 402 | -5998 | L1MEc | L1 | LINE |
| 76097844 | 76097949 | + | 14.4 | 2 | 106 | -450 | MLT2B4 | ERVL | LTR |
| 76097954 | 76098268 | - | 11.9 | 1 | 312 | -1 | AluSq | Alu | SINE |
| 76098298 | 76098511 | + | 21.3 | 555 | 775 | -5625 | L1MEc | L1 | LINE |
| 76098512 | 76098822 | + | 5.8 | 1 | 311 | 0 | AluY | Alu | SINE |
| 76098823 | 76099416 | + | 27.1 | 776 | 1645 | -4670 | L1MEc | L1 | LINE |
| 76099423 | 76099524 | + | 16.2 | 35 | 125 | -187 | AluJb | Alu | SINE |
| 76099525 | 76099827 | + | 4.0 | 1 | 302 | -9 | AluY | Alu | SINE |
| 76099828 | 76100042 | + | 16.2 | 126 | 299 | -13 | AluJb | Alu | SINE |
| 76100043 | 76100228 | + | 18.3 | 1730 | 1928 | -4472 | L1MEc | L1 | LINE |

  

|  |  |  |  |  |  |  |  |  |  |
| --- | --- | --- | --- | --- | --- | --- | --- | --- | --- |
| chr7:76108632-76112020 | | | 3019 bp | | |  |  |  |  |
| Genome Start | Genome End | Strand | Div | Repeat Start | Repeat End | Repeat Left | Name | Family | Class |
| 76108632 | 76108728 | + | 30.2 | 18 | 115 | -6432 | L1MD2 | L1 | LINE |
| 76108729 | 76109010 | + | 13.1 | 1 | 284 | -28 | AluSx | Alu | SINE |
| 76109112 | 76109419 | + | 31.4 | 179 | 470 | -6170 | L1MD2 | L1 | LINE |
| 76109596 | 76109740 | + | 8.3 | 1 | 135 | -108 | MER75B | PiggyBac | DNA |
| 76109741 | 76109832 | - | 10.8 | 522 | 604 | -130 | MER50 | ERV1 | LTR |
| 76109827 | 76109961 | + | 11.1 | 369 | 504 | 0 | MER75 | PiggyBac | DNA |
| 76109963 | 76110095 | + | 27.0 | 743 | 871 | -5676 | L1MD2 | L1 | LINE |
| 76110096 | 76110401 | + | 16.7 | 1 | 308 | -4 | AluJo | Alu | SINE |
| 76110402 | 76111137 | + | 23.7 | 872 | 1560 | -4987 | L1MD2 | L1 | LINE |
| 76111166 | 76111475 | + | 9.7 | 1 | 310 | -2 | AluSx | Alu | SINE |
| 76111546 | 76112020 | + | 25.1 | 2079 | 2577 | -4063 | L1MD2 | L1 | LINE |

  

|  |  |  |  |  |  |  |  |  |  |
| --- | --- | --- | --- | --- | --- | --- | --- | --- | --- |
| chr7:76130234-76131297 | | | 1064 bp | | |  |  |  |  |
| Genome Start | Genome End | Strand | Div | Repeat Start | Repeat End | Repeat Left | Name | Family | Class |
| 76130234 | 76130628 | - | 28.2 | 2532 | 2958 | -3188 | L1M5 | L1 | LINE |
| 76130629 | 76130921 | + | 5.1 | 1 | 293 | -18 | AluY | Alu | SINE |
| 76130922 | 76131297 | - | 28.2 | 2111 | 2531 | -3615 | L1M5 | L1 | LINE |

  

|  |  |  |  |  |  |  |  |  |  |
| --- | --- | --- | --- | --- | --- | --- | --- | --- | --- |
| chr7:76141891-76143021 | | | 1131 bp | | |  |  |  |  |
| Genome Start | Genome End | Strand | Div | Repeat Start | Repeat End | Repeat Left | Name | Family | Class |
| 76141891 | 76141975 | - | 34.5 | 5738 | 5825 | -296 | L1ME4a | L1 | LINE |
| 76141976 | 76142513 | - | 19.2 | 1 | 520 | 0 | MLT2B1 | ERVL | LTR |
| 76142514 | 76142671 | - | 34.5 | 5593 | 5737 | -384 | L1ME4a | L1 | LINE |
| 76142672 | 76142966 | + | 19.6 | 1 | 292 | -20 | AluJb | Alu | SINE |
| 76142967 | 76143021 | - | 34.5 | 5538 | 5592 | -529 | L1ME4a | L1 | LINE |

  

|  |  |  |  |  |  |  |  |  |  |
| --- | --- | --- | --- | --- | --- | --- | --- | --- | --- |
| chr7:76269938-76270438 | | | 501 bp | | |  |  |  |  |
| Genome Start | Genome End | Strand | Div | Repeat Start | Repeat End | Repeat Left | Name | Family | Class |
| 76269938 | 76269999 | - | 30.3 | 181 | 248 | -14 | MIRb | MIR | SINE |
| 76270000 | 76270344 | + | 11.9 | 1 | 364 | 0 | THE1B | MaLR | LTR |
| 76270345 | 76270438 | - | 30.6 | 93 | 184 | -84 | MIRb | MIR | SINE |

  

|  |  |  |  |  |  |  |  |  |  |
| --- | --- | --- | --- | --- | --- | --- | --- | --- | --- |
| chr7:76289892-76291501 | | | 1303 bp | | |  |  |  |  |
| Genome Start | Genome End | Strand | Div | Repeat Start | Repeat End | Repeat Left | Name | Family | Class |
| 76289892 | 76290491 | - | 31.0 | 5385 | 5970 | -1906 | L1MC4a | L1 | LINE |
| 76290615 | 76291074 | - | 20.4 | 5859 | 6343 | -16 | L1MD2 | L1 | LINE |
| 76291259 | 76291501 | - | 27.8 | 2103 | 2358 | -3857 | L1MC4a | L1 | LINE |

  

|  |  |  |  |  |  |  |  |  |  |
| --- | --- | --- | --- | --- | --- | --- | --- | --- | --- |
| chr7:76299188-76301336 | | | 1973 bp | | |  |  |  |  |
| Genome Start | Genome End | Strand | Div | Repeat Start | Repeat End | Repeat Left | Name | Family | Class |
| 76299188 | 76299620 | + | 29.2 | 1 | 426 | -2518 | BLACKJACK | AcHobo | DNA |
| 76299669 | 76300449 | + | 23.2 | 1390 | 2122 | -822 | BLACKJACK | AcHobo | DNA |
| 76300498 | 76301106 | - | 6.6 | 5546 | 6154 | 0 | L1PA7 | L1 | LINE |
| 76301187 | 76301336 | + | 25.7 | 2781 | 2944 | 0 | BLACKJACK | AcHobo | DNA |

  

|  |  |  |  |  |  |  |  |  |  |
| --- | --- | --- | --- | --- | --- | --- | --- | --- | --- |
| chr7:76317452-76319417 | | | 1482 bp | | |  |  |  |  |
| Genome Start | Genome End | Strand | Div | Repeat Start | Repeat End | Repeat Left | Name | Family | Class |
| 76317452 | 76317775 | + | 34.7 | 731 | 1097 | -3392 | L3 | CR1 | LINE |
| 76318177 | 76318390 | + | 30.1 | 1891 | 2106 | -2383 | L3 | CR1 | LINE |
| 76318393 | 76318994 | - | 22.5 | 1 | 624 | -2 | MLT1E2 | MaLR | LTR |
| 76319076 | 76319417 | + | 37.8 | 3931 | 4297 | -192 | L3 | CR1 | LINE |

  

|  |  |  |  |  |  |  |  |  |  |
| --- | --- | --- | --- | --- | --- | --- | --- | --- | --- |
| chr7:76397360-76398016 | | | 657 bp | | |  |  |  |  |
| Genome Start | Genome End | Strand | Div | Repeat Start | Repeat End | Repeat Left | Name | Family | Class |
| 76397360 | 76397592 | - | 29.1 | 5518 | 5737 | -2071 | L1MC | L1 | LINE |
| 76397593 | 76397909 | - | 11.3 | 1 | 304 | -9 | AluSq | Alu | SINE |
| 76397910 | 76398016 | - | 29.1 | 5409 | 5517 | -2291 | L1MC | L1 | LINE |

  

|  |  |  |  |  |  |  |  |  |  |
| --- | --- | --- | --- | --- | --- | --- | --- | --- | --- |
| chr7:76403275-76405540 | | | 2266 bp | | |  |  |  |  |
| Genome Start | Genome End | Strand | Div | Repeat Start | Repeat End | Repeat Left | Name | Family | Class |
| 76403275 | 76403427 | + | 30.1 | 1222 | 1396 | -2023 | L2 | L2 | LINE |
| 76403428 | 76403612 | - | 21.7 | 2 | 189 | 0 | MER5A | MER1\_type | DNA |
| 76403613 | 76405540 | + | 30.7 | 1397 | 3411 | -8 | L2 | L2 | LINE |

  

|  |  |  |  |  |  |  |  |  |  |
| --- | --- | --- | --- | --- | --- | --- | --- | --- | --- |
| chr7:76424320-76424932 | | | 613 bp | | |  |  |  |  |
| Genome Start | Genome End | Strand | Div | Repeat Start | Repeat End | Repeat Left | Name | Family | Class |
| 76424320 | 76424457 | + | 11.6 | 1 | 138 | -175 | AluSq | Alu | SINE |
| 76424458 | 76424736 | + | 10.0 | 3 | 285 | -25 | AluSg | Alu | SINE |
| 76424737 | 76424932 | + | 11.6 | 139 | 313 | 0 | AluSq | Alu | SINE |

  

|  |  |  |  |  |  |  |  |  |  |
| --- | --- | --- | --- | --- | --- | --- | --- | --- | --- |
| chr7:76434561-76435129 | | | 569 bp | | |  |  |  |  |
| Genome Start | Genome End | Strand | Div | Repeat Start | Repeat End | Repeat Left | Name | Family | Class |
| 76434561 | 76434723 | - | 29.2 | 121 | 289 | -2128 | Charlie8 | MER1\_type | DNA |
| 76434724 | 76435044 | - | 11.3 | 1 | 310 | -1 | AluY | Alu | SINE |
| 76435045 | 76435129 | - | 29.2 | 12 | 120 | -2297 | Charlie8 | MER1\_type | DNA |

  

|  |  |  |  |  |  |  |  |  |  |
| --- | --- | --- | --- | --- | --- | --- | --- | --- | --- |
| chr7:76455266-76455969 | | | 704 bp | | |  |  |  |  |
| Genome Start | Genome End | Strand | Div | Repeat Start | Repeat End | Repeat Left | Name | Family | Class |
| 76455266 | 76455401 | - | 24.5 | 7848 | 7980 | -15 | L1MD3 | L1 | LINE |
| 76455402 | 76455703 | + | 9.9 | 1 | 292 | -17 | AluSc | Alu | SINE |
| 76455704 | 76455969 | - | 24.5 | 7569 | 7847 | -148 | L1MD3 | L1 | LINE |

  

|  |  |  |  |  |  |  |  |  |  |
| --- | --- | --- | --- | --- | --- | --- | --- | --- | --- |
| chr7:76492582-76493193 | | | 568 bp | | |  |  |  |  |
| Genome Start | Genome End | Strand | Div | Repeat Start | Repeat End | Repeat Left | Name | Family | Class |
| 76492582 | 76492726 | + | 16.1 | 1 | 143 | -169 | AluJo | Alu | SINE |
| 76492727 | 76492990 | + | 18.2 | 12 | 276 | -36 | AluSx | Alu | SINE |
| 76493035 | 76493193 | + | 16.1 | 144 | 294 | -18 | AluJo | Alu | SINE |

  

|  |  |  |  |  |  |  |  |  |  |
| --- | --- | --- | --- | --- | --- | --- | --- | --- | --- |
| chr7:76499587-76500285 | | | 699 bp | | |  |  |  |  |
| Genome Start | Genome End | Strand | Div | Repeat Start | Repeat End | Repeat Left | Name | Family | Class |
| 76499587 | 76499919 | - | 37.0 | 3989 | 4324 | -165 | L3 | CR1 | LINE |
| 76499920 | 76500225 | + | 15.4 | 3 | 301 | -11 | AluSx | Alu | SINE |
| 76500226 | 76500285 | - | 37.0 | 3933 | 3988 | -501 | L3 | CR1 | LINE |

  

|  |  |  |  |  |  |  |  |  |  |
| --- | --- | --- | --- | --- | --- | --- | --- | --- | --- |
| chr7:76504270-76504863 | | | 594 bp | | |  |  |  |  |
| Genome Start | Genome End | Strand | Div | Repeat Start | Repeat End | Repeat Left | Name | Family | Class |
| 76504270 | 76504402 | + | 16.4 | 1 | 131 | -181 | AluJo | Alu | SINE |
| 76504403 | 76504694 | + | 11.7 | 1 | 292 | -20 | AluSx | Alu | SINE |
| 76504695 | 76504863 | + | 16.4 | 132 | 294 | -18 | AluJo | Alu | SINE |

  

|  |  |  |  |  |  |  |  |  |  |
| --- | --- | --- | --- | --- | --- | --- | --- | --- | --- |
| chr7:76514420-76515611 | | | 912 bp | | |  |  |  |  |
| Genome Start | Genome End | Strand | Div | Repeat Start | Repeat End | Repeat Left | Name | Family | Class |
| 76514420 | 76514547 | - | 29.0 | 5398 | 5522 | -2914 | HERV9 | ERV1 | LTR |
| 76514595 | 76514895 | + | 13.0 | 1 | 300 | -13 | AluSq | Alu | SINE |
| 76514898 | 76515198 | + | 14.3 | 3 | 303 | -7 | AluSg | Alu | SINE |
| 76515430 | 76515611 | - | 26.4 | 4474 | 4650 | -1889 | HERV9 | ERV1 | LTR |

  

|  |  |  |  |  |  |  |  |  |  |
| --- | --- | --- | --- | --- | --- | --- | --- | --- | --- |
| chr7:76520674-76521276 | | | 603 bp | | |  |  |  |  |
| Genome Start | Genome End | Strand | Div | Repeat Start | Repeat End | Repeat Left | Name | Family | Class |
| 76520674 | 76520811 | + | 16.6 | 1 | 134 | -178 | AluJo | Alu | SINE |
| 76520812 | 76521113 | + | 18.2 | 2 | 304 | -8 | AluSx | Alu | SINE |
| 76521114 | 76521276 | + | 16.6 | 135 | 298 | -14 | AluJo | Alu | SINE |

  

|  |  |  |  |  |  |  |  |  |  |
| --- | --- | --- | --- | --- | --- | --- | --- | --- | --- |
| chr7:76530594-76531211 | | | 618 bp | | |  |  |  |  |
| Genome Start | Genome End | Strand | Div | Repeat Start | Repeat End | Repeat Left | Name | Family | Class |
| 76530594 | 76530726 | + | 14.4 | 1 | 128 | -184 | AluJo | Alu | SINE |
| 76530727 | 76531034 | + | 11.0 | 1 | 309 | -4 | AluSq | Alu | SINE |
| 76531035 | 76531211 | + | 14.4 | 129 | 302 | -10 | AluJo | Alu | SINE |

  

|  |  |  |  |  |  |  |  |  |  |
| --- | --- | --- | --- | --- | --- | --- | --- | --- | --- |
| chr7:76541703-76543236 | | | 1517 bp | | |  |  |  |  |
| Genome Start | Genome End | Strand | Div | Repeat Start | Repeat End | Repeat Left | Name | Family | Class |
| 76541703 | 76541982 | - | 16.6 | 809 | 1125 | -5275 | L1MEc | L1 | LINE |
| 76541983 | 76542707 | + | 15.3 | 1 | 691 | 0 | LTR8 | ERV1 | LTR |
| 76542708 | 76543158 | - | 18.9 | 175 | 808 | -5592 | L1MEc | L1 | LINE |
| 76543176 | 76543236 | - | 19.7 | 44 | 105 | -6295 | L1MEc | L1 | LINE |

  

|  |  |  |  |  |  |  |  |  |  |
| --- | --- | --- | --- | --- | --- | --- | --- | --- | --- |
| chr7:76548204-76549966 | | | 1763 bp | | |  |  |  |  |
| Genome Start | Genome End | Strand | Div | Repeat Start | Repeat End | Repeat Left | Name | Family | Class |
| 76548204 | 76548883 | + | 13.5 | 1 | 694 | -537 | Tigger3b | MER2\_type | DNA |
| 76548884 | 76549189 | - | 11.5 | 1 | 309 | -4 | AluSq | Alu | SINE |
| 76549190 | 76549232 | + | 13.5 | 695 | 729 | -502 | Tigger3b | MER2\_type | DNA |
| 76549233 | 76549492 | + | 21.0 | 37 | 303 | -9 | AluJo | Alu | SINE |
| 76549493 | 76549966 | + | 10.8 | 728 | 1231 | 0 | Tigger3b | MER2\_type | DNA |

  

|  |  |  |  |  |  |  |  |  |  |
| --- | --- | --- | --- | --- | --- | --- | --- | --- | --- |
| chr7:76591968-76592724 | | | 757 bp | | |  |  |  |  |
| Genome Start | Genome End | Strand | Div | Repeat Start | Repeat End | Repeat Left | Name | Family | Class |
| 76591968 | 76592163 | + | 27.4 | 5526 | 5750 | -371 | L1ME4a | L1 | LINE |
| 76592164 | 76592457 | + | 12.6 | 1 | 293 | -20 | AluSq | Alu | SINE |
| 76592458 | 76592724 | + | 27.4 | 5751 | 6037 | -84 | L1ME4a | L1 | LINE |

  

|  |  |  |  |  |  |  |  |  |  |
| --- | --- | --- | --- | --- | --- | --- | --- | --- | --- |
| chr7:76641070-76642228 | | | 988 bp | | |  |  |  |  |
| Genome Start | Genome End | Strand | Div | Repeat Start | Repeat End | Repeat Left | Name | Family | Class |
| 76641070 | 76641152 | - | 24.1 | 6075 | 6161 | -1 | L1ME3 | L1 | LINE |
| 76641324 | 76641537 | - | 26.9 | 5798 | 6011 | -151 | L1ME3 | L1 | LINE |
| 76641538 | 76641984 | - | 16.5 | 1 | 486 | 0 | MER66B | ERV1 | LTR |
| 76641985 | 76642228 | - | 26.9 | 5544 | 5797 | -365 | L1ME3 | L1 | LINE |

  

|  |  |  |  |  |  |  |  |  |  |
| --- | --- | --- | --- | --- | --- | --- | --- | --- | --- |
| chr7:89564610-89565195 | | | 511 bp | | |  |  |  |  |
| Genome Start | Genome End | Strand | Div | Repeat Start | Repeat End | Repeat Left | Name | Family | Class |
| 89564610 | 89564752 | - | 21.0 | 35 | 178 | 0 | MER5B | MER1\_type | DNA |
| 89564828 | 89565169 | - | 14.7 | 1 | 354 | -27 | THE1D | MaLR | LTR |
| 89565170 | 89565195 | - | 11.5 | 15 | 40 | -138 | MER5B | MER1\_type | DNA |

  

|  |  |  |  |  |  |  |  |  |  |
| --- | --- | --- | --- | --- | --- | --- | --- | --- | --- |
| chr7:97342324-97342944 | | | 621 bp | | |  |  |  |  |
| Genome Start | Genome End | Strand | Div | Repeat Start | Repeat End | Repeat Left | Name | Family | Class |
| 97342324 | 97342398 | - | 25.1 | 340 | 407 | -2 | MLT1I | MaLR | LTR |
| 97342399 | 97342686 | - | 12.9 | 1 | 295 | -17 | AluSx | Alu | SINE |
| 97342687 | 97342944 | - | 25.1 | 87 | 339 | -70 | MLT1I | MaLR | LTR |

  

|  |  |  |  |  |  |  |  |  |  |
| --- | --- | --- | --- | --- | --- | --- | --- | --- | --- |
| chr7:97348031-97348981 | | | 936 bp | | |  |  |  |  |
| Genome Start | Genome End | Strand | Div | Repeat Start | Repeat End | Repeat Left | Name | Family | Class |
| 97348031 | 97348157 | + | 20.2 | 5 | 129 | -49 | MER5B | MER1\_type | DNA |
| 97348183 | 97348488 | - | 15.0 | 2 | 306 | 0 | AluSx | Alu | SINE |
| 97348494 | 97348776 | - | 18.7 | 5902 | 6174 | 0 | L1MB5 | L1 | LINE |
| 97348747 | 97348922 | + | 22.7 | 5684 | 5887 | -287 | L1MB5 | L1 | LINE |
| 97348938 | 97348981 | + | 6.8 | 135 | 178 | 0 | MER5B | MER1\_type | DNA |

  

|  |  |  |  |  |  |  |  |  |  |
| --- | --- | --- | --- | --- | --- | --- | --- | --- | --- |
| chr7:97351957-97352712 | | | 753 bp | | |  |  |  |  |
| Genome Start | Genome End | Strand | Div | Repeat Start | Repeat End | Repeat Left | Name | Family | Class |
| 97351957 | 97352160 | - | 21.1 | 5974 | 6184 | 0 | L1MB7 | L1 | LINE |
| 97352164 | 97352336 | - | 8.1 | 104 | 289 | -23 | AluSg/x | Alu | SINE |
| 97352337 | 97352712 | - | 17.8 | 5575 | 5959 | -219 | L1MB7 | L1 | LINE |

  

|  |  |  |  |  |  |  |  |  |  |
| --- | --- | --- | --- | --- | --- | --- | --- | --- | --- |
| chr7:97367182-97367802 | | | 621 bp | | |  |  |  |  |
| Genome Start | Genome End | Strand | Div | Repeat Start | Repeat End | Repeat Left | Name | Family | Class |
| 97367182 | 97367325 | + | 18.0 | 5348 | 5490 | -688 | L1M4 | L1 | LINE |
| 97367326 | 97367638 | + | 11.2 | 1 | 312 | 0 | AluSx | Alu | SINE |
| 97367639 | 97367802 | + | 18.0 | 5491 | 5653 | -525 | L1M4 | L1 | LINE |

  

|  |  |  |  |  |  |  |  |  |  |
| --- | --- | --- | --- | --- | --- | --- | --- | --- | --- |
| chr7:97369590-97370421 | | | 809 bp | | |  |  |  |  |
| Genome Start | Genome End | Strand | Div | Repeat Start | Repeat End | Repeat Left | Name | Family | Class |
| 97369590 | 97369677 | - | 26.4 | 6079 | 6170 | 0 | L1ME3A | L1 | LINE |
| 97369678 | 97369970 | + | 15.6 | 1 | 284 | -28 | AluJo | Alu | SINE |
| 97369994 | 97370145 | - | 26.4 | 5923 | 6078 | -92 | L1ME3A | L1 | LINE |
| 97370146 | 97370366 | + | 22.9 | 1 | 219 | 0 | MER20 | MER1\_type | DNA |
| 97370367 | 97370421 | - | 26.4 | 5875 | 5922 | -248 | L1ME3A | L1 | LINE |

  

|  |  |  |  |  |  |  |  |  |  |
| --- | --- | --- | --- | --- | --- | --- | --- | --- | --- |
| chr7:97380549-97381037 | | | 372 bp | | |  |  |  |  |
| Genome Start | Genome End | Strand | Div | Repeat Start | Repeat End | Repeat Left | Name | Family | Class |
| 97380549 | 97380659 | + | 38.2 | 3096 | 3209 | -169 | L2 | L2 | LINE |
| 97380795 | 97380978 | - | 26.9 | 12 | 210 | -52 | MIR | MIR | SINE |
| 97380961 | 97381037 | + | 33.3 | 3347 | 3418 | -1 | L2 | L2 | LINE |

  

|  |  |  |  |  |  |  |  |  |  |
| --- | --- | --- | --- | --- | --- | --- | --- | --- | --- |
| chr7:97389155-97389963 | | | 809 bp | | |  |  |  |  |
| Genome Start | Genome End | Strand | Div | Repeat Start | Repeat End | Repeat Left | Name | Family | Class |
| 97389155 | 97389330 | + | 13.1 | 5775 | 5950 | -341 | L1MA8 | L1 | LINE |
| 97389331 | 97389643 | + | 16.8 | 1 | 312 | 0 | AluJo | Alu | SINE |
| 97389644 | 97389963 | + | 13.1 | 5951 | 6288 | -3 | L1MA8 | L1 | LINE |

  

|  |  |  |  |  |  |  |  |  |  |
| --- | --- | --- | --- | --- | --- | --- | --- | --- | --- |
| chr7:97397180-97397991 | | | 791 bp | | |  |  |  |  |
| Genome Start | Genome End | Strand | Div | Repeat Start | Repeat End | Repeat Left | Name | Family | Class |
| 97397180 | 97397307 | - | 25.8 | 99 | 233 | -29 | MIR | MIR | SINE |
| 97397324 | 97397926 | + | 19.2 | 1473 | 2104 | -3610 | ERVL-B4 | ERVL | LTR |
| 97397932 | 97397991 | - | 32.2 | 15 | 75 | -187 | MIR | MIR | SINE |

  

|  |  |  |  |  |  |  |  |  |  |
| --- | --- | --- | --- | --- | --- | --- | --- | --- | --- |
| chr7:97410331-97411756 | | | 1426 bp | | |  |  |  |  |
| Genome Start | Genome End | Strand | Div | Repeat Start | Repeat End | Repeat Left | Name | Family | Class |
| 97410331 | 97410462 | - | 24.6 | 5814 | 5907 | -276 | L1MB3 | L1 | LINE |
| 97410463 | 97410757 | - | 15.0 | 7 | 300 | -12 | AluSx | Alu | SINE |
| 97410758 | 97411756 | - | 24.3 | 4818 | 5834 | -349 | L1MB3 | L1 | LINE |

  

|  |  |  |  |  |  |  |  |  |  |
| --- | --- | --- | --- | --- | --- | --- | --- | --- | --- |
| chr7:97412149-97412851 | | | 703 bp | | |  |  |  |  |
| Genome Start | Genome End | Strand | Div | Repeat Start | Repeat End | Repeat Left | Name | Family | Class |
| 97412149 | 97412388 | - | 25.9 | 5941 | 6162 | -138 | L1MA4 | L1 | LINE |
| 97412389 | 97412669 | - | 17.5 | 1 | 280 | -32 | AluSx | Alu | SINE |
| 97412670 | 97412851 | - | 24.2 | 5767 | 5940 | -360 | L1MA4 | L1 | LINE |

  

|  |  |  |  |  |  |  |  |  |  |
| --- | --- | --- | --- | --- | --- | --- | --- | --- | --- |
| chr7:97461450-97462051 | | | 602 bp | | |  |  |  |  |
| Genome Start | Genome End | Strand | Div | Repeat Start | Repeat End | Repeat Left | Name | Family | Class |
| 97461450 | 97461592 | + | 19.2 | 5 | 139 | -173 | AluJo | Alu | SINE |
| 97461593 | 97461889 | + | 8.5 | 1 | 296 | -16 | AluSx | Alu | SINE |
| 97461890 | 97462051 | + | 19.2 | 140 | 295 | -17 | AluJo | Alu | SINE |

  

|  |  |  |  |  |  |  |  |  |  |
| --- | --- | --- | --- | --- | --- | --- | --- | --- | --- |
| chr7:97462874-97463446 | | | 573 bp | | |  |  |  |  |
| Genome Start | Genome End | Strand | Div | Repeat Start | Repeat End | Repeat Left | Name | Family | Class |
| 97462874 | 97463108 | + | 13.6 | 5891 | 6133 | -50 | L1MB3 | L1 | LINE |
| 97463109 | 97463412 | + | 16.7 | 1 | 300 | -12 | AluJo | Alu | SINE |
| 97463413 | 97463446 | + | 13.6 | 6134 | 6165 | -18 | L1MB3 | L1 | LINE |

  

|  |  |  |  |  |  |  |  |  |  |
| --- | --- | --- | --- | --- | --- | --- | --- | --- | --- |
| chr7:97466145-97466737 | | | 593 bp | | |  |  |  |  |
| Genome Start | Genome End | Strand | Div | Repeat Start | Repeat End | Repeat Left | Name | Family | Class |
| 97466145 | 97466248 | + | 15.9 | 1 | 116 | -197 | AluSq | Alu | SINE |
| 97466249 | 97466558 | - | 9.9 | 1 | 302 | -10 | AluJo | Alu | SINE |
| 97466559 | 97466737 | + | 15.9 | 117 | 301 | -12 | AluSq | Alu | SINE |

  

|  |  |  |  |  |  |  |  |  |  |
| --- | --- | --- | --- | --- | --- | --- | --- | --- | --- |
| chr7:97479205-97481011 | | | 1807 bp | | |  |  |  |  |
| Genome Start | Genome End | Strand | Div | Repeat Start | Repeat End | Repeat Left | Name | Family | Class |
| 97479205 | 97479356 | - | 23.8 | 838 | 1006 | -5394 | L1MEc | L1 | LINE |
| 97479357 | 97479648 | - | 17.7 | 1 | 288 | -24 | AluJo | Alu | SINE |
| 97479649 | 97479783 | - | 23.8 | 702 | 837 | -5563 | L1MEc | L1 | LINE |
| 97479784 | 97480085 | - | 10.3 | 3 | 303 | -9 | AluSx | Alu | SINE |
| 97480086 | 97480448 | - | 23.8 | 320 | 701 | -5699 | L1MEc | L1 | LINE |
| 97480449 | 97480752 | - | 9.6 | 1 | 302 | -10 | AluSx | Alu | SINE |
| 97480753 | 97481011 | - | 23.8 | 28 | 319 | -6081 | L1MEc | L1 | LINE |

  

|  |  |  |  |  |  |  |  |  |  |
| --- | --- | --- | --- | --- | --- | --- | --- | --- | --- |
| chr7:99670837-99672426 | | | 1590 bp | | |  |  |  |  |
| Genome Start | Genome End | Strand | Div | Repeat Start | Repeat End | Repeat Left | Name | Family | Class |
| 99670837 | 99670928 | + | 25.4 | 5491 | 5583 | -583 | L1ME1 | L1 | LINE |
| 99670929 | 99671095 | - | 18.1 | 134 | 299 | -13 | AluJo | Alu | SINE |
| 99671096 | 99671391 | - | 9.9 | 3 | 299 | -13 | AluSx | Alu | SINE |
| 99671392 | 99671540 | - | 18.1 | 1 | 133 | -179 | AluJo | Alu | SINE |
| 99671541 | 99672037 | + | 25.4 | 5584 | 6091 | -75 | L1ME1 | L1 | LINE |
| 99672038 | 99672327 | + | 12.4 | 1 | 290 | -23 | AluSq | Alu | SINE |
| 99672328 | 99672426 | + | 25.4 | 6092 | 6161 | -5 | L1ME1 | L1 | LINE |

  

|  |  |  |  |  |  |  |  |  |  |
| --- | --- | --- | --- | --- | --- | --- | --- | --- | --- |
| chr7:99681196-99681793 | | | 598 bp | | |  |  |  |  |
| Genome Start | Genome End | Strand | Div | Repeat Start | Repeat End | Repeat Left | Name | Family | Class |
| 99681196 | 99681363 | - | 15.1 | 134 | 298 | -14 | AluJo | Alu | SINE |
| 99681364 | 99681660 | - | 6.8 | 1 | 296 | -15 | AluY | Alu | SINE |
| 99681661 | 99681793 | - | 15.1 | 6 | 133 | -179 | AluJo | Alu | SINE |

  

|  |  |  |  |  |  |  |  |  |  |
| --- | --- | --- | --- | --- | --- | --- | --- | --- | --- |
| chr7:99685047-99685650 | | | 604 bp | | |  |  |  |  |
| Genome Start | Genome End | Strand | Div | Repeat Start | Repeat End | Repeat Left | Name | Family | Class |
| 99685047 | 99685215 | - | 15.6 | 137 | 310 | -2 | AluJo | Alu | SINE |
| 99685216 | 99685521 | - | 13.7 | 1 | 306 | -6 | AluSx | Alu | SINE |
| 99685522 | 99685650 | - | 15.6 | 6 | 136 | -176 | AluJo | Alu | SINE |

  

|  |  |  |  |  |  |  |  |  |  |
| --- | --- | --- | --- | --- | --- | --- | --- | --- | --- |
| chr7:99691325-99691860 | | | 536 bp | | |  |  |  |  |
| Genome Start | Genome End | Strand | Div | Repeat Start | Repeat End | Repeat Left | Name | Family | Class |
| 99691325 | 99691349 | + | 32.6 | 2 | 24 | -244 | MIRb | MIR | SINE |
| 99691350 | 99691648 | - | 12.9 | 5 | 298 | -14 | AluSx | Alu | SINE |
| 99691649 | 99691860 | + | 32.6 | 25 | 266 | -2 | MIRb | MIR | SINE |

  

|  |  |  |  |  |  |  |  |  |  |
| --- | --- | --- | --- | --- | --- | --- | --- | --- | --- |
| chr7:99696204-99696587 | | | 384 bp | | |  |  |  |  |
| Genome Start | Genome End | Strand | Div | Repeat Start | Repeat End | Repeat Left | Name | Family | Class |
| 99696204 | 99696236 | - | 24.7 | 114 | 149 | -113 | MIR | MIR | SINE |
| 99696237 | 99696512 | + | 20.7 | 7 | 277 | -35 | AluJo | Alu | SINE |
| 99696513 | 99696587 | - | 27.7 | 46 | 120 | -148 | MIR | MIR | SINE |

  

|  |  |  |  |  |  |  |  |  |  |
| --- | --- | --- | --- | --- | --- | --- | --- | --- | --- |
| chr7:99712339-99712907 | | | 569 bp | | |  |  |  |  |
| Genome Start | Genome End | Strand | Div | Repeat Start | Repeat End | Repeat Left | Name | Family | Class |
| 99712339 | 99712403 | - | 23.9 | 68 | 162 | -433 | MLT1G1 | MaLR | LTR |
| 99712404 | 99712800 | + | 15.6 | 1 | 432 | 0 | MSTB1 | MaLR | LTR |
| 99712801 | 99712907 | - | 23.2 | 3 | 108 | -487 | MLT1G1 | MaLR | LTR |

  

|  |  |  |  |  |  |  |  |  |  |
| --- | --- | --- | --- | --- | --- | --- | --- | --- | --- |
| chr7:99713812-99714437 | | | 626 bp | | |  |  |  |  |
| Genome Start | Genome End | Strand | Div | Repeat Start | Repeat End | Repeat Left | Name | Family | Class |
| 99713812 | 99714032 | - | 19.3 | 7572 | 7783 | -2 | L1MC3 | L1 | LINE |
| 99714033 | 99714344 | - | 6.8 | 1 | 313 | 0 | AluSp | Alu | SINE |
| 99714345 | 99714437 | - | 19.3 | 7479 | 7571 | -214 | L1MC3 | L1 | LINE |

  

|  |  |  |  |  |  |  |  |  |  |
| --- | --- | --- | --- | --- | --- | --- | --- | --- | --- |
| chr7:99717172-99717644 | | | 473 bp | | |  |  |  |  |
| Genome Start | Genome End | Strand | Div | Repeat Start | Repeat End | Repeat Left | Name | Family | Class |
| 99717172 | 99717279 | + | 33.8 | 104 | 222 | -40 | MIR | MIR | SINE |
| 99717280 | 99717607 | + | 6.7 | 1 | 297 | -12 | AluSc | Alu | SINE |
| 99717608 | 99717644 | + | 33.8 | 223 | 257 | -5 | MIR | MIR | SINE |

  

|  |  |  |  |  |  |  |  |  |  |
| --- | --- | --- | --- | --- | --- | --- | --- | --- | --- |
| chr7:99740959-99741657 | | | 699 bp | | |  |  |  |  |
| Genome Start | Genome End | Strand | Div | Repeat Start | Repeat End | Repeat Left | Name | Family | Class |
| 99740959 | 99741018 | + | 38.1 | 3933 | 3988 | -501 | L3 | CR1 | LINE |
| 99741019 | 99741320 | - | 15.2 | 3 | 297 | -15 | AluSx | Alu | SINE |
| 99741321 | 99741657 | + | 38.1 | 3989 | 4324 | -165 | L3 | CR1 | LINE |

  

|  |  |  |  |  |  |  |  |  |  |
| --- | --- | --- | --- | --- | --- | --- | --- | --- | --- |
| chr7:99754285-99754906 | | | 574 bp | | |  |  |  |  |
| Genome Start | Genome End | Strand | Div | Repeat Start | Repeat End | Repeat Left | Name | Family | Class |
| 99754285 | 99754446 | - | 16.6 | 137 | 297 | -15 | AluJo | Alu | SINE |
| 99754495 | 99754768 | - | 19.0 | 2 | 276 | -36 | AluSx | Alu | SINE |
| 99754769 | 99754906 | - | 16.6 | 1 | 136 | -176 | AluJo | Alu | SINE |

  

|  |  |  |  |  |  |  |  |  |  |
| --- | --- | --- | --- | --- | --- | --- | --- | --- | --- |
| chr7:99759840-99761003 | | | 884 bp | | |  |  |  |  |
| Genome Start | Genome End | Strand | Div | Repeat Start | Repeat End | Repeat Left | Name | Family | Class |
| 99759840 | 99759994 | + | 26.9 | 4496 | 4650 | -1889 | MER84-int | ERV1 | LTR |
| 99760226 | 99760526 | - | 14.3 | 3 | 303 | -7 | AluSg | Alu | SINE |
| 99760529 | 99760825 | - | 12.8 | 1 | 296 | -17 | AluSq | Alu | SINE |
| 99760873 | 99761003 | + | 28.3 | 5398 | 5527 | -2909 | MER84-int | ERV1 | LTR |

  

|  |  |  |  |  |  |  |  |  |  |
| --- | --- | --- | --- | --- | --- | --- | --- | --- | --- |
| chr7:99762247-99762964 | | | 718 bp | | |  |  |  |  |
| Genome Start | Genome End | Strand | Div | Repeat Start | Repeat End | Repeat Left | Name | Family | Class |
| 99762247 | 99762277 | + | 16.9 | 3 | 32 | -457 | LTR45B | ERV1 | LTR |
| 99762278 | 99762574 | - | 9.5 | 4 | 302 | -11 | AluSq | Alu | SINE |
| 99762575 | 99762964 | + | 16.9 | 33 | 414 | -75 | LTR45B | ERV1 | LTR |

  

|  |  |  |  |  |  |  |  |  |  |
| --- | --- | --- | --- | --- | --- | --- | --- | --- | --- |
| chr7:99766460-99766934 | | | 475 bp | | |  |  |  |  |
| Genome Start | Genome End | Strand | Div | Repeat Start | Repeat End | Repeat Left | Name | Family | Class |
| 99766460 | 99766484 | + | 27.2 | 7 | 30 | -232 | MIR | MIR | SINE |
| 99766485 | 99766794 | + | 10.3 | 1 | 311 | -2 | AluSp | Alu | SINE |
| 99766795 | 99766934 | + | 27.2 | 31 | 187 | -75 | MIR | MIR | SINE |

  

|  |  |  |  |  |  |  |  |  |  |
| --- | --- | --- | --- | --- | --- | --- | --- | --- | --- |
| chr7:99775514-99775951 | | | 438 bp | | |  |  |  |  |
| Genome Start | Genome End | Strand | Div | Repeat Start | Repeat End | Repeat Left | Name | Family | Class |
| 99775514 | 99775615 | - | 25.2 | 2762 | 2864 | -3282 | L1M5 | L1 | LINE |
| 99775616 | 99775916 | + | 12.6 | 1 | 301 | -11 | AluSx | Alu | SINE |
| 99775917 | 99775951 | - | 25.2 | 2728 | 2761 | -3385 | L1M5 | L1 | LINE |

  

|  |  |  |  |  |  |  |  |  |  |
| --- | --- | --- | --- | --- | --- | --- | --- | --- | --- |
| chr7:101081463-101081931 | | | 469 bp | | |  |  |  |  |
| Genome Start | Genome End | Strand | Div | Repeat Start | Repeat End | Repeat Left | Name | Family | Class |
| 101081463 | 101081520 | - | 16.0 | 6124 | 6178 | 0 | L1MB8 | L1 | LINE |
| 101081521 | 101081756 | - | 11.9 | 1 | 230 | 0 | MER30 | MER1\_type | DNA |
| 101081757 | 101081931 | - | 16.0 | 5959 | 6123 | -55 | L1MB8 | L1 | LINE |

  

|  |  |  |  |  |  |  |  |  |  |
| --- | --- | --- | --- | --- | --- | --- | --- | --- | --- |
| chr7:101082847-101084466 | | | 1539 bp | | |  |  |  |  |
| Genome Start | Genome End | Strand | Div | Repeat Start | Repeat End | Repeat Left | Name | Family | Class |
| 101082847 | 101083122 | + | 24.6 | 1041 | 1338 | -1169 | HAL1 | L1 | LINE |
| 101083155 | 101083290 | + | 16.6 | 1 | 133 | -10 | FLAM\_C | Alu | SINE |
| 101083300 | 101083609 | - | 11.0 | 1 | 310 | -2 | AluSx | Alu | SINE |
| 101083650 | 101083759 | + | 21.8 | 1390 | 1512 | -995 | HAL1 | L1 | LINE |
| 101083760 | 101084061 | + | 10.9 | 1 | 302 | -10 | AluSx | Alu | SINE |
| 101084062 | 101084098 | + | 21.8 | 1513 | 1559 | -948 | HAL1 | L1 | LINE |
| 101084099 | 101084420 | - | 6.5 | 1 | 308 | -3 | AluY | Alu | SINE |
| 101084421 | 101084466 | + | 21.8 | 1560 | 1606 | -901 | HAL1 | L1 | LINE |

  

|  |  |  |  |  |  |  |  |  |  |
| --- | --- | --- | --- | --- | --- | --- | --- | --- | --- |
| chr7:101769684-101770878 | | | 911 bp | | |  |  |  |  |
| Genome Start | Genome End | Strand | Div | Repeat Start | Repeat End | Repeat Left | Name | Family | Class |
| 101769684 | 101769810 | - | 32.2 | 5682 | 5802 | -3117 | HUERS-P3 | ERV1 | LTR |
| 101769862 | 101770162 | + | 14.3 | 1 | 300 | -13 | AluSq | Alu | SINE |
| 101770165 | 101770465 | + | 14.3 | 3 | 303 | -7 | AluSg | Alu | SINE |
| 101770697 | 101770878 | - | 23.9 | 4474 | 4650 | -1889 | HUERS-P3 | ERV1 | LTR |

  

|  |  |  |  |  |  |  |  |  |  |
| --- | --- | --- | --- | --- | --- | --- | --- | --- | --- |
| chr7:101776055-101776657 | | | 603 bp | | |  |  |  |  |
| Genome Start | Genome End | Strand | Div | Repeat Start | Repeat End | Repeat Left | Name | Family | Class |
| 101776055 | 101776192 | + | 16.9 | 1 | 134 | -178 | AluJo | Alu | SINE |
| 101776193 | 101776495 | + | 17.5 | 2 | 305 | 0 | AluSx | Alu | SINE |
| 101776496 | 101776657 | + | 16.9 | 135 | 298 | -14 | AluJo | Alu | SINE |

  

|  |  |  |  |  |  |  |  |  |  |
| --- | --- | --- | --- | --- | --- | --- | --- | --- | --- |
| chr7:101784885-101785600 | | | 716 bp | | |  |  |  |  |
| Genome Start | Genome End | Strand | Div | Repeat Start | Repeat End | Repeat Left | Name | Family | Class |
| 101784885 | 101785228 | - | 35.4 | 3990 | 4333 | -156 | L3 | CR1 | LINE |
| 101785229 | 101785540 | + | 12.6 | 3 | 312 | 0 | AluSx | Alu | SINE |
| 101785541 | 101785600 | - | 35.4 | 3933 | 3989 | -500 | L3 | CR1 | LINE |

  

|  |  |  |  |  |  |  |  |  |  |
| --- | --- | --- | --- | --- | --- | --- | --- | --- | --- |
| chr7:101786833-101788428 | | | 1596 bp | | |  |  |  |  |
| Genome Start | Genome End | Strand | Div | Repeat Start | Repeat End | Repeat Left | Name | Family | Class |
| 101786833 | 101787015 | + | 22.1 | 21 | 201 | -510 | MER67C | ERV1 | LTR |
| 101787016 | 101787313 | - | 13.1 | 1 | 300 | -12 | AluSx | Alu | SINE |
| 101787314 | 101787561 | + | 22.1 | 202 | 452 | -259 | MER67C | ERV1 | LTR |
| 101787562 | 101787886 | + | 14.5 | 1 | 304 | -8 | AluSx | Alu | SINE |
| 101787887 | 101787986 | + | 22.1 | 453 | 543 | -168 | MER67C | ERV1 | LTR |
| 101787987 | 101788290 | + | 10.3 | 1 | 302 | -8 | AluSg | Alu | SINE |
| 101788291 | 101788428 | + | 22.1 | 544 | 681 | -30 | MER67C | ERV1 | LTR |

  

|  |  |  |  |  |  |  |  |  |  |
| --- | --- | --- | --- | --- | --- | --- | --- | --- | --- |
| chr7:101795361-101796146 | | | 786 bp | | |  |  |  |  |
| Genome Start | Genome End | Strand | Div | Repeat Start | Repeat End | Repeat Left | Name | Family | Class |
| 101795361 | 101795607 | - | 23.7 | 7133 | 7351 | -610 | L1MC5 | L1 | LINE |
| 101795608 | 101795889 | - | 17.0 | 1 | 299 | -13 | AluJo | Alu | SINE |
| 101795890 | 101796146 | - | 23.8 | 6852 | 7132 | -829 | L1MC5 | L1 | LINE |

  

|  |  |  |  |  |  |  |  |  |  |
| --- | --- | --- | --- | --- | --- | --- | --- | --- | --- |
| chr7:101797047-101799816 | | | 2687 bp | | |  |  |  |  |
| Genome Start | Genome End | Strand | Div | Repeat Start | Repeat End | Repeat Left | Name | Family | Class |
| 101797047 | 101797981 | - | 22.4 | 1929 | 2815 | -3404 | L1MEc | L1 | LINE |
| 101797982 | 101798313 | - | 12.0 | 1 | 332 | 0 | AluSx | Alu | SINE |
| 101798314 | 101799204 | - | 20.5 | 847 | 1928 | -4472 | L1MEc | L1 | LINE |
| 101799239 | 101799372 | - | 19.4 | 119 | 273 | -6127 | L1MEc | L1 | LINE |
| 101799375 | 101799681 | + | 11.1 | 3 | 308 | -2 | AluSg | Alu | SINE |
| 101799729 | 101799816 | - | 25.0 | 36 | 115 | -6285 | L1MEc | L1 | LINE |

  

|  |  |  |  |  |  |  |  |  |  |
| --- | --- | --- | --- | --- | --- | --- | --- | --- | --- |
| chr7:101905195-101905831 | | | 629 bp | | |  |  |  |  |
| Genome Start | Genome End | Strand | Div | Repeat Start | Repeat End | Repeat Left | Name | Family | Class |
| 101905195 | 101905271 | - | 34.6 | 184 | 262 | 0 | MIR | MIR | SINE |
| 101905272 | 101905430 | - | 18.9 | 1 | 159 | -7 | FRAM | Alu | SINE |
| 101905439 | 101905734 | - | 7.4 | 1 | 297 | -16 | AluSp | Alu | SINE |
| 101905735 | 101905831 | - | 34.6 | 79 | 177 | -85 | MIR | MIR | SINE |

  

|  |  |  |  |  |  |  |  |  |  |
| --- | --- | --- | --- | --- | --- | --- | --- | --- | --- |
| chr7:101908075-101908452 | | | 378 bp | | |  |  |  |  |
| Genome Start | Genome End | Strand | Div | Repeat Start | Repeat End | Repeat Left | Name | Family | Class |
| 101908075 | 101908238 | - | 20.7 | 7677 | 7820 | -141 | L1MC5 | L1 | LINE |
| 101908239 | 101908370 | - | 14.5 | 1 | 131 | -2 | FLAM\_A | Alu | SINE |
| 101908371 | 101908452 | - | 20.7 | 7604 | 7676 | -285 | L1MC5 | L1 | LINE |

  

|  |  |  |  |  |  |  |  |  |  |
| --- | --- | --- | --- | --- | --- | --- | --- | --- | --- |
| chr7:101914025-101914844 | | | 820 bp | | |  |  |  |  |
| Genome Start | Genome End | Strand | Div | Repeat Start | Repeat End | Repeat Left | Name | Family | Class |
| 101914025 | 101914113 | - | 26.6 | 151 | 209 | 0 | MER3 | MER1\_type | DNA |
| 101914114 | 101914428 | - | 11.0 | 1 | 312 | 0 | AluSx | Alu | SINE |
| 101914429 | 101914458 | - | 26.6 | 126 | 150 | -59 | MER3 | MER1\_type | DNA |
| 101914459 | 101914767 | + | 14.7 | 1 | 302 | -10 | AluJo | Alu | SINE |
| 101914768 | 101914844 | - | 26.6 | 48 | 125 | -84 | MER3 | MER1\_type | DNA |

  

|  |  |  |  |  |  |  |  |  |  |
| --- | --- | --- | --- | --- | --- | --- | --- | --- | --- |
| chr7:101916793-101917676 | | | 827 bp | | |  |  |  |  |
| Genome Start | Genome End | Strand | Div | Repeat Start | Repeat End | Repeat Left | Name | Family | Class |
| 101916793 | 101916927 | + | 27.6 | 3010 | 3167 | -252 | L2 | L2 | LINE |
| 101916948 | 101917123 | - | 10.8 | 120 | 295 | -14 | AluSc | Alu | SINE |
| 101917161 | 101917227 | + | 29.3 | 3175 | 3247 | -172 | L2 | L2 | LINE |
| 101917228 | 101917522 | - | 13.9 | 14 | 312 | 0 | AluJo | Alu | SINE |
| 101917523 | 101917676 | + | 29.3 | 3248 | 3418 | -1 | L2 | L2 | LINE |

  

|  |  |  |  |  |  |  |  |  |  |
| --- | --- | --- | --- | --- | --- | --- | --- | --- | --- |
| chr7:101928927-101929892 | | | 966 bp | | |  |  |  |  |
| Genome Start | Genome End | Strand | Div | Repeat Start | Repeat End | Repeat Left | Name | Family | Class |
| 101928927 | 101929114 | + | 6.3 | 5506 | 5692 | -459 | L1PB1 | L1 | LINE |
| 101929115 | 101929423 | - | 9.6 | 1 | 311 | 0 | AluSx | Alu | SINE |
| 101929424 | 101929892 | + | 6.3 | 5693 | 6148 | -3 | L1PB1 | L1 | LINE |

  

|  |  |  |  |  |  |  |  |  |  |
| --- | --- | --- | --- | --- | --- | --- | --- | --- | --- |
| chr7:101953722-101954784 | | | 1063 bp | | |  |  |  |  |
| Genome Start | Genome End | Strand | Div | Repeat Start | Repeat End | Repeat Left | Name | Family | Class |
| 101953722 | 101953910 | - | 29.4 | 5949 | 6131 | -31 | L1ME3 | L1 | LINE |
| 101953911 | 101954196 | - | 11.0 | 5 | 288 | -24 | AluJo | Alu | SINE |
| 101954197 | 101954436 | - | 29.4 | 5684 | 5948 | -214 | L1ME3 | L1 | LINE |
| 101954437 | 101954703 | - | 9.0 | 38 | 304 | -8 | AluSx | Alu | SINE |
| 101954704 | 101954784 | - | 29.4 | 5606 | 5683 | -479 | L1ME3 | L1 | LINE |

  

|  |  |  |  |  |  |  |  |  |  |
| --- | --- | --- | --- | --- | --- | --- | --- | --- | --- |
| chr7:101962974-101963589 | | | 616 bp | | |  |  |  |  |
| Genome Start | Genome End | Strand | Div | Repeat Start | Repeat End | Repeat Left | Name | Family | Class |
| 101962974 | 101963126 | - | 13.6 | 151 | 286 | -26 | AluJo | Alu | SINE |
| 101963127 | 101963428 | - | 12.7 | 1 | 302 | -10 | AluSx | Alu | SINE |
| 101963429 | 101963589 | - | 13.6 | 1 | 150 | -162 | AluJo | Alu | SINE |

  

|  |  |  |  |  |  |  |  |  |  |
| --- | --- | --- | --- | --- | --- | --- | --- | --- | --- |
| chr7:101973217-101973830 | | | 614 bp | | |  |  |  |  |
| Genome Start | Genome End | Strand | Div | Repeat Start | Repeat End | Repeat Left | Name | Family | Class |
| 101973217 | 101973393 | - | 17.4 | 144 | 311 | -1 | AluJb | Alu | SINE |
| 101973394 | 101973687 | - | 11.9 | 1 | 297 | -15 | AluSx | Alu | SINE |
| 101973688 | 101973830 | - | 17.4 | 1 | 143 | -169 | AluJb | Alu | SINE |

  

|  |  |  |  |  |  |  |  |  |  |
| --- | --- | --- | --- | --- | --- | --- | --- | --- | --- |
| chr7:101987325-101987927 | | | 589 bp | | |  |  |  |  |
| Genome Start | Genome End | Strand | Div | Repeat Start | Repeat End | Repeat Left | Name | Family | Class |
| 101987325 | 101987486 | - | 17.2 | 137 | 298 | -14 | AluJo | Alu | SINE |
| 101987501 | 101987789 | - | 17.6 | 2 | 291 | -21 | AluSx | Alu | SINE |
| 101987790 | 101987927 | - | 17.2 | 1 | 136 | -176 | AluJo | Alu | SINE |

  

|  |  |  |  |  |  |  |  |  |  |
| --- | --- | --- | --- | --- | --- | --- | --- | --- | --- |
| chr7:101993107-101994299 | | | 1029 bp | | |  |  |  |  |
| Genome Start | Genome End | Strand | Div | Repeat Start | Repeat End | Repeat Left | Name | Family | Class |
| 101993107 | 101993409 | + | 31.7 | 3999 | 4650 | -1889 | MER84-int | ERV1 | LTR |
| 101993521 | 101993822 | - | 11.9 | 3 | 304 | 0 | AluSg | Alu | SINE |
| 101993825 | 101994124 | - | 14.7 | 1 | 299 | -14 | AluSq | Alu | SINE |
| 101994176 | 101994299 | + | 33.9 | 5682 | 5802 | -3117 | MER84-int | ERV1 | LTR |

  

|  |  |  |  |  |  |  |  |  |  |
| --- | --- | --- | --- | --- | --- | --- | --- | --- | --- |
| chr7:102003229-102004194 | | | 966 bp | | |  |  |  |  |
| Genome Start | Genome End | Strand | Div | Repeat Start | Repeat End | Repeat Left | Name | Family | Class |
| 102003229 | 102003341 | + | 19.8 | 113 | 226 | -242 | LTR37B | ERV1 | LTR |
| 102003342 | 102003651 | + | 8.7 | 1 | 310 | 0 | AluSg | Alu | SINE |
| 102003652 | 102003733 | + | 19.8 | 227 | 312 | -156 | LTR37B | ERV1 | LTR |
| 102003734 | 102004036 | + | 17.9 | 1 | 305 | -7 | AluJo | Alu | SINE |
| 102004037 | 102004194 | + | 19.8 | 313 | 466 | -2 | LTR37B | ERV1 | LTR |

  

|  |  |  |  |  |  |  |  |  |  |
| --- | --- | --- | --- | --- | --- | --- | --- | --- | --- |
| chr7:102015384-102015910 | | | 393 bp | | |  |  |  |  |
| Genome Start | Genome End | Strand | Div | Repeat Start | Repeat End | Repeat Left | Name | Family | Class |
| 102015384 | 102015491 | - | 29.0 | 81 | 191 | -5 | MER91A | Tip100 | DNA |
| 102015526 | 102015650 | - | 15.2 | 23 | 151 | -161 | AluJo | Alu | SINE |
| 102015656 | 102015734 | + | 15.2 | 36 | 114 | -29 | FLAM\_C | Alu | SINE |
| 102015830 | 102015910 | - | 22.2 | 1 | 85 | -111 | MER91A | Tip100 | DNA |

  

|  |  |  |  |  |  |  |  |  |  |
| --- | --- | --- | --- | --- | --- | --- | --- | --- | --- |
| chr7:102016028-102016911 | | | 831 bp | | |  |  |  |  |
| Genome Start | Genome End | Strand | Div | Repeat Start | Repeat End | Repeat Left | Name | Family | Class |
| 102016028 | 102016162 | + | 27.6 | 3010 | 3167 | -252 | L2 | L2 | LINE |
| 102016179 | 102016358 | - | 10.0 | 120 | 299 | -10 | AluSc | Alu | SINE |
| 102016396 | 102016462 | + | 29.3 | 3175 | 3247 | -172 | L2 | L2 | LINE |
| 102016463 | 102016757 | - | 13.9 | 14 | 312 | 0 | AluJo | Alu | SINE |
| 102016758 | 102016911 | + | 29.3 | 3248 | 3418 | -1 | L2 | L2 | LINE |

  

|  |  |  |  |  |  |  |  |  |  |
| --- | --- | --- | --- | --- | --- | --- | --- | --- | --- |
| chr7:102024685-102025537 | | | 852 bp | | |  |  |  |  |
| Genome Start | Genome End | Strand | Div | Repeat Start | Repeat End | Repeat Left | Name | Family | Class |
| 102024685 | 102024754 | + | 17.7 | 4 | 68 | -125 | MER53 | DNA | DNA |
| 102024755 | 102024879 | - | 9.6 | 1 | 125 | -8 | FLAM\_C | Alu | SINE |
| 102024880 | 102024931 | + | 17.7 | 69 | 109 | -84 | MER53 | DNA | DNA |
| 102024932 | 102025239 | - | 11.0 | 1 | 309 | -1 | AluSg | Alu | SINE |
| 102025241 | 102025510 | - | 11.1 | 2 | 290 | -23 | AluSq | Alu | SINE |
| 102025511 | 102025537 | + | 17.7 | 110 | 135 | -58 | MER53 | DNA | DNA |

  

|  |  |  |  |  |  |  |  |  |  |
| --- | --- | --- | --- | --- | --- | --- | --- | --- | --- |
| chr7:102062312-102062927 | | | 616 bp | | |  |  |  |  |
| Genome Start | Genome End | Strand | Div | Repeat Start | Repeat End | Repeat Left | Name | Family | Class |
| 102062312 | 102062464 | - | 13.6 | 151 | 286 | -26 | AluJo | Alu | SINE |
| 102062465 | 102062766 | - | 13.0 | 1 | 302 | -10 | AluSx | Alu | SINE |
| 102062767 | 102062927 | - | 13.6 | 1 | 150 | -162 | AluJo | Alu | SINE |

  

|  |  |  |  |  |  |  |  |  |  |
| --- | --- | --- | --- | --- | --- | --- | --- | --- | --- |
| chr7:102076681-102077223 | | | 543 bp | | |  |  |  |  |
| Genome Start | Genome End | Strand | Div | Repeat Start | Repeat End | Repeat Left | Name | Family | Class |
| 102076681 | 102076779 | - | 15.4 | 136 | 224 | -88 | AluJo | Alu | SINE |
| 102076780 | 102077090 | - | 10.9 | 1 | 312 | -1 | AluSq | Alu | SINE |
| 102077091 | 102077223 | - | 15.4 | 1 | 135 | -177 | AluJo | Alu | SINE |

  

|  |  |  |  |  |  |  |  |  |  |
| --- | --- | --- | --- | --- | --- | --- | --- | --- | --- |
| chr7:102086553-102087155 | | | 580 bp | | |  |  |  |  |
| Genome Start | Genome End | Strand | Div | Repeat Start | Repeat End | Repeat Left | Name | Family | Class |
| 102086553 | 102086714 | - | 17.2 | 137 | 298 | -14 | AluJo | Alu | SINE |
| 102086738 | 102087017 | - | 17.5 | 2 | 282 | -30 | AluSx | Alu | SINE |
| 102087018 | 102087155 | - | 17.2 | 1 | 136 | -176 | AluJo | Alu | SINE |

  

|  |  |  |  |  |  |  |  |  |  |
| --- | --- | --- | --- | --- | --- | --- | --- | --- | --- |
| chr7:102098164-102098805 | | | 634 bp | | |  |  |  |  |
| Genome Start | Genome End | Strand | Div | Repeat Start | Repeat End | Repeat Left | Name | Family | Class |
| 102098164 | 102098240 | - | 34.1 | 184 | 262 | 0 | MIR | MIR | SINE |
| 102098241 | 102098404 | - | 18.2 | 1 | 159 | -7 | FRAM | Alu | SINE |
| 102098413 | 102098708 | - | 7.4 | 1 | 297 | -16 | AluSp | Alu | SINE |
| 102098709 | 102098805 | - | 34.1 | 79 | 177 | -85 | MIR | MIR | SINE |

  

|  |  |  |  |  |  |  |  |  |  |
| --- | --- | --- | --- | --- | --- | --- | --- | --- | --- |
| chr7:102099960-102101869 | | | 1556 bp | | |  |  |  |  |
| Genome Start | Genome End | Strand | Div | Repeat Start | Repeat End | Repeat Left | Name | Family | Class |
| 102099960 | 102100236 | - | 16.4 | 34 | 310 | -2 | AluJo | Alu | SINE |
| 102100247 | 102101217 | + | 3.5 | 1 | 1007 | 0 | LTR13 | ERVK | LTR |
| 102101562 | 102101826 | + | 6.2 | 1 | 257 | -56 | AluSq | Alu | SINE |
| 102101827 | 102101869 | - | 13.9 | 1 | 43 | -259 | AluJo | Alu | SINE |

  

|  |  |  |  |  |  |  |  |  |  |
| --- | --- | --- | --- | --- | --- | --- | --- | --- | --- |
| chr7:102106529-102106906 | | | 378 bp | | |  |  |  |  |
| Genome Start | Genome End | Strand | Div | Repeat Start | Repeat End | Repeat Left | Name | Family | Class |
| 102106529 | 102106692 | - | 18.8 | 7678 | 7820 | -141 | L1MC5 | L1 | LINE |
| 102106693 | 102106824 | - | 14.5 | 1 | 131 | -2 | FLAM\_A | Alu | SINE |
| 102106825 | 102106906 | - | 18.8 | 7604 | 7677 | -284 | L1MC5 | L1 | LINE |

  

|  |  |  |  |  |  |  |  |  |  |
| --- | --- | --- | --- | --- | --- | --- | --- | --- | --- |
| chr7:102112496-102113321 | | | 805 bp | | |  |  |  |  |
| Genome Start | Genome End | Strand | Div | Repeat Start | Repeat End | Repeat Left | Name | Family | Class |
| 102112496 | 102112557 | - | 21.0 | 142 | 209 | 0 | MER3 | MER1\_type | DNA |
| 102112564 | 102112916 | - | 10.0 | 1 | 350 | 0 | AluSx | Alu | SINE |
| 102112932 | 102112946 | - | 26.4 | 126 | 140 | -69 | MER3 | MER1\_type | DNA |
| 102112947 | 102113250 | + | 14.1 | 1 | 300 | -12 | AluJo | Alu | SINE |
| 102113251 | 102113321 | - | 26.4 | 54 | 125 | -84 | MER3 | MER1\_type | DNA |

  

|  |  |  |  |  |  |  |  |  |  |
| --- | --- | --- | --- | --- | --- | --- | --- | --- | --- |
| chr7:102131856-102133885 | | | 2030 bp | | |  |  |  |  |
| Genome Start | Genome End | Strand | Div | Repeat Start | Repeat End | Repeat Left | Name | Family | Class |
| 102131856 | 102131993 | + | 36.5 | 825 | 967 | -993 | L4 | RTE | LINE |
| 102131994 | 102133604 | - | 20.3 | 1 | 1581 | 0 | MER45R | Tip100 | DNA |
| 102133605 | 102133885 | + | 36.5 | 968 | 1280 | -680 | L4 | RTE | LINE |

  

|  |  |  |  |  |  |  |  |  |  |
| --- | --- | --- | --- | --- | --- | --- | --- | --- | --- |
| chr7:102221321-102221826 | | | 506 bp | | |  |  |  |  |
| Genome Start | Genome End | Strand | Div | Repeat Start | Repeat End | Repeat Left | Name | Family | Class |
| 102221321 | 102221439 | - | 19.7 | 80 | 209 | -6 | MER58C | MER1\_type | DNA |
| 102221440 | 102221748 | + | 8.1 | 1 | 312 | 0 | AluSx | Alu | SINE |
| 102221749 | 102221826 | - | 19.7 | 2 | 79 | -136 | MER58C | MER1\_type | DNA |

  

|  |  |  |  |  |  |  |  |  |  |
| --- | --- | --- | --- | --- | --- | --- | --- | --- | --- |
| chr7:102608116-102611135 | | | 2970 bp | | |  |  |  |  |
| Genome Start | Genome End | Strand | Div | Repeat Start | Repeat End | Repeat Left | Name | Family | Class |
| 102608116 | 102608669 | - | 25.8 | 2926 | 3382 | -3624 | L1MEb | L1 | LINE |
| 102608684 | 102609308 | - | 17.6 | 5593 | 6242 | 0 | L1MD1 | L1 | LINE |
| 102609314 | 102609765 | - | 14.6 | 5845 | 6300 | 0 | L1MA5 | L1 | LINE |
| 102609797 | 102611135 | - | 27.4 | 1332 | 2945 | -4242 | L1MEb | L1 | LINE |

  

|  |  |  |  |  |  |  |  |  |  |
| --- | --- | --- | --- | --- | --- | --- | --- | --- | --- |
| chr7:102613385-102613806 | | | 415 bp | | |  |  |  |  |
| Genome Start | Genome End | Strand | Div | Repeat Start | Repeat End | Repeat Left | Name | Family | Class |
| 102613385 | 102613507 | - | 36.9 | 139 | 267 | -1 | MIRb | MIR | SINE |
| 102613515 | 102613681 | + | 15.8 | 172 | 339 | 0 | MER1B | MER1\_type | DNA |
| 102613682 | 102613806 | - | 25.8 | 10 | 145 | -123 | MIRb | MIR | SINE |

  

|  |  |  |  |  |  |  |  |  |  |
| --- | --- | --- | --- | --- | --- | --- | --- | --- | --- |
| chr7:102697153-102698346 | | | 1190 bp | | |  |  |  |  |
| Genome Start | Genome End | Strand | Div | Repeat Start | Repeat End | Repeat Left | Name | Family | Class |
| 102697153 | 102697792 | + | 23.3 | 4168 | 4894 | -1252 | L1M5 | L1 | LINE |
| 102697783 | 102697948 | + | 27.9 | 5989 | 6161 | -5 | L1ME1 | L1 | LINE |
| 102697963 | 102698346 | + | 28.7 | 5329 | 5739 | -501 | L1M5 | L1 | LINE |

  

|  |  |  |  |  |  |  |  |  |  |
| --- | --- | --- | --- | --- | --- | --- | --- | --- | --- |
| chr7:122956896-122957498 | | | 603 bp | | |  |  |  |  |
| Genome Start | Genome End | Strand | Div | Repeat Start | Repeat End | Repeat Left | Name | Family | Class |
| 122956896 | 122957043 | + | 16.5 | 2 | 131 | -179 | AluSg | Alu | SINE |
| 122957044 | 122957349 | + | 11.1 | 1 | 307 | -2 | AluSc | Alu | SINE |
| 122957350 | 122957498 | + | 16.5 | 132 | 282 | -28 | AluSg | Alu | SINE |

  

|  |  |  |  |  |  |  |  |  |  |
| --- | --- | --- | --- | --- | --- | --- | --- | --- | --- |
| chr7:123902250-123902651 | | | 402 bp | | |  |  |  |  |
| Genome Start | Genome End | Strand | Div | Repeat Start | Repeat End | Repeat Left | Name | Family | Class |
| 123902250 | 123902312 | - | 9.9 | 616 | 676 | -189 | MER6 | MER2\_type | DNA |
| 123902313 | 123902611 | + | 12.8 | 1 | 302 | -10 | AluSx | Alu | SINE |
| 123902612 | 123902651 | - | 9.9 | 576 | 615 | -250 | MER6 | MER2\_type | DNA |

  

|  |  |  |  |  |  |  |  |  |  |
| --- | --- | --- | --- | --- | --- | --- | --- | --- | --- |
| chr7:127899415-127900123 | | | 709 bp | | |  |  |  |  |
| Genome Start | Genome End | Strand | Div | Repeat Start | Repeat End | Repeat Left | Name | Family | Class |
| 127899415 | 127899522 | + | 25.3 | 5609 | 5711 | -410 | L1ME1 | L1 | LINE |
| 127899523 | 127899832 | - | 10.3 | 1 | 310 | 0 | AluSg | Alu | SINE |
| 127899833 | 127900123 | + | 25.8 | 5712 | 6138 | -28 | L1ME1 | L1 | LINE |

  

|  |  |  |  |  |  |  |  |  |  |
| --- | --- | --- | --- | --- | --- | --- | --- | --- | --- |
| chr7:127910846-127911449 | | | 579 bp | | |  |  |  |  |
| Genome Start | Genome End | Strand | Div | Repeat Start | Repeat End | Repeat Left | Name | Family | Class |
| 127910846 | 127911008 | - | 9.4 | 126 | 288 | -24 | AluSx | Alu | SINE |
| 127911034 | 127911313 | - | 8.9 | 1 | 281 | -32 | AluSp | Alu | SINE |
| 127911314 | 127911449 | - | 9.4 | 1 | 125 | -187 | AluSx | Alu | SINE |

  

|  |  |  |  |  |  |  |  |  |  |
| --- | --- | --- | --- | --- | --- | --- | --- | --- | --- |
| chr7:127912471-127913249 | | | 759 bp | | |  |  |  |  |
| Genome Start | Genome End | Strand | Div | Repeat Start | Repeat End | Repeat Left | Name | Family | Class |
| 127912471 | 127912531 | + | 26.2 | 4 | 64 | -281 | MER2 | MER2\_type | DNA |
| 127912541 | 127912672 | + | 15.9 | 1 | 139 | -173 | AluJb | Alu | SINE |
| 127912673 | 127912972 | + | 6.7 | 2 | 298 | -13 | AluY | Alu | SINE |
| 127912973 | 127913121 | + | 15.9 | 140 | 302 | -10 | AluJb | Alu | SINE |
| 127913133 | 127913249 | + | 16.1 | 151 | 278 | -67 | MER2 | MER2\_type | DNA |

  

|  |  |  |  |  |  |  |  |  |  |
| --- | --- | --- | --- | --- | --- | --- | --- | --- | --- |
| chr7:127931978-127932585 | | | 608 bp | | |  |  |  |  |
| Genome Start | Genome End | Strand | Div | Repeat Start | Repeat End | Repeat Left | Name | Family | Class |
| 127931978 | 127932106 | + | 10.8 | 1 | 129 | -184 | AluSq | Alu | SINE |
| 127932107 | 127932408 | + | 8.3 | 1 | 304 | -9 | AluSp | Alu | SINE |
| 127932409 | 127932585 | + | 10.8 | 130 | 311 | -2 | AluSq | Alu | SINE |

  

|  |  |  |  |  |  |  |  |  |  |
| --- | --- | --- | --- | --- | --- | --- | --- | --- | --- |
| chr7:127947997-127948381 | | | 385 bp | | |  |  |  |  |
| Genome Start | Genome End | Strand | Div | Repeat Start | Repeat End | Repeat Left | Name | Family | Class |
| 127947997 | 127948027 | + | 4.3 | 478 | 507 | -127 | MER41B | ERV1 | LTR |
| 127948028 | 127948329 | - | 6.4 | 2 | 299 | -10 | AluSg1 | Alu | SINE |
| 127948330 | 127948381 | + | 4.3 | 508 | 546 | -88 | MER41B | ERV1 | LTR |

  

|  |  |  |  |  |  |  |  |  |  |
| --- | --- | --- | --- | --- | --- | --- | --- | --- | --- |
| chr7:127953740-127954665 | | | 926 bp | | |  |  |  |  |
| Genome Start | Genome End | Strand | Div | Repeat Start | Repeat End | Repeat Left | Name | Family | Class |
| 127953740 | 127953919 | - | 18.6 | 126 | 307 | -5 | AluJb | Alu | SINE |
| 127953920 | 127954230 | - | 8.4 | 1 | 311 | 0 | AluY | Alu | SINE |
| 127954231 | 127954254 | - | 18.6 | 111 | 125 | -187 | AluJb | Alu | SINE |
| 127954255 | 127954548 | - | 8.9 | 1 | 296 | -15 | AluY | Alu | SINE |
| 127954549 | 127954665 | - | 18.6 | 1 | 110 | -202 | AluJb | Alu | SINE |

  

|  |  |  |  |  |  |  |  |  |  |
| --- | --- | --- | --- | --- | --- | --- | --- | --- | --- |
| chr7:127968285-127969967 | | | 1500 bp | | |  |  |  |  |
| Genome Start | Genome End | Strand | Div | Repeat Start | Repeat End | Repeat Left | Name | Family | Class |
| 127968285 | 127968410 | - | 25.4 | 1866 | 1998 | -4151 | L1MEe | L1 | LINE |
| 127968416 | 127968601 | - | 14.5 | 120 | 305 | -7 | AluJb | Alu | SINE |
| 127968631 | 127968707 | - | 17.8 | 78 | 150 | -152 | AluJb | Alu | SINE |
| 127968788 | 127969093 | - | 12.1 | 1 | 298 | -14 | AluSx | Alu | SINE |
| 127969098 | 127969199 | - | 13.7 | 6206 | 6322 | -22 | L1MC2 | L1 | LINE |
| 127969232 | 127969388 | - | 13.4 | 6135 | 6322 | -22 | L1MC2 | L1 | LINE |
| 127969415 | 127969718 | - | 11.6 | 1 | 310 | -3 | AluSq | Alu | SINE |
| 127969726 | 127969967 | - | 31.1 | 946 | 1196 | -4923 | L1MEe | L1 | LINE |

  

|  |  |  |  |  |  |  |  |  |  |
| --- | --- | --- | --- | --- | --- | --- | --- | --- | --- |
| chr7:127972363-127973135 | | | 773 bp | | |  |  |  |  |
| Genome Start | Genome End | Strand | Div | Repeat Start | Repeat End | Repeat Left | Name | Family | Class |
| 127972363 | 127972419 | - | 16.8 | 6005 | 6060 | -123 | L1MB3 | L1 | LINE |
| 127972420 | 127972729 | + | 14.3 | 1 | 309 | -1 | AluSg | Alu | SINE |
| 127972730 | 127973135 | - | 16.8 | 5616 | 6004 | -179 | L1MB3 | L1 | LINE |

  

|  |  |  |  |  |  |  |  |  |  |
| --- | --- | --- | --- | --- | --- | --- | --- | --- | --- |
| chr7:127990828-127991523 | | | 696 bp | | |  |  |  |  |
| Genome Start | Genome End | Strand | Div | Repeat Start | Repeat End | Repeat Left | Name | Family | Class |
| 127990828 | 127991195 | + | 15.4 | 1 | 370 | -38 | MLT1A1 | MaLR | LTR |
| 127991196 | 127991477 | + | 13.1 | 2 | 290 | -22 | AluJb | Alu | SINE |
| 127991478 | 127991523 | + | 15.4 | 371 | 405 | -3 | MLT1A1 | MaLR | LTR |

  

|  |  |  |  |  |  |  |  |  |  |
| --- | --- | --- | --- | --- | --- | --- | --- | --- | --- |
| chr7:127991524-127992109 | | | 586 bp | | |  |  |  |  |
| Genome Start | Genome End | Strand | Div | Repeat Start | Repeat End | Repeat Left | Name | Family | Class |
| 127991524 | 127991683 | + | 28.6 | 2 | 166 | -96 | MIR | MIR | SINE |
| 127991684 | 127992038 | - | 18.6 | 1 | 377 | -13 | MLT1B | MaLR | LTR |
| 127992039 | 127992109 | + | 22.5 | 170 | 262 | 0 | MIR | MIR | SINE |

  

|  |  |  |  |  |  |  |  |  |  |
| --- | --- | --- | --- | --- | --- | --- | --- | --- | --- |
| chr7:127993698-127994856 | | | 1109 bp | | |  |  |  |  |
| Genome Start | Genome End | Strand | Div | Repeat Start | Repeat End | Repeat Left | Name | Family | Class |
| 127993698 | 127994015 | - | 21.3 | 4993 | 5365 | -994 | L1M | L1 | LINE |
| 127994016 | 127994044 | - | 8.7 | 286 | 309 | -4 | AluSq | Alu | SINE |
| 127994074 | 127994358 | - | 8.7 | 2 | 285 | -28 | AluSq | Alu | SINE |
| 127994359 | 127994477 | - | 21.7 | 4886 | 4992 | -1154 | L1M | L1 | LINE |
| 127994499 | 127994798 | - | 16.9 | 14 | 299 | -13 | AluJo | Alu | SINE |
| 127994799 | 127994856 | - | 15.5 | 4830 | 4887 | -1259 | L1M | L1 | LINE |

  

|  |  |  |  |  |  |  |  |  |  |
| --- | --- | --- | --- | --- | --- | --- | --- | --- | --- |
| chr7:128000418-128001028 | | | 576 bp | | |  |  |  |  |
| Genome Start | Genome End | Strand | Div | Repeat Start | Repeat End | Repeat Left | Name | Family | Class |
| 128000418 | 128000762 | - | 16.0 | 4606 | 4995 | -1151 | L1M4 | L1 | LINE |
| 128000763 | 128000870 | + | 9.3 | 1 | 108 | -204 | AluSq/x | Alu | SINE |
| 128000906 | 128001028 | - | 15.4 | 4277 | 4409 | -1737 | L1M4 | L1 | LINE |

  

|  |  |  |  |  |  |  |  |  |  |
| --- | --- | --- | --- | --- | --- | --- | --- | --- | --- |
| chr7:128060641-128061231 | | | 591 bp | | |  |  |  |  |
| Genome Start | Genome End | Strand | Div | Repeat Start | Repeat End | Repeat Left | Name | Family | Class |
| 128060641 | 128060767 | + | 18.7 | 10 | 136 | -176 | AluJb | Alu | SINE |
| 128060768 | 128061069 | + | 12.2 | 1 | 306 | -3 | AluSg1 | Alu | SINE |
| 128061070 | 128061231 | + | 18.7 | 137 | 298 | -14 | AluJb | Alu | SINE |

  

|  |  |  |  |  |  |  |  |  |  |
| --- | --- | --- | --- | --- | --- | --- | --- | --- | --- |
| chr7:128065032-128065506 | | | 432 bp | | |  |  |  |  |
| Genome Start | Genome End | Strand | Div | Repeat Start | Repeat End | Repeat Left | Name | Family | Class |
| 128065032 | 128065087 | + | 23.2 | 24 | 79 | -223 | AluJo | Alu | SINE |
| 128065131 | 128065404 | - | 12.8 | 1 | 279 | -33 | AluSx | Alu | SINE |
| 128065405 | 128065506 | + | 22.9 | 72 | 167 | -145 | AluJo | Alu | SINE |

  

|  |  |  |  |  |  |  |  |  |  |
| --- | --- | --- | --- | --- | --- | --- | --- | --- | --- |
| chr7:128070248-128071734 | | | 1487 bp | | |  |  |  |  |
| Genome Start | Genome End | Strand | Div | Repeat Start | Repeat End | Repeat Left | Name | Family | Class |
| 128070248 | 128070549 | - | 21.1 | 604 | 935 | 0 | MER21C | ERV1 | LTR |
| 128070550 | 128070852 | - | 20.9 | 1 | 302 | -10 | AluJo | Alu | SINE |
| 128070853 | 128070929 | - | 21.1 | 526 | 603 | -332 | MER21C | ERV1 | LTR |
| 128070930 | 128071229 | + | 20.0 | 9 | 294 | -18 | AluJb | Alu | SINE |
| 128071230 | 128071734 | - | 21.1 | 18 | 525 | -410 | MER21C | ERV1 | LTR |

  

|  |  |  |  |  |  |  |  |  |  |
| --- | --- | --- | --- | --- | --- | --- | --- | --- | --- |
| chr7:141886077-141886929 | | | 853 bp | | |  |  |  |  |
| Genome Start | Genome End | Strand | Div | Repeat Start | Repeat End | Repeat Left | Name | Family | Class |
| 141886077 | 141886260 | - | 27.2 | 5974 | 6163 | -1 | L1ME2 | L1 | LINE |
| 141886261 | 141886558 | - | 12.5 | 1 | 297 | -14 | AluY | Alu | SINE |
| 141886559 | 141886929 | - | 27.2 | 5604 | 5973 | -191 | L1ME2 | L1 | LINE |

  

|  |  |  |  |  |  |  |  |  |  |
| --- | --- | --- | --- | --- | --- | --- | --- | --- | --- |
| chr7:142088199-142089637 | | | 1426 bp | | |  |  |  |  |
| Genome Start | Genome End | Strand | Div | Repeat Start | Repeat End | Repeat Left | Name | Family | Class |
| 142088199 | 142088589 | + | 20.7 | 24 | 408 | -150 | MER74A | ERVL | LTR |
| 142088590 | 142089174 | - | 19.8 | 42 | 644 | 0 | MER73 | ERVL | LTR |
| 142089183 | 142089492 | + | 15.5 | 1 | 309 | -3 | AluSx | Alu | SINE |
| 142089498 | 142089637 | + | 24.5 | 420 | 558 | 0 | MER74A | ERVL | LTR |

  

|  |  |  |  |  |  |  |  |  |  |
| --- | --- | --- | --- | --- | --- | --- | --- | --- | --- |
| chr7:142956546-142964028 | | | 7480 bp | | |  |  |  |  |
| Genome Start | Genome End | Strand | Div | Repeat Start | Repeat End | Repeat Left | Name | Family | Class |
| 142956546 | 142957143 | - | 15.2 | 7132 | 7722 | 0 | L1MA4A | L1 | LINE |
| 142957147 | 142960144 | - | 16.5 | 3928 | 6943 | -779 | L1MA4A | L1 | LINE |
| 142960145 | 142961220 | - | 12.3 | 1 | 1232 | -4 | MER11B | ERVK | LTR |
| 142961221 | 142961613 | - | 17.0 | 3551 | 3927 | -3635 | L1MA4A | L1 | LINE |
| 142961614 | 142961925 | - | 9.1 | 1 | 309 | -3 | AluSx | Alu | SINE |
| 142961926 | 142964028 | - | 19.9 | 1399 | 3551 | -4015 | L1MA4A | L1 | LINE |

  

|  |  |  |  |  |  |  |  |  |  |
| --- | --- | --- | --- | --- | --- | --- | --- | --- | --- |
| chr7:142976682-142977141 | | | 460 bp | | |  |  |  |  |
| Genome Start | Genome End | Strand | Div | Repeat Start | Repeat End | Repeat Left | Name | Family | Class |
| 142976682 | 142976743 | + | 21.4 | 504 | 561 | -5966 | L1MEa | L1 | LINE |
| 142976744 | 142977127 | - | 11.3 | 1 | 364 | 0 | THE1B | MaLR | LTR |
| 142977128 | 142977141 | + | 21.4 | 562 | 573 | -5954 | L1MEa | L1 | LINE |

  

|  |  |  |  |  |  |  |  |  |  |
| --- | --- | --- | --- | --- | --- | --- | --- | --- | --- |
| chr7:143031954-143035647 | | | 3162 bp | | |  |  |  |  |
| Genome Start | Genome End | Strand | Div | Repeat Start | Repeat End | Repeat Left | Name | Family | Class |
| 143031954 | 143032197 | - | 35.1 | 3145 | 3394 | -25 | L2 | L2 | LINE |
| 143032240 | 143032691 | - | 28.5 | 2 | 472 | -51 | LTR33A | ERVL | LTR |
| 143032705 | 143032851 | - | 30.3 | 3010 | 3156 | -263 | L2 | L2 | LINE |
| 143032854 | 143033265 | + | 13.6 | 36 | 458 | 0 | LTR22A | ERVK | LTR |
| 143033308 | 143033777 | - | 28.7 | 2434 | 2921 | -498 | L2 | L2 | LINE |
| 143033794 | 143034136 | - | 29.7 | 142 | 497 | -14 | MLT1J | MaLR | LTR |
| 143034137 | 143034328 | - | 12.0 | 2501 | 2702 | -8 | Charlie3 | MER1\_type | DNA |
| 143034741 | 143035077 | - | 25.6 | 7575 | 7931 | -30 | L1MC5 | L1 | LINE |
| 143035078 | 143035373 | + | 14.6 | 1 | 300 | -12 | AluSx | Alu | SINE |
| 143035374 | 143035547 | - | 25.6 | 7409 | 7574 | -387 | L1MC5 | L1 | LINE |
| 143035553 | 143035647 | - | 29.0 | 3030 | 3122 | -297 | L2 | L2 | LINE |

  

|  |  |  |  |  |  |  |  |  |  |
| --- | --- | --- | --- | --- | --- | --- | --- | --- | --- |
| chr7:143042840-143043626 | | | 787 bp | | |  |  |  |  |
| Genome Start | Genome End | Strand | Div | Repeat Start | Repeat End | Repeat Left | Name | Family | Class |
| 143042840 | 143042868 | + | 29.0 | 35 | 62 | -206 | MIRb | MIR | SINE |
| 143042869 | 143043441 | - | 12.6 | 1 | 579 | -1 | LTR19B | ERV1 | LTR |
| 143043442 | 143043626 | + | 29.0 | 63 | 268 | 0 | MIRb | MIR | SINE |

  

|  |  |  |  |  |  |  |  |  |  |
| --- | --- | --- | --- | --- | --- | --- | --- | --- | --- |
| chr7:143075804-143076351 | | | 546 bp | | |  |  |  |  |
| Genome Start | Genome End | Strand | Div | Repeat Start | Repeat End | Repeat Left | Name | Family | Class |
| 143075804 | 143076100 | + | 14.5 | 1 | 298 | -68 | Tigger5a | MER2\_type | DNA |
| 143076101 | 143076272 | + | 20.4 | 1 | 167 | -18 | FAM | Alu | SINE |
| 143076275 | 143076351 | + | 24.7 | 372 | 448 | -1 | Tigger5a | MER2\_type | DNA |

  

|  |  |  |  |  |  |  |  |  |  |
| --- | --- | --- | --- | --- | --- | --- | --- | --- | --- |
| chr7:143121940-143123517 | | | 1367 bp | | |  |  |  |  |
| Genome Start | Genome End | Strand | Div | Repeat Start | Repeat End | Repeat Left | Name | Family | Class |
| 143121940 | 143122162 | + | 29.3 | 45 | 284 | -2223 | HAL1 | L1 | LINE |
| 143122343 | 143122975 | + | 28.9 | 530 | 1203 | -1304 | HAL1 | L1 | LINE |
| 143122978 | 143123271 | + | 10.9 | 1 | 310 | 0 | AluSg | Alu | SINE |
| 143123301 | 143123517 | + | 22.7 | 2104 | 2303 | -204 | HAL1 | L1 | LINE |

  

|  |  |  |  |  |  |  |  |  |  |
| --- | --- | --- | --- | --- | --- | --- | --- | --- | --- |
| chr7:143169972-143170816 | | | 845 bp | | |  |  |  |  |
| Genome Start | Genome End | Strand | Div | Repeat Start | Repeat End | Repeat Left | Name | Family | Class |
| 143169972 | 143170223 | + | 25.4 | 1 | 325 | -289 | MER90a | ERV1 | LTR |
| 143170224 | 143170525 | - | 8.3 | 1 | 303 | -10 | AluSp | Alu | SINE |
| 143170526 | 143170816 | + | 26.7 | 326 | 614 | 0 | MER90a | ERV1 | LTR |

  

|  |  |  |  |  |  |  |  |  |  |
| --- | --- | --- | --- | --- | --- | --- | --- | --- | --- |
| chr7:143180420-143181457 | | | 1038 bp | | |  |  |  |  |
| Genome Start | Genome End | Strand | Div | Repeat Start | Repeat End | Repeat Left | Name | Family | Class |
| 143180420 | 143181101 | - | 25.7 | 1041 | 1782 | -725 | HAL1 | L1 | LINE |
| 143181102 | 143181344 | - | 15.6 | 68 | 305 | -7 | AluJo | Alu | SINE |
| 143181345 | 143181457 | - | 25.7 | 916 | 1040 | -1467 | HAL1 | L1 | LINE |

  

|  |  |  |  |  |  |  |  |  |  |
| --- | --- | --- | --- | --- | --- | --- | --- | --- | --- |
| chr7:143508305-143508852 | | | 523 bp | | |  |  |  |  |
| Genome Start | Genome End | Strand | Div | Repeat Start | Repeat End | Repeat Left | Name | Family | Class |
| 143508305 | 143508601 | + | 15.5 | 1 | 298 | -68 | Tigger5a | MER2\_type | DNA |
| 143508602 | 143508746 | + | 22.1 | 1 | 140 | -45 | FAM | Alu | SINE |
| 143508772 | 143508852 | + | 24.7 | 368 | 448 | -1 | Tigger5a | MER2\_type | DNA |

  

|  |  |  |  |  |  |  |  |  |  |
| --- | --- | --- | --- | --- | --- | --- | --- | --- | --- |
| chr7:143548609-143549336 | | | 728 bp | | |  |  |  |  |
| Genome Start | Genome End | Strand | Div | Repeat Start | Repeat End | Repeat Left | Name | Family | Class |
| 143548609 | 143548962 | - | 23.8 | 5478 | 5887 | -1921 | L1MC4a | L1 | LINE |
| 143548963 | 143549239 | - | 15.2 | 1 | 294 | -18 | AluJb | Alu | SINE |
| 143549240 | 143549336 | - | 23.8 | 5395 | 5477 | -2331 | L1MC4a | L1 | LINE |

  

|  |  |  |  |  |  |  |  |  |  |
| --- | --- | --- | --- | --- | --- | --- | --- | --- | --- |
| chr7:143571448-143572657 | | | 1210 bp | | |  |  |  |  |
| Genome Start | Genome End | Strand | Div | Repeat Start | Repeat End | Repeat Left | Name | Family | Class |
| 143571448 | 143571549 | + | 20.1 | 3 | 96 | -767 | MER21B | ERV1 | LTR |
| 143571550 | 143571909 | + | 15.3 | 3 | 395 | -1 | MSTD | MaLR | LTR |
| 143571910 | 143572657 | + | 20.1 | 97 | 863 | 0 | MER21B | ERV1 | LTR |

  

|  |  |  |  |  |  |  |  |  |  |
| --- | --- | --- | --- | --- | --- | --- | --- | --- | --- |
| chr7:143599180-143599651 | | | 472 bp | | |  |  |  |  |
| Genome Start | Genome End | Strand | Div | Repeat Start | Repeat End | Repeat Left | Name | Family | Class |
| 143599180 | 143599193 | - | 27.4 | 562 | 573 | -5954 | L1MEc | L1 | LINE |
| 143599194 | 143599575 | + | 12.6 | 1 | 364 | 0 | THE1B | MaLR | LTR |
| 143599576 | 143599651 | - | 27.4 | 488 | 561 | -5966 | L1MEc | L1 | LINE |

  

|  |  |  |  |  |  |  |  |  |  |
| --- | --- | --- | --- | --- | --- | --- | --- | --- | --- |
| chr7:143606538-143608047 | | | 1279 bp | | |  |  |  |  |
| Genome Start | Genome End | Strand | Div | Repeat Start | Repeat End | Repeat Left | Name | Family | Class |
| 143606538 | 143606602 | - | 27.7 | 3294 | 3358 | -20 | L2 | L2 | LINE |
| 143606758 | 143606870 | + | 20.5 | 6160 | 6274 | -38 | L1MA9 | L1 | LINE |
| 143606897 | 143607192 | + | 16.9 | 1 | 298 | -14 | AluJb | Alu | SINE |
| 143607243 | 143607389 | - | 34.5 | 2797 | 2958 | -461 | L2 | L2 | LINE |
| 143607390 | 143607693 | - | 10.0 | 1 | 300 | -12 | AluSx | Alu | SINE |
| 143607694 | 143608047 | - | 34.5 | 2455 | 2796 | -623 | L2 | L2 | LINE |

  

|  |  |  |  |  |  |  |  |  |  |
| --- | --- | --- | --- | --- | --- | --- | --- | --- | --- |
| chr7:143632083-143632969 | | | 705 bp | | |  |  |  |  |
| Genome Start | Genome End | Strand | Div | Repeat Start | Repeat End | Repeat Left | Name | Family | Class |
| 143632083 | 143632193 | + | 34.5 | 3259 | 3374 | -4 | L2 | L2 | LINE |
| 143632281 | 143632608 | + | 11.2 | 1 | 326 | 0 | AluSg | Alu | SINE |
| 143632661 | 143632855 | - | 29.4 | 34 | 247 | -15 | MIR | MIR | SINE |
| 143632899 | 143632969 | + | 28.2 | 3348 | 3419 | 0 | L2 | L2 | LINE |

  

|  |  |  |  |  |  |  |  |  |  |
| --- | --- | --- | --- | --- | --- | --- | --- | --- | --- |
| chr7:143670016-143671758 | | | 1615 bp | | |  |  |  |  |
| Genome Start | Genome End | Strand | Div | Repeat Start | Repeat End | Repeat Left | Name | Family | Class |
| 143670016 | 143670242 | + | 13.7 | 4 | 230 | -380 | MER4B | ERV1 | LTR |
| 143670243 | 143670328 | + | 9.3 | 3908 | 3993 | -3720 | HERVH | ERV1 | LTR |
| 143670331 | 143670573 | + | 13.1 | 4488 | 4723 | -2990 | HERVH | ERV1 | LTR |
| 143670552 | 143670718 | + | 6.2 | 7550 | 7713 | 0 | HERVH | ERV1 | LTR |
| 143670719 | 143671199 | + | 5.4 | 1 | 448 | -2 | LTR7 | ERV1 | LTR |
| 143671348 | 143671758 | + | 13.8 | 206 | 610 | 0 | MER4B | ERV1 | LTR |

  

|  |  |  |  |  |  |  |  |  |  |
| --- | --- | --- | --- | --- | --- | --- | --- | --- | --- |
| chr7:149242663-149243973 | | | 985 bp | | |  |  |  |  |
| Genome Start | Genome End | Strand | Div | Repeat Start | Repeat End | Repeat Left | Name | Family | Class |
| 149242663 | 149242901 | + | 32.8 | 3951 | 4200 | -1946 | L1M5 | L1 | LINE |
| 149242902 | 149243196 | - | 9.8 | 2 | 301 | -11 | AluSx | Alu | SINE |
| 149243197 | 149243409 | + | 33.7 | 4200 | 4422 | -1724 | L1M5 | L1 | LINE |
| 149243736 | 149243973 | + | 27.9 | 5581 | 5822 | -299 | L1M5 | L1 | LINE |

  

|  |  |  |  |  |  |  |  |  |  |
| --- | --- | --- | --- | --- | --- | --- | --- | --- | --- |
| chr7:149251118-149254867 | | | 3487 bp | | |  |  |  |  |
| Genome Start | Genome End | Strand | Div | Repeat Start | Repeat End | Repeat Left | Name | Family | Class |
| 149251118 | 149251630 | + | 24.2 | 2037 | 2555 | -3660 | L1MC4 | L1 | LINE |
| 149251740 | 149252034 | + | 10.9 | 3 | 296 | -16 | AluSx | Alu | SINE |
| 149252116 | 149252404 | + | 11.1 | 1 | 290 | -23 | AluSq | Alu | SINE |
| 149252478 | 149252654 | + | 31.5 | 4126 | 4292 | -1922 | L1MC4 | L1 | LINE |
| 149252655 | 149252950 | - | 12.3 | 1 | 293 | -19 | AluSx | Alu | SINE |
| 149252951 | 149253801 | + | 31.5 | 4293 | 5121 | -1093 | L1MC4 | L1 | LINE |
| 149253802 | 149254112 | + | 13.9 | 1 | 310 | -2 | AluSx | Alu | SINE |
| 149254113 | 149254293 | + | 31.5 | 5122 | 5330 | -884 | L1MC4 | L1 | LINE |
| 149254294 | 149254800 | + | 20.9 | 1 | 559 | -4 | MER68 | ERVL | LTR |
| 149254801 | 149254867 | + | 31.5 | 5331 | 5391 | -823 | L1MC4 | L1 | LINE |

  

|  |  |  |  |  |  |  |  |  |  |
| --- | --- | --- | --- | --- | --- | --- | --- | --- | --- |
| chr7:149264727-149265193 | | | 467 bp | | |  |  |  |  |
| Genome Start | Genome End | Strand | Div | Repeat Start | Repeat End | Repeat Left | Name | Family | Class |
| 149264727 | 149264801 | + | 28.8 | 47 | 122 | -140 | MIR | MIR | SINE |
| 149264802 | 149265111 | - | 16.9 | 1 | 309 | -3 | AluJo | Alu | SINE |
| 149265112 | 149265193 | + | 28.8 | 123 | 207 | -55 | MIR | MIR | SINE |

  

|  |  |  |  |  |  |  |  |  |  |
| --- | --- | --- | --- | --- | --- | --- | --- | --- | --- |
| chr7:149326364-149327218 | | | 855 bp | | |  |  |  |  |
| Genome Start | Genome End | Strand | Div | Repeat Start | Repeat End | Repeat Left | Name | Family | Class |
| 149326364 | 149326401 | + | 23.9 | 5 | 40 | -527 | MLT1F | MaLR | LTR |
| 149326402 | 149326773 | - | 9.8 | 1 | 375 | 0 | THE1C | MaLR | LTR |
| 149326774 | 149327218 | + | 24.0 | 41 | 523 | -19 | MLT1F | MaLR | LTR |

  

|  |  |  |  |  |  |  |  |  |  |
| --- | --- | --- | --- | --- | --- | --- | --- | --- | --- |
| chr7:149336011-149336786 | | | 638 bp | | |  |  |  |  |
| Genome Start | Genome End | Strand | Div | Repeat Start | Repeat End | Repeat Left | Name | Family | Class |
| 149336011 | 149336272 | - | 37.2 | 4156 | 4423 | -66 | L3 | CR1 | LINE |
| 149336385 | 149336551 | + | 23.9 | 37 | 209 | 0 | MER3 | MER1\_type | DNA |
| 149336578 | 149336786 | - | 32.4 | 3769 | 4021 | -468 | L3 | CR1 | LINE |

  

|  |  |  |  |  |  |  |  |  |  |
| --- | --- | --- | --- | --- | --- | --- | --- | --- | --- |
| chr7:149395952-149397346 | | | 1054 bp | | |  |  |  |  |
| Genome Start | Genome End | Strand | Div | Repeat Start | Repeat End | Repeat Left | Name | Family | Class |
| 149395952 | 149396207 | - | 28.6 | 1652 | 1935 | -26 | Charlie4 | MER1\_type | DNA |
| 149396250 | 149396483 | + | 28.8 | 2871 | 3134 | -244 | L2 | L2 | LINE |
| 149396530 | 149396833 | + | 6.4 | 16 | 310 | -1 | AluY | Alu | SINE |
| 149397087 | 149397346 | - | 27.7 | 1 | 288 | -1673 | Charlie4 | MER1\_type | DNA |

  

|  |  |  |  |  |  |  |  |  |  |
| --- | --- | --- | --- | --- | --- | --- | --- | --- | --- |
| chr7:149494250-149495359 | | | 1110 bp | | |  |  |  |  |
| Genome Start | Genome End | Strand | Div | Repeat Start | Repeat End | Repeat Left | Name | Family | Class |
| 149494250 | 149494438 | - | 14.6 | 6108 | 6289 | -11 | L1MA4 | L1 | LINE |
| 149494439 | 149494730 | + | 12.0 | 1 | 294 | -18 | AluSx | Alu | SINE |
| 149494731 | 149495359 | - | 14.6 | 5480 | 6107 | -193 | L1MA4 | L1 | LINE |

  

|  |  |  |  |  |  |  |  |  |  |
| --- | --- | --- | --- | --- | --- | --- | --- | --- | --- |
| chr7:149498831-149500180 | | | 1350 bp | | |  |  |  |  |
| Genome Start | Genome End | Strand | Div | Repeat Start | Repeat End | Repeat Left | Name | Family | Class |
| 149498831 | 149499835 | + | 25.8 | 2313 | 3322 | -2824 | L1MD | L1 | LINE |
| 149499836 | 149500130 | - | 11.2 | 1 | 298 | -11 | AluSc | Alu | SINE |
| 149500131 | 149500180 | + | 25.8 | 3323 | 3364 | -2782 | L1MD | L1 | LINE |

  

|  |  |  |  |  |  |  |  |  |  |
| --- | --- | --- | --- | --- | --- | --- | --- | --- | --- |
| chr7:149578023-149578983 | | | 961 bp | | |  |  |  |  |
| Genome Start | Genome End | Strand | Div | Repeat Start | Repeat End | Repeat Left | Name | Family | Class |
| 149578023 | 149578224 | - | 29.0 | 2819 | 3026 | -393 | L2 | L2 | LINE |
| 149578225 | 149578524 | - | 3.3 | 1 | 300 | -11 | AluY | Alu | SINE |
| 149578525 | 149578983 | - | 29.0 | 2272 | 2818 | -601 | L2 | L2 | LINE |

  

|  |  |  |  |  |  |  |  |  |  |
| --- | --- | --- | --- | --- | --- | --- | --- | --- | --- |
| chr7:149587748-149588282 | | | 535 bp | | |  |  |  |  |
| Genome Start | Genome End | Strand | Div | Repeat Start | Repeat End | Repeat Left | Name | Family | Class |
| 149587748 | 149588026 | + | 32.9 | 3065 | 3359 | -60 | L2 | L2 | LINE |
| 149588027 | 149588239 | + | 22.5 | 1 | 224 | 0 | MER58A | MER1\_type | DNA |
| 149588240 | 149588282 | + | 32.9 | 3360 | 3403 | -16 | L2 | L2 | LINE |

  

|  |  |  |  |  |  |  |  |  |  |
| --- | --- | --- | --- | --- | --- | --- | --- | --- | --- |
| chr7:151538069-151539493 | | | 1425 bp | | |  |  |  |  |
| Genome Start | Genome End | Strand | Div | Repeat Start | Repeat End | Repeat Left | Name | Family | Class |
| 151538069 | 151538466 | + | 24.5 | 528 | 867 | -1640 | HAL1 | L1 | LINE |
| 151538467 | 151538671 | + | 11.0 | 1 | 230 | 0 | MER30 | MER1\_type | DNA |
| 151538672 | 151539493 | + | 24.5 | 868 | 1825 | -682 | HAL1 | L1 | LINE |

  

|  |  |  |  |  |  |  |  |  |  |
| --- | --- | --- | --- | --- | --- | --- | --- | --- | --- |
| chr7:151540345-151543083 | | | 2739 bp | | |  |  |  |  |
| Genome Start | Genome End | Strand | Div | Repeat Start | Repeat End | Repeat Left | Name | Family | Class |
| 151540345 | 151540776 | + | 16.6 | 4032 | 4484 | -1656 | L1MA9 | L1 | LINE |
| 151540777 | 151541082 | + | 9.7 | 1 | 299 | -13 | AluSx | Alu | SINE |
| 151541083 | 151542416 | + | 16.8 | 4485 | 5787 | -525 | L1MA9 | L1 | LINE |
| 151542417 | 151542618 | + | 22.3 | 4 | 206 | -4 | MER6B | MER2\_type | DNA |
| 151542619 | 151543083 | + | 17.1 | 5788 | 6274 | -38 | L1MA9 | L1 | LINE |

  

|  |  |  |  |  |  |  |  |  |  |
| --- | --- | --- | --- | --- | --- | --- | --- | --- | --- |
| chr7:151553345-151554606 | | | 1262 bp | | |  |  |  |  |
| Genome Start | Genome End | Strand | Div | Repeat Start | Repeat End | Repeat Left | Name | Family | Class |
| 151553345 | 151554237 | + | 27.8 | 1404 | 2438 | -69 | HAL1 | L1 | LINE |
| 151554238 | 151554549 | - | 13.7 | 1 | 308 | -4 | AluSx | Alu | SINE |
| 151554550 | 151554606 | + | 27.8 | 2439 | 2497 | -10 | HAL1 | L1 | LINE |

  

|  |  |  |  |  |  |  |  |  |  |
| --- | --- | --- | --- | --- | --- | --- | --- | --- | --- |
| chr7:151594869-151596491 | | | 1500 bp | | |  |  |  |  |
| Genome Start | Genome End | Strand | Div | Repeat Start | Repeat End | Repeat Left | Name | Family | Class |
| 151594869 | 151595547 | + | 35.0 | 1942 | 2701 | -718 | L2 | L2 | LINE |
| 151595548 | 151595627 | + | 11.2 | 1 | 80 | 0 | MADE1 | Mariner | DNA |
| 151595628 | 151596015 | + | 33.8 | 2702 | 3110 | -268 | L2 | L2 | LINE |
| 151596016 | 151596305 | + | 12.4 | 1 | 291 | -21 | AluSx | Alu | SINE |
| 151596429 | 151596491 | + | 32.3 | 3261 | 3326 | -52 | L2 | L2 | LINE |

  

|  |  |  |  |  |  |  |  |  |  |
| --- | --- | --- | --- | --- | --- | --- | --- | --- | --- |
| chr7:151605306-151605867 | | | 367 bp | | |  |  |  |  |
| Genome Start | Genome End | Strand | Div | Repeat Start | Repeat End | Repeat Left | Name | Family | Class |
| 151605306 | 151605424 | + | 26.5 | 1134 | 1254 | -1253 | HAL1 | L1 | LINE |
| 151605508 | 151605547 | - | 25.0 | 117 | 156 | -120 | MIRm | MIR | SINE |
| 151605660 | 151605867 | + | 36.1 | 1405 | 1616 | -891 | HAL1 | L1 | LINE |

  

|  |  |  |  |  |  |  |  |  |  |
| --- | --- | --- | --- | --- | --- | --- | --- | --- | --- |
| chr7:151614613-151617212 | | | 2574 bp | | |  |  |  |  |
| Genome Start | Genome End | Strand | Div | Repeat Start | Repeat End | Repeat Left | Name | Family | Class |
| 151614613 | 151615759 | + | 26.4 | 17 | 1225 | -5408 | L1MDa | L1 | LINE |
| 151615760 | 151616063 | + | 12.2 | 1 | 305 | -7 | AluSx | Alu | SINE |
| 151616064 | 151616269 | + | 26.4 | 1226 | 1490 | -5143 | L1MDa | L1 | LINE |
| 151616270 | 151616546 | + | 13.4 | 1 | 278 | -34 | AluSx | Alu | SINE |
| 151616573 | 151617212 | + | 26.4 | 1491 | 2170 | -4463 | L1MDa | L1 | LINE |

  

|  |  |  |  |  |  |  |  |  |  |
| --- | --- | --- | --- | --- | --- | --- | --- | --- | --- |
| chr7:151617213-151618763 | | | 1434 bp | | |  |  |  |  |
| Genome Start | Genome End | Strand | Div | Repeat Start | Repeat End | Repeat Left | Name | Family | Class |
| 151617213 | 151617798 | + | 22.8 | 1443 | 2069 | -692 | Charlie1 | MER1\_type | DNA |
| 151617799 | 151618096 | + | 14.6 | 1 | 320 | 0 | AluSx | Alu | SINE |
| 151618097 | 151618230 | + | 22.8 | 2070 | 2201 | -560 | Charlie1 | MER1\_type | DNA |
| 151618317 | 151618625 | - | 12.0 | 1 | 309 | 0 | AluSg1 | Alu | SINE |
| 151618657 | 151618763 | + | 17.5 | 2218 | 2314 | -447 | Charlie1 | MER1\_type | DNA |

  

|  |  |  |  |  |  |  |  |  |  |
| --- | --- | --- | --- | --- | --- | --- | --- | --- | --- |
| chr7:151629099-151629707 | | | 609 bp | | |  |  |  |  |
| Genome Start | Genome End | Strand | Div | Repeat Start | Repeat End | Repeat Left | Name | Family | Class |
| 151629099 | 151629240 | + | 20.8 | 1 | 133 | -169 | AluJo | Alu | SINE |
| 151629241 | 151629554 | + | 12.7 | 1 | 309 | -1 | AluSg | Alu | SINE |
| 151629555 | 151629707 | + | 20.8 | 134 | 289 | -13 | AluJo | Alu | SINE |

  

|  |  |  |  |  |  |  |  |  |  |
| --- | --- | --- | --- | --- | --- | --- | --- | --- | --- |
| chr7:151776013-151776639 | | | 627 bp | | |  |  |  |  |
| Genome Start | Genome End | Strand | Div | Repeat Start | Repeat End | Repeat Left | Name | Family | Class |
| 151776013 | 151776164 | + | 13.9 | 2 | 146 | -166 | AluJo | Alu | SINE |
| 151776165 | 151776468 | + | 9.9 | 1 | 303 | -10 | AluSp | Alu | SINE |
| 151776469 | 151776639 | + | 13.9 | 147 | 304 | -8 | AluJo | Alu | SINE |

  

|  |  |  |  |  |  |  |  |  |  |
| --- | --- | --- | --- | --- | --- | --- | --- | --- | --- |
| chr7:151777400-151778002 | | | 603 bp | | |  |  |  |  |
| Genome Start | Genome End | Strand | Div | Repeat Start | Repeat End | Repeat Left | Name | Family | Class |
| 151777400 | 151777565 | - | 9.0 | 134 | 305 | -8 | AluSq | Alu | SINE |
| 151777566 | 151777875 | - | 11.1 | 1 | 307 | -3 | AluSg | Alu | SINE |
| 151777876 | 151778002 | - | 9.0 | 11 | 133 | -180 | AluSq | Alu | SINE |

  

|  |  |  |  |  |  |  |  |  |  |
| --- | --- | --- | --- | --- | --- | --- | --- | --- | --- |
| chr7:152099563-152100768 | | | 1206 bp | | |  |  |  |  |
| Genome Start | Genome End | Strand | Div | Repeat Start | Repeat End | Repeat Left | Name | Family | Class |
| 152099563 | 152099811 | + | 34.5 | 749 | 1001 | -1008 | HAL1b | L1 | LINE |
| 152099812 | 152100102 | + | 10.0 | 1 | 302 | -11 | AluSp | Alu | SINE |
| 152100103 | 152100254 | + | 34.5 | 1002 | 1137 | -872 | HAL1b | L1 | LINE |
| 152100255 | 152100554 | + | 18.4 | 1 | 294 | -18 | AluJo | Alu | SINE |
| 152100555 | 152100768 | + | 34.5 | 1138 | 1345 | -664 | HAL1b | L1 | LINE |

  

|  |  |  |  |  |  |  |  |  |  |
| --- | --- | --- | --- | --- | --- | --- | --- | --- | --- |
| chr7:152101790-152102565 | | | 776 bp | | |  |  |  |  |
| Genome Start | Genome End | Strand | Div | Repeat Start | Repeat End | Repeat Left | Name | Family | Class |
| 152101790 | 152102151 | - | 28.3 | 96 | 487 | -2020 | HAL1 | L1 | LINE |
| 152102152 | 152102453 | - | 9.9 | 1 | 303 | -10 | AluSp | Alu | SINE |
| 152102454 | 152102565 | - | 28.3 | 14 | 95 | -2412 | HAL1 | L1 | LINE |

  

|  |  |  |  |  |  |  |  |  |  |
| --- | --- | --- | --- | --- | --- | --- | --- | --- | --- |
| chr7:152120205-152121981 | | | 1777 bp | | |  |  |  |  |
| Genome Start | Genome End | Strand | Div | Repeat Start | Repeat End | Repeat Left | Name | Family | Class |
| 152120205 | 152120485 | - | 18.6 | 1991 | 2235 | -4398 | L1MDa | L1 | LINE |
| 152120486 | 152120795 | + | 9.1 | 1 | 309 | 0 | AluSc | Alu | SINE |
| 152120796 | 152121981 | - | 18.6 | 853 | 1990 | -4643 | L1MDa | L1 | LINE |

  

|  |  |  |  |  |  |  |  |  |  |
| --- | --- | --- | --- | --- | --- | --- | --- | --- | --- |
| chr7:153125649-153126330 | | | 682 bp | | |  |  |  |  |
| Genome Start | Genome End | Strand | Div | Repeat Start | Repeat End | Repeat Left | Name | Family | Class |
| 153125649 | 153125851 | - | 22.1 | 161 | 363 | -11 | MLT1A | MaLR | LTR |
| 153125852 | 153126159 | - | 9.8 | 1 | 307 | -4 | AluY | Alu | SINE |
| 153126160 | 153126330 | - | 22.1 | 1 | 160 | -214 | MLT1A | MaLR | LTR |

  

|  |  |  |  |  |  |  |  |  |  |
| --- | --- | --- | --- | --- | --- | --- | --- | --- | --- |
| chr7:153344984-153345435 | | | 452 bp | | |  |  |  |  |
| Genome Start | Genome End | Strand | Div | Repeat Start | Repeat End | Repeat Left | Name | Family | Class |
| 153344984 | 153345095 | + | 13.0 | 5999 | 6109 | -191 | L1MA4 | L1 | LINE |
| 153345096 | 153345397 | - | 15.9 | 1 | 290 | -22 | AluJb | Alu | SINE |
| 153345398 | 153345435 | + | 13.0 | 6110 | 6140 | -160 | L1MA4 | L1 | LINE |

  

|  |  |  |  |  |  |  |  |  |  |
| --- | --- | --- | --- | --- | --- | --- | --- | --- | --- |
| chr7:153447116-153448009 | | | 894 bp | | |  |  |  |  |
| Genome Start | Genome End | Strand | Div | Repeat Start | Repeat End | Repeat Left | Name | Family | Class |
| 153447116 | 153447273 | + | 26.4 | 1 | 161 | -467 | MLT2E | ERVL | LTR |
| 153447274 | 153447871 | - | 16.2 | 1 | 610 | 0 | LTR38B | ERV1 | LTR |
| 153447872 | 153448009 | + | 26.4 | 162 | 304 | -324 | MLT2E | ERVL | LTR |

  

|  |  |  |  |  |  |  |  |  |  |
| --- | --- | --- | --- | --- | --- | --- | --- | --- | --- |
| chr7:153448827-153449773 | | | 947 bp | | |  |  |  |  |
| Genome Start | Genome End | Strand | Div | Repeat Start | Repeat End | Repeat Left | Name | Family | Class |
| 153448827 | 153448896 | - | 19.7 | 3191 | 3248 | -2898 | L1M4 | L1 | LINE |
| 153448897 | 153449244 | + | 15.7 | 1 | 372 | -3 | THE1C | MaLR | LTR |
| 153449245 | 153449773 | - | 19.7 | 2672 | 3190 | -2956 | L1M4 | L1 | LINE |

  

|  |  |  |  |  |  |  |  |  |  |
| --- | --- | --- | --- | --- | --- | --- | --- | --- | --- |
| chr7:153457499-153458145 | | | 647 bp | | |  |  |  |  |
| Genome Start | Genome End | Strand | Div | Repeat Start | Repeat End | Repeat Left | Name | Family | Class |
| 153457499 | 153457635 | + | 14.8 | 1 | 138 | -174 | AluSx | Alu | SINE |
| 153457636 | 153457944 | + | 8.8 | 1 | 308 | -3 | AluY | Alu | SINE |
| 153457945 | 153458145 | + | 14.8 | 139 | 312 | 0 | AluSx | Alu | SINE |

  


|  |  |  |  |  |  |  |  |  |  |
| --- | --- | --- | --- | --- | --- | --- | --- | --- | --- |
| chr8:14534-16654 | | | 2089 bp | | |  |  |  |  |
| Genome Start | Genome End | Strand | Div | Repeat Start | Repeat End | Repeat Left | Name | Family | Class |
| 14534 | 15352 | + | 17.1 | 4760 | 5599 | -701 | L1MA4 | L1 | LINE |
| 15353 | 15628 | + | 11.3 | 1 | 275 | -35 | AluSg | Alu | SINE |
| 15661 | 15973 | + | 8.6 | 1 | 304 | -5 | AluSc | Alu | SINE |
| 15974 | 16654 | + | 18.7 | 5600 | 6300 | 0 | L1MA4 | L1 | LINE |

  

|  |  |  |  |  |  |  |  |  |  |
| --- | --- | --- | --- | --- | --- | --- | --- | --- | --- |
| chr8:39783-40805 | | | 972 bp | | |  |  |  |  |
| Genome Start | Genome End | Strand | Div | Repeat Start | Repeat End | Repeat Left | Name | Family | Class |
| 39783 | 39876 | + | 27.1 | 4 | 98 | -493 | MLT1K | MaLR | LTR |
| 39877 | 40533 | + | 11.4 | 1 | 644 | 0 | LTR9B | ERV1 | LTR |
| 40534 | 40583 | + | 27.1 | 99 | 145 | -446 | MLT1K | MaLR | LTR |
| 40635 | 40805 | + | 25.4 | 398 | 575 | -16 | MLT1K | MaLR | LTR |

  

|  |  |  |  |  |  |  |  |  |  |
| --- | --- | --- | --- | --- | --- | --- | --- | --- | --- |
| chr8:41818-42888 | | | 1071 bp | | |  |  |  |  |
| Genome Start | Genome End | Strand | Div | Repeat Start | Repeat End | Repeat Left | Name | Family | Class |
| 41818 | 42167 | + | 6.9 | 1 | 333 | -448 | MER4E1 | ERV1 | LTR |
| 42168 | 42466 | - | 11.4 | 1 | 297 | -13 | AluSg | Alu | SINE |
| 42467 | 42888 | + | 6.9 | 334 | 781 | 0 | MER4E1 | ERV1 | LTR |

  

|  |  |  |  |  |  |  |  |  |  |
| --- | --- | --- | --- | --- | --- | --- | --- | --- | --- |
| chr8:50440-50860 | | | 383 bp | | |  |  |  |  |
| Genome Start | Genome End | Strand | Div | Repeat Start | Repeat End | Repeat Left | Name | Family | Class |
| 50440 | 50485 | + | 13.5 | 319 | 361 | -67 | MSTA | MaLR | LTR |
| 50486 | 50638 | - | 8.8 | 156 | 308 | -2 | AluSg | Alu | SINE |
| 50677 | 50796 | - | 8.8 | 2 | 155 | -155 | AluSg | Alu | SINE |
| 50797 | 50860 | + | 13.5 | 362 | 428 | 0 | MSTA | MaLR | LTR |

  

|  |  |  |  |  |  |  |  |  |  |
| --- | --- | --- | --- | --- | --- | --- | --- | --- | --- |
| chr8:53528-54706 | | | 1179 bp | | |  |  |  |  |
| Genome Start | Genome End | Strand | Div | Repeat Start | Repeat End | Repeat Left | Name | Family | Class |
| 53528 | 53841 | - | 18.3 | 460 | 782 | -1 | LTR39 | ERV1 | LTR |
| 53842 | 54140 | + | 15.1 | 1 | 299 | -13 | AluSx | Alu | SINE |
| 54141 | 54442 | - | 18.3 | 191 | 459 | -324 | LTR39 | ERV1 | LTR |
| 54443 | 54562 | - | 3.3 | 6034 | 6155 | 0 | L1PA4 | L1 | LINE |
| 54563 | 54706 | - | 18.3 | 51 | 190 | -593 | LTR39 | ERV1 | LTR |

  

|  |  |  |  |  |  |  |  |  |  |
| --- | --- | --- | --- | --- | --- | --- | --- | --- | --- |
| chr8:58594-59255 | | | 662 bp | | |  |  |  |  |
| Genome Start | Genome End | Strand | Div | Repeat Start | Repeat End | Repeat Left | Name | Family | Class |
| 58594 | 58889 | - | 26.7 | 93 | 375 | -167 | MLT1H | MaLR | LTR |
| 58890 | 59209 | - | 9.1 | 1 | 309 | -2 | AluY | Alu | SINE |
| 59210 | 59255 | - | 28.3 | 62 | 107 | -442 | MLT1H | MaLR | LTR |

  

|  |  |  |  |  |  |  |  |  |  |
| --- | --- | --- | --- | --- | --- | --- | --- | --- | --- |
| chr8:65861-66336 | | | 476 bp | | |  |  |  |  |
| Genome Start | Genome End | Strand | Div | Repeat Start | Repeat End | Repeat Left | Name | Family | Class |
| 65861 | 65963 | + | 31.1 | 3044 | 3144 | -3002 | L1M5 | L1 | LINE |
| 65964 | 66264 | + | 13.6 | 1 | 301 | -11 | AluSx | Alu | SINE |
| 66265 | 66336 | + | 31.1 | 3145 | 3217 | -2929 | L1M5 | L1 | LINE |

  

|  |  |  |  |  |  |  |  |  |  |
| --- | --- | --- | --- | --- | --- | --- | --- | --- | --- |
| chr8:76684-77687 | | | 801 bp | | |  |  |  |  |
| Genome Start | Genome End | Strand | Div | Repeat Start | Repeat End | Repeat Left | Name | Family | Class |
| 76684 | 76985 | - | 37.5 | 3030 | 3355 | -23 | L2 | L2 | LINE |
| 77186 | 77451 | - | 4.1 | 5881 | 6150 | -4 | L1PA5 | L1 | LINE |
| 77455 | 77687 | - | 37.7 | 2572 | 2813 | -606 | L2 | L2 | LINE |

  

|  |  |  |  |  |  |  |  |  |  |
| --- | --- | --- | --- | --- | --- | --- | --- | --- | --- |
| chr8:78125-83587 | | | 5321 bp | | |  |  |  |  |
| Genome Start | Genome End | Strand | Div | Repeat Start | Repeat End | Repeat Left | Name | Family | Class |
| 78125 | 78709 | - | 30.3 | 5508 | 6122 | -118 | L1ME3B | L1 | LINE |
| 78768 | 79781 | - | 17.1 | 3687 | 4697 | -1449 | L1P4 | L1 | LINE |
| 79832 | 80572 | - | 25.0 | 3352 | 4119 | -2027 | L1ME3B | L1 | LINE |
| 80607 | 80730 | - | 15.8 | 6109 | 6238 | -53 | L1MA8 | L1 | LINE |
| 80731 | 81466 | + | 10.3 | 5556 | 6303 | -1 | L1MA2 | L1 | LINE |
| 81467 | 83448 | - | 15.8 | 4080 | 6108 | -183 | L1MA8 | L1 | LINE |
| 83449 | 83587 | - | 26.1 | 3222 | 3366 | -2780 | L1ME3B | L1 | LINE |

  

|  |  |  |  |  |  |  |  |  |  |
| --- | --- | --- | --- | --- | --- | --- | --- | --- | --- |
| chr8:116509-117082 | | | 423 bp | | |  |  |  |  |
| Genome Start | Genome End | Strand | Div | Repeat Start | Repeat End | Repeat Left | Name | Family | Class |
| 116509 | 116637 | + | 28.4 | 866 | 995 | -1014 | HAL1b | L1 | LINE |
| 116718 | 116862 | + | 14.3 | 1 | 133 | 0 | FLAM\_C | Alu | SINE |
| 116934 | 117082 | + | 28.9 | 1398 | 1552 | -457 | HAL1b | L1 | LINE |

  

|  |  |  |  |  |  |  |  |  |  |
| --- | --- | --- | --- | --- | --- | --- | --- | --- | --- |
| chr8:118589-121388 | | | 2796 bp | | |  |  |  |  |
| Genome Start | Genome End | Strand | Div | Repeat Start | Repeat End | Repeat Left | Name | Family | Class |
| 118589 | 118829 | + | 29.3 | 1 | 249 | -306 | MLT1H1 | MaLR | LTR |
| 118830 | 119187 | - | 18.6 | 1 | 364 | 0 | THE1B | MaLR | LTR |
| 119188 | 120747 | - | 30.7 | 4 | 1735 | 0 | MLT-int | MaLR | LTR |
| 120752 | 121111 | - | 15.5 | 3 | 364 | 0 | THE1B | MaLR | LTR |
| 121112 | 121388 | + | 29.3 | 250 | 522 | -33 | MLT1H1 | MaLR | LTR |

  

|  |  |  |  |  |  |  |  |  |  |
| --- | --- | --- | --- | --- | --- | --- | --- | --- | --- |
| chr8:128394-130502 | | | 2114 bp | | |  |  |  |  |
| Genome Start | Genome End | Strand | Div | Repeat Start | Repeat End | Repeat Left | Name | Family | Class |
| 128394 | 128590 | + | 23.9 | 3794 | 3982 | -2164 | L1ME3B | L1 | LINE |
| 128584 | 128970 | + | 32.3 | 4505 | 4926 | -1220 | L1ME3B | L1 | LINE |
| 128973 | 129462 | + | 23.0 | 1 | 495 | -10 | MLT1D | MaLR | LTR |
| 129463 | 130502 | + | 27.2 | 4923 | 6059 | -181 | L1ME3B | L1 | LINE |

  

|  |  |  |  |  |  |  |  |  |  |
| --- | --- | --- | --- | --- | --- | --- | --- | --- | --- |
| chr8:139825-140356 | | | 532 bp | | |  |  |  |  |
| Genome Start | Genome End | Strand | Div | Repeat Start | Repeat End | Repeat Left | Name | Family | Class |
| 139825 | 139932 | - | 31.9 | 3313 | 3414 | -5 | L2 | L2 | LINE |
| 139933 | 140231 | - | 11.7 | 1 | 299 | -14 | AluSq | Alu | SINE |
| 140232 | 140356 | - | 31.9 | 3190 | 3312 | -107 | L2 | L2 | LINE |

  

|  |  |  |  |  |  |  |  |  |  |
| --- | --- | --- | --- | --- | --- | --- | --- | --- | --- |
| chr8:148103-149475 | | | 1363 bp | | |  |  |  |  |
| Genome Start | Genome End | Strand | Div | Repeat Start | Repeat End | Repeat Left | Name | Family | Class |
| 148103 | 148713 | + | 21.8 | 4898 | 5546 | -2239 | L1MC | L1 | LINE |
| 148719 | 149279 | + | 16.6 | 1 | 583 | -2 | MER34C\_ | ERV1 | LTR |
| 149285 | 149475 | + | 28.6 | 5577 | 5768 | -2017 | L1MC | L1 | LINE |

  

|  |  |  |  |  |  |  |  |  |  |
| --- | --- | --- | --- | --- | --- | --- | --- | --- | --- |
| chr8:149686-150685 | | | 1000 bp | | |  |  |  |  |
| Genome Start | Genome End | Strand | Div | Repeat Start | Repeat End | Repeat Left | Name | Family | Class |
| 149686 | 150332 | + | 15.5 | 1 | 683 | -39 | MER72 | ERV1 | LTR |
| 150333 | 150632 | + | 11.0 | 11 | 309 | -4 | AluSq | Alu | SINE |
| 150633 | 150685 | + | 15.5 | 684 | 722 | 0 | MER72 | ERV1 | LTR |

  

|  |  |  |  |  |  |  |  |  |  |
| --- | --- | --- | --- | --- | --- | --- | --- | --- | --- |
| chr8:385403-386240 | | | 838 bp | | |  |  |  |  |
| Genome Start | Genome End | Strand | Div | Repeat Start | Repeat End | Repeat Left | Name | Family | Class |
| 385403 | 385570 | - | 29.2 | 359 | 523 | 0 | Charlie1b | MER1\_type | DNA |
| 385571 | 385890 | + | 10.0 | 1 | 302 | -11 | AluSq | Alu | SINE |
| 385891 | 386240 | - | 29.2 | 4 | 358 | -165 | Charlie1b | MER1\_type | DNA |

  

|  |  |  |  |  |  |  |  |  |  |
| --- | --- | --- | --- | --- | --- | --- | --- | --- | --- |
| chr8:2178034-2178750 | | | 717 bp | | |  |  |  |  |
| Genome Start | Genome End | Strand | Div | Repeat Start | Repeat End | Repeat Left | Name | Family | Class |
| 2178034 | 2178205 | + | 15.6 | 1 | 196 | -232 | MSTA | MaLR | LTR |
| 2178206 | 2178513 | + | 16.2 | 1 | 297 | -15 | AluSx | Alu | SINE |
| 2178514 | 2178750 | + | 15.6 | 197 | 427 | -1 | MSTA | MaLR | LTR |

  

|  |  |  |  |  |  |  |  |  |  |
| --- | --- | --- | --- | --- | --- | --- | --- | --- | --- |
| chr8:6828931-6829522 | | | 592 bp | | |  |  |  |  |
| Genome Start | Genome End | Strand | Div | Repeat Start | Repeat End | Repeat Left | Name | Family | Class |
| 6828931 | 6829112 | + | 31.3 | 10 | 193 | -368 | MLT1G3 | MaLR | LTR |
| 6829113 | 6829475 | + | 24.4 | 1 | 363 | -2 | MLT1A0 | MaLR | LTR |
| 6829476 | 6829522 | + | 32.2 | 194 | 236 | -354 | MLT1G3 | MaLR | LTR |

  

|  |  |  |  |  |  |  |  |  |  |
| --- | --- | --- | --- | --- | --- | --- | --- | --- | --- |
| chr8:6923686-6924356 | | | 671 bp | | |  |  |  |  |
| Genome Start | Genome End | Strand | Div | Repeat Start | Repeat End | Repeat Left | Name | Family | Class |
| 6923686 | 6923816 | + | 27.8 | 5832 | 5907 | -282 | L1MB1 | L1 | LINE |
| 6923817 | 6924110 | + | 13.3 | 1 | 296 | -16 | AluSx | Alu | SINE |
| 6924111 | 6924356 | + | 23.9 | 5908 | 6148 | -35 | L1MB1 | L1 | LINE |

  

|  |  |  |  |  |  |  |  |  |  |
| --- | --- | --- | --- | --- | --- | --- | --- | --- | --- |
| chr8:6930841-6932014 | | | 1174 bp | | |  |  |  |  |
| Genome Start | Genome End | Strand | Div | Repeat Start | Repeat End | Repeat Left | Name | Family | Class |
| 6930841 | 6931527 | + | 30.3 | 2348 | 3193 | -226 | L2 | L2 | LINE |
| 6931528 | 6931830 | - | 14.3 | 1 | 303 | -10 | AluSq | Alu | SINE |
| 6931831 | 6932014 | + | 33.9 | 3194 | 3376 | -43 | L2 | L2 | LINE |

  

|  |  |  |  |  |  |  |  |  |  |
| --- | --- | --- | --- | --- | --- | --- | --- | --- | --- |
| chr8:6934534-6935341 | | | 787 bp | | |  |  |  |  |
| Genome Start | Genome End | Strand | Div | Repeat Start | Repeat End | Repeat Left | Name | Family | Class |
| 6934534 | 6934593 | + | 36.7 | 15 | 75 | -187 | MIR | MIR | SINE |
| 6934599 | 6935201 | - | 19.0 | 1473 | 2104 | -3610 | ERVL-B4 | ERVL | LTR |
| 6935218 | 6935341 | + | 30.6 | 99 | 224 | -38 | MIR | MIR | SINE |

  

|  |  |  |  |  |  |  |  |  |  |
| --- | --- | --- | --- | --- | --- | --- | --- | --- | --- |
| chr8:6943537-6944342 | | | 806 bp | | |  |  |  |  |
| Genome Start | Genome End | Strand | Div | Repeat Start | Repeat End | Repeat Left | Name | Family | Class |
| 6943537 | 6943857 | - | 12.8 | 5951 | 6288 | -3 | L1MA8 | L1 | LINE |
| 6943858 | 6944166 | - | 17.5 | 1 | 312 | 0 | AluJo | Alu | SINE |
| 6944167 | 6944342 | - | 12.8 | 5775 | 5950 | -341 | L1MA8 | L1 | LINE |

  

|  |  |  |  |  |  |  |  |  |  |
| --- | --- | --- | --- | --- | --- | --- | --- | --- | --- |
| chr8:6952462-6952951 | | | 379 bp | | |  |  |  |  |
| Genome Start | Genome End | Strand | Div | Repeat Start | Repeat End | Repeat Left | Name | Family | Class |
| 6952462 | 6952538 | - | 31.9 | 3347 | 3418 | -1 | L2 | L2 | LINE |
| 6952521 | 6952704 | + | 26.9 | 12 | 210 | -52 | MIR | MIR | SINE |
| 6952834 | 6952951 | - | 37.1 | 3096 | 3215 | -163 | L2 | L2 | LINE |

  

|  |  |  |  |  |  |  |  |  |  |
| --- | --- | --- | --- | --- | --- | --- | --- | --- | --- |
| chr8:6964100-6964586 | | | 487 bp | | |  |  |  |  |
| Genome Start | Genome End | Strand | Div | Repeat Start | Repeat End | Repeat Left | Name | Family | Class |
| 6964100 | 6964189 | + | 22.2 | 5996 | 6078 | -92 | L1ME3A | L1 | LINE |
| 6964190 | 6964498 | - | 15.7 | 1 | 301 | -11 | AluJo | Alu | SINE |
| 6964499 | 6964586 | + | 22.2 | 6079 | 6170 | 0 | L1ME3A | L1 | LINE |

  

|  |  |  |  |  |  |  |  |  |  |
| --- | --- | --- | --- | --- | --- | --- | --- | --- | --- |
| chr8:6966385-6967012 | | | 628 bp | | |  |  |  |  |
| Genome Start | Genome End | Strand | Div | Repeat Start | Repeat End | Repeat Left | Name | Family | Class |
| 6966385 | 6966547 | - | 18.4 | 5491 | 5653 | -521 | L1M4 | L1 | LINE |
| 6966548 | 6966845 | - | 13.5 | 6 | 301 | -11 | AluSx | Alu | SINE |
| 6966846 | 6967012 | - | 17.1 | 5333 | 5490 | -684 | L1M4 | L1 | LINE |

  

|  |  |  |  |  |  |  |  |  |  |
| --- | --- | --- | --- | --- | --- | --- | --- | --- | --- |
| chr8:6976865-6985823 | | | 8981 bp | | |  |  |  |  |
| Genome Start | Genome End | Strand | Div | Repeat Start | Repeat End | Repeat Left | Name | Family | Class |
| 6976865 | 6977453 | - | 14.9 | 6037 | 6629 | -376 | L1MA4A | L1 | LINE |
| 6977428 | 6977688 | - | 11.9 | 4958 | 5219 | -927 | L1PREC2 | L1 | LINE |
| 6977691 | 6978152 | - | 16.6 | 5437 | 5903 | -942 | L1MA4A | L1 | LINE |
| 6978153 | 6978424 | - | 8.7 | 1 | 304 | 0 | AluY | Alu | SINE |
| 6978425 | 6978914 | - | 16.6 | 4977 | 5436 | -1409 | L1MA4A | L1 | LINE |
| 6978915 | 6979213 | - | 15.4 | 1 | 302 | -10 | AluSx | Alu | SINE |
| 6979214 | 6981393 | - | 16.7 | 2780 | 4976 | -1869 | L1MA4A | L1 | LINE |
| 6981394 | 6981746 | + | 15.6 | 1 | 364 | 0 | THE1B | MaLR | LTR |
| 6981749 | 6983298 | + | 31.6 | 2 | 1735 | 0 | MLT-int | MaLR | LTR |
| 6983299 | 6983656 | + | 14.4 | 1 | 364 | 0 | THE1B | MaLR | LTR |
| 6983657 | 6983895 | - | 20.8 | 2527 | 2779 | -4070 | L1MA4A | L1 | LINE |
| 6983896 | 6984054 | - | 17.2 | 5 | 161 | -5 | FRAM | Alu | SINE |
| 6984055 | 6985099 | - | 20.8 | 1441 | 2526 | -4323 | L1MA4A | L1 | LINE |
| 6985100 | 6985404 | - | 12.5 | 1 | 306 | -4 | AluSg | Alu | SINE |
| 6985405 | 6985823 | - | 20.8 | 978 | 1440 | -5409 | L1MA4A | L1 | LINE |

  

|  |  |  |  |  |  |  |  |  |  |
| --- | --- | --- | --- | --- | --- | --- | --- | --- | --- |
| chr8:6989162-6989891 | | | 727 bp | | |  |  |  |  |
| Genome Start | Genome End | Strand | Div | Repeat Start | Repeat End | Repeat Left | Name | Family | Class |
| 6989162 | 6989537 | + | 17.6 | 5575 | 5959 | -219 | L1MB7 | L1 | LINE |
| 6989538 | 6989706 | + | 10.7 | 104 | 285 | -27 | AluSg/x | Alu | SINE |
| 6989710 | 6989891 | + | 24.7 | 5974 | 6158 | -26 | L1MB7 | L1 | LINE |

  

|  |  |  |  |  |  |  |  |  |  |
| --- | --- | --- | --- | --- | --- | --- | --- | --- | --- |
| chr8:6989908-6991221 | | | 1218 bp | | |  |  |  |  |
| Genome Start | Genome End | Strand | Div | Repeat Start | Repeat End | Repeat Left | Name | Family | Class |
| 6989908 | 6990059 | - | 34.6 | 3002 | 3157 | -221 | L2 | L2 | LINE |
| 6990060 | 6990352 | - | 13.3 | 1 | 293 | -20 | AluSq | Alu | SINE |
| 6990353 | 6990454 | - | 34.6 | 2910 | 3001 | -377 | L2 | L2 | LINE |
| 6990495 | 6990585 | + | 27.5 | 95 | 206 | -62 | MIRb | MIR | SINE |
| 6990610 | 6990911 | - | 6.6 | 1 | 302 | -9 | AluY | Alu | SINE |
| 6990944 | 6991221 | - | 32.5 | 2563 | 2860 | -559 | L2 | L2 | LINE |

  

|  |  |  |  |  |  |  |  |  |  |
| --- | --- | --- | --- | --- | --- | --- | --- | --- | --- |
| chr8:7005160-7005782 | | | 623 bp | | |  |  |  |  |
| Genome Start | Genome End | Strand | Div | Repeat Start | Repeat End | Repeat Left | Name | Family | Class |
| 7005160 | 7005234 | - | 26.4 | 340 | 407 | -2 | MLT1I | MaLR | LTR |
| 7005235 | 7005526 | - | 14.1 | 1 | 299 | -13 | AluSx | Alu | SINE |
| 7005527 | 7005782 | - | 26.4 | 87 | 339 | -70 | MLT1I | MaLR | LTR |

  

|  |  |  |  |  |  |  |  |  |  |
| --- | --- | --- | --- | --- | --- | --- | --- | --- | --- |
| chr8:7012991-7014308 | | | 1166 bp | | |  |  |  |  |
| Genome Start | Genome End | Strand | Div | Repeat Start | Repeat End | Repeat Left | Name | Family | Class |
| 7012991 | 7013255 | + | 28.8 | 2563 | 2832 | -587 | L2 | L2 | LINE |
| 7013304 | 7013605 | + | 6.0 | 1 | 300 | -11 | AluY | Alu | SINE |
| 7013669 | 7013722 | - | 27.8 | 95 | 148 | -128 | MIRm | MIR | SINE |
| 7013764 | 7013865 | + | 33.5 | 2910 | 3015 | -404 | L2 | L2 | LINE |
| 7013866 | 7014156 | + | 12.0 | 1 | 291 | -22 | AluSq | Alu | SINE |
| 7014157 | 7014308 | + | 33.5 | 3016 | 3158 | -261 | L2 | L2 | LINE |

  

|  |  |  |  |  |  |  |  |  |  |
| --- | --- | --- | --- | --- | --- | --- | --- | --- | --- |
| chr8:7018401-7027450 | | | 9055 bp | | |  |  |  |  |
| Genome Start | Genome End | Strand | Div | Repeat Start | Repeat End | Repeat Left | Name | Family | Class |
| 7018401 | 7018818 | + | 19.8 | 978 | 1440 | -5409 | L1MA4A | L1 | LINE |
| 7018819 | 7019129 | + | 11.3 | 1 | 310 | 0 | AluSg | Alu | SINE |
| 7019130 | 7020174 | + | 19.8 | 1441 | 2522 | -4327 | L1MA4A | L1 | LINE |
| 7020175 | 7020346 | + | 20.6 | 1 | 172 | 0 | FRAM | Alu | SINE |
| 7020357 | 7020567 | + | 19.8 | 2523 | 2768 | -4081 | L1MA4A | L1 | LINE |
| 7020568 | 7020925 | - | 13.3 | 1 | 364 | 0 | THE1B | MaLR | LTR |
| 7020926 | 7022496 | - | 31.2 | 2 | 1735 | 0 | MLT-int | MaLR | LTR |
| 7022499 | 7022852 | - | 15.0 | 1 | 364 | 0 | THE1B | MaLR | LTR |
| 7022853 | 7025043 | + | 17.2 | 2769 | 4969 | -1876 | L1MA4A | L1 | LINE |
| 7025044 | 7025351 | + | 13.0 | 1 | 311 | -1 | AluSx | Alu | SINE |
| 7025352 | 7025845 | + | 17.1 | 4970 | 5450 | -1395 | L1MA4A | L1 | LINE |
| 7025846 | 7026165 | + | 8.0 | 1 | 303 | -8 | AluY | Alu | SINE |
| 7026166 | 7026624 | + | 17.1 | 5451 | 5905 | -940 | L1MA4A | L1 | LINE |
| 7026625 | 7026870 | + | 11.4 | 4958 | 5204 | -942 | L1PREC2 | L1 | LINE |
| 7026854 | 7027450 | + | 15.9 | 6029 | 6633 | -372 | L1MA4A | L1 | LINE |

  

|  |  |  |  |  |  |  |  |  |  |
| --- | --- | --- | --- | --- | --- | --- | --- | --- | --- |
| chr8:7027541-7030017 | | | 2435 bp | | |  |  |  |  |
| Genome Start | Genome End | Strand | Div | Repeat Start | Repeat End | Repeat Left | Name | Family | Class |
| 7027541 | 7027717 | + | 16.5 | 6632 | 6827 | -175 | L1MA4 | L1 | LINE |
| 7027719 | 7028368 | - | 10.1 | 5493 | 6151 | 0 | L1PB1 | L1 | LINE |
| 7028401 | 7028686 | - | 10.5 | 1 | 286 | -24 | AluSg | Alu | SINE |
| 7028687 | 7029836 | - | 9.6 | 4294 | 5492 | -659 | L1PB1 | L1 | LINE |
| 7029846 | 7030017 | + | 16.3 | 6814 | 6990 | -12 | L1MA4 | L1 | LINE |

  

|  |  |  |  |  |  |  |  |  |  |
| --- | --- | --- | --- | --- | --- | --- | --- | --- | --- |
| chr8:7037288-7037920 | | | 633 bp | | |  |  |  |  |
| Genome Start | Genome End | Strand | Div | Repeat Start | Repeat End | Repeat Left | Name | Family | Class |
| 7037288 | 7037449 | + | 17.8 | 5333 | 5490 | -684 | L1M4 | L1 | LINE |
| 7037450 | 7037757 | + | 12.1 | 1 | 306 | -6 | AluSx | Alu | SINE |
| 7037758 | 7037920 | + | 18.7 | 5491 | 5653 | -521 | L1M4 | L1 | LINE |

  

|  |  |  |  |  |  |  |  |  |  |
| --- | --- | --- | --- | --- | --- | --- | --- | --- | --- |
| chr8:7059601-7060412 | | | 812 bp | | |  |  |  |  |
| Genome Start | Genome End | Strand | Div | Repeat Start | Repeat End | Repeat Left | Name | Family | Class |
| 7059601 | 7059776 | + | 12.2 | 5775 | 5950 | -341 | L1MA8 | L1 | LINE |
| 7059777 | 7060085 | + | 17.8 | 1 | 310 | -2 | AluJo | Alu | SINE |
| 7060086 | 7060412 | + | 12.2 | 5951 | 6288 | -3 | L1MA8 | L1 | LINE |

  

|  |  |  |  |  |  |  |  |  |  |
| --- | --- | --- | --- | --- | --- | --- | --- | --- | --- |
| chr8:7077983-7079166 | | | 1184 bp | | |  |  |  |  |
| Genome Start | Genome End | Strand | Div | Repeat Start | Repeat End | Repeat Left | Name | Family | Class |
| 7077983 | 7078163 | - | 35.6 | 3194 | 3374 | -45 | L2 | L2 | LINE |
| 7078164 | 7078476 | + | 14.5 | 1 | 312 | 0 | AluSx | Alu | SINE |
| 7078477 | 7079166 | - | 30.9 | 2344 | 3193 | -226 | L2 | L2 | LINE |

  

|  |  |  |  |  |  |  |  |  |  |
| --- | --- | --- | --- | --- | --- | --- | --- | --- | --- |
| chr8:7082121-7083017 | | | 721 bp | | |  |  |  |  |
| Genome Start | Genome End | Strand | Div | Repeat Start | Repeat End | Repeat Left | Name | Family | Class |
| 7082121 | 7082240 | + | 31.9 | 2424 | 2547 | -3599 | L1M5 | L1 | LINE |
| 7082255 | 7082632 | + | 24.3 | 1 | 448 | -2 | MLT1J2 | MaLR | LTR |
| 7082795 | 7083017 | + | 24.2 | 2716 | 2945 | -3201 | L1M5 | L1 | LINE |

  

|  |  |  |  |  |  |  |  |  |  |
| --- | --- | --- | --- | --- | --- | --- | --- | --- | --- |
| chr8:7087114-7089070 | | | 1957 bp | | |  |  |  |  |
| Genome Start | Genome End | Strand | Div | Repeat Start | Repeat End | Repeat Left | Name | Family | Class |
| 7087114 | 7087365 | - | 24.5 | 5907 | 6153 | -30 | L1MB3 | L1 | LINE |
| 7087366 | 7087658 | - | 13.0 | 3 | 295 | -17 | AluSx | Alu | SINE |
| 7087659 | 7087770 | - | 24.5 | 5825 | 5906 | -277 | L1MB3 | L1 | LINE |
| 7087771 | 7088084 | - | 15.7 | 1 | 312 | 0 | AluSx | Alu | SINE |
| 7088085 | 7089070 | - | 25.5 | 4818 | 5824 | -359 | L1MB3 | L1 | LINE |

  

|  |  |  |  |  |  |  |  |  |  |
| --- | --- | --- | --- | --- | --- | --- | --- | --- | --- |
| chr8:7089448-7090148 | | | 701 bp | | |  |  |  |  |
| Genome Start | Genome End | Strand | Div | Repeat Start | Repeat End | Repeat Left | Name | Family | Class |
| 7089448 | 7089681 | - | 25.2 | 5941 | 6160 | -140 | L1MA4 | L1 | LINE |
| 7089682 | 7089965 | - | 17.3 | 1 | 283 | -29 | AluSx | Alu | SINE |
| 7089966 | 7090148 | - | 25.2 | 5767 | 5940 | -360 | L1MA4 | L1 | LINE |

  

|  |  |  |  |  |  |  |  |  |  |
| --- | --- | --- | --- | --- | --- | --- | --- | --- | --- |
| chr8:7243007-7246143 | | | 2855 bp | | |  |  |  |  |
| Genome Start | Genome End | Strand | Div | Repeat Start | Repeat End | Repeat Left | Name | Family | Class |
| 7243007 | 7244023 | - | 28.8 | 2278 | 3341 | -2878 | L1MEc | L1 | LINE |
| 7244089 | 7244390 | + | 7.0 | 8 | 310 | -1 | AluY | Alu | SINE |
| 7244497 | 7244591 | - | 21.3 | 1425 | 1525 | -4875 | L1MEc | L1 | LINE |
| 7244592 | 7245014 | + | 18.3 | 1 | 422 | -6 | MSTA | MaLR | LTR |
| 7245126 | 7245617 | - | 28.4 | 800 | 1303 | -5097 | L1MEc | L1 | LINE |
| 7245618 | 7245904 | - | 17.2 | 3 | 298 | -14 | AluSx | Alu | SINE |
| 7245905 | 7246143 | - | 28.4 | 564 | 799 | -5601 | L1MEc | L1 | LINE |

  

|  |  |  |  |  |  |  |  |  |  |
| --- | --- | --- | --- | --- | --- | --- | --- | --- | --- |
| chr8:7270581-7272700 | | | 2120 bp | | |  |  |  |  |
| Genome Start | Genome End | Strand | Div | Repeat Start | Repeat End | Repeat Left | Name | Family | Class |
| 7270581 | 7270870 | - | 28.1 | 1480 | 1850 | -4550 | L1MEc | L1 | LINE |
| 7270871 | 7271181 | - | 12.1 | 1 | 305 | -7 | AluSx | Alu | SINE |
| 7271182 | 7271375 | - | 28.1 | 1263 | 1479 | -4921 | L1MEc | L1 | LINE |
| 7271376 | 7271672 | - | 9.8 | 1 | 296 | -14 | AluSg | Alu | SINE |
| 7271673 | 7272700 | - | 28.1 | 184 | 1262 | -5138 | L1MEc | L1 | LINE |

  

|  |  |  |  |  |  |  |  |  |  |
| --- | --- | --- | --- | --- | --- | --- | --- | --- | --- |
| chr8:7299807-7300851 | | | 1045 bp | | |  |  |  |  |
| Genome Start | Genome End | Strand | Div | Repeat Start | Repeat End | Repeat Left | Name | Family | Class |
| 7299807 | 7300066 | + | 20.0 | 5227 | 5484 | -690 | L1MB5 | L1 | LINE |
| 7300067 | 7300374 | + | 7.8 | 9 | 304 | -6 | AluSg | Alu | SINE |
| 7300375 | 7300851 | + | 18.5 | 5485 | 5951 | -223 | L1MB5 | L1 | LINE |

  

|  |  |  |  |  |  |  |  |  |  |
| --- | --- | --- | --- | --- | --- | --- | --- | --- | --- |
| chr8:7322202-7323143 | | | 942 bp | | |  |  |  |  |
| Genome Start | Genome End | Strand | Div | Repeat Start | Repeat End | Repeat Left | Name | Family | Class |
| 7322202 | 7322774 | + | 27.6 | 3 | 573 | -5472 | L1MCb | L1 | LINE |
| 7322775 | 7323056 | + | 14.3 | 1 | 291 | -19 | AluSg | Alu | SINE |
| 7323057 | 7323143 | + | 27.6 | 574 | 659 | -5386 | L1MCb | L1 | LINE |

  

|  |  |  |  |  |  |  |  |  |  |
| --- | --- | --- | --- | --- | --- | --- | --- | --- | --- |
| chr8:7439294-7439994 | | | 701 bp | | |  |  |  |  |
| Genome Start | Genome End | Strand | Div | Repeat Start | Repeat End | Repeat Left | Name | Family | Class |
| 7439294 | 7439476 | + | 24.9 | 5767 | 5940 | -360 | L1MA4 | L1 | LINE |
| 7439477 | 7439760 | + | 17.3 | 1 | 283 | -29 | AluSx | Alu | SINE |
| 7439761 | 7439994 | + | 24.9 | 5941 | 6160 | -140 | L1MA4 | L1 | LINE |

  

|  |  |  |  |  |  |  |  |  |  |
| --- | --- | --- | --- | --- | --- | --- | --- | --- | --- |
| chr8:7440372-7442322 | | | 1951 bp | | |  |  |  |  |
| Genome Start | Genome End | Strand | Div | Repeat Start | Repeat End | Repeat Left | Name | Family | Class |
| 7440372 | 7441356 | + | 25.4 | 4818 | 5824 | -359 | L1MB3 | L1 | LINE |
| 7441357 | 7441670 | + | 16.4 | 1 | 312 | 0 | AluSx | Alu | SINE |
| 7441671 | 7441778 | + | 24.4 | 5825 | 5906 | -277 | L1MB3 | L1 | LINE |
| 7441779 | 7442075 | + | 13.1 | 3 | 299 | -13 | AluSx | Alu | SINE |
| 7442076 | 7442322 | + | 24.4 | 5907 | 6153 | -30 | L1MB3 | L1 | LINE |

  

|  |  |  |  |  |  |  |  |  |  |
| --- | --- | --- | --- | --- | --- | --- | --- | --- | --- |
| chr8:7446395-7447287 | | | 893 bp | | |  |  |  |  |
| Genome Start | Genome End | Strand | Div | Repeat Start | Repeat End | Repeat Left | Name | Family | Class |
| 7446395 | 7446775 | - | 29.8 | 2558 | 2945 | -3201 | L1M5 | L1 | LINE |
| 7446776 | 7447153 | - | 25.2 | 1 | 448 | -2 | MLT1J2 | MaLR | LTR |
| 7447154 | 7447287 | - | 29.8 | 2424 | 2557 | -3589 | L1M5 | L1 | LINE |

  

|  |  |  |  |  |  |  |  |  |  |
| --- | --- | --- | --- | --- | --- | --- | --- | --- | --- |
| chr8:7450243-7451427 | | | 1185 bp | | |  |  |  |  |
| Genome Start | Genome End | Strand | Div | Repeat Start | Repeat End | Repeat Left | Name | Family | Class |
| 7450243 | 7450935 | + | 31.8 | 2344 | 3193 | -226 | L2 | L2 | LINE |
| 7450936 | 7451248 | - | 15.2 | 1 | 312 | 0 | AluSx | Alu | SINE |
| 7451249 | 7451427 | + | 34.7 | 3194 | 3374 | -45 | L2 | L2 | LINE |

  

|  |  |  |  |  |  |  |  |  |  |
| --- | --- | --- | --- | --- | --- | --- | --- | --- | --- |
| chr8:7568200-7569008 | | | 809 bp | | |  |  |  |  |
| Genome Start | Genome End | Strand | Div | Repeat Start | Repeat End | Repeat Left | Name | Family | Class |
| 7568200 | 7568375 | + | 11.8 | 5775 | 5950 | -341 | L1MA8 | L1 | LINE |
| 7568376 | 7568685 | + | 17.1 | 1 | 312 | 0 | AluJo | Alu | SINE |
| 7568686 | 7569008 | + | 11.8 | 5951 | 6288 | -3 | L1MA8 | L1 | LINE |

  

|  |  |  |  |  |  |  |  |  |  |
| --- | --- | --- | --- | --- | --- | --- | --- | --- | --- |
| chr8:7596203-7597614 | | | 1412 bp | | |  |  |  |  |
| Genome Start | Genome End | Strand | Div | Repeat Start | Repeat End | Repeat Left | Name | Family | Class |
| 7596203 | 7596334 | - | 24.5 | 5814 | 5906 | -277 | L1MB3 | L1 | LINE |
| 7596335 | 7596628 | - | 16.4 | 1 | 300 | -12 | AluSx | Alu | SINE |
| 7596629 | 7597614 | - | 26.2 | 4818 | 5831 | -352 | L1MB3 | L1 | LINE |

  

|  |  |  |  |  |  |  |  |  |  |
| --- | --- | --- | --- | --- | --- | --- | --- | --- | --- |
| chr8:7704527-7705124 | | | 598 bp | | |  |  |  |  |
| Genome Start | Genome End | Strand | Div | Repeat Start | Repeat End | Repeat Left | Name | Family | Class |
| 7704527 | 7704682 | - | 15.4 | 137 | 312 | 0 | AluJb | Alu | SINE |
| 7704683 | 7704988 | - | 11.8 | 1 | 305 | -5 | AluSg | Alu | SINE |
| 7704989 | 7705124 | - | 15.4 | 1 | 136 | -176 | AluJb | Alu | SINE |

  

|  |  |  |  |  |  |  |  |  |  |
| --- | --- | --- | --- | --- | --- | --- | --- | --- | --- |
| chr8:7726124-7726898 | | | 775 bp | | |  |  |  |  |
| Genome Start | Genome End | Strand | Div | Repeat Start | Repeat End | Repeat Left | Name | Family | Class |
| 7726124 | 7726357 | - | 22.1 | 4717 | 4947 | -1199 | L1MC | L1 | LINE |
| 7726358 | 7726715 | - | 17.6 | 1 | 364 | 0 | THE1B | MaLR | LTR |
| 7726716 | 7726898 | - | 22.1 | 4506 | 4716 | -1430 | L1MC | L1 | LINE |

  

|  |  |  |  |  |  |  |  |  |  |
| --- | --- | --- | --- | --- | --- | --- | --- | --- | --- |
| chr8:7729545-7730265 | | | 721 bp | | |  |  |  |  |
| Genome Start | Genome End | Strand | Div | Repeat Start | Repeat End | Repeat Left | Name | Family | Class |
| 7729545 | 7729861 | + | 28.7 | 35 | 371 | -93 | LTR16B | ERVL | LTR |
| 7729862 | 7730174 | - | 17.8 | 1 | 307 | -5 | AluJo | Alu | SINE |
| 7730175 | 7730265 | + | 28.7 | 372 | 464 | 0 | LTR16B | ERVL | LTR |

  

|  |  |  |  |  |  |  |  |  |  |
| --- | --- | --- | --- | --- | --- | --- | --- | --- | --- |
| chr8:7797198-7804706 | | | 7511 bp | | |  |  |  |  |
| Genome Start | Genome End | Strand | Div | Repeat Start | Repeat End | Repeat Left | Name | Family | Class |
| 7797198 | 7798348 | - | 19.7 | 477 | 1651 | 0 | MSTB-int | MaLR | LTR |
| 7798351 | 7798803 | - | 6.5 | 1 | 448 | -2 | LTR7 | ERV1 | LTR |
| 7798804 | 7799183 | - | 7.7 | 7322 | 7713 | 0 | HERVH | ERV1 | LTR |
| 7799182 | 7799397 | - | 13.9 | 5600 | 5819 | -1894 | HERVH | ERV1 | LTR |
| 7799399 | 7800058 | - | 14.8 | 4488 | 5149 | -2564 | HERVH | ERV1 | LTR |
| 7800061 | 7800551 | - | 11.0 | 3500 | 3993 | -3720 | HERVH | ERV1 | LTR |
| 7800551 | 7800696 | - | 17.9 | 3140 | 3290 | -4423 | HERVH | ERV1 | LTR |
| 7800691 | 7803783 | - | 9.9 | 1 | 2987 | -4726 | HERVH | ERV1 | LTR |
| 7803786 | 7804236 | - | 8.3 | 1 | 448 | -2 | LTR7 | ERV1 | LTR |
| 7804237 | 7804706 | - | 20.9 | 1 | 481 | -1170 | MSTB-int | MaLR | LTR |

  

|  |  |  |  |  |  |  |  |  |  |
| --- | --- | --- | --- | --- | --- | --- | --- | --- | --- |
| chr8:7820564-7821035 | | | 472 bp | | |  |  |  |  |
| Genome Start | Genome End | Strand | Div | Repeat Start | Repeat End | Repeat Left | Name | Family | Class |
| 7820564 | 7820600 | + | 21.3 | 1666 | 1700 | -1719 | L2 | L2 | LINE |
| 7820601 | 7820956 | - | 13.1 | 1 | 380 | -1 | THE1D | MaLR | LTR |
| 7820957 | 7821035 | + | 21.3 | 1701 | 1780 | -1639 | L2 | L2 | LINE |

  

|  |  |  |  |  |  |  |  |  |  |
| --- | --- | --- | --- | --- | --- | --- | --- | --- | --- |
| chr8:7858838-7861340 | | | 2468 bp | | |  |  |  |  |
| Genome Start | Genome End | Strand | Div | Repeat Start | Repeat End | Repeat Left | Name | Family | Class |
| 7858838 | 7859680 | - | 8.1 | 5315 | 6168 | 0 | L1PA10 | L1 | LINE |
| 7859690 | 7859980 | - | 16.9 | 5 | 294 | -18 | AluSx | Alu | SINE |
| 7860007 | 7861340 | - | 11.3 | 3693 | 5040 | -1106 | L1PA10 | L1 | LINE |

  

|  |  |  |  |  |  |  |  |  |  |
| --- | --- | --- | --- | --- | --- | --- | --- | --- | --- |
| chr8:7937290-7937990 | | | 701 bp | | |  |  |  |  |
| Genome Start | Genome End | Strand | Div | Repeat Start | Repeat End | Repeat Left | Name | Family | Class |
| 7937290 | 7937472 | + | 24.9 | 5767 | 5940 | -360 | L1MA4 | L1 | LINE |
| 7937473 | 7937756 | + | 17.3 | 1 | 283 | -29 | AluSx | Alu | SINE |
| 7937757 | 7937990 | + | 24.9 | 5941 | 6160 | -140 | L1MA4 | L1 | LINE |

  

|  |  |  |  |  |  |  |  |  |  |
| --- | --- | --- | --- | --- | --- | --- | --- | --- | --- |
| chr8:7938368-7940316 | | | 1949 bp | | |  |  |  |  |
| Genome Start | Genome End | Strand | Div | Repeat Start | Repeat End | Repeat Left | Name | Family | Class |
| 7938368 | 7939352 | + | 25.4 | 4818 | 5824 | -359 | L1MB3 | L1 | LINE |
| 7939353 | 7939666 | + | 16.1 | 1 | 312 | 0 | AluSx | Alu | SINE |
| 7939667 | 7939774 | + | 24.4 | 5825 | 5906 | -277 | L1MB3 | L1 | LINE |
| 7939775 | 7940072 | + | 13.4 | 3 | 300 | -12 | AluSx | Alu | SINE |
| 7940073 | 7940316 | + | 24.4 | 5907 | 6151 | -32 | L1MB3 | L1 | LINE |

  

|  |  |  |  |  |  |  |  |  |  |
| --- | --- | --- | --- | --- | --- | --- | --- | --- | --- |
| chr8:7944376-7945272 | | | 897 bp | | |  |  |  |  |
| Genome Start | Genome End | Strand | Div | Repeat Start | Repeat End | Repeat Left | Name | Family | Class |
| 7944376 | 7944760 | - | 29.6 | 2558 | 2945 | -3201 | L1M5 | L1 | LINE |
| 7944761 | 7945138 | - | 25.2 | 1 | 448 | -2 | MLT1J2 | MaLR | LTR |
| 7945139 | 7945272 | - | 29.6 | 2424 | 2557 | -3589 | L1M5 | L1 | LINE |

  

|  |  |  |  |  |  |  |  |  |  |
| --- | --- | --- | --- | --- | --- | --- | --- | --- | --- |
| chr8:7948232-7949415 | | | 1184 bp | | |  |  |  |  |
| Genome Start | Genome End | Strand | Div | Repeat Start | Repeat End | Repeat Left | Name | Family | Class |
| 7948232 | 7948921 | + | 31.9 | 2348 | 3208 | -211 | L2 | L2 | LINE |
| 7948922 | 7949234 | - | 14.8 | 1 | 312 | 0 | AluSx | Alu | SINE |
| 7949235 | 7949415 | + | 34.4 | 3209 | 3374 | -45 | L2 | L2 | LINE |

  

|  |  |  |  |  |  |  |  |  |  |
| --- | --- | --- | --- | --- | --- | --- | --- | --- | --- |
| chr8:7958087-7958893 | | | 793 bp | | |  |  |  |  |
| Genome Start | Genome End | Strand | Div | Repeat Start | Repeat End | Repeat Left | Name | Family | Class |
| 7958087 | 7958146 | + | 33.3 | 15 | 75 | -187 | MIR | MIR | SINE |
| 7958152 | 7958753 | - | 19.6 | 1473 | 2104 | -3610 | ERVL-B4 | ERVL | LTR |
| 7958763 | 7958893 | + | 28.2 | 92 | 224 | -38 | MIR | MIR | SINE |

  

|  |  |  |  |  |  |  |  |  |  |
| --- | --- | --- | --- | --- | --- | --- | --- | --- | --- |
| chr8:7987106-7987646 | | | 521 bp | | |  |  |  |  |
| Genome Start | Genome End | Strand | Div | Repeat Start | Repeat End | Repeat Left | Name | Family | Class |
| 7987106 | 7987252 | + | 27.0 | 5925 | 6080 | -90 | L1ME3A | L1 | LINE |
| 7987273 | 7987569 | - | 16.8 | 6 | 292 | -20 | AluJo | Alu | SINE |
| 7987570 | 7987646 | + | 27.0 | 6081 | 6158 | -12 | L1ME3A | L1 | LINE |

  

|  |  |  |  |  |  |  |  |  |  |
| --- | --- | --- | --- | --- | --- | --- | --- | --- | --- |
| chr8:7989454-7990085 | | | 632 bp | | |  |  |  |  |
| Genome Start | Genome End | Strand | Div | Repeat Start | Repeat End | Repeat Left | Name | Family | Class |
| 7989454 | 7989616 | - | 18.4 | 5491 | 5653 | -521 | L1M4 | L1 | LINE |
| 7989617 | 7989923 | - | 12.5 | 1 | 305 | -7 | AluSx | Alu | SINE |
| 7989924 | 7990085 | - | 17.7 | 5333 | 5490 | -684 | L1M4 | L1 | LINE |

  

|  |  |  |  |  |  |  |  |  |  |
| --- | --- | --- | --- | --- | --- | --- | --- | --- | --- |
| chr8:7997347-7999823 | | | 2435 bp | | |  |  |  |  |
| Genome Start | Genome End | Strand | Div | Repeat Start | Repeat End | Repeat Left | Name | Family | Class |
| 7997347 | 7997518 | - | 15.7 | 6112 | 6288 | -12 | L1MA4 | L1 | LINE |
| 7997528 | 7998677 | + | 9.5 | 4294 | 5489 | -662 | L1PB1 | L1 | LINE |
| 7998678 | 7998963 | + | 10.1 | 1 | 286 | -24 | AluSg | Alu | SINE |
| 7998996 | 7999645 | + | 10.0 | 5490 | 6148 | -3 | L1PB1 | L1 | LINE |
| 7999647 | 7999823 | - | 15.9 | 5930 | 6125 | -175 | L1MA4 | L1 | LINE |

  

|  |  |  |  |  |  |  |  |  |  |
| --- | --- | --- | --- | --- | --- | --- | --- | --- | --- |
| chr8:7999914-8008945 | | | 9014 bp | | |  |  |  |  |
| Genome Start | Genome End | Strand | Div | Repeat Start | Repeat End | Repeat Left | Name | Family | Class |
| 7999914 | 8000511 | - | 15.9 | 6029 | 6633 | -372 | L1MA4A | L1 | LINE |
| 8000495 | 8000740 | - | 11.4 | 4958 | 5204 | -942 | L1PREC2 | L1 | LINE |
| 8000741 | 8001199 | - | 17.4 | 5451 | 5905 | -940 | L1MA4A | L1 | LINE |
| 8001200 | 8001514 | - | 8.0 | 1 | 303 | -8 | AluY | Alu | SINE |
| 8001515 | 8002007 | - | 17.4 | 4970 | 5450 | -1395 | L1MA4A | L1 | LINE |
| 8002008 | 8002315 | - | 12.7 | 1 | 311 | -1 | AluSx | Alu | SINE |
| 8002316 | 8004495 | - | 17.4 | 2775 | 4969 | -1876 | L1MA4A | L1 | LINE |
| 8004496 | 8004849 | + | 15.6 | 1 | 364 | 0 | THE1B | MaLR | LTR |
| 8004852 | 8006422 | + | 31.3 | 2 | 1735 | 0 | MLT-int | MaLR | LTR |
| 8006423 | 8006780 | + | 13.6 | 1 | 364 | 0 | THE1B | MaLR | LTR |
| 8006781 | 8006991 | - | 19.8 | 2534 | 2774 | -4075 | L1MA4A | L1 | LINE |
| 8007025 | 8007173 | - | 20.6 | 2 | 150 | -16 | FRAM | Alu | SINE |
| 8007174 | 8008217 | - | 19.8 | 1451 | 2533 | -4316 | L1MA4A | L1 | LINE |
| 8008218 | 8008527 | - | 11.0 | 1 | 309 | -1 | AluSg | Alu | SINE |
| 8008528 | 8008945 | - | 19.8 | 985 | 1450 | -5399 | L1MA4A | L1 | LINE |

  

|  |  |  |  |  |  |  |  |  |  |
| --- | --- | --- | --- | --- | --- | --- | --- | --- | --- |
| chr8:8012283-8013038 | | | 753 bp | | |  |  |  |  |
| Genome Start | Genome End | Strand | Div | Repeat Start | Repeat End | Repeat Left | Name | Family | Class |
| 8012283 | 8012658 | + | 18.6 | 5575 | 5959 | -219 | L1MB7 | L1 | LINE |
| 8012659 | 8012831 | + | 9.2 | 104 | 289 | -23 | AluSg/x | Alu | SINE |
| 8012835 | 8013038 | + | 21.6 | 5974 | 6184 | 0 | L1MB7 | L1 | LINE |

  

|  |  |  |  |  |  |  |  |  |  |
| --- | --- | --- | --- | --- | --- | --- | --- | --- | --- |
| chr8:8021561-8022182 | | | 622 bp | | |  |  |  |  |
| Genome Start | Genome End | Strand | Div | Repeat Start | Repeat End | Repeat Left | Name | Family | Class |
| 8021561 | 8021816 | + | 26.7 | 87 | 339 | -70 | MLT1I | MaLR | LTR |
| 8021817 | 8022107 | + | 14.1 | 1 | 299 | -13 | AluSx | Alu | SINE |
| 8022108 | 8022182 | + | 26.7 | 340 | 407 | -2 | MLT1I | MaLR | LTR |

  

|  |  |  |  |  |  |  |  |  |  |
| --- | --- | --- | --- | --- | --- | --- | --- | --- | --- |
| chr8:8041865-8043164 | | | 1300 bp | | |  |  |  |  |
| Genome Start | Genome End | Strand | Div | Repeat Start | Repeat End | Repeat Left | Name | Family | Class |
| 8041865 | 8042225 | - | 26.0 | 194 | 549 | 0 | MLT1H | MaLR | LTR |
| 8042226 | 8042672 | - | 13.2 | 45 | 470 | -2 | MER4A1 | ERV1 | LTR |
| 8042673 | 8042953 | - | 12.1 | 1 | 280 | -32 | AluSx | Alu | SINE |
| 8042954 | 8042996 | - | 13.2 | 2 | 44 | -428 | MER4A1 | ERV1 | LTR |
| 8042997 | 8043164 | - | 27.5 | 12 | 193 | -356 | MLT1H | MaLR | LTR |

  

|  |  |  |  |  |  |  |  |  |  |
| --- | --- | --- | --- | --- | --- | --- | --- | --- | --- |
| chr8:8043933-8045033 | | | 942 bp | | |  |  |  |  |
| Genome Start | Genome End | Strand | Div | Repeat Start | Repeat End | Repeat Left | Name | Family | Class |
| 8043933 | 8044197 | + | 28.3 | 176 | 429 | -162 | MLT1K | MaLR | LTR |
| 8044321 | 8044626 | + | 12.4 | 1 | 308 | -2 | AluSg | Alu | SINE |
| 8044632 | 8044935 | + | 28.6 | 164 | 474 | -17 | LTR16C | ERVL | LTR |
| 8044967 | 8045033 | + | 21.2 | 521 | 590 | -1 | MLT1K | MaLR | LTR |

  

|  |  |  |  |  |  |  |  |  |  |
| --- | --- | --- | --- | --- | --- | --- | --- | --- | --- |
| chr8:8056564-8057412 | | | 849 bp | | |  |  |  |  |
| Genome Start | Genome End | Strand | Div | Repeat Start | Repeat End | Repeat Left | Name | Family | Class |
| 8056564 | 8056833 | - | 19.9 | 322 | 654 | 0 | MLT1E1 | MaLR | LTR |
| 8056834 | 8057145 | + | 10.9 | 1 | 312 | 0 | AluSx | Alu | SINE |
| 8057146 | 8057412 | - | 19.9 | 4 | 321 | -333 | MLT1E1 | MaLR | LTR |

  

|  |  |  |  |  |  |  |  |  |  |
| --- | --- | --- | --- | --- | --- | --- | --- | --- | --- |
| chr8:8058006-8058888 | | | 877 bp | | |  |  |  |  |
| Genome Start | Genome End | Strand | Div | Repeat Start | Repeat End | Repeat Left | Name | Family | Class |
| 8058006 | 8058385 | - | 23.9 | 82 | 467 | 0 | MLT1C | MaLR | LTR |
| 8058391 | 8058795 | - | 10.7 | 1 | 406 | 0 | AluSp | Alu | SINE |
| 8058797 | 8058888 | - | 17.4 | 3 | 97 | -370 | MLT1C | MaLR | LTR |

  

|  |  |  |  |  |  |  |  |  |  |
| --- | --- | --- | --- | --- | --- | --- | --- | --- | --- |
| chr8:8063068-8079294 | | | 16193 bp | | |  |  |  |  |
| Genome Start | Genome End | Strand | Div | Repeat Start | Repeat End | Repeat Left | Name | Family | Class |
| 8063068 | 8063133 | - | 25.1 | 393 | 484 | 0 | MLT1D | MaLR | LTR |
| 8063134 | 8063442 | + | 15.3 | 2 | 308 | -4 | AluSx | Alu | SINE |
| 8063443 | 8063637 | - | 25.1 | 193 | 432 | -73 | MLT1D | MaLR | LTR |
| 8063655 | 8064006 | + | 8.7 | 1 | 448 | -2 | LTR7 | ERV1 | LTR |
| 8064009 | 8066902 | + | 9.8 | 1 | 2993 | -4720 | HERVH | ERV1 | LTR |
| 8066888 | 8067192 | + | 11.1 | 3140 | 3441 | -4272 | HERVH | ERV1 | LTR |
| 8067193 | 8075019 | + | 6.9 | 3 | 7812 | -1 | HERVE | ERV1 | LTR |
| 8075020 | 8075516 | + | 9.5 | 1 | 501 | 0 | LTR2C | ERV1 | LTR |
| 8075517 | 8076070 | + | 8.9 | 3442 | 3993 | -3720 | HERVH | ERV1 | LTR |
| 8076073 | 8076675 | + | 14.1 | 4488 | 5089 | -2624 | HERVH | ERV1 | LTR |
| 8076676 | 8077742 | + | 4.7 | 1 | 1071 | 0 | MER11C | ERVK | LTR |
| 8077743 | 8077808 | + | 14.1 | 5090 | 5149 | -2564 | HERVH | ERV1 | LTR |
| 8077810 | 8078016 | + | 12.1 | 5600 | 5819 | -1894 | HERVH | ERV1 | LTR |
| 8078017 | 8078393 | + | 9.1 | 7324 | 7713 | 0 | HERVH | ERV1 | LTR |
| 8078395 | 8078788 | + | 9.2 | 2 | 444 | -6 | LTR7 | ERV1 | LTR |
| 8078805 | 8079103 | - | 10.0 | 3 | 302 | -11 | AluSp | Alu | SINE |
| 8079114 | 8079294 | - | 24.0 | 1 | 185 | -320 | MLT1D | MaLR | LTR |

  

|  |  |  |  |  |  |  |  |  |  |
| --- | --- | --- | --- | --- | --- | --- | --- | --- | --- |
| chr8:8083951-8084184 | | | 234 bp | | |  |  |  |  |
| Genome Start | Genome End | Strand | Div | Repeat Start | Repeat End | Repeat Left | Name | Family | Class |
| 8083951 | 8084007 | - | 30.9 | 42 | 97 | -81 | MER5B | MER1\_type | DNA |
| 8084008 | 8084140 | + | 15.8 | 1 | 133 | 0 | FLAM\_A | Alu | SINE |
| 8084141 | 8084184 | - | 30.9 | 4 | 41 | -137 | MER5B | MER1\_type | DNA |

  

|  |  |  |  |  |  |  |  |  |  |
| --- | --- | --- | --- | --- | --- | --- | --- | --- | --- |
| chr8:8085032-8085649 | | | 618 bp | | |  |  |  |  |
| Genome Start | Genome End | Strand | Div | Repeat Start | Repeat End | Repeat Left | Name | Family | Class |
| 8085032 | 8085196 | - | 13.8 | 145 | 299 | -13 | AluSx | Alu | SINE |
| 8085197 | 8085503 | - | 7.8 | 1 | 307 | -4 | AluY | Alu | SINE |
| 8085504 | 8085649 | - | 13.8 | 1 | 144 | -168 | AluSx | Alu | SINE |

  

|  |  |  |  |  |  |  |  |  |  |
| --- | --- | --- | --- | --- | --- | --- | --- | --- | --- |
| chr8:8087431-8088136 | | | 706 bp | | |  |  |  |  |
| Genome Start | Genome End | Strand | Div | Repeat Start | Repeat End | Repeat Left | Name | Family | Class |
| 8087431 | 8087502 | + | 24.4 | 6 | 89 | -378 | MLT1C | MaLR | LTR |
| 8087503 | 8087800 | + | 14.4 | 1 | 300 | -12 | AluSx | Alu | SINE |
| 8087801 | 8088136 | + | 24.4 | 90 | 451 | -16 | MLT1C | MaLR | LTR |

  

|  |  |  |  |  |  |  |  |  |  |
| --- | --- | --- | --- | --- | --- | --- | --- | --- | --- |
| chr8:8089212-8090558 | | | 1313 bp | | |  |  |  |  |
| Genome Start | Genome End | Strand | Div | Repeat Start | Repeat End | Repeat Left | Name | Family | Class |
| 8089212 | 8089268 | + | 26.3 | 1 | 58 | -244 | AluJo | Alu | SINE |
| 8089301 | 8089743 | + | 13.9 | 1 | 442 | -343 | LTR1 | ERV1 | LTR |
| 8089744 | 8089973 | + | 6.5 | 16 | 244 | -67 | AluY | Alu | SINE |
| 8089974 | 8090270 | + | 13.9 | 443 | 784 | -1 | LTR1 | ERV1 | LTR |
| 8090273 | 8090558 | + | 16.9 | 40 | 299 | -13 | AluJo | Alu | SINE |

  

|  |  |  |  |  |  |  |  |  |  |
| --- | --- | --- | --- | --- | --- | --- | --- | --- | --- |
| chr8:8103723-8104239 | | | 517 bp | | |  |  |  |  |
| Genome Start | Genome End | Strand | Div | Repeat Start | Repeat End | Repeat Left | Name | Family | Class |
| 8103723 | 8103868 | + | 21.3 | 8 | 148 | -61 | MER3 | MER1\_type | DNA |
| 8103869 | 8104167 | - | 12.0 | 5 | 306 | -6 | AluSx | Alu | SINE |
| 8104168 | 8104239 | + | 21.3 | 149 | 209 | 0 | MER3 | MER1\_type | DNA |

  

|  |  |  |  |  |  |  |  |  |  |
| --- | --- | --- | --- | --- | --- | --- | --- | --- | --- |
| chr8:8110167-8110729 | | | 563 bp | | |  |  |  |  |
| Genome Start | Genome End | Strand | Div | Repeat Start | Repeat End | Repeat Left | Name | Family | Class |
| 8110167 | 8110310 | + | 18.1 | 1 | 137 | -175 | AluJo | Alu | SINE |
| 8110311 | 8110619 | + | 18.8 | 1 | 309 | -3 | AluSx | Alu | SINE |
| 8110620 | 8110729 | + | 18.1 | 138 | 248 | -64 | AluJo | Alu | SINE |

  

|  |  |  |  |  |  |  |  |  |  |
| --- | --- | --- | --- | --- | --- | --- | --- | --- | --- |
| chr8:8112964-8115133 | | | 2170 bp | | |  |  |  |  |
| Genome Start | Genome End | Strand | Div | Repeat Start | Repeat End | Repeat Left | Name | Family | Class |
| 8112964 | 8113212 | + | 18.7 | 1 | 246 | -66 | AluJb | Alu | SINE |
| 8113213 | 8113650 | + | 16.2 | 1 | 453 | -33 | MER66B | ERV1 | LTR |
| 8113651 | 8115039 | + | 8.7 | 2 | 1578 | 0 | LTR12C | ERV1 | LTR |
| 8115040 | 8115075 | + | 16.2 | 454 | 486 | 0 | MER66B | ERV1 | LTR |
| 8115076 | 8115133 | + | 18.0 | 232 | 286 | -14 | AluJb | Alu | SINE |

  

|  |  |  |  |  |  |  |  |  |  |
| --- | --- | --- | --- | --- | --- | --- | --- | --- | --- |
| chr8:8129796-8130383 | | | 588 bp | | |  |  |  |  |
| Genome Start | Genome End | Strand | Div | Repeat Start | Repeat End | Repeat Left | Name | Family | Class |
| 8129796 | 8129928 | + | 10.2 | 1 | 133 | -177 | AluSg | Alu | SINE |
| 8129929 | 8130228 | + | 9.7 | 1 | 300 | -13 | AluSq | Alu | SINE |
| 8130229 | 8130383 | + | 10.2 | 134 | 298 | -12 | AluSg | Alu | SINE |

  

|  |  |  |  |  |  |  |  |  |  |
| --- | --- | --- | --- | --- | --- | --- | --- | --- | --- |
| chr8:11818996-11820968 | | | 1973 bp | | |  |  |  |  |
| Genome Start | Genome End | Strand | Div | Repeat Start | Repeat End | Repeat Left | Name | Family | Class |
| 11818996 | 11819247 | - | 23.0 | 5908 | 6153 | -30 | L1MB3 | L1 | LINE |
| 11819248 | 11819551 | - | 13.5 | 1 | 304 | -8 | AluSx | Alu | SINE |
| 11819552 | 11819666 | - | 23.0 | 5825 | 5907 | -276 | L1MB3 | L1 | LINE |
| 11819667 | 11819976 | - | 14.0 | 6 | 312 | 0 | AluSx | Alu | SINE |
| 11819977 | 11820968 | - | 23.9 | 4818 | 5824 | -359 | L1MB3 | L1 | LINE |

  

|  |  |  |  |  |  |  |  |  |  |
| --- | --- | --- | --- | --- | --- | --- | --- | --- | --- |
| chr8:11821402-11822072 | | | 671 bp | | |  |  |  |  |
| Genome Start | Genome End | Strand | Div | Repeat Start | Repeat End | Repeat Left | Name | Family | Class |
| 11821402 | 11821605 | - | 22.6 | 5941 | 6130 | -170 | L1MA4 | L1 | LINE |
| 11821606 | 11821889 | - | 15.5 | 1 | 283 | -29 | AluSx | Alu | SINE |
| 11821890 | 11822072 | - | 22.6 | 5771 | 5940 | -360 | L1MA4 | L1 | LINE |

  

|  |  |  |  |  |  |  |  |  |  |
| --- | --- | --- | --- | --- | --- | --- | --- | --- | --- |
| chr8:11900393-11901293 | | | 901 bp | | |  |  |  |  |
| Genome Start | Genome End | Strand | Div | Repeat Start | Repeat End | Repeat Left | Name | Family | Class |
| 11900393 | 11900497 | - | 21.1 | 1628 | 1732 | -4776 | L1MCa | L1 | LINE |
| 11900498 | 11900763 | + | 10.9 | 1 | 291 | -21 | AluSx | Alu | SINE |
| 11900764 | 11901293 | - | 21.1 | 1066 | 1627 | -4881 | L1MCa | L1 | LINE |

  

|  |  |  |  |  |  |  |  |  |  |
| --- | --- | --- | --- | --- | --- | --- | --- | --- | --- |
| chr8:11914938-11916108 | | | 1171 bp | | |  |  |  |  |
| Genome Start | Genome End | Strand | Div | Repeat Start | Repeat End | Repeat Left | Name | Family | Class |
| 11914938 | 11915120 | - | 33.6 | 3195 | 3376 | -43 | L2 | L2 | LINE |
| 11915121 | 11915421 | + | 15.5 | 1 | 299 | -14 | AluSq | Alu | SINE |
| 11915422 | 11916108 | - | 30.9 | 2348 | 3194 | -225 | L2 | L2 | LINE |

  

|  |  |  |  |  |  |  |  |  |  |
| --- | --- | --- | --- | --- | --- | --- | --- | --- | --- |
| chr8:11924615-11926036 | | | 1422 bp | | |  |  |  |  |
| Genome Start | Genome End | Strand | Div | Repeat Start | Repeat End | Repeat Left | Name | Family | Class |
| 11924615 | 11924764 | - | 25.6 | 5814 | 5906 | -277 | L1MB3 | L1 | LINE |
| 11924765 | 11925043 | - | 14.0 | 1 | 279 | -33 | AluSx | Alu | SINE |
| 11925044 | 11926036 | - | 25.1 | 4818 | 5831 | -352 | L1MB3 | L1 | LINE |

  

|  |  |  |  |  |  |  |  |  |  |
| --- | --- | --- | --- | --- | --- | --- | --- | --- | --- |
| chr8:11926480-11927154 | | | 675 bp | | |  |  |  |  |
| Genome Start | Genome End | Strand | Div | Repeat Start | Repeat End | Repeat Left | Name | Family | Class |
| 11926480 | 11926687 | - | 24.0 | 5934 | 6130 | -170 | L1MA4 | L1 | LINE |
| 11926688 | 11926971 | - | 19.8 | 1 | 283 | -29 | AluSx | Alu | SINE |
| 11926972 | 11927154 | - | 24.0 | 5767 | 5933 | -367 | L1MA4 | L1 | LINE |

  

|  |  |  |  |  |  |  |  |  |  |
| --- | --- | --- | --- | --- | --- | --- | --- | --- | --- |
| chr8:11940343-11941250 | | | 908 bp | | |  |  |  |  |
| Genome Start | Genome End | Strand | Div | Repeat Start | Repeat End | Repeat Left | Name | Family | Class |
| 11940343 | 11940447 | - | 21.2 | 1629 | 1732 | -4776 | L1MCa | L1 | LINE |
| 11940448 | 11940716 | + | 12.7 | 1 | 294 | -18 | AluSx | Alu | SINE |
| 11940717 | 11941250 | - | 21.2 | 1066 | 1628 | -4880 | L1MCa | L1 | LINE |

  

|  |  |  |  |  |  |  |  |  |  |
| --- | --- | --- | --- | --- | --- | --- | --- | --- | --- |
| chr8:11948362-11949497 | | | 1136 bp | | |  |  |  |  |
| Genome Start | Genome End | Strand | Div | Repeat Start | Repeat End | Repeat Left | Name | Family | Class |
| 11948362 | 11948661 | + | 35.3 | 2348 | 2641 | -778 | L2 | L2 | LINE |
| 11948662 | 11948974 | - | 14.5 | 1 | 304 | -8 | AluSx | Alu | SINE |
| 11948975 | 11949497 | + | 35.7 | 2642 | 3163 | -256 | L2 | L2 | LINE |

  

|  |  |  |  |  |  |  |  |  |  |
| --- | --- | --- | --- | --- | --- | --- | --- | --- | --- |
| chr8:11952552-11955415 | | | 2864 bp | | |  |  |  |  |
| Genome Start | Genome End | Strand | Div | Repeat Start | Repeat End | Repeat Left | Name | Family | Class |
| 11952552 | 11952771 | + | 17.4 | 1393 | 1612 | -5173 | L1PA13 | L1 | LINE |
| 11952772 | 11953104 | + | 14.7 | 1 | 364 | 0 | THE1B | MaLR | LTR |
| 11953105 | 11953480 | + | 17.4 | 1613 | 1982 | -4803 | L1PA13 | L1 | LINE |
| 11953481 | 11953950 | + | 7.5 | 5685 | 6154 | 0 | L1PA5 | L1 | LINE |
| 11953951 | 11955415 | + | 16.1 | 1983 | 3513 | -3253 | L1PA13 | L1 | LINE |

  

|  |  |  |  |  |  |  |  |  |  |
| --- | --- | --- | --- | --- | --- | --- | --- | --- | --- |
| chr8:11955916-11956894 | | | 979 bp | | |  |  |  |  |
| Genome Start | Genome End | Strand | Div | Repeat Start | Repeat End | Repeat Left | Name | Family | Class |
| 11955916 | 11956175 | + | 30.2 | 2202 | 2469 | -2178 | MER110-int | ERV1 | LTR |
| 11956176 | 11956467 | - | 9.9 | 1 | 310 | -2 | AluSx | Alu | SINE |
| 11956468 | 11956894 | + | 30.2 | 2470 | 2887 | -1760 | MER110-int | ERV1 | LTR |

  

|  |  |  |  |  |  |  |  |  |  |
| --- | --- | --- | --- | --- | --- | --- | --- | --- | --- |
| chr8:11968356-11970615 | | | 2260 bp | | |  |  |  |  |
| Genome Start | Genome End | Strand | Div | Repeat Start | Repeat End | Repeat Left | Name | Family | Class |
| 11968356 | 11968551 | - | 16.5 | 5937 | 6127 | -38 | L1PA15 | L1 | LINE |
| 11968552 | 11968813 | - | 10.7 | 39 | 300 | -11 | AluY | Alu | SINE |
| 11968814 | 11970615 | - | 14.3 | 4139 | 5936 | -229 | L1PA15 | L1 | LINE |

  

|  |  |  |  |  |  |  |  |  |  |
| --- | --- | --- | --- | --- | --- | --- | --- | --- | --- |
| chr8:11982010-11988103 | | | 5899 bp | | |  |  |  |  |
| Genome Start | Genome End | Strand | Div | Repeat Start | Repeat End | Repeat Left | Name | Family | Class |
| 11982010 | 11982202 | - | 28.6 | 4480 | 4687 | -2521 | L1M2 | L1 | LINE |
| 11982381 | 11982535 | - | 30.1 | 3938 | 4104 | -3104 | L1M2 | L1 | LINE |
| 11982543 | 11982933 | - | 31.7 | 3485 | 3879 | -3329 | L1M2 | L1 | LINE |
| 11982915 | 11983110 | - | 11.7 | 4596 | 4796 | -1350 | L1PB | L1 | LINE |
| 11983118 | 11983423 | - | 22.3 | 5551 | 5859 | -325 | L1MB7 | L1 | LINE |
| 11983421 | 11983507 | - | 8.1 | 1003 | 1088 | -5524 | L1PBa1 | L1 | LINE |
| 11983508 | 11984514 | + | 11.2 | 1 | 1130 | -124 | LTR12D | ERV1 | LTR |
| 11984515 | 11985503 | - | 11.6 | 2 | 1005 | -5607 | L1PBa1 | L1 | LINE |
| 11985513 | 11985911 | - | 11.3 | 4993 | 5398 | -748 | L1PREC2 | L1 | LINE |
| 11985925 | 11986031 | - | 18.9 | 2277 | 2382 | -4827 | L1M2 | L1 | LINE |
| 11986035 | 11986531 | - | 26.4 | 1611 | 2158 | -5051 | L1M2 | L1 | LINE |
| 11986532 | 11986837 | - | 8.8 | 13 | 299 | -14 | AluSp | Alu | SINE |
| 11986838 | 11988103 | - | 24.9 | 1 | 1610 | -5599 | L1M2 | L1 | LINE |

  

|  |  |  |  |  |  |  |  |  |  |
| --- | --- | --- | --- | --- | --- | --- | --- | --- | --- |
| chr8:12019579-12022084 | | | 2470 bp | | |  |  |  |  |
| Genome Start | Genome End | Strand | Div | Repeat Start | Repeat End | Repeat Left | Name | Family | Class |
| 12019579 | 12020422 | - | 9.0 | 5315 | 6168 | 0 | L1PA10 | L1 | LINE |
| 12020432 | 12020726 | - | 17.7 | 1 | 294 | -18 | AluSx | Alu | SINE |
| 12020754 | 12022084 | - | 11.3 | 3693 | 5035 | -1111 | L1PA10 | L1 | LINE |

  

|  |  |  |  |  |  |  |  |  |  |
| --- | --- | --- | --- | --- | --- | --- | --- | --- | --- |
| chr8:12049838-12050706 | | | 766 bp | | |  |  |  |  |
| Genome Start | Genome End | Strand | Div | Repeat Start | Repeat End | Repeat Left | Name | Family | Class |
| 12049838 | 12050196 | + | 29.1 | 19 | 378 | -6045 | L1MCc | L1 | LINE |
| 12050197 | 12050207 | - | 13.2 | 282 | 312 | 0 | AluSx | Alu | SINE |
| 12050311 | 12050587 | - | 13.2 | 2 | 281 | -31 | AluSx | Alu | SINE |
| 12050588 | 12050706 | + | 29.1 | 379 | 497 | -5926 | L1MCc | L1 | LINE |

  

|  |  |  |  |  |  |  |  |  |  |
| --- | --- | --- | --- | --- | --- | --- | --- | --- | --- |
| chr8:12082115-12082710 | | | 596 bp | | |  |  |  |  |
| Genome Start | Genome End | Strand | Div | Repeat Start | Repeat End | Repeat Left | Name | Family | Class |
| 12082115 | 12082277 | - | 12.3 | 137 | 308 | -4 | AluSx | Alu | SINE |
| 12082278 | 12082577 | - | 12.3 | 1 | 300 | -13 | AluSq | Alu | SINE |
| 12082578 | 12082710 | - | 12.3 | 1 | 136 | -176 | AluSx | Alu | SINE |

  

|  |  |  |  |  |  |  |  |  |  |
| --- | --- | --- | --- | --- | --- | --- | --- | --- | --- |
| chr8:12095759-12096370 | | | 612 bp | | |  |  |  |  |
| Genome Start | Genome End | Strand | Div | Repeat Start | Repeat End | Repeat Left | Name | Family | Class |
| 12095759 | 12095948 | - | 19.4 | 137 | 305 | -7 | AluJb | Alu | SINE |
| 12095949 | 12096235 | - | 12.6 | 1 | 298 | -14 | AluSx | Alu | SINE |
| 12096236 | 12096370 | - | 19.4 | 1 | 136 | -176 | AluJb | Alu | SINE |

  

|  |  |  |  |  |  |  |  |  |  |
| --- | --- | --- | --- | --- | --- | --- | --- | --- | --- |
| chr8:12096382-12097265 | | | 822 bp | | |  |  |  |  |
| Genome Start | Genome End | Strand | Div | Repeat Start | Repeat End | Repeat Left | Name | Family | Class |
| 12096382 | 12096451 | - | 18.8 | 6120 | 6184 | 0 | L1MB7 | L1 | LINE |
| 12096452 | 12096751 | + | 17.1 | 1 | 298 | -14 | AluJb | Alu | SINE |
| 12096752 | 12096950 | - | 18.8 | 5932 | 6119 | -65 | L1MB7 | L1 | LINE |
| 12097013 | 12097265 | - | 18.8 | 5653 | 5931 | -253 | L1MB7 | L1 | LINE |

  

|  |  |  |  |  |  |  |  |  |  |
| --- | --- | --- | --- | --- | --- | --- | --- | --- | --- |
| chr8:12097439-12099276 | | | 1838 bp | | |  |  |  |  |
| Genome Start | Genome End | Strand | Div | Repeat Start | Repeat End | Repeat Left | Name | Family | Class |
| 12097439 | 12097474 | - | 17.2 | 454 | 486 | 0 | MER66B | ERV1 | LTR |
| 12097475 | 12098838 | - | 8.7 | 2 | 1578 | 0 | LTR12C | ERV1 | LTR |
| 12098839 | 12099276 | - | 17.2 | 1 | 453 | -33 | MER66B | ERV1 | LTR |

  

|  |  |  |  |  |  |  |  |  |  |
| --- | --- | --- | --- | --- | --- | --- | --- | --- | --- |
| chr8:12272791-12275293 | | | 2468 bp | | |  |  |  |  |
| Genome Start | Genome End | Strand | Div | Repeat Start | Repeat End | Repeat Left | Name | Family | Class |
| 12272791 | 12273633 | - | 7.8 | 5315 | 6168 | 0 | L1PA10 | L1 | LINE |
| 12273643 | 12273933 | - | 16.9 | 5 | 294 | -18 | AluSx | Alu | SINE |
| 12273960 | 12275293 | - | 11.4 | 3693 | 5040 | -1106 | L1PA10 | L1 | LINE |

  

|  |  |  |  |  |  |  |  |  |  |
| --- | --- | --- | --- | --- | --- | --- | --- | --- | --- |
| chr8:12331358-12331950 | | | 593 bp | | |  |  |  |  |
| Genome Start | Genome End | Strand | Div | Repeat Start | Repeat End | Repeat Left | Name | Family | Class |
| 12331358 | 12331517 | - | 12.4 | 137 | 305 | -7 | AluSx | Alu | SINE |
| 12331518 | 12331817 | - | 12.3 | 1 | 300 | -13 | AluSq | Alu | SINE |
| 12331818 | 12331950 | - | 12.4 | 1 | 136 | -176 | AluSx | Alu | SINE |

  

|  |  |  |  |  |  |  |  |  |  |
| --- | --- | --- | --- | --- | --- | --- | --- | --- | --- |
| chr8:12344999-12345613 | | | 615 bp | | |  |  |  |  |
| Genome Start | Genome End | Strand | Div | Repeat Start | Repeat End | Repeat Left | Name | Family | Class |
| 12344999 | 12345188 | - | 18.8 | 137 | 305 | -7 | AluJb | Alu | SINE |
| 12345189 | 12345478 | - | 12.1 | 1 | 301 | -11 | AluSx | Alu | SINE |
| 12345479 | 12345613 | - | 18.8 | 1 | 136 | -176 | AluJb | Alu | SINE |

  

|  |  |  |  |  |  |  |  |  |  |
| --- | --- | --- | --- | --- | --- | --- | --- | --- | --- |
| chr8:12345625-12346501 | | | 821 bp | | |  |  |  |  |
| Genome Start | Genome End | Strand | Div | Repeat Start | Repeat End | Repeat Left | Name | Family | Class |
| 12345625 | 12345694 | - | 18.6 | 6120 | 6184 | 0 | L1MB7 | L1 | LINE |
| 12345695 | 12345994 | + | 17.4 | 1 | 298 | -14 | AluJb | Alu | SINE |
| 12345995 | 12346192 | - | 18.6 | 5932 | 6119 | -65 | L1MB7 | L1 | LINE |
| 12346249 | 12346501 | - | 18.6 | 5653 | 5931 | -253 | L1MB7 | L1 | LINE |

  

|  |  |  |  |  |  |  |  |  |  |
| --- | --- | --- | --- | --- | --- | --- | --- | --- | --- |
| chr8:12346618-12348761 | | | 2144 bp | | |  |  |  |  |
| Genome Start | Genome End | Strand | Div | Repeat Start | Repeat End | Repeat Left | Name | Family | Class |
| 12346618 | 12346674 | - | 18.3 | 244 | 297 | -5 | AluJb | Alu | SINE |
| 12346675 | 12346710 | - | 17.0 | 454 | 486 | 0 | MER66B | ERV1 | LTR |
| 12346711 | 12348074 | - | 8.8 | 2 | 1578 | 0 | LTR12C | ERV1 | LTR |
| 12348075 | 12348512 | - | 17.0 | 1 | 453 | -33 | MER66B | ERV1 | LTR |
| 12348513 | 12348761 | - | 17.5 | 1 | 246 | -66 | AluJb | Alu | SINE |

  

|  |  |  |  |  |  |  |  |  |  |
| --- | --- | --- | --- | --- | --- | --- | --- | --- | --- |
| chr8:12351773-12352331 | | | 559 bp | | |  |  |  |  |
| Genome Start | Genome End | Strand | Div | Repeat Start | Repeat End | Repeat Left | Name | Family | Class |
| 12351773 | 12351885 | - | 18.5 | 138 | 249 | -63 | AluJo | Alu | SINE |
| 12351886 | 12352185 | - | 17.7 | 1 | 300 | -12 | AluSx | Alu | SINE |
| 12352186 | 12352331 | - | 18.5 | 1 | 137 | -175 | AluJo | Alu | SINE |

  

|  |  |  |  |  |  |  |  |  |  |
| --- | --- | --- | --- | --- | --- | --- | --- | --- | --- |
| chr8:12358267-12358774 | | | 508 bp | | |  |  |  |  |
| Genome Start | Genome End | Strand | Div | Repeat Start | Repeat End | Repeat Left | Name | Family | Class |
| 12358267 | 12358334 | - | 23.0 | 138 | 209 | 0 | MER3 | MER1\_type | DNA |
| 12358335 | 12358643 | + | 14.6 | 1 | 312 | 0 | AluSx | Alu | SINE |
| 12358644 | 12358774 | - | 23.0 | 9 | 137 | -72 | MER3 | MER1\_type | DNA |

  

|  |  |  |  |  |  |  |  |  |  |
| --- | --- | --- | --- | --- | --- | --- | --- | --- | --- |
| chr8:12376829-12377437 | | | 609 bp | | |  |  |  |  |
| Genome Start | Genome End | Strand | Div | Repeat Start | Repeat End | Repeat Left | Name | Family | Class |
| 12376829 | 12376974 | + | 13.4 | 1 | 144 | -168 | AluSx | Alu | SINE |
| 12376975 | 12377272 | + | 8.1 | 1 | 298 | -13 | AluY | Alu | SINE |
| 12377273 | 12377437 | + | 13.4 | 145 | 299 | -13 | AluSx | Alu | SINE |

  

|  |  |  |  |  |  |  |  |  |  |
| --- | --- | --- | --- | --- | --- | --- | --- | --- | --- |
| chr8:12378285-12378518 | | | 234 bp | | |  |  |  |  |
| Genome Start | Genome End | Strand | Div | Repeat Start | Repeat End | Repeat Left | Name | Family | Class |
| 12378285 | 12378328 | + | 30.9 | 4 | 41 | -137 | MER5B | MER1\_type | DNA |
| 12378329 | 12378461 | - | 15.8 | 1 | 133 | 0 | FLAM\_A | Alu | SINE |
| 12378462 | 12378518 | + | 30.9 | 42 | 97 | -81 | MER5B | MER1\_type | DNA |

  

|  |  |  |  |  |  |  |  |  |  |
| --- | --- | --- | --- | --- | --- | --- | --- | --- | --- |
| chr8:12383179-12399410 | | | 16198 bp | | |  |  |  |  |
| Genome Start | Genome End | Strand | Div | Repeat Start | Repeat End | Repeat Left | Name | Family | Class |
| 12383179 | 12383359 | + | 23.4 | 1 | 185 | -320 | MLT1D | MaLR | LTR |
| 12383370 | 12383670 | + | 10.0 | 3 | 304 | -9 | AluSp | Alu | SINE |
| 12383687 | 12384080 | - | 9.2 | 2 | 444 | -6 | LTR7 | ERV1 | LTR |
| 12384082 | 12384458 | - | 9.4 | 7324 | 7713 | 0 | HERVH | ERV1 | LTR |
| 12384459 | 12384665 | - | 11.6 | 5600 | 5819 | -1894 | HERVH | ERV1 | LTR |
| 12384667 | 12384732 | - | 14.8 | 5091 | 5149 | -2564 | HERVH | ERV1 | LTR |
| 12384733 | 12385797 | - | 4.4 | 3 | 1071 | 0 | MER11C | ERVK | LTR |
| 12385798 | 12386402 | - | 14.8 | 4488 | 5090 | -2623 | HERVH | ERV1 | LTR |
| 12386405 | 12386958 | - | 9.4 | 3442 | 3993 | -3720 | HERVH | ERV1 | LTR |
| 12386959 | 12387455 | - | 9.1 | 1 | 501 | 0 | LTR2C | ERV1 | LTR |
| 12387456 | 12395282 | - | 6.8 | 3 | 7812 | -1 | HERVE | ERV1 | LTR |
| 12395283 | 12395587 | - | 11.1 | 3140 | 3441 | -4272 | HERVH | ERV1 | LTR |
| 12395573 | 12398469 | - | 9.9 | 1 | 2993 | -4720 | HERVH | ERV1 | LTR |
| 12398472 | 12398823 | - | 9.0 | 1 | 448 | -2 | LTR7 | ERV1 | LTR |
| 12398841 | 12399035 | + | 25.1 | 193 | 431 | -74 | MLT1D | MaLR | LTR |
| 12399036 | 12399344 | - | 14.7 | 2 | 308 | -4 | AluSx | Alu | SINE |
| 12399345 | 12399410 | + | 25.1 | 393 | 483 | 0 | MLT1D | MaLR | LTR |

  

|  |  |  |  |  |  |  |  |  |  |
| --- | --- | --- | --- | --- | --- | --- | --- | --- | --- |
| chr8:12403594-12404464 | | | 865 bp | | |  |  |  |  |
| Genome Start | Genome End | Strand | Div | Repeat Start | Repeat End | Repeat Left | Name | Family | Class |
| 12403594 | 12403685 | + | 17.4 | 3 | 97 | -370 | MLT1C | MaLR | LTR |
| 12403687 | 12404079 | + | 10.6 | 1 | 394 | 0 | AluSp | Alu | SINE |
| 12404085 | 12404464 | + | 24.2 | 82 | 467 | 0 | MLT1C | MaLR | LTR |

  

|  |  |  |  |  |  |  |  |  |  |
| --- | --- | --- | --- | --- | --- | --- | --- | --- | --- |
| chr8:12405058-12405906 | | | 849 bp | | |  |  |  |  |
| Genome Start | Genome End | Strand | Div | Repeat Start | Repeat End | Repeat Left | Name | Family | Class |
| 12405058 | 12405324 | + | 19.7 | 3 | 320 | -334 | MLT1E1 | MaLR | LTR |
| 12405325 | 12405636 | - | 11.2 | 1 | 312 | 0 | AluSx | Alu | SINE |
| 12405637 | 12405906 | + | 19.7 | 321 | 653 | -1 | MLT1E1 | MaLR | LTR |

  

|  |  |  |  |  |  |  |  |  |  |
| --- | --- | --- | --- | --- | --- | --- | --- | --- | --- |
| chr8:12430885-12439982 | | | 9068 bp | | |  |  |  |  |
| Genome Start | Genome End | Strand | Div | Repeat Start | Repeat End | Repeat Left | Name | Family | Class |
| 12430885 | 12431089 | + | 33.7 | 2261 | 2465 | -954 | L2 | L2 | LINE |
| 12431090 | 12431418 | + | 18.6 | 1 | 301 | -11 | AluJo | Alu | SINE |
| 12431419 | 12432087 | + | 33.1 | 2466 | 3188 | -190 | L2 | L2 | LINE |
| 12432111 | 12432579 | + | 5.2 | 1 | 578 | -688 | MER11A | ERVK | LTR |
| 12432577 | 12432772 | + | 11.7 | 1067 | 1266 | 0 | MER11A | ERVK | LTR |
| 12432800 | 12432884 | + | 35.3 | 3242 | 3327 | -51 | L2 | L2 | LINE |
| 12432885 | 12433950 | - | 5.1 | 1 | 1071 | 0 | MER11C | ERVK | LTR |
| 12433951 | 12434953 | - | 7.2 | 6954 | 7953 | 0 | HERVK11 | ERVK | LTR |
| 12434938 | 12436165 | - | 7.8 | 5525 | 6765 | -1188 | HERVK11 | ERVK | LTR |
| 12436164 | 12438073 | - | 7.4 | 1165 | 4601 | -3352 | HERVK11 | ERVK | LTR |
| 12438074 | 12438863 | - | 16.7 | 1 | 851 | -7102 | HERVK11 | ERVK | LTR |
| 12438864 | 12439922 | - | 4.7 | 1 | 1071 | 0 | MER11C | ERVK | LTR |
| 12439924 | 12439982 | + | 21.4 | 3364 | 3419 | 0 | L2 | L2 | LINE |

  

|  |  |  |  |  |  |  |  |  |  |
| --- | --- | --- | --- | --- | --- | --- | --- | --- | --- |
| chr8:12440313-12440938 | | | 626 bp | | |  |  |  |  |
| Genome Start | Genome End | Strand | Div | Repeat Start | Repeat End | Repeat Left | Name | Family | Class |
| 12440313 | 12440387 | - | 27.5 | 340 | 407 | -2 | MLT1I | MaLR | LTR |
| 12440388 | 12440679 | - | 14.1 | 1 | 299 | -13 | AluSx | Alu | SINE |
| 12440680 | 12440938 | - | 27.5 | 87 | 339 | -70 | MLT1I | MaLR | LTR |

  

|  |  |  |  |  |  |  |  |  |  |
| --- | --- | --- | --- | --- | --- | --- | --- | --- | --- |
| chr8:12446061-12447004 | | | 933 bp | | |  |  |  |  |
| Genome Start | Genome End | Strand | Div | Repeat Start | Repeat End | Repeat Left | Name | Family | Class |
| 12446061 | 12446188 | + | 16.3 | 5 | 129 | -49 | MER5B | MER1\_type | DNA |
| 12446210 | 12446512 | - | 13.9 | 2 | 304 | -8 | AluSx | Alu | SINE |
| 12446518 | 12446799 | - | 18.7 | 5902 | 6174 | 0 | L1MB5 | L1 | LINE |
| 12446770 | 12446945 | + | 23.3 | 5684 | 5887 | -287 | L1MB5 | L1 | LINE |
| 12446961 | 12447004 | + | 9.1 | 135 | 178 | 0 | MER5B | MER1\_type | DNA |

  

|  |  |  |  |  |  |  |  |  |  |
| --- | --- | --- | --- | --- | --- | --- | --- | --- | --- |
| chr8:12448584-12449901 | | | 1158 bp | | |  |  |  |  |
| Genome Start | Genome End | Strand | Div | Repeat Start | Repeat End | Repeat Left | Name | Family | Class |
| 12448584 | 12448826 | + | 27.2 | 2563 | 2810 | -609 | L2 | L2 | LINE |
| 12448897 | 12449206 | + | 7.8 | 1 | 309 | -2 | AluY | Alu | SINE |
| 12449256 | 12449313 | - | 29.3 | 95 | 152 | -124 | MIRm | MIR | SINE |
| 12449355 | 12449456 | + | 34.9 | 2910 | 3014 | -364 | L2 | L2 | LINE |
| 12449457 | 12449749 | + | 13.0 | 1 | 293 | -20 | AluSq | Alu | SINE |
| 12449750 | 12449901 | + | 34.9 | 3015 | 3157 | -221 | L2 | L2 | LINE |

  

|  |  |  |  |  |  |  |  |  |  |
| --- | --- | --- | --- | --- | --- | --- | --- | --- | --- |
| chr8:12449902-12450658 | | | 752 bp | | |  |  |  |  |
| Genome Start | Genome End | Strand | Div | Repeat Start | Repeat End | Repeat Left | Name | Family | Class |
| 12449902 | 12450105 | - | 21.6 | 5974 | 6184 | 0 | L1MB7 | L1 | LINE |
| 12450109 | 12450282 | - | 9.2 | 104 | 290 | -22 | AluSg/x | Alu | SINE |
| 12450285 | 12450658 | - | 17.4 | 5575 | 5957 | -221 | L1MB7 | L1 | LINE |

  

|  |  |  |  |  |  |  |  |  |  |
| --- | --- | --- | --- | --- | --- | --- | --- | --- | --- |
| chr8:12453987-12455754 | | | 1768 bp | | |  |  |  |  |
| Genome Start | Genome End | Strand | Div | Repeat Start | Repeat End | Repeat Left | Name | Family | Class |
| 12453987 | 12454394 | + | 21.4 | 1327 | 1800 | -5409 | L1M2b | L1 | LINE |
| 12454395 | 12454691 | + | 10.9 | 1 | 293 | -17 | AluSg | Alu | SINE |
| 12454692 | 12455754 | + | 18.6 | 1793 | 2882 | -4327 | L1M2b | L1 | LINE |

  

|  |  |  |  |  |  |  |  |  |  |
| --- | --- | --- | --- | --- | --- | --- | --- | --- | --- |
| chr8:12473825-12474452 | | | 628 bp | | |  |  |  |  |
| Genome Start | Genome End | Strand | Div | Repeat Start | Repeat End | Repeat Left | Name | Family | Class |
| 12473825 | 12473968 | + | 18.7 | 5348 | 5482 | -692 | L1M4 | L1 | LINE |
| 12473969 | 12474281 | + | 10.0 | 1 | 312 | 0 | AluSx | Alu | SINE |
| 12474282 | 12474452 | + | 18.7 | 5483 | 5653 | -521 | L1M4 | L1 | LINE |

  

|  |  |  |  |  |  |  |  |  |  |
| --- | --- | --- | --- | --- | --- | --- | --- | --- | --- |
| chr8:12476254-12477061 | | | 808 bp | | |  |  |  |  |
| Genome Start | Genome End | Strand | Div | Repeat Start | Repeat End | Repeat Left | Name | Family | Class |
| 12476254 | 12476341 | - | 26.3 | 6079 | 6170 | 0 | L1ME3A | L1 | LINE |
| 12476342 | 12476658 | + | 17.9 | 1 | 308 | -4 | AluJo | Alu | SINE |
| 12476659 | 12476806 | - | 26.3 | 5927 | 6078 | -92 | L1ME3A | L1 | LINE |
| 12476807 | 12477027 | + | 23.4 | 1 | 219 | 0 | MER20 | MER1\_type | DNA |
| 12477028 | 12477061 | - | 26.3 | 5893 | 5926 | -244 | L1ME3A | L1 | LINE |

  

|  |  |  |  |  |  |  |  |  |  |
| --- | --- | --- | --- | --- | --- | --- | --- | --- | --- |
| chr8:12495996-12496806 | | | 811 bp | | |  |  |  |  |
| Genome Start | Genome End | Strand | Div | Repeat Start | Repeat End | Repeat Left | Name | Family | Class |
| 12495996 | 12496171 | + | 12.4 | 5775 | 5950 | -341 | L1MA8 | L1 | LINE |
| 12496172 | 12496481 | + | 18.1 | 1 | 312 | 0 | AluJo | Alu | SINE |
| 12496482 | 12496806 | + | 12.4 | 5951 | 6288 | -3 | L1MA8 | L1 | LINE |

  

|  |  |  |  |  |  |  |  |  |  |
| --- | --- | --- | --- | --- | --- | --- | --- | --- | --- |
| chr8:12504909-12505699 | | | 786 bp | | |  |  |  |  |
| Genome Start | Genome End | Strand | Div | Repeat Start | Repeat End | Repeat Left | Name | Family | Class |
| 12504909 | 12505073 | - | 24.0 | 45 | 207 | -55 | MIR | MIR | SINE |
| 12505074 | 12505634 | + | 19.6 | 1511 | 2104 | -3610 | ERVL-B4 | ERVL | LTR |
| 12505640 | 12505699 | - | 33.3 | 15 | 75 | -187 | MIR | MIR | SINE |

  

|  |  |  |  |  |  |  |  |  |  |
| --- | --- | --- | --- | --- | --- | --- | --- | --- | --- |
| chr8:12531929-12532560 | | | 632 bp | | |  |  |  |  |
| Genome Start | Genome End | Strand | Div | Repeat Start | Repeat End | Repeat Left | Name | Family | Class |
| 12531929 | 12532090 | + | 18.9 | 5333 | 5482 | -692 | L1M4 | L1 | LINE |
| 12532091 | 12532388 | + | 12.2 | 1 | 294 | -18 | AluSx | Alu | SINE |
| 12532389 | 12532560 | + | 18.8 | 5483 | 5653 | -521 | L1M4 | L1 | LINE |

  

|  |  |  |  |  |  |  |  |  |  |
| --- | --- | --- | --- | --- | --- | --- | --- | --- | --- |
| chr8:12534370-12534916 | | | 515 bp | | |  |  |  |  |
| Genome Start | Genome End | Strand | Div | Repeat Start | Repeat End | Repeat Left | Name | Family | Class |
| 12534370 | 12534446 | - | 25.9 | 6079 | 6160 | -10 | L1ME3A | L1 | LINE |
| 12534447 | 12534740 | + | 17.6 | 1 | 284 | -28 | AluJo | Alu | SINE |
| 12534773 | 12534916 | - | 25.9 | 5925 | 6078 | -92 | L1ME3A | L1 | LINE |

  

|  |  |  |  |  |  |  |  |  |  |
| --- | --- | --- | --- | --- | --- | --- | --- | --- | --- |
| chr8:12545340-12545828 | | | 379 bp | | |  |  |  |  |
| Genome Start | Genome End | Strand | Div | Repeat Start | Repeat End | Repeat Left | Name | Family | Class |
| 12545340 | 12545457 | + | 37.1 | 3096 | 3215 | -163 | L2 | L2 | LINE |
| 12545586 | 12545769 | - | 28.6 | 12 | 210 | -52 | MIR | MIR | SINE |
| 12545752 | 12545828 | + | 31.9 | 3347 | 3418 | -1 | L2 | L2 | LINE |

  

|  |  |  |  |  |  |  |  |  |  |
| --- | --- | --- | --- | --- | --- | --- | --- | --- | --- |
| chr8:12553916-12554722 | | | 807 bp | | |  |  |  |  |
| Genome Start | Genome End | Strand | Div | Repeat Start | Repeat End | Repeat Left | Name | Family | Class |
| 12553916 | 12554091 | + | 13.4 | 5775 | 5950 | -341 | L1MA8 | L1 | LINE |
| 12554092 | 12554401 | + | 19.0 | 1 | 312 | 0 | AluJo | Alu | SINE |
| 12554402 | 12554722 | + | 13.4 | 5951 | 6288 | -3 | L1MA8 | L1 | LINE |

  

|  |  |  |  |  |  |  |  |  |  |
| --- | --- | --- | --- | --- | --- | --- | --- | --- | --- |
| chr8:12576824-12577723 | | | 900 bp | | |  |  |  |  |
| Genome Start | Genome End | Strand | Div | Repeat Start | Repeat End | Repeat Left | Name | Family | Class |
| 12576824 | 12576957 | + | 29.3 | 2424 | 2557 | -3589 | L1M5 | L1 | LINE |
| 12576958 | 12577340 | + | 24.8 | 1 | 448 | -2 | MLT1J2 | MaLR | LTR |
| 12577341 | 12577723 | + | 29.3 | 2558 | 2957 | -3189 | L1M5 | L1 | LINE |

  

|  |  |  |  |  |  |  |  |  |  |
| --- | --- | --- | --- | --- | --- | --- | --- | --- | --- |
| chr8:12581823-12583791 | | | 1969 bp | | |  |  |  |  |
| Genome Start | Genome End | Strand | Div | Repeat Start | Repeat End | Repeat Left | Name | Family | Class |
| 12581823 | 12582074 | - | 23.7 | 5907 | 6153 | -30 | L1MB3 | L1 | LINE |
| 12582075 | 12582367 | - | 13.7 | 3 | 294 | -18 | AluSx | Alu | SINE |
| 12582368 | 12582477 | - | 23.7 | 5825 | 5906 | -277 | L1MB3 | L1 | LINE |
| 12582478 | 12582797 | - | 16.1 | 1 | 312 | 0 | AluSx | Alu | SINE |
| 12582798 | 12583791 | - | 24.4 | 4818 | 5824 | -359 | L1MB3 | L1 | LINE |

  

|  |  |  |  |  |  |  |  |  |  |
| --- | --- | --- | --- | --- | --- | --- | --- | --- | --- |
| chr8:12583937-12584641 | | | 705 bp | | |  |  |  |  |
| Genome Start | Genome End | Strand | Div | Repeat Start | Repeat End | Repeat Left | Name | Family | Class |
| 12583937 | 12584174 | - | 24.3 | 5934 | 6160 | -140 | L1MA4 | L1 | LINE |
| 12584175 | 12584458 | - | 20.8 | 1 | 283 | -29 | AluSx | Alu | SINE |
| 12584459 | 12584641 | - | 23.7 | 5767 | 5933 | -367 | L1MA4 | L1 | LINE |

  

|  |  |  |  |  |  |  |  |  |  |
| --- | --- | --- | --- | --- | --- | --- | --- | --- | --- |
| chr8:12602260-12602899 | | | 640 bp | | |  |  |  |  |
| Genome Start | Genome End | Strand | Div | Repeat Start | Repeat End | Repeat Left | Name | Family | Class |
| 12602260 | 12602506 | - | 22.2 | 5908 | 6151 | -32 | L1MB3 | L1 | LINE |
| 12602507 | 12602809 | - | 15.5 | 1 | 303 | -9 | AluSx | Alu | SINE |
| 12602810 | 12602899 | - | 22.2 | 5818 | 5907 | -276 | L1MB3 | L1 | LINE |

  

|  |  |  |  |  |  |  |  |  |  |
| --- | --- | --- | --- | --- | --- | --- | --- | --- | --- |
| chr8:37485406-37485954 | | | 421 bp | | |  |  |  |  |
| Genome Start | Genome End | Strand | Div | Repeat Start | Repeat End | Repeat Left | Name | Family | Class |
| 37485406 | 37485483 | - | 24.1 | 184 | 261 | -7 | MIRb | MIR | SINE |
| 37485484 | 37485620 | + | 27.9 | 2 | 139 | -123 | MIR | MIR | SINE |
| 37485662 | 37485822 | - | 31.2 | 6 | 185 | -77 | MIR | MIR | SINE |
| 37485910 | 37485954 | - | 22.2 | 51 | 95 | -173 | MIRb | MIR | SINE |

  

|  |  |  |  |  |  |  |  |  |  |
| --- | --- | --- | --- | --- | --- | --- | --- | --- | --- |
| chr8:47606873-47607584 | | | 712 bp | | |  |  |  |  |
| Genome Start | Genome End | Strand | Div | Repeat Start | Repeat End | Repeat Left | Name | Family | Class |
| 47606873 | 47606986 | + | 28.1 | 25 | 163 | -99 | MIR | MIR | SINE |
| 47606987 | 47607526 | - | 30.1 | 1 | 595 | 0 | MLT1G1 | MaLR | LTR |
| 47607527 | 47607584 | + | 28.1 | 164 | 229 | -33 | MIR | MIR | SINE |

  

|  |  |  |  |  |  |  |  |  |  |
| --- | --- | --- | --- | --- | --- | --- | --- | --- | --- |
| chr8:48151105-48151446 | | | 322 bp | | |  |  |  |  |
| Genome Start | Genome End | Strand | Div | Repeat Start | Repeat End | Repeat Left | Name | Family | Class |
| 48151105 | 48151199 | + | 22.1 | 1 | 96 | -11 | MER58A | MER1\_type | DNA |
| 48151216 | 48151377 | + | 19.8 | 132 | 293 | -19 | AluJb | Alu | SINE |
| 48151382 | 48151446 | + | 15.4 | 160 | 224 | 0 | MER58A | MER1\_type | DNA |

  

|  |  |  |  |  |  |  |  |  |  |
| --- | --- | --- | --- | --- | --- | --- | --- | --- | --- |
| chr8:86678322-86679139 | | | 818 bp | | |  |  |  |  |
| Genome Start | Genome End | Strand | Div | Repeat Start | Repeat End | Repeat Left | Name | Family | Class |
| 86678322 | 86678734 | - | 14.8 | 5887 | 6286 | -5 | L1MA8 | L1 | LINE |
| 86678735 | 86679028 | - | 16.3 | 2 | 296 | -16 | AluJb | Alu | SINE |
| 86679029 | 86679139 | - | 14.8 | 5778 | 5886 | -405 | L1MA8 | L1 | LINE |

  

|  |  |  |  |  |  |  |  |  |  |
| --- | --- | --- | --- | --- | --- | --- | --- | --- | --- |
| chr8:86740042-86740863 | | | 822 bp | | |  |  |  |  |
| Genome Start | Genome End | Strand | Div | Repeat Start | Repeat End | Repeat Left | Name | Family | Class |
| 86740042 | 86740151 | + | 14.2 | 5778 | 5886 | -405 | L1MA8 | L1 | LINE |
| 86740152 | 86740451 | + | 20.3 | 1 | 296 | -16 | AluJb | Alu | SINE |
| 86740452 | 86740863 | + | 14.2 | 5887 | 6286 | -5 | L1MA8 | L1 | LINE |

  

|  |  |  |  |  |  |  |  |  |  |
| --- | --- | --- | --- | --- | --- | --- | --- | --- | --- |
| chr8:86748048-86748870 | | | 823 bp | | |  |  |  |  |
| Genome Start | Genome End | Strand | Div | Repeat Start | Repeat End | Repeat Left | Name | Family | Class |
| 86748048 | 86748157 | + | 17.4 | 5778 | 5886 | -405 | L1MA8 | L1 | LINE |
| 86748158 | 86748453 | + | 16.4 | 1 | 292 | -20 | AluJb | Alu | SINE |
| 86748454 | 86748870 | + | 17.4 | 5887 | 6286 | -5 | L1MA8 | L1 | LINE |

  

|  |  |  |  |  |  |  |  |  |  |
| --- | --- | --- | --- | --- | --- | --- | --- | --- | --- |
| chr8:86853870-86854692 | | | 823 bp | | |  |  |  |  |
| Genome Start | Genome End | Strand | Div | Repeat Start | Repeat End | Repeat Left | Name | Family | Class |
| 86853870 | 86853979 | + | 17.8 | 5778 | 5886 | -405 | L1MA8 | L1 | LINE |
| 86853980 | 86854278 | + | 16.6 | 1 | 295 | -17 | AluJb | Alu | SINE |
| 86854279 | 86854692 | + | 17.8 | 5887 | 6286 | -5 | L1MA8 | L1 | LINE |

  

|  |  |  |  |  |  |  |  |  |  |
| --- | --- | --- | --- | --- | --- | --- | --- | --- | --- |
| chr8:86866061-86866883 | | | 823 bp | | |  |  |  |  |
| Genome Start | Genome End | Strand | Div | Repeat Start | Repeat End | Repeat Left | Name | Family | Class |
| 86866061 | 86866170 | + | 17.4 | 5778 | 5886 | -405 | L1MA8 | L1 | LINE |
| 86866171 | 86866469 | + | 16.6 | 1 | 295 | -17 | AluJb | Alu | SINE |
| 86866470 | 86866883 | + | 17.4 | 5887 | 6286 | -5 | L1MA8 | L1 | LINE |

  

|  |  |  |  |  |  |  |  |  |  |
| --- | --- | --- | --- | --- | --- | --- | --- | --- | --- |
| chr8:86878252-86879074 | | | 823 bp | | |  |  |  |  |
| Genome Start | Genome End | Strand | Div | Repeat Start | Repeat End | Repeat Left | Name | Family | Class |
| 86878252 | 86878361 | + | 17.6 | 5778 | 5886 | -405 | L1MA8 | L1 | LINE |
| 86878362 | 86878660 | + | 16.9 | 1 | 295 | -17 | AluJb | Alu | SINE |
| 86878661 | 86879074 | + | 17.6 | 5887 | 6286 | -5 | L1MA8 | L1 | LINE |

  

|  |  |  |  |  |  |  |  |  |  |
| --- | --- | --- | --- | --- | --- | --- | --- | --- | --- |
| chr8:86890166-86890988 | | | 817 bp | | |  |  |  |  |
| Genome Start | Genome End | Strand | Div | Repeat Start | Repeat End | Repeat Left | Name | Family | Class |
| 86890166 | 86890269 | + | 20.2 | 5778 | 5884 | -407 | L1MA8 | L1 | LINE |
| 86890276 | 86890571 | + | 16.4 | 1 | 292 | -20 | AluJb | Alu | SINE |
| 86890572 | 86890988 | + | 17.6 | 5887 | 6286 | -5 | L1MA8 | L1 | LINE |

  

|  |  |  |  |  |  |  |  |  |  |
| --- | --- | --- | --- | --- | --- | --- | --- | --- | --- |
| chr8:143911814-143912577 | | | 764 bp | | |  |  |  |  |
| Genome Start | Genome End | Strand | Div | Repeat Start | Repeat End | Repeat Left | Name | Family | Class |
| 143911814 | 143912154 | + | 18.2 | 1 | 324 | -137 | MER65C | ERV1 | LTR |
| 143912155 | 143912438 | - | 13.0 | 8 | 291 | -21 | AluSx | Alu | SINE |
| 143912439 | 143912577 | + | 18.2 | 325 | 461 | 0 | MER65C | ERV1 | LTR |

  

|  |  |  |  |  |  |  |  |  |  |
| --- | --- | --- | --- | --- | --- | --- | --- | --- | --- |
| chr8:145277683-145278765 | | | 1083 bp | | |  |  |  |  |
| Genome Start | Genome End | Strand | Div | Repeat Start | Repeat End | Repeat Left | Name | Family | Class |
| 145277683 | 145277856 | + | 13.5 | 7 | 192 | -593 | LTR1 | ERV1 | LTR |
| 145277857 | 145278165 | + | 9.1 | 1 | 309 | -3 | AluSx | Alu | SINE |
| 145278166 | 145278765 | + | 13.5 | 193 | 778 | -7 | LTR1 | ERV1 | LTR |

  

|  |  |  |  |  |  |  |  |  |  |
| --- | --- | --- | --- | --- | --- | --- | --- | --- | --- |
| chr8:145279738-145280640 | | | 903 bp | | |  |  |  |  |
| Genome Start | Genome End | Strand | Div | Repeat Start | Repeat End | Repeat Left | Name | Family | Class |
| 145279738 | 145280085 | - | 15.6 | 5827 | 6181 | -110 | L1MA8 | L1 | LINE |
| 145280086 | 145280387 | - | 8.9 | 1 | 302 | -11 | AluSq | Alu | SINE |
| 145280388 | 145280640 | - | 15.6 | 5612 | 5826 | -465 | L1MA8 | L1 | LINE |

  

|  |  |  |  |  |  |  |  |  |  |
| --- | --- | --- | --- | --- | --- | --- | --- | --- | --- |
| chr8:145405483-145406668 | | | 1186 bp | | |  |  |  |  |
| Genome Start | Genome End | Strand | Div | Repeat Start | Repeat End | Repeat Left | Name | Family | Class |
| 145405483 | 145405898 | - | 17.5 | 5607 | 6023 | -161 | L1MB7 | L1 | LINE |
| 145405899 | 145406201 | - | 13.9 | 2 | 304 | -6 | AluSg | Alu | SINE |
| 145406202 | 145406333 | - | 17.5 | 5487 | 5606 | -578 | L1MB7 | L1 | LINE |
| 145406334 | 145406634 | + | 8.4 | 1 | 298 | -15 | AluSp | Alu | SINE |
| 145406635 | 145406668 | - | 17.5 | 5454 | 5486 | -698 | L1MB7 | L1 | LINE |

  

|  |  |  |  |  |  |  |  |  |  |
| --- | --- | --- | --- | --- | --- | --- | --- | --- | --- |
| chr8:145425231-145426519 | | | 1289 bp | | |  |  |  |  |
| Genome Start | Genome End | Strand | Div | Repeat Start | Repeat End | Repeat Left | Name | Family | Class |
| 145425231 | 145425581 | - | 17.6 | 341 | 725 | -1993 | Tigger2 | MER2\_type | DNA |
| 145425582 | 145425749 | - | 10.6 | 135 | 304 | -9 | AluSp | Alu | SINE |
| 145425750 | 145426033 | - | 9.5 | 1 | 288 | -22 | AluSg | Alu | SINE |
| 145426034 | 145426168 | - | 10.6 | 1 | 134 | -179 | AluSp | Alu | SINE |
| 145426169 | 145426519 | - | 17.6 | 1 | 340 | -2378 | Tigger2 | MER2\_type | DNA |

  

|  |  |  |  |  |  |  |  |  |  |
| --- | --- | --- | --- | --- | --- | --- | --- | --- | --- |
| chr8:145430615-145432200 | | | 1586 bp | | |  |  |  |  |
| Genome Start | Genome End | Strand | Div | Repeat Start | Repeat End | Repeat Left | Name | Family | Class |
| 145430615 | 145431222 | - | 30.7 | 7219 | 7900 | -142 | L1MC4 | L1 | LINE |
| 145431223 | 145431540 | + | 7.7 | 1 | 316 | 0 | AluSx | Alu | SINE |
| 145431541 | 145431660 | - | 30.7 | 7098 | 7218 | -824 | L1MC4 | L1 | LINE |
| 145431661 | 145431968 | - | 6.2 | 1 | 308 | -3 | AluY | Alu | SINE |
| 145431969 | 145432200 | - | 30.7 | 6843 | 7097 | -945 | L1MC4 | L1 | LINE |

  

|  |  |  |  |  |  |  |  |  |  |
| --- | --- | --- | --- | --- | --- | --- | --- | --- | --- |
| chr8:145447006-145447930 | | | 893 bp | | |  |  |  |  |
| Genome Start | Genome End | Strand | Div | Repeat Start | Repeat End | Repeat Left | Name | Family | Class |
| 145447006 | 145447150 | + | 12.2 | 1 | 135 | -177 | AluSx | Alu | SINE |
| 145447151 | 145447446 | + | 6.8 | 1 | 295 | -16 | AluY | Alu | SINE |
| 145447449 | 145447735 | + | 10.4 | 1 | 298 | -15 | AluSp | Alu | SINE |
| 145447766 | 145447930 | + | 12.2 | 143 | 293 | -19 | AluSx | Alu | SINE |

  


|  |  |  |  |  |  |  |  |  |  |
| --- | --- | --- | --- | --- | --- | --- | --- | --- | --- |
| chr9:13938-14561 | | | 546 bp | | |  |  |  |  |
| Genome Start | Genome End | Strand | Div | Repeat Start | Repeat End | Repeat Left | Name | Family | Class |
| 13938 | 14119 | + | 28.9 | 2957 | 3178 | -200 | L2 | L2 | LINE |
| 14185 | 14363 | + | 27.1 | 33 | 260 | -2 | MIR | MIR | SINE |
| 14377 | 14561 | + | 34.6 | 3232 | 3418 | -1 | L2 | L2 | LINE |

  

|  |  |  |  |  |  |  |  |  |  |
| --- | --- | --- | --- | --- | --- | --- | --- | --- | --- |
| chr9:27841-30777 | | | 2810 bp | | |  |  |  |  |
| Genome Start | Genome End | Strand | Div | Repeat Start | Repeat End | Repeat Left | Name | Family | Class |
| 27841 | 27973 | + | 17.4 | 2705 | 2846 | -573 | L2 | L2 | LINE |
| 28038 | 29246 | + | 22.9 | 131 | 1489 | 0 | MLT1E-int | MaLR | LTR |
| 29247 | 29405 | + | 24.5 | 1 | 168 | -425 | MLT1E | MaLR | LTR |
| 29406 | 29706 | + | 12.6 | 1 | 301 | -11 | AluSx | Alu | SINE |
| 29707 | 30076 | + | 24.5 | 169 | 579 | -14 | MLT1E | MaLR | LTR |
| 30115 | 30404 | + | 32.4 | 2924 | 3231 | -188 | L2 | L2 | LINE |
| 30408 | 30681 | - | 20.9 | 40 | 311 | -1 | AluJb | Alu | SINE |
| 30704 | 30777 | + | 21.6 | 3327 | 3419 | 0 | L2 | L2 | LINE |

  

|  |  |  |  |  |  |  |  |  |  |
| --- | --- | --- | --- | --- | --- | --- | --- | --- | --- |
| chr9:43530-44158 | | | 410 bp | | |  |  |  |  |
| Genome Start | Genome End | Strand | Div | Repeat Start | Repeat End | Repeat Left | Name | Family | Class |
| 43530 | 43591 | + | 16.1 | 1 | 73 | -87 | MER5A1 | MER1\_type | DNA |
| 43592 | 43871 | + | 13.6 | 1 | 281 | -31 | AluSx | Alu | SINE |
| 44091 | 44158 | + | 16.2 | 58 | 125 | -35 | MER5A1 | MER1\_type | DNA |

  

|  |  |  |  |  |  |  |  |  |  |
| --- | --- | --- | --- | --- | --- | --- | --- | --- | --- |
| chr9:44570-46410 | | | 1682 bp | | |  |  |  |  |
| Genome Start | Genome End | Strand | Div | Repeat Start | Repeat End | Repeat Left | Name | Family | Class |
| 44570 | 44641 | - | 29.6 | 2821 | 2891 | -528 | L2 | L2 | LINE |
| 44666 | 45263 | + | 26.7 | 2786 | 3419 | 0 | L2 | L2 | LINE |
| 45288 | 45488 | - | 34.3 | 2584 | 2793 | -626 | L2 | L2 | LINE |
| 45493 | 45939 | + | 23.2 | 1 | 509 | -58 | MLT1F | MaLR | LTR |
| 45945 | 46098 | + | 22.7 | 3236 | 3419 | 0 | L2 | L2 | LINE |
| 46187 | 46261 | + | 10.7 | 282 | 357 | -185 | MLT1F | MaLR | LTR |
| 46267 | 46318 | + | 13.5 | 490 | 541 | -1 | MLT1F | MaLR | LTR |
| 46328 | 46410 | - | 31.3 | 2500 | 2586 | -833 | L2 | L2 | LINE |

  

|  |  |  |  |  |  |  |  |  |  |
| --- | --- | --- | --- | --- | --- | --- | --- | --- | --- |
| chr9:47635-48451 | | | 806 bp | | |  |  |  |  |
| Genome Start | Genome End | Strand | Div | Repeat Start | Repeat End | Repeat Left | Name | Family | Class |
| 47635 | 47794 | + | 17.0 | 3954 | 4121 | -1593 | ERVL-B4 | ERVL | LTR |
| 47795 | 48204 | + | 16.3 | 1 | 416 | -10 | MSTB | MaLR | LTR |
| 48216 | 48451 | + | 14.8 | 4118 | 4355 | -1359 | ERVL-B4 | ERVL | LTR |

  

|  |  |  |  |  |  |  |  |  |  |
| --- | --- | --- | --- | --- | --- | --- | --- | --- | --- |
| chr9:59176-59857 | | | 682 bp | | |  |  |  |  |
| Genome Start | Genome End | Strand | Div | Repeat Start | Repeat End | Repeat Left | Name | Family | Class |
| 59176 | 59375 | + | 29.6 | 7507 | 7703 | -339 | L1MC4 | L1 | LINE |
| 59376 | 59627 | + | 15.2 | 41 | 293 | -19 | AluJo | Alu | SINE |
| 59628 | 59857 | + | 29.6 | 7704 | 7953 | -89 | L1MC4 | L1 | LINE |

  

|  |  |  |  |  |  |  |  |  |  |
| --- | --- | --- | --- | --- | --- | --- | --- | --- | --- |
| chr9:74274-74779 | | | 506 bp | | |  |  |  |  |
| Genome Start | Genome End | Strand | Div | Repeat Start | Repeat End | Repeat Left | Name | Family | Class |
| 74274 | 74333 | - | 31.6 | 153 | 211 | -57 | MIRb | MIR | SINE |
| 74334 | 74684 | - | 14.1 | 1 | 366 | 0 | Tigger5a | MER2\_type | DNA |
| 74685 | 74779 | - | 31.6 | 60 | 152 | -116 | MIRb | MIR | SINE |

  

|  |  |  |  |  |  |  |  |  |  |
| --- | --- | --- | --- | --- | --- | --- | --- | --- | --- |
| chr9:75507-76756 | | | 1211 bp | | |  |  |  |  |
| Genome Start | Genome End | Strand | Div | Repeat Start | Repeat End | Repeat Left | Name | Family | Class |
| 75507 | 76451 | + | 16.5 | 3278 | 4268 | -1875 | L1M2 | L1 | LINE |
| 76454 | 76654 | + | 12.3 | 5955 | 6150 | -16 | L1PA17 | L1 | LINE |
| 76692 | 76756 | + | 11.7 | 4254 | 4313 | -1830 | L1M2 | L1 | LINE |

  

|  |  |  |  |  |  |  |  |  |  |
| --- | --- | --- | --- | --- | --- | --- | --- | --- | --- |
| chr9:88943-89454 | | | 512 bp | | |  |  |  |  |
| Genome Start | Genome End | Strand | Div | Repeat Start | Repeat End | Repeat Left | Name | Family | Class |
| 88943 | 89103 | - | 18.6 | 68 | 219 | 0 | MER20 | MER1\_type | DNA |
| 89104 | 89373 | + | 14.1 | 5906 | 6176 | -7 | L1MB3 | L1 | LINE |
| 89374 | 89454 | - | 18.6 | 1 | 67 | -152 | MER20 | MER1\_type | DNA |

  

|  |  |  |  |  |  |  |  |  |  |
| --- | --- | --- | --- | --- | --- | --- | --- | --- | --- |
| chr9:93299-94084 | | | 786 bp | | |  |  |  |  |
| Genome Start | Genome End | Strand | Div | Repeat Start | Repeat End | Repeat Left | Name | Family | Class |
| 93299 | 93748 | + | 23.9 | 38 | 467 | -5933 | L1MEc | L1 | LINE |
| 93749 | 94049 | + | 13.1 | 1 | 307 | -2 | AluSc | Alu | SINE |
| 94050 | 94084 | + | 23.9 | 468 | 493 | -5907 | L1MEc | L1 | LINE |

  

|  |  |  |  |  |  |  |  |  |  |
| --- | --- | --- | --- | --- | --- | --- | --- | --- | --- |
| chr9:176823-177334 | | | 512 bp | | |  |  |  |  |
| Genome Start | Genome End | Strand | Div | Repeat Start | Repeat End | Repeat Left | Name | Family | Class |
| 176823 | 176941 | - | 29.8 | 5826 | 5935 | -1873 | L1MC4a | L1 | LINE |
| 176942 | 177244 | - | 11.9 | 1 | 303 | -10 | AluSq | Alu | SINE |
| 177245 | 177334 | - | 29.8 | 5735 | 5825 | -1983 | L1MC4a | L1 | LINE |

  

|  |  |  |  |  |  |  |  |  |  |
| --- | --- | --- | --- | --- | --- | --- | --- | --- | --- |
| chr9:189220-189789 | | | 550 bp | | |  |  |  |  |
| Genome Start | Genome End | Strand | Div | Repeat Start | Repeat End | Repeat Left | Name | Family | Class |
| 189220 | 189319 | - | 26.1 | 178 | 262 | -6 | MIRb | MIR | SINE |
| 189320 | 189603 | + | 8.4 | 1 | 284 | -28 | AluSx | Alu | SINE |
| 189624 | 189789 | - | 26.1 | 7 | 177 | -91 | MIRb | MIR | SINE |

  

|  |  |  |  |  |  |  |  |  |  |
| --- | --- | --- | --- | --- | --- | --- | --- | --- | --- |
| chr9:28280381-28281232 | | | 852 bp | | |  |  |  |  |
| Genome Start | Genome End | Strand | Div | Repeat Start | Repeat End | Repeat Left | Name | Family | Class |
| 28280381 | 28280442 | + | 24.0 | 100 | 162 | -329 | LTR16C | ERVL | LTR |
| 28280443 | 28280895 | - | 15.7 | 1 | 461 | 0 | LOR1b | ERV1 | LTR |
| 28280896 | 28281232 | + | 24.0 | 163 | 491 | 0 | LTR16C | ERVL | LTR |

  

|  |  |  |  |  |  |  |  |  |  |
| --- | --- | --- | --- | --- | --- | --- | --- | --- | --- |
| chr9:33378850-33379472 | | | 623 bp | | |  |  |  |  |
| Genome Start | Genome End | Strand | Div | Repeat Start | Repeat End | Repeat Left | Name | Family | Class |
| 33378850 | 33379021 | - | 17.1 | 143 | 305 | -7 | AluJb | Alu | SINE |
| 33379022 | 33379322 | - | 10.7 | 5 | 304 | -8 | AluSx | Alu | SINE |
| 33379323 | 33379472 | - | 17.1 | 1 | 142 | -170 | AluJb | Alu | SINE |

  

|  |  |  |  |  |  |  |  |  |  |
| --- | --- | --- | --- | --- | --- | --- | --- | --- | --- |
| chr9:33379708-33380392 | | | 685 bp | | |  |  |  |  |
| Genome Start | Genome End | Strand | Div | Repeat Start | Repeat End | Repeat Left | Name | Family | Class |
| 33379708 | 33379752 | - | 27.9 | 1926 | 1968 | -1451 | L2 | L2 | LINE |
| 33379753 | 33380053 | + | 10.0 | 1 | 310 | -2 | AluSx | Alu | SINE |
| 33380054 | 33380392 | - | 27.9 | 1546 | 1925 | -1494 | L2 | L2 | LINE |

  

|  |  |  |  |  |  |  |  |  |  |
| --- | --- | --- | --- | --- | --- | --- | --- | --- | --- |
| chr9:33389285-33390125 | | | 777 bp | | |  |  |  |  |
| Genome Start | Genome End | Strand | Div | Repeat Start | Repeat End | Repeat Left | Name | Family | Class |
| 33389285 | 33389310 | - | 30.6 | 3359 | 3378 | 0 | L2 | L2 | LINE |
| 33389311 | 33389610 | + | 14.6 | 1 | 294 | -18 | AluSx | Alu | SINE |
| 33389611 | 33389768 | - | 30.6 | 3179 | 3358 | -20 | L2 | L2 | LINE |
| 33389833 | 33390125 | - | 33.2 | 2351 | 2691 | -728 | L2 | L2 | LINE |

  

|  |  |  |  |  |  |  |  |  |  |
| --- | --- | --- | --- | --- | --- | --- | --- | --- | --- |
| chr9:33390169-33390928 | | | 760 bp | | |  |  |  |  |
| Genome Start | Genome End | Strand | Div | Repeat Start | Repeat End | Repeat Left | Name | Family | Class |
| 33390169 | 33390497 | - | 31.2 | 2993 | 3345 | -74 | L2 | L2 | LINE |
| 33390498 | 33390829 | + | 12.5 | 1 | 305 | -7 | AluSx | Alu | SINE |
| 33390830 | 33390928 | - | 20.0 | 2872 | 2992 | -427 | L2 | L2 | LINE |

  

|  |  |  |  |  |  |  |  |  |  |
| --- | --- | --- | --- | --- | --- | --- | --- | --- | --- |
| chr9:33527633-33528854 | | | 1222 bp | | |  |  |  |  |
| Genome Start | Genome End | Strand | Div | Repeat Start | Repeat End | Repeat Left | Name | Family | Class |
| 33527633 | 33527652 | - | 23.4 | 518 | 536 | -2988 | Zaphod2 | Tip100 | DNA |
| 33527653 | 33528087 | - | 19.5 | 1 | 434 | 0 | Tigger2a | MER2\_type | DNA |
| 33528088 | 33528454 | - | 23.4 | 179 | 517 | -3007 | Zaphod2 | Tip100 | DNA |
| 33528455 | 33528742 | + | 12.5 | 1 | 293 | -19 | AluSx | Alu | SINE |
| 33528743 | 33528854 | - | 23.4 | 80 | 178 | -3346 | Zaphod2 | Tip100 | DNA |

  

|  |  |  |  |  |  |  |  |  |  |
| --- | --- | --- | --- | --- | --- | --- | --- | --- | --- |
| chr9:33549788-33551904 | | | 2117 bp | | |  |  |  |  |
| Genome Start | Genome End | Strand | Div | Repeat Start | Repeat End | Repeat Left | Name | Family | Class |
| 33549788 | 33550108 | - | 22.4 | 5833 | 6173 | -5 | L1MB8 | L1 | LINE |
| 33550109 | 33550537 | - | 14.1 | 1 | 428 | 0 | MER61C | ERV1 | LTR |
| 33550538 | 33550676 | - | 19.2 | 5703 | 5836 | -342 | L1MB8 | L1 | LINE |
| 33550677 | 33550969 | + | 9.8 | 1 | 287 | -23 | AluSg | Alu | SINE |
| 33550970 | 33551904 | - | 19.3 | 4766 | 5702 | -476 | L1MB8 | L1 | LINE |

  

|  |  |  |  |  |  |  |  |  |  |
| --- | --- | --- | --- | --- | --- | --- | --- | --- | --- |
| chr9:33553648-33555354 | | | 1633 bp | | |  |  |  |  |
| Genome Start | Genome End | Strand | Div | Repeat Start | Repeat End | Repeat Left | Name | Family | Class |
| 33553648 | 33553927 | - | 16.7 | 6009 | 6294 | -6 | L1MA5 | L1 | LINE |
| 33554001 | 33554259 | - | 22.2 | 1 | 264 | -48 | AluJo | Alu | SINE |
| 33554261 | 33555354 | - | 19.2 | 4805 | 5946 | -354 | L1MA5 | L1 | LINE |

  

|  |  |  |  |  |  |  |  |  |  |
| --- | --- | --- | --- | --- | --- | --- | --- | --- | --- |
| chr9:33587041-33587718 | | | 591 bp | | |  |  |  |  |
| Genome Start | Genome End | Strand | Div | Repeat Start | Repeat End | Repeat Left | Name | Family | Class |
| 33587041 | 33587135 | + | 18.9 | 1 | 96 | -330 | MSTA | MaLR | LTR |
| 33587229 | 33587284 | + | 17.9 | 911 | 967 | -613 | THE1-int | MaLR | LTR |
| 33587273 | 33587514 | - | 24.1 | 909 | 1191 | -389 | THE1-int | MaLR | LTR |
| 33587521 | 33587718 | + | 26.0 | 76 | 287 | -141 | MSTA | MaLR | LTR |

  

|  |  |  |  |  |  |  |  |  |  |
| --- | --- | --- | --- | --- | --- | --- | --- | --- | --- |
| chr9:33713153-33713785 | | | 633 bp | | |  |  |  |  |
| Genome Start | Genome End | Strand | Div | Repeat Start | Repeat End | Repeat Left | Name | Family | Class |
| 33713153 | 33713504 | + | 21.7 | 5710 | 6082 | -82 | L1ME2 | L1 | LINE |
| 33713505 | 33713711 | + | 22.6 | 1 | 210 | 0 | MER63A | AcHobo | DNA |
| 33713712 | 33713785 | + | 21.7 | 6083 | 6154 | -10 | L1ME2 | L1 | LINE |

  

|  |  |  |  |  |  |  |  |  |  |
| --- | --- | --- | --- | --- | --- | --- | --- | --- | --- |
| chr9:33726367-33727191 | | | 825 bp | | |  |  |  |  |
| Genome Start | Genome End | Strand | Div | Repeat Start | Repeat End | Repeat Left | Name | Family | Class |
| 33726367 | 33726837 | + | 9.6 | 5632 | 6106 | -45 | L1PB1 | L1 | LINE |
| 33726838 | 33727135 | + | 7.4 | 1 | 299 | -12 | AluY | Alu | SINE |
| 33727136 | 33727191 | + | 9.6 | 6107 | 6148 | -3 | L1PB1 | L1 | LINE |

  

|  |  |  |  |  |  |  |  |  |  |
| --- | --- | --- | --- | --- | --- | --- | --- | --- | --- |
| chr9:33751096-33751973 | | | 875 bp | | |  |  |  |  |
| Genome Start | Genome End | Strand | Div | Repeat Start | Repeat End | Repeat Left | Name | Family | Class |
| 33751096 | 33751419 | - | 15.0 | 2755 | 3027 | -2 | Tigger3(Golem) | MER2\_type | DNA |
| 33751423 | 33751727 | + | 9.9 | 1 | 306 | 0 | AluSp | Alu | SINE |
| 33751728 | 33751895 | + | 14.3 | 134 | 301 | -11 | AluSg/x | Alu | SINE |
| 33751896 | 33751973 | - | 20.5 | 1 | 78 | -2951 | Tigger3(Golem) | MER2\_type | DNA |

  

|  |  |  |  |  |  |  |  |  |  |
| --- | --- | --- | --- | --- | --- | --- | --- | --- | --- |
| chr9:33767950-33771797 | | | 3842 bp | | |  |  |  |  |
| Genome Start | Genome End | Strand | Div | Repeat Start | Repeat End | Repeat Left | Name | Family | Class |
| 33767950 | 33768708 | + | 23.6 | 3727 | 4531 | -1615 | L1M4 | L1 | LINE |
| 33768707 | 33769583 | + | 14.4 | 940 | 1801 | -4702 | L1PA16 | L1 | LINE |
| 33769585 | 33769894 | + | 7.1 | 1 | 309 | -2 | AluY | Alu | SINE |
| 33769895 | 33771061 | + | 14.0 | 1802 | 3003 | -3480 | L1PA16 | L1 | LINE |
| 33771062 | 33771655 | + | 13.5 | 5854 | 6494 | -9 | L1PA16 | L1 | LINE |
| 33771663 | 33771797 | + | 25.6 | 4533 | 4668 | -1478 | L1M4 | L1 | LINE |

  

|  |  |  |  |  |  |  |  |  |  |
| --- | --- | --- | --- | --- | --- | --- | --- | --- | --- |
| chr9:37045474-37045913 | | | 440 bp | | |  |  |  |  |
| Genome Start | Genome End | Strand | Div | Repeat Start | Repeat End | Repeat Left | Name | Family | Class |
| 37045474 | 37045666 | + | 23.6 | 2 | 195 | -67 | MIR | MIR | SINE |
| 37045667 | 37045832 | + | 19.8 | 1 | 162 | -4 | FRAM | Alu | SINE |
| 37045833 | 37045913 | + | 23.6 | 196 | 262 | 0 | MIR | MIR | SINE |

  

|  |  |  |  |  |  |  |  |  |  |
| --- | --- | --- | --- | --- | --- | --- | --- | --- | --- |
| chr9:38522744-38523531 | | | 788 bp | | |  |  |  |  |
| Genome Start | Genome End | Strand | Div | Repeat Start | Repeat End | Repeat Left | Name | Family | Class |
| 38522744 | 38523128 | + | 21.1 | 6616 | 6974 | -987 | L1MC5 | L1 | LINE |
| 38523129 | 38523419 | + | 15.9 | 1 | 293 | -19 | AluJo | Alu | SINE |
| 38523420 | 38523531 | + | 21.1 | 6975 | 7078 | -883 | L1MC5 | L1 | LINE |

  

|  |  |  |  |  |  |  |  |  |  |
| --- | --- | --- | --- | --- | --- | --- | --- | --- | --- |
| chr9:38529134-38529709 | | | 573 bp | | |  |  |  |  |
| Genome Start | Genome End | Strand | Div | Repeat Start | Repeat End | Repeat Left | Name | Family | Class |
| 38529134 | 38529267 | - | 11.9 | 6159 | 6305 | -7 | L1MA9 | L1 | LINE |
| 38529268 | 38529388 | - | 11.0 | 173 | 292 | -20 | AluSg/x | Alu | SINE |
| 38529392 | 38529709 | - | 17.6 | 5819 | 6155 | -157 | L1MA9 | L1 | LINE |

  

|  |  |  |  |  |  |  |  |  |  |
| --- | --- | --- | --- | --- | --- | --- | --- | --- | --- |
| chr9:38569007-38570730 | | | 1650 bp | | |  |  |  |  |
| Genome Start | Genome End | Strand | Div | Repeat Start | Repeat End | Repeat Left | Name | Family | Class |
| 38569007 | 38570117 | + | 19.9 | 4805 | 5946 | -354 | L1MA5 | L1 | LINE |
| 38570119 | 38570377 | + | 23.4 | 1 | 264 | -48 | AluJo | Alu | SINE |
| 38570451 | 38570730 | + | 17.0 | 6009 | 6294 | -6 | L1MA5 | L1 | LINE |

  

|  |  |  |  |  |  |  |  |  |  |
| --- | --- | --- | --- | --- | --- | --- | --- | --- | --- |
| chr9:38593799-38594600 | | | 757 bp | | |  |  |  |  |
| Genome Start | Genome End | Strand | Div | Repeat Start | Repeat End | Repeat Left | Name | Family | Class |
| 38593799 | 38594014 | + | 32.1 | 5 | 243 | -372 | MLT1L | MaLR | LTR |
| 38594015 | 38594308 | + | 14.6 | 1 | 294 | -15 | AluSc | Alu | SINE |
| 38594309 | 38594495 | + | 32.1 | 244 | 448 | -167 | MLT1L | MaLR | LTR |
| 38594541 | 38594600 | + | 21.7 | 546 | 609 | -6 | MLT1L | MaLR | LTR |

  

|  |  |  |  |  |  |  |  |  |  |
| --- | --- | --- | --- | --- | --- | --- | --- | --- | --- |
| chr9:38775510-38776235 | | | 726 bp | | |  |  |  |  |
| Genome Start | Genome End | Strand | Div | Repeat Start | Repeat End | Repeat Left | Name | Family | Class |
| 38775510 | 38775547 | + | 18.6 | 4298 | 4327 | -2049 | L1M4c | L1 | LINE |
| 38775548 | 38775861 | - | 5.0 | 1 | 304 | -7 | AluY | Alu | SINE |
| 38775862 | 38776235 | + | 18.6 | 4328 | 4700 | -1676 | L1M4c | L1 | LINE |

  

|  |  |  |  |  |  |  |  |  |  |
| --- | --- | --- | --- | --- | --- | --- | --- | --- | --- |
| chr9:38784495-38795018 | | | 10478 bp | | |  |  |  |  |
| Genome Start | Genome End | Strand | Div | Repeat Start | Repeat End | Repeat Left | Name | Family | Class |
| 38784495 | 38786650 | - | 18.1 | 4922 | 7128 | -6 | L1MB5 | L1 | LINE |
| 38786651 | 38786959 | - | 12.5 | 1 | 295 | -15 | AluSg | Alu | SINE |
| 38786960 | 38787366 | - | 18.8 | 4520 | 4921 | -2185 | L1MB5 | L1 | LINE |
| 38787373 | 38787945 | - | 9.0 | 5591 | 6149 | -2 | L1PB2 | L1 | LINE |
| 38787946 | 38788258 | + | 8.0 | 1 | 311 | 0 | AluY | Alu | SINE |
| 38788259 | 38789524 | - | 11.2 | 4380 | 5590 | -561 | L1PB2 | L1 | LINE |
| 38789525 | 38789832 | - | 13.7 | 1 | 307 | -5 | AluSx | Alu | SINE |
| 38789833 | 38790285 | - | 12.1 | 3929 | 4379 | -1767 | L1PB2 | L1 | LINE |
| 38790287 | 38791795 | + | 12.8 | 2398 | 3910 | -2236 | L1PB2 | L1 | LINE |
| 38791792 | 38792017 | - | 16.7 | 4312 | 4538 | -2568 | L1MB5 | L1 | LINE |
| 38792019 | 38792315 | - | 9.1 | 1 | 297 | -15 | AluSx | Alu | SINE |
| 38792318 | 38792625 | - | 18.5 | 3625 | 3953 | -3153 | L1MB5 | L1 | LINE |
| 38792626 | 38792933 | - | 6.0 | 1 | 302 | -9 | AluY | Alu | SINE |
| 38792934 | 38793045 | - | 18.5 | 3515 | 3624 | -3482 | L1MB5 | L1 | LINE |
| 38793048 | 38793381 | - | 19.2 | 10 | 432 | 0 | MSTB1 | MaLR | LTR |
| 38793412 | 38793622 | - | 21.7 | 3306 | 3518 | -3582 | L1MB5 | L1 | LINE |
| 38793597 | 38794415 | - | 27.2 | 2565 | 3427 | -4138 | L1M1 | L1 | LINE |
| 38794422 | 38794837 | - | 28.1 | 649 | 1100 | -5930 | L1MB5 | L1 | LINE |
| 38794866 | 38795018 | - | 15.2 | 7 | 184 | -6846 | L1MB5 | L1 | LINE |

  

|  |  |  |  |  |  |  |  |  |  |
| --- | --- | --- | --- | --- | --- | --- | --- | --- | --- |
| chr9:38799250-38799871 | | | 622 bp | | |  |  |  |  |
| Genome Start | Genome End | Strand | Div | Repeat Start | Repeat End | Repeat Left | Name | Family | Class |
| 38799250 | 38799288 | - | 26.6 | 335 | 371 | -38 | MLT1I | MaLR | LTR |
| 38799289 | 38799590 | + | 10.6 | 1 | 303 | -7 | AluSg | Alu | SINE |
| 38799591 | 38799871 | - | 26.6 | 54 | 334 | -75 | MLT1I | MaLR | LTR |

  

|  |  |  |  |  |  |  |  |  |  |
| --- | --- | --- | --- | --- | --- | --- | --- | --- | --- |
| chr9:38808473-38811175 | | | 2681 bp | | |  |  |  |  |
| Genome Start | Genome End | Strand | Div | Repeat Start | Repeat End | Repeat Left | Name | Family | Class |
| 38808473 | 38808990 | - | 19.8 | 5692 | 6184 | 0 | L1MB7 | L1 | LINE |
| 38808991 | 38809281 | - | 9.0 | 1 | 292 | -18 | AluSg | Alu | SINE |
| 38809282 | 38810368 | - | 19.8 | 4536 | 5691 | -493 | L1MB7 | L1 | LINE |
| 38810369 | 38810493 | + | 10.7 | 174 | 295 | -18 | AluSp/q | Alu | SINE |
| 38810516 | 38810682 | - | 19.8 | 4387 | 4533 | -1613 | L1MB7 | L1 | LINE |
| 38810683 | 38810934 | - | 10.3 | 5876 | 6151 | -3 | L1PB3 | L1 | LINE |
| 38810935 | 38811175 | - | 19.8 | 4146 | 4386 | -1760 | L1MB7 | L1 | LINE |

  

|  |  |  |  |  |  |  |  |  |  |
| --- | --- | --- | --- | --- | --- | --- | --- | --- | --- |
| chr9:38855462-38857888 | | | 2427 bp | | |  |  |  |  |
| Genome Start | Genome End | Strand | Div | Repeat Start | Repeat End | Repeat Left | Name | Family | Class |
| 38855462 | 38855659 | + | 24.2 | 4351 | 4523 | -1856 | L1MB2 | L1 | LINE |
| 38855660 | 38855943 | + | 6.0 | 1 | 290 | -21 | AluY | Alu | SINE |
| 38855944 | 38857888 | + | 21.6 | 4524 | 6406 | -1 | L1MB2 | L1 | LINE |

  

|  |  |  |  |  |  |  |  |  |  |
| --- | --- | --- | --- | --- | --- | --- | --- | --- | --- |
| chr9:38864586-38865052 | | | 467 bp | | |  |  |  |  |
| Genome Start | Genome End | Strand | Div | Repeat Start | Repeat End | Repeat Left | Name | Family | Class |
| 38864586 | 38864699 | - | 32.9 | 64 | 178 | 0 | MER5B | MER1\_type | DNA |
| 38864700 | 38864991 | - | 12.7 | 1 | 293 | -19 | AluSx | Alu | SINE |
| 38864992 | 38865052 | - | 32.9 | 1 | 63 | -115 | MER5B | MER1\_type | DNA |

  

|  |  |  |  |  |  |  |  |  |  |
| --- | --- | --- | --- | --- | --- | --- | --- | --- | --- |
| chr9:38896891-38897691 | | | 775 bp | | |  |  |  |  |
| Genome Start | Genome End | Strand | Div | Repeat Start | Repeat End | Repeat Left | Name | Family | Class |
| 38896891 | 38897210 | - | 20.8 | 5833 | 6168 | -6 | L1MB5 | L1 | LINE |
| 38897211 | 38897231 | - | 15.3 | 287 | 307 | -5 | AluSx | Alu | SINE |
| 38897258 | 38897546 | - | 15.3 | 2 | 286 | -26 | AluSx | Alu | SINE |
| 38897547 | 38897691 | - | 21.9 | 5683 | 5832 | -342 | L1MB5 | L1 | LINE |

  

|  |  |  |  |  |  |  |  |  |  |
| --- | --- | --- | --- | --- | --- | --- | --- | --- | --- |
| chr9:38901114-38904193 | | | 3072 bp | | |  |  |  |  |
| Genome Start | Genome End | Strand | Div | Repeat Start | Repeat End | Repeat Left | Name | Family | Class |
| 38901114 | 38901992 | - | 23.5 | 3235 | 4149 | -42 | MER21C-int | ERV1 | LTR |
| 38901993 | 38902172 | - | 11.2 | 134 | 313 | 0 | AluSp | Alu | SINE |
| 38902173 | 38902475 | - | 8.7 | 1 | 300 | -11 | AluY | Alu | SINE |
| 38902476 | 38902611 | - | 11.2 | 1 | 133 | -180 | AluSp | Alu | SINE |
| 38902612 | 38903217 | - | 25.6 | 2565 | 3234 | -957 | MER21C-int | ERV1 | LTR |
| 38903226 | 38903298 | - | 29.9 | 2222 | 2284 | -1907 | MER21C-int | ERV1 | LTR |
| 38903299 | 38903451 | + | 21.6 | 1 | 151 | -257 | MLT1A1 | MaLR | LTR |
| 38903452 | 38903742 | + | 10.3 | 6 | 302 | -9 | AluY | Alu | SINE |
| 38903743 | 38903991 | + | 21.6 | 152 | 408 | 0 | MLT1A1 | MaLR | LTR |
| 38903992 | 38904193 | - | 29.9 | 2013 | 2221 | -1970 | MER21C-int | ERV1 | LTR |

  

|  |  |  |  |  |  |  |  |  |  |
| --- | --- | --- | --- | --- | --- | --- | --- | --- | --- |
| chr9:38912803-38913564 | | | 761 bp | | |  |  |  |  |
| Genome Start | Genome End | Strand | Div | Repeat Start | Repeat End | Repeat Left | Name | Family | Class |
| 38912803 | 38912941 | - | 17.3 | 5597 | 5735 | -431 | L1P4 | L1 | LINE |
| 38912943 | 38913255 | - | 7.1 | 1 | 309 | -2 | AluY | Alu | SINE |
| 38913256 | 38913564 | - | 9.1 | 4759 | 5067 | -1079 | L1P4 | L1 | LINE |

  

|  |  |  |  |  |  |  |  |  |  |
| --- | --- | --- | --- | --- | --- | --- | --- | --- | --- |
| chr9:38913565-38914275 | | | 711 bp | | |  |  |  |  |
| Genome Start | Genome End | Strand | Div | Repeat Start | Repeat End | Repeat Left | Name | Family | Class |
| 38913565 | 38913658 | + | 12.4 | 5736 | 5829 | -337 | L1PA16 | L1 | LINE |
| 38913659 | 38913954 | + | 7.8 | 1 | 294 | -17 | AluY | Alu | SINE |
| 38913955 | 38914275 | + | 12.4 | 5830 | 6156 | -10 | L1PA16 | L1 | LINE |

  

|  |  |  |  |  |  |  |  |  |  |
| --- | --- | --- | --- | --- | --- | --- | --- | --- | --- |
| chr9:38920040-38922279 | | | 2223 bp | | |  |  |  |  |
| Genome Start | Genome End | Strand | Div | Repeat Start | Repeat End | Repeat Left | Name | Family | Class |
| 38920040 | 38920562 | - | 19.5 | 5786 | 6295 | -39 | L1MA10 | L1 | LINE |
| 38920563 | 38921203 | - | 9.5 | 1 | 600 | 0 | MER4A1\_ | ERV1 | LTR |
| 38921204 | 38921393 | - | 19.5 | 5609 | 5785 | -549 | L1MA10 | L1 | LINE |
| 38921394 | 38921695 | - | 3.7 | 1 | 298 | -13 | AluY | Alu | SINE |
| 38921696 | 38921936 | - | 19.5 | 5364 | 5608 | -726 | L1MA10 | L1 | LINE |
| 38921954 | 38922133 | - | 6.7 | 135 | 314 | 0 | AluY | Alu | SINE |
| 38922134 | 38922279 | - | 16.5 | 5227 | 5380 | -766 | L1MA10 | L1 | LINE |

  

|  |  |  |  |  |  |  |  |  |  |
| --- | --- | --- | --- | --- | --- | --- | --- | --- | --- |
| chr9:38938985-38939966 | | | 982 bp | | |  |  |  |  |
| Genome Start | Genome End | Strand | Div | Repeat Start | Repeat End | Repeat Left | Name | Family | Class |
| 38938985 | 38939299 | + | 23.3 | 1 | 234 | -376 | LTR38B | ERV1 | LTR |
| 38939300 | 38939601 | + | 3.0 | 1 | 302 | -9 | AluY | Alu | SINE |
| 38939602 | 38939966 | + | 23.5 | 235 | 591 | 0 | LTR38B | ERV1 | LTR |

  

|  |  |  |  |  |  |  |  |  |  |
| --- | --- | --- | --- | --- | --- | --- | --- | --- | --- |
| chr9:38948151-38949834 | | | 1684 bp | | |  |  |  |  |
| Genome Start | Genome End | Strand | Div | Repeat Start | Repeat End | Repeat Left | Name | Family | Class |
| 38948151 | 38948492 | + | 14.3 | 1 | 395 | -505 | MER4D1 | ERV1 | LTR |
| 38948493 | 38948780 | + | 13.5 | 1 | 299 | -13 | AluSx | Alu | SINE |
| 38948781 | 38949277 | + | 14.3 | 396 | 855 | -45 | MER4D1 | ERV1 | LTR |
| 38949278 | 38949783 | - | 11.1 | 1 | 511 | 0 | MER9 | ERVK | LTR |
| 38949784 | 38949834 | + | 14.3 | 856 | 900 | 0 | MER4D1 | ERV1 | LTR |

  

|  |  |  |  |  |  |  |  |  |  |
| --- | --- | --- | --- | --- | --- | --- | --- | --- | --- |
| chr9:38964167-38965338 | | | 1172 bp | | |  |  |  |  |
| Genome Start | Genome End | Strand | Div | Repeat Start | Repeat End | Repeat Left | Name | Family | Class |
| 38964167 | 38964366 | + | 35.6 | 2092 | 2302 | -1117 | L2 | L2 | LINE |
| 38964367 | 38964517 | - | 24.5 | 3 | 156 | -4 | MER5A1 | MER1\_type | DNA |
| 38964518 | 38964825 | + | 35.6 | 2303 | 2617 | -802 | L2 | L2 | LINE |
| 38964826 | 38965129 | + | 7.2 | 1 | 304 | -6 | AluSg | Alu | SINE |
| 38965130 | 38965338 | + | 35.6 | 2618 | 2831 | -588 | L2 | L2 | LINE |

  

|  |  |  |  |  |  |  |  |  |  |
| --- | --- | --- | --- | --- | --- | --- | --- | --- | --- |
| chr9:38967027-38967930 | | | 904 bp | | |  |  |  |  |
| Genome Start | Genome End | Strand | Div | Repeat Start | Repeat End | Repeat Left | Name | Family | Class |
| 38967027 | 38967550 | - | 25.6 | 65 | 642 | -2 | LTR48B | ERV1 | LTR |
| 38967551 | 38967854 | + | 6.2 | 1 | 304 | -7 | AluY | Alu | SINE |
| 38967855 | 38967930 | - | 25.6 | 1 | 64 | -580 | LTR48B | ERV1 | LTR |

  

|  |  |  |  |  |  |  |  |  |  |
| --- | --- | --- | --- | --- | --- | --- | --- | --- | --- |
| chr9:38977227-38977978 | | | 707 bp | | |  |  |  |  |
| Genome Start | Genome End | Strand | Div | Repeat Start | Repeat End | Repeat Left | Name | Family | Class |
| 38977227 | 38977343 | - | 24.0 | 344 | 462 | 0 | LTR40b | ERVL | LTR |
| 38977389 | 38977675 | - | 9.4 | 1 | 287 | -24 | AluY | Alu | SINE |
| 38977676 | 38977978 | - | 24.0 | 1 | 343 | -119 | LTR40b | ERVL | LTR |

  

|  |  |  |  |  |  |  |  |  |  |
| --- | --- | --- | --- | --- | --- | --- | --- | --- | --- |
| chr9:38984676-38986664 | | | 1989 bp | | |  |  |  |  |
| Genome Start | Genome End | Strand | Div | Repeat Start | Repeat End | Repeat Left | Name | Family | Class |
| 38984676 | 38985803 | + | 16.8 | 4443 | 5587 | -757 | L1MC2 | L1 | LINE |
| 38985804 | 38986099 | - | 8.4 | 1 | 296 | -13 | AluSc | Alu | SINE |
| 38986100 | 38986664 | + | 15.6 | 5588 | 6235 | -109 | L1MC2 | L1 | LINE |

  

|  |  |  |  |  |  |  |  |  |  |
| --- | --- | --- | --- | --- | --- | --- | --- | --- | --- |
| chr9:38995003-38995809 | | | 807 bp | | |  |  |  |  |
| Genome Start | Genome End | Strand | Div | Repeat Start | Repeat End | Repeat Left | Name | Family | Class |
| 38995003 | 38995330 | - | 28.4 | 5851 | 6156 | -10 | L1ME1 | L1 | LINE |
| 38995331 | 38995623 | - | 7.9 | 1 | 292 | -19 | AluY | Alu | SINE |
| 38995624 | 38995809 | - | 28.4 | 5671 | 5850 | -316 | L1ME1 | L1 | LINE |

  

|  |  |  |  |  |  |  |  |  |  |
| --- | --- | --- | --- | --- | --- | --- | --- | --- | --- |
| chr9:39003877-39004376 | | | 500 bp | | |  |  |  |  |
| Genome Start | Genome End | Strand | Div | Repeat Start | Repeat End | Repeat Left | Name | Family | Class |
| 39003877 | 39003963 | + | 29.6 | 3225 | 3312 | -107 | L2 | L2 | LINE |
| 39003964 | 39004260 | - | 12.9 | 1 | 295 | -18 | AluSq | Alu | SINE |
| 39004261 | 39004376 | + | 29.6 | 3313 | 3418 | -1 | L2 | L2 | LINE |

  

|  |  |  |  |  |  |  |  |  |  |
| --- | --- | --- | --- | --- | --- | --- | --- | --- | --- |
| chr9:39014466-39015193 | | | 728 bp | | |  |  |  |  |
| Genome Start | Genome End | Strand | Div | Repeat Start | Repeat End | Repeat Left | Name | Family | Class |
| 39014466 | 39014799 | + | 26.1 | 8 | 304 | -187 | MER110 | ERV1 | LTR |
| 39014800 | 39015093 | - | 8.5 | 1 | 296 | -15 | AluY | Alu | SINE |
| 39015094 | 39015193 | + | 25.5 | 305 | 390 | -101 | MER110 | ERV1 | LTR |

  

|  |  |  |  |  |  |  |  |  |  |
| --- | --- | --- | --- | --- | --- | --- | --- | --- | --- |
| chr9:39034957-39036192 | | | 1166 bp | | |  |  |  |  |
| Genome Start | Genome End | Strand | Div | Repeat Start | Repeat End | Repeat Left | Name | Family | Class |
| 39034957 | 39035344 | - | 25.5 | 63 | 459 | -6 | LTR40c | ERVL | LTR |
| 39035408 | 39036118 | - | 18.9 | 5447 | 6181 | -2 | L1MB3 | L1 | LINE |
| 39036126 | 39036192 | - | 20.3 | 5 | 70 | -395 | LTR40c | ERVL | LTR |

  

|  |  |  |  |  |  |  |  |  |  |
| --- | --- | --- | --- | --- | --- | --- | --- | --- | --- |
| chr9:39036550-39042228 | | | 5679 bp | | |  |  |  |  |
| Genome Start | Genome End | Strand | Div | Repeat Start | Repeat End | Repeat Left | Name | Family | Class |
| 39036550 | 39038797 | + | 17.7 | 1301 | 3597 | -3169 | L1PA15 | L1 | LINE |
| 39038798 | 39039067 | - | 6.3 | 1 | 294 | -17 | AluY | Alu | SINE |
| 39039068 | 39042228 | + | 15.5 | 3598 | 6784 | -1 | L1PA15 | L1 | LINE |

  

|  |  |  |  |  |  |  |  |  |  |
| --- | --- | --- | --- | --- | --- | --- | --- | --- | --- |
| chr9:39044457-39045166 | | | 710 bp | | |  |  |  |  |
| Genome Start | Genome End | Strand | Div | Repeat Start | Repeat End | Repeat Left | Name | Family | Class |
| 39044457 | 39044812 | + | 24.8 | 1 | 339 | -35 | MLT1A | MaLR | LTR |
| 39044813 | 39045128 | - | 8.0 | 1 | 311 | 0 | AluY | Alu | SINE |
| 39045129 | 39045166 | + | 24.8 | 340 | 368 | -6 | MLT1A | MaLR | LTR |

  

|  |  |  |  |  |  |  |  |  |  |
| --- | --- | --- | --- | --- | --- | --- | --- | --- | --- |
| chr9:39047118-39047838 | | | 721 bp | | |  |  |  |  |
| Genome Start | Genome End | Strand | Div | Repeat Start | Repeat End | Repeat Left | Name | Family | Class |
| 39047118 | 39047212 | + | 27.5 | 2 | 83 | -179 | MIR | MIR | SINE |
| 39047213 | 39047698 | + | 14.4 | 1 | 489 | 0 | LTR45B | ERV1 | LTR |
| 39047699 | 39047838 | + | 27.5 | 84 | 225 | -37 | MIR | MIR | SINE |

  

|  |  |  |  |  |  |  |  |  |  |
| --- | --- | --- | --- | --- | --- | --- | --- | --- | --- |
| chr9:39071738-39072271 | | | 534 bp | | |  |  |  |  |
| Genome Start | Genome End | Strand | Div | Repeat Start | Repeat End | Repeat Left | Name | Family | Class |
| 39071738 | 39071793 | - | 19.5 | 162 | 209 | 0 | MER3 | MER1\_type | DNA |
| 39071794 | 39072103 | + | 8.3 | 1 | 301 | -10 | AluY | Alu | SINE |
| 39072104 | 39072271 | - | 19.5 | 5 | 161 | -48 | MER3 | MER1\_type | DNA |

  

|  |  |  |  |  |  |  |  |  |  |
| --- | --- | --- | --- | --- | --- | --- | --- | --- | --- |
| chr9:39094579-39096070 | | | 1492 bp | | |  |  |  |  |
| Genome Start | Genome End | Strand | Div | Repeat Start | Repeat End | Repeat Left | Name | Family | Class |
| 39094579 | 39095142 | - | 24.1 | 7100 | 7678 | -364 | L1MC4 | L1 | LINE |
| 39095143 | 39095449 | + | 8.5 | 1 | 307 | 0 | AluY | Alu | SINE |
| 39095450 | 39096070 | - | 24.1 | 6459 | 7099 | -943 | L1MC4 | L1 | LINE |

  

|  |  |  |  |  |  |  |  |  |  |
| --- | --- | --- | --- | --- | --- | --- | --- | --- | --- |
| chr9:39109642-39111392 | | | 1775 bp | | |  |  |  |  |
| Genome Start | Genome End | Strand | Div | Repeat Start | Repeat End | Repeat Left | Name | Family | Class |
| 39109642 | 39109820 | + | 24.8 | 3 | 298 | -6242 | L1MDa | L1 | LINE |
| 39109797 | 39110389 | + | 22.7 | 1327 | 1949 | -4684 | L1MDa | L1 | LINE |
| 39110390 | 39110690 | + | 7.0 | 1 | 305 | -6 | AluY | Alu | SINE |
| 39110691 | 39111392 | + | 20.4 | 1950 | 2888 | -3649 | L1MDa | L1 | LINE |

  

|  |  |  |  |  |  |  |  |  |  |
| --- | --- | --- | --- | --- | --- | --- | --- | --- | --- |
| chr9:39116757-39120986 | | | 4230 bp | | |  |  |  |  |
| Genome Start | Genome End | Strand | Div | Repeat Start | Repeat End | Repeat Left | Name | Family | Class |
| 39116757 | 39117821 | + | 20.1 | 2490 | 3550 | -2596 | L1MD1 | L1 | LINE |
| 39117822 | 39118135 | - | 10.9 | 1 | 312 | 0 | AluSx | Alu | SINE |
| 39118136 | 39120489 | + | 19.0 | 3551 | 6012 | -230 | L1MD1 | L1 | LINE |
| 39120490 | 39120799 | - | 6.2 | 1 | 306 | -5 | AluY | Alu | SINE |
| 39120800 | 39120986 | + | 16.7 | 6013 | 6221 | -21 | L1MD1 | L1 | LINE |

  

|  |  |  |  |  |  |  |  |  |  |
| --- | --- | --- | --- | --- | --- | --- | --- | --- | --- |
| chr9:39126357-39128969 | | | 2550 bp | | |  |  |  |  |
| Genome Start | Genome End | Strand | Div | Repeat Start | Repeat End | Repeat Left | Name | Family | Class |
| 39126357 | 39126417 | - | 16.4 | 2582 | 2647 | -3674 | L1MCa | L1 | LINE |
| 39126412 | 39126459 | - | 12.7 | 1605 | 1672 | -4836 | L1MCa | L1 | LINE |
| 39126480 | 39126614 | - | 12.7 | 1449 | 1604 | -4904 | L1MCa | L1 | LINE |
| 39126615 | 39126904 | + | 11.8 | 1 | 290 | -23 | AluSp | Alu | SINE |
| 39126905 | 39127497 | - | 12.7 | 860 | 1448 | -5060 | L1MCa | L1 | LINE |
| 39127498 | 39127801 | - | 8.6 | 10 | 311 | 0 | AluY | Alu | SINE |
| 39127802 | 39127849 | - | 12.7 | 817 | 859 | -5649 | L1MCa | L1 | LINE |
| 39127854 | 39128167 | - | 18.6 | 22 | 303 | -9 | AluJo | Alu | SINE |
| 39128174 | 39128449 | - | 18.1 | 530 | 804 | -5704 | L1MCa | L1 | LINE |
| 39128489 | 39128969 | - | 18.1 | 30 | 529 | -5979 | L1MCa | L1 | LINE |

  

|  |  |  |  |  |  |  |  |  |  |
| --- | --- | --- | --- | --- | --- | --- | --- | --- | --- |
| chr9:39246405-39247012 | | | 515 bp | | |  |  |  |  |
| Genome Start | Genome End | Strand | Div | Repeat Start | Repeat End | Repeat Left | Name | Family | Class |
| 39246405 | 39246507 | + | 21.0 | 24 | 127 | -88 | MER58C | MER1\_type | DNA |
| 39246510 | 39246817 | + | 20.9 | 1 | 306 | -6 | AluJo | Alu | SINE |
| 39246909 | 39247012 | + | 24.0 | 106 | 213 | -2 | MER58C | MER1\_type | DNA |

  

|  |  |  |  |  |  |  |  |  |  |
| --- | --- | --- | --- | --- | --- | --- | --- | --- | --- |
| chr9:39286369-39287283 | | | 915 bp | | |  |  |  |  |
| Genome Start | Genome End | Strand | Div | Repeat Start | Repeat End | Repeat Left | Name | Family | Class |
| 39286369 | 39286595 | + | 11.8 | 5522 | 5787 | -378 | L1PA15 | L1 | LINE |
| 39286596 | 39286903 | + | 9.1 | 1 | 308 | -3 | AluY | Alu | SINE |
| 39286904 | 39287283 | + | 11.8 | 5788 | 6158 | -7 | L1PA15 | L1 | LINE |

  

|  |  |  |  |  |  |  |  |  |  |
| --- | --- | --- | --- | --- | --- | --- | --- | --- | --- |
| chr9:39291050-39298366 | | | 7288 bp | | |  |  |  |  |
| Genome Start | Genome End | Strand | Div | Repeat Start | Repeat End | Repeat Left | Name | Family | Class |
| 39291050 | 39291128 | + | 24.0 | 1324 | 1787 | -4983 | L1M4c | L1 | LINE |
| 39291150 | 39291213 | + | 25.0 | 1595 | 1658 | -4726 | L1M4c | L1 | LINE |
| 39291215 | 39291448 | + | 17.9 | 3 | 228 | -6280 | L1MC2 | L1 | LINE |
| 39291449 | 39291753 | + | 13.4 | 1 | 308 | -5 | AluSq | Alu | SINE |
| 39291754 | 39294046 | + | 17.9 | 229 | 2470 | -3851 | L1MC2 | L1 | LINE |
| 39294047 | 39294618 | + | 12.3 | 1 | 572 | 0 | LTR43B | ERV1 | LTR |
| 39294619 | 39296441 | + | 19.0 | 2471 | 4273 | -2048 | L1MC2 | L1 | LINE |
| 39296442 | 39296750 | + | 1.3 | 1 | 309 | -1 | AluYa5 | Alu | SINE |
| 39296751 | 39297456 | + | 19.0 | 4274 | 4896 | -1425 | L1MC2 | L1 | LINE |
| 39297467 | 39297884 | + | 18.4 | 6082 | 6518 | -1 | L1MC2 | L1 | LINE |
| 39297882 | 39298366 | + | 22.9 | 2390 | 2889 | -3493 | L1M4c | L1 | LINE |

  

|  |  |  |  |  |  |  |  |  |  |
| --- | --- | --- | --- | --- | --- | --- | --- | --- | --- |
| chr9:39307674-39309562 | | | 1889 bp | | |  |  |  |  |
| Genome Start | Genome End | Strand | Div | Repeat Start | Repeat End | Repeat Left | Name | Family | Class |
| 39307674 | 39307794 | + | 29.9 | 2 | 124 | -1611 | MLT-int | MaLR | LTR |
| 39307795 | 39308101 | + | 12.3 | 1 | 308 | -5 | AluSq | Alu | SINE |
| 39308102 | 39309562 | + | 29.9 | 125 | 1734 | -1 | MLT-int | MaLR | LTR |

  

|  |  |  |  |  |  |  |  |  |  |
| --- | --- | --- | --- | --- | --- | --- | --- | --- | --- |
| chr9:39314665-39316429 | | | 1774 bp | | |  |  |  |  |
| Genome Start | Genome End | Strand | Div | Repeat Start | Repeat End | Repeat Left | Name | Family | Class |
| 39314665 | 39314876 | + | 21.1 | 2 | 211 | -872 | Tigger6a | MER2\_type | DNA |
| 39314877 | 39315332 | - | 22.8 | 11 | 467 | 0 | MLT1C | MaLR | LTR |
| 39315324 | 39315948 | + | 19.5 | 216 | 883 | -200 | Tigger6a | MER2\_type | DNA |
| 39315949 | 39316233 | + | 10.5 | 3 | 300 | -11 | AluY | Alu | SINE |
| 39316234 | 39316429 | + | 19.5 | 884 | 1083 | 0 | Tigger6a | MER2\_type | DNA |

  

|  |  |  |  |  |  |  |  |  |  |
| --- | --- | --- | --- | --- | --- | --- | --- | --- | --- |
| chr9:39322105-39323433 | | | 1217 bp | | |  |  |  |  |
| Genome Start | Genome End | Strand | Div | Repeat Start | Repeat End | Repeat Left | Name | Family | Class |
| 39322105 | 39322323 | - | 33.3 | 4851 | 5064 | -1082 | L1M5 | L1 | LINE |
| 39322437 | 39322510 | - | 24.3 | 4633 | 4709 | -1437 | L1M5 | L1 | LINE |
| 39322510 | 39322679 | - | 27.7 | 3928 | 4104 | -2042 | L1M5 | L1 | LINE |
| 39322680 | 39322981 | - | 8.3 | 1 | 301 | -10 | AluY | Alu | SINE |
| 39322982 | 39323433 | - | 27.7 | 3375 | 3927 | -2219 | L1M5 | L1 | LINE |

  

|  |  |  |  |  |  |  |  |  |  |
| --- | --- | --- | --- | --- | --- | --- | --- | --- | --- |
| chr9:39323458-39327481 | | | 3943 bp | | |  |  |  |  |
| Genome Start | Genome End | Strand | Div | Repeat Start | Repeat End | Repeat Left | Name | Family | Class |
| 39323458 | 39323727 | - | 26.9 | 5890 | 6204 | -19 | L1ME3B | L1 | LINE |
| 39323728 | 39324031 | - | 7.3 | 1 | 301 | -10 | AluY | Alu | SINE |
| 39324032 | 39324127 | - | 26.9 | 5828 | 5889 | -334 | L1ME3B | L1 | LINE |
| 39324128 | 39324605 | - | 14.9 | 5818 | 6322 | -11 | L1MC1 | L1 | LINE |
| 39324606 | 39324921 | - | 10.3 | 1 | 311 | 0 | AluY | Alu | SINE |
| 39324922 | 39325299 | - | 14.9 | 5435 | 5817 | -516 | L1MC1 | L1 | LINE |
| 39325300 | 39326016 | - | 27.4 | 5043 | 5827 | -396 | L1ME3B | L1 | LINE |
| 39326017 | 39326315 | + | 7.5 | 1 | 295 | -16 | AluY | Alu | SINE |
| 39326316 | 39326505 | - | 30.3 | 4839 | 5042 | -1087 | L1ME3B | L1 | LINE |
| 39326578 | 39327004 | - | 15.2 | 1 | 436 | -231 | LTR12B | ERV1 | LTR |
| 39327014 | 39327481 | - | 33.7 | 64 | 559 | -5560 | L1ME3B | L1 | LINE |

  

|  |  |  |  |  |  |  |  |  |  |
| --- | --- | --- | --- | --- | --- | --- | --- | --- | --- |
| chr9:39337885-39340931 | | | 3040 bp | | |  |  |  |  |
| Genome Start | Genome End | Strand | Div | Repeat Start | Repeat End | Repeat Left | Name | Family | Class |
| 39337885 | 39338279 | + | 23.8 | 2917 | 3348 | -4217 | L1M1 | L1 | LINE |
| 39338280 | 39338615 | - | 18.8 | 4 | 345 | -1 | MER7A | MER2\_type | DNA |
| 39338616 | 39339864 | + | 18.7 | 3349 | 4638 | -2921 | L1M1 | L1 | LINE |
| 39339865 | 39340175 | + | 0.3 | 1 | 310 | 0 | AluYa5 | Alu | SINE |
| 39340183 | 39340483 | + | 9.4 | 1 | 308 | 0 | AluY | Alu | SINE |
| 39340484 | 39340931 | + | 17.6 | 4642 | 5099 | -2460 | L1M1 | L1 | LINE |

  

|  |  |  |  |  |  |  |  |  |  |
| --- | --- | --- | --- | --- | --- | --- | --- | --- | --- |
| chr9:39362196-39363005 | | | 810 bp | | |  |  |  |  |
| Genome Start | Genome End | Strand | Div | Repeat Start | Repeat End | Repeat Left | Name | Family | Class |
| 39362196 | 39362266 | - | 9.9 | 320 | 390 | 0 | MLT1B | MaLR | LTR |
| 39362267 | 39362628 | - | 1.2 | 5815 | 6176 | 0 | L1PA3 | L1 | LINE |
| 39362629 | 39362673 | + | 0.0 | 5770 | 5814 | -358 | L1P3 | L1 | LINE |
| 39362674 | 39363005 | - | 18.0 | 1 | 331 | -59 | MLT1B | MaLR | LTR |

  

|  |  |  |  |  |  |  |  |  |  |
| --- | --- | --- | --- | --- | --- | --- | --- | --- | --- |
| chr9:39378160-39380367 | | | 2208 bp | | |  |  |  |  |
| Genome Start | Genome End | Strand | Div | Repeat Start | Repeat End | Repeat Left | Name | Family | Class |
| 39378160 | 39379314 | - | 18.2 | 4190 | 5331 | -815 | L1MC | L1 | LINE |
| 39379315 | 39379616 | - | 7.3 | 1 | 302 | -9 | AluY | Alu | SINE |
| 39379617 | 39379860 | - | 18.2 | 3965 | 4189 | -1957 | L1MC | L1 | LINE |
| 39379861 | 39380188 | - | 13.0 | 1 | 308 | -2 | AluSg | Alu | SINE |
| 39380189 | 39380367 | - | 18.2 | 3776 | 3964 | -2182 | L1MC | L1 | LINE |

  

|  |  |  |  |  |  |  |  |  |  |
| --- | --- | --- | --- | --- | --- | --- | --- | --- | --- |
| chr9:39380852-39381858 | | | 864 bp | | |  |  |  |  |
| Genome Start | Genome End | Strand | Div | Repeat Start | Repeat End | Repeat Left | Name | Family | Class |
| 39380852 | 39381068 | + | 33.8 | 3523 | 3773 | -716 | L3 | CR1 | LINE |
| 39381072 | 39381381 | + | 10.3 | 1 | 308 | 0 | AluSg | Alu | SINE |
| 39381522 | 39381858 | + | 33.6 | 3966 | 4333 | -156 | L3 | CR1 | LINE |

  

|  |  |  |  |  |  |  |  |  |  |
| --- | --- | --- | --- | --- | --- | --- | --- | --- | --- |
| chr9:39389585-39392245 | | | 2661 bp | | |  |  |  |  |
| Genome Start | Genome End | Strand | Div | Repeat Start | Repeat End | Repeat Left | Name | Family | Class |
| 39389585 | 39390981 | + | 10.5 | 3147 | 4544 | -1602 | L1PREC2 | L1 | LINE |
| 39390982 | 39391282 | + | 10.0 | 1 | 301 | -8 | AluSc | Alu | SINE |
| 39391283 | 39392245 | + | 9.8 | 4545 | 5480 | -680 | L1PREC2 | L1 | LINE |

  

|  |  |  |  |  |  |  |  |  |  |
| --- | --- | --- | --- | --- | --- | --- | --- | --- | --- |
| chr9:39396729-39397427 | | | 699 bp | | |  |  |  |  |
| Genome Start | Genome End | Strand | Div | Repeat Start | Repeat End | Repeat Left | Name | Family | Class |
| 39396729 | 39396908 | - | 18.5 | 138 | 316 | 0 | AluJo | Alu | SINE |
| 39396909 | 39397271 | - | 9.9 | 1 | 364 | 0 | THE1B | MaLR | LTR |
| 39397272 | 39397427 | - | 18.5 | 1 | 137 | -175 | AluJo | Alu | SINE |

  

|  |  |  |  |  |  |  |  |  |  |
| --- | --- | --- | --- | --- | --- | --- | --- | --- | --- |
| chr9:39403831-39405220 | | | 1390 bp | | |  |  |  |  |
| Genome Start | Genome End | Strand | Div | Repeat Start | Repeat End | Repeat Left | Name | Family | Class |
| 39403831 | 39404238 | + | 30.7 | 4475 | 4915 | -1231 | L1M5 | L1 | LINE |
| 39404239 | 39404574 | + | 8.5 | 1 | 322 | 0 | AluSx | Alu | SINE |
| 39404575 | 39404585 | + | 8.5 | 284 | 294 | -18 | AluSx | Alu | SINE |
| 39404586 | 39404807 | + | 30.7 | 4916 | 5170 | -976 | L1M5 | L1 | LINE |
| 39404808 | 39405099 | - | 7.5 | 1 | 294 | -17 | AluY | Alu | SINE |
| 39405100 | 39405220 | + | 30.7 | 5171 | 5283 | -863 | L1M5 | L1 | LINE |

  

|  |  |  |  |  |  |  |  |  |  |
| --- | --- | --- | --- | --- | --- | --- | --- | --- | --- |
| chr9:39427358-39429797 | | | 2420 bp | | |  |  |  |  |
| Genome Start | Genome End | Strand | Div | Repeat Start | Repeat End | Repeat Left | Name | Family | Class |
| 39427358 | 39428814 | + | 20.9 | 50 | 1538 | -5796 | L1M4b | L1 | LINE |
| 39428805 | 39428974 | + | 11.8 | 5979 | 6139 | -163 | L1MA1 | L1 | LINE |
| 39429002 | 39429049 | + | 15.2 | 459 | 504 | 0 | MER75 | PiggyBac | DNA |
| 39429050 | 39429212 | + | 5.5 | 6140 | 6302 | 0 | L1MA1 | L1 | LINE |
| 39429216 | 39429797 | + | 19.0 | 1509 | 2173 | -5161 | L1M4b | L1 | LINE |

  

|  |  |  |  |  |  |  |  |  |  |
| --- | --- | --- | --- | --- | --- | --- | --- | --- | --- |
| chr9:39441666-39442860 | | | 1113 bp | | |  |  |  |  |
| Genome Start | Genome End | Strand | Div | Repeat Start | Repeat End | Repeat Left | Name | Family | Class |
| 39441666 | 39441797 | - | 29.3 | 879 | 1015 | -5618 | L1MDa | L1 | LINE |
| 39441798 | 39442101 | - | 9.9 | 1 | 308 | -5 | AluSq | Alu | SINE |
| 39442102 | 39442168 | - | 29.3 | 822 | 878 | -5755 | L1MDa | L1 | LINE |
| 39442233 | 39442478 | - | 16.8 | 1 | 246 | -66 | AluSx | Alu | SINE |
| 39442479 | 39442577 | + | 13.1 | 45 | 143 | -2275 | Tigger1 | MER2\_type | DNA |
| 39442583 | 39442792 | + | 16.8 | 2205 | 2417 | -1 | Tigger1 | MER2\_type | DNA |
| 39442806 | 39442860 | - | 25.9 | 31 | 84 | -6549 | L1MDa | L1 | LINE |

  

|  |  |  |  |  |  |  |  |  |  |
| --- | --- | --- | --- | --- | --- | --- | --- | --- | --- |
| chr9:39442862-39444159 | | | 1105 bp | | |  |  |  |  |
| Genome Start | Genome End | Strand | Div | Repeat Start | Repeat End | Repeat Left | Name | Family | Class |
| 39442862 | 39443086 | - | 31.3 | 4836 | 5119 | -1027 | L1M5 | L1 | LINE |
| 39443087 | 39443372 | - | 9.1 | 1 | 309 | -2 | AluY | Alu | SINE |
| 39443373 | 39443560 | - | 31.3 | 4660 | 4835 | -1311 | L1M5 | L1 | LINE |
| 39443754 | 39444159 | - | 34.2 | 2578 | 3015 | -3131 | L1M5 | L1 | LINE |

  

|  |  |  |  |  |  |  |  |  |  |
| --- | --- | --- | --- | --- | --- | --- | --- | --- | --- |
| chr9:39445444-39446442 | | | 997 bp | | |  |  |  |  |
| Genome Start | Genome End | Strand | Div | Repeat Start | Repeat End | Repeat Left | Name | Family | Class |
| 39445444 | 39446065 | + | 21.1 | 5421 | 6084 | -94 | L1MB8 | L1 | LINE |
| 39446068 | 39446365 | - | 18.2 | 42 | 345 | 0 | MER2 | MER2\_type | DNA |
| 39446366 | 39446442 | + | 12.0 | 6103 | 6178 | 0 | L1MB8 | L1 | LINE |

  

|  |  |  |  |  |  |  |  |  |  |
| --- | --- | --- | --- | --- | --- | --- | --- | --- | --- |
| chr9:39448304-39450500 | | | 2197 bp | | |  |  |  |  |
| Genome Start | Genome End | Strand | Div | Repeat Start | Repeat End | Repeat Left | Name | Family | Class |
| 39448304 | 39449875 | + | 25.1 | 37 | 1682 | -5290 | L1M4 | L1 | LINE |
| 39449876 | 39450182 | + | 15.4 | 2 | 312 | 0 | AluSx | Alu | SINE |
| 39450183 | 39450500 | + | 24.8 | 1683 | 2056 | -4916 | L1M4 | L1 | LINE |

  

|  |  |  |  |  |  |  |  |  |  |
| --- | --- | --- | --- | --- | --- | --- | --- | --- | --- |
| chr9:39453126-39453628 | | | 498 bp | | |  |  |  |  |
| Genome Start | Genome End | Strand | Div | Repeat Start | Repeat End | Repeat Left | Name | Family | Class |
| 39453126 | 39453194 | + | 27.5 | 2 | 70 | -2542 | Charlie5 | MER1\_type | DNA |
| 39453200 | 39453282 | + | 23.8 | 2453 | 2539 | -73 | Charlie5 | MER1\_type | DNA |
| 39453283 | 39453576 | - | 9.2 | 1 | 297 | -15 | AluSx | Alu | SINE |
| 39453577 | 39453628 | + | 23.8 | 2540 | 2583 | -29 | Charlie5 | MER1\_type | DNA |

  

|  |  |  |  |  |  |  |  |  |  |
| --- | --- | --- | --- | --- | --- | --- | --- | --- | --- |
| chr9:39455484-39456062 | | | 579 bp | | |  |  |  |  |
| Genome Start | Genome End | Strand | Div | Repeat Start | Repeat End | Repeat Left | Name | Family | Class |
| 39455484 | 39455660 | - | 14.8 | 134 | 312 | 0 | AluJb | Alu | SINE |
| 39455661 | 39455953 | - | 10.7 | 1 | 299 | -14 | AluSq | Alu | SINE |
| 39455954 | 39456062 | - | 14.8 | 29 | 133 | -179 | AluJb | Alu | SINE |

  

|  |  |  |  |  |  |  |  |  |  |
| --- | --- | --- | --- | --- | --- | --- | --- | --- | --- |
| chr9:39456789-39458636 | | | 1848 bp | | |  |  |  |  |
| Genome Start | Genome End | Strand | Div | Repeat Start | Repeat End | Repeat Left | Name | Family | Class |
| 39456789 | 39457844 | - | 14.5 | 3855 | 4925 | -1221 | L1MC | L1 | LINE |
| 39457845 | 39458155 | - | 11.3 | 1 | 310 | 0 | AluSg | Alu | SINE |
| 39458156 | 39458636 | - | 14.5 | 3353 | 3854 | -2292 | L1MC | L1 | LINE |

  

|  |  |  |  |  |  |  |  |  |  |
| --- | --- | --- | --- | --- | --- | --- | --- | --- | --- |
| chr9:39458632-39460682 | | | 2051 bp | | |  |  |  |  |
| Genome Start | Genome End | Strand | Div | Repeat Start | Repeat End | Repeat Left | Name | Family | Class |
| 39458632 | 39458685 | - | 22.7 | 1572 | 1621 | -4887 | L1MCa | L1 | LINE |
| 39458686 | 39458979 | - | 7.6 | 9 | 299 | -12 | AluY | Alu | SINE |
| 39458980 | 39460682 | - | 22.7 | 1 | 1571 | -4937 | L1MCa | L1 | LINE |

  

|  |  |  |  |  |  |  |  |  |  |
| --- | --- | --- | --- | --- | --- | --- | --- | --- | --- |
| chr9:39468543-39469559 | | | 1017 bp | | |  |  |  |  |
| Genome Start | Genome End | Strand | Div | Repeat Start | Repeat End | Repeat Left | Name | Family | Class |
| 39468543 | 39468574 | + | 25.5 | 1 | 29 | -533 | LTR75 | ERVL | LTR |
| 39468575 | 39468857 | - | 9.8 | 12 | 292 | -17 | AluSc | Alu | SINE |
| 39468858 | 39468913 | + | 25.5 | 30 | 81 | -481 | LTR75 | ERVL | LTR |
| 39468914 | 39469095 | + | 8.2 | 1 | 296 | -4 | AluSg | Alu | SINE |
| 39469096 | 39469559 | + | 25.5 | 82 | 561 | -1 | LTR75 | ERVL | LTR |

  

|  |  |  |  |  |  |  |  |  |  |
| --- | --- | --- | --- | --- | --- | --- | --- | --- | --- |
| chr9:39470019-39472161 | | | 2120 bp | | |  |  |  |  |
| Genome Start | Genome End | Strand | Div | Repeat Start | Repeat End | Repeat Left | Name | Family | Class |
| 39470019 | 39470308 | + | 25.0 | 3498 | 3782 | -2361 | L1M2 | L1 | LINE |
| 39470309 | 39470619 | + | 10.0 | 1 | 310 | 0 | AluSg | Alu | SINE |
| 39470620 | 39471381 | + | 25.0 | 3783 | 4541 | -1602 | L1M2 | L1 | LINE |
| 39471405 | 39472161 | + | 25.0 | 4542 | 5196 | -947 | L1M2 | L1 | LINE |

  

|  |  |  |  |  |  |  |  |  |  |
| --- | --- | --- | --- | --- | --- | --- | --- | --- | --- |
| chr9:39476122-39477470 | | | 1296 bp | | |  |  |  |  |
| Genome Start | Genome End | Strand | Div | Repeat Start | Repeat End | Repeat Left | Name | Family | Class |
| 39476122 | 39476893 | + | 22.9 | 4181 | 5021 | -1125 | L1MD | L1 | LINE |
| 39476894 | 39477154 | - | 11.6 | 1 | 260 | -53 | AluSq | Alu | SINE |
| 39477155 | 39477214 | + | 22.9 | 5022 | 5079 | -1067 | L1MD | L1 | LINE |
| 39477268 | 39477470 | + | 21.2 | 5593 | 5791 | -568 | L1MD | L1 | LINE |

  

|  |  |  |  |  |  |  |  |  |  |
| --- | --- | --- | --- | --- | --- | --- | --- | --- | --- |
| chr9:39483476-39485690 | | | 2183 bp | | |  |  |  |  |
| Genome Start | Genome End | Strand | Div | Repeat Start | Repeat End | Repeat Left | Name | Family | Class |
| 39483476 | 39484295 | + | 22.2 | 2062 | 3043 | -3698 | L1MB5 | L1 | LINE |
| 39484326 | 39485127 | + | 22.2 | 3044 | 3869 | -2872 | L1MB5 | L1 | LINE |
| 39485130 | 39485433 | + | 5.6 | 1 | 303 | -8 | AluY | Alu | SINE |
| 39485434 | 39485690 | + | 20.1 | 4612 | 4867 | -1874 | L1MB5 | L1 | LINE |

  

|  |  |  |  |  |  |  |  |  |  |
| --- | --- | --- | --- | --- | --- | --- | --- | --- | --- |
| chr9:39498050-39499685 | | | 1640 bp | | |  |  |  |  |
| Genome Start | Genome End | Strand | Div | Repeat Start | Repeat End | Repeat Left | Name | Family | Class |
| 39498050 | 39498791 | + | 18.2 | 3165 | 3925 | -2218 | L1M2 | L1 | LINE |
| 39498788 | 39498853 | + | 16.7 | 4894 | 4961 | -1185 | L1M2 | L1 | LINE |
| 39498854 | 39499344 | + | 11.5 | 1 | 445 | -222 | LTR12B | ERV1 | LTR |
| 39499345 | 39499685 | + | 17.4 | 4962 | 5303 | -840 | L1M2 | L1 | LINE |

  

|  |  |  |  |  |  |  |  |  |  |
| --- | --- | --- | --- | --- | --- | --- | --- | --- | --- |
| chr9:39514979-39515722 | | | 540 bp | | |  |  |  |  |
| Genome Start | Genome End | Strand | Div | Repeat Start | Repeat End | Repeat Left | Name | Family | Class |
| 39514979 | 39515055 | + | 30.3 | 2347 | 2423 | -996 | L2 | L2 | LINE |
| 39515115 | 39515376 | - | 8.6 | 18 | 263 | -49 | AluSx | Alu | SINE |
| 39515522 | 39515722 | + | 40.2 | 2613 | 2822 | -597 | L2 | L2 | LINE |

  

|  |  |  |  |  |  |  |  |  |  |
| --- | --- | --- | --- | --- | --- | --- | --- | --- | --- |
| chr9:39520146-39521112 | | | 967 bp | | |  |  |  |  |
| Genome Start | Genome End | Strand | Div | Repeat Start | Repeat End | Repeat Left | Name | Family | Class |
| 39520146 | 39520693 | + | 21.6 | 1 | 523 | -101 | MLT1E2 | MaLR | LTR |
| 39520694 | 39521005 | - | 9.6 | 1 | 312 | -1 | AluSq | Alu | SINE |
| 39521006 | 39521112 | + | 21.2 | 523 | 626 | 0 | MLT1E2 | MaLR | LTR |

  

|  |  |  |  |  |  |  |  |  |  |
| --- | --- | --- | --- | --- | --- | --- | --- | --- | --- |
| chr9:39533274-39534937 | | | 1621 bp | | |  |  |  |  |
| Genome Start | Genome End | Strand | Div | Repeat Start | Repeat End | Repeat Left | Name | Family | Class |
| 39533274 | 39533543 | + | 25.0 | 1 | 272 | -439 | MER67C | ERV1 | LTR |
| 39533551 | 39533669 | + | 14.4 | 1 | 108 | -502 | MER4B | ERV1 | LTR |
| 39533670 | 39534009 | + | 13.6 | 1 | 343 | 0 | MER61A | ERV1 | LTR |
| 39534010 | 39534483 | + | 14.4 | 109 | 571 | -39 | MER4B | ERV1 | LTR |
| 39534520 | 39534937 | + | 19.5 | 276 | 711 | 0 | MER67C | ERV1 | LTR |

  

|  |  |  |  |  |  |  |  |  |  |
| --- | --- | --- | --- | --- | --- | --- | --- | --- | --- |
| chr9:39535376-39537853 | | | 2410 bp | | |  |  |  |  |
| Genome Start | Genome End | Strand | Div | Repeat Start | Repeat End | Repeat Left | Name | Family | Class |
| 39535376 | 39535436 | + | 19.7 | 1 | 61 | -592 | MER82 | MER2\_type | DNA |
| 39535438 | 39535996 | - | 16.9 | 219 | 787 | 0 | LTR48 | ERV1 | LTR |
| 39535997 | 39536302 | + | 6.9 | 1 | 303 | -8 | AluY | Alu | SINE |
| 39536305 | 39536614 | + | 9.7 | 1 | 308 | -2 | AluSg | Alu | SINE |
| 39536680 | 39536793 | - | 24.1 | 138 | 229 | -558 | LTR48 | ERV1 | LTR |
| 39536794 | 39537163 | + | 15.5 | 1 | 364 | 0 | THE1B | MaLR | LTR |
| 39537164 | 39537308 | - | 24.1 | 1 | 137 | -650 | LTR48 | ERV1 | LTR |
| 39537309 | 39537853 | + | 21.1 | 59 | 628 | -25 | MER82 | MER2\_type | DNA |

  

|  |  |  |  |  |  |  |  |  |  |
| --- | --- | --- | --- | --- | --- | --- | --- | --- | --- |
| chr9:39538697-39539593 | | | 897 bp | | |  |  |  |  |
| Genome Start | Genome End | Strand | Div | Repeat Start | Repeat End | Repeat Left | Name | Family | Class |
| 39538697 | 39538717 | - | 27.3 | 437 | 456 | -12 | MER110A | ERV1 | LTR |
| 39538718 | 39539216 | + | 20.4 | 1 | 505 | 0 | MLT1D | MaLR | LTR |
| 39539217 | 39539593 | - | 27.3 | 27 | 436 | -32 | MER110A | ERV1 | LTR |

  

|  |  |  |  |  |  |  |  |  |  |
| --- | --- | --- | --- | --- | --- | --- | --- | --- | --- |
| chr9:39545276-39545926 | | | 651 bp | | |  |  |  |  |
| Genome Start | Genome End | Strand | Div | Repeat Start | Repeat End | Repeat Left | Name | Family | Class |
| 39545276 | 39545350 | - | 26.9 | 5949 | 6047 | -193 | L1ME3B | L1 | LINE |
| 39545351 | 39545657 | - | 7.5 | 1 | 308 | -3 | AluY | Alu | SINE |
| 39545658 | 39545926 | - | 26.9 | 5689 | 5948 | -292 | L1ME3B | L1 | LINE |

  

|  |  |  |  |  |  |  |  |  |  |
| --- | --- | --- | --- | --- | --- | --- | --- | --- | --- |
| chr9:39547360-39548083 | | | 724 bp | | |  |  |  |  |
| Genome Start | Genome End | Strand | Div | Repeat Start | Repeat End | Repeat Left | Name | Family | Class |
| 39547360 | 39547548 | + | 19.3 | 6 | 195 | -231 | MSTB | MaLR | LTR |
| 39547549 | 39547862 | + | 13.9 | 1 | 314 | 0 | AluSx | Alu | SINE |
| 39547863 | 39548083 | + | 19.3 | 196 | 426 | 0 | MSTB | MaLR | LTR |

  

|  |  |  |  |  |  |  |  |  |  |
| --- | --- | --- | --- | --- | --- | --- | --- | --- | --- |
| chr9:39551435-39552662 | | | 1228 bp | | |  |  |  |  |
| Genome Start | Genome End | Strand | Div | Repeat Start | Repeat End | Repeat Left | Name | Family | Class |
| 39551435 | 39551752 | + | 16.6 | 2 | 300 | -66 | Tigger5a | MER2\_type | DNA |
| 39551753 | 39552136 | + | 8.0 | 1 | 387 | -167 | MER41A | ERV1 | LTR |
| 39552137 | 39552435 | + | 11.0 | 1 | 299 | -11 | AluSg | Alu | SINE |
| 39552436 | 39552610 | + | 8.0 | 388 | 554 | 0 | MER41A | ERV1 | LTR |
| 39552611 | 39552662 | + | 16.6 | 301 | 361 | -5 | Tigger5a | MER2\_type | DNA |

  

|  |  |  |  |  |  |  |  |  |  |
| --- | --- | --- | --- | --- | --- | --- | --- | --- | --- |
| chr9:39563425-39564684 | | | 1288 bp | | |  |  |  |  |
| Genome Start | Genome End | Strand | Div | Repeat Start | Repeat End | Repeat Left | Name | Family | Class |
| 39563425 | 39563735 | - | 16.4 | 398 | 705 | -2 | MER39 | ERV1 | LTR |
| 39563736 | 39564441 | - | 13.4 | 1 | 691 | 0 | LTR8 | ERV1 | LTR |
| 39564442 | 39564585 | - | 16.4 | 260 | 397 | -310 | MER39 | ERV1 | LTR |
| 39564558 | 39564684 | - | 22.2 | 1 | 138 | -569 | MER39 | ERV1 | LTR |

  

|  |  |  |  |  |  |  |  |  |  |
| --- | --- | --- | --- | --- | --- | --- | --- | --- | --- |
| chr9:39580702-39581757 | | | 1056 bp | | |  |  |  |  |
| Genome Start | Genome End | Strand | Div | Repeat Start | Repeat End | Repeat Left | Name | Family | Class |
| 39580702 | 39580849 | - | 14.1 | 618 | 765 | -18 | LTR39 | ERV1 | LTR |
| 39580850 | 39581152 | - | 6.3 | 1 | 303 | -8 | AluY | Alu | SINE |
| 39581153 | 39581757 | - | 14.1 | 1 | 617 | -166 | LTR39 | ERV1 | LTR |

  

|  |  |  |  |  |  |  |  |  |  |
| --- | --- | --- | --- | --- | --- | --- | --- | --- | --- |
| chr9:39586972-39588416 | | | 1445 bp | | |  |  |  |  |
| Genome Start | Genome End | Strand | Div | Repeat Start | Repeat End | Repeat Left | Name | Family | Class |
| 39586972 | 39587239 | - | 17.8 | 5161 | 5437 | -741 | L1M4 | L1 | LINE |
| 39587240 | 39587529 | + | 13.8 | 1 | 290 | -22 | AluSx | Alu | SINE |
| 39587530 | 39588416 | - | 17.6 | 4316 | 5160 | -986 | L1M4 | L1 | LINE |

  

|  |  |  |  |  |  |  |  |  |  |
| --- | --- | --- | --- | --- | --- | --- | --- | --- | --- |
| chr9:39594514-39595373 | | | 626 bp | | |  |  |  |  |
| Genome Start | Genome End | Strand | Div | Repeat Start | Repeat End | Repeat Left | Name | Family | Class |
| 39594514 | 39594745 | - | 19.1 | 4094 | 4321 | -1825 | L1M4 | L1 | LINE |
| 39594747 | 39595084 | + | 18.6 | 1 | 393 | -508 | MER54B | ERVL | LTR |
| 39595318 | 39595373 | - | 21.4 | 4039 | 4097 | -2049 | L1M4 | L1 | LINE |

  

|  |  |  |  |  |  |  |  |  |  |
| --- | --- | --- | --- | --- | --- | --- | --- | --- | --- |
| chr9:39599861-39600418 | | | 558 bp | | |  |  |  |  |
| Genome Start | Genome End | Strand | Div | Repeat Start | Repeat End | Repeat Left | Name | Family | Class |
| 39599861 | 39600045 | + | 20.5 | 5608 | 5819 | -345 | L1ME2 | L1 | LINE |
| 39600046 | 39600347 | - | 8.7 | 1 | 303 | -9 | AluSx | Alu | SINE |
| 39600348 | 39600418 | + | 20.5 | 5820 | 5886 | -278 | L1ME2 | L1 | LINE |

  

|  |  |  |  |  |  |  |  |  |  |
| --- | --- | --- | --- | --- | --- | --- | --- | --- | --- |
| chr9:39600699-39601847 | | | 1149 bp | | |  |  |  |  |
| Genome Start | Genome End | Strand | Div | Repeat Start | Repeat End | Repeat Left | Name | Family | Class |
| 39600699 | 39600754 | + | 24.1 | 208 | 255 | -86 | MER58B | MER1\_type | DNA |
| 39600755 | 39601238 | - | 15.6 | 3 | 489 | 0 | LTR45B | ERV1 | LTR |
| 39601239 | 39601290 | + | 24.1 | 256 | 303 | -38 | MER58B | MER1\_type | DNA |
| 39601291 | 39601800 | - | 17.3 | 2 | 550 | 0 | MER44B | MER2\_type | DNA |
| 39601801 | 39601847 | + | 24.1 | 304 | 340 | -1 | MER58B | MER1\_type | DNA |

  

|  |  |  |  |  |  |  |  |  |  |
| --- | --- | --- | --- | --- | --- | --- | --- | --- | --- |
| chr9:39627285-39632234 | | | 4625 bp | | |  |  |  |  |
| Genome Start | Genome End | Strand | Div | Repeat Start | Repeat End | Repeat Left | Name | Family | Class |
| 39627285 | 39627509 | - | 33.9 | 2949 | 3176 | -243 | L2 | L2 | LINE |
| 39627724 | 39628044 | + | 30.0 | 4 | 354 | -241 | MLT1H | MaLR | LTR |
| 39628140 | 39628936 | - | 30.4 | 1564 | 2565 | -854 | L2 | L2 | LINE |
| 39628937 | 39629139 | - | 20.7 | 137 | 396 | 0 | MLT1A1 | MaLR | LTR |
| 39629140 | 39629489 | + | 21.0 | 1 | 396 | 0 | MSTD | MaLR | LTR |
| 39629490 | 39629623 | - | 19.1 | 1 | 137 | -271 | MLT1A1 | MaLR | LTR |
| 39629629 | 39631372 | - | 21.6 | 2 | 1729 | -6 | MLT1A1-int | MaLR | LTR |
| 39631375 | 39631440 | - | 19.1 | 343 | 407 | -1 | MLT1A1 | MaLR | LTR |
| 39631441 | 39631749 | + | 13.6 | 1 | 311 | -2 | AluSq | Alu | SINE |
| 39631750 | 39632031 | - | 19.1 | 2 | 342 | -66 | MLT1A1 | MaLR | LTR |
| 39632041 | 39632234 | - | 30.4 | 1351 | 1560 | -1859 | L2 | L2 | LINE |

  

|  |  |  |  |  |  |  |  |  |  |
| --- | --- | --- | --- | --- | --- | --- | --- | --- | --- |
| chr9:39756152-39756712 | | | 561 bp | | |  |  |  |  |
| Genome Start | Genome End | Strand | Div | Repeat Start | Repeat End | Repeat Left | Name | Family | Class |
| 39756152 | 39756219 | - | 25.0 | 3315 | 3376 | -2 | L2 | L2 | LINE |
| 39756220 | 39756662 | - | 22.1 | 1 | 463 | -4 | MLT1C | MaLR | LTR |
| 39756663 | 39756712 | - | 25.0 | 3262 | 3314 | -64 | L2 | L2 | LINE |

  

|  |  |  |  |  |  |  |  |  |  |
| --- | --- | --- | --- | --- | --- | --- | --- | --- | --- |
| chr9:39775649-39776257 | | | 516 bp | | |  |  |  |  |
| Genome Start | Genome End | Strand | Div | Repeat Start | Repeat End | Repeat Left | Name | Family | Class |
| 39775649 | 39775752 | + | 22.6 | 24 | 127 | -88 | MER58C | MER1\_type | DNA |
| 39775755 | 39776062 | + | 20.9 | 1 | 306 | -6 | AluJo | Alu | SINE |
| 39776154 | 39776257 | + | 24.0 | 106 | 213 | -2 | MER58C | MER1\_type | DNA |

  

|  |  |  |  |  |  |  |  |  |  |
| --- | --- | --- | --- | --- | --- | --- | --- | --- | --- |
| chr9:39789338-39789993 | | | 546 bp | | |  |  |  |  |
| Genome Start | Genome End | Strand | Div | Repeat Start | Repeat End | Repeat Left | Name | Family | Class |
| 39789338 | 39789515 | + | 28.3 | 91 | 268 | -323 | MLT1K | MaLR | LTR |
| 39789571 | 39789717 | - | 24.5 | 3266 | 3419 | 0 | L2 | L2 | LINE |
| 39789773 | 39789993 | + | 31.1 | 312 | 576 | -15 | MLT1K | MaLR | LTR |

  

|  |  |  |  |  |  |  |  |  |  |
| --- | --- | --- | --- | --- | --- | --- | --- | --- | --- |
| chr9:39815620-39816533 | | | 914 bp | | |  |  |  |  |
| Genome Start | Genome End | Strand | Div | Repeat Start | Repeat End | Repeat Left | Name | Family | Class |
| 39815620 | 39815846 | + | 11.6 | 5522 | 5787 | -378 | L1PA15 | L1 | LINE |
| 39815847 | 39816154 | + | 9.1 | 1 | 308 | -3 | AluY | Alu | SINE |
| 39816155 | 39816533 | + | 11.6 | 5788 | 6158 | -7 | L1PA15 | L1 | LINE |

  

|  |  |  |  |  |  |  |  |  |  |
| --- | --- | --- | --- | --- | --- | --- | --- | --- | --- |
| chr9:39816809-39818448 | | | 1640 bp | | |  |  |  |  |
| Genome Start | Genome End | Strand | Div | Repeat Start | Repeat End | Repeat Left | Name | Family | Class |
| 39816809 | 39817931 | + | 24.3 | 1576 | 2606 | -3613 | L1MEc | L1 | LINE |
| 39817932 | 39818233 | - | 10.3 | 1 | 301 | -12 | AluSq | Alu | SINE |
| 39818234 | 39818448 | + | 27.8 | 2607 | 2825 | -3394 | L1MEc | L1 | LINE |

  

|  |  |  |  |  |  |  |  |  |  |
| --- | --- | --- | --- | --- | --- | --- | --- | --- | --- |
| chr9:39827616-39830720 | | | 3098 bp | | |  |  |  |  |
| Genome Start | Genome End | Strand | Div | Repeat Start | Repeat End | Repeat Left | Name | Family | Class |
| 39827616 | 39827791 | + | 22.2 | 2115 | 2335 | -4999 | L1M4b | L1 | LINE |
| 39827792 | 39828090 | + | 7.7 | 1 | 299 | -12 | AluY | Alu | SINE |
| 39828091 | 39828567 | + | 22.2 | 2336 | 2873 | -4461 | L1M4b | L1 | LINE |
| 39828563 | 39829328 | + | 8.7 | 4692 | 5461 | -711 | L1P3 | L1 | LINE |
| 39829334 | 39829674 | - | 3.3 | 1 | 341 | 0 | AluY | Alu | SINE |
| 39829675 | 39829855 | + | 11.3 | 156 | 364 | 0 | THE1B | MaLR | LTR |
| 39829858 | 39830661 | + | 21.3 | 3240 | 4271 | -2735 | L1M4b | L1 | LINE |
| 39830667 | 39830720 | + | 11.1 | 4402 | 4460 | -2540 | L1M4b | L1 | LINE |

  

|  |  |  |  |  |  |  |  |  |  |
| --- | --- | --- | --- | --- | --- | --- | --- | --- | --- |
| chr9:39843914-39845675 | | | 1771 bp | | |  |  |  |  |
| Genome Start | Genome End | Strand | Div | Repeat Start | Repeat End | Repeat Left | Name | Family | Class |
| 39843914 | 39844122 | + | 20.5 | 5 | 211 | -872 | Tigger6a | MER2\_type | DNA |
| 39844123 | 39844578 | - | 22.8 | 11 | 467 | 0 | MLT1C | MaLR | LTR |
| 39844570 | 39845194 | + | 19.2 | 216 | 883 | -200 | Tigger6a | MER2\_type | DNA |
| 39845195 | 39845479 | + | 10.5 | 3 | 300 | -11 | AluY | Alu | SINE |
| 39845480 | 39845675 | + | 19.2 | 884 | 1083 | 0 | Tigger6a | MER2\_type | DNA |

  

|  |  |  |  |  |  |  |  |  |  |
| --- | --- | --- | --- | --- | --- | --- | --- | --- | --- |
| chr9:39865101-39865760 | | | 660 bp | | |  |  |  |  |
| Genome Start | Genome End | Strand | Div | Repeat Start | Repeat End | Repeat Left | Name | Family | Class |
| 39865101 | 39865396 | + | 13.4 | 1 | 299 | -65 | THE1B | MaLR | LTR |
| 39865397 | 39865689 | - | 8.9 | 1 | 293 | -18 | AluY | Alu | SINE |
| 39865690 | 39865760 | + | 13.4 | 300 | 364 | 0 | THE1B | MaLR | LTR |

  

|  |  |  |  |  |  |  |  |  |  |
| --- | --- | --- | --- | --- | --- | --- | --- | --- | --- |
| chr9:39865761-39867143 | | | 1383 bp | | |  |  |  |  |
| Genome Start | Genome End | Strand | Div | Repeat Start | Repeat End | Repeat Left | Name | Family | Class |
| 39865761 | 39866625 | - | 20.1 | 4379 | 5306 | -834 | L1M3 | L1 | LINE |
| 39866626 | 39866888 | - | 8.4 | 1 | 287 | -24 | AluY | Alu | SINE |
| 39866889 | 39867143 | - | 20.1 | 4120 | 4378 | -1762 | L1M3 | L1 | LINE |

  

|  |  |  |  |  |  |  |  |  |  |
| --- | --- | --- | --- | --- | --- | --- | --- | --- | --- |
| chr9:39867139-39870185 | | | 3040 bp | | |  |  |  |  |
| Genome Start | Genome End | Strand | Div | Repeat Start | Repeat End | Repeat Left | Name | Family | Class |
| 39867139 | 39867533 | + | 23.8 | 2917 | 3348 | -4217 | L1M1 | L1 | LINE |
| 39867534 | 39867869 | - | 18.8 | 4 | 345 | -1 | MER7A | MER2\_type | DNA |
| 39867870 | 39869118 | + | 18.7 | 3349 | 4638 | -2921 | L1M1 | L1 | LINE |
| 39869119 | 39869429 | + | 0.3 | 1 | 310 | 0 | AluYa5 | Alu | SINE |
| 39869437 | 39869737 | + | 9.4 | 1 | 308 | 0 | AluY | Alu | SINE |
| 39869738 | 39870185 | + | 17.6 | 4642 | 5099 | -2460 | L1M1 | L1 | LINE |

  

|  |  |  |  |  |  |  |  |  |  |
| --- | --- | --- | --- | --- | --- | --- | --- | --- | --- |
| chr9:39891480-39892289 | | | 810 bp | | |  |  |  |  |
| Genome Start | Genome End | Strand | Div | Repeat Start | Repeat End | Repeat Left | Name | Family | Class |
| 39891480 | 39891543 | - | 9.4 | 327 | 390 | 0 | MLT1B | MaLR | LTR |
| 39891544 | 39891912 | - | 0.9 | 5815 | 6183 | 0 | L1PA3 | L1 | LINE |
| 39891913 | 39891957 | + | 0.0 | 5770 | 5814 | -358 | L1P3 | L1 | LINE |
| 39891958 | 39892289 | - | 18.4 | 1 | 331 | -59 | MLT1B | MaLR | LTR |

  

|  |  |  |  |  |  |  |  |  |  |
| --- | --- | --- | --- | --- | --- | --- | --- | --- | --- |
| chr9:39910155-39911161 | | | 1007 bp | | |  |  |  |  |
| Genome Start | Genome End | Strand | Div | Repeat Start | Repeat End | Repeat Left | Name | Family | Class |
| 39910155 | 39910374 | + | 35.6 | 3523 | 3776 | -713 | L3 | CR1 | LINE |
| 39910375 | 39910684 | + | 10.3 | 1 | 308 | 0 | AluSg | Alu | SINE |
| 39910685 | 39911161 | + | 35.6 | 3777 | 4333 | -156 | L3 | CR1 | LINE |

  

|  |  |  |  |  |  |  |  |  |  |
| --- | --- | --- | --- | --- | --- | --- | --- | --- | --- |
| chr9:39918877-39921536 | | | 2660 bp | | |  |  |  |  |
| Genome Start | Genome End | Strand | Div | Repeat Start | Repeat End | Repeat Left | Name | Family | Class |
| 39918877 | 39920272 | + | 10.4 | 3147 | 4544 | -1602 | L1PREC2 | L1 | LINE |
| 39920273 | 39920573 | + | 10.3 | 1 | 301 | -8 | AluSc | Alu | SINE |
| 39920574 | 39921536 | + | 9.8 | 4545 | 5480 | -680 | L1PREC2 | L1 | LINE |

  

|  |  |  |  |  |  |  |  |  |  |
| --- | --- | --- | --- | --- | --- | --- | --- | --- | --- |
| chr9:39933065-39934457 | | | 1393 bp | | |  |  |  |  |
| Genome Start | Genome End | Strand | Div | Repeat Start | Repeat End | Repeat Left | Name | Family | Class |
| 39933065 | 39933472 | + | 30.7 | 4475 | 4915 | -1231 | L1M5 | L1 | LINE |
| 39933473 | 39933812 | + | 8.5 | 1 | 326 | 0 | AluSx | Alu | SINE |
| 39933813 | 39933823 | + | 8.5 | 284 | 294 | -18 | AluSx | Alu | SINE |
| 39933824 | 39934045 | + | 30.7 | 4916 | 5170 | -976 | L1M5 | L1 | LINE |
| 39934046 | 39934336 | - | 7.6 | 1 | 293 | -18 | AluY | Alu | SINE |
| 39934337 | 39934457 | + | 30.7 | 5171 | 5283 | -863 | L1M5 | L1 | LINE |

  

|  |  |  |  |  |  |  |  |  |  |
| --- | --- | --- | --- | --- | --- | --- | --- | --- | --- |
| chr9:40037533-40038233 | | | 701 bp | | |  |  |  |  |
| Genome Start | Genome End | Strand | Div | Repeat Start | Repeat End | Repeat Left | Name | Family | Class |
| 40037533 | 40037570 | - | 25.1 | 340 | 368 | -6 | MLT1A | MaLR | LTR |
| 40037571 | 40037880 | + | 7.2 | 1 | 305 | -6 | AluY | Alu | SINE |
| 40037881 | 40038233 | - | 25.1 | 1 | 339 | -35 | MLT1A | MaLR | LTR |

  

|  |  |  |  |  |  |  |  |  |  |
| --- | --- | --- | --- | --- | --- | --- | --- | --- | --- |
| chr9:40046512-40047746 | | | 1169 bp | | |  |  |  |  |
| Genome Start | Genome End | Strand | Div | Repeat Start | Repeat End | Repeat Left | Name | Family | Class |
| 40046512 | 40046582 | + | 22.1 | 5 | 74 | -391 | LTR40c | ERVL | LTR |
| 40046586 | 40047295 | + | 18.8 | 5447 | 6181 | -2 | L1MB3 | L1 | LINE |
| 40047359 | 40047746 | + | 25.8 | 63 | 459 | -6 | LTR40c | ERVL | LTR |

  

|  |  |  |  |  |  |  |  |  |  |
| --- | --- | --- | --- | --- | --- | --- | --- | --- | --- |
| chr9:40078921-40083697 | | | 4715 bp | | |  |  |  |  |
| Genome Start | Genome End | Strand | Div | Repeat Start | Repeat End | Repeat Left | Name | Family | Class |
| 40078921 | 40078946 | - | 15.8 | 7220 | 7237 | -35 | L1MA9 | L1 | LINE |
| 40078947 | 40079249 | + | 10.6 | 1 | 306 | -7 | AluSq | Alu | SINE |
| 40079250 | 40079344 | - | 15.8 | 7113 | 7219 | -53 | L1MA9 | L1 | LINE |
| 40079380 | 40080166 | - | 15.8 | 6356 | 7112 | -160 | L1MA9 | L1 | LINE |
| 40080205 | 40080928 | - | 18.6 | 5615 | 6349 | -754 | L1MA9 | L1 | LINE |
| 40080929 | 40081233 | - | 9.0 | 1 | 301 | -12 | AluSp | Alu | SINE |
| 40081234 | 40081698 | - | 18.6 | 5005 | 5614 | -1489 | L1MA9 | L1 | LINE |
| 40081688 | 40083697 | - | 22.9 | 1 | 2508 | -4935 | L1MA9 | L1 | LINE |

  

|  |  |  |  |  |  |  |  |  |  |
| --- | --- | --- | --- | --- | --- | --- | --- | --- | --- |
| chr9:40086621-40087441 | | | 821 bp | | |  |  |  |  |
| Genome Start | Genome End | Strand | Div | Repeat Start | Repeat End | Repeat Left | Name | Family | Class |
| 40086621 | 40086806 | + | 26.7 | 5671 | 5870 | -296 | L1ME1 | L1 | LINE |
| 40086807 | 40087123 | + | 8.6 | 1 | 316 | 0 | AluY | Alu | SINE |
| 40087124 | 40087441 | + | 26.8 | 5871 | 6333 | -1 | L1ME1 | L1 | LINE |

  

|  |  |  |  |  |  |  |  |  |  |
| --- | --- | --- | --- | --- | --- | --- | --- | --- | --- |
| chr9:40087543-40088027 | | | 390 bp | | |  |  |  |  |
| Genome Start | Genome End | Strand | Div | Repeat Start | Repeat End | Repeat Left | Name | Family | Class |
| 40087543 | 40087600 | + | 29.3 | 2406 | 2464 | -955 | L2 | L2 | LINE |
| 40087654 | 40087733 | - | 22.1 | 956 | 1032 | -30 | MER63 | AcHobo | DNA |
| 40087776 | 40088027 | + | 36.3 | 3126 | 3407 | -12 | L2 | L2 | LINE |

  

|  |  |  |  |  |  |  |  |  |  |
| --- | --- | --- | --- | --- | --- | --- | --- | --- | --- |
| chr9:40088011-40089319 | | | 1309 bp | | |  |  |  |  |
| Genome Start | Genome End | Strand | Div | Repeat Start | Repeat End | Repeat Left | Name | Family | Class |
| 40088011 | 40088669 | - | 34.2 | 2762 | 3369 | -9 | L2 | L2 | LINE |
| 40088670 | 40089054 | + | 15.7 | 1 | 427 | -1 | MSTA | MaLR | LTR |
| 40089055 | 40089319 | - | 30.6 | 2445 | 2761 | -658 | L2 | L2 | LINE |

  

|  |  |  |  |  |  |  |  |  |  |
| --- | --- | --- | --- | --- | --- | --- | --- | --- | --- |
| chr9:40095775-40097757 | | | 1983 bp | | |  |  |  |  |
| Genome Start | Genome End | Strand | Div | Repeat Start | Repeat End | Repeat Left | Name | Family | Class |
| 40095775 | 40096340 | - | 15.9 | 5591 | 6235 | -109 | L1MC2 | L1 | LINE |
| 40096341 | 40096632 | + | 9.9 | 1 | 292 | -17 | AluSc | Alu | SINE |
| 40096633 | 40097757 | - | 16.5 | 4443 | 5590 | -754 | L1MC2 | L1 | LINE |

  

|  |  |  |  |  |  |  |  |  |  |
| --- | --- | --- | --- | --- | --- | --- | --- | --- | --- |
| chr9:40110070-40112274 | | | 2216 bp | | |  |  |  |  |
| Genome Start | Genome End | Strand | Div | Repeat Start | Repeat End | Repeat Left | Name | Family | Class |
| 40110070 | 40110301 | + | 18.1 | 4863 | 5097 | -1049 | L1MC1 | L1 | LINE |
| 40110297 | 40110476 | + | 15.6 | 5615 | 5794 | -539 | L1MC | L1 | LINE |
| 40110471 | 40110527 | + | 19.3 | 5108 | 5164 | -982 | L1MC1 | L1 | LINE |
| 40110528 | 40110823 | + | 13.2 | 1 | 297 | -15 | AluSx | Alu | SINE |
| 40110824 | 40111244 | + | 16.0 | 5165 | 5601 | -732 | L1MC1 | L1 | LINE |
| 40111245 | 40111547 | + | 4.7 | 1 | 301 | -10 | AluY | Alu | SINE |
| 40111548 | 40112274 | + | 15.3 | 5602 | 6327 | -6 | L1MC1 | L1 | LINE |

  

|  |  |  |  |  |  |  |  |  |  |
| --- | --- | --- | --- | --- | --- | --- | --- | --- | --- |
| chr9:40114527-40115431 | | | 905 bp | | |  |  |  |  |
| Genome Start | Genome End | Strand | Div | Repeat Start | Repeat End | Repeat Left | Name | Family | Class |
| 40114527 | 40114602 | + | 25.9 | 1 | 64 | -580 | LTR48B | ERV1 | LTR |
| 40114603 | 40114907 | - | 6.6 | 1 | 305 | -6 | AluY | Alu | SINE |
| 40114908 | 40115431 | + | 25.9 | 65 | 642 | -2 | LTR48B | ERV1 | LTR |

  

|  |  |  |  |  |  |  |  |  |  |
| --- | --- | --- | --- | --- | --- | --- | --- | --- | --- |
| chr9:40132964-40134647 | | | 1684 bp | | |  |  |  |  |
| Genome Start | Genome End | Strand | Div | Repeat Start | Repeat End | Repeat Left | Name | Family | Class |
| 40132964 | 40133014 | - | 14.4 | 856 | 900 | 0 | MER4D1 | ERV1 | LTR |
| 40133015 | 40133520 | + | 11.3 | 1 | 511 | 0 | MER9 | ERVK | LTR |
| 40133521 | 40134017 | - | 14.4 | 396 | 855 | -45 | MER4D1 | ERV1 | LTR |
| 40134018 | 40134305 | - | 12.8 | 1 | 299 | -13 | AluSx | Alu | SINE |
| 40134306 | 40134647 | - | 14.4 | 1 | 395 | -505 | MER4D1 | ERV1 | LTR |

  

|  |  |  |  |  |  |  |  |  |  |
| --- | --- | --- | --- | --- | --- | --- | --- | --- | --- |
| chr9:40136670-40137391 | | | 722 bp | | |  |  |  |  |
| Genome Start | Genome End | Strand | Div | Repeat Start | Repeat End | Repeat Left | Name | Family | Class |
| 40136670 | 40136805 | - | 13.6 | 173 | 310 | -2 | AluSx | Alu | SINE |
| 40136806 | 40137205 | + | 10.1 | 1 | 448 | -2 | LTR7 | ERV1 | LTR |
| 40137206 | 40137391 | - | 13.6 | 1 | 172 | -140 | AluSx | Alu | SINE |

  

|  |  |  |  |  |  |  |  |  |  |
| --- | --- | --- | --- | --- | --- | --- | --- | --- | --- |
| chr9:40140605-40141380 | | | 776 bp | | |  |  |  |  |
| Genome Start | Genome End | Strand | Div | Repeat Start | Repeat End | Repeat Left | Name | Family | Class |
| 40140605 | 40140897 | + | 22.1 | 250 | 537 | -170 | MER39 | ERV1 | LTR |
| 40140898 | 40141212 | - | 5.7 | 1 | 314 | 0 | AluY | Alu | SINE |
| 40141213 | 40141380 | + | 22.1 | 538 | 707 | 0 | MER39 | ERV1 | LTR |

  

|  |  |  |  |  |  |  |  |  |  |
| --- | --- | --- | --- | --- | --- | --- | --- | --- | --- |
| chr9:40142836-40143817 | | | 982 bp | | |  |  |  |  |
| Genome Start | Genome End | Strand | Div | Repeat Start | Repeat End | Repeat Left | Name | Family | Class |
| 40142836 | 40143200 | - | 23.4 | 235 | 591 | 0 | LTR38B | ERV1 | LTR |
| 40143201 | 40143502 | - | 3.6 | 1 | 302 | -9 | AluY | Alu | SINE |
| 40143503 | 40143817 | - | 23.1 | 1 | 234 | -376 | LTR38B | ERV1 | LTR |

  

|  |  |  |  |  |  |  |  |  |  |
| --- | --- | --- | --- | --- | --- | --- | --- | --- | --- |
| chr9:40145196-40147750 | | | 2546 bp | | |  |  |  |  |
| Genome Start | Genome End | Strand | Div | Repeat Start | Repeat End | Repeat Left | Name | Family | Class |
| 40145196 | 40145864 | + | 25.2 | 3172 | 3839 | -352 | MER31A-int | ERV1 | LTR |
| 40145865 | 40146172 | + | 9.8 | 1 | 308 | -3 | AluY | Alu | SINE |
| 40146173 | 40146270 | + | 25.2 | 3840 | 3930 | -261 | MER31A-int | ERV1 | LTR |
| 40146271 | 40146847 | + | 21.9 | 1 | 558 | 0 | MER74A | ERVL | LTR |
| 40146857 | 40147170 | + | 13.5 | 1 | 312 | 0 | AluSx | Alu | SINE |
| 40147171 | 40147222 | + | 25.2 | 3931 | 3962 | -229 | MER31A-int | ERV1 | LTR |
| 40147223 | 40147638 | - | 11.6 | 1 | 445 | 0 | LTR56 | ERV1 | LTR |
| 40147639 | 40147750 | + | 25.2 | 3963 | 4081 | -110 | MER31A-int | ERV1 | LTR |

  

|  |  |  |  |  |  |  |  |  |  |
| --- | --- | --- | --- | --- | --- | --- | --- | --- | --- |
| chr9:40160493-40162736 | | | 2227 bp | | |  |  |  |  |
| Genome Start | Genome End | Strand | Div | Repeat Start | Repeat End | Repeat Left | Name | Family | Class |
| 40160493 | 40160638 | + | 16.5 | 5227 | 5380 | -912 | L1MA10 | L1 | LINE |
| 40160639 | 40160822 | + | 6.7 | 135 | 318 | 0 | AluY | Alu | SINE |
| 40160840 | 40161080 | + | 19.4 | 5364 | 5608 | -726 | L1MA10 | L1 | LINE |
| 40161081 | 40161382 | + | 3.7 | 1 | 298 | -13 | AluY | Alu | SINE |
| 40161383 | 40161572 | + | 19.4 | 5609 | 5785 | -549 | L1MA10 | L1 | LINE |
| 40161573 | 40162213 | + | 9.5 | 1 | 600 | 0 | MER4A1\_ | ERV1 | LTR |
| 40162214 | 40162736 | + | 19.4 | 5786 | 6295 | -39 | L1MA10 | L1 | LINE |

  

|  |  |  |  |  |  |  |  |  |  |
| --- | --- | --- | --- | --- | --- | --- | --- | --- | --- |
| chr9:40168504-40169222 | | | 719 bp | | |  |  |  |  |
| Genome Start | Genome End | Strand | Div | Repeat Start | Repeat End | Repeat Left | Name | Family | Class |
| 40168504 | 40168824 | - | 12.1 | 5830 | 6156 | -10 | L1PA16 | L1 | LINE |
| 40168825 | 40169128 | - | 7.7 | 1 | 302 | -9 | AluY | Alu | SINE |
| 40169129 | 40169222 | - | 12.1 | 5736 | 5829 | -337 | L1PA16 | L1 | LINE |

  

|  |  |  |  |  |  |  |  |  |  |
| --- | --- | --- | --- | --- | --- | --- | --- | --- | --- |
| chr9:40178575-40181654 | | | 3072 bp | | |  |  |  |  |
| Genome Start | Genome End | Strand | Div | Repeat Start | Repeat End | Repeat Left | Name | Family | Class |
| 40178575 | 40178776 | + | 29.6 | 2011 | 2221 | -1970 | MER21C-int | ERV1 | LTR |
| 40178777 | 40179025 | - | 21.6 | 152 | 408 | 0 | MLT1A1 | MaLR | LTR |
| 40179026 | 40179316 | - | 10.3 | 6 | 302 | -9 | AluY | Alu | SINE |
| 40179317 | 40179469 | - | 21.6 | 1 | 151 | -257 | MLT1A1 | MaLR | LTR |
| 40179470 | 40179542 | + | 29.6 | 2222 | 2284 | -1907 | MER21C-int | ERV1 | LTR |
| 40179551 | 40180157 | + | 25.5 | 2565 | 3234 | -957 | MER21C-int | ERV1 | LTR |
| 40180158 | 40180293 | + | 11.5 | 1 | 133 | -180 | AluSp | Alu | SINE |
| 40180294 | 40180595 | + | 8.7 | 1 | 299 | -12 | AluY | Alu | SINE |
| 40180596 | 40180775 | + | 11.5 | 134 | 313 | 0 | AluSp | Alu | SINE |
| 40180776 | 40181654 | + | 22.5 | 3235 | 4149 | -42 | MER21C-int | ERV1 | LTR |

  

|  |  |  |  |  |  |  |  |  |  |
| --- | --- | --- | --- | --- | --- | --- | --- | --- | --- |
| chr9:40194351-40195160 | | | 810 bp | | |  |  |  |  |
| Genome Start | Genome End | Strand | Div | Repeat Start | Repeat End | Repeat Left | Name | Family | Class |
| 40194351 | 40194764 | + | 12.0 | 1 | 513 | -313 | LTR12 | ERV1 | LTR |
| 40194765 | 40195073 | + | 9.0 | 1 | 300 | -11 | AluY | Alu | SINE |
| 40195074 | 40195160 | + | 12.0 | 514 | 595 | -231 | LTR12 | ERV1 | LTR |

  

|  |  |  |  |  |  |  |  |  |  |
| --- | --- | --- | --- | --- | --- | --- | --- | --- | --- |
| chr9:40201956-40202758 | | | 713 bp | | |  |  |  |  |
| Genome Start | Genome End | Strand | Div | Repeat Start | Repeat End | Repeat Left | Name | Family | Class |
| 40201956 | 40202101 | - | 28.1 | 3267 | 3419 | 0 | L2 | L2 | LINE |
| 40202118 | 40202535 | + | 20.6 | 11 | 459 | -56 | MLT2B2 | ERVL | LTR |
| 40202575 | 40202605 | + | 20.6 | 507 | 550 | -12 | MLT2B2 | ERVL | LTR |
| 40202641 | 40202758 | - | 33.0 | 3012 | 3128 | -291 | L2 | L2 | LINE |

  

|  |  |  |  |  |  |  |  |  |  |
| --- | --- | --- | --- | --- | --- | --- | --- | --- | --- |
| chr9:40274489-40278781 | | | 4314 bp | | |  |  |  |  |
| Genome Start | Genome End | Strand | Div | Repeat Start | Repeat End | Repeat Left | Name | Family | Class |
| 40274489 | 40274877 | - | 15.2 | 3906 | 4312 | -2252 | L1MCb | L1 | LINE |
| 40274880 | 40275658 | - | 19.9 | 2593 | 3356 | -3208 | L1MCb | L1 | LINE |
| 40275659 | 40276717 | - | 5.1 | 5094 | 6151 | -3 | L1PA5 | L1 | LINE |
| 40276695 | 40277779 | - | 20.2 | 1442 | 2599 | -4152 | L1MCb | L1 | LINE |
| 40277780 | 40278080 | + | 5.4 | 1 | 299 | -12 | AluY | Alu | SINE |
| 40278081 | 40278781 | - | 20.9 | 634 | 1441 | -5310 | L1MCb | L1 | LINE |

  

|  |  |  |  |  |  |  |  |  |  |
| --- | --- | --- | --- | --- | --- | --- | --- | --- | --- |
| chr9:40292696-40294407 | | | 1332 bp | | |  |  |  |  |
| Genome Start | Genome End | Strand | Div | Repeat Start | Repeat End | Repeat Left | Name | Family | Class |
| 40292696 | 40292792 | - | 30.2 | 1046 | 1143 | -866 | HAL1b | L1 | LINE |
| 40293004 | 40293320 | + | 21.8 | 152 | 462 | 0 | LTR40b | ERVL | LTR |
| 40293327 | 40293826 | - | 26.0 | 432 | 1002 | -1007 | HAL1b | L1 | LINE |
| 40293990 | 40294407 | - | 31.6 | 7 | 411 | -1598 | HAL1b | L1 | LINE |

  

|  |  |  |  |  |  |  |  |  |  |
| --- | --- | --- | --- | --- | --- | --- | --- | --- | --- |
| chr9:40295192-40296681 | | | 1490 bp | | |  |  |  |  |
| Genome Start | Genome End | Strand | Div | Repeat Start | Repeat End | Repeat Left | Name | Family | Class |
| 40295192 | 40295822 | + | 23.4 | 6459 | 7099 | -943 | L1MC4 | L1 | LINE |
| 40295823 | 40296120 | - | 7.4 | 1 | 298 | -13 | AluY | Alu | SINE |
| 40296121 | 40296681 | + | 23.4 | 7100 | 7678 | -364 | L1MC4 | L1 | LINE |

  

|  |  |  |  |  |  |  |  |  |  |
| --- | --- | --- | --- | --- | --- | --- | --- | --- | --- |
| chr9:40303319-40307935 | | | 4594 bp | | |  |  |  |  |
| Genome Start | Genome End | Strand | Div | Repeat Start | Repeat End | Repeat Left | Name | Family | Class |
| 40303319 | 40304166 | + | 26.9 | 10 | 913 | -6421 | L1MB4 | L1 | LINE |
| 40304151 | 40304646 | + | 18.5 | 1708 | 2316 | -5018 | L1MB4 | L1 | LINE |
| 40304647 | 40304873 | + | 12.4 | 6072 | 6299 | -1 | L1MA4 | L1 | LINE |
| 40304874 | 40305560 | + | 18.5 | 2317 | 3123 | -4211 | L1MB4 | L1 | LINE |
| 40305577 | 40307237 | + | 20.1 | 4630 | 6333 | -707 | L1MB4 | L1 | LINE |
| 40307261 | 40307935 | + | 20.0 | 6334 | 7032 | -8 | L1MB4 | L1 | LINE |

  

|  |  |  |  |  |  |  |  |  |  |
| --- | --- | --- | --- | --- | --- | --- | --- | --- | --- |
| chr9:40319051-40319598 | | | 548 bp | | |  |  |  |  |
| Genome Start | Genome End | Strand | Div | Repeat Start | Repeat End | Repeat Left | Name | Family | Class |
| 40319051 | 40319218 | + | 20.5 | 5 | 161 | -48 | MER3 | MER1\_type | DNA |
| 40319219 | 40319542 | - | 7.9 | 1 | 304 | -7 | AluY | Alu | SINE |
| 40319543 | 40319598 | + | 20.5 | 162 | 209 | 0 | MER3 | MER1\_type | DNA |

  

|  |  |  |  |  |  |  |  |  |  |
| --- | --- | --- | --- | --- | --- | --- | --- | --- | --- |
| chr9:40332339-40336323 | | | 3976 bp | | |  |  |  |  |
| Genome Start | Genome End | Strand | Div | Repeat Start | Repeat End | Repeat Left | Name | Family | Class |
| 40332339 | 40332773 | + | 21.8 | 39 | 493 | -5907 | L1ME2 | L1 | LINE |
| 40332780 | 40332889 | - | 24.5 | 978 | 1059 | 0 | MER106B | MER1\_type | DNA |
| 40332890 | 40333206 | - | 10.5 | 1 | 296 | -15 | AluY | Alu | SINE |
| 40333207 | 40333283 | - | 24.5 | 898 | 977 | -82 | MER106B | MER1\_type | DNA |
| 40333308 | 40333371 | - | 27.9 | 1 | 61 | -998 | MER106B | MER1\_type | DNA |
| 40333373 | 40333624 | + | 22.2 | 526 | 785 | -5615 | L1ME2 | L1 | LINE |
| 40333625 | 40333911 | + | 8.0 | 1 | 290 | -21 | AluY | Alu | SINE |
| 40333912 | 40335328 | + | 22.2 | 786 | 2285 | -4115 | L1ME2 | L1 | LINE |
| 40335307 | 40336323 | + | 23.3 | 5167 | 6228 | -9 | L1ME2 | L1 | LINE |

  

|  |  |  |  |  |  |  |  |  |  |
| --- | --- | --- | --- | --- | --- | --- | --- | --- | --- |
| chr9:40343581-40344300 | | | 720 bp | | |  |  |  |  |
| Genome Start | Genome End | Strand | Div | Repeat Start | Repeat End | Repeat Left | Name | Family | Class |
| 40343581 | 40343720 | - | 27.5 | 84 | 225 | -37 | MIR | MIR | SINE |
| 40343721 | 40344205 | - | 14.9 | 1 | 489 | 0 | LTR45B | ERV1 | LTR |
| 40344206 | 40344300 | - | 27.5 | 2 | 83 | -179 | MIR | MIR | SINE |

  

|  |  |  |  |  |  |  |  |  |  |
| --- | --- | --- | --- | --- | --- | --- | --- | --- | --- |
| chr9:40355233-40356468 | | | 1166 bp | | |  |  |  |  |
| Genome Start | Genome End | Strand | Div | Repeat Start | Repeat End | Repeat Left | Name | Family | Class |
| 40355233 | 40355299 | + | 20.3 | 5 | 70 | -395 | LTR40c | ERVL | LTR |
| 40355307 | 40356017 | + | 18.9 | 5447 | 6181 | -2 | L1MB3 | L1 | LINE |
| 40356081 | 40356468 | + | 26.0 | 63 | 459 | -6 | LTR40c | ERVL | LTR |

  

|  |  |  |  |  |  |  |  |  |  |
| --- | --- | --- | --- | --- | --- | --- | --- | --- | --- |
| chr9:40365236-40369208 | | | 3531 bp | | |  |  |  |  |
| Genome Start | Genome End | Strand | Div | Repeat Start | Repeat End | Repeat Left | Name | Family | Class |
| 40365236 | 40365506 | + | 32.2 | 55 | 343 | -5776 | L1MEd | L1 | LINE |
| 40365507 | 40365804 | - | 7.4 | 2 | 308 | -3 | AluY | Alu | SINE |
| 40365805 | 40366290 | + | 32.2 | 344 | 854 | -5265 | L1MEd | L1 | LINE |
| 40366430 | 40366790 | - | 14.9 | 206 | 564 | -8 | LTR73 | ERV1 | LTR |
| 40366791 | 40367077 | - | 12.6 | 27 | 311 | -1 | AluSx | Alu | SINE |
| 40367078 | 40367384 | - | 21.6 | 1 | 297 | -15 | AluJo | Alu | SINE |
| 40367385 | 40367555 | - | 14.9 | 26 | 205 | -367 | LTR73 | ERV1 | LTR |
| 40367811 | 40368173 | + | 29.6 | 2483 | 2889 | -3240 | L1MEd | L1 | LINE |
| 40368222 | 40368252 | + | 31.4 | 3420 | 3446 | -2683 | L1MEd | L1 | LINE |
| 40368253 | 40368293 | - | 15.5 | 390 | 428 | 0 | MSTA | MaLR | LTR |
| 40368294 | 40368598 | + | 5.6 | 1 | 305 | -6 | AluY | Alu | SINE |
| 40368599 | 40368945 | - | 15.5 | 1 | 389 | -39 | MSTA | MaLR | LTR |
| 40368946 | 40369208 | + | 31.4 | 3447 | 3731 | -2398 | L1MEd | L1 | LINE |

  

|  |  |  |  |  |  |  |  |  |  |
| --- | --- | --- | --- | --- | --- | --- | --- | --- | --- |
| chr9:40378182-40378947 | | | 721 bp | | |  |  |  |  |
| Genome Start | Genome End | Strand | Div | Repeat Start | Repeat End | Repeat Left | Name | Family | Class |
| 40378182 | 40378559 | + | 13.7 | 1 | 389 | -39 | MSTA | MaLR | LTR |
| 40378560 | 40378852 | + | 13.9 | 1 | 276 | -36 | AluSx | Alu | SINE |
| 40378898 | 40378947 | + | 13.7 | 390 | 428 | 0 | MSTA | MaLR | LTR |

  

|  |  |  |  |  |  |  |  |  |  |
| --- | --- | --- | --- | --- | --- | --- | --- | --- | --- |
| chr9:40383151-40386317 | | | 3165 bp | | |  |  |  |  |
| Genome Start | Genome End | Strand | Div | Repeat Start | Repeat End | Repeat Left | Name | Family | Class |
| 40383151 | 40383232 | + | 18.3 | 3742 | 3823 | -2323 | L1MC2 | L1 | LINE |
| 40383233 | 40384069 | + | 14.8 | 1 | 983 | 0 | LTR1D | ERV1 | LTR |
| 40384070 | 40385309 | + | 19.6 | 3820 | 5070 | -1076 | L1MC2 | L1 | LINE |
| 40385310 | 40385452 | - | 19.0 | 22 | 163 | -13 | FRAM | Alu | SINE |
| 40385455 | 40386317 | + | 20.2 | 5063 | 6000 | -344 | L1MC2 | L1 | LINE |

  

|  |  |  |  |  |  |  |  |  |  |
| --- | --- | --- | --- | --- | --- | --- | --- | --- | --- |
| chr9:40387051-40387551 | | | 501 bp | | |  |  |  |  |
| Genome Start | Genome End | Strand | Div | Repeat Start | Repeat End | Repeat Left | Name | Family | Class |
| 40387051 | 40387166 | - | 29.6 | 3314 | 3419 | 0 | L2 | L2 | LINE |
| 40387167 | 40387464 | + | 12.5 | 1 | 296 | -17 | AluSq | Alu | SINE |
| 40387465 | 40387551 | - | 29.6 | 3226 | 3313 | -106 | L2 | L2 | LINE |

  

|  |  |  |  |  |  |  |  |  |  |
| --- | --- | --- | --- | --- | --- | --- | --- | --- | --- |
| chr9:40396729-40398037 | | | 1309 bp | | |  |  |  |  |
| Genome Start | Genome End | Strand | Div | Repeat Start | Repeat End | Repeat Left | Name | Family | Class |
| 40396729 | 40397387 | - | 34.4 | 2762 | 3369 | -9 | L2 | L2 | LINE |
| 40397388 | 40397772 | + | 16.0 | 1 | 427 | -1 | MSTA | MaLR | LTR |
| 40397773 | 40398037 | - | 31.9 | 2445 | 2761 | -658 | L2 | L2 | LINE |

  

|  |  |  |  |  |  |  |  |  |  |
| --- | --- | --- | --- | --- | --- | --- | --- | --- | --- |
| chr9:40404488-40406471 | | | 1984 bp | | |  |  |  |  |
| Genome Start | Genome End | Strand | Div | Repeat Start | Repeat End | Repeat Left | Name | Family | Class |
| 40404488 | 40405053 | - | 16.0 | 5591 | 6235 | -109 | L1MC2 | L1 | LINE |
| 40405054 | 40405346 | + | 10.2 | 1 | 293 | -16 | AluSc | Alu | SINE |
| 40405347 | 40406471 | - | 16.6 | 4443 | 5590 | -754 | L1MC2 | L1 | LINE |

  

|  |  |  |  |  |  |  |  |  |  |
| --- | --- | --- | --- | --- | --- | --- | --- | --- | --- |
| chr9:40413169-40413920 | | | 707 bp | | |  |  |  |  |
| Genome Start | Genome End | Strand | Div | Repeat Start | Repeat End | Repeat Left | Name | Family | Class |
| 40413169 | 40413471 | + | 23.5 | 1 | 343 | -119 | LTR40b | ERVL | LTR |
| 40413472 | 40413758 | + | 9.4 | 1 | 287 | -24 | AluY | Alu | SINE |
| 40413804 | 40413920 | + | 23.5 | 344 | 462 | 0 | LTR40b | ERVL | LTR |

  

|  |  |  |  |  |  |  |  |  |  |
| --- | --- | --- | --- | --- | --- | --- | --- | --- | --- |
| chr9:40483607-40484289 | | | 683 bp | | |  |  |  |  |
| Genome Start | Genome End | Strand | Div | Repeat Start | Repeat End | Repeat Left | Name | Family | Class |
| 40483607 | 40483692 | + | 18.9 | 18 | 86 | -340 | LTR37A | ERV1 | LTR |
| 40483693 | 40484002 | - | 7.2 | 1 | 308 | -3 | AluY | Alu | SINE |
| 40484003 | 40484289 | + | 18.9 | 87 | 426 | 0 | LTR37A | ERV1 | LTR |

  

|  |  |  |  |  |  |  |  |  |  |
| --- | --- | --- | --- | --- | --- | --- | --- | --- | --- |
| chr9:40484857-40485770 | | | 890 bp | | |  |  |  |  |
| Genome Start | Genome End | Strand | Div | Repeat Start | Repeat End | Repeat Left | Name | Family | Class |
| 40484857 | 40485023 | + | 18.6 | 111 | 323 | -103 | LTR37A | ERV1 | LTR |
| 40485024 | 40485153 | + | 18.7 | 1 | 146 | -565 | MER67C | ERV1 | LTR |
| 40485178 | 40485679 | + | 18.7 | 147 | 711 | 0 | MER67C | ERV1 | LTR |
| 40485680 | 40485770 | + | 18.6 | 324 | 413 | -13 | LTR37A | ERV1 | LTR |

  

|  |  |  |  |  |  |  |  |  |  |
| --- | --- | --- | --- | --- | --- | --- | --- | --- | --- |
| chr9:40531048-40531577 | | | 530 bp | | |  |  |  |  |
| Genome Start | Genome End | Strand | Div | Repeat Start | Repeat End | Repeat Left | Name | Family | Class |
| 40531048 | 40531285 | - | 21.4 | 75 | 297 | -15 | AluJo | Alu | SINE |
| 40531286 | 40531515 | + | 10.4 | 72 | 301 | -9 | AluSg/x | Alu | SINE |
| 40531516 | 40531577 | - | 21.4 | 15 | 74 | -238 | AluJo | Alu | SINE |

  

|  |  |  |  |  |  |  |  |  |  |
| --- | --- | --- | --- | --- | --- | --- | --- | --- | --- |
| chr9:40550732-40551705 | | | 546 bp | | |  |  |  |  |
| Genome Start | Genome End | Strand | Div | Repeat Start | Repeat End | Repeat Left | Name | Family | Class |
| 40550732 | 40550967 | - | 29.2 | 5621 | 5889 | -1919 | L1MC | L1 | LINE |
| 40551203 | 40551422 | + | 29.7 | 13 | 244 | -18 | MIR | MIR | SINE |
| 40551616 | 40551705 | - | 23.6 | 5383 | 5473 | -2312 | L1MC | L1 | LINE |

  

|  |  |  |  |  |  |  |  |  |  |
| --- | --- | --- | --- | --- | --- | --- | --- | --- | --- |
| chr9:40589663-40590274 | | | 508 bp | | |  |  |  |  |
| Genome Start | Genome End | Strand | Div | Repeat Start | Repeat End | Repeat Left | Name | Family | Class |
| 40589663 | 40589758 | + | 22.6 | 24 | 118 | -97 | MER58C | MER1\_type | DNA |
| 40589768 | 40590075 | + | 20.6 | 1 | 306 | -6 | AluJo | Alu | SINE |
| 40590171 | 40590274 | + | 24.0 | 106 | 213 | -2 | MER58C | MER1\_type | DNA |

  

|  |  |  |  |  |  |  |  |  |  |
| --- | --- | --- | --- | --- | --- | --- | --- | --- | --- |
| chr9:40606439-40607094 | | | 546 bp | | |  |  |  |  |
| Genome Start | Genome End | Strand | Div | Repeat Start | Repeat End | Repeat Left | Name | Family | Class |
| 40606439 | 40606616 | + | 28.3 | 91 | 268 | -323 | MLT1K | MaLR | LTR |
| 40606672 | 40606818 | - | 25.2 | 3266 | 3419 | 0 | L2 | L2 | LINE |
| 40606874 | 40607094 | + | 31.5 | 312 | 576 | -15 | MLT1K | MaLR | LTR |

  

|  |  |  |  |  |  |  |  |  |  |
| --- | --- | --- | --- | --- | --- | --- | --- | --- | --- |
| chr9:40632812-40634451 | | | 1640 bp | | |  |  |  |  |
| Genome Start | Genome End | Strand | Div | Repeat Start | Repeat End | Repeat Left | Name | Family | Class |
| 40632812 | 40633934 | + | 24.5 | 1576 | 2606 | -3613 | L1MEc | L1 | LINE |
| 40633935 | 40634236 | - | 10.3 | 1 | 301 | -12 | AluSq | Alu | SINE |
| 40634237 | 40634451 | + | 27.9 | 2607 | 2825 | -3394 | L1MEc | L1 | LINE |

  

|  |  |  |  |  |  |  |  |  |  |
| --- | --- | --- | --- | --- | --- | --- | --- | --- | --- |
| chr9:40652592-40654481 | | | 1890 bp | | |  |  |  |  |
| Genome Start | Genome End | Strand | Div | Repeat Start | Repeat End | Repeat Left | Name | Family | Class |
| 40652592 | 40652712 | + | 29.6 | 2 | 134 | -1601 | MLT-int | MaLR | LTR |
| 40652713 | 40653010 | + | 11.1 | 1 | 299 | -14 | AluSq | Alu | SINE |
| 40653011 | 40654481 | + | 29.6 | 135 | 1735 | 0 | MLT-int | MaLR | LTR |

  

|  |  |  |  |  |  |  |  |  |  |
| --- | --- | --- | --- | --- | --- | --- | --- | --- | --- |
| chr9:40659573-40661326 | | | 1767 bp | | |  |  |  |  |
| Genome Start | Genome End | Strand | Div | Repeat Start | Repeat End | Repeat Left | Name | Family | Class |
| 40659573 | 40659784 | + | 22.1 | 2 | 211 | -872 | Tigger6a | MER2\_type | DNA |
| 40659785 | 40660243 | - | 22.6 | 7 | 467 | 0 | MLT1C | MaLR | LTR |
| 40660231 | 40660852 | + | 19.1 | 216 | 883 | -200 | Tigger6a | MER2\_type | DNA |
| 40660853 | 40661130 | + | 11.2 | 3 | 293 | -18 | AluY | Alu | SINE |
| 40661131 | 40661326 | + | 19.1 | 884 | 1083 | 0 | Tigger6a | MER2\_type | DNA |

  

|  |  |  |  |  |  |  |  |  |  |
| --- | --- | --- | --- | --- | --- | --- | --- | --- | --- |
| chr9:40666993-40668318 | | | 1214 bp | | |  |  |  |  |
| Genome Start | Genome End | Strand | Div | Repeat Start | Repeat End | Repeat Left | Name | Family | Class |
| 40666993 | 40667211 | - | 31.4 | 4851 | 5064 | -1082 | L1M5 | L1 | LINE |
| 40667325 | 40667398 | - | 23.0 | 4633 | 4709 | -1437 | L1M5 | L1 | LINE |
| 40667398 | 40667567 | - | 27.1 | 3928 | 4104 | -2042 | L1M5 | L1 | LINE |
| 40667568 | 40667866 | - | 9.1 | 1 | 296 | -15 | AluY | Alu | SINE |
| 40667867 | 40668318 | - | 27.1 | 3375 | 3927 | -2219 | L1M5 | L1 | LINE |

  

|  |  |  |  |  |  |  |  |  |  |
| --- | --- | --- | --- | --- | --- | --- | --- | --- | --- |
| chr9:40668343-40672348 | | | 3925 bp | | |  |  |  |  |
| Genome Start | Genome End | Strand | Div | Repeat Start | Repeat End | Repeat Left | Name | Family | Class |
| 40668343 | 40668612 | - | 27.2 | 5890 | 6204 | -19 | L1ME3B | L1 | LINE |
| 40668613 | 40668915 | - | 7.0 | 1 | 300 | -11 | AluY | Alu | SINE |
| 40668916 | 40668999 | - | 27.2 | 5828 | 5889 | -334 | L1ME3B | L1 | LINE |
| 40669000 | 40669475 | - | 15.1 | 5818 | 6322 | -11 | L1MC1 | L1 | LINE |
| 40669476 | 40669791 | - | 10.3 | 1 | 311 | 0 | AluY | Alu | SINE |
| 40669792 | 40670169 | - | 15.1 | 5435 | 5817 | -516 | L1MC1 | L1 | LINE |
| 40670170 | 40670887 | - | 27.5 | 5043 | 5827 | -396 | L1ME3B | L1 | LINE |
| 40670888 | 40671184 | + | 7.5 | 1 | 295 | -16 | AluY | Alu | SINE |
| 40671185 | 40671374 | - | 31.9 | 4839 | 5042 | -1087 | L1ME3B | L1 | LINE |
| 40671447 | 40671873 | - | 15.2 | 1 | 436 | -231 | LTR12B | ERV1 | LTR |
| 40671883 | 40672348 | - | 33.2 | 64 | 559 | -5560 | L1ME3B | L1 | LINE |

  

|  |  |  |  |  |  |  |  |  |  |
| --- | --- | --- | --- | --- | --- | --- | --- | --- | --- |
| chr9:40681399-40682776 | | | 1378 bp | | |  |  |  |  |
| Genome Start | Genome End | Strand | Div | Repeat Start | Repeat End | Repeat Left | Name | Family | Class |
| 40681399 | 40682257 | - | 20.4 | 4377 | 5283 | -857 | L1M3 | L1 | LINE |
| 40682258 | 40682520 | - | 9.9 | 1 | 287 | -24 | AluY | Alu | SINE |
| 40682521 | 40682776 | - | 20.4 | 4117 | 4376 | -1764 | L1M3 | L1 | LINE |

  

|  |  |  |  |  |  |  |  |  |  |
| --- | --- | --- | --- | --- | --- | --- | --- | --- | --- |
| chr9:40682772-40685498 | | | 2727 bp | | |  |  |  |  |
| Genome Start | Genome End | Strand | Div | Repeat Start | Repeat End | Repeat Left | Name | Family | Class |
| 40682772 | 40683166 | + | 23.6 | 2917 | 3348 | -4217 | L1M1 | L1 | LINE |
| 40683167 | 40683502 | - | 18.4 | 4 | 345 | -1 | MER7A | MER2\_type | DNA |
| 40683503 | 40684750 | + | 18.4 | 3349 | 4644 | -2915 | L1M1 | L1 | LINE |
| 40684751 | 40685060 | + | 9.4 | 1 | 317 | 0 | AluY | Alu | SINE |
| 40685061 | 40685498 | + | 17.4 | 4645 | 5099 | -2460 | L1M1 | L1 | LINE |

  

|  |  |  |  |  |  |  |  |  |  |
| --- | --- | --- | --- | --- | --- | --- | --- | --- | --- |
| chr9:40722908-40725118 | | | 2186 bp | | |  |  |  |  |
| Genome Start | Genome End | Strand | Div | Repeat Start | Repeat End | Repeat Left | Name | Family | Class |
| 40722908 | 40724062 | - | 18.2 | 4190 | 5331 | -815 | L1MC | L1 | LINE |
| 40724063 | 40724365 | - | 7.9 | 1 | 303 | -8 | AluY | Alu | SINE |
| 40724366 | 40724610 | - | 18.2 | 3965 | 4189 | -1957 | L1MC | L1 | LINE |
| 40724636 | 40724939 | - | 12.2 | 1 | 287 | -24 | AluY | Alu | SINE |
| 40724940 | 40725118 | - | 18.2 | 3776 | 3964 | -2182 | L1MC | L1 | LINE |

  

|  |  |  |  |  |  |  |  |  |  |
| --- | --- | --- | --- | --- | --- | --- | --- | --- | --- |
| chr9:40734349-40737011 | | | 2663 bp | | |  |  |  |  |
| Genome Start | Genome End | Strand | Div | Repeat Start | Repeat End | Repeat Left | Name | Family | Class |
| 40734349 | 40735746 | + | 10.4 | 3147 | 4544 | -1602 | L1PREC2 | L1 | LINE |
| 40735747 | 40736047 | + | 11.0 | 1 | 301 | -8 | AluSc | Alu | SINE |
| 40736048 | 40737011 | + | 9.8 | 4545 | 5480 | -680 | L1PREC2 | L1 | LINE |

  

|  |  |  |  |  |  |  |  |  |  |
| --- | --- | --- | --- | --- | --- | --- | --- | --- | --- |
| chr9:40741495-40742188 | | | 694 bp | | |  |  |  |  |
| Genome Start | Genome End | Strand | Div | Repeat Start | Repeat End | Repeat Left | Name | Family | Class |
| 40741495 | 40741669 | - | 18.9 | 138 | 311 | 0 | AluJo | Alu | SINE |
| 40741670 | 40742032 | - | 9.6 | 1 | 364 | 0 | THE1B | MaLR | LTR |
| 40742033 | 40742188 | - | 18.9 | 1 | 137 | -175 | AluJo | Alu | SINE |

  

|  |  |  |  |  |  |  |  |  |  |
| --- | --- | --- | --- | --- | --- | --- | --- | --- | --- |
| chr9:40770459-40771753 | | | 1102 bp | | |  |  |  |  |
| Genome Start | Genome End | Strand | Div | Repeat Start | Repeat End | Repeat Left | Name | Family | Class |
| 40770459 | 40770678 | - | 31.6 | 4838 | 5119 | -1027 | L1M5 | L1 | LINE |
| 40770679 | 40770966 | - | 9.4 | 1 | 311 | 0 | AluY | Alu | SINE |
| 40770967 | 40771154 | - | 31.6 | 4660 | 4837 | -1309 | L1M5 | L1 | LINE |
| 40771348 | 40771753 | - | 34.7 | 2578 | 3015 | -3131 | L1M5 | L1 | LINE |

  

|  |  |  |  |  |  |  |  |  |  |
| --- | --- | --- | --- | --- | --- | --- | --- | --- | --- |
| chr9:40775901-40778090 | | | 2190 bp | | |  |  |  |  |
| Genome Start | Genome End | Strand | Div | Repeat Start | Repeat End | Repeat Left | Name | Family | Class |
| 40775901 | 40777472 | + | 25.1 | 37 | 1697 | -5275 | L1M4 | L1 | LINE |
| 40777473 | 40777777 | + | 13.8 | 2 | 312 | 0 | AluSx | Alu | SINE |
| 40777778 | 40778090 | + | 24.9 | 1698 | 2057 | -4915 | L1M4 | L1 | LINE |

  

|  |  |  |  |  |  |  |  |  |  |
| --- | --- | --- | --- | --- | --- | --- | --- | --- | --- |
| chr9:40784378-40786224 | | | 1847 bp | | |  |  |  |  |
| Genome Start | Genome End | Strand | Div | Repeat Start | Repeat End | Repeat Left | Name | Family | Class |
| 40784378 | 40785433 | - | 14.3 | 3855 | 4925 | -1221 | L1MC | L1 | LINE |
| 40785434 | 40785743 | - | 10.7 | 1 | 309 | -1 | AluSg | Alu | SINE |
| 40785744 | 40786224 | - | 14.3 | 3353 | 3854 | -2292 | L1MC | L1 | LINE |

  

|  |  |  |  |  |  |  |  |  |  |
| --- | --- | --- | --- | --- | --- | --- | --- | --- | --- |
| chr9:40786220-40788268 | | | 2049 bp | | |  |  |  |  |
| Genome Start | Genome End | Strand | Div | Repeat Start | Repeat End | Repeat Left | Name | Family | Class |
| 40786220 | 40786273 | - | 22.8 | 1572 | 1621 | -4887 | L1MCa | L1 | LINE |
| 40786274 | 40786565 | - | 8.0 | 9 | 297 | -14 | AluY | Alu | SINE |
| 40786566 | 40788268 | - | 22.8 | 1 | 1571 | -4937 | L1MCa | L1 | LINE |

  

|  |  |  |  |  |  |  |  |  |  |
| --- | --- | --- | --- | --- | --- | --- | --- | --- | --- |
| chr9:40819055-40821060 | | | 1927 bp | | |  |  |  |  |
| Genome Start | Genome End | Strand | Div | Repeat Start | Repeat End | Repeat Left | Name | Family | Class |
| 40819055 | 40819765 | + | 21.0 | 5104 | 5833 | -908 | L1MB5 | L1 | LINE |
| 40819815 | 40819997 | + | 14.2 | 5834 | 6035 | -749 | L1MB5 | L1 | LINE |
| 40820005 | 40820092 | + | 13.6 | 6151 | 6240 | -544 | L1MB5 | L1 | LINE |
| 40820094 | 40820505 | + | 21.6 | 5 | 492 | -64 | MLT2B4 | ERVL | LTR |
| 40820528 | 40821060 | + | 20.2 | 6243 | 6774 | -1 | L1MB5 | L1 | LINE |

  

|  |  |  |  |  |  |  |  |  |  |
| --- | --- | --- | --- | --- | --- | --- | --- | --- | --- |
| chr9:40838644-40839255 | | | 612 bp | | |  |  |  |  |
| Genome Start | Genome End | Strand | Div | Repeat Start | Repeat End | Repeat Left | Name | Family | Class |
| 40838644 | 40838776 | + | 13.0 | 3 | 127 | -185 | AluSx | Alu | SINE |
| 40838777 | 40839084 | + | 7.5 | 1 | 305 | -6 | AluY | Alu | SINE |
| 40839085 | 40839255 | + | 13.0 | 128 | 295 | -17 | AluSx | Alu | SINE |

  

|  |  |  |  |  |  |  |  |  |  |
| --- | --- | --- | --- | --- | --- | --- | --- | --- | --- |
| chr9:40842502-40843249 | | | 707 bp | | |  |  |  |  |
| Genome Start | Genome End | Strand | Div | Repeat Start | Repeat End | Repeat Left | Name | Family | Class |
| 40842502 | 40842600 | + | 36.8 | 2347 | 2439 | -980 | L2 | L2 | LINE |
| 40842642 | 40842903 | - | 9.0 | 18 | 263 | -49 | AluSx | Alu | SINE |
| 40842904 | 40843249 | + | 36.8 | 2440 | 2822 | -597 | L2 | L2 | LINE |

  

|  |  |  |  |  |  |  |  |  |  |
| --- | --- | --- | --- | --- | --- | --- | --- | --- | --- |
| chr9:40843924-40844632 | | | 709 bp | | |  |  |  |  |
| Genome Start | Genome End | Strand | Div | Repeat Start | Repeat End | Repeat Left | Name | Family | Class |
| 40843924 | 40843986 | + | 33.9 | 2912 | 2975 | -403 | L2 | L2 | LINE |
| 40843987 | 40844298 | - | 5.2 | 1 | 311 | 0 | AluY | Alu | SINE |
| 40844299 | 40844632 | + | 33.9 | 2976 | 3340 | -38 | L2 | L2 | LINE |

  

|  |  |  |  |  |  |  |  |  |  |
| --- | --- | --- | --- | --- | --- | --- | --- | --- | --- |
| chr9:40847686-40848650 | | | 965 bp | | |  |  |  |  |
| Genome Start | Genome End | Strand | Div | Repeat Start | Repeat End | Repeat Left | Name | Family | Class |
| 40847686 | 40848230 | + | 23.3 | 1 | 523 | -101 | MLT1E3 | MaLR | LTR |
| 40848231 | 40848543 | - | 9.9 | 1 | 313 | 0 | AluSq | Alu | SINE |
| 40848544 | 40848650 | + | 23.3 | 524 | 624 | 0 | MLT1E3 | MaLR | LTR |

  

|  |  |  |  |  |  |  |  |  |  |
| --- | --- | --- | --- | --- | --- | --- | --- | --- | --- |
| chr9:40849027-40849644 | | | 618 bp | | |  |  |  |  |
| Genome Start | Genome End | Strand | Div | Repeat Start | Repeat End | Repeat Left | Name | Family | Class |
| 40849027 | 40849195 | + | 11.0 | 1 | 183 | -181 | THE1B | MaLR | LTR |
| 40849196 | 40849427 | + | 9.3 | 5950 | 6181 | 0 | L1PA8 | L1 | LINE |
| 40849428 | 40849644 | + | 11.0 | 184 | 364 | 0 | THE1B | MaLR | LTR |

  

|  |  |  |  |  |  |  |  |  |  |
| --- | --- | --- | --- | --- | --- | --- | --- | --- | --- |
| chr9:40863231-40865704 | | | 2417 bp | | |  |  |  |  |
| Genome Start | Genome End | Strand | Div | Repeat Start | Repeat End | Repeat Left | Name | Family | Class |
| 40863231 | 40863291 | + | 21.3 | 1 | 61 | -592 | MER82 | MER2\_type | DNA |
| 40863293 | 40863851 | - | 17.7 | 219 | 787 | 0 | LTR48 | ERV1 | LTR |
| 40863852 | 40864155 | + | 7.0 | 1 | 301 | -10 | AluY | Alu | SINE |
| 40864158 | 40864467 | + | 11.0 | 1 | 308 | -2 | AluSg | Alu | SINE |
| 40864522 | 40864642 | - | 24.7 | 138 | 236 | -551 | LTR48 | ERV1 | LTR |
| 40864643 | 40865012 | + | 15.5 | 1 | 364 | 0 | THE1B | MaLR | LTR |
| 40865013 | 40865157 | - | 24.7 | 1 | 137 | -650 | LTR48 | ERV1 | LTR |
| 40865158 | 40865704 | + | 21.4 | 59 | 625 | -28 | MER82 | MER2\_type | DNA |

  

|  |  |  |  |  |  |  |  |  |  |
| --- | --- | --- | --- | --- | --- | --- | --- | --- | --- |
| chr9:40866551-40867447 | | | 897 bp | | |  |  |  |  |
| Genome Start | Genome End | Strand | Div | Repeat Start | Repeat End | Repeat Left | Name | Family | Class |
| 40866551 | 40866571 | - | 27.3 | 437 | 456 | -12 | MER110A | ERV1 | LTR |
| 40866572 | 40867070 | + | 21.3 | 1 | 505 | 0 | MLT1D | MaLR | LTR |
| 40867071 | 40867447 | - | 27.3 | 27 | 436 | -32 | MER110A | ERV1 | LTR |

  

|  |  |  |  |  |  |  |  |  |  |
| --- | --- | --- | --- | --- | --- | --- | --- | --- | --- |
| chr9:40875229-40875954 | | | 726 bp | | |  |  |  |  |
| Genome Start | Genome End | Strand | Div | Repeat Start | Repeat End | Repeat Left | Name | Family | Class |
| 40875229 | 40875415 | + | 19.4 | 8 | 195 | -231 | MSTB | MaLR | LTR |
| 40875416 | 40875733 | + | 14.9 | 1 | 318 | 0 | AluSx | Alu | SINE |
| 40875734 | 40875954 | + | 19.4 | 196 | 426 | 0 | MSTB | MaLR | LTR |

  

|  |  |  |  |  |  |  |  |  |  |
| --- | --- | --- | --- | --- | --- | --- | --- | --- | --- |
| chr9:40879291-40880524 | | | 1234 bp | | |  |  |  |  |
| Genome Start | Genome End | Strand | Div | Repeat Start | Repeat End | Repeat Left | Name | Family | Class |
| 40879291 | 40879608 | + | 15.7 | 2 | 300 | -66 | Tigger5a | MER2\_type | DNA |
| 40879609 | 40879992 | + | 7.6 | 1 | 387 | -167 | MER41A | ERV1 | LTR |
| 40879993 | 40880296 | + | 9.9 | 1 | 304 | -6 | AluSg | Alu | SINE |
| 40880297 | 40880472 | + | 7.6 | 388 | 554 | 0 | MER41A | ERV1 | LTR |
| 40880473 | 40880524 | + | 15.7 | 301 | 361 | -5 | Tigger5a | MER2\_type | DNA |

  

|  |  |  |  |  |  |  |  |  |  |
| --- | --- | --- | --- | --- | --- | --- | --- | --- | --- |
| chr9:40903288-40904734 | | | 1447 bp | | |  |  |  |  |
| Genome Start | Genome End | Strand | Div | Repeat Start | Repeat End | Repeat Left | Name | Family | Class |
| 40903288 | 40903555 | - | 17.4 | 5163 | 5437 | -741 | L1M4 | L1 | LINE |
| 40903556 | 40903842 | + | 12.5 | 1 | 290 | -22 | AluSx | Alu | SINE |
| 40903843 | 40904734 | - | 17.3 | 4316 | 5162 | -984 | L1M4 | L1 | LINE |

  

|  |  |  |  |  |  |  |  |  |  |
| --- | --- | --- | --- | --- | --- | --- | --- | --- | --- |
| chr9:40910505-40911368 | | | 626 bp | | |  |  |  |  |
| Genome Start | Genome End | Strand | Div | Repeat Start | Repeat End | Repeat Left | Name | Family | Class |
| 40910505 | 40910736 | - | 19.1 | 4094 | 4321 | -1825 | L1M4 | L1 | LINE |
| 40910738 | 40911075 | + | 18.6 | 1 | 393 | -508 | MER54B | ERVL | LTR |
| 40911313 | 40911368 | - | 23.2 | 4039 | 4097 | -2049 | L1M4 | L1 | LINE |

  

|  |  |  |  |  |  |  |  |  |  |
| --- | --- | --- | --- | --- | --- | --- | --- | --- | --- |
| chr9:40915859-40916415 | | | 557 bp | | |  |  |  |  |
| Genome Start | Genome End | Strand | Div | Repeat Start | Repeat End | Repeat Left | Name | Family | Class |
| 40915859 | 40916043 | + | 20.5 | 5608 | 5819 | -345 | L1ME2 | L1 | LINE |
| 40916044 | 40916344 | - | 9.4 | 1 | 302 | -10 | AluSx | Alu | SINE |
| 40916345 | 40916415 | + | 20.5 | 5820 | 5886 | -278 | L1ME2 | L1 | LINE |

  

|  |  |  |  |  |  |  |  |  |  |
| --- | --- | --- | --- | --- | --- | --- | --- | --- | --- |
| chr9:40916696-40917845 | | | 1150 bp | | |  |  |  |  |
| Genome Start | Genome End | Strand | Div | Repeat Start | Repeat End | Repeat Left | Name | Family | Class |
| 40916696 | 40916751 | + | 23.3 | 208 | 255 | -86 | MER58B | MER1\_type | DNA |
| 40916752 | 40917236 | - | 15.8 | 3 | 489 | 0 | LTR45B | ERV1 | LTR |
| 40917237 | 40917288 | + | 23.3 | 256 | 303 | -38 | MER58B | MER1\_type | DNA |
| 40917289 | 40917798 | - | 17.1 | 2 | 550 | 0 | MER44B | MER2\_type | DNA |
| 40917799 | 40917845 | + | 23.3 | 304 | 340 | -1 | MER58B | MER1\_type | DNA |

  

|  |  |  |  |  |  |  |  |  |  |
| --- | --- | --- | --- | --- | --- | --- | --- | --- | --- |
| chr9:40987686-40988737 | | | 1056 bp | | |  |  |  |  |
| Genome Start | Genome End | Strand | Div | Repeat Start | Repeat End | Repeat Left | Name | Family | Class |
| 40987686 | 40987915 | - | 29.6 | 5907 | 6132 | -34 | L1ME1 | L1 | LINE |
| 40987915 | 40988336 | + | 17.0 | 2295 | 2722 | -3654 | L1M4c | L1 | LINE |
| 40988334 | 40988737 | - | 23.6 | 4631 | 5091 | -1055 | L1ME1 | L1 | LINE |

  

|  |  |  |  |  |  |  |  |  |  |
| --- | --- | --- | --- | --- | --- | --- | --- | --- | --- |
| chr9:40996672-40997425 | | | 639 bp | | |  |  |  |  |
| Genome Start | Genome End | Strand | Div | Repeat Start | Repeat End | Repeat Left | Name | Family | Class |
| 40996672 | 40996794 | - | 29.4 | 114 | 241 | -21 | MIR | MIR | SINE |
| 40996802 | 40997231 | + | 17.1 | 1 | 511 | -45 | MLT2B4 | ERVL | LTR |
| 40997340 | 40997425 | - | 18.6 | 21 | 130 | -132 | MIR | MIR | SINE |

  

|  |  |  |  |  |  |  |  |  |  |
| --- | --- | --- | --- | --- | --- | --- | --- | --- | --- |
| chr9:40997487-40998022 | | | 536 bp | | |  |  |  |  |
| Genome Start | Genome End | Strand | Div | Repeat Start | Repeat End | Repeat Left | Name | Family | Class |
| 40997487 | 40997646 | + | 14.5 | 2 | 161 | -41 | MER6C | MER2\_type | DNA |
| 40997647 | 40997971 | - | 7.1 | 1 | 309 | -2 | AluY | Alu | SINE |
| 40997972 | 40998022 | + | 14.5 | 162 | 201 | -1 | MER6C | MER2\_type | DNA |

  

|  |  |  |  |  |  |  |  |  |  |
| --- | --- | --- | --- | --- | --- | --- | --- | --- | --- |
| chr9:41001708-41002752 | | | 1045 bp | | |  |  |  |  |
| Genome Start | Genome End | Strand | Div | Repeat Start | Repeat End | Repeat Left | Name | Family | Class |
| 41001708 | 41001833 | + | 15.8 | 1 | 114 | -312 | MSTB | MaLR | LTR |
| 41001834 | 41002143 | + | 4.2 | 1 | 308 | -3 | AluY | Alu | SINE |
| 41002144 | 41002397 | + | 15.8 | 115 | 375 | -51 | MSTB | MaLR | LTR |
| 41002398 | 41002702 | + | 4.3 | 1 | 299 | -12 | AluY | Alu | SINE |
| 41002703 | 41002752 | + | 15.8 | 376 | 426 | 0 | MSTB | MaLR | LTR |

  

|  |  |  |  |  |  |  |  |  |  |
| --- | --- | --- | --- | --- | --- | --- | --- | --- | --- |
| chr9:41017666-41018805 | | | 1140 bp | | |  |  |  |  |
| Genome Start | Genome End | Strand | Div | Repeat Start | Repeat End | Repeat Left | Name | Family | Class |
| 41017666 | 41017720 | + | 19.2 | 4177 | 4216 | -1930 | L1PREC2 | L1 | LINE |
| 41017721 | 41018017 | - | 12.5 | 1 | 296 | -16 | AluSx | Alu | SINE |
| 41018018 | 41018145 | + | 19.2 | 4217 | 4337 | -1809 | L1PREC2 | L1 | LINE |
| 41018146 | 41018683 | + | 16.8 | 1 | 556 | 0 | LTR38 | ERV1 | LTR |
| 41018684 | 41018805 | + | 19.2 | 4338 | 4454 | -1692 | L1PREC2 | L1 | LINE |

  

|  |  |  |  |  |  |  |  |  |  |
| --- | --- | --- | --- | --- | --- | --- | --- | --- | --- |
| chr9:41039717-41040628 | | | 853 bp | | |  |  |  |  |
| Genome Start | Genome End | Strand | Div | Repeat Start | Repeat End | Repeat Left | Name | Family | Class |
| 41039717 | 41039793 | + | 26.6 | 4865 | 4938 | -1208 | L1M5 | L1 | LINE |
| 41039794 | 41039997 | + | 21.3 | 1 | 194 | -220 | MLT2D | ERVL | LTR |
| 41039998 | 41040279 | + | 6.0 | 1 | 285 | -26 | AluY | Alu | SINE |
| 41040302 | 41040472 | + | 21.3 | 195 | 363 | -51 | MLT2D | ERVL | LTR |
| 41040510 | 41040560 | + | 21.3 | 510 | 560 | 0 | MLT2D | ERVL | LTR |
| 41040561 | 41040628 | + | 26.6 | 4939 | 5003 | -1143 | L1M5 | L1 | LINE |

  

|  |  |  |  |  |  |  |  |  |  |
| --- | --- | --- | --- | --- | --- | --- | --- | --- | --- |
| chr9:41052101-41059458 | | | 6689 bp | | |  |  |  |  |
| Genome Start | Genome End | Strand | Div | Repeat Start | Repeat End | Repeat Left | Name | Family | Class |
| 41052101 | 41052245 | - | 22.8 | 6649 | 6801 | 0 | MER89-int | ERV1 | LTR |
| 41052247 | 41052682 | + | 11.6 | 1 | 436 | -231 | LTR12B | ERV1 | LTR |
| 41052872 | 41053024 | - | 27.2 | 1603 | 1755 | 0 | MER52A | ERV1 | LTR |
| 41053033 | 41053659 | - | 24.8 | 54 | 1470 | -162 | MER52A | ERV1 | LTR |
| 41053660 | 41054140 | - | 20.4 | 6052 | 6559 | -242 | MER89-int | ERV1 | LTR |
| 41054240 | 41054539 | + | 9.2 | 1 | 293 | -18 | AluY | Alu | SINE |
| 41054684 | 41056093 | - | 24.5 | 2549 | 4047 | -2904 | MER89-int | ERV1 | LTR |
| 41056322 | 41056529 | - | 27.3 | 2315 | 2536 | -4265 | MER89-int | ERV1 | LTR |
| 41056530 | 41056843 | - | 15.9 | 1 | 296 | -16 | AluSx | Alu | SINE |
| 41056844 | 41057074 | - | 27.3 | 2077 | 2314 | -4487 | MER89-int | ERV1 | LTR |
| 41057075 | 41057397 | + | 14.9 | 1 | 311 | -1 | AluSx | Alu | SINE |
| 41057398 | 41059458 | - | 27.3 | 2 | 2076 | -4725 | MER89-int | ERV1 | LTR |

  

|  |  |  |  |  |  |  |  |  |  |
| --- | --- | --- | --- | --- | --- | --- | --- | --- | --- |
| chr9:41065297-41066443 | | | 1147 bp | | |  |  |  |  |
| Genome Start | Genome End | Strand | Div | Repeat Start | Repeat End | Repeat Left | Name | Family | Class |
| 41065297 | 41065343 | - | 23.3 | 304 | 340 | -1 | MER58B | MER1\_type | DNA |
| 41065344 | 41065850 | + | 16.8 | 2 | 550 | 0 | MER44B | MER2\_type | DNA |
| 41065851 | 41065902 | - | 23.3 | 253 | 303 | -38 | MER58B | MER1\_type | DNA |
| 41065903 | 41066387 | + | 16.2 | 3 | 489 | 0 | LTR45B | ERV1 | LTR |
| 41066388 | 41066443 | - | 23.3 | 208 | 252 | -89 | MER58B | MER1\_type | DNA |

  

|  |  |  |  |  |  |  |  |  |  |
| --- | --- | --- | --- | --- | --- | --- | --- | --- | --- |
| chr9:41066724-41067269 | | | 546 bp | | |  |  |  |  |
| Genome Start | Genome End | Strand | Div | Repeat Start | Repeat End | Repeat Left | Name | Family | Class |
| 41066724 | 41066794 | - | 19.7 | 5820 | 5886 | -278 | L1ME2 | L1 | LINE |
| 41066795 | 41067084 | + | 9.7 | 1 | 291 | -21 | AluSx | Alu | SINE |
| 41067085 | 41067269 | - | 19.7 | 5608 | 5819 | -345 | L1ME2 | L1 | LINE |

  

|  |  |  |  |  |  |  |  |  |  |
| --- | --- | --- | --- | --- | --- | --- | --- | --- | --- |
| chr9:41071759-41072591 | | | 618 bp | | |  |  |  |  |
| Genome Start | Genome End | Strand | Div | Repeat Start | Repeat End | Repeat Left | Name | Family | Class |
| 41071759 | 41071814 | + | 21.4 | 4039 | 4097 | -2049 | L1M4 | L1 | LINE |
| 41072029 | 41072358 | - | 18.6 | 1 | 386 | -515 | MER54B | ERVL | LTR |
| 41072360 | 41072591 | + | 19.6 | 4094 | 4321 | -1825 | L1M4 | L1 | LINE |

  

|  |  |  |  |  |  |  |  |  |  |
| --- | --- | --- | --- | --- | --- | --- | --- | --- | --- |
| chr9:41075683-41076282 | | | 355 bp | | |  |  |  |  |
| Genome Start | Genome End | Strand | Div | Repeat Start | Repeat End | Repeat Left | Name | Family | Class |
| 41075683 | 41075814 | - | 23.8 | 110 | 262 | 0 | MIRb | MIR | SINE |
| 41076019 | 41076183 | - | 27.9 | 42 | 222 | -262 | MLT1H2 | MaLR | LTR |
| 41076225 | 41076282 | - | 34.5 | 34 | 92 | -176 | MIRb | MIR | SINE |

  

|  |  |  |  |  |  |  |  |  |  |
| --- | --- | --- | --- | --- | --- | --- | --- | --- | --- |
| chr9:41078350-41079793 | | | 1444 bp | | |  |  |  |  |
| Genome Start | Genome End | Strand | Div | Repeat Start | Repeat End | Repeat Left | Name | Family | Class |
| 41078350 | 41079235 | + | 17.6 | 4316 | 5160 | -986 | L1M4 | L1 | LINE |
| 41079236 | 41079525 | - | 13.1 | 1 | 290 | -22 | AluSx | Alu | SINE |
| 41079526 | 41079793 | + | 17.8 | 5161 | 5437 | -741 | L1M4 | L1 | LINE |

  

|  |  |  |  |  |  |  |  |  |  |
| --- | --- | --- | --- | --- | --- | --- | --- | --- | --- |
| chr9:41099321-41100580 | | | 1288 bp | | |  |  |  |  |
| Genome Start | Genome End | Strand | Div | Repeat Start | Repeat End | Repeat Left | Name | Family | Class |
| 41099321 | 41099447 | + | 22.2 | 1 | 138 | -569 | MER39 | ERV1 | LTR |
| 41099420 | 41099563 | + | 16.6 | 260 | 397 | -310 | MER39 | ERV1 | LTR |
| 41099564 | 41100269 | + | 13.1 | 1 | 691 | 0 | LTR8 | ERV1 | LTR |
| 41100270 | 41100580 | + | 16.6 | 398 | 705 | -2 | MER39 | ERV1 | LTR |

  

|  |  |  |  |  |  |  |  |  |  |
| --- | --- | --- | --- | --- | --- | --- | --- | --- | --- |
| chr9:41111707-41112934 | | | 1228 bp | | |  |  |  |  |
| Genome Start | Genome End | Strand | Div | Repeat Start | Repeat End | Repeat Left | Name | Family | Class |
| 41111707 | 41111758 | - | 15.7 | 302 | 364 | -2 | Tigger5a | MER2\_type | DNA |
| 41111759 | 41111933 | - | 7.6 | 388 | 554 | 0 | MER41A | ERV1 | LTR |
| 41111934 | 41112232 | - | 10.4 | 1 | 299 | -11 | AluSg | Alu | SINE |
| 41112233 | 41112616 | - | 7.6 | 1 | 387 | -167 | MER41A | ERV1 | LTR |
| 41112617 | 41112934 | - | 15.7 | 5 | 301 | -65 | Tigger5a | MER2\_type | DNA |

  

|  |  |  |  |  |  |  |  |  |  |
| --- | --- | --- | --- | --- | --- | --- | --- | --- | --- |
| chr9:41116276-41117010 | | | 735 bp | | |  |  |  |  |
| Genome Start | Genome End | Strand | Div | Repeat Start | Repeat End | Repeat Left | Name | Family | Class |
| 41116276 | 41116496 | - | 17.7 | 195 | 426 | 0 | MSTB | MaLR | LTR |
| 41116497 | 41116818 | - | 14.9 | 1 | 322 | 0 | AluSx | Alu | SINE |
| 41116819 | 41117010 | - | 17.7 | 3 | 194 | -232 | MSTB | MaLR | LTR |

  

|  |  |  |  |  |  |  |  |  |  |
| --- | --- | --- | --- | --- | --- | --- | --- | --- | --- |
| chr9:41124741-41125637 | | | 897 bp | | |  |  |  |  |
| Genome Start | Genome End | Strand | Div | Repeat Start | Repeat End | Repeat Left | Name | Family | Class |
| 41124741 | 41125117 | + | 27.3 | 27 | 436 | -32 | MER110A | ERV1 | LTR |
| 41125118 | 41125616 | - | 21.5 | 1 | 505 | 0 | MLT1D | MaLR | LTR |
| 41125617 | 41125637 | + | 27.3 | 437 | 456 | -12 | MER110A | ERV1 | LTR |

  

|  |  |  |  |  |  |  |  |  |  |
| --- | --- | --- | --- | --- | --- | --- | --- | --- | --- |
| chr9:41126484-41128963 | | | 2410 bp | | |  |  |  |  |
| Genome Start | Genome End | Strand | Div | Repeat Start | Repeat End | Repeat Left | Name | Family | Class |
| 41126484 | 41127030 | - | 21.6 | 59 | 625 | -28 | MER82 | MER2\_type | DNA |
| 41127031 | 41127175 | + | 23.4 | 1 | 137 | -650 | LTR48 | ERV1 | LTR |
| 41127176 | 41127545 | - | 15.5 | 1 | 364 | 0 | THE1B | MaLR | LTR |
| 41127546 | 41127657 | + | 23.4 | 138 | 227 | -560 | LTR48 | ERV1 | LTR |
| 41127725 | 41128034 | - | 9.7 | 1 | 308 | -2 | AluSg | Alu | SINE |
| 41128037 | 41128342 | - | 6.6 | 1 | 303 | -8 | AluY | Alu | SINE |
| 41128343 | 41128901 | + | 17.1 | 219 | 787 | 0 | LTR48 | ERV1 | LTR |
| 41128903 | 41128963 | - | 19.7 | 1 | 61 | -592 | MER82 | MER2\_type | DNA |

  

|  |  |  |  |  |  |  |  |  |  |
| --- | --- | --- | --- | --- | --- | --- | --- | --- | --- |
| chr9:41129402-41131065 | | | 1657 bp | | |  |  |  |  |
| Genome Start | Genome End | Strand | Div | Repeat Start | Repeat End | Repeat Left | Name | Family | Class |
| 41129402 | 41129819 | - | 19.2 | 276 | 711 | 0 | MER67C | ERV1 | LTR |
| 41129820 | 41130329 | - | 14.4 | 109 | 900 | 0 | MER4B | ERV1 | LTR |
| 41130330 | 41130669 | - | 13.6 | 1 | 343 | 0 | MER61A | ERV1 | LTR |
| 41130670 | 41130788 | - | 14.4 | 1 | 108 | -502 | MER4B | ERV1 | LTR |
| 41130796 | 41131065 | - | 25.0 | 1 | 272 | -439 | MER67C | ERV1 | LTR |

  

|  |  |  |  |  |  |  |  |  |  |
| --- | --- | --- | --- | --- | --- | --- | --- | --- | --- |
| chr9:41196129-41197038 | | | 910 bp | | |  |  |  |  |
| Genome Start | Genome End | Strand | Div | Repeat Start | Repeat End | Repeat Left | Name | Family | Class |
| 41196129 | 41196518 | + | 26.7 | 1 | 436 | -32 | MER110A | ERV1 | LTR |
| 41196519 | 41197017 | - | 21.5 | 1 | 505 | 0 | MLT1D | MaLR | LTR |
| 41197018 | 41197038 | + | 26.7 | 437 | 456 | -12 | MER110A | ERV1 | LTR |

  

|  |  |  |  |  |  |  |  |  |  |
| --- | --- | --- | --- | --- | --- | --- | --- | --- | --- |
| chr9:41197882-41200357 | | | 2417 bp | | |  |  |  |  |
| Genome Start | Genome End | Strand | Div | Repeat Start | Repeat End | Repeat Left | Name | Family | Class |
| 41197882 | 41198431 | - | 21.1 | 59 | 628 | -25 | MER82 | MER2\_type | DNA |
| 41198432 | 41198576 | + | 23.6 | 1 | 137 | -650 | LTR48 | ERV1 | LTR |
| 41198577 | 41198946 | - | 15.5 | 1 | 364 | 0 | THE1B | MaLR | LTR |
| 41198947 | 41199065 | + | 23.6 | 138 | 235 | -552 | LTR48 | ERV1 | LTR |
| 41199122 | 41199431 | - | 10.7 | 1 | 308 | -2 | AluSg | Alu | SINE |
| 41199434 | 41199736 | - | 7.0 | 1 | 300 | -11 | AluY | Alu | SINE |
| 41199737 | 41200295 | + | 17.7 | 219 | 787 | 0 | LTR48 | ERV1 | LTR |
| 41200297 | 41200357 | - | 19.7 | 1 | 61 | -592 | MER82 | MER2\_type | DNA |

  

|  |  |  |  |  |  |  |  |  |  |
| --- | --- | --- | --- | --- | --- | --- | --- | --- | --- |
| chr9:41200787-41202449 | | | 1620 bp | | |  |  |  |  |
| Genome Start | Genome End | Strand | Div | Repeat Start | Repeat End | Repeat Left | Name | Family | Class |
| 41200787 | 41201204 | - | 18.7 | 276 | 711 | 0 | MER67C | ERV1 | LTR |
| 41201241 | 41201714 | - | 14.4 | 109 | 571 | -39 | MER4B | ERV1 | LTR |
| 41201715 | 41202053 | - | 13.6 | 1 | 343 | 0 | MER61A | ERV1 | LTR |
| 41202054 | 41202172 | - | 14.4 | 1 | 108 | -502 | MER4B | ERV1 | LTR |
| 41202180 | 41202449 | - | 25.4 | 1 | 272 | -439 | MER67C | ERV1 | LTR |

  

|  |  |  |  |  |  |  |  |  |  |
| --- | --- | --- | --- | --- | --- | --- | --- | --- | --- |
| chr9:41214284-41214896 | | | 613 bp | | |  |  |  |  |
| Genome Start | Genome End | Strand | Div | Repeat Start | Repeat End | Repeat Left | Name | Family | Class |
| 41214284 | 41214510 | - | 17.0 | 184 | 375 | 0 | THE1C | MaLR | LTR |
| 41214511 | 41214727 | - | 8.8 | 5950 | 6167 | -5 | L1PA8 | L1 | LINE |
| 41214728 | 41214896 | - | 17.0 | 1 | 183 | -192 | THE1C | MaLR | LTR |

  

|  |  |  |  |  |  |  |  |  |  |
| --- | --- | --- | --- | --- | --- | --- | --- | --- | --- |
| chr9:41218662-41219330 | | | 669 bp | | |  |  |  |  |
| Genome Start | Genome End | Strand | Div | Repeat Start | Repeat End | Repeat Left | Name | Family | Class |
| 41218662 | 41218999 | - | 32.4 | 2956 | 3340 | -38 | L2 | L2 | LINE |
| 41219000 | 41219308 | + | 5.8 | 1 | 308 | -3 | AluY | Alu | SINE |
| 41219309 | 41219330 | - | 32.4 | 2933 | 2955 | -423 | L2 | L2 | LINE |

  

|  |  |  |  |  |  |  |  |  |  |
| --- | --- | --- | --- | --- | --- | --- | --- | --- | --- |
| chr9:41220035-41220760 | | | 726 bp | | |  |  |  |  |
| Genome Start | Genome End | Strand | Div | Repeat Start | Repeat End | Repeat Left | Name | Family | Class |
| 41220035 | 41220374 | - | 35.1 | 2438 | 2822 | -597 | L2 | L2 | LINE |
| 41220375 | 41220656 | + | 10.4 | 18 | 297 | -15 | AluSx | Alu | SINE |
| 41220657 | 41220760 | - | 35.1 | 2347 | 2437 | -982 | L2 | L2 | LINE |

  

|  |  |  |  |  |  |  |  |  |  |
| --- | --- | --- | --- | --- | --- | --- | --- | --- | --- |
| chr9:41224005-41224620 | | | 616 bp | | |  |  |  |  |
| Genome Start | Genome End | Strand | Div | Repeat Start | Repeat End | Repeat Left | Name | Family | Class |
| 41224005 | 41224176 | - | 13.3 | 128 | 295 | -17 | AluSx | Alu | SINE |
| 41224177 | 41224487 | - | 7.1 | 1 | 308 | -3 | AluY | Alu | SINE |
| 41224488 | 41224620 | - | 13.3 | 3 | 127 | -185 | AluSx | Alu | SINE |

  

|  |  |  |  |  |  |  |  |  |  |
| --- | --- | --- | --- | --- | --- | --- | --- | --- | --- |
| chr9:41236018-41238885 | | | 2868 bp | | |  |  |  |  |
| Genome Start | Genome End | Strand | Div | Repeat Start | Repeat End | Repeat Left | Name | Family | Class |
| 41236018 | 41236359 | - | 16.5 | 4962 | 5300 | -856 | L1P | L1 | LINE |
| 41236360 | 41236859 | - | 10.3 | 1 | 445 | -222 | LTR12B | ERV1 | LTR |
| 41236860 | 41237266 | - | 17.8 | 4558 | 4960 | -1186 | L1P | L1 | LINE |
| 41237267 | 41237519 | - | 7.7 | 35 | 297 | -14 | AluY | Alu | SINE |
| 41237520 | 41238885 | - | 17.8 | 3167 | 4557 | -1589 | L1P | L1 | LINE |

  

|  |  |  |  |  |  |  |  |  |  |
| --- | --- | --- | --- | --- | --- | --- | --- | --- | --- |
| chr9:41258549-41259924 | | | 1349 bp | | |  |  |  |  |
| Genome Start | Genome End | Strand | Div | Repeat Start | Repeat End | Repeat Left | Name | Family | Class |
| 41258549 | 41258668 | - | 31.1 | 5168 | 5283 | -863 | L1M5 | L1 | LINE |
| 41258669 | 41258961 | + | 6.8 | 1 | 295 | -16 | AluY | Alu | SINE |
| 41258962 | 41259181 | - | 31.1 | 4913 | 5167 | -979 | L1M5 | L1 | LINE |
| 41259182 | 41259192 | - | 8.1 | 285 | 312 | 0 | AluSx | Alu | SINE |
| 41259220 | 41259516 | - | 8.1 | 2 | 284 | -28 | AluSx | Alu | SINE |
| 41259517 | 41259924 | - | 31.1 | 4478 | 4912 | -1234 | L1M5 | L1 | LINE |

  

|  |  |  |  |  |  |  |  |  |  |
| --- | --- | --- | --- | --- | --- | --- | --- | --- | --- |
| chr9:41270790-41273450 | | | 2661 bp | | |  |  |  |  |
| Genome Start | Genome End | Strand | Div | Repeat Start | Repeat End | Repeat Left | Name | Family | Class |
| 41270790 | 41271752 | - | 9.9 | 4545 | 5480 | -680 | L1PREC2 | L1 | LINE |
| 41271753 | 41272053 | - | 10.0 | 1 | 301 | -8 | AluSc | Alu | SINE |
| 41272054 | 41273450 | - | 10.5 | 3147 | 4544 | -1602 | L1PREC2 | L1 | LINE |

  

|  |  |  |  |  |  |  |  |  |  |
| --- | --- | --- | --- | --- | --- | --- | --- | --- | --- |
| chr9:41281185-41282191 | | | 1007 bp | | |  |  |  |  |
| Genome Start | Genome End | Strand | Div | Repeat Start | Repeat End | Repeat Left | Name | Family | Class |
| 41281185 | 41281661 | - | 35.9 | 3777 | 4333 | -156 | L3 | CR1 | LINE |
| 41281662 | 41281971 | - | 10.0 | 1 | 308 | 0 | AluSg | Alu | SINE |
| 41281972 | 41282191 | - | 35.9 | 3523 | 3776 | -713 | L3 | CR1 | LINE |

  

|  |  |  |  |  |  |  |  |  |  |
| --- | --- | --- | --- | --- | --- | --- | --- | --- | --- |
| chr9:41282676-41284875 | | | 2200 bp | | |  |  |  |  |
| Genome Start | Genome End | Strand | Div | Repeat Start | Repeat End | Repeat Left | Name | Family | Class |
| 41282676 | 41282854 | + | 18.1 | 3776 | 3964 | -2182 | L1MC | L1 | LINE |
| 41282855 | 41283171 | + | 13.7 | 1 | 308 | -2 | AluSg | Alu | SINE |
| 41283172 | 41283415 | + | 18.1 | 3965 | 4189 | -1957 | L1MC | L1 | LINE |
| 41283416 | 41283720 | + | 7.5 | 1 | 305 | -6 | AluY | Alu | SINE |
| 41283721 | 41284875 | + | 18.1 | 4190 | 5331 | -815 | L1MC | L1 | LINE |

  

|  |  |  |  |  |  |  |  |  |  |
| --- | --- | --- | --- | --- | --- | --- | --- | --- | --- |
| chr9:41300041-41300851 | | | 793 bp | | |  |  |  |  |
| Genome Start | Genome End | Strand | Div | Repeat Start | Repeat End | Repeat Left | Name | Family | Class |
| 41300041 | 41300373 | + | 18.0 | 1 | 331 | -59 | MLT1B | MaLR | LTR |
| 41300374 | 41300418 | - | 0.0 | 5770 | 5814 | -358 | L1P3 | L1 | LINE |
| 41300419 | 41300759 | + | 1.5 | 5815 | 6155 | 0 | L1PA3 | L1 | LINE |
| 41300778 | 41300851 | + | 10.8 | 317 | 390 | 0 | MLT1B | MaLR | LTR |

  

|  |  |  |  |  |  |  |  |  |  |
| --- | --- | --- | --- | --- | --- | --- | --- | --- | --- |
| chr9:41324878-41326254 | | | 1377 bp | | |  |  |  |  |
| Genome Start | Genome End | Strand | Div | Repeat Start | Repeat End | Repeat Left | Name | Family | Class |
| 41324878 | 41325132 | + | 20.1 | 4117 | 4376 | -1764 | L1M3 | L1 | LINE |
| 41325133 | 41325395 | + | 9.9 | 1 | 287 | -24 | AluY | Alu | SINE |
| 41325396 | 41326254 | + | 20.1 | 4377 | 5283 | -857 | L1M3 | L1 | LINE |

  

|  |  |  |  |  |  |  |  |  |  |
| --- | --- | --- | --- | --- | --- | --- | --- | --- | --- |
| chr9:41326265-41326921 | | | 657 bp | | |  |  |  |  |
| Genome Start | Genome End | Strand | Div | Repeat Start | Repeat End | Repeat Left | Name | Family | Class |
| 41326265 | 41326335 | - | 14.8 | 300 | 364 | 0 | THE1B | MaLR | LTR |
| 41326336 | 41326625 | + | 10.7 | 1 | 290 | -21 | AluY | Alu | SINE |
| 41326626 | 41326921 | - | 14.8 | 1 | 299 | -65 | THE1B | MaLR | LTR |

  

|  |  |  |  |  |  |  |  |  |  |
| --- | --- | --- | --- | --- | --- | --- | --- | --- | --- |
| chr9:41335306-41339321 | | | 3935 bp | | |  |  |  |  |
| Genome Start | Genome End | Strand | Div | Repeat Start | Repeat End | Repeat Left | Name | Family | Class |
| 41335306 | 41335773 | + | 33.9 | 64 | 559 | -5560 | L1ME3B | L1 | LINE |
| 41335783 | 41336209 | + | 16.0 | 1 | 436 | -231 | LTR12B | ERV1 | LTR |
| 41336282 | 41336471 | + | 31.6 | 4839 | 5042 | -1087 | L1ME3B | L1 | LINE |
| 41336472 | 41336770 | - | 7.5 | 1 | 295 | -16 | AluY | Alu | SINE |
| 41336771 | 41337487 | + | 27.7 | 5043 | 5827 | -396 | L1ME3B | L1 | LINE |
| 41337488 | 41337865 | + | 15.1 | 5435 | 5817 | -516 | L1MC1 | L1 | LINE |
| 41337866 | 41338181 | + | 10.0 | 1 | 311 | 0 | AluY | Alu | SINE |
| 41338182 | 41338658 | + | 15.1 | 5818 | 6322 | -11 | L1MC1 | L1 | LINE |
| 41338659 | 41338748 | + | 27.8 | 5828 | 5890 | -333 | L1ME3B | L1 | LINE |
| 41338749 | 41339051 | + | 6.7 | 1 | 300 | -11 | AluY | Alu | SINE |
| 41339052 | 41339321 | + | 27.8 | 5891 | 6203 | -20 | L1ME3B | L1 | LINE |

  

|  |  |  |  |  |  |  |  |  |  |
| --- | --- | --- | --- | --- | --- | --- | --- | --- | --- |
| chr9:41339346-41340676 | | | 1219 bp | | |  |  |  |  |
| Genome Start | Genome End | Strand | Div | Repeat Start | Repeat End | Repeat Left | Name | Family | Class |
| 41339346 | 41339797 | + | 27.2 | 3375 | 3927 | -2219 | L1M5 | L1 | LINE |
| 41339798 | 41340101 | + | 8.6 | 1 | 301 | -10 | AluY | Alu | SINE |
| 41340102 | 41340271 | + | 27.2 | 3928 | 4104 | -2042 | L1M5 | L1 | LINE |
| 41340271 | 41340344 | + | 24.3 | 4633 | 4709 | -1437 | L1M5 | L1 | LINE |
| 41340458 | 41340676 | + | 33.3 | 4851 | 5064 | -1082 | L1M5 | L1 | LINE |

  

|  |  |  |  |  |  |  |  |  |  |
| --- | --- | --- | --- | --- | --- | --- | --- | --- | --- |
| chr9:41346344-41348094 | | | 1758 bp | | |  |  |  |  |
| Genome Start | Genome End | Strand | Div | Repeat Start | Repeat End | Repeat Left | Name | Family | Class |
| 41346344 | 41346539 | - | 19.4 | 884 | 1083 | 0 | Tigger6a | MER2\_type | DNA |
| 41346540 | 41346817 | - | 10.8 | 3 | 293 | -18 | AluY | Alu | SINE |
| 41346818 | 41347433 | - | 19.4 | 220 | 883 | -200 | Tigger6a | MER2\_type | DNA |
| 41347427 | 41347885 | + | 23.5 | 7 | 467 | 0 | MLT1C | MaLR | LTR |
| 41347886 | 41348094 | - | 21.0 | 5 | 211 | -872 | Tigger6a | MER2\_type | DNA |

  

|  |  |  |  |  |  |  |  |  |  |
| --- | --- | --- | --- | --- | --- | --- | --- | --- | --- |
| chr9:41353196-41355086 | | | 1891 bp | | |  |  |  |  |
| Genome Start | Genome End | Strand | Div | Repeat Start | Repeat End | Repeat Left | Name | Family | Class |
| 41353196 | 41354656 | - | 29.5 | 126 | 1735 | 0 | MLT-int | MaLR | LTR |
| 41354657 | 41354965 | - | 12.0 | 1 | 310 | -3 | AluSq | Alu | SINE |
| 41354966 | 41355086 | - | 29.5 | 3 | 125 | -1610 | MLT-int | MaLR | LTR |

  

|  |  |  |  |  |  |  |  |  |  |
| --- | --- | --- | --- | --- | --- | --- | --- | --- | --- |
| chr9:41431654-41432567 | | | 914 bp | | |  |  |  |  |
| Genome Start | Genome End | Strand | Div | Repeat Start | Repeat End | Repeat Left | Name | Family | Class |
| 41431654 | 41431880 | + | 12.0 | 5522 | 5787 | -378 | L1PA15 | L1 | LINE |
| 41431881 | 41432188 | + | 9.1 | 1 | 308 | -3 | AluY | Alu | SINE |
| 41432189 | 41432567 | + | 12.0 | 5788 | 6158 | -7 | L1PA15 | L1 | LINE |

  

|  |  |  |  |  |  |  |  |  |  |
| --- | --- | --- | --- | --- | --- | --- | --- | --- | --- |
| chr9:41432909-41434486 | | | 1578 bp | | |  |  |  |  |
| Genome Start | Genome End | Strand | Div | Repeat Start | Repeat End | Repeat Left | Name | Family | Class |
| 41432909 | 41433965 | + | 24.4 | 2836 | 3952 | -3613 | L1M1 | L1 | LINE |
| 41433966 | 41434271 | - | 10.5 | 1 | 305 | -8 | AluSq | Alu | SINE |
| 41434272 | 41434486 | + | 28.4 | 3953 | 4171 | -3394 | L1M1 | L1 | LINE |

  

|  |  |  |  |  |  |  |  |  |  |
| --- | --- | --- | --- | --- | --- | --- | --- | --- | --- |
| chr9:41443348-41446929 | | | 3556 bp | | |  |  |  |  |
| Genome Start | Genome End | Strand | Div | Repeat Start | Repeat End | Repeat Left | Name | Family | Class |
| 41443348 | 41443523 | + | 22.2 | 2115 | 2335 | -4999 | L1MB8 | L1 | LINE |
| 41443524 | 41443823 | + | 7.0 | 1 | 300 | -11 | AluY | Alu | SINE |
| 41443824 | 41444300 | + | 22.2 | 2336 | 2873 | -4461 | L1MB8 | L1 | LINE |
| 41444296 | 41445061 | + | 8.7 | 4692 | 5461 | -711 | L1P3 | L1 | LINE |
| 41445089 | 41445396 | - | 3.9 | 1 | 308 | -3 | AluY | Alu | SINE |
| 41445397 | 41445577 | + | 12.4 | 156 | 364 | 0 | THE1B | MaLR | LTR |
| 41445580 | 41446448 | + | 20.4 | 3240 | 4463 | -2540 | L1MB8 | L1 | LINE |
| 41446451 | 41446929 | + | 23.1 | 6554 | 7032 | -6 | L1MB8 | L1 | LINE |

  

|  |  |  |  |  |  |  |  |  |  |
| --- | --- | --- | --- | --- | --- | --- | --- | --- | --- |
| chr9:41452657-41454548 | | | 1892 bp | | |  |  |  |  |
| Genome Start | Genome End | Strand | Div | Repeat Start | Repeat End | Repeat Left | Name | Family | Class |
| 41452657 | 41452777 | + | 29.8 | 2 | 124 | -1611 | MLT-int | MaLR | LTR |
| 41452778 | 41453087 | + | 11.9 | 1 | 311 | -2 | AluSq | Alu | SINE |
| 41453088 | 41454548 | + | 29.8 | 125 | 1734 | -1 | MLT-int | MaLR | LTR |

  

|  |  |  |  |  |  |  |  |  |  |
| --- | --- | --- | --- | --- | --- | --- | --- | --- | --- |
| chr9:41459656-41461414 | | | 1762 bp | | |  |  |  |  |
| Genome Start | Genome End | Strand | Div | Repeat Start | Repeat End | Repeat Left | Name | Family | Class |
| 41459656 | 41459864 | + | 20.5 | 5 | 211 | -872 | Tigger6a | MER2\_type | DNA |
| 41459865 | 41460319 | - | 24.1 | 11 | 467 | 0 | MLT1C | MaLR | LTR |
| 41460317 | 41460932 | + | 19.0 | 220 | 883 | -200 | Tigger6a | MER2\_type | DNA |
| 41460933 | 41461218 | + | 10.8 | 3 | 301 | -10 | AluY | Alu | SINE |
| 41461219 | 41461414 | + | 19.0 | 884 | 1083 | 0 | Tigger6a | MER2\_type | DNA |

  

|  |  |  |  |  |  |  |  |  |  |
| --- | --- | --- | --- | --- | --- | --- | --- | --- | --- |
| chr9:41467086-41468415 | | | 1218 bp | | |  |  |  |  |
| Genome Start | Genome End | Strand | Div | Repeat Start | Repeat End | Repeat Left | Name | Family | Class |
| 41467086 | 41467304 | - | 33.3 | 4851 | 5064 | -1082 | L1M5 | L1 | LINE |
| 41467418 | 41467491 | - | 23.0 | 4633 | 4709 | -1437 | L1M5 | L1 | LINE |
| 41467491 | 41467660 | - | 27.0 | 3928 | 4104 | -2042 | L1M5 | L1 | LINE |
| 41467661 | 41467963 | - | 8.9 | 1 | 302 | -9 | AluY | Alu | SINE |
| 41467964 | 41468415 | - | 27.0 | 3375 | 3927 | -2219 | L1M5 | L1 | LINE |

  

|  |  |  |  |  |  |  |  |  |  |
| --- | --- | --- | --- | --- | --- | --- | --- | --- | --- |
| chr9:41468436-41472444 | | | 3928 bp | | |  |  |  |  |
| Genome Start | Genome End | Strand | Div | Repeat Start | Repeat End | Repeat Left | Name | Family | Class |
| 41468436 | 41468705 | - | 27.5 | 5890 | 6204 | -19 | L1ME3B | L1 | LINE |
| 41468706 | 41469008 | - | 6.7 | 1 | 300 | -11 | AluY | Alu | SINE |
| 41469009 | 41469086 | - | 27.5 | 5828 | 5889 | -334 | L1ME3B | L1 | LINE |
| 41469087 | 41469565 | - | 14.9 | 5818 | 6322 | -11 | L1MC1 | L1 | LINE |
| 41469566 | 41469880 | - | 9.7 | 1 | 310 | -1 | AluY | Alu | SINE |
| 41469881 | 41470258 | - | 14.9 | 5435 | 5817 | -516 | L1MC1 | L1 | LINE |
| 41470259 | 41470976 | - | 28.0 | 5043 | 5827 | -396 | L1ME3B | L1 | LINE |
| 41470977 | 41471275 | + | 7.5 | 1 | 295 | -16 | AluY | Alu | SINE |
| 41471276 | 41471465 | - | 32.8 | 4839 | 5042 | -1087 | L1ME3B | L1 | LINE |
| 41471538 | 41471963 | - | 16.3 | 1 | 436 | -231 | LTR12B | ERV1 | LTR |
| 41471973 | 41472444 | - | 33.1 | 64 | 559 | -5560 | L1ME3B | L1 | LINE |

  

|  |  |  |  |  |  |  |  |  |  |
| --- | --- | --- | --- | --- | --- | --- | --- | --- | --- |
| chr9:41480812-41481471 | | | 660 bp | | |  |  |  |  |
| Genome Start | Genome End | Strand | Div | Repeat Start | Repeat End | Repeat Left | Name | Family | Class |
| 41480812 | 41481107 | + | 13.4 | 1 | 299 | -65 | THE1B | MaLR | LTR |
| 41481108 | 41481400 | - | 8.9 | 1 | 293 | -18 | AluY | Alu | SINE |
| 41481401 | 41481471 | + | 13.4 | 300 | 364 | 0 | THE1B | MaLR | LTR |

  

|  |  |  |  |  |  |  |  |  |  |
| --- | --- | --- | --- | --- | --- | --- | --- | --- | --- |
| chr9:41523244-41525451 | | | 2208 bp | | |  |  |  |  |
| Genome Start | Genome End | Strand | Div | Repeat Start | Repeat End | Repeat Left | Name | Family | Class |
| 41523244 | 41524398 | - | 18.2 | 4190 | 5331 | -815 | L1MC | L1 | LINE |
| 41524399 | 41524703 | - | 7.5 | 1 | 305 | -6 | AluY | Alu | SINE |
| 41524704 | 41524947 | - | 18.2 | 3965 | 4189 | -1957 | L1MC | L1 | LINE |
| 41524948 | 41525272 | - | 13.4 | 1 | 308 | -2 | AluSg | Alu | SINE |
| 41525273 | 41525451 | - | 18.2 | 3776 | 3964 | -2182 | L1MC | L1 | LINE |

  

|  |  |  |  |  |  |  |  |  |  |
| --- | --- | --- | --- | --- | --- | --- | --- | --- | --- |
| chr9:41525936-41526942 | | | 1007 bp | | |  |  |  |  |
| Genome Start | Genome End | Strand | Div | Repeat Start | Repeat End | Repeat Left | Name | Family | Class |
| 41525936 | 41526155 | + | 35.6 | 3523 | 3776 | -713 | L3 | CR1 | LINE |
| 41526156 | 41526465 | + | 10.3 | 1 | 308 | 0 | AluSg | Alu | SINE |
| 41526466 | 41526942 | + | 35.6 | 3777 | 4333 | -156 | L3 | CR1 | LINE |

  

|  |  |  |  |  |  |  |  |  |  |
| --- | --- | --- | --- | --- | --- | --- | --- | --- | --- |
| chr9:41541808-41542501 | | | 694 bp | | |  |  |  |  |
| Genome Start | Genome End | Strand | Div | Repeat Start | Repeat End | Repeat Left | Name | Family | Class |
| 41541808 | 41541982 | - | 18.9 | 138 | 311 | 0 | AluJo | Alu | SINE |
| 41541983 | 41542345 | - | 9.9 | 1 | 364 | 0 | THE1B | MaLR | LTR |
| 41542346 | 41542501 | - | 18.9 | 1 | 137 | -175 | AluJo | Alu | SINE |

  

|  |  |  |  |  |  |  |  |  |  |
| --- | --- | --- | --- | --- | --- | --- | --- | --- | --- |
| chr9:41572376-41574816 | | | 2421 bp | | |  |  |  |  |
| Genome Start | Genome End | Strand | Div | Repeat Start | Repeat End | Repeat Left | Name | Family | Class |
| 41572376 | 41573832 | + | 21.0 | 50 | 1538 | -5796 | L1M4b | L1 | LINE |
| 41573823 | 41573993 | + | 11.8 | 5979 | 6139 | -163 | L1MA1 | L1 | LINE |
| 41574021 | 41574068 | + | 15.2 | 459 | 504 | 0 | MER75 | PiggyBac | DNA |
| 41574069 | 41574231 | + | 5.5 | 6140 | 6302 | 0 | L1MA1 | L1 | LINE |
| 41574235 | 41574816 | + | 19.0 | 1509 | 2173 | -5161 | L1M4b | L1 | LINE |

  

|  |  |  |  |  |  |  |  |  |  |
| --- | --- | --- | --- | --- | --- | --- | --- | --- | --- |
| chr9:41583073-41586683 | | | 2797 bp | | |  |  |  |  |
| Genome Start | Genome End | Strand | Div | Repeat Start | Repeat End | Repeat Left | Name | Family | Class |
| 41583073 | 41583287 | + | 25.6 | 576 | 820 | -6282 | L1M | L1 | LINE |
| 41583748 | 41584087 | + | 24.0 | 3529 | 3882 | -3124 | L1M | L1 | LINE |
| 41584088 | 41584363 | + | 11.6 | 1 | 288 | -22 | AluSg | Alu | SINE |
| 41584364 | 41584627 | + | 24.0 | 3883 | 4223 | -2783 | L1M | L1 | LINE |
| 41584628 | 41584859 | - | 24.4 | 127 | 365 | 0 | MLT1A0 | MaLR | LTR |
| 41585214 | 41586683 | + | 24.8 | 4363 | 6019 | -987 | L1M | L1 | LINE |

  

|  |  |  |  |  |  |  |  |  |  |
| --- | --- | --- | --- | --- | --- | --- | --- | --- | --- |
| chr9:41586686-41587880 | | | 1113 bp | | |  |  |  |  |
| Genome Start | Genome End | Strand | Div | Repeat Start | Repeat End | Repeat Left | Name | Family | Class |
| 41586686 | 41586817 | - | 29.3 | 879 | 1015 | -5618 | L1MDa | L1 | LINE |
| 41586818 | 41587121 | - | 9.5 | 1 | 308 | -5 | AluSq | Alu | SINE |
| 41587122 | 41587188 | - | 29.3 | 822 | 878 | -5755 | L1MDa | L1 | LINE |
| 41587253 | 41587498 | - | 16.8 | 1 | 246 | -66 | AluSx | Alu | SINE |
| 41587499 | 41587597 | + | 13.1 | 45 | 143 | -2275 | Tigger1 | MER2\_type | DNA |
| 41587603 | 41587812 | + | 16.8 | 2205 | 2417 | -1 | Tigger1 | MER2\_type | DNA |
| 41587826 | 41587880 | - | 25.9 | 31 | 84 | -6549 | L1MDa | L1 | LINE |

  

|  |  |  |  |  |  |  |  |  |  |
| --- | --- | --- | --- | --- | --- | --- | --- | --- | --- |
| chr9:41598144-41598646 | | | 498 bp | | |  |  |  |  |
| Genome Start | Genome End | Strand | Div | Repeat Start | Repeat End | Repeat Left | Name | Family | Class |
| 41598144 | 41598212 | + | 27.5 | 2 | 70 | -2542 | Charlie5 | MER1\_type | DNA |
| 41598218 | 41598300 | + | 23.8 | 2453 | 2539 | -73 | Charlie5 | MER1\_type | DNA |
| 41598301 | 41598594 | - | 9.2 | 1 | 297 | -15 | AluSx | Alu | SINE |
| 41598595 | 41598646 | + | 23.8 | 2540 | 2583 | -29 | Charlie5 | MER1\_type | DNA |

  

|  |  |  |  |  |  |  |  |  |  |
| --- | --- | --- | --- | --- | --- | --- | --- | --- | --- |
| chr9:41600502-41601080 | | | 579 bp | | |  |  |  |  |
| Genome Start | Genome End | Strand | Div | Repeat Start | Repeat End | Repeat Left | Name | Family | Class |
| 41600502 | 41600678 | - | 14.8 | 134 | 312 | 0 | AluJb | Alu | SINE |
| 41600679 | 41600971 | - | 11.0 | 1 | 299 | -14 | AluSq | Alu | SINE |
| 41600972 | 41601080 | - | 14.8 | 29 | 133 | -179 | AluJb | Alu | SINE |

  

|  |  |  |  |  |  |  |  |  |  |
| --- | --- | --- | --- | --- | --- | --- | --- | --- | --- |
| chr9:41613561-41614578 | | | 1018 bp | | |  |  |  |  |
| Genome Start | Genome End | Strand | Div | Repeat Start | Repeat End | Repeat Left | Name | Family | Class |
| 41613561 | 41613592 | + | 25.5 | 1 | 29 | -533 | LTR75 | ERVL | LTR |
| 41613593 | 41613875 | - | 9.1 | 12 | 292 | -17 | AluSc | Alu | SINE |
| 41613876 | 41613931 | + | 25.5 | 30 | 81 | -481 | LTR75 | ERVL | LTR |
| 41613932 | 41614114 | + | 8.2 | 1 | 297 | -3 | AluSg | Alu | SINE |
| 41614115 | 41614578 | + | 25.5 | 82 | 561 | -1 | LTR75 | ERVL | LTR |

  

|  |  |  |  |  |  |  |  |  |  |
| --- | --- | --- | --- | --- | --- | --- | --- | --- | --- |
| chr9:41615038-41617180 | | | 2120 bp | | |  |  |  |  |
| Genome Start | Genome End | Strand | Div | Repeat Start | Repeat End | Repeat Left | Name | Family | Class |
| 41615038 | 41615327 | + | 25.4 | 3498 | 3782 | -2361 | L1M2 | L1 | LINE |
| 41615328 | 41615638 | + | 10.0 | 1 | 310 | 0 | AluSg | Alu | SINE |
| 41615639 | 41616400 | + | 25.4 | 3783 | 4572 | -1571 | L1M2 | L1 | LINE |
| 41616424 | 41617180 | + | 25.4 | 4573 | 5196 | -947 | L1M2 | L1 | LINE |

  

|  |  |  |  |  |  |  |  |  |  |
| --- | --- | --- | --- | --- | --- | --- | --- | --- | --- |
| chr9:41621191-41622488 | | | 1245 bp | | |  |  |  |  |
| Genome Start | Genome End | Strand | Div | Repeat Start | Repeat End | Repeat Left | Name | Family | Class |
| 41621191 | 41621911 | + | 23.2 | 4265 | 5021 | -1125 | L1MD | L1 | LINE |
| 41621912 | 41622172 | - | 11.2 | 1 | 260 | -53 | AluSq | Alu | SINE |
| 41622173 | 41622232 | + | 23.2 | 5022 | 5079 | -1067 | L1MD | L1 | LINE |
| 41622286 | 41622488 | + | 21.2 | 5593 | 5791 | -568 | L1MD | L1 | LINE |

  

|  |  |  |  |  |  |  |  |  |  |
| --- | --- | --- | --- | --- | --- | --- | --- | --- | --- |
| chr9:41628494-41630708 | | | 2183 bp | | |  |  |  |  |
| Genome Start | Genome End | Strand | Div | Repeat Start | Repeat End | Repeat Left | Name | Family | Class |
| 41628494 | 41629313 | + | 22.2 | 2062 | 3043 | -3698 | L1MB5 | L1 | LINE |
| 41629344 | 41630145 | + | 22.2 | 3044 | 3869 | -2872 | L1MB5 | L1 | LINE |
| 41630148 | 41630451 | + | 5.6 | 1 | 303 | -8 | AluY | Alu | SINE |
| 41630452 | 41630708 | + | 20.1 | 4612 | 4867 | -1874 | L1MB5 | L1 | LINE |

  

|  |  |  |  |  |  |  |  |  |  |
| --- | --- | --- | --- | --- | --- | --- | --- | --- | --- |
| chr9:41636561-41638550 | | | 1927 bp | | |  |  |  |  |
| Genome Start | Genome End | Strand | Div | Repeat Start | Repeat End | Repeat Left | Name | Family | Class |
| 41636561 | 41637271 | + | 22.9 | 5104 | 5833 | -908 | L1MB5 | L1 | LINE |
| 41637305 | 41637487 | + | 14.2 | 5834 | 6035 | -749 | L1MB5 | L1 | LINE |
| 41637495 | 41637582 | + | 13.6 | 6151 | 6240 | -544 | L1MB5 | L1 | LINE |
| 41637584 | 41637995 | + | 21.2 | 5 | 492 | -64 | MLT2B4 | ERVL | LTR |
| 41638018 | 41638550 | + | 20.2 | 6243 | 6774 | -1 | L1MB5 | L1 | LINE |

  

|  |  |  |  |  |  |  |  |  |  |
| --- | --- | --- | --- | --- | --- | --- | --- | --- | --- |
| chr9:41643065-41644712 | | | 1652 bp | | |  |  |  |  |
| Genome Start | Genome End | Strand | Div | Repeat Start | Repeat End | Repeat Left | Name | Family | Class |
| 41643065 | 41643806 | + | 18.2 | 3165 | 3925 | -2218 | L1M2 | L1 | LINE |
| 41643803 | 41643868 | + | 16.7 | 4894 | 4961 | -1185 | L1M2 | L1 | LINE |
| 41643869 | 41644359 | + | 11.5 | 1 | 445 | -222 | LTR12B | ERV1 | LTR |
| 41644360 | 41644712 | + | 17.0 | 4962 | 5305 | -851 | L1M2 | L1 | LINE |

  

|  |  |  |  |  |  |  |  |  |  |
| --- | --- | --- | --- | --- | --- | --- | --- | --- | --- |
| chr9:41656134-41656745 | | | 612 bp | | |  |  |  |  |
| Genome Start | Genome End | Strand | Div | Repeat Start | Repeat End | Repeat Left | Name | Family | Class |
| 41656134 | 41656266 | + | 13.0 | 3 | 127 | -185 | AluSx | Alu | SINE |
| 41656267 | 41656574 | + | 7.5 | 1 | 305 | -6 | AluY | Alu | SINE |
| 41656575 | 41656745 | + | 13.0 | 128 | 295 | -17 | AluSx | Alu | SINE |

  

|  |  |  |  |  |  |  |  |  |  |
| --- | --- | --- | --- | --- | --- | --- | --- | --- | --- |
| chr9:41666503-41667120 | | | 618 bp | | |  |  |  |  |
| Genome Start | Genome End | Strand | Div | Repeat Start | Repeat End | Repeat Left | Name | Family | Class |
| 41666503 | 41666671 | + | 11.3 | 1 | 183 | -181 | THE1B | MaLR | LTR |
| 41666672 | 41666903 | + | 9.3 | 5950 | 6181 | 0 | L1PA8 | L1 | LINE |
| 41666904 | 41667120 | + | 11.3 | 184 | 364 | 0 | THE1B | MaLR | LTR |

  

|  |  |  |  |  |  |  |  |  |  |
| --- | --- | --- | --- | --- | --- | --- | --- | --- | --- |
| chr9:41678292-41679955 | | | 1657 bp | | |  |  |  |  |
| Genome Start | Genome End | Strand | Div | Repeat Start | Repeat End | Repeat Left | Name | Family | Class |
| 41678292 | 41678561 | + | 25.0 | 1 | 272 | -439 | MER67C | ERV1 | LTR |
| 41678569 | 41678687 | + | 14.4 | 1 | 108 | -502 | MER4B | ERV1 | LTR |
| 41678688 | 41679027 | + | 13.6 | 1 | 343 | 0 | MER61A | ERV1 | LTR |
| 41679028 | 41679537 | + | 14.4 | 109 | 900 | 0 | MER4B | ERV1 | LTR |
| 41679538 | 41679955 | + | 19.5 | 276 | 711 | 0 | MER67C | ERV1 | LTR |

  

|  |  |  |  |  |  |  |  |  |  |
| --- | --- | --- | --- | --- | --- | --- | --- | --- | --- |
| chr9:41680394-41682868 | | | 2405 bp | | |  |  |  |  |
| Genome Start | Genome End | Strand | Div | Repeat Start | Repeat End | Repeat Left | Name | Family | Class |
| 41680394 | 41680454 | + | 19.7 | 1 | 61 | -592 | MER82 | MER2\_type | DNA |
| 41680456 | 41681014 | - | 16.9 | 219 | 787 | 0 | LTR48 | ERV1 | LTR |
| 41681015 | 41681320 | + | 6.9 | 1 | 303 | -8 | AluY | Alu | SINE |
| 41681323 | 41681632 | + | 9.7 | 1 | 308 | -2 | AluSg | Alu | SINE |
| 41681700 | 41681811 | - | 23.9 | 138 | 227 | -560 | LTR48 | ERV1 | LTR |
| 41681812 | 41682181 | + | 15.5 | 1 | 364 | 0 | THE1B | MaLR | LTR |
| 41682182 | 41682326 | - | 23.9 | 1 | 137 | -650 | LTR48 | ERV1 | LTR |
| 41682327 | 41682868 | + | 21.4 | 59 | 625 | -28 | MER82 | MER2\_type | DNA |

  

|  |  |  |  |  |  |  |  |  |  |
| --- | --- | --- | --- | --- | --- | --- | --- | --- | --- |
| chr9:41683715-41684611 | | | 897 bp | | |  |  |  |  |
| Genome Start | Genome End | Strand | Div | Repeat Start | Repeat End | Repeat Left | Name | Family | Class |
| 41683715 | 41683735 | - | 27.3 | 437 | 456 | -12 | MER110A | ERV1 | LTR |
| 41683736 | 41684234 | + | 21.5 | 1 | 505 | 0 | MLT1D | MaLR | LTR |
| 41684235 | 41684611 | - | 27.3 | 27 | 436 | -32 | MER110A | ERV1 | LTR |

  

|  |  |  |  |  |  |  |  |  |  |
| --- | --- | --- | --- | --- | --- | --- | --- | --- | --- |
| chr9:41692390-41693121 | | | 732 bp | | |  |  |  |  |
| Genome Start | Genome End | Strand | Div | Repeat Start | Repeat End | Repeat Left | Name | Family | Class |
| 41692390 | 41692578 | + | 19.3 | 6 | 195 | -231 | MSTB | MaLR | LTR |
| 41692579 | 41692900 | + | 13.9 | 1 | 322 | 0 | AluSx | Alu | SINE |
| 41692901 | 41693121 | + | 19.3 | 196 | 426 | 0 | MSTB | MaLR | LTR |

  

|  |  |  |  |  |  |  |  |  |  |
| --- | --- | --- | --- | --- | --- | --- | --- | --- | --- |
| chr9:41696475-41697702 | | | 1228 bp | | |  |  |  |  |
| Genome Start | Genome End | Strand | Div | Repeat Start | Repeat End | Repeat Left | Name | Family | Class |
| 41696475 | 41696792 | + | 16.6 | 2 | 300 | -66 | Tigger5a | MER2\_type | DNA |
| 41696793 | 41697176 | + | 8.0 | 1 | 387 | -167 | MER41A | ERV1 | LTR |
| 41697177 | 41697475 | + | 11.0 | 1 | 299 | -11 | AluSg | Alu | SINE |
| 41697476 | 41697650 | + | 8.0 | 388 | 554 | 0 | MER41A | ERV1 | LTR |
| 41697651 | 41697702 | + | 16.6 | 301 | 361 | -5 | Tigger5a | MER2\_type | DNA |

  

|  |  |  |  |  |  |  |  |  |  |
| --- | --- | --- | --- | --- | --- | --- | --- | --- | --- |
| chr9:41708453-41709712 | | | 1288 bp | | |  |  |  |  |
| Genome Start | Genome End | Strand | Div | Repeat Start | Repeat End | Repeat Left | Name | Family | Class |
| 41708453 | 41708763 | - | 16.4 | 398 | 705 | -2 | MER39 | ERV1 | LTR |
| 41708764 | 41709469 | - | 13.4 | 1 | 691 | 0 | LTR8 | ERV1 | LTR |
| 41709470 | 41709613 | - | 16.4 | 260 | 397 | -310 | MER39 | ERV1 | LTR |
| 41709586 | 41709712 | - | 22.2 | 1 | 138 | -569 | MER39 | ERV1 | LTR |

  

|  |  |  |  |  |  |  |  |  |  |
| --- | --- | --- | --- | --- | --- | --- | --- | --- | --- |
| chr9:41725719-41726774 | | | 1056 bp | | |  |  |  |  |
| Genome Start | Genome End | Strand | Div | Repeat Start | Repeat End | Repeat Left | Name | Family | Class |
| 41725719 | 41725866 | - | 14.4 | 618 | 765 | -18 | LTR39 | ERV1 | LTR |
| 41725867 | 41726169 | - | 4.6 | 1 | 303 | 0 | AluY | Alu | SINE |
| 41726170 | 41726774 | - | 14.4 | 1 | 617 | -166 | LTR39 | ERV1 | LTR |

  

|  |  |  |  |  |  |  |  |  |  |
| --- | --- | --- | --- | --- | --- | --- | --- | --- | --- |
| chr9:41752762-41760140 | | | 6809 bp | | |  |  |  |  |
| Genome Start | Genome End | Strand | Div | Repeat Start | Repeat End | Repeat Left | Name | Family | Class |
| 41752762 | 41754825 | + | 26.8 | 2 | 2076 | -4725 | MER89-int | ERV1 | LTR |
| 41754826 | 41755149 | - | 14.9 | 1 | 311 | -1 | AluSx | Alu | SINE |
| 41755150 | 41755380 | + | 26.8 | 2077 | 2314 | -4487 | MER89-int | ERV1 | LTR |
| 41755381 | 41755694 | + | 16.3 | 1 | 296 | -16 | AluSx | Alu | SINE |
| 41755695 | 41755902 | + | 26.8 | 2315 | 2536 | -4265 | MER89-int | ERV1 | LTR |
| 41756027 | 41757543 | + | 25.6 | 2549 | 4047 | -2904 | MER89-int | ERV1 | LTR |
| 41757694 | 41757993 | - | 8.5 | 1 | 293 | -18 | AluY | Alu | SINE |
| 41758093 | 41758602 | + | 20.4 | 6052 | 6559 | -242 | MER89-int | ERV1 | LTR |
| 41758603 | 41759229 | + | 23.4 | 54 | 1470 | -162 | MER52A | ERV1 | LTR |
| 41759238 | 41759390 | + | 27.8 | 1603 | 1755 | 0 | MER52A | ERV1 | LTR |
| 41759579 | 41759990 | - | 12.2 | 1 | 436 | -231 | LTR12B | ERV1 | LTR |
| 41759992 | 41760140 | + | 22.1 | 6649 | 6801 | 0 | MER89-int | ERV1 | LTR |

  

|  |  |  |  |  |  |  |  |  |  |
| --- | --- | --- | --- | --- | --- | --- | --- | --- | --- |
| chr9:41768996-41769904 | | | 909 bp | | |  |  |  |  |
| Genome Start | Genome End | Strand | Div | Repeat Start | Repeat End | Repeat Left | Name | Family | Class |
| 41768996 | 41769063 | - | 26.6 | 4939 | 5005 | -1141 | L1M5 | L1 | LINE |
| 41769064 | 41769339 | - | 21.9 | 195 | 414 | 0 | MLT2D | ERVL | LTR |
| 41769340 | 41769623 | - | 6.7 | 1 | 287 | -24 | AluY | Alu | SINE |
| 41769624 | 41769827 | - | 21.9 | 1 | 194 | -220 | MLT2D | ERVL | LTR |
| 41769828 | 41769904 | - | 26.6 | 4865 | 4938 | -1208 | L1M5 | L1 | LINE |

  

|  |  |  |  |  |  |  |  |  |  |
| --- | --- | --- | --- | --- | --- | --- | --- | --- | --- |
| chr9:41772314-41777260 | | | 4627 bp | | |  |  |  |  |
| Genome Start | Genome End | Strand | Div | Repeat Start | Repeat End | Repeat Left | Name | Family | Class |
| 41772314 | 41772538 | - | 34.4 | 2949 | 3176 | -243 | L2 | L2 | LINE |
| 41772753 | 41773073 | + | 29.5 | 4 | 354 | -241 | MLT1H | MaLR | LTR |
| 41773169 | 41773964 | - | 30.3 | 1564 | 2565 | -854 | L2 | L2 | LINE |
| 41773965 | 41774167 | - | 20.8 | 137 | 396 | 0 | MLT1A1 | MaLR | LTR |
| 41774168 | 41774517 | + | 20.8 | 1 | 396 | 0 | MSTD | MaLR | LTR |
| 41774518 | 41774651 | - | 19.1 | 1 | 137 | -271 | MLT1A1 | MaLR | LTR |
| 41774652 | 41776400 | - | 21.5 | 2 | 1735 | 0 | MLT1A1-int | MaLR | LTR |
| 41776403 | 41776468 | - | 19.4 | 343 | 407 | -1 | MLT1A1 | MaLR | LTR |
| 41776469 | 41776778 | + | 13.6 | 1 | 312 | -1 | AluSq | Alu | SINE |
| 41776779 | 41777057 | - | 19.4 | 2 | 342 | -66 | MLT1A1 | MaLR | LTR |
| 41777067 | 41777260 | - | 29.9 | 1351 | 1560 | -1859 | L2 | L2 | LINE |

  

|  |  |  |  |  |  |  |  |  |  |
| --- | --- | --- | --- | --- | --- | --- | --- | --- | --- |
| chr9:41801265-41802623 | | | 1130 bp | | |  |  |  |  |
| Genome Start | Genome End | Strand | Div | Repeat Start | Repeat End | Repeat Left | Name | Family | Class |
| 41801265 | 41801375 | - | 30.6 | 3251 | 3379 | -21 | L2 | L2 | LINE |
| 41801555 | 41801838 | + | 12.0 | 1 | 283 | -30 | AluSq | Alu | SINE |
| 41801863 | 41802355 | - | 34.7 | 1571 | 2074 | -1345 | L2 | L2 | LINE |
| 41802338 | 41802440 | + | 30.6 | 3274 | 3378 | 0 | L2 | L2 | LINE |
| 41802485 | 41802623 | - | 34.8 | 1284 | 1434 | -1985 | L2 | L2 | LINE |

  

|  |  |  |  |  |  |  |  |  |  |
| --- | --- | --- | --- | --- | --- | --- | --- | --- | --- |
| chr9:41806593-41807632 | | | 1040 bp | | |  |  |  |  |
| Genome Start | Genome End | Strand | Div | Repeat Start | Repeat End | Repeat Left | Name | Family | Class |
| 41806593 | 41806642 | - | 16.3 | 376 | 426 | 0 | MSTB | MaLR | LTR |
| 41806643 | 41806949 | - | 4.6 | 1 | 303 | -8 | AluY | Alu | SINE |
| 41806950 | 41807201 | - | 16.3 | 127 | 375 | -51 | MSTB | MaLR | LTR |
| 41807202 | 41807506 | - | 5.3 | 1 | 303 | -8 | AluY | Alu | SINE |
| 41807507 | 41807632 | - | 16.3 | 1 | 126 | -300 | MSTB | MaLR | LTR |

  

|  |  |  |  |  |  |  |  |  |  |
| --- | --- | --- | --- | --- | --- | --- | --- | --- | --- |
| chr9:41811318-41811847 | | | 530 bp | | |  |  |  |  |
| Genome Start | Genome End | Strand | Div | Repeat Start | Repeat End | Repeat Left | Name | Family | Class |
| 41811318 | 41811368 | - | 14.0 | 162 | 201 | -1 | MER6C | MER2\_type | DNA |
| 41811369 | 41811687 | + | 5.6 | 1 | 304 | -7 | AluY | Alu | SINE |
| 41811688 | 41811847 | - | 14.0 | 2 | 161 | -41 | MER6C | MER2\_type | DNA |

  

|  |  |  |  |  |  |  |  |  |  |
| --- | --- | --- | --- | --- | --- | --- | --- | --- | --- |
| chr9:41811909-41812664 | | | 639 bp | | |  |  |  |  |
| Genome Start | Genome End | Strand | Div | Repeat Start | Repeat End | Repeat Left | Name | Family | Class |
| 41811909 | 41811994 | + | 19.8 | 21 | 130 | -132 | MIR | MIR | SINE |
| 41812105 | 41812534 | - | 17.1 | 1 | 511 | -45 | MLT2B4 | ERVL | LTR |
| 41812542 | 41812664 | + | 29.4 | 114 | 241 | -21 | MIR | MIR | SINE |

  

|  |  |  |  |  |  |  |  |  |  |
| --- | --- | --- | --- | --- | --- | --- | --- | --- | --- |
| chr9:41842043-41848756 | | | 6682 bp | | |  |  |  |  |
| Genome Start | Genome End | Strand | Div | Repeat Start | Repeat End | Repeat Left | Name | Family | Class |
| 41842043 | 41843206 | + | 17.2 | 4 | 1217 | -4497 | ERVL-B4 | ERVL | LTR |
| 41843208 | 41843605 | - | 14.3 | 2007 | 2405 | 0 | Tigger5 | MER2\_type | DNA |
| 41843606 | 41843813 | + | 12.1 | 5936 | 6151 | 0 | L1PB1 | L1 | LINE |
| 41843841 | 41844082 | - | 17.4 | 1 | 251 | -2072 | Tigger5 | MER2\_type | DNA |
| 41844083 | 41844506 | + | 19.2 | 1216 | 1662 | -4052 | ERVL-B4 | ERVL | LTR |
| 41844511 | 41844905 | - | 13.7 | 1 | 422 | -6 | MSTA | MaLR | LTR |
| 41844906 | 41848756 | + | 16.9 | 1656 | 5714 | 0 | ERVL-B4 | ERVL | LTR |

  

|  |  |  |  |  |  |  |  |  |  |
| --- | --- | --- | --- | --- | --- | --- | --- | --- | --- |
| chr9:41859894-41861253 | | | 1234 bp | | |  |  |  |  |
| Genome Start | Genome End | Strand | Div | Repeat Start | Repeat End | Repeat Left | Name | Family | Class |
| 41859894 | 41860193 | + | 38.2 | 248 | 566 | -5553 | L1MEd | L1 | LINE |
| 41860318 | 41860482 | + | 17.1 | 5986 | 6167 | -4 | L1MB2 | L1 | LINE |
| 41860485 | 41860702 | + | 30.1 | 2534 | 2745 | -3384 | L1MEd | L1 | LINE |
| 41860703 | 41860992 | + | 10.3 | 1 | 290 | -19 | AluSc | Alu | SINE |
| 41860993 | 41861253 | + | 30.1 | 2746 | 3032 | -3097 | L1MEd | L1 | LINE |

  

|  |  |  |  |  |  |  |  |  |  |
| --- | --- | --- | --- | --- | --- | --- | --- | --- | --- |
| chr9:41876618-41877719 | | | 1102 bp | | |  |  |  |  |
| Genome Start | Genome End | Strand | Div | Repeat Start | Repeat End | Repeat Left | Name | Family | Class |
| 41876618 | 41876843 | + | 23.7 | 7260 | 7510 | -451 | L1MC5 | L1 | LINE |
| 41876844 | 41877320 | - | 7.6 | 1 | 486 | 0 | LTR19A | ERV1 | LTR |
| 41877321 | 41877719 | + | 23.7 | 7511 | 7926 | -35 | L1MC5 | L1 | LINE |

  

|  |  |  |  |  |  |  |  |  |  |
| --- | --- | --- | --- | --- | --- | --- | --- | --- | --- |
| chr9:41885358-41887027 | | | 1673 bp | | |  |  |  |  |
| Genome Start | Genome End | Strand | Div | Repeat Start | Repeat End | Repeat Left | Name | Family | Class |
| 41885358 | 41886261 | + | 27.3 | 283 | 1237 | -5303 | L1MD2 | L1 | LINE |
| 41886262 | 41886555 | - | 9.9 | 1 | 293 | -16 | AluSc | Alu | SINE |
| 41886556 | 41886937 | + | 26.6 | 1232 | 1630 | -5003 | L1MD2 | L1 | LINE |
| 41886935 | 41887027 | + | 20.0 | 2878 | 2968 | -3569 | L1MD2 | L1 | LINE |

  

|  |  |  |  |  |  |  |  |  |  |
| --- | --- | --- | --- | --- | --- | --- | --- | --- | --- |
| chr9:41900652-41901503 | | | 852 bp | | |  |  |  |  |
| Genome Start | Genome End | Strand | Div | Repeat Start | Repeat End | Repeat Left | Name | Family | Class |
| 41900652 | 41900766 | - | 7.6 | 6065 | 6170 | -2 | L1PA8 | L1 | LINE |
| 41900767 | 41901063 | - | 8.1 | 1 | 297 | -14 | AluY | Alu | SINE |
| 41901064 | 41901503 | - | 7.6 | 5627 | 6064 | -108 | L1PA8 | L1 | LINE |

  

|  |  |  |  |  |  |  |  |  |  |
| --- | --- | --- | --- | --- | --- | --- | --- | --- | --- |
| chr9:41904231-41904895 | | | 665 bp | | |  |  |  |  |
| Genome Start | Genome End | Strand | Div | Repeat Start | Repeat End | Repeat Left | Name | Family | Class |
| 41904231 | 41904281 | - | 13.7 | 391 | 428 | 0 | MSTA | MaLR | LTR |
| 41904282 | 41904571 | - | 12.4 | 3 | 292 | -17 | AluSc | Alu | SINE |
| 41904572 | 41904895 | - | 13.7 | 1 | 390 | -38 | MSTA | MaLR | LTR |

  

|  |  |  |  |  |  |  |  |  |  |
| --- | --- | --- | --- | --- | --- | --- | --- | --- | --- |
| chr9:41918233-41918698 | | | 466 bp | | |  |  |  |  |
| Genome Start | Genome End | Strand | Div | Repeat Start | Repeat End | Repeat Left | Name | Family | Class |
| 41918233 | 41918290 | + | 16.4 | 4322 | 4379 | -1764 | L1M2 | L1 | LINE |
| 41918291 | 41918512 | + | 7.9 | 1 | 217 | -96 | AluSq | Alu | SINE |
| 41918513 | 41918698 | + | 16.4 | 4380 | 4540 | -1603 | L1M2 | L1 | LINE |

  

|  |  |  |  |  |  |  |  |  |  |
| --- | --- | --- | --- | --- | --- | --- | --- | --- | --- |
| chr9:41924001-41925775 | | | 1775 bp | | |  |  |  |  |
| Genome Start | Genome End | Strand | Div | Repeat Start | Repeat End | Repeat Left | Name | Family | Class |
| 41924001 | 41925436 | - | 18.1 | 1652 | 3136 | -3185 | L1MCa | L1 | LINE |
| 41925437 | 41925740 | + | 10.8 | 2 | 298 | -14 | AluSx | Alu | SINE |
| 41925741 | 41925775 | - | 17.3 | 1620 | 1651 | -4857 | L1MCa | L1 | LINE |

  

|  |  |  |  |  |  |  |  |  |  |
| --- | --- | --- | --- | --- | --- | --- | --- | --- | --- |
| chr9:41936191-41937815 | | | 1625 bp | | |  |  |  |  |
| Genome Start | Genome End | Strand | Div | Repeat Start | Repeat End | Repeat Left | Name | Family | Class |
| 41936191 | 41936316 | + | 17.0 | 3 | 126 | -1454 | MSTA-int | MaLR | LTR |
| 41936317 | 41936627 | + | 17.3 | 1 | 307 | -5 | AluSx | Alu | SINE |
| 41936628 | 41937815 | + | 17.0 | 127 | 1231 | -349 | MSTA-int | MaLR | LTR |

  

|  |  |  |  |  |  |  |  |  |  |
| --- | --- | --- | --- | --- | --- | --- | --- | --- | --- |
| chr9:41939764-41941513 | | | 1399 bp | | |  |  |  |  |
| Genome Start | Genome End | Strand | Div | Repeat Start | Repeat End | Repeat Left | Name | Family | Class |
| 41939764 | 41939835 | + | 15.4 | 5102 | 5178 | -968 | L1MB7 | L1 | LINE |
| 41939836 | 41940211 | + | 11.6 | 1 | 364 | 0 | THE1B | MaLR | LTR |
| 41940212 | 41940742 | + | 16.3 | 5179 | 5739 | -439 | L1MB7 | L1 | LINE |
| 41940748 | 41940873 | + | 27.1 | 4018 | 4136 | -55 | MER21-int | ERV1 | LTR |
| 41941220 | 41941513 | + | 16.6 | 5735 | 6014 | -170 | L1MB7 | L1 | LINE |

  

|  |  |  |  |  |  |  |  |  |  |
| --- | --- | --- | --- | --- | --- | --- | --- | --- | --- |
| chr9:41941647-41942796 | | | 893 bp | | |  |  |  |  |
| Genome Start | Genome End | Strand | Div | Repeat Start | Repeat End | Repeat Left | Name | Family | Class |
| 41941647 | 41941868 | + | 29.1 | 4478 | 4702 | -1444 | L1M5 | L1 | LINE |
| 41941963 | 41942062 | + | 23.6 | 39 | 171 | -378 | MLT1H | MaLR | LTR |
| 41942073 | 41942369 | - | 11.7 | 1 | 292 | -17 | AluSc | Alu | SINE |
| 41942439 | 41942615 | + | 23.0 | 155 | 454 | -113 | MLT1G1 | MaLR | LTR |
| 41942700 | 41942796 | + | 29.2 | 4720 | 4818 | -1328 | L1M5 | L1 | LINE |

  

|  |  |  |  |  |  |  |  |  |  |
| --- | --- | --- | --- | --- | --- | --- | --- | --- | --- |
| chr9:41946279-41948669 | | | 2391 bp | | |  |  |  |  |
| Genome Start | Genome End | Strand | Div | Repeat Start | Repeat End | Repeat Left | Name | Family | Class |
| 41946279 | 41947952 | - | 18.5 | 3349 | 5099 | -2460 | L1M1 | L1 | LINE |
| 41947953 | 41948278 | + | 19.3 | 4 | 346 | 0 | MER7A | MER2\_type | DNA |
| 41948279 | 41948669 | - | 24.3 | 2917 | 3348 | -4217 | L1M1 | L1 | LINE |

  

|  |  |  |  |  |  |  |  |  |  |
| --- | --- | --- | --- | --- | --- | --- | --- | --- | --- |
| chr9:41958666-41959994 | | | 1329 bp | | |  |  |  |  |
| Genome Start | Genome End | Strand | Div | Repeat Start | Repeat End | Repeat Left | Name | Family | Class |
| 41958666 | 41958840 | + | 17.8 | 5224 | 5420 | -924 | L1MC2 | L1 | LINE |
| 41958841 | 41959146 | + | 11.8 | 5842 | 6157 | -9 | L1PA16 | L1 | LINE |
| 41959147 | 41959994 | + | 18.2 | 5421 | 6288 | -56 | L1MC2 | L1 | LINE |

  

|  |  |  |  |  |  |  |  |  |  |
| --- | --- | --- | --- | --- | --- | --- | --- | --- | --- |
| chr9:41963761-41964647 | | | 887 bp | | |  |  |  |  |
| Genome Start | Genome End | Strand | Div | Repeat Start | Repeat End | Repeat Left | Name | Family | Class |
| 41963761 | 41964003 | + | 23.9 | 7349 | 7598 | -363 | L1MC5 | L1 | LINE |
| 41964004 | 41964314 | + | 11.4 | 1 | 298 | -14 | AluSx | Alu | SINE |
| 41964315 | 41964647 | + | 23.9 | 7599 | 7947 | -14 | L1MC5 | L1 | LINE |

  

|  |  |  |  |  |  |  |  |  |  |
| --- | --- | --- | --- | --- | --- | --- | --- | --- | --- |
| chr9:41983914-41985674 | | | 1770 bp | | |  |  |  |  |
| Genome Start | Genome End | Strand | Div | Repeat Start | Repeat End | Repeat Left | Name | Family | Class |
| 41983914 | 41984122 | + | 20.5 | 5 | 211 | -872 | Tigger6a | MER2\_type | DNA |
| 41984123 | 41984577 | - | 23.5 | 11 | 467 | 0 | MLT1C | MaLR | LTR |
| 41984569 | 41985185 | + | 19.9 | 216 | 887 | -196 | Tigger6a | MER2\_type | DNA |
| 41985186 | 41985478 | + | 13.3 | 1 | 293 | -18 | AluY | Alu | SINE |
| 41985479 | 41985674 | + | 19.9 | 888 | 1082 | -1 | Tigger6a | MER2\_type | DNA |

  

|  |  |  |  |  |  |  |  |  |  |
| --- | --- | --- | --- | --- | --- | --- | --- | --- | --- |
| chr9:41991359-41995583 | | | 3992 bp | | |  |  |  |  |
| Genome Start | Genome End | Strand | Div | Repeat Start | Repeat End | Repeat Left | Name | Family | Class |
| 41991359 | 41991577 | - | 32.4 | 4851 | 5064 | -1082 | L1ME3B | L1 | LINE |
| 41991682 | 41991764 | - | 21.5 | 4633 | 4715 | -1431 | L1ME3B | L1 | LINE |
| 41991764 | 41992130 | - | 27.4 | 3709 | 4104 | -2042 | L1ME3B | L1 | LINE |
| 41992140 | 41992385 | - | 25.9 | 3375 | 3660 | -2486 | L1ME3B | L1 | LINE |
| 41992393 | 41992745 | - | 26.4 | 5831 | 6223 | 0 | L1ME3B | L1 | LINE |
| 41992746 | 41993581 | - | 16.2 | 5435 | 6322 | -11 | L1MC1 | L1 | LINE |
| 41993582 | 41994274 | - | 27.0 | 5088 | 5824 | -323 | L1ME3B | L1 | LINE |
| 41994339 | 41994504 | - | 34.2 | 4818 | 4983 | -1146 | L1ME3B | L1 | LINE |
| 41994544 | 41995099 | - | 19.1 | 3 | 608 | -218 | LTR12 | ERV1 | LTR |
| 41995111 | 41995583 | - | 33.9 | 64 | 559 | -5560 | L1ME3B | L1 | LINE |

  

|  |  |  |  |  |  |  |  |  |  |
| --- | --- | --- | --- | --- | --- | --- | --- | --- | --- |
| chr9:42000657-42005331 | | | 4630 bp | | |  |  |  |  |
| Genome Start | Genome End | Strand | Div | Repeat Start | Repeat End | Repeat Left | Name | Family | Class |
| 42000657 | 42001165 | - | 14.7 | 5783 | 6312 | 0 | L1MA9 | L1 | LINE |
| 42001201 | 42001640 | - | 14.7 | 5332 | 5782 | -530 | L1MA9 | L1 | LINE |
| 42001641 | 42002000 | + | 16.1 | 1 | 364 | 0 | THE1B | MaLR | LTR |
| 42002001 | 42003568 | + | 12.7 | 1 | 1580 | 0 | THE1B-int | MaLR | LTR |
| 42003569 | 42003932 | + | 14.0 | 1 | 364 | 0 | THE1B | MaLR | LTR |
| 42003943 | 42004789 | - | 19.1 | 4380 | 5286 | -857 | L1MA9 | L1 | LINE |
| 42004790 | 42005066 | - | 8.3 | 1 | 287 | -24 | AluY | Alu | SINE |
| 42005067 | 42005331 | - | 19.1 | 4120 | 4379 | -1764 | L1MA9 | L1 | LINE |

  

|  |  |  |  |  |  |  |  |  |  |
| --- | --- | --- | --- | --- | --- | --- | --- | --- | --- |
| chr9:42005327-42007714 | | | 2388 bp | | |  |  |  |  |
| Genome Start | Genome End | Strand | Div | Repeat Start | Repeat End | Repeat Left | Name | Family | Class |
| 42005327 | 42005711 | + | 25.5 | 2917 | 3348 | -4217 | L1M1 | L1 | LINE |
| 42005712 | 42006037 | - | 18.4 | 4 | 346 | 0 | MER7A | MER2\_type | DNA |
| 42006038 | 42007714 | + | 18.3 | 3349 | 5099 | -2460 | L1M1 | L1 | LINE |

  

|  |  |  |  |  |  |  |  |  |  |
| --- | --- | --- | --- | --- | --- | --- | --- | --- | --- |
| chr9:42012487-42013778 | | | 941 bp | | |  |  |  |  |
| Genome Start | Genome End | Strand | Div | Repeat Start | Repeat End | Repeat Left | Name | Family | Class |
| 42012487 | 42012780 | - | 17.3 | 5735 | 6014 | -170 | L1MB7 | L1 | LINE |
| 42013127 | 42013252 | - | 28.0 | 4018 | 4136 | -55 | MER21-int | ERV1 | LTR |
| 42013258 | 42013778 | - | 14.9 | 5188 | 5739 | -439 | L1MB7 | L1 | LINE |

  

|  |  |  |  |  |  |  |  |  |  |
| --- | --- | --- | --- | --- | --- | --- | --- | --- | --- |
| chr9:42031003-42032813 | | | 1738 bp | | |  |  |  |  |
| Genome Start | Genome End | Strand | Div | Repeat Start | Repeat End | Repeat Left | Name | Family | Class |
| 42031003 | 42031298 | + | 29.8 | 3297 | 3627 | -2519 | L1M5 | L1 | LINE |
| 42031330 | 42032093 | - | 1.7 | 5389 | 6152 | -3 | L1PA2 | L1 | LINE |
| 42032094 | 42032540 | + | 2.0 | 3557 | 4003 | -2143 | L1P1 | L1 | LINE |
| 42032537 | 42032694 | + | 26.5 | 3612 | 3763 | -2383 | L1MC | L1 | LINE |
| 42032741 | 42032813 | + | 29.2 | 3783 | 3854 | -2292 | L1M5 | L1 | LINE |

  

|  |  |  |  |  |  |  |  |  |  |
| --- | --- | --- | --- | --- | --- | --- | --- | --- | --- |
| chr9:42035931-42037231 | | | 891 bp | | |  |  |  |  |
| Genome Start | Genome End | Strand | Div | Repeat Start | Repeat End | Repeat Left | Name | Family | Class |
| 42035931 | 42036283 | - | 29.2 | 1359 | 1728 | -4421 | L1MEe | L1 | LINE |
| 42036318 | 42036530 | - | 36.0 | 3189 | 3415 | -4 | L2 | L2 | LINE |
| 42036907 | 42037231 | - | 34.5 | 500 | 835 | -5284 | L1MEe | L1 | LINE |

  

|  |  |  |  |  |  |  |  |  |  |
| --- | --- | --- | --- | --- | --- | --- | --- | --- | --- |
| chr9:42043255-42046928 | | | 3055 bp | | |  |  |  |  |
| Genome Start | Genome End | Strand | Div | Repeat Start | Repeat End | Repeat Left | Name | Family | Class |
| 42043255 | 42043630 | + | 34.6 | 428 | 812 | -5588 | L1MEc | L1 | LINE |
| 42043740 | 42044181 | + | 28.1 | 1004 | 1509 | -4891 | L1MEc | L1 | LINE |
| 42044203 | 42044290 | + | 28.1 | 1510 | 1610 | -4790 | L1MEc | L1 | LINE |
| 42044366 | 42044549 | + | 32.4 | 2 | 175 | 0 | MER96 | MER1\_type? | DNA |
| 42044674 | 42045087 | + | 24.4 | 2051 | 2441 | -3959 | L1MEc | L1 | LINE |
| 42045101 | 42045249 | + | 33.6 | 2374 | 2524 | -3700 | L1MEc | L1 | LINE |
| 42045243 | 42045374 | + | 22.9 | 6163 | 6305 | 0 | L1MA3 | L1 | LINE |
| 42045486 | 42045831 | + | 24.4 | 1 | 345 | -63 | MLT1A0 | MaLR | LTR |
| 42045933 | 42046449 | + | 28.1 | 3194 | 3731 | -2488 | L1MEc | L1 | LINE |
| 42046518 | 42046723 | + | 27.7 | 3961 | 4184 | -2038 | L1MEc | L1 | LINE |
| 42046728 | 42046928 | + | 23.3 | 4529 | 4731 | -1488 | L1MEc | L1 | LINE |

  

|  |  |  |  |  |  |  |  |  |  |
| --- | --- | --- | --- | --- | --- | --- | --- | --- | --- |
| chr9:42063000-42063897 | | | 898 bp | | |  |  |  |  |
| Genome Start | Genome End | Strand | Div | Repeat Start | Repeat End | Repeat Left | Name | Family | Class |
| 42063000 | 42063086 | - | 16.4 | 252 | 337 | -2 | MER1B | MER1\_type | DNA |
| 42063087 | 42063655 | + | 24.1 | 1 | 554 | 0 | MLT2B3 | ERVL | LTR |
| 42063656 | 42063897 | - | 16.4 | 1 | 251 | -88 | MER1B | MER1\_type | DNA |

  

|  |  |  |  |  |  |  |  |  |  |
| --- | --- | --- | --- | --- | --- | --- | --- | --- | --- |
| chr9:42064581-42065366 | | | 786 bp | | |  |  |  |  |
| Genome Start | Genome End | Strand | Div | Repeat Start | Repeat End | Repeat Left | Name | Family | Class |
| 42064581 | 42064757 | - | 17.1 | 309 | 500 | -1 | MER61D | ERV1 | LTR |
| 42064758 | 42065046 | - | 14.5 | 2 | 291 | -19 | AluSg | Alu | SINE |
| 42065047 | 42065366 | - | 17.1 | 1 | 308 | -193 | MER61D | ERV1 | LTR |

  

|  |  |  |  |  |  |  |  |  |  |
| --- | --- | --- | --- | --- | --- | --- | --- | --- | --- |
| chr9:42092796-42094016 | | | 1221 bp | | |  |  |  |  |
| Genome Start | Genome End | Strand | Div | Repeat Start | Repeat End | Repeat Left | Name | Family | Class |
| 42092796 | 42092904 | - | 18.2 | 194 | 298 | -14 | AluJb | Alu | SINE |
| 42092905 | 42093810 | - | 17.5 | 1 | 983 | 0 | LTR1D | ERV1 | LTR |
| 42093811 | 42094016 | - | 18.2 | 1 | 193 | -119 | AluJb | Alu | SINE |

  

|  |  |  |  |  |  |  |  |  |  |
| --- | --- | --- | --- | --- | --- | --- | --- | --- | --- |
| chr9:42108893-42111559 | | | 2555 bp | | |  |  |  |  |
| Genome Start | Genome End | Strand | Div | Repeat Start | Repeat End | Repeat Left | Name | Family | Class |
| 42108893 | 42109321 | + | 31.0 | 1203 | 1614 | -1805 | L2 | L2 | LINE |
| 42109322 | 42109551 | - | 24.6 | 1 | 219 | 0 | MER20 | MER1\_type | DNA |
| 42109552 | 42110344 | + | 31.0 | 1615 | 2425 | -994 | L2 | L2 | LINE |
| 42110457 | 42110845 | + | 29.4 | 87 | 517 | -6 | LTR33A | ERVL | LTR |
| 42110846 | 42111559 | + | 31.9 | 2415 | 3172 | -247 | L2 | L2 | LINE |

  

|  |  |  |  |  |  |  |  |  |  |
| --- | --- | --- | --- | --- | --- | --- | --- | --- | --- |
| chr9:42121880-42122957 | | | 1078 bp | | |  |  |  |  |
| Genome Start | Genome End | Strand | Div | Repeat Start | Repeat End | Repeat Left | Name | Family | Class |
| 42121880 | 42122113 | - | 25.4 | 244 | 477 | -28 | MLT1D | MaLR | LTR |
| 42122114 | 42122320 | + | 15.5 | 6087 | 6294 | -6 | L1MA6 | L1 | LINE |
| 42122321 | 42122573 | - | 25.4 | 32 | 243 | -262 | MLT1D | MaLR | LTR |
| 42122574 | 42122926 | - | 16.7 | 1 | 364 | 0 | THE1B | MaLR | LTR |
| 42122927 | 42122957 | - | 25.4 | 1 | 31 | -474 | MLT1D | MaLR | LTR |

  

|  |  |  |  |  |  |  |  |  |  |
| --- | --- | --- | --- | --- | --- | --- | --- | --- | --- |
| chr9:42126796-42130842 | | | 3936 bp | | |  |  |  |  |
| Genome Start | Genome End | Strand | Div | Repeat Start | Repeat End | Repeat Left | Name | Family | Class |
| 42126796 | 42127209 | + | 29.8 | 4755 | 5173 | -973 | L1ME1 | L1 | LINE |
| 42127218 | 42129249 | - | 8.1 | 4089 | 6144 | -10 | L1PA7 | L1 | LINE |
| 42129353 | 42129932 | + | 24.6 | 62 | 607 | 0 | MER77 | ERVL | LTR |
| 42129933 | 42130842 | + | 27.4 | 5243 | 6161 | -5 | L1ME1 | L1 | LINE |

  

|  |  |  |  |  |  |  |  |  |  |
| --- | --- | --- | --- | --- | --- | --- | --- | --- | --- |
| chr9:42138755-42140007 | | | 1010 bp | | |  |  |  |  |
| Genome Start | Genome End | Strand | Div | Repeat Start | Repeat End | Repeat Left | Name | Family | Class |
| 42138755 | 42139052 | - | 29.0 | 5506 | 5801 | -439 | L1M5 | L1 | LINE |
| 42139122 | 42139426 | + | 11.2 | 1 | 305 | -7 | AluSx | Alu | SINE |
| 42139518 | 42139818 | - | 34.8 | 5009 | 5342 | -804 | L1M5 | L1 | LINE |
| 42139902 | 42140007 | - | 20.8 | 4260 | 4378 | -1768 | L1M5 | L1 | LINE |

  

|  |  |  |  |  |  |  |  |  |  |
| --- | --- | --- | --- | --- | --- | --- | --- | --- | --- |
| chr9:42141515-42144954 | | | 3440 bp | | |  |  |  |  |
| Genome Start | Genome End | Strand | Div | Repeat Start | Repeat End | Repeat Left | Name | Family | Class |
| 42141515 | 42142451 | + | 15.8 | 2983 | 3942 | -2204 | L1PA16 | L1 | LINE |
| 42142452 | 42142749 | + | 5.5 | 1 | 310 | 0 | AluY | Alu | SINE |
| 42142750 | 42144954 | + | 16.0 | 3943 | 6157 | -9 | L1PA16 | L1 | LINE |

  

|  |  |  |  |  |  |  |  |  |  |
| --- | --- | --- | --- | --- | --- | --- | --- | --- | --- |
| chr9:42170766-42171264 | | | 499 bp | | |  |  |  |  |
| Genome Start | Genome End | Strand | Div | Repeat Start | Repeat End | Repeat Left | Name | Family | Class |
| 42170766 | 42170789 | - | 30.0 | 2683 | 2705 | -714 | L2 | L2 | LINE |
| 42170790 | 42171160 | + | 27.1 | 1 | 365 | 0 | MLT1A0 | MaLR | LTR |
| 42171161 | 42171264 | - | 30.0 | 2581 | 2682 | -737 | L2 | L2 | LINE |

  

|  |  |  |  |  |  |  |  |  |  |
| --- | --- | --- | --- | --- | --- | --- | --- | --- | --- |
| chr9:42172558-42173391 | | | 834 bp | | |  |  |  |  |
| Genome Start | Genome End | Strand | Div | Repeat Start | Repeat End | Repeat Left | Name | Family | Class |
| 42172558 | 42172846 | + | 25.1 | 3850 | 4088 | -2058 | L1M5 | L1 | LINE |
| 42172847 | 42173354 | - | 22.1 | 1 | 503 | -2 | MLT1D | MaLR | LTR |
| 42173355 | 42173391 | + | 25.1 | 4089 | 4124 | -2022 | L1M5 | L1 | LINE |

  

|  |  |  |  |  |  |  |  |  |  |
| --- | --- | --- | --- | --- | --- | --- | --- | --- | --- |
| chr9:42199432-42199849 | | | 339 bp | | |  |  |  |  |
| Genome Start | Genome End | Strand | Div | Repeat Start | Repeat End | Repeat Left | Name | Family | Class |
| 42199432 | 42199545 | + | 28.7 | 457 | 565 | -5554 | L1MEd | L1 | LINE |
| 42199623 | 42199670 | - | 20.8 | 2358 | 2405 | -15 | MER58 | MER1\_type | DNA |
| 42199673 | 42199849 | + | 31.2 | 965 | 1150 | -4969 | L1MEd | L1 | LINE |

  

|  |  |  |  |  |  |  |  |  |  |
| --- | --- | --- | --- | --- | --- | --- | --- | --- | --- |
| chr9:42205923-42206729 | | | 807 bp | | |  |  |  |  |
| Genome Start | Genome End | Strand | Div | Repeat Start | Repeat End | Repeat Left | Name | Family | Class |
| 42205923 | 42206090 | - | 35.9 | 85 | 251 | -17 | MIRb | MIR | SINE |
| 42206091 | 42206679 | + | 19.0 | 2 | 604 | -1 | MER6A | MER2\_type | DNA |
| 42206680 | 42206729 | - | 35.9 | 38 | 84 | -184 | MIRb | MIR | SINE |

  

|  |  |  |  |  |  |  |  |  |  |
| --- | --- | --- | --- | --- | --- | --- | --- | --- | --- |
| chr9:42211386-42212619 | | | 1234 bp | | |  |  |  |  |
| Genome Start | Genome End | Strand | Div | Repeat Start | Repeat End | Repeat Left | Name | Family | Class |
| 42211386 | 42211501 | + | 19.4 | 5661 | 5784 | -372 | L1PB4 | L1 | LINE |
| 42211502 | 42212208 | + | 7.9 | 5441 | 6147 | -7 | L1PA7 | L1 | LINE |
| 42212209 | 42212619 | + | 19.4 | 5785 | 6153 | -3 | L1PB4 | L1 | LINE |

  

|  |  |  |  |  |  |  |  |  |  |
| --- | --- | --- | --- | --- | --- | --- | --- | --- | --- |
| chr9:42220424-42222816 | | | 2393 bp | | |  |  |  |  |
| Genome Start | Genome End | Strand | Div | Repeat Start | Repeat End | Repeat Left | Name | Family | Class |
| 42220424 | 42220839 | - | 28.1 | 5556 | 5973 | -191 | L1ME2 | L1 | LINE |
| 42220840 | 42221145 | - | 9.9 | 1 | 294 | -17 | AluY | Alu | SINE |
| 42221146 | 42222056 | - | 26.9 | 4583 | 5555 | -609 | L1ME2 | L1 | LINE |
| 42222057 | 42222365 | - | 11.6 | 1 | 301 | -10 | AluY | Alu | SINE |
| 42222366 | 42222816 | - | 26.6 | 4157 | 4582 | -1564 | L1ME2 | L1 | LINE |

  

|  |  |  |  |  |  |  |  |  |  |
| --- | --- | --- | --- | --- | --- | --- | --- | --- | --- |
| chr9:42226282-42228589 | | | 2258 bp | | |  |  |  |  |
| Genome Start | Genome End | Strand | Div | Repeat Start | Repeat End | Repeat Left | Name | Family | Class |
| 42226282 | 42226713 | + | 25.0 | 1423 | 2039 | -4463 | L1MCa | L1 | LINE |
| 42226716 | 42226889 | + | 23.7 | 2 | 186 | -38 | MER58A | MER1\_type | DNA |
| 42226917 | 42227214 | + | 11.7 | 11 | 309 | 0 | AluSc | Alu | SINE |
| 42227236 | 42227562 | + | 21.9 | 2034 | 2365 | -4137 | L1MCa | L1 | LINE |
| 42227563 | 42228115 | - | 18.2 | 1 | 541 | -1 | MER34 | ERV1 | LTR |
| 42228116 | 42228164 | + | 21.9 | 2366 | 2411 | -4091 | L1MCa | L1 | LINE |
| 42228165 | 42228473 | + | 10.4 | 3 | 310 | 0 | AluSg | Alu | SINE |
| 42228474 | 42228589 | + | 21.9 | 2412 | 2520 | -3982 | L1MCa | L1 | LINE |

  

|  |  |  |  |  |  |  |  |  |  |
| --- | --- | --- | --- | --- | --- | --- | --- | --- | --- |
| chr9:42232203-42233009 | | | 807 bp | | |  |  |  |  |
| Genome Start | Genome End | Strand | Div | Repeat Start | Repeat End | Repeat Left | Name | Family | Class |
| 42232203 | 42232386 | + | 25.8 | 3 | 163 | -342 | MLT1D | MaLR | LTR |
| 42232387 | 42232691 | - | 12.2 | 1 | 303 | -9 | AluSx | Alu | SINE |
| 42232692 | 42233009 | + | 25.8 | 164 | 505 | 0 | MLT1D | MaLR | LTR |

  

|  |  |  |  |  |  |  |  |  |  |
| --- | --- | --- | --- | --- | --- | --- | --- | --- | --- |
| chr9:42237261-42240135 | | | 2824 bp | | |  |  |  |  |
| Genome Start | Genome End | Strand | Div | Repeat Start | Repeat End | Repeat Left | Name | Family | Class |
| 42237261 | 42237947 | - | 32.0 | 5297 | 6020 | -220 | L1ME3B | L1 | LINE |
| 42237944 | 42239529 | - | 31.9 | 3312 | 5026 | -1120 | L1ME3B | L1 | LINE |
| 42239564 | 42239714 | + | 23.8 | 164 | 325 | -16 | MER58B | MER1\_type | DNA |
| 42239736 | 42240135 | - | 28.6 | 2866 | 3319 | -2827 | L1ME3B | L1 | LINE |

  

|  |  |  |  |  |  |  |  |  |  |
| --- | --- | --- | --- | --- | --- | --- | --- | --- | --- |
| chr9:42257768-42258758 | | | 962 bp | | |  |  |  |  |
| Genome Start | Genome End | Strand | Div | Repeat Start | Repeat End | Repeat Left | Name | Family | Class |
| 42257768 | 42258063 | - | 26.8 | 1361 | 1660 | -5141 | MER89-int | ERV1 | LTR |
| 42258093 | 42258424 | - | 22.7 | 1 | 340 | -25 | MLT1A0 | MaLR | LTR |
| 42258425 | 42258758 | - | 28.1 | 1030 | 1357 | -5444 | MER89-int | ERV1 | LTR |

  

|  |  |  |  |  |  |  |  |  |  |
| --- | --- | --- | --- | --- | --- | --- | --- | --- | --- |
| chr9:42271363-42273727 | | | 2365 bp | | |  |  |  |  |
| Genome Start | Genome End | Strand | Div | Repeat Start | Repeat End | Repeat Left | Name | Family | Class |
| 42271363 | 42271505 | - | 25.8 | 2217 | 2349 | -4504 | L1PA15-16 | L1 | LINE |
| 42271506 | 42271799 | + | 15.3 | 1 | 294 | -18 | AluSx | Alu | SINE |
| 42271800 | 42273727 | - | 25.8 | 3 | 2216 | -4637 | L1PA15-16 | L1 | LINE |

  

|  |  |  |  |  |  |  |  |  |  |
| --- | --- | --- | --- | --- | --- | --- | --- | --- | --- |
| chr9:42275239-42277577 | | | 2294 bp | | |  |  |  |  |
| Genome Start | Genome End | Strand | Div | Repeat Start | Repeat End | Repeat Left | Name | Family | Class |
| 42275239 | 42276108 | - | 18.1 | 725 | 1651 | 0 | MSTA-int | MaLR | LTR |
| 42276111 | 42276604 | + | 7.9 | 1 | 487 | -6 | LTR15 | ERV1 | LTR |
| 42276613 | 42276910 | + | 11.7 | 3 | 301 | -10 | AluY | Alu | SINE |
| 42276946 | 42277577 | - | 23.1 | 3 | 667 | -913 | MSTA-int | MaLR | LTR |

  

|  |  |  |  |  |  |  |  |  |  |
| --- | --- | --- | --- | --- | --- | --- | --- | --- | --- |
| chr9:42297599-42300583 | | | 2857 bp | | |  |  |  |  |
| Genome Start | Genome End | Strand | Div | Repeat Start | Repeat End | Repeat Left | Name | Family | Class |
| 42297599 | 42297782 | + | 27.4 | 2330 | 2513 | -906 | L2 | L2 | LINE |
| 42297784 | 42297843 | + | 26.3 | 2488 | 2546 | -873 | L2 | L2 | LINE |
| 42297889 | 42298362 | - | 6.8 | 5680 | 6154 | 0 | L1PA7 | L1 | LINE |
| 42298363 | 42298459 | + | 7.7 | 5308 | 5406 | -748 | L1P | L1 | LINE |
| 42298460 | 42298927 | - | 20.2 | 1 | 458 | 0 | LTR22A | ERVK | LTR |
| 42298928 | 42299200 | + | 7.7 | 5407 | 5673 | -481 | L1P3 | L1 | LINE |
| 42299202 | 42299492 | - | 13.8 | 1 | 291 | -22 | AluSq | Alu | SINE |
| 42299494 | 42299791 | + | 27.1 | 2576 | 2871 | -548 | L2 | L2 | LINE |
| 42299794 | 42300228 | - | 22.6 | 6 | 466 | -1 | MLT1C | MaLR | LTR |
| 42300278 | 42300421 | + | 22.2 | 1 | 156 | -4 | MER5A1 | MER1\_type | DNA |
| 42300451 | 42300583 | + | 36.2 | 2923 | 3060 | -318 | L2 | L2 | LINE |

  

|  |  |  |  |  |  |  |  |  |  |
| --- | --- | --- | --- | --- | --- | --- | --- | --- | --- |
| chr9:42316247-42317083 | | | 837 bp | | |  |  |  |  |
| Genome Start | Genome End | Strand | Div | Repeat Start | Repeat End | Repeat Left | Name | Family | Class |
| 42316247 | 42316604 | - | 30.2 | 134 | 511 | 0 | MLT1J | MaLR | LTR |
| 42316605 | 42316974 | - | 12.9 | 1 | 381 | 0 | THE1D | MaLR | LTR |
| 42316975 | 42317083 | - | 30.2 | 26 | 133 | -378 | MLT1J | MaLR | LTR |

  

|  |  |  |  |  |  |  |  |  |  |
| --- | --- | --- | --- | --- | --- | --- | --- | --- | --- |
| chr9:42341935-42342433 | | | 390 bp | | |  |  |  |  |
| Genome Start | Genome End | Strand | Div | Repeat Start | Repeat End | Repeat Left | Name | Family | Class |
| 42341935 | 42342018 | - | 20.0 | 1656 | 1735 | -10 | Kanga1 | Tc2 | DNA |
| 42342128 | 42342345 | + | 33.6 | 425 | 651 | -5468 | L1MEd | L1 | LINE |
| 42342346 | 42342433 | - | 22.1 | 4 | 91 | -1830 | Kanga1 | Tc2 | DNA |

  

|  |  |  |  |  |  |  |  |  |  |
| --- | --- | --- | --- | --- | --- | --- | --- | --- | --- |
| chr9:42351353-42352860 | | | 1508 bp | | |  |  |  |  |
| Genome Start | Genome End | Strand | Div | Repeat Start | Repeat End | Repeat Left | Name | Family | Class |
| 42351353 | 42351639 | - | 24.5 | 602 | 916 | -19 | MER21C | ERV1 | LTR |
| 42351640 | 42351930 | - | 16.3 | 9 | 297 | -15 | AluJo | Alu | SINE |
| 42351931 | 42352008 | - | 24.5 | 529 | 601 | -334 | MER21C | ERV1 | LTR |
| 42352009 | 42352341 | + | 19.3 | 2 | 301 | -11 | AluJb | Alu | SINE |
| 42352342 | 42352860 | - | 24.5 | 18 | 528 | -407 | MER21C | ERV1 | LTR |

  

|  |  |  |  |  |  |  |  |  |  |
| --- | --- | --- | --- | --- | --- | --- | --- | --- | --- |
| chr9:42410342-42412080 | | | 1730 bp | | |  |  |  |  |
| Genome Start | Genome End | Strand | Div | Repeat Start | Repeat End | Repeat Left | Name | Family | Class |
| 42410342 | 42410664 | - | 20.4 | 5833 | 6173 | -5 | L1MB8 | L1 | LINE |
| 42410665 | 42411076 | - | 13.9 | 11 | 428 | 0 | MER61C | ERV1 | LTR |
| 42411086 | 42412080 | - | 19.1 | 4766 | 5836 | -342 | L1MB8 | L1 | LINE |

  

|  |  |  |  |  |  |  |  |  |  |
| --- | --- | --- | --- | --- | --- | --- | --- | --- | --- |
| chr9:42413825-42415547 | | | 1648 bp | | |  |  |  |  |
| Genome Start | Genome End | Strand | Div | Repeat Start | Repeat End | Repeat Left | Name | Family | Class |
| 42413825 | 42414100 | - | 17.0 | 5997 | 6294 | -6 | L1MA5 | L1 | LINE |
| 42414168 | 42414425 | - | 22.7 | 1 | 264 | -48 | AluJo | Alu | SINE |
| 42414434 | 42415547 | - | 20.6 | 4805 | 5941 | -359 | L1MA5 | L1 | LINE |

  

|  |  |  |  |  |  |  |  |  |  |
| --- | --- | --- | --- | --- | --- | --- | --- | --- | --- |
| chr9:42522158-42525556 | | | 3200 bp | | |  |  |  |  |
| Genome Start | Genome End | Strand | Div | Repeat Start | Repeat End | Repeat Left | Name | Family | Class |
| 42522158 | 42523607 | - | 30.1 | 3774 | 5277 | -1664 | L1MCc | L1 | LINE |
| 42523608 | 42523805 | - | 14.7 | 5942 | 6144 | -10 | L1PB3 | L1 | LINE |
| 42523806 | 42523857 | - | 30.1 | 3734 | 3773 | -3168 | L1MCc | L1 | LINE |
| 42523858 | 42524168 | - | 4.8 | 1 | 310 | -1 | AluY | Alu | SINE |
| 42524169 | 42524997 | - | 29.7 | 2834 | 3733 | -3208 | L1MCc | L1 | LINE |
| 42525197 | 42525556 | - | 29.1 | 2162 | 2576 | -4385 | L1MCc | L1 | LINE |

  

|  |  |  |  |  |  |  |  |  |  |
| --- | --- | --- | --- | --- | --- | --- | --- | --- | --- |
| chr9:42546326-42546871 | | | 465 bp | | |  |  |  |  |
| Genome Start | Genome End | Strand | Div | Repeat Start | Repeat End | Repeat Left | Name | Family | Class |
| 42546326 | 42546436 | - | 27.5 | 69 | 177 | -1 | MER5B | MER1\_type | DNA |
| 42546518 | 42546827 | - | 4.2 | 1 | 310 | -1 | AluY | Alu | SINE |
| 42546828 | 42546871 | - | 27.3 | 1 | 44 | -134 | MER5B | MER1\_type | DNA |

  

|  |  |  |  |  |  |  |  |  |  |
| --- | --- | --- | --- | --- | --- | --- | --- | --- | --- |
| chr9:42547742-42548193 | | | 452 bp | | |  |  |  |  |
| Genome Start | Genome End | Strand | Div | Repeat Start | Repeat End | Repeat Left | Name | Family | Class |
| 42547742 | 42547797 | - | 29.5 | 116 | 172 | -6 | MER5B | MER1\_type | DNA |
| 42547798 | 42548106 | + | 12.7 | 1 | 299 | -13 | AluSx | Alu | SINE |
| 42548107 | 42548193 | - | 29.5 | 27 | 115 | -63 | MER5B | MER1\_type | DNA |

  

|  |  |  |  |  |  |  |  |  |  |
| --- | --- | --- | --- | --- | --- | --- | --- | --- | --- |
| chr9:42570241-42570885 | | | 645 bp | | |  |  |  |  |
| Genome Start | Genome End | Strand | Div | Repeat Start | Repeat End | Repeat Left | Name | Family | Class |
| 42570241 | 42570497 | + | 32.0 | 5517 | 5802 | -438 | L1M5 | L1 | LINE |
| 42570498 | 42570801 | + | 11.2 | 1 | 304 | -6 | AluSg | Alu | SINE |
| 42570802 | 42570885 | + | 34.8 | 5803 | 5874 | -366 | L1M5 | L1 | LINE |

  

|  |  |  |  |  |  |  |  |  |  |
| --- | --- | --- | --- | --- | --- | --- | --- | --- | --- |
| chr9:42572477-42574078 | | | 1602 bp | | |  |  |  |  |
| Genome Start | Genome End | Strand | Div | Repeat Start | Repeat End | Repeat Left | Name | Family | Class |
| 42572477 | 42573166 | + | 9.1 | 4743 | 5443 | -711 | L1PA7 | L1 | LINE |
| 42573167 | 42573503 | + | 3.9 | 5815 | 6153 | -2 | L1PA4 | L1 | LINE |
| 42573504 | 42574078 | + | 8.5 | 5444 | 6006 | -148 | L1PA7 | L1 | LINE |

  

|  |  |  |  |  |  |  |  |  |  |
| --- | --- | --- | --- | --- | --- | --- | --- | --- | --- |
| chr9:42655059-42658458 | | | 3297 bp | | |  |  |  |  |
| Genome Start | Genome End | Strand | Div | Repeat Start | Repeat End | Repeat Left | Name | Family | Class |
| 42655059 | 42655171 | - | 22.7 | 1892 | 2009 | 0 | HAL1b | L1 | LINE |
| 42655172 | 42655446 | + | 12.0 | 1 | 275 | -37 | AluSx | Alu | SINE |
| 42655506 | 42655540 | - | 22.7 | 1859 | 1891 | -118 | HAL1b | L1 | LINE |
| 42655568 | 42655699 | + | 16.0 | 32 | 166 | -23 | MER5A | MER1\_type | DNA |
| 42655704 | 42655818 | - | 19.1 | 2304 | 2418 | 0 | Tigger1 | MER2\_type | DNA |
| 42655823 | 42655986 | + | 14.2 | 1 | 172 | -4 | FRAM | Alu | SINE |
| 42655987 | 42657271 | - | 9.7 | 964 | 2309 | -109 | Tigger1 | MER2\_type | DNA |
| 42657272 | 42657581 | + | 9.3 | 1 | 310 | 0 | AluSg | Alu | SINE |
| 42657582 | 42658134 | - | 9.7 | 398 | 963 | -1455 | Tigger1 | MER2\_type | DNA |
| 42658144 | 42658458 | - | 24.2 | 1095 | 1425 | -584 | HAL1b | L1 | LINE |

  

|  |  |  |  |  |  |  |  |  |  |
| --- | --- | --- | --- | --- | --- | --- | --- | --- | --- |
| chr9:42713448-42714128 | | | 681 bp | | |  |  |  |  |
| Genome Start | Genome End | Strand | Div | Repeat Start | Repeat End | Repeat Left | Name | Family | Class |
| 42713448 | 42713829 | - | 17.9 | 37 | 427 | -168 | LTR49 | ERV1 | LTR |
| 42713830 | 42714104 | + | 12.8 | 14 | 289 | -24 | AluSq | Alu | SINE |
| 42714105 | 42714128 | - | 17.9 | 12 | 36 | -559 | LTR49 | ERV1 | LTR |

  

|  |  |  |  |  |  |  |  |  |  |
| --- | --- | --- | --- | --- | --- | --- | --- | --- | --- |
| chr9:42718481-42719673 | | | 1146 bp | | |  |  |  |  |
| Genome Start | Genome End | Strand | Div | Repeat Start | Repeat End | Repeat Left | Name | Family | Class |
| 42718481 | 42718895 | + | 25.1 | 1 | 430 | -471 | MER54A | ERVL | LTR |
| 42718934 | 42719271 | - | 11.1 | 7 | 364 | 0 | THE1B | MaLR | LTR |
| 42719281 | 42719673 | + | 21.7 | 490 | 901 | 0 | MER54A | ERVL | LTR |

  

|  |  |  |  |  |  |  |  |  |  |
| --- | --- | --- | --- | --- | --- | --- | --- | --- | --- |
| chr9:42722147-42722960 | | | 814 bp | | |  |  |  |  |
| Genome Start | Genome End | Strand | Div | Repeat Start | Repeat End | Repeat Left | Name | Family | Class |
| 42722147 | 42722388 | - | 28.7 | 275 | 503 | -2 | MLT1D | MaLR | LTR |
| 42722389 | 42722689 | - | 12.6 | 1 | 303 | -8 | AluY | Alu | SINE |
| 42722690 | 42722960 | - | 28.7 | 5 | 274 | -231 | MLT1D | MaLR | LTR |

  

|  |  |  |  |  |  |  |  |  |  |
| --- | --- | --- | --- | --- | --- | --- | --- | --- | --- |
| chr9:42723755-42725249 | | | 1135 bp | | |  |  |  |  |
| Genome Start | Genome End | Strand | Div | Repeat Start | Repeat End | Repeat Left | Name | Family | Class |
| 42723755 | 42724150 | - | 26.4 | 3035 | 3438 | -3503 | L1MCa | L1 | LINE |
| 42724208 | 42724354 | - | 30.3 | 1851 | 1995 | -5133 | L1MCa | L1 | LINE |
| 42724588 | 42724881 | - | 24.0 | 73 | 365 | 0 | MLT1A0 | MaLR | LTR |
| 42724952 | 42725249 | - | 26.1 | 54 | 388 | -6035 | L1MCa | L1 | LINE |

  

|  |  |  |  |  |  |  |  |  |  |
| --- | --- | --- | --- | --- | --- | --- | --- | --- | --- |
| chr9:42750975-42753221 | | | 2246 bp | | |  |  |  |  |
| Genome Start | Genome End | Strand | Div | Repeat Start | Repeat End | Repeat Left | Name | Family | Class |
| 42750975 | 42751716 | - | 11.9 | 5512 | 6293 | -4 | L1MA5A | L1 | LINE |
| 42751717 | 42751833 | + | 15.8 | 168 | 292 | -20 | AluSg/x | Alu | SINE |
| 42751835 | 42753221 | - | 15.1 | 4106 | 5523 | -774 | L1MA5A | L1 | LINE |

  

|  |  |  |  |  |  |  |  |  |  |
| --- | --- | --- | --- | --- | --- | --- | --- | --- | --- |
| chr9:42759174-42761181 | | | 2005 bp | | |  |  |  |  |
| Genome Start | Genome End | Strand | Div | Repeat Start | Repeat End | Repeat Left | Name | Family | Class |
| 42759174 | 42759460 | + | 14.6 | 4771 | 5045 | -1098 | L1MA4 | L1 | LINE |
| 42759461 | 42759674 | + | 7.4 | 1 | 203 | -108 | AluY | Alu | SINE |
| 42759675 | 42759970 | + | 14.6 | 5046 | 5322 | -821 | L1MA4 | L1 | LINE |
| 42759971 | 42760007 | + | 18.0 | 33 | 65 | -111 | FRAM/FAM | Alu | SINE |
| 42760008 | 42760320 | - | 6.2 | 1 | 311 | 0 | AluY | Alu | SINE |
| 42760321 | 42760424 | + | 18.0 | 202 | 290 | -22 | AluJo/FRAM | Alu | SINE |
| 42760428 | 42761181 | + | 17.8 | 5288 | 6022 | -278 | L1MA4 | L1 | LINE |

  

|  |  |  |  |  |  |  |  |  |  |
| --- | --- | --- | --- | --- | --- | --- | --- | --- | --- |
| chr9:42763299-42764181 | | | 883 bp | | |  |  |  |  |
| Genome Start | Genome End | Strand | Div | Repeat Start | Repeat End | Repeat Left | Name | Family | Class |
| 42763299 | 42763490 | - | 22.3 | 5603 | 5796 | -370 | L1M5 | L1 | LINE |
| 42763491 | 42763796 | - | 5.9 | 1 | 307 | -4 | AluY | Alu | SINE |
| 42763797 | 42764181 | - | 22.4 | 5188 | 5602 | -564 | L1M5 | L1 | LINE |

  

|  |  |  |  |  |  |  |  |  |  |
| --- | --- | --- | --- | --- | --- | --- | --- | --- | --- |
| chr9:42804906-42806358 | | | 1382 bp | | |  |  |  |  |
| Genome Start | Genome End | Strand | Div | Repeat Start | Repeat End | Repeat Left | Name | Family | Class |
| 42804906 | 42805373 | - | 22.9 | 761 | 1227 | -5941 | L1M2a | L1 | LINE |
| 42805436 | 42805867 | + | 22.8 | 1 | 432 | 0 | MSTB1 | MaLR | LTR |
| 42805877 | 42806358 | - | 23.4 | 1 | 597 | -6571 | L1M2a | L1 | LINE |

  

|  |  |  |  |  |  |  |  |  |  |
| --- | --- | --- | --- | --- | --- | --- | --- | --- | --- |
| chr9:42849672-42850467 | | | 796 bp | | |  |  |  |  |
| Genome Start | Genome End | Strand | Div | Repeat Start | Repeat End | Repeat Left | Name | Family | Class |
| 42849672 | 42849794 | + | 20.8 | 2872 | 3003 | -416 | L2 | L2 | LINE |
| 42849795 | 42850140 | - | 12.5 | 1 | 312 | 0 | AluSx | Alu | SINE |
| 42850141 | 42850467 | + | 31.6 | 3004 | 3345 | -74 | L2 | L2 | LINE |

  

|  |  |  |  |  |  |  |  |  |  |
| --- | --- | --- | --- | --- | --- | --- | --- | --- | --- |
| chr9:42850511-42851346 | | | 770 bp | | |  |  |  |  |
| Genome Start | Genome End | Strand | Div | Repeat Start | Repeat End | Repeat Left | Name | Family | Class |
| 42850511 | 42850803 | + | 31.4 | 2351 | 2691 | -728 | L2 | L2 | LINE |
| 42850870 | 42851021 | + | 27.8 | 3182 | 3358 | -20 | L2 | L2 | LINE |
| 42851022 | 42851320 | - | 16.9 | 1 | 296 | -16 | AluSx | Alu | SINE |
| 42851321 | 42851346 | + | 27.8 | 3359 | 3378 | 0 | L2 | L2 | LINE |

  

|  |  |  |  |  |  |  |  |  |  |
| --- | --- | --- | --- | --- | --- | --- | --- | --- | --- |
| chr9:42861257-42861877 | | | 621 bp | | |  |  |  |  |
| Genome Start | Genome End | Strand | Div | Repeat Start | Repeat End | Repeat Left | Name | Family | Class |
| 42861257 | 42861406 | + | 17.8 | 1 | 142 | -170 | AluJb | Alu | SINE |
| 42861407 | 42861701 | + | 10.9 | 5 | 300 | -12 | AluSx | Alu | SINE |
| 42861702 | 42861877 | + | 17.8 | 143 | 309 | -3 | AluJb | Alu | SINE |

  

|  |  |  |  |  |  |  |  |  |  |
| --- | --- | --- | --- | --- | --- | --- | --- | --- | --- |
| chr9:42907834-42909437 | | | 1604 bp | | |  |  |  |  |
| Genome Start | Genome End | Strand | Div | Repeat Start | Repeat End | Repeat Left | Name | Family | Class |
| 42907834 | 42908410 | - | 8.4 | 5444 | 6006 | -148 | L1PA7 | L1 | LINE |
| 42908411 | 42908747 | - | 3.9 | 5815 | 6153 | -2 | L1PA4 | L1 | LINE |
| 42908748 | 42909437 | - | 9.1 | 4743 | 5443 | -711 | L1PA7 | L1 | LINE |

  

|  |  |  |  |  |  |  |  |  |  |
| --- | --- | --- | --- | --- | --- | --- | --- | --- | --- |
| chr9:42925479-42927205 | | | 1727 bp | | |  |  |  |  |
| Genome Start | Genome End | Strand | Div | Repeat Start | Repeat End | Repeat Left | Name | Family | Class |
| 42925479 | 42926643 | - | 15.8 | 427 | 1645 | -6 | THE1B-int | MaLR | LTR |
| 42926644 | 42926801 | - | 11.5 | 134 | 293 | -19 | AluSg/x | Alu | SINE |
| 42926802 | 42927205 | - | 15.8 | 5 | 426 | -1225 | THE1B-int | MaLR | LTR |

  

|  |  |  |  |  |  |  |  |  |  |
| --- | --- | --- | --- | --- | --- | --- | --- | --- | --- |
| chr9:42933710-42934161 | | | 452 bp | | |  |  |  |  |
| Genome Start | Genome End | Strand | Div | Repeat Start | Repeat End | Repeat Left | Name | Family | Class |
| 42933710 | 42933796 | + | 29.5 | 27 | 115 | -63 | MER5B | MER1\_type | DNA |
| 42933797 | 42934105 | - | 12.7 | 1 | 299 | -13 | AluSx | Alu | SINE |
| 42934106 | 42934161 | + | 29.5 | 116 | 172 | -6 | MER5B | MER1\_type | DNA |

  

|  |  |  |  |  |  |  |  |  |  |
| --- | --- | --- | --- | --- | --- | --- | --- | --- | --- |
| chr9:42965419-42972197 | | | 6725 bp | | |  |  |  |  |
| Genome Start | Genome End | Strand | Div | Repeat Start | Repeat End | Repeat Left | Name | Family | Class |
| 42965419 | 42965769 | - | 29.6 | 223 | 577 | -49 | MLT1E2 | MaLR | LTR |
| 42965802 | 42970310 | - | 19.7 | 2059 | 6686 | -1 | L1PREC2 | L1 | LINE |
| 42970308 | 42971210 | + | 18.8 | 2081 | 3005 | -5150 | L1PREC2 | L1 | LINE |
| 42971236 | 42971440 | + | 18.8 | 3006 | 3224 | -4931 | L1PREC2 | L1 | LINE |
| 42971441 | 42971802 | - | 13.1 | 1 | 381 | 0 | THE1D | MaLR | LTR |
| 42971803 | 42972019 | + | 18.8 | 3225 | 3433 | -4722 | L1PREC2 | L1 | LINE |
| 42972020 | 42972197 | - | 21.7 | 1 | 257 | -397 | MLT1E2 | MaLR | LTR |

  

|  |  |  |  |  |  |  |  |  |  |
| --- | --- | --- | --- | --- | --- | --- | --- | --- | --- |
| chr9:42979147-42980866 | | | 1720 bp | | |  |  |  |  |
| Genome Start | Genome End | Strand | Div | Repeat Start | Repeat End | Repeat Left | Name | Family | Class |
| 42979147 | 42979502 | - | 23.6 | 2019 | 2389 | -4119 | L1MCa | L1 | LINE |
| 42979503 | 42979858 | + | 22.4 | 1 | 365 | 0 | MLT1A0 | MaLR | LTR |
| 42979859 | 42980227 | - | 23.6 | 1602 | 2018 | -4490 | L1MCa | L1 | LINE |
| 42980228 | 42980484 | + | 11.8 | 1 | 256 | -55 | AluY | Alu | SINE |
| 42980485 | 42980866 | - | 23.6 | 1240 | 1601 | -4907 | L1MCa | L1 | LINE |

  

|  |  |  |  |  |  |  |  |  |  |
| --- | --- | --- | --- | --- | --- | --- | --- | --- | --- |
| chr9:43069814-43071552 | | | 1730 bp | | |  |  |  |  |
| Genome Start | Genome End | Strand | Div | Repeat Start | Repeat End | Repeat Left | Name | Family | Class |
| 43069814 | 43070808 | + | 19.1 | 4766 | 5836 | -342 | L1MB8 | L1 | LINE |
| 43070818 | 43071229 | + | 13.9 | 11 | 428 | 0 | MER61C | ERV1 | LTR |
| 43071230 | 43071552 | + | 20.4 | 5833 | 6173 | -5 | L1MB8 | L1 | LINE |

  

|  |  |  |  |  |  |  |  |  |  |
| --- | --- | --- | --- | --- | --- | --- | --- | --- | --- |
| chr9:43129042-43130543 | | | 1502 bp | | |  |  |  |  |
| Genome Start | Genome End | Strand | Div | Repeat Start | Repeat End | Repeat Left | Name | Family | Class |
| 43129042 | 43129558 | + | 24.5 | 18 | 528 | -407 | MER21C | ERV1 | LTR |
| 43129559 | 43129885 | - | 19.7 | 2 | 295 | -17 | AluJb | Alu | SINE |
| 43129886 | 43129963 | + | 24.5 | 529 | 601 | -334 | MER21C | ERV1 | LTR |
| 43129964 | 43130256 | + | 15.9 | 9 | 299 | -13 | AluJo | Alu | SINE |
| 43130257 | 43130543 | + | 24.5 | 602 | 916 | -19 | MER21C | ERV1 | LTR |

  

|  |  |  |  |  |  |  |  |  |  |
| --- | --- | --- | --- | --- | --- | --- | --- | --- | --- |
| chr9:43144994-43148609 | | | 3616 bp | | |  |  |  |  |
| Genome Start | Genome End | Strand | Div | Repeat Start | Repeat End | Repeat Left | Name | Family | Class |
| 43144994 | 43145849 | + | 17.4 | 3305 | 4175 | -3387 | L1M1 | L1 | LINE |
| 43145850 | 43146085 | - | 5.5 | 39 | 296 | -15 | AluY | Alu | SINE |
| 43146086 | 43148609 | + | 18.6 | 4176 | 6732 | -830 | L1M1 | L1 | LINE |

  

|  |  |  |  |  |  |  |  |  |  |
| --- | --- | --- | --- | --- | --- | --- | --- | --- | --- |
| chr9:43164742-43165578 | | | 837 bp | | |  |  |  |  |
| Genome Start | Genome End | Strand | Div | Repeat Start | Repeat End | Repeat Left | Name | Family | Class |
| 43164742 | 43164850 | + | 30.0 | 26 | 133 | -378 | MLT1J | MaLR | LTR |
| 43164851 | 43165220 | + | 13.2 | 1 | 381 | 0 | THE1D | MaLR | LTR |
| 43165221 | 43165578 | + | 30.0 | 134 | 511 | 0 | MLT1J | MaLR | LTR |

  

|  |  |  |  |  |  |  |  |  |  |
| --- | --- | --- | --- | --- | --- | --- | --- | --- | --- |
| chr9:43181224-43184214 | | | 2863 bp | | |  |  |  |  |
| Genome Start | Genome End | Strand | Div | Repeat Start | Repeat End | Repeat Left | Name | Family | Class |
| 43181224 | 43181361 | - | 35.5 | 2923 | 3066 | -312 | L2 | L2 | LINE |
| 43181391 | 43181534 | - | 22.2 | 1 | 156 | -4 | MER5A1 | MER1\_type | DNA |
| 43181584 | 43182018 | + | 22.6 | 6 | 466 | -1 | MLT1C | MaLR | LTR |
| 43182021 | 43182318 | - | 26.4 | 2576 | 2871 | -548 | L2 | L2 | LINE |
| 43182320 | 43182611 | + | 13.7 | 1 | 292 | -21 | AluSq | Alu | SINE |
| 43182613 | 43182885 | - | 8.0 | 5407 | 5673 | -481 | L1P3 | L1 | LINE |
| 43182886 | 43183353 | + | 19.9 | 1 | 458 | 0 | LTR22A | ERVK | LTR |
| 43183354 | 43183450 | - | 8.0 | 5308 | 5406 | -748 | L1P | L1 | LINE |
| 43183451 | 43183924 | + | 7.0 | 5680 | 6154 | 0 | L1PA7 | L1 | LINE |
| 43183970 | 43184029 | - | 26.3 | 2488 | 2546 | -873 | L2 | L2 | LINE |
| 43184031 | 43184214 | - | 27.4 | 2330 | 2513 | -906 | L2 | L2 | LINE |

  

|  |  |  |  |  |  |  |  |  |  |
| --- | --- | --- | --- | --- | --- | --- | --- | --- | --- |
| chr9:43267961-43269345 | | | 1114 bp | | |  |  |  |  |
| Genome Start | Genome End | Strand | Div | Repeat Start | Repeat End | Repeat Left | Name | Family | Class |
| 43267961 | 43268078 | - | 23.1 | 2747 | 3032 | -3097 | L1MEd | L1 | LINE |
| 43268224 | 43268512 | - | 10.4 | 1 | 289 | -20 | AluSc | Alu | SINE |
| 43268513 | 43268730 | - | 29.5 | 2534 | 2746 | -3383 | L1MEd | L1 | LINE |
| 43268733 | 43268897 | - | 16.5 | 5986 | 6167 | -4 | L1MB2 | L1 | LINE |
| 43269022 | 43269345 | - | 37.9 | 223 | 566 | -5553 | L1MEd | L1 | LINE |

  

|  |  |  |  |  |  |  |  |  |  |
| --- | --- | --- | --- | --- | --- | --- | --- | --- | --- |
| chr9:43280399-43287133 | | | 6703 bp | | |  |  |  |  |
| Genome Start | Genome End | Strand | Div | Repeat Start | Repeat End | Repeat Left | Name | Family | Class |
| 43280399 | 43284278 | - | 20.6 | 1656 | 5654 | 0 | HERVL-B4 | ERVL | LTR |
| 43284279 | 43284671 | + | 13.8 | 1 | 422 | -6 | MSTA | MaLR | LTR |
| 43284676 | 43285098 | - | 19.5 | 1216 | 1662 | -4052 | HERVL-B4 | ERVL | LTR |
| 43285099 | 43285340 | + | 16.1 | 1 | 251 | -2072 | Tigger5 | MER2\_type | DNA |
| 43285368 | 43285575 | - | 12.6 | 5936 | 6151 | 0 | L1PB1 | L1 | LINE |
| 43285576 | 43285968 | + | 14.5 | 2007 | 2405 | 0 | Tigger5 | MER2\_type | DNA |
| 43285970 | 43287133 | - | 17.1 | 4 | 1217 | -4497 | HERVL-B4 | ERVL | LTR |

  

|  |  |  |  |  |  |  |  |  |  |
| --- | --- | --- | --- | --- | --- | --- | --- | --- | --- |
| chr9:43309182-43310226 | | | 1049 bp | | |  |  |  |  |
| Genome Start | Genome End | Strand | Div | Repeat Start | Repeat End | Repeat Left | Name | Family | Class |
| 43309182 | 43309411 | - | 29.6 | 5907 | 6132 | -34 | L1ME1 | L1 | LINE |
| 43309411 | 43309832 | + | 17.7 | 2295 | 2722 | -3654 | L1M4c | L1 | LINE |
| 43309830 | 43310226 | - | 24.1 | 4631 | 5091 | -1055 | L1ME1 | L1 | LINE |

  

|  |  |  |  |  |  |  |  |  |  |
| --- | --- | --- | --- | --- | --- | --- | --- | --- | --- |
| chr9:43318194-43318949 | | | 643 bp | | |  |  |  |  |
| Genome Start | Genome End | Strand | Div | Repeat Start | Repeat End | Repeat Left | Name | Family | Class |
| 43318194 | 43318316 | - | 30.0 | 114 | 241 | -21 | MIR | MIR | SINE |
| 43318324 | 43318757 | + | 17.2 | 1 | 511 | -45 | MLT2B4 | ERVL | LTR |
| 43318864 | 43318949 | - | 18.6 | 21 | 130 | -132 | MIR | MIR | SINE |

  

|  |  |  |  |  |  |  |  |  |  |
| --- | --- | --- | --- | --- | --- | --- | --- | --- | --- |
| chr9:43319010-43319538 | | | 529 bp | | |  |  |  |  |
| Genome Start | Genome End | Strand | Div | Repeat Start | Repeat End | Repeat Left | Name | Family | Class |
| 43319010 | 43319166 | + | 13.6 | 1 | 161 | -41 | MER6C | MER2\_type | DNA |
| 43319167 | 43319486 | - | 9.5 | 1 | 305 | -6 | AluY | Alu | SINE |
| 43319487 | 43319538 | + | 13.6 | 162 | 202 | 0 | MER6C | MER2\_type | DNA |

  

|  |  |  |  |  |  |  |  |  |  |
| --- | --- | --- | --- | --- | --- | --- | --- | --- | --- |
| chr9:43327927-43329295 | | | 1128 bp | | |  |  |  |  |
| Genome Start | Genome End | Strand | Div | Repeat Start | Repeat End | Repeat Left | Name | Family | Class |
| 43327927 | 43328065 | + | 36.2 | 1284 | 1434 | -1985 | L2 | L2 | LINE |
| 43328110 | 43328212 | - | 30.6 | 3274 | 3378 | 0 | L2 | L2 | LINE |
| 43328195 | 43328685 | + | 35.6 | 1571 | 2075 | -1344 | L2 | L2 | LINE |
| 43328708 | 43328991 | - | 13.4 | 1 | 283 | -30 | AluSq | Alu | SINE |
| 43329185 | 43329295 | + | 30.6 | 3251 | 3379 | -21 | L2 | L2 | LINE |

  

|  |  |  |  |  |  |  |  |  |  |
| --- | --- | --- | --- | --- | --- | --- | --- | --- | --- |
| chr9:43330353-43330964 | | | 612 bp | | |  |  |  |  |
| Genome Start | Genome End | Strand | Div | Repeat Start | Repeat End | Repeat Left | Name | Family | Class |
| 43330353 | 43330498 | - | 20.5 | 6019 | 6170 | -8 | L1MB8 | L1 | LINE |
| 43330499 | 43330766 | + | 9.7 | 1 | 305 | -5 | AluSg | Alu | SINE |
| 43330767 | 43330964 | - | 20.5 | 5815 | 6018 | -160 | L1MB8 | L1 | LINE |

  

|  |  |  |  |  |  |  |  |  |  |
| --- | --- | --- | --- | --- | --- | --- | --- | --- | --- |
| chr9:43353554-43358504 | | | 4619 bp | | |  |  |  |  |
| Genome Start | Genome End | Strand | Div | Repeat Start | Repeat End | Repeat Left | Name | Family | Class |
| 43353554 | 43353747 | + | 29.9 | 1351 | 1560 | -1859 | L2 | L2 | LINE |
| 43353757 | 43354034 | + | 19.0 | 2 | 341 | -67 | MLT1A1 | MaLR | LTR |
| 43354035 | 43354345 | - | 12.7 | 1 | 313 | 0 | AluSq | Alu | SINE |
| 43354346 | 43354412 | + | 19.0 | 342 | 407 | -1 | MLT1A1 | MaLR | LTR |
| 43354415 | 43356156 | + | 22.1 | 2 | 1729 | -6 | MLT1A1-int | MaLR | LTR |
| 43356162 | 43356295 | + | 19.6 | 1 | 137 | -271 | MLT1A1 | MaLR | LTR |
| 43356296 | 43356645 | - | 20.2 | 1 | 396 | 0 | MSTD | MaLR | LTR |
| 43356646 | 43356848 | + | 20.6 | 138 | 396 | 0 | MLT1A1 | MaLR | LTR |
| 43356849 | 43357680 | + | 30.3 | 1564 | 2598 | -821 | L2 | L2 | LINE |
| 43357783 | 43358065 | - | 30.6 | 4 | 290 | -259 | MLT1H | MaLR | LTR |
| 43358280 | 43358504 | + | 33.5 | 2949 | 3176 | -243 | L2 | L2 | LINE |

  

|  |  |  |  |  |  |  |  |  |  |
| --- | --- | --- | --- | --- | --- | --- | --- | --- | --- |
| chr9:43360601-43361509 | | | 872 bp | | |  |  |  |  |
| Genome Start | Genome End | Strand | Div | Repeat Start | Repeat End | Repeat Left | Name | Family | Class |
| 43360601 | 43360677 | + | 26.6 | 4865 | 4938 | -1208 | L1M5 | L1 | LINE |
| 43360678 | 43360881 | + | 21.8 | 1 | 194 | -220 | MLT2D | ERVL | LTR |
| 43360882 | 43361165 | + | 6.7 | 1 | 287 | -24 | AluY | Alu | SINE |
| 43361166 | 43361353 | + | 21.8 | 195 | 363 | -51 | MLT2D | ERVL | LTR |
| 43361391 | 43361441 | + | 21.8 | 510 | 560 | 0 | MLT2D | ERVL | LTR |
| 43361442 | 43361509 | + | 26.6 | 4939 | 5004 | -1142 | L1M5 | L1 | LINE |

  

|  |  |  |  |  |  |  |  |  |  |
| --- | --- | --- | --- | --- | --- | --- | --- | --- | --- |
| chr9:43372993-43380349 | | | 6684 bp | | |  |  |  |  |
| Genome Start | Genome End | Strand | Div | Repeat Start | Repeat End | Repeat Left | Name | Family | Class |
| 43372993 | 43373137 | - | 22.1 | 6649 | 6801 | 0 | MER89-int | ERV1 | LTR |
| 43373139 | 43373574 | + | 11.3 | 1 | 436 | -231 | LTR12B | ERV1 | LTR |
| 43373764 | 43373916 | - | 27.8 | 1603 | 1755 | 0 | MER52A | ERV1 | LTR |
| 43373925 | 43374551 | - | 24.9 | 54 | 1470 | -162 | MER52A | ERV1 | LTR |
| 43374552 | 43375032 | - | 20.8 | 6052 | 6559 | -242 | MER89-int | ERV1 | LTR |
| 43375132 | 43375427 | + | 10.9 | 1 | 293 | -18 | AluY | Alu | SINE |
| 43375576 | 43376985 | - | 24.5 | 2549 | 4047 | -2904 | MER89-int | ERV1 | LTR |
| 43377214 | 43377421 | - | 27.1 | 2315 | 2536 | -4265 | MER89-int | ERV1 | LTR |
| 43377422 | 43377736 | - | 15.6 | 1 | 296 | -16 | AluSx | Alu | SINE |
| 43377737 | 43377967 | - | 27.1 | 2077 | 2314 | -4487 | MER89-int | ERV1 | LTR |
| 43377968 | 43378292 | + | 15.3 | 1 | 311 | -1 | AluSx | Alu | SINE |
| 43378293 | 43380349 | - | 27.1 | 2 | 2076 | -4725 | MER89-int | ERV1 | LTR |

  

|  |  |  |  |  |  |  |  |  |  |
| --- | --- | --- | --- | --- | --- | --- | --- | --- | --- |
| chr9:43386199-43387345 | | | 1147 bp | | |  |  |  |  |
| Genome Start | Genome End | Strand | Div | Repeat Start | Repeat End | Repeat Left | Name | Family | Class |
| 43386199 | 43386245 | - | 23.3 | 304 | 340 | -1 | MER58B | MER1\_type | DNA |
| 43386246 | 43386752 | + | 17.4 | 2 | 550 | 0 | MER44B | MER2\_type | DNA |
| 43386753 | 43386804 | - | 23.3 | 256 | 303 | -38 | MER58B | MER1\_type | DNA |
| 43386805 | 43387289 | + | 15.8 | 3 | 489 | 0 | LTR45B | ERV1 | LTR |
| 43387290 | 43387345 | - | 23.3 | 208 | 255 | -86 | MER58B | MER1\_type | DNA |

  

|  |  |  |  |  |  |  |  |  |  |
| --- | --- | --- | --- | --- | --- | --- | --- | --- | --- |
| chr9:43387626-43388171 | | | 546 bp | | |  |  |  |  |
| Genome Start | Genome End | Strand | Div | Repeat Start | Repeat End | Repeat Left | Name | Family | Class |
| 43387626 | 43387696 | - | 20.5 | 5820 | 5886 | -278 | L1ME2 | L1 | LINE |
| 43387697 | 43387986 | + | 9.7 | 1 | 291 | -21 | AluSx | Alu | SINE |
| 43387987 | 43388171 | - | 20.5 | 5608 | 5819 | -345 | L1ME2 | L1 | LINE |

  

|  |  |  |  |  |  |  |  |  |  |
| --- | --- | --- | --- | --- | --- | --- | --- | --- | --- |
| chr9:43392667-43393497 | | | 618 bp | | |  |  |  |  |
| Genome Start | Genome End | Strand | Div | Repeat Start | Repeat End | Repeat Left | Name | Family | Class |
| 43392667 | 43392722 | + | 21.4 | 4039 | 4097 | -2049 | L1M4 | L1 | LINE |
| 43392935 | 43393264 | - | 18.3 | 1 | 386 | -515 | MER54B | ERVL | LTR |
| 43393266 | 43393497 | + | 19.1 | 4094 | 4321 | -1825 | L1M4 | L1 | LINE |

  

|  |  |  |  |  |  |  |  |  |  |
| --- | --- | --- | --- | --- | --- | --- | --- | --- | --- |
| chr9:43399247-43400701 | | | 1455 bp | | |  |  |  |  |
| Genome Start | Genome End | Strand | Div | Repeat Start | Repeat End | Repeat Left | Name | Family | Class |
| 43399247 | 43400144 | + | 19.1 | 4317 | 5158 | -988 | L1M4 | L1 | LINE |
| 43400145 | 43400433 | - | 14.9 | 1 | 290 | -22 | AluSx | Alu | SINE |
| 43400434 | 43400701 | + | 22.0 | 5159 | 5437 | -741 | L1M4 | L1 | LINE |

  

|  |  |  |  |  |  |  |  |  |  |
| --- | --- | --- | --- | --- | --- | --- | --- | --- | --- |
| chr9:43405609-43406664 | | | 1056 bp | | |  |  |  |  |
| Genome Start | Genome End | Strand | Div | Repeat Start | Repeat End | Repeat Left | Name | Family | Class |
| 43405609 | 43406213 | + | 13.4 | 1 | 617 | -166 | LTR39 | ERV1 | LTR |
| 43406214 | 43406516 | + | 5.0 | 1 | 303 | 0 | AluY | Alu | SINE |
| 43406517 | 43406664 | + | 13.4 | 618 | 765 | -18 | LTR39 | ERV1 | LTR |

  

|  |  |  |  |  |  |  |  |  |  |
| --- | --- | --- | --- | --- | --- | --- | --- | --- | --- |
| chr9:43422737-43423967 | | | 1259 bp | | |  |  |  |  |
| Genome Start | Genome End | Strand | Div | Repeat Start | Repeat End | Repeat Left | Name | Family | Class |
| 43422737 | 43422863 | + | 21.4 | 1 | 138 | -569 | MER39 | ERV1 | LTR |
| 43422836 | 43422979 | + | 16.8 | 260 | 397 | -310 | MER39 | ERV1 | LTR |
| 43422980 | 43423648 | + | 13.7 | 1 | 691 | 0 | LTR8 | ERV1 | LTR |
| 43423649 | 43423967 | + | 16.8 | 398 | 705 | -2 | MER39 | ERV1 | LTR |

  

|  |  |  |  |  |  |  |  |  |  |
| --- | --- | --- | --- | --- | --- | --- | --- | --- | --- |
| chr9:43435058-43436287 | | | 1230 bp | | |  |  |  |  |
| Genome Start | Genome End | Strand | Div | Repeat Start | Repeat End | Repeat Left | Name | Family | Class |
| 43435058 | 43435109 | - | 16.9 | 302 | 364 | -2 | Tigger5a | MER2\_type | DNA |
| 43435110 | 43435284 | - | 7.6 | 388 | 554 | 0 | MER41A | ERV1 | LTR |
| 43435285 | 43435585 | - | 11.3 | 1 | 301 | -9 | AluSg | Alu | SINE |
| 43435586 | 43435969 | - | 7.6 | 1 | 387 | -167 | MER41A | ERV1 | LTR |
| 43435970 | 43436287 | - | 16.9 | 5 | 301 | -65 | Tigger5a | MER2\_type | DNA |

  

|  |  |  |  |  |  |  |  |  |  |
| --- | --- | --- | --- | --- | --- | --- | --- | --- | --- |
| chr9:43439650-43440372 | | | 723 bp | | |  |  |  |  |
| Genome Start | Genome End | Strand | Div | Repeat Start | Repeat End | Repeat Left | Name | Family | Class |
| 43439650 | 43439868 | - | 17.7 | 195 | 426 | 0 | MSTB | MaLR | LTR |
| 43439869 | 43440185 | - | 14.9 | 1 | 317 | 0 | AluSx | Alu | SINE |
| 43440186 | 43440372 | - | 17.7 | 8 | 194 | -232 | MSTB | MaLR | LTR |

  

|  |  |  |  |  |  |  |  |  |  |
| --- | --- | --- | --- | --- | --- | --- | --- | --- | --- |
| chr9:43441815-43442479 | | | 665 bp | | |  |  |  |  |
| Genome Start | Genome End | Strand | Div | Repeat Start | Repeat End | Repeat Left | Name | Family | Class |
| 43441815 | 43442082 | + | 28.0 | 5689 | 5948 | -292 | L1ME3B | L1 | LINE |
| 43442083 | 43442390 | + | 7.5 | 1 | 307 | -4 | AluY | Alu | SINE |
| 43442391 | 43442479 | + | 28.0 | 5949 | 6048 | -192 | L1ME3B | L1 | LINE |

  

|  |  |  |  |  |  |  |  |  |  |
| --- | --- | --- | --- | --- | --- | --- | --- | --- | --- |
| chr9:43449745-43450655 | | | 911 bp | | |  |  |  |  |
| Genome Start | Genome End | Strand | Div | Repeat Start | Repeat End | Repeat Left | Name | Family | Class |
| 43449745 | 43450135 | + | 26.6 | 1 | 436 | -32 | MER110A | ERV1 | LTR |
| 43450136 | 43450634 | - | 22.1 | 1 | 505 | 0 | MLT1D | MaLR | LTR |
| 43450635 | 43450655 | + | 26.6 | 437 | 456 | -12 | MER110A | ERV1 | LTR |

  

|  |  |  |  |  |  |  |  |  |  |
| --- | --- | --- | --- | --- | --- | --- | --- | --- | --- |
| chr9:43451499-43453976 | | | 2419 bp | | |  |  |  |  |
| Genome Start | Genome End | Strand | Div | Repeat Start | Repeat End | Repeat Left | Name | Family | Class |
| 43451499 | 43452043 | - | 21.3 | 59 | 628 | -25 | MER82 | MER2\_type | DNA |
| 43452044 | 43452188 | + | 23.6 | 1 | 137 | -650 | LTR48 | ERV1 | LTR |
| 43452189 | 43452558 | - | 15.2 | 1 | 364 | 0 | THE1B | MaLR | LTR |
| 43452559 | 43452677 | + | 23.6 | 138 | 235 | -552 | LTR48 | ERV1 | LTR |
| 43452734 | 43453043 | - | 10.4 | 1 | 308 | -2 | AluSg | Alu | SINE |
| 43453046 | 43453355 | - | 7.2 | 1 | 307 | -4 | AluY | Alu | SINE |
| 43453356 | 43453914 | + | 17.3 | 219 | 787 | 0 | LTR48 | ERV1 | LTR |
| 43453916 | 43453976 | - | 19.7 | 1 | 61 | -592 | MER82 | MER2\_type | DNA |

  

|  |  |  |  |  |  |  |  |  |  |
| --- | --- | --- | --- | --- | --- | --- | --- | --- | --- |
| chr9:43454404-43456065 | | | 1619 bp | | |  |  |  |  |
| Genome Start | Genome End | Strand | Div | Repeat Start | Repeat End | Repeat Left | Name | Family | Class |
| 43454404 | 43454821 | - | 19.0 | 276 | 711 | 0 | MER67C | ERV1 | LTR |
| 43454858 | 43455330 | - | 13.8 | 109 | 571 | -39 | MER4B | ERV1 | LTR |
| 43455331 | 43455669 | - | 13.3 | 1 | 343 | 0 | MER61A | ERV1 | LTR |
| 43455670 | 43455788 | - | 13.8 | 1 | 108 | -502 | MER4B | ERV1 | LTR |
| 43455796 | 43456065 | - | 25.0 | 1 | 272 | -439 | MER67C | ERV1 | LTR |

  

|  |  |  |  |  |  |  |  |  |  |
| --- | --- | --- | --- | --- | --- | --- | --- | --- | --- |
| chr9:43468564-43469520 | | | 957 bp | | |  |  |  |  |
| Genome Start | Genome End | Strand | Div | Repeat Start | Repeat End | Repeat Left | Name | Family | Class |
| 43468564 | 43468670 | - | 22.0 | 522 | 626 | 0 | MLT1E2 | MaLR | LTR |
| 43468671 | 43468983 | + | 10.2 | 1 | 313 | 0 | AluSq | Alu | SINE |
| 43468984 | 43469520 | - | 22.2 | 1 | 523 | -101 | MLT1E2 | MaLR | LTR |

  

|  |  |  |  |  |  |  |  |  |  |
| --- | --- | --- | --- | --- | --- | --- | --- | --- | --- |
| chr9:43473327-43474052 | | | 726 bp | | |  |  |  |  |
| Genome Start | Genome End | Strand | Div | Repeat Start | Repeat End | Repeat Left | Name | Family | Class |
| 43473327 | 43473666 | - | 35.6 | 2438 | 2822 | -597 | L2 | L2 | LINE |
| 43473667 | 43473948 | + | 10.4 | 18 | 297 | -15 | AluSx | Alu | SINE |
| 43473949 | 43474052 | - | 35.6 | 2347 | 2437 | -982 | L2 | L2 | LINE |

  

|  |  |  |  |  |  |  |  |  |  |
| --- | --- | --- | --- | --- | --- | --- | --- | --- | --- |
| chr9:43477291-43477906 | | | 616 bp | | |  |  |  |  |
| Genome Start | Genome End | Strand | Div | Repeat Start | Repeat End | Repeat Left | Name | Family | Class |
| 43477291 | 43477462 | - | 13.3 | 128 | 295 | -17 | AluSx | Alu | SINE |
| 43477463 | 43477773 | - | 8.1 | 1 | 308 | -3 | AluY | Alu | SINE |
| 43477774 | 43477906 | - | 13.3 | 3 | 127 | -185 | AluSx | Alu | SINE |

  

|  |  |  |  |  |  |  |  |  |  |
| --- | --- | --- | --- | --- | --- | --- | --- | --- | --- |
| chr9:43511839-43513214 | | | 1349 bp | | |  |  |  |  |
| Genome Start | Genome End | Strand | Div | Repeat Start | Repeat End | Repeat Left | Name | Family | Class |
| 43511839 | 43511958 | - | 30.8 | 5168 | 5283 | -863 | L1M5 | L1 | LINE |
| 43511959 | 43512251 | + | 6.8 | 1 | 295 | -16 | AluY | Alu | SINE |
| 43512252 | 43512471 | - | 30.8 | 4913 | 5167 | -979 | L1M5 | L1 | LINE |
| 43512472 | 43512482 | - | 7.8 | 285 | 312 | 0 | AluSx | Alu | SINE |
| 43512510 | 43512806 | - | 7.8 | 2 | 284 | -28 | AluSx | Alu | SINE |
| 43512807 | 43513214 | - | 30.8 | 4478 | 4912 | -1234 | L1M5 | L1 | LINE |

  

|  |  |  |  |  |  |  |  |  |  |
| --- | --- | --- | --- | --- | --- | --- | --- | --- | --- |
| chr9:43524088-43526705 | | | 2618 bp | | |  |  |  |  |
| Genome Start | Genome End | Strand | Div | Repeat Start | Repeat End | Repeat Left | Name | Family | Class |
| 43524088 | 43525051 | - | 9.5 | 4545 | 5480 | -680 | L1PREC2 | L1 | LINE |
| 43525052 | 43525352 | - | 11.0 | 1 | 301 | -8 | AluSc | Alu | SINE |
| 43525353 | 43526705 | - | 10.3 | 3192 | 4544 | -1602 | L1PREC2 | L1 | LINE |

  

|  |  |  |  |  |  |  |  |  |  |
| --- | --- | --- | --- | --- | --- | --- | --- | --- | --- |
| chr9:43535935-43538140 | | | 2206 bp | | |  |  |  |  |
| Genome Start | Genome End | Strand | Div | Repeat Start | Repeat End | Repeat Left | Name | Family | Class |
| 43535935 | 43536113 | + | 18.6 | 3776 | 3964 | -2182 | L1MC | L1 | LINE |
| 43536114 | 43536432 | + | 13.7 | 1 | 308 | -2 | AluSg | Alu | SINE |
| 43536433 | 43536676 | + | 18.6 | 3965 | 4189 | -1957 | L1MC | L1 | LINE |
| 43536677 | 43536986 | + | 8.4 | 1 | 310 | -1 | AluY | Alu | SINE |
| 43536987 | 43538140 | + | 18.6 | 4190 | 5331 | -815 | L1MC | L1 | LINE |

  

|  |  |  |  |  |  |  |  |  |  |
| --- | --- | --- | --- | --- | --- | --- | --- | --- | --- |
| chr9:43553322-43554127 | | | 792 bp | | |  |  |  |  |
| Genome Start | Genome End | Strand | Div | Repeat Start | Repeat End | Repeat Left | Name | Family | Class |
| 43553322 | 43553653 | + | 17.7 | 1 | 331 | -59 | MLT1B | MaLR | LTR |
| 43553654 | 43553698 | - | 0.0 | 5770 | 5814 | -358 | L1P3 | L1 | LINE |
| 43553699 | 43554039 | + | 1.2 | 5815 | 6155 | 0 | L1PA3 | L1 | LINE |
| 43554054 | 43554127 | + | 10.8 | 317 | 390 | 0 | MLT1B | MaLR | LTR |

  

|  |  |  |  |  |  |  |  |  |  |
| --- | --- | --- | --- | --- | --- | --- | --- | --- | --- |
| chr9:43575516-43578245 | | | 2730 bp | | |  |  |  |  |
| Genome Start | Genome End | Strand | Div | Repeat Start | Repeat End | Repeat Left | Name | Family | Class |
| 43575516 | 43575954 | - | 17.6 | 4645 | 5099 | -2460 | L1M1 | L1 | LINE |
| 43575955 | 43576264 | - | 9.8 | 1 | 317 | 0 | AluY | Alu | SINE |
| 43576265 | 43577514 | - | 18.8 | 3349 | 4644 | -2915 | L1M1 | L1 | LINE |
| 43577515 | 43577850 | + | 18.4 | 4 | 345 | -1 | MER7A | MER2\_type | DNA |
| 43577851 | 43578245 | - | 24.8 | 2917 | 3348 | -4217 | L1M1 | L1 | LINE |

  

|  |  |  |  |  |  |  |  |  |  |
| --- | --- | --- | --- | --- | --- | --- | --- | --- | --- |
| chr9:43614253-43615821 | | | 1569 bp | | |  |  |  |  |
| Genome Start | Genome End | Strand | Div | Repeat Start | Repeat End | Repeat Left | Name | Family | Class |
| 43614253 | 43614469 | - | 27.2 | 3953 | 4171 | -3394 | L1M1 | L1 | LINE |
| 43614470 | 43614770 | + | 10.3 | 1 | 300 | -13 | AluSq | Alu | SINE |
| 43614771 | 43615821 | - | 23.6 | 2836 | 3952 | -3613 | L1M1 | L1 | LINE |

  

|  |  |  |  |  |  |  |  |  |  |
| --- | --- | --- | --- | --- | --- | --- | --- | --- | --- |
| chr9:43616145-43617056 | | | 912 bp | | |  |  |  |  |
| Genome Start | Genome End | Strand | Div | Repeat Start | Repeat End | Repeat Left | Name | Family | Class |
| 43616145 | 43616523 | - | 12.3 | 5788 | 6158 | -7 | L1PA15 | L1 | LINE |
| 43616524 | 43616829 | - | 10.8 | 1 | 306 | -5 | AluY | Alu | SINE |
| 43616830 | 43617056 | - | 12.3 | 5522 | 5787 | -378 | L1PA15 | L1 | LINE |

  

|  |  |  |  |  |  |  |  |  |  |
| --- | --- | --- | --- | --- | --- | --- | --- | --- | --- |
| chr9:43642700-43643355 | | | 585 bp | | |  |  |  |  |
| Genome Start | Genome End | Strand | Div | Repeat Start | Repeat End | Repeat Left | Name | Family | Class |
| 43642700 | 43642920 | - | 31.1 | 312 | 576 | -15 | MLT1K | MaLR | LTR |
| 43642937 | 43643122 | + | 25.9 | 3218 | 3419 | 0 | L2 | L2 | LINE |
| 43643178 | 43643355 | - | 28.3 | 91 | 268 | -323 | MLT1K | MaLR | LTR |

  

|  |  |  |  |  |  |  |  |  |  |
| --- | --- | --- | --- | --- | --- | --- | --- | --- | --- |
| chr9:43712144-43717924 | | | 5781 bp | | |  |  |  |  |
| Genome Start | Genome End | Strand | Div | Repeat Start | Repeat End | Repeat Left | Name | Family | Class |
| 43712144 | 43717102 | - | 5.9 | 1181 | 6149 | -5 | L1PA5 | L1 | LINE |
| 43717103 | 43717147 | - | 2.2 | 6110 | 6154 | -1 | L1PA2 | L1 | LINE |
| 43717148 | 43717924 | - | 7.3 | 384 | 1180 | -1095 | L1PA5 | L1 | LINE |

  

|  |  |  |  |  |  |  |  |  |  |
| --- | --- | --- | --- | --- | --- | --- | --- | --- | --- |
| chr9:43765549-43766232 | | | 684 bp | | |  |  |  |  |
| Genome Start | Genome End | Strand | Div | Repeat Start | Repeat End | Repeat Left | Name | Family | Class |
| 43765549 | 43765835 | - | 18.6 | 87 | 426 | 0 | LTR37A | ERV1 | LTR |
| 43765836 | 43766146 | + | 7.5 | 1 | 309 | -2 | AluY | Alu | SINE |
| 43766147 | 43766232 | - | 18.6 | 18 | 86 | -340 | LTR37A | ERV1 | LTR |

  

|  |  |  |  |  |  |  |  |  |  |
| --- | --- | --- | --- | --- | --- | --- | --- | --- | --- |
| chr9:43797882-43798535 | | | 654 bp | | |  |  |  |  |
| Genome Start | Genome End | Strand | Div | Repeat Start | Repeat End | Repeat Left | Name | Family | Class |
| 43797882 | 43797978 | + | 17.8 | 1 | 111 | -255 | Tigger5a | MER2\_type | DNA |
| 43797979 | 43798280 | - | 10.3 | 1 | 300 | -9 | AluSc | Alu | SINE |
| 43798281 | 43798535 | + | 17.8 | 112 | 366 | 0 | Tigger5a | MER2\_type | DNA |

  

|  |  |  |  |  |  |  |  |  |  |
| --- | --- | --- | --- | --- | --- | --- | --- | --- | --- |
| chr9:43807442-43811740 | | | 4308 bp | | |  |  |  |  |
| Genome Start | Genome End | Strand | Div | Repeat Start | Repeat End | Repeat Left | Name | Family | Class |
| 43807442 | 43807838 | - | 15.6 | 3898 | 4312 | -2252 | L1MCb | L1 | LINE |
| 43807833 | 43808611 | - | 20.0 | 2593 | 3356 | -3208 | L1MCb | L1 | LINE |
| 43808612 | 43809650 | - | 4.6 | 5107 | 6151 | -3 | L1PA5 | L1 | LINE |
| 43809648 | 43810731 | - | 20.1 | 1442 | 2599 | -4152 | L1MCb | L1 | LINE |
| 43810732 | 43811039 | + | 5.2 | 1 | 306 | -5 | AluY | Alu | SINE |
| 43811040 | 43811740 | - | 21.0 | 634 | 1441 | -5310 | L1MCb | L1 | LINE |

  

|  |  |  |  |  |  |  |  |  |  |
| --- | --- | --- | --- | --- | --- | --- | --- | --- | --- |
| chr9:43812848-43814572 | | | 1734 bp | | |  |  |  |  |
| Genome Start | Genome End | Strand | Div | Repeat Start | Repeat End | Repeat Left | Name | Family | Class |
| 43812848 | 43813546 | - | 21.0 | 1942 | 2888 | -3649 | L1MDa | L1 | LINE |
| 43813547 | 43813847 | - | 7.0 | 1 | 305 | -6 | AluY | Alu | SINE |
| 43813848 | 43814435 | - | 22.5 | 1327 | 1941 | -4692 | L1MDa | L1 | LINE |
| 43814427 | 43814572 | - | 24.1 | 140 | 295 | -6245 | L1MDa | L1 | LINE |

  

|  |  |  |  |  |  |  |  |  |  |
| --- | --- | --- | --- | --- | --- | --- | --- | --- | --- |
| chr9:43820613-43821488 | | | 876 bp | | |  |  |  |  |
| Genome Start | Genome End | Strand | Div | Repeat Start | Repeat End | Repeat Left | Name | Family | Class |
| 43820613 | 43820996 | + | 27.8 | 4154 | 4544 | -1602 | L1M5 | L1 | LINE |
| 43820997 | 43821212 | + | 21.1 | 1 | 218 | -1 | MER20 | MER1\_type | DNA |
| 43821213 | 43821488 | + | 27.8 | 4545 | 4828 | -1318 | L1M5 | L1 | LINE |

  

|  |  |  |  |  |  |  |  |  |  |
| --- | --- | --- | --- | --- | --- | --- | --- | --- | --- |
| chr9:43825646-43827356 | | | 1331 bp | | |  |  |  |  |
| Genome Start | Genome End | Strand | Div | Repeat Start | Repeat End | Repeat Left | Name | Family | Class |
| 43825646 | 43825742 | - | 30.2 | 1046 | 1143 | -866 | HAL1b | L1 | LINE |
| 43825954 | 43826270 | + | 21.8 | 152 | 462 | 0 | LTR40b | ERVL | LTR |
| 43826277 | 43826776 | - | 26.6 | 432 | 1002 | -1007 | HAL1b | L1 | LINE |
| 43826940 | 43827356 | - | 31.4 | 7 | 411 | -1598 | HAL1b | L1 | LINE |

  

|  |  |  |  |  |  |  |  |  |  |
| --- | --- | --- | --- | --- | --- | --- | --- | --- | --- |
| chr9:43828141-43829634 | | | 1494 bp | | |  |  |  |  |
| Genome Start | Genome End | Strand | Div | Repeat Start | Repeat End | Repeat Left | Name | Family | Class |
| 43828141 | 43828770 | + | 23.3 | 6459 | 7099 | -943 | L1MC4 | L1 | LINE |
| 43828771 | 43829073 | - | 6.7 | 1 | 303 | 0 | AluY | Alu | SINE |
| 43829074 | 43829634 | + | 23.3 | 7100 | 7678 | -364 | L1MC4 | L1 | LINE |

  

|  |  |  |  |  |  |  |  |  |  |
| --- | --- | --- | --- | --- | --- | --- | --- | --- | --- |
| chr9:43836260-43840877 | | | 4595 bp | | |  |  |  |  |
| Genome Start | Genome End | Strand | Div | Repeat Start | Repeat End | Repeat Left | Name | Family | Class |
| 43836260 | 43837107 | + | 26.6 | 10 | 913 | -6421 | L1MB4 | L1 | LINE |
| 43837092 | 43837587 | + | 18.6 | 1708 | 2316 | -5018 | L1MB4 | L1 | LINE |
| 43837588 | 43837814 | + | 12.0 | 6072 | 6299 | -1 | L1MA4 | L1 | LINE |
| 43837815 | 43838501 | + | 18.6 | 2317 | 3123 | -4211 | L1MB4 | L1 | LINE |
| 43838518 | 43840179 | + | 20.2 | 4630 | 6333 | -707 | L1MB4 | L1 | LINE |
| 43840203 | 43840877 | + | 19.9 | 6334 | 7032 | -8 | L1MB4 | L1 | LINE |

  

|  |  |  |  |  |  |  |  |  |  |
| --- | --- | --- | --- | --- | --- | --- | --- | --- | --- |
| chr9:43840886-43845237 | | | 4345 bp | | |  |  |  |  |
| Genome Start | Genome End | Strand | Div | Repeat Start | Repeat End | Repeat Left | Name | Family | Class |
| 43840886 | 43843897 | + | 20.2 | 2226 | 5292 | -1090 | L1MB8 | L1 | LINE |
| 43843905 | 43844005 | + | 22.8 | 5432 | 5524 | -890 | L1MB8 | L1 | LINE |
| 43844006 | 43844311 | + | 6.2 | 2 | 307 | -4 | AluY | Alu | SINE |
| 43844312 | 43845237 | + | 22.2 | 5525 | 6414 | 0 | L1MB8 | L1 | LINE |

  

|  |  |  |  |  |  |  |  |  |  |
| --- | --- | --- | --- | --- | --- | --- | --- | --- | --- |
| chr9:43849739-43850171 | | | 433 bp | | |  |  |  |  |
| Genome Start | Genome End | Strand | Div | Repeat Start | Repeat End | Repeat Left | Name | Family | Class |
| 43849739 | 43849767 | - | 36.3 | 3349 | 3378 | 0 | L2 | L2 | LINE |
| 43849768 | 43850067 | + | 5.0 | 1 | 304 | -7 | AluY | Alu | SINE |
| 43850068 | 43850171 | - | 36.3 | 3253 | 3348 | -30 | L2 | L2 | LINE |

  

|  |  |  |  |  |  |  |  |  |  |
| --- | --- | --- | --- | --- | --- | --- | --- | --- | --- |
| chr9:43851987-43852532 | | | 546 bp | | |  |  |  |  |
| Genome Start | Genome End | Strand | Div | Repeat Start | Repeat End | Repeat Left | Name | Family | Class |
| 43851987 | 43852154 | + | 20.0 | 5 | 161 | -48 | MER3 | MER1\_type | DNA |
| 43852155 | 43852476 | - | 7.6 | 1 | 302 | -9 | AluY | Alu | SINE |
| 43852477 | 43852532 | + | 20.0 | 162 | 209 | 0 | MER3 | MER1\_type | DNA |

  

|  |  |  |  |  |  |  |  |  |  |
| --- | --- | --- | --- | --- | --- | --- | --- | --- | --- |
| chr9:43865270-43869251 | | | 3997 bp | | |  |  |  |  |
| Genome Start | Genome End | Strand | Div | Repeat Start | Repeat End | Repeat Left | Name | Family | Class |
| 43865270 | 43865704 | + | 21.8 | 39 | 493 | -5907 | L1ME2 | L1 | LINE |
| 43865711 | 43865800 | - | 25.8 | 978 | 1059 | 0 | MER106B | MER1\_type | DNA |
| 43865801 | 43866132 | - | 10.7 | 1 | 316 | 0 | AluY | Alu | SINE |
| 43866133 | 43866297 | - | 26.9 | 1 | 977 | -82 | MER106B | MER1\_type | DNA |
| 43866299 | 43866554 | + | 22.0 | 526 | 785 | -5615 | L1ME2 | L1 | LINE |
| 43866555 | 43866841 | + | 8.0 | 1 | 290 | -21 | AluY | Alu | SINE |
| 43866842 | 43868256 | + | 22.0 | 786 | 2285 | -4115 | L1ME2 | L1 | LINE |
| 43868235 | 43869251 | + | 23.2 | 5167 | 6228 | -9 | L1ME2 | L1 | LINE |

  

|  |  |  |  |  |  |  |  |  |  |
| --- | --- | --- | --- | --- | --- | --- | --- | --- | --- |
| chr9:43876469-43877188 | | | 720 bp | | |  |  |  |  |
| Genome Start | Genome End | Strand | Div | Repeat Start | Repeat End | Repeat Left | Name | Family | Class |
| 43876469 | 43876608 | - | 27.5 | 84 | 225 | -37 | MIR | MIR | SINE |
| 43876609 | 43877093 | - | 15.1 | 1 | 489 | 0 | LTR45B | ERV1 | LTR |
| 43877094 | 43877188 | - | 27.5 | 2 | 83 | -179 | MIR | MIR | SINE |

  

|  |  |  |  |  |  |  |  |  |  |
| --- | --- | --- | --- | --- | --- | --- | --- | --- | --- |
| chr9:43948096-43948774 | | | 648 bp | | |  |  |  |  |
| Genome Start | Genome End | Strand | Div | Repeat Start | Repeat End | Repeat Left | Name | Family | Class |
| 43948096 | 43948398 | - | 17.8 | 125 | 426 | 0 | MSTB | MaLR | LTR |
| 43948399 | 43948526 | + | 16.5 | 1 | 126 | -104 | MER30 | MER1\_type | DNA |
| 43948558 | 43948652 | + | 16.5 | 127 | 230 | 0 | MER30 | MER1\_type | DNA |
| 43948653 | 43948774 | - | 17.8 | 3 | 124 | -302 | MSTB | MaLR | LTR |

  

|  |  |  |  |  |  |  |  |  |  |
| --- | --- | --- | --- | --- | --- | --- | --- | --- | --- |
| chr9:43964543-43965185 | | | 544 bp | | |  |  |  |  |
| Genome Start | Genome End | Strand | Div | Repeat Start | Repeat End | Repeat Left | Name | Family | Class |
| 43964543 | 43964686 | - | 25.2 | 128 | 274 | -154 | MSTA | MaLR | LTR |
| 43964715 | 43964956 | + | 23.6 | 976 | 1256 | -395 | MST-int | MaLR | LTR |
| 43964945 | 43965000 | - | 19.6 | 911 | 967 | -613 | THE1-int | MaLR | LTR |
| 43965084 | 43965185 | - | 21.4 | 1 | 106 | -322 | MSTA | MaLR | LTR |

  

|  |  |  |  |  |  |  |  |  |  |
| --- | --- | --- | --- | --- | --- | --- | --- | --- | --- |
| chr9:43979071-43982077 | | | 2716 bp | | |  |  |  |  |
| Genome Start | Genome End | Strand | Div | Repeat Start | Repeat End | Repeat Left | Name | Family | Class |
| 43979071 | 43979178 | - | 22.4 | 58 | 180 | -248 | MSTA | MaLR | LTR |
| 43979224 | 43979322 | - | 28.3 | 1 | 100 | -328 | MSTA | MaLR | LTR |
| 43979323 | 43979400 | - | 17.3 | 1574 | 1651 | 0 | MSTA-int | MaLR | LTR |
| 43979403 | 43979463 | + | 18.0 | 317 | 377 | -51 | MSTA | MaLR | LTR |
| 43979536 | 43981070 | - | 22.4 | 15 | 1583 | -68 | MSTA-int | MaLR | LTR |
| 43981174 | 43981286 | + | 20.3 | 506 | 661 | -963 | MST-int | MaLR | LTR |
| 43981303 | 43981593 | - | 23.9 | 66 | 376 | -52 | MSTA | MaLR | LTR |
| 43981598 | 43981876 | + | 23.4 | 907 | 1218 | -362 | THE1-int | MaLR | LTR |
| 43981830 | 43981885 | - | 21.4 | 911 | 967 | -613 | THE1-int | MaLR | LTR |
| 43981982 | 43982077 | - | 20.8 | 1 | 97 | -331 | MSTA | MaLR | LTR |

  

|  |  |  |  |  |  |  |  |  |  |
| --- | --- | --- | --- | --- | --- | --- | --- | --- | --- |
| chr9:43984262-43984959 | | | 678 bp | | |  |  |  |  |
| Genome Start | Genome End | Strand | Div | Repeat Start | Repeat End | Repeat Left | Name | Family | Class |
| 43984262 | 43984412 | + | 27.9 | 466 | 617 | -963 | THE1-int | MaLR | LTR |
| 43984429 | 43984714 | - | 21.5 | 76 | 376 | -52 | MSTA | MaLR | LTR |
| 43984719 | 43984959 | + | 22.0 | 907 | 1191 | -389 | THE1-int | MaLR | LTR |

  

|  |  |  |  |  |  |  |  |  |  |
| --- | --- | --- | --- | --- | --- | --- | --- | --- | --- |
| chr9:44001245-44002967 | | | 1723 bp | | |  |  |  |  |
| Genome Start | Genome End | Strand | Div | Repeat Start | Repeat End | Repeat Left | Name | Family | Class |
| 44001245 | 44002366 | + | 19.6 | 4805 | 5947 | -353 | L1MA5 | L1 | LINE |
| 44002367 | 44002685 | + | 23.0 | 1 | 299 | -3 | AluJo | Alu | SINE |
| 44002686 | 44002967 | + | 20.1 | 5948 | 6294 | -6 | L1MA5 | L1 | LINE |

  

|  |  |  |  |  |  |  |  |  |  |
| --- | --- | --- | --- | --- | --- | --- | --- | --- | --- |
| chr9:44064063-44065555 | | | 1493 bp | | |  |  |  |  |
| Genome Start | Genome End | Strand | Div | Repeat Start | Repeat End | Repeat Left | Name | Family | Class |
| 44064063 | 44064570 | + | 24.4 | 18 | 528 | -407 | MER21C | ERV1 | LTR |
| 44064571 | 44064899 | - | 18.7 | 2 | 301 | -11 | AluJb | Alu | SINE |
| 44064900 | 44064977 | + | 24.4 | 529 | 601 | -334 | MER21C | ERV1 | LTR |
| 44064978 | 44065268 | + | 16.0 | 9 | 297 | -15 | AluJo | Alu | SINE |
| 44065269 | 44065555 | + | 24.4 | 602 | 916 | -19 | MER21C | ERV1 | LTR |

  

|  |  |  |  |  |  |  |  |  |  |
| --- | --- | --- | --- | --- | --- | --- | --- | --- | --- |
| chr9:44074487-44074977 | | | 382 bp | | |  |  |  |  |
| Genome Start | Genome End | Strand | Div | Repeat Start | Repeat End | Repeat Left | Name | Family | Class |
| 44074487 | 44074574 | + | 22.1 | 4 | 91 | -1830 | Kanga1 | Tc2 | DNA |
| 44074575 | 44074779 | - | 33.0 | 425 | 651 | -5468 | L1MEd | L1 | LINE |
| 44074889 | 44074977 | + | 20.0 | 1656 | 1735 | -10 | Kanga1 | Tc2 | DNA |

  

|  |  |  |  |  |  |  |  |  |  |
| --- | --- | --- | --- | --- | --- | --- | --- | --- | --- |
| chr9:44079999-44083614 | | | 3616 bp | | |  |  |  |  |
| Genome Start | Genome End | Strand | Div | Repeat Start | Repeat End | Repeat Left | Name | Family | Class |
| 44079999 | 44080852 | + | 17.3 | 3307 | 4175 | -3387 | L1M1 | L1 | LINE |
| 44080853 | 44081090 | - | 9.2 | 39 | 294 | -17 | AluY | Alu | SINE |
| 44081091 | 44083614 | + | 18.6 | 4176 | 6732 | -830 | L1M1 | L1 | LINE |

  

|  |  |  |  |  |  |  |  |  |  |
| --- | --- | --- | --- | --- | --- | --- | --- | --- | --- |
| chr9:44099734-44100569 | | | 836 bp | | |  |  |  |  |
| Genome Start | Genome End | Strand | Div | Repeat Start | Repeat End | Repeat Left | Name | Family | Class |
| 44099734 | 44099842 | + | 29.4 | 26 | 133 | -378 | MLT1J | MaLR | LTR |
| 44099843 | 44100211 | + | 13.1 | 1 | 381 | 0 | THE1D | MaLR | LTR |
| 44100212 | 44100569 | + | 29.4 | 134 | 511 | 0 | MLT1J | MaLR | LTR |

  

|  |  |  |  |  |  |  |  |  |  |
| --- | --- | --- | --- | --- | --- | --- | --- | --- | --- |
| chr9:44136265-44137173 | | | 909 bp | | |  |  |  |  |
| Genome Start | Genome End | Strand | Div | Repeat Start | Repeat End | Repeat Left | Name | Family | Class |
| 44136265 | 44136705 | + | 23.2 | 7360 | 7801 | -194 | L1MD3 | L1 | LINE |
| 44136706 | 44136999 | + | 15.0 | 1 | 294 | -18 | AluSx | Alu | SINE |
| 44137000 | 44137173 | + | 23.2 | 7802 | 7973 | -22 | L1MD3 | L1 | LINE |

  

|  |  |  |  |  |  |  |  |  |  |
| --- | --- | --- | --- | --- | --- | --- | --- | --- | --- |
| chr9:44144923-44147772 | | | 2846 bp | | |  |  |  |  |
| Genome Start | Genome End | Strand | Div | Repeat Start | Repeat End | Repeat Left | Name | Family | Class |
| 44144923 | 44145023 | - | 30.5 | 106 | 202 | -6 | MIR3 | MIR | SINE |
| 44145024 | 44145074 | - | 15.4 | 391 | 428 | 0 | MSTA | MaLR | LTR |
| 44145075 | 44145367 | - | 13.4 | 3 | 294 | -15 | AluSc | Alu | SINE |
| 44145368 | 44145690 | - | 15.4 | 1 | 390 | -38 | MSTA | MaLR | LTR |
| 44145693 | 44147295 | - | 31.2 | 2 | 1733 | -2 | MLT-int | MaLR | LTR |
| 44147298 | 44147672 | - | 13.8 | 1 | 428 | 0 | MSTA | MaLR | LTR |
| 44147673 | 44147772 | - | 30.5 | 8 | 105 | -103 | MIR3 | MIR | SINE |

  

|  |  |  |  |  |  |  |  |  |  |
| --- | --- | --- | --- | --- | --- | --- | --- | --- | --- |
| chr9:44155514-44156972 | | | 1409 bp | | |  |  |  |  |
| Genome Start | Genome End | Strand | Div | Repeat Start | Repeat End | Repeat Left | Name | Family | Class |
| 44155514 | 44156122 | - | 26.0 | 5556 | 6159 | -7 | L1ME1 | L1 | LINE |
| 44156173 | 44156270 | - | 22.9 | 3674 | 3769 | -2377 | L1ME1 | L1 | LINE |
| 44156271 | 44156568 | + | 9.8 | 1 | 297 | -13 | AluSg | Alu | SINE |
| 44156569 | 44156972 | - | 22.9 | 3254 | 3673 | -2473 | L1ME1 | L1 | LINE |

  

|  |  |  |  |  |  |  |  |  |  |
| --- | --- | --- | --- | --- | --- | --- | --- | --- | --- |
| chr9:44159038-44159502 | | | 465 bp | | |  |  |  |  |
| Genome Start | Genome End | Strand | Div | Repeat Start | Repeat End | Repeat Left | Name | Family | Class |
| 44159038 | 44159095 | + | 17.4 | 4322 | 4379 | -1764 | L1M2 | L1 | LINE |
| 44159096 | 44159317 | + | 9.7 | 1 | 217 | -96 | AluSq | Alu | SINE |
| 44159318 | 44159502 | + | 17.4 | 4380 | 4540 | -1603 | L1M2 | L1 | LINE |

  

|  |  |  |  |  |  |  |  |  |  |
| --- | --- | --- | --- | --- | --- | --- | --- | --- | --- |
| chr9:44177226-44178841 | | | 1616 bp | | |  |  |  |  |
| Genome Start | Genome End | Strand | Div | Repeat Start | Repeat End | Repeat Left | Name | Family | Class |
| 44177226 | 44177353 | + | 18.2 | 1 | 126 | -1525 | MSTA-int | MaLR | LTR |
| 44177354 | 44177671 | + | 15.7 | 1 | 312 | 0 | AluSx | Alu | SINE |
| 44177672 | 44178841 | + | 18.2 | 127 | 1297 | -354 | MSTA-int | MaLR | LTR |

  

|  |  |  |  |  |  |  |  |  |  |
| --- | --- | --- | --- | --- | --- | --- | --- | --- | --- |
| chr9:44180775-44182523 | | | 1402 bp | | |  |  |  |  |
| Genome Start | Genome End | Strand | Div | Repeat Start | Repeat End | Repeat Left | Name | Family | Class |
| 44180775 | 44180846 | + | 13.7 | 5102 | 5178 | -968 | L1MB7 | L1 | LINE |
| 44180847 | 44181222 | + | 14.1 | 1 | 364 | 0 | THE1B | MaLR | LTR |
| 44181223 | 44181753 | + | 15.6 | 5179 | 5739 | -445 | L1MB7 | L1 | LINE |
| 44181759 | 44181884 | + | 28.8 | 4018 | 4136 | -55 | MER21-int | ERV1 | LTR |
| 44182227 | 44182523 | + | 15.7 | 5731 | 6014 | -170 | L1MB7 | L1 | LINE |

  

|  |  |  |  |  |  |  |  |  |  |
| --- | --- | --- | --- | --- | --- | --- | --- | --- | --- |
| chr9:44187271-44189677 | | | 2407 bp | | |  |  |  |  |
| Genome Start | Genome End | Strand | Div | Repeat Start | Repeat End | Repeat Left | Name | Family | Class |
| 44187271 | 44188952 | - | 18.5 | 3349 | 5101 | -2458 | L1M1 | L1 | LINE |
| 44188953 | 44189287 | + | 19.1 | 4 | 345 | -1 | MER7A | MER2\_type | DNA |
| 44189288 | 44189677 | - | 24.4 | 2917 | 3348 | -4217 | L1M1 | L1 | LINE |

  

|  |  |  |  |  |  |  |  |  |  |
| --- | --- | --- | --- | --- | --- | --- | --- | --- | --- |
| chr9:44189673-44194361 | | | 4634 bp | | |  |  |  |  |
| Genome Start | Genome End | Strand | Div | Repeat Start | Repeat End | Repeat Left | Name | Family | Class |
| 44189673 | 44189936 | + | 19.5 | 4120 | 4378 | -1765 | L1MA9 | L1 | LINE |
| 44189937 | 44190212 | + | 9.1 | 1 | 287 | -24 | AluY | Alu | SINE |
| 44190213 | 44191060 | + | 19.5 | 4379 | 5286 | -857 | L1MA9 | L1 | LINE |
| 44191071 | 44191434 | - | 12.5 | 1 | 364 | 0 | THE1B | MaLR | LTR |
| 44191435 | 44192997 | - | 12.2 | 1 | 1580 | 0 | THE1B-int | MaLR | LTR |
| 44192998 | 44193356 | - | 17.2 | 1 | 364 | 0 | THE1B | MaLR | LTR |
| 44193357 | 44193800 | + | 15.4 | 5332 | 5782 | -530 | L1MA9 | L1 | LINE |
| 44193846 | 44194361 | + | 15.4 | 5783 | 6311 | -1 | L1MA9 | L1 | LINE |

  

|  |  |  |  |  |  |  |  |  |  |
| --- | --- | --- | --- | --- | --- | --- | --- | --- | --- |
| chr9:44209358-44211122 | | | 1768 bp | | |  |  |  |  |
| Genome Start | Genome End | Strand | Div | Repeat Start | Repeat End | Repeat Left | Name | Family | Class |
| 44209358 | 44209553 | - | 19.6 | 889 | 1083 | 0 | Tigger6a | MER2\_type | DNA |
| 44209554 | 44209850 | - | 12.8 | 1 | 297 | -14 | AluY | Alu | SINE |
| 44209851 | 44210461 | - | 19.6 | 221 | 888 | -195 | Tigger6a | MER2\_type | DNA |
| 44210459 | 44210913 | + | 22.8 | 11 | 467 | 0 | MLT1C | MaLR | LTR |
| 44210914 | 44211122 | - | 21.0 | 5 | 211 | -872 | Tigger6a | MER2\_type | DNA |

  

|  |  |  |  |  |  |  |  |  |  |
| --- | --- | --- | --- | --- | --- | --- | --- | --- | --- |
| chr9:44218541-44219755 | | | 1215 bp | | |  |  |  |  |
| Genome Start | Genome End | Strand | Div | Repeat Start | Repeat End | Repeat Left | Name | Family | Class |
| 44218541 | 44219121 | + | 13.7 | 5237 | 5795 | -359 | L1PB3 | L1 | LINE |
| 44219122 | 44219417 | + | 12.5 | 1 | 296 | -13 | AluSg1 | Alu | SINE |
| 44219418 | 44219755 | + | 14.2 | 5796 | 6152 | -2 | L1PB3 | L1 | LINE |

  

|  |  |  |  |  |  |  |  |  |  |
| --- | --- | --- | --- | --- | --- | --- | --- | --- | --- |
| chr9:44220092-44224074 | | | 3983 bp | | |  |  |  |  |
| Genome Start | Genome End | Strand | Div | Repeat Start | Repeat End | Repeat Left | Name | Family | Class |
| 44220092 | 44222831 | - | 15.4 | 3306 | 6161 | 0 | L1PA14 | L1 | LINE |
| 44222832 | 44223139 | - | 15.9 | 1 | 310 | -2 | AluSx | Alu | SINE |
| 44223140 | 44224074 | - | 17.7 | 2377 | 3305 | -2841 | L1PA14 | L1 | LINE |

  

|  |  |  |  |  |  |  |  |  |  |
| --- | --- | --- | --- | --- | --- | --- | --- | --- | --- |
| chr9:44231455-44232339 | | | 885 bp | | |  |  |  |  |
| Genome Start | Genome End | Strand | Div | Repeat Start | Repeat End | Repeat Left | Name | Family | Class |
| 44231455 | 44231785 | - | 24.2 | 7599 | 7947 | -14 | L1MC5 | L1 | LINE |
| 44231786 | 44232098 | - | 10.8 | 1 | 296 | -16 | AluSx | Alu | SINE |
| 44232099 | 44232339 | - | 24.2 | 7349 | 7598 | -363 | L1MC5 | L1 | LINE |

  

|  |  |  |  |  |  |  |  |  |  |
| --- | --- | --- | --- | --- | --- | --- | --- | --- | --- |
| chr9:44249397-44252558 | | | 3129 bp | | |  |  |  |  |
| Genome Start | Genome End | Strand | Div | Repeat Start | Repeat End | Repeat Left | Name | Family | Class |
| 44249397 | 44251527 | + | 10.0 | 2280 | 4397 | -1749 | L1PB | L1 | LINE |
| 44251561 | 44251642 | + | 10.0 | 4398 | 4478 | -1668 | L1PB | L1 | LINE |
| 44251643 | 44251820 | + | 14.6 | 136 | 313 | 0 | AluSp | Alu | SINE |
| 44251821 | 44252558 | + | 10.0 | 4479 | 5215 | -931 | L1PB | L1 | LINE |

  

|  |  |  |  |  |  |  |  |  |  |
| --- | --- | --- | --- | --- | --- | --- | --- | --- | --- |
| chr9:44295570-44296454 | | | 885 bp | | |  |  |  |  |
| Genome Start | Genome End | Strand | Div | Repeat Start | Repeat End | Repeat Left | Name | Family | Class |
| 44295570 | 44295810 | + | 24.2 | 7349 | 7598 | -363 | L1MC5 | L1 | LINE |
| 44295811 | 44296123 | + | 10.8 | 1 | 296 | -16 | AluSx | Alu | SINE |
| 44296124 | 44296454 | + | 24.2 | 7599 | 7947 | -14 | L1MC5 | L1 | LINE |

  

|  |  |  |  |  |  |  |  |  |  |
| --- | --- | --- | --- | --- | --- | --- | --- | --- | --- |
| chr9:44298421-44300315 | | | 1895 bp | | |  |  |  |  |
| Genome Start | Genome End | Strand | Div | Repeat Start | Repeat End | Repeat Left | Name | Family | Class |
| 44298421 | 44298541 | + | 30.4 | 2 | 124 | -1611 | MLT-int | MaLR | LTR |
| 44298542 | 44298851 | + | 12.6 | 1 | 311 | -2 | AluSq | Alu | SINE |
| 44298852 | 44300315 | + | 30.4 | 125 | 1733 | -2 | MLT-int | MaLR | LTR |

  

|  |  |  |  |  |  |  |  |  |  |
| --- | --- | --- | --- | --- | --- | --- | --- | --- | --- |
| chr9:44300918-44301399 | | | 482 bp | | |  |  |  |  |
| Genome Start | Genome End | Strand | Div | Repeat Start | Repeat End | Repeat Left | Name | Family | Class |
| 44300918 | 44301036 | + | 27.4 | 81 | 202 | -60 | MIR | MIR | SINE |
| 44301037 | 44301339 | - | 11.6 | 1 | 303 | -6 | AluSc | Alu | SINE |
| 44301340 | 44301399 | + | 27.4 | 203 | 260 | -2 | MIR | MIR | SINE |

  

|  |  |  |  |  |  |  |  |  |  |
| --- | --- | --- | --- | --- | --- | --- | --- | --- | --- |
| chr9:44303842-44307828 | | | 3987 bp | | |  |  |  |  |
| Genome Start | Genome End | Strand | Div | Repeat Start | Repeat End | Repeat Left | Name | Family | Class |
| 44303842 | 44304776 | + | 17.7 | 2377 | 3296 | -2850 | L1PA14 | L1 | LINE |
| 44304777 | 44305083 | + | 15.3 | 1 | 309 | -3 | AluSx | Alu | SINE |
| 44305084 | 44307828 | + | 15.5 | 3297 | 6161 | 0 | L1PA14 | L1 | LINE |

  

|  |  |  |  |  |  |  |  |  |  |
| --- | --- | --- | --- | --- | --- | --- | --- | --- | --- |
| chr9:44308166-44309380 | | | 1194 bp | | |  |  |  |  |
| Genome Start | Genome End | Strand | Div | Repeat Start | Repeat End | Repeat Left | Name | Family | Class |
| 44308166 | 44308499 | - | 13.0 | 5796 | 6149 | -5 | L1PB3 | L1 | LINE |
| 44308500 | 44308795 | - | 11.5 | 1 | 296 | -13 | AluSg1 | Alu | SINE |
| 44308796 | 44309107 | - | 13.0 | 5491 | 5795 | -359 | L1PB3 | L1 | LINE |
| 44309129 | 44309380 | - | 13.0 | 5237 | 5490 | -664 | L1PB3 | L1 | LINE |

  

|  |  |  |  |  |  |  |  |  |  |
| --- | --- | --- | --- | --- | --- | --- | --- | --- | --- |
| chr9:44316760-44318520 | | | 1764 bp | | |  |  |  |  |
| Genome Start | Genome End | Strand | Div | Repeat Start | Repeat End | Repeat Left | Name | Family | Class |
| 44316760 | 44316968 | + | 20.5 | 5 | 211 | -872 | Tigger6a | MER2\_type | DNA |
| 44316969 | 44317423 | - | 23.8 | 11 | 467 | 0 | MLT1C | MaLR | LTR |
| 44317421 | 44318031 | + | 19.9 | 220 | 887 | -196 | Tigger6a | MER2\_type | DNA |
| 44318032 | 44318324 | + | 13.3 | 1 | 293 | -18 | AluY | Alu | SINE |
| 44318325 | 44318520 | + | 19.9 | 888 | 1082 | -1 | Tigger6a | MER2\_type | DNA |

  

|  |  |  |  |  |  |  |  |  |  |
| --- | --- | --- | --- | --- | --- | --- | --- | --- | --- |
| chr9:44333510-44338186 | | | 4640 bp | | |  |  |  |  |
| Genome Start | Genome End | Strand | Div | Repeat Start | Repeat End | Repeat Left | Name | Family | Class |
| 44333510 | 44334025 | - | 15.1 | 5784 | 6312 | 0 | L1MA9 | L1 | LINE |
| 44334053 | 44334492 | - | 15.1 | 5333 | 5783 | -529 | L1MA9 | L1 | LINE |
| 44334493 | 44334852 | + | 17.2 | 1 | 364 | 0 | THE1B | MaLR | LTR |
| 44334853 | 44336419 | + | 12.7 | 1 | 1580 | 0 | THE1B-int | MaLR | LTR |
| 44336420 | 44336783 | + | 14.3 | 1 | 364 | 0 | THE1B | MaLR | LTR |
| 44336794 | 44337635 | - | 19.5 | 4380 | 5286 | -857 | L1MA9 | L1 | LINE |
| 44337636 | 44337912 | - | 9.8 | 1 | 287 | -24 | AluY | Alu | SINE |
| 44337913 | 44338186 | - | 19.5 | 4120 | 4379 | -1764 | L1MA9 | L1 | LINE |

  

|  |  |  |  |  |  |  |  |  |  |
| --- | --- | --- | --- | --- | --- | --- | --- | --- | --- |
| chr9:44345318-44347067 | | | 1403 bp | | |  |  |  |  |
| Genome Start | Genome End | Strand | Div | Repeat Start | Repeat End | Repeat Left | Name | Family | Class |
| 44345318 | 44345615 | - | 17.1 | 5731 | 6014 | -170 | L1MB7 | L1 | LINE |
| 44345958 | 44346083 | - | 27.1 | 4018 | 4136 | -55 | MER21-int | ERV1 | LTR |
| 44346089 | 44346619 | - | 16.5 | 5179 | 5739 | -439 | L1MB7 | L1 | LINE |
| 44346620 | 44346994 | - | 14.1 | 2 | 364 | 0 | THE1B | MaLR | LTR |
| 44346995 | 44347067 | - | 14.8 | 5102 | 5178 | -968 | L1MB7 | L1 | LINE |

  

|  |  |  |  |  |  |  |  |  |  |
| --- | --- | --- | --- | --- | --- | --- | --- | --- | --- |
| chr9:44349001-44350616 | | | 1616 bp | | |  |  |  |  |
| Genome Start | Genome End | Strand | Div | Repeat Start | Repeat End | Repeat Left | Name | Family | Class |
| 44349001 | 44350170 | - | 18.6 | 127 | 1297 | -354 | MSTA-int | MaLR | LTR |
| 44350171 | 44350488 | - | 15.7 | 1 | 312 | 0 | AluSx | Alu | SINE |
| 44350489 | 44350616 | - | 18.6 | 1 | 126 | -1525 | MSTA-int | MaLR | LTR |

  

|  |  |  |  |  |  |  |  |  |  |
| --- | --- | --- | --- | --- | --- | --- | --- | --- | --- |
| chr9:44368077-44368541 | | | 465 bp | | |  |  |  |  |
| Genome Start | Genome End | Strand | Div | Repeat Start | Repeat End | Repeat Left | Name | Family | Class |
| 44368077 | 44368260 | - | 17.4 | 4380 | 4540 | -1603 | L1M2 | L1 | LINE |
| 44368261 | 44368483 | - | 7.8 | 1 | 217 | -96 | AluSq | Alu | SINE |
| 44368484 | 44368541 | - | 17.4 | 4322 | 4379 | -1764 | L1M2 | L1 | LINE |

  

|  |  |  |  |  |  |  |  |  |  |
| --- | --- | --- | --- | --- | --- | --- | --- | --- | --- |
| chr9:44385320-44386171 | | | 852 bp | | |  |  |  |  |
| Genome Start | Genome End | Strand | Div | Repeat Start | Repeat End | Repeat Left | Name | Family | Class |
| 44385320 | 44385759 | + | 7.3 | 5627 | 6064 | -108 | L1PA8 | L1 | LINE |
| 44385760 | 44386054 | + | 8.8 | 1 | 295 | -16 | AluY | Alu | SINE |
| 44386055 | 44386171 | + | 7.3 | 6065 | 6172 | 0 | L1PA8 | L1 | LINE |

  

|  |  |  |  |  |  |  |  |  |  |
| --- | --- | --- | --- | --- | --- | --- | --- | --- | --- |
| chr9:44401175-44402837 | | | 1666 bp | | |  |  |  |  |
| Genome Start | Genome End | Strand | Div | Repeat Start | Repeat End | Repeat Left | Name | Family | Class |
| 44401175 | 44401267 | - | 21.1 | 2885 | 2975 | -3569 | L1MD2 | L1 | LINE |
| 44401265 | 44401646 | - | 25.2 | 1239 | 1637 | -5003 | L1MD2 | L1 | LINE |
| 44401647 | 44401939 | + | 11.0 | 1 | 293 | -16 | AluSc | Alu | SINE |
| 44401940 | 44402837 | - | 27.0 | 290 | 1242 | -5305 | L1MD2 | L1 | LINE |

  

|  |  |  |  |  |  |  |  |  |  |
| --- | --- | --- | --- | --- | --- | --- | --- | --- | --- |
| chr9:44426887-44428270 | | | 1113 bp | | |  |  |  |  |
| Genome Start | Genome End | Strand | Div | Repeat Start | Repeat End | Repeat Left | Name | Family | Class |
| 44426887 | 44427004 | - | 23.1 | 2747 | 3032 | -3097 | L1MEd | L1 | LINE |
| 44427150 | 44427437 | - | 10.4 | 1 | 288 | -21 | AluSc | Alu | SINE |
| 44427438 | 44427655 | - | 29.5 | 2534 | 2746 | -3383 | L1MEd | L1 | LINE |
| 44427658 | 44427822 | - | 16.5 | 5986 | 6167 | -4 | L1MB2 | L1 | LINE |
| 44427947 | 44428270 | - | 37.9 | 223 | 566 | -5553 | L1MEd | L1 | LINE |

  

|  |  |  |  |  |  |  |  |  |  |
| --- | --- | --- | --- | --- | --- | --- | --- | --- | --- |
| chr9:44468110-44469154 | | | 1049 bp | | |  |  |  |  |
| Genome Start | Genome End | Strand | Div | Repeat Start | Repeat End | Repeat Left | Name | Family | Class |
| 44468110 | 44468339 | - | 29.6 | 5907 | 6132 | -34 | L1ME1 | L1 | LINE |
| 44468339 | 44468760 | + | 17.7 | 2295 | 2722 | -3654 | L1M4c | L1 | LINE |
| 44468758 | 44469154 | - | 24.1 | 4631 | 5091 | -1055 | L1ME1 | L1 | LINE |

  

|  |  |  |  |  |  |  |  |  |  |
| --- | --- | --- | --- | --- | --- | --- | --- | --- | --- |
| chr9:44477114-44477869 | | | 643 bp | | |  |  |  |  |
| Genome Start | Genome End | Strand | Div | Repeat Start | Repeat End | Repeat Left | Name | Family | Class |
| 44477114 | 44477236 | - | 30.0 | 114 | 241 | -21 | MIR | MIR | SINE |
| 44477244 | 44477677 | + | 17.2 | 1 | 511 | -45 | MLT2B4 | ERVL | LTR |
| 44477784 | 44477869 | - | 18.6 | 21 | 130 | -132 | MIR | MIR | SINE |

  

|  |  |  |  |  |  |  |  |  |  |
| --- | --- | --- | --- | --- | --- | --- | --- | --- | --- |
| chr9:44477930-44478458 | | | 529 bp | | |  |  |  |  |
| Genome Start | Genome End | Strand | Div | Repeat Start | Repeat End | Repeat Left | Name | Family | Class |
| 44477930 | 44478086 | + | 13.6 | 1 | 161 | -41 | MER6C | MER2\_type | DNA |
| 44478087 | 44478406 | - | 9.2 | 1 | 305 | -6 | AluY | Alu | SINE |
| 44478407 | 44478458 | + | 13.6 | 162 | 202 | 0 | MER6C | MER2\_type | DNA |

  

|  |  |  |  |  |  |  |  |  |  |
| --- | --- | --- | --- | --- | --- | --- | --- | --- | --- |
| chr9:44482149-44482869 | | | 721 bp | | |  |  |  |  |
| Genome Start | Genome End | Strand | Div | Repeat Start | Repeat End | Repeat Left | Name | Family | Class |
| 44482149 | 44482516 | + | 15.6 | 1 | 375 | -51 | MSTB | MaLR | LTR |
| 44482517 | 44482819 | + | 4.7 | 1 | 300 | -11 | AluY | Alu | SINE |
| 44482820 | 44482869 | + | 15.6 | 376 | 426 | 0 | MSTB | MaLR | LTR |

  

|  |  |  |  |  |  |  |  |  |  |
| --- | --- | --- | --- | --- | --- | --- | --- | --- | --- |
| chr9:44486883-44488251 | | | 1128 bp | | |  |  |  |  |
| Genome Start | Genome End | Strand | Div | Repeat Start | Repeat End | Repeat Left | Name | Family | Class |
| 44486883 | 44487021 | + | 36.2 | 1284 | 1434 | -1985 | L2 | L2 | LINE |
| 44487066 | 44487168 | - | 30.6 | 3274 | 3378 | 0 | L2 | L2 | LINE |
| 44487151 | 44487641 | + | 35.6 | 1571 | 2075 | -1344 | L2 | L2 | LINE |
| 44487664 | 44487947 | - | 13.4 | 1 | 283 | -30 | AluSq | Alu | SINE |
| 44488141 | 44488251 | + | 30.6 | 3251 | 3379 | -21 | L2 | L2 | LINE |

  

|  |  |  |  |  |  |  |  |  |  |
| --- | --- | --- | --- | --- | --- | --- | --- | --- | --- |
| chr9:44489309-44489920 | | | 612 bp | | |  |  |  |  |
| Genome Start | Genome End | Strand | Div | Repeat Start | Repeat End | Repeat Left | Name | Family | Class |
| 44489309 | 44489454 | - | 20.0 | 6019 | 6170 | -8 | L1MB8 | L1 | LINE |
| 44489455 | 44489722 | + | 9.7 | 1 | 305 | -5 | AluSg | Alu | SINE |
| 44489723 | 44489920 | - | 20.0 | 5815 | 6018 | -160 | L1MB8 | L1 | LINE |

  

|  |  |  |  |  |  |  |  |  |  |
| --- | --- | --- | --- | --- | --- | --- | --- | --- | --- |
| chr9:44492212-44495476 | | | 3265 bp | | |  |  |  |  |
| Genome Start | Genome End | Strand | Div | Repeat Start | Repeat End | Repeat Left | Name | Family | Class |
| 44492212 | 44494641 | - | 18.5 | 1838 | 4480 | -1896 | L1M4c | L1 | LINE |
| 44494642 | 44494952 | - | 11.8 | 1 | 296 | -15 | AluY | Alu | SINE |
| 44494953 | 44495476 | - | 22.7 | 1297 | 1837 | -4547 | L1M4c | L1 | LINE |

  

|  |  |  |  |  |  |  |  |  |  |
| --- | --- | --- | --- | --- | --- | --- | --- | --- | --- |
| chr9:44512832-44517772 | | | 4652 bp | | |  |  |  |  |
| Genome Start | Genome End | Strand | Div | Repeat Start | Repeat End | Repeat Left | Name | Family | Class |
| 44512832 | 44513025 | + | 30.4 | 1351 | 1560 | -1859 | L2 | L2 | LINE |
| 44513035 | 44513312 | + | 18.7 | 2 | 341 | -67 | MLT1A1 | MaLR | LTR |
| 44513313 | 44513613 | - | 12.6 | 1 | 303 | -10 | AluSq | Alu | SINE |
| 44513614 | 44513680 | + | 18.7 | 342 | 407 | -1 | MLT1A1 | MaLR | LTR |
| 44513683 | 44515429 | + | 22.2 | 2 | 1735 | 0 | MLT1A1-int | MaLR | LTR |
| 44515430 | 44515563 | + | 19.6 | 1 | 137 | -271 | MLT1A1 | MaLR | LTR |
| 44515564 | 44515913 | - | 20.5 | 1 | 396 | 0 | MSTD | MaLR | LTR |
| 44515914 | 44516116 | + | 20.6 | 138 | 396 | 0 | MLT1A1 | MaLR | LTR |
| 44516117 | 44516948 | + | 30.7 | 1564 | 2598 | -821 | L2 | L2 | LINE |
| 44517013 | 44517333 | - | 29.1 | 4 | 354 | -241 | MLT1H | MaLR | LTR |
| 44517548 | 44517772 | + | 33.5 | 2949 | 3176 | -243 | L2 | L2 | LINE |

  

|  |  |  |  |  |  |  |  |  |  |
| --- | --- | --- | --- | --- | --- | --- | --- | --- | --- |
| chr9:44532246-44539600 | | | 6686 bp | | |  |  |  |  |
| Genome Start | Genome End | Strand | Div | Repeat Start | Repeat End | Repeat Left | Name | Family | Class |
| 44532246 | 44532390 | - | 22.8 | 6649 | 6801 | 0 | MER89-int | ERV1 | LTR |
| 44532392 | 44532827 | + | 11.6 | 1 | 436 | -231 | LTR12B | ERV1 | LTR |
| 44533017 | 44533169 | - | 27.2 | 1603 | 1755 | 0 | MER52A | ERV1 | LTR |
| 44533178 | 44533804 | - | 24.7 | 54 | 1470 | -162 | MER52A | ERV1 | LTR |
| 44533805 | 44534285 | - | 20.4 | 6052 | 6559 | -242 | MER89-int | ERV1 | LTR |
| 44534385 | 44534684 | + | 9.6 | 1 | 293 | -18 | AluY | Alu | SINE |
| 44534829 | 44536238 | - | 24.7 | 2549 | 4047 | -2904 | MER89-int | ERV1 | LTR |
| 44536467 | 44536674 | - | 27.2 | 2315 | 2536 | -4265 | MER89-int | ERV1 | LTR |
| 44536675 | 44536987 | - | 15.9 | 1 | 296 | -16 | AluSx | Alu | SINE |
| 44536988 | 44537218 | - | 27.2 | 2077 | 2314 | -4487 | MER89-int | ERV1 | LTR |
| 44537219 | 44537540 | + | 15.3 | 1 | 311 | -1 | AluSx | Alu | SINE |
| 44537541 | 44539600 | - | 27.2 | 2 | 2076 | -4725 | MER89-int | ERV1 | LTR |

  

|  |  |  |  |  |  |  |  |  |  |
| --- | --- | --- | --- | --- | --- | --- | --- | --- | --- |
| chr9:44545439-44546585 | | | 1147 bp | | |  |  |  |  |
| Genome Start | Genome End | Strand | Div | Repeat Start | Repeat End | Repeat Left | Name | Family | Class |
| 44545439 | 44545485 | - | 23.3 | 304 | 340 | -1 | MER58B | MER1\_type | DNA |
| 44545486 | 44545992 | + | 17.0 | 2 | 550 | 0 | MER44B | MER2\_type | DNA |
| 44545993 | 44546044 | - | 23.3 | 253 | 303 | -38 | MER58B | MER1\_type | DNA |
| 44546045 | 44546529 | + | 16.2 | 3 | 489 | 0 | LTR45B | ERV1 | LTR |
| 44546530 | 44546585 | - | 23.3 | 208 | 252 | -89 | MER58B | MER1\_type | DNA |

  

|  |  |  |  |  |  |  |  |  |  |
| --- | --- | --- | --- | --- | --- | --- | --- | --- | --- |
| chr9:44546866-44547411 | | | 546 bp | | |  |  |  |  |
| Genome Start | Genome End | Strand | Div | Repeat Start | Repeat End | Repeat Left | Name | Family | Class |
| 44546866 | 44546936 | - | 19.7 | 5820 | 5886 | -278 | L1ME2 | L1 | LINE |
| 44546937 | 44547226 | + | 9.7 | 1 | 291 | -21 | AluSx | Alu | SINE |
| 44547227 | 44547411 | - | 19.7 | 5608 | 5819 | -345 | L1ME2 | L1 | LINE |

  

|  |  |  |  |  |  |  |  |  |  |
| --- | --- | --- | --- | --- | --- | --- | --- | --- | --- |
| chr9:44551900-44552732 | | | 618 bp | | |  |  |  |  |
| Genome Start | Genome End | Strand | Div | Repeat Start | Repeat End | Repeat Left | Name | Family | Class |
| 44551900 | 44551955 | + | 21.4 | 4039 | 4097 | -2049 | L1M4 | L1 | LINE |
| 44552170 | 44552499 | - | 18.6 | 1 | 386 | -515 | MER54B | ERVL | LTR |
| 44552501 | 44552732 | + | 19.6 | 4094 | 4321 | -1825 | L1M4 | L1 | LINE |

  

|  |  |  |  |  |  |  |  |  |  |
| --- | --- | --- | --- | --- | --- | --- | --- | --- | --- |
| chr9:44558496-44559939 | | | 1444 bp | | |  |  |  |  |
| Genome Start | Genome End | Strand | Div | Repeat Start | Repeat End | Repeat Left | Name | Family | Class |
| 44558496 | 44559381 | + | 17.7 | 4316 | 5160 | -986 | L1M4 | L1 | LINE |
| 44559382 | 44559671 | - | 13.4 | 1 | 290 | -22 | AluSx | Alu | SINE |
| 44559672 | 44559939 | + | 17.9 | 5161 | 5437 | -741 | L1M4 | L1 | LINE |

  

|  |  |  |  |  |  |  |  |  |  |
| --- | --- | --- | --- | --- | --- | --- | --- | --- | --- |
| chr9:44564842-44565897 | | | 1056 bp | | |  |  |  |  |
| Genome Start | Genome End | Strand | Div | Repeat Start | Repeat End | Repeat Left | Name | Family | Class |
| 44564842 | 44565446 | + | 14.0 | 1 | 617 | -166 | LTR39 | ERV1 | LTR |
| 44565447 | 44565749 | + | 4.6 | 1 | 303 | 0 | AluY | Alu | SINE |
| 44565750 | 44565897 | + | 14.0 | 618 | 765 | -18 | LTR39 | ERV1 | LTR |

  

|  |  |  |  |  |  |  |  |  |  |
| --- | --- | --- | --- | --- | --- | --- | --- | --- | --- |
| chr9:44579455-44580714 | | | 1288 bp | | |  |  |  |  |
| Genome Start | Genome End | Strand | Div | Repeat Start | Repeat End | Repeat Left | Name | Family | Class |
| 44579455 | 44579581 | + | 21.4 | 1 | 138 | -569 | MER39 | ERV1 | LTR |
| 44579554 | 44579697 | + | 16.4 | 260 | 397 | -310 | MER39 | ERV1 | LTR |
| 44579698 | 44580403 | + | 13.4 | 1 | 691 | 0 | LTR8 | ERV1 | LTR |
| 44580404 | 44580714 | + | 15.3 | 394 | 705 | -2 | MER39 | ERV1 | LTR |

  

|  |  |  |  |  |  |  |  |  |  |
| --- | --- | --- | --- | --- | --- | --- | --- | --- | --- |
| chr9:44596399-44597128 | | | 730 bp | | |  |  |  |  |
| Genome Start | Genome End | Strand | Div | Repeat Start | Repeat End | Repeat Left | Name | Family | Class |
| 44596399 | 44596619 | - | 19.1 | 196 | 426 | 0 | MSTB | MaLR | LTR |
| 44596620 | 44596941 | - | 14.9 | 1 | 322 | 0 | AluSx | Alu | SINE |
| 44596942 | 44597128 | - | 19.1 | 8 | 195 | -231 | MSTB | MaLR | LTR |

  

|  |  |  |  |  |  |  |  |  |  |
| --- | --- | --- | --- | --- | --- | --- | --- | --- | --- |
| chr9:44604871-44605767 | | | 897 bp | | |  |  |  |  |
| Genome Start | Genome End | Strand | Div | Repeat Start | Repeat End | Repeat Left | Name | Family | Class |
| 44604871 | 44605247 | + | 27.3 | 27 | 436 | -32 | MER110A | ERV1 | LTR |
| 44605248 | 44605746 | - | 21.5 | 1 | 505 | 0 | MLT1D | MaLR | LTR |
| 44605747 | 44605767 | + | 27.3 | 437 | 456 | -12 | MER110A | ERV1 | LTR |

  

|  |  |  |  |  |  |  |  |  |  |
| --- | --- | --- | --- | --- | --- | --- | --- | --- | --- |
| chr9:44678068-44678598 | | | 531 bp | | |  |  |  |  |
| Genome Start | Genome End | Strand | Div | Repeat Start | Repeat End | Repeat Left | Name | Family | Class |
| 44678068 | 44678096 | + | 34.1 | 2306 | 2328 | -1091 | L2 | L2 | LINE |
| 44678097 | 44678389 | - | 13.0 | 1 | 295 | -14 | AluSc | Alu | SINE |
| 44678390 | 44678598 | + | 34.1 | 2329 | 2536 | -883 | L2 | L2 | LINE |

  

|  |  |  |  |  |  |  |  |  |  |
| --- | --- | --- | --- | --- | --- | --- | --- | --- | --- |
| chr9:44689795-44690605 | | | 432 bp | | |  |  |  |  |
| Genome Start | Genome End | Strand | Div | Repeat Start | Repeat End | Repeat Left | Name | Family | Class |
| 44689795 | 44690009 | - | 24.2 | 134 | 369 | -4 | MER83C | ERV1 | LTR |
| 44690043 | 44690116 | - | 25.7 | 121 | 197 | -181 | MER83B | ERV1 | LTR |
| 44690463 | 44690605 | - | 21.8 | 49 | 192 | -181 | MER83C | ERV1 | LTR |

  

|  |  |  |  |  |  |  |  |  |  |
| --- | --- | --- | --- | --- | --- | --- | --- | --- | --- |
| chr9:44698820-44699711 | | | 892 bp | | |  |  |  |  |
| Genome Start | Genome End | Strand | Div | Repeat Start | Repeat End | Repeat Left | Name | Family | Class |
| 44698820 | 44699262 | - | 24.8 | 707 | 2632 | -8 | MER69B | Tip100 | DNA |
| 44699263 | 44699538 | + | 16.3 | 1 | 291 | -21 | AluJb | Alu | SINE |
| 44699539 | 44699711 | - | 24.8 | 520 | 706 | -520 | MER69B | Tip100 | DNA |

  

|  |  |  |  |  |  |  |  |  |  |
| --- | --- | --- | --- | --- | --- | --- | --- | --- | --- |
| chr9:44705497-44707398 | | | 1773 bp | | |  |  |  |  |
| Genome Start | Genome End | Strand | Div | Repeat Start | Repeat End | Repeat Left | Name | Family | Class |
| 44705497 | 44705618 | - | 32.5 | 3269 | 3406 | -13 | L2 | L2 | LINE |
| 44705659 | 44705752 | + | 24.7 | 4080 | 4174 | -315 | L3 | CR1 | LINE |
| 44705769 | 44705898 | - | 24.2 | 984 | 1123 | -2296 | L2 | L2 | LINE |
| 44705899 | 44705940 | - | 9.5 | 445 | 486 | 0 | LTR19A | ERV1 | LTR |
| 44705941 | 44706158 | - | 6.0 | 7 | 241 | -245 | LTR19A | ERV1 | LTR |
| 44706163 | 44706366 | - | 25.8 | 756 | 969 | -2450 | L2 | L2 | LINE |
| 44706436 | 44707040 | - | 13.4 | 1 | 766 | 0 | MER4E | ERV1 | LTR |
| 44707041 | 44707398 | - | 24.5 | 8 | 426 | -2993 | L2 | L2 | LINE |

  

|  |  |  |  |  |  |  |  |  |  |
| --- | --- | --- | --- | --- | --- | --- | --- | --- | --- |
| chr9:44713795-44714617 | | | 823 bp | | |  |  |  |  |
| Genome Start | Genome End | Strand | Div | Repeat Start | Repeat End | Repeat Left | Name | Family | Class |
| 44713795 | 44714227 | + | 13.4 | 4730 | 5161 | -985 | L1P4 | L1 | LINE |
| 44714228 | 44714493 | + | 12.0 | 40 | 310 | 0 | AluSg | Alu | SINE |
| 44714494 | 44714617 | + | 13.4 | 5162 | 5286 | -860 | L1P4 | L1 | LINE |

  

|  |  |  |  |  |  |  |  |  |  |
| --- | --- | --- | --- | --- | --- | --- | --- | --- | --- |
| chr9:44716226-44716726 | | | 501 bp | | |  |  |  |  |
| Genome Start | Genome End | Strand | Div | Repeat Start | Repeat End | Repeat Left | Name | Family | Class |
| 44716226 | 44716443 | - | 22.6 | 6699 | 6892 | -916 | L1MC4a | L1 | LINE |
| 44716444 | 44716583 | + | 16.7 | 1 | 132 | -1 | FLAM\_C | Alu | SINE |
| 44716584 | 44716726 | - | 23.0 | 6599 | 6783 | -1110 | L1MC4a | L1 | LINE |

  

|  |  |  |  |  |  |  |  |  |  |
| --- | --- | --- | --- | --- | --- | --- | --- | --- | --- |
| chr9:44717040-44718109 | | | 1070 bp | | |  |  |  |  |
| Genome Start | Genome End | Strand | Div | Repeat Start | Repeat End | Repeat Left | Name | Family | Class |
| 44717040 | 44717139 | + | 18.9 | 4 | 108 | -951 | MER106B | MER1\_type | DNA |
| 44717140 | 44717435 | - | 13.2 | 1 | 296 | -16 | AluSx | Alu | SINE |
| 44717436 | 44717532 | + | 18.9 | 109 | 212 | -847 | MER106B | MER1\_type | DNA |
| 44717533 | 44717828 | + | 16.5 | 1 | 294 | -18 | AluSx | Alu | SINE |
| 44717829 | 44718109 | + | 18.9 | 213 | 532 | -527 | MER106B | MER1\_type | DNA |

  

|  |  |  |  |  |  |  |  |  |  |
| --- | --- | --- | --- | --- | --- | --- | --- | --- | --- |
| chr9:44720944-44721906 | | | 963 bp | | |  |  |  |  |
| Genome Start | Genome End | Strand | Div | Repeat Start | Repeat End | Repeat Left | Name | Family | Class |
| 44720944 | 44721183 | + | 16.1 | 22 | 354 | -417 | LTR25 | ERV1 | LTR |
| 44721184 | 44721511 | - | 15.8 | 1 | 460 | 0 | LTR46 | ERV1 | LTR |
| 44721512 | 44721906 | + | 15.4 | 355 | 779 | -65 | LTR25 | ERV1 | LTR |

  

|  |  |  |  |  |  |  |  |  |  |
| --- | --- | --- | --- | --- | --- | --- | --- | --- | --- |
| chr9:44722207-44723329 | | | 1123 bp | | |  |  |  |  |
| Genome Start | Genome End | Strand | Div | Repeat Start | Repeat End | Repeat Left | Name | Family | Class |
| 44722207 | 44722294 | + | 18.1 | 1 | 86 | -777 | MER21B | ERV1 | LTR |
| 44722295 | 44722595 | - | 12.4 | 3 | 298 | -14 | AluSx | Alu | SINE |
| 44722596 | 44723329 | + | 18.1 | 87 | 862 | -1 | MER21B | ERV1 | LTR |

  

|  |  |  |  |  |  |  |  |  |  |
| --- | --- | --- | --- | --- | --- | --- | --- | --- | --- |
| chr9:44757875-44760651 | | | 2775 bp | | |  |  |  |  |
| Genome Start | Genome End | Strand | Div | Repeat Start | Repeat End | Repeat Left | Name | Family | Class |
| 44757875 | 44758044 | + | 25.7 | 5298 | 5496 | -744 | L1M5 | L1 | LINE |
| 44758045 | 44758407 | - | 14.7 | 1 | 355 | 0 | THE1A | MaLR | LTR |
| 44758408 | 44759982 | - | 31.7 | 2 | 1735 | 0 | MLT-int | MaLR | LTR |
| 44759985 | 44760354 | - | 9.0 | 1 | 355 | 0 | THE1A | MaLR | LTR |
| 44760355 | 44760651 | + | 25.7 | 5497 | 5776 | -464 | L1M5 | L1 | LINE |

  

|  |  |  |  |  |  |  |  |  |  |
| --- | --- | --- | --- | --- | --- | --- | --- | --- | --- |
| chr9:44778096-44778803 | | | 708 bp | | |  |  |  |  |
| Genome Start | Genome End | Strand | Div | Repeat Start | Repeat End | Repeat Left | Name | Family | Class |
| 44778096 | 44778327 | - | 32.8 | 3169 | 3411 | -8 | L2 | L2 | LINE |
| 44778328 | 44778704 | + | 10.5 | 1 | 377 | 0 | AluSp | Alu | SINE |
| 44778705 | 44778803 | - | 32.8 | 3065 | 3168 | -251 | L2 | L2 | LINE |

  

|  |  |  |  |  |  |  |  |  |  |
| --- | --- | --- | --- | --- | --- | --- | --- | --- | --- |
| chr9:44793770-44794781 | | | 1012 bp | | |  |  |  |  |
| Genome Start | Genome End | Strand | Div | Repeat Start | Repeat End | Repeat Left | Name | Family | Class |
| 44793770 | 44793946 | + | 24.3 | 2642 | 2819 | -600 | L2 | L2 | LINE |
| 44793947 | 44794238 | + | 14.7 | 1 | 292 | -18 | AluSg | Alu | SINE |
| 44794239 | 44794781 | + | 26.4 | 2820 | 3401 | -18 | L2 | L2 | LINE |

  

|  |  |  |  |  |  |  |  |  |  |
| --- | --- | --- | --- | --- | --- | --- | --- | --- | --- |
| chr9:44800124-44800789 | | | 666 bp | | |  |  |  |  |
| Genome Start | Genome End | Strand | Div | Repeat Start | Repeat End | Repeat Left | Name | Family | Class |
| 44800124 | 44800306 | - | 24.8 | 81 | 260 | -2 | MIR | MIR | SINE |
| 44800307 | 44800720 | + | 15.1 | 1 | 365 | -1 | Tigger5a | MER2\_type | DNA |
| 44800721 | 44800789 | - | 24.8 | 13 | 80 | -182 | MIR | MIR | SINE |

  

|  |  |  |  |  |  |  |  |  |  |
| --- | --- | --- | --- | --- | --- | --- | --- | --- | --- |
| chr9:44802040-44802523 | | | 484 bp | | |  |  |  |  |
| Genome Start | Genome End | Strand | Div | Repeat Start | Repeat End | Repeat Left | Name | Family | Class |
| 44802040 | 44802171 | + | 16.3 | 8 | 134 | -44 | MER5B | MER1\_type | DNA |
| 44802172 | 44802481 | + | 12.3 | 1 | 310 | -3 | AluSq | Alu | SINE |
| 44802482 | 44802523 | + | 16.3 | 135 | 177 | -1 | MER5B | MER1\_type | DNA |

  

|  |  |  |  |  |  |  |  |  |  |
| --- | --- | --- | --- | --- | --- | --- | --- | --- | --- |
| chr9:44901954-44904491 | | | 2481 bp | | |  |  |  |  |
| Genome Start | Genome End | Strand | Div | Repeat Start | Repeat End | Repeat Left | Name | Family | Class |
| 44901954 | 44902381 | + | 31.9 | 745 | 1237 | -5548 | L1PA13 | L1 | LINE |
| 44902437 | 44902455 | + | 20.8 | 1 | 18 | -491 | LTR22C | ERVK | LTR |
| 44902456 | 44903108 | - | 12.8 | 1 | 675 | 0 | LTR76 | ERV1 | LTR |
| 44903109 | 44903551 | + | 22.4 | 19 | 458 | 0 | LTR22C | ERVK | LTR |
| 44903554 | 44904491 | + | 16.2 | 1423 | 2351 | -4434 | L1PA13 | L1 | LINE |

  

|  |  |  |  |  |  |  |  |  |  |
| --- | --- | --- | --- | --- | --- | --- | --- | --- | --- |
| chr9:44907546-44909877 | | | 2293 bp | | |  |  |  |  |
| Genome Start | Genome End | Strand | Div | Repeat Start | Repeat End | Repeat Left | Name | Family | Class |
| 44907546 | 44908139 | - | 16.1 | 588 | 1231 | 0 | Tigger3b | MER2\_type | DNA |
| 44908140 | 44909493 | + | 3.2 | 4801 | 6155 | 0 | L1PA3 | L1 | LINE |
| 44909510 | 44909758 | - | 14.9 | 332 | 587 | -644 | Tigger3b | MER2\_type | DNA |
| 44909782 | 44909877 | - | 21.5 | 1 | 94 | -1137 | Tigger3b | MER2\_type | DNA |

  

|  |  |  |  |  |  |  |  |  |  |
| --- | --- | --- | --- | --- | --- | --- | --- | --- | --- |
| chr9:44926487-44929831 | | | 3292 bp | | |  |  |  |  |
| Genome Start | Genome End | Strand | Div | Repeat Start | Repeat End | Repeat Left | Name | Family | Class |
| 44926487 | 44927546 | + | 25.9 | 1589 | 2661 | -3745 | L1MD2 | L1 | LINE |
| 44927547 | 44928140 | + | 14.0 | 5 | 622 | 0 | LTR77 | ERV1 | LTR |
| 44928141 | 44928376 | + | 22.7 | 2662 | 2903 | -3503 | L1MD2 | L1 | LINE |
| 44928423 | 44929071 | + | 22.7 | 2904 | 3537 | -2869 | L1MD2 | L1 | LINE |
| 44929049 | 44929396 | + | 22.2 | 5523 | 5867 | -635 | L1MD2 | L1 | LINE |
| 44929397 | 44929486 | - | 20.5 | 5987 | 6069 | -233 | L1MA1 | L1 | LINE |
| 44929517 | 44929831 | + | 26.3 | 5831 | 6150 | -469 | L1MD2 | L1 | LINE |

  

|  |  |  |  |  |  |  |  |  |  |
| --- | --- | --- | --- | --- | --- | --- | --- | --- | --- |
| chr9:44949321-44949986 | | | 666 bp | | |  |  |  |  |
| Genome Start | Genome End | Strand | Div | Repeat Start | Repeat End | Repeat Left | Name | Family | Class |
| 44949321 | 44949389 | + | 25.1 | 13 | 80 | -182 | MIR | MIR | SINE |
| 44949390 | 44949803 | - | 15.7 | 1 | 365 | -1 | Tigger5a | MER2\_type | DNA |
| 44949804 | 44949986 | + | 25.1 | 81 | 260 | -2 | MIR | MIR | SINE |

  

|  |  |  |  |  |  |  |  |  |  |
| --- | --- | --- | --- | --- | --- | --- | --- | --- | --- |
| chr9:44955330-44956341 | | | 1012 bp | | |  |  |  |  |
| Genome Start | Genome End | Strand | Div | Repeat Start | Repeat End | Repeat Left | Name | Family | Class |
| 44955330 | 44955870 | - | 27.4 | 2822 | 3401 | -18 | L2 | L2 | LINE |
| 44955871 | 44956164 | - | 13.9 | 1 | 294 | -16 | AluSg | Alu | SINE |
| 44956165 | 44956341 | - | 25.3 | 2643 | 2821 | -598 | L2 | L2 | LINE |

  

|  |  |  |  |  |  |  |  |  |  |
| --- | --- | --- | --- | --- | --- | --- | --- | --- | --- |
| chr9:44971701-44972374 | | | 672 bp | | |  |  |  |  |
| Genome Start | Genome End | Strand | Div | Repeat Start | Repeat End | Repeat Left | Name | Family | Class |
| 44971701 | 44971797 | + | 25.8 | 3065 | 3166 | -253 | L2 | L2 | LINE |
| 44971800 | 44972142 | - | 9.1 | 1 | 343 | 0 | AluSp | Alu | SINE |
| 44972143 | 44972374 | + | 34.5 | 3169 | 3411 | -8 | L2 | L2 | LINE |

  

|  |  |  |  |  |  |  |  |  |  |
| --- | --- | --- | --- | --- | --- | --- | --- | --- | --- |
| chr9:44994497-44997073 | | | 2125 bp | | |  |  |  |  |
| Genome Start | Genome End | Strand | Div | Repeat Start | Repeat End | Repeat Left | Name | Family | Class |
| 44994497 | 44995177 | + | 15.1 | 1066 | 1773 | -645 | Tigger1 | MER2\_type | DNA |
| 44995178 | 44995478 | - | 12.3 | 1 | 303 | -10 | AluSp | Alu | SINE |
| 44995536 | 44995754 | + | 26.3 | 445 | 643 | -50 | MER115 | Tip100 | DNA |
| 44996150 | 44996656 | + | 14.6 | 1801 | 2302 | -116 | Tigger1 | MER2\_type | DNA |
| 44996657 | 44996936 | - | 12.1 | 2 | 292 | -20 | AluSx | Alu | SINE |
| 44996937 | 44997073 | + | 14.6 | 2303 | 2418 | 0 | Tigger1 | MER2\_type | DNA |

  

|  |  |  |  |  |  |  |  |  |  |
| --- | --- | --- | --- | --- | --- | --- | --- | --- | --- |
| chr9:45004438-45006357 | | | 1769 bp | | |  |  |  |  |
| Genome Start | Genome End | Strand | Div | Repeat Start | Repeat End | Repeat Left | Name | Family | Class |
| 45004438 | 45004770 | + | 29.9 | 5421 | 5769 | -471 | L1ME3B | L1 | LINE |
| 45004847 | 45005132 | - | 15.7 | 3 | 288 | -24 | AluSx | Alu | SINE |
| 45005135 | 45005307 | - | 13.2 | 138 | 308 | -4 | AluSx | Alu | SINE |
| 45005308 | 45005590 | - | 15.9 | 8 | 292 | -19 | AluY | Alu | SINE |
| 45005591 | 45005731 | - | 13.2 | 1 | 137 | -175 | AluSx | Alu | SINE |
| 45005772 | 45005887 | + | 26.3 | 5852 | 5972 | -268 | L1ME3B | L1 | LINE |
| 45005913 | 45006150 | + | 14.7 | 66 | 297 | -15 | AluSg/x | Alu | SINE |
| 45006159 | 45006357 | + | 26.6 | 5972 | 6164 | -76 | L1ME3B | L1 | LINE |

  

|  |  |  |  |  |  |  |  |  |  |
| --- | --- | --- | --- | --- | --- | --- | --- | --- | --- |
| chr9:45027673-45028804 | | | 1132 bp | | |  |  |  |  |
| Genome Start | Genome End | Strand | Div | Repeat Start | Repeat End | Repeat Left | Name | Family | Class |
| 45027673 | 45028403 | - | 18.5 | 88 | 862 | -1 | MER21B | ERV1 | LTR |
| 45028404 | 45028716 | + | 11.6 | 3 | 310 | -2 | AluSx | Alu | SINE |
| 45028717 | 45028804 | - | 18.5 | 1 | 87 | -776 | MER21B | ERV1 | LTR |

  

|  |  |  |  |  |  |  |  |  |  |
| --- | --- | --- | --- | --- | --- | --- | --- | --- | --- |
| chr9:45032889-45033967 | | | 1079 bp | | |  |  |  |  |
| Genome Start | Genome End | Strand | Div | Repeat Start | Repeat End | Repeat Left | Name | Family | Class |
| 45032889 | 45033173 | - | 19.4 | 213 | 532 | -527 | MER106B | MER1\_type | DNA |
| 45033174 | 45033468 | - | 16.0 | 1 | 296 | -16 | AluSx | Alu | SINE |
| 45033469 | 45033566 | - | 19.4 | 109 | 212 | -847 | MER106B | MER1\_type | DNA |
| 45033567 | 45033867 | + | 13.0 | 1 | 301 | -11 | AluSx | Alu | SINE |
| 45033868 | 45033967 | - | 19.4 | 4 | 108 | -951 | MER106B | MER1\_type | DNA |

  

|  |  |  |  |  |  |  |  |  |  |
| --- | --- | --- | --- | --- | --- | --- | --- | --- | --- |
| chr9:45034282-45034782 | | | 501 bp | | |  |  |  |  |
| Genome Start | Genome End | Strand | Div | Repeat Start | Repeat End | Repeat Left | Name | Family | Class |
| 45034282 | 45034424 | + | 23.4 | 6646 | 6783 | -1259 | L1MC4 | L1 | LINE |
| 45034425 | 45034564 | - | 15.9 | 1 | 132 | -1 | FLAM\_C | Alu | SINE |
| 45034565 | 45034782 | + | 23.4 | 6784 | 6977 | -1065 | L1MC4 | L1 | LINE |

  

|  |  |  |  |  |  |  |  |  |  |
| --- | --- | --- | --- | --- | --- | --- | --- | --- | --- |
| chr9:45036392-45037215 | | | 824 bp | | |  |  |  |  |
| Genome Start | Genome End | Strand | Div | Repeat Start | Repeat End | Repeat Left | Name | Family | Class |
| 45036392 | 45036515 | - | 13.6 | 5162 | 5286 | -860 | L1P4 | L1 | LINE |
| 45036516 | 45036780 | - | 11.0 | 40 | 307 | -3 | AluSg | Alu | SINE |
| 45036781 | 45037215 | - | 13.6 | 4730 | 5161 | -985 | L1P4 | L1 | LINE |

  

|  |  |  |  |  |  |  |  |  |  |
| --- | --- | --- | --- | --- | --- | --- | --- | --- | --- |
| chr9:45051280-45052164 | | | 885 bp | | |  |  |  |  |
| Genome Start | Genome End | Strand | Div | Repeat Start | Repeat End | Repeat Left | Name | Family | Class |
| 45051280 | 45051452 | + | 25.7 | 520 | 706 | -520 | MER69B | Tip100 | DNA |
| 45051453 | 45051731 | - | 16.2 | 1 | 292 | -20 | AluJb | Alu | SINE |
| 45051732 | 45052164 | + | 25.7 | 707 | 2598 | -42 | MER69B | Tip100 | DNA |

  

|  |  |  |  |  |  |  |  |  |  |
| --- | --- | --- | --- | --- | --- | --- | --- | --- | --- |
| chr9:45055837-45056446 | | | 610 bp | | |  |  |  |  |
| Genome Start | Genome End | Strand | Div | Repeat Start | Repeat End | Repeat Left | Name | Family | Class |
| 45055837 | 45056119 | + | 18.4 | 2 | 329 | -67 | MSTD | MaLR | LTR |
| 45056120 | 45056428 | - | 11.7 | 1 | 309 | -3 | AluSx | Alu | SINE |
| 45056429 | 45056446 | + | 16.8 | 330 | 345 | -51 | MSTD | MaLR | LTR |

  

|  |  |  |  |  |  |  |  |  |  |
| --- | --- | --- | --- | --- | --- | --- | --- | --- | --- |
| chr9:45057595-45058222 | | | 628 bp | | |  |  |  |  |
| Genome Start | Genome End | Strand | Div | Repeat Start | Repeat End | Repeat Left | Name | Family | Class |
| 45057595 | 45057774 | - | 18.7 | 134 | 307 | -5 | AluJo | Alu | SINE |
| 45057775 | 45058105 | - | 17.9 | 1 | 301 | -11 | AluJb | Alu | SINE |
| 45058106 | 45058222 | - | 18.7 | 22 | 133 | -179 | AluJo | Alu | SINE |

  

|  |  |  |  |  |  |  |  |  |  |
| --- | --- | --- | --- | --- | --- | --- | --- | --- | --- |
| chr9:45063789-45064905 | | | 1117 bp | | |  |  |  |  |
| Genome Start | Genome End | Strand | Div | Repeat Start | Repeat End | Repeat Left | Name | Family | Class |
| 45063789 | 45063807 | + | 24.5 | 5 | 22 | -253 | ORSL | Tip100 | DNA |
| 45063808 | 45063942 | - | 11.8 | 468 | 600 | 0 | MER4A1\_ | ERV1 | LTR |
| 45063943 | 45064244 | - | 11.0 | 7 | 307 | 0 | AluSg1 | Alu | SINE |
| 45064245 | 45064719 | - | 11.8 | 1 | 467 | -133 | MER4A1\_ | ERV1 | LTR |
| 45064720 | 45064905 | + | 24.9 | 23 | 244 | -31 | ORSL | Tip100 | DNA |

  

|  |  |  |  |  |  |  |  |  |  |
| --- | --- | --- | --- | --- | --- | --- | --- | --- | --- |
| chr9:45072360-45072891 | | | 532 bp | | |  |  |  |  |
| Genome Start | Genome End | Strand | Div | Repeat Start | Repeat End | Repeat Left | Name | Family | Class |
| 45072360 | 45072568 | - | 34.2 | 2329 | 2536 | -883 | L2 | L2 | LINE |
| 45072569 | 45072860 | + | 13.8 | 1 | 293 | -16 | AluSc | Alu | SINE |
| 45072861 | 45072891 | - | 34.2 | 2304 | 2328 | -1091 | L2 | L2 | LINE |

  

|  |  |  |  |  |  |  |  |  |  |
| --- | --- | --- | --- | --- | --- | --- | --- | --- | --- |
| chr9:45090280-45090715 | | | 436 bp | | |  |  |  |  |
| Genome Start | Genome End | Strand | Div | Repeat Start | Repeat End | Repeat Left | Name | Family | Class |
| 45090280 | 45090312 | + | 25.6 | 5637 | 5665 | -456 | L1M5 | L1 | LINE |
| 45090313 | 45090617 | + | 6.3 | 1 | 301 | -10 | AluY | Alu | SINE |
| 45090618 | 45090715 | + | 19.3 | 5666 | 5767 | -395 | L1M5 | L1 | LINE |

  

|  |  |  |  |  |  |  |  |  |  |
| --- | --- | --- | --- | --- | --- | --- | --- | --- | --- |
| chr9:45109813-45110434 | | | 622 bp | | |  |  |  |  |
| Genome Start | Genome End | Strand | Div | Repeat Start | Repeat End | Repeat Left | Name | Family | Class |
| 45109813 | 45109851 | - | 26.2 | 335 | 371 | -38 | MLT1I | MaLR | LTR |
| 45109852 | 45110153 | + | 9.6 | 1 | 303 | -7 | AluSg | Alu | SINE |
| 45110154 | 45110434 | - | 26.2 | 54 | 334 | -75 | MLT1I | MaLR | LTR |

  

|  |  |  |  |  |  |  |  |  |  |
| --- | --- | --- | --- | --- | --- | --- | --- | --- | --- |
| chr9:45116701-45117933 | | | 742 bp | | |  |  |  |  |
| Genome Start | Genome End | Strand | Div | Repeat Start | Repeat End | Repeat Left | Name | Family | Class |
| 45116701 | 45116888 | - | 32.0 | 2031 | 2216 | -291 | HAL1 | L1 | LINE |
| 45116942 | 45117087 | - | 30.1 | 347 | 489 | -2 | LTR16C | ERVL | LTR |
| 45117090 | 45117317 | + | 10.5 | 67 | 295 | -14 | AluSc | Alu | SINE |
| 45117754 | 45117933 | - | 28.7 | 8 | 196 | -2311 | HAL1 | L1 | LINE |

  

|  |  |  |  |  |  |  |  |  |  |
| --- | --- | --- | --- | --- | --- | --- | --- | --- | --- |
| chr9:45121865-45127887 | | | 5668 bp | | |  |  |  |  |
| Genome Start | Genome End | Strand | Div | Repeat Start | Repeat End | Repeat Left | Name | Family | Class |
| 45121865 | 45122698 | - | 28.5 | 635 | 1616 | -3218 | L1MEe | L1 | LINE |
| 45122734 | 45123051 | - | 17.4 | 1 | 339 | -217 | MLT2B4 | ERVL | LTR |
| 45123227 | 45123360 | - | 19.8 | 6064 | 6180 | 0 | L1MB4 | L1 | LINE |
| 45123412 | 45123689 | - | 7.2 | 1 | 278 | -33 | AluY | Alu | SINE |
| 45123690 | 45124473 | - | 19.8 | 5286 | 6060 | -120 | L1MB4 | L1 | LINE |
| 45124474 | 45124857 | - | 14.8 | 1 | 428 | 0 | MSTA | MaLR | LTR |
| 45124858 | 45124908 | - | 20.1 | 5237 | 5284 | -862 | L1MD | L1 | LINE |
| 45124923 | 45124973 | - | 18.0 | 5472 | 5521 | -861 | L1M | L1 | LINE |
| 45124957 | 45125242 | - | 15.5 | 107 | 393 | -5991 | L1M | L1 | LINE |
| 45125243 | 45125542 | + | 9.4 | 1 | 298 | -13 | AluY | Alu | SINE |
| 45125543 | 45125646 | - | 15.5 | 1 | 106 | -6278 | L1M | L1 | LINE |
| 45125638 | 45126285 | - | 18.3 | 1515 | 2169 | -2665 | L1MEe | L1 | LINE |
| 45126287 | 45126412 | - | 21.7 | 2416 | 2535 | -77 | Charlie5 | MER1\_type | DNA |
| 45126425 | 45127212 | + | 16.8 | 11 | 785 | 0 | LTR1 | ERV1 | LTR |
| 45127273 | 45127657 | - | 21.4 | 1140 | 1523 | -3311 | L1MEe | L1 | LINE |
| 45127691 | 45127887 | - | 29.2 | 241 | 442 | -4412 | L1MEe | L1 | LINE |

  

|  |  |  |  |  |  |  |  |  |  |
| --- | --- | --- | --- | --- | --- | --- | --- | --- | --- |
| chr9:45128333-45129219 | | | 865 bp | | |  |  |  |  |
| Genome Start | Genome End | Strand | Div | Repeat Start | Repeat End | Repeat Left | Name | Family | Class |
| 45128333 | 45128671 | - | 17.7 | 2222 | 2555 | -4078 | L1MDa | L1 | LINE |
| 45128672 | 45128951 | + | 7.9 | 1 | 284 | -27 | AluY | Alu | SINE |
| 45128974 | 45129219 | - | 17.4 | 1853 | 2221 | -4412 | L1MDa | L1 | LINE |

  

|  |  |  |  |  |  |  |  |  |  |
| --- | --- | --- | --- | --- | --- | --- | --- | --- | --- |
| chr9:45161714-45165803 | | | 4090 bp | | |  |  |  |  |
| Genome Start | Genome End | Strand | Div | Repeat Start | Repeat End | Repeat Left | Name | Family | Class |
| 45161714 | 45163649 | + | 21.4 | 976 | 3113 | -3263 | L1M3d | L1 | LINE |
| 45163650 | 45163939 | - | 6.2 | 1 | 290 | -21 | AluY | Alu | SINE |
| 45163940 | 45164169 | + | 17.0 | 3114 | 3335 | -3041 | L1M3d | L1 | LINE |
| 45164170 | 45164480 | + | 8.7 | 1 | 310 | -1 | AluY | Alu | SINE |
| 45164481 | 45164735 | + | 17.0 | 3336 | 3588 | -2788 | L1M3d | L1 | LINE |
| 45164736 | 45165041 | + | 8.5 | 1 | 306 | -5 | AluY | Alu | SINE |
| 45165042 | 45165803 | + | 17.0 | 3589 | 4350 | -2029 | L1M3d | L1 | LINE |

  

|  |  |  |  |  |  |  |  |  |  |
| --- | --- | --- | --- | --- | --- | --- | --- | --- | --- |
| chr9:45248030-45251982 | | | 3953 bp | | |  |  |  |  |
| Genome Start | Genome End | Strand | Div | Repeat Start | Repeat End | Repeat Left | Name | Family | Class |
| 45248030 | 45248768 | - | 10.4 | 5194 | 5935 | -927 | L1PBa | L1 | LINE |
| 45248769 | 45248946 | - | 15.2 | 136 | 313 | 0 | AluSp | Alu | SINE |
| 45248947 | 45251982 | - | 10.4 | 2142 | 5193 | -1669 | L1PBa | L1 | LINE |

  

|  |  |  |  |  |  |  |  |  |  |
| --- | --- | --- | --- | --- | --- | --- | --- | --- | --- |
| chr9:45294331-45296660 | | | 2285 bp | | |  |  |  |  |
| Genome Start | Genome End | Strand | Div | Repeat Start | Repeat End | Repeat Left | Name | Family | Class |
| 45294331 | 45294951 | + | 23.0 | 3 | 667 | -913 | MSTA-int | MaLR | LTR |
| 45294987 | 45295284 | - | 11.7 | 3 | 301 | -10 | AluY | Alu | SINE |
| 45295293 | 45295786 | - | 8.3 | 1 | 487 | -6 | LTR15 | ERV1 | LTR |
| 45295789 | 45296660 | + | 18.8 | 725 | 1651 | 0 | MSTA-int | MaLR | LTR |

  

|  |  |  |  |  |  |  |  |  |  |
| --- | --- | --- | --- | --- | --- | --- | --- | --- | --- |
| chr9:45301604-45302199 | | | 596 bp | | |  |  |  |  |
| Genome Start | Genome End | Strand | Div | Repeat Start | Repeat End | Repeat Left | Name | Family | Class |
| 45301604 | 45301700 | + | 34.5 | 5542 | 5639 | -2169 | L1MC4a | L1 | LINE |
| 45301701 | 45301987 | - | 13.3 | 21 | 306 | 0 | AluSx | Alu | SINE |
| 45301988 | 45302199 | + | 34.5 | 5640 | 5882 | -1926 | L1MC4a | L1 | LINE |

  

|  |  |  |  |  |  |  |  |  |  |
| --- | --- | --- | --- | --- | --- | --- | --- | --- | --- |
| chr9:45312864-45313838 | | | 946 bp | | |  |  |  |  |
| Genome Start | Genome End | Strand | Div | Repeat Start | Repeat End | Repeat Left | Name | Family | Class |
| 45312864 | 45313181 | + | 25.4 | 1030 | 1357 | -5444 | MER89-int | ERV1 | LTR |
| 45313182 | 45313513 | + | 22.4 | 1 | 340 | -25 | MLT1A0 | MaLR | LTR |
| 45313543 | 45313838 | + | 26.4 | 1361 | 1660 | -5141 | MER89-int | ERV1 | LTR |

  

|  |  |  |  |  |  |  |  |  |  |
| --- | --- | --- | --- | --- | --- | --- | --- | --- | --- |
| chr9:45315139-45315711 | | | 537 bp | | |  |  |  |  |
| Genome Start | Genome End | Strand | Div | Repeat Start | Repeat End | Repeat Left | Name | Family | Class |
| 45315139 | 45315317 | - | 13.5 | 132 | 303 | -7 | AluSg | Alu | SINE |
| 45315354 | 45315576 | - | 7.1 | 63 | 282 | -29 | AluY | Alu | SINE |
| 45315577 | 45315711 | - | 13.5 | 1 | 131 | -179 | AluSg | Alu | SINE |

  

|  |  |  |  |  |  |  |  |  |  |
| --- | --- | --- | --- | --- | --- | --- | --- | --- | --- |
| chr9:45342416-45347232 | | | 4758 bp | | |  |  |  |  |
| Genome Start | Genome End | Strand | Div | Repeat Start | Repeat End | Repeat Left | Name | Family | Class |
| 45342416 | 45342873 | - | 26.5 | 5637 | 6102 | -239 | L1ME1 | L1 | LINE |
| 45342874 | 45343180 | - | 11.4 | 1 | 308 | -5 | AluSq | Alu | SINE |
| 45343183 | 45343492 | - | 17.5 | 5 | 296 | -16 | AluJo | Alu | SINE |
| 45343493 | 45344915 | - | 27.8 | 4168 | 5636 | -705 | L1ME1 | L1 | LINE |
| 45344923 | 45345044 | - | 22.0 | 2412 | 2520 | -3982 | L1ME1 | L1 | LINE |
| 45345045 | 45345353 | - | 10.1 | 3 | 310 | 0 | AluSg | Alu | SINE |
| 45345354 | 45345402 | - | 22.0 | 2366 | 2411 | -4091 | L1ME1 | L1 | LINE |
| 45345403 | 45345954 | + | 18.4 | 1 | 541 | -1 | MER34 | ERV1 | LTR |
| 45345955 | 45346279 | - | 22.0 | 2034 | 2365 | -4137 | L1ME1 | L1 | LINE |
| 45346301 | 45346598 | - | 11.7 | 11 | 309 | 0 | AluSc | Alu | SINE |
| 45346626 | 45346799 | - | 23.7 | 2 | 186 | -38 | MER58A | MER1\_type | DNA |
| 45346802 | 45347232 | - | 25.2 | 1423 | 2039 | -4463 | L1ME1 | L1 | LINE |

  

|  |  |  |  |  |  |  |  |  |  |
| --- | --- | --- | --- | --- | --- | --- | --- | --- | --- |
| chr9:45360908-45362141 | | | 1234 bp | | |  |  |  |  |
| Genome Start | Genome End | Strand | Div | Repeat Start | Repeat End | Repeat Left | Name | Family | Class |
| 45360908 | 45361318 | - | 19.2 | 5785 | 6153 | -3 | L1PB4 | L1 | LINE |
| 45361319 | 45362025 | - | 9.1 | 5441 | 6147 | -7 | L1PA7 | L1 | LINE |
| 45362026 | 45362141 | - | 19.2 | 5661 | 5784 | -372 | L1PB4 | L1 | LINE |

  

|  |  |  |  |  |  |  |  |  |  |
| --- | --- | --- | --- | --- | --- | --- | --- | --- | --- |
| chr9:45366799-45367605 | | | 807 bp | | |  |  |  |  |
| Genome Start | Genome End | Strand | Div | Repeat Start | Repeat End | Repeat Left | Name | Family | Class |
| 45366799 | 45366848 | + | 36.8 | 38 | 84 | -184 | MIRb | MIR | SINE |
| 45366849 | 45367437 | - | 19.0 | 2 | 604 | -1 | MER6A | MER2\_type | DNA |
| 45367438 | 45367605 | + | 36.8 | 85 | 251 | -17 | MIRb | MIR | SINE |

  

|  |  |  |  |  |  |  |  |  |  |
| --- | --- | --- | --- | --- | --- | --- | --- | --- | --- |
| chr9:45402015-45402513 | | | 499 bp | | |  |  |  |  |
| Genome Start | Genome End | Strand | Div | Repeat Start | Repeat End | Repeat Left | Name | Family | Class |
| 45402015 | 45402118 | + | 31.7 | 2581 | 2682 | -737 | L2 | L2 | LINE |
| 45402119 | 45402489 | - | 26.8 | 1 | 365 | 0 | MLT1A0 | MaLR | LTR |
| 45402490 | 45402513 | + | 31.7 | 2683 | 2705 | -714 | L2 | L2 | LINE |

  

|  |  |  |  |  |  |  |  |  |  |
| --- | --- | --- | --- | --- | --- | --- | --- | --- | --- |
| chr9:45410917-45412346 | | | 1430 bp | | |  |  |  |  |
| Genome Start | Genome End | Strand | Div | Repeat Start | Repeat End | Repeat Left | Name | Family | Class |
| 45410917 | 45411775 | + | 23.2 | 6607 | 7597 | -292 | L1MC4a | L1 | LINE |
| 45411776 | 45412080 | - | 8.3 | 4 | 306 | -5 | AluY | Alu | SINE |
| 45412081 | 45412346 | + | 23.2 | 7517 | 7807 | -1 | L1MC4a | L1 | LINE |

  

|  |  |  |  |  |  |  |  |  |  |
| --- | --- | --- | --- | --- | --- | --- | --- | --- | --- |
| chr9:45423264-45425212 | | | 1659 bp | | |  |  |  |  |
| Genome Start | Genome End | Strand | Div | Repeat Start | Repeat End | Repeat Left | Name | Family | Class |
| 45423264 | 45424420 | - | 30.4 | 5004 | 6139 | -27 | L1ME1 | L1 | LINE |
| 45424498 | 45424623 | + | 19.2 | 1 | 131 | -45 | FRAM | Alu | SINE |
| 45424837 | 45425212 | - | 29.3 | 4326 | 4700 | -1446 | L1ME1 | L1 | LINE |

  

|  |  |  |  |  |  |  |  |  |  |
| --- | --- | --- | --- | --- | --- | --- | --- | --- | --- |
| chr9:45427753-45431195 | | | 3443 bp | | |  |  |  |  |
| Genome Start | Genome End | Strand | Div | Repeat Start | Repeat End | Repeat Left | Name | Family | Class |
| 45427753 | 45429957 | - | 16.0 | 3943 | 6157 | -9 | L1PA16 | L1 | LINE |
| 45429958 | 45430259 | - | 4.8 | 1 | 314 | 0 | AluY | Alu | SINE |
| 45430260 | 45431195 | - | 15.7 | 2983 | 3942 | -2204 | L1PA16 | L1 | LINE |

  

|  |  |  |  |  |  |  |  |  |  |
| --- | --- | --- | --- | --- | --- | --- | --- | --- | --- |
| chr9:45441516-45445568 | | | 3953 bp | | |  |  |  |  |
| Genome Start | Genome End | Strand | Div | Repeat Start | Repeat End | Repeat Left | Name | Family | Class |
| 45441516 | 45442424 | - | 27.3 | 5244 | 6161 | -5 | L1ME1 | L1 | LINE |
| 45442426 | 45443006 | - | 24.2 | 62 | 607 | 0 | MER77 | ERVL | LTR |
| 45443110 | 45445158 | + | 8.1 | 4089 | 6152 | -2 | L1PA7 | L1 | LINE |
| 45445155 | 45445568 | - | 30.1 | 4755 | 5173 | -973 | L1ME1 | L1 | LINE |

  

|  |  |  |  |  |  |  |  |  |  |
| --- | --- | --- | --- | --- | --- | --- | --- | --- | --- |
| chr9:45449436-45450514 | | | 1079 bp | | |  |  |  |  |
| Genome Start | Genome End | Strand | Div | Repeat Start | Repeat End | Repeat Left | Name | Family | Class |
| 45449436 | 45449466 | + | 25.1 | 1 | 31 | -474 | MLT1D | MaLR | LTR |
| 45449467 | 45449819 | + | 16.7 | 1 | 364 | 0 | THE1B | MaLR | LTR |
| 45449820 | 45450072 | + | 25.1 | 32 | 243 | -262 | MLT1D | MaLR | LTR |
| 45450073 | 45450279 | - | 15.5 | 6087 | 6294 | -6 | L1MA6 | L1 | LINE |
| 45450280 | 45450514 | + | 25.1 | 244 | 477 | -28 | MLT1D | MaLR | LTR |

  

|  |  |  |  |  |  |  |  |  |  |
| --- | --- | --- | --- | --- | --- | --- | --- | --- | --- |
| chr9:45460002-45462664 | | | 2551 bp | | |  |  |  |  |
| Genome Start | Genome End | Strand | Div | Repeat Start | Repeat End | Repeat Left | Name | Family | Class |
| 45460002 | 45460715 | - | 32.4 | 2415 | 3172 | -247 | L2 | L2 | LINE |
| 45460716 | 45461104 | - | 29.4 | 87 | 517 | -6 | LTR33A | ERVL | LTR |
| 45461217 | 45462005 | - | 31.3 | 1615 | 2425 | -994 | L2 | L2 | LINE |
| 45462006 | 45462235 | + | 24.6 | 1 | 219 | 0 | MER20 | MER1\_type | DNA |
| 45462236 | 45462664 | - | 31.3 | 1203 | 1614 | -1805 | L2 | L2 | LINE |

  

|  |  |  |  |  |  |  |  |  |  |
| --- | --- | --- | --- | --- | --- | --- | --- | --- | --- |
| chr9:45477568-45478788 | | | 1221 bp | | |  |  |  |  |
| Genome Start | Genome End | Strand | Div | Repeat Start | Repeat End | Repeat Left | Name | Family | Class |
| 45477568 | 45477773 | + | 18.2 | 1 | 193 | -119 | AluJb | Alu | SINE |
| 45477774 | 45478679 | + | 17.4 | 1 | 983 | 0 | LTR1D | ERV1 | LTR |
| 45478680 | 45478788 | + | 18.2 | 194 | 298 | -14 | AluJb | Alu | SINE |

  

|  |  |  |  |  |  |  |  |  |  |
| --- | --- | --- | --- | --- | --- | --- | --- | --- | --- |
| chr9:45487046-45487861 | | | 816 bp | | |  |  |  |  |
| Genome Start | Genome End | Strand | Div | Repeat Start | Repeat End | Repeat Left | Name | Family | Class |
| 45487046 | 45487251 | - | 35.5 | 732 | 962 | -5157 | L1MEd | L1 | LINE |
| 45487252 | 45487552 | - | 12.3 | 2 | 302 | -11 | AluSq | Alu | SINE |
| 45487553 | 45487861 | - | 35.5 | 447 | 731 | -5388 | L1MEd | L1 | LINE |

  

|  |  |  |  |  |  |  |  |  |  |
| --- | --- | --- | --- | --- | --- | --- | --- | --- | --- |
| chr9:45500254-45503161 | | | 2909 bp | | |  |  |  |  |
| Genome Start | Genome End | Strand | Div | Repeat Start | Repeat End | Repeat Left | Name | Family | Class |
| 45500254 | 45500763 | + | 20.7 | 195 | 886 | -2596 | MER93B-int | ERV1 | LTR |
| 45500763 | 45501390 | + | 24.0 | 4205 | 4830 | -387 | MER93B-int | ERV1 | LTR |
| 45501391 | 45501710 | + | 6.9 | 1 | 306 | -5 | AluY | Alu | SINE |
| 45501711 | 45503161 | + | 16.8 | 609 | 4911 | -306 | MER93B-int | ERV1 | LTR |

  

|  |  |  |  |  |  |  |  |  |  |
| --- | --- | --- | --- | --- | --- | --- | --- | --- | --- |
| chr9:45506287-45507075 | | | 789 bp | | |  |  |  |  |
| Genome Start | Genome End | Strand | Div | Repeat Start | Repeat End | Repeat Left | Name | Family | Class |
| 45506287 | 45506606 | + | 17.3 | 1 | 308 | -193 | MER61D | ERV1 | LTR |
| 45506607 | 45506897 | + | 14.8 | 2 | 293 | -17 | AluSg | Alu | SINE |
| 45506898 | 45507075 | + | 17.3 | 309 | 501 | 0 | MER61D | ERV1 | LTR |

  

|  |  |  |  |  |  |  |  |  |  |
| --- | --- | --- | --- | --- | --- | --- | --- | --- | --- |
| chr9:45507760-45508657 | | | 898 bp | | |  |  |  |  |
| Genome Start | Genome End | Strand | Div | Repeat Start | Repeat End | Repeat Left | Name | Family | Class |
| 45507760 | 45508001 | + | 16.7 | 1 | 251 | -88 | MER1B | MER1\_type | DNA |
| 45508002 | 45508570 | - | 23.9 | 1 | 554 | 0 | MLT2B3 | ERVL | LTR |
| 45508571 | 45508657 | + | 16.7 | 252 | 337 | -2 | MER1B | MER1\_type | DNA |

  

|  |  |  |  |  |  |  |  |  |  |
| --- | --- | --- | --- | --- | --- | --- | --- | --- | --- |
| chr9:45537213-45538514 | | | 881 bp | | |  |  |  |  |
| Genome Start | Genome End | Strand | Div | Repeat Start | Repeat End | Repeat Left | Name | Family | Class |
| 45537213 | 45537515 | + | 35.0 | 500 | 807 | -5312 | L1MEd | L1 | LINE |
| 45537903 | 45538127 | + | 35.0 | 3179 | 3415 | -4 | L2 | L2 | LINE |
| 45538162 | 45538514 | + | 28.6 | 1359 | 1728 | -4421 | L1MEd | L1 | LINE |

  

|  |  |  |  |  |  |  |  |  |  |
| --- | --- | --- | --- | --- | --- | --- | --- | --- | --- |
| chr9:45543145-45544089 | | | 945 bp | | |  |  |  |  |
| Genome Start | Genome End | Strand | Div | Repeat Start | Repeat End | Repeat Left | Name | Family | Class |
| 45543145 | 45543437 | - | 30.4 | 3302 | 3624 | -2522 | L1MD | L1 | LINE |
| 45543438 | 45543745 | - | 13.0 | 1 | 308 | -4 | AluSx | Alu | SINE |
| 45543746 | 45544089 | - | 30.4 | 2948 | 3302 | -2844 | L1MD | L1 | LINE |

  

|  |  |  |  |  |  |  |  |  |  |
| --- | --- | --- | --- | --- | --- | --- | --- | --- | --- |
| chr9:45563687-45566345 | | | 2659 bp | | |  |  |  |  |
| Genome Start | Genome End | Strand | Div | Repeat Start | Repeat End | Repeat Left | Name | Family | Class |
| 45563687 | 45565717 | + | 15.6 | 24 | 2083 | -335 | Tigger1 | MER2\_type | DNA |
| 45565718 | 45566020 | + | 13.5 | 7 | 312 | 0 | AluSx | Alu | SINE |
| 45566021 | 45566345 | + | 15.6 | 2084 | 2417 | -1 | Tigger1 | MER2\_type | DNA |

  

|  |  |  |  |  |  |  |  |  |  |
| --- | --- | --- | --- | --- | --- | --- | --- | --- | --- |
| chr9:45574228-45578518 | | | 4291 bp | | |  |  |  |  |
| Genome Start | Genome End | Strand | Div | Repeat Start | Repeat End | Repeat Left | Name | Family | Class |
| 45574228 | 45575976 | + | 25.6 | 1762 | 3454 | -4701 | L1PREC2 | L1 | LINE |
| 45575977 | 45576267 | + | 11.6 | 1 | 311 | 0 | AluY | Alu | SINE |
| 45576268 | 45578518 | + | 21.5 | 3455 | 5808 | -2333 | L1PREC2 | L1 | LINE |

  

|  |  |  |  |  |  |  |  |  |  |
| --- | --- | --- | --- | --- | --- | --- | --- | --- | --- |
| chr9:45588741-45591278 | | | 2481 bp | | |  |  |  |  |
| Genome Start | Genome End | Strand | Div | Repeat Start | Repeat End | Repeat Left | Name | Family | Class |
| 45588741 | 45589168 | + | 32.5 | 745 | 1237 | -5548 | L1PA13 | L1 | LINE |
| 45589224 | 45589242 | + | 20.4 | 1 | 18 | -491 | LTR22C | ERVK | LTR |
| 45589243 | 45589895 | - | 13.0 | 1 | 675 | 0 | LTR76 | ERV1 | LTR |
| 45589896 | 45590338 | + | 22.3 | 19 | 458 | 0 | LTR22C | ERVK | LTR |
| 45590341 | 45591278 | + | 16.0 | 1423 | 2351 | -4434 | L1PA13 | L1 | LINE |

  

|  |  |  |  |  |  |  |  |  |  |
| --- | --- | --- | --- | --- | --- | --- | --- | --- | --- |
| chr9:45594333-45596663 | | | 2292 bp | | |  |  |  |  |
| Genome Start | Genome End | Strand | Div | Repeat Start | Repeat End | Repeat Left | Name | Family | Class |
| 45594333 | 45594930 | - | 16.0 | 588 | 1231 | 0 | Tigger3b | MER2\_type | DNA |
| 45594931 | 45596283 | + | 2.9 | 4801 | 6155 | 0 | L1PA3 | L1 | LINE |
| 45596302 | 45596546 | - | 15.9 | 332 | 587 | -644 | Tigger3b | MER2\_type | DNA |
| 45596568 | 45596663 | - | 20.4 | 1 | 94 | -1137 | Tigger3b | MER2\_type | DNA |

  

|  |  |  |  |  |  |  |  |  |  |
| --- | --- | --- | --- | --- | --- | --- | --- | --- | --- |
| chr9:45601705-45611318 | | | 9549 bp | | |  |  |  |  |
| Genome Start | Genome End | Strand | Div | Repeat Start | Repeat End | Repeat Left | Name | Family | Class |
| 45601705 | 45602446 | + | 31.2 | 9 | 848 | -5561 | L1MD2 | L1 | LINE |
| 45602447 | 45602691 | + | 23.2 | 1 | 245 | -227 | MER70A | ERVL | LTR |
| 45602692 | 45602994 | + | 8.7 | 1 | 305 | -8 | AluSq | Alu | SINE |
| 45602995 | 45603224 | + | 23.2 | 246 | 472 | 0 | MER70A | ERVL | LTR |
| 45603234 | 45604224 | + | 20.1 | 10 | 1076 | -3947 | MER70A-int | ERVL | LTR |
| 45604244 | 45604327 | + | 17.9 | 2300 | 2384 | -2639 | MER70A-int | ERVL | LTR |
| 45604328 | 45604721 | - | 11.7 | 1 | 450 | 0 | LTR7 | ERV1 | LTR |
| 45604722 | 45605154 | - | 9.6 | 7283 | 7713 | 0 | HERVH | ERV1 | LTR |
| 45605174 | 45606666 | - | 12.6 | 3582 | 5286 | -1805 | HERVH | ERV1 | LTR |
| 45606669 | 45606785 | - | 12.4 | 2613 | 2725 | -4988 | HERVH | ERV1 | LTR |
| 45606793 | 45607183 | - | 11.6 | 1 | 450 | 0 | LTR7 | ERV1 | LTR |
| 45607184 | 45609333 | + | 19.0 | 2380 | 4592 | -431 | MER70A-int | ERVL | LTR |
| 45609334 | 45609632 | - | 13.1 | 1 | 292 | -21 | AluSp | Alu | SINE |
| 45609633 | 45610062 | + | 19.0 | 4593 | 5022 | -1 | MER70A-int | ERVL | LTR |
| 45610071 | 45610574 | + | 24.0 | 1 | 472 | 0 | MER70A | ERVL | LTR |
| 45610576 | 45611318 | + | 24.1 | 845 | 1603 | -4899 | L1MD2 | L1 | LINE |

  

|  |  |  |  |  |  |  |  |  |  |
| --- | --- | --- | --- | --- | --- | --- | --- | --- | --- |
| chr9:45634774-45635439 | | | 666 bp | | |  |  |  |  |
| Genome Start | Genome End | Strand | Div | Repeat Start | Repeat End | Repeat Left | Name | Family | Class |
| 45634774 | 45634842 | + | 24.8 | 13 | 80 | -182 | MIR | MIR | SINE |
| 45634843 | 45635256 | - | 15.7 | 1 | 365 | -1 | Tigger5a | MER2\_type | DNA |
| 45635257 | 45635439 | + | 24.8 | 81 | 260 | -2 | MIR | MIR | SINE |

  

|  |  |  |  |  |  |  |  |  |  |
| --- | --- | --- | --- | --- | --- | --- | --- | --- | --- |
| chr9:45640778-45641789 | | | 1012 bp | | |  |  |  |  |
| Genome Start | Genome End | Strand | Div | Repeat Start | Repeat End | Repeat Left | Name | Family | Class |
| 45640778 | 45641320 | - | 26.4 | 2820 | 3401 | -18 | L2 | L2 | LINE |
| 45641321 | 45641612 | - | 13.0 | 1 | 292 | -18 | AluSg | Alu | SINE |
| 45641613 | 45641789 | - | 24.3 | 2642 | 2819 | -600 | L2 | L2 | LINE |

  

|  |  |  |  |  |  |  |  |  |  |
| --- | --- | --- | --- | --- | --- | --- | --- | --- | --- |
| chr9:45655246-45655767 | | | 522 bp | | |  |  |  |  |
| Genome Start | Genome End | Strand | Div | Repeat Start | Repeat End | Repeat Left | Name | Family | Class |
| 45655246 | 45655337 | - | 26.6 | 185 | 263 | -5 | MIRb | MIR | SINE |
| 45655338 | 45655598 | - | 14.6 | 1 | 280 | -32 | AluJb | Alu | SINE |
| 45655599 | 45655767 | - | 26.6 | 20 | 184 | -84 | MIRb | MIR | SINE |

  

|  |  |  |  |  |  |  |  |  |  |
| --- | --- | --- | --- | --- | --- | --- | --- | --- | --- |
| chr9:45656790-45657453 | | | 664 bp | | |  |  |  |  |
| Genome Start | Genome End | Strand | Div | Repeat Start | Repeat End | Repeat Left | Name | Family | Class |
| 45656790 | 45656888 | + | 33.8 | 3065 | 3168 | -251 | L2 | L2 | LINE |
| 45656889 | 45657221 | - | 9.1 | 1 | 333 | 0 | AluSp | Alu | SINE |
| 45657222 | 45657453 | + | 33.8 | 3169 | 3411 | -8 | L2 | L2 | LINE |

  

|  |  |  |  |  |  |  |  |  |  |
| --- | --- | --- | --- | --- | --- | --- | --- | --- | --- |
| chr9:45679256-45681831 | | | 2127 bp | | |  |  |  |  |
| Genome Start | Genome End | Strand | Div | Repeat Start | Repeat End | Repeat Left | Name | Family | Class |
| 45679256 | 45679936 | + | 14.9 | 1066 | 1773 | -645 | Tigger1 | MER2\_type | DNA |
| 45679937 | 45680239 | - | 12.5 | 1 | 305 | -8 | AluSp | Alu | SINE |
| 45680297 | 45680515 | + | 24.5 | 445 | 643 | -50 | MER115 | Tip100 | DNA |
| 45680908 | 45681414 | + | 14.8 | 1801 | 2302 | -116 | Tigger1 | MER2\_type | DNA |
| 45681415 | 45681694 | - | 11.8 | 2 | 292 | -20 | AluSx | Alu | SINE |
| 45681695 | 45681831 | + | 14.8 | 2303 | 2418 | 0 | Tigger1 | MER2\_type | DNA |

  

|  |  |  |  |  |  |  |  |  |  |
| --- | --- | --- | --- | --- | --- | --- | --- | --- | --- |
| chr9:45689197-45691110 | | | 1762 bp | | |  |  |  |  |
| Genome Start | Genome End | Strand | Div | Repeat Start | Repeat End | Repeat Left | Name | Family | Class |
| 45689197 | 45689529 | + | 29.9 | 5421 | 5769 | -471 | L1ME3B | L1 | LINE |
| 45689606 | 45689891 | - | 15.7 | 3 | 288 | -24 | AluSx | Alu | SINE |
| 45689894 | 45690062 | - | 13.0 | 138 | 304 | -8 | AluSx | Alu | SINE |
| 45690063 | 45690345 | - | 15.6 | 8 | 292 | -19 | AluY | Alu | SINE |
| 45690346 | 45690486 | - | 13.0 | 1 | 137 | -175 | AluSx | Alu | SINE |
| 45690527 | 45690640 | + | 25.0 | 5852 | 5972 | -268 | L1ME3B | L1 | LINE |
| 45690667 | 45690903 | + | 14.8 | 67 | 297 | -15 | AluSg/x | Alu | SINE |
| 45690912 | 45691110 | + | 27.2 | 5972 | 6164 | -76 | L1ME3B | L1 | LINE |

  

|  |  |  |  |  |  |  |  |  |  |
| --- | --- | --- | --- | --- | --- | --- | --- | --- | --- |
| chr9:45759937-45760354 | | | 339 bp | | |  |  |  |  |
| Genome Start | Genome End | Strand | Div | Repeat Start | Repeat End | Repeat Left | Name | Family | Class |
| 45759937 | 45760113 | - | 31.2 | 965 | 1150 | -4969 | L1MEd | L1 | LINE |
| 45760116 | 45760163 | + | 20.8 | 2358 | 2405 | -15 | MER58 | MER1\_type | DNA |
| 45760241 | 45760354 | - | 26.9 | 457 | 565 | -5554 | L1MEd | L1 | LINE |

  

|  |  |  |  |  |  |  |  |  |  |
| --- | --- | --- | --- | --- | --- | --- | --- | --- | --- |
| chr9:45779470-45780240 | | | 771 bp | | |  |  |  |  |
| Genome Start | Genome End | Strand | Div | Repeat Start | Repeat End | Repeat Left | Name | Family | Class |
| 45779470 | 45779559 | + | 33.3 | 2 | 89 | -2772 | Charlie2 | MER1\_type | DNA |
| 45779560 | 45780066 | - | 27.0 | 1 | 519 | 0 | LTR40a | ERVL | LTR |
| 45780067 | 45780240 | + | 33.3 | 90 | 258 | -2603 | Charlie2 | MER1\_type | DNA |

  

|  |  |  |  |  |  |  |  |  |  |
| --- | --- | --- | --- | --- | --- | --- | --- | --- | --- |
| chr9:45788256-45788754 | | | 499 bp | | |  |  |  |  |
| Genome Start | Genome End | Strand | Div | Repeat Start | Repeat End | Repeat Left | Name | Family | Class |
| 45788256 | 45788359 | + | 31.7 | 2581 | 2682 | -737 | L2 | L2 | LINE |
| 45788360 | 45788730 | - | 26.8 | 1 | 365 | 0 | MLT1A0 | MaLR | LTR |
| 45788731 | 45788754 | + | 31.7 | 2683 | 2705 | -714 | L2 | L2 | LINE |

  

|  |  |  |  |  |  |  |  |  |  |
| --- | --- | --- | --- | --- | --- | --- | --- | --- | --- |
| chr9:45797149-45798578 | | | 1430 bp | | |  |  |  |  |
| Genome Start | Genome End | Strand | Div | Repeat Start | Repeat End | Repeat Left | Name | Family | Class |
| 45797149 | 45798007 | + | 22.9 | 6614 | 7516 | -292 | L1MC4a | L1 | LINE |
| 45798008 | 45798312 | - | 8.6 | 4 | 306 | -5 | AluY | Alu | SINE |
| 45798313 | 45798578 | + | 22.9 | 7517 | 7807 | -1 | L1MC4a | L1 | LINE |

  

|  |  |  |  |  |  |  |  |  |  |
| --- | --- | --- | --- | --- | --- | --- | --- | --- | --- |
| chr9:45809509-45811457 | | | 1659 bp | | |  |  |  |  |
| Genome Start | Genome End | Strand | Div | Repeat Start | Repeat End | Repeat Left | Name | Family | Class |
| 45809509 | 45810665 | - | 30.5 | 5004 | 6139 | -27 | L1ME1 | L1 | LINE |
| 45810743 | 45810868 | + | 19.2 | 1 | 131 | -45 | FRAM | Alu | SINE |
| 45811082 | 45811457 | - | 29.6 | 4326 | 4700 | -1446 | L1ME1 | L1 | LINE |

  

|  |  |  |  |  |  |  |  |  |  |
| --- | --- | --- | --- | --- | --- | --- | --- | --- | --- |
| chr9:45814000-45817442 | | | 3443 bp | | |  |  |  |  |
| Genome Start | Genome End | Strand | Div | Repeat Start | Repeat End | Repeat Left | Name | Family | Class |
| 45814000 | 45816204 | - | 16.0 | 3943 | 6157 | -9 | L1PA16 | L1 | LINE |
| 45816205 | 45816506 | - | 4.8 | 1 | 314 | 0 | AluY | Alu | SINE |
| 45816507 | 45817442 | - | 15.6 | 2983 | 3942 | -2204 | L1PA16 | L1 | LINE |

  

|  |  |  |  |  |  |  |  |  |  |
| --- | --- | --- | --- | --- | --- | --- | --- | --- | --- |
| chr9:45818946-45820193 | | | 1157 bp | | |  |  |  |  |
| Genome Start | Genome End | Strand | Div | Repeat Start | Repeat End | Repeat Left | Name | Family | Class |
| 45818946 | 45819051 | + | 19.8 | 4260 | 4378 | -1768 | L1M5 | L1 | LINE |
| 45819135 | 45819404 | + | 34.7 | 5009 | 5304 | -842 | L1M5 | L1 | LINE |
| 45819413 | 45819526 | + | 30.8 | 5319 | 5437 | -803 | L1M5 | L1 | LINE |
| 45819527 | 45819830 | - | 12.2 | 1 | 304 | -8 | AluSx | Alu | SINE |
| 45819831 | 45820193 | + | 30.8 | 5438 | 5801 | -439 | L1M5 | L1 | LINE |

  

|  |  |  |  |  |  |  |  |  |  |
| --- | --- | --- | --- | --- | --- | --- | --- | --- | --- |
| chr9:45827767-45831819 | | | 3953 bp | | |  |  |  |  |
| Genome Start | Genome End | Strand | Div | Repeat Start | Repeat End | Repeat Left | Name | Family | Class |
| 45827767 | 45828675 | - | 27.3 | 5244 | 6161 | -5 | L1ME1 | L1 | LINE |
| 45828677 | 45829257 | - | 24.0 | 62 | 607 | 0 | MER77 | ERVL | LTR |
| 45829361 | 45831409 | + | 8.1 | 4089 | 6152 | -2 | L1PA7 | L1 | LINE |
| 45831406 | 45831819 | - | 30.1 | 4755 | 5173 | -973 | L1ME1 | L1 | LINE |

  

|  |  |  |  |  |  |  |  |  |  |
| --- | --- | --- | --- | --- | --- | --- | --- | --- | --- |
| chr9:45835682-45836760 | | | 1079 bp | | |  |  |  |  |
| Genome Start | Genome End | Strand | Div | Repeat Start | Repeat End | Repeat Left | Name | Family | Class |
| 45835682 | 45835712 | + | 25.1 | 1 | 31 | -474 | MLT1D | MaLR | LTR |
| 45835713 | 45836065 | + | 16.7 | 1 | 364 | 0 | THE1B | MaLR | LTR |
| 45836066 | 45836318 | + | 25.1 | 32 | 243 | -262 | MLT1D | MaLR | LTR |
| 45836319 | 45836525 | - | 15.5 | 6087 | 6294 | -6 | L1MA6 | L1 | LINE |
| 45836526 | 45836760 | + | 25.1 | 244 | 477 | -28 | MLT1D | MaLR | LTR |

  

|  |  |  |  |  |  |  |  |  |  |
| --- | --- | --- | --- | --- | --- | --- | --- | --- | --- |
| chr9:45846250-45848912 | | | 2551 bp | | |  |  |  |  |
| Genome Start | Genome End | Strand | Div | Repeat Start | Repeat End | Repeat Left | Name | Family | Class |
| 45846250 | 45846963 | - | 32.2 | 2415 | 3172 | -247 | L2 | L2 | LINE |
| 45846964 | 45847352 | - | 29.3 | 87 | 517 | -6 | LTR33A | ERVL | LTR |
| 45847465 | 45848253 | - | 31.3 | 1615 | 2425 | -994 | L2 | L2 | LINE |
| 45848254 | 45848483 | + | 24.6 | 1 | 219 | 0 | MER20 | MER1\_type | DNA |
| 45848484 | 45848912 | - | 31.3 | 1203 | 1614 | -1805 | L2 | L2 | LINE |

  

|  |  |  |  |  |  |  |  |  |  |
| --- | --- | --- | --- | --- | --- | --- | --- | --- | --- |
| chr9:45863820-45865040 | | | 1221 bp | | |  |  |  |  |
| Genome Start | Genome End | Strand | Div | Repeat Start | Repeat End | Repeat Left | Name | Family | Class |
| 45863820 | 45864025 | + | 18.2 | 1 | 193 | -119 | AluJb | Alu | SINE |
| 45864026 | 45864931 | + | 17.5 | 1 | 983 | 0 | LTR1D | ERV1 | LTR |
| 45864932 | 45865040 | + | 18.2 | 194 | 298 | -14 | AluJb | Alu | SINE |

  

|  |  |  |  |  |  |  |  |  |  |
| --- | --- | --- | --- | --- | --- | --- | --- | --- | --- |
| chr9:45873298-45874113 | | | 816 bp | | |  |  |  |  |
| Genome Start | Genome End | Strand | Div | Repeat Start | Repeat End | Repeat Left | Name | Family | Class |
| 45873298 | 45873504 | - | 35.5 | 732 | 962 | -5157 | L1MEd | L1 | LINE |
| 45873505 | 45873804 | - | 12.0 | 2 | 301 | -12 | AluSq | Alu | SINE |
| 45873805 | 45874113 | - | 35.5 | 447 | 731 | -5388 | L1MEd | L1 | LINE |

  

|  |  |  |  |  |  |  |  |  |  |
| --- | --- | --- | --- | --- | --- | --- | --- | --- | --- |
| chr9:45886430-45889341 | | | 2913 bp | | |  |  |  |  |
| Genome Start | Genome End | Strand | Div | Repeat Start | Repeat End | Repeat Left | Name | Family | Class |
| 45886430 | 45886939 | + | 21.2 | 195 | 886 | -2596 | MER93B-int | ERV1 | LTR |
| 45886939 | 45887566 | + | 24.3 | 4205 | 4830 | -387 | MER93B-int | ERV1 | LTR |
| 45887567 | 45887890 | + | 6.5 | 1 | 309 | -2 | AluY | Alu | SINE |
| 45887891 | 45889341 | + | 16.7 | 609 | 4911 | -306 | MER93B-int | ERV1 | LTR |

  

|  |  |  |  |  |  |  |  |  |  |
| --- | --- | --- | --- | --- | --- | --- | --- | --- | --- |
| chr9:45892467-45893255 | | | 789 bp | | |  |  |  |  |
| Genome Start | Genome End | Strand | Div | Repeat Start | Repeat End | Repeat Left | Name | Family | Class |
| 45892467 | 45892786 | + | 17.3 | 1 | 308 | -193 | MER61D | ERV1 | LTR |
| 45892787 | 45893077 | + | 15.1 | 2 | 293 | -17 | AluSg | Alu | SINE |
| 45893078 | 45893255 | + | 17.3 | 309 | 501 | 0 | MER61D | ERV1 | LTR |

  

|  |  |  |  |  |  |  |  |  |  |
| --- | --- | --- | --- | --- | --- | --- | --- | --- | --- |
| chr9:45915580-45917394 | | | 1507 bp | | |  |  |  |  |
| Genome Start | Genome End | Strand | Div | Repeat Start | Repeat End | Repeat Left | Name | Family | Class |
| 45915580 | 45915624 | - | 25.6 | 2098 | 2132 | -4268 | L1MEc | L1 | LINE |
| 45915625 | 45915931 | - | 1.3 | 1 | 307 | -3 | AluYa5 | Alu | SINE |
| 45915932 | 45915978 | - | 25.6 | 2051 | 2097 | -4303 | L1MEc | L1 | LINE |
| 45916103 | 45916288 | - | 32.4 | 2 | 175 | 0 | MER96 | MER1\_type? | DNA |
| 45916364 | 45916910 | - | 25.7 | 1004 | 1611 | -4789 | L1MEc | L1 | LINE |
| 45917020 | 45917394 | - | 34.8 | 428 | 812 | -5588 | L1MEc | L1 | LINE |

  

|  |  |  |  |  |  |  |  |  |  |
| --- | --- | --- | --- | --- | --- | --- | --- | --- | --- |
| chr9:45923403-45924704 | | | 903 bp | | |  |  |  |  |
| Genome Start | Genome End | Strand | Div | Repeat Start | Repeat End | Repeat Left | Name | Family | Class |
| 45923403 | 45923727 | + | 35.1 | 500 | 835 | -5284 | L1MEd | L1 | LINE |
| 45924093 | 45924317 | + | 34.7 | 3179 | 3415 | -4 | L2 | L2 | LINE |
| 45924352 | 45924704 | + | 28.6 | 1359 | 1728 | -4421 | L1MEd | L1 | LINE |

  

|  |  |  |  |  |  |  |  |  |  |
| --- | --- | --- | --- | --- | --- | --- | --- | --- | --- |
| chr9:45929335-45930279 | | | 945 bp | | |  |  |  |  |
| Genome Start | Genome End | Strand | Div | Repeat Start | Repeat End | Repeat Left | Name | Family | Class |
| 45929335 | 45929627 | - | 30.4 | 3302 | 3624 | -2522 | L1MD | L1 | LINE |
| 45929628 | 45929935 | - | 13.0 | 1 | 308 | -4 | AluSx | Alu | SINE |
| 45929936 | 45930279 | - | 30.4 | 2948 | 3302 | -2844 | L1MD | L1 | LINE |

  

|  |  |  |  |  |  |  |  |  |  |
| --- | --- | --- | --- | --- | --- | --- | --- | --- | --- |
| chr9:45949861-45952520 | | | 2660 bp | | |  |  |  |  |
| Genome Start | Genome End | Strand | Div | Repeat Start | Repeat End | Repeat Left | Name | Family | Class |
| 45949861 | 45951892 | + | 15.7 | 24 | 2083 | -335 | Tigger1 | MER2\_type | DNA |
| 45951893 | 45952195 | + | 13.2 | 7 | 312 | 0 | AluSx | Alu | SINE |
| 45952196 | 45952520 | + | 15.6 | 2084 | 2417 | -1 | Tigger1 | MER2\_type | DNA |

  

|  |  |  |  |  |  |  |  |  |  |
| --- | --- | --- | --- | --- | --- | --- | --- | --- | --- |
| chr9:45966395-45968427 | | | 2011 bp | | |  |  |  |  |
| Genome Start | Genome End | Strand | Div | Repeat Start | Repeat End | Repeat Left | Name | Family | Class |
| 45966395 | 45966550 | - | 27.1 | 1421 | 1562 | -4728 | L1P4e | L1 | LINE |
| 45966551 | 45966578 | - | 14.2 | 283 | 310 | -2 | AluSx | Alu | SINE |
| 45966601 | 45966881 | - | 14.2 | 3 | 282 | -30 | AluSx | Alu | SINE |
| 45966882 | 45968427 | - | 23.4 | 1 | 1420 | -4870 | L1P4e | L1 | LINE |

  

|  |  |  |  |  |  |  |  |  |  |
| --- | --- | --- | --- | --- | --- | --- | --- | --- | --- |
| chr9:45989422-45996249 | | | 6781 bp | | |  |  |  |  |
| Genome Start | Genome End | Strand | Div | Repeat Start | Repeat End | Repeat Left | Name | Family | Class |
| 45989422 | 45990410 | + | 20.0 | 10 | 1076 | -3947 | MER70A-int | ERVL | LTR |
| 45990430 | 45990513 | + | 19.1 | 2300 | 2384 | -2639 | MER70A-int | ERVL | LTR |
| 45990514 | 45990907 | - | 11.4 | 1 | 450 | 0 | LTR7 | ERV1 | LTR |
| 45990908 | 45991340 | - | 9.8 | 7283 | 7713 | 0 | HERVH | ERV1 | LTR |
| 45991360 | 45992852 | - | 12.3 | 3582 | 5010 | -2703 | HERVH | ERV1 | LTR |
| 45992855 | 45992971 | - | 11.5 | 2613 | 2725 | -4988 | HERVH | ERV1 | LTR |
| 45992979 | 45993369 | - | 12.1 | 1 | 450 | 0 | LTR7 | ERV1 | LTR |
| 45993370 | 45995519 | + | 19.0 | 2380 | 4592 | -431 | MER70A-int | ERVL | LTR |
| 45995520 | 45995819 | - | 13.7 | 1 | 292 | -21 | AluSp | Alu | SINE |
| 45995820 | 45996249 | + | 19.0 | 4593 | 5022 | -1 | MER70A-int | ERVL | LTR |

  

|  |  |  |  |  |  |  |  |  |  |
| --- | --- | --- | --- | --- | --- | --- | --- | --- | --- |
| chr9:46021099-46021764 | | | 666 bp | | |  |  |  |  |
| Genome Start | Genome End | Strand | Div | Repeat Start | Repeat End | Repeat Left | Name | Family | Class |
| 46021099 | 46021167 | + | 24.8 | 13 | 80 | -182 | MIR | MIR | SINE |
| 46021168 | 46021581 | - | 15.4 | 1 | 365 | -1 | Tigger5a | MER2\_type | DNA |
| 46021582 | 46021764 | + | 24.8 | 81 | 260 | -2 | MIR | MIR | SINE |

  

|  |  |  |  |  |  |  |  |  |  |
| --- | --- | --- | --- | --- | --- | --- | --- | --- | --- |
| chr9:46027108-46028119 | | | 1012 bp | | |  |  |  |  |
| Genome Start | Genome End | Strand | Div | Repeat Start | Repeat End | Repeat Left | Name | Family | Class |
| 46027108 | 46027650 | - | 26.1 | 2820 | 3401 | -18 | L2 | L2 | LINE |
| 46027651 | 46027942 | - | 13.0 | 1 | 292 | -18 | AluSg | Alu | SINE |
| 46027943 | 46028119 | - | 24.0 | 2642 | 2819 | -600 | L2 | L2 | LINE |

  

|  |  |  |  |  |  |  |  |  |  |
| --- | --- | --- | --- | --- | --- | --- | --- | --- | --- |
| chr9:46041579-46042100 | | | 522 bp | | |  |  |  |  |
| Genome Start | Genome End | Strand | Div | Repeat Start | Repeat End | Repeat Left | Name | Family | Class |
| 46041579 | 46041670 | - | 26.2 | 185 | 263 | -5 | MIRb | MIR | SINE |
| 46041671 | 46041931 | - | 15.3 | 1 | 280 | -32 | AluJb | Alu | SINE |
| 46041932 | 46042100 | - | 26.2 | 20 | 184 | -84 | MIRb | MIR | SINE |

  

|  |  |  |  |  |  |  |  |  |  |
| --- | --- | --- | --- | --- | --- | --- | --- | --- | --- |
| chr9:46076826-46077411 | | | 553 bp | | |  |  |  |  |
| Genome Start | Genome End | Strand | Div | Repeat Start | Repeat End | Repeat Left | Name | Family | Class |
| 46076826 | 46076941 | + | 27.2 | 5852 | 5972 | -268 | L1ME3B | L1 | LINE |
| 46076967 | 46077204 | + | 14.7 | 66 | 297 | -15 | AluSg/x | Alu | SINE |
| 46077213 | 46077411 | + | 26.6 | 5972 | 6164 | -76 | L1ME3B | L1 | LINE |

  

|  |  |  |  |  |  |  |  |  |  |
| --- | --- | --- | --- | --- | --- | --- | --- | --- | --- |
| chr9:46098738-46099868 | | | 1131 bp | | |  |  |  |  |
| Genome Start | Genome End | Strand | Div | Repeat Start | Repeat End | Repeat Left | Name | Family | Class |
| 46098738 | 46099468 | - | 18.4 | 88 | 862 | -1 | MER21B | ERV1 | LTR |
| 46099469 | 46099780 | + | 10.9 | 3 | 309 | -3 | AluSx | Alu | SINE |
| 46099781 | 46099868 | - | 18.4 | 1 | 87 | -776 | MER21B | ERV1 | LTR |

  

|  |  |  |  |  |  |  |  |  |  |
| --- | --- | --- | --- | --- | --- | --- | --- | --- | --- |
| chr9:46100170-46101124 | | | 955 bp | | |  |  |  |  |
| Genome Start | Genome End | Strand | Div | Repeat Start | Repeat End | Repeat Left | Name | Family | Class |
| 46100170 | 46100566 | - | 15.3 | 356 | 780 | -64 | LTR25 | ERV1 | LTR |
| 46100567 | 46100894 | + | 14.3 | 1 | 460 | 0 | LTR46 | ERV1 | LTR |
| 46100895 | 46101124 | - | 15.9 | 33 | 355 | -417 | LTR25 | ERV1 | LTR |

  

|  |  |  |  |  |  |  |  |  |  |
| --- | --- | --- | --- | --- | --- | --- | --- | --- | --- |
| chr9:46103954-46105033 | | | 1080 bp | | |  |  |  |  |
| Genome Start | Genome End | Strand | Div | Repeat Start | Repeat End | Repeat Left | Name | Family | Class |
| 46103954 | 46104238 | - | 19.2 | 213 | 532 | -527 | MER106B | MER1\_type | DNA |
| 46104239 | 46104535 | - | 15.9 | 1 | 298 | -14 | AluSx | Alu | SINE |
| 46104536 | 46104633 | - | 19.2 | 109 | 212 | -847 | MER106B | MER1\_type | DNA |
| 46104634 | 46104933 | + | 13.3 | 1 | 300 | -12 | AluSx | Alu | SINE |
| 46104934 | 46105033 | - | 19.2 | 4 | 108 | -951 | MER106B | MER1\_type | DNA |

  

|  |  |  |  |  |  |  |  |  |  |
| --- | --- | --- | --- | --- | --- | --- | --- | --- | --- |
| chr9:46156868-46157477 | | | 610 bp | | |  |  |  |  |
| Genome Start | Genome End | Strand | Div | Repeat Start | Repeat End | Repeat Left | Name | Family | Class |
| 46156868 | 46156885 | - | 16.1 | 330 | 345 | -51 | MSTD | MaLR | LTR |
| 46156886 | 46157194 | + | 12.0 | 1 | 309 | -3 | AluSx | Alu | SINE |
| 46157195 | 46157477 | - | 19.1 | 2 | 329 | -67 | MSTD | MaLR | LTR |

  

|  |  |  |  |  |  |  |  |  |  |
| --- | --- | --- | --- | --- | --- | --- | --- | --- | --- |
| chr9:46167720-46169557 | | | 1753 bp | | |  |  |  |  |
| Genome Start | Genome End | Strand | Div | Repeat Start | Repeat End | Repeat Left | Name | Family | Class |
| 46167720 | 46167841 | - | 30.8 | 3269 | 3406 | -13 | L2 | L2 | LINE |
| 46167817 | 46167974 | + | 30.3 | 4013 | 4174 | -315 | L3 | CR1 | LINE |
| 46167991 | 46168141 | - | 26.4 | 964 | 1123 | -2296 | L2 | L2 | LINE |
| 46168163 | 46168316 | - | 5.8 | 7 | 241 | -245 | LTR19A | ERV1 | LTR |
| 46168321 | 46168524 | - | 27.9 | 756 | 969 | -2450 | L2 | L2 | LINE |
| 46168594 | 46169198 | - | 13.2 | 1 | 766 | 0 | MER4E | ERV1 | LTR |
| 46169199 | 46169557 | - | 24.6 | 8 | 426 | -2993 | L2 | L2 | LINE |

  

|  |  |  |  |  |  |  |  |  |  |
| --- | --- | --- | --- | --- | --- | --- | --- | --- | --- |
| chr9:46175990-46176814 | | | 825 bp | | |  |  |  |  |
| Genome Start | Genome End | Strand | Div | Repeat Start | Repeat End | Repeat Left | Name | Family | Class |
| 46175990 | 46176424 | + | 14.5 | 4730 | 5161 | -985 | L1P4 | L1 | LINE |
| 46176425 | 46176690 | + | 11.7 | 40 | 310 | 0 | AluSg | Alu | SINE |
| 46176691 | 46176814 | + | 14.5 | 5162 | 5286 | -860 | L1P4 | L1 | LINE |

  

|  |  |  |  |  |  |  |  |  |  |
| --- | --- | --- | --- | --- | --- | --- | --- | --- | --- |
| chr9:46178429-46178929 | | | 501 bp | | |  |  |  |  |
| Genome Start | Genome End | Strand | Div | Repeat Start | Repeat End | Repeat Left | Name | Family | Class |
| 46178429 | 46178646 | - | 22.6 | 6699 | 6892 | -916 | L1MC4a | L1 | LINE |
| 46178647 | 46178786 | + | 15.9 | 1 | 132 | -1 | FLAM\_C | Alu | SINE |
| 46178787 | 46178929 | - | 22.9 | 6602 | 6783 | -1110 | L1MC4a | L1 | LINE |

  

|  |  |  |  |  |  |  |  |  |  |
| --- | --- | --- | --- | --- | --- | --- | --- | --- | --- |
| chr9:46179245-46180320 | | | 1076 bp | | |  |  |  |  |
| Genome Start | Genome End | Strand | Div | Repeat Start | Repeat End | Repeat Left | Name | Family | Class |
| 46179245 | 46179344 | + | 19.1 | 4 | 108 | -951 | MER106B | MER1\_type | DNA |
| 46179345 | 46179644 | - | 13.3 | 1 | 300 | -12 | AluSx | Alu | SINE |
| 46179645 | 46179742 | + | 19.1 | 109 | 212 | -847 | MER106B | MER1\_type | DNA |
| 46179743 | 46180037 | + | 16.0 | 1 | 296 | -16 | AluSx | Alu | SINE |
| 46180038 | 46180320 | + | 19.1 | 213 | 532 | -527 | MER106B | MER1\_type | DNA |

  

|  |  |  |  |  |  |  |  |  |  |
| --- | --- | --- | --- | --- | --- | --- | --- | --- | --- |
| chr9:46183151-46184105 | | | 955 bp | | |  |  |  |  |
| Genome Start | Genome End | Strand | Div | Repeat Start | Repeat End | Repeat Left | Name | Family | Class |
| 46183151 | 46183380 | + | 16.0 | 33 | 354 | -417 | LTR25 | ERV1 | LTR |
| 46183381 | 46183708 | - | 14.3 | 1 | 460 | 0 | LTR46 | ERV1 | LTR |
| 46183709 | 46184105 | + | 15.5 | 355 | 779 | -65 | LTR25 | ERV1 | LTR |

  

|  |  |  |  |  |  |  |  |  |  |
| --- | --- | --- | --- | --- | --- | --- | --- | --- | --- |
| chr9:46184407-46185529 | | | 1123 bp | | |  |  |  |  |
| Genome Start | Genome End | Strand | Div | Repeat Start | Repeat End | Repeat Left | Name | Family | Class |
| 46184407 | 46184494 | + | 18.2 | 1 | 87 | -776 | MER21B | ERV1 | LTR |
| 46184495 | 46184798 | - | 11.6 | 3 | 301 | -11 | AluSx | Alu | SINE |
| 46184799 | 46185529 | + | 18.2 | 88 | 862 | -1 | MER21B | ERV1 | LTR |

  

|  |  |  |  |  |  |  |  |  |  |
| --- | --- | --- | --- | --- | --- | --- | --- | --- | --- |
| chr9:46206836-46207421 | | | 553 bp | | |  |  |  |  |
| Genome Start | Genome End | Strand | Div | Repeat Start | Repeat End | Repeat Left | Name | Family | Class |
| 46206836 | 46207034 | - | 26.1 | 5972 | 6164 | -76 | L1ME3B | L1 | LINE |
| 46207043 | 46207280 | - | 14.7 | 66 | 297 | -15 | AluSg/x | Alu | SINE |
| 46207306 | 46207421 | - | 26.3 | 5852 | 5972 | -268 | L1ME3B | L1 | LINE |

  

|  |  |  |  |  |  |  |  |  |  |
| --- | --- | --- | --- | --- | --- | --- | --- | --- | --- |
| chr9:46207462-46208052 | | | 591 bp | | |  |  |  |  |
| Genome Start | Genome End | Strand | Div | Repeat Start | Repeat End | Repeat Left | Name | Family | Class |
| 46207462 | 46207602 | + | 13.6 | 1 | 137 | -175 | AluSx | Alu | SINE |
| 46207603 | 46207887 | + | 15.1 | 8 | 294 | -17 | AluY | Alu | SINE |
| 46207888 | 46208052 | + | 13.6 | 138 | 300 | -12 | AluSx | Alu | SINE |

  

|  |  |  |  |  |  |  |  |  |  |
| --- | --- | --- | --- | --- | --- | --- | --- | --- | --- |
| chr9:46216098-46218649 | | | 2103 bp | | |  |  |  |  |
| Genome Start | Genome End | Strand | Div | Repeat Start | Repeat End | Repeat Left | Name | Family | Class |
| 46216098 | 46216213 | - | 14.8 | 2303 | 2418 | 0 | Tigger1 | MER2\_type | DNA |
| 46216214 | 46216493 | + | 12.9 | 2 | 292 | -20 | AluSx | Alu | SINE |
| 46216494 | 46217000 | - | 14.8 | 1801 | 2302 | -116 | Tigger1 | MER2\_type | DNA |
| 46217393 | 46217611 | - | 23.9 | 445 | 643 | -50 | MER115 | Tip100 | DNA |
| 46217669 | 46217968 | + | 12.3 | 1 | 302 | -11 | AluSp | Alu | SINE |
| 46217969 | 46218649 | - | 14.7 | 1066 | 1773 | -645 | Tigger1 | MER2\_type | DNA |

  

|  |  |  |  |  |  |  |  |  |  |
| --- | --- | --- | --- | --- | --- | --- | --- | --- | --- |
| chr9:46240452-46241130 | | | 679 bp | | |  |  |  |  |
| Genome Start | Genome End | Strand | Div | Repeat Start | Repeat End | Repeat Left | Name | Family | Class |
| 46240452 | 46240683 | - | 34.5 | 3169 | 3411 | -8 | L2 | L2 | LINE |
| 46240684 | 46241031 | + | 9.4 | 1 | 348 | 0 | AluSp | Alu | SINE |
| 46241032 | 46241130 | - | 34.5 | 3065 | 3168 | -251 | L2 | L2 | LINE |

  

|  |  |  |  |  |  |  |  |  |  |
| --- | --- | --- | --- | --- | --- | --- | --- | --- | --- |
| chr9:46242149-46242622 | | | 474 bp | | |  |  |  |  |
| Genome Start | Genome End | Strand | Div | Repeat Start | Repeat End | Repeat Left | Name | Family | Class |
| 46242149 | 46242317 | + | 23.3 | 20 | 184 | -84 | MIRb | MIR | SINE |
| 46242318 | 46242578 | + | 15.3 | 1 | 280 | -32 | AluJb | Alu | SINE |
| 46242579 | 46242622 | + | 23.3 | 185 | 216 | -52 | MIRb | MIR | SINE |

  

|  |  |  |  |  |  |  |  |  |  |
| --- | --- | --- | --- | --- | --- | --- | --- | --- | --- |
| chr9:46262511-46263176 | | | 666 bp | | |  |  |  |  |
| Genome Start | Genome End | Strand | Div | Repeat Start | Repeat End | Repeat Left | Name | Family | Class |
| 46262511 | 46262693 | - | 24.8 | 81 | 260 | -2 | MIR | MIR | SINE |
| 46262694 | 46263107 | + | 15.7 | 1 | 365 | -1 | Tigger5a | MER2\_type | DNA |
| 46263108 | 46263176 | - | 24.8 | 13 | 80 | -182 | MIR | MIR | SINE |

  

|  |  |  |  |  |  |  |  |  |  |
| --- | --- | --- | --- | --- | --- | --- | --- | --- | --- |
| chr9:46264426-46265490 | | | 1065 bp | | |  |  |  |  |
| Genome Start | Genome End | Strand | Div | Repeat Start | Repeat End | Repeat Left | Name | Family | Class |
| 46264426 | 46264480 | + | 5.5 | 8 | 64 | -114 | MER5B | MER1\_type | DNA |
| 46264481 | 46264555 | - | 1.3 | 5721 | 5795 | -360 | L1P2 | L1 | LINE |
| 46264556 | 46265053 | + | 8.4 | 5657 | 6153 | -2 | L1PA2 | L1 | LINE |
| 46265054 | 46265138 | + | 18.7 | 55 | 136 | -42 | MER5B | MER1\_type | DNA |
| 46265139 | 46265448 | + | 12.3 | 1 | 310 | -3 | AluSq | Alu | SINE |
| 46265449 | 46265490 | + | 18.7 | 137 | 177 | -1 | MER5B | MER1\_type | DNA |

  

|  |  |  |  |  |  |  |  |  |  |
| --- | --- | --- | --- | --- | --- | --- | --- | --- | --- |
| chr9:46301471-46303800 | | | 2293 bp | | |  |  |  |  |
| Genome Start | Genome End | Strand | Div | Repeat Start | Repeat End | Repeat Left | Name | Family | Class |
| 46301471 | 46301566 | + | 21.5 | 1 | 94 | -1137 | Tigger3b | MER2\_type | DNA |
| 46301588 | 46301836 | + | 14.9 | 332 | 587 | -644 | Tigger3b | MER2\_type | DNA |
| 46301853 | 46303206 | - | 3.0 | 4801 | 6155 | 0 | L1PA3 | L1 | LINE |
| 46303207 | 46303800 | + | 15.9 | 588 | 1231 | 0 | Tigger3b | MER2\_type | DNA |

  

|  |  |  |  |  |  |  |  |  |  |
| --- | --- | --- | --- | --- | --- | --- | --- | --- | --- |
| chr9:46315881-46317912 | | | 2032 bp | | |  |  |  |  |
| Genome Start | Genome End | Strand | Div | Repeat Start | Repeat End | Repeat Left | Name | Family | Class |
| 46315881 | 46317426 | + | 23.1 | 1 | 1407 | -4883 | L1P4e | L1 | LINE |
| 46317427 | 46317747 | + | 13.2 | 2 | 312 | 0 | AluSx | Alu | SINE |
| 46317748 | 46317912 | + | 26.7 | 1408 | 1562 | -4728 | L1P4e | L1 | LINE |

  

|  |  |  |  |  |  |  |  |  |  |
| --- | --- | --- | --- | --- | --- | --- | --- | --- | --- |
| chr9:46319608-46323898 | | | 4291 bp | | |  |  |  |  |
| Genome Start | Genome End | Strand | Div | Repeat Start | Repeat End | Repeat Left | Name | Family | Class |
| 46319608 | 46321858 | - | 21.5 | 3455 | 5808 | -2333 | L1PREC2 | L1 | LINE |
| 46321859 | 46322149 | - | 11.6 | 1 | 311 | 0 | AluY | Alu | SINE |
| 46322150 | 46323898 | - | 25.6 | 1762 | 3454 | -4701 | L1PREC2 | L1 | LINE |

  

|  |  |  |  |  |  |  |  |  |  |
| --- | --- | --- | --- | --- | --- | --- | --- | --- | --- |
| chr9:46443018-46444238 | | | 1221 bp | | |  |  |  |  |
| Genome Start | Genome End | Strand | Div | Repeat Start | Repeat End | Repeat Left | Name | Family | Class |
| 46443018 | 46443223 | + | 18.2 | 1 | 193 | -119 | AluJb | Alu | SINE |
| 46443224 | 46444129 | + | 17.5 | 1 | 983 | 0 | LTR1D | ERV1 | LTR |
| 46444130 | 46444238 | + | 18.2 | 194 | 298 | -14 | AluJb | Alu | SINE |

  

|  |  |  |  |  |  |  |  |  |  |
| --- | --- | --- | --- | --- | --- | --- | --- | --- | --- |
| chr9:46452498-46453314 | | | 817 bp | | |  |  |  |  |
| Genome Start | Genome End | Strand | Div | Repeat Start | Repeat End | Repeat Left | Name | Family | Class |
| 46452498 | 46452704 | - | 35.7 | 732 | 962 | -5157 | L1MEd | L1 | LINE |
| 46452705 | 46453005 | - | 13.3 | 2 | 302 | -11 | AluSq | Alu | SINE |
| 46453006 | 46453314 | - | 35.7 | 447 | 731 | -5388 | L1MEd | L1 | LINE |

  

|  |  |  |  |  |  |  |  |  |  |
| --- | --- | --- | --- | --- | --- | --- | --- | --- | --- |
| chr9:46473142-46474039 | | | 898 bp | | |  |  |  |  |
| Genome Start | Genome End | Strand | Div | Repeat Start | Repeat End | Repeat Left | Name | Family | Class |
| 46473142 | 46473383 | + | 16.4 | 1 | 251 | -88 | MER1B | MER1\_type | DNA |
| 46473384 | 46473952 | - | 24.1 | 1 | 554 | 0 | MLT2B3 | ERVL | LTR |
| 46473953 | 46474039 | + | 16.4 | 252 | 337 | -2 | MER1B | MER1\_type | DNA |

  

|  |  |  |  |  |  |  |  |  |  |
| --- | --- | --- | --- | --- | --- | --- | --- | --- | --- |
| chr9:46490111-46493784 | | | 3029 bp | | |  |  |  |  |
| Genome Start | Genome End | Strand | Div | Repeat Start | Repeat End | Repeat Left | Name | Family | Class |
| 46490111 | 46490311 | - | 23.9 | 4529 | 4731 | -1488 | L1MEc | L1 | LINE |
| 46490316 | 46490521 | - | 27.7 | 3961 | 4184 | -2029 | L1MEc | L1 | LINE |
| 46490590 | 46491106 | - | 28.1 | 3194 | 3731 | -2488 | L1MEc | L1 | LINE |
| 46491208 | 46491553 | - | 24.4 | 1 | 345 | -63 | MLT1A0 | MaLR | LTR |
| 46491665 | 46491796 | - | 22.9 | 6163 | 6305 | 0 | L1MA3 | L1 | LINE |
| 46491790 | 46491912 | - | 30.2 | 2374 | 2524 | -3695 | L1MEc | L1 | LINE |
| 46491952 | 46492365 | - | 24.4 | 2051 | 2441 | -3959 | L1MEc | L1 | LINE |
| 46492490 | 46492673 | - | 32.4 | 2 | 175 | 0 | MER96 | MER1\_type? | DNA |
| 46492749 | 46492836 | - | 28.1 | 1511 | 1611 | -4789 | L1MEc | L1 | LINE |
| 46492858 | 46493299 | - | 28.1 | 1005 | 1510 | -4890 | L1MEc | L1 | LINE |
| 46493409 | 46493784 | - | 34.6 | 428 | 812 | -5588 | L1MEc | L1 | LINE |

  

|  |  |  |  |  |  |  |  |  |  |
| --- | --- | --- | --- | --- | --- | --- | --- | --- | --- |
| chr9:46524687-46525842 | | | 903 bp | | |  |  |  |  |
| Genome Start | Genome End | Strand | Div | Repeat Start | Repeat End | Repeat Left | Name | Family | Class |
| 46524687 | 46524908 | + | 28.8 | 4478 | 4702 | -1444 | L1M5 | L1 | LINE |
| 46525003 | 46525104 | + | 21.4 | 39 | 171 | -378 | MLT1H | MaLR | LTR |
| 46525113 | 46525417 | - | 12.4 | 1 | 299 | -10 | AluSc | Alu | SINE |
| 46525485 | 46525661 | + | 22.2 | 155 | 454 | -113 | MLT1G1 | MaLR | LTR |
| 46525746 | 46525842 | + | 29.2 | 4720 | 4818 | -1328 | L1M5 | L1 | LINE |

  

|  |  |  |  |  |  |  |  |  |  |
| --- | --- | --- | --- | --- | --- | --- | --- | --- | --- |
| chr9:46531707-46536369 | | | 4630 bp | | |  |  |  |  |
| Genome Start | Genome End | Strand | Div | Repeat Start | Repeat End | Repeat Left | Name | Family | Class |
| 46531707 | 46531971 | + | 19.2 | 4120 | 4379 | -1764 | L1MA9 | L1 | LINE |
| 46531972 | 46532248 | + | 8.3 | 1 | 287 | -24 | AluY | Alu | SINE |
| 46532249 | 46533095 | + | 19.2 | 4380 | 5286 | -857 | L1MA9 | L1 | LINE |
| 46533106 | 46533469 | - | 14.0 | 1 | 364 | 0 | THE1B | MaLR | LTR |
| 46533470 | 46535037 | - | 12.7 | 1 | 1580 | 0 | THE1B-int | MaLR | LTR |
| 46535038 | 46535397 | - | 16.1 | 1 | 364 | 0 | THE1B | MaLR | LTR |
| 46535398 | 46535837 | + | 14.7 | 5332 | 5782 | -530 | L1MA9 | L1 | LINE |
| 46535861 | 46536369 | + | 14.7 | 5783 | 6312 | 0 | L1MA9 | L1 | LINE |

  

|  |  |  |  |  |  |  |  |  |  |
| --- | --- | --- | --- | --- | --- | --- | --- | --- | --- |
| chr9:46541452-46545676 | | | 3992 bp | | |  |  |  |  |
| Genome Start | Genome End | Strand | Div | Repeat Start | Repeat End | Repeat Left | Name | Family | Class |
| 46541452 | 46541924 | + | 33.6 | 64 | 559 | -5560 | L1ME3B | L1 | LINE |
| 46541936 | 46542491 | + | 19.3 | 3 | 608 | -218 | LTR12 | ERV1 | LTR |
| 46542531 | 46542696 | + | 33.5 | 4818 | 4983 | -1146 | L1ME3B | L1 | LINE |
| 46542761 | 46543453 | + | 27.0 | 5088 | 5830 | -393 | L1ME3B | L1 | LINE |
| 46543454 | 46544289 | + | 16.2 | 5435 | 6322 | -11 | L1MC1 | L1 | LINE |
| 46544290 | 46544642 | + | 26.4 | 5831 | 6223 | 0 | L1ME3B | L1 | LINE |
| 46544650 | 46544895 | + | 25.9 | 3375 | 3660 | -2486 | L1ME3B | L1 | LINE |
| 46544905 | 46545271 | + | 27.7 | 3709 | 4104 | -2042 | L1ME3B | L1 | LINE |
| 46545271 | 46545353 | + | 21.5 | 4633 | 4715 | -1431 | L1ME3B | L1 | LINE |
| 46545458 | 46545676 | + | 32.4 | 4851 | 5064 | -1082 | L1ME3B | L1 | LINE |

  

|  |  |  |  |  |  |  |  |  |  |
| --- | --- | --- | --- | --- | --- | --- | --- | --- | --- |
| chr9:46551362-46553123 | | | 1771 bp | | |  |  |  |  |
| Genome Start | Genome End | Strand | Div | Repeat Start | Repeat End | Repeat Left | Name | Family | Class |
| 46551362 | 46551557 | - | 19.9 | 889 | 1083 | 0 | Tigger6a | MER2\_type | DNA |
| 46551558 | 46551851 | - | 13.3 | 1 | 294 | -17 | AluY | Alu | SINE |
| 46551852 | 46552468 | - | 19.9 | 217 | 888 | -195 | Tigger6a | MER2\_type | DNA |
| 46552460 | 46552914 | + | 23.3 | 11 | 467 | 0 | MLT1C | MaLR | LTR |
| 46552915 | 46553123 | - | 20.5 | 5 | 211 | -872 | Tigger6a | MER2\_type | DNA |

  

|  |  |  |  |  |  |  |  |  |  |
| --- | --- | --- | --- | --- | --- | --- | --- | --- | --- |
| chr9:46562050-46566038 | | | 3989 bp | | |  |  |  |  |
| Genome Start | Genome End | Strand | Div | Repeat Start | Repeat End | Repeat Left | Name | Family | Class |
| 46562050 | 46564794 | - | 15.6 | 3297 | 6161 | 0 | L1PA14 | L1 | LINE |
| 46564795 | 46565103 | - | 15.2 | 1 | 311 | -1 | AluSx | Alu | SINE |
| 46565104 | 46566038 | - | 17.7 | 2377 | 3296 | -2850 | L1PA14 | L1 | LINE |

  

|  |  |  |  |  |  |  |  |  |  |
| --- | --- | --- | --- | --- | --- | --- | --- | --- | --- |
| chr9:46568481-46568962 | | | 482 bp | | |  |  |  |  |
| Genome Start | Genome End | Strand | Div | Repeat Start | Repeat End | Repeat Left | Name | Family | Class |
| 46568481 | 46568540 | - | 27.4 | 203 | 260 | -2 | MIR | MIR | SINE |
| 46568541 | 46568843 | + | 11.2 | 1 | 303 | -6 | AluSc | Alu | SINE |
| 46568844 | 46568962 | - | 27.4 | 81 | 202 | -60 | MIR | MIR | SINE |

  

|  |  |  |  |  |  |  |  |  |  |
| --- | --- | --- | --- | --- | --- | --- | --- | --- | --- |
| chr9:46573430-46574315 | | | 886 bp | | |  |  |  |  |
| Genome Start | Genome End | Strand | Div | Repeat Start | Repeat End | Repeat Left | Name | Family | Class |
| 46573430 | 46573760 | - | 24.0 | 7599 | 7947 | -14 | L1MC5 | L1 | LINE |
| 46573761 | 46574073 | - | 11.2 | 1 | 296 | -16 | AluSx | Alu | SINE |
| 46574074 | 46574315 | - | 24.0 | 7349 | 7598 | -363 | L1MC5 | L1 | LINE |

  

|  |  |  |  |  |  |  |  |  |  |
| --- | --- | --- | --- | --- | --- | --- | --- | --- | --- |
| chr9:46591288-46594438 | | | 3121 bp | | |  |  |  |  |
| Genome Start | Genome End | Strand | Div | Repeat Start | Repeat End | Repeat Left | Name | Family | Class |
| 46591288 | 46593410 | + | 9.8 | 2280 | 4397 | -1749 | L1PB | L1 | LINE |
| 46593440 | 46593520 | + | 9.8 | 4398 | 4491 | -1655 | L1PB | L1 | LINE |
| 46593522 | 46593698 | + | 16.9 | 136 | 312 | -1 | AluSp | Alu | SINE |
| 46593699 | 46594438 | + | 8.6 | 4480 | 5219 | -927 | L1PB | L1 | LINE |

  

|  |  |  |  |  |  |  |  |  |  |
| --- | --- | --- | --- | --- | --- | --- | --- | --- | --- |
| chr9:46597697-46601655 | | | 3954 bp | | |  |  |  |  |
| Genome Start | Genome End | Strand | Div | Repeat Start | Repeat End | Repeat Left | Name | Family | Class |
| 46597697 | 46598307 | + | 21.2 | 1954 | 2564 | -4289 | L1PA15-16 | L1 | LINE |
| 46598308 | 46598600 | - | 13.7 | 1 | 295 | -17 | AluSx | Alu | SINE |
| 46598601 | 46600175 | + | 18.4 | 2565 | 4154 | -2695 | L1PA15-16 | L1 | LINE |
| 46600177 | 46600792 | + | 19.1 | 1020 | 1651 | 0 | MSTB-int | MaLR | LTR |
| 46600793 | 46601163 | + | 23.7 | 1 | 377 | -49 | MSTB | MaLR | LTR |
| 46601168 | 46601655 | + | 18.7 | 4152 | 4646 | -2203 | L1PA15-16 | L1 | LINE |

  

|  |  |  |  |  |  |  |  |  |  |
| --- | --- | --- | --- | --- | --- | --- | --- | --- | --- |
| chr9:46617304-46620465 | | | 3129 bp | | |  |  |  |  |
| Genome Start | Genome End | Strand | Div | Repeat Start | Repeat End | Repeat Left | Name | Family | Class |
| 46617304 | 46618041 | - | 9.9 | 4494 | 5219 | -927 | L1PB | L1 | LINE |
| 46618042 | 46618219 | - | 14.6 | 136 | 313 | 0 | AluSp | Alu | SINE |
| 46618220 | 46618301 | - | 9.9 | 4414 | 4493 | -1653 | L1PB | L1 | LINE |
| 46618335 | 46620465 | - | 9.9 | 2281 | 4411 | -1735 | L1PB | L1 | LINE |

  

|  |  |  |  |  |  |  |  |  |  |
| --- | --- | --- | --- | --- | --- | --- | --- | --- | --- |
| chr9:46632353-46633680 | | | 1328 bp | | |  |  |  |  |
| Genome Start | Genome End | Strand | Div | Repeat Start | Repeat End | Repeat Left | Name | Family | Class |
| 46632353 | 46632529 | + | 17.8 | 5224 | 5420 | -924 | L1MC2 | L1 | LINE |
| 46632530 | 46632835 | + | 11.2 | 5842 | 6157 | -9 | L1PA16 | L1 | LINE |
| 46632836 | 46633680 | + | 17.8 | 5421 | 6288 | -56 | L1MC2 | L1 | LINE |

  

|  |  |  |  |  |  |  |  |  |  |
| --- | --- | --- | --- | --- | --- | --- | --- | --- | --- |
| chr9:46637473-46638358 | | | 886 bp | | |  |  |  |  |
| Genome Start | Genome End | Strand | Div | Repeat Start | Repeat End | Repeat Left | Name | Family | Class |
| 46637473 | 46637714 | + | 24.0 | 7349 | 7598 | -363 | L1MC5 | L1 | LINE |
| 46637715 | 46638027 | + | 11.2 | 1 | 296 | -16 | AluSx | Alu | SINE |
| 46638028 | 46638358 | + | 24.0 | 7599 | 7947 | -14 | L1MC5 | L1 | LINE |

  

|  |  |  |  |  |  |  |  |  |  |
| --- | --- | --- | --- | --- | --- | --- | --- | --- | --- |
| chr9:46640329-46642224 | | | 1896 bp | | |  |  |  |  |
| Genome Start | Genome End | Strand | Div | Repeat Start | Repeat End | Repeat Left | Name | Family | Class |
| 46640329 | 46640449 | + | 30.5 | 2 | 124 | -1611 | MLT-int | MaLR | LTR |
| 46640450 | 46640760 | + | 12.5 | 1 | 312 | -1 | AluSq | Alu | SINE |
| 46640761 | 46642224 | + | 30.5 | 125 | 1733 | -2 | MLT-int | MaLR | LTR |

  

|  |  |  |  |  |  |  |  |  |  |
| --- | --- | --- | --- | --- | --- | --- | --- | --- | --- |
| chr9:46642827-46643308 | | | 482 bp | | |  |  |  |  |
| Genome Start | Genome End | Strand | Div | Repeat Start | Repeat End | Repeat Left | Name | Family | Class |
| 46642827 | 46642945 | + | 27.4 | 81 | 202 | -60 | MIR | MIR | SINE |
| 46642946 | 46643248 | - | 11.2 | 1 | 303 | -6 | AluSc | Alu | SINE |
| 46643249 | 46643308 | + | 27.4 | 203 | 260 | -2 | MIR | MIR | SINE |

  

|  |  |  |  |  |  |  |  |  |  |
| --- | --- | --- | --- | --- | --- | --- | --- | --- | --- |
| chr9:46650075-46651284 | | | 1210 bp | | |  |  |  |  |
| Genome Start | Genome End | Strand | Div | Repeat Start | Repeat End | Repeat Left | Name | Family | Class |
| 46650075 | 46650412 | - | 13.5 | 5796 | 6152 | -2 | L1PB3 | L1 | LINE |
| 46650413 | 46650708 | - | 12.8 | 1 | 296 | -13 | AluSg1 | Alu | SINE |
| 46650709 | 46651284 | - | 13.2 | 5237 | 5795 | -359 | L1PB3 | L1 | LINE |

  

|  |  |  |  |  |  |  |  |  |  |
| --- | --- | --- | --- | --- | --- | --- | --- | --- | --- |
| chr9:46658665-46660427 | | | 1766 bp | | |  |  |  |  |
| Genome Start | Genome End | Strand | Div | Repeat Start | Repeat End | Repeat Left | Name | Family | Class |
| 46658665 | 46658873 | + | 20.5 | 5 | 211 | -872 | Tigger6a | MER2\_type | DNA |
| 46658874 | 46659328 | - | 23.3 | 11 | 467 | 0 | MLT1C | MaLR | LTR |
| 46659326 | 46659936 | + | 20.1 | 220 | 887 | -196 | Tigger6a | MER2\_type | DNA |
| 46659937 | 46660231 | + | 13.6 | 1 | 295 | -16 | AluY | Alu | SINE |
| 46660232 | 46660427 | + | 20.1 | 888 | 1082 | -1 | Tigger6a | MER2\_type | DNA |

  

|  |  |  |  |  |  |  |  |  |  |
| --- | --- | --- | --- | --- | --- | --- | --- | --- | --- |
| chr9:46687216-46688965 | | | 1403 bp | | |  |  |  |  |
| Genome Start | Genome End | Strand | Div | Repeat Start | Repeat End | Repeat Left | Name | Family | Class |
| 46687216 | 46687513 | - | 16.7 | 5731 | 6014 | -170 | L1MB7 | L1 | LINE |
| 46687856 | 46687981 | - | 27.1 | 4018 | 4136 | -55 | MER21-int | ERV1 | LTR |
| 46687987 | 46688517 | - | 16.3 | 5179 | 5739 | -439 | L1MB7 | L1 | LINE |
| 46688518 | 46688892 | - | 14.1 | 2 | 364 | 0 | THE1B | MaLR | LTR |
| 46688893 | 46688965 | - | 14.4 | 5102 | 5178 | -968 | L1MB7 | L1 | LINE |

  

|  |  |  |  |  |  |  |  |  |  |
| --- | --- | --- | --- | --- | --- | --- | --- | --- | --- |
| chr9:46710006-46710469 | | | 464 bp | | |  |  |  |  |
| Genome Start | Genome End | Strand | Div | Repeat Start | Repeat End | Repeat Left | Name | Family | Class |
| 46710006 | 46710188 | - | 16.9 | 4380 | 4540 | -1603 | L1M2 | L1 | LINE |
| 46710189 | 46710411 | - | 7.8 | 1 | 217 | -96 | AluSq | Alu | SINE |
| 46710412 | 46710469 | - | 16.9 | 4322 | 4379 | -1764 | L1M2 | L1 | LINE |

  

|  |  |  |  |  |  |  |  |  |  |
| --- | --- | --- | --- | --- | --- | --- | --- | --- | --- |
| chr9:46727224-46728076 | | | 853 bp | | |  |  |  |  |
| Genome Start | Genome End | Strand | Div | Repeat Start | Repeat End | Repeat Left | Name | Family | Class |
| 46727224 | 46727663 | + | 7.9 | 5627 | 6064 | -108 | L1PA8 | L1 | LINE |
| 46727664 | 46727959 | + | 8.1 | 1 | 296 | -15 | AluY | Alu | SINE |
| 46727960 | 46728076 | + | 7.9 | 6065 | 6172 | 0 | L1PA8 | L1 | LINE |

  

|  |  |  |  |  |  |  |  |  |  |
| --- | --- | --- | --- | --- | --- | --- | --- | --- | --- |
| chr9:46751015-46752116 | | | 1102 bp | | |  |  |  |  |
| Genome Start | Genome End | Strand | Div | Repeat Start | Repeat End | Repeat Left | Name | Family | Class |
| 46751015 | 46751413 | - | 23.7 | 7511 | 7926 | -35 | L1MC5 | L1 | LINE |
| 46751414 | 46751890 | + | 7.8 | 1 | 486 | 0 | LTR19A | ERV1 | LTR |
| 46751891 | 46752116 | - | 23.7 | 7260 | 7510 | -451 | L1MC5 | L1 | LINE |

  

|  |  |  |  |  |  |  |  |  |  |
| --- | --- | --- | --- | --- | --- | --- | --- | --- | --- |
| chr9:46806905-46807956 | | | 1056 bp | | |  |  |  |  |
| Genome Start | Genome End | Strand | Div | Repeat Start | Repeat End | Repeat Left | Name | Family | Class |
| 46806905 | 46807134 | - | 29.1 | 5907 | 6132 | -34 | L1ME1 | L1 | LINE |
| 46807134 | 46807555 | + | 17.2 | 2295 | 2722 | -3654 | L1M4c | L1 | LINE |
| 46807553 | 46807956 | - | 23.6 | 4631 | 5091 | -1055 | L1ME1 | L1 | LINE |

  

|  |  |  |  |  |  |  |  |  |  |
| --- | --- | --- | --- | --- | --- | --- | --- | --- | --- |
| chr9:46815903-46816656 | | | 639 bp | | |  |  |  |  |
| Genome Start | Genome End | Strand | Div | Repeat Start | Repeat End | Repeat Left | Name | Family | Class |
| 46815903 | 46816025 | - | 29.4 | 114 | 241 | -21 | MIR | MIR | SINE |
| 46816033 | 46816462 | + | 17.1 | 1 | 511 | -45 | MLT2B4 | ERVL | LTR |
| 46816571 | 46816656 | - | 18.6 | 21 | 130 | -132 | MIR | MIR | SINE |

  

|  |  |  |  |  |  |  |  |  |  |
| --- | --- | --- | --- | --- | --- | --- | --- | --- | --- |
| chr9:46816718-46817252 | | | 535 bp | | |  |  |  |  |
| Genome Start | Genome End | Strand | Div | Repeat Start | Repeat End | Repeat Left | Name | Family | Class |
| 46816718 | 46816877 | + | 14.5 | 2 | 161 | -41 | MER6C | MER2\_type | DNA |
| 46816878 | 46817201 | - | 6.5 | 1 | 309 | -2 | AluY | Alu | SINE |
| 46817202 | 46817252 | + | 14.5 | 162 | 201 | -1 | MER6C | MER2\_type | DNA |

  

|  |  |  |  |  |  |  |  |  |  |
| --- | --- | --- | --- | --- | --- | --- | --- | --- | --- |
| chr9:46820938-46821977 | | | 1040 bp | | |  |  |  |  |
| Genome Start | Genome End | Strand | Div | Repeat Start | Repeat End | Repeat Left | Name | Family | Class |
| 46820938 | 46821063 | + | 15.8 | 1 | 117 | -309 | MSTB | MaLR | LTR |
| 46821064 | 46821367 | + | 4.6 | 1 | 302 | -9 | AluY | Alu | SINE |
| 46821368 | 46821619 | + | 15.8 | 118 | 375 | -51 | MSTB | MaLR | LTR |
| 46821620 | 46821927 | + | 4.6 | 1 | 304 | -7 | AluY | Alu | SINE |
| 46821928 | 46821977 | + | 15.8 | 376 | 426 | 0 | MSTB | MaLR | LTR |

  

|  |  |  |  |  |  |  |  |  |  |
| --- | --- | --- | --- | --- | --- | --- | --- | --- | --- |
| chr9:46828378-46828989 | | | 612 bp | | |  |  |  |  |
| Genome Start | Genome End | Strand | Div | Repeat Start | Repeat End | Repeat Left | Name | Family | Class |
| 46828378 | 46828523 | - | 21.4 | 6025 | 6170 | -8 | L1MB8 | L1 | LINE |
| 46828524 | 46828791 | + | 9.7 | 1 | 305 | -5 | AluSg | Alu | SINE |
| 46828792 | 46828989 | - | 21.4 | 5815 | 6024 | -154 | L1MB8 | L1 | LINE |

  

|  |  |  |  |  |  |  |  |  |  |
| --- | --- | --- | --- | --- | --- | --- | --- | --- | --- |
| chr9:46828989-46833710 | | | 4677 bp | | |  |  |  |  |
| Genome Start | Genome End | Strand | Div | Repeat Start | Repeat End | Repeat Left | Name | Family | Class |
| 46828989 | 46830203 | - | 18.6 | 5223 | 6398 | -9 | L1MB2 | L1 | LINE |
| 46830204 | 46830488 | - | 14.4 | 1 | 297 | -15 | AluJo | Alu | SINE |
| 46830489 | 46831235 | - | 17.7 | 4481 | 5222 | -1154 | L1MB2 | L1 | LINE |
| 46831281 | 46833710 | - | 18.6 | 1838 | 4480 | -1896 | L1MB2 | L1 | LINE |

  

|  |  |  |  |  |  |  |  |  |  |
| --- | --- | --- | --- | --- | --- | --- | --- | --- | --- |
| chr9:46851315-46856261 | | | 4622 bp | | |  |  |  |  |
| Genome Start | Genome End | Strand | Div | Repeat Start | Repeat End | Repeat Left | Name | Family | Class |
| 46851315 | 46851508 | + | 30.4 | 1351 | 1560 | -1859 | L2 | L2 | LINE |
| 46851518 | 46851796 | + | 19.1 | 2 | 342 | -66 | MLT1A1 | MaLR | LTR |
| 46851797 | 46852106 | - | 13.9 | 1 | 312 | -1 | AluSq | Alu | SINE |
| 46852107 | 46852172 | + | 19.1 | 343 | 407 | -1 | MLT1A1 | MaLR | LTR |
| 46852175 | 46853918 | + | 21.6 | 2 | 1729 | -6 | MLT1A1-int | MaLR | LTR |
| 46853924 | 46854057 | + | 19.1 | 1 | 137 | -271 | MLT1A1 | MaLR | LTR |
| 46854058 | 46854407 | - | 20.5 | 1 | 396 | 0 | MSTD | MaLR | LTR |
| 46854408 | 46854610 | + | 20.4 | 138 | 396 | 0 | MLT1A1 | MaLR | LTR |
| 46854611 | 46855406 | + | 30.6 | 1564 | 2565 | -854 | L2 | L2 | LINE |
| 46855502 | 46855822 | - | 30.0 | 4 | 354 | -241 | MLT1H | MaLR | LTR |
| 46856037 | 46856261 | + | 34.8 | 2949 | 3176 | -243 | L2 | L2 | LINE |

  

|  |  |  |  |  |  |  |  |  |  |
| --- | --- | --- | --- | --- | --- | --- | --- | --- | --- |
| chr9:46858676-46859584 | | | 909 bp | | |  |  |  |  |
| Genome Start | Genome End | Strand | Div | Repeat Start | Repeat End | Repeat Left | Name | Family | Class |
| 46858676 | 46858752 | + | 26.6 | 4865 | 4938 | -1208 | L1M5 | L1 | LINE |
| 46858753 | 46858956 | + | 22.1 | 1 | 194 | -220 | MLT2D | ERVL | LTR |
| 46858957 | 46859240 | + | 7.0 | 1 | 287 | -24 | AluY | Alu | SINE |
| 46859241 | 46859516 | + | 22.1 | 195 | 414 | 0 | MLT2D | ERVL | LTR |
| 46859517 | 46859584 | + | 26.6 | 4939 | 5005 | -1141 | L1M5 | L1 | LINE |

  

|  |  |  |  |  |  |  |  |  |  |
| --- | --- | --- | --- | --- | --- | --- | --- | --- | --- |
| chr9:46868456-46875845 | | | 6943 bp | | |  |  |  |  |
| Genome Start | Genome End | Strand | Div | Repeat Start | Repeat End | Repeat Left | Name | Family | Class |
| 46868456 | 46868604 | - | 21.5 | 6649 | 6801 | 0 | MER89-int | ERV1 | LTR |
| 46868606 | 46869017 | + | 12.4 | 1 | 436 | -231 | LTR12B | ERV1 | LTR |
| 46869206 | 46869358 | - | 27.8 | 1603 | 1755 | 0 | MER52A | ERV1 | LTR |
| 46869367 | 46869993 | - | 23.8 | 54 | 1470 | -162 | MER52A | ERV1 | LTR |
| 46869994 | 46870503 | - | 20.4 | 6052 | 6559 | -242 | MER89-int | ERV1 | LTR |
| 46870603 | 46870905 | + | 8.5 | 1 | 293 | -18 | AluY | Alu | SINE |
| 46870933 | 46872567 | - | 26.2 | 2549 | 4047 | -2778 | MER89-int | ERV1 | LTR |
| 46872692 | 46872899 | - | 27.0 | 2316 | 2536 | -4265 | MER89-int | ERV1 | LTR |
| 46872900 | 46873215 | - | 16.6 | 1 | 296 | -16 | AluSx | Alu | SINE |
| 46873216 | 46873446 | - | 27.0 | 2077 | 2315 | -4486 | MER89-int | ERV1 | LTR |
| 46873447 | 46873762 | + | 15.3 | 1 | 311 | -1 | AluSx | Alu | SINE |
| 46873763 | 46875845 | - | 27.0 | 2 | 2076 | -4725 | MER89-int | ERV1 | LTR |

  

|  |  |  |  |  |  |  |  |  |  |
| --- | --- | --- | --- | --- | --- | --- | --- | --- | --- |
| chr9:46881701-46882850 | | | 1150 bp | | |  |  |  |  |
| Genome Start | Genome End | Strand | Div | Repeat Start | Repeat End | Repeat Left | Name | Family | Class |
| 46881701 | 46881747 | - | 23.3 | 304 | 340 | -1 | MER58B | MER1\_type | DNA |
| 46881748 | 46882257 | + | 17.1 | 2 | 550 | 0 | MER44B | MER2\_type | DNA |
| 46882258 | 46882309 | - | 23.3 | 256 | 303 | -38 | MER58B | MER1\_type | DNA |
| 46882310 | 46882794 | + | 15.6 | 3 | 489 | 0 | LTR45B | ERV1 | LTR |
| 46882795 | 46882850 | - | 23.3 | 208 | 255 | -86 | MER58B | MER1\_type | DNA |

  

|  |  |  |  |  |  |  |  |  |  |
| --- | --- | --- | --- | --- | --- | --- | --- | --- | --- |
| chr9:46883131-46883687 | | | 557 bp | | |  |  |  |  |
| Genome Start | Genome End | Strand | Div | Repeat Start | Repeat End | Repeat Left | Name | Family | Class |
| 46883131 | 46883201 | - | 20.5 | 5820 | 5886 | -278 | L1ME2 | L1 | LINE |
| 46883202 | 46883502 | + | 9.0 | 1 | 302 | -10 | AluSx | Alu | SINE |
| 46883503 | 46883687 | - | 20.5 | 5608 | 5819 | -345 | L1ME2 | L1 | LINE |

  

|  |  |  |  |  |  |  |  |  |  |
| --- | --- | --- | --- | --- | --- | --- | --- | --- | --- |
| chr9:46888165-46888999 | | | 627 bp | | |  |  |  |  |
| Genome Start | Genome End | Strand | Div | Repeat Start | Repeat End | Repeat Left | Name | Family | Class |
| 46888165 | 46888220 | + | 21.4 | 4039 | 4097 | -2049 | L1M4 | L1 | LINE |
| 46888428 | 46888766 | - | 18.4 | 1 | 393 | -508 | MER54B | ERVL | LTR |
| 46888768 | 46888999 | + | 19.1 | 4094 | 4321 | -1825 | L1M4 | L1 | LINE |

  

|  |  |  |  |  |  |  |  |  |  |
| --- | --- | --- | --- | --- | --- | --- | --- | --- | --- |
| chr9:46895095-46896538 | | | 1444 bp | | |  |  |  |  |
| Genome Start | Genome End | Strand | Div | Repeat Start | Repeat End | Repeat Left | Name | Family | Class |
| 46895095 | 46895980 | + | 17.7 | 4316 | 5160 | -986 | L1M4 | L1 | LINE |
| 46895981 | 46896270 | - | 13.8 | 1 | 290 | -22 | AluSx | Alu | SINE |
| 46896271 | 46896538 | + | 17.9 | 5161 | 5437 | -741 | L1M4 | L1 | LINE |

  

|  |  |  |  |  |  |  |  |  |  |
| --- | --- | --- | --- | --- | --- | --- | --- | --- | --- |
| chr9:46958325-46959226 | | | 902 bp | | |  |  |  |  |
| Genome Start | Genome End | Strand | Div | Repeat Start | Repeat End | Repeat Left | Name | Family | Class |
| 46958325 | 46958491 | + | 25.8 | 520 | 699 | -527 | MER69B | Tip100 | DNA |
| 46958492 | 46958792 | - | 16.7 | 1 | 308 | -4 | AluJb | Alu | SINE |
| 46958793 | 46959226 | + | 25.8 | 700 | 2598 | -42 | MER69B | Tip100 | DNA |

  

|  |  |  |  |  |  |  |  |  |  |
| --- | --- | --- | --- | --- | --- | --- | --- | --- | --- |
| chr9:46965137-46965737 | | | 601 bp | | |  |  |  |  |
| Genome Start | Genome End | Strand | Div | Repeat Start | Repeat End | Repeat Left | Name | Family | Class |
| 46965137 | 46965311 | - | 21.9 | 134 | 306 | -6 | AluJo | Alu | SINE |
| 46965312 | 46965620 | - | 16.3 | 1 | 301 | -11 | AluJb | Alu | SINE |
| 46965621 | 46965737 | - | 21.9 | 25 | 133 | -179 | AluJo | Alu | SINE |

  

|  |  |  |  |  |  |  |  |  |  |
| --- | --- | --- | --- | --- | --- | --- | --- | --- | --- |
| chr9:46989221-46990254 | | | 1034 bp | | |  |  |  |  |
| Genome Start | Genome End | Strand | Div | Repeat Start | Repeat End | Repeat Left | Name | Family | Class |
| 46989221 | 46989448 | - | 35.5 | 5664 | 5915 | -206 | L1ME4a | L1 | LINE |
| 46989449 | 46989647 | + | 9.1 | 4 | 203 | -106 | AluSc | Alu | SINE |
| 46989648 | 46989659 | - | 35.5 | 5662 | 5663 | -458 | L1ME4a | L1 | LINE |
| 46989660 | 46989951 | + | 9.4 | 3 | 298 | -13 | AluY | Alu | SINE |
| 46989952 | 46990254 | - | 35.5 | 5348 | 5661 | -460 | L1ME4a | L1 | LINE |

  

|  |  |  |  |  |  |  |  |  |  |
| --- | --- | --- | --- | --- | --- | --- | --- | --- | --- |
| chr9:46992579-46993726 | | | 1148 bp | | |  |  |  |  |
| Genome Start | Genome End | Strand | Div | Repeat Start | Repeat End | Repeat Left | Name | Family | Class |
| 46992579 | 46992775 | + | 35.4 | 2092 | 2302 | -1117 | L2 | L2 | LINE |
| 46992776 | 46992926 | - | 25.2 | 3 | 156 | -4 | MER5A1 | MER1\_type | DNA |
| 46992927 | 46993233 | + | 35.4 | 2303 | 2617 | -802 | L2 | L2 | LINE |
| 46993234 | 46993539 | + | 7.8 | 1 | 306 | -4 | AluSg | Alu | SINE |
| 46993540 | 46993726 | + | 35.4 | 2618 | 2810 | -609 | L2 | L2 | LINE |

  

|  |  |  |  |  |  |  |  |  |  |
| --- | --- | --- | --- | --- | --- | --- | --- | --- | --- |
| chr9:46996593-46997433 | | | 836 bp | | |  |  |  |  |
| Genome Start | Genome End | Strand | Div | Repeat Start | Repeat End | Repeat Left | Name | Family | Class |
| 46996593 | 46997256 | - | 28.0 | 1375 | 2061 | -5067 | L1MCa | L1 | LINE |
| 46997262 | 46997383 | + | 8.2 | 174 | 295 | -15 | Alu | Alu | SINE |
| 46997384 | 46997433 | - | 22.0 | 1233 | 1282 | -5141 | L1MCa | L1 | LINE |

  

|  |  |  |  |  |  |  |  |  |  |
| --- | --- | --- | --- | --- | --- | --- | --- | --- | --- |
| chr9:46998156-47000359 | | | 2215 bp | | |  |  |  |  |
| Genome Start | Genome End | Strand | Div | Repeat Start | Repeat End | Repeat Left | Name | Family | Class |
| 46998156 | 46998883 | - | 15.2 | 5602 | 6327 | -6 | L1MC1 | L1 | LINE |
| 46998884 | 46999184 | - | 5.0 | 1 | 302 | -9 | AluY | Alu | SINE |
| 46999185 | 46999605 | - | 16.1 | 5166 | 5601 | -732 | L1MC1 | L1 | LINE |
| 46999606 | 46999901 | - | 14.2 | 1 | 297 | -15 | AluSx | Alu | SINE |
| 46999902 | 46999958 | - | 20.0 | 5109 | 5165 | -981 | L1MC1 | L1 | LINE |
| 46999953 | 47000132 | - | 16.1 | 5615 | 5794 | -539 | L1MC | L1 | LINE |
| 47000128 | 47000359 | - | 17.7 | 4863 | 5097 | -1049 | L1MC1 | L1 | LINE |

  

|  |  |  |  |  |  |  |  |  |  |
| --- | --- | --- | --- | --- | --- | --- | --- | --- | --- |
| chr9:47006109-47006860 | | | 707 bp | | |  |  |  |  |
| Genome Start | Genome End | Strand | Div | Repeat Start | Repeat End | Repeat Left | Name | Family | Class |
| 47006109 | 47006225 | - | 24.3 | 344 | 462 | 0 | LTR40b | ERVL | LTR |
| 47006271 | 47006557 | - | 9.1 | 1 | 287 | -24 | AluY | Alu | SINE |
| 47006558 | 47006860 | - | 24.3 | 1 | 343 | -119 | LTR40b | ERVL | LTR |

  

|  |  |  |  |  |  |  |  |  |  |
| --- | --- | --- | --- | --- | --- | --- | --- | --- | --- |
| chr9:47021969-47023277 | | | 1309 bp | | |  |  |  |  |
| Genome Start | Genome End | Strand | Div | Repeat Start | Repeat End | Repeat Left | Name | Family | Class |
| 47021969 | 47022233 | + | 31.0 | 2445 | 2761 | -658 | L2 | L2 | LINE |
| 47022234 | 47022618 | - | 13.2 | 1 | 427 | -1 | MSTA | MaLR | LTR |
| 47022619 | 47023277 | + | 32.0 | 2762 | 3410 | -9 | L2 | L2 | LINE |

  

|  |  |  |  |  |  |  |  |  |  |
| --- | --- | --- | --- | --- | --- | --- | --- | --- | --- |
| chr9:47032432-47032933 | | | 502 bp | | |  |  |  |  |
| Genome Start | Genome End | Strand | Div | Repeat Start | Repeat End | Repeat Left | Name | Family | Class |
| 47032432 | 47032518 | + | 28.0 | 3225 | 3312 | -107 | L2 | L2 | LINE |
| 47032519 | 47032817 | - | 13.1 | 1 | 297 | -16 | AluSq | Alu | SINE |
| 47032818 | 47032933 | + | 28.0 | 3313 | 3418 | -1 | L2 | L2 | LINE |

  

|  |  |  |  |  |  |  |  |  |  |
| --- | --- | --- | --- | --- | --- | --- | --- | --- | --- |
| chr9:47039549-47040310 | | | 721 bp | | |  |  |  |  |
| Genome Start | Genome End | Strand | Div | Repeat Start | Repeat End | Repeat Left | Name | Family | Class |
| 47039549 | 47039598 | - | 13.9 | 390 | 428 | 0 | MSTA | MaLR | LTR |
| 47039640 | 47039932 | - | 13.5 | 1 | 276 | -36 | AluSx | Alu | SINE |
| 47039933 | 47040310 | - | 13.9 | 1 | 389 | -39 | MSTA | MaLR | LTR |

  

|  |  |  |  |  |  |  |  |  |  |
| --- | --- | --- | --- | --- | --- | --- | --- | --- | --- |
| chr9:47041451-47042576 | | | 1075 bp | | |  |  |  |  |
| Genome Start | Genome End | Strand | Div | Repeat Start | Repeat End | Repeat Left | Name | Family | Class |
| 47041451 | 47041818 | + | 27.7 | 8 | 304 | -187 | MER110 | ERV1 | LTR |
| 47041819 | 47042112 | - | 7.1 | 1 | 296 | -15 | AluY | Alu | SINE |
| 47042113 | 47042235 | + | 27.3 | 305 | 413 | -78 | MER110 | ERV1 | LTR |
| 47042265 | 47042437 | + | 12.1 | 132 | 304 | -8 | AluSg/x | Alu | SINE |
| 47042438 | 47042488 | + | 15.7 | 378 | 428 | 0 | MSTA | MaLR | LTR |
| 47042511 | 47042576 | + | 24.2 | 410 | 482 | -9 | MER110 | ERV1 | LTR |

  

|  |  |  |  |  |  |  |  |  |  |
| --- | --- | --- | --- | --- | --- | --- | --- | --- | --- |
| chr9:47060361-47061594 | | | 1166 bp | | |  |  |  |  |
| Genome Start | Genome End | Strand | Div | Repeat Start | Repeat End | Repeat Left | Name | Family | Class |
| 47060361 | 47060748 | - | 25.8 | 63 | 459 | -6 | LTR40c | ERVL | LTR |
| 47060810 | 47061520 | - | 20.2 | 5447 | 6181 | -2 | L1MB3 | L1 | LINE |
| 47061528 | 47061594 | - | 20.3 | 5 | 70 | -395 | LTR40c | ERVL | LTR |

  

|  |  |  |  |  |  |  |  |  |  |
| --- | --- | --- | --- | --- | --- | --- | --- | --- | --- |
| chr9:47072566-47073285 | | | 720 bp | | |  |  |  |  |
| Genome Start | Genome End | Strand | Div | Repeat Start | Repeat End | Repeat Left | Name | Family | Class |
| 47072566 | 47072660 | + | 27.5 | 2 | 83 | -179 | MIR | MIR | SINE |
| 47072661 | 47073145 | + | 15.1 | 1 | 489 | 0 | LTR45B | ERV1 | LTR |
| 47073146 | 47073285 | + | 27.5 | 84 | 225 | -37 | MIR | MIR | SINE |

  

|  |  |  |  |  |  |  |  |  |  |
| --- | --- | --- | --- | --- | --- | --- | --- | --- | --- |
| chr9:47080453-47084448 | | | 4011 bp | | |  |  |  |  |
| Genome Start | Genome End | Strand | Div | Repeat Start | Repeat End | Repeat Left | Name | Family | Class |
| 47080453 | 47081469 | - | 23.3 | 5167 | 6228 | -9 | L1ME2 | L1 | LINE |
| 47081448 | 47082862 | - | 22.0 | 786 | 2285 | -4115 | L1ME2 | L1 | LINE |
| 47082863 | 47083149 | - | 8.0 | 1 | 290 | -21 | AluY | Alu | SINE |
| 47083150 | 47083405 | - | 22.0 | 526 | 785 | -5615 | L1ME2 | L1 | LINE |
| 47083407 | 47083571 | + | 26.9 | 1 | 977 | -82 | MER106B | MER1\_type | DNA |
| 47083572 | 47083917 | + | 11.4 | 1 | 331 | 0 | AluY | Alu | SINE |
| 47083918 | 47084007 | + | 25.8 | 978 | 1059 | 0 | MER106B | MER1\_type | DNA |
| 47084014 | 47084448 | - | 22.0 | 39 | 493 | -5907 | L1ME2 | L1 | LINE |

  

|  |  |  |  |  |  |  |  |  |  |
| --- | --- | --- | --- | --- | --- | --- | --- | --- | --- |
| chr9:47099569-47100009 | | | 441 bp | | |  |  |  |  |
| Genome Start | Genome End | Strand | Div | Repeat Start | Repeat End | Repeat Left | Name | Family | Class |
| 47099569 | 47099673 | + | 36.3 | 3252 | 3348 | -30 | L2 | L2 | LINE |
| 47099674 | 47099980 | - | 4.3 | 1 | 311 | 0 | AluY | Alu | SINE |
| 47099981 | 47100009 | + | 36.3 | 3349 | 3378 | 0 | L2 | L2 | LINE |

  

|  |  |  |  |  |  |  |  |  |  |
| --- | --- | --- | --- | --- | --- | --- | --- | --- | --- |
| chr9:65213433-65214439 | | | 864 bp | | |  |  |  |  |
| Genome Start | Genome End | Strand | Div | Repeat Start | Repeat End | Repeat Left | Name | Family | Class |
| 65213433 | 65213769 | - | 34.0 | 3966 | 4333 | -156 | L3 | CR1 | LINE |
| 65213910 | 65214219 | - | 10.0 | 1 | 308 | 0 | AluSg | Alu | SINE |
| 65214223 | 65214439 | - | 33.3 | 3523 | 3773 | -716 | L3 | CR1 | LINE |

  

|  |  |  |  |  |  |  |  |  |  |
| --- | --- | --- | --- | --- | --- | --- | --- | --- | --- |
| chr9:65254223-65256948 | | | 2726 bp | | |  |  |  |  |
| Genome Start | Genome End | Strand | Div | Repeat Start | Repeat End | Repeat Left | Name | Family | Class |
| 65254223 | 65254660 | - | 17.4 | 4645 | 5099 | -2460 | L1M1 | L1 | LINE |
| 65254661 | 65254970 | - | 9.4 | 1 | 317 | 0 | AluY | Alu | SINE |
| 65254971 | 65256216 | - | 18.4 | 3349 | 4644 | -2915 | L1M1 | L1 | LINE |
| 65256217 | 65256552 | + | 18.4 | 4 | 345 | -1 | MER7A | MER2\_type | DNA |
| 65256553 | 65256948 | - | 23.6 | 2917 | 3348 | -4217 | L1M1 | L1 | LINE |

  

|  |  |  |  |  |  |  |  |  |  |
| --- | --- | --- | --- | --- | --- | --- | --- | --- | --- |
| chr9:65256944-65258311 | | | 1368 bp | | |  |  |  |  |
| Genome Start | Genome End | Strand | Div | Repeat Start | Repeat End | Repeat Left | Name | Family | Class |
| 65256944 | 65257198 | + | 20.2 | 4117 | 4376 | -1764 | L1M3 | L1 | LINE |
| 65257199 | 65257461 | + | 9.9 | 1 | 287 | -24 | AluY | Alu | SINE |
| 65257462 | 65258311 | + | 20.2 | 4377 | 5274 | -866 | L1M3 | L1 | LINE |

  

|  |  |  |  |  |  |  |  |  |  |
| --- | --- | --- | --- | --- | --- | --- | --- | --- | --- |
| chr9:65258331-65258987 | | | 657 bp | | |  |  |  |  |
| Genome Start | Genome End | Strand | Div | Repeat Start | Repeat End | Repeat Left | Name | Family | Class |
| 65258331 | 65258401 | - | 14.8 | 300 | 364 | 0 | THE1B | MaLR | LTR |
| 65258402 | 65258691 | + | 10.7 | 1 | 290 | -21 | AluY | Alu | SINE |
| 65258692 | 65258987 | - | 14.8 | 1 | 299 | -65 | THE1B | MaLR | LTR |

  

|  |  |  |  |  |  |  |  |  |  |
| --- | --- | --- | --- | --- | --- | --- | --- | --- | --- |
| chr9:65267374-65271396 | | | 3942 bp | | |  |  |  |  |
| Genome Start | Genome End | Strand | Div | Repeat Start | Repeat End | Repeat Left | Name | Family | Class |
| 65267374 | 65267841 | + | 33.9 | 64 | 559 | -5560 | L1ME3B | L1 | LINE |
| 65267851 | 65268277 | + | 15.5 | 1 | 436 | -231 | LTR12B | ERV1 | LTR |
| 65268350 | 65268539 | + | 32.1 | 4839 | 5042 | -1087 | L1ME3B | L1 | LINE |
| 65268540 | 65268836 | - | 7.9 | 1 | 295 | -16 | AluY | Alu | SINE |
| 65268837 | 65269554 | + | 27.7 | 5043 | 5827 | -396 | L1ME3B | L1 | LINE |
| 65269555 | 65269932 | + | 14.8 | 5435 | 5817 | -516 | L1MC1 | L1 | LINE |
| 65269933 | 65270248 | + | 10.0 | 1 | 311 | 0 | AluY | Alu | SINE |
| 65270249 | 65270725 | + | 14.8 | 5818 | 6322 | -11 | L1MC1 | L1 | LINE |
| 65270726 | 65270823 | + | 27.6 | 5828 | 5890 | -333 | L1ME3B | L1 | LINE |
| 65270824 | 65271126 | + | 6.7 | 1 | 300 | -11 | AluY | Alu | SINE |
| 65271127 | 65271396 | + | 27.6 | 5891 | 6203 | -20 | L1ME3B | L1 | LINE |

  

|  |  |  |  |  |  |  |  |  |  |
| --- | --- | --- | --- | --- | --- | --- | --- | --- | --- |
| chr9:65307581-65308494 | | | 914 bp | | |  |  |  |  |
| Genome Start | Genome End | Strand | Div | Repeat Start | Repeat End | Repeat Left | Name | Family | Class |
| 65307581 | 65307959 | - | 12.0 | 5788 | 6158 | -7 | L1PA15 | L1 | LINE |
| 65307960 | 65308267 | - | 9.1 | 1 | 308 | -3 | AluY | Alu | SINE |
| 65308268 | 65308494 | - | 12.0 | 5522 | 5787 | -378 | L1PA15 | L1 | LINE |

  

|  |  |  |  |  |  |  |  |  |  |
| --- | --- | --- | --- | --- | --- | --- | --- | --- | --- |
| chr9:65339278-65339956 | | | 631 bp | | |  |  |  |  |
| Genome Start | Genome End | Strand | Div | Repeat Start | Repeat End | Repeat Left | Name | Family | Class |
| 65339278 | 65339369 | + | 16.6 | 1 | 107 | -259 | Tigger5a | MER2\_type | DNA |
| 65339418 | 65339700 | - | 8.5 | 1 | 281 | -28 | AluSc | Alu | SINE |
| 65339701 | 65339956 | + | 16.6 | 108 | 366 | 0 | Tigger5a | MER2\_type | DNA |

  

|  |  |  |  |  |  |  |  |  |  |
| --- | --- | --- | --- | --- | --- | --- | --- | --- | --- |
| chr9:65344571-65348798 | | | 4228 bp | | |  |  |  |  |
| Genome Start | Genome End | Strand | Div | Repeat Start | Repeat End | Repeat Left | Name | Family | Class |
| 65344571 | 65344759 | - | 16.8 | 6036 | 6223 | -19 | L1MD1 | L1 | LINE |
| 65344760 | 65345060 | + | 5.8 | 1 | 302 | -9 | AluY | Alu | SINE |
| 65345061 | 65347419 | - | 18.9 | 3551 | 6035 | -207 | L1MD1 | L1 | LINE |
| 65347420 | 65347733 | + | 11.2 | 1 | 312 | 0 | AluSx | Alu | SINE |
| 65347734 | 65348798 | - | 19.9 | 2490 | 3550 | -2596 | L1MD1 | L1 | LINE |

  

|  |  |  |  |  |  |  |  |  |  |
| --- | --- | --- | --- | --- | --- | --- | --- | --- | --- |
| chr9:65362039-65362907 | | | 869 bp | | |  |  |  |  |
| Genome Start | Genome End | Strand | Div | Repeat Start | Repeat End | Repeat Left | Name | Family | Class |
| 65362039 | 65362415 | + | 27.9 | 4147 | 4544 | -1602 | L1M5 | L1 | LINE |
| 65362416 | 65362631 | + | 21.6 | 1 | 218 | -1 | MER20 | MER1\_type | DNA |
| 65362632 | 65362907 | + | 27.9 | 4545 | 4828 | -1318 | L1M5 | L1 | LINE |

  

|  |  |  |  |  |  |  |  |  |  |
| --- | --- | --- | --- | --- | --- | --- | --- | --- | --- |
| chr9:65382283-65386638 | | | 4349 bp | | |  |  |  |  |
| Genome Start | Genome End | Strand | Div | Repeat Start | Repeat End | Repeat Left | Name | Family | Class |
| 65382283 | 65385298 | + | 20.2 | 2226 | 5292 | -1090 | L1MB8 | L1 | LINE |
| 65385306 | 65385406 | + | 22.2 | 5432 | 5530 | -890 | L1MB8 | L1 | LINE |
| 65385407 | 65385712 | + | 6.9 | 2 | 307 | -4 | AluY | Alu | SINE |
| 65385713 | 65386638 | + | 22.2 | 5525 | 6414 | 0 | L1MB8 | L1 | LINE |

  

|  |  |  |  |  |  |  |  |  |  |
| --- | --- | --- | --- | --- | --- | --- | --- | --- | --- |
| chr9:65391134-65391573 | | | 440 bp | | |  |  |  |  |
| Genome Start | Genome End | Strand | Div | Repeat Start | Repeat End | Repeat Left | Name | Family | Class |
| 65391134 | 65391162 | - | 36.3 | 3349 | 3378 | 0 | L2 | L2 | LINE |
| 65391163 | 65391469 | + | 4.3 | 1 | 311 | 0 | AluY | Alu | SINE |
| 65391470 | 65391573 | - | 36.3 | 3253 | 3348 | -30 | L2 | L2 | LINE |

  

|  |  |  |  |  |  |  |  |  |  |
| --- | --- | --- | --- | --- | --- | --- | --- | --- | --- |
| chr9:65393389-65393939 | | | 551 bp | | |  |  |  |  |
| Genome Start | Genome End | Strand | Div | Repeat Start | Repeat End | Repeat Left | Name | Family | Class |
| 65393389 | 65393556 | + | 20.5 | 5 | 161 | -48 | MER3 | MER1\_type | DNA |
| 65393557 | 65393883 | - | 7.9 | 1 | 304 | -7 | AluY | Alu | SINE |
| 65393884 | 65393939 | + | 20.5 | 162 | 209 | 0 | MER3 | MER1\_type | DNA |

  

|  |  |  |  |  |  |  |  |  |  |
| --- | --- | --- | --- | --- | --- | --- | --- | --- | --- |
| chr9:65406694-65410689 | | | 4011 bp | | |  |  |  |  |
| Genome Start | Genome End | Strand | Div | Repeat Start | Repeat End | Repeat Left | Name | Family | Class |
| 65406694 | 65407128 | + | 22.0 | 39 | 493 | -5907 | L1ME2 | L1 | LINE |
| 65407135 | 65407224 | - | 25.8 | 978 | 1059 | 0 | MER106B | MER1\_type | DNA |
| 65407225 | 65407570 | - | 11.4 | 1 | 331 | 0 | AluY | Alu | SINE |
| 65407571 | 65407735 | - | 26.9 | 1 | 977 | -82 | MER106B | MER1\_type | DNA |
| 65407737 | 65407992 | + | 22.0 | 526 | 785 | -5615 | L1ME2 | L1 | LINE |
| 65407993 | 65408279 | + | 8.0 | 1 | 290 | -21 | AluY | Alu | SINE |
| 65408280 | 65409694 | + | 22.0 | 786 | 2285 | -4115 | L1ME2 | L1 | LINE |
| 65409673 | 65410689 | + | 23.3 | 5167 | 6228 | -9 | L1ME2 | L1 | LINE |

  

|  |  |  |  |  |  |  |  |  |  |
| --- | --- | --- | --- | --- | --- | --- | --- | --- | --- |
| chr9:65417856-65418575 | | | 720 bp | | |  |  |  |  |
| Genome Start | Genome End | Strand | Div | Repeat Start | Repeat End | Repeat Left | Name | Family | Class |
| 65417856 | 65417995 | - | 27.5 | 84 | 225 | -37 | MIR | MIR | SINE |
| 65417996 | 65418480 | - | 15.3 | 1 | 489 | 0 | LTR45B | ERV1 | LTR |
| 65418481 | 65418575 | - | 27.5 | 2 | 83 | -179 | MIR | MIR | SINE |

  

|  |  |  |  |  |  |  |  |  |  |
| --- | --- | --- | --- | --- | --- | --- | --- | --- | --- |
| chr9:65450831-65451592 | | | 721 bp | | |  |  |  |  |
| Genome Start | Genome End | Strand | Div | Repeat Start | Repeat End | Repeat Left | Name | Family | Class |
| 65450831 | 65451208 | + | 13.9 | 1 | 389 | -39 | MSTA | MaLR | LTR |
| 65451209 | 65451501 | + | 13.5 | 1 | 276 | -36 | AluSx | Alu | SINE |
| 65451543 | 65451592 | + | 13.9 | 390 | 428 | 0 | MSTA | MaLR | LTR |

  

|  |  |  |  |  |  |  |  |  |  |
| --- | --- | --- | --- | --- | --- | --- | --- | --- | --- |
| chr9:65455625-65457492 | | | 1868 bp | | |  |  |  |  |
| Genome Start | Genome End | Strand | Div | Repeat Start | Repeat End | Repeat Left | Name | Family | Class |
| 65455625 | 65455649 | + | 22.1 | 3718 | 3744 | -2402 | L1MC2 | L1 | LINE |
| 65455650 | 65455810 | + | 19.5 | 6149 | 6312 | 0 | L1MA9 | L1 | LINE |
| 65455811 | 65455888 | + | 22.1 | 3745 | 3822 | -2324 | L1MC2 | L1 | LINE |
| 65455889 | 65456726 | + | 15.1 | 1 | 983 | 0 | LTR1D | ERV1 | LTR |
| 65456727 | 65457492 | + | 20.7 | 3820 | 6034 | -343 | L1MC2 | L1 | LINE |

  

|  |  |  |  |  |  |  |  |  |  |
| --- | --- | --- | --- | --- | --- | --- | --- | --- | --- |
| chr9:65458197-65458698 | | | 502 bp | | |  |  |  |  |
| Genome Start | Genome End | Strand | Div | Repeat Start | Repeat End | Repeat Left | Name | Family | Class |
| 65458197 | 65458312 | - | 28.0 | 3314 | 3419 | 0 | L2 | L2 | LINE |
| 65458313 | 65458611 | + | 13.1 | 1 | 297 | -16 | AluSq | Alu | SINE |
| 65458612 | 65458698 | - | 28.0 | 3226 | 3313 | -106 | L2 | L2 | LINE |

  

|  |  |  |  |  |  |  |  |  |  |
| --- | --- | --- | --- | --- | --- | --- | --- | --- | --- |
| chr9:65458789-65463562 | | | 4708 bp | | |  |  |  |  |
| Genome Start | Genome End | Strand | Div | Repeat Start | Repeat End | Repeat Left | Name | Family | Class |
| 65458789 | 65458814 | - | 16.2 | 7220 | 7237 | -35 | L1MA9 | L1 | LINE |
| 65458815 | 65459117 | + | 10.6 | 1 | 306 | -7 | AluSq | Alu | SINE |
| 65459118 | 65459212 | - | 16.2 | 7113 | 7219 | -53 | L1MA9 | L1 | LINE |
| 65459246 | 65460031 | - | 16.2 | 6356 | 7112 | -160 | L1MA9 | L1 | LINE |
| 65460076 | 65460802 | - | 18.4 | 5615 | 6349 | -754 | L1MA9 | L1 | LINE |
| 65460803 | 65461098 | - | 8.9 | 1 | 292 | -21 | AluSp | Alu | SINE |
| 65461099 | 65461563 | - | 18.4 | 5005 | 5614 | -1489 | L1MA9 | L1 | LINE |
| 65461553 | 65463562 | - | 22.9 | 1 | 2508 | -4935 | L1MA9 | L1 | LINE |

  

|  |  |  |  |  |  |  |  |  |  |
| --- | --- | --- | --- | --- | --- | --- | --- | --- | --- |
| chr9:65466468-65467283 | | | 816 bp | | |  |  |  |  |
| Genome Start | Genome End | Strand | Div | Repeat Start | Repeat End | Repeat Left | Name | Family | Class |
| 65466468 | 65466653 | + | 27.3 | 5671 | 5864 | -302 | L1ME1 | L1 | LINE |
| 65466654 | 65466959 | + | 9.2 | 1 | 305 | -6 | AluY | Alu | SINE |
| 65466960 | 65467283 | + | 27.3 | 5865 | 6333 | -1 | L1ME1 | L1 | LINE |

  

|  |  |  |  |  |  |  |  |  |  |
| --- | --- | --- | --- | --- | --- | --- | --- | --- | --- |
| chr9:65467853-65469161 | | | 1309 bp | | |  |  |  |  |
| Genome Start | Genome End | Strand | Div | Repeat Start | Repeat End | Repeat Left | Name | Family | Class |
| 65467853 | 65468511 | - | 32.0 | 2762 | 3410 | -9 | L2 | L2 | LINE |
| 65468512 | 65468896 | + | 13.2 | 1 | 427 | -1 | MSTA | MaLR | LTR |
| 65468897 | 65469161 | - | 31.0 | 2445 | 2761 | -658 | L2 | L2 | LINE |

  

|  |  |  |  |  |  |  |  |  |  |
| --- | --- | --- | --- | --- | --- | --- | --- | --- | --- |
| chr9:65475593-65477576 | | | 1984 bp | | |  |  |  |  |
| Genome Start | Genome End | Strand | Div | Repeat Start | Repeat End | Repeat Left | Name | Family | Class |
| 65475593 | 65476158 | - | 15.6 | 5591 | 6235 | -109 | L1MC2 | L1 | LINE |
| 65476159 | 65476451 | + | 9.6 | 1 | 293 | -16 | AluSc | Alu | SINE |
| 65476452 | 65477576 | - | 16.6 | 4443 | 5590 | -754 | L1MC2 | L1 | LINE |

  

|  |  |  |  |  |  |  |  |  |  |
| --- | --- | --- | --- | --- | --- | --- | --- | --- | --- |
| chr9:65484269-65485020 | | | 707 bp | | |  |  |  |  |
| Genome Start | Genome End | Strand | Div | Repeat Start | Repeat End | Repeat Left | Name | Family | Class |
| 65484269 | 65484571 | + | 24.0 | 1 | 343 | -119 | LTR40b | ERVL | LTR |
| 65484572 | 65484858 | + | 9.4 | 1 | 287 | -24 | AluY | Alu | SINE |
| 65484904 | 65485020 | + | 24.0 | 344 | 462 | 0 | LTR40b | ERVL | LTR |

  

|  |  |  |  |  |  |  |  |  |  |
| --- | --- | --- | --- | --- | --- | --- | --- | --- | --- |
| chr9:65489884-65492088 | | | 2216 bp | | |  |  |  |  |
| Genome Start | Genome End | Strand | Div | Repeat Start | Repeat End | Repeat Left | Name | Family | Class |
| 65489884 | 65490115 | + | 18.1 | 4863 | 5097 | -1049 | L1MC1 | L1 | LINE |
| 65490111 | 65490290 | + | 15.6 | 5615 | 5794 | -539 | L1MC | L1 | LINE |
| 65490285 | 65490341 | + | 19.6 | 5108 | 5164 | -982 | L1MC1 | L1 | LINE |
| 65490342 | 65490636 | + | 13.9 | 1 | 296 | -16 | AluSx | Alu | SINE |
| 65490637 | 65491057 | + | 16.1 | 5165 | 5601 | -732 | L1MC1 | L1 | LINE |
| 65491058 | 65491361 | + | 4.6 | 1 | 302 | -9 | AluY | Alu | SINE |
| 65491362 | 65492088 | + | 15.3 | 5602 | 6327 | -6 | L1MC1 | L1 | LINE |

  

|  |  |  |  |  |  |  |  |  |  |
| --- | --- | --- | --- | --- | --- | --- | --- | --- | --- |
| chr9:65494314-65495219 | | | 906 bp | | |  |  |  |  |
| Genome Start | Genome End | Strand | Div | Repeat Start | Repeat End | Repeat Left | Name | Family | Class |
| 65494314 | 65494389 | + | 25.9 | 1 | 64 | -580 | LTR48B | ERV1 | LTR |
| 65494390 | 65494695 | - | 6.5 | 1 | 306 | -5 | AluY | Alu | SINE |
| 65494696 | 65495219 | + | 25.9 | 65 | 642 | -2 | LTR48B | ERV1 | LTR |

  

|  |  |  |  |  |  |  |  |  |  |
| --- | --- | --- | --- | --- | --- | --- | --- | --- | --- |
| chr9:65496906-65498078 | | | 1173 bp | | |  |  |  |  |
| Genome Start | Genome End | Strand | Div | Repeat Start | Repeat End | Repeat Left | Name | Family | Class |
| 65496906 | 65497114 | - | 35.4 | 2618 | 2831 | -588 | L2 | L2 | LINE |
| 65497115 | 65497419 | - | 7.5 | 1 | 305 | -5 | AluSg | Alu | SINE |
| 65497420 | 65497727 | - | 35.4 | 2303 | 2617 | -802 | L2 | L2 | LINE |
| 65497728 | 65497878 | + | 24.5 | 3 | 156 | -4 | MER5A1 | MER1\_type | DNA |
| 65497879 | 65498078 | - | 35.4 | 2092 | 2302 | -1117 | L2 | L2 | LINE |

  

|  |  |  |  |  |  |  |  |  |  |
| --- | --- | --- | --- | --- | --- | --- | --- | --- | --- |
| chr9:65516129-65516850 | | | 722 bp | | |  |  |  |  |
| Genome Start | Genome End | Strand | Div | Repeat Start | Repeat End | Repeat Left | Name | Family | Class |
| 65516129 | 65516264 | - | 14.3 | 173 | 310 | -2 | AluSx | Alu | SINE |
| 65516265 | 65516664 | + | 9.8 | 1 | 448 | -2 | LTR7 | ERV1 | LTR |
| 65516665 | 65516850 | - | 14.3 | 1 | 172 | -140 | AluSx | Alu | SINE |

  

|  |  |  |  |  |  |  |  |  |  |
| --- | --- | --- | --- | --- | --- | --- | --- | --- | --- |
| chr9:65520063-65520835 | | | 773 bp | | |  |  |  |  |
| Genome Start | Genome End | Strand | Div | Repeat Start | Repeat End | Repeat Left | Name | Family | Class |
| 65520063 | 65520356 | + | 22.9 | 250 | 540 | -167 | MER39 | ERV1 | LTR |
| 65520357 | 65520667 | - | 5.7 | 1 | 310 | 0 | AluY | Alu | SINE |
| 65520668 | 65520835 | + | 22.9 | 541 | 707 | 0 | MER39 | ERV1 | LTR |

  

|  |  |  |  |  |  |  |  |  |  |
| --- | --- | --- | --- | --- | --- | --- | --- | --- | --- |
| chr9:65548722-65549482 | | | 760 bp | | |  |  |  |  |
| Genome Start | Genome End | Strand | Div | Repeat Start | Repeat End | Repeat Left | Name | Family | Class |
| 65548722 | 65549030 | + | 9.1 | 4759 | 5067 | -1079 | L1P4 | L1 | LINE |
| 65549031 | 65549342 | + | 7.8 | 1 | 308 | -3 | AluY | Alu | SINE |
| 65549344 | 65549482 | + | 18.0 | 5597 | 5735 | -431 | L1P4 | L1 | LINE |

  

|  |  |  |  |  |  |  |  |  |  |
| --- | --- | --- | --- | --- | --- | --- | --- | --- | --- |
| chr9:65564574-65565389 | | | 769 bp | | |  |  |  |  |
| Genome Start | Genome End | Strand | Div | Repeat Start | Repeat End | Repeat Left | Name | Family | Class |
| 65564574 | 65564718 | + | 21.9 | 5683 | 5832 | -342 | L1MB5 | L1 | LINE |
| 65564719 | 65565007 | + | 15.9 | 1 | 285 | -27 | AluSx | Alu | SINE |
| 65565055 | 65565069 | + | 15.9 | 286 | 300 | -12 | AluSx | Alu | SINE |
| 65565070 | 65565389 | + | 20.8 | 5833 | 6168 | -6 | L1MB5 | L1 | LINE |

  

|  |  |  |  |  |  |  |  |  |  |
| --- | --- | --- | --- | --- | --- | --- | --- | --- | --- |
| chr9:65573866-65574674 | | | 809 bp | | |  |  |  |  |
| Genome Start | Genome End | Strand | Div | Repeat Start | Repeat End | Repeat Left | Name | Family | Class |
| 65573866 | 65574279 | + | 11.8 | 1 | 513 | -313 | LTR12 | ERV1 | LTR |
| 65574280 | 65574587 | + | 9.1 | 1 | 299 | -12 | AluY | Alu | SINE |
| 65574588 | 65574674 | + | 11.8 | 514 | 595 | -231 | LTR12 | ERV1 | LTR |

  

|  |  |  |  |  |  |  |  |  |  |
| --- | --- | --- | --- | --- | --- | --- | --- | --- | --- |
| chr9:65581474-65582280 | | | 713 bp | | |  |  |  |  |
| Genome Start | Genome End | Strand | Div | Repeat Start | Repeat End | Repeat Left | Name | Family | Class |
| 65581474 | 65581619 | - | 28.1 | 3267 | 3419 | 0 | L2 | L2 | LINE |
| 65581636 | 65582053 | + | 20.6 | 11 | 459 | -56 | MLT2B2 | ERVL | LTR |
| 65582097 | 65582127 | + | 20.6 | 507 | 550 | -12 | MLT2B2 | ERVL | LTR |
| 65582163 | 65582280 | - | 33.0 | 3012 | 3128 | -291 | L2 | L2 | LINE |

  

|  |  |  |  |  |  |  |  |  |  |
| --- | --- | --- | --- | --- | --- | --- | --- | --- | --- |
| chr9:65597221-65597686 | | | 466 bp | | |  |  |  |  |
| Genome Start | Genome End | Strand | Div | Repeat Start | Repeat End | Repeat Left | Name | Family | Class |
| 65597221 | 65597281 | + | 32.9 | 1 | 63 | -115 | MER5B | MER1\_type | DNA |
| 65597282 | 65597572 | + | 13.1 | 1 | 292 | -20 | AluSx | Alu | SINE |
| 65597573 | 65597686 | + | 32.9 | 64 | 178 | 0 | MER5B | MER1\_type | DNA |

  

|  |  |  |  |  |  |  |  |  |  |
| --- | --- | --- | --- | --- | --- | --- | --- | --- | --- |
| chr9:65643641-65644527 | | | 865 bp | | |  |  |  |  |
| Genome Start | Genome End | Strand | Div | Repeat Start | Repeat End | Repeat Left | Name | Family | Class |
| 65643641 | 65643886 | + | 17.7 | 1853 | 2221 | -4412 | L1MDa | L1 | LINE |
| 65643909 | 65644188 | - | 7.5 | 1 | 284 | -27 | AluY | Alu | SINE |
| 65644189 | 65644527 | + | 17.7 | 2222 | 2555 | -4078 | L1MDa | L1 | LINE |

  

|  |  |  |  |  |  |  |  |  |  |
| --- | --- | --- | --- | --- | --- | --- | --- | --- | --- |
| chr9:65655250-65656482 | | | 742 bp | | |  |  |  |  |
| Genome Start | Genome End | Strand | Div | Repeat Start | Repeat End | Repeat Left | Name | Family | Class |
| 65655250 | 65655429 | + | 28.7 | 8 | 196 | -2311 | HAL1 | L1 | LINE |
| 65655866 | 65656093 | - | 11.0 | 67 | 295 | -14 | AluSc | Alu | SINE |
| 65656096 | 65656241 | + | 29.4 | 347 | 489 | -2 | LTR16C | ERVL | LTR |
| 65656295 | 65656482 | + | 32.0 | 2031 | 2216 | -291 | HAL1 | L1 | LINE |

  

|  |  |  |  |  |  |  |  |  |  |
| --- | --- | --- | --- | --- | --- | --- | --- | --- | --- |
| chr9:65711471-65712572 | | | 1102 bp | | |  |  |  |  |
| Genome Start | Genome End | Strand | Div | Repeat Start | Repeat End | Repeat Left | Name | Family | Class |
| 65711471 | 65711869 | - | 23.4 | 7511 | 7926 | -35 | L1MC5 | L1 | LINE |
| 65711870 | 65712346 | + | 7.8 | 1 | 486 | 0 | LTR19A | ERV1 | LTR |
| 65712347 | 65712572 | - | 23.4 | 7260 | 7510 | -451 | L1MC5 | L1 | LINE |

  

|  |  |  |  |  |  |  |  |  |  |
| --- | --- | --- | --- | --- | --- | --- | --- | --- | --- |
| chr9:65727929-65729310 | | | 1256 bp | | |  |  |  |  |
| Genome Start | Genome End | Strand | Div | Repeat Start | Repeat End | Repeat Left | Name | Family | Class |
| 65727929 | 65728189 | - | 30.1 | 2746 | 3032 | -3097 | L1MEd | L1 | LINE |
| 65728190 | 65728479 | - | 10.7 | 1 | 290 | -19 | AluSc | Alu | SINE |
| 65728480 | 65728697 | - | 30.1 | 2534 | 2745 | -3384 | L1MEd | L1 | LINE |
| 65728700 | 65728864 | - | 17.1 | 5986 | 6167 | -4 | L1MB2 | L1 | LINE |
| 65728989 | 65729310 | - | 38.1 | 223 | 566 | -5553 | L1MEd | L1 | LINE |

  

|  |  |  |  |  |  |  |  |  |  |
| --- | --- | --- | --- | --- | --- | --- | --- | --- | --- |
| chr9:65740423-65747136 | | | 6682 bp | | |  |  |  |  |
| Genome Start | Genome End | Strand | Div | Repeat Start | Repeat End | Repeat Left | Name | Family | Class |
| 65740423 | 65744273 | - | 16.9 | 1656 | 5714 | 0 | ERVL-B4 | ERVL | LTR |
| 65744274 | 65744668 | + | 13.9 | 1 | 422 | -6 | MSTA | MaLR | LTR |
| 65744673 | 65745096 | - | 19.2 | 1216 | 1662 | -4052 | ERVL-B4 | ERVL | LTR |
| 65745097 | 65745338 | + | 17.0 | 1 | 251 | -2072 | Tigger5 | MER2\_type | DNA |
| 65745366 | 65745573 | - | 12.1 | 5936 | 6151 | 0 | L1PB1 | L1 | LINE |
| 65745574 | 65745971 | + | 14.6 | 2007 | 2405 | 0 | Tigger5 | MER2\_type | DNA |
| 65745973 | 65747136 | - | 16.8 | 4 | 1217 | -4497 | ERVL-B4 | ERVL | LTR |

  

|  |  |  |  |  |  |  |  |  |  |
| --- | --- | --- | --- | --- | --- | --- | --- | --- | --- |
| chr9:65786581-65787954 | | | 1130 bp | | |  |  |  |  |
| Genome Start | Genome End | Strand | Div | Repeat Start | Repeat End | Repeat Left | Name | Family | Class |
| 65786581 | 65786719 | + | 34.8 | 1284 | 1434 | -1985 | L2 | L2 | LINE |
| 65786764 | 65786866 | - | 29.6 | 3274 | 3378 | 0 | L2 | L2 | LINE |
| 65786849 | 65787341 | + | 34.9 | 1571 | 2074 | -1345 | L2 | L2 | LINE |
| 65787367 | 65787650 | - | 11.7 | 1 | 283 | -30 | AluSq | Alu | SINE |
| 65787844 | 65787954 | + | 30.6 | 3251 | 3379 | -21 | L2 | L2 | LINE |

  

|  |  |  |  |  |  |  |  |  |  |
| --- | --- | --- | --- | --- | --- | --- | --- | --- | --- |
| chr9:65789015-65789626 | | | 612 bp | | |  |  |  |  |
| Genome Start | Genome End | Strand | Div | Repeat Start | Repeat End | Repeat Left | Name | Family | Class |
| 65789015 | 65789160 | - | 21.4 | 6025 | 6170 | -8 | L1MB8 | L1 | LINE |
| 65789161 | 65789428 | + | 9.3 | 1 | 305 | -5 | AluSg | Alu | SINE |
| 65789429 | 65789626 | - | 21.4 | 5815 | 6024 | -154 | L1MB8 | L1 | LINE |

  

|  |  |  |  |  |  |  |  |  |  |
| --- | --- | --- | --- | --- | --- | --- | --- | --- | --- |
| chr9:65789626-65794342 | | | 4682 bp | | |  |  |  |  |
| Genome Start | Genome End | Strand | Div | Repeat Start | Repeat End | Repeat Left | Name | Family | Class |
| 65789626 | 65790843 | - | 18.7 | 5223 | 6398 | -9 | L1MB2 | L1 | LINE |
| 65790844 | 65791128 | - | 14.4 | 1 | 297 | -15 | AluJo | Alu | SINE |
| 65791129 | 65791877 | - | 17.9 | 4481 | 5222 | -1154 | L1MB2 | L1 | LINE |
| 65791913 | 65794342 | - | 18.7 | 1838 | 4480 | -1896 | L1MB2 | L1 | LINE |

  

|  |  |  |  |  |  |  |  |  |  |
| --- | --- | --- | --- | --- | --- | --- | --- | --- | --- |
| chr9:65797521-65798661 | | | 1141 bp | | |  |  |  |  |
| Genome Start | Genome End | Strand | Div | Repeat Start | Repeat End | Repeat Left | Name | Family | Class |
| 65797521 | 65797576 | + | 19.2 | 4175 | 4216 | -1930 | L1PREC2 | L1 | LINE |
| 65797577 | 65797873 | - | 12.5 | 1 | 296 | -16 | AluSx | Alu | SINE |
| 65797874 | 65798001 | + | 19.2 | 4217 | 4337 | -1809 | L1PREC2 | L1 | LINE |
| 65798002 | 65798539 | + | 16.9 | 1 | 556 | 0 | LTR38 | ERV1 | LTR |
| 65798540 | 65798661 | + | 19.2 | 4338 | 4454 | -1692 | L1PREC2 | L1 | LINE |

  

|  |  |  |  |  |  |  |  |  |  |
| --- | --- | --- | --- | --- | --- | --- | --- | --- | --- |
| chr9:65811953-65816898 | | | 4621 bp | | |  |  |  |  |
| Genome Start | Genome End | Strand | Div | Repeat Start | Repeat End | Repeat Left | Name | Family | Class |
| 65811953 | 65812146 | + | 29.9 | 1351 | 1560 | -1859 | L2 | L2 | LINE |
| 65812156 | 65812434 | + | 19.6 | 2 | 342 | -66 | MLT1A1 | MaLR | LTR |
| 65812435 | 65812743 | - | 13.6 | 1 | 311 | -2 | AluSq | Alu | SINE |
| 65812744 | 65812809 | + | 19.6 | 343 | 407 | -1 | MLT1A1 | MaLR | LTR |
| 65812812 | 65814555 | + | 21.6 | 2 | 1729 | -6 | MLT1A1-int | MaLR | LTR |
| 65814561 | 65814694 | + | 19.1 | 1 | 137 | -271 | MLT1A1 | MaLR | LTR |
| 65814695 | 65815044 | - | 20.8 | 1 | 396 | 0 | MSTD | MaLR | LTR |
| 65815045 | 65815247 | + | 20.4 | 138 | 396 | 0 | MLT1A1 | MaLR | LTR |
| 65815248 | 65816043 | + | 30.3 | 1564 | 2565 | -854 | L2 | L2 | LINE |
| 65816139 | 65816459 | - | 30.0 | 4 | 354 | -241 | MLT1H | MaLR | LTR |
| 65816674 | 65816898 | + | 33.5 | 2949 | 3176 | -243 | L2 | L2 | LINE |

  

|  |  |  |  |  |  |  |  |  |  |
| --- | --- | --- | --- | --- | --- | --- | --- | --- | --- |
| chr9:65829074-65836449 | | | 6806 bp | | |  |  |  |  |
| Genome Start | Genome End | Strand | Div | Repeat Start | Repeat End | Repeat Left | Name | Family | Class |
| 65829074 | 65829222 | - | 21.5 | 6649 | 6801 | 0 | MER89-int | ERV1 | LTR |
| 65829224 | 65829635 | + | 11.9 | 1 | 436 | -231 | LTR12B | ERV1 | LTR |
| 65829824 | 65829976 | - | 27.8 | 1603 | 1755 | 0 | MER52A | ERV1 | LTR |
| 65829985 | 65830611 | - | 23.5 | 54 | 1470 | -162 | MER52A | ERV1 | LTR |
| 65830612 | 65831121 | - | 20.4 | 6052 | 6559 | -242 | MER89-int | ERV1 | LTR |
| 65831221 | 65831523 | + | 8.5 | 1 | 293 | -18 | AluY | Alu | SINE |
| 65831674 | 65833190 | - | 25.6 | 2549 | 4047 | -2904 | MER89-int | ERV1 | LTR |
| 65833315 | 65833522 | - | 27.0 | 2315 | 2536 | -4265 | MER89-int | ERV1 | LTR |
| 65833523 | 65833832 | - | 16.6 | 1 | 296 | -16 | AluSx | Alu | SINE |
| 65833833 | 65834063 | - | 27.0 | 2077 | 2314 | -4487 | MER89-int | ERV1 | LTR |
| 65834064 | 65834384 | + | 14.9 | 1 | 311 | -1 | AluSx | Alu | SINE |
| 65834385 | 65836449 | - | 27.0 | 2 | 2076 | -4725 | MER89-int | ERV1 | LTR |

  

|  |  |  |  |  |  |  |  |  |  |
| --- | --- | --- | --- | --- | --- | --- | --- | --- | --- |
| chr9:65842305-65843454 | | | 1150 bp | | |  |  |  |  |
| Genome Start | Genome End | Strand | Div | Repeat Start | Repeat End | Repeat Left | Name | Family | Class |
| 65842305 | 65842351 | - | 23.3 | 304 | 340 | -1 | MER58B | MER1\_type | DNA |
| 65842352 | 65842861 | + | 17.5 | 2 | 550 | 0 | MER44B | MER2\_type | DNA |
| 65842862 | 65842913 | - | 23.3 | 256 | 303 | -38 | MER58B | MER1\_type | DNA |
| 65842914 | 65843398 | + | 15.8 | 3 | 489 | 0 | LTR45B | ERV1 | LTR |
| 65843399 | 65843454 | - | 23.3 | 208 | 255 | -86 | MER58B | MER1\_type | DNA |

  

|  |  |  |  |  |  |  |  |  |  |
| --- | --- | --- | --- | --- | --- | --- | --- | --- | --- |
| chr9:65843735-65844291 | | | 557 bp | | |  |  |  |  |
| Genome Start | Genome End | Strand | Div | Repeat Start | Repeat End | Repeat Left | Name | Family | Class |
| 65843735 | 65843805 | - | 20.5 | 5820 | 5886 | -278 | L1ME2 | L1 | LINE |
| 65843806 | 65844106 | + | 9.0 | 1 | 302 | -10 | AluSx | Alu | SINE |
| 65844107 | 65844291 | - | 20.5 | 5608 | 5819 | -345 | L1ME2 | L1 | LINE |

  

|  |  |  |  |  |  |  |  |  |  |
| --- | --- | --- | --- | --- | --- | --- | --- | --- | --- |
| chr9:65848769-65849603 | | | 627 bp | | |  |  |  |  |
| Genome Start | Genome End | Strand | Div | Repeat Start | Repeat End | Repeat Left | Name | Family | Class |
| 65848769 | 65848824 | + | 21.4 | 4039 | 4097 | -2049 | L1M4 | L1 | LINE |
| 65849032 | 65849370 | - | 20.1 | 1 | 393 | -508 | MER54B | ERVL | LTR |
| 65849372 | 65849603 | + | 19.1 | 4094 | 4321 | -1825 | L1M4 | L1 | LINE |

  

|  |  |  |  |  |  |  |  |  |  |
| --- | --- | --- | --- | --- | --- | --- | --- | --- | --- |
| chr9:65855364-65856807 | | | 1444 bp | | |  |  |  |  |
| Genome Start | Genome End | Strand | Div | Repeat Start | Repeat End | Repeat Left | Name | Family | Class |
| 65855364 | 65856252 | + | 17.3 | 4316 | 5162 | -984 | L1M4 | L1 | LINE |
| 65856253 | 65856539 | - | 12.5 | 1 | 290 | -22 | AluSx | Alu | SINE |
| 65856540 | 65856807 | + | 17.5 | 5163 | 5437 | -741 | L1M4 | L1 | LINE |

  

|  |  |  |  |  |  |  |  |  |  |
| --- | --- | --- | --- | --- | --- | --- | --- | --- | --- |
| chr9:65867551-65868810 | | | 1288 bp | | |  |  |  |  |
| Genome Start | Genome End | Strand | Div | Repeat Start | Repeat End | Repeat Left | Name | Family | Class |
| 65867551 | 65867677 | + | 22.2 | 1 | 138 | -569 | MER39 | ERV1 | LTR |
| 65867650 | 65867793 | + | 16.4 | 260 | 397 | -310 | MER39 | ERV1 | LTR |
| 65867794 | 65868499 | + | 13.0 | 1 | 691 | 0 | LTR8 | ERV1 | LTR |
| 65868500 | 65868810 | + | 16.4 | 398 | 705 | -2 | MER39 | ERV1 | LTR |

  

|  |  |  |  |  |  |  |  |  |  |
| --- | --- | --- | --- | --- | --- | --- | --- | --- | --- |
| chr9:65879576-65880806 | | | 1231 bp | | |  |  |  |  |
| Genome Start | Genome End | Strand | Div | Repeat Start | Repeat End | Repeat Left | Name | Family | Class |
| 65879576 | 65879627 | - | 15.7 | 302 | 364 | -2 | Tigger5a | MER2\_type | DNA |
| 65879628 | 65879803 | - | 7.4 | 388 | 554 | 0 | MER41A | ERV1 | LTR |
| 65879804 | 65880104 | - | 9.6 | 1 | 301 | -9 | AluSg | Alu | SINE |
| 65880105 | 65880488 | - | 7.4 | 1 | 387 | -167 | MER41A | ERV1 | LTR |
| 65880489 | 65880806 | - | 15.7 | 5 | 301 | -65 | Tigger5a | MER2\_type | DNA |

  

|  |  |  |  |  |  |  |  |  |  |
| --- | --- | --- | --- | --- | --- | --- | --- | --- | --- |
| chr9:65884144-65884873 | | | 730 bp | | |  |  |  |  |
| Genome Start | Genome End | Strand | Div | Repeat Start | Repeat End | Repeat Left | Name | Family | Class |
| 65884144 | 65884364 | - | 19.4 | 196 | 426 | 0 | MSTB | MaLR | LTR |
| 65884365 | 65884686 | - | 14.9 | 1 | 322 | 0 | AluSx | Alu | SINE |
| 65884687 | 65884873 | - | 19.4 | 8 | 195 | -231 | MSTB | MaLR | LTR |

  

|  |  |  |  |  |  |  |  |  |  |
| --- | --- | --- | --- | --- | --- | --- | --- | --- | --- |
| chr9:65894362-65896839 | | | 2419 bp | | |  |  |  |  |
| Genome Start | Genome End | Strand | Div | Repeat Start | Repeat End | Repeat Left | Name | Family | Class |
| 65894362 | 65894911 | - | 21.1 | 59 | 628 | -25 | MER82 | MER2\_type | DNA |
| 65894912 | 65895056 | + | 24.0 | 1 | 137 | -650 | LTR48 | ERV1 | LTR |
| 65895057 | 65895426 | - | 15.5 | 1 | 364 | 0 | THE1B | MaLR | LTR |
| 65895427 | 65895545 | + | 24.0 | 138 | 235 | -552 | LTR48 | ERV1 | LTR |
| 65895602 | 65895911 | - | 10.7 | 1 | 308 | -2 | AluSg | Alu | SINE |
| 65895914 | 65896218 | - | 7.3 | 1 | 302 | -9 | AluY | Alu | SINE |
| 65896219 | 65896777 | + | 17.3 | 219 | 787 | 0 | LTR48 | ERV1 | LTR |
| 65896779 | 65896839 | - | 19.7 | 1 | 61 | -592 | MER82 | MER2\_type | DNA |

  

|  |  |  |  |  |  |  |  |  |  |
| --- | --- | --- | --- | --- | --- | --- | --- | --- | --- |
| chr9:65897269-65898931 | | | 1620 bp | | |  |  |  |  |
| Genome Start | Genome End | Strand | Div | Repeat Start | Repeat End | Repeat Left | Name | Family | Class |
| 65897269 | 65897686 | - | 18.5 | 276 | 711 | 0 | MER67C | ERV1 | LTR |
| 65897723 | 65898196 | - | 14.3 | 109 | 571 | -39 | MER4B | ERV1 | LTR |
| 65898197 | 65898535 | - | 13.6 | 1 | 343 | 0 | MER61A | ERV1 | LTR |
| 65898536 | 65898654 | - | 14.3 | 1 | 108 | -502 | MER4B | ERV1 | LTR |
| 65898662 | 65898931 | - | 25.4 | 1 | 272 | -439 | MER67C | ERV1 | LTR |

  

|  |  |  |  |  |  |  |  |  |  |
| --- | --- | --- | --- | --- | --- | --- | --- | --- | --- |
| chr9:65910425-65911042 | | | 618 bp | | |  |  |  |  |
| Genome Start | Genome End | Strand | Div | Repeat Start | Repeat End | Repeat Left | Name | Family | Class |
| 65910425 | 65910641 | - | 11.0 | 184 | 364 | 0 | THE1B | MaLR | LTR |
| 65910642 | 65910873 | - | 9.8 | 5950 | 6181 | 0 | L1PA8 | L1 | LINE |
| 65910874 | 65911042 | - | 11.0 | 1 | 183 | -181 | THE1B | MaLR | LTR |

  

|  |  |  |  |  |  |  |  |  |  |
| --- | --- | --- | --- | --- | --- | --- | --- | --- | --- |
| chr9:65911419-65912382 | | | 964 bp | | |  |  |  |  |
| Genome Start | Genome End | Strand | Div | Repeat Start | Repeat End | Repeat Left | Name | Family | Class |
| 65911419 | 65911525 | - | 21.2 | 523 | 626 | 0 | MLT1E2 | MaLR | LTR |
| 65911526 | 65911838 | + | 9.9 | 1 | 313 | 0 | AluSq | Alu | SINE |
| 65911839 | 65912382 | - | 21.6 | 1 | 523 | -101 | MLT1E2 | MaLR | LTR |

  

|  |  |  |  |  |  |  |  |  |  |
| --- | --- | --- | --- | --- | --- | --- | --- | --- | --- |
| chr9:65920810-65921421 | | | 612 bp | | |  |  |  |  |
| Genome Start | Genome End | Strand | Div | Repeat Start | Repeat End | Repeat Left | Name | Family | Class |
| 65920810 | 65920980 | - | 13.0 | 128 | 295 | -17 | AluSx | Alu | SINE |
| 65920981 | 65921288 | - | 7.5 | 1 | 305 | -6 | AluY | Alu | SINE |
| 65921289 | 65921421 | - | 13.0 | 3 | 127 | -185 | AluSx | Alu | SINE |

  

|  |  |  |  |  |  |  |  |  |  |
| --- | --- | --- | --- | --- | --- | --- | --- | --- | --- |
| chr9:66005607-66006862 | | | 944 bp | | |  |  |  |  |
| Genome Start | Genome End | Strand | Div | Repeat Start | Repeat End | Repeat Left | Name | Family | Class |
| 66005607 | 66006099 | - | 31.5 | 5357 | 5913 | -1895 | L1MC4a | L1 | LINE |
| 66006109 | 66006453 | - | 7.6 | 1 | 311 | 0 | AluY | Alu | SINE |
| 66006757 | 66006862 | - | 33.0 | 4981 | 5086 | -1060 | L1MC4a | L1 | LINE |

  

|  |  |  |  |  |  |  |  |  |  |
| --- | --- | --- | --- | --- | --- | --- | --- | --- | --- |
| chr9:66010638-66012367 | | | 1730 bp | | |  |  |  |  |
| Genome Start | Genome End | Strand | Div | Repeat Start | Repeat End | Repeat Left | Name | Family | Class |
| 66010638 | 66011802 | - | 15.8 | 427 | 1645 | -6 | THE1B-int | MaLR | LTR |
| 66011803 | 66011963 | - | 15.1 | 134 | 292 | -20 | AluSg/x | Alu | SINE |
| 66011964 | 66012367 | - | 15.8 | 5 | 426 | -1225 | THE1B-int | MaLR | LTR |

  

|  |  |  |  |  |  |  |  |  |  |
| --- | --- | --- | --- | --- | --- | --- | --- | --- | --- |
| chr9:66020173-66020709 | | | 460 bp | | |  |  |  |  |
| Genome Start | Genome End | Strand | Div | Repeat Start | Repeat End | Repeat Left | Name | Family | Class |
| 66020173 | 66020216 | + | 27.3 | 1 | 44 | -134 | MER5B | MER1\_type | DNA |
| 66020217 | 66020521 | + | 4.3 | 1 | 305 | -6 | AluY | Alu | SINE |
| 66020599 | 66020709 | + | 29.4 | 69 | 177 | -1 | MER5B | MER1\_type | DNA |

  

|  |  |  |  |  |  |  |  |  |  |
| --- | --- | --- | --- | --- | --- | --- | --- | --- | --- |
| chr9:66045390-66047044 | | | 1627 bp | | |  |  |  |  |
| Genome Start | Genome End | Strand | Div | Repeat Start | Repeat End | Repeat Left | Name | Family | Class |
| 66045390 | 66046532 | - | 10.0 | 5016 | 6145 | -6 | L1PB1 | L1 | LINE |
| 66046561 | 66046576 | - | 9.4 | 5016 | 5015 | -1131 | L1PB1 | L1 | LINE |
| 66046577 | 66046843 | - | 13.2 | 1 | 276 | -36 | AluSx | Alu | SINE |
| 66046844 | 66047044 | - | 9.4 | 4815 | 5015 | -1131 | L1PB1 | L1 | LINE |

  

|  |  |  |  |  |  |  |  |  |  |
| --- | --- | --- | --- | --- | --- | --- | --- | --- | --- |
| chr9:66050540-66057314 | | | 6746 bp | | |  |  |  |  |
| Genome Start | Genome End | Strand | Div | Repeat Start | Repeat End | Repeat Left | Name | Family | Class |
| 66050540 | 66050890 | - | 29.3 | 223 | 577 | -49 | MLT1E2 | MaLR | LTR |
| 66050923 | 66055431 | - | 18.8 | 2059 | 6686 | -1 | L1PREC2 | L1 | LINE |
| 66055429 | 66056557 | + | 18.9 | 2081 | 3225 | -4930 | L1PREC2 | L1 | LINE |
| 66056558 | 66056919 | - | 13.1 | 1 | 381 | 0 | THE1D | MaLR | LTR |
| 66056920 | 66057136 | + | 18.9 | 3226 | 3434 | -4721 | L1PREC2 | L1 | LINE |
| 66057137 | 66057314 | - | 21.1 | 1 | 257 | -397 | MLT1E2 | MaLR | LTR |

  

|  |  |  |  |  |  |  |  |  |  |
| --- | --- | --- | --- | --- | --- | --- | --- | --- | --- |
| chr9:66064252-66065970 | | | 1719 bp | | |  |  |  |  |
| Genome Start | Genome End | Strand | Div | Repeat Start | Repeat End | Repeat Left | Name | Family | Class |
| 66064252 | 66064607 | - | 23.7 | 2019 | 2389 | -4119 | L1MCa | L1 | LINE |
| 66064608 | 66064963 | + | 23.0 | 1 | 365 | 0 | MLT1A0 | MaLR | LTR |
| 66064964 | 66065332 | - | 23.7 | 1602 | 2018 | -4490 | L1MCa | L1 | LINE |
| 66065333 | 66065589 | + | 11.4 | 1 | 256 | -55 | AluY | Alu | SINE |
| 66065590 | 66065970 | - | 23.7 | 1240 | 1601 | -4907 | L1MCa | L1 | LINE |

  

|  |  |  |  |  |  |  |  |  |  |
| --- | --- | --- | --- | --- | --- | --- | --- | --- | --- |
| chr9:66095358-66096036 | | | 648 bp | | |  |  |  |  |
| Genome Start | Genome End | Strand | Div | Repeat Start | Repeat End | Repeat Left | Name | Family | Class |
| 66095358 | 66095660 | - | 17.8 | 125 | 426 | 0 | MSTB | MaLR | LTR |
| 66095661 | 66095788 | + | 16.5 | 1 | 126 | -104 | MER30 | MER1\_type | DNA |
| 66095820 | 66095914 | + | 16.5 | 127 | 230 | 0 | MER30 | MER1\_type | DNA |
| 66095915 | 66096036 | - | 17.8 | 3 | 124 | -302 | MSTB | MaLR | LTR |

  

|  |  |  |  |  |  |  |  |  |  |
| --- | --- | --- | --- | --- | --- | --- | --- | --- | --- |
| chr9:66227151-66229055 | | | 1908 bp | | |  |  |  |  |
| Genome Start | Genome End | Strand | Div | Repeat Start | Repeat End | Repeat Left | Name | Family | Class |
| 66227151 | 66227232 | - | 18.3 | 6003 | 6076 | -90 | L1PA16 | L1 | LINE |
| 66227233 | 66227541 | - | 12.4 | 1 | 310 | 0 | AluSx | Alu | SINE |
| 66227542 | 66228983 | - | 16.0 | 4550 | 6002 | -164 | L1PA16 | L1 | LINE |
| 66228981 | 66229055 | - | 21.9 | 2623 | 2706 | -3440 | L1PA16 | L1 | LINE |

  

|  |  |  |  |  |  |  |  |  |  |
| --- | --- | --- | --- | --- | --- | --- | --- | --- | --- |
| chr9:66235017-66238970 | | | 3954 bp | | |  |  |  |  |
| Genome Start | Genome End | Strand | Div | Repeat Start | Repeat End | Repeat Left | Name | Family | Class |
| 66235017 | 66235750 | - | 10.3 | 5199 | 5935 | -927 | L1PBa | L1 | LINE |
| 66235751 | 66235927 | - | 16.9 | 136 | 312 | -1 | AluSp | Alu | SINE |
| 66235928 | 66238970 | - | 10.3 | 2142 | 5198 | -1664 | L1PBa | L1 | LINE |

  

|  |  |  |  |  |  |  |  |  |  |
| --- | --- | --- | --- | --- | --- | --- | --- | --- | --- |
| chr9:66295331-66296481 | | | 903 bp | | |  |  |  |  |
| Genome Start | Genome End | Strand | Div | Repeat Start | Repeat End | Repeat Left | Name | Family | Class |
| 66295331 | 66295427 | - | 29.2 | 4720 | 4818 | -1328 | L1M5 | L1 | LINE |
| 66295512 | 66295688 | - | 22.2 | 155 | 454 | -113 | MLT1G1 | MaLR | LTR |
| 66295756 | 66296055 | + | 11.6 | 1 | 295 | -14 | AluSc | Alu | SINE |
| 66296065 | 66296171 | - | 24.0 | 37 | 171 | -378 | MLT1H | MaLR | LTR |
| 66296260 | 66296481 | - | 29.9 | 4478 | 4702 | -1444 | L1M5 | L1 | LINE |

  

|  |  |  |  |  |  |  |  |  |  |
| --- | --- | --- | --- | --- | --- | --- | --- | --- | --- |
| chr9:66300312-66301936 | | | 1625 bp | | |  |  |  |  |
| Genome Start | Genome End | Strand | Div | Repeat Start | Repeat End | Repeat Left | Name | Family | Class |
| 66300312 | 66301499 | - | 17.1 | 127 | 1231 | -349 | MSTA-int | MaLR | LTR |
| 66301500 | 66301810 | - | 16.9 | 1 | 307 | -5 | AluSx | Alu | SINE |
| 66301811 | 66301936 | - | 17.1 | 3 | 126 | -1454 | MSTA-int | MaLR | LTR |

  

|  |  |  |  |  |  |  |  |  |  |
| --- | --- | --- | --- | --- | --- | --- | --- | --- | --- |
| chr9:66312320-66314112 | | | 1793 bp | | |  |  |  |  |
| Genome Start | Genome End | Strand | Div | Repeat Start | Repeat End | Repeat Left | Name | Family | Class |
| 66312320 | 66312372 | + | 19.3 | 1609 | 1712 | -4857 | L1ME1 | L1 | LINE |
| 66312373 | 66312676 | - | 10.8 | 2 | 298 | -14 | AluSx | Alu | SINE |
| 66312677 | 66314112 | + | 18.1 | 1663 | 3197 | -3185 | L1ME1 | L1 | LINE |

  

|  |  |  |  |  |  |  |  |  |  |
| --- | --- | --- | --- | --- | --- | --- | --- | --- | --- |
| chr9:66321956-66323433 | | | 1428 bp | | |  |  |  |  |
| Genome Start | Genome End | Strand | Div | Repeat Start | Repeat End | Repeat Left | Name | Family | Class |
| 66321956 | 66322363 | + | 21.6 | 3490 | 3909 | -2473 | L1ME1 | L1 | LINE |
| 66322364 | 66322660 | - | 10.5 | 1 | 296 | -14 | AluSg | Alu | SINE |
| 66322661 | 66322758 | + | 22.8 | 3910 | 4005 | -2377 | L1ME1 | L1 | LINE |
| 66322809 | 66323433 | + | 25.2 | 5792 | 6570 | 0 | L1ME1 | L1 | LINE |

  

|  |  |  |  |  |  |  |  |  |  |
| --- | --- | --- | --- | --- | --- | --- | --- | --- | --- |
| chr9:66333237-66333903 | | | 667 bp | | |  |  |  |  |
| Genome Start | Genome End | Strand | Div | Repeat Start | Repeat End | Repeat Left | Name | Family | Class |
| 66333237 | 66333559 | + | 14.0 | 1 | 390 | -38 | MSTA | MaLR | LTR |
| 66333560 | 66333852 | + | 13.3 | 3 | 295 | -14 | AluSc | Alu | SINE |
| 66333853 | 66333903 | + | 14.0 | 391 | 428 | 0 | MSTA | MaLR | LTR |

  

|  |  |  |  |  |  |  |  |  |  |
| --- | --- | --- | --- | --- | --- | --- | --- | --- | --- |
| chr9:66336625-66337477 | | | 853 bp | | |  |  |  |  |
| Genome Start | Genome End | Strand | Div | Repeat Start | Repeat End | Repeat Left | Name | Family | Class |
| 66336625 | 66337064 | + | 7.0 | 5627 | 6064 | -108 | L1PA8 | L1 | LINE |
| 66337065 | 66337360 | + | 8.8 | 1 | 296 | -15 | AluY | Alu | SINE |
| 66337361 | 66337477 | + | 7.0 | 6065 | 6172 | 0 | L1PA8 | L1 | LINE |

  

|  |  |  |  |  |  |  |  |  |  |
| --- | --- | --- | --- | --- | --- | --- | --- | --- | --- |
| chr9:66337819-66341791 | | | 3979 bp | | |  |  |  |  |
| Genome Start | Genome End | Strand | Div | Repeat Start | Repeat End | Repeat Left | Name | Family | Class |
| 66337819 | 66338574 | - | 18.4 | 4546 | 5283 | -860 | L1M2 | L1 | LINE |
| 66338569 | 66339539 | - | 23.3 | 2916 | 3987 | -3575 | L1M2 | L1 | LINE |
| 66339540 | 66339851 | - | 11.0 | 1 | 309 | 0 | AluSc | Alu | SINE |
| 66339852 | 66341791 | - | 27.4 | 1216 | 2980 | -4650 | L1M2 | L1 | LINE |

  

|  |  |  |  |  |  |  |  |  |  |
| --- | --- | --- | --- | --- | --- | --- | --- | --- | --- |
| chr9:66341794-66342711 | | | 918 bp | | |  |  |  |  |
| Genome Start | Genome End | Strand | Div | Repeat Start | Repeat End | Repeat Left | Name | Family | Class |
| 66341794 | 66341967 | - | 22.4 | 7802 | 7973 | -22 | L1MD3 | L1 | LINE |
| 66341968 | 66342261 | - | 14.6 | 1 | 294 | -18 | AluSx | Alu | SINE |
| 66342262 | 66342711 | - | 22.4 | 7360 | 7801 | -194 | L1MD3 | L1 | LINE |

  

|  |  |  |  |  |  |  |  |  |  |
| --- | --- | --- | --- | --- | --- | --- | --- | --- | --- |
| chr9:66409449-66411928 | | | 2420 bp | | |  |  |  |  |
| Genome Start | Genome End | Strand | Div | Repeat Start | Repeat End | Repeat Left | Name | Family | Class |
| 66409449 | 66409623 | - | 22.4 | 6100 | 6287 | -4 | L1MA8 | L1 | LINE |
| 66409684 | 66411430 | - | 24.6 | 4102 | 5941 | -359 | L1MA8 | L1 | LINE |
| 66411431 | 66411725 | + | 9.5 | 1 | 297 | -14 | AluY | Alu | SINE |
| 66411726 | 66411928 | - | 24.7 | 3894 | 4101 | -2042 | L1MA8 | L1 | LINE |

  

|  |  |  |  |  |  |  |  |  |  |
| --- | --- | --- | --- | --- | --- | --- | --- | --- | --- |
| chr9:66432022-66432951 | | | 930 bp | | |  |  |  |  |
| Genome Start | Genome End | Strand | Div | Repeat Start | Repeat End | Repeat Left | Name | Family | Class |
| 66432022 | 66432476 | + | 20.6 | 5023 | 5497 | -687 | L1M4 | L1 | LINE |
| 66432477 | 66432734 | + | 8.2 | 1 | 258 | -53 | AluY | Alu | SINE |
| 66432735 | 66432951 | + | 20.4 | 5498 | 5719 | -465 | L1M4 | L1 | LINE |

  

|  |  |  |  |  |  |  |  |  |  |
| --- | --- | --- | --- | --- | --- | --- | --- | --- | --- |
| chr9:66440252-66447659 | | | 7422 bp | | |  |  |  |  |
| Genome Start | Genome End | Strand | Div | Repeat Start | Repeat End | Repeat Left | Name | Family | Class |
| 66440252 | 66442173 | - | 15.8 | 5804 | 7730 | -7 | HERVIP10F | ERV1 | LTR |
| 66442160 | 66445463 | - | 14.0 | 1786 | 5108 | -2629 | HERVIP10F | ERV1 | LTR |
| 66445464 | 66445761 | + | 12.8 | 1 | 298 | -13 | AluY | Alu | SINE |
| 66445762 | 66447659 | - | 15.8 | 1 | 1949 | -5836 | HERVIP10F | ERV1 | LTR |

  

|  |  |  |  |  |  |  |  |  |  |
| --- | --- | --- | --- | --- | --- | --- | --- | --- | --- |
| chr9:66462328-66462721 | | | 386 bp | | |  |  |  |  |
| Genome Start | Genome End | Strand | Div | Repeat Start | Repeat End | Repeat Left | Name | Family | Class |
| 66462328 | 66462570 | + | 23.8 | 1 | 244 | -68 | AluJo | Alu | SINE |
| 66462572 | 66462648 | - | 15.6 | 5463 | 5539 | -794 | L1MC | L1 | LINE |
| 66462656 | 66462721 | + | 18.5 | 233 | 299 | -3 | AluJo | Alu | SINE |

  

|  |  |  |  |  |  |  |  |  |  |
| --- | --- | --- | --- | --- | --- | --- | --- | --- | --- |
| chr9:66462994-66466890 | | | 3859 bp | | |  |  |  |  |
| Genome Start | Genome End | Strand | Div | Repeat Start | Repeat End | Repeat Left | Name | Family | Class |
| 66462994 | 66463414 | - | 21.8 | 6473 | 6899 | -1061 | L1MC3 | L1 | LINE |
| 66463415 | 66463725 | - | 10.5 | 9 | 313 | 0 | AluSq | Alu | SINE |
| 66463726 | 66463866 | - | 21.8 | 6329 | 6472 | -1488 | L1MC3 | L1 | LINE |
| 66463887 | 66463952 | - | 21.8 | 6271 | 6328 | -1632 | L1MC3 | L1 | LINE |
| 66463953 | 66464261 | + | 5.2 | 1 | 307 | -4 | AluY | Alu | SINE |
| 66464262 | 66464591 | - | 21.8 | 5811 | 6270 | -1690 | L1MC3 | L1 | LINE |
| 66464592 | 66464917 | + | 15.8 | 1 | 298 | -14 | AluSx | Alu | SINE |
| 66464918 | 66465037 | - | 21.8 | 5702 | 5810 | -2150 | L1MC3 | L1 | LINE |
| 66465054 | 66466440 | - | 20.8 | 2805 | 4265 | -2056 | L1MC3 | L1 | LINE |
| 66466443 | 66466729 | - | 15.6 | 1 | 306 | 0 | AluSx | Alu | SINE |
| 66466730 | 66466890 | - | 28.3 | 2062 | 2218 | -4290 | L1MC3 | L1 | LINE |

  

|  |  |  |  |  |  |  |  |  |  |
| --- | --- | --- | --- | --- | --- | --- | --- | --- | --- |
| chr9:66470484-66472900 | | | 2202 bp | | |  |  |  |  |
| Genome Start | Genome End | Strand | Div | Repeat Start | Repeat End | Repeat Left | Name | Family | Class |
| 66470484 | 66470623 | + | 25.2 | 130 | 268 | -200 | LTR37B | ERV1 | LTR |
| 66470822 | 66471823 | - | 19.5 | 5179 | 6180 | 0 | L1MB4 | L1 | LINE |
| 66471824 | 66472113 | - | 11.4 | 1 | 290 | -20 | AluSg | Alu | SINE |
| 66472114 | 66472395 | - | 21.8 | 4905 | 5178 | -968 | L1MB4 | L1 | LINE |
| 66472413 | 66472718 | - | 7.0 | 1 | 308 | -3 | AluY | Alu | SINE |
| 66472719 | 66472900 | + | 23.6 | 262 | 444 | -24 | LTR37B | ERV1 | LTR |

  

|  |  |  |  |  |  |  |  |  |  |
| --- | --- | --- | --- | --- | --- | --- | --- | --- | --- |
| chr9:66475419-66475947 | | | 529 bp | | |  |  |  |  |
| Genome Start | Genome End | Strand | Div | Repeat Start | Repeat End | Repeat Left | Name | Family | Class |
| 66475419 | 66475554 | + | 14.4 | 1 | 134 | -178 | AluSx | Alu | SINE |
| 66475555 | 66475769 | + | 10.7 | 1 | 215 | -95 | AluSg | Alu | SINE |
| 66475770 | 66475947 | + | 14.4 | 135 | 312 | 0 | AluSx | Alu | SINE |

  

|  |  |  |  |  |  |  |  |  |  |
| --- | --- | --- | --- | --- | --- | --- | --- | --- | --- |
| chr9:66478314-66479825 | | | 1512 bp | | |  |  |  |  |
| Genome Start | Genome End | Strand | Div | Repeat Start | Repeat End | Repeat Left | Name | Family | Class |
| 66478314 | 66478598 | + | 16.8 | 1 | 300 | -931 | Tigger3b | MER2\_type | DNA |
| 66478599 | 66478911 | - | 14.1 | 1 | 312 | 0 | AluSx | Alu | SINE |
| 66478912 | 66479825 | + | 16.8 | 301 | 1231 | 0 | Tigger3b | MER2\_type | DNA |

  

|  |  |  |  |  |  |  |  |  |  |
| --- | --- | --- | --- | --- | --- | --- | --- | --- | --- |
| chr9:66491563-66493206 | | | 1559 bp | | |  |  |  |  |
| Genome Start | Genome End | Strand | Div | Repeat Start | Repeat End | Repeat Left | Name | Family | Class |
| 66491563 | 66492688 | + | 35.4 | 2980 | 4197 | -1049 | ERVL-E | ERVL | LTR |
| 66492773 | 66492825 | + | 17.3 | 431 | 484 | -83 | MLT1F1 | MaLR | LTR |
| 66492827 | 66493206 | + | 36.3 | 4389 | 4779 | -888 | ERVL-E | ERVL | LTR |

  

|  |  |  |  |  |  |  |  |  |  |
| --- | --- | --- | --- | --- | --- | --- | --- | --- | --- |
| chr9:66655539-66656218 | | | 652 bp | | |  |  |  |  |
| Genome Start | Genome End | Strand | Div | Repeat Start | Repeat End | Repeat Left | Name | Family | Class |
| 66655539 | 66655724 | + | 20.8 | 4834 | 5024 | -1122 | L1MD | L1 | LINE |
| 66655728 | 66655886 | + | 21.4 | 126 | 287 | -25 | AluJo | Alu | SINE |
| 66655912 | 66656218 | + | 19.6 | 5032 | 5328 | -818 | L1MD | L1 | LINE |

  

|  |  |  |  |  |  |  |  |  |  |
| --- | --- | --- | --- | --- | --- | --- | --- | --- | --- |
| chr9:66698490-66699863 | | | 1278 bp | | |  |  |  |  |
| Genome Start | Genome End | Strand | Div | Repeat Start | Repeat End | Repeat Left | Name | Family | Class |
| 66698490 | 66699473 | - | 27.0 | 1614 | 2920 | -499 | L2 | L2 | LINE |
| 66699480 | 66699653 | + | 20.1 | 5 | 183 | -26 | MER3 | MER1\_type | DNA |
| 66699744 | 66699863 | - | 24.1 | 1383 | 1508 | -1911 | L2 | L2 | LINE |

  

|  |  |  |  |  |  |  |  |  |  |
| --- | --- | --- | --- | --- | --- | --- | --- | --- | --- |
| chr9:66706652-66708097 | | | 1335 bp | | |  |  |  |  |
| Genome Start | Genome End | Strand | Div | Repeat Start | Repeat End | Repeat Left | Name | Family | Class |
| 66706652 | 66707112 | - | 23.0 | 761 | 1227 | -5941 | L1M2a | L1 | LINE |
| 66707215 | 66707606 | + | 24.6 | 25 | 396 | 0 | MSTD | MaLR | LTR |
| 66707616 | 66708097 | - | 23.6 | 1 | 597 | -6571 | L1M2a | L1 | LINE |

  

|  |  |  |  |  |  |  |  |  |  |
| --- | --- | --- | --- | --- | --- | --- | --- | --- | --- |
| chr9:66709202-66709766 | | | 565 bp | | |  |  |  |  |
| Genome Start | Genome End | Strand | Div | Repeat Start | Repeat End | Repeat Left | Name | Family | Class |
| 66709202 | 66709365 | - | 36.7 | 1410 | 1607 | -353 | L4 | RTE | LINE |
| 66709366 | 66709561 | + | 10.2 | 106 | 301 | -10 | AluY | Alu | SINE |
| 66709562 | 66709766 | - | 36.7 | 1185 | 1409 | -551 | L4 | RTE | LINE |

  

|  |  |  |  |  |  |  |  |  |  |
| --- | --- | --- | --- | --- | --- | --- | --- | --- | --- |
| chr9:66753923-66754891 | | | 969 bp | | |  |  |  |  |
| Genome Start | Genome End | Strand | Div | Repeat Start | Repeat End | Repeat Left | Name | Family | Class |
| 66753923 | 66753996 | + | 21.2 | 495 | 568 | -295 | MER21B | ERV1 | LTR |
| 66753997 | 66754176 | - | 10.8 | 137 | 305 | -7 | AluSx | Alu | SINE |
| 66754177 | 66754480 | - | 10.6 | 3 | 306 | -5 | AluY | Alu | SINE |
| 66754481 | 66754618 | - | 10.8 | 1 | 136 | -176 | AluSx | Alu | SINE |
| 66754619 | 66754891 | + | 21.2 | 569 | 852 | -11 | MER21B | ERV1 | LTR |

  

|  |  |  |  |  |  |  |  |  |  |
| --- | --- | --- | --- | --- | --- | --- | --- | --- | --- |
| chr9:66772483-66773291 | | | 809 bp | | |  |  |  |  |
| Genome Start | Genome End | Strand | Div | Repeat Start | Repeat End | Repeat Left | Name | Family | Class |
| 66772483 | 66772608 | + | 21.9 | 2872 | 3009 | -410 | L2 | L2 | LINE |
| 66772609 | 66772964 | - | 11.6 | 1 | 310 | -2 | AluSx | Alu | SINE |
| 66772965 | 66773291 | + | 31.1 | 3008 | 3345 | -74 | L2 | L2 | LINE |

  

|  |  |  |  |  |  |  |  |  |  |
| --- | --- | --- | --- | --- | --- | --- | --- | --- | --- |
| chr9:66776352-66777005 | | | 654 bp | | |  |  |  |  |
| Genome Start | Genome End | Strand | Div | Repeat Start | Repeat End | Repeat Left | Name | Family | Class |
| 66776352 | 66776455 | - | 34.0 | 3272 | 3405 | -14 | L2 | L2 | LINE |
| 66776456 | 66776758 | - | 15.9 | 1 | 302 | -10 | AluJb | Alu | SINE |
| 66776759 | 66777005 | - | 34.0 | 3005 | 3271 | -148 | L2 | L2 | LINE |

  

|  |  |  |  |  |  |  |  |  |  |
| --- | --- | --- | --- | --- | --- | --- | --- | --- | --- |
| chr9:66783180-66783848 | | | 669 bp | | |  |  |  |  |
| Genome Start | Genome End | Strand | Div | Repeat Start | Repeat End | Repeat Left | Name | Family | Class |
| 66783180 | 66783512 | + | 29.5 | 1546 | 1919 | -1500 | L2 | L2 | LINE |
| 66783513 | 66783803 | - | 11.3 | 1 | 300 | -12 | AluSx | Alu | SINE |
| 66783804 | 66783848 | + | 29.5 | 1920 | 1967 | -1452 | L2 | L2 | LINE |

  

|  |  |  |  |  |  |  |  |  |  |
| --- | --- | --- | --- | --- | --- | --- | --- | --- | --- |
| chr9:66784084-66784706 | | | 623 bp | | |  |  |  |  |
| Genome Start | Genome End | Strand | Div | Repeat Start | Repeat End | Repeat Left | Name | Family | Class |
| 66784084 | 66784233 | + | 18.1 | 1 | 142 | -170 | AluJb | Alu | SINE |
| 66784234 | 66784530 | + | 10.8 | 5 | 302 | -10 | AluSx | Alu | SINE |
| 66784531 | 66784706 | + | 18.1 | 143 | 309 | -3 | AluJb | Alu | SINE |

  

|  |  |  |  |  |  |  |  |  |  |
| --- | --- | --- | --- | --- | --- | --- | --- | --- | --- |
| chr9:66830657-66832261 | | | 1605 bp | | |  |  |  |  |
| Genome Start | Genome End | Strand | Div | Repeat Start | Repeat End | Repeat Left | Name | Family | Class |
| 66830657 | 66831234 | - | 8.6 | 5444 | 6006 | -148 | L1PA7 | L1 | LINE |
| 66831235 | 66831571 | - | 4.2 | 5815 | 6153 | -2 | L1PA4 | L1 | LINE |
| 66831572 | 66832261 | - | 9.4 | 4743 | 5443 | -711 | L1PA7 | L1 | LINE |

  

|  |  |  |  |  |  |  |  |  |  |
| --- | --- | --- | --- | --- | --- | --- | --- | --- | --- |
| chr9:66903001-66904727 | | | 1727 bp | | |  |  |  |  |
| Genome Start | Genome End | Strand | Div | Repeat Start | Repeat End | Repeat Left | Name | Family | Class |
| 66903001 | 66903404 | + | 15.8 | 3 | 424 | -1227 | THE1B-int | MaLR | LTR |
| 66903405 | 66903562 | + | 11.5 | 134 | 293 | -19 | AluSg/x | Alu | SINE |
| 66903563 | 66904727 | + | 15.8 | 425 | 1643 | -8 | THE1B-int | MaLR | LTR |

  

|  |  |  |  |  |  |  |  |  |  |
| --- | --- | --- | --- | --- | --- | --- | --- | --- | --- |
| chr9:66917779-66919383 | | | 1605 bp | | |  |  |  |  |
| Genome Start | Genome End | Strand | Div | Repeat Start | Repeat End | Repeat Left | Name | Family | Class |
| 66917779 | 66918469 | + | 9.0 | 4743 | 5443 | -711 | L1PA7 | L1 | LINE |
| 66918470 | 66918806 | + | 3.9 | 5815 | 6153 | -2 | L1PA4 | L1 | LINE |
| 66918807 | 66919383 | + | 8.7 | 5444 | 6006 | -148 | L1PA7 | L1 | LINE |

  

|  |  |  |  |  |  |  |  |  |  |
| --- | --- | --- | --- | --- | --- | --- | --- | --- | --- |
| chr9:66965350-66965971 | | | 622 bp | | |  |  |  |  |
| Genome Start | Genome End | Strand | Div | Repeat Start | Repeat End | Repeat Left | Name | Family | Class |
| 66965350 | 66965525 | - | 17.8 | 143 | 309 | -3 | AluJb | Alu | SINE |
| 66965526 | 66965821 | - | 10.8 | 5 | 301 | -11 | AluSx | Alu | SINE |
| 66965822 | 66965971 | - | 17.8 | 1 | 142 | -170 | AluJb | Alu | SINE |

  

|  |  |  |  |  |  |  |  |  |  |
| --- | --- | --- | --- | --- | --- | --- | --- | --- | --- |
| chr9:66975879-66976714 | | | 770 bp | | |  |  |  |  |
| Genome Start | Genome End | Strand | Div | Repeat Start | Repeat End | Repeat Left | Name | Family | Class |
| 66975879 | 66975904 | - | 32.1 | 3359 | 3378 | 0 | L2 | L2 | LINE |
| 66975905 | 66976203 | + | 16.2 | 1 | 296 | -16 | AluSx | Alu | SINE |
| 66976204 | 66976355 | - | 32.1 | 3182 | 3358 | -20 | L2 | L2 | LINE |
| 66976422 | 66976714 | - | 31.7 | 2351 | 2691 | -728 | L2 | L2 | LINE |

  

|  |  |  |  |  |  |  |  |  |  |
| --- | --- | --- | --- | --- | --- | --- | --- | --- | --- |
| chr9:66995135-66996103 | | | 969 bp | | |  |  |  |  |
| Genome Start | Genome End | Strand | Div | Repeat Start | Repeat End | Repeat Left | Name | Family | Class |
| 66995135 | 66995407 | - | 22.2 | 569 | 852 | -11 | MER21B | ERV1 | LTR |
| 66995408 | 66995543 | + | 10.8 | 1 | 136 | -176 | AluSx | Alu | SINE |
| 66995544 | 66995849 | + | 11.6 | 1 | 306 | -5 | AluY | Alu | SINE |
| 66995850 | 66996029 | + | 10.8 | 137 | 305 | -7 | AluSx | Alu | SINE |
| 66996030 | 66996103 | - | 22.2 | 497 | 568 | -295 | MER21B | ERV1 | LTR |

  

|  |  |  |  |  |  |  |  |  |  |
| --- | --- | --- | --- | --- | --- | --- | --- | --- | --- |
| chr9:67011801-67012366 | | | 566 bp | | |  |  |  |  |
| Genome Start | Genome End | Strand | Div | Repeat Start | Repeat End | Repeat Left | Name | Family | Class |
| 67011801 | 67012005 | + | 36.7 | 1185 | 1409 | -551 | L4 | RTE | LINE |
| 67012006 | 67012202 | - | 10.7 | 106 | 302 | -9 | AluY | Alu | SINE |
| 67012203 | 67012366 | + | 36.7 | 1410 | 1607 | -353 | L4 | RTE | LINE |

  

|  |  |  |  |  |  |  |  |  |  |
| --- | --- | --- | --- | --- | --- | --- | --- | --- | --- |
| chr9:67014972-67017068 | | | 2033 bp | | |  |  |  |  |
| Genome Start | Genome End | Strand | Div | Repeat Start | Repeat End | Repeat Left | Name | Family | Class |
| 67014972 | 67015065 | + | 17.1 | 3577 | 3670 | -2470 | L1M3 | L1 | LINE |
| 67015130 | 67016061 | + | 17.1 | 3671 | 4622 | -1518 | L1M3 | L1 | LINE |
| 67016062 | 67016370 | + | 13.3 | 1 | 309 | -1 | AluSg | Alu | SINE |
| 67016371 | 67017068 | + | 17.1 | 4623 | 5323 | -817 | L1M3 | L1 | LINE |

  

|  |  |  |  |  |  |  |  |  |  |
| --- | --- | --- | --- | --- | --- | --- | --- | --- | --- |
| chr9:67021707-67023112 | | | 1310 bp | | |  |  |  |  |
| Genome Start | Genome End | Strand | Div | Repeat Start | Repeat End | Repeat Left | Name | Family | Class |
| 67021707 | 67021858 | + | 25.2 | 1346 | 1508 | -1911 | L2 | L2 | LINE |
| 67021949 | 67022122 | - | 20.7 | 5 | 183 | -26 | MER3 | MER1\_type | DNA |
| 67022129 | 67023112 | + | 26.8 | 1614 | 2920 | -499 | L2 | L2 | LINE |

  

|  |  |  |  |  |  |  |  |  |  |
| --- | --- | --- | --- | --- | --- | --- | --- | --- | --- |
| chr9:67026289-67029419 | | | 3020 bp | | |  |  |  |  |
| Genome Start | Genome End | Strand | Div | Repeat Start | Repeat End | Repeat Left | Name | Family | Class |
| 67026289 | 67026594 | + | 26.1 | 5 | 337 | -6072 | L1MDb | L1 | LINE |
| 67026607 | 67026859 | + | 18.6 | 654 | 907 | -5502 | L1MDb | L1 | LINE |
| 67026861 | 67028378 | + | 21.8 | 1028 | 2652 | -3754 | L1MDb | L1 | LINE |
| 67028393 | 67028896 | + | 25.6 | 20 | 519 | 0 | LTR53 | ERVL | LTR |
| 67028981 | 67029248 | + | 21.3 | 1 | 274 | -38 | AluJo | Alu | SINE |
| 67029249 | 67029419 | + | 26.0 | 2840 | 3020 | -3386 | L1MDb | L1 | LINE |

  

|  |  |  |  |  |  |  |  |  |  |
| --- | --- | --- | --- | --- | --- | --- | --- | --- | --- |
| chr9:67107352-67110434 | | | 3075 bp | | |  |  |  |  |
| Genome Start | Genome End | Strand | Div | Repeat Start | Repeat End | Repeat Left | Name | Family | Class |
| 67107352 | 67107553 | + | 29.6 | 2011 | 2221 | -1970 | MER21C-int | ERV1 | LTR |
| 67107554 | 67107802 | - | 21.6 | 152 | 408 | 0 | MLT1A1 | MaLR | LTR |
| 67107803 | 67108093 | - | 10.0 | 6 | 302 | -9 | AluY | Alu | SINE |
| 67108094 | 67108246 | - | 21.6 | 1 | 151 | -257 | MLT1A1 | MaLR | LTR |
| 67108247 | 67108319 | + | 29.6 | 2222 | 2284 | -1907 | MER21C-int | ERV1 | LTR |
| 67108328 | 67108934 | + | 25.7 | 2565 | 3234 | -957 | MER21C-int | ERV1 | LTR |
| 67108935 | 67109070 | + | 11.5 | 1 | 133 | -180 | AluSp | Alu | SINE |
| 67109071 | 67109375 | + | 8.0 | 1 | 302 | -9 | AluY | Alu | SINE |
| 67109376 | 67109555 | + | 11.5 | 134 | 313 | 0 | AluSp | Alu | SINE |
| 67109556 | 67110434 | + | 22.7 | 3235 | 4149 | -42 | MER21C-int | ERV1 | LTR |

  

|  |  |  |  |  |  |  |  |  |  |
| --- | --- | --- | --- | --- | --- | --- | --- | --- | --- |
| chr9:67113857-67114651 | | | 769 bp | | |  |  |  |  |
| Genome Start | Genome End | Strand | Div | Repeat Start | Repeat End | Repeat Left | Name | Family | Class |
| 67113857 | 67114001 | + | 21.9 | 5683 | 5832 | -342 | L1MB5 | L1 | LINE |
| 67114002 | 67114290 | + | 15.6 | 1 | 285 | -27 | AluSx | Alu | SINE |
| 67114317 | 67114331 | + | 15.6 | 286 | 300 | -12 | AluSx | Alu | SINE |
| 67114332 | 67114651 | + | 20.8 | 5833 | 6168 | -6 | L1MB5 | L1 | LINE |

  

|  |  |  |  |  |  |  |  |  |  |
| --- | --- | --- | --- | --- | --- | --- | --- | --- | --- |
| chr9:67123125-67123953 | | | 829 bp | | |  |  |  |  |
| Genome Start | Genome End | Strand | Div | Repeat Start | Repeat End | Repeat Left | Name | Family | Class |
| 67123125 | 67123538 | + | 11.8 | 1 | 513 | -313 | LTR12 | ERV1 | LTR |
| 67123539 | 67123858 | + | 8.7 | 1 | 311 | 0 | AluY | Alu | SINE |
| 67123859 | 67123953 | + | 11.8 | 514 | 595 | -231 | LTR12 | ERV1 | LTR |

  

|  |  |  |  |  |  |  |  |  |  |
| --- | --- | --- | --- | --- | --- | --- | --- | --- | --- |
| chr9:67130748-67131548 | | | 713 bp | | |  |  |  |  |
| Genome Start | Genome End | Strand | Div | Repeat Start | Repeat End | Repeat Left | Name | Family | Class |
| 67130748 | 67130893 | - | 28.1 | 3267 | 3419 | 0 | L2 | L2 | LINE |
| 67130910 | 67131327 | + | 20.0 | 11 | 459 | -56 | MLT2B2 | ERVL | LTR |
| 67131365 | 67131395 | + | 20.0 | 507 | 550 | -12 | MLT2B2 | ERVL | LTR |
| 67131431 | 67131548 | - | 33.0 | 3012 | 3128 | -291 | L2 | L2 | LINE |

  

|  |  |  |  |  |  |  |  |  |  |
| --- | --- | --- | --- | --- | --- | --- | --- | --- | --- |
| chr9:67192905-67193792 | | | 866 bp | | |  |  |  |  |
| Genome Start | Genome End | Strand | Div | Repeat Start | Repeat End | Repeat Left | Name | Family | Class |
| 67192905 | 67193150 | + | 17.7 | 1853 | 2221 | -4412 | L1MDa | L1 | LINE |
| 67193173 | 67193452 | - | 7.9 | 1 | 284 | -27 | AluY | Alu | SINE |
| 67193453 | 67193792 | + | 17.7 | 2222 | 2555 | -4078 | L1MDa | L1 | LINE |

  

|  |  |  |  |  |  |  |  |  |  |
| --- | --- | --- | --- | --- | --- | --- | --- | --- | --- |
| chr9:67249236-67249767 | | | 532 bp | | |  |  |  |  |
| Genome Start | Genome End | Strand | Div | Repeat Start | Repeat End | Repeat Left | Name | Family | Class |
| 67249236 | 67249266 | + | 34.2 | 2304 | 2328 | -1091 | L2 | L2 | LINE |
| 67249267 | 67249558 | - | 13.8 | 1 | 293 | -16 | AluSc | Alu | SINE |
| 67249559 | 67249767 | + | 34.2 | 2329 | 2536 | -883 | L2 | L2 | LINE |

  

|  |  |  |  |  |  |  |  |  |  |
| --- | --- | --- | --- | --- | --- | --- | --- | --- | --- |
| chr9:67256243-67257368 | | | 1126 bp | | |  |  |  |  |
| Genome Start | Genome End | Strand | Div | Repeat Start | Repeat End | Repeat Left | Name | Family | Class |
| 67256243 | 67256428 | - | 25.4 | 23 | 244 | -31 | ORSL | Tip100 | DNA |
| 67256429 | 67256903 | + | 11.8 | 1 | 467 | -133 | MER4A1\_ | ERV1 | LTR |
| 67256904 | 67257210 | + | 11.3 | 7 | 312 | 0 | AluSg1 | Alu | SINE |
| 67257211 | 67257345 | + | 11.8 | 468 | 600 | 0 | MER4A1\_ | ERV1 | LTR |
| 67257346 | 67257368 | - | 25.5 | 3 | 22 | -253 | ORSL | Tip100 | DNA |

  

|  |  |  |  |  |  |  |  |  |  |
| --- | --- | --- | --- | --- | --- | --- | --- | --- | --- |
| chr9:67260524-67261128 | | | 418 bp | | |  |  |  |  |
| Genome Start | Genome End | Strand | Div | Repeat Start | Repeat End | Repeat Left | Name | Family | Class |
| 67260524 | 67260746 | - | 24.7 | 130 | 373 | 0 | MER83C | ERV1 | LTR |
| 67260756 | 67260807 | - | 19.2 | 134 | 187 | -191 | MER83B | ERV1 | LTR |
| 67260986 | 67261128 | - | 21.8 | 49 | 192 | -181 | MER83C | ERV1 | LTR |

  

|  |  |  |  |  |  |  |  |  |  |
| --- | --- | --- | --- | --- | --- | --- | --- | --- | --- |
| chr9:67269883-67270773 | | | 891 bp | | |  |  |  |  |
| Genome Start | Genome End | Strand | Div | Repeat Start | Repeat End | Repeat Left | Name | Family | Class |
| 67269883 | 67270316 | - | 26.0 | 700 | 2598 | -42 | MER69B | Tip100 | DNA |
| 67270317 | 67270606 | + | 17.5 | 1 | 300 | -12 | AluJb | Alu | SINE |
| 67270607 | 67270773 | - | 26.0 | 520 | 699 | -527 | MER69B | Tip100 | DNA |

  

|  |  |  |  |  |  |  |  |  |  |
| --- | --- | --- | --- | --- | --- | --- | --- | --- | --- |
| chr9:67276517-67278353 | | | 1773 bp | | |  |  |  |  |
| Genome Start | Genome End | Strand | Div | Repeat Start | Repeat End | Repeat Left | Name | Family | Class |
| 67276517 | 67276638 | - | 30.8 | 3269 | 3406 | -13 | L2 | L2 | LINE |
| 67276614 | 67276771 | + | 33.1 | 4013 | 4174 | -315 | L3 | CR1 | LINE |
| 67276788 | 67276917 | - | 23.4 | 984 | 1123 | -2296 | L2 | L2 | LINE |
| 67276918 | 67276959 | - | 9.5 | 445 | 486 | 0 | LTR19A | ERV1 | LTR |
| 67276960 | 67277113 | - | 7.1 | 7 | 241 | -245 | LTR19A | ERV1 | LTR |
| 67277118 | 67277321 | - | 28.4 | 756 | 969 | -2450 | L2 | L2 | LINE |
| 67277391 | 67277995 | - | 13.1 | 1 | 766 | 0 | MER4E | ERV1 | LTR |
| 67277996 | 67278353 | - | 25.1 | 8 | 426 | -2993 | L2 | L2 | LINE |

  

|  |  |  |  |  |  |  |  |  |  |
| --- | --- | --- | --- | --- | --- | --- | --- | --- | --- |
| chr9:67282627-67283282 | | | 656 bp | | |  |  |  |  |
| Genome Start | Genome End | Strand | Div | Repeat Start | Repeat End | Repeat Left | Name | Family | Class |
| 67282627 | 67282705 | - | 24.3 | 5489 | 5571 | -595 | L1M5 | L1 | LINE |
| 67282706 | 67283251 | + | 19.0 | 2 | 607 | 0 | MER77 | ERVL | LTR |
| 67283252 | 67283282 | - | 24.3 | 5457 | 5488 | -678 | L1M5 | L1 | LINE |

  

|  |  |  |  |  |  |  |  |  |  |
| --- | --- | --- | --- | --- | --- | --- | --- | --- | --- |
| chr9:67284743-67285564 | | | 822 bp | | |  |  |  |  |
| Genome Start | Genome End | Strand | Div | Repeat Start | Repeat End | Repeat Left | Name | Family | Class |
| 67284743 | 67285177 | + | 14.1 | 4730 | 5161 | -985 | L1P4 | L1 | LINE |
| 67285178 | 67285440 | + | 12.2 | 40 | 307 | -3 | AluSg | Alu | SINE |
| 67285441 | 67285564 | + | 14.1 | 5162 | 5286 | -860 | L1P4 | L1 | LINE |

  

|  |  |  |  |  |  |  |  |  |  |
| --- | --- | --- | --- | --- | --- | --- | --- | --- | --- |
| chr9:67287174-67287674 | | | 502 bp | | |  |  |  |  |
| Genome Start | Genome End | Strand | Div | Repeat Start | Repeat End | Repeat Left | Name | Family | Class |
| 67287174 | 67287391 | - | 24.6 | 6784 | 6977 | -1065 | L1MC4 | L1 | LINE |
| 67287392 | 67287531 | + | 15.9 | 1 | 133 | 0 | FLAM\_C | Alu | SINE |
| 67287531 | 67287674 | - | 24.6 | 6646 | 6783 | -1259 | L1MC4 | L1 | LINE |

  

|  |  |  |  |  |  |  |  |  |  |
| --- | --- | --- | --- | --- | --- | --- | --- | --- | --- |
| chr9:67287992-67289067 | | | 1073 bp | | |  |  |  |  |
| Genome Start | Genome End | Strand | Div | Repeat Start | Repeat End | Repeat Left | Name | Family | Class |
| 67287992 | 67288088 | + | 22.7 | 4 | 100 | -959 | MER106B | MER1\_type | DNA |
| 67288092 | 67288390 | - | 13.4 | 1 | 299 | -13 | AluSx | Alu | SINE |
| 67288391 | 67288487 | + | 19.0 | 109 | 212 | -847 | MER106B | MER1\_type | DNA |
| 67288488 | 67288782 | + | 16.4 | 1 | 296 | -16 | AluSx | Alu | SINE |
| 67288783 | 67289067 | + | 19.0 | 213 | 532 | -527 | MER106B | MER1\_type | DNA |

  

|  |  |  |  |  |  |  |  |  |  |
| --- | --- | --- | --- | --- | --- | --- | --- | --- | --- |
| chr9:67291899-67292853 | | | 955 bp | | |  |  |  |  |
| Genome Start | Genome End | Strand | Div | Repeat Start | Repeat End | Repeat Left | Name | Family | Class |
| 67291899 | 67292128 | + | 15.9 | 33 | 354 | -417 | LTR25 | ERV1 | LTR |
| 67292129 | 67292456 | - | 13.7 | 1 | 460 | 0 | LTR46 | ERV1 | LTR |
| 67292457 | 67292853 | + | 15.3 | 355 | 779 | -65 | LTR25 | ERV1 | LTR |

  

|  |  |  |  |  |  |  |  |  |  |
| --- | --- | --- | --- | --- | --- | --- | --- | --- | --- |
| chr9:67293155-67294242 | | | 1088 bp | | |  |  |  |  |
| Genome Start | Genome End | Strand | Div | Repeat Start | Repeat End | Repeat Left | Name | Family | Class |
| 67293155 | 67293242 | + | 17.7 | 1 | 87 | -776 | MER21B | ERV1 | LTR |
| 67293243 | 67293552 | - | 11.3 | 3 | 307 | -5 | AluSx | Alu | SINE |
| 67293553 | 67294242 | + | 17.7 | 88 | 822 | -41 | MER21B | ERV1 | LTR |

  

|  |  |  |  |  |  |  |  |  |  |
| --- | --- | --- | --- | --- | --- | --- | --- | --- | --- |
| chr9:67341883-67342566 | | | 684 bp | | |  |  |  |  |
| Genome Start | Genome End | Strand | Div | Repeat Start | Repeat End | Repeat Left | Name | Family | Class |
| 67341883 | 67342114 | - | 33.8 | 3169 | 3411 | -8 | L2 | L2 | LINE |
| 67342115 | 67342467 | + | 9.1 | 1 | 353 | 0 | AluSp | Alu | SINE |
| 67342468 | 67342566 | - | 33.8 | 3065 | 3168 | -251 | L2 | L2 | LINE |

  

|  |  |  |  |  |  |  |  |  |  |
| --- | --- | --- | --- | --- | --- | --- | --- | --- | --- |
| chr9:67343583-67344104 | | | 522 bp | | |  |  |  |  |
| Genome Start | Genome End | Strand | Div | Repeat Start | Repeat End | Repeat Left | Name | Family | Class |
| 67343583 | 67343751 | + | 26.6 | 20 | 184 | -84 | MIRb | MIR | SINE |
| 67343752 | 67344012 | + | 14.9 | 1 | 280 | -32 | AluJb | Alu | SINE |
| 67344013 | 67344104 | + | 26.6 | 185 | 263 | -5 | MIRb | MIR | SINE |

  

|  |  |  |  |  |  |  |  |  |  |
| --- | --- | --- | --- | --- | --- | --- | --- | --- | --- |
| chr9:67357901-67358912 | | | 1012 bp | | |  |  |  |  |
| Genome Start | Genome End | Strand | Div | Repeat Start | Repeat End | Repeat Left | Name | Family | Class |
| 67357901 | 67358077 | + | 24.6 | 2642 | 2819 | -600 | L2 | L2 | LINE |
| 67358078 | 67358369 | + | 13.7 | 1 | 292 | -18 | AluSg | Alu | SINE |
| 67358370 | 67358912 | + | 26.5 | 2820 | 3401 | -18 | L2 | L2 | LINE |

  

|  |  |  |  |  |  |  |  |  |  |
| --- | --- | --- | --- | --- | --- | --- | --- | --- | --- |
| chr9:67366175-67367171 | | | 1125 bp | | |  |  |  |  |
| Genome Start | Genome End | Strand | Div | Repeat Start | Repeat End | Repeat Left | Name | Family | Class |
| 67366175 | 67366229 | + | 5.5 | 8 | 64 | -114 | MER5B | MER1\_type | DNA |
| 67366230 | 67366432 | - | 0.5 | 5593 | 5795 | -360 | L1P1 | L1 | LINE |
| 67366305 | 67366739 | + | 10.4 | 5657 | 6090 | -65 | L1PA2 | L1 | LINE |
| 67366740 | 67366819 | + | 18.6 | 60 | 136 | -42 | MER5B | MER1\_type | DNA |
| 67366820 | 67367129 | + | 12.6 | 1 | 310 | -3 | AluSq | Alu | SINE |
| 67367130 | 67367171 | + | 18.6 | 137 | 177 | -1 | MER5B | MER1\_type | DNA |

  

|  |  |  |  |  |  |  |  |  |  |
| --- | --- | --- | --- | --- | --- | --- | --- | --- | --- |
| chr9:67384410-67390463 | | | 5911 bp | | |  |  |  |  |
| Genome Start | Genome End | Strand | Div | Repeat Start | Repeat End | Repeat Left | Name | Family | Class |
| 67384410 | 67384692 | - | 25.9 | 5855 | 6150 | -469 | L1MD2 | L1 | LINE |
| 67384755 | 67384844 | + | 20.5 | 5987 | 6069 | -233 | L1MA1 | L1 | LINE |
| 67384845 | 67385192 | - | 22.2 | 5523 | 5867 | -635 | L1MD2 | L1 | LINE |
| 67385170 | 67385818 | - | 22.7 | 2904 | 3537 | -2869 | L1MD2 | L1 | LINE |
| 67385867 | 67386102 | - | 22.7 | 2662 | 2903 | -3503 | L1MD2 | L1 | LINE |
| 67386103 | 67386696 | - | 14.0 | 5 | 622 | 0 | LTR77 | ERV1 | LTR |
| 67386697 | 67387756 | - | 26.0 | 1589 | 2661 | -3745 | L1MD2 | L1 | LINE |
| 67387756 | 67387924 | + | 16.0 | 4866 | 5035 | -1108 | L1MA5 | L1 | LINE |
| 67387926 | 67388705 | + | 16.0 | 5530 | 6300 | 0 | L1MA5 | L1 | LINE |
| 67388728 | 67389184 | - | 40.8 | 2795 | 3292 | -1197 | L3 | CR1 | LINE |
| 67389211 | 67389712 | + | 25.2 | 1 | 465 | -40 | MLT1D | MaLR | LTR |
| 67389721 | 67390463 | - | 24.1 | 845 | 1603 | -4899 | L1MD2 | L1 | LINE |

  

|  |  |  |  |  |  |  |  |  |  |
| --- | --- | --- | --- | --- | --- | --- | --- | --- | --- |
| chr9:67397811-67398589 | | | 779 bp | | |  |  |  |  |
| Genome Start | Genome End | Strand | Div | Repeat Start | Repeat End | Repeat Left | Name | Family | Class |
| 67397811 | 67398040 | - | 23.2 | 246 | 472 | 0 | MER70A | ERVL | LTR |
| 67398041 | 67398344 | - | 10.2 | 1 | 306 | -7 | AluSq | Alu | SINE |
| 67398345 | 67398589 | - | 23.2 | 1 | 245 | -227 | MER70A | ERVL | LTR |

  

|  |  |  |  |  |  |  |  |  |  |
| --- | --- | --- | --- | --- | --- | --- | --- | --- | --- |
| chr9:67404374-67406705 | | | 2286 bp | | |  |  |  |  |
| Genome Start | Genome End | Strand | Div | Repeat Start | Repeat End | Repeat Left | Name | Family | Class |
| 67404374 | 67404469 | + | 21.5 | 1 | 94 | -1137 | Tigger3b | MER2\_type | DNA |
| 67404497 | 67404741 | + | 14.3 | 336 | 587 | -644 | Tigger3b | MER2\_type | DNA |
| 67404761 | 67406111 | - | 3.1 | 4801 | 6152 | -3 | L1PA3 | L1 | LINE |
| 67406112 | 67406705 | + | 15.9 | 588 | 1231 | 0 | Tigger3b | MER2\_type | DNA |

  

|  |  |  |  |  |  |  |  |  |  |
| --- | --- | --- | --- | --- | --- | --- | --- | --- | --- |
| chr9:67409760-67412297 | | | 2481 bp | | |  |  |  |  |
| Genome Start | Genome End | Strand | Div | Repeat Start | Repeat End | Repeat Left | Name | Family | Class |
| 67409760 | 67410697 | - | 16.5 | 1423 | 2351 | -4434 | L1PA13 | L1 | LINE |
| 67410700 | 67411142 | - | 22.3 | 19 | 458 | 0 | LTR22C | ERVK | LTR |
| 67411143 | 67411795 | + | 12.6 | 1 | 675 | 0 | LTR76 | ERV1 | LTR |
| 67411796 | 67411814 | - | 20.4 | 1 | 18 | -491 | LTR22C | ERVK | LTR |
| 67411870 | 67412297 | - | 31.3 | 745 | 1237 | -5548 | L1PA13 | L1 | LINE |

  

|  |  |  |  |  |  |  |  |  |  |
| --- | --- | --- | --- | --- | --- | --- | --- | --- | --- |
| chr9:67422508-67426805 | | | 4298 bp | | |  |  |  |  |
| Genome Start | Genome End | Strand | Div | Repeat Start | Repeat End | Repeat Left | Name | Family | Class |
| 67422508 | 67424766 | - | 21.5 | 3446 | 5808 | -2333 | L1PREC2 | L1 | LINE |
| 67424767 | 67425056 | - | 11.3 | 1 | 311 | 0 | AluY | Alu | SINE |
| 67425057 | 67426805 | - | 25.5 | 1763 | 3445 | -4710 | L1PREC2 | L1 | LINE |

  

|  |  |  |  |  |  |  |  |  |  |
| --- | --- | --- | --- | --- | --- | --- | --- | --- | --- |
| chr9:67434697-67437351 | | | 2655 bp | | |  |  |  |  |
| Genome Start | Genome End | Strand | Div | Repeat Start | Repeat End | Repeat Left | Name | Family | Class |
| 67434697 | 67435020 | - | 15.5 | 2084 | 2417 | -1 | Tigger1 | MER2\_type | DNA |
| 67435021 | 67435319 | - | 13.0 | 7 | 308 | -4 | AluSx | Alu | SINE |
| 67435320 | 67437351 | - | 15.5 | 24 | 2083 | -335 | Tigger1 | MER2\_type | DNA |

  

|  |  |  |  |  |  |  |  |  |  |
| --- | --- | --- | --- | --- | --- | --- | --- | --- | --- |
| chr9:67491547-67495138 | | | 3592 bp | | |  |  |  |  |
| Genome Start | Genome End | Strand | Div | Repeat Start | Repeat End | Repeat Left | Name | Family | Class |
| 67491547 | 67494056 | - | 18.5 | 4176 | 6732 | -830 | L1M1 | L1 | LINE |
| 67494057 | 67494291 | + | 5.5 | 39 | 295 | -16 | AluY | Alu | SINE |
| 67494292 | 67495138 | - | 17.2 | 3305 | 4175 | -3387 | L1M1 | L1 | LINE |

  

|  |  |  |  |  |  |  |  |  |  |
| --- | --- | --- | --- | --- | --- | --- | --- | --- | --- |
| chr9:67500144-67500642 | | | 438 bp | | |  |  |  |  |
| Genome Start | Genome End | Strand | Div | Repeat Start | Repeat End | Repeat Left | Name | Family | Class |
| 67500144 | 67500227 | - | 18.8 | 1656 | 1735 | -10 | Kanga1 | Tc2 | DNA |
| 67500277 | 67500529 | + | 29.9 | 367 | 626 | -5493 | L1MEd | L1 | LINE |
| 67500542 | 67500642 | - | 25.2 | 4 | 104 | -1794 | Kanga1 | Tc2 | DNA |

  

|  |  |  |  |  |  |  |  |  |  |
| --- | --- | --- | --- | --- | --- | --- | --- | --- | --- |
| chr9:67509575-67511081 | | | 1507 bp | | |  |  |  |  |
| Genome Start | Genome End | Strand | Div | Repeat Start | Repeat End | Repeat Left | Name | Family | Class |
| 67509575 | 67509858 | - | 24.5 | 602 | 916 | -19 | MER21C | ERV1 | LTR |
| 67509859 | 67510155 | - | 16.0 | 9 | 303 | -9 | AluJo | Alu | SINE |
| 67510156 | 67510233 | - | 24.5 | 529 | 601 | -334 | MER21C | ERV1 | LTR |
| 67510234 | 67510564 | + | 19.8 | 2 | 299 | -13 | AluJb | Alu | SINE |
| 67510565 | 67511081 | - | 24.5 | 18 | 528 | -407 | MER21C | ERV1 | LTR |

  

|  |  |  |  |  |  |  |  |  |  |
| --- | --- | --- | --- | --- | --- | --- | --- | --- | --- |
| chr9:67568587-67570325 | | | 1730 bp | | |  |  |  |  |
| Genome Start | Genome End | Strand | Div | Repeat Start | Repeat End | Repeat Left | Name | Family | Class |
| 67568587 | 67568909 | - | 20.4 | 5833 | 6173 | -5 | L1MB8 | L1 | LINE |
| 67568910 | 67569321 | - | 13.9 | 11 | 428 | 0 | MER61C | ERV1 | LTR |
| 67569331 | 67570325 | - | 19.2 | 4766 | 5836 | -342 | L1MB8 | L1 | LINE |

  

|  |  |  |  |  |  |  |  |  |  |
| --- | --- | --- | --- | --- | --- | --- | --- | --- | --- |
| chr9:67572070-67573792 | | | 1648 bp | | |  |  |  |  |
| Genome Start | Genome End | Strand | Div | Repeat Start | Repeat End | Repeat Left | Name | Family | Class |
| 67572070 | 67572345 | - | 17.0 | 5997 | 6294 | -6 | L1MA5 | L1 | LINE |
| 67572413 | 67572670 | - | 23.4 | 1 | 264 | -48 | AluJo | Alu | SINE |
| 67572679 | 67573792 | - | 20.6 | 4805 | 5941 | -359 | L1MA5 | L1 | LINE |

  

|  |  |  |  |  |  |  |  |  |  |
| --- | --- | --- | --- | --- | --- | --- | --- | --- | --- |
| chr9:67632389-67633204 | | | 816 bp | | |  |  |  |  |
| Genome Start | Genome End | Strand | Div | Repeat Start | Repeat End | Repeat Left | Name | Family | Class |
| 67632389 | 67632697 | + | 35.5 | 447 | 731 | -5388 | L1MEd | L1 | LINE |
| 67632698 | 67632997 | + | 12.4 | 2 | 301 | -12 | AluSq | Alu | SINE |
| 67632998 | 67633204 | + | 35.5 | 732 | 962 | -5157 | L1MEd | L1 | LINE |

  

|  |  |  |  |  |  |  |  |  |  |
| --- | --- | --- | --- | --- | --- | --- | --- | --- | --- |
| chr9:67669858-67670935 | | | 1078 bp | | |  |  |  |  |
| Genome Start | Genome End | Strand | Div | Repeat Start | Repeat End | Repeat Left | Name | Family | Class |
| 67669858 | 67670091 | - | 25.8 | 244 | 477 | -28 | MLT1D | MaLR | LTR |
| 67670092 | 67670298 | + | 15.5 | 6087 | 6294 | -6 | L1MA6 | L1 | LINE |
| 67670299 | 67670551 | - | 25.8 | 32 | 243 | -262 | MLT1D | MaLR | LTR |
| 67670552 | 67670904 | - | 15.9 | 1 | 364 | 0 | THE1B | MaLR | LTR |
| 67670905 | 67670935 | - | 25.8 | 1 | 31 | -474 | MLT1D | MaLR | LTR |

  

|  |  |  |  |  |  |  |  |  |  |
| --- | --- | --- | --- | --- | --- | --- | --- | --- | --- |
| chr9:67686394-67687643 | | | 1159 bp | | |  |  |  |  |
| Genome Start | Genome End | Strand | Div | Repeat Start | Repeat End | Repeat Left | Name | Family | Class |
| 67686394 | 67686756 | - | 31.3 | 5438 | 5801 | -439 | L1M5 | L1 | LINE |
| 67686757 | 67687062 | + | 11.8 | 1 | 306 | -6 | AluSx | Alu | SINE |
| 67687063 | 67687176 | - | 31.3 | 5319 | 5437 | -803 | L1M5 | L1 | LINE |
| 67687185 | 67687454 | - | 34.7 | 5009 | 5304 | -842 | L1M5 | L1 | LINE |
| 67687538 | 67687643 | - | 19.8 | 4260 | 4378 | -1768 | L1M5 | L1 | LINE |

  

|  |  |  |  |  |  |  |  |  |  |
| --- | --- | --- | --- | --- | --- | --- | --- | --- | --- |
| chr9:67708056-67709485 | | | 1430 bp | | |  |  |  |  |
| Genome Start | Genome End | Strand | Div | Repeat Start | Repeat End | Repeat Left | Name | Family | Class |
| 67708056 | 67708321 | - | 23.4 | 7529 | 7808 | 0 | L1MC4a | L1 | LINE |
| 67708322 | 67708627 | + | 7.3 | 4 | 306 | -5 | AluY | Alu | SINE |
| 67708628 | 67709485 | - | 23.4 | 6608 | 7528 | -280 | L1MC4a | L1 | LINE |

  

|  |  |  |  |  |  |  |  |  |  |
| --- | --- | --- | --- | --- | --- | --- | --- | --- | --- |
| chr9:67726384-67727154 | | | 771 bp | | |  |  |  |  |
| Genome Start | Genome End | Strand | Div | Repeat Start | Repeat End | Repeat Left | Name | Family | Class |
| 67726384 | 67726557 | - | 32.9 | 90 | 258 | -2603 | Charlie2 | MER1\_type | DNA |
| 67726558 | 67727064 | + | 25.6 | 1 | 519 | 0 | LTR40a | ERVL | LTR |
| 67727065 | 67727154 | - | 32.9 | 2 | 89 | -2772 | Charlie2 | MER1\_type | DNA |

  

|  |  |  |  |  |  |  |  |  |  |
| --- | --- | --- | --- | --- | --- | --- | --- | --- | --- |
| chr9:67745966-67746383 | | | 335 bp | | |  |  |  |  |
| Genome Start | Genome End | Strand | Div | Repeat Start | Repeat End | Repeat Left | Name | Family | Class |
| 67745966 | 67746075 | + | 27.9 | 457 | 561 | -5558 | L1MEd | L1 | LINE |
| 67746157 | 67746204 | - | 20.8 | 2358 | 2405 | -15 | MER58 | MER1\_type | DNA |
| 67746207 | 67746383 | + | 31.2 | 965 | 1150 | -4969 | L1MEd | L1 | LINE |

  

|  |  |  |  |  |  |  |  |  |  |
| --- | --- | --- | --- | --- | --- | --- | --- | --- | --- |
| chr9:67757909-67759142 | | | 1234 bp | | |  |  |  |  |
| Genome Start | Genome End | Strand | Div | Repeat Start | Repeat End | Repeat Left | Name | Family | Class |
| 67757909 | 67758024 | + | 19.2 | 5661 | 5784 | -372 | L1PB4 | L1 | LINE |
| 67758025 | 67758731 | + | 8.3 | 5441 | 6147 | -7 | L1PA7 | L1 | LINE |
| 67758732 | 67759142 | + | 19.2 | 5785 | 6153 | -3 | L1PB4 | L1 | LINE |

  

|  |  |  |  |  |  |  |  |  |  |
| --- | --- | --- | --- | --- | --- | --- | --- | --- | --- |
| chr9:67768214-67770745 | | | 2532 bp | | |  |  |  |  |
| Genome Start | Genome End | Strand | Div | Repeat Start | Repeat End | Repeat Left | Name | Family | Class |
| 67768214 | 67768629 | - | 27.1 | 5559 | 5973 | -191 | L1ME2 | L1 | LINE |
| 67768630 | 67768936 | - | 10.1 | 1 | 296 | -15 | AluY | Alu | SINE |
| 67768937 | 67769981 | - | 27.9 | 4583 | 5558 | -606 | L1ME2 | L1 | LINE |
| 67769982 | 67770290 | - | 12.3 | 1 | 300 | -11 | AluY | Alu | SINE |
| 67770291 | 67770745 | - | 28.1 | 4157 | 4592 | -1554 | L1ME2 | L1 | LINE |

  

|  |  |  |  |  |  |  |  |  |  |
| --- | --- | --- | --- | --- | --- | --- | --- | --- | --- |
| chr9:67774216-67776515 | | | 2259 bp | | |  |  |  |  |
| Genome Start | Genome End | Strand | Div | Repeat Start | Repeat End | Repeat Left | Name | Family | Class |
| 67774216 | 67774647 | + | 24.9 | 1423 | 2039 | -4463 | L1MCa | L1 | LINE |
| 67774650 | 67774823 | + | 23.7 | 2 | 186 | -38 | MER58A | MER1\_type | DNA |
| 67774843 | 67775148 | + | 13.1 | 3 | 309 | 0 | AluSc | Alu | SINE |
| 67775169 | 67775496 | + | 21.4 | 2034 | 2365 | -4137 | L1MCa | L1 | LINE |
| 67775497 | 67776048 | - | 18.4 | 1 | 541 | -1 | MER34 | ERV1 | LTR |
| 67776049 | 67776097 | + | 21.4 | 2366 | 2406 | -4096 | L1MCa | L1 | LINE |
| 67776098 | 67776403 | + | 9.8 | 3 | 307 | -3 | AluSg | Alu | SINE |
| 67776404 | 67776515 | + | 21.4 | 2407 | 2517 | -3985 | L1MCa | L1 | LINE |

  

|  |  |  |  |  |  |  |  |  |  |
| --- | --- | --- | --- | --- | --- | --- | --- | --- | --- |
| chr9:67785052-67787926 | | | 2824 bp | | |  |  |  |  |
| Genome Start | Genome End | Strand | Div | Repeat Start | Repeat End | Repeat Left | Name | Family | Class |
| 67785052 | 67785735 | - | 32.5 | 5297 | 6020 | -220 | L1ME3B | L1 | LINE |
| 67785732 | 67787321 | - | 31.4 | 3312 | 5026 | -1120 | L1ME3B | L1 | LINE |
| 67787356 | 67787506 | + | 21.9 | 164 | 325 | -16 | MER58B | MER1\_type | DNA |
| 67787528 | 67787926 | - | 29.7 | 2866 | 3319 | -2827 | L1ME3B | L1 | LINE |

  

|  |  |  |  |  |  |  |  |  |  |
| --- | --- | --- | --- | --- | --- | --- | --- | --- | --- |
| chr9:67818186-67818781 | | | 596 bp | | |  |  |  |  |
| Genome Start | Genome End | Strand | Div | Repeat Start | Repeat End | Repeat Left | Name | Family | Class |
| 67818186 | 67818397 | - | 34.2 | 5640 | 5882 | -1926 | L1MC4a | L1 | LINE |
| 67818398 | 67818684 | + | 14.0 | 21 | 306 | 0 | AluSx | Alu | SINE |
| 67818685 | 67818781 | - | 34.2 | 5542 | 5639 | -2169 | L1MC4a | L1 | LINE |

  

|  |  |  |  |  |  |  |  |  |  |
| --- | --- | --- | --- | --- | --- | --- | --- | --- | --- |
| chr9:67822214-67822596 | | | 419 bp | | |  |  |  |  |
| Genome Start | Genome End | Strand | Div | Repeat Start | Repeat End | Repeat Left | Name | Family | Class |
| 67822214 | 67822388 | + | 27.6 | 1875 | 2052 | -2819 | MER65C-int | ERV1 | LTR |
| 67822291 | 67822419 | + | 25.8 | 1024 | 1160 | -4183 | LTR57-int | ERVL | LTR |
| 67822482 | 67822596 | + | 22.6 | 4768 | 4871 | 0 | MER65C-int | ERV1 | LTR |

  

|  |  |  |  |  |  |  |  |  |  |
| --- | --- | --- | --- | --- | --- | --- | --- | --- | --- |
| chr9:67870956-67881084 | | | 10124 bp | | |  |  |  |  |
| Genome Start | Genome End | Strand | Div | Repeat Start | Repeat End | Repeat Left | Name | Family | Class |
| 67870956 | 67871263 | - | 25.9 | 5009 | 5324 | -1052 | L1M4c | L1 | LINE |
| 67871264 | 67871586 | - | 22.9 | 1 | 311 | -1 | AluJb | Alu | SINE |
| 67871587 | 67872337 | - | 25.9 | 4243 | 5008 | -1368 | L1M4c | L1 | LINE |
| 67872338 | 67872635 | - | 17.8 | 15 | 312 | 0 | AluSx | Alu | SINE |
| 67872636 | 67872826 | - | 25.9 | 4071 | 4242 | -2134 | L1M4c | L1 | LINE |
| 67872836 | 67878749 | + | 9.7 | 5 | 6151 | -3 | L1PA6 | L1 | LINE |
| 67878746 | 67879952 | - | 25.6 | 2744 | 4085 | -2291 | L1M4c | L1 | LINE |
| 67879953 | 67880104 | - | 18.3 | 1 | 143 | 0 | FLAM\_C | Alu | SINE |
| 67880105 | 67881084 | - | 23.7 | 1686 | 2753 | -3623 | L1M4c | L1 | LINE |

  

|  |  |  |  |  |  |  |  |  |  |
| --- | --- | --- | --- | --- | --- | --- | --- | --- | --- |
| chr9:67925853-67926243 | | | 391 bp | | |  |  |  |  |
| Genome Start | Genome End | Strand | Div | Repeat Start | Repeat End | Repeat Left | Name | Family | Class |
| 67925853 | 67925910 | - | 21.2 | 40 | 80 | -244 | MER33 | MER1\_type | DNA |
| 67925911 | 67926204 | + | 17.2 | 9 | 300 | -12 | AluJo | Alu | SINE |
| 67926205 | 67926243 | - | 21.2 | 1 | 39 | -285 | MER33 | MER1\_type | DNA |

  

|  |  |  |  |  |  |  |  |  |  |
| --- | --- | --- | --- | --- | --- | --- | --- | --- | --- |
| chr9:67943017-67943901 | | | 866 bp | | |  |  |  |  |
| Genome Start | Genome End | Strand | Div | Repeat Start | Repeat End | Repeat Left | Name | Family | Class |
| 67943017 | 67943165 | + | 29.1 | 17 | 171 | -91 | MIR | MIR | SINE |
| 67943167 | 67943800 | + | 17.0 | 5497 | 6168 | -16 | L1MB7 | L1 | LINE |
| 67943819 | 67943901 | + | 27.5 | 181 | 262 | 0 | MIR | MIR | SINE |

  

|  |  |  |  |  |  |  |  |  |  |
| --- | --- | --- | --- | --- | --- | --- | --- | --- | --- |
| chr9:67948274-67949827 | | | 1554 bp | | |  |  |  |  |
| Genome Start | Genome End | Strand | Div | Repeat Start | Repeat End | Repeat Left | Name | Family | Class |
| 67948274 | 67948676 | + | 13.1 | 5393 | 5788 | -378 | L1PA16 | L1 | LINE |
| 67948677 | 67949081 | - | 15.8 | 1 | 426 | -2 | MSTA | MaLR | LTR |
| 67949082 | 67949335 | + | 13.1 | 5789 | 6051 | -115 | L1PA16 | L1 | LINE |
| 67949336 | 67949717 | - | 16.4 | 1 | 428 | 0 | MSTA | MaLR | LTR |
| 67949718 | 67949827 | + | 13.1 | 6052 | 6157 | -9 | L1PA16 | L1 | LINE |

  

|  |  |  |  |  |  |  |  |  |  |
| --- | --- | --- | --- | --- | --- | --- | --- | --- | --- |
| chr9:67980593-67982815 | | | 1800 bp | | |  |  |  |  |
| Genome Start | Genome End | Strand | Div | Repeat Start | Repeat End | Repeat Left | Name | Family | Class |
| 67980593 | 67980966 | + | 37.8 | 1616 | 2004 | -1415 | L2 | L2 | LINE |
| 67980975 | 67981305 | - | 21.2 | 9 | 380 | -10 | MLT1B | MaLR | LTR |
| 67981665 | 67981782 | - | 23.9 | 6028 | 6153 | -3 | L1PB4 | L1 | LINE |
| 67981828 | 67981942 | + | 31.2 | 2164 | 2269 | -1150 | L2 | L2 | LINE |
| 67981943 | 67982137 | + | 11.3 | 4 | 230 | 0 | MER30 | MER1\_type | DNA |
| 67982138 | 67982703 | + | 31.2 | 2270 | 2864 | -555 | L2 | L2 | LINE |
| 67982715 | 67982815 | + | 33.7 | 3293 | 3400 | -19 | L2 | L2 | LINE |

  

|  |  |  |  |  |  |  |  |  |  |
| --- | --- | --- | --- | --- | --- | --- | --- | --- | --- |
| chr9:67987289-67992470 | | | 5154 bp | | |  |  |  |  |
| Genome Start | Genome End | Strand | Div | Repeat Start | Repeat End | Repeat Left | Name | Family | Class |
| 67987289 | 67987470 | - | 15.0 | 3123 | 3246 | 0 | Ricksha | MuDR | DNA |
| 67987471 | 67987768 | + | 14.8 | 1 | 298 | -13 | AluY | Alu | SINE |
| 67987769 | 67989659 | - | 15.0 | 1190 | 3122 | -124 | Ricksha | MuDR | DNA |
| 67989662 | 67989988 | - | 14.9 | 79 | 1691 | -1547 | Ricksha | MuDR | DNA |
| 67989992 | 67990098 | + | 14.9 | 5 | 111 | -32 | FLAM\_C | Alu | SINE |
| 67990101 | 67990473 | + | 10.1 | 2 | 375 | 0 | THE1C | MaLR | LTR |
| 67990474 | 67992000 | + | 12.9 | 1 | 1580 | 0 | THE1C-int | MaLR | LTR |
| 67992001 | 67992361 | + | 13.5 | 1 | 375 | 0 | THE1C | MaLR | LTR |
| 67992383 | 67992470 | - | 13.6 | 7 | 94 | -1954 | Ricksha | MuDR | DNA |

  

|  |  |  |  |  |  |  |  |  |  |
| --- | --- | --- | --- | --- | --- | --- | --- | --- | --- |
| chr9:68056498-68057938 | | | 1441 bp | | |  |  |  |  |
| Genome Start | Genome End | Strand | Div | Repeat Start | Repeat End | Repeat Left | Name | Family | Class |
| 68056498 | 68056599 | + | 5.4 | 5033 | 5134 | -1012 | L1PA4 | L1 | LINE |
| 68056600 | 68056909 | + | 1.3 | 1 | 310 | 0 | AluYa5 | Alu | SINE |
| 68056910 | 68057938 | + | 3.7 | 5135 | 6147 | -8 | L1PA4 | L1 | LINE |

  

|  |  |  |  |  |  |  |  |  |  |
| --- | --- | --- | --- | --- | --- | --- | --- | --- | --- |
| chr9:68069285-68074482 | | | 5171 bp | | |  |  |  |  |
| Genome Start | Genome End | Strand | Div | Repeat Start | Repeat End | Repeat Left | Name | Family | Class |
| 68069285 | 68069372 | + | 15.9 | 7 | 94 | -1954 | Ricksha | MuDR | DNA |
| 68069396 | 68069756 | - | 12.4 | 1 | 375 | 0 | THE1C | MaLR | LTR |
| 68069757 | 68071283 | - | 12.6 | 1 | 1580 | 0 | THE1C-int | MaLR | LTR |
| 68071284 | 68071661 | - | 10.5 | 1 | 375 | 0 | THE1C | MaLR | LTR |
| 68071663 | 68071771 | - | 14.7 | 3 | 111 | -32 | FLAM\_C | Alu | SINE |
| 68071773 | 68072100 | + | 14.8 | 79 | 1691 | -1547 | Ricksha | MuDR | DNA |
| 68072103 | 68074002 | + | 14.6 | 1190 | 3122 | -124 | Ricksha | MuDR | DNA |
| 68074003 | 68074300 | - | 12.8 | 1 | 298 | -13 | AluY | Alu | SINE |
| 68074301 | 68074482 | + | 14.6 | 3123 | 3246 | 0 | Ricksha | MuDR | DNA |

  

|  |  |  |  |  |  |  |  |  |  |
| --- | --- | --- | --- | --- | --- | --- | --- | --- | --- |
| chr9:68076398-68077093 | | | 626 bp | | |  |  |  |  |
| Genome Start | Genome End | Strand | Div | Repeat Start | Repeat End | Repeat Left | Name | Family | Class |
| 68076398 | 68076647 | - | 15.3 | 5483 | 5700 | -483 | L1MB4 | L1 | LINE |
| 68076648 | 68076835 | - | 14.7 | 123 | 302 | -10 | AluSx | Alu | SINE |
| 68076860 | 68076965 | - | 14.7 | 1 | 122 | -190 | AluSx | Alu | SINE |
| 68076966 | 68077016 | - | 15.3 | 5451 | 5482 | -701 | L1MB4 | L1 | LINE |
| 68077063 | 68077093 | - | 20.6 | 5421 | 5448 | -726 | L1MB4 | L1 | LINE |

  

|  |  |  |  |  |  |  |  |  |  |
| --- | --- | --- | --- | --- | --- | --- | --- | --- | --- |
| chr9:68078778-68081001 | | | 1841 bp | | |  |  |  |  |
| Genome Start | Genome End | Strand | Div | Repeat Start | Repeat End | Repeat Left | Name | Family | Class |
| 68078778 | 68078878 | - | 33.7 | 3293 | 3400 | -19 | L2 | L2 | LINE |
| 68078890 | 68079454 | - | 31.9 | 2270 | 2864 | -555 | L2 | L2 | LINE |
| 68079455 | 68079671 | - | 12.0 | 4 | 230 | 0 | MER30 | MER1\_type | DNA |
| 68079672 | 68079786 | - | 31.9 | 2164 | 2269 | -1150 | L2 | L2 | LINE |
| 68079832 | 68079949 | + | 25.6 | 6028 | 6153 | -3 | L1PB4 | L1 | LINE |
| 68080277 | 68080616 | + | 20.9 | 9 | 389 | -1 | MLT1B | MaLR | LTR |
| 68080617 | 68081001 | - | 37.7 | 1603 | 2003 | -1416 | L2 | L2 | LINE |

  

|  |  |  |  |  |  |  |  |  |  |
| --- | --- | --- | --- | --- | --- | --- | --- | --- | --- |
| chr9:68088453-68093168 | | | 4716 bp | | |  |  |  |  |
| Genome Start | Genome End | Strand | Div | Repeat Start | Repeat End | Repeat Left | Name | Family | Class |
| 68088453 | 68089127 | - | 18.4 | 5849 | 6541 | -7 | L1MA9 | L1 | LINE |
| 68089128 | 68089415 | - | 17.4 | 1 | 300 | -12 | AluJo | Alu | SINE |
| 68089416 | 68090774 | - | 19.0 | 4474 | 5848 | -700 | L1MA9 | L1 | LINE |
| 68090775 | 68090836 | - | 19.4 | 1 | 63 | -282 | MER2 | MER2\_type | DNA |
| 68090837 | 68090928 | + | 22.0 | 242 | 335 | -1 | MER2B | MER2\_type | DNA |
| 68090929 | 68093168 | - | 19.0 | 2207 | 4473 | -2075 | L1MA9 | L1 | LINE |

  

|  |  |  |  |  |  |  |  |  |  |
| --- | --- | --- | --- | --- | --- | --- | --- | --- | --- |
| chr9:68102260-68107674 | | | 5362 bp | | |  |  |  |  |
| Genome Start | Genome End | Strand | Div | Repeat Start | Repeat End | Repeat Left | Name | Family | Class |
| 68102260 | 68102364 | + | 25.2 | 466 | 568 | -6921 | L1M3e | L1 | LINE |
| 68102403 | 68103299 | + | 32.9 | 1385 | 2299 | -4266 | L1M3e | L1 | LINE |
| 68103315 | 68105225 | + | 17.9 | 2977 | 4963 | -2756 | L1M3e | L1 | LINE |
| 68105226 | 68105515 | + | 22.5 | 8 | 307 | -5 | AluJo | Alu | SINE |
| 68105516 | 68107674 | + | 17.9 | 4964 | 7207 | -512 | L1M3e | L1 | LINE |

  

|  |  |  |  |  |  |  |  |  |  |
| --- | --- | --- | --- | --- | --- | --- | --- | --- | --- |
| chr9:68111772-68113327 | | | 1556 bp | | |  |  |  |  |
| Genome Start | Genome End | Strand | Div | Repeat Start | Repeat End | Repeat Left | Name | Family | Class |
| 68111772 | 68111881 | - | 12.6 | 6052 | 6157 | -9 | L1PA16 | L1 | LINE |
| 68111882 | 68112263 | + | 15.9 | 1 | 428 | 0 | MSTA | MaLR | LTR |
| 68112264 | 68112518 | - | 12.6 | 5789 | 6051 | -115 | L1PA16 | L1 | LINE |
| 68112519 | 68112924 | + | 16.7 | 1 | 426 | -2 | MSTA | MaLR | LTR |
| 68112925 | 68113327 | - | 12.6 | 5393 | 5788 | -378 | L1PA16 | L1 | LINE |

  

|  |  |  |  |  |  |  |  |  |  |
| --- | --- | --- | --- | --- | --- | --- | --- | --- | --- |
| chr9:68119420-68122885 | | | 3470 bp | | |  |  |  |  |
| Genome Start | Genome End | Strand | Div | Repeat Start | Repeat End | Repeat Left | Name | Family | Class |
| 68119420 | 68119595 | - | 19.6 | 5477 | 5655 | -509 | L1M5 | L1 | LINE |
| 68119592 | 68119652 | - | 25.3 | 5168 | 5224 | -922 | L1M5 | L1 | LINE |
| 68119653 | 68119875 | - | 9.9 | 81 | 303 | -8 | AluY | Alu | SINE |
| 68119876 | 68121679 | - | 25.3 | 3193 | 5167 | -979 | L1M5 | L1 | LINE |
| 68121680 | 68121888 | - | 12.9 | 1 | 230 | 0 | MER30 | MER1\_type | DNA |
| 68121889 | 68122300 | - | 25.3 | 2666 | 3192 | -2954 | L1M5 | L1 | LINE |
| 68122301 | 68122609 | - | 10.9 | 3 | 305 | -7 | AluSx | Alu | SINE |
| 68122610 | 68122885 | - | 25.3 | 2366 | 2665 | -3481 | L1M5 | L1 | LINE |

  

|  |  |  |  |  |  |  |  |  |  |
| --- | --- | --- | --- | --- | --- | --- | --- | --- | --- |
| chr9:68135193-68135580 | | | 388 bp | | |  |  |  |  |
| Genome Start | Genome End | Strand | Div | Repeat Start | Repeat End | Repeat Left | Name | Family | Class |
| 68135193 | 68135231 | + | 22.5 | 1 | 39 | -285 | MER33 | MER1\_type | DNA |
| 68135232 | 68135522 | - | 17.1 | 9 | 297 | -15 | AluJo | Alu | SINE |
| 68135523 | 68135580 | + | 22.5 | 40 | 80 | -244 | MER33 | MER1\_type | DNA |

  

|  |  |  |  |  |  |  |  |  |  |
| --- | --- | --- | --- | --- | --- | --- | --- | --- | --- |
| chr9:68149398-68150092 | | | 583 bp | | |  |  |  |  |
| Genome Start | Genome End | Strand | Div | Repeat Start | Repeat End | Repeat Left | Name | Family | Class |
| 68149398 | 68149451 | - | 25.6 | 207 | 262 | 0 | MIR | MIR | SINE |
| 68149564 | 68149593 | - | 25.6 | 143 | 206 | -56 | MIR | MIR | SINE |
| 68149594 | 68150002 | + | 11.5 | 1 | 428 | 0 | MSTC | MaLR | LTR |
| 68150003 | 68150092 | - | 25.6 | 31 | 142 | -120 | MIR | MIR | SINE |

  

|  |  |  |  |  |  |  |  |  |  |
| --- | --- | --- | --- | --- | --- | --- | --- | --- | --- |
| chr9:68165957-68168365 | | | 2396 bp | | |  |  |  |  |
| Genome Start | Genome End | Strand | Div | Repeat Start | Repeat End | Repeat Left | Name | Family | Class |
| 68165957 | 68166274 | + | 23.1 | 638 | 1000 | -1507 | HAL1 | L1 | LINE |
| 68166278 | 68166975 | + | 18.3 | 5457 | 6161 | -5 | L1ME1 | L1 | LINE |
| 68166986 | 68168365 | + | 25.1 | 993 | 2488 | -19 | HAL1 | L1 | LINE |

  

|  |  |  |  |  |  |  |  |  |  |
| --- | --- | --- | --- | --- | --- | --- | --- | --- | --- |
| chr9:68180785-68182625 | | | 1666 bp | | |  |  |  |  |
| Genome Start | Genome End | Strand | Div | Repeat Start | Repeat End | Repeat Left | Name | Family | Class |
| 68180785 | 68180856 | - | 29.6 | 2821 | 2891 | -528 | L2 | L2 | LINE |
| 68180881 | 68181476 | + | 27.8 | 2786 | 3419 | 0 | L2 | L2 | LINE |
| 68181501 | 68181688 | - | 35.5 | 2599 | 2793 | -626 | L2 | L2 | LINE |
| 68181706 | 68182152 | + | 23.2 | 1 | 509 | -58 | MLT1F | MaLR | LTR |
| 68182158 | 68182313 | + | 22.4 | 3236 | 3419 | 0 | L2 | L2 | LINE |
| 68182405 | 68182476 | + | 9.7 | 285 | 357 | -185 | MLT1F | MaLR | LTR |
| 68182482 | 68182533 | + | 13.5 | 490 | 541 | -1 | MLT1F | MaLR | LTR |
| 68182543 | 68182625 | - | 30.1 | 2500 | 2586 | -833 | L2 | L2 | LINE |

  

|  |  |  |  |  |  |  |  |  |  |
| --- | --- | --- | --- | --- | --- | --- | --- | --- | --- |
| chr9:68183847-68184664 | | | 807 bp | | |  |  |  |  |
| Genome Start | Genome End | Strand | Div | Repeat Start | Repeat End | Repeat Left | Name | Family | Class |
| 68183847 | 68184006 | + | 15.1 | 3954 | 4121 | -1593 | ERVL-B4 | ERVL | LTR |
| 68184007 | 68184416 | + | 15.4 | 1 | 416 | -10 | MSTB | MaLR | LTR |
| 68184428 | 68184664 | + | 14.3 | 4118 | 4356 | -1358 | ERVL-B4 | ERVL | LTR |

  

|  |  |  |  |  |  |  |  |  |  |
| --- | --- | --- | --- | --- | --- | --- | --- | --- | --- |
| chr9:68209456-68209961 | | | 506 bp | | |  |  |  |  |
| Genome Start | Genome End | Strand | Div | Repeat Start | Repeat End | Repeat Left | Name | Family | Class |
| 68209456 | 68209515 | - | 30.9 | 153 | 211 | -57 | MIRb | MIR | SINE |
| 68209516 | 68209866 | - | 14.4 | 1 | 366 | 0 | Tigger5a | MER2\_type | DNA |
| 68209867 | 68209961 | - | 30.9 | 60 | 152 | -116 | MIRb | MIR | SINE |

  

|  |  |  |  |  |  |  |  |  |  |
| --- | --- | --- | --- | --- | --- | --- | --- | --- | --- |
| chr9:68296986-68298035 | | | 1050 bp | | |  |  |  |  |
| Genome Start | Genome End | Strand | Div | Repeat Start | Repeat End | Repeat Left | Name | Family | Class |
| 68296986 | 68297243 | - | 29.8 | 2800 | 3117 | -302 | L2 | L2 | LINE |
| 68297244 | 68297481 | + | 11.3 | 58 | 299 | -13 | AluSx | Alu | SINE |
| 68297482 | 68298035 | - | 29.6 | 2213 | 2799 | -620 | L2 | L2 | LINE |

  

|  |  |  |  |  |  |  |  |  |  |
| --- | --- | --- | --- | --- | --- | --- | --- | --- | --- |
| chr9:68308309-68313504 | | | 5172 bp | | |  |  |  |  |
| Genome Start | Genome End | Strand | Div | Repeat Start | Repeat End | Repeat Left | Name | Family | Class |
| 68308309 | 68308399 | + | 17.6 | 7 | 97 | -1951 | Ricksha | MuDR | DNA |
| 68308420 | 68308780 | - | 12.4 | 1 | 375 | 0 | THE1C | MaLR | LTR |
| 68308781 | 68310306 | - | 12.6 | 1 | 1580 | 0 | THE1C-int | MaLR | LTR |
| 68310307 | 68310684 | - | 10.5 | 1 | 375 | 0 | THE1C | MaLR | LTR |
| 68310686 | 68310794 | - | 15.6 | 3 | 111 | -32 | FLAM\_C | Alu | SINE |
| 68310796 | 68311122 | + | 14.7 | 79 | 1697 | -1547 | Ricksha | MuDR | DNA |
| 68311125 | 68313024 | + | 15.3 | 1190 | 3122 | -124 | Ricksha | MuDR | DNA |
| 68313025 | 68313312 | - | 11.5 | 11 | 298 | -13 | AluY | Alu | SINE |
| 68313313 | 68313504 | + | 17.5 | 3123 | 3254 | -13 | Ricksha | MuDR | DNA |

  

|  |  |  |  |  |  |  |  |  |  |
| --- | --- | --- | --- | --- | --- | --- | --- | --- | --- |
| chr9:68350875-68352352 | | | 1477 bp | | |  |  |  |  |
| Genome Start | Genome End | Strand | Div | Repeat Start | Repeat End | Repeat Left | Name | Family | Class |
| 68350875 | 68350906 | - | 12.5 | 6052 | 6078 | -88 | L1PA16 | L1 | LINE |
| 68350907 | 68351288 | + | 15.9 | 1 | 428 | 0 | MSTA | MaLR | LTR |
| 68351289 | 68351542 | - | 13.1 | 5789 | 6051 | -115 | L1PA16 | L1 | LINE |
| 68351544 | 68351949 | + | 16.7 | 1 | 426 | -2 | MSTA | MaLR | LTR |
| 68351950 | 68352352 | - | 12.5 | 5393 | 5788 | -378 | L1PA16 | L1 | LINE |

  

|  |  |  |  |  |  |  |  |  |  |
| --- | --- | --- | --- | --- | --- | --- | --- | --- | --- |
| chr9:68356603-68357487 | | | 865 bp | | |  |  |  |  |
| Genome Start | Genome End | Strand | Div | Repeat Start | Repeat End | Repeat Left | Name | Family | Class |
| 68356603 | 68356682 | - | 29.5 | 181 | 262 | 0 | MIR | MIR | SINE |
| 68356701 | 68357337 | - | 17.3 | 5497 | 6168 | -16 | L1MB7 | L1 | LINE |
| 68357340 | 68357487 | - | 27.9 | 17 | 170 | -92 | MIR | MIR | SINE |

  

|  |  |  |  |  |  |  |  |  |  |
| --- | --- | --- | --- | --- | --- | --- | --- | --- | --- |
| chr9:68374186-68374572 | | | 387 bp | | |  |  |  |  |
| Genome Start | Genome End | Strand | Div | Repeat Start | Repeat End | Repeat Left | Name | Family | Class |
| 68374186 | 68374224 | + | 22.5 | 1 | 39 | -285 | MER33 | MER1\_type | DNA |
| 68374225 | 68374514 | - | 17.1 | 9 | 296 | -16 | AluJo | Alu | SINE |
| 68374515 | 68374572 | + | 22.5 | 40 | 80 | -244 | MER33 | MER1\_type | DNA |

  

|  |  |  |  |  |  |  |  |  |  |
| --- | --- | --- | --- | --- | --- | --- | --- | --- | --- |
| chr9:68388386-68389024 | | | 583 bp | | |  |  |  |  |
| Genome Start | Genome End | Strand | Div | Repeat Start | Repeat End | Repeat Left | Name | Family | Class |
| 68388386 | 68388439 | - | 25.6 | 207 | 262 | 0 | MIR | MIR | SINE |
| 68388496 | 68388525 | - | 25.6 | 143 | 206 | -56 | MIR | MIR | SINE |
| 68388526 | 68388934 | + | 11.5 | 1 | 428 | 0 | MSTC | MaLR | LTR |
| 68388935 | 68389024 | - | 25.6 | 31 | 142 | -120 | MIR | MIR | SINE |

  

|  |  |  |  |  |  |  |  |  |  |
| --- | --- | --- | --- | --- | --- | --- | --- | --- | --- |
| chr9:68392831-68393356 | | | 497 bp | | |  |  |  |  |
| Genome Start | Genome End | Strand | Div | Repeat Start | Repeat End | Repeat Left | Name | Family | Class |
| 68392831 | 68392869 | - | 20.5 | 6135 | 6161 | -5 | L1ME1 | L1 | LINE |
| 68392870 | 68393179 | - | 9.7 | 1 | 309 | -3 | AluSx | Alu | SINE |
| 68393180 | 68393233 | - | 20.5 | 6091 | 6134 | -32 | L1ME1 | L1 | LINE |
| 68393263 | 68393356 | - | 20.5 | 6000 | 6090 | -76 | L1ME1 | L1 | LINE |

  

|  |  |  |  |  |  |  |  |  |  |
| --- | --- | --- | --- | --- | --- | --- | --- | --- | --- |
| chr9:68421912-68422776 | | | 854 bp | | |  |  |  |  |
| Genome Start | Genome End | Strand | Div | Repeat Start | Repeat End | Repeat Left | Name | Family | Class |
| 68421912 | 68422118 | + | 16.9 | 3847 | 4121 | -1593 | ERVL-B4 | ERVL | LTR |
| 68422119 | 68422528 | + | 15.8 | 1 | 416 | -10 | MSTB | MaLR | LTR |
| 68422540 | 68422776 | + | 13.5 | 4118 | 4356 | -1358 | ERVL-B4 | ERVL | LTR |

  

|  |  |  |  |  |  |  |  |  |  |
| --- | --- | --- | --- | --- | --- | --- | --- | --- | --- |
| chr9:68433411-68434015 | | | 605 bp | | |  |  |  |  |
| Genome Start | Genome End | Strand | Div | Repeat Start | Repeat End | Repeat Left | Name | Family | Class |
| 68433411 | 68433610 | + | 30.8 | 7507 | 7703 | -339 | L1MC4 | L1 | LINE |
| 68433611 | 68433862 | + | 15.6 | 41 | 293 | -19 | AluJo | Alu | SINE |
| 68433863 | 68434015 | + | 30.8 | 7704 | 7854 | -188 | L1MC4 | L1 | LINE |

  

|  |  |  |  |  |  |  |  |  |  |
| --- | --- | --- | --- | --- | --- | --- | --- | --- | --- |
| chr9:68441215-68441975 | | | 262 bp | | |  |  |  |  |
| Genome Start | Genome End | Strand | Div | Repeat Start | Repeat End | Repeat Left | Name | Family | Class |
| 68441215 | 68441307 | - | 25.8 | 67 | 166 | 0 | MER103 | DNA | DNA |
| 68441329 | 68441388 | - | 10.0 | 238 | 297 | -13 | Alu | Alu | SINE |
| 68441867 | 68441975 | - | 15.7 | 3 | 116 | -50 | MER103 | DNA | DNA |

  

|  |  |  |  |  |  |  |  |  |  |
| --- | --- | --- | --- | --- | --- | --- | --- | --- | --- |
| chr9:68448611-68449868 | | | 1258 bp | | |  |  |  |  |
| Genome Start | Genome End | Strand | Div | Repeat Start | Repeat End | Repeat Left | Name | Family | Class |
| 68448611 | 68449562 | + | 16.5 | 3278 | 4268 | -1875 | L1M2 | L1 | LINE |
| 68449563 | 68449786 | + | 11.4 | 5955 | 6166 | 0 | L1PA17 | L1 | LINE |
| 68449787 | 68449868 | + | 16.5 | 4269 | 4313 | -1830 | L1M2 | L1 | LINE |

  

|  |  |  |  |  |  |  |  |  |  |
| --- | --- | --- | --- | --- | --- | --- | --- | --- | --- |
| chr9:68462097-68462607 | | | 511 bp | | |  |  |  |  |
| Genome Start | Genome End | Strand | Div | Repeat Start | Repeat End | Repeat Left | Name | Family | Class |
| 68462097 | 68462257 | - | 19.1 | 68 | 219 | 0 | MER20 | MER1\_type | DNA |
| 68462258 | 68462526 | + | 13.4 | 5906 | 6176 | -7 | L1MB3 | L1 | LINE |
| 68462527 | 68462607 | - | 19.1 | 1 | 67 | -152 | MER20 | MER1\_type | DNA |

  

|  |  |  |  |  |  |  |  |  |  |
| --- | --- | --- | --- | --- | --- | --- | --- | --- | --- |
| chr9:68463748-68464972 | | | 1031 bp | | |  |  |  |  |
| Genome Start | Genome End | Strand | Div | Repeat Start | Repeat End | Repeat Left | Name | Family | Class |
| 68463748 | 68463936 | + | 37.0 | 1648 | 1845 | -1574 | L2 | L2 | LINE |
| 68463984 | 68464417 | + | 19.0 | 8 | 458 | -9 | MLT1C | MaLR | LTR |
| 68464565 | 68464972 | + | 31.5 | 2172 | 2594 | -825 | L2 | L2 | LINE |

  

|  |  |  |  |  |  |  |  |  |  |
| --- | --- | --- | --- | --- | --- | --- | --- | --- | --- |
| chr9:68484799-68485339 | | | 541 bp | | |  |  |  |  |
| Genome Start | Genome End | Strand | Div | Repeat Start | Repeat End | Repeat Left | Name | Family | Class |
| 68484799 | 68485079 | + | 24.9 | 7409 | 7698 | -263 | L1MC5 | L1 | LINE |
| 68485080 | 68485207 | - | 19.5 | 1 | 128 | -5 | FLAM\_C | Alu | SINE |
| 68485208 | 68485339 | + | 24.9 | 7699 | 7826 | -135 | L1MC5 | L1 | LINE |

  

|  |  |  |  |  |  |  |  |  |  |
| --- | --- | --- | --- | --- | --- | --- | --- | --- | --- |
| chr9:68556257-68557567 | | | 1289 bp | | |  |  |  |  |
| Genome Start | Genome End | Strand | Div | Repeat Start | Repeat End | Repeat Left | Name | Family | Class |
| 68556257 | 68556376 | - | 20.0 | 2294 | 2416 | -4 | MER58 | MER1\_type | DNA |
| 68556398 | 68556666 | + | 21.8 | 1 | 279 | -219 | LTR54B | ERV1 | LTR |
| 68556667 | 68556932 | + | 14.3 | 1 | 267 | -45 | AluSx | Alu | SINE |
| 68556933 | 68557023 | + | 21.8 | 280 | 377 | -121 | LTR54B | ERV1 | LTR |
| 68557024 | 68557151 | + | 18.9 | 6074 | 6185 | -2 | MER57A-int | ERV1 | LTR |
| 68557153 | 68557463 | + | 16.9 | 1 | 403 | 0 | MER57A | ERV1 | LTR |
| 68557464 | 68557567 | - | 22.4 | 38 | 144 | -197 | MER58 | MER1\_type | DNA |

  

|  |  |  |  |  |  |  |  |  |  |
| --- | --- | --- | --- | --- | --- | --- | --- | --- | --- |
| chr9:68646780-68650370 | | | 3591 bp | | |  |  |  |  |
| Genome Start | Genome End | Strand | Div | Repeat Start | Repeat End | Repeat Left | Name | Family | Class |
| 68646780 | 68649287 | - | 18.6 | 4176 | 6732 | -830 | L1M1 | L1 | LINE |
| 68649288 | 68649522 | + | 6.0 | 39 | 295 | -16 | AluY | Alu | SINE |
| 68649523 | 68650370 | - | 17.4 | 3305 | 4175 | -3387 | L1M1 | L1 | LINE |

  

|  |  |  |  |  |  |  |  |  |  |
| --- | --- | --- | --- | --- | --- | --- | --- | --- | --- |
| chr9:68655375-68655873 | | | 438 bp | | |  |  |  |  |
| Genome Start | Genome End | Strand | Div | Repeat Start | Repeat End | Repeat Left | Name | Family | Class |
| 68655375 | 68655458 | - | 20.0 | 1656 | 1735 | -10 | Kanga1 | Tc2 | DNA |
| 68655508 | 68655760 | + | 30.3 | 367 | 626 | -5493 | L1MEd | L1 | LINE |
| 68655773 | 68655873 | - | 25.2 | 4 | 104 | -1794 | Kanga1 | Tc2 | DNA |

  

|  |  |  |  |  |  |  |  |  |  |
| --- | --- | --- | --- | --- | --- | --- | --- | --- | --- |
| chr9:68664807-68666309 | | | 1503 bp | | |  |  |  |  |
| Genome Start | Genome End | Strand | Div | Repeat Start | Repeat End | Repeat Left | Name | Family | Class |
| 68664807 | 68665093 | - | 24.5 | 602 | 916 | -19 | MER21C | ERV1 | LTR |
| 68665094 | 68665384 | - | 16.3 | 9 | 297 | -15 | AluJo | Alu | SINE |
| 68665385 | 68665462 | - | 24.5 | 529 | 601 | -334 | MER21C | ERV1 | LTR |
| 68665463 | 68665792 | + | 19.5 | 2 | 298 | -14 | AluJb | Alu | SINE |
| 68665793 | 68666309 | - | 24.5 | 18 | 528 | -407 | MER21C | ERV1 | LTR |

  

|  |  |  |  |  |  |  |  |  |  |
| --- | --- | --- | --- | --- | --- | --- | --- | --- | --- |
| chr9:68723783-68725520 | | | 1729 bp | | |  |  |  |  |
| Genome Start | Genome End | Strand | Div | Repeat Start | Repeat End | Repeat Left | Name | Family | Class |
| 68723783 | 68724104 | - | 20.4 | 5833 | 6173 | -5 | L1MB8 | L1 | LINE |
| 68724105 | 68724516 | - | 14.4 | 11 | 428 | 0 | MER61C | ERV1 | LTR |
| 68724526 | 68725520 | - | 19.4 | 4766 | 5836 | -342 | L1MB8 | L1 | LINE |

  

|  |  |  |  |  |  |  |  |  |  |
| --- | --- | --- | --- | --- | --- | --- | --- | --- | --- |
| chr9:68727263-68728986 | | | 1659 bp | | |  |  |  |  |
| Genome Start | Genome End | Strand | Div | Repeat Start | Repeat End | Repeat Left | Name | Family | Class |
| 68727263 | 68727549 | - | 18.6 | 5981 | 6294 | -6 | L1MA5 | L1 | LINE |
| 68727607 | 68727864 | - | 23.1 | 1 | 264 | -48 | AluJo | Alu | SINE |
| 68727873 | 68728986 | - | 20.5 | 4805 | 5941 | -359 | L1MA5 | L1 | LINE |

  

|  |  |  |  |  |  |  |  |  |  |
| --- | --- | --- | --- | --- | --- | --- | --- | --- | --- |
| chr9:68743161-68744454 | | | 1160 bp | | |  |  |  |  |
| Genome Start | Genome End | Strand | Div | Repeat Start | Repeat End | Repeat Left | Name | Family | Class |
| 68743161 | 68743391 | + | 16.4 | 13 | 247 | -1404 | MSTA-int | MaLR | LTR |
| 68743395 | 68743636 | + | 18.4 | 1404 | 1651 | 0 | MSTA-int | MaLR | LTR |
| 68743637 | 68744010 | + | 23.9 | 1 | 376 | -52 | MSTA | MaLR | LTR |
| 68744026 | 68744176 | - | 21.8 | 490 | 657 | -959 | MST-int | MaLR | LTR |
| 68744174 | 68744279 | + | 23.6 | 28 | 133 | -1518 | MSTA-int | MaLR | LTR |
| 68744399 | 68744454 | + | 21.4 | 911 | 967 | -613 | MSTA-int | MaLR | LTR |

  

|  |  |  |  |  |  |  |  |  |  |
| --- | --- | --- | --- | --- | --- | --- | --- | --- | --- |
| chr9:68744443-68761008 | | | 15029 bp | | |  |  |  |  |
| Genome Start | Genome End | Strand | Div | Repeat Start | Repeat End | Repeat Left | Name | Family | Class |
| 68744443 | 68744683 | - | 21.6 | 907 | 1191 | -389 | THE1-int | MaLR | LTR |
| 68744688 | 68744973 | + | 22.5 | 76 | 376 | -52 | MSTA | MaLR | LTR |
| 68745008 | 68745140 | - | 22.0 | 507 | 661 | -990 | MST-int | MaLR | LTR |
| 68745138 | 68745776 | + | 25.0 | 27 | 696 | -955 | MSTA-int | MaLR | LTR |
| 68745783 | 68746346 | + | 21.1 | 1107 | 1650 | -1 | MSTA-int | MaLR | LTR |
| 68746348 | 68746446 | + | 21.2 | 1 | 100 | -328 | MSTA | MaLR | LTR |
| 68746550 | 68746617 | + | 19.1 | 95 | 166 | -262 | MSTA | MaLR | LTR |
| 68746622 | 68746967 | + | 23.0 | 1318 | 1649 | -2 | MSTA-int | MaLR | LTR |
| 68746970 | 68747065 | + | 20.8 | 1 | 97 | -331 | MSTA | MaLR | LTR |
| 68747162 | 68747217 | + | 19.6 | 911 | 967 | -613 | THE1-int | MaLR | LTR |
| 68747209 | 68747449 | - | 23.7 | 907 | 1188 | -392 | THE1-int | MaLR | LTR |
| 68747475 | 68747743 | + | 23.6 | 96 | 376 | -52 | MSTA | MaLR | LTR |
| 68747778 | 68747889 | - | 21.1 | 491 | 661 | -990 | MST-int | MaLR | LTR |
| 68747976 | 68749512 | + | 23.0 | 15 | 1583 | -68 | MST-int | MaLR | LTR |
| 68749585 | 68749645 | - | 18.0 | 317 | 377 | -51 | MSTA | MaLR | LTR |
| 68749648 | 68749725 | + | 20.0 | 1574 | 1651 | 0 | MST-int | MaLR | LTR |
| 68749867 | 68749969 | + | 23.5 | 58 | 171 | -257 | MSTA | MaLR | LTR |
| 68749968 | 68750314 | + | 21.6 | 1317 | 1651 | 0 | MSTA-int | MaLR | LTR |
| 68750315 | 68750410 | + | 15.6 | 1 | 97 | -331 | MSTA | MaLR | LTR |
| 68750507 | 68750562 | + | 19.6 | 911 | 967 | -613 | THE1-int | MaLR | LTR |
| 68750551 | 68750785 | - | 20.0 | 907 | 1191 | -389 | THE1-int | MaLR | LTR |
| 68750811 | 68751078 | + | 26.0 | 96 | 374 | -54 | MSTA | MaLR | LTR |
| 68751108 | 68751245 | - | 22.6 | 507 | 670 | -981 | MST-int | MaLR | LTR |
| 68751252 | 68751317 | + | 19.7 | 36 | 102 | -1549 | MST-int | MaLR | LTR |
| 68751367 | 68751436 | - | 27.1 | 907 | 977 | -603 | THE1-int | MaLR | LTR |
| 68751490 | 68751744 | + | 25.4 | 114 | 385 | -43 | MSTA | MaLR | LTR |
| 68751753 | 68751906 | - | 20.1 | 463 | 661 | -963 | MST-int | MaLR | LTR |
| 68751902 | 68753479 | + | 24.1 | 28 | 1651 | 0 | MSTA-int | MaLR | LTR |
| 68753480 | 68753576 | + | 22.7 | 1 | 99 | -329 | MSTA | MaLR | LTR |
| 68753638 | 68753727 | + | 28.9 | 75 | 171 | -257 | MSTA | MaLR | LTR |
| 68753726 | 68754065 | + | 22.8 | 1317 | 1651 | 0 | MST-int | MaLR | LTR |
| 68754066 | 68754128 | + | 20.6 | 1 | 65 | -363 | MSTA | MaLR | LTR |
| 68754248 | 68754301 | + | 18.9 | 914 | 967 | -613 | THE1-int | MaLR | LTR |
| 68754290 | 68754531 | - | 25.3 | 907 | 1191 | -389 | THE1-int | MaLR | LTR |
| 68754558 | 68754814 | + | 26.7 | 103 | 372 | -56 | MSTA | MaLR | LTR |
| 68754844 | 68754984 | - | 24.1 | 508 | 670 | -981 | MST-int | MaLR | LTR |
| 68754988 | 68756305 | + | 25.8 | 32 | 1389 | -262 | MST-int | MaLR | LTR |
| 68756302 | 68756417 | + | 15.7 | 1535 | 1651 | 0 | MST-int | MaLR | LTR |
| 68756418 | 68756515 | + | 20.6 | 1 | 99 | -329 | MSTA | MaLR | LTR |
| 68756565 | 68756665 | + | 19.8 | 60 | 171 | -257 | MSTA | MaLR | LTR |
| 68756664 | 68757000 | + | 19.9 | 1317 | 1651 | 0 | MST-int | MaLR | LTR |
| 68757001 | 68757095 | + | 17.9 | 1 | 97 | -331 | MSTA | MaLR | LTR |
| 68757247 | 68757480 | - | 17.9 | 904 | 1178 | -402 | THE1-int | MaLR | LTR |
| 68757480 | 68757764 | + | 23.4 | 74 | 376 | -52 | MSTA | MaLR | LTR |
| 68757793 | 68757934 | - | 29.1 | 464 | 608 | -972 | THE1-int | MaLR | LTR |
| 68757937 | 68759504 | + | 23.6 | 34 | 1651 | 0 | MST-int | MaLR | LTR |
| 68759520 | 68759599 | + | 18.8 | 16 | 97 | -331 | MSTA | MaLR | LTR |
| 68759650 | 68759743 | + | 28.6 | 65 | 159 | -269 | MSTA | MaLR | LTR |
| 68759751 | 68760090 | + | 21.8 | 1318 | 1644 | -7 | MSTA-int | MaLR | LTR |
| 68760097 | 68760191 | + | 18.9 | 1 | 97 | -331 | MSTA | MaLR | LTR |
| 68760319 | 68760562 | - | 20.1 | 907 | 1191 | -389 | THE1-int | MaLR | LTR |
| 68760567 | 68760832 | + | 20.8 | 76 | 368 | -60 | MSTA | MaLR | LTR |
| 68760869 | 68761008 | - | 25.9 | 466 | 608 | -972 | THE1-int | MaLR | LTR |

  

|  |  |  |  |  |  |  |  |  |  |
| --- | --- | --- | --- | --- | --- | --- | --- | --- | --- |
| chr9:68762588-68763792 | | | 1059 bp | | |  |  |  |  |
| Genome Start | Genome End | Strand | Div | Repeat Start | Repeat End | Repeat Left | Name | Family | Class |
| 68762588 | 68762685 | + | 21.6 | 1 | 99 | -329 | MSTA | MaLR | LTR |
| 68762735 | 68762831 | + | 24.7 | 60 | 171 | -257 | MSTA | MaLR | LTR |
| 68762830 | 68763149 | + | 24.8 | 1317 | 1650 | -1 | MSTA-int | MaLR | LTR |
| 68763150 | 68763251 | + | 22.4 | 1 | 106 | -322 | MSTA | MaLR | LTR |
| 68763335 | 68763390 | + | 19.6 | 911 | 967 | -613 | THE1-int | MaLR | LTR |
| 68763379 | 68763620 | - | 23.2 | 976 | 1256 | -395 | MST-int | MaLR | LTR |
| 68763649 | 68763792 | + | 25.9 | 128 | 274 | -154 | MSTA | MaLR | LTR |

  

|  |  |  |  |  |  |  |  |  |  |
| --- | --- | --- | --- | --- | --- | --- | --- | --- | --- |
| chr9:68778331-68779019 | | | 614 bp | | |  |  |  |  |
| Genome Start | Genome End | Strand | Div | Repeat Start | Repeat End | Repeat Left | Name | Family | Class |
| 68778331 | 68778452 | + | 12.2 | 1 | 124 | -302 | MSTB | MaLR | LTR |
| 68778453 | 68778546 | - | 14.7 | 115 | 230 | 0 | MER30 | MER1\_type | DNA |
| 68778622 | 68778718 | - | 14.7 | 3 | 105 | -125 | MER30 | MER1\_type | DNA |
| 68778719 | 68779019 | + | 18.6 | 117 | 426 | 0 | MSTB | MaLR | LTR |

  

|  |  |  |  |  |  |  |  |  |  |
| --- | --- | --- | --- | --- | --- | --- | --- | --- | --- |
| chr9:68808334-68810047 | | | 1714 bp | | |  |  |  |  |
| Genome Start | Genome End | Strand | Div | Repeat Start | Repeat End | Repeat Left | Name | Family | Class |
| 68808334 | 68808709 | + | 23.5 | 1239 | 1600 | -4908 | L1MCa | L1 | LINE |
| 68808710 | 68808966 | - | 12.2 | 1 | 255 | -54 | AluSc | Alu | SINE |
| 68808967 | 68809335 | + | 23.5 | 1601 | 2017 | -4491 | L1MCa | L1 | LINE |
| 68809336 | 68809691 | - | 23.0 | 1 | 365 | 0 | MLT1A0 | MaLR | LTR |
| 68809692 | 68810047 | + | 23.5 | 2018 | 2388 | -4120 | L1MCa | L1 | LINE |

  

|  |  |  |  |  |  |  |  |  |  |
| --- | --- | --- | --- | --- | --- | --- | --- | --- | --- |
| chr9:68816995-68823756 | | | 6733 bp | | |  |  |  |  |
| Genome Start | Genome End | Strand | Div | Repeat Start | Repeat End | Repeat Left | Name | Family | Class |
| 68816995 | 68817172 | + | 23.0 | 1 | 257 | -397 | MLT1E2 | MaLR | LTR |
| 68817173 | 68817389 | - | 18.8 | 3226 | 3434 | -4721 | L1PREC2 | L1 | LINE |
| 68817390 | 68817751 | + | 13.1 | 1 | 381 | 0 | THE1D | MaLR | LTR |
| 68817752 | 68818880 | - | 18.8 | 2081 | 3225 | -4930 | L1PREC2 | L1 | LINE |
| 68818878 | 68823373 | + | 19.3 | 2059 | 6686 | -1 | L1PREC2 | L1 | LINE |
| 68823406 | 68823756 | + | 29.9 | 223 | 577 | -49 | MLT1E2 | MaLR | LTR |

  

|  |  |  |  |  |  |  |  |  |  |
| --- | --- | --- | --- | --- | --- | --- | --- | --- | --- |
| chr9:68827253-68828916 | | | 1620 bp | | |  |  |  |  |
| Genome Start | Genome End | Strand | Div | Repeat Start | Repeat End | Repeat Left | Name | Family | Class |
| 68827253 | 68827453 | + | 8.9 | 4815 | 5015 | -1131 | L1PB1 | L1 | LINE |
| 68827454 | 68827727 | + | 13.2 | 1 | 276 | -36 | AluSx | Alu | SINE |
| 68827772 | 68828916 | + | 9.7 | 5016 | 6145 | -6 | L1PB1 | L1 | LINE |

  

|  |  |  |  |  |  |  |  |  |  |
| --- | --- | --- | --- | --- | --- | --- | --- | --- | --- |
| chr9:68829446-68832846 | | | 3202 bp | | |  |  |  |  |
| Genome Start | Genome End | Strand | Div | Repeat Start | Repeat End | Repeat Left | Name | Family | Class |
| 68829446 | 68830897 | - | 30.2 | 3774 | 5277 | -1664 | L1MCc | L1 | LINE |
| 68830898 | 68831095 | - | 14.7 | 5942 | 6144 | -10 | L1PB3 | L1 | LINE |
| 68831096 | 68831147 | - | 30.2 | 3734 | 3773 | -3168 | L1MCc | L1 | LINE |
| 68831148 | 68831458 | - | 6.2 | 1 | 310 | -1 | AluY | Alu | SINE |
| 68831459 | 68832287 | - | 29.6 | 2834 | 3733 | -3208 | L1MCc | L1 | LINE |
| 68832487 | 68832846 | - | 29.2 | 2162 | 2576 | -4385 | L1MCc | L1 | LINE |

  

|  |  |  |  |  |  |  |  |  |  |
| --- | --- | --- | --- | --- | --- | --- | --- | --- | --- |
| chr9:68853614-68854162 | | | 465 bp | | |  |  |  |  |
| Genome Start | Genome End | Strand | Div | Repeat Start | Repeat End | Repeat Left | Name | Family | Class |
| 68853614 | 68853724 | - | 27.5 | 69 | 177 | -1 | MER5B | MER1\_type | DNA |
| 68853809 | 68854118 | - | 4.9 | 1 | 310 | -1 | AluY | Alu | SINE |
| 68854119 | 68854162 | - | 27.3 | 1 | 44 | -134 | MER5B | MER1\_type | DNA |

  

|  |  |  |  |  |  |  |  |  |  |
| --- | --- | --- | --- | --- | --- | --- | --- | --- | --- |
| chr9:68855033-68855484 | | | 452 bp | | |  |  |  |  |
| Genome Start | Genome End | Strand | Div | Repeat Start | Repeat End | Repeat Left | Name | Family | Class |
| 68855033 | 68855088 | - | 28.8 | 116 | 172 | -6 | MER5B | MER1\_type | DNA |
| 68855089 | 68855397 | + | 12.7 | 1 | 299 | -13 | AluSx | Alu | SINE |
| 68855398 | 68855484 | - | 28.8 | 27 | 115 | -63 | MER5B | MER1\_type | DNA |

  

|  |  |  |  |  |  |  |  |  |  |
| --- | --- | --- | --- | --- | --- | --- | --- | --- | --- |
| chr9:68861984-68863710 | | | 1727 bp | | |  |  |  |  |
| Genome Start | Genome End | Strand | Div | Repeat Start | Repeat End | Repeat Left | Name | Family | Class |
| 68861984 | 68862387 | + | 15.7 | 3 | 424 | -1227 | THE1B-int | MaLR | LTR |
| 68862388 | 68862545 | + | 11.5 | 134 | 293 | -19 | AluSg/x | Alu | SINE |
| 68862546 | 68863710 | + | 15.7 | 425 | 1643 | -8 | THE1B-int | MaLR | LTR |

  

|  |  |  |  |  |  |  |  |  |  |
| --- | --- | --- | --- | --- | --- | --- | --- | --- | --- |
| chr9:68879765-68881369 | | | 1605 bp | | |  |  |  |  |
| Genome Start | Genome End | Strand | Div | Repeat Start | Repeat End | Repeat Left | Name | Family | Class |
| 68879765 | 68880454 | + | 9.4 | 4743 | 5443 | -711 | L1PA7 | L1 | LINE |
| 68880455 | 68880791 | + | 4.5 | 5815 | 6153 | -2 | L1PA4 | L1 | LINE |
| 68880792 | 68881369 | + | 8.7 | 5444 | 6006 | -148 | L1PA7 | L1 | LINE |

  

|  |  |  |  |  |  |  |  |  |  |
| --- | --- | --- | --- | --- | --- | --- | --- | --- | --- |
| chr9:68928142-68928810 | | | 669 bp | | |  |  |  |  |
| Genome Start | Genome End | Strand | Div | Repeat Start | Repeat End | Repeat Left | Name | Family | Class |
| 68928142 | 68928186 | - | 29.2 | 1921 | 1968 | -1451 | L2 | L2 | LINE |
| 68928187 | 68928477 | + | 11.0 | 1 | 300 | -12 | AluSx | Alu | SINE |
| 68928478 | 68928810 | - | 29.2 | 1547 | 1920 | -1499 | L2 | L2 | LINE |

  

|  |  |  |  |  |  |  |  |  |  |
| --- | --- | --- | --- | --- | --- | --- | --- | --- | --- |
| chr9:68934991-68935633 | | | 643 bp | | |  |  |  |  |
| Genome Start | Genome End | Strand | Div | Repeat Start | Repeat End | Repeat Left | Name | Family | Class |
| 68934991 | 68935226 | + | 34.0 | 3022 | 3271 | -148 | L2 | L2 | LINE |
| 68935227 | 68935529 | + | 15.5 | 1 | 302 | -10 | AluJb | Alu | SINE |
| 68935530 | 68935633 | + | 34.0 | 3272 | 3405 | -14 | L2 | L2 | LINE |

  

|  |  |  |  |  |  |  |  |  |  |
| --- | --- | --- | --- | --- | --- | --- | --- | --- | --- |
| chr9:68957089-68958062 | | | 974 bp | | |  |  |  |  |
| Genome Start | Genome End | Strand | Div | Repeat Start | Repeat End | Repeat Left | Name | Family | Class |
| 68957089 | 68957370 | - | 21.1 | 569 | 859 | -4 | MER21B | ERV1 | LTR |
| 68957371 | 68957508 | + | 10.6 | 1 | 136 | -176 | AluSx | Alu | SINE |
| 68957509 | 68957812 | + | 11.0 | 3 | 306 | -5 | AluY | Alu | SINE |
| 68957813 | 68957988 | + | 10.6 | 137 | 301 | -11 | AluSx | Alu | SINE |
| 68957989 | 68958062 | - | 21.1 | 497 | 568 | -295 | MER21B | ERV1 | LTR |

  

|  |  |  |  |  |  |  |  |  |  |
| --- | --- | --- | --- | --- | --- | --- | --- | --- | --- |
| chr9:69002215-69002780 | | | 566 bp | | |  |  |  |  |
| Genome Start | Genome End | Strand | Div | Repeat Start | Repeat End | Repeat Left | Name | Family | Class |
| 69002215 | 69002419 | + | 36.7 | 1185 | 1409 | -551 | L4 | RTE | LINE |
| 69002420 | 69002616 | - | 10.2 | 106 | 302 | -9 | AluY | Alu | SINE |
| 69002617 | 69002780 | + | 36.7 | 1410 | 1607 | -353 | L4 | RTE | LINE |

  

|  |  |  |  |  |  |  |  |  |  |
| --- | --- | --- | --- | --- | --- | --- | --- | --- | --- |
| chr9:69005381-69007459 | | | 2029 bp | | |  |  |  |  |
| Genome Start | Genome End | Strand | Div | Repeat Start | Repeat End | Repeat Left | Name | Family | Class |
| 69005381 | 69005474 | + | 17.1 | 3577 | 3670 | -2470 | L1M3 | L1 | LINE |
| 69005525 | 69006456 | + | 17.1 | 3671 | 4625 | -1515 | L1M3 | L1 | LINE |
| 69006457 | 69006766 | + | 13.9 | 1 | 310 | 0 | AluSg | Alu | SINE |
| 69006767 | 69007459 | + | 17.2 | 4626 | 5341 | -815 | L1M3 | L1 | LINE |

  

|  |  |  |  |  |  |  |  |  |  |
| --- | --- | --- | --- | --- | --- | --- | --- | --- | --- |
| chr9:69012118-69013491 | | | 1278 bp | | |  |  |  |  |
| Genome Start | Genome End | Strand | Div | Repeat Start | Repeat End | Repeat Left | Name | Family | Class |
| 69012118 | 69012237 | + | 24.1 | 1383 | 1508 | -1911 | L2 | L2 | LINE |
| 69012328 | 69012501 | - | 20.1 | 5 | 183 | -26 | MER3 | MER1\_type | DNA |
| 69012508 | 69013491 | + | 27.0 | 1614 | 2920 | -499 | L2 | L2 | LINE |

  

|  |  |  |  |  |  |  |  |  |  |
| --- | --- | --- | --- | --- | --- | --- | --- | --- | --- |
| chr9:69045129-69046009 | | | 881 bp | | |  |  |  |  |
| Genome Start | Genome End | Strand | Div | Repeat Start | Repeat End | Repeat Left | Name | Family | Class |
| 69045129 | 69045513 | + | 22.4 | 5188 | 5602 | -564 | L1M5 | L1 | LINE |
| 69045514 | 69045817 | + | 5.9 | 1 | 305 | -6 | AluY | Alu | SINE |
| 69045818 | 69046009 | + | 22.3 | 5603 | 5796 | -370 | L1M5 | L1 | LINE |

  

|  |  |  |  |  |  |  |  |  |  |
| --- | --- | --- | --- | --- | --- | --- | --- | --- | --- |
| chr9:69048127-69050125 | | | 1996 bp | | |  |  |  |  |
| Genome Start | Genome End | Strand | Div | Repeat Start | Repeat End | Repeat Left | Name | Family | Class |
| 69048127 | 69048880 | - | 17.9 | 5288 | 6022 | -278 | L1MA4 | L1 | LINE |
| 69048884 | 69048987 | - | 19.7 | 197 | 290 | -22 | AluJo/FRAM | Alu | SINE |
| 69048988 | 69049298 | + | 6.2 | 1 | 311 | 0 | AluY | Alu | SINE |
| 69049299 | 69049326 | - | 19.7 | 33 | 60 | -116 | FRAM/FAM | Alu | SINE |
| 69049327 | 69049623 | - | 14.6 | 5046 | 5322 | -821 | L1MA4 | L1 | LINE |
| 69049624 | 69049837 | - | 7.9 | 1 | 203 | -108 | AluY | Alu | SINE |
| 69049838 | 69050125 | - | 14.6 | 4771 | 5045 | -1098 | L1MA4 | L1 | LINE |

  

|  |  |  |  |  |  |  |  |  |  |
| --- | --- | --- | --- | --- | --- | --- | --- | --- | --- |
| chr9:69054156-69054844 | | | 671 bp | | |  |  |  |  |
| Genome Start | Genome End | Strand | Div | Repeat Start | Repeat End | Repeat Left | Name | Family | Class |
| 69054156 | 69054471 | - | 19.6 | 5032 | 5328 | -818 | L1MD | L1 | LINE |
| 69054478 | 69054633 | - | 18.6 | 10 | 168 | -8 | FRAM | Alu | SINE |
| 69054646 | 69054844 | - | 20.4 | 4834 | 5037 | -1109 | L1MD | L1 | LINE |

  

|  |  |  |  |  |  |  |  |  |  |
| --- | --- | --- | --- | --- | --- | --- | --- | --- | --- |
| chr9:69056076-69058323 | | | 2247 bp | | |  |  |  |  |
| Genome Start | Genome End | Strand | Div | Repeat Start | Repeat End | Repeat Left | Name | Family | Class |
| 69056076 | 69057461 | + | 15.1 | 4106 | 5523 | -774 | L1MA5A | L1 | LINE |
| 69057463 | 69057581 | - | 15.5 | 168 | 294 | -18 | AluSg/x | Alu | SINE |
| 69057582 | 69058323 | + | 11.9 | 5512 | 6293 | -4 | L1MA5A | L1 | LINE |

  

|  |  |  |  |  |  |  |  |  |  |
| --- | --- | --- | --- | --- | --- | --- | --- | --- | --- |
| chr9:69083219-69084705 | | | 1487 bp | | |  |  |  |  |
| Genome Start | Genome End | Strand | Div | Repeat Start | Repeat End | Repeat Left | Name | Family | Class |
| 69083219 | 69084044 | - | 23.5 | 2624 | 3354 | -3688 | L1M4b | L1 | LINE |
| 69084045 | 69084329 | - | 11.7 | 1 | 283 | -28 | AluY | Alu | SINE |
| 69084330 | 69084705 | - | 22.3 | 2146 | 2623 | -4711 | L1M4b | L1 | LINE |

  

|  |  |  |  |  |  |  |  |  |  |
| --- | --- | --- | --- | --- | --- | --- | --- | --- | --- |
| chr9:69091186-69092378 | | | 1146 bp | | |  |  |  |  |
| Genome Start | Genome End | Strand | Div | Repeat Start | Repeat End | Repeat Left | Name | Family | Class |
| 69091186 | 69091578 | - | 21.7 | 490 | 901 | 0 | MER54A | ERVL | LTR |
| 69091588 | 69091925 | + | 11.4 | 7 | 364 | 0 | THE1B | MaLR | LTR |
| 69091964 | 69092378 | - | 25.3 | 1 | 430 | -471 | MER54A | ERVL | LTR |

  

|  |  |  |  |  |  |  |  |  |  |
| --- | --- | --- | --- | --- | --- | --- | --- | --- | --- |
| chr9:69096728-69097409 | | | 682 bp | | |  |  |  |  |
| Genome Start | Genome End | Strand | Div | Repeat Start | Repeat End | Repeat Left | Name | Family | Class |
| 69096728 | 69096751 | + | 17.8 | 12 | 36 | -559 | LTR49 | ERV1 | LTR |
| 69096752 | 69097026 | - | 12.4 | 14 | 289 | -24 | AluSq | Alu | SINE |
| 69097027 | 69097409 | + | 17.8 | 37 | 427 | -168 | LTR49 | ERV1 | LTR |

  

|  |  |  |  |  |  |  |  |  |  |
| --- | --- | --- | --- | --- | --- | --- | --- | --- | --- |
| chr9:69142312-69144793 | | | 2422 bp | | |  |  |  |  |
| Genome Start | Genome End | Strand | Div | Repeat Start | Repeat End | Repeat Left | Name | Family | Class |
| 69142312 | 69142486 | - | 23.6 | 6100 | 6287 | -4 | L1MA8 | L1 | LINE |
| 69142547 | 69144293 | - | 24.7 | 4102 | 5941 | -359 | L1MA8 | L1 | LINE |
| 69144294 | 69144590 | + | 9.8 | 1 | 299 | -12 | AluY | Alu | SINE |
| 69144591 | 69144793 | - | 24.8 | 3894 | 4101 | -2042 | L1MA8 | L1 | LINE |

  

|  |  |  |  |  |  |  |  |  |  |
| --- | --- | --- | --- | --- | --- | --- | --- | --- | --- |
| chr9:69151491-69151945 | | | 455 bp | | |  |  |  |  |
| Genome Start | Genome End | Strand | Div | Repeat Start | Repeat End | Repeat Left | Name | Family | Class |
| 69151491 | 69151603 | + | 34.4 | 9 | 130 | -138 | MIRb | MIR | SINE |
| 69151604 | 69151896 | - | 10.9 | 1 | 293 | -20 | AluSq | Alu | SINE |
| 69151897 | 69151945 | + | 34.4 | 131 | 184 | -84 | MIRb | MIR | SINE |

  

|  |  |  |  |  |  |  |  |  |  |
| --- | --- | --- | --- | --- | --- | --- | --- | --- | --- |
| chr9:69162286-69163469 | | | 1154 bp | | |  |  |  |  |
| Genome Start | Genome End | Strand | Div | Repeat Start | Repeat End | Repeat Left | Name | Family | Class |
| 69162286 | 69162759 | + | 10.6 | 2808 | 3304 | -2842 | L1P3 | L1 | LINE |
| 69162790 | 69162970 | + | 13.8 | 114 | 294 | -18 | AluSg/x | Alu | SINE |
| 69162971 | 69163469 | + | 11.4 | 3294 | 3793 | -2353 | L1P3 | L1 | LINE |

  

|  |  |  |  |  |  |  |  |  |  |
| --- | --- | --- | --- | --- | --- | --- | --- | --- | --- |
| chr9:69165021-69165951 | | | 931 bp | | |  |  |  |  |
| Genome Start | Genome End | Strand | Div | Repeat Start | Repeat End | Repeat Left | Name | Family | Class |
| 69165021 | 69165475 | + | 20.6 | 5023 | 5497 | -687 | L1M4 | L1 | LINE |
| 69165476 | 69165734 | + | 7.8 | 1 | 258 | -53 | AluY | Alu | SINE |
| 69165735 | 69165951 | + | 20.4 | 5498 | 5719 | -465 | L1M4 | L1 | LINE |

  

|  |  |  |  |  |  |  |  |  |  |
| --- | --- | --- | --- | --- | --- | --- | --- | --- | --- |
| chr9:69173249-69180654 | | | 7420 bp | | |  |  |  |  |
| Genome Start | Genome End | Strand | Div | Repeat Start | Repeat End | Repeat Left | Name | Family | Class |
| 69173249 | 69175170 | - | 16.1 | 5804 | 7730 | -7 | HERVIP10F | ERV1 | LTR |
| 69175157 | 69178457 | - | 13.9 | 1778 | 5108 | -2629 | HERVIP10F | ERV1 | LTR |
| 69178458 | 69178755 | + | 12.4 | 1 | 298 | -13 | AluY | Alu | SINE |
| 69178756 | 69180654 | - | 15.6 | 1 | 1941 | -5844 | HERVIP10F | ERV1 | LTR |

  

|  |  |  |  |  |  |  |  |  |  |
| --- | --- | --- | --- | --- | --- | --- | --- | --- | --- |
| chr9:69200664-69203085 | | | 2207 bp | | |  |  |  |  |
| Genome Start | Genome End | Strand | Div | Repeat Start | Repeat End | Repeat Left | Name | Family | Class |
| 69200664 | 69200803 | + | 25.9 | 130 | 268 | -200 | LTR37B | ERV1 | LTR |
| 69201002 | 69202003 | - | 19.5 | 5187 | 6180 | 0 | L1MB4 | L1 | LINE |
| 69202004 | 69202295 | - | 11.3 | 1 | 292 | -18 | AluSg | Alu | SINE |
| 69202296 | 69202577 | - | 22.0 | 4905 | 5186 | -960 | L1MB4 | L1 | LINE |
| 69202595 | 69202903 | - | 7.2 | 1 | 311 | 0 | AluY | Alu | SINE |
| 69202904 | 69203085 | + | 23.6 | 262 | 444 | -24 | LTR37B | ERV1 | LTR |

  

|  |  |  |  |  |  |  |  |  |  |
| --- | --- | --- | --- | --- | --- | --- | --- | --- | --- |
| chr9:69205604-69206132 | | | 529 bp | | |  |  |  |  |
| Genome Start | Genome End | Strand | Div | Repeat Start | Repeat End | Repeat Left | Name | Family | Class |
| 69205604 | 69205739 | + | 14.4 | 1 | 134 | -178 | AluSx | Alu | SINE |
| 69205740 | 69205954 | + | 9.8 | 1 | 215 | -95 | AluSg | Alu | SINE |
| 69205955 | 69206132 | + | 14.4 | 135 | 312 | 0 | AluSx | Alu | SINE |

  

|  |  |  |  |  |  |  |  |  |  |
| --- | --- | --- | --- | --- | --- | --- | --- | --- | --- |
| chr9:69367669-69368234 | | | 566 bp | | |  |  |  |  |
| Genome Start | Genome End | Strand | Div | Repeat Start | Repeat End | Repeat Left | Name | Family | Class |
| 69367669 | 69367873 | + | 36.6 | 1185 | 1408 | -552 | L4 | RTE | LINE |
| 69367874 | 69368070 | - | 10.2 | 106 | 302 | -9 | AluY | Alu | SINE |
| 69368071 | 69368234 | + | 36.6 | 1409 | 1606 | -354 | L4 | RTE | LINE |

  

|  |  |  |  |  |  |  |  |  |  |
| --- | --- | --- | --- | --- | --- | --- | --- | --- | --- |
| chr9:69369339-69370791 | | | 1453 bp | | |  |  |  |  |
| Genome Start | Genome End | Strand | Div | Repeat Start | Repeat End | Repeat Left | Name | Family | Class |
| 69369339 | 69369829 | + | 22.7 | 1 | 618 | -6550 | L1M2a | L1 | LINE |
| 69369830 | 69370261 | - | 23.0 | 1 | 432 | 0 | MSTB1 | MaLR | LTR |
| 69370262 | 69370791 | + | 22.7 | 619 | 1227 | -5941 | L1M2a | L1 | LINE |

  

|  |  |  |  |  |  |  |  |  |  |
| --- | --- | --- | --- | --- | --- | --- | --- | --- | --- |
| chr9:69370834-69372908 | | | 2029 bp | | |  |  |  |  |
| Genome Start | Genome End | Strand | Div | Repeat Start | Repeat End | Repeat Left | Name | Family | Class |
| 69370834 | 69370927 | + | 17.1 | 3577 | 3670 | -2470 | L1M3 | L1 | LINE |
| 69370974 | 69371905 | + | 17.1 | 3671 | 4628 | -1512 | L1M3 | L1 | LINE |
| 69371906 | 69372215 | + | 13.9 | 1 | 310 | 0 | AluSg | Alu | SINE |
| 69372216 | 69372908 | + | 17.2 | 4629 | 5341 | -815 | L1M3 | L1 | LINE |

  

|  |  |  |  |  |  |  |  |  |  |
| --- | --- | --- | --- | --- | --- | --- | --- | --- | --- |
| chr9:69377564-69378937 | | | 1278 bp | | |  |  |  |  |
| Genome Start | Genome End | Strand | Div | Repeat Start | Repeat End | Repeat Left | Name | Family | Class |
| 69377564 | 69377683 | + | 24.1 | 1383 | 1508 | -1911 | L2 | L2 | LINE |
| 69377774 | 69377947 | - | 19.5 | 5 | 183 | -26 | MER3 | MER1\_type | DNA |
| 69377954 | 69378937 | + | 26.8 | 1614 | 2920 | -499 | L2 | L2 | LINE |

  

|  |  |  |  |  |  |  |  |  |  |
| --- | --- | --- | --- | --- | --- | --- | --- | --- | --- |
| chr9:69401554-69402013 | | | 460 bp | | |  |  |  |  |
| Genome Start | Genome End | Strand | Div | Repeat Start | Repeat End | Repeat Left | Name | Family | Class |
| 69401554 | 69401633 | - | 38.3 | 2442 | 2524 | -895 | L2 | L2 | LINE |
| 69401634 | 69401949 | + | 19.7 | 1 | 345 | 0 | MER2 | MER2\_type | DNA |
| 69401950 | 69402013 | - | 38.3 | 2378 | 2441 | -978 | L2 | L2 | LINE |

  

|  |  |  |  |  |  |  |  |  |  |
| --- | --- | --- | --- | --- | --- | --- | --- | --- | --- |
| chr9:69411817-69412696 | | | 880 bp | | |  |  |  |  |
| Genome Start | Genome End | Strand | Div | Repeat Start | Repeat End | Repeat Left | Name | Family | Class |
| 69411817 | 69412201 | + | 22.4 | 5188 | 5602 | -564 | L1M5 | L1 | LINE |
| 69412202 | 69412504 | + | 5.3 | 1 | 304 | -7 | AluY | Alu | SINE |
| 69412505 | 69412696 | + | 22.3 | 5603 | 5796 | -370 | L1M5 | L1 | LINE |

  

|  |  |  |  |  |  |  |  |  |  |
| --- | --- | --- | --- | --- | --- | --- | --- | --- | --- |
| chr9:69414810-69416805 | | | 1962 bp | | |  |  |  |  |
| Genome Start | Genome End | Strand | Div | Repeat Start | Repeat End | Repeat Left | Name | Family | Class |
| 69414810 | 69415563 | - | 17.8 | 5288 | 6022 | -278 | L1MA4 | L1 | LINE |
| 69415567 | 69415670 | - | 19.7 | 196 | 290 | -22 | AluJo/FRAM | Alu | SINE |
| 69415671 | 69415974 | + | 7.3 | 1 | 304 | -7 | AluY | Alu | SINE |
| 69415975 | 69416001 | - | 19.7 | 33 | 59 | -117 | FRAM/FAM | Alu | SINE |
| 69416002 | 69416273 | - | 14.4 | 5046 | 5322 | -821 | L1MA4 | L1 | LINE |
| 69416305 | 69416517 | - | 7.5 | 1 | 201 | -110 | AluY | Alu | SINE |
| 69416518 | 69416805 | - | 14.4 | 4771 | 5045 | -1098 | L1MA4 | L1 | LINE |

  

|  |  |  |  |  |  |  |  |  |  |
| --- | --- | --- | --- | --- | --- | --- | --- | --- | --- |
| chr9:69420836-69421520 | | | 648 bp | | |  |  |  |  |
| Genome Start | Genome End | Strand | Div | Repeat Start | Repeat End | Repeat Left | Name | Family | Class |
| 69420836 | 69421146 | - | 20.0 | 5032 | 5328 | -818 | L1MD | L1 | LINE |
| 69421172 | 69421308 | - | 17.9 | 10 | 151 | -25 | FRAM | Alu | SINE |
| 69421321 | 69421520 | - | 19.9 | 4834 | 5037 | -1109 | L1MD | L1 | LINE |

  

|  |  |  |  |  |  |  |  |  |  |
| --- | --- | --- | --- | --- | --- | --- | --- | --- | --- |
| chr9:69422775-69425001 | | | 2226 bp | | |  |  |  |  |
| Genome Start | Genome End | Strand | Div | Repeat Start | Repeat End | Repeat Left | Name | Family | Class |
| 69422775 | 69424141 | + | 15.0 | 4123 | 5523 | -774 | L1MA5A | L1 | LINE |
| 69424143 | 69424259 | - | 15.8 | 168 | 292 | -20 | AluSg/x | Alu | SINE |
| 69424260 | 69425001 | + | 12.1 | 5512 | 6293 | -4 | L1MA5A | L1 | LINE |

  

|  |  |  |  |  |  |  |  |  |  |
| --- | --- | --- | --- | --- | --- | --- | --- | --- | --- |
| chr9:69428417-69432966 | | | 4529 bp | | |  |  |  |  |
| Genome Start | Genome End | Strand | Div | Repeat Start | Repeat End | Repeat Left | Name | Family | Class |
| 69428417 | 69428483 | - | 9.4 | 490 | 552 | 0 | MLT2A1 | ERVL | LTR |
| 69428484 | 69428848 | + | 9.6 | 1 | 404 | -40 | MLT2A1 | ERVL | LTR |
| 69428888 | 69428950 | + | 17.3 | 493 | 561 | 0 | MLT2A2 | ERVL | LTR |
| 69428951 | 69429333 | - | 10.2 | 1 | 386 | -58 | MLT2A1 | ERVL | LTR |
| 69429334 | 69431776 | - | 11.5 | 3162 | 5654 | 0 | HERVL-A1 | ERVL | LTR |
| 69431770 | 69432267 | - | 10.7 | 1317 | 1814 | -3840 | HERVL-A1 | ERVL | LTR |
| 69432257 | 69432519 | - | 11.8 | 1 | 263 | -5391 | HERVL-A1 | ERVL | LTR |
| 69432520 | 69432966 | - | 10.4 | 1 | 444 | 0 | MLT2A1 | ERVL | LTR |

  

|  |  |  |  |  |  |  |  |  |  |
| --- | --- | --- | --- | --- | --- | --- | --- | --- | --- |
| chr9:69448234-69449701 | | | 1468 bp | | |  |  |  |  |
| Genome Start | Genome End | Strand | Div | Repeat Start | Repeat End | Repeat Left | Name | Family | Class |
| 69448234 | 69449044 | - | 20.5 | 2624 | 3651 | -3688 | L1M4b | L1 | LINE |
| 69449045 | 69449327 | - | 10.2 | 1 | 283 | -28 | AluY | Alu | SINE |
| 69449328 | 69449701 | - | 20.1 | 2146 | 2623 | -4711 | L1M4b | L1 | LINE |

  

|  |  |  |  |  |  |  |  |  |  |
| --- | --- | --- | --- | --- | --- | --- | --- | --- | --- |
| chr9:69450604-69452098 | | | 1135 bp | | |  |  |  |  |
| Genome Start | Genome End | Strand | Div | Repeat Start | Repeat End | Repeat Left | Name | Family | Class |
| 69450604 | 69450901 | + | 26.1 | 54 | 388 | -6035 | L1MCc | L1 | LINE |
| 69450972 | 69451265 | + | 24.0 | 73 | 365 | 0 | MLT1A0 | MaLR | LTR |
| 69451499 | 69451645 | + | 30.3 | 1851 | 1995 | -5133 | L1MCc | L1 | LINE |
| 69451703 | 69452098 | + | 26.7 | 3035 | 3438 | -3503 | L1MCc | L1 | LINE |

  

|  |  |  |  |  |  |  |  |  |  |
| --- | --- | --- | --- | --- | --- | --- | --- | --- | --- |
| chr9:69452902-69453714 | | | 813 bp | | |  |  |  |  |
| Genome Start | Genome End | Strand | Div | Repeat Start | Repeat End | Repeat Left | Name | Family | Class |
| 69452902 | 69453172 | + | 28.5 | 5 | 274 | -231 | MLT1D | MaLR | LTR |
| 69453173 | 69453472 | + | 12.7 | 1 | 302 | -9 | AluY | Alu | SINE |
| 69453473 | 69453714 | + | 28.5 | 275 | 503 | -2 | MLT1D | MaLR | LTR |

  

|  |  |  |  |  |  |  |  |  |  |
| --- | --- | --- | --- | --- | --- | --- | --- | --- | --- |
| chr9:69456188-69457380 | | | 1146 bp | | |  |  |  |  |
| Genome Start | Genome End | Strand | Div | Repeat Start | Repeat End | Repeat Left | Name | Family | Class |
| 69456188 | 69456580 | - | 20.9 | 490 | 901 | 0 | MER54A | ERVL | LTR |
| 69456590 | 69456927 | + | 11.1 | 7 | 364 | 0 | THE1B | MaLR | LTR |
| 69456966 | 69457380 | - | 24.8 | 1 | 430 | -471 | MER54A | ERVL | LTR |

  

|  |  |  |  |  |  |  |  |  |  |
| --- | --- | --- | --- | --- | --- | --- | --- | --- | --- |
| chr9:69568410-69568976 | | | 567 bp | | |  |  |  |  |
| Genome Start | Genome End | Strand | Div | Repeat Start | Repeat End | Repeat Left | Name | Family | Class |
| 69568410 | 69568614 | + | 36.7 | 1185 | 1409 | -551 | L4 | RTE | LINE |
| 69568615 | 69568812 | - | 10.1 | 106 | 303 | -8 | AluY | Alu | SINE |
| 69568813 | 69568976 | + | 36.7 | 1410 | 1607 | -353 | L4 | RTE | LINE |

  

|  |  |  |  |  |  |  |  |  |  |
| --- | --- | --- | --- | --- | --- | --- | --- | --- | --- |
| chr9:69570081-69571533 | | | 1382 bp | | |  |  |  |  |
| Genome Start | Genome End | Strand | Div | Repeat Start | Repeat End | Repeat Left | Name | Family | Class |
| 69570081 | 69570562 | + | 23.4 | 1 | 597 | -6571 | L1M2a | L1 | LINE |
| 69570572 | 69571003 | - | 22.8 | 1 | 432 | 0 | MSTB1 | MaLR | LTR |
| 69571066 | 69571533 | + | 22.9 | 761 | 1227 | -5941 | L1M2a | L1 | LINE |

  

|  |  |  |  |  |  |  |  |  |  |
| --- | --- | --- | --- | --- | --- | --- | --- | --- | --- |
| chr9:69571576-69573656 | | | 2029 bp | | |  |  |  |  |
| Genome Start | Genome End | Strand | Div | Repeat Start | Repeat End | Repeat Left | Name | Family | Class |
| 69571576 | 69571669 | + | 17.1 | 3577 | 3670 | -2470 | L1M3 | L1 | LINE |
| 69571722 | 69572653 | + | 17.1 | 3671 | 4628 | -1512 | L1M3 | L1 | LINE |
| 69572654 | 69572963 | + | 13.9 | 1 | 310 | 0 | AluSg | Alu | SINE |
| 69572964 | 69573656 | + | 17.2 | 4629 | 5341 | -815 | L1M3 | L1 | LINE |

  

|  |  |  |  |  |  |  |  |  |  |
| --- | --- | --- | --- | --- | --- | --- | --- | --- | --- |
| chr9:69578311-69579684 | | | 1278 bp | | |  |  |  |  |
| Genome Start | Genome End | Strand | Div | Repeat Start | Repeat End | Repeat Left | Name | Family | Class |
| 69578311 | 69578430 | + | 24.1 | 1383 | 1508 | -1911 | L2 | L2 | LINE |
| 69578521 | 69578694 | - | 19.5 | 5 | 183 | -26 | MER3 | MER1\_type | DNA |
| 69578701 | 69579684 | + | 27.0 | 1614 | 2920 | -499 | L2 | L2 | LINE |

  

|  |  |  |  |  |  |  |  |  |  |
| --- | --- | --- | --- | --- | --- | --- | --- | --- | --- |
| chr9:69601977-69602512 | | | 536 bp | | |  |  |  |  |
| Genome Start | Genome End | Strand | Div | Repeat Start | Repeat End | Repeat Left | Name | Family | Class |
| 69601977 | 69602056 | - | 39.0 | 2442 | 2524 | -895 | L2 | L2 | LINE |
| 69602057 | 69602372 | + | 20.1 | 1 | 345 | 0 | MER2 | MER2\_type | DNA |
| 69602373 | 69602512 | - | 39.0 | 2301 | 2441 | -978 | L2 | L2 | LINE |

  

|  |  |  |  |  |  |  |  |  |  |
| --- | --- | --- | --- | --- | --- | --- | --- | --- | --- |
| chr9:69612267-69613149 | | | 883 bp | | |  |  |  |  |
| Genome Start | Genome End | Strand | Div | Repeat Start | Repeat End | Repeat Left | Name | Family | Class |
| 69612267 | 69612651 | + | 22.4 | 5188 | 5602 | -564 | L1M5 | L1 | LINE |
| 69612652 | 69612957 | + | 5.9 | 1 | 307 | -4 | AluY | Alu | SINE |
| 69612958 | 69613149 | + | 22.3 | 5603 | 5796 | -370 | L1M5 | L1 | LINE |

  

|  |  |  |  |  |  |  |  |  |  |
| --- | --- | --- | --- | --- | --- | --- | --- | --- | --- |
| chr9:69615267-69617274 | | | 2005 bp | | |  |  |  |  |
| Genome Start | Genome End | Strand | Div | Repeat Start | Repeat End | Repeat Left | Name | Family | Class |
| 69615267 | 69616020 | - | 17.8 | 5288 | 6022 | -278 | L1MA4 | L1 | LINE |
| 69616024 | 69616127 | - | 18.0 | 202 | 290 | -22 | AluJo/FRAM | Alu | SINE |
| 69616128 | 69616440 | + | 6.2 | 1 | 311 | 0 | AluY | Alu | SINE |
| 69616441 | 69616477 | - | 18.0 | 33 | 65 | -111 | FRAM/FAM | Alu | SINE |
| 69616478 | 69616773 | - | 14.8 | 5046 | 5322 | -821 | L1MA4 | L1 | LINE |
| 69616774 | 69616987 | - | 7.4 | 1 | 203 | -108 | AluY | Alu | SINE |
| 69616988 | 69617274 | - | 14.8 | 4771 | 5045 | -1098 | L1MA4 | L1 | LINE |

  

|  |  |  |  |  |  |  |  |  |  |
| --- | --- | --- | --- | --- | --- | --- | --- | --- | --- |
| chr9:69621307-69621991 | | | 657 bp | | |  |  |  |  |
| Genome Start | Genome End | Strand | Div | Repeat Start | Repeat End | Repeat Left | Name | Family | Class |
| 69621307 | 69621617 | - | 20.0 | 5032 | 5328 | -818 | L1MD | L1 | LINE |
| 69621643 | 69621801 | - | 21.4 | 126 | 287 | -25 | AluJo | Alu | SINE |
| 69621805 | 69621991 | - | 20.2 | 4834 | 5024 | -1122 | L1MD | L1 | LINE |

  

|  |  |  |  |  |  |  |  |  |  |
| --- | --- | --- | --- | --- | --- | --- | --- | --- | --- |
| chr9:69623246-69625472 | | | 2226 bp | | |  |  |  |  |
| Genome Start | Genome End | Strand | Div | Repeat Start | Repeat End | Repeat Left | Name | Family | Class |
| 69623246 | 69624612 | + | 15.0 | 4123 | 5523 | -774 | L1MA5A | L1 | LINE |
| 69624614 | 69624730 | - | 15.8 | 168 | 292 | -20 | AluSg/x | Alu | SINE |
| 69624731 | 69625472 | + | 11.9 | 5512 | 6293 | -4 | L1MA5A | L1 | LINE |

  

|  |  |  |  |  |  |  |  |  |  |
| --- | --- | --- | --- | --- | --- | --- | --- | --- | --- |
| chr9:69649162-69650629 | | | 1468 bp | | |  |  |  |  |
| Genome Start | Genome End | Strand | Div | Repeat Start | Repeat End | Repeat Left | Name | Family | Class |
| 69649162 | 69649972 | - | 20.3 | 2624 | 3668 | -3688 | L1M4b | L1 | LINE |
| 69649973 | 69650255 | - | 10.2 | 1 | 283 | -28 | AluY | Alu | SINE |
| 69650256 | 69650629 | - | 19.9 | 2146 | 2623 | -4711 | L1M4b | L1 | LINE |

  

|  |  |  |  |  |  |  |  |  |  |
| --- | --- | --- | --- | --- | --- | --- | --- | --- | --- |
| chr9:69651532-69653026 | | | 1121 bp | | |  |  |  |  |
| Genome Start | Genome End | Strand | Div | Repeat Start | Repeat End | Repeat Left | Name | Family | Class |
| 69651532 | 69651829 | + | 24.7 | 54 | 388 | -6035 | L1MCc | L1 | LINE |
| 69651914 | 69652193 | + | 22.9 | 86 | 365 | 0 | MLT1A0 | MaLR | LTR |
| 69652427 | 69652573 | + | 30.3 | 1851 | 1995 | -5133 | L1MCc | L1 | LINE |
| 69652631 | 69653026 | + | 26.4 | 3035 | 3438 | -3503 | L1MCc | L1 | LINE |

  

|  |  |  |  |  |  |  |  |  |  |
| --- | --- | --- | --- | --- | --- | --- | --- | --- | --- |
| chr9:69653821-69654634 | | | 814 bp | | |  |  |  |  |
| Genome Start | Genome End | Strand | Div | Repeat Start | Repeat End | Repeat Left | Name | Family | Class |
| 69653821 | 69654091 | + | 28.7 | 5 | 274 | -231 | MLT1D | MaLR | LTR |
| 69654092 | 69654392 | + | 12.6 | 1 | 303 | -8 | AluY | Alu | SINE |
| 69654393 | 69654634 | + | 28.7 | 275 | 503 | -2 | MLT1D | MaLR | LTR |

  

|  |  |  |  |  |  |  |  |  |  |
| --- | --- | --- | --- | --- | --- | --- | --- | --- | --- |
| chr9:69657108-69658300 | | | 1146 bp | | |  |  |  |  |
| Genome Start | Genome End | Strand | Div | Repeat Start | Repeat End | Repeat Left | Name | Family | Class |
| 69657108 | 69657500 | - | 21.7 | 490 | 901 | 0 | MER54A | ERVL | LTR |
| 69657510 | 69657847 | + | 11.1 | 7 | 364 | 0 | THE1B | MaLR | LTR |
| 69657886 | 69658300 | - | 25.1 | 1 | 430 | -471 | MER54A | ERVL | LTR |

  

|  |  |  |  |  |  |  |  |  |  |
| --- | --- | --- | --- | --- | --- | --- | --- | --- | --- |
| chr9:69662642-69663333 | | | 692 bp | | |  |  |  |  |
| Genome Start | Genome End | Strand | Div | Repeat Start | Repeat End | Repeat Left | Name | Family | Class |
| 69662642 | 69662676 | + | 17.6 | 1 | 36 | -559 | LTR49 | ERV1 | LTR |
| 69662677 | 69662951 | - | 12.8 | 14 | 289 | -24 | AluSq | Alu | SINE |
| 69662952 | 69663333 | + | 17.6 | 37 | 427 | -168 | LTR49 | ERV1 | LTR |

  

|  |  |  |  |  |  |  |  |  |  |
| --- | --- | --- | --- | --- | --- | --- | --- | --- | --- |
| chr9:69718324-69721723 | | | 3297 bp | | |  |  |  |  |
| Genome Start | Genome End | Strand | Div | Repeat Start | Repeat End | Repeat Left | Name | Family | Class |
| 69718324 | 69718638 | + | 24.2 | 1095 | 1425 | -584 | HAL1b | L1 | LINE |
| 69718648 | 69719200 | + | 9.7 | 398 | 963 | -1455 | Tigger1 | MER2\_type | DNA |
| 69719201 | 69719510 | - | 9.3 | 1 | 310 | 0 | AluSg | Alu | SINE |
| 69719511 | 69720795 | + | 9.7 | 964 | 2309 | -109 | Tigger1 | MER2\_type | DNA |
| 69720796 | 69720959 | - | 14.2 | 1 | 172 | -4 | FRAM | Alu | SINE |
| 69720964 | 69721078 | + | 19.1 | 2304 | 2418 | 0 | Tigger1 | MER2\_type | DNA |
| 69721083 | 69721214 | - | 16.0 | 32 | 166 | -23 | MER5A | MER1\_type | DNA |
| 69721242 | 69721276 | + | 22.7 | 1856 | 1888 | -121 | HAL1b | L1 | LINE |
| 69721336 | 69721610 | - | 12.0 | 1 | 275 | -37 | AluSx | Alu | SINE |
| 69721611 | 69721723 | + | 22.7 | 1889 | 2006 | -3 | HAL1b | L1 | LINE |

  

|  |  |  |  |  |  |  |  |  |  |
| --- | --- | --- | --- | --- | --- | --- | --- | --- | --- |
| chr9:69733835-69735147 | | | 1291 bp | | |  |  |  |  |
| Genome Start | Genome End | Strand | Div | Repeat Start | Repeat End | Repeat Left | Name | Family | Class |
| 69733835 | 69733954 | - | 20.0 | 2294 | 2416 | -4 | MER58 | MER1\_type | DNA |
| 69733976 | 69734245 | + | 21.5 | 1 | 279 | -219 | LTR54B | ERV1 | LTR |
| 69734246 | 69734511 | + | 14.3 | 1 | 267 | -45 | AluSx | Alu | SINE |
| 69734512 | 69734603 | + | 21.5 | 280 | 377 | -121 | LTR54B | ERV1 | LTR |
| 69734604 | 69734731 | + | 18.9 | 6074 | 6185 | -2 | MER57A-int | ERV1 | LTR |
| 69734733 | 69735043 | + | 17.3 | 1 | 403 | 0 | MER57A | ERV1 | LTR |
| 69735044 | 69735147 | - | 22.4 | 38 | 144 | -197 | MER58 | MER1\_type | DNA |

  

|  |  |  |  |  |  |  |  |  |  |
| --- | --- | --- | --- | --- | --- | --- | --- | --- | --- |
| chr9:69805521-69806949 | | | 1429 bp | | |  |  |  |  |
| Genome Start | Genome End | Strand | Div | Repeat Start | Repeat End | Repeat Left | Name | Family | Class |
| 69805521 | 69805786 | - | 22.9 | 7518 | 7808 | 0 | L1MC4a | L1 | LINE |
| 69805787 | 69806090 | + | 8.0 | 4 | 305 | -6 | AluY | Alu | SINE |
| 69806091 | 69806949 | - | 22.9 | 6615 | 7517 | -291 | L1MC4a | L1 | LINE |

  

|  |  |  |  |  |  |  |  |  |  |
| --- | --- | --- | --- | --- | --- | --- | --- | --- | --- |
| chr9:69817122-69817955 | | | 834 bp | | |  |  |  |  |
| Genome Start | Genome End | Strand | Div | Repeat Start | Repeat End | Repeat Left | Name | Family | Class |
| 69817122 | 69817410 | + | 25.1 | 3850 | 4088 | -2058 | L1M5 | L1 | LINE |
| 69817411 | 69817918 | - | 22.1 | 1 | 503 | -2 | MLT1D | MaLR | LTR |
| 69817919 | 69817955 | + | 25.1 | 4089 | 4124 | -2022 | L1M5 | L1 | LINE |

  

|  |  |  |  |  |  |  |  |  |  |
| --- | --- | --- | --- | --- | --- | --- | --- | --- | --- |
| chr9:69850588-69851394 | | | 807 bp | | |  |  |  |  |
| Genome Start | Genome End | Strand | Div | Repeat Start | Repeat End | Repeat Left | Name | Family | Class |
| 69850588 | 69850755 | - | 35.9 | 85 | 251 | -17 | MIRb | MIR | SINE |
| 69850756 | 69851344 | + | 18.8 | 2 | 604 | -1 | MER6A | MER2\_type | DNA |
| 69851345 | 69851394 | - | 35.9 | 38 | 84 | -184 | MIRb | MIR | SINE |

  

|  |  |  |  |  |  |  |  |  |  |
| --- | --- | --- | --- | --- | --- | --- | --- | --- | --- |
| chr9:69873242-69875751 | | | 2508 bp | | |  |  |  |  |
| Genome Start | Genome End | Strand | Div | Repeat Start | Repeat End | Repeat Left | Name | Family | Class |
| 69873242 | 69874664 | + | 27.9 | 3993 | 5457 | -709 | L1ME1 | L1 | LINE |
| 69874665 | 69874974 | + | 17.5 | 5 | 296 | -16 | AluJo | Alu | SINE |
| 69874977 | 69875288 | + | 10.9 | 1 | 313 | 0 | AluSq | Alu | SINE |
| 69875289 | 69875751 | + | 26.4 | 5458 | 5927 | -239 | L1ME1 | L1 | LINE |

  

|  |  |  |  |  |  |  |  |  |  |
| --- | --- | --- | --- | --- | --- | --- | --- | --- | --- |
| chr9:69876930-69877657 | | | 728 bp | | |  |  |  |  |
| Genome Start | Genome End | Strand | Div | Repeat Start | Repeat End | Repeat Left | Name | Family | Class |
| 69876930 | 69877033 | + | 25.1 | 58 | 163 | -342 | MLT1D | MaLR | LTR |
| 69877034 | 69877339 | - | 12.9 | 1 | 304 | -8 | AluSx | Alu | SINE |
| 69877340 | 69877657 | + | 25.1 | 164 | 505 | 0 | MLT1D | MaLR | LTR |

  

|  |  |  |  |  |  |  |  |  |  |
| --- | --- | --- | --- | --- | --- | --- | --- | --- | --- |
| chr9:69881897-69884770 | | | 2823 bp | | |  |  |  |  |
| Genome Start | Genome End | Strand | Div | Repeat Start | Repeat End | Repeat Left | Name | Family | Class |
| 69881897 | 69882583 | - | 32.5 | 5297 | 6020 | -220 | L1ME3B | L1 | LINE |
| 69882580 | 69884164 | - | 31.8 | 3312 | 5026 | -1120 | L1ME3B | L1 | LINE |
| 69884199 | 69884349 | + | 23.8 | 164 | 325 | -16 | MER58B | MER1\_type | DNA |
| 69884371 | 69884770 | - | 29.1 | 2866 | 3319 | -2827 | L1ME3B | L1 | LINE |

  

|  |  |  |  |  |  |  |  |  |  |
| --- | --- | --- | --- | --- | --- | --- | --- | --- | --- |
| chr9:69903087-69904077 | | | 962 bp | | |  |  |  |  |
| Genome Start | Genome End | Strand | Div | Repeat Start | Repeat End | Repeat Left | Name | Family | Class |
| 69903087 | 69903382 | - | 26.4 | 1361 | 1660 | -5141 | MER89-int | ERV1 | LTR |
| 69903412 | 69903743 | - | 22.4 | 1 | 340 | -25 | MLT1A0 | MaLR | LTR |
| 69903744 | 69904077 | - | 28.1 | 1030 | 1357 | -5444 | MER89-int | ERV1 | LTR |

  

|  |  |  |  |  |  |  |  |  |  |
| --- | --- | --- | --- | --- | --- | --- | --- | --- | --- |
| chr9:69914823-69915417 | | | 595 bp | | |  |  |  |  |
| Genome Start | Genome End | Strand | Div | Repeat Start | Repeat End | Repeat Left | Name | Family | Class |
| 69914823 | 69915033 | - | 34.3 | 5640 | 5882 | -1926 | L1MC4a | L1 | LINE |
| 69915034 | 69915320 | + | 13.6 | 21 | 306 | 0 | AluSx | Alu | SINE |
| 69915321 | 69915417 | - | 34.3 | 5542 | 5639 | -2169 | L1MC4a | L1 | LINE |

  

|  |  |  |  |  |  |  |  |  |  |
| --- | --- | --- | --- | --- | --- | --- | --- | --- | --- |
| chr9:69916484-69918847 | | | 2364 bp | | |  |  |  |  |
| Genome Start | Genome End | Strand | Div | Repeat Start | Repeat End | Repeat Left | Name | Family | Class |
| 69916484 | 69916626 | - | 25.7 | 2217 | 2349 | -4504 | L1PA15-16 | L1 | LINE |
| 69916627 | 69916921 | + | 14.9 | 1 | 295 | -17 | AluSx | Alu | SINE |
| 69916922 | 69918847 | - | 25.7 | 5 | 2216 | -4637 | L1PA15-16 | L1 | LINE |

  

|  |  |  |  |  |  |  |  |  |  |
| --- | --- | --- | --- | --- | --- | --- | --- | --- | --- |
| chr9:69920361-69922699 | | | 2311 bp | | |  |  |  |  |
| Genome Start | Genome End | Strand | Div | Repeat Start | Repeat End | Repeat Left | Name | Family | Class |
| 69920361 | 69921230 | - | 18.5 | 725 | 1651 | 0 | MSTA-int | MaLR | LTR |
| 69921233 | 69921726 | + | 8.1 | 1 | 487 | -6 | LTR15 | ERV1 | LTR |
| 69921735 | 69922049 | + | 11.1 | 3 | 318 | 0 | AluY | Alu | SINE |
| 69922068 | 69922699 | - | 23.4 | 3 | 667 | -913 | MSTA-int | MaLR | LTR |

  

|  |  |  |  |  |  |  |  |  |  |
| --- | --- | --- | --- | --- | --- | --- | --- | --- | --- |
| chr9:69961287-69962123 | | | 837 bp | | |  |  |  |  |
| Genome Start | Genome End | Strand | Div | Repeat Start | Repeat End | Repeat Left | Name | Family | Class |
| 69961287 | 69961644 | - | 30.2 | 134 | 511 | 0 | MLT1J | MaLR | LTR |
| 69961645 | 69962014 | - | 13.4 | 1 | 381 | 0 | THE1D | MaLR | LTR |
| 69962015 | 69962123 | - | 30.2 | 26 | 133 | -378 | MLT1J | MaLR | LTR |

  

|  |  |  |  |  |  |  |  |  |  |
| --- | --- | --- | --- | --- | --- | --- | --- | --- | --- |
| chr9:70025949-70026518 | | | 548 bp | | |  |  |  |  |
| Genome Start | Genome End | Strand | Div | Repeat Start | Repeat End | Repeat Left | Name | Family | Class |
| 70025949 | 70026114 | + | 26.1 | 7 | 177 | -91 | MIRb | MIR | SINE |
| 70026137 | 70026418 | - | 7.1 | 1 | 282 | -30 | AluSx | Alu | SINE |
| 70026419 | 70026518 | + | 26.1 | 178 | 262 | -6 | MIRb | MIR | SINE |

  

|  |  |  |  |  |  |  |  |  |  |
| --- | --- | --- | --- | --- | --- | --- | --- | --- | --- |
| chr9:70026873-70031327 | | | 4167 bp | | |  |  |  |  |
| Genome Start | Genome End | Strand | Div | Repeat Start | Repeat End | Repeat Left | Name | Family | Class |
| 70026873 | 70027787 | + | 27.5 | 1 | 962 | -1545 | HAL1 | L1 | LINE |
| 70027788 | 70028478 | + | 10.8 | 5555 | 6251 | -54 | L1MA3 | L1 | LINE |
| 70028479 | 70028774 | + | 8.1 | 2 | 300 | -13 | AluSp | Alu | SINE |
| 70028775 | 70028827 | + | 10.8 | 6252 | 6304 | -1 | L1MA3 | L1 | LINE |
| 70028828 | 70029310 | + | 27.5 | 963 | 1419 | -1088 | HAL1 | L1 | LINE |
| 70029311 | 70029715 | - | 17.4 | 3 | 390 | 0 | MLT1B | MaLR | LTR |
| 70029716 | 70029876 | + | 27.5 | 1420 | 1572 | -935 | HAL1 | L1 | LINE |
| 70029877 | 70030008 | + | 9.8 | 1 | 133 | -177 | AluSg | Alu | SINE |
| 70030009 | 70030318 | + | 12.3 | 1 | 310 | 0 | AluSg | Alu | SINE |
| 70030599 | 70030700 | + | 27.8 | 324 | 426 | -6 | MLT1I | MaLR | LTR |
| 70030709 | 70031327 | + | 25.9 | 1597 | 2221 | -286 | HAL1 | L1 | LINE |

  

|  |  |  |  |  |  |  |  |  |  |
| --- | --- | --- | --- | --- | --- | --- | --- | --- | --- |
| chr9:70041511-70042822 | | | 1290 bp | | |  |  |  |  |
| Genome Start | Genome End | Strand | Div | Repeat Start | Repeat End | Repeat Left | Name | Family | Class |
| 70041511 | 70041614 | + | 22.4 | 38 | 144 | -197 | MER58 | MER1\_type | DNA |
| 70041615 | 70041925 | - | 17.3 | 1 | 403 | 0 | MER57A | ERV1 | LTR |
| 70041927 | 70042054 | - | 19.8 | 6074 | 6185 | -2 | MER57A-int | ERV1 | LTR |
| 70042055 | 70042145 | - | 21.2 | 280 | 377 | -121 | LTR54B | ERV1 | LTR |
| 70042146 | 70042411 | - | 13.9 | 1 | 267 | -45 | AluSx | Alu | SINE |
| 70042412 | 70042681 | - | 21.2 | 1 | 279 | -219 | LTR54B | ERV1 | LTR |
| 70042703 | 70042822 | + | 20.0 | 2294 | 2416 | -4 | MER58 | MER1\_type | DNA |

  

|  |  |  |  |  |  |  |  |  |  |
| --- | --- | --- | --- | --- | --- | --- | --- | --- | --- |
| chr9:70054915-70058333 | | | 3318 bp | | |  |  |  |  |
| Genome Start | Genome End | Strand | Div | Repeat Start | Repeat End | Repeat Left | Name | Family | Class |
| 70054915 | 70055027 | - | 22.7 | 1892 | 2009 | 0 | HAL1b | L1 | LINE |
| 70055028 | 70055302 | + | 13.1 | 1 | 275 | -37 | AluSx | Alu | SINE |
| 70055366 | 70055400 | - | 22.7 | 1859 | 1891 | -118 | HAL1b | L1 | LINE |
| 70055428 | 70055559 | + | 16.0 | 32 | 166 | -23 | MER5A | MER1\_type | DNA |
| 70055564 | 70055678 | - | 18.3 | 2304 | 2418 | 0 | Tigger1 | MER2\_type | DNA |
| 70055683 | 70055839 | + | 15.5 | 1 | 169 | -7 | FRAM | Alu | SINE |
| 70055840 | 70057145 | - | 9.6 | 972 | 2309 | -109 | Tigger1 | MER2\_type | DNA |
| 70057146 | 70057455 | + | 9.6 | 1 | 310 | 0 | AluSg | Alu | SINE |
| 70057456 | 70058014 | - | 9.6 | 388 | 971 | -1447 | Tigger1 | MER2\_type | DNA |
| 70058018 | 70058333 | - | 24.8 | 1095 | 1425 | -584 | HAL1b | L1 | LINE |

  

|  |  |  |  |  |  |  |  |  |  |
| --- | --- | --- | --- | --- | --- | --- | --- | --- | --- |
| chr9:70113790-70114330 | | | 541 bp | | |  |  |  |  |
| Genome Start | Genome End | Strand | Div | Repeat Start | Repeat End | Repeat Left | Name | Family | Class |
| 70113790 | 70113921 | - | 25.1 | 7699 | 7826 | -135 | L1MC5 | L1 | LINE |
| 70113922 | 70114049 | + | 19.5 | 1 | 128 | -5 | FLAM\_C | Alu | SINE |
| 70114050 | 70114330 | - | 25.1 | 7409 | 7698 | -263 | L1MC5 | L1 | LINE |

  

|  |  |  |  |  |  |  |  |  |  |
| --- | --- | --- | --- | --- | --- | --- | --- | --- | --- |
| chr9:70132073-70132862 | | | 790 bp | | |  |  |  |  |
| Genome Start | Genome End | Strand | Div | Repeat Start | Repeat End | Repeat Left | Name | Family | Class |
| 70132073 | 70132112 | - | 25.3 | 454 | 493 | -5907 | L1MEc | L1 | LINE |
| 70132113 | 70132412 | - | 12.7 | 1 | 308 | -1 | AluSc | Alu | SINE |
| 70132413 | 70132862 | - | 25.3 | 38 | 453 | -5947 | L1MEc | L1 | LINE |

  

|  |  |  |  |  |  |  |  |  |  |
| --- | --- | --- | --- | --- | --- | --- | --- | --- | --- |
| chr9:70136600-70137111 | | | 512 bp | | |  |  |  |  |
| Genome Start | Genome End | Strand | Div | Repeat Start | Repeat End | Repeat Left | Name | Family | Class |
| 70136600 | 70136680 | + | 18.6 | 1 | 67 | -152 | MER20 | MER1\_type | DNA |
| 70136681 | 70136950 | - | 13.8 | 5906 | 6176 | -7 | L1MB3 | L1 | LINE |
| 70136951 | 70137111 | + | 18.6 | 68 | 219 | 0 | MER20 | MER1\_type | DNA |

  

|  |  |  |  |  |  |  |  |  |  |
| --- | --- | --- | --- | --- | --- | --- | --- | --- | --- |
| chr9:70149492-70150744 | | | 1253 bp | | |  |  |  |  |
| Genome Start | Genome End | Strand | Div | Repeat Start | Repeat End | Repeat Left | Name | Family | Class |
| 70149492 | 70149573 | - | 16.1 | 4269 | 4313 | -1830 | L1M2 | L1 | LINE |
| 70149574 | 70149797 | - | 11.8 | 5955 | 6166 | 0 | L1PA17 | L1 | LINE |
| 70149798 | 70150744 | - | 16.1 | 3278 | 4268 | -1875 | L1M2 | L1 | LINE |

  

|  |  |  |  |  |  |  |  |  |  |
| --- | --- | --- | --- | --- | --- | --- | --- | --- | --- |
| chr9:70151466-70151971 | | | 506 bp | | |  |  |  |  |
| Genome Start | Genome End | Strand | Div | Repeat Start | Repeat End | Repeat Left | Name | Family | Class |
| 70151466 | 70151560 | + | 30.2 | 60 | 152 | -116 | MIRb | MIR | SINE |
| 70151561 | 70151911 | + | 15.0 | 1 | 366 | 0 | Tigger5a | MER2\_type | DNA |
| 70151912 | 70151971 | + | 30.2 | 153 | 211 | -57 | MIRb | MIR | SINE |

  

|  |  |  |  |  |  |  |  |  |  |
| --- | --- | --- | --- | --- | --- | --- | --- | --- | --- |
| chr9:70166058-70167046 | | | 1120 bp | | |  |  |  |  |
| Genome Start | Genome End | Strand | Div | Repeat Start | Repeat End | Repeat Left | Name | Family | Class |
| 70166058 | 70166180 | - | 22.3 | 7333 | 7463 | -498 | L1MC5 | L1 | LINE |
| 70166182 | 70166540 | - | 19.3 | 1 | 359 | -6 | MLT1A0 | MaLR | LTR |
| 70166541 | 70166612 | - | 26.4 | 7257 | 7331 | -630 | L1MC5 | L1 | LINE |
| 70166611 | 70166899 | - | 26.7 | 7096 | 7396 | -646 | L1MC4 | L1 | LINE |
| 70166770 | 70167046 | - | 28.8 | 6960 | 7228 | -733 | L1MC5 | L1 | LINE |

  

|  |  |  |  |  |  |  |  |  |  |
| --- | --- | --- | --- | --- | --- | --- | --- | --- | --- |
| chr9:70179162-70181003 | | | 1658 bp | | |  |  |  |  |
| Genome Start | Genome End | Strand | Div | Repeat Start | Repeat End | Repeat Left | Name | Family | Class |
| 70179162 | 70179244 | + | 31.3 | 2500 | 2586 | -833 | L2 | L2 | LINE |
| 70179254 | 70179305 | - | 13.5 | 490 | 541 | -1 | MLT1F | MaLR | LTR |
| 70179311 | 70179385 | - | 10.7 | 282 | 357 | -185 | MLT1F | MaLR | LTR |
| 70179474 | 70179628 | - | 23.2 | 3236 | 3419 | 0 | L2 | L2 | LINE |
| 70179634 | 70180082 | - | 23.4 | 1 | 509 | -58 | MLT1F | MaLR | LTR |
| 70180100 | 70180275 | + | 33.0 | 2599 | 2780 | -639 | L2 | L2 | LINE |
| 70180312 | 70180907 | - | 27.5 | 2786 | 3419 | 0 | L2 | L2 | LINE |
| 70180932 | 70181003 | + | 29.6 | 2821 | 2891 | -528 | L2 | L2 | LINE |

  

|  |  |  |  |  |  |  |  |  |  |
| --- | --- | --- | --- | --- | --- | --- | --- | --- | --- |
| chr9:70192587-70194992 | | | 2393 bp | | |  |  |  |  |
| Genome Start | Genome End | Strand | Div | Repeat Start | Repeat End | Repeat Left | Name | Family | Class |
| 70192587 | 70193967 | - | 24.8 | 993 | 2488 | -19 | HAL1 | L1 | LINE |
| 70193978 | 70194671 | - | 18.7 | 5457 | 6161 | -5 | L1ME1 | L1 | LINE |
| 70194675 | 70194992 | - | 24.3 | 638 | 1000 | -1507 | HAL1 | L1 | LINE |

  

|  |  |  |  |  |  |  |  |  |  |
| --- | --- | --- | --- | --- | --- | --- | --- | --- | --- |
| chr9:70206532-70207056 | | | 484 bp | | |  |  |  |  |
| Genome Start | Genome End | Strand | Div | Repeat Start | Repeat End | Repeat Left | Name | Family | Class |
| 70206532 | 70206625 | + | 21.1 | 6000 | 6090 | -76 | L1ME1 | L1 | LINE |
| 70206667 | 70206716 | + | 21.1 | 6091 | 6134 | -32 | L1ME1 | L1 | LINE |
| 70206717 | 70207017 | + | 9.7 | 1 | 300 | -12 | AluSx | Alu | SINE |
| 70207018 | 70207056 | + | 21.1 | 6135 | 6161 | -5 | L1ME1 | L1 | LINE |

  

|  |  |  |  |  |  |  |  |  |  |
| --- | --- | --- | --- | --- | --- | --- | --- | --- | --- |
| chr9:72527975-72528420 | | | 446 bp | | |  |  |  |  |
| Genome Start | Genome End | Strand | Div | Repeat Start | Repeat End | Repeat Left | Name | Family | Class |
| 72527975 | 72528013 | + | 28.3 | 69 | 108 | -154 | MIR | MIR | SINE |
| 72528014 | 72528319 | - | 16.5 | 1 | 303 | -9 | AluJo | Alu | SINE |
| 72528320 | 72528420 | + | 28.3 | 109 | 231 | -31 | MIR | MIR | SINE |

  

|  |  |  |  |  |  |  |  |  |  |
| --- | --- | --- | --- | --- | --- | --- | --- | --- | --- |
| chr9:85645602-85646121 | | | 520 bp | | |  |  |  |  |
| Genome Start | Genome End | Strand | Div | Repeat Start | Repeat End | Repeat Left | Name | Family | Class |
| 85645602 | 85645809 | + | 28.2 | 24 | 250 | -18 | MIRb | MIR | SINE |
| 85645810 | 85646105 | - | 9.2 | 1 | 296 | -17 | AluSq | Alu | SINE |
| 85646106 | 85646121 | + | 29.8 | 251 | 266 | -2 | MIRb | MIR | SINE |

  

|  |  |  |  |  |  |  |  |  |  |
| --- | --- | --- | --- | --- | --- | --- | --- | --- | --- |
| chr9:87571916-87572517 | | | 602 bp | | |  |  |  |  |
| Genome Start | Genome End | Strand | Div | Repeat Start | Repeat End | Repeat Left | Name | Family | Class |
| 87571916 | 87572083 | - | 9.6 | 137 | 304 | -8 | AluSx | Alu | SINE |
| 87572084 | 87572380 | - | 9.1 | 1 | 297 | -12 | AluSc | Alu | SINE |
| 87572381 | 87572517 | - | 9.6 | 1 | 136 | -176 | AluSx | Alu | SINE |

  

|  |  |  |  |  |  |  |  |  |  |
| --- | --- | --- | --- | --- | --- | --- | --- | --- | --- |
| chr9:87589090-87589371 | | | 282 bp | | |  |  |  |  |
| Genome Start | Genome End | Strand | Div | Repeat Start | Repeat End | Repeat Left | Name | Family | Class |
| 87589090 | 87589150 | - | 26.2 | 67 | 137 | -41 | MER5B | MER1\_type | DNA |
| 87589151 | 87589306 | - | 17.3 | 1 | 156 | -10 | FRAM | Alu | SINE |
| 87589307 | 87589371 | - | 26.2 | 1 | 66 | -112 | MER5B | MER1\_type | DNA |

  

|  |  |  |  |  |  |  |  |  |  |
| --- | --- | --- | --- | --- | --- | --- | --- | --- | --- |
| chr9:87624205-87625454 | | | 1228 bp | | |  |  |  |  |
| Genome Start | Genome End | Strand | Div | Repeat Start | Repeat End | Repeat Left | Name | Family | Class |
| 87624205 | 87624454 | - | 20.9 | 5915 | 6174 | -6 | L1MB4 | L1 | LINE |
| 87624455 | 87624734 | + | 12.6 | 1 | 293 | -19 | AluSx | Alu | SINE |
| 87624757 | 87625052 | + | 12.2 | 1 | 299 | -10 | AluSc | Alu | SINE |
| 87625053 | 87625224 | + | 15.1 | 134 | 305 | -7 | AluJb | Alu | SINE |
| 87625225 | 87625454 | - | 17.1 | 5664 | 5920 | -263 | L1MB4 | L1 | LINE |

  

|  |  |  |  |  |  |  |  |  |  |
| --- | --- | --- | --- | --- | --- | --- | --- | --- | --- |
| chr9:87631867-87632661 | | | 795 bp | | |  |  |  |  |
| Genome Start | Genome End | Strand | Div | Repeat Start | Repeat End | Repeat Left | Name | Family | Class |
| 87631867 | 87632079 | - | 17.0 | 2534 | 2756 | -5 | Charlie1 | MER1\_type | DNA |
| 87632080 | 87632370 | - | 8.2 | 1 | 293 | -16 | AluSc | Alu | SINE |
| 87632371 | 87632661 | - | 17.0 | 2231 | 2533 | -228 | Charlie1 | MER1\_type | DNA |

  

|  |  |  |  |  |  |  |  |  |  |
| --- | --- | --- | --- | --- | --- | --- | --- | --- | --- |
| chr9:89731067-89731743 | | | 578 bp | | |  |  |  |  |
| Genome Start | Genome End | Strand | Div | Repeat Start | Repeat End | Repeat Left | Name | Family | Class |
| 89731067 | 89731264 | - | 26.5 | 76 | 287 | -141 | MSTA | MaLR | LTR |
| 89731270 | 89731499 | + | 20.0 | 908 | 1175 | -405 | THE1-int | MaLR | LTR |
| 89731502 | 89731557 | - | 25.0 | 910 | 966 | -614 | THE1-int | MaLR | LTR |
| 89731650 | 89731743 | - | 18.1 | 1 | 97 | -331 | MSTA | MaLR | LTR |

  

|  |  |  |  |  |  |  |  |  |  |
| --- | --- | --- | --- | --- | --- | --- | --- | --- | --- |
| chr9:89734056-89734770 | | | 618 bp | | |  |  |  |  |
| Genome Start | Genome End | Strand | Div | Repeat Start | Repeat End | Repeat Left | Name | Family | Class |
| 89734056 | 89734299 | - | 22.4 | 96 | 351 | -77 | MSTA | MaLR | LTR |
| 89734320 | 89734541 | + | 18.5 | 907 | 1191 | -389 | THE1-int | MaLR | LTR |
| 89734530 | 89734585 | - | 21.4 | 911 | 967 | -613 | THE1-int | MaLR | LTR |
| 89734675 | 89734770 | - | 20.8 | 1 | 97 | -331 | MSTA | MaLR | LTR |

  

|  |  |  |  |  |  |  |  |  |  |
| --- | --- | --- | --- | --- | --- | --- | --- | --- | --- |
| chr9:92012838-92014796 | | | 1926 bp | | |  |  |  |  |
| Genome Start | Genome End | Strand | Div | Repeat Start | Repeat End | Repeat Left | Name | Family | Class |
| 92012838 | 92013829 | + | 23.7 | 4818 | 5824 | -359 | L1MB3 | L1 | LINE |
| 92013830 | 92014108 | + | 11.2 | 1 | 279 | -33 | AluSx | Alu | SINE |
| 92014142 | 92014242 | + | 22.4 | 5825 | 5907 | -276 | L1MB3 | L1 | LINE |
| 92014243 | 92014546 | + | 15.8 | 1 | 304 | -8 | AluSx | Alu | SINE |
| 92014547 | 92014796 | + | 22.4 | 5908 | 6153 | -30 | L1MB3 | L1 | LINE |

  

|  |  |  |  |  |  |  |  |  |  |
| --- | --- | --- | --- | --- | --- | --- | --- | --- | --- |
| chr9:92551266-92551932 | | | 667 bp | | |  |  |  |  |
| Genome Start | Genome End | Strand | Div | Repeat Start | Repeat End | Repeat Left | Name | Family | Class |
| 92551266 | 92551472 | - | 22.0 | 5934 | 6130 | -170 | L1MA4 | L1 | LINE |
| 92551473 | 92551749 | - | 14.4 | 1 | 280 | -32 | AluSx | Alu | SINE |
| 92551750 | 92551932 | - | 22.0 | 5771 | 5933 | -367 | L1MA4 | L1 | LINE |

  

|  |  |  |  |  |  |  |  |  |  |
| --- | --- | --- | --- | --- | --- | --- | --- | --- | --- |
| chr9:93901492-93902152 | | | 661 bp | | |  |  |  |  |
| Genome Start | Genome End | Strand | Div | Repeat Start | Repeat End | Repeat Left | Name | Family | Class |
| 93901492 | 93901583 | + | 16.0 | 1 | 91 | -255 | MER7A | MER2\_type | DNA |
| 93901584 | 93901884 | - | 17.4 | 1 | 299 | -13 | AluJb | Alu | SINE |
| 93901885 | 93902152 | + | 16.0 | 92 | 346 | 0 | MER7A | MER2\_type | DNA |

  

|  |  |  |  |  |  |  |  |  |  |
| --- | --- | --- | --- | --- | --- | --- | --- | --- | --- |
| chr9:93958274-93959047 | | | 739 bp | | |  |  |  |  |
| Genome Start | Genome End | Strand | Div | Repeat Start | Repeat End | Repeat Left | Name | Family | Class |
| 93958274 | 93958572 | - | 15.0 | 157 | 445 | 0 | MER65A | ERV1 | LTR |
| 93958573 | 93958852 | + | 15.4 | 1 | 280 | -32 | AluJo | Alu | SINE |
| 93958888 | 93959047 | - | 15.0 | 1 | 156 | -289 | MER65A | ERV1 | LTR |

  

|  |  |  |  |  |  |  |  |  |  |
| --- | --- | --- | --- | --- | --- | --- | --- | --- | --- |
| chr9:94004580-94005276 | | | 697 bp | | |  |  |  |  |
| Genome Start | Genome End | Strand | Div | Repeat Start | Repeat End | Repeat Left | Name | Family | Class |
| 94004580 | 94004923 | + | 13.0 | 1 | 397 | -31 | MSTA | MaLR | LTR |
| 94004924 | 94005235 | - | 12.5 | 1 | 312 | 0 | AluSx | Alu | SINE |
| 94005236 | 94005276 | + | 13.0 | 398 | 428 | 0 | MSTA | MaLR | LTR |

  

|  |  |  |  |  |  |  |  |  |  |
| --- | --- | --- | --- | --- | --- | --- | --- | --- | --- |
| chr9:94625035-94626308 | | | 1239 bp | | |  |  |  |  |
| Genome Start | Genome End | Strand | Div | Repeat Start | Repeat End | Repeat Left | Name | Family | Class |
| 94625035 | 94625090 | - | 23.1 | 519 | 581 | -2943 | Zaphod2 | Tip100 | DNA |
| 94625091 | 94625524 | - | 17.7 | 1 | 434 | 0 | Tigger2a | MER2\_type | DNA |
| 94625525 | 94625856 | - | 23.1 | 155 | 518 | -3006 | Zaphod2 | Tip100 | DNA |
| 94625892 | 94626188 | + | 14.1 | 1 | 298 | -14 | AluSx | Alu | SINE |
| 94626189 | 94626308 | - | 22.5 | 83 | 193 | -3331 | Zaphod2 | Tip100 | DNA |

  

|  |  |  |  |  |  |  |  |  |  |
| --- | --- | --- | --- | --- | --- | --- | --- | --- | --- |
| chr9:94627227-94628028 | | | 757 bp | | |  |  |  |  |
| Genome Start | Genome End | Strand | Div | Repeat Start | Repeat End | Repeat Left | Name | Family | Class |
| 94627227 | 94627286 | - | 25.0 | 546 | 609 | -6 | MLT1L | MaLR | LTR |
| 94627332 | 94627517 | - | 31.9 | 245 | 448 | -167 | MLT1L | MaLR | LTR |
| 94627518 | 94627812 | - | 13.9 | 1 | 295 | -14 | AluSc | Alu | SINE |
| 94627813 | 94628028 | - | 31.9 | 6 | 244 | -371 | MLT1L | MaLR | LTR |

  

|  |  |  |  |  |  |  |  |  |  |
| --- | --- | --- | --- | --- | --- | --- | --- | --- | --- |
| chr9:96154744-96156608 | | | 1865 bp | | |  |  |  |  |
| Genome Start | Genome End | Strand | Div | Repeat Start | Repeat End | Repeat Left | Name | Family | Class |
| 96154744 | 96155323 | + | 22.3 | 4844 | 5440 | -919 | L1MD2 | L1 | LINE |
| 96155324 | 96155647 | + | 16.6 | 1 | 303 | -9 | AluJo | Alu | SINE |
| 96155648 | 96155873 | + | 22.7 | 5441 | 5654 | -705 | L1MD2 | L1 | LINE |
| 96155874 | 96156181 | + | 6.5 | 1 | 308 | -3 | AluY | Alu | SINE |
| 96156182 | 96156608 | + | 22.7 | 5655 | 6105 | -254 | L1MD2 | L1 | LINE |

  

|  |  |  |  |  |  |  |  |  |  |
| --- | --- | --- | --- | --- | --- | --- | --- | --- | --- |
| chr9:96156964-96157667 | | | 704 bp | | |  |  |  |  |
| Genome Start | Genome End | Strand | Div | Repeat Start | Repeat End | Repeat Left | Name | Family | Class |
| 96156964 | 96157276 | - | 21.7 | 97 | 428 | -95 | Charlie1b | MER1\_type | DNA |
| 96157277 | 96157565 | - | 11.4 | 1 | 302 | -11 | AluSq | Alu | SINE |
| 96157566 | 96157667 | - | 21.7 | 1 | 96 | -427 | Charlie1b | MER1\_type | DNA |

  

|  |  |  |  |  |  |  |  |  |  |
| --- | --- | --- | --- | --- | --- | --- | --- | --- | --- |
| chr9:96167171-96167959 | | | 697 bp | | |  |  |  |  |
| Genome Start | Genome End | Strand | Div | Repeat Start | Repeat End | Repeat Left | Name | Family | Class |
| 96167171 | 96167217 | - | 22.6 | 2815 | 2862 | 0 | Charlie2 | MER1\_type | DNA |
| 96167218 | 96167334 | + | 16.5 | 4 | 117 | -195 | AluJo | Alu | SINE |
| 96167363 | 96167527 | + | 16.5 | 118 | 297 | -15 | AluJo | Alu | SINE |
| 96167528 | 96167628 | - | 22.6 | 2714 | 2814 | -48 | Charlie2 | MER1\_type | DNA |
| 96167693 | 96167959 | - | 24.8 | 4 | 285 | -2576 | Charlie2 | MER1\_type | DNA |

  

|  |  |  |  |  |  |  |  |  |  |
| --- | --- | --- | --- | --- | --- | --- | --- | --- | --- |
| chr9:96203577-96204983 | | | 1297 bp | | |  |  |  |  |
| Genome Start | Genome End | Strand | Div | Repeat Start | Repeat End | Repeat Left | Name | Family | Class |
| 96203577 | 96203647 | - | 18.6 | 5324 | 5396 | -937 | L1M | L1 | LINE |
| 96203676 | 96203819 | - | 17.7 | 361 | 543 | -72 | MLT1L | MaLR | LTR |
| 96203821 | 96204366 | - | 7.8 | 1 | 553 | -1 | MER41A | ERV1 | LTR |
| 96204450 | 96204631 | - | 21.1 | 5986 | 6165 | -19 | L1MB7 | L1 | LINE |
| 96204630 | 96204983 | - | 21.5 | 4978 | 5336 | -810 | L1M | L1 | LINE |

  

|  |  |  |  |  |  |  |  |  |  |
| --- | --- | --- | --- | --- | --- | --- | --- | --- | --- |
| chr9:96236722-96238150 | | | 1392 bp | | |  |  |  |  |
| Genome Start | Genome End | Strand | Div | Repeat Start | Repeat End | Repeat Left | Name | Family | Class |
| 96236722 | 96236876 | + | 23.5 | 771 | 945 | -2474 | L2 | L2 | LINE |
| 96236874 | 96237034 | - | 28.1 | 1 | 169 | -93 | MIR | MIR | SINE |
| 96237045 | 96237310 | + | 29.2 | 963 | 1255 | -2164 | L2 | L2 | LINE |
| 96237315 | 96237722 | + | 25.6 | 1453 | 1915 | -1504 | L2 | L2 | LINE |
| 96237748 | 96238053 | - | 7.2 | 1 | 310 | -3 | AluSq | Alu | SINE |
| 96238055 | 96238150 | + | 29.2 | 2090 | 2187 | -1232 | L2 | L2 | LINE |

  

|  |  |  |  |  |  |  |  |  |  |
| --- | --- | --- | --- | --- | --- | --- | --- | --- | --- |
| chr9:96290040-96291496 | | | 1457 bp | | |  |  |  |  |
| Genome Start | Genome End | Strand | Div | Repeat Start | Repeat End | Repeat Left | Name | Family | Class |
| 96290040 | 96290326 | - | 23.5 | 605 | 916 | -19 | MER21C | ERV1 | LTR |
| 96290327 | 96290619 | - | 18.1 | 6 | 298 | -14 | AluJo | Alu | SINE |
| 96290620 | 96290697 | - | 23.5 | 532 | 604 | -331 | MER21C | ERV1 | LTR |
| 96290698 | 96291005 | + | 16.8 | 5 | 312 | 0 | AluJb | Alu | SINE |
| 96291006 | 96291496 | - | 23.5 | 21 | 531 | -404 | MER21C | ERV1 | LTR |

  

|  |  |  |  |  |  |  |  |  |  |
| --- | --- | --- | --- | --- | --- | --- | --- | --- | --- |
| chr9:98700604-98701554 | | | 951 bp | | |  |  |  |  |
| Genome Start | Genome End | Strand | Div | Repeat Start | Repeat End | Repeat Left | Name | Family | Class |
| 98700604 | 98700647 | - | 16.4 | 6134 | 6176 | -7 | L1MB3 | L1 | LINE |
| 98700648 | 98700976 | - | 9.3 | 1 | 302 | -10 | AluSx | Alu | SINE |
| 98700977 | 98701294 | - | 16.4 | 5812 | 6133 | -50 | L1MB3 | L1 | LINE |
| 98701295 | 98701363 | + | 10.1 | 223 | 291 | -20 | Alu | Alu | SINE |
| 98701364 | 98701554 | - | 15.8 | 5265 | 5473 | -707 | L1MB3 | L1 | LINE |

  

|  |  |  |  |  |  |  |  |  |  |
| --- | --- | --- | --- | --- | --- | --- | --- | --- | --- |
| chr9:98716102-98717171 | | | 1067 bp | | |  |  |  |  |
| Genome Start | Genome End | Strand | Div | Repeat Start | Repeat End | Repeat Left | Name | Family | Class |
| 98716102 | 98716231 | - | 25.6 | 3653 | 3812 | -219 | Zaphod | Tip100 | DNA |
| 98716232 | 98716522 | - | 8.9 | 1 | 291 | -19 | AluSg | Alu | SINE |
| 98716523 | 98716576 | - | 25.6 | 3599 | 3652 | -379 | Zaphod | Tip100 | DNA |
| 98716580 | 98717171 | - | 27.0 | 1 | 637 | -3394 | Zaphod | Tip100 | DNA |

  

|  |  |  |  |  |  |  |  |  |  |
| --- | --- | --- | --- | --- | --- | --- | --- | --- | --- |
| chr9:98777919-98778754 | | | 767 bp | | |  |  |  |  |
| Genome Start | Genome End | Strand | Div | Repeat Start | Repeat End | Repeat Left | Name | Family | Class |
| 98777919 | 98778258 | + | 27.3 | 5258 | 5634 | -2174 | L1MC5 | L1 | LINE |
| 98778325 | 98778620 | + | 10.5 | 1 | 296 | -17 | AluSq | Alu | SINE |
| 98778624 | 98778754 | + | 26.7 | 7130 | 7259 | -783 | L1MC5 | L1 | LINE |

  

|  |  |  |  |  |  |  |  |  |  |
| --- | --- | --- | --- | --- | --- | --- | --- | --- | --- |
| chr9:98811494-98812148 | | | 653 bp | | |  |  |  |  |
| Genome Start | Genome End | Strand | Div | Repeat Start | Repeat End | Repeat Left | Name | Family | Class |
| 98811494 | 98811673 | + | 20.6 | 2 | 181 | -245 | MSTB | MaLR | LTR |
| 98811675 | 98811895 | - | 13.6 | 55 | 303 | -9 | AluSx | Alu | SINE |
| 98811897 | 98812148 | + | 14.4 | 165 | 426 | 0 | MSTB | MaLR | LTR |

  

|  |  |  |  |  |  |  |  |  |  |
| --- | --- | --- | --- | --- | --- | --- | --- | --- | --- |
| chr9:98875487-98877727 | | | 2241 bp | | |  |  |  |  |
| Genome Start | Genome End | Strand | Div | Repeat Start | Repeat End | Repeat Left | Name | Family | Class |
| 98875487 | 98877018 | + | 22.1 | 276 | 1912 | -1078 | MER68-int | ERVL | LTR |
| 98877019 | 98877318 | - | 5.4 | 1 | 300 | -11 | AluY | Alu | SINE |
| 98877319 | 98877727 | + | 22.1 | 1913 | 2326 | -664 | MER68-int | ERVL | LTR |

  

|  |  |  |  |  |  |  |  |  |  |
| --- | --- | --- | --- | --- | --- | --- | --- | --- | --- |
| chr9:98965811-98967511 | | | 1628 bp | | |  |  |  |  |
| Genome Start | Genome End | Strand | Div | Repeat Start | Repeat End | Repeat Left | Name | Family | Class |
| 98965811 | 98966901 | + | 19.4 | 4805 | 5946 | -354 | L1MA5 | L1 | LINE |
| 98966903 | 98967160 | + | 23.5 | 1 | 264 | -48 | AluJo | Alu | SINE |
| 98967233 | 98967511 | + | 16.5 | 6013 | 6294 | -6 | L1MA5 | L1 | LINE |

  

|  |  |  |  |  |  |  |  |  |  |
| --- | --- | --- | --- | --- | --- | --- | --- | --- | --- |
| chr9:98991913-98993182 | | | 1270 bp | | |  |  |  |  |
| Genome Start | Genome End | Strand | Div | Repeat Start | Repeat End | Repeat Left | Name | Family | Class |
| 98991913 | 98992026 | + | 22.7 | 80 | 178 | -3346 | Zaphod2 | Tip100 | DNA |
| 98992027 | 98992323 | - | 12.1 | 1 | 300 | -12 | AluSx | Alu | SINE |
| 98992324 | 98992690 | + | 22.7 | 179 | 517 | -3007 | Zaphod2 | Tip100 | DNA |
| 98992691 | 98993125 | + | 21.2 | 1 | 434 | 0 | Tigger2a | MER2\_type | DNA |
| 98993126 | 98993182 | + | 22.7 | 518 | 581 | -2943 | Zaphod2 | Tip100 | DNA |

  

|  |  |  |  |  |  |  |  |  |  |
| --- | --- | --- | --- | --- | --- | --- | --- | --- | --- |
| chr9:99029465-99030451 | | | 956 bp | | |  |  |  |  |
| Genome Start | Genome End | Strand | Div | Repeat Start | Repeat End | Repeat Left | Name | Family | Class |
| 99029465 | 99029670 | - | 23.1 | 2122 | 2334 | -4066 | L1MEc | L1 | LINE |
| 99029671 | 99029966 | + | 11.2 | 1 | 297 | -15 | AluSx | Alu | SINE |
| 99029967 | 99030001 | - | 23.1 | 2086 | 2121 | -4279 | L1MEc | L1 | LINE |
| 99030033 | 99030337 | - | 6.2 | 1 | 305 | -6 | AluY | Alu | SINE |
| 99030338 | 99030451 | - | 23.1 | 1971 | 2085 | -4315 | L1MEc | L1 | LINE |

  

|  |  |  |  |  |  |  |  |  |  |
| --- | --- | --- | --- | --- | --- | --- | --- | --- | --- |
| chr9:99031336-99031850 | | | 516 bp | | |  |  |  |  |
| Genome Start | Genome End | Strand | Div | Repeat Start | Repeat End | Repeat Left | Name | Family | Class |
| 99031336 | 99031449 | + | 10.5 | 5713 | 5829 | -327 | L1PB4 | L1 | LINE |
| 99031459 | 99031522 | + | 13.8 | 6105 | 6163 | -3 | L1PA17 | L1 | LINE |
| 99031513 | 99031850 | + | 14.8 | 5813 | 6154 | -2 | L1PB4 | L1 | LINE |

  

|  |  |  |  |  |  |  |  |  |  |
| --- | --- | --- | --- | --- | --- | --- | --- | --- | --- |
| chr9:99056893-99057289 | | | 397 bp | | |  |  |  |  |
| Genome Start | Genome End | Strand | Div | Repeat Start | Repeat End | Repeat Left | Name | Family | Class |
| 99056893 | 99056956 | - | 19.6 | 131 | 193 | 0 | MER53 | DNA | DNA |
| 99056957 | 99057252 | - | 12.8 | 1 | 297 | -15 | AluSx | Alu | SINE |
| 99057253 | 99057289 | - | 19.6 | 97 | 130 | -63 | MER53 | DNA | DNA |

  

|  |  |  |  |  |  |  |  |  |  |
| --- | --- | --- | --- | --- | --- | --- | --- | --- | --- |
| chr9:100520328-100520994 | | | 667 bp | | |  |  |  |  |
| Genome Start | Genome End | Strand | Div | Repeat Start | Repeat End | Repeat Left | Name | Family | Class |
| 100520328 | 100520647 | - | 15.5 | 40 | 408 | 0 | MLT1A1 | MaLR | LTR |
| 100520648 | 100520947 | - | 11.7 | 1 | 299 | -13 | AluSx | Alu | SINE |
| 100520948 | 100520994 | - | 15.5 | 1 | 39 | -369 | MLT1A1 | MaLR | LTR |

  

|  |  |  |  |  |  |  |  |  |  |
| --- | --- | --- | --- | --- | --- | --- | --- | --- | --- |
| chr9:131250984-131251445 | | | 353 bp | | |  |  |  |  |
| Genome Start | Genome End | Strand | Div | Repeat Start | Repeat End | Repeat Left | Name | Family | Class |
| 131250984 | 131251139 | - | 33.3 | 102 | 255 | -7 | MIR | MIR | SINE |
| 131251247 | 131251362 | - | 22.4 | 3 | 124 | -200 | MER5C | MER1\_type | DNA |
| 131251365 | 131251445 | - | 24.4 | 13 | 90 | -172 | MIR | MIR | SINE |

  

|  |  |  |  |  |  |  |  |  |  |
| --- | --- | --- | --- | --- | --- | --- | --- | --- | --- |
| chr9:134934864-134935440 | | | 577 bp | | |  |  |  |  |
| Genome Start | Genome End | Strand | Div | Repeat Start | Repeat End | Repeat Left | Name | Family | Class |
| 134934864 | 134934896 | - | 32.7 | 1871 | 1900 | -60 | L4 | RTE | LINE |
| 134934897 | 134935213 | + | 15.7 | 1 | 312 | 0 | AluSx | Alu | SINE |
| 134935214 | 134935440 | - | 32.7 | 1611 | 1870 | -90 | L4 | RTE | LINE |

  

|  |  |  |  |  |  |  |  |  |  |
| --- | --- | --- | --- | --- | --- | --- | --- | --- | --- |
| chr9:134947973-134948472 | | | 500 bp | | |  |  |  |  |
| Genome Start | Genome End | Strand | Div | Repeat Start | Repeat End | Repeat Left | Name | Family | Class |
| 134947973 | 134948077 | - | 20.5 | 86 | 187 | -6 | MER53 | DNA | DNA |
| 134948078 | 134948386 | + | 10.4 | 1 | 309 | -3 | AluSx | Alu | SINE |
| 134948387 | 134948472 | - | 20.5 | 1 | 85 | -108 | MER53 | DNA | DNA |

  

|  |  |  |  |  |  |  |  |  |  |
| --- | --- | --- | --- | --- | --- | --- | --- | --- | --- |
| chr9:137566551-137567485 | | | 935 bp | | |  |  |  |  |
| Genome Start | Genome End | Strand | Div | Repeat Start | Repeat End | Repeat Left | Name | Family | Class |
| 137566551 | 137566870 | - | 19.9 | 307 | 652 | -9 | MER67B | ERV1 | LTR |
| 137566871 | 137567192 | - | 12.2 | 1 | 312 | 0 | AluSx | Alu | SINE |
| 137567193 | 137567485 | - | 19.9 | 1 | 306 | -355 | MER67B | ERV1 | LTR |

  

|  |  |  |  |  |  |  |  |  |  |
| --- | --- | --- | --- | --- | --- | --- | --- | --- | --- |
| chr9:140146751-140148516 | | | 1764 bp | | |  |  |  |  |
| Genome Start | Genome End | Strand | Div | Repeat Start | Repeat End | Repeat Left | Name | Family | Class |
| 140146751 | 140146928 | - | 14.4 | 2108 | 2304 | -116 | Cheshire | MER1\_type | DNA |
| 140146925 | 140147043 | - | 18.7 | 1254 | 1360 | -1060 | Cheshire | MER1\_type | DNA |
| 140147050 | 140147221 | + | 14.0 | 4 | 175 | -137 | AluSx | Alu | SINE |
| 140147222 | 140148199 | + | 12.8 | 1 | 1002 | 0 | LTR5B | ERVK | LTR |
| 140148200 | 140148326 | + | 14.0 | 176 | 296 | -16 | AluSx | Alu | SINE |
| 140148327 | 140148516 | - | 20.5 | 1065 | 1267 | -1153 | Cheshire | MER1\_type | DNA |

  

|  |  |  |  |  |  |  |  |  |  |
| --- | --- | --- | --- | --- | --- | --- | --- | --- | --- |
| chr9:140163180-140163811 | | | 632 bp | | |  |  |  |  |
| Genome Start | Genome End | Strand | Div | Repeat Start | Repeat End | Repeat Left | Name | Family | Class |
| 140163180 | 140163367 | - | 20.0 | 124 | 303 | -9 | AluJo | Alu | SINE |
| 140163368 | 140163686 | + | 10.3 | 1 | 282 | -31 | AluSq | Alu | SINE |
| 140163687 | 140163811 | - | 20.0 | 1 | 123 | -189 | AluJo | Alu | SINE |

  

|  |  |  |  |  |  |  |  |  |  |
| --- | --- | --- | --- | --- | --- | --- | --- | --- | --- |
| chr9:140174288-140184649 | | | 10353 bp | | |  |  |  |  |
| Genome Start | Genome End | Strand | Div | Repeat Start | Repeat End | Repeat Left | Name | Family | Class |
| 140174288 | 140175187 | + | 16.0 | 1591 | 2560 | -5005 | L1MA2 | L1 | LINE |
| 140175188 | 140175484 | + | 6.5 | 1 | 296 | -15 | AluY | Alu | SINE |
| 140175485 | 140177310 | + | 15.5 | 2561 | 4427 | -3138 | L1MA2 | L1 | LINE |
| 140177313 | 140178304 | - | 1.8 | 5164 | 6155 | 0 | L1PA3 | L1 | LINE |
| 140178302 | 140179063 | + | 16.4 | 4420 | 5299 | -2266 | L1MA2 | L1 | LINE |
| 140179064 | 140179364 | + | 12.6 | 1 | 301 | -11 | AluSx | Alu | SINE |
| 140179365 | 140179724 | + | 16.4 | 5300 | 5669 | -1896 | L1MA2 | L1 | LINE |
| 140179725 | 140180138 | + | 19.6 | 1 | 428 | 0 | MSTA | MaLR | LTR |
| 140180141 | 140181770 | + | 31.6 | 2 | 1735 | 0 | MLT-int | MaLR | LTR |
| 140181771 | 140182177 | + | 21.5 | 1 | 428 | 0 | MSTA | MaLR | LTR |
| 140182178 | 140183164 | + | 16.4 | 5670 | 6679 | -886 | L1MA2 | L1 | LINE |
| 140183169 | 140183496 | + | 15.3 | 1 | 335 | -40 | THE1C | MaLR | LTR |
| 140183497 | 140183776 | + | 12.9 | 2 | 281 | -28 | AluSg1 | Alu | SINE |
| 140183777 | 140183844 | + | 12.1 | 343 | 381 | 0 | THE1C | MaLR | LTR |
| 140183849 | 140184068 | + | 14.6 | 6684 | 6915 | -809 | L1MA2 | L1 | LINE |
| 140184069 | 140184649 | + | 11.9 | 7130 | 7721 | -2 | L1MA2 | L1 | LINE |

  

|  |  |  |  |  |  |  |  |  |  |
| --- | --- | --- | --- | --- | --- | --- | --- | --- | --- |
| chr9:140186658-140189182 | | | 2525 bp | | |  |  |  |  |
| Genome Start | Genome End | Strand | Div | Repeat Start | Repeat End | Repeat Left | Name | Family | Class |
| 140186658 | 140186940 | + | 28.4 | 4242 | 4517 | -3239 | MER31-int | ERV1 | LTR |
| 140186941 | 140187148 | - | 11.6 | 165 | 364 | 0 | THE1B | MaLR | LTR |
| 140187149 | 140187428 | + | 12.9 | 23 | 303 | -9 | AluSx | Alu | SINE |
| 140187429 | 140187591 | - | 11.6 | 1 | 164 | -200 | THE1B | MaLR | LTR |
| 140187592 | 140189182 | + | 28.4 | 4518 | 6163 | -1593 | MER31-int | ERV1 | LTR |

  

|  |  |  |  |  |  |  |  |  |  |
| --- | --- | --- | --- | --- | --- | --- | --- | --- | --- |
| chr9:140203286-140205923 | | | 2638 bp | | |  |  |  |  |
| Genome Start | Genome End | Strand | Div | Repeat Start | Repeat End | Repeat Left | Name | Family | Class |
| 140203286 | 140203545 | - | 5.7 | 5895 | 6149 | -5 | L1PA7 | L1 | LINE |
| 140203546 | 140203859 | - | 13.1 | 1 | 313 | 0 | AluSp | Alu | SINE |
| 140203860 | 140205923 | - | 7.3 | 3784 | 5894 | -260 | L1PA7 | L1 | LINE |

  

|  |  |  |  |  |  |  |  |  |  |
| --- | --- | --- | --- | --- | --- | --- | --- | --- | --- |
| chr9:140232575-140233496 | | | 925 bp | | |  |  |  |  |
| Genome Start | Genome End | Strand | Div | Repeat Start | Repeat End | Repeat Left | Name | Family | Class |
| 140232575 | 140232798 | - | 19.3 | 2799 | 3028 | -1 | Tigger3(Golem) | MER2\_type | DNA |
| 140232799 | 140233379 | - | 7.9 | 3 | 600 | 0 | MER4A1\_ | ERV1 | LTR |
| 140233380 | 140233403 | - | 19.3 | 2772 | 2798 | -231 | Tigger3(Golem) | MER2\_type | DNA |
| 140233401 | 140233496 | - | 15.9 | 40 | 142 | -2887 | Tigger3(Golem) | MER2\_type | DNA |

  

|  |  |  |  |  |  |  |  |  |  |
| --- | --- | --- | --- | --- | --- | --- | --- | --- | --- |
| chr9:140234654-140235259 | | | 569 bp | | |  |  |  |  |
| Genome Start | Genome End | Strand | Div | Repeat Start | Repeat End | Repeat Left | Name | Family | Class |
| 140234654 | 140234783 | - | 15.4 | 6106 | 7537 | 0 | MER57A-int | ERV1 | LTR |
| 140234820 | 140235136 | - | 21.6 | 15 | 392 | -106 | LTR54B | ERV1 | LTR |
| 140235138 | 140235259 | - | 24.0 | 5686 | 5806 | -381 | MER57A-int | ERV1 | LTR |

  

|  |  |  |  |  |  |  |  |  |  |
| --- | --- | --- | --- | --- | --- | --- | --- | --- | --- |
| chr9:140238364-140239359 | | | 993 bp | | |  |  |  |  |
| Genome Start | Genome End | Strand | Div | Repeat Start | Repeat End | Repeat Left | Name | Family | Class |
| 140238364 | 140238740 | + | 17.9 | 8 | 417 | -518 | MER21C | ERV1 | LTR |
| 140238744 | 140239013 | - | 23.3 | 1 | 282 | -83 | MLT1A0 | MaLR | LTR |
| 140239014 | 140239359 | + | 20.9 | 582 | 933 | -2 | MER21C | ERV1 | LTR |

  

|  |  |  |  |  |  |  |  |  |  |
| --- | --- | --- | --- | --- | --- | --- | --- | --- | --- |
| chr9:140244304-140246867 | | | 2491 bp | | |  |  |  |  |
| Genome Start | Genome End | Strand | Div | Repeat Start | Repeat End | Repeat Left | Name | Family | Class |
| 140244304 | 140244734 | - | 19.2 | 5202 | 5689 | -485 | L1M4 | L1 | LINE |
| 140244754 | 140245350 | + | 27.1 | 19 | 645 | -218 | MER21B | ERV1 | LTR |
| 140245351 | 140245856 | + | 20.8 | 1 | 586 | -25 | LTR36 | ERV1 | LTR |
| 140245875 | 140246036 | + | 22.2 | 648 | 826 | -37 | MER21B | ERV1 | LTR |
| 140246073 | 140246867 | - | 26.4 | 4315 | 5205 | -941 | L1M4 | L1 | LINE |

  

|  |  |  |  |  |  |  |  |  |  |
| --- | --- | --- | --- | --- | --- | --- | --- | --- | --- |
| chr9:140252831-140253371 | | | 541 bp | | |  |  |  |  |
| Genome Start | Genome End | Strand | Div | Repeat Start | Repeat End | Repeat Left | Name | Family | Class |
| 140252831 | 140252931 | - | 13.7 | 137 | 301 | -1 | AluSx | Alu | SINE |
| 140252932 | 140253235 | - | 10.5 | 1 | 305 | -8 | AluSp | Alu | SINE |
| 140253236 | 140253371 | - | 13.7 | 1 | 136 | -166 | AluSx | Alu | SINE |

  

|  |  |  |  |  |  |  |  |  |  |
| --- | --- | --- | --- | --- | --- | --- | --- | --- | --- |
| chr9:140270012-140270357 | | | 346 bp | | |  |  |  |  |
| Genome Start | Genome End | Strand | Div | Repeat Start | Repeat End | Repeat Left | Name | Family | Class |
| 140270012 | 140270134 | - | 31.0 | 5576 | 5690 | -2118 | L1MC4 | L1 | LINE |
| 140270135 | 140270257 | + | 16.5 | 4 | 126 | -7 | FLAM\_C | Alu | SINE |
| 140270258 | 140270357 | - | 31.0 | 5483 | 5575 | -2233 | L1MC4 | L1 | LINE |

  


|  |  |  |  |  |  |  |  |  |  |
| --- | --- | --- | --- | --- | --- | --- | --- | --- | --- |
| chr10:50964-51712 | | | 749 bp | | |  |  |  |  |
| Genome Start | Genome End | Strand | Div | Repeat Start | Repeat End | Repeat Left | Name | Family | Class |
| 50964 | 51180 | - | 22.2 | 3779 | 4016 | -2130 | L1M4 | L1 | LINE |
| 51181 | 51345 | - | 22.4 | 1 | 165 | -1 | FRAM | Alu | SINE |
| 51346 | 51712 | - | 22.2 | 3398 | 3778 | -2368 | L1M4 | L1 | LINE |

  

|  |  |  |  |  |  |  |  |  |  |
| --- | --- | --- | --- | --- | --- | --- | --- | --- | --- |
| chr10:53293-59192 | | | 5900 bp | | |  |  |  |  |
| Genome Start | Genome End | Strand | Div | Repeat Start | Repeat End | Repeat Left | Name | Family | Class |
| 53293 | 57260 | + | 11.8 | 934 | 4882 | -1750 | L1PA17 | L1 | LINE |
| 57261 | 57438 | + | 13.5 | 136 | 313 | 0 | AluSp | Alu | SINE |
| 57439 | 59192 | + | 11.8 | 4883 | 6628 | -4 | L1PA17 | L1 | LINE |

  

|  |  |  |  |  |  |  |  |  |  |
| --- | --- | --- | --- | --- | --- | --- | --- | --- | --- |
| chr10:65252-67890 | | | 2639 bp | | |  |  |  |  |
| Genome Start | Genome End | Strand | Div | Repeat Start | Repeat End | Repeat Left | Name | Family | Class |
| 65252 | 67315 | + | 8.1 | 3784 | 5894 | -260 | L1PA7 | L1 | LINE |
| 67316 | 67627 | + | 14.2 | 1 | 311 | -2 | AluSp | Alu | SINE |
| 67628 | 67890 | + | 6.4 | 5895 | 6149 | -5 | L1PA7 | L1 | LINE |

  

|  |  |  |  |  |  |  |  |  |  |
| --- | --- | --- | --- | --- | --- | --- | --- | --- | --- |
| chr10:85311-87817 | | | 2507 bp | | |  |  |  |  |
| Genome Start | Genome End | Strand | Div | Repeat Start | Repeat End | Repeat Left | Name | Family | Class |
| 85311 | 86908 | - | 28.6 | 4518 | 6163 | -1593 | MER31-int | ERV1 | LTR |
| 86909 | 87071 | + | 12.4 | 1 | 164 | -200 | THE1B | MaLR | LTR |
| 87072 | 87325 | - | 13.0 | 23 | 303 | -9 | AluSx | Alu | SINE |
| 87326 | 87534 | + | 12.4 | 165 | 364 | 0 | THE1B | MaLR | LTR |
| 87535 | 87817 | - | 28.6 | 4243 | 4517 | -3239 | MER31-int | ERV1 | LTR |

  

|  |  |  |  |  |  |  |  |  |  |
| --- | --- | --- | --- | --- | --- | --- | --- | --- | --- |
| chr10:101316-102306 | | | 991 bp | | |  |  |  |  |
| Genome Start | Genome End | Strand | Div | Repeat Start | Repeat End | Repeat Left | Name | Family | Class |
| 101316 | 101382 | + | 28.2 | 3676 | 3728 | -2418 | L1M4 | L1 | LINE |
| 101383 | 101679 | + | 10.1 | 1 | 297 | -16 | AluSq | Alu | SINE |
| 101680 | 102306 | + | 28.2 | 3729 | 4383 | -1763 | L1M4 | L1 | LINE |

  

|  |  |  |  |  |  |  |  |  |  |
| --- | --- | --- | --- | --- | --- | --- | --- | --- | --- |
| chr10:102931-103722 | | | 792 bp | | |  |  |  |  |
| Genome Start | Genome End | Strand | Div | Repeat Start | Repeat End | Repeat Left | Name | Family | Class |
| 102931 | 103355 | - | 23.2 | 4445 | 4854 | -1292 | L1M4 | L1 | LINE |
| 103356 | 103664 | - | 14.9 | 1 | 308 | -3 | AluY | Alu | SINE |
| 103665 | 103722 | - | 23.2 | 4396 | 4444 | -1702 | L1M4 | L1 | LINE |

  

|  |  |  |  |  |  |  |  |  |  |
| --- | --- | --- | --- | --- | --- | --- | --- | --- | --- |
| chr10:113061-114260 | | | 1182 bp | | |  |  |  |  |
| Genome Start | Genome End | Strand | Div | Repeat Start | Repeat End | Repeat Left | Name | Family | Class |
| 113061 | 113084 | + | 26.3 | 4305 | 4326 | -1820 | L1MC | L1 | LINE |
| 113085 | 113447 | - | 19.6 | 1 | 302 | 0 | AluJb | Alu | SINE |
| 113448 | 113645 | + | 26.3 | 4327 | 4522 | -1624 | L1MC | L1 | LINE |
| 113664 | 114260 | + | 20.9 | 4730 | 5373 | -773 | L1MC | L1 | LINE |

  

|  |  |  |  |  |  |  |  |  |  |
| --- | --- | --- | --- | --- | --- | --- | --- | --- | --- |
| chr10:114564-115372 | | | 576 bp | | |  |  |  |  |
| Genome Start | Genome End | Strand | Div | Repeat Start | Repeat End | Repeat Left | Name | Family | Class |
| 114564 | 114648 | + | 24.4 | 278 | 371 | -134 | MLT1C | MaLR | LTR |
| 114649 | 114761 | + | 28.6 | 1 | 118 | -500 | LTR35 | ERV1 | LTR |
| 114888 | 115029 | + | 28.1 | 184 | 322 | -178 | LTR72B | ERV1 | LTR |
| 115137 | 115254 | + | 24.6 | 495 | 618 | 0 | LTR35 | ERV1 | LTR |
| 115255 | 115372 | + | 17.4 | 351 | 467 | 0 | MLT1C | MaLR | LTR |

  

|  |  |  |  |  |  |  |  |  |  |
| --- | --- | --- | --- | --- | --- | --- | --- | --- | --- |
| chr10:5285695-5286454 | | | 760 bp | | |  |  |  |  |
| Genome Start | Genome End | Strand | Div | Repeat Start | Repeat End | Repeat Left | Name | Family | Class |
| 5285695 | 5285822 | + | 19.4 | 12 | 139 | -226 | MLT1A0 | MaLR | LTR |
| 5285823 | 5286231 | - | 15.5 | 1 | 430 | -2 | MSTB1 | MaLR | LTR |
| 5286232 | 5286454 | + | 19.4 | 140 | 360 | -5 | MLT1A0 | MaLR | LTR |

  

|  |  |  |  |  |  |  |  |  |  |
| --- | --- | --- | --- | --- | --- | --- | --- | --- | --- |
| chr10:13511516-13512583 | | | 1068 bp | | |  |  |  |  |
| Genome Start | Genome End | Strand | Div | Repeat Start | Repeat End | Repeat Left | Name | Family | Class |
| 13511516 | 13511731 | + | 16.3 | 5384 | 5603 | -581 | L1MB7 | L1 | LINE |
| 13511732 | 13512015 | + | 14.8 | 1 | 298 | -14 | AluJo | Alu | SINE |
| 13512016 | 13512583 | + | 16.3 | 5604 | 6169 | -15 | L1MB7 | L1 | LINE |

  

|  |  |  |  |  |  |  |  |  |  |
| --- | --- | --- | --- | --- | --- | --- | --- | --- | --- |
| chr10:15085395-15087362 | | | 1968 bp | | |  |  |  |  |
| Genome Start | Genome End | Strand | Div | Repeat Start | Repeat End | Repeat Left | Name | Family | Class |
| 15085395 | 15085641 | - | 23.5 | 5907 | 6151 | -32 | L1MB3 | L1 | LINE |
| 15085642 | 15085931 | - | 13.4 | 12 | 304 | -8 | AluSx | Alu | SINE |
| 15085932 | 15086061 | - | 23.5 | 5819 | 5906 | -277 | L1MB3 | L1 | LINE |
| 15086062 | 15086367 | - | 12.5 | 6 | 310 | -2 | AluSx | Alu | SINE |
| 15086368 | 15087362 | - | 24.8 | 4818 | 5818 | -365 | L1MB3 | L1 | LINE |

  

|  |  |  |  |  |  |  |  |  |  |
| --- | --- | --- | --- | --- | --- | --- | --- | --- | --- |
| chr10:15087779-15088461 | | | 683 bp | | |  |  |  |  |
| Genome Start | Genome End | Strand | Div | Repeat Start | Repeat End | Repeat Left | Name | Family | Class |
| 15087779 | 15088002 | - | 23.4 | 5941 | 6147 | -153 | L1MA4 | L1 | LINE |
| 15088003 | 15088282 | - | 16.1 | 1 | 280 | -32 | AluSx | Alu | SINE |
| 15088283 | 15088461 | - | 23.4 | 5771 | 5940 | -360 | L1MA4 | L1 | LINE |

  

|  |  |  |  |  |  |  |  |  |  |
| --- | --- | --- | --- | --- | --- | --- | --- | --- | --- |
| chr10:15102934-15103606 | | | 673 bp | | |  |  |  |  |
| Genome Start | Genome End | Strand | Div | Repeat Start | Repeat End | Repeat Left | Name | Family | Class |
| 15102934 | 15103155 | + | 22.8 | 1 | 224 | -141 | MLT1A0 | MaLR | LTR |
| 15103156 | 15103458 | + | 11.2 | 1 | 296 | -16 | AluSx | Alu | SINE |
| 15103459 | 15103606 | + | 22.8 | 225 | 365 | 0 | MLT1A0 | MaLR | LTR |

  

|  |  |  |  |  |  |  |  |  |  |
| --- | --- | --- | --- | --- | --- | --- | --- | --- | --- |
| chr10:16923303-16924412 | | | 1062 bp | | |  |  |  |  |
| Genome Start | Genome End | Strand | Div | Repeat Start | Repeat End | Repeat Left | Name | Family | Class |
| 16923303 | 16923765 | + | 28.0 | 2646 | 3138 | -281 | L2 | L2 | LINE |
| 16923772 | 16923935 | + | 12.8 | 137 | 302 | -10 | AluSg/x | Alu | SINE |
| 16923972 | 16924052 | + | 22.8 | 3187 | 3268 | -151 | L2 | L2 | LINE |
| 16924055 | 16924262 | - | 16.4 | 87 | 295 | -17 | AluJo | Alu | SINE |
| 16924267 | 16924412 | + | 29.8 | 3274 | 3418 | -1 | L2 | L2 | LINE |

  

|  |  |  |  |  |  |  |  |  |  |
| --- | --- | --- | --- | --- | --- | --- | --- | --- | --- |
| chr10:17846516-17847298 | | | 783 bp | | |  |  |  |  |
| Genome Start | Genome End | Strand | Div | Repeat Start | Repeat End | Repeat Left | Name | Family | Class |
| 17846516 | 17846835 | - | 18.4 | 188 | 467 | 0 | MLT1C | MaLR | LTR |
| 17846836 | 17847124 | - | 12.5 | 1 | 299 | -10 | AluSc | Alu | SINE |
| 17847125 | 17847298 | - | 18.4 | 4 | 187 | -280 | MLT1C | MaLR | LTR |

  

|  |  |  |  |  |  |  |  |  |  |
| --- | --- | --- | --- | --- | --- | --- | --- | --- | --- |
| chr10:17856813-17857249 | | | 437 bp | | |  |  |  |  |
| Genome Start | Genome End | Strand | Div | Repeat Start | Repeat End | Repeat Left | Name | Family | Class |
| 17856813 | 17856831 | - | 30.7 | 4174 | 4192 | -1954 | L1PREC2 | L1 | LINE |
| 17856832 | 17857105 | - | 13.7 | 1 | 273 | -39 | AluSx | Alu | SINE |
| 17857106 | 17857249 | - | 30.7 | 4027 | 4173 | -1973 | L1PREC2 | L1 | LINE |

  

|  |  |  |  |  |  |  |  |  |  |
| --- | --- | --- | --- | --- | --- | --- | --- | --- | --- |
| chr10:17887573-17889078 | | | 1505 bp | | |  |  |  |  |
| Genome Start | Genome End | Strand | Div | Repeat Start | Repeat End | Repeat Left | Name | Family | Class |
| 17887573 | 17887797 | - | 27.9 | 113 | 362 | -6022 | L1M4c | L1 | LINE |
| 17887798 | 17887995 | + | 17.4 | 1 | 198 | -168 | Tigger5a | MER2\_type | DNA |
| 17887996 | 17888805 | - | 18.2 | 1 | 785 | 0 | LTR1 | ERV1 | LTR |
| 17888806 | 17888976 | + | 17.4 | 199 | 366 | 0 | Tigger5a | MER2\_type | DNA |
| 17888978 | 17889078 | - | 32.0 | 14 | 113 | -6271 | L1M4c | L1 | LINE |

  

|  |  |  |  |  |  |  |  |  |  |
| --- | --- | --- | --- | --- | --- | --- | --- | --- | --- |
| chr10:17892775-17893314 | | | 540 bp | | |  |  |  |  |
| Genome Start | Genome End | Strand | Div | Repeat Start | Repeat End | Repeat Left | Name | Family | Class |
| 17892775 | 17892799 | - | 23.2 | 228 | 252 | -72 | MER33 | MER1\_type | DNA |
| 17892800 | 17893097 | - | 13.1 | 1 | 298 | -14 | AluSx | Alu | SINE |
| 17893098 | 17893314 | - | 23.2 | 1 | 227 | -97 | MER33 | MER1\_type | DNA |

  

|  |  |  |  |  |  |  |  |  |  |
| --- | --- | --- | --- | --- | --- | --- | --- | --- | --- |
| chr10:17940154-17942619 | | | 1935 bp | | |  |  |  |  |
| Genome Start | Genome End | Strand | Div | Repeat Start | Repeat End | Repeat Left | Name | Family | Class |
| 17940154 | 17940403 | - | 25.4 | 1345 | 1582 | -4567 | L1MEe | L1 | LINE |
| 17940510 | 17940800 | - | 14.1 | 2 | 292 | -18 | AluSg | Alu | SINE |
| 17940856 | 17941143 | - | 31.6 | 919 | 1191 | -4928 | L1MEe | L1 | LINE |
| 17941347 | 17941633 | - | 17.1 | 1 | 287 | -25 | AluSx | Alu | SINE |
| 17941801 | 17942284 | - | 31.7 | 73 | 558 | -5561 | L1MEe | L1 | LINE |
| 17942285 | 17942593 | - | 16.6 | 1 | 303 | -9 | AluJb | Alu | SINE |
| 17942594 | 17942619 | - | 31.7 | 48 | 72 | -6047 | L1MEe | L1 | LINE |

  

|  |  |  |  |  |  |  |  |  |  |
| --- | --- | --- | --- | --- | --- | --- | --- | --- | --- |
| chr10:17946460-17946985 | | | 526 bp | | |  |  |  |  |
| Genome Start | Genome End | Strand | Div | Repeat Start | Repeat End | Repeat Left | Name | Family | Class |
| 17946460 | 17946562 | - | 30.8 | 136 | 237 | -31 | MIRb | MIR | SINE |
| 17946563 | 17946864 | + | 11.6 | 1 | 304 | -9 | AluSq | Alu | SINE |
| 17946865 | 17946985 | - | 30.8 | 2 | 135 | -133 | MIRb | MIR | SINE |

  

|  |  |  |  |  |  |  |  |  |  |
| --- | --- | --- | --- | --- | --- | --- | --- | --- | --- |
| chr10:18009692-18010721 | | | 1030 bp | | |  |  |  |  |
| Genome Start | Genome End | Strand | Div | Repeat Start | Repeat End | Repeat Left | Name | Family | Class |
| 18009692 | 18010032 | - | 17.4 | 5828 | 6177 | -6 | L1MB3 | L1 | LINE |
| 18010033 | 18010329 | - | 10.3 | 1 | 313 | 0 | AluSp | Alu | SINE |
| 18010330 | 18010721 | - | 17.4 | 5446 | 5827 | -356 | L1MB3 | L1 | LINE |

  

|  |  |  |  |  |  |  |  |  |  |
| --- | --- | --- | --- | --- | --- | --- | --- | --- | --- |
| chr10:18011335-18012004 | | | 647 bp | | |  |  |  |  |
| Genome Start | Genome End | Strand | Div | Repeat Start | Repeat End | Repeat Left | Name | Family | Class |
| 18011335 | 18011381 | + | 15.2 | 1 | 47 | -548 | LTR49 | ERV1 | LTR |
| 18011382 | 18011461 | - | 10.0 | 1 | 80 | 0 | MADE1 | Mariner | DNA |
| 18011462 | 18011931 | + | 15.2 | 48 | 545 | -50 | LTR49 | ERV1 | LTR |
| 18011955 | 18012004 | + | 15.2 | 546 | 595 | 0 | LTR49 | ERV1 | LTR |

  

|  |  |  |  |  |  |  |  |  |  |
| --- | --- | --- | --- | --- | --- | --- | --- | --- | --- |
| chr10:18013566-18014411 | | | 846 bp | | |  |  |  |  |
| Genome Start | Genome End | Strand | Div | Repeat Start | Repeat End | Repeat Left | Name | Family | Class |
| 18013566 | 18014047 | + | 20.7 | 1 | 462 | -58 | MLT2B1 | ERVL | LTR |
| 18014048 | 18014350 | + | 14.8 | 1 | 303 | -9 | AluSx | Alu | SINE |
| 18014351 | 18014411 | + | 20.7 | 510 | 567 | 0 | MLT2B1 | ERVL | LTR |

  

|  |  |  |  |  |  |  |  |  |  |
| --- | --- | --- | --- | --- | --- | --- | --- | --- | --- |
| chr10:18079857-18080488 | | | 632 bp | | |  |  |  |  |
| Genome Start | Genome End | Strand | Div | Repeat Start | Repeat End | Repeat Left | Name | Family | Class |
| 18079857 | 18080034 | - | 10.0 | 137 | 312 | 0 | AluSx | Alu | SINE |
| 18080035 | 18080350 | - | 11.8 | 1 | 306 | -7 | AluSq | Alu | SINE |
| 18080351 | 18080488 | - | 10.0 | 1 | 136 | -176 | AluSx | Alu | SINE |

  

|  |  |  |  |  |  |  |  |  |  |
| --- | --- | --- | --- | --- | --- | --- | --- | --- | --- |
| chr10:18086152-18086653 | | | 502 bp | | |  |  |  |  |
| Genome Start | Genome End | Strand | Div | Repeat Start | Repeat End | Repeat Left | Name | Family | Class |
| 18086152 | 18086303 | - | 28.0 | 78 | 226 | -42 | MIRb | MIR | SINE |
| 18086304 | 18086602 | + | 17.2 | 1 | 298 | -14 | AluJo | Alu | SINE |
| 18086603 | 18086653 | - | 28.0 | 40 | 77 | -191 | MIRb | MIR | SINE |

  

|  |  |  |  |  |  |  |  |  |  |
| --- | --- | --- | --- | --- | --- | --- | --- | --- | --- |
| chr10:18096613-18097555 | | | 797 bp | | |  |  |  |  |
| Genome Start | Genome End | Strand | Div | Repeat Start | Repeat End | Repeat Left | Name | Family | Class |
| 18096613 | 18096882 | + | 23.3 | 35 | 294 | -1667 | Charlie4 | MER1\_type | DNA |
| 18096897 | 18097214 | - | 14.1 | 1 | 297 | -15 | AluJb | Alu | SINE |
| 18097347 | 18097555 | + | 21.6 | 1754 | 1955 | -6 | Charlie4 | MER1\_type | DNA |

  

|  |  |  |  |  |  |  |  |  |  |
| --- | --- | --- | --- | --- | --- | --- | --- | --- | --- |
| chr10:18097557-18098562 | | | 931 bp | | |  |  |  |  |
| Genome Start | Genome End | Strand | Div | Repeat Start | Repeat End | Repeat Left | Name | Family | Class |
| 18097557 | 18097703 | - | 31.2 | 3231 | 3372 | -47 | L2 | L2 | LINE |
| 18097776 | 18097879 | - | 36.6 | 2841 | 2947 | -472 | L2 | L2 | LINE |
| 18097880 | 18098067 | - | 18.3 | 5997 | 6183 | 0 | L1MB3 | L1 | LINE |
| 18098068 | 18098248 | - | 36.6 | 2656 | 2840 | -579 | L2 | L2 | LINE |
| 18098252 | 18098562 | - | 27.4 | 2196 | 2524 | -895 | L2 | L2 | LINE |

  

|  |  |  |  |  |  |  |  |  |  |
| --- | --- | --- | --- | --- | --- | --- | --- | --- | --- |
| chr10:18118592-18119244 | | | 653 bp | | |  |  |  |  |
| Genome Start | Genome End | Strand | Div | Repeat Start | Repeat End | Repeat Left | Name | Family | Class |
| 18118592 | 18118733 | + | 18.5 | 1 | 126 | -186 | AluJo | Alu | SINE |
| 18118734 | 18119045 | + | 8.1 | 1 | 309 | -2 | AluY | Alu | SINE |
| 18119046 | 18119244 | + | 18.5 | 127 | 306 | -6 | AluJo | Alu | SINE |

  

|  |  |  |  |  |  |  |  |  |  |
| --- | --- | --- | --- | --- | --- | --- | --- | --- | --- |
| chr10:18119962-18120775 | | | 777 bp | | |  |  |  |  |
| Genome Start | Genome End | Strand | Div | Repeat Start | Repeat End | Repeat Left | Name | Family | Class |
| 18119962 | 18120016 | + | 32.7 | 2867 | 2921 | -498 | L2 | L2 | LINE |
| 18120044 | 18120157 | + | 24.5 | 43 | 158 | -110 | MIR | MIR | SINE |
| 18120158 | 18120457 | + | 17.8 | 1 | 287 | -25 | AluJo | Alu | SINE |
| 18120458 | 18120561 | + | 23.8 | 147 | 246 | -16 | MIR | MIR | SINE |
| 18120572 | 18120775 | + | 34.3 | 2943 | 3166 | -212 | L2 | L2 | LINE |

  

|  |  |  |  |  |  |  |  |  |  |
| --- | --- | --- | --- | --- | --- | --- | --- | --- | --- |
| chr10:18141382-18141884 | | | 501 bp | | |  |  |  |  |
| Genome Start | Genome End | Strand | Div | Repeat Start | Repeat End | Repeat Left | Name | Family | Class |
| 18141382 | 18141560 | - | 21.0 | 896 | 1061 | -1 | MER63 | AcHobo | DNA |
| 18141563 | 18141862 | - | 9.7 | 1 | 299 | -11 | AluSg | Alu | SINE |
| 18141863 | 18141884 | - | 21.9 | 28 | 48 | -162 | MER63 | AcHobo | DNA |

  

|  |  |  |  |  |  |  |  |  |  |
| --- | --- | --- | --- | --- | --- | --- | --- | --- | --- |
| chr10:18147273-18149352 | | | 1768 bp | | |  |  |  |  |
| Genome Start | Genome End | Strand | Div | Repeat Start | Repeat End | Repeat Left | Name | Family | Class |
| 18147273 | 18147515 | - | 31.1 | 3147 | 3418 | -1 | L2 | L2 | LINE |
| 18147516 | 18147802 | + | 13.6 | 1 | 297 | -15 | AluSx | Alu | SINE |
| 18147803 | 18147926 | - | 31.1 | 2981 | 3146 | -273 | L2 | L2 | LINE |
| 18148030 | 18148235 | - | 21.5 | 507 | 711 | 0 | MER67C | ERV1 | LTR |
| 18148237 | 18148341 | - | 23.1 | 228 | 338 | -373 | MER67C | ERV1 | LTR |
| 18148505 | 18148637 | - | 18.5 | 388 | 512 | -48 | MLT2D | ERVL | LTR |
| 18148638 | 18148950 | + | 15.4 | 1 | 311 | -1 | AluJo | Alu | SINE |
| 18148951 | 18149208 | - | 18.5 | 6 | 241 | -173 | MLT2D | ERVL | LTR |
| 18149254 | 18149352 | - | 36.4 | 2497 | 2596 | -823 | L2 | L2 | LINE |

  

|  |  |  |  |  |  |  |  |  |  |
| --- | --- | --- | --- | --- | --- | --- | --- | --- | --- |
| chr10:18217485-18221538 | | | 4054 bp | | |  |  |  |  |
| Genome Start | Genome End | Strand | Div | Repeat Start | Repeat End | Repeat Left | Name | Family | Class |
| 18217485 | 18218362 | - | 17.2 | 2132 | 3045 | -3276 | L1MCa | L1 | LINE |
| 18218363 | 18218500 | - | 16.5 | 1 | 133 | 0 | FLAM\_C | Alu | SINE |
| 18218501 | 18219693 | - | 20.3 | 934 | 2131 | -4377 | L1MCa | L1 | LINE |
| 18219694 | 18220066 | + | 12.2 | 1 | 366 | -163 | MER1A | MER1\_type | DNA |
| 18220067 | 18220398 | - | 6.4 | 3 | 334 | 0 | AluY | Alu | SINE |
| 18220399 | 18220562 | + | 12.2 | 367 | 529 | 0 | MER1A | MER1\_type | DNA |
| 18220563 | 18221538 | - | 23.8 | 12 | 933 | -5575 | L1MCa | L1 | LINE |

  

|  |  |  |  |  |  |  |  |  |  |
| --- | --- | --- | --- | --- | --- | --- | --- | --- | --- |
| chr10:18242506-18243028 | | | 523 bp | | |  |  |  |  |
| Genome Start | Genome End | Strand | Div | Repeat Start | Repeat End | Repeat Left | Name | Family | Class |
| 18242506 | 18242570 | - | 20.9 | 3874 | 3940 | 0 | Arthur1 | Tip100 | DNA |
| 18242571 | 18242873 | + | 16.4 | 1 | 299 | -13 | AluJo | Alu | SINE |
| 18242874 | 18243028 | - | 20.9 | 3703 | 3873 | -67 | Arthur1 | Tip100 | DNA |

  

|  |  |  |  |  |  |  |  |  |  |
| --- | --- | --- | --- | --- | --- | --- | --- | --- | --- |
| chr10:18244331-18247334 | | | 2429 bp | | |  |  |  |  |
| Genome Start | Genome End | Strand | Div | Repeat Start | Repeat End | Repeat Left | Name | Family | Class |
| 18244331 | 18245125 | - | 23.4 | 2863 | 3657 | -283 | Arthur1 | Tip100 | DNA |
| 18245257 | 18245303 | - | 31.9 | 594 | 633 | -593 | Arthur1 | Tip100 | DNA |
| 18245304 | 18245541 | - | 8.9 | 76 | 313 | 0 | AluSq | Alu | SINE |
| 18245542 | 18245778 | - | 31.9 | 371 | 593 | -633 | Arthur1 | Tip100 | DNA |
| 18245795 | 18245847 | + | 26.4 | 17 | 213 | -182 | MLT2C1 | ERVL | LTR |
| 18245848 | 18245986 | + | 20.0 | 87 | 222 | -402 | MLT1E3 | MaLR | LTR |
| 18246148 | 18246457 | - | 20.1 | 1 | 309 | -3 | AluJo | Alu | SINE |
| 18246705 | 18246998 | - | 7.8 | 1 | 293 | -20 | AluSq | Alu | SINE |
| 18247019 | 18247334 | - | 18.0 | 9 | 320 | -3620 | Arthur1 | Tip100 | DNA |

  

|  |  |  |  |  |  |  |  |  |  |
| --- | --- | --- | --- | --- | --- | --- | --- | --- | --- |
| chr10:27230701-27231994 | | | 1294 bp | | |  |  |  |  |
| Genome Start | Genome End | Strand | Div | Repeat Start | Repeat End | Repeat Left | Name | Family | Class |
| 27230701 | 27231300 | + | 7.7 | 5184 | 5789 | -383 | L1PA8 | L1 | LINE |
| 27231301 | 27231607 | + | 9.2 | 1 | 304 | -7 | AluY | Alu | SINE |
| 27231608 | 27231994 | + | 7.7 | 5790 | 6163 | -9 | L1PA8 | L1 | LINE |

  

|  |  |  |  |  |  |  |  |  |  |
| --- | --- | --- | --- | --- | --- | --- | --- | --- | --- |
| chr10:27248492-27248925 | | | 434 bp | | |  |  |  |  |
| Genome Start | Genome End | Strand | Div | Repeat Start | Repeat End | Repeat Left | Name | Family | Class |
| 27248492 | 27248586 | + | 31.9 | 108 | 219 | -49 | MIRb | MIR | SINE |
| 27248587 | 27248873 | + | 10.1 | 13 | 299 | -11 | AluSg | Alu | SINE |
| 27248874 | 27248925 | + | 31.9 | 220 | 268 | 0 | MIRb | MIR | SINE |

  

|  |  |  |  |  |  |  |  |  |  |
| --- | --- | --- | --- | --- | --- | --- | --- | --- | --- |
| chr10:27253620-27256149 | | | 2503 bp | | |  |  |  |  |
| Genome Start | Genome End | Strand | Div | Repeat Start | Repeat End | Repeat Left | Name | Family | Class |
| 27253620 | 27255201 | + | 16.2 | 4240 | 5825 | -519 | L1MC1 | L1 | LINE |
| 27255202 | 27255283 | + | 16.4 | 227 | 305 | -6 | AluS | Alu | SINE |
| 27255311 | 27255672 | + | 24.0 | 1 | 447 | -10 | MSTB2 | MaLR | LTR |
| 27255673 | 27256149 | + | 18.1 | 5843 | 6333 | 0 | L1MC1 | L1 | LINE |

  

|  |  |  |  |  |  |  |  |  |  |
| --- | --- | --- | --- | --- | --- | --- | --- | --- | --- |
| chr10:27259090-27259554 | | | 465 bp | | |  |  |  |  |
| Genome Start | Genome End | Strand | Div | Repeat Start | Repeat End | Repeat Left | Name | Family | Class |
| 27259090 | 27259192 | - | 17.3 | 93 | 230 | 0 | MER30 | MER1\_type | DNA |
| 27259193 | 27259477 | - | 14.8 | 5 | 289 | -23 | AluSx | Alu | SINE |
| 27259478 | 27259554 | - | 17.3 | 18 | 92 | -138 | MER30 | MER1\_type | DNA |

  

|  |  |  |  |  |  |  |  |  |  |
| --- | --- | --- | --- | --- | --- | --- | --- | --- | --- |
| chr10:27491735-27492280 | | | 546 bp | | |  |  |  |  |
| Genome Start | Genome End | Strand | Div | Repeat Start | Repeat End | Repeat Left | Name | Family | Class |
| 27491735 | 27491847 | + | 26.7 | 1 | 132 | -206 | MER46C | MER2\_type | DNA |
| 27491848 | 27492151 | - | 9.9 | 1 | 304 | -9 | AluSq | Alu | SINE |
| 27492152 | 27492280 | + | 25.8 | 133 | 293 | -45 | MER46C | MER2\_type | DNA |

  

|  |  |  |  |  |  |  |  |  |  |
| --- | --- | --- | --- | --- | --- | --- | --- | --- | --- |
| chr10:27655770-27657221 | | | 1406 bp | | |  |  |  |  |
| Genome Start | Genome End | Strand | Div | Repeat Start | Repeat End | Repeat Left | Name | Family | Class |
| 27655770 | 27655873 | - | 11.4 | 6066 | 6172 | -2 | L1PA11 | L1 | LINE |
| 27655920 | 27656206 | - | 13.6 | 5 | 284 | -28 | AluSx | Alu | SINE |
| 27656207 | 27657221 | - | 10.9 | 5037 | 6065 | -109 | L1PA11 | L1 | LINE |

  

|  |  |  |  |  |  |  |  |  |  |
| --- | --- | --- | --- | --- | --- | --- | --- | --- | --- |
| chr10:28291908-28292346 | | | 439 bp | | |  |  |  |  |
| Genome Start | Genome End | Strand | Div | Repeat Start | Repeat End | Repeat Left | Name | Family | Class |
| 28291908 | 28291946 | - | 25.9 | 223 | 265 | -3 | MIRb | MIR | SINE |
| 28291947 | 28292241 | - | 10.2 | 1 | 294 | -16 | AluSg | Alu | SINE |
| 28292242 | 28292346 | - | 25.9 | 110 | 222 | -46 | MIRb | MIR | SINE |

  

|  |  |  |  |  |  |  |  |  |  |
| --- | --- | --- | --- | --- | --- | --- | --- | --- | --- |
| chr10:28295732-28297326 | | | 1595 bp | | |  |  |  |  |
| Genome Start | Genome End | Strand | Div | Repeat Start | Repeat End | Repeat Left | Name | Family | Class |
| 28295732 | 28296543 | + | 26.7 | 22 | 1021 | -5612 | L1MDa | L1 | LINE |
| 28296544 | 28296839 | + | 12.8 | 1 | 297 | -15 | AluSx | Alu | SINE |
| 28296840 | 28297326 | + | 25.9 | 1022 | 1529 | -5104 | L1MDa | L1 | LINE |

  

|  |  |  |  |  |  |  |  |  |  |
| --- | --- | --- | --- | --- | --- | --- | --- | --- | --- |
| chr10:30713343-30714262 | | | 920 bp | | |  |  |  |  |
| Genome Start | Genome End | Strand | Div | Repeat Start | Repeat End | Repeat Left | Name | Family | Class |
| 30713343 | 30713530 | - | 15.2 | 110 | 297 | -15 | AluSx | Alu | SINE |
| 30713531 | 30714149 | - | 12.7 | 1 | 614 | 0 | LTR18B | ERVL | LTR |
| 30714150 | 30714262 | - | 15.2 | 1 | 109 | -203 | AluSx | Alu | SINE |

  

|  |  |  |  |  |  |  |  |  |  |
| --- | --- | --- | --- | --- | --- | --- | --- | --- | --- |
| chr10:31019178-31019724 | | | 547 bp | | |  |  |  |  |
| Genome Start | Genome End | Strand | Div | Repeat Start | Repeat End | Repeat Left | Name | Family | Class |
| 31019178 | 31019227 | + | 21.0 | 4 | 46 | -178 | MER58A | MER1\_type | DNA |
| 31019228 | 31019541 | - | 16.3 | 1 | 306 | -6 | AluJo | Alu | SINE |
| 31019542 | 31019724 | + | 21.0 | 47 | 223 | -1 | MER58A | MER1\_type | DNA |

  

|  |  |  |  |  |  |  |  |  |  |
| --- | --- | --- | --- | --- | --- | --- | --- | --- | --- |
| chr10:36326871-36328565 | | | 1695 bp | | |  |  |  |  |
| Genome Start | Genome End | Strand | Div | Repeat Start | Repeat End | Repeat Left | Name | Family | Class |
| 36326871 | 36327498 | - | 20.0 | 744 | 1453 | -2 | Charlie1a | MER1\_type | DNA |
| 36327499 | 36327793 | + | 17.4 | 1 | 291 | -21 | AluJo | Alu | SINE |
| 36327794 | 36328565 | - | 20.0 | 1 | 743 | -712 | Charlie1a | MER1\_type | DNA |

  

|  |  |  |  |  |  |  |  |  |  |
| --- | --- | --- | --- | --- | --- | --- | --- | --- | --- |
| chr10:37177118-37177670 | | | 553 bp | | |  |  |  |  |
| Genome Start | Genome End | Strand | Div | Repeat Start | Repeat End | Repeat Left | Name | Family | Class |
| 37177118 | 37177204 | + | 9.2 | 4936 | 5022 | -1124 | L1P3 | L1 | LINE |
| 37177205 | 37177504 | + | 9.4 | 1 | 308 | -2 | AluSg | Alu | SINE |
| 37177505 | 37177670 | + | 9.2 | 5023 | 5187 | -959 | L1P3 | L1 | LINE |

  

|  |  |  |  |  |  |  |  |  |  |
| --- | --- | --- | --- | --- | --- | --- | --- | --- | --- |
| chr10:37231140-37231736 | | | 597 bp | | |  |  |  |  |
| Genome Start | Genome End | Strand | Div | Repeat Start | Repeat End | Repeat Left | Name | Family | Class |
| 37231140 | 37231221 | + | 33.8 | 3242 | 3320 | -99 | L2 | L2 | LINE |
| 37231222 | 37231660 | - | 13.6 | 1 | 428 | 0 | MSTA | MaLR | LTR |
| 37231661 | 37231736 | + | 33.8 | 3321 | 3407 | -12 | L2 | L2 | LINE |

  

|  |  |  |  |  |  |  |  |  |  |
| --- | --- | --- | --- | --- | --- | --- | --- | --- | --- |
| chr10:37315957-37316604 | | | 648 bp | | |  |  |  |  |
| Genome Start | Genome End | Strand | Div | Repeat Start | Repeat End | Repeat Left | Name | Family | Class |
| 37315957 | 37316014 | + | 39.0 | 3012 | 3071 | -307 | L2 | L2 | LINE |
| 37316015 | 37316323 | - | 14.9 | 1 | 308 | -1 | AluSc | Alu | SINE |
| 37316324 | 37316604 | + | 39.0 | 3072 | 3376 | -2 | L2 | L2 | LINE |

  

|  |  |  |  |  |  |  |  |  |  |
| --- | --- | --- | --- | --- | --- | --- | --- | --- | --- |
| chr10:37360874-37361858 | | | 985 bp | | |  |  |  |  |
| Genome Start | Genome End | Strand | Div | Repeat Start | Repeat End | Repeat Left | Name | Family | Class |
| 37360874 | 37361026 | + | 20.8 | 5638 | 5787 | -504 | L1MA7 | L1 | LINE |
| 37361027 | 37361337 | + | 12.6 | 1 | 309 | -1 | AluSg | Alu | SINE |
| 37361338 | 37361858 | + | 20.8 | 5788 | 6289 | -2 | L1MA7 | L1 | LINE |

  

|  |  |  |  |  |  |  |  |  |  |
| --- | --- | --- | --- | --- | --- | --- | --- | --- | --- |
| chr10:37386861-37387818 | | | 522 bp | | |  |  |  |  |
| Genome Start | Genome End | Strand | Div | Repeat Start | Repeat End | Repeat Left | Name | Family | Class |
| 37386861 | 37386981 | - | 37.0 | 107 | 229 | -39 | MIRb | MIR | SINE |
| 37386983 | 37387290 | + | 9.7 | 1 | 309 | 0 | AluSc | Alu | SINE |
| 37387726 | 37387818 | - | 35.5 | 5 | 99 | -169 | MIRb | MIR | SINE |

  

|  |  |  |  |  |  |  |  |  |  |
| --- | --- | --- | --- | --- | --- | --- | --- | --- | --- |
| chr10:37707146-37707546 | | | 401 bp | | |  |  |  |  |
| Genome Start | Genome End | Strand | Div | Repeat Start | Repeat End | Repeat Left | Name | Family | Class |
| 37707146 | 37707292 | + | 24.1 | 28 | 180 | -81 | MER112 | MER1\_type | DNA |
| 37707293 | 37707469 | - | 21.2 | 3 | 180 | 0 | MER104 | Tc2 | DNA |
| 37707470 | 37707546 | + | 24.1 | 181 | 257 | -4 | MER112 | MER1\_type | DNA |

  

|  |  |  |  |  |  |  |  |  |  |
| --- | --- | --- | --- | --- | --- | --- | --- | --- | --- |
| chr10:38361277-38362645 | | | 1361 bp | | |  |  |  |  |
| Genome Start | Genome End | Strand | Div | Repeat Start | Repeat End | Repeat Left | Name | Family | Class |
| 38361277 | 38361377 | - | 22.8 | 6066 | 6165 | -13 | L1MB8 | L1 | LINE |
| 38361378 | 38361684 | - | 11.8 | 1 | 299 | -13 | AluSx | Alu | SINE |
| 38361693 | 38361981 | - | 11.1 | 1 | 289 | -23 | AluSx | Alu | SINE |
| 38361982 | 38362645 | - | 22.8 | 5404 | 6064 | -114 | L1MB8 | L1 | LINE |

  

|  |  |  |  |  |  |  |  |  |  |
| --- | --- | --- | --- | --- | --- | --- | --- | --- | --- |
| chr10:38422090-38422662 | | | 573 bp | | |  |  |  |  |
| Genome Start | Genome End | Strand | Div | Repeat Start | Repeat End | Repeat Left | Name | Family | Class |
| 38422090 | 38422205 | - | 13.3 | 6032 | 6145 | -9 | L1PB3 | L1 | LINE |
| 38422206 | 38422504 | - | 12.4 | 1 | 302 | -11 | AluSq | Alu | SINE |
| 38422505 | 38422662 | - | 13.3 | 5878 | 6031 | -123 | L1PB3 | L1 | LINE |

  

|  |  |  |  |  |  |  |  |  |  |
| --- | --- | --- | --- | --- | --- | --- | --- | --- | --- |
| chr10:38552373-38552776 | | | 395 bp | | |  |  |  |  |
| Genome Start | Genome End | Strand | Div | Repeat Start | Repeat End | Repeat Left | Name | Family | Class |
| 38552373 | 38552447 | + | 28.8 | 1 | 75 | -114 | MER5A | MER1\_type | DNA |
| 38552448 | 38552648 | + | 23.0 | 7730 | 7951 | -44 | L1MD3 | L1 | LINE |
| 38552658 | 38552776 | + | 31.9 | 55 | 176 | -13 | MER5A | MER1\_type | DNA |

  

|  |  |  |  |  |  |  |  |  |  |
| --- | --- | --- | --- | --- | --- | --- | --- | --- | --- |
| chr10:38560474-38562686 | | | 2189 bp | | |  |  |  |  |
| Genome Start | Genome End | Strand | Div | Repeat Start | Repeat End | Repeat Left | Name | Family | Class |
| 38560474 | 38560869 | - | 24.9 | 3102 | 3504 | -2715 | L1MEc | L1 | LINE |
| 38560870 | 38561180 | - | 14.2 | 1 | 312 | 0 | AluSx | Alu | SINE |
| 38561187 | 38561557 | - | 26.1 | 2469 | 2842 | -3377 | L1MEc | L1 | LINE |
| 38561576 | 38562138 | - | 25.7 | 738 | 1341 | -5059 | L1MEc | L1 | LINE |
| 38562139 | 38562212 | - | 9.5 | 104 | 177 | -8 | FAM | Alu | SINE |
| 38562213 | 38562686 | - | 25.7 | 229 | 737 | -5663 | L1MEc | L1 | LINE |

  

|  |  |  |  |  |  |  |  |  |  |
| --- | --- | --- | --- | --- | --- | --- | --- | --- | --- |
| chr10:38574324-38574733 | | | 410 bp | | |  |  |  |  |
| Genome Start | Genome End | Strand | Div | Repeat Start | Repeat End | Repeat Left | Name | Family | Class |
| 38574324 | 38574404 | - | 30.7 | 3332 | 3415 | -4 | L2 | L2 | LINE |
| 38574405 | 38574713 | + | 12.1 | 1 | 310 | -3 | AluSp | Alu | SINE |
| 38574714 | 38574733 | - | 30.7 | 3311 | 3331 | -88 | L2 | L2 | LINE |

  

|  |  |  |  |  |  |  |  |  |  |
| --- | --- | --- | --- | --- | --- | --- | --- | --- | --- |
| chr10:38604644-38605476 | | | 755 bp | | |  |  |  |  |
| Genome Start | Genome End | Strand | Div | Repeat Start | Repeat End | Repeat Left | Name | Family | Class |
| 38604644 | 38604773 | - | 23.9 | 3223 | 3377 | -1 | L2 | L2 | LINE |
| 38604836 | 38605191 | - | 13.3 | 1 | 381 | 0 | THE1D | MaLR | LTR |
| 38605208 | 38605476 | - | 32.7 | 2966 | 3269 | -150 | L2 | L2 | LINE |

  

|  |  |  |  |  |  |  |  |  |  |
| --- | --- | --- | --- | --- | --- | --- | --- | --- | --- |
| chr10:38610348-38611112 | | | 765 bp | | |  |  |  |  |
| Genome Start | Genome End | Strand | Div | Repeat Start | Repeat End | Repeat Left | Name | Family | Class |
| 38610348 | 38610414 | + | 9.2 | 5685 | 5758 | -405 | L1PA13 | L1 | LINE |
| 38610415 | 38610711 | + | 12.5 | 1 | 297 | -15 | AluSx | Alu | SINE |
| 38610712 | 38611112 | + | 9.2 | 5759 | 6155 | -8 | L1PA13 | L1 | LINE |

  

|  |  |  |  |  |  |  |  |  |  |
| --- | --- | --- | --- | --- | --- | --- | --- | --- | --- |
| chr10:38640562-38641940 | | | 1206 bp | | |  |  |  |  |
| Genome Start | Genome End | Strand | Div | Repeat Start | Repeat End | Repeat Left | Name | Family | Class |
| 38640562 | 38640651 | - | 30.4 | 1391 | 1482 | -7 | MLT1H-int | MaLR | LTR |
| 38640652 | 38641227 | - | 12.1 | 1 | 579 | 0 | MER34D | ERV1 | LTR |
| 38641228 | 38641324 | - | 26.8 | 1261 | 1390 | -99 | MLT1H-int | MaLR | LTR |
| 38641408 | 38641690 | - | 14.5 | 1 | 282 | -30 | AluSx | Alu | SINE |
| 38641781 | 38641940 | - | 29.4 | 183 | 346 | -1126 | MLT1H-int | MaLR | LTR |

  

|  |  |  |  |  |  |  |  |  |  |
| --- | --- | --- | --- | --- | --- | --- | --- | --- | --- |
| chr10:38683119-38684648 | | | 1530 bp | | |  |  |  |  |
| Genome Start | Genome End | Strand | Div | Repeat Start | Repeat End | Repeat Left | Name | Family | Class |
| 38683119 | 38683179 | + | 14.8 | 54 | 120 | -204 | MER5C | MER1\_type | DNA |
| 38683180 | 38683563 | - | 11.5 | 254 | 640 | 0 | MER51C | ERV1 | LTR |
| 38683564 | 38684193 | + | 7.0 | 1 | 660 | 0 | PABL\_A | ERV1 | LTR |
| 38684194 | 38684471 | - | 11.6 | 1 | 253 | -387 | MER51C | ERV1 | LTR |
| 38684472 | 38684648 | + | 20.1 | 117 | 324 | 0 | MER5C | MER1\_type | DNA |

  

|  |  |  |  |  |  |  |  |  |  |
| --- | --- | --- | --- | --- | --- | --- | --- | --- | --- |
| chr10:38735428-38736045 | | | 618 bp | | |  |  |  |  |
[truncated: 1,727,124 more chars]
